# Nucleotide sequence analysis reveals the presence of PVY-Tam isolates affecting tamarillo in Colombia

**DOI:** 10.1186/s12985-026-03166-6

**Published:** 2026-04-20

**Authors:** Carolina Martínez-Moncayo, Tulio César Lagos-Burbano, Clara Ontañón, Inmaculada Ferriol, Juan José López-Moya, Mireia Uranga

**Affiliations:** 1 Universidad de Nariño, Ciudad Universitaria Torobajo, 520002 Pasto, Colombia; 2 Centre for Research in Agricultural Genomics (CRAG), CSIC-IRTA-UAB-UB, Campus UAB, Cerdanyola del Vallès, 08193 Bellaterra, Spain; 3 Instituto de Ciencias Agrarias (ICA)-CSIC, 28006 Madrid, Spain; 4 Present Address: Institute for Integrative Systems Biology (I2SysBio), Universitat de València-CSIC, 46908 Paterna, Spain

**Keywords:** Tamarillo, High-throughput sequencing, Virus identification, Torradovirus, Potyvirus, Potato virus Y

## Abstract

**Background:**

Tamarillo or tree tomato (*Solanum betaceum* Cav.) is a fruit tree species of Andean origin with cultural and economic relevance in Colombia. The high incidence of complex viral diseases termed “virosis” in all tamarillo-growing regions of the country leads to huge production losses and seriously threatens its cultivation.

**Methods:**

RNA-seq libraries were constructed for symptomatic samples from eight tamarillo-growing locations across the Department of Nariño (Colombia). Their virus diversity was characterized using the Genome Detective software. The identified PVY isolate was subsequently subjected to RT-PCR validation and phylogenetic analysis.

**Results:**

Several virus species belonging to the genera *Torradovirus*, *Potyvirus* and *Polerovirus* were identified in mixed infections in tamarillo. Full- or nearly full-length genomes were generated for tomato torrado virus (ToTV), physalis torrado virus (PhyTV), potato leafroll virus (PLRV), and a novel isolate of potato virus Y-Tamarillo (PVY-Tam). When present, PVY-Tam seems to synergistically boost the accumulation of unrelated viruses, therefore contributing to the severity of the infections. Nucleotide sequence analysis suggests that the PVY-Tam from Nariño originated in South America by a recent divergence of the PVY^N^ lineage, and possibly by the recombination of geographically close isolates. Moreover, P3N-PIPO, a frameshift product from the potyviral P3 gene, shows variations in protein lenght between PVY isolates that might be involved with host-specific adaptations.

**Conclusions:**

We provide evidence of a novel PVY-Tam isolate in the Andean region that might promote the severity of unrelated virus partners in field-grown tamarillo. Our findings contribute to understanding tamarillo virosis for the development of effective diagnostic and control strategies.

**Supplementary Information:**

The online version contains supplementary material available at 10.1186/s12985-026-03166-6.

## Introduction

Tamarillo, or tree tomato (*Solanum betaceum* Cav., syn *Cyphomandra betacea* Sendt.), is a fruit tree of Andean origin with economic relevance in Colombia and Ecuador, where it is cultivated for the fresh fruit market and food processing industry [1]. The fruit is egg-shaped with purple-red to golden-yellow skin, yellow-orange firm flesh and jelly with small seeds [2]. Regarding its nutritional properties, it is relatively low in carbohydrates and a good source of dietary fiber, vitamins A, B_6_, C, and E, minerals (mainly potassium, phosphorus and magnesium) and a variety of functional bioactives with antioxidant activity like phenolics, anthocyanins, and carotenoids [3, 4]. Although the exact origin of tamarillo is unknown, it is possibly native to South American countries since wild relatives are found in Colombia, Peru, Ecuador, Bolivia, Brazil and Chile [5]. In the late nineteenth century, tamarillo was globally introduced to Australia, New Zealand, Southeast Asia, and some countries in Europe and Africa. Nowadays, it is commercially grown only in Colombia, Peru, Ecuador, New Zealand, and Australia [6, 7].

In Colombia, over 140,228 tons of tamarillo are produced annually in a total cultivated area of about 9,223 hectares distributed across 18 provinces, representing a relevant amount of the national fruit production [8]. Tamarillo cultivation in Colombia is threatened by the high incidence of complex viral diseases known as "tamarillo virosis" [9]. Symptoms include mosaics, vein outgrowth, leaf deformation, yellowing, ringspots and severe reductions in fruit production and plant longevity. This disease was initially observed in 1991 in the department of Antioquia and has since been reported in all tamarillo-growing regions of the country, including Nariño, Cundinamarca and Antioquia [10–15], causing significant production losses ranging 50–80% annually. The lack of effective treatments means that eradication is the only alternative in severe virosis cases. This significantly reduces the crop’s lifespan and increases the economic costs by the need to replace orchards with healthy plants.

Several studies have reported that virosis in tamarillo can be caused by combinations of viruses from different genera, including members of *Alfamovirus* (alfalfa mosaic virus, AMV), *Cucumovirus* (cucumber mosaic virus, CMV), *Nepovirus* (tomato ringspot virus, ToRSV), *Polerovirus* (potato leafroll virus, PLRV), *Potexvirus* (potato aucuba mosaic virus, PAMV), *Potyvirus* (potato virus Y, PVY; tamarillo leaf malformation virus, TLMV; potato virus A, PVA), *Tobamovirus* (tobacco mosaic virus, TMV; and tomato mosaic virus, ToMV), and *Tospovirus* (tomato spotted wilt virus, TSWV) [16–20]. Some reports have identified up to eight viruses in a single infected plant, of which at least two were potyviruses able to establish synergistic interactions with unrelated viruses [17, 21, 22].

In this work, we aimed to characterize the virome of symptomatic tamarillo plants cultivated across the Department of Nariño, Colombia. First, we generated RNA libraries from symptomatic plants collected in eight tamarillo-growing locations and submitted them to Next Generation Sequencing (NGS). Next, we studied the virus diversity of each sample using an innovative, web-based diagnostic tool named Genome Detective, confirming the presence of several viruses belonging to the genera *Torradovirus, Potyvirus*, and *Polerovirus*. Additionally, in-depth sequence analysis of the viral sequences enabled the identification of a novel isolate of potato virus Y-Tamarillo (PVY-Tam) that seems to synergistically boost the accumulation of unrelated viruses, thus promoting the severity of the infections. Using two virus-specific primer pairs, we validated the presence of PVY-Tam in five of the eight sites. Phylogenetic analysis indicated that PVY-Tam originated in South America by a recent divergence of the PVY^N^ lineage, possibly driven by the recombination of geographically closer isolates. Additionally, we observed clade-specific variations in the protein length of P3N-terminal half fused to the Pretty Interesting Potyvirus ORF (P3N-PIPO), which might be involved with host-specific adaptations of PVY isolates. Our findings contribute to a better understanding of tamarillo virosis in the Andean region, and overall, in other tamarillo-cultivating areas worldwide.

## Materials and methods

### Sample collection

**Fig. 1:**
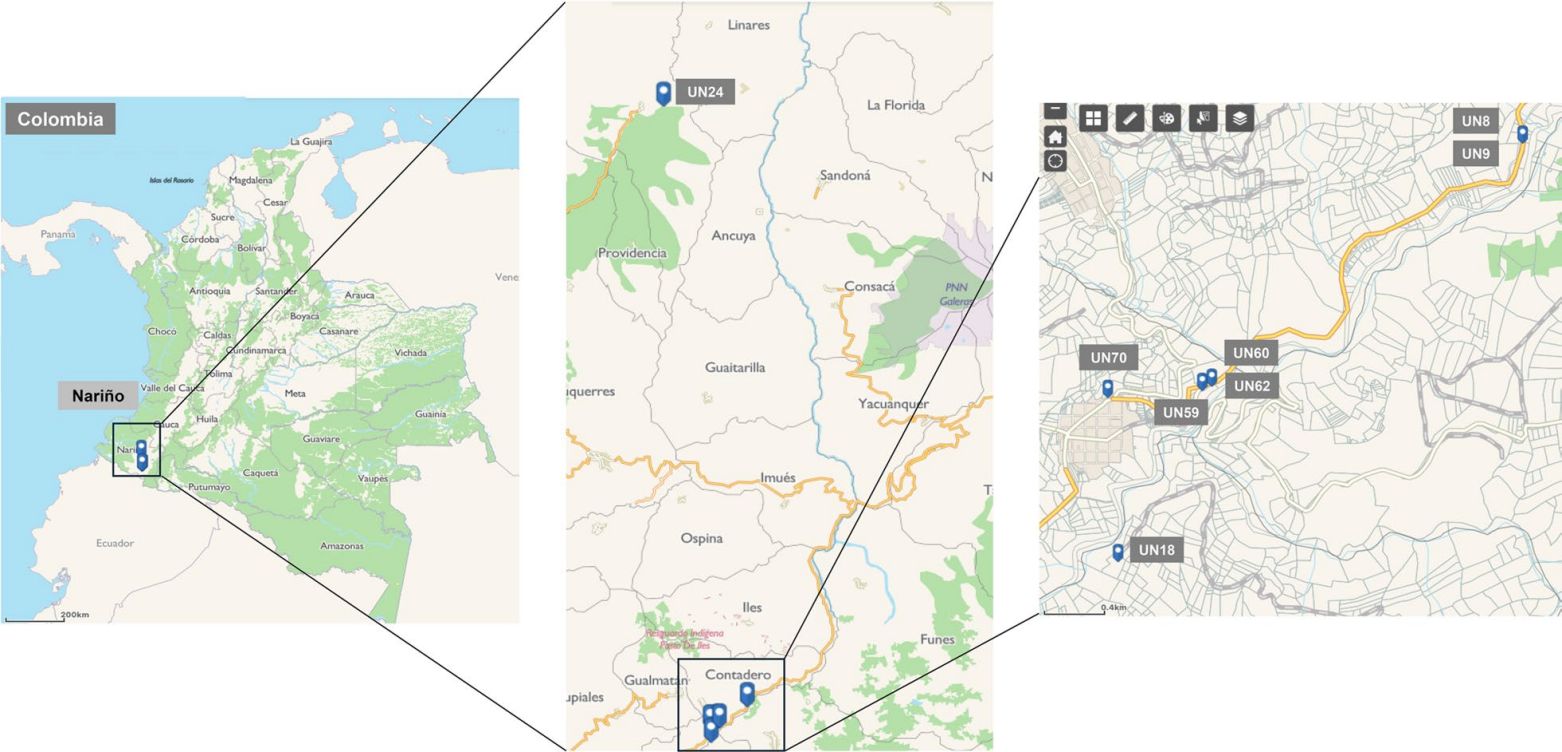
Geographic localization of tamarillo-growing orchards in Nariño (Colombia) sampled in the study**.** Samples of tamarillo plants showing symptoms of virosis were collected in a total of eight locations distributed in an altitude range of 2000–2500 m across the department. All locations except UN24 were situated in Contadero region

The sampling for this study was conducted in municipalities with the highest tamarillo production across the department of Nariño (Colombia) to ensure an accurate representation of the region’s phytosanitary situation. (Fig. 1 and Supplementary Table S1). Representative plots were chosen at each municipality to assess the spatial variability of the viral genomes, and orchards in the productive stage were identified. The sampled orchards corresponded to the to the limited number of tamarillo plants currently in production in Nariño.

Symptomatic tamarillo plants from each location were recorded during the study and sampled accordingly. Each sample consisted of young apical shoots from tamarillo plants showing symptoms of viral infections (Supplementary Table S2), which were collected and transported in polypropylene coolers containing ice packs to the Molecular Biology Laboratory at the University of Nariño. Next, shoots were individually wrapped with paper towels, placed inside labeled paper bags, and immediately processed to minimize the risk of degradation. Part of the plant material was stored at -80 °C for subsequent analysis.

### Total RNA extraction and RNA-seq library construction

Symptomatic shoots (50 mg) were frozen in liquid nitrogen and ground using a TissueLyser (Qiagen). Total RNA extraction was performed using TRIzol reagent (Life Technologies, USA) following the manufacturer’s instructions. RNA quality and concentration were estimated using a NanoDrop One (Thermo Fisher Scientific, USA) spectrophotometer and electrophoresis visualization on 1% agarose gels in 1X TAE buffer (40 mM Tris–acetate, 1 mM EDTA, pH 8.0) and staining with GelRed (Biotium, USA). Purified RNA samples were stored at -80 °C until use.

200 ng of total RNA were used for RNA-Seq library construction. Messenger RNA (mRNA) was purified from total RNA using poly-T oligo-conjugated magnetic beads (New England Biolabs, USA). Fragmented RNA was employed for the synthesis of first-strand complementary DNA (cDNA) using random hexamer primers, followed by second-strand cDNA synthesis. Library construction was performed using the TruSeq RNA Sample Preparation kit (Illumina, USA) as described [23]. Library quality and concentration were estimated using a Qubit (Thermo Fisher Scientific) fluorometer, and fragment size distribution was assessed by real-time PCR along with a Bioanalyzer. Finally, library sequencing was conducted at Novogene (USA) using the NovaSeq 6000 sequencer platform (Illumina) to generate pooled, paired-end (read size: 50–150 bp) libraries.

### Identification of viral sequences using genome detective software

The obtained raw reads were submitted to the web-based software application Genome Detective (https://www.genomedetective.com/app/typingtool/virus/) to identify viral sequences. By using a novel reference-based linking alignment method that combines nucleotide and amino acid scores, Genome Detective allows a rapid and accurate assembly of RNA-seq reads into annotated viral contigs [24]. For torradovirus samples, an additional analysis was performed calculating the nucleotide and amino acid p-distance of the RNA1 CG to GDD (Protease-Pol) conserved region from each contig to selected torradovirus reference sequences (Table 1) using the software MEGA 12 [25].

**Table 1:** Nucleotide (nt) and amino acid (aa) sequence identities comparison of the Pro-Pol (CG to GDD) conserved region of each torradovirus-like RNA1 contig to the RNA1 reference genome of selected torradovirus species. Amino acid identities > 80% in the Pro-Pol region are highlighted in grey. Accession numbers used for the Pro-Pol analysis: ToTV, tomato torrado virus (DQ388879); PhyTV, physalis torrado virus (MZ357183) ToMarV, tomato marchitez virus (EF681764); ToChSV, tomato chocolate spot virus (NC_013075); ToNDV, tomato necrotic dwarf virus (NCBI: NC_027926). Sequence identities were calculated based on two-sequence alignment using Blastn (for nucleotide) and Blastp (for amino acid)

Location	Contig name	ToTV (DQ388879)		PhyTV (MZ357183)		ToMarV (EF681764)		ToChSV (NC_013075.1)		ToNDV (NC_027926.1)	
		nt	aa	nt	aa	nt	aa	nt	aa	nt	aa
UN8	UN8-NC_009013.1–60-1	96.36	99.56	69.65	78.82	71.69	78.60	70.16	78.17	69.80	77.29
UN9	UN9-NC_009013.1–60-1	92.14	99.34	69.94	78.60	71.32	78.38	69.51	78.60	70.01	77.07
UN18	UN18_val-NC_009013.1–45-1	91.05	97.82	70.01	78.82	73.14	79.04	70.09	78.38	70.23	77.73
	UN18_val-NC_010987.1–46-1	68.92	78.60	71.62	82.53	69.29	78.60	68.70	76.86	69.72	76.20
	UN18_val-NC_027926.1–47-0	69.65	79.04	98.84	99.34	69.80	77.51	69.21	77.51	69.94	77.95
UN59	UN59-NC_010987.1–45-1	69.87	79.26	98.76	99.34	69.80	77.51	69.07	77.51	70.01	77.95
	UN59-NC_009013.1–44-1	93.52	99.34	69.80	78.60	71.47	78.38	69.72	78.17	70.31	77.07
UN60	UN60-NC_009013.1–63-1	93.30	99.78	70.23	79.04	71.76	78.38	70.01	78.60	70.38	77.07
	UN60-NC_010987.1–66-1	69.00	78.82	71.69	82.75	69.21	78.82	68.63	77.07	69.80	76.42
	UN60-NC_027926.1–64-0	69.51	79.04	98.98	99.56	69.72	77.51	68.85	77.51	69.94	78.17
UN62	UN62-NC_009013.1–38-1	89.52	96.94	70.09	78.38	72.56	78.60	70.89	77.95	71.03	77.29
UN70	UN70-NC_010987.1–1-2	69.80	79.26	98.98	99.34	69.87	77.51	69.07	77.51	70.01	77.95
	UN70-NC_009013.1–43-1	93.01	99.56	69.51	78.82	71.40	78.60	69.87	78.82	69.65	77.29

### Validation of PVY-Tam by RT-PCR

Total RNA extraction was performed using TRIzol reagent (Life Technologies, USA) following the manufacturer’s instructions. A RT-PCR assay was performed to confirm the presence of PVY-Tam in the total RNA of the samples collected in the eight tamarillo-growing locations, as described [26]. The M-MLV Reverse Transcriptase kit and *Taq* DNA polymerase (both from Invitrogen, USA), were used for reverse transcription and PCR, respectively, following the manufacturer’s instructions, with a modification of the annealing temperature to 53 °C for primers PVY-T CP and PVY-T P3. First, the consensus sequence of the polyprotein gene of PVY-Tam Nariño isolate was inferred by multiple alignment of the corresponding viral contigs from different locations. Then, specific primers were designed to amplify a 404-nt fragment in the P3 cistron, and a 323-nucleotide (nt) fragment between the nuclear inclusion b (NIb) and the coat protein (CP) cistrons, respectively (Supplementary Table S3). RT-PCR products were visualized by electrophoresis on 1% agarose gels in 1X TAE buffer (40 mM Tris–acetate, 1 mM EDTA, pH 8.0) and stained with GelRed and then sent for Sanger sequencing to the National University of Colombia (Bogotá Campus).

### Phylogenetic analysis

The polyprotein gene of the PVY-Tam Nariño isolate was translated into its corresponding amino acid sequence and used to infer phylogenetic relationships with 19 selected potyvirus species belonging to the PVY clade [27]. Phylogenetic and geographic criteria were applied to select potyvirus species based on the most recently available phylogenetic tree generated by the International Committee on Taxonomy of Viruses (ICTV) *Potyviridae* Study Group (available at https://ictv.global/taxonomy/taxondetails?taxnode_id=202404562&taxon_name=Potyvirus), choosing one representative per main branch or node, with preference for species reported in South America. Additional phylogenetic analyses were performed with type members of the major PVY clades, Colombian PVY isolates with potato and tomato as isolation hosts, and three PVY-Tam isolates previously reported in Ecuador [20]. A list of the potyvirus species and PVY isolates used in the study is included in Supplementary Table S4. Tree construction was performed using the maximum likelihood method with 1000 bootstraps interactions available in MEGA12. CLUSTAL W with default parameters was used to generate multiple sequence alignments for the P3N-PIPO sequences. To detect putative recombination breakpoints within available PVY-Tam sequences, we employed the Genetic Algorithm for Recombination Detection (GARD) hosted in the Datamonkey server [28, 29] with default configuration, using nucleotide sequence of PYV-Tam in a faster run mode, genetic universal code and no site-to-site rate variation.

## Results

### Symptomatology of complex viral infections in tamarillo

**Fig. 2:**
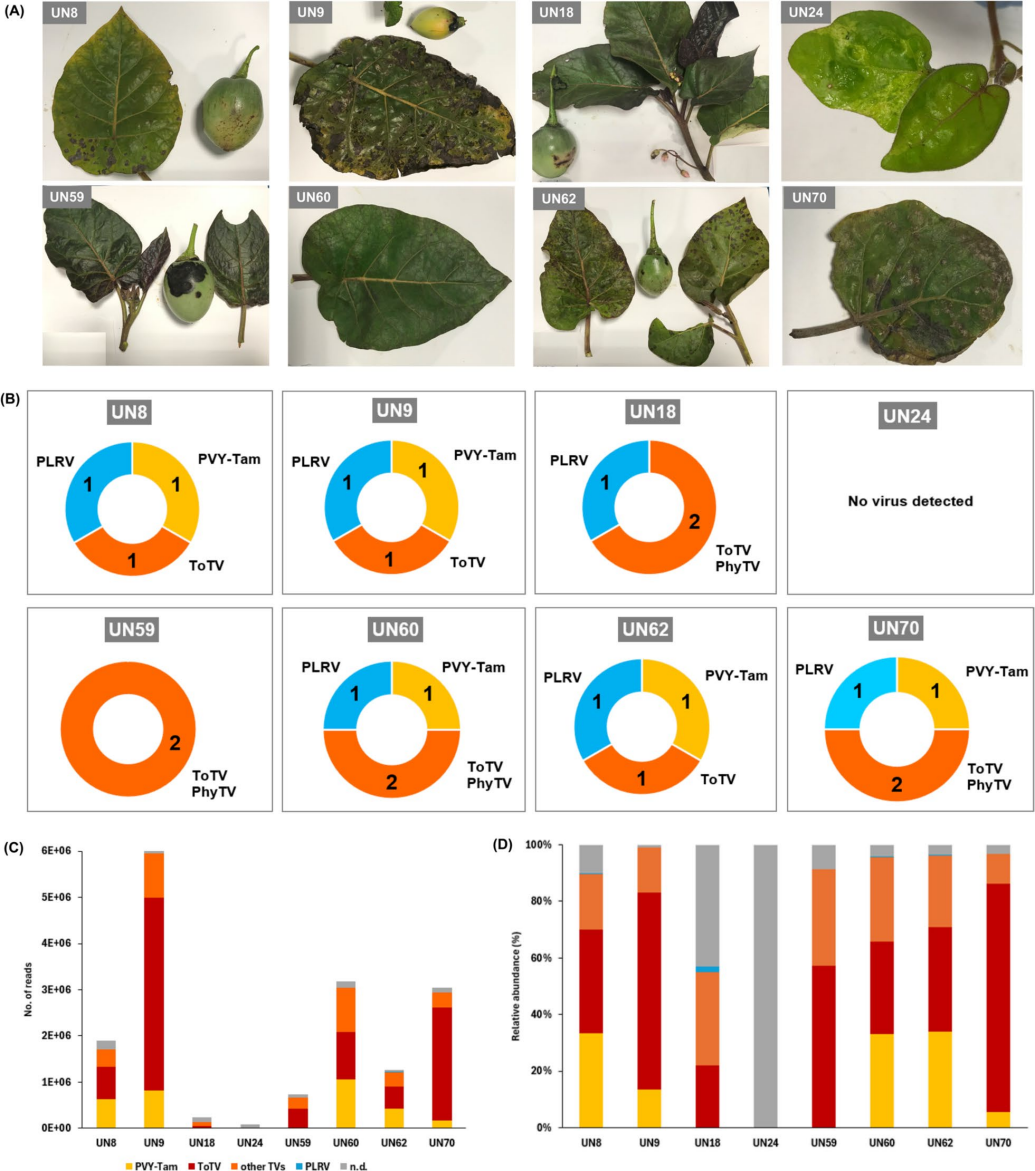
Characterization of virus diversity in tamarillo-growing orchards across Nariño. (**A**) Symptomatology of leaves and fruits from tamarillo plants collected in different geographic locations across Nariño. Symptoms include mosaicism, irregular mottling, chlorotic blisters, concentric ringspots and deformation in leaves, as well as early breaking and pulp hardness in fruits. (**B**) Ring chart representation of virus diversity in tamarillo plants showing virosis symptoms collected in the eight locations. The identified virus species are indicated with numbers and grouped to their corresponding genera (Potyvirus in yellow, Torradovirus in orange, and Polerovirus in blue). No viruses were detected on site UN24. (**C**) Number of RNA-seq reads and (**D**) relative abundance of the viral species identified in the eight geographic locations. The abundance percentage of each virus is normalized to the total numbers of RNA-seq reads counted in each sample. N.d., non-determined assignments

The Department of Nariño has previously reported to be affected by cases of tamarillo virosis [10, 30]. The high incidence and severity of viral diseases in southern Colombia have drastically reduced tamarillo-growing areas in Nariño, so plants in commercial production are scarce. To better understand the complex interactions between viral species in tamarillo, we selected a total of eight tamarillo-growing orchards located across the department of Nariño (from now on, referred to as UN8 to UN70), considered as the few tamarillo-producing sites still active in the region (Fig. 1 and Supplementary Table S1). Tamarillo plants collected in each location showed symptoms of viral infection, including general mosaic with irregular mottling, chlorotic blisters, concentric ringspots, and deformation in leaves, as well as early breaking and pulp hardness in fruits (Fig. 2A, Supplementary Table S2 and Supplementary Figure S1). Some plants also exhibited severe necrotic lesions in both leaves and fruits, possibly because of advanced viral infections or secondary co-infections with fungal pathogens. Conversely, tamarillo plants in UN24 showed a slightly different appearance consisting of partial chlorosis, mild leaf deformation and blistering.

### Characterization of virus diversity in tamarillo plants affected with virosis

To unravel the complex interactions between different viruses that can lead to tamarillo virosis, we generated RNA-seq libraries for symptomatic plants collected in the eight geographic locations. Analysis of RNA-seq reads using the Genome Detective software revealed that all tested samples, except for UN24, were positive for virus infections (Supplementary Files). Tomato torrado virus (ToTV) was found in all infected samples, where the assembled contigs showed > 90% amino acid sequence identities to its reference genome (Supplementary Table S5). Other assignments of members from the *Torradovirus* genera included tomato marchitez virus (ToMarV), tomato chocolate spot virus (ToChSV), and tomato necrotic dwarf virus (ToNDV), but amino acid identity levels were considerably lower (63.2–71%). To clarify the torradovirus assignments of the Genome detective software, we performed a Blastn analysis of the RNA1 nucleotide contigs of each sample, which confirmed that ToTV and/or physalis torrado virus (PhyTV) were the only torradoviruses present in the different locations. The taxonomy criterion for members of the family *Secoviridae* is usually based on the conserved protease-polymerase (Pro-Pol) region (from CG motif of the protease to the GDD motif of the Pol), and species demarcation is defined by amino acid sequence identities of > 75% in the coat protein(s) (CP) or > 80% in the Pro-Pol region, respectively [31]. However, CP cleavage sites are determined only for two torradoviruses (i.e. ToChSV and ToMarV) out of the twelve viral species recognized in this genus [32]. Hence, for our study, we defined the species demarcation based on the Pro-Pol conserved region from RNA1. We identified seven RNA1 contigs for ToTV in samples from different locations. The Pro-Pol region for these sequences showed 68.92–96.36% and 78.60–99.78% nucleotide and amino acid identity compared to that of ToTV isolate PRI-0301 (GenBank: DQ388879). Additionally, we found PhyTV in four out of eight locations. When using PhyTV isolate BPP2 (GenBank: MZ357183) as a reference, the Pro-Pol nucleotide and amino acid identities ranged between 69.51–98.98% and 78.38–99.56%, respectively (Table 1). Our findings constitute the very first evidence on the capacity of torradoviruses to infect tamarillo as a host species.

In addition to torradoviruses, we identified two viruses from unrelated genera in tamarillo plants: the potyvirus potato virus Y-Tamarillo (PVY-Tam) and the polerovirus potato leafroll virus (PLRV) appeared in five and six out of the eight locations, respectively (Fig. 2B). Remarkably, five locations showed simultaneous infections with multiple viruses from these three different genera (*Potyvirus*, *Torradovirus* and *Polerovirus*). Regarding the total number of identified reads, UN9 contained by far the highest viral load of all samples (5.8 × 10^6^ reads), while the load of infection was significantly reduced in samples UN18 and UN59 where PVY-Tam was absent (1 × 10^5^ and 6 × 10^5^ reads, respectively) (Fig. 2C). The predominant viral species in all infected samples was ToTV with a relative abundance of 34–86%, while PVY-Tam appeared in varying abundance (6–39.5%) and PLRV was marginally present (0.02–4%) (Fig. 2D). Unfortunately, we could not determine the abundance of PhyTV in our samples since there were not assigned hits to the reference sequence of PhyTV using the Genome Detective software (Supplementary Files**)**.

### Confirmation of a novel PVY-Tam isolate in Colombia

PVY is considered one of the most economically relevant viruses worldwide, but there is limited knowledge of the predominant tamarillo-infecting PVY isolates in the Andean region and their effects on this crop. First, we determined the consensus nucleotide sequence of our PVY-Tam Nariño isolate by performing a multiple alignment of the viral contigs identified as this virus in all the PVY-positive locations. Then, we designed and validated two primer pairs to amplify specific regions within the potyviral P3 cistron, and between NIb and CP (Supplementary Table S3). RT-PCR analysis of PVY-Tam P3 and CP using these primer pairs confirmed the presence of the virus in all the samples regarded as PVY-infected in the RNA-seq (Supplementary Figure S2), and we further validated the identity of PVY-Tam by Sanger sequencing of the RT-PCR products. Altogether, our results constitute the report of a novel PVY-Tam isolate in Colombia.

### Unraveling PVY diversification in the andean region

PVY belongs to a large clade of up to 19 virus species of the genus *Potyvirus* that are mostly present in South America, with at least 11 virus species being exclusively present on this continent [27]. Phylogenetic analysis of the polyproteins from selected potyviruses confirmed that the PVY-Tam isolate from Nariño conforms a well-defined clade with PVY and other potyviruses originated in neighboring South American countries, such as alstroemeria mosaic virus (AlMV) from Ecuador and bidens mosaic virus (BiMV) from Brazil (Supplementary Figure S3). Accordingly, PVY-Tam seems more distantly related to other potyviruses also previously reported in cases of virosis in tamarillo (i.e. colombian datura virus, CDV; TLMV; and PVA).

**Fig. 3:**
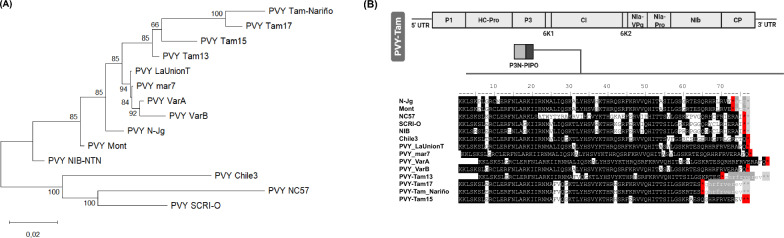
Phylogeny and molecular evolution of PVY-Tam. (**A**) Maximum likelihood tree of retrieved PVY isolates. The tree is drawn to scale, with branch lengths measured in the number of substitutions per site and bootstrap values indicated for each node. All positions containing gaps and missing data were eliminated. (**B**) Amino acid alignment of P3N-PIPO selected PVY isolates. A schematic representation of the PVY genome map is shown, indicating the mature gene products derived from the polyprotein and the additional P3N-PIPO generated by polymerase slippage at the conserved G2A6 sequence. The predominant residues are highlighted in black. The first stop codon in each P3N-PIPO is marked in red, with downstream sequences displayed in lowercase and shaded in light or dark grey (stop codon and amino acids, respectively)

PVY is classified into five major clades according to molecular, serological and biological properties [33]. The C (common), O (ordinary) N (necrotic) and NTN (N x O recombinants) clades show a worldwide distribution and cause severe diseases in potato and other solanaceous crops, while the Chilean clade has been reported only in this country and is non-infectious in potato. To study the evolutionary origin of PVY-Tam from Nariño, we carried out a phylogenetic analysis using type members for each major PVY clade, other Colombian isolates found in potato (LaUnionT) [34] and tomato (mar7, VarA, VarB) [35], as well as PVY-Tam isolates previously reported in Ecuador (Tam13, Tam15, Tam17) [20] (Supplementary Table S4). The latter are of special interest since they were collected from tamarillo orchards in Pichincha, a neighboring region about 350 km south of Nariño. Comparison of the polyprotein sequences indicated that all PVY-Tam isolates conform a distinct clade within the PVY^N^ lineage and are closely related to the clade formed by other PVY isolates from Colombia (Fig. 3A). Moreover, the fact that PVY-Tam from Nariño and Tam17 cluster together reflects a close evolutionary relationship between both isolates.

Because recombinants are common within the PVY^N^ lineage, we subjected all retrieved PVY isolates to an exhaustive recombination analysis. Following the approach by [36], we divided the viral genomes into three large regions generally considered recombination-free, based on the infrequent occurrence of predicted putative recombination events: R1 (310–2202 nt), R2 (2227–5628 nt) and R3 (5656–8993 nt). Phylogenetic analyses of R1 and R2 regions suggested that PVY-Tam from Nariño carries a long genomic section closely related to Tam17, while the R3 region is associated with that of Tam15 (Supplementary Figure S4). Thus, we conclude that the Nariño isolate is likely a recombinant arisen from genomic exchanges between relatives of Tam17 and Tam15. To further confirm this result, we applied a GARD analysis to the nucleotide sequences of PVY-Tam Nariño, Tam 13, Tam 15 and Tam 17. We identified up to four potential recombination breakpoints (Supplementary Figure S5), reinforcing the differences observed in the phylogenetic analysis of the three large regions.

### Variability in P3N-PIPO length among PVY isolates

Next, we studied the variability of individual PVY proteins and their biological significance. Most proteins were highly conserved among all PVY representatives (> 85% amino acid sequence similarities), except for P1 protein that is known for its variability among potyviruses, and P3N-PIPO (Supplementary Table S5). Multiple alignment of P3N-PIPO sequences confirmed the presence of the canonical UAA stop codon at position 77 in all the retrieved PVY isolates. Additionally, we observed clade-specific stop codons (Fig. 3B): UGA at position 73 for PVY^N−NA^ and PVY^N−Eu^; UAA at position 76 for PVY^C^, PVY^O^, PVY^NTN^ and PVY^N−SA^. Colombian PVY isolates reported in potato and tomato share a common P3N-PIPO amino acid sequence with the canonical UAA at position 77. Strikingly, the tamarillo-infecting isolate from Nariño and two from Ecuador (Tam13 and Tam 17) present an identical P3N-PIPO protein with a premature UAA stop codon at position 65 plus two more UAAs at positions 76 and 77 (Fig. 3B). This generates a P3N-PIPO protein of only 64 amino acids (aa), the shortest among all the isolates analyzed here. Conversely, Tam15 lacks the stop codon at position 65, which, together with some amino acid changes, evokes a diverse evolutionary origin among PVY-Tam isolates in geographic proximities.

## Discussion

Viral diseases are the most devastating phytosanitary problem in tamarillo production in Colombia. The wide variety of symptoms is commonly associated with viral complexes from various genera that establish intricated yet unknown interactions, hindering the development of effective control or mitigation strategies. In this work, we used the innovative, web-based Genome Detective software that allows straightforward analysis of RNA-seq data to characterize the virome of tamarillo plants from diverse locations across the Department of Nariño, Colombia (Fig. 1). Six out of the eight locations exhibited simultaneous infection with multiple viruses, where those belonging to the genus *Torradovirus* were the most prevalent (Fig. 2). Torradoviruses are an emerging plant virus genus with a similar genome to that of picorna-like viruses in the *Secoviridae* family. They were first described in the early 2000s, when ToTV was found as the causal agent of a new disease in tomato [37]. Of the 12 torradovirus species recognized by ICTV and other tentative members, many of them have been reported in South America. This includes ToTV, ToChSV, ToMarV, PhyTV [38–42], tomato chocolate virus (ToChV) [43], cassava torrado-like virus (CTLV) [44], and potato rugose stunting virus (PotRSV) [45]. We found that ToTV, the type member of the genus, was the prevailing virus species in all the virus-infected locations (Figs. 2C and 2D). This is the first evidence of ToTV in tamarillo plants in Colombia, since all previous reports corresponded to infections in tomato [39]. Based on sequence similarities of the torradovirus RNA1 Pro-Pol region, we also identified PhyTV in four out of the eight locations. PhyTV was first described in Colombia in plants of cape gooseberry (*Physalis peruviana*) infected with several plant viruses, including PVY [42]. Altogether, our results provide strong evidence that tamarillo is a natural host of torradoviruses and open new possibilities for the identification of new viral species of this emerging genus in the upcoming years.

Potyviruses are frequent players in tamarillo virosis [14, 17, 22]. PVY isolates infecting this crop (hereafter named PVY-Tam) were initially described in New Zealand [46] and India [47], but later reports indicated that it is also widespread in tamarillo orchards in Ecuador [18]. Serological and molecular analysis provided the first evidence for PVY-Tam infection in the main tamarillo-growing regions of Colombia [22]. In our study, we detected PVY-Tam in five out of the seven virus-infected locations, which interestingly burst in total viral load compared to that of the potyvirus-free locations (Fig. 2C). It is well known that the potyviral sequence P1/HC-Pro promotes the pathogenicity and replication of a broad range of unrelated viruses, leading to an enhanced severity of disease symptoms [48, 49]. We speculate that a similar PVY-Tam-associated synergism might lead to an increase in the accumulation of ToTV and other torradovirus partners, with negligible changes in the level of the potyvirus. The enhanced levels of torradovirus load and possible inter-species interactions would eventually worsen disease severity and result in total yield losses, as appreciated in the aggressive symptomatology (i.e. leaf blistering, necrosis and burnt leaves) of tamarillo plants at some of the analyzed locations (Fig. 2A). Moreover, potyviruses are known to be naturally transmitted by aphids, while torradoviruses disperse through whiteflies. Future efforts should focus on investigating the interactions between different viral genera, as well as the influence of insect vectors in the transmission of viruses infecting tamarillo.

The agronomical relevance of PVY infection in many solanaceous crops has motivated an extensive study of its biological and genetic diversity. For many years, phylogenetic studies were mainly restricted to potato, pepper and tobacco as for being the main crops affected by this virus [50]. However, recent findings propose that other solanaceous species can allow the replication of host-specific isolates with extraordinary genetic diversity [51, 52]. Our phylogenetic analysis indicates that the PVY^N^ lineage contains two well-defined clades: the first is composed of Colombian PVY isolates infecting potato or tomato, and the second clade exclusively contains PVY-Tam isolates from the Andean region (Fig. 3A). These results support the hypothesis that South America is the centre of origin and diversification for PVY [53]. The short evolutionary distances also highlight that PVY diversification is very recent. Interestingly, the novel PVY-Tam from Nariño appears to be a recombinant of the Ecuadorian isolates Tam17 (P1 to 6K2 cistrons) and Tam15 (VPg to CP cistrons), as inferred from the phylogenetic trees and GARD analysis (Supplementary Figures S3 and S4). Previous works [20] already suggested the recombinant nature of Ecuadorian PVY-Tam isolates by identifying specific genome segments from tomato isolates. Thus, it seems that specific mutations and gene rearrangements are major drivers of PVY diversification, generating novel isolates able to cross the host barrier [54]. The co-existence of solanaceous species acting as reservoirs, together with agricultural malpractices, might significantly favor the passage of PVY-Tam between geographical proximities.

P3N-PIPO, the only dedicated movement protein of potyviruses, interacts with other viral proteins for the cell-to-cell trafficking of virions in infected plants [55]. It is the product of a transcriptional slippage on a conserved GA6 motif of the P3 cistron and shows diverse lengths among potyviruses [56], but the effects of such genetic variation are still poorly understood [57]. In PVY, it has been postulated that P3N-PIPO encodes a 76-aa protein as determined by a canonical UAA stop codon at position 76 [58]. Although this attribute is present in all PVY isolates analyzed here, we also observed clade-specific stop codons that could promote differences in P3N-PIPO length (Fig. 3B). Interestingly, a stop codon at position 65 encoding seems to be the hallmark for PVY-Tam in the Andean region, since it is present in most of the isolates characterized in tamarillo so far (except for Tam15). The resulting 64-aa protein is much shorter than that of other PVY isolates and could be associated with specific adaptations to tamarillo. Our findings support the theory that differences in P3N-PIPO length might influence the host range and infectivity of PVY isolates, as previously proposed [36, 59]. Further experimental evidence and sequence analysis of P3N-PIPO from other PVY isolates are required to shed light on the biological implications of this protein.

## Conclusions

In summary, our study significantly contributes to the knowledge of complex viral infections in tamarillo in the Andean region. We provide the first evidence on the capacity of torradoviruses to infect tamarillo. Moreover, we characterize a novel PVY-Tam isolate in Colombia that might contribute through synergism to the severity of torradoviruses and other unrelated viral partners in field-grown tamarillo. The PVY-Tam from Nariño appears to be a recombinant form arising from geographically closer isolates and carries host-driven adaptations in P3N-PIPO. Further studies should address the interactions between different virus genera in tamarillo with a special focus on the torradovirus-potyvirus synergism, and the biological implications of P3N-PIPO on host-driven adaptations of PVY isolates. A better understanding of the epidemiological variables influencing the establishment and expansion of tamarillo virosis will enable us to develop effective and durable control methods.

## Supplementary Information

Additional file 1.

Additional file 2.

Additional file 3.

Additional file 4.

Additional file 5.

Additional file 6.

Additional file 7.

Additional file 8.

Additional file 9.

## Data Availability

All datasets generated for this study are included in the manuscript and/or the Supplementary files. GenBank accession numbers of full-length or near-full length contigs of viruses are provided in Supplementary Table S4.

## Abbreviations

RNA-seq: RNA sequencing
ToTV: Tomato torrado virus
PhyTV: Physalis torrado virus PVY-tam, potato virus Y-tamarillo
PLRV: Potato leafroll virus
CP: Coat protein
NIb: Nuclear inclusion b
P3N-PIPO: P3 N-terminal half fused to the pretty Interesting potyvirus ORF
Pro-Pol: Protease-polymerase
N-Eu: European N clade
N-NA: North American N clade
N-SA: South American N clade
Aa: Amino acid
