# Supplementary material for: Nucleotide sequence analysis reveals the presence of PVY-Tam isolates affecting tamarillo in Colombia
Source: Virol J. 2026 Apr 20;23:145. doi: 10.1186/s12985-026-03166-6 (PMC13234967; doi:10.1186/s12985-026-03166-6)
Supplement: Supplementary file 1 — Additional file 1. [file 12985_2026_3166_MOESM1_ESM.pdf]

Analysis of UN9

**Technology**	Paired-end short reads
**Input Files**	UN9_R1.fq.gz (1.41 GB), UN9_R2.fq.gz (1.45 GB)
**Submitted On**	2023-09-26 13:10:39 UTC
**Duration**	2h 48m 21s
**Tool Version**	panviral2.64
**Location**	https://www.genomedetective.com/db/ui/analysis/c136d63b-9925-4a37-b229-014ac43e54d9

Statistics

**Original Read Length**	20 - 150
**Trimmed Read Length**	50 - 135

	# Reads	% of Reads
**Input file**	44538018	100.0%
**After QC**	44242186	99.3%
**After filtering**	10664852	23.9%
**Mapped back to contigs**	6008674	13.5%

Assignments

Assignment	No. of Reads	Depth of Coverage	Identity		Genome Coverage	
			NT	AA		
Torradovirus lycopersici (2 segments out of 2)	4175010	42283.7	91.6%	97.1%	99.8%	
Torradovirus lycopersici (segment RNA 2)	2524050	60857.9	94.8%	96.5%	99.6%	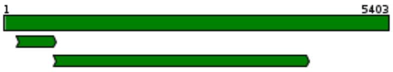
Torradovirus lycopersici (segment RNA 1)	1650960	29487.7	89.5%	97.5%	100.0%	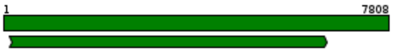
Potato virus Y	812319	11166.5	82.6%	90.0%	99.9%	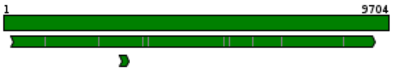
Tomato chocolate spot virus (2 segments out of 2)	490370	17490.7	68.2%	69.6%	26.5%	
Tomato chocolate spot virus (segment RNA2)	328101	19230.5	64.0%	66.1%	40.8%	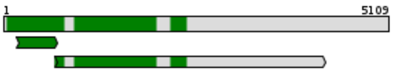
Tomato chocolate spot virus (segment RNA 1)	162269	14600.6	75.1%	76.9%	16.7%	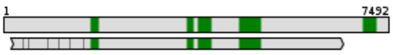
Torradovirus marchitezum (2 segments out of 2)	401174	16110.8	63.8%	63.2%	23.5%	

Assignment	No. of Reads	Depth of Coverage	Identity		Genome Coverage	
			NT	AA		
Torradovirus marchitezum (segment RNA 1)	77945	7049.3	65.4%	66.6%	17.9%	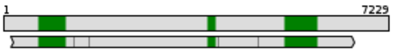
Torradovirus marchitezum (segment RNA 2)	323229	23655.4	62.3%	59.9%	31.7%	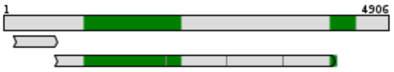
Diachasmimorpha longicaudata entomopoxvirus (segment NC_043455.1)	7050	776.0	59.3%	55.9%	83.4%	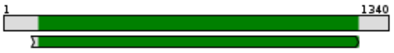
Bracoviriform glomeratae (segment NC_043292.1)	6038	2248.3	72.2%	85.0%	70.9%	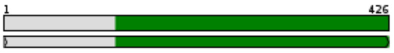
Potato leafroll virus	4587	104.8	97.6%	96.6%	97.3%	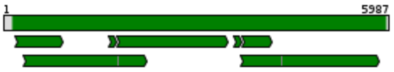
Solendovirus venanicotianae	233	11.9	79.9%	76.3%	28.5%	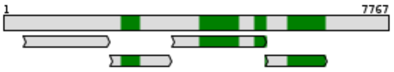
Duamitovirus soch1	163	11.3	69.7%	67.9%	62.1%	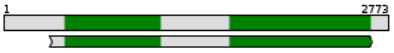
Harvey murine sarcoma virus	6	3.2	70.2%	83.3%	21.6%	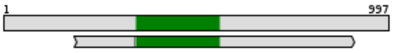

Discoveries

Similar to	No. of Reads	Depth of Coverage	Identity		Genome Coverage	
			NT	AA		
Tomato necrotic dwarf virus (segment RNA1)	65373	9641.5	71.6%	77.5%	11.0%	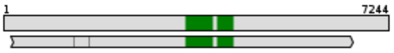
Brazilian marseillevirus	19918	9135.3	76.8%	94.4%	0.1%	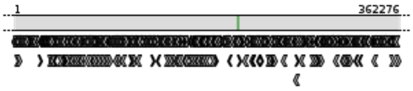
Tokyovirus A1	10543	6408.0	81.1%	94.4%	0.0%	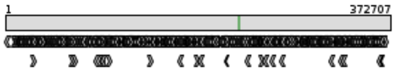
Lausannevirus	3601	2010.8	83.6%	98.0%	0.0%	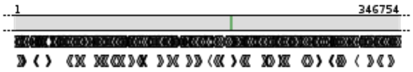
Betabaculovirus disaccharalis	2310	1356.2	84.7%	92.0%	0.2%	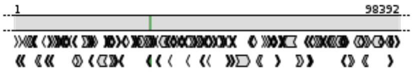
Lausannevirus	2249	390.8	78.4%	91.0%	0.1%	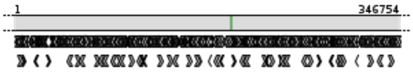
Makelovirus prm1	1984	253.9	76.6%	87.5%	0.7%	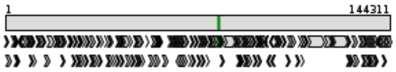

Similar to	No. of Reads	Depth of Coverage	Identity		Genome Coverage	
			NT	AA		
Golden Marseillevirus	1782	1085.8	85.8%	97.4%	0.0%	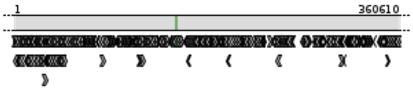
Cladosporium fulvum T-1 virus	808	54.0	52.5%	47.0%	23.2%	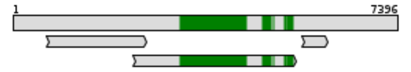
Lausannevirus	604	394.0	81.5%	92.2%	0.0%	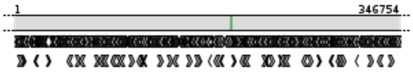
Errantivirus	402	65.5	53.6%	43.9%	7.6%	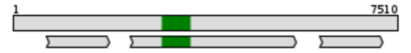
Epiphyllum badnavirus 1	324	124.8	60.3%	57.6%	3.5%	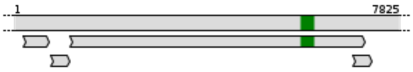
Lowelvirus tuscon4d	311	36.3	73.3%	87.1%	0.6%	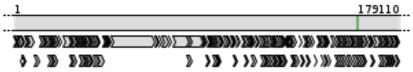
Hibiscus bacilliform virus GD1	204	73.4	59.5%	51.6%	3.8%	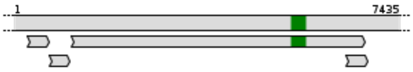
Cassava brown streak virus	197	45.5	60.5%	57.5%	5.8%	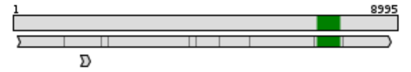
Badnavirus maculaucubae	137	52.7	56.1%	52.6%	3.1%	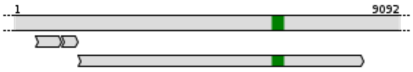
Errantivirus	111	19.5	52.9%	41.2%	8.0%	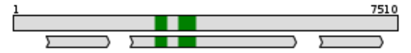
Badnavirus maculaucubae	81	26.2	55.8%	46.5%	3.6%	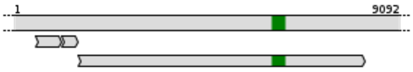
Nodensvirus spm2	67	39.2	80.7%	86.3%	0.1%	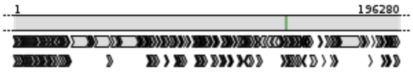
Epiphyllum badnavirus 1	66	15.2	52.2%	44.4%	5.4%	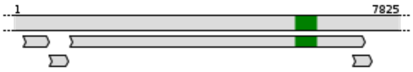
Caulimovirus venafragariae	60	21.2	54.2%	42.1%	4.1%	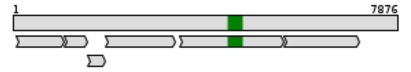
Epiphyllum badnavirus 1	55	18.6	52.9%	45.1%	3.9%	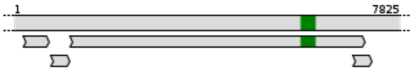
Caulimovirus glycinis	44	13.0	57.2%	43.3%	5.4%	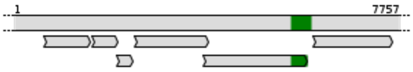
Caulimovirus venafragariae	41	6.4	56.2%	54.5%	10.1%	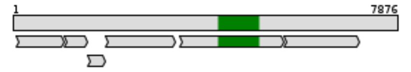

Similar to	No. of Reads	Depth of Coverage	Identity		Genome Coverage	
			NT	AA		
Pinus nigra virus 1	41	19.8	56.3%	50.0%	3.4%	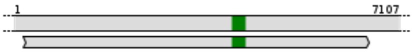
Hibiscus bacilliform virus GD1	37	9.2	52.2%	43.0%	6.7%	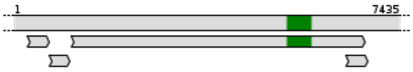
Dioscovirus dioscoreae	27	4.9	64.1%	56.1%	9.1%	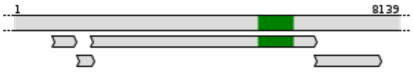
Badnavirus venatheobromae	25	11.2	50.6%	44.8%	3.5%	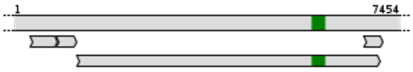
Epiphyllum badnavirus 1	25	10.2	57.0%	43.0%	3.3%	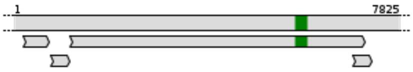
Badnavirus tessellocastaneae	22	6.4	57.2%	47.9%	5.8%	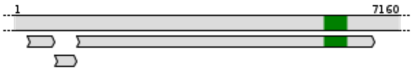
Ruflodivirus deformatiorudbeckiae	21	4.7	57.3%	45.5%	7.4%	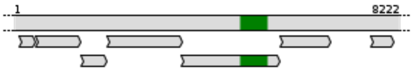
Caulimovirus latensarmoraciae	21	7.3	58.8%	56.2%	4.6%	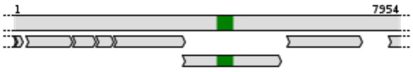
Badnavirus occultipomeae	18	5.4	54.9%	46.2%	4.3%	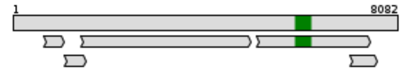
Badnavirus occultipomeae	15	6.7	61.5%	47.2%	3.2%	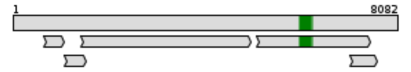
Badnavirus maculaucubae	15	5.3	52.7%	43.2%	3.8%	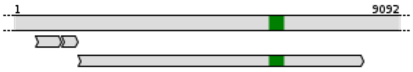
Bracoviriform congregatae (segment Circle 7)	14	9.0	78.4%	91.3%	0.6%	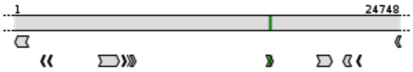
Badnavirus epsiloninflatheobromae	14	5.0	59.0%	49.5%	4.1%	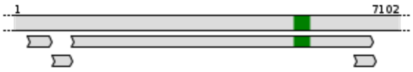
Badnavirus maculasmallanthi	14	4.2	58.0%	45.9%	4.2%	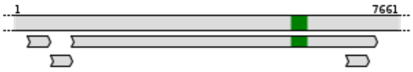
Arhar cryptic virus-I (segment RNA 3)	14	5.6	57.7%	42.6%	21.7%	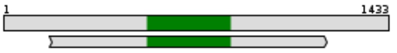
Ichnoviriform fugitivi (2 segments out of 56)	12	3.2	60.4%	50.0%	0.1%	
Ichnoviriform fugitivi (segment B17)	10	5.2	61.0%	50.9%	4.0%	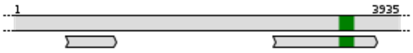
Ichnoviriform fugitivi (segment C16)	2	1.3	59.8%	49.2%	3.8%	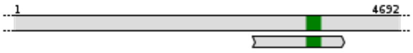

Similar to	No. of Reads	Depth of Coverage	Identity		Genome Coverage	
			NT	AA		
Dioscovirus dioscoreae	11	4.2	57.0%	49.5%	4.1%	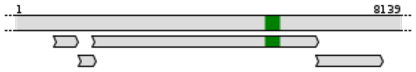
Dioscovirus dioscoreae	11	4.1	66.3%	55.6%	4.0%	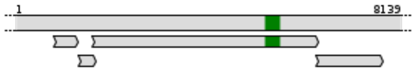
Badnavirus maculasmallanthi	10	4.2	53.0%	48.5%	3.8%	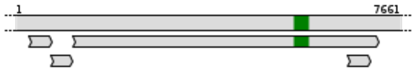
Pinus nigra virus 1	10	6.0	57.7%	47.4%	3.4%	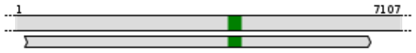
Badnavirus occultipomeae	9	4.2	58.7%	51.8%	3.1%	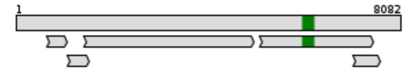
Epiphyllum badnavirus 1	9	4.0	58.0%	51.9%	3.1%	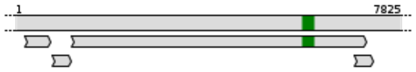
Colombian datura virus	8	2.3	75.5%	79.0%	4.5%	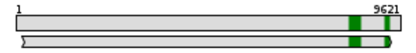
Pinus nigra virus 1	8	2.7	55.3%	50.4%	5.0%	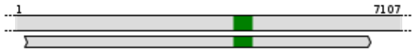
Badnavirus rutilanscamelliae	7	2.6	55.3%	45.1%	4.1%	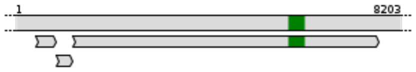
Fig cryptic virus (segment RNA 2)	6	3.1	63.4%	61.8%	11.6%	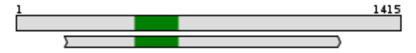
Caulimovirus venafragariae	6	2.8	61.3%	63.0%	3.1%	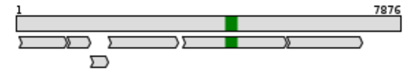
Tomato associated geminivirus 1	6	2.5	67.5%	62.0%	10.5%	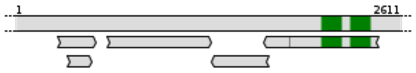
Moloney murine leukemia virus	2	2.0	79.1%	97.7%	1.6%	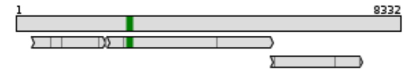
Friend murine leukemia virus	2	2.0	78.5%	97.8%	1.6%	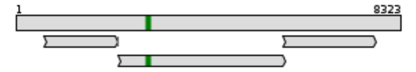
Tokyovirus A1	3	2.4	80.0%	92.9%	0.0%	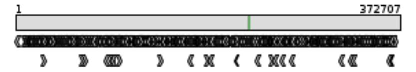
Nepovirus aeonii (segment RNA2)	2	1.1	71.2%	72.3%	6.2%	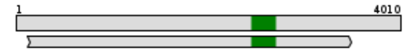
Duamitovirus peex1	1	1.0	71.9%	66.7%	5.0%	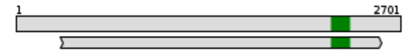
Plantago lanceolata latent virus	1	1.0	67.4%	54.1%	4.8%	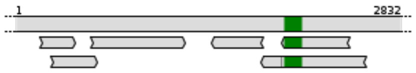

Similar to	No. of Reads	Depth of Coverage	Identity		Genome Coverage	
			NT	AA		
Duamitovirus dapi1	1	1.0	68.7%	68.2%	4.8%	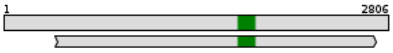

NGS Details (UN9): Torradovirus marchitezum (segment RNA 1)

Assembly

**Coverage Length**	1293 (3 contig(s))
**Depth Of Coverage**	7049.3
**Number Of Reads**	77945
**Reads Per Million**	1761.78 rpm (after QC)
**Ambiguities**	0
**Assembly Method**	de novo + reference guided assembly
**Consensus Caller**	Bcf Tools

Coverage Map

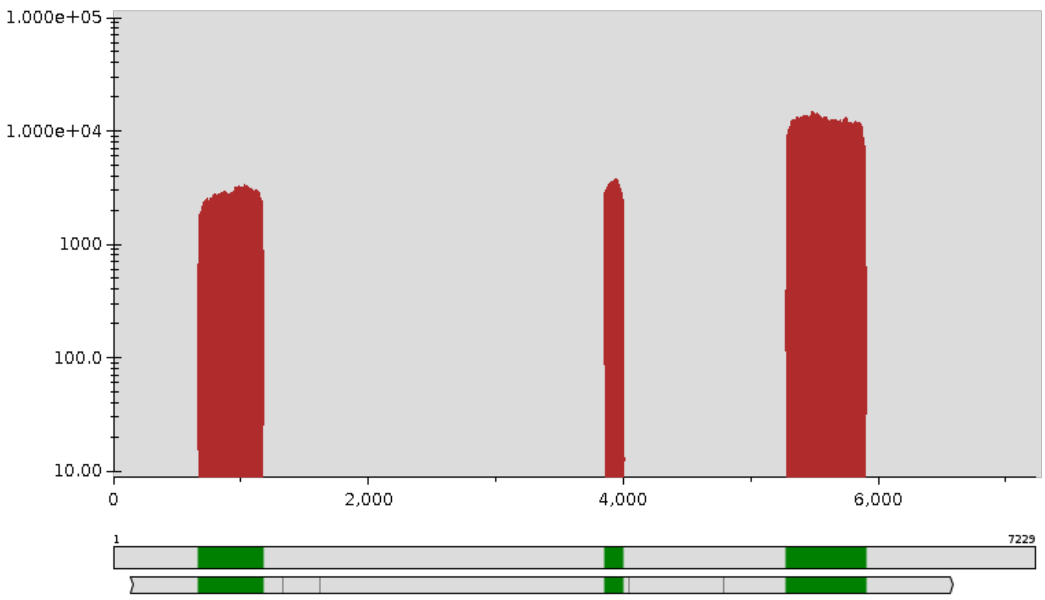

Assignment

**Type**	Torradovirus marchitezum (Taxonomy ID: 3048376)
**Reference Genome**	NC_010987.1
**NT Identity (%)**	65.4349
**AA Identity (%)**	66.5899
**Number Of Stop Codons**	0
**Number Of CDS**	1

Alignment

**Alignment Score**	781.0 (NT) + 2123.0 (AA) = 2904.0
**Concordance (%)**	52.071

Alignment Method	Global, seeded, nucleotide + amino acids (AGA)

Genome Region

Sequence starts at position 666 and ends at position 5905 relative to NC_010987.1 reference sequence.

Alignment Detailed Statistics

	Begin	End	Coverage	Score	Concordance	Matches	Identities	I/D/M/F*	Stop Codons
**NT**	**666**	**5905**	**17.9%**	**781**	**30.4%**	**1290 (99.1%)**	**850 (65.3%)**	**9/3**	
Mutations:	677C>T, 681T>A, 683A>T, 684G>A, 685A>G, 695A>C, 696A>C, 697A>T, 698A>C, 702G>T, 704C>T, 707A>G, 708C>A, 709G>A, 713C>T, 714A>T, 716C>T, 717A>C, 718T>A, 720G>A, 725C>A, 728A>G, 732A>T, 733G>C, 735A>G, 740T>A, 746G>T, 749A>G, 750T>A, 751C>G, 752A>C, 753G>T, 755T>G, 756T>A, 757C>A, 758A>T, 770G>C, 771T>A, 772C>G, 776G>A, 780A>C, 785C>T, 786A>T, 788A>T, 789A>T, 791A>G, 792T>A, 794G>A, 797T>A, 803G>C, 806T>C, 809G>A, 818G>A, 821C>T, 822A>G, 824T>G, 830G>A, 839A>G, 849T>C, 851G>T, 853G>C, 854C>A, 860A>G, 861T>C, 863G>A, 873C>T, 875C>G, 881G>A, 882A>G, 884A>G, 887A>C, 893T>A, 899G>A, 914A>C, 918A>C, 919C>A, 920T>A, 921C>T, 923T>G, 926C>T, 927C>A, 928G>A, 929T>G, 932T>C, 938A>G, 947C>A, 962A>T, 968T>G, 969C>T, 971T>G, 977A>T, 978A>G, 980A>T, 982T>C, 983C>T, 985G>A, 986C>T, 988C>T, 989C>A, 992T>C, 995A>G, 998C>T, 1001A>G, 1004T>C, 1010G>T, 1011T>C, 1016A>T, 1019T>A, 1022A>T, 1031G>A, 1032C>A, 1034G>T, 1046G>C, 1049C>T, 1059C>A, 1060A>C, 1061G>T, 1064G>A, 1068G>T, 1069A>C, 1077T>G, 1078G>C, 1091G>A, 1092C>A, 1094C>G, 1100G>A, 1109C>T, 1113A>C, 1114A>C, 1115G>T, 1121C>T, 1122C>T, 1124T>A, 1126C>T, 1142G>T, 1144G>A, 1145G>A, 1148A>G, 1149G>C, 1151A>G, 1154T>A, 1163C>A, 1169T>C, 3863G>C, 3872T>A, 3875C>T, 3881T>C, 3884C>T, 3890C>T, 3893G>A, 3894A>C, 3899G>T, 3900A>T, 3905G>T, 3907T>C, 3908G>C, 3911C>T, 3914G>A, 3917A>G, 3923T>C, 3926G>T, 3927C>G, 3930T>G, 3932C>A, 3933A>C, 3934C>A, 3935T>G, 3937G>A, 3938C>G, 3939G>A, 3940A>G, 3941A>T, 3942G>A, 3943G>C, 3944A>C, 3945G>C, 3947G>A, 3950A>G, 3953G>A, 3954G>C, 3955A>T, 3962A>G, 3966G>A, 3968C>T, 3969A>T, 3977A>T, 3980T>C, 3981G>C, 3992A>G, 5288T>A, 5294T>G, 5300T>G, 5303A>T, 5309C>T, 5333G>A, 5339G>C, 5346A>T, 5349T>G, 5354T>A, 5357A>C, 5358A>C, 5359T>A, 5364G>C, 5365C>A, 5366C>T, 5370A>C, 5371A>G, 5372G>C, 5375T>G, 5376G>A, 5377T>G, 5378G>T, 5379T>C, 5383C>A, 5386G>T, 5387A>T, 5390A>T, 5391C>G, 5395T>G, 5396A>G, 5399A>T, 5403A>T, 5404A>C, 5409G>A, 5410T>C, 5411T>C, 5417G>T, 5423A>T, 5429A>T, 5435T>A, 5436G>A, 5438C>A, 5440G>C, 5441C>T, 5447T>C, 5456G>A, 5457C>T, 5460A>T, 5462G>T, 5465C>A, 5468A>G, 5471A>C, 5474A>T, 5475A>C, 5476G>A, 5477G>T, 5483T>C, 5484T>A, 5486T>G, 5488T>C, 5489G>C, 5490G>A, 5491A>C, 5492G>A, 5496A>C, 5498A>T, 5501A>T, 5504T>G, 5505A>C, 5507A>C, 5508T>C, 5510G>A, 5513C>A, 5516A>C, 5519T>G, 5521G>C, 5522A>T, 5523A>G, 5524A>G, 5525T>G, 5531T>G, 5534C>T, 5537C>G, 5540T>C, 5546T>C, 5555C>T, 5560T>C, 5564G>A, 5567T>C, 5570T>G, 5573T>C, 5574C>A, 5582G>T, 5583C>G, 5584C>A, 5585T>A, 5586C>G, 5588T>C, 5588_5589insACC, 5589G>T, 5590G>C, 5592G>C, 5597A>G, 5598G>T, 5602C>G, 5603T>A, 5606A>G, 5612T>C, 5613G>A, 5617C>A, 5618G>T, 5619C>G, 5620T>G, 5622_5624delCAC, 5626G>C, 5627T>C, 5629G>C, 5635T>A, 5636G>C, 5642A>T, 5642_5643insCACAGT, 5644G>C, 5645G>A, 5649G>T, 5650A>C, 5652T>G, 5654G>T, 5657C>A, 5658A>C, 5659A>T, 5660C>T, 5663C>A, 5664C>A, 5666C>G, 5669T>A, 5670A>G, 5672A>T, 5674A>C, 5676G>T, 5677G>C, 5681T>C, 5682T>C, 5684A>C, 5685G>T, 5687A>T, 5688A>G, 5691C>A, 5695C>T, 5696A>G, 5700A>G, 5702G>T, 5705G>T, 5706A>T, 5714T>C, 5715G>A, 5716T>C, 5717G>A, 5718G>A, 5720A>T, 5723A>T, 5726A>T, 5727C>G, 5729T>A, 5735A>T, 5740G>C, 5741A>C, 5742A>C, 5744T>G, 5746T>G, 5747T>C, 5750C>A, 5753A>G, 5754C>A, 5757G>A, 5759T>C, 5762T>G, 5763G>A, 5766A>C, 5767G>A, 5769T>G, 5770T>C, 5771G>T, 5772C>T, 5774A>G, 5775C>G, 5777C>G, 5778T>A, 5779A>C, 5780T>A, 5784G>T, 5785A>C, 5786G>T, 5787C>G, 5788A>T, 5789G>C, 5790G>A, 5792A>T, 5798A>T, 5799G>A, 5800A>C, 5801G>T, 5802T>C, 5804A>T, 5805A>G, 5806T>C, 5807T>C, 5808C>G, 5809A>C, 5812A>G, 5813G>A, 5814A>G, 5815A>G, 5816T>A, 5817A>G, 5818C>T, 5819A>C, 5823C>G, 5825A>G, 5828T>A, 5829C>T, 5837C>T, 5843G>A, 5844C>T, 5846G>C, 5847A>T, 5848G>C, 5849T>C, 5850A>C, 5851T>A, 5852G>A, 5853G>C, 5855A>C, 5856A>G, 5859G>T, 5860T>G, 5861G>T, 5862C>G, 5865C>A, 5866A>T, 5867A>G, 5868C>A, 5870G>A, 5871A>C, 5873A>T, 5876T>G, 5880G>C, 5882T>G, 5883A>G, 5885G>A, 5886C>A, 5887T>G, 5891G>A, 5895T>G								
**CDS**									
ToMarV_RNA1gp1	176	1922	20.1%	2123	70.1%	431 (99.1%)	289 (66.4%)	3/1/0/0	0
Protein mutations:	L181I (681T>A 683A>T), D182S (684G>A 685A>G), K186L (696A>C 697A>T 698A>C), A188S (702G>T 704C>T), R190K (708C>A 709G>A), T192S (714A>T 716C>T), M193Q (717A>C 718T>A), V194M (720G>A), M199V (735A>G), V205L (753G>T 755T>G), S206N (756T>A 757C>A 758A>T), M214L (780A>C), T216S (786A>T 788A>T), I217L (789A>T 791A>G), L218I (792T>A 794G>A), I228V (822A>G 824T>G), S238T (853G>C 854C>A), T248A (882A>G 884A>G), T260Q (918A>C 919C>A 920T>A), R263K (927C>A 928G>A 929T>G), I280V (978A>G 980A>T), V281A (982T>C 983C>T), G282D (985G>A 986C>T), T283I (988C>T 989C>A), L298I (1032C>A 1034G>T), Q307T (1059C>A 1060A>C 1061G>T), D310S (1068G>T 1069A>C), C313A (1077T>G 1078G>C), K325P (1113A>C 1114A>C 1115G>T), P328S (1122C>T 1124T>A), T329I (1126C>T), R335K (1144G>A 1145G>A), E337Q (1149G>C 1151A>G), M1252L (3894A>C), M1254L (3900A>T), M1256T (3907T>C 3908G>C), Q1263E (3927C>G), S1264A (3930T>G 3932C>A), T1265Q (3933A>C 3934C>A 3935T>G), G1266E (3937G>A 3938C>G), E1267S (3939G>A 3940A>G 3941A>T), G1268T (3942G>A 3943G>C 3944A>C), E1269Q (3945G>C 3947G>A), E1272L (3954G>C 3955A>T), D1276N (3966G>A 3968C>T), T1277S (3969A>T), E1281Q (3981G>C), N1716K (5288T>A), M1731I (5333G>A), M1736L (5346A>T), S1737A (5349T>G), M1740Q (5358A>C 5359T>A), A1742H (5364G>C 5365C>A 5366C>T), K1744R (5370A>C 5371A>G 5372G>C), N1745K (5375T>G), V1746S (5376G>A 5377T>G 5378G>T), A1748E (5383C>A), G1749V (5386G>T 5387A>T), H1751D (5391C>G), V1752G (5395T>G 5396A>G), N1755S (5403A>T 5404A>C), V1757T (5409G>A 5410T>C 5411T>C), V1766I (5436G>A 5438C>A), G1767A (5440G>C 5441C>T), M1774F (5460A>T 5462G>T), R1779H (5475A>C 5476G>A 5477G>T), C1782R (5484T>A 5486T>G), V1783A (5488T>C 5489G>C), E1784T (5490G>A 5491A>C 5492G>A), I1786L (5496A>C 5498A>T), I1793M (5519T>G), G1794A (5521G>C 5522A>T), N1795G (5523A>G 5524A>G 5525T>G), V1807A (5560T>C), F1810L (5570T>G), Q1812K (5574C>A), P1815E (5583C>G 5584C>A 5585T>A), H1816D (5586C>G 5588T>C), H1816_G1817insT (5588_5589insACC), G1817S (5589G>T 5590G>C), D1818H (5592G>C), V1820L (5598G>T), A1821G (5602C>G 5603T>A), A1825T (5613G>A), S1826Y (5617C>A 5618G>T), L1827G (5619C>G 5620T>G), H1828del (5622_5624delCAC), S1829T (5626G>C 5627T>C), S1830T (5629G>C), M1832N (5635T>A 5636G>C), Q1834H (5642A>T), Q1834_G1835insHS (5642_5643insCACAGT), G1835A (5644G>C 5645G>A), E1837S (5649G>T 5650A>C), L1838V (5652T>G 5654G>T), N1840L (5658A>C 5659A>T 5660C>T), I1844V (5670A>G 5672A>T), H1845P (5674A>C), G1846S (5676G>T 5677G>C), A1849S (5685G>T 5687A>T), K1850E (5688A>G), Q1851K (5691C>A), T1852M (5695C>T 5696A>G), M1854V (5700A>G 5702G>T), M1856L (5706A>T), V1859T (5715G>A 5716T>C 5717G>A), V1860I (5718G>A 5720A>T), H1863E (5727C>G 5729T>A), G1867A (5740G>C 5741A>C), N1868Q (5742A>C 5744T>G), F1869C (5746T>G 5747T>C), Q1872K (5754C>A), D1873N (5757G>A 5759T>C), E1875K (5763G>A), S1876H (5766A>C 5767G>A), L1877A (5769T>G 5770T>C 5771G>T), H1879E (5775C>G 5777C>G), Y1880T (5778T>A 5779A>C 5780T>A), E1882S (5784G>T 5785A>C 5786G>T), Q1883V (5787C>G 5788A>T 5789G>C), G1884S (5790G>A 5792A>T), E1887T (5799G>A 5800A>C 5801G>T), I1889A (5805A>G 5806T>C 5807T>C), Q1890A (5808C>G 5809A>C), K1891R (5812A>G 5813G>A), N1892G (5814A>G 5815A>G 5816T>A), T1893V (5817A>G 5818C>T 5819A>C), L1895V (5823C>G 5825A>G), L1902F (5844C>T 5846G>C), M1904Q (5850A>C 5851T>A 5852G>A), E1905H (5853G>C 5855A>C), N1906D (5856A>G), V1907C (5859G>T 5860T>G 5861G>T), Q1908E (5862C>G), Q1909M (5865C>A 5866A>T 5867A>G), L1910I (5868C>A 5870G>A), I1911L (5871A>C 5873A>T), D1912E (5876T>G), V1914L (5880G>C 5882T>G), K1915E (5883A>G 5885G>A), L1916S (5886C>A 5887T>G), F1919V (5895T>G)								

	Begin	End	Coverage	Score	Concordance	Matches	Identities	I/D/M/F*	Stop Codons
**NT**	**666**	**5905**	**17.9%**	**781**	**30.4%**	**1290 (99.1%)**	**850 (65.3%)**	**9/3**	

Codon mutations: TAC179TAT (677C>T), TTA181ATT (681T>A 683A>T), GAT182AGT (684G>A 685A>G), CCA185CCC (695A>C), AAA186CTC (696A>C 697A>T 698A>C), GCC188TCT (702G>T 704C>T), GAA189GAG (707A>G), CGA190AAA (708C>A 709G>A), TTC191TTT (713C>T), ACC192TCT (714A>T 716C>T), ATG193CAG (717A>C 718T>A), GTG194ATG (720G>A), GCC195GCA (725C>A), CAA196CAG (728A>G), AGT198TCT (732A>T 733G>C), ATG199GTG (735A>G), CTT200CTA (740T>A), GGG202GGT (746G>T), GAA203GAG (749A>G), TCA204AGC (750T>A 751C>G 752A>C), GTT205TTG (753G>T 755T>G), TCA206AAT (756T>A 757C>A 758A>T), ACG210ACC (770G>C), TCT211AGT (771T>A 772C>G), CAG212CAA (776G>A), ATG214CTG (780A>C), TCC215TCT (785C>T), ACA216TCT (786A>T 788A>T), ATA217TTG (789A>T 791A>G), TTG218ATA (792T>A 794G>A), GCT219GCA (797T>A), GGG221GGC (803G>C), ATT222ATC (806T>C), TCG223TCA (809G>A), GTG226GTA (818G>A), TTC227TTT (821C>T), ATT228GTG (822A>G 824T>G), GTG230GTA (830G>A), AGA233AGG (839A>G), TTG237CTT (849T>C 851G>T), AGC238ACA (853G>C 854C>A), CCA240CCG (860A>G), TTG241CTA (861T>C 863G>A), CTC245TTG (873C>T 875C>G), TCG247TCA (881G>A), ACA248GCG (882A>G 884A>G), GGA249GGC (887A>C), GGT251GGA (893T>A), CAG253CAA (899G>A), CTA258CTC (914A>C), ACT260CAA (918A>C 919C>A 920T>A), CTT261TTG (921C>T 923T>G), TAC262TAT (926C>T), CGT263AAG (927C>A 928G>A 929T>G), AAT264AAC (932T>C), AAA266AAG (938A>G), ACC269ACA (947C>A), TCA274TCT (962A>T), GTT276GTG (968T>G), CTT277TTG (969C>T 971T>G), ATA279ATT (977A>T), ATA280GTT (978A>G 980A>T), GTC281GCT (982T>C 983C>T), GGC282GAT (985G>A 986C>T), ACC283ATA (988C>T 989C>A), TTT284TTC (992T>C), GGA285GGG (995A>G), TTC286TTT (998C>T), AAA287AAG (1001A>G), AAT288AAC (1004T>C), GTG290GTT (1010G>T), TTG291CTG (1011T>C), TCA292TCT (1016A>T), GCT293GCA (1019T>A), ATA294ATT (1022A>T), ACG297ACA (1031G>A), CTG298ATT (1032C>A 1034G>T), CTG302CTC (1046G>C), TTC303TTT (1049C>T), CAG307ACT (1059C>A 1060A>C 1061G>T), GAG308GAA (1064G>A), GAT310TCT (1068G>T 1069A>C), TGT313GCT (1077T>G 1078G>C), ACG317ACA (1091G>A), CGC318AGG (1092C>A 1094C>G), GAG320GAA (1100G>A), GCC323GCT (1109C>T), AAG325CCT (1113A>C 1114A>C 1115G>T), TTC327TTT (1121C>T), CCT328TCA (1122C>T 1124T>A), ACC329ATC (1126C>T), CTG334CTT (1142G>T), AGG335AAA (1144G>A 1145G>A), GAA336GAG (1148A>G), GAA337CAG (1149G>C 1151A>G), GCT338GCA (1154T>A), ATC341ATA (1163C>A), GCT343GCC (1169T>C), CTG1241CTC (3863G>C), TCT1244TCA (3872T>A), TGC1245TGT (3875C>T), TAT1247TAC (3881T>C), CCC1248CCT (3884C>T), AAC1250AAT (3890C>T), AAG1251AAA (3893G>A), ATG1252CTG (3894A>C), GGG1253GGT (3899G>T), ATG1254TTG (3900A>T), GGG1255GGT (3905G>T), ATG1256ACC (3907T>C 3908G>C), AAC1257AAT (3911C>T), AAG1258AAA (3914G>A), AGA1259AGG (3917A>G), TTT1261TTC (3923T>C), GTG1262GTT (3926G>T), CAA1263GAA (3927C>G), TCC1264GCA (3930T>G 3932C>A), ACT1265CAG (3933A>C 3934C>A 3935T>G), GGC1266GAG (3937G>A 3938C>G), GAA1267AGT (3939G>A 3940A>G 3941A>T), GGA1268ACC (3942G>A 3943G>C 3944A>C), GAG1269CAA (3945G>C 3947G>A), AGA1270AGG (3950A>G), GTG1271GTA (3953G>A), GAA1272CTA (3954G>C 3955A>T), AAA1274AAG (3962A>G), GAC1276AAT (3966G>A 3968C>T), ACT1277TCT (3969A>T), GTA1279GTT (3977A>T), TTT1280TTC (3980T>C), GAA1281CAA (3981G>C), GAA1284GAG (3992A>G), AAT1716AAA (5288T>A), GTT1718GTG (5294T>G), GGT1720GGG (5300T>G), CCA1721CCT (5303A>T), AAC1723AAT (5309C>T), ATG1731ATA (5333G>A), CCG1733CCC (5339G>C), ATG1736TTG (5346A>T), TCA1737GCA (5349T>G), GCT1738GCA (5354T>A), ATA1739ATC (5357A>C), ATG1740CAG (5358A>C 5359T>A), GCC1742CAT (5364G>C 5365C>A 5366C>T), AAG1744CGC (5370A>C 5371A>G 5372G>C), AAT1745AAG (5375T>G), GTG1746AGT (5376G>A 5377T>G 5378G>T), TTG1747CTG (5379T>C), GCA1748GAA (5383C>A), GGA1749GTT (5386G>T 5387A>T), GGA1750GGT (5390A>T), CAT1751GAT (5391C>G), GTA1752GGG (5395T>G 5396A>G), GCA1753GCT (5399A>T), AAT1755TCT (5403A>T 5404A>C), GTT1757ACC (5409G>A 5410T>C 5411T>C), GTG1759GTT (5417G>T), GGA1761GGT (5423A>T), GGA1763GGT (5429A>T), GCT1765GCA (5435T>A), GTC1766ATA (5436G>A 5438C>A), GGC1767GCT (5440G>C 5441C>T), ATT1769ATC (5447T>C), GCG1772GCA (5456G>A), CTG1773TTG (5457C>T), ATG1774TTT (5460A>T 5462G>T), GCC1775GCA (5465C>A), TTA1776TTG (5468A>G), GCA1777GCC (5471A>C), ACA1778ACT (5474A>T), AGG1779CAT (5475A>C 5476G>A 5477G>T), TAT1781TAC (5483T>C), TGT1782AGG (5484T>A 5486T>G), GTG1783GCC (5488T>C 5489G>C), GAG1784ACA (5490G>A 5491A>C 5492G>A), ATA1786CTT (5496A>C 5498A>T), ATA1787ATT (5501A>T), GTT1788GTG (5504T>G), AGA1789CGC (5505A>C 5507A>C), TTG1790CTA (5508T>C 5510G>A), CGC1791CGA (5513C>A), ACA1792ACC (5516A>C), ATT1793ATG (5519T>G), GGA1794GCT (5521G>C 5522A>T), AAT1795GGG (5523A>G 5524A>G 5525T>G), CTT1797CTG (5531T>G), GAC1798GAT (5534C>T), CTC1799CTG (5537C>G), AAT1800AAC (5540T>C), TAT1802TAC (5546T>C), GGC1805GGT (5555C>T), GTG1807GCG (5560T>C), CAG1808CAA (5564G>A), TAT1809TAC (5567T>C), TTT1810TTG (5570T>G), CTT1811CTC (5573T>C), CAA1812AAA (5574C>A), GTG1814GTT (5582G>T), CCT1815GAA (5583C>G 5584C>A 5585T>A), CAT1816GAC (5586C>G 5588T>C), CAT1816_GGA1817insACC (5588_5589insACC), GGA1817TCA (5589G>T 5590G>C), GAC1818CAC (5592G>C), AAA1819AAG (5597A>G), GTG1820TTG (5598G>T), GCT1821GGA (5602C>G 5603T>A), CAA1822CAG (5606A>G), GGT1824GGC (5612T>C), GCA1825ACA (5613G>A), TCG1826TAT (5617C>A 5618G>T), CTC1827GGC (5619C>G 5620T>G), CAC1828del (5622_5624delCAC), AGT1829ACC (5626G>C 5627T>C), AGT1830ACT (5629G>C), ATG1832AAC (5635T>A 5636G>C), CAA1834CAT (5642A>T), CAA1834_GGG1835insCACAGT (5642_5643insCACAGT), GGG1835GCA (5644G>C 5645G>A), GAA1837TCA (5649G>T 5650A>C), TTG1838GTT (5652T>G 5654G>T), GGC1839GGA (5657C>A), AAC1840CTT (5658A>C 5659A>T 5660C>T), CTC1841CTA (5663C>A), CGC1842AGG (5664C>A 5666C>G), ATT1843ATA (5669T>A), ATA1844GTT (5670A>G 5672A>T), CAT1845CCT (5674A>C), GGT1846TCT (5676G>T 5677G>C), GAT1847GAC (5681T>C), TTA1848CTC (5682T>C 5684A>C), GCA1849TCT (5685G>T 5687A>T), AAA1850GAA (5688A>G), CAG1851AAG (5691C>A), ACA1852ATG (5695C>T 5696A>G), ATG1854GTT (5700A>G 5702G>T), CGG1855CGT (5705G>T), ATG1856TTG (5706A>T), TAT1858TAC (5714T>C), GTG1859ACA (5715G>A 5716T>C 5717G>A), GTA1860ATT (5718G>A 5720A>T), GGA1861GGT (5723A>T), CCA1862CCT (5726A>T), CAT1863GAA (5727C>G 5729T>A), GGA1865GGT (5735A>T), GGA1867GCC (5740G>C 5741A>C), AAT1868CAG (5742A>C 5744T>G), TTT1869TGC (5746T>G 5747T>C), TCC1870TCA (5750C>A), ACA1871ACG (5753A>G), CAG1872AAG (5754C>A), GAT1873AAC (5757G>A 5759T>C), CTT1874CTG (5762T>G), GAG1875AAG (5763G>A), AGT1876CAT (5766A>C 5767G>A), TTG1877GCT (5769T>G 5770T>C 5771G>T), CTA1878TTG (5772C>T 5774A>G), CAC1879GAG (5775C>G 5777C>G), TAT1880ACA (5778T>A 5779A>C 5780T>A), GAG1882TCT (5784G>T 5785A>C 5786G>T), CAG1883GTC (5787C>G 5788A>T 5789G>C), GGA1884AGT (5790G>A 5792A>T), GCA1886GCT (5798A>T), GAG1887ACT (5799G>A 5800A>C 5801G>T), TTA1888CTT (5802T>C 5804A>T), ATT1889GCC (5805A>G 5806T>C 5807T>C), CAA1890GCA (5808C>G 5809A>C), AAG1891AGA (5812A>G 5813G>A), AAT1892GGA (5814A>G 5815A>G 5816T>A), ACA1893GTC (5817A>G 5818C>T 5819A>C), CTA1895GTG (5823C>G 5825A>G), ACT1896ACA (5828T>A), CTG1897TTG (5829C>T), TTC1899TTT (5837C>T), GAG1901GAA (5843G>A), CTG1902TTC (5844C>T 5846G>C), AGT1903TCC (5847A>T 5848G>C 5849T>C), ATG1904CAA (5850A>C 5851T>A 5852G>A), GAA1905CAC (5853G>C 5855A>C), AAT1906GAT (5856A>G), GTG1907TGT (5859G>T 5860T>G 5861G>T), CAA1908GAA (5862C>G), CAA1909ATG (5865C>A 5866A>T 5867A>G), CTG1910ATA (5868C>A 5870G>A), ATA1911CTT (5871A>C 5873A>T), GAT1912GAG (5876T>G), GTT1914CTG (5880G>C 5882T>G), AAG1915GAA (5883A>G 5885G>A), CTT1916AGT (5886C>A 5887T>G), CAG1917CAA (5891G>A), TTT1919GTT (5895T>G)

Proteins

	Begin	End	Coverage	Score	Concordance	Matches	Identities	I/D/M/F*	Stop Codons
polyprotein (YP_001976147.1)	176	1922	20.1%	2123	70.1%	431 (99.1%)	289 (66.4%)	3/1/0/0	0

Protein mutations: L181I (681T>A 683A>T), D182S (684G>A 685A>G), K186L (696A>C 697A>T 698A>C), A188S (702G>T 704C>T), R190K (708C>A 709G>A), T192S (714A>T 716C>T), M193Q (717A>C 718T>A), V194M (720G>A), M199V (735A>G), V205L (753G>T 755T>G), S206N (756T>A 757C>A 758A>T), M214L (780A>C), T216S (786A>T 788A>T), I217L (789A>T 791A>G), L218I (792T>A 794G>A), I228V (822A>G 824T>G), S238T (853G>C 854C>A), T248A (882A>G 884A>G), T260Q (918A>C 919C>A 920T>A), R263K (927C>A 928G>A 929T>G), I280V (978A>G 980A>T), V281A (982T>C 983C>T), G282D (985G>A 986C>T), T283I (988C>T 989C>A), L298I (1032C>A 1034G>T), Q307T (1059C>A 1060A>C 1061G>T), D310S (1068G>T 1069A>C), C313A (1077T>G 1078G>C), K325P (1113A>C 1114A>C 1115G>T), P328S (1122C>T 1124T>A), T329I (1126C>T), R335K (1144G>A 1145G>A), E337Q (1149G>C 1151A>G), M1252L (3894A>C), M1254L (3900A>T), M1256T (3907T>C 3908G>C), Q1263E (3927C>G), S1264A (3930T>G 3932C>A), T1265Q (3933A>C 3934C>A 3935T>G), G1266E (3937G>A 3938C>G), E1267S (3939G>A 3940A>G 3941A>T), G1268T (3942G>A 3943G>C 3944A>C), E1269Q (3945G>C 3947G>A), E1272L (3954G>C 3955A>T), D1276N (3966G>A 3968C>T), T1277S (3969A>T), E1281Q (3981G>C), N1716K (5288T>A), M1731I (5333G>A), M1736L (5346A>T), S1737A (5349T>G), M1740Q (5358A>C 5359T>A), A1742H (5364G>C 5365C>A 5366C>T), K1744R (5370A>C 5371A>G 5372G>C), N1745K (5375T>G), V1746S (5376G>A 5377T>G 5378G>T), A1748E (5383C>A), G1749V (5386G>T 5387A>T), H1751D (5391C>G), V1752G (5395T>G 5396A>G), N1755S (5403A>T 5404A>C), V1757T (5409G>A 5410T>C 5411T>C), V1766I (5436G>A 5438C>A), G1767A (5440G>C 5441C>T), M1774F (5460A>T 5462G>T), R1779H (5475A>C 5476G>A 5477G>T), C1782R (5484T>A 5486T>G), V1783A (5488T>C 5489G>C), E1784T (5490G>A 5491A>C 5492G>A), I1786L (5496A>C 5498A>T), I1793M (5519T>G), G1794A (5521G>C 5522A>T), N1795G (5523A>G 5524A>G 5525T>G), V1807A (5560T>C), F1810L (5570T>G), Q1812K (5574C>A), P1815E (5583C>G 5584C>A 5585T>A), H1816D (5586C>G 5588T>C), H1816_G1817insT (5588_5589insACC), G1817S (5589G>T 5590G>C), D1818H (5592G>C), V1820L (5598G>T), A1821G (5602C>G 5603T>A), A1825T (5613G>A), S1826Y (5617C>A 5618G>T), L1827G (5619C>G 5620T>G), H1828del (5622_5624delCAC), S1829T (5626G>C 5627T>C), S1830T (5629G>C), M1832N (5635T>A 5636G>C), Q1834H (5642A>T), Q1834_G1835insHS (5642_5643insCACAGT), G1835A (5644G>C 5645G>A), E1837S (5649G>T 5650A>C), L1838V (5652T>G 5654G>T), N1840L (5658A>C 5659A>T 5660C>T), I1844V (5670A>G 5672A>T), H1845P (5674A>C), G1846S (5676G>T 5677G>C), A1849S (5685G>T 5687A>T), K1850E (5688A>G), Q1851K (5691C>A), T1852M (5695C>T 5696A>G), M1854V (5700A>G 5702G>T), M1856L (5706A>T), V1859T (5715G>A 5716T>C 5717G>A), V1860I (5718G>A 5720A>T), H1863E (5727C>G 5729T>A), G1867A (5740G>C 5741A>C), N1868Q (5742A>C 5744T>G), F1869C (5746T>G 5747T>C), Q1872K (5754C>A), D1873N (5757G>A 5759T>C), E1875K (5763G>A), S1876H (5766A>C 5767G>A), L1877A (5769T>G 5770T>C 5771G>T), H1879E (5775C>G 5777C>G), Y1880T (5778T>A 5779A>C 5780T>A), E1882S (5784G>T 5785A>C 5786G>T), Q1883V (5787C>G 5788A>T 5789G>C), G1884S (5790G>A 5792A>T), E1887T (5799G>A 5800A>C 5801G>T), I1889A (5805A>G 5806T>C 5807T>C), Q1890A (5808C>G 5809A>C), K1891R (5812A>G 5813G>A), N1892G (5814A>G 5815A>G 5816T>A), T1893V (5817A>G 5818C>T 5819A>C), L1895V (5823C>G 5825A>G), L1902F (5844C>T 5846G>C), M1904Q (5850A>C 5851T>A 5852G>A), E1905H (5853G>C 5855A>C), N1906D (5856A>G), V1907C (5859G>T 5860T>G 5861G>T), Q1908E (5862C>G), Q1909M (5865C>A 5866A>T 5867A>G), L1910I (5868C>A 5870G>A), I1911L (5871A>C 5873A>T), D1912E (5876T>G), V1914L (5880G>C 5882T>G), K1915E (5883A>G 5885G>A), L1916S (5886C>A 5887T>G), F1919V (5895T>G)

	Begin	End	Coverage	Score	Concordance	Matches	Identities	I/D/M/F*	Stop Codons
**NT**	**666**	**5905**	**17.9%**	**781**	**30.4%**	**1290 (99.1%)**	**850 (65.3%)**	**9/3**	

Codon mutations:

TAC179TAT (677C>T), TTA181ATT (681T>A 683A>T), GAT182AGT (684G>A 685A>G), CCA185CCC (695A>C), AAA186CTC (696A>C 697A>T 698A>C), GCC188TCT (702G>T 704C>T), GAA189GAG (707A>G), CGA190AAA (708C>A 709G>A), TTC191TTT (713C>T), ACC192TCT (714A>T 716C>T), ATG193CAG (717A>C 718T>A), GTG194ATG (720G>A), GCC195GCA (725C>A), CAA196CAG (728A>G), AGT198TCT (732A>T 733G>C), ATG199GTG (735A>G), CTT200CTA (740T>A), GGG202GGT (746G>T), GAA203GAG (749A>G), TCA204AGC (750T>A 751C>G 752A>C), GTT205TTG (753G>T 755T>G), TCA206AAT (756T>A 757C>A 758A>T), ACG210ACC (770G>C), TCT211AGT (771T>A 772C>G), CAG212CAA (776G>A), ATG214CTG (780A>C), TCC215TCT (785C>T), ACA216TCT (786A>T 788A>T), ATA217TTG (789A>T 791A>G), TTG218ATA (792T>A 794G>A), GCT219GCA (797T>A), GGG221GGC (803G>C), ATT222ATC (806T>C), TCG223TCA (809G>A), GTG226GTA (818G>A), TTC227TTT (821C>T), ATT228GTG (822A>G 824T>G), GTG230GTA (830G>A), AGA233AGG (839A>G), TTG237CTT (849T>C 851G>T), AGC238ACA (853G>C 854C>A), CCA240CCG (860A>G), TTG241CTA (861T>C 863G>A), CTC245TTG (873C>T 875C>G), TCG247TCA (881G>A), ACA248GCG (882A>G 884A>G), GGA249GGC (887A>C), GGT251GGA (893T>A), CAG253CAA (899G>A), CTA258CTC (914A>C), ACT260CAA (918A>C 919C>A 920T>A), CTT261TTG (921C>T 923T>G), TAC262TAT (926C>T), CGT263AAG (927C>A 928G>A 929T>G), AAT264AAC (932T>C), AAA266AAG (938A>G), ACC269ACA (947C>A), TCA274TCT (962A>T), GTT276GTG (968T>G), CTT277TTG (969C>T 971T>G), ATA279ATT (977A>T), ATA280GTT (978A>G 980A>T), GTC281GCT (982T>C 983C>T), GGC282GAT (985G>A 986C>T), ACC283ATA (988C>T 989C>A), TTT284TTC (992T>C), GGA285GGG (995A>G), TTC286TTT (998C>T), AAA287AAG (1001A>G), AAT288AAC (1004T>C), GTG290GTT (1010G>T), TTG291CTG (1011T>C), TCA292TCT (1016A>T), GCT293GCA (1019T>A), ATA294ATT (1022A>T), ACG297ACA (1031G>A), CTG298ATT (1032C>A 1034G>T), CTG302CTC (1046G>C), TTC303TTT (1049C>T), CAG307ACT (1059C>A 1060A>C 1061G>T), GAG308GAA (1064G>A), GAT310TCT (1068G>T 1069A>C), TGT313GCT (1077T>G 1078G>C), ACG317ACA (1091G>A), CGC318AGG (1092C>A 1094C>G), GAG320GAA (1100G>A), GCC323GCT (1109C>T), AAG325CCT (1113A>C 1114A>C 1115G>T), TTC327TTT (1121C>T), CCT328TCA (1122C>T 1124T>A), ACC329ATC (1126C>T), CTG334CTT (1142G>T), AGG335AAA (1144G>A 1145G>A), GAA336GAG (1148A>G), GAA337CAG (1149G>C 1151A>G), GCT338GCA (1154T>A), ATC341ATA (1163C>A), GCT343GCC (1169T>C), CTG1241CTC (3863G>C), TCT1244TCA (3872T>A), TGC1245TGT (3875C>T), TAT1247TAC (3881T>C), CCC1248CCT (3884C>T), AAC1250AAT (3890C>T), AAG1251AAA (3893G>A), ATG1252CTG (3894A>C), GGG1253GGT (3899G>T), ATG1254TTG (3900A>T), GGG1255GGT (3905G>T), ATG1256ACC (3907T>C 3908G>C), AAC1257AAT (3911C>T), AAG1258AAA (3914G>A), AGA1259AGG (3917A>G), TTT1261TTC (3923T>C), GTG1262GTT (3926G>T), CAA1263GAA (3927C>G), TCC1264GCA (3930T>G 3932C>A), ACT1265CAG (3933A>C 3934C>A 3935T>G), GGC1266GAG (3937G>A 3938C>G), GAA1267AGT (3939G>A 3940A>G 3941A>T), GGA1268ACC (3942G>A 3943G>C 3944A>C), GAG1269CAA (3945G>C 3947G>A), AGA1270AGG (3950A>G), GTG1271GTA (3953G>A), GAA1272CTA (3954G>C 3955A>T), AAA1274AAG (3962A>G), GAC1276AAT (3966G>A 3968C>T), ACT1277TCT (3969A>T), GTA1279GTT (3977A>T), TTT1280TTC (3980T>C), GAA1281CAA (3981G>C), GAA1284GAG (3992A>G), AAT1716AAA (5288T>A), GTT1718GTG (5294T>G), GGT1720GGG (5300T>G), CCA1721CCT (5303A>T), AAC1723AAT (5309C>T), ATG1731ATA (5333G>A), CCG1733CCC (5339G>C), ATG1736TTG (5346A>T), TCA1737GCA (5349T>G), GCT1738GCA (5354T>A), ATA1739ATC (5357A>C), ATG1740CAG (5358A>C 5359T>A), GCC1742CAT (5364G>C 5365C>A 5366C>T), AAG1744CGC (5370A>C 5371A>G 5372G>C), AAT1745AAG (5375T>G), GTG1746AGT (5376G>A 5377T>G 5378G>T), TTG1747CTG (5379T>C), GCA1748GAA (5383C>A), GGA1749GTT (5386G>T 5387A>T), GGA1750GGT (5390A>T), CAT1751GAT (5391C>G), GTA1752GGG (5395T>G 5396A>G), GCA1753GCT (5399A>T), AAT1755TCT (5403A>T 5404A>C), GTT1757ACC (5409G>A 5410T>C 5411T>C), GTG1759GTT (5417G>T), GGA1761GGT (5423A>T), GGA1763GGT (5429A>T), GCT1765GCA (5435T>A), GTC1766ATA (5436G>A 5438C>A), GGC1767GCT (5440G>C 5441C>T), ATT1769ATC (5447T>C), GCG1772GCA (5456G>A), CTG1773TTG (5457C>T), ATG1774TTT (5460A>T 5462G>T), GCC1775GCA (5465C>A), TTA1776TTG (5468A>G), GCA1777GCC (5471A>C), ACA1778ACT (5474A>T), AGG1779CAT (5475A>C 5476G>A 5477G>T), TAT1781TAC (5483T>C), TGT1782AGG (5484T>A 5486T>G), GTG1783GCC (5488T>C 5489G>C), GAG1784ACA (5490G>A 5491A>C 5492G>A), ATA1786CTT (5496A>C 5498A>T), ATA1787ATT (5501A>T), GTT1788GTG (5504T>G), AGA1789CGC (5505A>C 5507A>C), TTG1790CTA (5508T>C 5510G>A), CGC1791CGA (5513C>A), ACA1792ACC (5516A>C), ATT1793ATG (5519T>G), GGA1794GCT (5521G>C 5522A>T), AAT1795GGG (5523A>G 5524A>G 5525T>G), CTT1797CTG (5531T>G), GAC1798GAT (5534C>T), CTC1799CTG (5537C>G), AAT1800AAC (5540T>C), TAT1802TAC (5546T>C), GGC1805GGT (5555C>T), GTG1807GCG (5560T>C), CAG1808CAA (5564G>A), TAT1809TAC (5567T>C), TTT1810TTG (5570T>G), CTT1811CTC (5573T>C), CAA1812AAA (5574C>A), GTG1814GTT (5582G>T), CCT1815GAA (5583C>G 5584C>A 5585T>A), CAT1816GAC (5586C>G 5588T>C), CAT1816_GGA1817insACC (5588_5589insACC), GGA1817TCA (5589G>T 5590G>C), GAC1818CAC (5592G>C), AAA1819AAG (5597A>G), GTG1820TTG (5598G>T), GCT1821GGA (5602C>G 5603T>A), CAA1822CAG (5606A>G), GGT1824GGC (5612T>C), GCA1825ACA (5613G>A), TCG1826TAT (5617C>A 5618G>T), CTC1827GGC (5619C>G 5620T>G), CAC1828del (5622_5624delCAC), AGT1829ACC (5626G>C 5627T>C), AGT1830ACT (5629G>C), ATG1832AAC (5635T>A 5636G>C), CAA1834CAT (5642A>T), CAA1834_GGG1835insCACAGT (5642_5643insCACAGT), GGG1835GCA (5644G>C 5645G>A), GAA1837TCA (5649G>T 5650A>C), TTG1838GTT (5652T>G 5654G>T), GGC1839GGA (5657C>A), AAC1840CTT (5658A>C 5659A>T 5660C>T), CTC1841CTA (5663C>A), CGC1842AGG (5664C>A 5666C>G), ATT1843ATA (5669T>A), ATA1844GTT (5670A>G 5672A>T), CAT1845CCT (5674A>C), GGT1846TCT (5676G>T 5677G>C), GAT1847GAC (5681T>C), TTA1848CTC (5682T>C 5684A>C), GCA1849TCT (5685G>T 5687A>T), AAA1850GAA (5688A>G), CAG1851AAG (5691C>A), ACA1852ATG (5695C>T 5696A>G), ATG1854GTT (5700A>G 5702G>T), CGG1855CGT (5705G>T), ATG1856TTG (5706A>T), TAT1858TAC (5714T>C), GTG1859ACA (5715G>A 5716T>C 5717G>A), GTA1860ATT (5718G>A 5720A>T), GGA1861GGT (5723A>T), CCA1862CCT (5726A>T), CAT1863GAA (5727C>G 5729T>A), GGA1865GGT (5735A>T), GGA1867GCC (5740G>C 5741A>C), AAT1868CAG (5742A>C 5744T>G), TTT1869TGC (5746T>G 5747T>C), TCC1870TCA (5750C>A), ACA1871ACG (5753A>G), CAG1872AAG (5754C>A), GAT1873AAC (5757G>A 5759T>C), CTT1874CTG (5762T>G), GAG1875AAG (5763G>A), AGT1876CAT (5766A>C 5767G>A), TTG1877GCT (5769T>G 5770T>C 5771G>T), CTA1878TTG (5772C>T 5774A>G), CAC1879GAG (5775C>G 5777C>G), TAT1880ACA (5778T>A 5779A>C 5780T>A), GAG1882TCT (5784G>T 5785A>C 5786G>T), CAG1883GTC (5787C>G 5788A>T 5789G>C), GGA1884AGT (5790G>A 5792A>T), GCA1886GCT (5798A>T), GAG1887ACT (5799G>A 5800A>C 5801G>T), TTA1888CTT (5802T>C 5804A>T), ATT1889GCC (5805A>G 5806T>C 5807T>C), CAA1890GCA (5808C>G 5809A>C), AAG1891AGA (5812A>G 5813G>A), AAT1892GGA (5814A>G 5815A>G 5816T>A), ACA1893GTC (5817A>G 5818C>T 5819A>C), CTA1895GTG (5823C>G 5825A>G), ACT1896ACA (5828T>A), CTG1897TTG (5829C>T), TTC1899TTT (5837C>T), GAG1901GAA (5843G>A), CTG1902TTC (5844C>T 5846G>C), AGT1903TCC (5847A>T 5848G>C 5849T>C), ATG1904CAA (5850A>C 5851T>A 5852G>A), GAA1905CAC (5853G>C 5855A>C), AAT1906GAT (5856A>G), GTG1907TGT (5859G>T 5860T>G 5861G>T), CAA1908GAA (5862C>G), CAA1909ATG (5865C>A 5866A>T 5867A>G), CTG1910ATA (5868C>A 5870G>A), ATA1911CTT (5871A>C 5873A>T), GAT1912GAG (5876T>G), GTT1914CTG (5880G>C 5882T>G), AAG1915GAA (5883A>G 5885G>A), CTT1916AGT (5886C>A 5887T>G), CAG1917CAA (5891G>A), TTT1919GTT (5895T>G)

*: Inserts / Deletes / Misaligned / Frameshifts

Analysis details

This analysis was performed with panviral2.64

NGS Details (UN9): Torradovirus marchitezum (segment RNA 2)

Assembly

**Coverage Length**	1553 (2 contig(s))
**Depth Of Coverage**	23655.4
**Number Of Reads**	323229
**Reads Per Million**	7305.90 rpm (after QC)
**Ambiguities**	0
**Assembly Method**	de novo + reference guided assembly
**Consensus Caller**	Bcf Tools

Coverage Map

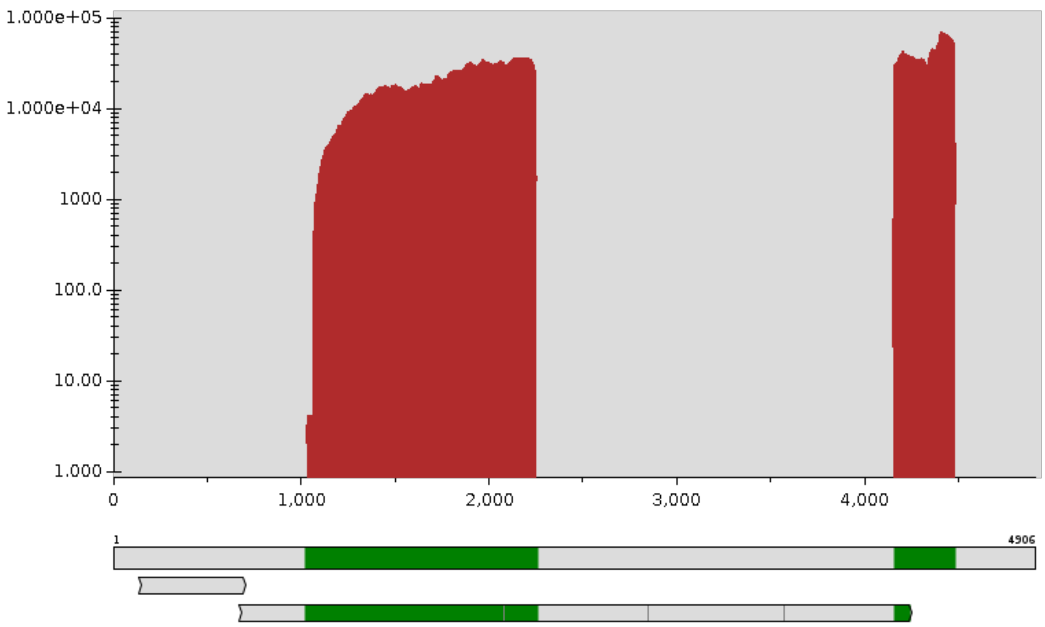

Assignment

**Type**	Torradovirus marchitezum (Taxonomy ID: 3048376)
**Reference Genome**	NC_010988.1
**NT Identity (%)**	62.3492
**AA Identity (%)**	59.9099
**Number Of Stop Codons**	1
**Number Of CDS**	2

Alignment

**Alignment Score**	701.0 (NT) + 1905.0 (AA) = 2606.0
**Concordance (%)**	43.2531

Alignment Method	Global, seeded, nucleotide + amino acids (AGA)

Genome Region

Sequence starts at position 1030 and ends at position 4484 relative to NC_010988.1 reference sequence.

Alignment Detailed Statistics

	Begin	End	Coverage	Score	Concordance	Matches	Identities	I/D/M/F*	Stop Codons
**NT**	**1030**	**4484**	**31.7%**	**701**	**23.0%**	**1541 (97.1%)**	**982 (61.9%)**	**34/12**	
Mutations:	1030T>C, 1039C>A, 1040C>A, 1041T>C, 1042C>T, 1043T>G, 1044C>A, 1045A>T, 1048G>T, 1051A>T, 1057A>G, 1065C>T, 1066A>G, 1067C>T, 1069G>A, 1071C>T, 1075G>T, 1076G>T, 1078C>A, 1080A>G, 1081T>G, 1087G>T, 1093A>T, 1096T>C, 1097C>T, 1099C>G, 1105C>T, 1108C>T, 1109A>G, 1110G>A, 1111C>A, 1114A>T, 1123C>T, 1127C>G, 1129C>T, 1132A>G, 1156C>T, 1159G>A, 1165C>T, 1167A>T, 1168A>T, 1171T>C, 1174C>A, 1175A>C, 1180A>T, 1182A>T, 1183G>A, 1189G>A, 1195C>T, 1198G>T, 1201T>G, 1204C>A, 1207A>T, 1213A>G, 1216A>G, 1222T>A, 1223G>A, 1224A>G, 1226A>C, 1231G>A, 1237A>T, 1244T>A, 1246A>G, 1247C>G, 1248A>C, 1249G>A, 1262T>A, 1263C>G, 1270C>A, 1273A>T, 1276A>G, 1279A>T, 1286C>A, 1288T>G, 1291G>A, 1294A>G, 1297T>C, 1300C>T, 1301C>T, 1302A>C, 1303G>T, 1312G>A, 1318T>A, 1321T>A, 1327T>C, 1330A>T, 1339T>C, 1342T>C, 1351G>A, 1352C>G, 1354A>G, 1357T>G, 1358A>G, 1360T>G, 1361C>T, 1362A>C, 1363A>T, 1364A>C, 1365T>A, 1366G>A, 1369T>C, 1372T>A, 1375A>G, 1378C>T, 1380A>G, 1382G>C, 1384C>T, 1390T>A, 1393A>T, 1395A>C, 1398A>T, 1399G>T, 1402T>C, 1406G>A, 1407G>A, 1411A>T, 1412A>T, 1413G>A, 1414A>C, 1418G>A, 1420C>T, 1423C>T, 1424A>C, 1425T>C, 1426G>C, 1427A>C, 1429T>G, 1432A>G, 1433A>T, 1434G>C, 1435C>T, 1436A>T, 1438T>A, 1439A>T, 1441G>A, 1444C>G, 1447G>T, 1448A>G, 1449G>C, 1450T>G, 1456T>G, 1457T>C, 1459G>T, 1460A>C, 1462A>C, 1463G>A, 1465C>T, 1471T>C, 1474C>A, 1477A>G, 1478G>A, 1480G>C, 1483G>A, 1490C>A, 1492C>G, 1495C>T, 1498G>T, 1501C>G, 1507A>G, 1513G>T, 1515C>A, 1517G>T, 1519A>T, 1520A>T, 1521G>C, 1522T>A, 1522_1523insAGGGCC, 1523A>T, 1531C>G, 1532T>A, 1533T>C, 1535C>G, 1537C>A, 1538A>G, 1540C>T, 1541A>G, 1542C>T, 1543A>G, 1544C>A, 1546T>A, 1549A>G, 1557T>G, 1558T>G, 1559C>G, 1560A>T, 1561T>A, 1565_1567delGAG, 1575G>C, 1576T>C, 1577G>A, 1578G>A, 1579G>T, 1580G>T, 1581G>T, 1583T>C, 1591A>T, 1593C>T, 1594A>C, 1595_1600delCCCAAG, 1603A>G, 1604A>G, 1608G>C, 1609C>A, 1612A>G, 1616T>A, 1618C>T, 1619A>G, 1621C>T, 1622A>T, 1624G>T, 1627T>C, 1628G>C, 1629C>A, 1630C>G, 1633T>C, 1639T>C, 1644T>A, 1645G>T, 1651G>C, 1652G>A, 1653A>C, 1654G>T, 1655T>G, 1656C>A, 1657G>T, 1660C>T, 1661G>A, 1662C>A, 1663A>T, 1664C>T, 1665A>C, 1666C>G, 1667A>T, 1684T>G, 1691G>A, 1692A>C, 1693A>C, 1694C>T, 1696A>G, 1697A>C, 1698C>A, 1699A>T, 1703G>T, 1705T>A, 1706C>A, 1707A>G, 1711G>A, 1712G>A, 1714A>T, 1715C>G, 1717A>T, 1721A>G, 1723C>G, 1724C>T, 1726C>G, 1728T>C, 1731G>C, 1732T>C, 1733G>C, 1734A>T, 1735G>C, 1736G>C, 1737C>T, 1738T>C, 1739A>C, 1740A>G, 1741A>C, 1742A>C, 1743G>A, 1744A>G, 1747A>G, 1749A>G, 1750C>A, 1751G>T, 1752T>G, 1754A>C, 1755T>A, 1757G>C, 1762A>C, 1764G>A, 1765A>G, 1768T>C, 1769G>A, 1773C>A, 1774A>G, 1776A>G, 1777G>A, 1780G>A, 1781C>G, 1783T>C, 1784C>T, 1785T>C, 1786G>C, 1789T>C, 1792T>C, 1795C>A, 1798T>C, 1799A>C, 1800G>T, 1801G>A, 1805C>T, 1810A>T, 1811A>G, 1812C>A, 1813C>T, 1817G>A, 1819C>T, 1822T>C, 1826G>C, 1829G>C, 1831A>G, 1832A>G, 1834A>G, 1837T>C, 1838G>A, 1840A>G, 1842G>A, 1843T>A, 1849T>C, 1850G>A, 1852T>C, 1855C>A, 1859A>G, 1861C>G, 1862C>G, 1863A>G, 1864A>T, 1865G>T, 1866G>C, 1868T>C, 1870G>A, 1871A>G, 1872C>A, 1873T>G, 1876C>T, 1881T>G, 1891T>C, 1892G>T, 1895A>T, 1897G>A, 1898T>A, 1905A>G, 1909G>A, 1912A>G, 1915T>G, 1916G>A, 1918G>A, 1921G>A, 1922C>T, 1924C>G, 1927C>G, 1933A>G, 1939T>C, 1943T>C, 1944C>A, 1945C>A, 1947T>A, 1951T>C, 1953T>G, 1954T>G, 1955C>T, 1957A>T, 1960G>T, 1961C>G, 1963G>T, 1964A>G, 1965C>A, 1966T>G, 1968A>C, 1969C>A, 1975A>C, 1981G>A, 1983A>G, 1984T>A, 1990C>T, 1996T>C, 2002A>C, 2003T>C, 2005A>C, 2008C>A, 2013A>T, 2014A>G, 2017G>C, 2018C>A, 2020T>A, 2023G>C, 2023_2024insAAGGGTGGCAGGGCTAAG, 2026G>A, 2027A>G, 2028T>A, 2032G>A, 2034A>C, 2035G>T, 2037A>G, 2038G>T, 2039C>G, 2040A>C, 2041A>C, 2042G>T, 2044G>A, 2047C>A, 2048_2050delCAA, 2053A>T, 2054A>T, 2055G>T, 2057T>C, 2059T>A, 2060G>C, 2063A>C, 2064G>C, 2065A>T, 2066C>A, 2067A>G, 2070C>T, 2071A>G, 2072C>A, 2078A>G, 2080A>C, 2080_2081insCAG, 2082C>A, 2084A>G, 2086G>A, 2089C>G, 2090A>G, 2091T>G, 2093G>A, 2094C>G, 2095T>C, 2096G>A, 2097A>G, 2098A>G, 2099A>T, 2100G>C, 2101T>C, 2102C>A, 2103A>C, 2104G>C, 2105C>A, 2110A>G, 2111G>A, 2112A>G, 2115G>A, 2116A>G, 2119G>T, 2120A>C, 2121C>T, 2122T>G, 2133T>C, 2134G>A, 2135T>A, 2137A>T, 2140T>A, 2143C>T, 2144A>G, 2146G>A, 2150G>C, 2152_2153insAGC, 2155T>G, 2156C>G, 2157C>G, 2158C>A, 2160C>T, 2164G>C, 2165C>A, 2166C>A, 2167A>G, 2170T>C, 2171A>C, 2173G>A, 2176A>T, 2178A>T, 2182T>A, 2184T>A, 2185T>G, 2187C>T, 2188A>T, 2190A>C, 2191G>T, 2193G>A, 2194A>C, 2195G>C, 2200A>G, 2201G>C, 2203A>G, 2207A>G, 2208C>T, 2209T>C, 2210C>T, 2211A>C, 2217G>A, 2218T>C, 2224T>C, 2225A>T, 2227T>C, 2239A>G, 2240C>G, 2241C>A, 2242A>G, 4180C>T, 4181A>G, 4192T>G, 4195C>T, 4198C>T, 4201T>C, 4204G>A, 4213A>T, 4219T>C, 4222T>A, 4225G>A, 4228A>G, 4229C>A, 4231T>A, 4234T>C, 4237T>C, 4238G>T, 4242T>A, 4243T>C, 4248A>C, 4252T>A, 4279A>T, 4279_4280insT, 4280A>T, 4280_4281insC, 4283A>G, 4286C>T, 4287T>A, 4288T>C, 4291C>A, 4294C>T, 4306G>A, 4309G>A, 4312G>T, 4313C>A, 4313_4314insG, 4315A>G, 4316A>T, 4318T>C, 4319G>T, 4320G>A, 4323T>G, 4324T>A, 4328T>C, 4330G>A, 4333A>G, 4335G>T, 4336A>C, 4338A>G, 4339A>G, 4340G>T, 4341G>C, 4342G>T, 4343A>T, 4345A>T, 4346G>C, 4349A>C, 4364C>T, 4365T>C, 4369A>G, 4372A>G, 4373G>A, 4392T>C, 4393C>T, 4419_4420insG, 4422A>G, 4426G>A, 4430G>A, 4437C>T, 4441T>C, 4449A>T, 4451T>G								
**CDS**									
ToMarV_RNA2gp2	121	1192	36.7%	1905	63.6%	434 (96.9%)	266 (59.4%)	10/4/0/0	1
Protein mutations:	L124T (1040C>A 1041T>C 1042C>T), S125D (1043T>G 1044C>A 1045A>T), T132M (1065C>T 1066A>G), S134F (1071C>T), L135F (1075G>T), A136S (1076G>T 1078C>A), D137G (1080A>G 1081T>G), S147E (1109A>G 1110G>A 1111C>A), L153V (1127C>G 1129C>T), Q166L (1167A>T 1168A>T), Q171L (1182A>T 1183G>A), D185S (1223G>A 1224A>G), T186P (1226A>C), S192T (1244T>A 1246A>G), Q193A (1247C>G 1248A>C 1249G>A), Q211S (1301C>T 1302A>C 1303G>T), Q228E (1352C>G 1354A>G), S229R (1357T>G), T230A (1358A>G 1360T>G), Q231S (1361C>T 1362A>C 1363A>T), M232Q (1364A>C 1365T>A 1366G>A), K237R (1380A>G), A238P (1382G>C 1384C>T), E242A (1395A>C), Q243L (1398A>T 1399G>T), G246N (1406G>A 1407G>A), R248Y (1412A>T 1413G>A 1414A>C), D250N (1418G>A 1420C>T), M252P (1424A>C 1425T>C 1426G>C), N253Q (1427A>C 1429T>G), T256S (1436A>T 1438T>A), M257L (1439A>T 1441G>A), I258M (1444C>G), S260A (1448A>G 1449G>C 1450T>G), V265I (1463G>A 1465C>T), V270I (1478G>A 1480G>C), A282D (1515C>A), V283F (1517G>T 1519A>T), S284_T285insRA (1522_1523insAGGGCC), T285S (1523A>T), L288T (1532T>A 1533T>C), L289V (1535C>G 1537C>A), S290G (1538A>G 1540C>T), T291V (1541A>G 1542C>T 1543A>G), V296G (1557T>G 1558T>G), H297V (1559C>G 1560A>T 1561T>A), E299del (1565_1567delGAG), C302S (1575G>C 1576T>C), G303N (1577G>A 1578G>A 1579G>T), G304L (1580G>T 1581G>T), A308V (1593C>T 1594A>C), P309_K310del (1595_1600delCCCAAG), K312E (1604A>G), G313A (1608G>C 1609C>A), F316I (1616T>A 1618C>T), N317D (1619A>G 1621C>T), M318F (1622A>T 1624G>T), A320Q (1628G>C 1629C>A 1630C>G), L325Y (1644T>A 1645G>T), M327I (1651G>C), E328T (1652G>A 1653A>C 1654G>T), S329D (1655T>G 1656C>A 1657G>T), A331N (1661G>A 1662C>A 1663A>T), H332S (1664C>T 1665A>C 1666C>G), I333F (1667A>T), D338E (1684T>G), E341T (1691G>A 1692A>C 1693A>C), R342W (1694C>T 1696A>G), T343H (1697A>C 1698C>A 1699A>T), A345S (1703G>T 1705T>A), Q346R (1706C>A 1707A>G), V348I (1712G>A 1714A>T), Q349D (1715C>G 1717A>T), I351V (1721A>G 1723C>G), F353S (1728T>C), S354T (1731G>C 1732T>C), E355L (1733G>C 1734A>T 1735G>C), A356L (1736G>C 1737C>T 1738T>C), K357R (1739A>C 1740A>G 1741A>C), R358Q (1742A>C 1743G>A 1744A>G), N360R (1749A>G 1750C>A), V361C (1751G>T 1752T>G), I362H (1754A>C 1755T>A), V363L (1757G>C), R365K (1764G>A 1765A>G), V367I (1769G>A), A368E (1773C>A 1774A>G), K369R (1776A>G 1777G>A), H371D (1781C>G 1783T>C), L372S (1784C>T 1785T>C 1786G>C), R377L (1799A>C 1800G>T 1801G>A), H379Y (1805C>T), T381D (1811A>G 1812C>A 1813C>T), D383N (1817G>A 1819C>T), V386L (1826G>C), E387Q (1829G>C 1831A>G), I388V (1832A>G 1834A>G), E390K (1838G>A 1840A>G), G391E (1842G>A 1843T>A), V394I (1850G>A 1852T>C), I397V (1859A>G 1861C>G), Q398G (1862C>G 1863A>G 1864A>T), G399S (1865G>T 1866G>C), T401E (1871A>G 1872C>A 1873T>G), F404C (1881T>G), A408S (1892G>T), M409L (1895A>T 1897G>A), S410T (1898T>A), K412R (1905A>G), V416I (1916G>A 1918G>A), S425Q (1943T>C 1944C>A 1945C>A), F426Y (1947T>A), I428R (1953T>G 1954T>G), Q429Y (1955C>T 1957A>T), Q431D (1961C>G 1963G>T), T432E (1964A>G 1965C>A 1966T>G), N433T (1968A>C 1969C>A), N438R (1983A>G 1984T>A), K448M (2013A>T 2014A>G), E449D (2017G>C), Q451H (2023G>C), Q451_L452insKGGRAK (2023_2024insAAGGGTGGCAGGGCTAAG), I453D (2027A>G 2028T>A), E455A (2034A>C 2035G>T), E456G (2037A>G 2038G>T), Q457A (2039C>G 2040A>C 2041A>C), V458L (2042G>T 2044G>A), Q460del (2048_2050delCAA), R461S (2053A>T), R462L (2054A>T 2055G>T), S463P (2057T>C 2059T>A), E464Q (2060G>C), R465P (2063A>C 2064G>C 2065A>T), Q466R (2066C>A 2067A>G), A467V (2070C>T 2071A>G), Q468K (2072C>A), T470A (2078A>G 2080A>C), T470_A471insQ (2080_2081insCAG), A471E (2082C>A), R472G (2084A>G 2086G>A), I474G (2090A>G 2091T>G), A475S (2093G>A 2094C>G 2095T>C), E476R (2096G>A 2097A>G 2098A>G), Q478T (2102C>A 2103A>C 2104G>C), P479T (2105C>A), D481S (2111G>A 2112A>G), R482K (2115G>A 2116A>G), T484L (2120A>C 2121C>T 2122T>G), V488A (2133T>C 2134G>A), S489T (2135T>A 2137A>T), T492A (2144A>G 2146G>A), E494Q (2150G>C), E494_D495insS (2152_2153insAGC), D495E (2155T>G), P496G (2156C>G 2157C>G 2158C>A), T497I (2160C>T), K498N (2164G>C), P499K (2165C>A 2166C>A 2167A>G), K501Q (2171A>C 2173G>A), E503V (2178A>T), V505E (2184T>A 2185T>G), A506V (2187C>T 2188A>T), E507A (2190A>C 2191G>T), G508D (2193G>A 2194A>C), A509P (2195G>C), E511Q (2201G>C 2203A>G), T513V (2207A>G 2208C>T 2209T>C), Q514S (2210C>T 2211A>C), G516D (2217G>A 2218T>C), I519F (2225A>T 2227T>C), P524E (2240C>G 2241C>A 2242A>G), N1171D (4181A>G), A1190S (4238G>T), I1191N (4242T>A 4243T>C)								

	Begin	End	Coverage	Score	Concordance	Matches	Identities	I/D/M/F*	Stop Codons
**NT**	**1030**	**4484**	**31.7%**	**701**	**23.0%**	**1541 (97.1%)**	**982 (61.9%)**	**34/12**	

Codon mutations:

AGT120..C (1030T>C), CCC123CCA (1039C>A), CTC124ACT (1040C>A 1041T>C 1042C>T), TCA125GAT (1043T>G 1044C>A 1045A>T), CCG126CCT (1048G>T), GTA127GTT (1051A>T), CAA129CAG (1057A>G), ACA132ATG (1065C>T 1066A>G), CTG133TTA (1067C>T 1069G>A), TCC134TTC (1071C>T), TTG135TTT (1075G>T), GCC136TCA (1076G>T 1078C>A), GAT137GGG (1080A>G 1081T>G), GCG139GCT (1087G>T), GCA141GCT (1093A>T), GTT142GTC (1096T>C), CTC143TTG (1097C>T 1099C>G), AGC145AGT (1105C>T), CCC146CCT (1108C>T), AGC147GAA (1109A>G 1110G>A 1111C>A), ATA148ATT (1114A>T), CCC151CCT (1123C>T), CTC153GTT (1127C>G 1129C>T), AAA154AAG (1132A>G), ACC162ACT (1156C>T), CTG163CTA (1159G>A), TGC165TGT (1165C>T), CAA166CTT (1167A>T 1168A>T), AAT167AAC (1171T>C), ACC168ACA (1174C>A), AGG169CGG (1175A>C), GCA170GCT (1180A>T), CAG171CTA (1182A>T 1183G>A), CCG173CCA (1189G>A), TCC175TCT (1195C>T), GTG176GTT (1198G>T), GTT177GTG (1201T>G), GCC178GCA (1204C>A), ATA179ATT (1207A>T), AAA181AAG (1213A>G), CAA182CAG (1216A>G), ATT184ATA (1222T>A), GAC185AGC (1223G>A 1224A>G), ACA186CCA (1226A>C), AGG187AGA (1231G>A), GCA189GCT (1237A>T), TCA192ACG (1244T>A 1246A>G), CAG193GCA (1247C>G 1248A>C 1249G>A), TCT198AGT (1262T>A 1263C>G), GCC200GCA (1270C>A), ATA201ATT (1273A>T), GAA202GAG (1276A>G), GCA203GCT (1279A>T), CGT206AGG (1286C>A 1288T>G), CCG207CCA (1291G>A), CAA208CAG (1294A>G), TTT209TTC (1297T>C), TTC210TTT (1300C>T), CAG211TCT (1301C>T 1302A>C 1303G>T), CAG214CAA (1312G>A), GCT216GCA (1318T>A), GCT217GCA (1321T>A), TAT219TAC (1327T>C), ATA220ATT (1330A>T), GTT223GTC (1339T>C), TAT224TAC (1342T>C), GGG227GGA (1351G>A), CAA228GAG (1352C>G 1354A>G), AGT229AGG (1357T>G), ACT230GCG (1358A>G 1360T>G), CAA231TCT (1361C>T 1362A>C 1363A>T), ATG232CAA (1364A>C 1365T>A 1366G>A), GCT233GCC (1369T>C), CTT234CTA (1372T>A), GAA235GAG (1375A>G), AAC236AAT (1378C>T), AAA237AGA (1380A>G), GCC238CCT (1382G>C 1384C>T), GTT240GTA (1390T>A), GGA241GGT (1393A>T), GAA242GCA (1395A>C), CAG243CTT (1398A>T 1399G>T), CTT244CTC (1402T>C), GGC246AAC (1406G>A 1407G>A), ACA247ACT (1411A>T), AGA248TAC (1412A>T 1413G>A 1414A>C), GAC250AAT (1418G>A 1420C>T), GTC251GTT (1423C>T), ATG252CCC (1424A>C 1425T>C 1426G>C), AAT253CAG (1427A>C 1429T>G), GAA254GAG (1432A>G), AGC255TCT (1433A>T 1434G>C 1435C>T), ACT256TCA (1436A>T 1438T>A), ATG257TTA (1439A>T 1441G>A), ATC258ATG (1444C>G), CGG259CGT (1447G>T), AGT260GCG (1448A>G 1449G>C 1450T>G), TCT262TCG (1456T>G), TTG263CTT (1457T>C 1459G>T), AGA264CGC (1460A>C 1462A>C), GTC265ATT (1463G>A 1465C>T), ACT267ACC (1471T>C), CTC268CTA (1474C>A), AAA269AAG (1477A>G), GTG270ATC (1478G>A 1480G>C), CAG271CAA (1483G>A), CGC274AGG (1490C>A 1492C>G), TTC275TTT (1495C>T), CCG276CCT (1498G>T), TCC277TCG (1501C>G), GAA279GAG (1507A>G), CCG281CCT (1513G>T), GCT282GAT (1515C>A), GTA283TTT (1517G>T 1519A>T), AGT284TCA (1520A>T 1521G>C 1522T>A), AGT284_ACA285insAGGGCC (1522_1523insAGGGCC), ACA285TCA (1523A>T), TCC287TCG (1531C>G), TTA288ACA (1532T>A 1533T>C), CTC289GTA (1535C>G 1537C>A), AGC290GGT (1538A>G 1540C>T), ACA291GTG (1541A>G 1542C>T 1543A>G), CGT292AGA (1544C>A 1546T>A), GAA293GAG (1549A>G), GTT296GGG (1557T>G 1558T>G), CAT297GTA (1559C>G 1560A>T 1561T>A), GAG299del (1565_1567delGAG), TGT302TCC (1575G>C 1576T>C), GGG303AAT (1577G>A 1578G>A 1579G>T), GGA304TTA (1580G>T 1581G>T), TTG305CTG (1583T>C), CCA307CCT (1591A>T), GCA308GTC (1593C>T 1594A>C), CCC309_AAG310del (1595_1600delCCCAAG), AAA311AAG (1603A>G), AAG312GAG (1604A>G), GGC313GCA (1608G>C 1609C>A), CAA314CAG (1612A>G), TTC316ATT (1616T>A 1618C>T), AAC317GAT (1619A>G 1621C>T), ATG318TTT (1622A>T 1624G>T), GGT319GGC (1627T>C), GCC320CAG (1628G>C 1629C>A 1630C>G), ATT321ATC (1633T>C), GAT323GAC (1639T>C), TTG325TAT (1644T>A 1645G>T), ATG327ATC (1651G>C), GAG328ACT (1652G>A 1653A>C 1654G>T), TCG329GAT (1655T>G 1656C>A 1657G>T), TTC330TTT (1660C>T), GCA331AAT (1661G>A 1662C>A 1663A>T), CAC332TCG (1664C>T 1665A>C 1666C>G), ATT333TTT (1667A>T), GAT338GAG (1684T>G), GAA341ACC (1691G>A 1692A>C 1693A>C), CGA342TGG (1694C>T 1696A>G), ACA343CAT (1697A>C 1698C>A 1699A>T), GCT345TCA (1703G>T 1705T>A), CAG346AGG (1706C>A 1707A>G), CAG347CAA (1711G>A), GTA348ATT (1712G>A 1714A>T), CAA349GAT (1715C>G 1717A>T), ATC351GTG (1721A>G 1723C>G), CTC352TTG (1724C>T 1726C>G), TTT353TCT (1728T>C), AGT354ACC (1731G>C 1732T>C), GAG355CTC (1733G>C 1734A>T 1735G>C), GCT356CTC (1736G>C 1737C>T 1738T>C), AAA357CGC (1739A>C 1740A>G 1741A>C), AGA358CAG (1742A>C 1743G>A 1744A>G), GGA359GGG (1747A>G), AAC360AGA (1749A>G 1750C>A), GTT361TGT (1751G>T 1752T>G), ATT362CAT (1754A>C 1755T>A), GTG363CTG (1757G>C), CCA364CCC (1762A>C), AGA365AAG (1764G>A 1765A>G), CAT366CAC (1768T>C), GTT367ATT (1769G>A), GCA368GAG (1773C>A 1774A>G), AAG369AGA (1776A>G 1777G>A), CGG370CGA (1780G>A), CAT371GAC (1781C>G 1783T>C), CTG372TCC (1784C>T 1785T>C 1786G>C), CAT373CAC (1789T>C), AAT374AAC (1792T>C), ATC375ATA (1795C>A), AAT376AAC (1798T>C), AGG377CTA (1799A>C 1800G>T 1801G>A), CAC379TAC (1805C>T), ATA380ATT (1810A>T), ACC381GAT (1811A>G 1812C>A 1813C>T), GAC383AAT (1817G>A 1819C>T), AAT384AAC (1822T>C), GTA386CTA (1826G>C), GAA387CAG (1829G>C 1831A>G), ATA388GTG (1832A>G 1834A>G), CTT389CTC (1837T>C), GAA390AAG (1838G>A 1840A>G), GGT391GAA (1842G>A 1843T>A), GGT393GGC (1849T>C), GTT394ATC (1850G>A 1852T>C), ACC395ACA (1855C>A), ATC397GTG (1859A>G 1861C>G), CAA398GGT (1862C>G 1863A>G 1864A>T), GGC399TCC (1865G>T 1866G>C), TTG400CTA (1868T>C 1870G>A), ACT401GAG (1871A>G 1872C>A 1873T>G), CGC402CGT (1876C>T), TTC404TGC (1881T>G), TAT407TAC (1891T>C), GCT408TCT (1892G>T), ATG409TTA (1895A>T 1897G>A), TCT410ACT (1898T>A), AAG412AGG (1905A>G), GAG413GAA (1909G>A), AGA414AGG (1912A>G), GTT415GTG (1915T>G), GTG416ATA (1916G>A 1918G>A), GAG417GAA (1921G>A), CTC418TTG (1922C>T 1924C>G), GTC419GTG (1927C>G), CAA421CAG (1933A>G), GAT423GAC (1939T>C), TCC425CAA (1943T>C 1944C>A 1945C>A), TTC426TAC (1947T>A), TAT427TAC (1951T>C), ATT428AGG (1953T>G 1954T>G), CAA429TAT (1955C>T 1957A>T), GGG430GGT (1960G>T), CAG431GAT (1961C>G 1963G>T), ACT432GAG (1964A>G 1965C>A 1966T>G), AAC433ACA (1968A>C 1969C>A), CCA435CCC (1975A>C), CCG437CCA (1981G>A), AAT438AGA (1983A>G 1984T>A), GAC440GAT (1990C>T), TAT442TAC (1996T>C), GGA444GGC (2002A>C), TTA445CTC (2003T>C 2005A>C), ACC446ACA (2008C>A), AAA448ATG (2013A>T 2014A>G), GAG449GAC (2017G>C), CGT450AGA (2018C>A 2020T>A), CAG451CAC (2023G>C), CAG451_TTG452insAAGGGTGGCAGGGCTAAG (2023_2024insAAGGGTGGCAGGGCTAAG), TTG452TTA (2026G>A), ATT453GAT (2027A>G 2028T>A), GTG454GTA (2032G>A), GAG455GCT (2034A>C 2035G>T), GAG456GGT (2037A>G 2038G>T), CAA457GCC (2039C>G 2040A>C 2041A>C), GTG458TTA (2042G>T 2044G>A), GCC459GCA (2047C>A), CAA460del (2048_2050delCAA), AGA461AGT (2053A>T), AGG462TTG (2054A>T 2055G>T), TCT463CCA (2057T>C 2059T>A), GAA464CAA (2060G>C), AGA465CCT (2063A>C 2064G>C 2065A>T), CAA466AGA (2066C>A 2067A>G), GCA467GTG (2070C>T 2071A>G), CAG468AAG (2072C>A), ACA470GCC (2078A>G 2080A>C), ACA470_GCA471insCAG (2080_2081insCAG), GCA471GAA (2082C>A), AGG472GGA (2084A>G 2086G>A), GGC473GGG (2089C>G), ATA474GGA (2090A>G 2091T>G), GCT475AGC (2093G>A 2094C>G 2095T>C), GAA476AGG (2096G>A 2097A>G 2098A>G), AGT477TCC (2099A>T 2100G>C 2101T>C), CAG478ACC (2102C>A 2103A>C 2104G>C), CCT479ACT (2105C>A), GAA480GAG (2110A>G), GAC481AGC (2111G>A 2112A>G), AGA482AAG (2115G>A 2116A>G), GTG483GTT (2119G>T), ACT484CTG (2120A>C 2121C>T 2122T>G), GTG488GCA (2133T>C 2134G>A), TCA489ACT (2135T>A 2137A>T), ACT490ACA (2140T>A), ACC491ACT (2143C>T), ACG492GCA (2144A>G 2146G>A), GAA494CAA (2150G>C), GAA494_GAT495insAGC (2152_2153insAGC), GAT495GAG (2155T>G), CCC496GGA (2156C>G 2157C>G 2158C>A), ACC497ATC (2160C>T), AAG498AAC (2164G>C), CCA499AAG (2165C>A 2166C>A 2167A>G), GAT500GAC (2170T>C), AAG501CAA (2171A>C 2173G>A), ATA502ATT (2176A>T), GAA503GTA (2178A>T), ATT504ATA (2182T>A), GTT505GAG (2184T>A 2185T>G), GCA506GTT (2187C>T 2188A>T), GAG507GCT (2190A>C 2191G>T), GGA508GAC (2193G>A 2194A>C), GCA509CCA (2195G>C), GAA510GAG (2200A>G), GAA511CAG (2201G>C 2203A>G), ACT513GTC (2207A>G 2208C>T 2209T>C), CAA514TCA (2210C>T 2211A>C), GGT516GAC (2217G>A 2218T>C), GTT518GTC (2224T>C), ATT519TTC (2225A>T 2227T>C), GGA523GGG (2239A>G), CCA524GAG (2240C>G 2241C>A 2242A>G), GGC1170GGT (4180C>T), AAT1171GAT (4181A>G), ACT1174ACG (4192T>G), CAC1175CAT (4195C>T), ACC1176ACT (4198C>T), TAT1177TAC (4201T>C), CAG1178CAA (4204G>A), TCA1181TCT (4213A>T), TTT1183TTC (4219T>C), TCT1184TCA (4222T>A), GAG1185GAA (4225G>A), CTA1186CTG (4228A>G), CGT1187AGA (4229C>A 4231T>A), GAT1188GAC (4234T>C), TTT1189TTC (4237T>C), GCG1190TCG (4238G>T), ATT1191AAC (4242T>A 4243T>C)

Proteins

	Begin	End	Coverage	Score	Concordance	Matches	Identities	I/D/M/F*	Stop Codons
polyprotein (YP_001976149.1)	121	1192	36.7%	1905	63.6%	434 (96.9%)	266 (59.4%)	10/4/0/0	1

Protein mutations:

L124T (1040C>A 1041T>C 1042C>T), S125D (1043T>G 1044C>A 1045A>T), T132M (1065C>T 1066A>G), S134F (1071C>T), L135F (1075G>T), A136S (1076G>T 1078C>A), D137G (1080A>G 1081T>G), S147E (1109A>G 1110G>A 1111C>A), L153V (1127C>G 1129C>T), Q166L (1167A>T 1168A>T), Q171L (1182A>T 1183G>A), D185S (1223G>A 1224A>G), T186P (1226A>C), S192T (1244T>A 1246A>G), Q193A (1247C>G 1248A>C 1249G>A), Q211S (1301C>T 1302A>C 1303G>T), Q228E (1352C>G 1354A>G), S229R (1357T>G), T230A (1358A>G 1360T>G), Q231S (1361C>T 1362A>C 1363A>T), M232Q (1364A>C 1365T>A 1366G>A), K237R (1380A>G), A238P (1382G>C 1384C>T), E242A (1395A>C), Q243L (1398A>T 1399G>T), G246N (1406G>A 1407G>A), R248Y (1412A>T 1413G>A 1414A>C), D250N (1418G>A 1420C>T), M252P (1424A>C 1425T>C 1426G>C), N253Q (1427A>C 1429T>G), T256S (1436A>T 1438T>A), M257L (1439A>T 1441G>A), I258M (1444C>G), S260A (1448A>G 1449G>C 1450T>G), V265I (1463G>A 1465C>T), V270I (1478G>A 1480G>C), A282D (1515C>A), V283F (1517G>T 1519A>T), S284_T285insRA (1522_1523insAGGGCC), T285S (1523A>T), L288T (1532T>A 1533T>C), L289V (1535C>G 1537C>A), S290G (1538A>G 1540C>T), T291V (1541A>G 1542C>T 1543A>G), V296G (1557T>G 1558T>G), H297V (1559C>G 1560A>T 1561T>A), E299del (1565_1567delGAG), C302S (1575G>C 1576T>C), G303N (1577G>A 1578G>A 1579G>T), G304L (1580G>T 1581G>T), A308V (1593C>T 1594A>C), P309_K310del (1595_1600delCCCAAG), K312E (1604A>G), G313A (1608G>C 1609C>A), F316I (1616T>A 1618C>T), N317D (1619A>G 1621C>T), M318F (1622A>T 1624G>T), A320Q (1628G>C 1629C>A 1630C>G), L325Y (1644T>A 1645G>T), M327I (1651G>C), E328T (1652G>A 1653A>C 1654G>T), S329D (1655T>G 1656C>A 1657G>T), A331N (1661G>A 1662C>A 1663A>T), H332S (1664C>T 1665A>C 1666C>G), I333F (1667A>T), D338E (1684T>G), E341T (1691G>A 1692A>C 1693A>C), R342W (1694C>T 1696A>G), T343H (1697A>C 1698C>A 1699A>T), A345S (1703G>T 1705T>A), Q346R (1706C>A 1707A>G), V348I (1712G>A 1714A>T), Q349D (1715C>G 1717A>T), I351V (1721A>G 1723C>G), F353S (1728T>C), S354T (1731G>C 1732T>C), E355L (1733G>C 1734A>T 1735G>C), A356L (1736G>C 1737C>T 1738T>C), K357R (1739A>C 1740A>G 1741A>C), R358Q (1742A>C 1743G>A 1744A>G), N360R (1749A>G 1750C>A), V361C (1751G>T 1752T>G), I362H (1754A>C 1755T>A), V363L (1757G>C), R365K (1764G>A 1765A>G), V367I (1769G>A), A368E (1773C>A 1774A>G), K369R (1776A>G 1777G>A), H371D (1781C>G 1783T>C), L372S (1784C>T 1785T>C 1786G>C), R377L (1799A>C 1800G>T 1801G>A), H379Y (1805C>T), T381D (1811A>G 1812C>A 1813C>T), D383N (1817G>A 1819C>T), V386L (1826G>C), E387Q (1829G>C 1831A>G), I388V (1832A>G 1834A>G), E390K (1838G>A 1840A>G), G391E (1842G>A 1843T>A), V394I (1850G>A 1852T>C), I397V (1859A>G 1861C>G), Q398G (1862C>G 1863A>G 1864A>T), G399S (1865G>T 1866G>C), T401E (1871A>G 1872C>A 1873T>G), F404C (1881T>G), A408S (1892G>T), M409L (1895A>T 1897G>A), S410T (1898T>A), K412R (1905A>G), V416I (1916G>A 1918G>A), S425Q (1943T>C 1944C>A 1945C>A), F426Y (1947T>A), I428R (1953T>G 1954T>G), Q429Y (1955C>T 1957A>T), Q431D (1961C>G 1963G>T), T432E (1964A>G 1965C>A 1966T>G), N433T (1968A>C 1969C>A), N438R (1983A>G 1984T>A), K448M (2013A>T 2014A>G), E449D (2017G>C), Q451H (2023G>C), Q451_L452insKGGRAK (2023_2024insAAGGGTGGCAGGGCTAAG), I453D (2027A>G 2028T>A), E455A (2034A>C 2035G>T), E456G (2037A>G 2038G>T), Q457A (2039C>G 2040A>C 2041A>C), V458L (2042G>T 2044G>A), Q460del (2048_2050delCAA), R461S (2053A>T), R462L (2054A>T 2055G>T), S463P (2057T>C 2059T>A), E464Q (2060G>C), R465P (2063A>C 2064G>C 2065A>T), Q466R (2066C>A 2067A>G), A467V (2070C>T 2071A>G), Q468K (2072C>A), T470A (2078A>G 2080A>C), T470_A471insQ (2080_2081insCAG), A471E (2082C>A), R472G (2084A>G 2086G>A), I474G (2090A>G 2091T>G), A475S (2093G>A 2094C>G 2095T>C), E476R (2096G>A 2097A>G 2098A>G), Q478T (2102C>A 2103A>C 2104G>C), P479T (2105C>A), D481S (2111G>A 2112A>G), R482K (2115G>A 2116A>G), T484L (2120A>C 2121C>T 2122T>G), V488A (2133T>C 2134G>A), S489T (2135T>A 2137A>T), T492A (2144A>G 2146G>A), E494Q (2150G>C), E494_D495insS (2152_2153insAGC), D495E (2155T>G), P496G (2156C>G 2157C>G 2158C>A), T497I (2160C>T), K498N (2164G>C), P499K (2165C>A 2166C>A 2167A>G), K501Q (2171A>C 2173G>A), E503V (2178A>T), V505E (2184T>A 2185T>G), A506V (2187C>T 2188A>T), E507A (2190A>C 2191G>T), G508D (2193G>A 2194A>C), A509P (2195G>C), E511Q (2201G>C 2203A>G), T513V (2207A>G 2208C>T 2209T>C), Q514S (2210C>T 2211A>C), G516D (2217G>A 2218T>C), I519F (2225A>T 2227T>C), P524E (2240C>G 2241C>A 2242A>G), N1171D (4181A>G), A1190S (4238G>T), I1191N (4242T>A 4243T>C)

	Begin	End	Coverage	Score	Concordance	Matches	Identities	I/D/M/F*	Stop Codons
**NT**	**1030**	**4484**	**31.7%**	**701**	**23.0%**	**1541 (97.1%)**	**982 (61.9%)**	**34/12**	
Codon mutations:	AGT120..C (1030T>C), CCC123CCA (1039C>A), CTC124ACT (1040C>A 1041T>C 1042C>T), TCA125GAT (1043T>G 1044C>A 1045A>T), CCG126CCT (1048G>T), GTA127GTT (1051A>T), CAA129CAG (1057A>G), ACA132ATG (1065C>T 1066A>G), CTG133TTA (1067C>T 1069G>A), TCC134TTC (1071C>T), TTG135TTT (1075G>T), GCC136TCA (1076G>T 1078C>A), GAT137GGG (1080A>G 1081T>G), GCG139GCT (1087G>T), GCA141GCT (1093A>T), GTT142GTC (1096T>C), CTC143TTG (1097C>T 1099C>G), AGC145AGT (1105C>T), CCC146CCT (1108C>T), AGC147GAA (1109A>G 1110G>A 1111C>A), ATA148ATT (1114A>T), CCC151CCT (1123C>T), CTC153GTT (1127C>G 1129C>T), AAA154AAG (1132A>G), ACC162ACT (1156C>T), CTG163CTA (1159G>A), TGC165TGT (1165C>T), CAA166CTT (1167A>T 1168A>T), AAT167AAC (1171T>C), ACC168ACA (1174C>A), AGG169CGG (1175A>C), GCA170GCT (1180A>T), CAG171CTA (1182A>T 1183G>A), CCG173CCA (1189G>A), TCC175TCT (1195C>T), GTG176GTT (1198G>T), GTT177GTG (1201T>G), GCC178GCA (1204C>A), ATA179ATT (1207A>T), AAA181AAG (1213A>G), CAA182CAG (1216A>G), ATT184ATA (1222T>A), GAC185AGC (1223G>A 1224A>G), ACA186CCA (1226A>C), AGG187AGA (1231G>A), GCA189GCT (1237A>T), TCA192ACG (1244T>A 1246A>G), CAG193GCA (1247C>G 1248A>C 1249G>A), TCT198AGT (1262T>A 1263C>G), GCC200GCA (1270C>A), ATA201ATT (1273A>T), GAA202GAG (1276A>G), GCA203GCT (1279A>T), CGT206AGG (1286C>A 1288T>G), CCG207CCA (1291G>A), CAA208CAG (1294A>G), TTT209TTC (1297T>C), TTC210TTT (1300C>T), CAG211TCT (1301C>T 1302A>C 1303G>T), CAG214CAA (1312G>A), GCT216GCA (1318T>A), GCT217GCA (1321T>A), TAT219TAC (1327T>C), ATA220ATT (1330A>T), GTT223GTC (1339T>C), TAT224TAC (1342T>C), GGG227GGA (1351G>A), CAA228GAG (1352C>G 1354A>G), AGT229AGG (1357T>G), ACT230GCG (1358A>G 1360T>G), CAA231TCT (1361C>T 1362A>C 1363A>T), ATG232CAA (1364A>C 1365T>A 1366G>A), GCT233GCC (1369T>C), CTT234CTA (1372T>A), GAA235GAG (1375A>G), AAC236AAT (1378C>T), AAA237AGA (1380A>G), GCC238CCT (1382G>C 1384C>T), GTT240GTA (1390T>A), GGA241GGT (1393A>T), GAA242GCA (1395A>C), CAG243CTT (1398A>T 1399G>T), CTT244CTC (1402T>C), GGC246AAC (1406G>A 1407G>A), ACA247ACT (1411A>T), AGA248TAC (1412A>T 1413G>A 1414A>C), GAC250AAT (1418G>A 1420C>T), GTC251GTT (1423C>T), ATG252CCC (1424A>C 1425T>C 1426G>C), AAT253CAG (1427A>C 1429T>G), GAA254GAG (1432A>G), AGC255TCT (1433A>T 1434G>C 1435C>T), ACT256TCA (1436A>T 1438T>A), ATG257TTA (1439A>T 1441G>A), ATC258ATG (1444C>G), CGG259CGT (1447G>T), AGT260GCG (1448A>G 1449G>C 1450T>G), TCT262TCG (1456T>G), TTG263CTT (1457T>C 1459G>T), AGA264CGC (1460A>C 1462A>C), GTC265ATT (1463G>A 1465C>T), ACT267ACC (1471T>C), CTC268CTA (1474C>A), AAA269AAG (1477A>G), GTG270ATC (1478G>A 1480G>C), CAG271CAA (1483G>A), CGC274AGG (1490C>A 1492C>G), TTC275TTT (1495C>T), CCG276CCT (1498G>T), TCC277TCG (1501C>G), GAA279GAG (1507A>G), CCG281CCT (1513G>T), GCT282GAT (1515C>A), GTA283TTT (1517G>T 1519A>T), AGT284TCA (1520A>T 1521G>C 1522T>A), AGT284_ACA285insAGGGCC (1522_1523insAGGGCC), ACA285TCA (1523A>T), TCC287TCG (1531C>G), TTA288ACA (1532T>A 1533T>C), CTC289GTA (1535C>G 1537C>A), AGC290GGT (1538A>G 1540C>T), ACA291GTG (1541A>G 1542C>T 1543A>G), CGT292AGA (1544C>A 1546T>A), GAA293GAG (1549A>G), GTT296GGG (1557T>G 1558T>G), CAT297GTA (1559C>G 1560A>T 1561T>A), GAG299del (1565_1567delGAG), TGT302TCC (1575G>C 1576T>C), GGG303AAT (1577G>A 1578G>A 1579G>T), GGA304TTA (1580G>T 1581G>T), TTG305CTG (1583T>C), CCA307CCT (1591A>T), GCA308GTC (1593C>T 1594A>C), CCC309_AAG310del (1595_1600delCCCAAG), AAA311AAG (1603A>G), AAG312GAG (1604A>G), GGC313GCA (1608G>C 1609C>A), CAA314CAG (1612A>G), TTC316ATT (1616T>A 1618C>T), AAC317GAT (1619A>G 1621C>T), ATG318TTT (1622A>T 1624G>T), GGT319GGC (1627T>C), GCC320CAG (1628G>C 1629C>A 1630C>G), ATT321ATC (1633T>C), GAT323GAC (1639T>C), TTG325TAT (1644T>A 1645G>T), ATG327ATC (1651G>C), GAG328ACT (1652G>A 1653A>C 1654G>T), TCG329GAT (1655T>G 1656C>A 1657G>T), TTC330TTT (1660C>T), GCA331AAT (1661G>A 1662C>A 1663A>T), CAC332TCG (1664C>T 1665A>C 1666C>G), ATT333TTT (1667A>T), GAT338GAG (1684T>G), GAA341ACC (1691G>A 1692A>C 1693A>C), CGA342TGG (1694C>T 1696A>G), ACA343CAT (1697A>C 1698C>A 1699A>T), GCT345TCA (1703G>T 1705T>A), CAG346AGG (1706C>A 1707A>G), CAG347CAA (1711G>A), GTA348ATT (1712G>A 1714A>T), CAA349GAT (1715C>G 1717A>T), ATC351GTG (1721A>G 1723C>G), CTC352TTG (1724C>T 1726C>G), TTT353TCT (1728T>C), AGT354ACC (1731G>C 1732T>C), GAG355CTC (1733G>C 1734A>T 1735G>C), GCT356CTC (1736G>C 1737C>T 1738T>C), AAA357CGC (1739A>C 1740A>G 1741A>C), AGA358CAG (1742A>C 1743G>A 1744A>G), GGA359GGG (1747A>G), AAC360AGA (1749A>G 1750C>A), GTT361TGT (1751G>T 1752T>G), ATT362CAT (1754A>C 1755T>A), GTG363CTG (1757G>C), CCA364CCC (1762A>C), AGA365AAG (1764G>A 1765A>G), CAT366CAC (1768T>C), GTT367ATT (1769G>A), GCA368GAG (1773C>A 1774A>G), AAG369AGA (1776A>G 1777G>A), CGG370CGA (1780G>A), CAT371GAC (1781C>G 1783T>C), CTG372TCC (1784C>T 1785T>C 1786G>C), CAT373CAC (1789T>C), AAT374AAC (1792T>C), ATC375ATA (1795C>A), AAT376AAC (1798T>C), AGG377CTA (1799A>C 1800G>T 1801G>A), CAC379TAC (1805C>T), ATA380ATT (1810A>T), ACC381GAT (1811A>G 1812C>A 1813C>T), GAC383AAT (1817G>A 1819C>T), AAT384AAC (1822T>C), GTA386CTA (1826G>C), GAA387CAG (1829G>C 1831A>G), ATA388GTG (1832A>G 1834A>G), CTT389CTC (1837T>C), GAA390AAG (1838G>A 1840A>G), GGT391GAA (1842G>A 1843T>A), GGT393GGC (1849T>C), GTT394ATC (1850G>A 1852T>C), ACC395ACA (1855C>A), ATC397GTG (1859A>G 1861C>G), CAA398GGT (1862C>G 1863A>G 1864A>T), GGC399TCC (1865G>T 1866G>C), TTG400CTA (1868T>C 1870G>A), ACT401GAG (1871A>G 1872C>A 1873T>G), CGC402CGT (1876C>T), TTC404TGC (1881T>G), TAT407TAC (1891T>C), GCT408TCT (1892G>T), ATG409TTA (1895A>T 1897G>A), TCT410ACT (1898T>A), AAG412AGG (1905A>G), GAG413GAA (1909G>A), AGA414AGG (1912A>G), GTT415GTG (1915T>G), GTG416ATA (1916G>A 1918G>A), GAG417GAA (1921G>A), CTC418TTG (1922C>T 1924C>G), GTC419GTG (1927C>G), CAA421CAG (1933A>G), GAT423GAC (1939T>C), TCC425CAA (1943T>C 1944C>A 1945C>A), TTC426TAC (1947T>A), TAT427TAC (1951T>C), ATT428AGG (1953T>G 1954T>G), CAA429TAT (1955C>T 1957A>T), GGG430GGT (1960G>T), CAG431GAT (1961C>G 1963G>T), ACT432GAG (1964A>G 1965C>A 1966T>G), AAC433ACA (1968A>C 1969C>A), CCA435CCC (1975A>C), CCG437CCA (1981G>A), AAT438AGA (1983A>G 1984T>A), GAC440GAT (1990C>T), TAT442TAC (1996T>C), GGA444GGC (2002A>C), TTA445CTC (2003T>C 2005A>C), ACC446ACA (2008C>A), AAA448ATG (2013A>T 2014A>G), GAG449GAC (2017G>C), CGT450AGA (2018C>A 2020T>A), CAG451CAC (2023G>C), CAG451_TTG452insAAGGGTGGCAGGGCTAAG (2023_2024insAAGGGTGGCAGGGCTAAG), TTG452TTA (2026G>A), ATT453GAT (2027A>G 2028T>A), GTG454GTA (2032G>A), GAG455GCT (2034A>C 2035G>T), GAG456GGT (2037A>G 2038G>T), CAA457GCC (2039C>G 2040A>C 2041A>C), GTG458TTA (2042G>T 2044G>A), GCC459GCA (2047C>A), CAA460del (2048_2050delCAA), AGA461AGT (2053A>T), AGG462TTG (2054A>T 2055G>T), TCT463CCA (2057T>C 2059T>A), GAA464CAA (2060G>C), AGA465CCT (2063A>C 2064G>C 2065A>T), CAA466AGA (2066C>A 2067A>G), GCA467GTG (2070C>T 2071A>G), CAG468AAG (2072C>A), ACA470GCC (2078A>G 2080A>C), ACA470_GCA471insCAG (2080_2081insCAG), GCA471GAA (2082C>A), AGG472GGA (2084A>G 2086G>A), GGC473GGG (2089C>G), ATA474GGA (2090A>G 2091T>G), GCT475AGC (2093G>A 2094C>G 2095T>C), GAA476AGG (2096G>A 2097A>G 2098A>G), AGT477TCC (2099A>T 2100G>C 2101T>C), CAG478ACC (2102C>A 2103A>C 2104G>C), CCT479ACT (2105C>A), GAA480GAG (2110A>G), GAC481AGC (2111G>A 2112A>G), AGA482AAG (2115G>A 2116A>G), GTG483GTT (2119G>T), ACT484CTG (2120A>C 2121C>T 2122T>G), GTG488GCA (2133T>C 2134G>A), TCA489ACT (2135T>A 2137A>T), ACT490ACA (2140T>A), ACC491ACT (2143C>T), ACG492GCA (2144A>G 2146G>A), GAA494CAA (2150G>C), GAA494_GAT495insAGC (2152_2153insAGC), GAT495GAG (2155T>G), CCC496GGA (2156C>G 2157C>G 2158C>A), ACC497ATC (2160C>T), AAG498AAC (2164G>C), CCA499AAG (2165C>A 2166C>A 2167A>G), GAT500GAC (2170T>C), AAG501CAA (2171A>C 2173G>A), ATA502ATT (2176A>T), GAA503GTA (2178A>T), ATT504ATA (2182T>A), GTT505GAG (2184T>A 2185T>G), GCA506GTT (2187C>T 2188A>T), GAG507GCT (2190A>C 2191G>T), GGA508GAC (2193G>A 2194A>C), GCA509CCA (2195G>C), GAA510GAG (2200A>G), GAA511CAG (2201G>C 2203A>G), ACT513GTC (2207A>G 2208C>T 2209T>C), CAA514TCA (2210C>T 2211A>C), GGT516GAC (2217G>A 2218T>C), GTT518GTC (2224T>C), ATT519TTC (2225A>T 2227T>C), GGA523GGG (2239A>G), CCA524GAG (2240C>G 2241C>A 2242A>G), GGC1170GGT (4180C>T), AAT1171GAT (4181A>G), ACT1174ACG (4192T>G), CAC1175CAT (4195C>T), ACC1176ACT (4198C>T), TAT1177TAC (4201T>C), CAG1178CAA (4204G>A), TCA1181TCT (4213A>T), TTT1183TTC (4219T>C), TCT1184TCA (4222T>A), GAG1185GAA (4225G>A), CTA1186CTG (4228A>G), CGT1187AGA (4229C>A 4231T>A), GAT1188GAC (4234T>C), TTT1189TTC (4237T>C), GCG1190TCG (4238G>T), ATT1191AAC (4242T>A 4243T>C)								
Coat protein A (YP_001976155.1)	1	60	23.6%	172	42.7%	60 (96.8%)	26 (41.9%)	2/0/0/0	0
Protein mutations:	T2A (2078A>G 2080A>C), T2_A3insQ (2080_2081insCAG), A3E (2082C>A), R4G (2084A>G 2086G>A), I6G (2090A>G 2091T>G), A7S (2093G>A 2094C>G 2095T>C), E8R (2096G>A 2097A>G 2098A>G), Q10T (2102C>A 2103A>C 2104G>C), P11T (2105C>A), D13S (2111G>A 2112A>G), R14K (2115G>A 2116A>G), T16L (2120A>C 2121C>T 2122T>G), V20A (2133T>C 2134G>A), S21T (2135T>A 2137A>T), T24A (2144A>G 2146G>A), E26Q (2150G>C), E26_D27insS (2152_2153insAGC), D27E (2155T>G), P28G (2156C>G 2157C>G 2158C>A), T29I (2160C>T), K30N (2164G>C), P31K (2165C>A 2166C>A 2167A>G), K33Q (2171A>C 2173G>A), E35V (2178A>T), V37E (2184T>A 2185T>G), A38V (2187C>T 2188A>T), E39A (2190A>C 2191G>T), G40D (2193G>A 2194A>C), A41P (2195G>C), E43Q (2201G>C 2203A>G), T45V (2207A>G 2208C>T 2209T>C), Q46S (2210C>T 2211A>C), G48D (2217G>A 2218T>C), I51F (2225A>T 2227T>C), P56E (2240C>G 2241C>A 2242A>G)								
Codon mutations:	ACA2GCC (2078A>G 2080A>C), ACA2_GCA3insCAG (2080_2081insCAG), GCA3GAA (2082C>A), AGG4GGA (2084A>G 2086G>A), GGC5GGG (2089C>G), ATA6GGA (2090A>G 2091T>G), GCT7AGC (2093G>A 2094C>G 2095T>C), GAA8AGG (2096G>A 2097A>G 2098A>G), AGT9TCC (2099A>T 2100G>C 2101T>C), CAG10ACC (2102C>A 2103A>C 2104G>C), CCT11ACT (2105C>A), GAA12GAG (2110A>G), GAC13AGC (2111G>A 2112A>G), AGA14AAG (2115G>A 2116A>G), GTG15GTT (2119G>T), ACT16CTG (2120A>C 2121C>T 2122T>G), GTG20GCA (2133T>C 2134G>A), TCA21ACT (2135T>A 2137A>T), ACT22ACA (2140T>A), ACC23ACT (2143C>T), ACG24GCA (2144A>G 2146G>A), GAA26CAA (2150G>C), GAA26_GAT27insAGC (2152_2153insAGC), GAT27GAG (2155T>G), CCC28GGA (2156C>G 2157C>G 2158C>A), ACC29ATC (2160C>T), AAG30AAC (2164G>C), CCA31AAG (2165C>A 2166C>A 2167A>G), GAT32GAC (2170T>C), AAG33CAA (2171A>C 2173G>A), ATA34ATT (2176A>T), GAA35GTA (2178A>T), ATT36ATA (2182T>A), GTT37GAG (2184T>A 2185T>G), GCA38GTT (2187C>T 2188A>T), GAG39GCT (2190A>C 2191G>T), GGA40GAC (2193G>A 2194A>C), GCA41CCA (2195G>C), GAA42GAG (2200A>G), GAA43CAG (2201G>C 2203A>G), ACT45GTC (2207A>G 2208C>T 2209T>C), CAA46TCA (2210C>T 2211A>C), GGT48GAC (2217G>A 2218T>C), GTT50GTC (2224T>C), ATT51TTC (2225A>T 2227T>C), GGA55GGG (2239A>G), CCA56GAG (2240C>G 2241C>A 2242A>G)								
Coat protein C (YP_001976157.1)	197	225	12.9%	192	90.6%	29 (100%)	26 (89.7%)	0/0/0/0	0
Protein mutations:	N205D (4181A>G), A224S (4238G>T), I225N (4242T>A 4243T>C)								
Codon mutations:	GGC204GGT (4180C>T), AAT205GAT (4181A>G), ACT208ACG (4192T>G), CAC209CAT (4195C>T), ACC210ACT (4198C>T), TAT211TAC (4201T>C), CAG212CAA (4204G>A), TCA215TCT (4213A>T), TTT217TTC (4219T>C), TCT218TCA (4222T>A), GAG219GAA (4225G>A), CTA220CTG (4228A>G), CGT221AGA (4229C>A 4231T>A), GAT222GAC (4234T>C), TTT223TTC (4237T>C), GCG224TCG (4238G>T), ATT225AAC (4242T>A 4243T>C)								

*: Inserts / Deletes / Misaligned / Frameshifts

Analysis details

This analysis was performed with panviral2.64

NGS Details (UN9): Torradovirus lycopersici (segment RNA 1)

Assembly

**Coverage Length**	7808 (1 contig(s))
**Depth Of Coverage**	29487.7
**Number Of Reads**	1650960
**Reads Per Million**	37316.42 rpm (after QC)
**Ambiguities**	0
**Assembly Method**	de novo + reference guided assembly
**Consensus Caller**	Bcf Tools

Coverage Map

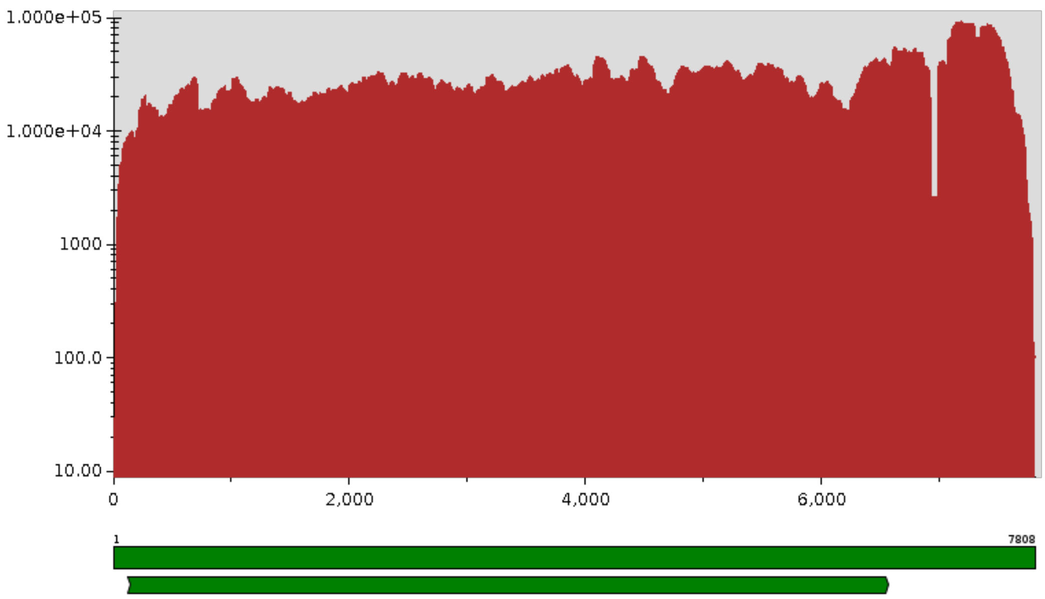

Assignment

**Type**	Torradovirus lycopersici (Taxonomy ID: 3048378)
**Reference Genome**	NC_009013.1
**NT Identity (%)**	89.4563
**AA Identity (%)**	97.5452
**Number Of Stop Codons**	1
**Number Of CDS**	1

Alignment

**Alignment Score**	11384.0 (NT) + 14810.0 (AA) = 26194.0
**Concordance (%)**	88.347

**Alignment Method**	Global, seeded, nucleotide + amino acids (AGA)

Genome Region

Sequence starts at position 1 and ends at position 7808 relative to NC_009013.1 reference sequence.

Alignment Detailed Statistics

	Begin	End	Coverage	Score	Concordance	Matches	Identities	I/D/M/F*	Stop Codons
**NT**	**1**	**7808**	**100%**	**11384**	**78.1%**	**7528 (96.0%)**	**6762 (86.3%)**	**31/280**	
Mutations:	22T>A, 24T>G, 26A>T, 65A>G, 72C>T, 77A>T, 78A>G, 83C>T, 83_84insTACTTTATATTGTTTTGTGCT, 88T>C, 90C>G, 92T>C, 93T>A, 101G>A, 102G>C, 103C>A, 104A>G, 115T>C, 118C>T, 121G>A, 122A>T, 124G>A, 128C>T, 130C>T, 134T>C, 135T>C, 136C>T, 138A>G, 140T>C, 142A>C, 144T>C, 145T>A, 147C>T, 148T>G, 151A>G, 155T>A, 157C>T, 159C>T, 161A>G, 178T>G, 187A>T, 196T>A, 197G>C, 198C>A, 199T>A, 208T>C, 211T>C, 214C>T, 220C>T, 223T>A, 226G>T, 247A>C, 253A>G, 256T>C, 262T>A, 265C>T, 271A>G, 275T>C, 328T>C, 334T>C, 340G>A, 349G>A, 373C>T, 376C>T, 445C>T, 487C>T, 502G>A, 541T>C, 559C>T, 578C>T, 628C>T, 655C>T, 676A>G, 694A>G, 707C>T, 727A>G, 760C>T, 781C>T, 790C>T, 799A>G, 805T>C, 818C>T, 820A>G, 832A>T, 835A>C, 841T>C, 842C>T, 847C>T, 883G>A, 886T>C, 892G>A, 901T>C, 913T>C, 925C>T, 952A>G, 964T>G, 970G>A, 997C>T, 1006T>C, 1033G>A, 1060T>C, 1063C>T, 1064T>C, 1078A>T, 1081C>T, 1090C>T, 1093T>C, 1105A>G, 1108T>C, 1109C>T, 1165C>T, 1180G>A, 1196A>G, 1231G>A, 1240C>T, 1255G>A, 1267T>C, 1276A>G, 1300G>A, 1303A>G, 1319C>T, 1361T>C, 1366C>T, 1408T>C, 1411T>C, 1417T>C, 1423T>C, 1426T>C, 1429C>T, 1430C>T, 1441C>T, 1450T>C, 1451C>T, 1480C>T, 1481A>G, 1489T>C, 1522C>T, 1552C>T, 1555C>T, 1570A>G, 1573T>C, 1582C>T, 1585A>T, 1591C>T, 1594T>C, 1607G>T, 1612G>A, 1618T>G, 1619C>T, 1624C>T, 1630C>T, 1639T>C, 1642G>A, 1645A>G, 1669C>T, 1672G>A, 1681T>A, 1684G>T, 1699T>C, 1705T>C, 1737A>G, 1738A>G, 1750A>T, 1756A>G, 1765T>G, 1781T>C, 1783G>A, 1784C>T, 1789A>G, 1810G>A, 1825C>T, 1897A>G, 1910T>C, 1954T>C, 1960A>G, 1984C>T, 2014A>G, 2056T>C, 2077C>A, 2082T>A, 2092A>G, 2107A>G, 2128T>C, 2138C>T, 2143T>C, 2149T>C, 2155T>C, 2174C>T, 2179A>C, 2182G>A, 2194T>A, 2200T>C, 2215G>A, 2218C>T, 2224C>T, 2236A>G, 2241A>G, 2250G>A, 2251C>T, 2257C>T, 2263G>A, 2275T>C, 2287G>A, 2290C>T, 2314C>T, 2323A>T, 2347C>T, 2350A>G, 2356C>T, 2359G>A, 2362C>T, 2377G>A, 2380T>C, 2392A>G, 2407T>C, 2409G>A, 2443T>C, 2446G>A, 2479G>A, 2485C>T, 2497G>A, 2500T>C, 2503G>A, 2509C>T, 2512T>C, 2542G>A, 2548C>T, 2557C>T, 2620G>A, 2626T>C, 2650G>A, 2665T>C, 2675T>C, 2683A>G, 2692G>A, 2702C>T, 2710A>G, 2719T>C, 2725T>C, 2731C>T, 2734C>T, 2743A>G, 2744T>C, 2752A>G, 2773A>G, 2785C>T, 2788C>T, 2809C>T, 2821G>T, 2827A>G, 2842T>C, 2860C>T, 2866T>A, 2869C>T, 2872A>T, 2893T>A, 2896C>A, 2926G>A, 2929C>T, 2930T>C, 2941A>G, 2944T>C, 2950C>T, 2951C>T, 2980C>T, 2983G>A, 2998A>G, 3007T>C, 3016T>C, 3028T>C, 3031T>C, 3034G>A, 3040T>C, 3058A>G, 3064A>G, 3073G>A, 3079T>C, 3109T>C, 3115G>A, 3145A>G, 3151C>T, 3163T>C, 3169C>T, 3172T>C, 3202T>C, 3241T>C, 3253C>T, 3256G>A, 3265A>G, 3298T>C, 3319C>T, 3406A>C, 3418C>A, 3421T>C, 3463A>G, 3466G>A, 3481C>T, 3484A>T, 3508G>A, 3559G>A, 3568A>G, 3574T>C, 3586T>C, 3591C>T, 3610A>G, 3613T>C, 3625C>T, 3628G>A, 3646C>T, 3661A>G, 3664T>C, 3673G>A, 3676C>T, 3686C>T, 3706T>C, 3716T>C, 3721T>A, 3739A>T, 3751A>G, 3754G>A, 3758G>A, 3766G>A, 3781G>A, 3805C>T, 3808T>C, 3814C>T, 3817G>C, 3824T>C, 3835T>C, 3844T>C, 3857T>C, 3859G>A, 3868C>T, 3871T>C, 3883G>A, 3898T>C, 3907A>T, 3916C>T, 3925A>G, 3928G>A, 3949C>T, 3955G>A, 3961G>A, 3970A>G, 3973T>C, 4034T>C, 4066T>C, 4075A>G, 4084C>T, 4100T>C, 4118C>T, 4129C>T, 4147C>A, 4159G>A, 4162A>T, 4168A>G, 4186A>T, 4195A>G, 4207G>A, 4210T>C, 4228C>T, 4294A>G, 4318C>T, 4324T>C, 4339T>C, 4343C>T, 4348C>T, 4354T>C, 4396T>C, 4408C>T, 4414T>G, 4417T>C, 4420G>A, 4459T>C, 4462C>T, 4465C>T, 4468C>T, 4471T>C, 4474T>C, 4486T>C, 4489T>C, 4504T>C, 4510T>A, 4511C>T, 4514T>C, 4519A>G, 4543T>C, 4555A>G, 4570C>T, 4574C>T, 4576A>G, 4588C>T, 4591T>C, 4612A>G, 4625T>C, 4630T>C, 4648T>C, 4660A>G, 4666G>A, 4672G>C, 4679A>G, 4684A>T, 4687G>A, 4696C>T, 4702G>A, 4705C>T, 4714T>C, 4730C>T, 4735C>T, 4747A>G, 4759C>T, 4768T>C, 4774G>A, 4777C>T, 4783A>G, 4786A>G, 4789C>T, 4813A>G, 4816T>C, 4843G>A, 4898G>A, 4906G>A, 4969G>A, 4988A>G, 4993A>C, 4999T>C, 5012T>C, 5017G>A, 5023C>T, 5044A>C, 5059G>A, 5071A>G, 5074T>C, 5077T>C, 5079T>C, 5086T>C, 5102T>C, 5104G>A, 5119T>C, 5120G>A, 5122T>C, 5123C>T, 5128T>C, 5132T>A, 5146T>C, 5176C>A, 5209C>T, 5244G>A, 5245A>G, 5251A>G, 5287A>G, 5290G>A, 5302T>C, 5332C>T, 5354G>A, 5356G>A, 5365A>T, 5392G>A, 5413C>T, 5419A>C, 5429C>T, 5437T>C, 5464T>C, 5470A>G, 5491T>G, 5492A>T, 5497T>C, 5518T>C, 5533T>C, 5537A>C, 5560A>G, 5575A>T, 5578T>C, 5591G>T, 5603A>G, 5608A>G, 5611A>G, 5617A>T, 5641T>C, 5647T>C, 5656G>T, 5674A>G, 5677G>A, 5686G>A, 5690T>C, 5702G>A, 5704G>A, 5710T>C, 5716G>A, 5719A>C, 5731C>T, 5734T>C, 5737T>C, 5739G>A, 5746T>C, 5750T>C, 5752G>A, 5758C>T, 5773G>A, 5782A>G, 5784T>C, 5786T>C, 5797A>G, 5806C>T, 5836A>T, 5849T>C, 5850T>A, 5851G>A, 5869G>A, 5875A>G, 5878G>C, 5884T>C, 5887C>A, 5890C>T, 5902G>T, 5908C>T, 5909T>C, 5914T>C, 5923T>C, 5927C>T, 5932C>T, 5955A>G, 5965G>A, 5998A>G, 6004C>T, 6016A>G, 6028C>T, 6031A>T, 6033C>T, 6035G>T, 6055T>A, 6067C>A, 6070T>C, 6094T>C, 6100T>C, 6103G>A, 6106G>A, 6109C>T, 6112A>T, 6131T>C, 6139T>C, 6143A>C, 6145T>C, 6154C>T, 6157C>T, 6163C>T, 6166C>T, 6167C>T, 6184A>G, 6187T>C, 6193C>T, 6196A>G, 6202G>A, 6208A>G, 6211A>G, 6217G>A, 6223A>G, 6226G>A, 6230A>G, 6232A>G, 6235G>A, 6244A>T, 6253A>G, 6256C>A, 6265G>A, 6268G>A, 6273G>A, 6274C>T, 6295T>C, 6296G>T, 6298A>G, 6304A>G, 6308T>C, 6315A>T, 6319A>T, 6322G>A, 6323A>G, 6327A>G, 6331T>C, 6344C>T, 6358T>C, 6382C>T, 6385C>T, 6406T>C, 6412C>T, 6414G>A, 6427A>G, 6442C>G, 6445C>T, 6448G>A, 6451T>C, 6457C>A, 6466T>C, 6484A>C, 6490A>G, 6493T>C, 6500A>T, 6517C>T, 6532C>T, 6545C>T, 6571C>T, 6587G>A, 6661T>C, 6670C>T, 6685A>G, 6742A>T, 6766delC, 6786T>C, 6798_6799insA, 6806_6852delTAACGGAGAGTACTGACTTTAACTAGTTGGGAGTCCGGCTCCATTTG, 6854A>T, 6857A>G, 6858C>A, 6859C>T, 6860A>G, 6861A>G, 6864A>T, 6867T>A, 6871_6894delATTGGTTAAAGAGATTTGCACGCC, 6897T>A, 6905T>G, 6907T>A, 6908G>T, 6911T>C, 6912C>G, 6915G>C, 6921T>G, 6923C>G, 6924T>A, 6927G>T, 6928T>G, 6931G>T, 6933_6935delAAG, 6938C>A, 6947A>C, 6948_6951delGGTT, 6957A>T, 6959_6968delTACGGGGAGC, 6975T>A, 6984_6985insG, 6988G>A, 6994G>A, 6997A>T, 6998T>C, 7002A>T, 7003T>A, 7005_7012delATAGTCCT, 7014T>A, 7020_7026delAAGGGTT, 7031T>A, 7033G>A, 7035T>G, 7038G>A, 7043T>C, 7045T>G, 7047_7060delGCCGTGATGAAAGA, 7063A>T, 7069C>T, 7071T>A, 7074C>A, 7077T>C, 7083G>T, 7085A>G, 7086G>T, 7087_7099delGCTGAAACTACAC, 7101T>A, 7108T>A, 7109G>T, 7113T>G, 7117C>T, 7118T>G, 7120A>T, 7123_7125delCCT, 7129_7133delATCAG, 7136C>A, 7137G>C, 7141G>A, 7144_7169delATTCGATAATTTTCCGTAGCTTGCGT, 7173_7177delGCTGC, 7179C>G, 7182G>A, 7183T>C, 7184T>C, 7185A>C, 7189G>A, 7194_7216delAGTGAAGATGCGCCGTACCACGT, 7221C>T, 7224_7270delGGCAATGCCAGTGGTTCAGAGCGGGCCCTCAGAATAGAGGTTAAAAC, 7275_7285delGTGATGGTGTA, 7288T>A, 7294T>G, 7295A>C, 7297A>C, 7300T>G, 7303C>A, 7304_7315delACCCGGGTTGTG, 7320delG, 7325_7326delGT, 7332C>G, 7333G>T, 7336C>T, 7337A>G, 7339_7348delAGGTCCCACC, 7350T>A, 7351A>T, 7353A>T, 7354G>A, 7358A>C, 7360G>A, 7361A>C, 7362A>C, 7364C>T, 7368G>A, 7372A>T, 7375T>G, 7376T>G, 7377T>A, 7379A>T, 7388C>T, 7397G>A, 7398C>A, 7399G>A, 7400A>T, 7404C>T, 7407A>T, 7408G>T, 7423A>T, 7425G>A, 7435T>C, 7436G>A, 7444C>T, 7445A>G, 7457A>G, 7458T>C, 7460G>A, 7498C>T, 7503C>T, 7505A>G, 7511_7512insC, 7513A>C, 7514A>T, 7515A>C, 7522T>C, 7525_7526insG, 7526T>A, 7526_7527insG, 7545T>A, 7554A>G, 7569G>A, 7589T>A, 7600T>G, 7601T>A, 7602T>G, 7603_7604insC, 7612G>A, 7615A>G, 7616A>C, 7617A>T, 7618C>T, 7625A>T, 7628_7631delGCTT, 7637G>T, 7641T>A, 7642T>A, 7644G>T, 7647T>G, 7648G>T, 7649T>C, 7654C>A, 7655T>A, 7657G>C, 7665G>T, 7666T>C, 7667G>T, 7678C>T, 7686T>G, 7687T>A, 7689G>T, 7698G>A, 7713G>A, 7717A>C, 7719T>A, 7739T>A, 7742_7743insC, 7749T>C, 7751T>C, 7752G>A, 7754_7755insG, 7756_7757insT, 7762C>T, 7763A>C, 7764A>T, 7767C>T, 7773G>T, 7775G>A, 7778T>A, 7782A>T, 7783A>C, 7784_7785insT									

CDS

	Begin	End	Coverage	Score	Concordance	Matches	Identities	I/D/M/F*	Stop Codons
ToTV_sRNA1gp1	1	2159	100%	14810	98.3%	2159 (100%)	2106 (97.5%)	0/0/0/0	1
Protein mutations:	M6L (122A>T 124G>A), P8S (128C>T 130C>T), F10P (134T>C 135T>C 136C>T), N11S (138A>G), S12P (140T>C 142A>C), V13A (144T>C 145T>A), T14M (147C>T 148T>G), C17S (155T>A 157C>T), A18V (159C>T), T19A (161A>G), A31Q (197G>C 198C>A 199T>A), T364A (1196A>G), I459V (1481A>G), A501S (1607G>T), E526D (1684G>T), K544R (1737A>G 1738A>G), F659Y (2082T>A), N712S (2241A>G), S715N (2250G>A 2251C>T), R768K (2409G>A), A1162V (3591C>T), V1218I (3758G>A), P1413S (4343C>T), I1525V (4679A>G), V1598I (4898G>A), T1628A (4988A>G), I1658T (5079T>C), V1672I (5120G>A 5122T>C), S1676T (5132T>A), R1713K (5244G>A 5245A>G), V1750I (5354G>A 5356G>A), T1796S (5492A>T), K1811Q (5537A>C), A1829S (5591G>T), T1833A (5603A>G), V1866I (5702G>A 5704G>A), R1878K (5739G>A), I1893T (5784T>C), L1915Q (5849T>C 5850T>A 5851G>A), N1950S (5955A>G), T1976I (6033C>T), A1977S (6035G>T), I2013L (6143A>C 6145T>C), M2032I (6202G>A), I2042V (6230A>G 6232A>G), S2056N (6273G>A 6274C>T), V2064L (6296G>T 6298A>G), N2070I (6315A>T), I2073V (6323A>G), Q2074R (6327A>G), R2103Q (6414G>A), I2132F (6500A>T), L2147F (6545C>T)									

	Begin	End	Coverage	Score	Concordance	Matches	Identities	I/D/M/F*	Stop Codons
**NT**	**1**	**7808**	**100%**	**11384**	**78.1%**	**7528 (96.0%)**	**6762 (86.3%)**	**31/280**	
Codon mutations:	TTT3TTC (115T>C), TCC4TCT (118C>T), AAG5AAA (121G>A), ATG6TTA (122A>T 124G>A), CCC8TCT (128C>T 130C>T), TTC10CCT (134T>C 135T>C 136C>T), AAC11AGC (138A>G), TCA12CCC (140T>C 142A>C), GTT13GCA (144T>C 145T>A), ACT14ATG (147C>T 148T>G), GAA15GAG (151A>G), TGC17AGT (155T>A 157C>T), GCT18GTT (159C>T), ACC19GCC (161A>G), TCT24TCG (178T>G), TCA27TCT (187A>T), ACT30ACA (196T>A), GCT31CAA (197G>C 198C>A 199T>A), AGT34AGC (208T>C), AAT35AAC (211T>C), TTC36TTT (214C>T), CGC38CGT (220C>T), ACT39ACA (223T>A), CTG40CTT (226G>T), TCA47TCC (247A>C), CAA49CAG (253A>G), ATT50ATC (256T>C), TCT52TCA (262T>A), CAC53CAT (265C>T), GAA55GAG (271A>G), TTG57CTG (275T>C), TCT74TCC (328T>C), ACT76ACC (334T>C), TTG78TTA (340G>A), AAG81AAA (349G>A), TTC89TTT (373C>T), ATC90ATT (376C>T), AGC113AGT (445C>T), GAC127GAT (487C>T), GTG132GTA (502G>A), TCT145TCC (541T>C), TTC151TTT (559C>T), CTA158TTA (578C>T), AAC174AAT (628C>T), GAC183GAT (655C>T), CAA190CAG (676A>G), GCA196GCG (694A>G), CTA201TTA (707C>T), TCA207TCG (727A>G), CTC218CTT (760C>T), ACC225ACT (781C>T), TTC228TTT (790C>T), GTA231GTG (799A>G), GGT233GGC (805T>C), CTA238TTG (818C>T 820A>G), CTA242CTT (832A>T), TCA243TCC (835A>C), ATT245ATC (841T>C), CTG246TTG (842C>T), TAC247TAT (847C>T), TTG259TTA (883G>A), TTT260TTC (886T>C), CTG262CTA (892G>A), AAT265AAC (901T>C), TGT269TGC (913T>C), GCC273GCT (925C>T), GTA282GTG (952A>G), GGT286GGG (964T>G), AAG288AAA (970G>A), GCC297GCT (997C>T), TCT300TCC (1006T>C), GAG309GAA (1033G>A), CAT318CAC (1060T>C), CGC319CGT (1063C>T), TTG320CTG (1064T>C), GCA324GCT (1078A>T), AAC325AAT (1081C>T), TTC328TTT (1090C>T), ACT329ACC (1093T>C), CAA333CAG (1105A>G), CAT334CAC (1108T>C), CTA335TTA (1109C>T), GCC353GCT (1165C>T), AGG358AGA (1180G>A), ACC364GCC (1196A>G), CAG375CAA (1231G>A), GGC378GGT (1240C>T), AGG383AGA (1255G>A), TTT387TTC (1267T>C), CAA390CAG (1276A>G), GGG398GGA (1300G>A), AAA399AAG (1303A>G), CTG405TTG (1319C>T), TTG419CTG (1361T>C), AAC420AAT (1366C>T), AAT434AAC (1408T>C), TAT435TAC (1411T>C), CAT437CAC (1417T>C), ACT439ACC (1423T>C), GCT440GCC (1426T>C), ATC441ATT (1429C>T), CTG442TTG (1430C>T), GAC445GAT (1441C>T), GCT448GCC (1450T>C), CTG449TTG (1451C>T), GAC458GAT (1480C>T), ATT459GTT (1481A>G), GAT461GAC (1489T>C), CCC472CCT (1522C>T), GGC482GGT (1552C>T), CGC483CGT (1555C>T), CGA488CGG (1570A>G), TAT489TAC (1573T>C), GCC492GCT (1582C>T), ACA493ACT (1585A>T), AAC495AAT (1591C>T), CTT496CTC (1594T>C), GCT501TCT (1607G>T), CAG502CAA (1612G>A), GGT504GGG (1618T>G), CTA505TTA (1619C>T), ACC506ACT (1624C>T), CCC508CCT (1630C>T), TTT511TTC (1639T>C), GAG512GAA (1642G>A), AGA513AGG (1645A>G), TGC521TGT (1669C>T), AGG522AGA (1672G>A), GGT525GGA (1681T>A), GAG526GAT (1684G>T), GCT531GCC (1699T>C), ACT533ACC (1705T>C), AAA544AGG (1737A>G 1738A>G), GCA548GCT (1750A>T), ACA550ACG (1756A>G), GGT553GGG (1765T>G), TTG559CTA (1781T>C 1783G>A), CTG560TTG (1784C>T), GAA561GAG (1789A>G), AAG568AAA (1810G>A), ATC573ATT (1825C>T), GCA597GCG (1897A>G), TTG602CTG (1910T>C), GTT616GTC (1954T>C), GTA618GTG (1960A>G), TAC626TAT (1984C>T), AAA636AAG (2014A>G), CCT650CCC (2056T>C), GCC657GCA (2077C>A), TTT659TAT (2082T>A), CTA662CTG (2092A>G), AAA667AAG (2107A>G), GTT674GTC (2128T>C), CTG678TTG (2138C>T), TAT679TAC (2143T>C), GGT681GGC (2149T>C), TGT683TGC (2155T>C), CTG690TTG (2174C>T), ATA691ATC (2179A>C), AAG692AAA (2182G>A), GCT696GCA (2194T>A), TAT698TAC (2200T>C), AAG703AAA (2215G>A), AGC704AGT (2218C>T), ATC706ATT (2224C>T), ACA710ACG (2236A>G), AAT712AGT (2241A>G), AGC715AAT (2250G>A 2251C>T), GAC717GAT (2257C>T), CAG719CAA (2263G>A), CCT723CCC (2275T>C), GCG727GCA (2287G>A), CAC728CAT (2290C>T), GAC736GAT (2314C>T), ATA739ATT (2323A>T), CAC747CAT (2347C>T), GTA748GTG (2350A>G), TTC750TTT (2356C>T), TTG751TTA (2359G>A), TAC752TAT (2362C>T), AGG757AGA (2377G>A), TAT758TAC (2380T>C), AGA762AGG (2392A>G), CCT767CCC (2407T>C), AGG768AAG (2409G>A), ATT779ATC (2443T>C), GTG780GTA (2446G>A), CCG791CCA (2479G>A), ATC793ATT (2485C>T), CTG797CTA (2497G>A), ATT798ATC (2500T>C), AAG799AAA (2503G>A), GGC801GGT (2509C>T), CTT802CTC (2512T>C), GGG812GGA (2542G>A), GTC814GTT (2548C>T), TTC817TTT (2557C>T), GAG838GAA (2620G>A), AAT840AAC (2626T>C), AAG848AAA (2650G>A), TTT853TTC (2665T>C), TTG857CTG (2675T>C), GCA859GCG (2683A>G), AAG862AAA (2692G>A), CTG866TTG (2702C>T), AAA868AAG (2710A>G), AGT871AGC (2719T>C), GAT873GAC (2725T>C), TTC875TTT (2731C>T), ATC876ATT (2734C>T), GGA879GGG (2743A>G), TTG880CTG (2744T>C), AAA882AAG (2752A>G), CTA889CTG (2773A>G), GTC893GTT (2785C>T), TAC894TAT (2788C>T), TGC901TGT (2809C>T), GTG905GTT (2821G>T), ACA907ACG (2827A>G), TTT912TTC (2842T>C), GGC918GGT (2860C>T), ACT920ACA (2866T>A), TTC921TTT (2869C>T), TCA922TCT (2872A>T), ATT929ATA (2893T>A), TCC930TCA (2896C>A), CGG940CGA (2926G>A), TTC941TTT (2929C>T), TTG942CTG (2930T>C), CAA945CAG (2941A>G), GAT946GAC (2944T>C), GTC948GTT (2950C>T), CTG949TTG (2951C>T), CCC958CCT (2980C>T), AAG959AAA (2983G>A), AAA964AAG (2998A>G), CCT967CCC (3007T>C), GAT970GAC (3016T>C), TTT974TTC (3028T>C), TCT975TCC (3031T>C), AAG976AAA (3034G>A), ACT978ACC (3040T>C), AGA984AGG (3058A>G), GTA986GTG (3064A>G), AAG989AAA (3073G>A), GCT991GCC (3079T>C), TTT1001TTC (3109T>C), GTG1003GTA (3115G>A), GTA1013GTG (3145A>G), TAC1015TAT (3151C>T), TCT1019TCC (3163T>C), TCC1021TCT (3169C>T), TTT1022TTC (3172T>C), TAT1032TAC (3202T>C), CTT1045CTC (3241T>C), AAC1049AAT (3253C>T), GTG1050GTA (3256G>A), AAA1053AAG (3265A>G), TCT1064TCC (3298T>C), CAC1071CAT (3319C>T), GTA1100GTC (3406A>C), CTC1104CTA (3418C>A), CTT1105CTC (3421T>C), AAA1119AAG (3463A>G), CAG1120CAA (3466G>A), GGC1125GGT (3481C>T), ACA1126ACT (3484A>T), ACG1134ACA (3508G>A), GGG1151GGA (3559G>A), GAA1154GAG (3568A>G), GCT1156GCC (3574T>C), ATT1160ATC (3586T>C), GCT1162GTT (3591C>T), GAA1168GAG (3610A>G), GGT1169GGC (3613T>C), TAC1173TAT (3625C>T), GTG1174GTA (3628G>A), TAC1180TAT (3646C>T), CAA1185CAG (3661A>G), AAT1186AAC (3664T>C), GAG1189GAA (3673G>A), GAC1190GAT (3676C>T), CTA1194TTA (3686C>T), CGT1200CGC (3706T>C), TTG1204CTG (3716T>C), CCT1205CCA (3721T>A), CGA1211CGT (3739A>T), GGA1215GGG (3751A>G), AAG1216AAA (3754G>A), GTC1218ATC (3758G>A), CAG1220CAA (3766G>A), GTG1225GTA (3781G>A), GAC1233GAT (3805C>T), TAT1234TAC (3808T>C), GAC1236GAT (3814C>T), GGG1237GGC (3817G>C), TTG1240CTG (3824T>C), TCT1243TCC (3835T>C), TAT1246TAC (3844T>C), TTG1251CTA (3857T>C 3859G>A), GGC1254GGT (3868C>T), ACT1255ACC (3871T>C), GAG1259GAA (3883G>A), AGT1264AGC (3898T>C), GGA1267GGT (3907A>T), GTC1270GTT (3916C>T), AAA1273AAG (3925A>G), AGG1274AGA (3928G>A), GAC1281GAT (3949C>T), GAG1283GAA (3955G>A), TTG1285TTA (3961G>A), GAA1288GAG (3970A>G), ATT1289ATC (3973T>C), TTG1310CTG (4034T>C), CGT1320CGC (4066T>C), GAA1323GAG (4075A>G), CCC1326CCT (4084C>T), TTG1332CTG (4100T>C), CTG1338TTG (4118C>T), TCC1341TCT (4129C>T), TCC1347TCA (4147C>A), CTG1351CTA (4159G>A), CCA1352CCT (4162A>T), AAA1354AAG (4168A>G), GGA1360GGT (4186A>T), GAA1363GAG (4195A>G), TTG1367TTA (4207G>A), GCT1368GCC (4210T>C), GTC1374GTT (4228C>T), CAA1396CAG (4294A>G), GAC1404GAT (4318C>T), TAT1406TAC (4324T>C), GGT1411GGC (4339T>C), CCA1413TCA (4343C>T), GAC1414GAT (4348C>T), AAT1416AAC (4354T>C), ACT1430ACC (4396T>C), AAC1434AAT (4408C>T), GTT1436GTG (4414T>G), TTT1437TTC (4417T>C), AGG1438AGA (4420G>A), GTT1451GTC (4459T>C), ATC1452ATT (4462C>T), TTC1453TTT (4465C>T), AAC1454AAT (4468C>T), TCT1455TCC (4471T>C), CTT1456CTC (4474T>C), TTT1460TTC (4486T>C), TAT1461TAC (4489T>C), TTT1466TTC (4504T>C), TCT1468TCA (4510T>A), CTA1469TTA (4511C>T), TTG1470CTG (4514T>C), AGA1471AGG (4519A>G), GCT1479GCC (4543T>C), AAA1483AAG (4555A>G), AAC1488AAT (4570C>T), CTA1490TTG (4574C>T 4576A>G), GTC1494GTT (4588C>T), TAT1495TAC (4591T>C), GCA1502GCG (4612A>G), TTG1507CTG (4625T>C), CAT1508CAC (4630T>C), GCT1514GCC (4648T>C), GAA1518GAG (4660A>G), GAG1520GAA (4666G>A), GTG1522GTC (4672G>C), ATT1525GTT (4679A>G), ATA1526ATT (4684A>T), AAG1527AAA (4687G>A), ATC1530ATT (4696C>T), AAG1532AAA (4702G>A), AAC1533AAT (4705C>T), GTT1536GTC (4714T>C), CTA1542TTA (4730C>T), GAC1543GAT (4735C>T), CTA1547CTG (4747A>G), AGC1551AGT (4759C>T), TTT1554TTC (4768T>C), AGG1556AGA (4774G>A), CAC1557CAT (4777C>T), CTA1559CTG (4783A>G), GGA1560GGG (4786A>G), TAC1561TAT (4789C>T), GTA1569GTG (4813A>G), TCT1570TCC (4816T>C), AGG1579AGA (4843G>A), GTT1598ATT (4898G>A), GAG1600GAA (4906G>A), AAG1621AAA (4969G>A), ACC1628GCC (4988A>G), ACA1629ACC (4993A>C), CCT1631CCC (4999T>C), TTG1636CTG (5012T>C), GAG1637GAA (5017G>A), GAC1639GAT (5023C>T), GGA1646GGC (5044A>C), GGG1651GGA (5059G>A), GAA1655GAG (5071A>G), CTT1656CTC (5074T>C), CAT1657CAC (5077T>C), ATT1658ACT (5079T>C), GCT1660GCC (5086T>C), TTG1666CTA (5102T>C 5104G>A), ACT1671ACC (5119T>C), GTT1672ATC (5120G>A 5122T>C), CTG1673TTG (5123C>T), AAT1674AAC (5128T>C), TCT1676ACT (5132T>A), CAT1680CAC (5146T>C), TCC1690TCA (5176C>A), ACC1701ACT (5209C>T), AGA1713AAG (5244G>A 5245A>G), ACA1715ACG (5251A>G), GAA1727GAG (5287A>G), GGG1728GGA (5290G>A), CCT1732CCC (5302T>C), ATC1742ATT (5332C>T), GTG1750ATA (5354G>A 5356G>A), CCA1753CCT (5365A>T), GGG1762GGA (5392G>A), TGC1769TGT (5413C>T), GCA1771GCC (5419A>C), CTG1775TTG (5429C>T), ACT1777ACC (5437T>C), GTT1786GTC (5464T>C), AGA1788AGG (5470A>G), GTT1795GTG (5491T>G), ACA1796TCA (5492A>T), GAT1797GAC (5497T>C), GGT1804GGC (5518T>C), CTT1809CTC (5533T>C), AAA1811CAA (5537A>C), GAA1818GAG (5560A>G), ACA1823ACT (5575A>T), TGT1824TGC (5578T>C), GCC1829TCC (5591G>T), ACA1833GCA (5603A>G), CCA1834CCG (5608A>G), GCA1835GCG (5611A>G), ATA1837ATT (5617A>T), GGT1845GGC (5641T>C), TTT1847TTC (5647T>C), GTG1850GTT (5656G>T), CTA1856CTG (5674A>G), CAG1857CAA (5677G>A), CAG1860CAA (5686G>A), TTG1862CTG (5690T>C), GTG1866ATA (5702G>A 5704G>A), CCT1868CCC (5710T>C), GGG1870GGA (5716G>A), GGA1871GGC (5719A>C), CAC1875CAT (5731C>T), ACT1876ACC (5734T>C), ACT1877ACC (5737T>C), AGA1878AAA (5739G>A), CTT1880CTC (5746T>C), TTG1882CTA (5750T>C 5752G>A), CTC1884CTT (5758C>T), AAG1889AAA (5773G>A), CAA1892CAG (5782A>G), ATT1893ACT (5784T>C), TTA1894CTA (5786T>C), AGA1897AGG (5797A>G), TTC1900TTT (5806C>T), TCA1910TCT (5836A>T), TTG1915CAA (5849T>C 5850T>A 5851G>A), AGG1921AGA (5869G>A), CAA1923CAG (5875A>G), GGG1924GGC (5878G>C), TTT1926TTC (5884T>C), CCC1927CCA (5887C>A), CGC1928CGT (5890C>T), GTG1932GTT (5902G>T), TAC1934TAT (5908C>T), TTG1935CTG (5909T>C), CTT1936CTC (5914T>C), TTT1939TTC (5923T>C), CTG1941TTG (5927C>T), AGC1942AGT (5932C>T), AAC1950AGC (5955A>G), ACG1953ACA (5965G>A), AAA1964AAG (5998A>G), ACC1966ACT (6004C>T), AAA1970AAG (6016A>G), TCC1974TCT (6028C>T), GCA1975GCT (6031A>T), ACA1976ATA (6033C>T), GCT1977TCT (6035G>T), GGT1983GGA (6055T>A), TCC1987TCA (6067C>A), GCT1988GCC (6070T>C), CTT1996CTC (6094T>C), CTT1998CTC (6100T>C), CAG1999CAA (6103G>A), AAG2000AAA (6106G>A), CCC2001CCT (6109C>T), ATA2002ATT (6112A>T), TTG2009CTG (6131T>C), TTT2011TTC (6139T>C), ATT2013CTC (6143A>C 6145T>C), GTC2016GTT (6154C>T), ATC2017ATT (6157C>T), GGC2019GGT (6163C>T), CAC2020CAT (6166C>T), CTG2021TTG (6167C>T), GTA2026GTG (6184A>G), ACT2027ACC (6187T>C), TTC2029TTT (6193C>T), CAA2030CAG (6196A>G), ATG2032ATA (6202G>A), CAA2034CAG (6208A>G), CAA2035CAG (6211A>G), CTG2037CTA (6217G>A), GAA2039GAG (6223A>G), GGG2040GGA (6226G>A), ATA2042GTG (6230A>G 6232A>G), GAG2043GAA (6235G>A), ATA2046ATT (6244A>T), TTA2049TTG (6253A>G), ACC2050ACA (6256C>A), CTG2053CTA (6265G>A), TCG2054TCA (6268G>A), AGC2056AAT (6273G>A 6274C>T), GAT2063GAC (6295T>C), GTA2064TTG (6296G>T 6298A>G), TTA2066TTG (6304A>G), TTG2068CTG (6308T>C), AAC2070ATC (6315A>T), ATA2071ATT (6319A>T), GAG2072GAA (6322G>A), ATC2073GTC (6323A>G), CAG2074CGG (6327A>G), GAT2075GAC (6331T>C), CTA2080TTA (6344C>T), AAT2084AAC (6358T>C), GCC2092GCT (6382C>T), ATC2093ATT (6385C>T), TGT2100TGC (6406T>C), AAC2102AAT (6412C>T), CGA2103CAA (6414G>A), AAA2107AAG (6427A>G), CGC2112CGG (6442C>G), TAC2113TAT (6445C>T), ACG2114ACA (6448G>A), ACT2115ACC (6451T>C), TCC2117TCA (6457C>A), GTT2120GTC (6466T>C), GCA2126GCC (6484A>C), AGA2128AGG (6490A>G), CAT2129CAC (6493T>C), ATT2132TTT (6500A>T), TCC2137TCT (6517C>T), CAC2142CAT (6532C>T), CTT2147TTT (6545C>T), TAC2155TAT (6571C>T)								

Proteins

	Begin	End	Coverage	Score	Concordance	Matches	Identities	I/D/M/F*	Stop Codons
polyprotein (YP_001039627.1)	1	2159	100%	14810	98.3%	2159 (100%)	2106 (97.5%)	0/0/0/0	1
Protein mutations:	M6L (122A>T 124G>A), P8S (128C>T 130C>T), F10P (134T>C 135T>C 136C>T), N11S (138A>G), S12P (140T>C 142A>C), V13A (144T>C 145T>A), T14M (147C>T 148T>G), C17S (155T>A 157C>T), A18V (159C>T), T19A (161A>G), A31Q (197G>C 198C>A 199T>A), T364A (1196A>G), I459V (1481A>G), A501S (1607G>T), E526D (1684G>T), K544R (1737A>G 1738A>G), F659Y (2082T>A), N712S (2241A>G), S715N (2250G>A 2251C>T), R768K (2409G>A), A1162V (3591C>T), V1218I (3758G>A), P1413S (4343C>T), I1525V (4679A>G), V1598I (4898G>A), T1628A (4988A>G), I1658T (5079T>C), V1672I (5120G>A 5122T>C), S1676T (5132T>A), R1713K (5244G>A 5245A>G), V1750I (5354G>A 5356G>A), T1796S (5492A>T), K1811Q (5537A>C), A1829S (5591G>T), T1833A (5603A>G), V1866I (5702G>A 5704G>A), R1878K (5739G>A), I1893T (5784T>C), L1915Q (5849T>C 5850T>A 5851G>A), N1950S (5955A>G), T1976I (6033C>T), A1977S (6035G>T), I2013L (6143A>C 6145T>C), M2032I (6202G>A), I2042V (6230A>G 6232A>G), S2056N (6273G>A 6274C>T), V2064L (6296G>T 6298A>G), N2070I (6315A>T), I2073V (6323A>G), Q2074R (6327A>G), R2103Q (6414G>A), I2132F (6500A>T), L2147F (6545C>T)								

	Begin	End	Coverage	Score	Concordance	Matches	Identities	I/D/M/F*	Stop Codons
**NT**	**1**	**7808**	**100%**	**11384**	**78.1%**	**7528 (96.0%)**	**6762 (86.3%)**	**31/280**	

Codon mutations: TTT3TTC (115T>C), TCC4TCT (118C>T), AAG5AAA (121G>A), ATG6TTA (122A>T 124G>A), CCC8TCT (128C>T 130C>T), TTC10CCT (134T>C 135T>C 136C>T), AAC11AGC (138A>G), TCA12CCC (140T>C 142A>C), GTT13GCA (144T>C 145T>A), ACT14ATG (147C>T 148T>G), GAA15GAG (151A>G), TGC17AGT (155T>A 157C>T), GCT18GTT (159C>T), ACC19GCC (161A>G), TCT24TCG (178T>G), TCA27TCT (187A>T), ACT30ACA (196T>A), GCT31CAA (197G>C 198C>A 199T>A), AGT34AGC (208T>C), AAT35AAC (211T>C), TTC36TTT (214C>T), CGC38CGT (220C>T), ACT39ACA (223T>A), CTG40CTT (226G>T), TCA47TCC (247A>C), CAA49CAG (253A>G), ATT50ATC (256T>C), TCT52TCA (262T>A), CAC53CAT (265C>T), GAA55GAG (271A>G), TTG57CTG (275T>C), TCT74TCC (328T>C), ACT76ACC (334T>C), TTG78TTA (340G>A), AAG81AAA (349G>A), TTC89TTT (373C>T), ATC90ATT (376C>T), AGC113AGT (445C>T), GAC127GAT (487C>T), GTG132GTA (502G>A), TCT145TCC (541T>C), TTC151TTT (559C>T), CTA158TTA (578C>T), AAC174AAT (628C>T), GAC183GAT (655C>T), CAA190CAG (676A>G), GCA196GCG (694A>G), CTA201TTA (707C>T), TCA207TCG (727A>G), CTC218CTT (760C>T), ACC225ACT (781C>T), TTC228TTT (790C>T), GTA231GTG (799A>G), GGT233GGC (805T>C), CTA238TTG (818C>T 820A>G), CTA242CTT (832A>T), TCA243TCC (835A>C), ATT245ATC (841T>C), CTG246TTG (842C>T), TAC247TAT (847C>T), TTG259TTA (883G>A), TTT260TTC (886T>C), CTG262CTA (892G>A), AAT265AAC (901T>C), TGT269TGC (913T>C), GCC273GCT (925C>T), GTA282GTG (952A>G), GGT286GGG (964T>G), AAG288AAA (970G>A), GCC297GCT (997C>T), TCT300TCC (1006T>C), GAG309GAA (1033G>A), CAT318CAC (1060T>C), CGC319CGT (1063C>T), TTG320CTG (1064T>C), GCA324GCT (1078A>T), AAC325AAT (1081C>T), TTC328TTT (1090C>T), ACT329ACC (1093T>C), CAA333CAG (1105A>G), CAT334CAC (1108T>C), CTA335TTA (1109C>T), GCC353GCT (1165C>T), AGG358AGA (1180G>A), ACC364GCC (1196A>G), CAG375CAA (1231G>A), GGC378GGT (1240C>T), AGG383AGA (1255G>A), TTT387TTC (1267T>C), CAA390CAG (1276A>G), GGG398GGA (1300G>A), AAA399AAG (1303A>G), CTG405TTG (1319C>T), TTG419CTG (1361T>C), AAC420AAT (1366C>T), AAT434AAC (1408T>C), TAT435TAC (1411T>C), CAT437CAC (1417T>C), ACT439ACC (1423T>C), GCT440GCC (1426T>C), ATC441ATT (1429C>T), CTG442TTG (1430C>T), GAC445GAT (1441C>T), GCT448GCC (1450T>C), CTG449TTG (1451C>T), GAC458GAT (1480C>T), ATT459GTT (1481A>G), GAT461GAC (1489T>C), CCC472CCT (1522C>T), GGC482GGT (1552C>T), CGC483CGT (1555C>T), CGA488CGG (1570A>G), TAT489TAC (1573T>C), GCC492GCT (1582C>T), ACA493ACT (1585A>T), AAC495AAT (1591C>T), CTT496CTC (1594T>C), GCT501TCT (1607G>T), CAG502CAA (1612G>A), GGT504GGG (1618T>G), CTA505TTA (1619C>T), ACC506ACT (1624C>T), CCC508CCT (1630C>T), TTT511TTC (1639T>C), GAG512GAA (1642G>A), AGA513AGG (1645A>G), TGC521TGT (1669C>T), AGG522AGA (1672G>A), GGT525GGA (1681T>A), GAG526GAT (1684G>T), GCT531GCC (1699T>C), ACT533ACC (1705T>C), AAA544AGG (1737A>G 1738A>G), GCA548GCT (1750A>T), ACA550ACG (1756A>G), GGT553GGG (1765T>G), TTG559CTA (1781T>C 1783G>A), CTG560TTG (1784C>T), GAA561GAG (1789A>G), AAG568AAA (1810G>A), ATC573ATT (1825C>T), GCA597GCG (1897A>G), TTG602CTG (1910T>C), GTT616GTC (1954T>C), GTA618GTG (1960A>G), TAC626TAT (1984C>T), AAA636AAG (2014A>G), CCT650CCC (2056T>C), GCC657GCA (2077C>A), TTT659TAT (2082T>A), CTA662CTG (2092A>G), AAA667AAG (2107A>G), GTT674GTC (2128T>C), CTG678TTG (2138C>T), TAT679TAC (2143T>C), GGT681GGC (2149T>C), TGT683TGC (2155T>C), CTG690TTG (2174C>T), ATA691ATC (2179A>C), AAG692AAA (2182G>A), GCT696GCA (2194T>A), TAT698TAC (2200T>C), AAG703AAA (2215G>A), AGC704AGT (2218C>T), ATC706ATT (2224C>T), ACA710ACG (2236A>G), AAT712AGT (2241A>G), AGC715AAT (2250G>A 2251C>T), GAC717GAT (2257C>T), CAG719CAA (2263G>A), CCT723CCC (2275T>C), GCG727GCA (2287G>A), CAC728CAT (2290C>T), GAC736GAT (2314C>T), ATA739ATT (2323A>T), CAC747CAT (2347C>T), GTA748GTG (2350A>G), TTC750TTT (2356C>T), TTG751TTA (2359G>A), TAC752TAT (2362C>T), AGG757AGA (2377G>A), TAT758TAC (2380T>C), AGA762AGG (2392A>G), CCT767CCC (2407T>C), AGG768AAG (2409G>A), ATT779ATC (2443T>C), GTG780GTA (2446G>A), CCG791CCA (2479G>A), ATC793ATT (2485C>T), CTG797CTA (2497G>A), ATT798ATC (2500T>C), AAG799AAA (2503G>A), GGC801GGT (2509C>T), CTT802CTC (2512T>C), GGG812GGA (2542G>A), GTC814GTT (2548C>T), TTC817TTT (2557C>T), GAG838GAA (2620G>A), AAT840AAC (2626T>C), AAG848AAA (2650G>A), TTT853TTC (2665T>C), TTG857CTG (2675T>C), GCA859GCG (2683A>G), AAG862AAA (2692G>A), CTG866TTG (2702C>T), AAA868AAG (2710A>G), AGT871AGC (2719T>C), GAT873GAC (2725T>C), TTC875TTT (2731C>T), ATC876ATT (2734C>T), GGA879GGG (2743A>G), TTG880CTG (2744T>C), AAA882AAG (2752A>G), CTA889CTG (2773A>G), GTC893GTT (2785C>T), TAC894TAT (2788C>T), TGC901TGT (2809C>T), GTG905GTT (2821G>T), ACA907ACG (2827A>G), TTT912TTC (2842T>C), GGC918GGT (2860C>T), ACT920ACA (2866T>A), TTC921TTT (2869C>T), TCA922TCT (2872A>T), ATT929ATA (2893T>A), TCC930TCA (2896C>A), CGG940CGA (2926G>A), TTC941TTT (2929C>T), TTG942CTG (2930T>C), CAA945CAG (2941A>G), GAT946GAC (2944T>C), GTC948GTT (2950C>T), CTG949TTG (2951C>T), CCC958CCT (2980C>T), AAG959AAA (2983G>A), AAA964AAG (2998A>G), CCT967CCC (3007T>C), GAT970GAC (3016T>C), TTT974TTC (3028T>C), TCT975TCC (3031T>C), AAG976AAA (3034G>A), ACT978ACC (3040T>C), AGA984AGG (3058A>G), GTA986GTG (3064A>G), AAG989AAA (3073G>A), GCT991GCC (3079T>C), TTT1001TTC (3109T>C), GTG1003GTA (3115G>A), GTA1013GTG (3145A>G), TAC1015TAT (3151C>T), TCT1019TCC (3163T>C), TCC1021TCT (3169C>T), TTT1022TTC (3172T>C), TAT1032TAC (3202T>C), CTT1045CTC (3241T>C), AAC1049AAT (3253C>T), GTG1050GTA (3256G>A), AAA1053AAG (3265A>G), TCT1064TCC (3298T>C), CAC1071CAT (3319C>T), GTA1100GTC (3406A>C), CTC1104CTA (3418C>A), CTT1105CTC (3421T>C), AAA1119AAG (3463A>G), CAG1120CAA (3466G>A), GGC1125GGT (3481C>T), ACA1126ACT (3484A>T), ACG1134ACA (3508G>A), GGG1151GGA (3559G>A), GAA1154GAG (3568A>G), GCT1156GCC (3574T>C), ATT1160ATC (3586T>C), GCT1162GTT (3591C>T), GAA1168GAG (3610A>G), GGT1169GGC (3613T>C), TAC1173TAT (3625C>T), GTG1174GTA (3628G>A), TAC1180TAT (3646C>T), CAA1185CAG (3661A>G), AAT1186AAC (3664T>C), GAG1189GAA (3673G>A), GAC1190GAT (3676C>T), CTA1194TTA (3686C>T), CGT1200CGC (3706T>C), TTG1204CTG (3716T>C), CCT1205CCA (3721T>A), CGA1211CGT (3739A>T), GGA1215GGG (3751A>G), AAG1216AAA (3754G>A), GTC1218ATC (3758G>A), CAG1220CAA (3766G>A), GTG1225GTA (3781G>A), GAC1233GAT (3805C>T), TAT1234TAC (3808T>C), GAC1236GAT (3814C>T), GGG1237GGC (3817G>C), TTG1240CTG (3824T>C), TCT1243TCC (3835T>C), TAT1246TAC (3844T>C), TTG1251CTA (3857T>C 3859G>A), GGC1254GGT (3868C>T), ACT1255ACC (3871T>C), GAG1259GAA (3883G>A), AGT1264AGC (3898T>C), GGA1267GGT (3907A>T), GTC1270GTT (3916C>T), AAA1273AAG (3925A>G), AGG1274AGA (3928G>A), GAC1281GAT (3949C>T), GAG1283GAA (3955G>A), TTG1285TTA (3961G>A), GAA1288GAG (3970A>G), ATT1289ATC (3973T>C), TTG1310CTG (4034T>C), CGT1320CGC (4066T>C), GAA1323GAG (4075A>G), CCC1326CCT (4084C>T), TTG1332CTG (4100T>C), CTG1338TTG (4118C>T), TCC1341TCT (4129C>T), TCC1347TCA (4147C>A), CTG1351CTA (4159G>A), CCA1352CCT (4162A>T), AAA1354AAG (4168A>G), GGA1360GGT (4186A>T), GAA1363GAG (4195A>G), TTG1367TTA (4207G>A), GCT1368GCC (4210T>C), GTC1374GTT (4228C>T), CAA1396CAG (4294A>G), GAC1404GAT (4318C>T), TAT1406TAC (4324T>C), GGT1411GGC (4339T>C), CCA1413TCA (4343C>T), GAC1414GAT (4348C>T), AAT1416AAC (4354T>C), ACT1430ACC (4396T>C), AAC1434AAT (4408C>T), GTT1436GTG (4414T>G), TTT1437TTC (4417T>C), AGG1438AGA (4420G>A), GTT1451GTC (4459T>C), ATC1452ATT (4462C>T), TTC1453TTT (4465C>T), AAC1454AAT (4468C>T), TCT1455TCC (4471T>C), CTT1456CTC (4474T>C), TTT1460TTC (4486T>C), TAT1461TAC (4489T>C), TTT1466TTC (4504T>C), TCT1468TCA (4510T>A), CTA1469TTA (4511C>T), TTG1470CTG (4514T>C), AGA1471AGG (4519A>G), GCT1479GCC (4543T>C), AAA1483AAG (4555A>G), AAC1488AAT (4570C>T), CTA1490TTG (4574C>T 4576A>G), GTC1494GTT (4588C>T), TAT1495TAC (4591T>C), GCA1502GCG (4612A>G), TTG1507CTG (4625T>C), CAT1508CAC (4630T>C), GCT1514GCC (4648T>C), GAA1518GAG (4660A>G), GAG1520GAA (4666G>A), GTG1522GTC (4672G>C), ATT1525GTT (4679A>G), ATA1526ATT (4684A>T), AAG1527AAA (4687G>A), ATC1530ATT (4696C>T), AAG1532AAA (4702G>A), AAC1533AAT (4705C>T), GTT1536GTC (4714T>C), CTA1542TTA (4730C>T), GAC1543GAT (4735C>T), CTA1547CTG (4747A>G), AGC1551AGT (4759C>T), TTT1554TTC (4768T>C), AGG1556AGA (4774G>A), CAC1557CAT (4777C>T), CTA1559CTG (4783A>G), GGA1560GGG (4786A>G), TAC1561TAT (4789C>T), GTA1569GTG (4813A>G), TCT1570TCC (4816T>C), AGG1579AGA (4843G>A), GTT1598ATT (4898G>A), GAG1600GAA (4906G>A), AAG1621AAA (4969G>A), ACC1628GCC (4988A>G), ACA1629ACC (4993A>C), CCT1631CCC (4999T>C), TTG1636CTG (5012T>C), GAG1637GAA (5017G>A), GAC1639GAT (5023C>T), GGA1646GGC (5044A>C), GGG1651GGA (5059G>A), GAA1655GAG (5071A>G), CTT1656CTC (5074T>C), CAT1657CAC (5077T>C), ATT1658ACT (5079T>C), GCT1660GCC (5086T>C), TTG1666CTA (5102T>C 5104G>A), ACT1671ACC (5119T>C), GTT1672ATC (5120G>A 5122T>C), CTG1673TTG (5123C>T), AAT1674AAC (5128T>C), TCT1676ACT (5132T>A), CAT1680CAC (5146T>C), TCC1690TCA (5176C>A), ACC1701ACT (5209C>T), AGA1713AAG (5244G>A 5245A>G), ACA1715ACG (5251A>G), GAA1727GAG (5287A>G), GGG1728GGA (5290G>A), CCT1732CCC (5302T>C), ATC1742ATT (5332C>T), GTG1750ATA (5354G>A 5356G>A), CCA1753CCT (5365A>T), GGG1762GGA (5392G>A), TGC1769TGT (5413C>T), GCA1771GCC (5419A>C), CTG1775TTG (5429C>T), ACT1777ACC (5437T>C), GTT1786GTC (5464T>C), AGA1788AGG (5470A>G), GTT1795GTG (5491T>G), ACA1796TCA (5492A>T), GAT1797GAC (5497T>C), GGT1804GGC (5518T>C), CTT1809CTC (5533T>C), AAA1811CAA (5537A>C), GAA1818GAG (5560A>G), ACA1823ACT (5575A>T), TGT1824TGC (5578T>C), GCC1829TCC (5591G>T), ACA1833GCA (5603A>G), CCA1834CCG (5608A>G), GCA1835GCG (5611A>G), ATA1837ATT (5617A>T), GGT1845GGC (5641T>C), TTT1847TTC (5647T>C), GTG1850GTT (5656G>T), CTA1856CTG (5674A>G), CAG1857CAA (5677G>A), CAG1860CAA (5686G>A), TTG1862CTG (5690T>C), GTG1866ATA (5702G>A 5704G>A), CCT1868CCC (5710T>C), GGG1870GGA (5716G>A), GGA1871GGC (5719A>C), CAC1875CAT (5731C>T), ACT1876ACC (5734T>C), ACT1877ACC (5737T>C), AGA1878AAA (5739G>A), CTT1880CTC (5746T>C), TTG1882CTA (5750T>C 5752G>A), CTC1884CTT (5758C>T), AAG1889AAA (5773G>A), CAA1892CAG (5782A>G), ATT1893ACT (5784T>C), TTA1894CTA (5786T>C), AGA1897AGG (5797A>G), TTC1900TTT (5806C>T), TCA1910TCT (5836A>T), TTG1915CAA (5849T>C 5850T>A 5851G>A), AGG1921AGA (5869G>A), CAA1923CAG (5875A>G), GGG1924GGC (5878G>C), TTT1926TTC (5884T>C), CCC1927CCA (5887C>A), CGC1928CGT (5890C>T), GTG1932GTT (5902G>T), TAC1934TAT (5908C>T), TTG1935CTG (5909T>C), CTT1936CTC (5914T>C), TTT1939TTC (5923T>C), CTG1941TTG (5927C>T), AGC1942AGT (5932C>T), AAC1950AGC (5955A>G), ACG1953ACA (5965G>A), AAA1964AAG (5998A>G), ACC1966ACT (6004C>T), AAA1970AAG (6016A>G), TCC1974TCT (6028C>T), GCA1975GCT (6031A>T), ACA1976ATA (6033C>T), GCT1977TCT (6035G>T), GGT1983GGA (6055T>A), TCC1987TCA (6067C>A), GCT1988GCC (6070T>C), CTT1996CTC (6094T>C), CTT1998CTC (6100T>C), CAG1999CAA (6103G>A), AAG2000AAA (6106G>A), CCC2001CCT (6109C>T), ATA2002ATT (6112A>T), TTG2009CTG (6131T>C), TTT2011TTC (6139T>C), ATT2013CTC (6143A>C 6145T>C), GTC2016GTT (6154C>T), ATC2017ATT (6157C>T), GGC2019GGT (6163C>T), CAC2020CAT (6166C>T), CTG2021TTG (6167C>T), GTA2026GTG (6184A>G), ACT2027ACC (6187T>C), TTC2029TTT (6193C>T), CAA2030CAG (6196A>G), ATG2032ATA (6202G>A), CAA2034CAG (6208A>G), CAA2035CAG (6211A>G), CTG2037CTA (6217G>A), GAA2039GAG (6223A>G), GGG2040GGA (6226G>A), ATA2042GTG (6230A>G 6232A>G), GAG2043GAA (6235G>A), ATA2046ATT (6244A>T), TTA2049TTG (6253A>G), ACC2050ACA (6256C>A), CTG2053CTA (6265G>A), TCG2054TCA (6268G>A), AGC2056AAT (6273G>A 6274C>T), GAT2063GAC (6295T>C), GTA2064TTG (6296G>T 6298A>G), TTA2066TTG (6304A>G), TTG2068CTG (6308T>C), AAC2070ATC (6315A>T), ATA2071ATT (6319A>T), GAG2072GAA (6322G>A), ATC2073GTC (6323A>G), CAG2074CGG (6327A>G), GAT2075GAC (6331T>C), CTA2080TTA (6344C>T), AAT2084AAC (6358T>C), GCC2092GCT (6382C>T), ATC2093ATT (6385C>T), TGT2100TGC (6406T>C), AAC2102AAT (6412C>T), CGA2103CAA (6414G>A), AAA2107AAG (6427A>G), CGC2112CGG (6442C>G), TAC2113TAT (6445C>T), ACG2114ACA (6448G>A), ACT2115ACC (6451T>C), TCC2117TCA (6457C>A), GTT2120GTC (6466T>C), GCA2126GCC (6484A>C), AGA2128AGG (6490A>G), CAT2129CAC (6493T>C), ATT2132TTT (6500A>T), TCC2137TCT (6517C>T), CAC2142CAT (6532C>T), CTT2147TTT (6545C>T), TAC2155TAT (6571C>T)

*: Inserts / Deletes / Misaligned / Frameshifts

Analysis details

This analysis was performed with panviral2.64

NGS Details (UN9): Torradovirus lycopersici (segment RNA 2)

Assembly

**Coverage Length**	5379 (1 contig(s))
**Depth Of Coverage**	60857.9
**Number Of Reads**	2524050
**Reads Per Million**	57050.75 rpm (after QC)
**Ambiguities**	0
**Assembly Method**	de novo + reference guided assembly
**Consensus Caller**	Bcf Tools

Coverage Map

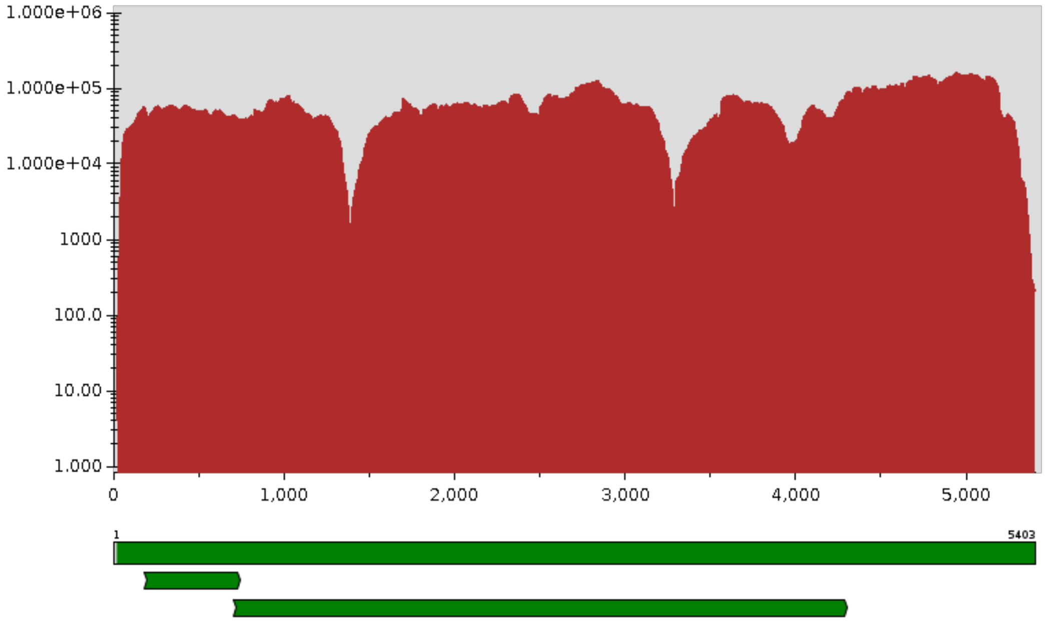

Assignment

**Type**	Torradovirus lycopersici (Taxonomy ID: 3048378)
**Reference Genome**	NC_009032.1
**NT Identity (%)**	94.8151
**AA Identity (%)**	96.4672
**Number Of Stop Codons**	3
**Number Of CDS**	2

Alignment

**Alignment Score**	9630.0 (NT) + 9213.0 (AA) = 18843.0
**Concordance (%)**	93.0748

**Alignment Method**	Global, seeded, nucleotide + amino acids (AGA)

Genome Region

Sequence starts at position 25 and ends at position 5403 relative to NC_009032.1 reference sequence.

Alignment Detailed Statistics

	Begin	End	Coverage	Score	Concordance	Matches	Identities	I/D/M/F*	Stop Codons
**NT**	**25**	**5403**	**99.6%**	**9630**	**89.5%**	**5379 (99.9%)**	**5102 (94.8%)**	**2/0**	
Mutations:	89G>A, 90C>T, 97C>T, 107G>A, 111C>A, 137T>C, 144A>G, 161A>G, 277C>T, 280T>C, 283C>T, 295T>C, 301A>C, 313C>T, 343T>C, 401T>C, 433C>T, 440C>T, 448C>A, 460T>C, 481C>A, 487A>G, 507G>A, 583A>T, 586A>G, 589A>G, 654T>A, 679G>A, 696T>C, 721C>T, 733A>G, 748A>G, 750A>G, 797T>C, 806C>T, 827A>G, 844A>T, 866A>G, 968A>G, 986G>A, 1004C>T, 1061T>C, 1064T>C, 1085G>A, 1106T>C, 1109C>T, 1139G>A, 1226A>G, 1271T>C, 1277A>G, 1300C>T, 1316C>T, 1337T>C, 1346G>A, 1355G>A, 1361A>T, 1367A>T, 1383A>G, 1384C>T, 1390C>G, 1394A>T, 1412G>T, 1421A>G, 1426A>C, 1436C>T, 1439A>C, 1457C>T, 1460A>G, 1465G>A, 1472C>G, 1475G>C, 1484G>A, 1487C>G, 1490G>A, 1502C>T, 1508G>A, 1547C>T, 1559C>A, 1608T>C, 1619G>A, 1637G>A, 1646T>C, 1651A>G, 1662C>T, 1685T>C, 1694T>C, 1724C>T, 1757A>G, 1794G>A, 1817A>T, 1819T>A, 1820C>G, 1847G>T, 1850G>A, 1859C>T, 1863C>T, 1881C>T, 1892C>T, 1896A>G, 1899G>A, 1900T>C, 1904T>G, 1906C>T, 1914C>T, 1967G>A, 1970A>G, 1994T>C, 2072G>A, 2084G>A, 2108A>G, 2128G>A, 2135G>A, 2138G>T, 2140A>C, 2159T>A, 2181A>G, 2182C>A, 2183C>T, 2189C>T, 2199G>A, 2200T>C, 2205A>G, 2207G>T, 2216C>G, 2225T>A, 2249T>C, 2263A>C, 2270A>G, 2285T>C, 2303A>G, 2312T>C, 2321T>C, 2324G>A, 2375C>T, 2405G>A, 2408A>G, 2414T>C, 2429G>A, 2432G>T, 2436T>G, 2459C>G, 2465T>C, 2468A>G, 2469T>A, 2471C>T, 2501A>C, 2503G>A, 2519A>G, 2570G>A, 2573T>C, 2612C>T, 2630C>T, 2660T>C, 2708A>G, 2720C>T, 2732T>C, 2792C>T, 2819G>A, 2840T>C, 2841C>T, 2885T>C, 2939A>G, 2969C>T, 3003T>C, 3050G>A, 3122T>C, 3128T>C, 3131G>A, 3168C>T, 3215T>C, 3227T>G, 3236C>T, 3239T>G, 3242G>A, 3253A>G, 3265G>A, 3266G>A, 3269T>C, 3270A>T, 3296T>C, 3303A>T, 3305C>T, 3311T>A, 3323T>C, 3324C>T, 3332G>A, 3335C>T, 3354A>C, 3368A>T, 3386A>T, 3395A>C, 3404A>G, 3413C>T, 3428G>A, 3429C>T, 3431T>G, 3437C>T, 3440A>T, 3447T>C, 3461C>T, 3470T>C, 3485C>T, 3491C>T, 3497T>C, 3503A>G, 3508A>G, 3524A>G, 3527C>T, 3533G>A, 3540T>C, 3543T>A, 3560T>A, 3569A>G, 3575G>A, 3581C>T, 3590A>C, 3605A>G, 3626A>G, 3638A>G, 3662A>T, 3674G>A, 3716C>T, 3722C>T, 3743G>A, 3767C>T, 3773C>T, 3800C>T, 3827C>T, 3863C>T, 3869A>T, 3872A>T, 3885A>G, 3893T>C, 3911T>C, 3914T>A, 3920A>T, 3932C>A, 3933A>G, 3941T>G, 3944C>A, 3950T>A, 3960G>A, 3984A>G, 3986G>T, 3990G>A, 3995C>T, 4001T>C, 4013G>C, 4040C>T, 4046C>T, 4059A>G, 4061A>G, 4069A>G, 4100A>T, 4112C>T, 4130C>T, 4157C>T, 4202T>C, 4205T>A, 4208A>T, 4224C>T, 4250C>T, 4457A>G, 4518G>A, 4556A>G, 4590G>A, 4690C>T, 4703G>A, 4749T>A, 4812C>T, 4823A>G, 4974_4975insT, 5089T>C, 5192C>T, 5206C>T, 5218C>T, 5235_5236insT, 5249A>G, 5262A>T								

CDS

	Begin	End	Coverage	Score	Concordance	Matches	Identities	I/D/M/F*	Stop Codons
ToTV_sRNA2gp1	1	188	100%	1189	98.3%	188 (100%)	185 (98.4%)	0/0/0/0	1
Protein mutations:	R109K (507G>A), L158Q (654T>A), L172P (696T>C)								
Codon mutations:	GGC32GGT (277C>T), TCT33TCC (280T>C), GGC34GGT (283C>T), AGT38AGC (295T>C), CCA40CCC (301A>C), TTC44TTT (313C>T), ATT54ATC (343T>C), TTA74CTA (401T>C), GGC84GGT (433C>T), CTA87TTA (440C>T), GGC89GGA (448C>A), CAT93CAC (460T>C), ACC100ACA (481C>A), AGA102AGG (487A>G), AGG109AAG (507G>A), ACA134ACT (583A>T), CAA135CAG (586A>G), TTA136TTG (589A>G), CTA158CAA (654T>A), TTG166TTA (679G>A), CTA172CCA (696T>C), GGC180GGT (721C>T), GAA184GAG (733A>G)								
ToTV_sRNA2gp2	1	1199	100%	8024	96.9%	1199 (100%)	1153 (96.2%)	0/0/0/0	2
Protein mutations:	A7V (721C>T), K11R (733A>G), K16R (748A>G), S17G (750A>G), Y48F (844A>T), A200V (1300C>T), T228V (1383A>G 1384C>T), S230* (1390C>G), Q242P (1426A>C), R255K (1465G>A), I257M (1472C>G), H286Q (1559C>A), Y303H (1608T>C), K317R (1651A>G), V365I (1794G>A), I373K (1819T>A 1820C>G), L382F (1847G>T), H388Y (1863C>T), I399V (1896A>G), V400T (1899G>A 1900T>C), N401K (1904T>G), A402V (1906C>T), R476K (2128G>A), K480T (2140A>C), T494D (2181A>G 2182C>A 2183C>T), V500T (2199G>A 2200T>C), M502V (2205A>G 2207G>T), D508E (2225T>A), E521A (2263A>C), S579A (2436T>G), S590T (2469T>A 2471C>T), R601K (2503G>A), K851R (3253A>G), R855K (3265G>A 3266G>A), T857S (3270A>T), T868S (3303A>T 3305C>T), N936S (3508A>G), S948T (3543T>A), T1062A (3885A>G), T1078A (3933A>G), A1087T (3960G>A), M1095V (3984A>G 3986G>T), D1097N (3990G>A), I1120V (4059A>G 4061A>G), N1123S (4069A>G), H1175Y (4224C>T)								
Codon mutations:	GCT7GTT (721C>T), AAG11AGG (733A>G), AAA16AGA (748A>G), AGT17GGT (750A>G), GTT32GTC (797T>C), GGC35GGT (806C>T), AAA42AAG (827A>G), TAT48TTT (844A>T), CAA55CAG (866A>G), GGA89GGG (968A>G), TTG95TTA (986G>A), TTC101TTT (1004C>T), AGT120AGC (1061T>C), AAT121AAC (1064T>C), CTG128CTA (1085G>A), TTT135TTC (1106T>C), TCC136TCT (1109C>T), CCG146CCA (1139G>A), TCA175TCG (1226A>G), ATT190ATC (1271T>C), TCA192TCG (1277A>G), GCT200GTT (1300C>T), CAC205CAT (1316C>T), GAT212GAC (1337T>C), GAG215GAA (1346G>A), CAG218CAA (1355G>A), ATA220ATT (1361A>T), TCA222TCT (1367A>T), ACC228GTC (1383A>G 1384C>T), TCA230TGA (1390C>G), GCA231GCT (1394A>T), CCG237CCT (1412G>T), GGA240GGG (1421A>G), CAG242CCG (1426A>C), GAC245GAT (1436C>T), ACA246ACC (1439A>C), AGC252AGT (1457C>T), GAA253GAG (1460A>G), AGA255AAA (1465G>A), ATC257ATG (1472C>G), CGG258CGC (1475G>C), TCG261TCA (1484G>A), CTC262CTG (1487C>G), CGG263CGA (1490G>A), CTC267CTT (1502C>T), GTG269GTA (1508G>A), ACC282ACT (1547C>T), CAC286CAA (1559C>A), TAC303CAC (1608T>C), GAG306GAA (1619G>A), TTG312TTA (1637G>A), GTT315GTC (1646T>C), AAG317AGG (1651A>G), CTG321TTG (1662C>T), GAT328GAC (1685T>C), GGT331GGC (1694T>C), CCC341CCT (1724C>T), CAA352CAG (1757A>G), GTT365ATT (1794G>A), ATA372ATT (1817A>T), ATC373AAG (1819T>A 1820C>G), TTG382TTT (1847G>T), GAG383GAA (1850G>A), ACC386ACT (1859C>T), CAC388TAC (1863C>T), CTG394TTG (1881C>T), TAC397TAT (1892C>T), ATT399GTT (1896A>G), GTG400ACG (1899G>A 1900T>C), AAT401AAG (1904T>G), GCC402GTC (1906C>T), CTG405TTG (1914C>T), GAG422GAA (1967G>A), CTA423CTG (1970A>G), TAT431TAC (1994T>C), GAG457GAA (2072G>A), GTG461GTA (2084G>A), AGA469AGG (2108A>G), AGG476AAG (2128G>A), CAG478CAA (2135G>A), GTG479GTT (2138G>T), AAA480ACA (2140A>C), ACT486ACA (2159T>A), ACC494GAT (2181A>G 2182C>A 2183C>T), TTC496TTT (2189C>T), GTT500ACT (2199G>A 2200T>C), ATG502GTT (2205A>G 2207G>T), GGC505GGG (2216C>G), GAT508GAA (2225T>A), GCT516GCC (2249T>C), GAA521GCA (2263A>C), GTA523GTG (2270A>G), GTT528GTC (2285T>C), CAA534CAG (2303A>G), AAT537AAC (2312T>C), GGT540GGC (2321T>C), GCG541GCA (2324G>A), TTC558TTT (2375C>T), AAG568AAA (2405G>A), AAA569AAG (2408A>G), AGT571AGC (2414T>C), GAG576GAA (2429G>A), GTG577GTT (2432G>T), TCA579GCA (2436T>G), CTC586CTG (2459C>G), GGT588GGC (2465T>C), AAA589AAG (2468A>G), TCC590ACT (2469T>A 2471C>T), CTA600CTC (2501A>C), AGG601AAG (2503G>A), GCA606GCG (2519A>G), TCG623TCA (2570G>A), GGT624GGC (2573T>C), AAC637AAT (2612C>T), ATC643ATT (2630C>T), CAT653CAC (2660T>C), ACA669ACG (2708A>G), ATC673ATT (2720C>T), GTT677GTC (2732T>C), CAC697CAT (2792C>T), GAG706GAA (2819G>A), CAT713CAC (2840T>C), CTG714TTG (2841C>T), CGT728CGC (2885T>C), CGA746CGG (2939A>G), TAC756TAT (2969C>T), TTA768CTA (3003T>C), ACG783ACA (3050G>A), ATT807ATC (3122T>C), ATT809ATC (3128T>C), GAG810GAA (3131G>A), CTG823TTG (3168C>T), AAT838AAC (3215T>C), GTT842GTG (3227T>G), AAC845AAT (3236C>T), CTT846CTG (3239T>G), GAG847GAA (3242G>A), AAG851AGG (3253A>G), AGG855AAA (3265G>A 3266G>A), TGT856TGC (3269T>C), ACG857TCG (3270A>T), AAT865AAC (3296T>C), ACC868TCT (3303A>T 3305C>T), GGT870GGA (3311T>A), TCT874TCC (3323T>C), CTA875TTA (3324C>T), CGG877CGA (3332G>A), CCC878CCT (3335C>T), AGG885CGG (3354A>C), ACA889ACT (3368A>T), GTA895GTT (3386A>T), TCA898TCC (3395A>C), CTA901CTG (3404A>G), GTC904GTT (3413C>T), CAG909CAA (3428G>A), CTT910TTG (3429C>T 3431T>G), TGC912TGT (3437C>T), ATA913ATT (3440A>T), TTG916CTG (3447T>C), GGC920GGT (3461C>T), TCT923TCC (3470T>C), TAC928TAT (3485C>T), CAC930CAT (3491C>T), ACT932ACC (3497T>C), GGA934GGG (3503A>G), AAT936AGT (3508A>G), CTA941CTG (3524A>G), TTC942TTT (3527C>T), GAG944GAA (3533G>A), TTG947CTG (3540T>C), TCT948ACT (3543T>A), ATT953ATA (3560T>A), TTA956TTG (3569A>G), AAG958AAA (3575G>A), AAC960AAT (3581C>T), ACA963ACC (3590A>C), GGA968GGG (3605A>G), CAA975CAG (3626A>G), AGA979AGG (3638A>G), GTA987GTT (3662A>T), GTG991GTA (3674G>A), TGC1005TGT (3716C>T), CCC1007CCT (3722C>T), CAG1014CAA (3743G>A), ATC1022ATT (3767C>T), TGC1024TGT (3773C>T), TTC1033TTT (3800C>T), TAC1042TAT (3827C>T), AAC1054AAT (3863C>T), GGA1056GGT (3869A>T), GGA1057GGT (3872A>T), ACC1062GCC (3885A>G), GAT1064GAC (3893T>C), TTT1070TTC (3911T>C), CCT1071CCA (3914T>A), GGA1073GGT (3920A>T), GGC1077GGA (3932C>A), ACC1078GCC (3933A>G), GTT1080GTG (3941T>G), GTC1081GTA (3944C>A), GCT1083GCA (3950T>A), GCA1087ACA (3960G>A), ATG1095GTT (3984A>G 3986G>T), GAC1097AAC (3990G>A), AAC1098AAT (3995C>T), TTT1100TTC (4001T>C), GTG1104GTC (4013G>C), CGC1113CGT (4040C>T), ACC1115ACT (4046C>T), ATA1120GTG (4059A>G 4061A>G), AAT1123AGT (4069A>G), CGA1133CGT (4100A>T), CTC1137CTT (4112C>T), GCC1143GCT (4130C>T), ATC1152ATT (4157C>T), GCT1167GCC (4202T>C), CCT1168CCA (4205T>A), TCA1169TCT (4208A>T), CAT1175TAT (4224C>T), ACC1183ACT (4250C>T)								

Proteins

	Begin	End	Coverage	Score	Concordance	Matches	Identities	I/D/M/F*	Stop Codons
Orf1 (YP_001040017.1)	1	188	100%	1189	98.3%	188 (100%)	185 (98.4%)	0/0/0/0	1
Protein mutations:	R109K (507G>A), L158Q (654T>A), L172P (696T>C)								
Codon mutations:	GGC32GGT (277C>T), TCT33TCC (280T>C), GGC34GGT (283C>T), AGT38AGC (295T>C), CCA40CCC (301A>C), TTC44TTT (313C>T), ATT54ATC (343T>C), TTA74CTA (401T>C), GGC84GGT (433C>T), CTA87TTA (440C>T), GGC89GGA (448C>A), CAT93CAC (460T>C), ACC100ACA (481C>A), AGA102AGG (487A>G), AGG109AAG (507G>A), ACA134ACT (583A>T), CAA135CAG (586A>G), TTA136TTG (589A>G), CTA158CAA (654T>A), TTG166TTA (679G>A), CTA172CCA (696T>C), GGC180GGT (721C>T), GAA184GAG (733A>G)								
polyprotein (YP_001040018.1)	1	1199	100%	8024	96.9%	1199 (100%)	1153 (96.2%)	0/0/0/0	2
Protein mutations:	A7V (721C>T), K11R (733A>G), K16R (748A>G), S17G (750A>G), Y48F (844A>T), A200V (1300C>T), T228V (1383A>G 1384C>T), S230* (1390C>G), Q242P (1426A>C), R255K (1465G>A), I257M (1472C>G), H286Q (1559C>A), Y303H (1608T>C), K317R (1651A>G), V365I (1794G>A), I373K (1819T>A 1820C>G), L382F (1847G>T), H388Y (1863C>T), I399V (1896A>G), V400T (1899G>A 1900T>C), N401K (1904T>G), A402V (1906C>T), R476K (2128G>A), K480T (2140A>C), T494D (2181A>G 2182C>A 2183C>T), V500T (2199G>A 2200T>C), M502V (2205A>G 2207G>T), D508E (2225T>A), E521A (2263A>C), S579A (2436T>G), S590T (2469T>A 2471C>T), R601K (2503G>A), K851R (3253A>G), R855K (3265G>A 3266G>A), T857S (3270A>T), T868S (3303A>T 3305C>T), N936S (3508A>G), S948T (3543T>A), T1062A (3885A>G), T1078A (3933A>G), A1087T (3960G>A), M1095V (3984A>G 3986G>T), D1097N (3990G>A), I1120V (4059A>G 4061A>G), N1123S (4069A>G), H1175Y (4224C>T)								

	Begin	End	Coverage	Score	Concordance	Matches	Identities	I/D/M/F*	Stop Codons
**NT**	**25**	**5403**	**99.6%**	**9630**	**89.5%**	**5379 (99.9%)**	**5102 (94.8%)**	**2/0**	
Codon mutations:	GCT7GTT (721C>T), AAG11AGG (733A>G), AAA16AGA (748A>G), AGT17GGT (750A>G), GTT32GTC (797T>C), GGC35GGT (806C>T), AAA42AAG (827A>G), TAT48TTT (844A>T), CAA55CAG (866A>G), GGA89GGG (968A>G), TTG95TTA (986G>A), TTC101TTT (1004C>T), AGT120AGC (1061T>C), AAT121AAC (1064T>C), CTG128CTA (1085G>A), TTT135TTC (1106T>C), TCC136TCT (1109C>T), CCG146CCA (1139G>A), TCA175TCG (1226A>G), ATT190ATC (1271T>C), TCA192TCG (1277A>G), GCT200GTT (1300C>T), CAC205CAT (1316C>T), GAT212GAC (1337T>C), GAG215GAA (1346G>A), CAG218CAA (1355G>A), ATA220ATT (1361A>T), TCA222TCT (1367A>T), ACC228GTC (1383A>G 1384C>T), TCA230TGA (1390C>G), GCA231GCT (1394A>T), CCG237CCT (1412G>T), GGA240GGG (1421A>G), CAG242CCG (1426A>C), GAC245GAT (1436C>T), ACA246ACC (1439A>C), AGC252AGT (1457C>T), GAA253GAG (1460A>G), AGA255AAA (1465G>A), ATC257ATG (1472C>G), CGG258CGC (1475G>C), TCG261TCA (1484G>A), CTC262CTG (1487C>G), CGG263CGA (1490G>A), CTC267CTT (1502C>T), GTG269GTA (1508G>A), ACC282ACT (1547C>T), CAC286CAA (1559C>A), TAC303CAC (1608T>C), GAG306GAA (1619G>A), TTG312TTA (1637G>A), GTT315GTC (1646T>C), AAG317AGG (1651A>G), CTG321TTG (1662C>T), GAT328GAC (1685T>C), GGT331GGC (1694T>C), CCC341CCT (1724C>T), CAA352CAG (1757A>G), GTT365ATT (1794G>A), ATA372ATT (1817A>T), ATC373AAG (1819T>A 1820C>G), TTG382TTT (1847G>T), GAG383GAA (1850G>A), ACC386ACT (1859C>T), CAC388TAC (1863C>T), CTG394TTG (1881C>T), TAC397TAT (1892C>T), ATT399GTT (1896A>G), GTG400ACG (1899G>A 1900T>C), AAT401AAG (1904T>G), GCC402GTC (1906C>T), CTG405TTG (1914C>T), GAG422GAA (1967G>A), CTA423CTG (1970A>G), TAT431TAC (1994T>C), GAG457GAA (2072G>A), GTG461GTA (2084G>A), AGA469AGG (2108A>G), AGG476AAG (2128G>A), CAG478CAA (2135G>A), GTG479GTT (2138G>T), AAA480ACA (2140A>C), ACT486ACA (2159T>A), ACC494GAT (2181A>G 2182C>A 2183C>T), TTC496TTT (2189C>T), GTT500ACT (2199G>A 2200T>C), ATG502GTT (2205A>G 2207G>T), GGC505GGG (2216C>G), GAT508GAA (2225T>A), GCT516GCC (2249T>C), GAA521GCA (2263A>C), GTA523GTG (2270A>G), GTT528GTC (2285T>C), CAA534CAG (2303A>G), AAT537AAC (2312T>C), GGT540GGC (2321T>C), GCG541GCA (2324G>A), TTC558TTT (2375C>T), AAG568AAA (2405G>A), AAA569AAG (2408A>G), AGT571AGC (2414T>C), GAG576GAA (2429G>A), GTG577GTT (2432G>T), TCA579GCA (2436T>G), CTC586CTG (2459C>G), GGT588GGC (2465T>C), AAA589AAG (2468A>G), TCC590ACT (2469T>A 2471C>T), CTA600CTC (2501A>C), AGG601AAG (2503G>A), GCA606GCG (2519A>G), TCG623TCA (2570G>A), GGT624GGC (2573T>C), AAC637AAT (2612C>T), ATC643ATT (2630C>T), CAT653CAC (2660T>C), ACA669ACG (2708A>G), ATC673ATT (2720C>T), GTT677GTC (2732T>C), CAC697CAT (2792C>T), GAG706GAA (2819G>A), CAT713CAC (2840T>C), CTG714TTG (2841C>T), CGT728CGC (2885T>C), CGA746CGG (2939A>G), TAC756TAT (2969C>T), TTA768CTA (3003T>C), ACG783ACA (3050G>A), ATT807ATC (3122T>C), ATT809ATC (3128T>C), GAG810GAA (3131G>A), CTG823TTG (3168C>T), AAT838AAC (3215T>C), GTT842GTG (3227T>G), AAC845AAT (3236C>T), CTT846CTG (3239T>G), GAG847GAA (3242G>A), AAG851AGG (3253A>G), AGG855AAA (3265G>A 3266G>A), TGT856TGC (3269T>C), ACG857TCG (3270A>T), AAT865AAC (3296T>C), ACC868TCT (3303A>T 3305C>T), GGT870GGA (3311T>A), TCT874TCC (3323T>C), CTA875TTA (3324C>T), CGG877CGA (3332G>A), CCC878CCT (3335C>T), AGG885CGG (3354A>C), ACA889ACT (3368A>T), GTA895GTT (3386A>T), TCA898TCC (3395A>C), CTA901CTG (3404A>G), GTC904GTT (3413C>T), CAG909CAA (3428G>A), CTT910TTG (3429C>T 3431T>G), TGC912TGT (3437C>T), ATA913ATT (3440A>T), TTG916CTG (3447T>C), GGC920GGT (3461C>T), TCT923TCC (3470T>C), TAC928TAT (3485C>T), CAC930CAT (3491C>T), ACT932ACC (3497T>C), GGA934GGG (3503A>G), AAT936AGT (3508A>G), CTA941CTG (3524A>G), TTC942TTT (3527C>T), GAG944GAA (3533G>A), TTG947CTG (3540T>C), TCT948ACT (3543T>A), ATT953ATA (3560T>A), TTA956TTG (3569A>G), AAG958AAA (3575G>A), AAC960AAT (3581C>T), ACA963ACC (3590A>C), GGA968GGG (3605A>G), CAA975CAG (3626A>G), AGA979AGG (3638A>G), GTA987GTT (3662A>T), GTG991GTA (3674G>A), TGC1005TGT (3716C>T), CCC1007CCT (3722C>T), CAG1014CAA (3743G>A), ATC1022ATT (3767C>T), TGC1024TGT (3773C>T), TTC1033TTT (3800C>T), TAC1042TAT (3827C>T), AAC1054AAT (3863C>T), GGA1056GGT (3869A>T), GGA1057GGT (3872A>T), ACC1062GCC (3885A>G), GAT1064GAC (3893T>C), TTT1070TTC (3911T>C), CCT1071CCA (3914T>A), GGA1073GGT (3920A>T), GGC1077GGA (3932C>A), ACC1078GCC (3933A>G), GTT1080GTG (3941T>G), GTC1081GTA (3944C>A), GCT1083GCA (3950T>A), GCA1087ACA (3960G>A), ATG1095GTT (3984A>G 3986G>T), GAC1097AAC (3990G>A), AAC1098AAT (3995C>T), TTT1100TTC (4001T>C), GTG1104GTC (4013G>C), CGC1113CGT (4040C>T), ACC1115ACT (4046C>T), ATA1120GTG (4059A>G 4061A>G), AAT1123AGT (4069A>G), CGA1133CGT (4100A>T), CTC1137CTT (4112C>T), GCC1143GCT (4130C>T), ATC1152ATT (4157C>T), GCT1167GCC (4202T>C), CCT1168CCA (4205T>A), TCA1169TCT (4208A>T), CAT1175TAT (4224C>T), ACC1183ACT (4250C>T)								

*: Inserts / Deletes / Misaligned / Frameshifts

Analysis details

This analysis was performed with panviral2.64

NGS Details (UN9): Potato virus Y

Assembly

**Coverage Length**	9700 (1 contig(s))
**Depth Of Coverage**	11166.5
**Number Of Reads**	812319
**Reads Per Million**	18360.73 rpm (after QC)
**Ambiguities**	0
**Assembly Method**	de novo + reference guided assembly
**Consensus Caller**	Bcf Tools

Coverage Map

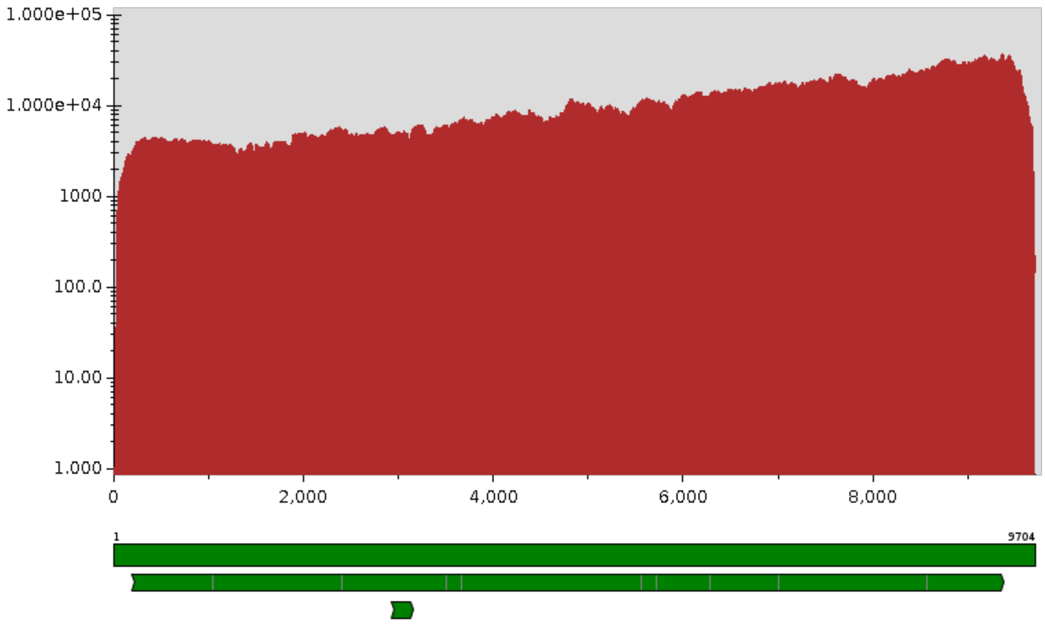

Assignment

**Type**	Potato virus Y (Taxonomy ID: 12216)
**Reference Genome**	NC_001616.1
**NT Identity (%)**	82.6033
**AA Identity (%)**	89.9682
**Number Of Stop Codons**	3
**Number Of CDS**	2

Alignment

**Alignment Score**	12522.0 (NT) + 19018.0 (AA) = 31540.0
**Concordance (%)**	78.0601

Alignment Method	Global, seeded, nucleotide + amino acids (AGA)

Genome Region

Sequence starts at position 5 and ends at position 9704 relative to NC_001616.1 reference sequence.

Alignment Detailed Statistics

	Begin	End	Coverage	Score	Concordance	Matches	Identities	I/D/M/F*	Stop Codons
NT	5	9704	99.9%	12522	64.8%	9688 (99.7%)	8015 (82.5%)	15/12	

Mutations:

5A>G, 7A>G, 12C>G, 13T>G, 14C>G, 21A>C, 22C>T, 23A>T, 32A>T, 42_43insGAAATAT, 45A>T, 47T>A, 50T>G, 53A>G, 54C>T, 55G>T, 56C>T, 58C>T, 60T>A, 60_61insA, 75C>T, 79C>T, 83G>A, 85T>C, 87C>T, 89G>A, 90T>C, 93A>T, 94A>G, 95A>T, 100T>C, 101T>A, 102C>T, 104T>C, 107C>T, 110T>G, 112C>T, 114C>T, 115T>C, 116A>T, 117G>C, 119A>G, 120C>T, 121A>T, 125T>G, 126T>C, 127G>A, 128G>T, 131A>G, 132C>T, 139A>T, 139_140insG, 141C>T, 143C>T, 146C>T, 150C>T, 150_151insT, 154T>C, 156C>A, 158T>A, 160A>C, 164C>T, 168C>A, 169C>G, 171A>C, 172T>C, 173T>A, 174T>G, 177G>C, 178A>G, 179T>C, 180C>T, 181C>A, 182T>G, 183C>T, 193T>C, 194T>C, 196C>A, 202A>G, 208C>T, 209T>C, 210G>T, 211T>G, 220G>A, 221T>A, 223T>G, 226A>G, 235A>T, 241C>T, 247A>T, 251T>C, 255G>T, 256C>T, 258A>G, 262T>C, 263A>G, 265T>G, 267T>C, 273A>T, 277A>T, 280A>G, 283G>T, 286G>A, 287G>A, 289T>C, 293G>A, 294T>C, 301T>C, 304C>T, 305G>A, 306C>T, 309A>G, 311C>T, 316A>T, 317A>G, 318C>T, 319A>G, 322A>G, 331A>T, 339T>A, 340C>A, 342A>G, 347A>G, 348A>C, 349A>G, 350T>C, 355T>A, 356A>G, 357C>T, 364T>C, 365G>A, 366T>C, 367G>A, 368C>T, 369T>C, 373A>G, 376C>T, 384T>G, 388G>A, 391C>T, 394A>G, 400G>A, 406T>C, 408C>T, 409C>T, 412G>A, 415A>T, 416A>G, 417T>A, 424T>C, 427G>A, 433A>G, 436G>C, 437G>A, 440A>G, 441G>A, 443A>C, 444A>G, 445G>A, 448T>A, 454A>G, 456A>C, 460T>C, 461C>A, 469A>G, 475C>G, 481T>A, 502T>C, 505A>T, 511C>T, 517T>A, 520T>A, 523A>T, 531C>T, 535G>A, 536C>T, 539C>T, 540A>G, 541A>G, 549G>A, 550A>G, 556C>T, 559T>C, 562T>C, 568T>C, 571A>G, 576T>C, 580T>C, 585T>C, 586C>G, 593C>T, 594G>A, 595C>T, 597C>G, 599A>G, 601A>G, 602A>C, 603T>C, 604A>G, 613A>G, 616A>G, 619C>G, 622G>A, 625G>A, 628T>C, 631C>T, 635A>G, 637T>C, 644A>G, 646A>G, 655T>C, 661G>A, 663A>C, 664G>C, 667A>G, 679C>T, 682C>A, 683T>C, 685A>G, 691T>C, 699C>G, 703T>A, 706T>A, 719A>G, 721G>A, 722A>G, 724A>G, 725C>T, 727T>G, 730T>A, 731G>T, 734T>C, 735A>C, 736C>T, 737T>C, 738C>G, 739C>T, 744C>T, 745G>C, 749C>T, 751A>C, 754T>C, 765T>A, 770T>C, 773C>A, 774G>A, 775A>G, 776C>A, 790C>T, 801T>A, 813G>C, 814A>G, 816T>A, 818T>C, 823A>G, 825G>A, 826T>A, 833C>A, 835G>A, 841C>T, 851A>G, 853T>C, 859C>T, 860C>T, 862C>T, 863A>G, 865T>C, 867T>C, 869A>G, 871C>T, 872A>T, 874A>G, 876G>A, 879G>A, 886T>C, 898C>T, 903A>G, 906C>A, 907A>C, 909A>C, 910A>C, 912G>A, 913C>T, 922C>A, 931T>A, 938T>C, 939C>T, 940A>T, 943A>G, 952C>T, 967A>G, 973A>G, 976G>C, 980T>A, 982G>C, 991A>G, 994T>C, 999G>A, 1003T>C, 1007C>A, 1008A>G, 1009G>A, 1010A>G, 1012T>G, 1013A>G, 1018A>G, 1019A>C, 1021C>G, 1030C>T, 1039G>A, 1042T>C, 1045C>A, 1048C>A, 1051T>C, 1060G>A, 1063T>A, 1064C>T, 1069C>T, 1083G>A, 1093T>C, 1097T>G, 1099G>A, 1105C>T, 1114A>G, 1117T>G, 1123A>G, 1126T>A, 1129C>T, 1143G>A, 1147A>T, 1150T>A, 1154T>A, 1156G>A, 1165C>T, 1168T>C, 1172C>T, 1174T>A, 1201C>T, 1221G>A, 1229G>A, 1230T>C, 1231T>C, 1233G>A, 1234C>T, 1240G>A, 1246G>A, 1250T>C, 1252G>A, 1255T>C, 1257A>C, 1258A>G, 1267A>G, 1273T>C, 1276G>A, 1278A>G, 1283T>C, 1290C>T, 1291G>A, 1298G>A, 1299A>C, 1300C>T, 1306T>C, 1307A>G, 1309A>G, 1312T>C, 1316A>G, 1318T>A, 1327G>A, 1329T>C, 1333G>T, 1336A>G, 1343C>T, 1345A>G, 1354G>A, 1357G>T, 1360C>T, 1367C>T, 1369C>G, 1373C>A, 1378C>T, 1384G>A, 1385A>G, 1393A>G, 1396C>T, 1405G>T, 1408A>G, 1411G>A, 1415G>T, 1417A>T, 1420G>C, 1429T>C, 1432A>G, 1436G>A, 1438C>T, 1439T>C, 1441A>G, 1444T>C, 1453C>T, 1456G>A, 1468A>G, 1492A>G, 1499T>C, 1501G>A, 1504T>C, 1507G>A, 1510C>T, 1513A>G, 1516A>G, 1519A>T, 1522G>A, 1525C>T, 1528G>A, 1534C>T, 1540T>A, 1546C>T, 1555A>G, 1558T>C, 1561T>C, 1564A>C, 1567T>A, 1573C>T, 1576A>G, 1583T>C, 1588T>A, 1594G>A, 1600C>T, 1609G>A, 1618G>A, 1621C>T, 1624C>T, 1631C>T, 1639A>G, 1651C>T, 1654C>G, 1675T>C, 1693A>G, 1702T>C, 1707A>G, 1718G>A, 1726A>G, 1729C>T, 1732A>C, 1738T>C, 1742A>G, 1744C>T, 1747C>T, 1753T>C, 1758G>A, 1768G>A, 1786C>T, 1789A>G, 1790G>A, 1795C>T, 1805T>C, 1807A>G, 1813G>A, 1819G>A, 1825G>A, 1837C>T, 1840T>C, 1849A>G, 1858C>G, 1864A>G, 1870C>T, 1876T>C, 1882A>G, 1888T>A, 1891T>C, 1906T>C, 1915A>T, 1930T>C, 1936C>T, 1937A>G, 1939T>C, 1945A>G, 1951C>T, 1960A>T, 1978A>G, 1981T>A, 1984C>T, 1987T>C, 1996C>T, 2004T>A, 2020A>G, 2023G>A, 2026T>C, 2029G>T, 2032G>A, 2059T>C, 2068T>G, 2071C>T, 2072G>A, 2074G>A, 2087C>T, 2089G>A, 2101C>T, 2104G>A, 2107G>A, 2116G>A, 2122C>T, 2137C>T, 2158T>C, 2164C>T, 2170A>G, 2182T>C, 2188G>A, 2191C>A, 2208G>A, 2218T>C, 2224C>T, 2227A>T, 2242G>A, 2245C>T, 2254G>A, 2257T>C, 2260C>T, 2266C>G, 2272A>G, 2275G>A, 2293A>G, 2299C>T, 2302G>A, 2305G>A, 2314A>T, 2317T>C, 2326A>G, 2341G>A, 2347A>G, 2350T>C, 2356G>A, 2365T>C, 2371A>G, 2374A>G, 2383T>C, 2386A>T, 2389A>G, 2392T>C, 2395T>C, 2414A>G, 2415A>G, 2416T>G, 2419T>A, 2420A>T, 2440A>T, 2467T>C, 2476T>C, 2482G>A, 2491G>A, 2497G>T, 2503G>A, 2518A>G, 2521T>C, 2523A>G, 2533A>G, 2539G>A, 2545A>G, 2551G>A, 2554T>A, 2558C>T, 2561T>C, 2563G>C, 2572A>G, 2584T>C, 2587C>T, 2593G>C, 2599T>C, 2608T>C, 2620T>C, 2629G>A, 2632G>A, 2634G>A, 2638G>A, 2644T>A, 2653A>G, 2665T>C, 2678C>T, 2683A>G, 2689T>C, 2698A>C, 2707A>G, 2710G>A, 2716A>G, 2719A>G, 2722C>T, 2723G>A, 2725C>T, 2728A>G, 2734G>A, 2737T>A, 2746T>C, 2747G>A, 2749T>A, 2752A>T, 2755T>A, 2761C>T, 2764C>T, 2779T>C, 2785G>T, 2791C>T, 2795C>A, 2806C>T, 2816T>C, 2818G>C, 2822G>A, 2824G>A, 2827G>A, 2836T>C, 2839G>A, 2842T>C, 2863C>A, 2864C>T, 2882C>A, 2885A>C, 2886G>A, 2893C>T, 2895C>T, 2902C>T, 2905A>G, 2911T>C, 2923T>C, 2931A>G, 2940A>G, 2947T>C, 2962T>C, 2971_2972insA, 2989T>C, 2991C>T, 2994A>G, 2999G>A, 3000A>G, 3016delC, 3021G>A, 3024A>T, 3025C>T, 3036C>T, 3039A>G, 3040A>C, 3042A>C, 3043G>A, 3052G>A, 3061A>G, 3082G>A, 3091C>T, 3099C>T, 3100C>A, 3104G>A, 3105T>G, 3106G>A, 3109C>T, 3112A>G, 3118T>C, 3121C>T, 3127A>G, 3132G>A, 3137C>A, 3138G>A, 3139A>G, 3151T>C, 3152T>A, 3154C>T, 3165G>A, 3169A>G, 3199C>T, 3203A>C, 3208T>C, 3214A>G, 3217C>T, 3218T>C, 3223C>A, 3225C>A, 3230G>A, 3235C>T, 3257T>C, 3262C>T, 3265C>T, 3266G>A, 3268A>G, 3271G>A, 3275G>A, 3284G>A, 3301G>A, 3315G>A, 3316A>G, 3318A>C, 3322C>T, 3325G>A, 3328G>A, 3337A>T, 3349G>A, 3355C>T, 3358C>T, 3359A>G, 3367A>G, 3370T>C, 3377T>C, 3379A>G, 3394A>G, 3397C>T, 3400T>G, 3406A>G, 3418C>T, 3421T>A, 3427T>C, 3433G>A, 3436A>T, 3439A>G, 3442C>T, 3445T>C, 3457G>A, 3460T>C, 3463T>A, 3469G>T, 3472G>A, 3496C>T, 3499G>A, 3505C>T, 3513T>G, 3520A>G, 3529A>G, 3532A>G, 3545A>G, 3548G>A, 3551T>C, 3554G>A, 3556C>T, 3583T>C, 3586T>C, 3589C>T, 3595C>T, 3607T>C, 3613T>C, 3616G>A, 3619T>C, 3623C>G, 3628C>T, 3632C>A, 3637T>C, 3638T>C, 3643A>G, 3661A>G, 3670G>A, 3682T>C, 3688G>A, 3697G>A, 3698A>G, 3706T>C, 3709T>C, 3713T>C, 3715G>A, 3717G>A, 3721G>A, 3731C>A, 3732G>A, 3736T>A, 3746C>T, 3748A>G, 3754A>G, 3763T>C, 3778A>G, 3781A>G, 3784C>T, 3796T>C, 3805A>T, 3811C>T, 3814A>G, 3829C>T, 3838T>C, 3844A>G, 3847A>C, 3856T>A, 3862G>T, 3871C>T, 3877C>A, 3883C>T, 3886A>G, 3892A>G, 3895C>T, 3898T>C, 3904A>G, 3905C>A, 3907G>A, 3913T>C, 3928G>A, 3931A>T, 3937G>A, 3955T>C, 3958G>A, 3961C>T, 3964A>G, 3967T>C, 3976A>G, 3979T>C, 3985A>T, 3997A>T, 4000G>A, 4006C>T, 4009T>G, 4012C>T, 4015A>G, 4024T>C, 4030A>G, 4033A>T, 4042G>A, 4052C>T, 4054G>A, 4057T>C, 4063T>A, 4066A>G, 4073A>G, 4078T>C, 4081C>T, 4084T>C, 4096C>T, 4108C>T, 4111A>T, 4117G>A, 4118C>T, 4120A>G, 4126C>T, 4128T>A, 4129C>T, 4132C>T, 4135T>C, 4147A>G, 4148T>C, 4151G>T, 4162C>T, 4166G>A, 4171A>C, 4180G>A, 4190C>T, 4198T>C, 4201C>T, 4210G>T, 4213C>T, 4219T>C, 4220C>T, 4223C>A, 4225G>A, 4231T>G, 4237T>C, 4252A>G, 4253T>C, 4255A>G, 4258A>G, 4270T>C, 4282A>G, 4285G>A, 4291A>G, 4303G>A, 4312C>T, 4319A>G, 4321A>G, 4324G>T, 4327G>A, 4336G>T, 4339T>C, 4348A>T, 4360A>G, 4366T>C, 4369T>A, 4375T>C, 4387T>C, 4395T>A, 4406G>A, 4411T>C, 4438T>C, 4444C>A, 4450C>T, 4456C>T, 4465C>T, 4475A>G, 4477G>T, 4480C>T, 4481A>T, 4483A>G, 4495C>T, 4507G>A, 4510C>T, 4513T>G, 4517C>T, 4528C>T, 4534A>G, 4540C>T, 4544G>T, 4545C>T, 4546G>A, 4548G>A, 4558T>C, 4564A>T, 4570C>T, 4576A>T, 4597G>A, 4600C>T, 4606C>T, 4609G>A, 4615G>A, 4618C>T, 4625T>C, 4627G>T, 4648C>T, 4651T>C, 4660G>A, 4663C>T, 4669T>A, 4679G>A, 4681G>C, 4684T>C, 4687T>G, 4699G>A, 4702A>G, 4708A>G, 4711G>A, 4712C>T, 4738A>G, 4744A>T, 4747A>G, 4748T>C, 4765T>C, 4796G>A, 4798C>T, 4803C>G, 4804T>C, 4807G>A, 4810G>T, 4813T>G, 4816T>C, 4831T>C, 4834C>T, 4840A>T, 4852A>G, 4855C>T, 4867T>C, 4870G>C, 4873T>A, 4882T>C, 4903A>T, 4909G>A, 4915T>C, 4918A>G, 4919T>C, 4921G>A, 4924T>C, 4927C>G, 4933T>C, 4936C>A, 4939G>A, 4942T>C, 4945C>T, 4963A>G, 4972T>C, 4990T>C, 5002A>G, 5023T>C, 5024T>C, 5029C>T, 5032T>C, 5038T>C, 5044A>T, 5053A>T, 5056G>A, 5057A>G, 5060A>G, 5071G>T, 5090C>A, 5095A>G, 5098G>T, 5100C>T, 5104A>G, 5107A>T, 5110T>C, 5113A>G, 5116G>A, 5119A>G, 5120G>T, 5122C>T, 5126A>G, 5137T>C, 5140C>T, 5143A>G, 5146G>A, 5161C>A, 5167A>G, 5173T>G, 5182G>A, 5183G>A, 5188C>T, 5191G>A, 5197A>G, 5200C>T, 5206C>T, 5209A>G, 5218C>T, 5230G>A, 5236C>T, 5239C>T, 5245C>T, 5263A>T, 5269C>T, 5272C>T, 5275C>A, 5278C>T, 5281A>C, 5284A>G, 5287T>C, 5288C>T, 5293A>T, 5294T>C, 5296G>A, 5302G>A, 5311T>A, 5314A>G, 5323A>T, 5347T>C, 5353C>T, 5365C>T, 5386T>C, 5395C>A, 5422A>G, 5425T>C, 5431C>T, 5437G>A, 5452T>C, 5458G>A, 5467T>C, 5470A>G, 5471C>T, 5473A>G, 5479A>G, 5482C>T, 5485A>C, 5488T>C, 5489T>C, 5491G>A, 5497T>C, 5506T>C, 5509G>A, 5512G>A, 5516T>C, 5530T>C, 5533G>A, 5536G>T, 5539G>A, 5548T>G, 5554C>T, 5560T>A, 5561G>A, 5563G>A, 5566G>C, 5569A>G, 5584C>G, 5593G>A, 5596G>A, 5597A>G, 5605C>A, 5617G>T, 5619C>T, 5620T>G, 5629G>A, 5630A>C, 5641C>T, 5650A>T, 5662A>T, 5665A>G, 5689A>G, 5693G>A, 5698G>A, 5713A>G, 5716G>C, 5719A>G, 5743C>A, 5755C>A, 5758T>C, 5764T>C, 5770A>G, 5779C>T, 5782T>C, 5791C>T, 5803C>T, 5836G>A, 5839A>G, 5845A>G, 5848A>G, 5853A>G, 5857T>C, 5860C>T, 5863A>T, 5869T>A, 5875T>C, 5884C>T, 5893C>T, 5894A>G, 5896C>G, 5899C>T, 5908G>A, 5911T>C, 5923G>A, 5938A>G, 5941C>T, 5944T>C, 5947T>C, 5959G>A, 5960C>G, 5961G>C, 5962G>C, 5974A>G, 5986T>A, 5989C>T, 5990A>G, 5998T>C, 6008A>C, 6010A>T, 6019A>T, 6022G>T, 6025A>T, 6030A>C, 6049C>A, 6058G>A, 6061A>G, 6064C>A, 6070T>C, 6072G>A, 6073T>C, 6074A>C, 6079G>A, 6081C>A, 6085A>T, 6091A>T, 6094C>T, 6111G>C, 6112T>C, 6118A>G, 6124G>A, 6127G>A, 6128A>G, 6130T>C, 6133T>C, 6136A>G, 6148C>T, 6157A>G, 6158G>A, 6163T>C, 6169A>G, 6170A>T, 6172A>G, 6178C>T, 6187A>G, 6190T>C, 6196G>A, 6203C>T, 6205C>T, 6208A>G, 6209C>T, 6211A>G, 6217G>A, 6229T>A, 6230G>A, 6238C>T, 6247G>A, 6253A>T, 6257G>A, 6258C>A, 6274T>C, 6282A>G, 6286G>A, 6298C>T, 6301G>A, 6304A>G, 6307C>T, 6322C>T, 6328A>T, 6331T>G, 6334T>C, 6337G>A, 6338C>T, 6358T>C, 6361G>A, 6362G>A, 6367A>T, 6370G>A, 6376C>T, 6379T>G, 6385A>C, 6391A>T, 6394A>G, 6397C>T, 6401G>A, 6405C>T, 6412C>T, 6418A>G, 6421T>C, 6429A>T, 6436T>A, 6445G>A, 6450A>G, 6454C>G, 6460C>T, 6463T>A, 6469C>T, 6472G>A, 6475G>A, 6481T>C, 6485C>A, 6487C>T, 6490T>C, 6496C>T, 6502G>A, 6514T>C, 6517G>A, 6527C>A, 6532C>A, 6541G>A, 6544A>G, 6556C>T, 6559T>C, 6565G>A, 6569T>C, 6571G>A, 6586T>A, 6588C>T, 6598A>G, 6601A>G, 6604T>C, 6607T>C, 6616A>G, 6622C>T, 6631G>A, 6634G>A, 6635T>C, 6640T>A, 6646G>T, 6647A>G, 6652C>T, 6664C>T, 6670T>G, 6677A>G, 6679A>G, 6685C>T, 6691A>T, 6694C>T, 6715A>G, 6716G>A, 6719A>G, 6724A>G, 6734C>T, 6742G>A, 6745G>A, 6754C>T, 6760A>G, 6764A>T, 6766A>G, 6775T>A, 6782C>T, 6787A>T, 6790C>T, 6795C>T, 6796A>G, 6799C>A, 6802C>A, 6808C>T, 6811C>T, 6814C>T, 6823C>T, 6829A>G, 6847C>T, 6853A>C, 6857A>G, 6862G>A, 6871A>G, 6875G>A, 6876T>C, 6877C>A, 6880G>A, 6883T>A, 6889T>G, 6901C>T, 6916C>T, 6919G>A, 6925A>G, 6928T>C, 6934C>A, 6949G>C, 6955C>T, 6958A>G, 6976A>G, 6980C>T, 6985C>T, 6997A>T, 7000G>T, 7001G>A, 7003G>A, 7012T>C, 7015G>A, 7018T>C, 7032T>A, 7039C>T, 7040T>C, 7042G>A, 7048A>G, 7060T>A, 7063C>A, 7078C>T, 7082T>C, 7084A>G, 7087A>G, 7090C>A, 7096T>C, 7099A>G, 7102T>C, 7111G>A, 7117A>G, 7125C>A, 7129A>G, 7132T>C, 7133C>T, 7135G>A, 7141G>A, 7144T>C, 7145G>T, 7147A>G, 7150G>A, 7159A>T, 7160_7165delGAGGCA, 7171C>T, 7189G>T, 7198A>G, 7201C>T, 7205C>T, 7210T>C, 7216T>A, 7219G>A, 7225C>A, 7228G>A, 7240G>A, 7243T>C, 7249A>G, 7252T>G, 7258T>A, 7261T>C, 7264T>A, 7267C>G, 7270G>A, 7273_7274insT, 7274C>G, 7275G>T, 7281A>T, 7284T>C, 7296C>T, 7311C>T, 7314C>T, 7317G>A, 7321T>A, 7322delG, 7327C>T, 7330C>T, 7333C>T, 7334A>C, 7342T>C, 7343G>A, 7344C>A, 7345A>C, 7354T>C, 7363A>G, 7369T>C, 7372C>T, 7378G>T, 7381C>T, 7396A>T, 7399C>T, 7405C>T, 7407G>T, 7408T>G, 7414G>T, 7415T>G, 7420A>G, 7429C>T, 7432T>C, 7441T>C, 7459G>A, 7462A>G, 7468A>T, 7471C>A, 7477A>G, 7480C>T, 7486G>T, 7495T>C, 7498A>G, 7501T>C, 7503T>C, 7504G>A, 7513T>A, 7519C>T, 7522A>G, 7531G>A, 7537G>A, 7540C>T, 7546T>C, 7570G>A, 7573G>A, 7579G>A, 7588T>C, 7595C>T, 7604T>C, 7606G>A, 7615T>A, 7624T>C, 7633C>T, 7636C>T, 7639T>C, 7648T>C, 7666A>G, 7672T>C, 7678A>T, 7684G>A, 7690T>C, 7696T>C, 7702T>G, 7709G>A, 7713A>G, 7714A>G, 7715C>T, 7726T>C, 7735G>A, 7744A>G, 7747C>T, 7762C>T, 7768G>A, 7771T>C, 7780A>G, 7783A>C, 7786T>C, 7793C>T, 7795A>G, 7798C>T, 7804T>C, 7812C>T, 7834A>G, 7837C>T, 7844G>T, 7846G>A, 7855G>A, 7861G>A, 7870A>G, 7873C>T, 7876T>A, 7879G>A, 7882T>A, 7883G>A, 7894A>T, 7897T>C, 7921C>T, 7927G>A, 7936T>C, 7939T>C, 7942T>C, 7966G>A, 7969T>C, 7975T>C, 7987C>T, 7990T>C, 8005T>C, 8032A>G, 8038C>T, 8041C>T, 8043G>A, 8080G>A, 8089G>A, 8095G>A, 8098G>A, 8107C>T, 8110T>C, 8116T>C, 8128A>G, 8146T>C, 8151A>G, 8152C>T, 8164G>A, 8178G>A, 8182G>A, 8191G>A, 8197C>T, 8203C>T, 8208G>A, 8213C>T, 8215G>A, 8218A>G, 8221C>T, 8224G>A, 8239A>G, 8254G>A, 8260T>C, 8263A>G, 8281T>C, 8284A>G, 8294C>G, 8296A>G, 8299G>A, 8302C>T, 8317T>C, 8320C>T, 8326T>A, 8335G>A, 8338C>T, 8349C>T, 8353A>G, 8362C>T, 8365A>G, 8368C>T, 8380C>T, 8383A>G, 8389A>G, 8404T>C, 8408G>T, 8413A>G, 8419G>T, 8434T>C, 8440T>C, 8452G>A, 8458A>G, 8460G>A, 8464A>G, 8467G>A, 8479G>A, 8480G>A, 8482T>A, 8497G>A, 8498C>T, 8500A>G, 8502G>A, 8503A>G, 8506C>T, 8515A>T, 8524C>T, 8527A>C, 8528T>C, 8530A>G, 8539G>C, 8546C>T, 8547T>G, 8552T>A, 8563A>G, 8574C>G, 8585A>G, 8598G>A, 8602C>T, 8603A>T, 8604A>C, 8605C>T, 8620A>G, 8622C>A, 8625A>G, 8626G>A, 8629G>A, 8632C>T, 8638C>T, 8641G>A, 8647C>T, 8648C>T, 8649C>T, 8650G>C, 8653C>T, 8656A>G, 8658G>A, 8664A>C, 8665T>G, 8674T>G, 8679C>T, 8680A>T, 8683C>A, 8692G>A, 8695A>T, 8707G>A, 8708A>C, 8716G>A, 8722C>T, 8725G>A, 8731A>G, 8737A>G, 8743C>T, 8745C>A, 8746A>G, 8752G>A, 8755A>T, 8770C>T, 8771T>C, 8776A>G, 8779T>C, 8782G>A, 8791T>C, 8797A>G, 8800A>G, 8803A>G, 8809T>C, 8812T>C, 8818T>C, 8824G>A, 8827A>G, 8848G>A, 8854T>C, 8856A>G, 8857G>A, 8863G>A, 8865G>A, 8867A>C, 8869G>T, 8872A>G, 8883G>A, 8888A>T, 8929C>T, 8954G>A, 8959C>T, 8962A>T, 8983G>A, 8984A>G, 8986T>C, 8988A>T, 8998G>A, 9004G>A, 9007G>A, 9016C>T, 9037C>A, 9085A>T, 9097C>T, 9131A>G, 9136A>T, 9145G>T, 9149A>G, 9150T>G, 9151G>A, 9152G>A, 9157A>G, 9160G>T, 9163T>C, 9184G>A, 9196A>G, 9214G>A, 9217A>G, 9220G>A, 9244A>T, 9247G>A, 9256C>T, 9260C>T, 9262T>C, 9274G>A, 9280C>T, 9289C>T, 9298A>C, 9304G>A, 9334C>T, 9364C>G, 9377_9378insT, 9387C>T, 9404G>A, 9411C>T, 9412A>G, 9414A>T, 9437C>T, 9454A>G, 9458T>C, 9467G>A, 9470_9471insT, 9482G>A, 9483C>T, 9498T>C, 9516T>G, 9517delA, 9533G>A, 9534G>T, 9548A>T, 9551C>T, 9553_9554delTA, 9556A>T, 9557T>A, 9558A>T, 9574T>A, 9581G>T, 9582T>G, 9583G>C, 9584_9585insG, 9590C>T, 9591T>C, 9607C>T, 9610T>C, 9615delC, 9620A>G, 9648A>G, 9674T>C, 9690C>T

	Begin	End	Coverage	Score	Concordance	Matches	Identities	I/D/M/F*	Stop Codons
**NT**	**5**	**9704**	**99.9%**	**12522**	**64.8%**	**9688 (99.7%)**	**8015 (82.5%)**	**15/12**	
**CDS**									
PVYgp1	1	3064	100%	18923	89.7%	3062 (99.9%)	2771 (90.4%)	2/2/4/4	1
Protein mutations:	Y4Q (194T>C 196C>A), C9L (209T>C 210G>T 211T>G), F13M (221T>A 223T>G), S23P (251T>C), C24F (255G>T 256C>T), E25G (258A>G), I27V (263A>G 265T>G), V28A (267T>C), E30V (273A>T), A35T (287G>A 289T>C), V37T (293G>A 294T>C), A41I (305G>A 306C>T), D42G (309A>G), E44D (316A>T), T45V (317A>G 318C>T 319A>G), L52Q (339T>A 340C>A), K53R (342A>G), K55A (347A>G 348A>C 349A>G), Y56H (350T>C), T58V (356A>G 357C>T), V61T (365G>A 366T>C 367G>A), L62S (368C>T 369T>C), F67C (384T>G), A75V (408C>T 409C>T), M78E (416A>G 417T>A), E85K (437G>A), R86E (440A>G 441G>A), K87R (443A>C 444A>G 445G>A), D88E (448T>A), E91A (456A>C), H93N (461C>A), D111E (517T>A), S116F (531C>T), P118S (536C>T), Q119W (539C>T 540A>G 541A>G), R122K (549G>A 550A>G), M131T (576T>C), V134A (585T>C 586C>G), R137Y (593C>T 594G>A 595C>T), P138R (597C>G), I139V (599A>G 601A>G), I140P (602A>C 603T>C 604A>G), M147I (625G>A), I151V (635A>G 637T>C), I154V (644A>G 646A>G), E160A (663A>C 664G>C), H166Q (682C>A), T172S (699C>G), H174Q (706T>A), K179E (719A>G 721G>A), I180V (722A>G 724A>G), A183S (731G>T), Y184P (734T>C 735A>C 736C>T), S185R (737T>C 738C>G 739C>T), A187V (744C>T 745G>C), R189C (749C>T 751A>C), M194K (765T>A), R197K (773C>A 774G>A 775A>G), M206K (801T>A), G210A (813G>C 814A>G), L211H (816T>A), R214Q (825G>A 826T>A), N223D (851A>G 853T>C), R226C (860C>T 862C>T), T227A (863A>G 865T>C), I228T (867T>C), N229D (869A>G 871C>T), I230L (872A>T 874A>G), R231Q (876G>A), R232K (879G>A), N240S (903A>G), T241N (906C>A 907A>C), K242T (909A>C 910A>C), S243N (912G>A 913C>T), S252L (938T>C 939C>T 940A>T), L266I (980T>A 982G>C), R272K (999G>A), Q275R (1007C>A 1008A>G 1009G>A), S276G (1010A>G 1012T>G), I277V (1013A>G), N279Q (1019A>C 1021C>G), D288E (1048C>A), R300Q (1083G>A), S305A (1097T>G 1099G>A), R320K (1143G>A), L324I (1154T>A 1156G>A), S346N (1221G>A), V349T (1229G>A 1230T>C 1231T>C), S350N (1233G>A 1234C>T), K358T (1257A>C 1258A>G), N365S (1278A>G), A369V (1290C>T 1291G>A), D372T (1298G>A 1299A>C 1300C>T), I375V (1307A>G 1309A>G), N378E (1316A>G 1318T>A), I382T (1329T>C), L397I (1373C>A), I401V (1385A>G), E407D (1405G>T), A411S (1415G>T 1417A>T), V418I (1436G>A 1438C>T), E508G (1707A>G), A512T (1718G>A), I520V (1742A>G 1744C>T), S525N (1758G>A), V536I (1790G>A), I585V (1937A>G 1939T>C), F607Y (2004T>A), I628M (2068T>G), V630I (2072G>A 2074G>A), R675K (2208G>A), D694E (2266C>G), N744G (2414A>G 2415A>G 2416T>G), S746C (2420A>T), K780R (2523A>G), R817K (2634G>A), V847I (2723G>A 2725C>T), A855T (2747G>A 2749T>A), H871N (2795C>A), V880I (2822G>A 2824G>A), P900T (2882C>A), S901H (2885A>C 2886G>A), T904M (2895C>T), N916S (2931A>G), N919S (2940A>G), E929_N930insX (2971_2972insA), T936I (2991C>T), H937R (2994A>G), E939R (2999G>A 3000A>G), R946Q (3021G>A), Y947F (3024A>T 3025C>T), T951I (3036C>T), E952G (3039A>G 3040A>C), K953T (3042A>C 3043G>A), A972V (3099C>T 3100C>A), V974R (3104G>A 3105T>G 3106G>A), S983N (3132G>A), R985K (3137C>A 3138G>A 3139A>G), F990I (3152T>A 3154C>T), C994Y (3165G>A), F1012L (3218T>C), T1014N (3225C>A), V1016I (3230G>A), V1028M (3266G>A 3268A>G), V1031M (3275G>A), A1034T (3284G>A), R1044K (3315G>A 3316A>G), E1045A (3318A>C), M1059V (3359A>G), D1072E (3400T>G), V1110G (3513T>G), M1121V (3545A>G), A1122T (3548G>A), V1124I (3554G>A 3556C>T), L1147V (3623C>G), L1150M (3632C>A), Y1152H (3638T>C), I1172V (3698A>G), S1178N (3717G>A), R1183K (3731C>A 3732G>A), I1297V (4073A>G), F1315Y (4128T>A 4129C>T), A1323S (4151G>T), V1328I (4166G>A), L1347I (4223C>A 4225G>A), I1379V (4319A>G 4321A>G), F1404Y (4395T>A), V1408I (4406G>A), M1431V (4475A>G 4477G>T), T1433S (4481A>T 4483A>G), A1454L (4544G>T 4545C>T 4546G>A), R1455K (4548G>A), V1499I (4679G>A 4681G>C), V1538I (4796G>A 4798C>T), T1540S (4803C>G 4804T>C), S1625G (5057A>G), T1626A (5060A>G), L1636I (5090C>A), A1639V (5100C>T), E1641D (5107A>T), V1646F (5120G>T 5122C>T), I1648V (5126A>G), V1667I (5183G>A), A1793T (5561G>A 5563G>A), I1805V (5597A>G), N1807K (5605C>A), A1812V (5619C>T 5620T>G), I1816L (5630A>C), V1837I (5693G>A), K1890R (5853A>G), I1904V (5894A>G 5896C>G), R1926A (5960C>G 5961G>C 5962G>C), I1936V (5990A>G), E1945D (6019A>T), K1949T (6030A>C), D1955E (6049C>A), M1958I (6058G>A), S1963N (6072G>A 6073T>C), N1964H (6074A>C), T1966N (6081C>A), C1976S (6111G>C 6112T>C), I1982V (6128A>G 6130T>C), V1992I (6158G>A), T1996S (6170A>T 6172A>G), L2007F (6203C>T 6205C>T), V2016I (6230G>A), A2025K (6257G>A 6258C>A), K2033R (6282A>G), A2060T (6362G>A), V2073I (6401G>A), A2074V (6405C>T), Y2082F (6429A>T), Q2089R (6450A>G), H2101N (6485C>A 6487C>T), L2115I (6527C>A), T2135I (6588C>T), Y2151H (6635T>C), I2155V (6647A>G), I2165V (6677A>G 6679A>G), D2178N (6716G>A), N2179D (6719A>G), I2194L (6764A>T 6766A>G), A2204V (6795C>T 6796A>G), H2205Q (6799C>A), N2225D (6857A>G), V2231T (6875G>A 6876T>C 6877C>A), D2250E (6934C>A), V2273I (7001G>A 7003G>A), F2283Y (7032T>A), T2314K (7125C>A), A2321S (7145G>T 7147A>G), E2326_A2327del (7160_7165delGAGGCA), D2344E (7216T>A), D2358E (7258T>A), D2363_R2364insX (7273_7274insT), R2364V (7274C>G 7275G>T), H2366L (7281A>T), L2367S (7284T>C), S2371L (7296C>T), S2376F (7311C>T), T2377I (7314C>T), C2378Y (7317G>A), N2379K (7321T>A), K2384Q (7334A>C), A2387N (7343G>A 7344C>A 7345A>C), S2408M (7407G>T 7408T>G), C2411G (7415T>G), L2440S (7503T>C 7504G>A), D2509N (7709G>A), K2510R (7713A>G 7714A>G), T2543I (7812C>T), V2554L (7844G>T 7846G>A), V2567I (7883G>A), S2620N (8043G>A), N2656S (8151A>G 8152C>T), R2665K (8178G>A), R2675K (8208G>A), P2704A (8294C>G 8296A>G), S2722F (8349C>T), A2742S (8408G>T), M2756I (8452G>A), R2759K (8460G>A), A2766T (8480G>A 8482T>A), R2773K (8502G>A 8503A>G), E2777D (8515A>T), E2785D (8539G>C), L2788C (8546C>T 8547T>G), S2790T (8552T>A), A2797G (8574C>G), I2801V (8585A>G), G2805E (8598G>A), N2807S (8603A>T 8604A>C 8605C>T), P2813Q (8622C>A), E2814G (8625A>G 8626G>A), P2822F (8648C>T 8649C>T 8650G>C), G2825E (8658G>A), D2827A (8664A>C 8665T>G), A2832V (8679C>T 8680A>T), T2854K (8745C>A 8746A>G), E2891G (8856A>G 8857G>A), R2894Q (8865G>A), M2895L (8867A>C 8869G>T), G2900E (8883G>A), T2902S (8888A>T), V2924I (8954G>A), N2934D (8984A>G 8986T>C), E2935V (8988A>T), I2983V (9131A>G), M2989G (9149A>G 9150T>G 9151G>A), G2990S (9152G>A), P3026S (9260C>T 9262T>C), Q3038H (9298A>C)								

	Begin	End	Coverage	Score	Concordance	Matches	Identities	I/D/M/F*	Stop Codons
NT	5	9704	99.9%	12522	64.8%	9688 (99.7%)	8015 (82.5%)	15/12	

Codon mutations:

ACT3ACC (193T>C), TAC4CAA (194T>C 196C>A), TCA6TCG (202A>G), ATC8ATT (208C>T), TGT9CTG (209T>C 210G>T 211T>G), TCG12TCA (220G>A), TTT13ATG (221T>A 223T>G), GAA14GAG (226A>G), CTA17CTT (235A>T), TAC19TAT (241C>T), CCA21CCT (247A>T), TCT23CCT (251T>C), TGC24TTT (255G>T 256C>T), GAG25GGG (258A>G), CAT26CAC (262T>C), ATT27GTG (263A>G 265T>G), GTG28GCG (267T>C), GAA30GTA (273A>T), CGA31CGT (277A>T), GAA32GAG (280A>G), GTG33GTT (283G>T), CCG34CCA (286G>A), GCT35ACC (287G>A 289T>C), GTT37ACT (293G>A 294T>C), CCT39CCC (301T>C), TTC40TTT (304C>T), GCA41ATA (305G>A 306C>T), GAT42GGT (309A>G), CTG43TTG (311C>T), GAA44GAT (316A>T), ACA45GTG (317A>G 318C>T 319A>G), CAA46CAG (322A>G), GCA49GCT (331A>T), CTC52CAA (339T>A 340C>A), AAG53AGG (342A>G), AAA55GCG (347A>G 348A>C 349A>G), TAT56CAT (350T>C), GCT57GCA (355T>A), ACT58GTT (356A>G 357C>T), CGT60CGC (364T>C), GTG61ACA (365G>A 366T>C 367G>A), CTC62TCC (368C>T 369T>C), AAA63AAG (373A>G), AAC64AAT (376C>T), TTT67TGT (384T>G), ACG68ACA (388G>A), TAC69TAT (391C>T), CGA70CGG (394A>G), AAG72AAA (400G>A), GAT74GAC (406T>C), GCC75GTT (408C>T 409C>T), CAG76CAA (412G>A), ATA77ATT (415A>T), ATG78GAG (416A>G 417T>A), ATT80ATC (424T>C), CAG81CAA (427G>A), AAA83AAG (433A>G), CTG84CTC (436G>C), GAG85AAG (437G>A), AGG86GAG (440A>G 441G>A), AAG87CGA (443A>C 444A>G 445G>A), GAT88GAA (448T>A), GAA90GAG (454A>G), GAA91GCA (456A>C), TAT92TAC (460T>C), CAC93AAC (461C>A), CAA95CAG (469A>G), GCC97GCG (475C>G), CCT99CCA (481T>A), ACT106ACC (502T>C), ATA107ATT (505A>T), GGC109GGT (511C>T), GAT111GAA (517T>A), CCT112CCA (520T>A), CCA113CCT (523A>T), TCT116TTT (531C>T), GAG117GAA (535G>A), CCA118TCA (536C>T), CAA119TGG (539C>T 540A>G 541A>G), AGA122AAG (549G>A 550A>G), ATC124ATT (556C>T), ATT125ATC (559T>C), CAT126CAC (562T>C), ACT128ACC (568T>C), CCA129CCG (571A>G), ATG131ACG (576T>C), CGT132CGC (580T>C), GTC134GCG (585T>C 586C>G), CGC137TAT (593C>T 594G>A 595C>T), CCC138CGC (597C>G), ATA139GTG (599A>G 601A>G), ATA140CCG (602A>C 603T>C 604A>G), ACA143ACG (613A>G), GAA144GAG (616A>G), GGC145GGG (619C>G), CAG146CAA (622G>A), ATG147ATA (625G>A), AAT148AAC (628T>C), CAC149CAT (631C>T), ATT151GTC (635A>G 637T>C), ATA154GTG (644A>G 646A>G), ATT157ATC (655T>C), TCG159TCA (661G>A), GAG160GCC (663A>C 664G>C), AAA161AAG (667A>G), GTC165GTT (679C>T), CAC166CAA (682C>A), TTA167CTG (683T>C 685A>G), AGT169AGC (691T>C), ACC172AGC (699C>G), ACT173ACA (703T>A), CAT174CAA (706T>A), AAG179GAA (719A>G 721G>A), ATA180GTG (722A>G 724A>G), CTT181TTG (725C>T 727T>G), GGT182GGA (730T>A), GCA183TCA (731G>T), TAC184CCT (734T>C 735A>C 736C>T), TCC185CGT (737T>C 738C>G 739C>T), GCG187GTC (744C>T 745G>C), CGA189TGC (749C>T 751A>C), ACT190ACC (754T>C), ATG194AAG (765T>A), TTG196CTG (770T>C), CGA197AAG (773C>A 774G>A 775A>G), CGG198AGG (776C>A), TTC202TTT (790C>T), ATG206AAG (801T>A), GGA210GCG (813G>C 814A>G), CTT211CAT (816T>A), TTG212CTG (818T>C), CAA213CAG (823A>G), CGT214CAA (825G>A 826T>A), CGG217AGA (833C>A 835G>A), GAC219GAT (841C>T), AAT223GAC (851A>G 853T>C), GTC225GTT (859C>T), CGC226TGT (860C>T 862C>T), ACT227GCC (863A>G 865T>C), ATC228ACC (867T>C), AAC229GAT (869A>G 871C>T), ATA230TTG (872A>T 874A>G), CGA231CAA (876G>A), AGG232AAG (879G>A), GAT234GAC (886T>C), ATC238ATT (898C>T), AAC240AGC (903A>G), ACA241AAC (906C>A 907A>C), AAA242ACC (909A>C 910A>C), AGC243AAT (912G>A 913C>T), GGC246GGA (922C>A), GGT249GGA (931T>A), TCA252CTT (938T>C 939C>T 940A>T), GGA253GGG (943A>G), TTC256TTT (952C>T), TCA261TCG (967A>G), GAA263GAG (973A>G), GGG264GGC (976G>C), TTG266ATC (980T>A 982G>C), GCA269GCG (991A>G), CGT270CGC (994T>C), AGA272AAA (999G>A), GTT273GTC (1003T>C), CAG275AGA (1007C>A 1008A>G 1009G>A), AGT276GGG (1010A>G 1012T>G), ATT277GTT (1013A>G), TTA278TTG (1018A>G), AAC279CAG (1019A>C 1021C>G), ATC282ATT (1030C>T), TCG285TCA (1039G>A), AAT286AAC (1042T>C), GCC287GCA (1045C>A), GAC288GAA (1048C>A), AAT289AAC (1051T>C), AAG292AAA (1060G>A), GGT293GGA (1063T>A), CTG294TTG (1064C>T), GAC295GAT (1069C>T), CGA300CAA (1083G>A), TAT303TAC (1093T>C), TCG305GCA (1097T>G 1099G>A), CAC307CAT (1105C>T), GTA310GTG (1114A>G), GCT311GCG (1117T>G), TTA313TTG (1123A>G), CCT314CCA (1126T>A), GTC315GTT (1129C>T), AGG320AAG (1143G>A), GTA321GTT (1147A>T), GCT322GCA (1150T>A), TTG324ATA (1154T>A 1156G>A), CAC327CAT (1165C>T), AGT328AGC (1168T>C), CTT330TTA (1172C>T 1174T>A), ACC339ACT (1201C>T), AGC346AAC (1221G>A), GTT349ACC (1229G>A 1230T>C 1231T>C), AGC350AAT (1233G>A 1234C>T), CTG352CTA (1240G>A), AAG354AAA (1246G>A), TTG356CTA (1250T>C 1252G>A), CAT357CAC (1255T>C), AAA358ACG (1257A>C 1258A>G), AGA361AGG (1267A>G), GGT363GGC (1273T>C), TTG364TTA (1276G>A), AAT365AGT (1278A>G), TTG367CTG (1283T>C), GCG369GTA (1290C>T 1291G>A), GAC372ACT (1298G>A 1299A>C 1300C>T), TTT374TTC (1306T>C), ATA375GTG (1307A>G 1309A>G), CAT376CAC (1312T>C), AAT378GAA (1316A>G 1318T>A), TTG381TTA (1327G>A), ATA382ACA (1329T>C), GCG383GCT (1333G>T), TTA384TTG (1336A>G), CTA387TTG (1343C>T 1345A>G), CCG390CCA (1354G>A), GTG391GTT (1357G>T), GAC392GAT (1360C>T), CTC395TTG (1367C>T 1369C>G), CTT397ATT (1373C>A), TTC398TTT (1378C>T), GAG400GAA (1384G>A), ATA401GTA (1385A>G), AAA403AAG (1393A>G), TCC404TCT (1396C>T), GAG407GAT (1405G>T), AAA408AAG (1408A>G), CAG409CAA (1411G>A), GCA411TCT (1415G>T 1417A>T), CCG412CCC (1420G>C), AAT415AAC (1429T>C), TTA416TTG (1432A>G), GTC418ATT (1436G>A 1438C>T), TTA419CTG (1439T>C 1441A>G), AAT420AAC (1444T>C), TTC423TTT (1453C>T), CTG424CTA (1456G>A), GAA428GAG (1468A>G), GTA436GTG (1492A>G), TTG439CTA (1499T>C 1501G>A), AGT440AGC (1504T>C), TTG441TTA (1507G>A), CTC442CTT (1510C>T), GAA443GAG (1513A>G), TTA444TTG (1516A>G), GCA445GCT (1519A>T), AGG446AGA (1522G>A), TTC447TTT (1525C>T), CAG448CAA (1528G>A), AAC450AAT (1534C>T), ACT452ACA (1540T>A), AAC454AAT (1546C>T), AAA457AAG (1555A>G), GGT458GGC (1558T>C), GAT459GAC (1561T>C), ATA460ATC (1564A>C), TCT461TCA (1567T>A), TTC463TTT (1573C>T), AGA464AGG (1576A>G), TTA467CTA (1583T>C), TCT468TCA (1588T>A), AAG470AAA (1594G>A), AAC472AAT (1600C>T), CTG475CTA (1609G>A), TCG478TCA (1618G>A), TGC479TGT (1621C>T), GAC480GAT (1624C>T), CTG483TTG (1631C>T), AAA485AAG (1639A>G), TTC489TTT (1651C>T), CTC490CTG (1654C>G), CAT497CAC (1675T>C), TCA503TCG (1693A>G), TTT506TTC (1702T>C), GAA508GGA (1707A>G), GCA512ACA (1718G>A), GGA514GGG (1726A>G), TAC515TAT (1729C>T), TCA516TCC (1732A>C), TAT518TAC (1738T>C), ATC520GTT (1742A>G 1744C>T), CGC521CGT (1747C>T), CAT523CAC (1753T>C), AGT525AAT (1758G>A), AGG528AGA (1768G>A), AAC534AAT (1786C>T), TTA535TTG (1789A>G), GTT536ATT (1790G>A), GTC537GTT (1795C>T), TTA541CTG (1805T>C 1807A>G), GAG543GAA (1813G>A), AGG545AGA (1819G>A), AAG547AAA (1825G>A), GAC551GAT (1837C>T), TAT552TAC (1840T>C), CAA555CAG (1849A>G), GTC558GTG (1858C>G), AAA560AAG (1864A>G), TGC562TGT (1870C>T), AGT564AGC (1876T>C), AAA566AAG (1882A>G), GGT568GGA (1888T>A), AAT569AAC (1891T>C), TGT574TGC (1906T>C), ACA577ACT (1915A>T), GGT582GGC (1930T>C), GCC584GCT (1936C>T), ATT585GTC (1937A>G 1939T>C), TCA587TCG (1945A>G), TTC589TTT (1951C>T), CCA592CCT (1960A>T), GTA598GTG (1978A>G), ATT599ATA (1981T>A), GGC600GGT (1984C>T), AAT601AAC (1987T>C), GAC604GAT (1996C>T), TTT607TAT (2004T>A), AAA612AAG (2020A>G), GGG613GGA (2023G>A), GAT614GAC (2026T>C), TCG615TCT (2029G>T), GAG616GAA (2032G>A), TAT625TAC (2059T>C), ATT628ATG (2068T>G), AAC629AAT (2071C>T), GTG630ATA (2072G>A 2074G>A), CTG635TTA (2087C>T 2089G>A), AGC639AGT (2101C>T), GAG640GAA (2104G>A), GAG641GAA (2107G>A), AAG644AAA (2116G>A), TTC646TTT (2122C>T), CGC651CGT (2137C>T), CTT658CTC (2158T>C), ACC660ACT (2164C>T), CCA662CCG (2170A>G), GAT666GAC (2182T>C), GCG668GCA (2188G>A), ACC669ACA (2191C>A), AGA675AAA (2208G>A), TAT678TAC (2218T>C), GAC680GAT (2224C>T), GTA681GTT (2227A>T), TTG686TTA (2242G>A), CCC687CCT (2245C>T), TTG690TTA (2254G>A), GTT691GTC (2257T>C), GAC692GAT (2260C>T), GAC694GAG (2266C>G), CAA696CAG (2272A>G), ACG697ACA (2275G>A), TCA703TCG (2293A>G), GGC705GGT (2299C>T), TCG706TCA (2302G>A), CAG707CAA (2305G>A), GGA710GGT (2314A>T), TAT711TAC (2317T>C), CTA714CTG (2326A>G), GTG719GTA (2341G>A), CAA721CAG (2347A>G), CTT722CTC (2350T>C), TTG724TTA (2356G>A), AAT727AAC (2365T>C), GAA729GAG (2371A>G), TTA730TTG (2374A>G), GAT733GAC (2383T>C), ATA734ATT (2386A>T), AAA735AAG (2389A>G), CAT736CAC (2392T>C), TAT737TAC (2395T>C), AAT744GGG (2414A>G 2415A>G 2416T>G), GCT745GCA (2419T>A), AGC746TGC (2420A>T), ACA752ACT (2440A>T), GTT761GTC (2467T>C), TCT764TCC (2476T>C), TCG766TCA (2482G>A), CTG769CTA (2491G>A), CTG771CTT (2497G>T), TTG773TTA (2503G>A), AGA778AGG (2518A>G), CCT779CCC (2521T>C), AAG780AGG (2523A>G), AGA783AGG (2533A>G), TTG785TTA (2539G>A), TTA787TTG (2545A>G), GAG789GAA (2551G>A), CCT790CCA (2554T>A), CTG792TTG (2558C>T), TTG793CTC (2561T>C 2563G>C), TCA796TCG (2572A>G), CCT800CCC (2584T>C), GGC801GGT (2587C>T), CTG803CTC (2593G>C), GCT805GCC (2599T>C), AAT808AAC (2608T>C), TTT812TTC (2620T>C), GCG815GCA (2629G>A), GTG816GTA (2632G>A), AGG817AAG (2634G>A), TTG818TTA (2638G>A), ATT820ATA (2644T>A), AAA823AAG (2653A>G), GCT827GCC (2665T>C), CTA832TTA (2678C>T), CTA833CTG (2683A>G), GCT835GCC (2689T>C), CTA838CTC (2698A>C), TCA841TCG (2707A>G), GCG842GCA (2710G>A), GAA844GAG (2716A>G), ACA845ACG (2719A>G), CTC846CTT (2722C>T), GTC847ATT (2723G>A 2725C>T), GCA848GCG (2728A>G), AGG850AGA (2734G>A), ATT851ATA (2737T>A), GAT854GAC (2746T>C), GCT855ACA (2747G>A 2749T>A), GCA856GCT (2752A>T), GCT857GCA (2755T>A), GAC859GAT (2761C>T), CTC860CTT (2764C>T), TGT865TGC (2779T>C), GGG867GGT (2785G>T), AAC869AAT (2791C>T), CAT871AAT (2795C>A), TAC874TAT (2806C>T), TTG878CTC (2816T>C 2818G>C), GTG880ATA (2822G>A 2824G>A), TTG881TTA (2827G>A), GTT884GTC (2836T>C), AAG885AAA (2839G>A), AAT886AAC (2842T>C), ACC893ACA (2863C>A), CTA894TTA (2864C>T), CCA900ACA (2882C>A), AGT901CAT (2885A>C 2886G>A), AAC903AAT (2893C>T), ACG904ATG (2895C>T), GTC906GTT (2902C>T), GTA907GTG (2905A>G), ATT909ATC (2911T>C), AAT913AAC (2923T>C), AAT916AGT (2931A>G), AAC919AGC (2940A>G), GCT921GCC (2947T>C), ACT926ACC (2962T>C), GAA929_AAT930insA-- (2971_2972insA), GGT935GGC (2989T>C), ACT936ATT (2991C>T), CAT937CGT (2994A>G), GAG939AGG (2999G>A 3000A>G), TCC944TC- (3016delC), CGG946CAG (3021G>A), TAC947TTT (3024A>T 3025C>T), ACA951ATA (3036C>T), GAA952GGC (3039A>G 3040A>C), AAG953ACA (3042A>C 3043G>A), TTG956TTA (3052G>A), TTA959TTG (3061A>G), GCG966GCA (3082G>A), GGC969GGT (3091C>T), GCC972GTA (3099C>T 3100C>A), GTG974AGA (3104G>A 3105T>G 3106G>A), GTC975GTT (3109C>T), AAA976AAG (3112A>G), ACT978ACC (3118T>C), GCC979GCT (3121C>T), GGA981GGG (3127A>G), AGC983AAC (3132G>A), CGA985AAG (3137C>A 3138G>A 3139A>G), TAT989TAC (3151T>C), TTC990ATT (3152T>A 3154C>T), TGT994TAT (3165G>A), GTA995GTG (3169A>G), ATC1005ATT (3199C>T), AGG1007CGG (3203A>C), CGT1008CGC (3208T>C), CCA1010CCG (3214A>G), ACC1011ACT (3217C>T), TTT1012CTT (3218T>C), GTC1013GTA (3223C>A), ACT1014AAT (3225C>A), GTT1016ATT (3230G>A), AAC1017AAT (3235C>T), TTA1025CTA (3257T>C), ACC1026ACT (3262C>T), AGC1027AGT (3265C>T), GTA1028ATG (3266G>A 3268A>G), GTG1029GTA (3271G>A), GTG1031ATG (3275G>A), GCA1034ACA (3284G>A), CAG1039CAA (3301G>A), AGA1044AAG (3315G>A 3316A>G), GAA1045GCA (3318A>C), ATC1046ATT (3322C>T), GAG1047GAA (3325G>A), TTG1048TTA (3328G>A), ATA1051ATT (3337A>T), GAG1055GAA (3349G>A), GTC1057GTT (3355C>T), TGC1058TGT (3358C>T), ATG1059GTG (3359A>G), CTA1061CTG (3367A>G), TAT1062TAC (3370T>C), TTA1065CTG (3377T>C 3379A>G), GAA1070GAG (3394A>G), CGC1071CGT (3397C>T), GAT1072GAG (3400T>G), ACA1074ACG (3406A>G), TAC1078TAT (3418C>T), ATT1079ATA (3421T>A), TAT1081TAC (3427T>C), AAG1083AAA (3433G>A), TCA1084TCT (3436A>T), GTA1085GTG (3439A>G), AAC1086AAT (3442C>T), CCT1087CCC (3445T>C), CAG1091CAA (3457G>A), TTT1092TTC (3460T>C), GCT1093GCA (3463T>A), GCG1095GCT (3469G>T), CAG1096CAA (3472G>A), CAC1104CAT (3496C>T), CAG1105CAA (3499G>A), TCC1107TCT (3505C>T), GTT1110GGT (3513T>G), AAA1112AAG (3520A>G), GAA1115GAG (3529A>G), CAA1116CAG (3532A>G), ATG1121GTG (3545A>G), GCT1122ACT (3548G>A), TTA1123CTA (3551T>C), GTC1124ATT (3554G>A 3556C>T), AGT1133AGC (3583T>C), GAT1134GAC (3586T>C), TGC1135TGT (3589C>T), TTC1137TTT (3595C>T), AAT1141AAC (3607T>C), TTT1143TTC (3613T>C), AAG1144AAA (3616G>A), GGT1145GGC (3619T>C), CTT1147GTT (3623C>G), TCC1148TCT (3628C>T), CTG1150ATG (3632C>A), GAT1151GAC (3637T>C), TAT1152CAT (3638T>C), GAA1153GAG (3643A>G), TTA1159TTG (3661A>G), GTG1162GTA (3670G>A), TTT1166TTC (3682T>C), GAG1168GAA (3688G>A), GAG1171GAA (3697G>A), ATT1172GTT (3698A>G), GAT1174GAC (3706T>C), TTT1175TTC (3709T>C), TTG1177CTA (3713T>C 3715G>A), AGT1178AAT (3717G>A), GAG1179GAA (3721G>A), CGA1183AAA (3731C>A 3732G>A), ACT1184ACA (3736T>A), CTA1188TTG (3746C>T 3748A>G), ACA1190ACG (3754A>G), AGT1193AGC (3763T>C), CGA1198CGG (3778A>G), CAA1199CAG (3781A>G), ATC1200ATT (3784C>T), CAT1204CAC (3796T>C), CCA1207CCT (3805A>T), TAC1209TAT (3811C>T), AGA1210AGG (3814A>G), TTC1215TTT (3829C>T), TTT1218TTC (3838T>C), AGA1220AGG (3844A>G), GCA1221GCC (3847A>C), GTT1224GTA (3856T>A), GTG1226GTT (3862G>T), GAC1229GAT (3871C>T), GCC1231GCA (3877C>A), AGC1233AGT (3883C>T), GAA1234GAG (3886A>G), CTA1236CTG (3892A>G), GAC1237GAT (3895C>T), TTT1238TTC (3898T>C), GTA1240GTG (3904A>G), CGG1241AGA (3905C>A 3907G>A), GCT1243GCC (3913T>C), AAG1248AAA (3928G>A), TCA1249TCT (3931A>T), GGG1251GGA (3937G>A), AGT1257AGC (3955T>C), GTG1258GTA (3958G>A), GCC1259GCT (3961C>T), GGA1260GGG (3964A>G), TCT1261TCC (3967T>C), TTA1264TTG (3976A>G), ATT1265ATC (3979T>C), CCA1267CCT (3985A>T), CTA1271CTT (3997A>T), GCG1272GCA (4000G>A), AAC1274AAT (4006C>T), GTT1275GTG (4009T>G), TTC1276TTT (4012C>T), AAA1277AAG (4015A>G), TCT1280TCC (4024T>C), GAA1282GAG (4030A>G), CCA1283CCT (4033A>T), AAG1286AAA (4042G>A), CTG1290TTA (4052C>T 4054G>A), CGT1291CGC (4057T>C), CGT1293CGA (4063T>A), GGA1294GGG (4066A>G), ATA1297GTA (4073A>G), TTT1298TTC (4078T>C), GGC1299GGT (4081C>T), TCT1300TCC (4084T>C), TCC1304TCT (4096C>T), AGC1308AGT (4108C>T), GGA1309GGT (4111A>T), GCG1311GCA (4117G>A), CTA1312TTG (4118C>T 4120A>G), TAC1314TAT (4126C>T), TTC1315TAT (4128T>A 4129C>T), GCC1316GCT (4132C>T), AAT1317AAC (4135T>C), CAA1321CAG (4147A>G), TTA1322CTA (4148T>C), GCT1323TCT (4151G>T), AAC1326AAT (4162C>T), GTA1328ATA (4166G>A), ATA1329ATC (4171A>C), GAG1332GAA (4180G>A), CTG1336TTG (4190C>T), CCT1338CCC (4198T>C), TCC1339TCT (4201C>T), GCG1342GCT (4210G>T), TTC1343TTT (4213C>T), AGT1345AGC (4219T>C), CTG1346TTG (4220C>T), CTG1347ATA (4223C>A 4225G>A), GTT1349GTG (4231T>G), CAT1351CAC (4237T>C), GTA1356GTG (4252A>G), TTA1357CTG (4253T>C 4255A>G), AAA1358AAG (4258A>G), ACT1362ACC (4270T>C), AGA1366AGG (4282A>G), GAG1367GAA (4285G>A), GAA1369GAG (4291A>G), CAG1373CAA (4303G>A), GTC1376GTT (4312C>T), ATA1379GTG (4319A>G 4321A>G), GTG1380GTT (4324G>T), GAG1381GAA (4327G>A), CTG1384CTT (4336G>T), TCT1385TCC (4339T>C), TCA1388TCT (4348A>T), GCA1392GCG (4360A>G), GGT1394GGC (4366T>C), TCT1395TCA (4369T>A), ACT1397ACC (4375T>C), GTT1401GTC (4387T>C), TTT1404TAT (4395T>A), GTA1408ATA (4406G>A), CTT1409CTC (4411T>C), GTT1418GTC (4438T>C), ACC1420ACA (4444C>A), GCC1422GCT (4450C>T), CTC1424CTT (4456C>T), GAC1427GAT (4465C>T), ATG1431GTT (4475A>G 4477G>T), GTC1432GTT (4480C>T), ACA1433TCG (4481A>T 4483A>G), GGC1437GGT (4495C>T), AAG1441AAA (4507G>A), CAC1442CAT (4510C>T), GGT1443GGG (4513T>G), CTA1445TTA (4517C>T), GTC1448GTT (4528C>T), AAA1450AAG (4534A>G), ACC1452ACT (4540C>T), GCG1454TTA (4544G>T 4545C>T 4546G>A), AGA1455AAA (4548G>A), TTT1458TTC (4558T>C), GTA1460GTT (4564A>T), ACC1462ACT (4570C>T), ATA1464ATT (4576A>T), TTG1471TTA (4597G>A), GAC1472GAT (4600C>T), GAC1474GAT (4606C>T), GTG1475GTA (4609G>A), GTG1477GTA (4615G>A), GAC1478GAT (4618C>T), TTG1481CTT (4625T>C 4627G>T), GAC1488GAT (4648C>T), ATT1489ATC (4651T>C), AGG1492AGA (4660G>A), AGC1493AGT (4663C>T), GCT1495GCA (4669T>A), GTG1499ATC (4679G>A 4681G>C), AGT1500AGC (4684T>C), GTT1501GTG (4687T>G), GAG1505GAA (4699G>A), AGA1506AGG (4702A>G), CAA1508CAG (4708A>G), AGG1509AGA (4711G>A), CTG1510TTG (4712C>T), AAA1518AAG (4738A>G), GTA1520GTT (4744A>T), GCA1521GCG (4747A>G), TTG1522CTG (4748T>C), ACT1527ACC (4765T>C), GTC1538ATT (4796G>A 4798C>T), ACT1540AGC (4803C>G 4804T>C), GAG1541GAA (4807G>A), GCG1542GCT (4810G>T), GCT1543GCG (4813T>G), CTT1544CTC (4816T>C), TAT1549TAC

	(4831T>C), AAC1550AAT (4834C>T), CCA1552CCT (4840A>T), GGA1556GGG (4852A>G), GGC1557GGT (4855C>T), AGT1561AGC (4867T>C), CTG1562CTC (4870G>C), ATT1563ATA (4873T>A), TGT1566TGC (4882T>C), ACA1573ACT (4903A>T), CAG1575CAA (4909G>A), TTT1577TTC (4915T>C), GAA1578GAG (4918A>G), TTG1579CTA (4919T>C 4921G>A), AGT1580AGC (4924T>C), CCC1581CCG (4927C>G), TTT1583TTC (4933T>C), ATC1584ATA (4936C>A), CAG1585CAA (4939G>A), AAT1586AAC (4942T>C), TTC1587TTT (4945C>T), TCA1593TCG (4963A>G), CCT1596CCC (4972T>C), CTT1602CTC (4990T>C), AAA1606AAG (5002A>G), CCT1613CCC (5023T>C), TTG1614CTG (5024T>C), TGC1615TGT (5029C>T), GAT1616GAC (5032T>C), TCT1618TCC (5038T>C), CCA1620CCT (5044A>T), GCA1623GCT (5053A>T), TCG1624TCA (5056G>A), AGT1625GGT (5057A>G), ACT1626GCT (5060A>G), TCG1629TCT (5071G>T), CTT1636ATT (5090C>A), GGA1637GGG (5095A>G), GTG1638GTT (5098G>T), GCC1639GTC (5100C>T), TTA1640TTG (5104A>G), GAA1641GAT (5107A>T), ATT1642ATC (5110T>C), CCA1643CCG (5113A>G), AAG1644AAA (5116G>A), CAA1645CAG (5119A>G), GTC1646TTT (5120G>T 5122C>T), ATT1648GTT (5126A>G), CAT1651CAC (5137T>C), ATC1652ATT (5140C>T), AAA1653AAG (5143A>G), GAG1654GAA (5146G>A), CTC1659CTA (5161C>A), GAA1661GAG (5167A>G), CTT1663CTG (5173T>G), ACG1666ACA (5182G>A), GTT1667ATT (5183G>A), GTC1668GTT (5188C>T), AAG1669AAA (5191G>A), AAA1671AAG (5197A>G), GAC1672GAT (5200C>T), TGC1674TGT (5206C>T), TTA1675TTG (5209A>G), AGC1678AGT (5218C>T), TCG1682TCA (5230G>A), ATC1684ATT (5236C>T), AGC1685AGT (5239C>T), ATC1687ATT (5245C>T), ACA1693ACT (5263A>T), CTC1695CTT (5269C>T), TTC1696TTT (5272C>T), GCC1697GCA (5275C>A), ATC1698ATT (5278C>T), CCA1699CCC (5281A>C), AGA1700AGG (5284A>G), ACT1701ACC (5287T>C), CTA1702TTA (5288C>T), ATA1703ATT (5293A>T), TTG1704CTA (5294T>C 5296G>A), GAG1706GAA (5302G>A), CTT1709CTA (5311T>A), GAA1710GAG (5314A>G), CGA1713CGT (5323A>T), AGT1721AGC (5347T>C), ATC1723ATT (5353C>T), TGC1727TGT (5365C>T), GTT1734GTC (5386T>C), ACC1737ACA (5395C>A), AAA1746AAG (5422A>G), GAT1747GAC (5425T>C), ACC1749ACT (5431C>T), GAG1751GAA (5437G>A), CTT1756CTC (5452T>C), AAG1758AAA (5458G>A), AGT1761AGC (5467T>C), CAA1762CAG (5470A>G), CTA1763TTG (5471C>T 5473A>G), GAA1765GAG (5479A>G), TTC1766TTT (5482C>T), TCA1767TCC (5485A>C), AAT1768AAC (5488T>C), TTG1769CTA (5489T>C 5491G>A), GGT1771GGC (5497T>C), TGT1774TGC (5506T>C), GAG1775GAA (5509G>A), GAG1776GAA (5512G>A), TTA1778CTA (5516T>C), TAT1782TAC (5530T>C), GAG1783GAA (5533G>A), TCG1784TCT (5536G>T), TTG1785TTA (5539G>A), GTT1788GTG (5548T>G), CAC1790CAT (5554C>T), GCT1792GCA (5560T>A), GCG1793ACA (5561G>A 5563G>A), ACG1794ACC (5566G>C), TCA1795TCG (5569A>G), CTC1800CTG (5584C>G), AAG1803AAA (5593G>A), GGG1804GGA (5596G>A), ATT1805GTT (5597A>G), AAC1807AAA (5605C>A), GTG1811GTT (5617G>T), GCT1812GTG (5619C>T 5620T>G), TTG1815TTA (5629G>A), ATC1816CTC (5630A>C), GGC1819GGT (5641C>T), GCA1822GCT (5650A>T), ATA1826ATT (5662A>T), GGA1827GGG (5665A>G), CAA1835CAG (5689A>G), GTT1837ATT (5693G>A), GAG1838GAA (5698G>A), CAA1843CAG (5713A>G), GGG1844GGC (5716G>C), AAA1845AAG (5719A>G), GCC1853GCA (5743C>A), CGC1857CGA (5755C>A), CAT1858CAC (5758T>C), CGT1860CGC (5764T>C), AAA1862AAG (5770A>G), GGC1865GGT (5779C>T), TTT1866TTC (5782T>C), GAC1869GAT (5791C>T), GAC1873GAT (5803C>T), AGG1884AGA (5836G>A), AAA1885AAG (5839A>G), GGA1887GGG (5845A>G), AAA1888AAG (5848A>G), AAA1890AGA (5853A>G), GGT1891GGC (5857T>C), ACC1892ACT (5860C>T), ACA1893ACT (5863A>T), GGT1895GGA (5869T>A), GGT1897GGC (5875T>C), AGC1900AGT (5884C>T), TTC1903TTT (5893C>T), ATC1904GTG (5894A>G 5896C>G), AAC1905AAT (5899C>T), GGG1908GGA (5908G>A), TTT1909TTC (5911T>C), GAG1913GAA (5923G>A), CAA1918CAG (5938A>G), TTC1919TTT (5941C>T), GTT1920GTC (5944T>C), GAT1921GAC (5947T>C), GGG1925GGA (5959G>A), CGG1926GCC (5960C>G 5961G>C 5962G>C), GAA1930GAG (5974A>G), GCT1934GCA (5986T>A), GAC1935GAT (5989C>T), ATT1936GTT (5990A>G), GAT1938GAC (5998T>C), AGA1942CGT (6008A>C 6010A>T), GAA1945GAT (6019A>T), GTG1946GTT (6022G>T), CGA1947CGT (6025A>T), AAA1949ACA (6030A>C), GAC1955GAA (6049C>A), ATG1958ATA (6058G>A), CAA1959CAG (6061A>G), GCC1960GCA (6064C>A), GGT1962GGC (6070T>C), AGT1963AAC (6072G>A 6073T>C), AAC1964CAC (6074A>C), ACG1965ACA (6079G>A), ACC1966AAC (6081C>A), ATA1967ATT (6085A>T), GCA1969GCT (6091A>T), TAC1970TAT (6094C>T), TGT1976TCC (6111G>C 6112T>C), AAA1978AAG (6118A>G), TTG1980TTA (6124G>A), AAG1981AAA (6127G>A), ATT1982GTC (6128A>G 6130T>C), GAT1983GAC (6133T>C), TTA1984TTG (6136A>G), AAC1988AAT (6148C>T), AAA1991AAG (6157A>G), GTT1992ATT (6158G>A), TGT1993TGC (6163T>C), AAA1995AAG (6169A>G), ACA1996TCG (6170A>T 6172A>G), GGC1998GGT (6178C>T), AAA2001AAG (6187A>G), TTT2002TTC (6190T>C), GAG2004GAA (6196G>A), CTC2007TTT (6203C>T 6205C>T), GAA2008GAG (6208A>G), CTA2009TTG (6209C>T 6211A>G), CAG2011CAA (6217G>A), GCT2015GCA (6229T>A), GTA2016ATA (6230G>A), GTC2018GTT (6238C>T), AAG2021AAA (6247G>A), ATA2023ATT (6253A>T), GCA2025AAA (6257G>A 6258C>A), CAT2030CAC (6274T>C), AAA2033AGA (6282A>G), TCG2034TCA (6286G>A), GGC2038GGT (6298C>T), TTG2039TTA (6301G>A), AGA2040AGG (6304A>G), GAC2041GAT (6307C>T), GCC2046GCT (6322C>T), ACA2048ACT (6328A>T), GTT2049GTG (6331T>G), TGT2050TGC (6334T>C), AGG2051AGA (6337G>A), CTG2052TTG (6338C>T), TAT2058TAC (6358T>C), GGG2059GGA (6361G>A), GCA2060ACA (6362G>A), TCA2061TCT (6367A>T), GAG2062GAA (6370G>A), TAC2064TAT (6376C>T), GGT2065GGG (6379T>G), GGA2067GGC (6385A>C), GGA2069GGT (6391A>T), GCA2070GCG (6394A>G), TAC2071TAT (6397C>T), GTA2073ATA (6401G>A), GCG2074GTG (6405C>T), CAC2076CAT (6412C>T), TTA2078TTG (6418A>G), TTT2079TTC (6421T>C), TAC2082TTC (6429A>T), GGT2084GGA (6436T>A), GAG2087GAA (6445G>A), CAA2089CGA (6450A>G), TCC2090TCG (6454C>G), CAC2092CAT (6460C>T), GGT2093GGA (6463T>A), TTC2095TTT (6469C>T), AGG2096AGA (6472G>A), GTG2097GTA (6475G>A), AAT2099AAC (6481T>C), CAC2101AAT (6485C>A 6487C>T), AGT2102AGC (6490T>C), AGC2104AGT (6496C>T), CTG2106CTA (6502G>A), GGT2110GGC (6514T>C), AGG2111AGA (6517G>A), CTC2115ATC (6527C>A), ATC2116ATA (6532C>A), CCG2119CCA (6541G>A), AAA2120AAG (6544A>G), GTC2124GTT (6556C>T), TTT2125TTC (6559T>C), CAG2127CAA (6565G>A), TTG2129CTA (6569T>C 6571G>A), CCT2134CCA (6586T>A), ACA2135ATA (6588C>T), GAA2138GAG (6598A>G), AGA2139AGG (6601A>G), ATT2140ATC (6604T>C), TGT2141TGC (6607T>C), GGA2144GGG (6616A>G), AAC2146AAT (6622C>T), GAG2149GAA (6631G>A), AAG2150AAA (6634G>A), TAT2151CAT (6635T>C), GCT2152GCA (6640T>A), TCG2154TCT (6646G>T), ATC2155GTC (6647A>G), ATC2156ATT (6652C>T), AGC2160AGT (6664C>T), ACT2162ACG (6670T>G), ATA2165GTG (6677A>G 6679A>G), GGC2167GGT (6685C>T), ACA2169ACT (6691A>T), TTC2170TTT (6694C>T), ACA2177ACG (6715A>G), GAT2178AAT (6716G>A), AAT2179GAT (6719A>G), GGA2180GGG (6724A>G), CTA2184TTA (6734C>T), GTG2186GTA (6742G>A), GTG2187GTA (6745G>A), GCC2190GCT (6754C>T), GGA2192GGG (6760A>G), ATA2194TTG (6764A>T 6766A>G), ATT2197ATA (6775T>A), CTG2200TTG (6782C>T), GCA2201GCT (6787A>T), AAC2202AAT (6790C>T), GCA2204GTG (6795C>T 6796A>G), CAC2205CAA (6799C>A), ACC2206ACA (6802C>A), AAC2208AAT (6808C>T), TAC2209TAT (6811C>T), TAC2210TAT (6814C>T), TTC2213TTT (6823C>T), GAA2215GAG (6829A>G), TAC2221TAT (6847C>T), CGA2223CGC (6853A>C), AAT2225GAT (6857A>G), GAG2226GAA (6862G>A), GAA2229GAG (6871A>G), GTC2231ACA (6875G>A 6876T>C 6877C>A), AAG2232AAA (6880G>A), TCT2233TCA (6883T>A), GTT2235GTG (6889T>G), GAC2239GAT (6901C>T), GGC2244GGT (6916C>T), CCG2245CCA (6919G>A), AAA2247AAG (6925A>G), CTT2248CTC (6928T>C), GAC2250GAA (6934C>A), GGG2255GGC (6949G>C), TTC2257TTT (6955C>T), AAA2258AAG (6958A>G), CAA2264CAG (6976A>G), CTA2266TTA (6980C>T), ATC2267ATT (6985C>T), GTA2271GTT (6997A>T), GTG2272GTT (7000G>T), GTG2273ATA (7001G>A 7003G>A), GCT2276GCC (7012T>C), AAG2277AAA (7015G>A), CAT2278CAC (7018T>C), TTT2283TAT (7032T>A), GCC2285GCT (7039C>T), TTG2286CTA (7040T>C 7042G>A), GGA2288GGG (7048A>G), GCT2292GCA (7060T>A), GTC2293GTA (7063C>A), AGC2298AGT (7078C>T), TTA2300CTG (7082T>C 7084A>G), GTA2301GTG (7087A>G), ACC2302ACA (7090C>A), CAT2304CAC (7096T>C), GTA2305GTG (7099A>G), GTT2306GTC (7102T>C), GAG2309GAA (7111G>A), CGA2311CGG (7117A>G), ACA2314AAA (7125C>A), GAA2315GAG (7129A>G), TTT2316TTC (7132T>C), CTG2317TTA (7133C>T 7135G>A), GTG2319GTA (7141G>A), GAT2320GAC (7144T>C), GCA2321TCG (7145G>T 7147A>G), GAG2322GAA (7150G>A), GCA2325GCT (7159A>T), GAG2326_GCA2327del (7160_7165delGAGGCA), TTC2329TTT (7171C>T), GCG2335GCT (7189G>T), AAA2338AAG (7198A>G), AGC2339AGT (7201C>T), CTA2341TTA (7205C>T), AAT2342AAC (7210T>C), GAT2344GAA (7216T>A), GCG2345GCA (7219G>A), ATC2347ATA (7225C>A), AAG2348AAA (7228G>A), AAG2352AAA (7240G>A), TAT2353TAC (7243T>C), AAA2355AAG (7249A>G), CCT2356CCG (7252T>G), GAT2358GAA (7258T>A), GTT2359GTC (7261T>C), GGT2360GGA (7264T>A), GTC2361GTG (7267C>G), GTG2362GTA (7270G>A), GAT2363_CGG2364insT-- (7273_7274insT), CGG2364GTG (7274C>G 7275G>T), CAT2366CTT (7281A>T), TTG2367TCG (7284T>C), TCA2371TTA (7296C>T), TCT2376TTT (7311C>T), ACC2377ATC (7314C>T), TGC2378TAC (7317G>A), AAT2379AAA (7321T>A), GTG2380-TG (7322delG), CAC2381CAT (7327C>T), GGC2382GGT (7330C>T), TTC2383TTT (7333C>T), AAG2384CAG (7334A>C), TGT2386TGC (7342T>C), GCA2387AAC (7343G>A 7344C>A 7345A>C), ACT2390ACC (7354T>C), CAA2393CAG (7363A>G), ATT2395ATC (7369T>C), TTC2396TTT (7372C>T), GCG2398GCT (7378G>T), CTC2399CTT (7381C>T), GCA2404GCT (7396A>T), GTC2405GTT (7399C>T), GCC2407GCT (7405C>T), AGT2408ATG (7407G>T 7408T>G), GGG2410GGT (7414G>T), TGC2411GGC (7415T>G), AAA2412AAG (7420A>G), GAC2415GAT (7429C>T), TAT2416TAC (7432T>C), CAT2419CAC (7441T>C), AAG2425AAA (7459G>A), GAA2426GAG (7462A>G), ATA2428ATT (7468A>T), GTC2429GTA (7471C>A), CAA2431CAG (7477A>G), AGC2432AGT (7480C>T), CTG2434CTT (7486G>T), TAT2437TAC (7495T>C), AAA2438AAG (7498A>G), GGT2439GGC (7501T>C), TTG2440TCA (7503T>C 7504G>A), ATT2443ATA (7513T>A), AAC2445AAT (7519C>T), GGA2446GGG (7522A>G), AAG2449AAA (7531G>A), GAG2451GAA (7537G>A), CTC2452CTT (7540C>T), TGT2454TGC (7546T>C), AAG2462AAA (7570G>A), ACG2463ACA (7573G>A), ACG2465ACA (7579G>A), GCT2468GCC (7588T>C), CTA2471TTA (7595C>T), TTG2474CTA (7604T>C 7606G>A), GGT2477GGA (7615T>A), TGT2480TGC (7624T>C), GAC2483GAT (7633C>T), TTC2484TTT (7636C>T), AAT2485AAC (7639T>C), TTT2488TTC (7648T>C), GAA2494GAG (7666A>G), TGT2496TGC (7672T>C), ACA2498ACT (7678A>T), GGG2500GGA (7684G>A), ACT2502ACC (7690T>C), TTT2504TTC (7696T>C), GGT2506GGG (7702T>G), GAT2509AAT (7709G>A), AAA2510AGG (7713A>G 7714A>G), CTG2511TTG (7715C>T), CGT2514CGC (7726T>C), GAG2517GAA (7735G>A), GTA2520GTG (7744A>G), TAC2521TAT (7747C>T), GGC2526GGT (7762C>T), CAG2528CAA (7768G>A), TTT2529TTC (7771T>C), TCA2532TCG (7780A>G), CTA2533CTC (7783A>C), ACT2534ACC (7786T>C), CTA2537TTG (7793C>T 7795A>G), ATC2538ATT (7798C>T), GCT2540GCC (7804T>C), ACC2543ATC (7812C>T), GAA2550GAG (7834A>G), GAC2551GAT (7837C>T), GTG2554TTA (7844G>T 7846G>A), CAG2557CAA (7855G>A), CTG2559CTA (7861G>A), TTA2562TTG (7870A>G), TAC2563TAT (7873C>T), ACT2564ACA (7876T>A), GAG2565GAA (7879G>A), ATT2566ATA (7882T>A), GTT2567ATT (7883G>A), CCA2570CCT (7894A>T), ATT2571ATC (7897T>C), GTC2579GTT (7921C>T), AAG2581AAA (7927G>A), GGT2584GGC (7936T>C), AAT2585AAC (7939T>C), AAT2586AAC (7942T>C), GTG2594GTA (7966G>A), GAT2595GAC (7969T>C), TCT2597TCC (7975T>C), GTC2601GTT (7987C>T), CTT2602CTC (7990T>C), GCT2607GCC (8005T>C), GAA2616GAG (8032A>G), ATC2618ATT (8038C>T), GAC2619GAT (8041C>T), AGC2620AAC (8043G>A), TTG2632TTA (8080G>A), GTG2635GTA (8089G>A), CCG2637CCA (8095G>A), GAG2638GAA (8098G>A), AGC2641AGT (8107C>T), ATT2642ATC (8110T>C), GAT2644GAC (8116T>C), CAA2648CAG (8128A>G), GGT2654GGC (8146T>C), AAC2656AGT (8151A>G 8152C>T), TCG2660TCA (8164G>A), AGG2665AAG (8178G>A), AAG2666AAA (8182G>A), TTG2669TTA (8191G>A), TTC2671TTT (8197C>T), TCC2673TCT (8203C>T), AGA2675AAA (8208G>A), CTG2677TTA (8213C>T 8215G>A), CTA2678CTG (8218A>G), ATC2679ATT (8221C>T), GAG2680GAA (8224G>A), CCA2685CCG (8239A>G), GAG2690GAA (8254G>A), ATT2692ATC (8260T>C), GTA2693GTG (8263A>G), GAT2699GAC (8281T>C), AGA2700AGG (8284A>G), CCA2704GCG (8294C>G 8296A>G), GAG2705GAA (8299G>A), CAC2706CAT (8302C>T), ATT2711ATC (8317T>C), TGC2712TGT (8320C>T), GCT2714GCA (8326T>A), GAG2717GAA (8335G>A), TCC2718TCT (8338C>T), TCT2722TTT (8349C>T), GAA2723GAG (8353A>G), CAC2726CAT (8362C>T), CAA2727CAG (8365A>G), ATC2728ATT (8368C>T), TAC2732TAT (8380C>T), TCA2733TCG (8383A>G), TTA2735TTG (8389A>G), CCT2740CCC (8404T>C), GCA2742TCA (8408G>T), ACA2743ACG (8413A>G), GCG2745GCT (8419G>T), GCT2750GCC (8434T>C), TAT2752TAC (8440T>C), ATG2756ATA (8452G>A), CTA2758CTG (8458A>G), AGG2759AAG (8460G>A), AAA2760AAG (8464A>G), CTG2761CTA (8467G>A), AGG2765AGA (8479G>A), GCT2766ACA (8480G>A 8482T>A), GAG2771GAA (8497G>A), CTA2772TTG (8498C>T 8500A>G), AGA2773AAG (8502G>A 8503A>G), GCC2774GCT (8506C>T), GAA2777GAT (8515A>T), GTC2780GTT (8524C>T), GCA2781GCC (8527A>C), TTA2782CTG (8528T>C 8530A>G), GAG2785GAC (8539G>C), CTT2788TGT (8546C>T 8547T>G), TCT2790ACT (8552T>A), GTA2793GTG (8563A>G), GCA2797GGA (8574C>G), ATT2801GTT (8585A>G), GGA2805GAA (8598G>A), AGC2806AGT (8602C>T), AAC2807TCT (8603A>T 8604A>C 8605C>T), AAA2812AAG (8620A>G), CCA2813CAA (8622C>A), GAG2814GGA (8625A>G 8626G>A), CAG2815CAA (8629G>A), GGC2816GGT (8632C>T), ATC2818ATT (8638C>T), CAG2819CAA (8641G>A), AAC2821AAT (8647C>T), CCG2822TTC (8648C>T 8649C>T 8650G>C), AAC2823AAT (8653C>T), AAA2824AAG (8656A>G), GGA2825GAA (8658G>A), GAT2827GCG (8664A>C 8665T>G), GTT2830GTG (8674T>G), GCA2832GTT (8679C>T 8680A>T), GGC2833GGA (8683C>A), GGG2836GGA (8692G>A), ACA2837ACT (8695A>T), CCG2841CCA (8707G>A), AGA2842CGA (8708A>C), AAG2844AAA (8716G>A), ATC2846ATT (8722C>T), ACG2847ACA (8725G>A), AAA2849AAG (8731A>G), AGA2851AGG (8737A>G), CCC2853CCT (8743C>T), ACA2854AAG (8745C>A 8746A>G), AAG2856AAA (8752G>A), GGA2857GGT (8755A>T), AAC2862AAT (8770C>T), TTA2863CTA (8771T>C), GAA2864GAG (8776A>G), CAT2865CAC (8779T>C), TTG2866TTA (8782G>A), TAT2869TAC (8791T>C), CCA2871CCG (8797A>G), CAA2872CAG (8800A>G), CAA2873CAG (8803A>G), GAT2875GAC (8809T>C), ATT2876ATC (8812T>C), AAT2878AAC (8818T>C), CGG2880CGA (8824G>A), GCA2881GCG (8827A>G), ACG2888ACA (8848G>A), TAT2890TAC (8854T>C), GAG2891GGA (8856A>G 8857G>A), GTG2893GTA (8863G>A), CGG2894CAG (8865G>A), ATG2895CTT (8867A>C 8869G>T), GCA2896GCG (8872A>G), GGA2900GAA (8883G>A), ACT2902TCT (8888A>T), TGC2915TGT (8929C>T), GTC2924ATC (8954G>A), AAC2925AAT (8959C>T), GGA2926GGT (8962A>T), GGG2933GGA (8983G>A), AAT2934GAC (8984A>G 8986T>C), GAA2935GTA (8988A>T), GAG2938GAA (8998G>A), CCG2940CCA (9004G>A), TTG2941TTA (9007G>A), ATC2944ATT (9016C>T), ACC2951ACA (9037C>A), ATA2967ATT (9085A>T), AAC2971AAT (9097C>T), ATT2983GTT (9131A>G), CGA2984CGT (9136A>T), CGG2987CGT (9145G>T), ATG2989GGA (9149A>G 9150T>G 9151G>A), GGT2990AGT (9152G>A), TTA2991TTG (9157A>G), GCG2992GCT (9160G>T), CGT2993CGC (9163T>C), GAG3000GAA (9184G>A), CGA3004CGG (9196A>G), AGG3010AGA (9214G>A), GAA3011GAG (9217A>G), GCG3012GCA (9220G>A), GCA3020GCT (9244A>T), TTG3021TTA (9247G>A), GCC3024GCT (9256C>T), CCT3026TCC (9260C>T 9262T>C), GGG3030GGA (9274G>A), GAC3032GAT (9280C>T), ATC3035ATT (9289C>T), CAA3038CAC (9298A>C), GAG3040GAA (9304G>A), GTC3050GTT (9334C>T), GTC3060GTG (9364C>G)							
PVYgp2	1	75	100%	95	18.1%	75 (98.7%)	54 (71.1%)	1/0/3/2	2
Protein mutations:	R17_K17insX (2971_2972insA), Y23H (2989T>C 2991C>T), R26K (2999G>A 3000A>G), H35Y (3025C>T), K40H (3040A>C 3042A>C), G41S (3043G>A), E44K (3052G>A), I47V (3061A>G), V54I (3082G>A), P57S (3091C>T), P60T (3100C>A), G61E (3104G>A 3105T>G), G62S (3106G>A), Q63* (3109C>T), R64G (3112A>G), C66R (3118T>C), L67F (3121C>T), I69V (3127A>G), A72E (3137C>A 3138G>A), I73V (3139A>G)								
Codon mutations:	TTA2CTA (2923T>C), AAA4AAG (2931A>G), GAA7GAG (2940A>G), TTG10CTG (2947T>C), TTG15CTG (2962T>C), AGA17_AAA17insAA- (2971_2972insA), TAC23CAT (2989T>C 2991C>T), TCA24TCG (2994A>G), AGA26AAG (2999G>A 3000A>G), CAC32-AC (3016delC), TCG33TCA (3021G>A), GTA34GTT (3024A>T), CAT35TAT (3025C>T), CAC38CAT (3036C>T), AGA39AGG (3039A>G), AAA40CAC (3040A>C 3042A>C), GGC41AGC (3043G>A), GAA44AAA (3052G>A), ATA47GTA (3061A>G), GTT54ATT (3082G>A), CCG57TCG (3091C>T), CGC59CGT (3099C>T), CCA60ACA (3100C>A), GGT61GAG (3104G>A 3105T>G), GGT62AGT (3106G>A), CAA63TAA (3109C>T), AGG64GGG (3112A>G), TGC66CGC (3118T>C), CTC67TTC (3121C>T), ATT69GTT (3127A>G), GAG70GAA (3132G>A), GCG72GAA (3137C>A 3138G>A), ATT73GTT (3139A>G)								

	Begin	End	Coverage	Score	Concordance	Matches	Identities	I/D/M/F*	Stop Codons
**NT**	**5**	**9704**	**99.9%**	**12522**	**64.8%**	**9688 (99.7%)**	**8015 (82.5%)**	**15/12**	
**Proteins**									
polyprotein (NP_056759.1)	1	3064	100%	18923	89.7%	3062 (99.9%)	2771 (90.4%)	2/2/4/4	1
Protein mutations:	Y4Q (194T>C 196C>A), C9L (209T>C 210G>T 211T>G), F13M (221T>A 223T>G), S23P (251T>C), C24F (255G>T 256C>T), E25G (258A>G), I27V (263A>G 265T>G), V28A (267T>C), E30V (273A>T), A35T (287G>A 289T>C), V37T (293G>A 294T>C), A41I (305G>A 306C>T), D42G (309A>G), E44D (316A>T), T45V (317A>G 318C>T 319A>G), L52Q (339T>A 340C>A), K53R (342A>G), K55A (347A>G 348A>C 349A>G), Y56H (350T>C), T58V (356A>G 357C>T), V61T (365G>A 366T>C 367G>A), L62S (368C>T 369T>C), F67C (384T>G), A75V (408C>T 409C>T), M78E (416A>G 417T>A), E85K (437G>A), R86E (440A>G 441G>A), K87R (443A>C 444A>G 445G>A), D88E (448T>A), E91A (456A>C), H93N (461C>A), D111E (517T>A), S116F (531C>T), P118S (536C>T), Q119W (539C>T 540A>G 541A>G), R122K (549G>A 550A>G), M131T (576T>C), V134A (585T>C 586C>G), R137Y (593C>T 594G>A 595C>T), P138R (597C>G), I139V (599A>G 601A>G), I140P (602A>C 603T>C 604A>G), M147I (625G>A), I151V (635A>G 637T>C), I154V (644A>G 646A>G), E160A (663A>C 664G>C), H166Q (682C>A), T172S (699C>G), H174Q (706T>A), K179E (719A>G 721G>A), I180V (722A>G 724A>G), A183S (731G>T), Y184P (734T>C 735A>C 736C>T), S185R (737T>C 738C>G 739C>T), A187V (744C>T 745G>C), R189C (749C>T 751A>C), M194K (765T>A), R197K (773C>A 774G>A 775A>G), M206K (801T>A), G210A (813G>C 814A>G), L211H (816T>A), R214Q (825G>A 826T>A), N223D (851A>G 853T>C), R226C (860C>T 862C>T), T227A (863A>G 865T>C), I228T (867T>C), N229D (869A>G 871C>T), I230L (872A>T 874A>G), R231Q (876G>A), R232K (879G>A), N240S (903A>G), T241N (906C>A 907A>C), K242T (909A>C 910A>C), S243N (912G>A 913C>T), S252L (938T>C 939C>T 940A>T), L266I (980T>A 982G>C), R272K (999G>A), Q275R (1007C>A 1008A>G 1009G>A), S276G (1010A>G 1012T>G), I277V (1013A>G), N279Q (1019A>C 1021C>G), D288E (1048C>A), R300Q (1083G>A), S305A (1097T>G 1099G>A), R320K (1143G>A), L324I (1154T>A 1156G>A), S346N (1221G>A), V349T (1229G>A 1230T>C 1231T>C), S350N (1233G>A 1234C>T), K358T (1257A>C 1258A>G), N365S (1278A>G), A369V (1290C>T 1291G>A), D372T (1298G>A 1299A>C 1300C>T), I375V (1307A>G 1309A>G), N378E (1316A>G 1318T>A), I382T (1329T>C), L397I (1373C>A), I401V (1385A>G), E407D (1405G>T), A411S (1415G>T 1417A>T), V418I (1436G>A 1438C>T), E508G (1707A>G), A512T (1718G>A), I520V (1742A>G 1744C>T), S525N (1758G>A), V536I (1790G>A), I585V (1937A>G 1939T>C), F607Y (2004T>A), I628M (2068T>G), V630I (2072G>A 2074G>A), R675K (2208G>A), D694E (2266C>G), N744G (2414A>G 2415A>G 2416T>G), S746C (2420A>T), K780R (2523A>G), R817K (2634G>A), V847I (2723G>A 2725C>T), A855T (2747G>A 2749T>A), H871N (2795C>A), V880I (2822G>A 2824G>A), P900T (2882C>A), S901H (2885A>C 2886G>A), T904M (2895C>T), N916S (2931A>G), N919S (2940A>G), E929_N930insX (2971_2972insA), T936I (2991C>T), H937R (2994A>G), E939R (2999G>A 3000A>G), R946Q (3021G>A), Y947F (3024A>T 3025C>T), T951I (3036C>T), E952G (3039A>G 3040A>C), K953T (3042A>C 3043G>A), A972V (3099C>T 3100C>A), V974R (3104G>A 3105T>G 3106G>A), S983N (3132G>A), R985K (3137C>A 3138G>A 3139A>G), F990I (3152T>A 3154C>T), C994Y (3165G>A), F1012L (3218T>C), T1014N (3225C>A), V1016I (3230G>A), V1028M (3266G>A 3268A>G), V1031M (3275G>A), A1034T (3284G>A), R1044K (3315G>A 3316A>G), E1045A (3318A>C), M1059V (3359A>G), D1072E (3400T>G), V1110G (3513T>G), M1121V (3545A>G), A1122T (3548G>A), V1124I (3554G>A 3556C>T), L1147V (3623C>G), L1150M (3632C>A), Y1152H (3638T>C), I1172V (3698A>G), S1178N (3717G>A), R1183K (3731C>A 3732G>A), I1297V (4073A>G), F1315Y (4128T>A 4129C>T), A1323S (4151G>T), V1328I (4166G>A), L1347I (4223C>A 4225G>A), I1379V (4319A>G 4321A>G), F1404Y (4395T>A), V1408I (4406G>A), M1431V (4475A>G 4477G>T), T1433S (4481A>T 4483A>G), A1454L (4544G>T 4545C>T 4546G>A), R1455K (4548G>A), V1499I (4679G>A 4681G>C), V1538I (4796G>A 4798C>T), T1540S (4803C>G 4804T>C), S1625G (5057A>G), T1626A (5060A>G), L1636I (5090C>A), A1639V (5100C>T), E1641D (5107A>T), V1646F (5120G>T 5122C>T), I1648V (5126A>G), V1667I (5183G>A), A1793T (5561G>A 5563G>A), I1805V (5597A>G), N1807K (5605C>A), A1812V (5619C>T 5620T>G), I1816L (5630A>C), V1837I (5693G>A), K1890R (5853A>G), I1904V (5894A>G 5896C>G), R1926A (5960C>G 5961G>C 5962G>C), I1936V (5990A>G), E1945D (6019A>T), K1949T (6030A>C), D1955E (6049C>A), M1958I (6058G>A), S1963N (6072G>A 6073T>C), N1964H (6074A>C), T1966N (6081C>A), C1976S (6111G>C 6112T>C), I1982V (6128A>G 6130T>C), V1992I (6158G>A), T1996S (6170A>T 6172A>G), L2007F (6203C>T 6205C>T), V2016I (6230G>A), A2025K (6257G>A 6258C>A), K2033R (6282A>G), A2060T (6362G>A), V2073I (6401G>A), A2074V (6405C>T), Y2082F (6429A>T), Q2089R (6450A>G), H2101N (6485C>A 6487C>T), L2115I (6527C>A), T2135I (6588C>T), Y2151H (6635T>C), I2155V (6647A>G), I2165V (6677A>G 6679A>G), D2178N (6716G>A), N2179D (6719A>G), I2194L (6764A>T 6766A>G), A2204V (6795C>T 6796A>G), H2205Q (6799C>A), N2225D (6857A>G), V2231T (6875G>A 6876T>C 6877C>A), D2250E (6934C>A), V2273I (7001G>A 7003G>A), F2283Y (7032T>A), T2314K (7125C>A), A2321S (7145G>T 7147A>G), E2326_A2327del (7160_7165delGAGGCA), D2344E (7216T>A), D2358E (7258T>A), D2363_R2364insX (7273_7274insT), R2364V (7274C>G 7275G>T), H2366L (7281A>T), L2367S (7284T>C), S2371L (7296C>T), S2376F (7311C>T), T2377I (7314C>T), C2378Y (7317G>A), N2379K (7321T>A), K2384Q (7334A>C), A2387N (7343G>A 7344C>A 7345A>C), S2408M (7407G>T 7408T>G), C2411G (7415T>G), L2440S (7503T>C 7504G>A), D2509N (7709G>A), K2510R (7713A>G 7714A>G), T2543I (7812C>T), V2554L (7844G>T 7846G>A), V2567I (7883G>A), S2620N (8043G>A), N2656S (8151A>G 8152C>T), R2665K (8178G>A), R2675K (8208G>A), P2704A (8294C>G 8296A>G), S2722F (8349C>T), A2742S (8408G>T), M2756I (8452G>A), R2759K (8460G>A), A2766T (8480G>A 8482T>A), R2773K (8502G>A 8503A>G), E2777D (8515A>T), E2785D (8539G>C), L2788C (8546C>T 8547T>G), S2790T (8552T>A), A2797G (8574C>G), I2801V (8585A>G), G2805E (8598G>A), N2807S (8603A>T 8604A>C 8605C>T), P2813Q (8622C>A), E2814G (8625A>G 8626G>A), P2822F (8648C>T 8649C>T 8650G>C), G2825E (8658G>A), D2827A (8664A>C 8665T>G), A2832V (8679C>T 8680A>T), T2854K (8745C>A 8746A>G), E2891G (8856A>G 8857G>A), R2894Q (8865G>A), M2895L (8867A>C 8869G>T), G2900E (8883G>A), T2902S (8888A>T), V2924I (8954G>A), N2934D (8984A>G 8986T>C), E2935V (8988A>T), I2983V (9131A>G), M2989G (9149A>G 9150T>G 9151G>A), G2990S (9152G>A), P3026S (9260C>T 9262T>C), Q3038H (9298A>C)								

	Begin	End	Coverage	Score	Concordance	Matches	Identities	I/D/M/F*	Stop Codons
NT	5	9704	99.9%	12522	64.8%	9688 (99.7%)	8015 (82.5%)	15/12	

Codon mutations:

ACT3ACC (193T>C), TAC4CAA (194T>C 196C>A), TCA6TCG (202A>G), ATC8ATT (208C>T), TGT9CTG (209T>C 210G>T 211T>G), TCG12TCA (220G>A), TTT13ATG (221T>A 223T>G), GAA14GAG (226A>G), CTA17CTT (235A>T), TAC19TAT (241C>T), CCA21CCT (247A>T), TCT23CCT (251T>C), TGC24TTT (255G>T 256C>T), GAG25GGG (258A>G), CAT26CAC (262T>C), ATT27GTG (263A>G 265T>G), GTG28GCG (267T>C), GAA30GTA (273A>T), CGA31CGT (277A>T), GAA32GAG (280A>G), GTG33GTT (283G>T), CCG34CCA (286G>A), GCT35ACC (287G>A 289T>C), GTT37ACT (293G>A 294T>C), CCT39CCC (301T>C), TTC40TTT (304C>T), GCA41ATA (305G>A 306C>T), GAT42GGT (309A>G), CTG43TTG (311C>T), GAA44GAT (316A>T), ACA45GTG (317A>G 318C>T 319A>G), CAA46CAG (322A>G), GCA49GCT (331A>T), CTC52CAA (339T>A 340C>A), AAG53AGG (342A>G), AAA55GCG (347A>G 348A>C 349A>G), TAT56CAT (350T>C), GCT57GCA (355T>A), ACT58GTT (356A>G 357C>T), CGT60CGC (364T>C), GTG61ACA (365G>A 366T>C 367G>A), CTC62TCC (368C>T 369T>C), AAA63AAG (373A>G), AAC64AAT (376C>T), TTT67TGT (384T>G), ACG68ACA (388G>A), TAC69TAT (391C>T), CGA70CGG (394A>G), AAG72AAA (400G>A), GAT74GAC (406T>C), GCC75GTT (408C>T 409C>T), CAG76CAA (412G>A), ATA77ATT (415A>T), ATG78GAG (416A>G 417T>A), ATT80ATC (424T>C), CAG81CAA (427G>A), AAA83AAG (433A>G), CTG84CTC (436G>C), GAG85AAG (437G>A), AGG86GAG (440A>G 441G>A), AAG87CGA (443A>C 444A>G 445G>A), GAT88GAA (448T>A), GAA90GAG (454A>G), GAA91GCA (456A>C), TAT92TAC (460T>C), CAC93AAC (461C>A), CAA95CAG (469A>G), GCC97GCG (475C>G), CCT99CCA (481T>A), ACT106ACC (502T>C), ATA107ATT (505A>T), GGC109GGT (511C>T), GAT111GAA (517T>A), CCT112CCA (520T>A), CCA113CCT (523A>T), TCT116TTT (531C>T), GAG117GAA (535G>A), CCA118TCA (536C>T), CAA119TGG (539C>T 540A>G 541A>G), AGA122AAG (549G>A 550A>G), ATC124ATT (556C>T), ATT125ATC (559T>C), CAT126CAC (562T>C), ACT128ACC (568T>C), CCA129CCG (571A>G), ATG131ACG (576T>C), CGT132CGC (580T>C), GTC134GCG (585T>C 586C>G), CGC137TAT (593C>T 594G>A 595C>T), CCC138CGC (597C>G), ATA139GTG (599A>G 601A>G), ATA140CCG (602A>C 603T>C 604A>G), ACA143ACG (613A>G), GAA144GAG (616A>G), GGC145GGG (619C>G), CAG146CAA (622G>A), ATG147ATA (625G>A), AAT148AAC (628T>C), CAC149CAT (631C>T), ATT151GTC (635A>G 637T>C), ATA154GTG (644A>G 646A>G), ATT157ATC (655T>C), TCG159TCA (661G>A), GAG160GCC (663A>C 664G>C), AAA161AAG (667A>G), GTC165GTT (679C>T), CAC166CAA (682C>A), TTA167CTG (683T>C 685A>G), AGT169AGC (691T>C), ACC172AGC (699C>G), ACT173ACA (703T>A), CAT174CAA (706T>A), AAG179GAA (719A>G 721G>A), ATA180GTG (722A>G 724A>G), CTT181TTG (725C>T 727T>G), GGT182GGA (730T>A), GCA183TCA (731G>T), TAC184CCT (734T>C 735A>C 736C>T), TCC185CGT (737T>C 738C>G 739C>T), GCG187GTC (744C>T 745G>C), CGA189TGC (749C>T 751A>C), ACT190ACC (754T>C), ATG194AAG (765T>A), TTG196CTG (770T>C), CGA197AAG (773C>A 774G>A 775A>G), CGG198AGG (776C>A), TTC202TTT (790C>T), ATG206AAG (801T>A), GGA210GCG (813G>C 814A>G), CTT211CAT (816T>A), TTG212CTG (818T>C), CAA213CAG (823A>G), CGT214CAA (825G>A 826T>A), CGG217AGA (833C>A 835G>A), GAC219GAT (841C>T), AAT223GAC (851A>G 853T>C), GTC225GTT (859C>T), CGC226TGT (860C>T 862C>T), ACT227GCC (863A>G 865T>C), ATC228ACC (867T>C), AAC229GAT (869A>G 871C>T), ATA230TTG (872A>T 874A>G), CGA231CAA (876G>A), AGG232AAG (879G>A), GAT234GAC (886T>C), ATC238ATT (898C>T), AAC240AGC (903A>G), ACA241AAC (906C>A 907A>C), AAA242ACC (909A>C 910A>C), AGC243AAT (912G>A 913C>T), GGC246GGA (922C>A), GGT249GGA (931T>A), TCA252CTT (938T>C 939C>T 940A>T), GGA253GGG (943A>G), TTC256TTT (952C>T), TCA261TCG (967A>G), GAA263GAG (973A>G), GGG264GGC (976G>C), TTG266ATC (980T>A 982G>C), GCA269GCG (991A>G), CGT270CGC (994T>C), AGA272AAA (999G>A), GTT273GTC (1003T>C), CAG275AGA (1007C>A 1008A>G 1009G>A), AGT276GGG (1010A>G 1012T>G), ATT277GTT (1013A>G), TTA278TTG (1018A>G), AAC279CAG (1019A>C 1021C>G), ATC282ATT (1030C>T), TCG285TCA (1039G>A), AAT286AAC (1042T>C), GCC287GCA (1045C>A), GAC288GAA (1048C>A), AAT289AAC (1051T>C), AAG292AAA (1060G>A), GGT293GGA (1063T>A), CTG294TTG (1064C>T), GAC295GAT (1069C>T), CGA300CAA (1083G>A), TAT303TAC (1093T>C), TCG305GCA (1097T>G 1099G>A), CAC307CAT (1105C>T), GTA310GTG (1114A>G), GCT311GCG (1117T>G), TTA313TTG (1123A>G), CCT314CCA (1126T>A), GTC315GTT (1129C>T), AGG320AAG (1143G>A), GTA321GTT (1147A>T), GCT322GCA (1150T>A), TTG324ATA (1154T>A 1156G>A), CAC327CAT (1165C>T), AGT328AGC (1168T>C), CTT330TTA (1172C>T 1174T>A), ACC339ACT (1201C>T), AGC346AAC (1221G>A), GTT349ACC (1229G>A 1230T>C 1231T>C), AGC350AAT (1233G>A 1234C>T), CTG352CTA (1240G>A), AAG354AAA (1246G>A), TTG356CTA (1250T>C 1252G>A), CAT357CAC (1255T>C), AAA358ACG (1257A>C 1258A>G), AGA361AGG (1267A>G), GGT363GGC (1273T>C), TTG364TTA (1276G>A), AAT365AGT (1278A>G), TTG367CTG (1283T>C), GCG369GTA (1290C>T 1291G>A), GAC372ACT (1298G>A 1299A>C 1300C>T), TTT374TTC (1306T>C), ATA375GTG (1307A>G 1309A>G), CAT376CAC (1312T>C), AAT378GAA (1316A>G 1318T>A), TTG381TTA (1327G>A), ATA382ACA (1329T>C), GCG383GCT (1333G>T), TTA384TTG (1336A>G), CTA387TTG (1343C>T 1345A>G), CCG390CCA (1354G>A), GTG391GTT (1357G>T), GAC392GAT (1360C>T), CTC395TTG (1367C>T 1369C>G), CTT397ATT (1373C>A), TTC398TTT (1378C>T), GAG400GAA (1384G>A), ATA401GTA (1385A>G), AAA403AAG (1393A>G), TCC404TCT (1396C>T), GAG407GAT (1405G>T), AAA408AAG (1408A>G), CAG409CAA (1411G>A), GCA411TCT (1415G>T 1417A>T), CCG412CCC (1420G>C), AAT415AAC (1429T>C), TTA416TTG (1432A>G), GTC418ATT (1436G>A 1438C>T), TTA419CTG (1439T>C 1441A>G), AAT420AAC (1444T>C), TTC423TTT (1453C>T), CTG424CTA (1456G>A), GAA428GAG (1468A>G), GTA436GTG (1492A>G), TTG439CTA (1499T>C 1501G>A), AGT440AGC (1504T>C), TTG441TTA (1507G>A), CTC442CTT (1510C>T), GAA443GAG (1513A>G), TTA444TTG (1516A>G), GCA445GCT (1519A>T), AGG446AGA (1522G>A), TTC447TTT (1525C>T), CAG448CAA (1528G>A), AAC450AAT (1534C>T), ACT452ACA (1540T>A), AAC454AAT (1546C>T), AAA457AAG (1555A>G), GGT458GGC (1558T>C), GAT459GAC (1561T>C), ATA460ATC (1564A>C), TCT461TCA (1567T>A), TTC463TTT (1573C>T), AGA464AGG (1576A>G), TTA467CTA (1583T>C), TCT468TCA (1588T>A), AAG470AAA (1594G>A), AAC472AAT (1600C>T), CTG475CTA (1609G>A), TCG478TCA (1618G>A), TGC479TGT (1621C>T), GAC480GAT (1624C>T), CTG483TTG (1631C>T), AAA485AAG (1639A>G), TTC489TTT (1651C>T), CTC490CTG (1654C>G), CAT497CAC (1675T>C), TCA503TCG (1693A>G), TTT506TTC (1702T>C), GAA508GGA (1707A>G), GCA512ACA (1718G>A), GGA514GGG (1726A>G), TAC515TAT (1729C>T), TCA516TCC (1732A>C), TAT518TAC (1738T>C), ATC520GTT (1742A>G 1744C>T), CGC521CGT (1747C>T), CAT523CAC (1753T>C), AGT525AAT (1758G>A), AGG528AGA (1768G>A), AAC534AAT (1786C>T), TTA535TTG (1789A>G), GTT536ATT (1790G>A), GTC537GTT (1795C>T), TTA541CTG (1805T>C 1807A>G), GAG543GAA (1813G>A), AGG545AGA (1819G>A), AAG547AAA (1825G>A), GAC551GAT (1837C>T), TAT552TAC (1840T>C), CAA555CAG (1849A>G), GTC558GTG (1858C>G), AAA560AAG (1864A>G), TGC562TGT (1870C>T), AGT564AGC (1876T>C), AAA566AAG (1882A>G), GGT568GGA (1888T>A), AAT569AAC (1891T>C), TGT574TGC (1906T>C), ACA577ACT (1915A>T), GGT582GGC (1930T>C), GCC584GCT (1936C>T), ATT585GTC (1937A>G 1939T>C), TCA587TCG (1945A>G), TTC589TTT (1951C>T), CCA592CCT (1960A>T), GTA598GTG (1978A>G), ATT599ATA (1981T>A), GGC600GGT (1984C>T), AAT601AAC (1987T>C), GAC604GAT (1996C>T), TTT607TAT (2004T>A), AAA612AAG (2020A>G), GGG613GGA (2023G>A), GAT614GAC (2026T>C), TCG615TCT (2029G>T), GAG616GAA (2032G>A), TAT625TAC (2059T>C), ATT628ATG (2068T>G), AAC629AAT (2071C>T), GTG630ATA (2072G>A 2074G>A), CTG635TTA (2087C>T 2089G>A), AGC639AGT (2101C>T), GAG640GAA (2104G>A), GAG641GAA (2107G>A), AAG644AAA (2116G>A), TTC646TTT (2122C>T), CGC651CGT (2137C>T), CTT658CTC (2158T>C), ACC660ACT (2164C>T), CCA662CCG (2170A>G), GAT666GAC (2182T>C), GCG668GCA (2188G>A), ACC669ACA (2191C>A), AGA675AAA (2208G>A), TAT678TAC (2218T>C), GAC680GAT (2224C>T), GTA681GTT (2227A>T), TTG686TTA (2242G>A), CCC687CCT (2245C>T), TTG690TTA (2254G>A), GTT691GTC (2257T>C), GAC692GAT (2260C>T), GAC694GAG (2266C>G), CAA696CAG (2272A>G), ACG697ACA (2275G>A), TCA703TCG (2293A>G), GGC705GGT (2299C>T), TCG706TCA (2302G>A), CAG707CAA (2305G>A), GGA710GGT (2314A>T), TAT711TAC (2317T>C), CTA714CTG (2326A>G), GTG719GTA (2341G>A), CAA721CAG (2347A>G), CTT722CTC (2350T>C), TTG724TTA (2356G>A), AAT727AAC (2365T>C), GAA729GAG (2371A>G), TTA730TTG (2374A>G), GAT733GAC (2383T>C), ATA734ATT (2386A>T), AAA735AAG (2389A>G), CAT736CAC (2392T>C), TAT737TAC (2395T>C), AAT744GGG (2414A>G 2415A>G 2416T>G), GCT745GCA (2419T>A), AGC746TGC (2420A>T), ACA752ACT (2440A>T), GTT761GTC (2467T>C), TCT764TCC (2476T>C), TCG766TCA (2482G>A), CTG769CTA (2491G>A), CTG771CTT (2497G>T), TTG773TTA (2503G>A), AGA778AGG (2518A>G), CCT779CCC (2521T>C), AAG780AGG (2523A>G), AGA783AGG (2533A>G), TTG785TTA (2539G>A), TTA787TTG (2545A>G), GAG789GAA (2551G>A), CCT790CCA (2554T>A), CTG792TTG (2558C>T), TTG793CTC (2561T>C 2563G>C), TCA796TCG (2572A>G), CCT800CCC (2584T>C), GGC801GGT (2587C>T), CTG803CTC (2593G>C), GCT805GCC (2599T>C), AAT808AAC (2608T>C), TTT812TTC (2620T>C), GCG815GCA (2629G>A), GTG816GTA (2632G>A), AGG817AAG (2634G>A), TTG818TTA (2638G>A), ATT820ATA (2644T>A), AAA823AAG (2653A>G), GCT827GCC (2665T>C), CTA832TTA (2678C>T), CTA833CTG (2683A>G), GCT835GCC (2689T>C), CTA838CTC (2698A>C), TCA841TCG (2707A>G), GCG842GCA (2710G>A), GAA844GAG (2716A>G), ACA845ACG (2719A>G), CTC846CTT (2722C>T), GTC847ATT (2723G>A 2725C>T), GCA848GCG (2728A>G), AGG850AGA (2734G>A), ATT851ATA (2737T>A), GAT854GAC (2746T>C), GCT855ACA (2747G>A 2749T>A), GCA856GCT (2752A>T), GCT857GCA (2755T>A), GAC859GAT (2761C>T), CTC860CTT (2764C>T), TGT865TGC (2779T>C), GGG867GGT (2785G>T), AAC869AAT (2791C>T), CAT871AAT (2795C>A), TAC874TAT (2806C>T), TTG878CTC (2816T>C 2818G>C), GTG880ATA (2822G>A 2824G>A), TTG881TTA (2827G>A), GTT884GTC (2836T>C), AAG885AAA (2839G>A), AAT886AAC (2842T>C), ACC893ACA (2863C>A), CTA894TTA (2864C>T), CCA900ACA (2882C>A), AGT901CAT (2885A>C 2886G>A), AAC903AAT (2893C>T), ACG904ATG (2895C>T), GTC906GTT (2902C>T), GTA907GTG (2905A>G), ATT909ATC (2911T>C), AAT913AAC (2923T>C), AAT916AGT (2931A>G), AAC919AGC (2940A>G), GCT921GCC (2947T>C), ACT926ACC (2962T>C), GAA929_AAT930insA-- (2971_2972insA), GGT935GGC (2989T>C), ACT936ATT (2991C>T), CAT937CGT (2994A>G), GAG939AGG (2999G>A 3000A>G), TCC944TC- (3016delC), CGG946CAG (3021G>A), TAC947TTT (3024A>T 3025C>T), ACA951ATA (3036C>T), GAA952GGC (3039A>G 3040A>C), AAG953ACA (3042A>C 3043G>A), TTG956TTA (3052G>A), TTA959TTG (3061A>G), GCG966GCA (3082G>A), GGC969GGT (3091C>T), GCC972GTA (3099C>T 3100C>A), GTG974AGA (3104G>A 3105T>G 3106G>A), GTC975GTT (3109C>T), AAA976AAG (3112A>G), ACT978ACC (3118T>C), GCC979GCT (3121C>T), GGA981GGG (3127A>G), AGC983AAC (3132G>A), CGA985AAG (3137C>A 3138G>A 3139A>G), TAT989TAC (3151T>C), TTC990ATT (3152T>A 3154C>T), TGT994TAT (3165G>A), GTA995GTG (3169A>G), ATC1005ATT (3199C>T), AGG1007CGG (3203A>C), CGT1008CGC (3208T>C), CCA1010CCG (3214A>G), ACC1011ACT (3217C>T), TTT1012CTT (3218T>C), GTC1013GTA (3223C>A), ACT1014AAT (3225C>A), GTT1016ATT (3230G>A), AAC1017AAT (3235C>T), TTA1025CTA (3257T>C), ACC1026ACT (3262C>T), AGC1027AGT (3265C>T), GTA1028ATG (3266G>A 3268A>G), GTG1029GTA (3271G>A), GTG1031ATG (3275G>A), GCA1034ACA (3284G>A), CAG1039CAA (3301G>A), AGA1044AAG (3315G>A 3316A>G), GAA1045GCA (3318A>C), ATC1046ATT (3322C>T), GAG1047GAA (3325G>A), TTG1048TTA (3328G>A), ATA1051ATT (3337A>T), GAG1055GAA (3349G>A), GTC1057GTT (3355C>T), TGC1058TGT (3358C>T), ATG1059GTG (3359A>G), CTA1061CTG (3367A>G), TAT1062TAC (3370T>C), TTA1065CTG (3377T>C 3379A>G), GAA1070GAG (3394A>G), CGC1071CGT (3397C>T), GAT1072GAG (3400T>G), ACA1074ACG (3406A>G), TAC1078TAT (3418C>T), ATT1079ATA (3421T>A), TAT1081TAC (3427T>C), AAG1083AAA (3433G>A), TCA1084TCT (3436A>T), GTA1085GTG (3439A>G), AAC1086AAT (3442C>T), CCT1087CCC (3445T>C), CAG1091CAA (3457G>A), TTT1092TTC (3460T>C), GCT1093GCA (3463T>A), GCG1095GCT (3469G>T), CAG1096CAA (3472G>A), CAC1104CAT (3496C>T), CAG1105CAA (3499G>A), TCC1107TCT (3505C>T), GTT1110GGT (3513T>G), AAA1112AAG (3520A>G), GAA1115GAG (3529A>G), CAA1116CAG (3532A>G), ATG1121GTG (3545A>G), GCT1122ACT (3548G>A), TTA1123CTA (3551T>C), GTC1124ATT (3554G>A 3556C>T), AGT1133AGC (3583T>C), GAT1134GAC (3586T>C), TGC1135TGT (3589C>T), TTC1137TTT (3595C>T), AAT1141AAC (3607T>C), TTT1143TTC (3613T>C), AAG1144AAA (3616G>A), GGT1145GGC (3619T>C), CTT1147GTT (3623C>G), TCC1148TCT (3628C>T), CTG1150ATG (3632C>A), GAT1151GAC (3637T>C), TAT1152CAT (3638T>C), GAA1153GAG (3643A>G), TTA1159TTG (3661A>G), GTG1162GTA (3670G>A), TTT1166TTC (3682T>C), GAG1168GAA (3688G>A), GAG1171GAA (3697G>A), ATT1172GTT (3698A>G), GAT1174GAC (3706T>C), TTT1175TTC (3709T>C), TTG1177CTA (3713T>C 3715G>A), AGT1178AAT (3717G>A), GAG1179GAA (3721G>A), CGA1183AAA (3731C>A 3732G>A), ACT1184ACA (3736T>A), CTA1188TTG (3746C>T 3748A>G), ACA1190ACG (3754A>G), AGT1193AGC (3763T>C), CGA1198CGG (3778A>G), CAA1199CAG (3781A>G), ATC1200ATT (3784C>T), CAT1204CAC (3796T>C), CCA1207CCT (3805A>T), TAC1209TAT (3811C>T), AGA1210AGG (3814A>G), TTC1215TTT (3829C>T), TTT1218TTC (3838T>C), AGA1220AGG (3844A>G), GCA1221GCC (3847A>C), GTT1224GTA (3856T>A), GTG1226GTT (3862G>T), GAC1229GAT (3871C>T), GCC1231GCA (3877C>A), AGC1233AGT (3883C>T), GAA1234GAG (3886A>G), CTA1236CTG (3892A>G), GAC1237GAT (3895C>T), TTT1238TTC (3898T>C), GTA1240GTG (3904A>G), CGG1241AGA (3905C>A 3907G>A), GCT1243GCC (3913T>C), AAG1248AAA (3928G>A), TCA1249TCT (3931A>T), GGG1251GGA (3937G>A), AGT1257AGC (3955T>C), GTG1258GTA (3958G>A), GCC1259GCT (3961C>T), GGA1260GGG (3964A>G), TCT1261TCC (3967T>C), TTA1264TTG (3976A>G), ATT1265ATC (3979T>C), CCA1267CCT (3985A>T), CTA1271CTT (3997A>T), GCG1272GCA (4000G>A), AAC1274AAT (4006C>T), GTT1275GTG (4009T>G), TTC1276TTT (4012C>T), AAA1277AAG (4015A>G), TCT1280TCC (4024T>C), GAA1282GAG (4030A>G), CCA1283CCT (4033A>T), AAG1286AAA (4042G>A), CTG1290TTA (4052C>T 4054G>A), CGT1291CGC (4057T>C), CGT1293CGA (4063T>A), GGA1294GGG (4066A>G), ATA1297GTA (4073A>G), TTT1298TTC (4078T>C), GGC1299GGT (4081C>T), TCT1300TCC (4084T>C), TCC1304TCT (4096C>T), AGC1308AGT (4108C>T), GGA1309GGT (4111A>T), GCG1311GCA (4117G>A), CTA1312TTG (4118C>T 4120A>G), TAC1314TAT (4126C>T), TTC1315TAT (4128T>A 4129C>T), GCC1316GCT (4132C>T), AAT1317AAC (4135T>C), CAA1321CAG (4147A>G), TTA1322CTA (4148T>C), GCT1323TCT (4151G>T), AAC1326AAT (4162C>T), GTA1328ATA (4166G>A), ATA1329ATC (4171A>C), GAG1332GAA (4180G>A), CTG1336TTG (4190C>T), CCT1338CCC (4198T>C), TCC1339TCT (4201C>T), GCG1342GCT (4210G>T), TTC1343TTT (4213C>T), AGT1345AGC (4219T>C), CTG1346TTG (4220C>T), CTG1347ATA (4223C>A 4225G>A), GTT1349GTG (4231T>G), CAT1351CAC (4237T>C), GTA1356GTG (4252A>G), TTA1357CTG (4253T>C 4255A>G), AAA1358AAG (4258A>G), ACT1362ACC (4270T>C), AGA1366AGG (4282A>G), GAG1367GAA (4285G>A), GAA1369GAG (4291A>G), CAG1373CAA (4303G>A), GTC1376GTT (4312C>T), ATA1379GTG (4319A>G 4321A>G), GTG1380GTT (4324G>T), GAG1381GAA (4327G>A), CTG1384CTT (4336G>T), TCT1385TCC (4339T>C), TCA1388TCT (4348A>T), GCA1392GCG (4360A>G), GGT1394GGC (4366T>C), TCT1395TCA (4369T>A), ACT1397ACC (4375T>C), GTT1401GTC (4387T>C), TTT1404TAT (4395T>A), GTA1408ATA (4406G>A), CTT1409CTC (4411T>C), GTT1418GTC (4438T>C), ACC1420ACA (4444C>A), GCC1422GCT (4450C>T), CTC1424CTT (4456C>T), GAC1427GAT (4465C>T), ATG1431GTT (4475A>G 4477G>T), GTC1432GTT (4480C>T), ACA1433TCG (4481A>T 4483A>G), GGC1437GGT (4495C>T), AAG1441AAA (4507G>A), CAC1442CAT (4510C>T), GGT1443GGG (4513T>G), CTA1445TTA (4517C>T), GTC1448GTT (4528C>T), AAA1450AAG (4534A>G), ACC1452ACT (4540C>T), GCG1454TTA (4544G>T 4545C>T 4546G>A), AGA1455AAA (4548G>A), TTT1458TTC (4558T>C), GTA1460GTT (4564A>T), ACC1462ACT (4570C>T), ATA1464ATT (4576A>T), TTG1471TTA (4597G>A), GAC1472GAT (4600C>T), GAC1474GAT (4606C>T), GTG1475GTA (4609G>A), GTG1477GTA (4615G>A), GAC1478GAT (4618C>T), TTG1481CTT (4625T>C 4627G>T), GAC1488GAT (4648C>T), ATT1489ATC (4651T>C), AGG1492AGA (4660G>A), AGC1493AGT (4663C>T), GCT1495GCA (4669T>A), GTG1499ATC (4679G>A 4681G>C), AGT1500AGC (4684T>C), GTT1501GTG (4687T>G), GAG1505GAA (4699G>A), AGA1506AGG (4702A>G), CAA1508CAG (4708A>G), AGG1509AGA (4711G>A), CTG1510TTG (4712C>T), AAA1518AAG (4738A>G), GTA1520GTT (4744A>T), GCA1521GCG (4747A>G), TTG1522CTG (4748T>C), ACT1527ACC (4765T>C), GTC1538ATT (4796G>A 4798C>T), ACT1540AGC (4803C>G 4804T>C), GAG1541GAA (4807G>A), GCG1542GCT (4810G>T), GCT1543GCG (4813T>G), CTT1544CTC (4816T>C), TAT1549TAC

	(4831T>C), AAC1550AAT (4834C>T), CCA1552CCT (4840A>T), GGA1556GGG (4852A>G), GGC1557GGT (4855C>T), AGT1561AGC (4867T>C), CTG1562CTC (4870G>C), ATT1563ATA (4873T>A), TGT1566TGC (4882T>C), ACA1573ACT (4903A>T), CAG1575CAA (4909G>A), TTT1577TTC (4915T>C), GAA1578GAG (4918A>G), TTG1579CTA (4919T>C 4921G>A), AGT1580AGC (4924T>C), CCC1581CCG (4927C>G), TTT1583TTC (4933T>C), ATC1584ATA (4936C>A), CAG1585CAA (4939G>A), AAT1586AAC (4942T>C), TTC1587TTT (4945C>T), TCA1593TCG (4963A>G), CCT1596CCC (4972T>C), CTT1602CTC (4990T>C), AAA1606AAG (5002A>G), CCT1613CCC (5023T>C), TTG1614CTG (5024T>C), TGC1615TGT (5029C>T), GAT1616GAC (5032T>C), TCT1618TCC (5038T>C), CCA1620CCT (5044A>T), GCA1623GCT (5053A>T), TCG1624TCA (5056G>A), AGT1625GGT (5057A>G), ACT1626GCT (5060A>G), TCG1629TCT (5071G>T), CTT1636ATT (5090C>A), GGA1637GGG (5095A>G), GTG1638GTT (5098G>T), GCC1639GTC (5100C>T), TTA1640TTG (5104A>G), GAA1641GAT (5107A>T), ATT1642ATC (5110T>C), CCA1643CCG (5113A>G), AAG1644AAA (5116G>A), CAA1645CAG (5119A>G), GTC1646TTT (5120G>T 5122C>T), ATT1648GTT (5126A>G), CAT1651CAC (5137T>C), ATC1652ATT (5140C>T), AAA1653AAG (5143A>G), GAG1654GAA (5146G>A), CTC1659CTA (5161C>A), GAA1661GAG (5167A>G), CTT1663CTG (5173T>G), ACG1666ACA (5182G>A), GTT1667ATT (5183G>A), GTC1668GTT (5188C>T), AAG1669AAA (5191G>A), AAA1671AAG (5197A>G), GAC1672GAT (5200C>T), TGC1674TGT (5206C>T), TTA1675TTG (5209A>G), AGC1678AGT (5218C>T), TCG1682TCA (5230G>A), ATC1684ATT (5236C>T), AGC1685AGT (5239C>T), ATC1687ATT (5245C>T), ACA1693ACT (5263A>T), CTC1695CTT (5269C>T), TTC1696TTT (5272C>T), GCC1697GCA (5275C>A), ATC1698ATT (5278C>T), CCA1699CCC (5281A>C), AGA1700AGG (5284A>G), ACT1701ACC (5287T>C), CTA1702TTA (5288C>T), ATA1703ATT (5293A>T), TTG1704CTA (5294T>C 5296G>A), GAG1706GAA (5302G>A), CTT1709CTA (5311T>A), GAA1710GAG (5314A>G), CGA1713CGT (5323A>T), AGT1721AGC (5347T>C), ATC1723ATT (5353C>T), TGC1727TGT (5365C>T), GTT1734GTC (5386T>C), ACC1737ACA (5395C>A), AAA1746AAG (5422A>G), GAT1747GAC (5425T>C), ACC1749ACT (5431C>T), GAG1751GAA (5437G>A), CTT1756CTC (5452T>C), AAG1758AAA (5458G>A), AGT1761AGC (5467T>C), CAA1762CAG (5470A>G), CTA1763TTG (5471C>T 5473A>G), GAA1765GAG (5479A>G), TTC1766TTT (5482C>T), TCA1767TCC (5485A>C), AAT1768AAC (5488T>C), TTG1769CTA (5489T>C 5491G>A), GGT1771GGC (5497T>C), TGT1774TGC (5506T>C), GAG1775GAA (5509G>A), GAG1776GAA (5512G>A), TTA1778CTA (5516T>C), TAT1782TAC (5530T>C), GAG1783GAA (5533G>A), TCG1784TCT (5536G>T), TTG1785TTA (5539G>A), GTT1788GTG (5548T>G), CAC1790CAT (5554C>T), GCT1792GCA (5560T>A), GCG1793ACA (5561G>A 5563G>A), ACG1794ACC (5566G>C), TCA1795TCG (5569A>G), CTC1800CTG (5584C>G), AAG1803AAA (5593G>A), GGG1804GGA (5596G>A), ATT1805GTT (5597A>G), AAC1807AAA (5605C>A), GTG1811GTT (5617G>T), GCT1812GTG (5619C>T 5620T>G), TTG1815TTA (5629G>A), ATC1816CTC (5630A>C), GGC1819GGT (5641C>T), GCA1822GCT (5650A>T), ATA1826ATT (5662A>T), GGA1827GGG (5665A>G), CAA1835CAG (5689A>G), GTT1837ATT (5693G>A), GAG1838GAA (5698G>A), CAA1843CAG (5713A>G), GGG1844GGC (5716G>C), AAA1845AAG (5719A>G), GCC1853GCA (5743C>A), CGC1857CGA (5755C>A), CAT1858CAC (5758T>C), CGT1860CGC (5764T>C), AAA1862AAG (5770A>G), GGC1865GGT (5779C>T), TTT1866TTC (5782T>C), GAC1869GAT (5791C>T), GAC1873GAT (5803C>T), AGG1884AGA (5836G>A), AAA1885AAG (5839A>G), GGA1887GGG (5845A>G), AAA1888AAG (5848A>G), AAA1890AGA (5853A>G), GGT1891GGC (5857T>C), ACC1892ACT (5860C>T), ACA1893ACT (5863A>T), GGT1895GGA (5869T>A), GGT1897GGC (5875T>C), AGC1900AGT (5884C>T), TTC1903TTT (5893C>T), ATC1904GTG (5894A>G 5896C>G), AAC1905AAT (5899C>T), GGG1908GGA (5908G>A), TTT1909TTC (5911T>C), GAG1913GAA (5923G>A), CAA1918CAG (5938A>G), TTC1919TTT (5941C>T), GTT1920GTC (5944T>C), GAT1921GAC (5947T>C), GGG1925GGA (5959G>A), CGG1926GCC (5960C>G 5961G>C 5962G>C), GAA1930GAG (5974A>G), GCT1934GCA (5986T>A), GAC1935GAT (5989C>T), ATT1936GTT (5990A>G), GAT1938GAC (5998T>C), AGA1942CGT (6008A>C 6010A>T), GAA1945GAT (6019A>T), GTG1946GTT (6022G>T), CGA1947CGT (6025A>T), AAA1949ACA (6030A>C), GAC1955GAA (6049C>A), ATG1958ATA (6058G>A), CAA1959CAG (6061A>G), GCC1960GCA (6064C>A), GGT1962GGC (6070T>C), AGT1963AAC (6072G>A 6073T>C), AAC1964CAC (6074A>C), ACG1965ACA (6079G>A), ACC1966AAC (6081C>A), ATA1967ATT (6085A>T), GCA1969GCT (6091A>T), TAC1970TAT (6094C>T), TGT1976TCC (6111G>C 6112T>C), AAA1978AAG (6118A>G), TTG1980TTA (6124G>A), AAG1981AAA (6127G>A), ATT1982GTC (6128A>G 6130T>C), GAT1983GAC (6133T>C), TTA1984TTG (6136A>G), AAC1988AAT (6148C>T), AAA1991AAG (6157A>G), GTT1992ATT (6158G>A), TGT1993TGC (6163T>C), AAA1995AAG (6169A>G), ACA1996TCG (6170A>T 6172A>G), GGC1998GGT (6178C>T), AAA2001AAG (6187A>G), TTT2002TTC (6190T>C), GAG2004GAA (6196G>A), CTC2007TTT (6203C>T 6205C>T), GAA2008GAG (6208A>G), CTA2009TTG (6209C>T 6211A>G), CAG2011CAA (6217G>A), GCT2015GCA (6229T>A), GTA2016ATA (6230G>A), GTC2018GTT (6238C>T), AAG2021AAA (6247G>A), ATA2023ATT (6253A>T), GCA2025AAA (6257G>A 6258C>A), CAT2030CAC (6274T>C), AAA2033AGA (6282A>G), TCG2034TCA (6286G>A), GGC2038GGT (6298C>T), TTG2039TTA (6301G>A), AGA2040AGG (6304A>G), GAC2041GAT (6307C>T), GCC2046GCT (6322C>T), ACA2048ACT (6328A>T), GTT2049GTG (6331T>G), TGT2050TGC (6334T>C), AGG2051AGA (6337G>A), CTG2052TTG (6338C>T), TAT2058TAC (6358T>C), GGG2059GGA (6361G>A), GCA2060ACA (6362G>A), TCA2061TCT (6367A>T), GAG2062GAA (6370G>A), TAC2064TAT (6376C>T), GGT2065GGG (6379T>G), GGA2067GGC (6385A>C), GGA2069GGT (6391A>T), GCA2070GCG (6394A>G), TAC2071TAT (6397C>T), GTA2073ATA (6401G>A), GCG2074GTG (6405C>T), CAC2076CAT (6412C>T), TTA2078TTG (6418A>G), TTT2079TTC (6421T>C), TAC2082TTC (6429A>T), GGT2084GGA (6436T>A), GAG2087GAA (6445G>A), CAA2089CGA (6450A>G), TCC2090TCG (6454C>G), CAC2092CAT (6460C>T), GGT2093GGA (6463T>A), TTC2095TTT (6469C>T), AGG2096AGA (6472G>A), GTG2097GTA (6475G>A), AAT2099AAC (6481T>C), CAC2101AAT (6485C>A 6487C>T), AGT2102AGC (6490T>C), AGC2104AGT (6496C>T), CTG2106CTA (6502G>A), GGT2110GGC (6514T>C), AGG2111AGA (6517G>A), CTC2115ATC (6527C>A), ATC2116ATA (6532C>A), CCG2119CCA (6541G>A), AAA2120AAG (6544A>G), GTC2124GTT (6556C>T), TTT2125TTC (6559T>C), CAG2127CAA (6565G>A), TTG2129CTA (6569T>C 6571G>A), CCT2134CCA (6586T>A), ACA2135ATA (6588C>T), GAA2138GAG (6598A>G), AGA2139AGG (6601A>G), ATT2140ATC (6604T>C), TGT2141TGC (6607T>C), GGA2144GGG (6616A>G), AAC2146AAT (6622C>T), GAG2149GAA (6631G>A), AAG2150AAA (6634G>A), TAT2151CAT (6635T>C), GCT2152GCA (6640T>A), TCG2154TCT (6646G>T), ATC2155GTC (6647A>G), ATC2156ATT (6652C>T), AGC2160AGT (6664C>T), ACT2162ACG (6670T>G), ATA2165GTG (6677A>G 6679A>G), GGC2167GGT (6685C>T), ACA2169ACT (6691A>T), TTC2170TTT (6694C>T), ACA2177ACG (6715A>G), GAT2178AAT (6716G>A), AAT2179GAT (6719A>G), GGA2180GGG (6724A>G), CTA2184TTA (6734C>T), GTG2186GTA (6742G>A), GTG2187GTA (6745G>A), GCC2190GCT (6754C>T), GGA2192GGG (6760A>G), ATA2194TTG (6764A>T 6766A>G), ATT2197ATA (6775T>A), CTG2200TTG (6782C>T), GCA2201GCT (6787A>T), AAC2202AAT (6790C>T), GCA2204GTG (6795C>T 6796A>G), CAC2205CAA (6799C>A), ACC2206ACA (6802C>A), AAC2208AAT (6808C>T), TAC2209TAT (6811C>T), TAC2210TAT (6814C>T), TTC2213TTT (6823C>T), GAA2215GAG (6829A>G), TAC2221TAT (6847C>T), CGA2223CGC (6853A>C), AAT2225GAT (6857A>G), GAG2226GAA (6862G>A), GAA2229GAG (6871A>G), GTC2231ACA (6875G>A 6876T>C 6877C>A), AAG2232AAA (6880G>A), TCT2233TCA (6883T>A), GTT2235GTG (6889T>G), GAC2239GAT (6901C>T), GGC2244GGT (6916C>T), CCG2245CCA (6919G>A), AAA2247AAG (6925A>G), CTT2248CTC (6928T>C), GAC2250GAA (6934C>A), GGG2255GGC (6949G>C), TTC2257TTT (6955C>T), AAA2258AAG (6958A>G), CAA2264CAG (6976A>G), CTA2266TTA (6980C>T), ATC2267ATT (6985C>T), GTA2271GTT (6997A>T), GTG2272GTT (7000G>T), GTG2273ATA (7001G>A 7003G>A), GCT2276GCC (7012T>C), AAG2277AAA (7015G>A), CAT2278CAC (7018T>C), TTT2283TAT (7032T>A), GCC2285GCT (7039C>T), TTG2286CTA (7040T>C 7042G>A), GGA2288GGG (7048A>G), GCT2292GCA (7060T>A), GTC2293GTA (7063C>A), AGC2298AGT (7078C>T), TTA2300CTG (7082T>C 7084A>G), GTA2301GTG (7087A>G), ACC2302ACA (7090C>A), CAT2304CAC (7096T>C), GTA2305GTG (7099A>G), GTT2306GTC (7102T>C), GAG2309GAA (7111G>A), CGA2311CGG (7117A>G), ACA2314AAA (7125C>A), GAA2315GAG (7129A>G), TTT2316TTC (7132T>C), CTG2317TTA (7133C>T 7135G>A), GTG2319GTA (7141G>A), GAT2320GAC (7144T>C), GCA2321TCG (7145G>T 7147A>G), GAG2322GAA (7150G>A), GCA2325GCT (7159A>T), GAG2326_GCA2327del (7160_7165delGAGGCA), TTC2329TTT (7171C>T), GCG2335GCT (7189G>T), AAA2338AAG (7198A>G), AGC2339AGT (7201C>T), CTA2341TTA (7205C>T), AAT2342AAC (7210T>C), GAT2344GAA (7216T>A), GCG2345GCA (7219G>A), ATC2347ATA (7225C>A), AAG2348AAA (7228G>A), AAG2352AAA (7240G>A), TAT2353TAC (7243T>C), AAA2355AAG (7249A>G), CCT2356CCG (7252T>G), GAT2358GAA (7258T>A), GTT2359GTC (7261T>C), GGT2360GGA (7264T>A), GTC2361GTG (7267C>G), GTG2362GTA (7270G>A), GAT2363_CGG2364insT-- (7273_7274insT), CGG2364GTG (7274C>G 7275G>T), CAT2366CTT (7281A>T), TTG2367TCG (7284T>C), TCA2371TTA (7296C>T), TCT2376TTT (7311C>T), ACC2377ATC (7314C>T), TGC2378TAC (7317G>A), AAT2379AAA (7321T>A), GTG2380-TG (7322delG), CAC2381CAT (7327C>T), GGC2382GGT (7330C>T), TTC2383TTT (7333C>T), AAG2384CAG (7334A>C), TGT2386TGC (7342T>C), GCA2387AAC (7343G>A 7344C>A 7345A>C), ACT2390ACC (7354T>C), CAA2393CAG (7363A>G), ATT2395ATC (7369T>C), TTC2396TTT (7372C>T), GCG2398GCT (7378G>T), CTC2399CTT (7381C>T), GCA2404GCT (7396A>T), GTC2405GTT (7399C>T), GCC2407GCT (7405C>T), AGT2408ATG (7407G>T 7408T>G), GGG2410GGT (7414G>T), TGC2411GGC (7415T>G), AAA2412AAG (7420A>G), GAC2415GAT (7429C>T), TAT2416TAC (7432T>C), CAT2419CAC (7441T>C), AAG2425AAA (7459G>A), GAA2426GAG (7462A>G), ATA2428ATT (7468A>T), GTC2429GTA (7471C>A), CAA2431CAG (7477A>G), AGC2432AGT (7480C>T), CTG2434CTT (7486G>T), TAT2437TAC (7495T>C), AAA2438AAG (7498A>G), GGT2439GGC (7501T>C), TTG2440TCA (7503T>C 7504G>A), ATT2443ATA (7513T>A), AAC2445AAT (7519C>T), GGA2446GGG (7522A>G), AAG2449AAA (7531G>A), GAG2451GAA (7537G>A), CTC2452CTT (7540C>T), TGT2454TGC (7546T>C), AAG2462AAA (7570G>A), ACG2463ACA (7573G>A), ACG2465ACA (7579G>A), GCT2468GCC (7588T>C), CTA2471TTA (7595C>T), TTG2474CTA (7604T>C 7606G>A), GGT2477GGA (7615T>A), TGT2480TGC (7624T>C), GAC2483GAT (7633C>T), TTC2484TTT (7636C>T), AAT2485AAC (7639T>C), TTT2488TTC (7648T>C), GAA2494GAG (7666A>G), TGT2496TGC (7672T>C), ACA2498ACT (7678A>T), GGG2500GGA (7684G>A), ACT2502ACC (7690T>C), TTT2504TTC (7696T>C), GGT2506GGG (7702T>G), GAT2509AAT (7709G>A), AAA2510AGG (7713A>G 7714A>G), CTG2511TTG (7715C>T), CGT2514CGC (7726T>C), GAG2517GAA (7735G>A), GTA2520GTG (7744A>G), TAC2521TAT (7747C>T), GGC2526GGT (7762C>T), CAG2528CAA (7768G>A), TTT2529TTC (7771T>C), TCA2532TCG (7780A>G), CTA2533CTC (7783A>C), ACT2534ACC (7786T>C), CTA2537TTG (7793C>T 7795A>G), ATC2538ATT (7798C>T), GCT2540GCC (7804T>C), ACC2543ATC (7812C>T), GAA2550GAG (7834A>G), GAC2551GAT (7837C>T), GTG2554TTA (7844G>T 7846G>A), CAG2557CAA (7855G>A), CTG2559CTA (7861G>A), TTA2562TTG (7870A>G), TAC2563TAT (7873C>T), ACT2564ACA (7876T>A), GAG2565GAA (7879G>A), ATT2566ATA (7882T>A), GTT2567ATT (7883G>A), CCA2570CCT (7894A>T), ATT2571ATC (7897T>C), GTC2579GTT (7921C>T), AAG2581AAA (7927G>A), GGT2584GGC (7936T>C), AAT2585AAC (7939T>C), AAT2586AAC (7942T>C), GTG2594GTA (7966G>A), GAT2595GAC (7969T>C), TCT2597TCC (7975T>C), GTC2601GTT (7987C>T), CTT2602CTC (7990T>C), GCT2607GCC (8005T>C), GAA2616GAG (8032A>G), ATC2618ATT (8038C>T), GAC2619GAT (8041C>T), AGC2620AAC (8043G>A), TTG2632TTA (8080G>A), GTG2635GTA (8089G>A), CCG2637CCA (8095G>A), GAG2638GAA (8098G>A), AGC2641AGT (8107C>T), ATT2642ATC (8110T>C), GAT2644GAC (8116T>C), CAA2648CAG (8128A>G), GGT2654GGC (8146T>C), AAC2656AGT (8151A>G 8152C>T), TCG2660TCA (8164G>A), AGG2665AAG (8178G>A), AAG2666AAA (8182G>A), TTG2669TTA (8191G>A), TTC2671TTT (8197C>T), TCC2673TCT (8203C>T), AGA2675AAA (8208G>A), CTG2677TTA (8213C>T 8215G>A), CTA2678CTG (8218A>G), ATC2679ATT (8221C>T), GAG2680GAA (8224G>A), CCA2685CCG (8239A>G), GAG2690GAA (8254G>A), ATT2692ATC (8260T>C), GTA2693GTG (8263A>G), GAT2699GAC (8281T>C), AGA2700AGG (8284A>G), CCA2704GCG (8294C>G 8296A>G), GAG2705GAA (8299G>A), CAC2706CAT (8302C>T), ATT2711ATC (8317T>C), TGC2712TGT (8320C>T), GCT2714GCA (8326T>A), GAG2717GAA (8335G>A), TCC2718TCT (8338C>T), TCT2722TTT (8349C>T), GAA2723GAG (8353A>G), CAC2726CAT (8362C>T), CAA2727CAG (8365A>G), ATC2728ATT (8368C>T), TAC2732TAT (8380C>T), TCA2733TCG (8383A>G), TTA2735TTG (8389A>G), CCT2740CCC (8404T>C), GCA2742TCA (8408G>T), ACA2743ACG (8413A>G), GCG2745GCT (8419G>T), GCT2750GCC (8434T>C), TAT2752TAC (8440T>C), ATG2756ATA (8452G>A), CTA2758CTG (8458A>G), AGG2759AAG (8460G>A), AAA2760AAG (8464A>G), CTG2761CTA (8467G>A), AGG2765AGA (8479G>A), GCT2766ACA (8480G>A 8482T>A), GAG2771GAA (8497G>A), CTA2772TTG (8498C>T 8500A>G), AGA2773AAG (8502G>A 8503A>G), GCC2774GCT (8506C>T), GAA2777GAT (8515A>T), GTC2780GTT (8524C>T), GCA2781GCC (8527A>C), TTA2782CTG (8528T>C 8530A>G), GAG2785GAC (8539G>C), CTT2788TGT (8546C>T 8547T>G), TCT2790ACT (8552T>A), GTA2793GTG (8563A>G), GCA2797GGA (8574C>G), ATT2801GTT (8585A>G), GGA2805GAA (8598G>A), AGC2806AGT (8602C>T), AAC2807TCT (8603A>T 8604A>C 8605C>T), AAA2812AAG (8620A>G), CCA2813CAA (8622C>A), GAG2814GGA (8625A>G 8626G>A), CAG2815CAA (8629G>A), GGC2816GGT (8632C>T), ATC2818ATT (8638C>T), CAG2819CAA (8641G>A), AAC2821AAT (8647C>T), CCG2822TTC (8648C>T 8649C>T 8650G>C), AAC2823AAT (8653C>T), AAA2824AAG (8656A>G), GGA2825GAA (8658G>A), GAT2827GCG (8664A>C 8665T>G), GTT2830GTG (8674T>G), GCA2832GTT (8679C>T 8680A>T), GGC2833GGA (8683C>A), GGG2836GGA (8692G>A), ACA2837ACT (8695A>T), CCG2841CCA (8707G>A), AGA2842CGA (8708A>C), AAG2844AAA (8716G>A), ATC2846ATT (8722C>T), ACG2847ACA (8725G>A), AAA2849AAG (8731A>G), AGA2851AGG (8737A>G), CCC2853CCT (8743C>T), ACA2854AAG (8745C>A 8746A>G), AAG2856AAA (8752G>A), GGA2857GGT (8755A>T), AAC2862AAT (8770C>T), TTA2863CTA (8771T>C), GAA2864GAG (8776A>G), CAT2865CAC (8779T>C), TTG2866TTA (8782G>A), TAT2869TAC (8791T>C), CCA2871CCG (8797A>G), CAA2872CAG (8800A>G), CAA2873CAG (8803A>G), GAT2875GAC (8809T>C), ATT2876ATC (8812T>C), AAT2878AAC (8818T>C), CGG2880CGA (8824G>A), GCA2881GCG (8827A>G), ACG2888ACA (8848G>A), TAT2890TAC (8854T>C), GAG2891GGA (8856A>G 8857G>A), GTG2893GTA (8863G>A), CGG2894CAG (8865G>A), ATG2895CTT (8867A>C 8869G>T), GCA2896GCG (8872A>G), GGA2900GAA (8883G>A), ACT2902TCT (8888A>T), TGC2915TGT (8929C>T), GTC2924ATC (8954G>A), AAC2925AAT (8959C>T), GGA2926GGT (8962A>T), GGG2933GGA (8983G>A), AAT2934GAC (8984A>G 8986T>C), GAA2935GTA (8988A>T), GAG2938GAA (8998G>A), CCG2940CCA (9004G>A), TTG2941TTA (9007G>A), ATC2944ATT (9016C>T), ACC2951ACA (9037C>A), ATA2967ATT (9085A>T), AAC2971AAT (9097C>T), ATT2983GTT (9131A>G), CGA2984CGT (9136A>T), CGG2987CGT (9145G>T), ATG2989GGA (9149A>G 9150T>G 9151G>A), GGT2990AGT (9152G>A), TTA2991TTG (9157A>G), GCG2992GCT (9160G>T), CGT2993CGC (9163T>C), GAG3000GAA (9184G>A), CGA3004CGG (9196A>G), AGG3010AGA (9214G>A), GAA3011GAG (9217A>G), GCG3012GCA (9220G>A), GCA3020GCT (9244A>T), TTG3021TTA (9247G>A), GCC3024GCT (9256C>T), CCT3026TCC (9260C>T 9262T>C), GGG3030GGA (9274G>A), GAC3032GAT (9280C>T), ATC3035ATT (9289C>T), CAA3038CAC (9298A>C), GAG3040GAA (9304G>A), GTC3050GTT (9334C>T), GTC3060GTG (9364C>G)								
P1 protein (NP_734243.1)	1	284	100%	1394	72.7%	284 (100%)	203 (71.5%)	0/0/0/0	0

	Begin	End	Coverage	Score	Concordance	Matches	Identities	I/D/M/F*	Stop Codons
**NT**	**5**	**9704**	**99.9%**	**12522**	**64.8%**	**9688 (99.7%)**	**8015 (82.5%)**	**15/12**	
Protein mutations:	Y4Q (194T>C 196C>A), C9L (209T>C 210G>T 211T>G), F13M (221T>A 223T>G), S23P (251T>C), C24F (255G>T 256C>T), E25G (258A>G), I27V (263A>G 265T>G), V28A (267T>C), E30V (273A>T), A35T (287G>A 289T>C), V37T (293G>A 294T>C), A41I (305G>A 306C>T), D42G (309A>G), E44D (316A>T), T45V (317A>G 318C>T 319A>G), L52Q (339T>A 340C>A), K53R (342A>G), K55A (347A>G 348A>C 349A>G), Y56H (350T>C), T58V (356A>G 357C>T), V61T (365G>A 366T>C 367G>A), L62S (368C>T 369T>C), F67C (384T>G), A75V (408C>T 409C>T), M78E (416A>G 417T>A), E85K (437G>A), R86E (440A>G 441G>A), K87R (443A>C 444A>G 445G>A), D88E (448T>A), E91A (456A>C), H93N (461C>A), D111E (517T>A), S116F (531C>T), P118S (536C>T), Q119W (539C>T 540A>G 541A>G), R122K (549G>A 550A>G), M131T (576T>C), V134A (585T>C 586C>G), R137Y (593C>T 594G>A 595C>T), P138R (597C>G), I139V (599A>G 601A>G), I140P (602A>C 603T>C 604A>G), M147I (625G>A), I151V (635A>G 637T>C), I154V (644A>G 646A>G), E160A (663A>C 664G>C), H166Q (682C>A), T172S (699C>G), H174Q (706T>A), K179E (719A>G 721G>A), I180V (722A>G 724A>G), A183S (731G>T), Y184P (734T>C 735A>C 736C>T), S185R (737T>C 738C>G 739C>T), A187V (744C>T 745G>C), R189C (749C>T 751A>C), M194K (765T>A), R197K (773C>A 774G>A 775A>G), M206K (801T>A), G210A (813G>C 814A>G), L211H (816T>A), R214Q (825G>A 826T>A), N223D (851A>G 853T>C), R226C (860C>T 862C>T), T227A (863A>G 865T>C), I228T (867T>C), N229D (869A>G 871C>T), I230L (872A>T 874A>G), R231Q (876G>A), R232K (879G>A), N240S (903A>G), T241N (906C>A 907A>C), K242T (909A>C 910A>C), S243N (912G>A 913C>T), S252L (938T>C 939C>T 940A>T), L266I (980T>A 982G>C), R272K (999G>A), Q275R (1007C>A 1008A>G 1009G>A), S276G (1010A>G 1012T>G), I277V (1013A>G), N279Q (1019A>C 1021C>G)								
Codon mutations:	ACT3ACC (193T>C), TAC4CAA (194T>C 196C>A), TCA6TCG (202A>G), ATC8ATT (208C>T), TGT9CTG (209T>C 210G>T 211T>G), TCG12TCA (220G>A), TTT13ATG (221T>A 223T>G), GAA14GAG (226A>G), CTA17CTT (235A>T), TAC19TAT (241C>T), CCA21CCT (247A>T), TCT23CCT (251T>C), TGC24TTT (255G>T 256C>T), GAG25GGG (258A>G), CAT26CAC (262T>C), ATT27GTG (263A>G 265T>G), GTG28GCG (267T>C), GAA30GTA (273A>T), CGA31CGT (277A>T), GAA32GAG (280A>G), GTG33GTT (283G>T), CCG34CCA (286G>A), GCT35ACC (287G>A 289T>C), GTT37ACT (293G>A 294T>C), CCT39CCC (301T>C), TTC40TTT (304C>T), GCA41ATA (305G>A 306C>T), GAT42GGT (309A>G), CTG43TTG (311C>T), GAA44GAT (316A>T), ACA45GTG (317A>G 318C>T 319A>G), CAA46CAG (322A>G), GCA49GCT (331A>T), CTC52CAA (339T>A 340C>A), AAG53AGG (342A>G), AAA55GCG (347A>G 348A>C 349A>G), TAT56CAT (350T>C), GCT57GCA (355T>A), ACT58GTT (356A>G 357C>T), CGT60CGC (364T>C), GTG61ACA (365G>A 366T>C 367G>A), CTC62TCC (368C>T 369T>C), AAA63AAG (373A>G), AAC64AAT (376C>T), TTT67TGT (384T>G), ACG68ACA (388G>A), TAC69TAT (391C>T), CGA70CGG (394A>G), AAG72AAA (400G>A), GAT74GAC (406T>C), GCC75GTT (408C>T 409C>T), CAG76CAA (412G>A), ATA77ATT (415A>T), ATG78GAG (416A>G 417T>A), ATT80ATC (424T>C), CAG81CAA (427G>A), AAA83AAG (433A>G), CTG84CTC (436G>C), GAG85AAG (437G>A), AGG86GAG (440A>G 441G>A), AAG87CGA (443A>C 444A>G 445G>A), GAT88GAA (448T>A), GAA90GAG (454A>G), GAA91GCA (456A>C), TAT92TAC (460T>C), CAC93AAC (461C>A), CAA95CAG (469A>G), GCC97GCG (475C>G), CCT99CCA (481T>A), ACT106ACC (502T>C), ATA107ATT (505A>T), GGC109GGT (511C>T), GAT111GAA (517T>A), CCT112CCA (520T>A), CCA113CCT (523A>T), TCT116TTT (531C>T), GAG117GAA (535G>A), CCA118TCA (536C>T), CAA119TGG (539C>T 540A>G 541A>G), AGA122AAG (549G>A 550A>G), ATC124ATT (556C>T), ATT125ATC (559T>C), CAT126CAC (562T>C), ACT128ACC (568T>C), CCA129CCG (571A>G), ATG131ACG (576T>C), CGT132CGC (580T>C), GTC134GCG (585T>C 586C>G), CGC137TAT (593C>T 594G>A 595C>T), CCC138CGC (597C>G), ATA139GTG (599A>G 601A>G), ATA140CCG (602A>C 603T>C 604A>G), ACA143ACG (613A>G), GAA144GAG (616A>G), GGC145GGG (619C>G), CAG146CAA (622G>A), ATG147ATA (625G>A), AAT148AAC (628T>C), CAC149CAT (631C>T), ATT151GTC (635A>G 637T>C), ATA154GTG (644A>G 646A>G), ATT157ATC (655T>C), TCG159TCA (661G>A), GAG160GCC (663A>C 664G>C), AAA161AAG (667A>G), GTC165GTT (679C>T), CAC166CAA (682C>A), TTA167CTG (683T>C 685A>G), AGT169AGC (691T>C), ACC172AGC (699C>G), ACT173ACA (703T>A), CAT174CAA (706T>A), AAG179GAA (719A>G 721G>A), ATA180GTG (722A>G 724A>G), CTT181TTG (725C>T 727T>G), GGT182GGA (730T>A), GCA183TCA (731G>T), TAC184CCT (734T>C 735A>C 736C>T), TCC185CGT (737T>C 738C>G 739C>T), GCG187GTC (744C>T 745G>C), CGA189TGC (749C>T 751A>C), ACT190ACC (754T>C), ATG194AAG (765T>A), TTG196CTG (770T>C), CGA197AAG (773C>A 774G>A 775A>G), CGG198AGG (776C>A), TTC202TTT (790C>T), ATG206AAG (801T>A), GGA210GCG (813G>C 814A>G), CTT211CAT (816T>A), TTG212CTG (818T>C), CAA213CAG (823A>G), CGT214CAA (825G>A 826T>A), CGG217AGA (833C>A 835G>A), GAC219GAT (841C>T), AAT223GAC (851A>G 853T>C), GTC225GTT (859C>T), CGC226TGT (860C>T 862C>T), ACT227GCC (863A>G 865T>C), ATC228ACC (867T>C), AAC229GAT (869A>G 871C>T), ATA230TTG (872A>T 874A>G), CGA231CAA (876G>A), AGG232AAG (879G>A), GAT234GAC (886T>C), ATC238ATT (898C>T), AAC240AGC (903A>G), ACA241AAC (906C>A 907A>C), AAA242ACC (909A>C 910A>C), AGC243AAT (912G>A 913C>T), GGC246GGA (922C>A), GGT249GGA (931T>A), TCA252CTT (938T>C 939C>T 940A>T), GGA253GGG (943A>G), TTC256TTT (952C>T), TCA261TCG (967A>G), GAA263GAG (973A>G), GGG264GGC (976G>C), TTG266ATC (980T>A 982G>C), GCA269GCG (991A>G), CGT270CGC (994T>C), AGA272AAA (999G>A), GTT273GTC (1003T>C), CAG275AGA (1007C>A 1008A>G 1009G>A), AGT276GGG (1010A>G 1012T>G), ATT277GTT (1013A>G), TTA278TTG (1018A>G), AAC279CAG (1019A>C 1021C>G), ATC282ATT (1030C>T)								
HC-Pro protein (NP_734244.1)	1	456	100%	3144	95.7%	456 (100%)	425 (93.2%)	0/0/0/0	0
Protein mutations:	D4E (1048C>A), R16Q (1083G>A), S21A (1097T>G 1099G>A), R36K (1143G>A), L40I (1154T>A 1156G>A), S62N (1221G>A), V65T (1229G>A 1230T>C 1231T>C), S66N (1233G>A 1234C>T), K74T (1257A>C 1258A>G), N81S (1278A>G), A85V (1290C>T 1291G>A), D88T (1298G>A 1299A>C 1300C>T), I91V (1307A>G 1309A>G), N94E (1316A>G 1318T>A), I98T (1329T>C), L113I (1373C>A), I117V (1385A>G), E123D (1405G>T), A127S (1415G>T 1417A>T), V134I (1436G>A 1438C>T), E224G (1707A>G), A228T (1718G>A), I236V (1742A>G 1744C>T), S241N (1758G>A), V252I (1790G>A), I301V (1937A>G 1939T>C), F323Y (2004T>A), I344M (2068T>G), V346I (2072G>A 2074G>A), R391K (2208G>A), D410E (2266C>G)								
Codon mutations:	TCG1TCA (1039G>A), AAT2AAC (1042T>C), GCC3GCA (1045C>A), GAC4GAA (1048C>A), AAT5AAC (1051T>C), AAG8AAA (1060G>A), GGT9GGA (1063T>A), CTG10TTG (1064C>T), GAC11GAT (1069C>T), CGA16CAA (1083G>A), TAT19TAC (1093T>C), TCG21GCA (1097T>G 1099G>A), CAC23CAT (1105C>T), GTA26GTG (1114A>G), GCT27GCG (1117T>G), TTA29TTG (1123A>G), CCT30CCA (1126T>A), GTC31GTT (1129C>T), AGG36AAG (1143G>A), GTA37GTT (1147A>T), GCT38GCA (1150T>A), TTG40ATA (1154T>A 1156G>A), CAC43CAT (1165C>T), AGT44AGC (1168T>C), CTT46TTA (1172C>T 1174T>A), ACC55ACT (1201C>T), AGC62AAC (1221G>A), GTT65ACC (1229G>A 1230T>C 1231T>C), AGC66AAT (1233G>A 1234C>T), CTG68CTA (1240G>A), AAG70AAA (1246G>A), TTG72CTA (1250T>C 1252G>A), CAT73CAC (1255T>C), AAA74ACG (1257A>C 1258A>G), AGA77AGG (1267A>G), GGT79GGC (1273T>C), TTG80TTA (1276G>A), AAT81AGT (1278A>G), TTG83CTG (1283T>C), GCG85GTA (1290C>T 1291G>A), GAC88ACT (1298G>A 1299A>C 1300C>T), TTT90TTC (1306T>C), ATA91GTG (1307A>G 1309A>G), CAT92CAC (1312T>C), AAT94GAA (1316A>G 1318T>A), TTG97TTA (1327G>A), ATA98ACA (1329T>C), GCG99GCT (1333G>T), TTA100TTG (1336A>G), CTA103TTG (1343C>T 1345A>G), CCG106CCA (1354G>A), GTG107GTT (1357G>T), GAC108GAT (1360C>T), CTC111TTG (1367C>T 1369C>G), CTT113ATT (1373C>A), TTC114TTT (1378C>T), GAG116GAA (1384G>A), ATA117GTA (1385A>G), AAA119AAG (1393A>G), TCC120TCT (1396C>T), GAG123GAT (1405G>T), AAA124AAG (1408A>G), CAG125CAA (1411G>A), GCA127TCT (1415G>T 1417A>T), CCG128CCC (1420G>C), AAT131AAC (1429T>C), TTA132TTG (1432A>G), GTC134ATT (1436G>A 1438C>T), TTA135CTG (1439T>C 1441A>G), AAT136AAC (1444T>C), TTC139TTT (1453C>T), CTG140CTA (1456G>A), GAA144GAG (1468A>G), GTA152GTG (1492A>G), TTG155CTA (1499T>C 1501G>A), AGT156AGC (1504T>C), TTG157TTA (1507G>A), CTC158CTT (1510C>T), GAA159GAG (1513A>G), TTA160TTG (1516A>G), GCA161GCT (1519A>T), AGG162AGA (1522G>A), TTC163TTT (1525C>T), CAG164CAA (1528G>A), AAC166AAT (1534C>T), ACT168ACA (1540T>A), AAC170AAT (1546C>T), AAA173AAG (1555A>G), GGT174GGC (1558T>C), GAT175GAC (1561T>C), ATA176ATC (1564A>C), TCT177TCA (1567T>A), TTC179TTT (1573C>T), AGA180AGG (1576A>G), TTA183CTA (1583T>C), TCT184TCA (1588T>A), AAG186AAA (1594G>A), AAC188AAT (1600C>T), CTG191CTA (1609G>A), TCG194TCA (1618G>A), TGC195TGT (1621C>T), GAC196GAT (1624C>T), CTG199TTG (1631C>T), AAA201AAG (1639A>G), TTC205TTT (1651C>T), CTC206CTG (1654C>G), CAT213CAC (1675T>C), TCA219TCG (1693A>G), TTT222TTC (1702T>C), GAA224GGA (1707A>G), GCA228ACA (1718G>A), GGA230GGG (1726A>G), TAC231TAT (1729C>T), TCA232TCC (1732A>C), TAT234TAC (1738T>C), ATC236GTT (1742A>G 1744C>T), CGC237CGT (1747C>T), CAT239CAC (1753T>C), AGT241AAT (1758G>A), AGG244AGA (1768G>A), AAC250AAT (1786C>T), TTA251TTG (1789A>G), GTT252ATT (1790G>A), GTC253GTT (1795C>T), TTA257CTG (1805T>C 1807A>G), GAG259GAA (1813G>A), AGG261AGA (1819G>A), AAG263AAA (1825G>A), GAC267GAT (1837C>T), TAT268TAC (1840T>C), CAA271CAG (1849A>G), GTC274GTG (1858C>G), AAA276AAG (1864A>G), TGC278TGT (1870C>T), AGT280AGC (1876T>C), AAA282AAG (1882A>G), GGT284GGA (1888T>A), AAT285AAC (1891T>C), TGT290TGC (1906T>C), ACA293ACT (1915A>T), GGT298GGC (1930T>C), GCC300GCT (1936C>T), ATT301GTC (1937A>G 1939T>C), TCA303TCG (1945A>G), TTC305TTT (1951C>T), CCA308CCT (1960A>T), GTA314GTG (1978A>G), ATT315ATA (1981T>A), GGC316GGT (1984C>T), AAT317AAC (1987T>C), GAC320GAT (1996C>T), TTT323TAT (2004T>A), AAA328AAG (2020A>G), GGG329GGA (2023G>A), GAT330GAC (2026T>C), TCG331TCT (2029G>T), GAG332GAA (2032G>A), TAT341TAC (2059T>C), ATT344ATG (2068T>G), AAC345AAT (2071C>T), GTG346ATA (2072G>A 2074G>A), CTG351TTA (2087C>T 2089G>A), AGC355AGT (2101C>T), GAG356GAA (2104G>A), GAG357GAA (2107G>A), AAG360AAA (2116G>A), TTC362TTT (2122C>T), CGC367CGT (2137C>T), CTT374CTC (2158T>C), ACC376ACT (2164C>T), CCA378CCG (2170A>G), GAT382GAC (2182T>C), GCG384GCA (2188G>A), ACC385ACA (2191C>A), AGA391AAA (2208G>A), TAT394TAC (2218T>C), CAG396GAT (2224C>T), GTA397GTT (2227A>T), TTG402TTA (2242G>A), CCC403CCT (2245C>T), TTG406TTA (2254G>A), GTT407GTC (2257T>C), GAC408GAT (2260C>T), GAC410GAG (2266C>G), CAA412CAG (2272A>G), ACG413ACA (2275G>A), TCA419TCG (2293A>G), GGC421GGT (2299C>T), TCG422TCA (2302G>A), CAG423CAA (2305G>A), GGA426GGT (2314A>T), TAT427TAC (2317T>C), CTA430CTG (2326A>G), GTG435GTA (2341G>A), CAA437CAG (2347A>G), CTT438CTC (2350T>C), TTG440TTA (2356G>A), AAT443AAC (2365T>C), GAA445GAG (2371A>G), TTA446TTG (2374A>G), GAT449GAC (2383T>C), ATA450ATT (2386A>T), AAA451AAG (2389A>G), CAT452CAC (2392T>C), TAT453TAC (2395T>C)								
P3 protein (NP_734251.1)	1	365	100%	1924	79.6%	365 (99.7%)	327 (89.3%)	1/0/2/2	0
Protein mutations:	N4G (2414A>G 2415A>G 2416T>G), S6C (2420A>T), K40R (2523A>G), R77K (2634G>A), V107I (2723G>A 2725C>T), A115T (2747G>A 2749T>A), H131N (2795C>A), V140I (2822G>A 2824G>A), P160T (2882C>A), S161H (2885A>C 2886G>A), T164M (2895C>T), N176S (2931A>G), N179S (2940A>G), E189_N190insX (2971_2972insA), T196I (2991C>T), H197R (2994A>G), E199R (2999G>A 3000A>G), R206Q (3021G>A), Y207F (3024A>T 3025C>T), T211I (3036C>T), E212G (3039A>G 3040A>C), K213T (3042A>C 3043G>A), A232V (3099C>T 3100C>A), V234R (3104G>A 3105T>G 3106G>A), S243N (3132G>A), R245K (3137C>A 3138G>A 3139A>G), F250I (3152T>A 3154C>T), C254Y (3165G>A), F272L (3218T>C), T274N (3225C>A), V276I (3230G>A), V288M (3266G>A 3268A>G), V291M (3275G>A), A294T (3284G>A), R304K (3315G>A 3316A>G), E305A (3318A>C), M319V (3359A>G), D332E (3400T>G)								

	Begin	End	Coverage	Score	Concordance	Matches	Identities	I/D/M/F*	Stop Codons
**NT**	**5**	**9704**	**99.9%**	**12522**	**64.8%**	**9688 (99.7%)**	**8015 (82.5%)**	**15/12**	
Codon mutations:	AAT4GGG (2414A>G 2415A>G 2416T>G), GCT5GCA (2419T>A), AGC6TGC (2420A>T), ACA12ACT (2440A>T), GTT21GTC (2467T>C), TCT24TCC (2476T>C), TCG26TCA (2482G>A), CTG29CTA (2491G>A), CTG31CTT (2497G>T), TTG33TTA (2503G>A), AGA38AGG (2518A>G), CCT39CCC (2521T>C), AAG40AGG (2523A>G), AGA43AGG (2533A>G), TTG45TTA (2539G>A), TTA47TTG (2545A>G), GAG49GAA (2551G>A), CCT50CCA (2554T>A), CTG52TTG (2558C>T), TTG53CTC (2561T>C 2563G>C), TCA56TCG (2572A>G), CCT60CCC (2584T>C), GGC61GGT (2587C>T), CTG63CTC (2593G>C), GCT65GCC (2599T>C), AAT68AAC (2608T>C), TTT72TTC (2620T>C), GCG75GCA (2629G>A), GTG76GTA (2632G>A), AGG77AAG (2634G>A), TTG78TTA (2638G>A), ATT80ATA (2644T>A), AAA83AAG (2653A>G), GCT87GCC (2665T>C), CTA92TTA (2678C>T), CTA93CTG (2683A>G), GCT95GCC (2689T>C), CTA98CTC (2698A>C), TCA101TCG (2707A>G), GCG102GCA (2710G>A), GAA104GAG (2716A>G), ACA105ACG (2719A>G), CTC106CTT (2722C>T), GTC107ATT (2723G>A 2725C>T), GCA108GCG (2728A>G), AGG110AGA (2734G>A), ATT111ATA (2737T>A), GAT114GAC (2746T>C), GCT115ACA (2747G>A 2749T>A), GCA116GCT (2752A>T), GCT117GCA (2755T>A), GAC119GAT (2761C>T), CTC120CTT (2764C>T), TGT125TGC (2779T>C), GGG127GGT (2785G>T), AAC129AAT (2791C>T), CAT131AAT (2795C>A), TAC134TAT (2806C>T), TTG138CTC (2816T>C 2818G>C), GTG140ATA (2822G>A 2824G>A), TTG141TTA (2827G>A), GTT144GTC (2836T>C), AAG145AAA (2839G>A), AAT146AAC (2842T>C), ACC153ACA (2863C>A), CTA154TTA (2864C>T), CCA160ACA (2882C>A), AGT161CAT (2885A>C 2886G>A), AAC163AAT (2893C>T), ACG164ATG (2895C>T), GTC166GTT (2902C>T), GTA167GTG (2905A>G), ATT169ATC (2911T>C), AAT173AAC (2923T>C), AAT176AGT (2931A>G), AAC179AGC (2940A>G), GCT181GCC (2947T>C), ACT186ACC (2962T>C), GAA189_AAT190insA-- (2971_2972insA), GGT195GGC (2989T>C), ACT196ATT (2991C>T), CAT197CGT (2994A>G), GAG199AGG (2999G>A 3000A>G), TCC204TC- (3016delC), CGG206CAG (3021G>A), TAC207TTT (3024A>T 3025C>T), ACA211ATA (3036C>T), GAA212GGC (3039A>G 3040A>C), AAG213ACA (3042A>C 3043G>A), TTG216TTA (3052G>A), TTA219TTG (3061A>G), GCG226GCA (3082G>A), GGC229GGT (3091C>T), GCC232GTA (3099C>T 3100C>A), GTG234AGA (3104G>A 3105T>G 3106G>A), GTC235GTT (3109C>T), AAA236AAG (3112A>G), ACT238ACC (3118T>C), GCC239GCT (3121C>T), GGA241GGG (3127A>G), AGC243AAC (3132G>A), CGA245AAG (3137C>A 3138G>A 3139A>G), TAT249TAC (3151T>C), TTC250ATT (3152T>A 3154C>T), TGT254TAT (3165G>A), GTA255GTG (3169A>G), ATC265ATT (3199C>T), AGG267CGG (3203A>C), CGT268CGC (3208T>C), CCA270CCG (3214A>G), ACC271ACT (3217C>T), TTT272CTT (3218T>C), GTC273GTA (3223C>A), ACT274AAT (3225C>A), GTT276ATT (3230G>A), AAC277AAT (3235C>T), TTA285CTA (3257T>C), ACC286ACT (3262C>T), AGC287AGT (3265C>T), GTA288ATG (3266G>A 3268A>G), GTG289GTA (3271G>A), GTG291ATG (3275G>A), GCA294ACA (3284G>A), CAG299CAA (3301G>A), AGA304AAG (3315G>A 3316A>G), GAA305GCA (3318A>C), ATC306ATT (3322C>T), GAG307GAA (3325G>A), TTG308TTA (3328G>A), ATA311ATT (3337A>T), GAG315GAA (3349G>A), GTC317GTT (3355C>T), TGC318TGT (3358C>T), ATG319GTG (3359A>G), CTA321CTG (3367A>G), TAT322TAC (3370T>C), TTA325CTG (3377T>C 3379A>G), GAA330GAG (3394A>G), CGC331CGT (3397C>T), GAT332GAG (3400T>G), ACA334ACG (3406A>G), TAC338TAT (3418C>T), ATT339ATA (3421T>A), TAT341TAC (3427T>C), AAG343AAA (3433G>A), TCA344TCT (3436A>T), GTA345GTG (3439A>G), AAC346AAT (3442C>T), CCT347CCC (3445T>C), CAG351CAA (3457G>A), TTT352TTC (3460T>C), GCT353GCA (3463T>A), GCG355GCT (3469G>T), CAG356CAA (3472G>A), CAC364CAT (3496C>T), CAG365CAA (3499G>A)								
6K1 protein (NP_734245.1)	1	52	100%	305	90.5%	52 (100%)	45 (86.5%)	0/0/0/0	0
Protein mutations:	V5G (3513T>G), M16V (3545A>G), A17T (3548G>A), V19I (3554G>A 3556C>T), L42V (3623C>G), L45M (3632C>A), Y47H (3638T>C)								
Codon mutations:	TCC2TCT (3505C>T), GTT5GGT (3513T>G), AAA7AAG (3520A>G), GAA10GAG (3529A>G), CAA11CAG (3532A>G), ATG16GTG (3545A>G), GCT17ACT (3548G>A), TTA18CTA (3551T>C), GTC19ATT (3554G>A 3556C>T), AGT28AGC (3583T>C), GAT29GAC (3586T>C), TGC30TGT (3589C>T), TTC32TTT (3595C>T), AAT36AAC (3607T>C), TTT38TTC (3613T>C), AAG39AAA (3616G>A), GGT40GGC (3619T>C), CTT42GTT (3623C>G), TCC43TCT (3628C>T), CTG45ATG (3632C>A), GAT46GAC (3637T>C), TAT47CAT (3638T>C), GAA48GAG (3643A>G)								
CI protein (NP_734246.1)	1	634	100%	4190	97.9%	634 (100%)	608 (95.9%)	0/0/0/0	0
Protein mutations:	I15V (3698A>G), S21N (3717G>A), R26K (3731C>A 3732G>A), I140V (4073A>G), F158Y (4128T>A 4129C>T), A166S (4151G>T), V171I (4166G>A), L190I (4223C>A 4225G>A), I222V (4319A>G 4321A>G), F247Y (4395T>A), V251I (4406G>A), M274V (4475A>G 4477G>T), T276S (4481A>T 4483A>G), A297L (4544G>T 4545C>T 4546G>A), R298K (4548G>A), V342I (4679G>A 4681G>C), V381I (4796G>A 4798C>T), T383S (4803C>G 4804T>C), S468G (5057A>G), T469A (5060A>G), L479I (5090C>A), A482V (5100C>T), E484D (5107A>T), V489F (5120G>T 5122C>T), I491V (5126A>G), V510I (5183G>A)								
Codon mutations:	TTA2TTG (3661A>G), GTG5GTA (3670G>A), TTT9TTC (3682T>C), GAG11GAA (3688G>A), GAG14GAA (3697G>A), ATT15GTT (3698A>G), GAT17GAC (3706T>C), TTT18TTC (3709T>C), TTG20CTA (3713T>C 3715G>A), AGT21AAT (3717G>A), GAG22GAA (3721G>A), CGA26AAA (3731C>A 3732G>A), ACT27ACA (3736T>A), CTA31TTG (3746C>T 3748A>G), ACA33ACG (3754A>G), AGT36AGC (3763T>C), CGA41CGG (3778A>G), CAA42CAG (3781A>G), ATC43ATT (3784C>T), CAT47CAC (3796T>C), CCA50CCT (3805A>T), TAC52TAT (3811C>T), AGA53AGG (3814A>G), TTC58TTT (3829C>T), TTT61TTC (3838T>C), AGA63AGG (3844A>G), GCA64GCC (3847A>C), GTT67GTA (3856T>A), GTG69GTT (3862G>T), GAC72GAT (3871C>T), GCC74GCA (3877C>A), AGC76AGT (3883C>T), GAA77GAG (3886A>G), CTA79CTG (3892A>G), GAC80GAT (3895C>T), TTT81TTC (3898T>C), GTA83GTG (3904A>G), CGG84AGA (3905C>A 3907G>A), GCT86GCC (3913T>C), AAG91AAA (3928G>A), TCA92TCT (3931A>T), GGG94GGA (3937G>A), AGT100AGC (3955T>C), GTG101GTA (3958G>A), GCC102GCT (3961C>T), GGA103GGG (3964A>G), TCT104TCC (3967T>C), TTA107TTG (3976A>G), ATT108ATC (3979T>C), CCA110CCT (3985A>T), CTA114CTT (3997A>T), GCG115GCA (4000G>A), AAC117AAT (4006C>T), GTT118GTG (4009T>G), TTC119TTT (4012C>T), AAA120AAG (4015A>G), TCT123TCC (4024T>C), GAA125GAG (4030A>G), CCA126CCT (4033A>T), AAG129AAA (4042G>A), CTG133TTA (4052C>T 4054G>A), CGT134CGC (4057T>C), CGT136CGA (4063T>A), GGA137GGG (4066A>G), ATA140GTA (4073A>G), TTT141TTC (4078T>C), GGC142GGT (4081C>T), TCT143TCC (4084T>C), TCC147TCT (4096C>T), AGC151AGT (4108C>T), GGA152GGT (4111A>T), GCG154GCA (4117G>A), CTA155TTG (4118C>T 4120A>G), TAC157TAT (4126C>T), TTC158TAT (4128T>A 4129C>T), GCC159GCT (4132C>T), AAT160AAC (4135T>C), CAA164CAG (4147A>G), TTA165CTA (4148T>C), GCT166TCT (4151G>T), AAC169AAT (4162C>T), GTA171ATA (4166G>A), ATA172ATC (4171A>C), GAG175GAA (4180G>A), CTG179TTG (4190C>T), CCT181CCC (4198T>C), TCC182TCT (4201C>T), GCG185GCT (4210G>T), TTC186TTT (4213C>T), AGT188AGC (4219T>C), CTG189TTG (4220C>T), CTG190ATA (4223C>A 4225G>A), GTT192GTG (4231T>G), CAT194CAC (4237T>C), GTA199GTG (4252A>G), TTA200CTG (4253T>C 4255A>G), AAA201AAG (4258A>G), ACT205ACC (4270T>C), AGA209AGG (4282A>G), GAG210GAA (4285G>A), GAA212GAG (4291A>G), CAG216CAA (4303G>A), GTC219GTT (4312C>T), ATA222GTG (4319A>G 4321A>G), GTG223GTT (4324G>T), GAG224GAA (4327G>A), CTG227CTT (4336G>T), TCT228TCC (4339T>C), TCA231TCT (4348A>T), GCA235GCG (4360A>G), GGT237GGC (4366T>C), TCT238TCA (4369T>A), ACT240ACC (4375T>C), GTT244GTC (4387T>C), TTT247TAT (4395T>A), GTA251ATA (4406G>A), CTT252CTC (4411T>C), GTT261GTC (4438T>C), ACC263ACA (4444C>A), GCC265GCT (4450C>T), CTC267CTT (4456C>T), GAC270GAT (4465C>T), ATG274GTT (4475A>G 4477G>T), GTC275GTT (4480C>T), ACA276TCG (4481A>T 4483A>G), GGC280GGT (4495C>T), AAG284AAA (4507G>A), CAC285CAT (4510C>T), GGT286GGG (4513T>G), CTA288TTA (4517C>T), GTC291GTT (4528C>T), AAA293AAG (4534A>G), ACC295ACT (4540C>T), GCG297TTA (4544G>T 4545C>T 4546G>A), AGA298AAA (4548G>A), TTT301TTC (4558T>C), GTA303GTT (4564A>T), ACC305ACT (4570C>T), ATA307ATT (4576A>T), TTG314TTA (4597G>A), GAC315GAT (4600C>T), GAC317GAT (4606C>T), GTG318GTA (4609G>A), GTG320GTA (4615G>A), GAC321GAT (4618C>T), TTG324CTT (4625T>C 4627G>T), GAC331GAT (4648C>T), ATT332ATC (4651T>C), AGG335AGA (4660G>A), AGC336AGT (4663C>T), GCT338GCA (4669T>A), GTG342ATC (4679G>A 4681G>C), AGT343AGC (4684T>C), GTT344GTG (4687T>G), GAG348GAA (4699G>A), AGA349AGG (4702A>G), CAA351CAG (4708A>G), AGG352AGA (4711G>A), CTG353TTG (4712C>T), AAA361AAG (4738A>G), GTA363GTT (4744A>T), GCA364GCG (4747A>G), TTG365CTG (4748T>C), ACT370ACC (4765T>C), GTC381ATT (4796G>A 4798C>T), ACT383AGC (4803C>G 4804T>C), GAG384GAA (4807G>A), GCG385GCT (4810G>T), GCT386GCG (4813T>G), CTT387CTC (4816T>C), TAT392TAC (4831T>C), AAC393AAT (4834C>T), CCA395CCT (4840A>T), GGA399GGG (4852A>G), GGC400GGT (4855C>T), AGT404AGC (4867T>C), CTG405CTC (4870G>C), ATT406ATA (4873T>A), TGT409TGC (4882T>C), ACA416ACT (4903A>T), CAG418CAA (4909G>A), TTT420TTC (4915T>C), GAA421GAG (4918A>G), TTG422CTA (4919T>C 4921G>A), AGT423AGC (4924T>C), CCC424CCG (4927C>G), TTT426TTC (4933T>C), ATC427ATA (4936C>A), CAG428CAA (4939G>A), AAT429AAC (4942T>C), TTC430TTT (4945C>T), TCA436TCG (4963A>G), CCT439CCC (4972T>C), CTT445CTC (4990T>C), AAA449AAG (5002A>G), CCT456CCC (5023T>C), TTG457CTG (5024T>C), TGC458TGT (5029C>T), GAT459GAC (5032T>C), TCT461TCC (5038T>C), CCA463CCT (5044A>T), GCA466GCT (5053A>T), TCG467TCA (5056G>A), AGT468GGT (5057A>G), ACT469GCT (5060A>G), TCG472TCT (5071G>T), CTT479ATT (5090C>A), GGA480GGG (5095A>G), GTG481GTT (5098G>T), GCC482GTC (5100C>T), TTA483TTG (5104A>G), GAA484GAT (5107A>T), ATT485ATC (5110T>C), CCA486CCG (5113A>G), AAG487AAA (5116G>A), CAA488CAG (5119A>G), GTC489TTT (5120G>T 5122C>T), ATT491GTT (5126A>G), CAT494CAC (5137T>C), ATC495ATT (5140C>T), AAA496AAG (5143A>G), GAG497GAA (5146G>A), CTC502CTA (5161C>A), GAA504GAG (5167A>G), CTT506CTG (5173T>G), ACG509ACA (5182G>A), GTT510ATT (5183G>A), GTC511GTT (5188C>T), AAG512AAA (5191G>A), AAA514AAG (5197A>G), GAC515GAT (5200C>T), TGC517TGT (5206C>T), TTA518TTG (5209A>G), AGC521AGT (5218C>T), TCG525TCA (5230G>A), ATC527ATT (5236C>T), AGC528AGT (5239C>T), ATC530ATT (5245C>T), ACA536ACT (5263A>T), CTC538CTT (5269C>T), TTC539TTT (5272C>T), GCC540GCA (5275C>A), ATC541ATT (5278C>T), CCA542CCC (5281A>C), AGA543AGG (5284A>G), ACT544ACC (5287T>C), CTA545TTA (5288C>T), ATA546ATT (5293A>T), TTG547CTA (5294T>C 5296G>A), GAG549GAA (5302G>A), CTT552CTA (5311T>A), GAA553GAG (5314A>G), CGA556CGT (5323A>T), AGT564AGC (5347T>C), ATC566ATT (5353C>T), TGC570TGT (5365C>T), GTT577GTC (5386T>C), ACC580ACA (5395C>A), AAA589AAG (5422A>G), GAT590GAC (5425T>C), ACC592ACT (5431C>T), GAG594GAA (5437G>A), CTT599CTC (5452T>C), AAG601AAA (5458G>A), AGT604AGC (5467T>C), CAA605CAG (5470A>G), CTA606TTG (5471C>T 5473A>G), GAA608GAG (5479A>G), TTC609TTT (5482C>T), TCA610TCC (5485A>C), AAT611AAC (5488T>C), TTG612CTA (5489T>C 5491G>A), GGT614GGC (5497T>C), TGT617TGC (5506T>C), GAG618GAA (5509G>A), GAG619GAA (5512G>A), TTA621CTA (5516T>C), TAT625TAC (5530T>C), GAG626GAA (5533G>A), TCG627TCT (5536G>T), TTG628TTA (5539G>A), GTT631GTG (5548T>G), CAC633CAT (5554C>T)								
6K2 protein (NP_734247.1)	1	52	100%	303	93.5%	52 (100%)	46 (88.5%)	0/0/0/0	0
Protein mutations:	A2T (5561G>A 5563G>A), I14V (5597A>G), N16K (5605C>A), A21V (5619C>T 5620T>G), I25L (5630A>C), V46I (5693G>A)								
Codon mutations:	GCT1GCA (5560T>A), GCG2ACA (5561G>A 5563G>A), ACG3ACC (5566G>C), TCA4TCG (5569A>G), CTC9CTG (5584C>G), AAG12AAA (5593G>A), GGG13GGA (5596G>A), ATT14GTT (5597A>G), AAC16AAA (5605C>A), GTG20GTT (5617G>T), GCT21GTG (5619C>T 5620T>G), TTG24TTA (5629G>A), ATC25CTC (5630A>C), GGC28GGT (5641C>T), GCA31GCT (5650A>T), ATA35ATT (5662A>T), GGA36GGG (5665A>G), CAA44CAG (5689A>G), GTT46ATT (5693G>A), GAG47GAA (5698G>A), CAA52CAG (5713A>G)								
NIa-VPg protein (NP_734252.1)	1	188	100%	1227	93.3%	188 (100%)	170 (90.4%)	0/0/0/0	0
Protein mutations:	K47R (5853A>G), I61V (5894A>G 5896C>G), R83A (5960C>G 5961G>C 5962G>C), I93V (5990A>G), E102D (6019A>T), K106T (6030A>C), D112E (6049C>A), M115I (6058G>A), S120N (6072G>A 6073T>C), N121H (6074A>C), T123N (6081C>A), C133S (6111G>C 6112T>C), I139V (6128A>G 6130T>C), V149I (6158G>A), T153S (6170A>T 6172A>G), L164F (6203C>T 6205C>T), V173I (6230G>A), A182K (6257G>A 6258C>A)								

	Begin	End	Coverage	Score	Concordance	Matches	Identities	I/D/M/F*	Stop Codons
**NT**	**5**	**9704**	**99.9%**	**12522**	**64.8%**	**9688 (99.7%)**	**8015 (82.5%)**	**15/12**	
Codon mutations:	GGG1GGC (5716G>C), AAA2AAG (5719A>G), GCC10GCA (5743C>A), CGC14CGA (5755C>A), CAT15CAC (5758T>C), CGT17CGC (5764T>C), AAA19AAG (5770A>G), GGC22GGT (5779C>T), TTT23TTC (5782T>C), GAC26GAT (5791C>T), GAC30GAT (5803C>T), AGG41AGA (5836G>A), AAA42AAG (5839A>G), GGA44GGG (5845A>G), AAA45AAG (5848A>G), AAA47AGA (5853A>G), GGT48GGC (5857T>C), ACC49ACT (5860C>T), ACA50ACT (5863A>T), GGT52GGA (5869T>A), GGT54GGC (5875T>C), AGC57AGT (5884C>T), TTC60TTT (5893C>T), ATC61GTG (5894A>G 5896C>G), AAC62AAT (5899C>T), GGG65GGA (5908G>A), TTT66TTC (5911T>C), GAG70GAA (5923G>A), CAA75CAG (5938A>G), TTC76TTT (5941C>T), GTT77GTC (5944T>C), GAT78GAC (5947T>C), GGG82GGA (5959G>A), CGG83GCC (5960C>G 5961G>C 5962G>C), GAA87GAG (5974A>G), GCT91GCA (5986T>A), GAC92GAT (5989C>T), ATT93GTT (5990A>G), GAT95GAC (5998T>C), AGA99CGT (6008A>C 6010A>T), GAA102GAT (6019A>T), GTG103GTT (6022G>T), CGA104CGT (6025A>T), AAA106ACA (6030A>C), GAC112GAA (6049C>A), ATG115ATA (6058G>A), CAA116CAG (6061A>G), GCC117GCA (6064C>A), GGT119GGC (6070T>C), AGT120AAC (6072G>A 6073T>C), AAC121CAC (6074A>C), ACG122ACA (6079G>A), ACC123AAC (6081C>A), ATA124ATT (6085A>T), GCA126GCT (6091A>T), TAC127TAT (6094C>T), TGT133TCC (6111G>C 6112T>C), AAA135AAG (6118A>G), TTG137TTA (6124G>A), AAG138AAA (6127G>A), ATT139GTC (6128A>G 6130T>C), GAT140GAC (6133T>C), TTA141TTG (6136A>G), AAC145AAT (6148C>T), AAA148AAG (6157A>G), GTT149ATT (6158G>A), TGT150TGC (6163T>C), AAA152AAG (6169A>G), ACA153TCG (6170A>T 6172A>G), GGC155GGT (6178C>T), AAA158AAG (6187A>G), TTT159TTC (6190T>C), GAG161GAA (6196G>A), CTC164TTT (6203C>T 6205C>T), GAA165GAG (6208A>G), CTA166TTG (6209C>T 6211A>G), CAG168CAA (6217G>A), GCT172GCA (6229T>A), GTA173ATA (6230G>A), GTC175GTT (6238C>T), AAG178AAA (6247G>A), ATA180ATT (6253A>T), GCA182AAA (6257G>A 6258C>A), CAT187CAC (6274T>C)								
NIa-Pro protein (NP_734248.1)	1	244	100%	1654	93.7%	244 (100%)	223 (91.4%)	0/0/0/0	0
Protein mutations:	K2R (6282A>G), A29T (6362G>A), V42I (6401G>A), A43V (6405C>T), Y51F (6429A>T), Q58R (6450A>G), H70N (6485C>A 6487C>T), L84I (6527C>A), T104I (6588C>T), Y120H (6635T>C), I124V (6647A>G), I134V (6677A>G 6679A>G), D147N (6716G>A), N148D (6719A>G), I163L (6764A>T 6766A>G), A173V (6795C>T 6796A>G), H174Q (6799C>A), N194D (6857A>G), V200T (6875G>A 6876T>C 6877C>A), D219E (6934C>A), V242I (7001G>A 7003G>A)								
Codon mutations:	AAA2AGA (6282A>G), TCG3TCA (6286G>A), GGC7GGT (6298C>T), TTG8TTA (6301G>A), AGA9AGG (6304A>G), GAC10GAT (6307C>T), GCC15GCT (6322C>T), ACA17ACT (6328A>T), GTT18GTG (6331T>G), TGT19TGC (6334T>C), AGG20AGA (6337G>A), CTG21TTG (6338C>T), TAT27TAC (6358T>C), GGG28GGA (6361G>A), GCA29ACA (6362G>A), TCA30TCT (6367A>T), GAG31GAA (6370G>A), TAC33TAT (6376C>T), GGT34GGG (6379T>G), GGA36GGC (6385A>C), GGA38GGT (6391A>T), GCA39GCG (6394A>G), TAC40TAT (6397C>T), GTA42ATA (6401G>A), GCG43GTG (6405C>T), CAC45CAT (6412C>T), TTA47TTG (6418A>G), TTT48TTC (6421T>C), TAC51TTC (6429A>T), GGT53GGA (6436T>A), GAG56GAA (6445G>A), CAA58CGA (6450A>G), TCC59TCG (6454C>G), CAC61CAT (6460C>T), GGT62GGA (6463T>A), TTC64TTT (6469C>T), AGG65AGA (6472G>A), GTG66GTA (6475G>A), AAT68AAC (6481T>C), CAC70AAT (6485C>A 6487C>T), AGT71AGC (6490T>C), AGC73AGT (6496C>T), CTG75CTA (6502G>A), GGT79GGC (6514T>C), AGG80AGA (6517G>A), CTC84ATC (6527C>A), ATC85ATA (6532C>A), CCG88CCA (6541G>A), AAA89AAG (6544A>G), GTC93GTT (6556C>T), TTT94TTC (6559T>C), CAG96CAA (6565G>A), TTG98CTA (6569T>C 6571G>A), CCT103CCA (6586T>A), ACA104ATA (6588C>T), GAA107GAG (6598A>G), AGA108AGG (6601A>G), ATT109ATC (6604T>C), TGT110TGC (6607T>C), GGA113GGG (6616A>G), AAC115AAT (6622C>T), GAG118GAA (6631G>A), AAG119AAA (6634G>A), TAT120CAT (6635T>C), GCT121GCA (6640T>A), TCG123TCT (6646G>T), ATC124GTC (6647A>G), ATC125ATT (6652C>T), AGC129AGT (6664C>T), ACT131ACG (6670T>G), ATA134GTG (6677A>G 6679A>G), GGC136GGT (6685C>T), ACA138ACT (6691A>T), TTC139TTT (6694C>T), ACA146ACG (6715A>G), GAT147AAT (6716G>A), AAT148GAT (6719A>G), GGA149GGG (6724A>G), CTA153TTA (6734C>T), GTG155GTA (6742G>A), GTG156GTA (6745G>A), GCC159GCT (6754C>T), GGA161GGG (6760A>G), ATA163TTG (6764A>T 6766A>G), ATT166ATA (6775T>A), CTG169TTG (6782C>T), GCA170GCT (6787A>T), AAC171AAT (6790C>T), GCA173GTG (6795C>T 6796A>G), CAC174CAA (6799C>A), ACC175ACA (6802C>A), AAC177AAT (6808C>T), TAC178TAT (6811C>T), TAC179TAT (6814C>T), TTC182TTT (6823C>T), GAA184GAG (6829A>G), TAC190TAT (6847C>T), CGA192CGC (6853A>C), AAT194GAT (6857A>G), GAG195GAA (6862G>A), GAA198GAG (6871A>G), GTC200ACA (6875G>A 6876T>C 6877C>A), AAG201AAA (6880G>A), TCT202TCA (6883T>A), GTT204GTG (6889T>G), GAC208GAT (6901C>T), GGC213GGT (6916C>T), CCG214CCA (6919G>A), AAA216AAG (6925A>G), CTT217CTC (6928T>C), GAC219GAA (6934C>A), GGG224GGC (6949G>C), TTC226TTT (6955C>T), AAA227AAG (6958A>G), CAA233CAG (6976A>G), CTA235TTA (6980C>T), ATC236ATT (6985C>T), GTA240GTT (6997A>T), GTG241GTT (7000G>T), GTG242ATA (7001G>A 7003G>A)								
NIb protein (NP_734249.1)	1	521	100%	3129	86.1%	519 (99.4%)	480 (92.0%)	1/2/2/2	0
Protein mutations:	F8Y (7032T>A), T39K (7125C>A), A46S (7145G>T 7147A>G), E51_A52del (7160_7165delGAGGCA), D69E (7216T>A), D83E (7258T>A), D88_R89insX (7273_7274insT), R89V (7274C>G 7275G>T), H91L (7281A>T), L92S (7284T>C), S96L (7296C>T), S101F (7311C>T), T102I (7314C>T), C103Y (7317G>A), N104K (7321T>A), K109Q (7334A>C), A112N (7343G>A 7344C>A 7345A>C), S133M (7407G>T 7408T>G), C136G (7415T>G), L165S (7503T>C 7504G>A), D234N (7709G>A), K235R (7713A>G 7714A>G), T268I (7812C>T), V279L (7844G>T 7846G>A), V292I (7883G>A), S345N (8043G>A), N381S (8151A>G 8152C>T), R390K (8178G>A), R400K (8208G>A), P429A (8294C>G 8296A>G), S447F (8349C>T), A467S (8408G>T), M481I (8452G>A), R484K (8460G>A), A491T (8480G>A 8482T>A), R498K (8502G>A 8503A>G), E502D (8515A>T), E510D (8539G>C), L513C (8546C>T 8547T>G), S515T (8552T>A)								
Codon mutations:	GCT1GCC (7012T>C), AAG2AAA (7015G>A), CAT3CAC (7018T>C), TTT8TAT (7032T>A), GCC10GCT (7039C>T), TTG11CTA (7040T>C 7042G>A), GGA13GGG (7048A>G), GCT17GCA (7060T>A), GTC18GTA (7063C>A), AGC23AGT (7078C>T), TTA25CTG (7082T>C 7084A>G), GTA26GTG (7087A>G), ACC27ACA (7090C>A), CAT29CAC (7096T>C), GTA30GTG (7099A>G), GTT31GTC (7102T>C), GAG34GAA (7111G>A), CGA36CGG (7117A>G), ACA39AAA (7125C>A), GAA40GAG (7129A>G), TTT41TTC (7132T>C), CTG42TTA (7133C>T 7135G>A), GTG44GTA (7141G>A), GAT45GAC (7144T>C), GCA46TCG (7145G>T 7147A>G), GAG47GAA (7150G>A), GCA50GCT (7159A>T), GAG51_GCA52del (7160_7165delGAGGCA), TTC54TTT (7171C>T), GCG60GCT (7189G>T), AAA63AAG (7198A>G), AGC64AGT (7201C>T), CTA66TTA (7205C>T), AAT67AAC (7210T>C), GAT69GAA (7216T>A), GCG70GCA (7219G>A), ATC72ATA (7225C>A), AAG73AAA (7228G>A), AAG77AAA (7240G>A), TAT78TAC (7243T>C), AAA80AAG (7249A>G), CCT81CCG (7252T>G), GAT83GAA (7258T>A), GTT84GTC (7261T>C), GGT85GGA (7264T>A), GTC86GTG (7267C>G), GTG87GTA (7270G>A), GAT88_CGG89insT-- (7273_7274insT), CGG89GTG (7274C>G 7275G>T), CAT91CTT (7281A>T), TTG92TCG (7284T>C), TCA96TTA (7296C>T), TCT101TTT (7311C>T), ACC102ATC (7314C>T), TGC103TAC (7317G>A), AAT104AAA (7321T>A), GTG105-TG (7322delG), CAC106CAT (7327C>T), GGC107GGT (7330C>T), TTC108TTT (7333C>T), AAG109CAG (7334A>C), TGT111TGC (7342T>C), GCA112AAC (7343G>A 7344C>A 7345A>C), ACT115ACC (7354T>C), CAA118CAG (7363A>G), ATT120ATC (7369T>C), TTC121TTT (7372C>T), GCG123GCT (7378G>T), CTC124CTT (7381C>T), GCA129GCT (7396A>T), GTC130GTT (7399C>T), GCC132GCT (7405C>T), AGT133ATG (7407G>T 7408T>G), GGG135GGT (7414G>T), TGC136GGC (7415T>G), AAA137AAG (7420A>G), GAC140GAT (7429C>T), TAT141TAC (7432T>C), CAT144CAC (7441T>C), AAG150AAA (7459G>A), GAA151GAG (7462A>G), ATA153ATT (7468A>T), GTC154GTA (7471C>A), CAA156CAG (7477A>G), AGC157AGT (7480C>T), CTG159CTT (7486G>T), TAT162TAC (7495T>C), AAA163AAG (7498A>G), GGT164GGC (7501T>C), TTG165TCA (7503T>C 7504G>A), ATT168ATA (7513T>A), AAC170AAT (7519C>T), GGA171GGG (7522A>G), AAG174AAA (7531G>A), GAG176GAA (7537G>A), CTC177CTT (7540C>T), TGT179TGC (7546T>C), AAG187AAA (7570G>A), ACG188ACA (7573G>A), ACG190ACA (7579G>A), GCT193GCC (7588T>C), CTA196TTA (7595C>T), TTG199CTA (7604T>C 7606G>A), GGT202GGA (7615T>A), TGT205TGC (7624T>C), GAC208GAT (7633C>T), TTC209TTT (7636C>T), AAT210AAC (7639T>C), TTT213TTC (7648T>C), GAA219GAG (7666A>G), TGT221TGC (7672T>C), ACA223ACT (7678A>T), GGG225GGA (7684G>A), ACT227ACC (7690T>C), TTT229TTC (7696T>C), GGT231GGG (7702T>G), GAT234AAT (7709G>A), AAA235AGG (7713A>G 7714A>G), CTG236TTG (7715C>T), CGT239CGC (7726T>C), GAG242GAA (7735G>A), GTA245GTG (7744A>G), TAC246TAT (7747C>T), GGC251GGT (7762C>T), CAG253CAA (7768G>A), TTT254TTC (7771T>C), TCA257TCG (7780A>G), CTA258CTC (7783A>C), ACT259ACC (7786T>C), CTA262TTG (7793C>T 7795A>G), ATC263ATT (7798C>T), GCT265GCC (7804T>C), ACC268ATC (7812C>T), GAA275GAG (7834A>G), GAC276GAT (7837C>T), GTG279TTA (7844G>T 7846G>A), CAG282CAA (7855G>A), CTG284CTA (7861G>A), TTA287TTG (7870A>G), TAC288TAT (7873C>T), ACT289ACA (7876T>A), GAG290GAA (7879G>A), ATT291ATA (7882T>A), GTT292ATT (7883G>A), CCA295CCT (7894A>T), ATT296ATC (7897T>C), GTC304GTT (7921C>T), AAG306AAA (7927G>A), GGT309GGC (7936T>C), AAT310AAC (7939T>C), AAT311AAC (7942T>C), GTG319GTA (7966G>A), GAT320GAC (7969T>C), TCT322TCC (7975T>C), GTC326GTT (7987C>T), CTT327CTC (7990T>C), GCT332GCC (8005T>C), GAA341GAG (8032A>G), ATC343ATT (8038C>T), GAC344GAT (8041C>T), AGC345AAC (8043G>A), TTG357TTA (8080G>A), GTG360GTA (8089G>A), CCG362CCA (8095G>A), GAG363GAA (8098G>A), AGC366AGT (8107C>T), ATT367ATC (8110T>C), GAT369GAC (8116T>C), CAA373CAG (8128A>G), GGT379GGC (8146T>C), AAC381AGT (8151A>G 8152C>T), TCG385TCA (8164G>A), AGG390AAG (8178G>A), AAG391AAA (8182G>A), TTG394TTA (8191G>A), TTC396TTT (8197C>T), TCC398TCT (8203C>T), AGA400AAA (8208G>A), CTG402TTA (8213C>T 8215G>A), CTA403CTG (8218A>G), ATC404ATT (8221C>T), GAG405GAA (8224G>A), CCA410CCG (8239A>G), GAG415GAA (8254G>A), ATT417ATC (8260T>C), GTA418GTG (8263A>G), GAT424GAC (8281T>C), AGA425AGG (8284A>G), CCA429GCG (8294C>G 8296A>G), GAG430GAA (8299G>A), CAC431CAT (8302C>T), ATT436ATC (8317T>C), TGC437TGT (8320C>T), GCT439GCA (8326T>A), GAG442GAA (8335G>A), TCC443TCT (8338C>T), TCT447TTT (8349C>T), GAA448GAG (8353A>G), CAC451CAT (8362C>T), CAA452CAG (8365A>G), ATC453ATT (8368C>T), TAC457TAT (8380C>T), TCA458TCG (8383A>G), TTA460TTG (8389A>G), CCT465CCC (8404T>C), GCA467TCA (8408G>T), ACA468ACG (8413A>G), GCG470GCT (8419G>T), GCT475GCC (8434T>C), TAT477TAC (8440T>C), ATG481ATA (8452G>A), CTA483CTG (8458A>G), AGG484AAG (8460G>A), AAA485AAG (8464A>G), CTG486CTA (8467G>A), AGG490AGA (8479G>A), GCT491ACA (8480G>A 8482T>A), GAG496GAA (8497G>A), CTA497TTG (8498C>T 8500A>G), AGA498AAG (8502G>A 8503A>G), GCC499GCT (8506C>T), GAA502GAT (8515A>T), GTC505GTT (8524C>T), GCA506GCC (8527A>C), TTA507CTG (8528T>C 8530A>G), GAG510GAC (8539G>C), CTT513TGT (8546C>T 8547T>G), TCT515ACT (8552T>A), GTA518GTG (8563A>G)								
coat protein (NP_734250.1)	1	267	100%	1652	90.6%	267 (100%)	243 (91.0%)	0/0/0/0	0
Protein mutations:	A1G (8574C>G), I5V (8585A>G), G9E (8598G>A), N11S (8603A>T 8604A>C 8605C>T), P17Q (8622C>A), E18G (8625A>G 8626G>A), P26F (8648C>T 8649C>T 8650G>C), G29E (8658G>A), D31A (8664A>C 8665T>G), A36V (8679C>T 8680A>T), T58K (8745C>A 8746A>G), E95G (8856A>G 8857G>A), R98Q (8865G>A), M99L (8867A>C 8869G>T), G104E (8883G>A), T106S (8888A>T), V128I (8954G>A), N138D (8984A>G 8986T>C), E139V (8988A>T), I187V (9131A>G), M193G (9149A>G 9150T>G 9151G>A), G194S (9152G>A), P230S (9260C>T 9262T>C), Q242H (9298A>C)								
Codon mutations:	GCA1GGA (8574C>G), ATT5GTT (8585A>G), GGA9GAA (8598G>A), AGC10AGT (8602C>T), AAC11TCT (8603A>T 8604A>C 8605C>T), AAA16AAG (8620A>G), CCA17CAA (8622C>A), GAG18GGA (8625A>G 8626G>A), CAG19CAA (8629G>A), GGC20GGT (8632C>T), ATC22ATT (8638C>T), CAG23CAA (8641G>A), AAC25AAT (8647C>T), CCG26TTC (8648C>T 8649C>T 8650G>C), AAC27AAT (8653C>T), AAA28AAG (8656A>G), GGA29GAA (8658G>A), GAT31GCG (8664A>C 8665T>G), GTT34GTG (8674T>G), GCA36GTT (8679C>T 8680A>T), GGC37GGA (8683C>A), GGG40GGA (8692G>A), ACA41ACT (8695A>T), CCG45CCA (8707G>A), AGA46CGA (8708A>C), AAG48AAA (8716G>A), ATC50ATT (8722C>T), ACG51ACA (8725G>A), AAA53AAG (8731A>G), AGA55AGG (8737A>G), CCC57CCT (8743C>T), ACA58AAG (8745C>A 8746A>G), AAG60AAA (8752G>A), GGA61GGT (8755A>T), AAC66AAT (8770C>T), TTA67CTA (8771T>C), GAA68GAG (8776A>G), CAT69CAC (8779T>C), TTG70TTA (8782G>A), TAT73TAC (8791T>C), CCA75CCG (8797A>G), CAA76CAG (8800A>G), CAA77CAG (8803A>G), GAT79GAC (8809T>C), ATT80ATC (8812T>C), AAT82AAC (8818T>C), CGG84CGA (8824G>A), GCA85GCG (8827A>G), ACG92ACA (8848G>A), TAT94TAC (8854T>C), GAG95GGA (8856A>G 8857G>A), GTG97GTA (8863G>A), CGG98CAG (8865G>A), ATG99CTT (8867A>C 8869G>T), GCA100GCG (8872A>G), GGA104GAA (8883G>A), ACT106TCT (8888A>T), TGC119TGT (8929C>T), GTC128ATC (8954G>A), AAC129AAT (8959C>T), GGA130GGT (8962A>T), GGG137GGA (8983G>A), AAT138GAC (8984A>G 8986T>C), GAA139GTA (8988A>T), GAG142GAA (8998G>A), CCG144CCA (9004G>A), TTG145TTA (9007G>A), ATC148ATT (9016C>T), ACC155ACA (9037C>A), ATA171ATT (9085A>T), AAC175AAT (9097C>T), ATT187GTT (9131A>G), CGA188CGT (9136A>T), CGG191CGT (9145G>T), ATG193GGA (9149A>G 9150T>G 9151G>A), GGT194AGT (9152G>A), TTA195TTG (9157A>G), GCG196GCT (9160G>T), CGT197CGC (9163T>C), GAG204GAA (9184G>A), CGA208CGG (9196A>G), AGG214AGA (9214G>A), GAA215GAG (9217A>G), GCG216GCA (9220G>A), GCA224GCT (9244A>T), TTG225TTA (9247G>A), GCC228GCT (9256C>T), CCT230TCC (9260C>T 9262T>C), GGG234GGA (9274G>A), GAC236GAT (9280C>T), ATC239ATT (9289C>T), CAA242CAC (9298A>C), GAG244GAA (9304G>A), GTC254GTT (9334C>T), GTC264GTG (9364C>G)								

	Begin	End	Coverage	Score	Concordance	Matches	Identities	I/D/M/F*	Stop Codons
**NT**	**5**	**9704**	**99.9%**	**12522**	**64.8%**	**9688 (99.7%)**	**8015 (82.5%)**	**15/12**	
PIPO (YP_006393460.1)	1	75	100%	95	18.1%	75 (98.7%)	54 (71.1%)	1/0/3/2	2
Protein mutations:	R17_K17insX (2971_2972insA), Y23H (2989T>C 2991C>T), R26K (2999G>A 3000A>G), H35Y (3025C>T), K40H (3040A>C 3042A>C), G41S (3043G>A), E44K (3052G>A), I47V (3061A>G), V54I (3082G>A), P57S (3091C>T), P60T (3100C>A), G61E (3104G>A 3105T>G), G62S (3106G>A), Q63* (3109C>T), R64G (3112A>G), C66R (3118T>C), L67F (3121C>T), I69V (3127A>G), A72E (3137C>A 3138G>A), I73V (3139A>G)								
Codon mutations:	TTA2CTA (2923T>C), AAA4AAG (2931A>G), GAA7GAG (2940A>G), TTG10CTG (2947T>C), TTG15CTG (2962T>C), AGA17_AAA17insAA- (2971_2972insA), TAC23CAT (2989T>C 2991C>T), TCA24TCG (2994A>G), AGA26AAG (2999G>A 3000A>G), CAC32-AC (3016delC), TCG33TCA (3021G>A), GTA34GTT (3024A>T), CAT35TAT (3025C>T), CAC38CAT (3036C>T), AGA39AGG (3039A>G), AAA40CAC (3040A>C 3042A>C), GGC41AGC (3043G>A), GAA44AAA (3052G>A), ATA47GTA (3061A>G), GTT54ATT (3082G>A), CCG57TCG (3091C>T), CGC59CGT (3099C>T), CCA60ACA (3100C>A), GGT61GAG (3104G>A 3105T>G), GGT62AGT (3106G>A), CAA63TAA (3109C>T), AGG64GGG (3112A>G), TGC66CGC (3118T>C), CTC67TTC (3121C>T), ATT69GTT (3127A>G), GAG70GAA (3132G>A), GCG72GAA (3137C>A 3138G>A), ATT73GTT (3139A>G)								

*: Inserts / Deletes / Misaligned / Frameshifts

Analysis details

This analysis was performed with panviral2.64

NGS Details (UN9): Tomato chocolate spot virus (segment RNA 1)

Assembly

**Coverage Length**	1254 (5 contig(s))
**Depth Of Coverage**	14600.6
**Number Of Reads**	162269
**Reads Per Million**	3667.74 rpm (after QC)
**Ambiguities**	0
**Assembly Method**	de novo + reference guided assembly
**Consensus Caller**	Bcf Tools

Coverage Map

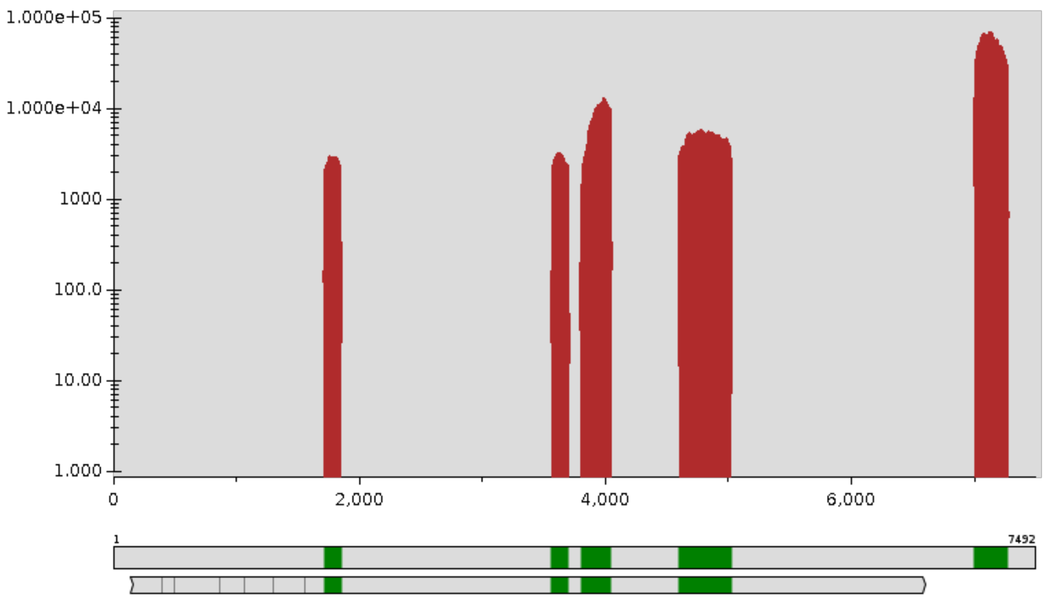

Assignment

**Type**	Tomato chocolate spot virus (Taxonomy ID: 661101)
**Reference Genome**	NC_013075.1
**NT Identity (%)**	75.1394
**AA Identity (%)**	76.9231
**Number Of Stop Codons**	0
**Number Of CDS**	1

Alignment

**Alignment Score**	1254.0 (NT) + 1858.0 (AA) = 3112.0
**Concordance (%)**	64.5509

Alignment Method	Global, seeded, nucleotide + amino acids (AGA)

Genome Region

Sequence starts at position 1710 and ends at position 7275 relative to NC_013075.1 reference sequence.

Alignment Detailed Statistics

	Begin	End	Coverage	Score	Concordance	Matches	Identities	I/D/M/F*	Stop Codons
**NT**	**1710**	**7275**	**16.7%**	**1254**	**50.0%**	**1254 (99.9%)**	**943 (75.1%)**	**1/0**	
Mutations:	1725T>A, 1728A>G, 1729G>A, 1734C>A, 1737T>A, 1739G>C, 1740C>A, 1748A>G, 1749A>C, 1755T>C, 1758T>C, 1759G>A, 1763A>G, 1764G>A, 1771C>A, 1773A>G, 1776A>T, 1785C>A, 1792C>A, 1794T>A, 1796G>A, 1797T>C, 1798T>C, 1800G>C, 1801T>A, 1806C>T, 1807A>T, 1808G>C, 1809C>T, 1812T>C, 1821C>A, 1824C>T, 1833A>C, 1839T>C, 1840C>A, 1845T>C, 3573A>G, 3575G>A, 3579C>T, 3581A>C, 3582G>A, 3594T>A, 3600A>T, 3601C>G, 3602C>A, 3603T>A, 3607G>C, 3612A>T, 3615A>T, 3618A>T, 3622A>C, 3624A>G, 3628A>T, 3630C>T, 3633C>G, 3636T>A, 3639C>T, 3642G>A, 3645G>A, 3648T>G, 3651G>T, 3654C>A, 3663A>T, 3675G>C, 3676G>A, 3678C>T, 3679A>G, 3680T>A, 3808G>A, 3810C>T, 3813A>G, 3819T>A, 3828C>T, 3831T>G, 3834T>A, 3840T>A, 3842G>T, 3843G>T, 3851A>T, 3852C>T, 3857G>C, 3858T>A, 3864G>A, 3865C>T, 3873T>G, 3888C>T, 3898A>T, 3903T>A, 3904A>C, 3906G>T, 3910C>T, 3911T>C, 3912C>T, 3921A>G, 3924G>A, 3934A>G, 3937A>C, 3938C>A, 3939T>A, 3940G>C, 3941G>C, 3942T>A, 3943G>A, 3944T>A, 3945G>C, 3946G>T, 3947G>C, 3949G>C, 3951C>G, 3952C>A, 3954C>G, 3958G>C, 3959A>T, 3960G>T, 3965A>T, 3966G>A, 3967C>A, 3969A>G, 3970T>A, 3971C>A, 3972A>C, 3981G>C, 3987A>T, 3990G>A, 3996G>A, 4005T>C, 4006G>T, 4007T>C, 4008T>G, 4011A>G, 4014G>A, 4017A>G, 4018A>C, 4024G>A, 4025T>C, 4026C>A, 4027C>T, 4028A>T, 4032G>T, 4038G>C, 4039T>G, 4611C>T, 4612C>G, 4614G>C, 4615C>T, 4617T>A, 4620C>T, 4621G>A, 4626T>A, 4635A>G, 4644C>T, 4650T>G, 4656T>G, 4659C>A, 4660T>A, 4666T>C, 4668G>T, 4671G>A, 4677T>C, 4678T>G, 4679C>A, 4680A>G, 4683G>A, 4686C>T, 4687A>G, 4688A>C, 4689C>A, 4692T>A, 4701A>G, 4704G>A, 4707T>A, 4708A>C, 4709T>A, 4710G>A, 4713A>G, 4716T>C, 4720G>A, 4722A>T, 4725T>C, 4728A>G, 4731C>T, 4734T>C, 4735T>C, 4737G>T, 4740C>T, 4744T>A, 4745C>A, 4746A>C, 4750G>A, 4755G>C, 4761G>T, 4764G>C, 4765C>A, 4767G>A, 4770T>C, 4785A>G, 4786C>T, 4791T>G, 4795C>T, 4806G>C, 4813A>C, 4815A>C, 4822C>T, 4827C>T, 4833T>A, 4834A>C, 4836G>A, 4839A>C, 4842G>C, 4845T>C, 4854A>T, 4860A>T, 4861A>G, 4863A>G, 4881A>C, 4882C>A, 4891C>G, 4892C>T, 4893C>T, 4896T>G, 4902T>A, 4906G>A, 4907C>T, 4908A>C, 4909C>A, 4910A>C, 4911G>T, 4914A>C, 4918A>T, 4919T>A, 4920G>T, 4929T>G, 4935T>G, 4938C>T, 4939A>G, 4950C>T, 4953T>C, 4956T>G, 4962T>A, 4965A>G, 4968T>C, 4970C>T, 4971A>T, 4977G>C, 4979A>C, 4984G>A, 4985T>C, 4986G>A, 4988C>A, 4989A>C, 4990G>A, 4995G>C, 4999G>A, 5005C>A, 5007G>A, 5009A>T, 5010T>G, 5011G>C, 5013A>G, 5016T>A, 5019A>C, 7007T>C, 7012T>G, 7018C>T, 7019A>G, 7020A>G, 7020_7021insT, 7023C>A, 7042G>T, 7044A>T, 7047G>A, 7048C>G, 7066G>A, 7082G>A, 7083T>C, 7098C>T, 7099T>C, 7104T>A, 7107G>A, 7116C>G, 7121G>A, 7127A>C, 7140C>T, 7151A>T, 7152T>C, 7177A>C, 7183C>T, 7184T>C, 7185G>T, 7186T>G, 7187T>C, 7188G>A, 7196C>T, 7197A>G, 7198A>C, 7199C>A, 7200A>G, 7201G>A, 7207A>T, 7208A>T, 7209T>G, 7210C>T, 7212T>A, 7219T>A, 7224T>C, 7242A>T, 7255A>T, 7257G>A, 7258G>C, 7260T>C, 7262C>G, 7264T>C								

CDS

	Begin	End	Coverage	Score	Concordance	Matches	Identities	I/D/M/F*	Stop Codons
ToChSV_s1_gp1	525	1631	15.1%	1858	78.7%	325 (100%)	250 (76.9%)	0/0/0/0	0
Protein mutations:	D531N (1729G>A), G534A (1739G>C 1740C>A), K537S (1748A>G 1749A>C), V541I (1759G>A), E542G (1763A>G 1764G>A), G553D (1796G>A 1797T>C), S555T (1801T>A), D561E (1821C>A), Q568K (1840C>A), R1146K (3575G>A), Q1148P (3581A>C 3582G>A), P1155E (3601C>G 3602C>A 3603T>A), E1157Q (3607G>C), I1162L (3622A>C 3624A>G), T1164S (3628A>T 3630C>T), N1166K (3636T>A), E1171D (3651G>T), V1180I (3676G>A 3678C>T), M1181E (3679A>G 3680T>A), D1224N (3808G>A 3810C>T), W1235F (3842G>T 3843G>T), Y1238F (3851A>T 3852C>T), G1240A (3857G>C 3858T>A), M1254L (3898A>T), M1256L (3904A>C 3906G>T), L1258S (3910C>T 3911T>C 3912C>T), T1266A (3934A>G), T1267Q (3937A>C 3938C>A 3939T>A), G1268P (3940G>C 3941G>C 3942T>A), V1269N (3943G>A 3944T>A 3945G>C), G1270S (3946G>T 3947G>C), D1271Q (3949G>C 3951C>G), E1274L (3958G>C 3959A>T 3960G>T), K1276I (3965A>T 3966G>A), S1278N (3970T>A 3971C>A 3972A>C), E1283D (3987A>T), V1290S (4006G>T 4007T>C 4008T>G), K1294Q (4018A>C), V1296T (4024G>A 4025T>C 4026C>A), H1297F (4027C>T 4028A>T), F1301V (4039T>G), Q1492D (4612C>G 4614G>C), V1495I (4621G>A), L1508M (4660T>A), S1514E (4678T>G 4679C>A 4680A>G), N1517A (4687A>G 4688A>C 4689C>A), D1523E (4707T>A), M1524Q (4708A>C 4709T>A 4710G>A), V1528I (4720G>A 4722A>T), S1536N (4744T>A 4745C>A 4746A>C), D1538N (4750G>A), E1541D (4761G>T), Q1543K (4765C>A 4767G>A), H1565Q (4833T>A), M1566L (4834A>C 4836G>A), I1575V (4861A>G 4863A>G), P1585V (4891C>G 4892C>T 4893C>T), A1590I (4906G>A 4907C>T 4908A>C), Q1591T (4909C>A 4910A>C 4911G>T), M1594Y (4918A>T 4919T>A 4920G>T), I1601V (4939A>G), H1606Q (4956T>G), A1611V (4970C>T 4971A>T), E1613D (4977G>C), K1614T (4979A>C), V1616T (4984G>A 4985T>C 4986G>A), A1617D (4988C>A 4989A>C), D1618N (4990G>A), E1621K (4999G>A), L1623I (5005C>A 5007G>A), N1624M (5009A>T 5010T>G), E1625Q (5011G>C 5013A>G)								
Codon mutations:	ACT529ACA (1725T>A), GAA530GAG (1728A>G), GAC531AAC (1729G>A), CCC532CCA (1734C>A), ACT533ACA (1737T>A), GGC534GCA (1739G>C 1740C>A), AAA537AGC (1748A>G 1749A>C), AAT539AAC (1755T>C), ATT540ATC (1758T>C), GTT541ATT (1759G>A), GAG542GGA (1763A>G 1764G>A), CGA545AGG (1771C>A 1773A>G), CCA546CCT (1776A>T), ATC549ATA (1785C>A), CGT552AGA (1792C>A 1794T>A), GGT553GAC (1796G>A 1797T>C), TTG554CTC (1798T>C 1800G>C), TCT555ACT (1801T>A), TAC556TAT (1806C>T), AGC557TCT (1807A>T 1808G>C 1809C>T), GAT558GAC (1812T>C), GAC561GAA (1821C>A), TAC562TAT (1824C>T), GCA565GCC (1833A>C), TGT567TGC (1839T>C), CAG568AAG (1840C>A), GTT569GTC (1845T>C), AGA1145AGG (3573A>G), AGA1146AAA (3575G>A), AGC1147AGT (3579C>T), CAG1148CCA (3581A>C 3582G>A), GCT1152GCA (3594T>A), GGA1154GGT (3600A>T), CCT1155GAA (3601C>G 3602C>A 3603T>A), GAA1157CAA (3607G>C), ACA1158ACT (3612A>T), GCA1159GCT (3615A>T), CCA1160CCT (3618A>T), ATA1162CTG (3622A>C 3624A>G), ACC1164TCT (3628A>T 3630C>T), GTC1165GTG (3633C>G), AAT1166AAA (3636T>A), GAC1167GAT (3639C>T), AAG1168AAA (3642G>A), AGG1169AGA (3645G>A), GGT1170GGG (3648T>G), GAG1171GAT (3651G>T), GGC1172GGA (3654C>A), CCA1175CCT (3663A>T), GGG1179GGC (3675G>C), GTC1180ATT (3676G>A 3678C>T), ATG1181GAG (3679A>G 3680T>A), GAC1224AAT (3808G>A 3810C>T), GAA1225GAG (3813A>G), GTT1227GTA (3819T>A), AAC1230AAT (3828C>T), GGT1231GGG (3831T>G), ATT1232ATA (3834T>A), GGT1234GGA (3840T>A), TGG1235TTT (3842G>T 3843G>T), TAC1238TTT (3851A>T 3852C>T), GGT1240GCA (3857G>C 3858T>A), GAG1242GAA (3864G>A), CTG1243TTG (3865C>T), ACT1245ACG (3873T>G), CCC1250CCT (3888C>T), ATG1254TTG (3898A>T), GGT1255GGA (3903T>A), ATG1256CTT (3904A>C 3906G>T), CTC1258TCT (3910C>T 3911T>C 3912C>T), AGA1261AGG (3921A>G), GAG1262GAA (3924G>A), ACC1266GCC (3934A>G), ACT1267CAA (3937A>C 3938C>A 3939T>A), GGT1268CCA (3940G>C 3941G>C 3942T>A), GTG1269AAC (3943G>A 3944T>A 3945G>C), GGG1270TCG (3946G>T 3947G>C), GAC1271CAG (3949G>C 3951C>G), CGC1272AGG (3952C>A 3954C>G), GAG1274CTT (3958G>C 3959A>T 3960G>T), AAG1276ATA (3965A>T 3966G>A), CGA1277AGG (3967C>A 3969A>G), TCA1278AAC (3970T>A 3971C>A 3972A>C), GTG1281GTC (3981G>C), GAA1283GAT (3987A>T), GCG1284GCA (3990G>A), GAG1286GAA (3996G>A), GAT1289GAC (4005T>C), GTT1290TCG (4006G>T 4007T>C 4008T>G), CAA1291CAG (4011A>G), GTG1292GTA (4014G>A), AGA1293AGG (4017A>G), AAA1294CAA (4018A>C), GTC1296ACA (4024G>A 4025T>C 4026C>A), CAT1297TTT (4027C>T 4028A>T), GTG1298GTT (4032G>T), CTG1300CTC (4038G>C), TTC1301GTC (4039T>G), AAC1491AAT (4611C>T), CAG1492GAC (4612C>G 4614G>C), CTT1493TTA (4615C>T 4617T>A), TTC1494TTT (4620C>T), GTT1495ATT (4621G>A), GCT1496GCA (4626T>A), GGA1499GGG (4635A>G), AAC1502AAT (4644C>T), GTT1504GTG (4650T>G), GTT1506GTG (4656T>G), CCC1507CCA (4659C>A), TTG1508ATG (4660T>A), TTG1510CTT (4666T>C 4668G>T), CAG1511CAA (4671G>A), TAT1513TAC (4677T>C), TCA1514GAG (4678T>G 4679C>A 4680A>G), TTG1515TTA (4683G>A), CCC1516CCT (4686C>T), AAC1517GCA (4687A>G 4688A>C 4689C>A), ATT1518ATA (4692T>A), GAA1521GAG (4701A>G), CTG1522CTA (4704G>A), GAT1523GAA (4707T>A), ATG1524CAA (4708A>C 4709T>A 4710G>A), GTA1525GTG (4713A>G), AAT1526AAC (4716T>C), GTA1528ATT (4720G>A 4722A>T), ATT1529ATC (4725T>C), AAA1530AAG (4728A>G), AAC1531AAT (4731C>T), GGT1532GGC (4734T>C), TTG1533CTT (4735T>C 4737G>T), GAC1534GAT (4740C>T), TCA1536AAC (4744T>A 4745C>A 4746A>C), GAT1538AAT (4750G>A), GTG1539GTC (4755G>C), GAG1541GAT (4761G>T), GTG1542GTC (4764G>C), CAG1543AAA (4765C>A 4767G>A), TTT1544TTC (4770T>C), GAA1549GAG (4785A>G), CTG1550TTG (4786C>T), ACT1551ACG (4791T>G), CTG1553TTG (4795C>T), GGG1556GGC (4806G>C), AGA1559CGC (4813A>C 4815A>C), CTG1562TTG (4822C>T), GGC1563GGT (4827C>T), CAT1565CAA (4833T>A), ATG1566CTA (4834A>C 4836G>A), GCA1567GCC (4839A>C), CCG1568CCC (4842G>C), CTT1569CTC (4845T>C), GTA1572GTT (4854A>T), ATA1574ATT (4860A>T), ATA1575GTG (4861A>G 4863A>G), ATA1581ATC (4881A>C), CGA1582AGA (4882C>A), CCC1585GTT (4891C>G 4892C>T 4893C>T), GGT1586GGG (4896T>G), CCT1588CCA (4902T>A), GCA1590ATC (4906G>A 4907C>T 4908A>C), CAG1591ACT (4909C>A 4910A>C 4911G>T), GCA1592GCC (4914A>C), ATG1594TAT (4918A>T 4919T>A 4920G>T), GTT1597GTG (4929T>G), ACT1599ACG (4935T>G), GGC1600GGT (4938C>T), ATA1601GTA (4939A>G), GCC1604GCT (4950C>T), TTT1605TTC (4953T>C), CAT1606CAG (4956T>G), GGT1608GGA (4962T>A), CAA1609CAG (4965A>G), GTT1610GTC (4968T>C), GCA1611GTT (4970C>T 4971A>T), GAG1613GAC (4977G>C), AAA1614ACA (4979A>C), GTG1616ACA (4984G>A 4985T>C 4986G>A), GCA1617GAC (4988C>A 4989A>C), GAT1618AAT (4990G>A), GTG1619GTC (4995G>C), GAA1621AAA (4999G>A), CTG1623ATA (5005C>A 5007G>A), AAT1624ATG (5009A>T 5010T>G), GAA1625CAG (5011G>C 5013A>G), CGT1626CGA (5016T>A), GGA1627GGC (5019A>C)								

Proteins

	Begin	End	Coverage	Score	Concordance	Matches	Identities	I/D/M/F*	Stop Codons
polyprotein (YP_003097229.1)	525	1631	15.1%	1858	78.7%	325 (100%)	250 (76.9%)	0/0/0/0	0
Protein mutations:	D531N (1729G>A), G534A (1739G>C 1740C>A), K537S (1748A>G 1749A>C), V541I (1759G>A), E542G (1763A>G 1764G>A), G553D (1796G>A 1797T>C), S555T (1801T>A), D561E (1821C>A), Q568K (1840C>A), R1146K (3575G>A), Q1148P (3581A>C 3582G>A), P1155E (3601C>G 3602C>A 3603T>A), E1157Q (3607G>C), I1162L (3622A>C 3624A>G), T1164S (3628A>T 3630C>T), N1166K (3636T>A), E1171D (3651G>T), V1180I (3676G>A 3678C>T), M1181E (3679A>G 3680T>A), D1224N (3808G>A 3810C>T), W1235F (3842G>T 3843G>T), Y1238F (3851A>T 3852C>T), G1240A (3857G>C 3858T>A), M1254L (3898A>T), M1256L (3904A>C 3906G>T), L1258S (3910C>T 3911T>C 3912C>T), T1266A (3934A>G), T1267Q (3937A>C 3938C>A 3939T>A), G1268P (3940G>C 3941G>C 3942T>A), V1269N (3943G>A 3944T>A 3945G>C), G1270S (3946G>T 3947G>C), D1271Q (3949G>C 3951C>G), E1274L (3958G>C 3959A>T 3960G>T), K1276I (3965A>T 3966G>A), S1278N (3970T>A 3971C>A 3972A>C), E1283D (3987A>T), V1290S (4006G>T 4007T>C 4008T>G), K1294Q (4018A>C), V1296T (4024G>A 4025T>C 4026C>A), H1297F (4027C>T 4028A>T), F1301V (4039T>G), Q1492D (4612C>G 4614G>C), V1495I (4621G>A), L1508M (4660T>A), S1514E (4678T>G 4679C>A 4680A>G), N1517A (4687A>G 4688A>C 4689C>A), D1523E (4707T>A), M1524Q (4708A>C 4709T>A 4710G>A), V1528I (4720G>A 4722A>T), S1536N (4744T>A 4745C>A 4746A>C), D1538N (4750G>A), E1541D (4761G>T), Q1543K (4765C>A 4767G>A), H1565Q (4833T>A), M1566L (4834A>C 4836G>A), I1575V (4861A>G 4863A>G), P1585V (4891C>G 4892C>T 4893C>T), A1590I (4906G>A 4907C>T 4908A>C), Q1591T (4909C>A 4910A>C 4911G>T), M1594Y (4918A>T 4919T>A 4920G>T), I1601V (4939A>G), H1606Q (4956T>G), A1611V (4970C>T 4971A>T), E1613D (4977G>C), K1614T (4979A>C), V1616T (4984G>A 4985T>C 4986G>A), A1617D (4988C>A 4989A>C), D1618N (4990G>A), E1621K (4999G>A), L1623I (5005C>A 5007G>A), N1624M (5009A>T 5010T>G), E1625Q (5011G>C 5013A>G)								

	Begin	End	Coverage	Score	Concordance	Matches	Identities	I/D/M/F*	Stop Codons
**NT**	**1710**	**7275**	**16.7%**	**1254**	**50.0%**	**1254 (99.9%)**	**943 (75.1%)**	**1/0**	
Codon mutations:	ACT529ACA (1725T>A), GAA530GAG (1728A>G), GAC531AAC (1729G>A), CCC532CCA (1734C>A), ACT533ACA (1737T>A), GGC534GCA (1739G>C 1740C>A), AAA537AGC (1748A>G 1749A>C), AAT539AAC (1755T>C), ATT540ATC (1758T>C), GTT541ATT (1759G>A), GAG542GGA (1763A>G 1764G>A), CGA545AGG (1771C>A 1773A>G), CCA546CCT (1776A>T), ATC549ATA (1785C>A), CGT552AGA (1792C>A 1794T>A), GGT553GAC (1796G>A 1797T>C), TTG554CTC (1798T>C 1800G>C), TCT555ACT (1801T>A), TAC556TAT (1806C>T), AGC557TCT (1807A>T 1808G>C 1809C>T), GAT558GAC (1812T>C), GAC561GAA (1821C>A), TAC562TAT (1824C>T), GCA565GCC (1833A>C), TGT567TGC (1839T>C), CAG568AAG (1840C>A), GTT569GTC (1845T>C), AGA1145AGG (3573A>G), AGA1146AAA (3575G>A), AGC1147AGT (3579C>T), CAG1148CCA (3581A>C 3582G>A), GCT1152GCA (3594T>A), GGA1154GGT (3600A>T), CCT1155GAA (3601C>G 3602C>A 3603T>A), GAA1157CAA (3607G>C), ACA1158ACT (3612A>T), GCA1159GCT (3615A>T), CCA1160CCT (3618A>T), ATA1162CTG (3622A>C 3624A>G), ACC1164TCT (3628A>T 3630C>T), GTC1165GTG (3633C>G), AAT1166AAA (3636T>A), GAC1167GAT (3639C>T), AAG1168AAA (3642G>A), AGG1169AGA (3645G>A), GGT1170GGG (3648T>G), GAG1171GAT (3651G>T), GGC1172GGA (3654C>A), CCA1175CCT (3663A>T), GGG1179GGC (3675G>C), GTC1180ATT (3676G>A 3678C>T), ATG1181GAG (3679A>G 3680T>A), GAC1224AAT (3808G>A 3810C>T), GAA1225GAG (3813A>G), GTT1227GTA (3819T>A), AAC1230AAT (3828C>T), GGT1231GGG (3831T>G), ATT1232ATA (3834T>A), GGT1234GGA (3840T>A), TGG1235TTT (3842G>T 3843G>T), TAC1238TTT (3851A>T 3852C>T), GGT1240GCA (3857G>C 3858T>A), GAG1242GAA (3864G>A), CTG1243TTG (3865C>T), ACT1245ACG (3873T>G), CCC1250CCT (3888C>T), ATG1254TTG (3898A>T), GGT1255GGA (3903T>A), ATG1256CTT (3904A>C 3906G>T), CTC1258TCT (3910C>T 3911T>C 3912C>T), AGA1261AGG (3921A>G), GAG1262GAA (3924G>A), ACC1266GCC (3934A>G), ACT1267CAA (3937A>C 3938C>A 3939T>A), GGT1268CCA (3940G>C 3941G>C 3942T>A), GTG1269AAC (3943G>A 3944T>A 3945G>C), GGG1270TCG (3946G>T 3947G>C), GAC1271CAG (3949G>C 3951C>G), CGC1272AGG (3952C>A 3954C>G), GAG1274CTT (3958G>C 3959A>T 3960G>T), AAG1276ATA (3965A>T 3966G>A), CGA1277AGG (3967C>A 3969A>G), TCA1278AAC (3970T>A 3971C>A 3972A>C), GTG1281GTC (3981G>C), GAA1283GAT (3987A>T), GCG1284GCA (3990G>A), GAG1286GAA (3996G>A), GAT1289GAC (4005T>C), GTT1290TCG (4006G>T 4007T>C 4008T>G), CAA1291CAG (4011A>G), GTG1292GTA (4014G>A), AGA1293AGG (4017A>G), AAA1294CAA (4018A>C), GTC1296ACA (4024G>A 4025T>C 4026C>A), CAT1297TTT (4027C>T 4028A>T), GTG1298GTT (4032G>T), CTG1300CTC (4038G>C), TTC1301GTC (4039T>G), AAC1491AAT (4611C>T), CAG1492GAC (4612C>G 4614G>C), CTT1493TTA (4615C>T 4617T>A), TTC1494TTT (4620C>T), GTT1495ATT (4621G>A), GCT1496GCA (4626T>A), GGA1499GGG (4635A>G), AAC1502AAT (4644C>T), GTT1504GTG (4650T>G), GTT1506GTG (4656T>G), CCC1507CCA (4659C>A), TTG1508ATG (4660T>A), TTG1510CTT (4666T>C 4668G>T), CAG1511CAA (4671G>A), TAT1513TAC (4677T>C), TCA1514GAG (4678T>G 4679C>A 4680A>G), TTG1515TTA (4683G>A), CCC1516CCT (4686C>T), AAC1517GCA (4687A>G 4688A>C 4689C>A), ATT1518ATA (4692T>A), GAA1521GAG (4701A>G), CTG1522CTA (4704G>A), GAT1523GAA (4707T>A), ATG1524CAA (4708A>C 4709T>A 4710G>A), GTA1525GTG (4713A>G), AAT1526AAC (4716T>C), GTA1528ATT (4720G>A 4722A>T), ATT1529ATC (4725T>C), AAA1530AAG (4728A>G), AAC1531AAT (4731C>T), GGT1532GGC (4734T>C), TTG1533CTT (4735T>C 4737G>T), GAC1534GAT (4740C>T), TCA1536AAC (4744T>A 4745C>A 4746A>C), GAT1538AAT (4750G>A), GTG1539GTC (4755G>C), GAG1541GAT (4761G>T), GTG1542GTC (4764G>C), CAG1543AAA (4765C>A 4767G>A), TTT1544TTC (4770T>C), GAA1549GAG (4785A>G), CTG1550TTG (4786C>T), ACT1551ACG (4791T>G), CTG1553TTG (4795C>T), GGG1556GGC (4806G>C), AGA1559CGC (4813A>C 4815A>C), CTG1562TTG (4822C>T), GGC1563GGT (4827C>T), CAT1565CAA (4833T>A), ATG1566CTA (4834A>C 4836G>A), GCA1567GCC (4839A>C), CCG1568CCC (4842G>C), CTT1569CTC (4845T>C), GTA1572GTT (4854A>T), ATA1574ATT (4860A>T), ATA1575GTG (4861A>G 4863A>G), ATA1581ATC (4881A>C), CGA1582AGA (4882C>A), CCC1585GTT (4891C>G 4892C>T 4893C>T), GGT1586GGG (4896T>G), CCT1588CCA (4902T>A), GCA1590ATC (4906G>A 4907C>T 4908A>C), CAG1591ACT (4909C>A 4910A>C 4911G>T), GCA1592GCC (4914A>C), ATG1594TAT (4918A>T 4919T>A 4920G>T), GTT1597GTG (4929T>G), ACT1599ACG (4935T>G), GGC1600GGT (4938C>T), ATA1601GTA (4939A>G), GCC1604GCT (4950C>T), TTT1605TTC (4953T>C), CAT1606CAG (4956T>G), GGT1608GGA (4962T>A), CAA1609CAG (4965A>G), GTT1610GTC (4968T>C), GCA1611GTT (4970C>T 4971A>T), GAG1613GAC (4977G>C), AAA1614ACA (4979A>C), GTG1616ACA (4984G>A 4985T>C 4986G>A), GCA1617GAC (4988C>A 4989A>C), GAT1618AAT (4990G>A), GTG1619GTC (4995G>C), GAA1621AAA (4999G>A), CTG1623ATA (5005C>A 5007G>A), AAT1624ATG (5009A>T 5010T>G), GAA1625CAG (5011G>C 5013A>G), CGT1626CGA (5016T>A), GGA1627GGC (5019A>C)								

*: Inserts / Deletes / Misaligned / Frameshifts

Analysis details

This analysis was performed with panviral2.64

NGS Details (UN9): Tomato chocolate spot virus (segment RNA2)

Assembly

**Coverage Length**	2083 (3 contig(s))
**Depth Of Coverage**	19230.5
**Number Of Reads**	328101
**Reads Per Million**	7416.02 rpm (after QC)
**Ambiguities**	0
**Assembly Method**	de novo + reference guided assembly
**Consensus Caller**	Bcf Tools

Coverage Map

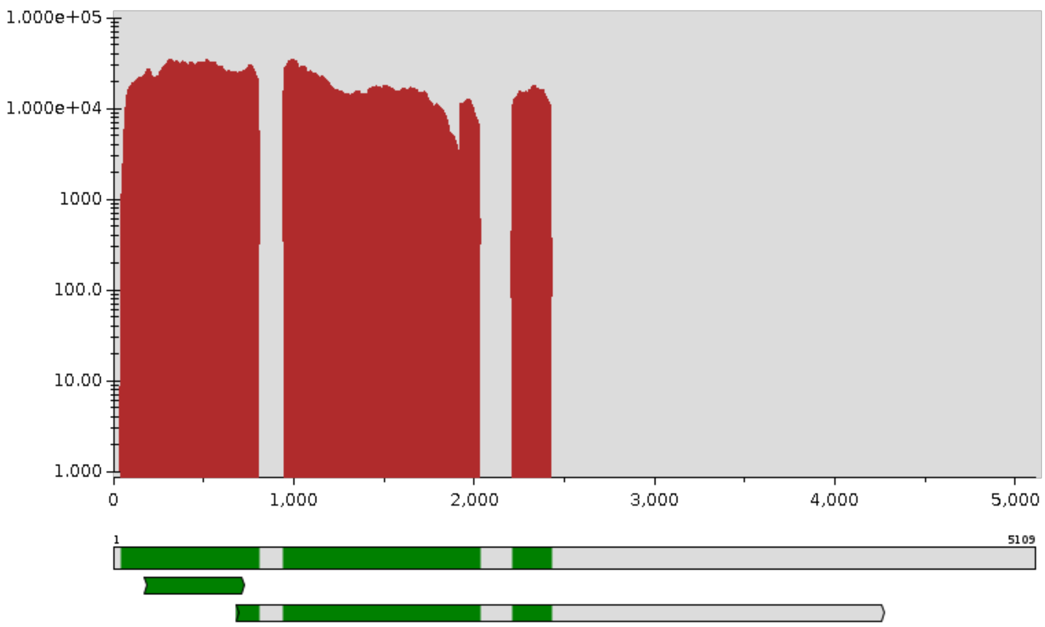

Assignment

**Type**	Tomato chocolate spot virus (Taxonomy ID: 661101)
**Reference Genome**	NC_013076.1
**NT Identity (%)**	63.9923
**AA Identity (%)**	66.0633
**Number Of Stop Codons**	1
**Number Of CDS**	2

Alignment

**Alignment Score**	1099.0 (NT) + 3139.0 (AA) = 4238.0
**Concordance (%)**	49.4978

Alignment Method	Global, seeded, nucleotide + amino acids (AGA)

Genome Region

Sequence starts at position 41 and ends at position 2430 relative to NC_013076.1 reference sequence.

Alignment Detailed Statistics

	Begin	End	Coverage	Score	Concordance	Matches	Identities	I/D/M/F*	Stop Codons
**NT**	**41**	**2430**	**40.8%**	**1099**	**27.0%**	**2063 (98.8%)**	**1324 (63.4%)**	**6/20**	
Mutations:	51A>T, 53C>T, 57T>C, 59T>A, 65A>C, 66A>G, 68C>A, 71G>C, 74T>C, 75T>G, 76T>A, 77A>C, 81G>C, 82A>C, 84A>C, 85T>A, 86C>A, 88T>A, 90G>T, 91T>A, 93T>C, 94T>C, 95G>A, 97G>T, 98T>A, 101T>C, 102delT, 106T>C, 107C>A, 110T>C, 111T>C, 112T>A, 114C>T, 116T>A, 117C>T, 118T>C, 120T>A, 123G>C, 124A>C, 125G>T, 127T>C, 130T>A, 132A>C, 134C>A, 135C>A, 138C>A, 139A>C, 140C>T, 141T>A, 149T>A, 151T>A, 152_158delGATAATC, 160C>T, 161A>C, 164C>A, 165C>T, 168G>T, 169T>C, 177T>C, 180T>C, 183T>C, 184G>T, 185G>C, 186A>T, 188G>A, 198A>T, 199G>C, 200C>A, 201A>G, 202G>T, 203A>T, 211A>C, 215C>A, 216T>A, 219C>T, 223C>A, 225A>T, 231C>A, 240C>A, 243T>G, 247A>G, 249T>G, 252C>T, 253T>A, 254C>G, 255G>T, 258G>T, 261C>T, 262G>A, 263T>C, 267A>T, 268T>G, 269C>T, 270A>T, 273T>A, 274G>A, 276A>T, 277A>G, 288C>T, 291C>G, 294T>G, 297A>T, 303T>C, 307G>A, 309G>C, 310A>T, 311T>G, 312T>C, 315C>G, 321A>T, 331G>A, 336A>T, 339G>C, 340C>A, 342A>C, 343A>T, 345T>C, 346G>A, 348T>C, 351G>C, 357G>A, 363A>T, 366A>T, 369A>G, 372G>A, 373T>C, 378T>A, 381G>T, 384C>T, 390T>C, 405T>A, 408T>G, 412C>G, 413C>A, 414T>C, 417T>C, 421A>G, 422A>C, 423C>T, 426G>C, 429G>A, 430T>C, 432A>C, 435T>A, 439C>T, 441C>G, 442T>A, 444G>A, 447G>T, 449A>G, 450T>G, 453T>A, 456T>A, 457G>A, 459T>C, 460C>A, 461G>A, 462T>A, 474C>A, 475A>C, 477G>C, 480A>G, 481G>C, 486A>T, 490C>G, 491A>C, 492G>A, 501T>A, 504A>T, 508_516delGCTCAACAA, 517A>T, 519T>A, 523A>G, 524A>C, 525T>G, 526T>C, 527C>A, 528T>A, 529G>T, 530T>C, 531A>C, 535G>T, 536A>C, 538A>C, 543G>T, 544A>C, 548C>A, 549A>G, 550A>C, 551C>T, 552C>T, 553G>T, 554A>C, 555A>T, 556A>C, 557C>T, 560C>T, 561T>A, 562T>A, 563G>A, 566A>G, 567G>T, 568T>C, 570C>T, 571A>G, 576G>A, 581T>A, 582T>C, 585T>A, 586C>G, 587A>C, 588T>A, 589C>T, 591C>A, 594C>G, 595G>A, 599A>C, 600G>T, 601C>T, 603T>G, 605A>C, 606G>T, 607C>G, 608G>T, 612A>T, 614G>A, 615A>G, 621A>G, 622A>T, 627A>C, 628G>C, 630A>G, 632A>G, 633A>C, 639A>T, 645A>C, 646T>A, 647C>T, 652T>C, 654G>T, 655C>A, 660T>G, 663G>A, 664C>A, 667T>C, 668C>A, 669C>G, 670A>G, 671A>C, 672T>A, 675G>A, 678T>A, 685A>G, 687T>A, 691A>G, 697A>T, 699A>G, 702T>C, 706T>A, 712G>A, 716G>A, 717G>T, 725A>G, 729G>A, 730G>T, 733G>T, 735A>G, 736G>C, 740_742delGAG, 745T>C, 752C>A, 753G>A, 754G>A, 755C>T, 760C>T, 761A>T, 763A>T, 769T>A, 775G>T, 778T>A, 781T>G, 786C>A, 787T>A, 790A>G, 802G>C, 958T>A, 967T>C, 970G>A, 976T>C, 979T>C, 980C>A, 982T>G, 985C>T, 994C>G, 997T>A, 1012G>A, 1019C>G, 1020G>C, 1021T>A, 1033T>C, 1034T>C, 1039T>C, 1042G>C, 1054C>A, 1056T>C, 1057C>T, 1058A>G, 1059G>A, 1060C>T, 1063G>T, 1066G>T, 1069T>A, 1072A>G, 1075G>A, 1079T>A, 1081C>G, 1084G>A, 1086C>T, 1087A>C, 1088C>T, 1090A>T, 1091G>T, 1093T>A, 1095A>G, 1096T>G, 1099T>A, 1102A>T, 1105A>G, 1108C>T, 1111G>C, 1112C>T, 1114C>G, 1124A>G, 1125G>A, 1126T>A, 1129C>T, 1138C>T, 1142C>G, 1144C>T, 1153A>G, 1156A>C, 1162C>T, 1181G>C, 1182A>T, 1183G>T, 1186T>C, 1192T>G, 1195A>T, 1197A>T, 1198G>A, 1201A>T, 1204T>A, 1210C>T, 1213G>T, 1216A>G, 1219G>A, 1222A>T, 1228A>G, 1231A>G, 1238G>A, 1239A>G, 1240T>C, 1241A>C, 1246G>A, 1252C>T, 1255A>T, 1258C>T, 1259T>A, 1261T>G, 1262C>G, 1263A>C, 1267G>A, 1273T>A, 1277T>A, 1278C>G, 1285C>A, 1291A>G, 1294A>T, 1295T>C, 1297G>C, 1300C>T, 1306T>A, 1309A>G, 1312T>C, 1315C>T, 1316C>T, 1317A>C, 1318A>T, 1327G>A, 1333T>A, 1336C>A, 1351A>T, 1354A>C, 1360T>G, 1366G>A, 1367C>G, 1369A>G, 1372T>G, 1373T>G, 1375C>G, 1376C>T, 1377A>C, 1378G>T, 1379A>C, 1380T>A, 1381G>A, 1385T>C, 1387G>A, 1394C>A, 1396T>A, 1397G>C, 1405T>A, 1408A>T, 1410A>C, 1411G>A, 1412A>C, 1413G>T, 1414G>T, 1423T>C, 1426A>T, 1427C>T, 1428G>A, 1429A>C, 1433G>A, 1438A>T, 1440T>C, 1441G>C, 1442A>C, 1443G>A, 1444T>G, 1448A>T, 1449G>C, 1450C>T, 1453C>A, 1454A>T, 1456G>A, 1458C>T, 1459T>G, 1463A>G, 1464G>C, 1465C>G, 1468T>A, 1471C>G, 1474G>T, 1477A>C, 1483G>A, 1489C>A, 1493G>A, 1495G>C, 1501T>C, 1503G>A, 1505C>A, 1513A>T, 1516C>G, 1522A>G, 1525A>G, 1531C>T, 1532G>T, 1534G>T, 1535C>T, 1537T>A, 1539A>G, 1540C>G, 1541A>G, 1547C>T, 1548G>C, 1551T>C, 1552A>G, 1555_1556insGTAGGT, 1556A>G, 1559C>A, 1572C>G, 1573T>G, 1574C>G, 1575A>T, 1576C>A, 1582A>T, 1585C>T, 1586A>T, 1588T>C, 1589G>A, 1590G>A, 1592G>T, 1593G>T, 1595T>C, 1603A>T, 1606T>C, 1610A>G, 1618A>G, 1620G>C, 1622T>A, 1624C>T, 1625A>G, 1627C>T, 1628A>T, 1630G>T, 1633T>C, 1634G>C, 1635T>A, 1636T>G, 1645T>C, 1649C>T, 1650T>A, 1651G>T, 1657A>C, 1658G>A, 1659A>C, 1660C>T, 1661T>G, 1662C>A, 1663C>T, 1668C>A, 1669A>T, 1670C>T, 1671A>C, 1672C>G, 1673A>T, 1678C>T, 1690T>G, 1696T>A, 1697G>A, 1698A>C, 1699G>C, 1700C>T, 1702C>G, 1703A>C, 1704C>A, 1705G>T, 1708T>A, 1709G>T, 1711G>A, 1712C>A, 1713A>G, 1714A>G, 1717G>A, 1718G>A, 1721C>G, 1723A>T, 1725A>T, 1726C>T, 1727A>G, 1729T>G, 1730C>T, 1734T>C, 1736G>A, 1737A>C, 1738T>C, 1739G>C, 1740A>T, 1741G>C, 1742G>C, 1743C>T, 1744T>C, 1745T>C, 1746C>G, 1747A>C, 1749G>A, 1750A>G, 1753C>G, 1755A>G, 1756T>A, 1757G>T, 1758T>G, 1759G>T, 1760A>C, 1761T>A, 1762C>T, 1763G>C, 1768G>C, 1771A>G, 1774T>C, 1775G>A, 1777A>T, 1778T>G, 1779C>A, 1780A>G, 1782A>G, 1783G>A, 1784A>C, 1786G>A, 1789G>C, 1790C>T, 1791A>C, 1792G>C, 1798T>C, 1801T>A, 1802T>A, 1805A>C, 1806A>T, 1807G>A, 1811C>T, 1816C>T, 1817A>G, 1818C>A, 1823G>A, 1825C>T, 1832G>C, 1834T>A, 1835G>C, 1837T>G, 1838A>G, 1840T>G, 1843T>C, 1844G>A, 1845C>A, 1846T>G, 1848C>A, 1861C>A, 1864G>T, 1867A>G, 1870A>T, 1871A>T, 1872A>C, 1873T>C, 1876C>A, 1877T>G, 1878C>A, 1879T>G, 1883T>A, 1885A>T, 1887T>G, 1888G>C, 1889G>A, 1897T>C, 1898G>T, 1899A>C, 1901A>T, 1903G>A, 1904T>A, 1906G>T, 1909T>C, 1910C>A, 1911A>G, 1912T>G, 1915G>A, 1918A>G, 1921C>G, 1924T>A, 1926C>A, 1927C>G, 1933G>C, 1937C>A, 1938A>T, 1939A>G, 1942A>G, 1945T>C, 1948C>T, 1952G>T, 1954G>T, 1957C>T, 1959T>G, 1960A>G, 1961A>T, 1962T>A, 1963G>T, 1964G>T, 1966T>G, 1972T>A, 1981C>G, 1982C>A, 1984A>G, 1988T>G, 1989C>A, 1990A>C, 1996T>C, 1997T>G, 1998G>T, 1999T>G, 2005C>T, 2011C>T, 2015A>C, 2017T>G, 2019G>C, 2020T>A, 2023G>T, 2218C>G, 2221A>T, 2224G>A, 2225C>G, 2228A>C, 2229T>C, 2230T>G, 2235A>G, 2236T>G, 2237G>A, 2239T>A, 2243A>G, 2244C>A, 2245T>G, 2249G>A, 2250G>A, 2254T>C, 2255G>A, 2258A>T, 2260T>C, 2266C>T, 2269C>T, 2272T>G, 2273T>G, 2274C>A, 2275A>G, 2278C>A, 2282G>A, 2283A>T, 2284C>G, 2285T>G, 2286C>G, 2287A>T, 2288A>G, 2290C>T, 2291C>G, 2293A>C, 2294G>C, 2296C>G, 2297A>T, 2300G>A, 2303T>C, 2305A>G, 2309A>T, 2317A>G, 2322T>C, 2332A>T, 2335C>T, 2341A>G, 2356T>G, 2357G>A, 2359G>T, 2365G>A, 2368C>T, 2369G>C, 2375G>A, 2386A>C, 2392G>T, 2395C>T, 2401A>G, 2404C>T, 2405A>C, 2407G>T, 2408T>G, 2411T>C, 2413G>A, 2414G>T, 2416A>C, 2419A>C									

CDS

	Begin	End	Coverage	Score	Concordance	Matches	Identities	I/D/M/F*	Stop Codons
ToChV_s2_gp1	1	185	100%	827	69.1%	182 (98.4%)	117 (63.2%)	0/3/0/0	1
Protein mutations:	G5S (184G>T 185G>C 186A>T), R6K (188G>A), A10Q (199G>C 200C>A 201A>G), E11L (202G>T 203A>T), K14Q (211A>C), A15E (215C>A 216T>A), Q18N (223C>A 225A>T), N24K (243T>G), I26V (247A>G 249T>G), V31T (262G>A 263T>C), S33V (268T>G 269C>T 270A>T), V35I (274G>A 276A>T), I36V (277A>G), V46I (307G>A 309G>C), I47C (310A>T 311T>G 312T>C), V54I (331G>A), L57I (340C>A 342A>C), T58S (343A>T 345T>C), V59I (346G>A 348T>C), P81D (412C>G 413C>A 414T>C), N84A (421A>G 422A>C 423C>T), L91I (442T>A 444G>A), H93R (449A>G 450T>G), V96I (457G>A 459T>C), R97K (460C>A 461G>A 462T>A), V104L (481G>C), Q107A (490C>G 491A>C 492G>A), A113_Q115del (508_516delGCTCAACAA), T116S (517A>T 519T>A), N118A (523A>G 524A>C 525T>G), S119Q (526T>C 527C>A 528T>A), V120S (529G>T 530T>C 531A>C), D122S (535G>T 536A>C), K123Q (538A>C), K125Q (544A>C), T126K (548C>A 549A>G), T127L (550A>C 551C>T 552C>T), E128S (553G>T 554A>C 555A>T), T129L (556A>C 557C>T), A130V (560C>T 561T>A), W131K (562T>A 563G>A), K132S (566A>G 567G>T), F133L (568T>C 570C>T), K134E (571A>G), I137N (581T>A 582T>C), H139A (586C>G 587A>C 588T>A), N141K (594C>G), A142T (595G>A), E143A (599A>C 600G>T), E145A (605A>C 606G>T), R146V (607C>G 608G>T), R148K (614G>A 615A>G), I151L (622A>T), E153Q (628G>C 630A>G), K154S (632A>G 633A>C), S159I (646T>A 647C>T), Q162K (655C>A), L165I (664C>A), S166Q (667T>C 668C>A 669C>G), N167A (670A>G 671A>C 672T>A), N172E (685A>G 687T>A), I174V (691A>G), T176S (697A>T 699A>G), S179T (706T>A), D181N (712G>A), G182D (716G>A 717G>T)									
Codon mutations:	TCT2TCC (177T>C), TTT3TTC (180T>C), ATT4ATC (183T>C), GGA5TCT (184G>T 185G>C 186A>T), AGG6AAG (188G>A), ACA9ACT (198A>T), GCA10CAG (199G>C 200C>A 201A>G), GAG11TTG (202G>T 203A>T), AAA14CAA (211A>C), GCT15GAA (215C>A 216T>A), TTC16TTT (219C>T), CAA18AAT (223C>A 225A>T), GTC20GTA (231C>A), TCC23TCA (240C>A), AAT24AAG (243T>G), ATT26GTG (247A>G 249T>G), TGC27TGT (252C>T), TCG28AGT (253T>A 254C>G 255G>T), GTG29GTT (258G>T), GAC30GAT (261C>T), GTA31ACA (262G>A 263T>C), GGA32GGT (267A>T), TCA33GTT (268T>G 269C>T 270A>T), GGT34GGA (273T>A), GTA35ATT (274G>A 276A>T), ATT36GTT (277A>G), AAC39AAT (288C>T), CCC40CCG (291C>G), ACT41ACG (294T>G), CTA42CTT (297A>T), TTT44TTC (303T>C), GTG46ATC (307G>A 309G>C), ATT47TGC (310A>T 311T>G 312T>C), CCC48CCG (315C>G), ACA50ACT (321A>T), GTT54ATT (331G>A), TCA55TCT (336A>T), GTG56GTC (339G>C), CTA57ATC (340C>A 342A>C), ACT58TCC (343A>T 345T>C), GTT59ATC (346G>A 348T>C), TCG60TCC (351G>C), GAG62GAA (357G>A), TCA64TCT (363A>T), ACA65ACT (366A>T), CCA66CCG (369A>G), CAG67CAA (372G>A), TTG68CTG (373T>C), GTT69GTA (378T>A), CCG70CCT (381G>T), GGC71GGT (384C>T), TAT73TAC (390T>C), GGT78GGA (405T>A), ACT79ACG (408T>G), CCT81GAC (412C>G 413C>A 414T>C), GTT82GTC (417T>C), AAC84GCT (421A>G 422A>C 423C>T), GTG85GTC (426G>C), AAG86AAA (429G>A), TTA87CTC (430T>C 432A>C), TCT88TCA (435T>A), CTC90TTG (439C>T 441C>G), TTG91ATA (442T>A 444G>A), GTG92GTT (447G>T), CAT93CGG (449A>G 450T>G), CGT94CGA (453T>A), TCT95TCA (456T>A), GTT96ATC (457G>A 459T>C), CGT97AAA (460C>A 461G>A 462T>A), ACC101ACA (474C>A), AGG102CGC (475A>C 477G>C), AAA103AAG (480A>G), GTT104CTT (481G>C), CTA105CTT (486A>T), CAG107GCA (490C>G 491A>C 492G>A), GTT110GTA (501T>A), TCA111TCT (504A>T), GCT113_CAA115del (508_516delGCTCAACAA), ACT116TCA (517A>T 519T>A), AAT118GCG (523A>G 524A>C 525T>G), TCT119CAA (526T>C 527C>A 528T>A), GTA120TCC (529G>T 530T>C 531A>C), GAC122TCC (535G>T 536A>C), AAA123CAA (538A>C), GGG124GGT (543G>T), AAA125CAA (544A>C), ACA126AAG (548C>A 549A>G), ACC127CTT (550A>C 551C>T 552C>T), GAA128TCT (553G>T 554A>C 555A>T), ACA129CTA (556A>C 557C>T), GCT130GTA (560C>T 561T>A), TGG131AAG (562T>A 563G>A), AAG132AGT (566A>G 567G>T), TTC133CTT (568T>C 570C>T), AAA134GAA (571A>G), GAG135GAA (576G>A), ATT137AAC (581T>A 582T>C), GCT138GCA (585T>A), CAT139GCA (586C>G 587A>C 588T>A), CTC140TTA (589C>T 591C>A), AAC141AAG (594C>G), GCT142ACT (595G>A), GAG143GCT (599A>C 600G>T), CTT144TTG (601C>T 603T>G), GAG145GCT (605A>C 606G>T), CGA146GTA (607C>G 608G>T), GCA147GCT (612A>T), AGA148AAG (614G>A 615A>G), GAA150GAG (621A>G), ATA151TTA (622A>T), GCA152GCC (627A>C), GAA153CAG (628G>C 630A>G), AAA154AGC (632A>G 633A>C), TCA156TCT (639A>T), ATA158ATC (645A>C), TCC159ATC (646T>A 647C>T), TTG161CTT (652T>C 654G>T), CAA162AAA (655C>A), CTT163CTG (660T>G), CAG164CAA (663G>A), CTC165ATC (664C>A), TCC166CAG (667T>C 668C>A 669C>G), AAT167GCA (670A>G 671A>C 672T>A), CAG168CAA (675G>A), CCT169CCA (678T>A), AAT172GAA (685A>G 687T>A), ATC174GTC (691A>G), ACA176TCG (697A>T 699A>G), GGT177GGC (702T>C), TCA179ACA (706T>A), GAT181AAT (712G>A), GGG182GAT (716G>A 717G>T), TAA185TGA (725A>G)									
ToChV_s2_gp2	1	582	40.1%	2312	69.6%	479 (99.4%)	321 (66.6%)	2/1/0/0	0

	Begin	End	Coverage	Score	Concordance	Matches	Identities	I/D/M/F*	Stop Codons
**NT**	**41**	**2430**	**40.8%**	**1099**	**27.0%**	**2063 (98.8%)**	**1324 (63.4%)**	**6/20**	

Protein mutations:

M1K (687T>A), I2M (691A>G), Q5R (699A>G), V6A (702T>C), G11I (716G>A 717G>T), N14D (725A>G), R15N (729G>A 730G>T), Q17R (735A>G 736G>C), E19del (740_742delGAG), R23K (752C>A 753G>A 754G>A), T26S (761A>T 763A>T), A34E (786C>A 787T>A), L99M (980C>A 982T>G), R112A (1019C>G 1020G>C 1021T>A), I124T (1056T>C 1057C>T), S125D (1058A>G 1059G>A 1060C>T), S132T (1079T>A 1081C>G), S134F (1086C>T 1087A>C), L135F (1088C>T 1090A>T), A136S (1091G>T 1093T>A), D137G (1095A>G 1096T>G), S147E (1124A>G 1125G>A 1126T>A), L153V (1142C>G 1144C>T), E166L (1181G>C 1182A>T 1183G>T), Q171L (1197A>T 1198G>A), D185S (1238G>A 1239A>G 1240T>C), T186P (1241A>C), S192T (1259T>A 1261T>G), Q193A (1262C>G 1263A>C), Q211S (1316C>T 1317A>C 1318A>T), Q228E (1367C>G 1369A>G), S229R (1372T>G), S230A (1373T>G 1375C>G), Q231S (1376C>T 1377A>C 1378G>T), M232Q (1379A>C 1380T>A 1381G>A), A238P (1397G>C), E242A (1410A>C 1411G>A), R243L (1412A>C 1413G>T 1414G>T), R248Y (1427C>T 1428G>A 1429A>C), D250N (1433G>A), L252P (1440T>C 1441G>C), S253Q (1442A>C 1443G>A 1444T>G), M257L (1454A>T 1456G>A), T258M (1458C>T 1459T>G), S260A (1463A>G 1464G>C 1465C>G), V270I (1493G>A 1495G>C), R273K (1503G>A), V283F (1532G>T 1534G>T), P284S (1535C>T 1537T>A), N285R (1539A>G 1540C>G), T286A (1541A>G), R288S (1547C>T 1548G>C), L289S (1551T>C 1552A>G), T290_M291insVG (1555_1556insGTAGGT), M291V (1556A>G), A296G (1572C>G 1573T>G), H297V (1574C>G 1575A>T 1576C>A), E299D (1582A>T), T301S (1586A>T 1588T>C), G302N (1589G>A 1590G>A), G303L (1592G>T 1593G>T), K309E (1610A>G), G312A (1620G>C), F313I (1622T>A 1624C>T), N314D (1625A>G 1627C>T), M315F (1628A>T 1630G>T), V317Q (1634G>C 1635T>A 1636T>G), L322Y (1649C>T 1650T>A 1651G>T), D325T (1658G>A 1659A>C 1660C>T), S326D (1661T>G 1662C>A 1663C>T), T328N (1668C>A 1669A>T), H329S (1670C>T 1671A>C 1672C>G), I330F (1673A>T), D335E (1690T>G), E338T (1697G>A 1698A>C 1699G>C), R339W (1700C>T 1702C>G), T340H (1703A>C 1704C>A 1705G>T), A342S (1709G>T 1711G>A), Q343R (1712C>A 1713A>G 1714A>G), V345I (1718G>A), Q346D (1721C>G 1723A>T), Y347F (1725A>T 1726C>T), I348V (1727A>G 1729T>G), F350S (1734T>C), D351T (1736G>A 1737A>C 1738T>C), E352L (1739G>C 1740A>T 1741G>C), A353L (1742G>C 1743C>T 1744T>C), S354R (1745T>C 1746C>G 1747A>C), R355Q (1749G>A 1750A>G), N357R (1755A>G 1756T>A), V358C (1757G>T 1758T>G 1759G>T), I359H (1760A>C 1761T>A 1762C>T), V360L (1763G>C), V364I (1775G>A 1777A>T), S365E (1778T>G 1779C>A 1780A>G), K366R (1782A>G 1783G>A), E368D (1789G>C), Q369S (1790C>T 1791A>C 1792G>C), Y373N (1802T>A), K374L (1805A>C 1806A>T 1807G>A), H376Y (1811C>T), T378D (1817A>G 1818C>A), D380N (1823G>A 1825C>T), V383L (1832G>C 1834T>A), D384Q (1835G>C 1837T>G), I385V (1838A>G 1840T>G), A387K (1844G>A 1845C>A 1846T>G), A388E (1848C>A), K393N (1864G>T), N396S (1871A>T 1872A>C 1873T>C), S398E (1877T>G 1878C>A 1879T>G), S400T (1883T>A 1885A>T), L401C (1887T>G 1888G>C), D402N (1889G>A), D405S (1898G>T 1899A>C), M406L (1901A>T 1903G>A), S407T (1904T>A 1906G>T), H409R (1910C>A 1911A>G 1912T>G), A414E (1926C>A 1927C>G), Q418M (1937C>A 1938A>T 1939A>G), V423F (1952G>T 1954G>T), I425R (1959T>G 1960A>G), M426Y (1961A>T 1962T>A 1963G>T), G427W (1964G>T 1966T>G), F429L (1972T>A), L433M (1982C>A 1984A>G), S435D (1988T>G 1989C>A 1990A>C), C438V (1997T>G 1998G>T 1999T>G), I444L (2015A>C 2017T>G), G445A (2019G>C 2020T>A), E446D (2023G>T), H514D (2225C>G), I515P (2228A>C 2229T>C 2230T>G), D517G (2235A>G 2236T>G), D518K (2237G>A 2239T>A), T520E (2243A>G 2244C>A 2245T>G), G522N (2249G>A 2250G>A), V524I (2255G>A), I525F (2258A>T 2260T>C), S530E (2273T>G 2274C>A 2275A>G), D531E (2278C>A), D533M (2282G>A 2283A>T 2284C>G), S534G (2285T>G 2286C>G 2287A>T), N535D (2288A>G 2290C>T), P536A (2291C>G 2293A>C), A537P (2294G>C 2296C>G), I538L (2297A>T), D539N (2300G>A), M542L (2309A>T), V546A (2322T>C), V558I (2357G>A 2359G>T), V562L (2369G>C), E564K (2375G>A), K567N (2386A>C), M574L (2405A>C 2407G>T), S575A (2408T>G), A577S (2414G>T 2416A>C)

Codon mutations:

ATG1AAG (687T>A), ATA2ATG (691A>G), TCA4TCT (697A>T), CAG5CGG (699A>G), GTT6GCT (702T>C), GGT7GGA (706T>A), AAG9AAA (712G>A), GGC11ATC (716G>A 717G>T), AAC14GAC (725A>G), AGG15AAT (729G>A 730G>T), GCG16GCT (733G>T), CAG17CGC (735A>G 736G>C), GAG19del (740_742delGAG), GCT20GCC (745T>C), CGG23AAA (752C>A 753G>A 754G>A), CTA24TTA (755C>T), ATC25ATT (760C>T), ACA26TCT (761A>T 763A>T), ATT28ATA (769T>A), GCG30GCT (775G>T), GCT31GCA (778T>A), GTT32GTG (781T>G), GCT34GAA (786C>A 787T>A), GGA35GGG (790A>G), CTG39CTC (802G>C), CTT91CTA (958T>A), CAT94CAC (967T>C), TTG95TTA (970G>A), GGT97GGC (976T>C), GTT98GTC (979T>C), CTT99ATG (980C>A 982T>G), TTC100TTT (985C>T), GTC103GTG (994C>G), CCT104CCA (997T>A), GGG109GGA (1012G>A), CGT112GCA (1019C>G 1020G>C 1021T>A), AGT116AGC (1033T>C), TTG117CTG (1034T>C), TGT118TGC (1039T>C), TCG119TCC (1042G>C), CCC123CCA (1054C>A), ATC124ACT (1056T>C 1057C>T), AGC125GAT (1058A>G 1059G>A 1060C>T), CCG126CCT (1063G>T), GTG127GTT (1066G>T), ATT128ATA (1069T>A), CAA129CAG (1072A>G), GAG130GAA (1075G>A), TCC132ACG (1079T>A 1081C>G), TTG133TTA (1084G>A), TCA134TTC (1086C>T 1087A>C), CTA135TTT (1088C>T 1090A>T), GCT136TCA (1091G>T 1093T>A), GAT137GGG (1095A>G 1096T>G), GGT138GGA (1099T>A), GCA139GCT (1102A>T), CAA140CAG (1105A>G), GCC141GCT (1108C>T), GTG142GTC (1111G>C), CTC143TTG (1112C>T 1114C>G), AGT147GAA (1124A>G 1125G>A 1126T>A), ATC148ATT (1129C>T), CCC151CCT (1138C>T), CTC153GTT (1142C>G 1144C>T), GGA156GGG (1153A>G), CCA157CCC (1156A>C), TTC159TTT (1162C>T), GAG166CTT (1181G>C 1182A>T 1183G>T), AAT167AAC (1186T>C), CGT169CGG (1192T>G), GCA170GCT (1195A>T), CAG171CTA (1197A>T 1198G>A), ATA172ATT (1201A>T), CCT173CCA (1204T>A), TCC175TCT (1210C>T), GTG176GTT (1213G>T), GTA177GTG (1216A>G), GCG178GCA (1219G>A), ATA179ATT (1222A>T), AAA181AAG (1228A>G), CAA182CAG (1231A>G), GAT185AGC (1238G>A 1239A>G 1240T>C), ACA186CCA (1241A>C), AGG187AGA (1246G>A), GCC189GCT (1252C>T), GTA190GTT (1255A>T), TAC191TAT (1258C>T), TCT192ACG (1259T>A 1261T>G), CAA193GCA (1262C>G 1263A>C), CAG194CAA (1267G>A), ACT196ACA (1273T>A), TCT198AGT (1277T>A 1278C>G), GCC200GCA (1285C>A), GAA202GAG (1291A>G), GCA203GCT (1294A>T), TTG204CTC (1295T>C 1297G>C), AAC205AAT (1300C>T), CCT207CCA (1306T>A), CAA208CAG (1309A>G), TTT209TTC (1312T>C), TTC210TTT (1315C>T), CAA211TCT (1316C>T 1317A>C 1318A>T), CAG214CAA (1327G>A), GCT216GCA (1333T>A), GCC217GCA (1336C>A), TCA222TCT (1351A>T), GTA223GTC (1354A>C), TCT225TCG (1360T>G), GGG227GGA (1366G>A), CAA228GAG (1367C>G 1369A>G), AGT229AGG (1372T>G), TCC230GCG (1373T>G 1375C>G), CAG231TCT (1376C>T 1377A>C 1378G>T), ATG232CAA (1379A>C 1380T>A 1381G>A), TTG234CTA (1385T>C 1387G>A), CGT237AGA (1394C>A 1396T>A), GCT238CCT (1397G>C), GTT240GTA (1405T>A), GGA241GGT (1408A>T), GAG242GCA (1410A>C 1411G>A), AGG243CTT (1412A>C 1413G>T 1414G>T), AAT246AAC (1423T>C), ACA247ACT (1426A>T), CGA248TAC (1427C>T 1428G>A 1429A>C), GAT250AAT (1433G>A), GTA251GTT (1438A>T), CTG252CCC (1440T>C 1441G>C), AGT253CAG (1442A>C 1443G>A 1444T>G), AGC255TCT (1448A>T 1449G>C 1450C>T), TCC256TCA (1453C>A), ATG257TTA (1454A>T 1456G>A), ACT258ATG (1458C>T 1459T>G), AGC260GCG (1463A>G 1464G>C 1465C>G), TCT261TCA (1468T>A), TCC262TCG (1471C>G), CTG263CTT (1474G>T), CGA264CGC (1477A>C), CCG266CCA (1483G>A), CTC268CTA (1489C>A), GTG270ATC (1493G>A 1495G>C), AGT272AGC (1501T>C), AGG273AAG (1503G>A), CGG274AGG (1505C>A), CCA276CCT (1513A>T), TCC277TCG (1516C>G), GAA279GAG (1522A>G), CTA280CTG (1525A>G), GAC282GAT (1531C>T), GTG283TTT (1532G>T 1534G>T), CCT284TCA (1535C>T 1537T>A), AAC285AGG (1539A>G 1540C>G), ACC286GCC (1541A>G), CGA288TCA (1547C>T 1548G>C), TTA289TCG (1551T>C 1552A>G), ACA290_ATG291insGTAGGT (1555_1556insGTAGGT), ATG291GTG (1556A>G), CGA292AGA (1559C>A), GCT296GGG (1572C>G 1573T>G), CAC297GTA (1574C>G 1575A>T 1576C>A), GAA299GAT (1582A>T), GAC300GAT (1585C>T), ACT301TCC (1586A>T 1588T>C), GGT302AAT (1589G>A 1590G>A), GGA303TTA (1592G>T 1593G>T), TTG304CTG (1595T>C), CCA306CCT (1603A>T), GTT307GTC (1606T>C), AAG309GAG (1610A>G), CAA311CAG (1618A>G), GGC312GCC (1620G>C), TTC313ATT (1622T>A 1624C>T), AAC314GAT (1625A>G 1627C>T), ATG315TTT (1628A>T 1630G>T), GGT316GGC (1633T>C), GTT317CAG (1634G>C 1635T>A 1636T>G), GAT320GAC (1645T>C), CTG322TAT (1649C>T 1650T>A 1651G>T), ATA324ATC (1657A>C), GAC325ACT (1658G>A 1659A>C 1660C>T), TCC326GAT (1661T>G 1662C>A 1663C>T), ACA328AAT (1668C>A 1669A>T), CAC329TCG (1670C>T 1671A>C 1672C>G), ATT330TTT (1673A>T), GAC331GAT (1678C>T), GAT335GAG (1690T>G), ACT337ACA (1696T>A), GAG338ACC (1697G>A 1698A>C 1699G>C), CGC339TGG (1700C>T 1702C>G), ACG340CAT (1703A>C 1704C>A 1705G>T), ATT341ATA (1708T>A), GCG342TCA (1709G>T 1711G>A), CAA343AGG (1712C>A 1713A>G 1714A>G), CAG344CAA (1717G>A), GTT345ATT (1718G>A), CAA346GAT (1721C>G 1723A>T), TAC347TTT (1725A>T 1726C>T), ATT348GTG (1727A>G 1729T>G), CTG349TTG (1730C>T), TTT350TCT (1734T>C), GAT351ACC (1736G>A 1737A>C 1738T>C), GAG352CTC (1739G>C 1740A>T 1741G>C), GCT353CTC (1742G>C 1743C>T 1744T>C), TCA354CGC (1745T>C 1746C>G 1747A>C), CGA355CAG (1749G>A 1750A>G), GGC356GGG (1753C>G), AAT357AGA (1755A>G 1756T>A), GTG358TGT (1757G>T 1758T>G 1759G>T), ATC359CAT (1760A>C 1761T>A 1762C>T), GTG360CTG (1763G>C), CCG361CCC (1768G>C), AAA362AAG (1771A>G), CAT363CAC (1774T>C), GTA364ATT (1775G>A 1777A>T), TCA365GAG (1778T>G 1779C>A 1780A>G), AAG366AGA (1782A>G 1783G>A), AGG367CGA (1784A>C 1786G>A), GAG368GAC (1789G>C), CAG369TCC (1790C>T 1791A>C 1792G>C), AAT371AAC (1798T>C), ATT372ATA (1801T>A), TAC373AAC (1802T>A), AAG374CTA (1805A>C 1806A>T 1807G>A), CAC376TAC (1811C>T), ATC377ATT (1816C>T), ACT378GAT (1817A>G 1818C>A), GAC380AAT (1823G>A 1825C>T), GTT383CTA (1832G>C 1834T>A), GAT384CAG (1835G>C 1837T>G), ATT385GTG (1838A>G 1840T>G), CTT386CTC (1843T>C), GCT387AAG (1844G>A 1845C>A 1846T>G), GCA388GAA (1848C>A), ACC392ACA (1861C>A), AAG393AAT (1864G>T), GTA394GTG (1867A>G), GGA395GGT (1870A>T), AAT396TCC (1871A>T 1872A>C 1873T>C), CTC397CTA (1876C>A), TCT398GAG (1877T>G 1878C>A 1879T>G), TCA400ACT (1883T>A 1885A>T), TTG401TGC (1887T>G 1888G>C), GAT402AAT (1889G>A), TAT404TAC (1897T>C), GAT405TCT (1898G>T 1899A>C), ATG406TTA (1901A>T 1903G>A), TCG407ACT (1904T>A 1906G>T), CTT408CTC (1909T>C), CAT409AGG (1910C>A 1911A>G 1912T>G), GAG410GAA (1915G>A), AGA411AGG (1918A>G), GTC412GTG (1921C>G), ATT413ATA (1924T>A), GCC414GAG (1926C>A 1927C>G), GTG416GTC (1933G>C), CAA418ATG (1937C>A 1938A>T 1939A>G), AGA419AGG (1942A>G), GAT420GAC (1945T>C), CAC421CAT (1948C>T), GTG423TTT (1952G>T 1954G>T), TAC424TAT (1957C>T), ATA425AGG (1959T>G 1960A>G), ATG426TAT (1961A>T 1962T>A 1963G>T), GGT427TGG (1964G>T 1966T>G), TTT429TTA (1972T>A), CCC432CCG (1981C>G), CTA433ATG (1982C>A 1984A>G), TCA435GAC (1988T>G 1989C>A 1990A>C), GAT437GAC (1996T>C), TGT438GTG (1997T>G 1998G>T 1999T>G), GAC440GAT (2005C>T), CTC442CTT (2011C>T), ATT444CTG (2015A>C 2017T>G), GGT445GCA (2019G>C 2020T>A), GAG446GAT (2023G>T), ACC511ACG (2218C>G), GCA512GCT (2221A>T), GAG513GAA (2224G>A), CAT514GAT (2225C>G), ATT515CCG (2228A>C 2229T>C 2230T>G), GAT517GGG (2235A>G 2236T>G), GAT518AAA (2237G>A 2239T>A), ACT520GAG (2243A>G 2244C>A 2245T>G), GGT522AAT (2249G>A 2250G>A), GAT523GAC (2254T>C), GTT524ATT (2255G>A), ATT525TTC (2258A>T 2260T>C), GAC527GAT (2266C>T), TTC528TTT (2269C>T), GGT529GGG (2272T>G), TCA530GAG (2273T>G 2274C>A 2275A>G), GAC531GAA (2278C>A), GAC533ATG (2282G>A 2283A>T 2284C>G), TCA534GGT (2285T>G 2286C>G 2287A>T), AAC535GAT (2288A>G 2290C>T), CCA536GCC (2291C>G 2293A>C), GCC537CCG (2294G>C 2296C>G), ATA538TTA (2297A>T), GAT539AAT (2300G>A), TTA540CTG (2303T>C 2305A>G), ATG542TTG (2309A>T), CAA544CAG (2317A>G), GTA546GCA (2322T>C), GCA549GCT (2332A>T), AGC550AGT (2335C>T), GAA552GAG (2341A>G), GGT557GGG (2356T>G), GTG558ATT (2357G>A 2359G>T), GAG560GAA (2365G>A), TTC561TTT (2368C>T), GTG562CTG (2369G>C), GAA564AAA (2375G>A), AAA567AAC (2386A>C), GTG569GTT (2392G>T), GCC570GCT (2395C>T), CAA572CAG (2401A>G), GTC573GTT (2404C>T), ATG574CTT (2405A>C 2407G>T), TCA575GCA (2408T>G), TTG576CTA (2411T>C 2413G>A), GCA577TCC (2414G>T 2416A>C), CTA578CTC (2419A>C)

Proteins

	Begin	End	Coverage	Score	Concordance	Matches	Identities	I/D/M/F*	Stop Codons
hypothetical protein (YP_003097230.1)	1	185	100%	827	69.1%	182 (98.4%)	117 (63.2%)	0/3/0/0	1

Protein mutations:

G5S (184G>T 185G>C 186A>T), R6K (188G>A), A10Q (199G>C 200C>A 201A>G), E11L (202G>T 203A>T), K14Q (211A>C), A15E (215C>A 216T>A), Q18N (223C>A 225A>T), N24K (243T>G), I26V (247A>G 249T>G), V31T (262G>A 263T>C), S33V (268T>G 269C>T 270A>T), V35I (274G>A 276A>T), I36V (277A>G), V46I (307G>A 309G>C), I47C (310A>T 311T>G 312T>C), V54I (331G>A), L57I (340C>A 342A>C), T58S (343A>T 345T>C), V59I (346G>A 348T>C), P81D (412C>G 413C>A 414T>C), N84A (421A>G 422A>C 423C>T), L91I (442T>A 444G>A), H93R (449A>G 450T>G), V96I (457G>A 459T>C), R97K (460C>A 461G>A 462T>A), V104L (481G>C), Q107A (490C>G 491A>C 492G>A), A113_Q115del (508_516delGCTCAACAA), T116S (517A>T 519T>A), N118A (523A>G 524A>C 525T>G), S119Q (526T>C 527C>A 528T>A), V120S (529G>T 530T>C 531A>C), D122S (535G>T 536A>C), K123Q (538A>C), K125Q (544A>C), T126K (548C>A 549A>G), T127L (550A>C 551C>T 552C>T), E128S (553G>T 554A>C 555A>T), T129L (556A>C 557C>T), A130V (560C>T 561T>A), W131K (562T>A 563G>A), K132S (566A>G 567G>T), F133L (568T>C 570C>T), K134E (571A>G), I137N (581T>A 582T>C), H139A (586C>G 587A>C 588T>A), N141K (594C>G), A142T (595G>A), E143A (599A>C 600G>T), E145A (605A>C 606G>T), R146V (607C>G 608G>T), R148K (614G>A 615A>G), I151L (622A>T), E153Q (628G>C 630A>G), K154S (632A>G 633A>C), S159I (646T>A 647C>T), Q162K (655C>A), L165I (664C>A), S166Q (667T>C 668C>A 669C>G), N167A (670A>G 671A>C 672T>A), N172E (685A>G 687T>A), I174V (691A>G), T176S (697A>T 699A>G), S179T (706T>A), D181N (712G>A), G182D (716G>A 717G>T)

	Begin	End	Coverage	Score	Concordance	Matches	Identities	I/D/M/F*	Stop Codons
**NT**	**41**	**2430**	**40.8%**	**1099**	**27.0%**	**2063 (98.8%)**	**1324 (63.4%)**	**6/20**	
Codon mutations:	TCT2TCC (177T>C), TTT3TTC (180T>C), ATT4ATC (183T>C), GGA5TCT (184G>T 185G>C 186A>T), AGG6AAG (188G>A), ACA9ACT (198A>T), GCA10CAG (199G>C 200C>A 201A>G), GAG11TTG (202G>T 203A>T), AAA14CAA (211A>C), GCT15GAA (215C>A 216T>A), TTC16TTT (219C>T), CAA18AAT (223C>A 225A>T), GTC20GTA (231C>A), TCC23TCA (240C>A), AAT24AAG (243T>G), ATT26GTG (247A>G 249T>G), TGC27TGT (252C>T), TCG28AGT (253T>A 254C>G 255G>T), GTG29GTT (258G>T), GAC30GAT (261C>T), GTA31ACA (262G>A 263T>C), GGA32GGT (267A>T), TCA33GTT (268T>G 269C>T 270A>T), GGT34GGA (273T>A), GTA35ATT (274G>A 276A>T), ATT36GTT (277A>G), AAC39AAT (288C>T), CCC40CCG (291C>G), ACT41ACG (294T>G), CTA42CTT (297A>T), TTT44TTC (303T>C), GTG46ATC (307G>A 309G>C), ATT47TGC (310A>T 311T>G 312T>C), CCC48CCG (315C>G), ACA50ACT (321A>T), GTT54ATT (331G>A), TCA55TCT (336A>T), GTG56GTC (339G>C), CTA57ATC (340C>A 342A>C), ACT58TCC (343A>T 345T>C), GTT59ATC (346G>A 348T>C), TCG60TCC (351G>C), GAG62GAA (357G>A), TCA64TCT (363A>T), ACA65ACT (366A>T), CCA66CCG (369A>G), CAG67CAA (372G>A), TTG68CTG (373T>C), GTT69GTA (378T>A), CCG70CCT (381G>T), GGC71GGT (384C>T), TAT73TAC (390T>C), GGT78GGA (405T>A), ACT79ACG (408T>G), CCT81GAC (412C>G 413C>A 414T>C), GTT82GTC (417T>C), AAC84GCT (421A>G 422A>C 423C>T), GTG85GTC (426G>C), AAG86AAA (429G>A), TTA87CTC (430T>C 432A>C), TCT88TCA (435T>A), CTC90TTG (439C>T 441C>G), TTG91ATA (442T>A 444G>A), GTG92GTT (447G>T), CAT93CGG (449A>G 450T>G), CGT94CGA (453T>A), TCT95TCA (456T>A), GTT96ATC (457G>A 459T>C), CGT97AAA (460C>A 461G>A 462T>A), ACC101ACA (474C>A), AGG102CGC (475A>C 477G>C), AAA103AAG (480A>G), GTT104CTT (481G>C), CTA105CTT (486A>T), CAG107GCA (490C>G 491A>C 492G>A), GTT110GTA (501T>A), TCA111TCT (504A>T), GCT113_CAA115del (508_516delGCTCAACAA), ACT116TCA (517A>T 519T>A), AAT118GCG (523A>G 524A>C 525T>G), TCT119CAA (526T>C 527C>A 528T>A), GTA120TCC (529G>T 530T>C 531A>C), GAC122TCC (535G>T 536A>C), AAA123CAA (538A>C), GGG124GGT (543G>T), AAA125CAA (544A>C), ACA126AAG (548C>A 549A>G), ACC127CTT (550A>C 551C>T 552C>T), GAA128TCT (553G>T 554A>C 555A>T), ACA129CTA (556A>C 557C>T), GCT130GTA (560C>T 561T>A), TGG131AAG (562T>A 563G>A), AAG132AGT (566A>G 567G>T), TTC133CTT (568T>C 570C>T), AAA134GAA (571A>G), GAG135GAA (576G>A), ATT137AAC (581T>A 582T>C), GCT138GCA (585T>A), CAT139GCA (586C>G 587A>C 588T>A), CTC140TTA (589C>T 591C>A), AAC141AAG (594C>G), GCT142ACT (595G>A), GAG143GCT (599A>C 600G>T), CTT144TTG (601C>T 603T>G), GAG145GCT (605A>C 606G>T), CGA146GTA (607C>G 608G>T), GCA147GCT (612A>T), AGA148AAG (614G>A 615A>G), GAA150GAG (621A>G), ATA151TTA (622A>T), GCA152GCC (627A>C), GAA153CAG (628G>C 630A>G), AAA154AGC (632A>G 633A>C), TCA156TCT (639A>T), ATA158ATC (645A>C), TCC159ATC (646T>A 647C>T), TTG161CTT (652T>C 654G>T), CAA162AAA (655C>A), CTT163CTG (660T>G), CAG164CAA (663G>A), CTC165ATC (664C>A), TCC166CAG (667T>C 668C>A 669C>G), AAT167GCA (670A>G 671A>C 672T>A), CAG168CAA (675G>A), CCT169CCA (678T>A), AAT172GAA (685A>G 687T>A), ATC174GTC (691A>G), ACA176TCG (697A>T 699A>G), GGT177GGC (702T>C), TCA179ACA (706T>A), GAT181AAT (712G>A), GGG182GAT (716G>A 717G>T), TAA185TGA (725A>G)								
polyprotein (YP_003097231.1)	1	582	40.1%	2312	69.6%	479 (99.4%)	321 (66.6%)	2/1/0/0	0
Protein mutations:	M1K (687T>A), I2M (691A>G), Q5R (699A>G), V6A (702T>C), G11I (716G>A 717G>T), N14D (725A>G), R15N (729G>A 730G>T), Q17R (735A>G 736G>C), E19del (740_742delGAG), R23K (752C>A 753G>A 754G>A), T26S (761A>T 763A>T), A34E (786C>A 787T>A), L99M (980C>A 982T>G), R112A (1019C>G 1020G>C 1021T>A), I124T (1056T>C 1057C>T), S125D (1058A>G 1059G>A 1060C>T), S132T (1079T>A 1081C>G), S134F (1086C>T 1087A>C), L135F (1088C>T 1090A>T), A136S (1091G>T 1093T>A), D137G (1095A>G 1096T>G), S147E (1124A>G 1125G>A 1126T>A), L153V (1142C>G 1144C>T), E166L (1181G>C 1182A>T 1183G>T), Q171L (1197A>T 1198G>A), D185S (1238G>A 1239A>G 1240T>C), T186P (1241A>C), S192T (1259T>A 1261T>G), Q193A (1262C>G 1263A>C), Q211S (1316C>T 1317A>C 1318A>T), Q228E (1367C>G 1369A>G), S229R (1372T>G), S230A (1373T>G 1375C>G), Q231S (1376C>T 1377A>C 1378G>T), M232Q (1379A>C 1380T>A 1381G>A), A238P (1397G>C), E242A (1410A>C 1411G>A), R243L (1412A>C 1413G>T 1414G>T), R248Y (1427C>T 1428G>A 1429A>C), D250N (1433G>A), L252P (1440T>C 1441G>C), S253Q (1442A>C 1443G>A 1444T>G), M257L (1454A>T 1456G>A), T258M (1458C>T 1459T>G), S260A (1463A>G 1464G>C 1465C>G), V270I (1493G>A 1495G>C), R273K (1503G>A), V283F (1532G>T 1534G>T), P284S (1535C>T 1537T>A), N285R (1539A>G 1540C>G), T286A (1541A>G), R288S (1547C>T 1548G>C), L289S (1551T>C 1552A>G), T290_M291insVG (1555_1556insGTAGGT), M291V (1556A>G), A296G (1572C>G 1573T>G), H297V (1574C>G 1575A>T 1576C>A), E299D (1582A>T), T301S (1586A>T 1588T>C), G302N (1589G>A 1590G>A), G303L (1592G>T 1593G>T), K309E (1610A>G), G312A (1620G>C), F313I (1622T>A 1624C>T), N314D (1625A>G 1627C>T), M315F (1628A>T 1630G>T), V317Q (1634G>C 1635T>A 1636T>G), L322Y (1649C>T 1650T>A 1651G>T), D325T (1658G>A 1659A>C 1660C>T), S326D (1661T>G 1662C>A 1663C>T), T328N (1668C>A 1669A>T), H329S (1670C>T 1671A>C 1672C>G), I330F (1673A>T), D335E (1690T>G), E338T (1697G>A 1698A>C 1699G>C), R339W (1700C>T 1702C>G), T340H (1703A>C 1704C>A 1705G>T), A342S (1709G>T 1711G>A), Q343R (1712C>A 1713A>G 1714A>G), V345I (1718G>A), Q346D (1721C>G 1723A>T), Y347F (1725A>T 1726C>T), I348V (1727A>G 1729T>G), F350S (1734T>C), D351T (1736G>A 1737A>C 1738T>C), E352L (1739G>C 1740A>T 1741G>C), A353L (1742G>C 1743C>T 1744T>C), S354R (1745T>C 1746C>G 1747A>C), R355Q (1749G>A 1750A>G), N357R (1755A>G 1756T>A), V358C (1757G>T 1758T>G 1759G>T), I359H (1760A>C 1761T>A 1762C>T), V360L (1763G>C), V364I (1775G>A 1777A>T), S365E (1778T>G 1779C>A 1780A>G), K366R (1782A>G 1783G>A), E368D (1789G>C), Q369S (1790C>T 1791A>C 1792G>C), Y373N (1802T>A), K374L (1805A>C 1806A>T 1807G>A), H376Y (1811C>T), T378D (1817A>G 1818C>A), D380N (1823G>A 1825C>T), V383L (1832G>C 1834T>A), D384Q (1835G>C 1837T>G), I385V (1838A>G 1840T>G), A387K (1844G>A 1845C>A 1846T>G), A388E (1848C>A), K393N (1864G>T), N396S (1871A>T 1872A>C 1873T>C), S398E (1877T>G 1878C>A 1879T>G), S400T (1883T>A 1885A>T), L401C (1887T>G 1888G>C), D402N (1889G>A), D405S (1898G>T 1899A>C), M406L (1901A>T 1903G>A), S407T (1904T>A 1906G>T), H409R (1910C>A 1911A>G 1912T>G), A414E (1926C>A 1927C>G), Q418M (1937C>A 1938A>T 1939A>G), V423F (1952G>T 1954G>T), I425R (1959T>G 1960A>G), M426Y (1961A>T 1962T>A 1963G>T), G427W (1964G>T 1966T>G), F429L (1972T>A), L433M (1982C>A 1984A>G), S435D (1988T>G 1989C>A 1990A>C), C438V (1997T>G 1998G>T 1999T>G), I444L (2015A>C 2017T>G), G445A (2019G>C 2020T>A), E446D (2023G>T), H514D (2225C>G), I515P (2228A>C 2229T>C 2230T>G), D517G (2235A>G 2236T>G), D518K (2237G>A 2239T>A), T520E (2243A>G 2244C>A 2245T>G), G522N (2249G>A 2250G>A), V524I (2255G>A), I525F (2258A>T 2260T>C), S530E (2273T>G 2274C>A 2275A>G), D531E (2278C>A), D533M (2282G>A 2283A>T 2284C>G), S534G (2285T>G 2286C>G 2287A>T), N535D (2288A>G 2290C>T), P536A (2291C>G 2293A>C), A537P (2294G>C 2296C>G), I538L (2297A>T), D539N (2300G>A), M542L (2309A>T), V546A (2322T>C), V558I (2357G>A 2359G>T), V562L (2369G>C), E564K (2375G>A), K567N (2386A>C), M574L (2405A>C 2407G>T), S575A (2408T>G), A577S (2414G>T 2416A>C)								
Codon mutations:	ATG1AAG (687T>A), ATA2ATG (691A>G), TCA4TCT (697A>T), CAG5CGG (699A>G), GTT6GCT (702T>C), GGT7GGA (706T>A), AAG9AAA (712G>A), GGC11ATC (716G>A 717G>T), AAC14GAC (725A>G), AGG15AAT (729G>A 730G>T), GCG16GCT (733G>T), CAG17CGC (735A>G 736G>C), GAG19del (740_742delGAG), GCT20GCC (745T>C), CGG23AAA (752C>A 753G>A 754G>A), CTA24TTA (755C>T), ATC25ATT (760C>T), ACA26TCT (761A>T 763A>T), ATT28ATA (769T>A), GCG30GCT (775G>T), GCT31GCA (778T>A), GTT32GTG (781T>G), GCT34GAA (786C>A 787T>A), GGA35GGG (790A>G), CTG39CTC (802G>C), CTT91CTA (958T>A), CAT94CAC (967T>C), TTG95TTA (970G>A), GGT97GGC (976T>C), GTT98GTC (979T>C), CTT99ATG (980C>A 982T>G), TTC100TTT (985C>T), GTC103GTG (994C>G), CCT104CCA (997T>A), GGG109GGA (1012G>A), CGT112GCA (1019C>G 1020G>C 1021T>A), AGT116AGC (1033T>C), TTG117CTG (1034T>C), TGT118TGC (1039T>C), TCG119TCC (1042G>C), CCC123CCA (1054C>A), ATC124ACT (1056T>C 1057C>T), AGC125GAT (1058A>G 1059G>A 1060C>T), CCG126CCT (1063G>T), GTG127GTT (1066G>T), ATT128ATA (1069T>A), CAA129CAG (1072A>G), GAG130GAA (1075G>A), TCC132ACG (1079T>A 1081C>G), TTG133TTA (1084G>A), TCA134TTC (1086C>T 1087A>C), CTA135TTT (1088C>T 1090A>T), GCT136TCA (1091G>T 1093T>A), GAT137GGG (1095A>G 1096T>G), GGT138GGA (1099T>A), GCA139GCT (1102A>T), CAA140CAG (1105A>G), GCC141GCT (1108C>T), GTG142GTC (1111G>C), CTC143TTG (1112C>T 1114C>G), AGT147GAA (1124A>G 1125G>A 1126T>A), ATC148ATT (1129C>T), CCC151CCT (1138C>T), CTC153GTT (1142C>G 1144C>T), GGA156GGG (1153A>G), CCA157CCC (1156A>C), TTC159TTT (1162C>T), GAG166CTT (1181G>C 1182A>T 1183G>T), AAT167AAC (1186T>C), CGT169CGG (1192T>G), GCA170GCT (1195A>T), CAG171CTA (1197A>T 1198G>A), ATA172ATT (1201A>T), CCT173CCA (1204T>A), TCC175TCT (1210C>T), GTG176GTT (1213G>T), GTA177GTG (1216A>G), GCG178GCA (1219G>A), ATA179ATT (1222A>T), AAA181AAG (1228A>G), CAA182CAG (1231A>G), GAT185AGC (1238G>A 1239A>G 1240T>C), ACA186CCA (1241A>C), AGG187AGA (1246G>A), GCC189GCT (1252C>T), GTA190GTT (1255A>T), TAC191TAT (1258C>T), TCT192ACG (1259T>A 1261T>G), CAA193GCA (1262C>G 1263A>C), CAG194CAA (1267G>A), ACT196ACA (1273T>A), TCT198AGT (1277T>A 1278C>G), GCC200GCA (1285C>A), GAA202GAG (1291A>G), GCA203GCT (1294A>T), TTG204CTC (1295T>C 1297G>C), AAC205AAT (1300C>T), CCT207CCA (1306T>A), CAA208CAG (1309A>G), TTT209TTC (1312T>C), TTC210TTT (1315C>T), CAA211TCT (1316C>T 1317A>C 1318A>T), CAG214CAA (1327G>A), GCT216GCA (1333T>A), GCC217GCA (1336C>A), TCA222TCT (1351A>T), GTA223GTC (1354A>C), TCT225TCG (1360T>G), GGG227GGA (1366G>A), CAA228GAG (1367C>G 1369A>G), AGT229AGG (1372T>G), TCC230GCG (1373T>G 1375C>G), CAG231TCT (1376C>T 1377A>C 1378G>T), ATG232CAA (1379A>C 1380T>A 1381G>A), TTG234CTA (1385T>C 1387G>A), CGT237AGA (1394C>A 1396T>A), GCT238CCT (1397G>C), GTT240GTA (1405T>A), GGA241GGT (1408A>T), GAG242GCA (1410A>C 1411G>A), AGG243CTT (1412A>C 1413G>T 1414G>T), AAT246AAC (1423T>C), ACA247ACT (1426A>T), CGA248TAC (1427C>T 1428G>A 1429A>C), GAT250AAT (1433G>A), GTA251GTT (1438A>T), CTG252CCC (1440T>C 1441G>C), AGT253CAG (1442A>C 1443G>A 1444T>G), AGC255TCT (1448A>T 1449G>C 1450C>T), TCC256TCA (1453C>A), ATG257TTA (1454A>T 1456G>A), ACT258ATG (1458C>T 1459T>G), AGC260GCG (1463A>G 1464G>C 1465C>G), TCT261TCA (1468T>A), TCC262TCG (1471C>G), CTG263CTT (1474G>T), CGA264CGC (1477A>C), CCG266CCA (1483G>A), CTC268CTA (1489C>A), GTG270ATC (1493G>A 1495G>C), AGT272AGC (1501T>C), AGG273AAG (1503G>A), CGG274AGG (1505C>A), CCA276CCT (1513A>T), TCC277TCG (1516C>G), GAA279GAG (1522A>G), CTA280CTG (1525A>G), GAC282GAT (1531C>T), GTG283TTT (1532G>T 1534G>T), CCT284TCA (1535C>T 1537T>A), AAC285AGG (1539A>G 1540C>G), ACC286GCC (1541A>G), CGA288TCA (1547C>T 1548G>C), TTA289TCG (1551T>C 1552A>G), ACA290_ATG291insGTAGGT (1555_1556insGTAGGT), ATG291GTG (1556A>G), CGA292AGA (1559C>A), GCT296GGG (1572C>G 1573T>G), CAC297GTA (1574C>G 1575A>T 1576C>A), GAA299GAT (1582A>T), GAC300GAT (1585C>T), ACT301TCC (1586A>T 1588T>C), GGT302AAT (1589G>A 1590G>A), GGA303TTA (1592G>T 1593G>T), TTG304CTG (1595T>C), CCA306CCT (1603A>T), GTT307GTC (1606T>C), AAG309GAG (1610A>G), CAA311CAG (1618A>G), GGC312GCC (1620G>C), TTC313ATT (1622T>A 1624C>T), AAC314GAT (1625A>G 1627C>T), ATG315TTT (1628A>T 1630G>T), GGT316GGC (1633T>C), GTT317CAG (1634G>C 1635T>A 1636T>G), GAT320GAC (1645T>C), CTG322TAT (1649C>T 1650T>A 1651G>T), ATA324ATC (1657A>C), GAC325ACT (1658G>A 1659A>C 1660C>T), TCC326GAT (1661T>G 1662C>A 1663C>T), ACA328AAT (1668C>A 1669A>T), CAC329TCG (1670C>T 1671A>C 1672C>G), ATT330TTT (1673A>T), GAC331GAT (1678C>T), GAT335GAG (1690T>G), ACT337ACA (1696T>A), GAG338ACC (1697G>A 1698A>C 1699G>C), CGC339TGG (1700C>T 1702C>G), ACG340CAT (1703A>C 1704C>A 1705G>T), ATT341ATA (1708T>A), GCG342TCA (1709G>T 1711G>A), CAA343AGG (1712C>A 1713A>G 1714A>G), CAG344CAA (1717G>A), GTT345ATT (1718G>A), CAA346GAT (1721C>G 1723A>T), TAC347TTT (1725A>T 1726C>T), ATT348GTG (1727A>G 1729T>G), CTG349TTG (1730C>T), TTT350TCT (1734T>C), GAT351ACC (1736G>A 1737A>C 1738T>C), GAG352CTC (1739G>C 1740A>T 1741G>C), GCT353CTC (1742G>C 1743C>T 1744T>C), TCA354CGC (1745T>C 1746C>G 1747A>C), CGA355CAG (1749G>A 1750A>G), GGC356GGG (1753C>G), AAT357AGA (1755A>G 1756T>A), GTG358TGT (1757G>T 1758T>G 1759G>T), ATC359CAT (1760A>C 1761T>A 1762C>T), GTG360CTG (1763G>C), CCG361CCC (1768G>C), AAA362AAG (1771A>G), CAT363CAC (1774T>C), GTA364ATT (1775G>A 1777A>T), TCA365GAG (1778T>G 1779C>A 1780A>G), AAG366AGA (1782A>G 1783G>A), AGG367CGA (1784A>C 1786G>A), GAG368GAC (1789G>C), CAG369TCC (1790C>T 1791A>C 1792G>C), AAT371AAC (1798T>C), ATT372ATA (1801T>A), TAC373AAC (1802T>A), AAG374CTA (1805A>C 1806A>T 1807G>A), CAC376TAC (1811C>T), ATC377ATT (1816C>T), ACT378GAT (1817A>G 1818C>A), GAC380AAT (1823G>A 1825C>T), GTT383CTA (1832G>C 1834T>A), GAT384CAG (1835G>C 1837T>G), ATT385GTG (1838A>G 1840T>G), CTT386CTC (1843T>C), GCT387AAG (1844G>A 1845C>A 1846T>G), GCA388GAA (1848C>A), ACC392ACA (1861C>A), AAG393AAT (1864G>T), GTA394GTG (1867A>G), GGA395GGT (1870A>T), AAT396TCC (1871A>T 1872A>C 1873T>C), CTC397CTA (1876C>A), TCT398GAG (1877T>G 1878C>A 1879T>G), TCA400ACT (1883T>A 1885A>T), TTG401TGC (1887T>G 1888G>C), GAT402AAT (1889G>A), TAT404TAC (1897T>C), GAT405TCT (1898G>T 1899A>C), ATG406TTA (1901A>T 1903G>A), TCG407ACT (1904T>A 1906G>T), CTT408CTC (1909T>C), CAT409AGG (1910C>A 1911A>G 1912T>G), GAG410GAA (1915G>A), AGA411AGG (1918A>G), GTC412GTG (1921C>G), ATT413ATA (1924T>A), GCC414GAG (1926C>A 1927C>G), GTG416GTC (1933G>C), CAA418ATG (1937C>A 1938A>T 1939A>G), AGA419AGG (1942A>G), GAT420GAC (1945T>C), CAC421CAT (1948C>T), GTG423TTT (1952G>T 1954G>T), TAC424TAT (1957C>T), ATA425AGG (1959T>G 1960A>G), ATG426TAT (1961A>T 1962T>A 1963G>T), GGT427TGG (1964G>T 1966T>G), TTT429TTA (1972T>A), CCC432CCG (1981C>G), CTA433ATG (1982C>A 1984A>G), TCA435GAC (1988T>G 1989C>A 1990A>C), GAT437GAC (1996T>C), TGT438GTG (1997T>G 1998G>T 1999T>G), GAC440GAT (2005C>T), CTC442CTT (2011C>T), ATT444CTG (2015A>C 2017T>G), GGT445GCA (2019G>C 2020T>A), GAG446GAT (2023G>T), ACC511ACG (2218C>G), GCA512GCT (2221A>T), GAG513GAA (2224G>A), CAT514GAT (2225C>G), ATT515CCG (2228A>C 2229T>C 2230T>G), GAT517GGG (2235A>G 2236T>G), GAT518AAA (2237G>A 2239T>A), ACT520GAG (2243A>G 2244C>A 2245T>G), GGT522AAT (2249G>A 2250G>A), GAT523GAC (2254T>C), GTT524ATT (2255G>A), ATT525TTC (2258A>T 2260T>C), GAC527GAT (2266C>T), TTC528TTT (2269C>T), GGT529GGG (2272T>G), TCA530GAG (2273T>G 2274C>A 2275A>G), GAC531GAA (2278C>A), GAC533ATG (2282G>A 2283A>T 2284C>G), TCA534GGT (2285T>G 2286C>G 2287A>T), AAC535GAT (2288A>G 2290C>T), CCA536GCC (2291C>G 2293A>C), GCC537CCG (2294G>C 2296C>G), ATA538TTA (2297A>T), GAT539AAT (2300G>A), TTA540CTG (2303T>C 2305A>G), ATG542TTG (2309A>T), CAA544CAG (2317A>G), GTA546GCA (2322T>C), GCA549GCT (2332A>T), AGC550AGT (2335C>T), GAA552GAG (2341A>G), GGT557GGG (2356T>G), GTG558ATT (2357G>A 2359G>T), GAG560GAA (2365G>A), TTC561TTT (2368C>T), GTG562CTG (2369G>C), GAA564AAA (2375G>A), AAA567AAC (2386A>C), GTG569GTT (2392G>T), GCC570GCT (2395C>T), CAA572CAG (2401A>G), GTC573GTT (2404C>T), ATG574CTT (2405A>C 2407G>T), TCA575GCA (2408T>G), TTG576CTA (2411T>C 2413G>A), GCA577TCC (2414G>T 2416A>C), CTA578CTC (2419A>C)								

*: Inserts / Deletes / Misaligned / Frameshifts

Analysis details

This analysis was performed with panviral2.64

NGS Details (UN9): Diachasmimorpha longicaudata entomopoxvirus (segment NC_043455.1)

Assembly

**Coverage Length**	1117 (1 contig(s))
**Depth Of Coverage**	776.0
**Number Of Reads**	7050
**Reads Per Million**	159.35 rpm (after QC)
**Ambiguities**	0
**Assembly Method**	de novo + reference guided assembly
**Consensus Caller**	Bcf Tools

Coverage Map

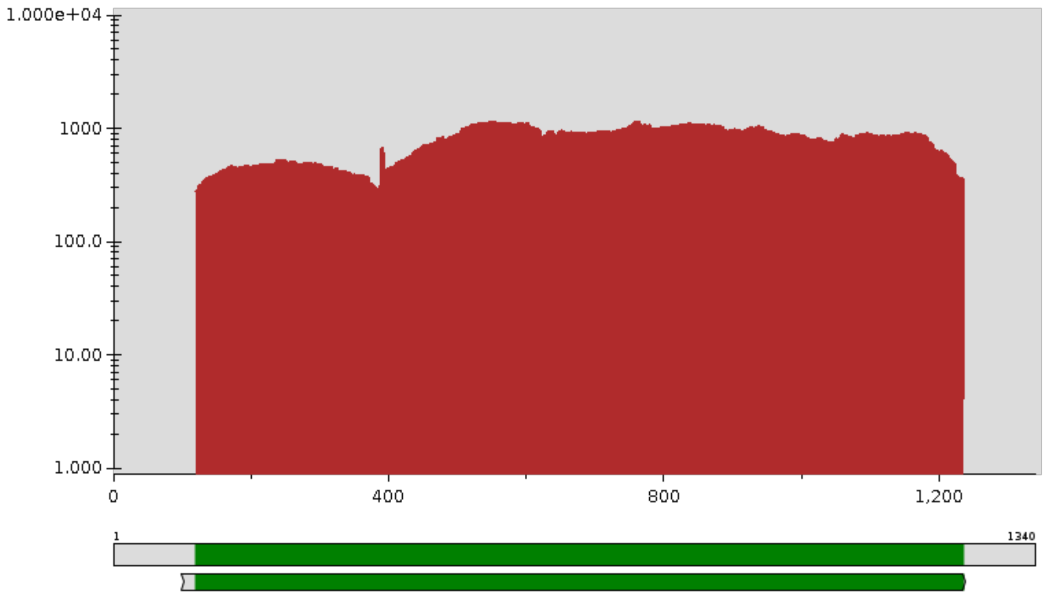

Assignment

**Type**	Diachasmimorpha longicaudata entomopoxvirus (Taxonomy ID: 109981)
**Reference Genome**	NC_043455.1
**NT Identity (%)**	59.2659
**AA Identity (%)**	55.914
**Number Of Stop Codons**	0
**Number Of CDS**	1

Alignment

**Alignment Score**	396.0 (NT) + 1471.0 (AA) = 1867.0
**Concordance (%)**	39.9188

Alignment Method	Global, seeded, nucleotide + amino acids (AGA)

Genome Region

Sequence starts at position 121 and ends at position 1237 relative to NC_043455.1 reference sequence.

Alignment Detailed Statistics

	Begin	End	Coverage	Score	Concordance	Matches	Identities	I/D/M/F*	Stop Codons
**NT**	**121**	**1237**	**83.4%**	**396**	**17.9%**	**1114 (99.5%)**	**662 (59.1%)**	**3/3**	
Mutations:	121A>T, 126A>C, 127C>T, 128C>G, 130T>G, 136C>G, 137C>A, 139C>T, 142G>A, 143A>G, 145A>T, 146A>G, 148T>C, 149A>C, 151C>T, 154A>T, 156A>G, 157G>A, 160T>G, 163A>T, 166T>C, 167T>C, 168C>A, 169T>G, 171G>A, 172T>C, 175A>G, 178T>C, 187T>A, 190A>T, 191A>G, 193T>C, 203A>C, 204A>G, 208C>T, 209A>G, 211T>A, 214T>G, 217A>T, 218T>A, 219G>T, 223T>A, 226T>G, 230A>C, 231A>G, 232A>T, 238G>C, 241A>T, 243T>C, 244C>T, 247A>G, 256G>C, 259A>T, 262A>C, 271A>C, 272G>T, 274A>C, 276C>T, 277A>G, 278T>A, 279A>T, 283A>T, 284A>C, 286C>T, 288G>C, 289T>C, 294T>G, 295A>C, 298G>A, 299C>G, 300A>T, 301A>T, 302A>G, 304A>G, 310T>C, 311T>A, 312C>A, 313T>A, 320A>G, 322T>G, 323A>G, 325C>A, 331T>A, 337C>A, 341A>T, 343C>T, 346A>T, 352T>G, 355A>G, 356T>C, 358G>T, 361T>A, 362C>G, 363T>C, 364C>T, 368G>A, 370T>G, 371C>G, 374C>A, 375G>A, 376T>G, 379A>G, 380T>A, 382G>T, 383C>T, 384A>T, 385A>G, 386A>G, 394G>T, 395A>G, 397T>C, 403A>T, 404_406delTAT, 407A>G, 408A>G, 410T>G, 412T>G, 415A>G, 416T>G, 417G>T, 421A>T, 423T>C, 424T>C, 428A>G, 430T>A, 436C>T, 439A>C, 443A>G, 446A>C, 447A>G, 448G>T, 451A>G, 452A>G, 453G>A, 454C>T, 458G>C, 459A>G, 460A>T, 462C>T, 463T>C, 466A>T, 467A>C, 469G>A, 471A>G, 472A>T, 472_473insGGT, 474C>T, 475C>T, 478G>T, 481T>G, 482T>G, 484G>T, 485A>G, 487T>C, 490A>T, 499A>C, 502A>T, 503A>G, 505G>A, 506A>T, 511T>C, 512C>A, 514A>G, 515C>T, 517C>G, 518A>C, 519C>G, 521C>A, 523A>G, 524A>C, 526A>G, 527A>T, 528G>C, 530A>C, 532C>T, 533G>C, 534A>G, 535T>C, 536A>C, 538C>T, 539A>G, 541A>C, 542G>C, 543C>A, 544A>C, 547T>C, 550A>G, 553A>G, 554G>T, 559A>T, 560A>T, 562A>G, 574T>C, 577G>A, 581T>C, 583G>C, 584A>T, 585T>C, 586A>T, 587G>A, 588A>G, 589T>A, 590A>G, 591A>G, 595T>C, 596T>A, 597T>A, 602A>C, 604A>G, 607A>T, 608C>T, 610A>T, 616T>A, 620G>C, 622A>G, 623T>C, 628T>G, 629G>C, 630A>C, 632A>C, 633G>C, 634T>A, 635C>A, 637T>A, 638G>A, 646T>G, 647A>G, 648T>G, 650T>G, 652A>T, 655A>C, 658A>T, 661A>C, 664C>T, 665G>A, 667T>G, 671T>C, 674A>G, 675G>A, 678T>C, 680A>C, 682C>T, 683A>G, 685T>G, 687C>T, 688A>T, 689T>A, 692C>A, 693A>G, 694G>A, 695G>A, 696T>A, 697C>G, 700T>C, 705G>A, 706A>C, 707G>A, 709T>G, 715A>G, 717A>G, 718A>G, 724G>T, 727A>G, 730A>G, 731A>C, 732A>G, 735C>A, 739A>G, 740T>C, 742G>C, 745A>T, 748C>T, 754C>T, 759G>A, 765A>T, 770A>G, 772A>C, 778A>C, 779A>G, 781G>T, 784A>G, 785A>G, 787T>A, 790T>A, 792T>G, 793T>G, 796A>G, 797G>C, 798C>T, 799A>T, 810T>G, 823C>G, 824C>A, 825A>C, 826C>T, 829A>G, 830A>G, 831G>C, 833C>A, 838T>C, 841A>G, 843C>G, 844A>T, 845C>G, 847A>C, 850A>C, 854T>G, 855G>T, 859T>C, 862A>T, 863C>A, 865T>G, 866A>C, 869C>A, 871A>G, 878G>T, 879T>G, 880A>G, 885T>C, 886G>T, 889A>T, 890T>A, 891G>A, 892T>G, 893T>A, 895A>G, 896A>C, 897C>G, 898A>C, 900A>G, 901T>C, 902A>C, 904A>T, 905A>G, 908T>C, 909T>A, 915C>T, 919A>T, 920A>G, 921G>C, 922C>A, 924T>C, 925C>T, 934T>C, 938T>G, 939C>A, 943A>G, 944C>A, 946A>C, 947G>A, 948A>C, 949A>T, 950C>A, 952T>A, 956T>A, 958T>C, 966A>G, 967A>G, 970A>G, 973T>C, 974A>C, 977G>T, 978A>C, 982T>C, 983A>T, 984A>C, 986A>T, 989A>C, 991A>T, 992A>G, 994A>G, 998C>A, 1000T>C, 1001T>A, 1007A>G, 1012A>C, 1013T>C, 1015A>T, 1018A>T, 1019A>C, 1021A>T, 1024C>T, 1033T>A, 1036A>G, 1040A>G, 1042A>C, 1045T>C, 1046T>C, 1048A>T, 1051T>C, 1054C>T, 1057T>C, 1063T>C, 1064T>C, 1070C>A, 1071A>C, 1073A>C, 1075T>A, 1076A>C, 1077G>C, 1087T>C, 1088A>C, 1090A>G, 1103A>C, 1105A>T, 1111T>A, 1112A>C, 1120C>A, 1121C>A, 1123A>G, 1126A>G, 1129G>T, 1130A>G, 1141T>C, 1145A>G, 1147A>C, 1150T>C, 1151G>A, 1153A>G, 1156C>T, 1159A>T, 1160T>G, 1161T>A, 1163C>A, 1164C>G, 1165A>G, 1167A>T, 1172G>T, 1173C>T, 1177A>C, 1178T>A, 1181G>C, 1183A>G, 1185C>A, 1186G>A, 1192T>C, 1195T>C, 1196A>G, 1197C>T, 1199A>G, 1200A>T, 1204A>C, 1207T>G, 1210A>G, 1211A>C, 1213G>C, 1218A>C, 1219G>C, 1223A>G, 1227T>C, 1232T>C, 1234G>C, 1235A>C, 1237A>G								

CDS

	Begin	End	Coverage	Score	Concordance	Matches	Identities	I/D/M/F*	Stop Codons
FLA14_p101	8	379	97.9%	1471	59.6%	371 (99.5%)	208 (55.8%)	1/1/0/0	0
Protein mutations:	D9A (126A>C 127C>T), H10E (128C>G 130T>G), L13I (137C>A 139C>T), K15D (143A>G 145A>T), N16D (146A>G 148T>C), I17L (149A>C 151C>T), K19R (156A>G 157G>A), S23Q (167T>C 168C>A 169T>G), C24Y (171G>A 172T>C), T31A (191A>G 193T>C), K35R (203A>C 204A>G), I37V (209A>G 211T>A), F38L (214T>G), C40I (218T>A 219G>T), K44R (230A>C 231A>G 232A>T), V48A (243T>C 244C>T), A58S (272G>T 274A>C), T59M (276C>T 277A>G), Y60I (278T>A 279A>T), I62L (284A>C 286C>T), S63T (288G>C 289T>C), L65C (294T>G 295A>C), Q67V (299C>G 300A>T 301A>T), I68V (302A>G 304A>G), S71K (311T>A 312C>A 313T>A), N74E (320A>G 322T>G), I75V (323A>G 325C>A), T81S (341A>T 343C>T), L88A (362C>G 363T>C 364C>T), A90T (368G>A 370T>G), Q91E (371C>G), R92K (374C>A 375G>A 376T>G), L94I (380T>A 382G>T), Q95L (383C>T 384A>T 385A>G), T96A (386A>G), N99D (395A>G 397T>C), Y102del (404_406delTAT), N103G (407A>G 408A>G), F104V (410T>G 412T>G), C106V (416T>G 417G>T), Q107H (421A>T), V108A (423T>C 424T>C), I110V (428A>G 430T>A), I115V (443A>G), K116R (446A>C 447A>G 448G>T), S118D (452A>G 453G>A 454C>T), E120R (458G>C 459A>G 460A>T), T121I (462C>T 463T>C), K123Q (467A>C 469G>A), K124S (471A>G 472A>T), K124_A125insG (472_473insGGT), A125V (474C>T 475C>T), Q126H (478G>T), L128V (482T>G 484G>T), I129V (485A>G 487T>C), M135V (503A>G 505G>A), I136F (506A>T), L138M (512C>A 514A>G), T140R (518A>C 519C>G), K142Q (524A>C 526A>G), I144L (530A>C 532C>T), D145R (533G>C 534A>G 535T>C), T146P (536A>C 538C>T), K147D (539A>G 541A>C), A148H (542G>C 543C>A 544A>C), I151M (553A>G), V152F (554G>T), I154L (560A>T 562A>G), I162S (584A>T 585T>C 586A>T), D163R (587G>A 588A>G 589T>A), N164G (590A>G 591A>G), L166K (596T>A 597T>A), K168Q (602A>C 604A>G), Q170Y (608C>T 610A>T), E174Q (620G>C 622A>G), F175L (623T>C), F176L (628T>G), E177P (629G>C 630A>C), S178P (632A>C 633G>C 634T>A), H179K (635C>A 637T>A), V180I (638G>A), I183G (647A>G 648T>G), L184V (650T>G 652A>T), L185F (655A>C), V189M (665G>A 667T>G), S191P (671T>C), R192E (674A>G 675G>A), V193A (678T>C), I194L (680A>C 682C>T), N195E (683A>G 685T>G), T196I (687C>T 688A>T), S197T (689T>A), Q198R (692C>A 693A>G 694G>A), V199K (695G>A 696T>A 697C>G), R202N (705G>A 706A>C), D203K (707G>A 709T>G), K206R (717A>G 718A>G), N211R (731A>C 732A>G), A212D (735C>A), R220K (759G>A), Y222F (765A>T), I224V (770A>G 772A>C), K227D (779A>G 781G>T), N229E (785A>G 787T>A), D230E (790T>A), F231W (792T>G 793T>G), A233L (797G>C 798C>T 799A>T), F237C (810T>G), D241E (823C>G), H242T (824C>A 825A>C 826C>T), S244A (830A>G 831G>C), L245I (833C>A), T248S (843C>G 844A>T), L249V (845C>G 847A>C), C252V (854T>G 855G>T), Q257K (869C>A 871A>G), V260W (878G>T 879T>G 880A>G), M262T (885T>C 886G>T), E263D (889A>T), C264K (890T>A 891G>A 892T>G), L265M (893T>A 895A>G), T266R (896A>C 897C>G 898A>C), N267S (900A>G 901T>C), N269D (905A>G), F270H (908T>C 909T>A), A272V (915C>T), S274A (920A>G 921G>C 922C>A), I275T (924T>C 925C>T), S280D (938T>G 939C>A), Q282N (944C>A 946A>C), E283T (947G>A 948A>C 949A>T), F286I (956T>A 958T>C), K289R (966A>G 967A>G), D293S (977G>T 978A>C), K295S (983A>T 984A>C), T296S (986A>T), I298V (992A>G 994A>G), L300I (998C>A 1000T>C), S301T (1001T>A), N303D (1007A>G), I314V (1040A>G 1042A>C), H324T (1070C>A 1071A>C), N325Q (1073A>C 1075T>A), R326P (1076A>C 1077G>C), I330L (1088A>C 1090A>G), I344V (1130A>G), I349V (1145A>G 1147A>C), E351K (1151G>A 1153A>G), E353D (1159A>T), L354E (1160T>G 1161T>A), P355R (1163C>A 1164C>G 1165A>G), K356M (1167A>T), A358F (1172G>T 1173C>T), E359D (1177A>C), L360I (1178T>A), E361Q (1181G>C 1183A>G), T362K (1185C>A 1186G>A), T366V (1196A>G 1197C>T), K367V (1199A>G 1200A>T), D369E (1207T>G), M371L (1211A>C 1213G>C), E373A (1218A>C 1219G>C), I375V (1223A>G), V376A (1227T>C), I379L (1235A>C 1237A>G)								

	Begin	End	Coverage	Score	Concordance	Matches	Identities	I/D/M/F*	Stop Codons
**NT**	**121**	**1237**	**83.4%**	**396**	**17.9%**	**1114 (99.5%)**	**662 (59.1%)**	**3/3**	
Codon mutations:	GAA7..T (121A>T), GAC9GCT (126A>C 127C>T), CAT10GAG (128C>G 130T>G), GGC12GGG (136C>G), CTC13ATT (137C>A 139C>T), AAG14AAA (142G>A), AAA15GAT (143A>G 145A>T), AAT16GAC (146A>G 148T>C), ATC17CTT (149A>C 151C>T), CTA18CTT (154A>T), AAG19AGA (156A>G 157G>A), GGT20GGG (160T>G), ATA21ATT (163A>T), TAT22TAC (166T>C), TCT23CAG (167T>C 168C>A 169T>G), TGT24TAC (171G>A 172T>C), GGA25GGG (175A>G), TTT26TTC (178T>C), CCT29CCA (187T>A), TCA30TCT (190A>T), ACT31GCC (191A>G 193T>C), AAA35CGA (203A>C 204A>G), GCC36GCT (208C>T), ATT37GTA (209A>G 211T>A), TTT38TTG (214T>G), CCA39CCT (217A>T), TGT40ATT (218T>A 219G>T), ATT41ATA (223T>A), TCT42TCG (226T>G), AAA44CGT (230A>C 231A>G 232A>T), GTG46GTC (238G>C), ATA47ATT (241A>T), GTC48GCT (243T>C 244C>T), CAA49CAG (247A>G), TCG52TCC (256G>C), GGA53GGT (259A>T), ACA54ACC (262A>C), ACA57ACC (271A>C), GCA58TCC (272G>T 274A>C), ACA59ATG (276C>T 277A>G), TAT60ATT (278T>A 279A>T), GCA61GCT (283A>T), ATC62CTT (284A>C 286C>T), AGT63ACC (288G>C 289T>C), TTA65TGC (294T>G 295A>C), CAG66CAA (298G>A), CAA67GTT (299C>G 300A>T 301A>T), ATA68GTG (302A>G 304A>G), ACT70ACC (310T>C), TCT71AAA (311T>A 312C>A 313T>A), AAT74GAG (320A>G 322T>G), ATC75GTA (323A>G 325C>A), GCT77GCA (331T>A), ATC79ATA (337C>A), ACC81TCT (341A>T 343C>T), CCA82CCT (346A>T), CGT84CGG (352T>G), GAA85GAG (355A>G), TTG86CTT (356T>C 358G>T), GCT87GCA (361T>A), CTC88GCT (362C>G 363T>C 364C>T), GCT90ACG (368G>A 370T>G), CAA91GAA (371C>G), CGT92AAG (374C>A 375G>A 376T>G), GTA93GTG (379A>G), TTG94ATT (380T>A 382G>T), CAA95TTG (383C>T 384A>T 385A>G), ACA96GCA (386A>G), GGG98GGT (394G>T), AAT99GAC (395A>G 397T>C), CTA101CTT (403A>T), TAT102del (404_406delTAT), AAT103GGT (407A>G 408A>G), TTT104GTG (410T>G 412T>G), AAA105AAG (415A>G), TGT106GTT (416T>G 417G>T), CAA107CAT (421A>T), GTT108GCC (423T>C 424T>C), ATT110GTA (428A>G 430T>A), GGC112GGT (436C>T), ACA113ACC (439A>C), ATC115GTC (443A>G), AAG116CGT (446A>C 447A>G 448G>T), GAA117GAG (451A>G), AGC118GAT (452A>G 453G>A 454C>T), GAA120CGT (458G>C 459A>G 460A>T), ACT121ATC (462C>T 463T>C), CTA122CTT (466A>T), AAG123CAA (467A>C 469G>A), AAA124AGT (471A>G 472A>T), AAA124_GCC125insGGT (472_473insGGT), GCC125GTT (474C>T 475C>T), CAG126CAT (478G>T), GTT127GTG (481T>G), TTG128GTT (482T>G 484G>T), ATT129GTC (485A>G 487T>C), GGA130GGT (490A>T), GGA133GGC (499A>C), CGA134CGT (502A>T), ATG135GTA (503A>G 505G>A), ATT136TTT (506A>T), GAT137GAC (511T>C), CTA138ATG (512C>A 514A>G), CTC139TTG (515C>T 517C>G), ACC140CGC (518A>C 519C>G), CGA141AGG (521C>A 523A>G), AAA142CAG (524A>C 526A>G), AGT143TCT (527A>T 528G>C), ATC144CTT (530A>C 532C>T), GAT145CGC (533G>C 534A>G 535T>C), ACC146CCT (536A>C 538C>T), AAA147GAC (539A>G 541A>C), GCA148CAC (542G>C 543C>A 544A>C), ATT149ATC (547T>C), AAA150AAG (550A>G), ATA151ATG (553A>G), GTT152TTT (554G>T), GTA153GTT (559A>T), ATA154TTG (560A>T 562A>G), GAT158GAC (574T>C), GAG159GAA (577G>A), TTG161CTC (581T>C 583G>C), ATA162TCT (584A>T 585T>C 586A>T), GAT163AGA (587G>A 588A>G 589T>A), AAT164GGT (590A>G 591A>G), TTT165TTC (595T>C), TTG166AAG (596T>A 597T>A), AAA168CAG (602A>C 604A>G), ATA169ATT (607A>T), CAA170TAT (608C>T 610A>T), ATT172ATA (616T>A), GAA174CAG (620G>C 622A>G), TTT175CTT (623T>C), TTT176TTG (628T>G), GAA177CCA (629G>C 630A>C), AGT178CCA (632A>C 633G>C 634T>A), CAT179AAA (635C>A 637T>A), GTC180ATC (638G>A), GTT182GTG (646T>G), ATT183GGT (647A>G 648T>G), TTA184GTT (650T>G 652A>T), TTA185TTC (655A>C), TCA186TCT (658A>T), GCA187GCC (661A>C), ACC188ACT (664C>T), GTT189ATG (665G>A 667T>G), TCA191CCA (671T>C), AGG192GAG (674A>G 675G>A), GTT193GCT (678T>C), ATC194CTT (680A>C 682C>T), AAT195GAG (683A>G 685T>G), ACA196ATT (687C>T 688A>T), TCT197ACT (689T>A), CAG198AGA (692C>A 693A>G 694G>A), GTC199AAG (695G>A 696T>A 697C>G), TTT200TTC (700T>C), AGA202AAC (705G>A 706A>C), GAT203AAG (707G>A 709T>G), GTA205GTG (715A>G), AAA206AGG (717A>G 718A>G), CTG208CTT (724G>T), GTA209GTG (727A>G), AAA210AAG (730A>G), AAT211CGT (731A>C 732A>G), GCT212GAT (735C>A), GAA213GAG (739A>G), TTG214CTC (740T>C 742G>C), ACA215ACT (745A>T), CTC216CTT (748C>T), GGC218GGT (754C>T), AGG220AAG (759G>A), TAT222TTT (765A>T), ATA224GTC (770A>G 772A>C), GTA226GTC (778A>C), AAG227GAT (779A>G 781G>T), AAA228AAG (784A>G), AAT229GAA (785A>G 787T>A), GAT230GAA (790T>A), TTT231TGG (792T>G 793T>G), AAA232AAG (796A>G), GCA233CTT (797G>C 798C>T 799A>T), TTT237TGT (810T>G), GAC241GAG (823C>G), CAC242ACT (824C>A 825A>C 826C>T), TTA243TTG (829A>G), AGC244GCC (830A>G 831G>C), CTC245ATC (833C>A), ACT246ACC (838T>C), CAA247CAG (841A>G), ACA248AGT (843C>G 844A>T), CTA249GTC (845C>G 847A>C), ATA250ATC (850A>C), TGT252GTT (854T>G 855G>T), AAT253AAC (859T>C), ACA254ACT (862A>T), CGT255AGG (863C>A 865T>G), AGA256CGA (866A>C), CAA257AAG (869C>A 871A>G), GTA260TGG (878G>T 879T>G 880A>G), ATG262ACT (885T>C 886G>T), GAA263GAT (889A>T), TGT264AAG (890T>A 891G>A 892T>G), TTA265ATG (893T>A 895A>G), ACA266CGC (896A>C 897C>G 898A>C), AAT267AGC (900A>G 901T>C), AGA268CGT (902A>C 904A>T), AAT269GAT (905A>G), TTC270CAC (908T>C 909T>A), GCA272GTA (915C>T), TCA273TCT (919A>T), AGC274GCA (920A>G 921G>C 922C>A), ATC275ACT (924T>C 925C>T), GAT278GAC (934T>C), TCC280GAC (938T>G 939C>A), CAA281CAG (943A>G), CAA282AAC (944C>A 946A>C), GAA283ACT (947G>A 948A>C 949A>T), CGT284AGA (950C>A 952T>A), TTT286ATC (956T>A 958T>C), AAA289AGG (966A>G 967A>G), GAA290GAG (970A>G), TTT291TTC (973T>C), AGA292CGA (974A>C), GAT293TCT (977G>T 978A>C), GGT294GGC (982T>C), AAA295TCA (983A>T 984A>C), ACT296TCT (986A>T), AGA297CGT (989A>C 991A>T), ATA298GTG (992A>G 994A>G), CTT300ATC (998C>A 1000T>C), TCA301ACA (1001T>A), AAT303GAT (1007A>G), CTA304CTC (1012A>C), TTA305CTT (1013T>C 1015A>T), GCA306GCT (1018A>T), AGA307CGT (1019A>C 1021A>T), GGC308GGT (1024C>T), GTT311GTA (1033T>A), CAA312CAG (1036A>G), ATA314GTC (1040A>G 1042A>C), TCT315TCC (1045T>C), TTA316CTT (1046T>C 1048A>T), GTT317GTC (1051T>C), ATC318ATT (1054C>T), AAT319AAC (1057T>C), GAT321GAC (1063T>C), TTG322CTG (1064T>C), CAT324ACT (1070C>A 1071A>C), AAT325CAA (1073A>C 1075T>A), AGA326CCA (1076A>C 1077G>C), TAT329TAC (1087T>C), ATA330CTG (1088A>C 1090A>G), AGA335CGT (1103A>C 1105A>T), GGT337GGA (1111T>A), AGA338CGA (1112A>C), GGC340GGA (1120C>A), CGA341AGG (1121C>A 1123A>G), AAA342AAG (1126A>G), GGG343GGT (1129G>T), ATT344GTT (1130A>G), AAT347AAC (1141T>C), ATA349GTC (1145A>G 1147A>C), ACT350ACC (1150T>C), GAA351AAG (1151G>A 1153A>G), GAC352GAT (1156C>T), GAA353GAT (1159A>T), TTA354GAA (1160T>G 1161T>A), CCA355AGG (1163C>A 1164C>G 1165A>G), AAG356ATG (1167A>T), GCT358TTT (1172G>T 1173C>T), GAA359GAC (1177A>C), TTA360ATA (1178T>A), GAA361CAG (1181G>C 1183A>G), ACG362AAA (1185C>A 1186G>A), TAT364TAC (1192T>C), AAT365AAC (1195T>C), ACC366GTC (1196A>G 1197C>T), AAA367GTA (1199A>G 1200A>T), ATA368ATC (1204A>C), GAT369GAG (1207T>G), GAA370GAG (1210A>G), ATG371CTC (1211A>C 1213G>C), GAG373GCC (1218A>C 1219G>C), ATT375GTT (1223A>G), GTT376GCT (1227T>C), TTG378CTC (1232T>C 1234G>C), ATA379CTG (1235A>C 1237A>G)								

Proteins

	Begin	End	Coverage	Score	Concordance	Matches	Identities	I/D/M/F*	Stop Codons
hypothetical protein (YP_009666148.1)	8	379	97.9%	1471	59.6%	371 (99.5%)	208 (55.8%)	1/1/0/0	0
Protein mutations:	D9A (126A>C 127C>T), H10E (128C>G 130T>G), L13I (137C>A 139C>T), K15D (143A>G 145A>T), N16D (146A>G 148T>C), I17L (149A>C 151C>T), K19R (156A>G 157G>A), S23Q (167T>C 168C>A 169T>G), C24Y (171G>A 172T>C), T31A (191A>G 193T>C), K35R (203A>C 204A>G), I37V (209A>G 211T>A), F38L (214T>G), C40I (218T>A 219G>T), K44R (230A>C 231A>G 232A>T), V48A (243T>C 244C>T), A58S (272G>T 274A>C), T59M (276C>T 277A>G), Y60I (278T>A 279A>T), I62L (284A>C 286C>T), S63T (288G>C 289T>C), L65C (294T>G 295A>C), Q67V (299C>G 300A>T 301A>T), I68V (302A>G 304A>G), S71K (311T>A 312C>A 313T>A), N74E (320A>G 322T>G), I75V (323A>G 325C>A), T81S (341A>T 343C>T), L88A (362C>G 363T>C 364C>T), A90T (368G>A 370T>G), Q91E (371C>G), R92K (374C>A 375G>A 376T>G), L94I (380T>A 382G>T), Q95L (383C>T 384A>T 385A>G), T96A (386A>G), N99D (395A>G 397T>C), Y102del (404_406delTAT), N103G (407A>G 408A>G), F104V (410T>G 412T>G), C106V (416T>G 417G>T), Q107H (421A>T), V108A (423T>C 424T>C), I110V (428A>G 430T>A), I115V (443A>G), K116R (446A>C 447A>G 448G>T), S118D (452A>G 453G>A 454C>T), E120R (458G>C 459A>G 460A>T), T121I (462C>T 463T>C), K123Q (467A>C 469G>A), K124S (471A>G 472A>T), K124_A125insG (472_473insGGT), A125V (474C>T 475C>T), Q126H (478G>T), L128V (482T>G 484G>T), I129V (485A>G 487T>C), M135V (503A>G 505G>A), I136F (506A>T), L138M (512C>A 514A>G), T140R (518A>C 519C>G), K142Q (524A>C 526A>G), I144L (530A>C 532C>T), D145R (533G>C 534A>G 535T>C), T146P (536A>C 538C>T), K147D (539A>G 541A>C), A148H (542G>C 543C>A 544A>C), I151M (553A>G), V152F (554G>T), I154L (560A>T 562A>G), I162S (584A>T 585T>C 586A>T), D163R (587G>A 588A>G 589T>A), N164G (590A>G 591A>G), L166K (596T>A 597T>A), K168Q (602A>C 604A>G), Q170Y (608C>T 610A>T), E174Q (620G>C 622A>G), F175L (623T>C), F176L (628T>G), E177P (629G>C 630A>C), S178P (632A>C 633G>C 634T>A), H179K (635C>A 637T>A), V180I (638G>A), I183G (647A>G 648T>G), L184V (650T>G 652A>T), L185F (655A>C), V189M (665G>A 667T>G), S191P (671T>C), R192E (674A>G 675G>A), V193A (678T>C), I194L (680A>C 682C>T), N195E (683A>G 685T>G), T196I (687C>T 688A>T), S197T (689T>A), Q198R (692C>A 693A>G 694G>A), V199K (695G>A 696T>A 697C>G), R202N (705G>A 706A>C), D203K (707G>A 709T>G), K206R (717A>G 718A>G), N211R (731A>C 732A>G), A212D (735C>A), R220K (759G>A), Y222F (765A>T), I224V (770A>G 772A>C), K227D (779A>G 781G>T), N229E (785A>G 787T>A), D230E (790T>A), F231W (792T>G 793T>G), A233L (797G>C 798C>T 799A>T), F237C (810T>G), D241E (823C>G), H242T (824C>A 825A>C 826C>T), S244A (830A>G 831G>C), L245I (833C>A), T248S (843C>G 844A>T), L249V (845C>G 847A>C), C252V (854T>G 855G>T), Q257K (869C>A 871A>G), V260W (878G>T 879T>G 880A>G), M262T (885T>C 886G>T), E263D (889A>T), C264K (890T>A 891G>A 892T>G), L265M (893T>A 895A>G), T266R (896A>C 897C>G 898A>C), N267S (900A>G 901T>C), N269D (905A>G), F270H (908T>C 909T>A), A272V (915C>T), S274A (920A>G 921G>C 922C>A), I275T (924T>C 925C>T), S280D (938T>G 939C>A), Q282N (944C>A 946A>C), E283T (947G>A 948A>C 949A>T), F286I (956T>A 958T>C), K289R (966A>G 967A>G), D293S (977G>T 978A>C), K295S (983A>T 984A>C), T296S (986A>T), I298V (992A>G 994A>G), L300I (998C>A 1000T>C), S301T (1001T>A), N303D (1007A>G), I314V (1040A>G 1042A>C), H324T (1070C>A 1071A>C), N325Q (1073A>C 1075T>A), R326P (1076A>C 1077G>C), I330L (1088A>C 1090A>G), I344V (1130A>G), I349V (1145A>G 1147A>C), E351K (1151G>A 1153A>G), E353D (1159A>T), L354E (1160T>G 1161T>A), P355R (1163C>A 1164C>G 1165A>G), K356M (1167A>T), A358F (1172G>T 1173C>T), E359D (1177A>C), L360I (1178T>A), E361Q (1181G>C 1183A>G), T362K (1185C>A 1186G>A), T366V (1196A>G 1197C>T), K367V (1199A>G 1200A>T), D369E (1207T>G), M371L (1211A>C 1213G>C), E373A (1218A>C 1219G>C), I375V (1223A>G), V376A (1227T>C), I379L (1235A>C 1237A>G)								

	Begin	End	Coverage	Score	Concordance	Matches	Identities	I/D/M/F*	Stop Codons
**NT**	**121**	**1237**	**83.4%**	**396**	**17.9%**	**1114 (99.5%)**	**662 (59.1%)**	**3/3**	
Codon mutations:	GAA7..T (121A>T), GAC9GCT (126A>C 127C>T), CAT10GAG (128C>G 130T>G), GGC12GGG (136C>G), CTC13ATT (137C>A 139C>T), AAG14AAA (142G>A), AAA15GAT (143A>G 145A>T), AAT16GAC (146A>G 148T>C), ATC17CTT (149A>C 151C>T), CTA18CTT (154A>T), AAG19AGA (156A>G 157G>A), GGT20GGG (160T>G), ATA21ATT (163A>T), TAT22TAC (166T>C), TCT23CAG (167T>C 168C>A 169T>G), TGT24TAC (171G>A 172T>C), GGA25GGG (175A>G), TTT26TTC (178T>C), CCT29CCA (187T>A), TCA30TCT (190A>T), ACT31GCC (191A>G 193T>C), AAA35CGA (203A>C 204A>G), GCC36GCT (208C>T), ATT37GTA (209A>G 211T>A), TTT38TTG (214T>G), CCA39CCT (217A>T), TGT40ATT (218T>A 219G>T), ATT41ATA (223T>A), TCT42TCG (226T>G), AAA44CGT (230A>C 231A>G 232A>T), GTG46GTC (238G>C), ATA47ATT (241A>T), GTC48GCT (243T>C 244C>T), CAA49CAG (247A>G), TCG52TCC (256G>C), GGA53GGT (259A>T), ACA54ACC (262A>C), ACA57ACC (271A>C), GCA58TCC (272G>T 274A>C), ACA59ATG (276C>T 277A>G), TAT60ATT (278T>A 279A>T), GCA61GCT (283A>T), ATC62CTT (284A>C 286C>T), AGT63ACC (288G>C 289T>C), TTA65TGC (294T>G 295A>C), CAG66CAA (298G>A), CAA67GTT (299C>G 300A>T 301A>T), ATA68GTG (302A>G 304A>G), ACT70ACC (310T>C), TCT71AAA (311T>A 312C>A 313T>A), AAT74GAG (320A>G 322T>G), ATC75GTA (323A>G 325C>A), GCT77GCA (331T>A), ATC79ATA (337C>A), ACC81TCT (341A>T 343C>T), CCA82CCT (346A>T), CGT84CGG (352T>G), GAA85GAG (355A>G), TTG86CTT (356T>C 358G>T), GCT87GCA (361T>A), CTC88GCT (362C>G 363T>C 364C>T), GCT90ACG (368G>A 370T>G), CAA91GAA (371C>G), CGT92AAG (374C>A 375G>A 376T>G), GTA93GTG (379A>G), TTG94ATT (380T>A 382G>T), CAA95TTG (383C>T 384A>T 385A>G), ACA96GCA (386A>G), GGG98GGT (394G>T), AAT99GAC (395A>G 397T>C), CTA101CTT (403A>T), TAT102del (404_406delTAT), AAT103GGT (407A>G 408A>G), TTT104GTG (410T>G 412T>G), AAA105AAG (415A>G), TGT106GTT (416T>G 417G>T), CAA107CAT (421A>T), GTT108GCC (423T>C 424T>C), ATT110GTA (428A>G 430T>A), GGC112GGT (436C>T), ACA113ACC (439A>C), ATC115GTC (443A>G), AAG116CGT (446A>C 447A>G 448G>T), GAA117GAG (451A>G), AGC118GAT (452A>G 453G>A 454C>T), GAA120CGT (458G>C 459A>G 460A>T), ACT121ATC (462C>T 463T>C), CTA122CTT (466A>T), AAG123CAA (467A>C 469G>A), AAA124AGT (471A>G 472A>T), AAA124_GCC125insGGT (472_473insGGT), GCC125GTT (474C>T 475C>T), CAG126CAT (478G>T), GTT127GTG (481T>G), TTG128GTT (482T>G 484G>T), ATT129GTC (485A>G 487T>C), GGA130GGT (490A>T), GGA133GGC (499A>C), CGA134CGT (502A>T), ATG135GTA (503A>G 505G>A), ATT136TTT (506A>T), GAT137GAC (511T>C), CTA138ATG (512C>A 514A>G), CTC139TTG (515C>T 517C>G), ACC140CGC (518A>C 519C>G), CGA141AGG (521C>A 523A>G), AAA142CAG (524A>C 526A>G), AGT143TCT (527A>T 528G>C), ATC144CTT (530A>C 532C>T), GAT145CGC (533G>C 534A>G 535T>C), ACC146CCT (536A>C 538C>T), AAA147GAC (539A>G 541A>C), GCA148CAC (542G>C 543C>A 544A>C), ATT149ATC (547T>C), AAA150AAG (550A>G), ATA151ATG (553A>G), GTT152TTT (554G>T), GTA153GTT (559A>T), ATA154TTG (560A>T 562A>G), GAT158GAC (574T>C), GAG159GAA (577G>A), TTG161CTC (581T>C 583G>C), ATA162TCT (584A>T 585T>C 586A>T), GAT163AGA (587G>A 588A>G 589T>A), AAT164GGT (590A>G 591A>G), TTT165TTC (595T>C), TTG166AAG (596T>A 597T>A), AAA168CAG (602A>C 604A>G), ATA169ATT (607A>T), CAA170TAT (608C>T 610A>T), ATT172ATA (616T>A), GAA174CAG (620G>C 622A>G), TTT175CTT (623T>C), TTT176TTG (628T>G), GAA177CCA (629G>C 630A>C), AGT178CCA (632A>C 633G>C 634T>A), CAT179AAA (635C>A 637T>A), GTC180ATC (638G>A), GTT182GTG (646T>G), ATT183GGT (647A>G 648T>G), TTA184GTT (650T>G 652A>T), TTA185TTC (655A>C), TCA186TCT (658A>T), GCA187GCC (661A>C), ACC188ACT (664C>T), GTT189ATG (665G>A 667T>G), TCA191CCA (671T>C), AGG192GAG (674A>G 675G>A), GTT193GCT (678T>C), ATC194CTT (680A>C 682C>T), AAT195GAG (683A>G 685T>G), ACA196ATT (687C>T 688A>T), TCT197ACT (689T>A), CAG198AGA (692C>A 693A>G 694G>A), GTC199AAG (695G>A 696T>A 697C>G), TTT200TTC (700T>C), AGA202AAC (705G>A 706A>C), GAT203AAG (707G>A 709T>G), GTA205GTG (715A>G), AAA206AGG (717A>G 718A>G), CTG208CTT (724G>T), GTA209GTG (727A>G), AAA210AAG (730A>G), AAT211CGT (731A>C 732A>G), GCT212GAT (735C>A), GAA213GAG (739A>G), TTG214CTC (740T>C 742G>C), ACA215ACT (745A>T), CTC216CTT (748C>T), GGC218GGT (754C>T), AGG220AAG (759G>A), TAT222TTT (765A>T), ATA224GTC (770A>G 772A>C), GTA226GTC (778A>C), AAG227GAT (779A>G 781G>T), AAA228AAG (784A>G), AAT229GAA (785A>G 787T>A), GAT230GAA (790T>A), TTT231TGG (792T>G 793T>G), AAA232AAG (796A>G), GCA233CTT (797G>C 798C>T 799A>T), TTT237TGT (810T>G), GAC241GAG (823C>G), CAC242ACT (824C>A 825A>C 826C>T), TTA243TTG (829A>G), AGC244GCC (830A>G 831G>C), CTC245ATC (833C>A), ACT246ACC (838T>C), CAA247CAG (841A>G), ACA248AGT (843C>G 844A>T), CTA249GTC (845C>G 847A>C), ATA250ATC (850A>C), TGT252GTT (854T>G 855G>T), AAT253AAC (859T>C), ACA254ACT (862A>T), CGT255AGG (863C>A 865T>G), AGA256CGA (866A>C), CAA257AAG (869C>A 871A>G), GTA260TGG (878G>T 879T>G 880A>G), ATG262ACT (885T>C 886G>T), GAA263GAT (889A>T), TGT264AAG (890T>A 891G>A 892T>G), TTA265ATG (893T>A 895A>G), ACA266CGC (896A>C 897C>G 898A>C), AAT267AGC (900A>G 901T>C), AGA268CGT (902A>C 904A>T), AAT269GAT (905A>G), TTC270CAC (908T>C 909T>A), GCA272GTA (915C>T), TCA273TCT (919A>T), AGC274GCA (920A>G 921G>C 922C>A), ATC275ACT (924T>C 925C>T), GAT278GAC (934T>C), TCC280GAC (938T>G 939C>A), CAA281CAG (943A>G), CAA282AAC (944C>A 946A>C), GAA283ACT (947G>A 948A>C 949A>T), CGT284AGA (950C>A 952T>A), TTT286ATC (956T>A 958T>C), AAA289AGG (966A>G 967A>G), GAA290GAG (970A>G), TTT291TTC (973T>C), AGA292CGA (974A>C), GAT293TCT (977G>T 978A>C), GGT294GGC (982T>C), AAA295TCA (983A>T 984A>C), ACT296TCT (986A>T), AGA297CGT (989A>C 991A>T), ATA298GTG (992A>G 994A>G), CTT300ATC (998C>A 1000T>C), TCA301ACA (1001T>A), AAT303GAT (1007A>G), CTA304CTC (1012A>C), TTA305CTT (1013T>C 1015A>T), GCA306GCT (1018A>T), AGA307CGT (1019A>C 1021A>T), GGC308GGT (1024C>T), GTT311GTA (1033T>A), CAA312CAG (1036A>G), ATA314GTC (1040A>G 1042A>C), TCT315TCC (1045T>C), TTA316CTT (1046T>C 1048A>T), GTT317GTC (1051T>C), ATC318ATT (1054C>T), AAT319AAC (1057T>C), GAT321GAC (1063T>C), TTG322CTG (1064T>C), CAT324ACT (1070C>A 1071A>C), AAT325CAA (1073A>C 1075T>A), AGA326CCA (1076A>C 1077G>C), TAT329TAC (1087T>C), ATA330CTG (1088A>C 1090A>G), AGA335CGT (1103A>C 1105A>T), GGT337GGA (1111T>A), AGA338CGA (1112A>C), GGC340GGA (1120C>A), CGA341AGG (1121C>A 1123A>G), AAA342AAG (1126A>G), GGG343GGT (1129G>T), ATT344GTT (1130A>G), AAT347AAC (1141T>C), ATA349GTC (1145A>G 1147A>C), ACT350ACC (1150T>C), GAA351AAG (1151G>A 1153A>G), GAC352GAT (1156C>T), GAA353GAT (1159A>T), TTA354GAA (1160T>G 1161T>A), CCA355AGG (1163C>A 1164C>G 1165A>G), AAG356ATG (1167A>T), GCT358TTT (1172G>T 1173C>T), GAA359GAC (1177A>C), TTA360ATA (1178T>A), GAA361CAG (1181G>C 1183A>G), ACG362AAA (1185C>A 1186G>A), TAT364TAC (1192T>C), AAT365AAC (1195T>C), ACC366GTC (1196A>G 1197C>T), AAA367GTA (1199A>G 1200A>T), ATA368ATC (1204A>C), GAT369GAG (1207T>G), GAA370GAG (1210A>G), ATG371CTC (1211A>C 1213G>C), GAG373GCC (1218A>C 1219G>C), ATT375GTT (1223A>G), GTT376GCT (1227T>C), TTG378CTC (1232T>C 1234G>C), ATA379CTG (1235A>C 1237A>G)								

*: Inserts / Deletes / Misaligned / Frameshifts

Analysis details

This analysis was performed with panviral2.64

NGS Details (UN9): Bracoviriform glomeratae (segment NC_043292.1)

Assembly

**Coverage Length**	302 (1 contig(s))
**Depth Of Coverage**	2248.3
**Number Of Reads**	6038
**Reads Per Million**	136.48 rpm (after QC)
**Ambiguities**	0
**Assembly Method**	de novo + reference guided assembly
**Consensus Caller**	Bcf Tools

Coverage Map

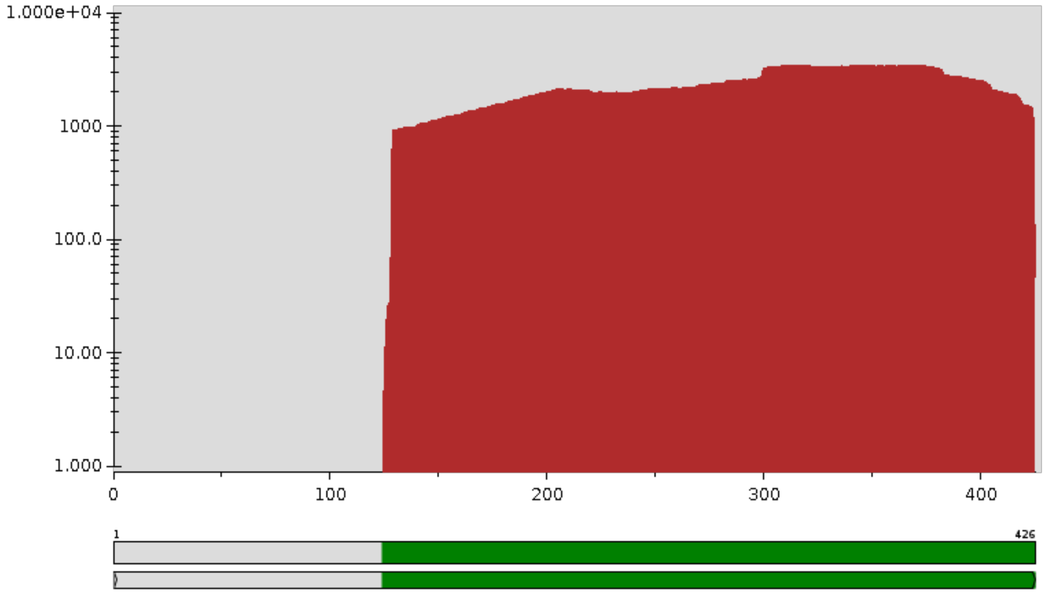

Assignment

**Type**	Bracoviriform glomeratae (Taxonomy ID: 257816)
**Reference Genome**	NC_043292.1
**NT Identity (%)**	72.1854
**AA Identity (%)**	85.0
**Number Of Stop Codons**	1
**Number Of CDS**	1

Alignment

**Alignment Score**	268.0 (NT) + 549.0 (AA) = 817.0
**Concordance (%)**	65.834

Alignment Method	Global, seeded, nucleotide + amino acids (AGA)

Genome Region

Sequence starts at position 125 and ends at position 426 relative to NC_043292.1 reference sequence.

Alignment Detailed Statistics

	Begin	End	Coverage	Score	Concordance	Matches	Identities	I/D/M/F*	Stop Codons
**NT**	**125**	**426**	**70.9%**	**268**	**44.4%**	**302 (100%)**	**218 (72.2%)**	**0/0**	
Mutations:	126G>A, 129C>A, 132A>G, 136A>G, 137C>G, 138G>C, 150A>C, 153A>G, 155C>G, 156G>A, 159C>A, 166C>A, 168T>G, 169T>C, 171T>C, 172C>A, 174C>G, 177A>G, 183T>A, 184C>A, 189T>C, 198A>G, 201A>C, 207C>T, 214A>G, 219C>T, 222T>C, 223C>A, 225T>G, 228G>T, 235C>A, 237T>G, 243A>T, 246C>G, 255C>T, 261A>G, 264A>G, 265G>A, 273A>G, 278T>C, 279T>C, 284A>G, 285T>A, 288T>G, 289T>C, 300C>T, 301C>T, 303C>G, 309C>T, 312T>G, 315C>T, 322G>T, 329T>C, 333T>C, 336C>T, 339A>G, 345A>C, 347A>G, 348A>G, 349C>A, 351T>A, 360C>T, 363C>T, 366C>T, 375C>T, 378C>T, 384A>C, 385T>C, 387G>T, 390A>G, 391C>A, 392A>G, 393T>A, 394A>C, 399C>A, 400C>A, 402T>G, 404T>C, 405G>T, 406A>C, 408C>T, 414T>A, 416A>T, 420A>G								

CDS

FK954_p501	43	142	70.4%	549	85.6%	100 (100%)	85 (85.0%)	0/0/0/0	1
Protein mutations:	T46G (136A>G 137C>G 138G>C), A52G (155C>G 156G>A), Y57H (169T>C 171T>C), T72A (214A>G), V89I (265G>A), I93T (278T>C 279T>C), D95G (284A>G 285T>A), A108S (322G>T), I110T (329T>C), K116R (347A>G 348A>G), H131R (391C>A 392A>G 393T>A), K132Q (394A>C), M135T (404T>C 405G>T), I136L (406A>C 408C>T), Y139F (416A>T)								
Codon mutations:	TTG42.TA (126G>A), GGC43GGA (129C>A), AAA44AAG (132A>G), ACG46GGC (136A>G 137C>G 138G>C), GGA50GGC (150A>C), AAA51AAG (153A>G), GCG52GGA (155C>G 156G>A), GGC53GGA (159C>A), CGT56AGG (166C>A 168T>G), TAT57CAC (169T>C 171T>C), CGC58AGG (172C>A 174C>G), AAA59AAG (177A>G), CTT61CTA (183T>A), CGA62AGA (184C>A), GAT63GAC (189T>C), CAA66CAG (198A>G), GGA67GGC (201A>C), ACC69ACT (207C>T), ACT72GCT (214A>G), ATC73ATT (219C>T), CGT74CGC (222T>C), CGT75AGG (223C>A 225T>G), CTG76CTT (228G>T), CGT79AGG (235C>A 237T>G), GGA81GGT (243A>T), GTC82GTG (246C>G), ATC85ATT (255C>T), GGA87GGG (261A>G), TTA88TTG (264A>G), GTC89ATC (265G>A), GAA91GAG (273A>G), ATT93ACC (278T>C 279T>C), GAT95GGA (284A>G 285T>A), GTT96GTG (288T>G), TTG97CTG (289T>C), TTC100TTT (300C>T), CTC101TTG (301C>T 303C>G), AAC103AAT (309C>T), GTT104GTG (312T>G), ATC105ATT (315C>T), GCT108TCT (322G>T), ATC110ACC (329T>C), TAT111TAC (333T>C), ACC112ACT (336C>T), GAA113GAG (339A>G), GCA115GCC (345A>C), AAA116AGG (347A>G 348A>G), CGT117AGA (349C>A 351T>A), GTC120GTT (360C>T), ACC121ACT (363C>T), GCC122GCT (366C>T), GTC125GTT (375C>T), GTC126GTT (378C>T), GCA128GCC (384A>C), TTG129CTT (385T>C 387G>T), AAA130AAG (390A>G), CAT131AGA (391C>A 392A>G 393T>A), AAA132CAA (394A>C), GGC133GGA (399C>A), CGT134AGG (400C>A 402T>G), ATG135ACT (404T>C 405G>T), ATC136CTT (406A>C 408C>T), GGT138GGA (414T>A), TAT139TTT (416A>T), GGA140GGG (420A>G)								

Proteins

putative histone 4 (YP_009665791.1)	43	142	70.4%	549	85.6%	100 (100%)	85 (85.0%)	0/0/0/0	1
Protein mutations:	T46G (136A>G 137C>G 138G>C), A52G (155C>G 156G>A), Y57H (169T>C 171T>C), T72A (214A>G), V89I (265G>A), I93T (278T>C 279T>C), D95G (284A>G 285T>A), A108S (322G>T), I110T (329T>C), K116R (347A>G 348A>G), H131R (391C>A 392A>G 393T>A), K132Q (394A>C), M135T (404T>C 405G>T), I136L (406A>C 408C>T), Y139F (416A>T)								
Codon mutations:	TTG42.TA (126G>A), GGC43GGA (129C>A), AAA44AAG (132A>G), ACG46GGC (136A>G 137C>G 138G>C), GGA50GGC (150A>C), AAA51AAG (153A>G), GCG52GGA (155C>G 156G>A), GGC53GGA (159C>A), CGT56AGG (166C>A 168T>G), TAT57CAC (169T>C 171T>C), CGC58AGG (172C>A 174C>G), AAA59AAG (177A>G), CTT61CTA (183T>A), CGA62AGA (184C>A), GAT63GAC (189T>C), CAA66CAG (198A>G), GGA67GGC (201A>C), ACC69ACT (207C>T), ACT72GCT (214A>G), ATC73ATT (219C>T), CGT74CGC (222T>C), CGT75AGG (223C>A 225T>G), CTG76CTT (228G>T), CGT79AGG (235C>A 237T>G), GGA81GGT (243A>T), GTC82GTG (246C>G), ATC85ATT (255C>T), GGA87GGG (261A>G), TTA88TTG (264A>G), GTC89ATC (265G>A), GAA91GAG (273A>G), ATT93ACC (278T>C 279T>C), GAT95GGA (284A>G 285T>A), GTT96GTG (288T>G), TTG97CTG (289T>C), TTC100TTT (300C>T), CTC101TTG (301C>T 303C>G), AAC103AAT (309C>T), GTT104GTG (312T>G), ATC105ATT (315C>T), GCT108TCT (322G>T), ATC110ACC (329T>C), TAT111TAC (333T>C), ACC112ACT (336C>T), GAA113GAG (339A>G), GCA115GCC (345A>C), AAA116AGG (347A>G 348A>G), CGT117AGA (349C>A 351T>A), GTC120GTT (360C>T), ACC121ACT (363C>T), GCC122GCT (366C>T), GTC125GTT (375C>T), GTC126GTT (378C>T), GCA128GCC (384A>C), TTG129CTT (385T>C 387G>T), AAA130AAG (390A>G), CAT131AGA (391C>A 392A>G 393T>A), AAA132CAA (394A>C), GGC133GGA (399C>A), CGT134AGG (400C>A 402T>G), ATG135ACT (404T>C 405G>T), ATC136CTT (406A>C 408C>T), GGT138GGA (414T>A), TAT139TTT (416A>T), GGA140GGG (420A>G)								

*: Inserts / Deletes / Misaligned / Frameshifts

Analysis details

This analysis was performed with panviral2.64

NGS Details (UN9): Potato leafroll virus

Assembly

**Coverage Length**	5825 (1 contig(s))
**Depth Of Coverage**	104.8
**Number Of Reads**	4587
**Reads Per Million**	103.68 rpm (after QC)
**Ambiguities**	0
**Assembly Method**	read mapping against reference + variant calling
**Consensus Caller**	Bcf Tools

Coverage Map

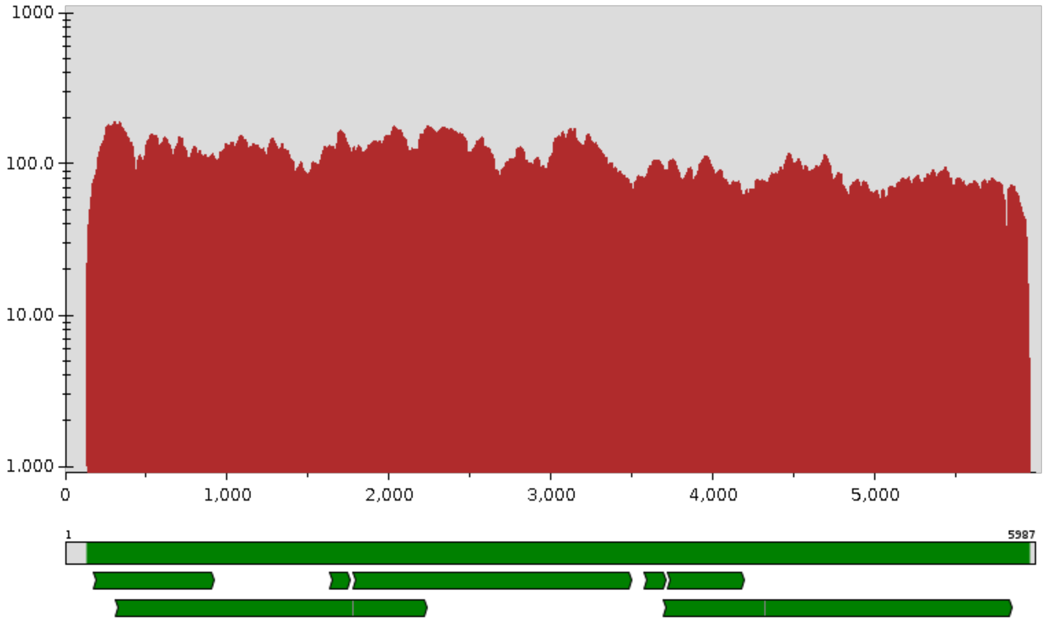

Assignment

**Type**	Potato leafroll virus (Taxonomy ID: 12045)
**Reference Genome**	NC_001747.1
**NT Identity (%)**	97.6481
**AA Identity (%)**	96.6091
**Number Of Stop Codons**	8
**Number Of CDS**	8

Alignment

**Alignment Score**	11084.0 (NT) + 20290.0 (AA) = 31374.0
**Concordance (%)**	95.6095

Alignment Method	Global, seeded, nucleotide + amino acids (AGA)

Genome Region

Sequence starts at position 135 and ends at position 5959 relative to NC_001747.1 reference sequence.

Alignment Detailed Statistics

	Begin	End	Coverage	Score	Concordance	Matches	Identities	I/D/M/F*	Stop Codons
**NT**	**135**	**5959**	**97.3%**	**11084**	**95.3%**	**5823 (99.9%)**	**5688 (97.6%)**	**2/2**	
Mutations:	191A>C, 221T>C, 228C>T, 269T>C, 276G>A, 300C>T, 343C>T, 462T>A, 476_477insTT, 479_480delTT, 492T>C, 525C>T, 561C>T, 600T>C, 665A>G, 697C>A, 699A>C, 822C>T, 825T>G, 922G>A, 931A>T, 958G>A, 1054C>T, 1132T>G, 1147C>T, 1171C>T, 1183T>C, 1204A>G, 1319G>A, 1356G>A, 1381T>C, 1411T>G, 1434A>G, 1465C>T, 1474G>A, 1534G>A, 1577G>T, 1593G>A, 1611A>G, 1618C>T, 1648T>C, 1662G>A, 1701A>G, 1716A>G, 1755T>C, 1757A>G, 1774T>C, 1814G>T, 1830C>T, 1894C>A, 1924G>A, 2007T>C, 2021A>G, 2047G>A, 2064A>G, 2079C>T, 2154C>T, 2187C>T, 2265A>G, 2283G>A, 2316T>G, 2400C>T, 2433T>C, 2470G>A, 2550G>A, 2574C>T, 2580A>G, 2586A>G, 2673C>T, 2704C>T, 2729A>G, 2850A>G, 2856G>A, 2869G>A, 2877C>T, 2898T>C, 2931T>C, 3000T>C, 3033C>T, 3036C>T, 3054T>C, 3096C>T, 3105C>T, 3117C>T, 3195C>T, 3321C>T, 3379A>G, 3420A>G, 3437A>G, 3441C>T, 3619C>T, 3652T>A, 3748T>G, 3750C>A, 3777G>T, 3788T>A, 3939A>T, 4013C>T, 4031A>G, 4034C>T, 4132A>T, 4151T>C, 4214T>G, 4265T>C, 4280C>T, 4379G>A, 4385C>T, 4421A>G, 4430G>A, 4433C>T, 4511T>C, 4520G>T, 4521T>G, 4661T>C, 4700C>T, 4733C>G, 4736A>G, 4817T>C, 4835C>T, 4883C>T, 4886T>C, 4949G>A, 4964A>G, 4979C>T, 5062C>T, 5072C>T, 5095G>A, 5129G>A, 5162C>T, 5375T>G, 5385G>A, 5432T>C, 5493G>A, 5517A>G, 5527C>T, 5530T>C, 5925T>C								
**CDS**									
PLRVgp1	1	248	100%	1428	82.4%	248 (99.6%)	241 (96.8%)	1/0/2/2	1
Protein mutations:	Q6P (191A>C), F16S (221T>C), L32P (269T>C), H57Y (343C>T), G101_L102insX (476_477insTT), Q164R (665A>G), Q175N (697C>A 699A>C)								
Codon mutations:	CAG6CCG (191A>C), TTT16TCT (221T>C), CTC18CTT (228C>T), CTT32CCT (269T>C), CTG34CTA (276G>A), GGC42GGT (300C>T), CAT57TAT (343C>T), GCT96GCA (462T>A), GGC101GGT (476_477insTT), GGC101_CTT102insT-C (476_477insTT), CTT102C-- (479_480delTT), ATT106ATC (492T>C), TAC117TAT (525C>T), TAC129TAT (561C>T), CAT142CAC (600T>C), CAA164CGA (665A>G), CAA175AAC (697C>A 699A>C), CGC216CGT (822C>T), GCT217GCG (825T>G)								
PLRVgp2	1	1063	100%	7004	93.9%	1063 (99.9%)	1029 (96.7%)	1/0/2/2	1
Protein mutations:	L52Q (462T>A), W56_A56insV (476_477insTT), L61S (492T>C), T72I (525C>T), T84I (561C>T), M97T (600T>C), K119E (665A>G), N130T (699A>C), A171V (822C>T), L172R (825T>G), E207D (931A>T), A337T (1319G>A), G349E (1356G>A), E375G (1434A>G), V423F (1577G>T), R428K (1593G>A), D434G (1611A>G), R451K (1662G>A), Q464R (1701A>G), E469G (1716A>G), L482P (1755T>C), T483A (1757A>G), W489R (1774T>C), R502L (1814G>T), Q529K (1894C>A), E539K (1924G>A), N571S (2021A>G), E580K (2047G>A), A721T (2470G>A), N807S (2729A>G), A854T (2869G>A), I1024V (3379A>G), E1043G (3437A>G)								
Codon mutations:	TTC12TTT (343C>T), CTA52CAA (462T>A), TGG56_GCC56insGTT (476_477insTT), TTA57--A (479_480delTT), TTA61TCA (492T>C), ACA72ATA (525C>T), ACA84ATA (561C>T), ATG97ACG (600T>C), AAA119GAA (665A>G), ATC129ATA (697C>A), AAC130ACC (699A>C), GCG171GTG (822C>T), CTA172CGA (825T>G), AGG204AGA (922G>A), GAA207GAT (931A>T), CAG216CAA (958G>A), GCC248GCT (1054C>T), ACT274ACG (1132T>G), TTC279TTT (1147C>T), TCC287TCT (1171C>T), GGT291GGC (1183T>C), CTA298CTG (1204A>G), GCC337ACC (1319G>A), GGA349GAA (1356G>A), TTT357TTC (1381T>C), CTT367CTG (1411T>G), GAG375GGG (1434A>G), CCC385CCT (1465C>T), CCG388CCA (1474G>A), TCG408TCA (1534G>A), GTC423TTC (1577G>T), AGA428AAA (1593G>A), GAC434GGC (1611A>G), AAC436AAT (1618C>T), GAT446GAC (1648T>C), AGA451AAA (1662G>A), CAA464CGA (1701A>G), GAG469GGG (1716A>G), CTA482CCA (1755T>C), ACA483GCA (1757A>G), AAT488AAC (1774T>C), TGG489CGG (1774T>C), CGA502CTA (1814G>T), CGC507CGT (1830C>T), CAA529AAA (1894C>A), GAG539AAG (1924G>A), TTT566TTC (2007T>C), AAC571AGC (2021A>G), GAA580AAA (2047G>A), CCA585CCG (2064A>G), TGC590TGT (2079C>T), CAC615CAT (2154C>T), GGC626GGT (2187C>T), TCA652TCG (2265A>G), GCG658GCA (2283G>A), GTT669GTG (2316T>G), ATC697ATT (2400C>T), GCT708GCC (2433T>C), GCA721ACA (2470G>A), TCG747TCA (2550G>A), AGC755AGT (2574C>T), GAA757GAG (2580A>G), CTA759CTG (2586A>G), CGC788CGT (2673C>T), CTG799TTG (2704C>T), AAT807AGT (2729A>G), CCA847CCG (2850A>G), GTG849GTA (2856G>A), GCA854ACA (2869G>A), CAC856CAT (2877C>T), ACT863ACC (2898T>C), TAT874TAC (2931T>C), CGT897CGC (3000T>C), AAC908AAT (3033C>T), TCC909TCT (3036C>T), GAT915GAC (3054T>C), AGC929AGT (3096C>T), TAC932TAT (3105C>T), TCC936TCT (3117C>T), CTC962CTT (3195C>T), AAC1004AAT (3321C>T), ATT1024GTT (3379A>G), GAA1037GAG (3420A>G), GAG1043GGG (3437A>G), CTC1044CTT (3441C>T)								
PLRVgp3	1	640	100%	3875	90.3%	640 (99.8%)	609 (95.0%)	1/0/2/2	1
Protein mutations:	L52Q (462T>A), W56_A56insV (476_477insTT), L61S (492T>C), T72I (525C>T), T84I (561C>T), M97T (600T>C), K119E (665A>G), N130T (699A>C), A171V (822C>T), L172R (825T>G), E207D (931A>T), A337T (1319G>A), G349E (1356G>A), E375G (1434A>G), V423F (1577G>T), R428K (1593G>A), D434G (1611A>G), R451K (1662G>A), Q464R (1701A>G), E469G (1716A>G), L482P (1755T>C), T483A (1757A>G), D502Y (1814G>T), A507V (1830C>T), L566S (2007T>C), T571A (2021A>G), Q585R (2064A>G), A590V (2079C>T), T615I (2154C>T), A626V (2187C>T)								
Codon mutations:	TTC12TTT (343C>T), CTA52CAA (462T>A), TGG56_GCC56insGTT (476_477insTT), TTA57--A (479_480delTT), TTA61TCA (492T>C), ACA72ATA (525C>T), ACA84ATA (561C>T), ATG97ACG (600T>C), AAA119GAA (665A>G), ATC129ATA (697C>A), AAC130ACC (699A>C), GCG171GTG (822C>T), CTA172CGA (825T>G), AGG204AGA (922G>A), GAA207GAT (931A>T), CAG216CAA (958G>A), GCC248GCT (1054C>T), ACT274ACG (1132T>G), TTC279TTT (1147C>T), TCC287TCT (1171C>T), GGT291GGC (1183T>C), CTA298CTG (1204A>G), GCC337ACC (1319G>A), GGA349GAA (1356G>A), TTT357TTC (1381T>C), CTT367CTG (1411T>G), GAG375GGG (1434A>G), CCC385CCT (1465C>T), CCG388CCA (1474G>A), TCG408TCA (1534G>A), GTC423TTC (1577G>T), AGA428AAA (1593G>A), GAC434GGC (1611A>G), AAC436AAT (1618C>T), GAT446GAC (1648T>C), AGA451AAA (1662G>A), CAA464CGA (1701A>G), GAG469GGG (1716A>G), CTA482CCA (1755T>C), ACA483GCA (1757A>G), AAT488AAC (1774T>C), GAC502TAC (1814G>T), GCT507GTT (1830C>T), ATC528ATA (1894C>A), GTG538GTA (1924G>A), TTA566TCA (2007T>C), ACG571GCG (2021A>G), AAG579AAA (2047G>A), CAG585CGG (2064A>G), GCG590GTG (2079C>T), ACC615ATC (2154C>T), GCT626GTT (2187C>T)								
Rap1	1	42	100%	257	89.5%	42 (100%)	37 (88.1%)	0/0/0/0	0
Protein mutations:	I6T (1648T>C), E11K (1662G>A), K24E (1701A>G), R29G (1716A>G), *42Q (1755T>C 1757A>G)								
Codon mutations:	ATT6ACT (1648T>C), GAG11AAG (1662G>A), AAA24GAA (1701A>G), AGA29GGA (1716A>G), TAA42CAG (1755T>C 1757A>G)								
ORF3a	1	46	100%	291	97.3%	46 (100%)	45 (97.8%)	0/0/0/0	1
Protein mutations:	F26L (3652T>A)								
Codon mutations:	TCC15TCT (3619C>T), TTT26TTA (3652T>A)								
PLRVgp4	1	718	100%	4966	98.6%	718 (100%)	704 (98.1%)	0/0/0/0	2
Protein mutations:	M19R (3748T>G), A29S (3777G>T), T83S (3939A>T), Y147F (4132A>T), K276N (4520G>T), F277V (4521T>G), A457V (5062C>T), G468E (5095G>A), N561K (5375T>G), E565K (5385G>A), V601I (5493G>A), K609E (5517A>G), S612L (5527C>T), L613P (5530T>C)								
Codon mutations:	ATG19AGG (3748T>G), CGA20AGA (3750C>A), GCT29TCT (3777G>T), GTT32GTA (3788T>A), ACC83TCC (3939A>T), TAC107TAT (4013C>T), ACA113ACG (4031A>G), AGC114AGT (4034C>T), TAC147TTC (4132A>T), ATT153ATC (4151T>C), TCT174TCG (4214T>G), GAT191GAC (4265T>C), TTC196TTT (4280C>T), CAG229CAA (4379G>A), CAC231CAT (4385C>T), CTA243CTG (4421A>G), CAG246CAA (4430G>A), GCC247GCT (4433C>T), AAT273AAC (4511T>C), AAG276AAT (4520G>T), TTT277GTT (4521T>G), GGT323GGC (4661T>C), TCC336TCT (4700C>T), GTC347GTG (4733C>G), AAA348AAG (4736A>G), GGT375GGC (4817T>C), GAC381GAT (4835C>T), TTC397TTT (4883C>T), CTT398CTC (4886T>C), ACG419ACA (4949G>A), GAA424GAG (4964A>G), ACC429ACT (4979C>T), GCC457GTC (5062C>T), CAC460CAT (5072C>T), GGA468GAA (5095G>A), ACG479ACA (5129G>A), GAC490GAT (5162C>T), AAT561AAG (5375T>G), GAA565AAA (5385G>A), ACT580ACC (5432T>C), GTC601ATC (5493G>A), AAG609GAG (5517A>G), TCA612TTA (5527C>T), CTT613CCT (5530T>C)								
PLRVgp5	1	209	100%	1417	98.7%	209 (100%)	205 (98.1%)	0/0/0/0	1
Protein mutations:	M19R (3748T>G), A29S (3777G>T), T83S (3939A>T), Y147F (4132A>T)								
Codon mutations:	ATG19AGG (3748T>G), CGA20AGA (3750C>A), GCT29TCT (3777G>T), GTT32GTA (3788T>A), ACC83TCC (3939A>T), TAC107TAT (4013C>T), ACA113ACG (4031A>G), AGC114AGT (4034C>T), TAC147TTC (4132A>T), ATT153ATC (4151T>C), TCT174TCG (4214T>G), GAT191GAC (4265T>C), TTC196TTT (4280C>T)								
PLRVgp6	1	157	100%	1052	95.5%	157 (100%)	150 (95.5%)	0/0/0/0	1
Protein mutations:	C11G (3748T>G 3750C>A), F24Y (3788T>A), T99I (4013C>T), Q105R (4031A>G), A106V (4034C>T), T139S (4132A>T), L145S (4151T>C)								
Codon mutations:	TGC11GGA (3748T>G 3750C>A), GCG20GCT (3777G>T), TTC24TAC (3788T>A), ACA74ACT (3939A>T), ACC99ATC (4013C>T), CAA105CGA (4031A>G), GCA106GTA (4034C>T), ACG139TCG (4132A>T), TTA145TCA (4151T>C)								

	Begin	End	Coverage	Score	Concordance	Matches	Identities	I/D/M/F*	Stop Codons
**NT**	**135**	**5959**	**97.3%**	**11084**	**95.3%**	**5823 (99.9%)**	**5688 (97.6%)**	**2/2**	

Proteins

	Begin	End	Coverage	Score	Concordance	Matches	Identities	I/D/M/F*	Stop Codons
P0 protein (NP_056746.1)	1	248	100%	1428	82.4%	248 (99.6%)	241 (96.8%)	1/0/2/2	1
Protein mutations:	Q6P (191A>C), F16S (221T>C), L32P (269T>C), H57Y (343C>T), G101_L102insX (476_477insTT), Q164R (665A>G), Q175N (697C>A 699A>C)								
Codon mutations:	CAG6CCG (191A>C), TTT16TCT (221T>C), CTC18CTT (228C>T), CTT32CCT (269T>C), CTG34CTA (276G>A), GGC42GGT (300C>T), CAT57TAT (343C>T), GCT96GCA (462T>A), GGC101GGT (476_477insTT), GGC101_CTT102insT-C (476_477insTT), CTT102C-- (479_480delTT), ATT106ATC (492T>C), TAC117TAT (525C>T), TAC129TAT (561C>T), CAT142CAC (600T>C), CAA164CGA (665A>G), CAA175AAC (697C>A 699A>C), CGC216CGT (822C>T), GCT217GCG (825T>G)								
RNA-dependent RNA polymerase (NP_056748.3)	1	1063	100%	7004	93.9%	1063 (99.9%)	1029 (96.7%)	1/0/2/2	1
Protein mutations:	L52Q (462T>A), W56_A56insV (476_477insTT), L61S (492T>C), T72I (525C>T), T84I (561C>T), M97T (600T>C), K119E (665A>G), N130T (699A>C), A171V (822C>T), L172R (825T>G), E207D (931A>T), A337T (1319G>A), G349E (1356G>A), E375G (1434A>G), V423F (1577G>T), R428K (1593G>A), D434G (1611A>G), R451K (1662G>A), Q464R (1701A>G), E469G (1716A>G), L482P (1755T>C), T483A (1757A>G), W489R (1774T>C), R502L (1814G>T), Q529K (1894C>A), E539K (1924G>A), N571S (2021A>G), E580K (2047G>A), A721T (2470G>A), N807S (2729A>G), A854T (2869G>A), I1024V (3379A>G), E1043G (3437A>G)								
Codon mutations:	TTC12TTT (343C>T), CTA52CAA (462T>A), TGG56_GCC56insGTT (476_477insTT), TTA57--A (479_480delTT), TTA61TCA (492T>C), ACA72ATA (525C>T), ACA84ATA (561C>T), ATG97ACG (600T>C), AAA119GAA (665A>G), ATC129ATA (697C>A), AAC130ACC (699A>C), GCG171GTG (822C>T), CTA172CGA (825T>G), AGG204AGA (922G>A), GAA207GAT (931A>T), CAG216CAA (958G>A), GCC248GCT (1054C>T), ACT274ACG (1132T>G), TTC279TTT (1147C>T), TCC287TCT (1171C>T), GGT291GGC (1183T>C), CTA298CTG (1204A>G), GCC337ACC (1319G>A), GGA349GAA (1356G>A), TTT357TTC (1381T>C), CTT367CTG (1411T>G), GAG375GGG (1434A>G), CCC385CCT (1465C>T), CCG388CCA (1474G>A), TCG408TCA (1534G>A), GTC423TTC (1577G>T), AGA428AAA (1593G>A), GAC434GGC (1611A>G), AAC436AAT (1618C>T), GAT446GAC (1648T>C), AGA451AAA (1662G>A), CAA464CGA (1701A>G), GAG469GGG (1716A>G), CTA482CCA (1755T>C), ACA483GCA (1757A>G), AAT488AAC (1774T>C), TGG489CGG (1774T>C), CGA502CTA (1814G>T), CGC507CGT (1830C>T), CAA529AAA (1894C>A), GAG539AAG (1924G>A), TTT566TTC (2007T>C), AAC571AGC (2021A>G), GAA580AAA (2047G>A), CCA585CCG (2064A>G), TGC590TGT (2079C>T), CAC615CAT (2154C>T), GGC626GGT (2187C>T), TCA652TCG (2265A>G), GCG658GCA (2283G>A), GTT669GTG (2316T>G), ATC697ATT (2400C>T), GCT708GCC (2433T>C), GCA721ACA (2470G>A), TCG747TCA (2550G>A), AGC755AGT (2574C>T), GAA757GAG (2580A>G), CTA759CTG (2586A>G), CGC788CGT (2673C>T), CTG799TTG (2704C>T), AAT807AGT (2729A>G), CCA847CCG (2850A>G), GTG849GTA (2856G>A), GCA854ACA (2869G>A), CAC856CAT (2877C>T), ACT863ACC (2898T>C), TAT874TAC (2931T>C), CGT897CGC (3000T>C), AAC908AAT (3033C>T), TCC909TCT (3036C>T), GAT915GAC (3054T>C), AGC929AGT (3096C>T), TAC932TAT (3105C>T), TCC936TCT (3117C>T), CTC962CTT (3195C>T), AAC1004AAT (3321C>T), ATT1024GTT (3379A>G), GAA1037GAG (3420A>G), GAG1043GGG (3437A>G), CTC1044CTT (3441C>T)								
P1 protein (NP_056747.1)	1	640	100%	3875	90.3%	640 (99.8%)	609 (95.0%)	1/0/2/2	1
Protein mutations:	L52Q (462T>A), W56_A56insV (476_477insTT), L61S (492T>C), T72I (525C>T), T84I (561C>T), M97T (600T>C), K119E (665A>G), N130T (699A>C), A171V (822C>T), L172R (825T>G), E207D (931A>T), A337T (1319G>A), G349E (1356G>A), E375G (1434A>G), V423F (1577G>T), R428K (1593G>A), D434G (1611A>G), R451K (1662G>A), Q464R (1701A>G), E469G (1716A>G), L482P (1755T>C), T483A (1757A>G), D502Y (1814G>T), A507V (1830C>T), L566S (2007T>C), T571A (2021A>G), Q585R (2064A>G), A590V (2079C>T), T615I (2154C>T), A626V (2187C>T)								
Codon mutations:	TTC12TTT (343C>T), CTA52CAA (462T>A), TGG56_GCC56insGTT (476_477insTT), TTA57--A (479_480delTT), TTA61TCA (492T>C), ACA72ATA (525C>T), ACA84ATA (561C>T), ATG97ACG (600T>C), AAA119GAA (665A>G), ATC129ATA (697C>A), AAC130ACC (699A>C), GCG171GTG (822C>T), CTA172CGA (825T>G), AGG204AGA (922G>A), GAA207GAT (931A>T), CAG216CAA (958G>A), GCC248GCT (1054C>T), ACT274ACG (1132T>G), TTC279TTT (1147C>T), TCC287TCT (1171C>T), GGT291GGC (1183T>C), CTA298CTG (1204A>G), GCC337ACC (1319G>A), GGA349GAA (1356G>A), TTT357TTC (1381T>C), CTT367CTG (1411T>G), GAG375GGG (1434A>G), CCC385CCT (1465C>T), CCG388CCA (1474G>A), TCG408TCA (1534G>A), GTC423TTC (1577G>T), AGA428AAA (1593G>A), GAC434GGC (1611A>G), AAC436AAT (1618C>T), GAT446GAC (1648T>C), AGA451AAA (1662G>A), CAA464CGA (1701A>G), GAG469GGG (1716A>G), CTA482CCA (1755T>C), ACA483GCA (1757A>G), AAT488AAC (1774T>C), GAC502TAC (1814G>T), GCT507GTT (1830C>T), ATC528ATA (1894C>A), GTG538GTA (1924G>A), TTA566TCA (2007T>C), ACG571GCG (2021A>G), AAG579AAA (2047G>A), CAG585CGG (2064A>G), GCG590GTG (2079C>T), ACC615ATC (2154C>T), GCT626GTT (2187C>T)								
Replication-associated protein (YP_006355442.1)	1	42	100%	257	89.5%	42 (100%)	37 (88.1%)	0/0/0/0	0
Protein mutations:	I6T (1648T>C), E11K (1662G>A), K24E (1701A>G), R29G (1716A>G), *42Q (1755T>C 1757A>G)								
Codon mutations:	ATT6ACT (1648T>C), GAG11AAG (1662G>A), AAA24GAA (1701A>G), AGA29GGA (1716A>G), TAA42CAG (1755T>C 1757A>G)								
protein 3a (YP_009179365.2)	1	46	100%	291	97.3%	46 (100%)	45 (97.8%)	0/0/0/0	1
Protein mutations:	F26L (3652T>A)								
Codon mutations:	TCC15TCT (3619C>T), TTT26TTA (3652T>A)								
CP read-through protein (NP_056751.2)	1	718	100%	4966	98.6%	718 (100%)	704 (98.1%)	0/0/0/0	2
Protein mutations:	M19R (3748T>G), A29S (3777G>T), T83S (3939A>T), Y147F (4132A>T), K276N (4520G>T), F277V (4521T>G), A457V (5062C>T), G468E (5095G>A), N561K (5375T>G), E565K (5385G>A), V601I (5493G>A), K609E (5517A>G), S612L (5527C>T), L613P (5530T>C)								
Codon mutations:	ATG19AGG (3748T>G), CGA20AGA (3750C>A), GCT29TCT (3777G>T), GTT32GTA (3788T>A), ACC83TCC (3939A>T), TAC107TAT (4013C>T), ACA113ACG (4031A>G), AGC114AGT (4034C>T), TAC147TTC (4132A>T), ATT153ATC (4151T>C), TCT174TCG (4214T>G), GAT191GAC (4265T>C), TTC196TTT (4280C>T), CAG229CAA (4379G>A), CAC231CAT (4385C>T), CTA243CTG (4421A>G), CAG246CAA (4430G>A), GCC247GCT (4433C>T), AAT273AAC (4511T>C), AAG276AAT (4520G>T), TTT277GTT (4521T>G), GGT323GGC (4661T>C), TCC336TCT (4700C>T), GTC347GTG (4733C>G), AAA348AAG (4736A>G), GGT375GGC (4817T>C), GAC381GAT (4835C>T), TTC397TTT (4883C>T), CTT398CTC (4886T>C), ACG419ACA (4949G>A), GAA424GAG (4964A>G), ACC429ACT (4979C>T), GCC457GTC (5062C>T), CAC460CAT (5072C>T), GGA468GAA (5095G>A), ACG479ACA (5129G>A), GAC490GAT (5162C>T), AAT561AAG (5375T>G), GAA565AAA (5385G>A), ACT580ACC (5432T>C), GTC601ATC (5493G>A), AAG609GAG (5517A>G), TCA612TTA (5527C>T), CTT613CCT (5530T>C)								
coat protein (NP_056749.1)	1	209	100%	1417	98.7%	209 (100%)	205 (98.1%)	0/0/0/0	1
Protein mutations:	M19R (3748T>G), A29S (3777G>T), T83S (3939A>T), Y147F (4132A>T)								
Codon mutations:	ATG19AGG (3748T>G), CGA20AGA (3750C>A), GCT29TCT (3777G>T), GTT32GTA (3788T>A), ACC83TCC (3939A>T), TAC107TAT (4013C>T), ACA113ACG (4031A>G), AGC114AGT (4034C>T), TAC147TTC (4132A>T), ATT153ATC (4151T>C), TCT174TCG (4214T>G), GAT191GAC (4265T>C), TTC196TTT (4280C>T)								
movement protein (NP_056750.1)	1	157	100%	1052	95.5%	157 (100%)	150 (95.5%)	0/0/0/0	1
Protein mutations:	C11G (3748T>G 3750C>A), F24Y (3788T>A), T99I (4013C>T), Q105R (4031A>G), A106V (4034C>T), T139S (4132A>T), L145S (4151T>C)								
Codon mutations:	TGC11GGA (3748T>G 3750C>A), GCG20GCT (3777G>T), TTC24TAC (3788T>A), ACA74ACT (3939A>T), ACC99ATC (4013C>T), CAA105CGA (4031A>G), GCA106GTA (4034C>T), ACG139TCG (4132A>T), TTA145TCA (4151T>C)								

*: Inserts / Deletes / Misaligned / Frameshifts

Analysis details

This analysis was performed with panviral2.64

NGS Details (UN9): Solendovirus venanicotianae

Assembly

**Coverage Length**	2217 (4 contig(s))
**Depth Of Coverage**	11.9
**Number Of Reads**	233
**Reads Per Million**	5.27 rpm (after QC)
**Ambiguities**	0
**Assembly Method**	de novo + reference guided assembly
**Consensus Caller**	Bcf Tools

Coverage Map

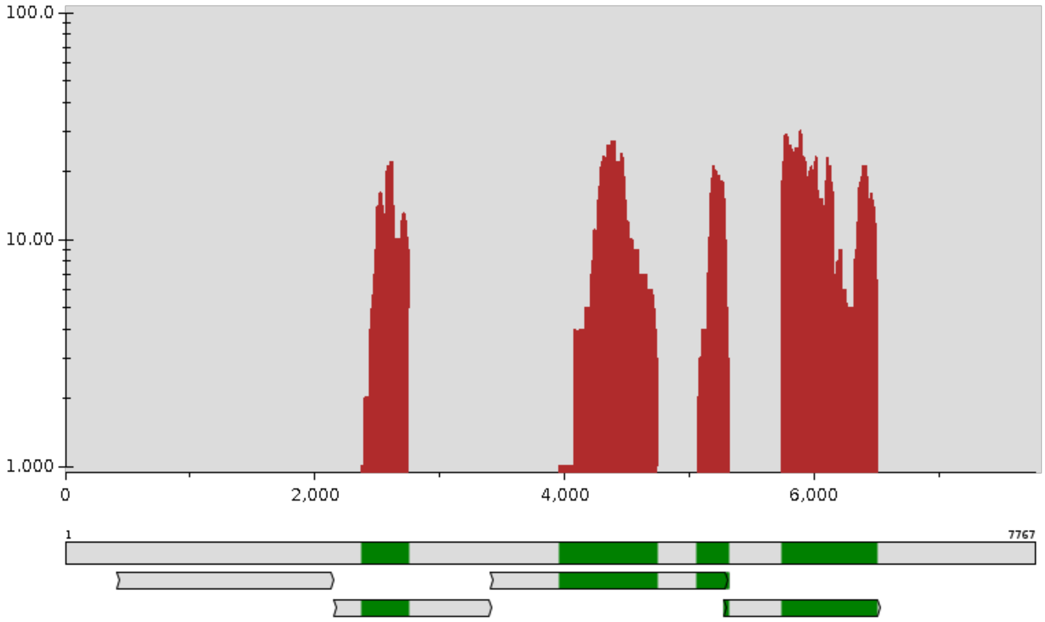

Assignment

**Type**	Solendovirus venanicotianae (Taxonomy ID: 3048371)
**Reference Genome**	NC_003378.1
**NT Identity (%)**	79.8918
**AA Identity (%)**	76.2599
**Number Of Stop Codons**	5
**Number Of CDS**	4

Alignment

**Alignment Score**	2644.0 (NT) + 3921.0 (AA) = 6565.0
**Concordance (%)**	68.8715

**Alignment Method**	Global, seeded, nucleotide + amino acids (AGA)

Genome Region

Sequence starts at position 2369 and ends at position 6509 relative to NC_003378.1 reference sequence.

Alignment Detailed Statistics

	Begin	End	Coverage	Score	Concordance	Matches	Identities	I/D/M/F*	Stop Codons
**NT**	**2369**	**6509**	**28.5%**	**2644**	**59.6%**	**2217 (99.9%)**	**1772 (79.9%)**	**1/0**	
Mutations:	2373T>A, 2376T>C, 2379C>T, 2388A>T, 2389G>A, 2390G>A, 2394T>A, 2397G>A, 2403A>T, 2410A>C, 2412A>T, 2417A>C, 2418C>T, 2420A>G, 2423G>A, 2425A>T, 2428A>G, 2433T>C, 2434A>C, 2443C>A, 2454G>A, 2461A>G, 2463C>A, 2469T>A, 2472G>A, 2481C>T, 2482T>C, 2493T>C, 2500T>C, 2501T>A, 2505A>T, 2535C>A, 2538T>G, 2545A>C, 2551C>T, 2565A>G, 2569A>G, 2571C>A, 2572C>G, 2589C>T, 2602A>G, 2613T>C, 2622T>C, 2628G>A, 2637T>C, 2638A>G, 2643A>C, 2646C>T, 2647G>C, 2649C>A, 2665C>T, 2668A>T, 2669A>C, 2670T>A, 2674A>G, 2675G>A, 2685T>A, 2688A>T, 2691A>T, 2700A>T, 2709G>A, 2712G>A, 2715A>G, 2722G>A, 2724G>A, 2725T>C, 2730A>T, 2733C>A, 2734G>T, 2738G>C, 2743G>A, 2748T>C, 2754C>A, 3957A>G, 3962T>A, 3983T>C, 3986A>G, 3990A>C, 3993G>A, 4013A>C, 4016G>A, 4019T>C, 4022T>C, 4037A>G, 4040T>A, 4045A>G, 4056A>G, 4059G>A, 4061T>C, 4065A>G, 4066C>A, 4076A>G, 4086A>G, 4088A>T, 4091T>A, 4100T>A, 4103T>A, 4104A>G, 4105A>G, 4115A>G, 4116G>A, 4127C>T, 4181A>T, 4187C>T, 4190G>A, 4196C>T, 4199A>T, 4212T>C, 4235T>C, 4241T>C, 4250C>T, 4265G>A, 4286T>C, 4292C>T, 4307C>T, 4313G>A, 4325T>C, 4328T>C, 4329T>C, 4331G>A, 4334A>G, 4340G>A, 4343T>A, 4353A>C, 4355A>G, 4362G>A, 4364T>A, 4388T>C, 4391A>G, 4393G>T, 4400T>A, 4403A>G, 4404C>T, 4411G>A, 4417A>G, 4421T>C, 4436C>T, 4451C>T, 4467C>T, 4470G>C, 4471A>C, 4475T>C, 4478T>C, 4482G>A, 4484A>T, 4497A>G, 4500C>A, 4503C>T, 4508T>C, 4513G>A, 4517A>T, 4518C>A, 4523T>A, 4530A>T, 4540T>C, 4545A>C, 4550C>T, 4551G>A, 4552C>T, 4554C>A, 4559T>A, 4560A>G, 4568C>A, 4571T>A, 4574T>A, 4581T>C, 4591C>A, 4592C>G, 4598A>T, 4601A>G, 4604T>A, 4605A>T, 4607G>T, 4626T>C, 4643T>C, 4653A>G, 4660T>C, 4665A>C, 4666C>A, 4667T>A, 4679A>G, 4687T>C, 4688C>A, 4691T>C, 4692C>T, 4693T>C, 4694T>A, 4697T>A, 4701A>G, 4703C>A, 4704A>T, 4706T>A, 4709T>C, 4721A>G, 4734C>T, 4740A>T, 5077T>A, 5078T>G, 5079G>T, 5080C>T, 5081A>G, 5094C>T, 5097T>C, 5102A>C, 5117A>T, 5120T>G, 5125C>A, 5126C>T, 5128G>A, 5132T>C, 5135T>A, 5136G>A, 5147C>T, 5156A>G, 5174T>A, 5180A>G, 5192A>G, 5195C>T, 5211C>A, 5217C>T, 5219G>A, 5237C>T, 5253G>A, 5256A>G, 5261T>A, 5263A>G, 5273G>A, 5280G>A, 5283G>A, 5286G>T, 5291T>C, 5295T>G, 5301C>T, 5310C>A, 5313A>G, 5745T>C, 5754T>A, 5755A>C, 5756C>A, 5757C>A, 5760T>C, 5763A>T, 5766C>A, 5781A>C, 5784G>A, 5786A>C, 5796T>A, 5797A>C, 5798A>T, 5799A>C, 5801A>C, 5802T>G, 5811C>T, 5812C>A, 5813A>G, 5814A>C, 5815T>A, 5820C>A, 5821C>G, 5826T>A, 5831A>C, 5838C>T, 5844T>A, 5847C>T, 5850C>A, 5851C>A, 5853T>A, 5854C>T, 5857A>C, 5858C>A, 5872T>C, 5874G>A, 5877A>T, 5880T>A, 5881T>C, 5882T>A, 5889T>A, 5892A>T, 5894G>A, 5897C>T, 5898A>C, 5901A>T, 5907A>T, 5908G>A, 5909C>G, 5910C>A, 5913C>A, 5916C>T, 5921A>G, 5932C>T, 5935G>A, 5940T>C, 5950C>T, 5952G>T, 5953A>G, 5954C>A, 5955A>C, 5959C>G, 5964G>A, 5968G>T, 5970C>A, 5971A>G, 5972A>C, 5979T>A, 5982G>A, 5983T>C, 5988C>T, 5991G>A, 5997C>T, 5999T>C, 6000G>A, 6001C>A, 6002A>C, 6009G>A, 6012G>A, 6015A>T, 6018T>A, 6024G>C, 6026C>A, 6027A>C, 6033C>G, 6036C>T, 6037G>A, 6040C>A, 6041G>A, 6042A>G, 6045C>T, 6049T>A, 6050C>T, 6051G>A, 6053C>T, 6054T>A, 6055A>C, 6059C>T, 6067C>T, 6071A>T, 6078G>A, 6078_6079insA, 6081A>T, 6084G>A, 6086C>T, 6087T>A, 6088A>G, 6090C>G, 6102C>T, 6108T>A, 6110G>A, 6114C>A, 6118A>C, 6120G>A, 6123A>T, 6129C>T, 6132A>T, 6135A>C, 6139A>G, 6141G>A, 6144A>T, 6154G>A, 6163G>A, 6164A>C, 6170T>A, 6172A>G, 6174T>G, 6176T>G, 6177T>A, 6180A>T, 6182A>T, 6183A>T, 6186C>T, 6201G>A, 6204A>T, 6211A>T, 6219T>A, 6222A>C, 6223C>A, 6225A>T, 6240C>T, 6243C>T, 6247C>A, 6253C>G, 6255G>A, 6259G>C, 6260C>T, 6264T>G, 6269G>T, 6273T>C, 6276T>C, 6277T>A, 6282G>A, 6285A>C, 6288G>A, 6290C>T, 6308G>C, 6309C>A, 6311G>A, 6315T>G, 6320G>T, 6321A>C, 6325G>A, 6326C>G, 6340C>G, 6341T>C, 6344G>T, 6345A>G, 6352G>A, 6354C>A, 6355T>A, 6357A>G, 6359G>C, 6360T>A, 6361C>T, 6363T>G, 6369A>T, 6375A>G, 6376C>A, 6380C>A, 6381T>A, 6382A>C, 6384A>T, 6386C>T, 6387C>T, 6401G>A, 6402G>A, 6404G>A, 6406A>G, 6407A>G, 6408T>A, 6411C>T, 6414C>T, 6417C>G, 6418C>G, 6419C>A, 6420A>T, 6423T>A, 6424C>T, 6426G>A, 6427T>C, 6446T>A, 6451A>G, 6453T>G, 6456C>A, 6462T>C, 6471C>T, 6477C>T, 6480A>G, 6489A>C								
**CDS**									
Tvvgp2	73	201	30.7%	701	81.3%	129 (100%)	100 (77.5%)	0/0/0/0	1
Protein mutations:	G79K (2389G>A 2390G>A), K83N (2403A>T), I86L (2410A>C 2412A>T), N88T (2417A>C 2418C>T), K89R (2420A>G), R90K (2423G>A), I91L (2425A>T), I92V (2428A>G), K94Q (2434A>C), Q97K (2443C>A), N103E (2461A>G 2463C>A), L116Q (2500T>C 2501T>A), I131L (2545A>C), I139V (2569A>G 2571C>A), Q140E (2572C>G), I150V (2602A>G), I162V (2638A>G), A165P (2647G>C 2649C>A), N172S (2668A>T 2669A>C 2670T>A), R174E (2674A>G 2675G>A), D177E (2685T>A), K178N (2688A>T), L182F (2700A>T), M186I (2712G>A), E190K (2722G>A 2724G>A), G194* (2734G>T), S195T (2738G>C), V197I (2743G>A)								
Codon mutations:	ATT73ATA (2373T>A), GAT74GAC (2376T>C), TGC75TGT (2379C>T), ACA78ACT (2388A>T), GGA79AAA (2389G>A 2390G>A), GGT80GGA (2394T>A), AAG81AAA (2397G>A), AAA83AAT (2403A>T), ATA86CTT (2410A>C 2412A>T), AAC88ACT (2417A>C 2418C>T), AAA89AGA (2420A>G), AGA90AAA (2423G>A), ATA91TTA (2425A>T), ATA92GTA (2428A>G), AAT93AAC (2433T>C), AAA94CAA (2434A>C), CAA97AAA (2443C>A), AAG100AAA (2454G>A), AAC103GAA (2461A>G 2463C>A), ATT105ATA (2469T>A), AAG106AAA (2472G>A), CAC109CAT (2481C>T), TTA110CTA (2482T>C), ACT113ACC (2493T>C), TTA116CAA (2500T>C 2501T>A), ATA117ATT (2505A>T), ACC127ACA (2535C>A), CCT128CCG (2538T>G), ATA131CTA (2545A>C), CTA133TTA (2551C>T), AGA137AGG (2565A>G), ATC139GTA (2569A>G 2571C>A), CAA140GAA (2572C>G), AGC145AGT (2589C>T), ATA150GTA (2602A>G), AAT153AAC (2613T>C), TAT156TAC (2622T>C), AAG158AAA (2628G>A), TTT161TTC (2637T>C), ATA162GTA (2638A>G), ATA163ATC (2643A>C), AGC164AGT (2646C>T), GCC165CCA (2647G>C 2649C>A), CTA171TTA (2665C>T), AAT172TCA (2668A>T 2669A>C 2670T>A), AGA174GAA (2674A>G 2675G>A), GAT177GAA (2685T>A), AAA178AAT (2688A>T), TCA179TCT (2691A>T), TTA182TTT (2700A>T), AAG185AAA (2709G>A), ATG186ATA (2712G>A), TCA187TCG (2715A>G), GAG190AAA (2722G>A 2724G>A), TTA191CTA (2725T>C), GCA192GCT (2730A>T), CCC193CCA (2733C>A), GGA194TGA (2734G>T), AGT195ACT (2738G>C), GTA197ATA (2743G>A), TTT198TTC (2748T>C), GCC200GCA (2754C>A)								
Tvvgp3	183	636	55.0%	2017	83.4%	350 (100%)	288 (82.3%)	0/0/0/0	3
Protein mutations:	N183D (3957A>G), K194Q (3990A>C), V195I (3993G>A), E201D (4013A>C), K212R (4045A>G), N216D (4056A>G), D217N (4059G>A 4061T>C), T219E (4065A>G 4066C>A), K226D (4086A>G 4088A>T), D227E (4091T>A), D230E (4100T>A), N231K (4103T>A), K232G (4104A>G 4105A>G), E236K (4116G>A), D311E (4343T>A), K315Q (4353A>C 4355A>G), A318T (4362G>A 4364T>A), W328L (4393G>T), P332S (4404C>T), G334E (4411G>A), K336R (4417A>G), E354P (4470G>C 4471A>C), V358I (4482G>A 4484A>T), I363V (4497A>G), L364I (4500C>A), R368K (4513G>A), Q370K (4518C>A), D371E (4523T>A), I374L (4530A>T), L377S (4540T>C), K379Q (4545A>C), A381I (4551G>A 4552C>T), H382N (4554C>A), I384V (4560A>G), N386K (4568C>A), T394K (4591C>A 4592C>G), M399F (4605A>T 4607G>T), I415V (4653A>G), M417T (4660T>C), T419Q (4665A>C 4666C>A 4667T>A), I426T (4687T>C 4688C>A), L428S (4692C>T 4693T>C 4694T>A), D429E (4697T>A), N431E (4701A>G 4703C>A), I432L (4704A>T 4706T>A), I444L (4740A>T), F556* (5077T>A 5078T>G), A557L (5079G>T 5080C>T 5081A>G), T572N (5125C>A 5126C>T), R573K (5128G>A), V576I (5136G>A), E615K (5253G>A), I616V (5256A>G), N618S (5263A>G), V624I (5280G>A), A625T (5283G>A), D626Y (5286G>T), S629A (5295T>G), Q631* (5301C>T), P634T (5310C>A), N635D (5313A>G)								
Codon mutations:	AAC183GAC (3957A>G), CCT184CCA (3962T>A), CAT191CAC (3983T>C), AAA192AAG (3986A>G), AAA194CAA (3990A>C), GTA195ATA (3993G>A), GAA201GAC (4013A>C), GAG202GAA (4016G>A), AAT203AAC (4019T>C), AGT204AGC (4022T>C), AAA209AAG (4037A>G), CCT210CCA (4040T>A), AAA212AGA (4045A>G), AAT216GAT (4056A>G), GAT217AAC (4059G>A 4061T>C), ACA219GAA (4065A>G 4066C>A), AAA222AAG (4076A>G), AAA226GAT (4086A>G 4088A>T), GAT227GAA (4091T>A), GAT230GAA (4100T>A), AAT231AAA (4103T>A), AAA232GGA (4104A>G 4105A>G), CAA235CAG (4115A>G), GAA236AAA (4116G>A), AGC239AGT (4127C>T), GGA257GGT (4181A>T), AGC259AGT (4187C>T), AGG260AGA (4190G>A), GTC262GTT (4196C>T), ATA263ATT (4199A>T), TTA268CTA (4212T>C), TAT275TAC (4235T>C), TAT277TAC (4241T>C), CCC280CCT (4250C>T), AAG285AAA (4265G>A), TAT292TAC (4286T>C), TAC294TAT (4292C>T), GAC299GAT (4307C>T), AAG301AAA (4313G>A), TAT305TAC (4325T>C), CAT306CAC (4328T>C), TTG307CTA (4329T>C 4331G>A), AAA308AAG (4334A>G), GAG310GAA (4340G>A), GAT311GAA (4343T>A), AAA315CAG (4353A>C 4355A>G), GCT318ACA (4362G>A 4364T>A), TAT326TAC (4388T>C), GAA327GAG (4391A>G), TGG328TTG (4393G>T), GTT330GTA (4400T>A), TTA331TTG (4403A>G), CCA332TCA (4404C>T), GGA334GAA (4411G>A), AAA336AGA (4417A>G), AAT337AAC (4421T>C), TAC342TAT (4436C>T), GAC347GAT (4451C>T), CTA353TTA (4467C>T), GAA354CCA (4470G>C 4471A>C), AAT355AAC (4475T>C), TGT356TGC (4478T>C), GTA358ATT (4482G>A 4484A>T), ATA363GTA (4497A>G), CTA364ATA (4500C>A), CTA365TTA (4503C>T), TAT366TAC (4508T>C), AGA368AAA (4513G>A), ACA369ACT (4517A>T), CAA370AAA (4518C>A), GAT371GAA (4523T>A), ATA374TTA (4530A>T), TTA377TCA (4540T>C), AAA379CAA (4545A>C), TTC380TTT (4550C>T), GCA381ATA (4551G>A 4552C>T), CAT382AAT (4554C>A), ATT383ATA (4559T>A), ATA384GTA (4560A>G), AAC386AAA (4568C>A), TCT387TCA (4571T>A), GGT388GGA (4574T>A), TTA391CTA (4581T>C), ACC394AAG (4591C>A 4592C>G), GCA396GCT (4598A>T), GAA397GAG (4601A>G), ATT398ATA (4604T>A), ATG399TTT (4605A>T 4607G>T), TTA406CTA (4626T>C), GAT411GAC (4643T>C), ATA415GTA (4653A>G), ATG417ACG (4660T>C), ACT419CAA (4665A>C 4666C>A 4667T>A), CAA423CAG (4679A>G), ATC426ACA (4687T>C 4688C>A), AAT427AAC (4691T>C), CTT428TCA (4692C>T 4693T>C 4694T>A), GAT429GAA (4697T>A), AAC431GAA (4701A>G 4703C>A), ATT432TTA (4704A>T 4706T>A), GAT433GAC (4709T>C), AAA437AAG (4721A>G), CTA442TTA (4734C>T), ATA444TTA (4740A>T), TTT556TAG (5077T>A 5078T>G), GCA557TTG (5079G>T 5080C>T 5081A>G), CTA562TTA (5094C>T), TTA563CTA (5097T>C), GCA564GCC (5102A>C), ATA569ATT (5117A>T), GTT570GTG (5120T>G), ACC572AAT (5125C>A 5126C>T), AGA573AAA (5128G>A), TTT574TTC (5132T>C), ATT575ATA (5135T>A), GTA576ATA (5136G>A), GAC579GAT (5147C>T), CAA582CAG (5156A>G), ACT588ACA (5174T>A), AAA590AAG (5180A>G), TCA594TCG (5192A>G), GTC595GTT (5195C>T), CGG601AGG (5211C>A), CTG603TTA (5217C>T 5219G>A), AAC609AAT (5237C>T), GAA615AAA (5253G>A), ATA616GTA (5256A>G), ATT617ATA (5261T>A), AAT618AGT (5263A>G), AAG621AAA (5273G>A), GTT624ATT (5280G>A), GCA625ACA (5283G>A), GAT626TAT (5286G>T), TAT627TAC (5291T>C), TCA629GCA (5295T>G), CAA631TAA (5301C>T), CCA634ACA (5310C>A), AAC635GAC (5313A>G)								
Tvvgp4	1	412	65.6%	1203	63.0%	274 (99.6%)	187 (68.0%)	1/0/1/1	1
Protein mutations:	Q4H (5286G>T), I6T (5291T>C), Y7* (5295T>G), T161Q (5755A>C 5756C>A 5757C>A), D164E (5766C>A), N171T (5786A>C), N174K (5796T>A), K175L (5797A>C 5798A>T 5799A>C), N176T (5801A>C 5802T>G), Q180S (5812C>A 5813A>G 5814A>C), S181T (5815T>A), Q183E (5821C>G), N184K (5826T>A), N186T (5831A>C), H192Q (5850C>A), H193K (5851C>A 5853T>A), T195Q (5857A>C 5858C>A), L203Q (5881T>C 5882T>A), N205K (5889T>A), S207N (5894G>A), A208V (5897C>T 5898A>C), K209N (5901A>T), A212R (5908G>A 5909C>G 5910C>A), N216S (5921A>G), D221N (5935G>A), Q226Y (5950C>T 5952G>T), T227D (5953A>G 5954C>A 5955A>C), Q229E (5959C>G), A232S (5968G>T 5970C>A), N233A (5971A>G 5972A>C), M242T (5999T>C 6000G>A), Q243T (6001C>A 6002A>C), T251N (6026C>A 6027A>C), F253L (6033C>G), V255I (6037G>A), R256K (6040C>A 6041G>A 6042A>G), S259I (6049T>A 6050C>T 6051G>A), A260V (6053C>T 6054T>A), T261P (6055A>C), A262V (6059C>T), Y266F (6071A>T), E268_I269insX (6078_6079insA), P271L (6086C>T 6087T>A), I272V (6088A>G 6090C>G), G279E (6110G>A), E283D (6123A>T), K289E (6139A>G 6141G>A), E294K (6154G>A), E297T (6163G>A 6164A>C), V299E (6170T>A), N300E (6172A>G 6174T>G), I301R (6176T>G 6177T>A), E303V (6182A>T 6183A>T), I313L (6211A>T), L317I (6223C>A 6225A>T), L325I (6247C>A), Q327E (6253C>G 6255G>A), A329L (6259G>C 6260C>T), I330M (6264T>G), S332I (6269G>T), S335T (6277T>A), E337D (6285A>C), T339I (6290C>T), C345S (6308G>C 6309C>A), R346K (6311G>A), D347E (6315T>G), R349I (6320G>T 6321A>C), A351R (6325G>A 6326C>G), L356A (6340C>G 6341T>C), R357M (6344G>T 6345A>G), V360I (6352G>A 6354C>A), L361M (6355T>A 6357A>G), S362T (6359G>C 6360T>A), K365N (6369A>T), Q368K (6376C>A), P369Q (6380C>A 6381T>A), T370P (6382A>C 6384A>T), T371I (6386C>T 6387C>T), R376K (6401G>A 6402G>A), R377K (6404G>A), N378G (6406A>G 6407A>G 6408T>A), P382D (6418C>G 6419C>A 6420A>T), D383E (6423T>A), L391Q (6446T>A), S393G (6451A>G 6453T>G), H394Q (6456C>A), K405N (6489A>C)								

	Begin	End	Coverage	Score	Concordance	Matches	Identities	I/D/M/F*	Stop Codons
**NT**	**2369**	**6509**	**28.5%**	**2644**	**59.6%**	**2217 (99.9%)**	**1772 (79.9%)**	**1/0**	
Codon mutations:	TTG2TTA (5280G>A), TTG3TTA (5283G>A), CAG4CAT (5286G>T), ATT6ACT (5291T>C), TAT7TAG (5295T>G), GAC9GAT (5301C>T), ACC12ACA (5310C>A), CAA13CAG (5313A>G), CAT157CAC (5745T>C), CGT160CGA (5754T>A), ACC161CAA (5755A>C 5756C>A 5757C>A), TAT162TAC (5760T>C), ATA163ATT (5763A>T), GAC164GAA (5766C>A), ATA169ATC (5781A>C), CAG170CAA (5784G>A), AAT171ACT (5786A>C), AAT174AAA (5796T>A), AAA175CTC (5797A>C 5798A>T 5799A>C), AAT176ACG (5801A>C 5802T>G), TCC179TCT (5811C>T), CAA180AGC (5812C>A 5813A>G 5814A>C), TCT181ACT (5815T>A), ACC182ACA (5820C>A), CAA183GAA (5821C>G), AAT184AAA (5826T>A), AAC186ACC (5831A>C), GAC188GAT (5838C>T), ATT190ATA (5844T>A), ACC191ACT (5847C>T), CAC192CAA (5850C>A), CAT193AAA (5851C>A 5853T>A), CTA194TTA (5854C>T), ACA195CAA (5857A>C 5858C>A), TTG200CTA (5872T>C 5874G>A), ATA201ATT (5877A>T), GCT202GCA (5880T>A), TTA203CAA (5881T>C 5882T>A), AAT205AAA (5889T>A), ACA206ACT (5892A>T), AGT207AAT (5894G>A), GCA208GTC (5897C>T 5898A>C), AAA209AAT (5901A>T), GTA211GTT (5907A>T), GCC212AGA (5908G>A 5909C>G 5910C>A), ACC213ACA (5913C>A), TGC214TGT (5916C>T), AAC216AGC (5921A>G), CTA220TTA (5932C>T), GAT221AAT (5935G>A), ACT222ACC (5940T>C), CAG226TAT (5950C>T 5952G>T), ACA227GAC (5953A>G 5954C>A 5955A>C), CAA229GAA (5959C>G), GAG230GAA (5964G>A), GCC232TCA (5968G>T 5970C>A), AAT233GCT (5971A>G 5972A>C), CCT235CCA (5979T>A), GAG236GAA (5982G>A), TTA237CTA (5983T>C), TAC238TAT (5988C>T), AAG239AAA (5991G>A), TTC241TTT (5997C>T), ATG242ACA (5999T>C 6000G>A), CAA243ACA (6001C>A 6002A>C), AAG245AAA (6009G>A), AGG246AGA (6012G>A), ATA247ATT (6015A>T), ACT248ACA (6018T>A), GGG250GGC (6024G>C), ACA251AAC (6026C>A 6027A>C), TTC253TTG (6033C>G), TAC254TAT (6036C>T), GTA255ATA (6037G>A), CGA256AAG (6040C>A 6041G>A 6042A>G), TTC257TTT (6045C>T), TCG259ATA (6049T>A 6050C>T 6051G>A), GCT260GTA (6053C>T 6054T>A), ACA261CCA (6055A>C), GCA262GTA (6059C>T), CTA265TTA (6067C>T), TAT266TTT (6071A>T), GAG268GAA (6078G>A), GAG268_ATA269insA-- (6078_6079insA), ATA269ATT (6081A>T), AAG270AAA (6084G>A), CCT271CTA (6086C>T 6087T>A), ATC272GTG (6088A>G 6090C>G), ATC276ATT (6102C>T), ATT278ATA (6108T>A), GGA279GAA (6110G>A), CTC280CTA (6114C>A), AGG282CGA (6118A>C 6120G>A), GAA283GAT (6123A>T), ATC285ATT (6129C>T), ATA286ATT (6132A>T), CCA287CCC (6135A>C), AAG289GAA (6139A>G 6141G>A), ATA290ATT (6144A>T), GAA294AAA (6154G>A), GAA297ACA (6163G>A 6164A>C), GTA299GAA (6170T>A), AAT300GAG (6172A>G 6174T>G), ATT301AGA (6176T>G 6177T>A), CCA302CCT (6180A>T), GAA303GTT (6182A>T 6183A>T), TTC304TTT (6186C>T), AGG309AGA (6201G>A), ATA310ATT (6204A>T), ATA313TTA (6211A>T), ACT315ACA (6219T>A), ATA316ATC (6222A>C), CTA317ATT (6223C>A 6225A>T), AAC322AAT (6240C>T), AAC323AAT (6243C>T), CTA325ATA (6247C>A), CAG327GAA (6253C>G 6255G>A), GCA329CTA (6259G>C 6260C>T), ATT330ATG (6264T>G), AGC332ATC (6269G>T), TAT333TAC (6273T>C), TAT334TAC (6276T>C), TCA335ACA (6277T>A), AGG336AGA (6282G>A), GAA337GAC (6285A>C), CAG338CAA (6288G>A), ACA339ATA (6290C>T), TGC345TCA (6308G>C 6309C>A), AGA346AAA (6311G>A), GAT347GAG (6315T>G), AGA349ATC (6320G>T 6321A>C), GCA351AGA (6325G>A 6326C>G), CTA356GCA (6340C>G 6341T>C), AGA357ATG (6344G>T 6345A>G), GTC360ATA (6352G>A 6354C>A), TTA361ATG (6355T>A 6357A>G), AGT362ACA (6359G>C 6360T>A), CTT363TTG (6361C>T 6363T>G), AAA365AAT (6369A>T), GAA367GAG (6375A>G), CAA368AAA (6376C>A), CCT369CAA (6380C>A 6381T>A), ACA370CCT (6382A>C 6384A>T), ACC371ATT (6386C>T 6387C>T), AGG376AAA (6401G>A 6402G>A), AGG377AAG (6404G>A), AAT378GGA (6406A>G 6407A>G 6408T>A), TTC379TTT (6411C>T), ATC380ATT (6414C>T), TCC381TCG (6417C>G), CCA382GAT (6418C>G 6419C>A 6420A>T), GAT383GAA (6423T>A), CTG384TTA (6424C>T 6426G>A), TTA385CTA (6427T>C), CTA391CAA (6446T>A), AGT393GGG (6451A>G 6453T>G), CAC394CAA (6456C>A), TAT396TAC (6462T>C), CAC399CAT (6471C>T), TGC401TGT (6477C>T), TCA402TCG (6480A>G), AAA405AAC (6489A>C)								
**Proteins**									
putative cell-to-cell movement protein (NP_569140.1)	73	201	30.7%	701	81.3%	129 (100%)	100 (77.5%)	0/0/0/0	1
Protein mutations:	G79K (2389G>A 2390G>A), K83N (2403A>T), I86L (2410A>C 2412A>T), N88T (2417A>C 2418C>T), K89R (2420A>G), R90K (2423G>A), I91L (2425A>T), I92V (2428A>G), K94Q (2434A>C), Q97K (2443C>A), N103E (2461A>G 2463C>A), L116Q (2500T>C 2501T>A), I131L (2545A>C), I139V (2569A>G 2571C>A), Q140E (2572C>G), I150V (2602A>G), I162V (2638A>G), A165P (2647G>C 2649C>A), N172S (2668A>T 2669A>C 2670T>A), R174E (2674A>G 2675G>A), D177E (2685T>A), K178N (2688A>T), L182F (2700A>T), M186I (2712G>A), E190K (2722G>A 2724G>A), G194* (2734G>T), S195T (2738G>C), V197I (2743G>A)								
Codon mutations:	ATT73ATA (2373T>A), GAT74GAC (2376T>C), TGC75TGT (2379C>T), ACA78ACT (2388A>T), GGA79AAA (2389G>A 2390G>A), GGT80GGA (2394T>A), AAG81AAA (2397G>A), AAA83AAT (2403A>T), ATA86CTT (2410A>C 2412A>T), AAC88ACT (2417A>C 2418C>T), AAA89AGA (2420A>G), AGA90AAA (2423G>A), ATA91TTA (2425A>T), ATA92GTA (2428A>G), AAT93AAC (2433T>C), AAA94CAA (2434A>C), CAA97AAA (2443C>A), AAG100AAA (2454G>A), AAC103GAA (2461A>G 2463C>A), ATT105ATA (2469T>A), AAG106AAA (2472G>A), CAC109CAT (2481C>T), TTA110CTA (2482T>C), ACT113ACC (2493T>C), TTA116CAA (2500T>C 2501T>A), ATA117ATT (2505A>T), ACC127ACA (2535C>A), CCT128CCG (2538T>G), ATA131CTA (2545A>C), CTA133TTA (2551C>T), AGA137AGG (2565A>G), ATC139GTA (2569A>G 2571C>A), CAA140GAA (2572C>G), AGC145AGT (2589C>T), ATA150GTA (2602A>G), AAT153AAC (2613T>C), TAT156TAC (2622T>C), AAG158AAA (2628G>A), TTT161TTC (2637T>C), ATA162GTA (2638A>G), ATA163ATC (2643A>C), AGC164AGT (2646C>T), GCC165CCA (2647G>C 2649C>A), CTA171TTA (2665C>T), AAT172TCA (2668A>T 2669A>C 2670T>A), AGA174GAA (2674A>G 2675G>A), GAT177GAA (2685T>A), AAA178AAT (2688A>T), TCA179TCT (2691A>T), TTA182TTT (2700A>T), AAG185AAA (2709G>A), ATG186ATA (2712G>A), TCA187TCG (2715A>G), GAG190AAA (2722G>A 2724G>A), TTA191CTA (2725T>C), GCA192GCT (2730A>T), CCC193CCA (2733C>A), GGA194TGA (2734G>T), AGT195ACT (2738G>C), GTA197ATA (2743G>A), TTT198TTC (2748T>C), GCC200GCA (2754C>A)								
polyprotein (NP_569141.1)	183	636	55.0%	2017	83.4%	350 (100%)	288 (82.3%)	0/0/0/0	3
Protein mutations:	N183D (3957A>G), K194Q (3990A>C), V195I (3993G>A), E201D (4013A>C), K212R (4045A>G), N216D (4056A>G), D217N (4059G>A 4061T>C), T219E (4065A>G 4066C>A), K226D (4086A>G 4088A>T), D227E (4091T>A), D230E (4100T>A), N231K (4103T>A), K232G (4104A>G 4105A>G), E236K (4116G>A), D311E (4343T>A), K315Q (4353A>C 4355A>G), A318T (4362G>A 4364T>A), W328L (4393G>T), P332S (4404C>T), G334E (4411G>A), K336R (4417A>G), E354P (4470G>C 4471A>C), V358I (4482G>A 4484A>T), I363V (4497A>G), L364I (4500C>A), R368K (4513G>A), Q370K (4518C>A), D371E (4523T>A), I374L (4530A>T), L377S (4540T>C), K379Q (4545A>C), A381I (4551G>A 4552C>T), H382N (4554C>A), I384V (4560A>G), N386K (4568C>A), T394K (4591C>A 4592C>G), M399F (4605A>T 4607G>T), I415V (4653A>G), M417T (4660T>C), T419Q (4665A>C 4666C>A 4667T>A), I426T (4687T>C 4688C>A), L428S (4692C>T 4693T>C 4694T>A), D429E (4697T>A), N431E (4701A>G 4703C>A), I432L (4704A>T 4706T>A), I444L (4740A>T), F556* (5077T>A 5078T>G), A557L (5079G>T 5080C>T 5081A>G), T572N (5125C>A 5126C>T), R573K (5128G>A), V576I (5136G>A), E615K (5253G>A), I616V (5256A>G), N618S (5263A>G), V624I (5280G>A), A625T (5283G>A), D626Y (5286G>T), S629A (5295T>G), Q631* (5301C>T), P634T (5310C>A), N635D (5313A>G)								
Codon mutations:	AAC183GAC (3957A>G), CCT184CCA (3962T>A), CAT191CAC (3983T>C), AAA192AAG (3986A>G), AAA194CAA (3990A>C), GTA195ATA (3993G>A), GAA201GAC (4013A>C), GAG202GAA (4016G>A), AAT203AAC (4019T>C), AGT204AGC (4022T>C), AAA209AAG (4037A>G), CCT210CCA (4040T>A), AAA212AGA (4045A>G), AAT216GAT (4056A>G), GAT217AAC (4059G>A 4061T>C), ACA219GAA (4065A>G 4066C>A), AAA222AAG (4076A>G), AAA226GAT (4086A>G 4088A>T), GAT227GAA (4091T>A), GAT230GAA (4100T>A), AAT231AAA (4103T>A), AAA232GGA (4104A>G 4105A>G), CAA235CAG (4115A>G), GAA236AAA (4116G>A), AGC239AGT (4127C>T), GGA257GGT (4181A>T), AGC259AGT (4187C>T), AGG260AGA (4190G>A), GTC262GTT (4196C>T), ATA263ATT (4199A>T), TTA268CTA (4212T>C), TAT275TAC (4235T>C), TAT277TAC (4241T>C), CCC280CCT (4250C>T), AAG285AAA (4265G>A), TAT292TAC (4286T>C), TAC294TAT (4292C>T), GAC299GAT (4307C>T), AAG301AAA (4313G>A), TAT305TAC (4325T>C), CAT306CAC (4328T>C), TTG307CTA (4329T>C 4331G>A), AAA308AAG (4334A>G), GAG310GAA (4340G>A), GAT311GAA (4343T>A), AAA315CAG (4353A>C 4355A>G), GCT318ACA (4362G>A 4364T>A), TAT326TAC (4388T>C), GAA327GAG (4391A>G), TGG328TTG (4393G>T), GTT330GTA (4400T>A), TTA331TTG (4403A>G), CCA332TCA (4404C>T), GGA334GAA (4411G>A), AAA336AGA (4417A>G), AAT337AAC (4421T>C), TAC342TAT (4436C>T), GAC347GAT (4451C>T), CTA353TTA (4467C>T), GAA354CCA (4470G>C 4471A>C), AAT355AAC (4475T>C), TGT356TGC (4478T>C), GTA358ATT (4482G>A 4484A>T), ATA363GTA (4497A>G), CTA364ATA (4500C>A), CTA365TTA (4503C>T), TAT366TAC (4508T>C), AGA368AAA (4513G>A), ACA369ACT (4517A>T), CAA370AAA (4518C>A), GAT371GAA (4523T>A), ATA374TTA (4530A>T), TTA377TCA (4540T>C), AAA379CAA (4545A>C), TTC380TTT (4550C>T), GCA381ATA (4551G>A 4552C>T), CAT382AAT (4554C>A), ATT383ATA (4559T>A), ATA384GTA (4560A>G), AAC386AAA (4568C>A), TCT387TCA (4571T>A), GGT388GGA (4574T>A), TTA391CTA (4581T>C), ACC394AAG (4591C>A 4592C>G), GCA396GCT (4598A>T), GAA397GAG (4601A>G), ATT398ATA (4604T>A), ATG399TTT (4605A>T 4607G>T), TTA406CTA (4626T>C), GAT411GAC (4643T>C), ATA415GTA (4653A>G), ATG417ACG (4660T>C), ACT419CAA (4665A>C 4666C>A 4667T>A), CAA423CAG (4679A>G), ATC426ACA (4687T>C 4688C>A), AAT427AAC (4691T>C), CTT428TCA (4692C>T 4693T>C 4694T>A), GAT429GAA (4697T>A), AAC431GAA (4701A>G 4703C>A), ATT432TTA (4704A>T 4706T>A), GAT433GAC (4709T>C), AAA437AAG (4721A>G), CTA442TTA (4734C>T), ATA444TTA (4740A>T), TTT556TAG (5077T>A 5078T>G), GCA557TTG (5079G>T 5080C>T 5081A>G), CTA562TTA (5094C>T), TTA563CTA (5097T>C), GCA564GCC (5102A>C), ATA569ATT (5117A>T), GTT570GTG (5120T>G), ACC572AAT (5125C>A 5126C>T), AGA573AAA (5128G>A), TTT574TTC (5132T>C), ATT575ATA (5135T>A), GTA576ATA (5136G>A), GAC579GAT (5147C>T), CAA582CAG (5156A>G), ACT588ACA (5174T>A), AAA590AAG (5180A>G), TCA594TCG (5192A>G), GTC595GTT (5195C>T), CGG601AGG (5211C>A), CTG603TTA (5217C>T 5219G>A), AAC609AAT (5237C>T), GAA615AAA (5253G>A), ATA616GTA (5256A>G), ATT617ATA (5261T>A), AAT618AGT (5263A>G), AAG621AAA (5273G>A), GTT624ATT (5280G>A), GCA625ACA (5283G>A), GAT626TAT (5286G>T), TAT627TAC (5291T>C), TCA629GCA (5295T>G), CAA631TAA (5301C>T), CCA634ACA (5310C>A), AAC635GAC (5313A>G)								
putative transactivator factor (NP_569142.1)	1	412	65.6%	1203	63.0%	274 (99.6%)	187 (68.0%)	1/0/1/1	1
Protein mutations:	Q4H (5286G>T), I6T (5291T>C), Y7* (5295T>G), T161Q (5755A>C 5756C>A 5757C>A), D164E (5766C>A), N171T (5786A>C), N174K (5796T>A), K175L (5797A>C 5798A>T 5799A>C), N176T (5801A>C 5802T>G), Q180S (5812C>A 5813A>G 5814A>C), S181T (5815T>A), Q183E (5821C>G), N184K (5826T>A), N186T (5831A>C), H192Q (5850C>A), H193K (5851C>A 5853T>A), T195Q (5857A>C 5858C>A), L203Q (5881T>C 5882T>A), N205K (5889T>A), S207N (5894G>A), A208V (5897C>T 5898A>C), K209N (5901A>T), A212R (5908G>A 5909C>G 5910C>A), N216S (5921A>G), D221N (5935G>A), Q226Y (5950C>T 5952G>T), T227D (5953A>G 5954C>A 5955A>C), Q229E (5959C>G), A232S (5968G>T 5970C>A), N233A (5971A>G 5972A>C), M242T (5999T>C 6000G>A), Q243T (6001C>A 6002A>C), T251N (6026C>A 6027A>C), F253L (6033C>G), V255I (6037G>A), R256K (6040C>A 6041G>A 6042A>G), S259I (6049T>A 6050C>T 6051G>A), A260V (6053C>T 6054T>A), T261P (6055A>C), A262V (6059C>T), Y266F (6071A>T), E268_I269insX (6078_6079insA), P271L (6086C>T 6087T>A), I272V (6088A>G 6090C>G), G279E (6110G>A), E283D (6123A>T), K289E (6139A>G 6141G>A), E294K (6154G>A), E297T (6163G>A 6164A>C), V299E (6170T>A), N300E (6172A>G 6174T>G), I301R (6176T>G 6177T>A), E303V (6182A>T 6183A>T), I313L (6211A>T), L317I (6223C>A 6225A>T), L325I (6247C>A), Q327E (6253C>G 6255G>A), A329L (6259G>C 6260C>T), I330M (6264T>G), S332I (6269G>T), S335T (6277T>A), E337D (6285A>C), T339I (6290C>T), C345S (6308G>C 6309C>A), R346K (6311G>A), D347E (6315T>G), R349I (6320G>T 6321A>C), A351R (6325G>A 6326C>G), L356A (6340C>G 6341T>C), R357M (6344G>T 6345A>G), V360I (6352G>A 6354C>A), L361M (6355T>A 6357A>G), S362T (6359G>C 6360T>A), K365N (6369A>T), Q368K (6376C>A), P369Q (6380C>A 6381T>A), T370P (6382A>C 6384A>T), T371I (6386C>T 6387C>T), R376K (6401G>A 6402G>A), R377K (6404G>A), N378G (6406A>G 6407A>G 6408T>A), P382D (6418C>G 6419C>A 6420A>T), D383E (6423T>A), L391Q (6446T>A), S393G (6451A>G 6453T>G), H394Q (6456C>A), K405N (6489A>C)								

	Begin	End	Coverage	Score	Concordance	Matches	Identities	I/D/M/F*	Stop Codons
**NT**	**2369**	**6509**	**28.5%**	**2644**	**59.6%**	**2217 (99.9%)**	**1772 (79.9%)**	**1/0**	
Codon mutations:	TTG2TTA (5280G>A), TTG3TTA (5283G>A), CAG4CAT (5286G>T), ATT6ACT (5291T>C), TAT7TAG (5295T>G), GAC9GAT (5301C>T), ACC12ACA (5310C>A), CAA13CAG (5313A>G), CAT157CAC (5745T>C), CGT160CGA (5754T>A), ACC161CAA (5755A>C 5756C>A 5757C>A), TAT162TAC (5760T>C), ATA163ATT (5763A>T), GAC164GAA (5766C>A), ATA169ATC (5781A>C), CAG170CAA (5784G>A), AAT171ACT (5786A>C), AAT174AAA (5796T>A), AAA175CTC (5797A>C 5798A>T 5799A>C), AAT176ACG (5801A>C 5802T>G), TCC179TCT (5811C>T), CAA180AGC (5812C>A 5813A>G 5814A>C), TCT181ACT (5815T>A), ACC182ACA (5820C>A), CAA183GAA (5821C>G), AAT184AAA (5826T>A), AAC186ACC (5831A>C), GAC188GAT (5838C>T), ATT190ATA (5844T>A), ACC191ACT (5847C>T), CAC192CAA (5850C>A), CAT193AAA (5851C>A 5853T>A), CTA194TTA (5854C>T), ACA195CAA (5857A>C 5858C>A), TTG200CTA (5872T>C 5874G>A), ATA201ATT (5877A>T), GCT202GCA (5880T>A), TTA203CAA (5881T>C 5882T>A), AAT205AAA (5889T>A), ACA206ACT (5892A>T), AGT207AAT (5894G>A), GCA208GTC (5897C>T 5898A>C), AAA209AAT (5901A>T), GTA211GTT (5907A>T), GCC212AGA (5908G>A 5909C>G 5910C>A), ACC213ACA (5913C>A), TGC214TGT (5916C>T), AAC216AGC (5921A>G), CTA220TTA (5932C>T), GAT221AAT (5935G>A), ACT222ACC (5940T>C), CAG226TAT (5950C>T 5952G>T), ACA227GAC (5953A>G 5954C>A 5955A>C), CAA229GAA (5959C>G), GAG230GAA (5964G>A), GCC232TCA (5968G>T 5970C>A), AAT233GCT (5971A>G 5972A>C), CCT235CCA (5979T>A), GAG236GAA (5982G>A), TTA237CTA (5983T>C), TAC238TAT (5988C>T), AAG239AAA (5991G>A), TTC241TTT (5997C>T), ATG242ACA (5999T>C 6000G>A), CAA243ACA (6001C>A 6002A>C), AAG245AAA (6009G>A), AGG246AGA (6012G>A), ATA247ATT (6015A>T), ACT248ACA (6018T>A), GGG250GGC (6024G>C), ACA251AAC (6026C>A 6027A>C), TTC253TTG (6033C>G), TAC254TAT (6036C>T), GTA255ATA (6037G>A), CGA256AAG (6040C>A 6041G>A 6042A>G), TTC257TTT (6045C>T), TCG259ATA (6049T>A 6050C>T 6051G>A), GCT260GTA (6053C>T 6054T>A), ACA261CCA (6055A>C), GCA262GTA (6059C>T), CTA265TTA (6067C>T), TAT266TTT (6071A>T), GAG268GAA (6078G>A), GAG268_ATA269insA-- (6078_6079insA), ATA269ATT (6081A>T), AAG270AAA (6084G>A), CCT271CTA (6086C>T 6087T>A), ATC272GTG (6088A>G 6090C>G), ATC276ATT (6102C>T), ATT278ATA (6108T>A), GGA279GAA (6110G>A), CTC280CTA (6114C>A), AGG282CGA (6118A>C 6120G>A), GAA283GAT (6123A>T), ATC285ATT (6129C>T), ATA286ATT (6132A>T), CCA287CCC (6135A>C), AAG289GAA (6139A>G 6141G>A), ATA290ATT (6144A>T), GAA294AAA (6154G>A), GAA297ACA (6163G>A 6164A>C), GTA299GAA (6170T>A), AAT300GAG (6172A>G 6174T>G), ATT301AGA (6176T>G 6177T>A), CCA302CCT (6180A>T), GAA303GTT (6182A>T 6183A>T), TTC304TTT (6186C>T), AGG309AGA (6201G>A), ATA310ATT (6204A>T), ATA313TTA (6211A>T), ACT315ACA (6219T>A), ATA316ATC (6222A>C), CTA317ATT (6223C>A 6225A>T), AAC322AAT (6240C>T), AAC323AAT (6243C>T), CTA325ATA (6247C>A), CAG327GAA (6253C>G 6255G>A), GCA329CTA (6259G>C 6260C>T), ATT330ATG (6264T>G), AGC332ATC (6269G>T), TAT333TAC (6273T>C), TAT334TAC (6276T>C), TCA335ACA (6277T>A), AGG336AGA (6282G>A), GAA337GAC (6285A>C), CAG338CAA (6288G>A), ACA339ATA (6290C>T), TGC345TCA (6308G>C 6309C>A), AGA346AAA (6311G>A), GAT347GAG (6315T>G), AGA349ATC (6320G>T 6321A>C), GCA351AGA (6325G>A 6326C>G), CTA356GCA (6340C>G 6341T>C), AGA357ATG (6344G>T 6345A>G), GTC360ATA (6352G>A 6354C>A), TTA361ATG (6355T>A 6357A>G), AGT362ACA (6359G>C 6360T>A), CTT363TTG (6361C>T 6363T>G), AAA365AAT (6369A>T), GAA367GAG (6375A>G), CAA368AAA (6376C>A), CCT369CAA (6380C>A 6381T>A), ACA370CCT (6382A>C 6384A>T), ACC371ATT (6386C>T 6387C>T), AGG376AAA (6401G>A 6402G>A), AGG377AAG (6404G>A), AAT378GGA (6406A>G 6407A>G 6408T>A), TTC379TTT (6411C>T), ATC380ATT (6414C>T), TCC381TCG (6417C>G), CCA382GAT (6418C>G 6419C>A 6420A>T), GAT383GAA (6423T>A), CTG384TTA (6424C>T 6426G>A), TTA385CTA (6427T>C), CTA391CAA (6446T>A), AGT393GGG (6451A>G 6453T>G), CAC394CAA (6456C>A), TAT396TAC (6462T>C), CAC399CAT (6471C>T), TGC401TGT (6477C>T), TCA402TCG (6480A>G), AAA405AAC (6489A>C)								

*: Inserts / Deletes / Misaligned / Frameshifts

Analysis details

This analysis was performed with panviral2.64

NGS Details (UN9): Duamitovirus soch1

Assembly

**Coverage Length**	1721 (2 contig(s))
**Depth Of Coverage**	11.3
**Number Of Reads**	163
**Reads Per Million**	3.68 rpm (after QC)
**Ambiguities**	0
**Assembly Method**	de novo + reference guided assembly
**Consensus Caller**	Bcf Tools

Coverage Map

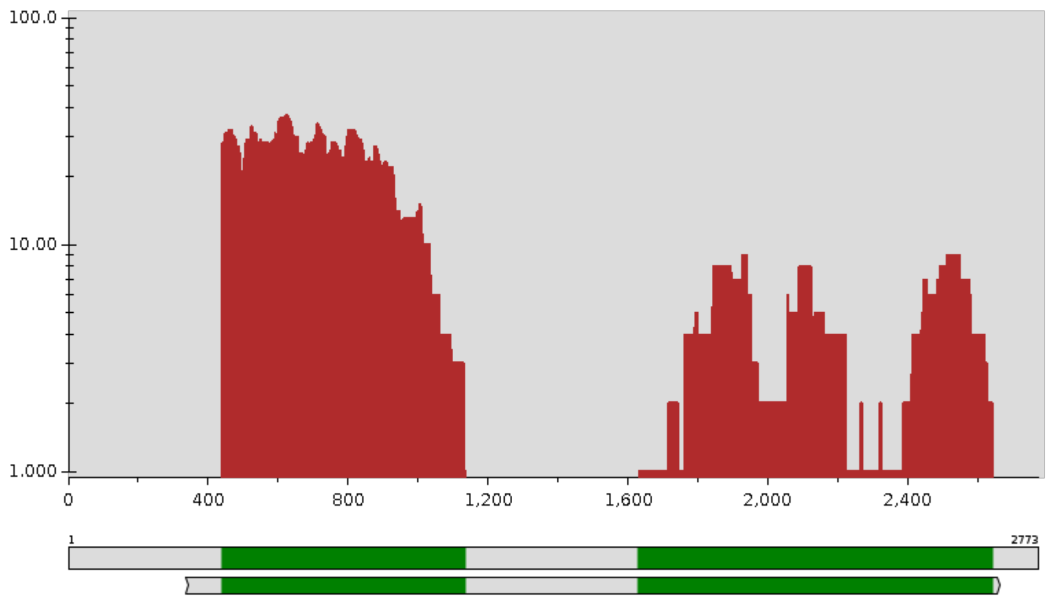

Assignment

**Type**	Duamitovirus soch1 (Taxonomy ID: 2955838)
**Reference Genome**	NC_076524.1
**NT Identity (%)**	69.7269
**AA Identity (%)**	67.9443
**Number Of Stop Codons**	7
**Number Of CDS**	1

Alignment

**Alignment Score**	1358.0 (NT) + 2762.0 (AA) = 4120.0
**Concordance (%)**	55.014

**Alignment Method**	Global, seeded, nucleotide + amino acids (AGA)

Genome Region

Sequence starts at position 437 and ends at position 2647 relative to NC_076524.1 reference sequence.

Alignment Detailed Statistics

	Begin	End	Coverage	Score	Concordance	Matches	Identities	I/D/M/F*	Stop Codons
**NT**	**437**	**2647**	**62.1%**	**1358**	**39.5%**	**1721 (100%)**	**1200 (69.7%)**	**0/0**	
Mutations:	437A>G, 441A>C, 443C>T, 447C>T, 459C>T, 466C>T, 470C>T, 471G>A, 472C>T, 473T>G, 477T>A, 480A>C, 481C>A, 482A>T, 485A>G, 489A>G, 494T>C, 497T>A, 500T>G, 503A>C, 506T>C, 509C>T, 512T>C, 513G>A, 516T>A, 517C>A, 518T>A, 520A>C, 524G>T, 525A>G, 526C>T, 527T>G, 529C>A, 530C>A, 531T>C, 533A>G, 534C>A, 535G>A, 536C>A, 539G>A, 540A>C, 541A>G, 542G>T, 545G>A, 548C>T, 550A>T, 556T>A, 557C>T, 560A>T, 569C>T, 575G>A, 581C>T, 582G>A, 587A>T, 589G>C, 591T>C, 593A>T, 599A>T, 602C>T, 612T>A, 613C>G, 614C>T, 616T>A, 618C>T, 619G>C, 620A>T, 624G>A, 626A>T, 629A>C, 630C>A, 632A>C, 633C>A, 635T>G, 638G>T, 641G>T, 644T>A, 647G>T, 651C>T, 653C>G, 656A>C, 657A>C, 661C>G, 662G>T, 665T>A, 668C>T, 672C>A, 673G>A, 674T>G, 680C>A, 683C>T, 684A>G, 685A>C, 686A>C, 692G>A, 695T>A, 699G>C, 700C>A, 701A>C, 702A>G, 705C>A, 707T>G, 708A>G, 709G>C, 710G>A, 713A>G, 714G>T, 715A>C, 719A>T, 722C>T, 737T>C, 746C>T, 752A>T, 761G>A, 765G>T, 767T>A, 770G>A, 771G>T, 772T>A, 773T>G, 774T>A, 776G>T, 777A>G, 778A>C, 779A>G, 782T>G, 796G>A, 797T>G, 803A>C, 809T>C, 810G>C, 815C>A, 818A>T, 821T>G, 824C>T, 827A>T, 831C>T, 833T>A, 841G>A, 842G>T, 850T>G, 851A>T, 857G>T, 860G>T, 862A>G, 869T>G, 875G>T, 880T>G, 881C>A, 885C>A, 887T>G, 892A>G, 905A>T, 908C>A, 909T>C, 910T>A, 916G>A, 918G>T, 919G>C, 923T>A, 926A>T, 927A>C, 929G>T, 934T>A, 935T>G, 938G>T, 942T>A, 943C>A, 948C>A, 971C>A, 972C>T, 975A>C, 976A>T, 977T>C, 983T>C, 990A>C, 992G>C, 993T>C, 994C>G, 995A>G, 997A>T, 998C>T, 999G>C, 1000G>A, 1001T>A, 1003T>A, 1004A>T, 1005G>T, 1007C>G, 1010A>G, 1016C>T, 1017A>G, 1018A>T, 1019A>T, 1022T>A, 1025T>C, 1026C>G, 1027A>G, 1030A>C, 1031G>A, 1034T>G, 1035A>G, 1036G>C, 1044T>G, 1046C>T, 1049G>A, 1050T>G, 1051T>A, 1052T>A, 1054C>A, 1055A>C, 1061T>C, 1067T>G, 1073G>A, 1076A>G, 1083C>A, 1088G>T, 1091C>G, 1099G>T, 1104A>G, 1105C>T, 1106A>G, 1115T>G, 1121A>C, 1122T>A, 1124C>T, 1127G>A, 1130C>T, 1629C>T, 1632T>A, 1634A>C, 1644G>A, 1664G>A, 1668G>A, 1670G>A, 1671C>A, 1673G>A, 1676T>A, 1679G>A, 1682C>T, 1686C>T, 1691T>A, 1696G>A, 1698G>T, 1703T>C, 1706C>T, 1707T>G, 1709G>T, 1710T>C, 1712G>A, 1724T>C, 1738C>G, 1742G>A, 1744G>A, 1745C>G, 1759G>A, 1760A>C, 1762G>A, 1766C>A, 1767T>A, 1769T>C, 1772A>C, 1775T>C, 1776G>A, 1777G>A, 1778G>A, 1781T>C, 1783A>C, 1784C>T, 1787T>C, 1790A>G, 1817T>C, 1819G>A, 1820T>G, 1823A>G, 1833G>C, 1840A>G, 1856A>G, 1859C>T, 1860A>G, 1861A>G, 1865C>T, 1867A>C, 1868A>C, 1874G>T, 1875A>T, 1878C>G, 1880A>T, 1886T>A, 1887T>A, 1889G>A, 1895T>C, 1898T>G, 1904A>T, 1907C>T, 1919G>C, 1922G>A, 1923A>T, 1925G>A, 1943G>T, 1947A>G, 1960C>T, 1967C>T, 1968C>G, 1970T>G, 1972G>A, 1973A>G, 1976T>A, 1980T>A, 1981T>C, 1984C>T, 1985T>G, 1991T>C, 1999T>G, 2003A>T, 2006T>A, 2010G>A, 2011C>A, 2013C>A, 2015C>T, 2020T>C, 2030T>C, 2036C>T, 2039A>G, 2049C>T, 2051A>G, 2052A>T, 2054A>G, 2063T>G, 2069G>A, 2071A>T, 2074A>G, 2075G>A, 2078A>T, 2082G>T, 2084G>T, 2087C>T, 2090A>G, 2091T>A, 2093C>G, 2094A>T, 2099C>T, 2103A>T, 2104G>C, 2105C>T, 2108G>A, 2117G>A, 2118C>A, 2120T>A, 2123A>G, 2129T>G, 2133C>G, 2134A>C, 2135T>A, 2136C>A, 2137T>G, 2138T>C, 2141G>A, 2145G>C, 2146T>A, 2147T>C, 2148C>A, 2156C>T, 2157T>C, 2160C>T, 2166T>C, 2167A>T, 2173G>A, 2174G>T, 2183A>G, 2186G>A, 2187G>T, 2188G>T, 2191T>A, 2192G>A, 2195C>T, 2204C>T, 2206A>T, 2213A>G, 2216G>A, 2223G>A, 2226A>G, 2227G>A, 2231C>T, 2236T>G, 2237T>G, 2240A>G, 2248A>C, 2250G>C, 2251T>C, 2259T>A, 2263A>G, 2264G>A, 2265A>C, 2271C>A, 2273A>T, 2274A>G, 2276G>A, 2280C>A, 2285G>A, 2288C>T, 2298G>T, 2299T>G, 2300T>G, 2303A>G, 2306A>C, 2309A>T, 2313C>T, 2315T>C, 2319G>A, 2322C>T, 2325C>G, 2326G>T, 2328C>A, 2330A>T, 2336A>T, 2337G>A, 2346T>C, 2348A>T, 2349A>C, 2350A>G, 2355C>G, 2357C>T, 2358A>T, 2359A>C, 2360C>T, 2362G>A, 2369T>C, 2372G>T, 2377G>T, 2378T>G, 2382A>T, 2383A>C, 2384C>T, 2385C>T, 2386C>T, 2387A>T, 2390T>A, 2396C>T, 2399T>C, 2400A>G, 2402G>A, 2404G>A, 2405T>A, 2406C>T, 2408C>G, 2411C>G, 2414C>T, 2415T>G, 2417C>T, 2420T>C, 2423A>T, 2425G>A, 2430A>T, 2431C>A, 2432A>G, 2435C>A, 2441A>G, 2442A>C, 2444G>A, 2445C>T, 2446T>C, 2447C>G, 2448G>A, 2450A>T, 2451A>G, 2452C>A, 2453T>A, 2456T>C, 2457T>C, 2459T>G, 2462T>A, 2465A>G, 2466A>G, 2477C>T, 2478C>T, 2489A>G, 2492T>C, 2495C>T, 2504T>A, 2510T>C, 2514A>G, 2518C>T, 2519C>G, 2524C>T, 2525C>T, 2528T>A, 2532C>A, 2533A>G, 2536G>A, 2537G>T, 2543T>G, 2551A>G, 2552C>T, 2555T>C, 2556G>T, 2557T>C, 2561T>C, 2568A>G, 2571A>C, 2572A>G, 2582A>T, 2585G>A, 2597G>A, 2598A>G, 2599A>C, 2600C>T, 2601G>A, 2609T>A, 2613T>G, 2615T>A, 2618T>A, 2622G>T, 2624A>T, 2626A>T, 2627C>A, 2629A>C, 2631A>T, 2634A>C, 2635C>T, 2638G>T, 2639A>G								
**CDS**									
RdRp	35	771	73.9%	2762	67.9%	574 (100%)	390 (67.9%)	0/0/0/0	7
Protein mutations:	I36L (441A>C 443C>T), L38F (447C>T), A44V (466C>T), A46M (471G>A 472C>T 473T>G), L48M (477T>A), T49H (480A>C 481C>A 482A>T), I52V (489A>G), V60I (513G>A), S61K (516T>A 517C>A 518T>A), K62T (520A>C), T64V (525A>G 526C>T 527T>G), T65K (529C>A 530C>A), R67K (534C>A 535G>A 536C>A), K69R (540A>C 541A>G 542G>T), Y72F (550A>T), F74Y (556T>A 557C>T), A83T (582G>A), C85S (589G>C), F94Y (616T>A), R95S (618C>T 619G>C 620A>T), E97N (624G>A 626A>T), E98D (629A>C), P99T (630C>A 632A>C), L100M (633C>A 635T>G), S109C (661C>G 662G>T), R113K (672C>A 673G>A 674T>G), K117A (684A>G 685A>C 686A>C), A122H (699G>C 700C>A 701A>C), I123V (702A>G), R125A (708A>G 709G>C 710G>A), D127S (714G>T 715A>C), E128D (719A>T), A144S (765G>T 767T>A), V146* (771G>T 772T>A 773T>G), L147I (774T>A 776G>T), K148A (777A>G 778A>C 779A>G), S154K (796G>A 797T>G), E159Q (810G>C), P166S (831C>T 833T>A), G169D (841G>A 842G>T), L172R (850T>G 851A>T), E176G (862A>G), I178M (869T>G), F182* (880T>G 881C>A), H184K (885C>A 887T>G), Q186R (892A>G), F192H (909T>C 910T>A), R194Q (916G>A), G195S (918G>T 919G>C), M198L (927A>C 929G>T), I200K (934T>A 935T>G), S203N (942T>A 943C>A), Q205K (948C>A), N214L (975A>C 976A>T 977T>C), S220R (993T>C 994C>G 995A>G), Y221F (997A>T 998C>T), G222Q (999G>C 1000G>A 1001T>A), L223Y (1003T>A 1004A>T), A224S (1005G>T 1007C>G), K228V (1017A>G 1018A>T 1019A>T), Q231G (1026C>G 1027A>G), E232A (1030A>C 1031G>A), S234A (1035A>G 1036G>C), F237V (1044T>G 1046C>T), F239E (1050T>G 1051T>A 1052T>A), P240H (1054C>A 1055A>C), L250I (1083C>A), W255L (1099G>T), T257V (1104A>G 1105C>T 1106A>G), I260M (1115T>G), F263I (1122T>A 1124C>T), P432S (1629C>T), L433I (1632T>A 1634A>C), V437M (1644G>A), G445R (1668G>A 1670G>A), Q446K (1671C>A 1673G>A), H451Y (1686C>T), W454* (1696G>A), A455S (1698G>T), S458A (1707T>G 1709G>T), A468G (1738C>G), S470K (1744G>A 1745C>G), G475D (1759G>A 1760A>C), C476Y (1762G>A), N477K (1766C>A), S478T (1767T>A 1769T>C), G481K (1776G>A 1777G>A 1778G>A), Y483S (1783A>C 1784C>T), S495K (1819G>A 1820T>G), E500Q (1833G>C), K502R (1840A>G), N509G (1860A>G 1861A>G), K511T (1867A>C 1868A>C), I514L (1875A>T), P515A (1878C>G 1880A>T), L518I (1887T>A 1889G>A), D521E (1898T>G), R530* (1923A>T 1925G>A), M536I (1943G>T), I538V (1947A>G), P542L (1960C>T), L545V (1968C>G 1970T>G), R546K (1972G>A 1973A>G), L549T (1980T>A 1981T>C), S550L (1984C>T 1985T>G), V555G (1999T>G), A559N (2010G>A 2011C>A), L560I (2013C>A 2015C>T), I562T (2020T>C), I568M (2039A>G), Q572* (2049C>T 2051A>G), R573W (2052A>T 2054A>G), Y579F (2071A>T), K580R (2074A>G 2075G>A), A583S (2082G>T 2084G>T), F586M (2091T>A 2093C>G), T587S (2094A>T), H600A (2133C>G 2134A>C 2135T>A), L601S (2136C>A 2137T>G 2138T>C), V604H (2145G>C 2146T>A 2147T>C), Y611L (2166T>C 2167A>T), W613Y (2173G>A 2174G>T), G618F (2187G>T 2188G>T), M619K (2191T>A 2192G>A), Y624F (2206A>T), V630I (2223G>A), S631D (2226A>G 2227G>A), L634R (2236T>G 2237T>G), K638T (2248A>C), V639P (2250G>C 2251T>C), L642I (2259T>A), Q643R (2263A>G 2264G>A), I644L (2265A>C), P646T (2271C>A 2273A>T), K647E (2274A>G 2276G>A), L649M (2280C>A), V655W (2298G>T 2299T>G 2300T>G), R660C (2313C>T 2315T>C), V662I (2319G>A), R664V (2325C>G 2326G>T), Q665N (2328C>A 2330A>T), E668K (2337G>A), K672R (2349A>C 2350A>G), L674V (2355C>G 2357C>T), N675S (2358A>T 2359A>C 2360C>T), W676* (2362G>A), C681L (2377G>T 2378T>G), N683S (2382A>T 2383A>C 2384C>T), P684F (2385C>T 2386C>T 2387A>T), D685E (2390T>A), K689E (2400A>G 2402G>A), S690K (2404G>A 2405T>A), I692M (2411C>G), S694A (2415T>G 2417C>T), C697Y (2425G>A), T699* (2430A>T 2431C>A 2432A>G), L704S (2445C>T 2446T>C 2447C>G), E705N (2448G>A 2450A>T), T706E (2451A>G 2452C>A 2453T>A), F708L (2457T>C 2459T>G), D709E (2462T>A), I711V (2466A>G), N727D (2514A>G), P728L (2518C>T 2519C>G), T730I (2524C>T 2525C>T), Q733R (2532C>A 2533A>G), W734Y (2536G>A 2537G>T), F736L (2543T>G), N739S (2551A>G 2552C>T), V741S (2556G>T 2557T>C), N745D (2568A>G), K746R (2571A>C 2572A>G), L749F (2582A>T), N755A (2598A>G 2599A>C 2600C>T), D756N (2601G>A), S760A (2613T>G 2615T>A), E763Y (2622G>T 2624A>T), Y764L (2626A>T 2627C>A), Q765P (2629A>C), T766S (2631A>T), T767L (2634A>C 2635C>T), G768V (2638G>T 2639A>G)								

	Begin	End	Coverage	Score	Concordance	Matches	Identities	I/D/M/F*	Stop Codons
**NT**	**437**	**2647**	**62.1%**	**1358**	**39.5%**	**1721 (100%)**	**1200 (69.7%)**	**0/0**	

Codon mutations:

AAA34..G (437A>G), ATC36CTT (441A>C 443C>T), CTT38TTT (447C>T), CTA42TTA (459C>T), GCC44GTC (466C>T), TCC45TCT (470C>T), GCT46ATG (471G>A 472C>T 473T>G), TTG48ATG (477T>A), ACA49CAT (480A>C 481C>A 482A>T), AAA50AAG (485A>G), ATC52GTC (489A>G), GCT53GCC (494T>C), CTT54CTA (497T>A), GCT55GCG (500T>G), GGA56GGC (503A>C), TGT57TGC (506T>C), GAC58GAT (509C>T), TTT59TTC (512T>C), GTA60ATA (513G>A), TCT61AAA (516T>A 517C>A 518T>A), AAA62ACA (520A>C), GTG63GTT (524G>T), ACT64GTG (525A>G 526C>T 527T>G), ACC65AAA (529C>A 530C>A), TTA66CTG (531T>C 533A>G), CGC67AAA (534C>A 535G>A 536C>A), CGG68CGA (539G>A), AAG69CGT (540A>C 541A>G 542G>T), TCG70TCA (545G>A), GGC71GGT (548C>T), TAT72TTT (550A>T), TTC74TAT (556T>A 557C>T), ACA75ACT (560A>T), TAC78TAT (569C>T), AAG80AAA (575G>A), TGC82TGT (581C>T), GCA83ACA (582G>A), GTA84GTT (587A>T), TGC85TCC (589G>C), TTA86CTT (591T>C 593A>T), CGA88CGT (599A>T), TAC89TAT (602C>T), TCC93AGT (612T>A 613C>G 614C>T), TTT94TAT (616T>A), CGA95TCT (618C>T 619G>C 620A>T), GAA97AAT (624G>A 626A>T), GAA98GAC (629A>C), CCA99ACC (630C>A 632A>C), CTT100ATG (633C>A 635T>G), TCG101TCT (638G>T), GTG102GTT (641G>T), CCT103CCA (644T>A), GTG104GTT (647G>T), CTC106TTG (651C>T 653C>G), ACA107ACC (656A>C), AGA108CGA (657A>C), TCG109TGT (661C>G 662G>T), GGT110GGA (665T>A), ATC111ATT (668C>T), CGT113AAG (672C>A 673G>A 674T>G), ATC115ATA (680C>A), CCC116CCT (683C>T), AAA117GCC (684A>G 685A>C 686A>C), TTG119TTA (692G>A), CGT120CGA (695T>A), GCA122CAC (699G>C 700C>A 701A>C), ATA123GTA (702A>G), CGT124AGG (705C>A 707T>G), AGG125GCA (708A>G 709G>C 710G>A), AAA126AAG (713A>G), GAT127TCT (714G>T 715A>C), GAA128GAT (719A>T), CAC129CAT (722C>T), GTT134GTC (737T>C), TAC137TAT (746C>T), TCA139TCT (752A>T), GGG142GGA (761G>A), GCT144TCA (765G>T 767T>A), AAG145AAA (770G>A), GTT146TAG (771G>T 772T>A 773T>G), TTG147ATT (774T>A 776G>T), AAA148GCG (777A>G 778A>C 779A>G), GTT149GTG (782T>G), AGT154AAG (796G>A 797T>G), GCA156GCC (803A>C), TTT158TTC (809T>C), GAA159CAA (810G>C), TCC160TCA (815C>A), ATA161ATT (818A>T), ACT162ACG (821T>G), ACC163ACT (824C>T), CCA164CCT (827A>T), CCT166TCA (831C>T 833T>A), GGG169GAT (841G>A 842G>T), CTA172CGT (850T>G 851A>T), GTG174GTT (857G>T), CTG175CTT (860G>T), GAA176GGA (862A>G), ATT178ATG (869T>G), ACG180ACT (875G>T), TTC182TGA (880T>G 881C>A), CAT184AAG (885C>A 887T>G), CAA186CGA (892A>G), CTA190CTT (905A>T), CCC191CCA (908C>A), TTT192CAT (909T>C 910T>A), CGA194CAA (916G>A), GGC195TCC (918G>T 919G>C), ATT196ATA (923T>A), CCA197CCT (926A>T), ATG198CTT (927A>C 929G>T), ATT200AAG (934T>A 935T>G), GGG201GGT (938G>T), TCT203AAT (942T>A 943C>A), CAA205AAA (948C>A), CCC212CCA (971C>A), CTA213TTA (972C>T), AAT214CTC (975A>C 976A>T 977T>C), AAT216AAC (983T>C), AGG219CGC (990A>C 992G>C), TCA220CGG (993T>C 994C>G 995A>G), TAC221TTT (997A>T 998C>T), GGT222CAA (999G>C 1000G>A 1001T>A), TTA223TAT (1003T>A 1004A>T), GCC224TCG (1005G>T 1007C>G), GTA225GTG (1010A>G), GAC227GAT (1016C>T), AAA228GTT (1017A>G 1018A>T 1019A>T), GTT229GTA (1022T>A), GAT230GAC (1025T>C), CAA231GGA (1026C>G 1027A>G), GAG232GCA (1030A>C 1031G>A), GCT233GCG (1034T>G), AGT234GCT (1035A>G 1036G>C), TTC237GTT (1044T>G 1046C>T), AAG238AAA (1049G>A), TTT239GAA (1050T>G 1051T>A 1052T>A), CCA240CAC (1054C>A 1055A>C), ATT242ATC (1061T>C), GTT244GTG (1067T>G), TTG246TTA (1073G>A), AAA247AAG (1076A>G), CTA250ATA (1083C>A), GCG251GCT (1088G>T), GCC252GCG (1091C>G), TGG255TTG (1099G>T), ACA257GTG (1104A>G 1105C>T 1106A>G), ATT260ATG (1115T>G), TCA262TCC (1121A>C), TTC263ATT (1122T>A 1124C>T), CAG264CAA (1127G>A), GAC265GAT (1130C>T), CCG432TCG (1629C>T), TTA433ATC (1632T>A 1634A>C), GTG437ATG (1644G>A), CTG443CTA (1664G>A), GGG445AGA (1668G>A 1670G>A), CAG446AAA (1671C>A 1673G>A), CCT447CCA (1676T>A), TTG448TTA (1679G>A), GGC449GGT (1682C>T), CAC451TAC (1686C>T), GGT452GGA (1691T>A), TGG454TAG (1696G>A), GCA455TCA (1698G>T), CTT456CTC (1703T>C), TTC457TTT (1706C>T), TCG458GCT (1707T>G 1709G>T), TTG459CTA (1710T>C 1712G>A), TAT463TAC (1724T>C), GCA468GGA (1738C>G), GCG469GCA (1742G>A), AGC470AAG (1744G>A 1745C>G), GGA475GAC (1759G>A 1760A>C), TGT476TAT (1762G>A), AAC477AAA (1766C>A), TCT478ACC (1767T>A 1769T>C), CCA479CCC (1772A>C), TTT480TTC (1775T>C), GGG481AAA (1776G>A 1777G>A 1778G>A), GAT482GAC (1781T>C), TAC483TCT (1783A>C 1784C>T), GCT484GCC (1787T>C), TTA485TTG (1790A>G), GAT494GAC (1817T>C), AGT495AAG (1819G>A 1820T>G), AAA496AAG (1823A>G), GAG500CAG (1833G>C), AAG502AGG (1840A>G), AGA507AGG (1856A>G), CTC508CTT (1859C>T), AAT509GGT (1860A>G 1861A>G), GTC510GTT (1865C>T), AAA511ACC (1867A>C 1868A>C), TCG513TCT (1874G>T), ATA514TTA (1875A>T), CCA515GCT (1878C>G 1880A>T), TCT517TCA (1886T>A), TTG518ATA (1887T>A 1889G>A), TCT520TCC (1895T>C), GAT521GAG (1898T>G), GGA523GGT (1904A>T), ACC524ACT (1907C>T), GCG528GCC (1919G>C), AAG529AAA (1922G>A), AGG530TGA (1923A>T 1925G>A), ATG536ATT (1943G>T), ATT538GTT (1947A>G), CCA542CTA (1960C>T), TCC544TCT (1967C>T), CTT545GTG (1968C>G 1970T>G), AGA546AAG (1972G>A 1973A>G), GCT547GCA (1976T>A), TTA549ACA (1980T>A 1981T>C), TCT550TTG (1984C>T 1985T>G), CGT552CGC (1991T>C), GTT555GGT (1999T>G), GGA556GGT (2003A>T), CTT557CTA (2006T>A), GCC559AAC (2010G>A 2011C>A), CTC560ATT (2013C>A 2015C>T), ATC562ACC (2020T>C), GAT565GAC (2030T>C), AAC567AAT (2036C>T), ATA568ATG (2039A>G), CAA572TAG (2049C>T 2051A>G), AGA573TGG (2052A>T 2054A>G), GGT576GGG (2063T>G), GGG578GGA (2069G>A), TAC579TTC (2071A>T), AAG580AGA (2074A>G 2075G>A), GTA581GTT (2078A>T), GCG583TCT (2082G>T 2084G>T), CGC584CGT (2087C>T), TTA585TTG (2090A>G), TTC586ATG (2091T>A 2093C>G), ACT587TCT (2094A>T), ACC588ACT (2099C>T), AGC590TCT (2103A>T 2104G>C 2105C>T), AAG591AAA (2108G>A), GAG594GAA (2117G>A), CGT595AGA (2118C>A 2120T>A), CTA596CTG (2123A>G), GCT598GCG (2129T>G), CAT600GCA (2133C>G 2134A>C 2135T>A), CTT601AGC (2136C>A 2137T>G 2138T>C), AAG602AAA (2141G>A), GTT604CAC (2145G>C 2146T>A 2147T>C), CGG605AGG (2148C>A), CAC607CAT (2156C>T), TTA608CTA (2157T>C), CTA609TTA (2160C>T), TAC611CTC (2166T>C 2167A>T), TGG613TAT (2173G>A 2174G>T), GGA616GGG (2183A>G), AGG617AGA (2186G>A), GGT618TTT (2187G>T 2188G>T), ATG619AAA (2191T>A 2192G>A), CCC620CCT (2195C>T), CCC623CCT (2204C>T), TAT624TTT (2206A>T), AAA626AAG (2213A>G), GGG627GGA (2216G>A), GTA630ATA (2223G>A), AGT631GAT (2226A>G 2227G>A), TAC632TAT (2231C>T), CTT634CGG (2236T>G 2237T>G), AAA635AAG (2240A>G), AAA638ACA (2248A>C), GTT639CCT (2250G>C 2251T>C), TTA642ATA (2259T>A), CAG643CGA (2263A>G 2264G>A), ATC644CTC (2265A>C), CCA646ACT (2271C>A 2273A>T), AAG647GAA (2274A>G 2276G>A), CTG649ATG (2280C>A), GTG650GTA (2285G>A), TTC651TTT (2288C>T), GTT655TGG (2298G>T 2299T>G 2300T>G), GAA656GAG (2303A>G), ATA657ATC (2306A>C), CTA658CTT (2309A>T), CGT660TGC (2313C>T 2315T>C), GTT662ATT (2319G>A), CTA663TTA (2322C>T), CGC664GTC (2325C>G 2326G>T), CAA665AAT (2328C>A 2330A>T), GTA667GTT (2336A>T), GAA668AAA (2337G>A), TTA671CTT (2346T>C 2348A>T), AAG672CGG (2349A>C 2350A>G), CTC674GTT (2355C>G 2357C>T), AAC675TCT (2358A>T 2359A>C 2360C>T), TGG676TAG (2362G>A), CAT678CAC (2369T>C), ACG679ACT (2372G>T), TGT681TTG (2377G>T 2378T>G), AAC683TCT (2382A>T 2383A>C 2384C>T), CCA684TTT (2385C>T 2386C>T 2387A>T), GAT685GAA (2390T>A), TCC687TCT (2396C>T), ATT688ATC (2399T>C), AAG689GAA (2400A>G 2402G>A), AGT690AAA (2404G>A 2405T>A), CTC691TTG (2406C>T 2408C>G), ATC692ATG (2411C>G), GAC693GAT (2414C>T), TCC694GCT (2415T>G 2417C>T), CCT695CCC (2420T>C), ATA696ATT (2423A>T), TGC697TAC (2425G>A), ACA699TAG (2430A>T 2431C>A 2432A>G), TCC700TCA (2435C>A), AAA702AAG (2441A>G), AGG703CGA (2442A>C 2444G>A), CTC704TCG (2445C>T 2446T>C 2447C>G), GAA705AAT (2448G>A 2450A>T), ACT706GAA (2451A>G 2452C>A 2453T>A), GAT707GAC (2456T>C), TTT708CTG (2457T>C 2459T>G), GAT709GAA (2462T>A), TTA710TTG (2465A>G), ATC711GTC (2466A>G), GGC714GGT (2477C>T), CTA715TTA (2478C>T), AAA718AAG (2489A>G), TGT719TGC (2492T>C), TAC720TAT (2495C>T), GGT723GGA (2504T>A), GGT725GGC (2510T>C), AAT727GAT (2514A>G), CCC728CTG (2518C>T 2519C>G), ACC730ATT (2524C>T 2525C>T), ACT731ACA (2528T>A), CAG733AGG (2532C>A 2533A>G), TGG734TAT (2536G>A 2537G>T), TTT736TTG (2543T>G), AAC739AGT (2551A>G 2552C>T), ACT740ACC (2555T>C), GTT741TCT (2556G>T 2557T>C), ATT742ATC (2561T>C), AAT745GAT (2568A>G), AAA746CGA (2571A>C 2572A>G), TTA749TTT (2582A>T), GGG750GGA (2585G>A), GGG754GGA (2597G>A), AAC755GCT (2598A>G 2599A>C 2600C>T), GAT756AAT (2601G>A), ATT758ATA (2609T>A), TCT760GCA (2613T>G 2615T>A), CCT761CCA (2618T>A), GAA763TAT (2622G>T 2624A>T), TAC764TTA (2626A>T 2627C>A), CAA765CCA (2629A>C), ACT766TCT (2631A>T), ACA767CTA (2634A>C 2635C>T), GGA768GTG (2638G>T 2639A>G)

Proteins

	Begin	End	Coverage	Score	Concordance	Matches	Identities	I/D/M/F*	Stop Codons
RNA-dependent RNA polymerase (YP_010798874.1)	35	771	73.9%	2762	67.9%	574 (100%)	390 (67.9%)	0/0/0/0	7

Protein mutations:

I36L (441A>C 443C>T), L38F (447C>T), A44V (466C>T), A46M (471G>A 472C>T 473T>G), L48M (477T>A), T49H (480A>C 481C>A 482A>T), I52V (489A>G), V60I (513G>A), S61K (516T>A 517C>A 518T>A), K62T (520A>C), T64V (525A>G 526C>T 527T>G), T65K (529C>A 530C>A), R67K (534C>A 535G>A 536C>A), K69R (540A>C 541A>G 542G>T), Y72F (550A>T), F74Y (556T>A 557C>T), A83T (582G>A), C85S (589G>C), F94Y (616T>A), R95S (618C>T 619G>C 620A>T), E97N (624G>A 626A>T), E98D (629A>C), P99T (630C>A 632A>C), L100M (633C>A 635T>G), S109C (661C>G 662G>T), R113K (672C>A 673G>A 674T>G), K117A (684A>G 685A>C 686A>C), A122H (699G>C 700C>A 701A>C), I123V (702A>G), R125A (708A>G 709G>C 710G>A), D127S (714G>T 715A>C), E128D (719A>T), A144S (765G>T 767T>A), V146* (771G>T 772T>A 773T>G), L147I (774T>A 776G>T), K148A (777A>G 778A>C 779A>G), S154K (796G>A 797T>G), E159Q (810G>C), P166S (831C>T 833T>A), G169D (841G>A 842G>T), L172R (850T>G 851A>T), E176G (862A>G), I178M (869T>G), F182* (880T>G 881C>A), H184K (885C>A 887T>G), Q186R (892A>G), F192H (909T>C 910T>A), R194Q (916G>A), G195S (918G>T 919G>C), M198L (927A>C 929G>T), I200K (934T>A 935T>G), S203N (942T>A 943C>A), Q205K (948C>A), N214L (975A>C 976A>T 977T>C), S220R (993T>C 994C>G 995A>G), Y221F (997A>T 998C>T), G222Q (999G>C 1000G>A 1001T>A), L223Y (1003T>A 1004A>T), A224S (1005G>T 1007C>G), K228V (1017A>G 1018A>T 1019A>T), Q231G (1026C>G 1027A>G), E232A (1030A>C 1031G>A), S234A (1035A>G 1036G>C), F237V (1044T>G 1046C>T), F239E (1050T>G 1051T>A 1052T>A), P240H (1054C>A 1055A>C), L250I (1083C>A), W255L (1099G>T), T257V (1104A>G 1105C>T 1106A>G), I260M (1115T>G), F263I (1122T>A 1124C>T), P432S (1629C>T), L433I (1632T>A 1634A>C), V437M (1644G>A), G445R (1668G>A 1670G>A), Q446K (1671C>A 1673G>A), H451Y (1686C>T), W454* (1696G>A), A455S (1698G>T), S458A (1707T>G 1709G>T), A468G (1738C>G), S470K (1744G>A 1745C>G), G475D (1759G>A 1760A>C), C476Y (1762G>A), N477K (1766C>A), S478T (1767T>A 1769T>C), G481K (1776G>A 1777G>A 1778G>A), Y483S (1783A>C 1784C>T), S495K (1819G>A 1820T>G), E500Q (1833G>C), K502R (1840A>G), N509G (1860A>G 1861A>G), K511T (1867A>C 1868A>C), I514L (1875A>T), P515A (1878C>G 1880A>T), L518I (1887T>A 1889G>A), D521E (1898T>G), R530* (1923A>T 1925G>A), M536I (1943G>T), I538V (1947A>G), P542L (1960C>T), L545V (1968C>G 1970T>G), R546K (1972G>A 1973A>G), L549T (1980T>A 1981T>C), S550L (1984C>T 1985T>G), V555G (1999T>G), A559N (2010G>A 2011C>A), L560I (2013C>A 2015C>T), I562T (2020T>C), I568M (2039A>G), Q572* (2049C>T 2051A>G), R573W (2052A>T 2054A>G), Y579F (2071A>T), K580R (2074A>G 2075G>A), A583S (2082G>T 2084G>T), F586M (2091T>A 2093C>G), T587S (2094A>T), H600A (2133C>G 2134A>C 2135T>A), L601S (2136C>A 2137T>G 2138T>C), V604H (2145G>C 2146T>A 2147T>C), Y611L (2166T>C 2167A>T), W613Y (2173G>A 2174G>T), G618F (2187G>T 2188G>T), M619K (2191T>A 2192G>A), Y624F (2206A>T), V630I (2223G>A), S631D (2226A>G 2227G>A), L634R (2236T>G 2237T>G), K638T (2248A>C), V639P (2250G>C 2251T>C), L642I (2259T>A), Q643R (2263A>G 2264G>A), I644L (2265A>C), P646T (2271C>A 2273A>T), K647E (2274A>G 2276G>A), L649M (2280C>A), V655W (2298G>T 2299T>G 2300T>G), R660C (2313C>T 2315T>C), V662I (2319G>A), R664V (2325C>G 2326G>T), Q665N (2328C>A 2330A>T), E668K (2337G>A), K672R (2349A>C 2350A>G), L674V (2355C>G 2357C>T), N675S (2358A>T 2359A>C 2360C>T), W676* (2362G>A), C681L (2377G>T 2378T>G), N683S (2382A>T 2383A>C 2384C>T), P684F (2385C>T 2386C>T 2387A>T), D685E (2390T>A), K689E (2400A>G 2402G>A), S690K (2404G>A 2405T>A), I692M (2411C>G), S694A (2415T>G 2417C>T), C697Y (2425G>A), T699* (2430A>T 2431C>A 2432A>G), L704S (2445C>T 2446T>C 2447C>G), E705N (2448G>A 2450A>T), T706E (2451A>G 2452C>A 2453T>A), F708L (2457T>C 2459T>G), D709E (2462T>A), I711V (2466A>G), N727D (2514A>G), P728L (2518C>T 2519C>G), T730I (2524C>T 2525C>T), Q733R (2532C>A 2533A>G), W734Y (2536G>A 2537G>T), F736L (2543T>G), N739S (2551A>G 2552C>T), V741S (2556G>T 2557T>C), N745D (2568A>G), K746R (2571A>C 2572A>G), L749F (2582A>T), N755A (2598A>G 2599A>C 2600C>T), D756N (2601G>A), S760A (2613T>G 2615T>A), E763Y (2622G>T 2624A>T), Y764L (2626A>T 2627C>A), Q765P (2629A>C), T766S (2631A>T), T767L (2634A>C 2635C>T), G768V (2638G>T 2639A>G)

	Begin	End	Coverage	Score	Concordance	Matches	Identities	I/D/M/F*	Stop Codons
**NT**	**437**	**2647**	**62.1%**	**1358**	**39.5%**	**1721 (100%)**	**1200 (69.7%)**	**0/0**	
Codon mutations:	AAA34..G (437A>G), ATC36CTT (441A>C 443C>T), CTT38TTT (447C>T), CTA42TTA (459C>T), GCC44GTC (466C>T), TCC45TCT (470C>T), GCT46ATG (471G>A 472C>T 473T>G), TTG48ATG (477T>A), ACA49CAT (480A>C 481C>A 482A>T), AAA50AAG (485A>G), ATC52GTC (489A>G), GCT53GCC (494T>C), CTT54CTA (497T>A), GCT55GCG (500T>G), GGA56GGC (503A>C), TGT57TGC (506T>C), GAC58GAT (509C>T), TTT59TTC (512T>C), GTA60ATA (513G>A), TCT61AAA (516T>A 517C>A 518T>A), AAA62ACA (520A>C), GTG63GTT (524G>T), ACT64GTG (525A>G 526C>T 527T>G), ACC65AAA (529C>A 530C>A), TTA66CTG (531T>C 533A>G), CGC67AAA (534C>A 535G>A 536C>A), CGG68CGA (539G>A), AAG69CGT (540A>C 541A>G 542G>T), TCG70TCA (545G>A), GGC71GGT (548C>T), TAT72TTT (550A>T), TTC74TAT (556T>A 557C>T), ACA75ACT (560A>T), TAC78TAT (569C>T), AAG80AAA (575G>A), TGC82TGT (581C>T), GCA83ACA (582G>A), GTA84GTT (587A>T), TGC85TCC (589G>C), TTA86CTT (591T>C 593A>T), CGA88CGT (599A>T), TAC89TAT (602C>T), TCC93AGT (612T>A 613C>G 614C>T), TTT94TAT (616T>A), CGA95TCT (618C>T 619G>C 620A>T), GAA97AAT (624G>A 626A>T), GAA98GAC (629A>C), CCA99ACC (630C>A 632A>C), CTT100ATG (633C>A 635T>G), TCG101TCT (638G>T), GTG102GTT (641G>T), CCT103CCA (644T>A), GTG104GTT (647G>T), CTC106TTG (651C>T 653C>G), ACA107ACC (656A>C), AGA108CGA (657A>C), TCG109TGT (661C>G 662G>T), GGT110GGA (665T>A), ATC111ATT (668C>T), CGT113AAG (672C>A 673G>A 674T>G), ATC115ATA (680C>A), CCC116CCT (683C>T), AAA117GCC (684A>G 685A>C 686A>C), TTG119TTA (692G>A), CGT120CGA (695T>A), GCA122CAC (699G>C 700C>A 701A>C), ATA123GTA (702A>G), CGT124AGG (705C>A 707T>G), AGG125GCA (708A>G 709G>C 710G>A), AAA126AAG (713A>G), GAT127TCT (714G>T 715A>C), GAA128GAT (719A>T), CAC129CAT (722C>T), GTT134GTC (737T>C), TAC137TAT (746C>T), TCA139TCT (752A>T), GGG142GGA (761G>A), GCT144TCA (765G>T 767T>A), AAG145AAA (770G>A), GTT146TAG (771G>T 772T>A 773T>G), TTG147ATT (774T>A 776G>T), AAA148GCG (777A>G 778A>C 779A>G), GTT149GTG (782T>G), AGT154AAG (796G>A 797T>G), GCA156GCC (803A>C), TTT158TTC (809T>C), GAA159CAA (810G>C), TCC160TCA (815C>A), ATA161ATT (818A>T), ACT162ACG (821T>G), ACC163ACT (824C>T), CCA164CCT (827A>T), CCT166TCA (831C>T 833T>A), GGG169GAT (841G>A 842G>T), CTA172CGT (850T>G 851A>T), GTG174GTT (857G>T), CTG175CTT (860G>T), GAA176GGA (862A>G), ATT178ATG (869T>G), ACG180ACT (875G>T), TTC182TGA (880T>G 881C>A), CAT184AAG (885C>A 887T>G), CAA186CGA (892A>G), CTA190CTT (905A>T), CCC191CCA (908C>A), TTT192CAT (909T>C 910T>A), CGA194CAA (916G>A), GGC195TCC (918G>T 919G>C), ATT196ATA (923T>A), CCA197CCT (926A>T), ATG198CTT (927A>C 929G>T), ATT200AAG (934T>A 935T>G), GGG201GGT (938G>T), TCT203AAT (942T>A 943C>A), CAA205AAA (948C>A), CCC212CCA (971C>A), CTA213TTA (972C>T), AAT214CTC (975A>C 976A>T 977T>C), AAT216AAC (983T>C), AGG219CGC (990A>C 992G>C), TCA220CGG (993T>C 994C>G 995A>G), TAC221TTT (997A>T 998C>T), GGT222CAA (999G>C 1000G>A 1001T>A), TTA223TAT (1003T>A 1004A>T), GCC224TCG (1005G>T 1007C>G), GTA225GTG (1010A>G), GAC227GAT (1016C>T), AAA228GTT (1017A>G 1018A>T 1019A>T), GTT229GTA (1022T>A), GAT230GAC (1025T>C), CAA231GGA (1026C>G 1027A>G), GAG232GCA (1030A>C 1031G>A), GCT233GCG (1034T>G), AGT234GCT (1035A>G 1036G>C), TTC237GTT (1044T>G 1046C>T), AAG238AAA (1049G>A), TTT239GAA (1050T>G 1051T>A 1052T>A), CCA240CAC (1054C>A 1055A>C), ATT242ATC (1061T>C), GTT244GTG (1067T>G), TTG246TTA (1073G>A), AAA247AAG (1076A>G), CTA250ATA (1083C>A), GCG251GCT (1088G>T), GCC252GCG (1091C>G), TGG255TTG (1099G>T), ACA257GTG (1104A>G 1105C>T 1106A>G), ATT260ATG (1115T>G), TCA262TCC (1121A>C), TTC263ATT (1122T>A 1124C>T), CAG264CAA (1127G>A), GAC265GAT (1130C>T), CCG432TCG (1629C>T), TTA433ATC (1632T>A 1634A>C), GTG437ATG (1644G>A), CTG443CTA (1664G>A), GGG445AGA (1668G>A 1670G>A), CAG446AAA (1671C>A 1673G>A), CCT447CCA (1676T>A), TTG448TTA (1679G>A), GGC449GGT (1682C>T), CAC451TAC (1686C>T), GGT452GGA (1691T>A), TGG454TAG (1696G>A), GCA455TCA (1698G>T), CTT456CTC (1703T>C), TTC457TTT (1706C>T), TCG458GCT (1707T>G 1709G>T), TTG459CTA (1710T>C 1712G>A), TAT463TAC (1724T>C), GCA468GGA (1738C>G), GCG469GCA (1742G>A), AGC470AAG (1744G>A 1745C>G), GGA475GAC (1759G>A 1760A>C), TGT476TAT (1762G>A), AAC477AAA (1766C>A), TCT478ACC (1767T>A 1769T>C), CCA479CCC (1772A>C), TTT480TTC (1775T>C), GGG481AAA (1776G>A 1777G>A 1778G>A), GAT482GAC (1781T>C), TAC483TCT (1783A>C 1784C>T), GCT484GCC (1787T>C), TTA485TTG (1790A>G), GAT494GAC (1817T>C), AGT495AAG (1819G>A 1820T>G), AAA496AAG (1823A>G), GAG500CAG (1833G>C), AAG502AGG (1840A>G), AGA507AGG (1856A>G), CTC508CTT (1859C>T), AAT509GGT (1860A>G 1861A>G), GTC510GTT (1865C>T), AAA511ACC (1867A>C 1868A>C), TCG513TCT (1874G>T), ATA514TTA (1875A>T), CCA515GCT (1878C>G 1880A>T), TCT517TCA (1886T>A), TTG518ATA (1887T>A 1889G>A), TCT520TCC (1895T>C), GAT521GAG (1898T>G), GGA523GGT (1904A>T), ACC524ACT (1907C>T), GCG528GCC (1919G>C), AAG529AAA (1922G>A), AGG530TGA (1923A>T 1925G>A), ATG536ATT (1943G>T), ATT538GTT (1947A>G), CCA542CTA (1960C>T), TCC544TCT (1967C>T), CTT545GTG (1968C>G 1970T>G), AGA546AAG (1972G>A 1973A>G), GCT547GCA (1976T>A), TTA549ACA (1980T>A 1981T>C), TCT550TTG (1984C>T 1985T>G), CGT552CGC (1991T>C), GTT555GGT (1999T>G), GGA556GGT (2003A>T), CTT557CTA (2006T>A), GCC559AAC (2010G>A 2011C>A), CTC560ATT (2013C>A 2015C>T), ATC562ACC (2020T>C), GAT565GAC (2030T>C), AAC567AAT (2036C>T), ATA568ATG (2039A>G), CAA572TAG (2049C>T 2051A>G), AGA573TGG (2052A>T 2054A>G), GGT576GGG (2063T>G), GGG578GGA (2069G>A), TAC579TTC (2071A>T), AAG580AGA (2074A>G 2075G>A), GTA581GTT (2078A>T), GCG583TCT (2082G>T 2084G>T), CGC584CGT (2087C>T), TTA585TTG (2090A>G), TTC586ATG (2091T>A 2093C>G), ACT587TCT (2094A>T), ACC588ACT (2099C>T), AGC590TCT (2103A>T 2104G>C 2105C>T), AAG591AAA (2108G>A), GAG594GAA (2117G>A), CGT595AGA (2118C>A 2120T>A), CTA596CTG (2123A>G), GCT598GCG (2129T>G), CAT600GCA (2133C>G 2134A>C 2135T>A), CTT601AGC (2136C>A 2137T>G 2138T>C), AAG602AAA (2141G>A), GTT604CAC (2145G>C 2146T>A 2147T>C), CGG605AGG (2148C>A), CAC607CAT (2156C>T), TTA608CTA (2157T>C), CTA609TTA (2160C>T), TAC611CTC (2166T>C 2167A>T), TGG613TAT (2173G>A 2174G>T), GGA616GGG (2183A>G), AGG617AGA (2186G>A), GGT618TTT (2187G>T 2188G>T), ATG619AAA (2191T>A 2192G>A), CCC620CCT (2195C>T), CCC623CCT (2204C>T), TAT624TTT (2206A>T), AAA626AAG (2213A>G), GGG627GGA (2216G>A), GTA630ATA (2223G>A), AGT631GAT (2226A>G 2227G>A), TAC632TAT (2231C>T), CTT634CGG (2236T>G 2237T>G), AAA635AAG (2240A>G), AAA638ACA (2248A>C), GTT639CCT (2250G>C 2251T>C), TTA642ATA (2259T>A), CAG643CGA (2263A>G 2264G>A), ATC644CTC (2265A>C), CCA646ACT (2271C>A 2273A>T), AAG647GAA (2274A>G 2276G>A), CTG649ATG (2280C>A), GTG650GTA (2285G>A), TTC651TTT (2288C>T), GTT655TGG (2298G>T 2299T>G 2300T>G), GAA656GAG (2303A>G), ATA657ATC (2306A>C), CTA658CTT (2309A>T), CGT660TGC (2313C>T 2315T>C), GTT662ATT (2319G>A), CTA663TTA (2322C>T), CGC664GTC (2325C>G 2326G>T), CAA665AAT (2328C>A 2330A>T), GTA667GTT (2336A>T), GAA668AAA (2337G>A), TTA671CTT (2346T>C 2348A>T), AAG672CGG (2349A>C 2350A>G), CTC674GTT (2355C>G 2357C>T), AAC675TCT (2358A>T 2359A>C 2360C>T), TGG676TAG (2362G>A), CAT678CAC (2369T>C), ACG679ACT (2372G>T), TGT681TTG (2377G>T 2378T>G), AAC683TCT (2382A>T 2383A>C 2384C>T), CCA684TTT (2385C>T 2386C>T 2387A>T), GAT685GAA (2390T>A), TCC687TCT (2396C>T), ATT688ATC (2399T>C), AAG689GAA (2400A>G 2402G>A), AGT690AAA (2404G>A 2405T>A), CTC691TTG (2406C>T 2408C>G), ATC692ATG (2411C>G), GAC693GAT (2414C>T), TCC694GCT (2415T>G 2417C>T), CCT695CCC (2420T>C), ATA696ATT (2423A>T), TGC697TAC (2425G>A), ACA699TAG (2430A>T 2431C>A 2432A>G), TCC700TCA (2435C>A), AAA702AAG (2441A>G), AGG703CGA (2442A>C 2444G>A), CTC704TCG (2445C>T 2446T>C 2447C>G), GAA705AAT (2448G>A 2450A>T), ACT706GAA (2451A>G 2452C>A 2453T>A), GAT707GAC (2456T>C), TTT708CTG (2457T>C 2459T>G), GAT709GAA (2462T>A), TTA710TTG (2465A>G), ATC711GTC (2466A>G), GGC714GGT (2477C>T), CTA715TTA (2478C>T), AAA718AAG (2489A>G), TGT719TGC (2492T>C), TAC720TAT (2495C>T), GGT723GGA (2504T>A), GGT725GGC (2510T>C), AAT727GAT (2514A>G), CCC728CTG (2518C>T 2519C>G), ACC730ATT (2524C>T 2525C>T), ACT731ACA (2528T>A), CAG733AGG (2532C>A 2533A>G), TGG734TAT (2536G>A 2537G>T), TTT736TTG (2543T>G), AAC739AGT (2551A>G 2552C>T), ACT740ACC (2555T>C), GTT741TCT (2556G>T 2557T>C), ATT742ATC (2561T>C), AAT745GAT (2568A>G), AAA746CGA (2571A>C 2572A>G), TTA749TTT (2582A>T), GGG750GGA (2585G>A), GGG754GGA (2597G>A), AAC755GCT (2598A>G 2599A>C 2600C>T), GAT756AAT (2601G>A), ATT758ATA (2609T>A), TCT760GCA (2613T>G 2615T>A), CCT761CCA (2618T>A), GAA763TAT (2622G>T 2624A>T), TAC764TTA (2626A>T 2627C>A), CAA765CCA (2629A>C), ACT766TCT (2631A>T), ACA767CTA (2634A>C 2635C>T), GGA768GTG (2638G>T 2639A>G)								

*: Inserts / Deletes / Misaligned / Frameshifts

Analysis details

This analysis was performed with panviral2.64

NGS Details (UN9): Harvey murine sarcoma virus

Assembly

**Coverage Length**	215 (1 contig(s))
**Depth Of Coverage**	3.2
**Number Of Reads**	6
**Reads Per Million**	0.14 rpm (after QC)
**Ambiguities**	0
**Assembly Method**	de novo + reference guided assembly
**Consensus Caller**	Bcf Tools

Coverage Map

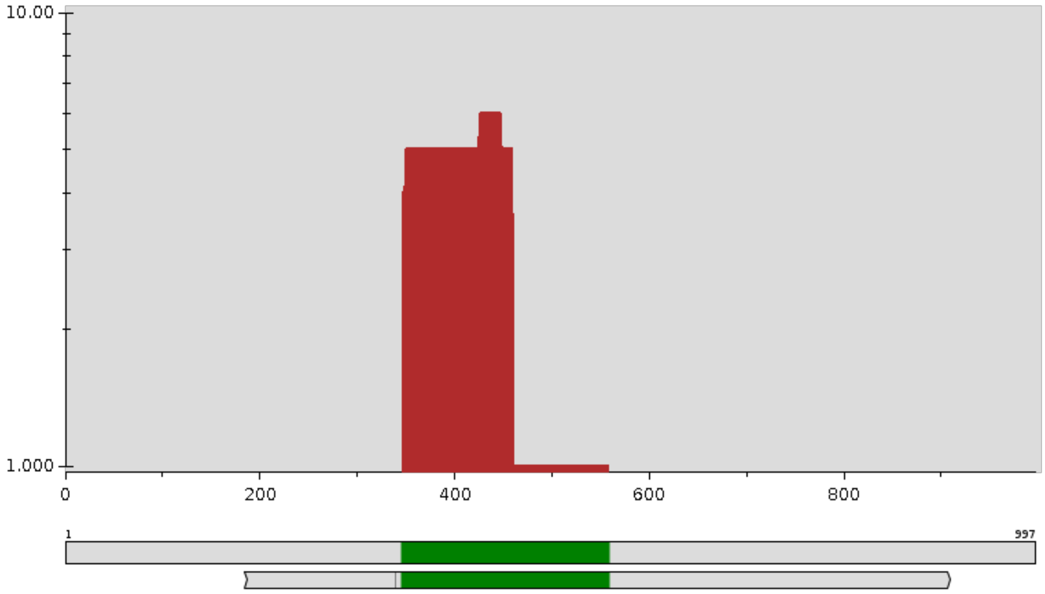

Assignment

**Type**	Harvey murine sarcoma virus (Taxonomy ID: 11807)
**Reference Genome**	NC_038668.1
**NT Identity (%)**	70.2326
**AA Identity (%)**	83.3333
**Number Of Stop Codons**	0
**Number Of CDS**	2

Alignment

**Alignment Score**	174.0 (NT) + 798.0 (AA) = 972.0
**Concordance (%)**	70.1299

Alignment Method	Global, seeded, nucleotide + amino acids (AGA)

Genome Region

Sequence starts at position 346 and ends at position 560 relative to NC_038668.1 reference sequence.

Alignment Detailed Statistics

	Begin	End	Coverage	Score	Concordance	Matches	Identities	I/D/M/F*	Stop Codons
**NT**	**346**	**560**	**21.6%**	**174**	**40.5%**	**215 (100%)**	**151 (70.2%)**	**0/0**	
Mutations:	346A>G, 355G>A, 358T>C, 361G>C, 364G>C, 367G>C, 372C>G, 374A>G, 376A>T, 379C>T, 382G>C, 389A>T, 390G>C, 391T>C, 394C>T, 397G>C, 400C>G, 403C>A, 406G>A, 407C>T, 409G>C, 412C>A, 415G>A, 416A>T, 417A>C, 418C>G, 424T>C, 427G>C, 430C>T, 439T>C, 442C>T, 448A>C, 451G>A, 457C>A, 463G>C, 469G>A, 470G>T, 471T>G, 472A>C, 475C>G, 478T>A, 481T>C, 483G>A, 484G>A, 487G>A, 488A>G, 489C>T, 490G>T, 491T>G, 492G>C, 493T>A, 503A>G, 511C>T, 515A>G, 517A>C, 520T>C, 529G>A, 532T>C, 533A>T, 534G>C, 542C>A, 547C>G, 550G>A, 559C>G								

CDS

	Begin	End	Coverage	Score	Concordance	Matches	Identities	I/D/M/F*	Stop Codons
D1R95_gp1	55	126	29.8%	399	81.8%	72 (100%)	60 (83.3%)	0/0/0/0	0
Protein mutations:	A63G (372C>G), R64G (374A>G 376A>T), L75F (407C>T 409G>C), N78S (416A>T 417A>C 418C>G), V96C (470G>T 471T>G 472A>C), G100E (483G>A 484G>A), T102V (488A>G 489C>T 490G>T), C103A (491T>G 492G>C 493T>A), I107V (503A>G), T111A (515A>G 517A>C), D121E (547C>G)								
Codon mutations:	ACA54..G (346A>G), AAG57AAA (355G>A), CTT58CTC (358T>C), GTG59GTC (361G>C), GTG60GTC (364G>C), GTG61GTC (367G>C), GCT63GGT (372C>G), AGA64GGT (374A>G 376A>T), GGC65GGT (379C>T), GTG66GTC (382G>C), AGT69TCC (389A>T 390G>C 391T>C), GCC70GCT (394C>T), CTG71CTC (397G>C), ACC72ACG (400C>G), ATC73ATA (403C>A), CAG74CAA (406G>A), CTG75TTC (407C>T 409G>C), ATC76ATA (412C>A), CAG77CAA (415G>A), AAC78TCG (416A>T 417A>C 418C>G), TTT80TTC (424T>C), GTG81GTC (427G>C), GAC82GAT (430C>T), GAT85GAC (439T>C), CCC86CCT (442C>T), ATA88ATC (448A>C), GAG89GAA (451G>A), TCC91TCA (457C>A), CGG93CGC (463G>C), CAG95CAA (469G>A), GTA96TGC (470G>T 471T>G 472A>C), GTC97GTG (475C>G), ATT98ATA (478T>A), GAT99GAC (481T>C), GGG100GAA (483G>A 484G>A), GAG101GAA (487G>A), ACG102GTT (488A>G 489C>T 490G>T), TGT103GCA (491T>G 492G>C 493T>A), ATC107GTC (503A>G), GAC109GAT (511C>T), ACA111GCC (515A>G 517A>C), GGT112GGC (520T>C), GAG115GAA (529G>A), TAT116TAC (532T>C), AGT117TCT (533A>T 534G>C), CGG120AGG (542C>A), GAC121GAG (547C>G), CAG122CAA (550G>A), CGC125CGG (559C>G)								
D1R95_gp2	3	74	37.9%	399	81.8%	72 (100%)	60 (83.3%)	0/0/0/0	0
Protein mutations:	A11G (372C>G), R12G (374A>G 376A>T), L23F (407C>T 409G>C), N26S (416A>T 417A>C 418C>G), V44C (470G>T 471T>G 472A>C), G48E (483G>A 484G>A), T50V (488A>G 489C>T 490G>T), C51A (491T>G 492G>C 493T>A), I55V (503A>G), T59A (515A>G 517A>C), D69E (547C>G)								
Codon mutations:	ACA2..G (346A>G), AAG5AAA (355G>A), CTT6CTC (358T>C), GTG7GTC (361G>C), GTG8GTC (364G>C), GTG9GTC (367G>C), GCT11GGT (372C>G), AGA12GGT (374A>G 376A>T), GGC13GGT (379C>T), GTG14GTC (382G>C), AGT17TCC (389A>T 390G>C 391T>C), GCC18GCT (394C>T), CTG19CTC (397G>C), ACC20ACG (400C>G), ATC21ATA (403C>A), CAG22CAA (406G>A), CTG23TTC (407C>T 409G>C), ATC24ATA (412C>A), CAG25CAA (415G>A), AAC26TCG (416A>T 417A>C 418C>G), TTT28TTC (424T>C), GTG29GTC (427G>C), GAC30GAT (430C>T), GAT33GAC (439T>C), CCC34CCT (442C>T), ATA36ATC (448A>C), GAG37GAA (451G>A), TCC39TCA (457C>A), CGG41CGC (463G>C), CAG43CAA (469G>A), GTA44TGC (470G>T 471T>G 472A>C), GTC45GTG (475C>G), ATT46ATA (478T>A), GAT47GAC (481T>C), GGG48GAA (483G>A 484G>A), GAG49GAA (487G>A), ACG50GTT (488A>G 489C>T 490G>T), TGT51GCA (491T>G 492G>C 493T>A), ATC55GTC (503A>G), GAC57GAT (511C>T), ACA59GCC (515A>G 517A>C), GGT60GGC (520T>C), GAG63GAA (529G>A), TAT64TAC (532T>C), AGT65TCT (533A>T 534G>C), CGG68AGG (542C>A), GAC69GAG (547C>G), CAG70CAA (550G>A), CGC73CGG (559C>G)								

Proteins

	Begin	End	Coverage	Score	Concordance	Matches	Identities	I/D/M/F*	Stop Codons
hypothetical protein (YP_009507788.1)	55	126	29.8%	399	81.8%	72 (100%)	60 (83.3%)	0/0/0/0	0
Protein mutations:	A63G (372C>G), R64G (374A>G 376A>T), L75F (407C>T 409G>C), N78S (416A>T 417A>C 418C>G), V96C (470G>T 471T>G 472A>C), G100E (483G>A 484G>A), T102V (488A>G 489C>T 490G>T), C103A (491T>G 492G>C 493T>A), I107V (503A>G), T111A (515A>G 517A>C), D121E (547C>G)								
Codon mutations:	ACA54..G (346A>G), AAG57AAA (355G>A), CTT58CTC (358T>C), GTG59GTC (361G>C), GTG60GTC (364G>C), GTG61GTC (367G>C), GCT63GGT (372C>G), AGA64GGT (374A>G 376A>T), GGC65GGT (379C>T), GTG66GTC (382G>C), AGT69TCC (389A>T 390G>C 391T>C), GCC70GCT (394C>T), CTG71CTC (397G>C), ACC72ACG (400C>G), ATC73ATA (403C>A), CAG74CAA (406G>A), CTG75TTC (407C>T 409G>C), ATC76ATA (412C>A), CAG77CAA (415G>A), AAC78TCG (416A>T 417A>C 418C>G), TTT80TTC (424T>C), GTG81GTC (427G>C), GAC82GAT (430C>T), GAT85GAC (439T>C), CCC86CCT (442C>T), ATA88ATC (448A>C), GAG89GAA (451G>A), TCC91TCA (457C>A), CGG93CGC (463G>C), CAG95CAA (469G>A), GTA96TGC (470G>T 471T>G 472A>C), GTC97GTG (475C>G), ATT98ATA (478T>A), GAT99GAC (481T>C), GGG100GAA (483G>A 484G>A), GAG101GAA (487G>A), ACG102GTT (488A>G 489C>T 490G>T), TGT103GCA (491T>G 492G>C 493T>A), ATC107GTC (503A>G), GAC109GAT (511C>T), ACA111GCC (515A>G 517A>C), GGT112GGC (520T>C), GAG115GAA (529G>A), TAT116TAC (532T>C), AGT117TCT (533A>T 534G>C), CGG120AGG (542C>A), GAC121GAG (547C>G), CAG122CAA (550G>A), CGC125CGG (559C>G)								
hypothetical protein (YP_009507789.1)	3	74	37.9%	399	81.8%	72 (100%)	60 (83.3%)	0/0/0/0	0
Protein mutations:	A11G (372C>G), R12G (374A>G 376A>T), L23F (407C>T 409G>C), N26S (416A>T 417A>C 418C>G), V44C (470G>T 471T>G 472A>C), G48E (483G>A 484G>A), T50V (488A>G 489C>T 490G>T), C51A (491T>G 492G>C 493T>A), I55V (503A>G), T59A (515A>G 517A>C), D69E (547C>G)								
Codon mutations:	ACA2..G (346A>G), AAG5AAA (355G>A), CTT6CTC (358T>C), GTG7GTC (361G>C), GTG8GTC (364G>C), GTG9GTC (367G>C), GCT11GGT (372C>G), AGA12GGT (374A>G 376A>T), GGC13GGT (379C>T), GTG14GTC (382G>C), AGT17TCC (389A>T 390G>C 391T>C), GCC18GCT (394C>T), CTG19CTC (397G>C), ACC20ACG (400C>G), ATC21ATA (403C>A), CAG22CAA (406G>A), CTG23TTC (407C>T 409G>C), ATC24ATA (412C>A), CAG25CAA (415G>A), AAC26TCG (416A>T 417A>C 418C>G), TTT28TTC (424T>C), GTG29GTC (427G>C), GAC30GAT (430C>T), GAT33GAC (439T>C), CCC34CCT (442C>T), ATA36ATC (448A>C), GAG37GAA (451G>A), TCC39TCA (457C>A), CGG41CGC (463G>C), CAG43CAA (469G>A), GTA44TGC (470G>T 471T>G 472A>C), GTC45GTG (475C>G), ATT46ATA (478T>A), GAT47GAC (481T>C), GGG48GAA (483G>A 484G>A), GAG49GAA (487G>A), ACG50GTT (488A>G 489C>T 490G>T), TGT51GCA (491T>G 492G>C 493T>A), ATC55GTC (503A>G), GAC57GAT (511C>T), ACA59GCC (515A>G 517A>C), GGT60GGC (520T>C), GAG63GAA (529G>A), TAT64TAC (532T>C), AGT65TCT (533A>T 534G>C), CGG68AGG (542C>A), GAC69GAG (547C>G), CAG70CAA (550G>A), CGC73CGG (559C>G)								

*: Inserts / Deletes / Misaligned / Frameshifts

Analysis details

This analysis was performed with panviral2.64

NGS Details (UN9): Tomato necrotic dwarf virus (segment RNA1)

Assembly

**Coverage Length**	799 (2 contig(s))
**Depth Of Coverage**	9641.5
**Number Of Reads**	65373
**Reads Per Million**	1477.62 rpm (after QC)
**Ambiguities**	0
**Assembly Method**	de novo + reference guided assembly
**Consensus Caller**	Bcf Tools

Coverage Map

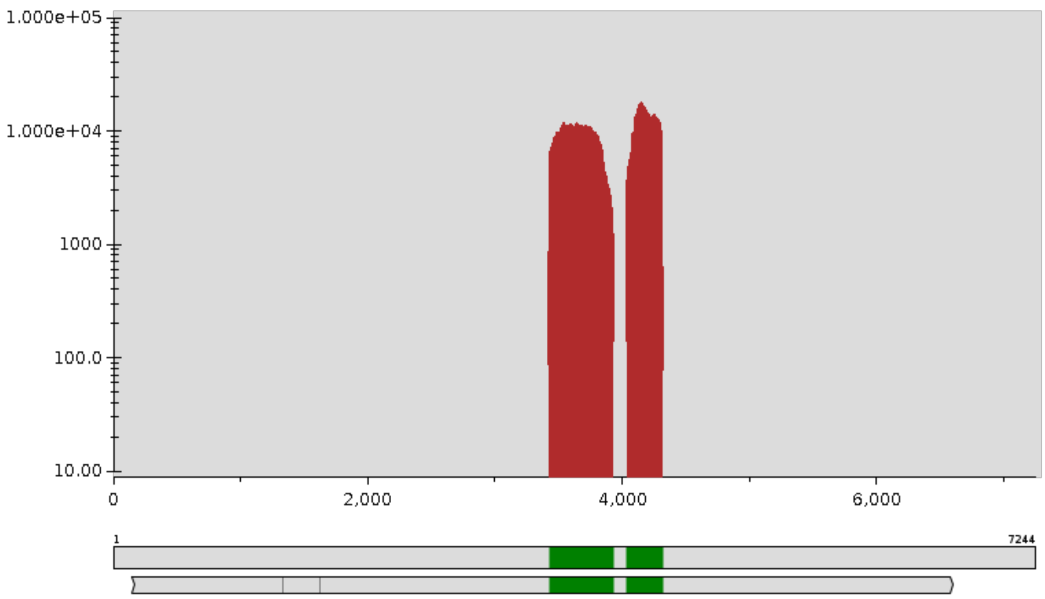

Assignment

**Type**	Tomato necrotic dwarf virus (Taxonomy ID: 1481465)
**Reference Genome**	NC_027926.1
**NT Identity (%)**	71.5895
**AA Identity (%)**	77.5281
**Number Of Stop Codons**	0
**Number Of CDS**	1

Alignment

**Alignment Score**	690.0 (NT) + 1456.0 (AA) = 2146.0
**Concordance (%)**	62.9326

Alignment Method	Global, seeded, nucleotide + amino acids (AGA)

Genome Region

Sequence starts at position 3424 and ends at position 4321 relative to NC_027926.1 reference sequence.

Alignment Detailed Statistics

	Begin	End	Coverage	Score	Concordance	Matches	Identities	I/D/M/F*	Stop Codons
**NT**	**3424**	**4321**	**11.0%**	**690**	**43.2%**	**799 (100%)**	**572 (71.6%)**	**0/0**	
Mutations:	3433G>A, 3440C>T, 3444A>T, 3453A>C, 3459C>A, 3460A>C, 3462T>G, 3463C>T, 3465C>G, 3468A>G, 3469G>C, 3470C>A, 3472A>C, 3474T>G, 3477G>A, 3478A>T, 3479G>C, 3480T>C, 3482G>A, 3483A>G, 3486A>G, 3487A>G, 3488C>A, 3489C>A, 3491C>G, 3492T>A, 3493C>T, 3495T>C, 3501A>G, 3507C>A, 3510A>G, 3513C>G, 3514T>G, 3516C>T, 3519T>C, 3520C>T, 3522T>G, 3531T>A, 3534T>G, 3535C>T, 3536A>T, 3537A>G, 3541T>A, 3546C>T, 3549A>T, 3555A>G, 3558C>G, 3561T>C, 3567T>A, 3570A>C, 3573C>T, 3575G>A, 3577A>C, 3578A>G, 3579G>C, 3584A>C, 3585A>C, 3586T>C, 3597A>T, 3603T>G, 3604C>G, 3605C>A, 3606T>C, 3610G>C, 3612A>G, 3615C>T, 3618T>C, 3621C>A, 3624A>C, 3627C>T, 3630C>A, 3631A>T, 3633C>T, 3635T>C, 3639T>A, 3646C>A, 3654G>A, 3657A>G, 3660C>T, 3666A>C, 3675A>T, 3681G>C, 3682A>G, 3683T>A, 3687A>G, 3690C>T, 3699T>A, 3703C>T, 3704A>C, 3705T>C, 3706G>A, 3707G>A, 3708T>C, 3714T>G, 3717T>G, 3718G>C, 3719A>C, 3720T>A, 3726T>A, 3729A>G, 3730T>C, 3733T>G, 3735C>T, 3741A>G, 3742A>G, 3743C>G, 3747C>T, 3751A>G, 3752G>C, 3753T>A, 3756A>C, 3757C>A, 3759G>T, 3762G>T, 3765A>T, 3766A>G, 3767C>T, 3768A>G, 3769A>C, 3771G>T, 3774A>G, 3776A>T, 3777C>G, 3780A>G, 3781A>C, 3782A>G, 3795C>G, 3801A>T, 3802C>G, 3803A>C, 3804G>A, 3805G>A, 3806A>T, 3807G>C, 3811G>A, 3822G>A, 3828G>C, 3837T>A, 3845G>T, 3854A>T, 3860G>C, 3861C>A, 3868C>T, 3870C>G, 3876A>G, 3879C>A, 3888C>T, 3894C>T, 3901A>T, 3906C>A, 3907A>C, 3909G>T, 3912A>T, 3913A>T, 3914T>C, 3915G>T, 3921G>A, 3922C>A, 4047C>A, 4050A>C, 4053A>G, 4059C>G, 4062G>A, 4068G>T, 4071C>G, 4072C>T, 4074T>G, 4077T>A, 4080T>G, 4083A>C, 4089T>A, 4092T>C, 4104A>G, 4107A>C, 4108A>C, 4110A>C, 4111T>C, 4113A>G, 4116T>C, 4119G>A, 4122A>C, 4137C>T, 4149C>G, 4150A>C, 4161T>C, 4173C>T, 4176C>T, 4177A>T, 4178C>G, 4179C>T, 4188A>T, 4189T>G, 4190T>C, 4197T>C, 4200C>A, 4201A>C, 4206A>C, 4209T>C, 4215T>A, 4218A>T, 4221A>C, 4222G>A, 4230C>G, 4233T>C, 4234T>C, 4235C>A, 4243T>A, 4245T>A, 4246C>G, 4248T>G, 4251T>G, 4254C>T, 4256A>G, 4257C>T, 4259A>G, 4263C>T, 4267G>T, 4269T>A, 4279A>G, 4280C>A, 4281T>G, 4284G>A, 4287T>C, 4293T>C, 4302A>G, 4305T>C, 4311C>T								

CDS

	Begin	End	Coverage	Score	Concordance	Matches	Identities	I/D/M/F*	Stop Codons
APL33_sRA11gp1	1092	1391	12.4%	1456	79.4%	267 (100%)	207 (77.5%)	0/0/0/0	0
Protein mutations:	V1095I (3433G>A), A1097V (3440C>T), D1103E (3459C>A), I1104L (3460A>C 3462T>G), A1107H (3469G>C 3470C>A), I1108L (3472A>C 3474T>G), R1111K (3482G>A 3483A>G), T1113E (3487A>G 3488C>A 3489C>A), T1114R (3491C>G 3492T>A), L1115F (3493C>T 3495T>C), N1119K (3507C>A), S1122A (3514T>G 3516C>T), Q1129L (3535C>T 3536A>T 3537A>G), L1131M (3541T>A), R1142K (3575G>A), K1143R (3577A>C 3578A>G 3579G>C), Q1145P (3584A>C 3585A>C), P1152D (3604C>G 3605C>A 3606T>C), E1154Q (3610G>C 3612A>G), T1161S (3631A>T 3633C>T), V1162A (3635T>C), N1163K (3639T>A), M1178E (3682A>G 3683T>A), H1185S (3703C>T 3704A>C 3705T>C), G1186N (3706G>A 3707G>A 3708T>C), D1188E (3714T>G), D1189E (3717T>G), D1190P (3718G>C 3719A>C 3720T>A), S1195A (3733T>G 3735C>T), T1198G (3742A>G 3743C>G), S1201A (3751A>G 3752G>C 3753T>A), L1203I (3757C>A 3759G>T), T1206V (3766A>G 3767C>T 3768A>G), M1207L (3769A>C 3771G>T), N1209M (3776A>T 3777C>G), K1211R (3781A>C 3782A>G), Q1218A (3802C>G 3803A>C 3804G>A), E1219I (3805G>A 3806A>T 3807G>C), D1221N (3811G>A), C1232F (3845G>T), Y1235F (3854A>T), G1237A (3860G>C 3861C>A), M1251L (3901A>T), M1253L (3907A>C 3909G>T), M1255S (3913A>T 3914T>C 3915G>T), E1306D (4068G>T), E1311D (4083A>C), T1343C (4177A>T 4178C>G 4179C>T), L1347A (4189T>G 4190T>C), I1351L (4201A>C), D1358N (4222G>A), S1362H (4234T>C 4235C>A), S1365T (4243T>A 4245T>A), L1366V (4246C>G 4248T>G), N1369S (4256A>G 4257C>T), N1370S (4259A>G), A1373S (4267G>T 4269T>A), T1377E (4279A>G 4280C>A 4281T>G)								
Codon mutations:	GTT1095ATT (3433G>A), GCG1097GTG (3440C>T), GCA1098GCT (3444A>T), CGA1101CGC (3453A>C), GAC1103GAA (3459C>A), ATT1104CTG (3460A>C 3462T>G), CTC1105TTG (3463C>T 3465C>G), AAA1106AAG (3468A>G), GCC1107CAC (3469G>C 3470C>A), ATT1108CTG (3472A>C 3474T>G), AGG1109AGA (3477G>A), AGT1110TCC (3478A>T 3479G>C 3480T>C), AGA1111AAG (3482G>A 3483A>G), GAA1112GAG (3486A>G), ACC1113GAA (3487A>G 3488C>A 3489C>A), ACT1114AGA (3491C>G 3492T>A), CTT1115TTC (3493C>T 3495T>C), GAA1117GAG (3501A>G), AAC1119AAA (3507C>A), CAA1120CAG (3510A>G), GTC1121GTG (3513C>G), TCC1122GCT (3514T>G 3516C>T), TTT1123TTC (3519T>C), CTT1124TTG (3520C>T 3522T>G), GTT1127GTA (3531T>A), CCT1128CCG (3534T>G), CAA1129TTG (3535C>T 3536A>T 3537A>G), TTG1131ATG (3541T>A), GCC1132GCT (3546C>T), GCA1133GCT (3549A>T), GTA1135GTG (3555A>G), CCC1136CCG (3558C>G), CAT1137CAC (3561T>C), ACT1139ACA (3567T>A), ACA1140ACC (3570A>C), CTC1141CTT (3573C>T), AGA1142AAA (3575G>A), AAG1143CGC (3577A>C 3578A>G 3579G>C), CAA1145CCC (3584A>C 3585A>C), TTG1146CTG (3586T>C), GCA1149GCT (3597A>T), GGT1151GGG (3603T>G), CCT1152GAC (3604C>G 3605C>A 3606T>C), GAA1154CAG (3610G>C 3612A>G), ACC1155ACT (3615C>T), GCT1156GCC (3618T>C), CCC1157CCA (3621C>A), TCA1158TCC (3624A>C), ATC1159ATT (3627C>T), CTC1160CTA (3630C>A), ACC1161TCT (3631A>T 3633C>T), GTC1162GCC (3635T>C), AAT1163AAA (3639T>A), CGG1166AGG (3646C>A), GAG1168GAA (3654G>A), GGA1169GGG (3657A>G), TTC1170TTT (3660C>T), CCA1172CCC (3666A>C), GCA1175GCT (3675A>T), GTG1177GTC (3681G>C), ATG1178GAG (3682A>G 3683T>A), AAA1179AAG (3687A>G), TAC1180TAT (3690C>T), ACT1183ACA (3699T>A), CAT1185TCC (3703C>T 3704A>C 3705T>C), GGT1186AAC (3706G>A 3707G>A 3708T>C), GAT1188GAG (3714T>G), GAT1189GAG (3717T>G), GAT1190CCA (3718G>C 3719A>C 3720T>A), GCT1192GCA (3726T>A), AAA1193AAG (3729A>G), TTG1194CTG (3730T>C), TCC1195GCT (3733T>G 3735C>T), GAA1197GAG (3741A>G), ACT1198GGT (3742A>G 3743C>G), CTC1199CTT (3747C>T), AGT1201GCA (3751A>G 3752G>C 3753T>A), TCA1202TCC (3756A>C), CTG1203ATT (3757C>A 3759G>T), CTG1204CTT (3762G>T), CCA1205CCT (3765A>T), ACA1206GTG (3766A>G 3767C>T 3768A>G), ATG1207CTT (3769A>C 3771G>T), AAA1208AAG (3774A>G), AAC1209ATG (3776A>T 3777C>G), CAA1210CAG (3780A>G), AAA1211CGA (3781A>C 3782A>G), GGC1215GGG (3795C>G), CCA1217CCT (3801A>T), CAG1218GCA (3802C>G 3803A>C 3804G>A), GAG1219ATC (3805G>A 3806A>T 3807G>C), GAT1221AAT (3811G>A), GTG1224GTA (3822G>A), CTG1226CTC (3828G>C), ATT1229ATA (3837T>A), TGT1232TTT (3845G>T), TAT1235TTT (3854A>T), GGC1237GCA (3860G>C 3861C>A), CTC1240TTG (3868C>T 3870C>G), ACA1242ACG (3876A>G), TCC1243TCA (3879C>A), TAC1246TAT (3888C>T), TTC1248TTT (3894C>T), ATG1251TTG (3901A>T), GGC1252GGA (3906C>A), ATG1253CTT (3907A>C 3909G>T), GGA1254GGT (3912A>T), ATG1255TCT (3913A>T 3914T>C 3915G>T), AAG1257AAA (3921G>A), CGG1258AGG (3922C>A), ACC1299ACA (4047C>A), ACA1300ACC (4050A>C), CAA1301CAG (4053A>G), GCC1303GCG (4059C>G), AAG1304AAA (4062G>A), GAG1306GAT (4068G>T), CGC1307CGG (4071C>G), CTT1308TTG (4072C>T 4074T>G), CCT1309CCA (4077T>A), CTT1310CTG (4080T>G), GAA1311GAC (4083A>C), ATT1313ATA (4089T>A), TAT1314TAC (4092T>C), AAA1318AAG (4104A>G), ACA1319ACC (4107A>C), AGA1320CGC (4108A>C 4110A>C), TTA1321CTG (4111T>C 4113A>G), TTT1322TTC (4116T>C), GAG1323GAA (4119G>A), ATA1324ATC (4122A>C), TAC1329TAT (4137C>T), GTC1333GTG (4149C>G), AGG1334CGG (4150A>C), TTT1337TTC (4161T>C), TCC1341TCT (4173C>T), GCC1342GCT (4176C>T), ACC1343TGT (4177A>T 4178C>G 4179C>T), GCA1346GCT (4188A>T), TTA1347GCA (4189T>G 4190T>C), AAT1349AAC (4197T>C), GTC1350GTA (4200C>A), ATC1351CTC (4201A>C), CCA1352CCC (4206A>C), TGT1353TGC (4209T>C), GTT1355GTA (4215T>A), GGA1356GGT (4218A>T), ATA1357ATC (4221A>C), GAT1358AAT (4222G>A), ACC1360ACG (4230C>G), AGT1361AGC (4233T>C), TCT1362CAT (4234T>C 4235C>A), TCT1365ACA (4243T>A 4245T>A), CTT1366GTG (4246C>G 4248T>G), CTT1367CTG (4251T>G), GCC1368GCT (4254C>T), AAC1369AGT (4256A>G 4257C>T), AAC1370AGC (4259A>G), TTC1371TTT (4263C>T), GCT1373TCA (4267G>T 4269T>A), ACT1377GAG (4279A>G 4280C>A 4281T>G), GGG1378GGA (4284G>A), TTT1379TTC (4287T>C), GCT1381GCC (4293T>C), TCA1384TCG (4302A>G), AGT1385AGC (4305T>C), GAC1387GAT (4311C>T)								

Proteins

	Begin	End	Coverage	Score	Concordance	Matches	Identities	I/D/M/F*	Stop Codons
polyprotein (YP_009165993.1)	1092	1391	12.4%	1456	79.4%	267 (100%)	207 (77.5%)	0/0/0/0	0
Protein mutations:	V1095I (3433G>A), A1097V (3440C>T), D1103E (3459C>A), I1104L (3460A>C 3462T>G), A1107H (3469G>C 3470C>A), I1108L (3472A>C 3474T>G), R1111K (3482G>A 3483A>G), T1113E (3487A>G 3488C>A 3489C>A), T1114R (3491C>G 3492T>A), L1115F (3493C>T 3495T>C), N1119K (3507C>A), S1122A (3514T>G 3516C>T), Q1129L (3535C>T 3536A>T 3537A>G), L1131M (3541T>A), R1142K (3575G>A), K1143R (3577A>C 3578A>G 3579G>C), Q1145P (3584A>C 3585A>C), P1152D (3604C>G 3605C>A 3606T>C), E1154Q (3610G>C 3612A>G), T1161S (3631A>T 3633C>T), V1162A (3635T>C), N1163K (3639T>A), M1178E (3682A>G 3683T>A), H1185S (3703C>T 3704A>C 3705T>C), G1186N (3706G>A 3707G>A 3708T>C), D1188E (3714T>G), D1189E (3717T>G), D1190P (3718G>C 3719A>C 3720T>A), S1195A (3733T>G 3735C>T), T1198G (3742A>G 3743C>G), S1201A (3751A>G 3752G>C 3753T>A), L1203I (3757C>A 3759G>T), T1206V (3766A>G 3767C>T 3768A>G), M1207L (3769A>C 3771G>T), N1209M (3776A>T 3777C>G), K1211R (3781A>C 3782A>G), Q1218A (3802C>G 3803A>C 3804G>A), E1219I (3805G>A 3806A>T 3807G>C), D1221N (3811G>A), C1232F (3845G>T), Y1235F (3854A>T), G1237A (3860G>C 3861C>A), M1251L (3901A>T), M1253L (3907A>C 3909G>T), M1255S (3913A>T 3914T>C 3915G>T), E1306D (4068G>T), E1311D (4083A>C), T1343C (4177A>T 4178C>G 4179C>T), L1347A (4189T>G 4190T>C), I1351L (4201A>C), D1358N (4222G>A), S1362H (4234T>C 4235C>A), S1365T (4243T>A 4245T>A), L1366V (4246C>G 4248T>G), N1369S (4256A>G 4257C>T), N1370S (4259A>G), A1373S (4267G>T 4269T>A), T1377E (4279A>G 4280C>A 4281T>G)								

	Begin	End	Coverage	Score	Concordance	Matches	Identities	I/D/M/F*	Stop Codons
**NT**	**3424**	**4321**	**11.0%**	**690**	**43.2%**	**799 (100%)**	**572 (71.6%)**	**0/0**	
Codon mutations:	GTT1095ATT (3433G>A), GCG1097GTG (3440C>T), GCA1098GCT (3444A>T), CGA1101CGC (3453A>C), GAC1103GAA (3459C>A), ATT1104CTG (3460A>C 3462T>G), CTC1105TTG (3463C>T 3465C>G), AAA1106AAG (3468A>G), GCC1107CAC (3469G>C 3470C>A), ATT1108CTG (3472A>C 3474T>G), AGG1109AGA (3477G>A), AGT1110TCC (3478A>T 3479G>C 3480T>C), AGA1111AAG (3482G>A 3483A>G), GAA1112GAG (3486A>G), ACC1113GAA (3487A>G 3488C>A 3489C>A), ACT1114AGA (3491C>G 3492T>A), CTT1115TTC (3493C>T 3495T>C), GAA1117GAG (3501A>G), AAC1119AAA (3507C>A), CAA1120CAG (3510A>G), GTC1121GTG (3513C>G), TCC1122GCT (3514T>G 3516C>T), TTT1123TTC (3519T>C), CTT1124TTG (3520C>T 3522T>G), GTT1127GTA (3531T>A), CCT1128CCG (3534T>G), CAA1129TTG (3535C>T 3536A>T 3537A>G), TTG1131ATG (3541T>A), GCC1132GCT (3546C>T), GCA1133GCT (3549A>T), GTA1135GTG (3555A>G), CCC1136CCG (3558C>G), CAT1137CAC (3561T>C), ACT1139ACA (3567T>A), ACA1140ACC (3570A>C), CTC1141CTT (3573C>T), AGA1142AAA (3575G>A), AAG1143CGC (3577A>C 3578A>G 3579G>C), CAA1145CCC (3584A>C 3585A>C), TTG1146CTG (3586T>C), GCA1149GCT (3597A>T), GGT1151GGG (3603T>G), CCT1152GAC (3604C>G 3605C>A 3606T>C), GAA1154CAG (3610G>C 3612A>G), ACC1155ACT (3615C>T), GCT1156GCC (3618T>C), CCC1157CCA (3621C>A), TCA1158TCC (3624A>C), ATC1159ATT (3627C>T), CTC1160CTA (3630C>A), ACC1161TCT (3631A>T 3633C>T), GTC1162GCC (3635T>C), AAT1163AAA (3639T>A), CGG1166AGG (3646C>A), GAG1168GAA (3654G>A), GGA1169GGG (3657A>G), TTC1170TTT (3660C>T), CCA1172CCC (3666A>C), GCA1175GCT (3675A>T), GTG1177GTC (3681G>C), ATG1178GAG (3682A>G 3683T>A), AAA1179AAG (3687A>G), TAC1180TAT (3690C>T), ACT1183ACA (3699T>A), CAT1185TCC (3703C>T 3704A>C 3705T>C), GGT1186AAC (3706G>A 3707G>A 3708T>C), GAT1188GAG (3714T>G), GAT1189GAG (3717T>G), GAT1190CCA (3718G>C 3719A>C 3720T>A), GCT1192GCA (3726T>A), AAA1193AAG (3729A>G), TTG1194CTG (3730T>C), TCC1195GCT (3733T>G 3735C>T), GAA1197GAG (3741A>G), ACT1198GGT (3742A>G 3743C>G), CTC1199CTT (3747C>T), AGT1201GCA (3751A>G 3752G>C 3753T>A), TCA1202TCC (3756A>C), CTG1203ATT (3757C>A 3759G>T), CTG1204CTT (3762G>T), CCA1205CCT (3765A>T), ACA1206GTG (3766A>G 3767C>T 3768A>G), ATG1207CTT (3769A>C 3771G>T), AAA1208AAG (3774A>G), AAC1209ATG (3776A>T 3777C>G), CAA1210CAG (3780A>G), AAA1211CGA (3781A>C 3782A>G), GGC1215GGG (3795C>G), CCA1217CCT (3801A>T), CAG1218GCA (3802C>G 3803A>C 3804G>A), GAG1219ATC (3805G>A 3806A>T 3807G>C), GAT1221AAT (3811G>A), GTG1224GTA (3822G>A), CTG1226CTC (3828G>C), ATT1229ATA (3837T>A), TGT1232TTT (3845G>T), TAT1235TTT (3854A>T), GGC1237GCA (3860G>C 3861C>A), CTC1240TTG (3868C>T 3870C>G), ACA1242ACG (3876A>G), TCC1243TCA (3879C>A), TAC1246TAT (3888C>T), TTC1248TTT (3894C>T), ATG1251TTG (3901A>T), GGC1252GGA (3906C>A), ATG1253CTT (3907A>C 3909G>T), GGA1254GGT (3912A>T), ATG1255TCT (3913A>T 3914T>C 3915G>T), AAG1257AAA (3921G>A), CGG1258AGG (3922C>A), ACC1299ACA (4047C>A), ACA1300ACC (4050A>C), CAA1301CAG (4053A>G), GCC1303GCG (4059C>G), AAG1304AAA (4062G>A), GAG1306GAT (4068G>T), CGC1307CGG (4071C>G), CTT1308TTG (4072C>T 4074T>G), CCT1309CCA (4077T>A), CTT1310CTG (4080T>G), GAA1311GAC (4083A>C), ATT1313ATA (4089T>A), TAT1314TAC (4092T>C), AAA1318AAG (4104A>G), ACA1319ACC (4107A>C), AGA1320CGC (4108A>C 4110A>C), TTA1321CTG (4111T>C 4113A>G), TTT1322TTC (4116T>C), GAG1323GAA (4119G>A), ATA1324ATC (4122A>C), TAC1329TAT (4137C>T), GTC1333GTG (4149C>G), AGG1334CGG (4150A>C), TTT1337TTC (4161T>C), TCC1341TCT (4173C>T), GCC1342GCT (4176C>T), ACC1343TGT (4177A>T 4178C>G 4179C>T), GCA1346GCT (4188A>T), TTA1347GCA (4189T>G 4190T>C), AAT1349AAC (4197T>C), GTC1350GTA (4200C>A), ATC1351CTC (4201A>C), CCA1352CCC (4206A>C), TGT1353TGC (4209T>C), GTT1355GTA (4215T>A), GGA1356GGT (4218A>T), ATA1357ATC (4221A>C), GAT1358AAT (4222G>A), ACC1360ACG (4230C>G), AGT1361AGC (4233T>C), TCT1362CAT (4234T>C 4235C>A), TCT1365ACA (4243T>A 4245T>A), CTT1366GTG (4246C>G 4248T>G), CTT1367CTG (4251T>G), GCC1368GCT (4254C>T), AAC1369AGT (4256A>G 4257C>T), AAC1370AGC (4259A>G), TTC1371TTT (4263C>T), GCT1373TCA (4267G>T 4269T>A), ACT1377GAG (4279A>G 4280C>A 4281T>G), GGG1378GGA (4284G>A), TTT1379TTC (4287T>C), GCT1381GCC (4293T>C), TCA1384TCG (4302A>G), AGT1385AGC (4305T>C), GAC1387GAT (4311C>T)								

*: Inserts / Deletes / Misaligned / Frameshifts

Analysis details

This analysis was performed with panviral2.64

NGS Details (UN9): Brazilian marseillevirus

Assembly

**Coverage Length**	224 (1 contig(s))
**Depth Of Coverage**	9135.3
**Number Of Reads**	19918
**Reads Per Million**	450.20 rpm (after QC)
**Ambiguities**	0
**Assembly Method**	de novo + reference guided assembly
**Consensus Caller**	Bcf Tools

Coverage Map

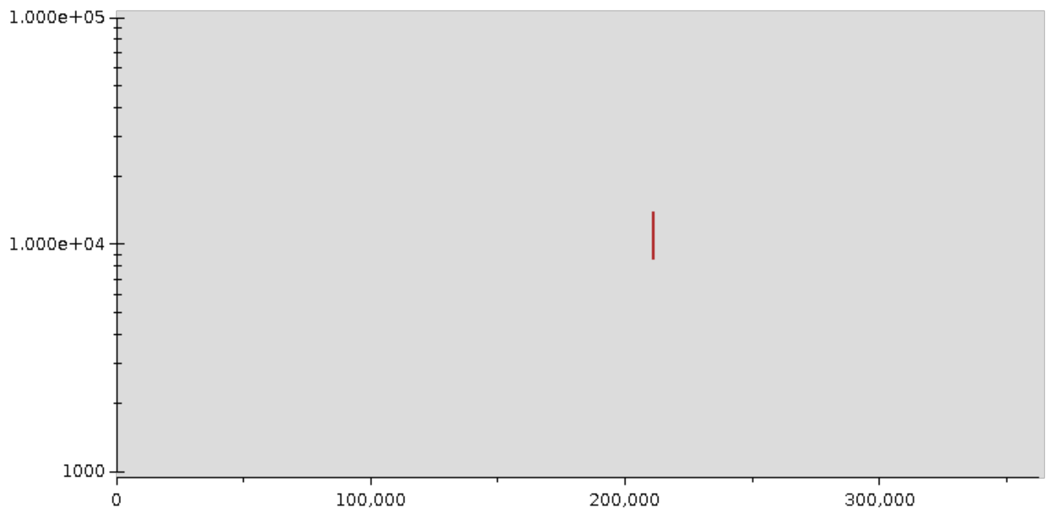

Assignment

**Type**	Brazilian marseillevirus (Taxonomy ID: 1813599)
**Reference Genome**	NC_029692.1
**NT Identity (%)**	76.7857
**AA Identity (%)**	94.4444
**Number Of Stop Codons**	0
**Number Of CDS**	491

Alignment

**Alignment Score**	240.0 (NT) + 428.0 (AA) = 668.0
**Concordance (%)**	74.4705
**Alignment Method**	Local, heuristic, nucleotide (BLASTN)

Genome Region

Sequence starts at position 210884 and ends at position 211107 relative to NC_029692.1 reference sequence.

Alignment Detailed Statistics

	Begin	End	Coverage	Score	Concordance	Matches	Identities	I/D/M/F*	Stop Codons
**NT**	**210884**	**211107**	**0.1%**	**240**	**53.6%**	**224 (100%)**	**172 (76.8%)**	**0/0**	
Mutations:	210884A>G, 210888T>C, 210889T>C, 210890T>A, 210892A>C, 210896A>G, 210899T>A, 210901T>G, 210902T>G, 210914A>C, 210920G>T, 210926T>C, 210929T>C, 210935G>A, 210938A>G, 210941A>G, 210953C>G, 210955T>G, 210956T>G, 210965C>G, 210974G>A, 210983G>T, 210986A>C, 210989G>C, 210991G>T, 210992T>C, 210995T>C, 211001A>T, 211004A>T, 211007A>G, 211009C>T, 211010T>C, 211013T>C, 211025G>A, 211031T>A, 211032T>G, 211033G>C, 211037C>A, 211040G>A, 211043T>G, 211046G>A, 211058G>A, 211064A>C, 211070G>A, 211073A>G, 211076G>A, 211079C>A, 211085T>G, 211088C>A, 211094A>T, 211097C>T, 211100T>G								

*: Inserts / Deletes / Misaligned / Frameshifts

Analysis details

This analysis was performed with panviral2.64

NGS Details (UN9): Tokyovirus A1

Assembly

**Coverage Length**	106 (1 contig(s))
**Depth Of Coverage**	6408.0
**Number Of Reads**	10543
**Reads Per Million**	238.30 rpm (after QC)
**Ambiguities**	0
**Assembly Method**	de novo + reference guided assembly
**Consensus Caller**	Bcf Tools

Coverage Map

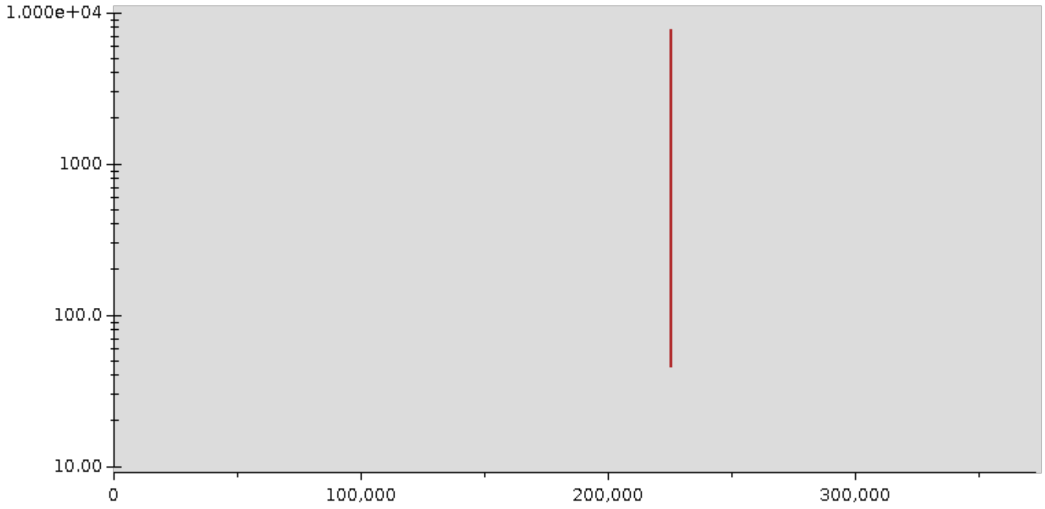

Assignment

**Type**	Tokyovirus A1 (Taxonomy ID: 1826170)
**Reference Genome**	NC_030230.1
**NT Identity (%)**	81.1321
**AA Identity (%)**	94.4444
**Number Of Stop Codons**	0
**Number Of CDS**	470

Alignment

**Alignment Score**	132.0 (NT) + 232.0 (AA) = 364.0
**Concordance (%)**	81.7978
**Alignment Method**	Local, heuristic, nucleotide (BLASTN)

Genome Region

Sequence starts at position 225497 and ends at position 225602 relative to NC_030230.1 reference sequence.

Alignment Detailed Statistics

	Begin	End	Coverage	Score	Concordance	Matches	Identities	I/D/M/F*	Stop Codons
**NT**	**225497**	**225602**	**0.1%**	**132**	**62.3%**	**106 (100%)**	**86 (81.1%)**	**0/0**	

Mutations: 225513G>C, 225519G>T, 225522T>C, 225525T>C, 225528T>C, 225533C>T, 225540A>G, 225543G>C, 225546C>A, 225548A>G, 225549A>G, 225552T>G, 225554T>G, 225555C>G, 225561T>C, 225564T>A, 225573C>A, 225585A>C, 225587G>A, 225588T>C

*: Inserts / Deletes / Misaligned / Frameshifts

Analysis details

This analysis was performed with panviral2.64

NGS Details (UN9): Lausannevirus

Assembly

**Coverage Length**	152 (1 contig(s))
**Depth Of Coverage**	2010.8
**Number Of Reads**	3601
**Reads Per Million**	81.39 rpm (after QC)
**Ambiguities**	1
**Assembly Method**	de novo + reference guided assembly
**Consensus Caller**	Bcf Tools

Coverage Map

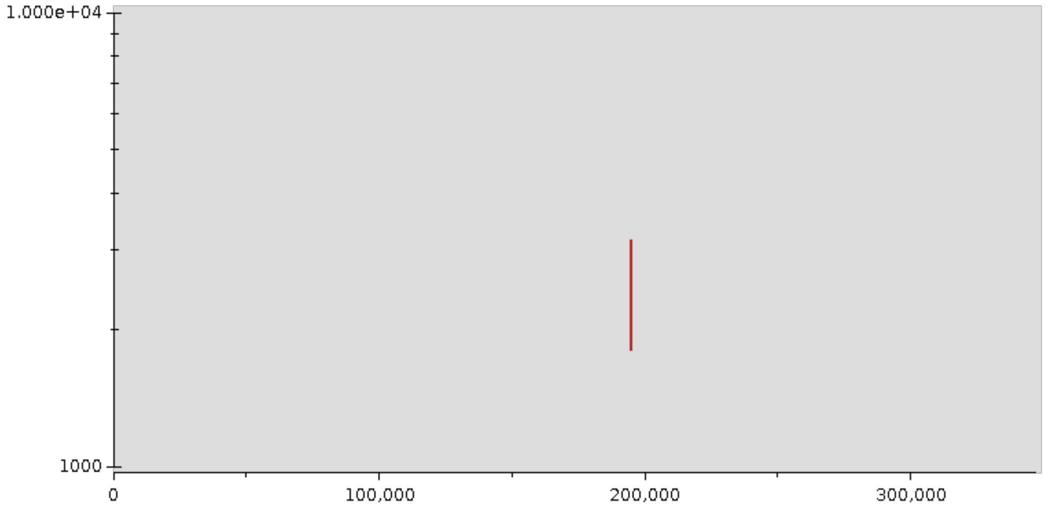

Assignment

**Type**	Lausannevirus (Taxonomy ID: 999883)
**Reference Genome**	NC_015326.1
**NT Identity (%)**	83.5526
**AA Identity (%)**	97.9592
**Number Of Stop Codons**	1
**Number Of CDS**	444

Alignment

**Alignment Score**	206.0 (NT) + 294.0 (AA) = 500.0
**Concordance (%)**	80.0
**Alignment Method**	Local, heuristic, nucleotide (BLASTN)

Genome Region

Sequence starts at position 194911 and ends at position 195062 relative to NC_015326.1 reference sequence.

Alignment Detailed Statistics

	Begin	End	Coverage	Score	Concordance	Matches	Identities	I/D/M/F*	Stop Codons
NT	194911	195062	0.1%	206	67.8%	152 (100%)	127 (83.6%)	0/0	

Mutations: 194925T>C, 194931A>C, 194937G>A, 194943A>G, 194946G>C, 194949C>T, 194955T>C, 194967A>G, 194970G>A, 194979T>C, 194982C>T, 194990C>M, 194991G>C, 194993G>A, 195003T>A, 195006G>A, 195009G>A, 195012A>C, 195015T>C, 195017G>T, 195021T>C, 195030A>G, 195039T>C, 195042T>C, 195048T>C

*: Inserts / Deletes / Misaligned / Frameshifts

Analysis details

This analysis was performed with panviral2.64

NGS Details (UN9): Betabaculovirus disaccharalis

Assembly

**Coverage Length**	150 (1 contig(s))
**Depth Of Coverage**	1356.2
**Number Of Reads**	2310
**Reads Per Million**	52.21 rpm (after QC)
**Ambiguities**	0
**Assembly Method**	de novo + reference guided assembly
**Consensus Caller**	Bcf Tools

Coverage Map

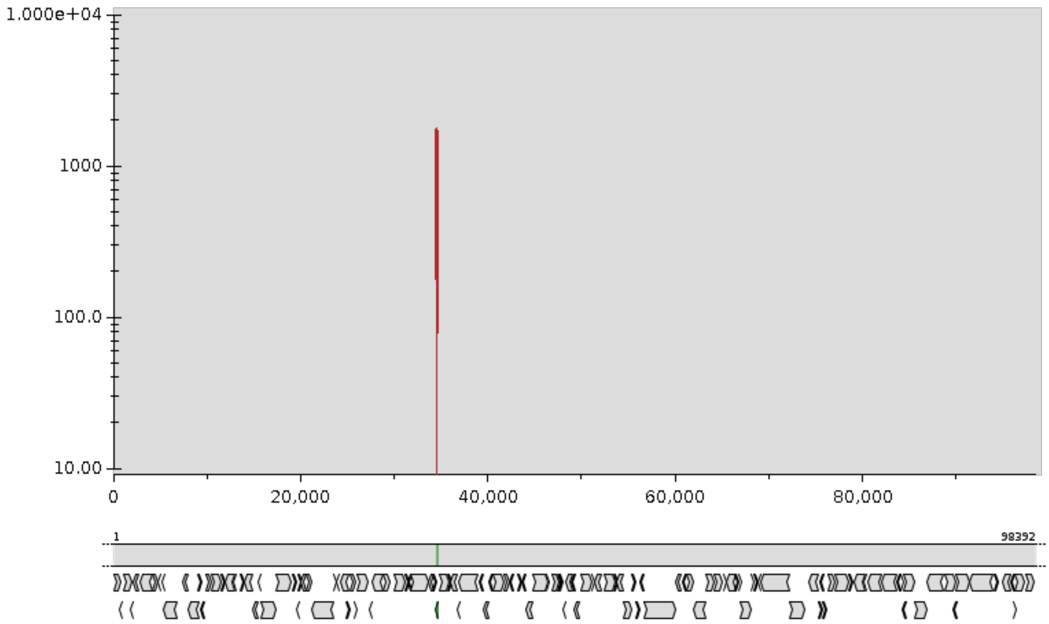

Assignment

**Type**	Betabaculovirus disaccharalis (Taxonomy ID: 3047795)
**Reference Genome**	NC_028491.1
**NT Identity (%)**	84.6667
**AA Identity (%)**	92.0
**Number Of Stop Codons**	0
**Number Of CDS**	125

Alignment

**Alignment Score**	208.0 (NT) + 314.0 (AA) = 522.0
**Concordance (%)**	83.3866

Alignment Method	Local, heuristic, nucleotide (BLASTN)

Genome Region

Sequence starts at position 34474 and ends at position 34623 relative to NC_028491.1 reference sequence.

Alignment Detailed Statistics

	Begin	End	Coverage	Score	Concordance	Matches	Identities	I/D/M/F*	Stop Codons
**NT**	**34474**	**34623**	**0.2%**	**208**	**69.3%**	**150 (100%)**	**127 (84.7%)**	**0/0**	
Mutations:	34485T>C, 34488A>C, 34490G>A, 34513C>G, 34514T>A, 34521T>C, 34524A>G, 34533A>G, 34538A>C, 34542T>A, 34545T>C, 34547G>T, 34549T>C, 34554T>C, 34569G>A, 34575T>G, 34578T>C, 34587T>C, 34593C>T, 34596C>G, 34599C>A, 34601C>T, 34602T>A								
**CDS**									
v-ubq	52	101	42.0%	314	91.3%	50 (100%)	46 (92.0%)	0/0/0/0	0
Protein mutations:	V59I (34599C>A 34601C>T), E76G (34549T>C), S80A (34538A>C)								
Codon mutations:	GGA58GGT (34602T>A), GTG59ATT (34599C>A 34601C>T), CCG60CCC (34596C>G), CCG61CCA (34593C>T), CAA63CAG (34587T>C), TTA66TTG (34578T>C), ATA67ATC (34575T>G), GCC69GCT (34569G>A), GAA74GAG (34554T>C), GAG76GGG (34549T>C), CGA77AGG (34545T>C 34547G>T), ACA78ACT (34542T>A), TCA80GCA (34538A>C), GAT81GAC (34533A>G), ATT84ATC (34524A>G), CAA85CAG (34521T>C), AGC88TCC (34513C>G 34514T>A), CTT96TTG (34488A>C 34490G>A), AGA97AGG (34485T>C)								
**Proteins**									
ubiquitin-like protein (YP_009182246.1)	52	101	42.0%	314	91.3%	50 (100%)	46 (92.0%)	0/0/0/0	0
Protein mutations:	V59I (34599C>A 34601C>T), E76G (34549T>C), S80A (34538A>C)								
Codon mutations:	GGA58GGT (34602T>A), GTG59ATT (34599C>A 34601C>T), CCG60CCC (34596C>G), CCG61CCA (34593C>T), CAA63CAG (34587T>C), TTA66TTG (34578T>C), ATA67ATC (34575T>G), GCC69GCT (34569G>A), GAA74GAG (34554T>C), GAG76GGG (34549T>C), CGA77AGG (34545T>C 34547G>T), ACA78ACT (34542T>A), TCA80GCA (34538A>C), GAT81GAC (34533A>G), ATT84ATC (34524A>G), CAA85CAG (34521T>C), AGC88TCC (34513C>G 34514T>A), CTT96TTG (34488A>C 34490G>A), AGA97AGG (34485T>C)								

*: Inserts / Deletes / Misaligned / Frameshifts

Analysis details

This analysis was performed with panviral2.64

NGS Details (UN9): Lausannevirus

Assembly

**Coverage Length**	208 (1 contig(s))
**Depth Of Coverage**	390.8
**Number Of Reads**	2249
**Reads Per Million**	50.83 rpm (after QC)
**Ambiguities**	0
**Assembly Method**	de novo + reference guided assembly
**Consensus Caller**	Bcf Tools

Coverage Map

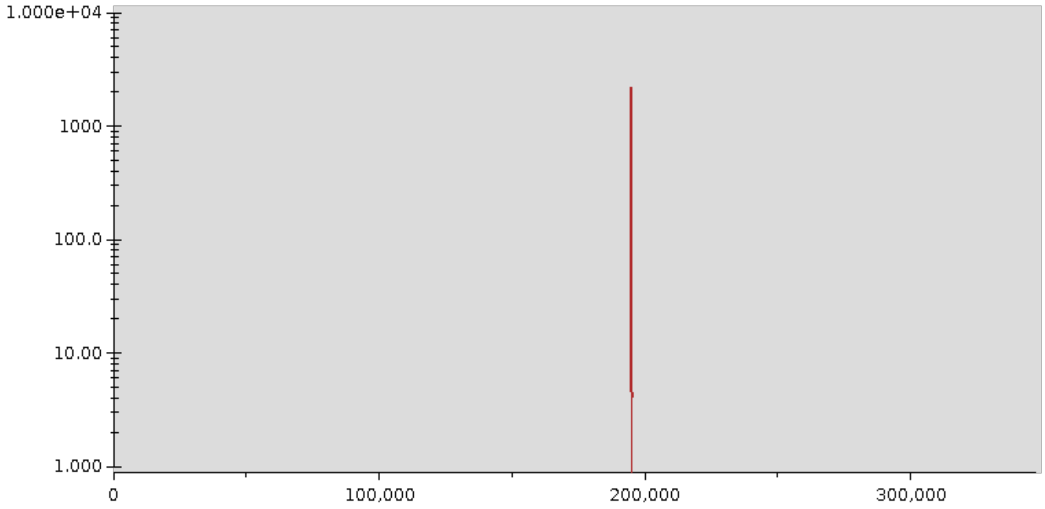

Assignment

**Type**	Lausannevirus (Taxonomy ID: 999883)
**Reference Genome**	NC_015326.1
**NT Identity (%)**	78.3654
**AA Identity (%)**	91.0448
**Number Of Stop Codons**	1
**Number Of CDS**	444

Alignment

**Alignment Score**	236.0 (NT) + 399.0 (AA) = 635.0
**Concordance (%)**	75.8662
**Alignment Method**	Local, heuristic, nucleotide (BLASTN)

Genome Region

Sequence starts at position 194910 and ends at position 195117 relative to NC_015326.1 reference sequence.

Alignment Detailed Statistics

	Begin	End	Coverage	Score	Concordance	Matches	Identities	I/D/M/F*	Stop Codons
**NT**	**194910**	**195117**	**0.1%**	**236**	**56.7%**	**208 (100%)**	**163 (78.4%)**	**0/0**	

Mutations: 194922G>C, 194928C>G, 194930A>G, 194931A>G, 194934A>C, 194936G>A, 194943A>G, 194952T>C, 194955T>C, 194961A>G, 194967A>G, 194970G>A, 194972C>A, 194973A>G, 194976A>G, 194982C>G, 194985A>G, 194994C>T, 195000A>T, 195003T>G, 195017G>T, 195021T>C, 195024G>A, 195027T>G, 195030A>T, 195033A>G, 195036G>A, 195042T>C, 195054T>C, 195057T>G, 195058T>G, 195059G>C, 195075G>A, 195077T>A, 195081C>A, 195084A>G, 195085C>G, 195086T>C, 195090C>G, 195093T>G, 195102A>G, 195104C>T, 195105C>G, 195111T>G, 195114T>A

*: Inserts / Deletes / Misaligned / Frameshifts

Analysis details

This analysis was performed with panviral2.64

NGS Details (UN9): Makelovirus prm1

Assembly

**Coverage Length**	1005 (1 contig(s))
**Depth Of Coverage**	253.9
**Number Of Reads**	1984
**Reads Per Million**	44.84 rpm (after QC)
**Ambiguities**	0
**Assembly Method**	de novo + reference guided assembly
**Consensus Caller**	Bcf Tools

Coverage Map

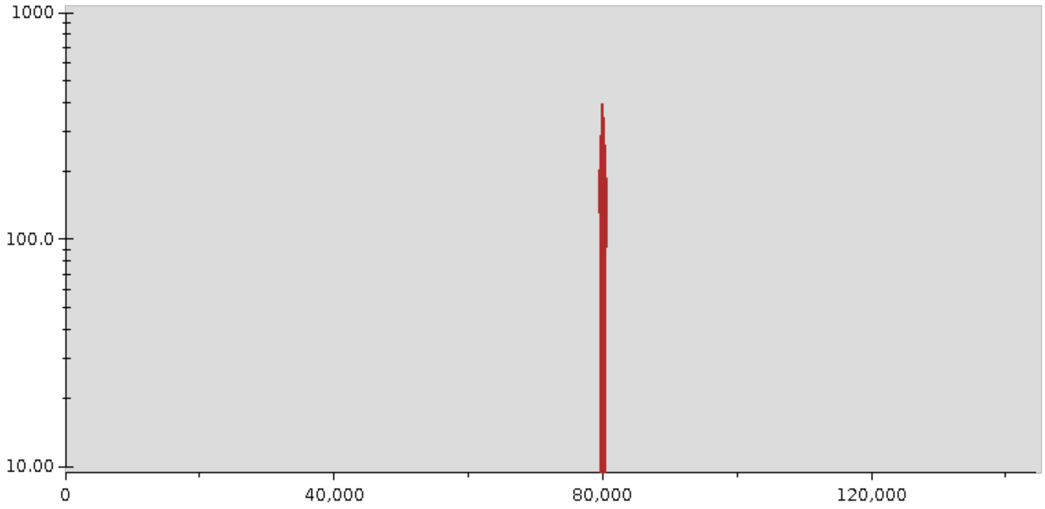

Assignment

**Type**	Makelovirus prm1 (Taxonomy ID: 2956181)
**Reference Genome**	NC_055761.1
**NT Identity (%)**	76.6169
**AA Identity (%)**	87.4627
**Number Of Stop Codons**	0
**Number Of CDS**	190

Alignment

**Alignment Score**	1070.0 (NT) + 2242.0 (AA) = 3312.0
**Concordance (%)**	74.2601
**Alignment Method**	Local, heuristic, nucleotide (BLASTN)

Genome Region

Sequence starts at position 79512 and ends at position 80516 relative to NC_055761.1 reference sequence.

Alignment Detailed Statistics

	Begin	End	Coverage	Score	Concordance	Matches	Identities	I/D/M/F*	Stop Codons
**NT**	**79512**	**80516**	**0.7%**	**1070**	**53.2%**	**1005 (100%)**	**770 (76.6%)**	**0/0**	

Mutations: 79519A>G, 79520A>T, 79522A>G, 79523A>C, 79529C>T, 79530G>A, 79532G>C, 79536G>A, 79541G>T, 79542T>A, 79543C>G, 79544T>C, 79547A>T, 79548A>G, 79550C>A, 79563G>A, 79565C>T, 79568T>A, 79574C>T, 79580G>T, 79581C>T, 79592A>T, 79595T>C, 79596C>T, 79598G>A, 79602G>A, 79604A>G, 79610C>T, 79611G>T, 79612T>C, 79613C>T, 79614T>G, 79615G>T, 79616C>A, 79619C>T, 79624C>T, 79628G>C, 79632G>A, 79643C>T, 79646T>A, 79649G>A, 79655C>T, 79658C>T, 79664C>T, 79673A>T, 79676C>T, 79677G>T, 79679T>A, 79682T>G, 79688G>A, 79689A>C, 79691G>T, 79694T>C, 79703C>T, 79712T>C, 79718T>C, 79719G>A, 79722G>A, 79724A>T, 79728T>A, 79733C>T, 79734A>G, 79735A>C, 79736C>A, 79739A>T, 79742T>A, 79746C>T, 79748T>A, 79751C>T, 79754C>T, 79760C>A, 79763T>C, 79772T>G, 79779C>G, 79791C>T, 79793C>A, 79799T>C, 79811C>T, 79812C>G, 79817G>A, 79818G>A, 79820C>T, 79823A>T, 79824T>C, 79826T>A, 79833C>T, 79835G>A, 79836A>C, 79838C>T, 79842A>G, 79844C>A, 79845T>G, 79846T>C, 79847C>T, 79850C>T, 79871A>G, 79874C>T, 79875T>A, 79876C>G, 79879A>T, 79880T>C, 79895T>A, 79898C>T, 79904C>T, 79905T>G, 79906G>C, 79907C>T, 79916C>T, 79919T>A, 79922A>T, 79931T>A, 79937A>T, 79938T>A, 79940T>C, 79946C>T, 79950C>T, 79953G>A, 79958T>C, 79961T>A, 79962T>A, 79973T>A, 79974T>A, 79975C>G, 79976G>T, 79979C>T, 79985C>T, 79994C>T, 79997C>A, 80000C>A, 80018C>T, 80025C>A, 80030C>A, 80039A>T, 80042A>G, 80054G>T, 80063C>A, 80066C>T, 80069T>C, 80073C>T, 80075T>A, 80078T>C, 80090C>A, 80096T>C, 80099T>C, 80105G>A, 80114A>T, 80120C>T, 80126T>C, 80127C>T, 80132T>A, 80139T>A, 80140C>G, 80141G>T, 80142C>T, 80145G>A, 80147T>C, 80148C>A, 80150T>G, 80159C>A, 80162G>A, 80165C>T, 80168G>A, 80171C>T, 80172C>G, 80173A>C, 80174G>T, 80177C>T, 80178T>G, 80180T>A, 80188A>G, 80189G>A, 80201A>G, 80207G>A, 80210C>T, 80213C>T, 80216C>T, 80222G>A, 80225T>C, 80237C>T, 80240C>T, 80243T>C, 80246C>A, 80247C>T, 80261C>T, 80265T>A, 80266C>G, 80267C>T, 80279C>T, 80286C>T, 80288G>A, 80291T>C, 80300T>A, 80306A>T, 80315T>A, 80318C>A, 80333T>C, 80336A>T, 80337C>T, 80339T>A, 80343G>A, 80346T>A, 80347C>G, 80351C>T, 80357A>T, 80366G>A, 80369C>T, 80378C>T, 80387G>A, 80390C>T, 80391A>G, 80393C>A, 80394A>G, 80396G>T, 80399T>C, 80400G>A, 80409A>C, 80410A>G, 80411A>T, 80414C>A, 80415C>A, 80417C>T, 80433G>A, 80435T>C, 80436C>A, 80438A>C, 80447C>T, 80453G>T, 80456G>T, 80462G>A, 80465A>T, 80471C>T, 80474G>A, 80486C>T, 80498G>A, 80504G>A

*: Inserts / Deletes / Misaligned / Frameshifts

Analysis details

This analysis was performed with panviral2.64

NGS Details (UN9): Golden Marseillevirus

Assembly

**Coverage Length**	120 (1 contig(s))
**Depth Of Coverage**	1085.8
**Number Of Reads**	1782
**Reads Per Million**	40.28 rpm (after QC)
**Ambiguities**	0
**Assembly Method**	de novo + reference guided assembly
**Consensus Caller**	Bcf Tools

Coverage Map

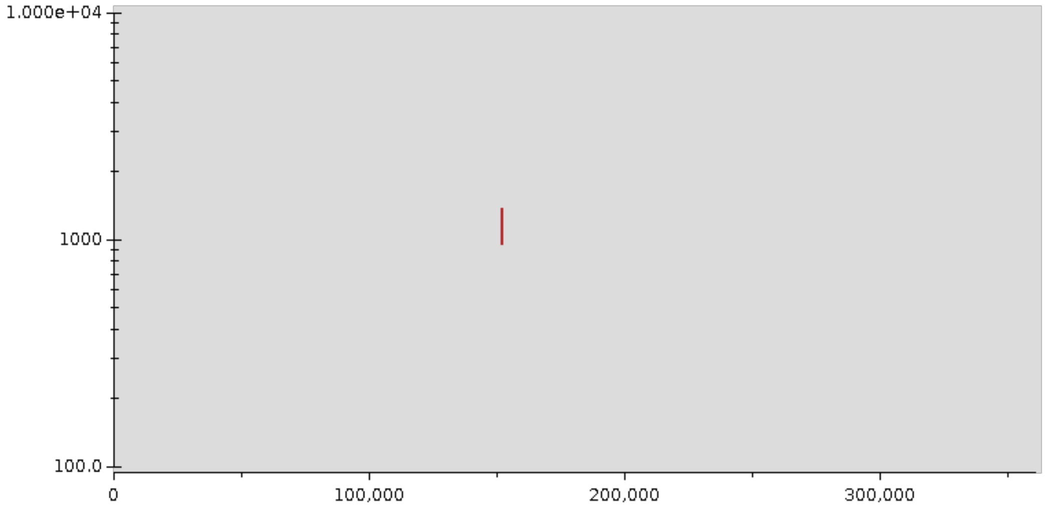

Assignment

**Type**	Golden Marseillevirus (Taxonomy ID: 1720526)
**Reference Genome**	NC_031465.1
**NT Identity (%)**	85.8333
**AA Identity (%)**	97.4359
**Number Of Stop Codons**	1
**Number Of CDS**	296

Alignment

**Alignment Score**	172.0 (NT) + 235.0 (AA) = 407.0
**Concordance (%)**	84.7917
**Alignment Method**	Local, heuristic, nucleotide (BLASTN)

Genome Region

Sequence starts at position 151878 and ends at position 151997 relative to NC_031465.1 reference sequence.

Alignment Detailed Statistics

	Begin	End	Coverage	Score	Concordance	Matches	Identities	I/D/M/F*	Stop Codons
**NT**	**151878**	**151997**	**0.1%**	**172**	**71.7%**	**120 (100%)**	**103 (85.8%)**	**0/0**	

Mutations: 151889T>G, 151891T>G, 151907A>G, 151910A>G, 151916T>C, 151931A>G, 151939A>G, 151940A>T, 151945T>G, 151946C>G, 151955T>A, 151958G>C, 151959C>T, 151960T>G, 151964C>T, 151970G>A, 151976A>C

*: Inserts / Deletes / Misaligned / Frameshifts

Analysis details

This analysis was performed with panviral2.64

NGS Details (UN9): Cladosporium fulvum T-1 virus

Assembly

**Coverage Length**	1715 (5 contig(s))
**Depth Of Coverage**	54.0
**Number Of Reads**	808
**Reads Per Million**	18.26 rpm (after QC)
**Ambiguities**	0
**Assembly Method**	de novo + reference guided assembly
**Consensus Caller**	Bcf Tools

Coverage Map

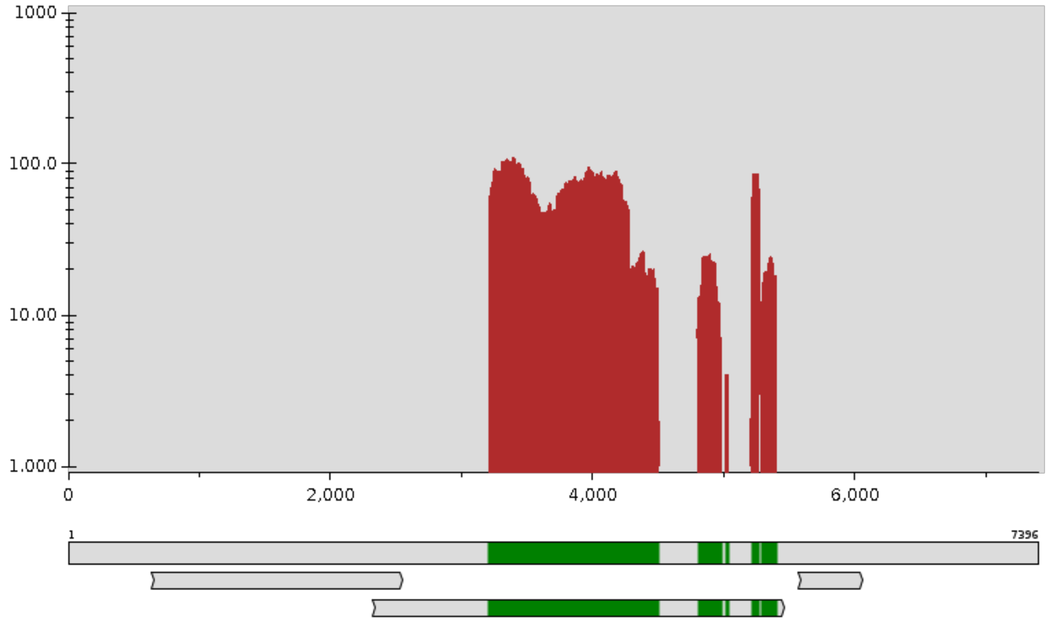

Assignment

**Type**	Cladosporium fulvum T-1 virus (Taxonomy ID: 2052899)
**Reference Genome**	NC_043491.1
**NT Identity (%)**	52.5117
**AA Identity (%)**	47.028
**Number Of Stop Codons**	1
**Number Of CDS**	3

Alignment

**Alignment Score**	115.0 (NT) + 1929.0 (AA) = 2044.0
**Concordance (%)**	28.0769

**Alignment Method**	Global, seeded, nucleotide + amino acids (AGA)

Genome Region

Sequence starts at position 3205 and ends at position 5406 relative to NC_043491.1 reference sequence.

Alignment Detailed Statistics

	Begin	End	Coverage	Score	Concordance	Matches	Identities	I/D/M/F*	Stop Codons
**NT**	**3205**	**5406**	**23.2%**	**115**	**3.4%**	**1697 (98.1%)**	**899 (52.0%)**	**15/18**	
Mutations:	3214G>T, 3216A>T, 3217A>G, 3219G>T, 3220A>G, 3221G>C, 3222G>C, 3223C>A, 3224A>C, 3225A>C, 3226G>T, 3229C>G, 3230G>T, 3231C>T, 3232C>G, 3233T>C, 3234T>C, 3236C>A, 3237C>A, 3238T>G, 3243A>T, 3244C>G, 3246A>C, 3251T>A, 3253_3254insAGGGATATT, 3261A>G, 3262G>A, 3265A>T, 3266A>G, 3268C>G, 3269A>T, 3271T>G, 3272C>A, 3273A>T, 3280G>T, 3281A>T, 3282A>C, 3283A>T, 3284G>A, 3285A>C, 3289T>G, 3292A>T, 3293T>G, 3294G>C, 3295G>A, 3296G>C, 3297G>A, 3299C>G, 3301C>T, 3303T>C, 3304A>T, 3307T>C, 3308C>A, 3309A>G, 3314T>G, 3317G>C, 3318A>C, 3319G>T, 3326C>T, 3328A>G, 3329C>G, 3330A>C, 3331G>T, 3332A>G, 3333C>A, 3334C>A, 3337A>G, 3338C>A, 3340A>G, 3341G>A, 3343A>G, 3344T>C, 3345G>A, 3352G>C, 3355G>A, 3356A>T, 3357A>T, 3361A>T, 3363C>A, 3364C>T, 3365A>T, 3366A>C, 3371T>C, 3372G>T, 3376A>T, 3378G>A, 3379A>G, 3381G>C, 3382A>T, 3385C>T, 3387C>A, 3388C>G, 3389T>G, 3391A>T, 3392A>C, 3393G>C, 3394T>C, 3395G>T, 3396C>A, 3397A>T, 3400A>T, 3401A>G, 3403T>A, 3406A>T, 3407T>G, 3408G>T, 3409C>T, 3410A>T, 3412G>A, 3416G>C, 3417T>A, 3418T>G, 3419C>A, 3420C>A, 3425G>C, 3426C>A, 3427A>G, 3428A>G, 3430C>T, 3434A>T, 3435A>C, 3437C>T, 3440C>A, 3442A>G, 3443C>A, 3445C>G, 3446G>T, 3447T>G, 3448A>C, 3449C>G, 3450A>T, 3451A>G, 3454C>T, 3457C>T, 3460A>G, 3461A>G, 3462A>C, 3464T>C, 3470G>A, 3473A>G, 3475C>A, 3478G>T, 3479A>G, 3481C>G, 3488C>A, 3489G>A, 3490A>G, 3493T>C, 3496G>A, 3497C>G, 3499A>T, 3502C>T, 3503A>T, 3504A>T, 3505C>G, 3506A>G, 3508C>G, 3509G>C, 3511A>G, 3514A>T, 3515G>C, 3516C>T, 3517A>G, 3518C>A, 3519A>T, 3520A>G, 3526A>G, 3529A>G, 3531C>G, 3533G>A, 3534G>A, 3536T>G, 3538A>G, 3539G>T, 3540A>G, 3546A>T, 3550G>T, 3554A>C, 3556T>A, 3559C>T, 3562A>T, 3563C>A, 3565A>G, 3566G>T, 3567A>C, 3568C>A, 3570C>G, 3573T>A, 3574C>T, 3576A>G, 3577T>G, 3578G>C, 3579C>A, 3580T>G, 3581A>G, 3583C>T, 3584C>A, 3586A>G, 3589G>A, 3598A>G, 3601A>T, 3604A>G, 3605T>C, 3606G>C, 3607G>T, 3610A>G, 3613C>T, 3614G>A, 3618T>G, 3619C>T, 3620A>G, 3621G>T, 3622G>A, 3625A>T, 3628A>G, 3631C>T, 3634A>G, 3635C>T, 3636T>C, 3637C>A, 3640C>T, 3647T>C, 3649G>T, 3652C>A, 3658A>G, 3659A>T, 3661G>T, 3664A>G, 3670C>T, 3673C>T, 3676A>C, 3679C>G, 3682A>T, 3683T>A, 3687G>T, 3688C>T, 3689C>T, 3690A>G, 3691G>C, 3692G>A, 3694C>T, 3695C>T, 3697T>A, 3698G>A, 3700C>G, 3703C>T, 3706A>C, 3707A>G, 3708C>T, 3712T>G, 3713A>T, 3714G>T, 3715A>T, 3719C>T, 3720T>A, 3721A>T, 3724C>T, 3727C>T, 3729T>A, 3730G>T, 3732G>T, 3733C>T, 3736C>A, 3739T>A, 3741C>T, 3742T>C, 3745C>T, 3746A>C, 3748G>A, 3751C>T, 3757A>T, 3758C>G, 3760G>C, 3761G>A, 3763C>A, 3766C>T, 3768C>G, 3769A>T, 3770A>C, 3773_3775delGGA, 3776T>A, 3778C>G, 3779C>T, 3781C>G, 3782C>G, 3784G>T, 3791A>G, 3792C>T, 3793C>T, 3795A>G, 3796G>C, 3799A>T, 3800G>T, 3802T>G, 3803C>A, 3804A>G, 3805A>T, 3806G>T, 3807A>T, 3808T>G, 3811G>A, 3814C>A, 3815G>T, 3816A>C, 3817A>T, 3820A>T, 3821C>T, 3823C>A, 3825C>G, 3827A>C, 3829G>A, 3831C>A, 3833G>A, 3834G>C, 3835A>T, 3836T>C, 3839A>T, 3841G>T, 3842A>G, 3843C>T, 3844A>T, 3845G>A, 3846C>A, 3848C>A, 3849C>T, 3850C>G, 3856A>G, 3859C>T, 3862A>G, 3865C>T, 3866C>G, 3867A>C, 3869A>C, 3871G>A, 3877A>G, 3878G>A, 3880C>A, 3886T>C, 3887T>C, 3892C>G, 3893T>C, 3894T>A, 3896A>C, 3898C>A, 3899A>G, 3901C>T, 3905A>G, 3906C>A, 3909C>A, 3910A>C, 3911G>C, 3912G>A, 3913G>A, 3914A>G, 3918C>G, 3922C>G, 3925C>T, 3929G>A, 3930C>A, 3931A>G, 3934G>A, 3935A>G, 3936C>T, 3937A>G, 3940G>A, 3941T>G, 3943A>C, 3946C>T, 3947A>G, 3948G>T, 3949A>T, 3952A>T, 3957C>A, 3960A>C, 3964G>C, 3967G>A, 3970A>T, 3973C>G, 3980G>C, 3982A>G, 3983C>A, 3984A>G, 3988A>T, 3992C>T, 3994T>G, 3997A>G, 4000C>A, 4003C>T, 4010A>T, 4012C>T, 4025A>G, 4026A>C, 4027G>T, 4029A>G, 4030C>A, 4033T>C, 4041C>A, 4048A>T, 4049C>G, 4052A>C, 4057G>T, 4058A>G, 4059T>A, 4060G>T, 4061C>T, 4063T>G, 4064A>C, 4065C>T, 4066A>G, 4068G>A, 4069A>G, 4072A>G, 4075C>A, 4081C>G, 4085A>G, 4086A>T, 4091G>T, 4092G>C, 4093A>G, 4094A>G, 4095A>T, 4097G>A, 4098A>G, 4100C>T, 4101A>G, 4102G>C, 4103A>G, 4104C>A, 4105C>A, 4111G>T, 4114C>T, 4115A>C, 4117A>G, 4120A>T, 4121C>T, 4123C>G, 4130C>G, 4131A>C, 4132G>T, 4133T>A, 4134G>T, 4135C>T, 4138T>A, 4143C>A, 4144C>A, 4149C>T, 4150G>A, 4153T>G, 4154C>A, 4155G>A, 4156A>G, 4159A>G, 4159_4160insCCAAAT, 4162C>T, 4165T>G, 4166G>C, 4167G>T, 4168T>A, 4169A>C, 4170G>C, 4171C>T, 4172A>T, 4173A>T, 4174G>T, 4177A>G, 4180C>A, 4184_4189delATCGAG, 4192C>T, 4195C>T, 4201T>G, 4204T>C, 4206T>A, 4207G>A, 4210A>T, 4211A>G, 4213A>G, 4216C>A, 4218C>G, 4219A>C, 4220T>G, 4221G>T, 4222T>A, 4223C>T, 4225A>G, 4226A>G, 4227C>T, 4228A>G, 4231G>A, 4233C>A, 4234A>T, 4235C>G, 4240T>A, 4243G>T, 4244_4249delAAAAGA, 4252C>T, 4255A>G, 4263A>T, 4265T>G, 4267T>G, 4268T>A, 4269C>G, 4271C>A, 4277A>T, 4281C>A, 4282C>T, 4283A>C, 4285A>G, 4286G>A, 4294G>A, 4296A>G, 4297C>A, 4300C>T, 4301G>T, 4302A>C, 4303C>T, 4304A>G, 4305T>C, 4306C>A, 4312C>A, 4319C>A, 4321T>G, 4322C>G, 4324A>G, 4327C>A, 4328A>G, 4330T>G, 4331G>C, 4333T>C, 4334G>C, 4335C>A, 4336C>T, 4337G>T, 4338C>G, 4339C>T, 4340A>C, 4342G>T, 4346C>A, 4347A>C, 4348T>A, 4354A>G, 4360C>T, 4361G>C, 4364G>T, 4365A>T, 4366G>A, 4369C>A, 4370C>A, 4373_4375delCCG, 4381A>T, 4382A>G, 4383C>T, 4384G>A, 4385A>G, 4387T>A, 4388C>A, 4389T>A, 4390T>G, 4391T>A, 4393A>G, 4397C>A, 4399C>T, 4400A>G, 4401A>T, 4403A>G, 4404A>C, 4406C>A, 4407T>A, 4408C>T, 4411G>T, 4419C>A, 4420G>A, 4423G>T, 4424A>C, 4425C>A, 4426G>A, 4427A>C, 4430G>A, 4433C>T, 4435T>G, 4437C>G, 4438C>T, 4439C>T, 4440G>T, 4441A>G, 4443G>A, 4444A>G, 4445C>T, 4447A>G, 4450C>G, 4451C>A, 4453C>A, 4457T>C, 4458C>A, 4459G>A, 4463C>A, 4465G>A, 4468T>A, 4470G>C, 4471G>A, 4472C>G, 4474G>A, 4477C>T, 4478A>G, 4480G>C, 4483C>T, 4485A>T, 4486A>G, 4487A>T, 4488T>G, 4489C>G, 4491A>T, 4492A>T, 4493T>C, 4495C>T, 4497C>A, 4498T>A, 4797G>T, 4800T>A, 4803C>A, 4804A>T, 4806T>G, 4807C>G, 4812A>G, 4813G>C, 4817A>C, 4819A>G, 4820C>G, 4821T>A, 4822T>G, 4823A>G, 4825G>T, 4826G>A, 4828G>A, 4829C>G, 4830T>A, 4838A>G, 4840C>G, 4841A>C, 4843G>A, 4845A>C, 4846A>T, 4849C>T, 4850G>C, 4852A>T, 4853C>G, 4854A>T, 4855C>G, 4865A>G, 4870A>G, 4872C>T, 4873T>A, 4875C>A, 4876A>G, 4878G>A, 4881A>G, 4882C>A, 4883G>C, 4884C>A, 4885G>A, 4886A>C, 4887A>C, 4888A>C, 4889T>G, 4890A>G, 4891C>A, 4894T>A, 4896A>T, 4897C>T, 4898C>T, 4901C>G, 4904T>C, 4905T>C, 4906C>A, 4907A>T, 4908G>T, 4909G>A, 4910A>C, 4911C>A, 4912A>T, 4913C>G, 4914C>T, 4916C>G, 4918A>C, 4919A>G, 4920C>A, 4922A>C, 4923A>G, 4924A>T, 4933C>G, 4934G>A, 4935A>G, 4936G>T, 4937G>A, 4939T>C, 4954T>A, 4957G>C, 4958A>T, 4959A>G, 4960A>T, 4961C>T, 4963C>G, 4966G>A, 4968G>A, 4972A>C, 4973A>G, 4975G>T, 5009C>T, 5010T>G, 5011A>G, 5014C>G, 5017G>T, 5020C>G, 5026A>G, 5027C>T, 5029C>G, 5032A>T, 5035A>G, 5037A>C, 5207C>T, 5208A>T, 5209C>G, 5212G>T, 5216T>A, 5217C>G, 5218A>T, 5226A>T, 5227C>T, 5233A>T, 5234G>C, 5239G>C, 5245G>C, 5248A>G, 5249A>T, 5251G>A, 5254A>G, 5257A>G, 5260G>T, 5262A>T, 5263C>T, 5269A>T, 5270C>T, 5278A>C, 5284A>T, 5285C>A, 5292A>T, 5294A>G, 5296C>G, 5298A>G, 5301A>C, 5302C>T, 5303G>C, 5304C>A, 5305A>T, 5309G>A, 5311C>A, 5312A>G, 5314C>T, 5319T>C, 5321T>A, 5322C>A, 5323A>G, 5326A>G, 5329G>A, 5330C>G, 5331C>A, 5332A>T, 5333A>G, 5338G>C, 5341G>A, 5342A>T, 5345G>T, 5348C>T, 5349T>A, 5350G>T, 5353C>T, 5354A>C, 5355A>T, 5356C>A, 5359C>G, 5360A>C, 5361A>G, 5362A>G, 5363T>A, 5364C>G, 5365A>T, 5368G>A, 5369A>G, 5371A>C, 5375T>G, 5376C>G, 5377G>C, 5379C>A, 5390A>G, 5391T>A, 5394G>T, 5396A>G, 5398T>C, 5399T>G, 5400T>C, 5401G>A, 5403C>G, 5404A>C, 5405A>C, 5406G>A								
**CDS**									
homologue_of_retroviral_POL_genes	294	1027	54.8%	1929	48.4%	567 (98.1%)	269 (46.5%)	5/6/0/0	1

	Begin	End	Coverage	Score	Concordance	Matches	Identities	I/D/M/F*	Stop Codons
**NT**	**3205**	**5406**	**23.2%**	**115**	**3.4%**	**1697 (98.1%)**	**899 (52.0%)**	**15/18**	
Protein mutations:	E296D (3214G>T), E297V (3216A>T 3217A>G), R298M (3219G>T 3220A>G), G299P (3221G>C 3222G>C 3223C>A), K300P (3224A>C 3225A>C 3226G>T), D301E (3229C>G), A302L (3230G>T 3231C>T 3232C>G), L303P (3233T>C 3234T>C), P304K (3236C>A 3237C>A 3238T>G), H306L (3243A>T 3244C>G), Q307P (3246A>C), W309R (3251T>A), W309_D310insRDI (3253_3254insAGGGATATT), K312R (3261A>G 3262G>A), N314E (3266A>G 3268C>G), I315L (3269A>T 3271T>G), Q316M (3272C>A 3273A>T), K319S (3281A>T 3282A>C 3283A>T), E320T (3284G>A 3285A>C), W323A (3293T>G 3294G>C 3295G>A), G324Q (3296G>C 3297G>A), P325A (3299C>G 3301C>T), L326P (3303T>C 3304A>T), Q328R (3308C>A 3309A>G), S330A (3314T>G), E331P (3317G>C 3318A>C 3319G>T), Q335A (3329C>G 3330A>C 3331G>T), T336E (3332A>G 3333C>A 3334C>A), E339K (3341G>A 3343A>G), W340Q (3344T>C 3345G>A), K342N (3352G>C), K344L (3356A>T 3357A>T), A346D (3363C>A 3364C>T), K347S (3365A>T 3366A>C), W349L (3371T>C 3372G>T), R351Q (3378G>A 3379A>G), R352P (3381G>C 3382A>T), T354K (3387C>A 3388C>G), S355A (3389T>G 3391A>T), S356P (3392A>C 3393G>C 3394T>C), A357Y (3395G>T 3396C>A 3397A>T), T359A (3401A>G 3403T>A), C361V (3407T>G 3408G>T 3409C>T), M362L (3410A>T 3412G>A), V364Q (3416G>C 3417T>A 3418T>G), P365K (3419C>A 3420C>A), A367Q (3425G>C 3426C>A 3427A>G), N368D (3428A>G 3430C>T), K370S (3434A>T 3435A>C), L373M (3443C>A 3445C>G), V374C (3446G>T 3447T>G 3448A>C), Q375V (3449C>G 3450A>T 3451A>G), K379A (3461A>G 3462A>C), E382K (3470G>A), I383V (3473A>G 3475C>A), I385V (3479A>G 3481C>G), R388K (3488C>A 3489G>A 3490A>G), L391V (3497C>G 3499A>T), N393L (3503A>T 3504A>T 3505C>G), I394V (3506A>G 3508C>G), E395Q (3509G>C 3511A>G), E396D (3514A>T), A397L (3515G>C 3516C>T 3517A>G), Q398M (3518C>A 3519A>T 3520A>G), T402S (3531C>G), G403K (3533G>A 3534G>A), S404A (3536T>G 3538A>G), D405C (3539G>T 3540A>G), Y407F (3546A>T), I410L (3554A>C 3556T>A), D414S (3566G>T 3567A>C 3568C>A), A415G (3570C>G), F416Y (3573T>A 3574C>T), Y417W (3576A>G 3577T>G), A418Q (3578G>C 3579C>A 3580T>G), I419V (3581A>G 3583C>T), M421I (3589G>A), E425D (3601A>T), W427P (3605T>C 3606G>C 3607G>T), A430T (3614G>A), F431C (3618T>G 3619C>T), R432V (3620A>G 3621G>T 3622G>A), L437S (3635C>T 3636T>C 3637C>A), M445F (3659A>T 3661G>T), S453T (3683T>A), C454F (3687G>T 3688C>T), Q455C (3689C>T 3690A>G 3691G>C), D456N (3692G>A 3694C>T), V458M (3698G>A 3700C>G), E460D (3706A>C), T461V (3707A>G 3708C>T), R463F (3713A>T 3714G>T 3715A>T), L465Y (3719C>T 3720T>A 3721A>T), V468D (3729T>A 3730G>T), C469F (3732G>T 3733C>T), A472V (3741C>T 3742T>C), M474L (3746A>C 3748G>A), L478V (3758C>G 3760G>C), V479I (3761G>A 3763C>A), T481S (3768C>G 3769A>T), K482Q (3770A>C), G483del (3773_3775delGGA), S484T (3776T>A 3778C>G), Q486D (3782C>G 3784G>T), T489V (3791A>G 3792C>T 3793C>T), K490S (3795A>G 3796G>C), Q491H (3799A>T), V492L (3800G>T 3802T>G), Q493S (3803C>A 3804A>G 3805A>T), D494L (3806G>T 3807A>T 3808T>G), F496L (3814C>A), E497S (3815G>T 3816A>C 3817A>T), T500R (3825C>G), K501Q (3827A>C 3829G>A), S502Y (3831C>A), G503T (3833G>A 3834G>C 3835A>T), F504L (3836T>C), K505Y (3839A>T 3841G>T), T506V (3842A>G 3843C>T 3844A>T), A507K (3845G>A 3846C>A), P508M (3848C>A 3849C>T 3850C>G), H514A (3866C>G 3867A>C), K515Q (3869A>C 3871G>A), V518I (3878G>A 3880C>A), F523H (3893T>C 3894T>A), I524L (3896A>C 3898C>A), I525V (3899A>G 3901C>T), T527E (3905A>G 3906C>A), T528N (3909C>A 3910A>C), G529Q (3911G>C 3912G>A 3913G>A), I530V (3914A>G), T531R (3918C>G), I532M (3922C>G), A535K (3929G>A 3930C>A 3931A>G), T537V (3935A>G 3936C>T 3937A>G), S539A (3941T>G 3943A>C), R541V (3947A>G 3948G>T 3949A>T), E542D (3952A>T), P544Q (3957C>A), E545A (3960A>C), V552L (3980G>C 3982A>G), Q553R (3983C>A 3984A>G), N562Y (4010A>T 4012C>T), K567A (4025A>G 4026A>C 4027G>T), D568G (4029A>G 4030C>A), T572K (4041C>A), P575A (4049C>G), M576L (4052A>C), M578D (4058A>G 4059T>A 4060G>T), T580L (4064A>C 4065C>T 4066A>G), R581K (4068G>A 4069A>G), D583E (4075C>A), N585K (4081C>G), K587V (4085A>G 4086A>T), G589S (4091G>T 4092G>C 4093A>G), K590V (4094A>G 4095A>T), E591R (4097G>A 4098A>G), Q592C (4100C>T 4101A>G 4102G>C), T593E (4103A>G 4104C>A 4105C>A), K597Q (4115A>C 4117A>G), R598S (4120A>T), Q602A (4130C>G 4131A>C 4132G>T), C603I (4133T>A 4134G>T 4135C>T), A606E (4143C>A 4144C>A), T608I (4149C>T 4150G>A), R610K (4154C>A 4155G>A 4156A>G), L611_F612insPN (4159_4160insCCAAAT), D613E (4165T>G), G614L (4166G>C 4167G>T 4168T>A), S615P (4169A>C 4170G>C 4171C>T), K616F (4172A>T 4173A>T 4174G>T), I620_E621del (4184_4189delATCGAG), M627K (4206T>A 4207G>A), I629V (4211A>G 4213A>G), A631G (4218C>G 4219A>C), C632V (4220T>G 4221G>T 4222T>A), T634V (4226A>G 4227C>T 4228A>G), T636N (4233C>A 4234A>T), H637D (4235C>G), D638E (4240T>A), K640_R641del (4244_4249delAAAAGA), Y646F (4263A>T), Y647E (4265T>G 4267T>G), M651L (4277A>T), T652N (4281C>A 4282C>T), T653P (4283A>C 4285A>G), A654T (4286G>A), N657R (4296A>G 4297C>A), D659S (4301G>T 4302A>C 4303C>T), I660A (4304A>G 4305T>C 4306C>A), D662E (4312C>A), L665M (4319C>A 4321T>G), L666V (4322C>G 4324A>G), I668V (4328A>G 4330T>G), V669L (4331G>C 4333T>C), A670H (4334G>C 4335C>A 4336C>T), A671C (4337G>T 4338C>G 4339C>T), M672L (4340A>C 4342G>T), H674T (4346C>A 4347A>C 4348T>A), V679L (4361G>C), E680L (4364G>T 4365A>T 4366G>A), P682T (4370C>A), P683del (4373_4375delCCG), L685F (4381A>T), T686V (4382A>G 4383C>T 4384G>A), I687V (4385A>G 4387T>A), L688K (4388C>A 4389T>A 4390T>G), S689T (4391T>A 4393A>G), H691N (4397C>A 4399C>T), K692V (4400A>G 4401A>T), N693A (4403A>G 4404A>C), L694N (4406C>A 4407T>A 4408C>T), T698K (4419C>A 4420G>A), T700Q (4424A>C 4425C>A 4426G>A), K701Q (4427A>C), E702K (4430G>A), T704S (4437C>G 4438C>T), R705L (4439C>T 4440G>T 4441A>G), R706K (4443G>A 4444A>G), Q707* (4445C>T 4447A>G), S711Q (4457T>C 4458C>A 4459G>A), L713I (4463C>A 4465G>A), G715A (4470G>C 4471G>A), Q716E (4472C>G 4474G>A), K718D (4478A>G 4480G>C), E720V (4485A>T 4486A>G), I721W (4487A>T 4488T>G 4489C>G), K722I (4491A>T 4492A>T), Y723H (4493T>C 4495C>T), T724K (4497C>A 4498T>A), F825Y (4800T>A), S826Y (4803C>A 4804A>T), F827W (4806T>G 4807C>G), Q829R (4812A>G 4813G>C), L832E (4820C>G 4821T>A 4822T>G), K833D (4823A>G 4825G>T), V834I (4826G>A 4828G>A), L835E (4829C>G 4830T>A), I838V (4838A>G 4840C>G), K839Q (4841A>C 4843G>A), K840T (4845A>C 4846A>T), V842L (4850G>C 4852A>T), H843V (4853C>G 4854A>T 4855C>G), N847D (4865A>G), A849V (4872C>T 4873T>A), A850E (4875C>A 4876A>G), R851Q (4878G>A), H852R (4881A>G 4882C>A), A853Q (4883G>C 4884C>A 4885G>A), K854P (4886A>C 4887A>C 4888A>C), Y855G (4889T>G 4890A>G 4891C>A), H857L (4896A>T 4897C>T), Q859E (4901C>G), F860P (4904T>C 4905T>C 4906C>A), R861L (4907A>T 4908G>T 4909G>A), T862H (4910A>C 4911C>A 4912A>T), P863V (4913C>G 4914C>T), P864A (4916C>G 4918A>C), T865E (4919A>G 4920C>A), K866R (4922A>C 4923A>G 4924A>T), D869E (4933C>G), E870S (4934G>A 4935A>G 4936G>T), V871I (4937G>A 4939T>C), K878C (4958A>T 4959A>G 4960A>T), R881K (4968G>A), K883D (4973A>G 4975G>T), L895W (5009C>T 5010T>G 5011A>G), M897I (5017G>T), H961L (5207C>T 5208A>T 5209C>G), K962N (5212G>T), Y967F (5226A>T 5227C>T), E970Q (5234G>C), T975S (5249A>T 5251G>A), N979I (5262A>T 5263C>T), Y989F (5292A>T), I990V (5294A>G 5296C>G), N991S (5298A>G), Y992S (5301A>C 5302C>T), A993H (5303G>C 5304C>A 5305A>T), D995K (5309G>A 5311C>A), N996D (5312A>G 5314C>T), V998A (5319T>C), S999K (5321T>A 5322C>A 5323A>G), P1002D (5330C>G 5331C>A 5332A>T), M1003V (5333A>G), I1006F (5342A>T), A1007S (5345G>T), L1008Y (5348C>T 5349T>A 5350G>T), N1010L (5354A>C 5355A>T 5356C>A), H1011Q (5359C>G), K1012R (5360A>C 5361A>G 5362A>G), T1015A (5369A>G 5371A>C), S1017G (5375T>G 5376C>G 5377G>C), T1018K (5379C>A), M1022E (5390A>G 5391T>A), R1023L (5394G>T), T1024A (5396A>G 5398T>C), L1025A (5399T>G 5400T>C 5401G>A), A1026G (5403C>G 5404A>C)								

	Begin	End	Coverage	Score	Concordance	Matches	Identities	I/D/M/F*	Stop Codons
**NT**	**3205**	**5406**	**23.2%**	**115**	**3.4%**	**1697 (98.1%)**	**899 (52.0%)**	**15/18**	
Codon mutations:	GAG296GAT (3214G>T), GAA297GTG (3216A>T 3217A>G), AGA298ATG (3219G>T 3220A>G), GGC299CCA (3221G>C 3222G>C 3223C>A), AAG300CCT (3224A>C 3225A>C 3226G>T), GAC301GAG (3229C>G), GCC302TTG (3230G>T 3231C>T 3232C>G), TTA303CCA (3233T>C 3234T>C), CCT304AAG (3236C>A 3237C>A 3238T>G), CAC306CTG (3243A>T 3244C>G), CAA307CCA (3246A>C), TGG309AGG (3251T>A), TGG309_GAT310insAGGGATATT (3253_3254insAGGGATATT), AAG312AGA (3261A>G 3262G>A), ATA313ATT (3265A>T), AAC314GAG (3266A>G 3268C>G), ATT315TTG (3269A>T 3271T>G), CAG316ATG (3272C>A 3273A>T), GGG318GGT (3280G>T), AAA319TCT (3281A>T 3282A>C 3283A>T), GAG320ACG (3284G>A 3285A>C), CCT321CCG (3289T>G), CCA322CCT (3292A>T), TGG323GCA (3293T>G 3294G>C 3295G>A), GGA324CAA (3296G>C 3297G>A), CCC325GCT (3299C>G 3301C>T), CTA326CCT (3303T>C 3304A>T), TAT327TAC (3307T>C), CAA328AGA (3308C>A 3309A>G), TCT330GCT (3314T>G), GAG331CCT (3317G>C 3318A>C 3319G>T), CTA334TTG (3326C>T 3328A>G), CAG335GCT (3329C>G 3330A>C 3331G>T), ACC336GAA (3332A>G 3333C>A 3334C>A), CTA337CTG (3337A>G), CGA338AGG (3338C>A 3340A>G), GAA339AAG (3341G>A 3343A>G), TGG340CAG (3344T>C 3345G>A), AAG342AAC (3352G>C), GAG343GAA (3355G>A), AAG344TTG (3356A>T 3357A>T), CTA345CTT (3361A>T), GCC346GAT (3363C>A 3364C>T), AAA347TCA (3365A>T 3366A>C), TGG349CTG (3371T>C 3372G>T), ATA350ATT (3376A>T), CGA351CAG (3378G>A 3379A>G), CGA352CCT (3381G>C 3382A>T), TCC353TCT (3385C>T), ACC354AAG (3387C>A 3388C>G), TCA355GCT (3389T>G 3391A>T), AGT356CCC (3392A>C 3393G>C 3394T>C), GCA357TAT (3395G>T 3396C>A 3397A>T), GGA358GGT (3400A>T), ACT359GCA (3401A>G 3403T>A), CCA360CCT (3406A>T), TGC361GTT (3407T>G 3408G>T 3409C>T), ATG362TTA (3410A>T 3412G>A), GTT364CAG (3416G>C 3417T>A 3418T>G), CCA365AAA (3419C>A 3420C>A), GCA367CAG (3425G>C 3426C>A 3427A>G), AAC368GAT (3428A>G 3430C>T), AAA370TCA (3434A>T 3435A>C), CTA371TTA (3437C>T), CGA372AGG (3440C>A 3442A>G), CTC373ATG (3443C>A 3445C>G), GTA374TGC (3446G>T 3447T>G 3448A>C), CAA375GTG (3449C>G 3450A>T 3451A>G), GAC376GAT (3454C>T), TAC377TAT (3457C>T), CGA378CGG (3460A>G), AAG379GCG (3461A>G 3462A>C), TTG380CTG (3464T>C), GAG382AAG (3470G>A), ATC383GTA (3473A>G 3475C>A), ACG384ACT (3478G>T), ATC385GTG (3479A>G 3481C>G), CGA388AAG (3488C>A 3489G>A 3490A>G), TAT389TAC (3493T>C), CCG390CCA (3496G>A), CTA391GTT (3497C>G 3499A>T), CCC392CCT (3502C>T), AAC393TTG (3503A>T 3504A>T 3505C>G), ATC394GTG (3506A>G 3508C>G), GAA395CAG (3509G>C 3511A>G), GAA396GAT (3514A>T), GCA397CTG (3515G>C 3516C>T 3517A>G), CAA398ATG (3518C>A 3519A>T 3520A>G), AGA400AGG (3526A>G), TTA401TTG (3529A>G), ACC402AGC (3531C>G), GGA403AAA (3533G>A 3534G>A), TCA404GCG (3536T>G 3538A>G), GAC405TGC (3539G>T 3540A>G), TAC407TTC (3546A>T), ACG408ACT (3550G>T), ATT410CTA (3554A>C 3556T>A), GAC411GAT (3559C>T), CTA412CTT (3562A>T), CGA413AGG (3563C>A 3565A>G), GAC414TCA (3566G>T 3567A>C 3568C>A), GCC415GGC (3570C>G), TTC416TAT (3573T>A 3574C>T), TAT417TGG (3576A>G 3577T>G), GCT418CAG (3578G>C 3579C>A 3580T>G), ATC419GTT (3581A>G 3583C>T), CGA420AGG (3584C>A 3586A>G), ATG421ATA (3589G>A), GGA424GGG (3598A>G), GAA425GAT (3601A>T), GAA426GAG (3604A>G), TGG427CCT (3605T>C 3606G>C 3607G>T), AAA428AAG (3610A>G), ACC429ACT (3613C>T), GCT430ACT (3614G>A), TTC431TGT (3618T>G 3619C>T), AGG432GTA (3620A>G 3621G>T 3622G>A), ACA433ACT (3625A>T), AGA434AGG (3628A>G), TAC435TAT (3631C>T), GGA436GGG (3634A>G), CTC437TCA (3635C>T 3636T>C 3637C>A), TAC438TAT (3640C>T), TTG441CTT (3647T>C 3649G>T), GTC442GTA (3652C>A), CCA444CCG (3658A>G), ATG445TTT (3659A>T 3661G>T), GGA446GGG (3664A>G), ACC448ACT (3670C>T), AAC449AAT (3673C>T), GCA450GCC (3676A>C), CCC451CCG (3679C>G), GCA452GCT (3682A>T), TCC453ACC (3683T>A), TGC454TTT (3687G>T 3688C>T), CAG455TGC (3689C>T 3690A>G 3691G>C), GAC456AAT (3692G>A 3694C>T), CTT457TTA (3695C>T 3697T>A), GTC458ATG (3698G>A 3700C>G), AAC459AAT (3703C>T), GAA460GAC (3706A>C), ACA461GTA (3707A>G 3708C>T), CTT462CTG (3712T>G), AGA463TTT (3713A>T 3714G>T 3715A>T), CTA465TAT (3719C>T 3720T>A 3721A>T), CTC466CTT (3724C>T), GAC467GAT (3727C>T), GTG468GAT (3729T>A 3730G>T), TGC469TTT (3732G>T 3733C>T), GTC470GTA (3736C>A), GTT471GTA (3739T>A), GCT472GTC (3741C>T 3742T>C), TAC473TAT (3745C>T), ATG474CTA (3746A>C 3748G>A), GAC475GAT (3751C>T), ATA477ATT (3757A>T), CTG478GTC (3758C>G 3760G>C), GTC479ATA (3761G>A 3763C>A), TAC480TAT (3766C>T), ACA481AGT (3768C>G 3769A>T), AAA482CAA (3770A>C), GGA483del (3773_3775delGGA), TCC484ACG (3776T>A 3778C>G), CTC485TTG (3779C>T 3781C>G), CAG486GAT (3782C>G 3784G>T), ACC489GTT (3791A>G 3792C>T 3793C>T), AAG490AGC (3795A>G 3796G>C), CAA491CAT (3799A>T), GTT492TTG (3800G>T 3802T>G), CAA493AGT (3803C>A 3804A>G 3805A>T), GAT494TTG (3806G>T 3807A>T 3808T>G), GTG495GTA (3811G>A), TTC496TTA (3814C>A), GAA497TCT (3815G>T 3816A>C 3817A>T), CGA498CGT (3820A>T), CTC499TTA (3821C>T 3823C>A), ACG500AGG (3825C>G), AAG501CAA (3827A>C 3829G>A), TCC502TAC (3831C>A), GGA503ACT (3833G>A 3834G>C 3835A>T), TTC504CTC (3836T>C), AAG505TAT (3839A>T 3841G>T), ACA506GTT (3842A>G 3843C>T 3844A>T), GCA507AAA (3845G>A 3846C>A), CCC508ATG (3848C>A 3849C>T 3850C>G), AAA510AAG (3856A>G), TGC511TGT (3859C>T), GAA512GAG (3862A>G), TTC513TTT (3865C>T), CAC514GCC (3866C>G 3867A>C), AAG515CAA (3869A>C 3871G>A), GAA517GAG (3877A>G), GTC518ATA (3878G>A 3880C>A), TTT520TTC (3886T>C), TTA521CTA (3887T>C), GGC522GGG (3892C>G), TTT523CAT (3893T>C 3894T>A), ATC524CTA (3896A>C 3898C>A), ATC525GTT (3899A>G 3901C>T), ACA527GAA (3905A>G 3906C>A), ACA528AAC (3909C>A 3910A>C), GGG529CAA (3911G>C 3912G>A 3913G>A), ATA530GTA (3914A>G), ACG531AGG (3918C>G), ATC532ATG (3922C>G), GAC533GAT (3925C>T), GCA535AAG (3929G>A 3930C>A 3931A>G), AAG536AAA (3934G>A), ACA537GTG (3935A>G 3936C>T 3937A>G), CAG538CAA (3940G>A), TCA539GCC (3941T>G 3943A>C), ATC540ATT (3946C>T), AGA541GTT (3947A>G 3948G>T 3949A>T), GAA542GAT (3952A>T), CCA544CAA (3957C>A), GAA545GCA (3960A>C), CCG546CCC (3964G>C), AAG547AAA (3967G>A), ACA548ACT (3970A>T), GTC549GTG (3973C>G), GTA552CTG (3980G>C 3982A>G), CAG553AGG (3983C>A 3984A>G), TCA554TCT (3988A>T), CTT556TTG (3992C>T 3994T>G), GGA557GGG (3997A>G), CTC558CTA (4000C>A), GCC559GCT (4003C>T), AAC562TAT (4010A>T 4012C>T), AAG567GCT (4025A>G 4026A>C 4027G>T), GAC568GGA (4029A>G 4030C>A), TAT569TAC (4033T>C), ACA572AAA (4041C>A), GCA574GCT (4048A>T), CCA575GCA (4049C>G), ATG576CTG (4052A>C), ACG577ACT (4057G>T), ATG578GAT (4058A>G 4059T>A 4060G>T), CTT579TTG (4061C>T 4063T>G), ACA580CTG (4064A>C 4065C>T 4066A>G), AGA581AAG (4068G>A 4069A>G), AAA582AAG (4072A>G), GAC583GAA (4075C>A), AAC585AAG (4081C>G), AAA587GTA (4085A>G 4086A>T), GGA589TCG (4091G>T 4092G>C 4093A>G), AAA590GTA (4094A>G 4095A>T), GAA591AGA (4097G>A 4098A>G), CAG592TGC (4100C>T 4101A>G 4102G>C), ACC593GAA (4103A>G 4104C>A 4105C>A), GCG595GCT (4111G>T), TTC596TTT (4114C>T), AAA597CAG (4115A>C 4117A>G), AGA598AGT (4120A>T), CTC599TTG (4121C>T 4123C>G), CAG602GCT (4130C>G 4131A>C 4132G>T), TGC603ATT (4133T>A 4134G>T 4135C>T), GCT604GCA (4138T>A), GCC606GAA (4143C>A 4144C>A), ACG608ATA (4149C>T 4150G>A), CTT609CTG (4153T>G), CGA610AAG (4154C>A 4155G>A 4156A>G), CTA611CTG (4159A>G), CTA611_TTC612insCCAAAT (4159_4160insCCAAAT), TTC612TTT (4162C>T), GAT613GAG (4165T>G), GGT614CTA (4166G>C 4167G>T 4168T>A), AGC615CCT (4169A>C 4170G>C 4171C>T), AAG616TTT (4172A>T 4173A>T 4174G>T), GAA617GAG (4177A>G), GTC618GTA (4180C>A), ATC620_GAG621del (4184_4189delATCGAG), ACC622ACT (4192C>T), GAC623GAT (4195C>T), TCT625TCG (4201T>G), GAT626GAC (4204T>C), ATG627AAA (4206T>A 4207G>A), GCA628GCT (4210A>T), ATA629GTG (4211A>G 4213A>G), GGC630GGA (4216C>A), GCA631GGC (4218C>G 4219A>C), TGT632GTA (4220T>G 4221G>T 4222T>A), CTA633TTG (4223C>T 4225A>G), ACA634GTG (4226A>G 4227C>T 4228A>G), CAG635CAA (4231G>A), ACA636AAT (4233C>A 4234A>T), CAC637GAC (4235C>G), GAT638GAA (4240T>A), GGG639GGT (4243G>T), AAA640_AGA641del (4244_4249delAAAAGA), CAC642CAT (4252C>T), CCA643CCG (4255A>G), TAT646TTT (4263A>T), TAT647GAG (4265T>G 4267T>G), TCC648AGC (4268T>A 4269C>G), CGG649AGG (4271C>A), ATG651TTG (4277A>T), ACC652AAT (4281C>A 4282C>T), ACA653CCG (4283A>C 4285A>G), GCG654ACG (4286G>A), CAG656CAA (4294G>A), AAC657AGA (4296A>G 4297C>A), TAC658TAT (4300C>T), GAC659TCT (4301G>T 4302A>C 4303C>T), ATC660GCA (4304A>G 4305T>C 4306C>A), GAC662GAA (4312C>A), CTT665ATG (4319C>A 4321T>G), CTA666GTG (4322C>G 4324A>G), GCC667GCA (4327C>A), ATT668GTG (4328A>G 4330T>G), GTT669CTC (4331G>C 4333T>C), GCC670CAT (4334G>C 4335C>A 4336C>T), GCC671TGT (4337G>T 4338C>G 4339C>T), ATG672CTT (4340A>C 4342G>T), CAT674ACA (4346C>A 4347A>C 4348T>A), AGA676AGG (4354A>G), TAC678TAT (4360C>T), GTC679CTC (4361G>C), GAG680TTA (4364G>T 4365A>T 4366G>A), GGC681GGA (4369C>A), CCA682ACA (4370C>A), CCG683del (4373_4375delCCG), TTA685TTT (4381A>T), ACG686GTA (4382A>G 4383C>T 4384G>A), ATT687GTA (4385A>G 4387T>A), CTT688AAG (4388C>A 4389T>A 4390T>G), TCA689ACG (4391T>A 4393A>G), CAC691AAT (4397C>A 4399C>T), AAG692GTG (4400A>G 4401A>T), AAT693GCT (4403A>G 4404A>C), CTC694AAT (4406C>A 4407T>A 4408C>T), ACG695ACT (4411G>T), ACG698AAA (4419C>A 4420G>A), ACG699ACT (4423G>T), ACG700CAA (4424A>C 4425C>A 4426G>A), AAG701CAG (4427A>C), GAA702AAA (4430G>A), CTT703TTG (4433C>T 4435T>G), ACC704AGT (4437C>G 4438C>T), CGA705TTG (4439C>T 4440G>T 4441A>G), AGA706AAG (4443G>A 4444A>G), CAA707TAG (4445C>T 4447A>G), GCC708GCG (4450C>G), CGC709AGA (4451C>A 4453C>A), TCG711CAA (4457T>C 4458C>A 4459G>A), CTG713ATA (4463C>A 4465G>A), CTT714CTA (4468T>A), GGG715GCA (4470G>C 4471G>A), CAG716GAA (4472C>G 4474G>A), TAC717TAT (4477C>T), AAG718GAC (4478A>G 4480G>C), TTC719TTT (4483C>T), GAA720GTG (4485A>T 4486A>G), ATC721TGG (4487A>T 4488T>G 4489C>G), AAA722ATT (4491A>T 4492A>T), TAC723CAT (4493T>C 4495C>T), ACT724AAA (4497C>A 4498T>A), AGC824.TC (4797G>T), TTC825TAC (4800T>A), TCA826TAT (4803C>A 4804A>T), TTC827TGG (4806T>G 4807C>G), CAG829CGC (4812A>G 4813G>C), AGA831CGG (4817A>C 4819A>G), CTT832GAG (4820C>G 4821T>A 4822T>G), AAG833GAT (4823A>G 4825G>T), GTG834ATA (4826G>A 4828G>A), CTA835GAA (4829C>G 4830T>A), ATC838GTG (4838A>G 4840C>G), AAG839CAA (4841A>C 4843G>A), AAA840ACT (4845A>C 4846A>T), TGC841TGT (4849C>T), GTA842CTT (4850G>C 4852A>T), CAC843GTG (4853C>G 4854A>T 4855C>G), AAC847GAC (4865A>G), AAA848AAG (4870A>G), GCT849GTA (4872C>T 4873T>A), GCA850GAG (4875C>A 4876A>G), CGG851CAG (4878G>A), CAC852CGA (4881A>G 4882C>A), GCG853CAA (4883G>C 4884C>A 4885G>A), AAA854CCC (4886A>C 4887A>C 4888A>C), TAC855GGA (4889T>G 4890A>G 4891C>A), GGT856GGA (4894T>A), CAC857CTT (4896A>T 4897C>T), CTA858TTA (4898C>T), CAG859GAG (4901C>G), TTC860CCA (4904T>C 4905T>C 4906C>A), AGG861TTA (4907A>T 4908G>T 4909G>A), ACA862CAT (4910A>C 4911C>A 4912A>T), CCA863GTA (4913C>G 4914C>T), CCA864GCC (4916C>G 4918A>C), ACG865GAG (4919A>G 4920C>A), AAA866CGT (4922A>C 4923A>G 4924A>T), GAC869GAG (4933C>G), GAG870AGT (4934G>A 4935A>G 4936G>T), GTT871ATC (4937G>A 4939T>C), ATT876ATA (4954T>A), ACG877ACC (4957G>C), AAA878TGT (4958A>T 4959A>G 4960A>T), CTC879TTG (4961C>T 4963C>G), CCG880CCA (4966G>A), AGG881AAG (4968G>A), TCA882TCC (4972A>C), AAG883GAT (4973A>G 4975G>T), CTA895TGG (5009C>T 5010T>G 5011A>G), GTC896GTG (5014C>G), ATG897ATT (5017G>T), GTC898GTG (5020C>G), AGA900AGG (5026A>G), CTC901TTG (5027C>T 5029C>G), ACA902ACT (5032A>T), AAA903AAG (5035A>G), TAT904TC. (5037A>C), CAC961TTG (5207C>T 5208A>T 5209C>G), AAG962AAT (5212G>T), TCA964AGT (5216T>A 5217C>G 5218A>T), TAC967TTT (5226A>T 5227C>T), CCA969CCT (5233A>T), GAG970CAG (5234G>C), ACG971ACC (5239G>C), GGG973GGC (5245G>C), CAA974CAG (5248A>G), ACG975TCA (5249A>T 5251G>A), GAA976GAG (5254A>G), AGA977AGG (5257A>G), ACG978ACT (5260G>T), AAC979ATT (5262A>T 5263C>T), ACA981ACT (5269A>T), CTC982T.. (5270C>T), CAA984..C (5278A>C), CTA986CTT (5284A>T), CGG987AGG (5285C>A), TAC989TTC (5292A>T), ATC990GTG (5294A>G 5296C>G), AAC991AGC (5298A>G), TAC992TCT (5301A>C 5302C>T), GCA993CAT (5303G>C 5304C>A 5305A>T), GAC995AAA (5309G>A 5311C>A), AAC996GAT (5312A>G 5314C>T), GTT998GCT (5319T>C), TCA999AAG (5321T>A 5322C>A 5323A>G), TTA1000TTG (5326A>G), CTG1001CTA (5329G>A), CCA1002GAT (5330C>G 5331C>A 5332A>T), ATG1003GTG (5333A>G), GCG1004GCC (5338G>C), CAG1005CAA (5341G>A), ATC1006TTC (5342A>T), GCA1007TCA (5345G>T), CTG1008TAT (5348C>T 5349T>A 5350G>T), AAC1009AAT (5353C>T), AAC1010CTA (5354A>C 5355A>T 5356C>A), CAC1011CAG (5359C>G), AAA1012CGG (5360A>C 5361A>G 5362A>G), TCA1013AGT (5363T>A 5364C>G 5365A>T), GAG1014GAA (5368G>A), ACA1015GCC (5369A>G 5371A>C), TCG1017GGC (5375T>G 5376C>G 5377G>C), ACG1018AAG (5379C>A), ATG1022GAG (5390A>G 5391T>A), CGA1023CTA (5394G>T), ACT1024GCC (5396A>G 5398T>C), TTG1025GCA (5399T>G 5400T>C 5401G>A), GCA1026GGC (5403C>G 5404A>C), AGG1027CA. (5405A>C 5406G>A)								

Proteins

	Begin	End	Coverage	Score	Concordance	Matches	Identities	I/D/M/F*	Stop Codons
**NT**	**3205**	**5406**	**23.2%**	**115**	**3.4%**	**1697 (98.1%)**	**899 (52.0%)**	**15/18**	
Reverse Transcriptase (YP_009666308.1)	294	1027	54.8%	1929	48.4%	567 (98.1%)	269 (46.5%)	5/6/0/0	1
Protein mutations:	E296D (3214G>T), E297V (3216A>T 3217A>G), R298M (3219G>T 3220A>G), G299P (3221G>C 3222G>C 3223C>A), K300P (3224A>C 3225A>C 3226G>T), D301E (3229C>G), A302L (3230G>T 3231C>T 3232C>G), L303P (3233T>C 3234T>C), P304K (3236C>A 3237C>A 3238T>G), H306L (3243A>T 3244C>G), Q307P (3246A>C), W309R (3251T>A), W309_D310insRDI (3253_3254insAGGGATATT), K312R (3261A>G 3262G>A), N314E (3266A>G 3268C>G), I315L (3269A>T 3271T>G), Q316M (3272C>A 3273A>T), K319S (3281A>T 3282A>C 3283A>T), E320T (3284G>A 3285A>C), W323A (3293T>G 3294G>C 3295G>A), G324Q (3296G>C 3297G>A), P325A (3299C>G 3301C>T), L326P (3303T>C 3304A>T), Q328R (3308C>A 3309A>G), S330A (3314T>G), E331P (3317G>C 3318A>C 3319G>T), Q335A (3329C>G 3330A>C 3331G>T), T336E (3332A>G 3333C>A 3334C>A), E339K (3341G>A 3343A>G), W340Q (3344T>C 3345G>A), K342N (3352G>C), K344L (3356A>T 3357A>T), A346D (3363C>A 3364C>T), K347S (3365A>T 3366A>C), W349L (3371T>C 3372G>T), R351Q (3378G>A 3379A>G), R352P (3381G>C 3382A>T), T354K (3387C>A 3388C>G), S355A (3389T>G 3391A>T), S356P (3392A>C 3393G>C 3394T>C), A357Y (3395G>T 3396C>A 3397A>T), T359A (3401A>G 3403T>A), C361V (3407T>G 3408G>T 3409C>T), M362L (3410A>T 3412G>A), V364Q (3416G>C 3417T>A 3418T>G), P365K (3419C>A 3420C>A), A367Q (3425G>C 3426C>A 3427A>G), N368D (3428A>G 3430C>T), K370S (3434A>T 3435A>C), L373M (3443C>A 3445C>G), V374C (3446G>T 3447T>G 3448A>C), Q375V (3449C>G 3450A>T 3451A>G), K379A (3461A>G 3462A>C), E382K (3470G>A), I383V (3473A>G 3475C>A), I385V (3479A>G 3481C>G), R388K (3488C>A 3489G>A 3490A>G), L391V (3497C>G 3499A>T), N393L (3503A>T 3504A>T 3505C>G), I394V (3506A>G 3508C>G), E395Q (3509G>C 3511A>G), E396D (3514A>T), A397L (3515G>C 3516C>T 3517A>G), Q398M (3518C>A 3519A>T 3520A>G), T402S (3531C>G), G403K (3533G>A 3534G>A), S404A (3536T>G 3538A>G), D405C (3539G>T 3540A>G), Y407F (3546A>T), I410L (3554A>C 3556T>A), D414S (3566G>T 3567A>C 3568C>A), A415G (3570C>G), F416Y (3573T>A 3574C>T), Y417W (3576A>G 3577T>G), A418Q (3578G>C 3579C>A 3580T>G), I419V (3581A>G 3583C>T), M421I (3589G>A), E425D (3601A>T), W427P (3605T>C 3606G>C 3607G>T), A430T (3614G>A), F431C (3618T>G 3619C>T), R432V (3620A>G 3621G>T 3622G>A), L437S (3635C>T 3636T>C 3637C>A), M445F (3659A>T 3661G>T), S453T (3683T>A), C454F (3687G>T 3688C>T), Q455C (3689C>T 3690A>G 3691G>C), D456N (3692G>A 3694C>T), V458M (3698G>A 3700C>G), E460D (3706A>C), T461V (3707A>G 3708C>T), R463F (3713A>T 3714G>T 3715A>T), L465Y (3719C>T 3720T>A 3721A>T), V468D (3729T>A 3730G>T), C469F (3732G>T 3733C>T), A472V (3741C>T 3742T>C), M474L (3746A>C 3748G>A), L478V (3758C>G 3760G>C), V479I (3761G>A 3763C>A), T481S (3768C>G 3769A>T), K482Q (3770A>C), G483del (3773_3775delGGA), S484T (3776T>A 3778C>G), Q486D (3782C>G 3784G>T), T489V (3791A>G 3792C>T 3793C>T), K490S (3795A>G 3796G>C), Q491H (3799A>T), V492L (3800G>T 3802T>G), Q493S (3803C>A 3804A>G 3805A>T), D494L (3806G>T 3807A>T 3808T>G), F496L (3814C>A), E497S (3815G>T 3816A>C 3817A>T), T500R (3825C>G), K501Q (3827A>C 3829G>A), S502Y (3831C>A), G503T (3833G>A 3834G>C 3835A>T), F504L (3836T>C), K505Y (3839A>T 3841G>T), T506V (3842A>G 3843C>T 3844A>T), A507K (3845G>A 3846C>A), P508M (3848C>A 3849C>T 3850C>G), H514A (3866C>G 3867A>C), K515Q (3869A>C 3871G>A), V518I (3878G>A 3880C>A), F523H (3893T>C 3894T>A), I524L (3896A>C 3898C>A), I525V (3899A>G 3901C>T), T527E (3905A>G 3906C>A), T528N (3909C>A 3910A>C), G529Q (3911G>C 3912G>A 3913G>A), I530V (3914A>G), T531R (3918C>G), I532M (3922C>G), A535K (3929G>A 3930C>A 3931A>G), T537V (3935A>G 3936C>T 3937A>G), S539A (3941T>G 3943A>C), R541V (3947A>G 3948G>T 3949A>T), E542D (3952A>T), P544Q (3957C>A), E545A (3960A>C), V552L (3980G>C 3982A>G), Q553R (3983C>A 3984A>G), N562Y (4010A>T 4012C>T), K567A (4025A>G 4026A>C 4027G>T), D568G (4029A>G 4030C>A), T572K (4041C>A), P575A (4049C>G), M576L (4052A>C), M578D (4058A>G 4059T>A 4060G>T), T580L (4064A>C 4065C>T 4066A>G), R581K (4068G>A 4069A>G), D583E (4075C>A), N585K (4081C>G), K587V (4085A>G 4086A>T), G589S (4091G>T 4092G>C 4093A>G), K590V (4094A>G 4095A>T), E591R (4097G>A 4098A>G), Q592C (4100C>T 4101A>G 4102G>C), T593E (4103A>G 4104C>A 4105C>A), K597Q (4115A>C 4117A>G), R598S (4120A>T), Q602A (4130C>G 4131A>C 4132G>T), C603I (4133T>A 4134G>T 4135C>T), A606E (4143C>A 4144C>A), T608I (4149C>T 4150G>A), R610K (4154C>A 4155G>A 4156A>G), L611_F612insPN (4159_4160insCCAAAT), D613E (4165T>G), G614L (4166G>C 4167G>T 4168T>A), S615P (4169A>C 4170G>C 4171C>T), K616F (4172A>T 4173A>T 4174G>T), I620_E621del (4184_4189delATCGAG), M627K (4206T>A 4207G>A), I629V (4211A>G 4213A>G), A631G (4218C>G 4219A>C), C632V (4220T>G 4221G>T 4222T>A), T634V (4226A>G 4227C>T 4228A>G), T636N (4233C>A 4234A>T), H637D (4235C>G), D638E (4240T>A), K640_R641del (4244_4249delAAAAGA), Y646F (4263A>T), Y647E (4265T>G 4267T>G), M651L (4277A>T), T652N (4281C>A 4282C>T), T653P (4283A>C 4285A>G), A654T (4286G>A), N657R (4296A>G 4297C>A), D659S (4301G>T 4302A>C 4303C>T), I660A (4304A>G 4305T>C 4306C>A), D662E (4312C>A), L665M (4319C>A 4321T>G), L666V (4322C>G 4324A>G), I668V (4328A>G 4330T>G), V669L (4331G>C 4333T>C), A670H (4334G>C 4335C>A 4336C>T), A671C (4337G>T 4338C>G 4339C>T), M672L (4340A>C 4342G>T), H674T (4346C>A 4347A>C 4348T>A), V679L (4361G>C), E680L (4364G>T 4365A>T 4366G>A), P682T (4370C>A), P683del (4373_4375delCCG), L685F (4381A>T), T686V (4382A>G 4383C>T 4384G>A), I687V (4385A>G 4387T>A), L688K (4388C>A 4389T>A 4390T>G), S689T (4391T>A 4393A>G), H691N (4397C>A 4399C>T), K692V (4400A>G 4401A>T), N693A (4403A>G 4404A>C), L694N (4406C>A 4407T>A 4408C>T), T698K (4419C>A 4420G>A), T700Q (4424A>C 4425C>A 4426G>A), K701Q (4427A>C), E702K (4430G>A), T704S (4437C>G 4438C>T), R705L (4439C>T 4440G>T 4441A>G), R706K (4443G>A 4444A>G), Q707* (4445C>T 4447A>G), S711Q (4457T>C 4458C>A 4459G>A), L713I (4463C>A 4465G>A), G715A (4470G>C 4471G>A), Q716E (4472C>G 4474G>A), K718D (4478A>G 4480G>C), E720V (4485A>T 4486A>G), I721W (4487A>T 4488T>G 4489C>G), K722I (4491A>T 4492A>T), Y723H (4493T>C 4495C>T), T724K (4497C>A 4498T>A), F825Y (4800T>A), S826Y (4803C>A 4804A>T), F827W (4806T>G 4807C>G), Q829R (4812A>G 4813G>C), L832E (4820C>G 4821T>A 4822T>G), K833D (4823A>G 4825G>T), V834I (4826G>A 4828G>A), L835E (4829C>G 4830T>A), I838V (4838A>G 4840C>G), K839Q (4841A>C 4843G>A), K840T (4845A>C 4846A>T), V842L (4850G>C 4852A>T), H843V (4853C>G 4854A>T 4855C>G), N847D (4865A>G), A849V (4872C>T 4873T>A), A850E (4875C>A 4876A>G), R851Q (4878G>A), H852R (4881A>G 4882C>A), A853Q (4883G>C 4884C>A 4885G>A), K854P (4886A>C 4887A>C 4888A>C), Y855G (4889T>G 4890A>G 4891C>A), H857L (4896A>T 4897C>T), Q859E (4901C>G), F860P (4904T>C 4905T>C 4906C>A), R861L (4907A>T 4908G>T 4909G>A), T862H (4910A>C 4911C>A 4912A>T), P863V (4913C>G 4914C>T), P864A (4916C>G 4918A>C), T865E (4919A>G 4920C>A), K866R (4922A>C 4923A>G 4924A>T), D869E (4933C>G), E870S (4934G>A 4935A>G 4936G>T), V871I (4937G>A 4939T>C), K878C (4958A>T 4959A>G 4960A>T), R881K (4968G>A), K883D (4973A>G 4975G>T), L895W (5009C>T 5010T>G 5011A>G), M897I (5017G>T), H961L (5207C>T 5208A>T 5209C>G), K962N (5212G>T), Y967F (5226A>T 5227C>T), E970Q (5234G>C), T975S (5249A>T 5251G>A), N979I (5262A>T 5263C>T), Y989F (5292A>T), I990V (5294A>G 5296C>G), N991S (5298A>G), Y992S (5301A>C 5302C>T), A993H (5303G>C 5304C>A 5305A>T), D995K (5309G>A 5311C>A), N996D (5312A>G 5314C>T), V998A (5319T>C), S999K (5321T>A 5322C>A 5323A>G), P1002D (5330C>G 5331C>A 5332A>T), M1003V (5333A>G), I1006F (5342A>T), A1007S (5345G>T), L1008Y (5348C>T 5349T>A 5350G>T), N1010L (5354A>C 5355A>T 5356C>A), H1011Q (5359C>G), K1012R (5360A>C 5361A>G 5362A>G), T1015A (5369A>G 5371A>C), S1017G (5375T>G 5376C>G 5377G>C), T1018K (5379C>A), M1022E (5390A>G 5391T>A), R1023L (5394G>T), T1024A (5396A>G 5398T>C), L1025A (5399T>G 5400T>C 5401G>A), A1026G (5403C>G 5404A>C)								

	Begin	End	Coverage	Score	Concordance	Matches	Identities	I/D/M/F*	Stop Codons
**NT**	**3205**	**5406**	**23.2%**	**115**	**3.4%**	**1697 (98.1%)**	**899 (52.0%)**	**15/18**	

Codon mutations:

GAG296GAT (3214G>T), GAA297GTG (3216A>T 3217A>G), AGA298ATG (3219G>T 3220A>G), GGC299CCA (3221G>C 3222G>C 3223C>A), AAG300CCT (3224A>C 3225A>C 3226G>T), GAC301GAG (3229C>G), GCC302TTG (3230G>T 3231C>T 3232C>G), TTA303CCA (3233T>C 3234T>C), CCT304AAG (3236C>A 3237C>A 3238T>G), CAC306CTG (3243A>T 3244C>G), CAA307CCA (3246A>C), TGG309AGG (3251T>A), TGG309_GAT310insAGGGATATT (3253_3254insAGGGATATT), AAG312AGA (3261A>G 3262G>A), ATA313ATT (3265A>T), AAC314GAG (3266A>G 3268C>G), ATT315TTG (3269A>T 3271T>G), CAG316ATG (3272C>A 3273A>T), GGG318GGT (3280G>T), AAA319TCT (3281A>T 3282A>C 3283A>T), GAG320ACG (3284G>A 3285A>C), CCT321CCG (3289T>G), CCA322CCT (3292A>T), TGG323GCA (3293T>G 3294G>C 3295G>A), GGA324CAA (3296G>C 3297G>A), CCC325GCT (3299C>G 3301C>T), CTA326CCT (3303T>C 3304A>T), TAT327TAC (3307T>C), CAA328AGA (3308C>A 3309A>G), TCT330GCT (3314T>G), GAG331CCT (3317G>C 3318A>C 3319G>T), CTA334TTG (3326C>T 3328A>G), CAG335GCT (3329C>G 3330A>C 3331G>T), ACC336GAA (3332A>G 3333C>A 3334C>A), CTA337CTG (3337A>G), CGA338AGG (3338C>A 3340A>G), GAA339AAG (3341G>A 3343A>G), TGG340CAG (3344T>C 3345G>A), AAG342AAC (3352G>C), GAG343GAA (3355G>A), AAG344TTG (3356A>T 3357A>T), CTA345CTT (3361A>T), GCC346GAT (3363C>A 3364C>T), AAA347TCA (3365A>T 3366A>C), TGG349CTG (3371T>C 3372G>T), ATA350ATT (3376A>T), CGA351CAG (3378G>A 3379A>G), CGA352CCT (3381G>C 3382A>T), TCC353TCT (3385C>T), ACC354AAG (3387C>A 3388C>G), TCA355GCT (3389T>G 3391A>T), AGT356CCC (3392A>C 3393G>C 3394T>C), GCA357TAT (3395G>T 3396C>A 3397A>T), GGA358GGT (3400A>T), ACT359GCA (3401A>G 3403T>A), CCA360CCT (3406A>T), TGC361GTT (3407T>G 3408G>T 3409C>T), ATG362TTA (3410A>T 3412G>A), GTT364CAG (3416G>C 3417T>A 3418T>G), CCA365AAA (3419C>A 3420C>A), GCA367CAG (3425G>C 3426C>A 3427A>G), AAC368GAT (3428A>G 3430C>T), AAA370TCA (3434A>T 3435A>C), CTA371TTA (3437C>T), CGA372AGG (3440C>A 3442A>G), CTC373ATG (3443C>A 3445C>G), GTA374TGC (3446G>T 3447T>G 3448A>C), CAA375GTG (3449C>G 3450A>T 3451A>G), GAC376GAT (3454C>T), TAC377TAT (3457C>T), CGA378CGG (3460A>G), AAG379GCG (3461A>G 3462A>C), TTG380CTG (3464T>C), GAG382AAG (3470G>A), ATC383GTA (3473A>G 3475C>A), ACG384ACT (3478G>T), ATC385GTG (3479A>G 3481C>G), CGA388AAG (3488C>A 3489G>A 3490A>G), TAT389TAC (3493T>C), CCG390CCA (3496G>A), CTA391GTT (3497C>G 3499A>T), CCC392CCT (3502C>T), AAC393TTG (3503A>T 3504A>T 3505C>G), ATC394GTG (3506A>G 3508C>G), GAA395CAG (3509G>C 3511A>G), GAA396GAT (3514A>T), GCA397CTG (3515G>C 3516C>T 3517A>G), CAA398ATG (3518C>A 3519A>T 3520A>G), AGA400AGG (3526A>G), TTA401TTG (3529A>G), ACC402AGC (3531C>G), GGA403AAA (3533G>A 3534G>A), TCA404GCG (3536T>G 3538A>G), GAC405TGC (3539G>T 3540A>G), TAC407TTC (3546A>T), ACG408ACT (3550G>T), ATT410CTA (3554A>C 3556T>A), GAC411GAT (3559C>T), CTA412CTT (3562A>T), CGA413AGG (3563C>A 3565A>G), GAC414TCA (3566G>T 3567A>C 3568C>A), GCC415GGC (3570C>G), TTC416TAT (3573T>A 3574C>T), TAT417TGG (3576A>G 3577T>G), GCT418CAG (3578G>C 3579C>A 3580T>G), ATC419GTT (3581A>G 3583C>T), CGA420AGG (3584C>A 3586A>G), ATG421ATA (3589G>A), GGA424GGG (3598A>G), GAA425GAT (3601A>T), GAA426GAG (3604A>G), TGG427CCT (3605T>C 3606G>C 3607G>T), AAA428AAG (3610A>G), ACC429ACT (3613C>T), GCT430ACT (3614G>A), TTC431TGT (3618T>G 3619C>T), AGG432GTA (3620A>G 3621G>T 3622G>A), ACA433ACT (3625A>T), AGA434AGG (3628A>G), TAC435TAT (3631C>T), GGA436GGG (3634A>G), CTC437TCA (3635C>T 3636T>C 3637C>A), TAC438TAT (3640C>T), TTG441CTT (3647T>C 3649G>T), GTC442GTA (3652C>A), CCA444CCG (3658A>G), ATG445TTT (3659A>T 3661G>T), GGA446GGG (3664A>G), ACC448ACT (3670C>T), AAC449AAT (3673C>T), GCA450GCC (3676A>C), CCC451CCG (3679C>G), GCA452GCT (3682A>T), TCC453ACC (3683T>A), TGC454TTT (3687G>T 3688C>T), CAG455TGC (3689C>T 3690A>G 3691G>C), GAC456AAT (3692G>A 3694C>T), CTT457TTA (3695C>T 3697T>A), GTC458ATG (3698G>A 3700C>G), AAC459AAT (3703C>T), GAA460GAC (3706A>C), ACA461GTA (3707A>G 3708C>T), CTT462CTG (3712T>G), AGA463TTT (3713A>T 3714G>T 3715A>T), CTA465TAT (3719C>T 3720T>A 3721A>T), CTC466CTT (3724C>T), GAC467GAT (3727C>T), GTG468GAT (3729T>A 3730G>T), TGC469TTT (3732G>T 3733C>T), GTC470GTA (3736C>A), GTT471GTA (3739T>A), GCT472GTC (3741C>T 3742T>C), TAC473TAT (3745C>T), ATG474CTA (3746A>C 3748G>A), GAC475GAT (3751C>T), ATA477ATT (3757A>T), CTG478GTC (3758C>G 3760G>C), GTC479ATA (3761G>A 3763C>A), TAC480TAT (3766C>T), ACA481AGT (3768C>G 3769A>T), AAA482CAA (3770A>C), GGA483del (3773_3775delGGA), TCC484ACG (3776T>A 3778C>G), CTC485TTG (3779C>T 3781C>G), CAG486GAT (3782C>G 3784G>T), ACC489GTT (3791A>G 3792C>T 3793C>T), AAG490AGC (3795A>G 3796G>C), CAA491CAT (3799A>T), GTT492TTG (3800G>T 3802T>G), CAA493AGT (3803C>A 3804A>G 3805A>T), GAT494TTG (3806G>T 3807A>T 3808T>G), GTG495GTA (3811G>A), TTC496TTA (3814C>A), GAA497TCT (3815G>T 3816A>C 3817A>T), CGA498CGT (3820A>T), CTC499TTA (3821C>T 3823C>A), ACG500AGG (3825C>G), AAG501CAA (3827A>C 3829G>A), TCC502TAC (3831C>A), GGA503ACT (3833G>A 3834G>C 3835A>T), TTC504CTC (3836T>C), AAG505TAT (3839A>T 3841G>T), ACA506GTT (3842A>G 3843C>T 3844A>T), GCA507AAA (3845G>A 3846C>A), CCC508ATG (3848C>A 3849C>T 3850C>G), AAA510AAG (3856A>G), TGC511TGT (3859C>T), GAA512GAG (3862A>G), TTC513TTT (3865C>T), CAC514GCC (3866C>G 3867A>C), AAG515CAA (3869A>C 3871G>A), GAA517GAG (3877A>G), GTC518ATA (3878G>A 3880C>A), TTT520TTC (3886T>C), TTA521CTA (3887T>C), GGC522GGG (3892C>G), TTT523CAT (3893T>C 3894T>A), ATC524CTA (3896A>C 3898C>A), ATC525GTT (3899A>G 3901C>T), ACA527GAA (3905A>G 3906C>A), ACA528AAC (3909C>A 3910A>C), GGG529CAA (3911G>C 3912G>A 3913G>A), ATA530GTA (3914A>G), ACG531AGG (3918C>G), ATC532ATG (3922C>G), GAC533GAT (3925C>T), GCA535AAG (3929G>A 3930C>A 3931A>G), AAG536AAA (3934G>A), ACA537GTG (3935A>G 3936C>T 3937A>G), CAG538CAA (3940G>A), TCA539GCC (3941T>G 3943A>C), ATC540ATT (3946C>T), AGA541GTT (3947A>G 3948G>T 3949A>T), GAA542GAT (3952A>T), CCA544CAA (3957C>A), GAA545GCA (3960A>C), CCG546CCC (3964G>C), AAG547AAA (3967G>A), ACA548ACT (3970A>T), GTC549GTG (3973C>G), GTA552CTG (3980G>C 3982A>G), CAG553AGG (3983C>A 3984A>G), TCA554TCT (3988A>T), CTT556TTG (3992C>T 3994T>G), GGA557GGG (3997A>G), CTC558CTA (4000C>A), GCC559GCT (4003C>T), AAC562TAT (4010A>T 4012C>T), AAG567GCT (4025A>G 4026A>C 4027G>T), GAC568GGA (4029A>G 4030C>A), TAT569TAC (4033T>C), ACA572AAA (4041C>A), GCA574GCT (4048A>T), CCA575GCA (4049C>G), ATG576CTG (4052A>C), ACG577ACT (4057G>T), ATG578GAT (4058A>G 4059T>A 4060G>T), CTT579TTG (4061C>T 4063T>G), ACA580CTG (4064A>C 4065C>T 4066A>G), AGA581AAG (4068G>A 4069A>G), AAA582AAG (4072A>G), GAC583GAA (4075C>A), AAC585AAG (4081C>G), AAA587GTA (4085A>G 4086A>T), GGA589TCG (4091G>T 4092G>C 4093A>G), AAA590GTA (4094A>G 4095A>T), GAA591AGA (4097G>A 4098A>G), CAG592TGC (4100C>T 4101A>G 4102G>C), ACC593GAA (4103A>G 4104C>A 4105C>A), GCG595GCT (4111G>T), TTC596TTT (4114C>T), AAA597CAG (4115A>C 4117A>G), AGA598AGT (4120A>T), CTC599TTG (4121C>T 4123C>G), CAG602GCT (4130C>G 4131A>C 4132G>T), TGC603ATT (4133T>A 4134G>T 4135C>T), GCT604GCA (4138T>A), GCC606GAA (4143C>A 4144C>A), ACG608ATA (4149C>T 4150G>A), CTT609CTG (4153T>G), CGA610AAG (4154C>A 4155G>A 4156A>G), CTA611CTG (4159A>G), CTA611_TTC612insCCAAAT (4159_4160insCCAAAT), TTC612TTT (4162C>T), GAT613GAG (4165T>G), GGT614CTA (4166G>C 4167G>T 4168T>A), AGC615CCT (4169A>C 4170G>C 4171C>T), AAG616TTT (4172A>T 4173A>T 4174G>T), GAA617GAG (4177A>G), GTC618GTA (4180C>A), ATC620_GAG621del (4184_4189delATCGAG), ACC622ACT (4192C>T), GAC623GAT (4195C>T), TCT625TCG (4201T>G), GAT626GAC (4204T>C), ATG627AAA (4206T>A 4207G>A), GCA628GCT (4210A>T), ATA629GTG (4211A>G 4213A>G), GGC630GGA (4216C>A), GCA631GGC (4218C>G 4219A>C), TGT632GTA (4220T>G 4221G>T 4222T>A), CTA633TTG (4223C>T 4225A>G), ACA634GTG (4226A>G 4227C>T 4228A>G), CAG635CAA (4231G>A), ACA636AAT (4233C>A 4234A>T), CAC637GAC (4235C>G), GAT638GAA (4240T>A), GGG639GGT (4243G>T), AAA640_AGA641del (4244_4249delAAAAGA), CAC642CAT (4252C>T), CCA643CCG (4255A>G), TAT646TTT (4263A>T), TAT647GAG (4265T>G 4267T>G), TCC648AGC (4268T>A 4269C>G), CGG649AGG (4271C>A), ATG651TTG (4277A>T), ACC652AAT (4281C>A 4282C>T), ACA653CCG (4283A>C 4285A>G), GCG654ACG (4286G>A), CAG656CAA (4294G>A), AAC657AGA (4296A>G 4297C>A), TAC658TAT (4300C>T), GAC659TCT (4301G>T 4302A>C 4303C>T), ATC660GCA (4304A>G 4305T>C 4306C>A), GAC662GAA (4312C>A), CTT665ATG (4319C>A 4321T>G), CTA666GTG (4322C>G 4324A>G), GCC667GCA (4327C>A), ATT668GTG (4328A>G 4330T>G), GTT669CTC (4331G>C 4333T>C), GCC670CAT (4334G>C 4335C>A 4336C>T), GCC671TGT (4337G>T 4338C>G 4339C>T), ATG672CTT (4340A>C 4342G>T), CAT674ACA (4346C>A 4347A>C 4348T>A), AGA676AGG (4354A>G), TAC678TAT (4360C>T), GTC679CTC (4361G>C), GAG680TTA (4364G>T 4365A>T 4366G>A), GGC681GGA (4369C>A), CCA682ACA (4370C>A), CCG683del (4373_4375delCCG), TTA685TTT (4381A>T), ACG686GTA (4382A>G 4383C>T 4384G>A), ATT687GTA (4385A>G 4387T>A), CTT688AAG (4388C>A 4389T>A 4390T>G), TCA689ACG (4391T>A 4393A>G), CAC691AAT (4397C>A 4399C>T), AAG692GTG (4400A>G 4401A>T), AAT693GCT (4403A>G 4404A>C), CTC694AAT (4406C>A 4407T>A 4408C>T), ACG695ACT (4411G>T), ACG698AAA (4419C>A 4420G>A), ACG699ACT (4423G>T), ACG700CAA (4424A>C 4425C>A 4426G>A), AAG701CAG (4427A>C), GAA702AAA (4430G>A), CTT703TTG (4433C>T 4435T>G), ACC704AGT (4437C>G 4438C>T), CGA705TTG (4439C>T 4440G>T 4441A>G), AGA706AAG (4443G>A 4444A>G), CAA707TAG (4445C>T 4447A>G), GCC708GCG (4450C>G), CGC709AGA (4451C>A 4453C>A), TCG711CAA (4457T>C 4458C>A 4459G>A), CTG713ATA (4463C>A 4465G>A), CTT714CTA (4468T>A), GGG715GCA (4470G>C 4471G>A), CAG716GAA (4472C>G 4474G>A), TAC717TAT (4477C>T), AAG718GAC (4478A>G 4480G>C), TTC719TTT (4483C>T), GAA720GTG (4485A>T 4486A>G), ATC721TGG (4487A>T 4488T>G 4489C>G), AAA722ATT (4491A>T 4492A>T), TAC723CAT (4493T>C 4495C>T), ACT724AAA (4497C>A 4498T>A), AGC824.TC (4797G>T), TTC825TAC (4800T>A), TCA826TAT (4803C>A 4804A>T), TTC827TGG (4806T>G 4807C>G), CAG829CGC (4812A>G 4813G>C), AGA831CGG (4817A>C 4819A>G), CTT832GAG (4820C>G 4821T>A 4822T>G), AAG833GAT (4823A>G 4825G>T), GTG834ATA (4826G>A 4828G>A), CTA835GAA (4829C>G 4830T>A), ATC838GTG (4838A>G 4840C>G), AAG839CAA (4841A>C 4843G>A), AAA840ACT (4845A>C 4846A>T), TGC841TGT (4849C>T), GTA842CTT (4850G>C 4852A>T), CAC843GTG (4853C>G 4854A>T 4855C>G), AAC847GAC (4865A>G), AAA848AAG (4870A>G), GCT849GTA (4872C>T 4873T>A), GCA850GAG (4875C>A 4876A>G), CGG851CAG (4878G>A), CAC852CGA (4881A>G 4882C>A), GCG853CAA (4883G>C 4884C>A 4885G>A), AAA854CCC (4886A>C 4887A>C 4888A>C), TAC855GGA (4889T>G 4890A>G 4891C>A), GGT856GGA (4894T>A), CAC857CTT (4896A>T 4897C>T), CTA858TTA (4898C>T), CAG859GAG (4901C>G), TTC860CCA (4904T>C 4905T>C 4906C>A), AGG861TTA (4907A>T 4908G>T 4909G>A), ACA862CAT (4910A>C 4911C>A 4912A>T), CCA863GTA (4913C>G 4914C>T), CCA864GCC (4916C>G 4918A>C), ACG865GAG (4919A>G 4920C>A), AAA866CGT (4922A>C 4923A>G 4924A>T), GAC869GAG (4933C>G), GAG870AGT (4934G>A 4935A>G 4936G>T), GTT871ATC (4937G>A 4939T>C), ATT876ATA (4954T>A), ACG877ACC (4957G>C), AAA878TGT (4958A>T 4959A>G 4960A>T), CTC879TTG (4961C>T 4963C>G), CCG880CCA (4966G>A), AGG881AAG (4968G>A), TCA882TCC (4972A>C), AAG883GAT (4973A>G 4975G>T), CTA895TGG (5009C>T 5010T>G 5011A>G), GTC896GTG (5014C>G), ATG897ATT (5017G>T), GTC898GTG (5020C>G), AGA900AGG (5026A>G), CTC901TTG (5027C>T 5029C>G), ACA902ACT (5032A>T), AAA903AAG (5035A>G), TAT904TC. (5037A>C), CAC961TTG (5207C>T 5208A>T 5209C>G), AAG962AAT (5212G>T), TCA964AGT (5216T>A 5217C>G 5218A>T), TAC967TTT (5226A>T 5227C>T), CCA969CCT (5233A>T), GAG970CAG (5234G>C), ACG971ACC (5239G>C), GGG973GGC (5245G>C), CAA974CAG (5248A>G), ACG975TCA (5249A>T 5251G>A), GAA976GAG (5254A>G), AGA977AGG (5257A>G), ACG978ACT (5260G>T), AAC979ATT (5262A>T 5263C>T), ACA981ACT (5269A>T), CTC982T.. (5270C>T), CAA984..C (5278A>C), CTA986CTT (5284A>T), CGG987AGG (5285C>A), TAC989TTC (5292A>T), ATC990GTG (5294A>G 5296C>G), AAC991AGC (5298A>G), TAC992TCT (5301A>C 5302C>T), GCA993CAT (5303G>C 5304C>A 5305A>T), GAC995AAA (5309G>A 5311C>A), AAC996GAT (5312A>G 5314C>T), GTT998GCT (5319T>C), TCA999AAG (5321T>A 5322C>A 5323A>G), TTA1000TTG (5326A>G), CTG1001CTA (5329G>A), CCA1002GAT (5330C>G 5331C>A 5332A>T), ATG1003GTG (5333A>G), GCG1004GCC (5338G>C), CAG1005CAA (5341G>A), ATC1006TTC (5342A>T), GCA1007TCA (5345G>T), CTG1008TAT (5348C>T 5349T>A 5350G>T), AAC1009AAT (5353C>T), AAC1010CTA (5354A>C 5355A>T 5356C>A), CAC1011CAG (5359C>G), AAA1012CGG (5360A>C 5361A>G 5362A>G), TCA1013AGT (5363T>A 5364C>G 5365A>T), GAG1014GAA (5368G>A), ACA1015GCC (5369A>G 5371A>C), TCG1017GGC (5375T>G 5376C>G 5377G>C), ACG1018AAG (5379C>A), ATG1022GAG (5390A>G 5391T>A), CGA1023CTA (5394G>T), ACT1024GCC (5396A>G 5398T>C), TTG1025GCA (5399T>G 5400T>C 5401G>A), GCA1026GGC (5403C>G 5404A>C), AGG1027CA. (5405A>C 5406G>A)

*: Inserts / Deletes / Misaligned / Frameshifts

Analysis details

This analysis was performed with panviral2.64

NGS Details (UN9): Lausannevirus

Assembly

**Coverage Length**	151 (1 contig(s))
**Depth Of Coverage**	394.0
**Number Of Reads**	604
**Reads Per Million**	13.65 rpm (after QC)
**Ambiguities**	0
**Assembly Method**	de novo + reference guided assembly
**Consensus Caller**	Bcf Tools

Coverage Map

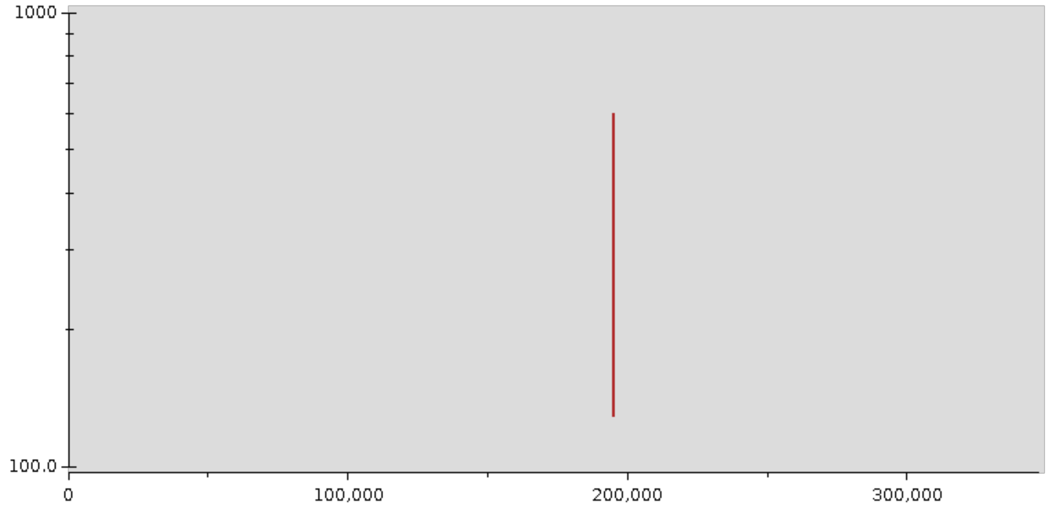

Assignment

**Type**	Lausannevirus (Taxonomy ID: 999883)
**Reference Genome**	NC_015326.1
**NT Identity (%)**	81.457
**AA Identity (%)**	92.1569
**Number Of Stop Codons**	1
**Number Of CDS**	444

Alignment

**Alignment Score**	190.0 (NT) + 308.0 (AA) = 498.0
**Concordance (%)**	78.3019
**Alignment Method**	Local, heuristic, nucleotide (BLASTN)

Genome Region

Sequence starts at position 194933 and ends at position 195083 relative to NC_015326.1 reference sequence.

Alignment Detailed Statistics

	Begin	End	Coverage	Score	Concordance	Matches	Identities	I/D/M/F*	Stop Codons
**NT**	**194933**	**195083**	**0.1%**	**190**	**62.9%**	**151 (100%)**	**123 (81.5%)**	**0/0**	

Mutations: 194943A>G, 194946G>C, 194949C>T, 194955T>C, 194967A>G, 194970G>A, 194979T>C, 194982C>T, 194990C>A, 194991G>C, 194993G>A, 195003T>A, 195006G>A, 195009G>A, 195015T>C, 195017G>T, 195021T>C, 195030A>G, 195039T>C, 195042T>C, 195043T>G, 195048T>C, 195054T>C, 195057T>G, 195058T>G, 195059G>T, 195063G>T, 195069C>T

*: Inserts / Deletes / Misaligned / Frameshifts

Analysis details

This analysis was performed with panviral2.64

NGS Details (UN9): Errantivirus

Assembly

**Coverage Length**	572 (1 contig(s))
**Depth Of Coverage**	65.5
**Number Of Reads**	402
**Reads Per Million**	9.09 rpm (after QC)
**Ambiguities**	0
**Assembly Method**	de novo + reference guided assembly
**Consensus Caller**	Bcf Tools

Coverage Map

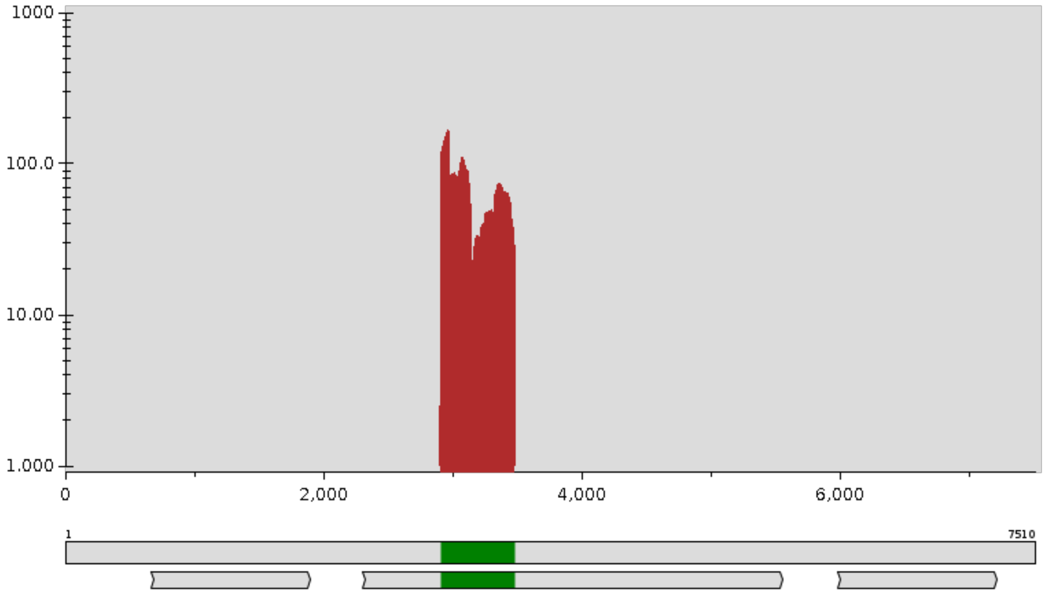

Assignment

**Type**	Errantivirus (Taxonomy ID: 186666)
**Reference Genome**	NC_038512.1
**NT Identity (%)**	53.5587
**AA Identity (%)**	43.8503
**Number Of Stop Codons**	0
**Number Of CDS**	3

Alignment

**Alignment Score**	61.0 (NT) + 478.0 (AA) = 539.0
**Concordance (%)**	23.8813

Alignment Method	Global, seeded, nucleotide + amino acids (AGA)

Genome Region

Sequence starts at position 2909 and ends at position 3480 relative to NC_038512.1 reference sequence.

Alignment Detailed Statistics

	Begin	End	Coverage	Score	Concordance	Matches	Identities	I/D/M/F*	Stop Codons
**NT**	**2909**	**3480**	**7.6%**	**61**	**5.5%**	**562 (98.3%)**	**301 (52.6%)**	**0/10**	
Mutations:	2919A>T, 2920G>C, 2924A>G, 2926C>G, 2927G>A, 2928A>G, 2929T>G, 2932C>T, 2934A>G, 2935A>G, 2937A>T, 2941G>A, 2944A>C, 2949A>C, 2950C>T, 2953A>T, 2954A>G, 2955G>A, 2959C>G, 2960G>C, 2963C>T, 2968C>T, 2969A>G, 2972T>G, 2975G>A, 2976G>A, 2978A>G, 2979A>G, 2980G>C, 2981T>G, 2982G>C, 2983C>T, 2984C>G, 2985A>T, 2986A>T, 2989C>T, 2993A>T, 2995C>A, 2997C>A, 2998C>G, 2999T>C, 3001A>T, 3008G>T, 3009C>T, 3011A>T, 3012G>C, 3016G>A, 3018T>A, 3019T>C, 3020T>G, 3025G>A, 3026G>A, 3028G>T, 3029G>C, 3030A>G, 3032A>G, 3034G>T, 3035G>A, 3037C>G, 3038C>G, 3039C>T, 3040T>G, 3041C>G, 3042A>C, 3050T>C, 3052G>C, 3058C>T, 3061G>T, 3066A>G, 3067C>G, 3068G>A, 3069T>C, 3071G>C, 3073A>C, 3074C>G, 3076C>T, 3079G>T, 3084T>A, 3085T>C, 3091C>T, 3092C>T, 3094T>A, 3095C>G, 3096G>T, 3097A>C, 3104A>T, 3106G>T, 3109A>G, 3112A>G, 3113A>T, 3114A>C, 3115A>T, 3119T>G, 3121A>G, 3122C>T, 3124A>G, 3127T>C, 3130T>A, 3135A>G, 3137_3146delAGAGTTATGG, 3150A>G, 3151T>G, 3154C>T, 3155C>T, 3157A>C, 3159G>A, 3160A>G, 3161G>T, 3162G>C, 3164C>T, 3168A>T, 3169A>C, 3170A>G, 3172T>C, 3174A>T, 3175C>G, 3176A>T, 3178C>T, 3179T>G, 3180G>T, 3181T>G, 3182C>G, 3184C>A, 3189A>T, 3191C>A, 3196C>T, 3200A>G, 3202T>A, 3203A>T, 3205T>G, 3206G>A, 3208C>A, 3212A>T, 3213G>C, 3215A>C, 3216C>G, 3217T>A, 3219C>G, 3220C>T, 3221C>A, 3222T>A, 3223A>G, 3224C>G, 3232C>T, 3233C>G, 3234T>A, 3238G>A, 3239A>C, 3241C>T, 3242C>T, 3245G>A, 3246A>G, 3247A>G, 3248C>A, 3249G>T, 3253T>A, 3254T>C, 3256C>G, 3259A>G, 3261G>C, 3263C>T, 3265T>A, 3268A>G, 3271A>T, 3272A>G, 3273G>A, 3274T>G, 3277C>G, 3280C>G, 3281A>T, 3283A>T, 3284A>G, 3285T>C, 3286T>C, 3287C>A, 3289A>G, 3290A>T, 3292G>T, 3295C>T, 3300C>G, 3307C>T, 3309T>G, 3311A>C, 3312A>T, 3314C>A, 3315T>A, 3316C>T, 3320A>G, 3321C>T, 3322T>G, 3325T>C, 3327A>T, 3328T>C, 3334T>G, 3337C>T, 3340C>A, 3341A>G, 3343A>T, 3344A>T, 3345G>C, 3346C>G, 3347A>G, 3349G>T, 3352C>T, 3355T>A, 3362C>G, 3363C>T, 3364T>G, 3365A>G, 3370T>G, 3371G>A, 3373T>G, 3376G>A, 3378T>C, 3380T>G, 3381C>A, 3382C>G, 3385T>G, 3386A>G, 3388T>C, 3389C>A, 3390A>G, 3391A>G, 3394A>T, 3396A>G, 3397T>G, 3399T>C, 3400G>A, 3402T>G, 3403T>G, 3406A>T, 3407A>T, 3408A>T, 3410A>T, 3412C>T, 3415T>G, 3417A>C, 3418G>T, 3421A>T, 3424A>T, 3426A>G, 3428C>A, 3429A>G, 3430A>C, 3436A>G, 3439C>A, 3440C>T, 3442T>G, 3443C>G, 3444T>C, 3445C>A, 3451T>C, 3454C>T, 3455C>A, 3457A>G, 3459A>G, 3464A>G, 3466T>A, 3467C>G, 3468C>A, 3471A>G, 3476G>T								

CDS

	Begin	End	Coverage	Score	Concordance	Matches	Identities	I/D/M/F*	Stop Codons
D1R33_gp2	202	392	17.5%	478	37.1%	187 (98.4%)	82 (43.2%)	0/3/1/1	0
Protein mutations:	K205I (2919A>T 2920G>C), I207V (2924A>G 2926C>G), D208R (2927G>A 2928A>G 2929T>G), K210R (2934A>G 2935A>G), Y211F (2937A>T), N215T (2949A>C 2950C>T), S217D (2954A>G 2955G>A), D218E (2959C>G), V219L (2960G>C), L220F (2963C>T), K222E (2969A>G), L223V (2972T>G), G224N (2975G>A 2976G>A), K225G (2978A>G 2979A>G 2980G>C), C226A (2981T>G 2982G>C 2983C>T), Q227V (2984C>G 2985A>T 2986A>T), T230S (2993A>T 2995C>A), T231K (2997C>A 2998C>G), A235L (3008G>T 3009C>T), F238Y (3018T>A 3019T>C), Y239D (3020T>G), V241I (3026G>A 3028G>T), E242R (3029G>C 3030A>G), M243V (3032A>G 3034G>T), D244K (3035G>A 3037C>G), P245V (3038C>G 3039C>T 3040T>G), Q246A (3041C>G 3042A>C), S249P (3050T>C 3052G>C), N254R (3066A>G 3067C>G), V255T (3068G>A 3069T>C), E256H (3071G>C 3073A>C), H257D (3074C>G 3076C>T), F260Y (3084T>A 3085T>C), R264V (3095C>G 3096G>T 3097A>C), M267F (3104A>T 3106G>T), K270S (3113A>T 3114A>C 3115A>T), S272A (3119T>G 3121A>G), P273S (3122C>T 3124A>G), Q277R (3135A>G), R278_M280del (3137_3146delAGAGTTATGG), N282R (3150A>G 3151T>G), L284F (3155C>T 3157A>C), R285K (3159G>A 3160A>G), G286S (3161G>T 3162G>C), L287F (3164C>T), Q288L (3168A>T 3169A>C), N289D (3170A>G 3172T>C), N290M (3174A>T 3175C>G), I291F (3176A>T 3178C>T), C292V (3179T>G 3180G>T 3181T>G), L293V (3182C>G 3184C>A), Y295F (3189A>T), L296I (3191C>A), I299V (3200A>G 3202T>A), I300L (3203A>T 3205T>G), V301I (3206G>A 3208C>A), T304R (3215A>C 3216C>G 3217T>A), S305C (3219C>G 3220C>T), L306K (3221C>A 3222T>A 3223A>G), Q307E (3224C>G), L310E (3233C>G 3234T>A), N312H (3239A>C 3241C>T), E314R (3245G>A 3246A>G 3247A>G), R315I (3248C>A 3249G>T), F317L (3254T>C 3256C>G), R319T (3261G>C), E322D (3271A>T), S323E (3272A>G 3273G>A 3274T>G), N324K (3277C>G), F325L (3280C>G), K326Y (3281A>T 3283A>T), I327A (3284A>G 3285T>C 3286T>C), Q328K (3287C>A 3289A>G), M329F (3290A>T 3292G>T), S332C (3300C>G), L335W (3309T>G), K336L (3311A>C 3312A>T), L337N (3314C>A 3315T>A 3316C>T), T339V (3320A>G 3321C>T 3322T>G), Y341F (3327A>T 3328T>C), I346V (3341A>G 3343A>T), R348G (3347A>G 3349G>T), P353V (3362C>G 3363C>T 3364T>G), N354D (3365A>G), D356K (3371G>A 3373T>G), I358T (3378T>C), S359E (3380T>G 3381C>A 3382C>G), I361V (3386A>G 3388T>C), Q362R (3389C>A 3390A>G 3391A>G), K363N (3394A>T), Y364W (3396A>G 3397T>G), L365P (3399T>C 3400G>A), I366R (3402T>G 3403T>G), K368L (3407A>T 3408A>T), T369S (3410A>T 3412C>T), K371T (3417A>C 3418G>T), E372D (3421A>T), K374R (3426A>G), Q375S (3428C>A 3429A>G 3430A>C), L380A (3443C>G 3444T>C 3445C>A), K385R (3459A>G), I387V (3464A>G 3466T>A), P388E (3467C>G 3468C>A), D389G (3471A>G), A391S (3476G>T)								
Codon mutations:	AAG205ATC (2919A>T 2920G>C), ATC207GTG (2924A>G 2926C>G), GAT208AGG (2927G>A 2928A>G 2929T>G), GAC209GAT (2932C>T), AAA210AGG (2934A>G 2935A>G), TAC211TTC (2937A>T), CCG212CCA (2941G>A), ATA213ATC (2944A>C), AAC215ACT (2949A>C 2950C>T), ATA216ATT (2953A>T), AGT217GAT (2954A>G 2955G>A), GAC218GAG (2959C>G), GTA219CTA (2960G>C), CTT220TTT (2963C>T), GAC221GAT (2968C>T), AAG222GAG (2969A>G), TTA223GTA (2972T>G), GGT224AAT (2975G>A 2976G>A), AAG225GGC (2978A>G 2979A>G 2980G>C), TGC226GCT (2981T>G 2982G>C 2983C>T), CAA227GTT (2984C>G 2985A>T 2986A>T), TAC228TAT (2989C>T), ACC230TCA (2993A>T 2995C>A), ACC231AAG (2997C>A 2998C>G), TTA232CTT (2999T>C 3001A>T), GCA235TTA (3008G>T 3009C>T), AGT236TCT (3011A>T 3012G>C), GGG237GGA (3016G>A), TTT238TAC (3018T>A 3019T>C), TAT239GAT (3020T>G), CAG240CAA (3025G>A), GTG241ATT (3026G>A 3028G>T), GAG242CGG (3029G>C 3030A>G), ATG243GTT (3032A>G 3034G>T), GAC244AAG (3035G>A 3037C>G), CCT245GTG (3038C>G 3039C>T 3040T>G), CAA246GCA (3041C>G 3042A>C), TCG249CCC (3050T>C 3052G>C), ACC251ACT (3058C>T), GCG252GCT (3061G>T), AAC254AGG (3066A>G 3067C>G), GTA255ACA (3068G>A 3069T>C), GAA256CAC (3071G>C 3073A>C), CAC257GAT (3074C>G 3076C>T), GGG258GGT (3079G>T), TTT260TAC (3084T>A 3085T>C), TTC262TTT (3091C>T), CTT263TTA (3092C>T 3094T>A), CGA264GTC (3095C>G 3096G>T 3097A>C), ATG267TTT (3104A>T 3106G>T), GGA268GGG (3109A>G), TTA269TTG (3112A>G), AAA270TCT (3113A>T 3114A>C 3115A>T), TCA272GCG (3119T>G 3121A>G), CCA273TCG (3122C>T 3124A>G), TCT274TCC (3127T>C), ACT275ACA (3130T>A), CAA277CGA (3135A>G), AGA278_ATG280del (3137_3146delAGAGTTATGG), GAC281-AC (3137_3146delAGAGTTATGG), AAT282AGG (3150A>G 3151T>G), GTC283GTT (3154C>T), CTA284TTC (3155C>T 3157A>C), AGA285AAG (3159G>A 3160A>G), GGT286TCT (3161G>T 3162G>C), CTC287TTC (3164C>T), CAA288CTC (3168A>T 3169A>C), AAT289GAC (3170A>G 3172T>C), AAC290ATG (3174A>T 3175C>G), ATC291TTT (3176A>T 3178C>T), TGT292GTG (3179T>G 3180G>T 3181T>G), CTC293GTA (3182C>G 3184C>A), TAC295TTC (3189A>T), CTT296ATT (3191C>A), GAC297GAT (3196C>T), ATT299GTA (3200A>G 3202T>A), ATT300TTG (3203A>T 3205T>G), GTC301ATA (3206G>A 3208C>A), AGT303TCT (3212A>T 3213G>C), ACT304CGA (3215A>C 3216C>G 3217T>A), TCC305TGT (3219C>G 3220C>T), CTA306AAG (3221C>A 3222T>A 3223A>G), CAG307GAG (3224C>G), CAC309CAT (3232C>T), CTG310GAG (3233C>G 3234T>A), GAG311GAA (3238G>A), AAC312CAT (3239A>C 3241C>T), CTG313TTG (3242C>T), GAA314AGG (3245G>A 3246A>G 3247A>G), CGA315ATA (3248C>A 3249G>T), GTT316GTA (3253T>A), TTC317CTG (3254T>C 3256C>G), CAA318CAG (3259A>G), AGA319ACA (3261G>C), CTT320TTA (3263C>T 3265T>A), AGA321AGG (3268A>G), GAA322GAT (3271A>T), AGT323GAG (3272A>G 3273G>A 3274T>G), AAC324AAG (3277C>G), TTC325TTG (3280C>G), AAA326TAT (3281A>T 3283A>T), ATT327GCC (3284A>G 3285T>C 3286T>C), CAA328AAG (3287C>A 3289A>G), ATG329TTT (3290A>T 3292G>T), GAC330GAT (3295C>T), TCC332TGC (3300C>G), TTC334TTT (3307C>T), TTG335TGG (3309T>G), AAG336CTG (3311A>C 3312A>T), CTC337AAT (3314C>A 3315T>A 3316C>T), ACT339GTG (3320A>G 3321C>T 3322T>G), GCT340GCC (3325T>C), TAT341TTC (3327A>T 3328T>C), GGT343GGG (3334T>G), CAC344CAT (3337C>T), ATC345ATA (3340C>A), ATA346GTT (3341A>G 3343A>T), AGC347TCG (3344A>T 3345G>C 3346C>G), AGG348GGT (3347A>G 3349G>T), GAC349GAT (3352C>T), GGT350GGA (3355T>A), CCT353GTG (3362C>G 3363C>T 3364T>G), AAC354GAC (3365A>G), CCT355CCG (3370T>G), GAT356AAG (3371G>A 3373T>G), AAG357AAA (3376G>A), ATT358ACT (3378T>C), TCC359GAG (3380T>G 3381C>A 3382C>G), GCT360GCG (3385T>G), ATT361GTC (3386A>G 3388T>C), CAA362AGG (3389C>A 3390A>G 3391A>G), AAA363AAT (3394A>T), TAT364TGG (3396A>G 3397T>G), CTG365CCA (3399T>C 3400G>A), ATT366AGG (3402T>G 3403T>G), CCA367CCT (3406A>T), AAG368TTG (3407A>T 3408A>T), ACC369TCT (3410A>T 3412C>T), CCT370CCG (3415T>G), AAG371ACT (3417A>C 3418G>T), GAA372GAT (3421A>T), ATA373ATT (3424A>T), AAA374AGA (3426A>G), CAA375AGC (3428C>A 3429A>G 3430A>C), TTA377TTG (3436A>G), GGC378GGA (3439C>A), CTT379TTG (3440C>T 3442T>G), CTC380GCA (3443C>G 3444T>C 3445C>A), TAT382TAC (3451T>C), TAC383TAT (3454C>T), CGA384AGG (3455C>A 3457A>G), AAA385AGA (3459A>G), ATT387GTA (3464A>G 3466T>A), CCA388GAA (3467C>G 3468C>A), GAT389GGT (3471A>G), GCA391TCA (3476G>T)								

Proteins

	Begin	End	Coverage	Score	Concordance	Matches	Identities	I/D/M/F*	Stop Codons
ORF B (YP_009507248.1)	202	392	17.5%	478	37.1%	187 (98.4%)	82 (43.2%)	0/3/1/1	0
Protein mutations:	K205I (2919A>T 2920G>C), I207V (2924A>G 2926C>G), D208R (2927G>A 2928A>G 2929T>G), K210R (2934A>G 2935A>G), Y211F (2937A>T), N215T (2949A>C 2950C>T), S217D (2954A>G 2955G>A), D218E (2959C>G), V219L (2960G>C), L220F (2963C>T), K222E (2969A>G), L223V (2972T>G), G224N (2975G>A 2976G>A), K225G (2978A>G 2979A>G 2980G>C), C226A (2981T>G 2982G>C 2983C>T), Q227V (2984C>G 2985A>T 2986A>T), T230S (2993A>T 2995C>A), T231K (2997C>A 2998C>G), A235L (3008G>T 3009C>T), F238Y (3018T>A 3019T>C), Y239D (3020T>G), V241I (3026G>A 3028G>T), E242R (3029G>C 3030A>G), M243V (3032A>G 3034G>T), D244K (3035G>A 3037C>G), P245V (3038C>G 3039C>T 3040T>G), Q246A (3041C>G 3042A>C), S249P (3050T>C 3052G>C), N254R (3066A>G 3067C>G), V255T (3068G>A 3069T>C), E256H (3071G>C 3073A>C), H257D (3074C>G 3076C>T), F260Y (3084T>A 3085T>C), R264V (3095C>G 3096G>T 3097A>C), M267F (3104A>T 3106G>T), K270S (3113A>T 3114A>C 3115A>T), S272A (3119T>G 3121A>G), P273S (3122C>T 3124A>G), Q277R (3135A>G), R278_M280del (3137_3146delAGAGTTATGG), N282R (3150A>G 3151T>G), L284F (3155C>T 3157A>C), R285K (3159G>A 3160A>G), G286S (3161G>T 3162G>C), L287F (3164C>T), Q288L (3168A>T 3169A>C), N289D (3170A>G 3172T>C), N290M (3174A>T 3175C>G), I291F (3176A>T 3178C>T), C292V (3179T>G 3180G>T 3181T>G), L293V (3182C>G 3184C>A), Y295F (3189A>T), L296I (3191C>A), I299V (3200A>G 3202T>A), I300L (3203A>T 3205T>G), V301I (3206G>A 3208C>A), T304R (3215A>C 3216C>G 3217T>A), S305C (3219C>G 3220C>T), L306K (3221C>A 3222T>A 3223A>G), Q307E (3224C>G), L310E (3233C>G 3234T>A), N312H (3239A>C 3241C>T), E314R (3245G>A 3246A>G 3247A>G), R315I (3248C>A 3249G>T), F317L (3254T>C 3256C>G), R319T (3261G>C), E322D (3271A>T), S323E (3272A>G 3273G>A 3274T>G), N324K (3277C>G), F325L (3280C>G), K326Y (3281A>T 3283A>T), I327A (3284A>G 3285T>C 3286T>C), Q328K (3287C>A 3289A>G), M329F (3290A>T 3292G>T), S332C (3300C>G), L335W (3309T>G), K336L (3311A>C 3312A>T), L337N (3314C>A 3315T>A 3316C>T), T339V (3320A>G 3321C>T 3322T>G), Y341F (3327A>T 3328T>C), I346V (3341A>G 3343A>T), R348G (3347A>G 3349G>T), P353V (3362C>G 3363C>T 3364T>G), N354D (3365A>G), D356K (3371G>A 3373T>G), I358T (3378T>C), S359E (3380T>G 3381C>A 3382C>G), I361V (3386A>G 3388T>C), Q362R (3389C>A 3390A>G 3391A>G), K363N (3394A>T), Y364W (3396A>G 3397T>G), L365P (3399T>C 3400G>A), I366R (3402T>G 3403T>G), K368L (3407A>T 3408A>T), T369S (3410A>T 3412C>T), K371T (3417A>C 3418G>T), E372D (3421A>T), K374R (3426A>G), Q375S (3428C>A 3429A>G 3430A>C), L380A (3443C>G 3444T>C 3445C>A), K385R (3459A>G), I387V (3464A>G 3466T>A), P388E (3467C>G 3468C>A), D389G (3471A>G), A391S (3476G>T)								

	Begin	End	Coverage	Score	Concordance	Matches	Identities	I/D/M/F*	Stop Codons
**NT**	**2909**	**3480**	**7.6%**	**61**	**5.5%**	**562 (98.3%)**	**301 (52.6%)**	**0/10**	
Codon mutations:	AAG205ATC (2919A>T 2920G>C), ATC207GTG (2924A>G 2926C>G), GAT208AGG (2927G>A 2928A>G 2929T>G), GAC209GAT (2932C>T), AAA210AGG (2934A>G 2935A>G), TAC211TTC (2937A>T), CCG212CCA (2941G>A), ATA213ATC (2944A>C), AAC215ACT (2949A>C 2950C>T), ATA216ATT (2953A>T), AGT217GAT (2954A>G 2955G>A), GAC218GAG (2959C>G), GTA219CTA (2960G>C), CTT220TTT (2963C>T), GAC221GAT (2968C>T), AAG222GAG (2969A>G), TTA223GTA (2972T>G), GGT224AAT (2975G>A 2976G>A), AAG225GGC (2978A>G 2979A>G 2980G>C), TGC226GCT (2981T>G 2982G>C 2983C>T), CAA227GTT (2984C>G 2985A>T 2986A>T), TAC228TAT (2989C>T), ACC230TCA (2993A>T 2995C>A), ACC231AAG (2997C>A 2998C>G), TTA232CTT (2999T>C 3001A>T), GCA235TTA (3008G>T 3009C>T), AGT236TCT (3011A>T 3012G>C), GGG237GGA (3016G>A), TTT238TAC (3018T>A 3019T>C), TAT239GAT (3020T>G), CAG240CAA (3025G>A), GTG241ATT (3026G>A 3028G>T), GAG242CGG (3029G>C 3030A>G), ATG243GTT (3032A>G 3034G>T), GAC244AAG (3035G>A 3037C>G), CCT245GTG (3038C>G 3039C>T 3040T>G), CAA246GCA (3041C>G 3042A>C), TCG249CCC (3050T>C 3052G>C), ACC251ACT (3058C>T), GCG252GCT (3061G>T), AAC254AGG (3066A>G 3067C>G), GTA255ACA (3068G>A 3069T>C), GAA256CAC (3071G>C 3073A>C), CAC257GAT (3074C>G 3076C>T), GGG258GGT (3079G>T), TTT260TAC (3084T>A 3085T>C), TTC262TTT (3091C>T), CTT263TTA (3092C>T 3094T>A), CGA264GTC (3095C>G 3096G>T 3097A>C), ATG267TTT (3104A>T 3106G>T), GGA268GGG (3109A>G), TTA269TTG (3112A>G), AAA270TCT (3113A>T 3114A>C 3115A>T), TCA272GCG (3119T>G 3121A>G), CCA273TCG (3122C>T 3124A>G), TCT274TCC (3127T>C), ACT275ACA (3130T>A), CAA277CGA (3135A>G), AGA278_ATG280del (3137_3146delAGAGTTATGG), GAC281-AC (3137_3146delAGAGTTATGG), AAT282AGG (3150A>G 3151T>G), GTC283GTT (3154C>T), CTA284TTC (3155C>T 3157A>C), AGA285AAG (3159G>A 3160A>G), GGT286TCT (3161G>T 3162G>C), CTC287TTC (3164C>T), CAA288CTC (3168A>T 3169A>C), AAT289GAC (3170A>G 3172T>C), AAC290ATG (3174A>T 3175C>G), ATC291TTT (3176A>T 3178C>T), TGT292GTG (3179T>G 3180G>T 3181T>G), CTC293GTA (3182C>G 3184C>A), TAC295TTC (3189A>T), CTT296ATT (3191C>A), GAC297GAT (3196C>T), ATT299GTA (3200A>G 3202T>A), ATT300TTG (3203A>T 3205T>G), GTC301ATA (3206G>A 3208C>A), AGT303TCT (3212A>T 3213G>C), ACT304CGA (3215A>C 3216C>G 3217T>A), TCC305TGT (3219C>G 3220C>T), CTA306AAG (3221C>A 3222T>A 3223A>G), CAG307GAG (3224C>G), CAC309CAT (3232C>T), CTG310GAG (3233C>G 3234T>A), GAG311GAA (3238G>A), AAC312CAT (3239A>C 3241C>T), CTG313TTG (3242C>T), GAA314AGG (3245G>A 3246A>G 3247A>G), CGA315ATA (3248C>A 3249G>T), GTT316GTA (3253T>A), TTC317CTG (3254T>C 3256C>G), CAA318CAG (3259A>G), AGA319ACA (3261G>C), CTT320TTA (3263C>T 3265T>A), AGA321AGG (3268A>G), GAA322GAT (3271A>T), AGT323GAG (3272A>G 3273G>A 3274T>G), AAC324AAG (3277C>G), TTC325TTG (3280C>G), AAA326TAT (3281A>T 3283A>T), ATT327GCC (3284A>G 3285T>C 3286T>C), CAA328AAG (3287C>A 3289A>G), ATG329TTT (3290A>T 3292G>T), GAC330GAT (3295C>T), TCC332TGC (3300C>G), TTC334TTT (3307C>T), TTG335TGG (3309T>G), AAG336CTG (3311A>C 3312A>T), CTC337AAT (3314C>A 3315T>A 3316C>T), ACT339GTG (3320A>G 3321C>T 3322T>G), GCT340GCC (3325T>C), TAT341TTC (3327A>T 3328T>C), GGT343GGG (3334T>G), CAC344CAT (3337C>T), ATC345ATA (3340C>A), ATA346GTT (3341A>G 3343A>T), AGC347TCG (3344A>T 3345G>C 3346C>G), AGG348GGT (3347A>G 3349G>T), GAC349GAT (3352C>T), GGT350GGA (3355T>A), CCT353GTG (3362C>G 3363C>T 3364T>G), AAC354GAC (3365A>G), CCT355CCG (3370T>G), GAT356AAG (3371G>A 3373T>G), AAG357AAA (3376G>A), ATT358ACT (3378T>C), TCC359GAG (3380T>G 3381C>A 3382C>G), GCT360GCG (3385T>G), ATT361GTC (3386A>G 3388T>C), CAA362AGG (3389C>A 3390A>G 3391A>G), AAA363AAT (3394A>T), TAT364TGG (3396A>G 3397T>G), CTG365CCA (3399T>C 3400G>A), ATT366AGG (3402T>G 3403T>G), CCA367CCT (3406A>T), AAG368TTG (3407A>T 3408A>T), ACC369TCT (3410A>T 3412C>T), CCT370CCG (3415T>G), AAG371ACT (3417A>C 3418G>T), GAA372GAT (3421A>T), ATA373ATT (3424A>T), AAA374AGA (3426A>G), CAA375AGC (3428C>A 3429A>G 3430A>C), TTA377TTG (3436A>G), GGC378GGA (3439C>A), CTT379TTG (3440C>T 3442T>G), CTC380GCA (3443C>G 3444T>C 3445C>A), TAT382TAC (3451T>C), TAC383TAT (3454C>T), CGA384AGG (3455C>A 3457A>G), AAA385AGA (3459A>G), ATT387GTA (3464A>G 3466T>A), CCA388GAA (3467C>G 3468C>A), GAT389GGT (3471A>G), GCA391TCA (3476G>T)								

*: Inserts / Deletes / Misaligned / Frameshifts

Analysis details

This analysis was performed with panviral2.64

NGS Details (UN9): Epiphyllum badnavirus 1

Assembly

**Coverage Length**	274 (1 contig(s))
**Depth Of Coverage**	124.8
**Number Of Reads**	324
**Reads Per Million**	7.32 rpm (after QC)
**Ambiguities**	0
**Assembly Method**	de novo + reference guided assembly
**Consensus Caller**	Bcf Tools

Coverage Map

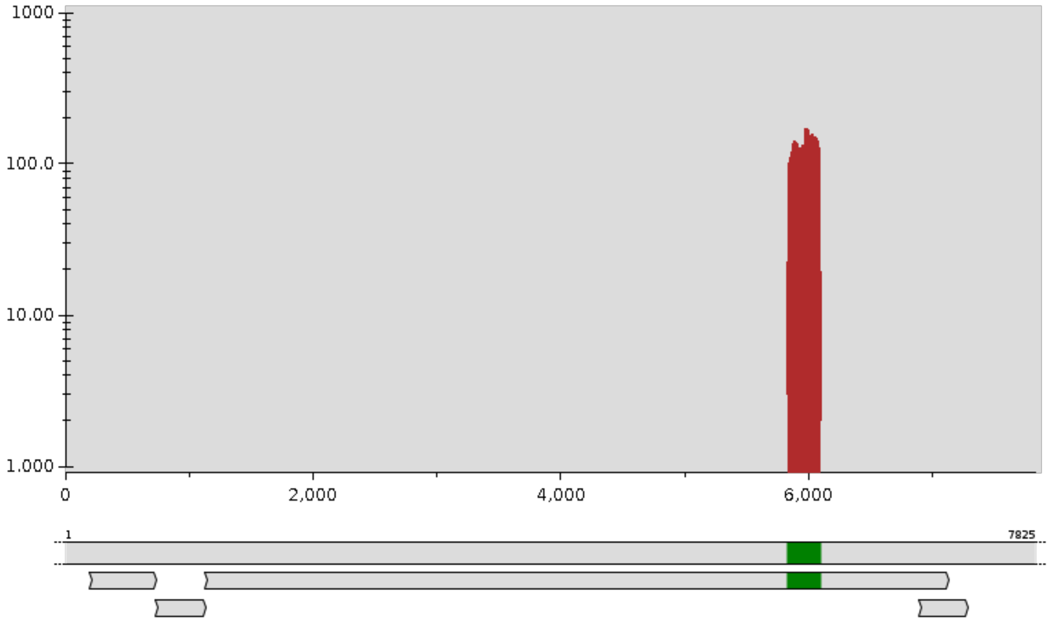

Assignment

**Type**	Epiphyllum badnavirus 1 (Taxonomy ID: 2518008)
**Reference Genome**	NC_076247.1
**NT Identity (%)**	60.2888
**AA Identity (%)**	57.6087
**Number Of Stop Codons**	0
**Number Of CDS**	4

Alignment

**Alignment Score**	108.0 (NT) + 362.0 (AA) = 470.0
**Concordance (%)**	39.0041

Alignment Method	Global, seeded, nucleotide + amino acids (AGA)

Genome Region

Sequence starts at position 5828 and ends at position 6101 relative to NC_076247.1 reference sequence.

Alignment Detailed Statistics

	Begin	End	Coverage	Score	Concordance	Matches	Identities	I/D/M/F*	Stop Codons
**NT**	**5828**	**6101**	**3.5%**	**108**	**19.7%**	**274 (98.9%)**	**167 (60.3%)**	**3/0**	
Mutations:	5837G>C, 5841C>G, 5847C>T, 5851T>A, 5853C>T, 5856T>C, 5857C>T, 5861A>G, 5862G>A, 5863A>T, 5864G>C, 5865C>G, 5868A>T, 5870T>A, 5871C>T, 5874C>T, 5878A>T, 5880T>G, 5882T>G, 5883G>A, 5884A>G, 5886G>T, 5887G>C, 5888A>G, 5889C>G, 5892A>G, 5893G>T, 5894A>C, 5896T>G, 5897C>A, 5898C>T, 5904T>G, 5905T>A, 5906G>A, 5910C>T, 5913C>G, 5917C>A, 5918T>G, 5924C>G, 5925A>C, 5926G>T, 5931A>T, 5933T>A, 5937C>T, 5940A>G, 5942G>T, 5943G>T, 5944T>G, 5946A>G, 5953C>T, 5955A>T, 5958C>T, 5961A>G, 5966A>C, 5967G>T, 5981T>C, 5982G>T, 5985C>T, 5986C>A, 5987A>T, 5988A>G, 5989C>G, 5990G>A, 5991A>G, 5992A>T, 5993A>T, 5998G>A, 6001A>C, 6002A>G, 6003C>A, 6004T>G, 6005G>T, 6006T>G, 6011G>A, 6013G>C, 6014A>C, 6015_6016insTAT, 6018G>T, 6019T>G, 6020C>A, 6021A>T, 6022G>C, 6023G>A, 6024C>G, 6030A>G, 6031G>A, 6032C>T, 6033A>T, 6036T>G, 6038A>T, 6042T>A, 6048C>T, 6052C>T, 6057C>T, 6061A>T, 6062G>C, 6063T>C, 6066C>G, 6068C>G, 6072A>G, 6073G>A, 6074A>G, 6078C>T, 6083C>T, 6084A>G, 6085C>A, 6090C>T, 6091C>T								

CDS

	Begin	End	Coverage	Score	Concordance	Matches	Identities	I/D/M/F*	Stop Codons
QKM20_gp3	1568	1658	4.5%	362	53.2%	91 (98.9%)	53 (57.6%)	1/0/0/0	0
Protein mutations:	S1570T (5837G>C), F1575I (5851T>A 5853C>T), K1578R (5861A>G 5862G>A), F1581Y (5870T>A 5871C>T), I1584L (5878A>T 5880T>G), M1585R (5882T>G 5883G>A), M1586V (5884A>G 5886G>T), D1587R (5887G>C 5888A>G 5889C>G), E1589S (5893G>T 5894A>C), S1590D (5896T>G 5897C>A 5898C>T), W1593K (5905T>A 5906G>A), L1597R (5917C>A 5918T>G), P1599R (5924C>G 5925A>C), D1600Y (5926G>T), L1602H (5933T>A), W1605F (5942G>T 5943G>T), L1606V (5944T>G 5946A>G), P1609S (5953C>T 5955A>T), K1613T (5966A>C 5967G>T), V1618A (5981T>C 5982G>T), Q1620M (5986C>A 5987A>T 5988A>G), R1621E (5989C>G 5990G>A 5991A>G), K1622L (5992A>T 5993A>T), D1624N (5998G>A), N1625R (6001A>C 6002A>G 6003C>A), C1626V (6004T>G 6005G>T 6006T>G), R1628K (6011G>A), D1629P (6013G>C 6014A>C), D1629_L1630insY (6015_6016insTAT), S1631D (6019T>G 6020C>A 6021A>T), G1632Q (6022G>C 6023G>A 6024C>G), A1635I (6031G>A 6032C>T 6033A>T), Y1637F (6038A>T), N1646K (6066C>G), T1647S (6068C>G), D1649S (6073G>A 6074A>G), A1652V (6083C>T 6084A>G), Q1653K (6085C>A)								
Codon mutations:	AGT1570ACT (5837G>C), GTC1571GTG (5841C>G), TCC1573TCT (5847C>T), TTC1575ATT (5851T>A 5853C>T), GAT1576GAC (5856T>C), CTG1577TTG (5857C>T), AAG1578AGA (5861A>G 5862G>A), AGC1579TCG (5863A>T 5864G>C 5865C>G), GGA1580GGT (5868A>T), TTC1581TAT (5870T>A 5871C>T), CAC1582CAT (5874C>T), ATT1584TTG (5878A>T 5880T>G), ATG1585AGA (5882T>G 5883G>A), ATG1586GTT (5884A>G 5886G>T), GAC1587CGG (5887G>C 5888A>G 5889C>G), GAA1588GAG (5892A>G), GAA1589TCA (5893G>T 5894A>C), TCC1590GAT (5896T>G 5897C>A 5898C>T), CCT1592CCG (5904T>G), TGG1593AAG (5905T>A 5906G>A), ACC1594ACT (5910C>T), GCC1595GCG (5913C>G), CTG1597AGG (5917C>A 5918T>G), CCA1599CGC (5924C>G 5925A>C), GAT1600TAT (5926G>T), GGA1601GGT (5931A>T), CTT1602CAT (5933T>A), TAC1603TAT (5937C>T), GAA1604GAG (5940A>G), TGG1605TTT (5942G>T 5943G>T), TTA1606GTG (5944T>G 5946A>G), CCA1609TCT (5953C>T 5955A>T), TTC1610TTT (5958C>T), GGA1611GGG (5961A>G), AAG1613ACT (5966A>C 5967G>T), GTG1618GCT (5981T>C 5982G>T), TTC1619TTT (5985C>T), CAA1620ATG (5986C>A 5987A>T 5988A>G), CGA1621GAG (5989C>G 5990G>A 5991A>G), AAG1622TTG (5992A>T 5993A>T), GAC1624AAC (5998G>A), AAC1625CGA (6001A>C 6002A>G 6003C>A), TGT1626GTG (6004T>G 6005G>T 6006T>G), AGA1628AAA (6011G>A), GAC1629CCC (6013G>C 6014A>C), GAC1629_CTG1630insTAT (6015_6016insTAT), CTG1630CTT (6018G>T), TCA1631GAT (6019T>G 6020C>A 6021A>T), GGC1632CAG (6022G>C 6023G>A 6024C>G), GTA1634GTG (6030A>G), GCA1635ATT (6031G>A 6032C>T 6033A>T), GTT1636GTG (6036T>G), TAC1637TTC (6038A>T), ATT1638ATA (6042T>A), GAC1640GAT (6048C>T), CTG1642TTG (6052C>T), GTC1643GTT (6057C>T), AGT1645TCC (6061A>T 6062G>C 6063T>C), AAC1646AAG (6066C>G), ACT1647AGT (6068C>G), GAA1648GAG (6072A>G), GAT1649AGT (6073G>A 6074A>G), GAC1650GAT (6078C>T), GCA1652GTG (6083C>T 6084A>G), CAG1653AAG (6085C>A), CAC1654CAT (6090C>T), CTG1655TTG (6091C>T)								

Proteins

	Begin	End	Coverage	Score	Concordance	Matches	Identities	I/D/M/F*	Stop Codons
polyprotein (YP_010797894.1)	1568	1658	4.5%	362	53.2%	91 (98.9%)	53 (57.6%)	1/0/0/0	0
Protein mutations:	S1570T (5837G>C), F1575I (5851T>A 5853C>T), K1578R (5861A>G 5862G>A), F1581Y (5870T>A 5871C>T), I1584L (5878A>T 5880T>G), M1585R (5882T>G 5883G>A), M1586V (5884A>G 5886G>T), D1587R (5887G>C 5888A>G 5889C>G), E1589S (5893G>T 5894A>C), S1590D (5896T>G 5897C>A 5898C>T), W1593K (5905T>A 5906G>A), L1597R (5917C>A 5918T>G), P1599R (5924C>G 5925A>C), D1600Y (5926G>T), L1602H (5933T>A), W1605F (5942G>T 5943G>T), L1606V (5944T>G 5946A>G), P1609S (5953C>T 5955A>T), K1613T (5966A>C 5967G>T), V1618A (5981T>C 5982G>T), Q1620M (5986C>A 5987A>T 5988A>G), R1621E (5989C>G 5990G>A 5991A>G), K1622L (5992A>T 5993A>T), D1624N (5998G>A), N1625R (6001A>C 6002A>G 6003C>A), C1626V (6004T>G 6005G>T 6006T>G), R1628K (6011G>A), D1629P (6013G>C 6014A>C), D1629_L1630insY (6015_6016insTAT), S1631D (6019T>G 6020C>A 6021A>T), G1632Q (6022G>C 6023G>A 6024C>G), A1635I (6031G>A 6032C>T 6033A>T), Y1637F (6038A>T), N1646K (6066C>G), T1647S (6068C>G), D1649S (6073G>A 6074A>G), A1652V (6083C>T 6084A>G), Q1653K (6085C>A)								
Codon mutations:	AGT1570ACT (5837G>C), GTC1571GTG (5841C>G), TCC1573TCT (5847C>T), TTC1575ATT (5851T>A 5853C>T), GAT1576GAC (5856T>C), CTG1577TTG (5857C>T), AAG1578AGA (5861A>G 5862G>A), AGC1579TCG (5863A>T 5864G>C 5865C>G), GGA1580GGT (5868A>T), TTC1581TAT (5870T>A 5871C>T), CAC1582CAT (5874C>T), ATT1584TTG (5878A>T 5880T>G), ATG1585AGA (5882T>G 5883G>A), ATG1586GTT (5884A>G 5886G>T), GAC1587CGG (5887G>C 5888A>G 5889C>G), GAA1588GAG (5892A>G), GAA1589TCA (5893G>T 5894A>C), TCC1590GAT (5896T>G 5897C>A 5898C>T), CCT1592CCG (5904T>G), TGG1593AAG (5905T>A 5906G>A), ACC1594ACT (5910C>T), GCC1595GCG (5913C>G), CTG1597AGG (5917C>A 5918T>G), CCA1599CGC (5924C>G 5925A>C), GAT1600TAT (5926G>T), GGA1601GGT (5931A>T), CTT1602CAT (5933T>A), TAC1603TAT (5937C>T), GAA1604GAG (5940A>G), TGG1605TTT (5942G>T 5943G>T), TTA1606GTG (5944T>G 5946A>G), CCA1609TCT (5953C>T 5955A>T), TTC1610TTT (5958C>T), GGA1611GGG (5961A>G), AAG1613ACT (5966A>C 5967G>T), GTG1618GCT (5981T>C 5982G>T), TTC1619TTT (5985C>T), CAA1620ATG (5986C>A 5987A>T 5988A>G), CGA1621GAG (5989C>G 5990G>A 5991A>G), AAG1622TTG (5992A>T 5993A>T), GAC1624AAC (5998G>A), AAC1625CGA (6001A>C 6002A>G 6003C>A), TGT1626GTG (6004T>G 6005G>T 6006T>G), AGA1628AAA (6011G>A), GAC1629CCC (6013G>C 6014A>C), GAC1629_CTG1630insTAT (6015_6016insTAT), CTG1630CTT (6018G>T), TCA1631GAT (6019T>G 6020C>A 6021A>T), GGC1632CAG (6022G>C 6023G>A 6024C>G), GTA1634GTG (6030A>G), GCA1635ATT (6031G>A 6032C>T 6033A>T), GTT1636GTG (6036T>G), TAC1637TTC (6038A>T), ATT1638ATA (6042T>A), GAC1640GAT (6048C>T), CTG1642TTG (6052C>T), GTC1643GTT (6057C>T), AGT1645TCC (6061A>T 6062G>C 6063T>C), AAC1646AAG (6066C>G), ACT1647AGT (6068C>G), GAA1648GAG (6072A>G), GAT1649AGT (6073G>A 6074A>G), GAC1650GAT (6078C>T), GCA1652GTG (6083C>T 6084A>G), CAG1653AAG (6085C>A), CAC1654CAT (6090C>T), CTG1655TTG (6091C>T)								

*: Inserts / Deletes / Misaligned / Frameshifts

Analysis details

This analysis was performed with panviral2.64

NGS Details (UN9): Lowelvirus tuscon4d

Assembly

**Coverage Length**	1030 (1 contig(s))
**Depth Of Coverage**	36.3
**Number Of Reads**	311
**Reads Per Million**	7.03 rpm (after QC)
**Ambiguities**	0
**Assembly Method**	de novo + reference guided assembly
**Consensus Caller**	Bcf Tools

Coverage Map

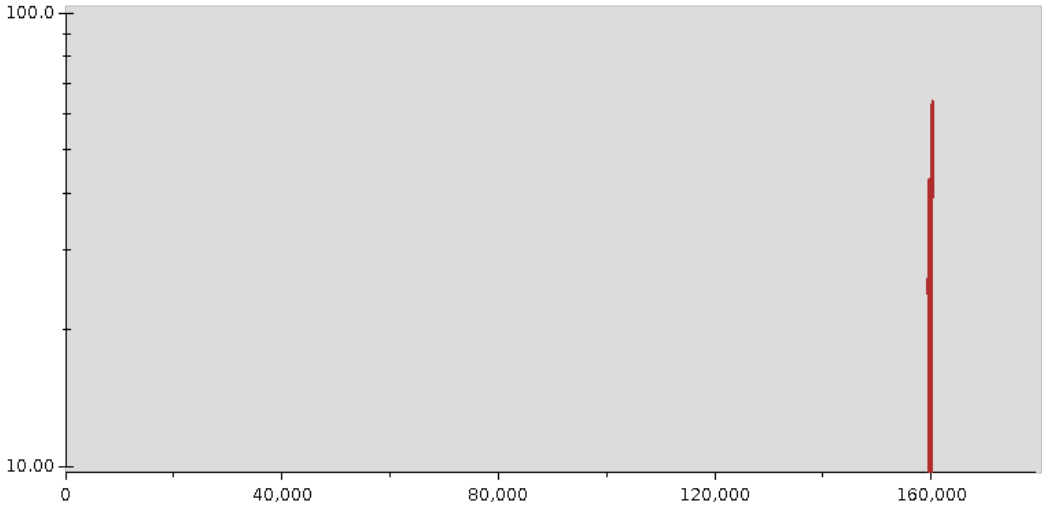

Assignment

**Type**	Lowelvirus tuscon4d (Taxonomy ID: 2956131)
**Reference Genome**	NC_026923.1
**NT Identity (%)**	73.301
**AA Identity (%)**	87.0588
**Number Of Stop Codons**	1
**Number Of CDS**	218

Alignment

**Alignment Score**	960.0 (NT) + 2288.0 (AA) = 3248.0
**Concordance (%)**	71.2593
**Alignment Method**	Local, heuristic, nucleotide (BLASTN)

Genome Region

Sequence starts at position 159375 and ends at position 160404 relative to NC_026923.1 reference sequence.

Alignment Detailed Statistics

	Begin	End	Coverage	Score	Concordance	Matches	Identities	I/D/M/F*	Stop Codons
**NT**	**159375**	**160404**	**0.6%**	**960**	**46.6%**	**1030 (100%)**	**755 (73.3%)**	**0/0**	
Mutations:	159385G>A, 159387C>T, 159388C>A, 159400C>T, 159402T>A, 159403A>C, 159404A>G, 159405A>G, 159406C>A, 159408A>G, 159411T>C, 159414C>T, 159417T>C, 159420C>T, 159426G>A, 159429T>C, 159435T>C, 159438A>T, 159441C>A, 159442C>T, 159444A>G, 159447T>C, 159448C>T, 159453C>T, 159454A>T, 159455C>G, 159459T>C, 159463A>T, 159465G>C, 159468C>T, 159469A>C, 159471T>A, 159474C>G, 159481C>T, 159483T>C, 159486T>A, 159489C>T, 159492G>A, 159495T>C, 159499A>G, 159501C>A, 159504G>T, 159505A>T, 159506G>C, 159507T>A, 159513C>T, 159519C>T, 159522T>A, 159523C>T, 159525T>G, 159528T>C, 159534C>T, 159537C>T, 159538C>T, 159540T>G, 159546T>C, 159547G>T, 159548C>G, 159549T>C, 159555T>C, 159556C>T, 159558C>A, 159561G>T, 159564A>C, 159567T>G, 159570C>T, 159573A>T, 159576G>T, 159583G>A, 159586G>A, 159587C>G, 159589A>T, 159591G>A, 159593G>C, 159594T>A, 159600T>G, 159601C>T, 159603T>G, 159604C>T, 159606T>G, 159607C>T, 159612T>A, 159624G>A, 159625T>G, 159627T>A, 159633C>A, 159636C>T, 159639C>T, 159640C>A, 159641A>C, 159642A>T, 159645C>T, 159651C>T, 159655C>T, 159657T>G, 159660A>G, 159663G>T, 159664A>C, 159666T>G, 159670T>A, 159672C>T, 159687C>T, 159690T>A, 159693A>T, 159696C>T, 159698C>G, 159699T>C, 159702C>A, 159711T>C, 159715C>T, 159720G>T, 159726T>C, 159729A>G, 159730A>C, 159738T>A, 159739C>T, 159740T>C, 159741A>T, 159745G>C, 159746G>A, 159747T>A, 159748A>T, 159750C>G, 159751C>A, 159753T>A, 159759C>T, 159765T>A, 159768T>C, 159771G>A, 159783T>A, 159798C>T, 159799A>T, 159800G>C, 159801C>T, 159807C>T, 159810T>G, 159813C>T, 159822G>A, 159832T>G, 159833C>G, 159834A>T, 159855C>T, 159858G>T, 159867A>T, 159870C>A, 159874A>C, 159880C>A, 159882G>C, 159883T>C, 159885G>A, 159888C>T, 159889C>T, 159894G>A, 159897T>G, 159900C>T, 159903C>T, 159906C>T, 159913C>T, 159915C>G, 159924C>T, 159942T>C, 159946A>G, 159949C>T, 159951A>G, 159954T>C, 159956G>C, 159957A>T, 159960A>T, 159961C>T, 159964T>C, 159966G>A, 159969T>C, 159972A>C, 159975C>T, 159987T>C, 159993G>A, 159996C>T, 159999A>T, 160002G>A, 160011T>C, 160014A>T, 160017A>T, 160018C>G, 160019A>G, 160020A>T, 160026C>T, 160032T>C, 160033A>C, 160034A>G, 160035G>T, 160038G>T, 160042G>A, 160044G>C, 160047T>A, 160055A>C, 160056A>C, 160062G>A, 160071T>A, 160080T>C, 160083A>T, 160092C>T, 160098G>C, 160101G>A, 160107C>T, 160110T>G, 160116A>T, 160122T>C, 160125C>T, 160128G>A, 160131G>T, 160135C>T, 160137T>A, 160143T>C, 160146C>T, 160152G>A, 160158G>A, 160161T>A, 160164T>A, 160166T>C, 160167G>C, 160171C>T, 160178C>T, 160179A>G, 160180T>A, 160181C>G, 160182C>T, 160183T>G, 160186A>C, 160191C>A, 160192A>G, 160194T>A, 160195A>G, 160197T>C, 160203T>A, 160207T>C, 160209C>G, 160215T>A, 160221T>C, 160224C>T, 160227T>C, 160233G>T, 160234A>T, 160235G>C, 160242G>A, 160246A>C, 160248A>C, 160254A>G, 160275C>T, 160287G>A, 160290C>T, 160293C>T, 160296A>C, 160299G>A, 160302T>C, 160308A>T, 160309C>A, 160314T>C, 160326A>G, 160327C>G, 160330G>C, 160331C>A, 160332T>A, 160338A>G, 160341A>T, 160351T>C, 160356C>A, 160368A>G, 160371A>T, 160377T>A, 160378A>C, 160380A>T, 160383T>A, 160389A>T, 160392G>T, 160394G>A								

*: Inserts / Deletes / Misaligned / Frameshifts

Analysis details

This analysis was performed with panviral2.64

NGS Details (UN9): Hibiscus bacilliform virus GD1

Assembly

**Coverage Length**	281 (1 contig(s))
**Depth Of Coverage**	73.4
**Number Of Reads**	204
**Reads Per Million**	4.61 rpm (after QC)
**Ambiguities**	0
**Assembly Method**	de novo + reference guided assembly
**Consensus Caller**	Bcf Tools

Coverage Map

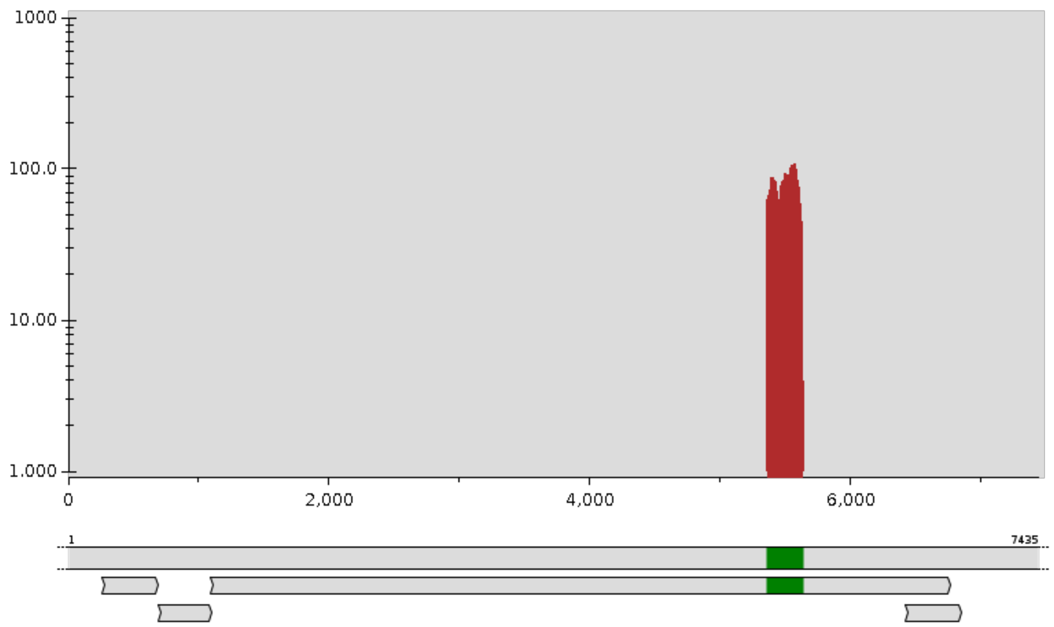

Assignment

**Type**	Hibiscus bacilliform virus GD1 (Taxonomy ID: 1459800)
**Reference Genome**	NC_023485.1
**NT Identity (%)**	59.507
**AA Identity (%)**	51.5789
**Number Of Stop Codons**	0
**Number Of CDS**	4

Alignment

**Alignment Score**	102.0 (NT) + 360.0 (AA) = 462.0
**Concordance (%)**	37.6835

**Alignment Method**	Global, seeded, nucleotide + amino acids (AGA)

Genome Region

Sequence starts at position 5357 and ends at position 5637 relative to NC_023485.1 reference sequence.

Alignment Detailed Statistics

	Begin	End	Coverage	Score	Concordance	Matches	Identities	I/D/M/F*	Stop Codons
**NT**	**5357**	**5637**	**3.8%**	**102**	**18.1%**	**281 (98.9%)**	**169 (59.5%)**	**3/0**	
Mutations:	5364A>G, 5366C>G, 5368A>T, 5372A>T, 5376T>A, 5382C>T, 5384C>G, 5386A>G, 5387G>A, 5388A>T, 5389G>T, 5390T>G, 5393A>T, 5395T>A, 5396T>C, 5403G>T, 5406G>A, 5407C>G, 5408T>A, 5409A>G, 5411G>T, 5414C>G, 5415C>G, 5416C>A, 5418G>T, 5419A>C, 5420G>A, 5421T>G, 5422C>A, 5427A>C, 5428A>C, 5430T>A, 5431G>A, 5438T>A, 5441C>T, 5445G>A, 5446T>C, 5447T>A, 5449C>G, 5450A>C, 5451G>T, 5456A>T, 5458T>A, 5459G>T, 5467G>T, 5468G>C, 5469C>G, 5471A>G, 5478C>T, 5480A>T, 5486A>G, 5491A>C, 5492A>C, 5495C>T, 5501A>T, 5504A>T, 5506T>C, 5510T>C, 5511C>A, 5512A>T, 5514C>G, 5515G>A, 5516A>G, 5517A>C, 5518A>T, 5523G>A, 5525C>A, 5526A>C, 5527A>G, 5528C>A, 5531C>G, 5534C>T, 5537A>G, 5538G>T, 5539G>C, 5541A>T, 5542C>A, 5543_5544insTTG, 5546A>T, 5547G>C, 5548C>A, 5549C>G, 5553A>G, 5555C>G, 5556G>A, 5557C>T, 5558T>C, 5561A>G, 5563A>T, 5564C>T, 5567T>A, 5576A>C, 5577C>T, 5579C>G, 5582A>T, 5584T>A, 5585T>C, 5589C>A, 5592T>A, 5593C>G, 5598G>A, 5599A>G, 5600G>T, 5603A>T, 5607A>G, 5609C>G, 5612G>A, 5616A>T, 5618A>G, 5619C>A, 5622G>A, 5624C>T, 5625A>G								

CDS

	Begin	End	Coverage	Score	Concordance	Matches	Identities	I/D/M/F*	Stop Codons
BU35_gp3	1423	1516	5.0%	360	51.6%	94 (98.9%)	49 (51.6%)	1/0/0/0	0
Protein mutations:	I1425V (5364A>G 5366C>G), Y1426F (5368A>T), F1429I (5376T>A), K1432R (5386A>G 5387G>A), S1433L (5388A>T 5389G>T 5390T>G), F1435Y (5395T>A 5396T>C), V1438L (5403G>T), A1439R (5406G>A 5407C>G 5408T>A), M1440V (5409A>G 5411G>T), H1441Q (5414C>G), P1442E (5415C>G 5416C>A), E1443S (5418G>T 5419A>C 5420G>A), S1444D (5421T>G 5422C>A), K1446P (5427A>C 5428A>C), W1447K (5430T>A 5431G>A), V1452T (5445G>A 5446T>C 5447T>A), P1453R (5449C>G 5450A>C), D1454Y (5451G>T), L1456H (5458T>A 5459G>T), W1459F (5467G>T 5468G>C), L1460V (5469C>G 5471A>G), P1463S (5478C>T 5480A>T), K1467T (5491A>C 5492A>C), V1472A (5506T>C), Q1474M (5511C>A 5512A>T), R1475E (5514C>G 5515G>A 5516A>G), K1476L (5517A>C 5518A>T), D1478K (5523G>A 5525C>A), N1479R (5526A>C 5527A>G 5528C>A), G1483S (5538G>T 5539G>C), T1484Y (5541A>T 5542C>A), T1484_E1485insL (5543_5544insTTG), E1485D (5546A>T), A1486Q (5547G>C 5548C>A 5549C>G), I1488V (5553A>G 5555C>G), A1489I (5556G>A 5557C>T 5558T>C), Y1491F (5563A>T 5564C>T), F1498Y (5584T>A 5585T>C), Q1500K (5589C>A), E1503S (5598G>A 5599A>G 5600G>T), E1504D (5603A>T), I1506V (5607A>G 5609C>G), I1509L (5616A>T 5618A>G), V1511I (5622G>A 5624C>T), M1512V (5625A>G)								
Codon mutations:	ATC1425GTG (5364A>G 5366C>G), TAC1426TTC (5368A>T), TCA1427TCT (5372A>T), TTT1429ATT (5376T>A), CTC1431TTG (5382C>T 5384C>G), AAG1432AGA (5386A>G 5387G>A), AGT1433TTG (5388A>T 5389G>T 5390T>G), GGA1434GGT (5393A>T), TTT1435TAC (5395T>A 5396T>C), GTG1438TTG (5403G>T), GCT1439AGA (5406G>A 5407C>G 5408T>A), ATG1440GTT (5409A>G 5411G>T), CAC1441CAG (5414C>G), CCG1442GAG (5415C>G 5416C>A), GAG1443TCA (5418G>T 5419A>C 5420G>A), TCT1444GAT (5421T>G 5422C>A), AAG1446CCG (5427A>C 5428A>C), TGG1447AAG (5430T>A 5431G>A), GCT1449GCA (5438T>A), TTC1450TTT (5441C>T), GTT1452ACA (5445G>A 5446T>C 5447T>A), CCA1453CGC (5449C>G 5450A>C), GAT1454TAT (5451G>T), GGA1455GGT (5456A>T), CTG1456CAT (5458T>A 5459G>T), TGG1459TTC (5467G>T 5468G>C), CTA1460GTG (5469C>G 5471A>G), CCA1463TCT (5478C>T 5480A>T), GGA1465GGG (5486A>G), AAA1467ACC (5491A>C 5492A>C), AAC1468AAT (5495C>T), CCA1470CCT (5501A>T), GCA1471GCT (5504A>T), GTA1472GCA (5506T>C), TTT1473TTC (5510T>C), CAG1474ATG (5511C>A 5512A>T), CGA1475GAG (5514C>G 5515G>A 5516A>G), AAG1476CTG (5517A>C 5518A>T), GAC1478AAA (5523G>A 5525C>A), AAC1479CGA (5526A>C 5527A>G 5528C>A), GTC1480GTG (5531C>G), TTC1481TTT (5534C>T), AAA1482AAG (5537A>G), GGT1483TCT (5538G>T 5539G>C), ACT1484TAT (5541A>T 5542C>A), ACT1484_GAA1485insTTG (5543_5544insTTG), GAA1485GAT (5546A>T), GCC1486CAG (5547G>C 5548C>A 5549C>G), ATC1488GTG (5553A>G 5555C>G), GCT1489ATC (5556G>A 5557C>T 5558T>C), GTA1490GTG (5561A>G), TAC1491TTT (5563A>T 5564C>T), ATT1492ATA (5567T>A), ATA1495ATC (5576A>C), CTC1496TTG (5577C>T 5579C>G), GTA1497GTT (5582A>T), TTT1498TAC (5584T>A 5585T>C), CAG1500AAG (5589C>A), TCT1501AGT (5592T>A 5593C>G), GAG1503AGT (5598G>A 5599A>G 5600G>T), GAA1504GAT (5603A>T), ATC1506GTG (5607A>G 5609C>G), AAG1507AAA (5612G>A), ATA1509TTG (5616A>T 5618A>G), CGG1510AGG (5619C>A), GTC1511ATT (5622G>A 5624C>T), ATG1512GTG (5625A>G)								

Proteins

	Begin	End	Coverage	Score	Concordance	Matches	Identities	I/D/M/F*	Stop Codons
polyprotein (YP_009002585.1)	1423	1516	5.0%	360	51.6%	94 (98.9%)	49 (51.6%)	1/0/0/0	0
Protein mutations:	I1425V (5364A>G 5366C>G), Y1426F (5368A>T), F1429I (5376T>A), K1432R (5386A>G 5387G>A), S1433L (5388A>T 5389G>T 5390T>G), F1435Y (5395T>A 5396T>C), V1438L (5403G>T), A1439R (5406G>A 5407C>G 5408T>A), M1440V (5409A>G 5411G>T), H1441Q (5414C>G), P1442E (5415C>G 5416C>A), E1443S (5418G>T 5419A>C 5420G>A), S1444D (5421T>G 5422C>A), K1446P (5427A>C 5428A>C), W1447K (5430T>A 5431G>A), V1452T (5445G>A 5446T>C 5447T>A), P1453R (5449C>G 5450A>C), D1454Y (5451G>T), L1456H (5458T>A 5459G>T), W1459F (5467G>T 5468G>C), L1460V (5469C>G 5471A>G), P1463S (5478C>T 5480A>T), K1467T (5491A>C 5492A>C), V1472A (5506T>C), Q1474M (5511C>A 5512A>T), R1475E (5514C>G 5515G>A 5516A>G), K1476L (5517A>C 5518A>T), D1478K (5523G>A 5525C>A), N1479R (5526A>C 5527A>G 5528C>A), G1483S (5538G>T 5539G>C), T1484Y (5541A>T 5542C>A), T1484_E1485insL (5543_5544insTTG), E1485D (5546A>T), A1486Q (5547G>C 5548C>A 5549C>G), I1488V (5553A>G 5555C>G), A1489I (5556G>A 5557C>T 5558T>C), Y1491F (5563A>T 5564C>T), F1498Y (5584T>A 5585T>C), Q1500K (5589C>A), E1503S (5598G>A 5599A>G 5600G>T), E1504D (5603A>T), I1506V (5607A>G 5609C>G), I1509L (5616A>T 5618A>G), V1511I (5622G>A 5624C>T), M1512V (5625A>G)								
Codon mutations:	ATC1425GTG (5364A>G 5366C>G), TAC1426TTC (5368A>T), TCA1427TCT (5372A>T), TTT1429ATT (5376T>A), CTC1431TTG (5382C>T 5384C>G), AAG1432AGA (5386A>G 5387G>A), AGT1433TTG (5388A>T 5389G>T 5390T>G), GGA1434GGT (5393A>T), TTT1435TAC (5395T>A 5396T>C), GTG1438TTG (5403G>T), GCT1439AGA (5406G>A 5407C>G 5408T>A), ATG1440GTT (5409A>G 5411G>T), CAC1441CAG (5414C>G), CCG1442GAG (5415C>G 5416C>A), GAG1443TCA (5418G>T 5419A>C 5420G>A), TCT1444GAT (5421T>G 5422C>A), AAG1446CCG (5427A>C 5428A>C), TGG1447AAG (5430T>A 5431G>A), GCT1449GCA (5438T>A), TTC1450TTT (5441C>T), GTT1452ACA (5445G>A 5446T>C 5447T>A), CCA1453CGC (5449C>G 5450A>C), GAT1454TAT (5451G>T), GGA1455GGT (5456A>T), CTG1456CAT (5458T>A 5459G>T), TGG1459TTC (5467G>T 5468G>C), CTA1460GTG (5469C>G 5471A>G), CCA1463TCT (5478C>T 5480A>T), GGA1465GGG (5486A>G), AAA1467ACC (5491A>C 5492A>C), AAC1468AAT (5495C>T), CCA1470CCT (5501A>T), GCA1471GCT (5504A>T), GTA1472GCA (5506T>C), TTT1473TTC (5510T>C), CAG1474ATG (5511C>A 5512A>T), CGA1475GAG (5514C>G 5515G>A 5516A>G), AAG1476CTG (5517A>C 5518A>T), GAC1478AAA (5523G>A 5525C>A), AAC1479CGA (5526A>C 5527A>G 5528C>A), GTC1480GTG (5531C>G), TTC1481TTT (5534C>T), AAA1482AAG (5537A>G), GGT1483TCT (5538G>T 5539G>C), ACT1484TAT (5541A>T 5542C>A), ACT1484_GAA1485insTTG (5543_5544insTTG), GAA1485GAT (5546A>T), GCC1486CAG (5547G>C 5548C>A 5549C>G), ATC1488GTG (5553A>G 5555C>G), GCT1489ATC (5556G>A 5557C>T 5558T>C), GTA1490GTG (5561A>G), TAC1491TTT (5563A>T 5564C>T), ATT1492ATA (5567T>A), ATA1495ATC (5576A>C), CTC1496TTG (5577C>T 5579C>G), GTA1497GTT (5582A>T), TTT1498TAC (5584T>A 5585T>C), CAG1500AAG (5589C>A), TCT1501AGT (5592T>A 5593C>G), GAG1503AGT (5598G>A 5599A>G 5600G>T), GAA1504GAT (5603A>T), ATC1506GTG (5607A>G 5609C>G), AAG1507AAA (5612G>A), ATA1509TTG (5616A>T 5618A>G), CGG1510AGG (5619C>A), GTC1511ATT (5622G>A 5624C>T), ATG1512GTG (5625A>G)								

*: Inserts / Deletes / Misaligned / Frameshifts

Analysis details

This analysis was performed with panviral2.64

NGS Details (UN9): Cassava brown streak virus

Assembly

**Coverage Length**	522 (1 contig(s))
**Depth Of Coverage**	45.5
**Number Of Reads**	197
**Reads Per Million**	4.45 rpm (after QC)
**Ambiguities**	0
**Assembly Method**	de novo + reference guided assembly
**Consensus Caller**	Bcf Tools

Coverage Map

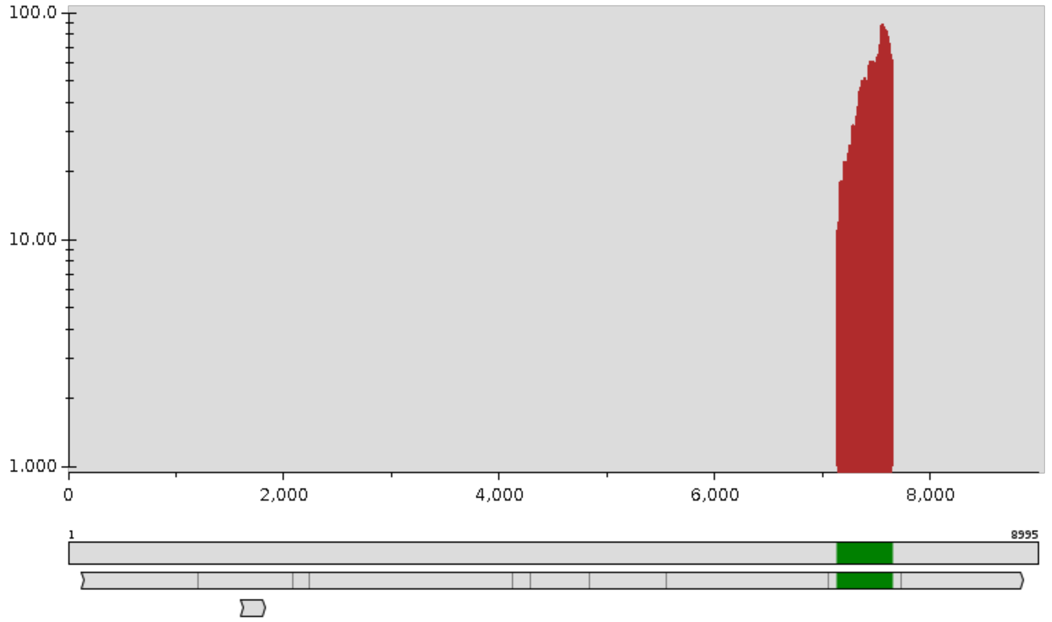

Assignment

**Type**	Cassava brown streak virus (Taxonomy ID: 137758)
**Reference Genome**	NC_012698.2
**NT Identity (%)**	60.5364
**AA Identity (%)**	57.4713
**Number Of Stop Codons**	0
**Number Of CDS**	2

Alignment

**Alignment Score**	220.0 (NT) + 732.0 (AA) = 952.0
**Concordance (%)**	42.2173

Alignment Method	Global, seeded, nucleotide + amino acids (AGA)

Genome Region

Sequence starts at position 7129 and ends at position 7650 relative to NC_012698.2 reference sequence.

Alignment Detailed Statistics

	Begin	End	Coverage	Score	Concordance	Matches	Identities	I/D/M/F*	Stop Codons
**NT**	**7129**	**7650**	**5.8%**	**220**	**21.1%**	**522 (100%)**	**316 (60.5%)**	**0/0**	
Mutations:	7130A>G, 7133A>G, 7134A>G, 7136T>G, 7142C>T, 7145A>T, 7148A>C, 7151G>A, 7157A>T, 7158C>A, 7163A>G, 7167A>G, 7175A>T, 7177A>G, 7178A>G, 7179C>G, 7180A>C, 7184A>T, 7185T>C, 7190T>C, 7192C>A, 7193C>G, 7194A>T, 7202A>C, 7203A>T, 7205C>T, 7206A>C, 7207T>A, 7208C>G, 7211T>C, 7213G>T, 7214G>T, 7217A>G, 7218G>C, 7229A>T, 7234C>T, 7235A>T, 7241A>G, 7242A>G, 7243C>A, 7244A>G, 7245G>C, 7246T>C, 7250G>A, 7253A>T, 7256T>A, 7257A>T, 7258T>C, 7270T>C, 7271A>T, 7272C>A, 7273G>A, 7274T>A, 7275G>A, 7277A>T, 7280G>T, 7281T>G, 7283T>C, 7284G>A, 7286G>A, 7287T>G, 7288T>A, 7290A>G, 7292T>G, 7293G>A, 7294G>A, 7295A>T, 7298G>A, 7304T>G, 7307C>A, 7310A>T, 7313A>G, 7319A>T, 7320A>T, 7323T>C, 7325G>C, 7331T>C, 7332G>A, 7334T>C, 7337T>C, 7340C>T, 7343T>G, 7355A>G, 7358A>T, 7361C>T, 7364T>C, 7374A>T, 7377G>C, 7380G>A, 7381G>A, 7385T>C, 7388A>T, 7390T>A, 7391A>T, 7397A>T, 7398T>C, 7400A>T, 7401T>A, 7403T>C, 7406G>C, 7409G>A, 7410G>T, 7413G>A, 7414A>T, 7416C>G, 7418G>T, 7422C>G, 7424A>G, 7425A>G, 7432T>C, 7433G>A, 7436T>A, 7437A>T, 7438G>A, 7442T>C, 7443C>A, 7445C>G, 7448C>T, 7449G>A, 7451G>C, 7455G>T, 7457T>A, 7458T>C, 7462T>C, 7463A>T, 7464A>C, 7465A>T, 7467A>G, 7468A>G, 7469A>G, 7470G>C, 7471T>C, 7472T>A, 7473G>A, 7474G>A, 7476G>A, 7477A>C, 7481T>G, 7484T>G, 7487A>G, 7489T>C, 7490C>T, 7494A>G, 7495A>T, 7496G>T, 7499T>A, 7500G>A, 7501T>A, 7502G>A, 7503C>A, 7504T>C, 7505A>G, 7506A>C, 7507G>T, 7508A>G, 7511T>A, 7512G>A, 7517T>A, 7520A>G, 7521A>C, 7522T>C, 7523G>A, 7524C>G, 7526A>T, 7527C>A, 7529C>G, 7535A>C, 7539T>A, 7540C>A, 7541A>T, 7544T>C, 7547G>A, 7553C>T, 7556A>G, 7559T>A, 7562C>T, 7568A>T, 7570T>A, 7572A>G, 7573A>G, 7576G>A, 7577G>T, 7578A>G, 7579G>A, 7580A>C, 7581A>C, 7586A>T, 7588T>A, 7589T>C, 7599A>C, 7600T>C, 7601G>C, 7602G>A, 7603C>A, 7604C>G, 7607G>A, 7610G>A, 7616T>C, 7618T>A, 7622A>T, 7628T>C, 7631A>C, 7632T>G, 7633T>G, 7635C>A, 7636G>A								

CDS

CBSV_gp1	2336	2509	6.0%	732	60.0%	174 (100%)	100 (57.5%)	0/0/0/0	0
Protein mutations:	I2337V (7134A>G 7136T>G), Q2345K (7158C>A), K2348E (7167A>G), K2351R (7177A>G 7178A>G), Q2352A (7179C>G 7180A>C), F2354L (7185T>C), P2356Q (7192C>A 7193C>G), T2357S (7194A>T), I2360F (7203A>T 7205C>T), I2361Q (7206A>C 7207T>A 7208C>G), R2363L (7213G>T 7214G>T), V2365L (7218G>C), P2370L (7234C>T 7235A>T), T2373E (7242A>G 7243C>A 7244A>G), V2374P (7245G>C 7246T>C), E2376D (7253A>T), I2378S (7257A>T 7258T>C), V2382A (7270T>C 7271A>T), R2383K (7272C>A 7273G>A 7274T>A), V2384I (7275G>A 7277A>T), S2386A (7281T>G 7283T>C), E2387K (7284G>A 7286G>A), L2388E (7287T>G 7288T>A), I2389V (7290A>G 7292T>G), G2390N (7293G>A 7294G>A 7295A>T), S2399C (7320A>T), D2403N (7332G>A 7334T>C), N2406K (7343T>G), M2417L (7374A>T), E2418Q (7377G>C), G2419K (7380G>A 7381G>A), L2422H (7390T>A 7391A>T), Y2426N (7401T>A 7403T>C), K2427N (7406G>C), V2429L (7410G>T), E2430M (7413G>A 7414A>T), P2431A (7416C>G 7418G>T), Q2433E (7422C>G 7424A>G), N2434D (7425A>G), M2436T (7432T>C 7433G>A), S2438Y (7437A>T 7438G>A), L2440M (7443C>A 7445C>G), V2442I (7449G>A 7451G>C), A2444S (7455G>T 7457T>A), F2445L (7458T>C), V2446A (7462T>C 7463A>T), N2447L (7464A>C 7465A>T), K2448G (7467A>G 7468A>G 7469A>G), V2449P (7470G>C 7471T>C 7472T>A), G2450N (7473G>A 7474G>A), D2451T (7476G>A 7477A>C), D2452E (7481T>G), I2454M (7487A>G), I2455T (7489T>C 7490C>T), K2457V (7494A>G 7495A>T 7496G>T), V2459K (7500G>A 7501T>A 7502G>A), L2460T (7503C>A 7504T>C 7505A>G), R2461L (7506A>C 7507G>T 7508A>G), E2463K (7512G>A), M2466P (7521A>C 7522T>C 7523G>A), P2467A (7524C>G 7526A>T), S2472N (7539T>A 7540C>A 7541A>T), L2482H (7570T>A), N2483G (7572A>G 7573A>G), W2484Y (7576G>A 7577G>T), R2485D (7578A>G 7579G>A 7580A>C), K2486Q (7581A>C), F2488Y (7588T>A 7589T>C), M2492P (7599A>C 7600T>C 7601G>C), A2493K (7602G>A 7603C>A 7604C>G), M2498K (7618T>A), F2503G (7632T>G 7633T>G), R2504K (7635C>A 7636G>A)								
Codon mutations:	GCA2335.CG (7130A>G), CCA2336CCG (7133A>G), ATT2337GTG (7134A>G 7136T>G), TTC2339TTT (7142C>T), GTA2340GTT (7145A>T), ACA2341ACC (7148A>C), GGG2342GGA (7151G>A), GCA2344GCT (7157A>T), CAA2345AAA (7158C>A), AAA2346AAG (7163A>G), AAG2348GAG (7167A>G), GTA2350GTT (7175A>T), AAA2351AGG (7177A>G 7178A>G), CAA2352GCA (7179C>G 7180A>C), ATA2353ATT (7184A>T), TTT2354CTT (7185T>C), GGT2355GGC (7190T>C), CCC2356CAG (7192C>A 7193C>G), ACT2357TCT (7194A>T), CCA2359CCC (7202A>C), ATC2360TTT (7203A>T 7205C>T), ATC2361CAG (7206A>C 7207T>A 7208C>G), TCT2362TCC (7211T>C), CGG2363CTT (7213G>T 7214G>T), AAA2364AAG (7217A>G), GTT2365CTT (7218G>C), CCA2368CCT (7229A>T), CCA2370CTT (7234C>T 7235A>T), GGA2372GGG (7241A>G), ACA2373GAG (7242A>G 7243C>A 7244A>G), GTT2374CCT (7245G>C 7246T>C), GAG2375GAA (7250G>A), GAA2376GAT (7253A>T), ATT2377ATA (7256T>A), ATC2378TCC (7257A>T 7258T>C), GTA2382GCT (7270T>C 7271A>T), CGT2383AAA (7272C>A 7273G>A 7274T>A), GTA2384ATT (7275G>A 7277A>T), GCG2385GCT (7280G>T), TCT2386GCC (7281T>G 7283T>C), GAG2387AAA (7284G>A 7286G>A), TTG2388GAG (7287T>G 7288T>A), ATT2389GTG (7290A>G 7292T>G), GGA2390AAT (7293G>A 7294G>A 7295A>T), GGG2391GGA (7298G>A), GTT2393GTG (7304T>G), CTC2394CTA (7307C>A), GTA2395GTT (7310A>T), GAA2396GAG (7313A>G), ACA2398ACT (7319A>T), AGT2399TGT (7320A>T), TTG2400CTC (7323T>C 7325G>C), TTT2402TTC (7331T>C), GAT2403AAC (7332G>A 7334T>C), GCT2404GCC (7337T>C), CTC2405CTT (7340C>T), AAT2406AAG (7343T>G), GGA2410GGG (7355A>G), CCA2411CCT (7358A>T), TAC2412TAT (7361C>T), ATT2413ATC (7364T>C), ATG2417TTG (7374A>T), GAA2418CAA (7377G>C), GGG2419AAG (7380G>A 7381G>A), ATT2420ATC (7385T>C), GGA2421GGT (7388A>T), CTA2422CAT (7390T>A 7391A>T), GGA2424GGT (7397A>T), TTA2425CTT (7398T>C 7400A>T), TAT2426AAC (7401T>A 7403T>C), AAG2427AAC (7406G>C), TTG2428TTA (7409G>A), GTG2429TTG (7410G>T), GAG2430ATG (7413G>A 7414A>T), CCG2431GCT (7416C>G 7418G>T), CAA2433GAG (7422C>G 7424A>G), AAT2434GAT (7425A>G), ATG2436ACA (7432T>C 7433G>A), GCT2437GCA (7436T>A), AGT2438TAT (7437A>T 7438G>A), GCT2439GCC (7442T>C), CTC2440ATG (7443C>A 7445C>G), TGC2441TGT (7448C>T), GTG2442ATC (7449G>A 7451G>C), GCT2444TCA (7455G>T 7457T>A), TTT2445CTT (7458T>C), GTA2446GCT (7462T>C 7463A>T), AAT2447CTT (7464A>C 7465A>T), AAA2448GGG (7467A>G 7468A>G 7469A>G), GTT2449CCA (7470G>C 7471T>C 7472T>A), GGT2450AAT (7473G>A 7474G>A), GAT2451ACT (7476G>A 7477A>C), GAT2452GAG (7481T>G), CCT2453CCG (7484T>G), ATA2454ATG (7487A>G), ATC2455ACT (7489T>C 7490C>T), AAG2457GTT (7494A>G 7495A>T 7496G>T), GGT2458GGA (7499T>A), GTG2459AAA (7500G>A 7501T>A 7502G>A), CTA2460ACG (7503C>A 7504T>C 7505A>G), AGA2461CTG (7506A>C 7507G>T 7508A>G), GGT2462GGA (7511T>A), GAG2463AAG (7512G>A), ATT2464ATA (7517T>A), GTA2465GTG (7520A>G), ATG2466CCA (7521A>C 7522T>C 7523G>A), CCA2467GCT (7524C>G 7526A>T), CGC2468AGG (7527C>A 7529C>G), CCA2470CCC (7535A>C), TCA2472AAT (7539T>A 7540C>A 7541A>T), TTT2473TTC (7544T>C), GGG2474GGA (7547G>A), GAC2476GAT (7553C>T), CCA2477CCG (7556A>G), ATT2478ATA (7559T>A), TTC2479TTT (7562C>T), CCA2481CCT (7568A>T), CTT2482CAT (7570T>A), AAC2483GGC (7572A>G 7573A>G), TGG2484TAT (7576G>A 7577G>T), AGA2485GAC (7578A>G 7579G>A 7580A>C), AAG2486CAG (7581A>C), ACA2487ACT (7586A>T), TTT2488TAC (7588T>A 7589T>C), ATG2492CCC (7599A>C 7600T>C 7601G>C), GCC2493AAG (7602G>A 7603C>A 7604C>G), GAG2494GAA (7607G>A), GAG2495GAA (7610G>A), AAT2497AAC (7616T>C), ATG2498AAG (7618T>A), ATA2499ATT (7622A>T), CAT2501CAC (7628T>C), CGA2502CGC (7631A>C), TTT2503GGT (7632T>G 7633T>G), CGA2504AAA (7635C>A 7636G>A)								

Proteins

polyprotein (YP_007027011.1)	2336	2509	6.0%	732	60.0%	174 (100%)	100 (57.5%)	0/0/0/0	0
Protein mutations:	I2337V (7134A>G 7136T>G), Q2345K (7158C>A), K2348E (7167A>G), K2351R (7177A>G 7178A>G), Q2352A (7179C>G 7180A>C), F2354L (7185T>C), P2356Q (7192C>A 7193C>G), T2357S (7194A>T), I2360F (7203A>T 7205C>T), I2361Q (7206A>C 7207T>A 7208C>G), R2363L (7213G>T 7214G>T), V2365L (7218G>C), P2370L (7234C>T 7235A>T), T2373E (7242A>G 7243C>A 7244A>G), V2374P (7245G>C 7246T>C), E2376D (7253A>T), I2378S (7257A>T 7258T>C), V2382A (7270T>C 7271A>T), R2383K (7272C>A 7273G>A 7274T>A), V2384I (7275G>A 7277A>T), S2386A (7281T>G 7283T>C), E2387K (7284G>A 7286G>A), L2388E (7287T>G 7288T>A), I2389V (7290A>G 7292T>G), G2390N (7293G>A 7294G>A 7295A>T), S2399C (7320A>T), D2403N (7332G>A 7334T>C), N2406K (7343T>G), M2417L (7374A>T), E2418Q (7377G>C), G2419K (7380G>A 7381G>A), L2422H (7390T>A 7391A>T), Y2426N (7401T>A 7403T>C), K2427N (7406G>C), V2429L (7410G>T), E2430M (7413G>A 7414A>T), P2431A (7416C>G 7418G>T), Q2433E (7422C>G 7424A>G), N2434D (7425A>G), M2436T (7432T>C 7433G>A), S2438Y (7437A>T 7438G>A), L2440M (7443C>A 7445C>G), V2442I (7449G>A 7451G>C), A2444S (7455G>T 7457T>A), F2445L (7458T>C), V2446A (7462T>C 7463A>T), N2447L (7464A>C 7465A>T), K2448G (7467A>G 7468A>G 7469A>G), V2449P (7470G>C 7471T>C 7472T>A), G2450N (7473G>A 7474G>A), D2451T (7476G>A 7477A>C), D2452E (7481T>G), I2454M (7487A>G), I2455T (7489T>C 7490C>T), K2457V (7494A>G 7495A>T 7496G>T), V2459K (7500G>A 7501T>A 7502G>A), L2460T (7503C>A 7504T>C 7505A>G), R2461L (7506A>C 7507G>T 7508A>G), E2463K (7512G>A), M2466P (7521A>C 7522T>C 7523G>A), P2467A (7524C>G 7526A>T), S2472N (7539T>A 7540C>A 7541A>T), L2482H (7570T>A), N2483G (7572A>G 7573A>G), W2484Y (7576G>A 7577G>T), R2485D (7578A>G 7579G>A 7580A>C), K2486Q (7581A>C), F2488Y (7588T>A 7589T>C), M2492P (7599A>C 7600T>C 7601G>C), A2493K (7602G>A 7603C>A 7604C>G), M2498K (7618T>A), F2503G (7632T>G 7633T>G), R2504K (7635C>A 7636G>A)								

	Begin	End	Coverage	Score	Concordance	Matches	Identities	I/D/M/F*	Stop Codons
**NT**	**7129**	**7650**	**5.8%**	**220**	**21.1%**	**522 (100%)**	**316 (60.5%)**	**0/0**	
Codon mutations:	GCA2335.CG (7130A>G), CCA2336CCG (7133A>G), ATT2337GTG (7134A>G 7136T>G), TTC2339TTT (7142C>T), GTA2340GTT (7145A>T), ACA2341ACC (7148A>C), GGG2342GGA (7151G>A), GCA2344GCT (7157A>T), CAA2345AAA (7158C>A), AAA2346AAG (7163A>G), AAG2348GAG (7167A>G), GTA2350GTT (7175A>T), AAA2351AGG (7177A>G 7178A>G), CAA2352GCA (7179C>G 7180A>C), ATA2353ATT (7184A>T), TTT2354CTT (7185T>C), GGT2355GGC (7190T>C), CCC2356CAG (7192C>A 7193C>G), ACT2357TCT (7194A>T), CCA2359CCC (7202A>C), ATC2360TTT (7203A>T 7205C>T), ATC2361CAG (7206A>C 7207T>A 7208C>G), TCT2362TCC (7211T>C), CGG2363CTT (7213G>T 7214G>T), AAA2364AAG (7217A>G), GTT2365CTT (7218G>C), CCA2368CCT (7229A>T), CCA2370CTT (7234C>T 7235A>T), GGA2372GGG (7241A>G), ACA2373GAG (7242A>G 7243C>A 7244A>G), GTT2374CCT (7245G>C 7246T>C), GAG2375GAA (7250G>A), GAA2376GAT (7253A>T), ATT2377ATA (7256T>A), ATC2378TCC (7257A>T 7258T>C), GTA2382GCT (7270T>C 7271A>T), CGT2383AAA (7272C>A 7273G>A 7274T>A), GTA2384ATT (7275G>A 7277A>T), GCG2385GCT (7280G>T), TCT2386GCC (7281T>G 7283T>C), GAG2387AAA (7284G>A 7286G>A), TTG2388GAG (7287T>G 7288T>A), ATT2389GTG (7290A>G 7292T>G), GGA2390AAT (7293G>A 7294G>A 7295A>T), GGG2391GGA (7298G>A), GTT2393GTG (7304T>G), CTC2394CTA (7307C>A), GTA2395GTT (7310A>T), GAA2396GAG (7313A>G), ACA2398ACT (7319A>T), AGT2399TGT (7320A>T), TTG2400CTC (7323T>C 7325G>C), TTT2402TTC (7331T>C), GAT2403AAC (7332G>A 7334T>C), GCT2404GCC (7337T>C), CTC2405CTT (7340C>T), AAT2406AAG (7343T>G), GGA2410GGG (7355A>G), CCA2411CCT (7358A>T), TAC2412TAT (7361C>T), ATT2413ATC (7364T>C), ATG2417TTG (7374A>T), GAA2418CAA (7377G>C), GGG2419AAG (7380G>A 7381G>A), ATT2420ATC (7385T>C), GGA2421GGT (7388A>T), CTA2422CAT (7390T>A 7391A>T), GGA2424GGT (7397A>T), TTA2425CTT (7398T>C 7400A>T), TAT2426AAC (7401T>A 7403T>C), AAG2427AAC (7406G>C), TTG2428TTA (7409G>A), GTG2429TTG (7410G>T), GAG2430ATG (7413G>A 7414A>T), CCG2431GCT (7416C>G 7418G>T), CAA2433GAG (7422C>G 7424A>G), AAT2434GAT (7425A>G), ATG2436ACA (7432T>C 7433G>A), GCT2437GCA (7436T>A), AGT2438TAT (7437A>T 7438G>A), GCT2439GCC (7442T>C), CTC2440ATG (7443C>A 7445C>G), TGC2441TGT (7448C>T), GTG2442ATC (7449G>A 7451G>C), GCT2444TCA (7455G>T 7457T>A), TTT2445CTT (7458T>C), GTA2446GCT (7462T>C 7463A>T), AAT2447CTT (7464A>C 7465A>T), AAA2448GGG (7467A>G 7468A>G 7469A>G), GTT2449CCA (7470G>C 7471T>C 7472T>A), GGT2450AAT (7473G>A 7474G>A), GAT2451ACT (7476G>A 7477A>C), GAT2452GAG (7481T>G), CCT2453CCG (7484T>G), ATA2454ATG (7487A>G), ATC2455ACT (7489T>C 7490C>T), AAG2457GTT (7494A>G 7495A>T 7496G>T), GGT2458GGA (7499T>A), GTG2459AAA (7500G>A 7501T>A 7502G>A), CTA2460ACG (7503C>A 7504T>C 7505A>G), AGA2461CTG (7506A>C 7507G>T 7508A>G), GGT2462GGA (7511T>A), GAG2463AAG (7512G>A), ATT2464ATA (7517T>A), GTA2465GTG (7520A>G), ATG2466CCA (7521A>C 7522T>C 7523G>A), CCA2467GCT (7524C>G 7526A>T), CGC2468AGG (7527C>A 7529C>G), CCA2470CCC (7535A>C), TCA2472AAT (7539T>A 7540C>A 7541A>T), TTT2473TTC (7544T>C), GGG2474GGA (7547G>A), GAC2476GAT (7553C>T), CCA2477CCG (7556A>G), ATT2478ATA (7559T>A), TTC2479TTT (7562C>T), CCA2481CCT (7568A>T), CTT2482CAT (7570T>A), AAC2483GGC (7572A>G 7573A>G), TGG2484TAT (7576G>A 7577G>T), AGA2485GAC (7578A>G 7579G>A 7580A>C), AAG2486CAG (7581A>C), ACA2487ACT (7586A>T), TTT2488TAC (7588T>A 7589T>C), ATG2492CCC (7599A>C 7600T>C 7601G>C), GCC2493AAG (7602G>A 7603C>A 7604C>G), GAG2494GAA (7607G>A), GAG2495GAA (7610G>A), AAT2497AAC (7616T>C), ATG2498AAG (7618T>A), ATA2499ATT (7622A>T), CAT2501CAC (7628T>C), CGA2502CGC (7631A>C), TTT2503GGT (7632T>G 7633T>G), CGA2504AAA (7635C>A 7636G>A)								
HAM1-like protein (YP_007032446.1)	28	201	77.0%	732	60.0%	174 (100%)	100 (57.5%)	0/0/0/0	0
Protein mutations:	I29V (7134A>G 7136T>G), Q37K (7158C>A), K40E (7167A>G), K43R (7177A>G 7178A>G), Q44A (7179C>G 7180A>C), F46L (7185T>C), P48Q (7192C>A 7193C>G), T49S (7194A>T), I52F (7203A>T 7205C>T), I53Q (7206A>C 7207T>A 7208C>G), R55L (7213G>T 7214G>T), V57L (7218G>C), P62L (7234C>T 7235A>T), T65E (7242A>G 7243C>A 7244A>G), V66P (7245G>C 7246T>C), E68D (7253A>T), I70S (7257A>T 7258T>C), V74A (7270T>C 7271A>T), R75K (7272C>A 7273G>A 7274T>A), V76I (7275G>A 7277A>T), S78A (7281T>G 7283T>C), E79K (7284G>A 7286G>A), L80E (7287T>G 7288T>A), I81V (7290A>G 7292T>G), G82N (7293G>A 7294G>A 7295A>T), S91C (7320A>T), D95N (7332G>A 7334T>C), N98K (7343T>G), M109L (7374A>T), E110Q (7377G>C), G111K (7380G>A 7381G>A), L114H (7390T>A 7391A>T), Y118N (7401T>A 7403T>C), K119N (7406G>C), V121L (7410G>T), E122M (7413G>A 7414A>T), P123A (7416C>G 7418G>T), Q125E (7422C>G 7424A>G), N126D (7425A>G), M128T (7432T>C 7433G>A), S130Y (7437A>T 7438G>A), L132M (7443C>A 7445C>G), V134I (7449G>A 7451G>C), A136S (7455G>T 7457T>A), F137L (7458T>C), V138A (7462T>C 7463A>T), N139L (7464A>C 7465A>T), K140G (7467A>G 7468A>G 7469A>G), V141P (7470G>C 7471T>C 7472T>A), G142N (7473G>A 7474G>A), D143T (7476G>A 7477A>C), D144E (7481T>G), I146M (7487A>G), I147T (7489T>C 7490C>T), K149V (7494A>G 7495A>T 7496G>T), V151K (7500G>A 7501T>A 7502G>A), L152T (7503C>A 7504T>C 7505A>G), R153L (7506A>C 7507G>T 7508A>G), E155K (7512G>A), M158P (7521A>C 7522T>C 7523G>A), P159A (7524C>G 7526A>T), S164N (7539T>A 7540C>A 7541A>T), L174H (7570T>A), N175G (7572A>G 7573A>G), W176Y (7576G>A 7577G>T), R177D (7578A>G 7579G>A 7580A>C), K178Q (7581A>C), F180Y (7588T>A 7589T>C), M184P (7599A>C 7600T>C 7601G>C), A185K (7602G>A 7603C>A 7604C>G), M190K (7618T>A), F195G (7632T>G 7633T>G), R196K (7635C>A 7636G>A)								
Codon mutations:	GCA27.CG (7130A>G), CCA28CCG (7133A>G), ATT29GTG (7134A>G 7136T>G), TTC31TTT (7142C>T), GTA32GTT (7145A>T), ACA33ACC (7148A>C), GGG34GGA (7151G>A), GCA36GCT (7157A>T), CAA37AAA (7158C>A), AAA38AAG (7163A>G), AAG40GAG (7167A>G), GTA42GTT (7175A>T), AAA43AGG (7177A>G 7178A>G), CAA44GCA (7179C>G 7180A>C), ATA45ATT (7184A>T), TTT46CTT (7185T>C), GGT47GGC (7190T>C), CCC48CAG (7192C>A 7193C>G), ACT49TCT (7194A>T), CCA51CCC (7202A>C), ATC52TTT (7203A>T 7205C>T), ATC53CAG (7206A>C 7207T>A 7208C>G), TCT54TCC (7211T>C), CGG55CTT (7213G>T 7214G>T), AAA56AAG (7217A>G), GTT57CTT (7218G>C), CCA60CCT (7229A>T), CCA62CTT (7234C>T 7235A>T), GGA64GGG (7241A>G), ACA65GAG (7242A>G 7243C>A 7244A>G), GTT66CCT (7245G>C 7246T>C), GAG67GAA (7250G>A), GAA68GAT (7253A>T), ATT69ATA (7256T>A), ATC70TCC (7257A>T 7258T>C), GTA74GCT (7270T>C 7271A>T), CGT75AAA (7272C>A 7273G>A 7274T>A), GTA76ATT (7275G>A 7277A>T), GCG77GCT (7280G>T), TCT78GCC (7281T>G 7283T>C), GAG79AAA (7284G>A 7286G>A), TTG80GAG (7287T>G 7288T>A), ATT81GTG (7290A>G 7292T>G), GGA82AAT (7293G>A 7294G>A 7295A>T), GGG83GGA (7298G>A), GTT85GTG (7304T>G), CTC86CTA (7307C>A), GTA87GTT (7310A>T), GAA88GAG (7313A>G), ACA90ACT (7319A>T), AGT91TGT (7320A>T), TTG92CTC (7323T>C 7325G>C), TTT94TTC (7331T>C), GAT95AAC (7332G>A 7334T>C), GCT96GCC (7337T>C), CTC97CTT (7340C>T), AAT98AAG (7343T>G), GGA102GGG (7355A>G), CCA103CCT (7358A>T), TAC104TAT (7361C>T), ATT105ATC (7364T>C), ATG109TTG (7374A>T), GAA110CAA (7377G>C), GGG111AAG (7380G>A 7381G>A), ATT112ATC (7385T>C), GGA113GGT (7388A>T), CTA114CAT (7390T>A 7391A>T), GGA116GGT (7397A>T), TTA117CTT (7398T>C 7400A>T), TAT118AAC (7401T>A 7403T>C), AAG119AAC (7406G>C), TTG120TTA (7409G>A), GTG121TTG (7410G>T), GAG122ATG (7413G>A 7414A>T), CCG123GCT (7416C>G 7418G>T), CAA125GAG (7422C>G 7424A>G), AAT126GAT (7425A>G), ATG128ACA (7432T>C 7433G>A), GCT129GCA (7436T>A), AGT130TAT (7437A>T 7438G>A), GCT131GCC (7442T>C), CTC132ATG (7443C>A 7445C>G), TGC133TGT (7448C>T), GTG134ATC (7449G>A 7451G>C), GCT136TCA (7455G>T 7457T>A), TTT137CTT (7458T>C), GTA138GCT (7462T>C 7463A>T), AAT139CTT (7464A>C 7465A>T), AAA140GGG (7467A>G 7468A>G 7469A>G), GTT141CCA (7470G>C 7471T>C 7472T>A), GGT142AAT (7473G>A 7474G>A), GAT143ACT (7476G>A 7477A>C), GAT144GAG (7481T>G), CCT145CCG (7484T>G), ATA146ATG (7487A>G), ATC147ACT (7489T>C 7490C>T), AAG149GTT (7494A>G 7495A>T 7496G>T), GGT150GGA (7499T>A), GTG151AAA (7500G>A 7501T>A 7502G>A), CTA152ACG (7503C>A 7504T>C 7505A>G), AGA153CTG (7506A>C 7507G>T 7508A>G), GGT154GGA (7511T>A), GAG155AAG (7512G>A), ATT156ATA (7517T>A), GTA157GTG (7520A>G), ATG158CCA (7521A>C 7522T>C 7523G>A), CCA159GCT (7524C>G 7526A>T), CGC160AGG (7527C>A 7529C>G), CCA162CCC (7535A>C), TCA164AAT (7539T>A 7540C>A 7541A>T), TTT165TTC (7544T>C), GGG166GGA (7547G>A), GAC168GAT (7553C>T), CCA169CCG (7556A>G), ATT170ATA (7559T>A), TTC171TTT (7562C>T), CCA173CCT (7568A>T), CTT174CAT (7570T>A), AAC175GGC (7572A>G 7573A>G), TGG176TAT (7576G>A 7577G>T), AGA177GAC (7578A>G 7579G>A 7580A>C), AAG178CAG (7581A>C), ACA179ACT (7586A>T), TTT180TAC (7588T>A 7589T>C), ATG184CCC (7599A>C 7600T>C 7601G>C), GCC185AAG (7602G>A 7603C>A 7604C>G), GAG186GAA (7607G>A), GAG187GAA (7610G>A), AAT189AAC (7616T>C), ATG190AAG (7618T>A), ATA191ATT (7622A>T), CAT193CAC (7628T>C), CGA194CGC (7631A>C), TTT195GGT (7632T>G 7633T>G), CGA196AAA (7635C>A 7636G>A)								

*: Inserts / Deletes / Misaligned / Frameshifts

Analysis details

This analysis was performed with panviral2.64

NGS Details (UN9): Badnavirus maculaucubae

Assembly

**Coverage Length**	282 (1 contig(s))
**Depth Of Coverage**	52.7
**Number Of Reads**	137
**Reads Per Million**	3.10 rpm (after QC)
**Ambiguities**	0
**Assembly Method**	de novo + reference guided assembly
**Consensus Caller**	Bcf Tools

Coverage Map

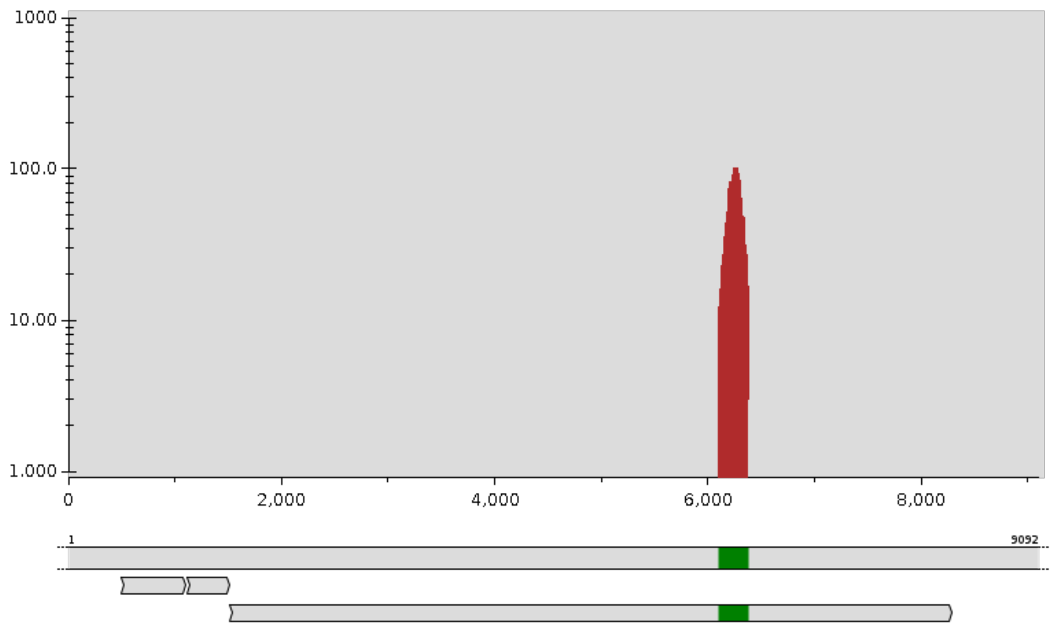

Assignment

**Type**	Badnavirus maculaucubae (Taxonomy ID: 3051986)
**Reference Genome**	NC_076606.1
**NT Identity (%)**	56.1404
**AA Identity (%)**	52.6316
**Number Of Stop Codons**	0
**Number Of CDS**	3

Alignment

**Alignment Score**	64.0 (NT) + 355.0 (AA) = 419.0
**Concordance (%)**	33.6276

Alignment Method	Global, seeded, nucleotide + amino acids (AGA)

Genome Region

Sequence starts at position 6095 and ends at position 6376 relative to NC_076606.1 reference sequence.

Alignment Detailed Statistics

	Begin	End	Coverage	Score	Concordance	Matches	Identities	I/D/M/F*	Stop Codons
**NT**	**6095**	**6376**	**3.1%**	**64**	**11.3%**	**282 (98.9%)**	**160 (56.1%)**	**3/0**	
Mutations:	6095T>G, 6100A>T, 6101G>C, 6102C>A, 6105A>G, 6106T>A, 6108T>A, 6112T>C, 6114G>T, 6115A>C, 6116A>G, 6120T>A, 6125T>A, 6133G>T, 6136G>A, 6137C>A, 6138A>G, 6139A>G, 6141G>C, 6142G>A, 6144A>G, 6147A>G, 6148T>A, 6149C>G, 6150A>T, 6151A>G, 6152G>A, 6159A>C, 6160T>A, 6161G>A, 6165G>T, 6168T>G, 6172A>C, 6173T>G, 6177T>A, 6178C>A, 6179C>G, 6180A>G, 6181G>T, 6186A>G, 6187C>A, 6188T>A, 6189A>T, 6191A>T, 6195A>G, 6197G>T, 6198G>T, 6201A>T, 6204A>C, 6208C>T, 6210A>C, 6217C>T, 6221A>G, 6222G>T, 6228A>T, 6231T>G, 6234A>T, 6237G>A, 6240C>T, 6241C>A, 6242A>T, 6243A>G, 6244A>G, 6245G>A, 6246G>T, 6247A>C, 6249A>T, 6253G>A, 6255C>T, 6258C>G, 6259T>A, 6260G>T, 6261T>A, 6266G>A, 6268G>C, 6269G>C, 6271A>T, 6272C>A, 6273A>C, 6274G>C, 6275A>T, 6276A>T, 6279T>C, 6279_6280insATG, 6281T>A, 6282C>T, 6283A>G, 6285C>G, 6287C>T, 6288T>G, 6291T>G, 6293A>T, 6294C>T, 6303T>C, 6306T>C, 6312A>C, 6314T>A, 6315C>T, 6318T>G, 6320A>G, 6322A>G, 6324T>C, 6325C>G, 6326T>A, 6327T>A, 6330A>G, 6333G>T, 6337G>A, 6338A>T, 6342G>A, 6346C>T, 6348C>G, 6350A>G, 6354A>T, 6355A>G, 6358C>T, 6361G>C, 6363A>G, 6364A>G, 6366A>T, 6368G>T, 6369C>G, 6373A>G								
**CDS**									
QKP79_gp3	1530	1623	4.2%	355	51.1%	94 (98.9%)	50 (52.6%)	1/0/0/0	0
Protein mutations:	F1533I (6106T>A 6108T>A), K1536R (6115A>C 6116A>G), F1539Y (6125T>A), V1542L (6133G>T), A1543K (6136G>A 6137C>A 6138A>G), M1544V (6139A>G 6141G>C), E1545K (6142G>A 6144A>G), S1548D (6151A>G 6152G>A), W1551K (6160T>A 6161G>A), I1555R (6172A>C 6173T>G), P1557R (6178C>A 6179C>G 6180A>G), D1558Y (6181G>T), L1560N (6187C>A 6188T>A 6189A>T), Y1561F (6191A>T), W1563F (6197G>T 6198G>T), P1567S (6208C>T 6210A>C), K1571S (6221A>G 6222G>T), Q1578M (6241C>A 6242A>T 6243A>G), R1579D (6244A>G 6245G>A 6246G>T), K1580H (6247A>C 6249A>T), D1582N (6253G>A 6255C>T), N1583K (6258C>G), C1584I (6259T>A 6260G>T 6261T>A), R1586K (6266G>A), G1587P (6268G>C 6269G>C), T1588Y (6271A>T 6272C>A 6273A>C), E1589L (6274G>C 6275A>T 6276A>T), D1590_F1591insM (6279_6280insATG), F1591Y (6281T>A 6282C>T), I1592V (6283A>G 6285C>G), A1593V (6287C>T 6288T>G), Y1595F (6293A>T 6294C>T), F1602Y (6314T>A 6315C>T), E1604G (6320A>G), N1605D (6322A>G 6324T>C), L1606E (6325C>G 6326T>A 6327T>A), E1608D (6333G>T), E1610M (6337G>A 6338A>T), K1614R (6350A>G), M1616V (6355A>G), E1618Q (6361G>C 6363A>G), I1619V (6364A>G 6366A>T), C1620L (6368G>T 6369C>G), K1622E (6373A>G)								
Codon mutations:	GTC1529.GC (6095T>G), AGC1531TCA (6100A>T 6101G>C 6102C>A), AAA1532AAG (6105A>G), TTT1533ATA (6106T>A 6108T>A), TTG1535CTT (6112T>C 6114G>T), AAA1536CGA (6115A>C 6116A>G), TCT1537TCA (6120T>A), TTT1539TAT (6125T>A), GTG1542TTG (6133G>T), GCA1543AAG (6136G>A 6137C>A 6138A>G), ATG1544GTC (6139A>G 6141G>C), GAA1545AAG (6142G>A 6144A>G), GAA1546GAG (6147A>G), TCA1547AGT (6148T>A 6149C>G 6150A>T), AGC1548GAC (6151A>G 6152G>A), CCA1550CCC (6159A>C), TGG1551AAG (6160T>A 6161G>A), ACG1552ACT (6165G>T), GCT1553GCG (6168T>G), ATA1555CGA (6172A>C 6173T>G), ACT1556ACA (6177T>A), CCA1557AGG (6178C>A 6179C>G 6180A>G), GAT1558TAT (6181G>T), GGA1559GGG (6186A>G), CTA1560AAT (6187C>A 6188T>A 6189A>T), TAC1561TTC (6191A>T), GAA1562GAG (6195A>G), TGG1563TTT (6197G>T 6198G>T), CTA1564CTT (6201A>T), GTA1565GTC (6204A>C), CCA1567TCC (6208C>T 6210A>C), CTA1570TTA (6217C>T), AAG1571AGT (6221A>G 6222G>T), GCA1573GCT (6228A>T), CCT1574CCG (6231T>G), GCA1575GCT (6234A>T), GTG1576GTA (6237G>A), TTC1577TTT (6240C>T), CAA1578ATG (6241C>A 6242A>T 6243A>G), AGG1579GAT (6244A>G 6245G>A 6246G>T), AAA1580CAT (6247A>C 6249A>T), GAC1582AAT (6253G>A 6255C>T), AAC1583AAG (6258C>G), TGT1584ATA (6259T>A 6260G>T 6261T>A), AGG1586AAG (6266G>A), GGT1587CCT (6268G>C 6269G>C), ACA1588TAC (6271A>T 6272C>A 6273A>C), GAA1589CTT (6274G>C 6275A>T 6276A>T), GAT1590GAC (6279T>C), GAT1590_TTC1591insATG (6279_6280insATG), TTC1591TAT (6281T>A 6282C>T), ATC1592GTG (6283A>G 6285C>G), GCT1593GTG (6287C>T 6288T>G), GTT1594GTG (6291T>G), TAC1595TTT (6293A>T 6294C>T), GAT1598GAC (6303T>C), ATT1599ATC (6306T>C), GTA1601GTC (6312A>C), TTC1602TAT (6314T>A 6315C>T), TCT1603TCG (6318T>G), GAA1604GGA (6320A>G), AAT1605GAC (6322A>G 6324T>C), CTT1606GAA (6325C>G 6326T>A 6327T>A), CAA1607CAG (6330A>G), GAG1608GAT (6333G>T), GAG1610ATG (6337G>A 6338A>T), GAG1611GAA (6342G>A), CTC1613TTG (6346C>T 6348C>G), AAA1614AGA (6350A>G), ATA1615ATT (6354A>T), ATG1616GTG (6355A>G), CTG1617TTG (6358C>T), GAA1618CAG (6361G>C 6363A>G), ATA1619GTT (6364A>G 6366A>T), TGC1620TTG (6368G>T 6369C>G), AAG1622GAG (6373A>G)								
**Proteins**									
hypothetical protein (YP_010799265.1)	1530	1623	4.2%	355	51.1%	94 (98.9%)	50 (52.6%)	1/0/0/0	0
Protein mutations:	F1533I (6106T>A 6108T>A), K1536R (6115A>C 6116A>G), F1539Y (6125T>A), V1542L (6133G>T), A1543K (6136G>A 6137C>A 6138A>G), M1544V (6139A>G 6141G>C), E1545K (6142G>A 6144A>G), S1548D (6151A>G 6152G>A), W1551K (6160T>A 6161G>A), I1555R (6172A>C 6173T>G), P1557R (6178C>A 6179C>G 6180A>G), D1558Y (6181G>T), L1560N (6187C>A 6188T>A 6189A>T), Y1561F (6191A>T), W1563F (6197G>T 6198G>T), P1567S (6208C>T 6210A>C), K1571S (6221A>G 6222G>T), Q1578M (6241C>A 6242A>T 6243A>G), R1579D (6244A>G 6245G>A 6246G>T), K1580H (6247A>C 6249A>T), D1582N (6253G>A 6255C>T), N1583K (6258C>G), C1584I (6259T>A 6260G>T 6261T>A), R1586K (6266G>A), G1587P (6268G>C 6269G>C), T1588Y (6271A>T 6272C>A 6273A>C), E1589L (6274G>C 6275A>T 6276A>T), D1590_F1591insM (6279_6280insATG), F1591Y (6281T>A 6282C>T), I1592V (6283A>G 6285C>G), A1593V (6287C>T 6288T>G), Y1595F (6293A>T 6294C>T), F1602Y (6314T>A 6315C>T), E1604G (6320A>G), N1605D (6322A>G 6324T>C), L1606E (6325C>G 6326T>A 6327T>A), E1608D (6333G>T), E1610M (6337G>A 6338A>T), K1614R (6350A>G), M1616V (6355A>G), E1618Q (6361G>C 6363A>G), I1619V (6364A>G 6366A>T), C1620L (6368G>T 6369C>G), K1622E (6373A>G)								
Codon mutations:	GTC1529.GC (6095T>G), AGC1531TCA (6100A>T 6101G>C 6102C>A), AAA1532AAG (6105A>G), TTT1533ATA (6106T>A 6108T>A), TTG1535CTT (6112T>C 6114G>T), AAA1536CGA (6115A>C 6116A>G), TCT1537TCA (6120T>A), TTT1539TAT (6125T>A), GTG1542TTG (6133G>T), GCA1543AAG (6136G>A 6137C>A 6138A>G), ATG1544GTC (6139A>G 6141G>C), GAA1545AAG (6142G>A 6144A>G), GAA1546GAG (6147A>G), TCA1547AGT (6148T>A 6149C>G 6150A>T), AGC1548GAC (6151A>G 6152G>A), CCA1550CCC (6159A>C), TGG1551AAG (6160T>A 6161G>A), ACG1552ACT (6165G>T), GCT1553GCG (6168T>G), ATA1555CGA (6172A>C 6173T>G), ACT1556ACA (6177T>A), CCA1557AGG (6178C>A 6179C>G 6180A>G), GAT1558TAT (6181G>T), GGA1559GGG (6186A>G), CTA1560AAT (6187C>A 6188T>A 6189A>T), TAC1561TTC (6191A>T), GAA1562GAG (6195A>G), TGG1563TTT (6197G>T 6198G>T), CTA1564CTT (6201A>T), GTA1565GTC (6204A>C), CCA1567TCC (6208C>T 6210A>C), CTA1570TTA (6217C>T), AAG1571AGT (6221A>G 6222G>T), GCA1573GCT (6228A>T), CCT1574CCG (6231T>G), GCA1575GCT (6234A>T), GTG1576GTA (6237G>A), TTC1577TTT (6240C>T), CAA1578ATG (6241C>A 6242A>T 6243A>G), AGG1579GAT (6244A>G 6245G>A 6246G>T), AAA1580CAT (6247A>C 6249A>T), GAC1582AAT (6253G>A 6255C>T), AAC1583AAG (6258C>G), TGT1584ATA (6259T>A 6260G>T 6261T>A), AGG1586AAG (6266G>A), GGT1587CCT (6268G>C 6269G>C), ACA1588TAC (6271A>T 6272C>A 6273A>C), GAA1589CTT (6274G>C 6275A>T 6276A>T), GAT1590GAC (6279T>C), GAT1590_TTC1591insATG (6279_6280insATG), TTC1591TAT (6281T>A 6282C>T), ATC1592GTG (6283A>G 6285C>G), GCT1593GTG (6287C>T 6288T>G), GTT1594GTG (6291T>G), TAC1595TTT (6293A>T 6294C>T), GAT1598GAC (6303T>C), ATT1599ATC (6306T>C), GTA1601GTC (6312A>C), TTC1602TAT (6314T>A 6315C>T), TCT1603TCG (6318T>G), GAA1604GGA (6320A>G), AAT1605GAC (6322A>G 6324T>C), CTT1606GAA (6325C>G 6326T>A 6327T>A), CAA1607CAG (6330A>G), GAG1608GAT (6333G>T), GAG1610ATG (6337G>A 6338A>T), GAG1611GAA (6342G>A), CTC1613TTG (6346C>T 6348C>G), AAA1614AGA (6350A>G), ATA1615ATT (6354A>T), ATG1616GTG (6355A>G), CTG1617TTG (6358C>T), GAA1618CAG (6361G>C 6363A>G), ATA1619GTT (6364A>G 6366A>T), TGC1620TTG (6368G>T 6369C>G), AAG1622GAG (6373A>G)								

*: Inserts / Deletes / Misaligned / Frameshifts

Analysis details

This analysis was performed with panviral2.64

NGS Details (UN9): Errantivirus

Assembly

**Coverage Length**	600 (2 contig(s))
**Depth Of Coverage**	19.5
**Number Of Reads**	111
**Reads Per Million**	2.51 rpm (after QC)
**Ambiguities**	0
**Assembly Method**	de novo + reference guided assembly
**Consensus Caller**	Bcf Tools

Coverage Map

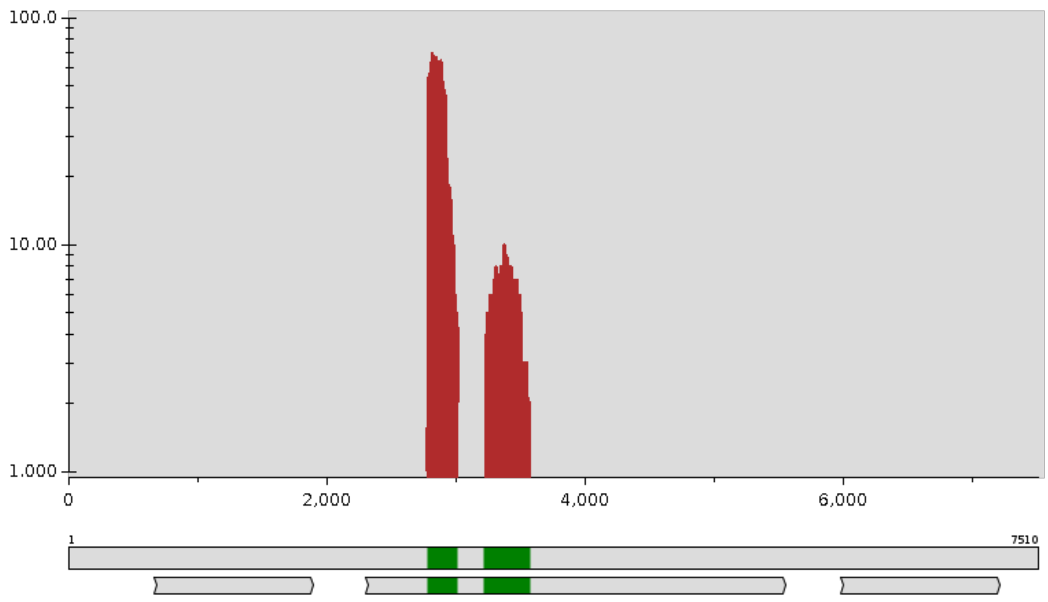

Assignment

**Type**	Errantivirus (Taxonomy ID: 186666)
**Reference Genome**	NC_038512.1
**NT Identity (%)**	52.921
**AA Identity (%)**	41.2371
**Number Of Stop Codons**	0
**Number Of CDS**	3

Alignment

**Alignment Score**	14.0 (NT) + 544.0 (AA) = 558.0
**Concordance (%)**	23.25

**Alignment Method**	Global, seeded, nucleotide + amino acids (AGA)

Genome Region

Sequence starts at position 2779 and ends at position 3581 relative to NC_038512.1 reference sequence.

Alignment Detailed Statistics

	Begin	End	Coverage	Score	Concordance	Matches	Identities	I/D/M/F*	Stop Codons
**NT**	**2779**	**3581**	**8.0%**	**14**	**1.3%**	**579 (96.0%)**	**308 (51.1%)**	**3/21**	
Mutations:	2786A>T, 2794C>G, 2795C>A, 2797A>G, 2800A>T, 2801A>T, 2809A>G, 2812A>T, 2813T>A, 2814C>G, 2815A>T, 2816G>A, 2818C>G, 2821T>C, 2822G>C, 2828A>G, 2830C>T, 2831T>G, 2832C>T, 2833A>G, 2836C>A, 2837A>G, 2841G>T, 2843G>T, 2849C>A, 2850C>G, 2851C>A, 2858_2860delATC, 2863C>T, 2865C>G, 2866T>C, 2869T>G, 2870_2881delGGGAAACAAAAG, 2883G>T, 2885C>A, 2887T>A, 2888C>A, 2890C>G, 2891G>T, 2892T>G, 2893A>T, 2894G>A, 2896T>A, 2901T>A, 2905T>G, 2908G>A, 2909T>C, 2911G>T, 2913A>G, 2914C>T, 2915G>A, 2918A>G, 2919A>T, 2920G>C, 2923T>C, 2926C>T, 2927G>A, 2929T>G, 2930G>A, 2932C>T, 2934A>G, 2935A>G, 2937A>T, 2944A>T, 2947A>C, 2949A>T, 2950C>G, 2953A>T, 2954A>G, 2955G>A, 2959C>G, 2960G>C, 2963C>T, 2968C>T, 2969A>G, 2974A>G, 2975G>C, 2976G>A, 2977T>A, 2978A>G, 2979A>G, 2981T>G, 2982G>C, 2983C>T, 2984C>A, 2985A>G, 2988A>T, 2993A>T, 2995C>T, 2997C>A, 2998C>A, 2999T>A, 3005T>C, 3007G>T, 3008G>A, 3009C>G, 3010A>T, 3011A>T, 3012G>C, 3013T>G, 3016G>C, 3018T>A, 3019T>C, 3020T>C, 3229A>T, 3233C>T, 3236G>T, 3237A>T, 3239A>C, 3242C>T, 3248C>G, 3249G>A, 3257C>A, 3258A>G, 3259A>G, 3261G>C, 3265T>G, 3269G>C, 3272A>C, 3273G>A, 3274T>A, 3275A>C, 3277C>T, 3280C>A, 3281A>T, 3282A>T, 3283A>G, 3284A>G, 3285T>C, 3286T>A, 3287C>A, 3290A>C, 3291T>A, 3292G>A, 3293G>T, 3294A>C, 3298G>A, 3300C>G, 3301C>T, 3302G>A, 3303A>T, 3304A>G, 3307C>T, 3308T>G, 3309T>G, 3310G>A, 3312A>T, 3313G>T, 3314C>T, 3315T>C, 3316C>T, 3317G>A, 3320A>G, 3321C>T, 3322T>G, 3324C>A, 3325T>G, 3329C>T, 3331T>A, 3334T>C, 3337C>T, 3338A>T, 3344A>T, 3345G>C, 3346C>T, 3347A>T, 3349G>T, 3352C>T, 3356A>G, 3358C>T, 3359A>T, 3360A>C, 3361G>T, 3362C>A, 3365A>G, 3367C>T, 3371G>A, 3372A>G, 3373T>G, 3376G>A, 3380T>C, 3381C>A, 3382C>A, 3384C>T, 3386A>G, 3388T>G, 3391A>G, 3392A>C, 3394A>C, 3396A>G, 3397T>G, 3399T>C, 3400G>T, 3402T>C, 3406A>T, 3408A>G, 3409G>C, 3411C>A, 3414C>T, 3415T>C, 3418G>A, 3419G>C, 3422A>C, 3424A>C, 3426A>G, 3428C>G, 3429A>G, 3430A>T, 3433T>C, 3442T>A, 3443C>G, 3444T>C, 3445C>A, 3448T>G, 3455C>A, 3457A>G, 3467C>A, 3468C>A, 3471A>G, 3472T>G, 3474T>A, 3477C>G, 3478A>T, 3479C>A, 3480G>T, 3481A>T, 3482C>A, 3484C>T, 3486C>G, 3487A>T, 3489A>G, 3490A>G, 3493C>A, 3499A>G, 3500C>G, 3502G>T, 3504G>T, 3505C>G, 3506T>C, 3508A>G, 3514A>G, 3516G>A, 3517T>C, 3519G>A, 3520T>C, 3521A>T, 3522A>T, 3523A>C, 3524G>A, 3527_3532delACTCTT, 3533A>T, 3535T>G, 3536C>T, 3538C>T, 3542T>A, 3543A>T, 3546T>C, 3547A>T, 3549A>C, 3550_3551insCAA, 3551G>C, 3557G>C, 3559A>T, 3560C>A, 3564G>T, 3565T>G, 3568A>G, 3569A>G, 3570C>T, 3571T>A, 3572C>G, 3573T>C, 3574G>A, 3580C>A, 3581A>T								

CDS

	Begin	End	Coverage	Score	Concordance	Matches	Identities	I/D/M/F*	Stop Codons
D1R33_gp2	159	426	18.4%	544	41.1%	193 (96.0%)	80 (39.8%)	1/7/0/0	0
Protein mutations:	M161L (2786A>T), D163E (2794C>G), Q164K (2795C>A 2797A>G), I166F (2801A>T), D171K (2816G>A 2818C>G), A173P (2822G>C), S175G (2828A>G 2830C>T), S176V (2831T>G 2832C>T 2833A>G), I178V (2837A>G), W179L (2841G>T), V180F (2843G>T), P182R (2849C>A 2850C>G 2851C>A), I185del (2858_2860delATC), A187G (2865C>G 2866T>C), G189_K192del (2870_2881delGGGAAACAAAAG), W193L (2883G>T), L195M (2888C>A 2890C>G), V196C (2891G>T 2892T>G 2893A>T), V197I (2894G>A 2896T>A), F199Y (2901T>A), N203S (2913A>G 2914C>T), E204K (2915G>A), K205V (2918A>G 2919A>T 2920G>C), D208K (2927G>A 2929T>G), D209N (2930G>A 2932C>T), K210R (2934A>G 2935A>G), Y211F (2937A>T), N215M (2949A>T 2950C>G), S217D (2954A>G 2955G>A), D218E (2959C>G), V219L (2960G>C), L220F (2963C>T), K222E (2969A>G), G224Q (2975G>C 2976G>A 2977T>A), K225G (2978A>G 2979A>G), C226A (2981T>G 2982G>C 2983C>T), Q227R (2984C>A 2985A>G), Y228F (2988A>T), T230S (2993A>T 2995C>T), T231K (2997C>A 2998C>A), L232I (2999T>A), A235S (3008G>A 3009C>G 3010A>T), F238Y (3018T>A 3019T>C), E308D (3229A>T), E311L (3236G>T 3237A>T), N312H (3239A>C), R315E (3248C>G 3249G>A), Q318R (3257C>A 3258A>G 3259A>G), R319T (3261G>C), E322Q (3269G>C), S323Q (3272A>C 3273G>A 3274T>A), N324H (3275A>C 3277C>T), F325L (3280C>A), K326L (3281A>T 3282A>T 3283A>G), I327A (3284A>G 3285T>C 3286T>A), Q328K (3287C>A), M329Q (3290A>C 3291T>A 3292G>A), D330S (3293G>T 3294A>C), S332C (3300C>G 3301C>T), E333M (3302G>A 3303A>T 3304A>G), L335G (3308T>G 3309T>G 3310G>A), K336I (3312A>T 3313G>T), L337S (3314C>T 3315T>C 3316C>T), E338K (3317G>A), T339V (3320A>G 3321C>T 3322T>G), A340E (3324C>A 3325T>G), I345F (3338A>T), R348C (3347A>T 3349G>T), I351V (3356A>G 3358C>T), K352S (3359A>T 3360A>C 3361G>T), P353T (3362C>A), N354D (3365A>G 3367C>T), D356R (3371G>A 3372A>G 3373T>G), S359Q (3380T>C 3381C>A 3382C>A), A360V (3384C>T), I361V (3386A>G 3388T>G), K363H (3392A>C 3394A>C), Y364W (3396A>G 3397T>G), L365P (3399T>C 3400G>T), I366T (3402T>C), K368S (3408A>G 3409G>C), T369N (3411C>A), P370L (3414C>T 3415T>C), E372Q (3419G>C), I373L (3422A>C 3424A>C), K374R (3426A>G), Q375G (3428C>G 3429A>G 3430A>T), L380A (3443C>G 3444T>C 3445C>A), P388K (3467C>A 3468C>A), D389G (3471A>G 3472T>G), F390Y (3474T>A), A391G (3477C>G 3478A>T), R392I (3479C>A 3480G>T 3481A>T), L393I (3482C>A 3484C>T), T394S (3486C>G 3487A>T), K395R (3489A>G 3490A>G), Q399D (3500C>G 3502G>T), C400L (3504G>T 3505C>G), G404D (3516G>A 3517T>C), S405N (3519G>A 3520T>C), K406F (3521A>T 3522A>T 3523A>C), V407I (3524G>A), T408_L409del (3527_3532delACTCTT), S410W (3533A>T 3535T>G), P411S (3536C>T 3538C>T), Y413I (3542T>A 3543A>T), V414A (3546T>C 3547A>T), N415T (3549A>C), N415_A416insQ (3550_3551insCAA), A416P (3551G>C), E418H (3557G>C 3559A>T), H419N (3560C>A), C420L (3564G>T 3565T>G), T422V (3569A>G 3570C>T 3571T>A), L423A (3572C>G 3573T>C 3574G>A)								
Codon mutations:	ATG161TTG (2786A>T), GAC163GAG (2794C>G), CAA164AAG (2795C>A 2797A>G), GGA165GGT (2800A>T), ATT166TTT (2801A>T), AGA168AGG (2809A>G), CCA169CCT (2812A>T), TCA170AGT (2813T>A 2814C>G 2815A>T), GAC171AAG (2816G>A 2818C>G), TCT172TCC (2821T>C), GCA173CCA (2822G>C), AGC175GGT (2828A>G 2830C>T), TCA176GTG (2831T>G 2832C>T 2833A>G), CCC177CCA (2836C>A), ATA178GTA (2837A>G), TGG179TTG (2841G>T), GTT180TTT (2843G>T), CCC182AGA (2849C>A 2850C>G 2851C>A), ATC185del (2858_2860delATC), GAC186GAT (2863C>T), GCT187GGC (2865C>G 2866T>C), TCT188TCG (2869T>G), GGG189_AAG192del (2870_2881delGGGAAACAAAAG), TGG193TTG (2883G>T), CGT194AGA (2885C>A 2887T>A), CTC195ATG (2888C>A 2890C>G), GTA196TGT (2891G>T 2892T>G 2893A>T), GTT197ATA (2894G>A 2896T>A), TTC199TAC (2901T>A), CGT200CGG (2905T>G), AAG201AAA (2908G>A), TTG202CTT (2909T>C 2911G>T), AAC203AGT (2913A>G 2914C>T), GAG204AAG (2915G>A), AAG205GTC (2918A>G 2919A>T 2920G>C), ACT206ACC (2923T>C), ATC207ATT (2926C>T), GAT208AAG (2927G>A 2929T>G), GAC209AAT (2930G>A 2932C>T), AAA210AGG (2934A>G 2935A>G), TAC211TTC (2937A>T), ATA213ATT (2944A>T), CCA214CCC (2947A>C), AAC215ATG (2949A>T 2950C>G), ATA216ATT (2953A>T), AGT217GAT (2954A>G 2955G>A), GAC218GAG (2959C>G), GTA219CTA (2960G>C), CTT220TTT (2963C>T), GAC221GAT (2968C>T), AAG222GAG (2969A>G), TTA223TTG (2974A>G), GGT224CAA (2975G>C 2976G>A 2977T>A), AAG225GGG (2978A>G 2979A>G), TGC226GCT (2981T>G 2982G>C 2983C>T), CAA227AGA (2984C>A 2985A>G), TAC228TTC (2988A>T), ACC230TCT (2993A>T 2995C>T), ACC231AAA (2997C>A 2998C>A), TTA232ATA (2999T>A), TTG234CTT (3005T>C 3007G>T), GCA235AGT (3008G>A 3009C>G 3010A>T), AGT236TCG (3011A>T 3012G>C 3013T>G), GGG237GGC (3016G>C), TTT238TAC (3018T>A 3019T>C), TAT239CA. (3020T>C), GAA308GAT (3229A>T), CTG310TTG (3233C>T), GAG311TTG (3236G>T 3237A>T), AAC312CAC (3239A>C), CTG313TTG (3242C>T), CGA315GAA (3248C>G 3249G>A), CAA318AGG (3257C>A 3258A>G 3259A>G), AGA319ACA (3261G>C), CTT320CTG (3265T>G), GAA322CAA (3269G>C), AGT323CAA (3272A>C 3273G>A 3274T>A), AAC324CAT (3275A>C 3277C>T), TTC325TTA (3280C>A), AAA326TTG (3281A>T 3282A>T 3283A>G), ATT327GCA (3284A>G 3285T>C 3286T>A), CAA328AAA (3287C>A), ATG329CAA (3290A>C 3291T>A 3292G>A), GAC330TCC (3293G>T 3294A>C), AAG331AAA (3298G>A), TCC332TGT (3300C>G 3301C>T), GAA333ATG (3302G>A 3303A>T 3304A>G), TTC334TTT (3307C>T), TTG335GGA (3308T>G 3309T>G 3310G>A), AAG336ATT (3312A>T 3313G>T), CTC337TCT (3314C>T 3315T>C 3316C>T), GAA338AAA (3317G>A), ACT339GTG (3320A>G 3321C>T 3322T>G), GCT340GAG (3324C>A 3325T>G), CTT342TTA (3329C>T 3331T>A), GGT343GGC (3334T>C), CAC344CAT (3337C>T), ATC345TTC (3338A>T), AGC347TCT (3344A>T 3345G>C 3346C>T), AGG348TGT (3347A>T 3349G>T), GAC349GAT (3352C>T), ATC351GTT (3356A>G 3358C>T), AAG352TCT (3359A>T 3360A>C 3361G>T), CCT353ACT (3362C>A), AAC354GAT (3365A>G 3367C>T), GAT356AGG (3371G>A 3372A>G 3373T>G), AAG357AAA (3376G>A), TCC359CAA (3380T>C 3381C>A 3382C>A), GCT360GTT (3384C>T), ATT361GTG (3386A>G 3388T>G), CAA362CAG (3391A>G), AAA363CAC (3392A>C 3394A>C), TAT364TGG (3396A>G 3397T>G), CTG365CCT (3399T>C 3400G>T), ATT366ACT (3402T>C), CCA367CCT (3406A>T), AAG368AGC (3408A>G 3409G>C), ACC369AAC (3411C>A), CCT370CTC (3414C>T 3415T>C), AAG371AAA (3418G>A), GAA372CAA (3419G>C), ATA373CTC (3422A>C 3424A>C), AAA374AGA (3426A>G), CAA375GGT (3428C>G 3429A>G 3430A>T), TTT376TTC (3433T>C), CTT379CTA (3442T>A), CTC380GCA (3443C>G 3444T>C 3445C>A), GGT381GGG (3448T>G), CGA384AGG (3455C>A 3457A>G), CCA388AAA (3467C>A 3468C>A), GAT389GGG (3471A>G 3472T>G), TTT390TAT (3474T>A), GCA391GGT (3477C>G 3478A>T), CGA392ATT (3479C>A 3480G>T 3481A>T), CTC393ATT (3482C>A 3484C>T), ACA394AGT (3486C>G 3487A>T), AAA395AGG (3489A>G 3490A>G), CCC396CCA (3493C>A), ACA398ACG (3499A>G), CAG399GAT (3500C>G 3502G>T), TGC400TTG (3504G>T 3505C>G), TTA401CTG (3506T>C 3508A>G), AAA403AAG (3514A>G), GGT404GAC (3516G>A 3517T>C), AGT405AAC (3519G>A 3520T>C), AAA406TTC (3521A>T 3522A>T 3523A>C), GTA407ATA (3524G>A), ACT408_CTT409del (3527_3532delACTCTT), AGT410TGG (3533A>T 3535T>G), CCC411TCT (3536C>T 3538C>T), TAT413ATT (3542T>A 3543A>T), GTA414GCT (3546T>C 3547A>T), AAT415ACT (3549A>C), AAT415_GCT416insCAA (3550_3551insCAA), GCT416CCT (3551G>C), GAA418CAT (3557G>C 3559A>T), CAC419AAC (3560C>A), TGT420TTG (3564G>T 3565T>G), AAA421AAG (3568A>G), ACT422GTA (3569A>G 3570C>T 3571T>A), CTG423GCA (3572C>G 3573T>C 3574G>A), ACC425ACA (3580C>A), AAC426T.. (3581A>T)								

Proteins

	Begin	End	Coverage	Score	Concordance	Matches	Identities	I/D/M/F*	Stop Codons
ORF B (YP_009507248.1)	159	426	18.4%	544	41.1%	193 (96.0%)	80 (39.8%)	1/7/0/0	0

	Begin	End	Coverage	Score	Concordance	Matches	Identities	I/D/M/F*	Stop Codons
**NT**	**2779**	**3581**	**8.0%**	**14**	**1.3%**	**579 (96.0%)**	**308 (51.1%)**	**3/21**	
Protein mutations:	M161L (2786A>T), D163E (2794C>G), Q164K (2795C>A 2797A>G), I166F (2801A>T), D171K (2816G>A 2818C>G), A173P (2822G>C), S175G (2828A>G 2830C>T), S176V (2831T>G 2832C>T 2833A>G), I178V (2837A>G), W179L (2841G>T), V180F (2843G>T), P182R (2849C>A 2850C>G 2851C>A), I185del (2858_2860delATC), A187G (2865C>G 2866T>C), G189_K192del (2870_2881delGGGAAACAAAAG), W193L (2883G>T), L195M (2888C>A 2890C>G), V196C (2891G>T 2892T>G 2893A>T), V197I (2894G>A 2896T>A), F199Y (2901T>A), N203S (2913A>G 2914C>T), E204K (2915G>A), K205V (2918A>G 2919A>T 2920G>C), D208K (2927G>A 2929T>G), D209N (2930G>A 2932C>T), K210R (2934A>G 2935A>G), Y211F (2937A>T), N215M (2949A>T 2950C>G), S217D (2954A>G 2955G>A), D218E (2959C>G), V219L (2960G>C), L220F (2963C>T), K222E (2969A>G), G224Q (2975G>C 2976G>A 2977T>A), K225G (2978A>G 2979A>G), C226A (2981T>G 2982G>C 2983C>T), Q227R (2984C>A 2985A>G), Y228F (2988A>T), T230S (2993A>T 2995C>T), T231K (2997C>A 2998C>A), L232I (2999T>A), A235S (3008G>A 3009C>G 3010A>T), F238Y (3018T>A 3019T>C), E308D (3229A>T), E311L (3236G>T 3237A>T), N312H (3239A>C), R315E (3248C>G 3249G>A), Q318R (3257C>A 3258A>G 3259A>G), R319T (3261G>C), E322Q (3269G>C), S323Q (3272A>C 3273G>A 3274T>A), N324H (3275A>C 3277C>T), F325L (3280C>A), K326L (3281A>T 3282A>T 3283A>G), I327A (3284A>G 3285T>C 3286T>A), Q328K (3287C>A), M329Q (3290A>C 3291T>A 3292G>A), D330S (3293G>T 3294A>C), S332C (3300C>G 3301C>T), E333M (3302G>A 3303A>T 3304A>G), L335G (3308T>G 3309T>G 3310G>A), K336I (3312A>T 3313G>T), L337S (3314C>T 3315T>C 3316C>T), E338K (3317G>A), T339V (3320A>G 3321C>T 3322T>G), A340E (3324C>A 3325T>G), I345F (3338A>T), R348C (3347A>T 3349G>T), I351V (3356A>G 3358C>T), K352S (3359A>T 3360A>C 3361G>T), P353T (3362C>A), N354D (3365A>G 3367C>T), D356R (3371G>A 3372A>G 3373T>G), S359Q (3380T>C 3381C>A 3382C>A), A360V (3384C>T), I361V (3386A>G 3388T>G), K363H (3392A>C 3394A>C), Y364W (3396A>G 3397T>G), L365P (3399T>C 3400G>T), I366T (3402T>C), K368S (3408A>G 3409G>C), T369N (3411C>A), P370L (3414C>T 3415T>C), E372Q (3419G>C), I373L (3422A>C 3424A>C), K374R (3426A>G), Q375G (3428C>G 3429A>G 3430A>T), L380A (3443C>G 3444T>C 3445C>A), P388K (3467C>A 3468C>A), D389G (3471A>G 3472T>G), F390Y (3474T>A), A391G (3477C>G 3478A>T), R392I (3479C>A 3480G>T 3481A>T), L393I (3482C>A 3484C>T), T394S (3486C>G 3487A>T), K395R (3489A>G 3490A>G), Q399D (3500C>G 3502G>T), C400L (3504G>T 3505C>G), G404D (3516G>A 3517T>C), S405N (3519G>A 3520T>C), K406F (3521A>T 3522A>T 3523A>C), V407I (3524G>A), T408_L409del (3527_3532delACTCTT), S410W (3533A>T 3535T>G), P411S (3536C>T 3538C>T), Y413I (3542T>A 3543A>T), V414A (3546T>C 3547A>T), N415T (3549A>C), N415_A416insQ (3550_3551insCAA), A416P (3551G>C), E418H (3557G>C 3559A>T), H419N (3560C>A), C420L (3564G>T 3565T>G), T422V (3569A>G 3570C>T 3571T>A), L423A (3572C>G 3573T>C 3574G>A)								
Codon mutations:	ATG161TTG (2786A>T), GAC163GAG (2794C>G), CAA164AAG (2795C>A 2797A>G), GGA165GGT (2800A>T), ATT166TTT (2801A>T), AGA168AGG (2809A>G), CCA169CCT (2812A>T), TCA170AGT (2813T>A 2814C>G 2815A>T), GAC171AAG (2816G>A 2818C>G), TCT172TCC (2821T>C), GCA173CCA (2822G>C), AGC175GGT (2828A>G 2830C>T), TCA176GTG (2831T>G 2832C>T 2833A>G), CCC177CCA (2836C>A), ATA178GTA (2837A>G), TGG179TTG (2841G>T), GTT180TTT (2843G>T), CCC182AGA (2849C>A 2850C>G 2851C>A), ATC185del (2858_2860delATC), GAC186GAT (2863C>T), GCT187GGC (2865C>G 2866T>C), TCT188TCG (2869T>G), GGG189_AAG192del (2870_2881delGGGAAACAAAAG), TGG193TTG (2883G>T), CGT194AGA (2885C>A 2887T>A), CTC195ATG (2888C>A 2890C>G), GTA196TGT (2891G>T 2892T>G 2893A>T), GTT197ATA (2894G>A 2896T>A), TTC199TAC (2901T>A), CGT200CGG (2905T>G), AAG201AAA (2908G>A), TTG202CTT (2909T>C 2911G>T), AAC203AGT (2913A>G 2914C>T), GAG204AAG (2915G>A), AAG205GTC (2918A>G 2919A>T 2920G>C), ACT206ACC (2923T>C), ATC207ATT (2926C>T), GAT208AAG (2927G>A 2929T>G), GAC209AAT (2930G>A 2932C>T), AAA210AGG (2934A>G 2935A>G), TAC211TTC (2937A>T), ATA213ATT (2944A>T), CCA214CCC (2947A>C), AAC215ATG (2949A>T 2950C>G), ATA216ATT (2953A>T), AGT217GAT (2954A>G 2955G>A), GAC218GAG (2959C>G), GTA219CTA (2960G>C), CTT220TTT (2963C>T), GAC221GAT (2968C>T), AAG222GAG (2969A>G), TTA223TTG (2974A>G), GGT224CAA (2975G>C 2976G>A 2977T>A), AAG225GGG (2978A>G 2979A>G), TGC226GCT (2981T>G 2982G>C 2983C>T), CAA227AGA (2984C>A 2985A>G), TAC228TTC (2988A>T), ACC230TCT (2993A>T 2995C>T), ACC231AAA (2997C>A 2998C>A), TTA232ATA (2999T>A), TTG234CTT (3005T>C 3007G>T), GCA235AGT (3008G>A 3009C>G 3010A>T), AGT236TCG (3011A>T 3012G>C 3013T>G), GGG237GGC (3016G>C), TTT238TAC (3018T>A 3019T>C), TAT239CA. (3020T>C), GAA308GAT (3229A>T), CTG310TTG (3233C>T), GAG311TTG (3236G>T 3237A>T), AAC312CAC (3239A>C), CTG313TTG (3242C>T), CGA315GAA (3248C>G 3249G>A), CAA318AGG (3257C>A 3258A>G 3259A>G), AGA319ACA (3261G>C), CTT320CTG (3265T>G), GAA322CAA (3269G>C), AGT323CAA (3272A>C 3273G>A 3274T>A), AAC324CAT (3275A>C 3277C>T), TTC325TTA (3280C>A), AAA326TTG (3281A>T 3282A>T 3283A>G), ATT327GCA (3284A>G 3285T>C 3286T>A), CAA328AAA (3287C>A), ATG329CAA (3290A>C 3291T>A 3292G>A), GAC330TCC (3293G>T 3294A>C), AAG331AAA (3298G>A), TCC332TGT (3300C>G 3301C>T), GAA333ATG (3302G>A 3303A>T 3304A>G), TTC334TTT (3307C>T), TTG335GGA (3308T>G 3309T>G 3310G>A), AAG336ATT (3312A>T 3313G>T), CTC337TCT (3314C>T 3315T>C 3316C>T), GAA338AAA (3317G>A), ACT339GTG (3320A>G 3321C>T 3322T>G), GCT340GAG (3324C>A 3325T>G), CTT342TTA (3329C>T 3331T>A), GGT343GGC (3334T>C), CAC344CAT (3337C>T), ATC345TTC (3338A>T), AGC347TCT (3344A>T 3345G>C 3346C>T), AGG348TGT (3347A>T 3349G>T), GAC349GAT (3352C>T), ATC351GTT (3356A>G 3358C>T), AAG352TCT (3359A>T 3360A>C 3361G>T), CCT353ACT (3362C>A), AAC354GAT (3365A>G 3367C>T), GAT356AGG (3371G>A 3372A>G 3373T>G), AAG357AAA (3376G>A), TCC359CAA (3380T>C 3381C>A 3382C>A), GCT360GTT (3384C>T), ATT361GTG (3386A>G 3388T>G), CAA362CAG (3391A>G), AAA363CAC (3392A>C 3394A>C), TAT364TGG (3396A>G 3397T>G), CTG365CCT (3399T>C 3400G>T), ATT366ACT (3402T>C), CCA367CCT (3406A>T), AAG368AGC (3408A>G 3409G>C), ACC369AAC (3411C>A), CCT370CTC (3414C>T 3415T>C), AAG371AAA (3418G>A), GAA372CAA (3419G>C), ATA373CTC (3422A>C 3424A>C), AAA374AGA (3426A>G), CAA375GGT (3428C>G 3429A>G 3430A>T), TTT376TTC (3433T>C), CTT379CTA (3442T>A), CTC380GCA (3443C>G 3444T>C 3445C>A), GGT381GGG (3448T>G), CGA384AGG (3455C>A 3457A>G), CCA388AAA (3467C>A 3468C>A), GAT389GGG (3471A>G 3472T>G), TTT390TAT (3474T>A), GCA391GGT (3477C>G 3478A>T), CGA392ATT (3479C>A 3480G>T 3481A>T), CTC393ATT (3482C>A 3484C>T), ACA394AGT (3486C>G 3487A>T), AAA395AGG (3489A>G 3490A>G), CCC396CCA (3493C>A), ACA398ACG (3499A>G), CAG399GAT (3500C>G 3502G>T), TGC400TTG (3504G>T 3505C>G), TTA401CTG (3506T>C 3508A>G), AAA403AAG (3514A>G), GGT404GAC (3516G>A 3517T>C), AGT405AAC (3519G>A 3520T>C), AAA406TTC (3521A>T 3522A>T 3523A>C), GTA407ATA (3524G>A), ACT408_CTT409del (3527_3532delACTCTT), AGT410TGG (3533A>T 3535T>G), CCC411TCT (3536C>T 3538C>T), TAT413ATT (3542T>A 3543A>T), GTA414GCT (3546T>C 3547A>T), AAT415ACT (3549A>C), AAT415_GCT416insCAA (3550_3551insCAA), GCT416CCT (3551G>C), GAA418CAT (3557G>C 3559A>T), CAC419AAC (3560C>A), TGT420TTG (3564G>T 3565T>G), AAA421AAG (3568A>G), ACT422GTA (3569A>G 3570C>T 3571T>A), CTG423GCA (3572C>G 3573T>C 3574G>A), ACC425ACA (3580C>A), AAC426T.. (3581A>T)								

*: Inserts / Deletes / Misaligned / Frameshifts

Analysis details

This analysis was performed with panviral2.64

NGS Details (UN9): Badnavirus maculaucubae

Assembly

**Coverage Length**	329 (1 contig(s))
**Depth Of Coverage**	26.2
**Number Of Reads**	81
**Reads Per Million**	1.83 rpm (after QC)
**Ambiguities**	0
**Assembly Method**	de novo + reference guided assembly
**Consensus Caller**	Bcf Tools

Coverage Map

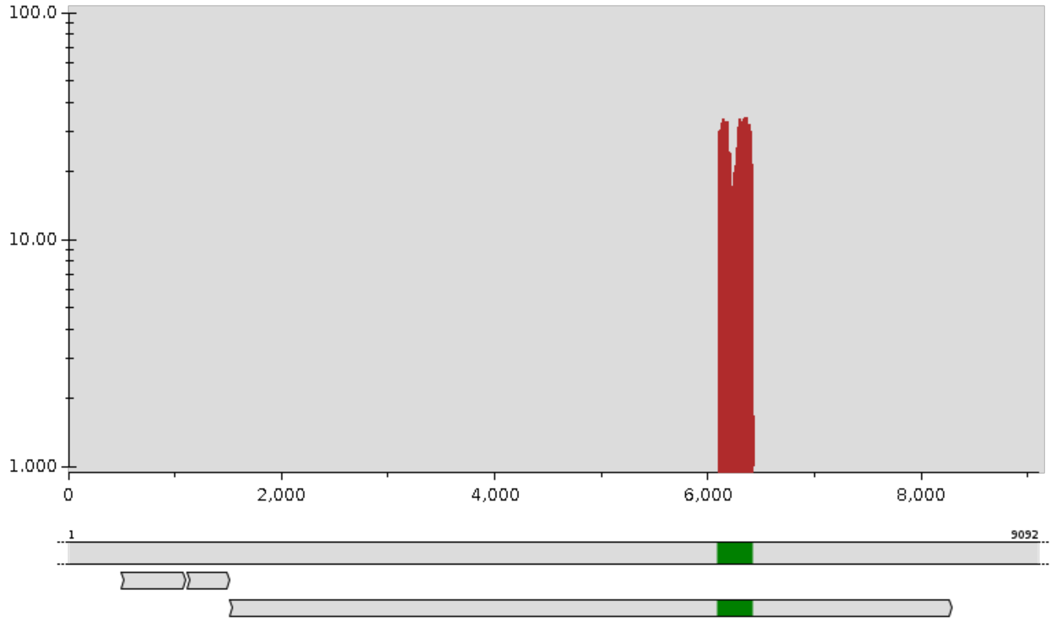

Assignment

**Type**	Badnavirus maculaucubae (Taxonomy ID: 3051986)
**Reference Genome**	NC_076606.1
**NT Identity (%)**	55.7522
**AA Identity (%)**	46.4912
**Number Of Stop Codons**	1
**Number Of CDS**	3

Alignment

**Alignment Score**	61.0 (NT) + 239.0 (AA) = 300.0
**Concordance (%)**	24.1119

**Alignment Method**	Global, seeded, nucleotide + amino acids (AGA)

Genome Region

Sequence starts at position 6094 and ends at position 6422 relative to NC_076606.1 reference sequence.

Alignment Detailed Statistics

	Begin	End	Coverage	Score	Concordance	Matches	Identities	I/D/M/F*	Stop Codons
**NT**	**6094**	**6422**	**3.6%**	**61**	**9.5%**	**327 (95.9%)**	**189 (55.4%)**	**12/2**	
Mutations:	6102C>T, 6105A>G, 6106T>A, 6112T>C, 6114G>T, 6115A>C, 6116A>G, 6120T>C, 6122G>A, 6125T>A, 6132G>A, 6133G>T, 6136G>A, 6137C>G, 6138A>G, 6139A>G, 6141G>A, 6142G>A, 6147A>G, 6149C>G, 6150A>T, 6151_6152delAG, 6153C>G, 6156T>C, 6159A>G, 6160T>A, 6161G>A, 6165G>T, 6171C>T, 6173T>G, 6179C>A, 6181G>T, 6184G>A, 6186A>T, 6188T>A, 6189A>C, 6191A>T, 6192C>T, 6197G>T, 6198G>T, 6199C>T, 6201A>G, 6204A>T, 6207G>A, 6208C>T, 6210A>T, 6216T>A, 6221A>C, 6222G>A, 6228A>T, 6231T>C, 6235G>A, 6236T>C, 6237G>T, 6240C>T, 6241C>A, 6242A>T, 6243A>G, 6244A>G, 6245G>A, 6246G>C, 6247A>T, 6248A>T, 6253G>A, 6257A>G, 6258C>G, 6259T>G, 6260G>T, 6261T>A, 6266G>A, 6268G>C, 6269G>C, 6270T>G, 6271A>T, 6272C>A, 6273A>T, 6274G>T, 6275A>T, 6276A>G, 6279T>C, 6279_6280insATG, 6282C>T, 6283A>G, 6285C>A, 6287C>T, 6288T>G, 6291T>C, 6292T>C, 6293A>T, 6297T>A, 6303T>C, 6306T>A, 6307C>T, 6312A>T, 6314T>A, 6319G>C, 6320A>G, 6321A>G, 6325C>G, 6326T>C, 6327T>C, 6328C>A, 6330A>G, 6333G>A, 6336C>T, 6342G>T, 6345T>C, 6346C>T, 6348C>G, 6350A>G, 6351A>G, 6354A>T, 6355A>G, 6358C>T, 6361G>C, 6365T>C, 6366A>T, 6371G>A, 6372G>A, 6373A>C, 6374A>T, 6375G>T, 6376A>T, 6377A>G, 6378T>A, 6382C>A, 6383T>C, 6384A>C, 6386T>A, 6387_6388insAATTGTTCG, 6390C>A, 6393C>T, 6394C>T, 6397A>G, 6398C>T, 6402A>T, 6403A>G, 6408A>T, 6409A>T, 6411A>T, 6413C>G								

CDS

	Begin	End	Coverage	Score	Concordance	Matches	Identities	I/D/M/F*	Stop Codons
QKP79_gp3	1529	1638	4.9%	239	30.4%	110 (96.5%)	53 (46.5%)	4/0/1/1	1
Protein mutations:	F1533I (6106T>A), K1536R (6115A>C 6116A>G), G1538D (6122G>A), F1539Y (6125T>A), V1542L (6133G>T), A1543R (6136G>A 6137C>G 6138A>G), M1544V (6139A>G 6141G>A), E1545K (6142G>A), S1547C (6149C>G 6150A>T), W1551K (6160T>A 6161G>A), I1555R (6173T>G), P1557Q (6179C>A), D1558Y (6181G>T), G1559S (6184G>A 6186A>T), L1560H (6188T>A 6189A>C), Y1561F (6191A>T 6192C>T), W1563F (6197G>T 6198G>T), M1566I (6207G>A), P1567S (6208C>T 6210A>T), K1571T (6221A>C 6222G>A), V1576T (6235G>A 6236T>C 6237G>T), Q1578M (6241C>A 6242A>T 6243A>G), R1579D (6244A>G 6245G>A 6246G>C), K1580L (6247A>T 6248A>T), D1582N (6253G>A), N1583R (6257A>G 6258C>G), C1584V (6259T>G 6260G>T 6261T>A), R1586K (6266G>A), G1587P (6268G>C 6269G>C 6270T>G), T1588Y (6271A>T 6272C>A 6273A>T), E1589L (6274G>T 6275A>T 6276A>G), D1590_F1591insM (6279_6280insATG), I1592V (6283A>G 6285C>A), A1593V (6287C>T 6288T>G), Y1595L (6292T>C 6293A>T), F1602Y (6314T>A), E1604R (6319G>C 6320A>G 6321A>G), L1606A (6325C>G 6326T>C 6327T>C), Q1607K (6328C>A 6330A>G), E1611D (6342G>T), K1614R (6350A>G 6351A>G), M1616V (6355A>G), E1618Q (6361G>C), I1619T (6365T>C 6366A>T), R1621K (6371G>A 6372G>A), K1622L (6373A>C 6374A>T 6375G>T), N1623* (6376A>T 6377A>G 6378T>A), L1625T (6382C>A 6383T>C 6384A>C), V1626D (6386T>A), V1626_L1627insNCS (6387_6388insAATTGTTCG), P1629S (6394C>T), T1630V (6397A>G 6398C>T), K1631N (6402A>T), M1632V (6403A>G), K1633N (6408A>T), I1634F (6409A>T 6411A>T), A1635G (6413C>G)								
Codon mutations:	AGC1531AGT (6102C>T), AAA1532AAG (6105A>G), TTT1533ATT (6106T>A), TTG1535CTT (6112T>C 6114G>T), AAA1536CGA (6115A>C 6116A>G), TCT1537TCC (6120T>C), GGT1538GAT (6122G>A), TTT1539TAT (6125T>A), CAG1541CAA (6132G>A), GTG1542TTG (6133G>T), GCA1543AGG (6136G>A 6137C>G 6138A>G), ATG1544GTA (6139A>G 6141G>A), GAA1545AAA (6142G>A), GAA1546GAG (6147A>G), TCA1547TGT (6149C>G 6150A>T), AGC1548--G (6151_6152delAG 6153C>G), ATT1549ATC (6156T>C), CCA1550CCG (6159A>G), TGG1551AAG (6160T>A 6161G>A), ACG1552ACT (6165G>T), TTC1554TTT (6171C>T), ATA1555AGA (6173T>G), CCA1557CAA (6179C>A), GAT1558TAT (6181G>T), GGA1559AGT (6184G>A 6186A>T), CTA1560CAC (6188T>A 6189A>C), TAC1561TTT (6191A>T 6192C>T), TGG1563TTT (6197G>T 6198G>T), CTA1564TTG (6199C>T 6201A>G), GTA1565GTT (6204A>T), ATG1566ATA (6207G>A), CCA1567TCT (6208C>T 6210A>T), GGT1569GGA (6216T>A), AAG1571ACA (6221A>C 6222G>A), GCA1573GCT (6228A>T), CCT1574CCC (6231T>C), GTG1576ACT (6235G>A 6236T>C 6237G>T), TTC1577TTT (6240C>T), CAA1578ATG (6241C>A 6242A>T 6243A>G), AGG1579GAC (6244A>G 6245G>A 6246G>C), AAA1580TTA (6247A>T 6248A>T), GAC1582AAC (6253G>A), AAC1583AGG (6257A>G 6258C>G), TGT1584GTA (6259T>G 6260G>T 6261T>A), AGG1586AAG (6266G>A), GGT1587CCG (6268G>C 6269G>C 6270T>G), ACA1588TAT (6271A>T 6272C>A 6273A>T), GAA1589TTG (6274G>T 6275A>T 6276A>G), GAT1590GAC (6279T>C), GAT1590_TTC1591insATG (6279_6280insATG), TTC1591TTT (6282C>T), ATC1592GTA (6283A>G 6285C>A), GCT1593GTG (6287C>T 6288T>G), GTT1594GTC (6291T>C), TAC1595CTC (6292T>C 6293A>T), ATT1596ATA (6297T>A), GAT1598GAC (6303T>C), ATT1599ATA (6306T>A), CTG1600TTG (6307C>T), GTA1601GTT (6312A>T), TTC1602TAC (6314T>A), GAA1604CGG (6319G>C 6320A>G 6321A>G), CTT1606GCC (6325C>G 6326T>C 6327T>C), CAA1607AAG (6328C>A 6330A>G), GAG1608GAA (6333G>A), CAC1609CAT (6336C>T), GAG1611GAT (6342G>T), CAT1612CAC (6345T>C), CTC1613TTG (6346C>T 6348C>G), AAA1614AGG (6350A>G 6351A>G), ATA1615ATT (6354A>T), ATG1616GTG (6355A>G), CTG1617TTG (6358C>T), GAA1618CAA (6361G>C), ATA1619ACT (6365T>C 6366A>T), AGG1621AAA (6371G>A 6372G>A), AAG1622CTT (6373A>C 6374A>T 6375G>T), AAT1623TGA (6376A>T 6377A>G 6378T>A), CTA1625ACC (6382C>A 6383T>C 6384A>C), GTC1626GAC (6386T>A), GTC1626_CTC1627insAATTGTTCG (6387_6388insAATTGTTCG), CTC1627CTA (6390C>A), AGC1628AGT (6393C>T), CCA1629TCA (6394C>T), ACA1630GTA (6397A>G 6398C>T), AAA1631AAT (6402A>T), ATG1632GTG (6403A>G), AAA1633AAT (6408A>T), ATA1634TTT (6409A>T 6411A>T), GCT1635GGT (6413C>G)								

Proteins

	Begin	End	Coverage	Score	Concordance	Matches	Identities	I/D/M/F*	Stop Codons
hypothetical protein (YP_010799265.1)	1529	1638	4.9%	239	30.4%	110 (96.5%)	53 (46.5%)	4/0/1/1	1
Protein mutations:	F1533I (6106T>A), K1536R (6115A>C 6116A>G), G1538D (6122G>A), F1539Y (6125T>A), V1542L (6133G>T), A1543R (6136G>A 6137C>G 6138A>G), M1544V (6139A>G 6141G>A), E1545K (6142G>A), S1547C (6149C>G 6150A>T), W1551K (6160T>A 6161G>A), I1555R (6173T>G), P1557Q (6179C>A), D1558Y (6181G>T), G1559S (6184G>A 6186A>T), L1560H (6188T>A 6189A>C), Y1561F (6191A>T 6192C>T), W1563F (6197G>T 6198G>T), M1566I (6207G>A), P1567S (6208C>T 6210A>T), K1571T (6221A>C 6222G>A), V1576T (6235G>A 6236T>C 6237G>T), Q1578M (6241C>A 6242A>T 6243A>G), R1579D (6244A>G 6245G>A 6246G>C), K1580L (6247A>T 6248A>T), D1582N (6253G>A), N1583R (6257A>G 6258C>G), C1584V (6259T>G 6260G>T 6261T>A), R1586K (6266G>A), G1587P (6268G>C 6269G>C 6270T>G), T1588Y (6271A>T 6272C>A 6273A>T), E1589L (6274G>T 6275A>T 6276A>G), D1590_F1591insM (6279_6280insATG), I1592V (6283A>G 6285C>A), A1593V (6287C>T 6288T>G), Y1595L (6292T>C 6293A>T), F1602Y (6314T>A), E1604R (6319G>C 6320A>G 6321A>G), L1606A (6325C>G 6326T>C 6327T>C), Q1607K (6328C>A 6330A>G), E1611D (6342G>T), K1614R (6350A>G 6351A>G), M1616V (6355A>G), E1618Q (6361G>C), I1619T (6365T>C 6366A>T), R1621K (6371G>A 6372G>A), K1622L (6373A>C 6374A>T 6375G>T), N1623* (6376A>T 6377A>G 6378T>A), L1625T (6382C>A 6383T>C 6384A>C), V1626D (6386T>A), V1626_L1627insNCS (6387_6388insAATTGTTCG), P1629S (6394C>T), T1630V (6397A>G 6398C>T), K1631N (6402A>T), M1632V (6403A>G), K1633N (6408A>T), I1634F (6409A>T 6411A>T), A1635G (6413C>G)								
Codon mutations:	AGC1531AGT (6102C>T), AAA1532AAG (6105A>G), TTT1533ATT (6106T>A), TTG1535CTT (6112T>C 6114G>T), AAA1536CGA (6115A>C 6116A>G), TCT1537TCC (6120T>C), GGT1538GAT (6122G>A), TTT1539TAT (6125T>A), CAG1541CAA (6132G>A), GTG1542TTG (6133G>T), GCA1543AGG (6136G>A 6137C>G 6138A>G), ATG1544GTA (6139A>G 6141G>A), GAA1545AAA (6142G>A), GAA1546GAG (6147A>G), TCA1547TGT (6149C>G 6150A>T), AGC1548--G (6151_6152delAG 6153C>G), ATT1549ATC (6156T>C), CCA1550CCG (6159A>G), TGG1551AAG (6160T>A 6161G>A), ACG1552ACT (6165G>T), TTC1554TTT (6171C>T), ATA1555AGA (6173T>G), CCA1557CAA (6179C>A), GAT1558TAT (6181G>T), GGA1559AGT (6184G>A 6186A>T), CTA1560CAC (6188T>A 6189A>C), TAC1561TTT (6191A>T 6192C>T), TGG1563TTT (6197G>T 6198G>T), CTA1564TTG (6199C>T 6201A>G), GTA1565GTT (6204A>T), ATG1566ATA (6207G>A), CCA1567TCT (6208C>T 6210A>T), GGT1569GGA (6216T>A), AAG1571ACA (6221A>C 6222G>A), GCA1573GCT (6228A>T), CCT1574CCC (6231T>C), GTG1576ACT (6235G>A 6236T>C 6237G>T), TTC1577TTT (6240C>T), CAA1578ATG (6241C>A 6242A>T 6243A>G), AGG1579GAC (6244A>G 6245G>A 6246G>C), AAA1580TTA (6247A>T 6248A>T), GAC1582AAC (6253G>A), AAC1583AGG (6257A>G 6258C>G), TGT1584GTA (6259T>G 6260G>T 6261T>A), AGG1586AAG (6266G>A), GGT1587CCG (6268G>C 6269G>C 6270T>G), ACA1588TAT (6271A>T 6272C>A 6273A>T), GAA1589TTG (6274G>T 6275A>T 6276A>G), GAT1590GAC (6279T>C), GAT1590_TTC1591insATG (6279_6280insATG), TTC1591TTT (6282C>T), ATC1592GTA (6283A>G 6285C>A), GCT1593GTG (6287C>T 6288T>G), GTT1594GTC (6291T>C), TAC1595CTC (6292T>C 6293A>T), ATT1596ATA (6297T>A), GAT1598GAC (6303T>C), ATT1599ATA (6306T>A), CTG1600TTG (6307C>T), GTA1601GTT (6312A>T), TTC1602TAC (6314T>A), GAA1604CGG (6319G>C 6320A>G 6321A>G), CTT1606GCC (6325C>G 6326T>C 6327T>C), CAA1607AAG (6328C>A 6330A>G), GAG1608GAA (6333G>A), CAC1609CAT (6336C>T), GAG1611GAT (6342G>T), CAT1612CAC (6345T>C), CTC1613TTG (6346C>T 6348C>G), AAA1614AGG (6350A>G 6351A>G), ATA1615ATT (6354A>T), ATG1616GTG (6355A>G), CTG1617TTG (6358C>T), GAA1618CAA (6361G>C), ATA1619ACT (6365T>C 6366A>T), AGG1621AAA (6371G>A 6372G>A), AAG1622CTT (6373A>C 6374A>T 6375G>T), AAT1623TGA (6376A>T 6377A>G 6378T>A), CTA1625ACC (6382C>A 6383T>C 6384A>C), GTC1626GAC (6386T>A), GTC1626_CTC1627insAATTGTTCG (6387_6388insAATTGTTCG), CTC1627CTA (6390C>A), AGC1628AGT (6393C>T), CCA1629TCA (6394C>T), ACA1630GTA (6397A>G 6398C>T), AAA1631AAT (6402A>T), ATG1632GTG (6403A>G), AAA1633AAT (6408A>T), ATA1634TTT (6409A>T 6411A>T), GCT1635GGT (6413C>G)								

*: Inserts / Deletes / Misaligned / Frameshifts

Analysis details

This analysis was performed with panviral2.64

NGS Details (UN9): Nodensvirus spm2

Assembly

**Coverage Length**	163 (1 contig(s))
**Depth Of Coverage**	39.2
**Number Of Reads**	67
**Reads Per Million**	1.51 rpm (after QC)
**Ambiguities**	0
**Assembly Method**	de novo + reference guided assembly
**Consensus Caller**	Bcf Tools

Coverage Map

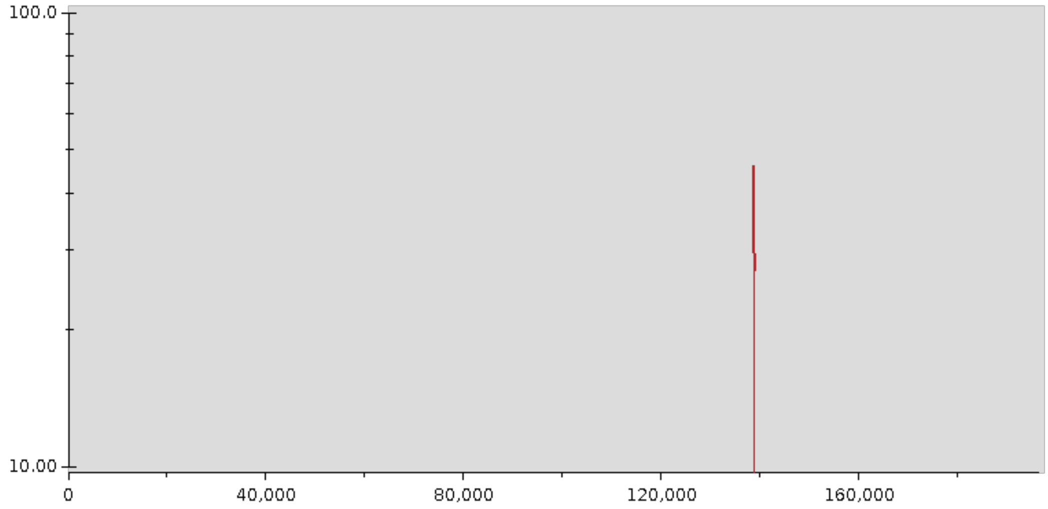

Assignment

**Type**	Nodensvirus spm2 (Taxonomy ID: 2734126)
**Reference Genome**	NC_006820.1
**NT Identity (%)**	80.7453
**AA Identity (%)**	86.2745
**Number Of Stop Codons**	1
**Number Of CDS**	243

Alignment

**Alignment Score**	187.0 (NT) + 209.0 (AA) = 396.0
**Concordance (%)**	72.7903
**Alignment Method**	Local, heuristic, nucleotide (BLASTN)

Genome Region

Sequence starts at position 138728 and ends at position 138890 relative to NC_006820.1 reference sequence.

Alignment Detailed Statistics

	Begin	End	Coverage	Score	Concordance	Matches	Identities	I/D/M/F*	Stop Codons
**NT**	**138728**	**138890**	**0.1%**	**187**	**60.1%**	**161 (98.8%)**	**130 (79.8%)**	**0/2**	
Mutations:	138734T>C, 138740A>G, 138755C>T, 138756G>A, 138761C>T, 138773G>A, 138776C>T, 138778T>C, 138783_138784delCT, 138785G>A, 138788T>C, 138795C>A, 138797G>T, 138803C>T, 138812T>G, 138813C>G, 138816G>C, 138817T>A, 138824A>G, 138830C>T, 138836C>T, 138840G>A, 138842T>A, 138851A>G, 138854A>G, 138857A>T, 138858T>C, 138860G>A, 138864A>C, 138866A>T, 138869C>A, 138878G>T								

*: Inserts / Deletes / Misaligned / Frameshifts

Analysis details

This analysis was performed with panviral2.64

NGS Details (UN9): Epiphyllum badnavirus 1

Assembly

**Coverage Length**	426 (1 contig(s))
**Depth Of Coverage**	15.2
**Number Of Reads**	66
**Reads Per Million**	1.49 rpm (after QC)
**Ambiguities**	0
**Assembly Method**	de novo + reference guided assembly
**Consensus Caller**	Bcf Tools

Coverage Map

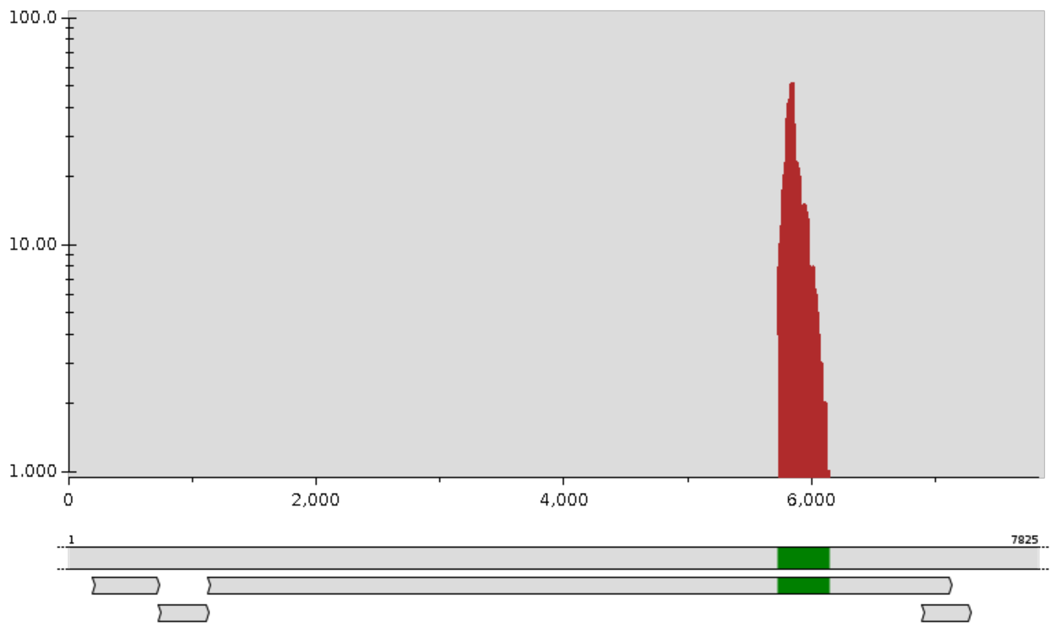

Assignment

**Type**	Epiphyllum badnavirus 1 (Taxonomy ID: 2518008)
**Reference Genome**	NC_076247.1
**NT Identity (%)**	52.2042
**AA Identity (%)**	44.4444
**Number Of Stop Codons**	0
**Number Of CDS**	4

Alignment

**Alignment Score**	25.0 (NT) + 292.0 (AA) = 317.0
**Concordance (%)**	18.4607

Alignment Method	Global, seeded, nucleotide + amino acids (AGA)

Genome Region

Sequence starts at position 5724 and ends at position 6149 relative to NC_076247.1 reference sequence.

Alignment Detailed Statistics

	Begin	End	Coverage	Score	Concordance	Matches	Identities	I/D/M/F*	Stop Codons
**NT**	**5724**	**6149**	**5.4%**	**25**	**2.9%**	**426 (98.8%)**	**225 (52.2%)**	**5/0**	
Mutations:	5724G>A, 5725A>G, 5727A>T, 5728G>A, 5731A>T, 5732A>C, 5734G>T, 5735A>T, 5739A>G, 5743G>T, 5744T>G, 5745C>T, 5746T>A, 5748C>A, 5749A>G, 5754C>T, 5755A>C, 5757A>G, 5758A>C, 5759C>A, 5760C>A, 5761T>C, 5763G>C, 5766C>T, 5767G>A, 5769C>G, 5770A>G, 5771A>T, 5772C>T, 5775G>T, 5776T>G, 5778C>T, 5782G>A, 5785C>A, 5791T>C, 5793T>A, 5794C>A, 5799T>C, 5800G>A, 5802C>T, 5806A>G, 5808C>T, 5809A>G, 5810C>A, 5811A>T, 5814C>G, 5815A>T, 5817A>T, 5818C>G, 5820G>C, 5821A>C, 5823G>A, 5824G>T, 5826C>A, 5827T>C, 5828G>A, 5829T>A, 5830A>G, 5831A>G, 5835C>T, 5839G>T, 5840T>G, 5847C>A, 5851T>A, 5853C>A, 5859G>C, 5860A>T, 5862G>T, 5863A>T, 5864G>C, 5868A>T, 5870T>A, 5871C>T, 5874C>T, 5878A>T, 5880T>G, 5882T>A, 5884A>G, 5887G>A, 5888A>G, 5889C>G, 5893G>C, 5894A>G, 5895A>T, 5896T>G, 5897C>A, 5898C>T, 5901C>T, 5905T>A, 5906G>A, 5907G>A, 5910C>G, 5913C>T, 5916C>T, 5918T>G, 5922C>A, 5923C>A, 5924C>G, 5926G>T, 5931A>G, 5933T>A, 5936A>T, 5937C>T, 5942G>T, 5943G>T, 5944T>C, 5946A>T, 5953C>T, 5955A>C, 5958C>T, 5961A>T, 5964G>A, 5966A>C, 5967G>T, 5973C>T, 5976T>G, 5977G>A, 5982G>A, 5985C>T, 5986C>A, 5987A>T, 5988A>G, 5989C>G, 5990G>A, 5991A>C, 5992A>C, 5993A>T, 5994G>C, 5998G>A, 6000C>T, 6002A>G, 6003C>G, 6003_6004insGTG, 6008T>C, 6009C>A, 6012A>C, 6012_6013insCC, 6013G>T, 6018G>T, 6019T>G, 6020C>A, 6021A>T, 6023G>T, 6024C>G, 6026T>A, 6030A>G, 6032C>T, 6033A>G, 6036T>G, 6038A>T, 6042T>C, 6048C>T, 6051C>T, 6059T>A, 6060C>T, 6061A>T, 6062G>C, 6063T>C, 6064A>C, 6065A>T, 6066C>A, 6067A>G, 6068C>G, 6069T>C, 6073G>C, 6075T>G, 6078C>T, 6081T>C, 6082G>A, 6083C>T, 6084A>G, 6085C>G, 6091C>T, 6095A>G, 6096G>A, 6097T>A, 6099C>G, 6102G>A, 6103C>T, 6108G>A, 6113G>T, 6114T>G, 6115G>A, 6116A>G, 6118A>G, 6119G>A, 6123G>A, 6124G>C, 6127C>T, 6130A>T, 6133C>G, 6134T>C, 6136T>A, 6137C>A, 6138T>A, 6139C>T, 6140C>A, 6142A>G, 6143C>A, 6144G>C, 6147G>A, 6148A>T, 6149T>G								

CDS

	Begin	End	Coverage	Score	Concordance	Matches	Identities	I/D/M/F*	Stop Codons
QKM20_gp3	1533	1674	7.1%	292	28.7%	142 (98.6%)	64 (44.4%)	2/0/1/1	0
Protein mutations:	K1533D (5725A>G 5727A>T), G1534R (5728G>A), K1535S (5731A>T 5732A>C), E1536L (5734G>T 5735A>T), V1539C (5743G>T 5744T>G 5745C>T), F1540I (5746T>A 5748C>A), N1541D (5749A>G), K1543Q (5755A>C 5757A>G), T1544Q (5758A>C 5759C>A 5760C>A), D1547K (5767G>A 5769C>G), N1548V (5770A>G 5771A>T 5772C>T), F1550V (5776T>G 5778C>T), D1552N (5782G>A), Q1553K (5785C>A), S1555P (5791T>C 5793T>A), L1556I (5794C>A), G1558S (5800G>A 5802C>T), N1560D (5806A>G 5808C>T), T1561D (5809A>G 5810C>A 5811A>T), I1562M (5814C>G), I1563F (5815A>T 5817A>T), Q1564D (5818C>G 5820G>C), K1565Q (5821A>C 5823G>A), V1566L (5824G>T 5826C>A), C1567Q (5827T>C 5828G>A 5829T>A), N1568G (5830A>G 5831A>G), V1571C (5839G>T 5840T>G), F1575I (5851T>A 5853C>A), K1578Y (5860A>T 5862G>T), F1581Y (5870T>A 5871C>T), I1584L (5878A>T 5880T>G), M1585K (5882T>A), M1586V (5884A>G), D1587R (5887G>A 5888A>G 5889C>G), E1589R (5893G>C 5894A>G 5895A>T), S1590D (5896T>G 5897C>A 5898C>T), W1593K (5905T>A 5906G>A 5907G>A), L1597R (5918T>G), P1599R (5923C>A 5924C>G), D1600Y (5926G>T), L1602H (5933T>A), Y1603F (5936A>T 5937C>T), W1605F (5942G>T 5943G>T), P1609S (5953C>T 5955A>C), K1613T (5966A>C 5967G>T), A1617T (5977G>A), Q1620M (5986C>A 5987A>T 5988A>G), R1621D (5989C>G 5990G>A 5991A>C), K1622L (5992A>C 5993A>T 5994G>C), D1624N (5998G>A 6000C>T), N1625R (6002A>G 6003C>G), N1625_C1626insV (6003_6004insGTG), F1627S (6008T>C 6009C>A), R1628S (6012A>C), R1628_D1629insX (6012_6013insCC), D1629Y (6013G>T), S1631D (6019T>G 6020C>A 6021A>T), G1632V (6023G>T 6024C>G), F1633Y (6026T>A), A1635V (6032C>T 6033A>G), Y1637F (6038A>T), F1644Y (6059T>A 6060C>T), N1646L (6064A>C 6065A>T 6066C>A), T1647G (6067A>G 6068C>G 6069T>C), D1649Q (6073G>C 6075T>G), A1652M (6082G>A 6083C>T 6084A>G), Q1653E (6085C>G), K1656R (6095A>G 6096G>A), S1657T (6097T>A 6099C>G), M1658I (6102G>A), C1662L (6113G>T 6114T>G), E1663R (6115G>A 6116A>G), R1664E (6118A>G 6119G>A), G1666R (6124G>C), I1668F (6130A>T), L1669A (6133C>G 6134T>C), S1670K (6136T>A 6137C>A 6138T>A), P1671Y (6139C>T 6140C>A), T1672D (6142A>G 6143C>A 6144G>C)								
Codon mutations:	AAG1532..A (5724G>A), AAA1533GAT (5725A>G 5727A>T), GGA1534AGA (5728G>A), AAG1535TCG (5731A>T 5732A>C), GAG1536TTG (5734G>T 5735A>T), AGA1537AGG (5739A>G), GTC1539TGT (5743G>T 5744T>G 5745C>T), TTC1540ATA (5746T>A 5748C>A), AAC1541GAC (5749A>G), TAC1542TAT (5754C>T), AAA1543CAG (5755A>C 5757A>G), ACC1544CAA (5758A>C 5759C>A 5760C>A), TTG1545CTC (5761T>C 5763G>C), AAC1546AAT (5766C>T), GAC1547AAG (5767G>A 5769C>G), AAC1548GTT (5770A>G 5771A>T 5772C>T), ACG1549ACT (5775G>T), TTC1550GTT (5776T>G 5778C>T), GAT1552AAT (5782G>A), CAG1553AAG (5785C>A), TCT1555CCA (5791T>C 5793T>A), CTA1556ATA (5794C>A), CCT1557CCC (5799T>C), GGC1558AGT (5800G>A 5802C>T), AAC1560GAT (5806A>G 5808C>T), ACA1561GAT (5809A>G 5810C>A 5811A>T), ATC1562ATG (5814C>G), ATA1563TTT (5815A>T 5817A>T), CAG1564GAC (5818C>G 5820G>C), AAG1565CAA (5821A>C 5823G>A), GTC1566TTA (5824G>T 5826C>A), TGT1567CAA (5827T>C 5828G>A 5829T>A), AAT1568GGT (5830A>G 5831A>G), GCC1569GCT (5835C>T), GTC1571TGC (5839G>T 5840T>G), TCC1573TCA (5847C>A), TTC1575ATA (5851T>A 5853C>A), CTG1577CTC (5859G>C), AAG1578TAT (5860A>T 5862G>T), AGC1579TCC (5863A>T 5864G>C), GGA1580GGT (5868A>T), TTC1581TAT (5870T>A 5871C>T), CAC1582CAT (5874C>T), ATT1584TTG (5878A>T 5880T>G), ATG1585AAG (5882T>A), ATG1586GTG (5884A>G), GAC1587AGG (5887G>A 5888A>G 5889C>G), GAA1589CGT (5893G>C 5894A>G 5895A>T), TCC1590GAT (5896T>G 5897C>A 5898C>T), ATC1591ATT (5901C>T), TGG1593AAA (5905T>A 5906G>A 5907G>A), ACC1594ACG (5910C>G), GCC1595GCT (5913C>T), TTC1596TTT (5916C>T), CTG1597CGG (5918T>G), ACC1598ACA (5922C>A), CCA1599AGA (5923C>A 5924C>G), GAT1600TAT (5926G>T), GGA1601GGG (5931A>G), CTT1602CAT (5933T>A), TAC1603TTT (5936A>T 5937C>T), TGG1605TTT (5942G>T 5943G>T), TTA1606CTT (5944T>C 5946A>T), CCA1609TCC (5953C>T 5955A>C), TTC1610TTT (5958C>T), GGA1611GGT (5961A>T), TTG1612TTA (5964G>A), AAG1613ACT (5966A>C 5967G>T), GCC1615GCT (5973C>T), CCT1616CCG (5976T>G), GCT1617ACT (5977G>A), GTG1618GTA (5982G>A), TTC1619TTT (5985C>T), CAA1620ATG (5986C>A 5987A>T 5988A>G), CGA1621GAC (5989C>G 5990G>A 5991A>C), AAG1622CTC (5992A>C 5993A>T 5994G>C), GAC1624AAT (5998G>A 6000C>T), AAC1625AGG (6002A>G 6003C>G), AAC1625_TGT1626insGTG (6003_6004insGTG), TTC1627TCA (6008T>C 6009C>A), AGA1628AGC (6012A>C), AGA1628_GAC1629insCC- (6012_6013insCC), GAC1629TAC (6013G>T), CTG1630CTT (6018G>T), TCA1631GAT (6019T>G 6020C>A 6021A>T), GGC1632GTG (6023G>T 6024C>G), TTT1633TAT (6026T>A), GTA1634GTG (6030A>G), GCA1635GTG (6032C>T 6033A>G), GTT1636GTG (6036T>G), TAC1637TTC (6038A>T), ATT1638ATC (6042T>C), GAC1640GAT (6048C>T), ATC1641ATT (6051C>T), TTC1644TAT (6059T>A 6060C>T), AGT1645TCC (6061A>T 6062G>C 6063T>C), AAC1646CTA (6064A>C 6065A>T 6066C>A), ACT1647GGC (6067A>G 6068C>G 6069T>C), GAT1649CAG (6073G>C 6075T>G), GAC1650GAT (6078C>T), CAT1651CAC (6081T>C), GCA1652ATG (6082G>A 6083C>T 6084A>G), CAG1653GAG (6085C>G), CTG1655TTG (6091C>T), AAG1656AGA (6095A>G 6096G>A), TCC1657ACG (6097T>A 6099C>G), ATG1658ATA (6102G>A), CTG1659TTG (6103C>T), CAG1660CAA (6108G>A), TGT1662TTG (6113G>T 6114T>G), GAG1663AGG (6115G>A 6116A>G), AGA1664GAA (6118A>G 6119G>A), GAG1665GAA (6123G>A), GGG1666CGG (6124G>C), CTG1667TTG (6127C>T), ATC1668TTC (6130A>T), CTC1669GCC (6133C>G 6134T>C), TCT1670AAA (6136T>A 6137C>A 6138T>A), CCT1671TAT (6139C>T 6140C>A), ACG1672GAC (6142A>G 6143C>A 6144G>C), AAG1673AAA (6147G>A), ATG1674TG. (6148A>T 6149T>G)								

Proteins

	Begin	End	Coverage	Score	Concordance	Matches	Identities	I/D/M/F*	Stop Codons
polyprotein (YP_010797894.1)	1533	1674	7.1%	292	28.7%	142 (98.6%)	64 (44.4%)	2/0/1/1	0
Protein mutations:	K1533D (5725A>G 5727A>T), G1534R (5728G>A), K1535S (5731A>T 5732A>C), E1536L (5734G>T 5735A>T), V1539C (5743G>T 5744T>G 5745C>T), F1540I (5746T>A 5748C>A), N1541D (5749A>G), K1543Q (5755A>C 5757A>G), T1544Q (5758A>C 5759C>A 5760C>A), D1547K (5767G>A 5769C>G), N1548V (5770A>G 5771A>T 5772C>T), F1550V (5776T>G 5778C>T), D1552N (5782G>A), Q1553K (5785C>A), S1555P (5791T>C 5793T>A), L1556I (5794C>A), G1558S (5800G>A 5802C>T), N1560D (5806A>G 5808C>T), T1561D (5809A>G 5810C>A 5811A>T), I1562M (5814C>G), I1563F (5815A>T 5817A>T), Q1564D (5818C>G 5820G>C), K1565Q (5821A>C 5823G>A), V1566L (5824G>T 5826C>A), C1567Q (5827T>C 5828G>A 5829T>A), N1568G (5830A>G 5831A>G), V1571C (5839G>T 5840T>G), F1575I (5851T>A 5853C>A), K1578Y (5860A>T 5862G>T), F1581Y (5870T>A 5871C>T), I1584L (5878A>T 5880T>G), M1585K (5882T>A), M1586V (5884A>G), D1587R (5887G>A 5888A>G 5889C>G), E1589R (5893G>C 5894A>G 5895A>T), S1590D (5896T>G 5897C>A 5898C>T), W1593K (5905T>A 5906G>A 5907G>A), L1597R (5918T>G), P1599R (5923C>A 5924C>G), D1600Y (5926G>T), L1602H (5933T>A), Y1603F (5936A>T 5937C>T), W1605F (5942G>T 5943G>T), P1609S (5953C>T 5955A>C), K1613T (5966A>C 5967G>T), A1617T (5977G>A), Q1620M (5986C>A 5987A>T 5988A>G), R1621D (5989C>G 5990G>A 5991A>C), K1622L (5992A>C 5993A>T 5994G>C), D1624N (5998G>A 6000C>T), N1625R (6002A>G 6003C>G), N1625_C1626insV (6003_6004insGTG), F1627S (6008T>C 6009C>A), R1628S (6012A>C), R1628_D1629insX (6012_6013insCC), D1629Y (6013G>T), S1631D (6019T>G 6020C>A 6021A>T), G1632V (6023G>T 6024C>G), F1633Y (6026T>A), A1635V (6032C>T 6033A>G), Y1637F (6038A>T), F1644Y (6059T>A 6060C>T), N1646L (6064A>C 6065A>T 6066C>A), T1647G (6067A>G 6068C>G 6069T>C), D1649Q (6073G>C 6075T>G), A1652M (6082G>A 6083C>T 6084A>G), Q1653E (6085C>G), K1656R (6095A>G 6096G>A), S1657T (6097T>A 6099C>G), M1658I (6102G>A), C1662L (6113G>T 6114T>G), E1663R (6115G>A 6116A>G), R1664E (6118A>G 6119G>A), G1666R (6124G>C), I1668F (6130A>T), L1669A (6133C>G 6134T>C), S1670K (6136T>A 6137C>A 6138T>A), P1671Y (6139C>T 6140C>A), T1672D (6142A>G 6143C>A 6144G>C)								

	Begin	End	Coverage	Score	Concordance	Matches	Identities	I/D/M/F*	Stop Codons
**NT**	**5724**	**6149**	**5.4%**	**25**	**2.9%**	**426 (98.8%)**	**225 (52.2%)**	**5/0**	
Codon mutations:	AAG1532..A (5724G>A), AAA1533GAT (5725A>G 5727A>T), GGA1534AGA (5728G>A), AAG1535TCG (5731A>T 5732A>C), GAG1536TTG (5734G>T 5735A>T), AGA1537AGG (5739A>G), GTC1539TGT (5743G>T 5744T>G 5745C>T), TTC1540ATA (5746T>A 5748C>A), AAC1541GAC (5749A>G), TAC1542TAT (5754C>T), AAA1543CAG (5755A>C 5757A>G), ACC1544CAA (5758A>C 5759C>A 5760C>A), TTG1545CTC (5761T>C 5763G>C), AAC1546AAT (5766C>T), GAC1547AAG (5767G>A 5769C>G), AAC1548GTT (5770A>G 5771A>T 5772C>T), ACG1549ACT (5775G>T), TTC1550GTT (5776T>G 5778C>T), GAT1552AAT (5782G>A), CAG1553AAG (5785C>A), TCT1555CCA (5791T>C 5793T>A), CTA1556ATA (5794C>A), CCT1557CCC (5799T>C), GGC1558AGT (5800G>A 5802C>T), AAC1560GAT (5806A>G 5808C>T), ACA1561GAT (5809A>G 5810C>A 5811A>T), ATC1562ATG (5814C>G), ATA1563TTT (5815A>T 5817A>T), CAG1564GAC (5818C>G 5820G>C), AAG1565CAA (5821A>C 5823G>A), GTC1566TTA (5824G>T 5826C>A), TGT1567CAA (5827T>C 5828G>A 5829T>A), AAT1568GGT (5830A>G 5831A>G), GCC1569GCT (5835C>T), GTC1571TGC (5839G>T 5840T>G), TCC1573TCA (5847C>A), TTC1575ATA (5851T>A 5853C>A), CTG1577CTC (5859G>C), AAG1578TAT (5860A>T 5862G>T), AGC1579TCC (5863A>T 5864G>C), GGA1580GGT (5868A>T), TTC1581TAT (5870T>A 5871C>T), CAC1582CAT (5874C>T), ATT1584TTG (5878A>T 5880T>G), ATG1585AAG (5882T>A), ATG1586GTG (5884A>G), GAC1587AGG (5887G>A 5888A>G 5889C>G), GAA1589CGT (5893G>C 5894A>G 5895A>T), TCC1590GAT (5896T>G 5897C>A 5898C>T), ATC1591ATT (5901C>T), TGG1593AAA (5905T>A 5906G>A 5907G>A), ACC1594ACG (5910C>G), GCC1595GCT (5913C>T), TTC1596TTT (5916C>T), CTG1597CGG (5918T>G), ACC1598ACA (5922C>A), CCA1599AGA (5923C>A 5924C>G), GAT1600TAT (5926G>T), GGA1601GGG (5931A>G), CTT1602CAT (5933T>A), TAC1603TTT (5936A>T 5937C>T), TGG1605TTT (5942G>T 5943G>T), TTA1606CTT (5944T>C 5946A>T), CCA1609TCC (5953C>T 5955A>C), TTC1610TTT (5958C>T), GGA1611GGT (5961A>T), TTG1612TTA (5964G>A), AAG1613ACT (5966A>C 5967G>T), GCC1615GCT (5973C>T), CCT1616CCG (5976T>G), GCT1617ACT (5977G>A), GTG1618GTA (5982G>A), TTC1619TTT (5985C>T), CAA1620ATG (5986C>A 5987A>T 5988A>G), CGA1621GAC (5989C>G 5990G>A 5991A>C), AAG1622CTC (5992A>C 5993A>T 5994G>C), GAC1624AAT (5998G>A 6000C>T), AAC1625AGG (6002A>G 6003C>G), AAC1625_TGT1626insGTG (6003_6004insGTG), TTC1627TCA (6008T>C 6009C>A), AGA1628AGC (6012A>C), AGA1628_GAC1629insCC- (6012_6013insCC), GAC1629TAC (6013G>T), CTG1630CTT (6018G>T), TCA1631GAT (6019T>G 6020C>A 6021A>T), GGC1632GTG (6023G>T 6024C>G), TTT1633TAT (6026T>A), GTA1634GTG (6030A>G), GCA1635GTG (6032C>T 6033A>G), GTT1636GTG (6036T>G), TAC1637TTC (6038A>T), ATT1638ATC (6042T>C), GAC1640GAT (6048C>T), ATC1641ATT (6051C>T), TTC1644TAT (6059T>A 6060C>T), AGT1645TCC (6061A>T 6062G>C 6063T>C), AAC1646CTA (6064A>C 6065A>T 6066C>A), ACT1647GGC (6067A>G 6068C>G 6069T>C), GAT1649CAG (6073G>C 6075T>G), GAC1650GAT (6078C>T), CAT1651CAC (6081T>C), GCA1652ATG (6082G>A 6083C>T 6084A>G), CAG1653GAG (6085C>G), CTG1655TTG (6091C>T), AAG1656AGA (6095A>G 6096G>A), TCC1657ACG (6097T>A 6099C>G), ATG1658ATA (6102G>A), CTG1659TTG (6103C>T), CAG1660CAA (6108G>A), TGT1662TTG (6113G>T 6114T>G), GAG1663AGG (6115G>A 6116A>G), AGA1664GAA (6118A>G 6119G>A), GAG1665GAA (6123G>A), GGG1666CGG (6124G>C), CTG1667TTG (6127C>T), ATC1668TTC (6130A>T), CTC1669GCC (6133C>G 6134T>C), TCT1670AAA (6136T>A 6137C>A 6138T>A), CCT1671TAT (6139C>T 6140C>A), ACG1672GAC (6142A>G 6143C>A 6144G>C), AAG1673AAA (6147G>A), ATG1674TG. (6148A>T 6149T>G)								

*: Inserts / Deletes / Misaligned / Frameshifts

Analysis details

This analysis was performed with panviral2.64

NGS Details (UN9): Caulimovirus venafragariae

Assembly

**Coverage Length**	321 (1 contig(s))
**Depth Of Coverage**	21.2
**Number Of Reads**	60
**Reads Per Million**	1.36 rpm (after QC)
**Ambiguities**	0
**Assembly Method**	de novo + reference guided assembly
**Consensus Caller**	Bcf Tools

Coverage Map

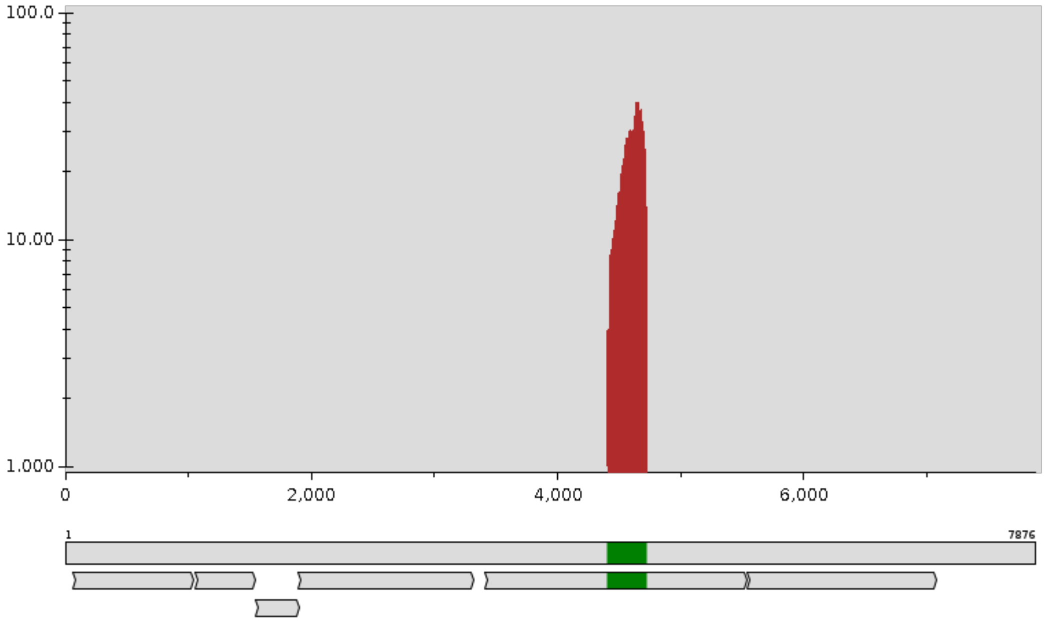

Assignment

**Type**	Caulimovirus venafragariae (Taxonomy ID: 3048344)
**Reference Genome**	NC_001725.1
**NT Identity (%)**	54.2056
**AA Identity (%)**	42.0561
**Number Of Stop Codons**	0
**Number Of CDS**	6

Alignment

**Alignment Score**	54.0 (NT) + 321.0 (AA) = 375.0
**Concordance (%)**	26.0236

Alignment Method	Global, seeded, nucleotide + amino acids (AGA)

Genome Region

Sequence starts at position 4405 and ends at position 4725 relative to NC_001725.1 reference sequence.

Alignment Detailed Statistics

	Begin	End	Coverage	Score	Concordance	Matches	Identities	I/D/M/F*	Stop Codons
**NT**	**4405**	**4725**	**4.1%**	**54**	**8.4%**	**321 (100%)**	**174 (54.2%)**	**0/0**	
Mutations:	4410A>C, 4413T>G, 4414A>G, 4419T>C, 4425G>A, 4426T>C, 4428A>T, 4434C>T, 4435C>G, 4436A>C, 4440A>C, 4441A>C, 4444G>A, 4445G>A, 4446A>G, 4449T>C, 4450G>C, 4451G>A, 4457T>C, 4459C>G, 4461T>G, 4464T>C, 4465A>T, 4466A>T, 4469A>T, 4470G>T, 4471G>A, 4473G>A, 4474C>A, 4477C>A, 4483C>G, 4485A>C, 4489A>T, 4491C>G, 4493G>C, 4494A>T, 4497T>G, 4498A>C, 4500G>A, 4501A>G, 4502C>A, 4503C>T, 4505T>A, 4509C>T, 4511C>G, 4512T>C, 4514C>T, 4518T>G, 4521C>T, 4522T>G, 4524T>G, 4525A>T, 4527A>C, 4535T>A, 4537T>A, 4538G>A, 4539G>T, 4542G>A, 4543G>A, 4545A>C, 4546C>A, 4547G>C, 4548C>A, 4549C>A, 4554T>A, 4560A>G, 4561A>G, 4562C>A, 4563A>C, 4564A>C, 4565T>A, 4566T>A, 4567C>G, 4570C>A, 4571T>A, 4572A>G, 4576G>A, 4583G>C, 4584C>A, 4587T>C, 4590C>T, 4591C>T, 4593A>T, 4596A>G, 4597C>A, 4598A>C, 4599C>A, 4602C>T, 4604A>C, 4607G>T, 4608G>C, 4609T>A, 4610T>A, 4611A>G, 4612G>C, 4613T>G, 4614C>G, 4626A>G, 4630A>T, 4631A>G, 4632A>C, 4633C>A, 4635A>T, 4638C>A, 4641T>G, 4642G>A, 4644T>A, 4646T>C, 4647T>A, 4650C>T, 4656G>A, 4657C>T, 4658A>G, 4663G>A, 4664A>T, 4665C>G, 4667A>C, 4668A>C, 4670G>T, 4671T>A, 4672C>T, 4675A>C, 4676G>A, 4678A>G, 4684T>G, 4685A>T, 4686T>A, 4687C>G, 4688C>A, 4690C>G, 4692G>T, 4696T>G, 4697G>T, 4700C>A, 4701T>G, 4704A>C, 4706A>T, 4707T>C, 4708G>A, 4710T>G, 4714G>A, 4716C>T, 4717A>T, 4719C>T, 4720A>T, 4721T>C, 4722C>A								

CDS

	Begin	End	Coverage	Score	Concordance	Matches	Identities	I/D/M/F*	Stop Codons
ORF_V	334	440	15.1%	321	40.2%	107 (100%)	45 (42.1%)	0/0/0/0	0
Protein mutations:	I336M (4413T>G), N337D (4414A>G), H344A (4435C>G 4436A>C), K346Q (4441A>C), G347K (4444G>A 4445G>A 4446A>G), G349H (4450G>C 4451G>A), L351P (4457T>C), L352V (4459C>G 4461T>G), N354F (4465A>T 4466A>T), K355I (4469A>T 4470G>T), E356K (4471G>A 4473G>A), Q357K (4474C>A), L358M (4477C>A), Q360D (4483C>G 4485A>C), I362L (4489A>T 4491C>G), G363A (4493G>C 4494A>T), K365Q (4498A>C 4500G>A), T366D (4501A>G 4502C>A 4503C>T), F367Y (4505T>A), S369C (4511C>G 4512T>C), S370F (4514C>T), F371L (4518T>G), C373G (4522T>G 4524T>G), K374Y (4525A>T 4527A>C), F377Y (4535T>A), W378N (4537T>A 4538G>A 4539G>T), V380I (4543G>A 4545A>C), R381T (4546C>A 4547G>C 4548C>A), L382I (4549C>A), T386D (4561A>G 4562C>A 4563A>C), I387Q (4564A>C 4565T>A 4566T>A), Q388E (4567C>G), L389K (4570C>A 4571T>A 4572A>G), A391T (4576G>A), S393T (4583G>C 4584C>A), Q396Y (4591C>T 4593A>T), H398T (4597C>A 4598A>C 4599C>A), E400A (4604A>C), W401F (4607G>T 4608G>C), L402K (4609T>A 4610T>A 4611A>G), V403R (4612G>C 4613T>G 4614C>G), K409C (4630A>T 4631A>G 4632A>C), Q410N (4633C>A 4635A>T), A413T (4642G>A 4644T>A), I414T (4646T>C 4647T>A), H418C (4657C>T 4658A>G), D420M (4663G>A 4664A>T 4665C>G), E421A (4667A>C 4668A>C), S422I (4670G>T 4671T>A), L423F (4672C>T), S424H (4675A>C 4676G>A), N425D (4678A>G), Y427V (4684T>G 4685A>T 4686T>A), P428E (4687C>G 4688C>A), Q429D (4690C>G 4692G>T), C431V (4696T>G 4697G>T), A432E (4700C>A 4701T>G), Y434F (4706A>T 4707T>C), V435M (4708G>A 4710T>G), D437N (4714G>A 4716C>T), I438F (4717A>T 4719C>T), I439S (4720A>T 4721T>C 4722C>A)								
Codon mutations:	GTA335GTC (4410A>C), ATT336ATG (4413T>G), AAC337GAC (4414A>G), TAT338TAC (4419T>C), AAG340AAA (4425G>A), TTA341CTT (4426T>C 4428A>T), GAC343GAT (4434C>T), CAT344GCT (4435C>G 4436A>C), ACA345ACC (4440A>C), AAG346CAG (4441A>C), GGA347AAG (4444G>A 4445G>A 4446A>G), GAT348GAC (4449T>C), GGC349CAC (4450G>C 4451G>A), CTA351CCA (4457T>C), CTT352GTG (4459C>G 4461T>G), CCT353CCC (4464T>C), AAC354TTC (4465A>T 4466A>T), AAG355ATT (4469A>T 4470G>T), GAG356AAA (4471G>A 4473G>A), CAA357AAA (4474C>A), CTG358ATG (4477C>A), CAA360GAC (4483C>G 4485A>C), ATC362TTG (4489A>T 4491C>G), GGA363GCT (4493G>C 4494A>T), GGT364GGG (4497T>G), AAG365CAA (4498A>C 4500G>A), ACC366GAT (4501A>G 4502C>A 4503C>T), TTT367TAT (4505T>A), TAC368TAT (4509C>T), TCT369TGC (4511C>G 4512T>C), TCC370TTC (4514C>T), TTT371TTG (4518T>G), GAC372GAT (4521C>T), TGT373GGG (4522T>G 4524T>G), AAA374TAC (4525A>T 4527A>C), TTT377TAT (4535T>A), TGG378AAT (4537T>A 4538G>A 4539G>T), CAG379CAA (4542G>A), GTA380ATC (4543G>A 4545A>C), CGC381ACA (4546C>A 4547G>C 4548C>A), CTT382ATT (4549C>A), GCT383GCA (4554T>A), GAA385GAG (4560A>G), ACA386GAC (4561A>G 4562C>A 4563A>C), ATT387CAA (4564A>C 4565T>A 4566T>A), CAG388GAG (4567C>G), CTA389AAG (4570C>A 4571T>A 4572A>G), GCT391ACT (4576G>A), AGC393ACA (4583G>C 4584C>A), TGT394TGC (4587T>C), CCC395CCT (4590C>T), CAA396TAT (4591C>T 4593A>T), GGA397GGG (4596A>G), CAC398ACA (4597C>A 4598A>C 4599C>A), TAC399TAT (4602C>T), GAA400GCA (4604A>C), TGG401TTC (4607G>T 4608G>C), TTA402AAG (4609T>A 4610T>A 4611A>G), GTC403CGG (4612G>C 4613T>G 4614C>G), GGA407GGG (4626A>G), AAA409TGC (4630A>T 4631A>G 4632A>C), CAA410AAT (4633C>A 4635A>T), GCC411GCA (4638C>A), CCT412CCG (4641T>G), GCT413ACA (4642G>A 4644T>A), ATT414ACA (4646T>C 4647T>A), TTC415TTT (4650C>T), AGG417AGA (4656G>A), CAC418TGC (4657C>T 4658A>G), GAC420ATG (4663G>A 4664A>T 4665C>G), GAA421GCC (4667A>C 4668A>C), AGT422ATA (4670G>T 4671T>A), CTC423TTC (4672C>T), AGC424CAC (4675A>C 4676G>A), AAC425GAC (4678A>G), TAT427GTA (4684T>G 4685A>T 4686T>A), CCA428GAA (4687C>G 4688C>A), CAG429GAT (4690C>G 4692G>T), TGT431GTT (4696T>G 4697G>T), GCT432GAG (4700C>A 4701T>G), GTA433GTC (4704A>C), TAT434TTC (4706A>T 4707T>C), GTT435ATG (4708G>A 4710T>G), GAC437AAT (4714G>A 4716C>T), ATC438TTT (4717A>T 4719C>T), ATC439TCA (4720A>T 4721T>C 4722C>A)								

Proteins

	Begin	End	Coverage	Score	Concordance	Matches	Identities	I/D/M/F*	Stop Codons
hypothetical protein (NP_043933.1)	334	440	15.1%	321	40.2%	107 (100%)	45 (42.1%)	0/0/0/0	0
Protein mutations:	I336M (4413T>G), N337D (4414A>G), H344A (4435C>G 4436A>C), K346Q (4441A>C), G347K (4444G>A 4445G>A 4446A>G), G349H (4450G>C 4451G>A), L351P (4457T>C), L352V (4459C>G 4461T>G), N354F (4465A>T 4466A>T), K355I (4469A>T 4470G>T), E356K (4471G>A 4473G>A), Q357K (4474C>A), L358M (4477C>A), Q360D (4483C>G 4485A>C), I362L (4489A>T 4491C>G), G363A (4493G>C 4494A>T), K365Q (4498A>C 4500G>A), T366D (4501A>G 4502C>A 4503C>T), F367Y (4505T>A), S369C (4511C>G 4512T>C), S370F (4514C>T), F371L (4518T>G), C373G (4522T>G 4524T>G), K374Y (4525A>T 4527A>C), F377Y (4535T>A), W378N (4537T>A 4538G>A 4539G>T), V380I (4543G>A 4545A>C), R381T (4546C>A 4547G>C 4548C>A), L382I (4549C>A), T386D (4561A>G 4562C>A 4563A>C), I387Q (4564A>C 4565T>A 4566T>A), Q388E (4567C>G), L389K (4570C>A 4571T>A 4572A>G), A391T (4576G>A), S393T (4583G>C 4584C>A), Q396Y (4591C>T 4593A>T), H398T (4597C>A 4598A>C 4599C>A), E400A (4604A>C), W401F (4607G>T 4608G>C), L402K (4609T>A 4610T>A 4611A>G), V403R (4612G>C 4613T>G 4614C>G), K409C (4630A>T 4631A>G 4632A>C), Q410N (4633C>A 4635A>T), A413T (4642G>A 4644T>A), I414T (4646T>C 4647T>A), H418C (4657C>T 4658A>G), D420M (4663G>A 4664A>T 4665C>G), E421A (4667A>C 4668A>C), S422I (4670G>T 4671T>A), L423F (4672C>T), S424H (4675A>C 4676G>A), N425D (4678A>G), Y427V (4684T>G 4685A>T 4686T>A), P428E (4687C>G 4688C>A), Q429D (4690C>G 4692G>T), C431V (4696T>G 4697G>T), A432E (4700C>A 4701T>G), Y434F (4706A>T 4707T>C), V435M (4708G>A 4710T>G), D437N (4714G>A 4716C>T), I438F (4717A>T 4719C>T), I439S (4720A>T 4721T>C 4722C>A)								
Codon mutations:	GTA335GTC (4410A>C), ATT336ATG (4413T>G), AAC337GAC (4414A>G), TAT338TAC (4419T>C), AAG340AAA (4425G>A), TTA341CTT (4426T>C 4428A>T), GAC343GAT (4434C>T), CAT344GCT (4435C>G 4436A>C), ACA345ACC (4440A>C), AAG346CAG (4441A>C), GGA347AAG (4444G>A 4445G>A 4446A>G), GAT348GAC (4449T>C), GGC349CAC (4450G>C 4451G>A), CTA351CCA (4457T>C), CTT352GTG (4459C>G 4461T>G), CCT353CCC (4464T>C), AAC354TTC (4465A>T 4466A>T), AAG355ATT (4469A>T 4470G>T), GAG356AAA (4471G>A 4473G>A), CAA357AAA (4474C>A), CTG358ATG (4477C>A), CAA360GAC (4483C>G 4485A>C), ATC362TTG (4489A>T 4491C>G), GGA363GCT (4493G>C 4494A>T), GGT364GGG (4497T>G), AAG365CAA (4498A>C 4500G>A), ACC366GAT (4501A>G 4502C>A 4503C>T), TTT367TAT (4505T>A), TAC368TAT (4509C>T), TCT369TGC (4511C>G 4512T>C), TCC370TTC (4514C>T), TTT371TTG (4518T>G), GAC372GAT (4521C>T), TGT373GGG (4522T>G 4524T>G), AAA374TAC (4525A>T 4527A>C), TTT377TAT (4535T>A), TGG378AAT (4537T>A 4538G>A 4539G>T), CAG379CAA (4542G>A), GTA380ATC (4543G>A 4545A>C), CGC381ACA (4546C>A 4547G>C 4548C>A), CTT382ATT (4549C>A), GCT383GCA (4554T>A), GAA385GAG (4560A>G), ACA386GAC (4561A>G 4562C>A 4563A>C), ATT387CAA (4564A>C 4565T>A 4566T>A), CAG388GAG (4567C>G), CTA389AAG (4570C>A 4571T>A 4572A>G), GCT391ACT (4576G>A), AGC393ACA (4583G>C 4584C>A), TGT394TGC (4587T>C), CCC395CCT (4590C>T), CAA396TAT (4591C>T 4593A>T), GGA397GGG (4596A>G), CAC398ACA (4597C>A 4598A>C 4599C>A), TAC399TAT (4602C>T), GAA400GCA (4604A>C), TGG401TTC (4607G>T 4608G>C), TTA402AAG (4609T>A 4610T>A 4611A>G), GTC403CGG (4612G>C 4613T>G 4614C>G), GGA407GGG (4626A>G), AAA409TGC (4630A>T 4631A>G 4632A>C), CAA410AAT (4633C>A 4635A>T), GCC411GCA (4638C>A), CCT412CCG (4641T>G), GCT413ACA (4642G>A 4644T>A), ATT414ACA (4646T>C 4647T>A), TTC415TTT (4650C>T), AGG417AGA (4656G>A), CAC418TGC (4657C>T 4658A>G), GAC420ATG (4663G>A 4664A>T 4665C>G), GAA421GCC (4667A>C 4668A>C), AGT422ATA (4670G>T 4671T>A), CTC423TTC (4672C>T), AGC424CAC (4675A>C 4676G>A), AAC425GAC (4678A>G), TAT427GTA (4684T>G 4685A>T 4686T>A), CCA428GAA (4687C>G 4688C>A), CAG429GAT (4690C>G 4692G>T), TGT431GTT (4696T>G 4697G>T), GCT432GAG (4700C>A 4701T>G), GTA433GTC (4704A>C), TAT434TTC (4706A>T 4707T>C), GTT435ATG (4708G>A 4710T>G), GAC437AAT (4714G>A 4716C>T), ATC438TTT (4717A>T 4719C>T), ATC439TCA (4720A>T 4721T>C 4722C>A)								

*: Inserts / Deletes / Misaligned / Frameshifts

Analysis details

This analysis was performed with panviral2.64

NGS Details (UN9): Epiphyllum badnavirus 1

Assembly

**Coverage Length**	306 (1 contig(s))
**Depth Of Coverage**	18.6
**Number Of Reads**	55
**Reads Per Million**	1.24 rpm (after QC)
**Ambiguities**	0
**Assembly Method**	de novo + reference guided assembly
**Consensus Caller**	Bcf Tools

Coverage Map

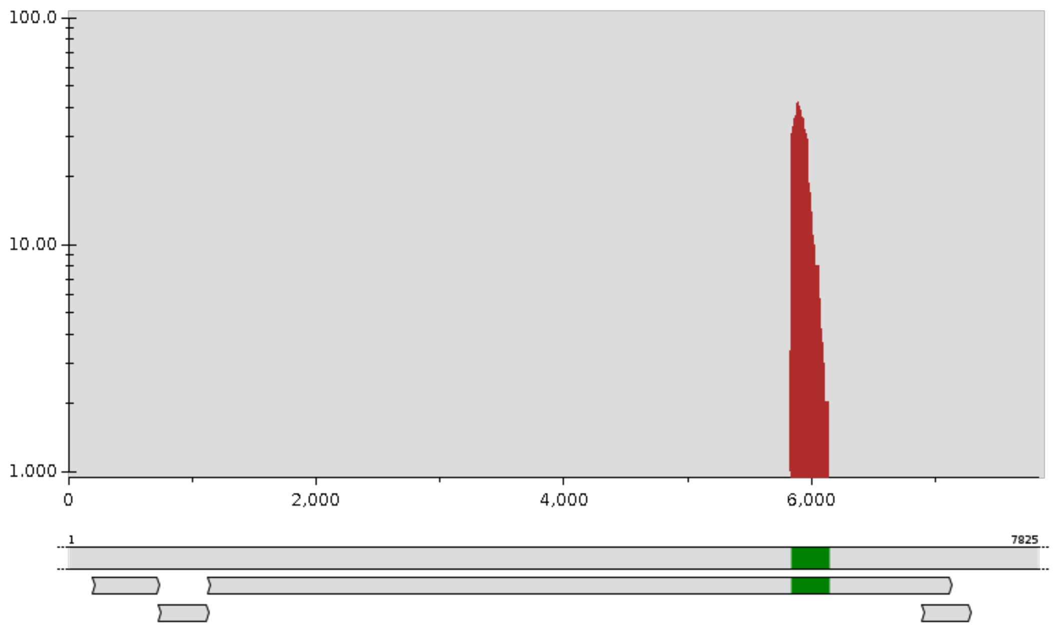

Assignment

**Type**	Epiphyllum badnavirus 1 (Taxonomy ID: 2518008)
**Reference Genome**	NC_076247.1
**NT Identity (%)**	52.9412
**AA Identity (%)**	45.098
**Number Of Stop Codons**	0
**Number Of CDS**	4

Alignment

**Alignment Score**	18.0 (NT) + 312.0 (AA) = 330.0
**Concordance (%)**	25.1524

Alignment Method	Global, seeded, nucleotide + amino acids (AGA)

Genome Region

Sequence starts at position 5835 and ends at position 6140 relative to NC_076247.1 reference sequence.

Alignment Detailed Statistics

	Begin	End	Coverage	Score	Concordance	Matches	Identities	I/D/M/F*	Stop Codons
**NT**	**5835**	**6140**	**3.9%**	**18**	**3.0%**	**303 (98.1%)**	**162 (52.4%)**	**3/3**	
Mutations:	5835C>G, 5839G>T, 5847C>A, 5851T>A, 5853C>A, 5859G>T, 5860A>C, 5861A>G, 5862G>T, 5863A>T, 5864G>C, 5865C>T, 5868A>G, 5870T>A, 5871C>T, 5878A>T, 5880T>G, 5882T>A, 5883G>A, 5884A>G, 5886G>T, 5887G>A, 5888A>G, 5889C>G, 5893G>A, 5894A>G, 5895A>T, 5896T>G, 5897C>A, 5898C>T, 5901C>T, 5904T>C, 5905T>A, 5906G>A, 5910C>A, 5911G>A, 5913C>A, 5918T>G, 5922C>A, 5923C>A, 5924C>G, 5925A>G, 5926G>T, 5933T>A, 5934T>C, 5936A>T, 5937C>T, 5938G>A, 5942G>T, 5943G>T, 5944T>C, 5946A>C, 5949T>C, 5953C>T, 5955A>C, 5958C>T, 5961A>T, 5964G>A, 5966A>C, 5967G>T, 5970T>C, 5971G>A, 5973C>T, 5982G>C, 5985C>T, 5986C>A, 5987A>T, 5988A>G, 5989C>G, 5990G>A, 5991A>T, 5992A>C, 5993A>T, 5994G>T, 5997G>A, 5998G>A, 6000C>T, 6002A>G, 6003C>G, 6004T>A, 6005G>T, 6006T>A, 6011G>A, 6012A>G, 6013G>C, 6014A>C, 6015_6016insCAT, 6018G>C, 6019T>G, 6020C>A, 6021A>T, 6022G>A, 6023G>T, 6024C>G, 6026T>A, 6030A>G, 6032C>T, 6033A>G, 6036T>G, 6038A>T, 6039C>T, 6059T>A, 6060C>T, 6061A>T, 6062G>C, 6063T>A, 6065A>G, 6066C>A, 6067A>G, 6068C>A, 6069T>C, 6070G>A, 6072A>G, 6073G>C, 6074A>G, 6075T>A, 6078C>G, 6081T>C, 6082G>A, 6083C>T, 6085C>G, 6088C>A, 6095A>G, 6096G>A, 6097T>A, 6099C>T, 6100A>G, 6113G>T, 6114T>G, 6115_6117delGAG, 6123G>A, 6125G>A, 6126G>A, 6128T>A, 6130A>T, 6132C>A, 6133C>T, 6134T>A, 6135C>T, 6136T>G, 6138T>A, 6139C>A, 6140C>A								

CDS

	Begin	End	Coverage	Score	Concordance	Matches	Identities	I/D/M/F*	Stop Codons
QKM20_gp3	1570	1671	5.1%	312	42.5%	101 (98.1%)	46 (44.7%)	1/1/0/0	0
Protein mutations:	V1571F (5839G>T), F1575I (5851T>A 5853C>A), K1578R (5860A>C 5861A>G 5862G>T), F1581Y (5870T>A 5871C>T), I1584L (5878A>T 5880T>G), M1585K (5882T>A 5883G>A), M1586V (5884A>G 5886G>T), D1587R (5887G>A 5888A>G 5889C>G), E1589S (5893G>A 5894A>G 5895A>T), S1590D (5896T>G 5897C>A 5898C>T), W1593K (5905T>A 5906G>A), A1595T (5911G>A 5913C>A), L1597R (5918T>G), P1599R (5923C>A 5924C>G 5925A>G), D1600Y (5926G>T), L1602H (5933T>A 5934T>C), Y1603F (5936A>T 5937C>T), E1604K (5938G>A), W1605F (5942G>T 5943G>T), P1609S (5953C>T 5955A>C), K1613T (5966A>C 5967G>T), A1615T (5971G>A 5973C>T), Q1620M (5986C>A 5987A>T 5988A>G), R1621D (5989C>G 5990G>A 5991A>T), K1622L (5992A>C 5993A>T 5994G>T), M1623I (5997G>A), D1624N (5998G>A 6000C>T), N1625R (6002A>G 6003C>G), C1626I (6004T>A 6005G>T 6006T>A), R1628K (6011G>A 6012A>G), D1629P (6013G>C 6014A>C), D1629_L1630insH (6015_6016insCAT), S1631D (6019T>G 6020C>A 6021A>T), G1632M (6022G>A 6023G>T 6024C>G), F1633Y (6026T>A), A1635V (6032C>T 6033A>G), Y1637F (6038A>T 6039C>T), F1644Y (6059T>A 6060C>T), N1646R (6065A>G 6066C>A), T1647D (6067A>G 6068C>A 6069T>C), E1648K (6070G>A 6072A>G), D1649R (6073G>C 6074A>G 6075T>A), D1650E (6078C>G), A1652I (6082G>A 6083C>T), Q1653E (6085C>G), H1654N (6088C>A), K1656R (6095A>G 6096G>A), S1657T (6097T>A 6099C>T), M1658V (6100A>G), C1662L (6113G>T 6114T>G), E1663del (6115_6117delGAG), G1666E (6125G>A 6126G>A), L1667Q (6128T>A), I1668L (6130A>T 6132C>A), L1669Y (6133C>T 6134T>A 6135C>T), S1670A (6136T>G 6138T>A)								
Codon mutations:	GCC1569..G (5835C>G), GTC1571TTC (5839G>T), TCC1573TCA (5847C>A), TTC1575ATA (5851T>A 5853C>A), CTG1577CTT (5859G>T), AAG1578CGT (5860A>C 5861A>G 5862G>T), AGC1579TCT (5863A>T 5864G>C 5865C>T), GGA1580GGG (5868A>G), TTC1581TAT (5870T>A 5871C>T), ATT1584TTG (5878A>T 5880T>G), ATG1585AAA (5882T>A 5883G>A), ATG1586GTT (5884A>G 5886G>T), GAC1587AGG (5887G>A 5888A>G 5889C>G), GAA1589AGT (5893G>A 5894A>G 5895A>T), TCC1590GAT (5896T>G 5897C>A 5898C>T), ATC1591ATT (5901C>T), CCT1592CCC (5904T>C), TGG1593AAG (5905T>A 5906G>A), ACC1594ACA (5910C>A), GCC1595ACA (5911G>A 5913C>A), CTG1597CGG (5918T>G), ACC1598ACA (5922C>A), CCA1599AGG (5923C>A 5924C>G 5925A>G), GAT1600TAT (5926G>T), CTT1602CAC (5933T>A 5934T>C), TAC1603TTT (5936A>T 5937C>T), GAA1604AAA (5938G>A), TGG1605TTT (5942G>T 5943G>T), TTA1606CTC (5944T>C 5946A>C), GTT1607GTC (5949T>C), CCA1609TCC (5953C>T 5955A>C), TTC1610TTT (5958C>T), GGA1611GGT (5961A>T), TTG1612TTA (5964G>A), AAG1613ACT (5966A>C 5967G>T), AAT1614AAC (5970T>C), GCC1615ACT (5971G>A 5973C>T), GTG1618GTC (5982G>C), TTC1619TTT (5985C>T), CAA1620ATG (5986C>A 5987A>T 5988A>G), CGA1621GAT (5989C>G 5990G>A 5991A>T), AAG1622CTT (5992A>C 5993A>T 5994G>T), ATG1623ATA (5997G>A), GAC1624AAT (5998G>A 6000C>T), AAC1625AGG (6002A>G 6003C>G), TGT1626ATA (6004T>A 6005G>T 6006T>A), AGA1628AAG (6011G>A 6012A>G), GAC1629CCC (6013G>C 6014A>C), GAC1629_CTG1630insCAT (6015_6016insCAT), CTG1630CTC (6018G>C), TCA1631GAT (6019T>G 6020C>A 6021A>T), GGC1632ATG (6022G>A 6023G>T 6024C>G), TTT1633TAT (6026T>A), GTA1634GTG (6030A>G), GCA1635GTG (6032C>T 6033A>G), GTT1636GTG (6036T>G), TAC1637TTT (6038A>T 6039C>T), TTC1644TAT (6059T>A 6060C>T), AGT1645TCA (6061A>T 6062G>C 6063T>A), AAC1646AGA (6065A>G 6066C>A), ACT1647GAC (6067A>G 6068C>A 6069T>C), GAA1648AAG (6070G>A 6072A>G), GAT1649CGA (6073G>C 6074A>G 6075T>A), GAC1650GAG (6078C>G), CAT1651CAC (6081T>C), GCA1652ATA (6082G>A 6083C>T), CAG1653GAG (6085C>G), CAC1654AAC (6088C>A), AAG1656AGA (6095A>G 6096G>A), TCC1657ACT (6097T>A 6099C>T), ATG1658GTG (6100A>G), TGT1662TTG (6113G>T 6114T>G), GAG1663del (6115_6117delGAG), GAG1665GAA (6123G>A), GGG1666GAA (6125G>A 6126G>A), CTG1667CAG (6128T>A), ATC1668TTA (6130A>T 6132C>A), CTC1669TAT (6133C>T 6134T>A 6135C>T), TCT1670GCA (6136T>G 6138T>A), CCT1671AA. (6139C>A 6140C>A)								

Proteins

	Begin	End	Coverage	Score	Concordance	Matches	Identities	I/D/M/F*	Stop Codons
polyprotein (YP_010797894.1)	1570	1671	5.1%	312	42.5%	101 (98.1%)	46 (44.7%)	1/1/0/0	0
Protein mutations:	V1571F (5839G>T), F1575I (5851T>A 5853C>A), K1578R (5860A>C 5861A>G 5862G>T), F1581Y (5870T>A 5871C>T), I1584L (5878A>T 5880T>G), M1585K (5882T>A 5883G>A), M1586V (5884A>G 5886G>T), D1587R (5887G>A 5888A>G 5889C>G), E1589S (5893G>A 5894A>G 5895A>T), S1590D (5896T>G 5897C>A 5898C>T), W1593K (5905T>A 5906G>A), A1595T (5911G>A 5913C>A), L1597R (5918T>G), P1599R (5923C>A 5924C>G 5925A>G), D1600Y (5926G>T), L1602H (5933T>A 5934T>C), Y1603F (5936A>T 5937C>T), E1604K (5938G>A), W1605F (5942G>T 5943G>T), P1609S (5953C>T 5955A>C), K1613T (5966A>C 5967G>T), A1615T (5971G>A 5973C>T), Q1620M (5986C>A 5987A>T 5988A>G), R1621D (5989C>G 5990G>A 5991A>T), K1622L (5992A>C 5993A>T 5994G>T), M1623I (5997G>A), D1624N (5998G>A 6000C>T), N1625R (6002A>G 6003C>G), C1626I (6004T>A 6005G>T 6006T>A), R1628K (6011G>A 6012A>G), D1629P (6013G>C 6014A>C), D1629_L1630insH (6015_6016insCAT), S1631D (6019T>G 6020C>A 6021A>T), G1632M (6022G>A 6023G>T 6024C>G), F1633Y (6026T>A), A1635V (6032C>T 6033A>G), Y1637F (6038A>T 6039C>T), F1644Y (6059T>A 6060C>T), N1646R (6065A>G 6066C>A), T1647D (6067A>G 6068C>A 6069T>C), E1648K (6070G>A 6072A>G), D1649R (6073G>C 6074A>G 6075T>A), D1650E (6078C>G), A1652I (6082G>A 6083C>T), Q1653E (6085C>G), H1654N (6088C>A), K1656R (6095A>G 6096G>A), S1657T (6097T>A 6099C>T), M1658V (6100A>G), C1662L (6113G>T 6114T>G), E1663del (6115_6117delGAG), G1666E (6125G>A 6126G>A), L1667Q (6128T>A), I1668L (6130A>T 6132C>A), L1669Y (6133C>T 6134T>A 6135C>T), S1670A (6136T>G 6138T>A)								
Codon mutations:	GCC1569..G (5835C>G), GTC1571TTC (5839G>T), TCC1573TCA (5847C>A), TTC1575ATA (5851T>A 5853C>A), CTG1577CTT (5859G>T), AAG1578CGT (5860A>C 5861A>G 5862G>T), AGC1579TCT (5863A>T 5864G>C 5865C>T), GGA1580GGG (5868A>G), TTC1581TAT (5870T>A 5871C>T), ATT1584TTG (5878A>T 5880T>G), ATG1585AAA (5882T>A 5883G>A), ATG1586GTT (5884A>G 5886G>T), GAC1587AGG (5887G>A 5888A>G 5889C>G), GAA1589AGT (5893G>A 5894A>G 5895A>T), TCC1590GAT (5896T>G 5897C>A 5898C>T), ATC1591ATT (5901C>T), CCT1592CCC (5904T>C), TGG1593AAG (5905T>A 5906G>A), ACC1594ACA (5910C>A), GCC1595ACA (5911G>A 5913C>A), CTG1597CGG (5918T>G), ACC1598ACA (5922C>A), CCA1599AGG (5923C>A 5924C>G 5925A>G), GAT1600TAT (5926G>T), CTT1602CAC (5933T>A 5934T>C), TAC1603TTT (5936A>T 5937C>T), GAA1604AAA (5938G>A), TGG1605TTT (5942G>T 5943G>T), TTA1606CTC (5944T>C 5946A>C), GTT1607GTC (5949T>C), CCA1609TCC (5953C>T 5955A>C), TTC1610TTT (5958C>T), GGA1611GGT (5961A>T), TTG1612TTA (5964G>A), AAG1613ACT (5966A>C 5967G>T), AAT1614AAC (5970T>C), GCC1615ACT (5971G>A 5973C>T), GTG1618GTC (5982G>C), TTC1619TTT (5985C>T), CAA1620ATG (5986C>A 5987A>T 5988A>G), CGA1621GAT (5989C>G 5990G>A 5991A>T), AAG1622CTT (5992A>C 5993A>T 5994G>T), ATG1623ATA (5997G>A), GAC1624AAT (5998G>A 6000C>T), AAC1625AGG (6002A>G 6003C>G), TGT1626ATA (6004T>A 6005G>T 6006T>A), AGA1628AAG (6011G>A 6012A>G), GAC1629CCC (6013G>C 6014A>C), GAC1629_CTG1630insCAT (6015_6016insCAT), CTG1630CTC (6018G>C), TCA1631GAT (6019T>G 6020C>A 6021A>T), GGC1632ATG (6022G>A 6023G>T 6024C>G), TTT1633TAT (6026T>A), GTA1634GTG (6030A>G), GCA1635GTG (6032C>T 6033A>G), GTT1636GTG (6036T>G), TAC1637TTT (6038A>T 6039C>T), TTC1644TAT (6059T>A 6060C>T), AGT1645TCA (6061A>T 6062G>C 6063T>A), AAC1646AGA (6065A>G 6066C>A), ACT1647GAC (6067A>G 6068C>A 6069T>C), GAA1648AAG (6070G>A 6072A>G), GAT1649CGA (6073G>C 6074A>G 6075T>A), GAC1650GAG (6078C>G), CAT1651CAC (6081T>C), GCA1652ATA (6082G>A 6083C>T), CAG1653GAG (6085C>G), CAC1654AAC (6088C>A), AAG1656AGA (6095A>G 6096G>A), TCC1657ACT (6097T>A 6099C>T), ATG1658GTG (6100A>G), TGT1662TTG (6113G>T 6114T>G), GAG1663del (6115_6117delGAG), GAG1665GAA (6123G>A), GGG1666GAA (6125G>A 6126G>A), CTG1667CAG (6128T>A), ATC1668TTA (6130A>T 6132C>A), CTC1669TAT (6133C>T 6134T>A 6135C>T), TCT1670GCA (6136T>G 6138T>A), CCT1671AA. (6139C>A 6140C>A)								

*: Inserts / Deletes / Misaligned / Frameshifts

Analysis details

This analysis was performed with panviral2.64

NGS Details (UN9): Caulimovirus glycinis

Assembly

**Coverage Length**	418 (1 contig(s))
**Depth Of Coverage**	13.0
**Number Of Reads**	44
**Reads Per Million**	0.99 rpm (after QC)
**Ambiguities**	0
**Assembly Method**	de novo + reference guided assembly
**Consensus Caller**	Bcf Tools

Coverage Map

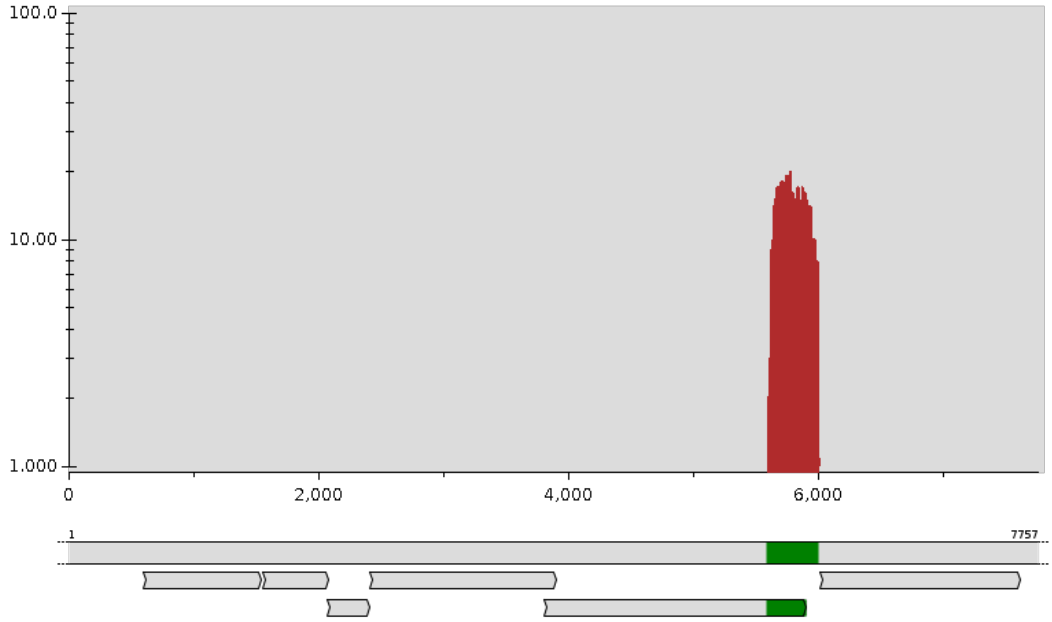

Assignment

**Type**	Caulimovirus glycinis (Taxonomy ID: 3048324)
**Reference Genome**	NC_018505.1
**NT Identity (%)**	57.2127
**AA Identity (%)**	43.2692
**Number Of Stop Codons**	2
**Number Of CDS**	6

Alignment

**Alignment Score**	73.0 (NT) + 321.0 (AA) = 394.0
**Concordance (%)**	26.041

Alignment Method	Global, seeded, nucleotide + amino acids (AGA)

Genome Region

Sequence starts at position 5592 and ends at position 6009 relative to NC_018505.1 reference sequence.

Alignment Detailed Statistics

	Begin	End	Coverage	Score	Concordance	Matches	Identities	I/D/M/F*	Stop Codons
**NT**	**5592**	**6009**	**5.4%**	**73**	**9.4%**	**405 (96.0%)**	**234 (55.5%)**	**4/13**	
Mutations:	5595T>A, 5599A>T, 5611A>T, 5612T>A, 5613C>G, 5617T>A, 5618T>A, 5620A>C, 5623C>T, 5626A>T, 5628C>A, 5630G>A, 5632T>A, 5636T>A, 5637T>C, 5638A>T, 5640A>G, 5641C>G, 5644T>C, 5645C>T, 5646A>T, 5647T>A, 5648T>A, 5650C>A, 5659G>A, 5663A>T, 5665T>A, 5668G>A, 5672G>A, 5674T>A, 5675A>G, 5677G>A, 5679A>G, 5680T>A, 5681A>G, 5682C>G, 5683A>C, 5686T>A, 5687A>C, 5688A>G, 5689G>A, 5692A>G, 5696A>T, 5700G>C, 5701T>A, 5703A>T, 5704T>C, 5705C>T, 5707T>A, 5708A>C, 5709C>T, 5713T>A, 5714G>A, 5715T>A, 5716A>G, 5717A>C, 5723T>A, 5725A>C, 5728A>C, 5729C>A, 5732A>T, 5734A>G, 5737T>C, 5742A>C, 5744A>C, 5746T>G, 5747T>G, 5749T>A, 5750A>T, 5752C>A, 5753C>G, 5754A>G, 5756T>C, 5758T>A, 5759C>A, 5761T>A, 5762A>T, 5763A>T, 5764A>C, 5766T>A, 5770C>A, 5774A>C, 5775A>C, 5777G>T, 5778G>C, 5782T>A, 5783T>C, 5784A>C, 5786A>C, 5788G>A, 5789C>T, 5791G>T, 5792G>A, 5794T>G, 5801G>C, 5802T>A, 5803A>T, 5809G>A, 5812A>G, 5813A>G, 5815G>A, 5817G>T, 5818G>A, 5821T>A, 5824A>T, 5825A>T, 5826G>T, 5827A>T, 5831G>A, 5832T>C, 5836T>C, 5837G>A, 5840G>A, 5842C>T, 5846C>A, 5849T>A, 5851G>T, 5852T>A, 5853C>A, 5858G>T, 5859A>C, 5860C>T, 5861A>G, 5863A>T, 5881T>C, 5882C>T, 5885A>T, 5887C>A, 5888C>A, 5890T>G, 5891G>A, 5893A>C, 5894T>G, 5899T>A, 5903T>G, 5906T>C, 5907T>A, 5908T>A, 5909C>T, 5911G>A, 5912G>A, 5913T>A, 5915T>G, 5915_5916insAAG, 5917C>T, 5918A>C, 5924T>A, 5925A>G, 5928G>A, 5931T>A, 5935C>T, 5940C>A, 5941T>A, 5944G>A, 5945_5956delAACCTTAACCGG, 5958T>G, 5959C>T, 5961A>T, 5962T>C, 5966G>T, 5969T>A, 5971A>G, 5972C>A, 5976_5977insA, 5980G>A, 5981G>T, 5984C>A, 5985G>A, 5987T>A, 5988T>A, 5989delT, 5993C>A, 5997C>T, 5998T>C, 6002G>T								
**CDS**									
C186_gp5	597	700	14.9%	321	43.4%	104 (100%)	45 (43.3%)	0/0/0/0	2
Protein mutations:	L597* (5595T>A), S605T (5618T>A 5620A>C), K607N (5626A>T), A608E (5628C>A), A609T (5630G>A 5632T>A), L611T (5636T>A 5637T>C 5638A>T), N612R (5640A>G 5641C>G), H614L (5645C>T 5646A>T 5647T>A), S615T (5648T>A 5650C>A), I620L (5663A>T 5665T>A), V623I (5672G>A 5674T>A), M624V (5675A>G 5677G>A), N625R (5679A>G 5680T>A), T626G (5681A>G 5682C>G 5683A>C), K628R (5687A>C 5688A>G 5689G>A), T631S (5696A>T), G632A (5700G>C 5701T>A), Y633F (5703A>T 5704T>C), T635L (5708A>C 5709C>T), V637K (5714G>A 5715T>A 5716A>G), K638Q (5717A>C), L640I (5723T>A 5725A>C), T643S (5732A>T 5734A>G), K646T (5742A>C), N647Q (5744A>C 5746T>G), F648V (5747T>G 5749T>A), T649S (5750A>T 5752C>A), H650G (5753C>G 5754A>G), F651L (5756T>C 5758T>A), L652I (5759C>A 5761T>A), K653F (5762A>T 5763A>T 5764A>C), I654N (5766T>A), N655K (5770C>A), K657P (5774A>C 5775A>C), G658S (5777G>T 5778G>C), D659E (5782T>A), Y660P (5783T>C 5784A>C), K661Q (5786A>C 5788G>A), Q662Y (5789C>T 5791G>T), G663R (5792G>A 5794T>G), V666H (5801G>C 5802T>A 5803A>T), W668* (5809G>A), M670V (5813A>G 5815G>A), W671L (5817G>T 5818G>A), F672L (5821T>A), R674F (5825A>T 5826G>T 5827A>T), V676T (5831G>A 5832T>C), E678K (5837G>A), V679I (5840G>A 5842C>T), H681N (5846C>A), L682I (5849T>A 5851G>T), S683K (5852T>A 5853C>A), D685S (5858G>T 5859A>C 5860C>T), K686D (5861A>G 5863A>T), T694S (5885A>T 5887C>A), E696N (5891G>A 5893A>C), F697V (5894T>G), H698Q (5899T>A), *700G (5903T>G)								
Codon mutations:	TTA597TAA (5595T>A), ATA598ATT (5599A>T), ACA602ACT (5611A>T), TCT603AGT (5612T>A 5613C>G), GGT604GGA (5617T>A), TCA605ACC (5618T>A 5620A>C), TTC606TTT (5623C>T), AAA607AAT (5626A>T), GCA608GAA (5628C>A), GCT609ACA (5630G>A 5632T>A), TTA611ACT (5636T>A 5637T>C 5638A>T), AAC612AGG (5640A>G 5641C>G), TAT613TAC (5644T>C), CAT614TTA (5645C>T 5646A>T 5647T>A), TCC615ACA (5648T>A 5650C>A), AAG618AAA (5659G>A), ATT620TTA (5663A>T 5665T>A), CTG621CTA (5668G>A), GTT623ATA (5672G>A 5674T>A), ATG624GTA (5675A>G 5677G>A), AAT625AGA (5679A>G 5680T>A), ACA626GGC (5681A>G 5682C>G 5683A>C), ATT627ATA (5686T>A), AAG628CGA (5687A>C 5688A>G 5689G>A), AAA629AAG (5692A>G), ACT631TCT (5696A>T), GGT632GCA (5700G>C 5701T>A), TAT633TTC (5703A>T 5704T>C), CTT634TTA (5705C>T 5707T>A), ACA635CTA (5708A>C 5709C>T), CCT636CCA (5713T>A), GTA637AAG (5714G>A 5715T>A 5716A>G), AAA638CAA (5717A>C), TTA640ATC (5723T>A 5725A>C), ATA641ATC (5728A>C), CGA642AGA (5729C>A), ACA643TCG (5732A>T 5734A>G), GAT644GAC (5737T>C), AAG646ACG (5742A>C), AAT647CAG (5744A>C 5746T>G), TTT648GTA (5747T>G 5749T>A), ACC649TCA (5750A>T 5752C>A), CAT650GGT (5753C>G 5754A>G), TTT651CTA (5756T>C 5758T>A), CTT652ATA (5759C>A 5761T>A), AAA653TTC (5762A>T 5763A>T 5764A>C), ATT654AAT (5766T>A), AAC655AAA (5770C>A), AAA657CCA (5774A>C 5775A>C), GGT658TCT (5777G>T 5778G>C), GAT659GAA (5782T>A), TAT660CCT (5783T>C 5784A>C), AAG661CAA (5786A>C 5788G>A), CAG662TAT (5789C>T 5791G>T), GGT663AGG (5792G>A 5794T>G), GTA666CAT (5801G>C 5802T>A 5803A>T), TGG668TGA (5809G>A), CAA669CAG (5812A>G), ATG670GTA (5813A>G 5815G>A), TGG671TTA (5817G>T 5818G>A), TTT672TTA (5821T>A), TCA673TCT (5824A>T), AGA674TTT (5825A>T 5826G>T 5827A>T), GTT676ACT (5831G>A 5832T>C), TTT677TTC (5836T>C), GAA678AAA (5837G>A), GTC679ATT (5840G>A 5842C>T), CAT681AAT (5846C>A), TTG682ATT (5849T>A 5851G>T), TCA683AAA (5852T>A 5853C>A), GAC685TCT (5858G>T 5859A>C 5860C>T), AAA686GAT (5861A>G 5863A>T), TTT692TTC (5881T>C), CTA693TTA (5882C>T), ACC694TCA (5885A>T 5887C>A), CGT695AGG (5888C>A 5890T>G), GAA696AAC (5891G>A 5893A>C), TTT697GTT (5894T>G), CAT698CAA (5899T>A), TGA700GGA (5903T>G)								
**Proteins**									
reverse transcriptase (YP_006607892.1)	597	700	14.9%	321	43.4%	104 (100%)	45 (43.3%)	0/0/0/0	2
Protein mutations:	L597* (5595T>A), S605T (5618T>A 5620A>C), K607N (5626A>T), A608E (5628C>A), A609T (5630G>A 5632T>A), L611T (5636T>A 5637T>C 5638A>T), N612R (5640A>G 5641C>G), H614L (5645C>T 5646A>T 5647T>A), S615T (5648T>A 5650C>A), I620L (5663A>T 5665T>A), V623I (5672G>A 5674T>A), M624V (5675A>G 5677G>A), N625R (5679A>G 5680T>A), T626G (5681A>G 5682C>G 5683A>C), K628R (5687A>C 5688A>G 5689G>A), T631S (5696A>T), G632A (5700G>C 5701T>A), Y633F (5703A>T 5704T>C), T635L (5708A>C 5709C>T), V637K (5714G>A 5715T>A 5716A>G), K638Q (5717A>C), L640I (5723T>A 5725A>C), T643S (5732A>T 5734A>G), K646T (5742A>C), N647Q (5744A>C 5746T>G), F648V (5747T>G 5749T>A), T649S (5750A>T 5752C>A), H650G (5753C>G 5754A>G), F651L (5756T>C 5758T>A), L652I (5759C>A 5761T>A), K653F (5762A>T 5763A>T 5764A>C), I654N (5766T>A), N655K (5770C>A), K657P (5774A>C 5775A>C), G658S (5777G>T 5778G>C), D659E (5782T>A), Y660P (5783T>C 5784A>C), K661Q (5786A>C 5788G>A), Q662Y (5789C>T 5791G>T), G663R (5792G>A 5794T>G), V666H (5801G>C 5802T>A 5803A>T), W668* (5809G>A), M670V (5813A>G 5815G>A), W671L (5817G>T 5818G>A), F672L (5821T>A), R674F (5825A>T 5826G>T 5827A>T), V676T (5831G>A 5832T>C), E678K (5837G>A), V679I (5840G>A 5842C>T), H681N (5846C>A), L682I (5849T>A 5851G>T), S683K (5852T>A 5853C>A), D685S (5858G>T 5859A>C 5860C>T), K686D (5861A>G 5863A>T), T694S (5885A>T 5887C>A), E696N (5891G>A 5893A>C), F697V (5894T>G), H698Q (5899T>A), *700G (5903T>G)								
Codon mutations:	TTA597TAA (5595T>A), ATA598ATT (5599A>T), ACA602ACT (5611A>T), TCT603AGT (5612T>A 5613C>G), GGT604GGA (5617T>A), TCA605ACC (5618T>A 5620A>C), TTC606TTT (5623C>T), AAA607AAT (5626A>T), GCA608GAA (5628C>A), GCT609ACA (5630G>A 5632T>A), TTA611ACT (5636T>A 5637T>C 5638A>T), AAC612AGG (5640A>G 5641C>G), TAT613TAC (5644T>C), CAT614TTA (5645C>T 5646A>T 5647T>A), TCC615ACA (5648T>A 5650C>A), AAG618AAA (5659G>A), ATT620TTA (5663A>T 5665T>A), CTG621CTA (5668G>A), GTT623ATA (5672G>A 5674T>A), ATG624GTA (5675A>G 5677G>A), AAT625AGA (5679A>G 5680T>A), ACA626GGC (5681A>G 5682C>G 5683A>C), ATT627ATA (5686T>A), AAG628CGA (5687A>C 5688A>G 5689G>A), AAA629AAG (5692A>G), ACT631TCT (5696A>T), GGT632GCA (5700G>C 5701T>A), TAT633TTC (5703A>T 5704T>C), CTT634TTA (5705C>T 5707T>A), ACA635CTA (5708A>C 5709C>T), CCT636CCA (5713T>A), GTA637AAG (5714G>A 5715T>A 5716A>G), AAA638CAA (5717A>C), TTA640ATC (5723T>A 5725A>C), ATA641ATC (5728A>C), CGA642AGA (5729C>A), ACA643TCG (5732A>T 5734A>G), GAT644GAC (5737T>C), AAG646ACG (5742A>C), AAT647CAG (5744A>C 5746T>G), TTT648GTA (5747T>G 5749T>A), ACC649TCA (5750A>T 5752C>A), CAT650GGT (5753C>G 5754A>G), TTT651CTA (5756T>C 5758T>A), CTT652ATA (5759C>A 5761T>A), AAA653TTC (5762A>T 5763A>T 5764A>C), ATT654AAT (5766T>A), AAC655AAA (5770C>A), AAA657CCA (5774A>C 5775A>C), GGT658TCT (5777G>T 5778G>C), GAT659GAA (5782T>A), TAT660CCT (5783T>C 5784A>C), AAG661CAA (5786A>C 5788G>A), CAG662TAT (5789C>T 5791G>T), GGT663AGG (5792G>A 5794T>G), GTA666CAT (5801G>C 5802T>A 5803A>T), TGG668TGA (5809G>A), CAA669CAG (5812A>G), ATG670GTA (5813A>G 5815G>A), TGG671TTA (5817G>T 5818G>A), TTT672TTA (5821T>A), TCA673TCT (5824A>T), AGA674TTT (5825A>T 5826G>T 5827A>T), GTT676ACT (5831G>A 5832T>C), TTT677TTC (5836T>C), GAA678AAA (5837G>A), GTC679ATT (5840G>A 5842C>T), CAT681AAT (5846C>A), TTG682ATT (5849T>A 5851G>T), TCA683AAA (5852T>A 5853C>A), GAC685TCT (5858G>T 5859A>C 5860C>T), AAA686GAT (5861A>G 5863A>T), TTT692TTC (5881T>C), CTA693TTA (5882C>T), ACC694TCA (5885A>T 5887C>A), CGT695AGG (5888C>A 5890T>G), GAA696AAC (5891G>A 5893A>C), TTT697GTT (5894T>G), CAT698CAA (5899T>A), TGA700GGA (5903T>G)								

*: Inserts / Deletes / Misaligned / Frameshifts

Analysis details

This analysis was performed with panviral2.64

NGS Details (UN9): Caulimovirus venafragariae

Assembly

**Coverage Length**	798 (2 contig(s))
**Depth Of Coverage**	6.4
**Number Of Reads**	41
**Reads Per Million**	0.93 rpm (after QC)
**Ambiguities**	0
**Assembly Method**	de novo + reference guided assembly
**Consensus Caller**	Bcf Tools

Coverage Map

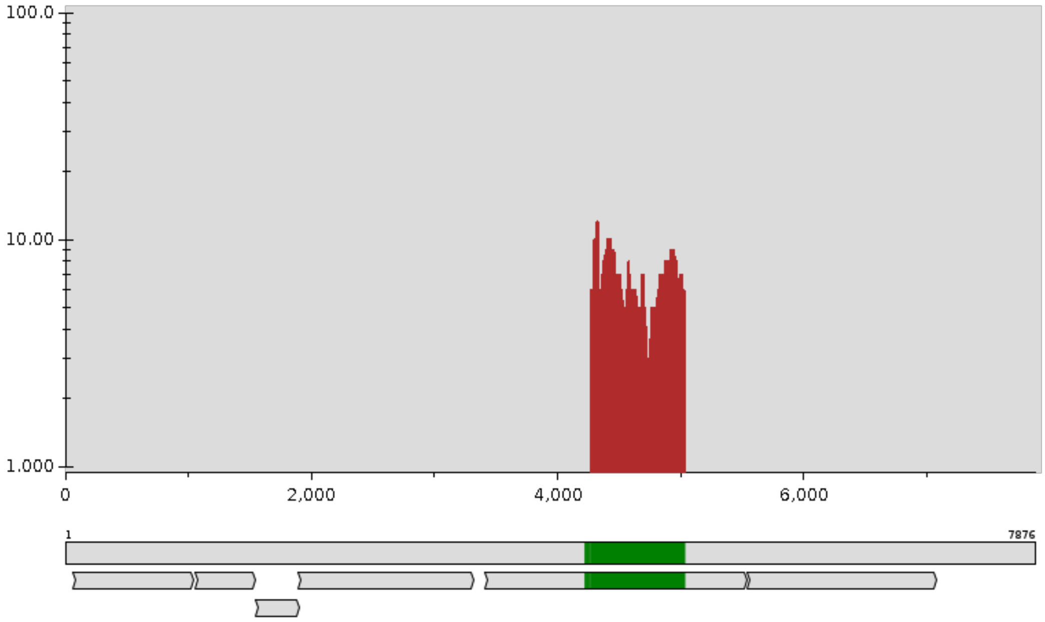

Assignment

**Type**	Caulimovirus venafragariae (Taxonomy ID: 3048344)
**Reference Genome**	NC_001725.1
**NT Identity (%)**	56.2025
**AA Identity (%)**	54.4747
**Number Of Stop Codons**	1
**Number Of CDS**	6

Alignment

**Alignment Score**	170.0 (NT) + 883.0 (AA) = 1053.0
**Concordance (%)**	32.9062

Alignment Method	Global, seeded, nucleotide + amino acids (AGA)

Genome Region

Sequence starts at position 4219 and ends at position 5042 relative to NC_001725.1 reference sequence.

Alignment Detailed Statistics

	Begin	End	Coverage	Score	Concordance	Matches	Identities	I/D/M/F*	Stop Codons
**NT**	**4219**	**5042**	**10.1%**	**170**	**10.9%**	**790 (99.0%)**	**444 (55.6%)**	**0/8**	
Mutations:	4219T>C, 4222T>A, 4223T>A, 4227C>T, 4228A>C, 4230T>C, 4232A>G, 4235A>T, 4236A>C, 4239T>A, 4242T>A, 4244A>G, 4245G>A, 4246T>G, 4247G>T, 4254A>T, 4259A>T, 4260G>A, 4263T>C, 4264T>A, 4265C>A, 4266A>T, 4267C>G, 4268C>A, 4269T>A, 4275C>T, 4276C>A, 4277G>A, 4279G>A, 4280A>C, 4281A>T, 4282G>A, 4284G>A, 4287T>C, 4289A>G, 4292C>T, 4299C>T, 4300G>C, 4308A>T, 4314G>A, 4315C>T, 4317T>A, 4318G>A, 4319G>A, 4320T>A, 4321A>T, 4322T>A, 4323C>A, 4326C>A, 4330C>G, 4331C>A, 4332T>A, 4335C>T, 4348T>A, 4349C>G, 4351T>A, 4352C>G, 4353A>T, 4356A>T, 4359C>A, 4362C>T, 4366G>A, 4371G>A, 4380C>G, 4383G>A, 4384A>C, 4385T>A, 4386C>A, 4390C>A, 4395C>A, 4398A>G, 4401A>T, 4402A>C, 4410A>T, 4413T>A, 4423A>G, 4426T>C, 4428A>T, 4432G>A, 4434C>A, 4435C>A, 4437T>C, 4438A>T, 4439C>G, 4440A>T, 4442A>T, 4443G>T, 4444G>T, 4445G>T, 4446A>T, 4449T>C, 4452C>A, 4456C>T, 4458A>T, 4459C>A, 4461T>C, 4467C>T, 4470G>A, 4471G>A, 4472A>C, 4473G>T, 4474C>A, 4476A>T, 4479G>T, 4480C>A, 4482T>A, 4483C>A, 4485A>T, 4486A>C, 4487G>T, 4489A>G, 4492G>A, 4493G>A, 4496G>T, 4497T>A, 4500G>A, 4505T>A, 4506T>C, 4508A>T, 4509C>T, 4512T>C, 4513T>A, 4514C>A, 4515C>A, 4521C>T, 4528T>A, 4529C>G, 4543G>A, 4546C>A, 4548C>A, 4551T>C, 4552G>T, 4554T>A, 4555C>G, 4556C>A, 4557A>G, 4561A>T, 4568A>C, 4569G>T, 4575C>A, 4578T>A, 4585T>A, 4586G>C, 4590C>T, 4591C>A, 4593A>T, 4599C>T, 4602C>T, 4614C>A, 4618C>T, 4620T>A, 4633C>A, 4635A>T, 4638C>A, 4641T>A, 4642G>C, 4643C>A, 4644T>A, 4650C>T, 4656G>A, 4657C>A, 4659C>A, 4663G>T, 4665C>T, 4666G>A, 4670G>T, 4671T>A, 4672C>T, 4674C>T, 4675_4679delAGCAA, 4680C>T, 4681A>C, 4684T>A, 4686T>A, 4687C>A, 4688C>A, 4689A>G, 4690C>A, 4692G>A, 4697G>T, 4698T>G, 4699G>A, 4700C>T, 4701T>A, 4707T>C, 4708G>A, 4710T>A, 4719C>T, 4720A>T, 4722C>G, 4725C>A, 4727T>G, 4729A>T, 4730G>C, 4731C>A, 4733A>C, 4737T>A, 4738G>T, 4740A>T, 4741G>C, 4743A>G, 4750T>C, 4752A>T, 4753G>A, 4754G>C, 4759G>C, 4761C>T, 4765A>G, 4766T>A, 4767T>A, 4768G>T, 4771C>T, 4772T>C, 4776C>A, 4777A>C, 4778G>T, 4779A>C, 4782C>T, 4783A>G, 4784A>T, 4785A>C, 4786G>A, 4787C>A, 4788C>T, 4789C>A, 4790T>A, 4791A>T, 4793G>A, 4794A>C, 4800G>A, 4801C>T, 4803T>A, 4806C>T, 4807A>C, 4809A>G, 4812G>A, 4818C>A, 4819C>G, 4821G>A, 4822T>A, 4824G>A, 4825T>A, 4826G>A, 4827C>T, 4832C>A, 4833A>T, 4835C>A, 4836T>A, 4839C>A, 4840A>G, 4842T>A, 4851T>A, 4852C>A, 4854A>G, 4855G>A, 4857A>T, 4860A>T, 4861G>T, 4862A>C, 4865G>A, 4867G>A, 4868G>A, 4869A>T, 4870A>G, 4871A>G, 4872T>A, 4873C>A, 4875C>A, 4876A>G, 4879G>T, 4881C>A, 4884G>A, 4886G>C, 4890T>C, 4893C>A, 4894G>T, 4895G>C, 4896A>C, 4897C>A, 4898T>A, 4900C>A, 4902C>G, 4903T>A, 4906G>T, 4908T>A, 4910C>A, 4911T>G, 4917A>T, 4920T>C, 4921C>A, 4923A>G, 4924C>T, 4926T>A, 4928G>T, 4929T>A, 4930G>A, 4931A>C, 4932T>C, 4934G>A, 4935G>A, 4936A>C, 4938T>A, 4940C>A, 4941C>A, 4942T>A, 4954T>C, 4956A>T, 4959C>A, 4960C>T, 4961T>G, 4963C>T, 4968T>C, 4972A>G, 4973T>C, 4974C>A, 4975T>G, 4976C>G, 4977C>A, 4979C>A, 4980T>A, 4982A>T, 4983T>C, 4984T>A, 4986T>A, 4990A>G, 4993A>T, 5001C>G, 5002C>A, 5003T>A, 5004T>G, 5007G>A, 5008T>A, 5009C>A, 5010A>T, 5012C>T, 5014T>C, 5016A>T, 5019G>A, 5020_5022delGTT, 5028T>C, 5030A>T, 5031G>C, 5033A>G, 5035G>A, 5037G>A, 5038A>T, 5039T>C, 5042C>A								

CDS

	Begin	End	Coverage	Score	Concordance	Matches	Identities	I/D/M/F*	Stop Codons
ORF_V	287	546	36.5%	883	47.1%	257 (99.2%)	140 (54.1%)	0/2/1/1	1
Protein mutations:	S287N (4264T>A 4265C>A 4266A>T), P288E (4267C>G 4268C>A 4269T>A), R291K (4276C>A 4277G>A), E292T (4279G>A 4280A>C 4281A>T), E293K (4282G>A 4284G>A), K295R (4289A>G), T296I (4292C>T), E299Q (4300G>C), G305K (4318G>A 4319G>A 4320T>A), I306* (4321A>T 4322T>A 4323C>A), P309E (4330C>G 4331C>A 4332T>A), V321I (4366G>A), I327Q (4384A>C 4385T>A 4386C>A), K340E (4423A>G), D343K (4432G>A 4434C>A), H344N (4435C>A 4437T>C), T345C (4438A>T 4439C>G 4440A>T), K346I (4442A>T 4443G>T), G347F (4444G>T 4445G>T 4446A>T), L351F (4456C>T 4458A>T), L352I (4459C>A 4461T>C), E356T (4471G>A 4472A>C 4473G>T), Q357N (4474C>A 4476A>T), L359I (4480C>A 4482T>A), Q360N (4483C>A 4485A>T), R361L (4486A>C 4487G>T), I362V (4489A>G), G363K (4492G>A 4493G>A), G364V (4496G>T 4497T>A), F367Y (4505T>A 4506T>C), Y368F (4508A>T 4509C>T), S370K (4513T>A 4514C>A 4515C>A), V380I (4543G>A), A383S (4552G>T 4554T>A), P384E (4555C>G 4556C>A 4557A>G), T386S (4561A>T), Q388P (4568A>C 4569G>T), C394T (4585T>A 4586G>C), Q396N (4591C>A 4593A>T), P405S (4618C>T 4620T>A), Q410N (4633C>A 4635A>T), A413Q (4642G>C 4643C>A 4644T>A), H418K (4657C>A 4659C>A), D420Y (4663G>T 4665C>T), E421K (4666G>A), S422I (4670G>T 4671T>A), L423F (4672C>T 4674C>T), S424del (4675_4679delAGCAA), M426L (4681A>C), Y427K (4684T>A 4686T>A), P428K (4687C>A 4688C>A 4689A>G), Q429K (4690C>A 4692G>A), C431L (4697G>T 4698T>G), A432I (4699G>A 4700C>T 4701T>A), V435I (4708G>A 4710T>A), I439L (4720A>T 4722C>G), F441C (4727T>G), K443T (4733A>C), E445Y (4738G>T 4740A>T), E446Q (4741G>C 4743A>G), G450T (4753G>A 4754G>C), V452L (4759G>C 4761C>T), I454E (4765A>G 4766T>A 4767T>A), V455F (4768G>T), L456S (4771C>T 4772T>C), N457K (4776C>A), R458L (4777A>C 4778G>T 4779A>C), K460V (4783A>G 4784A>T 4785A>C), A461N (4786G>A 4787C>A 4788C>T), L462N (4789C>A 4790T>A 4791A>T), G463D (4793G>A 4794A>C), K468Q (4807A>C 4809A>G), Q472E (4819C>G 4821G>A), L473I (4822T>A 4824G>A), C474N (4825T>A 4826G>A 4827C>T), T476N (4832C>A 4833A>T), T477K (4835C>A 4836T>A), N479E (4840A>G 4842T>A), L483M (4852C>A 4854A>G), V484I (4855G>A 4857A>T), E486S (4861G>T 4862A>C), R487K (4865G>A), G488N (4867G>A 4868G>A 4869A>T), N489G (4870A>G 4871A>G 4872T>A), L490I (4873C>A 4875C>A), K491E (4876A>G), V492L (4879G>T 4881C>A), S494T (4886G>C), G497S (4894G>T 4895G>C 4896A>C), L498K (4897C>A 4898T>A), H499K (4900C>A 4902C>G), L500I (4903T>A), V501L (4906G>T 4908T>A), A502E (4910C>A 4911T>G), Q506K (4921C>A 4923A>G), S508I (4928G>T 4929T>A), D509T (4930G>A 4931A>C 4932T>C), R510K (4934G>A 4935G>A), N511Q (4936A>C 4938T>A), A512E (4940C>A 4941C>A), L513I (4942T>A), L519C (4960C>T 4961T>G), I523A (4972A>G 4973T>C 4974C>A), S524G (4975T>G 4976C>G 4977C>A), A525E (4979C>A 4980T>A), Y526F (4982A>T 4983T>C), F527I (4984T>A 4986T>A), K529E (4990A>G), I530L (4993A>T), N532K (5001C>G), L533K (5002C>A 5003T>A 5004T>G), S535N (5008T>A 5009C>A 5010A>T), P536L (5012C>T), V539del (5020_5022delGTT), K542I (5030A>T 5031G>C), K543R (5033A>G), E544K (5035G>A 5037G>A), I545S (5038A>T 5039T>C)								
Codon mutations:	TTA272C.. (4219T>C), TTA273AA. (4222T>A 4223T>A), GAC274..T (4227C>T), ACT275C.C (4228A>C 4230T>C), AAC276.G. (4232A>G), AAA277.TC (4235A>T 4236A>C), ATT278..A (4239T>A), ATT279..A (4242T>A), AAG280.GA (4244A>G 4245G>A), TGT281GT. (4246T>G 4247G>T), CCA283..T (4254A>T), CAG285.TA (4259A>T 4260G>A), TAT286..C (4263T>C), TCA287AAT (4264T>A 4265C>A 4266A>T), CCT288GAA (4267C>G 4268C>A 4269T>A), GAC290GAT (4275C>T), CGA291AAA (4276C>A 4277G>A), GAA292ACT (4279G>A 4280A>C 4281A>T), GAG293AAA (4282G>A 4284G>A), TTT294TTC (4287T>C), AAA295AGA (4289A>G), ACT296ATT (4292C>T), ATC298ATT (4299C>T), GAA299CAA (4300G>C), CTA301CTT (4308A>T), AAG303AAA (4314G>A), CTT304TTA (4315C>T 4317T>A), GGT305AAA (4318G>A 4319G>A 4320T>A), ATC306TAA (4321A>T 4322T>A 4323C>A), ATC307ATA (4326C>A), CCT309GAA (4330C>G 4331C>A 4332T>A), AGC310AGT (4335C>T), TCC315AGC (4348T>A 4349C>G), TCA316AGT (4351T>A 4352C>G 4353A>T), CCA317CCT (4356A>T), GCC318GCA (4359C>A), TTC319TTT (4362C>T), GTA321ATA (4366G>A), AGG322AGA (4371G>A), GCC325GCG (4380C>G), GAG326GAA (4383G>A), ATC327CAA (4384A>C 4385T>A 4386C>A), CGA329AGA (4390C>A), GGC330GGA (4395C>A), AAA331AAG (4398A>G), GCA332GCT (4401A>T), AGA333CGA (4402A>C), GTA335GTT (4410A>T), ATT336ATA (4413T>A), AAG340GAG (4423A>G), TTA341CTT (4426T>C 4428A>T), GAC343AAA (4432G>A 4434C>A), CAT344AAC (4435C>A 4437T>C), ACA345TGT (4438A>T 4439C>G 4440A>T), AAG346ATT (4442A>T 4443G>T), GGA347TTT (4444G>T 4445G>T 4446A>T), GAT348GAC (4449T>C), GGC349GGA (4452C>A), CTA351TTT (4456C>T 4458A>T), CTT352ATC (4459C>A 4461T>C), AAC354AAT (4467C>T), AAG355AAA (4470G>A), GAG356ACT (4471G>A 4472A>C 4473G>T), CAA357AAT (4474C>A 4476A>T), CTG358CTT (4479G>T), CTT359ATA (4480C>A 4482T>A), CAA360AAT (4483C>A 4485A>T), AGA361CTA (4486A>C 4487G>T), ATC362GTC (4489A>G), GGA363AAA (4492G>A 4493G>A), GGT364GTA (4496G>T 4497T>A), AAG365AAA (4500G>A), TTT367TAC (4505T>A 4506T>C), TAC368TTT (4508A>T 4509C>T), TCT369TCC (4512T>C), TCC370AAA (4513T>A 4514C>A 4515C>A), GAC372GAT (4521C>T), TCT375AGT (4528T>A 4529C>G), GTA380ATA (4543G>A), CGC381AGA (4546C>A 4548C>A), CTT382CTC (4551T>C), GCT383TCA (4552G>T 4554T>A), CCA384GAG (4555C>G 4556C>A 4557A>G), ACA386TCA (4561A>T), CAG388CCT (4568A>C 4569G>T), ACC390ACA (4575C>A), GCT391GCA (4578T>A), TGT394ACT (4585T>A 4586G>C), CCC395CCT (4590C>T), CAA396AAT (4591C>A 4593A>T), CAC398CAT (4599C>T), TAC399TAT (4602C>T), GTC403GTA (4614C>A), CCT405TCA (4618C>T 4620T>A), CAA410AAT (4633C>A 4635A>T), GCC411GCA (4638C>A), CCT412CCA (4641T>A), GCT413CAA (4642G>C 4643C>A 4644T>A), TTC415TTT (4650C>T), AGG417AGA (4656G>A), CAC418AAA (4657C>A 4659C>A), GAC420TAT (4663G>T 4665C>T), GAA421AAA (4666G>A), AGT422ATA (4670G>T 4671T>A), CTC423TTT (4672C>T 4674C>T), AGC424del (4675_4679delAGCAA), AAC425--T (4675_4679delAGCAA 4680C>T), ATG426CTG (4681A>C), TAT427AAA (4684T>A 4686T>A), CCA428AAG (4687C>A 4688C>A 4689A>G), CAG429AAA (4690C>A 4692G>A), TGT431TTG (4697G>T 4698T>G), GCT432ATA (4699G>A 4700C>T 4701T>A), TAT434TAC (4707T>C), GTT435ATA (4708G>A 4710T>A), ATC438ATT (4719C>T), ATC439TTG (4720A>T 4722C>G), GTC440GTA (4725C>A), TTC441TGC (4727T>G), AGC442TCA (4729A>T 4730G>C 4731C>A), AAA443ACA (4733A>C), ACT444ACA (4737T>A), GAA445TAT (4738G>T 4740A>T), GAA446CAG (4741G>C 4743A>G), TTA449CTT (4750T>C 4752A>T), GGA450ACA (4753G>A 4754G>C), GTC452CTT (4759G>C 4761C>T), ATT454GAA (4765A>G 4766T>A 4767T>A), GTT455TTT (4768G>T), CTA456TCA (4771C>T 4772T>C), AAC457AAA (4776C>A), AGA458CTC (4777A>C 4778G>T 4779A>C), TGC459TGT (4782C>T), AAA460GTC (4783A>G 4784A>T 4785A>C), GCC461AAT (4786G>A 4787C>A 4788C>T), CTA462AAT (4789C>A 4790T>A 4791A>T), GGA463GAC (4793G>A 4794A>C), GTG465GTA (4800G>A), CTT466TTA (4801C>T 4803T>A), AGC467AGT (4806C>T), AAA468CAG (4807A>C 4809A>G), AAG469AAA (4812G>A), GCC471GCA (4818C>A), CAG472GAA (4819C>G 4821G>A), TTG473ATA (4822T>A 4824G>A), TGC474AAT (4825T>A 4826G>A 4827C>T), ACA476AAT (4832C>A 4833A>T), ACT477AAA (4835C>A 4836T>A), ATC478ATA (4839C>A), AAT479GAA (4840A>G 4842T>A), GGT482GGA (4851T>A), CTA483ATG (4852C>A 4854A>G), GTA484ATT (4855G>A 4857A>T), ATA485ATT (4860A>T), GAA486TCA (4861G>T 4862A>C), AGA487AAA (4865G>A), GGA488AAT (4867G>A 4868G>A 4869A>T), AAT489GGA (4870A>G 4871A>G 4872T>A), CTC490ATA (4873C>A 4875C>A), AAA491GAA (4876A>G), GTC492TTA (4879G>T 4881C>A), CAG493CAA (4884G>A), AGT494ACT (4886G>C), CAT495CAC (4890T>C), ATC496ATA (4893C>A), GGA497TCC (4894G>T 4895G>C 4896A>C), CTA498AAA (4897C>A 4898T>A), CAC499AAG (4900C>A 4902C>G), TTA500ATA (4903T>A), GTT501TTA (4906G>T 4908T>A), GCT502GAG (4910C>A 4911T>G), CCA504CCT (4917A>T), GAT505GAC (4920T>C), CAA506AAG (4921C>A 4923A>G), CTT507TTA (4924C>T 4926T>A), AGT508ATA (4928G>T 4929T>A), GAT509ACC (4930G>A 4931A>C 4932T>C), AGG510AAA (4934G>A 4935G>A), AAT511CAA (4936A>C 4938T>A), GCC512GAA (4940C>A 4941C>A), TTA513ATA (4942T>A), TTA517CTT (4954T>C 4956A>T), GGC518GGA (4959C>A), CTC519TGC (4960C>T 4961T>G), CTA520TTA (4963C>T), AAT521AAC (4968T>C), ATC523GCA (4972A>G 4973T>C 4974C>A), TCC524GGA (4975T>G 4976C>G 4977C>A), GCT525GAA (4979C>A 4980T>A), TAT526TTC (4982A>T 4983T>C), TTT527ATA (4984T>A 4986T>A), AAA529GAA (4990A>G), ATA530TTA (4993A>T), AAC532AAG (5001C>G), CTT533AAG (5002C>A 5003T>A 5004T>G), AGG534AGA (5007G>A), TCA535AAT (5008T>A 5009C>A 5010A>T), CCT536CTT (5012C>T), TTA537CTT (5014T>C 5016A>T), CAG538CAA (5019G>A), GTT539del (5020_5022delGTT), CTT541CTC (5028T>C), AAG542ATC (5030A>T 5031G>C), AAA543AGA (5033A>G), GAG544AAA (5035G>A 5037G>A), ATA545TCA (5038A>T 5039T>C), ACT546AA. (5042C>A)								

Proteins

	Begin	End	Coverage	Score	Concordance	Matches	Identities	I/D/M/F*	Stop Codons
hypothetical protein (NP_043933.1)	287	546	36.5%	883	47.1%	257 (99.2%)	140 (54.1%)	0/2/1/1	1

	Begin	End	Coverage	Score	Concordance	Matches	Identities	I/D/M/F*	Stop Codons
**NT**	**4219**	**5042**	**10.1%**	**170**	**10.9%**	**790 (99.0%)**	**444 (55.6%)**	**0/8**	
Protein mutations:	S287N (4264T>A 4265C>A 4266A>T), P288E (4267C>G 4268C>A 4269T>A), R291K (4276C>A 4277G>A), E292T (4279G>A 4280A>C 4281A>T), E293K (4282G>A 4284G>A), K295R (4289A>G), T296I (4292C>T), E299Q (4300G>C), G305K (4318G>A 4319G>A 4320T>A), I306* (4321A>T 4322T>A 4323C>A), P309E (4330C>G 4331C>A 4332T>A), V321I (4366G>A), I327Q (4384A>C 4385T>A 4386C>A), K340E (4423A>G), D343K (4432G>A 4434C>A), H344N (4435C>A 4437T>C), T345C (4438A>T 4439C>G 4440A>T), K346I (4442A>T 4443G>T), G347F (4444G>T 4445G>T 4446A>T), L351F (4456C>T 4458A>T), L352I (4459C>A 4461T>C), E356T (4471G>A 4472A>C 4473G>T), Q357N (4474C>A 4476A>T), L359I (4480C>A 4482T>A), Q360N (4483C>A 4485A>T), R361L (4486A>C 4487G>T), I362V (4489A>G), G363K (4492G>A 4493G>A), G364V (4496G>T 4497T>A), F367Y (4505T>A 4506T>C), Y368F (4508A>T 4509C>T), S370K (4513T>A 4514C>A 4515C>A), V380I (4543G>A), A383S (4552G>T 4554T>A), P384E (4555C>G 4556C>A 4557A>G), T386S (4561A>T), Q388P (4568A>C 4569G>T), C394T (4585T>A 4586G>C), Q396N (4591C>A 4593A>T), P405S (4618C>T 4620T>A), Q410N (4633C>A 4635A>T), A413Q (4642G>C 4643C>A 4644T>A), H418K (4657C>A 4659C>A), D420Y (4663G>T 4665C>T), E421K (4666G>A), S422I (4670G>T 4671T>A), L423F (4672C>T 4674C>T), S424del (4675_4679delAGCAA), M426L (4681A>C), Y427K (4684T>A 4686T>A), P428K (4687C>A 4688C>A 4689A>G), Q429K (4690C>A 4692G>A), C431L (4697G>T 4698T>G), A432I (4699G>A 4700C>T 4701T>A), V435I (4708G>A 4710T>A), I439L (4720A>T 4722C>G), F441C (4727T>G), K443T (4733A>C), E445Y (4738G>T 4740A>T), E446Q (4741G>C 4743A>G), G450T (4753G>A 4754G>C), V452L (4759G>C 4761C>T), I454E (4765A>G 4766T>A 4767T>A), V455F (4768G>T), L456S (4771C>T 4772T>C), N457K (4776C>A), R458L (4777A>C 4778G>T 4779A>C), K460V (4783A>G 4784A>T 4785A>C), A461N (4786G>A 4787C>A 4788C>T), L462N (4789C>A 4790T>A 4791A>T), G463D (4793G>A 4794A>C), K468Q (4807A>C 4809A>G), Q472E (4819C>G 4821G>A), L473I (4822T>A 4824G>A), C474N (4825T>A 4826G>A 4827C>T), T476N (4832C>A 4833A>T), T477K (4835C>A 4836T>A), N479E (4840A>G 4842T>A), L483M (4852C>A 4854A>G), V484I (4855G>A 4857A>T), E486S (4861G>T 4862A>C), R487K (4865G>A), G488N (4867G>A 4868G>A 4869A>T), N489G (4870A>G 4871A>G 4872T>A), L490I (4873C>A 4875C>A), K491E (4876A>G), V492L (4879G>T 4881C>A), S494T (4886G>C), G497S (4894G>T 4895G>C 4896A>C), L498K (4897C>A 4898T>A), H499K (4900C>A 4902C>G), L500I (4903T>A), V501L (4906G>T 4908T>A), A502E (4910C>A 4911T>G), Q506K (4921C>A 4923A>G), S508I (4928G>T 4929T>A), D509T (4930G>A 4931A>C 4932T>C), R510K (4934G>A 4935G>A), N511Q (4936A>C 4938T>A), A512E (4940C>A 4941C>A), L513I (4942T>A), L519C (4960C>T 4961T>G), I523A (4972A>G 4973T>C 4974C>A), S524G (4975T>G 4976C>G 4977C>A), A525E (4979C>A 4980T>A), Y526F (4982A>T 4983T>C), F527I (4984T>A 4986T>A), K529E (4990A>G), I530L (4993A>T), N532K (5001C>G), L533K (5002C>A 5003T>A 5004T>G), S535N (5008T>A 5009C>A 5010A>T), P536L (5012C>T), V539del (5020_5022delGTT), K542I (5030A>T 5031G>C), K543R (5033A>G), E544K (5035G>A 5037G>A), I545S (5038A>T 5039T>C)								
Codon mutations:	TTA272C.. (4219T>C), TTA273AA. (4222T>A 4223T>A), GAC274..T (4227C>T), ACT275C.C (4228A>C 4230T>C), AAC276.G. (4232A>G), AAA277.TC (4235A>T 4236A>C), ATT278..A (4239T>A), ATT279..A (4242T>A), AAG280.GA (4244A>G 4245G>A), TGT281GT. (4246T>G 4247G>T), CCA283..T (4254A>T), CAG285.TA (4259A>T 4260G>A), TAT286..C (4263T>C), TCA287AAT (4264T>A 4265C>A 4266A>T), CCT288GAA (4267C>G 4268C>A 4269T>A), GAC290GAT (4275C>T), CGA291AAA (4276C>A 4277G>A), GAA292ACT (4279G>A 4280A>C 4281A>T), GAG293AAA (4282G>A 4284G>A), TTT294TTC (4287T>C), AAA295AGA (4289A>G), ACT296ATT (4292C>T), ATC298ATT (4299C>T), GAA299CAA (4300G>C), CTA301CTT (4308A>T), AAG303AAA (4314G>A), CTT304TTA (4315C>T 4317T>A), GGT305AAA (4318G>A 4319G>A 4320T>A), ATC306TAA (4321A>T 4322T>A 4323C>A), ATC307ATA (4326C>A), CCT309GAA (4330C>G 4331C>A 4332T>A), AGC310AGT (4335C>T), TCC315AGC (4348T>A 4349C>G), TCA316AGT (4351T>A 4352C>G 4353A>T), CCA317CCT (4356A>T), GCC318GCA (4359C>A), TTC319TTT (4362C>T), GTA321ATA (4366G>A), AGG322AGA (4371G>A), GCC325GCG (4380C>G), GAG326GAA (4383G>A), ATC327CAA (4384A>C 4385T>A 4386C>A), CGA329AGA (4390C>A), GGC330GGA (4395C>A), AAA331AAG (4398A>G), GCA332GCT (4401A>T), AGA333CGA (4402A>C), GTA335GTT (4410A>T), ATT336ATA (4413T>A), AAG340GAG (4423A>G), TTA341CTT (4426T>C 4428A>T), GAC343AAA (4432G>A 4434C>A), CAT344AAC (4435C>A 4437T>C), ACA345TGT (4438A>T 4439C>G 4440A>T), AAG346ATT (4442A>T 4443G>T), GGA347TTT (4444G>T 4445G>T 4446A>T), GAT348GAC (4449T>C), GGC349GGA (4452C>A), CTA351TTT (4456C>T 4458A>T), CTT352ATC (4459C>A 4461T>C), AAC354AAT (4467C>T), AAG355AAA (4470G>A), GAG356ACT (4471G>A 4472A>C 4473G>T), CAA357AAT (4474C>A 4476A>T), CTG358CTT (4479G>T), CTT359ATA (4480C>A 4482T>A), CAA360AAT (4483C>A 4485A>T), AGA361CTA (4486A>C 4487G>T), ATC362GTC (4489A>G), GGA363AAA (4492G>A 4493G>A), GGT364GTA (4496G>T 4497T>A), AAG365AAA (4500G>A), TTT367TAC (4505T>A 4506T>C), TAC368TTT (4508A>T 4509C>T), TCT369TCC (4512T>C), TCC370AAA (4513T>A 4514C>A 4515C>A), GAC372GAT (4521C>T), TCT375AGT (4528T>A 4529C>G), GTA380ATA (4543G>A), CGC381AGA (4546C>A 4548C>A), CTT382CTC (4551T>C), GCT383TCA (4552G>T 4554T>A), CCA384GAG (4555C>G 4556C>A 4557A>G), ACA386TCA (4561A>T), CAG388CCT (4568A>C 4569G>T), ACC390ACA (4575C>A), GCT391GCA (4578T>A), TGT394ACT (4585T>A 4586G>C), CCC395CCT (4590C>T), CAA396AAT (4591C>A 4593A>T), CAC398CAT (4599C>T), TAC399TAT (4602C>T), GTC403GTA (4614C>A), CCT405TCA (4618C>T 4620T>A), CAA410AAT (4633C>A 4635A>T), GCC411GCA (4638C>A), CCT412CCA (4641T>A), GCT413CAA (4642G>C 4643C>A 4644T>A), TTC415TTT (4650C>T), AGG417AGA (4656G>A), CAC418AAA (4657C>A 4659C>A), GAC420TAT (4663G>T 4665C>T), GAA421AAA (4666G>A), AGT422ATA (4670G>T 4671T>A), CTC423TTT (4672C>T 4674C>T), AGC424del (4675_4679delAGCAA), AAC425--T (4675_4679delAGCAA 4680C>T), ATG426CTG (4681A>C), TAT427AAA (4684T>A 4686T>A), CCA428AAG (4687C>A 4688C>A 4689A>G), CAG429AAA (4690C>A 4692G>A), TGT431TTG (4697G>T 4698T>G), GCT432ATA (4699G>A 4700C>T 4701T>A), TAT434TAC (4707T>C), GTT435ATA (4708G>A 4710T>A), ATC438ATT (4719C>T), ATC439TTG (4720A>T 4722C>G), GTC440GTA (4725C>A), TTC441TGC (4727T>G), AGC442TCA (4729A>T 4730G>C 4731C>A), AAA443ACA (4733A>C), ACT444ACA (4737T>A), GAA445TAT (4738G>T 4740A>T), GAA446CAG (4741G>C 4743A>G), TTA449CTT (4750T>C 4752A>T), GGA450ACA (4753G>A 4754G>C), GTC452CTT (4759G>C 4761C>T), ATT454GAA (4765A>G 4766T>A 4767T>A), GTT455TTT (4768G>T), CTA456TCA (4771C>T 4772T>C), AAC457AAA (4776C>A), AGA458CTC (4777A>C 4778G>T 4779A>C), TGC459TGT (4782C>T), AAA460GTC (4783A>G 4784A>T 4785A>C), GCC461AAT (4786G>A 4787C>A 4788C>T), CTA462AAT (4789C>A 4790T>A 4791A>T), GGA463GAC (4793G>A 4794A>C), GTG465GTA (4800G>A), CTT466TTA (4801C>T 4803T>A), AGC467AGT (4806C>T), AAA468CAG (4807A>C 4809A>G), AAG469AAA (4812G>A), GCC471GCA (4818C>A), CAG472GAA (4819C>G 4821G>A), TTG473ATA (4822T>A 4824G>A), TGC474AAT (4825T>A 4826G>A 4827C>T), ACA476AAT (4832C>A 4833A>T), ACT477AAA (4835C>A 4836T>A), ATC478ATA (4839C>A), AAT479GAA (4840A>G 4842T>A), GGT482GGA (4851T>A), CTA483ATG (4852C>A 4854A>G), GTA484ATT (4855G>A 4857A>T), ATA485ATT (4860A>T), GAA486TCA (4861G>T 4862A>C), AGA487AAA (4865G>A), GGA488AAT (4867G>A 4868G>A 4869A>T), AAT489GGA (4870A>G 4871A>G 4872T>A), CTC490ATA (4873C>A 4875C>A), AAA491GAA (4876A>G), GTC492TTA (4879G>T 4881C>A), CAG493CAA (4884G>A), AGT494ACT (4886G>C), CAT495CAC (4890T>C), ATC496ATA (4893C>A), GGA497TCC (4894G>T 4895G>C 4896A>C), CTA498AAA (4897C>A 4898T>A), CAC499AAG (4900C>A 4902C>G), TTA500ATA (4903T>A), GTT501TTA (4906G>T 4908T>A), GCT502GAG (4910C>A 4911T>G), CCA504CCT (4917A>T), GAT505GAC (4920T>C), CAA506AAG (4921C>A 4923A>G), CTT507TTA (4924C>T 4926T>A), AGT508ATA (4928G>T 4929T>A), GAT509ACC (4930G>A 4931A>C 4932T>C), AGG510AAA (4934G>A 4935G>A), AAT511CAA (4936A>C 4938T>A), GCC512GAA (4940C>A 4941C>A), TTA513ATA (4942T>A), TTA517CTT (4954T>C 4956A>T), GGC518GGA (4959C>A), CTC519TGC (4960C>T 4961T>G), CTA520TTA (4963C>T), AAT521AAC (4968T>C), ATC523GCA (4972A>G 4973T>C 4974C>A), TCC524GGA (4975T>G 4976C>G 4977C>A), GCT525GAA (4979C>A 4980T>A), TAT526TTC (4982A>T 4983T>C), TTT527ATA (4984T>A 4986T>A), AAA529GAA (4990A>G), ATA530TTA (4993A>T), AAC532AAG (5001C>G), CTT533AAG (5002C>A 5003T>A 5004T>G), AGG534AGA (5007G>A), TCA535AAT (5008T>A 5009C>A 5010A>T), CCT536CTT (5012C>T), TTA537CTT (5014T>C 5016A>T), CAG538CAA (5019G>A), GTT539del (5020_5022delGTT), CTT541CTC (5028T>C), AAG542ATC (5030A>T 5031G>C), AAA543AGA (5033A>G), GAG544AAA (5035G>A 5037G>A), ATA545TCA (5038A>T 5039T>C), ACT546AA. (5042C>A)								

*: Inserts / Deletes / Misaligned / Frameshifts

Analysis details

This analysis was performed with panviral2.64

NGS Details (UN9): Pinus nigra virus 1

Assembly

**Coverage Length**	241 (1 contig(s))
**Depth Of Coverage**	19.8
**Number Of Reads**	41
**Reads Per Million**	0.93 rpm (after QC)
**Ambiguities**	0
**Assembly Method**	de novo + reference guided assembly
**Consensus Caller**	Bcf Tools

Coverage Map

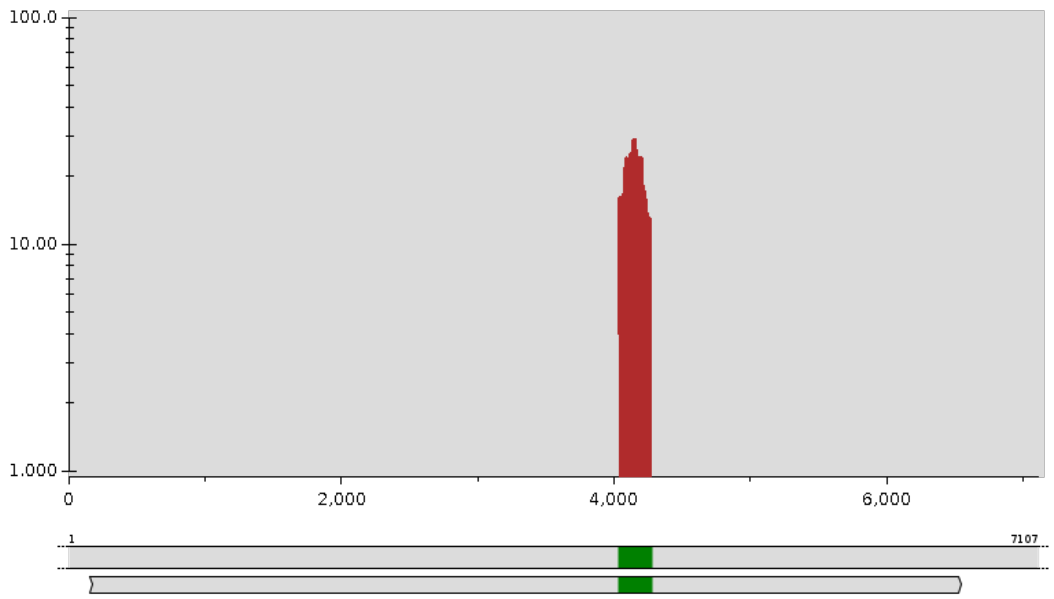

Assignment

**Type**	Pinus nigra virus 1 (Taxonomy ID: 2267679)
**Reference Genome**	NC_040841.1
**NT Identity (%)**	56.2753
**AA Identity (%)**	50.0
**Number Of Stop Codons**	0
**Number Of CDS**	1

Alignment

**Alignment Score**	59.0 (NT) + 311.0 (AA) = 370.0
**Concordance (%)**	33.6364

Alignment Method	Global, seeded, nucleotide + amino acids (AGA)

Genome Region

Sequence starts at position 4036 and ends at position 4276 relative to NC_040841.1 reference sequence.

Alignment Detailed Statistics

	Begin	End	Coverage	Score	Concordance	Matches	Identities	I/D/M/F*	Stop Codons
**NT**	**4036**	**4276**	**3.4%**	**59**	**12.2%**	**241 (97.6%)**	**139 (56.3%)**	**6/0**	
Mutations:	4040G>A, 4045T>C, 4046T>C, 4051G>A, 4054A>T, 4056A>G, 4057A>T, 4058A>G, 4060T>C, 4068A>C, 4073A>G, 4074G>C, 4075A>C, 4078C>T, 4090C>A, 4091A>C, 4092T>G, 4094T>A, 4095G>C, 4098C>A, 4099A>T, 4100G>T, 4102A>T, 4105A>G, 4106T>C, 4107T>A, 4108C>T, 4112C>G, 4114G>A, 4116G>T, 4117G>C, 4118A>C, 4119A>T, 4120A>T, 4123T>A, 4124G>A, 4126T>G, 4129A>T, 4132T>C, 4135T>A, 4136T>C, 4137T>A, 4138G>C, 4140A>C, 4141G>T, 4147G>T, 4150A>C, 4154T>A, 4155T>C, 4156C>T, 4159T>C, 4163A>G, 4164G>C, 4165A>C, 4167G>C, 4168G>T, 4172G>A, 4175T>G, 4177T>C, 4178A>C, 4180C>T, 4183T>C, 4184G>A, 4185C>G, 4186T>A, 4187A>C, 4188A>C, 4192_4193insCTCAGA, 4193G>A, 4195T>A, 4198T>C, 4201A>C, 4202G>T, 4204T>G, 4205A>G, 4206C>T, 4207A>T, 4209A>T, 4211A>T, 4213A>T, 4214G>A, 4225A>G, 4228T>C, 4229C>T, 4237A>T, 4238G>A, 4239A>C, 4240T>C, 4241G>C, 4243A>T, 4247A>C, 4249T>G, 4252T>C, 4253T>C, 4254T>A, 4255G>A, 4258G>C, 4261C>T, 4262T>C, 4264A>C, 4265G>C, 4267A>G, 4270T>A								

CDS

	Begin	End	Coverage	Score	Concordance	Matches	Identities	I/D/M/F*	Stop Codons
EXL67_gp1	1295	1374	3.8%	311	50.0%	80 (97.6%)	41 (50.0%)	2/0/0/0	0
Protein mutations:	G1296R (4040G>A), Y1298H (4046T>C), Q1301R (4056A>G 4057A>T), I1302V (4058A>G 4060T>C), E1305A (4068A>C), R1307A (4073A>G 4074G>C 4075A>C), F1312L (4090C>A), I1313R (4091A>C 4092T>G), C1314T (4094T>A 4095G>C), P1315H (4098C>A 4099A>T), A1316S (4100G>T 4102A>T), F1318H (4106T>C 4107T>A 4108C>T), Q1320E (4112C>G 4114G>A), W1321F (4116G>T 4117G>C), K1322L (4118A>C 4119A>T 4120A>T), V1324M (4124G>A 4126T>G), L1328H (4136T>C 4137T>A 4138G>C), K1329T (4140A>C 4141G>T), F1334T (4154T>A 4155T>C 4156C>T), R1337A (4163A>G 4164G>C 4165A>C), R1338T (4167G>C 4168G>T), D1340N (4172G>A), Y1341D (4175T>G 4177T>C), I1342L (4178A>C 4180C>T), A1344R (4184G>A 4185C>G 4186T>A), K1345P (4187A>C 4188A>C), Y1346_D1347insLR (4192_4193insCTCAGA), D1347K (4193G>A 4195T>A), V1350L (4202G>T 4204T>G), T1351V (4205A>G 4206C>T 4207A>T), Y1352F (4209A>T), I1353F (4211A>T 4213A>T), D1354N (4214G>A), H1359Y (4229C>T), D1362T (4238G>A 4239A>C 4240T>C), V1363L (4241G>C 4243A>T), N1365Q (4247A>C 4249T>G), L1367Q (4253T>C 4254T>A 4255G>A), K1368N (4258G>C), E1371Q (4265G>C 4267A>G)								
Codon mutations:	GGG1296AGG (4040G>A), TAT1297TAC (4045T>C), TAT1298CAT (4046T>C), CAG1299CAA (4051G>A), ATA1300ATT (4054A>T), CAA1301CGT (4056A>G 4057A>T), ATT1302GTC (4058A>G 4060T>C), GAA1305GCA (4068A>C), AGA1307GCC (4073A>G 4074G>C 4075A>C), CAC1308CAT (4078C>T), TTC1312TTA (4090C>A), ATA1313CGA (4091A>C 4092T>G), TGT1314ACT (4094T>A 4095G>C), CCA1315CAT (4098C>A 4099A>T), GCA1316TCT (4100G>T 4102A>T), GGA1317GGG (4105A>G), TTC1318CAT (4106T>C 4107T>A 4108C>T), CAG1320GAA (4112C>G 4114G>A), TGG1321TTC (4116G>T 4117G>C), AAA1322CTT (4118A>C 4119A>T 4120A>T), GTT1323GTA (4123T>A), GTT1324ATG (4124G>A 4126T>G), CCA1325CCT (4129A>T), TTT1326TTC (4132T>C), GGT1327GGA (4135T>A), TTG1328CAC (4136T>C 4137T>A 4138G>C), AAG1329ACT (4140A>C 4141G>T), GCG1331GCT (4147G>T), CCA1332CCC (4150A>C), TTC1334ACT (4154T>A 4155T>C 4156C>T), TTT1335TTC (4159T>C), AGA1337GCC (4163A>G 4164G>C 4165A>C), AGG1338ACT (4167G>C 4168G>T), GAT1340AAT (4172G>A), TAT1341GAC (4175T>G 4177T>C), ATC1342CTT (4178A>C 4180C>T), TTT1343TTC (4183T>C), GCT1344AGA (4184G>A 4185C>G 4186T>A), AAA1345CCA (4187A>C 4188A>C), TAT1346_GAT1347insCTCAGA (4192_4193insCTCAGA), GAT1347AAA (4193G>A 4195T>A), TTT1348TTC (4198T>C), ATA1349ATC (4201A>C), GTT1350TTG (4202G>T 4204T>G), ACA1351GTT (4205A>G 4206C>T 4207A>T), TAC1352TTC (4209A>T), ATA1353TTT (4211A>T 4213A>T), GAT1354AAT (4214G>A), TTA1357TTG (4225A>G), ATT1358ATC (4228T>C), CAT1359TAT (4229C>T), CCA1361CCT (4237A>T), GAT1362ACC (4238G>A 4239A>C 4240T>C), GTA1363CTT (4241G>C 4243A>T), AAT1365CAG (4247A>C 4249T>G), CAT1366CAC (4252T>C), TTG1367CAA (4253T>C 4254T>A 4255G>A), AAG1368AAC (4258G>C), CAC1369CAT (4261C>T), TTA1370CTC (4262T>C 4264A>C), GAA1371CAG (4265G>C 4267A>G), ATT1372ATA (4270T>A)								

Proteins

	Begin	End	Coverage	Score	Concordance	Matches	Identities	I/D/M/F*	Stop Codons
polyprotein (YP_009553669.1)	1295	1374	3.8%	311	50.0%	80 (97.6%)	41 (50.0%)	2/0/0/0	0
Protein mutations:	G1296R (4040G>A), Y1298H (4046T>C), Q1301R (4056A>G 4057A>T), I1302V (4058A>G 4060T>C), E1305A (4068A>C), R1307A (4073A>G 4074G>C 4075A>C), F1312L (4090C>A), I1313R (4091A>C 4092T>G), C1314T (4094T>A 4095G>C), P1315H (4098C>A 4099A>T), A1316S (4100G>T 4102A>T), F1318H (4106T>C 4107T>A 4108C>T), Q1320E (4112C>G 4114G>A), W1321F (4116G>T 4117G>C), K1322L (4118A>C 4119A>T 4120A>T), V1324M (4124G>A 4126T>G), L1328H (4136T>C 4137T>A 4138G>C), K1329T (4140A>C 4141G>T), F1334T (4154T>A 4155T>C 4156C>T), R1337A (4163A>G 4164G>C 4165A>C), R1338T (4167G>C 4168G>T), D1340N (4172G>A), Y1341D (4175T>G 4177T>C), I1342L (4178A>C 4180C>T), A1344R (4184G>A 4185C>G 4186T>A), K1345P (4187A>C 4188A>C), Y1346_D1347insLR (4192_4193insCTCAGA), D1347K (4193G>A 4195T>A), V1350L (4202G>T 4204T>G), T1351V (4205A>G 4206C>T 4207A>T), Y1352F (4209A>T), I1353F (4211A>T 4213A>T), D1354N (4214G>A), H1359Y (4229C>T), D1362T (4238G>A 4239A>C 4240T>C), V1363L (4241G>C 4243A>T), N1365Q (4247A>C 4249T>G), L1367Q (4253T>C 4254T>A 4255G>A), K1368N (4258G>C), E1371Q (4265G>C 4267A>G)								
Codon mutations:	GGG1296AGG (4040G>A), TAT1297TAC (4045T>C), TAT1298CAT (4046T>C), CAG1299CAA (4051G>A), ATA1300ATT (4054A>T), CAA1301CGT (4056A>G 4057A>T), ATT1302GTC (4058A>G 4060T>C), GAA1305GCA (4068A>C), AGA1307GCC (4073A>G 4074G>C 4075A>C), CAC1308CAT (4078C>T), TTC1312TTA (4090C>A), ATA1313CGA (4091A>C 4092T>G), TGT1314ACT (4094T>A 4095G>C), CCA1315CAT (4098C>A 4099A>T), GCA1316TCT (4100G>T 4102A>T), GGA1317GGG (4105A>G), TTC1318CAT (4106T>C 4107T>A 4108C>T), CAG1320GAA (4112C>G 4114G>A), TGG1321TTC (4116G>T 4117G>C), AAA1322CTT (4118A>C 4119A>T 4120A>T), GTT1323GTA (4123T>A), GTT1324ATG (4124G>A 4126T>G), CCA1325CCT (4129A>T), TTT1326TTC (4132T>C), GGT1327GGA (4135T>A), TTG1328CAC (4136T>C 4137T>A 4138G>C), AAG1329ACT (4140A>C 4141G>T), GCG1331GCT (4147G>T), CCA1332CCC (4150A>C), TTC1334ACT (4154T>A 4155T>C 4156C>T), TTT1335TTC (4159T>C), AGA1337GCC (4163A>G 4164G>C 4165A>C), AGG1338ACT (4167G>C 4168G>T), GAT1340AAT (4172G>A), TAT1341GAC (4175T>G 4177T>C), ATC1342CTT (4178A>C 4180C>T), TTT1343TTC (4183T>C), GCT1344AGA (4184G>A 4185C>G 4186T>A), AAA1345CCA (4187A>C 4188A>C), TAT1346_GAT1347insCTCAGA (4192_4193insCTCAGA), GAT1347AAA (4193G>A 4195T>A), TTT1348TTC (4198T>C), ATA1349ATC (4201A>C), GTT1350TTG (4202G>T 4204T>G), ACA1351GTT (4205A>G 4206C>T 4207A>T), TAC1352TTC (4209A>T), ATA1353TTT (4211A>T 4213A>T), GAT1354AAT (4214G>A), TTA1357TTG (4225A>G), ATT1358ATC (4228T>C), CAT1359TAT (4229C>T), CCA1361CCT (4237A>T), GAT1362ACC (4238G>A 4239A>C 4240T>C), GTA1363CTT (4241G>C 4243A>T), AAT1365CAG (4247A>C 4249T>G), CAT1366CAC (4252T>C), TTG1367CAA (4253T>C 4254T>A 4255G>A), AAG1368AAC (4258G>C), CAC1369CAT (4261C>T), TTA1370CTC (4262T>C 4264A>C), GAA1371CAG (4265G>C 4267A>G), ATT1372ATA (4270T>A)								

*: Inserts / Deletes / Misaligned / Frameshifts

Analysis details

This analysis was performed with panviral2.64

NGS Details (UN9): Hibiscus bacilliform virus GD1

Assembly

**Coverage Length**	501 (1 contig(s))
**Depth Of Coverage**	9.2
**Number Of Reads**	37
**Reads Per Million**	0.84 rpm (after QC)
**Ambiguities**	0
**Assembly Method**	de novo + reference guided assembly
**Consensus Caller**	Bcf Tools

Coverage Map

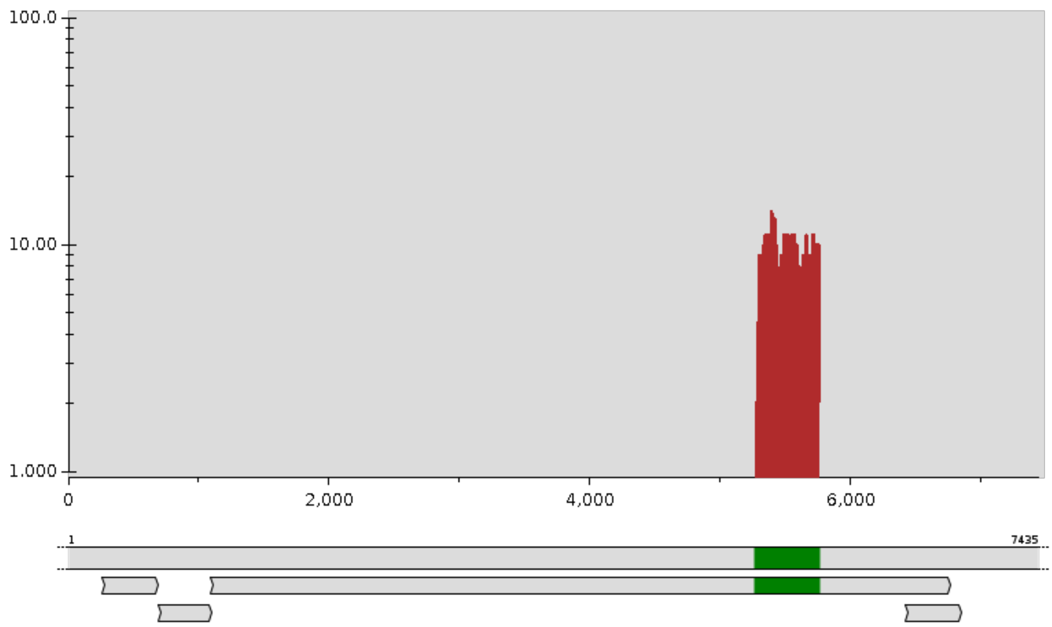

Assignment

**Type**	Hibiscus bacilliform virus GD1 (Taxonomy ID: 1459800)
**Reference Genome**	NC_023485.1
**NT Identity (%)**	52.2358
**AA Identity (%)**	43.0303
**Number Of Stop Codons**	1
**Number Of CDS**	4

Alignment

**Alignment Score**	8.0 (NT) + 194.0 (AA) = 202.0
**Concordance (%)**	13.0457

Alignment Method	Global, seeded, nucleotide + amino acids (AGA)

Genome Region

Sequence starts at position 5261 and ends at position 5761 relative to NC_023485.1 reference sequence.

Alignment Detailed Statistics

	Begin	End	Coverage	Score	Concordance	Matches	Identities	I/D/M/F*	Stop Codons
**NT**	**5261**	**5761**	**6.7%**	**8**	**0.8%**	**490 (97.4%)**	**257 (51.1%)**	**2/11**	
Mutations:	5267G>A, 5268G>T, 5269T>G, 5270G>T, 5271T>A, 5281A>G, 5282A>G, 5283A>T, 5284G>A, 5292G>A, 5294T>G, 5295C>G, 5296T>C, 5297C>A, 5300T>A, 5301G>A, 5302A>T, 5303G>C, 5306A>G, 5307G>A, 5310C>A, 5312G>A, 5316A>C, 5317G>T, 5318T>A, 5319C>T, 5324A>T, 5325G>C, 5327C>T, 5330C>T, 5331A>G, 5333C>T, 5335C>A, 5336C>T, 5337A>C, 5339T>A, 5340A>T, 5342G>C, 5345G>T, 5346A>C, 5347G>A, 5348G>A, 5349G>T, 5351T>A, 5352_5353delGG, 5355C>A, 5356A>G, 5357T>G, 5362A>C, 5363G>T, 5364A>T, 5365T>A, 5367T>A, 5368A>T, 5369C>T, 5372A>C, 5375G>A, 5376T>A, 5384C>G, 5386A>G, 5387G>A, 5388A>T, 5389G>C, 5390T>A, 5393A>T, 5395T>A, 5403G>C, 5406G>A, 5407C>G, 5408T>A, 5411G>T, 5412C>A, 5414C>G, 5415C>A, 5417G>T, 5420G>A, 5422C>A, 5427A>T, 5428A>C, 5429G>A, 5430T>A, 5431G>A, 5435T>A, 5438T>C, 5441C>T, 5445G>A, 5446T>C, 5447T>C, 5448C>A, 5449C>G, 5450A>G, 5451G>T, 5456A>G, 5458T>A, 5459G>C, 5461A>T, 5467G>T, 5468G>T, 5469C>T, 5471A>G, 5474T>G, 5478C>T, 5480A>T, 5487C>T, 5489T>G, 5491A>C, 5492A>T, 5495C>T, 5498C>A, 5501A>T, 5503C>T, 5506T>A, 5511C>A, 5512A>T, 5514C>G, 5515G>A, 5516A>C, 5517A>C, 5518A>T, 5519G>C, 5523G>A, 5525C>T, 5527A>G, 5528C>G, 5531C>A, 5533T>C, 5537A>G, 5538G>C, 5539G>C, 5540T>A, 5541A>T, 5542C>A, 5543T>C, 5544G>T, 5545A>T, 5548C>A, 5549_5550insCA, 5553A>T, 5555C>A, 5556G>A, 5557C>T, 5561A>G, 5563A>T, 5573C>T, 5576A>T, 5577C>T, 5579C>G, 5582A>C, 5584T>A, 5585T>C, 5588C>T, 5591G>A, 5592T>G, 5593C>A, 5599A>C, 5600G>C, 5603A>G, 5607A>G, 5608T>A, 5609C>A, 5610A>C, 5612G>T, 5615C>T, 5616A>T, 5618A>G, 5619C>A, 5621G>A, 5624C>T, 5625A>G, 5627G>A, 5630A>G, 5631G>A, 5633A>G, 5635A>C, 5637T>C, 5638G>T, 5639C>T, 5640C>A, 5642G>A, 5645G>A, 5646A>C, 5647A>C, 5648T>A, 5649G>A, 5650G>A, 5655G>T, 5656T>A, 5658C>G, 5659T>C, 5662G>A, 5663C>G, 5664C>T, 5665C>T, 5666A>T, 5667A>T, 5673A>T, 5674T>G, 5675G>T, 5676A>G, 5679A>T, 5681A>T, 5682G>T, 5683C>G, 5684C>G, 5685C>T, 5686A>T, 5688A>G, 5690G>C, 5692A>T, 5693G>A, 5696G>T, 5697G>T, 5698A>C, 5699A>C, 5703C>T, 5705G>A, 5709G>C, 5710C>A, 5711T>C, 5714T>G, 5715C>G, 5718G>T, 5719A>C, 5721G>C, 5722C>G, 5725G>A, 5726C>T, 5727C>G, 5729T>A, 5735G>A, 5736C>G, 5738G>T, 5739C>G, 5741G>C, 5744C>A, 5745_5753delCATATCATA									

CDS

	Begin	End	Coverage	Score	Concordance	Matches	Identities	I/D/M/F*	Stop Codons
BU35_gp3	1391	1557	8.8%	194	17.2%	164 (97.6%)	71 (42.3%)	1/3/2/2	1
Protein mutations:	M1392I (5267G>A), V1393C (5268G>T 5269T>G 5270G>T), F1394I (5271T>A), K1397R (5281A>G 5282A>G), R1398* (5283A>T 5284G>A), D1401K (5292G>A 5294T>G), L1402A (5295C>G 5296T>C 5297C>A), E1404I (5301G>A 5302A>T 5303G>C), D1406N (5307G>A), Q1407K (5310C>A 5312G>A), S1409L (5316A>C 5317G>T 5318T>A), G1412R (5325G>C 5327C>T), N1414D (5331A>G 5333C>T), T1415N (5335C>A 5336C>T), I1416L (5337A>C 5339T>A), M1417F (5340A>T 5342G>C), K1418N (5345G>T), R1419Q (5346A>C 5347G>A 5348G>A), V1420L (5349G>T 5351T>A), H1422R (5355C>A 5356A>G 5357T>G), K1424T (5362A>C 5363G>T), I1425Y (5364A>T 5365T>A), Y1426I (5367T>A 5368A>T 5369C>T), F1429I (5376T>A), K1432R (5386A>G 5387G>A), F1435Y (5395T>A), V1438L (5403G>C), A1439R (5406G>A 5407C>G 5408T>A), M1440I (5411G>T), H1441K (5412C>A 5414C>G), P1442T (5415C>A 5417G>T), S1444Y (5422C>A), K1446S (5427A>T 5428A>C 5429G>A), W1447K (5430T>A 5431G>A), V1452T (5445G>A 5446T>C 5447T>C), P1453R (5448C>A 5449C>G 5450A>G), D1454Y (5451G>T), L1456H (5458T>A 5459G>C), Y1457F (5461A>T), W1459F (5467G>T 5468G>T), P1463S (5478C>T 5480A>T), K1467T (5491A>C 5492A>T), A1471V (5503C>T), V1472E (5506T>A), Q1474M (5511C>A 5512A>T), R1475D (5514C>G 5515G>A 5516A>C), K1476L (5517A>C 5518A>T 5519G>C), D1478N (5523G>A 5525C>T), N1479R (5527A>G 5528C>G), F1481S (5533T>C), G1483P (5538G>C 5539G>C 5540T>A), T1484Y (5541A>T 5542C>A 5543T>C), E1485L (5544G>T 5545A>T), A1486D (5548C>A), A1486_F1487insX (5549_5550insCA), I1488L (5553A>T 5555C>A), A1489I (5556G>A 5557C>T), Y1491F (5563A>T), F1498Y (5584T>A 5585T>C), S1501D (5592T>G 5593C>A), E1503A (5599A>C 5600G>C), I1506E (5607A>G 5608T>A 5609C>A), K1507H (5610A>C 5612G>T), I1509L (5616A>T 5618A>G), M1512V (5625A>G 5627G>A), E1514K (5631G>A 5633A>G), K1515T (5635A>C), C1516L (5637T>C 5638G>T 5639C>T), N1519P (5646A>C 5647A>C 5648T>A), G1520K (5649G>A 5650G>A), V1522Y (5655G>T 5656T>A), L1523A (5658C>G 5659T>C), S1524K (5662G>A 5663C>G), P1525F (5664C>T 5665C>T 5666A>T), T1526S (5667A>T), M1528C (5673A>T 5674T>G 5675G>T), K1529E (5676A>G), I1530F (5679A>T 5681A>T), A1531W (5682G>T 5683C>G 5684C>G), Q1532L (5685C>T 5686A>T), R1533G (5688A>G 5690G>C), K1534I (5692A>T 5693G>A), E1536S (5697G>T 5698A>C 5699A>C), A1540H (5709G>C 5710C>A 5711T>C), I1541M (5714T>G), L1542V (5715C>G), E1543S (5718G>T 5719A>C), A1544R (5721G>C 5722C>G), G1545D (5725G>A 5726C>T), R1546G (5727C>G 5729T>A), L1549V (5736C>G 5738G>T), Q1550D (5739C>G 5741G>C), H1552_I1554del (5745_5753delCATATCATA)									
Codon mutations:	ATG1392ATA (5267G>A), GTG1393TGT (5268G>T 5269T>G 5270G>T), TTC1394ATC (5271T>A), AAA1397AGG (5281A>G 5282A>G), AGG1398TAG (5283A>T 5284G>A), GAT1401AAG (5292G>A 5294T>G), CTC1402GCA (5295C>G 5296T>C 5297C>A), ACT1403ACA (5300T>A), GAG1404ATC (5301G>A 5302A>T 5303G>C), AAA1405AAG (5306A>G), GAT1406AAT (5307G>A), CAG1407AAA (5310C>A 5312G>A), AGT1409CTA (5316A>C 5317G>T 5318T>A), CTG1410TTG (5319C>T), CCA1411CCT (5324A>T), GGC1412CGT (5325G>C 5327C>T), ATC1413ATT (5330C>T), AAC1414GAT (5331A>G 5333C>T), ACC1415AAT (5335C>A 5336C>T), ATT1416CTA (5337A>C 5339T>A), ATG1417TTC (5340A>T 5342G>C), AAG1418AAT (5345G>T), AGG1419CAA (5346A>C 5347G>A 5348G>A), GTT1420TTA (5349G>T 5351T>A), GGC1421--C (5352_5353delGG), CAT1422AGG (5355C>A 5356A>G 5357T>G), AAG1424ACT (5362A>C 5363G>T), ATC1425TAC (5364A>T 5365T>A), TAC1426ATT (5367T>A 5368A>T 5369C>T), TCA1427TCC (5372A>C), AAG1428AAA (5375G>A), TTT1429ATT (5376T>A), CTC1431CTG (5384C>G), AAG1432AGA (5386A>G 5387G>A), AGT1433TCA (5388A>T 5389G>C 5390T>A), GGA1434GGT (5393A>T), TTT1435TAT (5395T>A), GTG1438CTG (5403G>C), GCT1439AGA (5406G>A 5407C>G 5408T>A), ATG1440ATT (5411G>T), CAC1441AAG (5412C>A 5414C>G), CCG1442ACT (5415C>A 5417G>T), GAG1443GAA (5420G>A), TCT1444TAT (5422C>A), AAG1446TCA (5427A>T 5428A>C 5429G>A), TGG1447AAG (5430T>A 5431G>A), ACT1448ACA (5435T>A), GCT1449GCC (5438T>C), TTC1450TTT (5441C>T), GTT1452ACC (5445G>A 5446T>C 5447T>C), CCA1453AGG (5448C>A 5449C>G 5450A>G), GAT1454TAT (5451G>T), GGA1455GGG (5456A>G), CTG1456CAC (5458T>A 5459G>C), TAT1457TTT (5461A>T), TGG1459TTT (5467G>T 5468G>T), CTA1460TTG (5469C>T 5471A>G), GTT1461GTG (5474T>G), CCA1463TCT (5478C>T 5480A>T), CTT1466TTG (5487C>T 5489T>G), AAA1467ACT (5491A>C 5492A>T), AAC1468AAT (5495C>T), GCC1469GCA (5498C>A), CCA1470CCT (5501A>T), GCA1471GTA (5503C>T), GTA1472GAA (5506T>A), CAG1474ATG (5511C>A 5512A>T), CGA1475GAC (5514C>G 5515G>A 5516A>C), AAG1476CTC (5517A>C 5518A>T 5519G>C), GAC1478AAT (5523G>A 5525C>T), AAC1479AGG (5527A>G 5528C>G), GTC1480GTA (5531C>A), TTC1481TCC (5533T>C), AAA1482AAG (5537A>G), GGT1483CCA (5538G>C 5539G>C 5540T>A), ACT1484TAC (5541A>T 5542C>A 5543T>C), GAA1485TTA (5544G>T 5545A>T), GCC1486GAC (5548C>A), GCC1486_TTT1487insCA- (5549_5550insCA), ATC1488TTA (5553A>T 5555C>A), GCT1489ATT (5556G>A 5557C>T), GTA1490GTG (5561A>G), TAC1491TTC (5563A>T), GAC1494GAT (5573C>T), ATA1495ATT (5576A>T), CTC1496TTG (5577C>T 5579C>G), GTA1497GTC (5582A>C), TTT1498TAC (5584T>A 5585T>C), TCC1499TCT (5588C>T), CAG1500CAA (5591G>A), TCT1501GAT (5592T>G 5593C>A), GAG1503GCC (5599A>C 5600G>C), GAA1504GAG (5603A>G), ATC1506GAA (5607A>G 5608T>A 5609C>A), AAG1507CAT (5610A>C 5612G>T), CAC1508CAT (5615C>T), ATA1509TTG (5616A>T 5618A>G), CGG1510AGA (5619C>A 5621G>A), GTC1511GTT (5624C>T), ATG1512GTA (5625A>G 5627G>A), TTA1513TTG (5630A>G), GAA1514AAG (5631G>A 5633A>G), AAA1515ACA (5635A>C), TGC1516CTT (5637T>C 5638G>T 5639C>T), CGG1517AGA (5640C>A 5642G>A), GAG1518GAA (5645G>A), AAT1519CCA (5646A>C 5647A>C 5648T>A), GGG1520AAG (5649G>A 5650G>A), GTT1522TAT (5655G>T 5656T>A), CTT1523GCT (5658C>G 5659T>C), AGC1524AAG (5662G>A 5663C>G), CCA1525TTT (5664C>T 5665C>T 5666A>T), ACA1526TCA (5667A>T), ATG1528TGT (5673A>T 5674T>G 5675G>T), AAG1529GAG (5676A>G), ATA1530TTT (5679A>T 5681A>T), GCC1531TGG (5682G>T 5683C>G 5684C>G), CAG1532TTG (5685C>T 5686A>T), AGG1533GGC (5688A>G 5690G>C), AAG1534ATA (5692A>T 5693G>A), GTG1535GTT (5696G>T), GAA1536TCC (5697G>T 5698A>C 5699A>C), CTG1538TTA (5703C>T 5705G>A), GCT1540CAC (5709G>C 5710C>A 5711T>C), ATT1541ATG (5714T>G), CTG1542GTG (5715C>G), GAA1543TCA (5718G>T 5719A>C), GCA1544CGA (5721G>C 5722C>G), GGC1545GAT (5725G>A 5726C>T), CGT1546GGA (5727C>G 5729T>A), CAG1548CAA (5735G>A), CTG1549GTT (5736C>G 5738G>T), CAG1550GAC (5739C>G 5741G>C), CCC1551CCA (5744C>A), CAT1552_ATA1554del (5745_5753delCATATCATA)									

Proteins

	Begin	End	Coverage	Score	Concordance	Matches	Identities	I/D/M/F*	Stop Codons
polyprotein (YP_009002585.1)	1391	1557	8.8%	194	17.2%	164 (97.6%)	71 (42.3%)	1/3/2/2	1
Protein mutations:	M1392I (5267G>A), V1393C (5268G>T 5269T>G 5270G>T), F1394I (5271T>A), K1397R (5281A>G 5282A>G), R1398* (5283A>T 5284G>A), D1401K (5292G>A 5294T>G), L1402A (5295C>G 5296T>C 5297C>A), E1404I (5301G>A 5302A>T 5303G>C), D1406N (5307G>A), Q1407K (5310C>A 5312G>A), S1409L (5316A>C 5317G>T 5318T>A), G1412R (5325G>C 5327C>T), N1414D (5331A>G 5333C>T), T1415N (5335C>A 5336C>T), I1416L (5337A>C 5339T>A), M1417F (5340A>T 5342G>C), K1418N (5345G>T), R1419Q (5346A>C 5347G>A 5348G>A), V1420L (5349G>T 5351T>A), H1422R (5355C>A 5356A>G 5357T>G), K1424T (5362A>C 5363G>T), I1425Y (5364A>T 5365T>A), Y1426I (5367T>A 5368A>T 5369C>T), F1429I (5376T>A), K1432R (5386A>G 5387G>A), F1435Y (5395T>A), V1438L (5403G>C), A1439R (5406G>A 5407C>G 5408T>A), M1440I (5411G>T), H1441K (5412C>A 5414C>G), P1442T (5415C>A 5417G>T), S1444Y (5422C>A), K1446S (5427A>T 5428A>C 5429G>A), W1447K (5430T>A 5431G>A), V1452T (5445G>A 5446T>C 5447T>C), P1453R (5448C>A 5449C>G 5450A>G), D1454Y (5451G>T), L1456H (5458T>A 5459G>C), Y1457F (5461A>T), W1459F (5467G>T 5468G>T), P1463S (5478C>T 5480A>T), K1467T (5491A>C 5492A>T), A1471V (5503C>T), V1472E (5506T>A), Q1474M (5511C>A 5512A>T), R1475D (5514C>G 5515G>A 5516A>C), K1476L (5517A>C 5518A>T 5519G>C), D1478N (5523G>A 5525C>T), N1479R (5527A>G 5528C>G), F1481S (5533T>C), G1483P (5538G>C 5539G>C 5540T>A), T1484Y (5541A>T 5542C>A 5543T>C), E1485L (5544G>T 5545A>T), A1486D (5548C>A), A1486_F1487insX (5549_5550insCA), I1488L (5553A>T 5555C>A), A1489I (5556G>A 5557C>T), Y1491F (5563A>T), F1498Y (5584T>A 5585T>C), S1501D (5592T>G 5593C>A), E1503A (5599A>C 5600G>C), I1506E (5607A>G 5608T>A 5609C>A), K1507H (5610A>C 5612G>T), I1509L (5616A>T 5618A>G), M1512V (5625A>G 5627G>A), E1514K (5631G>A 5633A>G), K1515T (5635A>C), C1516L (5637T>C 5638G>T 5639C>T), N1519P (5646A>C 5647A>C 5648T>A), G1520K (5649G>A 5650G>A), V1522Y (5655G>T 5656T>A), L1523A (5658C>G 5659T>C), S1524K (5662G>A 5663C>G), P1525F (5664C>T 5665C>T 5666A>T), T1526S (5667A>T), M1528C (5673A>T 5674T>G 5675G>T), K1529E (5676A>G), I1530F (5679A>T 5681A>T), A1531W (5682G>T 5683C>G 5684C>G), Q1532L (5685C>T 5686A>T), R1533G (5688A>G 5690G>C), K1534I (5692A>T 5693G>A), E1536S (5697G>T 5698A>C 5699A>C), A1540H (5709G>C 5710C>A 5711T>C), I1541M (5714T>G), L1542V (5715C>G), E1543S (5718G>T 5719A>C), A1544R (5721G>C 5722C>G), G1545D (5725G>A 5726C>T), R1546G (5727C>G 5729T>A), L1549V (5736C>G 5738G>T), Q1550D (5739C>G 5741G>C), H1552_I1554del (5745_5753delCATATCATA)									

	Begin	End	Coverage	Score	Concordance	Matches	Identities	I/D/M/F*	Stop Codons
**NT**	**5261**	**5761**	**6.7%**	**8**	**0.8%**	**490 (97.4%)**	**257 (51.1%)**	**2/11**	
Codon mutations:	ATG1392ATA (5267G>A), GTG1393TGT (5268G>T 5269T>G 5270G>T), TTC1394ATC (5271T>A), AAA1397AGG (5281A>G 5282A>G), AGG1398TAG (5283A>T 5284G>A), GAT1401AAG (5292G>A 5294T>G), CTC1402GCA (5295C>G 5296T>C 5297C>A), ACT1403ACA (5300T>A), GAG1404ATC (5301G>A 5302A>T 5303G>C), AAA1405AAG (5306A>G), GAT1406AAT (5307G>A), CAG1407AAA (5310C>A 5312G>A), AGT1409CTA (5316A>C 5317G>T 5318T>A), CTG1410TTG (5319C>T), CCA1411CCT (5324A>T), GGC1412CGT (5325G>C 5327C>T), ATC1413ATT (5330C>T), AAC1414GAT (5331A>G 5333C>T), ACC1415AAT (5335C>A 5336C>T), ATT1416CTA (5337A>C 5339T>A), ATG1417TTC (5340A>T 5342G>C), AAG1418AAT (5345G>T), AGG1419CAA (5346A>C 5347G>A 5348G>A), GTT1420TTA (5349G>T 5351T>A), GGC1421--C (5352_5353delGG), CAT1422AGG (5355C>A 5356A>G 5357T>G), AAG1424ACT (5362A>C 5363G>T), ATC1425TAC (5364A>T 5365T>A), TAC1426ATT (5367T>A 5368A>T 5369C>T), TCA1427TCC (5372A>C), AAG1428AAA (5375G>A), TTT1429ATT (5376T>A), CTC1431CTG (5384C>G), AAG1432AGA (5386A>G 5387G>A), AGT1433TCA (5388A>T 5389G>C 5390T>A), GGA1434GGT (5393A>T), TTT1435TAT (5395T>A), GTG1438CTG (5403G>C), GCT1439AGA (5406G>A 5407C>G 5408T>A), ATG1440ATT (5411G>T), CAC1441AAG (5412C>A 5414C>G), CCG1442ACT (5415C>A 5417G>T), GAG1443GAA (5420G>A), TCT1444TAT (5422C>A), AAG1446TCA (5427A>T 5428A>C 5429G>A), TGG1447AAG (5430T>A 5431G>A), ACT1448ACA (5435T>A), GCT1449GCC (5438T>C), TTC1450TTT (5441C>T), GTT1452ACC (5445G>A 5446T>C 5447T>C), CCA1453AGG (5448C>A 5449C>G 5450A>G), GAT1454TAT (5451G>T), GGA1455GGG (5456A>G), CTG1456CAC (5458T>A 5459G>C), TAT1457TTT (5461A>T), TGG1459TTT (5467G>T 5468G>T), CTA1460TTG (5469C>T 5471A>G), GTT1461GTG (5474T>G), CCA1463TCT (5478C>T 5480A>T), CTT1466TTG (5487C>T 5489T>G), AAA1467ACT (5491A>C 5492A>T), AAC1468AAT (5495C>T), GCC1469GCA (5498C>A), CCA1470CCT (5501A>T), GCA1471GTA (5503C>T), GTA1472GAA (5506T>A), CAG1474ATG (5511C>A 5512A>T), CGA1475GAC (5514C>G 5515G>A 5516A>C), AAG1476CTC (5517A>C 5518A>T 5519G>C), GAC1478AAT (5523G>A 5525C>T), AAC1479AGG (5527A>G 5528C>G), GTC1480GTA (5531C>A), TTC1481TCC (5533T>C), AAA1482AAG (5537A>G), GGT1483CCA (5538G>C 5539G>C 5540T>A), ACT1484TAC (5541A>T 5542C>A 5543T>C), GAA1485TTA (5544G>T 5545A>T), GCC1486GAC (5548C>A), GCC1486_TTT1487insCA- (5549_5550insCA), ATC1488TTA (5553A>T 5555C>A), GCT1489ATT (5556G>A 5557C>T), GTA1490GTG (5561A>G), TAC1491TTC (5563A>T), GAC1494GAT (5573C>T), ATA1495ATT (5576A>T), CTC1496TTG (5577C>T 5579C>G), GTA1497GTC (5582A>C), TTT1498TAC (5584T>A 5585T>C), TCC1499TCT (5588C>T), CAG1500CAA (5591G>A), TCT1501GAT (5592T>G 5593C>A), GAG1503GCC (5599A>C 5600G>C), GAA1504GAG (5603A>G), ATC1506GAA (5607A>G 5608T>A 5609C>A), AAG1507CAT (5610A>C 5612G>T), CAC1508CAT (5615C>T), ATA1509TTG (5616A>T 5618A>G), CGG1510AGA (5619C>A 5621G>A), GTC1511GTT (5624C>T), ATG1512GTA (5625A>G 5627G>A), TTA1513TTG (5630A>G), GAA1514AAG (5631G>A 5633A>G), AAA1515ACA (5635A>C), TGC1516CTT (5637T>C 5638G>T 5639C>T), CGG1517AGA (5640C>A 5642G>A), GAG1518GAA (5645G>A), AAT1519CCA (5646A>C 5647A>C 5648T>A), GGG1520AAG (5649G>A 5650G>A), GTT1522TAT (5655G>T 5656T>A), CTT1523GCT (5658C>G 5659T>C), AGC1524AAG (5662G>A 5663C>G), CCA1525TTT (5664C>T 5665C>T 5666A>T), ACA1526TCA (5667A>T), ATG1528TGT (5673A>T 5674T>G 5675G>T), AAG1529GAG (5676A>G), ATA1530TTT (5679A>T 5681A>T), GCC1531TGG (5682G>T 5683C>G 5684C>G), CAG1532TTG (5685C>T 5686A>T), AGG1533GGC (5688A>G 5690G>C), AAG1534ATA (5692A>T 5693G>A), GTG1535GTT (5696G>T), GAA1536TCC (5697G>T 5698A>C 5699A>C), CTG1538TTA (5703C>T 5705G>A), GCT1540CAC (5709G>C 5710C>A 5711T>C), ATT1541ATG (5714T>G), CTG1542GTG (5715C>G), GAA1543TCA (5718G>T 5719A>C), GCA1544CGA (5721G>C 5722C>G), GGC1545GAT (5725G>A 5726C>T), CGT1546GGA (5727C>G 5729T>A), CAG1548CAA (5735G>A), CTG1549GTT (5736C>G 5738G>T), CAG1550GAC (5739C>G 5741G>C), CCC1551CCA (5744C>A), CAT1552_ATA1554del (5745_5753delCATATCATA)								

*: Inserts / Deletes / Misaligned / Frameshifts

Analysis details

This analysis was performed with panviral2.64

NGS Details (UN9): Dioscovirus dioscoreae

Assembly

**Coverage Length**	740 (1 contig(s))
**Depth Of Coverage**	4.9
**Number Of Reads**	27
**Reads Per Million**	0.61 rpm (after QC)
**Ambiguities**	0
**Assembly Method**	de novo + reference guided assembly
**Consensus Caller**	Bcf Tools

Coverage Map

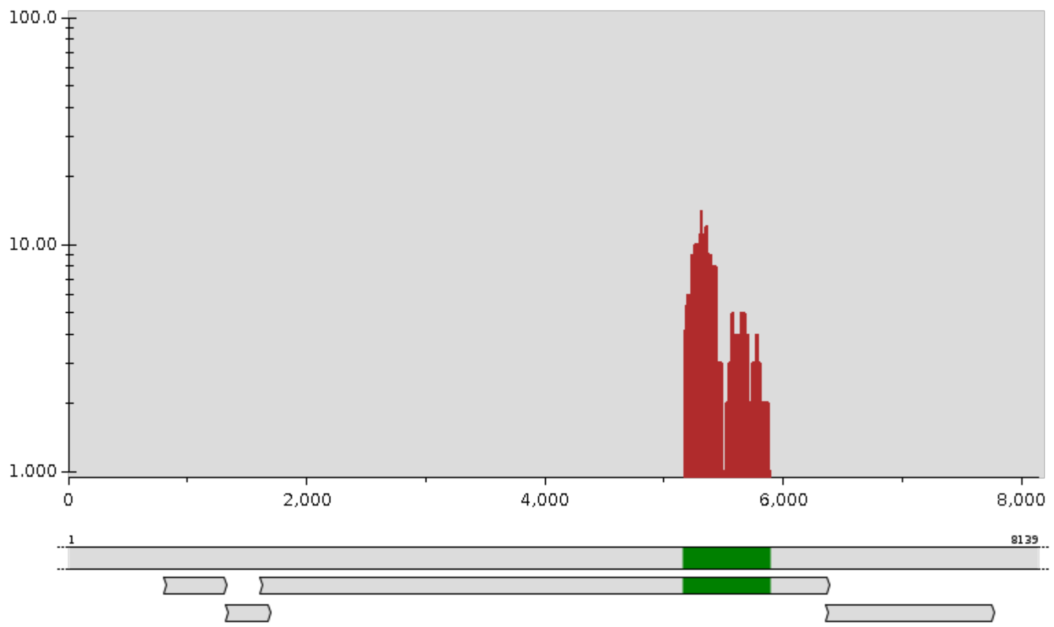

Assignment

**Type**	Dioscovirus dioscoreae (Taxonomy ID: 3052184)
**Reference Genome**	NC_040712.1
**NT Identity (%)**	64.1304
**AA Identity (%)**	56.0976
**Number Of Stop Codons**	0
**Number Of CDS**	4

Alignment

**Alignment Score**	394.0 (NT) + 914.0 (AA) = 1308.0
**Concordance (%)**	43.484

Alignment Method	Global, seeded, nucleotide + amino acids (AGA)

Genome Region

Sequence starts at position 5162 and ends at position 5901 relative to NC_040712.1 reference sequence.

Alignment Detailed Statistics

	Begin	End	Coverage	Score	Concordance	Matches	Identities	I/D/M/F*	Stop Codons
**NT**	**5162**	**5901**	**9.1%**	**394**	**27.2%**	**736 (99.5%)**	**472 (63.8%)**	**0/4**	
Mutations:	5168G>A, 5171T>A, 5174C>A, 5178C>A, 5179C>G, 5180T>A, 5183C>A, 5184C>A, 5185A>C, 5187A>T, 5188G>C, 5190C>A, 5192C>A, 5195T>C, 5198G>A, 5199A>T, 5207T>A, 5213T>G, 5216C>A, 5219C>A, 5220delA, 5222A>C, 5226T>A, 5227C>A, 5228T>C, 5232C>A, 5233A>T, 5237A>C, 5243T>A, 5247A>G, 5248G>C, 5249T>C, 5250C>A, 5252T>A, 5259T>A, 5260A>T, 5261C>A, 5271C>A, 5276A>C, 5288A>T, 5289T>A, 5290A>G, 5291C>A, 5293A>T, 5294A>G, 5297C>T, 5298C>G, 5299A>C, 5300A>T, 5303C>T, 5305C>A, 5307C>T, 5309C>A, 5313A>G, 5314G>A, 5315T>C, 5317T>A, 5318T>A, 5319G>A, 5321C>T, 5322T>G, 5324T>A, 5327C>A, 5331C>A, 5332T>A, 5335A>G, 5339T>A, 5340A>C, 5344A>G, 5345C>A, 5348G>A, 5350T>G, 5351T>A, 5352G>A, 5354C>T, 5356A>T, 5360C>T, 5369T>C, 5370C>T, 5371T>G, 5372A>T, 5375G>A, 5381A>G, 5383T>A, 5384C>T, 5385C>T, 5386A>G, 5387C>G, 5390G>A, 5391A>G, 5393T>A, 5395T>A, 5396G>A, 5400G>C, 5402T>C, 5405A>T, 5406C>G, 5408G>T, 5424G>A, 5430G>A, 5431T>C, 5432C>T, 5435C>T, 5439C>G, 5441G>A, 5444T>A, 5457A>T, 5461T>C, 5466C>T, 5471C>T, 5475C>T, 5477C>G, 5480A>G, 5482A>C, 5483T>A, 5489T>A, 5490T>C, 5492A>T, 5493G>A, 5502C>A, 5507A>G, 5513T>C, 5519A>T, 5523A>G, 5524A>G, 5526A>G, 5532T>A, 5533C>A, 5535G>A, 5538T>A, 5543C>A, 5544T>C, 5545G>T, 5546T>A, 5549T>C, 5553A>G, 5558C>T, 5561T>C, 5564T>C, 5565T>C, 5567G>A, 5568A>G, 5573T>C, 5574T>A, 5575C>G, 5577G>A, 5578A>G, 5580A>G, 5581G>A, 5585A>G, 5589C>G, 5595G>C, 5598C>G, 5607T>C, 5608T>A, 5610C>A, 5611A>C, 5612G>A, 5613T>A, 5615C>T, 5618C>T, 5619C>A, 5620A>G, 5621A>G, 5622G>T, 5626G>T, 5627T>C, 5628A>G, 5629A>T, 5630G>A, 5631G>A, 5632A>G, 5633A>T, 5634G>A, 5639G>A, 5640C>A, 5642T>A, 5645T>A, 5646T>A, 5648G>A, 5649T>A, 5651A>C, 5656C>A, 5657A>G, 5661T>A, 5664A>G, 5667A>T, 5670G>T, 5672A>T, 5673G>A, 5674T>A, 5676G>A, 5679A>C, 5685G>A, 5687A>C, 5691C>T, 5693T>A, 5696C>A, 5697C>G, 5700G>A, 5701A>C, 5702A>T, 5703A>T, 5711A>T, 5714A>G, 5717A>G, 5718G>A, 5720T>A, 5721C>A, 5726C>A, 5739T>G, 5740T>C, 5741A>T, 5748A>G, 5756A>T, 5757T>A, 5759T>G, 5762T>A, 5763_5765delGAA, 5768C>T, 5769C>A, 5777A>T, 5779C>A, 5781T>A, 5782T>C, 5786G>A, 5788G>A, 5790C>T, 5795G>A, 5798G>A, 5807G>A, 5808A>T, 5811T>G, 5813A>G, 5816C>T, 5822C>T, 5828T>C, 5830A>T, 5834C>A, 5836C>A, 5843G>A, 5844A>G, 5846T>A, 5850T>A, 5851A>T, 5853A>G, 5855T>A, 5856A>G, 5859A>C, 5860T>C, 5864T>A, 5868A>G, 5869A>C, 5870T>A, 5873G>A, 5874T>A, 5875G>T, 5876T>A, 5877T>G, 5878C>G, 5879C>A, 5882C>T, 5889G>C, 5893G>A, 5895A>C, 5897A>C								

CDS

	Begin	End	Coverage	Score	Concordance	Matches	Identities	I/D/M/F*	Stop Codons
EXK67_gp3	1185	1431	15.5%	914	53.3%	246 (99.6%)	138 (55.9%)	0/1/1/1	0
Protein mutations:	P1190R (5178C>A 5179C>G 5180T>A), Q1192T (5184C>A 5185A>C), T1197S (5199A>T), I1201M (5213T>G), N1203K (5219C>A), S1206N (5226T>A 5227C>A 5228T>C), Q1208I (5232C>A 5233A>T), S1213A (5247A>G 5248G>C 5249T>C), Y1217I (5259T>A 5260A>T 5261C>A), Y1227R (5289T>A 5290A>G 5291C>A), K1228M (5293A>T 5294A>G), Q1230A (5298C>G 5299A>C 5300A>T), T1232K (5305C>A), S1235D (5313A>G 5314G>A 5315T>C), I1236K (5317T>A 5318T>A), D1237N (5319G>A 5321C>T), Y1238E (5322T>G 5324T>A), L1241N (5331C>A 5332T>A), K1242R (5335A>G), K1244Q (5340A>C), D1245G (5344A>G 5345C>A), I1247R (5350T>G 5351T>A), V1248I (5352G>A 5354C>T), Y1249F (5356A>T), L1254C (5370C>T 5371T>G 5372A>T), F1258Y (5383T>A 5384C>T), H1259W (5385C>T 5386A>G 5387C>G), I1261V (5391A>G 5393T>A), M1262K (5395T>A 5396G>A), D1264H (5400G>C 5402T>C), Q1266D (5406C>G 5408G>T), A1272T (5424G>A), V1274T (5430G>A 5431T>C 5432C>T), Q1277E (5439C>G 5441G>A), I1283L (5457A>T), V1284A (5461T>C), P1286S (5466C>T), N1291T (5482A>C 5483T>A), S1294P (5490T>C 5492A>T), V1295I (5493G>A), K1305G (5523A>G 5524A>G), K1306E (5526A>G), S1308K (5532T>A 5533C>A), E1309K (5535G>A), F1310I (5538T>A), C1312L (5544T>C 5545G>T 5546T>A), I1315V (5553A>G), I1320V (5568A>G), E1323R (5577G>A 5578A>G), S1324D (5580A>G 5581G>A), I1325M (5585A>G), Q1327E (5589C>G), V1329L (5595G>C), Q1330E (5598C>G), L1333Q (5607T>C 5608T>A), Q1334T (5610C>A 5611A>C 5612G>A), F1335I (5613T>A 5615C>T), Q1337R (5619C>A 5620A>G 5621A>G), V1338L (5622G>T), C1339F (5626G>T 5627T>C), K1340V (5628A>G 5629A>T 5630G>A), E1341S (5631G>A 5632A>G 5633A>T), E1342K (5634G>A), L1344I (5640C>A 5642T>A), L1346I (5646T>A 5648G>A), S1347T (5649T>A 5651A>C), T1349K (5656C>A 5657A>G), L1351M (5661T>A), K1352E (5664A>G), I1353L (5667A>T), G1354C (5670G>T 5672A>T), V1355K (5673G>A 5674T>A), A1356T (5676G>A), N1357H (5679A>C), E1359N (5685G>A 5687A>C), L1363V (5697C>G), E1364T (5700G>A 5701A>C 5702A>T), I1365L (5703A>T), E1367D (5711A>T), V1370I (5718G>A 5720T>A), Q1371K (5721C>A), L1377A (5739T>G 5740T>C 5741A>T), I1380V (5748A>G), E1382D (5756A>T), F1383M (5757T>A 5759T>G), E1385del (5763_5765delGAA), Q1387K (5769C>A), E1389D (5777A>T), T1390K (5779C>A), L1391T (5781T>A 5782T>C), G1393D (5788G>A), I1400L (5808A>T), L1401V (5811T>G 5813A>G), Y1407F (5830A>T), P1409Q (5836C>A), S1412G (5844A>G 5846T>A), Y1414I (5850T>A 5851A>T), T1415A (5853A>G 5855T>A), R1416G (5856A>G), I1417P (5859A>C 5860T>C), F1418L (5864T>A), N1420A (5868A>G 5869A>C 5870T>A), C1422I (5874T>A 5875G>T 5876T>A), S1423G (5877T>G 5878C>G 5879C>A), E1427Q (5889G>C), R1428K (5893G>A), K1429H (5895A>C 5897A>C)								
Codon mutations:	AAG1186AAA (5168G>A), GTT1187GTA (5171T>A), ATC1188ATA (5174C>A), CCT1190AGA (5178C>A 5179C>G 5180T>A), TCC1191TCA (5183C>A), CAA1192ACA (5184C>A 5185A>C), AGT1193TCT (5187A>T 5188G>C), CGC1194AGA (5190C>A 5192C>A), CAT1195CAC (5195T>C), AGG1196AGA (5198G>A), ACA1197TCA (5199A>T), GCT1199GCA (5207T>A), ATT1201ATG (5213T>G), GTC1202GTA (5216C>A), AAC1203AAA (5219C>A), AAA1204-AC (5220delA 5222A>C), TCT1206AAC (5226T>A 5227C>A 5228T>C), CAA1208ATA (5232C>A 5233A>T), GTA1209GTC (5237A>C), GGT1211GGA (5243T>A), AGT1213GCC (5247A>G 5248G>C 5249T>C), CGT1214AGA (5250C>A 5252T>A), TAC1217ATA (5259T>A 5260A>T 5261C>A), CGA1221AGA (5271C>A), CTA1222CTC (5276A>C), ACA1226ACT (5288A>T), TAC1227AGA (5289T>A 5290A>G 5291C>A), AAA1228ATG (5293A>T 5294A>G), GAC1229GAT (5297C>T), CAA1230GCT (5298C>G 5299A>C 5300A>T), TAC1231TAT (5303C>T), ACA1232AAA (5305C>A), CTC1233TTA (5307C>T 5309C>A), AGT1235GAC (5313A>G 5314G>A 5315T>C), ATT1236AAA (5317T>A 5318T>A), GAC1237AAT (5319G>A 5321C>T), TAT1238GAA (5322T>G 5324T>A), CTC1239CTA (5327C>A), CTT1241AAT (5331C>A 5332T>A), AAA1242AGA (5335A>G), ATT1243ATA (5339T>A), AAA1244CAA (5340A>C), GAC1245GGA (5344A>G 5345C>A), AAG1246AAA (5348G>A), ATT1247AGA (5350T>G 5351T>A), GTC1248ATT (5352G>A 5354C>T), TAC1249TTC (5356A>T), AGC1250AGT (5360C>T), GAT1253GAC (5369T>C), CTA1254TGT (5370C>T 5371T>G 5372A>T), AAG1255AAA (5375G>A), GGA1257GGG (5381A>G), TTC1258TAT (5383T>A 5384C>T), CAC1259TGG (5385C>T 5386A>G 5387C>G), CAG1260CAA (5390G>A), ATT1261GTA (5391A>G 5393T>A), ATG1262AAA (5395T>A 5396G>A), GAT1264CAC (5400G>C 5402T>C), CCA1265CCT (5405A>T), CAG1266GAT (5406C>G 5408G>T), GCA1272ACA (5424G>A), GTC1274ACT (5430G>A 5431T>C 5432C>T), TGC1275TGT (5435C>T), CAG1277GAA (5439C>G 5441G>A), GGT1278GGA (5444T>A), ATA1283TTA (5457A>T), GTA1284GCA (5461T>C), CCA1286TCA (5466C>T), TTC1287TTT (5471C>T), CTC1289TTG (5475C>T 5477C>G), AAA1290AAG (5480A>G), AAT1291ACA (5482A>C 5483T>A), CCT1293CCA (5489T>A), TCA1294CCT (5490T>C 5492A>T), GTA1295ATA (5493G>A), CGA1298AGA (5502C>A), AAA1299AAG (5507A>G), GAT1301GAC (5513T>C), ATA1303ATT (5519A>T), AAA1305GGA (5523A>G 5524A>G), AAA1306GAA (5526A>G), TCA1308AAA (5532T>A 5533C>A), GAA1309AAA (5535G>A), TTT1310ATT (5538T>A), GTC1311GTA (5543C>A), TGT1312CTA (5544T>C 5545G>T 5546T>A), GTT1313GTC (5549T>C), ATA1315GTA (5553A>G), GAC1316GAT (5558C>T), GAT1317GAC (5561T>C), ATT1318ATC (5564T>C), TTG1319CTA (5565T>C 5567G>A), ATA1320GTA (5568A>G), TTT1321TTC (5573T>C), TCT1322AGT (5574T>A 5575C>G), GAA1323AGA (5577G>A 5578A>G), AGT1324GAT (5580A>G 5581G>A), ATA1325ATG (5585A>G), CAA1327GAA (5589C>G), GTA1329CTA (5595G>C), CAA1330GAA (5598C>G), TTA1333CAA (5607T>C 5608T>A), CAG1334ACA (5610C>A 5611A>C 5612G>A), TTC1335ATT (5613T>A 5615C>T), TTC1336TTT (5618C>T), CAA1337AGG (5619C>A 5620A>G 5621A>G), GTA1338TTA (5622G>T), TGT1339TTC (5626G>T 5627T>C), AAG1340GTA (5628A>G 5629A>T 5630G>A), GAA1341AGT (5631G>A 5632A>G 5633A>T), GAA1342AAA (5634G>A), GGG1343GGA (5639G>A), CTT1344ATA (5640C>A 5642T>A), ATT1345ATA (5645T>A), TTG1346ATA (5646T>A 5648G>A), TCA1347ACC (5649T>A 5651A>C), ACA1349AAG (5656C>A 5657A>G), TTG1351ATG (5661T>A), AAA1352GAA (5664A>G), ATA1353TTA (5667A>T), GGA1354TGT (5670G>T 5672A>T), GTA1355AAA (5673G>A 5674T>A), GCA1356ACA (5676G>A), AAT1357CAT (5679A>C), GAA1359AAC (5685G>A 5687A>C), CTT1361TTA (5691C>T 5693T>A), GGC1362GGA (5696C>A), CTA1363GTA (5697C>G), GAA1364ACT (5700G>A 5701A>C 5702A>T), ATA1365TTA (5703A>T), GAA1367GAT (5711A>T), GGA1368GGG (5714A>G), AAA1369AAG (5717A>G), GTT1370ATA (5718G>A 5720T>A), CAA1371AAA (5721C>A), CTC1372CTA (5726C>A), TTA1377GCT (5739T>G 5740T>C 5741A>T), ATA1380GTA (5748A>G), GAA1382GAT (5756A>T), TTT1383ATG (5757T>A 5759T>G), CCT1384CCA (5762T>A), GAA1385del (5763_5765delGAA), GAC1386GAT (5768C>T), CAA1387AAA (5769C>A), GAA1389GAT (5777A>T), ACA1390AAA (5779C>A), TTA1391ACA (5781T>A 5782T>C), AAG1392AAA (5786G>A), GGT1393GAT (5788G>A), CTA1394TTA (5790C>T), CAG1395CAA (5795G>A), AAG1396AAA (5798G>A), GGG1399GGA (5807G>A), ATA1400TTA (5808A>T), TTA1401GTG (5811T>G 5813A>G), AAC1402AAT (5816C>T), GCC1404GCT (5822C>T), AAT1406AAC (5828T>C), TAT1407TTT (5830A>T), ATC1408ATA (5834C>A), CCA1409CAA (5836C>A), TTG1411TTA (5843G>A), AGT1412GGA (5844A>G 5846T>A), TAT1414ATT (5850T>A 5851A>T), ACT1415GCA (5853A>G 5855T>A), AGA1416GGA (5856A>G), ATA1417CCA (5859A>C 5860T>C), TTT1418TTA (5864T>A), AAT1420GCA (5868A>G 5869A>C 5870T>A), AAG1421AAA (5873G>A), TGT1422ATA (5874T>A 5875G>T 5876T>A), TCC1423GGA (5877T>G 5878C>G 5879C>A), AGC1424AGT (5882C>T), GAA1427CAA (5889G>C), AGA1428AAA (5893G>A), AAA1429CAC (5895A>C 5897A>C)								

Proteins

	Begin	End	Coverage	Score	Concordance	Matches	Identities	I/D/M/F*	Stop Codons
ORF3 (YP_009553219.1)	1185	1431	15.5%	914	53.3%	246 (99.6%)	138 (55.9%)	0/1/1/1	0
Protein mutations:	P1190R (5178C>A 5179C>G 5180T>A), Q1192T (5184C>A 5185A>C), T1197S (5199A>T), I1201M (5213T>G), N1203K (5219C>A), S1206N (5226T>A 5227C>A 5228T>C), Q1208I (5232C>A 5233A>T), S1213A (5247A>G 5248G>C 5249T>C), Y1217I (5259T>A 5260A>T 5261C>A), Y1227R (5289T>A 5290A>G 5291C>A), K1228M (5293A>T 5294A>G), Q1230A (5298C>G 5299A>C 5300A>T), T1232K (5305C>A), S1235D (5313A>G 5314G>A 5315T>C), I1236K (5317T>A 5318T>A), D1237N (5319G>A 5321C>T), Y1238E (5322T>G 5324T>A), L1241N (5331C>A 5332T>A), K1242R (5335A>G), K1244Q (5340A>C), D1245G (5344A>G 5345C>A), I1247R (5350T>G 5351T>A), V1248I (5352G>A 5354C>T), Y1249F (5356A>T), L1254C (5370C>T 5371T>G 5372A>T), F1258Y (5383T>A 5384C>T), H1259W (5385C>T 5386A>G 5387C>G), I1261V (5391A>G 5393T>A), M1262K (5395T>A 5396G>A), D1264H (5400G>C 5402T>C), Q1266D (5406C>G 5408G>T), A1272T (5424G>A), V1274T (5430G>A 5431T>C 5432C>T), Q1277E (5439C>G 5441G>A), I1283L (5457A>T), V1284A (5461T>C), P1286S (5466C>T), N1291T (5482A>C 5483T>A), S1294P (5490T>C 5492A>T), V1295I (5493G>A), K1305G (5523A>G 5524A>G), K1306E (5526A>G), S1308K (5532T>A 5533C>A), E1309K (5535G>A), F1310I (5538T>A), C1312L (5544T>C 5545G>T 5546T>A), I1315V (5553A>G), I1320V (5568A>G), E1323R (5577G>A 5578A>G), S1324D (5580A>G 5581G>A), I1325M (5585A>G), Q1327E (5589C>G), V1329L (5595G>C), Q1330E (5598C>G), L1333Q (5607T>C 5608T>A), Q1334T (5610C>A 5611A>C 5612G>A), F1335I (5613T>A 5615C>T), Q1337R (5619C>A 5620A>G 5621A>G), V1338L (5622G>T), C1339F (5626G>T 5627T>C), K1340V (5628A>G 5629A>T 5630G>A), E1341S (5631G>A 5632A>G 5633A>T), E1342K (5634G>A), L1344I (5640C>A 5642T>A), L1346I (5646T>A 5648G>A), S1347T (5649T>A 5651A>C), T1349K (5656C>A 5657A>G), L1351M (5661T>A), K1352E (5664A>G), I1353L (5667A>T), G1354C (5670G>T 5672A>T), V1355K (5673G>A 5674T>A), A1356T (5676G>A), N1357H (5679A>C), E1359N (5685G>A 5687A>C), L1363V (5697C>G), E1364T (5700G>A 5701A>C 5702A>T), I1365L (5703A>T), E1367D (5711A>T), V1370I (5718G>A 5720T>A), Q1371K (5721C>A), L1377A (5739T>G 5740T>C 5741A>T), I1380V (5748A>G), E1382D (5756A>T), F1383M (5757T>A 5759T>G), E1385del (5763_5765delGAA), Q1387K (5769C>A), E1389D (5777A>T), T1390K (5779C>A), L1391T (5781T>A 5782T>C), G1393D (5788G>A), I1400L (5808A>T), L1401V (5811T>G 5813A>G), Y1407F (5830A>T), P1409Q (5836C>A), S1412G (5844A>G 5846T>A), Y1414I (5850T>A 5851A>T), T1415A (5853A>G 5855T>A), R1416G (5856A>G), I1417P (5859A>C 5860T>C), F1418L (5864T>A), N1420A (5868A>G 5869A>C 5870T>A), C1422I (5874T>A 5875G>T 5876T>A), S1423G (5877T>G 5878C>G 5879C>A), E1427Q (5889G>C), R1428K (5893G>A), K1429H (5895A>C 5897A>C)								

	Begin	End	Coverage	Score	Concordance	Matches	Identities	I/D/M/F*	Stop Codons
**NT**	**5162**	**5901**	**9.1%**	**394**	**27.2%**	**736 (99.5%)**	**472 (63.8%)**	**0/4**	
Codon mutations:	AAG1186AAA (5168G>A), GTT1187GTA (5171T>A), ATC1188ATA (5174C>A), CCT1190AGA (5178C>A 5179C>G 5180T>A), TCC1191TCA (5183C>A), CAA1192ACA (5184C>A 5185A>C), AGT1193TCT (5187A>T 5188G>C), CGC1194AGA (5190C>A 5192C>A), CAT1195CAC (5195T>C), AGG1196AGA (5198G>A), ACA1197TCA (5199A>T), GCT1199GCA (5207T>A), ATT1201ATG (5213T>G), GTC1202GTA (5216C>A), AAC1203AAA (5219C>A), AAA1204-AC (5220delA 5222A>C), TCT1206AAC (5226T>A 5227C>A 5228T>C), CAA1208ATA (5232C>A 5233A>T), GTA1209GTC (5237A>C), GGT1211GGA (5243T>A), AGT1213GCC (5247A>G 5248G>C 5249T>C), CGT1214AGA (5250C>A 5252T>A), TAC1217ATA (5259T>A 5260A>T 5261C>A), CGA1221AGA (5271C>A), CTA1222CTC (5276A>C), ACA1226ACT (5288A>T), TAC1227AGA (5289T>A 5290A>G 5291C>A), AAA1228ATG (5293A>T 5294A>G), GAC1229GAT (5297C>T), CAA1230GCT (5298C>G 5299A>C 5300A>T), TAC1231TAT (5303C>T), ACA1232AAA (5305C>A), CTC1233TTA (5307C>T 5309C>A), AGT1235GAC (5313A>G 5314G>A 5315T>C), ATT1236AAA (5317T>A 5318T>A), GAC1237AAT (5319G>A 5321C>T), TAT1238GAA (5322T>G 5324T>A), CTC1239CTA (5327C>A), CTT1241AAT (5331C>A 5332T>A), AAA1242AGA (5335A>G), ATT1243ATA (5339T>A), AAA1244CAA (5340A>C), GAC1245GGA (5344A>G 5345C>A), AAG1246AAA (5348G>A), ATT1247AGA (5350T>G 5351T>A), GTC1248ATT (5352G>A 5354C>T), TAC1249TTC (5356A>T), AGC1250AGT (5360C>T), GAT1253GAC (5369T>C), CTA1254TGT (5370C>T 5371T>G 5372A>T), AAG1255AAA (5375G>A), GGA1257GGG (5381A>G), TTC1258TAT (5383T>A 5384C>T), CAC1259TGG (5385C>T 5386A>G 5387C>G), CAG1260CAA (5390G>A), ATT1261GTA (5391A>G 5393T>A), ATG1262AAA (5395T>A 5396G>A), GAT1264CAC (5400G>C 5402T>C), CCA1265CCT (5405A>T), CAG1266GAT (5406C>G 5408G>T), GCA1272ACA (5424G>A), GTC1274ACT (5430G>A 5431T>C 5432C>T), TGC1275TGT (5435C>T), CAG1277GAA (5439C>G 5441G>A), GGT1278GGA (5444T>A), ATA1283TTA (5457A>T), GTA1284GCA (5461T>C), CCA1286TCA (5466C>T), TTC1287TTT (5471C>T), CTC1289TTG (5475C>T 5477C>G), AAA1290AAG (5480A>G), AAT1291ACA (5482A>C 5483T>A), CCT1293CCA (5489T>A), TCA1294CCT (5490T>C 5492A>T), GTA1295ATA (5493G>A), CGA1298AGA (5502C>A), AAA1299AAG (5507A>G), GAT1301GAC (5513T>C), ATA1303ATT (5519A>T), AAA1305GGA (5523A>G 5524A>G), AAA1306GAA (5526A>G), TCA1308AAA (5532T>A 5533C>A), GAA1309AAA (5535G>A), TTT1310ATT (5538T>A), GTC1311GTA (5543C>A), TGT1312CTA (5544T>C 5545G>T 5546T>A), GTT1313GTC (5549T>C), ATA1315GTA (5553A>G), GAC1316GAT (5558C>T), GAT1317GAC (5561T>C), ATT1318ATC (5564T>C), TTG1319CTA (5565T>C 5567G>A), ATA1320GTA (5568A>G), TTT1321TTC (5573T>C), TCT1322AGT (5574T>A 5575C>G), GAA1323AGA (5577G>A 5578A>G), AGT1324GAT (5580A>G 5581G>A), ATA1325ATG (5585A>G), CAA1327GAA (5589C>G), GTA1329CTA (5595G>C), CAA1330GAA (5598C>G), TTA1333CAA (5607T>C 5608T>A), CAG1334ACA (5610C>A 5611A>C 5612G>A), TTC1335ATT (5613T>A 5615C>T), TTC1336TTT (5618C>T), CAA1337AGG (5619C>A 5620A>G 5621A>G), GTA1338TTA (5622G>T), TGT1339TTC (5626G>T 5627T>C), AAG1340GTA (5628A>G 5629A>T 5630G>A), GAA1341AGT (5631G>A 5632A>G 5633A>T), GAA1342AAA (5634G>A), GGG1343GGA (5639G>A), CTT1344ATA (5640C>A 5642T>A), ATT1345ATA (5645T>A), TTG1346ATA (5646T>A 5648G>A), TCA1347ACC (5649T>A 5651A>C), ACA1349AAG (5656C>A 5657A>G), TTG1351ATG (5661T>A), AAA1352GAA (5664A>G), ATA1353TTA (5667A>T), GGA1354TGT (5670G>T 5672A>T), GTA1355AAA (5673G>A 5674T>A), GCA1356ACA (5676G>A), AAT1357CAT (5679A>C), GAA1359AAC (5685G>A 5687A>C), CTT1361TTA (5691C>T 5693T>A), GGC1362GGA (5696C>A), CTA1363GTA (5697C>G), GAA1364ACT (5700G>A 5701A>C 5702A>T), ATA1365TTA (5703A>T), GAA1367GAT (5711A>T), GGA1368GGG (5714A>G), AAA1369AAG (5717A>G), GTT1370ATA (5718G>A 5720T>A), CAA1371AAA (5721C>A), CTC1372CTA (5726C>A), TTA1377GCT (5739T>G 5740T>C 5741A>T), ATA1380GTA (5748A>G), GAA1382GAT (5756A>T), TTT1383ATG (5757T>A 5759T>G), CCT1384CCA (5762T>A), GAA1385del (5763_5765delGAA), GAC1386GAT (5768C>T), CAA1387AAA (5769C>A), GAA1389GAT (5777A>T), ACA1390AAA (5779C>A), TTA1391ACA (5781T>A 5782T>C), AAG1392AAA (5786G>A), GGT1393GAT (5788G>A), CTA1394TTA (5790C>T), CAG1395CAA (5795G>A), AAG1396AAA (5798G>A), GGG1399GGA (5807G>A), ATA1400TTA (5808A>T), TTA1401GTG (5811T>G 5813A>G), AAC1402AAT (5816C>T), GCC1404GCT (5822C>T), AAT1406AAC (5828T>C), TAT1407TTT (5830A>T), ATC1408ATA (5834C>A), CCA1409CAA (5836C>A), TTG1411TTA (5843G>A), AGT1412GGA (5844A>G 5846T>A), TAT1414ATT (5850T>A 5851A>T), ACT1415GCA (5853A>G 5855T>A), AGA1416GGA (5856A>G), ATA1417CCA (5859A>C 5860T>C), TTT1418TTA (5864T>A), AAT1420GCA (5868A>G 5869A>C 5870T>A), AAG1421AAA (5873G>A), TGT1422ATA (5874T>A 5875G>T 5876T>A), TCC1423GGA (5877T>G 5878C>G 5879C>A), AGC1424AGT (5882C>T), GAA1427CAA (5889G>C), AGA1428AAA (5893G>A), AAA1429CAC (5895A>C 5897A>C)								

*: Inserts / Deletes / Misaligned / Frameshifts

Analysis details

This analysis was performed with panviral2.64

NGS Details (UN9): Badnavirus venatheobromae

Assembly

**Coverage Length**	258 (1 contig(s))
**Depth Of Coverage**	11.2
**Number Of Reads**	25
**Reads Per Million**	0.57 rpm (after QC)
**Ambiguities**	0
**Assembly Method**	de novo + reference guided assembly
**Consensus Caller**	Bcf Tools

Coverage Map

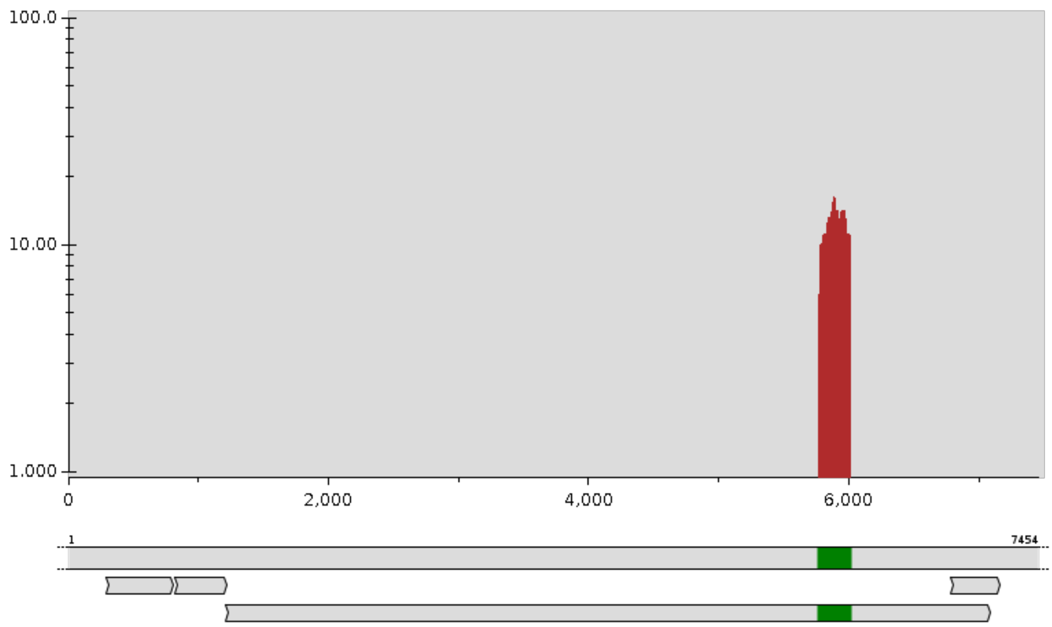

Assignment

**Type**	Badnavirus venatheobromae (Taxonomy ID: 3047715)
**Reference Genome**	NC_033739.1
**NT Identity (%)**	50.5747
**AA Identity (%)**	44.8276
**Number Of Stop Codons**	0
**Number Of CDS**	4

Alignment

**Alignment Score**	0.0 (NT) + 287.0 (AA) = 287.0
**Concordance (%)**	25.4658

Alignment Method	Global, seeded, nucleotide + amino acids (AGA)

Genome Region

Sequence starts at position 5764 and ends at position 6021 relative to NC_033739.1 reference sequence.

Alignment Detailed Statistics

	Begin	End	Coverage	Score	Concordance	Matches	Identities	I/D/M/F*	Stop Codons
**NT**	**5764**	**6021**	**3.5%**	**0**	**0%**	**258 (98.9%)**	**132 (50.6%)**	**3/0**	
Mutations:	5765T>C, 5768A>G, 5770G>T, 5771G>C, 5772C>A, 5773T>A, 5774A>G, 5777C>G, 5786C>T, 5789A>C, 5790T>C, 5792A>T, 5794A>C, 5795G>A, 5807C>T, 5809T>C, 5813T>C, 5817A>G, 5818G>C, 5819A>C, 5820A>T, 5821A>T, 5822G>C, 5826G>A, 5828C>T, 5829A>C, 5831C>G, 5832T>G, 5833G>T, 5834C>T, 5837T>C, 5838A>G, 5841G>C, 5842G>C, 5843A>T, 5843_5844insTTT, 5844A>C, 5845C>T, 5846A>G, 5847G>A, 5848A>G, 5850G>A, 5852A>G, 5856A>G, 5858A>T, 5859G>T, 5860C>T, 5861A>G, 5864T>A, 5866A>T, 5868A>T, 5879C>A, 5882A>T, 5885C>T, 5887T>A, 5889T>A, 5890C>G, 5891A>T, 5892C>T, 5893A>T, 5897T>A, 5899A>T, 5900G>A, 5901G>A, 5902A>G, 5903A>T, 5906A>T, 5912C>T, 5914G>A, 5915T>G, 5918T>C, 5919C>T, 5922C>A, 5923A>G, 5924A>G, 5925A>G, 5926G>T, 5927G>T, 5928T>G, 5933C>T, 5934C>G, 5938T>C, 5939A>T, 5940T>C, 5941G>T, 5943C>A, 5946A>T, 5947A>G, 5948G>T, 5952G>T, 5953G>C, 5954G>A, 5955T>C, 5957A>C, 5958G>T, 5959T>A, 5960C>T, 5961C>G, 5962T>C, 5963G>T, 5965G>A, 5966T>G, 5968A>G, 5970G>T, 5971A>C, 5972T>A, 5976A>T, 5977T>G, 5978G>T, 5979G>T, 5981A>C, 5982A>T, 5984A>T, 5986C>G, 5987A>C, 5988G>C, 5989T>A, 5990C>G, 5997A>G, 6004T>A, 6006C>T, 6008T>A, 6011A>T, 6012G>C, 6013C>A, 6017T>A, 6021G>A								

CDS

	Begin	End	Coverage	Score	Concordance	Matches	Identities	I/D/M/F*	Stop Codons
B1U04_gp3	1519	1604	4.4%	287	45.6%	86 (98.9%)	39 (44.8%)	1/0/0/0	0
Protein mutations:	W1520F (5770G>T 5771G>C), L1521K (5772C>A 5773T>A 5774A>G), K1528T (5794A>C 5795G>A), I1533T (5809T>C), R1536A (5817A>G 5818G>C 5819A>C), K1537F (5820A>T 5821A>T 5822G>C), D1539N (5826G>A 5828C>T), N1540Q (5829A>C 5831C>G), C1541V (5832T>G 5833G>T 5834C>T), K1543E (5838A>G), G1544P (5841G>C 5842G>C 5843A>T), G1544_T1545insF (5843_5844insTTT), T1545L (5844A>C 5845C>T 5846A>G), E1546R (5847G>A 5848A>G), E1547K (5850G>A 5852A>G), I1549V (5856A>G 5858A>T), A1550L (5859G>T 5860C>T 5861A>G), Y1552F (5866A>T), I1553F (5868A>T), F1559Y (5887T>A), Q1561L (5892C>T 5893A>T), E1563V (5899A>T 5900G>A), E1564S (5901G>A 5902A>G 5903A>T), E1565D (5906A>T), R1568Q (5914G>A 5915T>G), Q1571R (5922C>A 5923A>G 5924A>G), R1572V (5925A>G 5926G>T 5927G>T), F1573V (5928T>G), Q1575E (5934C>G), I1576T (5938T>C 5939A>T), C1577L (5940T>C 5941G>T), Q1578K (5943C>A), K1579C (5946A>T 5947A>G 5948G>T), G1581S (5952G>T 5953G>C 5954G>A), V1583Y (5958G>T 5959T>A 5960C>T), L1584A (5961C>G 5962T>C 5963G>T), S1585K (5965G>A 5966T>G), K1586R (5968A>G), D1587S (5970G>T 5971A>C 5972T>A), M1589C (5976A>T 5977T>G 5978G>T), A1590S (5979G>T 5981A>C), I1591F (5982A>T 5984A>T), A1592G (5986C>G 5987A>C), V1593Q (5988G>C 5989T>A 5990C>G), I1596V (5997A>G), F1598Y (6004T>A), A1601H (6012G>C 6013C>A)								
Codon mutations:	TAT1518.AC (5765T>C), GAA1519GAG (5768A>G), TGG1520TTC (5770G>T 5771G>C), CTA1521AAG (5772C>A 5773T>A 5774A>G), GTC1522GTG (5777C>G), TTC1525TTT (5786C>T), GGA1526GGC (5789A>C), TTA1527CTT (5790T>C 5792A>T), AAG1528ACA (5794A>C 5795G>A), GCC1532GCT (5807C>T), ATT1533ACT (5809T>C), TTT1534TTC (5813T>C), AGA1536GCC (5817A>G 5818G>C 5819A>C), AAG1537TTC (5820A>T 5821A>T 5822G>C), GAC1539AAT (5826G>A 5828C>T), AAC1540CAG (5829A>C 5831C>G), TGC1541GTT (5832T>G 5833G>T 5834C>T), TTT1542TTC (5837T>C), AAA1543GAA (5838A>G), GGA1544CCT (5841G>C 5842G>C 5843A>T), GGA1544_ACA1545insTTT (5843_5844insTTT), ACA1545CTG (5844A>C 5845C>T 5846A>G), GAA1546AGA (5847G>A 5848A>G), GAA1547AAG (5850G>A 5852A>G), ATA1549GTT (5856A>G 5858A>T), GCA1550TTG (5859G>T 5860C>T 5861A>G), GTT1551GTA (5864T>A), TAT1552TTT (5866A>T), ATT1553TTT (5868A>T), ATC1556ATA (5879C>A), CTA1557CTT (5882A>T), GTC1558GTT (5885C>T), TTC1559TAC (5887T>A), TCA1560AGT (5889T>A 5890C>G 5891A>T), CAG1561TTG (5892C>T 5893A>T), ACT1562ACA (5897T>A), GAG1563GTA (5899A>T 5900G>A), GAA1564AGT (5901G>A 5902A>G 5903A>T), GAA1565GAT (5906A>T), GTC1567GTT (5912C>T), CGT1568CAG (5914G>A 5915T>G), CAT1569CAC (5918T>C), CTA1570TTA (5919C>T), CAA1571AGG (5922C>A 5923A>G 5924A>G), AGG1572GTT (5925A>G 5926G>T 5927G>T), TTC1573GTC (5928T>G), TTC1574TTT (5933C>T), CAG1575GAG (5934C>G), ATA1576ACT (5938T>C 5939A>T), TGC1577CTC (5940T>C 5941G>T), CAG1578AAG (5943C>A), AAG1579TGT (5946A>T 5947A>G 5948G>T), GGG1581TCA (5952G>T 5953G>C 5954G>A), TTA1582CTC (5955T>C 5957A>C), GTC1583TAT (5958G>T 5959T>A 5960C>T), CTG1584GCT (5961C>G 5962T>C 5963G>T), AGT1585AAG (5965G>A 5966T>G), AAG1586AGG (5968A>G), GAT1587TCA (5970G>T 5971A>C 5972T>A), ATG1589TGT (5976A>T 5977T>G 5978G>T), GCA1590TCC (5979G>T 5981A>C), ATA1591TTT (5982A>T 5984A>T), GCA1592GGC (5986C>G 5987A>C), GTC1593CAG (5988G>C 5989T>A 5990C>G), ATT1596GTT (5997A>G), TTC1598TAC (6004T>A), CTT1599TTA (6006C>T 6008T>A), GGA1600GGT (6011A>T), GCC1601CAC (6012G>C 6013C>A), ATT1602ATA (6017T>A), GGT1604A.. (6021G>A)								

Proteins

	Begin	End	Coverage	Score	Concordance	Matches	Identities	I/D/M/F*	Stop Codons
polyprotein (YP_009345075.1)	1519	1604	4.4%	287	45.6%	86 (98.9%)	39 (44.8%)	1/0/0/0	0
Protein mutations:	W1520F (5770G>T 5771G>C), L1521K (5772C>A 5773T>A 5774A>G), K1528T (5794A>C 5795G>A), I1533T (5809T>C), R1536A (5817A>G 5818G>C 5819A>C), K1537F (5820A>T 5821A>T 5822G>C), D1539N (5826G>A 5828C>T), N1540Q (5829A>C 5831C>G), C1541V (5832T>G 5833G>T 5834C>T), K1543E (5838A>G), G1544P (5841G>C 5842G>C 5843A>T), G1544_T1545insF (5843_5844insTTT), T1545L (5844A>C 5845C>T 5846A>G), E1546R (5847G>A 5848A>G), E1547K (5850G>A 5852A>G), I1549V (5856A>G 5858A>T), A1550L (5859G>T 5860C>T 5861A>G), Y1552F (5866A>T), I1553F (5868A>T), F1559Y (5887T>A), Q1561L (5892C>T 5893A>T), E1563V (5899A>T 5900G>A), E1564S (5901G>A 5902A>G 5903A>T), E1565D (5906A>T), R1568Q (5914G>A 5915T>G), Q1571R (5922C>A 5923A>G 5924A>G), R1572V (5925A>G 5926G>T 5927G>T), F1573V (5928T>G), Q1575E (5934C>G), I1576T (5938T>C 5939A>T), C1577L (5940T>C 5941G>T), Q1578K (5943C>A), K1579C (5946A>T 5947A>G 5948G>T), G1581S (5952G>T 5953G>C 5954G>A), V1583Y (5958G>T 5959T>A 5960C>T), L1584A (5961C>G 5962T>C 5963G>T), S1585K (5965G>A 5966T>G), K1586R (5968A>G), D1587S (5970G>T 5971A>C 5972T>A), M1589C (5976A>T 5977T>G 5978G>T), A1590S (5979G>T 5981A>C), I1591F (5982A>T 5984A>T), A1592G (5986C>G 5987A>C), V1593Q (5988G>C 5989T>A 5990C>G), I1596V (5997A>G), F1598Y (6004T>A), A1601H (6012G>C 6013C>A)								
Codon mutations:	TAT1518.AC (5765T>C), GAA1519GAG (5768A>G), TGG1520TTC (5770G>T 5771G>C), CTA1521AAG (5772C>A 5773T>A 5774A>G), GTC1522GTG (5777C>G), TTC1525TTT (5786C>T), GGA1526GGC (5789A>C), TTA1527CTT (5790T>C 5792A>T), AAG1528ACA (5794A>C 5795G>A), GCC1532GCT (5807C>T), ATT1533ACT (5809T>C), TTT1534TTC (5813T>C), AGA1536GCC (5817A>G 5818G>C 5819A>C), AAG1537TTC (5820A>T 5821A>T 5822G>C), GAC1539AAT (5826G>A 5828C>T), AAC1540CAG (5829A>C 5831C>G), TGC1541GTT (5832T>G 5833G>T 5834C>T), TTT1542TTC (5837T>C), AAA1543GAA (5838A>G), GGA1544CCT (5841G>C 5842G>C 5843A>T), GGA1544_ACA1545insTTT (5843_5844insTTT), ACA1545CTG (5844A>C 5845C>T 5846A>G), GAA1546AGA (5847G>A 5848A>G), GAA1547AAG (5850G>A 5852A>G), ATA1549GTT (5856A>G 5858A>T), GCA1550TTG (5859G>T 5860C>T 5861A>G), GTT1551GTA (5864T>A), TAT1552TTT (5866A>T), ATT1553TTT (5868A>T), ATC1556ATA (5879C>A), CTA1557CTT (5882A>T), GTC1558GTT (5885C>T), TTC1559TAC (5887T>A), TCA1560AGT (5889T>A 5890C>G 5891A>T), CAG1561TTG (5892C>T 5893A>T), ACT1562ACA (5897T>A), GAG1563GTA (5899A>T 5900G>A), GAA1564AGT (5901G>A 5902A>G 5903A>T), GAA1565GAT (5906A>T), GTC1567GTT (5912C>T), CGT1568CAG (5914G>A 5915T>G), CAT1569CAC (5918T>C), CTA1570TTA (5919C>T), CAA1571AGG (5922C>A 5923A>G 5924A>G), AGG1572GTT (5925A>G 5926G>T 5927G>T), TTC1573GTC (5928T>G), TTC1574TTT (5933C>T), CAG1575GAG (5934C>G), ATA1576ACT (5938T>C 5939A>T), TGC1577CTC (5940T>C 5941G>T), CAG1578AAG (5943C>A), AAG1579TGT (5946A>T 5947A>G 5948G>T), GGG1581TCA (5952G>T 5953G>C 5954G>A), TTA1582CTC (5955T>C 5957A>C), GTC1583TAT (5958G>T 5959T>A 5960C>T), CTG1584GCT (5961C>G 5962T>C 5963G>T), AGT1585AAG (5965G>A 5966T>G), AAG1586AGG (5968A>G), GAT1587TCA (5970G>T 5971A>C 5972T>A), ATG1589TGT (5976A>T 5977T>G 5978G>T), GCA1590TCC (5979G>T 5981A>C), ATA1591TTT (5982A>T 5984A>T), GCA1592GGC (5986C>G 5987A>C), GTC1593CAG (5988G>C 5989T>A 5990C>G), ATT1596GTT (5997A>G), TTC1598TAC (6004T>A), CTT1599TTA (6006C>T 6008T>A), GGA1600GGT (6011A>T), GCC1601CAC (6012G>C 6013C>A), ATT1602ATA (6017T>A), GGT1604A.. (6021G>A)								

*: Inserts / Deletes / Misaligned / Frameshifts

Analysis details

This analysis was performed with panviral2.64

NGS Details (UN9): Epiphyllum badnavirus 1

Assembly

**Coverage Length**	258 (1 contig(s))
**Depth Of Coverage**	10.2
**Number Of Reads**	25
**Reads Per Million**	0.57 rpm (after QC)
**Ambiguities**	0
**Assembly Method**	de novo + reference guided assembly
**Consensus Caller**	Bcf Tools

Coverage Map

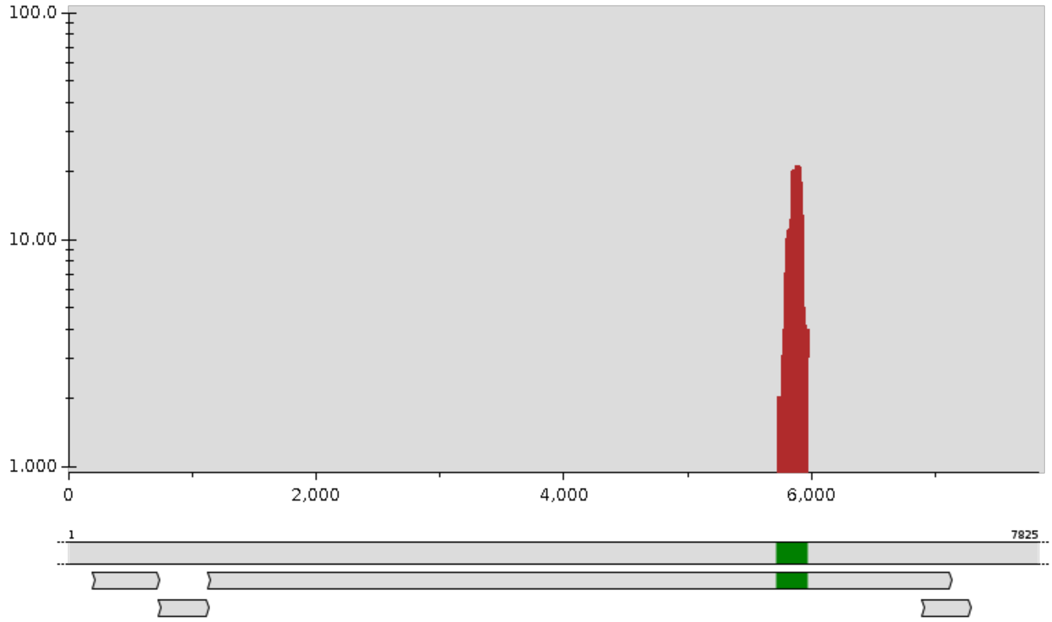

Assignment

**Type**	Epiphyllum badnavirus 1 (Taxonomy ID: 2518008)
**Reference Genome**	NC_076247.1
**NT Identity (%)**	56.9767
**AA Identity (%)**	43.0233
**Number Of Stop Codons**	0
**Number Of CDS**	4

Alignment

**Alignment Score**	72.0 (NT) + 240.0 (AA) = 312.0
**Concordance (%)**	27.932

Alignment Method	Global, seeded, nucleotide + amino acids (AGA)

Genome Region

Sequence starts at position 5717 and ends at position 5974 relative to NC_076247.1 reference sequence.

Alignment Detailed Statistics

	Begin	End	Coverage	Score	Concordance	Matches	Identities	I/D/M/F*	Stop Codons
**NT**	**5717**	**5974**	**3.3%**	**72**	**14.0%**	**258 (100%)**	**147 (57.0%)**	**0/0**	
Mutations:	5719G>A, 5725A>G, 5727A>T, 5730A>C, 5732A>C, 5734G>T, 5735A>T, 5737A>C, 5739A>C, 5743G>T, 5744T>G, 5746T>A, 5748C>T, 5749A>G, 5751C>T, 5754C>T, 5755A>T, 5756A>G, 5757A>G, 5758A>C, 5759C>A, 5760C>G, 5766C>T, 5767G>A, 5769C>G, 5770A>G, 5771A>T, 5772C>G, 5773A>C, 5776T>G, 5778C>G, 5781A>G, 5782G>A, 5784T>C, 5786A>G, 5787G>T, 5789A>G, 5790C>T, 5791T>C, 5794C>A, 5796A>G, 5800G>C, 5802C>T, 5806A>G, 5808C>T, 5809A>G, 5810C>A, 5811A>C, 5812A>C, 5814C>G, 5815A>T, 5817A>T, 5818C>G, 5820G>T, 5821A>C, 5824G>C, 5827T>C, 5828G>A, 5829T>G, 5830A>G, 5837G>C, 5841C>G, 5844C>T, 5847C>T, 5851T>A, 5853C>T, 5856T>C, 5857C>T, 5861A>G, 5863A>T, 5864G>C, 5868A>T, 5870T>A, 5871C>T, 5878A>T, 5880T>G, 5882T>G, 5886G>C, 5887G>A, 5888A>G, 5889C>G, 5891A>T, 5895A>T, 5896T>G, 5897C>A, 5898C>T, 5901C>A, 5905T>A, 5906G>A, 5910C>T, 5916C>T, 5918T>G, 5924C>G, 5926G>T, 5931A>C, 5933T>A, 5934T>C, 5936A>T, 5937C>T, 5940A>G, 5942G>T, 5943G>T, 5949T>A, 5953C>T, 5955A>T, 5958C>T, 5961A>G, 5962T>C, 5964G>T, 5966A>C, 5967G>T								
**CDS**									
QKM20_gp3	1531	1616	4.3%	240	38.8%	86 (100%)	37 (43.0%)	0/0/0/0	0
Protein mutations:	E1531K (5719G>A), K1533D (5725A>G 5727A>T), K1535T (5732A>C), E1536L (5734G>T 5735A>T), V1539C (5743G>T 5744T>G), F1540I (5746T>A 5748C>T), N1541D (5749A>G 5751C>T), K1543W (5755A>T 5756A>G 5757A>G), T1544Q (5758A>C 5759C>A 5760C>G), D1547K (5767G>A 5769C>G), N1548V (5770A>G 5771A>T 5772C>G), T1549P (5773A>C), F1550V (5776T>G 5778C>G), D1552N (5782G>A 5784T>C), Q1553R (5786A>G 5787G>T), Y1554C (5789A>G 5790C>T), S1555P (5791T>C), L1556M (5794C>A 5796A>G), G1558R (5800G>C 5802C>T), N1560D (5806A>G 5808C>T), T1561D (5809A>G 5810C>A 5811A>C), I1562L (5812A>C 5814C>G), I1563F (5815A>T 5817A>T), Q1564D (5818C>G 5820G>T), K1565Q (5821A>C), V1566L (5824G>C), C1567Q (5827T>C 5828G>A 5829T>G), N1568D (5830A>G), S1570T (5837G>C), F1575I (5851T>A 5853C>T), K1578R (5861A>G), F1581Y (5870T>A 5871C>T), I1584L (5878A>T 5880T>G), M1585R (5882T>G), M1586I (5886G>C), D1587R (5887G>A 5888A>G 5889C>G), E1588V (5891A>T), E1589D (5895A>T), S1590D (5896T>G 5897C>A 5898C>T), W1593K (5905T>A 5906G>A), L1597R (5918T>G), P1599R (5924C>G), D1600Y (5926G>T), L1602H (5933T>A 5934T>C), Y1603F (5936A>T 5937C>T), W1605F (5942G>T 5943G>T), P1609S (5953C>T 5955A>T), K1613T (5966A>C 5967G>T)								
Codon mutations:	GAG1531AAG (5719G>A), AAA1533GAT (5725A>G 5727A>T), GGA1534GGC (5730A>C), AAG1535ACG (5732A>C), GAG1536TTG (5734G>T 5735A>T), AGA1537CGC (5737A>C 5739A>C), GTC1539TGC (5743G>T 5744T>G), TTC1540ATT (5746T>A 5748C>T), AAC1541GAT (5749A>G 5751C>T), TAC1542TAT (5754C>T), AAA1543TGG (5755A>T 5756A>G 5757A>G), ACC1544CAG (5758A>C 5759C>A 5760C>G), AAC1546AAT (5766C>T), GAC1547AAG (5767G>A 5769C>G), AAC1548GTG (5770A>G 5771A>T 5772C>G), ACG1549CCG (5773A>C), TTC1550GTG (5776T>G 5778C>G), AAA1551AAG (5781A>G), GAT1552AAC (5782G>A 5784T>C), CAG1553CGT (5786A>G 5787G>T), TAC1554TGT (5789A>G 5790C>T), TCT1555CCT (5791T>C), CTA1556ATG (5794C>A 5796A>G), GGC1558CGT (5800G>C 5802C>T), AAC1560GAT (5806A>G 5808C>T), ACA1561GAC (5809A>G 5810C>A 5811A>C), ATC1562CTG (5812A>C 5814C>G), ATA1563TTT (5815A>T 5817A>T), CAG1564GAT (5818C>G 5820G>T), AAG1565CAG (5821A>C), GTC1566CTC (5824G>C), TGT1567CAG (5827T>C 5828G>A 5829T>G), AAT1568GAT (5830A>G), AGT1570ACT (5837G>C), GTC1571GTG (5841C>G), TTC1572TTT (5844C>T), TCC1573TCT (5847C>T), TTC1575ATT (5851T>A 5853C>T), GAT1576GAC (5856T>C), CTG1577TTG (5857C>T), AAG1578AGG (5861A>G), AGC1579TCC (5863A>T 5864G>C), GGA1580GGT (5868A>T), TTC1581TAT (5870T>A 5871C>T), ATT1584TTG (5878A>T 5880T>G), ATG1585AGG (5882T>G), ATG1586ATC (5886G>C), GAC1587AGG (5887G>A 5888A>G 5889C>G), GAA1588GTA (5891A>T), GAA1589GAT (5895A>T), TCC1590GAT (5896T>G 5897C>A 5898C>T), ATC1591ATA (5901C>A), TGG1593AAG (5905T>A 5906G>A), ACC1594ACT (5910C>T), TTC1596TTT (5916C>T), CTG1597CGG (5918T>G), CCA1599CGA (5924C>G), GAT1600TAT (5926G>T), GGA1601GGC (5931A>C), CTT1602CAC (5933T>A 5934T>C), TAC1603TTT (5936A>T 5937C>T), GAA1604GAG (5940A>G), TGG1605TTT (5942G>T 5943G>T), GTT1607GTA (5949T>A), CCA1609TCT (5953C>T 5955A>T), TTC1610TTT (5958C>T), GGA1611GGG (5961A>G), TTG1612CTT (5962T>C 5964G>T), AAG1613ACT (5966A>C 5967G>T)								
**Proteins**									
polyprotein (YP_010797894.1)	1531	1616	4.3%	240	38.8%	86 (100%)	37 (43.0%)	0/0/0/0	0
Protein mutations:	E1531K (5719G>A), K1533D (5725A>G 5727A>T), K1535T (5732A>C), E1536L (5734G>T 5735A>T), V1539C (5743G>T 5744T>G), F1540I (5746T>A 5748C>T), N1541D (5749A>G 5751C>T), K1543W (5755A>T 5756A>G 5757A>G), T1544Q (5758A>C 5759C>A 5760C>G), D1547K (5767G>A 5769C>G), N1548V (5770A>G 5771A>T 5772C>G), T1549P (5773A>C), F1550V (5776T>G 5778C>G), D1552N (5782G>A 5784T>C), Q1553R (5786A>G 5787G>T), Y1554C (5789A>G 5790C>T), S1555P (5791T>C), L1556M (5794C>A 5796A>G), G1558R (5800G>C 5802C>T), N1560D (5806A>G 5808C>T), T1561D (5809A>G 5810C>A 5811A>C), I1562L (5812A>C 5814C>G), I1563F (5815A>T 5817A>T), Q1564D (5818C>G 5820G>T), K1565Q (5821A>C), V1566L (5824G>C), C1567Q (5827T>C 5828G>A 5829T>G), N1568D (5830A>G), S1570T (5837G>C), F1575I (5851T>A 5853C>T), K1578R (5861A>G), F1581Y (5870T>A 5871C>T), I1584L (5878A>T 5880T>G), M1585R (5882T>G), M1586I (5886G>C), D1587R (5887G>A 5888A>G 5889C>G), E1588V (5891A>T), E1589D (5895A>T), S1590D (5896T>G 5897C>A 5898C>T), W1593K (5905T>A 5906G>A), L1597R (5918T>G), P1599R (5924C>G), D1600Y (5926G>T), L1602H (5933T>A 5934T>C), Y1603F (5936A>T 5937C>T), W1605F (5942G>T 5943G>T), P1609S (5953C>T 5955A>T), K1613T (5966A>C 5967G>T)								
Codon mutations:	GAG1531AAG (5719G>A), AAA1533GAT (5725A>G 5727A>T), GGA1534GGC (5730A>C), AAG1535ACG (5732A>C), GAG1536TTG (5734G>T 5735A>T), AGA1537CGC (5737A>C 5739A>C), GTC1539TGC (5743G>T 5744T>G), TTC1540ATT (5746T>A 5748C>T), AAC1541GAT (5749A>G 5751C>T), TAC1542TAT (5754C>T), AAA1543TGG (5755A>T 5756A>G 5757A>G), ACC1544CAG (5758A>C 5759C>A 5760C>G), AAC1546AAT (5766C>T), GAC1547AAG (5767G>A 5769C>G), AAC1548GTG (5770A>G 5771A>T 5772C>G), ACG1549CCG (5773A>C), TTC1550GTG (5776T>G 5778C>G), AAA1551AAG (5781A>G), GAT1552AAC (5782G>A 5784T>C), CAG1553CGT (5786A>G 5787G>T), TAC1554TGT (5789A>G 5790C>T), TCT1555CCT (5791T>C), CTA1556ATG (5794C>A 5796A>G), GGC1558CGT (5800G>C 5802C>T), AAC1560GAT (5806A>G 5808C>T), ACA1561GAC (5809A>G 5810C>A 5811A>C), ATC1562CTG (5812A>C 5814C>G), ATA1563TTT (5815A>T 5817A>T), CAG1564GAT (5818C>G 5820G>T), AAG1565CAG (5821A>C), GTC1566CTC (5824G>C), TGT1567CAG (5827T>C 5828G>A 5829T>G), AAT1568GAT (5830A>G), AGT1570ACT (5837G>C), GTC1571GTG (5841C>G), TTC1572TTT (5844C>T), TCC1573TCT (5847C>T), TTC1575ATT (5851T>A 5853C>T), GAT1576GAC (5856T>C), CTG1577TTG (5857C>T), AAG1578AGG (5861A>G), AGC1579TCC (5863A>T 5864G>C), GGA1580GGT (5868A>T), TTC1581TAT (5870T>A 5871C>T), ATT1584TTG (5878A>T 5880T>G), ATG1585AGG (5882T>G), ATG1586ATC (5886G>C), GAC1587AGG (5887G>A 5888A>G 5889C>G), GAA1588GTA (5891A>T), GAA1589GAT (5895A>T), TCC1590GAT (5896T>G 5897C>A 5898C>T), ATC1591ATA (5901C>A), TGG1593AAG (5905T>A 5906G>A), ACC1594ACT (5910C>T), TTC1596TTT (5916C>T), CTG1597CGG (5918T>G), CCA1599CGA (5924C>G), GAT1600TAT (5926G>T), GGA1601GGC (5931A>C), CTT1602CAC (5933T>A 5934T>C), TAC1603TTT (5936A>T 5937C>T), GAA1604GAG (5940A>G), TGG1605TTT (5942G>T 5943G>T), GTT1607GTA (5949T>A), CCA1609TCT (5953C>T 5955A>T), TTC1610TTT (5958C>T), GGA1611GGG (5961A>G), TTG1612CTT (5962T>C 5964G>T), AAG1613ACT (5966A>C 5967G>T)								

*: Inserts / Deletes / Misaligned / Frameshifts

Analysis details

This analysis was performed with panviral2.64

NGS Details (UN9): Badnavirus tessellocastaneae

Assembly

**Coverage Length**	415 (1 contig(s))
**Depth Of Coverage**	6.4
**Number Of Reads**	22
**Reads Per Million**	0.50 rpm (after QC)
**Ambiguities**	0
**Assembly Method**	de novo + reference guided assembly
**Consensus Caller**	Bcf Tools

Coverage Map

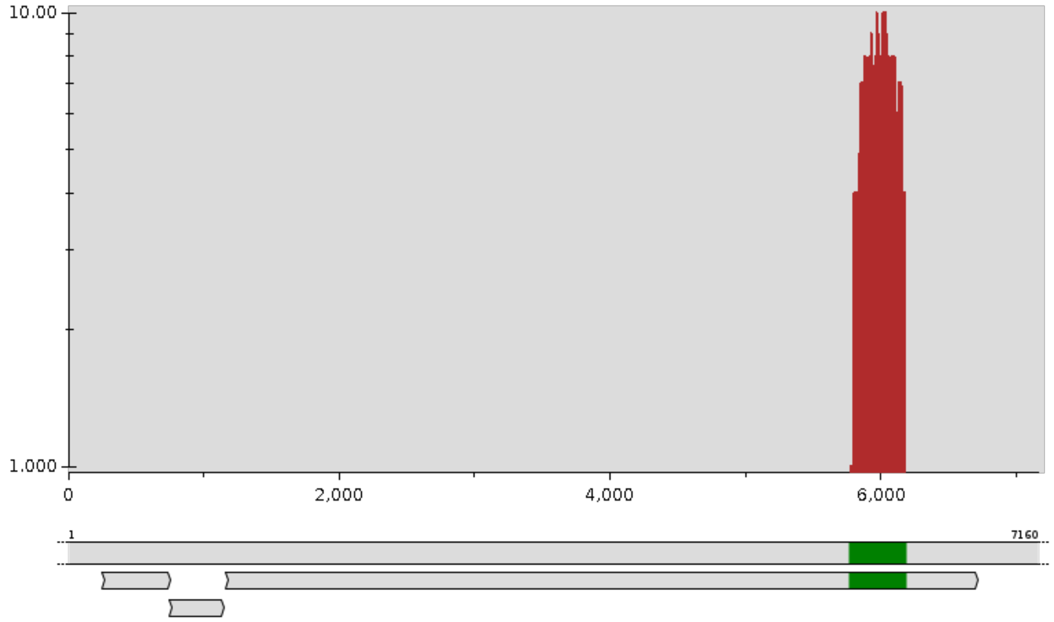

Assignment

**Type**	Badnavirus tessellocastaneae (Taxonomy ID: 3051987)
**Reference Genome**	NC_076885.1
**NT Identity (%)**	57.177
**AA Identity (%)**	47.8571
**Number Of Stop Codons**	0
**Number Of CDS**	3

Alignment

**Alignment Score**	105.0 (NT) + 490.0 (AA) = 595.0
**Concordance (%)**	33.1661

Alignment Method	Global, seeded, nucleotide + amino acids (AGA)

Genome Region

Sequence starts at position 5774 and ends at position 6188 relative to NC_076885.1 reference sequence.

Alignment Detailed Statistics

	Begin	End	Coverage	Score	Concordance	Matches	Identities	I/D/M/F*	Stop Codons
**NT**	**5774**	**6188**	**5.8%**	**105**	**12.9%**	**412 (97.9%)**	**239 (56.8%)**	**6/3**	
Mutations:	5774C>A, 5777C>A, 5779G>C, 5780C>A, 5782C>A, 5789C>T, 5792G>A, 5793A>G, 5794A>C, 5800C>A, 5802C>T, 5807G>T, 5808G>C, 5810A>G, 5811G>A, 5812C>T, 5815C>T, 5816A>C, 5822C>A, 5837T>A, 5840A>C, 5842G>C, 5843G>C, 5854T>A, 5856G>C, 5857A>T, 5862G>T, 5863T>A, 5867T>G, 5868G>C, 5869G>A, 5870G>A, 5871C>G, 5875G>A, 5876T>A, 5877T>A, 5882G>T, 5883G>A, 5884A>C, 5885G>A, 5886C>G, 5887C>T, 5888A>C, 5889A>C, 5890G>T, 5895G>A, 5896T>A, 5902G>A, 5905G>A, 5906G>A, 5908C>T, 5917T>C, 5918G>C, 5919C>A, 5921T>A, 5922C>G, 5923A>T, 5926A>T, 5929G>A, 5931T>A, 5933C>A, 5934C>A, 5935C>A, 5936A>G, 5937C>A, 5938_5939insAAGGGT, 5939C>A, 5940T>A, 5943A>T, 5944A>G, 5947C>T, 5949C>G, 5950C>T, 5953C>A, 5956T>C, 5958C>T, 5959A>C, 5963A>G, 5965C>A, 5967A>T, 5968T>A, 5971G>T, 5972G>A, 5974C>A, 5977G>A, 5978G>T, 5980A>T, 5981A>G, 5982C>G, 5983C>A, 5984A>C, 5986G>A, 5987G>A, 5989A>T, 5990T>A, 5991C>G, 5995G>C, 5999A>T, 6001C>A, 6005T>A, 6006A>T, 6007C>T, 6011G>A, 6013T>C, 6014C>A, 6016G>A, 6018A>G, 6019G>T, 6021C>A, 6026A>G, 6027C>T, 6034G>A, 6040C>T, 6044C>G, 6049A>T, 6052C>A, 6053A>G, 6056G>A, 6057C>A, 6061C>T, 6064C>T, 6065G>C, 6067C>A, 6071C>A, 6072A>T, 6074C>G, 6075T>A, 6076T>C, 6079C>G, 6085G>T, 6086C>A, 6087C>G, 6088C>T, 6089T>A, 6090C>G, 6091A>T, 6093G>A, 6095G>A, 6096T>G, 6097G>A, 6098C>A, 6104A>C, 6106C>A, 6109C>T, 6113T>A, 6114G>T, 6115T>A, 6119T>A, 6120A>C, 6121T>C, 6124A>T, 6125A>T, 6127G>T, 6128G>A, 6129G>T, 6131A>T, 6132C>A, 6134G>C, 6135G>C, 6136G>T, 6137_6139delGTC, 6140G>A, 6142A>T, 6146A>G, 6147A>T, 6148A>T, 6155C>T, 6160C>A, 6167G>A, 6170A>G, 6172C>T, 6175C>T, 6176C>A, 6178G>T, 6187C>T								

CDS

	Begin	End	Coverage	Score	Concordance	Matches	Identities	I/D/M/F*	Stop Codons
ORF3	1538	1676	7.5%	490	49.1%	138 (97.9%)	67 (47.5%)	2/1/0/0	0
Protein mutations:	Q1538K (5774C>A), Q1539N (5777C>A 5779G>C), L1540I (5780C>A 5782C>A), E1544S (5792G>A 5793A>G 5794A>C), P1547L (5802C>T), G1549S (5807G>T 5808G>C), S1550D (5810A>G 5811G>A 5812C>T), I1552L (5816A>C), L1554I (5822C>A), C1559S (5837T>A), M1560L (5840A>C 5842G>C), E1561Q (5843G>C), G1565A (5856G>C 5857A>T), C1567L (5862G>T 5863T>A), W1569A (5867T>G 5868G>C 5869G>A), A1570R (5870G>A 5871C>G), F1572N (5876T>A 5877T>A), G1574Y (5882G>T 5883G>A 5884A>C), A1575S (5885G>A 5886C>G 5887C>T), K1576P (5888A>C 5889A>C 5890G>T), S1578K (5895G>A 5896T>A), V1582I (5906G>A 5908C>T), A1586Q (5918G>C 5919C>A), F1590Y (5931T>A), P1591K (5933C>A 5934C>A 5935C>A), T1592E (5936A>G 5937C>A), T1592_L1593insKG (5938_5939insAAGGGT), L1593N (5939C>A 5940T>A), K1594M (5943A>T 5944A>G), T1596S (5949C>G 5950C>T), A1599V (5958C>T 5959A>C), I1601V (5963A>G 5965C>A), Y1602L (5967A>T 5968T>A), V1604I (5972G>A 5974C>A), M1605I (5977G>A), E1606Y (5978G>T 5980A>T), T1607G (5981A>G 5982C>G 5983C>A), M1608L (5984A>C 5986G>A), E1609N (5987G>A 5989A>T), L1611F (5995G>C), I1613L (5999A>T 6001C>A), Y1615I (6005T>A 6006A>T 6007C>T), D1617N (6011G>A 6013T>C), Q1618K (6014C>A 6016G>A), K1619S (6018A>G 6019G>T), A1620E (6021C>A), T1622V (6026A>G 6027C>T), Q1628E (6044C>G), I1631V (6053A>G), A1632K (6056G>A 6057C>A), D1635Q (6065G>C 6067C>A), Q1637I (6071C>A 6072A>T), L1638D (6074C>G 6075T>A 6076T>C), H1639Q (6079C>G), K1641N (6085G>T), P1642S (6086C>A 6087C>G 6088C>T), R1644K (6093G>A), V1645R (6095G>A 6096T>G 6097G>A), I1648L (6104A>C 6106C>A), C1651I (6113T>A 6114G>T 6115T>A), Y1653T (6119T>A 6120A>C 6121T>C), T1655S (6125A>T 6127G>T), G1656I (6128G>A 6129G>T), T1657Y (6131A>T 6132C>A), G1658P (6134G>C 6135G>C 6136G>T), V1659del (6137_6139delGTC), E1660N (6140G>A 6142A>T), K1662V (6146A>G 6147A>T 6148A>T), H1665Y (6155C>T), E1669K (6167G>A), N1670D (6170A>G 6172C>T), Q1672N (6176C>A 6178G>T)								
Codon mutations:	CAA1538AAA (5774C>A), CAG1539AAC (5777C>A 5779G>C), CTC1540ATA (5780C>A 5782C>A), CTA1543TTA (5789C>T), GAA1544AGC (5792G>A 5793A>G 5794A>C), CCC1546CCA (5800C>A), CCA1547CTA (5802C>T), GGA1549TCA (5807G>T 5808G>C), AGC1550GAT (5810A>G 5811G>A 5812C>T), TAC1551TAT (5815C>T), ATA1552CTA (5816A>C), CTA1554ATA (5822C>A), TGC1559AGC (5837T>A), ATG1560CTC (5840A>C 5842G>C), GAA1561CAA (5843G>C), GGT1564GGA (5854T>A), GGA1565GCT (5856G>C 5857A>T), TGT1567TTA (5862G>T 5863T>A), TGG1569GCA (5867T>G 5868G>C 5869G>A), GCA1570AGA (5870G>A 5871C>G), CCG1571CCA (5875G>A), TTC1572AAC (5876T>A 5877T>A), GGA1574TAC (5882G>T 5883G>A 5884A>C), GCC1575AGT (5885G>A 5886C>G 5887C>T), AAG1576CCT (5888A>C 5889A>C 5890G>T), AGT1578AAA (5895G>A 5896T>A), GAG1580GAA (5902G>A), AAG1581AAA (5905G>A), GTC1582ATT (5906G>A 5908C>T), TAT1585TAC (5917T>C), GCA1586CAA (5918G>C 5919C>A), TCA1587AGT (5921T>A 5922C>G 5923A>T), GGA1588GGT (5926A>T), AAG1589AAA (5929G>A), TTC1590TAC (5931T>A), CCC1591AAA (5933C>A 5934C>A 5935C>A), ACA1592GAA (5936A>G 5937C>A), ACA1592_CTC1593insAAGGGT (5938_5939insAAGGGT), CTC1593AAC (5939C>A 5940T>A), AAA1594ATG (5943A>T 5944A>G), AGC1595AGT (5947C>T), ACC1596AGT (5949C>G 5950C>T), ATC1597ATA (5953C>A), GAT1598GAC (5956T>C), GCA1599GTC (5958C>T 5959A>C), ATC1601GTA (5963A>G 5965C>A), TAT1602TTA (5967A>T 5968T>A), GCG1603GCT (5971G>T), GTC1604ATA (5972G>A 5974C>A), ATG1605ATA (5977G>A), GAA1606TAT (5978G>T 5980A>T), ACC1607GGA (5981A>G 5982C>G 5983C>A), ATG1608CTA (5984A>C 5986G>A), GAA1609AAT (5987G>A 5989A>T), TCT1610AGT (5990T>A 5991C>G), TTG1611TTC (5995G>C), ATC1613TTA (5999A>T 6001C>A), TAC1615ATT (6005T>A 6006A>T 6007C>T), GAT1617AAC (6011G>A 6013T>C), CAG1618AAA (6014C>A 6016G>A), AAG1619AGT (6018A>G 6019G>T), GCA1620GAA (6021C>A), ACA1622GTA (6026A>G 6027C>T), AGG1624AGA (6034G>A), GAC1626GAT (6040C>T), CAA1628GAA (6044C>G), GCA1629GCT (6049A>T), ATC1630ATA (6052C>A), ATA1631GTA (6053A>G), GCA1632AAA (6056G>A 6057C>A), TTC1633TTT (6061C>T), CAC1634CAT (6064C>T), GAC1635CAA (6065G>C 6067C>A), CAA1637ATA (6071C>A 6072A>T), CTT1638GAC (6074C>G 6075T>A 6076T>C), CAC1639CAG (6079C>G), AAG1641AAT (6085G>T), CCC1642AGT (6086C>A 6087C>G 6088C>T), TCA1643AGT (6089T>A 6090C>G 6091A>T), AGA1644AAA (6093G>A), GTG1645AGA (6095G>A 6096T>G 6097G>A), CGA1646AGA (6098C>A), ATC1648CTA (6104A>C 6106C>A), AAC1649AAT (6109C>T), TGT1651ATA (6113T>A 6114G>T 6115T>A), TAT1653ACC (6119T>A 6120A>C 6121T>C), ATA1654ATT (6124A>T), ACG1655TCT (6125A>T 6127G>T), GGA1656ATA (6128G>A 6129G>T), ACC1657TAC (6131A>T 6132C>A), GGG1658CCT (6134G>C 6135G>C 6136G>T), GTC1659del (6137_6139delGTC), GAA1660AAT (6140G>A 6142A>T), AAA1662GTT (6146A>G 6147A>T 6148A>T), CAT1665TAT (6155C>T), ATC1666ATA (6160C>A), GAA1669AAA (6167G>A), AAC1670GAT (6170A>G 6172C>T), AAC1671AAT (6175C>T), CAG1672AAT (6176C>A 6178G>T), GAC1675GAT (6187C>T)								

Proteins

	Begin	End	Coverage	Score	Concordance	Matches	Identities	I/D/M/F*	Stop Codons
polyprotein (YP_010800602.1)	1538	1676	7.5%	490	49.1%	138 (97.9%)	67 (47.5%)	2/1/0/0	0
Protein mutations:	Q1538K (5774C>A), Q1539N (5777C>A 5779G>C), L1540I (5780C>A 5782C>A), E1544S (5792G>A 5793A>G 5794A>C), P1547L (5802C>T), G1549S (5807G>T 5808G>C), S1550D (5810A>G 5811G>A 5812C>T), I1552L (5816A>C), L1554I (5822C>A), C1559S (5837T>A), M1560L (5840A>C 5842G>C), E1561Q (5843G>C), G1565A (5856G>C 5857A>T), C1567L (5862G>T 5863T>A), W1569A (5867T>G 5868G>C 5869G>A), A1570R (5870G>A 5871C>G), F1572N (5876T>A 5877T>A), G1574Y (5882G>T 5883G>A 5884A>C), A1575S (5885G>A 5886C>G 5887C>T), K1576P (5888A>C 5889A>C 5890G>T), S1578K (5895G>A 5896T>A), V1582I (5906G>A 5908C>T), A1586Q (5918G>C 5919C>A), F1590Y (5931T>A), P1591K (5933C>A 5934C>A 5935C>A), T1592E (5936A>G 5937C>A), T1592_L1593insKG (5938_5939insAAGGGT), L1593N (5939C>A 5940T>A), K1594M (5943A>T 5944A>G), T1596S (5949C>G 5950C>T), A1599V (5958C>T 5959A>C), I1601V (5963A>G 5965C>A), Y1602L (5967A>T 5968T>A), V1604I (5972G>A 5974C>A), M1605I (5977G>A), E1606Y (5978G>T 5980A>T), T1607G (5981A>G 5982C>G 5983C>A), M1608L (5984A>C 5986G>A), E1609N (5987G>A 5989A>T), L1611F (5995G>C), I1613L (5999A>T 6001C>A), Y1615I (6005T>A 6006A>T 6007C>T), D1617N (6011G>A 6013T>C), Q1618K (6014C>A 6016G>A), K1619S (6018A>G 6019G>T), A1620E (6021C>A), T1622V (6026A>G 6027C>T), Q1628E (6044C>G), I1631V (6053A>G), A1632K (6056G>A 6057C>A), D1635Q (6065G>C 6067C>A), Q1637I (6071C>A 6072A>T), L1638D (6074C>G 6075T>A 6076T>C), H1639Q (6079C>G), K1641N (6085G>T), P1642S (6086C>A 6087C>G 6088C>T), R1644K (6093G>A), V1645R (6095G>A 6096T>G 6097G>A), I1648L (6104A>C 6106C>A), C1651I (6113T>A 6114G>T 6115T>A), Y1653T (6119T>A 6120A>C 6121T>C), T1655S (6125A>T 6127G>T), G1656I (6128G>A 6129G>T), T1657Y (6131A>T 6132C>A), G1658P (6134G>C 6135G>C 6136G>T), V1659del (6137_6139delGTC), E1660N (6140G>A 6142A>T), K1662V (6146A>G 6147A>T 6148A>T), H1665Y (6155C>T), E1669K (6167G>A), N1670D (6170A>G 6172C>T), Q1672N (6176C>A 6178G>T)								
Codon mutations:	CAA1538AAA (5774C>A), CAG1539AAC (5777C>A 5779G>C), CTC1540ATA (5780C>A 5782C>A), CTA1543TTA (5789C>T), GAA1544AGC (5792G>A 5793A>G 5794A>C), CCC1546CCA (5800C>A), CCA1547CTA (5802C>T), GGA1549TCA (5807G>T 5808G>C), AGC1550GAT (5810A>G 5811G>A 5812C>T), TAC1551TAT (5815C>T), ATA1552CTA (5816A>C), CTA1554ATA (5822C>A), TGC1559AGC (5837T>A), ATG1560CTC (5840A>C 5842G>C), GAA1561CAA (5843G>C), GGT1564GGA (5854T>A), GGA1565GCT (5856G>C 5857A>T), TGT1567TTA (5862G>T 5863T>A), TGG1569GCA (5867T>G 5868G>C 5869G>A), GCA1570AGA (5870G>A 5871C>G), CCG1571CCA (5875G>A), TTC1572AAC (5876T>A 5877T>A), GGA1574TAC (5882G>T 5883G>A 5884A>C), GCC1575AGT (5885G>A 5886C>G 5887C>T), AAG1576CCT (5888A>C 5889A>C 5890G>T), AGT1578AAA (5895G>A 5896T>A), GAG1580GAA (5902G>A), AAG1581AAA (5905G>A), GTC1582ATT (5906G>A 5908C>T), TAT1585TAC (5917T>C), GCA1586CAA (5918G>C 5919C>A), TCA1587AGT (5921T>A 5922C>G 5923A>T), GGA1588GGT (5926A>T), AAG1589AAA (5929G>A), TTC1590TAC (5931T>A), CCC1591AAA (5933C>A 5934C>A 5935C>A), ACA1592GAA (5936A>G 5937C>A), ACA1592_CTC1593insAAGGGT (5938_5939insAAGGGT), CTC1593AAC (5939C>A 5940T>A), AAA1594ATG (5943A>T 5944A>G), AGC1595AGT (5947C>T), ACC1596AGT (5949C>G 5950C>T), ATC1597ATA (5953C>A), GAT1598GAC (5956T>C), GCA1599GTC (5958C>T 5959A>C), ATC1601GTA (5963A>G 5965C>A), TAT1602TTA (5967A>T 5968T>A), GCG1603GCT (5971G>T), GTC1604ATA (5972G>A 5974C>A), ATG1605ATA (5977G>A), GAA1606TAT (5978G>T 5980A>T), ACC1607GGA (5981A>G 5982C>G 5983C>A), ATG1608CTA (5984A>C 5986G>A), GAA1609AAT (5987G>A 5989A>T), TCT1610AGT (5990T>A 5991C>G), TTG1611TTC (5995G>C), ATC1613TTA (5999A>T 6001C>A), TAC1615ATT (6005T>A 6006A>T 6007C>T), GAT1617AAC (6011G>A 6013T>C), CAG1618AAA (6014C>A 6016G>A), AAG1619AGT (6018A>G 6019G>T), GCA1620GAA (6021C>A), ACA1622GTA (6026A>G 6027C>T), AGG1624AGA (6034G>A), GAC1626GAT (6040C>T), CAA1628GAA (6044C>G), GCA1629GCT (6049A>T), ATC1630ATA (6052C>A), ATA1631GTA (6053A>G), GCA1632AAA (6056G>A 6057C>A), TTC1633TTT (6061C>T), CAC1634CAT (6064C>T), GAC1635CAA (6065G>C 6067C>A), CAA1637ATA (6071C>A 6072A>T), CTT1638GAC (6074C>G 6075T>A 6076T>C), CAC1639CAG (6079C>G), AAG1641AAT (6085G>T), CCC1642AGT (6086C>A 6087C>G 6088C>T), TCA1643AGT (6089T>A 6090C>G 6091A>T), AGA1644AAA (6093G>A), GTG1645AGA (6095G>A 6096T>G 6097G>A), CGA1646AGA (6098C>A), ATC1648CTA (6104A>C 6106C>A), AAC1649AAT (6109C>T), TGT1651ATA (6113T>A 6114G>T 6115T>A), TAT1653ACC (6119T>A 6120A>C 6121T>C), ATA1654ATT (6124A>T), ACG1655TCT (6125A>T 6127G>T), GGA1656ATA (6128G>A 6129G>T), ACC1657TAC (6131A>T 6132C>A), GGG1658CCT (6134G>C 6135G>C 6136G>T), GTC1659del (6137_6139delGTC), GAA1660AAT (6140G>A 6142A>T), AAA1662GTT (6146A>G 6147A>T 6148A>T), CAT1665TAT (6155C>T), ATC1666ATA (6160C>A), GAA1669AAA (6167G>A), AAC1670GAT (6170A>G 6172C>T), AAC1671AAT (6175C>T), CAG1672AAT (6176C>A 6178G>T), GAC1675GAT (6187C>T)								

*: Inserts / Deletes / Misaligned / Frameshifts

Analysis details

This analysis was performed with panviral2.64

NGS Details (UN9): Ruflodivirus deformatiorudbeckiae

Assembly

**Coverage Length**	605 (1 contig(s))
**Depth Of Coverage**	4.7
**Number Of Reads**	21
**Reads Per Million**	0.47 rpm (after QC)
**Ambiguities**	0
**Assembly Method**	de novo + reference guided assembly
**Consensus Caller**	Bcf Tools

Coverage Map

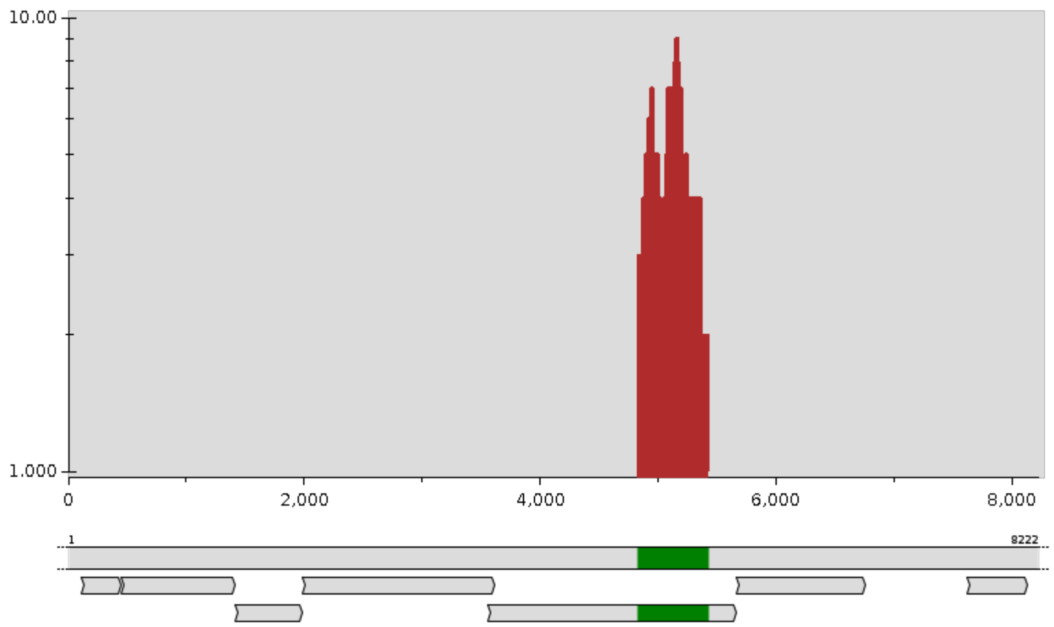

Assignment

**Type**	Ruflodivirus deformatiorudbeckiae (Taxonomy ID: 3048307)
**Reference Genome**	NC_011920.1
**NT Identity (%)**	57.3356
**AA Identity (%)**	45.4545
**Number Of Stop Codons**	0
**Number Of CDS**	7

Alignment

**Alignment Score**	144.0 (NT) + 646.0 (AA) = 790.0
**Concordance (%)**	31.6887

**Alignment Method**	Global, seeded, nucleotide + amino acids (AGA)

Genome Region

Sequence starts at position 4826 and ends at position 5430 relative to NC_011920.1 reference sequence.

Alignment Detailed Statistics

	Begin	End	Coverage	Score	Concordance	Matches	Identities	I/D/M/F*	Stop Codons
**NT**	**4826**	**5430**	**7.4%**	**144**	**12.5%**	**593 (98.0%)**	**340 (56.2%)**	**0/12**	

Mutations: 4835G>A, 4843C>T, 4847C>T, 4849T>A, 4850G>A, 4852C>A, 4854T>G, 4855C>T, 4858A>C, 4861A>C, 4862A>T, 4865C>T, 4866T>A, 4868G>C, 4870T>A, 4878A>T, 4879T>A, 4880G>A, 4881A>C, 4888A>T, 4889G>A, 4892A>C, 4893C>A, 4894T>A, 4895G>T, 4897G>T, 4898C>T, 4899T>C, 4901A>C, 4902G>A, 4905A>T, 4906G>A, 4909C>T, 4910A>T, 4911T>G, 4912T>C, 4917C>A, 4921C>A, 4924A>T, 4925A>G, 4927C>G, 4928C>T, 4930T>A, 4931T>A, 4932C>G, 4933T>C, 4934A>C, 4936A>G, 4939G>A, 4942A>G, 4945T>A, 4948G>C, 4949G>A, 4951T>A, 4952G>A, 4953C>A, 4959C>A, 4961A>G, 4966C>T, 4967A>G, 4969T>A, 4971A>T, 4975T>C, 4978T>A, 4979T>A, 4981T>G, 4983C>T, 4984T>A, 4987C>T, 4990C>A, 4993T>A, 4994G>A, 4995G>A, 4996A>T, 4998A>G, 4999A>C, 5006C>T, 5008T>A, 5012T>A, 5020C>A, 5021C>T, 5022T>C, 5023T>A, 5024G>A, 5026G>A, 5029T>G, 5032A>C, 5033A>T, 5034A>G, 5035A>T, 5036C>G, 5037T>A, 5038T>A, 5045A>G, 5046G>A, 5049G>A, 5051A>C, 5053T>A, 5054C>A, 5055C>A, 5057G>A, 5058A>C, 5059T>A, 5063A>C, 5066T>G, 5067C>A, 5069C>A, 5071A>C, 5087A>T, 5088T>G, 5089A>T, 5090C>T, 5094C>A, 5095G>T, 5098T>C, 5102G>A, 5103A>G, 5105C>G, 5109A>T, 5110C>T, 5115G>C, 5117A>G, 5119A>C, 5122T>A, 5125C>A, 5126G>A, 5129T>A, 5130G>A, 5132C>A, 5137A>T, 5138C>T, 5139C>T, 5141C>T, 5143T>A, 5146G>A, 5149A>G, 5150A>C, 5151A>T, 5152A>T, 5153T>A, 5155G>T, 5156A>C, 5157A>G, 5158G>A, 5162G>T, 5163A>C, 5164T>C, 5166C>A, 5167T>C, 5168G>C, 5169T>G, 5171T>A, 5172G>T, 5175A>G, 5181A>C, 5182T>A, 5186T>G, 5187C>A, 5188C>A, 5189G>C, 5194T>C, 5195C>A, 5197G>A, 5199A>G, 5200C>T, 5203G>T, 5204G>C, 5208A>G, 5209A>C, 5210T>C, 5212T>A, 5215G>A, 5216A>C, 5217G>A, 5219A>G, 5221T>A, 5222C>T, 5223T>G, 5225A>T, 5226A>C, 5227A>C, 5228G>A, 5233C>A, 5239A>G, 5240T>C, 5243C>A, 5244A>G, 5245C>A, 5247A>T, 5251T>A, 5252T>G, 5253T>A, 5257G>A, 5260T>A, 5263A>C, 5264T>A, 5269G>A, 5270A>G, 5272C>T, 5273A>T, 5275C>A, 5276G>C, 5281T>A, 5287A>T, 5291C>G, 5294G>T, 5296A>C, 5299C>T, 5304C>G, 5305T>A, 5307G>C, 5308T>A, 5309G>T, 5311C>A, 5312C>T, 5315A>C, 5317G>A, 5318_5326delTCCCGAGGG, 5333A>C, 5334A>T, 5335C>T, 5336T>A, 5337T>A, 5338A>T, 5342_5344delCGA, 5345C>A, 5347A>T, 5351C>A, 5353T>G, 5362C>T, 5365A>T, 5366T>A, 5371C>T, 5374A>T, 5375C>G, 5376C>A, 5377C>A, 5378G>A, 5380T>C, 5385T>C, 5386C>T, 5389C>A, 5392T>C, 5393C>T, 5394A>C, 5395C>A, 5397G>C, 5398C>A, 5401C>T, 5411G>T, 5413G>A, 5416T>A, 5420G>A, 5423A>G, 5424A>T

CDS

	Begin	End	Coverage	Score	Concordance	Matches	Identities	I/D/M/F*	Stop Codons
RuFDV_gp5	424	625	28.8%	646	48.1%	198 (98.0%)	90 (44.6%)	0/4/0/0	0

Protein mutations: V427I (4835G>A), V432I (4850G>A 4852C>A), F433C (4854T>G 4855C>T), K435N (4861A>C), T436S (4862A>T), L437Y (4865C>T 4866T>A), D438Q (4868G>C 4870T>A), Y441L (4878A>T 4879T>A), D442T (4880G>A 4881A>C), E445K (4889G>A), T446Q (4892A>C 4893C>A 4894T>A), V447F (4895G>T 4897G>T), L448S (4898C>T 4899T>C), R449Q (4901A>C 4902G>A), K450I (4905A>T 4906G>A), I452C (4910A>T 4911T>G 4912T>C), T454N (4917C>A), I457V (4925A>G 4927C>G), K460Q (4934A>C 4936A>G), E464D (4948G>C), V465I (4949G>A 4951T>A), A466N (4952G>A 4953C>A), T468N (4959C>A), K469E (4961A>G), N471E (4967A>G 4969T>A), Y472F (4971A>T), F475M (4979T>A 4981T>G), T476I (4983C>T 4984T>A), N479K (4993T>A), G480N (4994G>A 4995G>A 4996A>T), E481G (4998A>G 4999A>C), S486T (5012T>A), L489S (5021C>T 5022T>C 5023T>A), E490K (5024G>A 5026G>A), N491K (5029T>G), K493C (5033A>T 5034A>G 5035A>T), L494E (5036C>G 5037T>A 5038T>A), S497D (5045A>G 5046G>A), R498K (5049G>A), I499L (5051A>C 5053T>A), P500N (5054C>A 5055C>A), D501T (5057G>A 5058A>C 5059T>A), K503Q (5063A>C), S504E (5066T>G 5067C>A), L505I (5069C>A 5071A>C), I511C (5087A>T 5088T>G 5089A>T), T513N (5094C>A 5095G>T), D516S (5102G>A 5103A>G), Q517E (5105C>G), Y518F (5109A>T 5110C>T), R520P (5115G>C), K521D (5117A>G 5119A>C), E524K (5126G>A), W525K (5129T>A 5130G>A), K527N (5137A>T), P528F (5138C>T 5139C>T), K532L (5150A>C 5151A>T 5152A>T), L533I (5153T>A 5155G>T), K534R (5156A>C 5157A>G 5158G>A), D536S (5162G>T 5163A>C 5164T>C), T537N (5166C>A 5167T>C), V538R (5168G>C 5169T>G), W539M (5171T>A 5172G>T), E540G (5175A>G), N542T (5181A>C 5182T>A), S544E (5186T>G 5187C>A 5188C>A), D545H (5189G>C), Q547K (5195C>A 5197G>A), Y548C (5199A>G 5200C>T), E550Q (5204G>C), K551S (5208A>G 5209A>C), F552L (5210T>C 5212T>A), R554Q (5216A>C 5217G>A), N555E (5219A>G 5221T>A), L556C (5222C>T 5223T>G), K557S (5225A>T 5226A>C 5227A>C), E558K (5228G>A), F559L (5233C>A), H563R (5243C>A 5244A>G 5245C>A), H564L (5247A>T), L566E (5252T>G 5253T>A), D568E (5260T>A), E569D (5263A>C), Y570N (5264T>A), I572V (5270A>G 5272C>T), I573L (5273A>T 5275C>A), E574Q (5276G>C), H579D (5291C>G), E580Y (5294G>T 5296A>C), A583G (5304C>G 5305T>A), G584A (5307G>C 5308T>A), V585L (5309G>T 5311C>A), K587Q (5315A>C 5317G>A), S588_G590del (5318_5326delTCCCGAGGG), N593L (5333A>C 5334A>T 5335C>T), L594N (5336T>A 5337T>A 5338A>T), R596del (5342_5344delCGA), L597I (5345C>A 5347A>T), S604T (5366T>A), K606N (5374A>T), P607E (5375C>G 5376C>A 5377C>A), A608T (5378G>A 5380T>C), I610T (5385T>C 5386C>T), N611K (5389C>A), H613S (5393C>T 5394A>C 5395C>A), S614T (5397G>C 5398C>A), V619L (5411G>T 5413G>A), V622I (5420G>A), K623V (5423A>G 5424A>T)

Codon mutations: GTA427ATA (4835G>A), GAC429GAT (4843C>T), CTT431TTA (4847C>T 4849T>A), GTC432ATA (4850G>A 4852C>A), TTC433TGT (4854T>G 4855C>T), TCA434TCC (4858A>C), AAA435AAC (4861A>C), ACA436TCA (4862A>T), CTT437TAT (4865C>T 4866T>A), GAT438CAA (4868G>C 4870T>A), TAT441TTA (4878A>T 4879T>A), GAT442ACT (4880G>A 4881A>C), CTA444CTT (4888A>T), GAA445AAA (4889G>A), ACT446CAA (4892A>C 4893C>A 4894T>A), GTG447TTT (4895G>T 4897G>T), CTT448TCT (4898C>T 4899T>C), AGA449CAA (4901A>C 4902G>A), AAG450ATA (4905A>T 4906G>A), TGC451TGT (4909C>T), ATT452TGC (4910A>T 4911T>G 4912T>C), ACT454AAT (4917C>A), GGC455GGA (4921C>A), ATA456ATT (4924A>T), ATC457GTG (4925A>G 4927C>G), CTT458TTA (4928C>T 4930T>A), TCT459AGC (4931T>A 4932C>G 4933T>C), AAA460CAG (4934A>C 4936A>G), AAG461AAA (4939G>A), AAA462AAG (4942A>G), GCT463GCA (4945T>A), GAG464GAC (4948G>C), GTT465ATA (4949G>A 4951T>A), GCT466AAT (4952G>A 4953C>A), ACT468AAT (4959C>A), AAA469GAA (4961A>G), ATC470ATT (4966C>T), AAT471GAA (4967A>G 4969T>A), TAT472TTT (4971A>T), CTT473CTC (4975T>C), GGT474GGA (4978T>A), TTT475ATG (4979T>A 4981T>G), ACT476ATA (4983C>T 4984T>A), ATC477ATT (4987C>T), TCC478TCA (4990C>A), AAT479AAA (4993T>A), GGA480AAT (4994G>A 4995G>A 4996A>T), GAA481GGC (4998A>G 4999A>C), CTT484TTA (5006C>T 5008T>A), TCT486ACT (5012T>A), ATC488ATA (5020C>A), CTT489TCA (5021C>T 5022T>C 5023T>A), GAG490AAA (5024G>A 5026G>A), AAT491AAG (5029T>G), ATA492ATC (5032A>C), AAA493TGT (5033A>T 5034A>G 5035A>T), CTT494GAA (5036C>G 5037T>A 5038T>A), AGT497GAT (5045A>G 5046G>A), AGA498AAA (5049G>A), ATT499CTA (5051A>C 5053T>A), CCT500AAT (5054C>A 5055C>A), GAT501ACA (5057G>A 5058A>C 5059T>A), AAA503CAA (5063A>C), TCA504GAA (5066T>G 5067C>A), CTA505ATC (5069C>A 5071A>C), ATA511TGT (5087A>T 5088T>G 5089A>T), CTA512TTA (5090C>T), ACG513AAT (5094C>A 5095G>T), TAT514TAC (5098T>C), GAT516AGT (5102G>A 5103A>G), CAA517GAA (5105C>G), TAC518TTT (5109A>T 5110C>T), CGA520CCA (5115G>C), AAA521GAC (5117A>G 5119A>C), CTT522CTA (5122T>A), GCC523GCA (5125C>A), GAA524AAA (5126G>A), TGG525AAG (5129T>A 5130G>A), CGA526AGA (5132C>A), AAA527AAT (5137A>T), CCT528TTT (5138C>T 5139C>T), CTT529TTA (5141C>T 5143T>A), CAG530CAA (5146G>A), AGA531AGG (5149A>G), AAA532CTT (5150A>C 5151A>T 5152A>T), TTG533ATT (5153T>A 5155G>T), AAG534CGA (5156A>C 5157A>G 5158G>A), GAT536TCC (5162G>T 5163A>C 5164T>C), ACT537AAC (5166C>A 5167T>C), GTA538CGA (5168G>C 5169T>G), TGG539ATG (5171T>A 5172G>T), GAA540GGA (5175A>G), AAT542ACA (5181A>C 5182T>A), TCC544GAA (5186T>G 5187C>A 5188C>A), GAT545CAT (5189G>C), ACT546ACC (5194T>C), CAG547AAA (5195C>A 5197G>A), TAC548TGT (5199A>G 5200C>T), GTG549GTT (5203G>T), GAA550CAA (5204G>C), AAA551AGC (5208A>G 5209A>C), TTT552CTA (5210T>C 5212T>A), AAG553AAA (5215G>A), AGA554CAA (5216A>C 5217G>A), AAT555GAA (5219A>G 5221T>A), CTT556TGT (5222C>T 5223T>G), AAA557TCC (5225A>T 5226A>C 5227A>C), GAA558AAA (5228G>A), TTC559TTA (5233C>A), AAA561AAG (5239A>G), TTA562CTA (5240T>C), CAC563AGA (5243C>A 5244A>G 5245C>A), CAT564CTT (5247A>T), CCT565CCA (5251T>A), TTA566GAA (5252T>G 5253T>A), CCG567CCA (5257G>A), GAT568GAA (5260T>A), GAA569GAC (5263A>C), TAC570AAC (5264T>A), TTG571TTA (5269G>A), ATC572GTT (5270A>G 5272C>T), ATC573TTA (5273A>T 5275C>A), GAA574CAA (5276G>C), ACT575ACA (5281T>A), GCA577GCT (5287A>T), CAT579GAT (5291C>G), GAA580TAC (5294G>T 5296A>C), CAC581CAT (5299C>T), GCT583GGA (5304C>G 5305T>A), GGT584GCA (5307G>C 5308T>A), GTC585TTA (5309G>T 5311C>A), CTA586TTA (5312C>T), AAG587CAA (5315A>C 5317G>A), TCC588_GGG590del (5318_5326delTCCCGAGGG), AAC593CTT (5333A>C 5334A>T 5335C>T), TTA594AAT (5336T>A 5337T>A 5338A>T), CGA596del (5342_5344delCGA), CTA597ATT (5345C>A 5347A>T), CGT599AGG (5351C>A 5353T>G), AGC602AGT (5362C>T), GGA603GGT (5365A>T), TCA604ACA (5366T>A), TTC605TTT (5371C>T), AAA606AAT (5374A>T), CCC607GAA (5375C>G 5376C>A 5377C>A), GCT608ACC (5378G>A 5380T>C), ATC610ACT (5385T>C 5386C>T), AAC611AAA (5389C>A), TAT612TAC (5392T>C), CAC613TCA (5393C>T 5394A>C 5395C>A), AGC614ACA (5397G>C 5398C>A), AAC615AAT (5401C>T), GTG619TTA (5411G>T 5413G>A), CTT620CTA (5416T>A), GTA622ATA (5420G>A), AAA623GTA (5423A>G 5424A>T)

Proteins

	Begin	End	Coverage	Score	Concordance	Matches	Identities	I/D/M/F*	Stop Codons
putative enzymatic polyprotein (YP_002519387.1)	424	625	28.8%	646	48.1%	198 (98.0%)	90 (44.6%)	0/4/0/0	0

Protein mutations: V427I (4835G>A), V432I (4850G>A 4852C>A), F433C (4854T>G 4855C>T), K435N (4861A>C), T436S (4862A>T), L437Y (4865C>T 4866T>A), D438Q (4868G>C 4870T>A), Y441L (4878A>T 4879T>A), D442T (4880G>A 4881A>C), E445K (4889G>A), T446Q (4892A>C 4893C>A 4894T>A), V447F (4895G>T 4897G>T), L448S (4898C>T 4899T>C), R449Q (4901A>C 4902G>A), K450I (4905A>T 4906G>A), I452C (4910A>T 4911T>G 4912T>C), T454N (4917C>A), I457V (4925A>G 4927C>G), K460Q (4934A>C 4936A>G), E464D (4948G>C), V465I (4949G>A 4951T>A), A466N (4952G>A 4953C>A), T468N (4959C>A), K469E (4961A>G), N471E (4967A>G 4969T>A), Y472F (4971A>T), F475M (4979T>A 4981T>G), T476I (4983C>T 4984T>A), N479K (4993T>A), G480N (4994G>A 4995G>A 4996A>T), E481G (4998A>G 4999A>C), S486T (5012T>A), L489S (5021C>T 5022T>C 5023T>A), E490K (5024G>A 5026G>A), N491K (5029T>G), K493C (5033A>T 5034A>G 5035A>T), L494E (5036C>G 5037T>A 5038T>A), S497D (5045A>G 5046G>A), R498K (5049G>A), I499L (5051A>C 5053T>A), P500N (5054C>A 5055C>A), D501T (5057G>A 5058A>C 5059T>A), K503Q (5063A>C), S504E (5066T>G 5067C>A), L505I (5069C>A 5071A>C), I511C (5087A>T 5088T>G 5089A>T), T513N (5094C>A 5095G>T), D516S (5102G>A 5103A>G), Q517E (5105C>G), Y518F (5109A>T 5110C>T), R520P (5115G>C), K521D (5117A>G 5119A>C), E524K (5126G>A), W525K (5129T>A 5130G>A), K527N (5137A>T), P528F (5138C>T 5139C>T), K532L (5150A>C 5151A>T 5152A>T), L533I (5153T>A 5155G>T), K534R (5156A>C 5157A>G 5158G>A), D536S (5162G>T 5163A>C 5164T>C), T537N (5166C>A 5167T>C), V538R (5168G>C 5169T>G), W539M (5171T>A 5172G>T), E540G (5175A>G), N542T (5181A>C 5182T>A), S544E (5186T>G 5187C>A 5188C>A), D545H (5189G>C), Q547K (5195C>A 5197G>A), Y548C (5199A>G 5200C>T), E550Q (5204G>C), K551S (5208A>G 5209A>C), F552L (5210T>C 5212T>A), R554Q (5216A>C 5217G>A), N555E (5219A>G 5221T>A), L556C (5222C>T 5223T>G), K557S (5225A>T 5226A>C 5227A>C), E558K (5228G>A), F559L (5233C>A), H563R (5243C>A 5244A>G 5245C>A), H564L (5247A>T), L566E (5252T>G 5253T>A), D568E (5260T>A), E569D (5263A>C), Y570N (5264T>A), I572V (5270A>G 5272C>T), I573L (5273A>T 5275C>A), E574Q (5276G>C), H579D (5291C>G), E580Y (5294G>T 5296A>C), A583G (5304C>G 5305T>A), G584A (5307G>C 5308T>A), V585L (5309G>T 5311C>A), K587Q (5315A>C 5317G>A), S588_G590del (5318_5326delTCCCGAGGG), N593L (5333A>C 5334A>T 5335C>T), L594N (5336T>A 5337T>A 5338A>T), R596del (5342_5344delCGA), L597I (5345C>A 5347A>T), S604T (5366T>A), K606N (5374A>T), P607E (5375C>G 5376C>A 5377C>A), A608T (5378G>A 5380T>C), I610T (5385T>C 5386C>T), N611K (5389C>A), H613S (5393C>T 5394A>C 5395C>A), S614T (5397G>C 5398C>A), V619L (5411G>T 5413G>A), V622I (5420G>A), K623V (5423A>G 5424A>T)

	Begin	End	Coverage	Score	Concordance	Matches	Identities	I/D/M/F*	Stop Codons
**NT**	**4826**	**5430**	**7.4%**	**144**	**12.5%**	**593 (98.0%)**	**340 (56.2%)**	**0/12**	
Codon mutations:	GTA427ATA (4835G>A), GAC429GAT (4843C>T), CTT431TTA (4847C>T 4849T>A), GTC432ATA (4850G>A 4852C>A), TTC433TGT (4854T>G 4855C>T), TCA434TCC (4858A>C), AAA435AAC (4861A>C), ACA436TCA (4862A>T), CTT437TAT (4865C>T 4866T>A), GAT438CAA (4868G>C 4870T>A), TAT441TTA (4878A>T 4879T>A), GAT442ACT (4880G>A 4881A>C), CTA444CTT (4888A>T), GAA445AAA (4889G>A), ACT446CAA (4892A>C 4893C>A 4894T>A), GTG447TTT (4895G>T 4897G>T), CTT448TCT (4898C>T 4899T>C), AGA449CAA (4901A>C 4902G>A), AAG450ATA (4905A>T 4906G>A), TGC451TGT (4909C>T), ATT452TGC (4910A>T 4911T>G 4912T>C), ACT454AAT (4917C>A), GGC455GGA (4921C>A), ATA456ATT (4924A>T), ATC457GTG (4925A>G 4927C>G), CTT458TTA (4928C>T 4930T>A), TCT459AGC (4931T>A 4932C>G 4933T>C), AAA460CAG (4934A>C 4936A>G), AAG461AAA (4939G>A), AAA462AAG (4942A>G), GCT463GCA (4945T>A), GAG464GAC (4948G>C), GTT465ATA (4949G>A 4951T>A), GCT466AAT (4952G>A 4953C>A), ACT468AAT (4959C>A), AAA469GAA (4961A>G), ATC470ATT (4966C>T), AAT471GAA (4967A>G 4969T>A), TAT472TTT (4971A>T), CTT473CTC (4975T>C), GGT474GGA (4978T>A), TTT475ATG (4979T>A 4981T>G), ACT476ATA (4983C>T 4984T>A), ATC477ATT (4987C>T), TCC478TCA (4990C>A), AAT479AAA (4993T>A), GGA480AAT (4994G>A 4995G>A 4996A>T), GAA481GGC (4998A>G 4999A>C), CTT484TTA (5006C>T 5008T>A), TCT486ACT (5012T>A), ATC488ATA (5020C>A), CTT489TCA (5021C>T 5022T>C 5023T>A), GAG490AAA (5024G>A 5026G>A), AAT491AAG (5029T>G), ATA492ATC (5032A>C), AAA493TGT (5033A>T 5034A>G 5035A>T), CTT494GAA (5036C>G 5037T>A 5038T>A), AGT497GAT (5045A>G 5046G>A), AGA498AAA (5049G>A), ATT499CTA (5051A>C 5053T>A), CCT500AAT (5054C>A 5055C>A), GAT501ACA (5057G>A 5058A>C 5059T>A), AAA503CAA (5063A>C), TCA504GAA (5066T>G 5067C>A), CTA505ATC (5069C>A 5071A>C), ATA511TGT (5087A>T 5088T>G 5089A>T), CTA512TTA (5090C>T), ACG513AAT (5094C>A 5095G>T), TAT514TAC (5098T>C), GAT516AGT (5102G>A 5103A>G), CAA517GAA (5105C>G), TAC518TTT (5109A>T 5110C>T), CGA520CCA (5115G>C), AAA521GAC (5117A>G 5119A>C), CTT522CTA (5122T>A), GCC523GCA (5125C>A), GAA524AAA (5126G>A), TGG525AAG (5129T>A 5130G>A), CGA526AGA (5132C>A), AAA527AAT (5137A>T), CCT528TTT (5138C>T 5139C>T), CTT529TTA (5141C>T 5143T>A), CAG530CAA (5146G>A), AGA531AGG (5149A>G), AAA532CTT (5150A>C 5151A>T 5152A>T), TTG533ATT (5153T>A 5155G>T), AAG534CGA (5156A>C 5157A>G 5158G>A), GAT536TCC (5162G>T 5163A>C 5164T>C), ACT537AAC (5166C>A 5167T>C), GTA538CGA (5168G>C 5169T>G), TGG539ATG (5171T>A 5172G>T), GAA540GGA (5175A>G), AAT542ACA (5181A>C 5182T>A), TCC544GAA (5186T>G 5187C>A 5188C>A), GAT545CAT (5189G>C), ACT546ACC (5194T>C), CAG547AAA (5195C>A 5197G>A), TAC548TGT (5199A>G 5200C>T), GTG549GTT (5203G>T), GAA550CAA (5204G>C), AAA551AGC (5208A>G 5209A>C), TTT552CTA (5210T>C 5212T>A), AAG553AAA (5215G>A), AGA554CAA (5216A>C 5217G>A), AAT555GAA (5219A>G 5221T>A), CTT556TGT (5222C>T 5223T>G), AAA557TCC (5225A>T 5226A>C 5227A>C), GAA558AAA (5228G>A), TTC559TTA (5233C>A), AAA561AAG (5239A>G), TTA562CTA (5240T>C), CAC563AGA (5243C>A 5244A>G 5245C>A), CAT564CTT (5247A>T), CCT565CCA (5251T>A), TTA566GAA (5252T>G 5253T>A), CCG567CCA (5257G>A), GAT568GAA (5260T>A), GAA569GAC (5263A>C), TAC570AAC (5264T>A), TTG571TTA (5269G>A), ATC572GTT (5270A>G 5272C>T), ATC573TTA (5273A>T 5275C>A), GAA574CAA (5276G>C), ACT575ACA (5281T>A), GCA577GCT (5287A>T), CAT579GAT (5291C>G), GAA580TAC (5294G>T 5296A>C), CAC581CAT (5299C>T), GCT583GGA (5304C>G 5305T>A), GGT584GCA (5307G>C 5308T>A), GTC585TTA (5309G>T 5311C>A), CTA586TTA (5312C>T), AAG587CAA (5315A>C 5317G>A), TCC588_GGG590del (5318_5326delTCCCGAGGG), AAC593CTT (5333A>C 5334A>T 5335C>T), TTA594AAT (5336T>A 5337T>A 5338A>T), CGA596del (5342_5344delCGA), CTA597ATT (5345C>A 5347A>T), CGT599AGG (5351C>A 5353T>G), AGC602AGT (5362C>T), GGA603GGT (5365A>T), TCA604ACA (5366T>A), TTC605TTT (5371C>T), AAA606AAT (5374A>T), CCC607GAA (5375C>G 5376C>A 5377C>A), GCT608ACC (5378G>A 5380T>C), ATC610ACT (5385T>C 5386C>T), AAC611AAA (5389C>A), TAT612TAC (5392T>C), CAC613TCA (5393C>T 5394A>C 5395C>A), AGC614ACA (5397G>C 5398C>A), AAC615AAT (5401C>T), GTG619TTA (5411G>T 5413G>A), CTT620CTA (5416T>A), GTA622ATA (5420G>A), AAA623GTA (5423A>G 5424A>T)								

*: Inserts / Deletes / Misaligned / Frameshifts

Analysis details

This analysis was performed with panviral2.64

NGS Details (UN9): Caulimovirus latensarmoraciae

Assembly

**Coverage Length**	364 (1 contig(s))
**Depth Of Coverage**	7.3
**Number Of Reads**	21
**Reads Per Million**	0.47 rpm (after QC)
**Ambiguities**	0
**Assembly Method**	de novo + reference guided assembly
**Consensus Caller**	Bcf Tools

Coverage Map

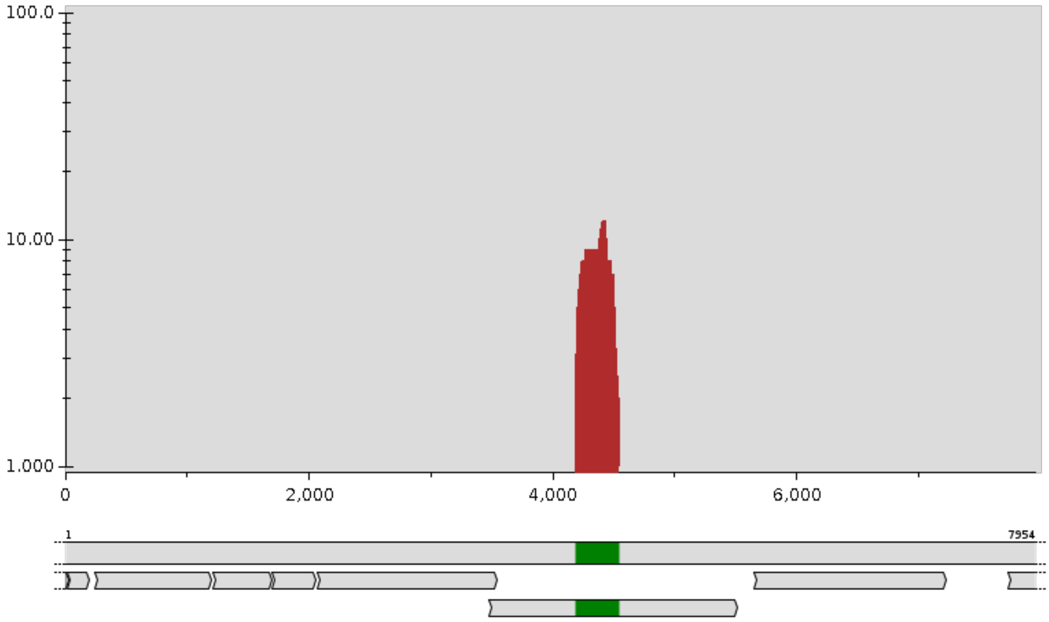

Assignment

**Type**	Caulimovirus latensarmoraciae (Taxonomy ID: 3047955)
**Reference Genome**	NC_018858.1
**NT Identity (%)**	58.7912
**AA Identity (%)**	56.1983
**Number Of Stop Codons**	0
**Number Of CDS**	8

Alignment

**Alignment Score**	128.0 (NT) + 500.0 (AA) = 628.0
**Concordance (%)**	41.5344

Alignment Method	Global, seeded, nucleotide + amino acids (AGA)

Genome Region

Sequence starts at position 4182 and ends at position 4545 relative to NC_018858.1 reference sequence.

Alignment Detailed Statistics

	Begin	End	Coverage	Score	Concordance	Matches	Identities	I/D/M/F*	Stop Codons
**NT**	**4182**	**4545**	**4.6%**	**128**	**17.6%**	**364 (100%)**	**214 (58.8%)**	**0/0**	
Mutations:	4182A>G, 4186C>A, 4189C>T, 4190A>T, 4192C>A, 4194A>C, 4195A>T, 4199T>A, 4200C>A, 4201G>A, 4209C>G, 4210G>T, 4212A>T, 4213G>C, 4214G>A, 4216T>A, 4219C>A, 4221A>G, 4222G>A, 4231C>T, 4235A>C, 4236A>T, 4240T>C, 4242G>A, 4243C>T, 4244C>G, 4245C>A, 4247A>C, 4248T>A, 4249G>A, 4252C>T, 4253C>A, 4254G>A, 4256G>A, 4257A>C, 4258A>T, 4265G>A, 4267G>A, 4269A>T, 4270G>T, 4273A>G, 4286C>T, 4288G>A, 4289G>A, 4291C>T, 4292C>A, 4294C>G, 4297G>A, 4298G>T, 4303C>A, 4306G>A, 4308C>A, 4309C>T, 4318C>T, 4326T>G, 4327G>T, 4328G>A, 4329C>G, 4330A>C, 4333A>C, 4336G>A, 4339C>T, 4340T>A, 4342A>G, 4345C>A, 4347A>G, 4348C>A, 4352G>C, 4354A>T, 4356C>G, 4357T>C, 4360G>A, 4361A>C, 4364C>A, 4365G>A, 4366C>A, 4367A>C, 4369A>T, 4375G>A, 4376A>G, 4377A>C, 4378G>T, 4381T>A, 4387G>A, 4388G>A, 4390C>A, 4396C>T, 4401C>A, 4402A>G, 4403A>C, 4405G>T, 4409G>A, 4411T>A, 4412G>A, 4413C>A, 4414G>T, 4415A>T, 4416C>G, 4420A>C, 4421G>T, 4422G>T, 4428C>G, 4432C>T, 4433A>T, 4434A>T, 4435T>C, 4436C>A, 4438T>C, 4441C>A, 4444C>T, 4447G>A, 4448G>A, 4451G>A, 4453A>C, 4456C>T, 4457C>A, 4459T>A, 4461C>A, 4462G>T, 4465C>T, 4466A>G, 4467T>C, 4468C>A, 4470G>A, 4477G>A, 4479A>C, 4480A>C, 4481A>T, 4482T>A, 4486C>T, 4489T>A, 4490T>A, 4491C>A, 4492C>A, 4498C>T, 4505T>A, 4506C>G, 4507A>T, 4513C>T, 4520G>A, 4522T>A, 4524G>A, 4525G>A, 4528T>A, 4531C>A, 4535G>A, 4537A>T, 4540A>T, 4542A>T, 4544T>G								

CDS

	Begin	End	Coverage	Score	Concordance	Matches	Identities	I/D/M/F*	Stop Codons
D306_gp7	236	356	17.8%	500	62.8%	121 (100%)	68 (56.2%)	0/0/0/0	0
Protein mutations:	I238L (4190A>T 4192C>A), K239T (4194A>C 4195A>T), S241K (4199T>A 4200C>A 4201G>A), T244S (4209C>G 4210G>T), K245I (4212A>T 4213G>C), V246I (4214G>A 4216T>A), K248R (4221A>G 4222G>A), K253L (4235A>C 4236A>T), S255N (4242G>A 4243C>T), P256E (4244C>G 4245C>A), M257Q (4247A>C 4248T>A 4249G>A), R259K (4253C>A 4254G>A), E260T (4256G>A 4257A>C 4258A>T), E263K (4265G>A 4267G>A), K264I (4269A>T 4270G>T), D271N (4289G>A 4291C>T), L272M (4292C>A 4294C>G), V274L (4298G>T), P277H (4308C>A 4309C>T), M283S (4326T>G 4327G>T), A284S (4328G>A 4329C>G 4330A>C), L288M (4340T>A 4342A>G), N290R (4347A>G 4348C>A), E292H (4352G>C 4354A>T), A293G (4356C>G 4357T>C), K295Q (4361A>C), R296K (4364C>A 4365G>A 4366C>A), K300A (4376A>G 4377A>C 4378G>T), V304I (4388G>A 4390C>A), A308E (4401C>A 4402A>G), M309L (4403A>C 4405G>T), D311K (4409G>A 4411T>A), A312N (4412G>A 4413C>A 4414G>T), T313C (4415A>T 4416C>G), G315F (4421G>T 4422G>T), A317G (4428C>G), N319F (4433A>T 4434A>T 4435T>C), L320I (4436C>A 4438T>C), D324N (4448G>A), E325N (4451G>A 4453A>C), L327I (4457C>A 4459T>A), T328N (4461C>A 4462G>T), I330A (4466A>G 4467T>C 4468C>A), R331K (4470G>A), K334T (4479A>C 4480A>C), I335Y (4481A>T 4482T>A), S338K (4490T>A 4491C>A 4492C>A), V348I (4520G>A 4522T>A), R349K (4524G>A 4525G>A), D351E (4531C>A), E353N (4535G>A 4537A>T), K355I (4542A>T)								
Codon mutations:	CAA235.GA (4182A>G), GCC236GCA (4186C>A), AGC237AGT (4189C>T), ATC238TTA (4190A>T 4192C>A), AAA239ACT (4194A>C 4195A>T), TCG241AAA (4199T>A 4200C>A 4201G>A), ACG244AGT (4209C>G 4210G>T), AAG245ATC (4212A>T 4213G>C), GTT246ATA (4214G>A 4216T>A), ATC247ATA (4219C>A), AAG248AGA (4221A>G 4222G>A), CCC251CCT (4231C>T), AAA253CTA (4235A>C 4236A>T), TAT254TAC (4240T>C), AGC255AAT (4242G>A 4243C>T), CCA256GAA (4244C>G 4245C>A), ATG257CAA (4247A>C 4248T>A 4249G>A), GAC258GAT (4252C>T), CGA259AAA (4253C>A 4254G>A), GAA260ACT (4256G>A 4257A>C 4258A>T), GAG263AAA (4265G>A 4267G>A), AAG264ATT (4269A>T 4270G>T), CAA265CAG (4273A>G), CTG270TTA (4286C>T 4288G>A), GAC271AAT (4289G>A 4291C>T), CTC272ATG (4292C>A 4294C>G), AAG273AAA (4297G>A), GTA274TTA (4298G>T), ATC275ATA (4303C>A), AGG276AGA (4306G>A), CCC277CAT (4308C>A 4309C>T), AGC280AGT (4318C>T), ATG283AGT (4326T>G 4327G>T), GCA284AGC (4328G>A 4329C>G 4330A>C), CCA285CCC (4333A>C), GCG286GCA (4336G>A), TTC287TTT (4339C>T), TTA288ATG (4340T>A 4342A>G), GTC289GTA (4345C>A), AAC290AGA (4347A>G 4348C>A), GAA292CAT (4352G>C 4354A>T), GCT293GGC (4356C>G 4357T>C), GAG294GAA (4360G>A), AAA295CAA (4361A>C), CGC296AAA (4364C>A 4365G>A 4366C>A), AGA297CGT (4367A>C 4369A>T), AAG299AAA (4375G>A), AAG300GCT (4376A>G 4377A>C 4378G>T), CGT301CGA (4381T>A), GTG303GTA (4387G>A), GTC304ATA (4388G>A 4390C>A), TAC306TAT (4396C>T), GCA308GAG (4401C>A 4402A>G), ATG309CTT (4403A>C 4405G>T), GAT311AAA (4409G>A 4411T>A), GCG312AAT (4412G>A 4413C>A 4414G>T), ACT313TGT (4415A>T 4416C>G), GTA314GTC (4420A>C), GGC315TTC (4421G>T 4422G>T), GCA317GGA (4428C>G), TAC318TAT (4432C>T), AAT319TTC (4433A>T 4434A>T 4435T>C), CTT320ATC (4436C>A 4438T>C), CCC321CCA (4441C>A), AAC322AAT (4444C>T), AAG323AAA (4447G>A), GAT324AAT (4448G>A), GAA325AAC (4451G>A 4453A>C), CTC326CTT (4456C>T), CTT327ATA (4457C>A 4459T>A), ACG328AAT (4461C>A 4462G>T), CTC329CTT (4465C>T), ATC330GCA (4466A>G 4467T>C 4468C>A), AGA331AAA (4470G>A), AAG333AAA (4477G>A), AAA334ACC (4479A>C 4480A>C), ATC335TAC (4481A>T 4482T>A), TTC336TTT (4486C>T), TCT337TCA (4489T>A), TCC338AAA (4490T>A 4491C>A 4492C>A), GAC340GAT (4498C>T), TCA343AGT (4505T>A 4506C>G 4507A>T), TTC345TTT (4513C>T), GTT348ATA (4520G>A 4522T>A), AGG349AAA (4524G>A 4525G>A), CTT350CTA (4528T>A), GAC351GAA (4531C>A), GAA353AAT (4535G>A 4537A>T), TCA354TCT (4540A>T), AAA355ATA (4542A>T), TCC356GC. (4544T>G)								

Proteins

	Begin	End	Coverage	Score	Concordance	Matches	Identities	I/D/M/F*	Stop Codons
polyprotein (YP_006907834.1)	236	356	17.8%	500	62.8%	121 (100%)	68 (56.2%)	0/0/0/0	0
Protein mutations:	I238L (4190A>T 4192C>A), K239T (4194A>C 4195A>T), S241K (4199T>A 4200C>A 4201G>A), T244S (4209C>G 4210G>T), K245I (4212A>T 4213G>C), V246I (4214G>A 4216T>A), K248R (4221A>G 4222G>A), K253L (4235A>C 4236A>T), S255N (4242G>A 4243C>T), P256E (4244C>G 4245C>A), M257Q (4247A>C 4248T>A 4249G>A), R259K (4253C>A 4254G>A), E260T (4256G>A 4257A>C 4258A>T), E263K (4265G>A 4267G>A), K264I (4269A>T 4270G>T), D271N (4289G>A 4291C>T), L272M (4292C>A 4294C>G), V274L (4298G>T), P277H (4308C>A 4309C>T), M283S (4326T>G 4327G>T), A284S (4328G>A 4329C>G 4330A>C), L288M (4340T>A 4342A>G), N290R (4347A>G 4348C>A), E292H (4352G>C 4354A>T), A293G (4356C>G 4357T>C), K295Q (4361A>C), R296K (4364C>A 4365G>A 4366C>A), K300A (4376A>G 4377A>C 4378G>T), V304I (4388G>A 4390C>A), A308E (4401C>A 4402A>G), M309L (4403A>C 4405G>T), D311K (4409G>A 4411T>A), A312N (4412G>A 4413C>A 4414G>T), T313C (4415A>T 4416C>G), G315F (4421G>T 4422G>T), A317G (4428C>G), N319F (4433A>T 4434A>T 4435T>C), L320I (4436C>A 4438T>C), D324N (4448G>A), E325N (4451G>A 4453A>C), L327I (4457C>A 4459T>A), T328N (4461C>A 4462G>T), I330A (4466A>G 4467T>C 4468C>A), R331K (4470G>A), K334T (4479A>C 4480A>C), I335Y (4481A>T 4482T>A), S338K (4490T>A 4491C>A 4492C>A), V348I (4520G>A 4522T>A), R349K (4524G>A 4525G>A), D351E (4531C>A), E353N (4535G>A 4537A>T), K355I (4542A>T)								
Codon mutations:	CAA235.GA (4182A>G), GCC236GCA (4186C>A), AGC237AGT (4189C>T), ATC238TTA (4190A>T 4192C>A), AAA239ACT (4194A>C 4195A>T), TCG241AAA (4199T>A 4200C>A 4201G>A), ACG244AGT (4209C>G 4210G>T), AAG245ATC (4212A>T 4213G>C), GTT246ATA (4214G>A 4216T>A), ATC247ATA (4219C>A), AAG248AGA (4221A>G 4222G>A), CCC251CCT (4231C>T), AAA253CTA (4235A>C 4236A>T), TAT254TAC (4240T>C), AGC255AAT (4242G>A 4243C>T), CCA256GAA (4244C>G 4245C>A), ATG257CAA (4247A>C 4248T>A 4249G>A), GAC258GAT (4252C>T), CGA259AAA (4253C>A 4254G>A), GAA260ACT (4256G>A 4257A>C 4258A>T), GAG263AAA (4265G>A 4267G>A), AAG264ATT (4269A>T 4270G>T), CAA265CAG (4273A>G), CTG270TTA (4286C>T 4288G>A), GAC271AAT (4289G>A 4291C>T), CTC272ATG (4292C>A 4294C>G), AAG273AAA (4297G>A), GTA274TTA (4298G>T), ATC275ATA (4303C>A), AGG276AGA (4306G>A), CCC277CAT (4308C>A 4309C>T), AGC280AGT (4318C>T), ATG283AGT (4326T>G 4327G>T), GCA284AGC (4328G>A 4329C>G 4330A>C), CCA285CCC (4333A>C), GCG286GCA (4336G>A), TTC287TTT (4339C>T), TTA288ATG (4340T>A 4342A>G), GTC289GTA (4345C>A), AAC290AGA (4347A>G 4348C>A), GAA292CAT (4352G>C 4354A>T), GCT293GGC (4356C>G 4357T>C), GAG294GAA (4360G>A), AAA295CAA (4361A>C), CGC296AAA (4364C>A 4365G>A 4366C>A), AGA297CGT (4367A>C 4369A>T), AAG299AAA (4375G>A), AAG300GCT (4376A>G 4377A>C 4378G>T), CGT301CGA (4381T>A), GTG303GTA (4387G>A), GTC304ATA (4388G>A 4390C>A), TAC306TAT (4396C>T), GCA308GAG (4401C>A 4402A>G), ATG309CTT (4403A>C 4405G>T), GAT311AAA (4409G>A 4411T>A), GCG312AAT (4412G>A 4413C>A 4414G>T), ACT313TGT (4415A>T 4416C>G), GTA314GTC (4420A>C), GGC315TTC (4421G>T 4422G>T), GCA317GGA (4428C>G), TAC318TAT (4432C>T), AAT319TTC (4433A>T 4434A>T 4435T>C), CTT320ATC (4436C>A 4438T>C), CCC321CCA (4441C>A), AAC322AAT (4444C>T), AAG323AAA (4447G>A), GAT324AAT (4448G>A), GAA325AAC (4451G>A 4453A>C), CTC326CTT (4456C>T), CTT327ATA (4457C>A 4459T>A), ACG328AAT (4461C>A 4462G>T), CTC329CTT (4465C>T), ATC330GCA (4466A>G 4467T>C 4468C>A), AGA331AAA (4470G>A), AAG333AAA (4477G>A), AAA334ACC (4479A>C 4480A>C), ATC335TAC (4481A>T 4482T>A), TTC336TTT (4486C>T), TCT337TCA (4489T>A), TCC338AAA (4490T>A 4491C>A 4492C>A), GAC340GAT (4498C>T), TCA343AGT (4505T>A 4506C>G 4507A>T), TTC345TTT (4513C>T), GTT348ATA (4520G>A 4522T>A), AGG349AAA (4524G>A 4525G>A), CTT350CTA (4528T>A), GAC351GAA (4531C>A), GAA353AAT (4535G>A 4537A>T), TCA354TCT (4540A>T), AAA355ATA (4542A>T), TCC356GC. (4544T>G)								

*: Inserts / Deletes / Misaligned / Frameshifts

Analysis details

This analysis was performed with panviral2.64

NGS Details (UN9): Badnavirus occultipomeae

Assembly

**Coverage Length**	347 (1 contig(s))
**Depth Of Coverage**	5.4
**Number Of Reads**	18
**Reads Per Million**	0.41 rpm (after QC)
**Ambiguities**	0
**Assembly Method**	de novo + reference guided assembly
**Consensus Caller**	Bcf Tools

Coverage Map

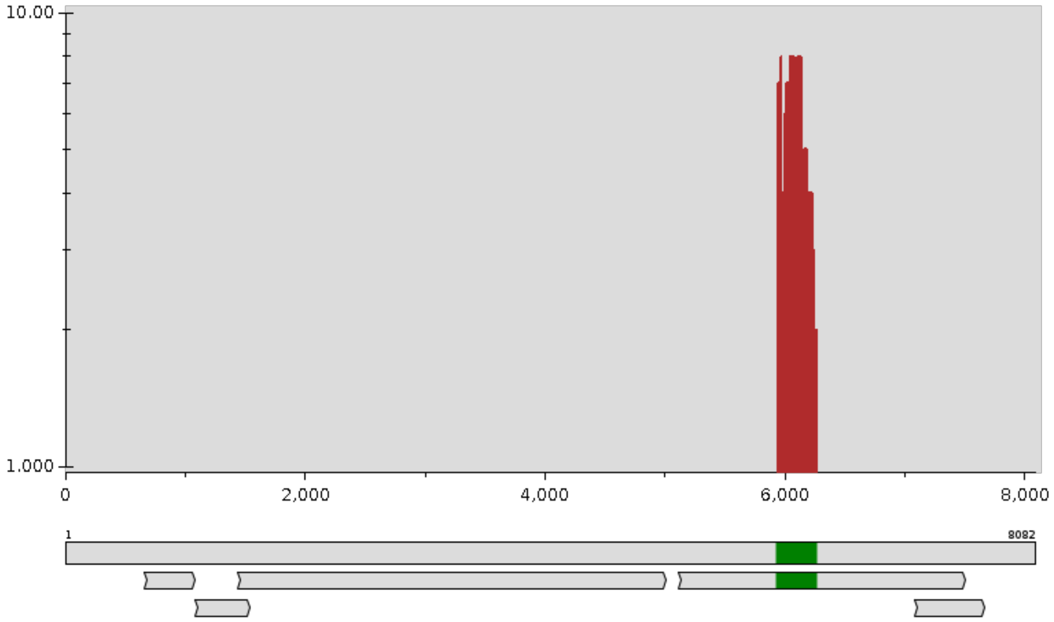

Assignment

**Type**	Badnavirus occultipomeae (Taxonomy ID: 3048353)
**Reference Genome**	NC_015655.1
**NT Identity (%)**	54.8571
**AA Identity (%)**	46.1538
**Number Of Stop Codons**	0
**Number Of CDS**	5

Alignment

**Alignment Score**	62.0 (NT) + 380.0 (AA) = 442.0
**Concordance (%)**	29.4079

**Alignment Method**	Global, seeded, nucleotide + amino acids (AGA)

Genome Region

Sequence starts at position 5926 and ends at position 6272 relative to NC_015655.1 reference sequence.

Alignment Detailed Statistics

	Begin	End	Coverage	Score	Concordance	Matches	Identities	I/D/M/F*	Stop Codons
**NT**	**5926**	**6272**	**4.3%**	**62**	**8.9%**	**347 (99.1%)**	**192 (54.9%)**	**3/0**	
Mutations:	5927A>T, 5928A>T, 5929A>C, 5935G>T, 5936T>G, 5938T>A, 5939A>T, 5940C>T, 5941A>G, 5943C>T, 5946C>T, 5947A>C, 5948A>G, 5949G>T, 5951G>A, 5952C>A, 5953C>T, 5955C>G, 5959G>A, 5961C>A, 5962A>G, 5963A>T, 5964T>C, 5967A>C, 5968G>A, 5969A>T, 5970A>C, 5973A>G, 5974G>A, 5976T>C, 5977C>A, 5985C>A, 5986C>A, 5988A>T, 5991G>T, 5992G>A, 5998A>G, 6002C>A, 6004A>T, 6006T>G, 6007G>T, 6009C>T, 6010A>G, 6011G>A, 6013A>C, 6014G>A, 6016A>T, 6018A>G, 6019T>C, 6020C>A, 6021T>A, 6024A>G, 6025A>G, 6026A>G, 6027A>T, 6029A>G, 6030G>T, 6031A>C, 6032T>A, 6033A>C, 6039A>C, 6042A>G, 6043T>A, 6048T>C, 6051C>T, 6052A>C, 6053A>G, 6054A>T, 6055G>T, 6056C>T, 6057A>C, 6060C>T, 6062T>A, 6069G>A, 6072A>G, 6075A>G, 6076A>G, 6078G>A, 6079G>A, 6080A>G, 6085A>T, 6086A>G, 6087G>T, 6088T>G, 6089C>A, 6090C>T, 6091A>G, 6092A>T, 6093A>T, 6096A>C, 6097T>A, 6098G>A, 6099G>A, 6102T>G, 6103G>A, 6109T>C, 6114T>A, 6115C>A, 6116C>G, 6117A>G, 6118G>T, 6120A>C, 6125T>A, 6128A>T, 6137A>T, 6138A>G, 6141C>G, 6145C>T, 6147C>T, 6153A>G, 6154C>T, 6156G>A, 6168A>G, 6171A>G, 6173A>T, 6174T>G, 6177T>C, 6178C>A, 6179A>T, 6182G>A, 6183A>T, 6184A>C, 6185A>T, 6186G>A, 6190G>A, 6194A>G, 6197C>T, 6198A>G, 6201T>C, 6203G>A, 6205G>C, 6206G>C, 6207A>T, 6207_6208insTAT, 6208A>C, 6209C>T, 6210A>T, 6214G>A, 6220A>G, 6222C>G, 6224C>T, 6225A>G, 6228A>C, 6230A>T, 6231T>C, 6234T>A, 6246G>A, 6247G>A, 6249A>C, 6251T>A, 6256G>C, 6257A>G, 6258A>G, 6259A>G, 6260A>G, 6270G>A								

CDS

	Begin	End	Coverage	Score	Concordance	Matches	Identities	I/D/M/F*	Stop Codons
SPBVa_gp4	271	386	14.6%	380	46.1%	116 (99.1%)	54 (46.2%)	1/0/0/0	0
Protein mutations:	Q271L (5927A>T 5928A>T), V274C (5935G>T 5936T>G), Y275I (5938T>A 5939A>T 5940C>T), N276D (5941A>G 5943C>T), K278R (5947A>C 5948A>G 5949G>T), R279Q (5951G>A 5952C>A), D282K (5959G>A 5961C>A), N283V (5962A>G 5963A>T 5964T>C), E285I (5968G>A 5969A>T 5970A>C), D287N (5974G>A 5976T>C), Q288K (5977C>A), L291I (5986C>A 5988A>T), G293R (5992G>A), N295D (5998A>G), T296N (6002C>A), I297L (6004A>T 6006T>G), V298F (6007G>T 6009C>T), S299D (6010A>G 6011G>A), R300Q (6013A>C 6014G>A), I301L (6016A>T 6018A>G), S302Q (6019T>C 6020C>A 6021T>A), K304G (6025A>G 6026A>G 6027A>T), K305S (6029A>G 6030G>T), I306H (6031A>C 6032T>A 6033A>C), F310I (6043T>A), K313R (6052A>C 6053A>G 6054A>T), A314F (6055G>T 6056C>T 6057A>C), F316Y (6062T>A), I319M (6072A>G), M321V (6076A>G 6078G>A), E322R (6079G>A 6080A>G), K324C (6085A>T 6086A>G 6087G>T), S325D (6088T>G 6089C>A 6090C>T), K326V (6091A>G 6092A>T 6093A>T), W328K (6097T>A 6098G>A 6099G>A), A330T (6103G>A), W332R (6109T>C), P334R (6115C>A 6116C>G 6117A>G), E335Y (6118G>T 6120A>C), L337H (6125T>A), Y338F (6128A>T), E341V (6137A>T 6138A>G), P344S (6145C>T 6147C>T), D353V (6173A>T 6174T>G), Q355M (6178C>A 6179A>T), R356N (6182G>A 6183A>T), K357L (6184A>C 6185A>T 6186G>A), D359N (6190G>A), N360S (6194A>G), A361V (6197C>T 6198A>G), R363K (6203G>A), G364P (6205G>C 6206G>C 6207A>T), G364_T365insY (6207_6208insTAT), T365L (6208A>C 6209C>T 6210A>T), A367T (6214G>A), I369V (6220A>G 6222C>G), A370V (6224C>T 6225A>G), Y372F (6230A>T 6231T>C), V378I (6247G>A 6249A>C), F379Y (6251T>A), E381R (6256G>C 6257A>G 6258A>G), N382G (6259A>G 6260A>G)								
Codon mutations:	CAA271CTT (5927A>T 5928A>T), AGG272CGG (5929A>C), GTT274TGT (5935G>T 5936T>G), TAC275ATT (5938T>A 5939A>T 5940C>T), AAC276GAT (5941A>G 5943C>T), TAC277TAT (5946C>T), AAG278CGT (5947A>C 5948A>G 5949G>T), CGC279CAA (5951G>A 5952C>A), CTC280TTG (5953C>T 5955C>G), GAC282AAA (5959G>A 5961C>A), AAT283GTC (5962A>G 5963A>T 5964T>C), ACA284ACC (5967A>C), GAA285ATC (5968G>A 5969A>T 5970A>C), AAA286AAG (5973A>G), GAT287AAC (5974G>A 5976T>C), CAG288AAG (5977C>A), TCC290TCA (5985C>A), CTA291ATT (5986C>A 5988A>T), CCG292CCT (5991G>T), GGA293AGA (5992G>A), AAC295GAC (5998A>G), ACT296AAT (6002C>A), ATT297TTG (6004A>T 6006T>G), GTC298TTT (6007G>T 6009C>T), AGT299GAT (6010A>G 6011G>A), AGA300CAA (6013A>C 6014G>A), ATA301TTG (6016A>T 6018A>G), TCT302CAA (6019T>C 6020C>A 6021T>A), GGA303GGG (6024A>G), AAA304GGT (6025A>G 6026A>G 6027A>T), AAG305AGT (6029A>G 6030G>T), ATA306CAC (6031A>C 6032T>A 6033A>C), TCA308TCC (6039A>C), AAA309AAG (6042A>G), TTT310ATT (6043T>A), GAT311GAC (6048T>C), CTC312CTT (6051C>T), AAA313CGT (6052A>C 6053A>G 6054A>T), GCA314TTC (6055G>T 6056C>T 6057A>C), GGC315GGT (6060C>T), TTT316TAT (6062T>A), CAG318CAA (6069G>A), ATA319ATG (6072A>G), AGA320AGG (6075A>G), ATG321GTA (6076A>G 6078G>A), GAG322AGG (6079G>A 6080A>G), AAG324TGT (6085A>T 6086A>G 6087G>T), TCC325GAT (6088T>G 6089C>A 6090C>T), AAA326GTT (6091A>G 6092A>T 6093A>T), CCA327CCC (6096A>C), TGG328AAA (6097T>A 6098G>A 6099G>A), ACT329ACG (6102T>G), GCT330ACT (6103G>A), TGG332CGG (6109T>C), ACT333ACA (6114T>A), CCA334AGG (6115C>A 6116C>G 6117A>G), GAA335TAC (6118G>T 6120A>C), CTC337CAC (6125T>A), TAT338TTT (6128A>T), GAA341GTG (6137A>T 6138A>G), GTC342GTG (6141C>G), CCC344TCT (6145C>T 6147C>T), GGA346GGG (6153A>G), CTG347TTA (6154C>T 6156G>A), CCA351CCG (6168A>G), GCA352GCG (6171A>G), GAT353GTG (6173A>T 6174T>G), TTT354TTC (6177T>C), CAG355ATG (6178C>A 6179A>T), AGA356AAT (6182G>A 6183A>T), AAG357CTA (6184A>C 6185A>T 6186G>A), GAT359AAT (6190G>A), AAT360AGT (6194A>G), GCA361GTG (6197C>T 6198A>G), TTT362TTC (6201T>C), AGG363AAG (6203G>A), GGA364CCT (6205G>C 6206G>C 6207A>T), GGA364_ACA365insTAT (6207_6208insTAT), ACA365CTT (6208A>C 6209C>T 6210A>T), GCA367ACA (6214G>A), ATC369GTG (6220A>G 6222C>G), GCA370GTG (6224C>T 6225A>G), GTA371GTC (6228A>C), TAT372TTC (6230A>T 6231T>C), ATT373ATA (6234T>A), TTG377TTA (6246G>A), GTA378ATC (6247G>A 6249A>C), TTC379TAC (6251T>A), GAA381CGG (6256G>C 6257A>G 6258A>G), AAT382GGT (6259A>G 6260A>G), GAG385GAA (6270G>A)								

Proteins

	Begin	End	Coverage	Score	Concordance	Matches	Identities	I/D/M/F*	Stop Codons
RNaseH/reverse transcriptase (YP_004581513.1)	271	386	14.6%	380	46.1%	116 (99.1%)	54 (46.2%)	1/0/0/0	0
Protein mutations:	Q271L (5927A>T 5928A>T), V274C (5935G>T 5936T>G), Y275I (5938T>A 5939A>T 5940C>T), N276D (5941A>G 5943C>T), K278R (5947A>C 5948A>G 5949G>T), R279Q (5951G>A 5952C>A), D282K (5959G>A 5961C>A), N283V (5962A>G 5963A>T 5964T>C), E285I (5968G>A 5969A>T 5970A>C), D287N (5974G>A 5976T>C), Q288K (5977C>A), L291I (5986C>A 5988A>T), G293R (5992G>A), N295D (5998A>G), T296N (6002C>A), I297L (6004A>T 6006T>G), V298F (6007G>T 6009C>T), S299D (6010A>G 6011G>A), R300Q (6013A>C 6014G>A), I301L (6016A>T 6018A>G), S302Q (6019T>C 6020C>A 6021T>A), K304G (6025A>G 6026A>G 6027A>T), K305S (6029A>G 6030G>T), I306H (6031A>C 6032T>A 6033A>C), F310I (6043T>A), K313R (6052A>C 6053A>G 6054A>T), A314F (6055G>T 6056C>T 6057A>C), F316Y (6062T>A), I319M (6072A>G), M321V (6076A>G 6078G>A), E322R (6079G>A 6080A>G), K324C (6085A>T 6086A>G 6087G>T), S325D (6088T>G 6089C>A 6090C>T), K326V (6091A>G 6092A>T 6093A>T), W328K (6097T>A 6098G>A 6099G>A), A330T (6103G>A), W332R (6109T>C), P334R (6115C>A 6116C>G 6117A>G), E335Y (6118G>T 6120A>C), L337H (6125T>A), Y338F (6128A>T), E341V (6137A>T 6138A>G), P344S (6145C>T 6147C>T), D353V (6173A>T 6174T>G), Q355M (6178C>A 6179A>T), R356N (6182G>A 6183A>T), K357L (6184A>C 6185A>T 6186G>A), D359N (6190G>A), N360S (6194A>G), A361V (6197C>T 6198A>G), R363K (6203G>A), G364P (6205G>C 6206G>C 6207A>T), G364_T365insY (6207_6208insTAT), T365L (6208A>C 6209C>T 6210A>T), A367T (6214G>A), I369V (6220A>G 6222C>G), A370V (6224C>T 6225A>G), Y372F (6230A>T 6231T>C), V378I (6247G>A 6249A>C), F379Y (6251T>A), E381R (6256G>C 6257A>G 6258A>G), N382G (6259A>G 6260A>G)								
Codon mutations:	CAA271CTT (5927A>T 5928A>T), AGG272CGG (5929A>C), GTT274TGT (5935G>T 5936T>G), TAC275ATT (5938T>A 5939A>T 5940C>T), AAC276GAT (5941A>G 5943C>T), TAC277TAT (5946C>T), AAG278CGT (5947A>C 5948A>G 5949G>T), CGC279CAA (5951G>A 5952C>A), CTC280TTG (5953C>T 5955C>G), GAC282AAA (5959G>A 5961C>A), AAT283GTC (5962A>G 5963A>T 5964T>C), ACA284ACC (5967A>C), GAA285ATC (5968G>A 5969A>T 5970A>C), AAA286AAG (5973A>G), GAT287AAC (5974G>A 5976T>C), CAG288AAG (5977C>A), TCC290TCA (5985C>A), CTA291ATT (5986C>A 5988A>T), CCG292CCT (5991G>T), GGA293AGA (5992G>A), AAC295GAC (5998A>G), ACT296AAT (6002C>A), ATT297TTG (6004A>T 6006T>G), GTC298TTT (6007G>T 6009C>T), AGT299GAT (6010A>G 6011G>A), AGA300CAA (6013A>C 6014G>A), ATA301TTG (6016A>T 6018A>G), TCT302CAA (6019T>C 6020C>A 6021T>A), GGA303GGG (6024A>G), AAA304GGT (6025A>G 6026A>G 6027A>T), AAG305AGT (6029A>G 6030G>T), ATA306CAC (6031A>C 6032T>A 6033A>C), TCA308TCC (6039A>C), AAA309AAG (6042A>G), TTT310ATT (6043T>A), GAT311GAC (6048T>C), CTC312CTT (6051C>T), AAA313CGT (6052A>C 6053A>G 6054A>T), GCA314TTC (6055G>T 6056C>T 6057A>C), GGC315GGT (6060C>T), TTT316TAT (6062T>A), CAG318CAA (6069G>A), ATA319ATG (6072A>G), AGA320AGG (6075A>G), ATG321GTA (6076A>G 6078G>A), GAG322AGG (6079G>A 6080A>G), AAG324TGT (6085A>T 6086A>G 6087G>T), TCC325GAT (6088T>G 6089C>A 6090C>T), AAA326GTT (6091A>G 6092A>T 6093A>T), CCA327CCC (6096A>C), TGG328AAA (6097T>A 6098G>A 6099G>A), ACT329ACG (6102T>G), GCT330ACT (6103G>A), TGG332CGG (6109T>C), ACT333ACA (6114T>A), CCA334AGG (6115C>A 6116C>G 6117A>G), GAA335TAC (6118G>T 6120A>C), CTC337CAC (6125T>A), TAT338TTT (6128A>T), GAA341GTG (6137A>T 6138A>G), GTC342GTG (6141C>G), CCC344TCT (6145C>T 6147C>T), GGA346GGG (6153A>G), CTG347TTA (6154C>T 6156G>A), CCA351CCG (6168A>G), GCA352GCG (6171A>G), GAT353GTG (6173A>T 6174T>G), TTT354TTC (6177T>C), CAG355ATG (6178C>A 6179A>T), AGA356AAT (6182G>A 6183A>T), AAG357CTA (6184A>C 6185A>T 6186G>A), GAT359AAT (6190G>A), AAT360AGT (6194A>G), GCA361GTG (6197C>T 6198A>G), TTT362TTC (6201T>C), AGG363AAG (6203G>A), GGA364CCT (6205G>C 6206G>C 6207A>T), GGA364_ACA365insTAT (6207_6208insTAT), ACA365CTT (6208A>C 6209C>T 6210A>T), GCA367ACA (6214G>A), ATC369GTG (6220A>G 6222C>G), GCA370GTG (6224C>T 6225A>G), GTA371GTC (6228A>C), TAT372TTC (6230A>T 6231T>C), ATT373ATA (6234T>A), TTG377TTA (6246G>A), GTA378ATC (6247G>A 6249A>C), TTC379TAC (6251T>A), GAA381CGG (6256G>C 6257A>G 6258A>G), AAT382GGT (6259A>G 6260A>G), GAG385GAA (6270G>A)								

*: Inserts / Deletes / Misaligned / Frameshifts

Analysis details

This analysis was performed with panviral2.64

NGS Details (UN9): Badnavirus occultipomeae

Assembly

**Coverage Length**	257 (1 contig(s))
**Depth Of Coverage**	6.7
**Number Of Reads**	15
**Reads Per Million**	0.34 rpm (after QC)
**Ambiguities**	0
**Assembly Method**	de novo + reference guided assembly
**Consensus Caller**	Bcf Tools

Coverage Map

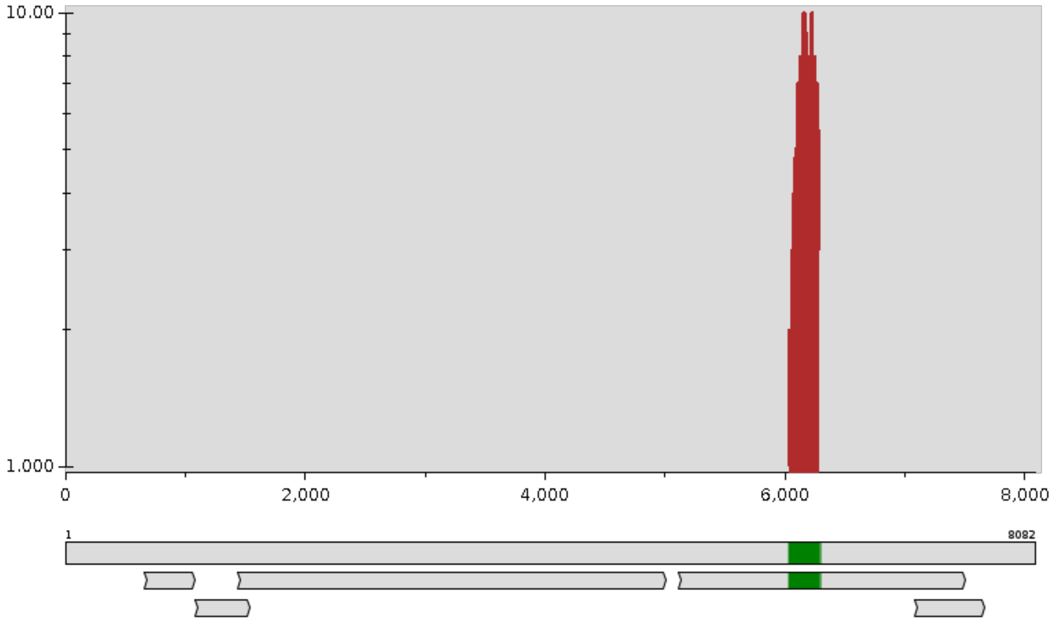

Assignment

**Type**	Badnavirus occultipomeae (Taxonomy ID: 3048353)
**Reference Genome**	NC_015655.1
**NT Identity (%)**	61.4504
**AA Identity (%)**	47.191
**Number Of Stop Codons**	0
**Number Of CDS**	5

Alignment

**Alignment Score**	107.0 (NT) + 177.0 (AA) = 284.0
**Concordance (%)**	27.6539

**Alignment Method**	Global, seeded, nucleotide + amino acids (AGA)

Genome Region

Sequence starts at position 6031 and ends at position 6289 relative to NC_015655.1 reference sequence.

Alignment Detailed Statistics

	Begin	End	Coverage	Score	Concordance	Matches	Identities	I/D/M/F*	Stop Codons
**NT**	**6031**	**6289**	**3.2%**	**107**	**20.8%**	**257 (98.1%)**	**161 (61.5%)**	**5/0**	
Mutations:	6031A>G, 6033A>G, 6043T>A, 6051C>A, 6053A>G, 6055G>T, 6057A>T, 6060C>T, 6062T>A, 6063T>C, 6070A>T, 6074G>A, 6075A>G, 6077T>A, 6078G>T, 6079G>A, 6082G>C, 6083A>C, 6088T>G, 6089C>A, 6090C>T, 6091A>G, 6092A>T, 6093A>G, 6096A>T, 6097T>A, 6109T>A, 6111G>A, 6116C>G, 6117A>G, 6118G>T, 6120A>T, 6125T>A, 6129T>C, 6135T>C, 6136G>T, 6137A>T, 6138A>G, 6141C>G, 6145C>T, 6147C>G, 6148T>A, 6153A>G, 6154C>T, 6158T>C, 6159G>C, 6165A>G, 6173A>C, 6174T>C, 6177T>C, 6178C>A, 6179A>T, 6183A>T, 6184A>T, 6185A>T, 6190G>A, 6193A>G, 6194A>G, 6195T>G, 6197C>T, 6198A>G, 6203G>A, 6205G>C, 6206G>C, 6207_6208insTTT, 6208A>T, 6209C>T, 6210A>G, 6214G>T, 6216A>G, 6220A>G, 6222C>G, 6223G>A, 6224C>T, 6225A>T, 6227T>G, 6228A>G, 6230A>T, 6243A>T, 6244T>C, 6246G>T, 6247G>A, 6249A>T, 6251T>A, 6252C>T, 6255T>A, 6256G>A, 6258_6259insAG, 6260A>G, 6262G>A, 6264A>G, 6275A>C, 6276G>A, 6277G>C, 6279T>C, 6285C>A, 6286T>A, 6289A>T								
**CDS**									
SPBVa_gp4	306	392	10.9%	177	27.7%	87 (97.8%)	42 (47.2%)	2/0/2/1	0
Protein mutations:	I306V (6031A>G 6033A>G), F310I (6043T>A), K313R (6053A>G), A314S (6055G>T 6057A>T), F316Y (6062T>A 6063T>C), I319L (6070A>T), R320K (6074G>A 6075A>G), M321N (6077T>A 6078G>T), E322K (6079G>A), E323P (6082G>C 6083A>C), S325D (6088T>G 6089C>A 6090C>T), K326V (6091A>G 6092A>T 6093A>G), W328R (6097T>A), W332R (6109T>A 6111G>A), P334R (6116C>G 6117A>G), E335Y (6118G>T 6120A>T), L337H (6125T>A), E341L (6136G>T 6137A>T 6138A>G), P344S (6145C>T 6147C>G), F345I (6148T>A), M348T (6158T>C 6159G>C), D353A (6173A>C 6174T>C), Q355M (6178C>A 6179A>T), R356S (6183A>T), K357L (6184A>T 6185A>T), D359N (6190G>A), N360G (6193A>G 6194A>G 6195T>G), A361V (6197C>T 6198A>G), R363K (6203G>A), G364P (6205G>C 6206G>C), G364_T365insF (6207_6208insTTT), T365L (6208A>T 6209C>T 6210A>G), A367S (6214G>T 6216A>G), I369V (6220A>G 6222C>G), A370I (6223G>A 6224C>T 6225A>T), V371G (6227T>G 6228A>G), Y372F (6230A>T), V378I (6247G>A 6249A>T), F379Y (6251T>A 6252C>T), E381K (6256G>A), E381_N382insX (6258_6259insAG), N382S (6260A>G), E383K (6262G>A 6264A>G), E387A (6275A>C 6276G>A), D388H (6277G>C 6279T>C)								
Codon mutations:	ATA306GTG (6031A>G 6033A>G), TTT310ATT (6043T>A), CTC312CTA (6051C>A), AAA313AGA (6053A>G), GCA314TCT (6055G>T 6057A>T), GGC315GGT (6060C>T), TTT316TAC (6062T>A 6063T>C), ATA319TTA (6070A>T), AGA320AAG (6074G>A 6075A>G), ATG321AAT (6077T>A 6078G>T), GAG322AAG (6079G>A), GAA323CCA (6082G>C 6083A>C), TCC325GAT (6088T>G 6089C>A 6090C>T), AAA326GTG (6091A>G 6092A>T 6093A>G), CCA327CCT (6096A>T), TGG328AGG (6097T>A), TGG332AGA (6109T>A 6111G>A), CCA334CGG (6116C>G 6117A>G), GAA335TAT (6118G>T 6120A>T), CTC337CAC (6125T>A), TAT338TAC (6129T>C), TTT340TTC (6135T>C), GAA341TTG (6136G>T 6137A>T 6138A>G), GTC342GTG (6141C>G), CCC344TCG (6145C>T 6147C>G), TTT345ATT (6148T>A), GGA346GGG (6153A>G), CTG347TTG (6154C>T), ATG348ACC (6158T>C 6159G>C), GCA350GCG (6165A>G), GAT353GCC (6173A>C 6174T>C), TTT354TTC (6177T>C), CAG355ATG (6178C>A 6179A>T), AGA356AGT (6183A>T), AAG357TTG (6184A>T 6185A>T), GAT359AAT (6190G>A), AAT360GGG (6193A>G 6194A>G 6195T>G), GCA361GTG (6197C>T 6198A>G), AGG363AAG (6203G>A), GGA364CCA (6205G>C 6206G>C), GGA364_ACA365insTTT (6207_6208insTTT), ACA365TTG (6208A>T 6209C>T 6210A>G), GCA367TCG (6214G>T 6216A>G), ATC369GTG (6220A>G 6222C>G), GCA370ATT (6223G>A 6224C>T 6225A>T), GTA371GGG (6227T>G 6228A>G), TAT372TTT (6230A>T), ATA376ATT (6243A>T), TTG377CTT (6244T>C 6246G>T), GTA378ATT (6247G>A 6249A>T), TTC379TAT (6251T>A 6252C>T), TCT380TCA (6255T>A), GAA381AAA (6256G>A), GAA381_AAT382insAG- (6258_6259insAG), AAT382AGT (6260A>G), GAA383AAG (6262G>A 6264A>G), GAG387GCA (6275A>C 6276G>A), GAT388CAC (6277G>C 6279T>C), CTC390CTA (6285C>A), TTA391A.. (6286T>A), AAT392T.. (6289A>T)								
**Proteins**									
RNaseH/reverse transcriptase (YP_004581513.1)	306	392	10.9%	177	27.7%	87 (97.8%)	42 (47.2%)	2/0/2/1	0
Protein mutations:	I306V (6031A>G 6033A>G), F310I (6043T>A), K313R (6053A>G), A314S (6055G>T 6057A>T), F316Y (6062T>A 6063T>C), I319L (6070A>T), R320K (6074G>A 6075A>G), M321N (6077T>A 6078G>T), E322K (6079G>A), E323P (6082G>C 6083A>C), S325D (6088T>G 6089C>A 6090C>T), K326V (6091A>G 6092A>T 6093A>G), W328R (6097T>A), W332R (6109T>A 6111G>A), P334R (6116C>G 6117A>G), E335Y (6118G>T 6120A>T), L337H (6125T>A), E341L (6136G>T 6137A>T 6138A>G), P344S (6145C>T 6147C>G), F345I (6148T>A), M348T (6158T>C 6159G>C), D353A (6173A>C 6174T>C), Q355M (6178C>A 6179A>T), R356S (6183A>T), K357L (6184A>T 6185A>T), D359N (6190G>A), N360G (6193A>G 6194A>G 6195T>G), A361V (6197C>T 6198A>G), R363K (6203G>A), G364P (6205G>C 6206G>C), G364_T365insF (6207_6208insTTT), T365L (6208A>T 6209C>T 6210A>G), A367S (6214G>T 6216A>G), I369V (6220A>G 6222C>G), A370I (6223G>A 6224C>T 6225A>T), V371G (6227T>G 6228A>G), Y372F (6230A>T), V378I (6247G>A 6249A>T), F379Y (6251T>A 6252C>T), E381K (6256G>A), E381_N382insX (6258_6259insAG), N382S (6260A>G), E383K (6262G>A 6264A>G), E387A (6275A>C 6276G>A), D388H (6277G>C 6279T>C)								
Codon mutations:	ATA306GTG (6031A>G 6033A>G), TTT310ATT (6043T>A), CTC312CTA (6051C>A), AAA313AGA (6053A>G), GCA314TCT (6055G>T 6057A>T), GGC315GGT (6060C>T), TTT316TAC (6062T>A 6063T>C), ATA319TTA (6070A>T), AGA320AAG (6074G>A 6075A>G), ATG321AAT (6077T>A 6078G>T), GAG322AAG (6079G>A), GAA323CCA (6082G>C 6083A>C), TCC325GAT (6088T>G 6089C>A 6090C>T), AAA326GTG (6091A>G 6092A>T 6093A>G), CCA327CCT (6096A>T), TGG328AGG (6097T>A), TGG332AGA (6109T>A 6111G>A), CCA334CGG (6116C>G 6117A>G), GAA335TAT (6118G>T 6120A>T), CTC337CAC (6125T>A), TAT338TAC (6129T>C), TTT340TTC (6135T>C), GAA341TTG (6136G>T 6137A>T 6138A>G), GTC342GTG (6141C>G), CCC344TCG (6145C>T 6147C>G), TTT345ATT (6148T>A), GGA346GGG (6153A>G), CTG347TTG (6154C>T), ATG348ACC (6158T>C 6159G>C), GCA350GCG (6165A>G), GAT353GCC (6173A>C 6174T>C), TTT354TTC (6177T>C), CAG355ATG (6178C>A 6179A>T), AGA356AGT (6183A>T), AAG357TTG (6184A>T 6185A>T), GAT359AAT (6190G>A), AAT360GGG (6193A>G 6194A>G 6195T>G), GCA361GTG (6197C>T 6198A>G), AGG363AAG (6203G>A), GGA364CCA (6205G>C 6206G>C), GGA364_ACA365insTTT (6207_6208insTTT), ACA365TTG (6208A>T 6209C>T 6210A>G), GCA367TCG (6214G>T 6216A>G), ATC369GTG (6220A>G 6222C>G), GCA370ATT (6223G>A 6224C>T 6225A>T), GTA371GGG (6227T>G 6228A>G), TAT372TTT (6230A>T), ATA376ATT (6243A>T), TTG377CTT (6244T>C 6246G>T), GTA378ATT (6247G>A 6249A>T), TTC379TAT (6251T>A 6252C>T), TCT380TCA (6255T>A), GAA381AAA (6256G>A), GAA381_AAT382insAG- (6258_6259insAG), AAT382AGT (6260A>G), GAA383AAG (6262G>A 6264A>G), GAG387GCA (6275A>C 6276G>A), GAT388CAC (6277G>C 6279T>C), CTC390CTA (6285C>A), TTA391A.. (6286T>A), AAT392T.. (6289A>T)								

*: Inserts / Deletes / Misaligned / Frameshifts

Analysis details

This analysis was performed with panviral2.64

NGS Details (UN9): Badnavirus maculaucubae

Assembly

**Coverage Length**	350 (1 contig(s))
**Depth Of Coverage**	5.3
**Number Of Reads**	15
**Reads Per Million**	0.34 rpm (after QC)
**Ambiguities**	0
**Assembly Method**	de novo + reference guided assembly
**Consensus Caller**	Bcf Tools

Coverage Map

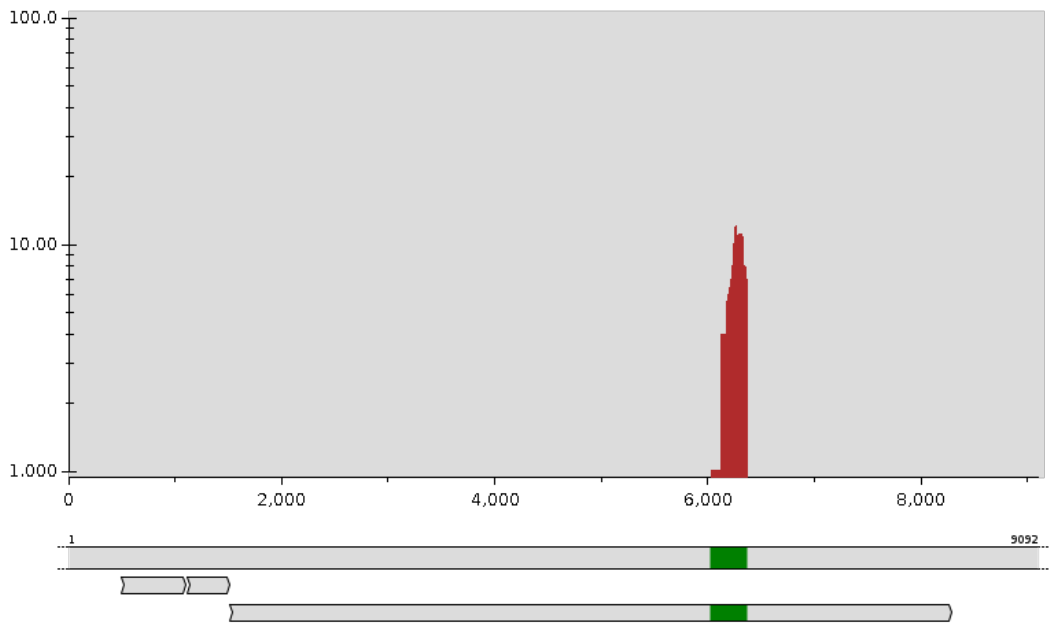

Assignment

**Type**	Badnavirus maculaucubae (Taxonomy ID: 3051986)
**Reference Genome**	NC_076606.1
**NT Identity (%)**	52.6912
**AA Identity (%)**	43.2203
**Number Of Stop Codons**	1
**Number Of CDS**	3

Alignment

**Alignment Score**	32.0 (NT) + 342.0 (AA) = 374.0
**Concordance (%)**	24.3648

Alignment Method	Global, seeded, nucleotide + amino acids (AGA)

Genome Region

Sequence starts at position 6024 and ends at position 6373 relative to NC_076606.1 reference sequence.

Alignment Detailed Statistics

	Begin	End	Coverage	Score	Concordance	Matches	Identities	I/D/M/F*	Stop Codons
**NT**	**6024**	**6373**	**3.8%**	**32**	**4.6%**	**350 (99.2%)**	**186 (52.7%)**	**3/0**	
Mutations:	6024T>G, 6025A>G, 6026A>T, 6027T>C, 6030T>G, 6031C>G, 6032A>T, 6033T>G, 6036A>G, 6037G>A, 6040C>A, 6041A>G, 6046A>T, 6047G>T, 6048C>G, 6049C>T, 6051T>G, 6055G>A, 6056G>A, 6060C>T, 6061A>G, 6063C>T, 6064T>G, 6065C>A, 6067A>C, 6069T>A, 6070A>T, 6072A>T, 6073A>G, 6075A>T, 6076G>C, 6077C>A, 6078C>A, 6079A>C, 6082G>T, 6083G>A, 6085A>G, 6087T>A, 6092A>G, 6093A>T, 6094G>T, 6095T>A, 6096C>T, 6100A>T, 6101G>C, 6102C>A, 6105A>G, 6106T>A, 6108T>C, 6112T>C, 6114G>T, 6115A>C, 6117A>T, 6123T>A, 6125T>A, 6132G>A, 6133G>C, 6135G>T, 6136G>A, 6137C>A, 6138A>G, 6139A>G, 6142G>A, 6144A>G, 6147A>G, 6148T>A, 6149C>G, 6150A>C, 6151A>G, 6152G>A, 6153C>T, 6154A>G, 6158C>T, 6159A>G, 6160T>A, 6161G>A, 6164C>T, 6167C>A, 6171C>T, 6172A>C, 6173T>G, 6174A>G, 6176C>T, 6177T>G, 6178C>A, 6179C>G, 6180A>G, 6181G>T, 6186A>T, 6188T>A, 6189A>T, 6191A>T, 6192C>T, 6197G>T, 6198G>C, 6201A>T, 6204A>C, 6208C>T, 6210A>T, 6216T>G, 6217C>T, 6219A>G, 6221A>C, 6222G>T, 6228A>C, 6231T>G, 6234A>T, 6239T>G, 6240C>T, 6241C>A, 6242A>T, 6243A>G, 6244A>G, 6245G>A, 6246G>T, 6247A>T, 6248A>T, 6249A>G, 6252G>A, 6253G>A, 6257A>G, 6258C>G, 6259T>G, 6260G>T, 6264C>T, 6266G>A, 6268G>C, 6269G>C, 6271A>T, 6272C>T, 6273A>C, 6274G>C, 6275A>T, 6276A>T, 6279_6280insATG, 6285C>A, 6287C>T, 6288T>G, 6291T>G, 6293A>T, 6294C>T, 6304A>G, 6306T>G, 6310G>A, 6314T>A, 6318T>C, 6319G>C, 6320A>G, 6322A>G, 6325C>A, 6326T>G, 6327T>G, 6328C>G, 6336C>T, 6345T>C, 6346C>T, 6348C>A, 6350A>G, 6355A>G, 6361G>C, 6362A>G, 6363A>G, 6365T>C, 6368G>T, 6369C>G								

CDS

	Begin	End	Coverage	Score	Concordance	Matches	Identities	I/D/M/F*	Stop Codons
QKP79_gp3	1506	1622	5.2%	342	40.3%	117 (99.2%)	51 (43.2%)	1/0/0/0	1
Protein mutations:	N1506V (6025A>G 6026A>T 6027T>C), H1508V (6031C>G 6032A>T 6033T>G), D1510N (6037G>A), Q1511R (6040C>A 6041A>G), S1513L (6046A>T 6047G>T 6048C>G), G1516K (6055G>A 6056G>A), N1518D (6061A>G 6063C>T), S1519D (6064T>G 6065C>A), I1520L (6067A>C 6069T>A), I1521F (6070A>T 6072A>T), K1522D (6073A>G 6075A>T), A1523Q (6076G>C 6077C>A 6078C>A), I1524L (6079A>C), G1525* (6082G>T 6083G>A), N1526E (6085A>G 6087T>A), K1528S (6092A>G 6093A>T), V1529Y (6094G>T 6095T>A 6096C>T), F1533I (6106T>A 6108T>C), K1536H (6115A>C 6117A>T), F1539Y (6125T>A), V1542L (6133G>C 6135G>T), A1543K (6136G>A 6137C>A 6138A>G), M1544V (6139A>G), E1545K (6142G>A 6144A>G), S1548D (6151A>G 6152G>A 6153C>T), I1549V (6154A>G), P1550L (6158C>T 6159A>G), W1551K (6160T>A 6161G>A), T1552M (6164C>T), A1553D (6167C>A), I1555R (6172A>C 6173T>G 6174A>G), T1556M (6176C>T 6177T>G), P1557R (6178C>A 6179C>G 6180A>G), D1558Y (6181G>T), L1560H (6188T>A 6189A>T), Y1561F (6191A>T 6192C>T), W1563F (6197G>T 6198G>C), P1567S (6208C>T 6210A>T), K1571T (6221A>C 6222G>T), F1577C (6239T>G 6240C>T), Q1578M (6241C>A 6242A>T 6243A>G), R1579D (6244A>G 6245G>A 6246G>T), K1580L (6247A>T 6248A>T 6249A>G), M1581I (6252G>A), D1582N (6253G>A), N1583R (6257A>G 6258C>G), C1584V (6259T>G 6260G>T), R1586K (6266G>A), G1587P (6268G>C 6269G>C), T1588F (6271A>T 6272C>T 6273A>C), E1589L (6274G>C 6275A>T 6276A>T), D1590_F1591insM (6279_6280insATG), A1593V (6287C>T 6288T>G), Y1595F (6293A>T 6294C>T), I1599V (6304A>G 6306T>G), V1601I (6310G>A), F1602Y (6314T>A), E1604R (6319G>C 6320A>G), N1605D (6322A>G), L1606R (6325C>A 6326T>G 6327T>G), Q1607E (6328C>G), K1614R (6350A>G), M1616V (6355A>G), E1618R (6361G>C 6362A>G 6363A>G), I1619T (6365T>C), C1620L (6368G>T 6369C>G)								
Codon mutations:	GAT1505..G (6024T>G), AAT1506GTC (6025A>G 6026A>T 6027T>C), ACT1507ACG (6030T>G), CAT1508GTG (6031C>G 6032A>T 6033T>G), AAA1509AAG (6036A>G), GAT1510AAT (6037G>A), CAA1511AGA (6040C>A 6041A>G), AGC1513TTG (6046A>T 6047G>T 6048C>G), CTT1514TTG (6049C>T 6051T>G), GGG1516AAG (6055G>A 6056G>A), ATC1517ATT (6060C>T), AAC1518GAT (6061A>G 6063C>T), TCC1519GAC (6064T>G 6065C>A), ATT1520CTA (6067A>C 6069T>A), ATA1521TTT (6070A>T 6072A>T), AAA1522GAT (6073A>G 6075A>T), GCC1523CAA (6076G>C 6077C>A 6078C>A), ATT1524CTT (6079A>C), GGA1525TAA (6082G>T 6083G>A), AAT1526GAA (6085A>G 6087T>A), AAA1528AGT (6092A>G 6093A>T), GTC1529TAT (6094G>T 6095T>A 6096C>T), AGC1531TCA (6100A>T 6101G>C 6102C>A), AAA1532AAG (6105A>G), TTT1533ATC (6106T>A 6108T>C), TTG1535CTT (6112T>C 6114G>T), AAA1536CAT (6115A>C 6117A>T), GGT1538GGA (6123T>A), TTT1539TAT (6125T>A), CAG1541CAA (6132G>A), GTG1542CTT (6133G>C 6135G>T), GCA1543AAG (6136G>A 6137C>A 6138A>G), ATG1544GTG (6139A>G), GAA1545AAG (6142G>A 6144A>G), GAA1546GAG (6147A>G), TCA1547AGC (6148T>A 6149C>G 6150A>C), AGC1548GAT (6151A>G 6152G>A 6153C>T), ATT1549GTT (6154A>G), CCA1550CTG (6158C>T 6159A>G), TGG1551AAG (6160T>A 6161G>A), ACG1552ATG (6164C>T), GCT1553GAT (6167C>A), TTC1554TTT (6171C>T), ATA1555CGG (6172A>C 6173T>G 6174A>G), ACT1556ATG (6176C>T 6177T>G), CCA1557AGG (6178C>A 6179C>G 6180A>G), GAT1558TAT (6181G>T), GGA1559GGT (6186A>T), CTA1560CAT (6188T>A 6189A>T), TAC1561TTT (6191A>T 6192C>T), TGG1563TTC (6197G>T 6198G>C), CTA1564CTT (6201A>T), GTA1565GTC (6204A>C), CCA1567TCT (6208C>T 6210A>T), GGT1569GGG (6216T>G), CTA1570TTG (6217C>T 6219A>G), AAG1571ACT (6221A>C 6222G>T), GCA1573GCC (6228A>C), CCT1574CCG (6231T>G), GCA1575GCT (6234A>T), TTC1577TGT (6239T>G 6240C>T), CAA1578ATG (6241C>A 6242A>T 6243A>G), AGG1579GAT (6244A>G 6245G>A 6246G>T), AAA1580TTG (6247A>T 6248A>T 6249A>G), ATG1581ATA (6252G>A), GAC1582AAC (6253G>A), AAC1583AGG (6257A>G 6258C>G), TGT1584GTT (6259T>G 6260G>T), TTC1585TTT (6264C>T), AGG1586AAG (6266G>A), GGT1587CCT (6268G>C 6269G>C), ACA1588TTC (6271A>T 6272C>T 6273A>C), GAA1589CTT (6274G>C 6275A>T 6276A>T), GAT1590_TTC1591insATG (6279_6280insATG), ATC1592ATA (6285C>A), GCT1593GTG (6287C>T 6288T>G), GTT1594GTG (6291T>G), TAC1595TTT (6293A>T 6294C>T), ATT1599GTG (6304A>G 6306T>G), GTA1601ATA (6310G>A), TTC1602TAC (6314T>A), TCT1603TCC (6318T>C), GAA1604CGA (6319G>C 6320A>G), AAT1605GAT (6322A>G), CTT1606AGG (6325C>A 6326T>G 6327T>G), CAA1607GAA (6328C>G), CAC1609CAT (6336C>T), CAT1612CAC (6345T>C), CTC1613TTA (6346C>T 6348C>A), AAA1614AGA (6350A>G), ATG1616GTG (6355A>G), GAA1618CGG (6361G>C 6362A>G 6363A>G), ATA1619ACA (6365T>C), TGC1620TTG (6368G>T 6369C>G)								

Proteins

	Begin	End	Coverage	Score	Concordance	Matches	Identities	I/D/M/F*	Stop Codons
hypothetical protein (YP_010799265.1)	1506	1622	5.2%	342	40.3%	117 (99.2%)	51 (43.2%)	1/0/0/0	1
Protein mutations:	N1506V (6025A>G 6026A>T 6027T>C), H1508V (6031C>G 6032A>T 6033T>G), D1510N (6037G>A), Q1511R (6040C>A 6041A>G), S1513L (6046A>T 6047G>T 6048C>G), G1516K (6055G>A 6056G>A), N1518D (6061A>G 6063C>T), S1519D (6064T>G 6065C>A), I1520L (6067A>C 6069T>A), I1521F (6070A>T 6072A>T), K1522D (6073A>G 6075A>T), A1523Q (6076G>C 6077C>A 6078C>A), I1524L (6079A>C), G1525* (6082G>T 6083G>A), N1526E (6085A>G 6087T>A), K1528S (6092A>G 6093A>T), V1529Y (6094G>T 6095T>A 6096C>T), F1533I (6106T>A 6108T>C), K1536H (6115A>C 6117A>T), F1539Y (6125T>A), V1542L (6133G>C 6135G>T), A1543K (6136G>A 6137C>A 6138A>G), M1544V (6139A>G), E1545K (6142G>A 6144A>G), S1548D (6151A>G 6152G>A 6153C>T), I1549V (6154A>G), P1550L (6158C>T 6159A>G), W1551K (6160T>A 6161G>A), T1552M (6164C>T), A1553D (6167C>A), I1555R (6172A>C 6173T>G 6174A>G), T1556M (6176C>T 6177T>G), P1557R (6178C>A 6179C>G 6180A>G), D1558Y (6181G>T), L1560H (6188T>A 6189A>T), Y1561F (6191A>T 6192C>T), W1563F (6197G>T 6198G>C), P1567S (6208C>T 6210A>T), K1571T (6221A>C 6222G>T), F1577C (6239T>G 6240C>T), Q1578M (6241C>A 6242A>T 6243A>G), R1579D (6244A>G 6245G>A 6246G>T), K1580L (6247A>T 6248A>T 6249A>G), M1581I (6252G>A), D1582N (6253G>A), N1583R (6257A>G 6258C>G), C1584V (6259T>G 6260G>T), R1586K (6266G>A), G1587P (6268G>C 6269G>C), T1588F (6271A>T 6272C>T 6273A>C), E1589L (6274G>C 6275A>T 6276A>T), D1590_F1591insM (6279_6280insATG), A1593V (6287C>T 6288T>G), Y1595F (6293A>T 6294C>T), I1599V (6304A>G 6306T>G), V1601I (6310G>A), F1602Y (6314T>A), E1604R (6319G>C 6320A>G), N1605D (6322A>G), L1606R (6325C>A 6326T>G 6327T>G), Q1607E (6328C>G), K1614R (6350A>G), M1616V (6355A>G), E1618R (6361G>C 6362A>G 6363A>G), I1619T (6365T>C), C1620L (6368G>T 6369C>G)								
Codon mutations:	GAT1505..G (6024T>G), AAT1506GTC (6025A>G 6026A>T 6027T>C), ACT1507ACG (6030T>G), CAT1508GTG (6031C>G 6032A>T 6033T>G), AAA1509AAG (6036A>G), GAT1510AAT (6037G>A), CAA1511AGA (6040C>A 6041A>G), AGC1513TTG (6046A>T 6047G>T 6048C>G), CTT1514TTG (6049C>T 6051T>G), GGG1516AAG (6055G>A 6056G>A), ATC1517ATT (6060C>T), AAC1518GAT (6061A>G 6063C>T), TCC1519GAC (6064T>G 6065C>A), ATT1520CTA (6067A>C 6069T>A), ATA1521TTT (6070A>T 6072A>T), AAA1522GAT (6073A>G 6075A>T), GCC1523CAA (6076G>C 6077C>A 6078C>A), ATT1524CTT (6079A>C), GGA1525TAA (6082G>T 6083G>A), AAT1526GAA (6085A>G 6087T>A), AAA1528AGT (6092A>G 6093A>T), GTC1529TAT (6094G>T 6095T>A 6096C>T), AGC1531TCA (6100A>T 6101G>C 6102C>A), AAA1532AAG (6105A>G), TTT1533ATC (6106T>A 6108T>C), TTG1535CTT (6112T>C 6114G>T), AAA1536CAT (6115A>C 6117A>T), GGT1538GGA (6123T>A), TTT1539TAT (6125T>A), CAG1541CAA (6132G>A), GTG1542CTT (6133G>C 6135G>T), GCA1543AAG (6136G>A 6137C>A 6138A>G), ATG1544GTG (6139A>G), GAA1545AAG (6142G>A 6144A>G), GAA1546GAG (6147A>G), TCA1547AGC (6148T>A 6149C>G 6150A>C), AGC1548GAT (6151A>G 6152G>A 6153C>T), ATT1549GTT (6154A>G), CCA1550CTG (6158C>T 6159A>G), TGG1551AAG (6160T>A 6161G>A), ACG1552ATG (6164C>T), GCT1553GAT (6167C>A), TTC1554TTT (6171C>T), ATA1555CGG (6172A>C 6173T>G 6174A>G), ACT1556ATG (6176C>T 6177T>G), CCA1557AGG (6178C>A 6179C>G 6180A>G), GAT1558TAT (6181G>T), GGA1559GGT (6186A>T), CTA1560CAT (6188T>A 6189A>T), TAC1561TTT (6191A>T 6192C>T), TGG1563TTC (6197G>T 6198G>C), CTA1564CTT (6201A>T), GTA1565GTC (6204A>C), CCA1567TCT (6208C>T 6210A>T), GGT1569GGG (6216T>G), CTA1570TTG (6217C>T 6219A>G), AAG1571ACT (6221A>C 6222G>T), GCA1573GCC (6228A>C), CCT1574CCG (6231T>G), GCA1575GCT (6234A>T), TTC1577TGT (6239T>G 6240C>T), CAA1578ATG (6241C>A 6242A>T 6243A>G), AGG1579GAT (6244A>G 6245G>A 6246G>T), AAA1580TTG (6247A>T 6248A>T 6249A>G), ATG1581ATA (6252G>A), GAC1582AAC (6253G>A), AAC1583AGG (6257A>G 6258C>G), TGT1584GTT (6259T>G 6260G>T), TTC1585TTT (6264C>T), AGG1586AAG (6266G>A), GGT1587CCT (6268G>C 6269G>C), ACA1588TTC (6271A>T 6272C>T 6273A>C), GAA1589CTT (6274G>C 6275A>T 6276A>T), GAT1590_TTC1591insATG (6279_6280insATG), ATC1592ATA (6285C>A), GCT1593GTG (6287C>T 6288T>G), GTT1594GTG (6291T>G), TAC1595TTT (6293A>T 6294C>T), ATT1599GTG (6304A>G 6306T>G), GTA1601ATA (6310G>A), TTC1602TAC (6314T>A), TCT1603TCC (6318T>C), GAA1604CGA (6319G>C 6320A>G), AAT1605GAT (6322A>G), CTT1606AGG (6325C>A 6326T>G 6327T>G), CAA1607GAA (6328C>G), CAC1609CAT (6336C>T), CAT1612CAC (6345T>C), CTC1613TTA (6346C>T 6348C>A), AAA1614AGA (6350A>G), ATG1616GTG (6355A>G), GAA1618CGG (6361G>C 6362A>G 6363A>G), ATA1619ACA (6365T>C), TGC1620TTG (6368G>T 6369C>G)								

*: Inserts / Deletes / Misaligned / Frameshifts

Analysis details

This analysis was performed with panviral2.64

NGS Details (UN9): Bracoviriform congregatae (segment Circle 7)

Assembly

**Coverage Length**	139 (1 contig(s))
**Depth Of Coverage**	9.0
**Number Of Reads**	14
**Reads Per Million**	0.32 rpm (after QC)
**Ambiguities**	0
**Assembly Method**	de novo + reference guided assembly
**Consensus Caller**	Bcf Tools

Coverage Map

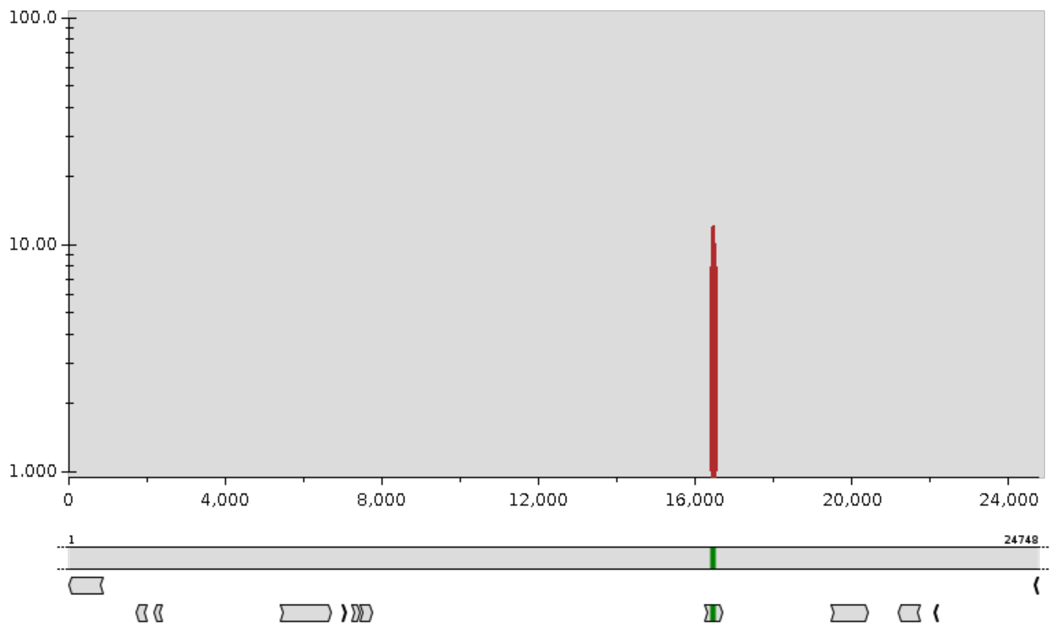

Assignment

**Type**	Bracoviriform congregatae (Taxonomy ID: 39640)
**Reference Genome**	NC_006639.1
**NT Identity (%)**	78.4173
**AA Identity (%)**	91.3043
**Number Of Stop Codons**	0
**Number Of CDS**	5

Alignment

**Alignment Score**	158.0 (NT) + 282.0 (AA) = 440.0
**Concordance (%)**	76.7888

Alignment Method	Global, seeded, nucleotide + amino acids (AGA)

Genome Region

Sequence starts at position 16401 and ends at position 16539 relative to NC_006639.1 reference sequence.

Alignment Detailed Statistics

	Begin	End	Coverage	Score	Concordance	Matches	Identities	I/D/M/F*	Stop Codons
**NT**	**16401**	**16539**	**0.6%**	**158**	**56.8%**	**139 (100%)**	**109 (78.4%)**	**0/0**	
Mutations:	16402A>T, 16408A>T, 16411G>A, 16414A>G, 16423A>C, 16426A>G, 16429G>C, 16435T>G, 16439C>A, 16441T>A, 16444T>C, 16449G>A, 16450A>G, 16451T>A, 16452C>T, 16453T>C, 16454C>T, 16456T>G, 16462T>C, 16471A>G, 16474A>T, 16493C>A, 16495T>A, 16501G>A, 16504T>A, 16507T>A, 16508C>A, 16510T>A, 16516A>T, 16526G>A								
**CDS**									
Histone	58	103	29.5%	282	92.5%	46 (100%)	42 (91.3%)	0/0/0/0	0
Protein mutations:	R73K (16449G>A 16450A>G), S74I (16451T>A 16452C>T 16453T>C), V99I (16526G>A)								
Codon mutations:	GTA57.TT (16402A>T), GGA59GGT (16408A>T), GGG60GGA (16411G>A), AAA61AAG (16414A>G), GGA64GGC (16423A>C), AAA65AAG (16426A>G), GGG66GGC (16429G>C), GCT68GCG (16435T>G), CGT70AGA (16439C>A 16441T>A), CAT71CAC (16444T>C), AGA73AAG (16449G>A 16450A>G), TCT74ATC (16451T>A 16452C>T 16453T>C), CTT75TTG (16454C>T 16456T>G), GAT77GAC (16462T>C), CAA80CAG (16471A>G), GGA81GGT (16474A>T), CGT88AGA (16493C>A 16495T>A), CTG90CTA (16501G>A), GCT91GCA (16504T>A), CGT92CGA (16507T>A), CGT93AGA (16508C>A 16510T>A), GGA95GGT (16516A>T), GTC99ATC (16526G>A)								
**Proteins**									
hypothetical protein (YP_184795.1)	58	103	29.5%	282	92.5%	46 (100%)	42 (91.3%)	0/0/0/0	0
Protein mutations:	R73K (16449G>A 16450A>G), S74I (16451T>A 16452C>T 16453T>C), V99I (16526G>A)								
Codon mutations:	GTA57.TT (16402A>T), GGA59GGT (16408A>T), GGG60GGA (16411G>A), AAA61AAG (16414A>G), GGA64GGC (16423A>C), AAA65AAG (16426A>G), GGG66GGC (16429G>C), GCT68GCG (16435T>G), CGT70AGA (16439C>A 16441T>A), CAT71CAC (16444T>C), AGA73AAG (16449G>A 16450A>G), TCT74ATC (16451T>A 16452C>T 16453T>C), CTT75TTG (16454C>T 16456T>G), GAT77GAC (16462T>C), CAA80CAG (16471A>G), GGA81GGT (16474A>T), CGT88AGA (16493C>A 16495T>A), CTG90CTA (16501G>A), GCT91GCA (16504T>A), CGT92CGA (16507T>A), CGT93AGA (16508C>A 16510T>A), GGA95GGT (16516A>T), GTC99ATC (16526G>A)								

*: Inserts / Deletes / Misaligned / Frameshifts

Analysis details

This analysis was performed with panviral2.64

NGS Details (UN9): Badnavirus epsiloninflatheobromae

Assembly

**Coverage Length**	290 (1 contig(s))
**Depth Of Coverage**	5.0
**Number Of Reads**	14
**Reads Per Million**	0.32 rpm (after QC)
**Ambiguities**	0
**Assembly Method**	de novo + reference guided assembly
**Consensus Caller**	Bcf Tools

Coverage Map

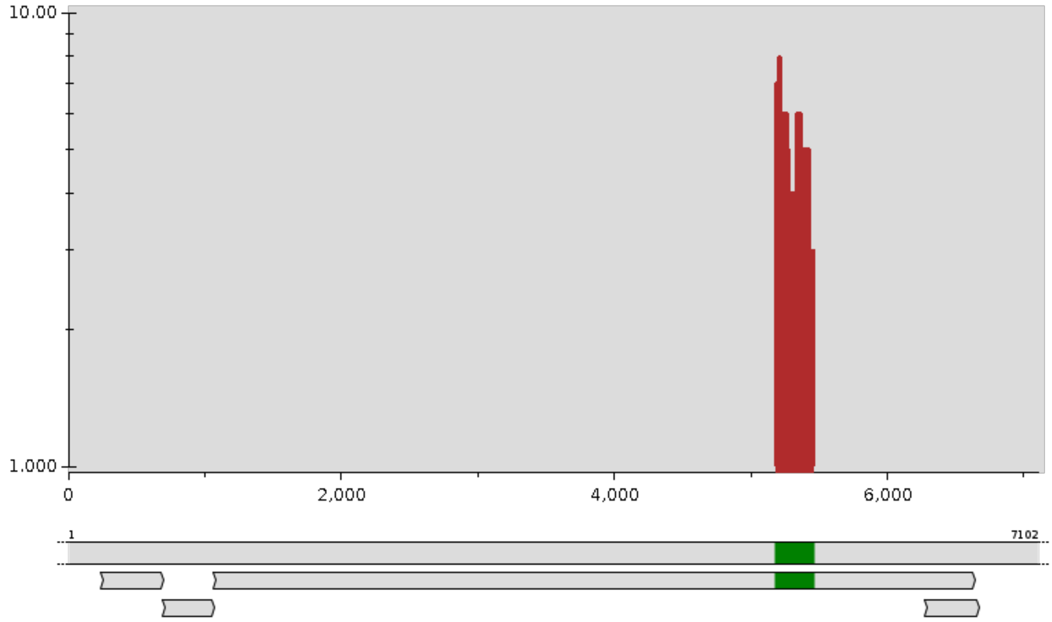

Assignment

**Type**	Badnavirus epsiloninflatheobromae (Taxonomy ID: 3047711)
**Reference Genome**	NC_043535.1
**NT Identity (%)**	59.0444
**AA Identity (%)**	49.4845
**Number Of Stop Codons**	1
**Number Of CDS**	4

Alignment

**Alignment Score**	100.0 (NT) + 347.0 (AA) = 447.0
**Concordance (%)**	34.2005

Alignment Method	Global, seeded, nucleotide + amino acids (AGA)

Genome Region

Sequence starts at position 5176 and ends at position 5465 relative to NC_043535.1 reference sequence.

Alignment Detailed Statistics

	Begin	End	Coverage	Score	Concordance	Matches	Identities	I/D/M/F*	Stop Codons
**NT**	**5176**	**5465**	**4.1%**	**100**	**17.2%**	**290 (99.0%)**	**173 (59.0%)**	**3/0**	
Mutations:	5179A>T, 5182C>A, 5183A>T, 5186G>A, 5187A>C, 5192C>A, 5198A>C, 5201A>G, 5202A>T, 5203T>A, 5209G>C, 5213A>G, 5214T>G, 5216C>G, 5220T>C, 5222G>C, 5225A>G, 5227G>A, 5228T>G, 5231A>C, 5233T>A, 5234T>C, 5235C>T, 5237T>C, 5241A>G, 5243C>G, 5244G>C, 5245C>G, 5246A>C, 5249G>A, 5250C>G, 5251A>C, 5252T>A, 5257A>G, 5259T>G, 5260C>A, 5261A>C, 5262A>G, 5263T>A, 5264C>G, 5267A>G, 5268T>A, 5269G>A, 5273C>G, 5274G>A, 5278T>A, 5280T>G, 5281G>T, 5283G>A, 5285A>G, 5286C>A, 5287C>G, 5288T>A, 5289C>T, 5291A>T, 5292G>A, 5294A>G, 5295C>G, 5296T>C, 5303A>G, 5307C>T, 5312T>G, 5318A>T, 5325C>T, 5327C>A, 5329A>C, 5330A>C, 5336C>A, 5339T>C, 5341C>T, 5344T>C, 5345C>T, 5349C>T, 5350A>G, 5351A>C, 5353G>C, 5355A>C, 5356A>T, 5361G>A, 5363T>C, 5364C>G, 5366T>G, 5367T>A, 5368G>T, 5369T>C, 5371T>A, 5372_5373insCAT, 5373G>C, 5375A>C, 5376G>T, 5377G>A, 5378A>C, 5379A>T, 5380C>T, 5381A>G, 5384A>T, 5385G>T, 5390T>C, 5393T>G, 5395C>T, 5396T>A, 5402C>T, 5403A>T, 5405T>G, 5415A>G, 5420T>C, 5422T>A, 5423C>T, 5429T>C, 5432A>C, 5433G>T, 5434A>C, 5439C>G, 5444T>C, 5446A>T, 5450A>G, 5454C>T, 5456G>A								

CDS

	Begin	End	Coverage	Score	Concordance	Matches	Identities	I/D/M/F*	Stop Codons
FLA70_gp3	1373	1468	5.2%	347	47.3%	96 (99.0%)	48 (49.5%)	1/0/0/0	1
Protein mutations:	H1373L (5179A>T), A1374D (5182C>A 5183A>T), I1376L (5187A>C), I1381Y (5202A>T 5203T>A), S1383T (5209G>C), F1385V (5214T>G 5216C>G), S1389K (5227G>A 5228T>G), F1391Y (5233T>A 5234T>C), H1392Y (5235C>T 5237T>C), I1394V (5241A>G 5243C>G), A1395R (5244G>C 5245C>G 5246A>C), M1396I (5249G>A), H1397A (5250C>G 5251A>C 5252T>A), E1399G (5257A>G), S1400D (5259T>G 5260C>A 5261A>C), I1401E (5262A>G 5263T>A 5264C>G), W1403K (5268T>A 5269G>A), A1405T (5274G>A), F1406Y (5278T>A), W1407V (5280T>G 5281G>T), V1408M (5283G>A 5285A>G), P1409R (5286C>A 5287C>G 5288T>A), Q1410Y (5289C>T 5291A>T), G1411R (5292G>A 5294A>G), L1412A (5295C>G 5296T>C), K1423T (5329A>C 5330A>C), A1427V (5341C>T), I1428T (5344T>C 5345C>T), Q1430C (5349C>T 5350A>G 5351A>C), R1431T (5353G>C), K1432L (5355A>C 5356A>T), D1434N (5361G>A 5363T>C), H1435E (5364C>G 5366T>G), C1436I (5367T>A 5368G>T 5369T>C), F1437Y (5371T>A), F1437_A1438insH (5372_5373insCAT), A1438P (5373G>C 5375A>C), G1439Y (5376G>T 5377G>A 5378A>C), T1440L (5379A>T 5380C>T 5381A>G), E1441D (5384A>T), E1442* (5385G>T), I1444M (5393T>G), A1445V (5395C>T 5396T>A), I1448L (5403A>T 5405T>G), I1452V (5415A>G), F1454Y (5422T>A 5423C>T), E1458S (5433G>T 5434A>C), Q1460E (5439C>G), K1462M (5446A>T)								
Codon mutations:	CAT1373CTT (5179A>T), GCA1374GAT (5182C>A 5183A>T), AGG1375AGA (5186G>A), ATT1376CTT (5187A>C), GGC1377GGA (5192C>A), GCA1379GCC (5198A>C), AAA1380AAG (5201A>G), ATC1381TAC (5202A>T 5203T>A), AGC1383ACC (5209G>C), AAA1384AAG (5213A>G), TTC1385GTG (5214T>G 5216C>G), TTG1387CTC (5220T>C 5222G>C), AAA1388AAG (5225A>G), AGT1389AAG (5227G>A 5228T>G), GGA1390GGC (5231A>C), TTT1391TAC (5233T>A 5234T>C), CAT1392TAC (5235C>T 5237T>C), ATC1394GTG (5241A>G 5243C>G), GCA1395CGC (5244G>C 5245C>G 5246A>C), ATG1396ATA (5249G>A), CAT1397GCA (5250C>G 5251A>C 5252T>A), GAG1399GGG (5257A>G), TCA1400GAC (5259T>G 5260C>A 5261A>C), ATC1401GAG (5262A>G 5263T>A 5264C>G), CCA1402CCG (5267A>G), TGG1403AAG (5268T>A 5269G>A), ACC1404ACG (5273C>G), GCG1405ACG (5274G>A), TTT1406TAT (5278T>A), TGG1407GTG (5280T>G 5281G>T), GTA1408ATG (5283G>A 5285A>G), CCT1409AGA (5286C>A 5287C>G 5288T>A), CAA1410TAT (5289C>T 5291A>T), GGA1411AGG (5292G>A 5294A>G), CTT1412GCT (5295C>G 5296T>C), GAA1414GAG (5303A>G), CTG1416TTG (5307C>T), GTT1417GTG (5312T>G), CCA1419CCT (5318A>T), CTC1422TTA (5325C>T 5327C>A), AAA1423ACC (5329A>C 5330A>C), GCC1425GCA (5336C>A), CCT1426CCC (5339T>C), GCC1427GTC (5341C>T), ATC1428ACT (5344T>C 5345C>T), CAA1430TGC (5349C>T 5350A>G 5351A>C), AGA1431ACA (5353G>C), AAA1432CTA (5355A>C 5356A>T), GAT1434AAC (5361G>A 5363T>C), CAT1435GAG (5364C>G 5366T>G), TGT1436ATC (5367T>A 5368G>T 5369T>C), TTT1437TAT (5371T>A), TTT1437_GCA1438insCAT (5372_5373insCAT), GCA1438CCC (5373G>C 5375A>C), GGA1439TAC (5376G>T 5377G>A 5378A>C), ACA1440TTG (5379A>T 5380C>T 5381A>G), GAA1441GAT (5384A>T), GAG1442TAG (5385G>T), TTT1443TTC (5390T>C), ATT1444ATG (5393T>G), GCT1445GTA (5395C>T 5396T>A), TAC1447TAT (5402C>T), ATT1448TTG (5403A>T 5405T>G), ATC1452GTC (5415A>G), ATT1453ATC (5420T>C), TTC1454TAT (5422T>A 5423C>T), AAT1456AAC (5429T>C), ACA1457ACC (5432A>C), GAG1458TCG (5433G>T 5434A>C), CAG1460GAG (5439C>G), CAT1461CAC (5444T>C), AAG1462ATG (5446A>T), GAA1463GAG (5450A>G), CTG1465TTA (5454C>T 5456G>A)								

Proteins

	Begin	End	Coverage	Score	Concordance	Matches	Identities	I/D/M/F*	Stop Codons
ORF3 polyprotein (YP_009666830.1)	1373	1468	5.2%	347	47.3%	96 (99.0%)	48 (49.5%)	1/0/0/0	1
Protein mutations:	H1373L (5179A>T), A1374D (5182C>A 5183A>T), I1376L (5187A>C), I1381Y (5202A>T 5203T>A), S1383T (5209G>C), F1385V (5214T>G 5216C>G), S1389K (5227G>A 5228T>G), F1391Y (5233T>A 5234T>C), H1392Y (5235C>T 5237T>C), I1394V (5241A>G 5243C>G), A1395R (5244G>C 5245C>G 5246A>C), M1396I (5249G>A), H1397A (5250C>G 5251A>C 5252T>A), E1399G (5257A>G), S1400D (5259T>G 5260C>A 5261A>C), I1401E (5262A>G 5263T>A 5264C>G), W1403K (5268T>A 5269G>A), A1405T (5274G>A), F1406Y (5278T>A), W1407V (5280T>G 5281G>T), V1408M (5283G>A 5285A>G), P1409R (5286C>A 5287C>G 5288T>A), Q1410Y (5289C>T 5291A>T), G1411R (5292G>A 5294A>G), L1412A (5295C>G 5296T>C), K1423T (5329A>C 5330A>C), A1427V (5341C>T), I1428T (5344T>C 5345C>T), Q1430C (5349C>T 5350A>G 5351A>C), R1431T (5353G>C), K1432L (5355A>C 5356A>T), D1434N (5361G>A 5363T>C), H1435E (5364C>G 5366T>G), C1436I (5367T>A 5368G>T 5369T>C), F1437Y (5371T>A), F1437_A1438insH (5372_5373insCAT), A1438P (5373G>C 5375A>C), G1439Y (5376G>T 5377G>A 5378A>C), T1440L (5379A>T 5380C>T 5381A>G), E1441D (5384A>T), E1442* (5385G>T), I1444M (5393T>G), A1445V (5395C>T 5396T>A), I1448L (5403A>T 5405T>G), I1452V (5415A>G), F1454Y (5422T>A 5423C>T), E1458S (5433G>T 5434A>C), Q1460E (5439C>G), K1462M (5446A>T)								
Codon mutations:	CAT1373CTT (5179A>T), GCA1374GAT (5182C>A 5183A>T), AGG1375AGA (5186G>A), ATT1376CTT (5187A>C), GGC1377GGA (5192C>A), GCA1379GCC (5198A>C), AAA1380AAG (5201A>G), ATC1381TAC (5202A>T 5203T>A), AGC1383ACC (5209G>C), AAA1384AAG (5213A>G), TTC1385GTG (5214T>G 5216C>G), TTG1387CTC (5220T>C 5222G>C), AAA1388AAG (5225A>G), AGT1389AAG (5227G>A 5228T>G), GGA1390GGC (5231A>C), TTT1391TAC (5233T>A 5234T>C), CAT1392TAC (5235C>T 5237T>C), ATC1394GTG (5241A>G 5243C>G), GCA1395CGC (5244G>C 5245C>G 5246A>C), ATG1396ATA (5249G>A), CAT1397GCA (5250C>G 5251A>C 5252T>A), GAG1399GGG (5257A>G), TCA1400GAC (5259T>G 5260C>A 5261A>C), ATC1401GAG (5262A>G 5263T>A 5264C>G), CCA1402CCG (5267A>G), TGG1403AAG (5268T>A 5269G>A), ACC1404ACG (5273C>G), GCG1405ACG (5274G>A), TTT1406TAT (5278T>A), TGG1407GTG (5280T>G 5281G>T), GTA1408ATG (5283G>A 5285A>G), CCT1409AGA (5286C>A 5287C>G 5288T>A), CAA1410TAT (5289C>T 5291A>T), GGA1411AGG (5292G>A 5294A>G), CTT1412GCT (5295C>G 5296T>C), GAA1414GAG (5303A>G), CTG1416TTG (5307C>T), GTT1417GTG (5312T>G), CCA1419CCT (5318A>T), CTC1422TTA (5325C>T 5327C>A), AAA1423ACC (5329A>C 5330A>C), GCC1425GCA (5336C>A), CCT1426CCC (5339T>C), GCC1427GTC (5341C>T), ATC1428ACT (5344T>C 5345C>T), CAA1430TGC (5349C>T 5350A>G 5351A>C), AGA1431ACA (5353G>C), AAA1432CTA (5355A>C 5356A>T), GAT1434AAC (5361G>A 5363T>C), CAT1435GAG (5364C>G 5366T>G), TGT1436ATC (5367T>A 5368G>T 5369T>C), TTT1437TAT (5371T>A), TTT1437_GCA1438insCAT (5372_5373insCAT), GCA1438CCC (5373G>C 5375A>C), GGA1439TAC (5376G>T 5377G>A 5378A>C), ACA1440TTG (5379A>T 5380C>T 5381A>G), GAA1441GAT (5384A>T), GAG1442TAG (5385G>T), TTT1443TTC (5390T>C), ATT1444ATG (5393T>G), GCT1445GTA (5395C>T 5396T>A), TAC1447TAT (5402C>T), ATT1448TTG (5403A>T 5405T>G), ATC1452GTC (5415A>G), ATT1453ATC (5420T>C), TTC1454TAT (5422T>A 5423C>T), AAT1456AAC (5429T>C), ACA1457ACC (5432A>C), GAG1458TCG (5433G>T 5434A>C), CAG1460GAG (5439C>G), CAT1461CAC (5444T>C), AAG1462ATG (5446A>T), GAA1463GAG (5450A>G), CTG1465TTA (5454C>T 5456G>A)								

*: Inserts / Deletes / Misaligned / Frameshifts

Analysis details

This analysis was performed with panviral2.64

NGS Details (UN9): Badnavirus maculasmallanthi

Assembly

**Coverage Length**	323 (1 contig(s))
**Depth Of Coverage**	4.2
**Number Of Reads**	14
**Reads Per Million**	0.32 rpm (after QC)
**Ambiguities**	0
**Assembly Method**	de novo + reference guided assembly
**Consensus Caller**	Bcf Tools

Coverage Map

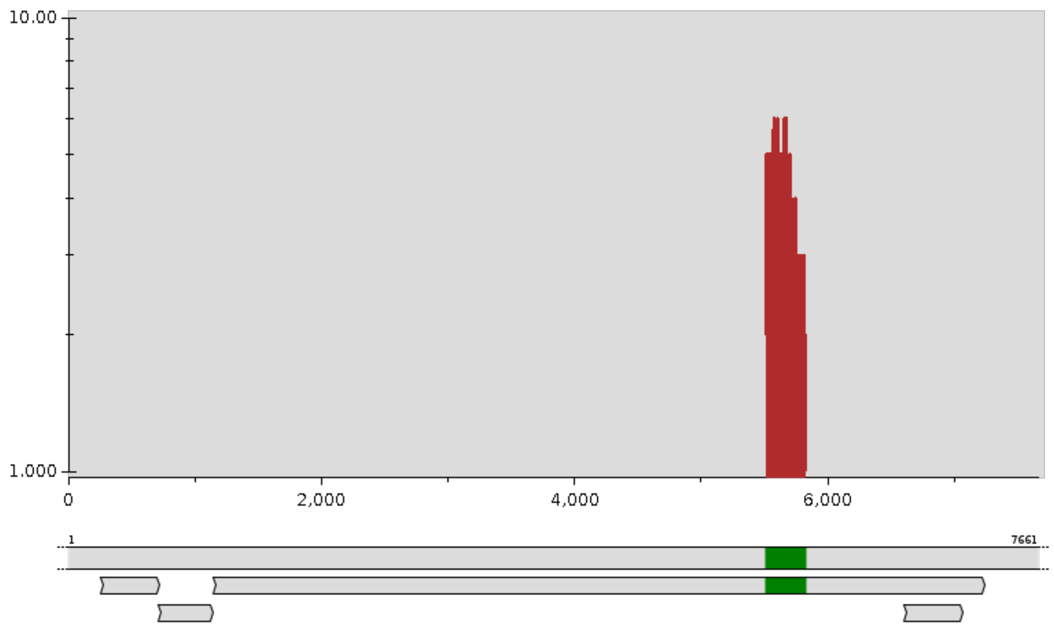

Assignment

**Type**	Badnavirus maculasmallanthi (Taxonomy ID: 3048453)
**Reference Genome**	NC_026472.1
**NT Identity (%)**	57.9755
**AA Identity (%)**	45.8716
**Number Of Stop Codons**	0
**Number Of CDS**	4

Alignment

**Alignment Score**	98.0 (NT) + 335.0 (AA) = 433.0
**Concordance (%)**	30.8845

Alignment Method	Global, seeded, nucleotide + amino acids (AGA)

Genome Region

Sequence starts at position 5509 and ends at position 5831 relative to NC_026472.1 reference sequence.

Alignment Detailed Statistics

	Begin	End	Coverage	Score	Concordance	Matches	Identities	I/D/M/F*	Stop Codons
**NT**	**5509**	**5831**	**4.2%**	**98**	**15.2%**	**323 (99.1%)**	**189 (58.0%)**	**3/0**	
Mutations:	5509G>A, 5512C>A, 5518T>C, 5521C>A, 5526C>G, 5527G>A, 5532A>T, 5533A>G, 5535C>T, 5536A>G, 5537C>A, 5538C>T, 5542C>T, 5544A>C, 5545A>G, 5547A>T, 5549G>A, 5551A>C, 5553A>T, 5554G>A, 5555G>A, 5556G>A, 5557A>G, 5558A>G, 5564A>G, 5565G>T, 5566A>T, 5567T>A, 5568A>C, 5574A>T, 5578T>A, 5586G>T, 5587A>C, 5588A>G, 5589G>T, 5592T>C, 5597T>A, 5600A>T, 5601T>C, 5605G>T, 5607T>G, 5608G>A, 5609C>G, 5610C>G, 5611A>G, 5614G>A, 5615A>G, 5616A>G, 5619A>G, 5620G>T, 5621A>G, 5622A>T, 5623T>G, 5624C>A, 5628A>T, 5631C>A, 5632T>A, 5633G>A, 5638G>A, 5640C>T, 5644T>A, 5645T>G, 5648T>C, 5651C>G, 5653G>T, 5654G>A, 5655A>T, 5658A>T, 5659T>C, 5660T>A, 5661A>T, 5663A>T, 5664C>T, 5669G>T, 5670G>T, 5671C>T, 5673T>G, 5676C>T, 5680C>T, 5682A>T, 5685C>T, 5686G>A, 5691C>A, 5692A>G, 5693A>C, 5700A>T, 5706T>A, 5708T>C, 5713C>A, 5714A>T, 5716A>G, 5717G>A, 5718A>C, 5719A>T, 5720A>T, 5721A>G, 5725G>A, 5727T>C, 5730A>G, 5731T>G, 5732G>T, 5733C>T, 5739_5740insCCG, 5740G>T, 5743A>T, 5744C>T, 5748A>T, 5749G>A, 5750A>T, 5755A>G, 5757C>G, 5759C>T, 5760C>G, 5763A>C, 5765A>T, 5769C>A, 5772C>T, 5778C>A, 5779C>T, 5784A>T, 5787C>T, 5791A>C, 5793C>A, 5801C>A, 5802G>A, 5805T>G, 5809G>A, 5810A>T, 5811A>G, 5812A>G, 5814G>T, 5820A>G, 5822A>G, 5827A>G, 5830C>T								

CDS

	Begin	End	Coverage	Score	Concordance	Matches	Identities	I/D/M/F*	Stop Codons
UF61_gp3	1456	1563	5.3%	335	44.1%	108 (99.1%)	50 (45.9%)	1/0/0/0	0
Protein mutations:	D1456N (5509G>A), Q1457K (5512C>A), S1459P (5518T>C), L1460I (5521C>A), G1462R (5527G>A), N1464D (5533A>G 5535C>T), T1465D (5536A>G 5537C>A 5538C>T), L1467F (5542C>T 5544A>C), K1468D (5545A>G 5547A>T), R1469K (5549G>A), I1470L (5551A>C 5553A>T), G1471K (5554G>A 5555G>A 5556G>A), N1472G (5557A>G 5558A>G), K1474S (5564A>G 5565G>T), I1475Y (5566A>T 5567T>A 5568A>C), F1479I (5578T>A), K1482R (5587A>C 5588A>G 5589G>T), F1485Y (5597T>A), H1486L (5600A>T 5601T>C), V1488L (5605G>T 5607T>G), A1489R (5608G>A 5609C>G 5610C>G), M1490V (5611A>G), E1491R (5614G>A 5615A>G 5616A>G), E1493C (5620G>T 5621A>G 5622A>T), S1494D (5623T>G 5624C>A), W1497K (5632T>A 5633G>A), A1499T (5638G>A 5640C>T), L1501R (5644T>A 5645T>G), I1502T (5648T>C), P1503R (5651C>G), G1504Y (5653G>T 5654G>A 5655A>T), L1506H (5659T>C 5660T>A 5661A>T), Y1507F (5663A>T 5664C>T), W1509F (5669G>T 5670G>T), P1513S (5680C>T 5682A>T), G1515R (5686G>A), K1517A (5692A>G 5693A>C), I1522T (5708T>C), Q1524M (5713C>A 5714A>T), R1525D (5716A>G 5717G>A 5718A>C), K1526L (5719A>T 5720A>T 5721A>G), D1528N (5725G>A 5727T>C), C1530V (5731T>G 5732G>T 5733C>T), K1532_D1533insP (5739_5740insCCG), D1533Y (5740G>T), T1534L (5743A>T 5744C>T), E1535D (5748A>T), E1536M (5749G>A 5750A>T), I1538V (5755A>G 5757C>G), A1539V (5759C>T 5760C>G), Y1541F (5765A>T), N1550Q (5791A>C 5793C>A), A1553E (5801C>A 5802G>A), D1554E (5805T>G), E1556M (5809G>A 5810A>T 5811A>G), R1557G (5812A>G 5814G>T), K1560R (5822A>G), M1562V (5827A>G)								
Codon mutations:	GAT1456AAT (5509G>A), CAG1457AAG (5512C>A), TCC1459CCC (5518T>C), CTT1460ATT (5521C>A), CCC1461CCG (5526C>G), GGG1462AGG (5527G>A), ATA1463ATT (5532A>T), AAC1464GAT (5533A>G 5535C>T), ACC1465GAT (5536A>G 5537C>A 5538C>T), CTA1467TTC (5542C>T 5544A>C), AAA1468GAT (5545A>G 5547A>T), AGA1469AAA (5549G>A), ATA1470CTT (5551A>C 5553A>T), GGG1471AAA (5554G>A 5555G>A 5556G>A), AAT1472GGT (5557A>G 5558A>G), AAG1474AGT (5564A>G 5565G>T), ATA1475TAC (5566A>T 5567T>A 5568A>C), TCA1477TCT (5574A>T), TTT1479ATT (5578T>A), CTG1481CTT (5586G>T), AAG1482CGT (5587A>C 5588A>G 5589G>T), TCT1483TCC (5592T>C), TTT1485TAT (5597T>A), CAT1486CTC (5600A>T 5601T>C), GTT1488TTG (5605G>T 5607T>G), GCC1489AGG (5608G>A 5609C>G 5610C>G), ATG1490GTG (5611A>G), GAA1491AGG (5614G>A 5615A>G 5616A>G), GAA1492GAG (5619A>G), GAA1493TGT (5620G>T 5621A>G 5622A>T), TCC1494GAC (5623T>G 5624C>A), ATA1495ATT (5628A>T), CCC1496CCA (5631C>A), TGG1497AAG (5632T>A 5633G>A), GCC1499ACT (5638G>A 5640C>T), TTA1501AGA (5644T>A 5645T>G), ATC1502ACC (5648T>C), CCA1503CGA (5651C>G), GGA1504TAT (5653G>T 5654G>A 5655A>T), GGA1505GGT (5658A>T), TTA1506CAT (5659T>C 5660T>A 5661A>T), TAC1507TTT (5663A>T 5664C>T), TGG1509TTT (5669G>T 5670G>T), CTT1510TTG (5671C>T 5673T>G), GTC1511GTT (5676C>T), CCA1513TCT (5680C>T 5682A>T), TTC1514TTT (5685C>T), GGA1515AGA (5686G>A), CTC1516CTA (5691C>A), AAA1517GCA (5692A>G 5693A>C), GCA1519GCT (5700A>T), GCT1521GCA (5706T>A), ATT1522ACT (5708T>C), CAG1524ATG (5713C>A 5714A>T), AGA1525GAC (5716A>G 5717G>A 5718A>C), AAA1526TTG (5719A>T 5720A>T 5721A>G), GAT1528AAC (5725G>A 5727T>C), AAA1529AAG (5730A>G), TGC1530GTT (5731T>G 5732G>T 5733C>T), AAA1532_GAC1533insCCG (5739_5740insCCG), GAC1533TAC (5740G>T), ACA1534TTA (5743A>T 5744C>T), GAA1535GAT (5748A>T), GAG1536ATG (5749G>A 5750A>T), ATC1538GTG (5755A>G 5757C>G), GCC1539GTG (5759C>T 5760C>G), GTA1540GTC (5763A>C), TAC1541TTC (5765A>T), ATC1542ATA (5769C>A), GAC1543GAT (5772C>T), ATC1545ATA (5778C>A), CTG1546TTG (5779C>T), GTA1547GTT (5784A>T), TAC1548TAT (5787C>T), AAC1550CAA (5791A>C 5793C>A), GCG1553GAA (5801C>A 5802G>A), GAT1554GAG (5805T>G), GAA1556ATG (5809G>A 5810A>T 5811A>G), AGG1557GGT (5812A>G 5814G>T), TTA1559TTG (5820A>G), AAG1560AGG (5822A>G), ATG1562GTG (5827A>G), CTG1563TT. (5830C>T)								

Proteins

	Begin	End	Coverage	Score	Concordance	Matches	Identities	I/D/M/F*	Stop Codons
ORF3 (YP_009121747.1)	1456	1563	5.3%	335	44.1%	108 (99.1%)	50 (45.9%)	1/0/0/0	0
Protein mutations:	D1456N (5509G>A), Q1457K (5512C>A), S1459P (5518T>C), L1460I (5521C>A), G1462R (5527G>A), N1464D (5533A>G 5535C>T), T1465D (5536A>G 5537C>A 5538C>T), L1467F (5542C>T 5544A>C), K1468D (5545A>G 5547A>T), R1469K (5549G>A), I1470L (5551A>C 5553A>T), G1471K (5554G>A 5555G>A 5556G>A), N1472G (5557A>G 5558A>G), K1474S (5564A>G 5565G>T), I1475Y (5566A>T 5567T>A 5568A>C), F1479I (5578T>A), K1482R (5587A>C 5588A>G 5589G>T), F1485Y (5597T>A), H1486L (5600A>T 5601T>C), V1488L (5605G>T 5607T>G), A1489R (5608G>A 5609C>G 5610C>G), M1490V (5611A>G), E1491R (5614G>A 5615A>G 5616A>G), E1493C (5620G>T 5621A>G 5622A>T), S1494D (5623T>G 5624C>A), W1497K (5632T>A 5633G>A), A1499T (5638G>A 5640C>T), L1501R (5644T>A 5645T>G), I1502T (5648T>C), P1503R (5651C>G), G1504Y (5653G>T 5654G>A 5655A>T), L1506H (5659T>C 5660T>A 5661A>T), Y1507F (5663A>T 5664C>T), W1509F (5669G>T 5670G>T), P1513S (5680C>T 5682A>T), G1515R (5686G>A), K1517A (5692A>G 5693A>C), I1522T (5708T>C), Q1524M (5713C>A 5714A>T), R1525D (5716A>G 5717G>A 5718A>C), K1526L (5719A>T 5720A>T 5721A>G), D1528N (5725G>A 5727T>C), C1530V (5731T>G 5732G>T 5733C>T), K1532_D1533insP (5739_5740insCCG), D1533Y (5740G>T), T1534L (5743A>T 5744C>T), E1535D (5748A>T), E1536M (5749G>A 5750A>T), I1538V (5755A>G 5757C>G), A1539V (5759C>T 5760C>G), Y1541F (5765A>T), N1550Q (5791A>C 5793C>A), A1553E (5801C>A 5802G>A), D1554E (5805T>G), E1556M (5809G>A 5810A>T 5811A>G), R1557G (5812A>G 5814G>T), K1560R (5822A>G), M1562V (5827A>G)								
Codon mutations:	GAT1456AAT (5509G>A), CAG1457AAG (5512C>A), TCC1459CCC (5518T>C), CTT1460ATT (5521C>A), CCC1461CCG (5526C>G), GGG1462AGG (5527G>A), ATA1463ATT (5532A>T), AAC1464GAT (5533A>G 5535C>T), ACC1465GAT (5536A>G 5537C>A 5538C>T), CTA1467TTC (5542C>T 5544A>C), AAA1468GAT (5545A>G 5547A>T), AGA1469AAA (5549G>A), ATA1470CTT (5551A>C 5553A>T), GGG1471AAA (5554G>A 5555G>A 5556G>A), AAT1472GGT (5557A>G 5558A>G), AAG1474AGT (5564A>G 5565G>T), ATA1475TAC (5566A>T 5567T>A 5568A>C), TCA1477TCT (5574A>T), TTT1479ATT (5578T>A), CTG1481CTT (5586G>T), AAG1482CGT (5587A>C 5588A>G 5589G>T), TCT1483TCC (5592T>C), TTT1485TAT (5597T>A), CAT1486CTC (5600A>T 5601T>C), GTT1488TTG (5605G>T 5607T>G), GCC1489AGG (5608G>A 5609C>G 5610C>G), ATG1490GTG (5611A>G), GAA1491AGG (5614G>A 5615A>G 5616A>G), GAA1492GAG (5619A>G), GAA1493TGT (5620G>T 5621A>G 5622A>T), TCC1494GAC (5623T>G 5624C>A), ATA1495ATT (5628A>T), CCC1496CCA (5631C>A), TGG1497AAG (5632T>A 5633G>A), GCC1499ACT (5638G>A 5640C>T), TTA1501AGA (5644T>A 5645T>G), ATC1502ACC (5648T>C), CCA1503CGA (5651C>G), GGA1504TAT (5653G>T 5654G>A 5655A>T), GGA1505GGT (5658A>T), TTA1506CAT (5659T>C 5660T>A 5661A>T), TAC1507TTT (5663A>T 5664C>T), TGG1509TTT (5669G>T 5670G>T), CTT1510TTG (5671C>T 5673T>G), GTC1511GTT (5676C>T), CCA1513TCT (5680C>T 5682A>T), TTC1514TTT (5685C>T), GGA1515AGA (5686G>A), CTC1516CTA (5691C>A), AAA1517GCA (5692A>G 5693A>C), GCA1519GCT (5700A>T), GCT1521GCA (5706T>A), ATT1522ACT (5708T>C), CAG1524ATG (5713C>A 5714A>T), AGA1525GAC (5716A>G 5717G>A 5718A>C), AAA1526TTG (5719A>T 5720A>T 5721A>G), GAT1528AAC (5725G>A 5727T>C), AAA1529AAG (5730A>G), TGC1530GTT (5731T>G 5732G>T 5733C>T), AAA1532_GAC1533insCCG (5739_5740insCCG), GAC1533TAC (5740G>T), ACA1534TTA (5743A>T 5744C>T), GAA1535GAT (5748A>T), GAG1536ATG (5749G>A 5750A>T), ATC1538GTG (5755A>G 5757C>G), GCC1539GTG (5759C>T 5760C>G), GTA1540GTC (5763A>C), TAC1541TTC (5765A>T), ATC1542ATA (5769C>A), GAC1543GAT (5772C>T), ATC1545ATA (5778C>A), CTG1546TTG (5779C>T), GTA1547GTT (5784A>T), TAC1548TAT (5787C>T), AAC1550CAA (5791A>C 5793C>A), GCG1553GAA (5801C>A 5802G>A), GAT1554GAG (5805T>G), GAA1556ATG (5809G>A 5810A>T 5811A>G), AGG1557GGT (5812A>G 5814G>T), TTA1559TTG (5820A>G), AAG1560AGG (5822A>G), ATG1562GTG (5827A>G), CTG1563TT. (5830C>T)								

*: Inserts / Deletes / Misaligned / Frameshifts

Analysis details

This analysis was performed with panviral2.64

NGS Details (UN9): Arhar cryptic virus-I (segment RNA 3)

Assembly

**Coverage Length**	311 (1 contig(s))
**Depth Of Coverage**	5.6
**Number Of Reads**	14
**Reads Per Million**	0.32 rpm (after QC)
**Ambiguities**	0
**Assembly Method**	de novo + reference guided assembly
**Consensus Caller**	Bcf Tools

Coverage Map

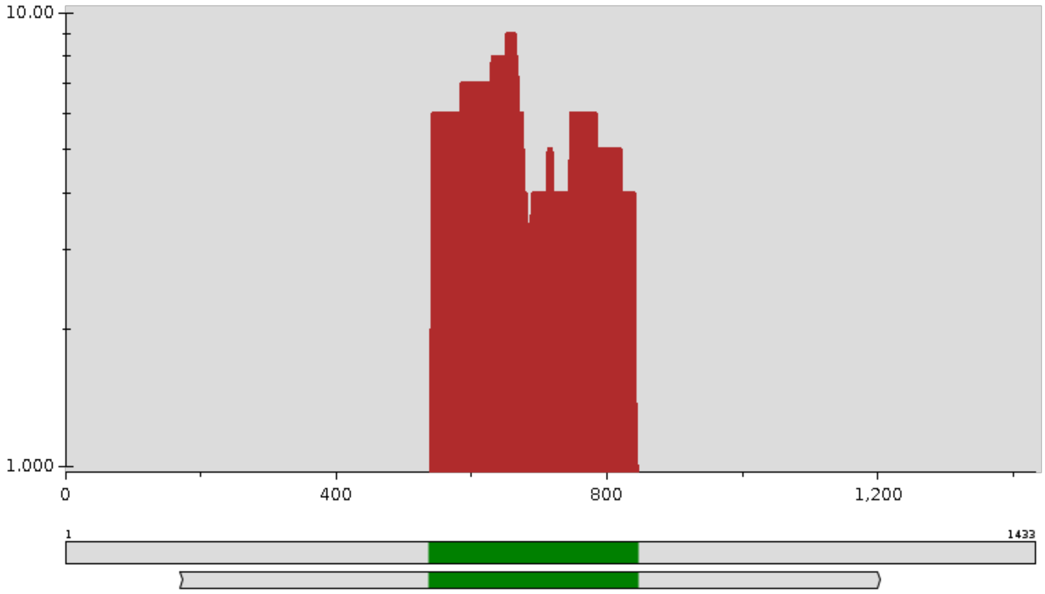

Assignment

**Type**	Arhar cryptic virus-I (Taxonomy ID: 1585924)
**Reference Genome**	NC_024010.1
**NT Identity (%)**	57.7049
**AA Identity (%)**	42.5743
**Number Of Stop Codons**	0
**Number Of CDS**	1

Alignment

**Alignment Score**	79.0 (NT) + 309.0 (AA) = 388.0
**Concordance (%)**	30.4553

**Alignment Method**	Global, seeded, nucleotide + amino acids (AGA)

Genome Region

Sequence starts at position 538 and ends at position 848 relative to NC_024010.1 reference sequence.

Alignment Detailed Statistics

	Begin	End	Coverage	Score	Concordance	Matches	Identities	I/D/M/F*	Stop Codons
**NT**	**538**	**848**	**21.7%**	**79**	**13.3%**	**305 (98.1%)**	**176 (56.6%)**	**0/6**	
Mutations:	543G>A, 544G>A, 549A>C, 553G>A, 557A>T, 559C>A, 560T>A, 561_566delGAAATT, 570A>G, 572G>T, 576A>G, 578A>T, 581T>G, 582A>G, 583C>A, 585T>C, 586T>A, 589T>C, 591C>A, 592C>T, 593G>T, 594C>A, 595A>T, 599T>G, 601T>C, 603A>G, 608T>G, 617T>A, 620T>A, 623T>C, 629T>G, 635T>G, 637G>A, 638C>T, 640G>A, 642C>A, 645A>C, 648A>C, 649A>G, 650A>T, 651T>G, 655A>T, 656C>T, 660C>A, 661G>C, 662C>T, 663C>T, 666G>T, 667G>C, 668A>C, 670C>A, 674A>G, 677C>T, 678G>A, 680C>A, 681A>G, 682C>A, 684G>C, 686C>T, 690G>C, 691T>G, 692C>A, 696A>G, 698C>A, 699A>G, 700A>T, 704T>C, 706T>C, 707C>T, 711A>T, 713A>G, 716A>C, 717T>G, 718T>C, 719A>C, 722G>T, 723C>T, 728T>A, 729C>A, 730T>A, 731C>G, 734A>G, 737A>C, 738C>T, 739A>C, 740A>T, 741G>A, 742A>G, 744C>A, 750T>G, 751C>T, 756C>A, 758G>T, 761C>T, 762C>G, 764G>A, 765A>T, 766A>T, 767T>G, 768C>A, 769T>C, 772A>T, 778A>T, 779C>T, 781T>A, 782C>G, 784T>C, 788G>T, 789A>G, 791A>G, 797C>A, 803A>C, 806A>G, 809T>A, 810G>A, 811T>C, 812G>T, 813C>G, 815G>A, 816C>A, 824A>T, 825C>T, 827C>T, 828A>T, 830A>C, 831G>A, 832C>G, 834G>C, 835C>A, 836T>G								
**CDS**									
CP	124	226	29.9%	309	45.2%	101 (98.1%)	43 (41.7%)	0/2/0/0	0
Protein mutations:	G125N (543G>A 544G>A), M127L (549A>C), R128H (553G>A), Q129H (557A>T), T130K (559C>A 560T>A), E131_I132del (561_566delGAAATT), T134A (570A>G 572G>T), I136V (576A>G 578A>T), T138D (582A>G 583C>A), F139H (585T>C 586T>A), V140A (589T>C), P141I (591C>A 592C>T 593G>T), H142I (594C>A 595A>T), I143M (599T>G), V144A (601T>C), N145D (603A>G), R156H (637G>A 638C>T), S157N (640G>A), L158I (642C>A), N159H (645A>C), K160R (648A>C 649A>G 650A>T), S161A (651T>G), Y162F (655A>T 656C>T), R164T (660C>A 661G>C 662C>T), H165Y (663C>T), G166S (666G>T 667G>C 668A>C), T167N (670C>A), A170T (678G>A 680C>A), T171D (681A>G 682C>A), A172P (684G>C 686C>T), V174R (690G>C 691T>G 692C>A), T176A (696A>G 698C>A), K177V (699A>G 700A>T), I179T (706T>C 707C>T), I181L (711A>T 713A>G), L183A (717T>G 718T>C 719A>C), L185F (723C>T), L187K (729C>A 730T>A 731C>G), Q190S (738C>T 739A>C 740A>T), E191R (741G>A 742A>G), L192I (744C>A), S194V (750T>G 751C>T), Q196N (756C>A 758G>T), Q198E (762C>G 764G>A), N199L (765A>T 766A>T 767T>G), L200T (768C>A 769T>C), E201V (772A>T), N203I (778A>T 779C>T), V204E (781T>A 782C>G), I205T (784T>C), M206I (788G>T), I207V (789A>G 791A>G), V214T (810G>A 811T>C 812G>T), Q215E (813C>G 815G>A), H216N (816C>A), R219C (825C>T 827C>T), T220S (828A>T 830A>C), A221S (831G>A 832C>G), A222Q (834G>C 835C>A 836T>G)								
Codon mutations:	GGC125AAC (543G>A 544G>A), ATG127CTG (549A>C), CGC128CAC (553G>A), CAA129CAT (557A>T), ACT130AAA (559C>A 560T>A), GAA131_ATT132del (561_566delGAAATT), ACG134GCT (570A>G 572G>T), ATA136GTT (576A>G 578A>T), GCT137GCG (581T>G), ACT138GAT (582A>G 583C>A), TTC139CAC (585T>C 586T>A), GTT140GCT (589T>C), CCG141ATT (591C>A 592C>T 593G>T), CAC142ATC (594C>A 595A>T), ATT143ATG (599T>G), GTT144GCT (601T>C), AAT145GAT (603A>G), GTT146GTG (608T>G), TCT149TCA (617T>A), GCT150GCA (620T>A), CTT151CTC (623T>C), GCT153GCG (629T>G), CTT155CTG (635T>G), CGC156CAT (637G>A 638C>T), AGC157AAC (640G>A), CTT158ATT (642C>A), AAC159CAC (645A>C), AAA160CGT (648A>C 649A>G 650A>T), TCC161GCC (651T>G), TAC162TTT (655A>T 656C>T), CGC164ACT (660C>A 661G>C 662C>T), CAT165TAT (663C>T), GGA166TCC (666G>T 667G>C 668A>C), ACC167AAC (670C>A), AGA168AGG (674A>G), TAC169TAT (677C>T), GCC170ACA (678G>A 680C>A), ACT171GAT (681A>G 682C>A), GCC172CCT (684G>C 686C>T), GTC174CGA (690G>C 691T>G 692C>A), ACC176GCA (696A>G 698C>A), AAG177GTG (699A>G 700A>T), GAT178GAC (704T>C), ATC179ACT (706T>C 707C>T), ATA181TTG (711A>T 713A>G), CCA182CCC (716A>C), TTA183GCC (717T>G 718T>C 719A>C), CCG184CCT (722G>T), CTT185TTT (723C>T), GCT186GCA (728T>A), CTC187AAG (729C>A 730T>A 731C>G), GCA188GCG (734A>G), ATA189ATC (737A>C), CAA190TCT (738C>T 739A>C 740A>T), GAA191AGA (741G>A 742A>G), CTT192ATT (744C>A), TCC194GTC (750T>G 751C>T), CAG196AAT (756C>A 758G>T), ACC197ACT (761C>T), CAG198GAA (762C>G 764G>A), AAT199TTG (765A>T 766A>T 767T>G), CTT200ACT (768C>A 769T>C), GAG201GTG (772A>T), AAC203ATT (778A>T 779C>T), GTC204GAG (781T>A 782C>G), ATT205ACT (784T>C), ATG206ATT (788G>T), ATA207GTG (789A>G 791A>G), ACC209ACA (797C>A), CCA211CCC (803A>C), GAA212GAG (806A>G), GGT213GGA (809T>A), GTG214ACT (810G>A 811T>C 812G>T), CAG215GAA (813C>G 815G>A), CAT216AAT (816C>A), GGA218GGT (824A>T), CGC219TGT (825C>T 827C>T), ACA220TCC (828A>T 830A>C), GCC221AGC (831G>A 832C>G), GCT222CAG (834G>C 835C>A 836T>G)								
**Proteins**									
Coat Protein (YP_009026398.1)	124	226	29.9%	309	45.2%	101 (98.1%)	43 (41.7%)	0/2/0/0	0
Protein mutations:	G125N (543G>A 544G>A), M127L (549A>C), R128H (553G>A), Q129H (557A>T), T130K (559C>A 560T>A), E131_I132del (561_566delGAAATT), T134A (570A>G 572G>T), I136V (576A>G 578A>T), T138D (582A>G 583C>A), F139H (585T>C 586T>A), V140A (589T>C), P141I (591C>A 592C>T 593G>T), H142I (594C>A 595A>T), I143M (599T>G), V144A (601T>C), N145D (603A>G), R156H (637G>A 638C>T), S157N (640G>A), L158I (642C>A), N159H (645A>C), K160R (648A>C 649A>G 650A>T), S161A (651T>G), Y162F (655A>T 656C>T), R164T (660C>A 661G>C 662C>T), H165Y (663C>T), G166S (666G>T 667G>C 668A>C), T167N (670C>A), A170T (678G>A 680C>A), T171D (681A>G 682C>A), A172P (684G>C 686C>T), V174R (690G>C 691T>G 692C>A), T176A (696A>G 698C>A), K177V (699A>G 700A>T), I179T (706T>C 707C>T), I181L (711A>T 713A>G), L183A (717T>G 718T>C 719A>C), L185F (723C>T), L187K (729C>A 730T>A 731C>G), Q190S (738C>T 739A>C 740A>T), E191R (741G>A 742A>G), L192I (744C>A), S194V (750T>G 751C>T), Q196N (756C>A 758G>T), Q198E (762C>G 764G>A), N199L (765A>T 766A>T 767T>G), L200T (768C>A 769T>C), E201V (772A>T), N203I (778A>T 779C>T), V204E (781T>A 782C>G), I205T (784T>C), M206I (788G>T), I207V (789A>G 791A>G), V214T (810G>A 811T>C 812G>T), Q215E (813C>G 815G>A), H216N (816C>A), R219C (825C>T 827C>T), T220S (828A>T 830A>C), A221S (831G>A 832C>G), A222Q (834G>C 835C>A 836T>G)								
Codon mutations:	GGC125AAC (543G>A 544G>A), ATG127CTG (549A>C), CGC128CAC (553G>A), CAA129CAT (557A>T), ACT130AAA (559C>A 560T>A), GAA131_ATT132del (561_566delGAAATT), ACG134GCT (570A>G 572G>T), ATA136GTT (576A>G 578A>T), GCT137GCG (581T>G), ACT138GAT (582A>G 583C>A), TTC139CAC (585T>C 586T>A), GTT140GCT (589T>C), CCG141ATT (591C>A 592C>T 593G>T), CAC142ATC (594C>A 595A>T), ATT143ATG (599T>G), GTT144GCT (601T>C), AAT145GAT (603A>G), GTT146GTG (608T>G), TCT149TCA (617T>A), GCT150GCA (620T>A), CTT151CTC (623T>C), GCT153GCG (629T>G), CTT155CTG (635T>G), CGC156CAT (637G>A 638C>T), AGC157AAC (640G>A), CTT158ATT (642C>A), AAC159CAC (645A>C), AAA160CGT (648A>C 649A>G 650A>T), TCC161GCC (651T>G), TAC162TTT (655A>T 656C>T), CGC164ACT (660C>A 661G>C 662C>T), CAT165TAT (663C>T), GGA166TCC (666G>T 667G>C 668A>C), ACC167AAC (670C>A), AGA168AGG (674A>G), TAC169TAT (677C>T), GCC170ACA (678G>A 680C>A), ACT171GAT (681A>G 682C>A), GCC172CCT (684G>C 686C>T), GTC174CGA (690G>C 691T>G 692C>A), ACC176GCA (696A>G 698C>A), AAG177GTG (699A>G 700A>T), GAT178GAC (704T>C), ATC179ACT (706T>C 707C>T), ATA181TTG (711A>T 713A>G), CCA182CCC (716A>C), TTA183GCC (717T>G 718T>C 719A>C), CCG184CCT (722G>T), CTT185TTT (723C>T), GCT186GCA (728T>A), CTC187AAG (729C>A 730T>A 731C>G), GCA188GCG (734A>G), ATA189ATC (737A>C), CAA190TCT (738C>T 739A>C 740A>T), GAA191AGA (741G>A 742A>G), CTT192ATT (744C>A), TCC194GTC (750T>G 751C>T), CAG196AAT (756C>A 758G>T), ACC197ACT (761C>T), CAG198GAA (762C>G 764G>A), AAT199TTG (765A>T 766A>T 767T>G), CTT200ACT (768C>A 769T>C), GAG201GTG (772A>T), AAC203ATT (778A>T 779C>T), GTC204GAG (781T>A 782C>G), ATT205ACT (784T>C), ATG206ATT (788G>T), ATA207GTG (789A>G 791A>G), ACC209ACA (797C>A), CCA211CCC (803A>C), GAA212GAG (806A>G), GGT213GGA (809T>A), GTG214ACT (810G>A 811T>C 812G>T), CAG215GAA (813C>G 815G>A), CAT216AAT (816C>A), GGA218GGT (824A>T), CGC219TGT (825C>T 827C>T), ACA220TCC (828A>T 830A>C), GCC221AGC (831G>A 832C>G), GCT222CAG (834G>C 835C>A 836T>G)								

*: Inserts / Deletes / Misaligned / Frameshifts

Analysis details

This analysis was performed with panviral2.64

NGS Details (UN9): Dioscovirus dioscoreae

Assembly

**Coverage Length**	332 (1 contig(s))
**Depth Of Coverage**	4.2
**Number Of Reads**	11
**Reads Per Million**	0.25 rpm (after QC)
**Ambiguities**	0
**Assembly Method**	de novo + reference guided assembly
**Consensus Caller**	Bcf Tools

Coverage Map

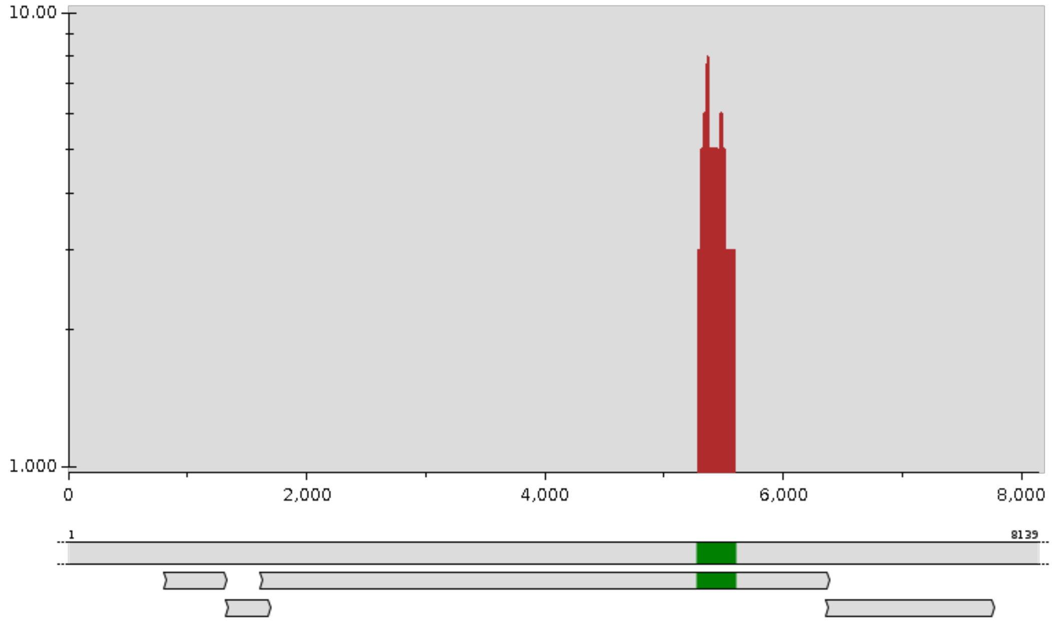

Assignment

**Type**	Dioscovirus dioscoreae (Taxonomy ID: 3052184)
**Reference Genome**	NC_040712.1
**NT Identity (%)**	57.0122
**AA Identity (%)**	49.5413
**Number Of Stop Codons**	0
**Number Of CDS**	4

Alignment

**Alignment Score**	70.0 (NT) + 258.0 (AA) = 328.0
**Concordance (%)**	25.4658

**Alignment Method**	Global, seeded, nucleotide + amino acids (AGA)

Genome Region

Sequence starts at position 5274 and ends at position 5605 relative to NC_040712.1 reference sequence.

Alignment Detailed Statistics

	Begin	End	Coverage	Score	Concordance	Matches	Identities	I/D/M/F*	Stop Codons
**NT**	**5274**	**5605**	**4.1%**	**70**	**11.0%**	**325 (97.0%)**	**187 (55.8%)**	**3/7**	
Mutations:	5274C>T, 5276A>G, 5280G>C, 5282T>C, 5284A>T, 5288A>C, 5289T>A, 5290A>T, 5298C>A, 5303C>T, 5304A>C, 5306A>T, 5307C>A, 5312A>T, 5314G>T, 5321C>A, 5322T>G, 5324T>A, 5327C>G, 5328C>T, 5330A>G, 5331C>G, 5332T>A, 5334A>G, 5337A>C, 5340A>G, 5341A>G, 5342A>G, 5345C>T, 5346A>G, 5347A>C, 5348G>A, 5349A>C, 5350T>A, 5351T>A, 5352G>C, 5353T>G, 5354C>G, 5356A>T, 5357C>T, 5358A>T, 5359G>C, 5363A>G, 5364T>G, 5372A>C, 5374A>G, 5378A>T, 5381A>G, 5383T>A, 5387C>T, 5393T>C, 5395T>G, 5401A>C, 5402T>C, 5403C>A, 5407A>C, 5408G>A, 5410G>T, 5414A>C, 5415G>C, 5417A>T, 5418T>A, 5419G>A, 5420G>A, 5423A>T, 5426A>C, 5430G>A, 5431T>G, 5432C>G, 5433T>A, 5434G>A, 5435C>A, 5437C>A, 5438A>T, 5439C>T, 5440A>C, 5441G>T, 5444T>C, 5447T>C, 5449T>A, 5451_5457delGAATGGA, 5458T>C, 5462A>G, 5468A>T, 5471C>T, 5477C>A, 5479A>C, 5480A>T, 5485C>A, 5486A>C, 5492A>T, 5493G>A, 5494T>G, 5495A>T, 5498C>T, 5501A>G, 5502C>A, 5504A>T, 5505A>T, 5506A>T, 5511G>A, 5514A>C, 5515A>G, 5516T>A, 5522C>T, 5523A>C, 5528A>G, 5529T>C, 5531T>C, 5532T>C, 5533C>T, 5534_5535insCGG, 5535G>A, 5536A>G, 5541G>A, 5545G>T, 5546T>G, 5549T>G, 5551A>T, 5553A>T, 5555A>T, 5558C>T, 5564T>C, 5565T>C, 5567G>C, 5570A>T, 5574T>A, 5575C>G, 5577G>A, 5580A>T, 5583A>C, 5585A>G, 5586C>G, 5589C>G, 5591A>T, 5597A>G, 5598C>G, 5599A>T, 5600A>T, 5603T>C								

CDS

	Begin	End	Coverage	Score	Concordance	Matches	Identities	I/D/M/F*	Stop Codons
EXK67_gp3	1222	1332	6.9%	258	32.9%	108 (97.3%)	54 (48.6%)	1/2/1/1	0
Protein mutations:	D1224H (5280G>C 5282T>C), N1225I (5284A>T), Y1227I (5289T>A 5290A>T), Q1230K (5298C>A), T1232P (5304A>C 5306A>T), L1233I (5307C>A), S1235I (5314G>T), D1237E (5321C>A), Y1238E (5322T>G 5324T>A), L1241D (5331C>G 5332T>A), K1242E (5334A>G), I1243L (5337A>C), K1244G (5340A>G 5341A>G 5342A>G), K1246A (5346A>G 5347A>C 5348G>A), I1247Q (5349A>C 5350T>A 5351T>A), V1248R (5352G>C 5353T>G 5354C>G), Y1249F (5356A>T 5357C>T), F1252V (5364T>G), K1255R (5374A>G), F1258Y (5383T>A), M1262R (5395T>G), D1264A (5401A>C 5402T>C), P1265T (5403C>A), Q1266P (5407A>C 5408G>A), S1267I (5410G>T), E1269H (5415G>C 5417A>T), W1270K (5418T>A 5419G>A 5420G>A), V1274R (5430G>A 5431T>G 5432C>G), C1275K (5433T>A 5434G>A 5435C>A), P1276H (5437C>A 5438A>T), Q1277S (5439C>T 5440A>C 5441G>T), F1280Y (5449T>A), E1281_W1282del (5451_5457delGAATGGA), K1290T (5479A>C 5480A>T), A1292D (5485C>A 5486A>C), V1295S (5493G>A 5494T>G 5495A>T), R1298S (5502C>A 5504A>T), K1299L (5505A>T 5506A>T), D1301N (5511G>A), N1302R (5514A>C 5515A>G 5516T>A), K1305Q (5523A>C), Y1307H (5529T>C 5531T>C), S1308L (5532T>C 5533C>T), S1308_E1309insR (5534_5535insCGG), E1309R (5535G>A 5536A>G), V1311I (5541G>A), C1312L (5545G>T 5546T>G), Y1314F (5551A>T), I1315F (5553A>T 5555A>T), E1323K (5577G>A), S1324C (5580A>T), I1325L (5583A>C 5585A>G), Q1326E (5586C>G), Q1327D (5589C>G 5591A>T), Q1330V (5598C>G 5599A>T 5600A>T)								
Codon mutations:	CTA1222TTG (5274C>T 5276A>G), GAT1224CAC (5280G>C 5282T>C), AAT1225ATT (5284A>T), ACA1226ACC (5288A>C), TAC1227ATC (5289T>A 5290A>T), CAA1230AAA (5298C>A), TAC1231TAT (5303C>T), ACA1232CCT (5304A>C 5306A>T), CTC1233ATC (5307C>A), CCA1234CCT (5312A>T), AGT1235ATT (5314G>T), GAC1237GAA (5321C>A), TAT1238GAA (5322T>G 5324T>A), CTC1239CTG (5327C>G), CTA1240TTG (5328C>T 5330A>G), CTT1241GAT (5331C>G 5332T>A), AAA1242GAA (5334A>G), ATT1243CTT (5337A>C), AAA1244GGG (5340A>G 5341A>G 5342A>G), GAC1245GAT (5345C>T), AAG1246GCA (5346A>G 5347A>C 5348G>A), ATT1247CAA (5349A>C 5350T>A 5351T>A), GTC1248CGG (5352G>C 5353T>G 5354C>G), TAC1249TTT (5356A>T 5357C>T), AGC1250TCC (5358A>T 5359G>C), AAA1251AAG (5363A>G), TTT1252GTT (5364T>G), CTA1254CTC (5372A>C), AAG1255AGG (5374A>G), TCA1256TCT (5378A>T), GGA1257GGG (5381A>G), TTC1258TAC (5383T>A), CAC1259CAT (5387C>T), ATT1261ATC (5393T>C), ATG1262AGG (5395T>G), GAT1264GCC (5401A>C 5402T>C), CCA1265ACA (5403C>A), CAG1266CCA (5407A>C 5408G>A), AGT1267ATT (5410G>T), ATA1268ATC (5414A>C), GAA1269CAT (5415G>C 5417A>T), TGG1270AAA (5418T>A 5419G>A 5420G>A), ACA1271ACT (5423A>T), GCA1272GCC (5426A>C), GTC1274AGG (5430G>A 5431T>G 5432C>G), TGC1275AAA (5433T>A 5434G>A 5435C>A), CCA1276CAT (5437C>A 5438A>T), CAG1277TCT (5439C>T 5440A>C 5441G>T), GGT1278GGC (5444T>C), CAT1279CAC (5447T>C), TTT1280TAT (5449T>A), GAA1281_TGG1282del (5451_5457delGAATGGA), ATA1283-CA (5451_5457delGAATGGA 5458T>C), GTA1284GTG (5462A>G), CCA1286CCT (5468A>T), TTC1287TTT (5471C>T), CTC1289CTA (5477C>A), AAA1290ACT (5479A>C 5480A>T), GCA1292GAC (5485C>A 5486A>C), TCA1294TCT (5492A>T), GTA1295AGT (5493G>A 5494T>G 5495A>T), TTC1296TTT (5498C>T), CAA1297CAG (5501A>G), CGA1298AGT (5502C>A 5504A>T), AAA1299TTA (5505A>T 5506A>T), GAT1301AAT (5511G>A), AAT1302CGA (5514A>C 5515A>G 5516T>A), TTC1304TTT (5522C>T), AAA1305CAA (5523A>C), AAA1306AAG (5528A>G), TAT1307CAC (5529T>C 5531T>C), TCA1308CTA (5532T>C 5533C>T), TCA1308_GAA1309insCGG (5534_5535insCGG), GAA1309AGA (5535G>A 5536A>G), GTC1311ATC (5541G>A), TGT1312TTG (5545G>T 5546T>G), GTT1313GTG (5549T>G), TAC1314TTC (5551A>T), ATA1315TTT (5553A>T 5555A>T), GAC1316GAT (5558C>T), ATT1318ATC (5564T>C), TTG1319CTC (5565T>C 5567G>C), ATA1320ATT (5570A>T), TCT1322AGT (5574T>A 5575C>G), GAA1323AAA (5577G>A), AGT1324TGT (5580A>T), ATA1325CTG (5583A>C 5585A>G), CAA1326GAA (5586C>G), CAA1327GAT (5589C>G 5591A>T), GTA1329GTG (5597A>G), CAA1330GTT (5598C>G 5599A>T 5600A>T), CAT1331CAC (5603T>C)								

Proteins

	Begin	End	Coverage	Score	Concordance	Matches	Identities	I/D/M/F*	Stop Codons
ORF3 (YP_009553219.1)	1222	1332	6.9%	258	32.9%	108 (97.3%)	54 (48.6%)	1/2/1/1	0
Protein mutations:	D1224H (5280G>C 5282T>C), N1225I (5284A>T), Y1227I (5289T>A 5290A>T), Q1230K (5298C>A), T1232P (5304A>C 5306A>T), L1233I (5307C>A), S1235I (5314G>T), D1237E (5321C>A), Y1238E (5322T>G 5324T>A), L1241D (5331C>G 5332T>A), K1242E (5334A>G), I1243L (5337A>C), K1244G (5340A>G 5341A>G 5342A>G), K1246A (5346A>G 5347A>C 5348G>A), I1247Q (5349A>C 5350T>A 5351T>A), V1248R (5352G>C 5353T>G 5354C>G), Y1249F (5356A>T 5357C>T), F1252V (5364T>G), K1255R (5374A>G), F1258Y (5383T>A), M1262R (5395T>G), D1264A (5401A>C 5402T>C), P1265T (5403C>A), Q1266P (5407A>C 5408G>A), S1267I (5410G>T), E1269H (5415G>C 5417A>T), W1270K (5418T>A 5419G>A 5420G>A), V1274R (5430G>A 5431T>G 5432C>G), C1275K (5433T>A 5434G>A 5435C>A), P1276H (5437C>A 5438A>T), Q1277S (5439C>T 5440A>C 5441G>T), F1280Y (5449T>A), E1281_W1282del (5451_5457delGAATGGA), K1290T (5479A>C 5480A>T), A1292D (5485C>A 5486A>C), V1295S (5493G>A 5494T>G 5495A>T), R1298S (5502C>A 5504A>T), K1299L (5505A>T 5506A>T), D1301N (5511G>A), N1302R (5514A>C 5515A>G 5516T>A), K1305Q (5523A>C), Y1307H (5529T>C 5531T>C), S1308L (5532T>C 5533C>T), S1308_E1309insR (5534_5535insCGG), E1309R (5535G>A 5536A>G), V1311I (5541G>A), C1312L (5545G>T 5546T>G), Y1314F (5551A>T), I1315F (5553A>T 5555A>T), E1323K (5577G>A), S1324C (5580A>T), I1325L (5583A>C 5585A>G), Q1326E (5586C>G), Q1327D (5589C>G 5591A>T), Q1330V (5598C>G 5599A>T 5600A>T)								
Codon mutations:	CTA1222TTG (5274C>T 5276A>G), GAT1224CAC (5280G>C 5282T>C), AAT1225ATT (5284A>T), ACA1226ACC (5288A>C), TAC1227ATC (5289T>A 5290A>T), CAA1230AAA (5298C>A), TAC1231TAT (5303C>T), ACA1232CCT (5304A>C 5306A>T), CTC1233ATC (5307C>A), CCA1234CCT (5312A>T), AGT1235ATT (5314G>T), GAC1237GAA (5321C>A), TAT1238GAA (5322T>G 5324T>A), CTC1239CTG (5327C>G), CTA1240TTG (5328C>T 5330A>G), CTT1241GAT (5331C>G 5332T>A), AAA1242GAA (5334A>G), ATT1243CTT (5337A>C), AAA1244GGG (5340A>G 5341A>G 5342A>G), GAC1245GAT (5345C>T), AAG1246GCA (5346A>G 5347A>C 5348G>A), ATT1247CAA (5349A>C 5350T>A 5351T>A), GTC1248CGG (5352G>C 5353T>G 5354C>G), TAC1249TTT (5356A>T 5357C>T), AGC1250TCC (5358A>T 5359G>C), AAA1251AAG (5363A>G), TTT1252GTT (5364T>G), CTA1254CTC (5372A>C), AAG1255AGG (5374A>G), TCA1256TCT (5378A>T), GGA1257GGG (5381A>G), TTC1258TAC (5383T>A), CAC1259CAT (5387C>T), ATT1261ATC (5393T>C), ATG1262AGG (5395T>G), GAT1264GCC (5401A>C 5402T>C), CCA1265ACA (5403C>A), CAG1266CCA (5407A>C 5408G>A), AGT1267ATT (5410G>T), ATA1268ATC (5414A>C), GAA1269CAT (5415G>C 5417A>T), TGG1270AAA (5418T>A 5419G>A 5420G>A), ACA1271ACT (5423A>T), GCA1272GCC (5426A>C), GTC1274AGG (5430G>A 5431T>G 5432C>G), TGC1275AAA (5433T>A 5434G>A 5435C>A), CCA1276CAT (5437C>A 5438A>T), CAG1277TCT (5439C>T 5440A>C 5441G>T), GGT1278GGC (5444T>C), CAT1279CAC (5447T>C), TTT1280TAT (5449T>A), GAA1281_TGG1282del (5451_5457delGAATGGA), ATA1283-CA (5451_5457delGAATGGA 5458T>C), GTA1284GTG (5462A>G), CCA1286CCT (5468A>T), TTC1287TTT (5471C>T), CTC1289CTA (5477C>A), AAA1290ACT (5479A>C 5480A>T), GCA1292GAC (5485C>A 5486A>C), TCA1294TCT (5492A>T), GTA1295AGT (5493G>A 5494T>G 5495A>T), TTC1296TTT (5498C>T), CAA1297CAG (5501A>G), CGA1298AGT (5502C>A 5504A>T), AAA1299TTA (5505A>T 5506A>T), GAT1301AAT (5511G>A), AAT1302CGA (5514A>C 5515A>G 5516T>A), TTC1304TTT (5522C>T), AAA1305CAA (5523A>C), AAA1306AAG (5528A>G), TAT1307CAC (5529T>C 5531T>C), TCA1308CTA (5532T>C 5533C>T), TCA1308_GAA1309insCGG (5534_5535insCGG), GAA1309AGA (5535G>A 5536A>G), GTC1311ATC (5541G>A), TGT1312TTG (5545G>T 5546T>G), GTT1313GTG (5549T>G), TAC1314TTC (5551A>T), ATA1315TTT (5553A>T 5555A>T), GAC1316GAT (5558C>T), ATT1318ATC (5564T>C), TTG1319CTC (5565T>C 5567G>C), ATA1320ATT (5570A>T), TCT1322AGT (5574T>A 5575C>G), GAA1323AAA (5577G>A), AGT1324TGT (5580A>T), ATA1325CTG (5583A>C 5585A>G), CAA1326GAA (5586C>G), CAA1327GAT (5589C>G 5591A>T), GTA1329GTG (5597A>G), CAA1330GTT (5598C>G 5599A>T 5600A>T), CAT1331CAC (5603T>C)								

*: Inserts / Deletes / Misaligned / Frameshifts

Analysis details

This analysis was performed with panviral2.64

NGS Details (UN9): Dioscovirus dioscoreae

Assembly

**Coverage Length**	323 (1 contig(s))
**Depth Of Coverage**	4.1
**Number Of Reads**	11
**Reads Per Million**	0.25 rpm (after QC)
**Ambiguities**	0
**Assembly Method**	de novo + reference guided assembly
**Consensus Caller**	Bcf Tools

Coverage Map

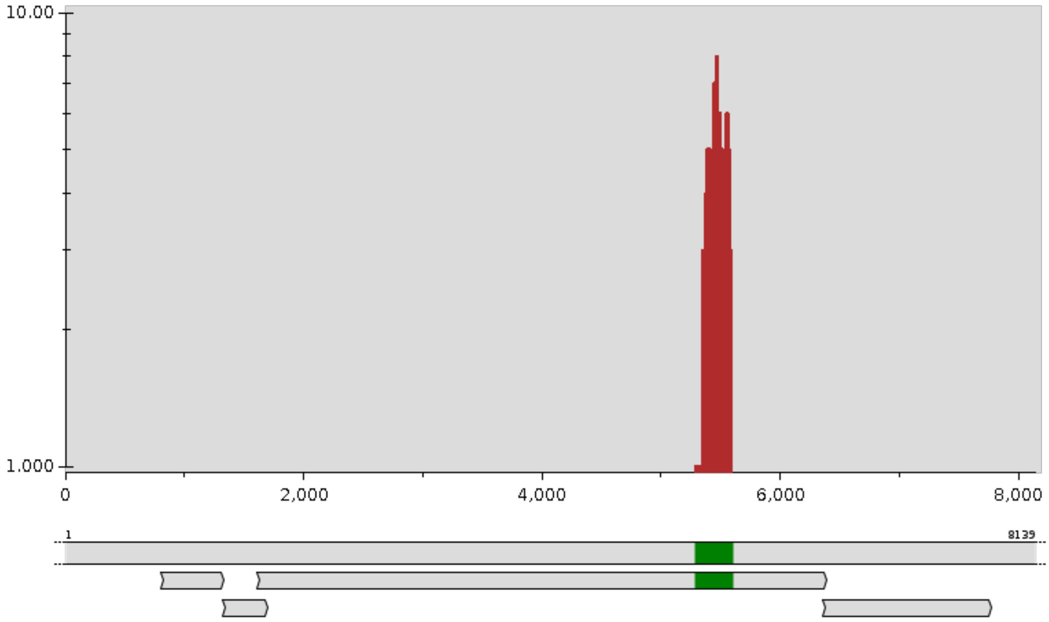

Assignment

**Type**	Dioscovirus dioscoreae (Taxonomy ID: 3052184)
**Reference Genome**	NC_040712.1
**NT Identity (%)**	66.2539
**AA Identity (%)**	55.5556
**Number Of Stop Codons**	1
**Number Of CDS**	4

Alignment

**Alignment Score**	210.0 (NT) + 482.0 (AA) = 692.0
**Concordance (%)**	48.089

Alignment Method	Global, seeded, nucleotide + amino acids (AGA)

Genome Region

Sequence starts at position 5283 and ends at position 5605 relative to NC_040712.1 reference sequence.

Alignment Detailed Statistics

	Begin	End	Coverage	Score	Concordance	Matches	Identities	I/D/M/F*	Stop Codons
**NT**	**5283**	**5605**	**4.0%**	**210**	**32.5%**	**323 (100%)**	**214 (66.3%)**	**0/0**	
Mutations:	5288A>T, 5289T>A, 5290A>G, 5291C>A, 5293A>C, 5297C>T, 5298C>G, 5299A>C, 5303C>T, 5305C>A, 5307C>T, 5309C>A, 5310C>T, 5313A>G, 5314G>A, 5317T>A, 5318T>A, 5322T>A, 5324T>A, 5325C>T, 5327C>G, 5328C>A, 5330A>T, 5331C>T, 5332T>G, 5335A>G, 5340A>C, 5344A>G, 5345C>T, 5348G>A, 5350T>A, 5351T>A, 5352G>A, 5356A>T, 5357C>T, 5360C>T, 5361A>G, 5370C>T, 5371T>G, 5372A>T, 5375G>A, 5378A>G, 5383T>A, 5385C>T, 5387C>G, 5390G>A, 5391A>G, 5393T>A, 5395T>A, 5396G>A, 5400G>C, 5402T>C, 5406C>G, 5408G>C, 5429T>C, 5430G>A, 5431T>C, 5434G>A, 5435C>T, 5437C>T, 5438A>T, 5439C>G, 5444T>A, 5457A>C, 5461T>C, 5462A>C, 5471C>T, 5474A>T, 5475C>T, 5477C>A, 5482A>C, 5483T>A, 5486A>G, 5492A>T, 5493G>A, 5498C>T, 5502C>A, 5511G>A, 5514A>G, 5519A>G, 5523A>G, 5524A>G, 5525A>C, 5526A>G, 5528A>C, 5532T>A, 5533C>A, 5535G>A, 5537A>C, 5543C>T, 5545G>T, 5546T>A, 5549T>C, 5553A>G, 5558C>T, 5567G>A, 5568A>G, 5574T>A, 5575C>G, 5576T>C, 5577G>A, 5580A>G, 5581G>A, 5589C>G, 5594T>C, 5595G>C, 5598C>G, 5599A>G, 5604T>C								

CDS

	Begin	End	Coverage	Score	Concordance	Matches	Identities	I/D/M/F*	Stop Codons
EXK67_gp3	1225	1332	6.8%	482	60.5%	108 (100%)	60 (55.6%)	0/0/0/0	1
Protein mutations:	Y1227R (5289T>A 5290A>G 5291C>A), K1228T (5293A>C), Q1230A (5298C>G 5299A>C), T1232K (5305C>A), P1234S (5310C>T), S1235D (5313A>G 5314G>A), I1236K (5317T>A 5318T>A), Y1238K (5322T>A 5324T>A), L1240I (5328C>A 5330A>T), L1241C (5331C>T 5332T>G), K1242R (5335A>G), K1244Q (5340A>C), D1245G (5344A>G 5345C>T), I1247K (5350T>A 5351T>A), V1248I (5352G>A), Y1249F (5356A>T 5357C>T), K1251E (5361A>G), L1254C (5370C>T 5371T>G 5372A>T), F1258Y (5383T>A), H1259* (5385C>T 5387C>G), I1261V (5391A>G 5393T>A), M1262K (5395T>A 5396G>A), D1264H (5400G>C 5402T>C), Q1266D (5406C>G 5408G>C), V1274T (5430G>A 5431T>C), C1275Y (5434G>A 5435C>T), P1276L (5437C>T 5438A>T), Q1277E (5439C>G), I1283L (5457A>C), V1284A (5461T>C 5462A>C), N1291T (5482A>C 5483T>A), V1295I (5493G>A), D1301N (5511G>A), N1302D (5514A>G), I1303M (5519A>G), K1305G (5523A>G 5524A>G 5525A>C), K1306D (5526A>G 5528A>C), S1308K (5532T>A 5533C>A), E1309N (5535G>A 5537A>C), C1312L (5545G>T 5546T>A), I1315V (5553A>G), I1320V (5568A>G), E1323K (5577G>A), S1324D (5580A>G 5581G>A), Q1327E (5589C>G), V1329L (5595G>C), Q1330G (5598C>G 5599A>G)								
Codon mutations:	ACA1226ACT (5288A>T), TAC1227AGA (5289T>A 5290A>G 5291C>A), AAA1228ACA (5293A>C), GAC1229GAT (5297C>T), CAA1230GCA (5298C>G 5299A>C), TAC1231TAT (5303C>T), ACA1232AAA (5305C>A), CTC1233TTA (5307C>T 5309C>A), CCA1234TCA (5310C>T), AGT1235GAT (5313A>G 5314G>A), ATT1236AAA (5317T>A 5318T>A), TAT1238AAA (5322T>A 5324T>A), CTC1239TTG (5325C>T 5327C>G), CTA1240ATT (5328C>A 5330A>T), CTT1241TGT (5331C>T 5332T>G), AAA1242AGA (5335A>G), AAA1244CAA (5340A>C), GAC1245GGT (5344A>G 5345C>T), AAG1246AAA (5348G>A), ATT1247AAA (5350T>A 5351T>A), GTC1248ATC (5352G>A), TAC1249TTT (5356A>T 5357C>T), AGC1250AGT (5360C>T), AAA1251GAA (5361A>G), CTA1254TGT (5370C>T 5371T>G 5372A>T), AAG1255AAA (5375G>A), TCA1256TCG (5378A>G), TTC1258TAC (5383T>A), CAC1259TAG (5385C>T 5387C>G), CAG1260CAA (5390G>A), ATT1261GTA (5391A>G 5393T>A), ATG1262AAA (5395T>A 5396G>A), GAT1264CAC (5400G>C 5402T>C), CAG1266GAC (5406C>G 5408G>C), TTT1273TTC (5429T>C), GTC1274ACC (5430G>A 5431T>C), TGC1275TAT (5434G>A 5435C>T), CCA1276CTT (5437C>T 5438A>T), CAG1277GAG (5439C>G), GGT1278GGA (5444T>A), ATA1283CTA (5457A>C), GTA1284GCC (5461T>C 5462A>C), TTC1287TTT (5471C>T), GGA1288GGT (5474A>T), CTC1289TTA (5475C>T 5477C>A), AAT1291ACA (5482A>C 5483T>A), GCA1292GCG (5486A>G), TCA1294TCT (5492A>T), GTA1295ATA (5493G>A), TTC1296TTT (5498C>T), CGA1298AGA (5502C>A), GAT1301AAT (5511G>A), AAT1302GAT (5514A>G), ATA1303ATG (5519A>G), AAA1305GGC (5523A>G 5524A>G 5525A>C), AAA1306GAC (5526A>G 5528A>C), TCA1308AAA (5532T>A 5533C>A), GAA1309AAC (5535G>A 5537A>C), GTC1311GTT (5543C>T), TGT1312TTA (5545G>T 5546T>A), GTT1313GTC (5549T>C), ATA1315GTA (5553A>G), GAC1316GAT (5558C>T), TTG1319TTA (5567G>A), ATA1320GTA (5568A>G), TCT1322AGC (5574T>A 5575C>G 5576T>C), GAA1323AAA (5577G>A), AGT1324GAT (5580A>G 5581G>A), CAA1327GAA (5589C>G), CAT1328CAC (5594T>C), GTA1329CTA (5595G>C), CAA1330GGA (5598C>G 5599A>G), TTA1332CT. (5604T>C)								

Proteins

	Begin	End	Coverage	Score	Concordance	Matches	Identities	I/D/M/F*	Stop Codons
ORF3 (YP_009553219.1)	1225	1332	6.8%	482	60.5%	108 (100%)	60 (55.6%)	0/0/0/0	1
Protein mutations:	Y1227R (5289T>A 5290A>G 5291C>A), K1228T (5293A>C), Q1230A (5298C>G 5299A>C), T1232K (5305C>A), P1234S (5310C>T), S1235D (5313A>G 5314G>A), I1236K (5317T>A 5318T>A), Y1238K (5322T>A 5324T>A), L1240I (5328C>A 5330A>T), L1241C (5331C>T 5332T>G), K1242R (5335A>G), K1244Q (5340A>C), D1245G (5344A>G 5345C>T), I1247K (5350T>A 5351T>A), V1248I (5352G>A), Y1249F (5356A>T 5357C>T), K1251E (5361A>G), L1254C (5370C>T 5371T>G 5372A>T), F1258Y (5383T>A), H1259* (5385C>T 5387C>G), I1261V (5391A>G 5393T>A), M1262K (5395T>A 5396G>A), D1264H (5400G>C 5402T>C), Q1266D (5406C>G 5408G>C), V1274T (5430G>A 5431T>C), C1275Y (5434G>A 5435C>T), P1276L (5437C>T 5438A>T), Q1277E (5439C>G), I1283L (5457A>C), V1284A (5461T>C 5462A>C), N1291T (5482A>C 5483T>A), V1295I (5493G>A), D1301N (5511G>A), N1302D (5514A>G), I1303M (5519A>G), K1305G (5523A>G 5524A>G 5525A>C), K1306D (5526A>G 5528A>C), S1308K (5532T>A 5533C>A), E1309N (5535G>A 5537A>C), C1312L (5545G>T 5546T>A), I1315V (5553A>G), I1320V (5568A>G), E1323K (5577G>A), S1324D (5580A>G 5581G>A), Q1327E (5589C>G), V1329L (5595G>C), Q1330G (5598C>G 5599A>G)								
Codon mutations:	ACA1226ACT (5288A>T), TAC1227AGA (5289T>A 5290A>G 5291C>A), AAA1228ACA (5293A>C), GAC1229GAT (5297C>T), CAA1230GCA (5298C>G 5299A>C), TAC1231TAT (5303C>T), ACA1232AAA (5305C>A), CTC1233TTA (5307C>T 5309C>A), CCA1234TCA (5310C>T), AGT1235GAT (5313A>G 5314G>A), ATT1236AAA (5317T>A 5318T>A), TAT1238AAA (5322T>A 5324T>A), CTC1239TTG (5325C>T 5327C>G), CTA1240ATT (5328C>A 5330A>T), CTT1241TGT (5331C>T 5332T>G), AAA1242AGA (5335A>G), AAA1244CAA (5340A>C), GAC1245GGT (5344A>G 5345C>T), AAG1246AAA (5348G>A), ATT1247AAA (5350T>A 5351T>A), GTC1248ATC (5352G>A), TAC1249TTT (5356A>T 5357C>T), AGC1250AGT (5360C>T), AAA1251GAA (5361A>G), CTA1254TGT (5370C>T 5371T>G 5372A>T), AAG1255AAA (5375G>A), TCA1256TCG (5378A>G), TTC1258TAC (5383T>A), CAC1259TAG (5385C>T 5387C>G), CAG1260CAA (5390G>A), ATT1261GTA (5391A>G 5393T>A), ATG1262AAA (5395T>A 5396G>A), GAT1264CAC (5400G>C 5402T>C), CAG1266GAC (5406C>G 5408G>C), TTT1273TTC (5429T>C), GTC1274ACC (5430G>A 5431T>C), TGC1275TAT (5434G>A 5435C>T), CCA1276CTT (5437C>T 5438A>T), CAG1277GAG (5439C>G), GGT1278GGA (5444T>A), ATA1283CTA (5457A>C), GTA1284GCC (5461T>C 5462A>C), TTC1287TTT (5471C>T), GGA1288GGT (5474A>T), CTC1289TTA (5475C>T 5477C>A), AAT1291ACA (5482A>C 5483T>A), GCA1292GCG (5486A>G), TCA1294TCT (5492A>T), GTA1295ATA (5493G>A), TTC1296TTT (5498C>T), CGA1298AGA (5502C>A), GAT1301AAT (5511G>A), AAT1302GAT (5514A>G), ATA1303ATG (5519A>G), AAA1305GGC (5523A>G 5524A>G 5525A>C), AAA1306GAC (5526A>G 5528A>C), TCA1308AAA (5532T>A 5533C>A), GAA1309AAC (5535G>A 5537A>C), GTC1311GTT (5543C>T), TGT1312TTA (5545G>T 5546T>A), GTT1313GTC (5549T>C), ATA1315GTA (5553A>G), GAC1316GAT (5558C>T), TTG1319TTA (5567G>A), ATA1320GTA (5568A>G), TCT1322AGC (5574T>A 5575C>G 5576T>C), GAA1323AAA (5577G>A), AGT1324GAT (5580A>G 5581G>A), CAA1327GAA (5589C>G), CAT1328CAC (5594T>C), GTA1329CTA (5595G>C), CAA1330GGA (5598C>G 5599A>G), TTA1332CT. (5604T>C)								

*: Inserts / Deletes / Misaligned / Frameshifts

Analysis details

This analysis was performed with panviral2.64

NGS Details (UN9): Ichnoviriform fugitivi (segment C16)

Assembly

**Coverage Length**	179 (1 contig(s))
**Depth Of Coverage**	1.3
**Number Of Reads**	2
**Reads Per Million**	0.05 rpm (after QC)
**Ambiguities**	0
**Assembly Method**	de novo + reference guided assembly
**Consensus Caller**	Bcf Tools

Coverage Map

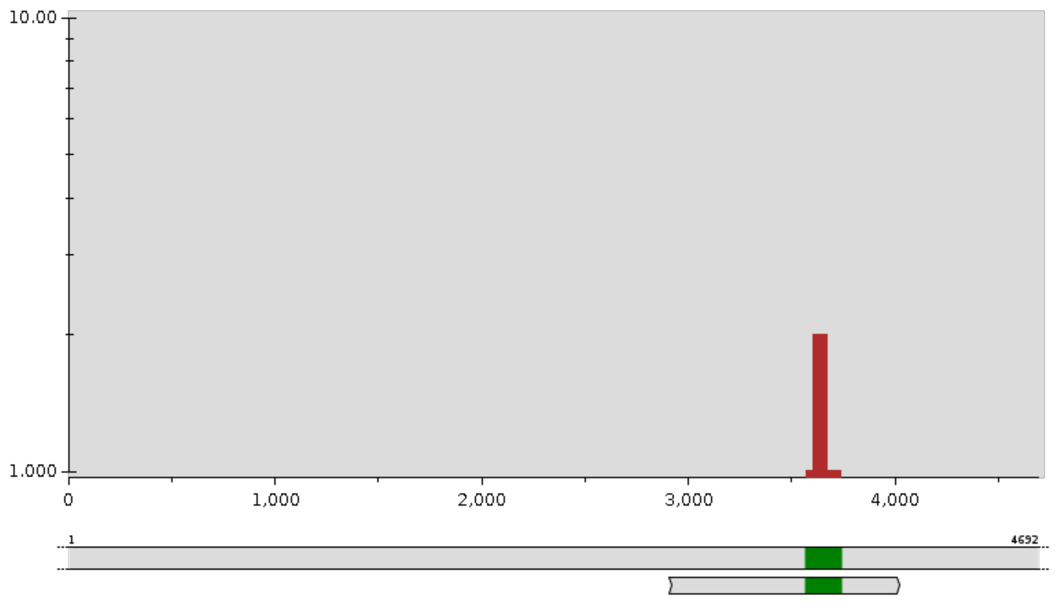

Assignment

**Type**	Ichnoviriform fugitivi (Taxonomy ID: 265522)
**Reference Genome**	NC_008946.1
**NT Identity (%)**	59.7765
**AA Identity (%)**	49.1525
**Number Of Stop Codons**	0
**Number Of CDS**	1

Alignment

**Alignment Score**	70.0 (NT) + 268.0 (AA) = 338.0
**Concordance (%)**	41.8835

Alignment Method	Global, seeded, nucleotide + amino acids (AGA)

Genome Region

Sequence starts at position 3567 and ends at position 3745 relative to NC_008946.1 reference sequence.

Alignment Detailed Statistics

	Begin	End	Coverage	Score	Concordance	Matches	Identities	I/D/M/F*	Stop Codons
**NT**	**3567**	**3745**	**3.8%**	**70**	**19.6%**	**179 (100%)**	**107 (59.8%)**	**0/0**	
Mutations:	3567G>C, 3570G>A, 3572A>G, 3576T>G, 3577G>A, 3581A>G, 3586T>A, 3591A>T, 3592A>T, 3595A>G, 3598G>T, 3601C>T, 3604A>T, 3605A>C, 3606C>G, 3607G>T, 3608A>G, 3610C>T, 3614C>A, 3621T>C, 3622T>C, 3624A>T, 3625T>C, 3626G>C, 3628A>C, 3634C>T, 3637A>T, 3638C>T, 3646G>A, 3647A>G, 3648C>A, 3650A>G, 3652G>T, 3653G>C, 3657G>A, 3658C>G, 3659A>C, 3660G>A, 3661G>T, 3664C>T, 3665G>T, 3666G>C, 3667A>T, 3668A>C, 3670C>T, 3673C>T, 3674G>A, 3676G>T, 3677A>C, 3679G>T, 3680G>C, 3683C>T, 3684A>T, 3689T>A, 3690C>T, 3691A>T, 3692G>A, 3694C>T, 3698T>G, 3699C>A, 3703G>A, 3706C>T, 3710G>A, 3712T>C, 3716T>C, 3718G>C, 3724C>T, 3731C>G, 3732A>T, 3733C>T, 3734A>G, 3743C>G								
**CDS**									
HfIV_sC16gp1	221	279	15.9%	268	58.6%	59 (100%)	29 (49.2%)	0/0/0/0	0
Protein mutations:	G221E (3570G>A), N222D (3572A>G), M223R (3576T>G 3577G>A), N225D (3581A>G), E228V (3591A>T 3592A>T), T233R (3605A>C 3606C>G 3607G>T), I234V (3608A>G 3610C>T), Q236K (3614C>A), I238T (3621T>C 3622T>C), Y239F (3624A>T 3625T>C), E240H (3626G>C 3628A>C), P244S (3638C>T), T247D (3647A>G 3648C>A), M248V (3650A>G 3652G>T), E249Q (3653G>C), S250K (3657G>A 3658C>G), R251H (3659A>C 3660G>A 3661G>T), G253S (3665G>T 3666G>C 3667A>T), I254L (3668A>C 3670C>T), V256I (3674G>A 3676G>T), M257L (3677A>C 3679G>T), A258P (3680G>C), Q259L (3683C>T 3684A>T), S261I (3689T>A 3690C>T 3691A>T), V262I (3692G>A 3694C>T), S264E (3698T>G 3699C>A), V268I (3710G>A 3712T>C), H275V (3731C>G 3732A>T 3733C>T), I276V (3734A>G), L279V (3743C>G)								
Codon mutations:	GGA220.CA (3567G>C), GGG221GAG (3570G>A), AAT222GAT (3572A>G), ATG223AGA (3576T>G 3577G>A), AAT225GAT (3581A>G), CCT226CCA (3586T>A), GAA228GTT (3591A>T 3592A>T), AAA229AAG (3595A>G), GTG230GTT (3598G>T), TTC231TTT (3601C>T), CCA232CCT (3604A>T), ACG233CGT (3605A>C 3606C>G 3607G>T), ATC234GTT (3608A>G 3610C>T), CAG236AAG (3614C>A), ATT238ACC (3621T>C 3622T>C), TAT239TTC (3624A>T 3625T>C), GAA240CAC (3626G>C 3628A>C), TAC242TAT (3634C>T), GGA243GGT (3637A>T), CCT244TCT (3638C>T), GGG246GGA (3646G>A), ACT247GAT (3647A>G 3648C>A), ATG248GTT (3650A>G 3652G>T), GAG249CAG (3653G>C), AGC250AAG (3657G>A 3658C>G), AGG251CAT (3659A>C 3660G>A 3661G>T), GAC252GAT (3664C>T), GGA253TCT (3665G>T 3666G>C 3667A>T), ATC254CTT (3668A>C 3670C>T), TGC255TGT (3673C>T), GTG256ATT (3674G>A 3676G>T), ATG257CTT (3677A>C 3679G>T), GCA258CCA (3680G>C), CAG259TTG (3683C>T 3684A>T), TCA261ATT (3689T>A 3690C>T 3691A>T), GTC262ATT (3692G>A 3694C>T), TCG264GAG (3698T>G 3699C>A), AAG265AAA (3703G>A), ATC266ATT (3706C>T), GTT268ATC (3710G>A 3712T>C), TTG270CTC (3716T>C 3718G>C), TTC272TTT (3724C>T), CAC275GTT (3731C>G 3732A>T 3733C>T), ATT276GTT (3734A>G), CTT279GTT (3743C>G)								
**Proteins**									
viral inexin-like protein (YP_001031223.1)	221	279	15.9%	268	58.6%	59 (100%)	29 (49.2%)	0/0/0/0	0
Protein mutations:	G221E (3570G>A), N222D (3572A>G), M223R (3576T>G 3577G>A), N225D (3581A>G), E228V (3591A>T 3592A>T), T233R (3605A>C 3606C>G 3607G>T), I234V (3608A>G 3610C>T), Q236K (3614C>A), I238T (3621T>C 3622T>C), Y239F (3624A>T 3625T>C), E240H (3626G>C 3628A>C), P244S (3638C>T), T247D (3647A>G 3648C>A), M248V (3650A>G 3652G>T), E249Q (3653G>C), S250K (3657G>A 3658C>G), R251H (3659A>C 3660G>A 3661G>T), G253S (3665G>T 3666G>C 3667A>T), I254L (3668A>C 3670C>T), V256I (3674G>A 3676G>T), M257L (3677A>C 3679G>T), A258P (3680G>C), Q259L (3683C>T 3684A>T), S261I (3689T>A 3690C>T 3691A>T), V262I (3692G>A 3694C>T), S264E (3698T>G 3699C>A), V268I (3710G>A 3712T>C), H275V (3731C>G 3732A>T 3733C>T), I276V (3734A>G), L279V (3743C>G)								
Codon mutations:	GGA220.CA (3567G>C), GGG221GAG (3570G>A), AAT222GAT (3572A>G), ATG223AGA (3576T>G 3577G>A), AAT225GAT (3581A>G), CCT226CCA (3586T>A), GAA228GTT (3591A>T 3592A>T), AAA229AAG (3595A>G), GTG230GTT (3598G>T), TTC231TTT (3601C>T), CCA232CCT (3604A>T), ACG233CGT (3605A>C 3606C>G 3607G>T), ATC234GTT (3608A>G 3610C>T), CAG236AAG (3614C>A), ATT238ACC (3621T>C 3622T>C), TAT239TTC (3624A>T 3625T>C), GAA240CAC (3626G>C 3628A>C), TAC242TAT (3634C>T), GGA243GGT (3637A>T), CCT244TCT (3638C>T), GGG246GGA (3646G>A), ACT247GAT (3647A>G 3648C>A), ATG248GTT (3650A>G 3652G>T), GAG249CAG (3653G>C), AGC250AAG (3657G>A 3658C>G), AGG251CAT (3659A>C 3660G>A 3661G>T), GAC252GAT (3664C>T), GGA253TCT (3665G>T 3666G>C 3667A>T), ATC254CTT (3668A>C 3670C>T), TGC255TGT (3673C>T), GTG256ATT (3674G>A 3676G>T), ATG257CTT (3677A>C 3679G>T), GCA258CCA (3680G>C), CAG259TTG (3683C>T 3684A>T), TCA261ATT (3689T>A 3690C>T 3691A>T), GTC262ATT (3692G>A 3694C>T), TCG264GAG (3698T>G 3699C>A), AAG265AAA (3703G>A), ATC266ATT (3706C>T), GTT268ATC (3710G>A 3712T>C), TTG270CTC (3716T>C 3718G>C), TTC272TTT (3724C>T), CAC275GTT (3731C>G 3732A>T 3733C>T), ATT276GTT (3734A>G), CTT279GTT (3743C>G)								

*: Inserts / Deletes / Misaligned / Frameshifts

Analysis details

This analysis was performed with panviral2.64

NGS Details (UN9): Ichnoviriform fugitivi (segment B17)

Assembly

**Coverage Length**	159 (1 contig(s))
**Depth Of Coverage**	5.2
**Number Of Reads**	10
**Reads Per Million**	0.23 rpm (after QC)
**Ambiguities**	0
**Assembly Method**	de novo + reference guided assembly
**Consensus Caller**	Bcf Tools

Coverage Map

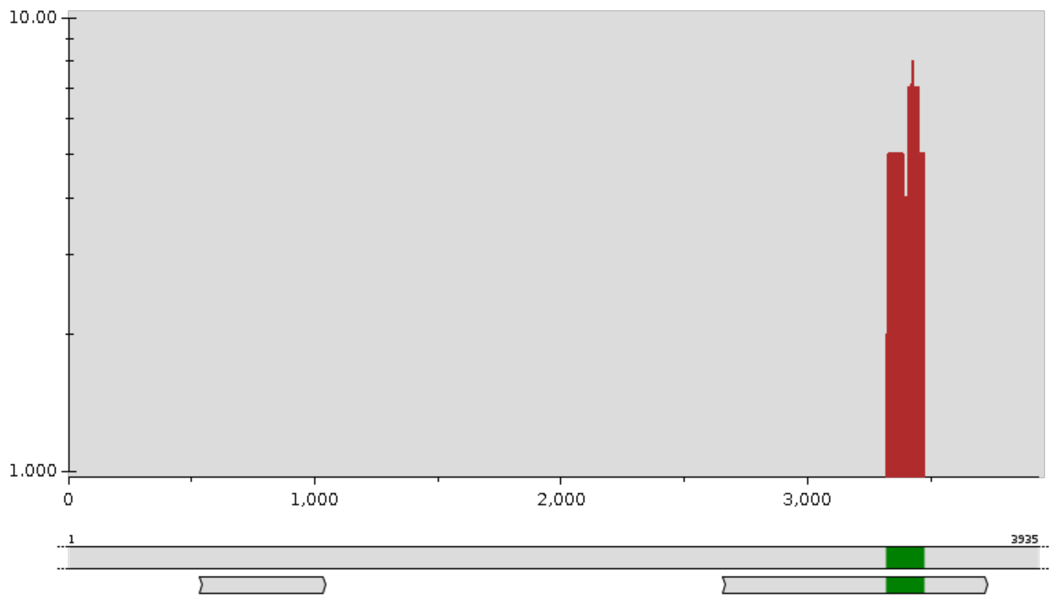

Assignment

**Type**	Ichnoviriform fugitivi (Taxonomy ID: 265522)
**Reference Genome**	NC_008953.1
**NT Identity (%)**	61.0063
**AA Identity (%)**	50.9434
**Number Of Stop Codons**	0
**Number Of CDS**	2

Alignment

**Alignment Score**	70.0 (NT) + 258.0 (AA) = 328.0
**Concordance (%)**	45.7462

Alignment Method	Global, seeded, nucleotide + amino acids (AGA)

Genome Region

Sequence starts at position 3318 and ends at position 3476 relative to NC_008953.1 reference sequence.

Alignment Detailed Statistics

	Begin	End	Coverage	Score	Concordance	Matches	Identities	I/D/M/F*	Stop Codons
**NT**	**3318**	**3476**	**4.0%**	**70**	**22.0%**	**159 (100%)**	**97 (61.0%)**	**0/0**	
Mutations:	3326T>A, 3329C>T, 3330G>C, 3336C>A, 3337A>T, 3339G>A, 3341A>C, 3342G>T, 3348C>G, 3354A>T, 3356C>A, 3360C>G, 3363T>C, 3364C>A, 3365G>A, 3366A>G, 3371C>A, 3372T>C, 3376C>A, 3378C>T, 3379A>C, 3381A>T, 3384C>T, 3388C>T, 3390T>C, 3393G>T, 3396C>T, 3397T>G, 3398C>A, 3399C>T, 3400C>G, 3402G>T, 3403G>C, 3407A>T, 3408C>T, 3411C>T, 3414G>T, 3415G>A, 3416G>A, 3417A>C, 3418C>T, 3419T>A, 3423C>T, 3424A>T, 3426C>A, 3429C>G, 3432G>A, 3433G>T, 3434A>T, 3439G>A, 3441G>T, 3442G>A, 3444C>T, 3447C>T, 3450G>A, 3456C>T, 3463T>G, 3464T>C, 3465C>T, 3466C>T, 3472T>C, 3474C>T								
**CDS**									
HfIV_sB17gp2	222	274	14.8%	258	59.3%	53 (100%)	27 (50.9%)	0/0/0/0	0
Protein mutations:	M224K (3326T>A), T225I (3329C>T 3330G>C), M228L (3337A>T 3339G>A), K229T (3341A>C 3342G>T), T234K (3356C>A), I235M (3360C>G), R237K (3364C>A 3365G>A 3366A>G), T239N (3371C>A 3372T>C), H241N (3376C>A 3378C>T), K242H (3379A>C 3381A>T), P245S (3388C>T 3390T>C), S248D (3397T>G 3398C>A 3399C>T), L249V (3400C>G 3402G>T), E250Q (3403G>C), N251I (3407A>T 3408C>T), E253D (3414G>T), G254N (3415G>A 3416G>A 3417A>C), L255Y (3418C>T 3419T>A), I257L (3424A>T 3426C>A), E260L (3433G>T 3434A>T), V262I (3439G>A 3441G>T), V263I (3442G>A 3444C>T), F270A (3463T>G 3464T>C 3465C>T), L271F (3466C>T), F273L (3472T>C 3474C>T)								
Codon mutations:	ATG224AAG (3326T>A), ACG225ATC (3329C>T 3330G>C), CCC227CCA (3336C>A), ATG228TTA (3337A>T 3339G>A), AAG229ACT (3341A>C 3342G>T), GTC231GTG (3348C>G), CCA233CCT (3354A>T), ACA234AAA (3356C>A), ATC235ATG (3360C>G), ACT236ACC (3363T>C), CGA237AAG (3364C>A 3365G>A 3366A>G), ACT239AAC (3371C>A 3372T>C), CAC241AAT (3376C>A 3378C>T), AAA242CAT (3379A>C 3381A>T), TAC243TAT (3384C>T), CCT245TCC (3388C>T 3390T>C), TCG246TCT (3393G>T), GGC247GGT (3396C>T), TCC248GAT (3397T>G 3398C>A 3399C>T), CTG249GTT (3400C>G 3402G>T), GAA250CAA (3403G>C), AAC251ATT (3407A>T 3408C>T), TAC252TAT (3411C>T), GAG253GAT (3414G>T), GGA254AAC (3415G>A 3416G>A 3417A>C), CTC255TAC (3418C>T 3419T>A), TGC256TGT (3423C>T), ATC257TTA (3424A>T 3426C>A), CTC258CTG (3429C>G), CCG259CCA (3432G>A), GAG260TTG (3433G>T 3434A>T), GTG262ATT (3439G>A 3441G>T), GTC263ATT (3442G>A 3444C>T), AAC264AAT (3447C>T), GAG265GAA (3450G>A), ATC267ATT (3456C>T), TTC270GCT (3463T>G 3464T>C 3465C>T), CTC271TTC (3466C>T), TTC273CTT (3472T>C 3474C>T)								
**Proteins**									
viral inexin-like protein (YP_001031233.1)	222	274	14.8%	258	59.3%	53 (100%)	27 (50.9%)	0/0/0/0	0
Protein mutations:	M224K (3326T>A), T225I (3329C>T 3330G>C), M228L (3337A>T 3339G>A), K229T (3341A>C 3342G>T), T234K (3356C>A), I235M (3360C>G), R237K (3364C>A 3365G>A 3366A>G), T239N (3371C>A 3372T>C), H241N (3376C>A 3378C>T), K242H (3379A>C 3381A>T), P245S (3388C>T 3390T>C), S248D (3397T>G 3398C>A 3399C>T), L249V (3400C>G 3402G>T), E250Q (3403G>C), N251I (3407A>T 3408C>T), E253D (3414G>T), G254N (3415G>A 3416G>A 3417A>C), L255Y (3418C>T 3419T>A), I257L (3424A>T 3426C>A), E260L (3433G>T 3434A>T), V262I (3439G>A 3441G>T), V263I (3442G>A 3444C>T), F270A (3463T>G 3464T>C 3465C>T), L271F (3466C>T), F273L (3472T>C 3474C>T)								
Codon mutations:	ATG224AAG (3326T>A), ACG225ATC (3329C>T 3330G>C), CCC227CCA (3336C>A), ATG228TTA (3337A>T 3339G>A), AAG229ACT (3341A>C 3342G>T), GTC231GTG (3348C>G), CCA233CCT (3354A>T), ACA234AAA (3356C>A), ATC235ATG (3360C>G), ACT236ACC (3363T>C), CGA237AAG (3364C>A 3365G>A 3366A>G), ACT239AAC (3371C>A 3372T>C), CAC241AAT (3376C>A 3378C>T), AAA242CAT (3379A>C 3381A>T), TAC243TAT (3384C>T), CCT245TCC (3388C>T 3390T>C), TCG246TCT (3393G>T), GGC247GGT (3396C>T), TCC248GAT (3397T>G 3398C>A 3399C>T), CTG249GTT (3400C>G 3402G>T), GAA250CAA (3403G>C), AAC251ATT (3407A>T 3408C>T), TAC252TAT (3411C>T), GAG253GAT (3414G>T), GGA254AAC (3415G>A 3416G>A 3417A>C), CTC255TAC (3418C>T 3419T>A), TGC256TGT (3423C>T), ATC257TTA (3424A>T 3426C>A), CTC258CTG (3429C>G), CCG259CCA (3432G>A), GAG260TTG (3433G>T 3434A>T), GTG262ATT (3439G>A 3441G>T), GTC263ATT (3442G>A 3444C>T), AAC264AAT (3447C>T), GAG265GAA (3450G>A), ATC267ATT (3456C>T), TTC270GCT (3463T>G 3464T>C 3465C>T), CTC271TTC (3466C>T), TTC273CTT (3472T>C 3474C>T)								

*: Inserts / Deletes / Misaligned / Frameshifts

Analysis details

This analysis was performed with panviral2.64

NGS Details (UN9): Badnavirus maculasmallanthi

Assembly

**Coverage Length**	293 (1 contig(s))
**Depth Of Coverage**	4.2
**Number Of Reads**	10
**Reads Per Million**	0.23 rpm (after QC)
**Ambiguities**	0
**Assembly Method**	de novo + reference guided assembly
**Consensus Caller**	Bcf Tools

Coverage Map

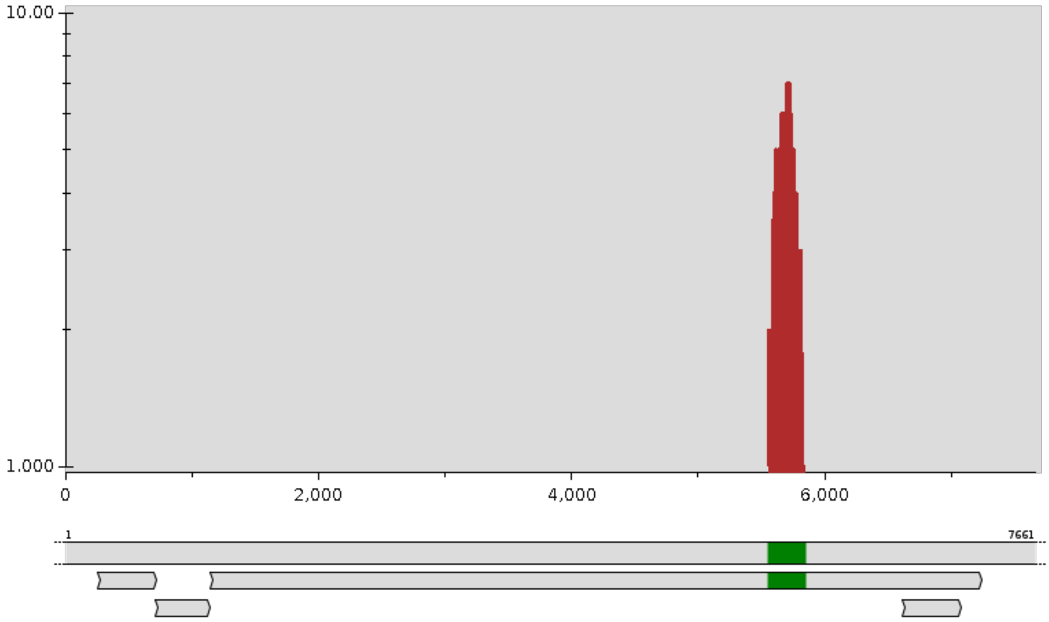

Assignment

**Type**	Badnavirus maculasmallanthi (Taxonomy ID: 3048453)
**Reference Genome**	NC_026472.1
**NT Identity (%)**	52.9801
**AA Identity (%)**	48.5149
**Number Of Stop Codons**	0
**Number Of CDS**	4

Alignment

**Alignment Score**	27.0 (NT) + 343.0 (AA) = 370.0
**Concordance (%)**	28.8387

Alignment Method	Global, seeded, nucleotide + amino acids (AGA)

Genome Region

Sequence starts at position 5557 and ends at position 5849 relative to NC_026472.1 reference sequence.

Alignment Detailed Statistics

	Begin	End	Coverage	Score	Concordance	Matches	Identities	I/D/M/F*	Stop Codons
**NT**	**5557**	**5849**	**3.8%**	**27**	**4.6%**	**293 (97.0%)**	**160 (53.0%)**	**9/0**	
Mutations:	5558A>G, 5562A>G, 5565G>A, 5566A>T, 5567T>A, 5568A>C, 5571C>T, 5574A>T, 5578T>C, 5580T>G, 5583T>C, 5586G>T, 5588A>G, 5592T>G, 5595A>G, 5597T>A, 5605G>A, 5608G>A, 5609C>G, 5610C>A, 5614G>C, 5616A>T, 5620G>C, 5621A>C, 5622A>C, 5623T>G, 5624C>A, 5628A>T, 5632T>A, 5633G>A, 5634G>A, 5637G>T, 5644T>A, 5645T>G, 5648T>C, 5649C>T, 5651C>A, 5652A>C, 5653G>C, 5654G>A, 5655A>T, 5658A>T, 5659T>C, 5660T>A, 5661A>T, 5664C>T, 5669G>T, 5670G>C, 5671C>T, 5673T>G, 5682A>T, 5685C>T, 5688A>C, 5693A>C, 5697T>C, 5700A>T, 5701C>T, 5703C>A, 5704G>T, 5706T>G, 5708T>C, 5709T>C, 5712T>C, 5715G>A, 5718A>C, 5719A>C, 5720A>T, 5721A>C, 5725G>A, 5727T>C, 5730A>G, 5731T>A, 5732G>T, 5736C>T, 5737A>C, 5739A>C, 5739_5740insTCT, 5740G>T, 5743A>C, 5744C>T, 5745A>C, 5746G>A, 5747A>G, 5749G>A, 5754T>C, 5757C>T, 5758G>T, 5759C>T, 5760C>G, 5765A>T, 5766C>T, 5767A>T, 5772C>T, 5775C>T, 5781G>C, 5782G>A, 5784A>C, 5787C>T, 5788T>A, 5789C>G, 5790C>T, 5791A>T, 5792A>C, 5793C>T, 5794A>G, 5795G>A, 5797G>T, 5798A>G, 5799A>G, 5802G>A, 5804A>G, 5805T>G, 5809G>T, 5810A>T, 5812A>T, 5813G>C, 5814G>T, 5821A>G, 5823G>A, 5823_5824insGAGGCT, 5824A>T, 5826T>C, 5828T>A, 5830C>G, 5832G>A, 5833G>C, 5834G>T, 5835T>G, 5837A>G, 5844A>C, 5845G>A, 5846A>C, 5847A>T, 5848A>T, 5849A>T								

CDS

	Begin	End	Coverage	Score	Concordance	Matches	Identities	I/D/M/F*	Stop Codons
UF61_gp3	1472	1569	4.8%	343	48.7%	98 (97.0%)	49 (48.5%)	3/0/0/0	0
Protein mutations:	N1472S (5558A>G), I1475Y (5566A>T 5567T>A 5568A>C), F1479L (5578T>C 5580T>G), K1482R (5588A>G), F1485Y (5597T>A), V1488I (5605G>A), A1489R (5608G>A 5609C>G 5610C>A), E1491H (5614G>C 5616A>T), E1493P (5620G>C 5621A>C 5622A>C), S1494D (5623T>G 5624C>A), W1497K (5632T>A 5633G>A 5634G>A), L1501R (5644T>A 5645T>G), I1502T (5648T>C 5649C>T), P1503H (5651C>A 5652A>C), G1504H (5653G>C 5654G>A 5655A>T), L1506H (5659T>C 5660T>A 5661A>T), W1509F (5669G>T 5670G>C), K1517T (5693A>C), P1520S (5701C>T 5703C>A), A1521S (5704G>T 5706T>G), I1522T (5708T>C 5709T>C), R1525S (5718A>C), K1526L (5719A>C 5720A>T 5721A>C), D1528N (5725G>A 5727T>C), C1530I (5731T>A 5732G>T), K1532H (5737A>C 5739A>C), K1532_D1533insS (5739_5740insTCT), D1533Y (5740G>T), T1534L (5743A>C 5744C>T 5745A>C), E1535R (5746G>A 5747A>G), E1536K (5749G>A), A1539L (5758G>T 5759C>T 5760C>G), Y1541F (5765A>T 5766C>T), I1542F (5767A>T), V1547I (5782G>A 5784A>C), N1550S (5791A>T 5792A>C 5793C>T), S1551D (5794A>G 5795G>A), E1552W (5797G>T 5798A>G 5799A>G), D1554G (5804A>G 5805T>G), E1556L (5809G>T 5810A>T), R1557S (5812A>T 5813G>C 5814G>T), K1560E (5821A>G 5823G>A), K1560_I1561insEA (5823_5824insGAGGCT), I1561F (5824A>T 5826T>C), M1562K (5828T>A), L1563V (5830C>G 5832G>A), G1564L (5833G>C 5834G>T 5835T>G), K1565R (5837A>G), Q1567H (5844A>C), E1568T (5845G>A 5846A>C 5847A>T)								
Codon mutations:	AAT1472AGT (5558A>G), GCA1473GCG (5562A>G), AAG1474AAA (5565G>A), ATA1475TAC (5566A>T 5567T>A 5568A>C), TTC1476TTT (5571C>T), TCA1477TCT (5574A>T), TTT1479CTG (5578T>C 5580T>G), GAT1480GAC (5583T>C), CTG1481CTT (5586G>T), AAG1482AGG (5588A>G), TCT1483TCG (5592T>G), GGA1484GGG (5595A>G), TTT1485TAT (5597T>A), GTT1488ATT (5605G>A), GCC1489AGA (5608G>A 5609C>G 5610C>A), GAA1491CAT (5614G>C 5616A>T), GAA1493CCC (5620G>C 5621A>C 5622A>C), TCC1494GAC (5623T>G 5624C>A), ATA1495ATT (5628A>T), TGG1497AAA (5632T>A 5633G>A 5634G>A), ACG1498ACT (5637G>T), TTA1501AGA (5644T>A 5645T>G), ATC1502ACT (5648T>C 5649C>T), CCA1503CAC (5651C>A 5652A>C), GGA1504CAT (5653G>C 5654G>A 5655A>T), GGA1505GGT (5658A>T), TTA1506CAT (5659T>C 5660T>A 5661A>T), TAC1507TAT (5664C>T), TGG1509TTC (5669G>T 5670G>C), CTT1510TTG (5671C>T 5673T>G), CCA1513CCT (5682A>T), TTC1514TTT (5685C>T), GGA1515GGC (5688A>C), AAA1517ACA (5693A>C), AAT1518AAC (5697T>C), GCA1519GCT (5700A>T), CCC1520TCA (5701C>T 5703C>A), GCT1521TCG (5704G>T 5706T>G), ATT1522ACC (5708T>C 5709T>C), TTT1523TTC (5712T>C), CAG1524CAA (5715G>A), AGA1525AGC (5718A>C), AAA1526CTC (5719A>C 5720A>T 5721A>C), GAT1528AAC (5725G>A 5727T>C), AAA1529AAG (5730A>G), TGC1530ATC (5731T>A 5732G>T), TTC1531TTT (5736C>T), AAA1532CAC (5737A>C 5739A>C), AAA1532_GAC1533insTCT (5739_5740insTCT), GAC1533TAC (5740G>T), ACA1534CTC (5743A>C 5744C>T 5745A>C), GAA1535AGA (5746G>A 5747A>G), GAG1536AAG (5749G>A), TTT1537TTC (5754T>C), ATC1538ATT (5757C>T), GCC1539TTG (5758G>T 5759C>T 5760C>G), TAC1541TTT (5765A>T 5766C>T), ATC1542TTC (5767A>T), GAC1543GAT (5772C>T), GAC1544GAT (5775C>T), CTG1546CTC (5781G>C), GTA1547ATC (5782G>A 5784A>C), TAC1548TAT (5787C>T), TCC1549AGT (5788T>A 5789C>G 5790C>T), AAC1550TCT (5791A>T 5792A>C 5793C>T), AGT1551GAT (5794A>G 5795G>A), GAA1552TGG (5797G>T 5798A>G 5799A>G), GCG1553GCA (5802G>A), GAT1554GGG (5804A>G 5805T>G), GAA1556TTA (5809G>T 5810A>T), AGG1557TCT (5812A>T 5813G>C 5814G>T), AAG1560GAA (5821A>G 5823G>A), AAG1560_ATT1561insGAGGCT (5823_5824insGAGGCT), ATT1561TTC (5824A>T 5826T>C), ATG1562AAG (5828T>A), CTG1563GTA (5830C>G 5832G>A), GGT1564CTG (5833G>C 5834G>T 5835T>G), AAA1565AGA (5837A>G), CAA1567CAC (5844A>C), GAA1568ACT (5845G>A 5846A>C 5847A>T), AAT1569TT. (5848A>T 5849A>T)								

Proteins

	Begin	End	Coverage	Score	Concordance	Matches	Identities	I/D/M/F*	Stop Codons
ORF3 (YP_009121747.1)	1472	1569	4.8%	343	48.7%	98 (97.0%)	49 (48.5%)	3/0/0/0	0
Protein mutations:	N1472S (5558A>G), I1475Y (5566A>T 5567T>A 5568A>C), F1479L (5578T>C 5580T>G), K1482R (5588A>G), F1485Y (5597T>A), V1488I (5605G>A), A1489R (5608G>A 5609C>G 5610C>A), E1491H (5614G>C 5616A>T), E1493P (5620G>C 5621A>C 5622A>C), S1494D (5623T>G 5624C>A), W1497K (5632T>A 5633G>A 5634G>A), L1501R (5644T>A 5645T>G), I1502T (5648T>C 5649C>T), P1503H (5651C>A 5652A>C), G1504H (5653G>C 5654G>A 5655A>T), L1506H (5659T>C 5660T>A 5661A>T), W1509F (5669G>T 5670G>C), K1517T (5693A>C), P1520S (5701C>T 5703C>A), A1521S (5704G>T 5706T>G), I1522T (5708T>C 5709T>C), R1525S (5718A>C), K1526L (5719A>C 5720A>T 5721A>C), D1528N (5725G>A 5727T>C), C1530I (5731T>A 5732G>T), K1532H (5737A>C 5739A>C), K1532_D1533insS (5739_5740insTCT), D1533Y (5740G>T), T1534L (5743A>C 5744C>T 5745A>C), E1535R (5746G>A 5747A>G), E1536K (5749G>A), A1539L (5758G>T 5759C>T 5760C>G), Y1541F (5765A>T 5766C>T), I1542F (5767A>T), V1547I (5782G>A 5784A>C), N1550S (5791A>T 5792A>C 5793C>T), S1551D (5794A>G 5795G>A), E1552W (5797G>T 5798A>G 5799A>G), D1554G (5804A>G 5805T>G), E1556L (5809G>T 5810A>T), R1557S (5812A>T 5813G>C 5814G>T), K1560E (5821A>G 5823G>A), K1560_I1561insEA (5823_5824insGAGGCT), I1561F (5824A>T 5826T>C), M1562K (5828T>A), L1563V (5830C>G 5832G>A), G1564L (5833G>C 5834G>T 5835T>G), K1565R (5837A>G), Q1567H (5844A>C), E1568T (5845G>A 5846A>C 5847A>T)								
Codon mutations:	AAT1472AGT (5558A>G), GCA1473GCG (5562A>G), AAG1474AAA (5565G>A), ATA1475TAC (5566A>T 5567T>A 5568A>C), TTC1476TTT (5571C>T), TCA1477TCT (5574A>T), TTT1479CTG (5578T>C 5580T>G), GAT1480GAC (5583T>C), CTG1481CTT (5586G>T), AAG1482AGG (5588A>G), TCT1483TCG (5592T>G), GGA1484GGG (5595A>G), TTT1485TAT (5597T>A), GTT1488ATT (5605G>A), GCC1489AGA (5608G>A 5609C>G 5610C>A), GAA1491CAT (5614G>C 5616A>T), GAA1493CCC (5620G>C 5621A>C 5622A>C), TCC1494GAC (5623T>G 5624C>A), ATA1495ATT (5628A>T), TGG1497AAA (5632T>A 5633G>A 5634G>A), ACG1498ACT (5637G>T), TTA1501AGA (5644T>A 5645T>G), ATC1502ACT (5648T>C 5649C>T), CCA1503CAC (5651C>A 5652A>C), GGA1504CAT (5653G>C 5654G>A 5655A>T), GGA1505GGT (5658A>T), TTA1506CAT (5659T>C 5660T>A 5661A>T), TAC1507TAT (5664C>T), TGG1509TTC (5669G>T 5670G>C), CTT1510TTG (5671C>T 5673T>G), CCA1513CCT (5682A>T), TTC1514TTT (5685C>T), GGA1515GGC (5688A>C), AAA1517ACA (5693A>C), AAT1518AAC (5697T>C), GCA1519GCT (5700A>T), CCC1520TCA (5701C>T 5703C>A), GCT1521TCG (5704G>T 5706T>G), ATT1522ACC (5708T>C 5709T>C), TTT1523TTC (5712T>C), CAG1524CAA (5715G>A), AGA1525AGC (5718A>C), AAA1526CTC (5719A>C 5720A>T 5721A>C), GAT1528AAC (5725G>A 5727T>C), AAA1529AAG (5730A>G), TGC1530ATC (5731T>A 5732G>T), TTC1531TTT (5736C>T), AAA1532CAC (5737A>C 5739A>C), AAA1532_GAC1533insTCT (5739_5740insTCT), GAC1533TAC (5740G>T), ACA1534CTC (5743A>C 5744C>T 5745A>C), GAA1535AGA (5746G>A 5747A>G), GAG1536AAG (5749G>A), TTT1537TTC (5754T>C), ATC1538ATT (5757C>T), GCC1539TTG (5758G>T 5759C>T 5760C>G), TAC1541TTT (5765A>T 5766C>T), ATC1542TTC (5767A>T), GAC1543GAT (5772C>T), GAC1544GAT (5775C>T), CTG1546CTC (5781G>C), GTA1547ATC (5782G>A 5784A>C), TAC1548TAT (5787C>T), TCC1549AGT (5788T>A 5789C>G 5790C>T), AAC1550TCT (5791A>T 5792A>C 5793C>T), AGT1551GAT (5794A>G 5795G>A), GAA1552TGG (5797G>T 5798A>G 5799A>G), GCG1553GCA (5802G>A), GAT1554GGG (5804A>G 5805T>G), GAA1556TTA (5809G>T 5810A>T), AGG1557TCT (5812A>T 5813G>C 5814G>T), AAG1560GAA (5821A>G 5823G>A), AAG1560_ATT1561insGAGGCT (5823_5824insGAGGCT), ATT1561TTC (5824A>T 5826T>C), ATG1562AAG (5828T>A), CTG1563GTA (5830C>G 5832G>A), GGT1564CTG (5833G>C 5834G>T 5835T>G), AAA1565AGA (5837A>G), CAA1567CAC (5844A>C), GAA1568ACT (5845G>A 5846A>C 5847A>T), AAT1569TT. (5848A>T 5849A>T)								

*: Inserts / Deletes / Misaligned / Frameshifts

Analysis details

This analysis was performed with panviral2.64

NGS Details (UN9): Pinus nigra virus 1

Assembly

**Coverage Length**	239 (1 contig(s))
**Depth Of Coverage**	6.0
**Number Of Reads**	10
**Reads Per Million**	0.23 rpm (after QC)
**Ambiguities**	0
**Assembly Method**	de novo + reference guided assembly
**Consensus Caller**	Bcf Tools

Coverage Map

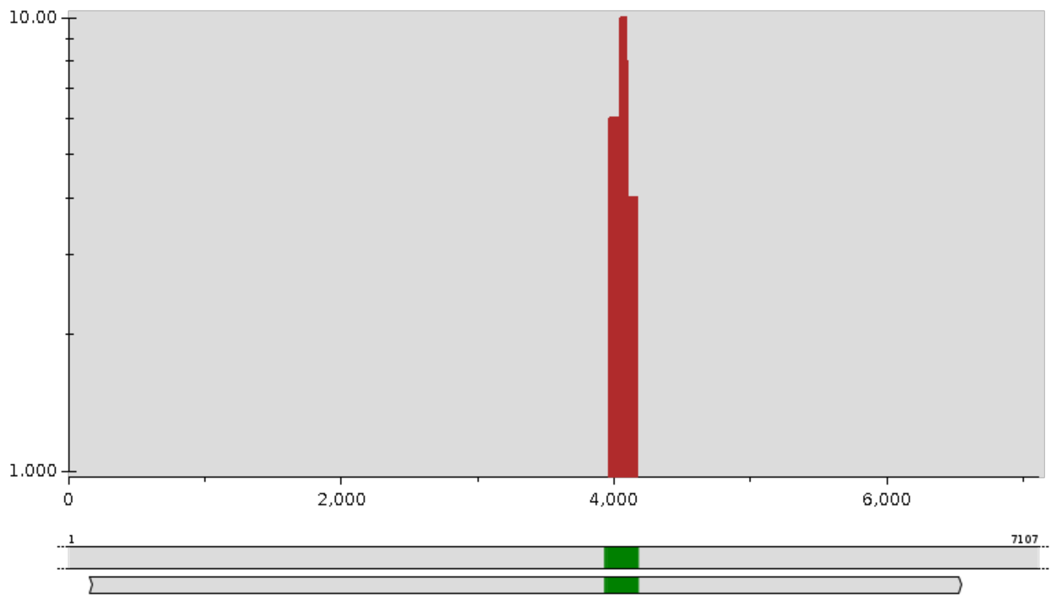

Assignment

**Type**	Pinus nigra virus 1 (Taxonomy ID: 2267679)
**Reference Genome**	NC_040841.1
**NT Identity (%)**	57.7406
**AA Identity (%)**	47.4359
**Number Of Stop Codons**	0
**Number Of CDS**	1

Alignment

**Alignment Score**	74.0 (NT) + 260.0 (AA) = 334.0
**Concordance (%)**	31.8702

Alignment Method	Global, seeded, nucleotide + amino acids (AGA)

Genome Region

Sequence starts at position 3935 and ends at position 4180 relative to NC_040841.1 reference sequence.

Alignment Detailed Statistics

	Begin	End	Coverage	Score	Concordance	Matches	Identities	I/D/M/F*	Stop Codons
**NT**	**3935**	**4180**	**3.4%**	**74**	**15.5%**	**239 (100%)**	**138 (57.7%)**	**0/0**	
Mutations:	3935C>T, 3937T>A, 3941G>T, 3943C>T, 3944A>G, 3946C>T, 3947A>T, 3948C>T, 3949T>A, 3950C>A, 3952G>A, 3953A>T, 3954A>G, 3955T>G, 3956T>A, 3960A>G, 3961T>A, 3967A>G, 3968T>A, 3970G>T, 3973A>T, 3975G>A, 3976A>T, 3977C>A, 3978C>A, 3980G>A, 3981A>T, 3982A>G, 3984T>A, 3985G>T, 3986A>C, 3989A>T, 3992C>A, 3996A>G, 3999T>C, 4001C>T, 4002A>T, 4003G>T, 4004C>A, 4006T>A, 4008G>C, 4009T>C, 4012G>T, 4013G>A, 4015G>T, 4017T>A, 4021C>T, 4024G>A, 4030C>T, 4036G>A, 4042G>T, 4044A>T, 4045T>C, 4047A>G, 4048T>G, 4051G>A, 4054A>T, 4061C>T, 4062A>C, 4064C>A, 4065C>A, 4067G>A, 4074G>A, 4076C>T, 4078C>T, 4084A>T, 4092T>A, 4093A>C, 4094T>G, 4095G>T, 4096T>C, 4099A>C, 4100G>T, 4101C>T, 4102A>C, 4106T>C, 4107T>A, 4108C>A, 4112C>G, 4114G>A, 4120A>T, 4124G>A, 4126T>G, 4129A>C, 4135T>A, 4138G>A, 4141G>A, 4147G>C, 4150A>T, 4151G>T, 4153C>T, 4154T>G, 4155T>A, 4156C>A, 4164G>A, 4165A>C, 4166A>G, 4167G>T, 4168G>T, 4172G>A, 4176A>C								

CDS

	Begin	End	Coverage	Score	Concordance	Matches	Identities	I/D/M/F*	Stop Codons
EXL67_gp1	1265	1342	3.7%	260	42.5%	78 (100%)	37 (47.4%)	0/0/1/0	0
Protein mutations:	T1265L (3947A>T 3948C>T 3949T>A), N1267W (3953A>T 3954A>G 3955T>G), F1268I (3956T>A), N1269R (3960A>G 3961T>A), L1272I (3968T>A 3970G>T), R1274N (3975G>A 3976A>T), P1275K (3977C>A 3978C>A), E1276M (3980G>A 3981A>T 3982A>G), V1277D (3984T>A 3985G>T), I1278L (3986A>C), M1279L (3989A>T), Q1280K (3992C>A), K1281R (3996A>G), I1282T (3999T>C), Q1283F (4001C>T 4002A>T 4003G>T), H1284K (4004C>A 4006T>A), S1285T (4008G>C 4009T>C), K1286N (4012G>T), V1287I (4013G>A 4015G>T), F1288Y (4017T>A), Y1297F (4044A>T 4045T>C), Y1298W (4047A>G 4048T>G), Q1303S (4061C>T 4062A>C), P1304K (4064C>A 4065C>A), E1305K (4067G>A), R1307K (4074G>A), H1308Y (4076C>T 4078C>T), I1313N (4092T>A 4093A>C), C1314V (4094T>G 4095G>T 4096T>C), A1316F (4100G>T 4101C>T 4102A>C), F1318Q (4106T>C 4107T>A 4108C>A), Q1320E (4112C>G 4114G>A), K1322N (4120A>T), V1324M (4124G>A 4126T>G), A1333S (4151G>T 4153C>T), F1334E (4154T>G 4155T>A 4156C>A), R1337N (4164G>A 4165A>C), R1338V (4166A>G 4167G>T 4168G>T), D1340N (4172G>A), Y1341S (4176A>C)								
Codon mutations:	CTT1261T.A (3935C>T 3937T>A), GCC1263T.T (3941G>T 3943C>T), ATC1264G.T (3944A>G 3946C>T), ACT1265TTA (3947A>T 3948C>T 3949T>A), CAG1266A.A (3950C>A 3952G>A), AAT1267TGG (3953A>T 3954A>G 3955T>G), TTT1268ATT (3956T>A), AAT1269AGA (3960A>G 3961T>A), CCA1271CCG (3967A>G), TTG1272ATT (3968T>A 3970G>T), CCA1273CCT (3973A>T), AGA1274AAT (3975G>A 3976A>T), CCA1275AAA (3977C>A 3978C>A), GAA1276ATG (3980G>A 3981A>T 3982A>G), GTG1277GAT (3984T>A 3985G>T), ATT1278CTT (3986A>C), ATG1279TTG (3989A>T), CAA1280AAA (3992C>A), AAA1281AGA (3996A>G), ATT1282ACT (3999T>C), CAG1283TTT (4001C>T 4002A>T 4003G>T), CAT1284AAA (4004C>A 4006T>A), AGT1285ACC (4008G>C 4009T>C), AAG1286AAT (4012G>T), GTG1287ATT (4013G>A 4015G>T), TTC1288TAC (4017T>A), AGC1289AGT (4021C>T), AAG1290AAA (4024G>A), GAC1292GAT (4030C>T), AAG1294AAA (4036G>A), GGG1296GGT (4042G>T), TAT1297TTC (4044A>T 4045T>C), TAT1298TGG (4047A>G 4048T>G), CAG1299CAA (4051G>A), ATA1300ATT (4054A>T), CAG1303TCG (4061C>T 4062A>C), CCA1304AAA (4064C>A 4065C>A), GAA1305AAA (4067G>A), AGA1307AAA (4074G>A), CAC1308TAT (4076C>T 4078C>T), ACA1310ACT (4084A>T), ATA1313AAC (4092T>A 4093A>C), TGT1314GTC (4094T>G 4095G>T 4096T>C), CCA1315CCC (4099A>C), GCA1316TTC (4100G>T 4101C>T 4102A>C), TTC1318CAA (4106T>C 4107T>A 4108C>A), CAG1320GAA (4112C>G 4114G>A), AAA1322AAT (4120A>T), GTT1324ATG (4124G>A 4126T>G), CCA1325CCC (4129A>C), GGT1327GGA (4135T>A), TTG1328TTA (4138G>A), AAG1329AAA (4141G>A), GCG1331GCC (4147G>C), CCA1332CCT (4150A>T), GCC1333TCT (4151G>T 4153C>T), TTC1334GAA (4154T>G 4155T>A 4156C>A), AGA1337AAC (4164G>A 4165A>C), AGG1338GTT (4166A>G 4167G>T 4168G>T), GAT1340AAT (4172G>A), TAT1341TCT (4176A>C)								

Proteins

	Begin	End	Coverage	Score	Concordance	Matches	Identities	I/D/M/F*	Stop Codons
polyprotein (YP_009553669.1)	1265	1342	3.7%	260	42.5%	78 (100%)	37 (47.4%)	0/0/1/0	0
Protein mutations:	T1265L (3947A>T 3948C>T 3949T>A), N1267W (3953A>T 3954A>G 3955T>G), F1268I (3956T>A), N1269R (3960A>G 3961T>A), L1272I (3968T>A 3970G>T), R1274N (3975G>A 3976A>T), P1275K (3977C>A 3978C>A), E1276M (3980G>A 3981A>T 3982A>G), V1277D (3984T>A 3985G>T), I1278L (3986A>C), M1279L (3989A>T), Q1280K (3992C>A), K1281R (3996A>G), I1282T (3999T>C), Q1283F (4001C>T 4002A>T 4003G>T), H1284K (4004C>A 4006T>A), S1285T (4008G>C 4009T>C), K1286N (4012G>T), V1287I (4013G>A 4015G>T), F1288Y (4017T>A), Y1297F (4044A>T 4045T>C), Y1298W (4047A>G 4048T>G), Q1303S (4061C>T 4062A>C), P1304K (4064C>A 4065C>A), E1305K (4067G>A), R1307K (4074G>A), H1308Y (4076C>T 4078C>T), I1313N (4092T>A 4093A>C), C1314V (4094T>G 4095G>T 4096T>C), A1316F (4100G>T 4101C>T 4102A>C), F1318Q (4106T>C 4107T>A 4108C>A), Q1320E (4112C>G 4114G>A), K1322N (4120A>T), V1324M (4124G>A 4126T>G), A1333S (4151G>T 4153C>T), F1334E (4154T>G 4155T>A 4156C>A), R1337N (4164G>A 4165A>C), R1338V (4166A>G 4167G>T 4168G>T), D1340N (4172G>A), Y1341S (4176A>C)								
Codon mutations:	CTT1261T.A (3935C>T 3937T>A), GCC1263T.T (3941G>T 3943C>T), ATC1264G.T (3944A>G 3946C>T), ACT1265TTA (3947A>T 3948C>T 3949T>A), CAG1266A.A (3950C>A 3952G>A), AAT1267TGG (3953A>T 3954A>G 3955T>G), TTT1268ATT (3956T>A), AAT1269AGA (3960A>G 3961T>A), CCA1271CCG (3967A>G), TTG1272ATT (3968T>A 3970G>T), CCA1273CCT (3973A>T), AGA1274AAT (3975G>A 3976A>T), CCA1275AAA (3977C>A 3978C>A), GAA1276ATG (3980G>A 3981A>T 3982A>G), GTG1277GAT (3984T>A 3985G>T), ATT1278CTT (3986A>C), ATG1279TTG (3989A>T), CAA1280AAA (3992C>A), AAA1281AGA (3996A>G), ATT1282ACT (3999T>C), CAG1283TTT (4001C>T 4002A>T 4003G>T), CAT1284AAA (4004C>A 4006T>A), AGT1285ACC (4008G>C 4009T>C), AAG1286AAT (4012G>T), GTG1287ATT (4013G>A 4015G>T), TTC1288TAC (4017T>A), AGC1289AGT (4021C>T), AAG1290AAA (4024G>A), GAC1292GAT (4030C>T), AAG1294AAA (4036G>A), GGG1296GGT (4042G>T), TAT1297TTC (4044A>T 4045T>C), TAT1298TGG (4047A>G 4048T>G), CAG1299CAA (4051G>A), ATA1300ATT (4054A>T), CAG1303TCG (4061C>T 4062A>C), CCA1304AAA (4064C>A 4065C>A), GAA1305AAA (4067G>A), AGA1307AAA (4074G>A), CAC1308TAT (4076C>T 4078C>T), ACA1310ACT (4084A>T), ATA1313AAC (4092T>A 4093A>C), TGT1314GTC (4094T>G 4095G>T 4096T>C), CCA1315CCC (4099A>C), GCA1316TTC (4100G>T 4101C>T 4102A>C), TTC1318CAA (4106T>C 4107T>A 4108C>A), CAG1320GAA (4112C>G 4114G>A), AAA1322AAT (4120A>T), GTT1324ATG (4124G>A 4126T>G), CCA1325CCC (4129A>C), GGT1327GGA (4135T>A), TTG1328TTA (4138G>A), AAG1329AAA (4141G>A), GCG1331GCC (4147G>C), CCA1332CCT (4150A>T), GCC1333TCT (4151G>T 4153C>T), TTC1334GAA (4154T>G 4155T>A 4156C>A), AGA1337AAC (4164G>A 4165A>C), AGG1338GTT (4166A>G 4167G>T 4168G>T), GAT1340AAT (4172G>A), TAT1341TCT (4176A>C)								

*: Inserts / Deletes / Misaligned / Frameshifts

Analysis details

This analysis was performed with panviral2.64

NGS Details (UN9): Badnavirus occultipomeae

Assembly

**Coverage Length**	251 (1 contig(s))
**Depth Of Coverage**	4.2
**Number Of Reads**	9
**Reads Per Million**	0.20 rpm (after QC)
**Ambiguities**	0
**Assembly Method**	de novo + reference guided assembly
**Consensus Caller**	Bcf Tools

Coverage Map

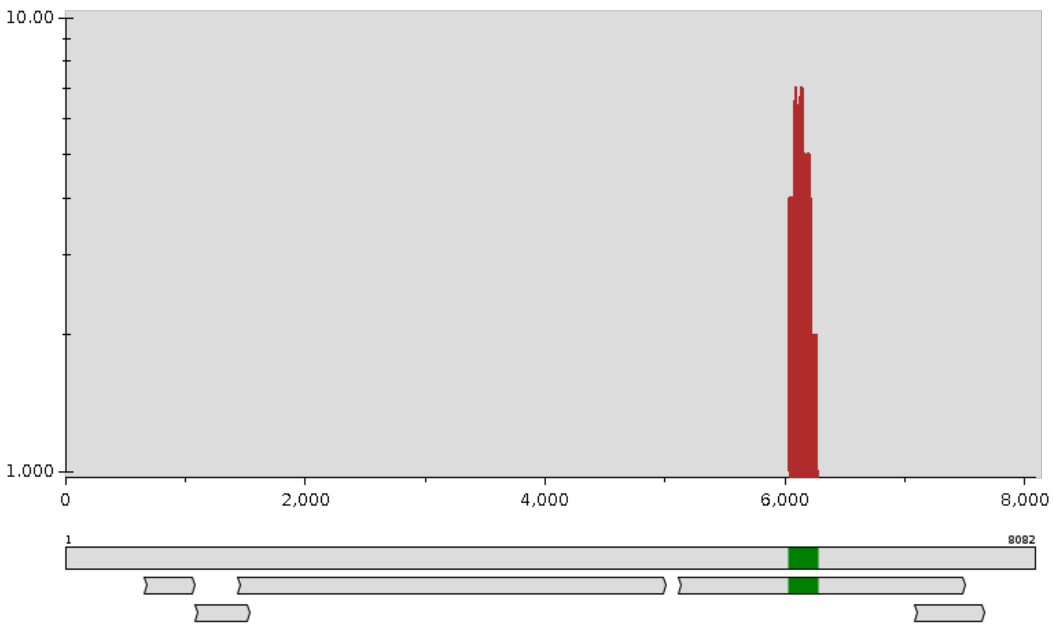

Assignment

**Type**	Badnavirus occultipomeae (Taxonomy ID: 3048353)
**Reference Genome**	NC_015655.1
**NT Identity (%)**	58.6614
**AA Identity (%)**	51.7647
**Number Of Stop Codons**	0
**Number Of CDS**	5

Alignment

**Alignment Score**	82.0 (NT) + 327.0 (AA) = 409.0
**Concordance (%)**	36.8468

**Alignment Method**	Global, seeded, nucleotide + amino acids (AGA)

Genome Region

Sequence starts at position 6031 and ends at position 6281 relative to NC_015655.1 reference sequence.

Alignment Detailed Statistics

	Begin	End	Coverage	Score	Concordance	Matches	Identities	I/D/M/F*	Stop Codons
**NT**	**6031**	**6281**	**3.1%**	**82**	**16.3%**	**251 (98.8%)**	**149 (58.7%)**	**3/0**	
Mutations:	6033A>T, 6039A>T, 6042A>G, 6043T>A, 6049C>T, 6051C>T, 6052A>T, 6053A>G, 6054A>G, 6055G>T, 6056C>T, 6057A>C, 6060C>G, 6062T>A, 6069G>A, 6070A>G, 6072A>G, 6075A>G, 6076A>G, 6078G>A, 6079G>A, 6080A>G, 6085A>T, 6086A>G, 6087G>T, 6088T>G, 6089C>A, 6090C>T, 6092A>T, 6093A>T, 6096A>C, 6097T>A, 6098G>A, 6099G>A, 6102T>G, 6108T>C, 6115C>A, 6116C>G, 6117A>G, 6118G>T, 6120A>T, 6125T>A, 6130G>A, 6137A>T, 6138A>T, 6141C>G, 6145C>T, 6147C>T, 6154C>T, 6158T>C, 6159G>C, 6168A>G, 6171A>C, 6172G>A, 6173A>T, 6174T>C, 6177T>C, 6178C>A, 6179A>T, 6181A>G, 6182G>A, 6183A>C, 6184A>C, 6185A>T, 6186G>T, 6190G>A, 6194A>G, 6195T>G, 6197C>T, 6198A>C, 6201T>C, 6203G>A, 6204G>A, 6205G>C, 6206G>C, 6207A>C, 6207_6208insTAC, 6208A>C, 6209C>T, 6210A>T, 6213T>C, 6214G>T, 6216A>T, 6220A>G, 6222C>T, 6224C>T, 6225A>G, 6228A>G, 6230A>T, 6231T>C, 6234T>C, 6243A>T, 6247G>A, 6249A>C, 6251T>A, 6256G>C, 6258A>T, 6260A>G, 6264A>G, 6267A>G, 6275A>T, 6278A>G, 6279T>C								
**CDS**									
SPBVa_gp4	306	389	10.6%	327	52.5%	84 (98.8%)	44 (51.8%)	1/0/0/0	0
Protein mutations:	F310I (6043T>A), L312F (6049C>T 6051C>T), K313W (6052A>T 6053A>G 6054A>G), A314F (6055G>T 6056C>T 6057A>C), F316Y (6062T>A), I319V (6070A>G 6072A>G), M321V (6076A>G 6078G>A), E322R (6079G>A 6080A>G), K324C (6085A>T 6086A>G 6087G>T), S325D (6088T>G 6089C>A 6090C>T), K326I (6092A>T 6093A>T), W328K (6097T>A 6098G>A 6099G>A), P334R (6115C>A 6116C>G 6117A>G), E335Y (6118G>T 6120A>T), L337H (6125T>A), E339K (6130G>A), E341V (6137A>T 6138A>T), P344S (6145C>T 6147C>T), M348T (6158T>C 6159G>C), D353I (6172G>A 6173A>T 6174T>C), Q355M (6178C>A 6179A>T), R356D (6181A>G 6182G>A 6183A>C), K357L (6184A>C 6185A>T 6186G>T), D359N (6190G>A), N360R (6194A>G 6195T>G), A361V (6197C>T 6198A>C), R363K (6203G>A 6204G>A), G364P (6205G>C 6206G>C 6207A>C), G364_T365insY (6207_6208insTAC), T365L (6208A>C 6209C>T 6210A>T), A367S (6214G>T 6216A>T), I369V (6220A>G 6222C>T), A370V (6224C>T 6225A>G), Y372F (6230A>T 6231T>C), V378I (6247G>A 6249A>C), F379Y (6251T>A), E381H (6256G>C 6258A>T), N382S (6260A>G), E387V (6275A>T), D388G (6278A>G 6279T>C)								
Codon mutations:	ATA306ATT (6033A>T), TCA308TCT (6039A>T), AAA309AAG (6042A>G), TTT310ATT (6043T>A), CTC312TTT (6049C>T 6051C>T), AAA313TGG (6052A>T 6053A>G 6054A>G), GCA314TTC (6055G>T 6056C>T 6057A>C), GGC315GGG (6060C>G), TTT316TAT (6062T>A), CAG318CAA (6069G>A), ATA319GTG (6070A>G 6072A>G), AGA320AGG (6075A>G), ATG321GTA (6076A>G 6078G>A), GAG322AGG (6079G>A 6080A>G), AAG324TGT (6085A>T 6086A>G 6087G>T), TCC325GAT (6088T>G 6089C>A 6090C>T), AAA326ATT (6092A>T 6093A>T), CCA327CCC (6096A>C), TGG328AAA (6097T>A 6098G>A 6099G>A), ACT329ACG (6102T>G), TTT331TTC (6108T>C), CCA334AGG (6115C>A 6116C>G 6117A>G), GAA335TAT (6118G>T 6120A>T), CTC337CAC (6125T>A), GAA339AAA (6130G>A), GAA341GTT (6137A>T 6138A>T), GTC342GTG (6141C>G), CCC344TCT (6145C>T 6147C>T), CTG347TTG (6154C>T), ATG348ACC (6158T>C 6159G>C), CCA351CCG (6168A>G), GCA352GCC (6171A>C), GAT353ATC (6172G>A 6173A>T 6174T>C), TTT354TTC (6177T>C), CAG355ATG (6178C>A 6179A>T), AGA356GAC (6181A>G 6182G>A 6183A>C), AAG357CTT (6184A>C 6185A>T 6186G>T), GAT359AAT (6190G>A), AAT360AGG (6194A>G 6195T>G), GCA361GTC (6197C>T 6198A>C), TTT362TTC (6201T>C), AGG363AAA (6203G>A 6204G>A), GGA364CCC (6205G>C 6206G>C 6207A>C), GGA364_ACA365insTAC (6207_6208insTAC), ACA365CTT (6208A>C 6209C>T 6210A>T), GAT366GAC (6213T>C), GCA367TCT (6214G>T 6216A>T), ATC369GTT (6220A>G 6222C>T), GCA370GTG (6224C>T 6225A>G), GTA371GTG (6228A>G), TAT372TTC (6230A>T 6231T>C), ATT373ATC (6234T>C), ATA376ATT (6243A>T), GTA378ATC (6247G>A 6249A>C), TTC379TAC (6251T>A), GAA381CAT (6256G>C 6258A>T), AAT382AGT (6260A>G), GAA383GAG (6264A>G), GAA384GAG (6267A>G), GAG387GTG (6275A>T), GAT388GGC (6278A>G 6279T>C)								
**Proteins**									
RNaseH/reverse transcriptase (YP_004581513.1)	306	389	10.6%	327	52.5%	84 (98.8%)	44 (51.8%)	1/0/0/0	0
Protein mutations:	F310I (6043T>A), L312F (6049C>T 6051C>T), K313W (6052A>T 6053A>G 6054A>G), A314F (6055G>T 6056C>T 6057A>C), F316Y (6062T>A), I319V (6070A>G 6072A>G), M321V (6076A>G 6078G>A), E322R (6079G>A 6080A>G), K324C (6085A>T 6086A>G 6087G>T), S325D (6088T>G 6089C>A 6090C>T), K326I (6092A>T 6093A>T), W328K (6097T>A 6098G>A 6099G>A), P334R (6115C>A 6116C>G 6117A>G), E335Y (6118G>T 6120A>T), L337H (6125T>A), E339K (6130G>A), E341V (6137A>T 6138A>T), P344S (6145C>T 6147C>T), M348T (6158T>C 6159G>C), D353I (6172G>A 6173A>T 6174T>C), Q355M (6178C>A 6179A>T), R356D (6181A>G 6182G>A 6183A>C), K357L (6184A>C 6185A>T 6186G>T), D359N (6190G>A), N360R (6194A>G 6195T>G), A361V (6197C>T 6198A>C), R363K (6203G>A 6204G>A), G364P (6205G>C 6206G>C 6207A>C), G364_T365insY (6207_6208insTAC), T365L (6208A>C 6209C>T 6210A>T), A367S (6214G>T 6216A>T), I369V (6220A>G 6222C>T), A370V (6224C>T 6225A>G), Y372F (6230A>T 6231T>C), V378I (6247G>A 6249A>C), F379Y (6251T>A), E381H (6256G>C 6258A>T), N382S (6260A>G), E387V (6275A>T), D388G (6278A>G 6279T>C)								
Codon mutations:	ATA306ATT (6033A>T), TCA308TCT (6039A>T), AAA309AAG (6042A>G), TTT310ATT (6043T>A), CTC312TTT (6049C>T 6051C>T), AAA313TGG (6052A>T 6053A>G 6054A>G), GCA314TTC (6055G>T 6056C>T 6057A>C), GGC315GGG (6060C>G), TTT316TAT (6062T>A), CAG318CAA (6069G>A), ATA319GTG (6070A>G 6072A>G), AGA320AGG (6075A>G), ATG321GTA (6076A>G 6078G>A), GAG322AGG (6079G>A 6080A>G), AAG324TGT (6085A>T 6086A>G 6087G>T), TCC325GAT (6088T>G 6089C>A 6090C>T), AAA326ATT (6092A>T 6093A>T), CCA327CCC (6096A>C), TGG328AAA (6097T>A 6098G>A 6099G>A), ACT329ACG (6102T>G), TTT331TTC (6108T>C), CCA334AGG (6115C>A 6116C>G 6117A>G), GAA335TAT (6118G>T 6120A>T), CTC337CAC (6125T>A), GAA339AAA (6130G>A), GAA341GTT (6137A>T 6138A>T), GTC342GTG (6141C>G), CCC344TCT (6145C>T 6147C>T), CTG347TTG (6154C>T), ATG348ACC (6158T>C 6159G>C), CCA351CCG (6168A>G), GCA352GCC (6171A>C), GAT353ATC (6172G>A 6173A>T 6174T>C), TTT354TTC (6177T>C), CAG355ATG (6178C>A 6179A>T), AGA356GAC (6181A>G 6182G>A 6183A>C), AAG357CTT (6184A>C 6185A>T 6186G>T), GAT359AAT (6190G>A), AAT360AGG (6194A>G 6195T>G), GCA361GTC (6197C>T 6198A>C), TTT362TTC (6201T>C), AGG363AAA (6203G>A 6204G>A), GGA364CCC (6205G>C 6206G>C 6207A>C), GGA364_ACA365insTAC (6207_6208insTAC), ACA365CTT (6208A>C 6209C>T 6210A>T), GAT366GAC (6213T>C), GCA367TCT (6214G>T 6216A>T), ATC369GTT (6220A>G 6222C>T), GCA370GTG (6224C>T 6225A>G), GTA371GTG (6228A>G), TAT372TTC (6230A>T 6231T>C), ATT373ATC (6234T>C), ATA376ATT (6243A>T), GTA378ATC (6247G>A 6249A>C), TTC379TAC (6251T>A), GAA381CAT (6256G>C 6258A>T), AAT382AGT (6260A>G), GAA383GAG (6264A>G), GAA384GAG (6267A>G), GAG387GTG (6275A>T), GAT388GGC (6278A>G 6279T>C)								

*: Inserts / Deletes / Misaligned / Frameshifts

Analysis details

This analysis was performed with panviral2.64

NGS Details (UN9): Epiphyllum badnavirus 1

Assembly

**Coverage Length**	240 (1 contig(s))
**Depth Of Coverage**	4.0
**Number Of Reads**	9
**Reads Per Million**	0.20 rpm (after QC)
**Ambiguities**	0
**Assembly Method**	de novo + reference guided assembly
**Consensus Caller**	Bcf Tools

Coverage Map

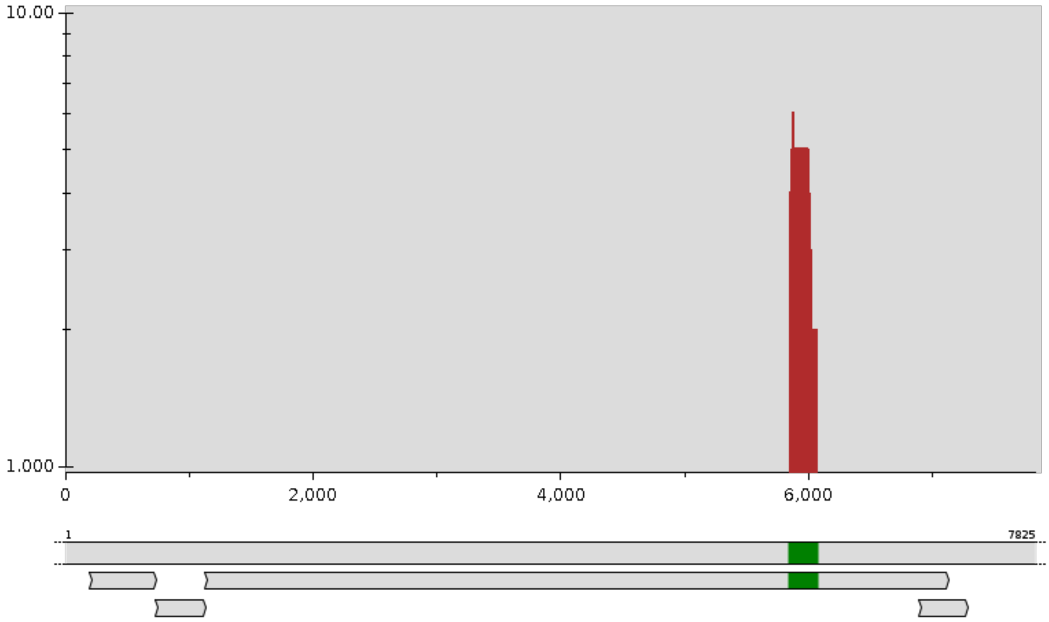

Assignment

**Type**	Epiphyllum badnavirus 1 (Taxonomy ID: 2518008)
**Reference Genome**	NC_076247.1
**NT Identity (%)**	58.0247
**AA Identity (%)**	51.8519
**Number Of Stop Codons**	0
**Number Of CDS**	4

Alignment

**Alignment Score**	72.0 (NT) + 288.0 (AA) = 360.0
**Concordance (%)**	33.9943

Alignment Method	Global, seeded, nucleotide + amino acids (AGA)

Genome Region

Sequence starts at position 5838 and ends at position 6077 relative to NC_076247.1 reference sequence.

Alignment Detailed Statistics

	Begin	End	Coverage	Score	Concordance	Matches	Identities	I/D/M/F*	Stop Codons
**NT**	**5838**	**6077**	**3.1%**	**72**	**15.0%**	**240 (98.8%)**	**141 (58.0%)**	**3/0**	
Mutations:	5838T>G, 5841C>G, 5851T>A, 5853C>A, 5857C>T, 5861A>G, 5863A>T, 5864G>C, 5865C>G, 5868A>G, 5870T>A, 5871C>T, 5874C>T, 5877G>A, 5878A>C, 5880T>G, 5882T>G, 5886G>T, 5887G>A, 5888A>G, 5889C>G, 5892A>G, 5895A>C, 5896T>G, 5897C>A, 5905T>A, 5906G>A, 5910C>T, 5912C>T, 5916C>T, 5917C>A, 5918T>G, 5922C>T, 5924C>G, 5925A>T, 5926G>T, 5931A>C, 5933T>A, 5934T>C, 5940A>G, 5942G>T, 5943G>C, 5949T>C, 5953C>T, 5955A>T, 5958C>T, 5966A>C, 5967G>T, 5976T>C, 5977G>A, 5979T>A, 5980G>A, 5981T>C, 5982G>A, 5985C>T, 5986C>A, 5987A>T, 5988A>G, 5989C>G, 5990G>A, 5991A>T, 5992A>T, 5993A>T, 5998G>A, 6001A>C, 6002A>G, 6003C>T, 6004T>G, 6005G>T, 6006T>A, 6009C>T, 6012_6013insTCT, 6013G>T, 6019T>G, 6020C>A, 6021A>C, 6022G>A, 6023G>T, 6030A>T, 6031G>A, 6032C>T, 6033A>T, 6036T>A, 6038A>T, 6042T>A, 6048C>T, 6051C>T, 6055G>A, 6059T>A, 6061A>T, 6062G>C, 6063T>C, 6065A>G, 6066C>G, 6068C>G, 6069T>C, 6070G>A, 6071A>G, 6075T>G, 6076G>C								
**CDS**									
QKM20_gp3	1571	1650	4.0%	288	48.6%	80 (98.8%)	42 (51.9%)	1/0/0/0	0
Protein mutations:	F1575I (5851T>A 5853C>A), K1578R (5861A>G), F1581Y (5870T>A 5871C>T), I1584L (5878A>C 5880T>G), M1585R (5882T>G), M1586I (5886G>T), D1587R (5887G>A 5888A>G 5889C>G), E1589D (5895A>C), S1590D (5896T>G 5897C>A), W1593K (5905T>A 5906G>A), A1595V (5912C>T), L1597R (5917C>A 5918T>G), P1599R (5924C>G 5925A>T), D1600Y (5926G>T), L1602H (5933T>A 5934T>C), W1605F (5942G>T 5943G>C), P1609S (5953C>T 5955A>T), K1613T (5966A>C 5967G>T), A1617T (5977G>A 5979T>A), V1618T (5980G>A 5981T>C 5982G>A), Q1620M (5986C>A 5987A>T 5988A>G), R1621D (5989C>G 5990G>A 5991A>T), K1622L (5992A>T 5993A>T), D1624N (5998G>A), N1625R (6001A>C 6002A>G 6003C>T), C1626V (6004T>G 6005G>T 6006T>A), R1628_D1629insS (6012_6013insTCT), D1629Y (6013G>T), S1631D (6019T>G 6020C>A 6021A>C), G1632I (6022G>A 6023G>T), A1635I (6031G>A 6032C>T 6033A>T), Y1637F (6038A>T), V1643I (6055G>A), F1644Y (6059T>A), N1646R (6065A>G 6066C>G), T1647S (6068C>G 6069T>C), E1648R (6070G>A 6071A>G), D1649E (6075T>G)								
Codon mutations:	AGT1570..G (5838T>G), GTC1571GTG (5841C>G), TTC1575ATA (5851T>A 5853C>A), CTG1577TTG (5857C>T), AAG1578AGG (5861A>G), AGC1579TCG (5863A>T 5864G>C 5865C>G), GGA1580GGG (5868A>G), TTC1581TAT (5870T>A 5871C>T), CAC1582CAT (5874C>T), CAG1583CAA (5877G>A), ATT1584CTG (5878A>C 5880T>G), ATG1585AGG (5882T>G), ATG1586ATT (5886G>T), GAC1587AGG (5887G>A 5888A>G 5889C>G), GAA1588GAG (5892A>G), GAA1589GAC (5895A>C), TCC1590GAC (5896T>G 5897C>A), TGG1593AAG (5905T>A 5906G>A), ACC1594ACT (5910C>T), GCC1595GTC (5912C>T), TTC1596TTT (5916C>T), CTG1597AGG (5917C>A 5918T>G), ACC1598ACT (5922C>T), CCA1599CGT (5924C>G 5925A>T), GAT1600TAT (5926G>T), GGA1601GGC (5931A>C), CTT1602CAC (5933T>A 5934T>C), GAA1604GAG (5940A>G), TGG1605TTC (5942G>T 5943G>C), GTT1607GTC (5949T>C), CCA1609TCT (5953C>T 5955A>T), TTC1610TTT (5958C>T), AAG1613ACT (5966A>C 5967G>T), CCT1616CCC (5976T>C), GCT1617ACA (5977G>A 5979T>A), GTG1618ACA (5980G>A 5981T>C 5982G>A), TTC1619TTT (5985C>T), CAA1620ATG (5986C>A 5987A>T 5988A>G), CGA1621GAT (5989C>G 5990G>A 5991A>T), AAG1622TTG (5992A>T 5993A>T), GAC1624AAC (5998G>A), AAC1625CGT (6001A>C 6002A>G 6003C>T), TGT1626GTA (6004T>G 6005G>T 6006T>A), TTC1627TTT (6009C>T), AGA1628_GAC1629insTCT (6012_6013insTCT), GAC1629TAC (6013G>T), TCA1631GAC (6019T>G 6020C>A 6021A>C), GGC1632ATC (6022G>A 6023G>T), GTA1634GTT (6030A>T), GCA1635ATT (6031G>A 6032C>T 6033A>T), GTT1636GTA (6036T>A), TAC1637TTC (6038A>T), ATT1638ATA (6042T>A), GAC1640GAT (6048C>T), ATC1641ATT (6051C>T), GTC1643ATC (6055G>A), TTC1644TAC (6059T>A), AGT1645TCC (6061A>T 6062G>C 6063T>C), AAC1646AGG (6065A>G 6066C>G), ACT1647AGC (6068C>G 6069T>C), GAA1648AGA (6070G>A 6071A>G), GAT1649GAG (6075T>G), GAC1650CA. (6076G>C)								
**Proteins**									
polyprotein (YP_010797894.1)	1571	1650	4.0%	288	48.6%	80 (98.8%)	42 (51.9%)	1/0/0/0	0
Protein mutations:	F1575I (5851T>A 5853C>A), K1578R (5861A>G), F1581Y (5870T>A 5871C>T), I1584L (5878A>C 5880T>G), M1585R (5882T>G), M1586I (5886G>T), D1587R (5887G>A 5888A>G 5889C>G), E1589D (5895A>C), S1590D (5896T>G 5897C>A), W1593K (5905T>A 5906G>A), A1595V (5912C>T), L1597R (5917C>A 5918T>G), P1599R (5924C>G 5925A>T), D1600Y (5926G>T), L1602H (5933T>A 5934T>C), W1605F (5942G>T 5943G>C), P1609S (5953C>T 5955A>T), K1613T (5966A>C 5967G>T), A1617T (5977G>A 5979T>A), V1618T (5980G>A 5981T>C 5982G>A), Q1620M (5986C>A 5987A>T 5988A>G), R1621D (5989C>G 5990G>A 5991A>T), K1622L (5992A>T 5993A>T), D1624N (5998G>A), N1625R (6001A>C 6002A>G 6003C>T), C1626V (6004T>G 6005G>T 6006T>A), R1628_D1629insS (6012_6013insTCT), D1629Y (6013G>T), S1631D (6019T>G 6020C>A 6021A>C), G1632I (6022G>A 6023G>T), A1635I (6031G>A 6032C>T 6033A>T), Y1637F (6038A>T), V1643I (6055G>A), F1644Y (6059T>A), N1646R (6065A>G 6066C>G), T1647S (6068C>G 6069T>C), E1648R (6070G>A 6071A>G), D1649E (6075T>G)								
Codon mutations:	AGT1570..G (5838T>G), GTC1571GTG (5841C>G), TTC1575ATA (5851T>A 5853C>A), CTG1577TTG (5857C>T), AAG1578AGG (5861A>G), AGC1579TCG (5863A>T 5864G>C 5865C>G), GGA1580GGG (5868A>G), TTC1581TAT (5870T>A 5871C>T), CAC1582CAT (5874C>T), CAG1583CAA (5877G>A), ATT1584CTG (5878A>C 5880T>G), ATG1585AGG (5882T>G), ATG1586ATT (5886G>T), GAC1587AGG (5887G>A 5888A>G 5889C>G), GAA1588GAG (5892A>G), GAA1589GAC (5895A>C), TCC1590GAC (5896T>G 5897C>A), TGG1593AAG (5905T>A 5906G>A), ACC1594ACT (5910C>T), GCC1595GTC (5912C>T), TTC1596TTT (5916C>T), CTG1597AGG (5917C>A 5918T>G), ACC1598ACT (5922C>T), CCA1599CGT (5924C>G 5925A>T), GAT1600TAT (5926G>T), GGA1601GGC (5931A>C), CTT1602CAC (5933T>A 5934T>C), GAA1604GAG (5940A>G), TGG1605TTC (5942G>T 5943G>C), GTT1607GTC (5949T>C), CCA1609TCT (5953C>T 5955A>T), TTC1610TTT (5958C>T), AAG1613ACT (5966A>C 5967G>T), CCT1616CCC (5976T>C), GCT1617ACA (5977G>A 5979T>A), GTG1618ACA (5980G>A 5981T>C 5982G>A), TTC1619TTT (5985C>T), CAA1620ATG (5986C>A 5987A>T 5988A>G), CGA1621GAT (5989C>G 5990G>A 5991A>T), AAG1622TTG (5992A>T 5993A>T), GAC1624AAC (5998G>A), AAC1625CGT (6001A>C 6002A>G 6003C>T), TGT1626GTA (6004T>G 6005G>T 6006T>A), TTC1627TTT (6009C>T), AGA1628_GAC1629insTCT (6012_6013insTCT), GAC1629TAC (6013G>T), TCA1631GAC (6019T>G 6020C>A 6021A>C), GGC1632ATC (6022G>A 6023G>T), GTA1634GTT (6030A>T), GCA1635ATT (6031G>A 6032C>T 6033A>T), GTT1636GTA (6036T>A), TAC1637TTC (6038A>T), ATT1638ATA (6042T>A), GAC1640GAT (6048C>T), ATC1641ATT (6051C>T), GTC1643ATC (6055G>A), TTC1644TAC (6059T>A), AGT1645TCC (6061A>T 6062G>C 6063T>C), AAC1646AGG (6065A>G 6066C>G), ACT1647AGC (6068C>G 6069T>C), GAA1648AGA (6070G>A 6071A>G), GAT1649GAG (6075T>G), GAC1650CA. (6076G>C)								

*: Inserts / Deletes / Misaligned / Frameshifts

Analysis details

This analysis was performed with panviral2.64

NGS Details (UN9): Colombian datura virus

Assembly

**Coverage Length**	429 (2 contig(s))
**Depth Of Coverage**	2.3
**Number Of Reads**	8
**Reads Per Million**	0.18 rpm (after QC)
**Ambiguities**	1
**Assembly Method**	de novo + reference guided assembly
**Consensus Caller**	Bcf Tools

Coverage Map

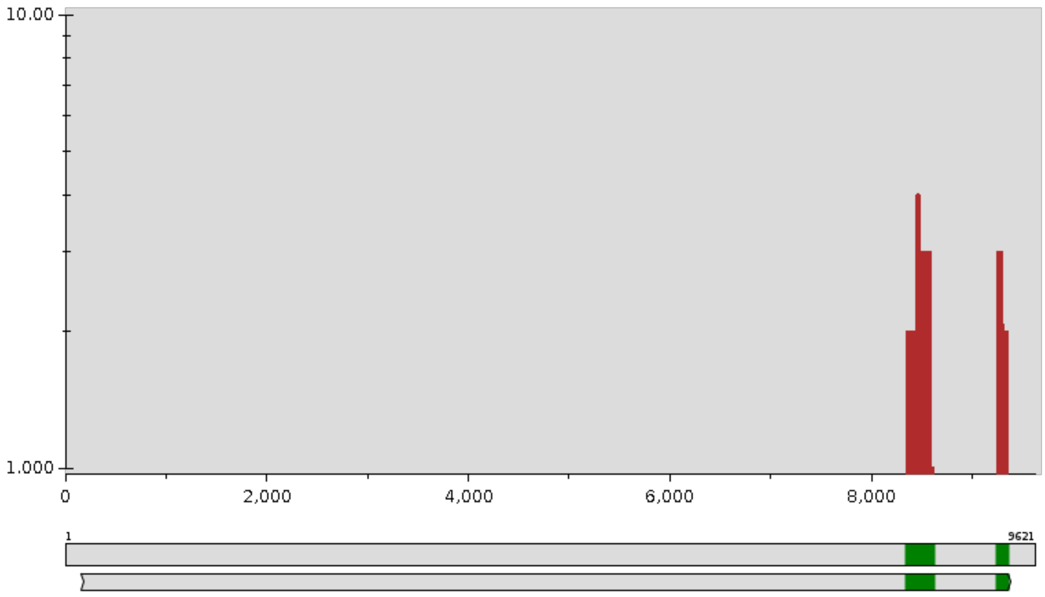

Assignment

**Type**	Colombian datura virus (Taxonomy ID: 91613)
**Reference Genome**	NC_020072.1
**NT Identity (%)**	75.5245
**AA Identity (%)**	79.021
**Number Of Stop Codons**	0
**Number Of CDS**	1

Alignment

**Alignment Score**	440.0 (NT) + 731.0 (AA) = 1171.0
**Concordance (%)**	66.0462

Alignment Method	Global, seeded, nucleotide + amino acids (AGA)

Genome Region

Sequence starts at position 8339 and ends at position 9368 relative to NC_020072.1 reference sequence.

Alignment Detailed Statistics

	Begin	End	Coverage	Score	Concordance	Matches	Identities	I/D/M/F*	Stop Codons
**NT**	**8339**	**9368**	**4.5%**	**440**	**51.3%**	**429 (100%)**	**324 (75.5%)**	**0/0**	
Mutations:	8341A>G, 8342C>T, 8347T>A, 8349A>T, 8353G>A, 8357A>C, 8369C>T, 8371T>G, 8383G>A, 8395C>T, 8411G>C, 8416G>A, 8418G>A, 8422T>A, 8428C>T, 8431A>G, 8434T>C, 8437A>G, 8440T>G, 8443T>A, 8444C>T, 8455A>G, 8461T>A, 8464T>C, 8465G>A, 8467T>C, 8473C>A, 8476T>G, 8479A>T, 8480T>A, 8481T>A, 8482G>A, 8485A>G, 8488G>A, 8489C>T, 8491T>G, 8499C>T, 8500A>T, 8503G>T, 8506C>T, 8507A>G, 8515T>A, 8518T>G, 8520A>C, 8524T>C, 8525G>T, 8526A>T, 8527T>G, 8528A>G, 8529G>A, 8530C>T, 8531T>G, 8533G>A, 8535C>A, 8537T>C, 8538C>A, 8539T>A, 8542A>G, 8543G>C, 8545T>C, 8548A>G, 8549T>A, 8550A>T, 8551T>A, 8552C>T, 8560T>A, 8563T>C, 8565C>A, 8578T>A, 8581T>C, 8587A>C, 8591G>A, 8596A>T, 8599G>A, 8601A>G, 8602C>T, 8606T>G, 8607C>G, 8624A>C, 8625G>T, 8626T>C, 8628T>C, 9244T>A, 9256A>T, 9261A>C, 9262C>A, 9265A>G, 9268G>C, 9271G>A, 9272T>C, 9274A>C, 9283G>C, 9307A>G, 9316C>A, 9325C>T, 9332C>A, 9333A>C, 9334A>T, 9339T>W, 9340T>G, 9346A>G, 9347G>A, 9348G>A, 9349T>C, 9364T>A								

CDS

	Begin	End	Coverage	Score	Concordance	Matches	Identities	I/D/M/F*	Stop Codons
G357_gp1	2729	3072	4.6%	731	78.8%	143 (100%)	113 (79.0%)	0/0/1/0	0
Protein mutations:	K2732M (8349A>T), E2753Q (8411G>C), R2755K (8418G>A), A2771T (8465G>A 8467T>C), E2775D (8479A>T), L2776K (8480T>A 8481T>A 8482G>A), A2782V (8499C>T 8500A>T), I2785V (8507A>G), D2788E (8518T>G), Y2789S (8520A>C), D2791L (8525G>T 8526A>T 8527T>G), S2792D (8528A>G 8529G>A 8530C>T), L2793V (8531T>G 8533G>A), A2794E (8535C>A), S2795Q (8537T>C 8538C>A 8539T>A), D2797H (8543G>C 8545T>C), I2798M (8548A>G), Y2799I (8549T>A 8550A>T 8551T>A), L2800F (8552C>T), T2804K (8565C>A), A2813T (8591G>A), N2816S (8601A>G 8602C>T), S2818G (8606T>G 8607C>G), S2824L (8624A>C 8625G>T 8626T>C), V2825A (8628T>C), N3036T (9261A>C 9262C>A), Q3060T (9332C>A 9333A>C 9334A>T), G3065N (9347G>A 9348G>A 9349T>C)								
Codon mutations:	GAA2729GAG (8341A>G), CTG2730TTG (8342C>T), ACT2731ACA (8347T>A), AAG2732ATG (8349A>T), AGG2733AGA (8353G>A), AGA2735CGA (8357A>C), CTT2739TTG (8369C>T 8371T>G), GAG2743GAA (8383G>A), TAC2747TAT (8395C>T), GAA2753CAA (8411G>C), GGG2754GGA (8416G>A), AGA2755AAA (8418G>A), GCT2756GCA (8422T>A), TAC2758TAT (8428C>T), CTA2759CTG (8431A>G), GCT2760GCC (8434T>C), GAA2761GAG (8437A>G), ACT2762ACG (8440T>G), GCT2763GCA (8443T>A), CTG2764TTG (8444C>T), TTA2767TTG (8455A>G), CTT2769CTA (8461T>A), GAT2770GAC (8464T>C), GCT2771ACC (8465G>A 8467T>C), CCC2773CCA (8473C>A), ACT2774ACG (8476T>G), GAA2775GAT (8479A>T), TTG2776AAA (8480T>A 8481T>A 8482G>A), GAA2777GAG (8485A>G), TTG2778TTA (8488G>A), CTT2779TTG (8489C>T 8491T>G), GCA2782GTT (8499C>T 8500A>T), GGG2783GGT (8503G>T), TGC2784TGT (8506C>T), ATC2785GTC (8507A>G), GGT2787GGA (8515T>A), GAT2788GAG (8518T>G), TAT2789TCT (8520A>C), GAT2790GAC (8524T>C), GAT2791TTG (8525G>T 8526A>T 8527T>G), AGC2792GAT (8528A>G 8529G>A 8530C>T), TTG2793GTA (8531T>G 8533G>A), GCA2794GAA (8535C>A), TCT2795CAA (8537T>C 8538C>A 8539T>A), GAA2796GAG (8542A>G), GAT2797CAC (8543G>C 8545T>C), ATA2798ATG (8548A>G), TAT2799ATA (8549T>A 8550A>T 8551T>A), CTC2800TTC (8552C>T), GCT2802GCA (8560T>A), GAT2803GAC (8563T>C), ACA2804AAA (8565C>A), GGT2808GGA (8578T>A), AGT2809AGC (8581T>C), ACA2811ACC (8587A>C), GCC2813ACC (8591G>A), GGA2814GGT (8596A>T), AAG2815AAA (8599G>A), AAC2816AGT (8601A>G 8602C>T), TCA2818GGA (8606T>G 8607C>G), AGT2824CTC (8624A>C 8625G>T 8626T>C), GTT2825GCT (8628T>C), GCT3030GCA (9244T>A), CGA3034CGT (9256A>T), AAC3036ACA (9261A>C 9262C>A), ACA3037ACG (9265A>G), ACG3038ACC (9268G>C), AAG3039AAA (9271G>A), TTA3040CTC (9272T>C 9274A>C), CTG3043CTC (9283G>C), GAA3051GAG (9307A>G), ACC3054ACA (9316C>A), CAC3057CAT (9325C>T), CAA3060ACT (9332C>A 9333A>C 9334A>T), GTT3062GWG (9339T>W 9340T>G), AGA3064AGG (9346A>G), GGT3065AAC (9347G>A 9348G>A 9349T>C), CTT3070CTA (9364T>A)								

Proteins

	Begin	End	Coverage	Score	Concordance	Matches	Identities	I/D/M/F*	Stop Codons
polyprotein (YP_007346986.1)	2729	3072	4.6%	731	78.8%	143 (100%)	113 (79.0%)	0/0/1/0	0
Protein mutations:	K2732M (8349A>T), E2753Q (8411G>C), R2755K (8418G>A), A2771T (8465G>A 8467T>C), E2775D (8479A>T), L2776K (8480T>A 8481T>A 8482G>A), A2782V (8499C>T 8500A>T), I2785V (8507A>G), D2788E (8518T>G), Y2789S (8520A>C), D2791L (8525G>T 8526A>T 8527T>G), S2792D (8528A>G 8529G>A 8530C>T), L2793V (8531T>G 8533G>A), A2794E (8535C>A), S2795Q (8537T>C 8538C>A 8539T>A), D2797H (8543G>C 8545T>C), I2798M (8548A>G), Y2799I (8549T>A 8550A>T 8551T>A), L2800F (8552C>T), T2804K (8565C>A), A2813T (8591G>A), N2816S (8601A>G 8602C>T), S2818G (8606T>G 8607C>G), S2824L (8624A>C 8625G>T 8626T>C), V2825A (8628T>C), N3036T (9261A>C 9262C>A), Q3060T (9332C>A 9333A>C 9334A>T), G3065N (9347G>A 9348G>A 9349T>C)								
Codon mutations:	GAA2729GAG (8341A>G), CTG2730TTG (8342C>T), ACT2731ACA (8347T>A), AAG2732ATG (8349A>T), AGG2733AGA (8353G>A), AGA2735CGA (8357A>C), CTT2739TTG (8369C>T 8371T>G), GAG2743GAA (8383G>A), TAC2747TAT (8395C>T), GAA2753CAA (8411G>C), GGG2754GGA (8416G>A), AGA2755AAA (8418G>A), GCT2756GCA (8422T>A), TAC2758TAT (8428C>T), CTA2759CTG (8431A>G), GCT2760GCC (8434T>C), GAA2761GAG (8437A>G), ACT2762ACG (8440T>G), GCT2763GCA (8443T>A), CTG2764TTG (8444C>T), TTA2767TTG (8455A>G), CTT2769CTA (8461T>A), GAT2770GAC (8464T>C), GCT2771ACC (8465G>A 8467T>C), CCC2773CCA (8473C>A), ACT2774ACG (8476T>G), GAA2775GAT (8479A>T), TTG2776AAA (8480T>A 8481T>A 8482G>A), GAA2777GAG (8485A>G), TTG2778TTA (8488G>A), CTT2779TTG (8489C>T 8491T>G), GCA2782GTT (8499C>T 8500A>T), GGG2783GGT (8503G>T), TGC2784TGT (8506C>T), ATC2785GTC (8507A>G), GGT2787GGA (8515T>A), GAT2788GAG (8518T>G), TAT2789TCT (8520A>C), GAT2790GAC (8524T>C), GAT2791TTG (8525G>T 8526A>T 8527T>G), AGC2792GAT (8528A>G 8529G>A 8530C>T), TTG2793GTA (8531T>G 8533G>A), GCA2794GAA (8535C>A), TCT2795CAA (8537T>C 8538C>A 8539T>A), GAA2796GAG (8542A>G), GAT2797CAC (8543G>C 8545T>C), ATA2798ATG (8548A>G), TAT2799ATA (8549T>A 8550A>T 8551T>A), CTC2800TTC (8552C>T), GCT2802GCA (8560T>A), GAT2803GAC (8563T>C), ACA2804AAA (8565C>A), GGT2808GGA (8578T>A), AGT2809AGC (8581T>C), ACA2811ACC (8587A>C), GCC2813ACC (8591G>A), GGA2814GGT (8596A>T), AAG2815AAA (8599G>A), AAC2816AGT (8601A>G 8602C>T), TCA2818GGA (8606T>G 8607C>G), AGT2824CTC (8624A>C 8625G>T 8626T>C), GTT2825GCT (8628T>C), GCT3030GCA (9244T>A), CGA3034CGT (9256A>T), AAC3036ACA (9261A>C 9262C>A), ACA3037ACG (9265A>G), ACG3038ACC (9268G>C), AAG3039AAA (9271G>A), TTA3040CTC (9272T>C 9274A>C), CTG3043CTC (9283G>C), GAA3051GAG (9307A>G), ACC3054ACA (9316C>A), CAC3057CAT (9325C>T), CAA3060ACT (9332C>A 9333A>C 9334A>T), GTT3062GWG (9339T>W 9340T>G), AGA3064AGG (9346A>G), GGT3065AAC (9347G>A 9348G>A 9349T>C), CTT3070CTA (9364T>A)								

*: Inserts / Deletes / Misaligned / Frameshifts

Analysis details

This analysis was performed with panviral2.64

NGS Details (UN9): Pinus nigra virus 1

Assembly

**Coverage Length**	356 (1 contig(s))
**Depth Of Coverage**	2.7
**Number Of Reads**	8
**Reads Per Million**	0.18 rpm (after QC)
**Ambiguities**	0
**Assembly Method**	de novo + reference guided assembly
**Consensus Caller**	Bcf Tools

Coverage Map

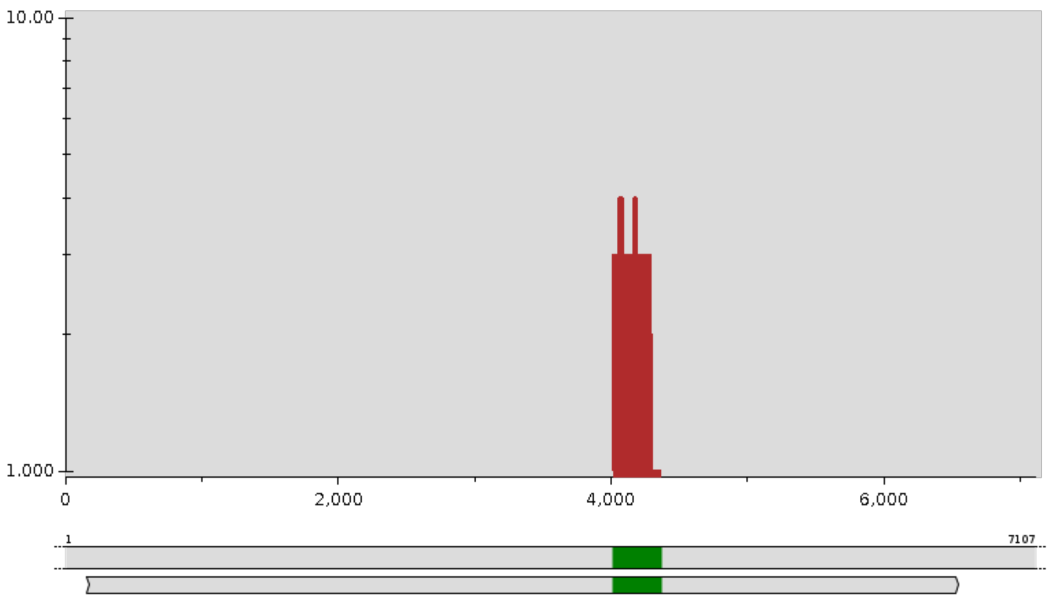

Assignment

**Type**	Pinus nigra virus 1 (Taxonomy ID: 2267679)
**Reference Genome**	NC_040841.1
**NT Identity (%)**	55.3073
**AA Identity (%)**	50.4202
**Number Of Stop Codons**	1
**Number Of CDS**	1

Alignment

**Alignment Score**	60.0 (NT) + 317.0 (AA) = 377.0
**Concordance (%)**	27.1175

Alignment Method	Global, seeded, nucleotide + amino acids (AGA)

Genome Region

Sequence starts at position 4017 and ends at position 4372 relative to NC_040841.1 reference sequence.

Alignment Detailed Statistics

	Begin	End	Coverage	Score	Concordance	Matches	Identities	I/D/M/F*	Stop Codons
**NT**	**4017**	**4372**	**5.0%**	**60**	**8.6%**	**355 (98.9%)**	**198 (55.2%)**	**3/1**	
Mutations:	4018C>T, 4021C>T, 4024G>A, 4036G>A, 4042G>C, 4044A>T, 4047A>G, 4048T>G, 4051G>A, 4054A>C, 4061C>T, 4062A>T, 4063G>C, 4064C>G, 4065C>A, 4067G>A, 4069A>G, 4074G>A, 4076C>T, 4078C>T, 4084A>T, 4092T>A, 4093A>T, 4094T>G, 4095G>T, 4096T>C, 4099A>G, 4100G>T, 4101C>T, 4102A>T, 4106T>C, 4107T>A, 4108C>A, 4112C>G, 4114G>A, 4120A>T, 4122T>C, 4124G>A, 4126T>G, 4135T>A, 4136T>C, 4138G>A, 4141G>A, 4144T>C, 4147G>C, 4150A>T, 4151G>T, 4152C>T, 4154T>G, 4155T>A, 4156C>G, 4159T>C, 4164G>A, 4165A>C, 4167G>T, 4168G>C, 4171G>A, 4172G>A, 4175T>A, 4180C>T, 4183T>C, 4184delG, 4185C>A, 4186T>A, 4187A>C, 4188A>C, 4193G>C, 4194A>G, 4195T>C, 4196T>A, 4197T>A, 4201A>T, 4201_4202insTCT, 4202G>A, 4204T>C, 4205A>G, 4206C>T, 4210C>T, 4213A>T, 4220A>G, 4222T>A, 4223T>C, 4228T>A, 4229C>T, 4230A>T, 4231T>C, 4232A>T, 4233G>C, 4235C>A, 4236C>A, 4237A>G, 4240T>C, 4241G>A, 4244C>G, 4246G>T, 4247A>T, 4248A>C, 4252T>C, 4255G>T, 4258G>A, 4261C>T, 4264A>G, 4265G>A, 4267A>C, 4269T>C, 4270T>C, 4273T>C, 4274C>T, 4276T>C, 4277G>A, 4280G>A, 4281A>T, 4282A>C, 4283G>A, 4285C>T, 4289A>C, 4291A>C, 4292C>A, 4297A>T, 4298A>C, 4300T>C, 4303T>C, 4304T>G, 4306G>T, 4307T>A, 4308C>G, 4309A>T, 4311A>C, 4312A>T, 4314G>A, 4315A>T, 4318G>A, 4322T>A, 4323C>A, 4324C>A, 4325T>A, 4327A>T, 4330T>C, 4333G>A, 4334G>A, 4335A>C, 4336T>A, 4339T>A, 4342T>C, 4343G>A, 4344A>G, 4345T>A, 4349T>A, 4351G>T, 4354A>G, 4355A>C, 4356T>A, 4358A>T, 4361G>T, 4363T>G, 4364G>T, 4365C>A, 4367A>C, 4369T>A								

CDS

	Begin	End	Coverage	Score	Concordance	Matches	Identities	I/D/M/F*	Stop Codons
EXL67_gp1	1289	1406	5.5%	317	36.3%	118 (99.2%)	60 (50.4%)	1/0/1/1	1
Protein mutations:	Y1297F (4044A>T), Y1298W (4047A>G 4048T>G), Q1303F (4061C>T 4062A>T 4063G>C), P1304E (4064C>G 4065C>A), E1305K (4067G>A 4069A>G), R1307K (4074G>A), H1308Y (4076C>T 4078C>T), I1313N (4092T>A 4093A>T), C1314V (4094T>G 4095G>T 4096T>C), A1316F (4100G>T 4101C>T 4102A>T), F1318Q (4106T>C 4107T>A 4108C>A), Q1320E (4112C>G 4114G>A), K1322N (4120A>T), V1323A (4122T>C), V1324M (4124G>A 4126T>G), A1333F (4151G>T 4152C>T), F1334E (4154T>G 4155T>A 4156C>G), R1337N (4164G>A 4165A>C), R1338I (4167G>T 4168G>C), M1339I (4171G>A), D1340N (4172G>A), Y1341N (4175T>A), K1345P (4187A>C 4188A>C), D1347R (4193G>C 4194A>G 4195T>C), F1348N (4196T>A 4197T>A), I1349_V1350insS (4201_4202insTCT), V1350I (4202G>A 4204T>C), T1351V (4205A>G 4206C>T), I1356V (4220A>G 4222T>A), H1359F (4229C>T 4230A>T 4231T>C), P1361K (4235C>A 4236C>A 4237A>G), V1363I (4241G>A), Q1364D (4244C>G 4246G>T), N1365S (4247A>T 4248A>C), L1367F (4255G>T), E1371N (4265G>A 4267A>C), I1372T (4269T>C 4270T>C), L1374F (4274C>T 4276T>C), E1375K (4277G>A), E1376I (4280G>A 4281A>T 4282A>C), V1377I (4283G>A 4285C>T), K1379H (4289A>C 4291A>C), H1380N (4292C>A), I1382L (4298A>C 4300T>C), L1384V (4304T>G 4306G>T), E1386A (4311A>C 4312A>T), R1387N (4314G>A 4315A>T), S1390K (4322T>A 4323C>A 4324C>A), L1391I (4325T>A 4327A>T), D1394T (4334G>A 4335A>C 4336T>A), N1395K (4339T>A), D1397R (4343G>A 4344A>G 4345T>A), L1399I (4349T>A 4351G>T), I1401H (4355A>C 4356T>A), N1402Y (4358A>T), V1403L (4361G>T 4363T>G), A1404* (4364G>T 4365C>A), N1405Q (4367A>C 4369T>A)								
Codon mutations:	TTC1288.TT (4018C>T), AGC1289AGT (4021C>T), AAG1290AAA (4024G>A), AAG1294AAA (4036G>A), GGG1296GGC (4042G>C), TAT1297TTT (4044A>T), TAT1298TGG (4047A>G 4048T>G), CAG1299CAA (4051G>A), ATA1300ATC (4054A>C), CAG1303TTC (4061C>T 4062A>T 4063G>C), CCA1304GAA (4064C>G 4065C>A), GAA1305AAG (4067G>A 4069A>G), AGA1307AAA (4074G>A), CAC1308TAT (4076C>T 4078C>T), ACA1310ACT (4084A>T), ATA1313AAT (4092T>A 4093A>T), TGT1314GTC (4094T>G 4095G>T 4096T>C), CCA1315CCG (4099A>G), GCA1316TTT (4100G>T 4101C>T 4102A>T), TTC1318CAA (4106T>C 4107T>A 4108C>A), CAG1320GAA (4112C>G 4114G>A), AAA1322AAT (4120A>T), GTT1323GCT (4122T>C), GTT1324ATG (4124G>A 4126T>G), GGT1327GGA (4135T>A), TTG1328CTA (4136T>C 4138G>A), AAG1329AAA (4141G>A), AAT1330AAC (4144T>C), GCG1331GCC (4147G>C), CCA1332CCT (4150A>T), GCC1333TTC (4151G>T 4152C>T), TTC1334GAG (4154T>G 4155T>A 4156C>G), TTT1335TTC (4159T>C), AGA1337AAC (4164G>A 4165A>C), AGG1338ATC (4167G>T 4168G>C), ATG1339ATA (4171G>A), GAT1340AAT (4172G>A), TAT1341AAT (4175T>A), ATC1342ATT (4180C>T), TTT1343TTC (4183T>C), GCT1344-AA (4184delG 4185C>A 4186T>A), AAA1345CCA (4187A>C 4188A>C), GAT1347CGC (4193G>C 4194A>G 4195T>C), TTT1348AAT (4196T>A 4197T>A), ATA1349ATT (4201A>T), ATA1349_GTT1350insTCT (4201_4202insTCT), GTT1350ATC (4202G>A 4204T>C), ACA1351GTA (4205A>G 4206C>T), TAC1352TAT (4210C>T), ATA1353ATT (4213A>T), ATT1356GTA (4220A>G 4222T>A), TTA1357CTA (4223T>C), ATT1358ATA (4228T>A), CAT1359TTC (4229C>T 4230A>T 4231T>C), AGT1360TCT (4232A>T 4233G>C), CCA1361AAG (4235C>A 4236C>A 4237A>G), GAT1362GAC (4240T>C), GTA1363ATA (4241G>A), CAG1364GAT (4244C>G 4246G>T), AAT1365TCT (4247A>T 4248A>C), CAT1366CAC (4252T>C), TTG1367TTT (4255G>T), AAG1368AAA (4258G>A), CAC1369CAT (4261C>T), TTA1370TTG (4264A>G), GAA1371AAC (4265G>A 4267A>C), ATT1372ACC (4269T>C 4270T>C), TTT1373TTC (4273T>C), CTT1374TTC (4274C>T 4276T>C), GAA1375AAA (4277G>A), GAA1376ATC (4280G>A 4281A>T 4282A>C), GTC1377ATT (4283G>A 4285C>T), AAA1379CAC (4289A>C 4291A>C), CAT1380AAT (4292C>A), GGA1381GGT (4297A>T), ATT1382CTC (4298A>C 4300T>C), GTT1383GTC (4303T>C), TTG1384GTT (4304T>G 4306G>T), TCA1385AGT (4307T>A 4308C>G 4309A>T), GAA1386GCT (4311A>C 4312A>T), AGA1387AAT (4314G>A 4315A>T), AAG1388AAA (4318G>A), TCC1390AAA (4322T>A 4323C>A 4324C>A), TTA1391ATT (4325T>A 4327A>T), TTT1392TTC (4330T>C), CAG1393CAA (4333G>A), GAT1394ACA (4334G>A 4335A>C 4336T>A), AAT1395AAA (4339T>A), ATT1396ATC (4342T>C), GAT1397AGA (4343G>A 4344A>G 4345T>A), TTG1399ATT (4349T>A 4351G>T), GGA1400GGG (4354A>G), ATT1401CAT (4355A>C 4356T>A), AAT1402TAT (4358A>T), GTT1403TTG (4361G>T 4363T>G), GCA1404TAA (4364G>T 4365C>A), AAT1405CAA (4367A>C 4369T>A)								

Proteins

	Begin	End	Coverage	Score	Concordance	Matches	Identities	I/D/M/F*	Stop Codons
polyprotein (YP_009553669.1)	1289	1406	5.5%	317	36.3%	118 (99.2%)	60 (50.4%)	1/0/1/1	1
Protein mutations:	Y1297F (4044A>T), Y1298W (4047A>G 4048T>G), Q1303F (4061C>T 4062A>T 4063G>C), P1304E (4064C>G 4065C>A), E1305K (4067G>A 4069A>G), R1307K (4074G>A), H1308Y (4076C>T 4078C>T), I1313N (4092T>A 4093A>T), C1314V (4094T>G 4095G>T 4096T>C), A1316F (4100G>T 4101C>T 4102A>T), F1318Q (4106T>C 4107T>A 4108C>A), Q1320E (4112C>G 4114G>A), K1322N (4120A>T), V1323A (4122T>C), V1324M (4124G>A 4126T>G), A1333F (4151G>T 4152C>T), F1334E (4154T>G 4155T>A 4156C>G), R1337N (4164G>A 4165A>C), R1338I (4167G>T 4168G>C), M1339I (4171G>A), D1340N (4172G>A), Y1341N (4175T>A), K1345P (4187A>C 4188A>C), D1347R (4193G>C 4194A>G 4195T>C), F1348N (4196T>A 4197T>A), I1349_V1350insS (4201_4202insTCT), V1350I (4202G>A 4204T>C), T1351V (4205A>G 4206C>T), I1356V (4220A>G 4222T>A), H1359F (4229C>T 4230A>T 4231T>C), P1361K (4235C>A 4236C>A 4237A>G), V1363I (4241G>A), Q1364D (4244C>G 4246G>T), N1365S (4247A>T 4248A>C), L1367F (4255G>T), E1371N (4265G>A 4267A>C), I1372T (4269T>C 4270T>C), L1374F (4274C>T 4276T>C), E1375K (4277G>A), E1376I (4280G>A 4281A>T 4282A>C), V1377I (4283G>A 4285C>T), K1379H (4289A>C 4291A>C), H1380N (4292C>A), I1382L (4298A>C 4300T>C), L1384V (4304T>G 4306G>T), E1386A (4311A>C 4312A>T), R1387N (4314G>A 4315A>T), S1390K (4322T>A 4323C>A 4324C>A), L1391I (4325T>A 4327A>T), D1394T (4334G>A 4335A>C 4336T>A), N1395K (4339T>A), D1397R (4343G>A 4344A>G 4345T>A), L1399I (4349T>A 4351G>T), I1401H (4355A>C 4356T>A), N1402Y (4358A>T), V1403L (4361G>T 4363T>G), A1404* (4364G>T 4365C>A), N1405Q (4367A>C 4369T>A)								
Codon mutations:	TTC1288.TT (4018C>T), AGC1289AGT (4021C>T), AAG1290AAA (4024G>A), AAG1294AAA (4036G>A), GGG1296GGC (4042G>C), TAT1297TTT (4044A>T), TAT1298TGG (4047A>G 4048T>G), CAG1299CAA (4051G>A), ATA1300ATC (4054A>C), CAG1303TTC (4061C>T 4062A>T 4063G>C), CCA1304GAA (4064C>G 4065C>A), GAA1305AAG (4067G>A 4069A>G), AGA1307AAA (4074G>A), CAC1308TAT (4076C>T 4078C>T), ACA1310ACT (4084A>T), ATA1313AAT (4092T>A 4093A>T), TGT1314GTC (4094T>G 4095G>T 4096T>C), CCA1315CCG (4099A>G), GCA1316TTT (4100G>T 4101C>T 4102A>T), TTC1318CAA (4106T>C 4107T>A 4108C>A), CAG1320GAA (4112C>G 4114G>A), AAA1322AAT (4120A>T), GTT1323GCT (4122T>C), GTT1324ATG (4124G>A 4126T>G), GGT1327GGA (4135T>A), TTG1328CTA (4136T>C 4138G>A), AAG1329AAA (4141G>A), AAT1330AAC (4144T>C), GCG1331GCC (4147G>C), CCA1332CCT (4150A>T), GCC1333TTC (4151G>T 4152C>T), TTC1334GAG (4154T>G 4155T>A 4156C>G), TTT1335TTC (4159T>C), AGA1337AAC (4164G>A 4165A>C), AGG1338ATC (4167G>T 4168G>C), ATG1339ATA (4171G>A), GAT1340AAT (4172G>A), TAT1341AAT (4175T>A), ATC1342ATT (4180C>T), TTT1343TTC (4183T>C), GCT1344-AA (4184delG 4185C>A 4186T>A), AAA1345CCA (4187A>C 4188A>C), GAT1347CGC (4193G>C 4194A>G 4195T>C), TTT1348AAT (4196T>A 4197T>A), ATA1349ATT (4201A>T), ATA1349_GTT1350insTCT (4201_4202insTCT), GTT1350ATC (4202G>A 4204T>C), ACA1351GTA (4205A>G 4206C>T), TAC1352TAT (4210C>T), ATA1353ATT (4213A>T), ATT1356GTA (4220A>G 4222T>A), TTA1357CTA (4223T>C), ATT1358ATA (4228T>A), CAT1359TTC (4229C>T 4230A>T 4231T>C), AGT1360TCT (4232A>T 4233G>C), CCA1361AAG (4235C>A 4236C>A 4237A>G), GAT1362GAC (4240T>C), GTA1363ATA (4241G>A), CAG1364GAT (4244C>G 4246G>T), AAT1365TCT (4247A>T 4248A>C), CAT1366CAC (4252T>C), TTG1367TTT (4255G>T), AAG1368AAA (4258G>A), CAC1369CAT (4261C>T), TTA1370TTG (4264A>G), GAA1371AAC (4265G>A 4267A>C), ATT1372ACC (4269T>C 4270T>C), TTT1373TTC (4273T>C), CTT1374TTC (4274C>T 4276T>C), GAA1375AAA (4277G>A), GAA1376ATC (4280G>A 4281A>T 4282A>C), GTC1377ATT (4283G>A 4285C>T), AAA1379CAC (4289A>C 4291A>C), CAT1380AAT (4292C>A), GGA1381GGT (4297A>T), ATT1382CTC (4298A>C 4300T>C), GTT1383GTC (4303T>C), TTG1384GTT (4304T>G 4306G>T), TCA1385AGT (4307T>A 4308C>G 4309A>T), GAA1386GCT (4311A>C 4312A>T), AGA1387AAT (4314G>A 4315A>T), AAG1388AAA (4318G>A), TCC1390AAA (4322T>A 4323C>A 4324C>A), TTA1391ATT (4325T>A 4327A>T), TTT1392TTC (4330T>C), CAG1393CAA (4333G>A), GAT1394ACA (4334G>A 4335A>C 4336T>A), AAT1395AAA (4339T>A), ATT1396ATC (4342T>C), GAT1397AGA (4343G>A 4344A>G 4345T>A), TTG1399ATT (4349T>A 4351G>T), GGA1400GGG (4354A>G), ATT1401CAT (4355A>C 4356T>A), AAT1402TAT (4358A>T), GTT1403TTG (4361G>T 4363T>G), GCA1404TAA (4364G>T 4365C>A), AAT1405CAA (4367A>C 4369T>A)								

*: Inserts / Deletes / Misaligned / Frameshifts

Analysis details

This analysis was performed with panviral2.64

NGS Details (UN9): Badnavirus rutilanscamelliae

Assembly

**Coverage Length**	335 (1 contig(s))
**Depth Of Coverage**	2.6
**Number Of Reads**	7
**Reads Per Million**	0.16 rpm (after QC)
**Ambiguities**	0
**Assembly Method**	de novo + reference guided assembly
**Consensus Caller**	Bcf Tools

Coverage Map

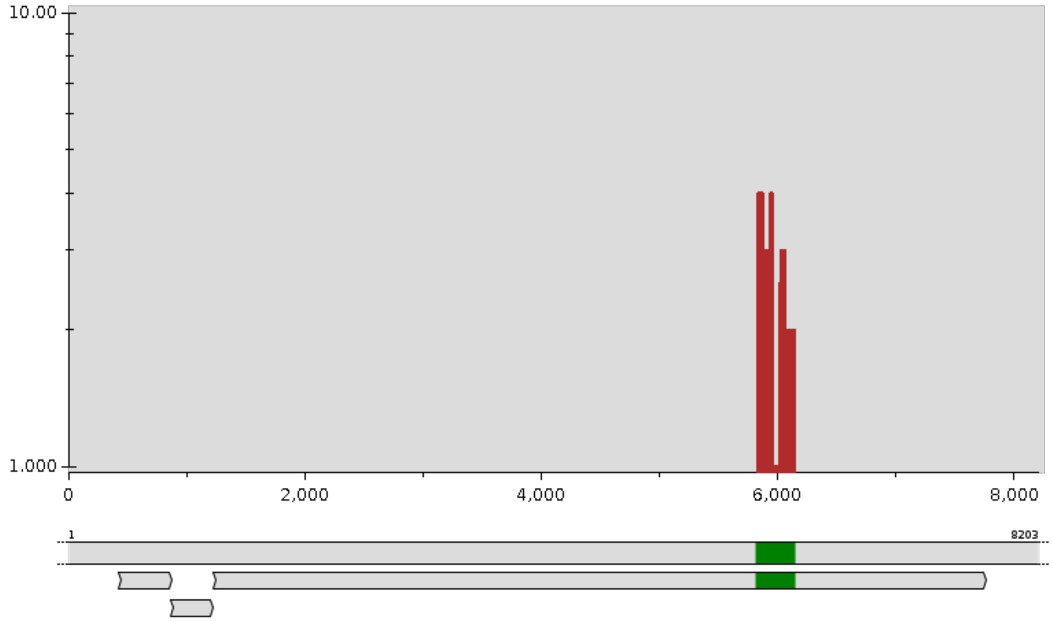

Assignment

**Type**	Badnavirus rutilanscamelliae (Taxonomy ID: 3047719)
**Reference Genome**	NC_055598.1
**NT Identity (%)**	55.3254
**AA Identity (%)**	45.1327
**Number Of Stop Codons**	0
**Number Of CDS**	3

Alignment

**Alignment Score**	66.0 (NT) + 368.0 (AA) = 434.0
**Concordance (%)**	28.972

Alignment Method	Global, seeded, nucleotide + amino acids (AGA)

Genome Region

Sequence starts at position 5822 and ends at position 6156 relative to NC_055598.1 reference sequence.

Alignment Detailed Statistics

	Begin	End	Coverage	Score	Concordance	Matches	Identities	I/D/M/F*	Stop Codons
**NT**	**5822**	**6156**	**4.1%**	**66**	**9.9%**	**335 (99.1%)**	**187 (55.3%)**	**3/0**	
Mutations:	5822G>A, 5824A>G, 5828A>G, 5830A>T, 5834A>T, 5835A>C, 5836G>A, 5837G>A, 5838A>T, 5842G>A, 5846G>T, 5847T>G, 5848C>T, 5849T>A, 5850G>T, 5851C>T, 5852A>G, 5854C>T, 5857C>T, 5859A>G, 5860G>A, 5861A>C, 5862G>A, 5864C>T, 5866T>G, 5870G>A, 5872C>G, 5873A>G, 5874A>T, 5875C>G, 5879C>G, 5880A>T, 5881C>A, 5884A>G, 5885G>A, 5888C>A, 5894A>C, 5895G>C, 5899T>C, 5902A>C, 5903G>C, 5905G>T, 5908T>C, 5911C>T, 5913C>A, 5915A>T, 5917T>A, 5918G>T, 5920G>T, 5922C>A, 5923G>T, 5924A>C, 5925G>A, 5927A>T, 5930G>C, 5931G>A, 5933C>G, 5934A>G, 5935T>A, 5936A>G, 5937G>C, 5938C>A, 5939A>G, 5940A>C, 5941A>C, 5944C>A, 5946A>T, 5947T>C, 5950G>T, 5954T>A, 5956C>T, 5959C>T, 5964A>G, 5965G>A, 5967C>T, 5968A>C, 5971G>A, 5973T>A, 5980A>G, 5981G>T, 5983T>G, 5984G>A, 5985C>A, 5986T>G, 5989G>T, 5990G>C, 5996C>T, 5997A>C, 5998A>T, 5999C>G, 6001C>T, 6005G>C, 6006A>C, 6007A>T, 6008T>A, 6009G>A, 6013C>A, 6019T>C, 6020T>C, 6025T>C, 6026C>A, 6027C>G, 6028A>G, 6029G>T, 6030T>A, 6031G>T, 6035T>C, 6036T>A, 6037A>T, 6043A>G, 6045G>T, 6046G>T, 6049G>T, 6056C>T, 6058A>G, 6065C>T, 6067T>G, 6069A>C, 6070G>T, 6079A>G, 6080G>A, 6084T>C, 6089C>A, 6090A>T, 6091A>G, 6092A>G, 6093G>A, 6094G>A, 6095A>T, 6096A>T, 6097G>A, 6101G>A, 6103C>T, 6104C>G, 6106A>G, 6107A>G, 6109C>G, 6113A>C, 6118A>G, 6121_6122insTTG, 6123C>A, 6125G>T, 6126G>C, 6131A>G, 6133T>G, 6134G>A, 6135C>T, 6139G>A, 6141A>T								

CDS

	Begin	End	Coverage	Score	Concordance	Matches	Identities	I/D/M/F*	Stop Codons
KM754_gp3	1534	1645	5.1%	368	44.2%	112 (99.1%)	51 (45.1%)	1/0/0/0	0
Protein mutations:	E1534K (5822G>A 5824A>G), K1536D (5828A>G 5830A>T), K1538S (5834A>T 5835A>C 5836G>A), E1539M (5837G>A 5838A>T), V1542C (5846G>T 5847T>G 5848C>T), C1543I (5849T>A 5850G>T 5851C>T), N1544D (5852A>G 5854C>T), K1546R (5859A>G 5860G>A), R1547Q (5861A>C 5862G>A), D1550K (5870G>A 5872C>G), N1551V (5873A>G 5874A>T 5875C>G), H1553V (5879C>G 5880A>T 5881C>A), D1555N (5885G>A), Q1556K (5888C>A), S1558P (5894A>C 5895G>C), G1561R (5903G>C 5905G>T), T1564N (5913C>A), I1565L (5915A>T 5917T>A), V1566F (5918G>T 5920G>T), A1567D (5922C>A 5923G>T), R1568Q (5924A>C 5925G>A), I1569L (5927A>T), G1570Q (5930G>C 5931G>A), H1571G (5933C>G 5934A>G 5935T>A), S1572A (5936A>G 5937G>C 5938C>A), K1573A (5939A>G 5940A>C 5941A>C), Y1575F (5946A>T 5947T>C), F1578I (5954T>A 5956C>T), K1581R (5964A>G 5965G>A), S1582F (5967C>T 5968A>C), F1584Y (5973T>A), V1587L (5981G>T 5983T>G), A1588K (5984G>A 5985C>A 5986T>G), M1589I (5989G>T), D1590H (5990G>C), Q1592S (5996C>T 5997A>C 5998A>T), H1593D (5999C>G 6001C>T), E1595P (6005G>C 6006A>C 6007A>T), W1596K (6008T>A 6009G>A), W1600R (6020T>C), P1602R (6026C>A 6027C>G 6028A>G), V1603Y (6029G>T 6030T>A 6031G>T), L1605H (6035T>C 6036T>A 6037A>T), W1608F (6045G>T 6046G>T), P1612S (6056C>T 6058A>G), K1616T (6069A>C 6070G>T), A1620T (6080G>A), V1621A (6084T>C), Q1623M (6089C>A 6090A>T 6091A>G), R1624E (6092A>G 6093G>A 6094G>A), K1625L (6095A>T 6096A>T 6097G>A), D1627N (6101G>A 6103C>T), R1628G (6104C>G 6106A>G), I1629V (6107A>G 6109C>G), N1631H (6113A>C), Y1633_A1634insL (6121_6122insTTG), A1634D (6123C>A), G1635S (6125G>T 6126G>C), I1637V (6131A>G 6133T>G), A1638I (6134G>A 6135C>T), Y1640F (6141A>T)								
Codon mutations:	GAA1534AAG (5822G>A 5824A>G), AAA1536GAT (5828A>G 5830A>T), AAG1538TCA (5834A>T 5835A>C 5836G>A), GAG1539ATG (5837G>A 5838A>T), AGG1540AGA (5842G>A), GTC1542TGT (5846G>T 5847T>G 5848C>T), TGC1543ATT (5849T>A 5850G>T 5851C>T), AAC1544GAT (5852A>G 5854C>T), TAC1545TAT (5857C>T), AAG1546AGA (5859A>G 5860G>A), AGG1547CAG (5861A>C 5862G>A), CTT1548TTG (5864C>T 5866T>G), GAC1550AAG (5870G>A 5872C>G), AAC1551GTG (5873A>G 5874A>T 5875C>G), CAC1553GTA (5879C>G 5880A>T 5881C>A), AAA1554AAG (5884A>G), GAC1555AAC (5885G>A), CAA1556AAA (5888C>A), AGC1558CCC (5894A>C 5895G>C), CTT1559CTC (5899T>C), CCA1560CCC (5902A>C), GGG1561CGT (5903G>C 5905G>T), ATT1562ATC (5908T>C), GAC1563GAT (5911C>T), ACT1564AAT (5913C>A), ATT1565TTA (5915A>T 5917T>A), GTG1566TTT (5918G>T 5920G>T), GCG1567GAT (5922C>A 5923G>T), AGG1568CAG (5924A>C 5925G>A), ATA1569TTA (5927A>T), GGG1570CAG (5930G>C 5931G>A), CAT1571GGA (5933C>G 5934A>G 5935T>A), AGC1572GCA (5936A>G 5937G>C 5938C>A), AAA1573GCC (5939A>G 5940A>C 5941A>C), ATC1574ATA (5944C>A), TAT1575TTC (5946A>T 5947T>C), TCG1576TCT (5950G>T), TTC1578ATT (5954T>A 5956C>T), GAC1579GAT (5959C>T), AAG1581AGA (5964A>G 5965G>A), TCA1582TTC (5967C>T 5968A>C), GGG1583GGA (5971G>A), TTT1584TAT (5973T>A), CAA1586CAG (5980A>G), GTT1587TTG (5981G>T 5983T>G), GCT1588AAG (5984G>A 5985C>A 5986T>G), ATG1589ATT (5989G>T), GAT1590CAT (5990G>C), CAA1592TCT (5996C>T 5997A>C 5998A>T), CAC1593GAT (5999C>G 6001C>T), GAA1595CCT (6005G>C 6006A>C 6007A>T), TGG1596AAG (6008T>A 6009G>A), ACC1597ACA (6013C>A), TTT1599TTC (6019T>C), TGG1600CGG (6020T>C), ACT1601ACC (6025T>C), CCA1602AGG (6026C>A 6027C>G 6028A>G), GTG1603TAT (6029G>T 6030T>A 6031G>T), TTA1605CAT (6035T>C 6036T>A 6037A>T), GAA1607GAG (6043A>G), TGG1608TTT (6045G>T 6046G>T), CTG1609CTT (6049G>T), CCA1612TCG (6056C>T 6058A>G), CTT1615TTG (6065C>T 6067T>G), AAG1616ACT (6069A>C 6070G>T), CCA1619CCG (6079A>G), GCA1620ACA (6080G>A), GTA1621GCA (6084T>C), CAA1623ATG (6089C>A 6090A>T 6091A>G), AGG1624GAA (6092A>G 6093G>A 6094G>A), AAG1625TTA (6095A>T 6096A>T 6097G>A), GAC1627AAT (6101G>A 6103C>T), CGA1628GGG (6104C>G 6106A>G), ATC1629GTG (6107A>G 6109C>G), AAT1631CAT (6113A>C), CCA1632CCG (6118A>G), TAC1633_GCT1634insTTG (6121_6122insTTG), GCT1634GAT (6123C>A), GGA1635TCA (6125G>T 6126G>C), ATT1637GTG (6131A>G 6133T>G), GCT1638ATT (6134G>A 6135C>T), GTG1639GTA (6139G>A), TAT1640TTT (6141A>T)								

Proteins

	Begin	End	Coverage	Score	Concordance	Matches	Identities	I/D/M/F*	Stop Codons
polyprotein (YP_010087856.1)	1534	1645	5.1%	368	44.2%	112 (99.1%)	51 (45.1%)	1/0/0/0	0
Protein mutations:	E1534K (5822G>A 5824A>G), K1536D (5828A>G 5830A>T), K1538S (5834A>T 5835A>C 5836G>A), E1539M (5837G>A 5838A>T), V1542C (5846G>T 5847T>G 5848C>T), C1543I (5849T>A 5850G>T 5851C>T), N1544D (5852A>G 5854C>T), K1546R (5859A>G 5860G>A), R1547Q (5861A>C 5862G>A), D1550K (5870G>A 5872C>G), N1551V (5873A>G 5874A>T 5875C>G), H1553V (5879C>G 5880A>T 5881C>A), D1555N (5885G>A), Q1556K (5888C>A), S1558P (5894A>C 5895G>C), G1561R (5903G>C 5905G>T), T1564N (5913C>A), I1565L (5915A>T 5917T>A), V1566F (5918G>T 5920G>T), A1567D (5922C>A 5923G>T), R1568Q (5924A>C 5925G>A), I1569L (5927A>T), G1570Q (5930G>C 5931G>A), H1571G (5933C>G 5934A>G 5935T>A), S1572A (5936A>G 5937G>C 5938C>A), K1573A (5939A>G 5940A>C 5941A>C), Y1575F (5946A>T 5947T>C), F1578I (5954T>A 5956C>T), K1581R (5964A>G 5965G>A), S1582F (5967C>T 5968A>C), F1584Y (5973T>A), V1587L (5981G>T 5983T>G), A1588K (5984G>A 5985C>A 5986T>G), M1589I (5989G>T), D1590H (5990G>C), Q1592S (5996C>T 5997A>C 5998A>T), H1593D (5999C>G 6001C>T), E1595P (6005G>C 6006A>C 6007A>T), W1596K (6008T>A 6009G>A), W1600R (6020T>C), P1602R (6026C>A 6027C>G 6028A>G), V1603Y (6029G>T 6030T>A 6031G>T), L1605H (6035T>C 6036T>A 6037A>T), W1608F (6045G>T 6046G>T), P1612S (6056C>T 6058A>G), K1616T (6069A>C 6070G>T), A1620T (6080G>A), V1621A (6084T>C), Q1623M (6089C>A 6090A>T 6091A>G), R1624E (6092A>G 6093G>A 6094G>A), K1625L (6095A>T 6096A>T 6097G>A), D1627N (6101G>A 6103C>T), R1628G (6104C>G 6106A>G), I1629V (6107A>G 6109C>G), N1631H (6113A>C), Y1633_A1634insL (6121_6122insTTG), A1634D (6123C>A), G1635S (6125G>T 6126G>C), I1637V (6131A>G 6133T>G), A1638I (6134G>A 6135C>T), Y1640F (6141A>T)								
Codon mutations:	GAA1534AAG (5822G>A 5824A>G), AAA1536GAT (5828A>G 5830A>T), AAG1538TCA (5834A>T 5835A>C 5836G>A), GAG1539ATG (5837G>A 5838A>T), AGG1540AGA (5842G>A), GTC1542TGT (5846G>T 5847T>G 5848C>T), TGC1543ATT (5849T>A 5850G>T 5851C>T), AAC1544GAT (5852A>G 5854C>T), TAC1545TAT (5857C>T), AAG1546AGA (5859A>G 5860G>A), AGG1547CAG (5861A>C 5862G>A), CTT1548TTG (5864C>T 5866T>G), GAC1550AAG (5870G>A 5872C>G), AAC1551GTG (5873A>G 5874A>T 5875C>G), CAC1553GTA (5879C>G 5880A>T 5881C>A), AAA1554AAG (5884A>G), GAC1555AAC (5885G>A), CAA1556AAA (5888C>A), AGC1558CCC (5894A>C 5895G>C), CTT1559CTC (5899T>C), CCA1560CCC (5902A>C), GGG1561CGT (5903G>C 5905G>T), ATT1562ATC (5908T>C), GAC1563GAT (5911C>T), ACT1564AAT (5913C>A), ATT1565TTA (5915A>T 5917T>A), GTG1566TTT (5918G>T 5920G>T), GCG1567GAT (5922C>A 5923G>T), AGG1568CAG (5924A>C 5925G>A), ATA1569TTA (5927A>T), GGG1570CAG (5930G>C 5931G>A), CAT1571GGA (5933C>G 5934A>G 5935T>A), AGC1572GCA (5936A>G 5937G>C 5938C>A), AAA1573GCC (5939A>G 5940A>C 5941A>C), ATC1574ATA (5944C>A), TAT1575TTC (5946A>T 5947T>C), TCG1576TCT (5950G>T), TTC1578ATT (5954T>A 5956C>T), GAC1579GAT (5959C>T), AAG1581AGA (5964A>G 5965G>A), TCA1582TTC (5967C>T 5968A>C), GGG1583GGA (5971G>A), TTT1584TAT (5973T>A), CAA1586CAG (5980A>G), GTT1587TTG (5981G>T 5983T>G), GCT1588AAG (5984G>A 5985C>A 5986T>G), ATG1589ATT (5989G>T), GAT1590CAT (5990G>C), CAA1592TCT (5996C>T 5997A>C 5998A>T), CAC1593GAT (5999C>G 6001C>T), GAA1595CCT (6005G>C 6006A>C 6007A>T), TGG1596AAG (6008T>A 6009G>A), ACC1597ACA (6013C>A), TTT1599TTC (6019T>C), TGG1600CGG (6020T>C), ACT1601ACC (6025T>C), CCA1602AGG (6026C>A 6027C>G 6028A>G), GTG1603TAT (6029G>T 6030T>A 6031G>T), TTA1605CAT (6035T>C 6036T>A 6037A>T), GAA1607GAG (6043A>G), TGG1608TTT (6045G>T 6046G>T), CTG1609CTT (6049G>T), CCA1612TCG (6056C>T 6058A>G), CTT1615TTG (6065C>T 6067T>G), AAG1616ACT (6069A>C 6070G>T), CCA1619CCG (6079A>G), GCA1620ACA (6080G>A), GTA1621GCA (6084T>C), CAA1623ATG (6089C>A 6090A>T 6091A>G), AGG1624GAA (6092A>G 6093G>A 6094G>A), AAG1625TTA (6095A>T 6096A>T 6097G>A), GAC1627AAT (6101G>A 6103C>T), CGA1628GGG (6104C>G 6106A>G), ATC1629GTG (6107A>G 6109C>G), AAT1631CAT (6113A>C), CCA1632CCG (6118A>G), TAC1633_GCT1634insTTG (6121_6122insTTG), GCT1634GAT (6123C>A), GGA1635TCA (6125G>T 6126G>C), ATT1637GTG (6131A>G 6133T>G), GCT1638ATT (6134G>A 6135C>T), GTG1639GTA (6139G>A), TAT1640TTT (6141A>T)								

*: Inserts / Deletes / Misaligned / Frameshifts

Analysis details

This analysis was performed with panviral2.64

NGS Details (UN9): Fig cryptic virus (segment RNA 2)

Assembly

**Coverage Length**	164 (1 contig(s))
**Depth Of Coverage**	3.1
**Number Of Reads**	6
**Reads Per Million**	0.14 rpm (after QC)
**Ambiguities**	0
**Assembly Method**	de novo + reference guided assembly
**Consensus Caller**	Bcf Tools

Coverage Map

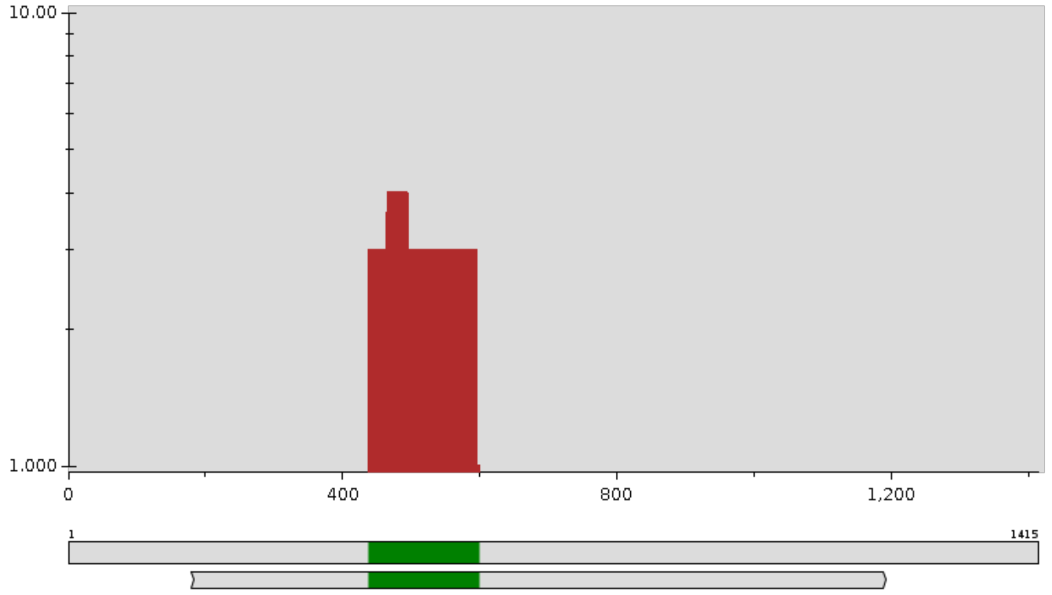

Assignment

**Type**	Fig cryptic virus (Taxonomy ID: 882768)
**Reference Genome**	NC_015495.1
**NT Identity (%)**	63.4146
**AA Identity (%)**	61.8182
**Number Of Stop Codons**	0
**Number Of CDS**	1

Alignment

**Alignment Score**	88.0 (NT) + 226.0 (AA) = 314.0
**Concordance (%)**	43.6718

Alignment Method	Global, seeded, nucleotide + amino acids (AGA)

Genome Region

Sequence starts at position 438 and ends at position 601 relative to NC_015495.1 reference sequence.

Alignment Detailed Statistics

	Begin	End	Coverage	Score	Concordance	Matches	Identities	I/D/M/F*	Stop Codons
**NT**	**438**	**601**	**11.6%**	**88**	**26.8%**	**164 (100%)**	**104 (63.4%)**	**0/0**	
Mutations:	438A>T, 441T>C, 444T>C, 447C>T, 450A>G, 456A>C, 457T>A, 460G>A, 468A>G, 469T>C, 471C>T, 475C>T, 476A>C, 477C>T, 479A>C, 480C>A, 481C>A, 483A>C, 486G>C, 487C>A, 489T>G, 493T>A, 495G>T, 498A>C, 504C>T, 505A>C, 507A>T, 516A>T, 517A>T, 519T>G, 520T>A, 521C>G, 522T>C, 529A>C, 538C>T, 540A>G, 541A>G, 542G>T, 547C>A, 549T>C, 555T>G, 557T>C, 558G>T, 561T>C, 562G>T, 563A>C, 564A>T, 567T>A, 571A>G, 574T>C, 575G>C, 576G>A, 580C>A, 581G>T, 582T>C, 585T>C, 588C>T, 590A>G, 591C>T, 592G>A								

CDS

	Begin	End	Coverage	Score	Concordance	Matches	Identities	I/D/M/F*	Stop Codons
CP	87	141	16.3%	226	55.3%	55 (100%)	34 (61.8%)	0/0/0/0	0
Protein mutations:	S93T (457T>A), V94I (460G>A), C97R (469T>C 471C>T), H99S (475C>T 476A>C 477C>T), H100P (479A>C 480C>A), P101T (481C>A 483A>C), S105T (493T>A 495G>T), T109P (505A>C 507A>T), I113L (517A>T 519T>G), M117L (529A>C), P120S (538C>T 540A>G), S121V (541A>G 542G>T), L123I (547C>A 549T>C), M126T (557T>C 558G>T), E128S (562G>T 563A>C 564A>T), S131G (571A>G), W132P (574T>C 575G>C 576G>A), R134I (580C>A 581G>T 582T>C), D137G (590A>G 591C>T), G138R (592G>A)								
Codon mutations:	CAA86..T (438A>T), CTT87CTC (441T>C), TAT88TAC (444T>C), AAC89AAT (447C>T), AGA90AGG (450A>G), CGA92CGC (456A>C), TCT93ACT (457T>A), GTT94ATT (460G>A), GAA96GAG (468A>G), TGC97CGT (469T>C 471C>T), CAC99TCT (475C>T 476A>C 477C>T), CAC100CCA (479A>C 480C>A), CCA101ACC (481C>A 483A>C), ACG102ACC (486G>C), CGT103AGG (487C>A 489T>G), TCG105ACT (493T>A 495G>T), CGA106CGC (498A>C), AAC108AAT (504C>T), ACA109CCT (505A>C 507A>T), CCA112CCT (516A>T), ATT113TTG (517A>T 519T>G), TCT114AGC (520T>A 521C>G 522T>C), ATG117CTG (529A>C), CCA120TCG (538C>T 540A>G), AGC121GTC (541A>G 542G>T), CTT123ATC (547C>A 549T>C), TCT125TCG (555T>G), ATG126ACT (557T>C 558G>T), CTT127CTC (561T>C), GAA128TCT (562G>T 563A>C 564A>T), TCT129TCA (567T>A), AGC131GGC (571A>G), TGG132CCA (574T>C 575G>C 576G>A), CGT134ATC (580C>A 581G>T 582T>C), ATT135ATC (585T>C), AAC136AAT (588C>T), GAC137GGT (590A>G 591C>T), GGA138AGA (592G>A)								

Proteins

	Begin	End	Coverage	Score	Concordance	Matches	Identities	I/D/M/F*	Stop Codons
putative capsid protein (YP_004429259.1)	87	141	16.3%	226	55.3%	55 (100%)	34 (61.8%)	0/0/0/0	0
Protein mutations:	S93T (457T>A), V94I (460G>A), C97R (469T>C 471C>T), H99S (475C>T 476A>C 477C>T), H100P (479A>C 480C>A), P101T (481C>A 483A>C), S105T (493T>A 495G>T), T109P (505A>C 507A>T), I113L (517A>T 519T>G), M117L (529A>C), P120S (538C>T 540A>G), S121V (541A>G 542G>T), L123I (547C>A 549T>C), M126T (557T>C 558G>T), E128S (562G>T 563A>C 564A>T), S131G (571A>G), W132P (574T>C 575G>C 576G>A), R134I (580C>A 581G>T 582T>C), D137G (590A>G 591C>T), G138R (592G>A)								
Codon mutations:	CAA86..T (438A>T), CTT87CTC (441T>C), TAT88TAC (444T>C), AAC89AAT (447C>T), AGA90AGG (450A>G), CGA92CGC (456A>C), TCT93ACT (457T>A), GTT94ATT (460G>A), GAA96GAG (468A>G), TGC97CGT (469T>C 471C>T), CAC99TCT (475C>T 476A>C 477C>T), CAC100CCA (479A>C 480C>A), CCA101ACC (481C>A 483A>C), ACG102ACC (486G>C), CGT103AGG (487C>A 489T>G), TCG105ACT (493T>A 495G>T), CGA106CGC (498A>C), AAC108AAT (504C>T), ACA109CCT (505A>C 507A>T), CCA112CCT (516A>T), ATT113TTG (517A>T 519T>G), TCT114AGC (520T>A 521C>G 522T>C), ATG117CTG (529A>C), CCA120TCG (538C>T 540A>G), AGC121GTC (541A>G 542G>T), CTT123ATC (547C>A 549T>C), TCT125TCG (555T>G), ATG126ACT (557T>C 558G>T), CTT127CTC (561T>C), GAA128TCT (562G>T 563A>C 564A>T), TCT129TCA (567T>A), AGC131GGC (571A>G), TGG132CCA (574T>C 575G>C 576G>A), CGT134ATC (580C>A 581G>T 582T>C), ATT135ATC (585T>C), AAC136AAT (588C>T), GAC137GGT (590A>G 591C>T), GGA138AGA (592G>A)								

*: Inserts / Deletes / Misaligned / Frameshifts

Analysis details

This analysis was performed with panviral2.64

NGS Details (UN9): Caulimovirus venafragariae

Assembly

**Coverage Length**	243 (1 contig(s))
**Depth Of Coverage**	2.8
**Number Of Reads**	6
**Reads Per Million**	0.14 rpm (after QC)
**Ambiguities**	0
**Assembly Method**	de novo + reference guided assembly
**Consensus Caller**	Bcf Tools

Coverage Map

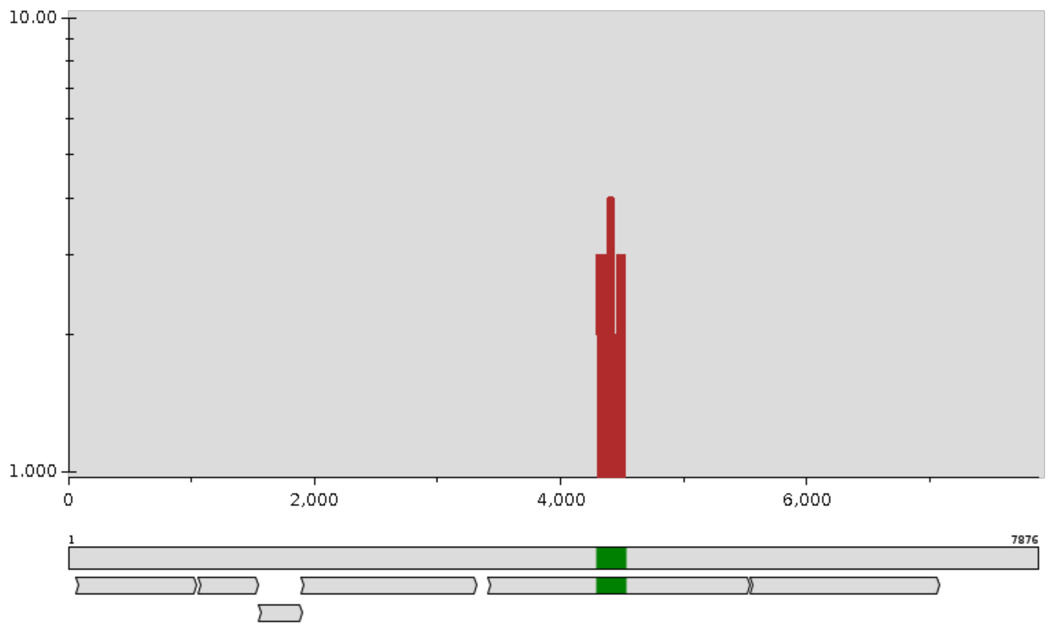

Assignment

**Type**	Caulimovirus venafragariae (Taxonomy ID: 3048344)
**Reference Genome**	NC_001725.1
**NT Identity (%)**	61.3169
**AA Identity (%)**	62.963
**Number Of Stop Codons**	0
**Number Of CDS**	6

Alignment

**Alignment Score**	110.0 (NT) + 359.0 (AA) = 469.0
**Concordance (%)**	45.1396

Alignment Method	Global, seeded, nucleotide + amino acids (AGA)

Genome Region

Sequence starts at position 4294 and ends at position 4536 relative to NC_001725.1 reference sequence.

Alignment Detailed Statistics

	Begin	End	Coverage	Score	Concordance	Matches	Identities	I/D/M/F*	Stop Codons
**NT**	**4294**	**4536**	**3.1%**	**110**	**22.6%**	**243 (100%)**	**149 (61.3%)**	**0/0**	
Mutations:	4296G>A, 4299C>T, 4300G>A, 4311C>A, 4314G>C, 4315C>T, 4317T>A, 4320T>A, 4321A>G, 4323C>T, 4326C>A, 4330C>A, 4331C>G, 4332T>A, 4333A>T, 4334G>C, 4335C>T, 4337A>C, 4338G>A, 4339A>T, 4340G>C, 4341T>A, 4342C>A, 4343C>A, 4348T>A, 4349C>G, 4350C>A, 4354C>G, 4362C>T, 4371G>A, 4378G>A, 4379C>G, 4383G>A, 4386C>A, 4387A>G, 4388A>T, 4390C>A, 4395C>T, 4402A>C, 4403G>A, 4408G>A, 4413T>A, 4419T>C, 4424A>G, 4425G>A, 4426T>C, 4428A>T, 4434C>T, 4435C>A, 4437T>C, 4440A>T, 4442A>G, 4443G>A, 4444G>A, 4445G>C, 4446A>G, 4447G>T, 4451G>C, 4452C>T, 4455C>T, 4456C>A, 4457T>A, 4458A>G, 4461T>A, 4464T>A, 4465A>G, 4467C>T, 4470G>A, 4471G>A, 4472A>G, 4473G>T, 4474C>G, 4477C>T, 4479G>A, 4480C>A, 4482T>A, 4483C>A, 4485A>T, 4491C>A, 4492G>C, 4493G>A, 4503C>A, 4504T>G, 4506T>A, 4508A>T, 4510T>A, 4511C>A, 4513T>A, 4514C>A, 4515C>A, 4521C>T, 4530T>A, 4533A>G, 4535T>A								
**CDS**									
ORF_V	297	377	11.4%	359	64.9%	81 (100%)	51 (63.0%)	0/0/0/0	0
Protein mutations:	E299K (4300G>A), K303N (4314G>C), I306V (4321A>G 4323C>T), P309R (4330C>A 4331C>G 4332T>A), K311T (4337A>C 4338G>A), P313K (4342C>A 4343C>A), S315R (4348T>A 4349C>G 4350C>A), P317A (4354C>G), A325S (4378G>A 4379C>G), K328V (4387A>G 4388A>T), R333Q (4402A>C 4403G>A), V335I (4408G>A), K340R (4424A>G 4425G>A), H344N (4435C>A 4437T>C), K346R (4442A>G 4443G>A), G347T (4444G>A 4445G>C 4446A>G), D348Y (4447G>T), G349A (4451G>C 4452C>T), L351K (4456C>A 4457T>A 4458A>G), N354D (4465A>G 4467C>T), E356S (4471G>A 4472A>G 4473G>T), Q357E (4474C>G), L359I (4480C>A 4482T>A), Q360N (4483C>A 4485A>T), G363Q (4492G>C 4493G>A), F367V (4504T>G 4506T>A), Y368F (4508A>T), S369N (4510T>A 4511C>A), S370K (4513T>A 4514C>A 4515C>A), F377Y (4535T>A)								
Codon mutations:	CAG297CAA (4296G>A), ATC298ATT (4299C>T), GAA299AAA (4300G>A), CTC302CTA (4311C>A), AAG303AAC (4314G>C), CTT304TTA (4315C>T 4317T>A), GGT305GGA (4320T>A), ATC306GTT (4321A>G 4323C>T), ATC307ATA (4326C>A), CCT309AGA (4330C>A 4331C>G 4332T>A), AGC310TCT (4333A>T 4334G>C 4335C>T), AAG311ACA (4337A>C 4338G>A), AGT312TCA (4339A>T 4340G>C 4341T>A), CCA313AAA (4342C>A 4343C>A), TCC315AGA (4348T>A 4349C>G 4350C>A), CCA317GCA (4354C>G), TTC319TTT (4362C>T), AGG322AGA (4371G>A), GCC325AGC (4378G>A 4379C>G), GAG326GAA (4383G>A), ATC327ATA (4386C>A), AAA328GTA (4387A>G 4388A>T), CGA329AGA (4390C>A), GGC330GGT (4395C>T), AGA333CAA (4402A>C 4403G>A), GTA335ATA (4408G>A), ATT336ATA (4413T>A), TAT338TAC (4419T>C), AAG340AGA (4424A>G 4425G>A), TTA341CTT (4426T>C 4428A>T), GAC343GAT (4434C>T), CAT344AAC (4435C>A 4437T>C), ACA345ACT (4440A>T), AAG346AGA (4442A>G 4443G>A), GGA347ACG (4444G>A 4445G>C 4446A>G), GAT348TAT (4447G>T), GGC349GCT (4451G>C 4452C>T), TAC350TAT (4455C>T), CTA351AAG (4456C>A 4457T>A 4458A>G), CTT352CTA (4461T>A), CCT353CCA (4464T>A), AAC354GAT (4465A>G 4467C>T), AAG355AAA (4470G>A), GAG356AGT (4471G>A 4472A>G 4473G>T), CAA357GAA (4474C>G), CTG358TTA (4477C>T 4479G>A), CTT359ATA (4480C>A 4482T>A), CAA360AAT (4483C>A 4485A>T), ATC362ATA (4491C>A), GGA363CAA (4492G>C 4493G>A), ACC366ACA (4503C>A), TTT367GTA (4504T>G 4506T>A), TAC368TTC (4508A>T), TCT369AAT (4510T>A 4511C>A), TCC370AAA (4513T>A 4514C>A 4515C>A), GAC372GAT (4521C>T), TCT375TCA (4530T>A), GGA376GGG (4533A>G), TTT377TAT (4535T>A)								
**Proteins**									
hypothetical protein (NP_043933.1)	297	377	11.4%	359	64.9%	81 (100%)	51 (63.0%)	0/0/0/0	0
Protein mutations:	E299K (4300G>A), K303N (4314G>C), I306V (4321A>G 4323C>T), P309R (4330C>A 4331C>G 4332T>A), K311T (4337A>C 4338G>A), P313K (4342C>A 4343C>A), S315R (4348T>A 4349C>G 4350C>A), P317A (4354C>G), A325S (4378G>A 4379C>G), K328V (4387A>G 4388A>T), R333Q (4402A>C 4403G>A), V335I (4408G>A), K340R (4424A>G 4425G>A), H344N (4435C>A 4437T>C), K346R (4442A>G 4443G>A), G347T (4444G>A 4445G>C 4446A>G), D348Y (4447G>T), G349A (4451G>C 4452C>T), L351K (4456C>A 4457T>A 4458A>G), N354D (4465A>G 4467C>T), E356S (4471G>A 4472A>G 4473G>T), Q357E (4474C>G), L359I (4480C>A 4482T>A), Q360N (4483C>A 4485A>T), G363Q (4492G>C 4493G>A), F367V (4504T>G 4506T>A), Y368F (4508A>T), S369N (4510T>A 4511C>A), S370K (4513T>A 4514C>A 4515C>A), F377Y (4535T>A)								
Codon mutations:	CAG297CAA (4296G>A), ATC298ATT (4299C>T), GAA299AAA (4300G>A), CTC302CTA (4311C>A), AAG303AAC (4314G>C), CTT304TTA (4315C>T 4317T>A), GGT305GGA (4320T>A), ATC306GTT (4321A>G 4323C>T), ATC307ATA (4326C>A), CCT309AGA (4330C>A 4331C>G 4332T>A), AGC310TCT (4333A>T 4334G>C 4335C>T), AAG311ACA (4337A>C 4338G>A), AGT312TCA (4339A>T 4340G>C 4341T>A), CCA313AAA (4342C>A 4343C>A), TCC315AGA (4348T>A 4349C>G 4350C>A), CCA317GCA (4354C>G), TTC319TTT (4362C>T), AGG322AGA (4371G>A), GCC325AGC (4378G>A 4379C>G), GAG326GAA (4383G>A), ATC327ATA (4386C>A), AAA328GTA (4387A>G 4388A>T), CGA329AGA (4390C>A), GGC330GGT (4395C>T), AGA333CAA (4402A>C 4403G>A), GTA335ATA (4408G>A), ATT336ATA (4413T>A), TAT338TAC (4419T>C), AAG340AGA (4424A>G 4425G>A), TTA341CTT (4426T>C 4428A>T), GAC343GAT (4434C>T), CAT344AAC (4435C>A 4437T>C), ACA345ACT (4440A>T), AAG346AGA (4442A>G 4443G>A), GGA347ACG (4444G>A 4445G>C 4446A>G), GAT348TAT (4447G>T), GGC349GCT (4451G>C 4452C>T), TAC350TAT (4455C>T), CTA351AAG (4456C>A 4457T>A 4458A>G), CTT352CTA (4461T>A), CCT353CCA (4464T>A), AAC354GAT (4465A>G 4467C>T), AAG355AAA (4470G>A), GAG356AGT (4471G>A 4472A>G 4473G>T), CAA357GAA (4474C>G), CTG358TTA (4477C>T 4479G>A), CTT359ATA (4480C>A 4482T>A), CAA360AAT (4483C>A 4485A>T), ATC362ATA (4491C>A), GGA363CAA (4492G>C 4493G>A), ACC366ACA (4503C>A), TTT367GTA (4504T>G 4506T>A), TAC368TTC (4508A>T), TCT369AAT (4510T>A 4511C>A), TCC370AAA (4513T>A 4514C>A 4515C>A), GAC372GAT (4521C>T), TCT375TCA (4530T>A), GGA376GGG (4533A>G), TTT377TAT (4535T>A)								

*: Inserts / Deletes / Misaligned / Frameshifts

Analysis details

This analysis was performed with panviral2.64

NGS Details (UN9): Tomato associated geminivirus 1

Assembly

**Coverage Length**	274 (2 contig(s))
**Depth Of Coverage**	2.5
**Number Of Reads**	6
**Reads Per Million**	0.14 rpm (after QC)
**Ambiguities**	0
**Assembly Method**	de novo + reference guided assembly
**Consensus Caller**	Bcf Tools

Coverage Map

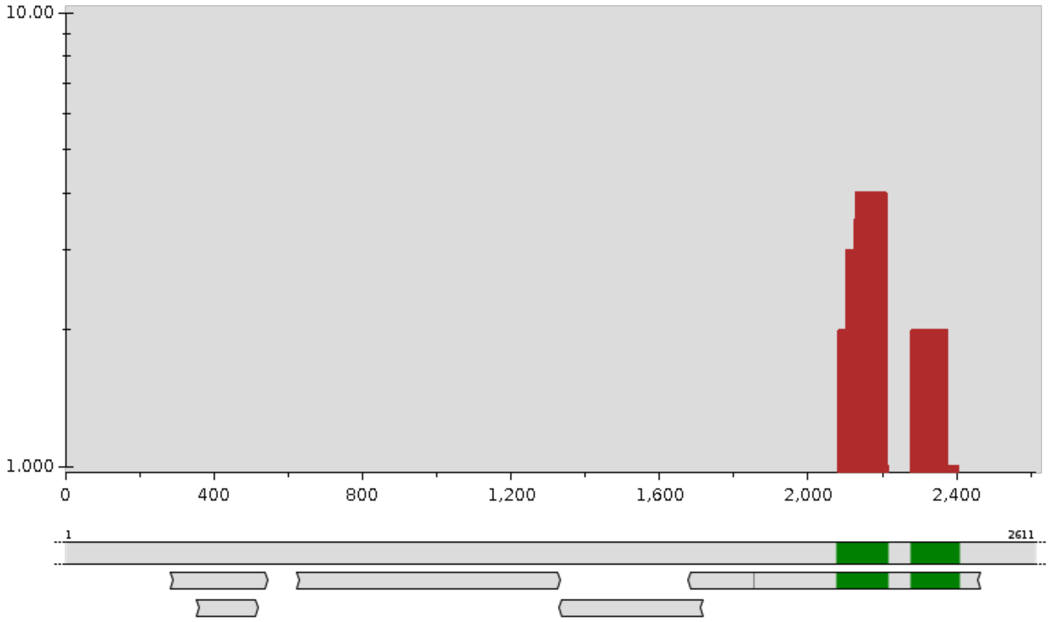

Assignment

**Type**	Tomato associated geminivirus 1 (Taxonomy ID: 2027645)
**Reference Genome**	NC_035676.1
**NT Identity (%)**	67.5182
**AA Identity (%)**	61.9565
**Number Of Stop Codons**	0
**Number Of CDS**	5

Alignment

**Alignment Score**	192.0 (NT) + 976.0 (AA) = 1168.0
**Concordance (%)**	60.5181

**Alignment Method**	Global, seeded, nucleotide + amino acids (AGA)

Genome Region

Sequence starts at position 2078 and ends at position 2408 relative to NC_035676.1 reference sequence.

Alignment Detailed Statistics

	Begin	End	Coverage	Score	Concordance	Matches	Identities	I/D/M/F*	Stop Codons
**NT**	**2078**	**2408**	**10.5%**	**192**	**35.0%**	**274 (100%)**	**185 (67.5%)**	**0/0**	
Mutations:	2082T>C, 2086T>G, 2090T>G, 2091C>T, 2094C>A, 2099T>C, 2102T>G, 2104C>T, 2105G>T, 2106G>T, 2108A>T, 2110G>T, 2111C>T, 2112T>C, 2113C>T, 2117G>A, 2118A>T, 2120T>C, 2121A>T, 2124T>A, 2127C>T, 2136G>T, 2137G>A, 2139A>T, 2142C>T, 2148G>T, 2151A>G, 2154G>A, 2157G>A, 2160C>G, 2162C>A, 2163A>G, 2166A>G, 2168C>G, 2170G>T, 2171C>T, 2172A>G, 2176C>G, 2177T>A, 2181T>C, 2184G>T, 2190G>A, 2191A>G, 2196T>G, 2199A>G, 2203T>A, 2205A>T, 2210C>T, 2280C>T, 2283A>G, 2289G>A, 2291T>G, 2292G>A, 2298C>T, 2301T>C, 2313G>T, 2314T>G, 2319C>T, 2322A>G, 2323C>G, 2324A>C, 2326G>A, 2333C>T, 2334G>A, 2337T>A, 2338G>T, 2340G>A, 2341A>G, 2342C>G, 2346A>G, 2354T>G, 2358T>A, 2360A>T, 2363G>T, 2364A>T, 2365A>T, 2366A>T, 2367A>G, 2369A>G, 2371T>A, 2373A>G, 2376G>T, 2379A>G, 2381T>C, 2385A>T, 2390G>C, 2391A>G, 2400A>G, 2406T>G								

CDS

	Begin	End	Coverage	Score	Concordance	Matches	Identities	I/D/M/F*	Stop Codons
CK833_gp4	20	130	27.6%	488	68.6%	92 (100%)	57 (62.0%)	0/0/0/0	0
Protein mutations:	P26A (2390G>C), I29V (2379A>G 2381T>C), Y32F (2371T>A), F33L (2367A>G 2369A>G), F34K (2364A>T 2365A>T 2366A>T), Q35K (2363G>T), L36I (2358T>A 2360A>T), K38Q (2354T>G), V42P (2340G>A 2341A>G 2342C>G), T43N (2337T>A 2338G>T), V45I (2333C>T), A47V (2326G>A), C48A (2322A>G 2323C>G 2324A>C), N51T (2313G>T 2314T>G), I59L (2289G>A 2291T>G), G86R (2210C>T), Y88F (2203T>A), I92T (2190G>A 2191A>G), R97S (2176C>G 2177T>A), A99K (2170G>T 2171C>T), D100H (2166A>G 2168C>G), A102S (2160C>G 2162C>A), T110I (2136G>T 2137G>A), R114S (2124T>A), I116V (2118A>T 2120T>C), L117F (2117G>A), R118K (2112T>C 2113C>T), A119N (2110G>T 2111C>T), S120T (2106G>T 2108A>T), R121K (2104C>T 2105G>T), S123G (2099T>C), K126Q (2090T>G), N127T (2086T>G)								
Codon mutations:	CCA20CCC (2406T>G), TGT22TGC (2400A>G), ATT25ATC (2391A>G), CCA26GCA (2390G>C), ATT27ATA (2385A>T), ATT29GTC (2379A>G 2381T>C), ATC30ATA (2376G>T), GAT31GAC (2373A>G), TAC32TTC (2371T>A), TTT33CTC (2367A>G 2369A>G), TTT34AAA (2364A>T 2365A>T 2366A>T), CAA35AAA (2363G>T), TTA36ATT (2358T>A 2360A>T), AAA38CAA (2354T>G), TAT40TAC (2346A>G), GTC42CCT (2340G>A 2341A>G 2342C>G), ACA43AAT (2337T>A 2338G>T), TAC44TAT (2334G>A), GTT45ATT (2333C>T), GCT47GTT (2326G>A), TGT48GCC (2322A>G 2323C>G 2324A>C), ACG49ACA (2319C>T), AAC51ACA (2313G>T 2314T>G), GGA55GGG (2301T>C), GAG56GAA (2298C>T), CAC58CAT (2292G>A), ATC59CTT (2289G>A 2291T>G), TGT61TGC (2283A>G), CTG62CTA (2280C>T), GGA86AGA (2210C>T), ATT87ATA (2205A>T), TAT88TTT (2203T>A), CAT89CAC (2199A>G), CCA90CCC (2196T>G), ATC92ACT (2190G>A 2191A>G), ATC94ATA (2184G>T), CCA95CCG (2181T>C), AGG97TCG (2176C>G 2177T>A), GAT98GAC (2172A>G), GCA99AAA (2170G>T 2171C>T), GAT100CAC (2166A>G 2168C>G), GTT101GTC (2163A>G), GCG102TCC (2160C>G 2162C>A), GAC103GAT (2157G>A), TAC104TAT (2154G>A), ATT105ATC (2151A>G), TCC106TCA (2148G>T), GGG108GGA (2142C>T), GGT109GGA (2139A>T), ACC110ATA (2136G>T 2137G>A), GAG113GAA (2127C>T), AGA114AGT (2124T>A), GGT115GGA (2121A>T), ATT116GTA (2118A>T 2120T>C), CTT117TTT (2117G>A), AGA118AAG (2112T>C 2113C>T), GCT119AAT (2110G>T 2111C>T), TCC120ACA (2106G>T 2108A>T), CGA121AAA (2104C>T 2105G>T), AGA122CGA (2102T>G), AGT123GGT (2099T>C), CCG124CCT (2094C>A), AAG125AAA (2091C>T), AAG126CAG (2090T>G), AAT127ACT (2086T>G), AGA128AGG (2082T>C)								
CK833_gp5	20	130	35.1%	488	68.6%	92 (100%)	57 (62.0%)	0/0/0/0	0
Protein mutations:	P26A (2390G>C), I29V (2379A>G 2381T>C), Y32F (2371T>A), F33L (2367A>G 2369A>G), F34K (2364A>T 2365A>T 2366A>T), Q35K (2363G>T), L36I (2358T>A 2360A>T), K38Q (2354T>G), V42P (2340G>A 2341A>G 2342C>G), T43N (2337T>A 2338G>T), V45I (2333C>T), A47V (2326G>A), C48A (2322A>G 2323C>G 2324A>C), N51T (2313G>T 2314T>G), I59L (2289G>A 2291T>G), G86R (2210C>T), Y88F (2203T>A), I92T (2190G>A 2191A>G), R97S (2176C>G 2177T>A), A99K (2170G>T 2171C>T), D100H (2166A>G 2168C>G), A102S (2160C>G 2162C>A), T110I (2136G>T 2137G>A), R114S (2124T>A), I116V (2118A>T 2120T>C), L117F (2117G>A), R118K (2112T>C 2113C>T), A119N (2110G>T 2111C>T), S120T (2106G>T 2108A>T), R121K (2104C>T 2105G>T), S123G (2099T>C), K126Q (2090T>G), N127T (2086T>G)								
Codon mutations:	CCA20CCC (2406T>G), TGT22TGC (2400A>G), ATT25ATC (2391A>G), CCA26GCA (2390G>C), ATT27ATA (2385A>T), ATT29GTC (2379A>G 2381T>C), ATC30ATA (2376G>T), GAT31GAC (2373A>G), TAC32TTC (2371T>A), TTT33CTC (2367A>G 2369A>G), TTT34AAA (2364A>T 2365A>T 2366A>T), CAA35AAA (2363G>T), TTA36ATT (2358T>A 2360A>T), AAA38CAA (2354T>G), TAT40TAC (2346A>G), GTC42CCT (2340G>A 2341A>G 2342C>G), ACA43AAT (2337T>A 2338G>T), TAC44TAT (2334G>A), GTT45ATT (2333C>T), GCT47GTT (2326G>A), TGT48GCC (2322A>G 2323C>G 2324A>C), ACG49ACA (2319C>T), AAC51ACA (2313G>T 2314T>G), GGA55GGG (2301T>C), GAG56GAA (2298C>T), CAC58CAT (2292G>A), ATC59CTT (2289G>A 2291T>G), TGT61TGC (2283A>G), CTG62CTA (2280C>T), GGA86AGA (2210C>T), ATT87ATA (2205A>T), TAT88TTT (2203T>A), CAT89CAC (2199A>G), CCA90CCC (2196T>G), ATC92ACT (2190G>A 2191A>G), ATC94ATA (2184G>T), CCA95CCG (2181T>C), AGG97TCG (2176C>G 2177T>A), GAT98GAC (2172A>G), GCA99AAA (2170G>T 2171C>T), GAT100CAC (2166A>G 2168C>G), GTT101GTC (2163A>G), GCG102TCC (2160C>G 2162C>A), GAC103GAT (2157G>A), TAC104TAT (2154G>A), ATT105ATC (2151A>G), TCC106TCA (2148G>T), GGG108GGA (2142C>T), GGT109GGA (2139A>T), ACC110ATA (2136G>T 2137G>A), GAG113GAA (2127C>T), AGA114AGT (2124T>A), GGT115GGA (2121A>T), ATT116GTA (2118A>T 2120T>C), CTT117TTT (2117G>A), AGA118AAG (2112T>C 2113C>T), GCT119AAT (2110G>T 2111C>T), TCC120ACA (2106G>T 2108A>T), CGA121AAA (2104C>T 2105G>T), AGA122CGA (2102T>G), AGT123GGT (2099T>C), CCG124CCT (2094C>A), AAG125AAA (2091C>T), AAG126CAG (2090T>G), AAT127ACT (2086T>G), AGA128AGG (2082T>C)								

Proteins

	Begin	End	Coverage	Score	Concordance	Matches	Identities	I/D/M/F*	Stop Codons
replication-associated protein (YP_009417307.1)	20	130	27.6%	488	68.6%	92 (100%)	57 (62.0%)	0/0/0/0	0
Protein mutations:	P26A (2390G>C), I29V (2379A>G 2381T>C), Y32F (2371T>A), F33L (2367A>G 2369A>G), F34K (2364A>T 2365A>T 2366A>T), Q35K (2363G>T), L36I (2358T>A 2360A>T), K38Q (2354T>G), V42P (2340G>A 2341A>G 2342C>G), T43N (2337T>A 2338G>T), V45I (2333C>T), A47V (2326G>A), C48A (2322A>G 2323C>G 2324A>C), N51T (2313G>T 2314T>G), I59L (2289G>A 2291T>G), G86R (2210C>T), Y88F (2203T>A), I92T (2190G>A 2191A>G), R97S (2176C>G 2177T>A), A99K (2170G>T 2171C>T), D100H (2166A>G 2168C>G), A102S (2160C>G 2162C>A), T110I (2136G>T 2137G>A), R114S (2124T>A), I116V (2118A>T 2120T>C), L117F (2117G>A), R118K (2112T>C 2113C>T), A119N (2110G>T 2111C>T), S120T (2106G>T 2108A>T), R121K (2104C>T 2105G>T), S123G (2099T>C), K126Q (2090T>G), N127T (2086T>G)								
Codon mutations:	CCA20CCC (2406T>G), TGT22TGC (2400A>G), ATT25ATC (2391A>G), CCA26GCA (2390G>C), ATT27ATA (2385A>T), ATT29GTC (2379A>G 2381T>C), ATC30ATA (2376G>T), GAT31GAC (2373A>G), TAC32TTC (2371T>A), TTT33CTC (2367A>G 2369A>G), TTT34AAA (2364A>T 2365A>T 2366A>T), CAA35AAA (2363G>T), TTA36ATT (2358T>A 2360A>T), AAA38CAA (2354T>G), TAT40TAC (2346A>G), GTC42CCT (2340G>A 2341A>G 2342C>G), ACA43AAT (2337T>A 2338G>T), TAC44TAT (2334G>A), GTT45ATT (2333C>T), GCT47GTT (2326G>A), TGT48GCC (2322A>G 2323C>G 2324A>C), ACG49ACA (2319C>T), AAC51ACA (2313G>T 2314T>G), GGA55GGG (2301T>C), GAG56GAA (2298C>T), CAC58CAT (2292G>A), ATC59CTT (2289G>A 2291T>G), TGT61TGC (2283A>G), CTG62CTA (2280C>T), GGA86AGA (2210C>T), ATT87ATA (2205A>T), TAT88TTT (2203T>A), CAT89CAC (2199A>G), CCA90CCC (2196T>G), ATC92ACT (2190G>A 2191A>G), ATC94ATA (2184G>T), CCA95CCG (2181T>C), AGG97TCG (2176C>G 2177T>A), GAT98GAC (2172A>G), GCA99AAA (2170G>T 2171C>T), GAT100CAC (2166A>G 2168C>G), GTT101GTC (2163A>G), GCG102TCC (2160C>G 2162C>A), GAC103GAT (2157G>A), TAC104TAT (2154G>A), ATT105ATC (2151A>G), TCC106TCA (2148G>T), GGG108GGA (2142C>T), GGT109GGA (2139A>T), ACC110ATA (2136G>T 2137G>A), GAG113GAA (2127C>T), AGA114AGT (2124T>A), GGT115GGA (2121A>T), ATT116GTA (2118A>T 2120T>C), CTT117TTT (2117G>A), AGA118AAG (2112T>C 2113C>T), GCT119AAT (2110G>T 2111C>T), TCC120ACA (2106G>T 2108A>T), CGA121AAA (2104C>T 2105G>T), AGA122CGA (2102T>G), AGT123GGT (2099T>C), CCG124CCT (2094C>A), AAG125AAA (2091C>T), AAG126CAG (2090T>G), AAT127ACT (2086T>G), AGA128AGG (2082T>C)								
RepA (YP_009417308.1)	20	130	35.1%	488	68.6%	92 (100%)	57 (62.0%)	0/0/0/0	0
Protein mutations:	P26A (2390G>C), I29V (2379A>G 2381T>C), Y32F (2371T>A), F33L (2367A>G 2369A>G), F34K (2364A>T 2365A>T 2366A>T), Q35K (2363G>T), L36I (2358T>A 2360A>T), K38Q (2354T>G), V42P (2340G>A 2341A>G 2342C>G), T43N (2337T>A 2338G>T), V45I (2333C>T), A47V (2326G>A), C48A (2322A>G 2323C>G 2324A>C), N51T (2313G>T 2314T>G), I59L (2289G>A 2291T>G), G86R (2210C>T), Y88F (2203T>A), I92T (2190G>A 2191A>G), R97S (2176C>G 2177T>A), A99K (2170G>T 2171C>T), D100H (2166A>G 2168C>G), A102S (2160C>G 2162C>A), T110I (2136G>T 2137G>A), R114S (2124T>A), I116V (2118A>T 2120T>C), L117F (2117G>A), R118K (2112T>C 2113C>T), A119N (2110G>T 2111C>T), S120T (2106G>T 2108A>T), R121K (2104C>T 2105G>T), S123G (2099T>C), K126Q (2090T>G), N127T (2086T>G)								

	Begin	End	Coverage	Score	Concordance	Matches	Identities	I/D/M/F*	Stop Codons
**NT**	**2078**	**2408**	**10.5%**	**192**	**35.0%**	**274 (100%)**	**185 (67.5%)**	**0/0**	
Codon mutations:	CCA20CCC (2406T>G), TGT22TGC (2400A>G), ATT25ATC (2391A>G), CCA26GCA (2390G>C), ATT27ATA (2385A>T), ATT29GTC (2379A>G 2381T>C), ATC30ATA (2376G>T), GAT31GAC (2373A>G), TAC32TTC (2371T>A), TTT33CTC (2367A>G 2369A>G), TTT34AAA (2364A>T 2365A>T 2366A>T), CAA35AAA (2363G>T), TTA36ATT (2358T>A 2360A>T), AAA38CAA (2354T>G), TAT40TAC (2346A>G), GTC42CCT (2340G>A 2341A>G 2342C>G), ACA43AAT (2337T>A 2338G>T), TAC44TAT (2334G>A), GTT45ATT (2333C>T), GCT47GTT (2326G>A), TGT48GCC (2322A>G 2323C>G 2324A>C), ACG49ACA (2319C>T), AAC51ACA (2313G>T 2314T>G), GGA55GGG (2301T>C), GAG56GAA (2298C>T), CAC58CAT (2292G>A), ATC59CTT (2289G>A 2291T>G), TGT61TGC (2283A>G), CTG62CTA (2280C>T), GGA86AGA (2210C>T), ATT87ATA (2205A>T), TAT88TTT (2203T>A), CAT89CAC (2199A>G), CCA90CCC (2196T>G), ATC92ACT (2190G>A 2191A>G), ATC94ATA (2184G>T), CCA95CCG (2181T>C), AGG97TCG (2176C>G 2177T>A), GAT98GAC (2172A>G), GCA99AAA (2170G>T 2171C>T), GAT100CAC (2166A>G 2168C>G), GTT101GTC (2163A>G), GCG102TCC (2160C>G 2162C>A), GAC103GAT (2157G>A), TAC104TAT (2154G>A), ATT105ATC (2151A>G), TCC106TCA (2148G>T), GGG108GGA (2142C>T), GGT109GGA (2139A>T), ACC110ATA (2136G>T 2137G>A), GAG113GAA (2127C>T), AGA114AGT (2124T>A), GGT115GGA (2121A>T), ATT116GTA (2118A>T 2120T>C), CTT117TTT (2117G>A), AGA118AAG (2112T>C 2113C>T), GCT119AAT (2110G>T 2111C>T), TCC120ACA (2106G>T 2108A>T), CGA121AAA (2104C>T 2105G>T), AGA122CGA (2102T>G), AGT123GGT (2099T>C), CCG124CCT (2094C>A), AAG125AAA (2091C>T), AAG126CAG (2090T>G), AAT127ACT (2086T>G), AGA128AGG (2082T>C)								

*: Inserts / Deletes / Misaligned / Frameshifts

Analysis details

This analysis was performed with panviral2.64

NGS Details (UN9): Tokyovirus A1

Assembly

**Coverage Length**	125 (1 contig(s))
**Depth Of Coverage**	2.4
**Number Of Reads**	3
**Reads Per Million**	0.07 rpm (after QC)
**Ambiguities**	0
**Assembly Method**	de novo + reference guided assembly
**Consensus Caller**	Bcf Tools

Coverage Map

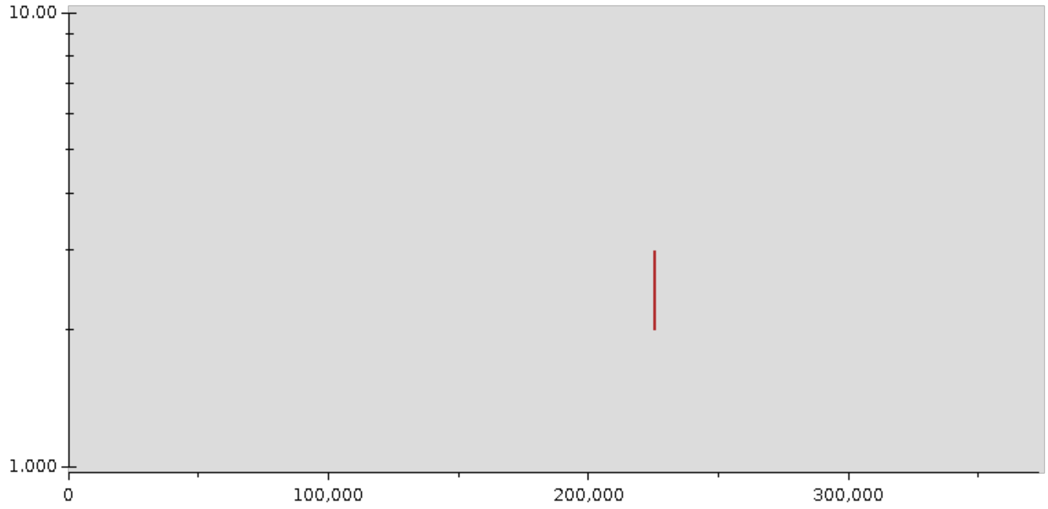

Assignment

**Type**	Tokyovirus A1 (Taxonomy ID: 1826170)
**Reference Genome**	NC_030230.1
**NT Identity (%)**	80.0
**AA Identity (%)**	92.8571
**Number Of Stop Codons**	0
**Number Of CDS**	470

Alignment

**Alignment Score**	150.0 (NT) + 273.0 (AA) = 423.0
**Concordance (%)**	80.2657
**Alignment Method**	Local, heuristic, nucleotide (BLASTN)

Genome Region

Sequence starts at position 225496 and ends at position 225620 relative to NC_030230.1 reference sequence.

Alignment Detailed Statistics

	Begin	End	Coverage	Score	Concordance	Matches	Identities	I/D/M/F*	Stop Codons
**NT**	**225496**	**225620**	**0.1%**	**150**	**60.0%**	**125 (100%)**	**100 (80.0%)**	**0/0**	

Mutations: 225501C>G, 225507A>C, 225509G>A, 225519G>A, 225525T>C, 225528T>C, 225533C>T, 225534A>G, 225540A>G, 225543G>A, 225545C>A, 225548A>G, 225555C>G, 225558A>G, 225564T>G, 225573C>G, 225579G>A, 225585A>G, 225590G>T, 225594T>C, 225597G>A, 225600G>A, 225603C>T, 225606A>G, 225609A>G

*: Inserts / Deletes / Misaligned / Frameshifts

Analysis details

This analysis was performed with panviral2.64

NGS Details (UN9): Nepovirus aeonii (segment RNA2)

Assembly

**Coverage Length**	250 (1 contig(s))
**Depth Of Coverage**	1.1
**Number Of Reads**	2
**Reads Per Million**	0.05 rpm (after QC)
**Ambiguities**	0
**Assembly Method**	de novo + reference guided assembly
**Consensus Caller**	Bcf Tools

Coverage Map

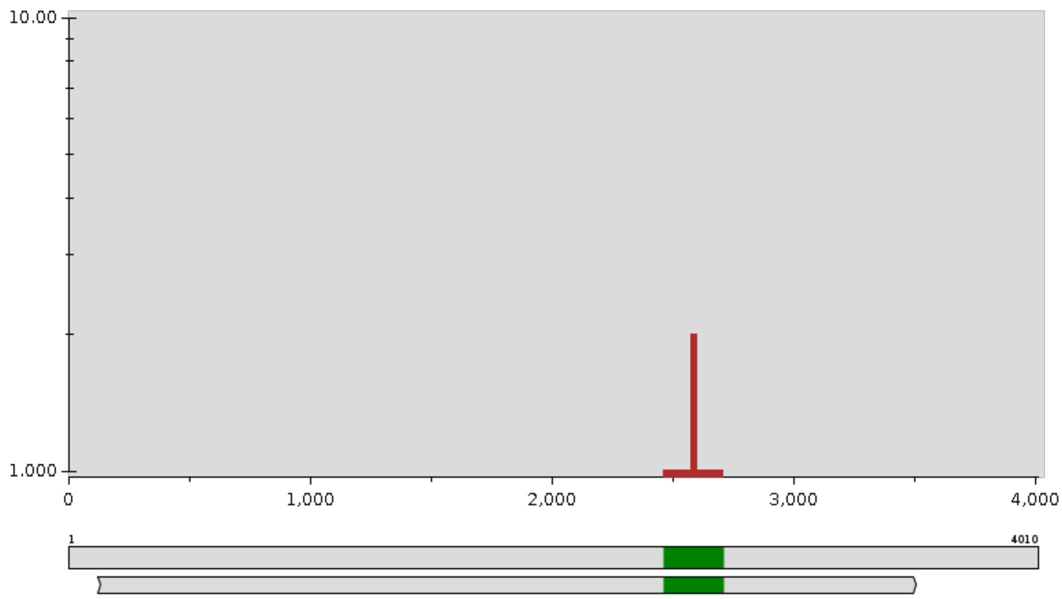

Assignment

**Type**	Nepovirus aeonii (Taxonomy ID: 3047350)
**Reference Genome**	NC_038761.1
**NT Identity (%)**	71.2
**AA Identity (%)**	72.2892
**Number Of Stop Codons**	0
**Number Of CDS**	1

Alignment

**Alignment Score**	212.0 (NT) + 425.0 (AA) = 637.0
**Concordance (%)**	61.6054

Alignment Method	Global, seeded, nucleotide + amino acids (AGA)

Genome Region

Sequence starts at position 2462 and ends at position 2711 relative to NC_038761.1 reference sequence.

Alignment Detailed Statistics

	Begin	End	Coverage	Score	Concordance	Matches	Identities	I/D/M/F*	Stop Codons
**NT**	**2462**	**2711**	**6.2%**	**212**	**42.4%**	**250 (100%)**	**178 (71.2%)**	**0/0**	
Mutations:	2470C>G, 2472G>A, 2474A>C, 2481G>A, 2482T>C, 2483C>A, 2484T>G, 2491C>T, 2493T>G, 2496C>T, 2499C>A, 2502G>A, 2503G>A, 2505T>G, 2514G>T, 2515C>A, 2517G>A, 2519G>C, 2520T>A, 2521G>C, 2522T>A, 2524G>A, 2526G>T, 2529T>C, 2530T>A, 2531C>G, 2532A>T, 2533C>T, 2535C>T, 2536A>G, 2537G>A, 2541T>G, 2542G>A, 2562A>G, 2565G>A, 2571G>A, 2580G>C, 2583T>C, 2592A>G, 2593A>T, 2607T>C, 2608A>G, 2622T>C, 2623A>G, 2629C>T, 2631C>T, 2632A>T, 2634T>A, 2635C>A, 2637A>G, 2643C>T, 2646G>A, 2649T>A, 2652T>G, 2658T>C, 2661T>A, 2664T>A, 2666T>C, 2667T>A, 2671G>T, 2673A>T, 2674C>A, 2676T>A, 2682A>T, 2685A>G, 2687T>G, 2688T>G, 2695T>A, 2696C>G, 2697T>A, 2698A>C, 2703A>G								

CDS

	Begin	End	Coverage	Score	Concordance	Matches	Identities	I/D/M/F*	Stop Codons
D1S54_s2gp1	781	863	7.4%	425	77.4%	83 (100%)	60 (72.3%)	0/0/0/0	0
Protein mutations:	Q783E (2470C>G 2472G>A), Q784P (2474A>C), S787Q (2482T>C 2483C>A 2484T>G), V794M (2503G>A 2505T>G), L798I (2515C>A 2517G>A), S799T (2519G>C 2520T>A), V800H (2521G>C 2522T>A), G801S (2524G>A 2526G>T), L804F (2533C>T 2535C>T), S805D (2536A>G 2537G>A), D807N (2542G>A), M824L (2593A>T), N829D (2608A>G), I834V (2623A>G), L836F (2629C>T 2631C>T), T837S (2632A>T 2634T>A), Q838K (2635C>A 2637A>G), V848A (2666T>C 2667T>A), A850S (2671G>T 2673A>T), F855W (2687T>G 2688T>G), S858R (2695T>A 2696C>G 2697T>A), I859L (2698A>C)								
Codon mutations:	CAG783GAA (2470C>G 2472G>A), CAA784CCA (2474A>C), GAG786GAA (2481G>A), TCT787CAG (2482T>C 2483C>A 2484T>G), CTT790TTG (2491C>T 2493T>G), GCC791GCT (2496C>T), CCC792CCA (2499C>A), ACG793ACA (2502G>A), GTT794ATG (2503G>A 2505T>G), CCG797CCT (2514G>T), CTG798ATA (2515C>A 2517G>A), AGT799ACA (2519G>C 2520T>A), GTT800CAT (2521G>C 2522T>A), GGG801AGT (2524G>A 2526G>T), GAT802GAC (2529T>C), TCA803AGT (2530T>A 2531C>G 2532A>T), CTC804TTT (2533C>T 2535C>T), AGT805GAT (2536A>G 2537G>A), GTT806GTG (2541T>G), GAT807AAT (2542G>A), AAA813AAG (2562A>G), GAG814GAA (2565G>A), AAG816AAA (2571G>A), GGG819GGC (2580G>C), ACT820ACC (2583T>C), TTA823TTG (2592A>G), ATG824TTG (2593A>T), TAT828TAC (2607T>C), AAT829GAT (2608A>G), CCT833CCC (2622T>C), ATC834GTC (2623A>G), CTC836TTT (2629C>T 2631C>T), ACT837TCA (2632A>T 2634T>A), CAA838AAG (2635C>A 2637A>G), AGC840AGT (2643C>T), TCG841TCA (2646G>A), ATT842ATA (2649T>A), TCT843TCG (2652T>G), CCT845CCC (2658T>C), CGT846CGA (2661T>A), GCT847GCA (2664T>A), GTT848GCA (2666T>C 2667T>A), GCA850TCT (2671G>T 2673A>T), CGT851AGA (2674C>A 2676T>A), ATA853ATT (2682A>T), GGA854GGG (2685A>G), TTT855TGG (2687T>G 2688T>G), TCT858AGA (2695T>A 2696C>G 2697T>A), ATC859CTC (2698A>C), AAA860AAG (2703A>G)								

Proteins

	Begin	End	Coverage	Score	Concordance	Matches	Identities	I/D/M/F*	Stop Codons
polyprotein P2 (YP_009507917.1)	781	863	7.4%	425	77.4%	83 (100%)	60 (72.3%)	0/0/0/0	0
Protein mutations:	Q783E (2470C>G 2472G>A), Q784P (2474A>C), S787Q (2482T>C 2483C>A 2484T>G), V794M (2503G>A 2505T>G), L798I (2515C>A 2517G>A), S799T (2519G>C 2520T>A), V800H (2521G>C 2522T>A), G801S (2524G>A 2526G>T), L804F (2533C>T 2535C>T), S805D (2536A>G 2537G>A), D807N (2542G>A), M824L (2593A>T), N829D (2608A>G), I834V (2623A>G), L836F (2629C>T 2631C>T), T837S (2632A>T 2634T>A), Q838K (2635C>A 2637A>G), V848A (2666T>C 2667T>A), A850S (2671G>T 2673A>T), F855W (2687T>G 2688T>G), S858R (2695T>A 2696C>G 2697T>A), I859L (2698A>C)								
Codon mutations:	CAG783GAA (2470C>G 2472G>A), CAA784CCA (2474A>C), GAG786GAA (2481G>A), TCT787CAG (2482T>C 2483C>A 2484T>G), CTT790TTG (2491C>T 2493T>G), GCC791GCT (2496C>T), CCC792CCA (2499C>A), ACG793ACA (2502G>A), GTT794ATG (2503G>A 2505T>G), CCG797CCT (2514G>T), CTG798ATA (2515C>A 2517G>A), AGT799ACA (2519G>C 2520T>A), GTT800CAT (2521G>C 2522T>A), GGG801AGT (2524G>A 2526G>T), GAT802GAC (2529T>C), TCA803AGT (2530T>A 2531C>G 2532A>T), CTC804TTT (2533C>T 2535C>T), AGT805GAT (2536A>G 2537G>A), GTT806GTG (2541T>G), GAT807AAT (2542G>A), AAA813AAG (2562A>G), GAG814GAA (2565G>A), AAG816AAA (2571G>A), GGG819GGC (2580G>C), ACT820ACC (2583T>C), TTA823TTG (2592A>G), ATG824TTG (2593A>T), TAT828TAC (2607T>C), AAT829GAT (2608A>G), CCT833CCC (2622T>C), ATC834GTC (2623A>G), CTC836TTT (2629C>T 2631C>T), ACT837TCA (2632A>T 2634T>A), CAA838AAG (2635C>A 2637A>G), AGC840AGT (2643C>T), TCG841TCA (2646G>A), ATT842ATA (2649T>A), TCT843TCG (2652T>G), CCT845CCC (2658T>C), CGT846CGA (2661T>A), GCT847GCA (2664T>A), GTT848GCA (2666T>C 2667T>A), GCA850TCT (2671G>T 2673A>T), CGT851AGA (2674C>A 2676T>A), ATA853ATT (2682A>T), GGA854GGG (2685A>G), TTT855TGG (2687T>G 2688T>G), TCT858AGA (2695T>A 2696C>G 2697T>A), ATC859CTC (2698A>C), AAA860AAG (2703A>G)								

*: Inserts / Deletes / Misaligned / Frameshifts

Analysis details

This analysis was performed with panviral2.64

NGS Details (UN9): Moloney murine leukemia virus

Assembly

**Coverage Length**	134 (1 contig(s))
**Depth Of Coverage**	2.0
**Number Of Reads**	2
**Reads Per Million**	0.05 rpm (after QC)
**Ambiguities**	0
**Assembly Method**	de novo + reference guided assembly
**Consensus Caller**	Bcf Tools

Coverage Map

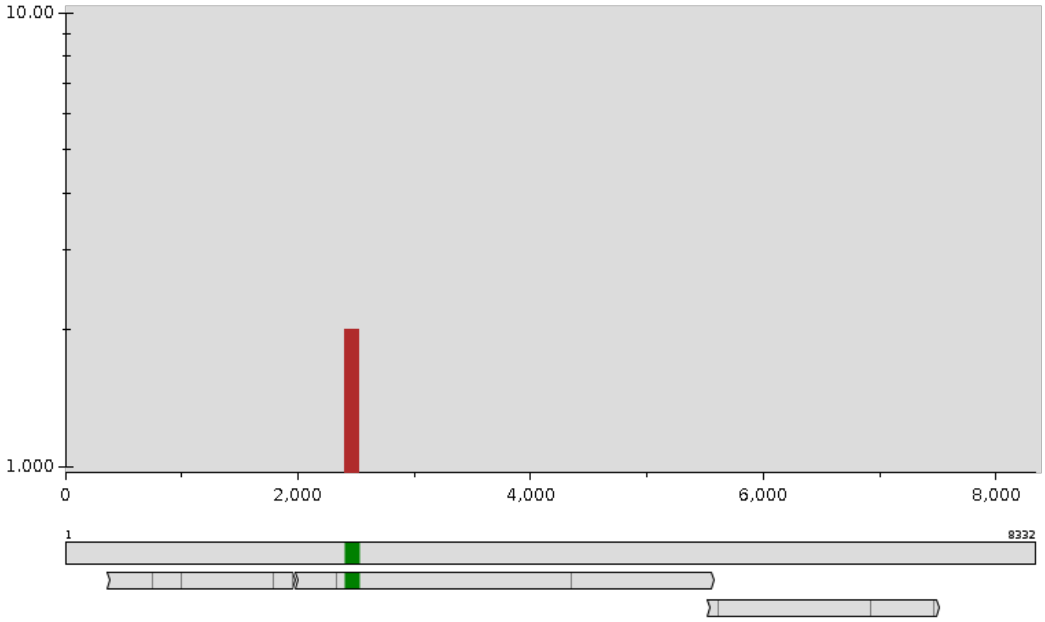

Assignment

**Type**	Moloney murine leukemia virus (Taxonomy ID: 11801)
**Reference Genome**	NC_001501.1
**NT Identity (%)**	79.1045
**AA Identity (%)**	97.7273
**Number Of Stop Codons**	0
**Number Of CDS**	3

Alignment

**Alignment Score**	156.0 (NT) + 299.0 (AA) = 455.0
**Concordance (%)**	79.4066

Alignment Method	Global, seeded, nucleotide + amino acids (AGA)

Genome Region

Sequence starts at position 2398 and ends at position 2531 relative to NC_001501.1 reference sequence.

Alignment Detailed Statistics

	Begin	End	Coverage	Score	Concordance	Matches	Identities	I/D/M/F*	Stop Codons
**NT**	**2398**	**2531**	**1.6%**	**156**	**58.2%**	**134 (100%)**	**106 (79.1%)**	**0/0**	
Mutations:	2401C>G, 2405A>C, 2412T>A, 2413C>G, 2414T>C, 2423T>G, 2429C>G, 2444G>C, 2453A>C, 2459A>G, 2462T>G, 2468A>G, 2471T>G, 2474T>G, 2480C>T, 2483A>T, 2486T>G, 2495A>G, 2499T>A, 2500C>G, 2501T>C, 2507C>G, 2511T>A, 2512C>G, 2516A>T, 2522A>G, 2525C>T, 2528C>G								
**CDS**									
gag-pol	681	724	2.5%	299	95.5%	44 (100%)	43 (97.7%)	0/0/0/0	0
Protein mutations:	S681C (2401C>G)								
Codon mutations:	TCC681TGC (2401C>G), ACA682ACC (2405A>C), TCT685AGC (2412T>A 2413C>G 2414T>C), CCT688CCG (2423T>G), GCC690GCG (2429C>G), GGG695GGC (2444G>C), GGA698GGC (2453A>C), GCA700GCG (2459A>G), GTT701GTG (2462T>G), CAA703CAG (2468A>G), GCT704GCG (2471T>G), CCT705CCG (2474T>G), ATC707ATT (2480C>T), ATA708ATT (2483A>T), CCT709CCG (2486T>G), GCA712GCG (2495A>G), TCT714AGC (2499T>A 2500C>G 2501T>C), CCC716CCG (2507C>G), TCC718AGC (2511T>A 2512C>G), ATA719ATT (2516A>T), CAA721CAG (2522A>G), TAC722TAT (2525C>T), CCC723CCG (2528C>G)								
**Proteins**									
Pr180 (NP_057933.2)	681	724	2.5%	299	95.5%	44 (100%)	43 (97.7%)	0/0/0/0	0
Protein mutations:	S681C (2401C>G)								
Codon mutations:	TCC681TGC (2401C>G), ACA682ACC (2405A>C), TCT685AGC (2412T>A 2413C>G 2414T>C), CCT688CCG (2423T>G), GCC690GCG (2429C>G), GGG695GGC (2444G>C), GGA698GGC (2453A>C), GCA700GCG (2459A>G), GTT701GTG (2462T>G), CAA703CAG (2468A>G), GCT704GCG (2471T>G), CCT705CCG (2474T>G), ATC707ATT (2480C>T), ATA708ATT (2483A>T), CCT709CCG (2486T>G), GCA712GCG (2495A>G), TCT714AGC (2499T>A 2500C>G 2501T>C), CCC716CCG (2507C>G), TCC718AGC (2511T>A 2512C>G), ATA719ATT (2516A>T), CAA721CAG (2522A>G), TAC722TAT (2525C>T), CCC723CCG (2528C>G)								
p80 RT (NP_955591.1)	22	65	6.6%	299	95.5%	44 (100%)	43 (97.7%)	0/0/0/0	0
Protein mutations:	S22C (2401C>G)								
Codon mutations:	TCC22TGC (2401C>G), ACA23ACC (2405A>C), TCT26AGC (2412T>A 2413C>G 2414T>C), CCT29CCG (2423T>G), GCC31GCG (2429C>G), GGG36GGC (2444G>C), GGA39GGC (2453A>C), GCA41GCG (2459A>G), GTT42GTG (2462T>G), CAA44CAG (2468A>G), GCT45GCG (2471T>G), CCT46CCG (2474T>G), ATC48ATT (2480C>T), ATA49ATT (2483A>T), CCT50CCG (2486T>G), GCA53GCG (2495A>G), TCT55AGC (2499T>A 2500C>G 2501T>C), CCC57CCG (2507C>G), TCC59AGC (2511T>A 2512C>G), ATA60ATT (2516A>T), CAA62CAG (2522A>G), TAC63TAT (2525C>T), CCC64CCG (2528C>G)								

*: Inserts / Deletes / Misaligned / Frameshifts

Analysis details

This analysis was performed with panviral2.64

NGS Details (UN9): Friend murine leukemia virus

Assembly

**Coverage Length**	135 (1 contig(s))
**Depth Of Coverage**	2.0
**Number Of Reads**	2
**Reads Per Million**	0.05 rpm (after QC)
**Ambiguities**	0
**Assembly Method**	de novo + reference guided assembly
**Consensus Caller**	Bcf Tools

Coverage Map

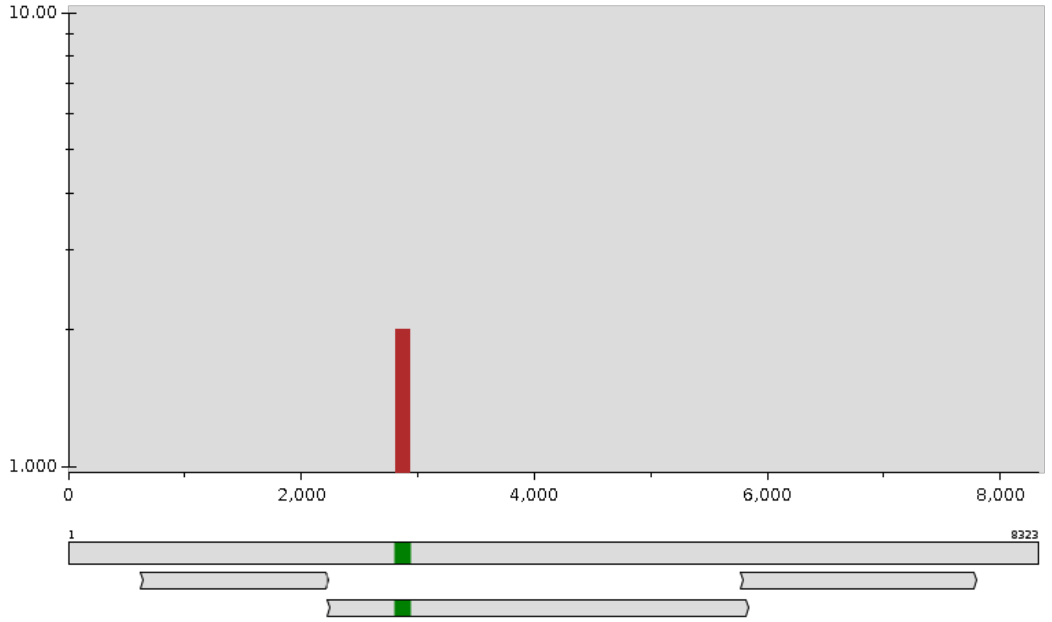

Assignment

**Type**	Friend murine leukemia virus (Taxonomy ID: 11795)
**Reference Genome**	NC_001362.1
**NT Identity (%)**	78.5185
**AA Identity (%)**	97.7778
**Number Of Stop Codons**	0
**Number Of CDS**	3

Alignment

**Alignment Score**	154.0 (NT) + 343.0 (AA) = 497.0
**Concordance (%)**	81.0767

Alignment Method	Global, seeded, nucleotide + amino acids (AGA)

Genome Region

Sequence starts at position 2805 and ends at position 2939 relative to NC_001362.1 reference sequence.

Alignment Detailed Statistics

	Begin	End	Coverage	Score	Concordance	Matches	Identities	I/D/M/F*	Stop Codons
**NT**	**2805**	**2939**	**1.6%**	**154**	**57.0%**	**135 (100%)**	**106 (78.5%)**	**0/0**	
Mutations:	2806A>C, 2808A>C, 2814G>C, 2817C>T, 2820G>A, 2823C>G, 2826C>T, 2829A>T, 2833A>C, 2835A>C, 2850A>C, 2859A>G, 2862C>G, 2869T>A, 2870C>G, 2874C>G, 2883G>C, 2886C>G, 2892A>G, 2895C>G, 2898T>G, 2901G>A, 2910G>C, 2913T>C, 2916T>C, 2923A>C, 2925G>C, 2928T>G, 2931C>G								
**CDS**									
pol	196	240	3.7%	343	97.7%	45 (100%)	44 (97.8%)	0/0/0/0	0
**Proteins**									
RNA-dependent DNA polymerase (NP_040333.1)	196	240	3.7%	343	97.7%	45 (100%)	44 (97.8%)	0/0/0/0	0
Protein mutations:	*none*								
Codon mutations:	AGA196CGC (2806A>C 2808A>C), GGG198GGC (2814G>C), ATC199ATT (2817C>T), AAG200AAA (2820G>A), CCC201CCG (2823C>G), CAC202CAT (2826C>T), ATA203ATT (2829A>T), AGA205CGC (2833A>C 2835A>C), GGA210GGC (2850A>C), GTA213GTG (2859A>G), CCC214CCG (2862C>G), TCC217AGC (2869T>A 2870C>G), CCC218CCG (2874C>G), ACG221ACC (2883G>C), CCC222CCG (2886C>G), CTA224CTG (2892A>G), CCC225CCG (2895C>G), GTT226GTG (2898T>G), AAG227AAA (2901G>A), GGG230GGC (2910G>C), ACT231ACC (2913T>C), AAT232AAC (2916T>C), AGG235CGC (2923A>C 2925G>C), CCT236CCG (2928T>G), GTC237GTG (2931C>G)								

*: Inserts / Deletes / Misaligned / Frameshifts

Analysis details

This analysis was performed with panviral2.64

NGS Details (UN9): Duamitovirus peex1

Assembly

**Coverage Length**	135 (1 contig(s))
**Depth Of Coverage**	1.0
**Number Of Reads**	1
**Reads Per Million**	0.02 rpm (after QC)
**Ambiguities**	0
**Assembly Method**	de novo + reference guided assembly
**Consensus Caller**	Bcf Tools

Coverage Map

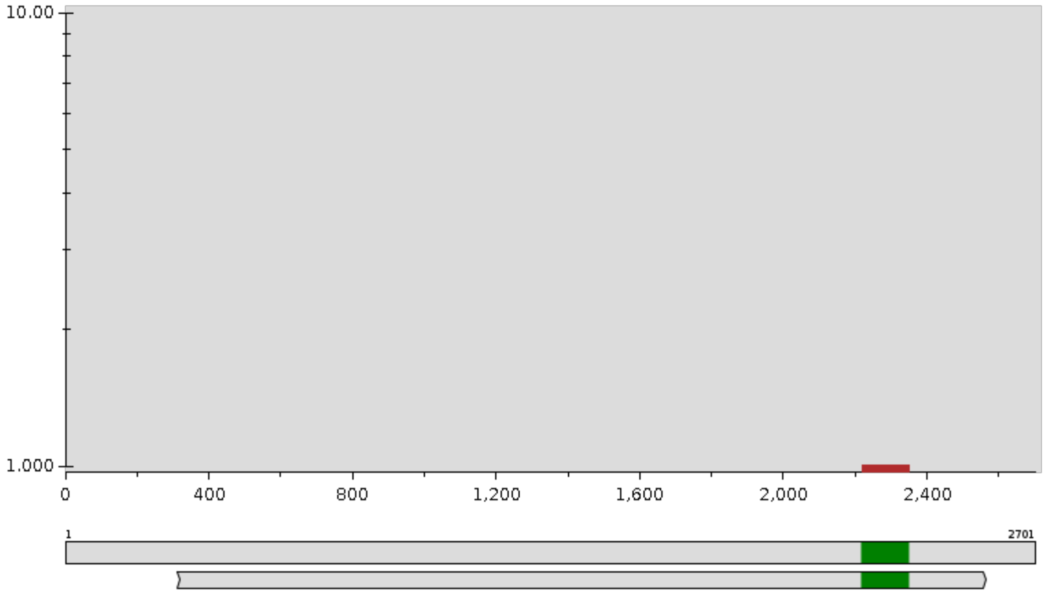

Assignment

**Type**	Duamitovirus peex1 (Taxonomy ID: 2955799)
**Reference Genome**	NC_076525.1
**NT Identity (%)**	71.8519
**AA Identity (%)**	66.6667
**Number Of Stop Codons**	0
**Number Of CDS**	1

Alignment

**Alignment Score**	118.0 (NT) + 249.0 (AA) = 367.0
**Concordance (%)**	59.3851

Alignment Method	Global, seeded, nucleotide + amino acids (AGA)

Genome Region

Sequence starts at position 2218 and ends at position 2352 relative to NC_076525.1 reference sequence.

Alignment Detailed Statistics

	Begin	End	Coverage	Score	Concordance	Matches	Identities	I/D/M/F*	Stop Codons
**NT**	**2218**	**2352**	**5.0%**	**118**	**43.7%**	**135 (100%)**	**97 (71.9%)**	**0/0**	
Mutations:	2222A>T, 2223C>G, 2225A>G, 2226C>T, 2234A>T, 2243A>G, 2244C>A, 2245A>G, 2246T>A, 2249G>A, 2255A>T, 2268G>T, 2272G>T, 2273T>C, 2274C>A, 2275A>G, 2276T>A, 2280A>G, 2283G>A, 2294T>G, 2295C>A, 2296G>A, 2301C>G, 2303T>C, 2306A>T, 2312T>C, 2315C>T, 2321G>T, 2322G>T, 2325A>G, 2326A>C, 2327T>A, 2330C>A, 2331C>G, 2333G>A, 2339G>A, 2345A>T, 2348C>T								
**CDS**									
RdRp	637	681	6.0%	249	68.6%	45 (100%)	30 (66.7%)	0/0/0/0	0
Protein mutations:	K637N (2222A>T), Q638E (2223C>G 2225A>G), L641F (2234A>T), H645R (2244C>A 2245A>G 2246T>A), V653L (2268G>T), R654L (2272G>T 2273T>C), H655R (2274C>A 2275A>G 2276T>A), M657V (2280A>G), E658K (2283G>A), R662K (2295C>A 2296G>A), L664V (2301C>G 2303T>C), A671S (2322G>T), N672A (2325A>G 2326A>C 2327T>A), P674A (2331C>G 2333G>A)								
Codon mutations:	AAA637AAT (2222A>T), CAA638GAG (2223C>G 2225A>G), CTG639TTG (2226C>T), TTA641TTT (2234A>T), GAA644GAG (2243A>G), CAT645AGA (2244C>A 2245A>G 2246T>A), GAG646GAA (2249G>A), CTA648CTT (2255A>T), GTA653TTA (2268G>T), CGT654CTC (2272G>T 2273T>C), CAT655AGA (2274C>A 2275A>G 2276T>A), ATG657GTG (2280A>G), GAA658AAA (2283G>A), CTT661CTG (2294T>G), CGA662AAA (2295C>A 2296G>A), CTT664GTC (2301C>G 2303T>C), TCA665TCT (2306A>T), TAT667TAC (2312T>C), CAC668CAT (2315C>T), GTG670GTT (2321G>T), GCA671TCA (2322G>T), AAT672GCA (2325A>G 2326A>C 2327T>A), TCC673TCA (2330C>A), CCG674GCA (2331C>G 2333G>A), GTG676GTA (2339G>A), ATA678ATT (2345A>T), GAC679GAT (2348C>T)								
**Proteins**									
RNA-dependent RNA polymerase (YP_010798875.1)	637	681	6.0%	249	68.6%	45 (100%)	30 (66.7%)	0/0/0/0	0
Protein mutations:	K637N (2222A>T), Q638E (2223C>G 2225A>G), L641F (2234A>T), H645R (2244C>A 2245A>G 2246T>A), V653L (2268G>T), R654L (2272G>T 2273T>C), H655R (2274C>A 2275A>G 2276T>A), M657V (2280A>G), E658K (2283G>A), R662K (2295C>A 2296G>A), L664V (2301C>G 2303T>C), A671S (2322G>T), N672A (2325A>G 2326A>C 2327T>A), P674A (2331C>G 2333G>A)								
Codon mutations:	AAA637AAT (2222A>T), CAA638GAG (2223C>G 2225A>G), CTG639TTG (2226C>T), TTA641TTT (2234A>T), GAA644GAG (2243A>G), CAT645AGA (2244C>A 2245A>G 2246T>A), GAG646GAA (2249G>A), CTA648CTT (2255A>T), GTA653TTA (2268G>T), CGT654CTC (2272G>T 2273T>C), CAT655AGA (2274C>A 2275A>G 2276T>A), ATG657GTG (2280A>G), GAA658AAA (2283G>A), CTT661CTG (2294T>G), CGA662AAA (2295C>A 2296G>A), CTT664GTC (2301C>G 2303T>C), TCA665TCT (2306A>T), TAT667TAC (2312T>C), CAC668CAT (2315C>T), GTG670GTT (2321G>T), GCA671TCA (2322G>T), AAT672GCA (2325A>G 2326A>C 2327T>A), TCC673TCA (2330C>A), CCG674GCA (2331C>G 2333G>A), GTG676GTA (2339G>A), ATA678ATT (2345A>T), GAC679GAT (2348C>T)								

*: Inserts / Deletes / Misaligned / Frameshifts

Analysis details

This analysis was performed with panviral2.64

NGS Details (UN9): Plantago lanceolata latent virus

Assembly

**Coverage Length**	135 (1 contig(s))
**Depth Of Coverage**	1.0
**Number Of Reads**	1
**Reads Per Million**	0.02 rpm (after QC)
**Ambiguities**	0
**Assembly Method**	de novo + reference guided assembly
**Consensus Caller**	Bcf Tools

Coverage Map

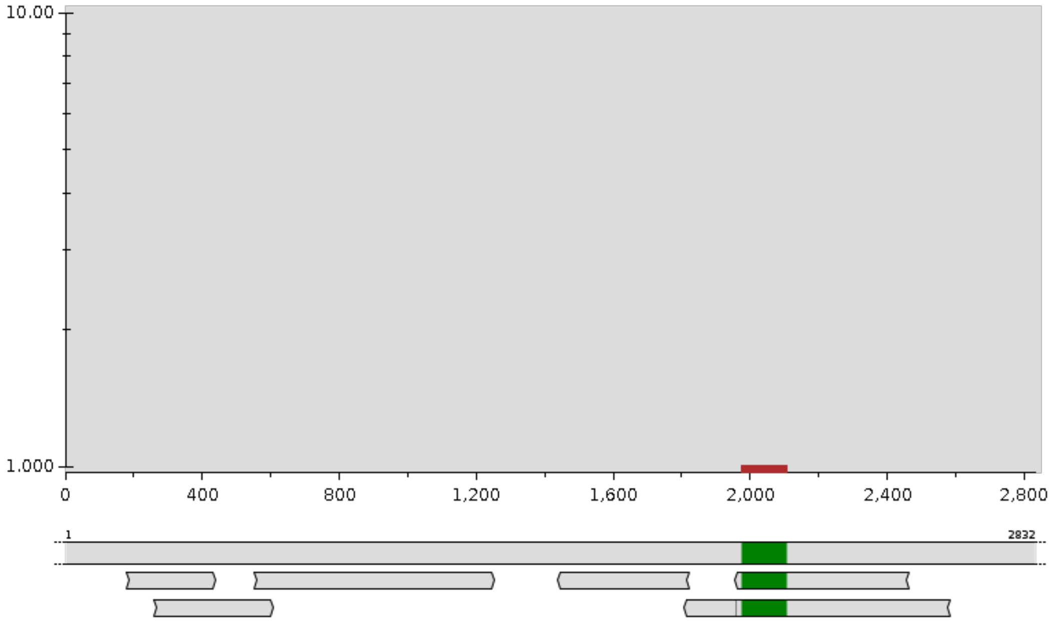

Assignment

**Type**	Plantago lanceolata latent virus (Taxonomy ID: 1830242)
**Reference Genome**	NC_038477.1
**NT Identity (%)**	67.4074
**AA Identity (%)**	54.0741
**Number Of Stop Codons**	4
**Number Of CDS**	6

Alignment

**Alignment Score**	94.0 (NT) + 589.0 (AA) = 683.0
**Concordance (%)**	51.4695

Alignment Method	Global, seeded, nucleotide + amino acids (AGA)

Genome Region

Sequence starts at position 1975 and ends at position 2109 relative to NC_038477.1 reference sequence.

Alignment Detailed Statistics

	Begin	End	Coverage	Score	Concordance	Matches	Identities	I/D/M/F*	Stop Codons
**NT**	**1975**	**2109**	**4.8%**	**94**	**34.8%**	**135 (100%)**	**91 (67.4%)**	**0/0**	
Mutations:	1976T>C, 1979G>T, 1980G>A, 1985T>G, 1987G>C, 1994G>T, 1995G>T, 1996G>C, 1997C>T, 2000A>G, 2005A>C, 2008T>G, 2010G>A, 2011G>T, 2012G>A, 2016G>T, 2017C>T, 2018C>T, 2019C>A, 2021G>C, 2022T>C, 2024T>C, 2027G>T, 2032C>G, 2042A>G, 2045G>T, 2046G>T, 2057T>C, 2058C>T, 2059T>G, 2060G>T, 2061G>T, 2062A>T, 2063A>G, 2075C>T, 2078C>A, 2084G>T, 2086G>A, 2089A>G, 2090C>T, 2102T>G, 2103G>A, 2106T>A, 2108G>C								
**CDS**									
D1Q99_gp4	160	204	13.4%	233	63.7%	45 (100%)	28 (62.2%)	0/0/0/0	1
Protein mutations:	Y160F (2106T>A), T161I (2102T>G 2103G>A), R167* (2084G>T 2086G>A), S175K (2060G>T 2061G>T 2062A>T), R176Q (2057T>C 2058C>T 2059T>G), P180Q (2045G>T 2046G>T), E185Q (2032C>G), Y188W (2021G>C 2022T>C), R189I (2018C>T 2019C>A), A190N (2016G>T 2017C>T), P192I (2010G>A 2011G>T), N193H (2008T>G), L194V (2005A>C), P197E (1994G>T 1995G>T 1996G>C), Q200D (1985T>G 1987G>C), A202V (1979G>T 1980G>A)								
Codon mutations:	CCC159.CG (2108G>C), TAC160TTC (2106T>A), ACA161ATC (2102T>G 2103G>A), CAG165CAA (2090C>T), TTA166CTA (2089A>G), CGC167TGA (2084G>T 2086G>A), CTG169CTT (2078C>A), GAG170GAA (2075C>T), AAT174AAC (2063A>G), TCC175AAA (2060G>T 2061G>T 2062A>T), AGA176CAG (2057T>C 2058C>T 2059T>G), CCC180CAA (2045G>T 2046G>T), CCT181CCC (2042A>G), GAA185CAA (2032C>G), CCC186CCA (2027G>T), AGA187AGG (2024T>C), TAC188TGG (2021G>C 2022T>C), AGG189ATA (2018C>T 2019C>A), GCT190AAT (2016G>T 2017C>T), TTC191TTT (2012G>A), CCT192ATT (2010G>A 2011G>T), AAT193CAT (2008T>G), TTA194GTA (2005A>C), CCT195CCC (2000A>G), GAG196GAA (1997C>T), CCC197GAA (1994G>T 1995G>T 1996G>C), CAA200GAC (1985T>G 1987G>C), GCC202GTA (1979G>T 1980G>A), GAA203GAG (1976T>C)								
D1Q99_gp5	160	204	17.4%	233	63.7%	45 (100%)	28 (62.2%)	0/0/0/0	1
Protein mutations:	Y160F (2106T>A), T161I (2102T>G 2103G>A), R167* (2084G>T 2086G>A), S175K (2060G>T 2061G>T 2062A>T), R176Q (2057T>C 2058C>T 2059T>G), P180Q (2045G>T 2046G>T), E185Q (2032C>G), Y188W (2021G>C 2022T>C), R189I (2018C>T 2019C>A), A190N (2016G>T 2017C>T), P192I (2010G>A 2011G>T), N193H (2008T>G), L194V (2005A>C), P197E (1994G>T 1995G>T 1996G>C), Q200D (1985T>G 1987G>C), A202V (1979G>T 1980G>A)								
Codon mutations:	CCC159.CG (2108G>C), TAC160TTC (2106T>A), ACA161ATC (2102T>G 2103G>A), CAG165CAA (2090C>T), TTA166CTA (2089A>G), CGC167TGA (2084G>T 2086G>A), CTG169CTT (2078C>A), GAG170GAA (2075C>T), AAT174AAC (2063A>G), TCC175AAA (2060G>T 2061G>T 2062A>T), AGA176CAG (2057T>C 2058C>T 2059T>G), CCC180CAA (2045G>T 2046G>T), CCT181CCC (2042A>G), GAA185CAA (2032C>G), CCC186CCA (2027G>T), AGA187AGG (2024T>C), TAC188TGG (2021G>C 2022T>C), AGG189ATA (2018C>T 2019C>A), GCT190AAT (2016G>T 2017C>T), TTC191TTT (2012G>A), CCT192ATT (2010G>A 2011G>T), AAT193CAT (2008T>G), TTA194GTA (2005A>C), CCT195CCC (2000A>G), GAG196GAA (1997C>T), CCC197GAA (1994G>T 1995G>T 1996G>C), CAA200GAC (1985T>G 1987G>C), GCC202GTA (1979G>T 1980G>A), GAA203GAG (1976T>C)								
D1Q99_gp6	120	164	26.5%	123	34.5%	45 (100%)	17 (37.8%)	0/0/0/0	2
Protein mutations:	P120R (2108G>C), T121S (2106T>A), H122S (2102T>G 2103G>A), S126N (2089A>G 2090C>T), A128E (2084G>T), W130L (2078C>A), S131N (2075C>T), I135T (2062A>T 2063A>G), P136N (2059T>G 2060G>T 2061G>T), D137S (2057T>C 2058C>T), P141N (2045G>T 2046G>T), L142P (2042A>G), M145I (2032C>G), P147Q (2027G>T), D148G (2024T>C), T149G (2021G>C 2022T>C), G150* (2017C>T 2018C>T 2019C>A), L151I (2016G>T), S152L (2011G>T 2012G>A), L153F (2008T>G 2010G>A), I154M (2005A>C), L156P (2000A>G), S157K (1996G>C 1997C>T), P158K (1994G>T 1995G>T), N160K (1987G>C), N161T (1985T>G), P163* (1979G>T 1980G>A), K164R (1976T>C)								
Codon mutations:	CCT120CGT (2108G>C), ACA121TCA (2106T>A), CAT122TCT (2102T>G 2103G>A), AGT126AAC (2089A>G 2090C>T), TAC127TAT (2086G>A), GCA128GAA (2084G>T), TGG130TTG (2078C>A), AGT131AAT (2075C>T), ATT135ACA (2062A>T 2063A>G), CCA136AAC (2059T>G 2060G>T 2061G>T), GAT137AGT (2057T>C 2058C>T), CCC141AAC (2045G>T 2046G>T), CTG142CCG (2042A>G), ATG145ATC (2032C>G), CCA147CAA (2027G>T), GAT148GGT (2024T>C), ACA149GGA (2021G>C 2022T>C), GGG150TAA (2017C>T 2018C>T 2019C>A), CTT151ATT (2016G>T), TCC152TTA (2011G>T 2012G>A), CTA153TTC (2008T>G 2010G>A), ATT154ATG (2005A>C), CTG156CCG (2000A>G), AGC157AAG (1996G>C 1997C>T), CCA158AAA (1994G>T 1995G>T), AAC160AAG (1987G>C), AAT161ACT (1985T>G), CCG163TAG (1979G>T 1980G>A), AAA164AGA (1976T>C)								
**Proteins**									
replication-associated protein (YP_009506572.1)	160	204	13.4%	233	63.7%	45 (100%)	28 (62.2%)	0/0/0/0	1
Protein mutations:	Y160F (2106T>A), T161I (2102T>G 2103G>A), R167* (2084G>T 2086G>A), S175K (2060G>T 2061G>T 2062A>T), R176Q (2057T>C 2058C>T 2059T>G), P180Q (2045G>T 2046G>T), E185Q (2032C>G), Y188W (2021G>C 2022T>C), R189I (2018C>T 2019C>A), A190N (2016G>T 2017C>T), P192I (2010G>A 2011G>T), N193H (2008T>G), L194V (2005A>C), P197E (1994G>T 1995G>T 1996G>C), Q200D (1985T>G 1987G>C), A202V (1979G>T 1980G>A)								
Codon mutations:	CCC159.CG (2108G>C), TAC160TTC (2106T>A), ACA161ATC (2102T>G 2103G>A), CAG165CAA (2090C>T), TTA166CTA (2089A>G), CGC167TGA (2084G>T 2086G>A), CTG169CTT (2078C>A), GAG170GAA (2075C>T), AAT174AAC (2063A>G), TCC175AAA (2060G>T 2061G>T 2062A>T), AGA176CAG (2057T>C 2058C>T 2059T>G), CCC180CAA (2045G>T 2046G>T), CCT181CCC (2042A>G), GAA185CAA (2032C>G), CCC186CCA (2027G>T), AGA187AGG (2024T>C), TAC188TGG (2021G>C 2022T>C), AGG189ATA (2018C>T 2019C>A), GCT190AAT (2016G>T 2017C>T), TTC191TTT (2012G>A), CCT192ATT (2010G>A 2011G>T), AAT193CAT (2008T>G), TTA194GTA (2005A>C), CCT195CCC (2000A>G), GAG196GAA (1997C>T), CCC197GAA (1994G>T 1995G>T 1996G>C), CAA200GAC (1985T>G 1987G>C), GCC202GTA (1979G>T 1980G>A), GAA203GAG (1976T>C)								
RepA (YP_009506573.1)	160	204	17.4%	233	63.7%	45 (100%)	28 (62.2%)	0/0/0/0	1
Protein mutations:	Y160F (2106T>A), T161I (2102T>G 2103G>A), R167* (2084G>T 2086G>A), S175K (2060G>T 2061G>T 2062A>T), R176Q (2057T>C 2058C>T 2059T>G), P180Q (2045G>T 2046G>T), E185Q (2032C>G), Y188W (2021G>C 2022T>C), R189I (2018C>T 2019C>A), A190N (2016G>T 2017C>T), P192I (2010G>A 2011G>T), N193H (2008T>G), L194V (2005A>C), P197E (1994G>T 1995G>T 1996G>C), Q200D (1985T>G 1987G>C), A202V (1979G>T 1980G>A)								
Codon mutations:	CCC159.CG (2108G>C), TAC160TTC (2106T>A), ACA161ATC (2102T>G 2103G>A), CAG165CAA (2090C>T), TTA166CTA (2089A>G), CGC167TGA (2084G>T 2086G>A), CTG169CTT (2078C>A), GAG170GAA (2075C>T), AAT174AAC (2063A>G), TCC175AAA (2060G>T 2061G>T 2062A>T), AGA176CAG (2057T>C 2058C>T 2059T>G), CCC180CAA (2045G>T 2046G>T), CCT181CCC (2042A>G), GAA185CAA (2032C>G), CCC186CCA (2027G>T), AGA187AGG (2024T>C), TAC188TGG (2021G>C 2022T>C), AGG189ATA (2018C>T 2019C>A), GCT190AAT (2016G>T 2017C>T), TTC191TTT (2012G>A), CCT192ATT (2010G>A 2011G>T), AAT193CAT (2008T>G), TTA194GTA (2005A>C), CCT195CCC (2000A>G), GAG196GAA (1997C>T), CCC197GAA (1994G>T 1995G>T 1996G>C), CAA200GAC (1985T>G 1987G>C), GCC202GTA (1979G>T 1980G>A), GAA203GAG (1976T>C)								
C3 (YP_009506574.1)	120	164	26.5%	123	34.5%	45 (100%)	17 (37.8%)	0/0/0/0	2
Protein mutations:	P120R (2108G>C), T121S (2106T>A), H122S (2102T>G 2103G>A), S126N (2089A>G 2090C>T), A128E (2084G>T), W130L (2078C>A), S131N (2075C>T), I135T (2062A>T 2063A>G), P136N (2059T>G 2060G>T 2061G>T), D137S (2057T>C 2058C>T), P141N (2045G>T 2046G>T), L142P (2042A>G), M145I (2032C>G), P147Q (2027G>T), D148G (2024T>C), T149G (2021G>C 2022T>C), G150* (2017C>T 2018C>T 2019C>A), L151I (2016G>T), S152L (2011G>T 2012G>A), L153F (2008T>G 2010G>A), I154M (2005A>C), L156P (2000A>G), S157K (1996G>C 1997C>T), P158K (1994G>T 1995G>T), N160K (1987G>C), N161T (1985T>G), P163* (1979G>T 1980G>A), K164R (1976T>C)								
Codon mutations:	CCT120CGT (2108G>C), ACA121TCA (2106T>A), CAT122TCT (2102T>G 2103G>A), AGT126AAC (2089A>G 2090C>T), TAC127TAT (2086G>A), GCA128GAA (2084G>T), TGG130TTG (2078C>A), AGT131AAT (2075C>T), ATT135ACA (2062A>T 2063A>G), CCA136AAC (2059T>G 2060G>T 2061G>T), GAT137AGT (2057T>C 2058C>T), CCC141AAC (2045G>T 2046G>T), CTG142CCG (2042A>G), ATG145ATC (2032C>G), CCA147CAA (2027G>T), GAT148GGT (2024T>C), ACA149GGA (2021G>C 2022T>C), GGG150TAA (2017C>T 2018C>T 2019C>A), CTT151ATT (2016G>T), TCC152TTA (2011G>T 2012G>A), CTA153TTC (2008T>G 2010G>A), ATT154ATG (2005A>C), CTG156CCG (2000A>G), AGC157AAG (1996G>C 1997C>T), CCA158AAA (1994G>T 1995G>T), AAC160AAG (1987G>C), AAT161ACT (1985T>G), CCG163TAG (1979G>T 1980G>A), AAA164AGA (1976T>C)								

*: Inserts / Deletes / Misaligned / Frameshifts

Analysis details

This analysis was performed with panviral2.64

NGS Details (UN9): Duamitovirus dapi1

Assembly

**Coverage Length**	134 (1 contig(s))
**Depth Of Coverage**	1.0
**Number Of Reads**	1
**Reads Per Million**	0.02 rpm (after QC)
**Ambiguities**	0
**Assembly Method**	de novo + reference guided assembly
**Consensus Caller**	Bcf Tools

Coverage Map

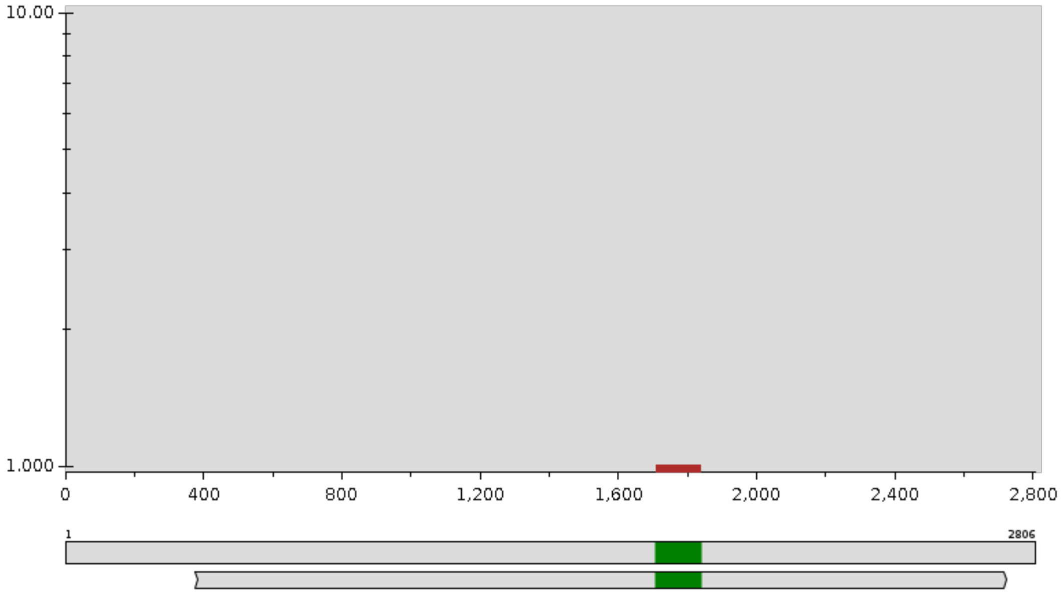

Assignment

**Type**	Duamitovirus dapi1 (Taxonomy ID: 2955778)
**Reference Genome**	NC_076528.1
**NT Identity (%)**	68.6567
**AA Identity (%)**	68.1818
**Number Of Stop Codons**	0
**Number Of CDS**	1

Alignment

**Alignment Score**	100.0 (NT) + 249.0 (AA) = 349.0
**Concordance (%)**	57.0261

Alignment Method	Global, seeded, nucleotide + amino acids (AGA)

Genome Region

Sequence starts at position 1709 and ends at position 1842 relative to NC_076528.1 reference sequence.

Alignment Detailed Statistics

	Begin	End	Coverage	Score	Concordance	Matches	Identities	I/D/M/F*	Stop Codons
**NT**	**1709**	**1842**	**4.8%**	**100**	**37.3%**	**134 (100%)**	**92 (68.7%)**	**0/0**	
Mutations:	1710C>T, 1713A>G, 1715G>A, 1725T>C, 1728G>C, 1734C>T, 1736A>T, 1737C>G, 1738T>A, 1739C>G, 1743C>T, 1749G>T, 1750T>C, 1751C>T, 1755C>T, 1756G>A, 1763C>A, 1774G>C, 1776A>T, 1783C>A, 1785G>A, 1788G>T, 1789G>A, 1791C>T, 1792T>G, 1793G>A, 1794G>A, 1800A>G, 1804C>G, 1805C>G, 1806T>C, 1809T>G, 1810C>A, 1811A>C, 1816T>C, 1819A>T, 1820A>G, 1821G>C, 1827C>T, 1830T>C, 1831C>A, 1839T>C								
**CDS**									
RdRp	446	489	5.6%	249	70.3%	44 (100%)	30 (68.2%)	0/0/0/0	0
Protein mutations:	R447K (1715G>A), Y454L (1736A>T 1737C>G), S459L (1750T>C 1751C>T), A461T (1756G>A), T463K (1763C>A), V467L (1774G>C 1776A>T), L470I (1783C>A 1785G>A), A472T (1789G>A 1791C>T), W473E (1792T>G 1793G>A 1794G>A), P477G (1804C>G 1805C>G 1806T>C), Q479T (1810C>A 1811A>C), F481L (1816T>C), K482C (1819A>T 1820A>G 1821G>C), L486I (1831C>A)								
Codon mutations:	TTC445.TT (1710C>T), ACA446ACG (1713A>G), AGG447AAG (1715G>A), CCT450CCC (1725T>C), CTG451CTC (1728G>C), TTC453TTT (1734C>T), TAC454TTG (1736A>T 1737C>G), TCT455AGT (1738T>A 1739C>G), TCC456TCT (1743C>T), CCG458CCT (1749G>T), TCA459CTA (1750T>C 1751C>T), TTC460TTT (1755C>T), GCA461ACA (1756G>A), ACA463AAA (1763C>A), GTA467CTT (1774G>C 1776A>T), CTG470ATA (1783C>A 1785G>A), GCG471GCT (1788G>T), GCC472ACT (1789G>A 1791C>T), TGG473GAA (1792T>G 1793G>A 1794G>A), GTA475GTG (1800A>G), CCT477GGC (1804C>G 1805C>G 1806T>C), ACT478ACG (1809T>G), CAG479ACG (1810C>A 1811A>C), TTT481CTT (1816T>C), AAG482TGC (1819A>T 1820A>G 1821G>C), TAC484TAT (1827C>T), GCT485GCC (1830T>C), CTT486ATT (1831C>A), GGT488GGC (1839T>C)								
**Proteins**									
RNA-dependent RNA polymerase (YP_010798878.1)	446	489	5.6%	249	70.3%	44 (100%)	30 (68.2%)	0/0/0/0	0
Protein mutations:	R447K (1715G>A), Y454L (1736A>T 1737C>G), S459L (1750T>C 1751C>T), A461T (1756G>A), T463K (1763C>A), V467L (1774G>C 1776A>T), L470I (1783C>A 1785G>A), A472T (1789G>A 1791C>T), W473E (1792T>G 1793G>A 1794G>A), P477G (1804C>G 1805C>G 1806T>C), Q479T (1810C>A 1811A>C), F481L (1816T>C), K482C (1819A>T 1820A>G 1821G>C), L486I (1831C>A)								
Codon mutations:	TTC445.TT (1710C>T), ACA446ACG (1713A>G), AGG447AAG (1715G>A), CCT450CCC (1725T>C), CTG451CTC (1728G>C), TTC453TTT (1734C>T), TAC454TTG (1736A>T 1737C>G), TCT455AGT (1738T>A 1739C>G), TCC456TCT (1743C>T), CCG458CCT (1749G>T), TCA459CTA (1750T>C 1751C>T), TTC460TTT (1755C>T), GCA461ACA (1756G>A), ACA463AAA (1763C>A), GTA467CTT (1774G>C 1776A>T), CTG470ATA (1783C>A 1785G>A), GCG471GCT (1788G>T), GCC472ACT (1789G>A 1791C>T), TGG473GAA (1792T>G 1793G>A 1794G>A), GTA475GTG (1800A>G), CCT477GGC (1804C>G 1805C>G 1806T>C), ACT478ACG (1809T>G), CAG479ACG (1810C>A 1811A>C), TTT481CTT (1816T>C), AAG482TGC (1819A>T 1820A>G 1821G>C), TAC484TAT (1827C>T), GCT485GCC (1830T>C), CTT486ATT (1831C>A), GGT488GGC (1839T>C)								

*: Inserts / Deletes / Misaligned / Frameshifts

Analysis details

This analysis was performed with panviral2.64
