# Supplementary material for: Nucleotide sequence analysis reveals the presence of PVY-Tam isolates affecting tamarillo in Colombia
Source: Virol J. 2026 Apr 20;23:145. doi: 10.1186/s12985-026-03166-6 (PMC13234967; doi:10.1186/s12985-026-03166-6)
Supplement: Supplementary file 2 — Additional file 2. [file 12985_2026_3166_MOESM2_ESM.pdf]

Analysis of UN8

**Technology**	Paired-end short reads
**Input Files**	UN8_R1.fq.gz (1.92 GB), UN8_R2.fq.gz (2 GB)
**Submitted On**	2023-09-26 13:08:53 UTC
**Duration**	3h 48m 49s
**Tool Version**	panviral2.64
**Location**	https://www.genomedetective.com/db/ui/analysis/35ae2222-bf0b-41be-887f-70156779792e

Statistics

**Original Read Length**	20 - 150
**Trimmed Read Length**	50 - 135

	# Reads	% of Reads
**Input file**	60259918	100.0%
**After QC**	59835436	99.3%
**After filtering**	4578960	7.6%
**Mapped back to contigs**	1898442	3.2%

Assignments

Assignment	No. of Reads	Depth of Coverage	Identity		Genome Coverage	
			NT	AA		
Torradovirus lycopersici (4 segments out of 2)	693606	3689.4	89.6%	95.7%	99.9%	
Torradovirus lycopersici (segment RNA 2)	313618	7518.4	96.6%	98.0%	99.6%	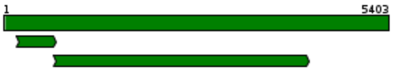
Torradovirus lycopersici (segment RNA 1)	186149	4265.8	94.3%	98.8%	99.4%	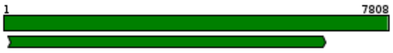
Torradovirus lycopersici (segment RNA 1)	114995	2254.7	90.5%	97.8%	87.0%	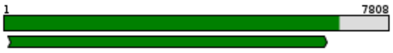
Torradovirus lycopersici (segment RNA 1)	78839	1527.7	78.6%	89.9%	86.9%	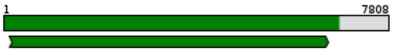
Potato virus Y	634281	8776.7	82.8%	90.0%	99.5%	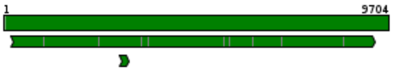
Torradovirus marchitezum (2 segments out of 2)	274810	4599.5	63.8%	64.4%	64.1%	
Torradovirus marchitezum (segment RNA 1)	176957	3512.7	63.0%	63.4%	93.0%	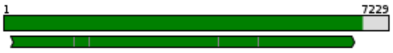

Assignment	No. of Reads	Depth of Coverage	Identity		Genome Coverage	
			NT	AA		
Torradovirus marchitezum (segment RNA 2)	97853	11498.6	69.3%	72.8%	21.6%	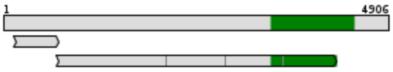
Diachasmimorpha longicaudata entomopoxvirus (segment NC_043455.1)	13895	1498.5	59.8%	55.3%	83.7%	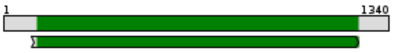
Bracoviriform glomeratae (segment NC_043292.1)	5742	2096.9	72.6%	84.5%	72.8%	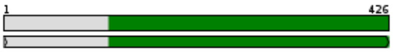
Potato leafroll virus	3146	72.1	98.3%	97.8%	97.3%	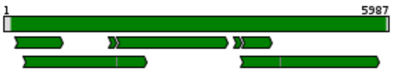
Duamitovirus soch1	508	25.7	69.1%	69.5%	81.3%	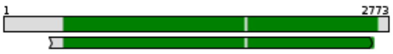
Solendovirus venanicotianae	430	21.1	79.8%	76.8%	30.0%	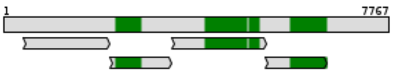
Harvey murine sarcoma virus	8	3.9	73.3%	90.0%	28.0%	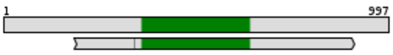

Discoveries

Similar to	No. of Reads	Depth of Coverage	Identity		Genome Coverage	
			NT	AA		
Tomato chocolate spot virus (3 segments out of 2)	101720	6454.9	65.8%	66.0%	15.3%	
Tomato chocolate spot virus (segment RNA2)	50187	5116.5	65.2%	70.5%	24.5%	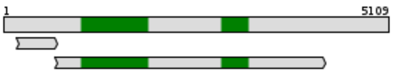
Tomato chocolate spot virus (segment RNA 1)	36615	15605.2	82.4%	0.0%	3.7%	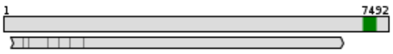
Tomato chocolate spot virus (segment RNA2)	14918	4272.8	56.2%	51.9%	7.7%	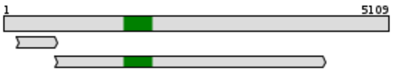
Lausannevirus	34961	23002.5	80.3%	93.5%	0.0%	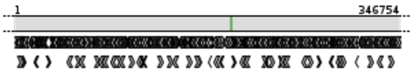
Yellowstone lake phycodnavirus 1	29336	15316.6	82.1%	93.1%	0.1%	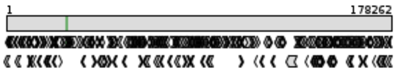
Euproctis digramma nucleopolyhedrovirus	26156	15809.8	79.5%	92.0%	0.1%	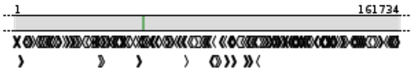
Betabaculovirus clanastomosis	22579	13042.5	83.6%	86.7%	0.1%	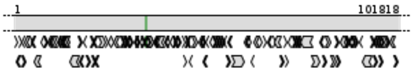
Lausannevirus	11975	7006.5	84.2%	100.0%	0.0%	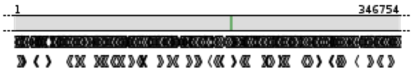

Similar to	No. of Reads	Depth of Coverage	Identity		Genome Coverage	
			NT	AA		
Yellowstone lake phycodnavirus 1	11161	6804.1	83.0%	90.2%	0.1%	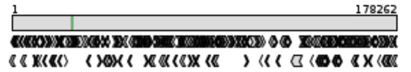
Lausannevirus	6209	3607.8	82.1%	94.1%	0.0%	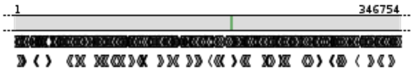
Synechococcus phage S-CRM01	6209	3685.1	78.8%	92.0%	0.1%	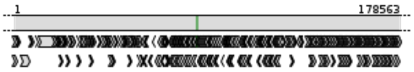
Marseillevirus marseillevirus	5547	3706.7	85.7%	97.5%	0.0%	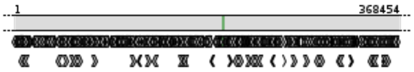
Lausannevirus	4798	3324.6	80.0%	95.2%	0.0%	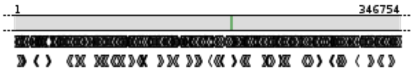
Betabaculovirus disaccharalis	2967	1794.9	82.4%	92.9%	0.1%	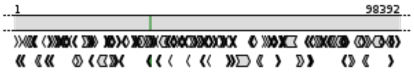
Lausannevirus	2902	1791.9	80.8%	94.1%	0.0%	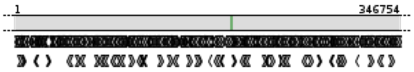
Lausannevirus	1319	631.3	83.8%	95.3%	0.0%	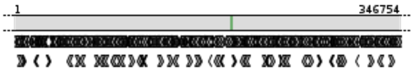
Cladosporium fulvum T-1 virus	1081	75.0	53.2%	48.1%	19.3%	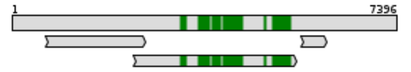
Errantivirus	891	139.9	53.1%	40.8%	9.3%	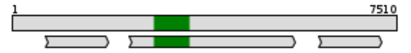
Human gammaherpesvirus 8 (subtype: Could not assign)	368	228.3	83.7%	84.4%	0.1%	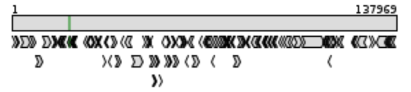
Epiphyllum badnavirus 1	360	148.9	57.5%	57.0%	3.3%	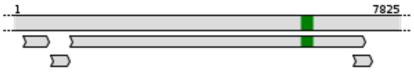
Cassava brown streak virus	308	70.8	60.9%	58.0%	5.9%	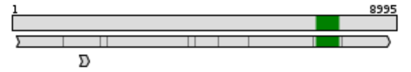
Epiphyllum badnavirus 1	137	50.7	57.6%	52.7%	3.5%	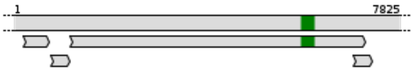
Errantivirus	133	22.2	52.4%	46.2%	9.5%	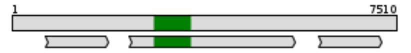
Dioscovirus dioscoreae	87	10.3	61.7%	49.9%	13.1%	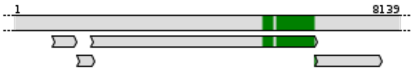
Badnavirus occultipomeae	84	29.0	51.1%	41.3%	4.0%	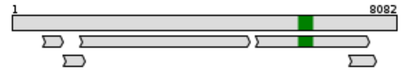

Similar to	No. of Reads	Depth of Coverage	Identity		Genome Coverage	
			NT	AA		
Errantivirus	73	22.1	54.6%	45.3%	5.1%	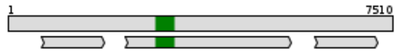
Errantivirus	64	20.6	56.9%	47.5%	4.7%	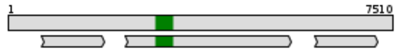
Errantivirus	59	18.4	53.1%	42.4%	5.0%	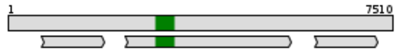
Badnavirus maculacommelinae	55	7.8	54.7%	47.9%	12.1%	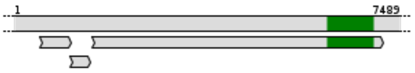
Epiphyllum badnavirus 1	55	16.6	52.4%	41.5%	5.2%	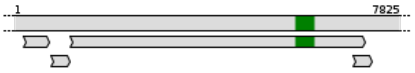
Petuvirus venapetuniae	50	7.5	51.7%	43.1%	11.4%	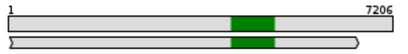
Badnavirus occultipomeae	40	11.3	55.0%	40.7%	4.5%	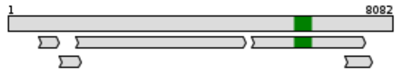
Caulimovirus tessellobrassicae	39	6.6	56.7%	46.4%	9.0%	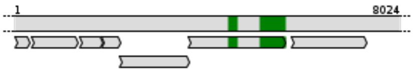
Caulimovirus venafragariae	39	11.4	64.1%	67.6%	5.4%	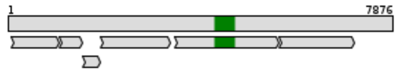
Caulimovirus deformatiolamii	27	5.8	54.5%	45.0%	7.5%	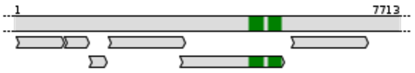
Petuvirus venapetuniae	25	5.8	55.2%	49.5%	7.6%	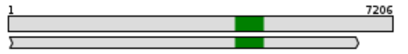
Errantivirus	23	7.6	55.4%	47.5%	4.8%	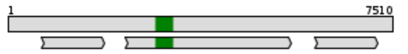
Badnavirus maculaucubae	20	8.4	57.2%	57.5%	3.7%	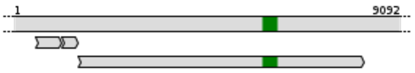
Hubei noda-like virus 9 (2 segments out of 2)	20	2.2	66.4%	69.7%	20.9%	
Hubei noda-like virus 9 (segment 1)	11	1.9	66.4%	69.9%	21.6%	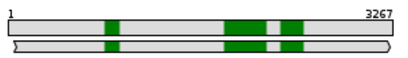
Hubei noda-like virus 9 (segment 2)	9	2.7	66.5%	69.1%	19.4%	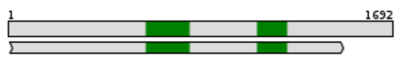
Caulimovirus maculatractylodei	18	4.7	56.7%	47.0%	6.3%	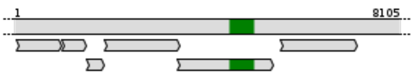
Cavemovirus venamanihotis	15	4.8	60.2%	50.0%	4.6%	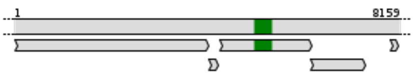

Similar to	No. of Reads	Depth of Coverage	Identity		Genome Coverage	
			NT	AA		
Errantivirus	14	5.4	56.4%	48.6%	4.2%	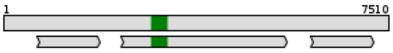
Caulimovirus venafragariae	14	6.4	60.9%	61.9%	3.2%	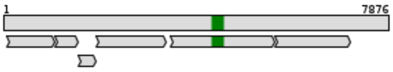
Badnavirus maculasmallanthi	13	4.7	51.1%	41.2%	4.0%	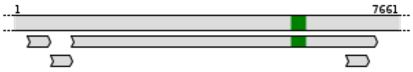
Caulimovirus venafragariae	9	4.2	63.6%	63.5%	3.2%	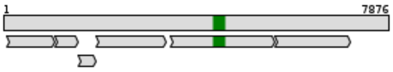
Cavemovirus venamanihotis	8	3.2	60.1%	51.6%	3.3%	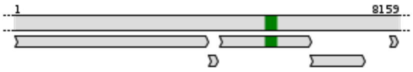
Alphabaculovirus chofumiferanae	7	4.5	78.7%	80.0%	0.1%	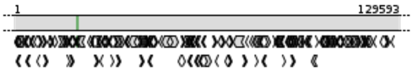
Badnavirus volubetulae	7	3.2	61.1%	47.5%	3.9%	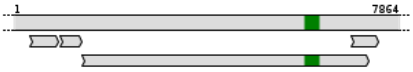
Epiphyllum badnavirus 1	6	2.2	52.8%	43.8%	3.9%	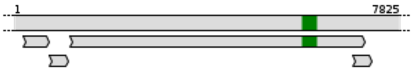
Potato virus X	4	2.3	96.6%	95.6%	3.7%	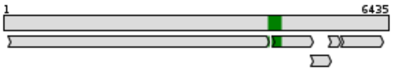
Petuvirus venapetuniae	4	2.4	68.4%	69.3%	3.1%	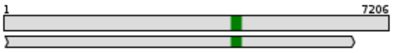
Tomato associated geminivirus 1	4	1.8	63.7%	59.0%	9.0%	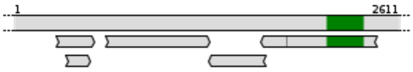
Bracoviriform congregatae (segment Circle 7)	3	2.5	75.6%	86.7%	0.5%	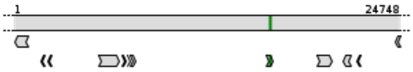
Tobacco streak virus (segment RNA 1)	2	1.0	74.3%	80.0%	7.7%	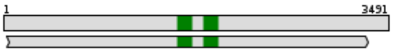
Nepovirus aeonii (segment RNA2)	2	1.1	69.0%	66.7%	6.0%	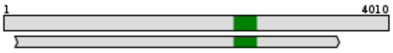
Duamitovirus peex1	2	1.5	70.4%	73.3%	5.0%	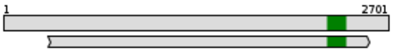
Moloney murine leukemia virus	2	1.3	81.2%	78.3%	2.7%	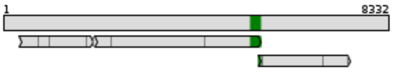
Teseptimavirus EG1	2	1.2	75.1%	98.6%	0.5%	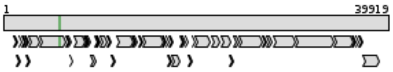
Duamitovirus peex1	1	1.0	80.0%	80.0%	5.0%	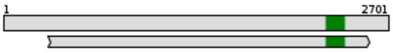

Similar to	No. of Reads	Depth of Coverage	Identity		Genome Coverage	
			NT	AA		
Cyvirus cyprinidallo3	1	1.0	83.3%	80.0%	0.0%	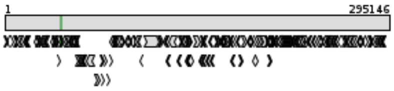
Diplodia scrobiculata RNA virus 1	1	1.0	80.8%	80.0%	2.4%	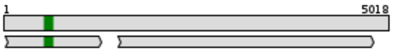

NGS Details (UN8): Potato virus Y

Assembly

**Coverage Length**	9653 (1 contig(s))
**Depth Of Coverage**	8776.7
**Number Of Reads**	634281
**Reads Per Million**	10600.42 rpm (after QC)
**Ambiguities**	0
**Assembly Method**	de novo + reference guided assembly
**Consensus Caller**	Bcf Tools

Coverage Map

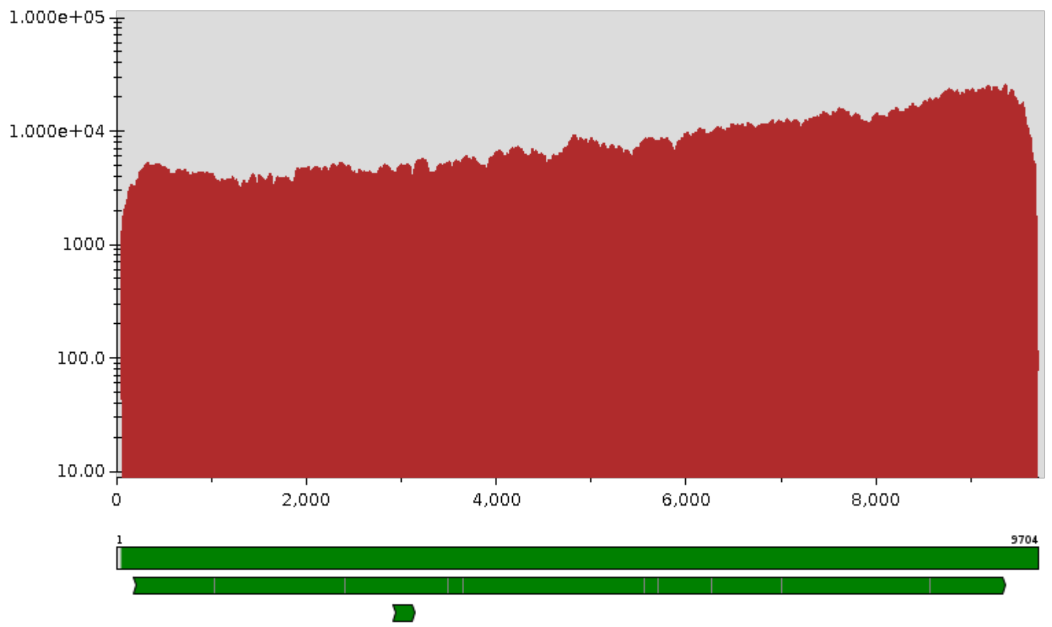

Assignment

**Type**	Potato virus Y (Taxonomy ID: 12216)
**Reference Genome**	NC_001616.1
**NT Identity (%)**	82.7547
**AA Identity (%)**	90.0
**Number Of Stop Codons**	3
**Number Of CDS**	2

Alignment

**Alignment Score**	12530.0 (NT) + 19272.0 (AA) = 31802.0
**Concordance (%)**	78.3324

Alignment Method	Global, seeded, nucleotide + amino acids (AGA)

Genome Region

Sequence starts at position 52 and ends at position 9704 relative to NC_001616.1 reference sequence.

Alignment Detailed Statistics

	Begin	End	Coverage	Score	Concordance	Matches	Identities	I/D/M/F*	Stop Codons
**NT**	**52**	**9704**	**99.5%**	**12530**	**65.2%**	**9642 (99.8%)**	**7985 (82.7%)**	**7/11**	

Mutations:

75C>T, 79C>T, 83G>A, 85T>C, 87C>T, 89G>A, 90T>C, 93A>T, 94A>G, 95A>T, 100T>C, 101T>A, 102C>T, 104T>C, 107C>T, 110T>G, 112C>T, 114C>T, 115T>C, 116A>T, 117G>C,
119A>G, 120C>T, 121A>T, 125T>G, 126T>C, 127G>A, 128G>T, 131A>G, 132C>T, 139A>T, 139_140insG, 141C>T, 143C>T, 146C>T, 150C>T, 150_151insT, 154T>C, 156C>A,
158T>A, 160A>C, 164C>T, 168C>A, 169C>G, 171A>C, 172T>C, 173T>A, 174T>G, 177G>C, 178A>G, 179T>C, 180C>T, 181C>A, 182T>G, 183C>T, 193T>C, 194T>C, 196C>A,
202A>G, 208C>T, 209T>C, 210G>T, 211T>G, 220G>A, 221T>A, 223T>G, 226A>G, 235A>T, 241C>T, 247A>T, 251T>C, 255G>T, 256C>T, 258A>G, 262T>C, 263A>G, 265T>G,
267T>C, 273A>T, 277A>T, 280A>G, 283G>T, 286G>A, 287G>A, 289T>C, 293G>A, 294T>C, 301T>C, 304C>T, 305G>A, 306C>T, 309A>G, 311C>T, 316A>T, 317A>G, 318C>T,
319A>G, 322A>G, 331A>T, 339T>A, 340C>A, 342A>G, 347A>G, 348A>C, 349A>G, 350T>C, 355T>A, 356A>G, 357C>T, 364T>C, 365G>A, 366T>C, 367G>A, 368C>T, 369T>C,
373A>G, 376C>T, 384T>G, 388G>A, 391C>T, 394A>G, 400G>A, 406T>C, 408C>T, 409C>T, 412G>A, 415A>T, 416A>G, 417T>A, 424T>C, 427G>A, 433A>G, 436G>C, 437G>A,
440A>G, 441G>A, 443A>C, 444A>G, 445G>A, 448T>A, 454A>G, 456A>C, 460T>C, 461C>A, 469A>G, 475C>G, 481T>A, 502T>C, 505A>T, 511C>T, 517T>A, 520T>A, 523A>T,
531C>T, 535G>A, 536C>T, 539C>T, 540A>G, 541A>G, 549G>A, 550A>G, 556C>T, 559T>C, 562T>C, 568T>C, 571A>G, 576T>C, 580T>C, 585T>C, 586C>G, 593C>T, 594G>A,
595C>T, 597C>G, 599A>G, 601A>G, 602A>C, 603T>C, 604A>G, 613A>G, 616A>G, 619C>G, 622G>A, 625G>A, 628T>C, 631C>T, 635A>G, 637T>C, 644A>G, 646A>G, 655T>C,
661G>A, 663A>C, 664G>C, 667A>G, 679C>T, 682C>A, 683T>C, 685A>G, 691T>C, 699C>G, 703T>A, 706T>A, 719A>G, 721G>A, 722A>G, 724A>G, 725C>T, 727T>G, 730T>A,
731G>T, 734T>C, 735A>C, 736C>T, 737T>C, 738C>G, 739C>T, 744C>T, 745G>C, 749C>T, 751A>C, 754T>C, 765T>A, 770T>C, 773C>A, 774G>A, 775A>G, 776C>A, 790C>T,
801T>A, 813G>C, 814A>G, 816T>A, 818T>C, 823A>G, 825G>A, 826T>A, 833C>A, 835G>A, 841C>T, 851A>G, 853T>C, 859C>T, 860C>T, 862C>T, 863A>G, 865T>C, 867T>C,
869A>G, 871C>T, 872A>T, 874A>G, 876G>A, 879G>A, 886T>C, 898C>T, 903A>G, 906C>A, 907A>C, 909A>C, 910A>C, 912G>A, 913C>T, 922C>A, 931T>A, 938T>C, 939C>T,
940A>T, 943A>G, 952C>T, 967A>G, 973A>G, 976G>C, 980T>A, 982G>C, 991A>G, 994T>C, 999G>A, 1003T>C, 1007C>A, 1008A>G, 1009G>A, 1010A>G, 1012T>G, 1013A>G,
1018A>G, 1019A>C, 1021C>G, 1030C>T, 1039G>A, 1042T>C, 1045C>A, 1048C>A, 1051T>C, 1060G>A, 1063T>A, 1064C>T, 1069C>T, 1083G>A, 1093T>C, 1097T>G, 1099G>A,
1105C>T, 1114A>G, 1117T>G, 1123A>G, 1126T>A, 1129C>T, 1143G>A, 1147A>T, 1150T>A, 1154T>A, 1156G>A, 1165C>T, 1168T>C, 1172C>T, 1174T>A, 1201C>T, 1221G>A,
1229G>A, 1230T>C, 1231T>C, 1233G>A, 1234C>T, 1240G>A, 1246G>A, 1250T>C, 1252G>A, 1255T>C, 1257A>C, 1258A>G, 1267A>G, 1273T>C, 1276G>A, 1278A>G, 1283T>C,
1290C>T, 1291G>A, 1298G>A, 1299A>C, 1300C>T, 1306T>C, 1307A>G, 1309A>G, 1312T>C, 1316A>G, 1318T>A, 1327G>A, 1329T>C, 1333G>T, 1336A>G, 1343C>T, 1345A>G,
1354G>A, 1357G>T, 1360C>T, 1367C>T, 1369C>G, 1373C>A, 1378C>T, 1384G>A, 1385A>G, 1393A>G, 1396C>T, 1405G>T, 1408A>G, 1411G>A, 1415G>T, 1417A>T, 1420G>C,
1429T>C, 1432A>G, 1436G>A, 1438C>T, 1439T>C, 1441A>G, 1444T>C, 1453C>T, 1456G>A, 1468A>G, 1492A>G, 1499T>C, 1501G>A, 1504T>C, 1507G>A, 1510C>T, 1513A>G,
1516A>G, 1519A>T, 1522G>A, 1525C>T, 1528G>A, 1534C>T, 1540T>A, 1546C>T, 1555A>G, 1558T>C, 1561T>C, 1564A>C, 1567T>A, 1573C>T, 1576A>G, 1583T>C, 1588T>A,
1594G>A, 1600C>T, 1609G>A, 1618G>A, 1621C>T, 1624C>T, 1631C>T, 1639A>G, 1651C>T, 1654C>G, 1675T>C, 1693A>G, 1702T>C, 1707A>G, 1718G>A, 1726A>G, 1729C>T,
1732A>C, 1738T>C, 1742A>G, 1744C>T, 1747C>T, 1753T>C, 1758G>A, 1768G>A, 1786C>T, 1789A>G, 1790G>A, 1795C>T, 1805T>C, 1807A>G, 1813G>A, 1819G>A, 1825G>A,
1837C>T, 1840T>C, 1849A>G, 1858C>G, 1864A>G, 1870C>T, 1876T>C, 1882A>G, 1888T>A, 1891T>C, 1906T>C, 1915A>T, 1930T>C, 1936C>T, 1937A>G, 1939T>C, 1945A>G,
1951C>T, 1960A>T, 1978A>G, 1981T>A, 1984C>T, 1987T>C, 1996C>T, 2004T>A, 2020A>G, 2023G>A, 2026T>C, 2029G>T, 2032G>A, 2059T>C, 2068T>G, 2071C>T, 2072G>A,
2074G>A, 2087C>T, 2089G>A, 2101C>T, 2104G>A, 2107G>A, 2116G>A, 2122C>T, 2137C>T, 2158T>C, 2164C>T, 2170A>G, 2182T>C, 2188G>A, 2191C>A, 2208G>A, 2218T>C,
2224C>T, 2227A>T, 2242G>A, 2245C>T, 2254G>A, 2257T>C, 2260C>T, 2266C>G, 2272A>G, 2275G>A, 2293A>G, 2299C>T, 2302G>A, 2305G>A, 2314A>T, 2317T>C, 2326A>G,
2341G>A, 2347A>G, 2350T>C, 2356G>A, 2365T>C, 2371A>G, 2374A>G, 2383T>C, 2386A>T, 2389A>G, 2392T>C, 2395T>C, 2414A>G, 2415A>G, 2416T>G, 2419T>A, 2420A>T,
2440A>T, 2467T>C, 2476T>C, 2482G>A, 2491G>A, 2497G>T, 2503G>A, 2518A>G, 2521T>C, 2523A>G, 2533A>G, 2539G>A, 2545A>G, 2551G>A, 2554T>A, 2558C>T, 2561T>C,
2563G>C, 2572A>G, 2584T>C, 2587C>T, 2593G>C, 2599T>C, 2608T>C, 2620T>C, 2629G>A, 2632G>A, 2634G>A, 2638G>A, 2644T>A, 2653A>G, 2665T>C, 2678C>T, 2683A>G,
2689T>C, 2698A>C, 2707A>G, 2710G>A, 2716A>G, 2719A>G, 2722C>T, 2723G>A, 2725C>T, 2728A>G, 2734G>A, 2737T>A, 2746T>C, 2747G>A, 2749T>A, 2752A>T, 2755T>A,
2761C>T, 2764C>T, 2779T>C, 2785G>T, 2791C>T, 2795C>A, 2806C>T, 2816T>C, 2818G>C, 2822G>A, 2824G>A, 2827G>A, 2836T>C, 2839G>A, 2842T>C, 2863C>A, 2864C>T,
2882C>A, 2885A>C, 2886G>A, 2893C>T, 2895C>T, 2902C>T, 2905A>G, 2911T>C, 2923T>C, 2931A>G, 2940A>G, 2947T>C, 2962T>C, 2971_2972insA, 2989T>C, 2991C>T,
2994A>G, 2999G>A, 3000A>G, 3015C>T, 3021G>A, 3024A>T, 3025C>T, 3036C>T, 3039A>G, 3040A>C, 3042A>C, 3043G>A, 3052G>A, 3061A>G, 3082G>A, 3091C>T, 3099C>T,
3100C>A, 3104G>A, 3105T>G, 3106G>A, 3109C>T, 3112A>G, 3118T>C, 3121C>T, 3127A>G, 3132G>A, 3137C>A, 3138G>A, 3139A>G, 3151T>C, 3152T>A, 3154C>T, 3165G>A,
3169A>G, 3199C>T, 3203A>C, 3208T>C, 3214A>G, 3217C>T, 3218T>C, 3223C>A, 3225C>A, 3230G>A, 3235C>T, 3257T>C, 3262C>T, 3265C>T, 3266G>A, 3268A>G, 3271G>A,
3275G>A, 3284G>A, 3301G>A, 3315G>A, 3316A>G, 3318A>C, 3322C>T, 3325G>A, 3328G>A, 3337A>T, 3349G>A, 3355C>T, 3358C>T, 3359A>G, 3367A>G, 3370T>C, 3377T>C,
3379A>G, 3394A>G, 3397C>T, 3400T>G, 3406A>G, 3418C>T, 3421T>A, 3427T>C, 3433G>A, 3436A>T, 3439A>G, 3442C>T, 3445T>C, 3457G>A, 3460T>C, 3463T>A, 3469G>T,
3472G>A, 3496C>T, 3499G>A, 3505C>T, 3513T>G, 3520A>G, 3529A>G, 3532A>G, 3545A>G, 3548G>A, 3551T>C, 3554G>A, 3556C>T, 3583T>C, 3586T>C, 3589C>T, 3595C>T,
3607T>C, 3613T>C, 3616G>A, 3619T>C, 3623C>G, 3628C>T, 3632C>A, 3637T>C, 3638T>C, 3643A>G, 3661A>G, 3670G>A, 3682T>C, 3688G>A, 3697G>A, 3698A>G, 3706T>C,
3709T>C, 3713T>C, 3715G>A, 3717G>A, 3721G>A, 3731C>A, 3732G>A, 3736T>A, 3746C>T, 3748A>G, 3754A>G, 3763T>C, 3778A>G, 3781A>G, 3784C>T, 3796T>C, 3805A>T,
3811C>T, 3814A>G, 3829C>T, 3838T>C, 3844A>G, 3847A>C, 3856T>A, 3862G>T, 3871C>T, 3877C>A, 3883C>T, 3886A>G, 3892A>G, 3895C>T, 3898T>C, 3904A>G, 3905C>A,
3907G>A, 3913T>C, 3928G>A, 3931A>T, 3937G>A, 3955T>C, 3958G>A, 3961C>T, 3964A>G, 3967T>C, 3976A>G, 3979T>C, 3985A>T, 3997A>T, 4000G>A, 4006C>T, 4009T>G,
4012C>T, 4015A>G, 4024T>C, 4030A>G, 4033A>T, 4042G>A, 4052C>T, 4054G>A, 4057T>C, 4063T>A, 4066A>G, 4073A>G, 4078T>C, 4081C>T, 4084T>C, 4096C>T, 4108C>T,
4111A>T, 4117G>A, 4118C>T, 4120A>G, 4126C>T, 4128T>A, 4129C>T, 4132C>T, 4135T>C, 4147A>G, 4148T>C, 4151G>T, 4162C>T, 4166G>A, 4171A>C, 4180G>A, 4190C>T,
4198T>C, 4201C>T, 4210G>T, 4213C>T, 4219T>C, 4220C>T, 4223C>A, 4225G>A, 4231T>G, 4237T>C, 4252A>G, 4253T>C, 4255A>G, 4258A>G, 4270T>C, 4282A>G, 4285G>A,
4291A>G, 4303G>A, 4312C>T, 4319A>G, 4321A>G, 4324G>T, 4327G>A, 4336G>T, 4339T>C, 4348A>T, 4360A>G, 4366T>C, 4369T>A, 4375T>C, 4387T>C, 4395T>A, 4406G>A,
4411T>C, 4438T>C, 4444C>A, 4450C>T, 4456C>T, 4465C>T, 4475A>G, 4477G>T, 4480C>T, 4481A>T, 4483A>G, 4495C>T, 4507G>A, 4510C>T, 4513T>G, 4517C>T, 4528C>T,
4534A>G, 4540C>T, 4544G>T, 4545C>T, 4546G>A, 4548G>A, 4558T>C, 4564A>T, 4570C>T, 4576A>T, 4597G>A, 4600C>T, 4606C>T, 4609G>A, 4615G>A, 4618C>T, 4625T>C,
4627G>T, 4648C>T, 4651T>C, 4660G>A, 4663C>T, 4669T>A, 4679G>A, 4681G>C, 4684T>C, 4687T>G, 4699G>A, 4702A>G, 4708A>G, 4711G>A, 4712C>T, 4738A>G, 4744A>T,
4747A>G, 4748T>C, 4765T>C, 4796G>A, 4798C>T, 4803C>G, 4804T>C, 4807G>A, 4810G>T, 4813T>G, 4816T>C, 4831T>C, 4834C>T, 4840A>T, 4852A>G, 4855C>T, 4867T>C,
4870G>C, 4873T>A, 4882T>C, 4903A>T, 4909G>A, 4915T>C, 4918A>G, 4919T>C, 4921G>A, 4924T>C, 4927C>G, 4933T>C, 4936C>A, 4939G>A, 4942T>C, 4945C>T, 4963A>G,
4972T>C, 4990T>C, 5002A>G, 5023T>C, 5024T>C, 5029C>T, 5032T>C, 5038T>C, 5044A>T, 5053A>T, 5056G>A, 5057A>G, 5060A>G, 5071G>T, 5090C>A, 5095A>G, 5098G>T,
5100C>T, 5104A>G, 5107A>T, 5110T>C, 5113A>G, 5116G>A, 5119A>G, 5120G>T, 5122C>T, 5126A>G, 5137T>C, 5140C>T, 5143A>G, 5146G>A, 5161C>A, 5167A>G, 5173T>G,
5182G>A, 5183G>A, 5188C>T, 5191G>A, 5197A>G, 5200C>T, 5206C>T, 5209A>G, 5218C>T, 5230G>A, 5236C>T, 5239C>T, 5245C>T, 5263A>T, 5269C>T, 5272C>T, 5275C>A,
5278C>T, 5281A>C, 5284A>G, 5287T>C, 5288C>T, 5293A>T, 5294T>C, 5296G>A, 5302G>A, 5311T>A, 5314A>G, 5323A>T, 5347T>C, 5353C>T, 5365C>T, 5386T>C, 5395C>A,
5422A>G, 5425T>C, 5431C>T, 5437G>A, 5452T>C, 5458G>A, 5467T>C, 5470A>G, 5471C>T, 5473A>G, 5479A>G, 5482C>T, 5485A>C, 5488T>C, 5489T>C, 5491G>A, 5497T>C,
5506T>C, 5509G>A, 5512G>A, 5516T>C, 5530T>C, 5533G>A, 5536G>T, 5539G>A, 5548T>G, 5554C>T, 5560T>A, 5561G>A, 5563G>A, 5566G>C, 5569A>G, 5584C>G, 5593G>A,
5596G>A, 5597A>G, 5605C>A, 5617G>T, 5619C>T, 5620T>G, 5629G>A, 5630A>C, 5641C>T, 5650A>T, 5662A>T, 5665A>G, 5689A>G, 5693G>A, 5698G>A, 5713A>G, 5716G>C,
5719A>G, 5743C>A, 5755C>A, 5758T>C, 5764T>C, 5770A>G, 5779C>T, 5782T>C, 5791C>T, 5803C>T, 5836G>A, 5839A>G, 5845A>G, 5848A>G, 5853A>G, 5857T>C, 5860C>T,
5863A>T, 5869T>A, 5875T>C, 5884C>T, 5893C>T, 5894A>G, 5896C>G, 5899C>T, 5908G>A, 5911T>C, 5923G>A, 5938A>G, 5941C>T, 5944T>C, 5947T>C, 5959G>A, 5960C>G,
5961G>C, 5962G>C, 5974A>G, 5986T>A, 5989C>T, 5990A>G, 5998T>C, 6008A>C, 6010A>T, 6019A>T, 6022G>T, 6025A>T, 6030A>C, 6049C>A, 6058G>A, 6061A>G, 6064C>A,
6070T>C, 6072G>A, 6073T>C, 6074A>C, 6079G>A, 6081C>A, 6085A>T, 6091A>T, 6094C>T, 6111G>C, 6112T>C, 6118A>G, 6124G>A, 6127G>A, 6128A>G, 6130T>C, 6133T>C,
6136A>G, 6148C>T, 6157A>G, 6158G>A, 6163T>C, 6169A>G, 6170A>T, 6172A>G, 6178C>T, 6187A>G, 6190T>C, 6196G>A, 6203C>T, 6205C>T, 6208A>G, 6209C>T, 6211A>G,
6217G>A, 6229T>A, 6230G>A, 6238C>T, 6247G>A, 6253A>T, 6257G>A, 6258C>A, 6265G>A, 6274T>C, 6282A>G, 6286G>A, 6298C>T, 6301G>A, 6304A>G, 6307C>T, 6322C>T,
6328A>T, 6331T>G, 6334T>C, 6337G>A, 6338C>T, 6358T>C, 6361G>A, 6362G>A, 6367A>T, 6370G>A, 6376C>T, 6379T>G, 6385A>C, 6391A>T, 6394A>G, 6397C>T, 6401G>A,
6405C>T, 6412C>T, 6418A>G, 6421T>C, 6429A>T, 6436T>A, 6445G>A, 6450A>G, 6454C>G, 6460C>T, 6463T>A, 6469C>T, 6472G>A, 6475G>A, 6481T>C, 6485C>A, 6487C>T,
6490T>C, 6496C>T, 6502G>A, 6514T>C, 6517G>A, 6527C>A, 6532C>A, 6541G>A, 6544A>G, 6556C>T, 6559T>C, 6565G>A, 6569T>C, 6571G>A, 6586T>A, 6588C>T, 6598A>G,
6601A>G, 6604T>C, 6607T>C, 6616A>G, 6622C>T, 6631G>A, 6634G>A, 6635T>C, 6640T>A, 6646G>T, 6647A>G, 6652C>T, 6664C>T, 6670T>G, 6677A>G, 6679A>G, 6685C>T,
6691A>T, 6694C>T, 6715A>G, 6716G>A, 6719A>G, 6724A>G, 6734C>T, 6742G>A, 6745G>A, 6754C>T, 6760A>G, 6764A>T, 6766A>G, 6775T>A, 6782C>T, 6787A>T, 6790C>T,
6795C>T, 6796A>G, 6799C>A, 6802C>A, 6808C>T, 6811C>T, 6814C>T, 6823C>T, 6829A>G, 6847C>T, 6853A>C, 6857A>G, 6862G>A, 6871A>G, 6875G>A, 6876T>C, 6877C>A,
6880G>A, 6883T>A, 6889T>G, 6901C>T, 6916C>T, 6919G>A, 6925A>G, 6928T>C, 6934C>A, 6949G>C, 6955C>T, 6958A>G, 6976A>G, 6980C>T, 6985C>T, 6997A>T, 7000G>T,
7001G>A, 7003G>A, 7012T>C, 7015G>A, 7018T>C, 7032T>A, 7039C>T, 7040T>C, 7042G>A, 7048A>G, 7060T>A, 7063C>A, 7078C>T, 7082T>C, 7084A>G, 7087A>G, 7090C>A,
7096T>C, 7099A>G, 7102T>C, 7111G>A, 7117A>G, 7125C>A, 7129A>G, 7132T>C, 7133C>T, 7135G>A, 7141G>A, 7144T>C, 7145G>T, 7147A>G, 7150G>A, 7159A>T,
7160_7165delGAGGCA, 7171C>T, 7189G>T, 7198A>G, 7201C>T, 7205C>T, 7210T>C, 7216T>A, 7219G>A, 7225C>A, 7228G>A, 7240G>A, 7243T>C, 7249A>G, 7252T>G,
7258T>A, 7261T>C, 7264T>A, 7267C>G, 7270G>A, 7273_7274insT, 7274C>G, 7275G>T, 7281A>T, 7284T>C, 7296C>T, 7311C>T, 7314C>T, 7317G>A, 7321T>A, 7322delG,
7327C>T, 7330C>T, 7333C>T, 7334A>C, 7342T>C, 7343G>A, 7344C>A, 7345A>C, 7354T>C, 7363A>G, 7369T>C, 7372C>T, 7378G>T, 7381C>T, 7396A>T, 7399C>T, 7405C>T,
7407G>T, 7408T>G, 7414G>T, 7415T>G, 7420A>G, 7429C>T, 7432T>C, 7441T>C, 7459G>A, 7462A>G, 7468A>T, 7471C>A, 7477A>G, 7480C>T, 7486G>T, 7495T>C, 7498A>G,
7501T>C, 7503T>C, 7504G>A, 7513T>A, 7519C>T, 7522A>G, 7531G>A, 7537G>A, 7540C>T, 7546T>C, 7570G>A, 7573G>A, 7579G>A, 7588T>C, 7595C>T, 7604T>C, 7606G>A,
7615T>A, 7624T>C, 7633C>T, 7636C>T, 7639T>C, 7648T>C, 7666A>G, 7672T>C, 7678A>T, 7684G>A, 7690T>C, 7696T>C, 7702T>G, 7709G>A, 7713A>G, 7714A>G, 7715C>T,
7726T>C, 7735G>A, 7744A>G, 7747C>T, 7762C>T, 7768G>A, 7771T>C, 7780A>G, 7783A>C, 7786T>C, 7793C>T, 7795A>G, 7798C>T, 7804T>C, 7812C>T, 7834A>G, 7837C>T,
7844G>T, 7846G>A, 7855G>A, 7861G>A, 7870A>G, 7873C>T, 7876T>A, 7879G>A, 7882T>A, 7883G>A, 7894A>T, 7897T>C, 7921C>T, 7927G>A, 7936T>C, 7939T>C, 7942T>C,
7966G>A, 7969T>C, 7975T>C, 7987C>T, 7990T>C, 8005T>C, 8032A>G, 8038C>T, 8041C>T, 8043G>A, 8080G>A, 8089G>A, 8095G>A, 8098G>A, 8107C>T, 8110T>C, 8116T>C,
8128A>G, 8146T>C, 8151A>G, 8152C>T, 8164G>A, 8178G>A, 8182G>A, 8191G>A, 8197C>T, 8203C>T, 8208G>A, 8213C>T, 8215G>A, 8218A>G, 8221C>T, 8224G>A, 8239A>G,
8254G>A, 8260T>C, 8263A>G, 8281T>C, 8284A>G, 8294C>G, 8296A>G, 8299G>A, 8302C>T, 8317T>C, 8320C>T, 8326T>A, 8335G>A, 8338C>T, 8349C>T, 8353A>G, 8362C>T,
8365A>G, 8368C>T, 8380C>T, 8383A>G, 8389A>G, 8404T>C, 8408G>T, 8413A>G, 8419G>T, 8434T>C, 8440T>C, 8452G>A, 8458A>G, 8460G>A, 8464A>G, 8467G>A, 8479G>A,
8480G>A, 8482T>A, 8497G>A, 8498C>T, 8500A>G, 8502G>A, 8503A>G, 8506C>T, 8515A>T, 8524C>T, 8527A>C, 8528T>C, 8530A>G, 8539G>C, 8546C>T, 8547T>G, 8552T>A,
8563A>G, 8574C>G, 8585A>G, 8598G>A, 8602C>T, 8603A>T, 8604A>C, 8605C>T, 8620A>G, 8622C>A, 8625A>G, 8626G>A, 8629G>A, 8632C>T, 8638C>T, 8641G>A, 8647C>T,
8648C>T, 8649C>T, 8650G>C, 8653C>T, 8656A>G, 8658G>A, 8664A>C, 8665T>G, 8674T>G, 8679C>T, 8680A>T, 8683C>A, 8692G>A, 8695A>T, 8707G>A, 8708A>C, 8716G>A,
8722C>T, 8725G>A, 8731A>G, 8737A>G, 8743C>T, 8745C>A, 8746A>G, 8752G>A, 8755A>T, 8770C>T, 8771T>C, 8776A>G, 8779T>C, 8782G>A, 8791T>C, 8797A>G, 8800A>G,
8803A>G, 8809T>C, 8812T>C, 8818T>C, 8824G>A, 8827A>G, 8848G>A, 8854T>C, 8856A>G, 8857G>A, 8863G>A, 8865G>A, 8867A>C, 8869G>T, 8872A>G, 8883G>A, 8888A>T,
8929C>T, 8954G>A, 8959C>T, 8962A>T, 8983G>A, 8984A>G, 8986T>C, 8988A>T, 8998G>A, 9004G>A, 9007G>A, 9016C>T, 9037C>A, 9085A>T, 9097C>T, 9131A>G, 9136A>T,
9145G>T, 9149A>G, 9150T>G, 9151G>A, 9152G>A, 9157A>G, 9160G>T, 9163T>C, 9184G>A, 9196A>G, 9214G>A, 9217A>G, 9220G>A, 9244A>T, 9247G>A, 9256C>T, 9260C>T,
9262T>C, 9274G>A, 9280C>T, 9289C>T, 9298A>C, 9304G>A, 9334C>T, 9364C>G, 9377_9378insT, 9387C>T, 9404G>A, 9411C>T, 9412A>G, 9414A>T, 9437C>T, 9454A>G,
9458T>C, 9467G>A, 9470_9471insT, 9482G>A, 9483C>T, 9498T>C, 9516T>G, 9517delA, 9533G>A, 9534G>T, 9548A>T, 9551C>T, 9553_9554delTA, 9556A>T, 9557T>A, 9558A>T,
9574T>A, 9581G>T, 9582T>G, 9583G>C, 9584_9585insG, 9590C>T, 9591T>C, 9607C>T, 9610T>C, 9615delC, 9620A>G, 9648A>G, 9674T>C, 9690C>T

	Begin	End	Coverage	Score	Concordance	Matches	Identities	I/D/M/F*	Stop Codons
**NT**	**52**	**9704**	**99.5%**	**12530**	**65.2%**	**9642 (99.8%)**	**7985 (82.7%)**	**7/11**	
**CDS**									
PVYgp1	1	3064	100%	19042	90.2%	3062 (99.9%)	2771 (90.4%)	2/2/3/3	1
Protein mutations:	Y4Q (194T>C 196C>A), C9L (209T>C 210G>T 211T>G), F13M (221T>A 223T>G), S23P (251T>C), C24F (255G>T 256C>T), E25G (258A>G), I27V (263A>G 265T>G), V28A (267T>C), E30V (273A>T), A35T (287G>A 289T>C), V37T (293G>A 294T>C), A41I (305G>A 306C>T), D42G (309A>G), E44D (316A>T), T45V (317A>G 318C>T 319A>G), L52Q (339T>A 340C>A), K53R (342A>G), K55A (347A>G 348A>C 349A>G), Y56H (350T>C), T58V (356A>G 357C>T), V61T (365G>A 366T>C 367G>A), L62S (368C>T 369T>C), F67C (384T>G), A75V (408C>T 409C>T), M78E (416A>G 417T>A), E85K (437G>A), R86E (440A>G 441G>A), K87R (443A>C 444A>G 445G>A), D88E (448T>A), E91A (456A>C), H93N (461C>A), D111E (517T>A), S116F (531C>T), P118S (536C>T), Q119W (539C>T 540A>G 541A>G), R122K (549G>A 550A>G), M131T (576T>C), V134A (585T>C 586C>G), R137Y (593C>T 594G>A 595C>T), P138R (597C>G), I139V (599A>G 601A>G), I140P (602A>C 603T>C 604A>G), M147I (625G>A), I151V (635A>G 637T>C), I154V (644A>G 646A>G), E160A (663A>C 664G>C), H166Q (682C>A), T172S (699C>G), H174Q (706T>A), K179E (719A>G 721G>A), I180V (722A>G 724A>G), A183S (731G>T), Y184P (734T>C 735A>C 736C>T), S185R (737T>C 738C>G 739C>T), A187V (744C>T 745G>C), R189C (749C>T 751A>C), M194K (765T>A), R197K (773C>A 774G>A 775A>G), M206K (801T>A), G210A (813G>C 814A>G), L211H (816T>A), R214Q (825G>A 826T>A), N223D (851A>G 853T>C), R226C (860C>T 862C>T), T227A (863A>G 865T>C), I228T (867T>C), N229D (869A>G 871C>T), I230L (872A>T 874A>G), R231Q (876G>A), R232K (879G>A), N240S (903A>G), T241N (906C>A 907A>C), K242T (909A>C 910A>C), S243N (912G>A 913C>T), S252L (938T>C 939C>T 940A>T), L266I (980T>A 982G>C), R272K (999G>A), Q275R (1007C>A 1008A>G 1009G>A), S276G (1010A>G 1012T>G), I277V (1013A>G), N279Q (1019A>C 1021C>G), D288E (1048C>A), R300Q (1083G>A), S305A (1097T>G 1099G>A), R320K (1143G>A), L324I (1154T>A 1156G>A), S346N (1221G>A), V349T (1229G>A 1230T>C 1231T>C), S350N (1233G>A 1234C>T), K358T (1257A>C 1258A>G), N365S (1278A>G), A369V (1290C>T 1291G>A), D372T (1298G>A 1299A>C 1300C>T), I375V (1307A>G 1309A>G), N378E (1316A>G 1318T>A), I382T (1329T>C), L397I (1373C>A), I401V (1385A>G), E407D (1405G>T), A411S (1415G>T 1417A>T), V418I (1436G>A 1438C>T), E508G (1707A>G), A512T (1718G>A), I520V (1742A>G 1744C>T), S525N (1758G>A), V536I (1790G>A), I585V (1937A>G 1939T>C), F607Y (2004T>A), I628M (2068T>G), V630I (2072G>A 2074G>A), R675K (2208G>A), D694E (2266C>G), N744G (2414A>G 2415A>G 2416T>G), S746C (2420A>T), K780R (2523A>G), R817K (2634G>A), V847I (2723G>A 2725C>T), A855T (2747G>A 2749T>A), H871N (2795C>A), V880I (2822G>A 2824G>A), P900T (2882C>A), S901H (2885A>C 2886G>A), T904M (2895C>T), N916S (2931A>G), N919S (2940A>G), E929_N930insX (2971_2972insA), T936I (2991C>T), H937R (2994A>G), E939R (2999G>A 3000A>G), S944F (3015C>T), R946Q (3021G>A), Y947F (3024A>T 3025C>T), T951I (3036C>T), E952G (3039A>G 3040A>C), K953T (3042A>C 3043G>A), A972V (3099C>T 3100C>A), V974R (3104G>A 3105T>G 3106G>A), S983N (3132G>A), R985K (3137C>A 3138G>A 3139A>G), F990I (3152T>A 3154C>T), C994Y (3165G>A), F1012L (3218T>C), T1014N (3225C>A), V1016I (3230G>A), V1028M (3266G>A 3268A>G), V1031M (3275G>A), A1034T (3284G>A), R1044K (3315G>A 3316A>G), E1045A (3318A>C), M1059V (3359A>G), D1072E (3400T>G), V1110G (3513T>G), M1121V (3545A>G), A1122T (3548G>A), V1124I (3554G>A 3556C>T), L1147V (3623C>G), L1150M (3632C>A), Y1152H (3638T>C), I1172V (3698A>G), S1178N (3717G>A), R1183K (3731C>A 3732G>A), I1297V (4073A>G), F1315Y (4128T>A 4129C>T), A1323S (4151G>T), V1328I (4166G>A), L1347I (4223C>A 4225G>A), I1379V (4319A>G 4321A>G), F1404Y (4395T>A), V1408I (4406G>A), M1431V (4475A>G 4477G>T), T1433S (4481A>T 4483A>G), A1454L (4544G>T 4545C>T 4546G>A), R1455K (4548G>A), V1499I (4679G>A 4681G>C), V1538I (4796G>A 4798C>T), T1540S (4803C>G 4804T>C), S1625G (5057A>G), T1626A (5060A>G), L1636I (5090C>A), A1639V (5100C>T), E1641D (5107A>T), V1646F (5120G>T 5122C>T), I1648V (5126A>G), V1667I (5183G>A), A1793T (5561G>A 5563G>A), I1805V (5597A>G), N1807K (5605C>A), A1812V (5619C>T 5620T>G), I1816L (5630A>C), V1837I (5693G>A), K1890R (5853A>G), I1904V (5894A>G 5896C>G), R1926A (5960C>G 5961G>C 5962G>C), I1936V (5990A>G), E1945D (6019A>T), K1949T (6030A>C), D1955E (6049C>A), M1958I (6058G>A), S1963N (6072G>A 6073T>C), N1964H (6074A>C), T1966N (6081C>A), C1976S (6111G>C 6112T>C), I1982V (6128A>G 6130T>C), V1992I (6158G>A), T1996S (6170A>T 6172A>G), L2007F (6203C>T 6205C>T), V2016I (6230G>A), A2025K (6257G>A 6258C>A), K2033R (6282A>G), A2060T (6362G>A), V2073I (6401G>A), A2074V (6405C>T), Y2082F (6429A>T), Q2089R (6450A>G), H2101N (6485C>A 6487C>T), L2115I (6527C>A), T2135I (6588C>T), Y2151H (6635T>C), I2155V (6647A>G), I2165V (6677A>G 6679A>G), D2178N (6716G>A), N2179D (6719A>G), I2194L (6764A>T 6766A>G), A2204V (6795C>T 6796A>G), H2205Q (6799C>A), N2225D (6857A>G), V2231T (6875G>A 6876T>C 6877C>A), D2250E (6934C>A), V2273I (7001G>A 7003G>A), F2283Y (7032T>A), T2314K (7125C>A), A2321S (7145G>T 7147A>G), E2326_A2327del (7160_7165delGAGGCA), D2344E (7216T>A), D2358E (7258T>A), D2363_R2364insX (7273_7274insT), R2364V (7274C>G 7275G>T), H2366L (7281A>T), L2367S (7284T>C), S2371L (7296C>T), S2376F (7311C>T), T2377I (7314C>T), C2378Y (7317G>A), N2379K (7321T>A), K2384Q (7334A>C), A2387N (7343G>A 7344C>A 7345A>C), S2408M (7407G>T 7408T>G), C2411G (7415T>G), L2440S (7503T>C 7504G>A), D2509N (7709G>A), K2510R (7713A>G 7714A>G), T2543I (7812C>T), V2554L (7844G>T 7846G>A), V2567I (7883G>A), S2620N (8043G>A), N2656S (8151A>G 8152C>T), R2665K (8178G>A), R2675K (8208G>A), P2704A (8294C>G 8296A>G), S2722F (8349C>T), A2742S (8408G>T), M2756I (8452G>A), R2759K (8460G>A), A2766T (8480G>A 8482T>A), R2773K (8502G>A 8503A>G), E2777D (8515A>T), E2785D (8539G>C), L2788C (8546C>T 8547T>G), S2790T (8552T>A), A2797G (8574C>G), I2801V (8585A>G), G2805E (8598G>A), N2807S (8603A>T 8604A>C 8605C>T), P2813Q (8622C>A), E2814G (8625A>G 8626G>A), P2822F (8648C>T 8649C>T 8650G>C), G2825E (8658G>A), D2827A (8664A>C 8665T>G), A2832V (8679C>T 8680A>T), T2854K (8745C>A 8746A>G), E2891G (8856A>G 8857G>A), R2894Q (8865G>A), M2895L (8867A>C 8869G>T), G2900E (8883G>A), T2902S (8888A>T), V2924I (8954G>A), N2934D (8984A>G 8986T>C), E2935V (8988A>T), I2983V (9131A>G), M2989G (9149A>G 9150T>G 9151G>A), G2990S (9152G>A), P3026S (9260C>T 9262T>C), Q3038H (9298A>C)								

	Begin	End	Coverage	Score	Concordance	Matches	Identities	I/D/M/F*	Stop Codons
**NT**	**52**	**9704**	**99.5%**	**12530**	**65.2%**	**9642 (99.8%)**	**7985 (82.7%)**	**7/11**	

Codon mutations:

ACT3ACC (193T>C), TAC4CAA (194T>C 196C>A), TCA6TCG (202A>G), ATC8ATT (208C>T), TGT9CTG (209T>C 210G>T 211T>G), TCG12TCA (220G>A), TTT13ATG (221T>A 223T>G), GAA14GAG (226A>G), CTA17CTT (235A>T), TAC19TAT (241C>T), CCA21CCT (247A>T), TCT23CCT (251T>C), TGC24TTT (255G>T 256C>T), GAG25GGG (258A>G), CAT26CAC (262T>C), ATT27GTG (263A>G 265T>G), GTG28GCG (267T>C), GAA30GTA (273A>T), CGA31CGT (277A>T), GAA32GAG (280A>G), GTG33GTT (283G>T), CCG34CCA (286G>A), GCT35ACC (287G>A 289T>C), GTT37ACT (293G>A 294T>C), CCT39CCC (301T>C), TTC40TTT (304C>T), GCA41ATA (305G>A 306C>T), GAT42GGT (309A>G), CTG43TTG (311C>T), GAA44GAT (316A>T), ACA45GTG (317A>G 318C>T 319A>G), CAA46CAG (322A>G), GCA49GCT (331A>T), CTC52CAA (339T>A 340C>A), AAG53AGG (342A>G), AAA55GCG (347A>G 348A>C 349A>G), TAT56CAT (350T>C), GCT57GCA (355T>A), ACT58GTT (356A>G 357C>T), CGT60CGC (364T>C), GTG61ACA (365G>A 366T>C 367G>A), CTC62TCC (368C>T 369T>C), AAA63AAG (373A>G), AAC64AAT (376C>T), TTT67TGT (384T>G), ACG68ACA (388G>A), TAC69TAT (391C>T), CGA70CGG (394A>G), AAG72AAA (400G>A), GAT74GAC (406T>C), GCC75GTT (408C>T 409C>T), CAG76CAA (412G>A), ATA77ATT (415A>T), ATG78GAG (416A>G 417T>A), ATT80ATC (424T>C), CAG81CAA (427G>A), AAA83AAG (433A>G), CTG84CTC (436G>C), GAG85AAG (437G>A), AGG86GAG (440A>G 441G>A), AAG87CGA (443A>C 444A>G 445G>A), GAT88GAA (448T>A), GAA90GAG (454A>G), GAA91GCA (456A>C), TAT92TAC (460T>C), CAC93AAC (461C>A), CAA95CAG (469A>G), GCC97GCG (475C>G), CCT99CCA (481T>A), ACT106ACC (502T>C), ATA107ATT (505A>T), GGC109GGT (511C>T), GAT111GAA (517T>A), CCT112CCA (520T>A), CCA113CCT (523A>T), TCT116TTT (531C>T), GAG117GAA (535G>A), CCA118TCA (536C>T), CAA119TGG (539C>T 540A>G 541A>G), AGA122AAG (549G>A 550A>G), ATC124ATT (556C>T), ATT125ATC (559T>C), CAT126CAC (562T>C), ACT128ACC (568T>C), CCA129CCG (571A>G), ATG131ACG (576T>C), CGT132CGC (580T>C), GTC134GCG (585T>C 586C>G), CGC137TAT (593C>T 594G>A 595C>T), CCC138CGC (597C>G), ATA139GTG (599A>G 601A>G), ATA140CCG (602A>C 603T>C 604A>G), ACA143ACG (613A>G), GAA144GAG (616A>G), GGC145GGG (619C>G), CAG146CAA (622G>A), ATG147ATA (625G>A), AAT148AAC (628T>C), CAC149CAT (631C>T), ATT151GTC (635A>G 637T>C), ATA154GTG (644A>G 646A>G), ATT157ATC (655T>C), TCG159TCA (661G>A), GAG160GCC (663A>C 664G>C), AAA161AAG (667A>G), GTC165GTT (679C>T), CAC166CAA (682C>A), TTA167CTG (683T>C 685A>G), AGT169AGC (691T>C), ACC172AGC (699C>G), ACT173ACA (703T>A), CAT174CAA (706T>A), AAG179GAA (719A>G 721G>A), ATA180GTG (722A>G 724A>G), CTT181TTG (725C>T 727T>G), GGT182GGA (730T>A), GCA183TCA (731G>T), TAC184CCT (734T>C 735A>C 736C>T), TCC185CGT (737T>C 738C>G 739C>T), GCG187GTC (744C>T 745G>C), CGA189TGC (749C>T 751A>C), ACT190ACC (754T>C), ATG194AAG (765T>A), TTG196CTG (770T>C), CGA197AAG (773C>A 774G>A 775A>G), CGG198AGG (776C>A), TTC202TTT (790C>T), ATG206AAG (801T>A), GGA210GCG (813G>C 814A>G), CTT211CAT (816T>A), TTG212CTG (818T>C), CAA213CAG (823A>G), CGT214CAA (825G>A 826T>A), CGG217AGA (833C>A 835G>A), GAC219GAT (841C>T), AAT223GAC (851A>G 853T>C), GTC225GTT (859C>T), CGC226TGT (860C>T 862C>T), ACT227GCC (863A>G 865T>C), ATC228ACC (867T>C), AAC229GAT (869A>G 871C>T), ATA230TTG (872A>T 874A>G), CGA231CAA (876G>A), AGG232AAG (879G>A), GAT234GAC (886T>C), ATC238ATT (898C>T), AAC240AGC (903A>G), ACA241AAC (906C>A 907A>C), AAA242ACC (909A>C 910A>C), AGC243AAT (912G>A 913C>T), GGC246GGA (922C>A), GGT249GGA (931T>A), TCA252CTT (938T>C 939C>T 940A>T), GGA253GGG (943A>G), TTC256TTT (952C>T), TCA261TCG (967A>G), GAA263GAG (973A>G), GGG264GGC (976G>C), TTG266ATC (980T>A 982G>C), GCA269GCG (991A>G), CGT270CGC (994T>C), AGA272AAA (999G>A), GTT273GTC (1003T>C), CAG275AGA (1007C>A 1008A>G 1009G>A), AGT276GGG (1010A>G 1012T>G), ATT277GTT (1013A>G), TTA278TTG (1018A>G), AAC279CAG (1019A>C 1021C>G), ATC282ATT (1030C>T), TCG285TCA (1039G>A), AAT286AAC (1042T>C), GCC287GCA (1045C>A), GAC288GAA (1048C>A), AAT289AAC (1051T>C), AAG292AAA (1060G>A), GGT293GGA (1063T>A), CTG294TTG (1064C>T), GAC295GAT (1069C>T), CGA300CAA (1083G>A), TAT303TAC (1093T>C), TCG305GCA (1097T>G 1099G>A), CAC307CAT (1105C>T), GTA310GTG (1114A>G), GCT311GCG (1117T>G), TTA313TTG (1123A>G), CCT314CCA (1126T>A), GTC315GTT (1129C>T), AGG320AAG (1143G>A), GTA321GTT (1147A>T), GCT322GCA (1150T>A), TTG324ATA (1154T>A 1156G>A), CAC327CAT (1165C>T), AGT328AGC (1168T>C), CTT330TTA (1172C>T 1174T>A), ACC339ACT (1201C>T), AGC346AAC (1221G>A), GTT349ACC (1229G>A 1230T>C 1231T>C), AGC350AAT (1233G>A 1234C>T), CTG352CTA (1240G>A), AAG354AAA (1246G>A), TTG356CTA (1250T>C 1252G>A), CAT357CAC (1255T>C), AAA358ACG (1257A>C 1258A>G), AGA361AGG (1267A>G), GGT363GGC (1273T>C), TTG364TTA (1276G>A), AAT365AGT (1278A>G), TTG367CTG (1283T>C), GCG369GTA (1290C>T 1291G>A), GAC372ACT (1298G>A 1299A>C 1300C>T), TTT374TTC (1306T>C), ATA375GTG (1307A>G 1309A>G), CAT376CAC (1312T>C), AAT378GAA (1316A>G 1318T>A), TTG381TTA (1327G>A), ATA382ACA (1329T>C), GCG383GCT (1333G>T), TTA384TTG (1336A>G), CTA387TTG (1343C>T 1345A>G), CCG390CCA (1354G>A), GTG391GTT (1357G>T), GAC392GAT (1360C>T), CTC395TTG (1367C>T 1369C>G), CTT397ATT (1373C>A), TTC398TTT (1378C>T), GAG400GAA (1384G>A), ATA401GTA (1385A>G), AAA403AAG (1393A>G), TCC404TCT (1396C>T), GAG407GAT (1405G>T), AAA408AAG (1408A>G), CAG409CAA (1411G>A), GCA411TCT (1415G>T 1417A>T), CCG412CCC (1420G>C), AAT415AAC (1429T>C), TTA416TTG (1432A>G), GTC418ATT (1436G>A 1438C>T), TTA419CTG (1439T>C 1441A>G), AAT420AAC (1444T>C), TTC423TTT (1453C>T), CTG424CTA (1456G>A), GAA428GAG (1468A>G), GTA436GTG (1492A>G), TTG439CTA (1499T>C 1501G>A), AGT440AGC (1504T>C), TTG441TTA (1507G>A), CTC442CTT (1510C>T), GAA443GAG (1513A>G), TTA444TTG (1516A>G), GCA445GCT (1519A>T), AGG446AGA (1522G>A), TTC447TTT (1525C>T), CAG448CAA (1528G>A), AAC450AAT (1534C>T), ACT452ACA (1540T>A), AAC454AAT (1546C>T), AAA457AAG (1555A>G), GGT458GGC (1558T>C), GAT459GAC (1561T>C), ATA460ATC (1564A>C), TCT461TCA (1567T>A), TTC463TTT (1573C>T), AGA464AGG (1576A>G), TTA467CTA (1583T>C), TCT468TCA (1588T>A), AAG470AAA (1594G>A), AAC472AAT (1600C>T), CTG475CTA (1609G>A), TCG478TCA (1618G>A), TGC479TGT (1621C>T), GAC480GAT (1624C>T), CTG483TTG (1631C>T), AAA485AAG (1639A>G), TTC489TTT (1651C>T), CTC490CTG (1654C>G), CAT497CAC (1675T>C), TCA503TCG (1693A>G), TTT506TTC (1702T>C), GAA508GGA (1707A>G), GCA512ACA (1718G>A), GGA514GGG (1726A>G), TAC515TAT (1729C>T), TCA516TCC (1732A>C), TAT518TAC (1738T>C), ATC520GTT (1742A>G 1744C>T), CGC521CGT (1747C>T), CAT523CAC (1753T>C), AGT525AAT (1758G>A), AGG528AGA (1768G>A), AAC534AAT (1786C>T), TTA535TTG (1789A>G), GTT536ATT (1790G>A), GTC537GTT (1795C>T), TTA541CTG (1805T>C 1807A>G), GAG543GAA (1813G>A), AGG545AGA (1819G>A), AAG547AAA (1825G>A), GAC551GAT (1837C>T), TAT552TAC (1840T>C), CAA555CAG (1849A>G), GTC558GTG (1858C>G), AAA560AAG (1864A>G), TGC562TGT (1870C>T), AGT564AGC (1876T>C), AAA566AAG (1882A>G), GGT568GGA (1888T>A), AAT569AAC (1891T>C), TGT574TGC (1906T>C), ACA577ACT (1915A>T), GGT582GGC (1930T>C), GCC584GCT (1936C>T), ATT585GTC (1937A>G 1939T>C), TCA587TCG (1945A>G), TTC589TTT (1951C>T), CCA592CCT (1960A>T), GTA598GTG (1978A>G), ATT599ATA (1981T>A), GGC600GGT (1984C>T), AAT601AAC (1987T>C), GAC604GAT (1996C>T), TTT607TAT (2004T>A), AAA612AAG (2020A>G), GGG613GGA (2023G>A), GAT614GAC (2026T>C), TCG615TCT (2029G>T), GAG616GAA (2032G>A), TAT625TAC (2059T>C), ATT628ATG (2068T>G), AAC629AAT (2071C>T), GTG630ATA (2072G>A 2074G>A), CTG635TTA (2087C>T 2089G>A), AGC639AGT (2101C>T), GAG640GAA (2104G>A), GAG641GAA (2107G>A), AAG644AAA (2116G>A), TTC646TTT (2122C>T), CGC651CGT (2137C>T), CTT658CTC (2158T>C), ACC660ACT (2164C>T), CCA662CCG (2170A>G), GAT666GAC (2182T>C), GCG668GCA (2188G>A), ACC669ACA (2191C>A), AGA675AAA (2208G>A), TAT678TAC (2218T>C), GAC680GAT (2224C>T), GTA681GTT (2227A>T), TTG686TTA (2242G>A), CCC687CCT (2245C>T), TTG690TTA (2254G>A), GTT691GTC (2257T>C), GAC692GAT (2260C>T), GAC694GAG (2266C>G), CAA696CAG (2272A>G), ACG697ACA (2275G>A), TCA703TCG (2293A>G), GGC705GGT (2299C>T), TCG706TCA (2302G>A), CAG707CAA (2305G>A), GGA710GGT (2314A>T), TAT711TAC (2317T>C), CTA714CTG (2326A>G), GTG719GTA (2341G>A), CAA721CAG (2347A>G), CTT722CTC (2350T>C), TTG724TTA (2356G>A), AAT727AAC (2365T>C), GAA729GAG (2371A>G), TTA730TTG (2374A>G), GAT733GAC (2383T>C), ATA734ATT (2386A>T), AAA735AAG (2389A>G), CAT736CAC (2392T>C), TAT737TAC (2395T>C), AAT744GGG (2414A>G 2415A>G 2416T>G), GCT745GCA (2419T>A), AGC746TGC (2420A>T), ACA752ACT (2440A>T), GTT761GTC (2467T>C), TCT764TCC (2476T>C), TCG766TCA (2482G>A), CTG769CTA (2491G>A), CTG771CTT (2497G>T), TTG773TTA (2503G>A), AGA778AGG (2518A>G), CCT779CCC (2521T>C), AAG780AGG (2523A>G), AGA783AGG (2533A>G), TTG785TTA (2539G>A), TTA787TTG (2545A>G), GAG789GAA (2551G>A), CCT790CCA (2554T>A), CTG792TTG (2558C>T), TTG793CTC (2561T>C 2563G>C), TCA796TCG (2572A>G), CCT800CCC (2584T>C), GGC801GGT (2587C>T), CTG803CTC (2593G>C), GCT805GCC (2599T>C), AAT808AAC (2608T>C), TTT812TTC (2620T>C), GCG815GCA (2629G>A), GTG816GTA (2632G>A), AGG817AAG (2634G>A), TTG818TTA (2638G>A), ATT820ATA (2644T>A), AAA823AAG (2653A>G), GCT827GCC (2665T>C), CTA832TTA (2678C>T), CTA833CTG (2683A>G), GCT835GCC (2689T>C), CTA838CTC (2698A>C), TCA841TCG (2707A>G), GCG842GCA (2710G>A), GAA844GAG (2716A>G), ACA845ACG (2719A>G), CTC846CTT (2722C>T), GTC847ATT (2723G>A 2725C>T), GCA848GCG (2728A>G), AGG850AGA (2734G>A), ATT851ATA (2737T>A), GAT854GAC (2746T>C), GCT855ACA (2747G>A 2749T>A), GCA856GCT (2752A>T), GCT857GCA (2755T>A), GAC859GAT (2761C>T), CTC860CTT (2764C>T), TGT865TGC (2779T>C), GGG867GGT (2785G>T), AAC869AAT (2791C>T), CAT871AAT (2795C>A), TAC874TAT (2806C>T), TTG878CTC (2816T>C 2818G>C), GTG880ATA (2822G>A 2824G>A), TTG881TTA (2827G>A), GTT884GTC (2836T>C), AAG885AAA (2839G>A), AAT886AAC (2842T>C), ACC893ACA (2863C>A), CTA894TTA (2864C>T), CCA900ACA (2882C>A), AGT901CAT (2885A>C 2886G>A), AAC903AAT (2893C>T), ACG904ATG (2895C>T), GTC906GTT (2902C>T), GTA907GTG (2905A>G), ATT909ATC (2911T>C), AAT913AAC (2923T>C), AAT916AGT (2931A>G), AAC919AGC (2940A>G), GCT921GCC (2947T>C), ACT926ACC (2962T>C), GAA929_AAT930insA-- (2971_2972insA), GGT935GGC (2989T>C), ACT936ATT (2991C>T), CAT937CGT (2994A>G), GAG939AGG (2999G>A 3000A>G), TCC944TTC (3015C>T), CGG946CAG (3021G>A), TAC947TTT (3024A>T 3025C>T), ACA951ATA (3036C>T), GAA952GGC (3039A>G 3040A>C), AAG953ACA (3042A>C 3043G>A), TTG956TTA (3052G>A), TTA959TTG (3061A>G), GCG966GCA (3082G>A), GGC969GGT (3091C>T), GCC972GTA (3099C>T 3100C>A), GTG974AGA (3104G>A 3105T>G 3106G>A), GTC975GTT (3109C>T), AAA976AAG (3112A>G), ACT978ACC (3118T>C), GCC979GCT (3121C>T), GGA981GGG (3127A>G), AGC983AAC (3132G>A), CGA985AAG (3137C>A 3138G>A 3139A>G), TAT989TAC (3151T>C), TTC990ATT (3152T>A 3154C>T), TGT994TAT (3165G>A), GTA995GTG (3169A>G), ATC1005ATT (3199C>T), AGG1007CGG (3203A>C), CGT1008CGC (3208T>C), CCA1010CCG (3214A>G), ACC1011ACT (3217C>T), TTT1012CTT (3218T>C), GTC1013GTA (3223C>A), ACT1014AAT (3225C>A), GTT1016ATT (3230G>A), AAC1017AAT (3235C>T), TTA1025CTA (3257T>C), ACC1026ACT (3262C>T), AGC1027AGT (3265C>T), GTA1028ATG (3266G>A 3268A>G), GTG1029GTA (3271G>A), GTG1031ATG (3275G>A), GCA1034ACA (3284G>A), CAG1039CAA (3301G>A), AGA1044AAG (3315G>A 3316A>G), GAA1045GCA (3318A>C), ATC1046ATT (3322C>T), GAG1047GAA (3325G>A), TTG1048TTA (3328G>A), ATA1051ATT (3337A>T), GAG1055GAA (3349G>A), GTC1057GTT (3355C>T), TGC1058TGT (3358C>T), ATG1059GTG (3359A>G), CTA1061CTG (3367A>G), TAT1062TAC (3370T>C), TTA1065CTG (3377T>C 3379A>G), GAA1070GAG (3394A>G), CGC1071CGT (3397C>T), GAT1072GAG (3400T>G), ACA1074ACG (3406A>G), TAC1078TAT (3418C>T), ATT1079ATA (3421T>A), TAT1081TAC (3427T>C), AAG1083AAA (3433G>A), TCA1084TCT (3436A>T), GTA1085GTG (3439A>G), AAC1086AAT (3442C>T), CCT1087CCC (3445T>C), CAG1091CAA (3457G>A), TTT1092TTC (3460T>C), GCT1093GCA (3463T>A), GCG1095GCT (3469G>T), CAG1096CAA (3472G>A), CAC1104CAT (3496C>T), CAG1105CAA (3499G>A), TCC1107TCT (3505C>T), GTT1110GGT (3513T>G), AAA1112AAG (3520A>G), GAA1115GAG (3529A>G), CAA1116CAG (3532A>G), ATG1121GTG (3545A>G), GCT1122ACT (3548G>A), TTA1123CTA (3551T>C), GTC1124ATT (3554G>A 3556C>T), AGT1133AGC (3583T>C), GAT1134GAC (3586T>C), TGC1135TGT (3589C>T), TTC1137TTT (3595C>T), AAT1141AAC (3607T>C), TTT1143TTC (3613T>C), AAG1144AAA (3616G>A), GGT1145GGC (3619T>C), CTT1147GTT (3623C>G), TCC1148TCT (3628C>T), CTG1150ATG (3632C>A), GAT1151GAC (3637T>C), TAT1152CAT (3638T>C), GAA1153GAG (3643A>G), TTA1159TTG (3661A>G), GTG1162GTA (3670G>A), TTT1166TTC (3682T>C), GAG1168GAA (3688G>A), GAG1171GAA (3697G>A), ATT1172GTT (3698A>G), GAT1174GAC (3706T>C), TTT1175TTC (3709T>C), TTG1177CTA (3713T>C 3715G>A), AGT1178AAT (3717G>A), GAG1179GAA (3721G>A), CGA1183AAA (3731C>A 3732G>A), ACT1184ACA (3736T>A), CTA1188TTG (3746C>T 3748A>G), ACA1190ACG (3754A>G), AGT1193AGC (3763T>C), CGA1198CGG (3778A>G), CAA1199CAG (3781A>G), ATC1200ATT (3784C>T), CAT1204CAC (3796T>C), CCA1207CCT (3805A>T), TAC1209TAT (3811C>T), AGA1210AGG (3814A>G), TTC1215TTT (3829C>T), TTT1218TTC (3838T>C), AGA1220AGG (3844A>G), GCA1221GCC (3847A>C), GTT1224GTA (3856T>A), GTG1226GTT (3862G>T), GAC1229GAT (3871C>T), GCC1231GCA (3877C>A), AGC1233AGT (3883C>T), GAA1234GAG (3886A>G), CTA1236CTG (3892A>G), GAC1237GAT (3895C>T), TTT1238TTC (3898T>C), GTA1240GTG (3904A>G), CGG1241AGA (3905C>A 3907G>A), GCT1243GCC (3913T>C), AAG1248AAA (3928G>A), TCA1249TCT (3931A>T), GGG1251GGA (3937G>A), AGT1257AGC (3955T>C), GTG1258GTA (3958G>A), GCC1259GCT (3961C>T), GGA1260GGG (3964A>G), TCT1261TCC (3967T>C), TTA1264TTG (3976A>G), ATT1265ATC (3979T>C), CCA1267CCT (3985A>T), CTA1271CTT (3997A>T), GCG1272GCA (4000G>A), AAC1274AAT (4006C>T), GTT1275GTG (4009T>G), TTC1276TTT (4012C>T), AAA1277AAG (4015A>G), TCT1280TCC (4024T>C), GAA1282GAG (4030A>G), CCA1283CCT (4033A>T), AAG1286AAA (4042G>A), CTG1290TTA (4052C>T 4054G>A), CGT1291CGC (4057T>C), CGT1293CGA (4063T>A), GGA1294GGG (4066A>G), ATA1297GTA (4073A>G), TTT1298TTC (4078T>C), GGC1299GGT (4081C>T), TCT1300TCC (4084T>C), TCC1304TCT (4096C>T), AGC1308AGT (4108C>T), GGA1309GGT (4111A>T), GCG1311GCA (4117G>A), CTA1312TTG (4118C>T 4120A>G), TAC1314TAT (4126C>T), TTC1315TAT (4128T>A 4129C>T), GCC1316GCT (4132C>T), AAT1317AAC (4135T>C), CAA1321CAG (4147A>G), TTA1322CTA (4148T>C), GCT1323TCT (4151G>T), AAC1326AAT (4162C>T), GTA1328ATA (4166G>A), ATA1329ATC (4171A>C), GAG1332GAA (4180G>A), CTG1336TTG (4190C>T), CCT1338CCC (4198T>C), TCC1339TCT (4201C>T), GCG1342GCT (4210G>T), TTC1343TTT (4213C>T), AGT1345AGC (4219T>C), CTG1346TTG (4220C>T), CTG1347ATA (4223C>A 4225G>A), GTT1349GTG (4231T>G), CAT1351CAC (4237T>C), GTA1356GTG (4252A>G), TTA1357CTG (4253T>C 4255A>G), AAA1358AAG (4258A>G), ACT1362ACC (4270T>C), AGA1366AGG (4282A>G), GAG1367GAA (4285G>A), GAA1369GAG (4291A>G), CAG1373CAA (4303G>A), GTC1376GTT (4312C>T), ATA1379GTG (4319A>G 4321A>G), GTG1380GTT (4324G>T), GAG1381GAA (4327G>A), CTG1384CTT (4336G>T), TCT1385TCC (4339T>C), TCA1388TCT (4348A>T), GCA1392GCG (4360A>G), GGT1394GGC (4366T>C), TCT1395TCA (4369T>A), ACT1397ACC (4375T>C), GTT1401GTC (4387T>C), TTT1404TAT (4395T>A), GTA1408ATA (4406G>A), CTT1409CTC (4411T>C), GTT1418GTC (4438T>C), ACC1420ACA (4444C>A), GCC1422GCT (4450C>T), CTC1424CTT (4456C>T), GAC1427GAT (4465C>T), ATG1431GTT (4475A>G 4477G>T), GTC1432GTT (4480C>T), ACA1433TCG (4481A>T 4483A>G), GGC1437GGT (4495C>T), AAG1441AAA (4507G>A), CAC1442CAT (4510C>T), GGT1443GGG (4513T>G), CTA1445TTA (4517C>T), GTC1448GTT (4528C>T), AAA1450AAG (4534A>G), ACC1452ACT (4540C>T), GCG1454TTA (4544G>T 4545C>T 4546G>A), AGA1455AAA (4548G>A), TTT1458TTC (4558T>C), GTA1460GTT (4564A>T), ACC1462ACT (4570C>T), ATA1464ATT (4576A>T), TTG1471TTA (4597G>A), GAC1472GAT (4600C>T), GAC1474GAT (4606C>T), GTG1475GTA (4609G>A), GTG1477GTA (4615G>A), GAC1478GAT (4618C>T), TTG1481CTT (4625T>C 4627G>T), GAC1488GAT (4648C>T), ATT1489ATC (4651T>C), AGG1492AGA (4660G>A), AGC1493AGT (4663C>T), GCT1495GCA (4669T>A), GTG1499ATC (4679G>A 4681G>C), AGT1500AGC (4684T>C), GTT1501GTG (4687T>G), GAG1505GAA (4699G>A), AGA1506AGG (4702A>G), CAA1508CAG (4708A>G), AGG1509AGA (4711G>A), CTG1510TTG (4712C>T), AAA1518AAG (4738A>G), GTA1520GTT (4744A>T), GCA1521GCG (4747A>G), TTG1522CTG (4748T>C), ACT1527ACC (4765T>C), GTC1538ATT (4796G>A 4798C>T), ACT1540AGC (4803C>G 4804T>C), GAG1541GAA (4807G>A), GCG1542GCT (4810G>T), GCT1543GCG (4813T>G), CTT1544CTC (4816T>C), TAT1549TAC

	(4831T>C), AAC1550AAT (4834C>T), CCA1552CCT (4840A>T), GGA1556GGG (4852A>G), GGC1557GGT (4855C>T), AGT1561AGC (4867T>C), CTG1562CTC (4870G>C), ATT1563ATA (4873T>A), TGT1566TGC (4882T>C), ACA1573ACT (4903A>T), CAG1575CAA (4909G>A), TTT1577TTC (4915T>C), GAA1578GAG (4918A>G), TTG1579CTA (4919T>C 4921G>A), AGT1580AGC (4924T>C), CCC1581CCG (4927C>G), TTT1583TTC (4933T>C), ATC1584ATA (4936C>A), CAG1585CAA (4939G>A), AAT1586AAC (4942T>C), TTC1587TTT (4945C>T), TCA1593TCG (4963A>G), CCT1596CCC (4972T>C), CTT1602CTC (4990T>C), AAA1606AAG (5002A>G), CCT1613CCC (5023T>C), TTG1614CTG (5024T>C), TGC1615TGT (5029C>T), GAT1616GAC (5032T>C), TCT1618TCC (5038T>C), CCA1620CCT (5044A>T), GCA1623GCT (5053A>T), TCG1624TCA (5056G>A), AGT1625GGT (5057A>G), ACT1626GCT (5060A>G), TCG1629TCT (5071G>T), CTT1636ATT (5090C>A), GGA1637GGG (5095A>G), GTG1638GTT (5098G>T), GCC1639GTC (5100C>T), TTA1640TTG (5104A>G), GAA1641GAT (5107A>T), ATT1642ATC (5110T>C), CCA1643CCG (5113A>G), AAG1644AAA (5116G>A), CAA1645CAG (5119A>G), GTC1646TTT (5120G>T 5122C>T), ATT1648GTT (5126A>G), CAT1651CAC (5137T>C), ATC1652ATT (5140C>T), AAA1653AAG (5143A>G), GAG1654GAA (5146G>A), CTC1659CTA (5161C>A), GAA1661GAG (5167A>G), CTT1663CTG (5173T>G), ACG1666ACA (5182G>A), GTT1667ATT (5183G>A), GTC1668GTT (5188C>T), AAG1669AAA (5191G>A), AAA1671AAG (5197A>G), GAC1672GAT (5200C>T), TGC1674TGT (5206C>T), TTA1675TTG (5209A>G), AGC1678AGT (5218C>T), TCG1682TCA (5230G>A), ATC1684ATT (5236C>T), AGC1685AGT (5239C>T), ATC1687ATT (5245C>T), ACA1693ACT (5263A>T), CTC1695CTT (5269C>T), TTC1696TTT (5272C>T), GCC1697GCA (5275C>A), ATC1698ATT (5278C>T), CCA1699CCC (5281A>C), AGA1700AGG (5284A>G), ACT1701ACC (5287T>C), CTA1702TTA (5288C>T), ATA1703ATT (5293A>T), TTG1704CTA (5294T>C 5296G>A), GAG1706GAA (5302G>A), CTT1709CTA (5311T>A), GAA1710GAG (5314A>G), CGA1713CGT (5323A>T), AGT1721AGC (5347T>C), ATC1723ATT (5353C>T), TGC1727TGT (5365C>T), GTT1734GTC (5386T>C), ACC1737ACA (5395C>A), AAA1746AAG (5422A>G), GAT1747GAC (5425T>C), ACC1749ACT (5431C>T), GAG1751GAA (5437G>A), CTT1756CTC (5452T>C), AAG1758AAA (5458G>A), AGT1761AGC (5467T>C), CAA1762CAG (5470A>G), CTA1763TTG (5471C>T 5473A>G), GAA1765GAG (5479A>G), TTC1766TTT (5482C>T), TCA1767TCC (5485A>C), AAT1768AAC (5488T>C), TTG1769CTA (5489T>C 5491G>A), GGT1771GGC (5497T>C), TGT1774TGC (5506T>C), GAG1775GAA (5509G>A), GAG1776GAA (5512G>A), TTA1778CTA (5516T>C), TAT1782TAC (5530T>C), GAG1783GAA (5533G>A), TCG1784TCT (5536G>T), TTG1785TTA (5539G>A), GTT1788GTG (5548T>G), CAC1790CAT (5554C>T), GCT1792GCA (5560T>A), GCG1793ACA (5561G>A 5563G>A), ACG1794ACC (5566G>C), TCA1795TCG (5569A>G), CTC1800CTG (5584C>G), AAG1803AAA (5593G>A), GGG1804GGA (5596G>A), ATT1805GTT (5597A>G), AAC1807AAA (5605C>A), GTG1811GTT (5617G>T), GCT1812GTG (5619C>T 5620T>G), TTG1815TTA (5629G>A), ATC1816CTC (5630A>C), GGC1819GGT (5641C>T), GCA1822GCT (5650A>T), ATA1826ATT (5662A>T), GGA1827GGG (5665A>G), CAA1835CAG (5689A>G), GTT1837ATT (5693G>A), GAG1838GAA (5698G>A), CAA1843CAG (5713A>G), GGG1844GGC (5716G>C), AAA1845AAG (5719A>G), GCC1853GCA (5743C>A), CGC1857CGA (5755C>A), CAT1858CAC (5758T>C), CGT1860CGC (5764T>C), AAA1862AAG (5770A>G), GGC1865GGT (5779C>T), TTT1866TTC (5782T>C), GAC1869GAT (5791C>T), GAC1873GAT (5803C>T), AGG1884AGA (5836G>A), AAA1885AAG (5839A>G), GGA1887GGG (5845A>G), AAA1888AAG (5848A>G), AAA1890AGA (5853A>G), GGT1891GGC (5857T>C), ACC1892ACT (5860C>T), ACA1893ACT (5863A>T), GGT1895GGA (5869T>A), GGT1897GGC (5875T>C), AGC1900AGT (5884C>T), TTC1903TTT (5893C>T), ATC1904GTG (5894A>G 5896C>G), AAC1905AAT (5899C>T), GGG1908GGA (5908G>A), TTT1909TTC (5911T>C), GAG1913GAA (5923G>A), CAA1918CAG (5938A>G), TTC1919TTT (5941C>T), GTT1920GTC (5944T>C), GAT1921GAC (5947T>C), GGG1925GGA (5959G>A), CGG1926GCC (5960C>G 5961G>C 5962G>C), GAA1930GAG (5974A>G), GCT1934GCA (5986T>A), GAC1935GAT (5989C>T), ATT1936GTT (5990A>G), GAT1938GAC (5998T>C), AGA1942CGT (6008A>C 6010A>T), GAA1945GAT (6019A>T), GTG1946GTT (6022G>T), CGA1947CGT (6025A>T), AAA1949ACA (6030A>C), GAC1955GAA (6049C>A), ATG1958ATA (6058G>A), CAA1959CAG (6061A>G), GCC1960GCA (6064C>A), GGT1962GGC (6070T>C), AGT1963AAC (6072G>A 6073T>C), AAC1964CAC (6074A>C), ACG1965ACA (6079G>A), ACC1966AAC (6081C>A), ATA1967ATT (6085A>T), GCA1969GCT (6091A>T), TAC1970TAT (6094C>T), TGT1976TCC (6111G>C 6112T>C), AAA1978AAG (6118A>G), TTG1980TTA (6124G>A), AAG1981AAA (6127G>A), ATT1982GTC (6128A>G 6130T>C), GAT1983GAC (6133T>C), TTA1984TTG (6136A>G), AAC1988AAT (6148C>T), AAA1991AAG (6157A>G), GTT1992ATT (6158G>A), TGT1993TGC (6163T>C), AAA1995AAG (6169A>G), ACA1996TCG (6170A>T 6172A>G), GGC1998GGT (6178C>T), AAA2001AAG (6187A>G), TTT2002TTC (6190T>C), GAG2004GAA (6196G>A), CTC2007TTT (6203C>T 6205C>T), GAA2008GAG (6208A>G), CTA2009TTG (6209C>T 6211A>G), CAG2011CAA (6217G>A), GCT2015GCA (6229T>A), GTA2016ATA (6230G>A), GTC2018GTT (6238C>T), AAG2021AAA (6247G>A), ATA2023ATT (6253A>T), GCA2025AAA (6257G>A 6258C>A), GAG2027GAA (6265G>A), CAT2030CAC (6274T>C), AAA2033AGA (6282A>G), TCG2034TCA (6286G>A), GGC2038GGT (6298C>T), TTG2039TTA (6301G>A), AGA2040AGG (6304A>G), GAC2041GAT (6307C>T), GCC2046GCT (6322C>T), ACA2048ACT (6328A>T), GTT2049GTG (6331T>G), TGT2050TGC (6334T>C), AGG2051AGA (6337G>A), CTG2052TTG (6338C>T), TAT2058TAC (6358T>C), GGG2059GGA (6361G>A), GCA2060ACA (6362G>A), TCA2061TCT (6367A>T), GAG2062GAA (6370G>A), TAC2064TAT (6376C>T), GGT2065GGG (6379T>G), GGA2067GGC (6385A>C), GGA2069GGT (6391A>T), GCA2070GCG (6394A>G), TAC2071TAT (6397C>T), GTA2073ATA (6401G>A), GCG2074GTG (6405C>T), CAC2076CAT (6412C>T), TTA2078TTG (6418A>G), TTT2079TTC (6421T>C), TAC2082TTC (6429A>T), GGT2084GGA (6436T>A), GAG2087GAA (6445G>A), CAA2089CGA (6450A>G), TCC2090TCG (6454C>G), CAC2092CAT (6460C>T), GGT2093GGA (6463T>A), TTC2095TTT (6469C>T), AGG2096AGA (6472G>A), GTG2097GTA (6475G>A), AAT2099AAC (6481T>C), CAC2101AAT (6485C>A 6487C>T), AGT2102AGC (6490T>C), AGC2104AGT (6496C>T), CTG2106CTA (6502G>A), GGT2110GGC (6514T>C), AGG2111AGA (6517G>A), CTC2115ATC (6527C>A), ATC2116ATA (6532C>A), CCG2119CCA (6541G>A), AAA2120AAG (6544A>G), GTC2124GTT (6556C>T), TTT2125TTC (6559T>C), CAG2127CAA (6565G>A), TTG2129CTA (6569T>C 6571G>A), CCT2134CCA (6586T>A), ACA2135ATA (6588C>T), GAA2138GAG (6598A>G), AGA2139AGG (6601A>G), ATT2140ATC (6604T>C), TGT2141TGC (6607T>C), GGA2144GGG (6616A>G), AAC2146AAT (6622C>T), GAG2149GAA (6631G>A), AAG2150AAA (6634G>A), TAT2151CAT (6635T>C), GCT2152GCA (6640T>A), TCG2154TCT (6646G>T), ATC2155GTC (6647A>G), ATC2156ATT (6652C>T), AGC2160AGT (6664C>T), ACT2162ACG (6670T>G), ATA2165GTG (6677A>G 6679A>G), GGC2167GGT (6685C>T), ACA2169ACT (6691A>T), TTC2170TTT (6694C>T), ACA2177ACG (6715A>G), GAT2178AAT (6716G>A), AAT2179GAT (6719A>G), GGA2180GGG (6724A>G), CTA2184TTA (6734C>T), GTG2186GTA (6742G>A), GTG2187GTA (6745G>A), GCC2190GCT (6754C>T), GGA2192GGG (6760A>G), ATA2194TTG (6764A>T 6766A>G), ATT2197ATA (6775T>A), CTG2200TTG (6782C>T), GCA2201GCT (6787A>T), AAC2202AAT (6790C>T), GCA2204GTG (6795C>T 6796A>G), CAC2205CAA (6799C>A), ACC2206ACA (6802C>A), AAC2208AAT (6808C>T), TAC2209TAT (6811C>T), TAC2210TAT (6814C>T), TTC2213TTT (6823C>T), GAA2215GAG (6829A>G), TAC2221TAT (6847C>T), CGA2223CGC (6853A>C), AAT2225GAT (6857A>G), GAG2226GAA (6862G>A), GAA2229GAG (6871A>G), GTC2231ACA (6875G>A 6876T>C 6877C>A), AAG2232AAA (6880G>A), TCT2233TCA (6883T>A), GTT2235GTG (6889T>G), GAC2239GAT (6901C>T), GGC2244GGT (6916C>T), CCG2245CCA (6919G>A), AAA2247AAG (6925A>G), CTT2248CTC (6928T>C), GAC2250GAA (6934C>A), GGG2255GGC (6949G>C), TTC2257TTT (6955C>T), AAA2258AAG (6958A>G), CAA2264CAG (6976A>G), CTA2266TTA (6980C>T), ATC2267ATT (6985C>T), GTA2271GTT (6997A>T), GTG2272GTT (7000G>T), GTG2273ATA (7001G>A 7003G>A), GCT2276GCC (7012T>C), AAG2277AAA (7015G>A), CAT2278CAC (7018T>C), TTT2283TAT (7032T>A), GCC2285GCT (7039C>T), TTG2286CTA (7040T>C 7042G>A), GGA2288GGG (7048A>G), GCT2292GCA (7060T>A), GTC2293GTA (7063C>A), AGC2298AGT (7078C>T), TTA2300CTG (7082T>C 7084A>G), GTA2301GTG (7087A>G), ACC2302ACA (7090C>A), CAT2304CAC (7096T>C), GTA2305GTG (7099A>G), GTT2306GTC (7102T>C), GAG2309GAA (7111G>A), CGA2311CGG (7117A>G), ACA2314AAA (7125C>A), GAA2315GAG (7129A>G), TTT2316TTC (7132T>C), CTG2317TTA (7133C>T 7135G>A), GTG2319GTA (7141G>A), GAT2320GAC (7144T>C), GCA2321TCG (7145G>T 7147A>G), GAG2322GAA (7150G>A), GCA2325GCT (7159A>T), GAG2326_GCA2327del (7160_7165delGAGGCA), TTC2329TTT (7171C>T), GCG2335GCT (7189G>T), AAA2338AAG (7198A>G), AGC2339AGT (7201C>T), CTA2341TTA (7205C>T), AAT2342AAC (7210T>C), GAT2344GAA (7216T>A), GCG2345GCA (7219G>A), ATC2347ATA (7225C>A), AAG2348AAA (7228G>A), AAG2352AAA (7240G>A), TAT2353TAC (7243T>C), AAA2355AAG (7249A>G), CCT2356CCG (7252T>G), GAT2358GAA (7258T>A), GTT2359GTC (7261T>C), GGT2360GGA (7264T>A), GTC2361GTG (7267C>G), GTG2362GTA (7270G>A), GAT2363_CGG2364insT-- (7273_7274insT), CGG2364GTG (7274C>G 7275G>T), CAT2366CTT (7281A>T), TTG2367TCG (7284T>C), TCA2371TTA (7296C>T), TCT2376TTT (7311C>T), ACC2377ATC (7314C>T), TGC2378TAC (7317G>A), AAT2379AAA (7321T>A), GTG2380-TG (7322delG), CAC2381CAT (7327C>T), GGC2382GGT (7330C>T), TTC2383TTT (7333C>T), AAG2384CAG (7334A>C), TGT2386TGC (7342T>C), GCA2387AAC (7343G>A 7344C>A 7345A>C), ACT2390ACC (7354T>C), CAA2393CAG (7363A>G), ATT2395ATC (7369T>C), TTC2396TTT (7372C>T), GCG2398GCT (7378G>T), CTC2399CTT (7381C>T), GCA2404GCT (7396A>T), GTC2405GTT (7399C>T), GCC2407GCT (7405C>T), AGT2408ATG (7407G>T 7408T>G), GGG2410GGT (7414G>T), TGC2411GGC (7415T>G), AAA2412AAG (7420A>G), GAC2415GAT (7429C>T), TAT2416TAC (7432T>C), CAT2419CAC (7441T>C), AAG2425AAA (7459G>A), GAA2426GAG (7462A>G), ATA2428ATT (7468A>T), GTC2429GTA (7471C>A), CAA2431CAG (7477A>G), AGC2432AGT (7480C>T), CTG2434CTT (7486G>T), TAT2437TAC (7495T>C), AAA2438AAG (7498A>G), GGT2439GGC (7501T>C), TTG2440TCA (7503T>C 7504G>A), ATT2443ATA (7513T>A), AAC2445AAT (7519C>T), GGA2446GGG (7522A>G), AAG2449AAA (7531G>A), GAG2451GAA (7537G>A), CTC2452CTT (7540C>T), TGT2454TGC (7546T>C), AAG2462AAA (7570G>A), ACG2463ACA (7573G>A), ACG2465ACA (7579G>A), GCT2468GCC (7588T>C), CTA2471TTA (7595C>T), TTG2474CTA (7604T>C 7606G>A), GGT2477GGA (7615T>A), TGT2480TGC (7624T>C), GAC2483GAT (7633C>T), TTC2484TTT (7636C>T), AAT2485AAC (7639T>C), TTT2488TTC (7648T>C), GAA2494GAG (7666A>G), TGT2496TGC (7672T>C), ACA2498ACT (7678A>T), GGG2500GGA (7684G>A), ACT2502ACC (7690T>C), TTT2504TTC (7696T>C), GGT2506GGG (7702T>G), GAT2509AAT (7709G>A), AAA2510AGG (7713A>G 7714A>G), CTG2511TTG (7715C>T), CGT2514CGC (7726T>C), GAG2517GAA (7735G>A), GTA2520GTG (7744A>G), TAC2521TAT (7747C>T), GGC2526GGT (7762C>T), CAG2528CAA (7768G>A), TTT2529TTC (7771T>C), TCA2532TCG (7780A>G), CTA2533CTC (7783A>C), ACT2534ACC (7786T>C), CTA2537TTG (7793C>T 7795A>G), ATC2538ATT (7798C>T), GCT2540GCC (7804T>C), ACC2543ATC (7812C>T), GAA2550GAG (7834A>G), GAC2551GAT (7837C>T), GTG2554TTA (7844G>T 7846G>A), CAG2557CAA (7855G>A), CTG2559CTA (7861G>A), TTA2562TTG (7870A>G), TAC2563TAT (7873C>T), ACT2564ACA (7876T>A), GAG2565GAA (7879G>A), ATT2566ATA (7882T>A), GTT2567ATT (7883G>A), CCA2570CCT (7894A>T), ATT2571ATC (7897T>C), GTC2579GTT (7921C>T), AAG2581AAA (7927G>A), GGT2584GGC (7936T>C), AAT2585AAC (7939T>C), AAT2586AAC (7942T>C), GTG2594GTA (7966G>A), GAT2595GAC (7969T>C), TCT2597TCC (7975T>C), GTC2601GTT (7987C>T), CTT2602CTC (7990T>C), GCT2607GCC (8005T>C), GAA2616GAG (8032A>G), ATC2618ATT (8038C>T), GAC2619GAT (8041C>T), AGC2620AAC (8043G>A), TTG2632TTA (8080G>A), GTG2635GTA (8089G>A), CCG2637CCA (8095G>A), GAG2638GAA (8098G>A), AGC2641AGT (8107C>T), ATT2642ATC (8110T>C), GAT2644GAC (8116T>C), CAA2648CAG (8128A>G), GGT2654GGC (8146T>C), AAC2656AGT (8151A>G 8152C>T), TCG2660TCA (8164G>A), AGG2665AAG (8178G>A), AAG2666AAA (8182G>A), TTG2669TTA (8191G>A), TTC2671TTT (8197C>T), TCC2673TCT (8203C>T), AGA2675AAA (8208G>A), CTG2677TTA (8213C>T 8215G>A), CTA2678CTG (8218A>G), ATC2679ATT (8221C>T), GAG2680GAA (8224G>A), CCA2685CCG (8239A>G), GAG2690GAA (8254G>A), ATT2692ATC (8260T>C), GTA2693GTG (8263A>G), GAT2699GAC (8281T>C), AGA2700AGG (8284A>G), CCA2704GCG (8294C>G 8296A>G), GAG2705GAA (8299G>A), CAC2706CAT (8302C>T), ATT2711ATC (8317T>C), TGC2712TGT (8320C>T), GCT2714GCA (8326T>A), GAG2717GAA (8335G>A), TCC2718TCT (8338C>T), TCT2722TTT (8349C>T), GAA2723GAG (8353A>G), CAC2726CAT (8362C>T), CAA2727CAG (8365A>G), ATC2728ATT (8368C>T), TAC2732TAT (8380C>T), TCA2733TCG (8383A>G), TTA2735TTG (8389A>G), CCT2740CCC (8404T>C), GCA2742TCA (8408G>T), ACA2743ACG (8413A>G), GCG2745GCT (8419G>T), GCT2750GCC (8434T>C), TAT2752TAC (8440T>C), ATG2756ATA (8452G>A), CTA2758CTG (8458A>G), AGG2759AAG (8460G>A), AAA2760AAG (8464A>G), CTG2761CTA (8467G>A), AGG2765AGA (8479G>A), GCT2766ACA (8480G>A 8482T>A), GAG2771GAA (8497G>A), CTA2772TTG (8498C>T 8500A>G), AGA2773AAG (8502G>A 8503A>G), GCC2774GCT (8506C>T), GAA2777GAT (8515A>T), GTC2780GTT (8524C>T), GCA2781GCC (8527A>C), TTA2782CTG (8528T>C 8530A>G), GAG2785GAC (8539G>C), CTT2788TGT (8546C>T 8547T>G), TCT2790ACT (8552T>A), GTA2793GTG (8563A>G), GCA2797GGA (8574C>G), ATT2801GTT (8585A>G), GGA2805GAA (8598G>A), AGC2806AGT (8602C>T), AAC2807TCT (8603A>T 8604A>C 8605C>T), AAA2812AAG (8620A>G), CCA2813CAA (8622C>A), GAG2814GGA (8625A>G 8626G>A), CAG2815CAA (8629G>A), GGC2816GGT (8632C>T), ATC2818ATT (8638C>T), CAG2819CAA (8641G>A), AAC2821AAT (8647C>T), CCG2822TTC (8648C>T 8649C>T 8650G>C), AAC2823AAT (8653C>T), AAA2824AAG (8656A>G), GGA2825GAA (8658G>A), GAT2827GCG (8664A>C 8665T>G), GTT2830GTG (8674T>G), GCA2832GTT (8679C>T 8680A>T), GGC2833GGA (8683C>A), GGG2836GGA (8692G>A), ACA2837ACT (8695A>T), CCG2841CCA (8707G>A), AGA2842CGA (8708A>C), AAG2844AAA (8716G>A), ATC2846ATT (8722C>T), ACG2847ACA (8725G>A), AAA2849AAG (8731A>G), AGA2851AGG (8737A>G), CCC2853CCT (8743C>T), ACA2854AAG (8745C>A 8746A>G), AAG2856AAA (8752G>A), GGA2857GGT (8755A>T), AAC2862AAT (8770C>T), TTA2863CTA (8771T>C), GAA2864GAG (8776A>G), CAT2865CAC (8779T>C), TTG2866TTA (8782G>A), TAT2869TAC (8791T>C), CCA2871CCG (8797A>G), CAA2872CAG (8800A>G), CAA2873CAG (8803A>G), GAT2875GAC (8809T>C), ATT2876ATC (8812T>C), AAT2878AAC (8818T>C), CGG2880CGA (8824G>A), GCA2881GCG (8827A>G), ACG2888ACA (8848G>A), TAT2890TAC (8854T>C), GAG2891GGA (8856A>G 8857G>A), GTG2893GTA (8863G>A), CGG2894CAG (8865G>A), ATG2895CTT (8867A>C 8869G>T), GCA2896GCG (8872A>G), GGA2900GAA (8883G>A), ACT2902TCT (8888A>T), TGC2915TGT (8929C>T), GTC2924ATC (8954G>A), AAC2925AAT (8959C>T), GGA2926GGT (8962A>T), GGG2933GGA (8983G>A), AAT2934GAC (8984A>G 8986T>C), GAA2935GTA (8988A>T), GAG2938GAA (8998G>A), CCG2940CCA (9004G>A), TTG2941TTA (9007G>A), ATC2944ATT (9016C>T), ACC2951ACA (9037C>A), ATA2967ATT (9085A>T), AAC2971AAT (9097C>T), ATT2983GTT (9131A>G), CGA2984CGT (9136A>T), CGG2987CGT (9145G>T), ATG2989GGA (9149A>G 9150T>G 9151G>A), GGT2990AGT (9152G>A), TTA2991TTG (9157A>G), GCG2992GCT (9160G>T), CGT2993CGC (9163T>C), GAG3000GAA (9184G>A), CGA3004CGG (9196A>G), AGG3010AGA (9214G>A), GAA3011GAG (9217A>G), GCG3012GCA (9220G>A), GCA3020GCT (9244A>T), TTG3021TTA (9247G>A), GCC3024GCT (9256C>T), CCT3026TCC (9260C>T 9262T>C), GGG3030GGA (9274G>A), GAC3032GAT (9280C>T), ATC3035ATT (9289C>T), CAA3038CAC (9298A>C), GAG3040GAA (9304G>A), GTC3050GTT (9334C>T), GTC3060GTG (9364C>G)								
PVYgp2	1	75	100%	230	43.7%	75 (98.7%)	55 (72.4%)	1/0/2/1	2
Protein mutations:	R17_K17insX (2971_2972insA), Y23H (2989T>C 2991C>T), R26K (2999G>A 3000A>G), H35Y (3025C>T), K40H (3040A>C 3042A>C), G41S (3043G>A), E44K (3052G>A), I47V (3061A>G), V54I (3082G>A), P57S (3091C>T), P60T (3100C>A), G61E (3104G>A 3105T>G), G62S (3106G>A), Q63* (3109C>T), R64G (3112A>G), C66R (3118T>C), L67F (3121C>T), I69V (3127A>G), A72E (3137C>A 3138G>A), I73V (3139A>G)								
Codon mutations:	TTA2CTA (2923T>C), AAA4AAG (2931A>G), GAA7GAG (2940A>G), TTG10CTG (2947T>C), TTG15CTG (2962T>C), AGA17_AAA17insAA- (2971_2972insA), TAC23CAT (2989T>C 2991C>T), TCA24TCG (2994A>G), AGA26AAG (2999G>A 3000A>G), ATC31ATT (3015C>T), TCG33TCA (3021G>A), GTA34GTT (3024A>T), CAT35TAT (3025C>T), CAC38CAT (3036C>T), AGA39AGG (3039A>G), AAA40CAC (3040A>C 3042A>C), GGC41AGC (3043G>A), GAA44AAA (3052G>A), ATA47GTA (3061A>G), GTT54ATT (3082G>A), CCG57TCG (3091C>T), CGC59CGT (3099C>T), CCA60ACA (3100C>A), GGT61GAG (3104G>A 3105T>G), GGT62AGT (3106G>A), CAA63TAA (3109C>T), AGG64GGG (3112A>G), TGC66CGC (3118T>C), CTC67TTC (3121C>T), ATT69GTT (3127A>G), GAG70GAA (3132G>A), GCG72GAA (3137C>A 3138G>A), ATT73GTT (3139A>G)								

	Begin	End	Coverage	Score	Concordance	Matches	Identities	I/D/M/F*	Stop Codons
**NT**	**52**	**9704**	**99.5%**	**12530**	**65.2%**	**9642 (99.8%)**	**7985 (82.7%)**	**7/11**	

Proteins

	Begin	End	Coverage	Score	Concordance	Matches	Identities	I/D/M/F*	Stop Codons
polyprotein (NP_056759.1)	1	3064	100%	19042	90.2%	3062 (99.9%)	2771 (90.4%)	2/2/3/3	1
Protein mutations:	Y4Q (194T>C 196C>A), C9L (209T>C 210G>T 211T>G), F13M (221T>A 223T>G), S23P (251T>C), C24F (255G>T 256C>T), E25G (258A>G), I27V (263A>G 265T>G), V28A (267T>C), E30V (273A>T), A35T (287G>A 289T>C), V37T (293G>A 294T>C), A41I (305G>A 306C>T), D42G (309A>G), E44D (316A>T), T45V (317A>G 318C>T 319A>G), L52Q (339T>A 340C>A), K53R (342A>G), K55A (347A>G 348A>C 349A>G), Y56H (350T>C), T58V (356A>G 357C>T), V61T (365G>A 366T>C 367G>A), L62S (368C>T 369T>C), F67C (384T>G), A75V (408C>T 409C>T), M78E (416A>G 417T>A), E85K (437G>A), R86E (440A>G 441G>A), K87R (443A>C 444A>G 445G>A), D88E (448T>A), E91A (456A>C), H93N (461C>A), D111E (517T>A), S116F (531C>T), P118S (536C>T), Q119W (539C>T 540A>G 541A>G), R122K (549G>A 550A>G), M131T (576T>C), V134A (585T>C 586C>G), R137Y (593C>T 594G>A 595C>T), P138R (597C>G), I139V (599A>G 601A>G), I140P (602A>C 603T>C 604A>G), M147I (625G>A), I151V (635A>G 637T>C), I154V (644A>G 646A>G), E160A (663A>C 664G>C), H166Q (682C>A), T172S (699C>G), H174Q (706T>A), K179E (719A>G 721G>A), I180V (722A>G 724A>G), A183S (731G>T), Y184P (734T>C 735A>C 736C>T), S185R (737T>C 738C>G 739C>T), A187V (744C>T 745G>C), R189C (749C>T 751A>C), M194K (765T>A), R197K (773C>A 774G>A 775A>G), M206K (801T>A), G210A (813G>C 814A>G), L211H (816T>A), R214Q (825G>A 826T>A), N223D (851A>G 853T>C), R226C (860C>T 862C>T), T227A (863A>G 865T>C), I228T (867T>C), N229D (869A>G 871C>T), I230L (872A>T 874A>G), R231Q (876G>A), R232K (879G>A), N240S (903A>G), T241N (906C>A 907A>C), K242T (909A>C 910A>C), S243N (912G>A 913C>T), S252L (938T>C 939C>T 940A>T), L266I (980T>A 982G>C), R272K (999G>A), Q275R (1007C>A 1008A>G 1009G>A), S276G (1010A>G 1012T>G), I277V (1013A>G), N279Q (1019A>C 1021C>G), D288E (1048C>A), R300Q (1083G>A), S305A (1097T>G 1099G>A), R320K (1143G>A), L324I (1154T>A 1156G>A), S346N (1221G>A), V349T (1229G>A 1230T>C 1231T>C), S350N (1233G>A 1234C>T), K358T (1257A>C 1258A>G), N365S (1278A>G), A369V (1290C>T 1291G>A), D372T (1298G>A 1299A>C 1300C>T), I375V (1307A>G 1309A>G), N378E (1316A>G 1318T>A), I382T (1329T>C), L397I (1373C>A), I401V (1385A>G), E407D (1405G>T), A411S (1415G>T 1417A>T), V418I (1436G>A 1438C>T), E508G (1707A>G), A512T (1718G>A), I520V (1742A>G 1744C>T), S525N (1758G>A), V536I (1790G>A), I585V (1937A>G 1939T>C), F607Y (2004T>A), I628M (2068T>G), V630I (2072G>A 2074G>A), R675K (2208G>A), D694E (2266C>G), N744G (2414A>G 2415A>G 2416T>G), S746C (2420A>T), K780R (2523A>G), R817K (2634G>A), V847I (2723G>A 2725C>T), A855T (2747G>A 2749T>A), H871N (2795C>A), V880I (2822G>A 2824G>A), P900T (2882C>A), S901H (2885A>C 2886G>A), T904M (2895C>T), N916S (2931A>G), N919S (2940A>G), E929_N930insX (2971_2972insA), T936I (2991C>T), H937R (2994A>G), E939R (2999G>A 3000A>G), S944F (3015C>T), R946Q (3021G>A), Y947F (3024A>T 3025C>T), T951I (3036C>T), E952G (3039A>G 3040A>C), K953T (3042A>C 3043G>A), A972V (3099C>T 3100C>A), V974R (3104G>A 3105T>G 3106G>A), S983N (3132G>A), R985K (3137C>A 3138G>A 3139A>G), F990I (3152T>A 3154C>T), C994Y (3165G>A), F1012L (3218T>C), T1014N (3225C>A), V1016I (3230G>A), V1028M (3266G>A 3268A>G), V1031M (3275G>A), A1034T (3284G>A), R1044K (3315G>A 3316A>G), E1045A (3318A>C), M1059V (3359A>G), D1072E (3400T>G), V1110G (3513T>G), M1121V (3545A>G), A1122T (3548G>A), V1124I (3554G>A 3556C>T), L1147V (3623C>G), L1150M (3632C>A), Y1152H (3638T>C), I1172V (3698A>G), S1178N (3717G>A), R1183K (3731C>A 3732G>A), I1297V (4073A>G), F1315Y (4128T>A 4129C>T), A1323S (4151G>T), V1328I (4166G>A), L1347I (4223C>A 4225G>A), I1379V (4319A>G 4321A>G), F1404Y (4395T>A), V1408I (4406G>A), M1431V (4475A>G 4477G>T), T1433S (4481A>T 4483A>G), A1454L (4544G>T 4545C>T 4546G>A), R1455K (4548G>A), V1499I (4679G>A 4681G>C), V1538I (4796G>A 4798C>T), T1540S (4803C>G 4804T>C), S1625G (5057A>G), T1626A (5060A>G), L1636I (5090C>A), A1639V (5100C>T), E1641D (5107A>T), V1646F (5120G>T 5122C>T), I1648V (5126A>G), V1667I (5183G>A), A1793T (5561G>A 5563G>A), I1805V (5597A>G), N1807K (5605C>A), A1812V (5619C>T 5620T>G), I1816L (5630A>C), V1837I (5693G>A), K1890R (5853A>G), I1904V (5894A>G 5896C>G), R1926A (5960C>G 5961G>C 5962G>C), I1936V (5990A>G), E1945D (6019A>T), K1949T (6030A>C), D1955E (6049C>A), M1958I (6058G>A), S1963N (6072G>A 6073T>C), N1964H (6074A>C), T1966N (6081C>A), C1976S (6111G>C 6112T>C), I1982V (6128A>G 6130T>C), V1992I (6158G>A), T1996S (6170A>T 6172A>G), L2007F (6203C>T 6205C>T), V2016I (6230G>A), A2025K (6257G>A 6258C>A), K2033R (6282A>G), A2060T (6362G>A), V2073I (6401G>A), A2074V (6405C>T), Y2082F (6429A>T), Q2089R (6450A>G), H2101N (6485C>A 6487C>T), L2115I (6527C>A), T2135I (6588C>T), Y2151H (6635T>C), I2155V (6647A>G), I2165V (6677A>G 6679A>G), D2178N (6716G>A), N2179D (6719A>G), I2194L (6764A>T 6766A>G), A2204V (6795C>T 6796A>G), H2205Q (6799C>A), N2225D (6857A>G), V2231T (6875G>A 6876T>C 6877C>A), D2250E (6934C>A), V2273I (7001G>A 7003G>A), F2283Y (7032T>A), T2314K (7125C>A), A2321S (7145G>T 7147A>G), E2326_A2327del (7160_7165delGAGGCA), D2344E (7216T>A), D2358E (7258T>A), D2363_R2364insX (7273_7274insT), R2364V (7274C>G 7275G>T), H2366L (7281A>T), L2367S (7284T>C), S2371L (7296C>T), S2376F (7311C>T), T2377I (7314C>T), C2378Y (7317G>A), N2379K (7321T>A), K2384Q (7334A>C), A2387N (7343G>A 7344C>A 7345A>C), S2408M (7407G>T 7408T>G), C2411G (7415T>G), L2440S (7503T>C 7504G>A), D2509N (7709G>A), K2510R (7713A>G 7714A>G), T2543I (7812C>T), V2554L (7844G>T 7846G>A), V2567I (7883G>A), S2620N (8043G>A), N2656S (8151A>G 8152C>T), R2665K (8178G>A), R2675K (8208G>A), P2704A (8294C>G 8296A>G), S2722F (8349C>T), A2742S (8408G>T), M2756I (8452G>A), R2759K (8460G>A), A2766T (8480G>A 8482T>A), R2773K (8502G>A 8503A>G), E2777D (8515A>T), E2785D (8539G>C), L2788C (8546C>T 8547T>G), S2790T (8552T>A), A2797G (8574C>G), I2801V (8585A>G), G2805E (8598G>A), N2807S (8603A>T 8604A>C 8605C>T), P2813Q (8622C>A), E2814G (8625A>G 8626G>A), P2822F (8648C>T 8649C>T 8650G>C), G2825E (8658G>A), D2827A (8664A>C 8665T>G), A2832V (8679C>T 8680A>T), T2854K (8745C>A 8746A>G), E2891G (8856A>G 8857G>A), R2894Q (8865G>A), M2895L (8867A>C 8869G>T), G2900E (8883G>A), T2902S (8888A>T), V2924I (8954G>A), N2934D (8984A>G 8986T>C), E2935V (8988A>T), I2983V (9131A>G), M2989G (9149A>G 9150T>G 9151G>A), G2990S (9152G>A), P3026S (9260C>T 9262T>C), Q3038H (9298A>C)								

	Begin	End	Coverage	Score	Concordance	Matches	Identities	I/D/M/F*	Stop Codons
**NT**	**52**	**9704**	**99.5%**	**12530**	**65.2%**	**9642 (99.8%)**	**7985 (82.7%)**	**7/11**	

Codon mutations:

ACT3ACC (193T>C), TAC4CAA (194T>C 196C>A), TCA6TCG (202A>G), ATC8ATT (208C>T), TGT9CTG (209T>C 210G>T 211T>G), TCG12TCA (220G>A), TTT13ATG (221T>A 223T>G), GAA14GAG (226A>G), CTA17CTT (235A>T), TAC19TAT (241C>T), CCA21CCT (247A>T), TCT23CCT (251T>C), TGC24TTT (255G>T 256C>T), GAG25GGG (258A>G), CAT26CAC (262T>C), ATT27GTG (263A>G 265T>G), GTG28GCG (267T>C), GAA30GTA (273A>T), CGA31CGT (277A>T), GAA32GAG (280A>G), GTG33GTT (283G>T), CCG34CCA (286G>A), GCT35ACC (287G>A 289T>C), GTT37ACT (293G>A 294T>C), CCT39CCC (301T>C), TTC40TTT (304C>T), GCA41ATA (305G>A 306C>T), GAT42GGT (309A>G), CTG43TTG (311C>T), GAA44GAT (316A>T), ACA45GTG (317A>G 318C>T 319A>G), CAA46CAG (322A>G), GCA49GCT (331A>T), CTC52CAA (339T>A 340C>A), AAG53AGG (342A>G), AAA55GCG (347A>G 348A>C 349A>G), TAT56CAT (350T>C), GCT57GCA (355T>A), ACT58GTT (356A>G 357C>T), CGT60CGC (364T>C), GTG61ACA (365G>A 366T>C 367G>A), CTC62TCC (368C>T 369T>C), AAA63AAG (373A>G), AAC64AAT (376C>T), TTT67TGT (384T>G), ACG68ACA (388G>A), TAC69TAT (391C>T), CGA70CGG (394A>G), AAG72AAA (400G>A), GAT74GAC (406T>C), GCC75GTT (408C>T 409C>T), CAG76CAA (412G>A), ATA77ATT (415A>T), ATG78GAG (416A>G 417T>A), ATT80ATC (424T>C), CAG81CAA (427G>A), AAA83AAG (433A>G), CTG84CTC (436G>C), GAG85AAG (437G>A), AGG86GAG (440A>G 441G>A), AAG87CGA (443A>C 444A>G 445G>A), GAT88GAA (448T>A), GAA90GAG (454A>G), GAA91GCA (456A>C), TAT92TAC (460T>C), CAC93AAC (461C>A), CAA95CAG (469A>G), GCC97GCG (475C>G), CCT99CCA (481T>A), ACT106ACC (502T>C), ATA107ATT (505A>T), GGC109GGT (511C>T), GAT111GAA (517T>A), CCT112CCA (520T>A), CCA113CCT (523A>T), TCT116TTT (531C>T), GAG117GAA (535G>A), CCA118TCA (536C>T), CAA119TGG (539C>T 540A>G 541A>G), AGA122AAG (549G>A 550A>G), ATC124ATT (556C>T), ATT125ATC (559T>C), CAT126CAC (562T>C), ACT128ACC (568T>C), CCA129CCG (571A>G), ATG131ACG (576T>C), CGT132CGC (580T>C), GTC134GCG (585T>C 586C>G), CGC137TAT (593C>T 594G>A 595C>T), CCC138CGC (597C>G), ATA139GTG (599A>G 601A>G), ATA140CCG (602A>C 603T>C 604A>G), ACA143ACG (613A>G), GAA144GAG (616A>G), GGC145GGG (619C>G), CAG146CAA (622G>A), ATG147ATA (625G>A), AAT148AAC (628T>C), CAC149CAT (631C>T), ATT151GTC (635A>G 637T>C), ATA154GTG (644A>G 646A>G), ATT157ATC (655T>C), TCG159TCA (661G>A), GAG160GCC (663A>C 664G>C), AAA161AAG (667A>G), GTC165GTT (679C>T), CAC166CAA (682C>A), TTA167CTG (683T>C 685A>G), AGT169AGC (691T>C), ACC172AGC (699C>G), ACT173ACA (703T>A), CAT174CAA (706T>A), AAG179GAA (719A>G 721G>A), ATA180GTG (722A>G 724A>G), CTT181TTG (725C>T 727T>G), GGT182GGA (730T>A), GCA183TCA (731G>T), TAC184CCT (734T>C 735A>C 736C>T), TCC185CGT (737T>C 738C>G 739C>T), GCG187GTC (744C>T 745G>C), CGA189TGC (749C>T 751A>C), ACT190ACC (754T>C), ATG194AAG (765T>A), TTG196CTG (770T>C), CGA197AAG (773C>A 774G>A 775A>G), CGG198AGG (776C>A), TTC202TTT (790C>T), ATG206AAG (801T>A), GGA210GCG (813G>C 814A>G), CTT211CAT (816T>A), TTG212CTG (818T>C), CAA213CAG (823A>G), CGT214CAA (825G>A 826T>A), CGG217AGA (833C>A 835G>A), GAC219GAT (841C>T), AAT223GAC (851A>G 853T>C), GTC225GTT (859C>T), CGC226TGT (860C>T 862C>T), ACT227GCC (863A>G 865T>C), ATC228ACC (867T>C), AAC229GAT (869A>G 871C>T), ATA230TTG (872A>T 874A>G), CGA231CAA (876G>A), AGG232AAG (879G>A), GAT234GAC (886T>C), ATC238ATT (898C>T), AAC240AGC (903A>G), ACA241AAC (906C>A 907A>C), AAA242ACC (909A>C 910A>C), AGC243AAT (912G>A 913C>T), GGC246GGA (922C>A), GGT249GGA (931T>A), TCA252CTT (938T>C 939C>T 940A>T), GGA253GGG (943A>G), TTC256TTT (952C>T), TCA261TCG (967A>G), GAA263GAG (973A>G), GGG264GGC (976G>C), TTG266ATC (980T>A 982G>C), GCA269GCG (991A>G), CGT270CGC (994T>C), AGA272AAA (999G>A), GTT273GTC (1003T>C), CAG275AGA (1007C>A 1008A>G 1009G>A), AGT276GGG (1010A>G 1012T>G), ATT277GTT (1013A>G), TTA278TTG (1018A>G), AAC279CAG (1019A>C 1021C>G), ATC282ATT (1030C>T), TCG285TCA (1039G>A), AAT286AAC (1042T>C), GCC287GCA (1045C>A), GAC288GAA (1048C>A), AAT289AAC (1051T>C), AAG292AAA (1060G>A), GGT293GGA (1063T>A), CTG294TTG (1064C>T), GAC295GAT (1069C>T), CGA300CAA (1083G>A), TAT303TAC (1093T>C), TCG305GCA (1097T>G 1099G>A), CAC307CAT (1105C>T), GTA310GTG (1114A>G), GCT311GCG (1117T>G), TTA313TTG (1123A>G), CCT314CCA (1126T>A), GTC315GTT (1129C>T), AGG320AAG (1143G>A), GTA321GTT (1147A>T), GCT322GCA (1150T>A), TTG324ATA (1154T>A 1156G>A), CAC327CAT (1165C>T), AGT328AGC (1168T>C), CTT330TTA (1172C>T 1174T>A), ACC339ACT (1201C>T), AGC346AAC (1221G>A), GTT349ACC (1229G>A 1230T>C 1231T>C), AGC350AAT (1233G>A 1234C>T), CTG352CTA (1240G>A), AAG354AAA (1246G>A), TTG356CTA (1250T>C 1252G>A), CAT357CAC (1255T>C), AAA358ACG (1257A>C 1258A>G), AGA361AGG (1267A>G), GGT363GGC (1273T>C), TTG364TTA (1276G>A), AAT365AGT (1278A>G), TTG367CTG (1283T>C), GCG369GTA (1290C>T 1291G>A), GAC372ACT (1298G>A 1299A>C 1300C>T), TTT374TTC (1306T>C), ATA375GTG (1307A>G 1309A>G), CAT376CAC (1312T>C), AAT378GAA (1316A>G 1318T>A), TTG381TTA (1327G>A), ATA382ACA (1329T>C), GCG383GCT (1333G>T), TTA384TTG (1336A>G), CTA387TTG (1343C>T 1345A>G), CCG390CCA (1354G>A), GTG391GTT (1357G>T), GAC392GAT (1360C>T), CTC395TTG (1367C>T 1369C>G), CTT397ATT (1373C>A), TTC398TTT (1378C>T), GAG400GAA (1384G>A), ATA401GTA (1385A>G), AAA403AAG (1393A>G), TCC404TCT (1396C>T), GAG407GAT (1405G>T), AAA408AAG (1408A>G), CAG409CAA (1411G>A), GCA411TCT (1415G>T 1417A>T), CCG412CCC (1420G>C), AAT415AAC (1429T>C), TTA416TTG (1432A>G), GTC418ATT (1436G>A 1438C>T), TTA419CTG (1439T>C 1441A>G), AAT420AAC (1444T>C), TTC423TTT (1453C>T), CTG424CTA (1456G>A), GAA428GAG (1468A>G), GTA436GTG (1492A>G), TTG439CTA (1499T>C 1501G>A), AGT440AGC (1504T>C), TTG441TTA (1507G>A), CTC442CTT (1510C>T), GAA443GAG (1513A>G), TTA444TTG (1516A>G), GCA445GCT (1519A>T), AGG446AGA (1522G>A), TTC447TTT (1525C>T), CAG448CAA (1528G>A), AAC450AAT (1534C>T), ACT452ACA (1540T>A), AAC454AAT (1546C>T), AAA457AAG (1555A>G), GGT458GGC (1558T>C), GAT459GAC (1561T>C), ATA460ATC (1564A>C), TCT461TCA (1567T>A), TTC463TTT (1573C>T), AGA464AGG (1576A>G), TTA467CTA (1583T>C), TCT468TCA (1588T>A), AAG470AAA (1594G>A), AAC472AAT (1600C>T), CTG475CTA (1609G>A), TCG478TCA (1618G>A), TGC479TGT (1621C>T), GAC480GAT (1624C>T), CTG483TTG (1631C>T), AAA485AAG (1639A>G), TTC489TTT (1651C>T), CTC490CTG (1654C>G), CAT497CAC (1675T>C), TCA503TCG (1693A>G), TTT506TTC (1702T>C), GAA508GGA (1707A>G), GCA512ACA (1718G>A), GGA514GGG (1726A>G), TAC515TAT (1729C>T), TCA516TCC (1732A>C), TAT518TAC (1738T>C), ATC520GTT (1742A>G 1744C>T), CGC521CGT (1747C>T), CAT523CAC (1753T>C), AGT525AAT (1758G>A), AGG528AGA (1768G>A), AAC534AAT (1786C>T), TTA535TTG (1789A>G), GTT536ATT (1790G>A), GTC537GTT (1795C>T), TTA541CTG (1805T>C 1807A>G), GAG543GAA (1813G>A), AGG545AGA (1819G>A), AAG547AAA (1825G>A), GAC551GAT (1837C>T), TAT552TAC (1840T>C), CAA555CAG (1849A>G), GTC558GTG (1858C>G), AAA560AAG (1864A>G), TGC562TGT (1870C>T), AGT564AGC (1876T>C), AAA566AAG (1882A>G), GGT568GGA (1888T>A), AAT569AAC (1891T>C), TGT574TGC (1906T>C), ACA577ACT (1915A>T), GGT582GGC (1930T>C), GCC584GCT (1936C>T), ATT585GTC (1937A>G 1939T>C), TCA587TCG (1945A>G), TTC589TTT (1951C>T), CCA592CCT (1960A>T), GTA598GTG (1978A>G), ATT599ATA (1981T>A), GGC600GGT (1984C>T), AAT601AAC (1987T>C), GAC604GAT (1996C>T), TTT607TAT (2004T>A), AAA612AAG (2020A>G), GGG613GGA (2023G>A), GAT614GAC (2026T>C), TCG615TCT (2029G>T), GAG616GAA (2032G>A), TAT625TAC (2059T>C), ATT628ATG (2068T>G), AAC629AAT (2071C>T), GTG630ATA (2072G>A 2074G>A), CTG635TTA (2087C>T 2089G>A), AGC639AGT (2101C>T), GAG640GAA (2104G>A), GAG641GAA (2107G>A), AAG644AAA (2116G>A), TTC646TTT (2122C>T), CGC651CGT (2137C>T), CTT658CTC (2158T>C), ACC660ACT (2164C>T), CCA662CCG (2170A>G), GAT666GAC (2182T>C), GCG668GCA (2188G>A), ACC669ACA (2191C>A), AGA675AAA (2208G>A), TAT678TAC (2218T>C), GAC680GAT (2224C>T), GTA681GTT (2227A>T), TTG686TTA (2242G>A), CCC687CCT (2245C>T), TTG690TTA (2254G>A), GTT691GTC (2257T>C), GAC692GAT (2260C>T), GAC694GAG (2266C>G), CAA696CAG (2272A>G), ACG697ACA (2275G>A), TCA703TCG (2293A>G), GGC705GGT (2299C>T), TCG706TCA (2302G>A), CAG707CAA (2305G>A), GGA710GGT (2314A>T), TAT711TAC (2317T>C), CTA714CTG (2326A>G), GTG719GTA (2341G>A), CAA721CAG (2347A>G), CTT722CTC (2350T>C), TTG724TTA (2356G>A), AAT727AAC (2365T>C), GAA729GAG (2371A>G), TTA730TTG (2374A>G), GAT733GAC (2383T>C), ATA734ATT (2386A>T), AAA735AAG (2389A>G), CAT736CAC (2392T>C), TAT737TAC (2395T>C), AAT744GGG (2414A>G 2415A>G 2416T>G), GCT745GCA (2419T>A), AGC746TGC (2420A>T), ACA752ACT (2440A>T), GTT761GTC (2467T>C), TCT764TCC (2476T>C), TCG766TCA (2482G>A), CTG769CTA (2491G>A), CTG771CTT (2497G>T), TTG773TTA (2503G>A), AGA778AGG (2518A>G), CCT779CCC (2521T>C), AAG780AGG (2523A>G), AGA783AGG (2533A>G), TTG785TTA (2539G>A), TTA787TTG (2545A>G), GAG789GAA (2551G>A), CCT790CCA (2554T>A), CTG792TTG (2558C>T), TTG793CTC (2561T>C 2563G>C), TCA796TCG (2572A>G), CCT800CCC (2584T>C), GGC801GGT (2587C>T), CTG803CTC (2593G>C), GCT805GCC (2599T>C), AAT808AAC (2608T>C), TTT812TTC (2620T>C), GCG815GCA (2629G>A), GTG816GTA (2632G>A), AGG817AAG (2634G>A), TTG818TTA (2638G>A), ATT820ATA (2644T>A), AAA823AAG (2653A>G), GCT827GCC (2665T>C), CTA832TTA (2678C>T), CTA833CTG (2683A>G), GCT835GCC (2689T>C), CTA838CTC (2698A>C), TCA841TCG (2707A>G), GCG842GCA (2710G>A), GAA844GAG (2716A>G), ACA845ACG (2719A>G), CTC846CTT (2722C>T), GTC847ATT (2723G>A 2725C>T), GCA848GCG (2728A>G), AGG850AGA (2734G>A), ATT851ATA (2737T>A), GAT854GAC (2746T>C), GCT855ACA (2747G>A 2749T>A), GCA856GCT (2752A>T), GCT857GCA (2755T>A), GAC859GAT (2761C>T), CTC860CTT (2764C>T), TGT865TGC (2779T>C), GGG867GGT (2785G>T), AAC869AAT (2791C>T), CAT871AAT (2795C>A), TAC874TAT (2806C>T), TTG878CTC (2816T>C 2818G>C), GTG880ATA (2822G>A 2824G>A), TTG881TTA (2827G>A), GTT884GTC (2836T>C), AAG885AAA (2839G>A), AAT886AAC (2842T>C), ACC893ACA (2863C>A), CTA894TTA (2864C>T), CCA900ACA (2882C>A), AGT901CAT (2885A>C 2886G>A), AAC903AAT (2893C>T), ACG904ATG (2895C>T), GTC906GTT (2902C>T), GTA907GTG (2905A>G), ATT909ATC (2911T>C), AAT913AAC (2923T>C), AAT916AGT (2931A>G), AAC919AGC (2940A>G), GCT921GCC (2947T>C), ACT926ACC (2962T>C), GAA929_AAT930insA-- (2971_2972insA), GGT935GGC (2989T>C), ACT936ATT (2991C>T), CAT937CGT (2994A>G), GAG939AGG (2999G>A 3000A>G), TCC944TTC (3015C>T), CGG946CAG (3021G>A), TAC947TTT (3024A>T 3025C>T), ACA951ATA (3036C>T), GAA952GGC (3039A>G 3040A>C), AAG953ACA (3042A>C 3043G>A), TTG956TTA (3052G>A), TTA959TTG (3061A>G), GCG966GCA (3082G>A), GGC969GGT (3091C>T), GCC972GTA (3099C>T 3100C>A), GTG974AGA (3104G>A 3105T>G 3106G>A), GTC975GTT (3109C>T), AAA976AAG (3112A>G), ACT978ACC (3118T>C), GCC979GCT (3121C>T), GGA981GGG (3127A>G), AGC983AAC (3132G>A), CGA985AAG (3137C>A 3138G>A 3139A>G), TAT989TAC (3151T>C), TTC990ATT (3152T>A 3154C>T), TGT994TAT (3165G>A), GTA995GTG (3169A>G), ATC1005ATT (3199C>T), AGG1007CGG (3203A>C), CGT1008CGC (3208T>C), CCA1010CCG (3214A>G), ACC1011ACT (3217C>T), TTT1012CTT (3218T>C), GTC1013GTA (3223C>A), ACT1014AAT (3225C>A), GTT1016ATT (3230G>A), AAC1017AAT (3235C>T), TTA1025CTA (3257T>C), ACC1026ACT (3262C>T), AGC1027AGT (3265C>T), GTA1028ATG (3266G>A 3268A>G), GTG1029GTA (3271G>A), GTG1031ATG (3275G>A), GCA1034ACA (3284G>A), CAG1039CAA (3301G>A), AGA1044AAG (3315G>A 3316A>G), GAA1045GCA (3318A>C), ATC1046ATT (3322C>T), GAG1047GAA (3325G>A), TTG1048TTA (3328G>A), ATA1051ATT (3337A>T), GAG1055GAA (3349G>A), GTC1057GTT (3355C>T), TGC1058TGT (3358C>T), ATG1059GTG (3359A>G), CTA1061CTG (3367A>G), TAT1062TAC (3370T>C), TTA1065CTG (3377T>C 3379A>G), GAA1070GAG (3394A>G), CGC1071CGT (3397C>T), GAT1072GAG (3400T>G), ACA1074ACG (3406A>G), TAC1078TAT (3418C>T), ATT1079ATA (3421T>A), TAT1081TAC (3427T>C), AAG1083AAA (3433G>A), TCA1084TCT (3436A>T), GTA1085GTG (3439A>G), AAC1086AAT (3442C>T), CCT1087CCC (3445T>C), CAG1091CAA (3457G>A), TTT1092TTC (3460T>C), GCT1093GCA (3463T>A), GCG1095GCT (3469G>T), CAG1096CAA (3472G>A), CAC1104CAT (3496C>T), CAG1105CAA (3499G>A), TCC1107TCT (3505C>T), GTT1110GGT (3513T>G), AAA1112AAG (3520A>G), GAA1115GAG (3529A>G), CAA1116CAG (3532A>G), ATG1121GTG (3545A>G), GCT1122ACT (3548G>A), TTA1123CTA (3551T>C), GTC1124ATT (3554G>A 3556C>T), AGT1133AGC (3583T>C), GAT1134GAC (3586T>C), TGC1135TGT (3589C>T), TTC1137TTT (3595C>T), AAT1141AAC (3607T>C), TTT1143TTC (3613T>C), AAG1144AAA (3616G>A), GGT1145GGC (3619T>C), CTT1147GTT (3623C>G), TCC1148TCT (3628C>T), CTG1150ATG (3632C>A), GAT1151GAC (3637T>C), TAT1152CAT (3638T>C), GAA1153GAG (3643A>G), TTA1159TTG (3661A>G), GTG1162GTA (3670G>A), TTT1166TTC (3682T>C), GAG1168GAA (3688G>A), GAG1171GAA (3697G>A), ATT1172GTT (3698A>G), GAT1174GAC (3706T>C), TTT1175TTC (3709T>C), TTG1177CTA (3713T>C 3715G>A), AGT1178AAT (3717G>A), GAG1179GAA (3721G>A), CGA1183AAA (3731C>A 3732G>A), ACT1184ACA (3736T>A), CTA1188TTG (3746C>T 3748A>G), ACA1190ACG (3754A>G), AGT1193AGC (3763T>C), CGA1198CGG (3778A>G), CAA1199CAG (3781A>G), ATC1200ATT (3784C>T), CAT1204CAC (3796T>C), CCA1207CCT (3805A>T), TAC1209TAT (3811C>T), AGA1210AGG (3814A>G), TTC1215TTT (3829C>T), TTT1218TTC (3838T>C), AGA1220AGG (3844A>G), GCA1221GCC (3847A>C), GTT1224GTA (3856T>A), GTG1226GTT (3862G>T), GAC1229GAT (3871C>T), GCC1231GCA (3877C>A), AGC1233AGT (3883C>T), GAA1234GAG (3886A>G), CTA1236CTG (3892A>G), GAC1237GAT (3895C>T), TTT1238TTC (3898T>C), GTA1240GTG (3904A>G), CGG1241AGA (3905C>A 3907G>A), GCT1243GCC (3913T>C), AAG1248AAA (3928G>A), TCA1249TCT (3931A>T), GGG1251GGA (3937G>A), AGT1257AGC (3955T>C), GTG1258GTA (3958G>A), GCC1259GCT (3961C>T), GGA1260GGG (3964A>G), TCT1261TCC (3967T>C), TTA1264TTG (3976A>G), ATT1265ATC (3979T>C), CCA1267CCT (3985A>T), CTA1271CTT (3997A>T), GCG1272GCA (4000G>A), AAC1274AAT (4006C>T), GTT1275GTG (4009T>G), TTC1276TTT (4012C>T), AAA1277AAG (4015A>G), TCT1280TCC (4024T>C), GAA1282GAG (4030A>G), CCA1283CCT (4033A>T), AAG1286AAA (4042G>A), CTG1290TTA (4052C>T 4054G>A), CGT1291CGC (4057T>C), CGT1293CGA (4063T>A), GGA1294GGG (4066A>G), ATA1297GTA (4073A>G), TTT1298TTC (4078T>C), GGC1299GGT (4081C>T), TCT1300TCC (4084T>C), TCC1304TCT (4096C>T), AGC1308AGT (4108C>T), GGA1309GGT (4111A>T), GCG1311GCA (4117G>A), CTA1312TTG (4118C>T 4120A>G), TAC1314TAT (4126C>T), TTC1315TAT (4128T>A 4129C>T), GCC1316GCT (4132C>T), AAT1317AAC (4135T>C), CAA1321CAG (4147A>G), TTA1322CTA (4148T>C), GCT1323TCT (4151G>T), AAC1326AAT (4162C>T), GTA1328ATA (4166G>A), ATA1329ATC (4171A>C), GAG1332GAA (4180G>A), CTG1336TTG (4190C>T), CCT1338CCC (4198T>C), TCC1339TCT (4201C>T), GCG1342GCT (4210G>T), TTC1343TTT (4213C>T), AGT1345AGC (4219T>C), CTG1346TTG (4220C>T), CTG1347ATA (4223C>A 4225G>A), GTT1349GTG (4231T>G), CAT1351CAC (4237T>C), GTA1356GTG (4252A>G), TTA1357CTG (4253T>C 4255A>G), AAA1358AAG (4258A>G), ACT1362ACC (4270T>C), AGA1366AGG (4282A>G), GAG1367GAA (4285G>A), GAA1369GAG (4291A>G), CAG1373CAA (4303G>A), GTC1376GTT (4312C>T), ATA1379GTG (4319A>G 4321A>G), GTG1380GTT (4324G>T), GAG1381GAA (4327G>A), CTG1384CTT (4336G>T), TCT1385TCC (4339T>C), TCA1388TCT (4348A>T), GCA1392GCG (4360A>G), GGT1394GGC (4366T>C), TCT1395TCA (4369T>A), ACT1397ACC (4375T>C), GTT1401GTC (4387T>C), TTT1404TAT (4395T>A), GTA1408ATA (4406G>A), CTT1409CTC (4411T>C), GTT1418GTC (4438T>C), ACC1420ACA (4444C>A), GCC1422GCT (4450C>T), CTC1424CTT (4456C>T), GAC1427GAT (4465C>T), ATG1431GTT (4475A>G 4477G>T), GTC1432GTT (4480C>T), ACA1433TCG (4481A>T 4483A>G), GGC1437GGT (4495C>T), AAG1441AAA (4507G>A), CAC1442CAT (4510C>T), GGT1443GGG (4513T>G), CTA1445TTA (4517C>T), GTC1448GTT (4528C>T), AAA1450AAG (4534A>G), ACC1452ACT (4540C>T), GCG1454TTA (4544G>T 4545C>T 4546G>A), AGA1455AAA (4548G>A), TTT1458TTC (4558T>C), GTA1460GTT (4564A>T), ACC1462ACT (4570C>T), ATA1464ATT (4576A>T), TTG1471TTA (4597G>A), GAC1472GAT (4600C>T), GAC1474GAT (4606C>T), GTG1475GTA (4609G>A), GTG1477GTA (4615G>A), GAC1478GAT (4618C>T), TTG1481CTT (4625T>C 4627G>T), GAC1488GAT (4648C>T), ATT1489ATC (4651T>C), AGG1492AGA (4660G>A), AGC1493AGT (4663C>T), GCT1495GCA (4669T>A), GTG1499ATC (4679G>A 4681G>C), AGT1500AGC (4684T>C), GTT1501GTG (4687T>G), GAG1505GAA (4699G>A), AGA1506AGG (4702A>G), CAA1508CAG (4708A>G), AGG1509AGA (4711G>A), CTG1510TTG (4712C>T), AAA1518AAG (4738A>G), GTA1520GTT (4744A>T), GCA1521GCG (4747A>G), TTG1522CTG (4748T>C), ACT1527ACC (4765T>C), GTC1538ATT (4796G>A 4798C>T), ACT1540AGC (4803C>G 4804T>C), GAG1541GAA (4807G>A), GCG1542GCT (4810G>T), GCT1543GCG (4813T>G), CTT1544CTC (4816T>C), TAT1549TAC

	(4831T>C), AAC1550AAT (4834C>T), CCA1552CCT (4840A>T), GGA1556GGG (4852A>G), GGC1557GGT (4855C>T), AGT1561AGC (4867T>C), CTG1562CTC (4870G>C), ATT1563ATA (4873T>A), TGT1566TGC (4882T>C), ACA1573ACT (4903A>T), CAG1575CAA (4909G>A), TTT1577TTC (4915T>C), GAA1578GAG (4918A>G), TTG1579CTA (4919T>C 4921G>A), AGT1580AGC (4924T>C), CCC1581CCG (4927C>G), TTT1583TTC (4933T>C), ATC1584ATA (4936C>A), CAG1585CAA (4939G>A), AAT1586AAC (4942T>C), TTC1587TTT (4945C>T), TCA1593TCG (4963A>G), CCT1596CCC (4972T>C), CTT1602CTC (4990T>C), AAA1606AAG (5002A>G), CCT1613CCC (5023T>C), TTG1614CTG (5024T>C), TGC1615TGT (5029C>T), GAT1616GAC (5032T>C), TCT1618TCC (5038T>C), CCA1620CCT (5044A>T), GCA1623GCT (5053A>T), TCG1624TCA (5056G>A), AGT1625GGT (5057A>G), ACT1626GCT (5060A>G), TCG1629TCT (5071G>T), CTT1636ATT (5090C>A), GGA1637GGG (5095A>G), GTG1638GTT (5098G>T), GCC1639GTC (5100C>T), TTA1640TTG (5104A>G), GAA1641GAT (5107A>T), ATT1642ATC (5110T>C), CCA1643CCG (5113A>G), AAG1644AAA (5116G>A), CAA1645CAG (5119A>G), GTC1646TTT (5120G>T 5122C>T), ATT1648GTT (5126A>G), CAT1651CAC (5137T>C), ATC1652ATT (5140C>T), AAA1653AAG (5143A>G), GAG1654GAA (5146G>A), CTC1659CTA (5161C>A), GAA1661GAG (5167A>G), CTT1663CTG (5173T>G), ACG1666ACA (5182G>A), GTT1667ATT (5183G>A), GTC1668GTT (5188C>T), AAG1669AAA (5191G>A), AAA1671AAG (5197A>G), GAC1672GAT (5200C>T), TGC1674TGT (5206C>T), TTA1675TTG (5209A>G), AGC1678AGT (5218C>T), TCG1682TCA (5230G>A), ATC1684ATT (5236C>T), AGC1685AGT (5239C>T), ATC1687ATT (5245C>T), ACA1693ACT (5263A>T), CTC1695CTT (5269C>T), TTC1696TTT (5272C>T), GCC1697GCA (5275C>A), ATC1698ATT (5278C>T), CCA1699CCC (5281A>C), AGA1700AGG (5284A>G), ACT1701ACC (5287T>C), CTA1702TTA (5288C>T), ATA1703ATT (5293A>T), TTG1704CTA (5294T>C 5296G>A), GAG1706GAA (5302G>A), CTT1709CTA (5311T>A), GAA1710GAG (5314A>G), CGA1713CGT (5323A>T), AGT1721AGC (5347T>C), ATC1723ATT (5353C>T), TGC1727TGT (5365C>T), GTT1734GTC (5386T>C), ACC1737ACA (5395C>A), AAA1746AAG (5422A>G), GAT1747GAC (5425T>C), ACC1749ACT (5431C>T), GAG1751GAA (5437G>A), CTT1756CTC (5452T>C), AAG1758AAA (5458G>A), AGT1761AGC (5467T>C), CAA1762CAG (5470A>G), CTA1763TTG (5471C>T 5473A>G), GAA1765GAG (5479A>G), TTC1766TTT (5482C>T), TCA1767TCC (5485A>C), AAT1768AAC (5488T>C), TTG1769CTA (5489T>C 5491G>A), GGT1771GGC (5497T>C), TGT1774TGC (5506T>C), GAG1775GAA (5509G>A), GAG1776GAA (5512G>A), TTA1778CTA (5516T>C), TAT1782TAC (5530T>C), GAG1783GAA (5533G>A), TCG1784TCT (5536G>T), TTG1785TTA (5539G>A), GTT1788GTG (5548T>G), CAC1790CAT (5554C>T), GCT1792GCA (5560T>A), GCG1793ACA (5561G>A 5563G>A), ACG1794ACC (5566G>C), TCA1795TCG (5569A>G), CTC1800CTG (5584C>G), AAG1803AAA (5593G>A), GGG1804GGA (5596G>A), ATT1805GTT (5597A>G), AAC1807AAA (5605C>A), GTG1811GTT (5617G>T), GCT1812GTG (5619C>T 5620T>G), TTG1815TTA (5629G>A), ATC1816CTC (5630A>C), GGC1819GGT (5641C>T), GCA1822GCT (5650A>T), ATA1826ATT (5662A>T), GGA1827GGG (5665A>G), CAA1835CAG (5689A>G), GTT1837ATT (5693G>A), GAG1838GAA (5698G>A), CAA1843CAG (5713A>G), GGG1844GGC (5716G>C), AAA1845AAG (5719A>G), GCC1853GCA (5743C>A), CGC1857CGA (5755C>A), CAT1858CAC (5758T>C), CGT1860CGC (5764T>C), AAA1862AAG (5770A>G), GGC1865GGT (5779C>T), TTT1866TTC (5782T>C), GAC1869GAT (5791C>T), GAC1873GAT (5803C>T), AGG1884AGA (5836G>A), AAA1885AAG (5839A>G), GGA1887GGG (5845A>G), AAA1888AAG (5848A>G), AAA1890AGA (5853A>G), GGT1891GGC (5857T>C), ACC1892ACT (5860C>T), ACA1893ACT (5863A>T), GGT1895GGA (5869T>A), GGT1897GGC (5875T>C), AGC1900AGT (5884C>T), TTC1903TTT (5893C>T), ATC1904GTG (5894A>G 5896C>G), AAC1905AAT (5899C>T), GGG1908GGA (5908G>A), TTT1909TTC (5911T>C), GAG1913GAA (5923G>A), CAA1918CAG (5938A>G), TTC1919TTT (5941C>T), GTT1920GTC (5944T>C), GAT1921GAC (5947T>C), GGG1925GGA (5959G>A), CGG1926GCC (5960C>G 5961G>C 5962G>C), GAA1930GAG (5974A>G), GCT1934GCA (5986T>A), GAC1935GAT (5989C>T), ATT1936GTT (5990A>G), GAT1938GAC (5998T>C), AGA1942CGT (6008A>C 6010A>T), GAA1945GAT (6019A>T), GTG1946GTT (6022G>T), CGA1947CGT (6025A>T), AAA1949ACA (6030A>C), GAC1955GAA (6049C>A), ATG1958ATA (6058G>A), CAA1959CAG (6061A>G), GCC1960GCA (6064C>A), GGT1962GGC (6070T>C), AGT1963AAC (6072G>A 6073T>C), AAC1964CAC (6074A>C), ACG1965ACA (6079G>A), ACC1966AAC (6081C>A), ATA1967ATT (6085A>T), GCA1969GCT (6091A>T), TAC1970TAT (6094C>T), TGT1976TCC (6111G>C 6112T>C), AAA1978AAG (6118A>G), TTG1980TTA (6124G>A), AAG1981AAA (6127G>A), ATT1982GTC (6128A>G 6130T>C), GAT1983GAC (6133T>C), TTA1984TTG (6136A>G), AAC1988AAT (6148C>T), AAA1991AAG (6157A>G), GTT1992ATT (6158G>A), TGT1993TGC (6163T>C), AAA1995AAG (6169A>G), ACA1996TCG (6170A>T 6172A>G), GGC1998GGT (6178C>T), AAA2001AAG (6187A>G), TTT2002TTC (6190T>C), GAG2004GAA (6196G>A), CTC2007TTT (6203C>T 6205C>T), GAA2008GAG (6208A>G), CTA2009TTG (6209C>T 6211A>G), CAG2011CAA (6217G>A), GCT2015GCA (6229T>A), GTA2016ATA (6230G>A), GTC2018GTT (6238C>T), AAG2021AAA (6247G>A), ATA2023ATT (6253A>T), GCA2025AAA (6257G>A 6258C>A), GAG2027GAA (6265G>A), CAT2030CAC (6274T>C), AAA2033AGA (6282A>G), TCG2034TCA (6286G>A), GGC2038GGT (6298C>T), TTG2039TTA (6301G>A), AGA2040AGG (6304A>G), GAC2041GAT (6307C>T), GCC2046GCT (6322C>T), ACA2048ACT (6328A>T), GTT2049GTG (6331T>G), TGT2050TGC (6334T>C), AGG2051AGA (6337G>A), CTG2052TTG (6338C>T), TAT2058TAC (6358T>C), GGG2059GGA (6361G>A), GCA2060ACA (6362G>A), TCA2061TCT (6367A>T), GAG2062GAA (6370G>A), TAC2064TAT (6376C>T), GGT2065GGG (6379T>G), GGA2067GGC (6385A>C), GGA2069GGT (6391A>T), GCA2070GCG (6394A>G), TAC2071TAT (6397C>T), GTA2073ATA (6401G>A), GCG2074GTG (6405C>T), CAC2076CAT (6412C>T), TTA2078TTG (6418A>G), TTT2079TTC (6421T>C), TAC2082TTC (6429A>T), GGT2084GGA (6436T>A), GAG2087GAA (6445G>A), CAA2089CGA (6450A>G), TCC2090TCG (6454C>G), CAC2092CAT (6460C>T), GGT2093GGA (6463T>A), TTC2095TTT (6469C>T), AGG2096AGA (6472G>A), GTG2097GTA (6475G>A), AAT2099AAC (6481T>C), CAC2101AAT (6485C>A 6487C>T), AGT2102AGC (6490T>C), AGC2104AGT (6496C>T), CTG2106CTA (6502G>A), GGT2110GGC (6514T>C), AGG2111AGA (6517G>A), CTC2115ATC (6527C>A), ATC2116ATA (6532C>A), CCG2119CCA (6541G>A), AAA2120AAG (6544A>G), GTC2124GTT (6556C>T), TTT2125TTC (6559T>C), CAG2127CAA (6565G>A), TTG2129CTA (6569T>C 6571G>A), CCT2134CCA (6586T>A), ACA2135ATA (6588C>T), GAA2138GAG (6598A>G), AGA2139AGG (6601A>G), ATT2140ATC (6604T>C), TGT2141TGC (6607T>C), GGA2144GGG (6616A>G), AAC2146AAT (6622C>T), GAG2149GAA (6631G>A), AAG2150AAA (6634G>A), TAT2151CAT (6635T>C), GCT2152GCA (6640T>A), TCG2154TCT (6646G>T), ATC2155GTC (6647A>G), ATC2156ATT (6652C>T), AGC2160AGT (6664C>T), ACT2162ACG (6670T>G), ATA2165GTG (6677A>G 6679A>G), GGC2167GGT (6685C>T), ACA2169ACT (6691A>T), TTC2170TTT (6694C>T), ACA2177ACG (6715A>G), GAT2178AAT (6716G>A), AAT2179GAT (6719A>G), GGA2180GGG (6724A>G), CTA2184TTA (6734C>T), GTG2186GTA (6742G>A), GTG2187GTA (6745G>A), GCC2190GCT (6754C>T), GGA2192GGG (6760A>G), ATA2194TTG (6764A>T 6766A>G), ATT2197ATA (6775T>A), CTG2200TTG (6782C>T), GCA2201GCT (6787A>T), AAC2202AAT (6790C>T), GCA2204GTG (6795C>T 6796A>G), CAC2205CAA (6799C>A), ACC2206ACA (6802C>A), AAC2208AAT (6808C>T), TAC2209TAT (6811C>T), TAC2210TAT (6814C>T), TTC2213TTT (6823C>T), GAA2215GAG (6829A>G), TAC2221TAT (6847C>T), CGA2223CGC (6853A>C), AAT2225GAT (6857A>G), GAG2226GAA (6862G>A), GAA2229GAG (6871A>G), GTC2231ACA (6875G>A 6876T>C 6877C>A), AAG2232AAA (6880G>A), TCT2233TCA (6883T>A), GTT2235GTG (6889T>G), GAC2239GAT (6901C>T), GGC2244GGT (6916C>T), CCG2245CCA (6919G>A), AAA2247AAG (6925A>G), CTT2248CTC (6928T>C), GAC2250GAA (6934C>A), GGG2255GGC (6949G>C), TTC2257TTT (6955C>T), AAA2258AAG (6958A>G), CAA2264CAG (6976A>G), CTA2266TTA (6980C>T), ATC2267ATT (6985C>T), GTA2271GTT (6997A>T), GTG2272GTT (7000G>T), GTG2273ATA (7001G>A 7003G>A), GCT2276GCC (7012T>C), AAG2277AAA (7015G>A), CAT2278CAC (7018T>C), TTT2283TAT (7032T>A), GCC2285GCT (7039C>T), TTG2286CTA (7040T>C 7042G>A), GGA2288GGG (7048A>G), GCT2292GCA (7060T>A), GTC2293GTA (7063C>A), AGC2298AGT (7078C>T), TTA2300CTG (7082T>C 7084A>G), GTA2301GTG (7087A>G), ACC2302ACA (7090C>A), CAT2304CAC (7096T>C), GTA2305GTG (7099A>G), GTT2306GTC (7102T>C), GAG2309GAA (7111G>A), CGA2311CGG (7117A>G), ACA2314AAA (7125C>A), GAA2315GAG (7129A>G), TTT2316TTC (7132T>C), CTG2317TTA (7133C>T 7135G>A), GTG2319GTA (7141G>A), GAT2320GAC (7144T>C), GCA2321TCG (7145G>T 7147A>G), GAG2322GAA (7150G>A), GCA2325GCT (7159A>T), GAG2326_GCA2327del (7160_7165delGAGGCA), TTC2329TTT (7171C>T), GCG2335GCT (7189G>T), AAA2338AAG (7198A>G), AGC2339AGT (7201C>T), CTA2341TTA (7205C>T), AAT2342AAC (7210T>C), GAT2344GAA (7216T>A), GCG2345GCA (7219G>A), ATC2347ATA (7225C>A), AAG2348AAA (7228G>A), AAG2352AAA (7240G>A), TAT2353TAC (7243T>C), AAA2355AAG (7249A>G), CCT2356CCG (7252T>G), GAT2358GAA (7258T>A), GTT2359GTC (7261T>C), GGT2360GGA (7264T>A), GTC2361GTG (7267C>G), GTG2362GTA (7270G>A), GAT2363_CGG2364insT-- (7273_7274insT), CGG2364GTG (7274C>G 7275G>T), CAT2366CTT (7281A>T), TTG2367TCG (7284T>C), TCA2371TTA (7296C>T), TCT2376TTT (7311C>T), ACC2377ATC (7314C>T), TGC2378TAC (7317G>A), AAT2379AAA (7321T>A), GTG2380-TG (7322delG), CAC2381CAT (7327C>T), GGC2382GGT (7330C>T), TTC2383TTT (7333C>T), AAG2384CAG (7334A>C), TGT2386TGC (7342T>C), GCA2387AAC (7343G>A 7344C>A 7345A>C), ACT2390ACC (7354T>C), CAA2393CAG (7363A>G), ATT2395ATC (7369T>C), TTC2396TTT (7372C>T), GCG2398GCT (7378G>T), CTC2399CTT (7381C>T), GCA2404GCT (7396A>T), GTC2405GTT (7399C>T), GCC2407GCT (7405C>T), AGT2408ATG (7407G>T 7408T>G), GGG2410GGT (7414G>T), TGC2411GGC (7415T>G), AAA2412AAG (7420A>G), GAC2415GAT (7429C>T), TAT2416TAC (7432T>C), CAT2419CAC (7441T>C), AAG2425AAA (7459G>A), GAA2426GAG (7462A>G), ATA2428ATT (7468A>T), GTC2429GTA (7471C>A), CAA2431CAG (7477A>G), AGC2432AGT (7480C>T), CTG2434CTT (7486G>T), TAT2437TAC (7495T>C), AAA2438AAG (7498A>G), GGT2439GGC (7501T>C), TTG2440TCA (7503T>C 7504G>A), ATT2443ATA (7513T>A), AAC2445AAT (7519C>T), GGA2446GGG (7522A>G), AAG2449AAA (7531G>A), GAG2451GAA (7537G>A), CTC2452CTT (7540C>T), TGT2454TGC (7546T>C), AAG2462AAA (7570G>A), ACG2463ACA (7573G>A), ACG2465ACA (7579G>A), GCT2468GCC (7588T>C), CTA2471TTA (7595C>T), TTG2474CTA (7604T>C 7606G>A), GGT2477GGA (7615T>A), TGT2480TGC (7624T>C), GAC2483GAT (7633C>T), TTC2484TTT (7636C>T), AAT2485AAC (7639T>C), TTT2488TTC (7648T>C), GAA2494GAG (7666A>G), TGT2496TGC (7672T>C), ACA2498ACT (7678A>T), GGG2500GGA (7684G>A), ACT2502ACC (7690T>C), TTT2504TTC (7696T>C), GGT2506GGG (7702T>G), GAT2509AAT (7709G>A), AAA2510AGG (7713A>G 7714A>G), CTG2511TTG (7715C>T), CGT2514CGC (7726T>C), GAG2517GAA (7735G>A), GTA2520GTG (7744A>G), TAC2521TAT (7747C>T), GGC2526GGT (7762C>T), CAG2528CAA (7768G>A), TTT2529TTC (7771T>C), TCA2532TCG (7780A>G), CTA2533CTC (7783A>C), ACT2534ACC (7786T>C), CTA2537TTG (7793C>T 7795A>G), ATC2538ATT (7798C>T), GCT2540GCC (7804T>C), ACC2543ATC (7812C>T), GAA2550GAG (7834A>G), GAC2551GAT (7837C>T), GTG2554TTA (7844G>T 7846G>A), CAG2557CAA (7855G>A), CTG2559CTA (7861G>A), TTA2562TTG (7870A>G), TAC2563TAT (7873C>T), ACT2564ACA (7876T>A), GAG2565GAA (7879G>A), ATT2566ATA (7882T>A), GTT2567ATT (7883G>A), CCA2570CCT (7894A>T), ATT2571ATC (7897T>C), GTC2579GTT (7921C>T), AAG2581AAA (7927G>A), GGT2584GGC (7936T>C), AAT2585AAC (7939T>C), AAT2586AAC (7942T>C), GTG2594GTA (7966G>A), GAT2595GAC (7969T>C), TCT2597TCC (7975T>C), GTC2601GTT (7987C>T), CTT2602CTC (7990T>C), GCT2607GCC (8005T>C), GAA2616GAG (8032A>G), ATC2618ATT (8038C>T), GAC2619GAT (8041C>T), AGC2620AAC (8043G>A), TTG2632TTA (8080G>A), GTG2635GTA (8089G>A), CCG2637CCA (8095G>A), GAG2638GAA (8098G>A), AGC2641AGT (8107C>T), ATT2642ATC (8110T>C), GAT2644GAC (8116T>C), CAA2648CAG (8128A>G), GGT2654GGC (8146T>C), AAC2656AGT (8151A>G 8152C>T), TCG2660TCA (8164G>A), AGG2665AAG (8178G>A), AAG2666AAA (8182G>A), TTG2669TTA (8191G>A), TTC2671TTT (8197C>T), TCC2673TCT (8203C>T), AGA2675AAA (8208G>A), CTG2677TTA (8213C>T 8215G>A), CTA2678CTG (8218A>G), ATC2679ATT (8221C>T), GAG2680GAA (8224G>A), CCA2685CCG (8239A>G), GAG2690GAA (8254G>A), ATT2692ATC (8260T>C), GTA2693GTG (8263A>G), GAT2699GAC (8281T>C), AGA2700AGG (8284A>G), CCA2704GCG (8294C>G 8296A>G), GAG2705GAA (8299G>A), CAC2706CAT (8302C>T), ATT2711ATC (8317T>C), TGC2712TGT (8320C>T), GCT2714GCA (8326T>A), GAG2717GAA (8335G>A), TCC2718TCT (8338C>T), TCT2722TTT (8349C>T), GAA2723GAG (8353A>G), CAC2726CAT (8362C>T), CAA2727CAG (8365A>G), ATC2728ATT (8368C>T), TAC2732TAT (8380C>T), TCA2733TCG (8383A>G), TTA2735TTG (8389A>G), CCT2740CCC (8404T>C), GCA2742TCA (8408G>T), ACA2743ACG (8413A>G), GCG2745GCT (8419G>T), GCT2750GCC (8434T>C), TAT2752TAC (8440T>C), ATG2756ATA (8452G>A), CTA2758CTG (8458A>G), AGG2759AAG (8460G>A), AAA2760AAG (8464A>G), CTG2761CTA (8467G>A), AGG2765AGA (8479G>A), GCT2766ACA (8480G>A 8482T>A), GAG2771GAA (8497G>A), CTA2772TTG (8498C>T 8500A>G), AGA2773AAG (8502G>A 8503A>G), GCC2774GCT (8506C>T), GAA2777GAT (8515A>T), GTC2780GTT (8524C>T), GCA2781GCC (8527A>C), TTA2782CTG (8528T>C 8530A>G), GAG2785GAC (8539G>C), CTT2788TGT (8546C>T 8547T>G), TCT2790ACT (8552T>A), GTA2793GTG (8563A>G), GCA2797GGA (8574C>G), ATT2801GTT (8585A>G), GGA2805GAA (8598G>A), AGC2806AGT (8602C>T), AAC2807TCT (8603A>T 8604A>C 8605C>T), AAA2812AAG (8620A>G), CCA2813CAA (8622C>A), GAG2814GGA (8625A>G 8626G>A), CAG2815CAA (8629G>A), GGC2816GGT (8632C>T), ATC2818ATT (8638C>T), CAG2819CAA (8641G>A), AAC2821AAT (8647C>T), CCG2822TTC (8648C>T 8649C>T 8650G>C), AAC2823AAT (8653C>T), AAA2824AAG (8656A>G), GGA2825GAA (8658G>A), GAT2827GCG (8664A>C 8665T>G), GTT2830GTG (8674T>G), GCA2832GTT (8679C>T 8680A>T), GGC2833GGA (8683C>A), GGG2836GGA (8692G>A), ACA2837ACT (8695A>T), CCG2841CCA (8707G>A), AGA2842CGA (8708A>C), AAG2844AAA (8716G>A), ATC2846ATT (8722C>T), ACG2847ACA (8725G>A), AAA2849AAG (8731A>G), AGA2851AGG (8737A>G), CCC2853CCT (8743C>T), ACA2854AAG (8745C>A 8746A>G), AAG2856AAA (8752G>A), GGA2857GGT (8755A>T), AAC2862AAT (8770C>T), TTA2863CTA (8771T>C), GAA2864GAG (8776A>G), CAT2865CAC (8779T>C), TTG2866TTA (8782G>A), TAT2869TAC (8791T>C), CCA2871CCG (8797A>G), CAA2872CAG (8800A>G), CAA2873CAG (8803A>G), GAT2875GAC (8809T>C), ATT2876ATC (8812T>C), AAT2878AAC (8818T>C), CGG2880CGA (8824G>A), GCA2881GCG (8827A>G), ACG2888ACA (8848G>A), TAT2890TAC (8854T>C), GAG2891GGA (8856A>G 8857G>A), GTG2893GTA (8863G>A), CGG2894CAG (8865G>A), ATG2895CTT (8867A>C 8869G>T), GCA2896GCG (8872A>G), GGA2900GAA (8883G>A), ACT2902TCT (8888A>T), TGC2915TGT (8929C>T), GTC2924ATC (8954G>A), AAC2925AAT (8959C>T), GGA2926GGT (8962A>T), GGG2933GGA (8983G>A), AAT2934GAC (8984A>G 8986T>C), GAA2935GTA (8988A>T), GAG2938GAA (8998G>A), CCG2940CCA (9004G>A), TTG2941TTA (9007G>A), ATC2944ATT (9016C>T), ACC2951ACA (9037C>A), ATA2967ATT (9085A>T), AAC2971AAT (9097C>T), ATT2983GTT (9131A>G), CGA2984CGT (9136A>T), CGG2987CGT (9145G>T), ATG2989GGA (9149A>G 9150T>G 9151G>A), GGT2990AGT (9152G>A), TTA2991TTG (9157A>G), GCG2992GCT (9160G>T), CGT2993CGC (9163T>C), GAG3000GAA (9184G>A), CGA3004CGG (9196A>G), AGG3010AGA (9214G>A), GAA3011GAG (9217A>G), GCG3012GCA (9220G>A), GCA3020GCT (9244A>T), TTG3021TTA (9247G>A), GCC3024GCT (9256C>T), CCT3026TCC (9260C>T 9262T>C), GGG3030GGA (9274G>A), GAC3032GAT (9280C>T), ATC3035ATT (9289C>T), CAA3038CAC (9298A>C), GAG3040GAA (9304G>A), GTC3050GTT (9334C>T), GTC3060GTG (9364C>G)								
P1 protein (NP_734243.1)	1	284	100%	1394	72.7%	284 (100%)	203 (71.5%)	0/0/0/0	0

	Begin	End	Coverage	Score	Concordance	Matches	Identities	I/D/M/F*	Stop Codons
**NT**	**52**	**9704**	**99.5%**	**12530**	**65.2%**	**9642 (99.8%)**	**7985 (82.7%)**	**7/11**	
Protein mutations:	Y4Q (194T>C 196C>A), C9L (209T>C 210G>T 211T>G), F13M (221T>A 223T>G), S23P (251T>C), C24F (255G>T 256C>T), E25G (258A>G), I27V (263A>G 265T>G), V28A (267T>C), E30V (273A>T), A35T (287G>A 289T>C), V37T (293G>A 294T>C), A41I (305G>A 306C>T), D42G (309A>G), E44D (316A>T), T45V (317A>G 318C>T 319A>G), L52Q (339T>A 340C>A), K53R (342A>G), K55A (347A>G 348A>C 349A>G), Y56H (350T>C), T58V (356A>G 357C>T), V61T (365G>A 366T>C 367G>A), L62S (368C>T 369T>C), F67C (384T>G), A75V (408C>T 409C>T), M78E (416A>G 417T>A), E85K (437G>A), R86E (440A>G 441G>A), K87R (443A>C 444A>G 445G>A), D88E (448T>A), E91A (456A>C), H93N (461C>A), D111E (517T>A), S116F (531C>T), P118S (536C>T), Q119W (539C>T 540A>G 541A>G), R122K (549G>A 550A>G), M131T (576T>C), V134A (585T>C 586C>G), R137Y (593C>T 594G>A 595C>T), P138R (597C>G), I139V (599A>G 601A>G), I140P (602A>C 603T>C 604A>G), M147I (625G>A), I151V (635A>G 637T>C), I154V (644A>G 646A>G), E160A (663A>C 664G>C), H166Q (682C>A), T172S (699C>G), H174Q (706T>A), K179E (719A>G 721G>A), I180V (722A>G 724A>G), A183S (731G>T), Y184P (734T>C 735A>C 736C>T), S185R (737T>C 738C>G 739C>T), A187V (744C>T 745G>C), R189C (749C>T 751A>C), M194K (765T>A), R197K (773C>A 774G>A 775A>G), M206K (801T>A), G210A (813G>C 814A>G), L211H (816T>A), R214Q (825G>A 826T>A), N223D (851A>G 853T>C), R226C (860C>T 862C>T), T227A (863A>G 865T>C), I228T (867T>C), N229D (869A>G 871C>T), I230L (872A>T 874A>G), R231Q (876G>A), R232K (879G>A), N240S (903A>G), T241N (906C>A 907A>C), K242T (909A>C 910A>C), S243N (912G>A 913C>T), S252L (938T>C 939C>T 940A>T), L266I (980T>A 982G>C), R272K (999G>A), Q275R (1007C>A 1008A>G 1009G>A), S276G (1010A>G 1012T>G), I277V (1013A>G), N279Q (1019A>C 1021C>G)								
Codon mutations:	ACT3ACC (193T>C), TAC4CAA (194T>C 196C>A), TCA6TCG (202A>G), ATC8ATT (208C>T), TGT9CTG (209T>C 210G>T 211T>G), TCG12TCA (220G>A), TTT13ATG (221T>A 223T>G), GAA14GAG (226A>G), CTA17CTT (235A>T), TAC19TAT (241C>T), CCA21CCT (247A>T), TCT23CCT (251T>C), TGC24TTT (255G>T 256C>T), GAG25GGG (258A>G), CAT26CAC (262T>C), ATT27GTG (263A>G 265T>G), GTG28GCG (267T>C), GAA30GTA (273A>T), CGA31CGT (277A>T), GAA32GAG (280A>G), GTG33GTT (283G>T), CCG34CCA (286G>A), GCT35ACC (287G>A 289T>C), GTT37ACT (293G>A 294T>C), CCT39CCC (301T>C), TTC40TTT (304C>T), GCA41ATA (305G>A 306C>T), GAT42GGT (309A>G), CTG43TTG (311C>T), GAA44GAT (316A>T), ACA45GTG (317A>G 318C>T 319A>G), CAA46CAG (322A>G), GCA49GCT (331A>T), CTC52CAA (339T>A 340C>A), AAG53AGG (342A>G), AAA55GCG (347A>G 348A>C 349A>G), TAT56CAT (350T>C), GCT57GCA (355T>A), ACT58GTT (356A>G 357C>T), CGT60CGC (364T>C), GTG61ACA (365G>A 366T>C 367G>A), CTC62TCC (368C>T 369T>C), AAA63AAG (373A>G), AAC64AAT (376C>T), TTT67TGT (384T>G), ACG68ACA (388G>A), TAC69TAT (391C>T), CGA70CGG (394A>G), AAG72AAA (400G>A), GAT74GAC (406T>C), GCC75GTT (408C>T 409C>T), CAG76CAA (412G>A), ATA77ATT (415A>T), ATG78GAG (416A>G 417T>A), ATT80ATC (424T>C), CAG81CAA (427G>A), AAA83AAG (433A>G), CTG84CTC (436G>C), GAG85AAG (437G>A), AGG86GAG (440A>G 441G>A), AAG87CGA (443A>C 444A>G 445G>A), GAT88GAA (448T>A), GAA90GAG (454A>G), GAA91GCA (456A>C), TAT92TAC (460T>C), CAC93AAC (461C>A), CAA95CAG (469A>G), GCC97GCG (475C>G), CCT99CCA (481T>A), ACT106ACC (502T>C), ATA107ATT (505A>T), GGC109GGT (511C>T), GAT111GAA (517T>A), CCT112CCA (520T>A), CCA113CCT (523A>T), TCT116TTT (531C>T), GAG117GAA (535G>A), CCA118TCA (536C>T), CAA119TGG (539C>T 540A>G 541A>G), AGA122AAG (549G>A 550A>G), ATC124ATT (556C>T), ATT125ATC (559T>C), CAT126CAC (562T>C), ACT128ACC (568T>C), CCA129CCG (571A>G), ATG131ACG (576T>C), CGT132CGC (580T>C), GTC134GCG (585T>C 586C>G), CGC137TAT (593C>T 594G>A 595C>T), CCC138CGC (597C>G), ATA139GTG (599A>G 601A>G), ATA140CCG (602A>C 603T>C 604A>G), ACA143ACG (613A>G), GAA144GAG (616A>G), GGC145GGG (619C>G), CAG146CAA (622G>A), ATG147ATA (625G>A), AAT148AAC (628T>C), CAC149CAT (631C>T), ATT151GTC (635A>G 637T>C), ATA154GTG (644A>G 646A>G), ATT157ATC (655T>C), TCG159TCA (661G>A), GAG160GCC (663A>C 664G>C), AAA161AAG (667A>G), GTC165GTT (679C>T), CAC166CAA (682C>A), TTA167CTG (683T>C 685A>G), AGT169AGC (691T>C), ACC172AGC (699C>G), ACT173ACA (703T>A), CAT174CAA (706T>A), AAG179GAA (719A>G 721G>A), ATA180GTG (722A>G 724A>G), CTT181TTG (725C>T 727T>G), GGT182GGA (730T>A), GCA183TCA (731G>T), TAC184CCT (734T>C 735A>C 736C>T), TCC185CGT (737T>C 738C>G 739C>T), GCG187GTC (744C>T 745G>C), CGA189TGC (749C>T 751A>C), ACT190ACC (754T>C), ATG194AAG (765T>A), TTG196CTG (770T>C), CGA197AAG (773C>A 774G>A 775A>G), CGG198AGG (776C>A), TTC202TTT (790C>T), ATG206AAG (801T>A), GGA210GCG (813G>C 814A>G), CTT211CAT (816T>A), TTG212CTG (818T>C), CAA213CAG (823A>G), CGT214CAA (825G>A 826T>A), CGG217AGA (833C>A 835G>A), GAC219GAT (841C>T), AAT223GAC (851A>G 853T>C), GTC225GTT (859C>T), CGC226TGT (860C>T 862C>T), ACT227GCC (863A>G 865T>C), ATC228ACC (867T>C), AAC229GAT (869A>G 871C>T), ATA230TTG (872A>T 874A>G), CGA231CAA (876G>A), AGG232AAG (879G>A), GAT234GAC (886T>C), ATC238ATT (898C>T), AAC240AGC (903A>G), ACA241AAC (906C>A 907A>C), AAA242ACC (909A>C 910A>C), AGC243AAT (912G>A 913C>T), GGC246GGA (922C>A), GGT249GGA (931T>A), TCA252CTT (938T>C 939C>T 940A>T), GGA253GGG (943A>G), TTC256TTT (952C>T), TCA261TCG (967A>G), GAA263GAG (973A>G), GGG264GGC (976G>C), TTG266ATC (980T>A 982G>C), GCA269GCG (991A>G), CGT270CGC (994T>C), AGA272AAA (999G>A), GTT273GTC (1003T>C), CAG275AGA (1007C>A 1008A>G 1009G>A), AGT276GGG (1010A>G 1012T>G), ATT277GTT (1013A>G), TTA278TTG (1018A>G), AAC279CAG (1019A>C 1021C>G), ATC282ATT (1030C>T)								
HC-Pro protein (NP_734244.1)	1	456	100%	3144	95.7%	456 (100%)	425 (93.2%)	0/0/0/0	0
Protein mutations:	D4E (1048C>A), R16Q (1083G>A), S21A (1097T>G 1099G>A), R36K (1143G>A), L40I (1154T>A 1156G>A), S62N (1221G>A), V65T (1229G>A 1230T>C 1231T>C), S66N (1233G>A 1234C>T), K74T (1257A>C 1258A>G), N81S (1278A>G), A85V (1290C>T 1291G>A), D88T (1298G>A 1299A>C 1300C>T), I91V (1307A>G 1309A>G), N94E (1316A>G 1318T>A), I98T (1329T>C), L113I (1373C>A), I117V (1385A>G), E123D (1405G>T), A127S (1415G>T 1417A>T), V134I (1436G>A 1438C>T), E224G (1707A>G), A228T (1718G>A), I236V (1742A>G 1744C>T), S241N (1758G>A), V252I (1790G>A), I301V (1937A>G 1939T>C), F323Y (2004T>A), I344M (2068T>G), V346I (2072G>A 2074G>A), R391K (2208G>A), D410E (2266C>G)								
Codon mutations:	TCG1TCA (1039G>A), AAT2AAC (1042T>C), GCC3GCA (1045C>A), GAC4GAA (1048C>A), AAT5AAC (1051T>C), AAG8AAA (1060G>A), GGT9GGA (1063T>A), CTG10TTG (1064C>T), GAC11GAT (1069C>T), CGA16CAA (1083G>A), TAT19TAC (1093T>C), TCG21GCA (1097T>G 1099G>A), CAC23CAT (1105C>T), GTA26GTG (1114A>G), GCT27GCG (1117T>G), TTA29TTG (1123A>G), CCT30CCA (1126T>A), GTC31GTT (1129C>T), AGG36AAG (1143G>A), GTA37GTT (1147A>T), GCT38GCA (1150T>A), TTG40ATA (1154T>A 1156G>A), CAC43CAT (1165C>T), AGT44AGC (1168T>C), CTT46TTA (1172C>T 1174T>A), ACC55ACT (1201C>T), AGC62AAC (1221G>A), GTT65ACC (1229G>A 1230T>C 1231T>C), AGC66AAT (1233G>A 1234C>T), CTG68CTA (1240G>A), AAG70AAA (1246G>A), TTG72CTA (1250T>C 1252G>A), CAT73CAC (1255T>C), AAA74ACG (1257A>C 1258A>G), AGA77AGG (1267A>G), GGT79GGC (1273T>C), TTG80TTA (1276G>A), AAT81AGT (1278A>G), TTG83CTG (1283T>C), GCG85GTA (1290C>T 1291G>A), GAC88ACT (1298G>A 1299A>C 1300C>T), TTT90TTC (1306T>C), ATA91GTG (1307A>G 1309A>G), CAT92CAC (1312T>C), AAT94GAA (1316A>G 1318T>A), TTG97TTA (1327G>A), ATA98ACA (1329T>C), GCG99GCT (1333G>T), TTA100TTG (1336A>G), CTA103TTG (1343C>T 1345A>G), CCG106CCA (1354G>A), GTG107GTT (1357G>T), GAC108GAT (1360C>T), CTC111TTG (1367C>T 1369C>G), CTT113ATT (1373C>A), TTC114TTT (1378C>T), GAG116GAA (1384G>A), ATA117GTA (1385A>G), AAA119AAG (1393A>G), TCC120TCT (1396C>T), GAG123GAT (1405G>T), AAA124AAG (1408A>G), CAG125CAA (1411G>A), GCA127TCT (1415G>T 1417A>T), CCG128CCC (1420G>C), AAT131AAC (1429T>C), TTA132TTG (1432A>G), GTC134ATT (1436G>A 1438C>T), TTA135CTG (1439T>C 1441A>G), AAT136AAC (1444T>C), TTC139TTT (1453C>T), CTG140CTA (1456G>A), GAA144GAG (1468A>G), GTA152GTG (1492A>G), TTG155CTA (1499T>C 1501G>A), AGT156AGC (1504T>C), TTG157TTA (1507G>A), CTC158CTT (1510C>T), GAA159GAG (1513A>G), TTA160TTG (1516A>G), GCA161GCT (1519A>T), AGG162AGA (1522G>A), TTC163TTT (1525C>T), CAG164CAA (1528G>A), AAC166AAT (1534C>T), ACT168ACA (1540T>A), AAC170AAT (1546C>T), AAA173AAG (1555A>G), GGT174GGC (1558T>C), GAT175GAC (1561T>C), ATA176ATC (1564A>C), TCT177TCA (1567T>A), TTC179TTT (1573C>T), AGA180AGG (1576A>G), TTA183CTA (1583T>C), TCT184TCA (1588T>A), AAG186AAA (1594G>A), AAC188AAT (1600C>T), CTG191CTA (1609G>A), TCG194TCA (1618G>A), TGC195TGT (1621C>T), GAC196GAT (1624C>T), CTG199TTG (1631C>T), AAA201AAG (1639A>G), TTC205TTT (1651C>T), CTC206CTG (1654C>G), CAT213CAC (1675T>C), TCA219TCG (1693A>G), TTT222TTC (1702T>C), GAA224GGA (1707A>G), GCA228ACA (1718G>A), GGA230GGG (1726A>G), TAC231TAT (1729C>T), TCA232TCC (1732A>C), TAT234TAC (1738T>C), ATC236GTT (1742A>G 1744C>T), CGC237CGT (1747C>T), CAT239CAC (1753T>C), AGT241AAT (1758G>A), AGG244AGA (1768G>A), AAC250AAT (1786C>T), TTA251TTG (1789A>G), GTT252ATT (1790G>A), GTC253GTT (1795C>T), TTA257CTG (1805T>C 1807A>G), GAG259GAA (1813G>A), AGG261AGA (1819G>A), AAG263AAA (1825G>A), GAC267GAT (1837C>T), TAT268TAC (1840T>C), CAA271CAG (1849A>G), GTC274GTG (1858C>G), AAA276AAG (1864A>G), TGC278TGT (1870C>T), AGT280AGC (1876T>C), AAA282AAG (1882A>G), GGT284GGA (1888T>A), AAT285AAC (1891T>C), TGT290TGC (1906T>C), ACA293ACT (1915A>T), GGT298GGC (1930T>C), GCC300GCT (1936C>T), ATT301GTC (1937A>G 1939T>C), TCA303TCG (1945A>G), TTC305TTT (1951C>T), CCA308CCT (1960A>T), GTA314GTG (1978A>G), ATT315ATA (1981T>A), GGC316GGT (1984C>T), AAT317AAC (1987T>C), GAC320GAT (1996C>T), TTT323TAT (2004T>A), AAA328AAG (2020A>G), GGG329GGA (2023G>A), GAT330GAC (2026T>C), TCG331TCT (2029G>T), GAG332GAA (2032G>A), TAT341TAC (2059T>C), ATT344ATG (2068T>G), AAC345AAT (2071C>T), GTG346ATA (2072G>A 2074G>A), CTG351TTA (2087C>T 2089G>A), AGC355AGT (2101C>T), GAG356GAA (2104G>A), GAG357GAA (2107G>A), AAG360AAA (2116G>A), TTC362TTT (2122C>T), CGC367CGT (2137C>T), CTT374CTC (2158T>C), ACC376ACT (2164C>T), CCA378CCG (2170A>G), GAT382GAC (2182T>C), GCG384GCA (2188G>A), ACC385ACA (2191C>A), AGA391AAA (2208G>A), TAT394TAC (2218T>C), GAC396GAT (2224C>T), GTA397GTT (2227A>T), TTG402TTA (2242G>A), CCC403CCT (2245C>T), TTG406TTA (2254G>A), GTT407GTC (2257T>C), GAC408GAT (2260C>T), GAC410GAG (2266C>G), CAA412CAG (2272A>G), ACG413ACA (2275G>A), TCA419TCG (2293A>G), GGC421GGT (2299C>T), TCG422TCA (2302G>A), CAG423CAA (2305G>A), GGA426GGT (2314A>T), TAT427TAC (2317T>C), CTA430CTG (2326A>G), GTG435GTA (2341G>A), CAA437CAG (2347A>G), CTT438CTC (2350T>C), TTG440TTA (2356G>A), AAT443AAC (2365T>C), GAA445GAG (2371A>G), TTA446TTG (2374A>G), GAT449GAC (2383T>C), ATA450ATT (2386A>T), AAA451AAG (2389A>G), CAT452CAC (2392T>C), TAT453TAC (2395T>C)								
P3 protein (NP_734251.1)	1	365	100%	2043	84.5%	365 (99.7%)	327 (89.3%)	1/0/1/1	0
Protein mutations:	N4G (2414A>G 2415A>G 2416T>G), S6C (2420A>T), K40R (2523A>G), R77K (2634G>A), V107I (2723G>A 2725C>T), A115T (2747G>A 2749T>A), H131N (2795C>A), V140I (2822G>A 2824G>A), P160T (2882C>A), S161H (2885A>C 2886G>A), T164M (2895C>T), N176S (2931A>G), N179S (2940A>G), E189_N190insX (2971_2972insA), T196I (2991C>T), H197R (2994A>G), E199R (2999G>A 3000A>G), S204F (3015C>T), R206Q (3021G>A), Y207F (3024A>T 3025C>T), T211I (3036C>T), E212G (3039A>G 3040A>C), K213T (3042A>C 3043G>A), A232V (3099C>T 3100C>A), V234R (3104G>A 3105T>G 3106G>A), S243N (3132G>A), R245K (3137C>A 3138G>A 3139A>G), F250I (3152T>A 3154C>T), C254Y (3165G>A), F272L (3218T>C), T274N (3225C>A), V276I (3230G>A), V288M (3266G>A 3268A>G), V291M (3275G>A), A294T (3284G>A), R304K (3315G>A 3316A>G), E305A (3318A>C), M319V (3359A>G), D332E (3400T>G)								

	Begin	End	Coverage	Score	Concordance	Matches	Identities	I/D/M/F*	Stop Codons
**NT**	**52**	**9704**	**99.5%**	**12530**	**65.2%**	**9642 (99.8%)**	**7985 (82.7%)**	**7/11**	
Codon mutations:	AAT4GGG (2414A>G 2415A>G 2416T>G), GCT5GCA (2419T>A), AGC6TGC (2420A>T), ACA12ACT (2440A>T), GTT21GTC (2467T>C), TCT24TCC (2476T>C), TCG26TCA (2482G>A), CTG29CTA (2491G>A), CTG31CTT (2497G>T), TTG33TTA (2503G>A), AGA38AGG (2518A>G), CCT39CCC (2521T>C), AAG40AGG (2523A>G), AGA43AGG (2533A>G), TTG45TTA (2539G>A), TTA47TTG (2545A>G), GAG49GAA (2551G>A), CCT50CCA (2554T>A), CTG52TTG (2558C>T), TTG53CTC (2561T>C 2563G>C), TCA56TCG (2572A>G), CCT60CCC (2584T>C), GGC61GGT (2587C>T), CTG63CTC (2593G>C), GCT65GCC (2599T>C), AAT68AAC (2608T>C), TTT72TTC (2620T>C), GCG75GCA (2629G>A), GTG76GTA (2632G>A), AGG77AAG (2634G>A), TTG78TTA (2638G>A), ATT80ATA (2644T>A), AAA83AAG (2653A>G), GCT87GCC (2665T>C), CTA92TTA (2678C>T), CTA93CTG (2683A>G), GCT95GCC (2689T>C), CTA98CTC (2698A>C), TCA101TCG (2707A>G), GCG102GCA (2710G>A), GAA104GAG (2716A>G), ACA105ACG (2719A>G), CTC106CTT (2722C>T), GTC107ATT (2723G>A 2725C>T), GCA108GCG (2728A>G), AGG110AGA (2734G>A), ATT111ATA (2737T>A), GAT114GAC (2746T>C), GCT115ACA (2747G>A 2749T>A), GCA116GCT (2752A>T), GCT117GCA (2755T>A), GAC119GAT (2761C>T), CTC120CTT (2764C>T), TGT125TGC (2779T>C), GGG127GGT (2785G>T), AAC129AAT (2791C>T), CAT131AAT (2795C>A), TAC134TAT (2806C>T), TTG138CTC (2816T>C 2818G>C), GTG140ATA (2822G>A 2824G>A), TTG141TTA (2827G>A), GTT144GTC (2836T>C), AAG145AAA (2839G>A), AAT146AAC (2842T>C), ACC153ACA (2863C>A), CTA154TTA (2864C>T), CCA160ACA (2882C>A), AGT161CAT (2885A>C 2886G>A), AAC163AAT (2893C>T), ACG164ATG (2895C>T), GTC166GTT (2902C>T), GTA167GTG (2905A>G), ATT169ATC (2911T>C), AAT173AAC (2923T>C), AAT176AGT (2931A>G), AAC179AGC (2940A>G), GCT181GCC (2947T>C), ACT186ACC (2962T>C), GAA189_AAT190insA-- (2971_2972insA), GGT195GGC (2989T>C), ACT196ATT (2991C>T), CAT197CGT (2994A>G), GAG199AGG (2999G>A 3000A>G), TCC204TTC (3015C>T), CGG206CAG (3021G>A), TAC207TTT (3024A>T 3025C>T), ACA211ATA (3036C>T), GAA212GGC (3039A>G 3040A>C), AAG213ACA (3042A>C 3043G>A), TTG216TTA (3052G>A), TTA219TTG (3061A>G), GCG226GCA (3082G>A), GGC229GGT (3091C>T), GCC232GTA (3099C>T 3100C>A), GTG234AGA (3104G>A 3105T>G 3106G>A), GTC235GTT (3109C>T), AAA236AAG (3112A>G), ACT238ACC (3118T>C), GCC239GCT (3121C>T), GGA241GGG (3127A>G), AGC243AAC (3132G>A), CGA245AAG (3137C>A 3138G>A 3139A>G), TAT249TAC (3151T>C), TTC250ATT (3152T>A 3154C>T), TGT254TAT (3165G>A), GTA255GTG (3169A>G), ATC265ATT (3199C>T), AGG267CGG (3203A>C), CGT268CGC (3208T>C), CCA270CCG (3214A>G), ACC271ACT (3217C>T), TTT272CTT (3218T>C), GTC273GTA (3223C>A), ACT274AAT (3225C>A), GTT276ATT (3230G>A), AAC277AAT (3235C>T), TTA285CTA (3257T>C), ACC286ACT (3262C>T), AGC287AGT (3265C>T), GTA288ATG (3266G>A 3268A>G), GTG289GTA (3271G>A), GTG291ATG (3275G>A), GCA294ACA (3284G>A), CAG299CAA (3301G>A), AGA304AAG (3315G>A 3316A>G), GAA305GCA (3318A>C), ATC306ATT (3322C>T), GAG307GAA (3325G>A), TTG308TTA (3328G>A), ATA311ATT (3337A>T), GAG315GAA (3349G>A), GTC317GTT (3355C>T), TGC318TGT (3358C>T), ATG319GTG (3359A>G), CTA321CTG (3367A>G), TAT322TAC (3370T>C), TTA325CTG (3377T>C 3379A>G), GAA330GAG (3394A>G), CGC331CGT (3397C>T), GAT332GAG (3400T>G), ACA334ACG (3406A>G), TAC338TAT (3418C>T), ATT339ATA (3421T>A), TAT341TAC (3427T>C), AAG343AAA (3433G>A), TCA344TCT (3436A>T), GTA345GTG (3439A>G), AAC346AAT (3442C>T), CCT347CCC (3445T>C), CAG351CAA (3457G>A), TTT352TTC (3460T>C), GCT353GCA (3463T>A), GCG355GCT (3469G>T), CAG356CAA (3472G>A), CAC364CAT (3496C>T), CAG365CAA (3499G>A)								
6K1 protein (NP_734245.1)	1	52	100%	305	90.5%	52 (100%)	45 (86.5%)	0/0/0/0	0
Protein mutations:	V5G (3513T>G), M16V (3545A>G), A17T (3548G>A), V19I (3554G>A 3556C>T), L42V (3623C>G), L45M (3632C>A), Y47H (3638T>C)								
Codon mutations:	TCC2TCT (3505C>T), GTT5GGT (3513T>G), AAA7AAG (3520A>G), GAA10GAG (3529A>G), CAA11CAG (3532A>G), ATG16GTG (3545A>G), GCT17ACT (3548G>A), TTA18CTA (3551T>C), GTC19ATT (3554G>A 3556C>T), AGT28AGC (3583T>C), GAT29GAC (3586T>C), TGC30TGT (3589C>T), TTC32TTT (3595C>T), AAT36AAC (3607T>C), TTT38TTC (3613T>C), AAG39AAA (3616G>A), GGT40GGC (3619T>C), CTT42GTT (3623C>G), TCC43TCT (3628C>T), CTG45ATG (3632C>A), GAT46GAC (3637T>C), TAT47CAT (3638T>C), GAA48GAG (3643A>G)								
CI protein (NP_734246.1)	1	634	100%	4190	97.9%	634 (100%)	608 (95.9%)	0/0/0/0	0
Protein mutations:	I15V (3698A>G), S21N (3717G>A), R26K (3731C>A 3732G>A), I140V (4073A>G), F158Y (4128T>A 4129C>T), A166S (4151G>T), V171I (4166G>A), L190I (4223C>A 4225G>A), I222V (4319A>G 4321A>G), F247Y (4395T>A), V251I (4406G>A), M274V (4475A>G 4477G>T), T276S (4481A>T 4483A>G), A297L (4544G>T 4545C>T 4546G>A), R298K (4548G>A), V342I (4679G>A 4681G>C), V381I (4796G>A 4798C>T), T383S (4803C>G 4804T>C), S468G (5057A>G), T469A (5060A>G), L479I (5090C>A), A482V (5100C>T), E484D (5107A>T), V489F (5120G>T 5122C>T), I491V (5126A>G), V510I (5183G>A)								
Codon mutations:	TTA2TTG (3661A>G), GTG5GTA (3670G>A), TTT9TTC (3682T>C), GAG11GAA (3688G>A), GAG14GAA (3697G>A), ATT15GTT (3698A>G), GAT17GAC (3706T>C), TTT18TTC (3709T>C), TTG20CTA (3713T>C 3715G>A), AGT21AAT (3717G>A), GAG22GAA (3721G>A), CGA26AAA (3731C>A 3732G>A), ACT27ACA (3736T>A), CTA31TTG (3746C>T 3748A>G), ACA33ACG (3754A>G), AGT36AGC (3763T>C), CGA41CGG (3778A>G), CAA42CAG (3781A>G), ATC43ATT (3784C>T), CAT47CAC (3796T>C), CCA50CCT (3805A>T), TAC52TAT (3811C>T), AGA53AGG (3814A>G), TTC58TTT (3829C>T), TTT61TTC (3838T>C), AGA63AGG (3844A>G), GCA64GCC (3847A>C), GTT67GTA (3856T>A), GTG69GTT (3862G>T), GAC72GAT (3871C>T), GCC74GCA (3877C>A), AGC76AGT (3883C>T), GAA77GAG (3886A>G), CTA79CTG (3892A>G), GAC80GAT (3895C>T), TTT81TTC (3898T>C), GTA83GTG (3904A>G), CGG84AGA (3905C>A 3907G>A), GCT86GCC (3913T>C), AAG91AAA (3928G>A), TCA92TCT (3931A>T), GGG94GGA (3937G>A), AGT100AGC (3955T>C), GTG101GTA (3958G>A), GCC102GCT (3961C>T), GGA103GGG (3964A>G), TCT104TCC (3967T>C), TTA107TTG (3976A>G), ATT108ATC (3979T>C), CCA110CCT (3985A>T), CTA114CTT (3997A>T), GCG115GCA (4000G>A), AAC117AAT (4006C>T), GTT118GTG (4009T>G), TTC119TTT (4012C>T), AAA120AAG (4015A>G), TCT123TCC (4024T>C), GAA125GAG (4030A>G), CCA126CCT (4033A>T), AAG129AAA (4042G>A), CTG133TTA (4052C>T 4054G>A), CGT134CGC (4057T>C), CGT136CGA (4063T>A), GGA137GGG (4066A>G), ATA140GTA (4073A>G), TTT141TTC (4078T>C), GGC142GGT (4081C>T), TCT143TCC (4084T>C), TCC147TCT (4096C>T), AGC151AGT (4108C>T), GGA152GGT (4111A>T), GCG154GCA (4117G>A), CTA155TTG (4118C>T 4120A>G), TAC157TAT (4126C>T), TTC158TAT (4128T>A 4129C>T), GCC159GCT (4132C>T), AAT160AAC (4135T>C), CAA164CAG (4147A>G), TTA165CTA (4148T>C), GCT166TCT (4151G>T), AAC169AAT (4162C>T), GTA171ATA (4166G>A), ATA172ATC (4171A>C), GAG175GAA (4180G>A), CTG179TTG (4190C>T), CCT181CCC (4198T>C), TCC182TCT (4201C>T), GCG185GCT (4210G>T), TTC186TTT (4213C>T), AGT188AGC (4219T>C), CTG189TTG (4220C>T), CTG190ATA (4223C>A 4225G>A), GTT192GTG (4231T>G), CAT194CAC (4237T>C), GTA199GTG (4252A>G), TTA200CTG (4253T>C 4255A>G), AAA201AAG (4258A>G), ACT205ACC (4270T>C), AGA209AGG (4282A>G), GAG210GAA (4285G>A), GAA212GAG (4291A>G), CAG216CAA (4303G>A), GTC219GTT (4312C>T), ATA222GTG (4319A>G 4321A>G), GTG223GTT (4324G>T), GAG224GAA (4327G>A), CTG227CTT (4336G>T), TCT228TCC (4339T>C), TCA231TCT (4348A>T), GCA235GCG (4360A>G), GGT237GGC (4366T>C), TCT238TCA (4369T>A), ACT240ACC (4375T>C), GTT244GTC (4387T>C), TTT247TAT (4395T>A), GTA251ATA (4406G>A), CTT252CTC (4411T>C), GTT261GTC (4438T>C), ACC263ACA (4444C>A), GCC265GCT (4450C>T), CTC267CTT (4456C>T), GAC270GAT (4465C>T), ATG274GTT (4475A>G 4477G>T), GTC275GTT (4480C>T), ACA276TCG (4481A>T 4483A>G), GGC280GGT (4495C>T), AAG284AAA (4507G>A), CAC285CAT (4510C>T), GGT286GGG (4513T>G), CTA288TTA (4517C>T), GTC291GTT (4528C>T), AAA293AAG (4534A>G), ACC295ACT (4540C>T), GCG297TTA (4544G>T 4545C>T 4546G>A), AGA298AAA (4548G>A), TTT301TTC (4558T>C), GTA303GTT (4564A>T), ACC305ACT (4570C>T), ATA307ATT (4576A>T), TTG314TTA (4597G>A), GAC315GAT (4600C>T), GAC317GAT (4606C>T), GTG318GTA (4609G>A), GTG320GTA (4615G>A), GAC321GAT (4618C>T), TTG324CTT (4625T>C 4627G>T), GAC331GAT (4648C>T), ATT332ATC (4651T>C), AGG335AGA (4660G>A), AGC336AGT (4663C>T), GCT338GCA (4669T>A), GTG342ATC (4679G>A 4681G>C), AGT343AGC (4684T>C), GTT344GTG (4687T>G), GAG348GAA (4699G>A), AGA349AGG (4702A>G), CAA351CAG (4708A>G), AGG352AGA (4711G>A), CTG353TTG (4712C>T), AAA361AAG (4738A>G), GTA363GTT (4744A>T), GCA364GCG (4747A>G), TTG365CTG (4748T>C), ACT370ACC (4765T>C), GTC381ATT (4796G>A 4798C>T), ACT383AGC (4803C>G 4804T>C), GAG384GAA (4807G>A), GCG385GCT (4810G>T), GCT386GCG (4813T>G), CTT387CTC (4816T>C), TAT392TAC (4831T>C), AAC393AAT (4834C>T), CCA395CCT (4840A>T), GGA399GGG (4852A>G), GGC400GGT (4855C>T), AGT404AGC (4867T>C), CTG405CTC (4870G>C), ATT406ATA (4873T>A), TGT409TGC (4882T>C), ACA416ACT (4903A>T), CAG418CAA (4909G>A), TTT420TTC (4915T>C), GAA421GAG (4918A>G), TTG422CTA (4919T>C 4921G>A), AGT423AGC (4924T>C), CCC424CCG (4927C>G), TTT426TTC (4933T>C), ATC427ATA (4936C>A), CAG428CAA (4939G>A), AAT429AAC (4942T>C), TTC430TTT (4945C>T), TCA436TCG (4963A>G), CCT439CCC (4972T>C), CTT445CTC (4990T>C), AAA449AAG (5002A>G), CCT456CCC (5023T>C), TTG457CTG (5024T>C), TGC458TGT (5029C>T), GAT459GAC (5032T>C), TCT461TCC (5038T>C), CCA463CCT (5044A>T), GCA466GCT (5053A>T), TCG467TCA (5056G>A), AGT468GGT (5057A>G), ACT469GCT (5060A>G), TCG472TCT (5071G>T), CTT479ATT (5090C>A), GGA480GGG (5095A>G), GTG481GTT (5098G>T), GCC482GTC (5100C>T), TTA483TTG (5104A>G), GAA484GAT (5107A>T), ATT485ATC (5110T>C), CCA486CCG (5113A>G), AAG487AAA (5116G>A), CAA488CAG (5119A>G), GTC489TTT (5120G>T 5122C>T), ATT491GTT (5126A>G), CAT494CAC (5137T>C), ATC495ATT (5140C>T), AAA496AAG (5143A>G), GAG497GAA (5146G>A), CTC502CTA (5161C>A), GAA504GAG (5167A>G), CTT506CTG (5173T>G), ACG509ACA (5182G>A), GTT510ATT (5183G>A), GTC511GTT (5188C>T), AAG512AAA (5191G>A), AAA514AAG (5197A>G), GAC515GAT (5200C>T), TGC517TGT (5206C>T), TTA518TTG (5209A>G), AGC521AGT (5218C>T), TCG525TCA (5230G>A), ATC527ATT (5236C>T), AGC528AGT (5239C>T), ATC530ATT (5245C>T), ACA536ACT (5263A>T), CTC538CTT (5269C>T), TTC539TTT (5272C>T), GCC540GCA (5275C>A), ATC541ATT (5278C>T), CCA542CCC (5281A>C), AGA543AGG (5284A>G), ACT544ACC (5287T>C), CTA545TTA (5288C>T), ATA546ATT (5293A>T), TTG547CTA (5294T>C 5296G>A), GAG549GAA (5302G>A), CTT552CTA (5311T>A), GAA553GAG (5314A>G), CGA556CGT (5323A>T), AGT564AGC (5347T>C), ATC566ATT (5353C>T), TGC570TGT (5365C>T), GTT577GTC (5386T>C), ACC580ACA (5395C>A), AAA589AAG (5422A>G), GAT590GAC (5425T>C), ACC592ACT (5431C>T), GAG594GAA (5437G>A), CTT599CTC (5452T>C), AAG601AAA (5458G>A), AGT604AGC (5467T>C), CAA605CAG (5470A>G), CTA606TTG (5471C>T 5473A>G), GAA608GAG (5479A>G), TTC609TTT (5482C>T), TCA610TCC (5485A>C), AAT611AAC (5488T>C), TTG612CTA (5489T>C 5491G>A), GGT614GGC (5497T>C), TGT617TGC (5506T>C), GAG618GAA (5509G>A), GAG619GAA (5512G>A), TTA621CTA (5516T>C), TAT625TAC (5530T>C), GAG626GAA (5533G>A), TCG627TCT (5536G>T), TTG628TTA (5539G>A), GTT631GTG (5548T>G), CAC633CAT (5554C>T)								
6K2 protein (NP_734247.1)	1	52	100%	303	93.5%	52 (100%)	46 (88.5%)	0/0/0/0	0
Protein mutations:	A2T (5561G>A 5563G>A), I14V (5597A>G), N16K (5605C>A), A21V (5619C>T 5620T>G), I25L (5630A>C), V46I (5693G>A)								
Codon mutations:	GCT1GCA (5560T>A), GCG2ACA (5561G>A 5563G>A), ACG3ACC (5566G>C), TCA4TCG (5569A>G), CTC9CTG (5584C>G), AAG12AAA (5593G>A), GGG13GGA (5596G>A), ATT14GTT (5597A>G), AAC16AAA (5605C>A), GTG20GTT (5617G>T), GCT21GTG (5619C>T 5620T>G), TTG24TTA (5629G>A), ATC25CTC (5630A>C), GGC28GGT (5641C>T), GCA31GCT (5650A>T), ATA35ATT (5662A>T), GGA36GGG (5665A>G), CAA44CAG (5689A>G), GTT46ATT (5693G>A), GAG47GAA (5698G>A), CAA52CAG (5713A>G)								
NIa-VPg protein (NP_734252.1)	1	188	100%	1227	93.3%	188 (100%)	170 (90.4%)	0/0/0/0	0
Protein mutations:	K47R (5853A>G), I61V (5894A>G 5896C>G), R83A (5960C>G 5961G>C 5962G>C), I93V (5990A>G), E102D (6019A>T), K106T (6030A>C), D112E (6049C>A), M115I (6058G>A), S120N (6072G>A 6073T>C), N121H (6074A>C), T123N (6081C>A), C133S (6111G>C 6112T>C), I139V (6128A>G 6130T>C), V149I (6158G>A), T153S (6170A>T 6172A>G), L164F (6203C>T 6205C>T), V173I (6230G>A), A182K (6257G>A 6258C>A)								

	Begin	End	Coverage	Score	Concordance	Matches	Identities	I/D/M/F*	Stop Codons
**NT**	**52**	**9704**	**99.5%**	**12530**	**65.2%**	**9642 (99.8%)**	**7985 (82.7%)**	**7/11**	
Codon mutations:	GGG1GGC (5716G>C), AAA2AAG (5719A>G), GCC10GCA (5743C>A), CGC14CGA (5755C>A), CAT15CAC (5758T>C), CGT17CGC (5764T>C), AAA19AAG (5770A>G), GGC22GGT (5779C>T), TTT23TTC (5782T>C), GAC26GAT (5791C>T), GAC30GAT (5803C>T), AGG41AGA (5836G>A), AAA42AAG (5839A>G), GGA44GGG (5845A>G), AAA45AAG (5848A>G), AAA47AGA (5853A>G), GGT48GGC (5857T>C), ACC49ACT (5860C>T), ACA50ACT (5863A>T), GGT52GGA (5869T>A), GGT54GGC (5875T>C), AGC57AGT (5884C>T), TTC60TTT (5893C>T), ATC61GTG (5894A>G 5896C>G), AAC62AAT (5899C>T), GGG65GGA (5908G>A), TTT66TTC (5911T>C), GAG70GAA (5923G>A), CAA75CAG (5938A>G), TTC76TTT (5941C>T), GTT77GTC (5944T>C), GAT78GAC (5947T>C), GGG82GGA (5959G>A), CGG83GCC (5960C>G 5961G>C 5962G>C), GAA87GAG (5974A>G), GCT91GCA (5986T>A), GAC92GAT (5989C>T), ATT93GTT (5990A>G), GAT95GAC (5998T>C), AGA99CGT (6008A>C 6010A>T), GAA102GAT (6019A>T), GTG103GTT (6022G>T), CGA104CGT (6025A>T), AAA106ACA (6030A>C), GAC112GAA (6049C>A), ATG115ATA (6058G>A), CAA116CAG (6061A>G), GCC117GCA (6064C>A), GGT119GGC (6070T>C), AGT120AAC (6072G>A 6073T>C), AAC121CAC (6074A>C), ACG122ACA (6079G>A), ACC123AAC (6081C>A), ATA124ATT (6085A>T), GCA126GCT (6091A>T), TAC127TAT (6094C>T), TGT133TCC (6111G>C 6112T>C), AAA135AAG (6118A>G), TTG137TTA (6124G>A), AAG138AAA (6127G>A), ATT139GTC (6128A>G 6130T>C), GAT140GAC (6133T>C), TTA141TTG (6136A>G), AAC145AAT (6148C>T), AAA148AAG (6157A>G), GTT149ATT (6158G>A), TGT150TGC (6163T>C), AAA152AAG (6169A>G), ACA153TCG (6170A>T 6172A>G), GGC155GGT (6178C>T), AAA158AAG (6187A>G), TTT159TTC (6190T>C), GAG161GAA (6196G>A), CTC164TTT (6203C>T 6205C>T), GAA165GAG (6208A>G), CTA166TTG (6209C>T 6211A>G), CAG168CAA (6217G>A), GCT172GCA (6229T>A), GTA173ATA (6230G>A), GTC175GTT (6238C>T), AAG178AAA (6247G>A), ATA180ATT (6253A>T), GCA182AAA (6257G>A 6258C>A), GAG184GAA (6265G>A), CAT187CAC (6274T>C)								
NIa-Pro protein (NP_734248.1)	1	244	100%	1654	93.7%	244 (100%)	223 (91.4%)	0/0/0/0	0
Protein mutations:	K2R (6282A>G), A29T (6362G>A), V42I (6401G>A), A43V (6405C>T), Y51F (6429A>T), Q58R (6450A>G), H70N (6485C>A 6487C>T), L84I (6527C>A), T104I (6588C>T), Y120H (6635T>C), I124V (6647A>G), I134V (6677A>G 6679A>G), D147N (6716G>A), N148D (6719A>G), I163L (6764A>T 6766A>G), A173V (6795C>T 6796A>G), H174Q (6799C>A), N194D (6857A>G), V200T (6875G>A 6876T>C 6877C>A), D219E (6934C>A), V242I (7001G>A 7003G>A)								
Codon mutations:	AAA2AGA (6282A>G), TCG3TCA (6286G>A), GGC7GGT (6298C>T), TTG8TTA (6301G>A), AGA9AGG (6304A>G), GAC10GAT (6307C>T), GCC15GCT (6322C>T), ACA17ACT (6328A>T), GTT18GTG (6331T>G), TGT19TGC (6334T>C), AGG20AGA (6337G>A), CTG21TTG (6338C>T), TAT27TAC (6358T>C), GGG28GGA (6361G>A), GCA29ACA (6362G>A), TCA30TCT (6367A>T), GAG31GAA (6370G>A), TAC33TAT (6376C>T), GGT34GGG (6379T>G), GGA36GGC (6385A>C), GGA38GGT (6391A>T), GCA39GCG (6394A>G), TAC40TAT (6397C>T), GTA42ATA (6401G>A), GCG43GTG (6405C>T), CAC45CAT (6412C>T), TTA47TTG (6418A>G), TTT48TTC (6421T>C), TAC51TTC (6429A>T), GGT53GGA (6436T>A), GAG56GAA (6445G>A), CAA58CGA (6450A>G), TCC59TCG (6454C>G), CAC61CAT (6460C>T), GGT62GGA (6463T>A), TTC64TTT (6469C>T), AGG65AGA (6472G>A), GTG66GTA (6475G>A), AAT68AAC (6481T>C), CAC70AAT (6485C>A 6487C>T), AGT71AGC (6490T>C), AGC73AGT (6496C>T), CTG75CTA (6502G>A), GGT79GGC (6514T>C), AGG80AGA (6517G>A), CTC84ATC (6527C>A), ATC85ATA (6532C>A), CCG88CCA (6541G>A), AAA89AAG (6544A>G), GTC93GTT (6556C>T), TTT94TTC (6559T>C), CAG96CAA (6565G>A), TTG98CTA (6569T>C 6571G>A), CCT103CCA (6586T>A), ACA104ATA (6588C>T), GAA107GAG (6598A>G), AGA108AGG (6601A>G), ATT109ATC (6604T>C), TGT110TGC (6607T>C), GGA113GGG (6616A>G), AAC115AAT (6622C>T), GAG118GAA (6631G>A), AAG119AAA (6634G>A), TAT120CAT (6635T>C), GCT121GCA (6640T>A), TCG123TCT (6646G>T), ATC124GTC (6647A>G), ATC125ATT (6652C>T), AGC129AGT (6664C>T), ACT131ACG (6670T>G), ATA134GTG (6677A>G 6679A>G), GGC136GGT (6685C>T), ACA138ACT (6691A>T), TTC139TTT (6694C>T), ACA146ACG (6715A>G), GAT147AAT (6716G>A), AAT148GAT (6719A>G), GGA149GGG (6724A>G), CTA153TTA (6734C>T), GTG155GTA (6742G>A), GTG156GTA (6745G>A), GCC159GCT (6754C>T), GGA161GGG (6760A>G), ATA163TTG (6764A>T 6766A>G), ATT166ATA (6775T>A), CTG169TTG (6782C>T), GCA170GCT (6787A>T), AAC171AAT (6790C>T), GCA173GTG (6795C>T 6796A>G), CAC174CAA (6799C>A), ACC175ACA (6802C>A), AAC177AAT (6808C>T), TAC178TAT (6811C>T), TAC179TAT (6814C>T), TTC182TTT (6823C>T), GAA184GAG (6829A>G), TAC190TAT (6847C>T), CGA192CGC (6853A>C), AAT194GAT (6857A>G), GAG195GAA (6862G>A), GAA198GAG (6871A>G), GTC200ACA (6875G>A 6876T>C 6877C>A), AAG201AAA (6880G>A), TCT202TCA (6883T>A), GTT204GTG (6889T>G), GAC208GAT (6901C>T), GGC213GGT (6916C>T), CCG214CCA (6919G>A), AAA216AAG (6925A>G), CTT217CTC (6928T>C), GAC219GAA (6934C>A), GGG224GGC (6949G>C), TTC226TTT (6955C>T), AAA227AAG (6958A>G), CAA233CAG (6976A>G), CTA235TTA (6980C>T), ATC236ATT (6985C>T), GTA240GTT (6997A>T), GTG241GTT (7000G>T), GTG242ATA (7001G>A 7003G>A)								
NIb protein (NP_734249.1)	1	521	100%	3129	86.1%	519 (99.4%)	480 (92.0%)	1/2/2/2	0
Protein mutations:	F8Y (7032T>A), T39K (7125C>A), A46S (7145G>T 7147A>G), E51_A52del (7160_7165delGAGGCA), D69E (7216T>A), D83E (7258T>A), D88_R89insX (7273_7274insT), R89V (7274C>G 7275G>T), H91L (7281A>T), L92S (7284T>C), S96L (7296C>T), S101F (7311C>T), T102I (7314C>T), C103Y (7317G>A), N104K (7321T>A), K109Q (7334A>C), A112N (7343G>A 7344C>A 7345A>C), S133M (7407G>T 7408T>G), C136G (7415T>G), L165S (7503T>C 7504G>A), D234N (7709G>A), K235R (7713A>G 7714A>G), T268I (7812C>T), V279L (7844G>T 7846G>A), V292I (7883G>A), S345N (8043G>A), N381S (8151A>G 8152C>T), R390K (8178G>A), R400K (8208G>A), P429A (8294C>G 8296A>G), S447F (8349C>T), A467S (8408G>T), M481I (8452G>A), R484K (8460G>A), A491T (8480G>A 8482T>A), R498K (8502G>A 8503A>G), E502D (8515A>T), E510D (8539G>C), L513C (8546C>T 8547T>G), S515T (8552T>A)								
Codon mutations:	GCT1GCC (7012T>C), AAG2AAA (7015G>A), CAT3CAC (7018T>C), TTT8TAT (7032T>A), GCC10GCT (7039C>T), TTG11CTA (7040T>C 7042G>A), GGA13GGG (7048A>G), GCT17GCA (7060T>A), GTC18GTA (7063C>A), AGC23AGT (7078C>T), TTA25CTG (7082T>C 7084A>G), GTA26GTG (7087A>G), ACC27ACA (7090C>A), CAT29CAC (7096T>C), GTA30GTG (7099A>G), GTT31GTC (7102T>C), GAG34GAA (7111G>A), CGA36CGG (7117A>G), ACA39AAA (7125C>A), GAA40GAG (7129A>G), TTT41TTC (7132T>C), CTG42TTA (7133C>T 7135G>A), GTG44GTA (7141G>A), GAT45GAC (7144T>C), GCA46TCG (7145G>T 7147A>G), GAG47GAA (7150G>A), GCA50GCT (7159A>T), GAG51_GCA52del (7160_7165delGAGGCA), TTC54TTT (7171C>T), GCG60GCT (7189G>T), AAA63AAG (7198A>G), AGC64AGT (7201C>T), CTA66TTA (7205C>T), AAT67AAC (7210T>C), GAT69GAA (7216T>A), GCG70GCA (7219G>A), ATC72ATA (7225C>A), AAG73AAA (7228G>A), AAG77AAA (7240G>A), TAT78TAC (7243T>C), AAA80AAG (7249A>G), CCT81CCG (7252T>G), GAT83GAA (7258T>A), GTT84GTC (7261T>C), GGT85GGA (7264T>A), GTC86GTG (7267C>G), GTG87GTA (7270G>A), GAT88_CGG89insT-- (7273_7274insT), CGG89GTG (7274C>G 7275G>T), CAT91CTT (7281A>T), TTG92TCG (7284T>C), TCA96TTA (7296C>T), TCT101TTT (7311C>T), ACC102ATC (7314C>T), TGC103TAC (7317G>A), AAT104AAA (7321T>A), GTG105-TG (7322delG), CAC106CAT (7327C>T), GGC107GGT (7330C>T), TTC108TTT (7333C>T), AAG109CAG (7334A>C), TGT111TGC (7342T>C), GCA112AAC (7343G>A 7344C>A 7345A>C), ACT115ACC (7354T>C), CAA118CAG (7363A>G), ATT120ATC (7369T>C), TTC121TTT (7372C>T), GCG123GCT (7378G>T), CTC124CTT (7381C>T), GCA129GCT (7396A>T), GTC130GTT (7399C>T), GCC132GCT (7405C>T), AGT133ATG (7407G>T 7408T>G), GGG135GGT (7414G>T), TGC136GGC (7415T>G), AAA137AAG (7420A>G), GAC140GAT (7429C>T), TAT141TAC (7432T>C), CAT144CAC (7441T>C), AAG150AAA (7459G>A), GAA151GAG (7462A>G), ATA153ATT (7468A>T), GTC154GTA (7471C>A), CAA156CAG (7477A>G), AGC157AGT (7480C>T), CTG159CTT (7486G>T), TAT162TAC (7495T>C), AAA163AAG (7498A>G), GGT164GGC (7501T>C), TTG165TCA (7503T>C 7504G>A), ATT168ATA (7513T>A), AAC170AAT (7519C>T), GGA171GGG (7522A>G), AAG174AAA (7531G>A), GAG176GAA (7537G>A), CTC177CTT (7540C>T), TGT179TGC (7546T>C), AAG187AAA (7570G>A), ACG188ACA (7573G>A), ACG190ACA (7579G>A), GCT193GCC (7588T>C), CTA196TTA (7595C>T), TTG199CTA (7604T>C 7606G>A), GGT202GGA (7615T>A), TGT205TGC (7624T>C), GAC208GAT (7633C>T), TTC209TTT (7636C>T), AAT210AAC (7639T>C), TTT213TTC (7648T>C), GAA219GAG (7666A>G), TGT221TGC (7672T>C), ACA223ACT (7678A>T), GGG225GGA (7684G>A), ACT227ACC (7690T>C), TTT229TTC (7696T>C), GGT231GGG (7702T>G), GAT234AAT (7709G>A), AAA235AGG (7713A>G 7714A>G), CTG236TTG (7715C>T), CGT239CGC (7726T>C), GAG242GAA (7735G>A), GTA245GTG (7744A>G), TAC246TAT (7747C>T), GGC251GGT (7762C>T), CAG253CAA (7768G>A), TTT254TTC (7771T>C), TCA257TCG (7780A>G), CTA258CTC (7783A>C), ACT259ACC (7786T>C), CTA262TTG (7793C>T 7795A>G), ATC263ATT (7798C>T), GCT265GCC (7804T>C), ACC268ATC (7812C>T), GAA275GAG (7834A>G), GAC276GAT (7837C>T), GTG279TTA (7844G>T 7846G>A), CAG282CAA (7855G>A), CTG284CTA (7861G>A), TTA287TTG (7870A>G), TAC288TAT (7873C>T), ACT289ACA (7876T>A), GAG290GAA (7879G>A), ATT291ATA (7882T>A), GTT292ATT (7883G>A), CCA295CCT (7894A>T), ATT296ATC (7897T>C), GTC304GTT (7921C>T), AAG306AAA (7927G>A), GGT309GGC (7936T>C), AAT310AAC (7939T>C), AAT311AAC (7942T>C), GTG319GTA (7966G>A), GAT320GAC (7969T>C), TCT322TCC (7975T>C), GTC326GTT (7987C>T), CTT327CTC (7990T>C), GCT332GCC (8005T>C), GAA341GAG (8032A>G), ATC343ATT (8038C>T), GAC344GAT (8041C>T), AGC345AAC (8043G>A), TTG357TTA (8080G>A), GTG360GTA (8089G>A), CCG362CCA (8095G>A), GAG363GAA (8098G>A), AGC366AGT (8107C>T), ATT367ATC (8110T>C), GAT369GAC (8116T>C), CAA373CAG (8128A>G), GGT379GGC (8146T>C), AAC381AGT (8151A>G 8152C>T), TCG385TCA (8164G>A), AGG390AAG (8178G>A), AAG391AAA (8182G>A), TTG394TTA (8191G>A), TTC396TTT (8197C>T), TCC398TCT (8203C>T), AGA400AAA (8208G>A), CTG402TTA (8213C>T 8215G>A), CTA403CTG (8218A>G), ATC404ATT (8221C>T), GAG405GAA (8224G>A), CCA410CCG (8239A>G), GAG415GAA (8254G>A), ATT417ATC (8260T>C), GTA418GTG (8263A>G), GAT424GAC (8281T>C), AGA425AGG (8284A>G), CCA429GCG (8294C>G 8296A>G), GAG430GAA (8299G>A), CAC431CAT (8302C>T), ATT436ATC (8317T>C), TGC437TGT (8320C>T), GCT439GCA (8326T>A), GAG442GAA (8335G>A), TCC443TCT (8338C>T), TCT447TTT (8349C>T), GAA448GAG (8353A>G), CAC451CAT (8362C>T), CAA452CAG (8365A>G), ATC453ATT (8368C>T), TAC457TAT (8380C>T), TCA458TCG (8383A>G), TTA460TTG (8389A>G), CCT465CCC (8404T>C), GCA467TCA (8408G>T), ACA468ACG (8413A>G), GCG470GCT (8419G>T), GCT475GCC (8434T>C), TAT477TAC (8440T>C), ATG481ATA (8452G>A), CTA483CTG (8458A>G), AGG484AAG (8460G>A), AAA485AAG (8464A>G), CTG486CTA (8467G>A), AGG490AGA (8479G>A), GCT491ACA (8480G>A 8482T>A), GAG496GAA (8497G>A), CTA497TTG (8498C>T 8500A>G), AGA498AAG (8502G>A 8503A>G), GCC499GCT (8506C>T), GAA502GAT (8515A>T), GTC505GTT (8524C>T), GCA506GCC (8527A>C), TTA507CTG (8528T>C 8530A>G), GAG510GAC (8539G>C), CTT513TGT (8546C>T 8547T>G), TCT515ACT (8552T>A), GTA518GTG (8563A>G)								
coat protein (NP_734250.1)	1	267	100%	1652	90.6%	267 (100%)	243 (91.0%)	0/0/0/0	0
Protein mutations:	A1G (8574C>G), I5V (8585A>G), G9E (8598G>A), N11S (8603A>T 8604A>C 8605C>T), P17Q (8622C>A), E18G (8625A>G 8626G>A), P26F (8648C>T 8649C>T 8650G>C), G29E (8658G>A), D31A (8664A>C 8665T>G), A36V (8679C>T 8680A>T), T58K (8745C>A 8746A>G), E95G (8856A>G 8857G>A), R98Q (8865G>A), M99L (8867A>C 8869G>T), G104E (8883G>A), T106S (8888A>T), V128I (8954G>A), N138D (8984A>G 8986T>C), E139V (8988A>T), I187V (9131A>G), M193G (9149A>G 9150T>G 9151G>A), G194S (9152G>A), P230S (9260C>T 9262T>C), Q242H (9298A>C)								
Codon mutations:	GCA1GGA (8574C>G), ATT5GTT (8585A>G), GGA9GAA (8598G>A), AGC10AGT (8602C>T), AAC11TCT (8603A>T 8604A>C 8605C>T), AAA16AAG (8620A>G), CCA17CAA (8622C>A), GAG18GGA (8625A>G 8626G>A), CAG19CAA (8629G>A), GGC20GGT (8632C>T), ATC22ATT (8638C>T), CAG23CAA (8641G>A), AAC25AAT (8647C>T), CCG26TTC (8648C>T 8649C>T 8650G>C), AAC27AAT (8653C>T), AAA28AAG (8656A>G), GGA29GAA (8658G>A), GAT31GCG (8664A>C 8665T>G), GTT34GTG (8674T>G), GCA36GTT (8679C>T 8680A>T), GGC37GGA (8683C>A), GGG40GGA (8692G>A), ACA41ACT (8695A>T), CCG45CCA (8707G>A), AGA46CGA (8708A>C), AAG48AAA (8716G>A), ATC50ATT (8722C>T), ACG51ACA (8725G>A), AAA53AAG (8731A>G), AGA55AGG (8737A>G), CCC57CCT (8743C>T), ACA58AAG (8745C>A 8746A>G), AAG60AAA (8752G>A), GGA61GGT (8755A>T), AAC66AAT (8770C>T), TTA67CTA (8771T>C), GAA68GAG (8776A>G), CAT69CAC (8779T>C), TTG70TTA (8782G>A), TAT73TAC (8791T>C), CCA75CCG (8797A>G), CAA76CAG (8800A>G), CAA77CAG (8803A>G), GAT79GAC (8809T>C), ATT80ATC (8812T>C), AAT82AAC (8818T>C), CGG84CGA (8824G>A), GCA85GCG (8827A>G), ACG92ACA (8848G>A), TAT94TAC (8854T>C), GAG95GGA (8856A>G 8857G>A), GTG97GTA (8863G>A), CGG98CAG (8865G>A), ATG99CTT (8867A>C 8869G>T), GCA100GCG (8872A>G), GGA104GAA (8883G>A), ACT106TCT (8888A>T), TGC119TGT (8929C>T), GTC128ATC (8954G>A), AAC129AAT (8959C>T), GGA130GGT (8962A>T), GGG137GGA (8983G>A), AAT138GAC (8984A>G 8986T>C), GAA139GTA (8988A>T), GAG142GAA (8998G>A), CCG144CCA (9004G>A), TTG145TTA (9007G>A), ATC148ATT (9016C>T), ACC155ACA (9037C>A), ATA171ATT (9085A>T), AAC175AAT (9097C>T), ATT187GTT (9131A>G), CGA188CGT (9136A>T), CGG191CGT (9145G>T), ATG193GGA (9149A>G 9150T>G 9151G>A), GGT194AGT (9152G>A), TTA195TTG (9157A>G), GCG196GCT (9160G>T), CGT197CGC (9163T>C), GAG204GAA (9184G>A), CGA208CGG (9196A>G), AGG214AGA (9214G>A), GAA215GAG (9217A>G), GCG216GCA (9220G>A), GCA224GCT (9244A>T), TTG225TTA (9247G>A), GCC228GCT (9256C>T), CCT230TCC (9260C>T 9262T>C), GGG234GGA (9274G>A), GAC236GAT (9280C>T), ATC239ATT (9289C>T), CAA242CAC (9298A>C), GAG244GAA (9304G>A), GTC254GTT (9334C>T), GTC264GTG (9364C>G)								

	Begin	End	Coverage	Score	Concordance	Matches	Identities	I/D/M/F*	Stop Codons
**NT**	**52**	**9704**	**99.5%**	**12530**	**65.2%**	**9642 (99.8%)**	**7985 (82.7%)**	**7/11**	
PIPO (YP_006393460.1)	1	75	100%	230	43.7%	75 (98.7%)	55 (72.4%)	1/0/2/1	2
Protein mutations:	R17_K17insX (2971_2972insA), Y23H (2989T>C 2991C>T), R26K (2999G>A 3000A>G), H35Y (3025C>T), K40H (3040A>C 3042A>C), G41S (3043G>A), E44K (3052G>A), I47V (3061A>G), V54I (3082G>A), P57S (3091C>T), P60T (3100C>A), G61E (3104G>A 3105T>G), G62S (3106G>A), Q63* (3109C>T), R64G (3112A>G), C66R (3118T>C), L67F (3121C>T), I69V (3127A>G), A72E (3137C>A 3138G>A), I73V (3139A>G)								
Codon mutations:	TTA2CTA (2923T>C), AAA4AAG (2931A>G), GAA7GAG (2940A>G), TTG10CTG (2947T>C), TTG15CTG (2962T>C), AGA17_AAA17insAA- (2971_2972insA), TAC23CAT (2989T>C 2991C>T), TCA24TCG (2994A>G), AGA26AAG (2999G>A 3000A>G), ATC31ATT (3015C>T), TCG33TCA (3021G>A), GTA34GTT (3024A>T), CAT35TAT (3025C>T), CAC38CAT (3036C>T), AGA39AGG (3039A>G), AAA40CAC (3040A>C 3042A>C), GGC41AGC (3043G>A), GAA44AAA (3052G>A), ATA47GTA (3061A>G), GTT54ATT (3082G>A), CCG57TCG (3091C>T), CGC59CGT (3099C>T), CCA60ACA (3100C>A), GGT61GAG (3104G>A 3105T>G), GGT62AGT (3106G>A), CAA63TAA (3109C>T), AGG64GGG (3112A>G), TGC66CGC (3118T>C), CTC67TTC (3121C>T), ATT69GTT (3127A>G), GAG70GAA (3132G>A), GCG72GAA (3137C>A 3138G>A), ATT73GTT (3139A>G)								

*: Inserts / Deletes / Misaligned / Frameshifts

Analysis details

This analysis was performed with panviral2.64

NGS Details (UN8): Torradovirus lycopersici (segment RNA 1)

Assembly

**Coverage Length**	6786 (1 contig(s))
**Depth Of Coverage**	1527.7
**Number Of Reads**	78839
**Reads Per Million**	1317.60 rpm (after QC)
**Ambiguities**	0
**Assembly Method**	de novo + reference guided assembly
**Consensus Caller**	Bcf Tools

Coverage Map

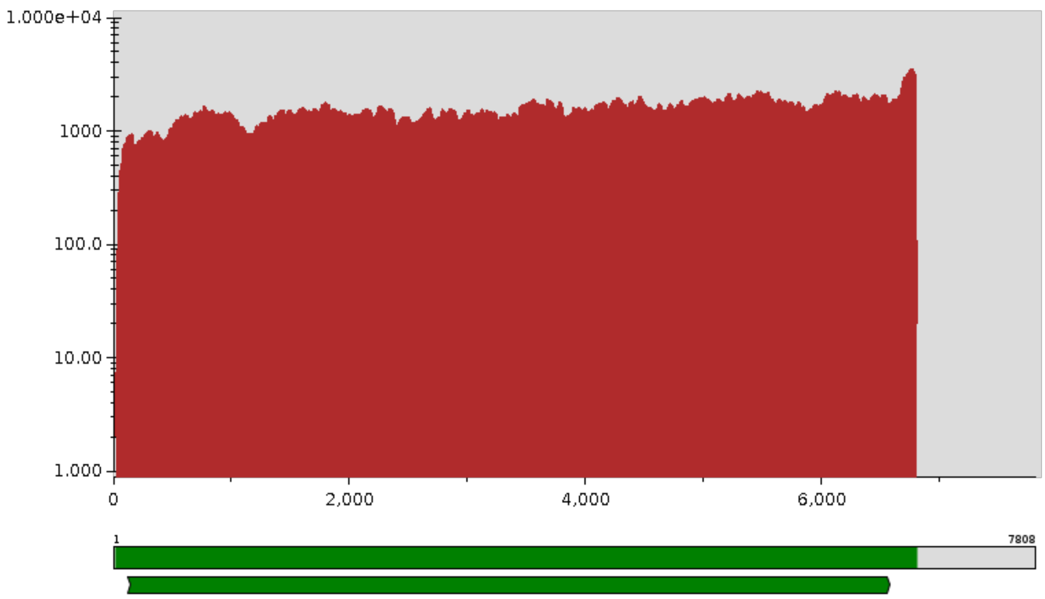

Assignment

**Type**	Torradovirus lycopersici (Taxonomy ID: 3048378)
**Reference Genome**	NC_009013.1
**NT Identity (%)**	78.5609
**AA Identity (%)**	89.9027
**Number Of Stop Codons**	1
**Number Of CDS**	1

Alignment

**Alignment Score**	7777.0 (NT) + 13837.0 (AA) = 21614.0
**Concordance (%)**	75.4916

**Alignment Method**	Global, seeded, nucleotide + amino acids (AGA)

Genome Region

Sequence starts at position 21 and ends at position 6806 relative to NC_009013.1 reference sequence.

Alignment Detailed Statistics

	Begin	End	Coverage	Score	Concordance	Matches	Identities	I/D/M/F*	Stop Codons
**NT**	**21**	**6806**	**86.9%**	**7777**	**57.4%**	**6785 (99.6%)**	**5350 (78.5%)**	**25/1**	
Mutations:	65A>G, 72C>T, 77A>T, 78A>G, 83C>T, 83_84insTACTTTATATTGTTTTGTGCT, 88T>C, 90C>G, 92T>C, 93T>A, 101G>A, 102G>C, 103C>A, 104A>G, 115T>C, 118C>T, 121G>A, 122A>T, 124G>A, 128C>T, 130C>T, 134T>C, 135T>C, 136C>T, 138A>G, 140T>C, 142A>C, 144T>C, 145T>A, 147C>T, 148T>G, 151A>G, 155T>A, 157C>T, 159C>T, 161A>G, 178T>G, 187A>T, 196T>A, 197G>C, 198C>A, 199T>A, 208T>C, 211T>C, 214C>T, 220C>T, 223T>A, 226G>T, 247A>C, 253A>G, 256T>C, 262T>A, 265C>T, 271A>G, 275T>C, 280G>A, 283T>C, 286A>G, 289T>G, 292A>T, 301T>A, 302T>A, 307C>T, 310T>C, 311A>T, 313C>T, 315G>A, 316C>T, 322T>C, 324A>G, 325C>T, 328T>A, 334T>C, 337A>G, 340G>A, 343A>G, 349G>A, 358T>A, 359T>C, 364T>A, 367A>G, 370C>T, 373C>T, 376C>A, 382T>A, 394C>T, 397A>C, 400C>T, 409A>G, 412A>C, 415T>C, 427T>G, 433G>A, 439T>C, 440G>A, 443A>G, 451A>G, 457G>A, 473A>G, 485G>A, 487C>T, 493T>C, 496G>A, 502G>T, 526T>C, 553T>A, 559C>T, 568A>T, 573G>A, 578C>T, 586A>T, 595G>A, 599A>T, 601T>A, 604C>T, 606T>C, 607A>T, 615A>G, 618C>T, 619A>T, 628C>T, 640T>C, 650T>C, 652A>G, 655C>G, 659T>C, 661A>G, 664G>C, 673C>T, 676A>G, 694A>G, 707C>T, 715G>A, 718A>G, 719T>G, 721C>T, 722A>G, 727A>T, 730C>T, 739T>G, 748G>T, 751A>T, 754A>G, 758C>T, 760C>A, 763A>T, 766C>T, 772G>C, 775T>A, 778T>A, 781C>A, 790C>T, 793G>A, 796T>A, 799A>T, 802T>G, 805T>C, 806A>C, 808A>T, 817T>A, 820A>C, 821T>A, 826C>T, 829G>A, 832A>C, 835A>C, 838A>G, 842C>T, 844G>A, 847C>T, 853G>A, 862T>A, 865T>C, 877T>C, 881T>C, 883G>T, 886T>C, 889C>A, 895C>T, 898G>A, 901T>C, 913T>C, 949A>T, 958T>A, 964T>A, 967T>C, 976A>T, 977G>A, 979T>A, 988T>A, 991T>C, 1001T>C, 1003G>T, 1004T>G, 1006T>A, 1009G>A, 1018C>T, 1019A>G, 1020C>T, 1033G>A, 1054A>C, 1060T>C, 1063C>A, 1064T>C, 1066G>T, 1072T>C, 1078A>G, 1081C>T, 1093T>C, 1096C>A, 1109C>T, 1111A>G, 1129A>G, 1135T>A, 1141C>T, 1147T>C, 1159A>G, 1165C>A, 1180G>A, 1183A>T, 1186G>A, 1189A>C, 1192A>C, 1195A>T, 1196A>T, 1198C>T, 1204T>G, 1207T>G, 1219G>C, 1225C>T, 1237T>A, 1240C>T, 1243G>T, 1246C>T, 1249A>G, 1255G>A, 1258A>T, 1264T>C, 1285T>A, 1291T>C, 1300G>A, 1309C>A, 1318G>A, 1319C>T, 1321G>A, 1327C>T, 1336T>G, 1337T>C, 1339G>T, 1343C>A, 1345C>G, 1351A>T, 1354G>A, 1357A>C, 1361T>C, 1363G>T, 1367A>C, 1372C>G, 1378T>C, 1381G>A, 1390A>T, 1405A>C, 1408T>C, 1411T>C, 1426T>A, 1432G>T, 1450T>A, 1451C>T, 1462G>T, 1465A>T, 1471A>G, 1480C>T, 1481A>G, 1486G>A, 1495A>T, 1501C>T, 1507C>A, 1510T>A, 1516A>T, 1522C>A, 1525G>A, 1528C>A, 1534A>T, 1553C>A, 1555C>G, 1568C>A, 1570A>G, 1573T>C, 1576A>T, 1582C>T, 1585A>C, 1588C>A, 1591C>T, 1600A>C, 1607G>T, 1609T>A, 1612G>A, 1618T>G, 1624C>A, 1643A>C, 1645A>G, 1649C>A, 1657T>G, 1660T>G, 1663G>A, 1669C>T, 1681T>A, 1684G>A, 1687T>C, 1691A>G, 1692C>T, 1693T>A, 1699T>A, 1702C>T, 1703A>G, 1705T>A, 1706T>G, 1708T>C, 1714T>G, 1715G>A, 1716A>G, 1726G>C, 1729T>G, 1732G>A, 1735C>T, 1737A>G, 1738A>G, 1739A>C, 1741A>T, 1744C>A, 1755C>T, 1756A>C, 1762G>A, 1769A>G, 1770G>A, 1771C>T, 1777T>C, 1780T>C, 1781T>C, 1783G>A, 1786G>T, 1810G>A, 1813T>G, 1819A>C, 1825C>A, 1828A>T, 1831A>C, 1837G>C, 1839A>T, 1844G>A, 1849A>C, 1850T>A, 1852A>C, 1858G>A, 1876G>C, 1877T>A, 1879A>T, 1885G>A, 1889A>G, 1891A>G, 1894A>T, 1906T>A, 1907A>C, 1908T>C, 1909G>A, 1911T>G, 1915T>C, 1918C>T, 1921A>G, 1924T>C, 1927C>A, 1936T>C, 1937G>A, 1939T>A, 1942G>A, 1945T>G, 1948C>G, 1951G>A, 1957T>G, 1960A>C, 1978G>A, 1984C>T, 1994T>C, 1996G>A, 2002G>T, 2005A>G, 2008T>C, 2014A>G, 2016A>T, 2020T>C, 2023T>C, 2044A>G, 2050T>C, 2053C>T, 2056T>A, 2062C>T, 2071A>G, 2082T>A, 2092A>T, 2099T>C, 2101C>T, 2110A>G, 2111A>T, 2113A>C, 2119C>T, 2120A>C, 2128T>C, 2130A>C, 2131T>A, 2134C>T, 2140G>C, 2141T>C, 2143T>A, 2146A>G, 2149T>A, 2155T>C, 2158T>C, 2160A>C, 2164C>T, 2176G>C, 2177A>G, 2179A>T, 2182G>A, 2188C>T, 2194T>A, 2200T>C, 2224C>A, 2227C>T, 2230T>G, 2240A>T, 2241A>G, 2245G>T, 2248T>C, 2250G>A, 2251C>A, 2257C>T, 2260T>G, 2263G>A, 2270C>T, 2272G>A, 2281A>C, 2282A>T, 2287G>A, 2290C>T, 2296C>T, 2299A>T, 2305T>A, 2311G>T, 2314C>T, 2318C>T, 2320G>A, 2323A>T, 2326C>T, 2327G>A, 2329G>C, 2332G>A, 2335G>A, 2341T>A, 2344C>A, 2347C>T, 2350A>G, 2356C>T, 2359G>A, 2365A>T, 2368A>T, 2371T>C, 2375A>C, 2377G>T, 2380T>C, 2387C>A, 2389C>A, 2392A>G, 2404G>A, 2407T>C, 2409G>A, 2410G>A, 2416C>T, 2476A>G, 2479G>A, 2485C>T, 2488G>A, 2491A>G, 2494T>G, 2497G>T, 2500T>C, 2503G>A, 2506T>A, 2509C>G, 2510C>T, 2512T>G, 2515T>C, 2516T>C, 2524A>C, 2536A>T, 2542G>T, 2554A>T, 2557C>T, 2563T>G, 2571A>G, 2572A>G, 2575C>A, 2587T>C, 2600A>C, 2602A>G, 2611T>C, 2614C>A, 2617C>T, 2626T>C, 2629C>T, 2639C>A, 2650G>A, 2662C>T, 2677G>A, 2678C>T, 2683A>G, 2692G>A, 2695T>A, 2704G>T, 2710A>G, 2716A>C, 2726G>A, 2731C>T, 2743A>C, 2744T>C, 2761C>G, 2765T>C, 2767G>C, 2768A>C, 2776T>G, 2788C>T, 2800C>A, 2806C>T, 2809C>T, 2812G>T, 2815C>A, 2819G>A, 2821G>A, 2827A>T, 2830G>A, 2842T>C, 2845A>C, 2848C>T, 2852C>A, 2854T>A, 2860C>G, 2863A>T, 2864A>G, 2865C>T, 2866T>G, 2869C>T, 2875A>T, 2878A>C, 2882G>T, 2884C>G, 2890A>C, 2908C>T, 2911T>C, 2926G>T, 2929C>T, 2930T>C, 2932G>A, 2935G>A, 2941A>G, 2947T>A, 2950C>G, 2953G>T, 2956C>G, 2959T>C, 2965A>T, 2971C>T, 2974T>C, 2980C>G, 2986T>G, 2992T>C, 2998A>G, 3011C>A, 3025T>C, 3031T>A, 3034G>A, 3037C>A, 3038A>T, 3046G>T, 3049A>C, 3052T>C, 3064A>T, 3073G>A, 3079T>C, 3083C>A, 3085A>G, 3101T>C, 3103G>T, 3115G>T, 3124C>A, 3136T>G, 3146A>T, 3148G>C, 3160C>T, 3166T>C, 3169C>A, 3178G>A, 3184T>A, 3196T>C, 3202T>C, 3226T>C, 3238T>G, 3239C>T, 3241T>A, 3242C>T, 3244T>G, 3247G>A, 3253C>T, 3256G>A, 3265A>G, 3274C>T, 3277T>A, 3286T>A, 3298T>A, 3310A>G, 3319C>T, 3325G>C, 3328T>C, 3331A>T, 3334A>C, 3346A>G, 3349T>G, 3352T>C, 3356C>A, 3365T>C, 3371G>A, 3373T>C, 3374C>T, 3376T>G, 3379G>A, 3382T>A, 3385T>G, 3388G>T, 3394A>G, 3400A>C, 3406A>G, 3412C>T, 3413C>G, 3416C>T, 3418C>G, 3421T>G, 3428C>T, 3430A>G, 3431C>A, 3439G>A, 3445G>T, 3457A>G, 3466G>A, 3474T>A, 3475T>C, 3476G>C, 3481C>A, 3484A>T, 3492C>A, 3505C>A, 3508G>T, 3514C>A, 3520A>G, 3523A>C, 3526G>A, 3529G>C, 3530C>A, 3532A>G, 3534G>A, 3541C>T, 3542T>C, 3544G>C, 3553T>A, 3559G>A, 3565A>T, 3580T>C, 3583C>A, 3586T>C, 3589A>G, 3591C>T, 3592T>C, 3593T>C, 3594C>A, 3602C>A, 3604T>A, 3607G>A, 3610A>G, 3613T>C, 3622G>A, 3625C>T, 3628G>C, 3629G>T, 3637A>C, 3640A>G, 3643A>G, 3646C>T, 3652A>G, 3658A>C, 3661A>G, 3664T>C, 3673G>A, 3676C>T, 3679T>C, 3682G>C, 3684G>A, 3685G>A, 3686C>T, 3688A>G, 3691C>T, 3693A>T, 3700G>A, 3701C>T, 3703A>G, 3706T>G, 3709A>G, 3710G>T, 3712A>T, 3713A>G, 3715T>G, 3716T>C, 3722G>A, 3724G>A, 3729A>G, 3733C>G, 3742T>G, 3752A>G, 3758G>A, 3763A>T, 3766G>A, 3776G>A, 3778G>T, 3781G>T, 3782C>T, 3784C>G, 3790T>A, 3793T>C, 3802T>C, 3808T>C, 3814C>T, 3817G>A, 3824T>C, 3841G>T, 3847G>A, 3850C>T, 3856G>A, 3868C>T, 3871T>C, 3883G>A, 3889G>A, 3895A>C, 3901A>C, 3907A>T, 3910T>C, 3911C>A, 3916C>G, 3920C>T, 3922C>G, 3925A>G, 3928G>A, 3934T>C, 3949C>T, 3955G>A, 3958T>A, 3959T>C, 3961G>A, 3973T>C, 3982A>T, 3995C>T, 4000C>T, 4003C>A, 4006C>A, 4009A>G, 4015C>A, 4018A>G, 4028T>C, 4030G>A, 4033G>T, 4034T>C, 4039A>G, 4045T>G, 4051G>C, 4055C>A, 4063C>A, 4067T>C, 4069G>A, 4075A>G, 4078T>A, 4081T>A, 4084C>T, 4093C>T, 4096T>C, 4118C>T, 4123T>C, 4129C>T, 4147C>G, 4157C>T, 4160C>G, 4162A>T, 4189T>A, 4192T>G, 4195A>G, 4202C>T, 4204G>A, 4205T>C, 4207G>A, 4210T>A, 4213T>C, 4214G>A, 4219C>T, 4220A>C, 4222A>G, 4225A>T, 4228C>T, 4237A>G, 4246T>C, 4249T>A, 4252C>T, 4255T>C, 4282C>T, 4288C>A, 4294A>G, 4303T>C, 4309T>G, 4318C>T, 4327A>C, 4335C>A, 4336T>A, 4339T>C, 4342T>C, 4348C>T, 4351C>A, 4354T>C, 4358A>C, 4360A>C, 4363T>C, 4366G>A, 4367C>T, 4369T>G, 4372T>G, 4381T>C, 4384A>T, 4387C>T, 4396T>C, 4399T>C, 4405G>A, 4414T>G, 4417T>C, 4418A>C, 4420G>C, 4421T>C, 4426G>T, 4429A>G, 4432T>C, 4438T>A, 4441A>T, 4444A>G, 4450A>T, 4453C>T, 4456C>T, 4459T>C, 4462C>T, 4465C>T, 4468C>T, 4495T>G, 4501A>T, 4507T>C, 4510T>A, 4513A>G, 4514T>C, 4522A>G, 4528T>C, 4534A>T, 4537T>A, 4540A>G, 4546A>T, 4549G>C, 4552C>T, 4555A>G, 4558C>A, 4570C>T, 4576A>T, 4582G>T, 4585A>T, 4588C>A, 4600C>T, 4606A>T, 4615T>A, 4618C>A, 4623A>G, 4624A>T, 4625T>C, 4627G>C, 4636T>C, 4637A>T, 4639T>G, 4648T>A, 4651T>A, 4660A>G, 4661T>C, 4663G>A, 4678T>C, 4684A>C, 4696C>A, 4702G>A, 4705C>T, 4714T>C, 4716G>A, 4717C>T, 4718A>C, 4719G>A, 4732A>G, 4735C>T, 4736T>C, 4738G>T, 4741T>A, 4745C>T, 4747A>G, 4753T>C, 4756A>T, 4759C>T, 4762A>G, 4765T>C, 4768T>C, 4771G>A, 4774G>A, 4786A>G, 4789C>T, 4801T>C, 4802C>T, 4813A>G, 4816T>C, 4825A>G, 4828A>C, 4834C>T, 4840A>T, 4843G>A, 4846T>C, 4850G>A, 4851T>C, 4867T>C, 4868C>T, 4870C>G, 4879G>A, 4885C>T, 4888A>T, 4891C>T, 4894G>T, 4897A>C, 4898G>A, 4903A>G, 4909A>C, 4915C>T, 4921G>T, 4927G>A, 4930T>G, 4936A>C, 4945G>T, 4947C>A, 4948A>T, 4954T>G, 4958A>C, 4963T>C, 4969G>A, 4975G>A, 4981C>T, 4984A>T, 4987C>T, 4988A>G, 4990C>A, 4999T>G, 5002A>T, 5005T>C, 5017G>A, 5020G>A, 5023C>T, 5029G>T, 5030G>A, 5035G>A, 5038T>A, 5041A>T, 5044A>C, 5050A>T, 5059G>T, 5062C>T, 5072C>T, 5074T>A, 5079T>C, 5080T>A, 5083A>T, 5089A>C, 5090G>T, 5092T>A, 5098G>T, 5101T>A, 5102T>C, 5105C>G, 5107C>T, 5110A>T, 5113C>T, 5114A>G, 5116T>A, 5119T>C, 5120G>A, 5122T>A, 5123C>T, 5131T>C, 5132T>A, 5141G>A, 5143G>T, 5146T>C, 5149T>A, 5152T>C, 5155T>A, 5159C>A, 5173C>T, 5176C>T, 5177T>G, 5179G>C, 5180C>A, 5182A>G, 5185A>T, 5194A>C, 5197G>T, 5200G>A, 5206C>T, 5207A>T, 5209C>A, 5212C>A, 5218T>C, 5227C>T, 5233T>C, 5239A>C, 5242G>A, 5257A>T, 5260A>G, 5263G>A, 5266C>A, 5269C>A, 5276A>C, 5278G>A, 5290G>T, 5293T>C, 5296T>C, 5302T>A, 5314T>C, 5326C>T, 5329C>T, 5336A>G, 5341T>C, 5342G>A, 5344A>C, 5347C>A, 5353G>A, 5357A>T, 5359C>T, 5360A>C, 5362A>C, 5366C>A, 5368T>C, 5369G>A, 5371G>T, 5374T>A, 5377T>C, 5386C>G, 5389T>C, 5392G>A, 5399A>G, 5401T>G, 5404T>A, 5407A>T, 5410G>T, 5413C>T, 5416T>A, 5419A>T, 5420T>C, 5422G>A, 5429C>T, 5431G>A, 5434C>T, 5437T>G, 5440C>A, 5443A>G, 5446T>C, 5449T>A, 5452T>C, 5459G>A, 5461T>C, 5462G>A, 5467G>C, 5479C>T, 5482A>T, 5485A>T, 5486G>A, 5491T>G, 5492A>C, 5494A>T, 5498C>T, 5500C>G, 5503T>C, 5506A>T, 5509T>C, 5512A>T, 5515A>T, 5524C>T, 5527G>A, 5530T>C, 5531C>T, 5533T>G, 5534C>G, 5537A>C, 5543G>T, 5548T>C, 5551A>C, 5558G>C, 5566T>A, 5569A>T, 5575A>G, 5578T>C, 5582A>G, 5584T>G, 5591G>T, 5593C>A, 5602G>A, 5603A>C, 5604C>T, 5608A>G, 5611A>T, 5613A>T, 5617A>T, 5620T>A, 5626A>T, 5632T>C, 5638T>G, 5641T>C, 5656G>A, 5659A>G, 5662G>A, 5664G>A, 5665A>T, 5672C>T, 5673T>G, 5674A>T, 5675C>A, 5676A>G, 5677G>T, 5686G>A, 5687C>A, 5689C>A, 5690T>C, 5692G>T, 5707A>T, 5710T>A, 5711T>C, 5716G>T, 5719A>G, 5734T>C, 5737T>C, 5739G>A, 5743G>A, 5746T>A, 5749G>A, 5751T>G, 5753C>T, 5767G>T, 5773G>C, 5774A>G, 5776A>G, 5779T>C, 5784T>C, 5785T>A, 5786T>C, 5788A>T, 5789G>T, 5791C>G, 5794A>G, 5797A>G, 5799A>G, 5800G>T, 5804T>A, 5805T>C, 5806C>T, 5812T>C, 5815C>G, 5818C>T, 5826A>T, 5830C>T, 5836A>C, 5843G>A, 5845G>A, 5846A>T, 5848G>C, 5849T>C, 5850T>A, 5851G>A, 5857T>G, 5860C>A, 5864C>T, 5866T>G, 5872C>A, 5878G>T, 5881T>C, 5884T>C, 5887C>T, 5890C>T, 5894A>C, 5902G>A, 5907A>T, 5914T>C, 5923T>C, 5929G>A, 5942C>T, 5943A>T, 5944C>T, 5950G>A, 5953A>T, 5955A>G, 5956C>T, 5965G>T, 5968T>G, 5971C>T, 5974T>C, 5975A>C, 5977A>T, 5980G>A, 5983T>C, 5986T>C, 5989C>T, 5990T>G, 5992A>C, 5998A>G, 6002A>G, 6003C>T, 6004C>T, 6009G>A, 6010G>A, 6013C>A, 6016A>G, 6022T>A, 6028C>T, 6031A>T, 6033C>T, 6034A>C, 6035G>T, 6040T>A, 6043G>A, 6044T>A, 6046A>G, 6049G>A, 6050T>C, 6055T>G, 6059G>A, 6062G>A, 6064T>C, 6067C>T, 6070T>C, 6079C>G, 6085T>C, 6088G>A, 6089C>T, 6092C>T, 6094T>A, 6097T>A, 6100T>G, 6103G>A, 6106G>A, 6112A>G, 6121A>T, 6124C>A, 6125A>C, 6129A>G, 6130G>T, 6139T>C, 6142A>G, 6145T>C, 6148T>C, 6150G>C, 6152G>T, 6154C>G, 6160G>A, 6163C>G, 6175G>A, 6177A>G, 6184A>G, 6185A>G, 6186C>A, 6187T>G, 6189A>G, 6193C>T, 6196A>G, 6199C>T, 6202G>C, 6208A>G, 6214T>G, 6215C>G, 6217G>T, 6218A>C, 6221G>T, 6222A>C, 6223A>T, 6225G>A, 6226G>A, 6227A>G, 6229C>G, 6230A>G, 6232A>G, 6235G>A, 6237A>G, 6238A>T, 6239G>A, 6240T>C, 6241G>A, 6242A>G, 6244A>G, 6251T>C, 6253A>C, 6256C>A, 6262A>T, 6263C>T, 6266T>A, 6268G>T, 6273G>T, 6274C>G, 6283G>A, 6287C>T, 6289T>G, 6291T>A, 6292T>A, 6294A>G, 6296G>C, 6298A>C, 6301T>A, 6302T>A, 6303T>C, 6304A>G, 6307C>A, 6308T>C, 6310G>T, 6311G>T, 6312A>C, 6313T>C, 6316C>T, 6317A>G, 6319A>G, 6322G>A, 6323A>G, 6325C>G, 6328G>A, 6334A>G, 6337T>C, 6344C>T, 6364T>C, 6371G>A, 6373A>T, 6382C>T, 6383A>G, 6385C>G, 6397C>A, 6404T>C, 6405G>A, 6412C>T, 6413C>G, 6414G>T, 6415A>G, 6416T>A, 6417C>A, 6418A>C, 6421A>T, 6433G>T, 6436C>T, 6437T>A, 6445C>T, 6448G>A, 6449A>T, 6455T>G, 6457C>A, 6458T>A, 6461C>T, 6463T>G, 6472C>T, 6478G>T, 6479C>T, 6481T>A, 6485C>A, 6490A>C, 6491C>A, 6494C>G, 6495A>T, 6496G>A, 6497A>C, 6499A>T, 6500A>G, 6501T>A, 6502T>G, 6503G>A, 6505T>C, 6514G>T, 6515T>C, 6517C>A, 6518T>C, 6520C>T, 6529A>T, 6541G>A, 6544G>A, 6545C>T, 6547T>G, 6548T>C, 6552C>T, 6553A>G, 6556A>C, 6562A>T, 6566G>A, 6567G>A, 6568C>T, 6571C>T, 6574A>G, 6587G>A, 6591T>A, 6592A>T, 6593A>T, 6595T>C, 6602G>A, 6603C>T, 6647_6648insGTTA, 6669G>A, 6670C>T, 6675C>T, 6685A>G, 6692A>C, 6715C>T, 6716G>A, 6721A>T, 6727T>C, 6742A>T, 6752T>C, 6759G>A, 6766delC, 6776C>A, 6784C>T, 6786T>C, 6787G>A								

CDS

ToTV_sRNA1gp1	1	2159	100%	13837	91.8%	2159 (100%)	1941 (89.9%)	0/0/0/0	1

	Begin	End	Coverage	Score	Concordance	Matches	Identities	I/D/M/F*	Stop Codons
**NT**	**21**	**6806**	**86.9%**	**7777**	**57.4%**	**6785 (99.6%)**	**5350 (78.5%)**	**25/1**	
Protein mutations:	M6L (122A>T 124G>A), P8S (128C>T 130C>T), F10P (134T>C 135T>C 136C>T), N11S (138A>G), S12P (140T>C 142A>C), V13A (144T>C 145T>A), T14M (147C>T 148T>G), C17S (155T>A 157C>T), A18V (159C>T), T19A (161A>G), A31Q (197G>C 198C>A 199T>A), S66T (302T>A), T69S (311A>T 313C>T), S70N (315G>A 316C>T), N73S (324A>G 325C>T), A112T (440G>A), S113G (443A>G), N123D (473A>G), D127N (485G>A 487C>T), S156N (573G>A), T165S (599A>T 601T>A), I167T (606T>C 607A>T), N170S (615A>G), T171I (618C>T 619A>T), D183E (655C>G), S205A (719T>G 721C>T), M206V (722A>G), S239T (821T>A), V291I (977G>A 979T>A), S300A (1004T>G 1006T>A), T305V (1019A>G 1020C>T), T364S (1196A>T 1198C>T), D367E (1207T>G), E415D (1351A>T), I459V (1481A>G), A501S (1607G>T 1609T>A), T529V (1691A>G 1692C>T 1693T>A), T533A (1703A>G 1705T>A), S534A (1706T>G 1708T>C), E537R (1715G>A 1716A>G), K544R (1737A>G 1738A>G), T550I (1755C>T 1756A>C), S555D (1769A>G 1770G>A 1771C>T), Y578F (1839A>T), A580T (1844G>A), S582T (1850T>A 1852A>C), S591T (1877T>A 1879A>T), I595V (1889A>G 1891A>G), D600E (1906T>A), M601P (1907A>C 1908T>C 1909G>A), L602W (1911T>G), A611T (1937G>A 1939T>A), Y637F (2016A>T), F659Y (2082T>A), S665P (2099T>C 2101C>T), T669S (2111A>T 2113A>C), N675T (2130A>C 2131T>A), Y679Q (2141T>C 2143T>A), E685A (2160A>C), I691V (2177A>G 2179A>T), N712C (2240A>T 2241A>G), E713D (2245G>T), S715K (2250G>A 2251C>A), M726L (2282A>T), V741I (2327G>A 2329G>C), R768K (2409G>A 2410G>A), Q822R (2571A>G 2572A>G), G874S (2726G>A), V905I (2819G>A 2821G>A), T920V (2864A>G 2865C>T 2866T>G), A926S (2882G>T 2884C>G), T978S (3038A>T), Q993K (3083C>A 3085A>G), T1014S (3146A>T 3148G>C), D1089N (3371G>A 3373T>C), Q1103E (3413C>G), F1123Y (3474T>A 3475T>C), V1124L (3476G>C), A1129D (3492C>A), R1143K (3534G>A), A1162V (3591C>T 3592T>C), S1163Q (3593T>C 3594C>A), A1175S (3629G>T), R1193K (3684G>A 3685G>A), Y1196F (3693A>T), A1202S (3710G>T 3712A>T), I1203V (3713A>G 3715T>G), V1206I (3722G>A 3724G>A), H1208R (3729A>G), K1216E (3752A>G), V1218I (3758G>A), V1224I (3776G>A 3778G>T), P1352A (4160C>G 4162A>T), I1362M (4192T>G), G1370S (4214G>A), P1410Q (4335C>A 4336T>A), E1506G (4623A>G 4624A>T), T1511S (4637A>T 4639T>G), S1537N (4716G>A 4717C>T), S1538H (4718A>C 4719G>A), V1582T (4850G>A 4851T>C), V1598I (4898G>A), E1610D (4936A>C), T1614N (4947C>A 4948A>T), N1618H (4958A>C), T1628A (4988A>G 4990C>A), V1642M (5030G>A), I1658T (5079T>C 5080T>A), A1662S (5090G>T 5092T>A), P1667A (5105C>G 5107C>T), N1670E (5114A>G 5116T>A), V1672I (5120G>A 5122T>A), S1676T (5132T>A), V1679I (5141G>A 5143G>T), S1691A (5177T>G 5179G>C), Q1697H (5197G>T), T1701S (5207A>T 5209C>A), S1744G (5336A>G), V1746I (5342G>A 5344A>C), T1751S (5357A>T 5359C>T), K1752H (5360A>C 5362A>C), H1754N (5366C>A 5368T>C), V1755I (5369G>A 5371G>T), I1765V (5399A>G 5401T>G), V1785I (5459G>A 5461T>C), V1786I (5462G>A), D1794N (5486G>A), T1796P (5492A>C 5494A>T), Q1800H (5506A>T), L1810V (5534C>G), K1811Q (5537A>C), A1813S (5543G>T), E1818Q (5558G>C), I1826V (5582A>G 5584T>G), A1829S (5591G>T 5593C>A), T1833L (5603A>C 5604C>T), Y1836F (5613A>T), R1853N (5664G>A 5665A>T), L1856C (5672C>T 5673T>G 5674A>T), Q1857S (5675C>A 5676A>G 5677G>T), Y1869H (5711T>C), R1878K (5739G>A), L1882W (5751T>G), M1887I (5767G>T), K1889N (5773G>C), I1890V (5774A>G 5776A>G), I1893T (5784T>C 5785T>A), V1895L (5789G>T 5791C>G), E1898G (5799A>G 5800G>T), F1900T (5804T>A 5805T>C 5806C>T), Y1907F (5826A>T), V1913I (5843G>A 5845G>A), M1914F (5846A>T 5848G>C), L1915Q (5849T>C 5850T>A 5851G>A), D1918E (5860C>A), N1930H (5894A>C), Y1934F (5907A>T), H1946F (5942C>T 5943A>T 5944C>T), E1949D (5953A>T), N1950S (5955A>G 5956C>T), S1962A (5990T>G 5992A>C), T1966V (6002A>G 6003C>T 6004C>T), G1968E (6009G>A 6010G>A), T1976I (6033C>T 6034A>C), A1977S (6035G>T), S1980T (6044T>A 6046A>G), F1982L (6050T>C), D1985N (6059G>A), V1986I (6062G>A 6064T>C), H1997Q (6097T>A), I2002M (6112A>G), M2007L (6125A>C), E2008G (6129A>G 6130G>T), C2015S (6150G>C), V2016L (6152G>T 6154C>G), E2024G (6177A>G), T2027E (6185A>G 6186C>A 6187T>G), H2028R (6189A>G), M2032I (6202G>C), D2036E (6214T>G), L2037V (6215C>G 6217G>T), T2038P (6218A>C), E2039S (6221G>T 6222A>C 6223A>T), G2040E (6225G>A 6226G>A), I2041V (6227A>G 6229C>G), I2042V (6230A>G 6232A>G), K2044S (6237A>G 6238A>T), V2045T (6239G>A 6240T>C 6241G>A), I2046V (6242A>G 6244A>G), S2054T (6266T>A 6268G>T), S2056M (6273G>T 6274C>G), V2062E (6291T>A 6292T>A), D2063G (6294A>G), V2064L (6296G>C 6298A>C), L2066T (6302T>A 6303T>C 6304A>G), D2069S (6311G>T 6312A>C 6313T>C), I2071V (6317A>G 6319A>G), I2073V (6323A>G 6325C>G), A2089T (6371G>A 6373A>T), I2093V (6383A>G 6385C>G), C2100H (6404T>C 6405G>A), R2103V (6413C>G 6414G>T 6415A>G), S2104N (6416T>A 6417C>A 6418A>C), C2111S (6437T>A), T2115S (6449A>T), S2117A (6455T>G 6457C>A), S2118T (6458T>A), H2127N (6485C>A), R2128S (6490A>C), H2129N (6491C>A), Q2130V (6494C>G 6495A>T 6496G>A), T2131P (6497A>C 6499A>T), I2132E (6500A>G 6501T>A 6502T>G), D2133N (6503G>A 6505T>C), S2137P (6515T>C 6517C>A), F2138L (6518T>C 6520C>T), T2149M (6552C>T 6553A>G), G2154N (6566G>A 6567G>A 6568C>T)									

	Begin	End	Coverage	Score	Concordance	Matches	Identities	I/D/M/F*	Stop Codons
**NT**	**21**	**6806**	**86.9%**	**7777**	**57.4%**	**6785 (99.6%)**	**5350 (78.5%)**	**25/1**	

Codon mutations:

TTT3TTC (115T>C), TCC4TCT (118C>T), AAG5AAA (121G>A), ATG6TTA (122A>T 124G>A), CCC8TCT (128C>T 130C>T), TTC10CCT (134T>C 135T>C 136C>T), AAC11AGC (138A>G), TCA12CCC (140T>C 142A>C), GTT13GCA (144T>C 145T>A), ACT14ATG (147C>T 148T>G), GAA15GAG (151A>G), TGC17AGT (155T>A 157C>T), GCT18GTT (159C>T), ACC19GCC (161A>G), TCT24TCG (178T>G), TCA27TCT (187A>T), ACT30ACA (196T>A), GCT31CAA (197G>C 198C>A 199T>A), AGT34AGC (208T>C), AAT35AAC (211T>C), TTC36TTT (214C>T), CGC38CGT (220C>T), ACT39ACA (223T>A), CTG40CTT (226G>T), TCA47TCC (247A>C), CAA49CAG (253A>G), ATT50ATC (256T>C), TCT52TCA (262T>A), CAC53CAT (265C>T), GAA55GAG (271A>G), TTG57CTG (275T>C), AAG58AAA (280G>A), CCT59CCC (283T>C), TCA60TCG (286A>G), GTT61GTG (289T>G), ACA62ACT (292A>T), GCT65GCA (301T>A), TCT66ACT (302T>A), TCC67TCT (307C>T), TTT68TTC (310T>C), ACC69TCT (311A>T 313C>T), AGC70AAT (315G>A 316C>T), TGT72TGC (322T>C), AAC73AGT (324A>G 325C>T), TCT74TCA (328T>A), ACT76ACC (334T>C), AAA77AAG (337A>G), TTG78TTA (340G>A), TTA79TTG (343A>G), AAG81AAA (349G>A), ACT84ACA (358T>A), TTA85CTA (359T>C), ATT86ATA (364T>A), GAA87GAG (367A>G), CCC88CCT (370C>T), TTC89TTT (373C>T), ATC90ATA (376C>A), GCT92GCA (382T>A), GTC96GTT (394C>T), GCA97GCC (397A>C), TCC98TCT (400C>T), AAA101AAG (409A>G), TCA102TCC (412A>C), ATT103ATC (415T>C), GTT107GTG (427T>G), AAG109AAA (433G>A), GTT111GTC (439T>C), GCA112ACA (440G>A), AGC113GGC (443A>G), AAA115AAG (451A>G), AAG117AAA (457G>A), AAC123GAC (473A>G), GAC127AAT (485G>A 487C>T), TCT129TCC (493T>C), GAG130GAA (496G>A), GTG132GTT (502G>T), CTT140CTC (526T>C), ATT149ATA (553T>A), TTC151TTT (559C>T), CCA154CCT (568A>T), AGT156AAT (573G>A), CTA158TTA (578C>T), GGA160GGT (586A>T), CAG163CAA (595G>A), ACT165TCA (599A>T 601T>A), CAC166CAT (604C>T), ATA167ACT (606T>C 607A>T), AAT170AGT (615A>G), ACA171ATT (618C>T 619A>T), AAC174AAT (628C>T), GCT178GCC (640T>C), TTA182CTG (650T>C 652A>G), GAC183GAG (655C>G), TTA185CTG (659T>C 661A>G), CCG186CCC (664G>C), TCC189TCT (673C>T), CAA190CAG (676A>G), GCA196GCG (694A>G), CTA201TTA (707C>T), GGG203GGA (715G>A), GAA204GAG (718A>G), TCC205GCT (719T>G 721C>T), ATG206GTG (722A>G), TCA207TCT (727A>T), CAC208CAT (730C>T), CTT211CTG (739T>G), GTG214GTT (748G>T), GTA215GTT (751A>T), GCA216GCG (754A>G), CTC218TTA (758C>T 760C>A), ATA219ATT (763A>T), GCC220GCT (766C>T), GGG222GGC (772G>C), ATT223ATA (775T>A), TCT224TCA (778T>A), ACC225ACA (781C>A), TTC228TTT (790C>T), GTG229GTA (793G>A), GCT230GCA (796T>A), GTA231GTT (799A>T), CCT232CCG (802T>G), GGT233GGC (805T>C), AGA234CGT (806A>C 808A>T), GGT237GGA (817T>A), CTA238CTC (820A>C), TCC239ACC (821T>A), AAC240AAT (826C>T), CCG241CCA (829G>A), CTA242CTC (832A>C), TCA243TCC (835A>C), AAA244AAG (838A>G), CTG246TTA (842C>T 844G>A), TAC247TAT (847C>T), GCG249GCA (853G>A), GGT252GGA (862T>A), GCT253GCC (865T>C), AAT257AAC (877T>C), TTG259CTT (881T>C 883G>T), TTT260TTC (886T>C), ACC261ACA (889C>A), TTC263TTT (895C>T), AGG264AGA (898G>A), AAT265AAC (901T>C), TGT269TGC (913T>C), ATA281ATT (949A>T), ATT284ATA (958T>A), GGT286GGA (964T>A), TTT287TTC (967T>C), CCA290CCT (976A>T), GTT291ATA (977G>A 979T>A), GCT294GCA (988T>A), ATT295ATC (991T>C), TTG299CTT (1001T>C 1003G>T), TCT300GCA (1004T>G 1006T>A), ACG301ACA (1009G>A), TTC304TTT (1018C>T), ACG305GTG (1019A>G 1020C>T), GAG309GAA (1033G>A), CCA316CCC (1054A>C), CAT318CAC (1060T>C), CGC319CGA (1063C>A), TTG320CTT (1064T>C 1066G>T), AAT322AAC (1072T>C), GCA324GCG (1078A>G), AAC325AAT (1081C>T), ACT329ACC (1093T>C), ATC330ATA (1096C>A), CTA335TTG (1109C>T 1111A>G), AAA341AAG (1129A>G), TCT343TCA (1135T>A), TAC345TAT (1141C>T), GCT347GCC (1147T>C), GTA351GTG (1159A>G), GCC353GCA (1165C>A), AGG358AGA (1180G>A), GTA359GTT (1183A>T), ACG360ACA (1186G>A), GCA361GCC (1189A>C), GCA362GCC (1192A>C), ATA363ATT (1195A>T), ACC364TCT (1196A>T 1198C>T), CTT366CTG (1204T>G), GAT367GAG (1207T>G), GGG371GGC (1219G>C), AAC373AAT (1225C>T), ACT377ACA (1237T>A), GGC378GGT (1240C>T), GTG379GTT (1243G>T), GGC380GGT (1246C>T), CAA381CAG (1249A>G), AGG383AGA (1255G>A), GCA384GCT (1258A>T), CCT386CCC (1264T>C), GGT393GGA (1285T>A), AGT395AGC (1291T>C), GGG398GGA (1300G>A), ACC401ACA (1309C>A), CGG404CGA (1318G>A), CTG405TTA (1319C>T 1321G>A), ATC407ATT (1327C>T), GTT410GTG (1336T>G), TTG411CTT (1337T>C 1339G>T), CGC413AGG (1343C>A 1345C>G), GAA415GAT (1351A>T), GAG416GAA (1354G>A), CCA417CCC (1357A>C), TTG419CTT (1361T>C 1363G>T), AGG421CGG (1367A>C), CTC422CTG (1372C>G), GCT424GCC (1378T>C), GTG425GTA (1381G>A), CGA428CGT (1390A>T), TCA433TCC (1405A>C), AAT434AAC (1408T>C), TAT435TAC (1411T>C), GCT440GCA (1426T>A), CTG442CTT (1432G>T), GCT448GCA (1450T>A), CTG449TTG (1451C>T), GGG452GGT (1462G>T), GCA453GCT (1465A>T), CAA455CAG (1471A>G), GAC458GAT (1480C>T), ATT459GTT (1481A>G), AAG460AAA (1486G>A), ATA463ATT (1495A>T), ATC465ATT (1501C>T), TCC467TCA (1507C>A), ACT468ACA (1510T>A), CCA470CCT (1516A>T), CCC472CCA (1522C>A), TTG473TTA (1525G>A), CCC474CCA (1528C>A), GCA476GCT (1534A>T), CGC483AGG (1553C>A 1555C>G), CGA488AGG (1568C>A 1570A>G), TAT489TAC (1573T>C), ATA490ATT (1576A>T), GCC492GCT (1582C>T), ACA493ACC (1585A>C), TCC494TCA (1588C>A), AAC495AAT (1591C>T), TCA498TCC (1600A>C), GCT501TCA (1607G>T 1609T>A), CAG502CAA (1612G>A), GGT504GGG (1618T>G), ACC506ACA (1624C>A), AGA513CGG (1643A>C 1645A>G), CGG515AGG (1649C>A), GTT517GTG (1657T>G), CTT518CTG (1660T>G), GTG519GTA (1663G>A), TGC521TGT (1669C>T), GGT525GGA (1681T>A), GAG526GAA (1684G>A), TTT527TTC (1687T>C), ACT529GTA (1691A>G 1692C>T 1693T>A), GCT531GCA (1699T>A), CCC532CCT (1702C>T), ACT533GCA (1703A>G 1705T>A), TCT534GCC (1706T>G 1708T>C), CTT536CTG (1714T>G), GAA537AGA (1715G>A 1716A>G), GTG540GTC (1726G>C), GTT541GTG (1729T>G), GAG542GAA (1732G>A), AGC543AGT (1735C>T), AAA544AGG (1737A>G 1738A>G), AGA545CGT (1739A>C 1741A>T), CCC546CCA (1744C>A), ACA550ATC (1755C>T 1756A>C), AGG552AGA (1762G>A), AGC555GAT (1769A>G 1770G>A 1771C>T), GAT557GAC (1777T>C), GAT558GAC (1780T>C), TTG559CTA (1781T>C 1783G>A), CTG560CTT (1786G>T), AAG568AAA (1810G>A), GTT569GTG (1813T>G), GCA571GCC (1819A>C), ATC573ATA (1825C>A), TCA574TCT (1828A>T), GGA575GGC (1831A>C), CTG577CTC (1837G>C), TAT578TTT (1839A>T), GCC580ACC (1844G>A), ACA581ACC (1849A>C), TCA582ACC (1850T>A 1852A>C), AAG584AAA (1858G>A), GTG590GTC (1876G>C), TCA591ACT (1877T>A 1879A>T), GAG593GAA (1885G>A), ATA595GTG (1889A>G 1891A>G), ATA596ATT (1894A>T), GAT600GAA (1906T>A), ATG601CCA (1907A>C 1908T>C 1909G>A), TTG602TGG (1911T>G), AAT603AAC (1915T>C), ATC604ATT (1918C>T), CAA605CAG (1921A>G), GAT606GAC (1924T>C), ACC607ACA (1927C>A), GAT610GAC (1936T>C), GCT611ACA (1937G>A 1939T>A), AAG612AAA (1942G>A), CTT613CTG (1945T>G), CCC614CCG (1948C>G), GTG615GTA (1951G>A), GTT617GTG (1957T>G), GTA618GTC (1960A>C), GTG624GTA (1978G>A), TAC626TAT (1984C>T), TTG630CTA (1994T>C 1996G>A), GTG632GTT (2002G>T), GAA633GAG (2005A>G), GCT634GCC (2008T>C), AAA636AAG (2014A>G), TAT637TTT (2016A>T), GCT638GCC (2020T>C), TAT639TAC (2023T>C), GCA646GCG (2044A>G), TAT648TAC (2050T>C), TTC649TTT (2053C>T), CCT650CCA (2056T>A), GAC652GAT (2062C>T), AAA655AAG (2071A>G), TTT659TAT (2082T>A), CTA662CTT (2092A>T), TCC665CCT (2099T>C 2101C>T), GAA668GAG (2110A>G), ACA669TCC (2111A>T 2113A>C), ACC671ACT (2119C>T), AGG672CGG (2120A>C), GTT674GTC (2128T>C), AAT675ACA (2130A>C 2131T>A), GAC676GAT (2134C>T), CTG678CTC (2140G>C), TAT679CAA (2141T>C 2143T>A), CAA680CAG (2146A>G), GGT681GGA (2149T>A), TGT683TGC (2155T>C), AAT684AAC (2158T>C), GAG685GCG (2160A>C), AAC686AAT (2164C>T), CTG690CTC (2176G>C), ATA691GTT (2177A>G 2179A>T), AAG692AAA (2182G>A), ATC694ATT (2188C>T), GCT696GCA (2194T>A), TAT698TAC (2200T>C), ATC706ATA (2224C>A), TAC707TAT (2227C>T), GCT708GCG (2230T>G), AAT712TGT (2240A>T 2241A>G), GAG713GAT (2245G>T), CGT714CGC (2248T>C), AGC715AAA (2250G>A 2251C>A), GAC717GAT (2257C>T), GTT718GTG (2260T>G), CAG719CAA (2263G>A), CTG722TTA (2270C>T 2272G>A), GGA725GGC (2281A>C), ATG726TTG (2282A>T), GCG727GCA (2287G>A), CAC728CAT (2290C>T), GCC730GCT (2296C>T), ATA731ATT (2299A>T), GCT733GCA (2305T>A), GTG735GTT (2311G>T), GAC736GAT (2314C>T), CTG738TTA (2318C>T 2320G>A), ATA739ATT (2323A>T), TGC740TGT (2326C>T), GTG741ATC (2327G>A 2329G>C), GAG742GAA (2332G>A), CAG743CAA (2335G>A), CCT745CCA (2341T>A), GCC746GCA (2344C>A), CAC747CAT (2347C>T), GTA748GTG (2350A>G), TTC750TTT (2356C>T), TTG751TTA (2359G>A), ACA753ACT (2365A>T), GCA754GCT (2368A>T), TTT755TTC (2371T>C), AGG757CGT (2375A>C 2377G>T), TAT758TAC (2380T>C), CGC761AGA (2387C>A 2389C>A), AGA762AGG (2392A>G), CAG766CAA (2404G>A), CCT767CCC (2407T>C), AGG768AAA (2409G>A 2410G>A), TCC770TCT (2416C>T), TTA790TTG (2476A>G), CCG791CCA (2479G>A), ATC793ATT (2485C>T), TTG794TTA (2488G>A), AGA795AGG (2491A>G), GTT796GTG (2494T>G), CTG797CTT (2497G>T), ATT798ATC (2500T>C), AAG799AAA (2503G>A), GCT800GCA (2506T>A), GGC801GGG (2509C>G), CTT802TTG (2510C>T 2512T>G), ATT803ATC (2515T>C), TTA804CTA (2516T>C), GCA806GCC (2524A>C), GCA810GCT (2536A>T), GGG812GGT (2542G>T), GCA816GCT (2554A>T), TTC817TTT (2557C>T), GCT819GCG (2563T>G), CAA822CGG (2571A>G 2572A>G), TCC823TCA (2575C>A), CCT827CCC (2587T>C), AGA832CGG (2600A>C 2602A>G), ATT835ATC (2611T>C), ACC836ACA (2614C>A), AAC837AAT (2617C>T), AAT840AAC (2626T>C), AGC841AGT (2629C>T), CGG845AGG (2639C>A), AAG848AAA (2650G>A), ATC852ATT (2662C>T), TTG857TTA (2677G>A), CTG858TTG (2678C>T), GCA859GCG (2683A>G), AAG862AAA (2692G>A), GGT863GGA (2695T>A), CTG866CTT (2704G>T), AAA868AAG (2710A>G), GCA870GCC (2716A>C), GGC874AGC (2726G>A), TTC875TTT (2731C>T), GGA879GGC (2743A>C), TTG880CTG (2744T>C), GTC885GTG (2761C>G), TTG887CTC (2765T>C 2767G>C), AGA888CGA (2768A>C), GGT890GGG (2776T>G), TAC894TAT (2788C>T), ACC898ACA (2800C>A), GTC900GTT (2806C>T), TGC901TGT (2809C>T), TCG902TCT (2812G>T), GGC903GGA (2815C>A), GTG905ATA (2819G>A 2821G>A), ACA907ACT (2827A>T), GTG908GTA (2830G>A), TTT912TTC (2842T>C), TCA913TCC (2845A>C), AGC914AGT (2848C>T), CGT916AGA (2852C>A 2854T>A), GGC918GGG (2860C>G), ACA919ACT (2863A>T), ACT920GTG (2864A>G 2865C>T 2866T>G), TTC921TTT (2869C>T), ATA923ATT (2875A>T), ATA924ATC (2878A>C), GCC926TCG (2882G>T 2884C>G), TCA928TCC (2890A>C), TAC934TAT (2908C>T), AAT935AAC (2911T>C), CGG940CGT (2926G>T), TTC941TTT (2929C>T), TTG942CTA (2930T>C 2932G>A), AAG943AAA (2935G>A), CAA945CAG (2941A>G), ATT947ATA (2947T>A), GTC948GTG (2950C>G), CTG949CTT (2953G>T), CTC950CTG (2956C>G), AAT951AAC (2959T>C), GGA953GGT (2965A>T), CCC955CCT (2971C>T), GAT956GAC (2974T>C), CCC958CCG (2980C>G), CCT960CCG (2986T>G), ATT962ATC (2992T>C), AAA964AAG (2998A>G), CGG969AGG (3011C>A), TGT973TGC (3025T>C), TCT975TCA (3031T>A), AAG976AAA (3034G>A), GGC977GGA (3037C>A), ACT978TCT (3038A>T), GGG980GGT (3046G>T), GTA981GTC (3049A>C), TGT982TGC (3052T>C), GTA986GTT (3064A>T), AAG989AAA (3073G>A), GCT991GCC (3079T>C), CAA993AAG (3083C>A 3085A>G), TTG999CTT (3101T>C 3103G>T), GTG1003GTT (3115G>T), TCC1006TCA (3124C>A), CTT1010CTG (3136T>G), ACG1014TCC (3146A>T 3148G>C), GAC1018GAT (3160C>T), AGT1020AGC (3166T>C), TCC1021TCA (3169C>A), CTG1024CTA (3178G>A), TCT1026TCA (3184T>A), TAT1030TAC (3196T>C), TAT1032TAC (3202T>C), GAT1040GAC (3226T>C), CTT1044CTG (3238T>G), CTT1045TTA (3239C>T 3241T>A), CTT1046TTG (3242C>T 3244T>G), TTG1047TTA (3247G>A), AAC1049AAT (3253C>T), GTG1050GTA (3256G>A), AAA1053AAG (3265A>G), TGC1056TGT (3274C>T), ATT1057ATA (3277T>A), ATT1060ATA (3286T>A), TCT1064TCA (3298T>A), GAA1068GAG (3310A>G), CAC1071CAT (3319C>T), GGG1073GGC (3325G>C), TTT1074TTC (3328T>C), GTA1075GTT (3331A>T), GCA1076GCC (3334A>C), ACA1080ACG (3346A>G), GCT1081GCG (3349T>G), ATT1082ATC (3352T>C), CGA1084AGA (3356C>A), TTG1087CTG (3365T>C), GAT1089AAC (3371G>A 3373T>C), CTT1090TTG (3374C>T 3376T>G), CCG1091CCA (3379G>A), ACT1092ACA (3382T>A), GGT1093GGG (3385T>G), CCG1094CCT (3388G>T), AAA1096AAG (3394A>G), GCA1098GCC (3400A>C), GTA1100GTG (3406A>G), TGC1102TGT (3412C>T), CAG1103GAG (3413C>G), CTC1104TTG (3416C>T 3418C>G), CTT1105CTG (3421T>G), CTA1108TTG (3428C>T 3430A>G), CGA1109AGA (3431C>A), AGG1111AGA (3439G>A), GCG1113GCT (3445G>T), GAA1117GAG (3457A>G), CAG1120CAA (3466G>A), TTT1123TAC (3474T>A 3475T>C), GTT1124CTT (3476G>C), GGC1125GGA (3481C>A), ACA1126ACT (3484A>T), GCT1129GAT (3492C>A), GCC1133GCA (3505C>A), ACG1134ACT (3508G>T), CCC1136CCA (3514C>A), AAA1138AAG (3520A>G), ACA1139ACC (3523A>C), ACG1140ACA (3526G>A), CTG1141CTC (3529G>C), CGA1142AGG (3530C>A 3532A>G), AGG1143AAG (3534G>A), GGC1145GGT (3541C>T), TTG1146CTC (3542T>C 3544G>C), GCT1149GCA (3553T>A), GGG1151GGA (3559G>A), GCA1153GCT (3565A>T), TCT1158TCC (3580T>C), ATC1159ATA (3583C>A), ATT1160ATC (3586T>C), TCA1161TCG (3589A>G), GCT1162GTC (3591C>T 3592T>C), TCA1163CAA (3593T>C 3594C>A), CGT1166AGA (3602C>A 3604T>A), GGG1167GGA (3607G>A), GAA1168GAG (3610A>G), GGT1169GGC (3613T>C), CCG1172CCA (3622G>A), TAC1173TAT (3625C>T), GTG1174GTC (3628G>C), GCT1175TCT (3629G>T), ATA1177ATC (3637A>C), CAA1178CAG (3640A>G), AAA1179AAG (3643A>G), TAC1180TAT (3646C>T), GAA1182GAG (3652A>G), GCA1184GCC (3658A>C), CAA1185CAG (3661A>G), AAT1186AAC (3664T>C), GAG1189GAA (3673G>A), GAC1190GAT (3676C>T), ATT1191ATC (3679T>C), GCG1192GCC (3682G>C), AGG1193AAA (3684G>A 3685G>A), CTA1194TTG (3686C>T 3688A>G), GCC1195GCT (3691C>T), TAT1196TTT (3693A>T), GGG1198GGA (3700G>A), CTA1199TTG (3701C>T 3703A>G), CGT1200CGG (3706T>G), CAA1201CAG (3709A>G), GCA1202TCT (3710G>T 3712A>T), ATT1203GTG (3713A>G 3715T>G), TTG1204CTG (3716T>C), GTG1206ATA (3722G>A 3724G>A), CAC1208CGC (3729A>G), TCC1209TCG (3733C>G), GTT1212GTG (3742T>G), AAG1216GAG (3752A>G), GTC1218ATC (3758G>A), ACA1219ACT (3763A>T), CAG1220CAA (3766G>A), GTG1224ATT (3776G>A 3778G>T), GTG1225GTT (3781G>T), CTC1226TTG (3782C>T 3784C>G), GGT1228GGA (3790T>A), GTT1229GTC (3793T>C), TTT1232TTC (3802T>C), TAT1234TAC (3808T>C), GAC1236GAT (3814C>T), GGG1237GGA (3817G>A), TTG1240CTG (3824T>C), GGG1245GGT (3841G>T), CCG1247CCA (3847G>A), TAC1248TAT (3850C>T), AAG1250AAA (3856G>A), GGC1254GGT (3868C>T), ACT1255ACC (3871T>C), GAG1259GAA (3883G>A), GTG1261GTA (3889G>A), CCA1263CCC (3895A>C), GGA1265GGC (3901A>C), GGA1267GGT (3907A>T), GAT1268GAC (3910T>C), CGA1269AGA (3911C>A), GTC1270GTG (3916C>G), CTC1272TTG (3920C>T 3922C>G), AAA1273AAG (3925A>G), AGG1274AGA (3928G>A), ACT1276ACC (3934T>C), GAC1281GAT (3949C>T), GAG1283GAA (3955G>A), GCT1284GCA (3958T>A), TTG1285CTA (3959T>C 3961G>A), ATT1289ATC (3973T>C), GGA1292GGT (3982A>T), CTG1297TTG (3995C>T), GTC1298GTT (4000C>T), ACC1299ACA (4003C>A), ACC1300ACA (4006C>A), CAA1301CAG (4009A>G), GCC1303GCA (4015C>A), AAA1304AAG (4018A>G), TTG1308CTA (4028T>C 4030G>A), CCG1309CCT (4033G>T), TTG1310CTG (4034T>C), GAA1311GAG (4039A>G), GTT1313GTG (4045T>G), GGG1315GGC (4051G>C), CGG1317AGG (4055C>A), ACC1319ACA (4063C>A), TTG1321CTA (4067T>C 4069G>A), GAA1323GAG (4075A>G), ATT1324ATA (4078T>A), CTT1325CTA (4081T>A), CCC1326CCT (4084C>T), TAC1329TAT (4093C>T), AAT1330AAC (4096T>C), CTG1338TTG (4118C>T), GAT1339GAC (4123T>C), TCC1341TCT (4129C>T), TCC1347TCG (4147C>G), CTG1351TTG (4157C>T), CCA1352GCT (4160C>G 4162A>T), GGT1361GGA (4189T>A), ATT1362ATG (4192T>G), GAA1363GAG (4195A>G), CTG1366TTA (4202C>T 4204G>A), TTG1367CTA (4205T>C 4207G>A), GCT1368GCA (4210T>A), AAT1369AAC (4213T>C), GGC1370AGC (4214G>A), TTC1371TTT (4219C>T), AGA1372CGG (4220A>C 4222A>G), GCA1373GCT (4225A>T), GTC1374GTT (4228C>T), ACA1377ACG (4237A>G), TCT1380TCC (4246T>C), GCT1381GCA (4249T>A), GAC1382GAT (4252C>T), TAT1383TAC (4255T>C), ATC1392ATT (4282C>T), GCC1394GCA (4288C>A), CAA1396CAG (4294A>G), TGT1399TGC (4303T>C), CTT1401CTG (4309T>G), GAC1404GAT (4318C>T), GGA1407GGC (4327A>C), CCT1410CAA (4335C>A 4336T>A), GGT1411GGC (4339T>C), TCT1412TCC (4342T>C), GAC1414GAT (4348C>T), TCC1415TCA (4351C>A), AAT1416AAC (4354T>C), AGA1418CGC (4358A>C 4360A>C), CAT1419CAC (4363T>C), GTG1420GTA (4366G>A), CTT1421TTG (4367C>T 4369T>G), CTT1422CTG (4372T>G), GCT1425GCC (4381T>C), TCA1426TCT (4384A>T), TGC1427TGT (4387C>T), ACT1430ACC (4396T>C), ATT1431ATC (4399T>C), GAG1433GAA (4405G>A), GTT1436GTG (4414T>G), TTT1437TTC (4417T>C), AGG1438CGC (4418A>C 4420G>C), TTG1439CTG (4421T>C), GTG1440GTT (4426G>T), GGA1441GGG (4429A>G), GGT1442GGC (4432T>C), CCT1444CCA (4438T>A), TCA1445TCT (4441A>T), GGA1446GGG (4444A>G), GCA1448GCT (4450A>T), CTC1449CTT (4453C>T), ACC1450ACT (4456C>T), GTT1451GTC (4459T>C),

	ATC1452ATT (4462C>T), TTC1453TTT (4465C>T), AAC1454AAT (4468C>T), CGT1463CGG (4495T>G), GCA1465GCT (4501A>T), ATT1467ATC (4507T>C), TCT1468TCA (4510T>A), CTA1469CTG (4513A>G), TTG1470CTG (4514T>C), AGA1472AGG (4522A>G), CAT1474CAC (4528T>C), GCA1476GCT (4534A>T), GCT1477GCA (4537T>A), CAA1478CAG (4540A>G), ATA1480ATT (4546A>T), GGG1481GGC (4549G>C), TGC1482TGT (4552C>T), AAA1483AAG (4555A>G), CCC1484CCA (4558C>A), AAC1488AAT (4570C>T), CTA1490CTT (4576A>T), GTG1492GTT (4582G>T), GCA1493GCT (4585A>T), GTC1494GTA (4588C>A), GAC1498GAT (4600C>T), CTA1500CTT (4606A>T), GTT1503GTA (4615T>A), CCC1504CCA (4618C>A), GAA1506GGT (4623A>G 4624A>T), TTG1507CTC (4625T>C 4627G>C), TAT1510TAC (4636T>C), ACT1511TCG (4637A>T 4639T>G), GCT1514GCA (4648T>A), ATT1515ATA (4651T>A), GAA1518GAG (4660A>G), TTG1519CTA (4661T>C 4663G>A), GTT1524GTC (4678T>C), ATA1526ATC (4684A>C), ATC1530ATA (4696C>A), AAG1532AAA (4702G>A), AAC1533AAT (4705C>T), GTT1536GTC (4714T>C), AGC1537AAT (4716G>A 4717C>T), AGT1538CAT (4718A>C 4719G>A), CTA1542CTG (4732A>G), GAC1543GAT (4735C>T), TTG1544CTT (4736T>C 4738G>T), TCT1545TCA (4741T>A), CTA1547TTG (4745C>T 4747A>G), TTT1549TTC (4753T>C), CTA1550CTT (4756A>T), AGC1551AGT (4759C>T), AGA1552AGG (4762A>G), GGT1553GGC (4765T>C), TTT1554TTC (4768T>C), AAG1555AAA (4771G>A), AGG1556AGA (4774G>A), GGA1560GGG (4786A>G), TAC1561TAT (4789C>T), CCT1565CCC (4801T>C), CTG1566TTG (4802C>T), GTA1569GTG (4813A>G), TCT1570TCC (4816T>C), GAA1573GAG (4825A>G), CCA1574CCC (4828A>C), TAC1576TAT (4834C>T), ATA1578ATT (4840A>T), AGG1579AGA (4843G>A), CCT1580CCC (4846T>C), GTT1582ACT (4850G>A 4851T>C), GCT1587GCC (4867T>C), CTC1588TTG (4868C>T 4870C>G), TTG1591TTA (4879G>A), AAC1593AAT (4885C>T), ATA1594ATT (4888A>T), GAC1595GAT (4891C>T), ACG1596ACT (4894G>T), GGA1597GGC (4897A>C), GTT1598ATT (4898G>A), AGA1599AGG (4903A>G), GCA1601GCC (4909A>C), CAC1603CAT (4915C>T), GGG1605GGT (4921G>T), CAG1607CAA (4927G>A), GTT1608GTG (4930T>G), GAA1610GAC (4936A>C), GTG1613GTT (4945G>T), ACA1614AAT (4947C>A 4948A>T), GTT1616GTG (4954T>G), AAC1618CAC (4958A>C), GCT1619GCC (4963T>C), AAG1621AAA (4969G>A), CGG1623CGA (4975G>A), TTC1625TTT (4981C>T), CCA1626CCT (4984A>T), GCC1627GCT (4987C>T), ACC1628GCA (4988A>G 4990C>A), CCT1631CCG (4999T>G), ACA1632ACT (5002A>T), TAT1633TAC (5005T>C), GAG1637GAA (5017G>A), CAG1638CAA (5020G>A), GAC1639GAT (5023C>T), CTG1641CTT (5029G>T), GTG1642ATG (5030G>A), GAG1643GAA (5035G>A), GTT1644GTA (5038T>A), ACA1645ACT (5041A>T), GGA1646GGC (5044A>C), CCA1648CCT (5050A>T), GGG1651GGT (5059G>T), CTC1652CTT (5062C>T), CTT1656TTA (5072C>T 5074T>A), ATT1658ACA (5079T>C 5080T>A), GCA1659GCT (5083A>T), TCA1661TCC (5089A>C), GCT1662TCA (5090G>T 5092T>A), GTG1664GTT (5098G>T), CCT1665CCA (5101T>A), TTG1666CTG (5102T>C), CCC1667GCT (5105C>G 5107C>T), CCA1668CCT (5110A>T), GGC1669GGT (5113C>T), AAT1670GAA (5114A>G 5116T>A), ACT1671ACC (5119T>C), GTT1672ATA (5120G>A 5122T>A), CTG1673TTG (5123C>T), TTT1675TTC (5131T>C), TCT1676ACT (5132T>A), GTG1679ATT (5141G>A 5143G>T), CAT1680CAC (5146T>C), ACT1681ACA (5149T>A), TTT1682TTC (5152T>C), GCT1683GCA (5155T>A), CGA1685AGA (5159C>A), TGC1689TGT (5173C>T), TCC1690TCT (5176C>T), TCG1691GCC (5177T>G 5179G>C), CGA1692AGG (5180C>A 5182A>G), ACA1693ACT (5185A>T), GCA1696GCC (5194A>C), CAG1697CAT (5197G>T), CAG1698CAA (5200G>A), GAC1700GAT (5206C>T), ACC1701TCA (5207A>T 5209C>A), ACC1702ACA (5212C>A), GTT1704GTC (5218T>C), AAC1707AAT (5227C>T), ACT1709ACC (5233T>C), GCA1711GCC (5239A>C), AAG1712AAA (5242G>A), GTA1717GTT (5257A>T), AGA1718AGG (5260A>G), GGG1719GGA (5263G>A), CCC1720CCA (5266C>A), ACC1721ACA (5269C>A), AGG1724CGA (5276A>C 5278G>A), GGG1728GGT (5290G>T), CTT1729CTC (5293T>C), ATT1730ATC (5296T>C), CCT1732CCA (5302T>A), GCT1736GCC (5314T>C), GAC1740GAT (5326C>T), CAC1741CAT (5329C>T), AGC1744GGC (5336A>G), ATT1745ATC (5341T>C), GTA1746ATC (5342G>A 5344A>C), ACC1747ACA (5347C>A), GGG1749GGA (5353G>A), ACC1751TCT (5357A>T 5359C>T), AAA1752CAC (5360A>C 5362A>C), CAT1754AAC (5366C>A 5368T>C), GTG1755ATT (5369G>A 5371G>T), GTT1756GTA (5374T>A), TTT1757TTC (5377T>C), GGC1760GGG (5386C>G), AAT1761AAC (5389T>C), GGG1762GGA (5392G>A), ATT1765GTG (5399A>G 5401T>G), GGT1766GGA (5404T>A), CCA1767CCT (5407A>T), GTG1768GTT (5410G>T), TGC1769TGT (5413C>T), GCT1770GCA (5416T>A), GCA1771GCT (5419A>T), TTG1772CTA (5420T>C 5422G>A), CTG1775TTA (5429C>T 5431G>A), TCC1776TCT (5434C>T), ACT1777ACG (5437T>G), GGC1778GGA (5440C>A), CAA1779CAG (5443A>G), TAT1780TAC (5446T>C), TCT1781TCA (5449T>A), TCT1782TCC (5452T>C), GTT1785ATC (5459G>A 5461T>C), GTT1786ATT (5462G>A), GTG1787GTC (5467G>C), ACC1791ACT (5479C>T), ATA1792ATT (5482A>T), GCA1793GCT (5485A>T), GAT1794AAT (5486G>A), GTT1795GTG (5491T>G), ACA1796CCT (5492A>C 5494A>T), CTC1798TTG (5498C>T 5500C>G), AGT1799AGC (5503T>C), CAA1800CAT (5506A>T), TAT1801TAC (5509T>C), CCA1802CCT (5512A>T), GGA1803GGT (5515A>T), GCC1806GCT (5524C>T), AAG1807AAA (5527G>A), TAT1808TAC (5530T>C), CTT1809TTG (5531C>T 5533T>G), CTG1810GTG (5534C>G), AAA1811CAA (5537A>C), GCT1813TCT (5543G>T), GAT1814GAC (5548T>C), ACA1815ACC (5551A>C), GAA1818CAA (5558G>C), CTT1820CTA (5566T>A), GCA1821GCT (5569A>T), ACA1823ACG (5575A>G), TGT1824TGC (5578T>C), ATT1826GTG (5582A>G 5584T>G), GCC1829TCA (5591G>T 5593C>A), GAG1832GAA (5602G>A), ACA1833CTA (5603A>C 5604C>T), CCA1834CCG (5608A>G), GCA1835GCT (5611A>T), TAC1836TTC (5613A>T), ATA1837ATT (5617A>T), CCT1838CCA (5620T>A), GGA1840GGT (5626A>T), TTT1842TTC (5632T>C), CTT1844CTG (5638T>G), GGT1845GGC (5641T>C), GTG1850GTA (5656G>A), CAA1851CAG (5659A>G), GGG1852GGA (5662G>A), AGA1853AAT (5664G>A 5665A>T), CTA1856TGT (5672C>T 5673T>G 5674A>T), CAG1857AGT (5675C>A 5676A>G 5677G>T), CAG1860CAA (5686G>A), CGC1861AGA (5687C>A 5689C>A), TTG1862CTT (5690T>C 5692G>T), GGA1867GGT (5707A>T), CCT1868CCA (5710T>A), TAT1869CAT (5711T>C), GGG1870GGT (5716G>T), GGA1871GGG (5719A>G), ACT1876ACC (5734T>C), ACT1877ACC (5737T>C), AGA1878AAA (5739G>A), GAG1879GAA (5743G>A), CTT1880CTA (5746T>A), AAG1881AAA (5749G>A), TTG1882TGG (5751T>G), CTG1883TTG (5753C>T), ATG1887ATT (5767G>T), AAG1889AAC (5773G>C), ATA1890GTG (5774A>G 5776A>G), TAT1891TAC (5779T>C), ATT1893ACA (5784T>C 5785T>A), TTA1894CTT (5786T>C 5788A>T), GTC1895TTG (5789G>T 5791C>G), CAA1896CAG (5794A>G), AGA1897AGG (5797A>G), GAG1898GGT (5799A>G 5800G>T), TTC1900ACT (5804T>A 5805T>C 5806C>T), ACT1902ACC (5812T>C), CTC1903CTG (5815C>G), TAC1904TAT (5818C>T), TAT1907TTT (5826A>T), CTC1908CTT (5830C>T), TCA1910TCC (5836A>C), GTG1913ATA (5843G>A 5845G>A), ATG1914TTC (5846A>T 5848G>C), TTG1915CAA (5849T>C 5850T>A 5851G>A), GTT1917GTG (5857T>G), GAC1918GAA (5860C>A), CTT1920TTG (5864C>T 5866T>G), CTC1922CTA (5872C>A), GGG1924GGT (5878G>T), TTT1925TTC (5881T>C), TTT1926TTC (5884T>C), CCC1927CCT (5887C>T), CGC1928CGT (5890C>T), AAT1930CAT (5894A>C), GTG1932GTA (5902G>A), TAC1934TTC (5907A>T), CTT1936CTC (5914T>C), TTT1939TTC (5923T>C), CTG1941CTA (5929G>A), CAC1946TTT (5942C>T 5943A>T 5944C>T), AAG1948AAA (5950G>A), GAA1949GAT (5953A>T), AAC1950AGT (5955A>G 5956C>T), ACG1953ACT (5965G>T), GTT1954GTG (5968T>G), TAC1955TAT (5971C>T), TTT1956TTC (5974T>C), AGA1957CGT (5975A>C 5977A>T), AAG1958AAA (5980G>A), CCT1959CCC (5983T>C), TTT1960TTC (5986T>C), CTC1961CTT (5989C>T), TCA1962GCC (5990T>G 5992A>C), AAA1964AAG (5998A>G), ACC1966GTT (6002A>G 6003C>T 6004C>T), GGG1968GAA (6009G>A 6010G>A), TCC1969TCA (6013C>A), AAA1970AAG (6016A>G), ATT1972ATA (6022T>A), TCC1974TCT (6028C>T), GCA1975GCT (6031A>T), ACA1976ATC (6033C>T 6034A>C), GCT1977TCT (6035G>T), GCT1978GCA (6040T>A), GAG1979GAA (6043G>A), TCA1980ACG (6044T>A 6046A>G), TTG1981TTA (6049G>A), TTT1982CTT (6050T>C), GGT1983GGG (6055T>G), GAT1985AAT (6059G>A), GTT1986ATC (6062G>A 6064T>C), TCC1987TCT (6067C>T), GCT1988GCC (6070T>C), CTC1991CTG (6079C>G), AGT1993AGC (6085T>C), AGG1994AGA (6088G>A), CTA1995TTA (6089C>T), CTT1996TTA (6092C>T 6094T>A), CAT1997CAA (6097T>A), CTT1998CTG (6100T>G), CAG1999CAA (6103G>A), AAG2000AAA (6106G>A), ATA2002ATG (6112A>G), TCA2005TCT (6121A>T), TCC2006TCA (6124C>A), ATG2007CTG (6125A>C), GAG2008GGT (6129A>G 6130G>T), TTT2011TTC (6139T>C), AAA2012AAG (6142A>G), ATT2013ATC (6145T>C), TAT2014TAC (6148T>C), TGT2015TCT (6150G>C), GTC2016TTG (6152G>T 6154C>G), CAG2018CAA (6160G>A), GGC2019GGG (6163C>G), AAG2023AAA (6175G>A), GAA2024GGA (6177A>G), GTA2026GTG (6184A>G), ACT2027GAG (6185A>G 6186C>A 6187T>G), CAC2028CGC (6189A>G), TTC2029TTT (6193C>T), CAA2030CAG (6196A>G), CGC2031CGT (6199C>T), ATG2032ATC (6202G>C), CAA2034CAG (6208A>G), GAT2036GAG (6214T>G), CTG2037GTT (6215C>G 6217G>T), ACA2038CCA (6218A>C), GAA2039TCT (6221G>T 6222A>C 6223A>T), GGG2040GAA (6225G>A 6226G>A), ATC2041GTG (6227A>G 6229C>G), ATA2042GTG (6230A>G 6232A>G), GAG2043GAA (6235G>A), AAA2044AGT (6237A>G 6238A>T), GTG2045ACA (6239G>A 6240T>C 6241G>A), ATA2046GTG (6242A>G 6244A>G), TTA2049CTC (6251T>C 6253A>C), ACC2050ACA (6256C>A), ACA2052ACT (6262A>T), CTG2053TTG (6263C>T), TCG2054ACT (6266T>A 6268G>T), AGC2056ATG (6273G>T 6274C>G), GTG2059GTA (6283G>A), CTT2061TTG (6287C>T 6289T>G), GTT2062GAA (6291T>A 6292T>A), GAT2063GGT (6294A>G), GTA2064CTC (6296G>C 6298A>C), CCT2065CCA (6301T>A), TTA2066ACG (6302T>A 6303T>C 6304A>G), GGC2067GGA (6307C>A), TTG2068CTT (6308T>C 6310G>T), GAT2069TCC (6311G>T 6312A>C 6313T>C), AAC2070AAT (6316C>T), ATA2071GTG (6317A>G 6319A>G), GAG2072GAA (6322G>A), ATC2073GTG (6323A>G 6325C>G), CAG2074CAA (6328G>A), AAA2076AAG (6334A>G), GGT2077GGC (6337T>C), CTA2080TTA (6344C>T), AAT2086AAC (6364T>C), GCA2089ACT (6371G>A 6373A>T), GCC2092GCT (6382C>T), ATC2093GTG (6383A>G 6385C>G), CTC2097CTA (6397C>A), TGT2100CAT (6404T>C 6405G>A), AAC2102AAT (6412C>T), CGA2103GTG (6413C>G 6414G>T 6415A>G), TCA2104AAC (6416T>A 6417C>A 6418A>C), ACA2105ACT (6421A>T), GTG2109GTT (6433G>T), TTC2110TTT (6436C>T), TGT2111AGT (6437T>A), TAC2113TAT (6445C>T), ACG2114ACA (6448G>A), ACT2115TCT (6449A>T), TCC2117GCA (6455T>G 6457C>A), TCT2118ACT (6458T>A), CTT2119TTG (6461C>T 6463T>G), GCC2122GCT (6472C>T), GTG2124GTT (6478G>T), CTT2125TTA (6479C>T 6481T>A), CAT2127AAT (6485C>A), AGA2128AGC (6490A>C), CAT2129AAT (6491C>A), CAG2130GTA (6494C>G 6495A>T 6496G>A), ACA2131CCT (6497A>C 6499A>T), ATT2132GAG (6500A>G 6501T>A 6502T>G), GAT2133AAC (6503G>A 6505T>C), CCG2136CCT (6514G>T), TCC2137CCA (6515T>C 6517C>A), TTC2138CTT (6518T>C 6520C>T), ACA2141ACT (6529A>T), GTG2145GTA (6541G>A), TTG2146TTA (6544G>A), CTT2147TTG (6545C>T 6547T>G), TTG2148CTG (6548T>C), ACA2149ATG (6552C>T 6553A>G), CCA2150CCC (6556A>C), CTA2152CTT (6562A>T), GGC2154AAT (6566G>A 6567G>A 6568C>T), TAC2155TAT (6571C>T), AAA2156AAG (6574A>G)

Proteins

polyprotein (YP_001039627.1)	1	2159	100%	13837	91.8%	2159 (100%)	1941 (89.9%)	0/0/0/0	1

Protein mutations:	M6L (122A>T 124G>A), P8S (128C>T 130C>T), F10P (134T>C 135T>C 136C>T), N11S (138A>G), S12P (140T>C 142A>C), V13A (144T>C 145T>A), T14M (147C>T 148T>G), C17S (155T>A 157C>T), A18V (159C>T), T19A (161A>G), A31Q (197G>C 198C>A 199T>A), S66T (302T>A), T69S (311A>T 313C>T), S70N (315G>A 316C>T), N73S (324A>G 325C>T), A112T (440G>A), S113G (443A>G), N123D (473A>G), D127N (485G>A 487C>T), S156N (573G>A), T165S (599A>T 601T>A), I167T (606T>C 607A>T), N170S (615A>G), T171I (618C>T 619A>T), D183E (655C>G), S205A (719T>G 721C>T), M206V (722A>G), S239T (821T>A), V291I (977G>A 979T>A), S300A (1004T>G 1006T>A), T305V (1019A>G 1020C>T), T364S (1196A>T 1198C>T), D367E (1207T>G), E415D (1351A>T), I459V (1481A>G), A501S (1607G>T 1609T>A), T529V (1691A>G 1692C>T 1693T>A), T533A (1703A>G 1705T>A), S534A (1706T>G 1708T>C), E537R (1715G>A 1716A>G), K544R (1737A>G 1738A>G), T550I (1755C>T 1756A>C), S555D (1769A>G 1770G>A 1771C>T), Y578F (1839A>T), A580T (1844G>A), S582T (1850T>A 1852A>C), S591T (1877T>A 1879A>T), I595V (1889A>G 1891A>G), D600E (1906T>A), M601P (1907A>C 1908T>C 1909G>A), L602W (1911T>G), A611T (1937G>A 1939T>A), Y637F (2016A>T), F659Y (2082T>A), S665P (2099T>C 2101C>T), T669S (2111A>T 2113A>C), N675T (2130A>C 2131T>A), Y679Q (2141T>C 2143T>A), E685A (2160A>C), I691V (2177A>G 2179A>T), N712C (2240A>T 2241A>G), E713D (2245G>T), S715K (2250G>A 2251C>A), M726L (2282A>T), V741I (2327G>A 2329G>C), R768K (2409G>A 2410G>A), Q822R (2571A>G 2572A>G), G874S (2726G>A), V905I (2819G>A 2821G>A), T920V (2864A>G 2865C>T 2866T>G), A926S (2882G>T 2884C>G), T978S (3038A>T), Q993K (3083C>A 3085A>G), T1014S (3146A>T 3148G>C), D1089N (3371G>A 3373T>C), Q1103E (3413C>G), F1123Y (3474T>A 3475T>C), V1124L (3476G>C), A1129D (3492C>A), R1143K (3534G>A), A1162V (3591C>T 3592T>C), S1163Q (3593T>C 3594C>A), A1175S (3629G>T), R1193K (3684G>A 3685G>A), Y1196F (3693A>T), A1202S (3710G>T 3712A>T), I1203V (3713A>G 3715T>G), V1206I (3722G>A 3724G>A), H1208R (3729A>G), K1216E (3752A>G), V1218I (3758G>A), V1224I (3776G>A 3778G>T), P1352A (4160C>G 4162A>T), I1362M (4192T>G), G1370S (4214G>A), P1410Q (4335C>A 4336T>A), E1506G (4623A>G 4624A>T), T1511S (4637A>T 4639T>G), S1537N (4716G>A 4717C>T), S1538H (4718A>C 4719G>A), V1582T (4850G>A 4851T>C), V1598I (4898G>A), E1610D (4936A>C), T1614N (4947C>A 4948A>T), N1618H (4958A>C), T1628A (4988A>G 4990C>A), V1642M (5030G>A), I1658T (5079T>C 5080T>A), A1662S (5090G>T 5092T>A), P1667A (5105C>G 5107C>T), N1670E (5114A>G 5116T>A), V1672I (5120G>A 5122T>A), S1676T (5132T>A), V1679I (5141G>A 5143G>T), S1691A (5177T>G 5179G>C), Q1697H (5197G>T), T1701S (5207A>T 5209C>A), S1744G (5336A>G), V1746I (5342G>A 5344A>C), T1751S (5357A>T 5359C>T), K1752H (5360A>C 5362A>C), H1754N (5366C>A 5368T>C), V1755I (5369G>A 5371G>T), I1765V (5399A>G 5401T>G), V1785I (5459G>A 5461T>C), V1786I (5462G>A), D1794N (5486G>A), T1796P (5492A>C 5494A>T), Q1800H (5506A>T), L1810V (5534C>G), K1811Q (5537A>C), A1813S (5543G>T), E1818Q (5558G>C), I1826V (5582A>G 5584T>G), A1829S (5591G>T 5593C>A), T1833L (5603A>C 5604C>T), Y1836F (5613A>T), R1853N (5664G>A 5665A>T), L1856C (5672C>T 5673T>G 5674A>T), Q1857S (5675C>A 5676A>G 5677G>T), Y1869H (5711T>C), R1878K (5739G>A), L1882W (5751T>G), M1887I (5767G>T), K1889N (5773G>C), I1890V (5774A>G 5776A>G), I1893T (5784T>C 5785T>A), V1895L (5789G>T 5791C>G), E1898G (5799A>G 5800G>T), F1900T (5804T>A 5805T>C 5806C>T), Y1907F (5826A>T), V1913I (5843G>A 5845G>A), M1914F (5846A>T 5848G>C), L1915Q (5849T>C 5850T>A 5851G>A), D1918E (5860C>A), N1930H (5894A>C), Y1934F (5907A>T), H1946F (5942C>T 5943A>T 5944C>T), E1949D (5953A>T), N1950S (5955A>G 5956C>T), S1962A (5990T>G 5992A>C), T1966V (6002A>G 6003C>T 6004C>T), G1968E (6009G>A 6010G>A), T1976I (6033C>T 6034A>C), A1977S (6035G>T), S1980T (6044T>A 6046A>G), F1982L (6050T>C), D1985N (6059G>A), V1986I (6062G>A 6064T>C), H1997Q (6097T>A), I2002M (6112A>G), M2007L (6125A>C), E2008G (6129A>G 6130G>T), C2015S (6150G>C), V2016L (6152G>T 6154C>G), E2024G (6177A>G), T2027E (6185A>G 6186C>A 6187T>G), H2028R (6189A>G), M2032I (6202G>C), D2036E (6214T>G), L2037V (6215C>G 6217G>T), T2038P (6218A>C), E2039S (6221G>T 6222A>C 6223A>T), G2040E (6225G>A 6226G>A), I2041V (6227A>G 6229C>G), I2042V (6230A>G 6232A>G), K2044S (6237A>G 6238A>T), V2045T (6239G>A 6240T>C 6241G>A), I2046V (6242A>G 6244A>G), S2054T (6266T>A 6268G>T), S2056M (6273G>T 6274C>G), V2062E (6291T>A 6292T>A), D2063G (6294A>G), V2064L (6296G>C 6298A>C), L2066T (6302T>A 6303T>C 6304A>G), D2069S (6311G>T 6312A>C 6313T>C), I2071V (6317A>G 6319A>G), I2073V (6323A>G 6325C>G), A2089T (6371G>A 6373A>T), I2093V (6383A>G 6385C>G), C2100H (6404T>C 6405G>A), R2103V (6413C>G 6414G>T 6415A>G), S2104N (6416T>A 6417C>A 6418A>C), C2111S (6437T>A), T2115S (6449A>T), S2117A (6455T>G 6457C>A), S2118T (6458T>A), H2127N (6485C>A), R2128S (6490A>C), H2129N (6491C>A), Q2130V (6494C>G 6495A>T 6496G>A), T2131P (6497A>C 6499A>T), I2132E (6500A>G 6501T>A 6502T>G), D2133N (6503G>A 6505T>C), S2137P (6515T>C 6517C>A), F2138L (6518T>C 6520C>T), T2149M (6552C>T 6553A>G), G2154N (6566G>A 6567G>A 6568C>T)

	Begin	End	Coverage	Score	Concordance	Matches	Identities	I/D/M/F*	Stop Codons
**NT**	**21**	**6806**	**86.9%**	**7777**	**57.4%**	**6785 (99.6%)**	**5350 (78.5%)**	**25/1**	

Codon mutations:

TTT3TTC (115T>C), TCC4TCT (118C>T), AAG5AAA (121G>A), ATG6TTA (122A>T 124G>A), CCC8TCT (128C>T 130C>T), TTC10CCT (134T>C 135T>C 136C>T), AAC11AGC (138A>G), TCA12CCC (140T>C 142A>C), GTT13GCA (144T>C 145T>A), ACT14ATG (147C>T 148T>G), GAA15GAG (151A>G), TGC17AGT (155T>A 157C>T), GCT18GTT (159C>T), ACC19GCC (161A>G), TCT24TCG (178T>G), TCA27TCT (187A>T), ACT30ACA (196T>A), GCT31CAA (197G>C 198C>A 199T>A), AGT34AGC (208T>C), AAT35AAC (211T>C), TTC36TTT (214C>T), CGC38CGT (220C>T), ACT39ACA (223T>A), CTG40CTT (226G>T), TCA47TCC (247A>C), CAA49CAG (253A>G), ATT50ATC (256T>C), TCT52TCA (262T>A), CAC53CAT (265C>T), GAA55GAG (271A>G), TTG57CTG (275T>C), AAG58AAA (280G>A), CCT59CCC (283T>C), TCA60TCG (286A>G), GTT61GTG (289T>G), ACA62ACT (292A>T), GCT65GCA (301T>A), TCT66ACT (302T>A), TCC67TCT (307C>T), TTT68TTC (310T>C), ACC69TCT (311A>T 313C>T), AGC70AAT (315G>A 316C>T), TGT72TGC (322T>C), AAC73AGT (324A>G 325C>T), TCT74TCA (328T>A), ACT76ACC (334T>C), AAA77AAG (337A>G), TTG78TTA (340G>A), TTA79TTG (343A>G), AAG81AAA (349G>A), ACT84ACA (358T>A), TTA85CTA (359T>C), ATT86ATA (364T>A), GAA87GAG (367A>G), CCC88CCT (370C>T), TTC89TTT (373C>T), ATC90ATA (376C>A), GCT92GCA (382T>A), GTC96GTT (394C>T), GCA97GCC (397A>C), TCC98TCT (400C>T), AAA101AAG (409A>G), TCA102TCC (412A>C), ATT103ATC (415T>C), GTT107GTG (427T>G), AAG109AAA (433G>A), GTT111GTC (439T>C), GCA112ACA (440G>A), AGC113GGC (443A>G), AAA115AAG (451A>G), AAG117AAA (457G>A), AAC123GAC (473A>G), GAC127AAT (485G>A 487C>T), TCT129TCC (493T>C), GAG130GAA (496G>A), GTG132GTT (502G>T), CTT140CTC (526T>C), ATT149ATA (553T>A), TTC151TTT (559C>T), CCA154CCT (568A>T), AGT156AAT (573G>A), CTA158TTA (578C>T), GGA160GGT (586A>T), CAG163CAA (595G>A), ACT165TCA (599A>T 601T>A), CAC166CAT (604C>T), ATA167ACT (606T>C 607A>T), AAT170AGT (615A>G), ACA171ATT (618C>T 619A>T), AAC174AAT (628C>T), GCT178GCC (640T>C), TTA182CTG (650T>C 652A>G), GAC183GAG (655C>G), TTA185CTG (659T>C 661A>G), CCG186CCC (664G>C), TCC189TCT (673C>T), CAA190CAG (676A>G), GCA196GCG (694A>G), CTA201TTA (707C>T), GGG203GGA (715G>A), GAA204GAG (718A>G), TCC205GCT (719T>G 721C>T), ATG206GTG (722A>G), TCA207TCT (727A>T), CAC208CAT (730C>T), CTT211CTG (739T>G), GTG214GTT (748G>T), GTA215GTT (751A>T), GCA216GCG (754A>G), CTC218TTA (758C>T 760C>A), ATA219ATT (763A>T), GCC220GCT (766C>T), GGG222GGC (772G>C), ATT223ATA (775T>A), TCT224TCA (778T>A), ACC225ACA (781C>A), TTC228TTT (790C>T), GTG229GTA (793G>A), GCT230GCA (796T>A), GTA231GTT (799A>T), CCT232CCG (802T>G), GGT233GGC (805T>C), AGA234CGT (806A>C 808A>T), GGT237GGA (817T>A), CTA238CTC (820A>C), TCC239ACC (821T>A), AAC240AAT (826C>T), CCG241CCA (829G>A), CTA242CTC (832A>C), TCA243TCC (835A>C), AAA244AAG (838A>G), CTG246TTA (842C>T 844G>A), TAC247TAT (847C>T), GCG249GCA (853G>A), GGT252GGA (862T>A), GCT253GCC (865T>C), AAT257AAC (877T>C), TTG259CTT (881T>C 883G>T), TTT260TTC (886T>C), ACC261ACA (889C>A), TTC263TTT (895C>T), AGG264AGA (898G>A), AAT265AAC (901T>C), TGT269TGC (913T>C), ATA281ATT (949A>T), ATT284ATA (958T>A), GGT286GGA (964T>A), TTT287TTC (967T>C), CCA290CCT (976A>T), GTT291ATA (977G>A 979T>A), GCT294GCA (988T>A), ATT295ATC (991T>C), TTG299CTT (1001T>C 1003G>T), TCT300GCA (1004T>G 1006T>A), ACG301ACA (1009G>A), TTC304TTT (1018C>T), ACG305GTG (1019A>G 1020C>T), GAG309GAA (1033G>A), CCA316CCC (1054A>C), CAT318CAC (1060T>C), CGC319CGA (1063C>A), TTG320CTT (1064T>C 1066G>T), AAT322AAC (1072T>C), GCA324GCG (1078A>G), AAC325AAT (1081C>T), ACT329ACC (1093T>C), ATC330ATA (1096C>A), CTA335TTG (1109C>T 1111A>G), AAA341AAG (1129A>G), TCT343TCA (1135T>A), TAC345TAT (1141C>T), GCT347GCC (1147T>C), GTA351GTG (1159A>G), GCC353GCA (1165C>A), AGG358AGA (1180G>A), GTA359GTT (1183A>T), ACG360ACA (1186G>A), GCA361GCC (1189A>C), GCA362GCC (1192A>C), ATA363ATT (1195A>T), ACC364TCT (1196A>T 1198C>T), CTT366CTG (1204T>G), GAT367GAG (1207T>G), GGG371GGC (1219G>C), AAC373AAT (1225C>T), ACT377ACA (1237T>A), GGC378GGT (1240C>T), GTG379GTT (1243G>T), GGC380GGT (1246C>T), CAA381CAG (1249A>G), AGG383AGA (1255G>A), GCA384GCT (1258A>T), CCT386CCC (1264T>C), GGT393GGA (1285T>A), AGT395AGC (1291T>C), GGG398GGA (1300G>A), ACC401ACA (1309C>A), CGG404CGA (1318G>A), CTG405TTA (1319C>T 1321G>A), ATC407ATT (1327C>T), GTT410GTG (1336T>G), TTG411CTT (1337T>C 1339G>T), CGC413AGG (1343C>A 1345C>G), GAA415GAT (1351A>T), GAG416GAA (1354G>A), CCA417CCC (1357A>C), TTG419CTT (1361T>C 1363G>T), AGG421CGG (1367A>C), CTC422CTG (1372C>G), GCT424GCC (1378T>C), GTG425GTA (1381G>A), CGA428CGT (1390A>T), TCA433TCC (1405A>C), AAT434AAC (1408T>C), TAT435TAC (1411T>C), GCT440GCA (1426T>A), CTG442CTT (1432G>T), GCT448GCA (1450T>A), CTG449TTG (1451C>T), GGG452GGT (1462G>T), GCA453GCT (1465A>T), CAA455CAG (1471A>G), GAC458GAT (1480C>T), ATT459GTT (1481A>G), AAG460AAA (1486G>A), ATA463ATT (1495A>T), ATC465ATT (1501C>T), TCC467TCA (1507C>A), ACT468ACA (1510T>A), CCA470CCT (1516A>T), CCC472CCA (1522C>A), TTG473TTA (1525G>A), CCC474CCA (1528C>A), GCA476GCT (1534A>T), CGC483AGG (1553C>A 1555C>G), CGA488AGG (1568C>A 1570A>G), TAT489TAC (1573T>C), ATA490ATT (1576A>T), GCC492GCT (1582C>T), ACA493ACC (1585A>C), TCC494TCA (1588C>A), AAC495AAT (1591C>T), TCA498TCC (1600A>C), GCT501TCA (1607G>T 1609T>A), CAG502CAA (1612G>A), GGT504GGG (1618T>G), ACC506ACA (1624C>A), AGA513CGG (1643A>C 1645A>G), CGG515AGG (1649C>A), GTT517GTG (1657T>G), CTT518CTG (1660T>G), GTG519GTA (1663G>A), TGC521TGT (1669C>T), GGT525GGA (1681T>A), GAG526GAA (1684G>A), TTT527TTC (1687T>C), ACT529GTA (1691A>G 1692C>T 1693T>A), GCT531GCA (1699T>A), CCC532CCT (1702C>T), ACT533GCA (1703A>G 1705T>A), TCT534GCC (1706T>G 1708T>C), CTT536CTG (1714T>G), GAA537AGA (1715G>A 1716A>G), GTG540GTC (1726G>C), GTT541GTG (1729T>G), GAG542GAA (1732G>A), AGC543AGT (1735C>T), AAA544AGG (1737A>G 1738A>G), AGA545CGT (1739A>C 1741A>T), CCC546CCA (1744C>A), ACA550ATC (1755C>T 1756A>C), AGG552AGA (1762G>A), AGC555GAT (1769A>G 1770G>A 1771C>T), GAT557GAC (1777T>C), GAT558GAC (1780T>C), TTG559CTA (1781T>C 1783G>A), CTG560CTT (1786G>T), AAG568AAA (1810G>A), GTT569GTG (1813T>G), GCA571GCC (1819A>C), ATC573ATA (1825C>A), TCA574TCT (1828A>T), GGA575GGC (1831A>C), CTG577CTC (1837G>C), TAT578TTT (1839A>T), GCC580ACC (1844G>A), ACA581ACC (1849A>C), TCA582ACC (1850T>A 1852A>C), AAG584AAA (1858G>A), GTG590GTC (1876G>C), TCA591ACT (1877T>A 1879A>T), GAG593GAA (1885G>A), ATA595GTG (1889A>G 1891A>G), ATA596ATT (1894A>T), GAT600GAA (1906T>A), ATG601CCA (1907A>C 1908T>C 1909G>A), TTG602TGG (1911T>G), AAT603AAC (1915T>C), ATC604ATT (1918C>T), CAA605CAG (1921A>G), GAT606GAC (1924T>C), ACC607ACA (1927C>A), GAT610GAC (1936T>C), GCT611ACA (1937G>A 1939T>A), AAG612AAA (1942G>A), CTT613CTG (1945T>G), CCC614CCG (1948C>G), GTG615GTA (1951G>A), GTT617GTG (1957T>G), GTA618GTC (1960A>C), GTG624GTA (1978G>A), TAC626TAT (1984C>T), TTG630CTA (1994T>C 1996G>A), GTG632GTT (2002G>T), GAA633GAG (2005A>G), GCT634GCC (2008T>C), AAA636AAG (2014A>G), TAT637TTT (2016A>T), GCT638GCC (2020T>C), TAT639TAC (2023T>C), GCA646GCG (2044A>G), TAT648TAC (2050T>C), TTC649TTT (2053C>T), CCT650CCA (2056T>A), GAC652GAT (2062C>T), AAA655AAG (2071A>G), TTT659TAT (2082T>A), CTA662CTT (2092A>T), TCC665CCT (2099T>C 2101C>T), GAA668GAG (2110A>G), ACA669TCC (2111A>T 2113A>C), ACC671ACT (2119C>T), AGG672CGG (2120A>C), GTT674GTC (2128T>C), AAT675ACA (2130A>C 2131T>A), GAC676GAT (2134C>T), CTG678CTC (2140G>C), TAT679CAA (2141T>C 2143T>A), CAA680CAG (2146A>G), GGT681GGA (2149T>A), TGT683TGC (2155T>C), AAT684AAC (2158T>C), GAG685GCG (2160A>C), AAC686AAT (2164C>T), CTG690CTC (2176G>C), ATA691GTT (2177A>G 2179A>T), AAG692AAA (2182G>A), ATC694ATT (2188C>T), GCT696GCA (2194T>A), TAT698TAC (2200T>C), ATC706ATA (2224C>A), TAC707TAT (2227C>T), GCT708GCG (2230T>G), AAT712TGT (2240A>T 2241A>G), GAG713GAT (2245G>T), CGT714CGC (2248T>C), AGC715AAA (2250G>A 2251C>A), GAC717GAT (2257C>T), GTT718GTG (2260T>G), CAG719CAA (2263G>A), CTG722TTA (2270C>T 2272G>A), GGA725GGC (2281A>C), ATG726TTG (2282A>T), GCG727GCA (2287G>A), CAC728CAT (2290C>T), GCC730GCT (2296C>T), ATA731ATT (2299A>T), GCT733GCA (2305T>A), GTG735GTT (2311G>T), GAC736GAT (2314C>T), CTG738TTA (2318C>T 2320G>A), ATA739ATT (2323A>T), TGC740TGT (2326C>T), GTG741ATC (2327G>A 2329G>C), GAG742GAA (2332G>A), CAG743CAA (2335G>A), CCT745CCA (2341T>A), GCC746GCA (2344C>A), CAC747CAT (2347C>T), GTA748GTG (2350A>G), TTC750TTT (2356C>T), TTG751TTA (2359G>A), ACA753ACT (2365A>T), GCA754GCT (2368A>T), TTT755TTC (2371T>C), AGG757CGT (2375A>C 2377G>T), TAT758TAC (2380T>C), CGC761AGA (2387C>A 2389C>A), AGA762AGG (2392A>G), CAG766CAA (2404G>A), CCT767CCC (2407T>C), AGG768AAA (2409G>A 2410G>A), TCC770TCT (2416C>T), TTA790TTG (2476A>G), CCG791CCA (2479G>A), ATC793ATT (2485C>T), TTG794TTA (2488G>A), AGA795AGG (2491A>G), GTT796GTG (2494T>G), CTG797CTT (2497G>T), ATT798ATC (2500T>C), AAG799AAA (2503G>A), GCT800GCA (2506T>A), GGC801GGG (2509C>G), CTT802TTG (2510C>T 2512T>G), ATT803ATC (2515T>C), TTA804CTA (2516T>C), GCA806GCC (2524A>C), GCA810GCT (2536A>T), GGG812GGT (2542G>T), GCA816GCT (2554A>T), TTC817TTT (2557C>T), GCT819GCG (2563T>G), CAA822CGG (2571A>G 2572A>G), TCC823TCA (2575C>A), CCT827CCC (2587T>C), AGA832CGG (2600A>C 2602A>G), ATT835ATC (2611T>C), ACC836ACA (2614C>A), AAC837AAT (2617C>T), AAT840AAC (2626T>C), AGC841AGT (2629C>T), CGG845AGG (2639C>A), AAG848AAA (2650G>A), ATC852ATT (2662C>T), TTG857TTA (2677G>A), CTG858TTG (2678C>T), GCA859GCG (2683A>G), AAG862AAA (2692G>A), GGT863GGA (2695T>A), CTG866CTT (2704G>T), AAA868AAG (2710A>G), GCA870GCC (2716A>C), GGC874AGC (2726G>A), TTC875TTT (2731C>T), GGA879GGC (2743A>C), TTG880CTG (2744T>C), GTC885GTG (2761C>G), TTG887CTC (2765T>C 2767G>C), AGA888CGA (2768A>C), GGT890GGG (2776T>G), TAC894TAT (2788C>T), ACC898ACA (2800C>A), GTC900GTT (2806C>T), TGC901TGT (2809C>T), TCG902TCT (2812G>T), GGC903GGA (2815C>A), GTG905ATA (2819G>A 2821G>A), ACA907ACT (2827A>T), GTG908GTA (2830G>A), TTT912TTC (2842T>C), TCA913TCC (2845A>C), AGC914AGT (2848C>T), CGT916AGA (2852C>A 2854T>A), GGC918GGG (2860C>G), ACA919ACT (2863A>T), ACT920GTG (2864A>G 2865C>T 2866T>G), TTC921TTT (2869C>T), ATA923ATT (2875A>T), ATA924ATC (2878A>C), GCC926TCG (2882G>T 2884C>G), TCA928TCC (2890A>C), TAC934TAT (2908C>T), AAT935AAC (2911T>C), CGG940CGT (2926G>T), TTC941TTT (2929C>T), TTG942CTA (2930T>C 2932G>A), AAG943AAA (2935G>A), CAA945CAG (2941A>G), ATT947ATA (2947T>A), GTC948GTG (2950C>G), CTG949CTT (2953G>T), CTC950CTG (2956C>G), AAT951AAC (2959T>C), GGA953GGT (2965A>T), CCC955CCT (2971C>T), GAT956GAC (2974T>C), CCC958CCG (2980C>G), CCT960CCG (2986T>G), ATT962ATC (2992T>C), AAA964AAG (2998A>G), CGG969AGG (3011C>A), TGT973TGC (3025T>C), TCT975TCA (3031T>A), AAG976AAA (3034G>A), GGC977GGA (3037C>A), ACT978TCT (3038A>T), GGG980GGT (3046G>T), GTA981GTC (3049A>C), TGT982TGC (3052T>C), GTA986GTT (3064A>T), AAG989AAA (3073G>A), GCT991GCC (3079T>C), CAA993AAG (3083C>A 3085A>G), TTG999CTT (3101T>C 3103G>T), GTG1003GTT (3115G>T), TCC1006TCA (3124C>A), CTT1010CTG (3136T>G), ACG1014TCC (3146A>T 3148G>C), GAC1018GAT (3160C>T), AGT1020AGC (3166T>C), TCC1021TCA (3169C>A), CTG1024CTA (3178G>A), TCT1026TCA (3184T>A), TAT1030TAC (3196T>C), TAT1032TAC (3202T>C), GAT1040GAC (3226T>C), CTT1044CTG (3238T>G), CTT1045TTA (3239C>T 3241T>A), CTT1046TTG (3242C>T 3244T>G), TTG1047TTA (3247G>A), AAC1049AAT (3253C>T), GTG1050GTA (3256G>A), AAA1053AAG (3265A>G), TGC1056TGT (3274C>T), ATT1057ATA (3277T>A), ATT1060ATA (3286T>A), TCT1064TCA (3298T>A), GAA1068GAG (3310A>G), CAC1071CAT (3319C>T), GGG1073GGC (3325G>C), TTT1074TTC (3328T>C), GTA1075GTT (3331A>T), GCA1076GCC (3334A>C), ACA1080ACG (3346A>G), GCT1081GCG (3349T>G), ATT1082ATC (3352T>C), CGA1084AGA (3356C>A), TTG1087CTG (3365T>C), GAT1089AAC (3371G>A 3373T>C), CTT1090TTG (3374C>T 3376T>G), CCG1091CCA (3379G>A), ACT1092ACA (3382T>A), GGT1093GGG (3385T>G), CCG1094CCT (3388G>T), AAA1096AAG (3394A>G), GCA1098GCC (3400A>C), GTA1100GTG (3406A>G), TGC1102TGT (3412C>T), CAG1103GAG (3413C>G), CTC1104TTG (3416C>T 3418C>G), CTT1105CTG (3421T>G), CTA1108TTG (3428C>T 3430A>G), CGA1109AGA (3431C>A), AGG1111AGA (3439G>A), GCG1113GCT (3445G>T), GAA1117GAG (3457A>G), CAG1120CAA (3466G>A), TTT1123TAC (3474T>A 3475T>C), GTT1124CTT (3476G>C), GGC1125GGA (3481C>A), ACA1126ACT (3484A>T), GCT1129GAT (3492C>A), GCC1133GCA (3505C>A), ACG1134ACT (3508G>T), CCC1136CCA (3514C>A), AAA1138AAG (3520A>G), ACA1139ACC (3523A>C), ACG1140ACA (3526G>A), CTG1141CTC (3529G>C), CGA1142AGG (3530C>A 3532A>G), AGG1143AAG (3534G>A), GGC1145GGT (3541C>T), TTG1146CTC (3542T>C 3544G>C), GCT1149GCA (3553T>A), GGG1151GGA (3559G>A), GCA1153GCT (3565A>T), TCT1158TCC (3580T>C), ATC1159ATA (3583C>A), ATT1160ATC (3586T>C), TCA1161TCG (3589A>G), GCT1162GTC (3591C>T 3592T>C), TCA1163CAA (3593T>C 3594C>A), CGT1166AGA (3602C>A 3604T>A), GGG1167GGA (3607G>A), GAA1168GAG (3610A>G), GGT1169GGC (3613T>C), CCG1172CCA (3622G>A), TAC1173TAT (3625C>T), GTG1174GTC (3628G>C), GCT1175TCT (3629G>T), ATA1177ATC (3637A>C), CAA1178CAG (3640A>G), AAA1179AAG (3643A>G), TAC1180TAT (3646C>T), GAA1182GAG (3652A>G), GCA1184GCC (3658A>C), CAA1185CAG (3661A>G), AAT1186AAC (3664T>C), GAG1189GAA (3673G>A), GAC1190GAT (3676C>T), ATT1191ATC (3679T>C), GCG1192GCC (3682G>C), AGG1193AAA (3684G>A 3685G>A), CTA1194TTG (3686C>T 3688A>G), GCC1195GCT (3691C>T), TAT1196TTT (3693A>T), GGG1198GGA (3700G>A), CTA1199TTG (3701C>T 3703A>G), CGT1200CGG (3706T>G), CAA1201CAG (3709A>G), GCA1202TCT (3710G>T 3712A>T), ATT1203GTG (3713A>G 3715T>G), TTG1204CTG (3716T>C), GTG1206ATA (3722G>A 3724G>A), CAC1208CGC (3729A>G), TCC1209TCG (3733C>G), GTT1212GTG (3742T>G), AAG1216GAG (3752A>G), GTC1218ATC (3758G>A), ACA1219ACT (3763A>T), CAG1220CAA (3766G>A), GTG1224ATT (3776G>A 3778G>T), GTG1225GTT (3781G>T), CTC1226TTG (3782C>T 3784C>G), GGT1228GGA (3790T>A), GTT1229GTC (3793T>C), TTT1232TTC (3802T>C), TAT1234TAC (3808T>C), GAC1236GAT (3814C>T), GGG1237GGA (3817G>A), TTG1240CTG (3824T>C), GGG1245GGT (3841G>T), CCG1247CCA (3847G>A), TAC1248TAT (3850C>T), AAG1250AAA (3856G>A), GGC1254GGT (3868C>T), ACT1255ACC (3871T>C), GAG1259GAA (3883G>A), GTG1261GTA (3889G>A), CCA1263CCC (3895A>C), GGA1265GGC (3901A>C), GGA1267GGT (3907A>T), GAT1268GAC (3910T>C), CGA1269AGA (3911C>A), GTC1270GTG (3916C>G), CTC1272TTG (3920C>T 3922C>G), AAA1273AAG (3925A>G), AGG1274AGA (3928G>A), ACT1276ACC (3934T>C), GAC1281GAT (3949C>T), GAG1283GAA (3955G>A), GCT1284GCA (3958T>A), TTG1285CTA (3959T>C 3961G>A), ATT1289ATC (3973T>C), GGA1292GGT (3982A>T), CTG1297TTG (3995C>T), GTC1298GTT (4000C>T), ACC1299ACA (4003C>A), ACC1300ACA (4006C>A), CAA1301CAG (4009A>G), GCC1303GCA (4015C>A), AAA1304AAG (4018A>G), TTG1308CTA (4028T>C 4030G>A), CCG1309CCT (4033G>T), TTG1310CTG (4034T>C), GAA1311GAG (4039A>G), GTT1313GTG (4045T>G), GGG1315GGC (4051G>C), CGG1317AGG (4055C>A), ACC1319ACA (4063C>A), TTG1321CTA (4067T>C 4069G>A), GAA1323GAG (4075A>G), ATT1324ATA (4078T>A), CTT1325CTA (4081T>A), CCC1326CCT (4084C>T), TAC1329TAT (4093C>T), AAT1330AAC (4096T>C), CTG1338TTG (4118C>T), GAT1339GAC (4123T>C), TCC1341TCT (4129C>T), TCC1347TCG (4147C>G), CTG1351TTG (4157C>T), CCA1352GCT (4160C>G 4162A>T), GGT1361GGA (4189T>A), ATT1362ATG (4192T>G), GAA1363GAG (4195A>G), CTG1366TTA (4202C>T 4204G>A), TTG1367CTA (4205T>C 4207G>A), GCT1368GCA (4210T>A), AAT1369AAC (4213T>C), GGC1370AGC (4214G>A), TTC1371TTT (4219C>T), AGA1372CGG (4220A>C 4222A>G), GCA1373GCT (4225A>T), GTC1374GTT (4228C>T), ACA1377ACG (4237A>G), TCT1380TCC (4246T>C), GCT1381GCA (4249T>A), GAC1382GAT (4252C>T), TAT1383TAC (4255T>C), ATC1392ATT (4282C>T), GCC1394GCA (4288C>A), CAA1396CAG (4294A>G), TGT1399TGC (4303T>C), CTT1401CTG (4309T>G), GAC1404GAT (4318C>T), GGA1407GGC (4327A>C), CCT1410CAA (4335C>A 4336T>A), GGT1411GGC (4339T>C), TCT1412TCC (4342T>C), GAC1414GAT (4348C>T), TCC1415TCA (4351C>A), AAT1416AAC (4354T>C), AGA1418CGC (4358A>C 4360A>C), CAT1419CAC (4363T>C), GTG1420GTA (4366G>A), CTT1421TTG (4367C>T 4369T>G), CTT1422CTG (4372T>G), GCT1425GCC (4381T>C), TCA1426TCT (4384A>T), TGC1427TGT (4387C>T), ACT1430ACC (4396T>C), ATT1431ATC (4399T>C), GAG1433GAA (4405G>A), GTT1436GTG (4414T>G), TTT1437TTC (4417T>C), AGG1438CGC (4418A>C 4420G>C), TTG1439CTG (4421T>C), GTG1440GTT (4426G>T), GGA1441GGG (4429A>G), GGT1442GGC (4432T>C), CCT1444CCA (4438T>A), TCA1445TCT (4441A>T), GGA1446GGG (4444A>G), GCA1448GCT (4450A>T), CTC1449CTT (4453C>T), ACC1450ACT (4456C>T), GTT1451GTC (4459T>C),

ATC1452ATT (4462C>T), TTC1453TTT (4465C>T), AAC1454AAT (4468C>T), CGT1463CGG (4495T>G), GCA1465GCT (4501A>T), ATT1467ATC (4507T>C), TCT1468TCA (4510T>A), CTA1469CTG (4513A>G), TTG1470CTG (4514T>C), AGA1472AGG (4522A>G), CAT1474CAC (4528T>C), GCA1476GCT (4534A>T), GCT1477GCA (4537T>A), CAA1478CAG (4540A>G), ATA1480ATT (4546A>T), GGG1481GGC (4549G>C), TGC1482TGT (4552C>T), AAA1483AAG (4555A>G), CCC1484CCA (4558C>A), AAC1488AAT (4570C>T), CTA1490CTT (4576A>T), GTG1492GTT (4582G>T), GCA1493GCT (4585A>T), GTC1494GTA (4588C>A), GAC1498GAT (4600C>T), CTA1500CTT (4606A>T), GTT1503GTA (4615T>A), CCC1504CCA (4618C>A), GAA1506GGT (4623A>G 4624A>T), TTG1507CTC (4625T>C 4627G>C), TAT1510TAC (4636T>C), ACT1511TCG (4637A>T 4639T>G), GCT1514GCA (4648T>A), ATT1515ATA (4651T>A), GAA1518GAG (4660A>G), TTG1519CTA (4661T>C 4663G>A), GTT1524GTC (4678T>C), ATA1526ATC (4684A>C), ATC1530ATA (4696C>A), AAG1532AAA (4702G>A), AAC1533AAT (4705C>T), GTT1536GTC (4714T>C), AGC1537AAT (4716G>A 4717C>T), AGT1538CAT (4718A>C 4719G>A), CTA1542CTG (4732A>G), GAC1543GAT (4735C>T), TTG1544CTT (4736T>C 4738G>T), TCT1545TCA (4741T>A), CTA1547TTG (4745C>T 4747A>G), TTT1549TTC (4753T>C), CTA1550CTT (4756A>T), AGC1551AGT (4759C>T), AGA1552AGG (4762A>G), GGT1553GGC (4765T>C), TTT1554TTC (4768T>C), AAG1555AAA (4771G>A), AGG1556AGA (4774G>A), GGA1560GGG (4786A>G), TAC1561TAT (4789C>T), CCT1565CCC (4801T>C), CTG1566TTG (4802C>T), GTA1569GTG (4813A>G), TCT1570TCC (4816T>C), GAA1573GAG (4825A>G), CCA1574CCC (4828A>C), TAC1576TAT (4834C>T), ATA1578ATT (4840A>T), AGG1579AGA (4843G>A), CCT1580CCC (4846T>C), GTT1582ACT (4850G>A 4851T>C), GCT1587GCC (4867T>C), CTC1588TTG (4868C>T 4870C>G), TTG1591TTA (4879G>A), AAC1593AAT (4885C>T), ATA1594ATT (4888A>T), GAC1595GAT (4891C>T), ACG1596ACT (4894G>T), GGA1597GGC (4897A>C), GTT1598ATT (4898G>A), AGA1599AGG (4903A>G), GCA1601GCC (4909A>C), CAC1603CAT (4915C>T), GGG1605GGT (4921G>T), CAG1607CAA (4927G>A), GTT1608GTG (4930T>G), GAA1610GAC (4936A>C), GTG1613GTT (4945G>T), ACA1614AAT (4947C>A 4948A>T), GTT1616GTG (4954T>G), AAC1618CAC (4958A>C), GCT1619GCC (4963T>C), AAG1621AAA (4969G>A), CGG1623CGA (4975G>A), TTC1625TTT (4981C>T), CCA1626CCT (4984A>T), GCC1627GCT (4987C>T), ACC1628GCA (4988A>G 4990C>A), CCT1631CCG (4999T>G), ACA1632ACT (5002A>T), TAT1633TAC (5005T>C), GAG1637GAA (5017G>A), CAG1638CAA (5020G>A), GAC1639GAT (5023C>T), CTG1641CTT (5029G>T), GTG1642ATG (5030G>A), GAG1643GAA (5035G>A), GTT1644GTA (5038T>A), ACA1645ACT (5041A>T), GGA1646GGC (5044A>C), CCA1648CCT (5050A>T), GGG1651GGT (5059G>T), CTC1652CTT (5062C>T), CTT1656TTA (5072C>T 5074T>A), ATT1658ACA (5079T>C 5080T>A), GCA1659GCT (5083A>T), TCA1661TCC (5089A>C), GCT1662TCA (5090G>T 5092T>A), GTG1664GTT (5098G>T), CCT1665CCA (5101T>A), TTG1666CTG (5102T>C), CCC1667GCT (5105C>G 5107C>T), CCA1668CCT (5110A>T), GGC1669GGT (5113C>T), AAT1670GAA (5114A>G 5116T>A), ACT1671ACC (5119T>C), GTT1672ATA (5120G>A 5122T>A), CTG1673TTG (5123C>T), TTT1675TTC (5131T>C), TCT1676ACT (5132T>A), GTG1679ATT (5141G>A 5143G>T), CAT1680CAC (5146T>C), ACT1681ACA (5149T>A), TTT1682TTC (5152T>C), GCT1683GCA (5155T>A), CGA1685AGA (5159C>A), TGC1689TGT (5173C>T), TCC1690TCT (5176C>T), TCG1691GCC (5177T>G 5179G>C), CGA1692AGG (5180C>A 5182A>G), ACA1693ACT (5185A>T), GCA1696GCC (5194A>C), CAG1697CAT (5197G>T), CAG1698CAA (5200G>A), GAC1700GAT (5206C>T), ACC1701TCA (5207A>T 5209C>A), ACC1702ACA (5212C>A), GTT1704GTC (5218T>C), AAC1707AAT (5227C>T), ACT1709ACC (5233T>C), GCA1711GCC (5239A>C), AAG1712AAA (5242G>A), GTA1717GTT (5257A>T), AGA1718AGG (5260A>G), GGG1719GGA (5263G>A), CCC1720CCA (5266C>A), ACC1721ACA (5269C>A), AGG1724CGA (5276A>C 5278G>A), GGG1728GGT (5290G>T), CTT1729CTC (5293T>C), ATT1730ATC (5296T>C), CCT1732CCA (5302T>A), GCT1736GCC (5314T>C), GAC1740GAT (5326C>T), CAC1741CAT (5329C>T), AGC1744GGC (5336A>G), ATT1745ATC (5341T>C), GTA1746ATC (5342G>A 5344A>C), ACC1747ACA (5347C>A), GGG1749GGA (5353G>A), ACC1751TCT (5357A>T 5359C>T), AAA1752CAC (5360A>C 5362A>C), CAT1754AAC (5366C>A 5368T>C), GTG1755ATT (5369G>A 5371G>T), GTT1756GTA (5374T>A), TTT1757TTC (5377T>C), GGC1760GGG (5386C>G), AAT1761AAC (5389T>C), GGG1762GGA (5392G>A), ATT1765GTG (5399A>G 5401T>G), GGT1766GGA (5404T>A), CCA1767CCT (5407A>T), GTG1768GTT (5410G>T), TGC1769TGT (5413C>T), GCT1770GCA (5416T>A), GCA1771GCT (5419A>T), TTG1772CTA (5420T>C 5422G>A), CTG1775TTA (5429C>T 5431G>A), TCC1776TCT (5434C>T), ACT1777ACG (5437T>G), GGC1778GGA (5440C>A), CAA1779CAG (5443A>G), TAT1780TAC (5446T>C), TCT1781TCA (5449T>A), TCT1782TCC (5452T>C), GTT1785ATC (5459G>A 5461T>C), GTT1786ATT (5462G>A), GTG1787GTC (5467G>C), ACC1791ACT (5479C>T), ATA1792ATT (5482A>T), GCA1793GCT (5485A>T), GAT1794AAT (5486G>A), GTT1795GTG (5491T>G), ACA1796CCT (5492A>C 5494A>T), CTC1798TTG (5498C>T 5500C>G), AGT1799AGC (5503T>C), CAA1800CAT (5506A>T), TAT1801TAC (5509T>C), CCA1802CCT (5512A>T), GGA1803GGT (5515A>T), GCC1806GCT (5524C>T), AAG1807AAA (5527G>A), TAT1808TAC (5530T>C), CTT1809TTG (5531C>T 5533T>G), CTG1810GTG (5534C>G), AAA1811CAA (5537A>C), GCT1813TCT (5543G>T), GAT1814GAC (5548T>C), ACA1815ACC (5551A>C), GAA1818CAA (5558G>C), CTT1820CTA (5566T>A), GCA1821GCT (5569A>T), ACA1823ACG (5575A>G), TGT1824TGC (5578T>C), ATT1826GTG (5582A>G 5584T>G), GCC1829TCA (5591G>T 5593C>A), GAG1832GAA (5602G>A), ACA1833CTA (5603A>C 5604C>T), CCA1834CCG (5608A>G), GCA1835GCT (5611A>T), TAC1836TTC (5613A>T), ATA1837ATT (5617A>T), CCT1838CCA (5620T>A), GGA1840GGT (5626A>T), TTT1842TTC (5632T>C), CTT1844CTG (5638T>G), GGT1845GGC (5641T>C), GTG1850GTA (5656G>A), CAA1851CAG (5659A>G), GGG1852GGA (5662G>A), AGA1853AAT (5664G>A 5665A>T), CTA1856TGT (5672C>T 5673T>G 5674A>T), CAG1857AGT (5675C>A 5676A>G 5677G>T), CAG1860CAA (5686G>A), CGC1861AGA (5687C>A 5689C>A), TTG1862CTT (5690T>C 5692G>T), GGA1867GGT (5707A>T), CCT1868CCA (5710T>A), TAT1869CAT (5711T>C), GGG1870GGT (5716G>T), GGA1871GGG (5719A>G), ACT1876ACC (5734T>C), ACT1877ACC (5737T>C), AGA1878AAA (5739G>A), GAG1879GAA (5743G>A), CTT1880CTA (5746T>A), AAG1881AAA (5749G>A), TTG1882TGG (5751T>G), CTG1883TTG (5753C>T), ATG1887ATT (5767G>T), AAG1889AAC (5773G>C), ATA1890GTG (5774A>G 5776A>G), TAT1891TAC (5779T>C), ATT1893ACA (5784T>C 5785T>A), TTA1894CTT (5786T>C 5788A>T), GTC1895TTG (5789G>T 5791C>G), CAA1896CAG (5794A>G), AGA1897AGG (5797A>G), GAG1898GGT (5799A>G 5800G>T), TTC1900ACT (5804T>A 5805T>C 5806C>T), ACT1902ACC (5812T>C), CTC1903CTG (5815C>G), TAC1904TAT (5818C>T), TAT1907TTT (5826A>T), CTC1908CTT (5830C>T), TCA1910TCC (5836A>C), GTG1913ATA (5843G>A 5845G>A), ATG1914TTC (5846A>T 5848G>C), TTG1915CAA (5849T>C 5850T>A 5851G>A), GTT1917GTG (5857T>G), GAC1918GAA (5860C>A), CTT1920TTG (5864C>T 5866T>G), CTC1922CTA (5872C>A), GGG1924GGT (5878G>T), TTT1925TTC (5881T>C), TTT1926TTC (5884T>C), CCC1927CCT (5887C>T), CGC1928CGT (5890C>T), AAT1930CAT (5894A>C), GTG1932GTA (5902G>A), TAC1934TTC (5907A>T), CTT1936CTC (5914T>C), TTT1939TTC (5923T>C), CTG1941CTA (5929G>A), CAC1946TTT (5942C>T 5943A>T 5944C>T), AAG1948AAA (5950G>A), GAA1949GAT (5953A>T), AAC1950AGT (5955A>G 5956C>T), ACG1953ACT (5965G>T), GTT1954GTG (5968T>G), TAC1955TAT (5971C>T), TTT1956TTC (5974T>C), AGA1957CGT (5975A>C 5977A>T), AAG1958AAA (5980G>A), CCT1959CCC (5983T>C), TTT1960TTC (5986T>C), CTC1961CTT (5989C>T), TCA1962GCC (5990T>G 5992A>C), AAA1964AAG (5998A>G), ACC1966GTT (6002A>G 6003C>T 6004C>T), GGG1968GAA (6009G>A 6010G>A), TCC1969TCA (6013C>A), AAA1970AAG (6016A>G), ATT1972ATA (6022T>A), TCC1974TCT (6028C>T), GCA1975GCT (6031A>T), ACA1976ATC (6033C>T 6034A>C), GCT1977TCT (6035G>T), GCT1978GCA (6040T>A), GAG1979GAA (6043G>A), TCA1980ACG (6044T>A 6046A>G), TTG1981TTA (6049G>A), TTT1982CTT (6050T>C), GGT1983GGG (6055T>G), GAT1985AAT (6059G>A), GTT1986ATC (6062G>A 6064T>C), TCC1987TCT (6067C>T), GCT1988GCC (6070T>C), CTC1991CTG (6079C>G), AGT1993AGC (6085T>C), AGG1994AGA (6088G>A), CTA1995TTA (6089C>T), CTT1996TTA (6092C>T 6094T>A), CAT1997CAA (6097T>A), CTT1998CTG (6100T>G), CAG1999CAA (6103G>A), AAG2000AAA (6106G>A), ATA2002ATG (6112A>G), TCA2005TCT (6121A>T), TCC2006TCA (6124C>A), ATG2007CTG (6125A>C), GAG2008GGT (6129A>G 6130G>T), TTT2011TTC (6139T>C), AAA2012AAG (6142A>G), ATT2013ATC (6145T>C), TAT2014TAC (6148T>C), TGT2015TCT (6150G>C), GTC2016TTG (6152G>T 6154C>G), CAG2018CAA (6160G>A), GGC2019GGG (6163C>G), AAG2023AAA (6175G>A), GAA2024GGA (6177A>G), GTA2026GTG (6184A>G), ACT2027GAG (6185A>G 6186C>A 6187T>G), CAC2028CGC (6189A>G), TTC2029TTT (6193C>T), CAA2030CAG (6196A>G), CGC2031CGT (6199C>T), ATG2032ATC (6202G>C), CAA2034CAG (6208A>G), GAT2036GAG (6214T>G), CTG2037GTT (6215C>G 6217G>T), ACA2038CCA (6218A>C), GAA2039TCT (6221G>T 6222A>C 6223A>T), GGG2040GAA (6225G>A 6226G>A), ATC2041GTG (6227A>G 6229C>G), ATA2042GTG (6230A>G 6232A>G), GAG2043GAA (6235G>A), AAA2044AGT (6237A>G 6238A>T), GTG2045ACA (6239G>A 6240T>C 6241G>A), ATA2046GTG (6242A>G 6244A>G), TTA2049CTC (6251T>C 6253A>C), ACC2050ACA (6256C>A), ACA2052ACT (6262A>T), CTG2053TTG (6263C>T), TCG2054ACT (6266T>A 6268G>T), AGC2056ATG (6273G>T 6274C>G), GTG2059GTA (6283G>A), CTT2061TTG (6287C>T 6289T>G), GTT2062GAA (6291T>A 6292T>A), GAT2063GGT (6294A>G), GTA2064CTC (6296G>C 6298A>C), CCT2065CCA (6301T>A), TTA2066ACG (6302T>A 6303T>C 6304A>G), GGC2067GGA (6307C>A), TTG2068CTT (6308T>C 6310G>T), GAT2069TCC (6311G>T 6312A>C 6313T>C), AAC2070AAT (6316C>T), ATA2071GTG (6317A>G 6319A>G), GAG2072GAA (6322G>A), ATC2073GTG (6323A>G 6325C>G), CAG2074CAA (6328G>A), AAA2076AAG (6334A>G), GGT2077GGC (6337T>C), CTA2080TTA (6344C>T), AAT2086AAC (6364T>C), GCA2089ACT (6371G>A 6373A>T), GCC2092GCT (6382C>T), ATC2093GTG (6383A>G 6385C>G), CTC2097CTA (6397C>A), TGT2100CAT (6404T>C 6405G>A), AAC2102AAT (6412C>T), CGA2103GTG (6413C>G 6414G>T 6415A>G), TCA2104AAC (6416T>A 6417C>A 6418A>C), ACA2105ACT (6421A>T), GTG2109GTT (6433G>T), TTC2110TTT (6436C>T), TGT2111AGT (6437T>A), TAC2113TAT (6445C>T), ACG2114ACA (6448G>A), ACT2115TCT (6449A>T), TCC2117GCA (6455T>G 6457C>A), TCT2118ACT (6458T>A), CTT2119TTG (6461C>T 6463T>G), GCC2122GCT (6472C>T), GTG2124GTT (6478G>T), CTT2125TTA (6479C>T 6481T>A), CAT2127AAT (6485C>A), AGA2128AGC (6490A>C), CAT2129AAT (6491C>A), CAG2130GTA (6494C>G 6495A>T 6496G>A), ACA2131CCT (6497A>C 6499A>T), ATT2132GAG (6500A>G 6501T>A 6502T>G), GAT2133AAC (6503G>A 6505T>C), CCG2136CCT (6514G>T), TCC2137CCA (6515T>C 6517C>A), TTC2138CTT (6518T>C 6520C>T), ACA2141ACT (6529A>T), GTG2145GTA (6541G>A), TTG2146TTA (6544G>A), CTT2147TTG (6545C>T 6547T>G), TTG2148CTG (6548T>C), ACA2149ATG (6552C>T 6553A>G), CCA2150CCC (6556A>C), CTA2152CTT (6562A>T), GGC2154AAT (6566G>A 6567G>A 6568C>T), TAC2155TAT (6571C>T), AAA2156AAG (6574A>G)

*: Inserts / Deletes / Misaligned / Frameshifts

Analysis details

This analysis was performed with panviral2.64

NGS Details (UN8): Torradovirus lycopersici (segment RNA 1)

Assembly

**Coverage Length**	6792 (1 contig(s))
**Depth Of Coverage**	2254.7
**Number Of Reads**	114995
**Reads Per Million**	1921.85 rpm (after QC)
**Ambiguities**	0
**Assembly Method**	de novo + reference guided assembly
**Consensus Caller**	Bcf Tools

Coverage Map

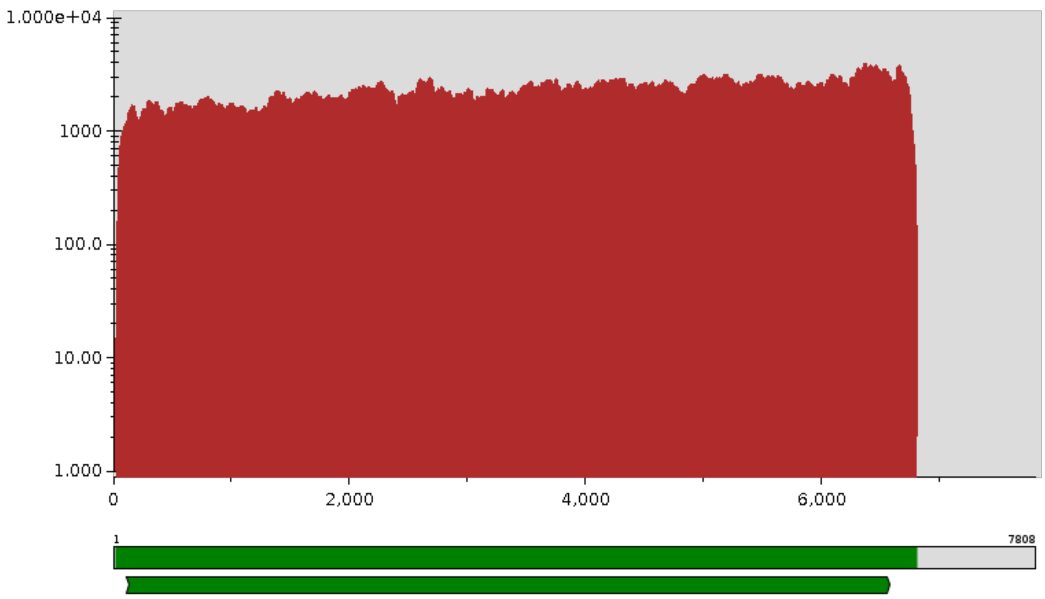

Assignment

**Type**	Torradovirus lycopersici (Taxonomy ID: 3048378)
**Reference Genome**	NC_009013.1
**NT Identity (%)**	90.4769
**AA Identity (%)**	97.8231
**Number Of Stop Codons**	1
**Number Of CDS**	1

Alignment

**Alignment Score**	10966.0 (NT) + 14853.0 (AA) = 25819.0
**Concordance (%)**	90.1407

Alignment Method	Global, seeded, nucleotide + amino acids (AGA)

Genome Region

Sequence starts at position 22 and ends at position 6813 relative to NC_009013.1 reference sequence.

Alignment Detailed Statistics

	Begin	End	Coverage	Score	Concordance	Matches	Identities	I/D/M/F*	Stop Codons
**NT**	**22**	**6813**	**87.0%**	**10966**	**80.8%**	**6791 (99.9%)**	**6147 (90.5%)**	**3/1**	
Mutations:	22T>A, 69C>T, 76_77insT, 83C>T, 83_84insT, 85T>C, 87C>T, 95A>G, 96C>T, 98T>C, 99T>C, 100T>C, 101G>A, 102G>A, 115T>C, 127C>T, 128C>T, 130C>T, 135T>C, 148T>C, 151A>G, 158G>T, 161A>G, 175T>C, 178T>C, 184T>C, 190G>A, 199T>A, 202T>C, 205T>A, 220C>T, 224C>T, 253A>G, 259T>C, 262T>A, 265C>T, 338T>C, 340G>A, 349G>A, 361A>G, 367A>G, 370C>T, 376C>T, 385C>T, 388T>C, 409A>G, 421T>C, 433G>A, 436T>A, 451A>G, 454T>C, 472T>C, 487C>T, 490A>G, 502G>A, 559C>T, 577A>G, 578C>T, 583T>A, 637A>C, 655C>T, 676A>G, 706T>C, 707C>T, 709A>G, 727A>G, 760C>T, 781C>T, 790C>T, 799A>G, 805T>C, 818C>T, 820A>G, 832A>T, 835A>C, 841T>C, 842C>T, 847C>T, 883G>A, 886T>C, 892G>A, 901T>C, 913T>C, 925C>T, 938C>T, 952A>G, 955C>T, 964T>G, 970G>A, 973T>C, 977G>A, 980T>C, 997C>T, 1006T>C, 1012C>T, 1013T>C, 1018C>T, 1033G>A, 1063C>T, 1064T>C, 1078A>T, 1081C>T, 1093T>C, 1105A>G, 1108T>C, 1109C>T, 1165C>T, 1180G>A, 1196A>G, 1231G>A, 1240C>T, 1255G>A, 1267T>C, 1276A>G, 1300G>A, 1303A>G, 1319C>T, 1324C>T, 1360G>A, 1361T>C, 1366C>T, 1408T>C, 1411T>C, 1417T>C, 1423T>C, 1426T>C, 1429C>T, 1430C>T, 1441C>T, 1450T>C, 1451C>T, 1480C>T, 1481A>G, 1489T>C, 1522C>T, 1552C>T, 1555C>T, 1570A>G, 1573T>C, 1582C>T, 1585A>T, 1591C>T, 1594T>C, 1607G>T, 1612G>A, 1618T>G, 1619C>T, 1624C>T, 1630C>T, 1639T>C, 1642G>A, 1645A>G, 1669C>T, 1672G>A, 1681T>A, 1684G>T, 1699T>C, 1705T>C, 1737A>G, 1738A>G, 1750A>T, 1756A>G, 1765T>G, 1781T>C, 1783G>A, 1784C>T, 1789A>G, 1810G>A, 1825C>T, 1834A>G, 1867G>A, 1879A>G, 1888A>G, 1910T>C, 1912G>A, 1960A>G, 1966A>G, 1984C>T, 1990G>A, 1993G>A, 2009T>C, 2023T>C, 2056T>C, 2077C>A, 2082T>A, 2092A>G, 2107A>G, 2128T>C, 2138C>T, 2143T>C, 2149T>C, 2155T>C, 2174C>T, 2179A>C, 2182G>A, 2194T>A, 2200T>C, 2215G>A, 2218C>T, 2224C>T, 2236A>G, 2241A>G, 2250G>A, 2251C>T, 2257C>T, 2263G>A, 2275T>C, 2287G>A, 2290C>T, 2314C>T, 2320G>A, 2323A>T, 2347C>T, 2350A>G, 2356C>T, 2359G>A, 2362C>T, 2377G>A, 2380T>C, 2392A>G, 2407T>C, 2409G>A, 2443T>C, 2446G>A, 2479G>A, 2485C>T, 2497G>A, 2500T>C, 2503G>A, 2509C>T, 2512T>C, 2542G>A, 2548C>T, 2557C>T, 2620G>A, 2626T>C, 2650G>A, 2665T>C, 2675T>C, 2683A>G, 2692G>A, 2702C>T, 2710A>G, 2719T>C, 2725T>C, 2731C>T, 2734C>T, 2743A>G, 2744T>C, 2752A>G, 2773A>G, 2785C>T, 2788C>T, 2809C>T, 2821G>T, 2827A>G, 2842T>C, 2860C>T, 2866T>A, 2869C>T, 2872A>T, 2893T>A, 2896C>A, 2926G>A, 2929C>T, 2930T>C, 2941A>G, 2944T>C, 2950C>T, 2951C>T, 2980C>T, 2983G>A, 2998A>G, 3007T>C, 3016T>C, 3028T>C, 3031T>C, 3034G>A, 3040T>C, 3058A>G, 3064A>G, 3073G>A, 3079T>C, 3109T>C, 3115G>A, 3145A>G, 3151C>T, 3163T>C, 3169C>T, 3172T>C, 3202T>C, 3241T>C, 3253C>T, 3256G>A, 3262C>T, 3283T>C, 3289T>C, 3298T>C, 3307A>G, 3319C>T, 3358A>T, 3406A>C, 3418C>A, 3421T>C, 3463A>G, 3466G>A, 3481C>T, 3484A>T, 3508G>A, 3559G>A, 3568A>G, 3574T>C, 3577A>G, 3586T>C, 3591C>T, 3625C>T, 3628G>A, 3646C>T, 3661A>G, 3664T>C, 3673G>A, 3676C>T, 3686C>T, 3706T>C, 3716T>C, 3721T>A, 3739A>T, 3751A>G, 3754G>A, 3758G>A, 3766G>A, 3781G>A, 3805C>T, 3808T>C, 3814C>T, 3817G>C, 3824T>C, 3835T>C, 3844T>C, 3857T>C, 3859G>A, 3868C>T, 3871T>C, 3883G>A, 3898T>C, 3907A>T, 3916C>T, 3925A>G, 3928G>A, 3949C>T, 3955G>A, 3961G>A, 3970A>G, 3973T>C, 4034T>C, 4066T>C, 4075A>G, 4084C>T, 4100T>C, 4118C>T, 4129C>T, 4147C>A, 4159G>A, 4162A>T, 4168A>G, 4186A>T, 4195A>G, 4207G>A, 4210T>C, 4228C>T, 4234T>C, 4243T>C, 4273A>G, 4282C>A, 4288C>T, 4291T>C, 4300T>C, 4318C>T, 4324T>C, 4339T>C, 4343C>T, 4348C>T, 4354T>C, 4396T>C, 4408C>T, 4414T>G, 4417T>C, 4420G>A, 4459T>C, 4462C>T, 4465C>T, 4468C>T, 4471T>C, 4474T>C, 4486T>C, 4489T>C, 4504T>C, 4510T>A, 4511C>T, 4514T>C, 4519A>G, 4543T>C, 4555A>G, 4570C>T, 4574C>T, 4576A>G, 4588C>T, 4591T>C, 4612A>G, 4625T>C, 4630T>C, 4648T>C, 4660A>G, 4666G>A, 4672G>C, 4679A>G, 4684A>T, 4687G>A, 4696C>T, 4702G>A, 4705C>T, 4714T>C, 4720T>C, 4723C>T, 4730C>T, 4735C>T, 4747A>G, 4756A>C, 4765T>G, 4772A>C, 4774G>A, 4780T>C, 4781C>T, 4786A>G, 4795A>G, 4802C>T, 4807A>G, 4813A>G, 4816T>C, 4834C>T, 4858T>C, 4861C>T, 4864T>C, 4903A>G, 4924T>C, 4946A>G, 4960C>T, 4975G>A, 4993A>C, 4999T>C, 5012T>C, 5017G>A, 5023C>T, 5044A>C, 5059G>A, 5071A>G, 5074T>C, 5077T>C, 5079T>C, 5086T>C, 5102T>C, 5104G>A, 5119T>C, 5120G>A, 5122T>C, 5123C>T, 5128T>C, 5132T>A, 5140A>G, 5146T>C, 5161A>G, 5167T>C, 5170C>T, 5176C>A, 5209C>T, 5244G>A, 5245A>G, 5251A>G, 5287A>G, 5290G>A, 5302T>C, 5332C>T, 5354G>A, 5356G>A, 5365A>T, 5392G>A, 5413C>T, 5419A>C, 5429C>T, 5437T>C, 5464T>C, 5470A>G, 5491T>G, 5492A>T, 5497T>C, 5518T>C, 5533T>C, 5537A>C, 5560A>G, 5563G>A, 5575A>T, 5578T>C, 5581A>G, 5590A>G, 5591G>T, 5599T>A, 5603A>G, 5608A>G, 5611A>G, 5617A>C, 5623A>G, 5635C>T, 5641T>A, 5644T>C, 5647T>C, 5656G>T, 5668T>C, 5674A>G, 5677G>A, 5678T>C, 5686G>A, 5690T>C, 5702G>A, 5704G>A, 5710T>C, 5716G>A, 5719A>C, 5731C>T, 5734T>C, 5737T>C, 5739G>A, 5746T>C, 5750T>C, 5752G>A, 5758C>T, 5773G>A, 5782A>G, 5784T>C, 5786T>C, 5797A>G, 5806C>T, 5836A>T, 5849T>C, 5850T>A, 5851G>A, 5869G>A, 5875A>G, 5878G>C, 5884T>C, 5887C>A, 5890C>T, 5902G>T, 5908C>T, 5909T>C, 5914T>C, 5923T>C, 5927C>T, 5932C>T, 5955A>G, 5965G>A, 5998A>G, 6004C>T, 6016A>G, 6028C>T, 6031A>T, 6033C>T, 6035G>T, 6055T>A, 6067C>A, 6070T>C, 6094T>C, 6100T>C, 6103G>A, 6106G>A, 6109C>T, 6112A>T, 6131T>C, 6139T>C, 6143A>C, 6145T>C, 6154C>T, 6157C>T, 6163C>T, 6166C>T, 6167C>T, 6187T>C, 6193C>T, 6196A>G, 6202G>A, 6208A>G, 6211A>G, 6217G>A, 6223A>G, 6226G>A, 6230A>G, 6232A>G, 6235G>A, 6244A>T, 6253A>G, 6256C>A, 6265G>A, 6268G>A, 6273G>A, 6274C>T, 6295T>C, 6296G>T, 6298A>G, 6304A>G, 6308T>C, 6315A>T, 6319A>T, 6322G>A, 6323A>G, 6327A>G, 6331T>C, 6344C>T, 6358T>C, 6382C>T, 6385C>T, 6406T>C, 6412C>T, 6414G>A, 6427A>G, 6442C>G, 6445C>T, 6448G>A, 6451T>C, 6457C>A, 6466T>C, 6484A>C, 6490A>G, 6493T>C, 6500A>T, 6517C>T, 6532C>T, 6545C>T, 6568C>T, 6571C>T, 6587G>A, 6643A>G, 6670C>T, 6685A>G, 6715C>T, 6721A>T, 6742A>T, 6766delC, 6786T>C, 6798_6799insA								
**CDS**									
ToTV_sRNA1gp1	1	2159	100%	14853	98.6%	2159 (100%)	2112 (97.8%)	0/0/0/0	1
Protein mutations:	P8S (128C>T 130C>T), F10S (135T>C), A18S (158G>T), T19A (161A>G), D159E (583T>A), V291I (977G>A), T364A (1196A>G), I459V (1481A>G), A501S (1607G>T), E526D (1684G>T), K544R (1737A>G 1738A>G), F659Y (2082T>A), N712S (2241A>G), S715N (2250G>A 2251C>T), R768K (2409G>A), A1162V (3591C>T), V1218I (3758G>A), P1413S (4343C>T), I1525V (4679A>G), T1614A (4946A>G), I1658T (5079T>C), V1672I (5120G>A 5122T>C), S1676T (5132T>A), R1713K (5244G>A 5245A>G), V1750I (5354G>A 5356G>A), T1796S (5492A>T), K1811Q (5537A>C), A1829S (5591G>T), T1833A (5603A>G), V1866I (5702G>A 5704G>A), R1878K (5739G>A), I1893T (5784T>C), L1915Q (5849T>C 5850T>A 5851G>A), N1950S (5955A>G), T1976I (6033C>T), A1977S (6035G>T), I2013L (6143A>C 6145T>C), M2032I (6202G>A), I2042V (6230A>G 6232A>G), S2056N (6273G>A 6274C>T), V2064L (6296G>T 6298A>G), N2070I (6315A>T), I2073V (6323A>G), Q2074R (6327A>G), R2103Q (6414G>A), I2132F (6500A>T), L2147F (6545C>T)								

	Begin	End	Coverage	Score	Concordance	Matches	Identities	I/D/M/F*	Stop Codons
**NT**	**22**	**6813**	**87.0%**	**10966**	**80.8%**	**6791 (99.9%)**	**6147 (90.5%)**	**3/1**	
Codon mutations:	TTT3TTC (115T>C), TTC7TTT (127C>T), CCC8TCT (128C>T 130C>T), TTC10TCC (135T>C), ACT14ACC (148T>C), GAA15GAG (151A>G), GCT18TCT (158G>T), ACC19GCC (161A>G), GGT23GGC (175T>C), TCT24TCC (178T>C), TTT26TTC (184T>C), GAG28GAA (190G>A), GCT31GCA (199T>A), AGT32AGC (202T>C), ATT33ATA (205T>A), CGC38CGT (220C>T), CTG40TTG (224C>T), CAA49CAG (253A>G), TCT51TCC (259T>C), TCT52TCA (262T>A), CAC53CAT (265C>T), TTG78CTA (338T>C 340G>A), AAG81AAA (349G>A), TTA85TTG (361A>G), GAA87GAG (367A>G), CCC88CCT (370C>T), ATC90ATT (376C>T), TAC93TAT (385C>T), TCT94TCC (388T>C), AAA101AAG (409A>G), GAT105GAC (421T>C), AAG109AAA (433G>A), ATT110ATA (436T>A), AAA115AAG (451A>G), GAT116GAC (454T>C), TTT122TTC (472T>C), GAC127GAT (487C>T), AAA128AAG (490A>G), GTG132GTA (502G>A), TTC151TTT (559C>T), GTA157GTG (577A>G), CTA158TTA (578C>T), GAT159GAA (583T>A), TCA177TCC (637A>C), GAC183GAT (655C>T), CAA190CAG (676A>G), GTT200GTC (706T>C), CTA201TTG (707C>T 709A>G), TCA207TCG (727A>G), CTC218CTT (760C>T), ACC225ACT (781C>T), TTC228TTT (790C>T), GTA231GTG (799A>G), GGT233GGC (805T>C), CTA238TTG (818C>T 820A>G), CTA242CTT (832A>T), TCA243TCC (835A>C), ATT245ATC (841T>C), CTG246TTG (842C>T), TAC247TAT (847C>T), TTG259TTA (883G>A), TTT260TTC (886T>C), CTG262CTA (892G>A), AAT265AAC (901T>C), TGT269TGC (913T>C), GCC273GCT (925C>T), CTG278TTG (938C>T), GTA282GTG (952A>G), GAC283GAT (955C>T), GGT286GGG (964T>G), AAG288AAA (970G>A), AAT289AAC (973T>C), GTT291ATT (977G>A), TTG292CTG (980T>C), GCC297GCT (997C>T), TCT300TCC (1006T>C), GAC302GAT (1012C>T), TTA303CTA (1013T>C), TTC304TTT (1018C>T), GAG309GAA (1033G>A), CGC319CGT (1063C>T), TTG320CTG (1064T>C), GCA324GCT (1078A>T), AAC325AAT (1081C>T), ACT329ACC (1093T>C), CAA333CAG (1105A>G), CAT334CAC (1108T>C), CTA335TTA (1109C>T), GCC353GCT (1165C>T), AGG358AGA (1180G>A), ACC364GCC (1196A>G), CAG375CAA (1231G>A), GGC378GGT (1240C>T), AGG383AGA (1255G>A), TTT387TTC (1267T>C), CAA390CAG (1276A>G), GGG398GGA (1300G>A), AAA399AAG (1303A>G), CTG405TTG (1319C>T), TTC406TTT (1324C>T), AAG418AAA (1360G>A), TTG419CTG (1361T>C), AAC420AAT (1366C>T), AAT434AAC (1408T>C), TAT435TAC (1411T>C), CAT437CAC (1417T>C), ACT439ACC (1423T>C), GCT440GCC (1426T>C), ATC441ATT (1429C>T), CTG442TTG (1430C>T), GAC445GAT (1441C>T), GCT448GCC (1450T>C), CTG449TTG (1451C>T), GAC458GAT (1480C>T), ATT459GTT (1481A>G), GAT461GAC (1489T>C), CCC472CCT (1522C>T), GGC482GGT (1552C>T), CGC483CGT (1555C>T), CGA488CGG (1570A>G), TAT489TAC (1573T>C), GCC492GCT (1582C>T), ACA493ACT (1585A>T), AAC495AAT (1591C>T), CTT496CTC (1594T>C), GCT501TCT (1607G>T), CAG502CAA (1612G>A), GGT504GGG (1618T>G), CTA505TTA (1619C>T), ACC506ACT (1624C>T), CCC508CCT (1630C>T), TTT511TTC (1639T>C), GAG512GAA (1642G>A), AGA513AGG (1645A>G), TGC521TGT (1669C>T), AGG522AGA (1672G>A), GGT525GGA (1681T>A), GAG526GAT (1684G>T), GCT531GCC (1699T>C), ACT533ACC (1705T>C), AAA544AGG (1737A>G 1738A>G), GCA548GCT (1750A>T), ACA550ACG (1756A>G), GGT553GGG (1765T>G), TTG559CTA (1781T>C 1783G>A), CTG560TTG (1784C>T), GAA561GAG (1789A>G), AAG568AAA (1810G>A), ATC573ATT (1825C>T), AAA576AAG (1834A>G), CAG587CAA (1867G>A), TCA591TCG (1879A>G), GAA594GAG (1888A>G), TTG602CTA (1910T>C 1912G>A), GTA618GTG (1960A>G), GAA620GAG (1966A>G), TAC626TAT (1984C>T), CAG628CAA (1990G>A), GAG629GAA (1993G>A), TTG635CTG (2009T>C), TAT639TAC (2023T>C), CCT650CCC (2056T>C), GCC657GCA (2077C>A), TTT659TAT (2082T>A), CTA662CTG (2092A>G), AAA667AAG (2107A>G), GTT674GTC (2128T>C), CTG678TTG (2138C>T), TAT679TAC (2143T>C), GGT681GGC (2149T>C), TGT683TGC (2155T>C), CTG690TTG (2174C>T), ATA691ATC (2179A>C), AAG692AAA (2182G>A), GCT696GCA (2194T>A), TAT698TAC (2200T>C), AAG703AAA (2215G>A), AGC704AGT (2218C>T), ATC706ATT (2224C>T), ACA710ACG (2236A>G), AAT712AGT (2241A>G), AGC715AAT (2250G>A 2251C>T), GAC717GAT (2257C>T), CAG719CAA (2263G>A), CCT723CCC (2275T>C), GCG727GCA (2287G>A), CAC728CAT (2290C>T), GAC736GAT (2314C>T), CTG738CTA (2320G>A), ATA739ATT (2323A>T), CAC747CAT (2347C>T), GTA748GTG (2350A>G), TTC750TTT (2356C>T), TTG751TTA (2359G>A), TAC752TAT (2362C>T), AGG757AGA (2377G>A), TAT758TAC (2380T>C), AGA762AGG (2392A>G), CCT767CCC (2407T>C), AGG768AAG (2409G>A), ATT779ATC (2443T>C), GTG780GTA (2446G>A), CCG791CCA (2479G>A), ATC793ATT (2485C>T), CTG797CTA (2497G>A), ATT798ATC (2500T>C), AAG799AAA (2503G>A), GGC801GGT (2509C>T), CTT802CTC (2512T>C), GGG812GGA (2542G>A), GTC814GTT (2548C>T), TTC817TTT (2557C>T), GAG838GAA (2620G>A), AAT840AAC (2626T>C), AAG848AAA (2650G>A), TTT853TTC (2665T>C), TTG857CTG (2675T>C), GCA859GCG (2683A>G), AAG862AAA (2692G>A), CTG866TTG (2702C>T), AAA868AAG (2710A>G), AGT871AGC (2719T>C), GAT873GAC (2725T>C), TTC875TTT (2731C>T), ATC876ATT (2734C>T), GGA879GGG (2743A>G), TTG880CTG (2744T>C), AAA882AAG (2752A>G), CTA889CTG (2773A>G), GTC893GTT (2785C>T), TAC894TAT (2788C>T), TGC901TGT (2809C>T), GTG905GTT (2821G>T), ACA907ACG (2827A>G), TTT912TTC (2842T>C), GGC918GGT (2860C>T), ACT920ACA (2866T>A), TTC921TTT (2869C>T), TCA922TCT (2872A>T), ATT929ATA (2893T>A), TCC930TCA (2896C>A), CGG940CGA (2926G>A), TTC941TTT (2929C>T), TTG942CTG (2930T>C), CAA945CAG (2941A>G), GAT946GAC (2944T>C), GTC948GTT (2950C>T), CTG949TTG (2951C>T), CCC958CCT (2980C>T), AAG959AAA (2983G>A), AAA964AAG (2998A>G), CCT967CCC (3007T>C), GAT970GAC (3016T>C), TTT974TTC (3028T>C), TCT975TCC (3031T>C), AAG976AAA (3034G>A), ACT978ACC (3040T>C), AGA984AGG (3058A>G), GTA986GTG (3064A>G), AAG989AAA (3073G>A), GCT991GCC (3079T>C), TTT1001TTC (3109T>C), GTG1003GTA (3115G>A), GTA1013GTG (3145A>G), TAC1015TAT (3151C>T), TCT1019TCC (3163T>C), TCC1021TCT (3169C>T), TTT1022TTC (3172T>C), TAT1032TAC (3202T>C), CTT1045CTC (3241T>C), AAC1049AAT (3253C>T), GTG1050GTA (3256G>A), GAC1052GAT (3262C>T), GGT1059GGC (3283T>C), CAT1061CAC (3289T>C), TCT1064TCC (3298T>C), GAA1067GAG (3307A>G), CAC1071CAT (3319C>T), CGA1084CGT (3358A>T), GTA1100GTC (3406A>C), CTC1104CTA (3418C>A), CTT1105CTC (3421T>C), AAA1119AAG (3463A>G), CAG1120CAA (3466G>A), GGC1125GGT (3481C>T), ACA1126ACT (3484A>T), ACG1134ACA (3508G>A), GGG1151GGA (3559G>A), GAA1154GAG (3568A>G), GCT1156GCC (3574T>C), CCA1157CCG (3577A>G), ATT1160ATC (3586T>C), GCT1162GTT (3591C>T), TAC1173TAT (3625C>T), GTG1174GTA (3628G>A), TAC1180TAT (3646C>T), CAA1185CAG (3661A>G), AAT1186AAC (3664T>C), GAG1189GAA (3673G>A), GAC1190GAT (3676C>T), CTA1194TTA (3686C>T), CGT1200CGC (3706T>C), TTG1204CTG (3716T>C), CCT1205CCA (3721T>A), CGA1211CGT (3739A>T), GGA1215GGG (3751A>G), AAG1216AAA (3754G>A), GTC1218ATC (3758G>A), CAG1220CAA (3766G>A), GTG1225GTA (3781G>A), GAC1233GAT (3805C>T), TAT1234TAC (3808T>C), GAC1236GAT (3814C>T), GGG1237GGC (3817G>C), TTG1240CTG (3824T>C), TCT1243TCC (3835T>C), TAT1246TAC (3844T>C), TTG1251CTA (3857T>C 3859G>A), GGC1254GGT (3868C>T), ACT1255ACC (3871T>C), GAG1259GAA (3883G>A), AGT1264AGC (3898T>C), GGA1267GGT (3907A>T), GTC1270GTT (3916C>T), AAA1273AAG (3925A>G), AGG1274AGA (3928G>A), GAC1281GAT (3949C>T), GAG1283GAA (3955G>A), TTG1285TTA (3961G>A), GAA1288GAG (3970A>G), ATT1289ATC (3973T>C), TTG1310CTG (4034T>C), CGT1320CGC (4066T>C), GAA1323GAG (4075A>G), CCC1326CCT (4084C>T), TTG1332CTG (4100T>C), CTG1338TTG (4118C>T), TCC1341TCT (4129C>T), TCC1347TCA (4147C>A), CTG1351CTA (4159G>A), CCA1352CCT (4162A>T), AAA1354AAG (4168A>G), GGA1360GGT (4186A>T), GAA1363GAG (4195A>G), TTG1367TTA (4207G>A), GCT1368GCC (4210T>C), GTC1374GTT (4228C>T), GAT1376GAC (4234T>C), TTT1379TTC (4243T>C), AGA1389AGG (4273A>G), ATC1392ATA (4282C>A), GCC1394GCT (4288C>T), TTT1395TTC (4291T>C), TTT1398TTC (4300T>C), GAC1404GAT (4318C>T), TAT1406TAC (4324T>C), GGT1411GGC (4339T>C), CCA1413TCA (4343C>T), GAC1414GAT (4348C>T), AAT1416AAC (4354T>C), ACT1430ACC (4396T>C), AAC1434AAT (4408C>T), GTT1436GTG (4414T>G), TTT1437TTC (4417T>C), AGG1438AGA (4420G>A), GTT1451GTC (4459T>C), ATC1452ATT (4462C>T), TTC1453TTT (4465C>T), AAC1454AAT (4468C>T), TCT1455TCC (4471T>C), CTT1456CTC (4474T>C), TTT1460TTC (4486T>C), TAT1461TAC (4489T>C), TTT1466TTC (4504T>C), TCT1468TCA (4510T>A), CTA1469TTA (4511C>T), TTG1470CTG (4514T>C), AGA1471AGG (4519A>G), GCT1479GCC (4543T>C), AAA1483AAG (4555A>G), AAC1488AAT (4570C>T), CTA1490TTG (4574C>T 4576A>G), GTC1494GTT (4588C>T), TAT1495TAC (4591T>C), GCA1502GCG (4612A>G), TTG1507CTG (4625T>C), CAT1508CAC (4630T>C), GCT1514GCC (4648T>C), GAA1518GAG (4660A>G), GAG1520GAA (4666G>A), GTG1522GTC (4672G>C), ATT1525GTT (4679A>G), ATA1526ATT (4684A>T), AAG1527AAA (4687G>A), ATC1530ATT (4696C>T), AAG1532AAA (4702G>A), AAC1533AAT (4705C>T), GTT1536GTC (4714T>C), AGT1538AGC (4720T>C), TCC1539TCT (4723C>T), CTA1542TTA (4730C>T), GAC1543GAT (4735C>T), CTA1547CTG (4747A>G), CTA1550CTC (4756A>C), GGT1553GGG (4765T>G), AGG1556CGA (4772A>C 4774G>A), CGT1558CGC (4780T>C), CTA1559TTA (4781C>T), GGA1560GGG (4786A>G), CAA1563CAG (4795A>G), CTG1566TTG (4802C>T), AAA1567AAG (4807A>G), GTA1569GTG (4813A>G), TCT1570TCC (4816T>C), TAC1576TAT (4834C>T), TGT1584TGC (4858T>C), CCC1585CCT (4861C>T), GAT1586GAC (4864T>C), AGA1599AGG (4903A>G), CCT1606CCC (4924T>C), ACA1614GCA (4946A>G), AAC1618AAT (4960C>T), CGG1623CGA (4975G>A), ACA1629ACC (4993A>C), CCT1631CCC (4999T>C), TTG1636CTG (5012T>C), GAG1637GAA (5017G>A), GAC1639GAT (5023C>T), GGA1646GGC (5044A>C), GGG1651GGA (5059G>A), GAA1655GAG (5071A>G), CTT1656CTC (5074T>C), CAT1657CAC (5077T>C), ATT1658ACT (5079T>C), GCT1660GCC (5086T>C), TTG1666CTA (5102T>C 5104G>A), ACT1671ACC (5119T>C), GTT1672ATC (5120G>A 5122T>C), CTG1673TTG (5123C>T), AAT1674AAC (5128T>C), TCT1676ACT (5132T>A), GGA1678GGG (5140A>G), CAT1680CAC (5146T>C), CGA1685CGG (5161A>G), AGT1687AGC (5167T>C), TTC1688TTT (5170C>T), TCC1690TCA (5176C>A), ACC1701ACT (5209C>T), AGA1713AAG (5244G>A 5245A>G), ACA1715ACG (5251A>G), GAA1727GAG (5287A>G), GGG1728GGA (5290G>A), CCT1732CCC (5302T>C), ATC1742ATT (5332C>T), GTG1750ATA (5354G>A 5356G>A), CCA1753CCT (5365A>T), GGG1762GGA (5392G>A), TGC1769TGT (5413C>T), GCA1771GCC (5419A>C), CTG1775TTG (5429C>T), ACT1777ACC (5437T>C), GTT1786GTC (5464T>C), AGA1788AGG (5470A>G), GTT1795GTG (5491T>G), ACA1796TCA (5492A>T), GAT1797GAC (5497T>C), GGT1804GGC (5518T>C), CTT1809CTC (5533T>C), AAA1811CAA (5537A>C), GAA1818GAG (5560A>G), GAG1819GAA (5563G>A), ACA1823ACT (5575A>T), TGT1824TGC (5578T>C), AAA1825AAG (5581A>G), CAA1828CAG (5590A>G), GCC1829TCC (5591G>T), GGT1831GGA (5599T>A), ACA1833GCA (5603A>G), CCA1834CCG (5608A>G), GCA1835GCG (5611A>G), ATA1837ATC (5617A>C), CAA1839CAG (5623A>G), TCC1843TCT (5635C>T), GGT1845GGA (5641T>A), AAT1846AAC (5644T>C), TTT1847TTC (5647T>C), GTG1850GTT (5656G>T), ATT1854ATC (5668T>C), CTA1856CTG (5674A>G), CAG1857CAA (5677G>A), TTG1858CTG (5678T>C), CAG1860CAA (5686G>A), TTG1862CTG (5690T>C), GTG1866ATA (5702G>A 5704G>A), CCT1868CCC (5710T>C), GGG1870GGA (5716G>A), GGA1871GGC (5719A>C), CAC1875CAT (5731C>T), ACT1876ACC (5734T>C), ACT1877ACC (5737T>C), AGA1878AAA (5739G>A), CTT1880CTC (5746T>C), TTG1882CTA (5750T>C 5752G>A), CTC1884CTT (5758C>T), AAG1889AAA (5773G>A), CAA1892CAG (5782A>G), ATT1893ACT (5784T>C), TTA1894CTA (5786T>C), AGA1897AGG (5797A>G), TTC1900TTT (5806C>T), TCA1910TCT (5836A>T), TTG1915CAA (5849T>C 5850T>A 5851G>A), AGG1921AGA (5869G>A), CAA1923CAG (5875A>G), GGG1924GGC (5878G>C), TTT1926TTC (5884T>C), CCC1927CCA (5887C>A), CGC1928CGT (5890C>T), GTG1932GTT (5902G>T), TAC1934TAT (5908C>T), TTG1935CTG (5909T>C), CTT1936CTC (5914T>C), TTT1939TTC (5923T>C), CTG1941TTG (5927C>T), AGC1942AGT (5932C>T), AAC1950AGC (5955A>G), ACG1953ACA (5965G>A), AAA1964AAG (5998A>G), ACC1966ACT (6004C>T), AAA1970AAG (6016A>G), TCC1974TCT (6028C>T), GCA1975GCT (6031A>T), ACA1976ATA (6033C>T), GCT1977TCT (6035G>T), GGT1983GGA (6055T>A), TCC1987TCA (6067C>A), GCT1988GCC (6070T>C), CTT1996CTC (6094T>C), CTT1998CTC (6100T>C), CAG1999CAA (6103G>A), AAG2000AAA (6106G>A), CCC2001CCT (6109C>T), ATA2002ATT (6112A>T), TTG2009CTG (6131T>C), TTT2011TTC (6139T>C), ATT2013CTC (6143A>C 6145T>C), GTC2016GTT (6154C>T), ATC2017ATT (6157C>T), GGC2019GGT (6163C>T), CAC2020CAT (6166C>T), CTG2021TTG (6167C>T), ACT2027ACC (6187T>C), TTC2029TTT (6193C>T), CAA2030CAG (6196A>G), ATG2032ATA (6202G>A), CAA2034CAG (6208A>G), CAA2035CAG (6211A>G), CTG2037CTA (6217G>A), GAA2039GAG (6223A>G), GGG2040GGA (6226G>A), ATA2042GTG (6230A>G 6232A>G), GAG2043GAA (6235G>A), ATA2046ATT (6244A>T), TTA2049TTG (6253A>G), ACC2050ACA (6256C>A), CTG2053CTA (6265G>A), TCG2054TCA (6268G>A), AGC2056AAT (6273G>A 6274C>T), GAT2063GAC (6295T>C), GTA2064TTG (6296G>T 6298A>G), TTA2066TTG (6304A>G), TTG2068CTG (6308T>C), AAC2070ATC (6315A>T), ATA2071ATT (6319A>T), GAG2072GAA (6322G>A), ATC2073GTC (6323A>G), CAG2074CGG (6327A>G), GAT2075GAC (6331T>C), CTA2080TTA (6344C>T), AAT2084AAC (6358T>C), GCC2092GCT (6382C>T), ATC2093ATT (6385C>T), TGT2100TGC (6406T>C), AAC2102AAT (6412C>T), CGA2103CAA (6414G>A), AAA2107AAG (6427A>G), CGC2112CGG (6442C>G), TAC2113TAT (6445C>T), ACG2114ACA (6448G>A), ACT2115ACC (6451T>C), TCC2117TCA (6457C>A), GTT2120GTC (6466T>C), GCA2126GCC (6484A>C), AGA2128AGG (6490A>G), CAT2129CAC (6493T>C), ATT2132TTT (6500A>T), TCC2137TCT (6517C>T), CAC2142CAT (6532C>T), CTT2147TTT (6545C>T), GGC2154GGT (6568C>T), TAC2155TAT (6571C>T)								

Proteins

polyprotein (YP_001039627.1)	1	2159	100%	14853	98.6%	2159 (100%)	2112 (97.8%)	0/0/0/0	1
Protein mutations:	P8S (128C>T 130C>T), F10S (135T>C), A18S (158G>T), T19A (161A>G), D159E (583T>A), V291I (977G>A), T364A (1196A>G), I459V (1481A>G), A501S (1607G>T), E526D (1684G>T), K544R (1737A>G 1738A>G), F659Y (2082T>A), N712S (2241A>G), S715N (2250G>A 2251C>T), R768K (2409G>A), A1162V (3591C>T), V1218I (3758G>A), P1413S (4343C>T), I1525V (4679A>G), T1614A (4946A>G), I1658T (5079T>C), V1672I (5120G>A 5122T>C), S1676T (5132T>A), R1713K (5244G>A 5245A>G), V1750I (5354G>A 5356G>A), T1796S (5492A>T), K1811Q (5537A>C), A1829S (5591G>T), T1833A (5603A>G), V1866I (5702G>A 5704G>A), R1878K (5739G>A), I1893T (5784T>C), L1915Q (5849T>C 5850T>A 5851G>A), N1950S (5955A>G), T1976I (6033C>T), A1977S (6035G>T), I2013L (6143A>C 6145T>C), M2032I (6202G>A), I2042V (6230A>G 6232A>G), S2056N (6273G>A 6274C>T), V2064L (6296G>T 6298A>G), N2070I (6315A>T), I2073V (6323A>G), Q2074R (6327A>G), R2103Q (6414G>A), I2132F (6500A>T), L2147F (6545C>T)								

	Begin	End	Coverage	Score	Concordance	Matches	Identities	I/D/M/F*	Stop Codons
**NT**	**22**	**6813**	**87.0%**	**10966**	**80.8%**	**6791 (99.9%)**	**6147 (90.5%)**	**3/1**	

Codon mutations: TTT3TTC (115T>C), TTC7TTT (127C>T), CCC8TCT (128C>T 130C>T), TTC10TCC (135T>C), ACT14ACC (148T>C), GAA15GAG (151A>G), GCT18TCT (158G>T), ACC19GCC (161A>G), GGT23GGC (175T>C), TCT24TCC (178T>C), TTT26TTC (184T>C), GAG28GAA (190G>A), GCT31GCA (199T>A), AGT32AGC (202T>C), ATT33ATA (205T>A), CGC38CGT (220C>T), CTG40TTG (224C>T), CAA49CAG (253A>G), TCT51TCC (259T>C), TCT52TCA (262T>A), CAC53CAT (265C>T), TTG78CTA (338T>C 340G>A), AAG81AAA (349G>A), TTA85TTG (361A>G), GAA87GAG (367A>G), CCC88CCT (370C>T), ATC90ATT (376C>T), TAC93TAT (385C>T), TCT94TCC (388T>C), AAA101AAG (409A>G), GAT105GAC (421T>C), AAG109AAA (433G>A), ATT110ATA (436T>A), AAA115AAG (451A>G), GAT116GAC (454T>C), TTT122TTC (472T>C), GAC127GAT (487C>T), AAA128AAG (490A>G), GTG132GTA (502G>A), TTC151TTT (559C>T), GTA157GTG (577A>G), CTA158TTA (578C>T), GAT159GAA (583T>A), TCA177TCC (637A>C), GAC183GAT (655C>T), CAA190CAG (676A>G), GTT200GTC (706T>C), CTA201TTG (707C>T 709A>G), TCA207TCG (727A>G), CTC218CTT (760C>T), ACC225ACT (781C>T), TTC228TTT (790C>T), GTA231GTG (799A>G), GGT233GGC (805T>C), CTA238TTG (818C>T 820A>G), CTA242CTT (832A>T), TCA243TCC (835A>C), ATT245ATC (841T>C), CTG246TTG (842C>T), TAC247TAT (847C>T), TTG259TTA (883G>A), TTT260TTC (886T>C), CTG262CTA (892G>A), AAT265AAC (901T>C), TGT269TGC (913T>C), GCC273GCT (925C>T), CTG278TTG (938C>T), GTA282GTG (952A>G), GAC283GAT (955C>T), GGT286GGG (964T>G), AAG288AAA (970G>A), AAT289AAC (973T>C), GTT291ATT (977G>A), TTG292CTG (980T>C), GCC297GCT (997C>T), TCT300TCC (1006T>C), GAC302GAT (1012C>T), TTA303CTA (1013T>C), TTC304TTT (1018C>T), GAG309GAA (1033G>A), CGC319CGT (1063C>T), TTG320CTG (1064T>C), GCA324GCT (1078A>T), AAC325AAT (1081C>T), ACT329ACC (1093T>C), CAA333CAG (1105A>G), CAT334CAC (1108T>C), CTA335TTA (1109C>T), GCC353GCT (1165C>T), AGG358AGA (1180G>A), ACC364GCC (1196A>G), CAG375CAA (1231G>A), GGC378GGT (1240C>T), AGG383AGA (1255G>A), TTT387TTC (1267T>C), CAA390CAG (1276A>G), GGG398GGA (1300G>A), AAA399AAG (1303A>G), CTG405TTG (1319C>T), TTC406TTT (1324C>T), AAG418AAA (1360G>A), TTG419CTG (1361T>C), AAC420AAT (1366C>T), AAT434AAC (1408T>C), TAT435TAC (1411T>C), CAT437CAC (1417T>C), ACT439ACC (1423T>C), GCT440GCC (1426T>C), ATC441ATT (1429C>T), CTG442TTG (1430C>T), GAC445GAT (1441C>T), GCT448GCC (1450T>C), CTG449TTG (1451C>T), GAC458GAT (1480C>T), ATT459GTT (1481A>G), GAT461GAC (1489T>C), CCC472CCT (1522C>T), GGC482GGT (1552C>T), CGC483CGT (1555C>T), CGA488CGG (1570A>G), TAT489TAC (1573T>C), GCC492GCT (1582C>T), ACA493ACT (1585A>T), AAC495AAT (1591C>T), CTT496CTC (1594T>C), GCT501TCT (1607G>T), CAG502CAA (1612G>A), GGT504GGG (1618T>G), CTA505TTA (1619C>T), ACC506ACT (1624C>T), CCC508CCT (1630C>T), TTT511TTC (1639T>C), GAG512GAA (1642G>A), AGA513AGG (1645A>G), TGC521TGT (1669C>T), AGG522AGA (1672G>A), GGT525GGA (1681T>A), GAG526GAT (1684G>T), GCT531GCC (1699T>C), ACT533ACC (1705T>C), AAA544AGG (1737A>G 1738A>G), GCA548GCT (1750A>T), ACA550ACG (1756A>G), GGT553GGG (1765T>G), TTG559CTA (1781T>C 1783G>A), CTG560TTG (1784C>T), GAA561GAG (1789A>G), AAG568AAA (1810G>A), ATC573ATT (1825C>T), AAA576AAG (1834A>G), CAG587CAA (1867G>A), TCA591TCG (1879A>G), GAA594GAG (1888A>G), TTG602CTA (1910T>C 1912G>A), GTA618GTG (1960A>G), GAA620GAG (1966A>G), TAC626TAT (1984C>T), CAG628CAA (1990G>A), GAG629GAA (1993G>A), TTG635CTG (2009T>C), TAT639TAC (2023T>C), CCT650CCC (2056T>C), GCC657GCA (2077C>A), TTT659TAT (2082T>A), CTA662CTG (2092A>G), AAA667AAG (2107A>G), GTT674GTC (2128T>C), CTG678TTG (2138C>T), TAT679TAC (2143T>C), GGT681GGC (2149T>C), TGT683TGC (2155T>C), CTG690TTG (2174C>T), ATA691ATC (2179A>C), AAG692AAA (2182G>A), GCT696GCA (2194T>A), TAT698TAC (2200T>C), AAG703AAA (2215G>A), AGC704AGT (2218C>T), ATC706ATT (2224C>T), ACA710ACG (2236A>G), AAT712AGT (2241A>G), AGC715AAT (2250G>A 2251C>T), GAC717GAT (2257C>T), CAG719CAA (2263G>A), CCT723CCC (2275T>C), GCG727GCA (2287G>A), CAC728CAT (2290C>T), GAC736GAT (2314C>T), CTG738CTA (2320G>A), ATA739ATT (2323A>T), CAC747CAT (2347C>T), GTA748GTG (2350A>G), TTC750TTT (2356C>T), TTG751TTA (2359G>A), TAC752TAT (2362C>T), AGG757AGA (2377G>A), TAT758TAC (2380T>C), AGA762AGG (2392A>G), CCT767CCC (2407T>C), AGG768AAG (2409G>A), ATT779ATC (2443T>C), GTG780GTA (2446G>A), CCG791CCA (2479G>A), ATC793ATT (2485C>T), CTG797CTA (2497G>A), ATT798ATC (2500T>C), AAG799AAA (2503G>A), GGC801GGT (2509C>T), CTT802CTC (2512T>C), GGG812GGA (2542G>A), GTC814GTT (2548C>T), TTC817TTT (2557C>T), GAG838GAA (2620G>A), AAT840AAC (2626T>C), AAG848AAA (2650G>A), TTT853TTC (2665T>C), TTG857CTG (2675T>C), GCA859GCG (2683A>G), AAG862AAA (2692G>A), CTG866TTG (2702C>T), AAA868AAG (2710A>G), AGT871AGC (2719T>C), GAT873GAC (2725T>C), TTC875TTT (2731C>T), ATC876ATT (2734C>T), GGA879GGG (2743A>G), TTG880CTG (2744T>C), AAA882AAG (2752A>G), CTA889CTG (2773A>G), GTC893GTT (2785C>T), TAC894TAT (2788C>T), TGC901TGT (2809C>T), GTG905GTT (2821G>T), ACA907ACG (2827A>G), TTT912TTC (2842T>C), GGC918GGT (2860C>T), ACT920ACA (2866T>A), TTC921TTT (2869C>T), TCA922TCT (2872A>T), ATT929ATA (2893T>A), TCC930TCA (2896C>A), CGG940CGA (2926G>A), TTC941TTT (2929C>T), TTG942CTG (2930T>C), CAA945CAG (2941A>G), GAT946GAC (2944T>C), GTC948GTT (2950C>T), CTG949TTG (2951C>T), CCC958CCT (2980C>T), AAG959AAA (2983G>A), AAA964AAG (2998A>G), CCT967CCC (3007T>C), GAT970GAC (3016T>C), TTT974TTC (3028T>C), TCT975TCC (3031T>C), AAG976AAA (3034G>A), ACT978ACC (3040T>C), AGA984AGG (3058A>G), GTA986GTG (3064A>G), AAG989AAA (3073G>A), GCT991GCC (3079T>C), TTT1001TTC (3109T>C), GTG1003GTA (3115G>A), GTA1013GTG (3145A>G), TAC1015TAT (3151C>T), TCT1019TCC (3163T>C), TCC1021TCT (3169C>T), TTT1022TTC (3172T>C), TAT1032TAC (3202T>C), CTT1045CTC (3241T>C), AAC1049AAT (3253C>T), GTG1050GTA (3256G>A), GAC1052GAT (3262C>T), GGT1059GGC (3283T>C), CAT1061CAC (3289T>C), TCT1064TCC (3298T>C), GAA1067GAG (3307A>G), CAC1071CAT (3319C>T), CGA1084CGT (3358A>T), GTA1100GTC (3406A>C), CTC1104CTA (3418C>A), CTT1105CTC (3421T>C), AAA1119AAG (3463A>G), CAG1120CAA (3466G>A), GGC1125GGT (3481C>T), ACA1126ACT (3484A>T), ACG1134ACA (3508G>A), GGG1151GGA (3559G>A), GAA1154GAG (3568A>G), GCT1156GCC (3574T>C), CCA1157CCG (3577A>G), ATT1160ATC (3586T>C), GCT1162GTT (3591C>T), TAC1173TAT (3625C>T), GTG1174GTA (3628G>A), TAC1180TAT (3646C>T), CAA1185CAG (3661A>G), AAT1186AAC (3664T>C), GAG1189GAA (3673G>A), GAC1190GAT (3676C>T), CTA1194TTA (3686C>T), CGT1200CGC (3706T>C), TTG1204CTG (3716T>C), CCT1205CCA (3721T>A), CGA1211CGT (3739A>T), GGA1215GGG (3751A>G), AAG1216AAA (3754G>A), GTC1218ATC (3758G>A), CAG1220CAA (3766G>A), GTG1225GTA (3781G>A), GAC1233GAT (3805C>T), TAT1234TAC (3808T>C), GAC1236GAT (3814C>T), GGG1237GGC (3817G>C), TTG1240CTG (3824T>C), TCT1243TCC (3835T>C), TAT1246TAC (3844T>C), TTG1251CTA (3857T>C 3859G>A), GGC1254GGT (3868C>T), ACT1255ACC (3871T>C), GAG1259GAA (3883G>A), AGT1264AGC (3898T>C), GGA1267GGT (3907A>T), GTC1270GTT (3916C>T), AAA1273AAG (3925A>G), AGG1274AGA (3928G>A), GAC1281GAT (3949C>T), GAG1283GAA (3955G>A), TTG1285TTA (3961G>A), GAA1288GAG (3970A>G), ATT1289ATC (3973T>C), TTG1310CTG (4034T>C), CGT1320CGC (4066T>C), GAA1323GAG (4075A>G), CCC1326CCT (4084C>T), TTG1332CTG (4100T>C), CTG1338TTG (4118C>T), TCC1341TCT (4129C>T), TCC1347TCA (4147C>A), CTG1351CTA (4159G>A), CCA1352CCT (4162A>T), AAA1354AAG (4168A>G), GGA1360GGT (4186A>T), GAA1363GAG (4195A>G), TTG1367TTA (4207G>A), GCT1368GCC (4210T>C), GTC1374GTT (4228C>T), GAT1376GAC (4234T>C), TTT1379TTC (4243T>C), AGA1389AGG (4273A>G), ATC1392ATA (4282C>A), GCC1394GCT (4288C>T), TTT1395TTC (4291T>C), TTT1398TTC (4300T>C), GAC1404GAT (4318C>T), TAT1406TAC (4324T>C), GGT1411GGC (4339T>C), CCA1413TCA (4343C>T), GAC1414GAT (4348C>T), AAT1416AAC (4354T>C), ACT1430ACC (4396T>C), AAC1434AAT (4408C>T), GTT1436GTG (4414T>G), TTT1437TTC (4417T>C), AGG1438AGA (4420G>A), GTT1451GTC (4459T>C), ATC1452ATT (4462C>T), TTC1453TTT (4465C>T), AAC1454AAT (4468C>T), TCT1455TCC (4471T>C), CTT1456CTC (4474T>C), TTT1460TTC (4486T>C), TAT1461TAC (4489T>C), TTT1466TTC (4504T>C), TCT1468TCA (4510T>A), CTA1469TTA (4511C>T), TTG1470CTG (4514T>C), AGA1471AGG (4519A>G), GCT1479GCC (4543T>C), AAA1483AAG (4555A>G), AAC1488AAT (4570C>T), CTA1490TTG (4574C>T 4576A>G), GTC1494GTT (4588C>T), TAT1495TAC (4591T>C), GCA1502GCG (4612A>G), TTG1507CTG (4625T>C), CAT1508CAC (4630T>C), GCT1514GCC (4648T>C), GAA1518GAG (4660A>G), GAG1520GAA (4666G>A), GTG1522GTC (4672G>C), ATT1525GTT (4679A>G), ATA1526ATT (4684A>T), AAG1527AAA (4687G>A), ATC1530ATT (4696C>T), AAG1532AAA (4702G>A), AAC1533AAT (4705C>T), GTT1536GTC (4714T>C), AGT1538AGC (4720T>C), TCC1539TCT (4723C>T), CTA1542TTA (4730C>T), GAC1543GAT (4735C>T), CTA1547CTG (4747A>G), CTA1550CTC (4756A>C), GGT1553GGG (4765T>G), AGG1556CGA (4772A>C 4774G>A), CGT1558CGC (4780T>C), CTA1559TTA (4781C>T), GGA1560GGG (4786A>G), CAA1563CAG (4795A>G), CTG1566TTG (4802C>T), AAA1567AAG (4807A>G), GTA1569GTG (4813A>G), TCT1570TCC (4816T>C), TAC1576TAT (4834C>T), TGT1584TGC (4858T>C), CCC1585CCT (4861C>T), GAT1586GAC (4864T>C), AGA1599AGG (4903A>G), CCT1606CCC (4924T>C), ACA1614GCA (4946A>G), AAC1618AAT (4960C>T), CGG1623CGA (4975G>A), ACA1629ACC (4993A>C), CCT1631CCC (4999T>C), TTG1636CTG (5012T>C), GAG1637GAA (5017G>A), GAC1639GAT (5023C>T), GGA1646GGC (5044A>C), GGG1651GGA (5059G>A), GAA1655GAG (5071A>G), CTT1656CTC (5074T>C), CAT1657CAC (5077T>C), ATT1658ACT (5079T>C), GCT1660GCC (5086T>C), TTG1666CTA (5102T>C 5104G>A), ACT1671ACC (5119T>C), GTT1672ATC (5120G>A 5122T>C), CTG1673TTG (5123C>T), AAT1674AAC (5128T>C), TCT1676ACT (5132T>A), GGA1678GGG (5140A>G), CAT1680CAC (5146T>C), CGA1685CGG (5161A>G), AGT1687AGC (5167T>C), TTC1688TTT (5170C>T), TCC1690TCA (5176C>A), ACC1701ACT (5209C>T), AGA1713AAG (5244G>A 5245A>G), ACA1715ACG (5251A>G), GAA1727GAG (5287A>G), GGG1728GGA (5290G>A), CCT1732CCC (5302T>C), ATC1742ATT (5332C>T), GTG1750ATA (5354G>A 5356G>A), CCA1753CCT (5365A>T), GGG1762GGA (5392G>A), TGC1769TGT (5413C>T), GCA1771GCC (5419A>C), CTG1775TTG (5429C>T), ACT1777ACC (5437T>C), GTT1786GTC (5464T>C), AGA1788AGG (5470A>G), GTT1795GTG (5491T>G), ACA1796TCA (5492A>T), GAT1797GAC (5497T>C), GGT1804GGC (5518T>C), CTT1809CTC (5533T>C), AAA1811CAA (5537A>C), GAA1818GAG (5560A>G), GAG1819GAA (5563G>A), ACA1823ACT (5575A>T), TGT1824TGC (5578T>C), AAA1825AAG (5581A>G), CAA1828CAG (5590A>G), GCC1829TCC (5591G>T), GGT1831GGA (5599T>A), ACA1833GCA (5603A>G), CCA1834CCG (5608A>G), GCA1835GCG (5611A>G), ATA1837ATC (5617A>C), CAA1839CAG (5623A>G), TCC1843TCT (5635C>T), GGT1845GGA (5641T>A), AAT1846AAC (5644T>C), TTT1847TTC (5647T>C), GTG1850GTT (5656G>T), ATT1854ATC (5668T>C), CTA1856CTG (5674A>G), CAG1857CAA (5677G>A), TTG1858CTG (5678T>C), CAG1860CAA (5686G>A), TTG1862CTG (5690T>C), GTG1866ATA (5702G>A 5704G>A), CCT1868CCC (5710T>C), GGG1870GGA (5716G>A), GGA1871GGC (5719A>C), CAC1875CAT (5731C>T), ACT1876ACC (5734T>C), ACT1877ACC (5737T>C), AGA1878AAA (5739G>A), CTT1880CTC (5746T>C), TTG1882CTA (5750T>C 5752G>A), CTC1884CTT (5758C>T), AAG1889AAA (5773G>A), CAA1892CAG (5782A>G), ATT1893ACT (5784T>C), TTA1894CTA (5786T>C), AGA1897AGG (5797A>G), TTC1900TTT (5806C>T), TCA1910TCT (5836A>T), TTG1915CAA (5849T>C 5850T>A 5851G>A), AGG1921AGA (5869G>A), CAA1923CAG (5875A>G), GGG1924GGC (5878G>C), TTT1926TTC (5884T>C), CCC1927CCA (5887C>A), CGC1928CGT (5890C>T), GTG1932GTT (5902G>T), TAC1934TAT (5908C>T), TTG1935CTG (5909T>C), CTT1936CTC (5914T>C), TTT1939TTC (5923T>C), CTG1941TTG (5927C>T), AGC1942AGT (5932C>T), AAC1950AGC (5955A>G), ACG1953ACA (5965G>A), AAA1964AAG (5998A>G), ACC1966ACT (6004C>T), AAA1970AAG (6016A>G), TCC1974TCT (6028C>T), GCA1975GCT (6031A>T), ACA1976ATA (6033C>T), GCT1977TCT (6035G>T), GGT1983GGA (6055T>A), TCC1987TCA (6067C>A), GCT1988GCC (6070T>C), CTT1996CTC (6094T>C), CTT1998CTC (6100T>C), CAG1999CAA (6103G>A), AAG2000AAA (6106G>A), CCC2001CCT (6109C>T), ATA2002ATT (6112A>T), TTG2009CTG (6131T>C), TTT2011TTC (6139T>C), ATT2013CTC (6143A>C 6145T>C), GTC2016GTT (6154C>T), ATC2017ATT (6157C>T), GGC2019GGT (6163C>T), CAC2020CAT (6166C>T), CTG2021TTG (6167C>T), ACT2027ACC (6187T>C), TTC2029TTT (6193C>T), CAA2030CAG (6196A>G), ATG2032ATA (6202G>A), CAA2034CAG (6208A>G), CAA2035CAG (6211A>G), CTG2037CTA (6217G>A), GAA2039GAG (6223A>G), GGG2040GGA (6226G>A), ATA2042GTG (6230A>G 6232A>G), GAG2043GAA (6235G>A), ATA2046ATT (6244A>T), TTA2049TTG (6253A>G), ACC2050ACA (6256C>A), CTG2053CTA (6265G>A), TCG2054TCA (6268G>A), AGC2056AAT (6273G>A 6274C>T), GAT2063GAC (6295T>C), GTA2064TTG (6296G>T 6298A>G), TTA2066TTG (6304A>G), TTG2068CTG (6308T>C), AAC2070ATC (6315A>T), ATA2071ATT (6319A>T), GAG2072GAA (6322G>A), ATC2073GTC (6323A>G), CAG2074CGG (6327A>G), GAT2075GAC (6331T>C), CTA2080TTA (6344C>T), AAT2084AAC (6358T>C), GCC2092GCT (6382C>T), ATC2093ATT (6385C>T), TGT2100TGC (6406T>C), AAC2102AAT (6412C>T), CGA2103CAA (6414G>A), AAA2107AAG (6427A>G), CGC2112CGG (6442C>G), TAC2113TAT (6445C>T), ACG2114ACA (6448G>A), ACT2115ACC (6451T>C), TCC2117TCA (6457C>A), GTT2120GTC (6466T>C), GCA2126GCC (6484A>C), AGA2128AGG (6490A>G), CAT2129CAC (6493T>C), ATT2132TTT (6500A>T), TCC2137TCT (6517C>T), CAC2142CAT (6532C>T), CTT2147TTT (6545C>T), GGC2154GGT (6568C>T), TAC2155TAT (6571C>T)

*: Inserts / Deletes / Misaligned / Frameshifts

Analysis details

This analysis was performed with panviral2.64

NGS Details (UN8): Torradovirus lycopersici (segment RNA 1)

Assembly

**Coverage Length**	7760 (1 contig(s))
**Depth Of Coverage**	4265.8
**Number Of Reads**	186149
**Reads Per Million**	3111.02 rpm (after QC)
**Ambiguities**	0
**Assembly Method**	de novo + reference guided assembly
**Consensus Caller**	Bcf Tools

Coverage Map

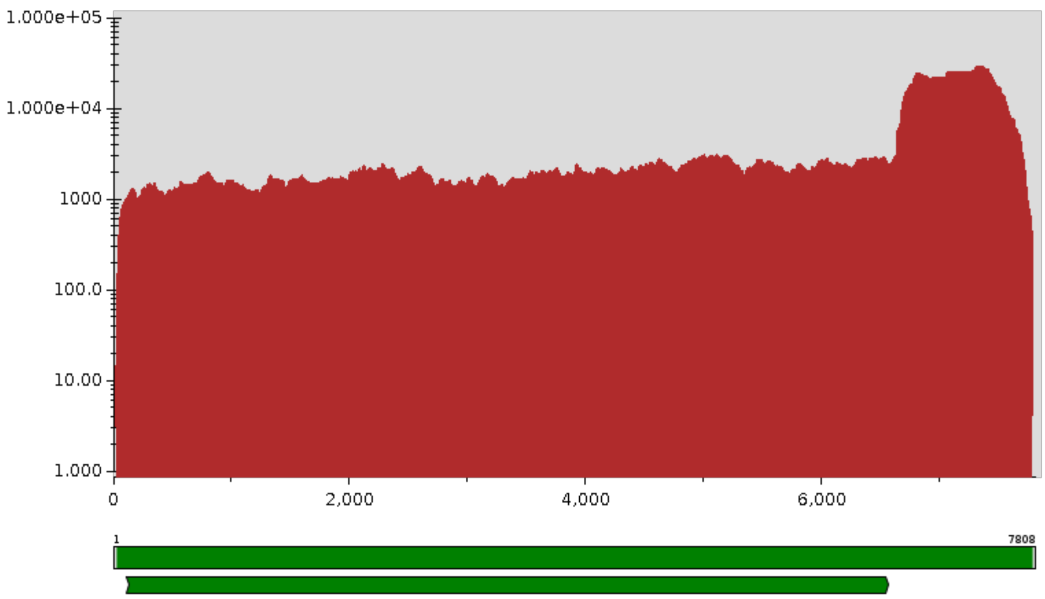

Assignment

**Type**	Torradovirus lycopersici (Taxonomy ID: 3048378)
**Reference Genome**	NC_009013.1
**NT Identity (%)**	94.2857
**AA Identity (%)**	98.8421
**Number Of Stop Codons**	1
**Number Of CDS**	1

Alignment

**Alignment Score**	12464.0 (NT) + 14956.0 (AA) = 27420.0
**Concordance (%)**	93.7051

**Alignment Method**	Global, seeded, nucleotide + amino acids (AGA)

Genome Region

Sequence starts at position 27 and ends at position 7786 relative to NC_009013.1 reference sequence.

Alignment Detailed Statistics

	Begin	End	Coverage	Score	Concordance	Matc
**NT**	**27**	**7786**	**99.4%**	**12464**	**87.8%**	**7359 (9**
Mutations:	28C>T, 76_77insT, 87C>T, 115T>C, 127C>T, 128C>T, 130C>T, 135T>C, 158G>A, 160T>C, 161A>G, 187A>G, 193T>C, 208T>C, 220C>T, 224C>T, 328T>C, 334T>C, 340G>A, 349G>A, 373C>T, 376C> 925C>T, 952A>G, 964T>G, 970G>A, 997C>T, 1006T>C, 1033G>A, 1060T>C, 1196A>G, 1207T>C, 1222G>A, 1234C>T, 1240C>T, 1255G>A, 1276A>G, 1300G>A, 1351A>G, 1372C>T, 1411T>C, 1417T 1789A>G, 1897A>G, 1910T>C, 1954T>C, 1960A>G, 1984C>T, 2014A>G, 2042G>A, 2092A>G, 2138C>T, 2149T>C, 2155T>C, 2174C>T, 2200T>C, 2227C>T, 2251C>T, 2257C>T, 2263G>A, 2287G>A, 2512T>C, 2516T>C, 2620G>A, 2662C>T, 2665T>C, 2728C>T, 2734C>T, 2743A>G, 2765T>C, 2773A>G, 2809C>T, 2812G>A, 2827A>G, 2869C>T, 2881T>C, 2914C>T, 2935G>A, 2950C>T, 2951C>T, 3451T>C, 3499A>G, 3534G>A, 3538T>C, 3541C>T, 3580T>C, 3583C>T, 3610A>G, 3613T>C, 3625C>T, 3628G>A, 3643A>G, 3676C>T, 3685G>A, 3701C>T, 3710G>A, 3739A>T, 3754G>A, 3784C>T, 4129C>T, 4150C>T, 4157C>T, 4159G>A, 4168A>G, 4210T>C, 4219C>T, 4294A>G, 4351C>T, 4357C>T, 4360A>G, 4393T>C, 4417T>C, 4421T>C, 4477C>T, 4543T>C, 4591T>C, 4636T>C, 4640C>T, 4813A>G, 4816T>C, 4843G>A, 4898G>A, 4906G>A, 4969G>A, 4988A>G, 4993A>T, 5005T>C, 5012T>C, 5029G>A, 5065C>T, 5074T>C, 5077T>C, 5079T>C, 5102T>C, 5118C>T, 5119T>C, 5120G>A, 5369G>A, 5371G>A, 5386C>T, 5392G>A, 5407A>G, 5437T>C, 5440C>T, 5461T>C, 5471T>C, 5482A>G, 5533T>A, 5572T>C, 5578T>C, 5584T>C, 5617A>T, 5641T>C, 5647T>C, 5695T>C, 5746T>C, 6049G>A, 6055T>A, 6073T>C, 6079C>T, 6091A>G, 6092C>A, 6106G>A, 6118T>C, 6121A>G, 6148T>C, 6178A>G, 6184A>G, 6193C>T, 6196A>G, 6200A>T, 6205C>T, 6247G>A, 6256C>A, 6262A>G, 6721A>T, 6742A>T, 6766delC, 6786T>C, 6798_6799insA, 6806T>G, 6807_6832delAACGGAGAGTACTGACTTTAACTAGT, 6835G>T, 6838_6850delGTCCGGCTCCATT, 6854A>T, 6858C>T, 6859C>A, 6912_6916delCGTGT, 6918C>T, 6921T>G, 6923C>G, 6924T>A, 6927_6941delGTTAGAAAGTTCCTT, 6947A>C, 6948G>T, 6949_6972delGTTTACAAAGTACGGGGAGCACTC, 6976G>A, 6984T>C, 69 7043T>C, 7045T>G, 7047_7060delGCCGTGATGAAAGA, 7063A>T, 7069C>A, 7071_7272delTCCCCCTTATTGGGAGGCTGAAACTACACATATGTAGTGGGTTTGACTGAGTCCTATAATCAGTCCGTTTGAAATTCGATAATTTTCCGTAGCTTGCGTCAAGCTGCTCACGTTAGGGGTGT 7292G>T, 7293A>T, 7297_7298insC, 7299G>A, 7311T>A, 7318G>T, 7320G>T, 7331A>T, 7333G>A, 7343C>A, 7344C>G, 7346A>T, 7349_7350insC, 7354_7355insGAGCACTGCCATTA, 7358A>C, 736 7398C>A, 7399G>A, 7400A>T, 7404C>T, 7407A>T, 7408G>T, 7423A>T, 7425G>A, 7435T>C, 7436G>A, 7444C>T, 7445A>G, 7457A>G, 7458T>C, 7460G>A, 7498C>T, 7503C>T, 7505A>G, 7511_7512 7585T>C, 7589T>A, 7600T>G, 7601T>A, 7602T>G, 7603_7604insC, 7610C>T, 7612G>A, 7615A>G, 7616A>C, 7617A>T, 7618C>T, 7625_7631delATTGCTT, 7637G>T, 7639T>A, 7641T>A, 7644_7650 7698G>A, 7717A>C, 7719T>A, 7723T>C, 7739T>A, 7742_7743insC, 7748A>G, 7749T>C, 7751T>C, 7752G>A, 7754_7755insG, 7756_7757insT, 7762C>T, 7763A>C, 7764A>T, 7767C>T, 7773G>T, 777

CDS

ToTV_sRNA1gp1	1	2159	100%	14956	99.2%	2159 (1
Protein mutations:	P8S (128C>T 130C>T), F10S (135T>C), A18T (158G>A 160T>C), T19A (161A>G), T364A (1196A>G), A646T (2042G>A), R768K (2409G>A), V773I (2423G>A), R1143K (3534G>A), A1202T (3710G>A), V1672I (5120G>A), T1681N (5148C>A), R1713K (5244G>A), V1755I (5369G>A 5371G>A), I1792M (5482A>G), N1947S (5946A>G), T1976I (6033C>T), L1996I (6092C>A), M2032L (6200A>T), A2089V (					
Codon mutations:	TTT3TTC (115T>C), TTC7TTT (127C>T), CCC8TCT (128C>T 130C>T), TTC10TCC (135T>C), GCT18ACC (158G>A 160T>C), ACC19GCC (161A>G), TCA27TCG (187A>G), CTT29CTC (193T>C), AGT (340G>A), AAG81AAA (349G>A), TTC89TTT (373C>T), ATC90ATT (376C>T), AGC113AGT (445C>T), GAC127GAT (487C>T), GTG132GTA (502G>A), TCT145TCC (541T>C), TTC151TTT (559C>T), C CCA210CCG (736A>G), CTG246TTG (842C>T), CAA258CAG (880A>G), GCC273GCT (925C>T), GTA282GTG (952A>G), GGT286GGG (964T>G), AAG288AAA (970G>A), GCC297GCT (997C>T), TC GAG372GAA (1222G>A), CAC376CAT (1234C>T), GGC378GGT (1240C>T), AGG383AGA (1255G>A), CAA390CAG (1276A>G), GGG398GGA (1300G>A), GAA415GAG (1351A>G), CTC422CTT (137 (1543G>A), AAA481AAG (1549A>G), GGC482GGT (1552C>T), AAC495AAT (1591C>T), CAG502CAA (1612G>A), GAT516GAC (1654T>C), TGC521TGT (1669C>T), AGC555AGT (1771C>T), TTG559 GTA618GTG (1960A>G), TAC626TAT (1984C>T), AAA636AAG (2014A>G), GCA646ACA (2042G>A), CTA662CTG (2092A>G), CTG678TTG (2138C>T), GGT681GGC (2149T>C), TGT683TGC (2155T> (2257C>T), CAG719CAA (2263G>A), GCG727GCA (2287G>A), CAC728CAT (2290C>T), CTG738TTG (2318C>T), TTC750TTT (2356C>T), TTG751TTA (2359G>A), TAT758TAC (2380T>C), AGG768A GTT796GTC (2494T>C), GGC801GGT (2509C>T), CTT802CTC (2512T>C), TTA804CTA (2516T>C), GAG838GAA (2620G>A), ATC852ATT (2662C>T), TTT853TTC (2665T>C), GGC874GGT (2728C> (2809C>T), TCG902TCA (2812G>A), ACA907ACG (2827A>G), TTC921TTT (2869C>T), CAT925CAC (2881T>C), GCC936GCT (2914C>T), AAG943AAA (2935G>A), GTC948GTT (2950C>T), CTG949T TTT1001TTC (3109T>C), TCT1019TCC (3163T>C), TCC1021TCT (3169C>T), AAA1023AAG (3175A>G), CTG1024TTG (3176C>T), TCT1029TCA (3193T>A), AAA1053AAG (3265A>G), CTC1104CTT (GGC1145GGT (3541C>T), TCT1158TCC (3580T>C), ATC1159ATT (3583C>T), GAA1168GAG (3610A>G), GGT1169GGC (3613T>C), TAC1173TAT (3625C>T), GTG1174GTA (3628G>A), AAA1179AA CGA1211CGT (3739A>T), AAG1216AAA (3754G>A), CTC1226CTT (3784C>T), GAC1236GAT (3814C>T), GAG1262GAA (3892G>A), GAC1281GAT (3949C>T), TTG1285CTG (3959T>C), AAA1304AA (4084C>T), CAT1328CAC (4090T>C), TAC1329TAT (4093C>T), TCC1341TCT (4129C>T), CAC1348CAT (4150C>T), CTG1351TTA (4157C>T 4159G>A), AAA1354AAG (4168A>G), GCT1368GCC (42 AGA1418AGG (4360A>G), TAT1429TAC (4393T>C), TTT1437TTC (4417T>C), TTG1439CTG (4421T>C), CTC1457CTT (4477C>T), GCT1479GCC (4543T>C), TAT1495TAC (4591T>C), TAT1510TAC ((4696C>T), GAC1531GAT (4699C>T), AGC1551AGT (4759C>T), TTT1554TTC (4768T>C), AGG1556AGA (4774G>A), CAC1557CAT (4777C>T), CTA1559CTG (4783A>G), GGA1560GGG (4786A>G), (4898G>A), GAG1600GAA (4906G>A), AAG1621AAA (4969G>A), ACC1628GCC (4988A>G), ACA1629ACT (4993A>T), TAT1633TAC (5005T>C), TTG1636CTG (5012T>C), CTG1641CTA (5029G>A), A (5102T>C), ACT1671ATC (5118C>T 5119T>C), GTT1672ATT (5120G>A), CTG1673CTC (5125G>C), CAT1680CAC (5146T>C), ACT1681AAT (5148C>A), CAG1698CAA (5200G>A), GTT1704GTG (52 ATT1745ATA (5341T>A), GTA1746GTG (5344A>G), ACC1747ACT (5347C>T), GTG1755ATA (5369G>A 5371G>A), GGC1760GGT (5386C>T), GGG1762GGA (5392G>A), CCA1767CCG (5407A>G), A (5482A>G), CTT1809CTA (5533T>A), GAT1822GAC (5572T>C), TGT1824TGC (5578T>C), ATT1826ATC (5584T>C), ATA1837ATT (5617A>T), GGT1845GGC (5641T>C), TTT1847TTC (5647T>C), CC (5797A>G), TAT1907TAC (5827T>C), GGG1924GGA (5878G>A), CCC1927CCT (5887C>T), CAG1944CAA (5938G>A), CAC1946CAT (5944C>T), AAT1947AGT (5946A>G), TTT1960TTC (5986T>C), T (6073T>C), CTC1991CTT (6079C>T), CTA1995CTG (6091A>G), CTT1996ATT (6092C>A), AAG2000AAA (6106G>A), TGT2004TGC (6118T>C), TCA2005TCG (6121A>G), TAT2014TAC (6148T>C), GA (6200A>T), TAC2033TAT (6205C>T), TTG2047TTA (6247G>A), ACC2050ACA (6256C>A), ACA2052ACG (6262A>G), CTG2053CTA (6265G>A), GTA2064GTG (6298A>G), GAT2069GAC (6313T>C), A (6493T>C)					

Proteins

polyprotein (YP_001039627.1)	1	2159	100%	14956	99.2%	2159 (1
Protein mutations:	P8S (128C>T 130C>T), F10S (135T>C), A18T (158G>A 160T>C), T19A (161A>G), T364A (1196A>G), A646T (2042G>A), R768K (2409G>A), V773I (2423G>A), R1143K (3534G>A), A1202T (3710G>A), V1672I (5120G>A), T1681N (5148C>A), R1713K (5244G>A), V1755I (5369G>A 5371G>A), I1792M (5482A>G), N1947S (5946A>G), T1976I (6033C>T), L1996I (6092C>A), M2032L (6200A>T), A2089V (					
Codon mutations:	TTT3TTC (115T>C), TTC7TTT (127C>T), CCC8TCT (128C>T 130C>T), TTC10TCC (135T>C), GCT18ACC (158G>A 160T>C), ACC19GCC (161A>G), TCA27TCG (187A>G), CTT29CTC (193T>C), AGT (340G>A), AAG81AAA (349G>A), TTC89TTT (373C>T), ATC90ATT (376C>T), AGC113AGT (445C>T), GAC127GAT (487C>T), GTG132GTA (502G>A), TCT145TCC (541T>C), TTC151TTT (559C>T), C CCA210CCG (736A>G), CTG246TTG (842C>T), CAA258CAG (880A>G), GCC273GCT (925C>T), GTA282GTG (952A>G), GGT286GGG (964T>G), AAG288AAA (970G>A), GCC297GCT (997C>T), TC GAG372GAA (1222G>A), CAC376CAT (1234C>T), GGC378GGT (1240C>T), AGG383AGA (1255G>A), CAA390CAG (1276A>G), GGG398GGA (1300G>A), GAA415GAG (1351A>G), CTC422CTT (137 (1543G>A), AAA481AAG (1549A>G), GGC482GGT (1552C>T), AAC495AAT (1591C>T), CAG502CAA (1612G>A), GAT516GAC (1654T>C), TGC521TGT (1669C>T), AGC555AGT (1771C>T), TTG559 GTA618GTG (1960A>G), TAC626TAT (1984C>T), AAA636AAG (2014A>G), GCA646ACA (2042G>A), CTA662CTG (2092A>G), CTG678TTG (2138C>T), GGT681GGC (2149T>C), TGT683TGC (2155T> (2257C>T), CAG719CAA (2263G>A), GCG727GCA (2287G>A), CAC728CAT (2290C>T), CTG738TTG (2318C>T), TTC750TTT (2356C>T), TTG751TTA (2359G>A), TAT758TAC (2380T>C), AGG768A GTT796GTC (2494T>C), GGC801GGT (2509C>T), CTT802CTC (2512T>C), TTA804CTA (2516T>C), GAG838GAA (2620G>A), ATC852ATT (2662C>T), TTT853TTC (2665T>C), GGC874GGT (2728C> (2809C>T), TCG902TCA (2812G>A), ACA907ACG (2827A>G), TTC921TTT (2869C>T), CAT925CAC (2881T>C), GCC936GCT (2914C>T), AAG943AAA (2935G>A), GTC948GTT (2950C>T), CTG949T TTT1001TTC (3109T>C), TCT1019TCC (3163T>C), TCC1021TCT (3169C>T), AAA1023AAG (3175A>G), CTG1024TTG (3176C>T), TCT1029TCA (3193T>A), AAA1053AAG (3265A>G), CTC1104CTT (GGC1145GGT (3541C>T), TCT1158TCC (3580T>C), ATC1159ATT (3583C>T), GAA1168GAG (3610A>G), GGT1169GGC (3613T>C), TAC1173TAT (3625C>T), GTG1174GTA (3628G>A), AAA1179AA CGA1211CGT (3739A>T), AAG1216AAA (3754G>A), CTC1226CTT (3784C>T), GAC1236GAT (3814C>T), GAG1262GAA (3892G>A), GAC1281GAT (3949C>T), TTG1285CTG (3959T>C), AAA1304AA (4084C>T), CAT1328CAC (4090T>C), TAC1329TAT (4093C>T), TCC1341TCT (4129C>T), CAC1348CAT (4150C>T), CTG1351TTA (4157C>T 4159G>A), AAA1354AAG (4168A>G), GCT1368GCC (42 AGA1418AGG (4360A>G), TAT1429TAC (4393T>C), TTT1437TTC (4417T>C), TTG1439CTG (4421T>C), CTC1457CTT (4477C>T), GCT1479GCC (4543T>C), TAT1495TAC (4591T>C), TAT1510TAC ((4696C>T), GAC1531GAT (4699C>T), AGC1551AGT (4759C>T), TTT1554TTC (4768T>C), AGG1556AGA (4774G>A), CAC1557CAT (4777C>T), CTA1559CTG (4783A>G), GGA1560GGG (4786A>G), (4898G>A), GAG1600GAA (4906G>A), AAG1621AAA (4969G>A), ACC1628GCC (4988A>G), ACA1629ACT (4993A>T), TAT1633TAC (5005T>C), TTG1636CTG (5012T>C), CTG1641CTA (5029G>A), A (5102T>C), ACT1671ATC (5118C>T 5119T>C), GTT1672ATT (5120G>A), CTG1673CTC (5125G>C), CAT1680CAC (5146T>C), ACT1681AAT (5148C>A), CAG1698CAA (5200G>A), GTT1704GTG (52 ATT1745ATA (5341T>A), GTA1746GTG (5344A>G), ACC1747ACT (5347C>T), GTG1755ATA (5369G>A 5371G>A), GGC1760GGT (5386C>T), GGG1762GGA (5392G>A), CCA1767CCG (5407A>G), A (5482A>G), CTT1809CTA (5533T>A), GAT1822GAC (5572T>C), TGT1824TGC (5578T>C), ATT1826ATC (5584T>C), ATA1837ATT (5617A>T), GGT1845GGC (5641T>C), TTT1847TTC (5647T>C), CC (5797A>G), TAT1907TAC (5827T>C), GGG1924GGA (5878G>A), CCC1927CCT (5887C>T), CAG1944CAA (5938G>A), CAC1946CAT (5944C>T), AAT1947AGT (5946A>G), TTT1960TTC (5986T>C), T (6073T>C), CTC1991CTT (6079C>T), CTA1995CTG (6091A>G), CTT1996ATT (6092C>A), AAG2000AAA (6106G>A), TGT2004TGC (6118T>C), TCA2005TCG (6121A>G), TAT2014TAC (6148T>C), GA (6200A>T), TAC2033TAT (6205C>T), TTG2047TTA (6247G>A), ACC2050ACA (6256C>A), ACA2052ACG (6262A>G), CTG2053CTA (6265G>A), GTA2064GTG (6298A>G), GAT2069GAC (6313T>C), A (6493T>C)					

*: Inserts / Deletes / Misaligned / Frameshifts

Analysis details

This analysis was performed with panviral2.64

NGS Details (UN8): Torradovirus lycopersici (segment RNA 2)

Assembly

**Coverage Length**	5382 (1 contig(s))
**Depth Of Coverage**	7518.4
**Number Of Reads**	313618
**Reads Per Million**	5241.34 rpm (after QC)
**Ambiguities**	0
**Assembly Method**	read mapping against reference + variant calling
**Consensus Caller**	Bcf Tools

Coverage Map

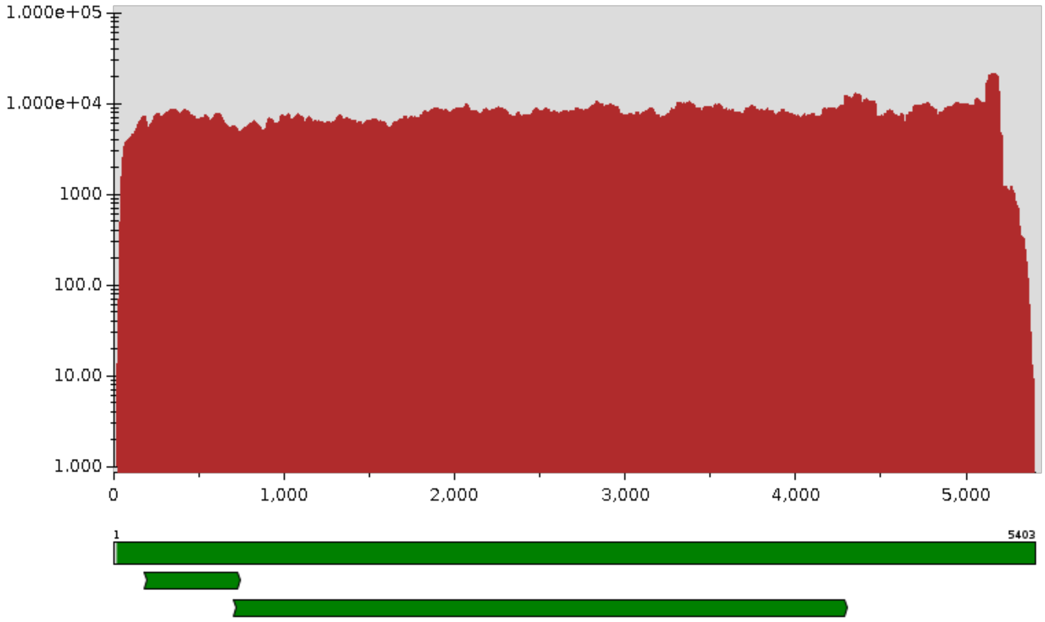

Assignment

**Type**	Torradovirus lycopersici (Taxonomy ID: 3048378)
**Reference Genome**	NC_009032.1
**NT Identity (%)**	96.5633
**AA Identity (%)**	97.9813
**Number Of Stop Codons**	2
**Number Of CDS**	2

Alignment

**Alignment Score**	10018.0 (NT) + 9318.0 (AA) = 19336.0
**Concordance (%)**	95.4817

Alignment Method	Global, seeded, nucleotide + amino acids (AGA)

Genome Region

Sequence starts at position 22 and ends at position 5403 relative to NC_009032.1 reference sequence.

Alignment Detailed Statistics

	Begin	End	Coverage	Score	Concordance	Matches	Identities	I/D/M/F*	Stop Codons
**NT**	**22**	**5403**	**99.6%**	**10018**	**93.1%**	**5382 (99.9%)**	**5198 (96.6%)**	**1/0**	
Mutations:	89G>A, 90C>T, 97C>T, 107G>A, 111C>A, 137T>C, 144A>G, 161A>G, 224G>A, 277C>T, 280T>C, 283C>T, 295T>C, 301A>C, 313C>T, 343T>C, 401T>C, 433C>T, 440C>T, 448C>A, 460T>C, 481C>A, 487A>G, 507G>A, 583A>T, 586A>G, 589A>G, 654T>A, 679G>A, 696T>C, 721C>T, 733A>G, 748A>G, 750A>G, 797T>C, 806C>T, 827A>G, 844A>T, 866A>G, 965T>C, 968A>G, 1010T>G, 1061T>C, 1085G>A, 1106T>C, 1109C>T, 1139G>A, 1226A>G, 1271T>C, 1277A>G, 1300C>T, 1316C>T, 1337T>C, 1418G>A, 1421A>G, 1457C>T, 1490G>A, 1502C>T, 1508G>A, 1547C>T, 1557C>T, 1608T>C, 1619G>A, 1637G>A, 1646T>C, 1651A>G, 1662C>T, 1685T>C, 1694T>C, 1724C>T, 1757A>G, 1796T>A, 1859C>T, 1863C>T, 1878A>G, 1892C>T, 1967G>A, 1970A>G, 1994T>C, 2072G>A, 2084G>A, 2108A>G, 2128G>A, 2140A>C, 2159T>A, 2189C>T, 2205A>G, 2249T>C, 2263A>C, 2270A>G, 2285T>C, 2303A>G, 2312T>C, 2321T>C, 2324G>A, 2375C>T, 2429G>A, 2439C>T, 2465T>C, 2471C>T, 2501A>C, 2503G>A, 2519A>G, 2570G>A, 2573T>C, 2612C>T, 2630C>T, 2660T>C, 2708A>G, 2720C>T, 2732T>C, 2792C>T, 2819G>A, 2840T>C, 2841C>T, 2885T>C, 2939A>G, 2969C>T, 3003T>C, 3050G>A, 3122T>C, 3128T>C, 3131G>A, 3168C>T, 3215T>C, 3236C>T, 3265G>A, 3269T>C, 3320T>C, 3323T>C, 3335C>T, 3368A>G, 3386A>G, 3413C>T, 3422A>G, 3431T>C, 3437C>T, 3446T>C, 3470T>C, 3512T>C, 3514T>C, 3543T>A, 3551C>T, 3560T>A, 3569A>G, 3595A>G, 3626A>G, 3638A>G, 3662A>T, 3674G>A, 3716C>T, 3722C>T, 3743G>A, 3767C>T, 3773C>T, 3800C>T, 3827C>T, 3893T>C, 3933A>G, 3995C>T, 4013G>C, 4040C>T, 4046C>T, 4059A>G, 4061A>G, 4069A>G, 4100A>T, 4112C>T, 4130C>T, 4190A>G, 4224C>T, 4250C>T, 4457A>G, 4518G>A, 4556A>G, 4590G>A, 4592T>C, 4812C>T, 5180T>G, 5192C>T, 5206C>T, 5218C>T, 5235_5236insT, 5249A>G, 5262A>T								

CDS

	Begin	End	Coverage	Score	Concordance	Matches	Identities	I/D/M/F*	Stop Codons
ToTV_sRNA2gp1	1	188	100%	1186	98.1%	188 (100%)	184 (97.9%)	0/0/0/0	1
Protein mutations:	A15T (224G>A), R109K (507G>A), L158Q (654T>A), L172P (696T>C)								
Codon mutations:	GCA15ACA (224G>A), GGC32GGT (277C>T), TCT33TCC (280T>C), GGC34GGT (283C>T), AGT38AGC (295T>C), CCA40CCC (301A>C), TTC44TTT (313C>T), ATT54ATC (343T>C), TTA74CTA (401T>C), GGC84GGT (433C>T), CTA87TTA (440C>T), GGC89GGA (448C>A), CAT93CAC (460T>C), ACC100ACA (481C>A), AGA102AGG (487A>G), AGG109AAG (507G>A), ACA134ACT (583A>T), CAA135CAG (586A>G), TTA136TTG (589A>G), CTA158CAA (654T>A), TTG166TTA (679G>A), CTA172CCA (696T>C), GGC180GGT (721C>T), GAA184GAG (733A>G)								
ToTV_sRNA2gp2	1	1199	100%	8132	98.2%	1199 (100%)	1175 (98.0%)	0/0/0/0	1
Protein mutations:	A7V (721C>T), K11R (733A>G), K16R (748A>G), S17G (750A>G), Y48F (844A>T), A200V (1300C>T), H286Y (1557C>T), Y303H (1608T>C), K317R (1651A>G), H388Y (1863C>T), I393V (1878A>G), R476K (2128G>A), K480T (2140A>C), M502V (2205A>G), E521A (2263A>C), R601K (2503G>A), R855K (3265G>A), V938A (3514T>C), S948T (3543T>A), D965G (3595A>G), T1078A (3933A>G), I1120V (4059A>G 4061A>G), N1123S (4069A>G), H1175Y (4224C>T)								
Codon mutations:	GCT7GTT (721C>T), AAG11AGG (733A>G), AAA16AGA (748A>G), AGT17GGT (750A>G), GTT32GTC (797T>C), GGC35GGT (806C>T), AAA42AAG (827A>G), TAT48TTT (844A>T), CAA55CAG (866A>G), CAT88CAC (965T>C), GGA89GGG (968A>G), GTT103GTG (1010T>G), AGT120AGC (1061T>C), CTG128CTA (1085G>A), TTT135TTC (1106T>C), TCC136TCT (1109C>T), CCG146CCA (1139G>A), TCA175TCG (1226A>G), ATT190ATC (1271T>C), TCA192TCG (1277A>G), GCT200GTT (1300C>T), CAC205CAT (1316C>T), GAT212GAC (1337T>C), GTG239GTA (1418G>A), GGA240GGG (1421A>G), AGC252AGT (1457C>T), CGG263CGA (1490G>A), CTC267CTT (1502C>T), GTG269GTA (1508G>A), ACC282ACT (1547C>T), CAC286TAC (1557C>T), TAC303CAC (1608T>C), GAG306GAA (1619G>A), TTG312TTA (1637G>A), GTT315GTC (1646T>C), AAG317AGG (1651A>G), CTG321TTG (1662C>T), GAT328GAC (1685T>C), GGT331GGC (1694T>C), CCC341CCT (1724C>T), CAA352CAG (1757A>G), GTT365GTA (1796T>A), ACC386ACT (1859C>T), CAC388TAC (1863C>T), ATA393GTA (1878A>G), TAC397TAT (1892C>T), GAG422GAA (1967G>A), CTA423CTG (1970A>G), TAT431TAC (1994T>C), GAG457GAA (2072G>A), GTG461GTA (2084G>A), AGA469AGG (2108A>G), AGG476AAG (2128G>A), AAA480ACA (2140A>C), ACT486ACA (2159T>A), TTC496TTT (2189C>T), ATG502GTG (2205A>G), GCT516GCC (2249T>C), GAA521GCA (2263A>C), GTA523GTG (2270A>G), GTT528GTC (2285T>C), CAA534CAG (2303A>G), AAT537AAC (2312T>C), GGT540GGC (2321T>C), GCG541GCA (2324G>A), TTC558TTT (2375C>T), GAG576GAA (2429G>A), CTG580TTG (2439C>T), GGT588GGC (2465T>C), TCC590TCT (2471C>T), CTA600CTC (2501A>C), AGG601AAG (2503G>A), GCA606GCG (2519A>G), TCG623TCA (2570G>A), GGT624GGC (2573T>C), AAC637AAT (2612C>T), ATC643ATT (2630C>T), CAT653CAC (2660T>C), ACA669ACG (2708A>G), ATC673ATT (2720C>T), GTT677GTC (2732T>C), CAC697CAT (2792C>T), GAG706GAA (2819G>A), CAT713CAC (2840T>C), CTG714TTG (2841C>T), CGT728CGC (2885T>C), CGA746CGG (2939A>G), TAC756TAT (2969C>T), TTA768CTA (3003T>C), ACG783ACA (3050G>A), ATT807ATC (3122T>C), ATT809ATC (3128T>C), GAG810GAA (3131G>A), CTG823TTG (3168C>T), AAT838AAC (3215T>C), AAC845AAT (3236C>T), AGG855AAG (3265G>A), TGT856TGC (3269T>C), AGT873AGC (3320T>C), TCT874TCC (3323T>C), CCC878CCT (3335C>T), ACA889ACG (3368A>G), GTA895GTG (3386A>G), GTC904GTT (3413C>T), ACA907ACG (3422A>G), CTT910CTC (3431T>C), TGC912TGT (3437C>T), GAT915GAC (3446T>C), TCT923TCC (3470T>C), GCT937GCC (3512T>C), GTG938GCG (3514T>C), TCT948ACT (3543T>A), CGC950CGT (3551C>T), ATT953ATA (3560T>A), TTA956TTG (3569A>G), GAT965GGT (3595A>G), CAA975CAG (3626A>G), AGA979AGG (3638A>G), GTA987GTT (3662A>T), GTG991GTA (3674G>A), TGC1005TGT (3716C>T), CCC1007CCT (3722C>T), CAG1014CAA (3743G>A), ATC1022ATT (3767C>T), TGC1024TGT (3773C>T), TTC1033TTT (3800C>T), TAC1042TAT (3827C>T), GAT1064GAC (3893T>C), ACC1078GCC (3933A>G), AAC1098AAT (3995C>T), GTG1104GTC (4013G>C), CGC1113CGT (4040C>T), ACC1115ACT (4046C>T), ATA1120GTG (4059A>G 4061A>G), AAT1123AGT (4069A>G), CGA1133CGT (4100A>T), CTC1137CTT (4112C>T), GCC1143GCT (4130C>T), CAA1163CAG (4190A>G), CAT1175TAT (4224C>T), ACC1183ACT (4250C>T)								

Proteins

	Begin	End	Coverage	Score	Concordance	Matches	Identities	I/D/M/F*	Stop Codons
Orf1 (YP_001040017.1)	1	188	100%	1186	98.1%	188 (100%)	184 (97.9%)	0/0/0/0	1
Protein mutations:	A15T (224G>A), R109K (507G>A), L158Q (654T>A), L172P (696T>C)								
Codon mutations:	GCA15ACA (224G>A), GGC32GGT (277C>T), TCT33TCC (280T>C), GGC34GGT (283C>T), AGT38AGC (295T>C), CCA40CCC (301A>C), TTC44TTT (313C>T), ATT54ATC (343T>C), TTA74CTA (401T>C), GGC84GGT (433C>T), CTA87TTA (440C>T), GGC89GGA (448C>A), CAT93CAC (460T>C), ACC100ACA (481C>A), AGA102AGG (487A>G), AGG109AAG (507G>A), ACA134ACT (583A>T), CAA135CAG (586A>G), TTA136TTG (589A>G), CTA158CAA (654T>A), TTG166TTA (679G>A), CTA172CCA (696T>C), GGC180GGT (721C>T), GAA184GAG (733A>G)								
polyprotein (YP_001040018.1)	1	1199	100%	8132	98.2%	1199 (100%)	1175 (98.0%)	0/0/0/0	1
Protein mutations:	A7V (721C>T), K11R (733A>G), K16R (748A>G), S17G (750A>G), Y48F (844A>T), A200V (1300C>T), H286Y (1557C>T), Y303H (1608T>C), K317R (1651A>G), H388Y (1863C>T), I393V (1878A>G), R476K (2128G>A), K480T (2140A>C), M502V (2205A>G), E521A (2263A>C), R601K (2503G>A), R855K (3265G>A), V938A (3514T>C), S948T (3543T>A), D965G (3595A>G), T1078A (3933A>G), I1120V (4059A>G 4061A>G), N1123S (4069A>G), H1175Y (4224C>T)								
Codon mutations:	GCT7GTT (721C>T), AAG11AGG (733A>G), AAA16AGA (748A>G), AGT17GGT (750A>G), GTT32GTC (797T>C), GGC35GGT (806C>T), AAA42AAG (827A>G), TAT48TTT (844A>T), CAA55CAG (866A>G), CAT88CAC (965T>C), GGA89GGG (968A>G), GTT103GTG (1010T>G), AGT120AGC (1061T>C), CTG128CTA (1085G>A), TTT135TTC (1106T>C), TCC136TCT (1109C>T), CCG146CCA (1139G>A), TCA175TCG (1226A>G), ATT190ATC (1271T>C), TCA192TCG (1277A>G), GCT200GTT (1300C>T), CAC205CAT (1316C>T), GAT212GAC (1337T>C), GTG239GTA (1418G>A), GGA240GGG (1421A>G), AGC252AGT (1457C>T), CGG263CGA (1490G>A), CTC267CTT (1502C>T), GTG269GTA (1508G>A), ACC282ACT (1547C>T), CAC286TAC (1557C>T), TAC303CAC (1608T>C), GAG306GAA (1619G>A), TTG312TTA (1637G>A), GTT315GTC (1646T>C), AAG317AGG (1651A>G), CTG321TTG (1662C>T), GAT328GAC (1685T>C), GGT331GGC (1694T>C), CCC341CCT (1724C>T), CAA352CAG (1757A>G), GTT365GTA (1796T>A), ACC386ACT (1859C>T), CAC388TAC (1863C>T), ATA393GTA (1878A>G), TAC397TAT (1892C>T), GAG422GAA (1967G>A), CTA423CTG (1970A>G), TAT431TAC (1994T>C), GAG457GAA (2072G>A), GTG461GTA (2084G>A), AGA469AGG (2108A>G), AGG476AAG (2128G>A), AAA480ACA (2140A>C), ACT486ACA (2159T>A), TTC496TTT (2189C>T), ATG502GTG (2205A>G), GCT516GCC (2249T>C), GAA521GCA (2263A>C), GTA523GTG (2270A>G), GTT528GTC (2285T>C), CAA534CAG (2303A>G), AAT537AAC (2312T>C), GGT540GGC (2321T>C), GCG541GCA (2324G>A), TTC558TTT (2375C>T), GAG576GAA (2429G>A), CTG580TTG (2439C>T), GGT588GGC (2465T>C), TCC590TCT (2471C>T), CTA600CTC (2501A>C), AGG601AAG (2503G>A), GCA606GCG (2519A>G), TCG623TCA (2570G>A), GGT624GGC (2573T>C), AAC637AAT (2612C>T), ATC643ATT (2630C>T), CAT653CAC (2660T>C), ACA669ACG (2708A>G), ATC673ATT (2720C>T), GTT677GTC (2732T>C), CAC697CAT (2792C>T), GAG706GAA (2819G>A), CAT713CAC (2840T>C), CTG714TTG (2841C>T), CGT728CGC (2885T>C), CGA746CGG (2939A>G), TAC756TAT (2969C>T), TTA768CTA (3003T>C), ACG783ACA (3050G>A), ATT807ATC (3122T>C), ATT809ATC (3128T>C), GAG810GAA (3131G>A), CTG823TTG (3168C>T), AAT838AAC (3215T>C), AAC845AAT (3236C>T), AGG855AAG (3265G>A), TGT856TGC (3269T>C), AGT873AGC (3320T>C), TCT874TCC (3323T>C), CCC878CCT (3335C>T), ACA889ACG (3368A>G), GTA895GTG (3386A>G), GTC904GTT (3413C>T), ACA907ACG (3422A>G), CTT910CTC (3431T>C), TGC912TGT (3437C>T), GAT915GAC (3446T>C), TCT923TCC (3470T>C), GCT937GCC (3512T>C), GTG938GCG (3514T>C), TCT948ACT (3543T>A), CGC950CGT (3551C>T), ATT953ATA (3560T>A), TTA956TTG (3569A>G), GAT965GGT (3595A>G), CAA975CAG (3626A>G), AGA979AGG (3638A>G), GTA987GTT (3662A>T), GTG991GTA (3674G>A), TGC1005TGT (3716C>T), CCC1007CCT (3722C>T), CAG1014CAA (3743G>A), ATC1022ATT (3767C>T), TGC1024TGT (3773C>T), TTC1033TTT (3800C>T), TAC1042TAT (3827C>T), GAT1064GAC (3893T>C), ACC1078GCC (3933A>G), AAC1098AAT (3995C>T), GTG1104GTC (4013G>C), CGC1113CGT (4040C>T), ACC1115ACT (4046C>T), ATA1120GTG (4059A>G 4061A>G), AAT1123AGT (4069A>G), CGA1133CGT (4100A>T), CTC1137CTT (4112C>T), GCC1143GCT (4130C>T), CAA1163CAG (4190A>G), CAT1175TAT (4224C>T), ACC1183ACT (4250C>T)								

*: Inserts / Deletes / Misaligned / Frameshifts

Analysis details

This analysis was performed with panviral2.64

NGS Details (UN8): Torradovirus marchitezum (segment RNA 2)

Assembly

**Coverage Length**	1059 (1 contig(s))
**Depth Of Coverage**	11498.6
**Number Of Reads**	97853
**Reads Per Million**	1635.37 rpm (after QC)
**Ambiguities**	0
**Assembly Method**	de novo + reference guided assembly
**Consensus Caller**	Bcf Tools

Coverage Map

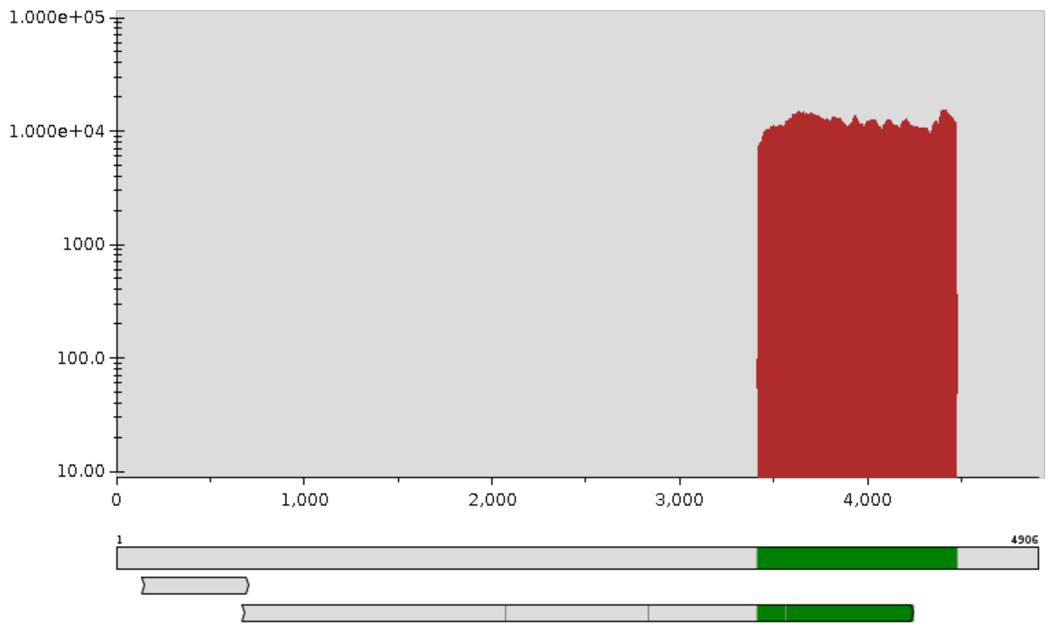

Assignment

**Type**	Torradovirus marchitezum (Taxonomy ID: 3048376)
**Reference Genome**	NC_010988.1
**NT Identity (%)**	69.2669
**AA Identity (%)**	72.7599
**Number Of Stop Codons**	1
**Number Of CDS**	2

Alignment

**Alignment Score**	771.0 (NT) + 1569.0 (AA) = 2340.0
**Concordance (%)**	57.1289

**Alignment Method**	Global, seeded, nucleotide + amino acids (AGA)

Genome Region

Sequence starts at position 3416 and ends at position 4474 relative to NC_010988.1 reference sequence.

Alignment Detailed Statistics

	Begin	End	Coverage	Score	Concordance	Matches	Identities	I/D/M/F*	Stop Codons
**NT**	**3416**	**4474**	**21.6%**	**771**	**36.8%**	**1054 (98.6%)**	**737 (68.9%)**	**10/5**	
Mutations:	3427G>T, 3436A>T, 3442C>G, 3445A>G, 3448C>T, 3449T>G, 3451A>C, 3457T>C, 3460C>T, 3461C>T, 3463T>G, 3466C>T, 3476G>A, 3478T>C, 3481A>G, 3482T>A, 3484A>T, 3493G>A, 3497A>C, 3506T>C, 3514G>A, 3517C>A, 3520C>T, 3521A>G, 3528A>T, 3529G>C, 3532C>T, 3533C>G, 3535G>T, 3538G>T, 3544C>G, 3544_3545insAAGAAG, 3550T>C, 3551C>A, 3552C>A, 3554C>G, 3555C>A, 3556C>G, 3559G>A, 3560A>G, 3561A>T, 3564C>G, 3568A>G, 3569A>G, 3570G>T, 3572A>G, 3573T>C, 3574T>A, 3577G>T, 3578T>G, 3579C>G, 3580C>A, 3581A>C, 3583A>T, 3584A>C, 3586A>G, 3589G>A, 3595T>C, 3598C>T, 3604T>A, 3610A>T, 3619C>T, 3622C>T, 3623A>T, 3624T>C, 3625G>T, 3627G>C, 3628T>C, 3638G>A, 3639A>C, 3641G>A, 3642A>G, 3643A>C, 3644A>C, 3646A>C, 3647T>C, 3650T>G, 3655A>T, 3656A>C, 3661T>A, 3667A>T, 3670A>G, 3676C>T, 3677A>G, 3680A>C, 3682G>A, 3685T>C, 3686G>T, 3687A>C, 3689G>C, 3690G>C, 3691G>C, 3692C>A, 3693A>C, 3700T>C, 3712A>C, 3715C>A, 3718T>G, 3724A>C, 3730A>C, 3739T>C, 3740A>T, 3742A>T, 3751T>C, 3760A>T, 3763T>C, 3767T>C, 3769G>T, 3770A>G, 3772T>G, 3775T>C, 3776T>A, 3778A>C, 3779A>C, 3781T>C, 3782G>A, 3784G>C, 3787A>C, 3790C>T, 3793A>G, 3796A>C, 3800T>G, 3801C>G, 3803A>T, 3804G>C, 3806C>A, 3807C>A, 3811T>C, 3812G>A, 3817A>T, 3826A>T, 3832T>G, 3838T>G, 3841T>C, 3842G>A, 3860C>T, 3862A>T, 3863G>T, 3864C>A, 3868G>T, 3869C>G, 3871T>G, 3872A>G, 3873A>C, 3874T>A, 3875A>G, 3877A>G, 3880A>G, 3881A>G, 3882A>C, 3883T>A, 3884T>C, 3890G>C, 3892A>T, 3901T>C, 3904A>T, 3907C>T, 3908A>T, 3910A>C, 3917G>A, 3922T>C, 3925A>C, 3931T>A, 3934G>T, 3935G>A, 3937A>C, 3940A>T, 3943T>C, 3945C>T, 3946G>T, 3949C>T, 3952A>C, 3955C>T, 3956A>G, 3957C>T, 3958T>G, 3967T>C, 3971G>A, 3973G>A, 3982A>T, 3983A>C, 3986C>A, 3989C>T, 3994A>T, 3996G>A, 3998A>G, 3999T>C, 4000G>A, 4001A>C, 4003G>A, 4009C>T, 4010T>G, 4011C>A, 4012A>C, 4014G>A, 4015C>T, 4019C>A, 4020A>G, 4021A>T, 4024C>T, 4027C>T, 4030C>T, 4031A>G, 4033G>T, 4036A>T, 4037C>T, 4039A>G, 4045T>C, 4047G>A, 4048G>A, 4049C>T, 4051T>G, 4054A>T, 4055A>C, 4056A>T, 4060G>T, 4061A>T, 4063C>G, 4066A>C, 4070T>C, 4072G>A, 4075T>A, 4078T>C, 4079T>G, 4080C>T, 4081C>A, 4083C>A, 4085A>T, 4088G>A, 4090G>T, 4093T>C, 4102G>A, 4105A>C, 4106C>A, 4108T>G, 4109A>G, 4111A>T, 4112T>A, 4113C>A, 4114T>G, 4117T>A, 4120A>G, 4122T>C, 4123T>A, 4129C>T, 4131A>G, 4135G>A, 4138G>A, 4139T>C, 4141G>T, 4154T>C, 4155C>T, 4156T>G, 4159C>T, 4162C>T, 4180C>T, 4181A>G, 4192T>G, 4195C>T, 4198C>T, 4201T>C, 4204G>A, 4213A>T, 4219T>C, 4222T>A, 4225G>A, 4228A>G, 4229C>A, 4231T>A, 4234T>C, 4237T>C, 4238G>T, 4242T>A, 4243T>C, 4248A>C, 4252T>A, 4279A>T, 4279_4280insT, 4280A>T, 4280_4281insC, 4283A>G, 4286C>T, 4287T>A, 4288T>C, 4291C>A, 4294C>T, 4306G>A, 4309G>A, 4312G>T, 4313C>A, 4313_4314insG, 4315A>G, 4316A>T, 4318T>C, 4319G>T, 4320G>A, 4323T>G, 4324T>A, 4328T>C, 4330G>A, 4333A>G, 4335G>T, 4336A>C, 4338A>G, 4339_4343delAGGGA, 4345A>T, 4346G>C, 4349A>C, 4364C>T, 4365T>C, 4369A>G, 4372A>G, 4373G>A, 4392T>C, 4393C>T, 4419_4420insG, 4422A>G, 4426G>A, 4430G>A, 4437C>T, 4441T>C, 4449A>T, 4451T>G								

CDS

	Begin	End	Coverage	Score	Concordance	Matches	Identities	I/D/M/F*	Stop Codons
ToMarV_RNA2gp2	916	1192	23.2%	1569	78.4%	277 (99.3%)	203 (72.8%)	2/0/0/0	1
Protein mutations:	S927A (3449T>G 3451A>C), D936N (3476G>A 3478T>C), S938T (3482T>A 3484A>T), I943L (3497A>C), D949E (3517C>A), I951V (3521A>G), E953V (3528A>T 3529G>C), Q955D (3533C>G 3535G>T), E956D (3538G>T), G958_K959insKK (3544_3545insAAGAAG), P961K (3551C>A 3552C>A), P962E (3554C>G 3555C>A 3556C>G), K964V (3560A>G 3561A>T), A965G (3564C>G), S967V (3569A>G 3570G>T), I968A (3572A>G 3573T>C 3574T>A), S970G (3578T>G 3579C>G 3580C>A), I971L (3581A>C 3583A>T), M985S (3623A>T 3624T>C 3625G>T), G986A (3627G>C 3628T>C), D990T (3638G>A 3639A>C), E991S (3641G>A 3642A>G 3643A>C), L994V (3650T>G), I996L (3656A>C), I1003V (3677A>G), E1006S (3686G>T 3687A>C), G1007P (3689G>C 3690G>C 3691G>C), H1008T (3692C>A 3693A>C), T1024S (3740A>T 3742A>T), N1034E (3770A>G 3772T>G), S1036T (3776T>A 3778A>C), I1037L (3779A>C 3781T>C), V1038I (3782G>A 3784G>C), S1044G (3800T>G 3801C>G), P1046N (3806C>A 3807C>A), V1048I (3812G>A), F1056L (3838T>G), A1058T (3842G>A), P1064S (3860C>T 3862A>T), A1065Y (3863G>T 3864C>A), L1067V (3869C>G 3871T>G), N1068A (3872A>G 3873A>C 3874T>A), K1069E (3875A>G 3877A>G), N1071A (3881A>G 3882A>C 3883T>A), Y1072H (3884T>C), V1074L (3890G>C 3892A>T), T1080S (3908A>T 3910A>C), D1083N (3917G>A), A1089T (3935G>A 3937A>C), T1092I (3945C>T 3946G>T), T1096V (3956A>G 3957C>T 3958T>G), E1101K (3971G>A 3973G>A), H1107Y (3989C>T), R1109K (3996G>A), M1110A (3998A>G 3999T>C 4000G>A), S1114D (4010T>G 4011C>A 4012A>C), S1115N (4014G>A 4015C>T), Q1117S (4019C>A 4020A>G 4021A>T), M1121V (4031A>G 4033G>T), R1126K (4047G>A 4048G>A), N1129L (4055A>C 4056A>T), I1131L (4061A>T 4063C>G), S1137V (4079T>G 4080C>T 4081C>A), A1138D (4083C>A), I1139L (4085A>T), V1140I (4088G>A 4090G>T), L1146M (4106C>A 4108T>G), I1147V (4109A>G 4111A>T), S1148K (4112T>A 4113C>A 4114T>G), L1151P (4122T>C 4123T>A), K1154R (4131A>G), S1162L (4154T>C 4155C>T 4156T>G), N1171D (4181A>G), A1190S (4238G>T), I1191N (4242T>A 4243T>C)								
Codon mutations:	GGG919GGT (3427G>T), ACA922ACT (3436A>T), GGC924GGG (3442C>G), AAA925AAG (3445A>G), AGC926AGT (3448C>T), TCA927GCC (3449T>G 3451A>C), GAT929GAC (3457T>C), TTC930TTT (3460C>T), CTT931TTG (3461C>T 3463T>G), TTC932TTT (3466C>T), GAT936AAC (3476G>A 3478T>C), TTA937TTG (3481A>G), TCA938ACT (3482T>A 3484A>T), AAG941AAA (3493G>A), ATT943CTT (3497A>C), TTG946CTG (3506T>C), AAG948AAA (3514G>A), GAC949GAA (3517C>A), AAC950AAT (3520C>T), ATT951GTT (3521A>G), GAG953GTC (3528A>T 3529G>C), TAC954TAT (3532C>T), CAG955GAT (3533C>G 3535G>T), GAG956GAT (3538G>T), GGC958GGG (3544C>G), GGC958_AAA959insAAGAAG (3544_3545insAAGAAG), GAT960GAC (3550T>C), CCG961AAG (3551C>A 3552C>A), CCC962GAG (3554C>G 3555C>A 3556C>G), AAG963AAA (3559G>A), AAG964GTG (3560A>G 3561A>T), GCT965GGT (3564C>G), CAA966CAG (3568A>G), AGT967GTT (3569A>G 3570G>T), ATT968GCA (3572A>G 3573T>C 3574T>A), CTG969CTT (3577G>T), TCC970GGA (3578T>G 3579C>G 3580C>A), ATA971CTT (3581A>C 3583A>T), AGA972CGG (3584A>C 3586A>G), GAG973GAA (3589G>A), TTT975TTC (3595T>C), TCC976TCT (3598C>T), GGT978GGA (3604T>A), GTA980GTT (3610A>T), TTC983TTT (3619C>T), TGC984TGT (3622C>T), ATG985TCT (3623A>T 3624T>C 3625G>T), GGT986GCC (3627G>C 3628T>C), GAC990ACC (3638G>A 3639A>C), GAA991AGC (3641G>A 3642A>G 3643A>C), AGA992CGC (3644A>C 3646A>C), TTG993CTG (3647T>C), TTG994GTG (3650T>G), GTA995GTT (3655A>T), ATT996CTT (3656A>C), CCT997CCA (3661T>A), GCA999GCT (3667A>T), CCA1000CCG (3670A>G), TCC1002TCT (3676C>T), ATA1003GTA (3677A>G), AGG1004CGA (3680A>C 3682G>A), TTT1005TTC (3685T>C), GAA1006TCA (3686G>T 3687A>C), GGG1007CCC (3689G>C 3690G>C 3691G>C), CAC1008ACC (3692C>A 3693A>C), CCT1010CCC (3700T>C), GCA1014GCC (3712A>C), ATC1015ATA (3715C>A), ACT1016ACG (3718T>G), CCA1018CCC (3724A>C), ATA1020ATC (3730A>C), TGT1023TGC (3739T>C), ACA1024TCT (3740A>T 3742A>T), TGT1027TGC (3751T>C), TCA1030TCT (3760A>T), GGT1031GGC (3763T>C), TTG1033CTT (3767T>C 3769G>T), AAT1034GAG (3770A>G 3772T>G), TAT1035TAC (3775T>C), TCA1036ACC (3776T>A 3778A>C), ATT1037CTC (3779A>C 3781T>C), GTG1038ATC (3782G>A 3784G>C), ATA1039ATC (3787A>C), CAC1040CAT (3790C>T), AGA1041AGG (3793A>G), GTA1042GTC (3796A>C), TCC1044GGC (3800T>G 3801C>G), AGT1045TCT (3803A>T 3804G>C), CCT1046AAT (3806C>A 3807C>A), AAT1047AAC (3811T>C), GTT1048ATT (3812G>A), GGA1049GGT (3817A>T), CTA1052CTT (3826A>T), GTT1054GTG (3832T>G), TTT1056TTG (3838T>G), GAT1057GAC (3841T>C), GCC1058ACC (3842G>A), CCA1064TCT (3860C>T 3862A>T), GCT1065TAT (3863G>T 3864C>A), GGG1066GGT (3868G>T), CTT1067GTG (3869C>G 3871T>G), AAT1068GCA (3872A>G 3873A>C 3874T>A), AAA1069GAG (3875A>G 3877A>G), GGA1070GGG (3880A>G), AAT1071GCA (3881A>G 3882A>C 3883T>A), TAT1072CAT (3884T>C), GTA1074CTT (3890G>C 3892A>T), GGT1077GGC (3901T>C), GGA1078GGT (3904A>T), GGC1079GGT (3907C>T), ACA1080TCC (3908A>T 3910A>C), GAT1083AAT (3917G>A), TTT1084TTC (3922T>C), TCA1085TCC (3925A>C), GGT1087GGA (3931T>A), GTG1088GTT (3934G>T), GCA1089ACC (3935G>A 3937A>C), ACA1090ACT (3940A>T), AAT1091AAC (3943T>C), ACG1092ATT (3945C>T 3946G>T), TTC1093TTT (3949C>T), TCA1094TCC (3952A>C), TTC1095TTT (3955C>T), ACT1096GTG (3956A>G 3957C>T 3958T>G), GAT1099GAC (3967T>C), GAG1101AAA (3971G>A 3973G>A), CCA1104CCT (3982A>T), AGG1105CGG (3983A>C), CGG1106AGG (3986C>A), CAT1107TAT (3989C>T), ACA1108ACT (3994A>T), AGG1109AAG (3996G>A), ATG1110GCA (3998A>G 3999T>C 4000G>A), AGG1111CGA (4001A>C 4003G>A), TTC1113TTT (4009C>T), TCA1114GAC (4010T>G 4011C>A 4012A>C), AGC1115AAT (4014G>A 4015C>T), CAA1117AGT (4019C>A 4020A>G 4021A>T), TCC1118TCT (4024C>T), CGC1119CGT (4027C>T), ATC1120ATT (4030C>T), ATG1121GTT (4031A>G 4033G>T), TCA1122TCT (4036A>T), CTA1123TTG (4037C>T 4039A>G), GAT1125GAC (4045T>C), AGG1126AAA (4047G>A 4048G>A), CTT1127TTG (4049C>T 4051T>G), GGA1128GGT (4054A>T), AAT1129CTT (4055A>C 4056A>T), CTG1130CTT (4060G>T), ATC1131TTG (4061A>T 4063C>G), ATA1132ATC (4066A>C), TTG1134CTA (4070T>C 4072G>A), CCT1135CCA (4075T>A), CCT1136CCC (4078T>C), TCC1137GTA (4079T>G 4080C>T 4081C>A), GCC1138GAC (4083C>A), ATA1139TTA (4085A>T), GTG1140ATT (4088G>A 4090G>T), AGT1141AGC (4093T>C), GAG1144GAA (4102G>A), ATA1145ATC (4105A>C), CTT1146ATG (4106C>A 4108T>G), ATA1147GTT (4109A>G 4111A>T), TCT1148AAG (4112T>A 4113C>A 4114T>G), CCT1149CCA (4117T>A), GGA1150GGG (4120A>G), CTT1151CCA (4122T>C 4123T>A), TTC1153TTT (4129C>T), AAG1154AGG (4131A>G), TTG1155TTA (4135G>A), GAG1156GAA (4138G>A), TTG1157CTT (4139T>C 4141G>T), TCT1162CTG (4154T>C 4155C>T 4156T>G), GCC1163GCT (4159C>T), AAC1164AAT (4162C>T), GGC1170GGT (4180C>T), AAT1171GAT (4181A>G), ACT1174ACG (4192T>G), CAC1175CAT (4195C>T), ACC1176ACT (4198C>T), TAT1177TAC (4201T>C), CAG1178CAA (4204G>A), TCA1181TCT (4213A>T), TTT1183TTC (4219T>C), TCT1184TCA (4222T>A), GAG1185GAA (4225G>A), CTA1186CTG (4228A>G), CGT1187AGA (4229C>A 4231T>A), GAT1188GAC (4234T>C), TTT1189TTC (4237T>C), GCG1190TCG (4238G>T), ATT1191AAC (4242T>A 4243T>C)								

Proteins

	Begin	End	Coverage	Score	Concordance	Matches	Identities	I/D/M/F*	Stop Codons
polyprotein (YP_001976149.1)	916	1192	23.2%	1569	78.4%	277 (99.3%)	203 (72.8%)	2/0/0/0	1
Protein mutations:	S927A (3449T>G 3451A>C), D936N (3476G>A 3478T>C), S938T (3482T>A 3484A>T), I943L (3497A>C), D949E (3517C>A), I951V (3521A>G), E953V (3528A>T 3529G>C), Q955D (3533C>G 3535G>T), E956D (3538G>T), G958_K959insKK (3544_3545insAAGAAG), P961K (3551C>A 3552C>A), P962E (3554C>G 3555C>A 3556C>G), K964V (3560A>G 3561A>T), A965G (3564C>G), S967V (3569A>G 3570G>T), I968A (3572A>G 3573T>C 3574T>A), S970G (3578T>G 3579C>G 3580C>A), I971L (3581A>C 3583A>T), M985S (3623A>T 3624T>C 3625G>T), G986A (3627G>C 3628T>C), D990T (3638G>A 3639A>C), E991S (3641G>A 3642A>G 3643A>C), L994V (3650T>G), I996L (3656A>C), I1003V (3677A>G), E1006S (3686G>T 3687A>C), G1007P (3689G>C 3690G>C 3691G>C), H1008T (3692C>A 3693A>C), T1024S (3740A>T 3742A>T), N1034E (3770A>G 3772T>G), S1036T (3776T>A 3778A>C), I1037L (3779A>C 3781T>C), V1038I (3782G>A 3784G>C), S1044G (3800T>G 3801C>G), P1046N (3806C>A 3807C>A), V1048I (3812G>A), F1056L (3838T>G), A1058T (3842G>A), P1064S (3860C>T 3862A>T), A1065Y (3863G>T 3864C>A), L1067V (3869C>G 3871T>G), N1068A (3872A>G 3873A>C 3874T>A), K1069E (3875A>G 3877A>G), N1071A (3881A>G 3882A>C 3883T>A), Y1072H (3884T>C), V1074L (3890G>C 3892A>T), T1080S (3908A>T 3910A>C), D1083N (3917G>A), A1089T (3935G>A 3937A>C), T1092I (3945C>T 3946G>T), T1096V (3956A>G 3957C>T 3958T>G), E1101K (3971G>A 3973G>A), H1107Y (3989C>T), R1109K (3996G>A), M1110A (3998A>G 3999T>C 4000G>A), S1114D (4010T>G 4011C>A 4012A>C), S1115N (4014G>A 4015C>T), Q1117S (4019C>A 4020A>G 4021A>T), M1121V (4031A>G 4033G>T), R1126K (4047G>A 4048G>A), N1129L (4055A>C 4056A>T), I1131L (4061A>T 4063C>G), S1137V (4079T>G 4080C>T 4081C>A), A1138D (4083C>A), I1139L (4085A>T), V1140I (4088G>A 4090G>T), L1146M (4106C>A 4108T>G), I1147V (4109A>G 4111A>T), S1148K (4112T>A 4113C>A 4114T>G), L1151P (4122T>C 4123T>A), K1154R (4131A>G), S1162L (4154T>C 4155C>T 4156T>G), N1171D (4181A>G), A1190S (4238G>T), I1191N (4242T>A 4243T>C)								

	Begin	End	Coverage	Score	Concordance	Matches	Identities	I/D/M/F*	Stop Codons
**NT**	**3416**	**4474**	**21.6%**	**771**	**36.8%**	**1054 (98.6%)**	**737 (68.9%)**	**10/5**	
Codon mutations:	GGG919GGT (3427G>T), ACA922ACT (3436A>T), GGC924GGG (3442C>G), AAA925AAG (3445A>G), AGC926AGT (3448C>T), TCA927GCC (3449T>G 3451A>C), GAT929GAC (3457T>C), TTC930TTT (3460C>T), CTT931TTG (3461C>T 3463T>G), TTC932TTT (3466C>T), GAT936AAC (3476G>A 3478T>C), TTA937TTG (3481A>G), TCA938ACT (3482T>A 3484A>T), AAG941AAA (3493G>A), ATT943CTT (3497A>C), TTG946CTG (3506T>C), AAG948AAA (3514G>A), GAC949GAA (3517C>A), AAC950AAT (3520C>T), ATT951GTT (3521A>G), GAG953GTC (3528A>T 3529G>C), TAC954TAT (3532C>T), CAG955GAT (3533C>G 3535G>T), GAG956GAT (3538G>T), GGC958GGG (3544C>G), GGC958_AAA959insAAGAAG (3544_3545insAAGAAG), GAT960GAC (3550T>C), CCG961AAG (3551C>A 3552C>A), CCC962GAG (3554C>G 3555C>A 3556C>G), AAG963AAA (3559G>A), AAG964GTG (3560A>G 3561A>T), GCT965GGT (3564C>G), CAA966CAG (3568A>G), AGT967GTT (3569A>G 3570G>T), ATT968GCA (3572A>G 3573T>C 3574T>A), CTG969CTT (3577G>T), TCC970GGA (3578T>G 3579C>G 3580C>A), ATA971CTT (3581A>C 3583A>T), AGA972CGG (3584A>C 3586A>G), GAG973GAA (3589G>A), TTT975TTC (3595T>C), TCC976TCT (3598C>T), GGT978GGA (3604T>A), GTA980GTT (3610A>T), TTC983TTT (3619C>T), TGC984TGT (3622C>T), ATG985TCT (3623A>T 3624T>C 3625G>T), GGT986GCC (3627G>C 3628T>C), GAC990ACC (3638G>A 3639A>C), GAA991AGC (3641G>A 3642A>G 3643A>C), AGA992CGC (3644A>C 3646A>C), TTG993CTG (3647T>C), TTG994GTG (3650T>G), GTA995GTT (3655A>T), ATT996CTT (3656A>C), CCT997CCA (3661T>A), GCA999GCT (3667A>T), CCA1000CCG (3670A>G), TCC1002TCT (3676C>T), ATA1003GTA (3677A>G), AGG1004CGA (3680A>C 3682G>A), TTT1005TTC (3685T>C), GAA1006TCA (3686G>T 3687A>C), GGG1007CCC (3689G>C 3690G>C 3691G>C), CAC1008ACC (3692C>A 3693A>C), CCT1010CCC (3700T>C), GCA1014GCC (3712A>C), ATC1015ATA (3715C>A), ACT1016ACG (3718T>G), CCA1018CCC (3724A>C), ATA1020ATC (3730A>C), TGT1023TGC (3739T>C), ACA1024TCT (3740A>T 3742A>T), TGT1027TGC (3751T>C), TCA1030TCT (3760A>T), GGT1031GGC (3763T>C), TTG1033CTT (3767T>C 3769G>T), AAT1034GAG (3770A>G 3772T>G), TAT1035TAC (3775T>C), TCA1036ACC (3776T>A 3778A>C), ATT1037CTC (3779A>C 3781T>C), GTG1038ATC (3782G>A 3784G>C), ATA1039ATC (3787A>C), CAC1040CAT (3790C>T), AGA1041AGG (3793A>G), GTA1042GTC (3796A>C), TCC1044GGC (3800T>G 3801C>G), AGT1045TCT (3803A>T 3804G>C), CCT1046AAT (3806C>A 3807C>A), AAT1047AAC (3811T>C), GTT1048ATT (3812G>A), GGA1049GGT (3817A>T), CTA1052CTT (3826A>T), GTT1054GTG (3832T>G), TTT1056TTG (3838T>G), GAT1057GAC (3841T>C), GCC1058ACC (3842G>A), CCA1064TCT (3860C>T 3862A>T), GCT1065TAT (3863G>T 3864C>A), GGG1066GGT (3868G>T), CTT1067GTG (3869C>G 3871T>G), AAT1068GCA (3872A>G 3873A>C 3874T>A), AAA1069GAG (3875A>G 3877A>G), GGA1070GGG (3880A>G), AAT1071GCA (3881A>G 3882A>C 3883T>A), TAT1072CAT (3884T>C), GTA1074CTT (3890G>C 3892A>T), GGT1077GGC (3901T>C), GGA1078GGT (3904A>T), GGC1079GGT (3907C>T), ACA1080TCC (3908A>T 3910A>C), GAT1083AAT (3917G>A), TTT1084TTC (3922T>C), TCA1085TCC (3925A>C), GGT1087GGA (3931T>A), GTG1088GTT (3934G>T), GCA1089ACC (3935G>A 3937A>C), ACA1090ACT (3940A>T), AAT1091AAC (3943T>C), ACG1092ATT (3945C>T 3946G>T), TTC1093TTT (3949C>T), TCA1094TCC (3952A>C), TTC1095TTT (3955C>T), ACT1096GTG (3956A>G 3957C>T 3958T>G), GAT1099GAC (3967T>C), GAG1101AAA (3971G>A 3973G>A), CCA1104CCT (3982A>T), AGG1105CGG (3983A>C), CGG1106AGG (3986C>A), CAT1107TAT (3989C>T), ACA1108ACT (3994A>T), AGG1109AAG (3996G>A), ATG1110GCA (3998A>G 3999T>C 4000G>A), AGG1111CGA (4001A>C 4003G>A), TTC1113TTT (4009C>T), TCA1114GAC (4010T>G 4011C>A 4012A>C), AGC1115AAT (4014G>A 4015C>T), CAA1117AGT (4019C>A 4020A>G 4021A>T), TCC1118TCT (4024C>T), CGC1119CGT (4027C>T), ATC1120ATT (4030C>T), ATG1121GTT (4031A>G 4033G>T), TCA1122TCT (4036A>T), CTA1123TTG (4037C>T 4039A>G), GAT1125GAC (4045T>C), AGG1126AAA (4047G>A 4048G>A), CTT1127TTG (4049C>T 4051T>G), GGA1128GGT (4054A>T), AAT1129CTT (4055A>C 4056A>T), CTG1130CTT (4060G>T), ATC1131TTG (4061A>T 4063C>G), ATA1132ATC (4066A>C), TTG1134CTA (4070T>C 4072G>A), CCT1135CCA (4075T>A), CCT1136CCC (4078T>C), TCC1137GTA (4079T>G 4080C>T 4081C>A), GCC1138GAC (4083C>A), ATA1139TTA (4085A>T), GTG1140ATT (4088G>A 4090G>T), AGT1141AGC (4093T>C), GAG1144GAA (4102G>A), ATA1145ATC (4105A>C), CTT1146ATG (4106C>A 4108T>G), ATA1147GTT (4109A>G 4111A>T), TCT1148AAG (4112T>A 4113C>A 4114T>G), CCT1149CCA (4117T>A), GGA1150GGG (4120A>G), CTT1151CCA (4122T>C 4123T>A), TTC1153TTT (4129C>T), AAG1154AGG (4131A>G), TTG1155TTA (4135G>A), GAG1156GAA (4138G>A), TTG1157CTT (4139T>C 4141G>T), TCT1162CTG (4154T>C 4155C>T 4156T>G), GCC1163GCT (4159C>T), AAC1164AAT (4162C>T), GGC1170GGT (4180C>T), AAT1171GAT (4181A>G), ACT1174ACG (4192T>G), CAC1175CAT (4195C>T), ACC1176ACT (4198C>T), TAT1177TAC (4201T>C), CAG1178CAA (4204G>A), TCA1181TCT (4213A>T), TTT1183TTC (4219T>C), TCT1184TCA (4222T>A), GAG1185GAA (4225G>A), CTA1186CTG (4228A>G), CGT1187AGA (4229C>A 4231T>A), GAT1188GAC (4234T>C), TTT1189TTC (4237T>C), GCG1190TCG (4238G>T), ATT1191AAC (4242T>A 4243T>C)								
Coat protein B (YP_001976156.1)	194	244	20.9%	263	74.9%	51 (96.2%)	38 (71.7%)	2/0/0/0	0
Protein mutations:	S205A (3449T>G 3451A>C), D214N (3476G>A 3478T>C), S216T (3482T>A 3484A>T), I221L (3497A>C), D227E (3517C>A), I229V (3521A>G), E231V (3528A>T 3529G>C), Q233D (3533C>G 3535G>T), E234D (3538G>T), G236_K237insKK (3544_3545insAAGAAG), P239K (3551C>A 3552C>A), P240E (3554C>G 3555C>A 3556C>G), K242V (3560A>G 3561A>T), A243G (3564C>G)								
Codon mutations:	GGG197GGT (3427G>T), ACA200ACT (3436A>T), GGC202GGG (3442C>G), AAA203AAG (3445A>G), AGC204AGT (3448C>T), TCA205GCC (3449T>G 3451A>C), GAT207GAC (3457T>C), TTC208TTT (3460C>T), CTT209TTG (3461C>T 3463T>G), TTC210TTT (3466C>T), GAT214AAC (3476G>A 3478T>C), TTA215TTG (3481A>G), TCA216ACT (3482T>A 3484A>T), AAG219AAA (3493G>A), ATT221CTT (3497A>C), TTG224CTG (3506T>C), AAG226AAA (3514G>A), GAC227GAA (3517C>A), AAC228AAT (3520C>T), ATT229GTT (3521A>G), GAG231GTC (3528A>T 3529G>C), TAC232TAT (3532C>T), CAG233GAT (3533C>G 3535G>T), GAG234GAT (3538G>T), GGC236GGG (3544C>G), GGC236_AAA237insAAGAAG (3544_3545insAAGAAG), GAT238GAC (3550T>C), CCG239AAG (3551C>A 3552C>A), CCC240GAG (3554C>G 3555C>A 3556C>G), AAG241AAA (3559G>A), AAG242GTG (3560A>G 3561A>T), GCT243GGT (3564C>G), CAA244CAG (3568A>G)								
Coat protein C (YP_001976157.1)	1	225	100%	1305	79.1%	225 (100%)	164 (72.9%)	0/0/0/0	0
Protein mutations:	S1V (3569A>G 3570G>T), I2A (3572A>G 3573T>C 3574T>A), S4G (3578T>G 3579C>G 3580C>A), I5L (3581A>C 3583A>T), M19S (3623A>T 3624T>C 3625G>T), G20A (3627G>C 3628T>C), D24T (3638G>A 3639A>C), E25S (3641G>A 3642A>G 3643A>C), L28V (3650T>G), I30L (3656A>C), I37V (3677A>G), E40S (3686G>T 3687A>C), G41P (3689G>C 3690G>C 3691G>C), H42T (3692C>A 3693A>C), T58S (3740A>T 3742A>T), N68E (3770A>G 3772T>G), S70T (3776T>A 3778A>C), I71L (3779A>C 3781T>C), V72I (3782G>A 3784G>C), S78G (3800T>G 3801C>G), P80N (3806C>A 3807C>A), V82I (3812G>A), F90L (3838T>G), A92T (3842G>A), P98S (3860C>T 3862A>T), A99Y (3863G>T 3864C>A), L101V (3869C>G 3871T>G), N102A (3872A>G 3873A>C 3874T>A), K103E (3875A>G 3877A>G), N105A (3881A>G 3882A>C 3883T>A), Y106H (3884T>C), V108L (3890G>C 3892A>T), T114S (3908A>T 3910A>C), D117N (3917G>A), A123T (3935G>A 3937A>C), T126I (3945C>T 3946G>T), T130V (3956A>G 3957C>T 3958T>G), E135K (3971G>A 3973G>A), H141Y (3989C>T), R143K (3996G>A), M144A (3998A>G 3999T>C 4000G>A), S148D (4010T>G 4011C>A 4012A>C), S149N (4014G>A 4015C>T), Q151S (4019C>A 4020A>G 4021A>T), M155V (4031A>G 4033G>T), R160K (4047G>A 4048G>A), N163L (4055A>C 4056A>T), I165L (4061A>T 4063C>G), S171V (4079T>G 4080C>T 4081C>A), A172D (4083C>A), I173L (4085A>T), V174I (4088G>A 4090G>T), L180M (4106C>A 4108T>G), I181V (4109A>G 4111A>T), S182K (4112T>A 4113C>A 4114T>G), L185P (4122T>C 4123T>A), K188R (4131A>G), S196L (4154T>C 4155C>T 4156T>G), N205D (4181A>G), A224S (4238G>T), I225N (4242T>A 4243T>C)								
Codon mutations:	AGT1GTT (3569A>G 3570G>T), ATT2GCA (3572A>G 3573T>C 3574T>A), CTG3CTT (3577G>T), TCC4GGA (3578T>G 3579C>G 3580C>A), ATA5CTT (3581A>C 3583A>T), AGA6CGG (3584A>C 3586A>G), GAG7GAA (3589G>A), TTT9TTC (3595T>C), TCC10TCT (3598C>T), GGT12GGA (3604T>A), GTA14GTT (3610A>T), TTC17TTT (3619C>T), TGC18TGT (3622C>T), ATG19TCT (3623A>T 3624T>C 3625G>T), GGT20GCC (3627G>C 3628T>C), GAC24ACC (3638G>A 3639A>C), GAA25AGC (3641G>A 3642A>G 3643A>C), AGA26CGC (3644A>C 3646A>C), TTG27CTG (3647T>C), TTG28GTG (3650T>G), GTA29GTT (3655A>T), ATT30CTT (3656A>C), CCT31CCA (3661T>A), GCA33GCT (3667A>T), CCA34CCG (3670A>G), TCC36TCT (3676C>T), ATA37GTA (3677A>G), AGG38CGA (3680A>C 3682G>A), TTT39TTC (3685T>C), GAA40TCA (3686G>T 3687A>C), GGG41CCC (3689G>C 3690G>C 3691G>C), CAC42ACC (3692C>A 3693A>C), CCT44CCC (3700T>C), GCA48GCC (3712A>C), ATC49ATA (3715C>A), ACT50ACG (3718T>G), CCA52CCC (3724A>C), ATA54ATC (3730A>C), TGT57TGC (3739T>C), ACA58TCT (3740A>T 3742A>T), TGT61TGC (3751T>C), TCA64TCT (3760A>T), GGT65GGC (3763T>C), TTG67CTT (3767T>C 3769G>T), AAT68GAG (3770A>G 3772T>G), TAT69TAC (3775T>C), TCA70ACC (3776T>A 3778A>C), ATT71CTC (3779A>C 3781T>C), GTG72ATC (3782G>A 3784G>C), ATA73ATC (3787A>C), CAC74CAT (3790C>T), AGA75AGG (3793A>G), GTA76GTC (3796A>C), TCC78GGC (3800T>G 3801C>G), AGT79TCT (3803A>T 3804G>C), CCT80AAT (3806C>A 3807C>A), AAT81AAC (3811T>C), GTT82ATT (3812G>A), GGA83GGT (3817A>T), CTA86CTT (3826A>T), GTT88GTG (3832T>G), TTT90TTG (3838T>G), GAT91GAC (3841T>C), GCC92ACC (3842G>A), CCA98TCT (3860C>T 3862A>T), GCT99TAT (3863G>T 3864C>A), GGG100GGT (3868G>T), CTT101GTG (3869C>G 3871T>G), AAT102GCA (3872A>G 3873A>C 3874T>A), AAA103GAG (3875A>G 3877A>G), GGA104GGG (3880A>G), AAT105GCA (3881A>G 3882A>C 3883T>A), TAT106CAT (3884T>C), GTA108CTT (3890G>C 3892A>T), GGT111GGC (3901T>C), GGA112GGT (3904A>T), GGC113GGT (3907C>T), ACA114TCC (3908A>T 3910A>C), GAT117AAT (3917G>A), TTT118TTC (3922T>C), TCA119TCC (3925A>C), GGT121GGA (3931T>A), GTG122GTT (3934G>T), GCA123ACC (3935G>A 3937A>C), ACA124ACT (3940A>T), AAT125AAC (3943T>C), ACG126ATT (3945C>T 3946G>T), TTC127TTT (3949C>T), TCA128TCC (3952A>C), TTC129TTT (3955C>T), ACT130GTG (3956A>G 3957C>T 3958T>G), GAT133GAC (3967T>C), GAG135AAA (3971G>A 3973G>A), CCA138CCT (3982A>T), AGG139CGG (3983A>C), CGG140AGG (3986C>A), CAT141TAT (3989C>T), ACA142ACT (3994A>T), AGG143AAG (3996G>A), ATG144GCA (3998A>G 3999T>C 4000G>A), AGG145CGA (4001A>C 4003G>A), TTC147TTT (4009C>T), TCA148GAC (4010T>G 4011C>A 4012A>C), AGC149AAT (4014G>A 4015C>T), CAA151AGT (4019C>A 4020A>G 4021A>T), TCC152TCT (4024C>T), CGC153CGT (4027C>T), ATC154ATT (4030C>T), ATG155GTT (4031A>G 4033G>T), TCA156TCT (4036A>T), CTA157TTG (4037C>T 4039A>G), GAT159GAC (4045T>C), AGG160AAA (4047G>A 4048G>A), CTT161TTG (4049C>T 4051T>G), GGA162GGT (4054A>T), AAT163CTT (4055A>C 4056A>T), CTG164CTT (4060G>T), ATC165TTG (4061A>T 4063C>G), ATA166ATC (4066A>C), TTG168CTA (4070T>C 4072G>A), CCT169CCA (4075T>A), CCT170CCC (4078T>C), TCC171GTA (4079T>G 4080C>T 4081C>A), GCC172GAC (4083C>A), ATA173TTA (4085A>T), GTG174ATT (4088G>A 4090G>T), AGT175AGC (4093T>C), GAG178GAA (4102G>A), ATA179ATC (4105A>C), CTT180ATG (4106C>A 4108T>G), ATA181GTT (4109A>G 4111A>T), TCT182AAG (4112T>A 4113C>A 4114T>G), CCT183CCA (4117T>A), GGA184GGG (4120A>G), CTT185CCA (4122T>C 4123T>A), TTC187TTT (4129C>T), AAG188AGG (4131A>G), TTG189TTA (4135G>A), GAG190GAA (4138G>A), TTG191CTT (4139T>C 4141G>T), TCT196CTG (4154T>C 4155C>T 4156T>G), GCC197GCT (4159C>T), AAC198AAT (4162C>T), GGC204GGT (4180C>T), AAT205GAT (4181A>G), ACT208ACG (4192T>G), CAC209CAT (4195C>T), ACC210ACT (4198C>T), TAT211TAC (4201T>C), CAG212CAA (4204G>A), TCA215TCT (4213A>T), TTT217TTC (4219T>C), TCT218TCA (4222T>A), GAG219GAA (4225G>A), CTA220CTG (4228A>G), CGT221AGA (4229C>A 4231T>A), GAT222GAC (4234T>C), TTT223TTC (4237T>C), GCG224TCG (4238G>T), ATT225AAC (4242T>A 4243T>C)								

*: Inserts / Deletes / Misaligned / Frameshifts

Analysis details

This analysis was performed with panviral2.64

NGS Details (UN8): Torradovirus marchitezum (segment RNA 1)

Assembly

**Coverage Length**	6723 (1 contig(s))
**Depth Of Coverage**	3512.7
**Number Of Reads**	176957
**Reads Per Million**	2957.39 rpm (after QC)
**Ambiguities**	0
**Assembly Method**	de novo + reference guided assembly
**Consensus Caller**	Bcf Tools

Coverage Map

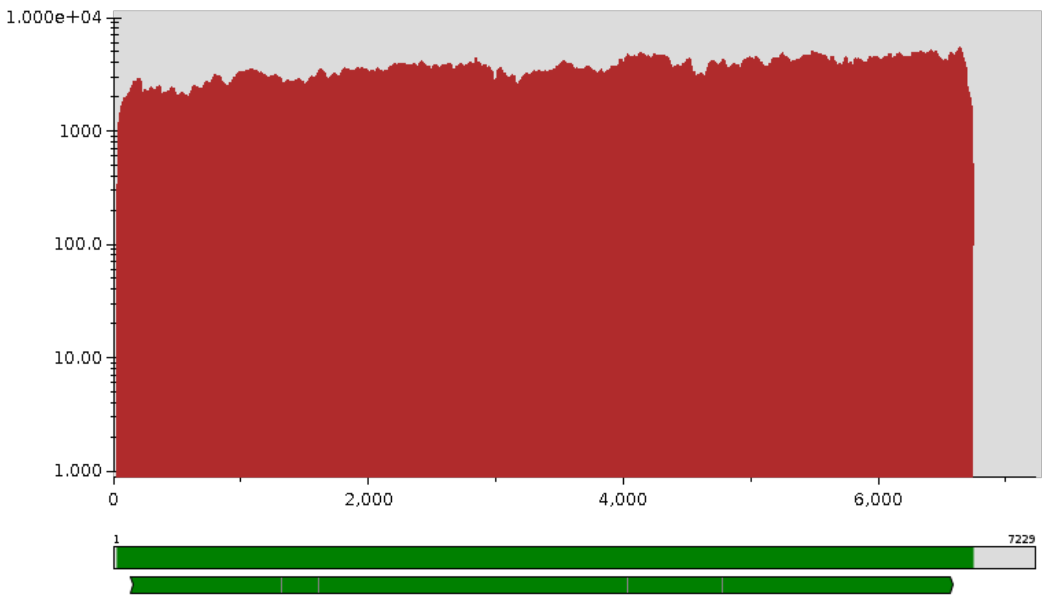

Assignment

**Type**	Torradovirus marchitezum (Taxonomy ID: 3048376)
**Reference Genome**	NC_010987.1
**NT Identity (%)**	62.9646
**AA Identity (%)**	63.3737
**Number Of Stop Codons**	0
**Number Of CDS**	1

Alignment

**Alignment Score**	3297.0 (NT) + 9863.0 (AA) = 13160.0
**Concordance (%)**	47.0084

Alignment Method	Global, seeded, nucleotide + amino acids (AGA)

Genome Region

Sequence starts at position 25 and ends at position 6747 relative to NC_010987.1 reference sequence.

Alignment Detailed Statistics

	Begin	End	Coverage	Score	Concordance	Matches	Identities	I/D/M/F*	Stop Codons
**NT**	**25**	**6747**	**93.0%**	**3297**	**25.1%**	**6658 (98.4%)**	**4218 (62.4%)**	**41/65**	

	Begin	End	Coverage	Score	Concordance	Matches	Identities	I/D/M/F*	Stop Codons
**NT**	**25**	**6747**	**93.0%**	**3297**	**25.1%**	**6658 (98.4%)**	**4218 (62.4%)**	**41/65**	

Mutations:

32T>C, 37G>A, 40C>T, 59A>C, 61T>C, 62T>A, 63G>C, 66A>C, 67T>A, 68T>A, 70G>A, 71A>C, 74C>T, 75T>G, 76T>C, 77G>T, 79A>T, 81A>C, 82C>T, 85T>C, 87C>A,
89_99delAACATCTTCTT, 108A>T, 112G>C, 114C>T, 115C>T, 116A>C, 118T>C, 119A>T, 122T>G, 125A>T, 128C>A, 129C>A, 130T>A, 133_134delAT, 136T>A, 146T>A, 160T>G,
161C>G, 162T>A, 163C>G, 164C>T, 165_188delAAGGTTGCTGGTGTTGATGACGCC, 189A>C, 190G>C, 192T>C, 195T>G, 200C>T, 201A>T, 203C>A, 204A>C, 217G>C, 218T>A,
222C>G, 227A>T, 228A>T, 229A>C, 234A>T, 239C>T, 240A>T, 241A>C, 242A>T, 243T>C, 251A>G, 252G>A, 254G>T, 255G>C, 257T>C, 258A>T, 259G>C, 260T>C, 264A>G,
268C>A, 269C>T, 271C>A, 272T>A, 273A>G, 275G>T, 283A>C, 284G>C, 288T>G, 289C>T, 296T>C, 297C>A, 299T>A, 306C>G, 308G>C, 314A>G, 315T>C, 317T>A, 320G>T,
322C>T, 323T>G, 325A>C, 326T>A, 328T>C, 329G>A, 330T>G, 332C>A, 338T>A, 343T>C, 344G>C, 345G>T, 347C>T, 350T>A, 353T>C, 355G>A, 357T>G, 359A>T, 360C>G,
362T>A, 363A>G, 365T>A, 367A>G, 374G>A, 377C>T, 380A>G, 385G>C, 386T>A, 387G>A, 388C>A, 390C>A, 392T>A, 395A>G, 398T>A, 401A>C, 404T>C, 405C>A, 407T>C,
410A>G, 412C>G, 413G>T, 416C>T, 417T>G, 418C>A, 422C>T, 423T>G, 425G>T, 426T>A, 427C>A, 428C>A, 431A>T, 441T>A, 446C>A, 447A>G, 448A>T, 449G>A, 453A>C,
455G>T, 458T>A, 459T>C, 461A>T, 465T>A, 468T>G, 471G>T, 472A>T, 473G>A, 477T>G, 479G>C, 480T>A, 481C>A, 482C>G, 483A>G, 485T>C, 489G>T, 491T>C, 493A>T,
494A>C, 495T>C, 502T>A, 503T>C, 505C>A, 506T>A, 510G>T, 512G>A, 516A>G, 518T>A, 522A>G, 523G>T, 527A>G, 528G>T, 529A>C, 530T>A, 531G>T, 542T>G, 543G>T,
545A>T, 546T>A, 547A>T, 548T>A, 550G>C, 555C>T, 557T>A, 560A>T, 567A>T, 569A>G, 570A>G, 572A>C, 573T>A, 575G>T, 578A>G, 579G>C, 581G>A, 582C>T, 584T>A,
587A>G, 589T>A, 593T>A, 596T>C, 600A>G, 601G>T, 602T>A, 611T>A, 612G>A, 613A>C, 615G>C, 616G>C, 618G>T, 621T>A, 622C>A, 626C>T, 627A>G, 628C>T, 629T>A,
632C>T, 633A>G, 634A>T, 635A>T, 637A>C, 639A>T, 641C>G, 646T>C, 647G>A, 648A>G, 650A>T, 653C>T, 659C>T, 660A>T, 662G>T, 665T>C, 668A>T, 669A>T, 670G>C,
671T>A, 672C>T, 674A>G, 677C>T, 697A>C, 702G>T, 704C>T, 706A>G, 707A>G, 708C>A, 709G>A, 710A>G, 713C>T, 714A>T, 717A>C, 718T>A, 720G>C, 725C>G, 731T>A,
732A>G, 733G>C, 734T>A, 735A>G, 737G>A, 746G>T, 750T>A, 751C>G, 752A>T, 753G>T, 755T>A, 756T>A, 758A>G, 770G>T, 771T>A, 772C>G, 773T>C, 776G>A, 777T>C,
779G>T, 780A>T, 782G>A, 786A>T, 789A>T, 791A>G, 803G>T, 806T>C, 809G>A, 812G>T, 815G>A, 818G>A, 821C>T, 822A>G, 827A>T, 830G>T, 836T>G, 848C>T, 849T>C,
851G>T, 852A>T, 853G>C, 854C>A, 861T>C, 866C>A, 872T>C, 873C>T, 875C>G, 878T>C, 881G>T, 882A>G, 884A>T, 896T>G, 899G>A, 911A>G, 914A>G, 918A>C, 919C>A,
920T>G, 921C>T, 923T>G, 927C>A, 929T>G, 932T>C, 938A>G, 944C>T, 947C>A, 950C>T, 953G>A, 956T>C, 974A>G, 978A>G, 980A>G, 983C>T, 984G>A, 988C>T, 989C>T,
995A>C, 998C>T, 1010G>A, 1019T>G, 1024G>A, 1028A>T, 1031G>C, 1032C>T, 1034G>A, 1037C>T, 1040T>C, 1046G>T, 1050G>C, 1059C>A, 1060A>C, 1064G>A, 1068G>T,
1069A>C, 1070T>A, 1072C>A, 1073A>G, 1076G>T, 1077T>G, 1078G>C, 1082T>A, 1085T>A, 1086G>T, 1088A>T, 1090C>T, 1100G>A, 1103C>T, 1109C>A, 1113A>C, 1114A>C,
1115G>T, 1118T>A, 1121C>T, 1124T>A, 1126C>T, 1127C>T, 1130A>G, 1134A>C, 1136C>A, 1140C>T, 1142G>A, 1144G>A, 1145G>A, 1148A>G, 1149G>C, 1151A>G, 1154T>C,
1155C>G, 1160G>A, 1163C>A, 1178A>C, 1193G>A, 1199C>T, 1200A>T, 1215A>T, 1216G>C, 1226T>A, 1227G>A, 1232G>A, 1233T>C, 1238A>T, 1242G>T, 1244T>G, 1245T>G,
1246A>T, 1253A>G, 1254A>G, 1255G>C, 1256T>C, 1257T>A, 1259T>C, 1260A>C, 1261G>A, 1262G>A, 1265C>T, 1266A>T, 1267T>C, 1268G>T, 1271C>T, 1280G>A, 1284C>A,
1286T>G, 1292A>G, 1298T>C, 1304A>G, 1313C>T, 1316G>A, 1317T>G, 1319C>T, 1325G>A, 1337T>A, 1340A>G, 1342C>G, 1350T>C, 1352A>T, 1355T>C, 1367G>T, 1368T>C,
1370G>C, 1374A>G, 1375G>C, 1385G>A, 1391A>G, 1394C>G, 1397T>C, 1400G>C, 1401C>T, 1403A>G, 1409A>G, 1418G>A, 1421G>A, 1445T>A, 1448C>T, 1457T>A, 1458A>G,
1460C>G, 1463G>T, 1472C>T, 1478A>T, 1481A>T, 1482T>C, 1484G>T, 1487A>G, 1496T>A, 1505C>T, 1508A>G, 1511T>C, 1514T>A, 1517A>G, 1529T>C, 1538A>T, 1541A>C,
1544A>G, 1547A>T, 1553C>T, 1554T>C, 1556A>T, 1565C>T, 1571T>C, 1577C>T, 1583C>A, 1589T>C, 1592C>T, 1595C>A, 1598C>A, 1604T>C, 1607A>C, 1616A>C, 1623C>T,
1628C>A, 1631A>T, 1637T>A, 1640C>T, 1643A>G, 1649T>G, 1652T>A, 1655A>G, 1661A>T, 1667T>A, 1673G>A, 1674C>A, 1679A>G, 1682G>A, 1685C>T, 1688C>T, 1689C>T,
1691G>A, 1694G>T, 1700T>C, 1702T>G, 1703G>A, 1708A>T, 1715G>T, 1719T>G, 1720C>A, 1721C>T, 1722A>C, 1724T>A, 1725G>A, 1727A>T, 1729A>C, 1733T>A, 1736G>A,
1738G>C, 1739A>C, 1742T>C, 1745C>A, 1746A>G, 1747G>T, 1748A>T, 1752A>G, 1754C>T, 1755A>G, 1757A>G, 1763G>A, 1781T>C, 1787T>A, 1790C>T, 1793G>A, 1794A>G,
1795A>C, 1799G>A, 1800A>T, 1806A>G, 1807G>A, 1811T>C, 1814T>C, 1818G>C, 1820G>A, 1829A>G, 1832C>A, 1835A>G, 1838T>C, 1840A>G, 1844A>G, 1847T>C, 1848G>T,
1849C>A, 1850A>T, 1853A>G, 1856A>G, 1861A>G, 1862G>C, 1866T>C, 1868G>T, 1870T>A, 1872G>A, 1874A>T, 1875G>A, 1878G>A, 1879A>C, 1883T>A, 1886C>A, 1887A>T,
1889A>G, 1890A>G, 1891G>C, 1893C>G, 1894C>A, 1895T>C, 1899A>G, 1901A>T, 1910T>C, 1911C>A, 1913A>T, 1917T>G, 1921C>T, 1922A>T, 1928T>A, 1929G>A, 1932A>C,
1934A>G, 1936C>A, 1937G>T, 1941G>T, 1943G>C, 1944G>C, 1946T>A, 1948G>A, 1949G>T, 1952T>C, 1953_1955delAGA, 1958G>A, 1960G>A, 1961C>A, 1962A>G, 1963C>T,
1964C>T, 1965A>T, 1966A>T, 1969A>C, 1970A>T, 1971G>C, 1972A>C, 1980A>G, 1981T>C, 1983T>A, 1984T>C, 1985G>T, 1986G>T, 1987T>A, 1988G>T, 1989C>G, 1990C>A,
1991C>G, 1992A>C, 1993C>A, 1994T>G, 1995G>A, 1996T>C, 1997T>A, 1998G>A, 2000G>T, 2002G>C, 2003T>A, 2007A>G, 2008A>C, 2012T>A, 2014G>A, 2016G>A, 2018G>T,
2019A>G, 2021C>T, 2024T>C, 2025G>T, 2027A>C, 2028A>C, 2029G>A, 2030A>G, 2033G>A, 2039G>A, 2042A>T, 2048A>C, 2049T>C, 2051G>C, 2060A>T, 2066G>A, 2069C>G,
2073T>A, 2076G>A, 2079C>A, 2080C>A, 2081T>A, 2082G>T, 2083A>T, 2084G>T, 2087A>G, 2088C>T, 2093T>C, 2096T>A, 2102C>T, 2104A>T, 2105T>C, 2106C>T, 2107A>C,
2108T>C, 2117C>T, 2120G>C, 2127T>G, 2128C>T, 2129T>G, 2132T>A, 2133G>T, 2134A>C, 2136G>T, 2137A>C, 2141A>T, 2142C>G, 2143A>C, 2144T>C, 2148G>C, 2152T>A,
2156C>T, 2157A>G, 2159C>A, 2161A>T, 2162A>G, 2166A>G, 2167G>T, 2169G>A, 2170T>A, 2171C>T, 2172A>G, 2174C>T, 2175A>T, 2181T>G, 2182A>T, 2183T>C, 2185G>A,
2193G>A, 2194A>G, 2196G>C, 2199G>C, 2201A>G, 2208C>G, 2209G>C, 2210A>T, 2219A>G, 2221A>G, 2237C>T, 2241A>G, 2243A>C, 2249C>T, 2252T>C, 2268G>A, 2269C>G,
2270A>C, 2271T>A, 2279C>T, 2285G>A, 2286A>G, 2289A>T, 2290A>T, 2291A>G, 2294A>G, 2296C>A, 2297A>T, 2300G>C, 2303G>A, 2304A>C, 2305C>G, 2306A>C, 2307G>A,
2309G>T, 2310A>C, 2312G>T, 2313A>G, 2314G>A, 2316A>C, 2317C>A, 2318T>A, 2321A>G, 2322T>A, 2323T>A, 2324T>A, 2327A>G, 2328C>T, 2331T>A, 2332C>A, 2333T>G,
2334T>G, 2335G>C, 2336C>G, 2337A>C, 2340G>C, 2342T>G, 2345A>T, 2350T>C, 2351T>A, 2354C>T, 2357C>A, 2360C>T, 2362T>C, 2363A>G, 2369C>T, 2372G>A, 2381A>G,
2385T>A, 2386T>G, 2387T>A, 2390T>A, 2396T>C, 2397T>C, 2399A>G, 2405A>T, 2408A>T, 2415A>C, 2417A>C, 2426C>T, 2428A>G, 2429C>G, 2435A>T, 2436T>G,
2438_2439insGGT, 2440A>C, 2441A>T, 2444G>C, 2445C>A, 2446C>A, 2447A>G, 2451G>A, 2452A>G, 2454T>A, 2455C>A, 2456A>T, 2462C>T, 2464A>T, 2465A>C, 2466A>G,
2467T>A, 2468T>A, 2472T>A, 2473A>T, 2474C>G, 2475C>A, 2476A>T, 2478A>G, 2479A>G, 2480A>T, 2484G>A, 2485T>A, 2486T>G, 2489A>C, 2490T>A, 2492T>A, 2495C>G,
2497A>T, 2498G>C, 2499G>C, 2501T>G, 2503C>T, 2504A>G, 2508T>A, 2509G>A, 2510G>T, 2511A>G, 2512G>A, 2514C>T, 2516T>G, 2521A>T, 2522T>C, 2523G>A, 2524C>T,
2525A>C, 2526C>T, 2530G>A, 2531A>G, 2537C>T, 2546A>C, 2549T>A, 2556A>T, 2558A>G, 2561G>A, 2564T>A, 2573A>C, 2574G>A, 2575T>C, 2576T>C, 2577T>A, 2579T>G,
2580G>A, 2581G>A, 2582A>T, 2584G>C, 2586A>G, 2591A>T, 2594A>T, 2597T>C, 2598T>A, 2600G>T, 2601G>A, 2603G>T, 2607T>C, 2608G>T, 2609G>C, 2610C>A, 2613A>G,
2614G>T, 2615T>A, 2616A>G, 2617A>C, 2618C>A, 2619T>A, 2623T>C, 2627C>T, 2628A>T, 2629A>T, 2630T>A, 2633A>T, 2634T>G, 2635C>A, 2636G>T, 2645A>G, 2648T>C,
2651G>A, 2660A>G, 2672A>C, 2678T>C, 2684G>A, 2687C>T, 2690G>A, 2693T>G, 2696T>G, 2698A>C, 2699G>T, 2699_2700insCAT, 2700C>T, 2703A>G, 2704G>C, 2708T>A,
2709C>T, 2710T>C, 2711T>C, 2712C>T, 2714C>G, 2715G>C, 2717T>A, 2720T>G, 2724A>G, 2725G>C, 2726T>G, 2727T>G, 2729T>A, 2730C>G, 2732A>C, 2733T>A, 2734C>A,
2735A>C, 2736T>A, 2737T>A, 2738G>C, 2739G>C, 2740C>A, 2741A>T, 2742C>G, 2743A>T, 2744A>G, 2747T>C, 2747_2748insCGA, 2753T>A, 2755C>A, 2756T>A, 2757G>C,
2759A>G, 2771A>T, 2775C>G, 2777G>A, 2780C>T, 2783C>G, 2784A>C, 2786G>C, 2792T>C, 2798G>T, 2801G>T, 2804A>G, 2808C>A, 2812G>C, 2822G>C, 2825C>T, 2828T>C,
2834C>T, 2837C>G, 2840C>T, 2843G>T, 2858A>T, 2859A>C, 2861G>C, 2864C>T, 2867A>G, 2870T>A, 2876A>C, 2879C>T, 2880C>A, 2881A>G, 2882C>A, 2885T>C, 2886G>A,
2887C>T, 2888T>G, 2889C>A, 2891A>G, 2897T>C, 2903A>T, 2907A>T, 2909A>C, 2915C>T, 2920C>A, 2921C>G, 2922A>C, 2927T>C, 2928C>A, 2929G>T, 2930A>T, 2932C>G,
2936A>T, 2939T>G, 2942A>G, 2944A>T, 2945C>T, 2948C>T, 2953G>C, 2954A>C, 2955G>C, 2964A>T, 2966T>G, 2969T>A, 2975C>T, 2981C>T, 2982A>G, 2984C>G, 2988C>T,
2994C>A, 2996C>G, 2999T>A, 3002T>G, 3017A>C, 3020G>A, 3021C>T, 3023T>A, 3026C>T, 3029C>A, 3030C>A, 3032T>A, 3038C>T, 3044A>T, 3045A>C, 3047A>C, 3050G>A,
3056G>T, 3062C>T, 3064T>A, 3066A>T, 3068A>C, 3078C>A, 3079A>T, 3080A>G, 3083A>C, 3090T>G, 3091G>T, 3092T>G, 3095A>G, 3101T>A, 3104G>T, 3107T>A, 3109C>A,
3110A>G, 3116A>C, 3117C>A, 3118G>A, 3119T>G, 3120C>A, 3121T>A, 3122T>A, 3123G>T, 3124G>C, 3131A>G, 3135T>G, 3137T>G, 3140C>T, 3143G>A, 3144A>T, 3145T>A,
3146G>T, 3167A>C, 3173A>C, 3175T>A, 3176T>A, 3182G>C, 3183G>A, 3184A>C, 3190A>G, 3192C>T, 3194G>A, 3195A>G, 3197C>T, 3200C>G, 3201T>A, 3202C>A, 3203C>T,
3204A>G, 3205G>C, 3206T>A, 3210A>G, 3211A>C, 3212G>T, 3213A>C, 3216T>A, 3221T>A, 3225C>G, 3226A>T, 3227A>T, 3228T>G, 3230T>A, 3236A>G, 3245A>T, 3249G>A,
3251G>T, 3254A>G, 3260T>A, 3263C>T, 3269C>T, 3272G>T, 3273T>C, 3275G>T, 3278A>C, 3279C>T, 3281G>A, 3284A>G, 3289G>C, 3293T>G, 3299T>C, 3305A>G, 3308T>G,
3309G>T, 3310T>G, 3311G>C, 3320C>A, 3323C>A, 3326C>T, 3335A>T, 3343G>A, 3344C>T, 3345G>A, 3346T>C, 3347A>C, 3350A>T, 3353T>A, 3356A>G, 3357C>A, 3358G>A,
3359T>G, 3362A>T, 3364T>A, 3365T>C, 3366A>G, 3368C>G, 3369T>G, 3377T>C, 3380T>A, 3386T>A, 3387A>G, 3389C>G, 3391A>T, 3392C>T, 3395G>A, 3398A>T, 3402C>T,
3404A>G, 3407G>A, 3416A>C, 3419T>G, 3422G>A, 3425G>C, 3426G>A, 3428C>T, 3433C>T, 3434A>G, 3437A>T, 3443A>G, 3452T>A, 3453A>C, 3455T>G, 3456C>T, 3462T>C,
3463C>A, 3464A>C, 3465A>C, 3467T>G, 3470A>G, 3471A>T, 3472G>C, 3475G>A, 3476A>G, 3479A>G, 3480A>G, 3481C>A, 3483C>A, 3484A>G, 3485G>A, 3486C>T, 3488T>C,
3497A>G, 3500C>A, 3503A>G, 3507T>G, 3508A>C, 3509C>T, 3511A>T, 3513C>T, 3515T>G, 3518A>C, 3521A>T, 3524T>A, 3527A>G, 3528C>T, 3529A>T, 3533G>A, 3534T>A,
3539C>T, 3542C>T, 3548T>G, 3551C>A, 3557G>A, 3560C>A, 3563T>C, 3566G>T, 3567C>A, 3568G>A, 3569G>A, 3570A>C, 3571A>G, 3572A>C, 3575C>T, 3577A>C, 3578A>C,
3579T>C, 3587A>G, 3590A>T, 3593C>T, 3596A>G, 3597C>G, 3598C>A, 3602A>T, 3603G>C, 3608A>T, 3611A>C, 3620T>C, 3624A>T, 3626A>T, 3628T>C, 3629T>C, 3630C>A,
3632T>A, 3641A>G, 3644T>A, 3647T>A, 3650T>G, 3656C>T, 3671G>T, 3674A>C, 3675A>G, 3676T>A, 3680A>G, 3683C>T, 3697G>C, 3698T>C, 3699G>A, 3700G>A, 3701A>C,
3707T>G, 3710T>G, 3711G>C, 3712A>C, 3713C>A, 3719C>A, 3722A>G, 3725A>G, 3728A>C, 3731C>T, 3734A>G, 3735A>G, 3736A>G, 3740C>T, 3743G>A, 3744T>G, 3745G>C,
3746C>A, 3749G>C, 3750C>A, 3752G>T, 3755A>T, 3759A>G, 3761C>G, 3762A>C, 3764G>T, 3765C>A, 3766G>A, 3767T>G, 3769G>T, 3770C>G, 3774A>C, 3775A>G, 3776G>A,
3777A>G, 3779C>G, 3782T>C, 3788A>G, 3789C>A, 3790G>A, 3791T>A, 3794A>T, 3795T>G, 3796G>C, 3797T>A, 3798G>A, 3799A>T, 3800A>T, 3803G>A, 3804G>A, 3827A>G,
3838G>T, 3839T>C, 3845C>T, 3847A>T, 3853G>C, 3854C>A, 3860G>A, 3861C>T, 3869A>G, 3872T>A, 3875C>T, 3878A>T, 3884C>T, 3887C>T, 3894A>T, 3898G>A, 3899G>A,
3900A>C, 3902G>T, 3905G>T, 3906A>T, 3907T>C, 3908G>T, 3914G>A, 3917A>G, 3927C>G, 3930T>G, 3933A>C, 3934C>A, 3935T>A, 3936G>C, 3937G>C, 3938C>A, 3941A>C,
3942G>T, 3943G>C, 3944A>G, 3945G>C, 3950A>G, 3953G>T, 3954G>C, 3955A>T, 3956A>T, 3965A>G, 3966G>A, 3971T>A, 3977A>C, 3983A>T, 3996C>T, 3998A>G, 4001T>C,
4002G>T, 4003T>C, 4008A>G, 4010T>A, 4014A>C, 4019C>A, 4021T>C, 4022C>A, 4023C>T, 4024A>T, 4028G>T, 4031T>C, 4034G>T, 4040C>A, 4046A>G, 4049C>T, 4052C>G,
4064C>G, 4065C>T, 4067C>G, 4079A>G, 4088C>G, 4094A>G, 4101A>C, 4103G>C, 4106C>G, 4109T>C, 4112G>A, 4115A>C, 4116C>T, 4118T>G, 4130C>T, 4137T>C, 4142C>G,
4143A>C, 4148G>A, 4151T>C, 4154T>C, 4160T>C, 4163C>T, 4166A>T, 4169C>T, 4170A>T, 4171C>G, 4172A>T, 4182T>G, 4183T>C, 4184G>A, 4190T>C, 4192C>T, 4193T>A,
4194A>C, 4196A>C, 4199A>T, 4205A>G, 4208T>A, 4214T>C, 4215G>A, 4220T>A, 4223A>G, 4226T>C, 4227T>C, 4228C>A, 4239T>G, 4242T>C, 4247A>T, 4249A>G, 4251G>A,
4253G>C, 4259A>G, 4260G>T, 4262T>A, 4265G>A, 4268A>T, 4271C>T, 4273T>A, 4280T>C, 4286T>C, 4292T>C, 4295C>G, 4316T>C, 4317G>A, 4319T>C, 4325T>A, 4331G>A,
4344T>C, 4346G>A, 4354A>C, 4355A>T, 4357A>T, 4358C>T, 4361C>T, 4364A>T, 4365T>G, 4366C>A, 4367A>T, 4368A>G, 4376C>G, 4385T>G, 4388C>A, 4395C>A, 4397A>G,
4400T>C, 4401G>A, 4404C>T, 4406T>A, 4418A>C, 4424T>C, 4430C>T, 4433A>C, 4436G>C, 4439C>T, 4443G>A, 4445T>C, 4448A>G, 4449G>T, 4455A>C, 4457G>C, 4458T>C,
4460G>A, 4463T>G, 4466G>T, 4469C>A, 4475A>C, 4478A>T, 4481A>T, 4487A>T, 4488C>T, 4490A>G, 4493G>T, 4499C>A, 4502C>T, 4508T>A, 4509C>T, 4511C>G, 4514C>A,
4530C>A, 4535T>C, 4538C>G, 4548T>C, 4553A>G, 4556A>G, 4562C>A, 4566A>G, 4568T>C, 4569G>A, 4570C>A, 4571T>A, 4576G>A, 4577G>A, 4578G>T, 4581A>G, 4583A>G,
4586A>C, 4587G>A, 4592A>G, 4597G>A, 4601T>C, 4611C>T, 4613A>G, 4622T>A, 4625T>A, 4631A>T, 4637C>T, 4640T>C, 4641C>T, 4643T>G, 4649T>G, 4652A>T, 4656T>A,
4658A>G, 4662C>T, 4664C>G, 4665C>A, 4666A>G, 4673T>C, 4674T>G, 4675C>A, 4679G>A, 4683A>T, 4684A>C, 4685T>C, 4688A>T, 4692C>A, 4693A>G, 4694T>A, 4697G>A,
4698T>C, 4700A>G, 4704C>G, 4705T>C, 4706G>T, 4709C>T, 4712T>C, 4715A>C, 4716A>G, 4731C>T, 4733T>G, 4739A>G, 4742G>A, 4751T>A, 4752A>G, 4754T>G, 4755G>A,
4758G>A, 4759T>C, 4761C>A, 4768A>T, 4769A>G, 4784A>C, 4787T>C, 4793G>C, 4799A>G, 4805T>C, 4808A>G, 4817T>A, 4818C>T, 4820T>G, 4826C>T, 4829C>A, 4835T>A,
4838T>A, 4844G>A, 4854A>G, 4868G>T, 4871T>C, 4877A>T, 4880A>G, 4883T>A, 4886C>A, 4887C>G, 4888C>T, 4889A>G, 4892T>A, 4901C>T, 4902A>G, 4903C>T, 4904T>A,
4907G>A, 4910C>T, 4914A>T, 4915T>A, 4916G>T, 4923G>A, 4925G>T, 4934A>C, 4937A>C, 4938C>A, 4940T>A, 4946T>C, 4955C>T, 4958T>G, 4960G>A, 4961T>G, 4963T>C,
4964G>C, 4965G>A, 4973C>A, 4975A>G, 4976G>T, 4977C>T, 4982C>A, 4983T>A, 4984T>A, 4985A>T, 4993A>T, 4994G>T, 4995A>G, 4996C>G, 4997G>A, 5000G>T, 5001T>C,
5003G>C, 5004G>A, 5006T>G, 5007G>C, 5009A>G, 5012G>A, 5013G>A, 5014G>A, 5015T>C, 5016T>C, 5019A>C, 5020G>C, 5021A>C, 5022G>A, 5023C>T, 5024T>C, 5025G>A,
5031T>A, 5033T>G, 5036T>C, 5037T>A, 5039C>T, 5042T>C, 5044T>A, 5045G>T, 5049G>C, 5051G>T, 5055C>A, 5056A>G, 5057G>T, 5058G>C, 5064A>T, 5066T>G, 5067G>A,
5070A>G, 5075A>C, 5081G>A, 5085T>G, 5086C>A, 5087A>G, 5091G>A, 5092C>T, 5093C>T, 5094C>G, 5095T>G, 5097A>G, 5098C>T, 5099A>T, 5100A>T, 5102T>G, 5103T>A,
5104G>T, 5105T>G, 5108A>G, 5109A>C, 5111G>A, 5112G>T, 5115A>G, 5117A>T, 5120A>T, 5121G>A, 5122C>A, 5123T>A, 5125T>A, 5127T>A, 5129C>A, 5132T>C, 5135G>C,
5138A>T, 5141G>A, 5142G>C, 5143A>C, 5144C>T, 5147A>T, 5150T>G, 5153G>A, 5159C>T, 5160_5162delACA, 5172C>G, 5173G>A, 5174T>G, 5176A>G, 5178C>G, 5180G>C,
5181A>C, 5184T>G, 5185G>A, 5186G>A, 5192A>G, 5195A>T, 5196A>G, 5198C>T, 5199A>G, 5201T>A, 5202G>T, 5203G>T, 5207T>C, 5211T>A, 5212C>G, 5213A>T, 5216A>T,
5219T>G, 5226G>T, 5228C>A, 5229C>A, 5232T>C, 5234C>A, 5235A>C, 5236C>A, 5237G>A, 5241G>T, 5242A>C, 5243T>A, 5244G>T, 5245A>C, 5249G>T, 5250T>A, 5255T>C,
5256A>C, 5258T>G, 5264C>T, 5267T>C, 5270A>T, 5273T>A, 5276A>C, 5277A>C, 5278A>G, 5279G>C, 5281A>G, 5282G>A, 5283T>A, 5285C>A, 5287A>C, 5288T>A, 5294T>G,
5300T>G, 5303A>T, 5309C>T, 5318C>T, 5333G>A, 5339G>C, 5346A>T, 5349T>G, 5354T>A, 5357A>C, 5358A>C, 5359T>A, 5364G>C, 5365C>A, 5366C>T, 5370A>C, 5371A>G,
5372G>C, 5375T>G, 5376G>A, 5377T>A, 5378G>T, 5379T>C, 5383C>A, 5386G>C, 5387A>T, 5390A>T, 5391C>G, 5395T>G, 5399A>T, 5403A>T, 5404A>C, 5409G>A, 5410T>C,
5411T>C, 5417G>T, 5423A>C, 5429A>T, 5435T>A, 5436G>A, 5438C>A, 5440G>C, 5441C>T, 5447T>C, 5456G>A, 5457C>T, 5460A>T, 5462G>T, 5465C>A, 5468A>G, 5471A>C,
5474A>T, 5475A>C, 5476G>A, 5477G>T, 5483T>C, 5484T>A, 5486T>G, 5488T>C, 5489G>C, 5490G>A, 5491A>C, 5492G>A, 5496A>C, 5498A>T, 5501A>T, 5504T>G, 5505A>C,
5507A>C, 5508T>C, 5510G>A, 5513C>A, 5516A>C, 5519T>G, 5521G>C, 5522A>T, 5523A>G, 5524A>G, 5525T>G, 5531T>G, 5534C>T, 5537C>G, 5540T>C, 5546T>C, 5549T>C,
5555C>T, 5560T>C, 5564G>A, 5567T>C, 5570T>G, 5573T>C, 5574C>A, 5576A>G, 5582G>T, 5583C>G, 5584C>A, 5585T>A, 5586C>G, 5588T>C, 5588_5589insACC, 5589G>T,
5590G>C, 5592G>C, 5597A>G, 5598G>T, 5602C>G, 5603T>A, 5606A>G, 5612T>C, 5613G>A, 5617C>A, 5618G>T, 5619C>G, 5620T>G, 5622_5624delCAC, 5626G>C, 5627T>C,
5629G>C, 5635T>A, 5636G>C, 5642A>T, 5642_5643insCACAGT, 5644G>C, 5645G>A, 5649G>T, 5650A>C, 5652T>G, 5654G>T, 5657C>A, 5658A>C, 5659A>T, 5660C>T,
5663C>A, 5664C>A, 5666C>G, 5669T>A, 5670A>G, 5672A>T, 5674A>C, 5676G>T, 5677G>C, 5678T>C, 5681T>C, 5682T>C, 5684A>C, 5685G>T, 5687A>T, 5688A>G, 5691C>A,

	5695C>T, 5696A>G, 5700A>G, 5701T>C, 5702G>T, 5705G>T, 5706A>T, 5714T>C, 5715G>A, 5716T>C, 5717G>A, 5718G>A, 5720A>T, 5723A>T, 5726A>T, 5727C>G, 5729T>A, 5735A>T, 5740G>C, 5741A>C, 5742A>C, 5744T>G, 5746T>G, 5747T>C, 5750C>A, 5753A>G, 5754C>A, 5757G>A, 5759T>C, 5762T>G, 5763G>A, 5766A>C, 5767G>A, 5769T>G, 5770T>C, 5771G>T, 5772C>T, 5774A>G, 5775C>G, 5777C>G, 5778T>A, 5779A>C, 5780T>A, 5783G>A, 5784G>T, 5785A>C, 5786G>T, 5787C>G, 5788A>T, 5789G>C, 5790G>A, 5792A>T, 5798A>T, 5799G>A, 5800A>C, 5801G>T, 5802T>C, 5804A>T, 5805A>G, 5806T>C, 5807T>C, 5808C>G, 5809A>C, 5812A>G, 5813G>A, 5814A>G, 5815A>G, 5816T>A, 5817A>G, 5818C>T, 5819A>C, 5823C>G, 5825A>G, 5828T>A, 5829C>T, 5837C>T, 5843G>A, 5844C>T, 5846G>C, 5847A>T, 5848G>C, 5849T>C, 5850A>C, 5851T>A, 5852G>A, 5853G>C, 5855A>C, 5856A>G, 5859G>T, 5860T>G, 5861G>T, 5862C>G, 5865C>A, 5866A>T, 5867A>G, 5868C>A, 5870G>A, 5871A>C, 5873A>T, 5876T>G, 5880G>C, 5882T>G, 5883A>G, 5885G>A, 5886C>A, 5887T>G, 5891G>A, 5895T>G, 5897T>A, 5898T>G, 5900C>A, 5901C>T, 5902C>T, 5903A>C, 5905A>T, 5906A>G, 5908A>G, 5909A>G, 5911C>G, 5912T>C, 5913A>G, 5914C>T, 5915C>T, 5917T>A, 5918T>A, 5919C>A, 5921G>A, 5922A>C, 5924G>A, 5925C>A, 5927C>G, 5928A>T, 5929A>T, 5930A>G, 5931A>T, 5932A>C, 5933T>C, 5936T>C, 5937G>A, 5939T>G, 5940G>A, 5943G>C, 5944C>A, 5945T>A, 5946G>C, 5948A>G, 5949T>A, 5950G>T, 5951T>G, 5952C>T, 5953T>C, 5956C>A, 5958T>A, 5959T>C, 5961A>C, 5965C>T, 5966A>T, 5967A>G, 5968G>C, 5969G>C, 5972_5973insCCATGTTGTGTCACA, 5978G>T, 5979C>A, 5981C>A, 5983T>C, 5985G>T, 5986T>G, 5987T>G, 5988T>C, 5990C>A, 5992A>C, 5993A>T, 5997A>G, 5999A>C, 6000C>A, 6003T>G, 6005G>C, 6007G>T, 6008T>G, 6009T>A, 6011C>A, 6013C>A, 6015T>G, 6017G>T, 6020A>G, 6022T>A, 6024T>C, 6025G>C, 6026T>A, 6029T>A, 6030_6032delGAA, 6033A>T, 6034G>C, 6039_6050delGTTGCTTCAAGG, 6051T>C, 6056A>T, 6059G>A, 6061G>C, 6062T>A, 6063C>G, 6065A>G, 6068C>T, 6070C>T, 6074C>G, 6078T>C, 6079T>C, 6080G>T, 6083T>A, 6086T>A, 6087T>C, 6088C>T, 6089C>A, 6092A>T, 6093T>G, 6097A>G, 6102T>A, 6104A>G, 6108G>C, 6109A>G, 6110A>C, 6112G>A, 6113A>T, 6114C>A, 6115A>G, 6116C>T, 6117A>G, 6118C>G, 6121G>A, 6122A>G, 6126A>T, 6127T>A, 6128G>C, 6129A>T, 6130G>C, 6131T>A, 6135C>A, 6136A>C, 6137G>A, 6139G>C, 6140T>A, 6141A>C, 6146G>A, 6153C>T, 6155G>T, 6157A>G, 6161A>G, 6164T>C, 6165C>A, 6167A>G, 6169T>G, 6170A>T, 6171A>C, 6173C>G, 6174T>G, 6175T>A, 6176C>A, 6177C>G, 6178A>G, 6179A>G, 6180G>T, 6181T>A, 6182A>T, 6183A>T, 6186A>G, 6187C>A, 6188A>T, 6190G>A, 6191T>A, 6193A>C, 6194G>C, 6198T>G, 6200A>G, 6201A>G, 6203G>C, 6204A>T, 6205A>G, 6206G>C, 6209T>C, 6210G>A, 6211A>G, 6212A>T, 6215T>G, 6219T>A, 6221T>G, 6222C>G, 6223A>G, 6224G>C, 6226A>T, 6227G>T, 6228A>T, 6229A>G, 6230G>T, 6231A>G, 6233A>G, 6234T>C, 6236C>G, 6237A>G, 6238C>A, 6239C>A, 6240A>C, 6241C>G, 6243C>A, 6245A>C, 6246C>T, 6248A>G, 6249A>G, 6251C>G, 6252A>C, 6253A>G, 6254A>C, 6255G>A, 6256T>C, 6258T>A, 6260C>T, 6261T>G, 6262T>C, 6263G>T, 6270G>T, 6271A>C, 6272G>C, 6273G>A, 6274A>C, 6276A>T, 6277G>C, 6278T>A, 6280A>T, 6281T>G, 6282C>A, 6284G>C, 6285A>G, 6286C>A, 6287T>G, 6288G>A, 6290G>T, 6294T>A, 6295G>C, 6297A>G, 6299A>G, 6300G>T, 6302T>C, 6303T>A, 6305T>A, 6306C>G, 6308G>T, 6310T>G, 6314C>T, 6315C>T, 6316A>T, 6317G>T, 6318A>G, 6319G>A, 6320G>T, 6321_6323delATA, 6324A>C, 6326C>T, 6328C>T, 6330C>A, 6332G>A, 6333A>G, 6334A>C, 6336T>G, 6338C>G, 6339A>G, 6340G>A, 6341A>C, 6342G>T, 6344G>C, 6345C>A, 6348G>C, 6349A>C, 6350A>T, 6353T>G, 6359T>C, 6361A>C, 6362T>C, 6364T>G, 6366C>G, 6368T>A, 6369C>A, 6370C>T, 6372G>A, 6374A>T, 6376G>A, 6377A>T, 6378A>G, 6379T>C, 6383C>T, 6389T>C, 6392C>T, 6394T>C, 6402A>G, 6404A>G, 6405A>T, 6407A>G, 6411T>G, 6412C>A, 6413T>A, 6416G>C, 6420G>A, 6421A>G, 6423T>C, 6424C>A, 6427A>G, 6428T>G, 6429A>T, 6430G>C, 6431C>A, 6432C>A, 6433G>A, 6434A>G, 6435G>T, 6436A>C, 6437T>A, 6438A>C, 6439G>A, 6440T>A, 6442A>G, 6444T>A, 6445C>A, 6446C>G, 6448A>T, 6449T>C, 6450T>A, 6453G>T, 6455G>T, 6456T>G, 6458A>C, 6460C>A, 6462C>G, 6463C>A, 6464C>A, 6465A>G, 6467C>T, 6468A>G, 6471C>A, 6473G>A, 6474G>T, 6475G>C, 6476G>A, 6477T>A, 6478G>A, 6479T>C, 6480T>C, 6482C>G, 6483A>C, 6488C>A, 6489C>G, 6490T>C, 6494C>T, 6495G>T, 6496T>A, 6497G>T, 6498C>T, 6503T>A, 6505G>A, 6507C>A, 6509A>G, 6510G>C, 6511G>T, 6514A>C, 6515G>A, 6517A>G, 6518A>T, 6519G>C, 6520A>T, 6521T>A, 6522C>G, 6524T>G, 6525C>T, 6526C>A, 6528G>T, 6529T>A, 6531C>G, 6532G>A, 6533G>T, 6534A>T, 6535G>C, 6536G>C, 6537T>A, 6539T>A, 6540T>G, 6541C>A, 6543A>T, 6545G>T, 6546G>T, 6548T>G, 6549T>G, 6550T>A, 6552C>T, 6554T>G, 6555A>T, 6556A>T, 6558A>C, 6560C>A, 6561T>A, 6562G>T, 6563T>A, 6566A>T, 6572T>A, 6576A>G, 6582T>G, 6583A>T, 6588G>T, 6590G>C, 6594T>G, 6596A>G, 6597G>T, 6598T>C, 6599C>G, 6600A>G, 6602G>A, 6603C>T, 6605T>A, 6608delT, 6612T>C, 6615A>T, 6618T>G, 6619G>C, 6620T>A, 6621G>C, 6622C>A, 6623G>T, 6624A>G, 6626A>T, 6629C>T, 6643G>A, 6647_6648insACAAACAT, 6649T>A, 6652A>G, 6654C>T, 6655G>T, 6657T>G, 6658A>T, 6659G>A, 6660G>T, 6661A>C, 6663G>A, 6664G>C, 6667T>G, 6669G>A, 6672A>C, 6683C>T, 6687C>T, 6688T>C, 6692A>G, 6695A>G, 6696G>A, 6715T>C

CDS

ToMarV_RNA1gp1	1	2152	100%	9863	66.5%	2135 (98.7%)	1360 (62.9%)	11/17/0/0	0

	Begin	End	Coverage	Score	Concordance	Matches	Identities	I/D/M/F*	Stop Codons
**NT**	**25**	**6747**	**93.0%**	**3297**	**25.1%**	**6658 (98.4%)**	**4218 (62.4%)**	**41/65**	

Protein mutations:

F7W (160T>G 161C>G), K9_A16del (165_188delAAGGTTGCTGGTGTTGATGACGCC), S17P (189A>C 190G>C), S18P (192T>C), S19A (195T>G), T21S (201A>T 203C>A), T22P (204A>C), G26A (217G>C 218T>A), L28V (222C>G), N30S (228A>T 229A>C), I32F (234A>T), K34S (240A>T 241A>C 242A>T), F35L (243T>C), A38T (252G>A 254G>T), V39L (255G>C 257T>C), I42V (264A>G), T43N (268C>A 269C>T), T44K (271C>A 272T>A), M45V (273A>G 275G>T), K48T (283A>C 284G>C), S50V (288T>G 289C>T), L53I (297C>A 299T>A), L56V (306C>G 308G>C), S59P (315T>C 317T>A), T61M (322C>T 323T>G), D62A (325A>C 326T>A), V63A (328T>C 329G>A), S64A (330T>G 332C>A), V68A (343T>C 344G>C), A69S (345G>T 347C>T), S72N (355G>A), S73A (357T>G 359A>T), L74V (360C>G 362T>A), N75E (363A>G 365T>A), K76R (367A>G), G82A (385G>C 386T>A), A83K (387G>A 388C>A), L84I (390C>A 392T>A), I85M (395A>G), D86E (398T>A), L89I (405C>A 407T>C), A91G (412C>G 413G>T), S93D (417T>G 418C>A), L95V (423T>G 425G>T), S96K (426T>A 427C>A 428C>A), S101T (441T>A), K103V (447A>G 448A>T 449G>A), M105L (453A>C 455G>T), L109M (465T>A), F110V (468T>G), E111L (471G>T 472A>T 473G>A), L113V (477T>G 479G>C), S114K (480T>A 481C>A 482C>G), N115D (483A>G 485T>C), V117F (489G>T 491T>C), K118I (493A>T 494A>C), F121Y (502T>A 503T>C), A122E (505C>A 506T>A), V124L (510G>T 512G>A), N126E (516A>G 518T>A), S128V (522A>G 523G>T), D130S (528G>T 529A>C 530T>A), V131L (531G>T), V135F (543G>T 545A>T), Y136I (546T>A 547A>T 548T>A), G137A (550G>C), I143L (567A>T 569A>G), T144A (570A>G 572A>C), L145I (573T>A 575G>T), V147L (579G>C 581G>A), F150Y (589T>A), S154V (600A>G 601G>T 602T>A), E158T (612G>A 613A>C), G159P (615G>C 616G>C), V160F (618G>T), S161N (621T>A 622C>A), T163V (627A>G 628C>T 629T>A), K165V (633A>G 634A>T 635A>T), N166T (637A>C), I167L (639A>T 641C>G), L169S (646T>C 647G>A), I170V (648A>G 650A>T), M174F (660A>T 662G>T), K186T (697A>C), A188S (702G>T 704C>T), E189G (706A>G 707A>G), R190K (708C>A 709G>A 710A>G), T192S (714A>T), M193Q (717A>C 718T>A), V194L (720G>C), S198A (732A>G 733G>C 734T>A), M199V (735A>G 737G>A), V205L (753G>T 755T>A), S206T (756T>A 758A>G), M214L (780A>T 782G>A), T216S (786A>T), I217L (789A>T 791A>G), I228V (822A>G), T248A (882A>G 884A>T), T260Q (918A>C 919C>A 920T>G), I280V (978A>G 980A>G), G282S (984G>A), T283I (988C>T 989C>T), S295N (1024G>A), E304Q (1050G>C), Q307T (1059C>A 1060A>C), D310S (1068G>T 1069A>C 1070T>A), A311E (1072C>A 1073A>G), C313A (1077T>G 1078G>C), D314E (1082T>A), A316S (1086G>T 1088A>T), T317M (1090C>T), K325P (1113A>C 1114A>C 1115G>T), T329I (1126C>T 1127C>T), N332Q (1134A>C 1136C>A), R335K (1144G>A 1145G>A), E337Q (1149G>C 1151A>G), L339V (1155C>G), M354L (1200A>T), A363T (1227G>A), E366D (1238A>T), V368L (1242G>T 1244T>G), Y369V (1245T>G 1246A>T), S372A (1254A>G 1255G>C 1256T>C), C373S (1257T>A 1259T>C), R374Q (1260A>C 1261G>A 1262G>A), M376S (1266A>T 1267T>C 1268G>T), S393A (1317T>G 1319C>T), S401C (1342C>G), R412A (1374A>G 1375G>C), I440V (1458A>G 1460C>G), M521R (1702T>G 1703G>A), E523V (1708A>T), E525D (1715G>T), S527D (1719T>G 1720C>A 1721C>T), T528P (1722A>C 1724T>A), E529N (1725G>A 1727A>T), D530A (1729A>C), G533A (1738G>C 1739A>C), R536V (1746A>G 1747G>T 1748A>T), N538D (1752A>G 1754C>T), I539V (1755A>G 1757A>G), N552A (1794A>G 1795A>C), T554S (1800A>T), S556D (1806A>G 1807G>A), E560Q (1818G>C 1820G>A), Q567R (1840A>G), A570Y (1848G>T 1849C>A 1850A>T), K574S (1861A>G 1862G>C), F577Y (1870T>A), E578N (1872G>A 1874A>T), A579T (1875G>A), E580T (1878G>A 1879A>C), I583L (1887A>T 1889A>G), S584A (1890A>G 1891G>C), P585D (1893C>G 1894C>A 1895T>C), I587V (1899A>G 1901A>T), Q591N (1911C>A 1913A>T), S593A (1917T>G), A594V (1921C>T 1922A>T), D596E (1928T>A), V597M (1929G>A), I598L (1932A>C 1934A>G), A599D (1936C>A 1937G>T), V601F (1941G>T 1943G>C), D602Q (1944G>C 1946T>A), G603D (1948G>A 1949G>T), R605del (1953_1955delAGA), R607Q (1960G>A 1961C>A), T608V (1962A>G 1963C>T 1964C>T), K609L (1965A>T 1966A>T), Q610P (1969A>C 1970A>T), D611P (1971G>C 1972A>C), I614A (1980A>G 1981T>C), L615T (1983T>A 1984T>C 1985G>T), V616Y (1986G>T 1987T>A 1988G>T), P617E (1989C>G 1990C>A 1991C>G), T618Q (1992A>C 1993C>A 1994T>G), V619T (1995G>A 1996T>C 1997T>A), V620I (1998G>A 2000G>T), S621T (2002G>C 2003T>A), N623A (2007A>G 2008A>C), D624E (2012T>A), R625K (2014G>A), V626I (2016G>A 2018G>T), I627V (2019A>G 2021C>T), A629S (2025G>T 2027A>C), R630Q (2028A>C 2029G>A 2030A>G), L645M (2073T>A), D646N (2076G>A), P647K (2079C>A 2080C>A 2081T>A), E648F (2082G>T 2083A>T 2084G>T), L650F (2088C>T), H655L (2104A>T 2105T>C), H656S (2106C>T 2107A>C 2108T>C), M660I (2120G>C), S663V (2127T>G 2128C>T 2129T>G), D665S (2133G>T 2134A>C), D666S (2136G>T 2137A>C), E667D (2141A>T), H668A (2142C>G 2143A>C 2144T>C), E670Q (2148G>C), I671N (2152T>A), N673E (2157A>G 2159C>A), K674M (2161A>T 2162A>G), R676V (2166A>G 2167G>T), V677N (2169G>A 2170T>A 2171C>T), N678D (2172A>G 2174C>T), M679L (2175A>T), Y681V (2181T>G 2182A>T 2183T>C), R682K (2185G>A), D685S (2193G>A 2194A>G), A686P (2196G>C), E687Q (2199G>C 2201A>G), R690A (2208C>G 2209G>C 2210A>T), Q694R (2221A>G), I701V (2241A>G 2243A>C), A710S (2268G>A 2269C>G 2270A>C), S711T (2271T>A), K716E (2286A>G), K717L (2289A>T 2290A>T 2291A>G), A719D (2296C>A 2297A>T), T722R (2304A>C 2305C>G 2306A>C), E723N (2307G>A 2309G>T), M724L (2310A>C 2312G>T), R725E (2313A>G 2314G>A), T726Q (2316A>C 2317C>A 2318T>A), F728K (2322T>A 2323T>A 2324T>A), H730Y (2328C>T), S731K (2331T>A 2332C>A 2333T>G), C732A (2334T>G 2335G>C 2336C>G), I733L (2337A>C), D734Q (2340G>C 2342T>G), V737A (2350T>C 2351T>A), I741T (2362T>C 2363A>G), F749R (2385T>A 2386T>G 2387T>A), N763R (2428A>G 2429C>G), S766A (2436T>G), S766_K767insG (2438_2439insGGT), K767T (2440A>C 2441A>T), E768D (2444G>C), P769K (2445C>A 2446C>A 2447A>G), E771R (2451G>A 2452A>G), S772N (2454T>A 2455C>A 2456A>T), K775I (2464A>T 2465A>C), I776E (2466A>G 2467T>A 2468T>A), Y778M (2472T>A 2473A>T 2474C>G), H779I (2475C>A 2476A>T), K780G (2478A>G 2479A>G 2480A>T), V782K (2484G>A 2485T>A 2486T>G), E783D (2489A>C), Y784K (2490T>A 2492T>A), I785M (2495C>G), K786I (2497A>T 2498G>C), D787Q (2499G>C 2501T>G), T788M (2503C>T 2504A>G), W790N (2508T>A 2509G>A 2510G>T), S791D (2511A>G 2512G>A), Y794F (2521A>T 2522T>C), A795I (2523G>A 2524C>T 2525A>C), R797K (2530G>A 2531A>G), I806L (2556A>T 2558A>G), M807I (2561G>A), V812T (2574G>A 2575T>C 2576T>C), F813M (2577T>A 2579T>G), G814N (2580G>A 2581G>A 2582A>T), G815A (2584G>C), I816V (2586A>G), L820I (2598T>A 2600G>T), A821T (2601G>A 2603G>T), W823L (2607T>C 2608G>T 2609G>C), Q824K (2610C>A), S825V (2613A>G 2614G>T 2615T>A), N826A (2616A>G 2617A>C 2618C>A), S827T (2619T>A), F828S (2623T>C), N830L (2628A>T 2629A>T 2630T>A), S832D (2634T>G 2635C>A 2636G>T), N852K (2696T>G), K853T (2698A>C 2699G>T), K853_L854insH (2699_2700insCAT), L854F (2700C>T), R855A (2703A>G 2704G>C), N856K (2708T>A), L857S (2709C>T 2710T>C 2711T>C), V859L (2715G>C 2717T>A), S862A (2724A>G 2725G>C 2726T>G), S863A (2727T>G 2729T>A), Q864D (2730C>G 2732A>C), S865N (2733T>A 2734C>A 2735A>C), L866N (2736T>A 2737T>A 2738G>C), A867H (2739G>C 2740C>A 2741A>T), Q868V (2742C>G 2743A>T 2744A>G), D869_W870insR (2747_2748insCGA), A872E (2755C>A 2756T>A), E873Q (2757G>C 2759A>G), Q879E (2775C>G 2777G>A), K882H (2784A>C 2786G>C), L890I (2808C>A), G891A (2812G>C), M907L (2859A>C 2861G>C), H914R (2880C>A 2881A>G 2882C>A), A916M (2886G>A 2887C>T 2888T>G), T923S (2907A>T 2909A>C), A927E (2920C>A 2921C>G), N928H (2922A>C), R930I (2928C>A 2929G>T 2930A>T), S931C (2932C>G), K932N (2936A>T), Y935F (2944A>T 2945C>T), R938T (2953G>C 2954A>C), E939Q (2955G>C), I942L (2964A>T 2966T>G), I948V (2982A>G 2984C>G), P961S (3021C>T 3023T>A), E972D (3056G>T), F975Y (3064T>A), T976S (3066A>T 3068A>C), Q980M (3078C>A 3079A>T 3080A>G), C984V (3090T>G 3091G>T 3092T>G), T990K (3109C>A 3110A>G), R993K (3117C>A 3118G>A 3119T>G), L994K (3120C>A 3121T>A 3122T>A), G995S (3123G>T 3124G>C), F999V (3135T>G 3137T>G), M1002Y (3144A>T 3145T>A 3146G>T), V1012E (3175T>A 3176T>A), E1015T (3183G>A 3184A>C), E1017G (3190A>G), N1019D (3195A>G 3197C>T), S1021N (3201T>A 3202C>A 3203C>T), S1022A (3204A>G 3205G>C 3206T>A), K1024A (3210A>G 3211A>C 3212G>T), I1025L (3213A>C), C1026S (3216T>A), Q1029V (3225C>G 3226A>T 3227A>T), S1030A (3228T>G 3230T>A), G1037S (3249G>A 3251G>T), S1050T (3289G>C), V1057C (3309G>T 3310T>G 3311G>C), G1068D (3343G>A 3344C>T), V1069T (3345G>A 3346T>C 3347A>C), R1073K (3357C>A 3358G>A 3359T>G), F1075Y (3364T>A 3365T>C), I1076V (3366A>G 3368C>G), S1077A (3369T>G), I1083V (3387A>G 3389C>G), Y1084F (3391A>T 3392C>T), E1086D (3398A>T), V1096I (3426G>A 3428C>T), A1098V (3433C>T 3434A>G), D1104E (3452T>A), I1105L (3453A>C 3455T>G), S1108H (3462T>C 3463C>A 3464A>C), I1109L (3465A>C 3467T>G), R1112K (3475G>A 3476A>G), T1114E (3480A>G 3481C>A), Q1115R (3483C>A 3484A>G 3485G>A), L1116F (3486C>T 3488T>C), N1120K (3500C>A), Y1123A (3507T>G 3508A>C 3509C>T), Y1124F (3511A>T), Q1130L (3528C>T 3529A>T), L1132M (3534T>A), R1143K (3567C>A 3568G>A 3569G>A), K1144R (3570A>C 3571A>G 3572A>C), Q1146P (3577A>C 3578A>C), P1153D (3597C>G 3598C>A), E1155Q (3603G>C), T1162S (3624A>T 3626A>T), V1163A (3628T>C 3629T>C), H1164K (3630C>A 3632T>A), D1169E (3647T>A), M1179E (3675A>G 3676T>A), C1186S (3697G>C 3698T>C), G1187N (3699G>A 3700G>A 3701A>C), D1189E (3707T>G), D1190E (3710T>G), D1191P (3711G>C 3712A>C 3713C>A), N1199G (3735A>G 3736A>G), C1202A (3744T>G 3745G>C 3746C>A), L1204I (3750C>A 3752G>T), I1207V (3759A>G 3761C>G), M1208L (3762A>C 3764G>T), R1209K (3765C>A 3766G>A 3767T>G), S1210M (3769G>T 3770C>G), K1212R (3774A>C 3775A>G 3776G>A), I1213V (3777A>G 3779C>G), R1217K (3789C>A 3790G>A 3791T>A), C1219A (3795T>G 3796G>C 3797T>A), E1220I (3798G>A 3799A>T 3800A>T), D1222N (3804G>A), C1233F (3838G>T 3839T>C), Y1236F (3847A>T), G1238A (3853G>C 3854C>A), M1252L (3894A>T), G1253E (3898G>A 3899G>A), M1254L (3900A>C 3902G>T), M1256S (3906A>T 3907T>C 3908G>T), Q1263E (3927C>G), S1264A (3930T>G), T1265Q (3933A>C 3934C>A 3935T>A), G1266P (3936G>C 3937G>C 3938C>A), E1267D (3941A>C), G1268S (3942G>T 3943G>C 3944A>G), E1269Q (3945G>C), E1272L (3954G>C 3955A>T 3956A>T), D1276N (3966G>A), E1281D (3983A>T), V1288S (4002G>T 4003T>C), I1290V (4008A>G 4010T>A), K1292Q (4014A>C), I1294T (4021T>C 4022C>A), H1295F (4023C>T 4024A>T), T1344C (4170A>T 4171C>G 4172A>T), L1348A (4182T>G 4183T>C 4184G>A), A1351V (4192C>T 4193T>A), I1352L (4194A>C 4196A>C), D1359N (4215G>A), S1363H (4227T>C 4228C>A), L1367V (4239T>G), N1370S (4249A>G), G1371S (4251G>A 4253G>C), A1374S (4260G>T 4262T>A), V1378E (4273T>A), V1393I (4317G>A 4319T>C), E1405A (4354A>C 4355A>T), Y1406F (4357A>T 4358C>T), S1409D (4365T>G 4366C>A 4367A>T), K1410E (4368A>G), D1415E (4385T>G), A1421T (4401G>A), D1435N (4443G>A 4445T>C), V1437L (4449G>T), I1476V (4566A>G 4568T>C), A1477K (4569G>A 4570C>A 4571T>A), R1479K (4576G>A 4577G>A), A1480S (4578G>T), I1481V (4581A>G 4583A>G), V1483I (4587G>A), S1486N (4597G>A), L1506M (4656T>A 4658A>G), Q1509R (4665C>A 4666A>G), S1512D (4674T>G 4675C>A), N1515S (4683A>T 4684A>C 4685T>C), H1518R (4692C>A 4693A>G 4694T>A), L1522A (4704C>G 4705T>C 4706G>T), I1526V (4716A>G), N1538E (4752A>G 4754T>G), E1539K (4755G>A), V1540T (4758G>A 4759T>C), Q1541K (4761C>A), Q1543L (4768A>T 4769A>G), H1563Q (4829C>A), I1572V (4854A>G), M1576I (4868G>T), P1583V (4887C>G 4888C>T 4889A>G), T1588V (4902A>G 4903C>T 4904T>A), M1592Y (4914A>T 4915T>A 4916G>T), V1595I (4923G>A 4925G>T), R1607Q (4960G>A 4961T>G), V1608A (4963T>C 4964G>C), A1609T (4965G>A), D1611E (4973C>A), K1612S (4975A>G 4976G>T), L1613F (4977C>T), L1615N (4983T>A 4984T>A 4985A>T), Q1618L (4993A>T 4994G>T), T1619G (4995A>G 4996C>G 4997G>A), D1622K (5004G>A 5006T>G), E1623Q (5007G>C 5009A>G), G1625N (5013G>A 5014G>A 5015T>C), F1626L (5016T>C), R1627P (5019A>C 5020G>C 5021A>C), A1628I (5022G>A 5023C>T 5024T>C), V1629M (5025G>A), F1631M (5031T>A 5033T>G), S1633T (5037T>A 5039C>T), L1635Y (5044T>A 5045G>T), V1637L (5049G>C 5051G>T), Q1639S (5055C>A 5056A>G 5057G>T), E1640Q (5058G>C), I1642L (5064A>T 5066T>G), A1643T (5067G>A), K1644E (5070A>G), S1649E (5085T>G 5086C>A 5087A>G), A1651I (5091G>A 5092C>T 5093C>T), L1652G (5094C>G 5095T>G), T1653V (5097A>G 5098C>T 5099A>T), I1654L (5100A>T 5102T>G), C1655M (5103T>A 5104G>T 5105T>G), M1657L (5109A>C 5111G>A), A1658S (5112G>T), K1659D (5115A>G 5117A>T), A1661K (5121G>A 5122C>A 5123T>A), I1662N (5125T>A), S1663T (5127T>A 5129C>A), D1668P (5142G>C 5143A>C 5144C>T), T1674del (5160_5162delACA), R1678E (5172C>G 5173G>A 5174T>G), D1679G (5176A>G), L1680V (5178C>G 5180G>C), N1681H (5181A>C), W1682E (5184T>G 5185G>A 5186G>A), N1686D (5196A>G 5198C>T), I1687V (5199A>G 5201T>A), G1688F (5202G>T 5203G>T), A1696S (5226G>T 5228C>A), H1697N (5229C>A), Y1698Q (5232T>C 5234C>A), T1699Q (5235A>C 5236C>A 5237G>A), D1701S (5241G>T 5242A>C 5243T>A), E1702S (5244G>T 5245A>C), Y1704N (5250T>A), I1706L (5256A>C 5258T>G), K1713R (5277A>C 5278A>G 5279G>C), K1714R (5281A>G 5282G>A), S1715T (5283T>A 5285C>A), N1716T (5287A>C 5288T>A), M1731I (5333G>A), M1736L (5346A>T), S1737A (5349T>G), M1740Q (5358A>C 5359T>A), A1742H (5364G>C 5365C>A 5366C>T), K1744R (5370A>C 5371A>G 5372G>C), N1745K (5375T>G), V1746N (5376G>A 5377T>A 5378G>T), A1748E (5383C>A), G1749A (5386G>C 5387A>T), H1751D (5391C>G), V1752G (5395T>G), N1755S (5403A>T 5404A>C), V1757T (5409G>A 5410T>C 5411T>C), V1766I (5436G>A 5438C>A), G1767A (5440G>C 5441C>T), M1774F (5460A>T 5462G>T), R1779H (5475A>C 5476G>A 5477G>T), C1782R (5484T>A 5486T>G), V1783A (5488T>C 5489G>C), E1784T (5490G>A 5491A>C 5492G>A), I1786L (5496A>C 5498A>T), I1793M (5519T>G), G1794A (5521G>C 5522A>T), N1795G (5523A>G 5524A>G 5525T>G), V1807A (5560T>C), F1810L (5570T>G), Q1812K (5574C>A 5576A>G), P1815E (5583C>G 5584C>A 5585T>A), H1816D (5586C>G 5588T>C), H1816_G1817insT (5588_5589insACC), G1817S (5589G>T 5590G>C), D1818H (5592G>C), V1820L (5598G>T), A1821G (5602C>G 5603T>A), A1825T (5613G>A), S1826Y (5617C>A 5618G>T), L1827G (5619C>G 5620T>G), H1828del (5622_5624delCAC), S1829T (5626G>C 5627T>C), S1830T (5629G>C), M1832N (5635T>A 5636G>C), Q1834H (5642A>T), Q1834_G1835insHS (5642_5643insCACAGT), G1835A (5644G>C 5645G>A), E1837S (5649G>T 5650A>C), L1838V (5652T>G 5654G>T), N1840L (5658A>C 5659A>T 5660C>T), I1844V (5670A>G 5672A>T), H1845P (5674A>C), G1846S (5676G>T 5677G>C 5678T>C), A1849S (5685G>T 5687A>T), K1850E (5688A>G), Q1851K (5691C>A), T1852M (5695C>T 5696A>G), M1854A (5700A>G 5701T>C 5702G>T), M1856L (5706A>T), V1859T (5715G>A 5716T>C 5717G>A), V1860I (5718G>A 5720A>T), H1863E (5727C>G 5729T>A), G1867A (5740G>C 5741A>C), N1868Q (5742A>C 5744T>G), F1869C (5746T>G 5747T>C), Q1872K (5754C>A), D1873N (5757G>A 5759T>C), E1875K (5763G>A), S1876H (5766A>C 5767G>A), L1877A (5769T>G 5770T>C 5771G>T), H1879E (5775C>G 5777C>G), Y1880T (5778T>A 5779A>C 5780T>A), E1882S (5784G>T 5785A>C 5786G>T), Q1883V (5787C>G 5788A>T 5789G>C), G1884S (5790G>A 5792A>T), E1887T (5799G>A 5800A>C 5801G>T), I1889A (5805A>G 5806T>C 5807T>C), Q1890A (5808C>G 5809A>C), K1891R (5812A>G 5813G>A), N1892G (5814A>G 5815A>G 5816T>A), T1893V (5817A>G 5818C>T 5819A>C), L1895V (5823C>G 5825A>G), L1902F (5844C>T 5846G>C), M1904Q (5850A>C 5851T>A 5852G>A), E1905H (5853G>C 5855A>C), N1906D (5856A>G), V1907C (5859G>T 5860T>G 5861G>T), Q1908E (5862C>G), Q1909M (5865C>A 5866A>T 5867A>G), L1910I (5868C>A 5870G>A), I1911L (5871A>C 5873A>T), D1912E (5876T>G), V1914L (5880G>C 5882T>G), K1915E (5883A>G 5885G>A), L1916S (5886C>A 5887T>G), F1919V (5895T>G 5897T>A), F1920V (5898T>G 5900C>A), P1921F (5901C>T 5902C>T 5903A>C), K1922M (5905A>T 5906A>G), E1923G (5908A>G 5909A>G), T1924S (5911C>G 5912T>C), T1925V (5913A>G 5914C>T 5915C>T), I1926K (5917T>A 5918T>A), Q1927K (5919C>A 5921G>A), K1928Q (5922A>C 5924G>A), L1929M (5925C>A 5927C>G), K1930L (5928A>T 5929A>T 5930A>G), N1931S (5931A>T 5932A>C 5933T>C), V1933M (5937G>A 5939T>G), D1934N (5940G>A), A1935Q (5943G>C 5944C>A 5945T>A), E1936Q (5946G>C 5948A>G), C1937M (5949T>A 5950G>T 5951T>G), L1938S (5952C>T 5953T>C), T1939K (5956C>A), F1940T (5958T>A 5959T>C), K1941Q (5961A>C), A1942V (5965C>T 5966A>T), R1943A (5967A>G 5968G>C 5969G>C), S1944_F1945insPCCVT (5972_5973insCCATGTTGTGTCACA), R1946S (5978G>T), H1947K (5979C>A 5981C>A), V1948A (5983T>C), V1949W (5985G>T 5986T>G 5987T>G), F1950L (5988T>C 5990C>A), K1951T (5992A>C 5993A>T), K1953D (5997A>G 5999A>C), L1954I (6000C>A), L1955V (6003T>G 6005G>C), S1956M (6007G>T 6008T>G), S1957T (6009T>A 6011C>A), T1958K (6013C>A), W1959G (6015T>G 6017G>T), M1961K (6022T>A), C1962P (6024T>C 6025G>C 6026T>A), E1964del (6030_6032delGAA), V1967_R1970del (6039_6050delGTTGCTTCAAGG), S1971P (6051T>C), S1974T (6061G>C 6062T>A), L1975V (6063C>G 6065A>G), P1977L (6070C>T), L1980P (6078T>C 6079T>C 6080G>T), S1983L (6087T>C 6088C>T 6089C>A), L1985V (6093T>G), K1986R (6097A>G), L1988M (6102T>A 6104A>G), E1990R (6108G>C 6109A>G 6110A>C), R1991N (6112G>A 6113A>T), H1992S (6114C>A 6115A>G 6116C>T), T1993G (6117A>G 6118C>G), R1994K (6121G>A 6122A>G), M1996Y (6126A>T 6127T>A

6128G>C), Q1999T (6135C>A 6136A>C 6137G>A), S2000T (6139G>C 6140T>A), M2001L (6141A>C), L2005F (6153C>T 6155G>T), K2006R (6157A>G), I2007M (6161A>G), L2009M (6165C>A 6167A>G), L2010C (6169T>G 6170A>T), N2011Q (6171A>C 6173C>G), F2012E (6174T>G 6175T>A 6176C>A), Q2013G (6177C>G 6178A>G 6179A>G), V2014Y (6180G>T 6181T>A 6182A>T), I2015L (6183A>T), T2016D (6186A>G 6187C>A 6188A>T), S2017K (6190G>A 6191T>A), E2018A (6193A>C 6194G>C), L2020V (6198T>G 6200A>G), K2021D (6201A>G 6203G>C), K2022C (6204A>T 6205A>G 6206G>C), E2024S (6210G>A 6211A>G 6212A>T), D2025E (6215T>G), F2027M (6219T>A 6221T>G), Q2028G (6222C>G 6223A>G 6224G>C), E2029V (6226A>T 6227G>T), K2030C (6228A>T 6229A>G 6230G>T), I2031V (6231A>G 6233A>G), S2032P (6234T>C 6236C>G), T2033E (6237A>G 6238C>A 6239C>A), T2034R (6240A>C 6241C>G), L2035I (6243C>A 6245A>C), I2037V (6249A>G 6251C>G), K2038R (6252A>C 6253A>G 6254A>C), V2039T (6255G>A 6256T>C), F2040I (6258T>A 6260C>T), L2041A (6261T>G 6262T>C 6263G>T), E2044S (6270G>T 6271A>C 6272G>C), E2045T (6273G>A 6274A>C), Y2047L (6280A>T 6281T>G), Q2048N (6282C>A 6284G>C), T2049E (6285A>G 6286C>A 6287T>G), E2050N (6288G>A 6290G>T), C2052T (6294T>A 6295G>C), I2053V (6297A>G 6299A>G), D2054Y (6300G>T 6302T>C), S2055T (6303T>A 6305T>A), Q2056D (6306C>G 6308G>T), V2057G (6310T>G), Q2059F (6315C>T 6316A>T 6317G>T), R2060D (6318A>G 6319G>A 6320G>T), I2061del (6321_6323delATA), N2062H (6324A>C 6326C>T), S2063F (6328C>T), Q2064K (6330C>A 6332G>A), K2065A (6333A>G 6334A>C), F2066V (6336T>G 6338C>G), R2067D (6339A>G 6340G>A 6341A>C), V2068F (6342G>T 6344G>C), Q2069K (6345C>A), E2070P (6348G>C 6349A>C 6350A>T), Y2074S (6361A>C 6362T>C), L2075W (6364T>G), H2076E (6366C>G 6368T>A), P2077I (6369C>A 6370C>T), E2078N (6372G>A 6374A>T), G2079D (6376G>A 6377A>T), I2080A (6378A>G 6379T>C), V2085A (6394T>C), I2088V (6402A>G 6404A>G), I2089L (6405A>T 6407A>G), S2091E (6411T>G 6412C>A 6413T>A), E2094R (6420G>A 6421A>G), S2095H (6423T>C 6424C>A), Y2096W (6427A>G 6428T>G), R2098K (6432C>A 6433G>A 6434A>G), D2099S (6435G>T 6436A>C 6437T>A), S2100Q (6438A>C 6439G>A 6440T>A), N2101S (6442A>G), S2102K (6444T>A 6445C>A 6446C>G), Y2103F (6448A>T 6449T>C), S2104T (6450T>A), V2105F (6453G>T 6455G>T), S2106A (6456T>G 6458A>C), T2107N (6460C>A), P2108E (6462C>G 6463C>A 6464C>A), I2109V (6465A>G 6467C>T), K2110E (6468A>G), L2111I (6471C>A 6473G>A), G2112S (6474G>T 6475G>C 6476G>A), C2113N (6477T>A 6478G>A 6479T>C), F2114L (6480T>C 6482C>G), I2115L (6483A>C), F2116L (6488C>A), L2117A (6489C>G 6490T>C), V2119Y (6495G>T 6496T>A 6497G>T), D2121E (6503T>A), S2122N (6505G>A), Q2123K (6507C>A 6509A>G), G2124L (6510G>C 6511G>T), K2125T (6514A>C 6515G>A), E2126G (6517A>G 6518A>T), D2127L (6519G>C 6520A>T 6521T>A), H2128E (6522C>G 6524T>G), P2129Y (6525C>T 6526C>A), V2130Y (6528G>T 6529T>A), R2131D (6531C>G 6532G>A 6533G>T), R2132S (6534A>T 6535G>C 6536G>C), F2133I (6537T>A 6539T>A), S2134E (6540T>G 6541C>A), T2135S (6543A>T 6545G>T), V2136L (6546G>T 6548T>G), F2137D (6549T>G 6550T>A), K2139L (6555A>T 6556A>T), N2140Q (6558A>C 6560C>A), C2141I (6561T>A 6562G>T 6563T>A), E2142D (6566A>T), T2146A (6576A>G), Y2148V (6582T>G 6583A>T), E2150Y (6588G>T 6590G>C), *2152G (6594T>G 6596A>G)

	Begin	End	Coverage	Score	Concordance	Matches	Identities	I/D/M/F*	Stop Codons
**NT**	**25**	**6747**	**93.0%**	**3297**	**25.1%**	**6658 (98.4%)**	**4218 (62.4%)**	**41/65**	

GCT2GCA (146T>A), TTC7TGG (160T>G 161C>G), TCC8AGT (162T>A 163C>G 164C>T), AAG9_GCC16del (165_188delAAGGTTGCTGGTGTTGATGACGCC), AGT17CCT (189A>C 190G>C), TCT18CCT (192T>C), TCT19GCT (195T>G), TCC20TCT (200C>T), ACC21TCA (201A>T 203C>A), ACT22CCT (204A>C), GGT26GCA (217G>C 218T>A), CTT28GTT (222C>G), TCA29TCT (227A>T), AAT30TCT (228A>T 229A>C), ATT32TTT (234A>T), TCC33TCT (239C>T), AAA34TCT (240A>T 241A>C 242A>T), TTT35CTT (243T>C), AAA37AAG (251A>G), GCG38ACT (252G>A 254G>T), GTT39CTC (255G>C 257T>C), AGT40TCC (258A>T 259G>C 260T>C), ATT42GTT (264A>G), ACC43AAT (268C>A 269C>T), ACT44AAA (271C>A 272T>A), ATG45GTT (273A>G 275G>T), AAG48ACC (283A>C 284G>C), TCA50GTA (288T>G 289C>T), CAT52CAC (296T>C), CTT53ATA (297C>A 299T>A), CTG56GTC (306C>G 308G>C), CCA58CCG (314A>G), TCT59CCA (315T>C 317T>A), GTG60GTT (320G>T), ACT61ATG (322C>T 323T>G), GAT62GCA (325A>C 326T>A), GTG63GCA (328T>C 329G>A), TCC64GCA (330T>G 332C>A), TCT66TCA (338T>A), GTG68GCC (343T>C 344G>C), GCC69TCT (345G>T 347C>T), ACT70ACA (350T>A), TGT71TGC (353T>C), AGT72AAT (355G>A), TCA73GCT (357T>G 359A>T), CTT74GTA (360C>G 362T>A), AAT75GAA (363A>G 365T>A), AAA76AGA (367A>G), TTG78TTA (374G>A), GAC79GAT (377C>T), AAA80AAG (380A>G), GGT82GCA (385G>C 386T>A), GCA83AAA (387G>A 388C>A), CTT84ATA (390C>A 392T>A), ATA85ATG (395A>G), GAT86GAA (398T>A), CCA87CCC (401A>C), TTT88TTC (404T>C), CTT89ATC (405C>A 407T>C), AAA90AAG (410A>G), GCG91GGT (412C>G 413G>T), TAC92TAT (416C>T), TCT93GAT (417T>G 418C>A), TTC94TTT (422C>T), TTG95GTT (423T>G 425G>T), TCC96AAA (426T>A 427C>A 428C>A), ACA97ACT (431A>T), TCT101ACT (441T>A), ATC102ATA (446C>A), AAG103GTA (447A>G 448A>T 449G>A), ATG105CTT (453A>C 455G>T), GTT106GTA (458T>A), TTA107CTT (459T>C 461A>T), TTG109ATG (465T>A), TTT110GTT (468T>G), GAG111TTA (471G>T 472A>T 473G>A), TTG113GTC (477T>G 479G>C), TCC114AAG (480T>A 481C>A 482C>G), AAT115GAC (483A>G 485T>C), GTT117TTC (489G>T 491T>C), AAA118ATC (493A>T 494A>C), TTA119CTA (495T>C), TTT121TAC (502T>A 503T>C), GCT122GAA (505C>A 506T>A), GTG124TTA (510G>T 512G>A), AAT126GAA (516A>G 518T>A), AGT128GTT (522A>G 523G>T), GAA129GAG (527A>G), GAT130TCA (528G>T 529A>C 530T>A), GTA131TTA (531G>T), GTT134GTG (542T>G), GTA135TTT (543G>T 545A>T), TAT136ATA (546T>A 547A>T 548T>A), GGT137GCT (550G>C), CTT139TTA (555C>T 557T>A), GTA140GTT (560A>T), ATA143TTG (567A>T 569A>G), ACA144GCC (570A>G 572A>C), TTG145ATT (573T>A 575G>T), TTA146TTG (578A>G), GTG147CTA (579G>C 581G>A), CTT148TTA (582C>T 584T>A), CTA149CTG (587A>G), TTT150TAT (589T>A), GTT151GTA (593T>A), TGT152TGC (596T>C), AGT154GTA (600A>G 601G>T 602T>A), GTT157GTA (611T>A), GAG158ACG (612G>A 613A>C), GGT159CCT (615G>C 616G>C), GTT160TTT (618G>T), TCT161AAT (621T>A 622C>A), AAC162AAT (626C>T), ACT163GTA (627A>G 628C>T 629T>A), GTC164GTT (632C>T), AAA165GTT (633A>G 634A>T 635A>T), AAT166ACT (637A>C), ATC167TTG (639A>T 641C>G), TTG169TCA (646T>C 647G>A), ATA170GTT (648A>G 650A>T), GTC171GTT (653C>T), AAC173AAT (659C>T), ATG174TTT (660A>T 662G>T), TTT175TTC (665T>C), TCA176TCT (668A>T), AGT177TCA (669A>T 670G>C 671T>A), CTA178TTG (672C>T 674A>G), TAC179TAT (677C>T), AAA186ACA (697A>C), GCC188TCT (702G>T 704C>T), GAA189GGG (706A>G 707A>G), CGA190AAG (708C>A 709G>A 710A>G), TTC191TTT (713C>T), ACC192TCC (714A>T), ATG193CAG (717A>C 718T>A), GTG194CTG (720G>C), GCC195GCG (725C>G), GCT197GCA (731T>A), AGT198GCA (732A>G 733G>C 734T>A), ATG199GTA (735A>G 737G>A), GGG202GGT (746G>T), TCA204AGT (750T>A 751C>G 752A>T), GTT205TTA (753G>T 755T>A), TCA206ACG (756T>A 758A>G), ACG210ACT (770G>T), TCT211AGC (771T>A 772C>G 773T>C), CAG212CAA (776G>A), TTG213CTT (777T>C 779G>T), ATG214TTA (780A>T 782G>A), ACA216TCA (786A>T), ATA217TTG (789A>T 791A>G), GGG221GGT (803G>T), ATT222ATC (806T>C), TCG223TCA (809G>A), ACG224ACT (812G>T), TTG225TTA (815G>A), GTG226GTA (818G>A), TTC227TTT (821C>T), ATT228GTT (822A>G), GCA229GCT (827A>T), GTG230GTT (830G>T), GGT232GGG (836T>G), GGC236GGT (848C>T), TTG237CTT (849T>C 851G>T), AGC238TCA (852A>T 853G>C 854C>A), TTG241CTG (861T>C), TCC242TCA (866C>A), ATT244ATC (872T>C), CTC245TTG (873C>T 875C>G), TAT246TAC (878T>C), TCG247TCT (881G>T), ACA248GCT (882A>G 884A>T), GCT252GCG (896T>G), CAG253CAA (899G>A), CAA257CAG (911A>G), CTA258CTG (914A>G), ACT260CAG (918A>C 919C>A 920T>G), CTT261TTG (921C>T 923T>G), CGT263AGG (927C>A 929T>G), AAT264AAC (932T>C), AAA266AAG (938A>G), TGC268TGT (944C>T), ACC269ACA (947C>A), TCC270TCT (950C>T), CAG271CAA (953G>A), GCT272GCC (956T>C), GAA278GAG (974A>G), ATA280GTG (978A>G 980A>G), GTC281GTT (983C>T), GGC282AGC (984G>A), ACC283ATT (988C>T 989C>T), GGA285GGC (995A>C), TTC286TTT (998C>T), GTG290GTA (1010G>A), GCT293GCG (1019T>G), AGT295AAT (1024G>A), GCA296GCT (1028A>T), ACG297ACC (1031G>C), CTG298TTA (1032C>T 1034G>A), TCC299TCT (1037C>T), ACT300ACC (1040T>C), CTG302CTT (1046G>T), GAG304CAG (1050G>C), CAG307ACG (1059C>A 1060A>C), GAG308GAA (1064G>A), GAT310TCA (1068G>T 1069A>C 1070T>A), GCA311GAG (1072C>A 1073A>G), GTG312GTT (1076G>T), TGT313GCT (1077T>G 1078G>C), GAT314GAA (1082T>A), CCT315CCA (1085T>A), GCA316TCT (1086G>T 1088A>T), ACG317ATG (1090C>T), GAG320GAA (1100G>A), AAC321AAT (1103C>T), GCC323GCA (1109C>A), AAG325CCT (1113A>C 1114A>C 1115G>T), GCT326GCA (1118T>A), TTC327TTT (1121C>T), CCT328CCA (1124T>A), ACC329ATT (1126C>T 1127C>T), AAA330AAG (1130A>G), AAC332CAA (1134A>C 1136C>A), CTG334TTA (1140C>T 1142G>A), AGG335AAA (1144G>A 1145G>A), GAA336GAG (1148A>G), GAA337CAG (1149G>C 1151A>G), GCT338GCC (1154T>C), CTC339GTC (1155C>G), AAG340AAA (1160G>A), ATC341ATA (1163C>A), GCA346GCC (1178A>C), GCG351GCA (1193G>A), TTC353TTT (1199C>T), ATG354TTG (1200A>T), AGT359TCT (1215A>T 1216G>C), ATT362ATA (1226T>A), GCG363ACG (1227G>A), CAG364CAA (1232G>A), TTA365CTA (1233T>C), GAA366GAT (1238A>T), GTT368TTG (1242G>T 1244T>G), TAT369GTT (1245T>G 1246A>T), GAA371GAG (1253A>G), AGT372GCC (1254A>G 1255G>C 1256T>C), TGT373AGC (1257T>A 1259T>C), AGG374CAA (1260A>C 1261G>A 1262G>A), CAC375CAT (1265C>T), ATG376TCT (1266A>T 1267T>C 1268G>T), GGC377GGT (1271C>T), CAG380CAA (1280G>A), CGT382AGG (1284C>A 1286T>G), GAA384GAG (1292A>G), TTT386TTC (1298T>C), GTA388GTG (1304A>G), TTC391TTT (1313C>T), GGG392GGA (1316G>A), TCC393GCT (1317T>G 1319C>T), GGG395GGA (1325G>A), TCT399TCA (1337T>A), ACA400ACG (1340A>G), TCC401TGC (1342C>G), TTA404CTT (1350T>C 1352A>T), TTT405TTC (1355T>C), GTG409GTT (1367G>T), TTG410CTC (1368T>C 1370G>C), AGA412GCA (1374A>G 1375G>C), GAG415GAA (1385G>A), AAA417AAG (1391A>G), CTC418CTG (1394C>G), AAT419AAC (1397T>C), CGG420CGC (1400G>C), CTA421TTG (1401C>T 1403A>G), GCA423GCG (1409A>G), AAG426AAA (1418G>A), AGG427AGA (1421G>A), GCT435GCA (1445T>A), CAC436CAT (1448C>T), GCT439GCA (1457T>A), ATC440GTG (1458A>G 1460C>G), CTG441CTT (1463G>T), GAC444GAT (1472C>T), GGA446GGT (1478A>T), GCA447GCT (1481A>T), TTG448CTT (1482T>C 1484G>T), CGA449CGG (1487A>G), GCT452GCA (1496T>A), TGC455TGT (1505C>T), CAA456CAG (1508A>G), GAT457GAC (1511T>C), ATT458ATA (1514T>A), AAA459AAG (1517A>G), GAT463GAC (1529T>C), TCA466TCT (1538A>T), ACA467ACC (1541A>C), CAA468CAG (1544A>G), CCA469CCT (1547A>T), CCC471CCT (1553C>T), TTA472CTT (1554T>C 1556A>T), GCC475GCT (1565C>T), GTT477GTC (1571T>C), GAC479GAT (1577C>T), GGC481GGA (1583C>A), CAT483CAC (1589T>C), TTC484TTT (1592C>T), ACC485ACA (1595C>A), TCC486TCA (1598C>A), TAT488TAC (1604T>C), ATA489ATC (1607A>C), ACA492ACC (1616A>C), CTG495TTG (1623C>T), ATC496ATA (1628C>A), TCA497TCT (1631A>T), CCT499CCA (1637T>A), GCC500GCT (1640C>T), CAA501CAG (1643A>G), GGT503GGG (1649T>G), CTT504CTA (1652T>A), ACA505ACG (1655A>G), CCA507CCT (1661A>T), GCT509GCA (1667T>A), GAG511GAA (1673G>A), CGG512AGG (1674C>A), AGA513AGG (1679A>G), AGG514AGA (1682G>A), GAC515GAT (1685C>T), ATC516ATT (1688C>T), CTG517TTA (1689C>T 1691G>A), GTG518GTT (1694G>T), TGT520TGC (1700T>C), ATG521AGA (1702T>G 1703G>A), GAG523GTG (1708A>T), GAG525GAT (1715G>T), TCC527GAT (1719T>G 1720C>A 1721C>T), ACT528CCA (1722A>C 1724T>A), GAA529AAT (1725G>A 1727A>T), GAT530GCT (1729A>C), CCT531CCA (1733T>A), ACG532ACA (1736G>A), GGA533GCC (1738G>C 1739A>C), CAT534CAC (1742T>C), CTC535CTA (1745C>A), AGA536GTT (1746A>G 1747G>T 1748A>T), AAC538GAT (1752A>G 1754C>T), ATA539GTG (1755A>G 1757A>G), GAG541GAA (1763G>A), GCT547GCC (1781T>C), ACT549ACA (1787T>A), CAC550CAT (1790C>T), AGG551AGA (1793G>A), AAT552GCT (1794A>G 1795A>C), TTG553TTA (1799G>A), ACC554TCC (1800A>T), AGT556GAT (1806A>G 1807G>A), GAT557GAC (1811T>C), CTT558CTC (1814T>C), GAG560CAA (1818G>C 1820G>A), GTA563GTG (1829A>G), GCC564GCA (1832C>A), AAA565AAG (1835A>G), TGT566TGC (1838T>C), CAG567CGG (1840A>G), GTA568GTG (1844A>G), CAT569CAC (1847T>C), GCA570TAT (1848G>T 1849C>A 1850A>T), GAA571GAG (1853A>G), GTA572GTG (1856A>G), AAG574AGC (1861A>G 1862G>C), TTG576CTT (1866T>C 1868G>T), TTT577TAT (1870T>A), GAA578AAT (1872G>A 1874A>T), GCT579ACT (1875G>A), GAA580ACA (1878G>A 1879A>C), TCT581TCA (1883T>A), GGC582GGA (1886C>A), ATA583TTG (1887A>T 1889A>G), AGT584GCT (1890A>G 1891G>C), CCT585GAC (1893C>G 1894C>A 1895T>C), ATA587GTT (1899A>G 1901A>T), GTT590GTC (1910T>C), CAA591AAT (1911C>A 1913A>T), TCT593GCT (1917T>G), GCA594GTT (1921C>T 1922A>T), GAT596GAA (1928T>A), GTG597ATG (1929G>A), ATA598CTG (1932A>C 1934A>G), GCG599GAT (1936C>A 1937G>T), GTG601TTC (1941G>T 1943G>C), GAT602CAA (1944G>C 1946T>A), GGG603GAT (1948G>A 1949G>T), GCT604GCC (1952T>C), AGA605del (1953_1955delAGA), TTG606TTA (1958G>A), CGC607CAA (1960G>A 1961C>A), ACC608GTT (1962A>G 1963C>T 1964C>T), AAG609TTG (1965A>T 1966A>T), CAA610CCT (1969A>C 1970A>T), GAT611CCT (1971G>C 1972A>C), ATA614GCA (1980A>G 1981T>C), TTG615ACT (1983T>A 1984T>C 1985G>T), GTG616TAT (1986G>T 1987T>A 1988G>T), CCC617GAG (1989C>G 1990C>A 1991C>G), ACT618CAG (1992A>C 1993C>A 1994T>G), GTT619ACA (1995G>A 1996T>C 1997T>A), GTG620ATT (1998G>A 2000G>T), AGT621ACA (2002G>C 2003T>A), AAT623GCT (2007A>G 2008A>C), GAT624GAA (2012T>A), AGA625AAA (2014G>A), GTG626ATT (2016G>A 2018G>T), ATC627GTT (2019A>G 2021C>T), TAT628TAC (2024T>C), GCA629TCC (2025G>T 2027A>C), AGA630CAG (2028A>C 2029G>A 2030A>G), GAG631GAA (2033G>A), ACG633ACA (2039G>A), GTA634GTT (2042A>T), GCA636GCC (2048A>C), TTG637CTC (2049T>C 2051G>C), GCA640GCT (2060A>T), CAG642CAA (2066G>A), GGC643GGG (2069C>G), TTG645ATG (2073T>A), GAC646AAC (2076G>A), CCT647AAA (2079C>A 2080C>A 2081T>A), GAG648TTT (2082G>T 2083A>T 2084G>T), GAA649GAG (2087A>G), CTT650TTT (2088C>T), TTT651TTC (2093T>C), CCT652CCA (2096T>A), GAC654GAT (2102C>T), CAT655CTC (2104A>T 2105T>C), CAT656TCC (2106C>T 2107A>C 2108T>C), GCC659GCT (2117C>T), ATG660ATC (2120G>C), TCT663GTG (2127T>G 2128C>T 2129T>G), CTT664CTA (2132T>A), GAT665TCT (2133G>T 2134A>C), GAT666TCT (2136G>T 2137A>C), GAA667GAT (2141A>T), CAT668GCC (2142C>G 2143A>C 2144T>C), GAG670CAG (2148G>C), ATT671AAT (2152T>A), TTC672TTT (2156C>T), AAC673GAA (2157A>G 2159C>A), AAA674ATG (2161A>T 2162A>G), AGA676GTA (2166A>G 2167G>T), GTC677AAT (2169G>A 2170T>A 2171C>T), AAC678GAT (2172A>G 2174C>T), ATG679TTG (2175A>T), TAT681GTC (2181T>G 2182A>T 2183T>C), AGA682AAA (2185G>A), GAT685AGT (2193G>A 2194A>G), GCT686CCT (2196G>C), GAA687CAG (2199G>C 2201A>G), CGA690GCT (2208C>G 2209G>C 2210A>T), GTA693GTG (2219A>G), CAA694CGA (2221A>G), GAC699GAT (2237C>T), ATA701GTC (2241A>G 2243A>C), CAC703CAT (2249C>T), TTT704TTC (2252T>C), GCA710AGC (2268G>A 2269C>G 2270A>C), TCA711ACA (2271T>A), ATC713ATT (2279C>T), GAG715GAA (2285G>A), AAG716GAG (2286A>G), AAA717TTG (2289A>T 2290A>T 2291A>G), CTA718CTG (2294A>G), GCA719GAT (2296C>A 2297A>T), GTG720GTC (2300G>C), CAG721CAA (2303G>A), ACA722CGC (2304A>C 2305C>G 2306A>C), GAG723AAT (2307G>A 2309G>T), ATG724CTT (2310A>C 2312G>T), AGG725GAG (2313A>G 2314G>A), ACT726CAA (2316A>C 2317C>A 2318T>A), GGA727GGG (2321A>G), TTT728AAA (2322T>A 2323T>A 2324T>A), GCA729GCG (2327A>G), CAT730TAT (2328C>T), TCT731AAG (2331T>A 2332C>A 2333T>G), TGC732GCG (2334T>G 2335G>C 2336C>G), ATT733CTT (2337A>C), GAT734CAG (2340G>C 2342T>G), GCA735GCT (2345A>T), GTT737GCA (2350T>C 2351T>A), GAC738GAT (2354C>T), ACC739ACA (2357C>A), CTC740CTT (2360C>T), ATA741ACG (2362T>C 2363A>G), ATC743ATT (2369C>T), GAG744GAA (2372G>A), CCA747CCG (2381A>G), TTT749AGA (2385T>A 2386T>G 2387T>A), GTT750GTA (2390T>A), TTT752TTC (2396T>C), TTA753CTG (2397T>C 2399A>G), ACA755ACT (2405A>T), GCA756GCT (2408A>T), AGA759CGC (2415A>C 2417A>C), TGC762TGT (2426C>T), AAC763AGG (2428A>G 2429C>G), GTA765GTT (2435A>T), TCA766GCA (2436T>G), TCA766_AAA767insGGT (2438_2439insGGT), AAA767ACT (2440A>C 2441A>T), GAG768GAC (2444G>C), CCA769AAG (2445C>A 2446C>A 2447A>G), GAA771AGA (2451G>A 2452A>G), TCA772AAT (2454T>A 2455C>A 2456A>T), ATC774ATT (2462C>T), AAA775ATC (2464A>T 2465A>C), ATT776GAA (2466A>G 2467T>A 2468T>A), TAC778ATG (2472T>A 2473A>T 2474C>G), CAT779ATT (2475C>A 2476A>T), AAA780GGT (2478A>G 2479A>G 2480A>T), GTT782AAG (2484G>A 2485T>A 2486T>G), GAA783GAC (2489A>C), TAT784AAA (2490T>A 2492T>A), ATC785ATG (2495C>G), AAG786ATC (2497A>T 2498G>C), GAT787CAG (2499G>C 2501T>G), ACA788ATG (2503C>T 2504A>G), TGG790AAT (2508T>A 2509G>A 2510G>T), AGC791GAC (2511A>G 2512G>A), CTT792TTG (2514C>T 2516T>G), TAT794TTC (2521A>T 2522T>C), GCA795ATC (2523G>A 2524C>T 2525A>C), CTG796TTG (2526C>T), AGA797AAG (2530G>A 2531A>G), CTC799CTT (2537C>T), GCA802GCC (2546A>C), GGT803GGA (2549T>A), ATA806TTG (2556A>T 2558A>G), ATG807ATA (2561G>A), GCT808GCA (2564T>A), GGA811GGC (2573A>C), GTT812ACC (2574G>A 2575T>C 2576T>C), TTT813ATG (2577T>A 2579T>G), GGA814AAT (2580G>A 2581G>A 2582A>T), GGC815GCC (2584G>C), ATT816GTT (2586A>G), ACA817ACT (2591A>T), GCA818GCT (2594A>T), TTT819TTC (2597T>C), TTG820ATT (2598T>A 2600G>T), GCG821ACT (2601G>A 2603G>T), TGG823CTC (2607T>C 2608G>T 2609G>C), CAG824AAG (2610C>A), AGT825GTA (2613A>G 2614G>T 2615T>A), AAC826GCA (2616A>G 2617A>C 2618C>A), TCT827ACT (2619T>A), TTC828TCC (2623T>C), CCC829CCT (2627C>T), AAT830TTA (2628A>T 2629A>T 2630T>A), GCA831GCT (2633A>T), TCG832GAT (2634T>G 2635C>A 2636G>T), GGA835GGG (2645A>G), GGT836GGC (2648T>C), GTG837GTA (2651G>A), GAA840GAG (2660A>G), ATA844ATC (2672A>C), AGT846AGC (2678T>C), AAG848AAA (2684G>A), AAC849AAT (2687C>T), AAG850AAA (2690G>A), GGT851GGG (2693T>G), AAT852AAG (2696T>G), AAG853ACT (2698A>C 2699G>T), AAG853_CTC854insCAT (2699_2700insCAT), CTC854TTC (2700C>T), AGA855GCA (2703A>G 2704G>C), AAT856AAA (2708T>A), CTT857TCC (2709C>T 2710T>C 2711T>C), CTC858TTG (2712C>T 2714C>G), GTT859CTA (2715G>C 2717T>A), GGT860GGG (2720T>G), AGT862GCG (2724A>G 2725G>C 2726T>G), TCT863GCA (2727T>G 2729T>A), CAA864GAC (2730C>G 2732A>C), TCA865AAC (2733T>A 2734C>A 2735A>C), TTG866AAC (2736T>A 2737T>A 2738G>C), GCA867CAT (2739G>C 2740C>A 2741A>T), CAA868GTG (2742C>G 2743A>T 2744A>G), GAT869GAC (2747T>C), GAT869_TGG870insCGA (2747_2748insCGA), GCT871GCA (2753T>A), GCT872GAA (2755C>A 2756T>A), GAA873CAG (2757G>C 2759A>G), GTA877GTT (2771A>T), CAG879GAA (2775C>G 2777G>A), AGC880AGT (2780C>T), CTC881CTG (2783C>G), AAG882CAC (2784A>C 2786G>C), AAT884AAC (2792T>C), GTG886GTT (2798G>T), GTG887GTT (2801G>T), TTA888TTG (2804A>G), CTT890ATT (2808C>A), GGT891GCT (2812G>C), GTG894GTC (2822G>C), TAC895TAT (2825C>T), TTT896TTC (2828T>C), GGC898GGT (2834C>T), ACC899ACG (2837C>G), TAC900TAT (2840C>T), GTG901GTT (2843G>T), ATA906ATT (2858A>T), ATG907CTC (2859A>C 2861G>C), ACC908ACT (2864C>T), GTA909GTG (2867A>G), GCT910GCA (2870T>A), GCA912GCC (2876A>C), TTC913TTT (2879C>T), CAC914AGA (2880C>A 2881A>G 2882C>A), AAT915AAC (2885T>C), GCT916ATG (2886G>A 2887C>T 2888T>G), CGA917AGG (2889C>A 2891A>G), GGT919GGC (2897T>C), CCA921CCT (2903A>T), ACA923TCC (2907A>T 2909A>C), ATC925ATT (2915C>T), GCC927GAG (2920C>A 2921C>G), AAT928CAT (2922A>C), TCT929TCC (2927T>C), CGA930ATT (2928C>A 2929G>T 2930A>T), TCT931TGT (2932C>G), AAA932AAT (2936A>T), GTT933GTG (2939T>G), CAA934CAG (2942A>G), TAC935TTT (2944A>T 2945C>T), AAC936AAT (2948C>T), AGA938ACC (2953G>C 2954A>C), GAA939CAA (2955G>C), ATT942TTG (2964A>T 2966T>G), ATT943ATA (2969T>A), GGC945GGT (2975C>T), GAC947GAT (2981C>T), ATC948GTG (2982A>G 2984C>G), CTG950TTG (2988C>T), CGC952AGG (2994C>A 2996C>G), GTT953GTA (2999T>A), GGT954GGG (3002T>G), CCA959CCC (3017A>C), AAG960AAA (3020G>A), CCT961TCA (3021C>T 3023T>A), GAC962GAT (3026C>T), ATC963ATA (3029C>A), CGT964AGA (3030C>A 3032T>A), CAC966CAT (3038C>T), CCA968CCT (3044A>T), AGA969CGC (3045A>C 3047A>C), AGG970AGA (3050G>A), GAG972GAT (3056G>T),

Codon mutations:	TGC974TGT (3062C>T), TTC975TAC (3064T>A), ACA976TCC (3066A>T 3068A>C), CAA980ATG (3078C>A 3079A>T 3080A>G), GGA981GGC (3083A>C), TGT984GTG (3090T>G 3091G>T 3092T>G), AGA985AGG (3095A>G), GTT987GTA (3101T>A), GCG988GCT (3104G>T), TCT989TCA (3107T>A), ACA990AAG (3109C>A 3110A>G), CCA992CCC (3116A>C), CGT993AAG (3117C>A 3118G>A 3119T>G), CTT994AAA (3120C>A 3121T>A 3122T>A), GGC995TCC (3123G>T 3124G>C), TTA997TTG (3131A>G), TTT999GTG (3135T>G 3137T>G), CTC1000CTT (3140C>T), AAG1001AAA (3143G>A), ATG1002TAT (3144A>T 3145T>A 3146G>T), GGA1009GGC (3167A>C), ACA1011ACC (3173A>C), GTT1012GAA (3175T>A 3176T>A), GTG1014GTC (3182G>C), GAA1015ACA (3183G>A 3184A>C), GAA1017GGA (3190A>G), CTG1018TTA (3192C>T 3194G>A), AAC1019GAT (3195A>G 3197C>T), TCC1020TCG (3200C>G), TCC1021AAT (3201T>A 3202C>A 3203C>T), AGT1022GCA (3204A>G 3205G>C 3206T>A), AAG1024GCT (3210A>G 3211A>C 3212G>T), ATT1025CTT (3213A>C), TGC1026AGC (3216T>A), TCT1027TCA (3221T>A), CAA1029GTT (3225C>G 3226A>T 3227A>T), TCT1030GCA (3228T>G 3230T>A), GAA1032GAG (3236A>G), ATA1035ATT (3245A>T), GGG1037AGT (3249G>A 3251G>T), GAA1038GAG (3254A>G), GGT1040GGA (3260T>A), GAC1041GAT (3263C>T), GGC1043GGT (3269C>T), ACG1044ACT (3272G>T), TTG1045CTT (3273T>C 3275G>T), CTA1046CTC (3278A>C), CTG1047TTA (3279C>T 3281G>A), TTA1048TTG (3284A>G), AGT1050ACT (3289G>C), GTT1051GTG (3293T>G), AAT1053AAC (3299T>C), CAA1055CAG (3305A>G), CCT1056CCG (3308T>G), GTG1057TGC (3309G>T 3310T>G 3311G>C), GGC1060GGA (3320C>A), ATC1061ATA (3323C>A), CAC1062CAT (3326C>T), TCA1065TCT (3335A>T), GGC1068GAT (3343G>A 3344C>T), GTA1069ACC (3345G>A 3346T>C 3347A>C), GCA1070GCT (3350A>T), GCT1071GCA (3353T>A), GAA1072GAG (3356A>G), CGT1073AAG (3357C>A 3358G>A 3359T>G), GGA1074GGT (3362A>T), TTT1075TAC (3364T>A 3365T>C), ATC1076GTG (3366A>G 3368C>G), TCT1077GCT (3369T>G), AAT1079AAC (3377T>C), GCT1080GCA (3380T>A), GCT1082GCA (3386T>A), ATC1083GTG (3387A>G 3389C>G), TAC1084TTT (3391A>T 3392C>T), AGG1085AGA (3395G>A), GAA1086GAT (3398A>T), CTA1088TTG (3402C>T 3404A>G), GAG1089GAA (3407G>A), CCA1092CCC (3416A>C), ACT1093ACG (3419T>G), GGG1094GGA (3422G>A), CCG1095CCC (3425G>C), GTC1096ATT (3426G>A 3428C>T), GCA1098GTG (3433C>T 3434A>G), GCA1099GCT (3437A>T), GTA1101GTG (3443A>G), GAT1104GAA (3452T>A), ATT1105CTG (3453A>C 3455T>G), CTG1106TTG (3456C>T), TCA1108CAC (3462T>C 3463C>A 3464A>C), ATT1109CTG (3465A>C 3467T>G), AGA1110AGG (3470A>G), AGC1111TCC (3471A>T 3472G>C), AGA1112AAG (3475G>A 3476A>G), GAA1113GAG (3479A>G), ACA1114GAA (3480A>G 3481C>A), CAG1115AGA (3483C>A 3484A>G 3485G>A), CTT1116TTC (3486C>T 3488T>C), GAA1119GAG (3497A>G), AAC1120AAA (3500C>A), CAA1121CAG (3503A>G), TAC1123GCT (3507T>G 3508A>C 3509C>T), TAC1124TTC (3511A>T), CTT1125TTG (3513C>T 3515T>G), GGA1126GGC (3518A>C), ACA1127ACT (3521A>T), GTT1128GTA (3524T>A), CCA1129CCG (3527A>G), CAG1130TTG (3528C>T 3529A>T), GAG1131GAA (3533G>A), TTG1132ATG (3534T>A), GCC1133GCT (3539C>T), GCC1134GCT (3542C>T), GTT1136GTG (3548T>G), CCC1137CCA (3551C>A), AAG1139AAA (3557G>A), ACC1140ACA (3560C>A), ACT1141ACC (3563T>C), CTG1142CTT (3566G>T), CGG1143AAA (3567C>A 3568G>A 3569G>A), AAA1144CGC (3570A>C 3571A>G 3572A>C), AGC1145AGT (3575C>T), CAA1146CCC (3577A>C 3578A>C), TTG1147CTG (3579T>C), GAA1149GAG (3587A>G), GCA1150GCT (3590A>T), TTC1151TTT (3593C>T), GGA1152GGG (3596A>G), CCT1153GAT (3597C>G 3598C>A), GCA1154GCT (3602A>T), GAG1155CAG (3603G>C), ACA1156ACT (3608A>T), GCA1157GCC (3611A>C), ATT1160ATC (3620T>C), ACA1162TCT (3624A>T 3626A>T), GTT1163GCC (3628T>C 3629T>C), CAT1164AAA (3630C>A 3632T>A), AGA1167AGG (3641A>G), GGT1168GGA (3644T>A), GAT1169GAA (3647T>A), GGT1170GGG (3650T>G), GAC1172GAT (3656C>T), GGG1177GGT (3671G>T), GTA1178GTC (3674A>C), ATG1179GAG (3675A>G 3676T>A), AAA1180AAG (3680A>G), TAC1181TAT (3683C>T), TGT1186TCC (3697G>C 3698T>C), GGA1187AAC (3699G>A 3700G>A 3701A>C), GAT1189GAG (3707T>G), GAT1190GAG (3710T>G), GAC1191CCA (3711G>C 3712A>C 3713C>A), GCC1193GCA (3719C>A), AAA1194AAG (3722A>G), CTA1195CTG (3725A>G), GCA1196GCC (3728A>C), TTC1197TTT (3731C>T), GAA1198GAG (3734A>G), AAT1199GGT (3735A>G 3736A>G), CTC1200CTT (3740C>T), AAG1201AAA (3743G>A), TGC1202GCA (3744T>G 3745G>C 3746C>A), TCG1203TCC (3749G>C), CTG1204ATT (3750C>A 3752G>T), CTA1205CTT (3755A>T), ATC1207GTG (3759A>G 3761C>G), ATG1208CTT (3762A>C 3764G>T), CGT1209AAG (3765C>A 3766G>A 3767T>G), AGC1210ATG (3769G>T 3770C>G), AAG1212CGA (3774A>C 3775A>G 3776G>A), ATC1213GTG (3777A>G 3779C>G), CCT1214CCC (3782T>C), GGA1216GGG (3788A>G), CGT1217AAA (3789C>A 3790G>A 3791T>A), CCA1218CCT (3794A>T), TGT1219GCA (3795T>G 3796G>C 3797T>A), GAA1220ATT (3798G>A 3799A>T 3800A>T), AGG1221AGA (3803G>A), GAT1222AAT (3804G>A), GGA1229GGG (3827A>G), TGT1233TTC (3838G>T 3839T>C), TAC1235TAT (3845C>T), TAT1236TTT (3847A>T), GGC1238GCA (3853G>C 3854C>A), GAG1240GAA (3860G>A), CTG1241TTG (3861C>T), ACA1243ACG (3869A>G), TCT1244TCA (3872T>A), TGC1245TGT (3875C>T), GGA1246GGT (3878A>T), CCC1248CCT (3884C>T), TTC1249TTT (3887C>T), ATG1252TTG (3894A>T), GGG1253GAA (3898G>A 3899G>A), ATG1254CTT (3900A>C 3902G>T), GGG1255GGT (3905G>T), ATG1256TCT (3906A>T 3907T>C 3908G>T), AAG1258AAA (3914G>A), AGA1259AGG (3917A>G), CAA1263GAA (3927C>G), TCC1264GCC (3930T>G), ACT1265CAA (3933A>C 3934C>A 3935T>A), GGC1266CCA (3936G>C 3937G>C 3938C>A), GAA1267GAC (3941A>C), GGA1268TCG (3942G>T 3943G>C 3944A>G), GAG1269CAG (3945G>C), AGA1270AGG (3950A>G), GTG1271GTT (3953G>T), GAA1272CTT (3954G>C 3955A>T 3956A>T), AGA1275AGG (3965A>G), GAC1276AAC (3966G>A), ACT1277ACA (3971T>A), GTA1279GTC (3977A>C), GAA1281GAT (3983A>T), CTA1286TTG (3996C>T 3998A>G), GAT1287GAC (4001T>C), GTG1288TCG (4002G>T 4003T>C), ATT1290GTA (4008A>G 4010T>A), AAA1292CAA (4014A>C), GGC1293GGA (4019C>A), ATC1294ACA (4021T>C 4022C>A), CAT1295TTT (4023C>T 4024A>T), GTG1296GTT (4028G>T), GAT1297GAC (4031T>C), CTG1298CTT (4034G>T), ACC1300ACA (4040C>A), CAA1302CAG (4046A>G), TGC1303TGT (4049C>T), GCC1304GCG (4052C>G), CGC1308CGG (4064C>G), CTC1309TTG (4065C>T 4067C>G), AAA1313AAG (4079A>G), GGC1316GGG (4088C>G), AGA1318AGG (4094A>G), AGG1321CGC (4101A>C 4103G>C), CTC1322CTG (4106C>G), TTT1323TTC (4109T>C), GAG1324GAA (4112G>A), ATA1325ATC (4115A>C), CTT1326TTG (4116C>T 4118T>G), TAC1330TAT (4130C>T), TTG1333CTG (4137T>C), GTC1334GTG (4142C>G), AGG1335CGG (4143A>C), AAG1336AAA (4148G>A), TAT1337TAC (4151T>C), TTT1338TTC (4154T>C), GAT1340GAC (4160T>C), TTC1341TTT (4163C>T), TCA1342TCT (4166A>T), GCC1343GCT (4169C>T), ACA1344TGT (4170A>T 4171C>G 4172A>T), TTG1348GCA (4182T>G 4183T>C 4184G>A), AAT1350AAC (4190T>C), GCT1351GTA (4192C>T 4193T>A), ATA1352CTC (4194A>C 4196A>C), CCA1353CCT (4199A>T), AAA1355AAG (4205A>G), GTT1356GTA (4208T>A), ATT1358ATC (4214T>C), GAT1359AAT (4215G>A), CCT1360CCA (4220T>A), ACA1361ACG (4223A>G), AGT1362AGC (4226T>C), TCT1363CAT (4227T>C 4228C>A), TTG1367GTG (4239T>G), TTG1368CTG (4242T>C), GCA1369GCT (4247A>T), AAT1370AGT (4249A>G), GGG1371AGC (4251G>A 4253G>C), AGA1373AGG (4259A>G), GCT1374TCA (4260G>T 4262T>A), GTG1375GTA (4265G>A), TCA1376TCT (4268A>T), GAC1377GAT (4271C>T), GTG1378GAG (4273T>A), TTT1380TTC (4280T>C), GCT1382GCC (4286T>C), TAT1384TAC (4292T>C), TCC1385TCG (4295C>G), CCT1392CCC (4316T>C), GTT1393ATC (4317G>A 4319T>C), GCT1395GCA (4325T>A), CAG1397CAA (4331G>A), TTG1402CTA (4344T>C 4346G>A), GAA1405GCT (4354A>C 4355A>T), TAC1406TTT (4357A>T 4358C>T), TAC1407TAT (4361C>T), GGA1408GGT (4364A>T), TCA1409GAT (4365T>G 4366C>A 4367A>T), AAG1410GAG (4368A>G), GGC1412GGG (4376C>G), GAT1415GAG (4385T>G), TCC1416TCA (4388C>A), CGA1419AGG (4395C>A 4397A>G), CAT1420CAC (4400T>C), GCA1421ACA (4401G>A), CTT1422TTA (4404C>T 4406T>A), GCA1426GCC (4418A>C), TGT1428TGC (4424T>C), TAC1430TAT (4430C>T), ACA1431ACC (4433A>C), CTG1432CTC (4436G>C), TGC1433TGT (4439C>T), GAT1435AAC (4443G>A 4445T>C), AAA1436AAG (4448A>G), GTG1437TTG (4449G>T), AGG1439CGC (4455A>C 4457G>C), TTG1440CTA (4458T>C 4460G>A), GTT1441GTG (4463T>G), GGG1442GGT (4466G>T), GGC1443GGA (4469C>A), CCA1445CCC (4475A>C), TCA1446TCT (4478A>T), GGA1447GGT (4481A>T), GCA1449GCT (4487A>T), CTA1450TTG (4488C>T 4490A>G), ACG1451ACT (4493G>T), ATC1453ATA (4499C>A), TTC1454TTT (4502C>T), TCT1456TCA (4508T>A), CTC1457TTG (4509C>T 4511C>G), CTC1458CTA (4514C>A), CGA1464AGA (4530C>A), TAT1465TAC (4535T>C), GCC1466GCG (4538C>G), TTG1470CTG (4548T>C), TTA1471TTG (4553A>G), AGA1472AGG (4556A>G), CCC1474CCA (4562C>A), ATT1476GTC (4566A>G 4568T>C), GCT1477AAA (4569G>A 4570C>A 4571T>A), AGG1479AAA (4576G>A 4577G>A), GCT1480TCT (4578G>T), ATA1481GTG (4581A>G 4583A>G), GGA1482GGC (4586A>C), GTT1483ATT (4587G>A), AAA1484AAG (4592A>G), AGT1486AAT (4597G>A), GAT1487GAC (4601T>C), CTA1491TTG (4611C>T 4613A>G), GCT1494GCA (4622T>A), GTT1495GTA (4625T>A), GGA1497GGT (4631A>T), GAC1499GAT (4637C>T), AAT1500AAC (4640T>C), CTT1501TTG (4641C>T 4643T>G), GCT1503GCG (4649T>G), GTA1504GTT (4652A>T), TTA1506ATG (4656T>A 4658A>G), CTC1508TTG (4662C>T 4664C>G), CAG1509AGG (4665C>A 4666A>G), TAT1511TAC (4673T>C), TCT1512GAT (4674T>G 4675C>A), CTG1513CTA (4679G>A), AAT1515TCC (4683A>T 4684A>C 4685T>C), ATA1516ATT (4688A>T), CAT1518AGA (4692C>A 4693A>G 4694T>A), GAG1519GAA (4697G>A), TTA1520CTG (4698T>C 4700A>G), CTG1522GCT (4704C>G 4705T>C 4706G>T), GTC1523GTT (4709C>T), AAT1524AAC (4712T>C), GTA1525GTC (4715A>C), ATC1526GTC (4716A>G), CTT1531TTG (4731C>T 4733T>G), AAA1533AAG (4739A>G), TCG1534TCA (4742G>A), GTT1537GTA (4751T>A), AAT1538GAG (4752A>G 4754T>G), GAG1539AAG (4755G>A), GTA1540ACA (4758G>A 4759T>C), CAA1541AAA (4761C>A), CAA1543CTG (4768A>T 4769A>G), CTA1548CTC (4784A>C), ACT1549ACC (4787T>C), CTG1551CTC (4793G>C), AGA1553AGG (4799A>G), TTT1555TTC (4805T>C), AAA1556AAG (4808A>G), GCT1559GCA (4817T>A), CTT1560TTG (4818C>T 4820T>G), TAC1562TAT (4826C>T), CAC1563CAA (4829C>A), GCT1565GCA (4835T>A), CCT1566CCA (4838T>A), AAG1568AAA (4844G>A), ATC1572GTC (4854A>G), ATG1576ATT (4868G>T), TAT1577TAC (4871T>C), ATA1579ATT (4877A>T), AGA1580AGG (4880A>G), CCT1581CCA (4883T>A), GCC1582GCA (4886C>A), CCA1583GTG (4887C>G 4888C>T 4889A>G), GGT1584GGA (4892T>A), GAC1587GAT (4901C>T), ACT1588GTA (4902A>G 4903C>T 4904T>A), CAG1589CAA (4907G>A), GCC1590GCT (4910C>T), ATG1592TAT (4914A>T 4915T>A 4916G>T), GTG1595ATT (4923G>A 4925G>T), GGA1598GGC (4934A>C), ATA1599ATC (4937A>C), CGT1600AGA (4938C>A 4940T>A), GCT1602GCC (4946T>C), CAC1605CAT (4955C>T), GGT1606GGG (4958T>G), CGT1607CAG (4960G>A 4961T>G), GTG1608GCC (4963T>C 4964G>C), GCT1609ACT (4965G>A), GAC1611GAA (4973C>A), AAG1612AGT (4975A>G 4976G>T), CTT1613TTT (4977C>T), GTC1614GTA (4982C>A), TTA1615AAT (4983T>A 4984T>A 4985A>T), CAG1618CTT (4993A>T 4994G>T), ACG1619GGA (4995A>G 4996C>G 4997G>A), GCG1620GCT (5000G>T), TTG1621CTC (5001T>C 5003G>C), GAT1622AAG (5004G>A 5006T>G), GAA1623CAG (5007G>C 5009A>G), AGG1624AGA (5012G>A), GGT1625AAC (5013G>A 5014G>A 5015T>C), TTC1626CTC (5016T>C), AGA1627CCC (5019A>C 5020G>C 5021A>C), GCT1628ATC (5022G>A 5023C>T 5024T>C), GTG1629ATG (5025G>A), TTT1631ATG (5031T>A 5033T>G), CCT1632CCC (5036T>C), TCC1633ACT (5037T>A 5039C>T), TAT1634TAC (5042T>C), TTG1635TAT (5044T>A 5045G>T), GTG1637CTT (5049G>C 5051G>T), CAG1639AGT (5055C>A 5056A>G 5057G>T), GAA1640CAA (5058G>C), ATT1642TTG (5064A>T 5066T>G), GCA1643ACA (5067G>A), AAG1644GAG (5070A>G), GTA1645GTC (5075A>C), GGG1647GGA (5081G>A), TCA1649GAG (5085T>G 5086C>A 5087A>G), GCC1651ATT (5091G>A 5092C>T 5093C>T), CTG1652GGG (5094C>G 5095T>G), ACA1653GTT (5097A>G 5098C>T 5099A>T), ATT1654TTG (5100A>T 5102T>G), TGT1655ATG (5103T>A 5104G>T 5105T>G), GAA1656GAG (5108A>G), ATG1657CTA (5109A>C 5111G>A), GCA1658TCA (5112G>T), AAA1659GAT (5115A>G 5117A>T), GCA1660GCT (5120A>T), GCT1661AAA (5121G>A 5122C>A 5123T>A), ATT1662AAT (5125T>A), TCC1663ACA (5127T>A 5129C>A), TAT1664TAC (5132T>C), ACG1665ACC (5135G>C), CCA1666CCT (5138A>T), TTG1667TTA (5141G>A), GAC1668CCT (5142G>C 5143A>C 5144C>T), GCA1669GCT (5147A>T), GGT1670GGG (5150T>G), GAG1671GAA (5153G>A), ATC1673ATT (5159C>T), ACA1674del (5160_5162delACA), CGT1678GAG (5172C>G 5173G>A 5174T>G), GAT1679GGT (5176A>G), CTG1680GTC (5178C>G 5180G>C), AAT1681CAT (5181A>C), TGG1682GAA (5184T>G 5185G>A 5186G>A), GCA1684GCG (5192A>G), CCA1685CCT (5195A>T), AAC1686GAT (5196A>G 5198C>T), ATT1687GTA (5199A>G 5201T>A), GGT1688TTT (5202G>T 5203G>T), TTT1689TTC (5207T>C), TCA1691AGT (5211T>A 5212C>G 5213A>T), GCA1692GCT (5216A>T), CGT1693CGG (5219T>G), GCC1696TCA (5226G>T 5228C>A), CAC1697AAC (5229C>A), TAC1698CAA (5232T>C 5234C>A), ACG1699CAA (5235A>C 5236C>A 5237G>A), GAT1701TCA (5241G>T 5242A>C 5243T>A), GAA1702TCA (5244G>T 5245A>C), GGG1703GGT (5249G>T), TAC1704AAC (5250T>A), ATT1705ATC (5255T>C), ATT1706CTG (5256A>C 5258T>G), AAC1708AAT (5264C>T), TGT1709TGC (5267T>C), ACA1710ACT (5270A>T), GGT1711GGA (5273T>A), GCA1712GCC (5276A>C), AAG1713CGC (5277A>C 5278A>G 5279G>C), AAG1714AGA (5281A>G 5282G>A), TCC1715ACA (5283T>A 5285C>A), AAT1716ACA (5287A>C 5288T>A), GTT1718GTG (5294T>G), GGT1720GGG (5300T>G), CCA1721CCT (5303A>T), AAC1723AAT (5309C>T), GAC1726GAT (5318C>T), ATG1731ATA (5333G>A), CCG1733CCC (5339G>C), ATG1736TTG (5346A>T), TCA1737GCA (5349T>G), GCT1738GCA (5354T>A), ATA1739ATC (5357A>C), ATG1740CAG (5358A>C 5359T>A), GCC1742CAT (5364G>C 5365C>A 5366C>T), AAG1744CGC (5370A>C 5371A>G 5372G>C), AAT1745AAG (5375T>G), GTG1746AAT (5376G>A 5377T>A 5378G>T), TTG1747CTG (5379T>C), GCA1748GAA (5383C>A), GGA1749GCT (5386G>C 5387A>T), GGA1750GGT (5390A>T), CAT1751GAT (5391C>G), GTA1752GGA (5395T>G), GCA1753GCT (5399A>T), AAT1755TCT (5403A>T 5404A>C), GTT1757ACC (5409G>A 5410T>C 5411T>C), GTG1759GTT (5417G>T), GGA1761GGC (5423A>C), GGA1763GGT (5429A>T), GCT1765GCA (5435T>A), GTC1766ATA (5436G>A 5438C>A), GGC1767GCT (5440G>C 5441C>T), ATT1769ATC (5447T>C), GCG1772GCA (5456G>A), CTG1773TTG (5457C>T), ATG1774TTT (5460A>T 5462G>T), GCC1775GCA (5465C>A), TTA1776TTG (5468A>G), GCA1777GCC (5471A>C), ACA1778ACT (5474A>T), AGG1779CAT (5475A>C 5476G>A 5477G>T), TAT1781TAC (5483T>C), TGT1782AGG (5484T>A 5486T>G), GTG1783GCC (5488T>C 5489G>C), GAG1784ACA (5490G>A 5491A>C 5492G>A), ATA1786CTT (5496A>C 5498A>T), ATA1787ATT (5501A>T), GTT1788GTG (5504T>G), AGA1789CGC (5505A>C 5507A>C), TTG1790CTA (5508T>C 5510G>A), CGC1791CGA (5513C>A), ACA1792ACC (5516A>C), ATT1793ATG (5519T>G), GGA1794GCT (5521G>C 5522A>T), AAT1795GGG (5523A>G 5524A>G 5525T>G), CTT1797CTG (5531T>G), GAC1798GAT (5534C>T), CTC1799CTG (5537C>G), AAT1800AAC (5540T>C), TAT1802TAC (5546T>C), CCT1803CCC (5549T>C), GGC1805GGT (5555C>T), GTG1807GCG (5560T>C), CAG1808CAA (5564G>A), TAT1809TAC (5567T>C), TTT1810TTG (5570T>G), CTT1811CTC (5573T>C), CAA1812AAG (5574C>A 5576A>G), GTG1814GTT (5582G>T), CCT1815GAA (5583C>G 5584C>A 5585T>A), CAT1816GAC (5586C>G 5588T>C), CAT1816_GGA1817insACC (5588_5589insACC), GGA1817TCA (5589G>T 5590G>C), GAC1818CAC (5592G>C), AAA1819AAG (5597A>G), GTG1820TTG (5598G>T), GCT1821GGA (5602C>G 5603T>A), CAA1822CAG (5606A>G), GGT1824GGC (5612T>C), GCA1825ACA (5613G>A), TCG1826TAT (5617C>A 5618G>T), CTC1827GGC (5619C>G 5620T>G), CAC1828del (5622_5624delCAC), AGT1829ACC (5626G>C 5627T>C), AGT1830ACT (5629G>C), ATG1832AAC (5635T>A 5636G>C), CAA1834CAT (5642A>T), CAA1834_GGG1835insCACAGT (5642_5643insCACAGT), GGG1835GCA (5644G>C 5645G>A), GAA1837TCA (5649G>T 5650A>C), TTG1838GTT (5652T>G 5654G>T), GGC1839GGA (5657C>A), AAC1840CTT (5658A>C 5659A>T 5660C>T), CTC1841CTA (5663C>A), CGC1842AGG (5664C>A 5666C>G), ATT1843ATA (5669T>A), ATA1844GTT (5670A>G 5672A>T), CAT1845CCT (5674A>C), GGT1846TCC (5676G>T 5677G>C 5678T>C), GAT1847GAC (5681T>C), TTA1848CTC (5682T>C 5684A>C), GCA1849TCT (5685G>T 5687A>T), AAA1850GAA (5688A>G), CAG1851AAG (5691C>A), ACA1852ATG (5695C>T 5696A>G), ATG1854GCT (5700A>G 5701T>C 5702G>T), CGG1855CGT (5705G>T), ATG1856TTG (5706A>T), TAT1858TAC (5714T>C), GTG1859ACA (5715G>A 5716T>C 5717G>A), GTA1860ATT (5718G>A 5720A>T), GGA1861GGT (5723A>T), CCA1862CCT (5726A>T), CAT1863GAA (5727C>G 5729T>A), GGA1865GGT (5735A>T), GGA1867GCC (5740G>C 5741A>C), AAT1868CAG (5742A>C 5744T>G), TTT1869TGC (5746T>G 5747T>C), TCC1870TCA (5750C>A), ACA1871ACG (5753A>G), CAG1872AAG (5754C>A), GAT1873AAC (5757G>A 5759T>C), CTT1874CTG (5762T>G), GAG1875AAG (5763G>A), AGT1876CAT (5766A>C 5767G>A), TTG1877GCT (5769T>G 5770T>C 5771G>T), CTA1878TTG (5772C>T 5774A>G), CAC1879GAG (5775C>G 5777C>G), TAT1880ACA (5778T>A 5779A>C 5780T>A), TTG1881TTA (5783G>A), GAG1882TCT (5784G>T 5785A>C 5786G>T), CAG1883GTC (5787C>G 5788A>T 5789G>C), GGA1884AGT (5790G>A 5792A>T), GCA1886GCT (5798A>T), GAG1887ACT (5799G>A 5800A>C 5801G>T), TTA1888CTT (5802T>C 5804A>T), ATT1889GCC (5805A>G 5806T>C 5807T>C), CAA1890GCA (5808C>G 5809A>C), AAG1891AGA (5812A>G 5813G>A), AAT1892GGA (5814A>G 5815A>G 5816T>A), ACA1893GTC (5817A>G 5818C>T 5819A>C), CTA1895GTG (5823C>G 5825A>G), ACT1896ACA (5828T>A), CTG1897TTG (5829C>T), TTC1899TTT (5837C>T), GAG1901GAA (5843G>A), CTG1902TTC (5844C>T 5846G>C), AGT1903TCC (5847A>T 5848G>C 5849T>C), ATG1904CAA (5850A>C 5851T>A 5852G>A), GAA1905CAC (5853G>C 5855A>C), AAT1906GAT (5856A>G), GTG1907TGT (5859G>T 5860T>G 5861G>T), CAA1908GAA (5862C>G), CAA1909ATG (5865C>A 5866A>T 5867A>G), CTG1910ATA (5868C>A 5870G>A), ATA1911CTT (5871A>C 5873A>T), GAT1912GAG (5876T>G), GTT1914CTG (5880G>C 5882T>G), AAG1915GAA (5883A>G 5885G>A), CTT1916AGT (5886C>A 5887T>G), CAG1917CAA (5891G>A), TTT1919GTA (5895T>G 5897T>A), TTC1920GTA (5898T>G 5900C>A), CCA1921TTC (5901C>T 5902C>T 5903A>C), AAA1922ATG (5905A>T 5906A>G), GAA1923GGG (5908A>G 5909A>G), ACT1924AGC

(5911C>G 5912T>C), ACC1925GTT (5913A>G 5914C>T 5915C>T), ATT1926AAA (5917T>A 5918T>A), CAG1927AAA (5919C>A 5921G>A), AAG1928CAA (5922A>C 5924G>A), CTC1929ATG (5925C>A 5927C>G), AAA1930TTG (5928A>T 5929A>T 5930A>G), AAT1931TCC (5931A>T 5932A>C 5933T>C), TTT1932TTC (5936T>C), GTT1933ATG (5937G>A 5939T>G), GAT1934AAT (5940G>A), GCT1935CAA (5943G>C 5944C>A 5945T>A), GAA1936CAG (5946G>C 5948A>G), TGT1937ATG (5949T>A 5950G>T 5951T>G), CTA1938TCA (5952C>T 5953T>C), ACA1939AAA (5956C>A), TTC1940ACC (5958T>A 5959T>C), AAA1941CAA (5961A>C), GCA1942GTT (5965C>T 5966A>T), AGG1943GCC (5967A>G 5968G>C 5969G>C), AGC1944_TTT1945insCCATGTTGTGTCACA (5972_5973insCCATGTTGTGTCACA), AGG1946AGT (5978G>T), CAC1947AAA (5979C>A 5981C>A), GTA1948GCA (5983T>C), GTT1949TGG (5985G>T 5986T>G 5987T>G), TTC1950CTA (5988T>C 5990C>A), AAA1951ACT (5992A>C 5993A>T), AAA1953GAC (5997A>G 5999A>C), CTT1954ATT (6000C>A), TTG1955GTC (6003T>G 6005G>C), AGT1956ATG (6007G>T 6008T>G), TCC1957ACA (6009T>A 6011C>A), ACA1958AAA (6013C>A), TGG1959GGT (6015T>G 6017G>T), AAA1960AAG (6020A>G), ATG1961AAG (6022T>A), TGT1962CCA (6024T>C 6025G>C 6026T>A), GGT1963GGA (6029T>A), GAA1964del (6030_6032delGAA), AGT1965TCT (6033A>T 6034G>C), GTT1967_AGG1970del (6039_6050delGTTGCTTCAAGG), TCT1971CCT (6051T>C), GCA1972GCT (6056A>T), GAG1973GAA (6059G>A), AGT1974ACA (6061G>C 6062T>A), CTA1975GTG (6063C>G 6065A>G), TTC1976TTT (6068C>T), CCT1977CTT (6070C>T), GGC1978GGG (6074C>G), TTG1980CCT (6078T>C 6079T>C 6080G>T), TCT1981TCA (6083T>A), GCT1982GCA (6086T>A), TCC1983CTA (6087T>C 6088C>T 6089C>A), GTA1984GTT (6092A>T), TTG1985GTG (6093T>G), AAA1986AGA (6097A>G), TTA1988ATG (6102T>A 6104A>G), GAA1990CGC (6108G>C 6109A>G 6110A>C), AGA1991AAT (6112G>A 6113A>T), CAC1992AGT (6114C>A 6115A>G 6116C>T), ACA1993GGA (6117A>G 6118C>G), AGA1994AAG (6121G>A 6122A>G), ATG1996TAC (6126A>T 6127T>A 6128G>C), AGT1997TCA (6129A>T 6130G>C 6131T>A), CAG1999ACA (6135C>A 6136A>C 6137G>A), AGT2000ACA (6139G>C 6140T>A), ATG2001CTG (6141A>C), GAG2002GAA (6146G>A), CTG2005TTT (6153C>T 6155G>T), AAA2006AGA (6157A>G), ATA2007ATG (6161A>G), TAT2008TAC (6164T>C), CTA2009ATG (6165C>A 6167A>G), TTA2010TGT (6169T>G 6170A>T), AAC2011CAG (6171A>C 6173C>G), TTC2012GAA (6174T>G 6175T>A 6176C>A), CAA2013GGG (6177C>G 6178A>G 6179A>G), GTA2014TAT (6180G>T 6181T>A 6182A>T), ATA2015TTA (6183A>T), ACA2016GAT (6186A>G 6187C>A 6188A>T), AGT2017AAA (6190G>A 6191T>A), GAG2018GCC (6193A>C 6194G>C), TTA2020GTG (6198T>G 6200A>G), AAG2021GAC (6201A>G 6203G>C), AAG2022TGC (6204A>T 6205A>G 6206G>C), TTT2023TTC (6209T>C), GAA2024AGT (6210G>A 6211A>G 6212A>T), GAT2025GAG (6215T>G), TTT2027ATG (6219T>A 6221T>G), CAG2028GGC (6222C>G 6223A>G 6224G>C), GAG2029GTT (6226A>T 6227G>T), AAG2030TGT (6228A>T 6229A>G 6230G>T), ATA2031GTG (6231A>G 6233A>G), TCC2032CCG (6234T>C 6236C>G), ACC2033GAA (6237A>G 6238C>A 6239C>A), ACC2034CGC (6240A>C 6241C>G), CTA2035ATC (6243C>A 6245A>C), CTA2036TTG (6246C>T 6248A>G), ATC2037GTG (6249A>G 6251C>G), AAA2038CGC (6252A>C 6253A>G 6254A>C), GTA2039ACA (6255G>A 6256T>C), TTC2040ATT (6258T>A 6260C>T), TTG2041GCT (6261T>G 6262T>C 6263G>T), GAG2044TCC (6270G>T 6271A>C 6272G>C), GAG2045ACG (6273G>A 6274A>C), AGT2046TCA (6276A>T 6277G>C 6278T>A), TAT2047TTG (6280A>T 6281T>G), CAG2048AAC (6282C>A 6284G>C), ACT2049GAG (6285A>G 6286C>A 6287T>G), GAG2050AAT (6288G>A 6290G>T), TGT2052ACT (6294T>A 6295G>C), ATA2053GTG (6297A>G 6299A>G), GAT2054TAC (6300G>T 6302T>C), TCT2055ACA (6303T>A 6305T>A), CAG2056GAT (6306C>G 6308G>T), GTG2057GGG (6310T>G), CTC2058CTT (6314C>T), CAG2059TTT (6315C>T 6316A>T 6317G>T), AGG2060GAT (6318A>G 6319G>A 6320G>T), ATA2061del (6321_6323delATA), AAC2062CAT (6324A>C 6326C>T), TCC2063TTC (6328C>T), CAG2064AAA (6330C>A 6332G>A), AAA2065GCA (6333A>G 6334A>C), TTC2066GTG (6336T>G 6338C>G), AGA2067GAC (6339A>G 6340G>A 6341A>C), GTG2068TTC (6342G>T 6344G>C), CAG2069AAG (6345C>A), GAA2070CCT (6348G>C 6349A>C 6350A>T), GGT2071GGG (6353T>G), TTT2073TTC (6359T>C), TAT2074TCC (6361A>C 6362T>C), TTG2075TGG (6364T>G), CAT2076GAA (6366C>G 6368T>A), CCT2077ATT (6369C>A 6370C>T), GAA2078AAT (6372G>A 6374A>T), GGA2079GAT (6376G>A 6377A>T), ATC2080GCC (6378A>G 6379T>C), AAC2081AAT (6383C>T), AAT2083AAC (6389T>C), GCC2084GCT (6392C>T), GTA2085GCA (6394T>C), ATA2088GTG (6402A>G 6404A>G), ATA2089TTG (6405A>T 6407A>G), TCT2091GAA (6411T>G 6412C>A 6413T>A), CTG2092CTC (6416G>C), GAG2094AGG (6420G>A 6421A>G), TCC2095CAC (6423T>C 6424C>A), TAT2096TGG (6427A>G 6428T>G), AGC2097TCA (6429A>T 6430G>C 6431C>A), CGA2098AAG (6432C>A 6433G>A 6434A>G), GAT2099TCA (6435G>T 6436A>C 6437T>A), AGT2100CAA (6438A>C 6439G>A 6440T>A), AAC2101AGC (6442A>G), TCC2102AAG (6444T>A 6445C>A 6446C>G), TAT2103TTC (6448A>T 6449T>C), TCT2104ACT (6450T>A), GTG2105TTT (6453G>T 6455G>T), TCA2106GCC (6456T>G 6458A>C), ACT2107AAT (6460C>A), CCC2108GAA (6462C>G 6463C>A 6464C>A), ATC2109GTT (6465A>G 6467C>T), AAA2110GAA (6468A>G), CTG2111ATA (6471C>A 6473G>A), GGG2112TCA (6474G>T 6475G>C 6476G>A), TGT2113AAC (6477T>A 6478G>A 6479T>C), TTC2114CTG (6480T>C 6482C>G), ATT2115CTT (6483A>C), TTC2116TTA (6488C>A), CTT2117GCT (6489C>G 6490T>C), GTC2118GTT (6494C>T), GTG2119TAT (6495G>T 6496T>A 6497G>T), CTA2120TTA (6498C>T), GAT2121GAA (6503T>A), AGT2122AAT (6505G>A), CAA2123AAG (6507C>A 6509A>G), GGC2124CTC (6510G>C 6511G>T), AAG2125ACA (6514A>C 6515G>A), GAA2126GGT (6517A>G 6518A>T), GAT2127CTA (6519G>C 6520A>T 6521T>A), CAT2128GAG (6522C>G 6524T>G), CCT2129TAT (6525C>T 6526C>A), GTT2130TAT (6528G>T 6529T>A), CGG2131GAT (6531C>G 6532G>A 6533G>T), AGG2132TCC (6534A>T 6535G>C 6536G>C), TTT2133ATA (6537T>A 6539T>A), TCA2134GAA (6540T>G 6541C>A), ACG2135TCT (6543A>T 6545G>T), GTT2136TTG (6546G>T 6548T>G), TTC2137GAC (6549T>G 6550T>A), CTT2138TTG (6552C>T 6554T>G), AAA2139TTA (6555A>T 6556A>T), AAC2140CAA (6558A>C 6560C>A), TGT2141ATA (6561T>A 6562G>T 6563T>A), GAA2142GAT (6566A>T), ATT2144ATA (6572T>A), ACA2146GCA (6576A>G), TAC2148GTC (6582T>G 6583A>T), GAG2150TAC (6588G>T 6590G>C), TGA2152GGG (6594T>G 6596A>G)

Proteins

polyprotein (YP_001976147.1)	1	2152	100%	9863	66.5%	2135 (98.7%)	1360 (62.9%)	11/17/0/0	0

	Begin	End	Coverage	Score	Concordance	Matches	Identities	I/D/M/F*	Stop Codons
**NT**	**25**	**6747**	**93.0%**	**3297**	**25.1%**	**6658 (98.4%)**	**4218 (62.4%)**	**41/65**	

Protein mutations:

F7W (160T>G 161C>G), K9_A16del (165_188delAAGGTTGCTGGTGTTGATGACGCC), S17P (189A>C 190G>C), S18P (192T>C), S19A (195T>G), T21S (201A>T 203C>A), T22P (204A>C), G26A (217G>C 218T>A), L28V (222C>G), N30S (228A>T 229A>C), I32F (234A>T), K34S (240A>T 241A>C 242A>T), F35L (243T>C), A38T (252G>A 254G>T), V39L (255G>C 257T>C), I42V (264A>G), T43N (268C>A 269C>T), T44K (271C>A 272T>A), M45V (273A>G 275G>T), K48T (283A>C 284G>C), S50V (288T>G 289C>T), L53I (297C>A 299T>A), L56V (306C>G 308G>C), S59P (315T>C 317T>A), T61M (322C>T 323T>G), D62A (325A>C 326T>A), V63A (328T>C 329G>A), S64A (330T>G 332C>A), V68A (343T>C 344G>C), A69S (345G>T 347C>T), S72N (355G>A), S73A (357T>G 359A>T), L74V (360C>G 362T>A), N75E (363A>G 365T>A), K76R (367A>G), G82A (385G>C 386T>A), A83K (387G>A 388C>A), L84I (390C>A 392T>A), I85M (395A>G), D86E (398T>A), L89I (405C>A 407T>C), A91G (412C>G 413G>T), S93D (417T>G 418C>A), L95V (423T>G 425G>T), S96K (426T>A 427C>A 428C>A), S101T (441T>A), K103V (447A>G 448A>T 449G>A), M105L (453A>C 455G>T), L109M (465T>A), F110V (468T>G), E111L (471G>T 472A>T 473G>A), L113V (477T>G 479G>C), S114K (480T>A 481C>A 482C>G), N115D (483A>G 485T>C), V117F (489G>T 491T>C), K118I (493A>T 494A>C), F121Y (502T>A 503T>C), A122E (505C>A 506T>A), V124L (510G>T 512G>A), N126E (516A>G 518T>A), S128V (522A>G 523G>T), D130S (528G>T 529A>C 530T>A), V131L (531G>T), V135F (543G>T 545A>T), Y136I (546T>A 547A>T 548T>A), G137A (550G>C), I143L (567A>T 569A>G), T144A (570A>G 572A>C), L145I (573T>A 575G>T), V147L (579G>C 581G>A), F150Y (589T>A), S154V (600A>G 601G>T 602T>A), E158T (612G>A 613A>C), G159P (615G>C 616G>C), V160F (618G>T), S161N (621T>A 622C>A), T163V (627A>G 628C>T 629T>A), K165V (633A>G 634A>T 635A>T), N166T (637A>C), I167L (639A>T 641C>G), L169S (646T>C 647G>A), I170V (648A>G 650A>T), M174F (660A>T 662G>T), K186T (697A>C), A188S (702G>T 704C>T), E189G (706A>G 707A>G), R190K (708C>A 709G>A 710A>G), T192S (714A>T), M193Q (717A>C 718T>A), V194L (720G>C), S198A (732A>G 733G>C 734T>A), M199V (735A>G 737G>A), V205L (753G>T 755T>A), S206T (756T>A 758A>G), M214L (780A>T 782G>A), T216S (786A>T), I217L (789A>T 791A>G), I228V (822A>G), T248A (882A>G 884A>T), T260Q (918A>C 919C>A 920T>G), I280V (978A>G 980A>G), G282S (984G>A), T283I (988C>T 989C>T), S295N (1024G>A), E304Q (1050G>C), Q307T (1059C>A 1060A>C), D310S (1068G>T 1069A>C 1070T>A), A311E (1072C>A 1073A>G), C313A (1077T>G 1078G>C), D314E (1082T>A), A316S (1086G>T 1088A>T), T317M (1090C>T), K325P (1113A>C 1114A>C 1115G>T), T329I (1126C>T 1127C>T), N332Q (1134A>C 1136C>A), R335K (1144G>A 1145G>A), E337Q (1149G>C 1151A>G), L339V (1155C>G), M354L (1200A>T), A363T (1227G>A), E366D (1238A>T), V368L (1242G>T 1244T>G), Y369V (1245T>G 1246A>T), S372A (1254A>G 1255G>C 1256T>C), C373S (1257T>A 1259T>C), R374Q (1260A>C 1261G>A 1262G>A), M376S (1266A>T 1267T>C 1268G>T), S393A (1317T>G 1319C>T), S401C (1342C>G), R412A (1374A>G 1375G>C), I440V (1458A>G 1460C>G), M521R (1702T>G 1703G>A), E523V (1708A>T), E525D (1715G>T), S527D (1719T>G 1720C>A 1721C>T), T528P (1722A>C 1724T>A), E529N (1725G>A 1727A>T), D530A (1729A>C), G533A (1738G>C 1739A>C), R536V (1746A>G 1747G>T 1748A>T), N538D (1752A>G 1754C>T), I539V (1755A>G 1757A>G), N552A (1794A>G 1795A>C), T554S (1800A>T), S556D (1806A>G 1807G>A), E560Q (1818G>C 1820G>A), Q567R (1840A>G), A570Y (1848G>T 1849C>A 1850A>T), K574S (1861A>G 1862G>C), F577Y (1870T>A), E578N (1872G>A 1874A>T), A579T (1875G>A), E580T (1878G>A 1879A>C), I583L (1887A>T 1889A>G), S584A (1890A>G 1891G>C), P585D (1893C>G 1894C>A 1895T>C), I587V (1899A>G 1901A>T), Q591N (1911C>A 1913A>T), S593A (1917T>G), A594V (1921C>T 1922A>T), D596E (1928T>A), V597M (1929G>A), I598L (1932A>C 1934A>G), A599D (1936C>A 1937G>T), V601F (1941G>T 1943G>C), D602Q (1944G>C 1946T>A), G603D (1948G>A 1949G>T), R605del (1953_1955delAGA), R607Q (1960G>A 1961C>A), T608V (1962A>G 1963C>T 1964C>T), K609L (1965A>T 1966A>T), Q610P (1969A>C 1970A>T), D611P (1971G>C 1972A>C), I614A (1980A>G 1981T>C), L615T (1983T>A 1984T>C 1985G>T), V616Y (1986G>T 1987T>A 1988G>T), P617E (1989C>G 1990C>A 1991C>G), T618Q (1992A>C 1993C>A 1994T>G), V619T (1995G>A 1996T>C 1997T>A), V620I (1998G>A 2000G>T), S621T (2002G>C 2003T>A), N623A (2007A>G 2008A>C), D624E (2012T>A), R625K (2014G>A), V626I (2016G>A 2018G>T), I627V (2019A>G 2021C>T), A629S (2025G>T 2027A>C), R630Q (2028A>C 2029G>A 2030A>G), L645M (2073T>A), D646N (2076G>A), P647K (2079C>A 2080C>A 2081T>A), E648F (2082G>T 2083A>T 2084G>T), L650F (2088C>T), H655L (2104A>T 2105T>C), H656S (2106C>T 2107A>C 2108T>C), M660I (2120G>C), S663V (2127T>G 2128C>T 2129T>G), D665S (2133G>T 2134A>C), D666S (2136G>T 2137A>C), E667D (2141A>T), H668A (2142C>G 2143A>C 2144T>C), E670Q (2148G>C), I671N (2152T>A), N673E (2157A>G 2159C>A), K674M (2161A>T 2162A>G), R676V (2166A>G 2167G>T), V677N (2169G>A 2170T>A 2171C>T), N678D (2172A>G 2174C>T), M679L (2175A>T), Y681V (2181T>G 2182A>T 2183T>C), R682K (2185G>A), D685S (2193G>A 2194A>G), A686P (2196G>C), E687Q (2199G>C 2201A>G), R690A (2208C>G 2209G>C 2210A>T), Q694R (2221A>G), I701V (2241A>G 2243A>C), A710S (2268G>A 2269C>G 2270A>C), S711T (2271T>A), K716E (2286A>G), K717L (2289A>T 2290A>T 2291A>G), A719D (2296C>A 2297A>T), T722R (2304A>C 2305C>G 2306A>C), E723N (2307G>A 2309G>T), M724L (2310A>C 2312G>T), R725E (2313A>G 2314G>A), T726Q (2316A>C 2317C>A 2318T>A), F728K (2322T>A 2323T>A 2324T>A), H730Y (2328C>T), S731K (2331T>A 2332C>A 2333T>G), C732A (2334T>G 2335G>C 2336C>G), I733L (2337A>C), D734Q (2340G>C 2342T>G), V737A (2350T>C 2351T>A), I741T (2362T>C 2363A>G), F749R (2385T>A 2386T>G 2387T>A), N763R (2428A>G 2429C>G), S766A (2436T>G), S766_K767insG (2438_2439insGGT), K767T (2440A>C 2441A>T), E768D (2444G>C), P769K (2445C>A 2446C>A 2447A>G), E771R (2451G>A 2452A>G), S772N (2454T>A 2455C>A 2456A>T), K775I (2464A>T 2465A>C), I776E (2466A>G 2467T>A 2468T>A), Y778M (2472T>A 2473A>T 2474C>G), H779I (2475C>A 2476A>T), K780G (2478A>G 2479A>G 2480A>T), V782K (2484G>A 2485T>A 2486T>G), E783D (2489A>C), Y784K (2490T>A 2492T>A), I785M (2495C>G), K786I (2497A>T 2498G>C), D787Q (2499G>C 2501T>G), T788M (2503C>T 2504A>G), W790N (2508T>A 2509G>A 2510G>T), S791D (2511A>G 2512G>A), Y794F (2521A>T 2522T>C), A795I (2523G>A 2524C>T 2525A>C), R797K (2530G>A 2531A>G), I806L (2556A>T 2558A>G), M807I (2561G>A), V812T (2574G>A 2575T>C 2576T>C), F813M (2577T>A 2579T>G), G814N (2580G>A 2581G>A 2582A>T), G815A (2584G>C), I816V (2586A>G), L820I (2598T>A 2600G>T), A821T (2601G>A 2603G>T), W823L (2607T>C 2608G>T 2609G>C), Q824K (2610C>A), S825V (2613A>G 2614G>T 2615T>A), N826A (2616A>G 2617A>C 2618C>A), S827T (2619T>A), F828S (2623T>C), N830L (2628A>T 2629A>T 2630T>A), S832D (2634T>G 2635C>A 2636G>T), N852K (2696T>G), K853T (2698A>C 2699G>T), K853_L854insH (2699_2700insCAT), L854F (2700C>T), R855A (2703A>G 2704G>C), N856K (2708T>A), L857S (2709C>T 2710T>C 2711T>C), V859L (2715G>C 2717T>A), S862A (2724A>G 2725G>C 2726T>G), S863A (2727T>G 2729T>A), Q864D (2730C>G 2732A>C), S865N (2733T>A 2734C>A 2735A>C), L866N (2736T>A 2737T>A 2738G>C), A867H (2739G>C 2740C>A 2741A>T), Q868V (2742C>G 2743A>T 2744A>G), D869_W870insR (2747_2748insCGA), A872E (2755C>A 2756T>A), E873Q (2757G>C 2759A>G), Q879E (2775C>G 2777G>A), K882H (2784A>C 2786G>C), L890I (2808C>A), G891A (2812G>C), M907L (2859A>C 2861G>C), H914R (2880C>A 2881A>G 2882C>A), A916M (2886G>A 2887C>T 2888T>G), T923S (2907A>T 2909A>C), A927E (2920C>A 2921C>G), N928H (2922A>C), R930I (2928C>A 2929G>T 2930A>T), S931C (2932C>G), K932N (2936A>T), Y935F (2944A>T 2945C>T), R938T (2953G>C 2954A>C), E939Q (2955G>C), I942L (2964A>T 2966T>G), I948V (2982A>G 2984C>G), P961S (3021C>T 3023T>A), E972D (3056G>T), F975Y (3064T>A), T976S (3066A>T 3068A>C), Q980M (3078C>A 3079A>T 3080A>G), C984V (3090T>G 3091G>T 3092T>G), T990K (3109C>A 3110A>G), R993K (3117C>A 3118G>A 3119T>G), L994K (3120C>A 3121T>A 3122T>A), G995S (3123G>T 3124G>C), F999V (3135T>G 3137T>G), M1002Y (3144A>T 3145T>A 3146G>T), V1012E (3175T>A 3176T>A), E1015T (3183G>A 3184A>C), E1017G (3190A>G), N1019D (3195A>G 3197C>T), S1021N (3201T>A 3202C>A 3203C>T), S1022A (3204A>G 3205G>C 3206T>A), K1024A (3210A>G 3211A>C 3212G>T), I1025L (3213A>C), C1026S (3216T>A), Q1029V (3225C>G 3226A>T 3227A>T), S1030A (3228T>G 3230T>A), G1037S (3249G>A 3251G>T), S1050T (3289G>C), V1057C (3309G>T 3310T>G 3311G>C), G1068D (3343G>A 3344C>T), V1069T (3345G>A 3346T>C 3347A>C), R1073K (3357C>A 3358G>A 3359T>G), F1075Y (3364T>A 3365T>C), I1076V (3366A>G 3368C>G), S1077A (3369T>G), I1083V (3387A>G 3389C>G), Y1084F (3391A>T 3392C>T), E1086D (3398A>T), V1096I (3426G>A 3428C>T), A1098V (3433C>T 3434A>G), D1104E (3452T>A), I1105L (3453A>C 3455T>G), S1108H (3462T>C 3463C>A 3464A>C), I1109L (3465A>C 3467T>G), R1112K (3475G>A 3476A>G), T1114E (3480A>G 3481C>A), Q1115R (3483C>A 3484A>G 3485G>A), L1116F (3486C>T 3488T>C), N1120K (3500C>A), Y1123A (3507T>G 3508A>C 3509C>T), Y1124F (3511A>T), Q1130L (3528C>T 3529A>T), L1132M (3534T>A), R1143K (3567C>A 3568G>A 3569G>A), K1144R (3570A>C 3571A>G 3572A>C), Q1146P (3577A>C 3578A>C), P1153D (3597C>G 3598C>A), E1155Q (3603G>C), T1162S (3624A>T 3626A>T), V1163A (3628T>C 3629T>C), H1164K (3630C>A 3632T>A), D1169E (3647T>A), M1179E (3675A>G 3676T>A), C1186S (3697G>C 3698T>C), G1187N (3699G>A 3700G>A 3701A>C), D1189E (3707T>G), D1190E (3710T>G), D1191P (3711G>C 3712A>C 3713C>A), N1199G (3735A>G 3736A>G), C1202A (3744T>G 3745G>C 3746C>A), L1204I (3750C>A 3752G>T), I1207V (3759A>G 3761C>G), M1208L (3762A>C 3764G>T), R1209K (3765C>A 3766G>A 3767T>G), S1210M (3769G>T 3770C>G), K1212R (3774A>C 3775A>G 3776G>A), I1213V (3777A>G 3779C>G), R1217K (3789C>A 3790G>A 3791T>A), C1219A (3795T>G 3796G>C 3797T>A), E1220I (3798G>A 3799A>T 3800A>T), D1222N (3804G>A), C1233F (3838G>T 3839T>C), Y1236F (3847A>T), G1238A (3853G>C 3854C>A), M1252L (3894A>T), G1253E (3898G>A 3899G>A), M1254L (3900A>C 3902G>T), M1256S (3906A>T 3907T>C 3908G>T), Q1263E (3927C>G), S1264A (3930T>G), T1265Q (3933A>C 3934C>A 3935T>A), G1266P (3936G>C 3937G>C 3938C>A), E1267D (3941A>C), G1268S (3942G>T 3943G>C 3944A>G), E1269Q (3945G>C), E1272L (3954G>C 3955A>T 3956A>T), D1276N (3966G>A), E1281D (3983A>T), V1288S (4002G>T 4003T>C), I1290V (4008A>G 4010T>A), K1292Q (4014A>C), I1294T (4021T>C 4022C>A), H1295F (4023C>T 4024A>T), T1344C (4170A>T 4171C>G 4172A>T), L1348A (4182T>G 4183T>C 4184G>A), A1351V (4192C>T 4193T>A), I1352L (4194A>C 4196A>C), D1359N (4215G>A), S1363H (4227T>C 4228C>A), L1367V (4239T>G), N1370S (4249A>G), G1371S (4251G>A 4253G>C), A1374S (4260G>T 4262T>A), V1378E (4273T>A), V1393I (4317G>A 4319T>C), E1405A (4354A>C 4355A>T), Y1406F (4357A>T 4358C>T), S1409D (4365T>G 4366C>A 4367A>T), K1410E (4368A>G), D1415E (4385T>G), A1421T (4401G>A), D1435N (4443G>A 4445T>C), V1437L (4449G>T), I1476V (4566A>G 4568T>C), A1477K (4569G>A 4570C>A 4571T>A), R1479K (4576G>A 4577G>A), A1480S (4578G>T), I1481V (4581A>G 4583A>G), V1483I (4587G>A), S1486N (4597G>A), L1506M (4656T>A 4658A>G), Q1509R (4665C>A 4666A>G), S1512D (4674T>G 4675C>A), N1515S (4683A>T 4684A>C 4685T>C), H1518R (4692C>A 4693A>G 4694T>A), L1522A (4704C>G 4705T>C 4706G>T), I1526V (4716A>G), N1538E (4752A>G 4754T>G), E1539K (4755G>A), V1540T (4758G>A 4759T>C), Q1541K (4761C>A), Q1543L (4768A>T 4769A>G), H1563Q (4829C>A), I1572V (4854A>G), M1576I (4868G>T), P1583V (4887C>G 4888C>T 4889A>G), T1588V (4902A>G 4903C>T 4904T>A), M1592Y (4914A>T 4915T>A 4916G>T), V1595I (4923G>A 4925G>T), R1607Q (4960G>A 4961T>G), V1608A (4963T>C 4964G>C), A1609T (4965G>A), D1611E (4973C>A), K1612S (4975A>G 4976G>T), L1613F (4977C>T), L1615N (4983T>A 4984T>A 4985A>T), Q1618L (4993A>T 4994G>T), T1619G (4995A>G 4996C>G 4997G>A), D1622K (5004G>A 5006T>G), E1623Q (5007G>C 5009A>G), G1625N (5013G>A 5014G>A 5015T>C), F1626L (5016T>C), R1627P (5019A>C 5020G>C 5021A>C), A1628I (5022G>A 5023C>T 5024T>C), V1629M (5025G>A), F1631M (5031T>A 5033T>G), S1633T (5037T>A 5039C>T), L1635Y (5044T>A 5045G>T), V1637L (5049G>C 5051G>T), Q1639S (5055C>A 5056A>G 5057G>T), E1640Q (5058G>C), I1642L (5064A>T 5066T>G), A1643T (5067G>A), K1644E (5070A>G), S1649E (5085T>G 5086C>A 5087A>G), A1651I (5091G>A 5092C>T 5093C>T), L1652G (5094C>G 5095T>G), T1653V (5097A>G 5098C>T 5099A>T), I1654L (5100A>T 5102T>G), C1655M (5103T>A 5104G>T 5105T>G), M1657L (5109A>C 5111G>A), A1658S (5112G>T), K1659D (5115A>G 5117A>T), A1661K (5121G>A 5122C>A 5123T>A), I1662N (5125T>A), S1663T (5127T>A 5129C>A), D1668P (5142G>C 5143A>C 5144C>T), T1674del (5160_5162delACA), R1678E (5172C>G 5173G>A 5174T>G), D1679G (5176A>G), L1680V (5178C>G 5180G>C), N1681H (5181A>C), W1682E (5184T>G 5185G>A 5186G>A), N1686D (5196A>G 5198C>T), I1687V (5199A>G 5201T>A), G1688F (5202G>T 5203G>T), A1696S (5226G>T 5228C>A), H1697N (5229C>A), Y1698Q (5232T>C 5234C>A), T1699Q (5235A>C 5236C>A 5237G>A), D1701S (5241G>T 5242A>C 5243T>A), E1702S (5244G>T 5245A>C), Y1704N (5250T>A), I1706L (5256A>C 5258T>G), K1713R (5277A>C 5278A>G 5279G>C), K1714R (5281A>G 5282G>A), S1715T (5283T>A 5285C>A), N1716T (5287A>C 5288T>A), M1731I (5333G>A), M1736L (5346A>T), S1737A (5349T>G), M1740Q (5358A>C 5359T>A), A1742H (5364G>C 5365C>A 5366C>T), K1744R (5370A>C 5371A>G 5372G>C), N1745K (5375T>G), V1746N (5376G>A 5377T>A 5378G>T), A1748E (5383C>A), G1749A (5386G>C 5387A>T), H1751D (5391C>G), V1752G (5395T>G), N1755S (5403A>T 5404A>C), V1757T (5409G>A 5410T>C 5411T>C), V1766I (5436G>A 5438C>A), G1767A (5440G>C 5441C>T), M1774F (5460A>T 5462G>T), R1779H (5475A>C 5476G>A 5477G>T), C1782R (5484T>A 5486T>G), V1783A (5488T>C 5489G>C), E1784T (5490G>A 5491A>C 5492G>A), I1786L (5496A>C 5498A>T), I1793M (5519T>G), G1794A (5521G>C 5522A>T), N1795G (5523A>G 5524A>G 5525T>G), V1807A (5560T>C), F1810L (5570T>G), Q1812K (5574C>A 5576A>G), P1815E (5583C>G 5584C>A 5585T>A), H1816D (5586C>G 5588T>C), H1816_G1817insT (5588_5589insACC), G1817S (5589G>T 5590G>C), D1818H (5592G>C), V1820L (5598G>T), A1821G (5602C>G 5603T>A), A1825T (5613G>A), S1826Y (5617C>A 5618G>T), L1827G (5619C>G 5620T>G), H1828del (5622_5624delCAC), S1829T (5626G>C 5627T>C), S1830T (5629G>C), M1832N (5635T>A 5636G>C), Q1834H (5642A>T), Q1834_G1835insHS (5642_5643insCACAGT), G1835A (5644G>C 5645G>A), E1837S (5649G>T 5650A>C), L1838V (5652T>G 5654G>T), N1840L (5658A>C 5659A>T 5660C>T), I1844V (5670A>G 5672A>T), H1845P (5674A>C), G1846S (5676G>T 5677G>C 5678T>C), A1849S (5685G>T 5687A>T), K1850E (5688A>G), Q1851K (5691C>A), T1852M (5695C>T 5696A>G), M1854A (5700A>G 5701T>C 5702G>T), M1856L (5706A>T), V1859T (5715G>A 5716T>C 5717G>A), V1860I (5718G>A 5720A>T), H1863E (5727C>G 5729T>A), G1867A (5740G>C 5741A>C), N1868Q (5742A>C 5744T>G), F1869C (5746T>G 5747T>C), Q1872K (5754C>A), D1873N (5757G>A 5759T>C), E1875K (5763G>A), S1876H (5766A>C 5767G>A), L1877A (5769T>G 5770T>C 5771G>T), H1879E (5775C>G 5777C>G), Y1880T (5778T>A 5779A>C 5780T>A), E1882S (5784G>T 5785A>C 5786G>T), Q1883V (5787C>G 5788A>T 5789G>C), G1884S (5790G>A 5792A>T), E1887T (5799G>A 5800A>C 5801G>T), I1889A (5805A>G 5806T>C 5807T>C), Q1890A (5808C>G 5809A>C), K1891R (5812A>G 5813G>A), N1892G (5814A>G 5815A>G 5816T>A), T1893V (5817A>G 5818C>T 5819A>C), L1895V (5823C>G 5825A>G), L1902F (5844C>T 5846G>C), M1904Q (5850A>C 5851T>A 5852G>A), E1905H (5853G>C 5855A>C), N1906D (5856A>G), V1907C (5859G>T 5860T>G 5861G>T), Q1908E (5862C>G), Q1909M (5865C>A 5866A>T 5867A>G), L1910I (5868C>A 5870G>A), I1911L (5871A>C 5873A>T), D1912E (5876T>G), V1914L (5880G>C 5882T>G), K1915E (5883A>G 5885G>A), L1916S (5886C>A 5887T>G), F1919V (5895T>G 5897T>A), F1920V (5898T>G 5900C>A), P1921F (5901C>T 5902C>T 5903A>C), K1922M (5905A>T 5906A>G), E1923G (5908A>G 5909A>G), T1924S (5911C>G 5912T>C), T1925V (5913A>G 5914C>T 5915C>T), I1926K (5917T>A 5918T>A), Q1927K (5919C>A 5921G>A), K1928Q (5922A>C 5924G>A), L1929M (5925C>A 5927C>G), K1930L (5928A>T 5929A>T 5930A>G), N1931S (5931A>T 5932A>C 5933T>C), V1933M (5937G>A 5939T>G), D1934N (5940G>A), A1935Q (5943G>C 5944C>A 5945T>A), E1936Q (5946G>C 5948A>G), C1937M (5949T>A 5950G>T 5951T>G), L1938S (5952C>T 5953T>C), T1939K (5956C>A), F1940T (5958T>A 5959T>C), K1941Q (5961A>C), A1942V (5965C>T 5966A>T), R1943A (5967A>G 5968G>C 5969G>C), S1944_F1945insPCCVT (5972_5973insCCATGTTGTGTCACA), R1946S (5978G>T), H1947K (5979C>A 5981C>A), V1948A (5983T>C), V1949W (5985G>T 5986T>G 5987T>G), F1950L (5988T>C 5990C>A), K1951T (5992A>C 5993A>T), K1953D (5997A>G 5999A>C), L1954I (6000C>A), L1955V (6003T>G 6005G>C), S1956M (6007G>T 6008T>G), S1957T (6009T>A 6011C>A), T1958K (6013C>A), W1959G (6015T>G 6017G>T), M1961K (6022T>A), C1962P (6024T>C 6025G>C 6026T>A), E1964del (6030_6032delGAA), V1967_R1970del (6039_6050delGTTGCTTCAAGG), S1971P (6051T>C), S1974T (6061G>C 6062T>A), L1975V (6063C>G 6065A>G), P1977L (6070C>T), L1980P (6078T>C 6079T>C 6080G>T), S1983L (6087T>C 6088C>T 6089C>A), L1985V (6093T>G), K1986R (6097A>G), L1988M (6102T>A 6104A>G), E1990R (6108G>C 6109A>G 6110A>C), R1991N (6112G>A 6113A>T), H1992S (6114C>A 6115A>G 6116C>T), T1993G (6117A>G 6118C>G), R1994K (6121G>A 6122A>G), M1996Y (6126A>T 6127T>A

6128G>C), Q1999T (6135C>A 6136A>C 6137G>A), S2000T (6139G>C 6140T>A), M2001L (6141A>C), L2005F (6153C>T 6155G>T), K2006R (6157A>G), I2007M (6161A>G), L2009M (6165C>A 6167A>G), L2010C (6169T>G 6170A>T), N2011Q (6171A>C 6173C>G), F2012E (6174T>G 6175T>A 6176C>A), Q2013G (6177C>G 6178A>G 6179A>G), V2014Y (6180G>T 6181T>A 6182A>T), I2015L (6183A>T), T2016D (6186A>G 6187C>A 6188A>T), S2017K (6190G>A 6191T>A), E2018A (6193A>C 6194G>C), L2020V (6198T>G 6200A>G), K2021D (6201A>G 6203G>C), K2022C (6204A>T 6205A>G 6206G>C), E2024S (6210G>A 6211A>G 6212A>T), D2025E (6215T>G), F2027M (6219T>A 6221T>G), Q2028G (6222C>G 6223A>G 6224G>C), E2029V (6226A>T 6227G>T), K2030C (6228A>T 6229A>G 6230G>T), I2031V (6231A>G 6233A>G), S2032P (6234T>C 6236C>G), T2033E (6237A>G 6238C>A 6239C>A), T2034R (6240A>C 6241C>G), L2035I (6243C>A 6245A>C), I2037V (6249A>G 6251C>G), K2038R (6252A>C 6253A>G 6254A>C), V2039T (6255G>A 6256T>C), F2040I (6258T>A 6260C>T), L2041A (6261T>G 6262T>C 6263G>T), E2044S (6270G>T 6271A>C 6272G>C), E2045T (6273G>A 6274A>C), Y2047L (6280A>T 6281T>G), Q2048N (6282C>A 6284G>C), T2049E (6285A>G 6286C>A 6287T>G), E2050N (6288G>A 6290G>T), C2052T (6294T>A 6295G>C), I2053V (6297A>G 6299A>G), D2054Y (6300G>T 6302T>C), S2055T (6303T>A 6305T>A), Q2056D (6306C>G 6308G>T), V2057G (6310T>G), Q2059F (6315C>T 6316A>T 6317G>T), R2060D (6318A>G 6319G>A 6320G>T), I2061del (6321_6323delATA), N2062H (6324A>C 6326C>T), S2063F (6328C>T), Q2064K (6330C>A 6332G>A), K2065A (6333A>G 6334A>C), F2066V (6336T>G 6338C>G), R2067D (6339A>G 6340G>A 6341A>C), V2068F (6342G>T 6344G>C), Q2069K (6345C>A), E2070P (6348G>C 6349A>C 6350A>T), Y2074S (6361A>C 6362T>C), L2075W (6364T>G), H2076E (6366C>G 6368T>A), P2077I (6369C>A 6370C>T), E2078N (6372G>A 6374A>T), G2079D (6376G>A 6377A>T), I2080A (6378A>G 6379T>C), V2085A (6394T>C), I2088V (6402A>G 6404A>G), I2089L (6405A>T 6407A>G), S2091E (6411T>G 6412C>A 6413T>A), E2094R (6420G>A 6421A>G), S2095H (6423T>C 6424C>A), Y2096W (6427A>G 6428T>G), R2098K (6432C>A 6433G>A 6434A>G), D2099S (6435G>T 6436A>C 6437T>A), S2100Q (6438A>C 6439G>A 6440T>A), N2101S (6442A>G), S2102K (6444T>A 6445C>A 6446C>G), Y2103F (6448A>T 6449T>C), S2104T (6450T>A), V2105F (6453G>T 6455G>T), S2106A (6456T>G 6458A>C), T2107N (6460C>A), P2108E (6462C>G 6463C>A 6464C>A), I2109V (6465A>G 6467C>T), K2110E (6468A>G), L2111I (6471C>A 6473G>A), G2112S (6474G>T 6475G>C 6476G>A), C2113N (6477T>A 6478G>A 6479T>C), F2114L (6480T>C 6482C>G), I2115L (6483A>C), F2116L (6488C>A), L2117A (6489C>G 6490T>C), V2119Y (6495G>T 6496T>A 6497G>T), D2121E (6503T>A), S2122N (6505G>A), Q2123K (6507C>A 6509A>G), G2124L (6510G>C 6511G>T), K2125T (6514A>C 6515G>A), E2126G (6517A>G 6518A>T), D2127L (6519G>C 6520A>T 6521T>A), H2128E (6522C>G 6524T>G), P2129Y (6525C>T 6526C>A), V2130Y (6528G>T 6529T>A), R2131D (6531C>G 6532G>A 6533G>T), R2132S (6534A>T 6535G>C 6536G>C), F2133I (6537T>A 6539T>A), S2134E (6540T>G 6541C>A), T2135S (6543A>T 6545G>T), V2136L (6546G>T 6548T>G), F2137D (6549T>G 6550T>A), K2139L (6555A>T 6556A>T), N2140Q (6558A>C 6560C>A), C2141I (6561T>A 6562G>T 6563T>A), E2142D (6566A>T), T2146A (6576A>G), Y2148V (6582T>G 6583A>T), E2150Y (6588G>T 6590G>C), *2152G (6594T>G 6596A>G)

	Begin	End	Coverage	Score	Concordance	Matches	Identities	I/D/M/F*	Stop Codons
**NT**	**25**	**6747**	**93.0%**	**3297**	**25.1%**	**6658 (98.4%)**	**4218 (62.4%)**	**41/65**	

GCT2GCA (146T>A), TTC7TGG (160T>G 161C>G), TCC8AGT (162T>A 163C>G 164C>T), AAG9_GCC16del (165_188delAAGGTTGCTGGTGTTGATGACGCC), AGT17CCT (189A>C 190G>C), TCT18CCT (192T>C), TCT19GCT (195T>G), TCC20TCT (200C>T), ACC21TCA (201A>T 203C>A), ACT22CCT (204A>C), GGT26GCA (217G>C 218T>A), CTT28GTT (222C>G), TCA29TCT (227A>T), AAT30TCT (228A>T 229A>C), ATT32TTT (234A>T), TCC33TCT (239C>T), AAA34TCT (240A>T 241A>C 242A>T), TTT35CTT (243T>C), AAA37AAG (251A>G), GCG38ACT (252G>A 254G>T), GTT39CTC (255G>C 257T>C), AGT40TCC (258A>T 259G>C 260T>C), ATT42GTT (264A>G), ACC43AAT (268C>A 269C>T), ACT44AAA (271C>A 272T>A), ATG45GTT (273A>G 275G>T), AAG48ACC (283A>C 284G>C), TCA50GTA (288T>G 289C>T), CAT52CAC (296T>C), CTT53ATA (297C>A 299T>A), CTG56GTC (306C>G 308G>C), CCA58CCG (314A>G), TCT59CCA (315T>C 317T>A), GTG60GTT (320G>T), ACT61ATG (322C>T 323T>G), GAT62GCA (325A>C 326T>A), GTG63GCA (328T>C 329G>A), TCC64GCA (330T>G 332C>A), TCT66TCA (338T>A), GTG68GCC (343T>C 344G>C), GCC69TCT (345G>T 347C>T), ACT70ACA (350T>A), TGT71TGC (353T>C), AGT72AAT (355G>A), TCA73GCT (357T>G 359A>T), CTT74GTA (360C>G 362T>A), AAT75GAA (363A>G 365T>A), AAA76AGA (367A>G), TTG78TTA (374G>A), GAC79GAT (377C>T), AAA80AAG (380A>G), GGT82GCA (385G>C 386T>A), GCA83AAA (387G>A 388C>A), CTT84ATA (390C>A 392T>A), ATA85ATG (395A>G), GAT86GAA (398T>A), CCA87CCC (401A>C), TTT88TTC (404T>C), CTT89ATC (405C>A 407T>C), AAA90AAG (410A>G), GCG91GGT (412C>G 413G>T), TAC92TAT (416C>T), TCT93GAT (417T>G 418C>A), TTC94TTT (422C>T), TTG95GTT (423T>G 425G>T), TCC96AAA (426T>A 427C>A 428C>A), ACA97ACT (431A>T), TCT101ACT (441T>A), ATC102ATA (446C>A), AAG103GTA (447A>G 448A>T 449G>A), ATG105CTT (453A>C 455G>T), GTT106GTA (458T>A), TTA107CTT (459T>C 461A>T), TTG109ATG (465T>A), TTT110GTT (468T>G), GAG111TTA (471G>T 472A>T 473G>A), TTG113GTC (477T>G 479G>C), TCC114AAG (480T>A 481C>A 482C>G), AAT115GAC (483A>G 485T>C), GTT117TTC (489G>T 491T>C), AAA118ATC (493A>T 494A>C), TTA119CTA (495T>C), TTT121TAC (502T>A 503T>C), GCT122GAA (505C>A 506T>A), GTG124TTA (510G>T 512G>A), AAT126GAA (516A>G 518T>A), AGT128GTT (522A>G 523G>T), GAA129GAG (527A>G), GAT130TCA (528G>T 529A>C 530T>A), GTA131TTA (531G>T), GTT134GTG (542T>G), GTA135TTT (543G>T 545A>T), TAT136ATA (546T>A 547A>T 548T>A), GGT137GCT (550G>C), CTT139TTA (555C>T 557T>A), GTA140GTT (560A>T), ATA143TTG (567A>T 569A>G), ACA144GCC (570A>G 572A>C), TTG145ATT (573T>A 575G>T), TTA146TTG (578A>G), GTG147CTA (579G>C 581G>A), CTT148TTA (582C>T 584T>A), CTA149CTG (587A>G), TTT150TAT (589T>A), GTT151GTA (593T>A), TGT152TGC (596T>C), AGT154GTA (600A>G 601G>T 602T>A), GTT157GTA (611T>A), GAG158ACG (612G>A 613A>C), GGT159CCT (615G>C 616G>C), GTT160TTT (618G>T), TCT161AAT (621T>A 622C>A), AAC162AAT (626C>T), ACT163GTA (627A>G 628C>T 629T>A), GTC164GTT (632C>T), AAA165GTT (633A>G 634A>T 635A>T), AAT166ACT (637A>C), ATC167TTG (639A>T 641C>G), TTG169TCA (646T>C 647G>A), ATA170GTT (648A>G 650A>T), GTC171GTT (653C>T), AAC173AAT (659C>T), ATG174TTT (660A>T 662G>T), TTT175TTC (665T>C), TCA176TCT (668A>T), AGT177TCA (669A>T 670G>C 671T>A), CTA178TTG (672C>T 674A>G), TAC179TAT (677C>T), AAA186ACA (697A>C), GCC188TCT (702G>T 704C>T), GAA189GGG (706A>G 707A>G), CGA190AAG (708C>A 709G>A 710A>G), TTC191TTT (713C>T), ACC192TCC (714A>T), ATG193CAG (717A>C 718T>A), GTG194CTG (720G>C), GCC195GCG (725C>G), GCT197GCA (731T>A), AGT198GCA (732A>G 733G>C 734T>A), ATG199GTA (735A>G 737G>A), GGG202GGT (746G>T), TCA204AGT (750T>A 751C>G 752A>T), GTT205TTA (753G>T 755T>A), TCA206ACG (756T>A 758A>G), ACG210ACT (770G>T), TCT211AGC (771T>A 772C>G 773T>C), CAG212CAA (776G>A), TTG213CTT (777T>C 779G>T), ATG214TTA (780A>T 782G>A), ACA216TCA (786A>T), ATA217TTG (789A>T 791A>G), GGG221GGT (803G>T), ATT222ATC (806T>C), TCG223TCA (809G>A), ACG224ACT (812G>T), TTG225TTA (815G>A), GTG226GTA (818G>A), TTC227TTT (821C>T), ATT228GTT (822A>G), GCA229GCT (827A>T), GTG230GTT (830G>T), GGT232GGG (836T>G), GGC236GGT (848C>T), TTG237CTT (849T>C 851G>T), AGC238TCA (852A>T 853G>C 854C>A), TTG241CTG (861T>C), TCC242TCA (866C>A), ATT244ATC (872T>C), CTC245TTG (873C>T 875C>G), TAT246TAC (878T>C), TCG247TCT (881G>T), ACA248GCT (882A>G 884A>T), GCT252GCG (896T>G), CAG253CAA (899G>A), CAA257CAG (911A>G), CTA258CTG (914A>G), ACT260CAG (918A>C 919C>A 920T>G), CTT261TTG (921C>T 923T>G), CGT263AGG (927C>A 929T>G), AAT264AAC (932T>C), AAA266AAG (938A>G), TGC268TGT (944C>T), ACC269ACA (947C>A), TCC270TCT (950C>T), CAG271CAA (953G>A), GCT272GCC (956T>C), GAA278GAG (974A>G), ATA280GTG (978A>G 980A>G), GTC281GTT (983C>T), GGC282AGC (984G>A), ACC283ATT (988C>T 989C>T), GGA285GGC (995A>C), TTC286TTT (998C>T), GTG290GTA (1010G>A), GCT293GCG (1019T>G), AGT295AAT (1024G>A), GCA296GCT (1028A>T), ACG297ACC (1031G>C), CTG298TTA (1032C>T 1034G>A), TCC299TCT (1037C>T), ACT300ACC (1040T>C), CTG302CTT (1046G>T), GAG304CAG (1050G>C), CAG307ACG (1059C>A 1060A>C), GAG308GAA (1064G>A), GAT310TCA (1068G>T 1069A>C 1070T>A), GCA311GAG (1072C>A 1073A>G), GTG312GTT (1076G>T), TGT313GCT (1077T>G 1078G>C), GAT314GAA (1082T>A), CCT315CCA (1085T>A), GCA316TCT (1086G>T 1088A>T), ACG317ATG (1090C>T), GAG320GAA (1100G>A), AAC321AAT (1103C>T), GCC323GCA (1109C>A), AAG325CCT (1113A>C 1114A>C 1115G>T), GCT326GCA (1118T>A), TTC327TTT (1121C>T), CCT328CCA (1124T>A), ACC329ATT (1126C>T 1127C>T), AAA330AAG (1130A>G), AAC332CAA (1134A>C 1136C>A), CTG334TTA (1140C>T 1142G>A), AGG335AAA (1144G>A 1145G>A), GAA336GAG (1148A>G), GAA337CAG (1149G>C 1151A>G), GCT338GCC (1154T>C), CTC339GTC (1155C>G), AAG340AAA (1160G>A), ATC341ATA (1163C>A), GCA346GCC (1178A>C), GCG351GCA (1193G>A), TTC353TTT (1199C>T), ATG354TTG (1200A>T), AGT359TCT (1215A>T 1216G>C), ATT362ATA (1226T>A), GCG363ACG (1227G>A), CAG364CAA (1232G>A), TTA365CTA (1233T>C), GAA366GAT (1238A>T), GTT368TTG (1242G>T 1244T>G), TAT369GTT (1245T>G 1246A>T), GAA371GAG (1253A>G), AGT372GCC (1254A>G 1255G>C 1256T>C), TGT373AGC (1257T>A 1259T>C), AGG374CAA (1260A>C 1261G>A 1262G>A), CAC375CAT (1265C>T), ATG376TCT (1266A>T 1267T>C 1268G>T), GGC377GGT (1271C>T), CAG380CAA (1280G>A), CGT382AGG (1284C>A 1286T>G), GAA384GAG (1292A>G), TTT386TTC (1298T>C), GTA388GTG (1304A>G), TTC391TTT (1313C>T), GGG392GGA (1316G>A), TCC393GCT (1317T>G 1319C>T), GGG395GGA (1325G>A), TCT399TCA (1337T>A), ACA400ACG (1340A>G), TCC401TGC (1342C>G), TTA404CTT (1350T>C 1352A>T), TTT405TTC (1355T>C), GTG409GTT (1367G>T), TTG410CTC (1368T>C 1370G>C), AGA412GCA (1374A>G 1375G>C), GAG415GAA (1385G>A), AAA417AAG (1391A>G), CTC418CTG (1394C>G), AAT419AAC (1397T>C), CGG420CGC (1400G>C), CTA421TTG (1401C>T 1403A>G), GCA423GCG (1409A>G), AAG426AAA (1418G>A), AGG427AGA (1421G>A), GCT435GCA (1445T>A), CAC436CAT (1448C>T), GCT439GCA (1457T>A), ATC440GTG (1458A>G 1460C>G), CTG441CTT (1463G>T), GAC444GAT (1472C>T), GGA446GGT (1478A>T), GCA447GCT (1481A>T), TTG448CTT (1482T>C 1484G>T), CGA449CGG (1487A>G), GCT452GCA (1496T>A), TGC455TGT (1505C>T), CAA456CAG (1508A>G), GAT457GAC (1511T>C), ATT458ATA (1514T>A), AAA459AAG (1517A>G), GAT463GAC (1529T>C), TCA466TCT (1538A>T), ACA467ACC (1541A>C), CAA468CAG (1544A>G), CCA469CCT (1547A>T), CCC471CCT (1553C>T), TTA472CTT (1554T>C 1556A>T), GCC475GCT (1565C>T), GTT477GTC (1571T>C), GAC479GAT (1577C>T), GGC481GGA (1583C>A), CAT483CAC (1589T>C), TTC484TTT (1592C>T), ACC485ACA (1595C>A), TCC486TCA (1598C>A), TAT488TAC (1604T>C), ATA489ATC (1607A>C), ACA492ACC (1616A>C), CTG495TTG (1623C>T), ATC496ATA (1628C>A), TCA497TCT (1631A>T), CCT499CCA (1637T>A), GCC500GCT (1640C>T), CAA501CAG (1643A>G), GGT503GGG (1649T>G), CTT504CTA (1652T>A), ACA505ACG (1655A>G), CCA507CCT (1661A>T), GCT509GCA (1667T>A), GAG511GAA (1673G>A), CGG512AGG (1674C>A), AGA513AGG (1679A>G), AGG514AGA (1682G>A), GAC515GAT (1685C>T), ATC516ATT (1688C>T), CTG517TTA (1689C>T 1691G>A), GTG518GTT (1694G>T), TGT520TGC (1700T>C), ATG521AGA (1702T>G 1703G>A), GAG523GTG (1708A>T), GAG525GAT (1715G>T), TCC527GAT (1719T>G 1720C>A 1721C>T), ACT528CCA (1722A>C 1724T>A), GAA529AAT (1725G>A 1727A>T), GAT530GCT (1729A>C), CCT531CCA (1733T>A), ACG532ACA (1736G>A), GGA533GCC (1738G>C 1739A>C), CAT534CAC (1742T>C), CTC535CTA (1745C>A), AGA536GTT (1746A>G 1747G>T 1748A>T), AAC538GAT (1752A>G 1754C>T), ATA539GTG (1755A>G 1757A>G), GAG541GAA (1763G>A), GCT547GCC (1781T>C), ACT549ACA (1787T>A), CAC550CAT (1790C>T), AGG551AGA (1793G>A), AAT552GCT (1794A>G 1795A>C), TTG553TTA (1799G>A), ACC554TCC (1800A>T), AGT556GAT (1806A>G 1807G>A), GAT557GAC (1811T>C), CTT558CTC (1814T>C), GAG560CAA (1818G>C 1820G>A), GTA563GTG (1829A>G), GCC564GCA (1832C>A), AAA565AAG (1835A>G), TGT566TGC (1838T>C), CAG567CGG (1840A>G), GTA568GTG (1844A>G), CAT569CAC (1847T>C), GCA570TAT (1848G>T 1849C>A 1850A>T), GAA571GAG (1853A>G), GTA572GTG (1856A>G), AAG574AGC (1861A>G 1862G>C), TTG576CTT (1866T>C 1868G>T), TTT577TAT (1870T>A), GAA578AAT (1872G>A 1874A>T), GCT579ACT (1875G>A), GAA580ACA (1878G>A 1879A>C), TCT581TCA (1883T>A), GGC582GGA (1886C>A), ATA583TTG (1887A>T 1889A>G), AGT584GCT (1890A>G 1891G>C), CCT585GAC (1893C>G 1894C>A 1895T>C), ATA587GTT (1899A>G 1901A>T), GTT590GTC (1910T>C), CAA591AAT (1911C>A 1913A>T), TCT593GCT (1917T>G), GCA594GTT (1921C>T 1922A>T), GAT596GAA (1928T>A), GTG597ATG (1929G>A), ATA598CTG (1932A>C 1934A>G), GCG599GAT (1936C>A 1937G>T), GTG601TTC (1941G>T 1943G>C), GAT602CAA (1944G>C 1946T>A), GGG603GAT (1948G>A 1949G>T), GCT604GCC (1952T>C), AGA605del (1953_1955delAGA), TTG606TTA (1958G>A), CGC607CAA (1960G>A 1961C>A), ACC608GTT (1962A>G 1963C>T 1964C>T), AAG609TTG (1965A>T 1966A>T), CAA610CCT (1969A>C 1970A>T), GAT611CCT (1971G>C 1972A>C), ATA614GCA (1980A>G 1981T>C), TTG615ACT (1983T>A 1984T>C 1985G>T), GTG616TAT (1986G>T 1987T>A 1988G>T), CCC617GAG (1989C>G 1990C>A 1991C>G), ACT618CAG (1992A>C 1993C>A 1994T>G), GTT619ACA (1995G>A 1996T>C 1997T>A), GTG620ATT (1998G>A 2000G>T), AGT621ACA (2002G>C 2003T>A), AAT623GCT (2007A>G 2008A>C), GAT624GAA (2012T>A), AGA625AAA (2014G>A), GTG626ATT (2016G>A 2018G>T), ATC627GTT (2019A>G 2021C>T), TAT628TAC (2024T>C), GCA629TCC (2025G>T 2027A>C), AGA630CAG (2028A>C 2029G>A 2030A>G), GAG631GAA (2033G>A), ACG633ACA (2039G>A), GTA634GTT (2042A>T), GCA636GCC (2048A>C), TTG637CTC (2049T>C 2051G>C), GCA640GCT (2060A>T), CAG642CAA (2066G>A), GGC643GGG (2069C>G), TTG645ATG (2073T>A), GAC646AAC (2076G>A), CCT647AAA (2079C>A 2080C>A 2081T>A), GAG648TTT (2082G>T 2083A>T 2084G>T), GAA649GAG (2087A>G), CTT650TTT (2088C>T), TTT651TTC (2093T>C), CCT652CCA (2096T>A), GAC654GAT (2102C>T), CAT655CTC (2104A>T 2105T>C), CAT656TCC (2106C>T 2107A>C 2108T>C), GCC659GCT (2117C>T), ATG660ATC (2120G>C), TCT663GTG (2127T>G 2128C>T 2129T>G), CTT664CTA (2132T>A), GAT665TCT (2133G>T 2134A>C), GAT666TCT (2136G>T 2137A>C), GAA667GAT (2141A>T), CAT668GCC (2142C>G 2143A>C 2144T>C), GAG670CAG (2148G>C), ATT671AAT (2152T>A), TTC672TTT (2156C>T), AAC673GAA (2157A>G 2159C>A), AAA674ATG (2161A>T 2162A>G), AGA676GTA (2166A>G 2167G>T), GTC677AAT (2169G>A 2170T>A 2171C>T), AAC678GAT (2172A>G 2174C>T), ATG679TTG (2175A>T), TAT681GTC (2181T>G 2182A>T 2183T>C), AGA682AAA (2185G>A), GAT685AGT (2193G>A 2194A>G), GCT686CCT (2196G>C), GAA687CAG (2199G>C 2201A>G), CGA690GCT (2208C>G 2209G>C 2210A>T), GTA693GTG (2219A>G), CAA694CGA (2221A>G), GAC699GAT (2237C>T), ATA701GTC (2241A>G 2243A>C), CAC703CAT (2249C>T), TTT704TTC (2252T>C), GCA710AGC (2268G>A 2269C>G 2270A>C), TCA711ACA (2271T>A), ATC713ATT (2279C>T), GAG715GAA (2285G>A), AAG716GAG (2286A>G), AAA717TTG (2289A>T 2290A>T 2291A>G), CTA718CTG (2294A>G), GCA719GAT (2296C>A 2297A>T), GTG720GTC (2300G>C), CAG721CAA (2303G>A), ACA722CGC (2304A>C 2305C>G 2306A>C), GAG723AAT (2307G>A 2309G>T), ATG724CTT (2310A>C 2312G>T), AGG725GAG (2313A>G 2314G>A), ACT726CAA (2316A>C 2317C>A 2318T>A), GGA727GGG (2321A>G), TTT728AAA (2322T>A 2323T>A 2324T>A), GCA729GCG (2327A>G), CAT730TAT (2328C>T), TCT731AAG (2331T>A 2332C>A 2333T>G), TGC732GCG (2334T>G 2335G>C 2336C>G), ATT733CTT (2337A>C), GAT734CAG (2340G>C 2342T>G), GCA735GCT (2345A>T), GTT737GCA (2350T>C 2351T>A), GAC738GAT (2354C>T), ACC739ACA (2357C>A), CTC740CTT (2360C>T), ATA741ACG (2362T>C 2363A>G), ATC743ATT (2369C>T), GAG744GAA (2372G>A), CCA747CCG (2381A>G), TTT749AGA (2385T>A 2386T>G 2387T>A), GTT750GTA (2390T>A), TTT752TTC (2396T>C), TTA753CTG (2397T>C 2399A>G), ACA755ACT (2405A>T), GCA756GCT (2408A>T), AGA759CGC (2415A>C 2417A>C), TGC762TGT (2426C>T), AAC763AGG (2428A>G 2429C>G), GTA765GTT (2435A>T), TCA766GCA (2436T>G), TCA766_AAA767insGGT (2438_2439insGGT), AAA767ACT (2440A>C 2441A>T), GAG768GAC (2444G>C), CCA769AAG (2445C>A 2446C>A 2447A>G), GAA771AGA (2451G>A 2452A>G), TCA772AAT (2454T>A 2455C>A 2456A>T), ATC774ATT (2462C>T), AAA775ATC (2464A>T 2465A>C), ATT776GAA (2466A>G 2467T>A 2468T>A), TAC778ATG (2472T>A 2473A>T 2474C>G), CAT779ATT (2475C>A 2476A>T), AAA780GGT (2478A>G 2479A>G 2480A>T), GTT782AAG (2484G>A 2485T>A 2486T>G), GAA783GAC (2489A>C), TAT784AAA (2490T>A 2492T>A), ATC785ATG (2495C>G), AAG786ATC (2497A>T 2498G>C), GAT787CAG (2499G>C 2501T>G), ACA788ATG (2503C>T 2504A>G), TGG790AAT (2508T>A 2509G>A 2510G>T), AGC791GAC (2511A>G 2512G>A), CTT792TTG (2514C>T 2516T>G), TAT794TTC (2521A>T 2522T>C), GCA795ATC (2523G>A 2524C>T 2525A>C), CTG796TTG (2526C>T), AGA797AAG (2530G>A 2531A>G), CTC799CTT (2537C>T), GCA802GCC (2546A>C), GGT803GGA (2549T>A), ATA806TTG (2556A>T 2558A>G), ATG807ATA (2561G>A), GCT808GCA (2564T>A), GGA811GGC (2573A>C), GTT812ACC (2574G>A 2575T>C 2576T>C), TTT813ATG (2577T>A 2579T>G), GGA814AAT (2580G>A 2581G>A 2582A>T), GGC815GCC (2584G>C), ATT816GTT (2586A>G), ACA817ACT (2591A>T), GCA818GCT (2594A>T), TTT819TTC (2597T>C), TTG820ATT (2598T>A 2600G>T), GCG821ACT (2601G>A 2603G>T), TGG823CTC (2607T>C 2608G>T 2609G>C), CAG824AAG (2610C>A), AGT825GTA (2613A>G 2614G>T 2615T>A), AAC826GCA (2616A>G 2617A>C 2618C>A), TCT827ACT (2619T>A), TTC828TCC (2623T>C), CCC829CCT (2627C>T), AAT830TTA (2628A>T 2629A>T 2630T>A), GCA831GCT (2633A>T), TCG832GAT (2634T>G 2635C>A 2636G>T), GGA835GGG (2645A>G), GGT836GGC (2648T>C), GTG837GTA (2651G>A), GAA840GAG (2660A>G), ATA844ATC (2672A>C), AGT846AGC (2678T>C), AAG848AAA (2684G>A), AAC849AAT (2687C>T), AAG850AAA (2690G>A), GGT851GGG (2693T>G), AAT852AAG (2696T>G), AAG853ACT (2698A>C 2699G>T), AAG853_CTC854insCAT (2699_2700insCAT), CTC854TTC (2700C>T), AGA855GCA (2703A>G 2704G>C), AAT856AAA (2708T>A), CTT857TCC (2709C>T 2710T>C 2711T>C), CTC858TTG (2712C>T 2714C>G), GTT859CTA (2715G>C 2717T>A), GGT860GGG (2720T>G), AGT862GCG (2724A>G 2725G>C 2726T>G), TCT863GCA (2727T>G 2729T>A), CAA864GAC (2730C>G 2732A>C), TCA865AAC (2733T>A 2734C>A 2735A>C), TTG866AAC (2736T>A 2737T>A 2738G>C), GCA867CAT (2739G>C 2740C>A 2741A>T), CAA868GTG (2742C>G 2743A>T 2744A>G), GAT869GAC (2747T>C), GAT869_TGG870insCGA (2747_2748insCGA), GCT871GCA (2753T>A), GCT872GAA (2755C>A 2756T>A), GAA873CAG (2757G>C 2759A>G), GTA877GTT (2771A>T), CAG879GAA (2775C>G 2777G>A), AGC880AGT (2780C>T), CTC881CTG (2783C>G), AAG882CAC (2784A>C 2786G>C), AAT884AAC (2792T>C), GTG886GTT (2798G>T), GTG887GTT (2801G>T), TTA888TTG (2804A>G), CTT890ATT (2808C>A), GGT891GCT (2812G>C), GTG894GTC (2822G>C), TAC895TAT (2825C>T), TTT896TTC (2828T>C), GGC898GGT (2834C>T), ACC899ACG (2837C>G), TAC900TAT (2840C>T), GTG901GTT (2843G>T), ATA906ATT (2858A>T), ATG907CTC (2859A>C 2861G>C), ACC908ACT (2864C>T), GTA909GTG (2867A>G), GCT910GCA (2870T>A), GCA912GCC (2876A>C), TTC913TTT (2879C>T), CAC914AGA (2880C>A 2881A>G 2882C>A), AAT915AAC (2885T>C), GCT916ATG (2886G>A 2887C>T 2888T>G), CGA917AGG (2889C>A 2891A>G), GGT919GGC (2897T>C), CCA921CCT (2903A>T), ACA923TCC (2907A>T 2909A>C), ATC925ATT (2915C>T), GCC927GAG (2920C>A 2921C>G), AAT928CAT (2922A>C), TCT929TCC (2927T>C), CGA930ATT (2928C>A 2929G>T 2930A>T), TCT931TGT (2932C>G), AAA932AAT (2936A>T), GTT933GTG (2939T>G), CAA934CAG (2942A>G), TAC935TTT (2944A>T 2945C>T), AAC936AAT (2948C>T), AGA938ACC (2953G>C 2954A>C), GAA939CAA (2955G>C), ATT942TTG (2964A>T 2966T>G), ATT943ATA (2969T>A), GGC945GGT (2975C>T), GAC947GAT (2981C>T), ATC948GTG (2982A>G 2984C>G), CTG950TTG (2988C>T), CGC952AGG (2994C>A 2996C>G), GTT953GTA (2999T>A), GGT954GGG (3002T>G), CCA959CCC (3017A>C), AAG960AAA (3020G>A), CCT961TCA (3021C>T 3023T>A), GAC962GAT (3026C>T), ATC963ATA (3029C>A), CGT964AGA (3030C>A 3032T>A), CAC966CAT (3038C>T), CCA968CCT (3044A>T), AGA969CGC (3045A>C 3047A>C), AGG970AGA (3050G>A), GAG972GAT (3056G>T),

Codon mutations: TGC974TGT (3062C>T), TTC975TAC (3064T>A), ACA976TCC (3066A>T 3068A>C), CAA980ATG (3078C>A 3079A>T 3080A>G), GGA981GGC (3083A>C), TGT984GTG (3090T>G 3091G>T 3092T>G), AGA985AGG (3095A>G), GTT987GTA (3101T>A), GCG988GCT (3104G>T), TCT989TCA (3107T>A), ACA990AAG (3109C>A 3110A>G), CCA992CCC (3116A>C), CGT993AAG (3117C>A 3118G>A 3119T>G), CTT994AAA (3120C>A 3121T>A 3122T>A), GGC995TCC (3123G>T 3124G>C), TTA997TTG (3131A>G), TTT999GTG (3135T>G 3137T>G), CTC1000CTT (3140C>T), AAG1001AAA (3143G>A), ATG1002TAT (3144A>T 3145T>A 3146G>T), GGA1009GGC (3167A>C), ACA1011ACC (3173A>C), GTT1012GAA (3175T>A 3176T>A), GTG1014GTC (3182G>C), GAA1015ACA (3183G>A 3184A>C), GAA1017GGA (3190A>G), CTG1018TTA (3192C>T 3194G>A), AAC1019GAT (3195A>G 3197C>T), TCC1020TCG (3200C>G), TCC1021AAT (3201T>A 3202C>A 3203C>T), AGT1022GCA (3204A>G 3205G>C 3206T>A), AAG1024GCT (3210A>G 3211A>C 3212G>T), ATT1025CTT (3213A>C), TGC1026AGC (3216T>A), TCT1027TCA (3221T>A), CAA1029GTT (3225C>G 3226A>T 3227A>T), TCT1030GCA (3228T>G 3230T>A), GAA1032GAG (3236A>G), ATA1035ATT (3245A>T), GGG1037AGT (3249G>A 3251G>T), GAA1038GAG (3254A>G), GGT1040GGA (3260T>A), GAC1041GAT (3263C>T), GGC1043GGT (3269C>T), ACG1044ACT (3272G>T), TTG1045CTT (3273T>C 3275G>T), CTA1046CTC (3278A>C), CTG1047TTA (3279C>T 3281G>A), TTA1048TTG (3284A>G), AGT1050ACT (3289G>C), GTT1051GTG (3293T>G), AAT1053AAC (3299T>C), CAA1055CAG (3305A>G), CCT1056CCG (3308T>G), GTG1057TGC (3309G>T 3310T>G 3311G>C), GGC1060GGA (3320C>A), ATC1061ATA (3323C>A), CAC1062CAT (3326C>T), TCA1065TCT (3335A>T), GGC1068GAT (3343G>A 3344C>T), GTA1069ACC (3345G>A 3346T>C 3347A>C), GCA1070GCT (3350A>T), GCT1071GCA (3353T>A), GAA1072GAG (3356A>G), CGT1073AAG (3357C>A 3358G>A 3359T>G), GGA1074GGT (3362A>T), TTT1075TAC (3364T>A 3365T>C), ATC1076GTG (3366A>G 3368C>G), TCT1077GCT (3369T>G), AAT1079AAC (3377T>C), GCT1080GCA (3380T>A), GCT1082GCA (3386T>A), ATC1083GTG (3387A>G 3389C>G), TAC1084TTT (3391A>T 3392C>T), AGG1085AGA (3395G>A), GAA1086GAT (3398A>T), CTA1088TTG (3402C>T 3404A>G), GAG1089GAA (3407G>A), CCA1092CCC (3416A>C), ACT1093ACG (3419T>G), GGG1094GGA (3422G>A), CCG1095CCC (3425G>C), GTC1096ATT (3426G>A 3428C>T), GCA1098GTG (3433C>T 3434A>G), GCA1099GCT (3437A>T), GTA1101GTG (3443A>G), GAT1104GAA (3452T>A), ATT1105CTG (3453A>C 3455T>G), CTG1106TTG (3456C>T), TCA1108CAC (3462T>C 3463C>A 3464A>C), ATT1109CTG (3465A>C 3467T>G), AGA1110AGG (3470A>G), AGC1111TCC (3471A>T 3472G>C), AGA1112AAG (3475G>A 3476A>G), GAA1113GAG (3479A>G), ACA1114GAA (3480A>G 3481C>A), CAG1115AGA (3483C>A 3484A>G 3485G>A), CTT1116TTC (3486C>T 3488T>C), GAA1119GAG (3497A>G), AAC1120AAA (3500C>A), CAA1121CAG (3503A>G), TAC1123GCT (3507T>G 3508A>C 3509C>T), TAC1124TTC (3511A>T), CTT1125TTG (3513C>T 3515T>G), GGA1126GGC (3518A>C), ACA1127ACT (3521A>T), GTT1128GTA (3524T>A), CCA1129CCG (3527A>G), CAG1130TTG (3528C>T 3529A>T), GAG1131GAA (3533G>A), TTG1132ATG (3534T>A), GCC1133GCT (3539C>T), GCC1134GCT (3542C>T), GTT1136GTG (3548T>G), CCC1137CCA (3551C>A), AAG1139AAA (3557G>A), ACC1140ACA (3560C>A), ACT1141ACC (3563T>C), CTG1142CTT (3566G>T), CGG1143AAA (3567C>A 3568G>A 3569G>A), AAA1144CGC (3570A>C 3571A>G 3572A>C), AGC1145AGT (3575C>T), CAA1146CCC (3577A>C 3578A>C), TTG1147CTG (3579T>C), GAA1149GAG (3587A>G), GCA1150GCT (3590A>T), TTC1151TTT (3593C>T), GGA1152GGG (3596A>G), CCT1153GAT (3597C>G 3598C>A), GCA1154GCT (3602A>T), GAG1155CAG (3603G>C), ACA1156ACT (3608A>T), GCA1157GCC (3611A>C), ATT1160ATC (3620T>C), ACA1162TCT (3624A>T 3626A>T), GTT1163GCC (3628T>C 3629T>C), CAT1164AAA (3630C>A 3632T>A), AGA1167AGG (3641A>G), GGT1168GGA (3644T>A), GAT1169GAA (3647T>A), GGT1170GGG (3650T>G), GAC1172GAT (3656C>T), GGG1177GGT (3671G>T), GTA1178GTC (3674A>C), ATG1179GAG (3675A>G 3676T>A), AAA1180AAG (3680A>G), TAC1181TAT (3683C>T), TGT1186TCC (3697G>C 3698T>C), GGA1187AAC (3699G>A 3700G>A 3701A>C), GAT1189GAG (3707T>G), GAT1190GAG (3710T>G), GAC1191CCA (3711G>C 3712A>C 3713C>A), GCC1193GCA (3719C>A), AAA1194AAG (3722A>G), CTA1195CTG (3725A>G), GCA1196GCC (3728A>C), TTC1197TTT (3731C>T), GAA1198GAG (3734A>G), AAT1199GGT (3735A>G 3736A>G), CTC1200CTT (3740C>T), AAG1201AAA (3743G>A), TGC1202GCA (3744T>G 3745G>C 3746C>A), TCG1203TCC (3749G>C), CTG1204ATT (3750C>A 3752G>T), CTA1205CTT (3755A>T), ATC1207GTG (3759A>G 3761C>G), ATG1208CTT (3762A>C 3764G>T), CGT1209AAG (3765C>A 3766G>A 3767T>G), AGC1210ATG (3769G>T 3770C>G), AAG1212CGA (3774A>C 3775A>G 3776G>A), ATC1213GTG (3777A>G 3779C>G), CCT1214CCC (3782T>C), GGA1216GGG (3788A>G), CGT1217AAA (3789C>A 3790G>A 3791T>A), CCA1218CCT (3794A>T), TGT1219GCA (3795T>G 3796G>C 3797T>A), GAA1220ATT (3798G>A 3799A>T 3800A>T), AGG1221AGA (3803G>A), GAT1222AAT (3804G>A), GGA1229GGG (3827A>G), TGT1233TTC (3838G>T 3839T>C), TAC1235TAT (3845C>T), TAT1236TTT (3847A>T), GGC1238GCA (3853G>C 3854C>A), GAG1240GAA (3860G>A), CTG1241TTG (3861C>T), ACA1243ACG (3869A>G), TCT1244TCA (3872T>A), TGC1245TGT (3875C>T), GGA1246GGT (3878A>T), CCC1248CCT (3884C>T), TTC1249TTT (3887C>T), ATG1252TTG (3894A>T), GGG1253GAA (3898G>A 3899G>A), ATG1254CTT (3900A>C 3902G>T), GGG1255GGT (3905G>T), ATG1256TCT (3906A>T 3907T>C 3908G>T), AAG1258AAA (3914G>A), AGA1259AGG (3917A>G), CAA1263GAA (3927C>G), TCC1264GCC (3930T>G), ACT1265CAA (3933A>C 3934C>A 3935T>A), GGC1266CCA (3936G>C 3937G>C 3938C>A), GAA1267GAC (3941A>C), GGA1268TCG (3942G>T 3943G>C 3944A>G), GAG1269CAG (3945G>C), AGA1270AGG (3950A>G), GTG1271GTT (3953G>T), GAA1272CTT (3954G>C 3955A>T 3956A>T), AGA1275AGG (3965A>G), GAC1276AAC (3966G>A), ACT1277ACA (3971T>A), GTA1279GTC (3977A>C), GAA1281GAT (3983A>T), CTA1286TTG (3996C>T 3998A>G), GAT1287GAC (4001T>C), GTG1288TCG (4002G>T 4003T>C), ATT1290GTA (4008A>G 4010T>A), AAA1292CAA (4014A>C), GGC1293GGA (4019C>A), ATC1294ACA (4021T>C 4022C>A), CAT1295TTT (4023C>T 4024A>T), GTG1296GTT (4028G>T), GAT1297GAC (4031T>C), CTG1298CTT (4034G>T), ACC1300ACA (4040C>A), CAA1302CAG (4046A>G), TGC1303TGT (4049C>T), GCC1304GCG (4052C>G), CGC1308CGG (4064C>G), CTC1309TTG (4065C>T 4067C>G), AAA1313AAG (4079A>G), GGC1316GGG (4088C>G), AGA1318AGG (4094A>G), AGG1321CGC (4101A>C 4103G>C), CTC1322CTG (4106C>G), TTT1323TTC (4109T>C), GAG1324GAA (4112G>A), ATA1325ATC (4115A>C), CTT1326TTG (4116C>T 4118T>G), TAC1330TAT (4130C>T), TTG1333CTG (4137T>C), GTC1334GTG (4142C>G), AGG1335CGG (4143A>C), AAG1336AAA (4148G>A), TAT1337TAC (4151T>C), TTT1338TTC (4154T>C), GAT1340GAC (4160T>C), TTC1341TTT (4163C>T), TCA1342TCT (4166A>T), GCC1343GCT (4169C>T), ACA1344TGT (4170A>T 4171C>G 4172A>T), TTG1348GCA (4182T>G 4183T>C 4184G>A), AAT1350AAC (4190T>C), GCT1351GTA (4192C>T 4193T>A), ATA1352CTC (4194A>C 4196A>C), CCA1353CCT (4199A>T), AAA1355AAG (4205A>G), GTT1356GTA (4208T>A), ATT1358ATC (4214T>C), GAT1359AAT (4215G>A), CCT1360CCA (4220T>A), ACA1361ACG (4223A>G), AGT1362AGC (4226T>C), TCT1363CAT (4227T>C 4228C>A), TTG1367GTG (4239T>G), TTG1368CTG (4242T>C), GCA1369GCT (4247A>T), AAT1370AGT (4249A>G), GGG1371AGC (4251G>A 4253G>C), AGA1373AGG (4259A>G), GCT1374TCA (4260G>T 4262T>A), GTG1375GTA (4265G>A), TCA1376TCT (4268A>T), GAC1377GAT (4271C>T), GTG1378GAG (4273T>A), TTT1380TTC (4280T>C), GCT1382GCC (4286T>C), TAT1384TAC (4292T>C), TCC1385TCG (4295C>G), CCT1392CCC (4316T>C), GTT1393ATC (4317G>A 4319T>C), GCT1395GCA (4325T>A), CAG1397CAA (4331G>A), TTG1402CTA (4344T>C 4346G>A), GAA1405GCT (4354A>C 4355A>T), TAC1406TTT (4357A>T 4358C>T), TAC1407TAT (4361C>T), GGA1408GGT (4364A>T), TCA1409GAT (4365T>G 4366C>A 4367A>T), AAG1410GAG (4368A>G), GGC1412GGG (4376C>G), GAT1415GAG (4385T>G), TCC1416TCA (4388C>A), CGA1419AGG (4395C>A 4397A>G), CAT1420CAC (4400T>C), GCA1421ACA (4401G>A), CTT1422TTA (4404C>T 4406T>A), GCA1426GCC (4418A>C), TGT1428TGC (4424T>C), TAC1430TAT (4430C>T), ACA1431ACC (4433A>C), CTG1432CTC (4436G>C), TGC1433TGT (4439C>T), GAT1435AAC (4443G>A 4445T>C), AAA1436AAG (4448A>G), GTG1437TTG (4449G>T), AGG1439CGC (4455A>C 4457G>C), TTG1440CTA (4458T>C 4460G>A), GTT1441GTG (4463T>G), GGG1442GGT (4466G>T), GGC1443GGA (4469C>A), CCA1445CCC (4475A>C), TCA1446TCT (4478A>T), GGA1447GGT (4481A>T), GCA1449GCT (4487A>T), CTA1450TTG (4488C>T 4490A>G), ACG1451ACT (4493G>T), ATC1453ATA (4499C>A), TTC1454TTT (4502C>T), TCT1456TCA (4508T>A), CTC1457TTG (4509C>T 4511C>G), CTC1458CTA (4514C>A), CGA1464AGA (4530C>A), TAT1465TAC (4535T>C), GCC1466GCG (4538C>G), TTG1470CTG (4548T>C), TTA1471TTG (4553A>G), AGA1472AGG (4556A>G), CCC1474CCA (4562C>A), ATT1476GTC (4566A>G 4568T>C), GCT1477AAA (4569G>A 4570C>A 4571T>A), AGG1479AAA (4576G>A 4577G>A), GCT1480TCT (4578G>T), ATA1481GTG (4581A>G 4583A>G), GGA1482GGC (4586A>C), GTT1483ATT (4587G>A), AAA1484AAG (4592A>G), AGT1486AAT (4597G>A), GAT1487GAC (4601T>C), CTA1491TTG (4611C>T 4613A>G), GCT1494GCA (4622T>A), GTT1495GTA (4625T>A), GGA1497GGT (4631A>T), GAC1499GAT (4637C>T), AAT1500AAC (4640T>C), CTT1501TTG (4641C>T 4643T>G), GCT1503GCG (4649T>G), GTA1504GTT (4652A>T), TTA1506ATG (4656T>A 4658A>G), CTC1508TTG (4662C>T 4664C>G), CAG1509AGG (4665C>A 4666A>G), TAT1511TAC (4673T>C), TCT1512GAT (4674T>G 4675C>A), CTG1513CTA (4679G>A), AAT1515TCC (4683A>T 4684A>C 4685T>C), ATA1516ATT (4688A>T), CAT1518AGA (4692C>A 4693A>G 4694T>A), GAG1519GAA (4697G>A), TTA1520CTG (4698T>C 4700A>G), CTG1522GCT (4704C>G 4705T>C 4706G>T), GTC1523GTT (4709C>T), AAT1524AAC (4712T>C), GTA1525GTC (4715A>C), ATC1526GTC (4716A>G), CTT1531TTG (4731C>T 4733T>G), AAA1533AAG (4739A>G), TCG1534TCA (4742G>A), GTT1537GTA (4751T>A), AAT1538GAG (4752A>G 4754T>G), GAG1539AAG (4755G>A), GTA1540ACA (4758G>A 4759T>C), CAA1541AAA (4761C>A), CAA1543CTG (4768A>T 4769A>G), CTA1548CTC (4784A>C), ACT1549ACC (4787T>C), CTG1551CTC (4793G>C), AGA1553AGG (4799A>G), TTT1555TTC (4805T>C), AAA1556AAG (4808A>G), GCT1559GCA (4817T>A), CTT1560TTG (4818C>T 4820T>G), TAC1562TAT (4826C>T), CAC1563CAA (4829C>A), GCT1565GCA (4835T>A), CCT1566CCA (4838T>A), AAG1568AAA (4844G>A), ATC1572GTC (4854A>G), ATG1576ATT (4868G>T), TAT1577TAC (4871T>C), ATA1579ATT (4877A>T), AGA1580AGG (4880A>G), CCT1581CCA (4883T>A), GCC1582GCA (4886C>A), CCA1583GTG (4887C>G 4888C>T 4889A>G), GGT1584GGA (4892T>A), GAC1587GAT (4901C>T), ACT1588GTA (4902A>G 4903C>T 4904T>A), CAG1589CAA (4907G>A), GCC1590GCT (4910C>T), ATG1592TAT (4914A>T 4915T>A 4916G>T), GTG1595ATT (4923G>A 4925G>T), GGA1598GGC (4934A>C), ATA1599ATC (4937A>C), CGT1600AGA (4938C>A 4940T>A), GCT1602GCC (4946T>C), CAC1605CAT (4955C>T), GGT1606GGG (4958T>G), CGT1607CAG (4960G>A 4961T>G), GTG1608GCC (4963T>C 4964G>C), GCT1609ACT (4965G>A), GAC1611GAA (4973C>A), AAG1612AGT (4975A>G 4976G>T), CTT1613TTT (4977C>T), GTC1614GTA (4982C>A), TTA1615AAT (4983T>A 4984T>A 4985A>T), CAG1618CTT (4993A>T 4994G>T), ACG1619GGA (4995A>G 4996C>G 4997G>A), GCG1620GCT (5000G>T), TTG1621CTC (5001T>C 5003G>C), GAT1622AAG (5004G>A 5006T>G), GAA1623CAG (5007G>C 5009A>G), AGG1624AGA (5012G>A), GGT1625AAC (5013G>A 5014G>A 5015T>C), TTC1626CTC (5016T>C), AGA1627CCC (5019A>C 5020G>C 5021A>C), GCT1628ATC (5022G>A 5023C>T 5024T>C), GTG1629ATG (5025G>A), TTT1631ATG (5031T>A 5033T>G), CCT1632CCC (5036T>C), TCC1633ACT (5037T>A 5039C>T), TAT1634TAC (5042T>C), TTG1635TAT (5044T>A 5045G>T), GTG1637CTT (5049G>C 5051G>T), CAG1639AGT (5055C>A 5056A>G 5057G>T), GAA1640CAA (5058G>C), ATT1642TTG (5064A>T 5066T>G), GCA1643ACA (5067G>A), AAG1644GAG (5070A>G), GTA1645GTC (5075A>C), GGG1647GGA (5081G>A), TCA1649GAG (5085T>G 5086C>A 5087A>G), GCC1651ATT (5091G>A 5092C>T 5093C>T), CTG1652GGG (5094C>G 5095T>G), ACA1653GTT (5097A>G 5098C>T 5099A>T), ATT1654TTG (5100A>T 5102T>G), TGT1655ATG (5103T>A 5104G>T 5105T>G), GAA1656GAG (5108A>G), ATG1657CTA (5109A>C 5111G>A), GCA1658TCA (5112G>T), AAA1659GAT (5115A>G 5117A>T), GCA1660GCT (5120A>T), GCT1661AAA (5121G>A 5122C>A 5123T>A), ATT1662AAT (5125T>A), TCC1663ACA (5127T>A 5129C>A), TAT1664TAC (5132T>C), ACG1665ACC (5135G>C), CCA1666CCT (5138A>T), TTG1667TTA (5141G>A), GAC1668CCT (5142G>C 5143A>C 5144C>T), GCA1669GCT (5147A>T), GGT1670GGG (5150T>G), GAG1671GAA (5153G>A), ATC1673ATT (5159C>T), ACA1674del (5160_5162delACA), CGT1678GAG (5172C>G 5173G>A 5174T>G), GAT1679GGT (5176A>G), CTG1680GTC (5178C>G 5180G>C), AAT1681CAT (5181A>C), TGG1682GAA (5184T>G 5185G>A 5186G>A), GCA1684GCG (5192A>G), CCA1685CCT (5195A>T), AAC1686GAT (5196A>G 5198C>T), ATT1687GTA (5199A>G 5201T>A), GGT1688TTT (5202G>T 5203G>T), TTT1689TTC (5207T>C), TCA1691AGT (5211T>A 5212C>G 5213A>T), GCA1692GCT (5216A>T), CGT1693CGG (5219T>G), GCC1696TCA (5226G>T 5228C>A), CAC1697AAC (5229C>A), TAC1698CAA (5232T>C 5234C>A), ACG1699CAA (5235A>C 5236C>A 5237G>A), GAT1701TCA (5241G>T 5242A>C 5243T>A), GAA1702TCA (5244G>T 5245A>C), GGG1703GGT (5249G>T), TAC1704AAC (5250T>A), ATT1705ATC (5255T>C), ATT1706CTG (5256A>C 5258T>G), AAC1708AAT (5264C>T), TGT1709TGC (5267T>C), ACA1710ACT (5270A>T), GGT1711GGA (5273T>A), GCA1712GCC (5276A>C), AAG1713CGC (5277A>C 5278A>G 5279G>C), AAG1714AGA (5281A>G 5282G>A), TCC1715ACA (5283T>A 5285C>A), AAT1716ACA (5287A>C 5288T>A), GTT1718GTG (5294T>G), GGT1720GGG (5300T>G), CCA1721CCT (5303A>T), AAC1723AAT (5309C>T), GAC1726GAT (5318C>T), ATG1731ATA (5333G>A), CCG1733CCC (5339G>C), ATG1736TTG (5346A>T), TCA1737GCA (5349T>G), GCT1738GCA (5354T>A), ATA1739ATC (5357A>C), ATG1740CAG (5358A>C 5359T>A), GCC1742CAT (5364G>C 5365C>A 5366C>T), AAG1744CGC (5370A>C 5371A>G 5372G>C), AAT1745AAG (5375T>G), GTG1746AAT (5376G>A 5377T>A 5378G>T), TTG1747CTG (5379T>C), GCA1748GAA (5383C>A), GGA1749GCT (5386G>C 5387A>T), GGA1750GGT (5390A>T), CAT1751GAT (5391C>G), GTA1752GGA (5395T>G), GCA1753GCT (5399A>T), AAT1755TCT (5403A>T 5404A>C), GTT1757ACC (5409G>A 5410T>C 5411T>C), GTG1759GTT (5417G>T), GGA1761GGC (5423A>C), GGA1763GGT (5429A>T), GCT1765GCA (5435T>A), GTC1766ATA (5436G>A 5438C>A), GGC1767GCT (5440G>C 5441C>T), ATT1769ATC (5447T>C), GCG1772GCA (5456G>A), CTG1773TTG (5457C>T), ATG1774TTT (5460A>T 5462G>T), GCC1775GCA (5465C>A), TTA1776TTG (5468A>G), GCA1777GCC (5471A>C), ACA1778ACT (5474A>T), AGG1779CAT (5475A>C 5476G>A 5477G>T), TAT1781TAC (5483T>C), TGT1782AGG (5484T>A 5486T>G), GTG1783GCC (5488T>C 5489G>C), GAG1784ACA (5490G>A 5491A>C 5492G>A), ATA1786CTT (5496A>C 5498A>T), ATA1787ATT (5501A>T), GTT1788GTG (5504T>G), AGA1789CGC (5505A>C 5507A>C), TTG1790CTA (5508T>C 5510G>A), CGC1791CGA (5513C>A), ACA1792ACC (5516A>C), ATT1793ATG (5519T>G), GGA1794GCT (5521G>C 5522A>T), AAT1795GGG (5523A>G 5524A>G 5525T>G), CTT1797CTG (5531T>G), GAC1798GAT (5534C>T), CTC1799CTG (5537C>G), AAT1800AAC (5540T>C), TAT1802TAC (5546T>C), CCT1803CCC (5549T>C), GGC1805GGT (5555C>T), GTG1807GCG (5560T>C), CAG1808CAA (5564G>A), TAT1809TAC (5567T>C), TTT1810TTG (5570T>G), CTT1811CTC (5573T>C), CAA1812AAG (5574C>A 5576A>G), GTG1814GTT (5582G>T), CCT1815GAA (5583C>G 5584C>A 5585T>A), CAT1816GAC (5586C>G 5588T>C), CAT1816_GGA1817insACC (5588_5589insACC), GGA1817TCA (5589G>T 5590G>C), GAC1818CAC (5592G>C), AAA1819AAG (5597A>G), GTG1820TTG (5598G>T), GCT1821GGA (5602C>G 5603T>A), CAA1822CAG (5606A>G), GGT1824GGC (5612T>C), GCA1825ACA (5613G>A), TCG1826TAT (5617C>A 5618G>T), CTC1827GGC (5619C>G 5620T>G), CAC1828del (5622_5624delCAC), AGT1829ACC (5626G>C 5627T>C), AGT1830ACT (5629G>C), ATG1832AAC (5635T>A 5636G>C), CAA1834CAT (5642A>T), CAA1834_GGG1835insCACAGT (5642_5643insCACAGT), GGG1835GCA (5644G>C 5645G>A), GAA1837TCA (5649G>T 5650A>C), TTG1838GTT (5652T>G 5654G>T), GGC1839GGA (5657C>A), AAC1840CTT (5658A>C 5659A>T 5660C>T), CTC1841CTA (5663C>A), CGC1842AGG (5664C>A 5666C>G), ATT1843ATA (5669T>A), ATA1844GTT (5670A>G 5672A>T), CAT1845CCT (5674A>C), GGT1846TCC (5676G>T 5677G>C 5678T>C), GAT1847GAC (5681T>C), TTA1848CTC (5682T>C 5684A>C), GCA1849TCT (5685G>T 5687A>T), AAA1850GAA (5688A>G), CAG1851AAG (5691C>A), ACA1852ATG (5695C>T 5696A>G), ATG1854GCT (5700A>G 5701T>C 5702G>T), CGG1855CGT (5705G>T), ATG1856TTG (5706A>T), TAT1858TAC (5714T>C), GTG1859ACA (5715G>A 5716T>C 5717G>A), GTA1860ATT (5718G>A 5720A>T), GGA1861GGT (5723A>T), CCA1862CCT (5726A>T), CAT1863GAA (5727C>G 5729T>A), GGA1865GGT (5735A>T), GGA1867GCC (5740G>C 5741A>C), AAT1868CAG (5742A>C 5744T>G), TTT1869TGC (5746T>G 5747T>C), TCC1870TCA (5750C>A), ACA1871ACG (5753A>G), CAG1872AAG (5754C>A), GAT1873AAC (5757G>A 5759T>C), CTT1874CTG (5762T>G), GAG1875AAG (5763G>A), AGT1876CAT (5766A>C 5767G>A), TTG1877GCT (5769T>G 5770T>C 5771G>T), CTA1878TTG (5772C>T 5774A>G), CAC1879GAG (5775C>G 5777C>G), TAT1880ACA (5778T>A 5779A>C 5780T>A), TTG1881TTA (5783G>A), GAG1882TCT (5784G>T 5785A>C 5786G>T), CAG1883GTC (5787C>G 5788A>T 5789G>C), GGA1884AGT (5790G>A 5792A>T), GCA1886GCT (5798A>T), GAG1887ACT (5799G>A 5800A>C 5801G>T), TTA1888CTT (5802T>C 5804A>T), ATT1889GCC (5805A>G 5806T>C 5807T>C), CAA1890GCA (5808C>G 5809A>C), AAG1891AGA (5812A>G 5813G>A), AAT1892GGA (5814A>G 5815A>G 5816T>A), ACA1893GTC (5817A>G 5818C>T 5819A>C), CTA1895GTG (5823C>G 5825A>G), ACT1896ACA (5828T>A), CTG1897TTG (5829C>T), TTC1899TTT (5837C>T), GAG1901GAA (5843G>A), CTG1902TTC (5844C>T 5846G>C), AGT1903TCC (5847A>T 5848G>C 5849T>C), ATG1904CAA (5850A>C 5851T>A 5852G>A), GAA1905CAC (5853G>C 5855A>C), AAT1906GAT (5856A>G), GTG1907TGT (5859G>T 5860T>G 5861G>T), CAA1908GAA (5862C>G), CAA1909ATG (5865C>A 5866A>T 5867A>G), CTG1910ATA (5868C>A 5870G>A), ATA1911CTT (5871A>C 5873A>T), GAT1912GAG (5876T>G), GTT1914CTG (5880G>C 5882T>G), AAG1915GAA (5883A>G 5885G>A), CTT1916AGT (5886C>A 5887T>G), CAG1917CAA (5891G>A), TTT1919GTA (5895T>G 5897T>A), TTC1920GTA (5898T>G 5900C>A), CCA1921TTC (5901C>T 5902C>T 5903A>C), AAA1922ATG (5905A>T 5906A>G), GAA1923GGG (5908A>G 5909A>G), ACT1924AGC

	(5911C>G 5912T>C), ACC1925GTT (5913A>G 5914C>T 5915C>T), ATT1926AAA (5917T>A 5918T>A), CAG1927AAA (5919C>A 5921G>A), AAG1928CAA (5922A>C 5924G>A), CTC1929ATG (5925C>A 5927C>G), AAA1930TTG (5928A>T 5929A>T 5930A>G), AAT1931TCC (5931A>T 5932A>C 5933T>C), TTT1932TTC (5936T>C), GTT1933ATG (5937G>A 5939T>G), GAT1934AAT (5940G>A), GCT1935CAA (5943G>C 5944C>A 5945T>A), GAA1936CAG (5946G>C 5948A>G), TGT1937ATG (5949T>A 5950G>T 5951T>G), CTA1938TCA (5952C>T 5953T>C), ACA1939AAA (5956C>A), TTC1940ACC (5958T>A 5959T>C), AAA1941CAA (5961A>C), GCA1942GTT (5965C>T 5966A>T), AGG1943GCC (5967A>G 5968G>C 5969G>C), AGC1944_TTT1945insCCATGTTGTGTCACA (5972_5973insCCATGTTGTGTCACA), AGG1946AGT (5978G>T), CAC1947AAA (5979C>A 5981C>A), GTA1948GCA (5983T>C), GTT1949TGG (5985G>T 5986T>G 5987T>G), TTC1950CTA (5988T>C 5990C>A), AAA1951ACT (5992A>C 5993A>T), AAA1953GAC (5997A>G 5999A>C), CTT1954ATT (6000C>A), TTG1955GTC (6003T>G 6005G>C), AGT1956ATG (6007G>T 6008T>G), TCC1957ACA (6009T>A 6011C>A), ACA1958AAA (6013C>A), TGG1959GGT (6015T>G 6017G>T), AAA1960AAG (6020A>G), ATG1961AAG (6022T>A), TGT1962CCA (6024T>C 6025G>C 6026T>A), GGT1963GGA (6029T>A), GAA1964del (6030_6032delGAA), AGT1965TCT (6033A>T 6034G>C), GTT1967_AGG1970del (6039_6050delGTTGCTTCAAGG), TCT1971CCT (6051T>C), GCA1972GCT (6056A>T), GAG1973GAA (6059G>A), AGT1974ACA (6061G>C 6062T>A), CTA1975GTG (6063C>G 6065A>G), TTC1976TTT (6068C>T), CCT1977CTT (6070C>T), GGC1978GGG (6074C>G), TTG1980CCT (6078T>C 6079T>C 6080G>T), TCT1981TCA (6083T>A), GCT1982GCA (6086T>A), TCC1983CTA (6087T>C 6088C>T 6089C>A), GTA1984GTT (6092A>T), TTG1985GTG (6093T>G), AAA1986AGA (6097A>G), TTA1988ATG (6102T>A 6104A>G), GAA1990CGC (6108G>C 6109A>G 6110A>C), AGA1991AAT (6112G>A 6113A>T), CAC1992AGT (6114C>A 6115A>G 6116C>T), ACA1993GGA (6117A>G 6118C>G), AGA1994AAG (6121G>A 6122A>G), ATG1996TAC (6126A>T 6127T>A 6128G>C), AGT1997TCA (6129A>T 6130G>C 6131T>A), CAG1999ACA (6135C>A 6136A>C 6137G>A), AGT2000ACA (6139G>C 6140T>A), ATG2001CTG (6141A>C), GAG2002GAA (6146G>A), CTG2005TTT (6153C>T 6155G>T), AAA2006AGA (6157A>G), ATA2007ATG (6161A>G), TAT2008TAC (6164T>C), CTA2009ATG (6165C>A 6167A>G), TTA2010TGT (6169T>G 6170A>T), AAC2011CAG (6171A>C 6173C>G), TTC2012GAA (6174T>G 6175T>A 6176C>A), CAA2013GGG (6177C>G 6178A>G 6179A>G), GTA2014TAT (6180G>T 6181T>A 6182A>T), ATA2015TTA (6183A>T), ACA2016GAT (6186A>G 6187C>A 6188A>T), AGT2017AAA (6190G>A 6191T>A), GAG2018GCC (6193A>C 6194G>C), TTA2020GTG (6198T>G 6200A>G), AAG2021GAC (6201A>G 6203G>C), AAG2022TGC (6204A>T 6205A>G 6206G>C), TTT2023TTC (6209T>C), GAA2024AGT (6210G>A 6211A>G 6212A>T), GAT2025GAG (6215T>G), TTT2027ATG (6219T>A 6221T>G), CAG2028GGC (6222C>G 6223A>G 6224G>C), GAG2029GTT (6226A>T 6227G>T), AAG2030TGT (6228A>T 6229A>G 6230G>T), ATA2031GTG (6231A>G 6233A>G), TCC2032CCG (6234T>C 6236C>G), ACC2033GAA (6237A>G 6238C>A 6239C>A), ACC2034CGC (6240A>C 6241C>G), CTA2035ATC (6243C>A 6245A>C), CTA2036TTG (6246C>T 6248A>G), ATC2037GTG (6249A>G 6251C>G), AAA2038CGC (6252A>C 6253A>G 6254A>C), GTA2039ACA (6255G>A 6256T>C), TTC2040ATT (6258T>A 6260C>T), TTG2041GCT (6261T>G 6262T>C 6263G>T), GAG2044TCC (6270G>T 6271A>C 6272G>C), GAG2045ACG (6273G>A 6274A>C), AGT2046TCA (6276A>T 6277G>C 6278T>A), TAT2047TTG (6280A>T 6281T>G), CAG2048AAC (6282C>A 6284G>C), ACT2049GAG (6285A>G 6286C>A 6287T>G), GAG2050AAT (6288G>A 6290G>T), TGT2052ACT (6294T>A 6295G>C), ATA2053GTG (6297A>G 6299A>G), GAT2054TAC (6300G>T 6302T>C), TCT2055ACA (6303T>A 6305T>A), CAG2056GAT (6306C>G 6308G>T), GTG2057GGG (6310T>G), CTC2058CTT (6314C>T), CAG2059TTT (6315C>T 6316A>T 6317G>T), AGG2060GAT (6318A>G 6319G>A 6320G>T), ATA2061del (6321_6323delATA), AAC2062CAT (6324A>C 6326C>T), TCC2063TTC (6328C>T), CAG2064AAA (6330C>A 6332G>A), AAA2065GCA (6333A>G 6334A>C), TTC2066GTG (6336T>G 6338C>G), AGA2067GAC (6339A>G 6340G>A 6341A>C), GTG2068TTC (6342G>T 6344G>C), CAG2069AAG (6345C>A), GAA2070CCT (6348G>C 6349A>C 6350A>T), GGT2071GGG (6353T>G), TTT2073TTC (6359T>C), TAT2074TCC (6361A>C 6362T>C), TTG2075TGG (6364T>G), CAT2076GAA (6366C>G 6368T>A), CCT2077ATT (6369C>A 6370C>T), GAA2078AAT (6372G>A 6374A>T), GGA2079GAT (6376G>A 6377A>T), ATC2080GCC (6378A>G 6379T>C), AAC2081AAT (6383C>T), AAT2083AAC (6389T>C), GCC2084GCT (6392C>T), GTA2085GCA (6394T>C), ATA2088GTG (6402A>G 6404A>G), ATA2089TTG (6405A>T 6407A>G), TCT2091GAA (6411T>G 6412C>A 6413T>A), CTG2092CTC (6416G>C), GAG2094AGG (6420G>A 6421A>G), TCC2095CAC (6423T>C 6424C>A), TAT2096TGG (6427A>G 6428T>G), AGC2097TCA (6429A>T 6430G>C 6431C>A), CGA2098AAG (6432C>A 6433G>A 6434A>G), GAT2099TCA (6435G>T 6436A>C 6437T>A), AGT2100CAA (6438A>C 6439G>A 6440T>A), AAC2101AGC (6442A>G), TCC2102AAG (6444T>A 6445C>A 6446C>G), TAT2103TTC (6448A>T 6449T>C), TCT2104ACT (6450T>A), GTG2105TTT (6453G>T 6455G>T), TCA2106GCC (6456T>G 6458A>C), ACT2107AAT (6460C>A), CCC2108GAA (6462C>G 6463C>A 6464C>A), ATC2109GTT (6465A>G 6467C>T), AAA2110GAA (6468A>G), CTG2111ATA (6471C>A 6473G>A), GGG2112TCA (6474G>T 6475G>C 6476G>A), TGT2113AAC (6477T>A 6478G>A 6479T>C), TTC2114CTG (6480T>C 6482C>G), ATT2115CTT (6483A>C), TTC2116TTA (6488C>A), CTT2117GCT (6489C>G 6490T>C), GTC2118GTT (6494C>T), GTG2119TAT (6495G>T 6496T>A 6497G>T), CTA2120TTA (6498C>T), GAT2121GAA (6503T>A), AGT2122AAT (6505G>A), CAA2123AAG (6507C>A 6509A>G), GGC2124CTC (6510G>C 6511G>T), AAG2125ACA (6514A>C 6515G>A), GAA2126GGT (6517A>G 6518A>T), GAT2127CTA (6519G>C 6520A>T 6521T>A), CAT2128GAG (6522C>G 6524T>G), CCT2129TAT (6525C>T 6526C>A), GTT2130TAT (6528G>T 6529T>A), CGG2131GAT (6531C>G 6532G>A 6533G>T), AGG2132TCC (6534A>T 6535G>C 6536G>C), TTT2133ATA (6537T>A 6539T>A), TCA2134GAA (6540T>G 6541C>A), ACG2135TCT (6543A>T 6545G>T), GTT2136TTG (6546G>T 6548T>G), TTC2137GAC (6549T>G 6550T>A), CTT2138TTG (6552C>T 6554T>G), AAA2139TTA (6555A>T 6556A>T), AAC2140CAA (6558A>C 6560C>A), TGT2141ATA (6561T>A 6562G>T 6563T>A), GAA2142GAT (6566A>T), ATT2144ATA (6572T>A), ACA2146GCA (6576A>G), TAC2148GTC (6582T>G 6583A>T), GAG2150TAC (6588G>T 6590G>C), TGA2152GGG (6594T>G 6596A>G)								
helicase (YP_001976154.1)	1	98	100%	654	97.5%	98 (100%)	95 (96.9%)	0/0/0/0	0
Protein mutations:	S5C (1342C>G), R16A (1374A>G 1375G>C), I44V (1458A>G 1460C>G)								
Codon mutations:	TCT3TCA (1337T>A), ACA4ACG (1340A>G), TCC5TGC (1342C>G), TTA8CTT (1350T>C 1352A>T), TTT9TTC (1355T>C), GTG13GTT (1367G>T), TTG14CTC (1368T>C 1370G>C), AGA16GCA (1374A>G 1375G>C), GAG19GAA (1385G>A), AAA21AAG (1391A>G), CTC22CTG (1394C>G), AAT23AAC (1397T>C), CGG24CGC (1400G>C), CTA25TTG (1401C>T 1403A>G), GCA27GCG (1409A>G), AAG30AAA (1418G>A), AGG31AGA (1421G>A), GCT39GCA (1445T>A), CAC40CAT (1448C>T), GCT43GCA (1457T>A), ATC44GTG (1458A>G 1460C>G), CTG45CTT (1463G>T), GAC48GAT (1472C>T), GGA50GGT (1478A>T), GCA51GCT (1481A>T), TTG52CTT (1482T>C 1484G>T), CGA53CGG (1487A>G), GCT56GCA (1496T>A), TGC59TGT (1505C>T), CAA60CAG (1508A>G), GAT61GAC (1511T>C), ATT62ATA (1514T>A), AAA63AAG (1517A>G), GAT67GAC (1529T>C), TCA70TCT (1538A>T), ACA71ACC (1541A>C), CAA72CAG (1544A>G), CCA73CCT (1547A>T), CCC75CCT (1553C>T), TTA76CTT (1554T>C 1556A>T), GCC79GCT (1565C>T), GTT81GTC (1571T>C), GAC83GAT (1577C>T), GGC85GGA (1583C>A), CAT87CAC (1589T>C), TTC88TTT (1592C>T), ACC89ACA (1595C>A), TCC90TCA (1598C>A), TAT92TAC (1604T>C), ATA93ATC (1607A>C), ACA96ACC (1616A>C)								
RNA-dependent RNA polymerase (YP_001976153.1)	1	249	100%	1552	88.4%	249 (100%)	210 (84.3%)	0/0/0/0	0
Protein mutations:	T40C (4170A>T 4171C>G 4172A>T), L44A (4182T>G 4183T>C 4184G>A), A47V (4192C>T 4193T>A), I48L (4194A>C 4196A>C), D55N (4215G>A), S59H (4227T>C 4228C>A), L63V (4239T>G), N66S (4249A>G), G67S (4251G>A 4253G>C), A70S (4260G>T 4262T>A), V74E (4273T>A), V89I (4317G>A 4319T>C), E101A (4354A>C 4355A>T), Y102F (4357A>T 4358C>T), S105D (4365T>G 4366C>A 4367A>T), K106E (4368A>G), D111E (4385T>G), A117T (4401G>A), D131N (4443G>A 4445T>C), V133L (4449G>T), I172V (4566A>G 4568T>C), A173K (4569G>A 4570C>A 4571T>A), R175K (4576G>A 4577G>A), A176S (4578G>T), I177V (4581A>G 4583A>G), V179I (4587G>A), S182N (4597G>A), L202M (4656T>A 4658A>G), Q205R (4665C>A 4666A>G), S208D (4674T>G 4675C>A), N211S (4683A>T 4684A>C 4685T>C), H214R (4692C>A 4693A>G 4694T>A), L218A (4704C>G 4705T>C 4706G>T), I222V (4716A>G), N234E (4752A>G 4754T>G), E235K (4755G>A), V236T (4758G>A 4759T>C), Q237K (4761C>A), Q239L (4768A>T 4769A>G)								
Codon mutations:	CGC4CGG (4064C>G), CTC5TTG (4065C>T 4067C>G), AAA9AAG (4079A>G), GGC12GGG (4088C>G), AGA14AGG (4094A>G), AGG17CGC (4101A>C 4103G>C), CTC18CTG (4106C>G), TTT19TTC (4109T>C), GAG20GAA (4112G>A), ATA21ATC (4115A>C), CTT22TTG (4116C>T 4118T>G), TAC26TAT (4130C>T), TTG29CTG (4137T>C), GTC30GTG (4142C>G), AGG31CGG (4143A>C), AAG32AAA (4148G>A), TAT33TAC (4151T>C), TTT34TTC (4154T>C), GAT36GAC (4160T>C), TTC37TTT (4163C>T), TCA38TCT (4166A>T), GCC39GCT (4169C>T), ACA40TGT (4170A>T 4171C>G 4172A>T), TTG44GCA (4182T>G 4183T>C 4184G>A), AAT46AAC (4190T>C), GCT47GTA (4192C>T 4193T>A), ATA48CTC (4194A>C 4196A>C), CCA49CCT (4199A>T), AAA51AAG (4205A>G), GTT52GTA (4208T>A), ATT54ATC (4214T>C), GAT55AAT (4215G>A), CCT56CCA (4220T>A), ACA57ACG (4223A>G), AGT58AGC (4226T>C), TCT59CAT (4227T>C 4228C>A), TTG63GTG (4239T>G), TTG64CTG (4242T>C), GCA65GCT (4247A>T), AAT66AGT (4249A>G), GGG67AGC (4251G>A 4253G>C), AGA69AGG (4259A>G), GCT70TCA (4260G>T 4262T>A), GTG71GTA (4265G>A), TCA72TCT (4268A>T), GAC73GAT (4271C>T), GTG74GAG (4273T>A), TTT76TTC (4280T>C), GCT78GCC (4286T>C), TAT80TAC (4292T>C), TCC81TCG (4295C>G), CCT88CCC (4316T>C), GTT89ATC (4317G>A 4319T>C), GCT91GCA (4325T>A), CAG93CAA (4331G>A), TTG98CTA (4344T>C 4346G>A), GAA101GCT (4354A>C 4355A>T), TAC102TTT (4357A>T 4358C>T), TAC103TAT (4361C>T), GGA104GGT (4364A>T), TCA105GAT (4365T>G 4366C>A 4367A>T), AAG106GAG (4368A>G), GGC108GGG (4376C>G), GAT111GAG (4385T>G), TCC112TCA (4388C>A), CGA115AGG (4395C>A 4397A>G), CAT116CAC (4400T>C), GCA117ACA (4401G>A), CTT118TTA (4404C>T 4406T>A), GCA122GCC (4418A>C), TGT124TGC (4424T>C), TAC126TAT (4430C>T), ACA127ACC (4433A>C), CTG128CTC (4436G>C), TGC129TGT (4439C>T), GAT131AAC (4443G>A 4445T>C), AAA132AAG (4448A>G), GTG133TTG (4449G>T), AGG135CGC (4455A>C 4457G>C), TTG136CTA (4458T>C 4460G>A), GTT137GTG (4463T>G), GGG138GGT (4466G>T), GGC139GGA (4469C>A), CCA141CCC (4475A>C), TCA142TCT (4478A>T), GGA143GGT (4481A>T), GCA145GCT (4487A>T), CTA146TTG (4488C>T 4490A>G), ACG147ACT (4493G>T), ATC149ATA (4499C>A), TTC150TTT (4502C>T), TCT152TCA (4508T>A), CTC153TTG (4509C>T 4511C>G), CTC154CTA (4514C>A), CGA160AGA (4530C>A), TAT161TAC (4535T>C), GCC162GCG (4538C>G), TTG166CTG (4548T>C), TTA167TTG (4553A>G), AGA168AGG (4556A>G), CCC170CCA (4562C>A), ATT172GTC (4566A>G 4568T>C), GCT173AAA (4569G>A 4570C>A 4571T>A), AGG175AAA (4576G>A 4577G>A), GCT176TCT (4578G>T), ATA177GTG (4581A>G 4583A>G), GGA178GGC (4586A>C), GTT179ATT (4587G>A), AAA180AAG (4592A>G), AGT182AAT (4597G>A), GAT183GAC (4601T>C), CTA187TTG (4611C>T 4613A>G), GCT190GCA (4622T>A), GTT191GTA (4625T>A), GGA193GGT (4631A>T), GAC195GAT (4637C>T), AAT196AAC (4640T>C), CTT197TTG (4641C>T 4643T>G), GCT199GCG (4649T>G), GTA200GTT (4652A>T), TTA202ATG (4656T>A 4658A>G), CTC204TTG (4662C>T 4664C>G), CAG205AGG (4665C>A 4666A>G), TAT207TAC (4673T>C), TCT208GAT (4674T>G 4675C>A), CTG209CTA (4679G>A), AAT211TCC (4683A>T 4684A>C 4685T>C), ATA212ATT (4688A>T), CAT214AGA (4692C>A 4693A>G 4694T>A), GAG215GAA (4697G>A), TTA216CTG (4698T>C 4700A>G), CTG218GCT (4704C>G 4705T>C 4706G>T), GTC219GTT (4709C>T), AAT220AAC (4712T>C), GTA221GTC (4715A>C), ATC222GTC (4716A>G), CTT227TTG (4731C>T 4733T>G), AAA229AAG (4739A>G), TCG230TCA (4742G>A), GTT233GTA (4751T>A), AAT234GAG (4752A>G 4754T>G), GAG235AAG (4755G>A), GTA236ACA (4758G>A 4759T>C), CAA237AAA (4761C>A), CAA239CTG (4768A>T 4769A>G), CTA244CTC (4784A>C), ACT245ACC (4787T>C), CTG247CTC (4793G>C), AGA249AGG (4799A>G)								

*: Inserts / Deletes / Misaligned / Frameshifts

Analysis details

This analysis was performed with panviral2.64

NGS Details (UN8): Diachasmimorpha longicaudata entomopoxvirus (segment NC_043455.1)

Assembly

**Coverage Length**	1121 (1 contig(s))
**Depth Of Coverage**	1498.5
**Number Of Reads**	13895
**Reads Per Million**	232.22 rpm (after QC)
**Ambiguities**	0
**Assembly Method**	de novo + reference guided assembly
**Consensus Caller**	Bcf Tools

Coverage Map

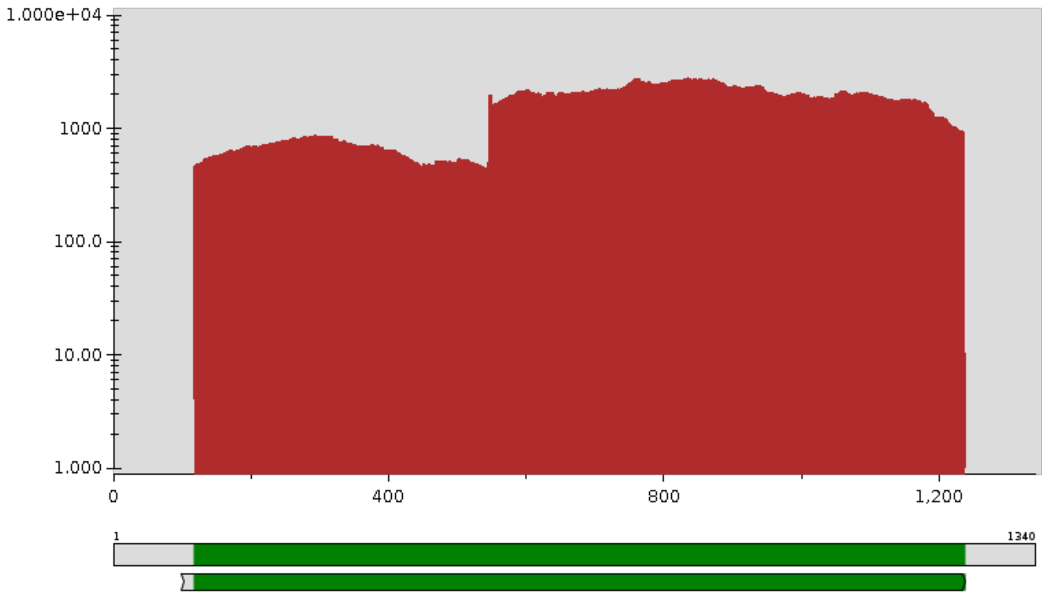

Assignment

**Type**	Diachasmimorpha longicaudata entomopoxvirus (Taxonomy ID: 109981)
**Reference Genome**	NC_043455.1
**NT Identity (%)**	59.7681
**AA Identity (%)**	55.3476
**Number Of Stop Codons**	0
**Number Of CDS**	1

Alignment

**Alignment Score**	420.0 (NT) + 1460.0 (AA) = 1880.0
**Concordance (%)**	40.1366

**Alignment Method**	Global, seeded, nucleotide + amino acids (AGA)

Genome Region

Sequence starts at position 118 and ends at position 1238 relative to NC_043455.1 reference sequence.

Alignment Detailed Statistics

	Begin	End	Coverage	Score	Concordance	Matches	Identities	I/D/M/F*	Stop Codons
**NT**	**118**	**1238**	**83.7%**	**420**	**18.9%**	**1118 (99.5%)**	**670 (59.6%)**	**3/3**	
Mutations:	118T>G, 119G>A, 120A>G, 121A>T, 126A>C, 127C>T, 128C>G, 130T>G, 136C>G, 137C>A, 139C>T, 142G>A, 143A>G, 145A>T, 146A>G, 148T>C, 149A>C, 151C>T, 154A>T, 156A>G, 157G>A, 160T>G, 163A>T, 166T>C, 167T>C, 168C>A, 169T>G, 171G>A, 172T>C, 175A>G, 178T>C, 187T>A, 190A>T, 191A>G, 193T>C, 203A>C, 204A>G, 208C>T, 209A>G, 211T>A, 214T>G, 217A>T, 218T>A, 219G>T, 223T>A, 226T>G, 230A>C, 231A>G, 232A>T, 238G>C, 241A>T, 243T>C, 244C>T, 247A>G, 256G>C, 259A>T, 262A>C, 271A>C, 272G>T, 274A>C, 276C>T, 277A>G, 278T>A, 279A>T, 283A>T, 284A>C, 286C>T, 288G>C, 289T>C, 294T>G, 295A>C, 298G>A, 299C>G, 300A>T, 301A>T, 302A>G, 304A>G, 310T>C, 311T>A, 312C>A, 313T>A, 320A>G, 322T>G, 323A>G, 325C>A, 331T>A, 337C>A, 341A>T, 343C>T, 346A>T, 352T>G, 355A>G, 356T>C, 358G>T, 361T>A, 362C>G, 363T>C, 364C>T, 368G>A, 370T>G, 371C>G, 374C>A, 375G>A, 376T>G, 379A>G, 380T>A, 382G>T, 383C>T, 384A>T, 385A>G, 386A>G, 394G>A, 395A>G, 400T>C, 401C>A, 403A>T, 404_406delTAT, 409T>C, 410T>A, 412T>A, 413A>C, 415A>G, 416T>G, 417G>C, 418T>A, 421A>T, 423T>C, 424T>G, 438C>A, 442T>C, 443A>G, 445C>G, 446A>G, 447A>G, 448G>T, 452A>G, 453G>A, 454C>T, 455C>A, 456A>T, 457G>C, 458G>A, 459A>G, 462C>A, 463T>G, 467A>G, 469_470insCAT, 470A>G, 471A>G, 474C>T, 475C>A, 478G>A, 482T>G, 484G>C, 485A>T, 486T>C, 500C>A, 503A>G, 505G>C, 506A>T, 507T>G, 511T>C, 512C>A, 514A>G, 515C>A, 517C>T, 519C>A, 520C>A, 521C>A, 523A>G, 525A>G, 528G>C, 530A>T, 532C>A, 533G>C, 534A>G, 535T>A, 540A>G, 541A>G, 544A>C, 547T>C, 550A>G, 553A>G, 554G>T, 559A>T, 560A>T, 562A>G, 574T>C, 577G>A, 581T>C, 583G>C, 584A>T, 585T>C, 586A>T, 587G>A, 588A>G, 589T>A, 590A>G, 591A>G, 595T>C, 596T>A, 597T>A, 602A>C, 604A>G, 607A>T, 608C>T, 610A>T, 616T>A, 620G>C, 622A>G, 623T>C, 628T>G, 629G>C, 630A>C, 632A>C, 633G>C, 634T>A, 635C>A, 637T>A, 638G>A, 646T>G, 647A>G, 648T>G, 650T>G, 652A>T, 655A>C, 658A>T, 661A>C, 664C>T, 665G>A, 667T>G, 671T>C, 674A>G, 675G>A, 678T>C, 680A>C, 682C>T, 683A>G, 685T>G, 687C>T, 688A>T, 689T>A, 692C>A, 693A>G, 694G>A, 695G>A, 696T>A, 697C>G, 700T>C, 705G>A, 706A>C, 707G>A, 709T>G, 715A>G, 717A>G, 718A>G, 724G>T, 727A>G, 730A>G, 731A>C, 732A>G, 735C>A, 739A>G, 740T>C, 742G>C, 745A>T, 748C>T, 754C>T, 759G>A, 765A>T, 770A>G, 772A>C, 778A>C, 779A>G, 781G>T, 784A>G, 785A>G, 787T>A, 790T>G, 792T>G, 793T>G, 796A>G, 797G>C, 798C>T, 799A>T, 810T>G, 820T>C, 823C>G, 824C>A, 825A>C, 826C>T, 829A>G, 830A>G, 831G>C, 833C>A, 838T>C, 841A>G, 843C>G, 844A>T, 845C>G, 847A>C, 850A>C, 854T>G, 855G>T, 859T>C, 862A>T, 863C>A, 865T>G, 866A>C, 869C>A, 871A>G, 878G>T, 879T>G, 880A>G, 885T>C, 886G>T, 889A>T, 890T>A, 891G>A, 892T>A, 893T>A, 895A>G, 896A>C, 897C>G, 898A>T, 900A>G, 901T>C, 902A>C, 904A>T, 905A>G, 908T>C, 909T>A, 915C>T, 919A>T, 920A>G, 921G>C, 922C>A, 924T>C, 925C>T, 934T>C, 938T>G, 939C>A, 940C>T, 943A>G, 944C>A, 946A>T, 947G>A, 948A>C, 950C>A, 952T>A, 956T>A, 958T>C, 966A>G, 967A>G, 970A>G, 973T>C, 974A>C, 977G>T, 978A>C, 982T>C, 983A>T, 984A>C, 986A>T, 989A>C, 991A>T, 992A>G, 994A>G, 998C>A, 1000T>C, 1001T>A, 1007A>G, 1012A>C, 1013T>C, 1015A>T, 1018A>T, 1019A>C, 1021A>T, 1024C>T, 1033T>A, 1036A>G, 1040A>G, 1042A>C, 1045T>C, 1046T>C, 1048A>T, 1051T>C, 1054C>T, 1057T>C, 1063T>C, 1064T>C, 1070C>A, 1071A>C, 1073A>C, 1075T>A, 1076A>C, 1077G>C, 1087T>C, 1088A>C, 1090A>G, 1103A>C, 1105A>T, 1111T>A, 1112A>C, 1120C>A, 1121C>A, 1123A>G, 1126A>G, 1129G>T, 1130A>G, 1141T>C, 1145A>G, 1147A>C, 1150T>C, 1151G>A, 1153A>G, 1156C>T, 1159A>T, 1160T>G, 1161T>A, 1163C>A, 1164C>G, 1165A>G, 1167A>T, 1172G>T, 1173C>T, 1177A>C, 1178T>A, 1181G>C, 1183A>G, 1185C>A, 1186G>A, 1192T>C, 1195T>C, 1196A>G, 1197C>T, 1199A>G, 1200A>T, 1204A>C, 1207T>G, 1210A>G, 1211A>C, 1213G>C, 1218A>C, 1219G>C, 1223A>G, 1227T>C, 1232T>C, 1234G>C, 1235A>C, 1237A>G								

CDS

	Begin	End	Coverage	Score	Concordance	Matches	Identities	I/D/M/F*	Stop Codons
FLA14_p101	7	380	98.4%	1460	59.1%	373 (99.5%)	207 (55.2%)	1/1/0/0	0
Protein mutations:	E7S (119G>A 120A>G 121A>T), D9A (126A>C 127C>T), H10E (128C>G 130T>G), L13I (137C>A 139C>T), K15D (143A>G 145A>T), N16D (146A>G 148T>C), I17L (149A>C 151C>T), K19R (156A>G 157G>A), S23Q (167T>C 168C>A 169T>G), C24Y (171G>A 172T>C), T31A (191A>G 193T>C), K35R (203A>C 204A>G), I37V (209A>G 211T>A), F38L (214T>G), C40I (218T>A 219G>T), K44R (230A>C 231A>G 232A>T), V48A (243T>C 244C>T), A58S (272G>T 274A>C), T59M (276C>T 277A>G), Y60I (278T>A 279A>T), I62L (284A>C 286C>T), S63T (288G>C 289T>C), L65C (294T>G 295A>C), Q67V (299C>G 300A>T 301A>T), I68V (302A>G 304A>G), S71K (311T>A 312C>A 313T>A), N74E (320A>G 322T>G), I75V (323A>G 325C>A), T81S (341A>T 343C>T), L88A (362C>G 363T>C 364C>T), A90T (368G>A 370T>G), Q91E (371C>G), R92K (374C>A 375G>A 376T>G), L94I (380T>A 382G>T), Q95L (383C>T 384A>T 385A>G), T96A (386A>G), N99D (395A>G), L101I (401C>A 403A>T), Y102del (404_406delTAT), F104I (410T>A 412T>A), K105Q (413A>C 415A>G), C106A (416T>G 417G>C 418T>A), Q107H (421A>T), V108A (423T>C 424T>G), T113K (438C>A), I115V (443A>G 445C>G), K116G (446A>G 447A>G 448G>T), S118D (452A>G 453G>A 454C>T), Q119I (455C>A 456A>T 457G>C), E120R (458G>A 459A>G), T121K (462C>A 463T>G), K123E (467A>G), K123_K124insH (469_470insCAT), K124G (470A>G 471A>G), A125V (474C>T 475C>A), L128V (482T>G 484G>C), I129S (485A>T 486T>C), M135V (503A>G 505G>C), I136C (506A>T 507T>G), L138M (512C>A 514A>G), L139I (515C>A 517C>T), T140K (519C>A 520C>A), K142R (525A>G), S143T (528G>C), I144L (530A>T 532C>A), D145R (533G>C 534A>G 535T>A), K147R (540A>G 541A>G), I151M (553A>G), V152F (554G>T), I154L (560A>T 562A>G), I162S (584A>T 585T>C 586A>T), D163R (587G>A 588A>G 589T>A), N164G (590A>G 591A>G), L166K (596T>A 597T>A), K168Q (602A>C 604A>G), Q170Y (608C>T 610A>T), E174Q (620G>C 622A>G), F175L (623T>C), F176L (628T>G), E177P (629G>C 630A>C), S178P (632A>C 633G>C 634T>A), H179K (635C>A 637T>A), V180I (638G>A), I183G (647A>G 648T>G), L184V (650T>G 652A>T), L185F (655A>C), V189M (665G>A 667T>G), S191P (671T>C), R192E (674A>G 675G>A), V193A (678T>C), I194L (680A>C 682C>T), N195E (683A>G 685T>G), T196I (687C>T 688A>T), S197T (689T>A), Q198R (692C>A 693A>G 694G>A), V199K (695G>A 696T>A 697C>G), R202N (705G>A 706A>C), D203K (707G>A 709T>G), K206R (717A>G 718A>G), N211R (731A>C 732A>G), A212D (735C>A), R220K (759G>A), Y222F (765A>T), I224V (770A>G 772A>C), K227D (779A>G 781G>T), N229E (785A>G 787T>A), D230E (790T>G), F231W (792T>G 793T>G), A233L (797G>C 798C>T 799A>T), F237C (810T>G), D241E (823C>G), H242T (824C>A 825A>C 826C>T), S244A (830A>G 831G>C), L245I (833C>A), T248S (843C>G 844A>T), L249V (845C>G 847A>C), C252V (854T>G 855G>T), Q257K (869C>A 871A>G), V260W (878G>T 879T>G 880A>G), M262T (885T>C 886G>T), E263D (889A>T), C264K (890T>A 891G>A 892T>A), L265M (893T>A 895A>G), T266R (896A>C 897C>G 898A>T), N267S (900A>G 901T>C), N269D (905A>G), F270H (908T>C 909T>A), A272V (915C>T), S274A (920A>G 921G>C 922C>A), I275T (924T>C 925C>T), S280D (938T>G 939C>A 940C>T), Q282N (944C>A 946A>T), E283T (947G>A 948A>C), F286I (956T>A 958T>C), K289R (966A>G 967A>G), D293S (977G>T 978A>C), K295S (983A>T 984A>C), T296S (986A>T), I298V (992A>G 994A>G), L300I (998C>A 1000T>C), S301T (1001T>A), N303D (1007A>G), I314V (1040A>G 1042A>C), H324T (1070C>A 1071A>C), N325Q (1073A>C 1075T>A), R326P (1076A>C 1077G>C), I330L (1088A>C 1090A>G), I344V (1130A>G), I349V (1145A>G 1147A>C), E351K (1151G>A 1153A>G), E353D (1159A>T), L354E (1160T>G 1161T>A), P355R (1163C>A 1164C>G 1165A>G), K356M (1167A>T), A358F (1172G>T 1173C>T), E359D (1177A>C), L360I (1178T>A), E361Q (1181G>C 1183A>G), T362K (1185C>A 1186G>A), T366V (1196A>G 1197C>T), K367V (1199A>G 1200A>T), D369E (1207T>G), M371L (1211A>C 1213G>C), E373A (1218A>C 1219G>C), I375V (1223A>G), V376A (1227T>C), I379L (1235A>C 1237A>G)								

	Begin	End	Coverage	Score	Concordance	Matches	Identities	I/D/M/F*	Stop Codons
**NT**	**118**	**1238**	**83.7%**	**420**	**18.9%**	**1118 (99.5%)**	**670 (59.6%)**	**3/3**	
Codon mutations:	GCT6..G (118T>G), GAA7AGT (119G>A 120A>G 121A>T), GAC9GCT (126A>C 127C>T), CAT10GAG (128C>G 130T>G), GGC12GGG (136C>G), CTC13ATT (137C>A 139C>T), AAG14AAA (142G>A), AAA15GAT (143A>G 145A>T), AAT16GAC (146A>G 148T>C), ATC17CTT (149A>C 151C>T), CTA18CTT (154A>T), AAG19AGA (156A>G 157G>A), GGT20GGG (160T>G), ATA21ATT (163A>T), TAT22TAC (166T>C), TCT23CAG (167T>C 168C>A 169T>G), TGT24TAC (171G>A 172T>C), GGA25GGG (175A>G), TTT26TTC (178T>C), CCT29CCA (187T>A), TCA30TCT (190A>T), ACT31GCC (191A>G 193T>C), AAA35CGA (203A>C 204A>G), GCC36GCT (208C>T), ATT37GTA (209A>G 211T>A), TTT38TTG (214T>G), CCA39CCT (217A>T), TGT40ATT (218T>A 219G>T), ATT41ATA (223T>A), TCT42TCG (226T>G), AAA44CGT (230A>C 231A>G 232A>T), GTG46GTC (238G>C), ATA47ATT (241A>T), GTC48GCT (243T>C 244C>T), CAA49CAG (247A>G), TCG52TCC (256G>C), GGA53GGT (259A>T), ACA54ACC (262A>C), ACA57ACC (271A>C), GCA58TCC (272G>T 274A>C), ACA59ATG (276C>T 277A>G), TAT60ATT (278T>A 279A>T), GCA61GCT (283A>T), ATC62CTT (284A>C 286C>T), AGT63ACC (288G>C 289T>C), TTA65TGC (294T>G 295A>C), CAG66CAA (298G>A), CAA67GTT (299C>G 300A>T 301A>T), ATA68GTG (302A>G 304A>G), ACT70ACC (310T>C), TCT71AAA (311T>A 312C>A 313T>A), AAT74GAG (320A>G 322T>G), ATC75GTA (323A>G 325C>A), GCT77GCA (331T>A), ATC79ATA (337C>A), ACC81TCT (341A>T 343C>T), CCA82CCT (346A>T), CGT84CGG (352T>G), GAA85GAG (355A>G), TTG86CTT (356T>C 358G>T), GCT87GCA (361T>A), CTC88GCT (362C>G 363T>C 364C>T), GCT90ACG (368G>A 370T>G), CAA91GAA (371C>G), CGT92AAG (374C>A 375G>A 376T>G), GTA93GTG (379A>G), TTG94ATT (380T>A 382G>T), CAA95TTG (383C>T 384A>T 385A>G), ACA96GCA (386A>G), GGG98GGA (394G>A), AAT99GAT (395A>G), TAT100TAC (400T>C), CTA101ATT (401C>A 403A>T), TAT102del (404_406delTAT), AAT103AAC (409T>C), TTT104ATA (410T>A 412T>A), AAA105CAG (413A>C 415A>G), TGT106GCA (416T>G 417G>C 418T>A), CAA107CAT (421A>T), GTT108GCG (423T>C 424T>G), ACA113AAA (438C>A), AGT114AGC (442T>C), ATC115GTG (443A>G 445C>G), AAG116GGT (446A>G 447A>G 448G>T), AGC118GAT (452A>G 453G>A 454C>T), CAG119ATC (455C>A 456A>T 457G>C), GAA120AGA (458G>A 459A>G), ACT121AAG (462C>A 463T>G), AAG123GAG (467A>G), AAG123_AAA124insCAT (469_470insCAT), AAA124GGA (470A>G 471A>G), GCC125GTA (474C>T 475C>A), CAG126CAA (478G>A), TTG128GTC (482T>G 484G>C), ATT129TCT (485A>T 486T>C), CGA134AGA (500C>A), ATG135GTC (503A>G 505G>C), ATT136TGT (506A>T 507T>G), GAT137GAC (511T>C), CTA138ATG (512C>A 514A>G), CTC139ATT (515C>A 517C>T), ACC140AAA (519C>A 520C>A), CGA141AGG (521C>A 523A>G), AAA142AGA (525A>G), AGT143ACT (528G>C), ATC144TTA (530A>T 532C>A), GAT145CGA (533G>C 534A>G 535T>A), AAA147AGG (540A>G 541A>G), GCA148GCC (544A>C), ATT149ATC (547T>C), AAA150AAG (550A>G), ATA151ATG (553A>G), GTT152TTT (554G>T), GTA153GTT (559A>T), ATA154TTG (560A>T 562A>G), GAT158GAC (574T>C), GAG159GAA (577G>A), TTG161CTC (581T>C 583G>C), ATA162TCT (584A>T 585T>C 586A>T), GAT163AGA (587G>A 588A>G 589T>A), AAT164GGT (590A>G 591A>G), TTT165TTC (595T>C), TTG166AAG (596T>A 597T>A), AAA168CAG (602A>C 604A>G), ATA169ATT (607A>T), CAA170TAT (608C>T 610A>T), ATT172ATA (616T>A), GAA174CAG (620G>C 622A>G), TTT175CTT (623T>C), TTT176TTG (628T>G), GAA177CCA (629G>C 630A>C), AGT178CCA (632A>C 633G>C 634T>A), CAT179AAA (635C>A 637T>A), GTC180ATC (638G>A), GTT182GTG (646T>G), ATT183GGT (647A>G 648T>G), TTA184GTT (650T>G 652A>T), TTA185TTC (655A>C), TCA186TCT (658A>T), GCA187GCC (661A>C), ACC188ACT (664C>T), GTT189ATG (665G>A 667T>G), TCA191CCA (671T>C), AGG192GAG (674A>G 675G>A), GTT193GCT (678T>C), ATC194CTT (680A>C 682C>T), AAT195GAG (683A>G 685T>G), ACA196ATT (687C>T 688A>T), TCT197ACT (689T>A), CAG198AGA (692C>A 693A>G 694G>A), GTC199AAG (695G>A 696T>A 697C>G), TTT200TTC (700T>C), AGA202AAC (705G>A 706A>C), GAT203AAG (707G>A 709T>G), GTA205GTG (715A>G), AAA206AGG (717A>G 718A>G), CTG208CTT (724G>T), GTA209GTG (727A>G), AAA210AAG (730A>G), AAT211CGT (731A>C 732A>G), GCT212GAT (735C>A), GAA213GAG (739A>G), TTG214CTC (740T>C 742G>C), ACA215ACT (745A>T), CTC216CTT (748C>T), GGC218GGT (754C>T), AGG220AAG (759G>A), TAT222TTT (765A>T), ATA224GTC (770A>G 772A>C), GTA226GTC (778A>C), AAG227GAT (779A>G 781G>T), AAA228AAG (784A>G), AAT229GAA (785A>G 787T>A), GAT230GAG (790T>G), TTT231TGG (792T>G 793T>G), AAA232AAG (796A>G), GCA233CTT (797G>C 798C>T 799A>T), TTT237TGT (810T>G), TAT240TAC (820T>C), GAC241GAG (823C>G), CAC242ACT (824C>A 825A>C 826C>T), TTA243TTG (829A>G), AGC244GCC (830A>G 831G>C), CTC245ATC (833C>A), ACT246ACC (838T>C), CAA247CAG (841A>G), ACA248AGT (843C>G 844A>T), CTA249GTC (845C>G 847A>C), ATA250ATC (850A>C), TGT252GTT (854T>G 855G>T), AAT253AAC (859T>C), ACA254ACT (862A>T), CGT255AGG (863C>A 865T>G), AGA256CGA (866A>C), CAA257AAG (869C>A 871A>G), GTA260TGG (878G>T 879T>G 880A>G), ATG262ACT (885T>C 886G>T), GAA263GAT (889A>T), TGT264AAA (890T>A 891G>A 892T>A), TTA265ATG (893T>A 895A>G), ACA266CGT (896A>C 897C>G 898A>T), AAT267AGC (900A>G 901T>C), AGA268CGT (902A>C 904A>T), AAT269GAT (905A>G), TTC270CAC (908T>C 909T>A), GCA272GTA (915C>T), TCA273TCT (919A>T), AGC274GCA (920A>G 921G>C 922C>A), ATC275ACT (924T>C 925C>T), GAT278GAC (934T>C), TCC280GAT (938T>G 939C>A 940C>T), CAA281CAG (943A>G), CAA282AAT (944C>A 946A>T), GAA283ACA (947G>A 948A>C), CGT284AGA (950C>A 952T>A), TTT286ATC (956T>A 958T>C), AAA289AGG (966A>G 967A>G), GAA290GAG (970A>G), TTT291TTC (973T>C), AGA292CGA (974A>C), GAT293TCT (977G>T 978A>C), GGT294GGC (982T>C), AAA295TCA (983A>T 984A>C), ACT296TCT (986A>T), AGA297CGT (989A>C 991A>T), ATA298GTG (992A>G 994A>G), CTT300ATC (998C>A 1000T>C), TCA301ACA (1001T>A), AAT303GAT (1007A>G), CTA304CTC (1012A>C), TTA305CTT (1013T>C 1015A>T), GCA306GCT (1018A>T), AGA307CGT (1019A>C 1021A>T), GGC308GGT (1024C>T), GTT311GTA (1033T>A), CAA312CAG (1036A>G), ATA314GTC (1040A>G 1042A>C), TCT315TCC (1045T>C), TTA316CTT (1046T>C 1048A>T), GTT317GTC (1051T>C), ATC318ATT (1054C>T), AAT319AAC (1057T>C), GAT321GAC (1063T>C), TTG322CTG (1064T>C), CAT324ACT (1070C>A 1071A>C), AAT325CAA (1073A>C 1075T>A), AGA326CCA (1076A>C 1077G>C), TAT329TAC (1087T>C), ATA330CTG (1088A>C 1090A>G), AGA335CGT (1103A>C 1105A>T), GGT337GGA (1111T>A), AGA338CGA (1112A>C), GGC340GGA (1120C>A), CGA341AGG (1121C>A 1123A>G), AAA342AAG (1126A>G), GGG343GGT (1129G>T), ATT344GTT (1130A>G), AAT347AAC (1141T>C), ATA349GTC (1145A>G 1147A>C), ACT350ACC (1150T>C), GAA351AAG (1151G>A 1153A>G), GAC352GAT (1156C>T), GAA353GAT (1159A>T), TTA354GAA (1160T>G 1161T>A), CCA355AGG (1163C>A 1164C>G 1165A>G), AAG356ATG (1167A>T), GCT358TTT (1172G>T 1173C>T), GAA359GAC (1177A>C), TTA360ATA (1178T>A), GAA361CAG (1181G>C 1183A>G), ACG362AAA (1185C>A 1186G>A), TAT364TAC (1192T>C), AAT365AAC (1195T>C), ACC366GTC (1196A>G 1197C>T), AAA367GTA (1199A>G 1200A>T), ATA368ATC (1204A>C), GAT369GAG (1207T>G), GAA370GAG (1210A>G), ATG371CTC (1211A>C 1213G>C), GAG373GCC (1218A>C 1219G>C), ATT375GTT (1223A>G), GTT376GCT (1227T>C), TTG378CTC (1232T>C 1234G>C), ATA379CTG (1235A>C 1237A>G)								

Proteins

	Begin	End	Coverage	Score	Concordance	Matches	Identities	I/D/M/F*	Stop Codons
hypothetical protein (YP_009666148.1)	7	380	98.4%	1460	59.1%	373 (99.5%)	207 (55.2%)	1/1/0/0	0
Protein mutations:	E7S (119G>A 120A>G 121A>T), D9A (126A>C 127C>T), H10E (128C>G 130T>G), L13I (137C>A 139C>T), K15D (143A>G 145A>T), N16D (146A>G 148T>C), I17L (149A>C 151C>T), K19R (156A>G 157G>A), S23Q (167T>C 168C>A 169T>G), C24Y (171G>A 172T>C), T31A (191A>G 193T>C), K35R (203A>C 204A>G), I37V (209A>G 211T>A), F38L (214T>G), C40I (218T>A 219G>T), K44R (230A>C 231A>G 232A>T), V48A (243T>C 244C>T), A58S (272G>T 274A>C), T59M (276C>T 277A>G), Y60I (278T>A 279A>T), I62L (284A>C 286C>T), S63T (288G>C 289T>C), L65C (294T>G 295A>C), Q67V (299C>G 300A>T 301A>T), I68V (302A>G 304A>G), S71K (311T>A 312C>A 313T>A), N74E (320A>G 322T>G), I75V (323A>G 325C>A), T81S (341A>T 343C>T), L88A (362C>G 363T>C 364C>T), A90T (368G>A 370T>G), Q91E (371C>G), R92K (374C>A 375G>A 376T>G), L94I (380T>A 382G>T), Q95L (383C>T 384A>T 385A>G), T96A (386A>G), N99D (395A>G), L101I (401C>A 403A>T), Y102del (404_406delTAT), F104I (410T>A 412T>A), K105Q (413A>C 415A>G), C106A (416T>G 417G>C 418T>A), Q107H (421A>T), V108A (423T>C 424T>G), T113K (438C>A), I115V (443A>G 445C>G), K116G (446A>G 447A>G 448G>T), S118D (452A>G 453G>A 454C>T), Q119I (455C>A 456A>T 457G>C), E120R (458G>A 459A>G), T121K (462C>A 463T>G), K123E (467A>G), K123_K124insH (469_470insCAT), K124G (470A>G 471A>G), A125V (474C>T 475C>A), L128V (482T>G 484G>C), I129S (485A>T 486T>C), M135V (503A>G 505G>C), I136C (506A>T 507T>G), L138M (512C>A 514A>G), L139I (515C>A 517C>T), T140K (519C>A 520C>A), K142R (525A>G), S143T (528G>C), I144L (530A>T 532C>A), D145R (533G>C 534A>G 535T>A), K147R (540A>G 541A>G), I151M (553A>G), V152F (554G>T), I154L (560A>T 562A>G), I162S (584A>T 585T>C 586A>T), D163R (587G>A 588A>G 589T>A), N164G (590A>G 591A>G), L166K (596T>A 597T>A), K168Q (602A>C 604A>G), Q170Y (608C>T 610A>T), E174Q (620G>C 622A>G), F175L (623T>C), F176L (628T>G), E177P (629G>C 630A>C), S178P (632A>C 633G>C 634T>A), H179K (635C>A 637T>A), V180I (638G>A), I183G (647A>G 648T>G), L184V (650T>G 652A>T), L185F (655A>C), V189M (665G>A 667T>G), S191P (671T>C), R192E (674A>G 675G>A), V193A (678T>C), I194L (680A>C 682C>T), N195E (683A>G 685T>G), T196I (687C>T 688A>T), S197T (689T>A), Q198R (692C>A 693A>G 694G>A), V199K (695G>A 696T>A 697C>G), R202N (705G>A 706A>C), D203K (707G>A 709T>G), K206R (717A>G 718A>G), N211R (731A>C 732A>G), A212D (735C>A), R220K (759G>A), Y222F (765A>T), I224V (770A>G 772A>C), K227D (779A>G 781G>T), N229E (785A>G 787T>A), D230E (790T>G), F231W (792T>G 793T>G), A233L (797G>C 798C>T 799A>T), F237C (810T>G), D241E (823C>G), H242T (824C>A 825A>C 826C>T), S244A (830A>G 831G>C), L245I (833C>A), T248S (843C>G 844A>T), L249V (845C>G 847A>C), C252V (854T>G 855G>T), Q257K (869C>A 871A>G), V260W (878G>T 879T>G 880A>G), M262T (885T>C 886G>T), E263D (889A>T), C264K (890T>A 891G>A 892T>A), L265M (893T>A 895A>G), T266R (896A>C 897C>G 898A>T), N267S (900A>G 901T>C), N269D (905A>G), F270H (908T>C 909T>A), A272V (915C>T), S274A (920A>G 921G>C 922C>A), I275T (924T>C 925C>T), S280D (938T>G 939C>A 940C>T), Q282N (944C>A 946A>T), E283T (947G>A 948A>C), F286I (956T>A 958T>C), K289R (966A>G 967A>G), D293S (977G>T 978A>C), K295S (983A>T 984A>C), T296S (986A>T), I298V (992A>G 994A>G), L300I (998C>A 1000T>C), S301T (1001T>A), N303D (1007A>G), I314V (1040A>G 1042A>C), H324T (1070C>A 1071A>C), N325Q (1073A>C 1075T>A), R326P (1076A>C 1077G>C), I330L (1088A>C 1090A>G), I344V (1130A>G), I349V (1145A>G 1147A>C), E351K (1151G>A 1153A>G), E353D (1159A>T), L354E (1160T>G 1161T>A), P355R (1163C>A 1164C>G 1165A>G), K356M (1167A>T), A358F (1172G>T 1173C>T), E359D (1177A>C), L360I (1178T>A), E361Q (1181G>C 1183A>G), T362K (1185C>A 1186G>A), T366V (1196A>G 1197C>T), K367V (1199A>G 1200A>T), D369E (1207T>G), M371L (1211A>C 1213G>C), E373A (1218A>C 1219G>C), I375V (1223A>G), V376A (1227T>C), I379L (1235A>C 1237A>G)								

	Begin	End	Coverage	Score	Concordance	Matches	Identities	I/D/M/F*	Stop Codons
**NT**	**118**	**1238**	**83.7%**	**420**	**18.9%**	**1118 (99.5%)**	**670 (59.6%)**	**3/3**	
Codon mutations:	GCT6..G (118T>G), GAA7AGT (119G>A 120A>G 121A>T), GAC9GCT (126A>C 127C>T), CAT10GAG (128C>G 130T>G), GGC12GGG (136C>G), CTC13ATT (137C>A 139C>T), AAG14AAA (142G>A), AAA15GAT (143A>G 145A>T), AAT16GAC (146A>G 148T>C), ATC17CTT (149A>C 151C>T), CTA18CTT (154A>T), AAG19AGA (156A>G 157G>A), GGT20GGG (160T>G), ATA21ATT (163A>T), TAT22TAC (166T>C), TCT23CAG (167T>C 168C>A 169T>G), TGT24TAC (171G>A 172T>C), GGA25GGG (175A>G), TTT26TTC (178T>C), CCT29CCA (187T>A), TCA30TCT (190A>T), ACT31GCC (191A>G 193T>C), AAA35CGA (203A>C 204A>G), GCC36GCT (208C>T), ATT37GTA (209A>G 211T>A), TTT38TTG (214T>G), CCA39CCT (217A>T), TGT40ATT (218T>A 219G>T), ATT41ATA (223T>A), TCT42TCG (226T>G), AAA44CGT (230A>C 231A>G 232A>T), GTG46GTC (238G>C), ATA47ATT (241A>T), GTC48GCT (243T>C 244C>T), CAA49CAG (247A>G), TCG52TCC (256G>C), GGA53GGT (259A>T), ACA54ACC (262A>C), ACA57ACC (271A>C), GCA58TCC (272G>T 274A>C), ACA59ATG (276C>T 277A>G), TAT60ATT (278T>A 279A>T), GCA61GCT (283A>T), ATC62CTT (284A>C 286C>T), AGT63ACC (288G>C 289T>C), TTA65TGC (294T>G 295A>C), CAG66CAA (298G>A), CAA67GTT (299C>G 300A>T 301A>T), ATA68GTG (302A>G 304A>G), ACT70ACC (310T>C), TCT71AAA (311T>A 312C>A 313T>A), AAT74GAG (320A>G 322T>G), ATC75GTA (323A>G 325C>A), GCT77GCA (331T>A), ATC79ATA (337C>A), ACC81TCT (341A>T 343C>T), CCA82CCT (346A>T), CGT84CGG (352T>G), GAA85GAG (355A>G), TTG86CTT (356T>C 358G>T), GCT87GCA (361T>A), CTC88GCT (362C>G 363T>C 364C>T), GCT90ACG (368G>A 370T>G), CAA91GAA (371C>G), CGT92AAG (374C>A 375G>A 376T>G), GTA93GTG (379A>G), TTG94ATT (380T>A 382G>T), CAA95TTG (383C>T 384A>T 385A>G), ACA96GCA (386A>G), GGG98GGA (394G>A), AAT99GAT (395A>G), TAT100TAC (400T>C), CTA101ATT (401C>A 403A>T), TAT102del (404_406delTAT), AAT103AAC (409T>C), TTT104ATA (410T>A 412T>A), AAA105CAG (413A>C 415A>G), TGT106GCA (416T>G 417G>C 418T>A), CAA107CAT (421A>T), GTT108GCG (423T>C 424T>G), ACA113AAA (438C>A), AGT114AGC (442T>C), ATC115GTG (443A>G 445C>G), AAG116GGT (446A>G 447A>G 448G>T), AGC118GAT (452A>G 453G>A 454C>T), CAG119ATC (455C>A 456A>T 457G>C), GAA120AGA (458G>A 459A>G), ACT121AAG (462C>A 463T>G), AAG123GAG (467A>G), AAG123_AAA124insCAT (469_470insCAT), AAA124GGA (470A>G 471A>G), GCC125GTA (474C>T 475C>A), CAG126CAA (478G>A), TTG128GTC (482T>G 484G>C), ATT129TCT (485A>T 486T>C), CGA134AGA (500C>A), ATG135GTC (503A>G 505G>C), ATT136TGT (506A>T 507T>G), GAT137GAC (511T>C), CTA138ATG (512C>A 514A>G), CTC139ATT (515C>A 517C>T), ACC140AAA (519C>A 520C>A), CGA141AGG (521C>A 523A>G), AAA142AGA (525A>G), AGT143ACT (528G>C), ATC144TTA (530A>T 532C>A), GAT145CGA (533G>C 534A>G 535T>A), AAA147AGG (540A>G 541A>G), GCA148GCC (544A>C), ATT149ATC (547T>C), AAA150AAG (550A>G), ATA151ATG (553A>G), GTT152TTT (554G>T), GTA153GTT (559A>T), ATA154TTG (560A>T 562A>G), GAT158GAC (574T>C), GAG159GAA (577G>A), TTG161CTC (581T>C 583G>C), ATA162TCT (584A>T 585T>C 586A>T), GAT163AGA (587G>A 588A>G 589T>A), AAT164GGT (590A>G 591A>G), TTT165TTC (595T>C), TTG166AAG (596T>A 597T>A), AAA168CAG (602A>C 604A>G), ATA169ATT (607A>T), CAA170TAT (608C>T 610A>T), ATT172ATA (616T>A), GAA174CAG (620G>C 622A>G), TTT175CTT (623T>C), TTT176TTG (628T>G), GAA177CCA (629G>C 630A>C), AGT178CCA (632A>C 633G>C 634T>A), CAT179AAA (635C>A 637T>A), GTC180ATC (638G>A), GTT182GTG (646T>G), ATT183GGT (647A>G 648T>G), TTA184GTT (650T>G 652A>T), TTA185TTC (655A>C), TCA186TCT (658A>T), GCA187GCC (661A>C), ACC188ACT (664C>T), GTT189ATG (665G>A 667T>G), TCA191CCA (671T>C), AGG192GAG (674A>G 675G>A), GTT193GCT (678T>C), ATC194CTT (680A>C 682C>T), AAT195GAG (683A>G 685T>G), ACA196ATT (687C>T 688A>T), TCT197ACT (689T>A), CAG198AGA (692C>A 693A>G 694G>A), GTC199AAG (695G>A 696T>A 697C>G), TTT200TTC (700T>C), AGA202AAC (705G>A 706A>C), GAT203AAG (707G>A 709T>G), GTA205GTG (715A>G), AAA206AGG (717A>G 718A>G), CTG208CTT (724G>T), GTA209GTG (727A>G), AAA210AAG (730A>G), AAT211CGT (731A>C 732A>G), GCT212GAT (735C>A), GAA213GAG (739A>G), TTG214CTC (740T>C 742G>C), ACA215ACT (745A>T), CTC216CTT (748C>T), GGC218GGT (754C>T), AGG220AAG (759G>A), TAT222TTT (765A>T), ATA224GTC (770A>G 772A>C), GTA226GTC (778A>C), AAG227GAT (779A>G 781G>T), AAA228AAG (784A>G), AAT229GAA (785A>G 787T>A), GAT230GAG (790T>G), TTT231TGG (792T>G 793T>G), AAA232AAG (796A>G), GCA233CTT (797G>C 798C>T 799A>T), TTT237TGT (810T>G), TAT240TAC (820T>C), GAC241GAG (823C>G), CAC242ACT (824C>A 825A>C 826C>T), TTA243TTG (829A>G), AGC244GCC (830A>G 831G>C), CTC245ATC (833C>A), ACT246ACC (838T>C), CAA247CAG (841A>G), ACA248AGT (843C>G 844A>T), CTA249GTC (845C>G 847A>C), ATA250ATC (850A>C), TGT252GTT (854T>G 855G>T), AAT253AAC (859T>C), ACA254ACT (862A>T), CGT255AGG (863C>A 865T>G), AGA256CGA (866A>C), CAA257AAG (869C>A 871A>G), GTA260TGG (878G>T 879T>G 880A>G), ATG262ACT (885T>C 886G>T), GAA263GAT (889A>T), TGT264AAA (890T>A 891G>A 892T>A), TTA265ATG (893T>A 895A>G), ACA266CGT (896A>C 897C>G 898A>T), AAT267AGC (900A>G 901T>C), AGA268CGT (902A>C 904A>T), AAT269GAT (905A>G), TTC270CAC (908T>C 909T>A), GCA272GTA (915C>T), TCA273TCT (919A>T), AGC274GCA (920A>G 921G>C 922C>A), ATC275ACT (924T>C 925C>T), GAT278GAC (934T>C), TCC280GAT (938T>G 939C>A 940C>T), CAA281CAG (943A>G), CAA282AAT (944C>A 946A>T), GAA283ACA (947G>A 948A>C), CGT284AGA (950C>A 952T>A), TTT286ATC (956T>A 958T>C), AAA289AGG (966A>G 967A>G), GAA290GAG (970A>G), TTT291TTC (973T>C), AGA292CGA (974A>C), GAT293TCT (977G>T 978A>C), GGT294GGC (982T>C), AAA295TCA (983A>T 984A>C), ACT296TCT (986A>T), AGA297CGT (989A>C 991A>T), ATA298GTG (992A>G 994A>G), CTT300ATC (998C>A 1000T>C), TCA301ACA (1001T>A), AAT303GAT (1007A>G), CTA304CTC (1012A>C), TTA305CTT (1013T>C 1015A>T), GCA306GCT (1018A>T), AGA307CGT (1019A>C 1021A>T), GGC308GGT (1024C>T), GTT311GTA (1033T>A), CAA312CAG (1036A>G), ATA314GTC (1040A>G 1042A>C), TCT315TCC (1045T>C), TTA316CTT (1046T>C 1048A>T), GTT317GTC (1051T>C), ATC318ATT (1054C>T), AAT319AAC (1057T>C), GAT321GAC (1063T>C), TTG322CTG (1064T>C), CAT324ACT (1070C>A 1071A>C), AAT325CAA (1073A>C 1075T>A), AGA326CCA (1076A>C 1077G>C), TAT329TAC (1087T>C), ATA330CTG (1088A>C 1090A>G), AGA335CGT (1103A>C 1105A>T), GGT337GGA (1111T>A), AGA338CGA (1112A>C), GGC340GGA (1120C>A), CGA341AGG (1121C>A 1123A>G), AAA342AAG (1126A>G), GGG343GGT (1129G>T), ATT344GTT (1130A>G), AAT347AAC (1141T>C), ATA349GTC (1145A>G 1147A>C), ACT350ACC (1150T>C), GAA351AAG (1151G>A 1153A>G), GAC352GAT (1156C>T), GAA353GAT (1159A>T), TTA354GAA (1160T>G 1161T>A), CCA355AGG (1163C>A 1164C>G 1165A>G), AAG356ATG (1167A>T), GCT358TTT (1172G>T 1173C>T), GAA359GAC (1177A>C), TTA360ATA (1178T>A), GAA361CAG (1181G>C 1183A>G), ACG362AAA (1185C>A 1186G>A), TAT364TAC (1192T>C), AAT365AAC (1195T>C), ACC366GTC (1196A>G 1197C>T), AAA367GTA (1199A>G 1200A>T), ATA368ATC (1204A>C), GAT369GAG (1207T>G), GAA370GAG (1210A>G), ATG371CTC (1211A>C 1213G>C), GAG373GCC (1218A>C 1219G>C), ATT375GTT (1223A>G), GTT376GCT (1227T>C), TTG378CTC (1232T>C 1234G>C), ATA379CTG (1235A>C 1237A>G)								

*: Inserts / Deletes / Misaligned / Frameshifts

Analysis details

This analysis was performed with panviral2.64

NGS Details (UN8): Bracoviriform glomeratae (segment NC_043292.1)

Assembly

**Coverage Length**	310 (1 contig(s))
**Depth Of Coverage**	2096.9
**Number Of Reads**	5742
**Reads Per Million**	95.96 rpm (after QC)
**Ambiguities**	0
**Assembly Method**	de novo + reference guided assembly
**Consensus Caller**	Bcf Tools

Coverage Map

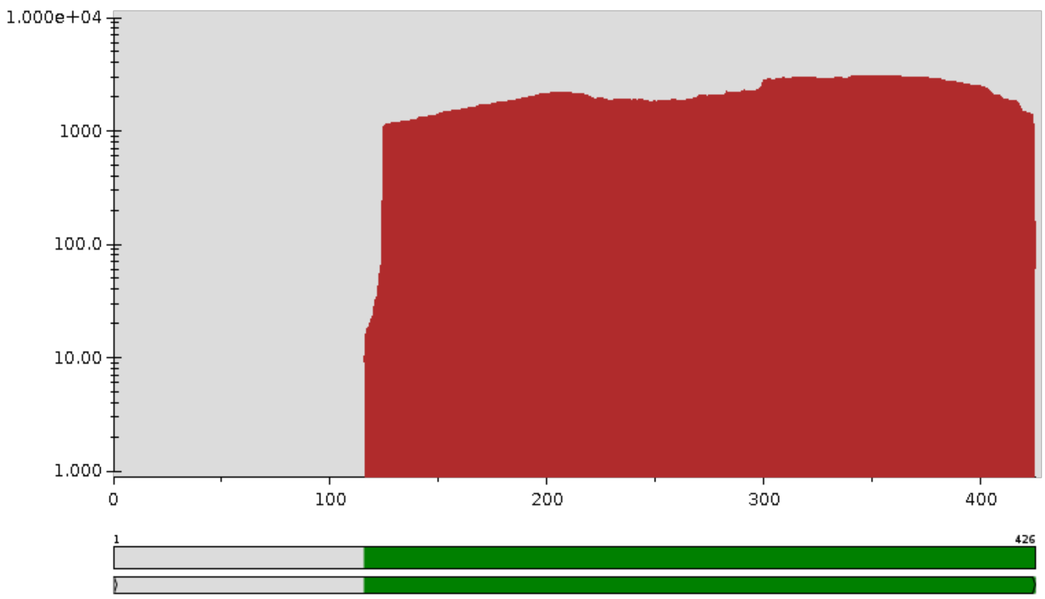

Assignment

**Type**	Bracoviriform glomeratae (Taxonomy ID: 257816)
**Reference Genome**	NC_043292.1
**NT Identity (%)**	72.5806
**AA Identity (%)**	84.466
**Number Of Stop Codons**	1
**Number Of CDS**	1

Alignment

**Alignment Score**	280.0 (NT) + 567.0 (AA) = 847.0
**Concordance (%)**	66.3273

Alignment Method	Global, seeded, nucleotide + amino acids (AGA)

Genome Region

Sequence starts at position 117 and ends at position 426 relative to NC_043292.1 reference sequence.

Alignment Detailed Statistics

	Begin	End	Coverage	Score	Concordance	Matches	Identities	I/D/M/F*	Stop Codons
**NT**	**117**	**426**	**72.8%**	**280**	**45.2%**	**310 (100%)**	**225 (72.6%)**	**0/0**	
Mutations:	126G>T, 129C>A, 132A>G, 136A>G, 137C>G, 138G>C, 150A>C, 153A>G, 155C>G, 156G>A, 159C>A, 166C>A, 168T>G, 169T>C, 171T>C, 172C>A, 174C>G, 177A>G, 183T>A, 184C>A, 189T>C, 198A>G, 201A>C, 207C>T, 214A>G, 219C>T, 222T>C, 223C>A, 225T>G, 228G>T, 235C>A, 237T>G, 243A>T, 246C>G, 255C>T, 261A>G, 262T>C, 264A>G, 265G>A, 273A>G, 278T>C, 279T>C, 284A>G, 285T>A, 288T>G, 289T>C, 300C>T, 301C>T, 303C>G, 309C>T, 312T>G, 315C>T, 322G>T, 329T>C, 333T>C, 336C>T, 339A>G, 345A>C, 347A>G, 348A>G, 349C>A, 351T>A, 360C>T, 363C>T, 366C>T, 375C>T, 378C>T, 384A>C, 385T>C, 387G>C, 390A>G, 391C>A, 392A>G, 393T>A, 394A>C, 396A>G, 399C>A, 400C>A, 402T>G, 404T>C, 405G>T, 406A>C, 414T>G, 416A>T, 420A>T								
**CDS**									
FK954_p501	40	142	72.5%	567	85.3%	103 (100%)	87 (84.5%)	0/0/0/0	1
Protein mutations:	L42F (126G>T), T46G (136A>G 137C>G 138G>C), A52G (155C>G 156G>A), Y57H (169T>C 171T>C), T72A (214A>G), V89I (265G>A), I93T (278T>C 279T>C), D95G (284A>G 285T>A), A108S (322G>T), I110T (329T>C), K116R (347A>G 348A>G), H131R (391C>A 392A>G 393T>A), K132Q (394A>C 396A>G), M135T (404T>C 405G>T), I136L (406A>C), Y139F (416A>T)								
Codon mutations:	TTG42TTT (126G>T), GGC43GGA (129C>A), AAA44AAG (132A>G), ACG46GGC (136A>G 137C>G 138G>C), GGA50GGC (150A>C), AAA51AAG (153A>G), GCG52GGA (155C>G 156G>A), GGC53GGA (159C>A), CGT56AGG (166C>A 168T>G), TAT57CAC (169T>C 171T>C), CGC58AGG (172C>A 174C>G), AAA59AAG (177A>G), CTT61CTA (183T>A), CGA62AGA (184C>A), GAT63GAC (189T>C), CAA66CAG (198A>G), GGA67GGC (201A>C), ACC69ACT (207C>T), ACT72GCT (214A>G), ATC73ATT (219C>T), CGT74CGC (222T>C), CGT75AGG (223C>A 225T>G), CTG76CTT (228G>T), CGT79AGG (235C>A 237T>G), GGA81GGT (243A>T), GTC82GTG (246C>G), ATC85ATT (255C>T), GGA87GGG (261A>G), TTA88CTG (262T>C 264A>G), GTC89ATC (265G>A), GAA91GAG (273A>G), ATT93ACC (278T>C 279T>C), GAT95GGA (284A>G 285T>A), GTT96GTG (288T>G), TTG97CTG (289T>C), TTC100TTT (300C>T), CTC101TTG (301C>T 303C>G), AAC103AAT (309C>T), GTT104GTG (312T>G), ATC105ATT (315C>T), GCT108TCT (322G>T), ATC110ACC (329T>C), TAT111TAC (333T>C), ACC112ACT (336C>T), GAA113GAG (339A>G), GCA115GCC (345A>C), AAA116AGG (347A>G 348A>G), CGT117AGA (349C>A 351T>A), GTC120GTT (360C>T), ACC121ACT (363C>T), GCC122GCT (366C>T), GTC125GTT (375C>T), GTC126GTT (378C>T), GCA128GCC (384A>C), TTG129CTC (385T>C 387G>C), AAA130AAG (390A>G), CAT131AGA (391C>A 392A>G 393T>A), AAA132CAG (394A>C 396A>G), GGC133GGA (399C>A), CGT134AGG (400C>A 402T>G), ATG135ACT (404T>C 405G>T), ATC136CTC (406A>C), GGT138GGG (414T>G), TAT139TTT (416A>T), GGA140GGT (420A>T)								
**Proteins**									
putative histone 4 (YP_009665791.1)	40	142	72.5%	567	85.3%	103 (100%)	87 (84.5%)	0/0/0/0	1
Protein mutations:	L42F (126G>T), T46G (136A>G 137C>G 138G>C), A52G (155C>G 156G>A), Y57H (169T>C 171T>C), T72A (214A>G), V89I (265G>A), I93T (278T>C 279T>C), D95G (284A>G 285T>A), A108S (322G>T), I110T (329T>C), K116R (347A>G 348A>G), H131R (391C>A 392A>G 393T>A), K132Q (394A>C 396A>G), M135T (404T>C 405G>T), I136L (406A>C), Y139F (416A>T)								
Codon mutations:	TTG42TTT (126G>T), GGC43GGA (129C>A), AAA44AAG (132A>G), ACG46GGC (136A>G 137C>G 138G>C), GGA50GGC (150A>C), AAA51AAG (153A>G), GCG52GGA (155C>G 156G>A), GGC53GGA (159C>A), CGT56AGG (166C>A 168T>G), TAT57CAC (169T>C 171T>C), CGC58AGG (172C>A 174C>G), AAA59AAG (177A>G), CTT61CTA (183T>A), CGA62AGA (184C>A), GAT63GAC (189T>C), CAA66CAG (198A>G), GGA67GGC (201A>C), ACC69ACT (207C>T), ACT72GCT (214A>G), ATC73ATT (219C>T), CGT74CGC (222T>C), CGT75AGG (223C>A 225T>G), CTG76CTT (228G>T), CGT79AGG (235C>A 237T>G), GGA81GGT (243A>T), GTC82GTG (246C>G), ATC85ATT (255C>T), GGA87GGG (261A>G), TTA88CTG (262T>C 264A>G), GTC89ATC (265G>A), GAA91GAG (273A>G), ATT93ACC (278T>C 279T>C), GAT95GGA (284A>G 285T>A), GTT96GTG (288T>G), TTG97CTG (289T>C), TTC100TTT (300C>T), CTC101TTG (301C>T 303C>G), AAC103AAT (309C>T), GTT104GTG (312T>G), ATC105ATT (315C>T), GCT108TCT (322G>T), ATC110ACC (329T>C), TAT111TAC (333T>C), ACC112ACT (336C>T), GAA113GAG (339A>G), GCA115GCC (345A>C), AAA116AGG (347A>G 348A>G), CGT117AGA (349C>A 351T>A), GTC120GTT (360C>T), ACC121ACT (363C>T), GCC122GCT (366C>T), GTC125GTT (375C>T), GTC126GTT (378C>T), GCA128GCC (384A>C), TTG129CTC (385T>C 387G>C), AAA130AAG (390A>G), CAT131AGA (391C>A 392A>G 393T>A), AAA132CAG (394A>C 396A>G), GGC133GGA (399C>A), CGT134AGG (400C>A 402T>G), ATG135ACT (404T>C 405G>T), ATC136CTC (406A>C), GGT138GGG (414T>G), TAT139TTT (416A>T), GGA140GGT (420A>T)								

*: Inserts / Deletes / Misaligned / Frameshifts

Analysis details

This analysis was performed with panviral2.64

NGS Details (UN8): Potato leafroll virus

Assembly

**Coverage Length**	5827 (1 contig(s))
**Depth Of Coverage**	72.1
**Number Of Reads**	3146
**Reads Per Million**	52.58 rpm (after QC)
**Ambiguities**	0
**Assembly Method**	read mapping against reference + variant calling
**Consensus Caller**	Bcf Tools

Coverage Map

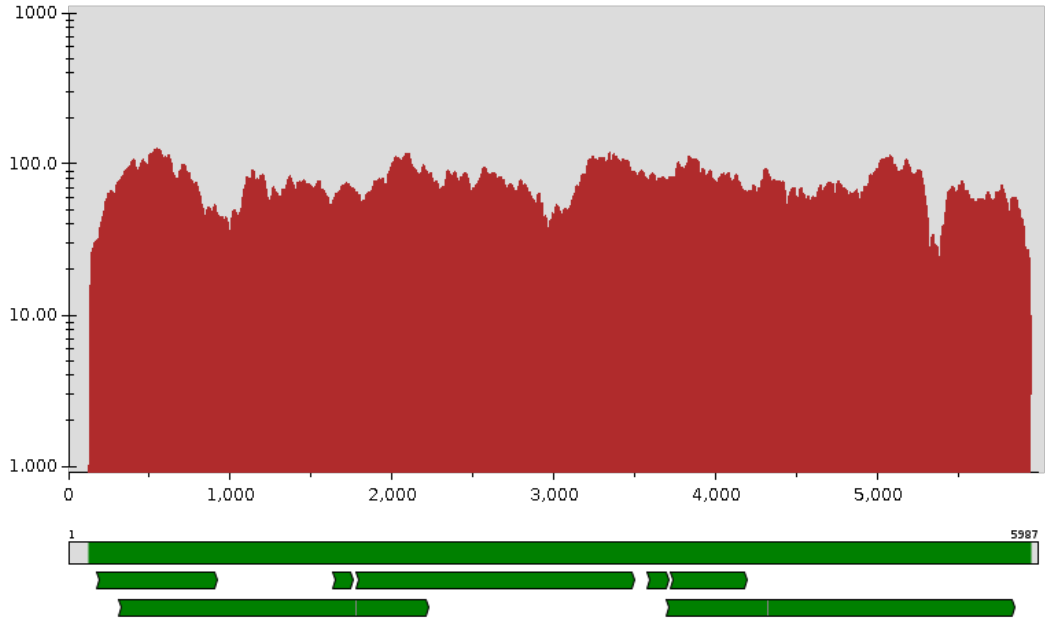

Assignment

**Type**	Potato leafroll virus (Taxonomy ID: 12045)
**Reference Genome**	NC_001747.1
**NT Identity (%)**	98.3173
**AA Identity (%)**	97.8212
**Number Of Stop Codons**	9
**Number Of CDS**	8

Alignment

**Alignment Score**	11226.0 (NT) + 20943.0 (AA) = 32169.0
**Concordance (%)**	97.2882

**Alignment Method**	Global, seeded, nucleotide + amino acids (AGA)

Genome Region

Sequence starts at position 122 and ends at position 5948 relative to NC_001747.1 reference sequence.

Alignment Detailed Statistics

	Begin	End	Coverage	Score	Concordance	Matches	Identities	I/D/M/F*	Stop Codons
**NT**	**122**	**5948**	**97.3%**	**11226**	**96.6%**	**5822 (99.9%)**	**5726 (98.2%)**	**2/5**	
Mutations:	656G>A, 665A>G, 699A>C, 822C>T, 825T>G, 903G>A, 931A>G, 958G>A, 1036G>A, 1126G>A, 1132T>G, 1147C>T, 1171C>T, 1204A>G, 1234A>T, 1300T>C, 1319G>A, 1327C>T, 1356G>A, 1577G>T, 1593G>A, 1611A>G, 1716A>G, 1754C>T, 1779A>G, 1894C>A, 1909A>C, 1915G>A, 1957C>A, 2007T>C, 2051G>A, 2154C>T, 2265A>G, 2283G>A, 2313A>G, 2316T>G, 2470G>A, 2555C>T, 2586A>G, 2640C>T, 2649C>T, 2729A>G, 2849C>T, 2856G>A, 2877C>T, 2898T>C, 2931T>C, 3000T>C, 3033C>T, 3036C>T, 3069C>T, 3117C>T, 3171A>G, 3186T>C, 3321C>T, 3378G>T, 3379A>G, 3387C>T, 3614T>A, 3619C>T, 3652T>A, 3721T>G, 3748_3750delTGC, 3788T>G, 3830C>T, 3939A>T, 4031A>G, 4094G>A, 4113G>A, 4166C>T, 4172T>C, 4208C>T, 4214T>G, 4658G>A, 4661T>C, 4733C>G, 4817T>C, 4883C>T, 4886T>C, 4913A>T, 4949G>A, 4964A>G, 4979C>T, 5038C>T, 5062C>T, 5071A>G, 5072C>T, 5129G>A, 5162C>T, 5189C>T, 5199C>T, 5216A>C, 5268A>G, 5689_5690insGA, 5692_5693delGT, 5741T>G, 5748A>G, 5781G>A, 5925T>C								

CDS

	Begin	End	Coverage	Score	Concordance	Matches	Identities	I/D/M/F*	Stop Codons
PLRVgp1	1	248	100%	1715	99.0%	248 (100%)	245 (98.8%)	0/0/0/0	1
Protein mutations:	S161N (656G>A), Q164R (665A>G), Q175H (699A>C)								
Codon mutations:	AGT161AAT (656G>A), CAA164CGA (665A>G), CAA175CAC (699A>C), CGC216CGT (822C>T), GCT217GCG (825T>G), AAG243AAA (903G>A)								
PLRVgp2	1	1063	100%	7327	98.2%	1063 (100%)	1041 (97.9%)	0/0/0/0	1
Protein mutations:	V117I (656G>A), K120E (665A>G), N131T (699A>C), A172V (822C>T), L173R (825T>G), R199K (903G>A), M273I (1126G>A), A338T (1319G>A), G350E (1356G>A), V424F (1577G>T), R429K (1593G>A), D435G (1611A>G), E470G (1716A>G), Q530K (1894C>A), E537K (1915G>A), Q551K (1957C>A), G582E (2051G>A), A722T (2470G>A), A750V (2555C>T), N808S (2729A>G), P848L (2849C>T), I1025V (3379A>G)								
Codon mutations:	GTA117ATA (656G>A), AAA120GAA (665A>G), AAC131ACC (699A>C), GCG172GTG (822C>T), CTA173CGA (825T>G), AGG199AAG (903G>A), GAA208GAG (931A>G), CAG217CAA (958G>A), TTG243TTA (1036G>A), ATG273ATA (1126G>A), ACT275ACG (1132T>G), TTC280TTT (1147C>T), TCC288TCT (1171C>T), CTA299CTG (1204A>G), ACA309ACT (1234A>T), CAT331CAC (1300T>C), GCC338ACC (1319G>A), CAC340CAT (1327C>T), GGA350GAA (1356G>A), GTC424TTC (1577G>T), AGA429AAA (1593G>A), GAC435GGC (1611A>G), GAG470GGG (1716A>G), CTA483TTA (1754C>T), GCA491GCG (1779A>G), CAA530AAA (1894C>A), AGA535CGA (1909A>C), GAA537AAA (1915G>A), CAA551AAA (1957C>A), TTT567TTC (2007T>C), GGA582GAA (2051G>A), CAC616CAT (2154C>T), TCA653TCG (2265A>G), GCG659GCA (2283G>A), ACA669ACG (2313A>G), GTT670GTG (2316T>G), GCA722ACA (2470G>A), GCC750GTC (2555C>T), CTA760CTG (2586A>G), CAC778CAT (2640C>T), AGC781AGT (2649C>T), AAT808AGT (2729A>G), CCA848CTA (2849C>T), GTG850GTA (2856G>A), CAC857CAT (2877C>T), ACT864ACC (2898T>C), TAT875TAC (2931T>C), CGT898CGC (3000T>C), AAC909AAT (3033C>T), TCC910TCT (3036C>T), GCC921GCT (3069C>T), TCC937TCT (3117C>T), GCA955GCG (3171A>G), GAT960GAC (3186T>C), AAC1005AAT (3321C>T), GTG1024GTT (3378G>T), ATT1025GTT (3379A>G), AAC1027AAT (3387C>T)								
PLRVgp3	1	640	100%	4199	97.8%	640 (100%)	623 (97.3%)	0/0/0/0	1
Protein mutations:	V117I (656G>A), K120E (665A>G), N131T (699A>C), A172V (822C>T), L173R (825T>G), R199K (903G>A), M273I (1126G>A), A338T (1319G>A), G350E (1356G>A), V424F (1577G>T), R429K (1593G>A), D435G (1611A>G), E470G (1716A>G), Q491R (1779A>G), L567S (2007T>C), E582K (2051G>A), T616I (2154C>T)								
Codon mutations:	GTA117ATA (656G>A), AAA120GAA (665A>G), AAC131ACC (699A>C), GCG172GTG (822C>T), CTA173CGA (825T>G), AGG199AAG (903G>A), GAA208GAG (931A>G), CAG217CAA (958G>A), TTG243TTA (1036G>A), ATG273ATA (1126G>A), ACT275ACG (1132T>G), TTC280TTT (1147C>T), TCC288TCT (1171C>T), CTA299CTG (1204A>G), ACA309ACT (1234A>T), CAT331CAC (1300T>C), GCC338ACC (1319G>A), CAC340CAT (1327C>T), GGA350GAA (1356G>A), GTC424TTC (1577G>T), AGA429AAA (1593G>A), GAC435GGC (1611A>G), GAG470GGG (1716A>G), CTA483TTA (1754C>T), CAA491CGA (1779A>G), ATC529ATA (1894C>A), ATA534ATC (1909A>C), GAG536GAA (1915G>A), CCC550CCA (1957C>A), TTA567TCA (2007T>C), GAG582AAG (2051G>A), ACC616ATC (2154C>T)								
Rap1	1	42	100%	277	96.5%	42 (100%)	41 (97.6%)	0/0/0/0	1
Protein mutations:	R29G (1716A>G)								
Codon mutations:	AGA29GGA (1716A>G), CTC41CTT (1754C>T)								
ORF3a	1	46	100%	289	96.7%	46 (100%)	44 (95.7%)	0/0/0/0	1
Protein mutations:	S14T (3614T>A), F26L (3652T>A)								
Codon mutations:	TCA14ACA (3614T>A), TCC15TCT (3619C>T), TTT26TTA (3652T>A)								
PLRVgp4	1	718	100%	4695	93.4%	717 (99.7%)	705 (98.1%)	1/1/2/2	2
Protein mutations:	V10G (3721T>G), M19del (3748_3750delTGC), T83S (3939A>T), V141I (4113G>A), P449L (5038C>T), A457V (5062C>T), H460R (5071A>G 5072C>T), P503S (5199C>T), T526A (5268A>G), E666_R667insX (5689_5690insGA), F683L (5741T>G), T686A (5748A>G), E697K (5781G>A)								
Codon mutations:	GTC10GGC (3721T>G), ATG19del (3748_3750delTGC), CGA20AGA (3748_3750delTGC), GTT32GTG (3788T>G), CGC46CGT (3830C>T), ACC83TCC (3939A>T), ACA113ACG (4031A>G), GAG134GAA (4094G>A), GTA141ATA (4113G>A), GCC158GCT (4166C>T), ACT160ACC (4172T>C), CAC172CAT (4208C>T), TCT174TCG (4214T>G), CGG322CGA (4658G>A), GGT323GGC (4661T>C), GTC347GTG (4733C>G), GGT375GGC (4817T>C), TTC397TTT (4883C>T), CTT398CTC (4886T>C), GCA407GCT (4913A>T), ACG419ACA (4949G>A), GAA424GAG (4964A>G), ACC429ACT (4979C>T), CCA449CTA (5038C>T), GCC457GTC (5062C>T), CAC460CGT (5071A>G 5072C>T), ACG479ACA (5129G>A), GAC490GAT (5162C>T), AAC499AAT (5189C>T), CCA503TCA (5199C>T), CGA508CGC (5216A>C), ACA526GCA (5268A>G), GAA666GAG (5689_5690insGA), GAA666_CGT667insA-A (5689_5690insGA), CGT667C-- (5692_5693delGT), TTT683TTG (5741T>G), ACT686GCT (5748A>G), GAA697AAA (5781G>A)								
PLRVgp5	1	209	100%	1411	99.2%	208 (99.5%)	205 (98.1%)	0/1/0/0	1
Protein mutations:	V10G (3721T>G), M19del (3748_3750delTGC), T83S (3939A>T), V141I (4113G>A)								
Codon mutations:	GTC10GGC (3721T>G), ATG19del (3748_3750delTGC), CGA20AGA (3748_3750delTGC), GTT32GTG (3788T>G), CGC46CGT (3830C>T), ACC83TCC (3939A>T), ACA113ACG (4031A>G), GAG134GAA (4094G>A), GTA141ATA (4113G>A), GCC158GCT (4166C>T), ACT160ACC (4172T>C), CAC172CAT (4208C>T), TCT174TCG (4214T>G)								
PLRVgp6	1	157	100%	1030	95.5%	156 (99.4%)	149 (94.9%)	0/1/0/0	1
Protein mutations:	S2A (3721T>G), C11del (3748_3750delTGC), F24C (3788T>G), A38V (3830C>T), Q105R (4031A>G), S126N (4094G>A), P150L (4166C>T), L152P (4172T>C)								
Codon mutations:	TCA2GCA (3721T>G), TGC11del (3748_3750delTGC), TTC24TGC (3788T>G), GCC38GTC (3830C>T), ACA74ACT (3939A>T), CAA105CGA (4031A>G), AGT126AAT (4094G>A), AAG132AAA (4113G>A), CCA150CTA (4166C>T), CTT152CCT (4172T>C)								

Proteins

	Begin	End	Coverage	Score	Concordance	Matches	Identities	I/D/M/F*	Stop Codons
P0 protein (NP_056746.1)	1	248	100%	1715	99.0%	248 (100%)	245 (98.8%)	0/0/0/0	1
Protein mutations:	S161N (656G>A), Q164R (665A>G), Q175H (699A>C)								
Codon mutations:	AGT161AAT (656G>A), CAA164CGA (665A>G), CAA175CAC (699A>C), CGC216CGT (822C>T), GCT217GCG (825T>G), AAG243AAA (903G>A)								

	Begin	End	Coverage	Score	Concordance	Matches	Identities	I/D/M/F*	Stop Codons
**NT**	**122**	**5948**	**97.3%**	**11226**	**96.6%**	**5822 (99.9%)**	**5726 (98.2%)**	**2/5**	
RNA-dependent RNA polymerase (NP_056748.3)	1	1063	100%	7327	98.2%	1063 (100%)	1041 (97.9%)	0/0/0/0	1
Protein mutations:	V117I (656G>A), K120E (665A>G), N131T (699A>C), A172V (822C>T), L173R (825T>G), R199K (903G>A), M273I (1126G>A), A338T (1319G>A), G350E (1356G>A), V424F (1577G>T), R429K (1593G>A), D435G (1611A>G), E470G (1716A>G), Q530K (1894C>A), E537K (1915G>A), Q551K (1957C>A), G582E (2051G>A), A722T (2470G>A), A750V (2555C>T), N808S (2729A>G), P848L (2849C>T), I1025V (3379A>G)								
Codon mutations:	GTA117ATA (656G>A), AAA120GAA (665A>G), AAC131ACC (699A>C), GCG172GTG (822C>T), CTA173CGA (825T>G), AGG199AAG (903G>A), GAA208GAG (931A>G), CAG217CAA (958G>A), TTG243TTA (1036G>A), ATG273ATA (1126G>A), ACT275ACG (1132T>G), TTC280TTT (1147C>T), TCC288TCT (1171C>T), CTA299CTG (1204A>G), ACA309ACT (1234A>T), CAT331CAC (1300T>C), GCC338ACC (1319G>A), CAC340CAT (1327C>T), GGA350GAA (1356G>A), GTC424TTC (1577G>T), AGA429AAA (1593G>A), GAC435GGC (1611A>G), GAG470GGG (1716A>G), CTA483TTA (1754C>T), GCA491GCG (1779A>G), CAA530AAA (1894C>A), AGA535CGA (1909A>C), GAA537AAA (1915G>A), CAA551AAA (1957C>A), TTT567TTC (2007T>C), GGA582GAA (2051G>A), CAC616CAT (2154C>T), TCA653TCG (2265A>G), GCG659GCA (2283G>A), ACA669ACG (2313A>G), GTT670GTG (2316T>G), GCA722ACA (2470G>A), GCC750GTC (2555C>T), CTA760CTG (2586A>G), CAC778CAT (2640C>T), AGC781AGT (2649C>T), AAT808AGT (2729A>G), CCA848CTA (2849C>T), GTG850GTA (2856G>A), CAC857CAT (2877C>T), ACT864ACC (2898T>C), TAT875TAC (2931T>C), CGT898CGC (3000T>C), AAC909AAT (3033C>T), TCC910TCT (3036C>T), GCC921GCT (3069C>T), TCC937TCT (3117C>T), GCA955GCG (3171A>G), GAT960GAC (3186T>C), AAC1005AAT (3321C>T), GTG1024GTT (3378G>T), ATT1025GTT (3379A>G), AAC1027AAT (3387C>T)								
P1 protein (NP_056747.1)	1	640	100%	4199	97.8%	640 (100%)	623 (97.3%)	0/0/0/0	1
Protein mutations:	V117I (656G>A), K120E (665A>G), N131T (699A>C), A172V (822C>T), L173R (825T>G), R199K (903G>A), M273I (1126G>A), A338T (1319G>A), G350E (1356G>A), V424F (1577G>T), R429K (1593G>A), D435G (1611A>G), E470G (1716A>G), Q491R (1779A>G), L567S (2007T>C), E582K (2051G>A), T616I (2154C>T)								
Codon mutations:	GTA117ATA (656G>A), AAA120GAA (665A>G), AAC131ACC (699A>C), GCG172GTG (822C>T), CTA173CGA (825T>G), AGG199AAG (903G>A), GAA208GAG (931A>G), CAG217CAA (958G>A), TTG243TTA (1036G>A), ATG273ATA (1126G>A), ACT275ACG (1132T>G), TTC280TTT (1147C>T), TCC288TCT (1171C>T), CTA299CTG (1204A>G), ACA309ACT (1234A>T), CAT331CAC (1300T>C), GCC338ACC (1319G>A), CAC340CAT (1327C>T), GGA350GAA (1356G>A), GTC424TTC (1577G>T), AGA429AAA (1593G>A), GAC435GGC (1611A>G), GAG470GGG (1716A>G), CTA483TTA (1754C>T), CAA491CGA (1779A>G), ATC529ATA (1894C>A), ATA534ATC (1909A>C), GAG536GAA (1915G>A), CCC550CCA (1957C>A), TTA567TCA (2007T>C), GAG582AAG (2051G>A), ACC616ATC (2154C>T)								
Replication-associated protein (YP_006355442.1)	1	42	100%	277	96.5%	42 (100%)	41 (97.6%)	0/0/0/0	1
Protein mutations:	R29G (1716A>G)								
Codon mutations:	AGA29GGA (1716A>G), CTC41CTT (1754C>T)								
protein 3a (YP_009179365.2)	1	46	100%	289	96.7%	46 (100%)	44 (95.7%)	0/0/0/0	1
Protein mutations:	S14T (3614T>A), F26L (3652T>A)								
Codon mutations:	TCA14ACA (3614T>A), TCC15TCT (3619C>T), TTT26TTA (3652T>A)								
CP read-through protein (NP_056751.2)	1	718	100%	4695	93.4%	717 (99.7%)	705 (98.1%)	1/1/2/2	2
Protein mutations:	V10G (3721T>G), M19del (3748_3750delTGC), T83S (3939A>T), V141I (4113G>A), P449L (5038C>T), A457V (5062C>T), H460R (5071A>G 5072C>T), P503S (5199C>T), T526A (5268A>G), E666_R667insX (5689_5690insGA), F683L (5741T>G), T686A (5748A>G), E697K (5781G>A)								
Codon mutations:	GTC10GGC (3721T>G), ATG19del (3748_3750delTGC), CGA20AGA (3748_3750delTGC), GTT32GTG (3788T>G), CGC46CGT (3830C>T), ACC83TCC (3939A>T), ACA113ACG (4031A>G), GAG134GAA (4094G>A), GTA141ATA (4113G>A), GCC158GCT (4166C>T), ACT160ACC (4172T>C), CAC172CAT (4208C>T), TCT174TCG (4214T>G), CGG322CGA (4658G>A), GGT323GGC (4661T>C), GTC347GTG (4733C>G), GGT375GGC (4817T>C), TTC397TTT (4883C>T), CTT398CTC (4886T>C), GCA407GCT (4913A>T), ACG419ACA (4949G>A), GAA424GAG (4964A>G), ACC429ACT (4979C>T), CCA449CTA (5038C>T), GCC457GTC (5062C>T), CAC460CGT (5071A>G 5072C>T), ACG479ACA (5129G>A), GAC490GAT (5162C>T), AAC499AAT (5189C>T), CCA503TCA (5199C>T), CGA508CGC (5216A>C), ACA526GCA (5268A>G), GAA666GAG (5689_5690insGA), GAA666_CGT667insA-A (5689_5690insGA), CGT667C-- (5692_5693delGT), TTT683TTG (5741T>G), ACT686GCT (5748A>G), GAA697AAA (5781G>A)								
coat protein (NP_056749.1)	1	209	100%	1411	99.2%	208 (99.5%)	205 (98.1%)	0/1/0/0	1
Protein mutations:	V10G (3721T>G), M19del (3748_3750delTGC), T83S (3939A>T), V141I (4113G>A)								
Codon mutations:	GTC10GGC (3721T>G), ATG19del (3748_3750delTGC), CGA20AGA (3748_3750delTGC), GTT32GTG (3788T>G), CGC46CGT (3830C>T), ACC83TCC (3939A>T), ACA113ACG (4031A>G), GAG134GAA (4094G>A), GTA141ATA (4113G>A), GCC158GCT (4166C>T), ACT160ACC (4172T>C), CAC172CAT (4208C>T), TCT174TCG (4214T>G)								
movement protein (NP_056750.1)	1	157	100%	1030	95.5%	156 (99.4%)	149 (94.9%)	0/1/0/0	1
Protein mutations:	S2A (3721T>G), C11del (3748_3750delTGC), F24C (3788T>G), A38V (3830C>T), Q105R (4031A>G), S126N (4094G>A), P150L (4166C>T), L152P (4172T>C)								
Codon mutations:	TCA2GCA (3721T>G), TGC11del (3748_3750delTGC), TTC24TGC (3788T>G), GCC38GTC (3830C>T), ACA74ACT (3939A>T), CAA105CGA (4031A>G), AGT126AAT (4094G>A), AAG132AAA (4113G>A), CCA150CTA (4166C>T), CTT152CCT (4172T>C)								

*: Inserts / Deletes / Misaligned / Frameshifts

Analysis details

This analysis was performed with panviral2.64

NGS Details (UN8): Duamitovirus soch1

Assembly

**Coverage Length**	2255 (2 contig(s))
**Depth Of Coverage**	25.7
**Number Of Reads**	508
**Reads Per Million**	8.49 rpm (after QC)
**Ambiguities**	0
**Assembly Method**	de novo + reference guided assembly
**Consensus Caller**	Bcf Tools

Coverage Map

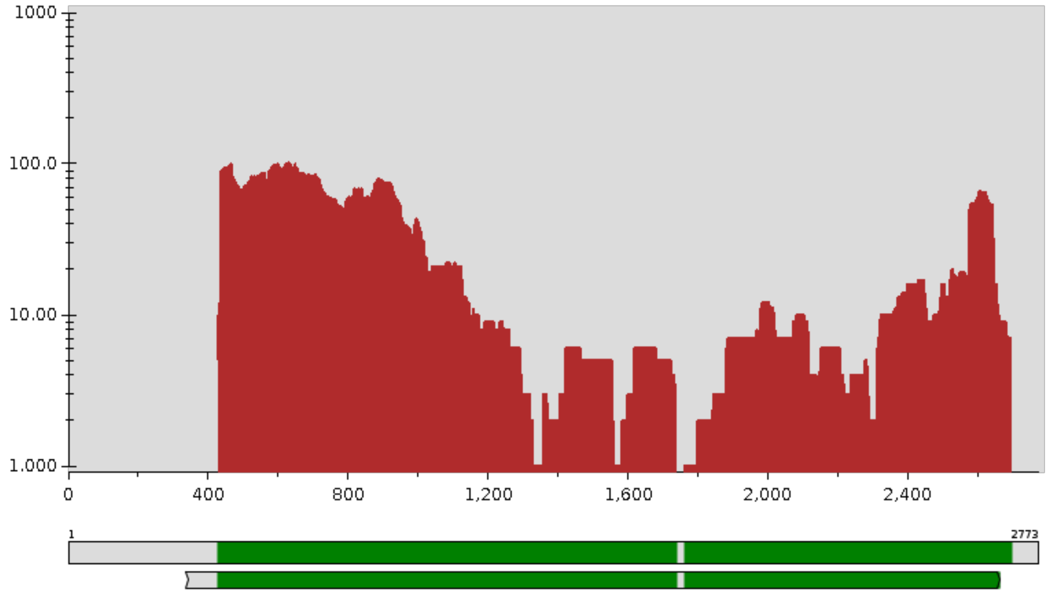

Assignment

**Type**	Duamitovirus soch1 (Taxonomy ID: 2955838)
**Reference Genome**	NC_076524.1
**NT Identity (%)**	69.0561
**AA Identity (%)**	69.4708
**Number Of Stop Codons**	5
**Number Of CDS**	1

Alignment

**Alignment Score**	1676.0 (NT) + 3439.0 (AA) = 5115.0
**Concordance (%)**	54.6692

Alignment Method	Global, seeded, nucleotide + amino acids (AGA)

Genome Region

Sequence starts at position 428 and ends at position 2700 relative to NC_076524.1 reference sequence.

Alignment Detailed Statistics

	Begin	End	Coverage	Score	Concordance	Matches	Identities	I/D/M/F*	Stop Codons
**NT**	**428**	**2700**	**81.3%**	**1676**	**37.6%**	**2246 (99.6%)**	**1551 (68.8%)**	**0/9**	

Mutations: 437A>G, 441A>C, 443C>T, 447C>T, 459C>T, 466C>T, 470C>T, 471G>A, 472C>T, 473T>G, 477T>A, 480A>C, 481C>A, 482A>T, 485A>G, 489A>G, 494T>C, 497T>A, 500T>G, 503A>C, 506T>C, 509C>T, 512T>C, 513G>A, 516T>A, 517C>A, 518T>A, 520A>C, 524G>T, 525A>G, 526C>T, 527T>G, 529C>A, 530C>A, 531T>C, 533A>G, 534C>A, 535G>A, 536C>A, 539G>A, 540A>C, 541A>G, 542G>T, 545G>A, 548C>T, 550A>T, 556T>A, 557C>T, 560A>T, 569C>T, 575G>A, 581C>T, 582G>A, 587A>T, 589G>C, 591T>C, 593A>T, 599A>T, 602C>T, 612T>A, 613C>G, 614C>T, 616T>A, 618C>T, 619G>C, 620A>T, 624G>A, 626A>T, 629A>C, 630C>A, 632A>C, 633C>A, 635T>G, 638G>T, 641G>T, 644T>A, 647G>T, 651C>T, 653C>G, 656A>C, 657A>C, 661C>G, 662G>T, 665T>A, 668C>T, 672C>A, 673G>A, 674T>G, 680C>A, 683C>T, 684A>G, 685A>C, 686A>C, 692G>A, 695T>A, 699G>C, 700C>A, 701A>C, 702A>G, 705C>A, 707T>G, 708A>G, 709G>C, 710G>A, 713A>G, 714G>T, 715A>C, 719A>T, 722C>T, 737T>C, 746C>T, 752A>T, 761G>A, 765G>T, 767T>A, 770G>A, 771G>T, 772T>A, 773T>G, 774T>A, 776G>T, 777A>G, 778A>C, 779A>G, 782T>G, 796G>A, 797T>G, 803A>C, 809T>C, 810G>C, 815C>A, 818A>T, 821T>G, 824C>T, 827A>T, 831C>T, 833T>A, 841G>A, 842G>T, 850T>G, 851A>T, 857G>T, 860G>T, 862A>G, 869T>G, 875G>T, 880T>G, 881C>A, 885C>A, 887T>G, 892A>G, 905A>T, 908C>A, 909T>C, 910T>A, 916G>A, 918G>T, 919G>C, 923T>A, 926A>T, 927A>C, 929G>T, 934T>A, 935T>G, 938G>T, 942T>A, 943C>A, 948C>A, 971C>A, 972C>T, 975A>C, 976A>T, 977T>C, 983T>C, 990A>C, 992G>C, 993T>C, 994C>G, 995A>G, 997A>T, 998C>T, 999G>C, 1000G>A, 1001T>A, 1003T>A, 1004A>T, 1005G>T, 1007C>G, 1010A>G, 1016C>T, 1017A>G, 1018A>T, 1019A>T, 1022T>A, 1025T>C, 1026C>G, 1027A>G, 1030A>C, 1031G>A, 1034T>G, 1035A>G, 1036G>C, 1044T>G, 1046C>T, 1049G>A, 1050T>G, 1051T>A, 1052T>A, 1054C>A, 1055A>C, 1061T>C, 1067T>G, 1073G>A, 1076A>G, 1083C>A, 1088G>T, 1091C>G, 1099G>T, 1104A>G, 1105C>T, 1106A>G, 1115T>G, 1121A>C, 1122T>A, 1124C>T, 1127G>A, 1130C>T, 1133C>G, 1142A>C, 1145C>T, 1148G>T, 1151G>T, 1152T>C, 1154G>T, 1160C>T, 1161A>C, 1164T>A, 1165C>T, 1166T>G, 1169C>T, 1172C>A, 1174T>A, 1175T>C, 1178T>A, 1181A>T, 1184C>T, 1185A>T, 1186G>C, 1187G>T, 1188G>A, 1189C>A, 1191G>A, 1192C>A, 1193A>T, 1196T>C, 1197_1199delGAA, 1205A>T, 1211C>T, 1212T>G, 1213T>A, 1217T>C, 1220C>T, 1223A>T, 1224C>T, 1225A>T, 1226C>A, 1232G>A, 1236A>C, 1237G>A, 1238T>A, 1241C>A, 1247A>C, 1248T>C, 1249G>T, 1250G>T, 1253T>C, 1256A>T, 1258C>G, 1265G>A, 1266A>G, 1268T>G, 1269C>G, 1271T>C, 1274T>C, 1276G>A, 1280G>A, 1283G>T, 1292T>G, 1301G>A, 1302C>T, 1304T>G, 1306G>C, 1308G>C, 1309C>A, 1310A>G, 1312T>C, 1313G>C, 1316A>T, 1319G>A, 1322C>T, 1328A>G, 1332A>C, 1334A>G, 1338G>A, 1340A>C, 1345T>C, 1346A>T, 1349G>A, 1355C>T, 1357A>C, 1358C>T, 1370A>G, 1371C>T, 1372T>G, 1373T>A, 1376C>G, 1377C>T, 1378A>T, 1379C>T, 1382T>C, 1385T>C, 1389G>C, 1391T>A, 1397T>A, 1402C>A, 1409G>A, 1413A>C, 1414A>G, 1415A>C, 1416A>C, 1418A>T, 1419A>C, 1420A>C, 1422A>G, 1424A>G, 1427C>T, 1430G>A, 1431A>G, 1433T>A, 1445G>A, 1446A>C, 1447A>G, 1448A>T, 1451T>G, 1455T>G, 1457G>A, 1458G>C, 1459C>G, 1460T>A, 1464A>C, 1465A>G, 1466G>A, 1467C>T, 1468A>C, 1469A>T, 1470A>C, 1472G>A, 1475C>G, 1476T>A, 1477T>A, 1478T>A, 1479A>G, 1480A>G, 1481A>T, 1486G>C, 1489A>T, 1490T>C, 1502A>C, 1505G>A, 1508A>G, 1526G>A, 1538G>A, 1541T>C, 1545C>T, 1547T>C, 1553T>C, 1554A>T, 1555T>G, 1556G>C, 1559A>G, 1566C>A, 1567C>G, 1571C>A, 1572T>C, 1580C>A, 1589T>C, 1593_1597delAGCTC, 1598T>A, 1607T>C, 1613G>T, 1614A>T, 1617C>T, 1619T>G, 1620G>A, 1622T>C, 1623A>G, 1624A>T, 1626A>C, 1627A>C, 1628A>T, 1632T>A, 1634A>G, 1635A>G, 1636C>T, 1637A>C, 1643A>G, 1647T>A, 1648A>G, 1655A>T, 1661T>C, 1662C>T, 1664G>A, 1667T>A, 1670G>A, 1676T>G, 1679G>A, 1682C>T, 1684T>A, 1686C>T, 1691T>A, 1694T>A, 1698G>T, 1700A>C, 1703T>A, 1706C>T, 1707T>G, 1710T>C, 1712G>T, 1715T>C, 1721C>T, 1727A>G, 1734T>C, 1739A>T, 1761T>A, 1765A>C, 1766C>T, 1767T>A, 1769T>C, 1772A>T, 1777G>C, 1778G>T, 1784C>T, 1787T>C, 1788T>C, 1790A>G, 1819G>C, 1820T>A, 1821A>G, 1823A>G, 1829C>G, 1830A>C, 1831A>G, 1832T>G, 1833G>C, 1840A>G, 1856A>G, 1859C>T, 1860A>G, 1861A>G, 1865C>T, 1867A>C, 1868A>C, 1874G>T, 1875A>T, 1878C>G, 1880A>T, 1883A>G, 1886T>A, 1887T>A, 1889G>T, 1892A>T, 1895T>G, 1898T>G, 1904A>T, 1907C>T, 1919G>C, 1922G>A, 1923A>C, 1940A>G, 1947A>G, 1967C>T, 1968C>G, 1970T>G, 1972G>A, 1973A>G, 1976T>A, 1980T>A, 1981T>C, 1984C>T, 1985T>C, 1991T>C, 2000T>G, 2003A>T, 2006T>G, 2010G>A, 2011C>A, 2013C>A, 2015C>T, 2020T>C, 2030T>C, 2036C>T, 2039A>G, 2052A>C, 2054A>G, 2060G>T, 2063T>A, 2066C>T, 2071A>T, 2074A>G, 2084G>C, 2087C>T, 2088T>C, 2090A>T, 2093C>T, 2102A>G, 2103A>T, 2104G>C, 2105C>A, 2108G>A, 2111T>A, 2117G>A, 2118C>A, 2120T>G, 2121C>T, 2128C>T, 2129T>G, 2133C>A, 2134A>G, 2135T>G, 2138T>C, 2141G>A, 2143C>T, 2146T>G, 2148C>A, 2152C>T, 2158T>C, 2167A>T, 2168C>A, 2173G>T, 2174G>T, 2177G>T, 2186G>A, 2191T>A, 2192G>A, 2195C>T, 2204C>T, 2210G>A, 2222A>G, 2225A>G, 2230A>T, 2231C>T, 2234C>T, 2237T>A, 2239A>G, 2240A>G, 2241G>A, 2243A>G, 2250G>C, 2251T>C, 2252T>C, 2264G>T, 2271C>A, 2273A>T, 2274A>G, 2276G>A, 2280C>A, 2285G>A, 2288C>T, 2298G>T, 2299T>G, 2300T>G, 2303A>G, 2306A>C, 2309A>T, 2313C>T, 2315T>C, 2322C>T, 2326G>T, 2328C>A, 2330A>T, 2336A>G, 2337G>A, 2346T>C, 2348A>T, 2349A>C, 2350A>G, 2355C>G, 2357C>T, 2358A>T, 2359A>C, 2360C>T, 2369T>C, 2372G>T, 2377G>T, 2378T>G, 2382A>T, 2383A>C, 2384C>T, 2385C>T, 2386C>T, 2387A>T, 2390T>A, 2396C>T, 2399T>C, 2400A>G, 2402G>A, 2403A>C, 2404G>A, 2405T>A, 2406C>T, 2408C>G, 2411C>G, 2414C>T, 2415T>G, 2417C>T, 2420T>C, 2423A>T, 2431C>A, 2432A>G, 2435C>A, 2441A>G, 2442A>C, 2444G>A, 2445C>T, 2446T>C, 2447C>G, 2448G>A, 2450A>T, 2451A>G, 2452C>A, 2453T>A, 2456T>C, 2457T>C, 2459T>G, 2460G>A, 2462T>A, 2465A>G, 2466A>G, 2477C>T, 2478C>T, 2489A>G, 2492T>C, 2495C>T, 2504T>A, 2510T>C, 2514A>G, 2518C>T, 2519C>G, 2525C>T, 2528T>A, 2532C>A, 2533A>G, 2536G>A, 2537G>T, 2541T>C, 2543T>G, 2551A>G, 2552C>T, 2555T>C, 2556G>T, 2557T>C, 2561T>C, 2568A>G, 2571A>C, 2572A>G, 2582A>G, 2585G>A, 2597G>A, 2598A>G, 2599A>C, 2600C>T, 2609T>A, 2613T>G, 2615T>A, 2618T>A, 2624A>T, 2626A>T, 2627C>A, 2629A>C, 2631A>T, 2634delA, 2636A>T, 2639A>T, 2640A>G, 2643G>A, 2644A>G, 2647A>T, 2648A>G, 2649G>T, 2650T>A, 2653G>T, 2655T>A, 2681G>A, 2686T>C, 2689T>C, 2690G>C, 2694A>G, 2695T>A

CDS

	Begin	End	Coverage	Score	Concordance	Matches	Identities	I/D/M/F*	Stop Codons
RdRp	32	777	95.1%	3439	66.5%	737 (99.7%)	512 (69.3%)	0/2/2/2	5

Protein mutations: I36L (441A>C 443C>T), L38F (447C>T), A44V (466C>T), A46M (471G>A 472C>T 473T>G), L48M (477T>A), T49H (480A>C 481C>A 482A>T), I52V (489A>G), V60I (513G>A), S61K (516T>A 517C>A 518T>A), K62T (520A>C), T64V (525A>G 526C>T 527T>G), T65K (529C>A 530C>A), R67K (534C>A 535G>A 536C>A), K69R (540A>C 541A>G 542G>T), Y72F (550A>T), F74Y (556T>A 557C>T), A83T (582G>A), C85S (589G>C), F94Y (616T>A), R95S (618C>T 619G>C 620A>T), E97N (624G>A 626A>T), E98D (629A>C), P99T (630C>A 632A>C), L100M (633C>A 635T>G), S109C (661C>G 662G>T), R113K (672C>A 673G>A 674T>G), K117A (684A>G 685A>C 686A>C), A122H (699G>C 700C>A 701A>C), I123V (702A>G), R125A (708A>G 709G>C 710G>A), D127S (714G>T 715A>C), E128D (719A>T), A144S (765G>T 767T>A), V146* (771G>T 772T>A 773T>G), L147I (774T>A 776G>T), K148A (777A>G 778A>C 779A>G), S154K (796G>A 797T>G), E159Q (810G>C), P166S (831C>T 833T>A), G169D (841G>A 842G>T), L172R (850T>G 851A>T), E176G (862A>G), I178M (869T>G), F182* (880T>G 881C>A), H184K (885C>A 887T>G), Q186R (892A>G), F192H (909T>C 910T>A), R194Q (916G>A), G195S (918G>T 919G>C), M198L (927A>C 929G>T), I200K (934T>A 935T>G), S203N (942T>A 943C>A), Q205K (948C>A), N214L (975A>C 976A>T 977T>C), S220R (993T>C 994C>G 995A>G), Y221F (997A>T 998C>T), G222Q (999G>C 1000G>A 1001T>A), L223Y (1003T>A 1004A>T), A224S (1005G>T 1007C>G), K228V (1017A>G 1018A>T 1019A>T), Q231G (1026C>G 1027A>G), E232A (1030A>C 1031G>A), S234A (1035A>G 1036G>C), F237V (1044T>G 1046C>T), F239E (1050T>G 1051T>A 1052T>A), P240H (1054C>A 1055A>C), L250I (1083C>A), W255L (1099G>T), T257V (1104A>G 1105C>T 1106A>G), I260M (1115T>G), F263I (1122T>A 1124C>T), M272I (1151G>T), K276Q (1161A>C), S277M (1164T>A 1165C>T 1166T>G), F279L (1172C>A), F280Y (1174T>A 1175T>C), L282F (1181A>T), R284S (1185A>T 1186G>C 1187G>T), A285N (1188G>A 1189C>A), A286N (1191G>A 1192C>A 1193A>T), E288del (1197_1199delGAA), L290F (1205A>T), L293E (1212T>G 1213T>A), E296D (1223A>T), H297L (1224C>T 1225A>T 1226C>A), S301Q (1236A>C 1237G>A 1238T>A), W305L (1248T>C 1249G>T 1250G>T), T308S (1258C>G), S311G (1266A>G 1268T>G), P312A (1269C>G 1271T>C), R314Q (1276G>A), G324A (1306G>C), A325Q (1308G>C 1309C>A 1310A>G), V326A (1312T>C 1313G>C), V335I (1338G>A 1340A>C), V337A (1345T>C 1346A>T), M338I (1349G>A), Y341S (1357A>C 1358C>T), L346* (1371C>T 1372T>G 1373T>A), H348F (1377C>T 1378A>T 1379C>T), D352Q (1389G>C 1391T>A), T356K (1402C>A), K360R (1413A>C 1414A>G 1415A>C), I361L (1416A>C 1418A>T), K362P (1419A>C 1420A>C), T363A (1422A>G 1424A>G), T366A (1431A>G 1433T>A), K371R (1446A>C 1447A>G 1448A>T), L374V (1455T>G 1457G>A), A375R (1458G>C 1459C>G 1460T>A), K377R (1464A>C 1465A>G 1466G>A), Q378S (1467C>T 1468A>C 1469A>T), K379Q (1470A>C 1472G>A), N380K (1475C>G), F381K (1476T>A 1477T>A 1478T>A), K382G (1479A>G 1480A>G 1481A>T), C384S (1486G>C), Y385F (1489A>T 1490T>C), L389F (1502A>C), M401I (1538G>A), L404F (1545C>T 1547T>C), M407C (1554A>T 1555T>G 1556G>C), I408M (1559A>G), P411S (1566C>A 1567C>G), S420del (1593_1597delAGCTC), I427F (1614A>T), V429I (1620G>A 1622T>C), N430V (1623A>G 1624A>T), K431P (1626A>C 1627A>C 1628A>T), L433M (1632T>A 1634A>G), T434V (1635A>G 1636C>T 1637A>C), Y438S (1647T>A 1648A>G), F450Y (1684T>A), H451Y (1686C>T), A455S (1698G>T 1700A>C), S458A (1707T>G), I464M (1727A>G), C476S (1761T>A), N477T (1765A>C 1766C>T), S478T (1767T>A 1769T>C), G481A (1777G>C 1778G>T), S495T (1819G>C 1820T>A), K496E (1821A>G 1823A>G), N499R (1830A>C 1831A>G 1832T>G), E500Q (1833G>C), K502R (1840A>G), N509G (1860A>G 1861A>G), K511T (1867A>C 1868A>C), I514L (1875A>T), P515A (1878C>G 1880A>T), L518I (1887T>A 1889G>T), D521E (1898T>G), I538V (1947A>G), L545V (1968C>G 1970T>G), R546K (1972G>A 1973A>G), L549T (1980T>A 1981T>C), S550F (1984C>T 1985T>C), A559N (2010G>A 2011C>A), L560I (2013C>A 2015C>T), I562T (2020T>C), I568M (2039A>G), Y579F (2071A>T), K580R (2074A>G), A598V (2128C>T 2129T>G), H600R (2133C>A 2134A>G 2135T>G), P603L (2143C>T), V604G (2146T>G), S606L (2152C>T), L608S (2158T>C), Y611L (2167A>T 2168C>A), W613F (2173G>T 2174G>T), W614C (2177G>T), M619K (2191T>A 2192G>A), I629M (2222A>G), Y632F (2230A>T 2231C>T), K635R (2239A>G 2240A>G), E636K (2241G>A 2243A>G), V639P (2250G>C 2251T>C 2252T>C), Q643H (2264G>T), P646T (2271C>A 2273A>T), K647E (2274A>G 2276G>A), L649M (2280C>A), V655W (2298G>T 2299T>G 2300T>G), R660C (2313C>T 2315T>C), R664L (2326G>T), Q665N (2328C>A 2330A>T), E668K (2337G>A), K672R (2349A>C 2350A>G), L674V (2355C>G 2357C>T), N675S (2358A>T 2359A>C 2360C>T), C681L (2377G>T 2378T>G), N683S (2382A>T 2383A>C 2384C>T), P684F (2385C>T 2386C>T 2387A>T), D685E (2390T>A), K689E (2400A>G 2402G>A), S690Q (2403A>C 2404G>A 2405T>A), I692M (2411C>G), S694A (2415T>G 2417C>T), T699K (2431C>A 2432A>G), L704S (2445C>T 2446T>C 2447C>G), E705N (2448G>A 2450A>T), T706E (2451A>G 2452C>A 2453T>A), F708L (2457T>C 2459T>G), D709K (2460G>A 2462T>A), I711V (2466A>G), N727D (2514A>G), P728L (2518C>T 2519C>G), Q733R (2532C>A 2533A>G), W734Y (2536G>A 2537G>T), F736L (2541T>C 2543T>G), N739S (2551A>G 2552C>T), V741S (2556G>T 2557T>C), N745D (2568A>G), K746R (2571A>C 2572A>G), N755A (2598A>G 2599A>C 2600C>T), S760A (2613T>G 2615T>A), E763D (2624A>T), Y764L (2626A>T 2627C>A), Q765P (2629A>C), T766S (2631A>T), K769E (2640A>G), E770R (2643G>A 2644A>G), E771V (2647A>T 2648A>G), V772* (2649G>T 2650T>A), G773V (2653G>T), L774I (2655T>A)

	Begin	End	Coverage	Score	Concordance	Matches	Identities	I/D/M/F*	Stop Codons
**NT**	**428**	**2700**	**81.3%**	**1676**	**37.6%**	**2246 (99.6%)**	**1551 (68.8%)**	**0/9**	

Codon mutations:

AAA34AAG (437A>G), ATC36CTT (441A>C 443C>T), CTT38TTT (447C>T), CTA42TTA (459C>T), GCC44GTC (466C>T), TCC45TCT (470C>T), GCT46ATG (471G>A 472C>T 473T>G), TTG48ATG (477T>A), ACA49CAT (480A>C 481C>A 482A>T), AAA50AAG (485A>G), ATC52GTC (489A>G), GCT53GCC (494T>C), CTT54CTA (497T>A), GCT55GCG (500T>G), GGA56GGC (503A>C), TGT57TGC (506T>C), GAC58GAT (509C>T), TTT59TTC (512T>C), GTA60ATA (513G>A), TCT61AAA (516T>A 517C>A 518T>A), AAA62ACA (520A>C), GTG63GTT (524G>T), ACT64GTG (525A>G 526C>T 527T>G), ACC65AAA (529C>A 530C>A), TTA66CTG (531T>C 533A>G), CGC67AAA (534C>A 535G>A 536C>A), CGG68CGA (539G>A), AAG69CGT (540A>C 541A>G 542G>T), TCG70TCA (545G>A), GGC71GGT (548C>T), TAT72TTT (550A>T), TTC74TAT (556T>A 557C>T), ACA75ACT (560A>T), TAC78TAT (569C>T), AAG80AAA (575G>A), TGC82TGT (581C>T), GCA83ACA (582G>A), GTA84GTT (587A>T), TGC85TCC (589G>C), TTA86CTT (591T>C 593A>T), CGA88CGT (599A>T), TAC89TAT (602C>T), TCC93AGT (612T>A 613C>G 614C>T), TTT94TAT (616T>A), CGA95TCT (618C>T 619G>C 620A>T), GAA97AAT (624G>A 626A>T), GAA98GAC (629A>C), CCA99ACC (630C>A 632A>C), CTT100ATG (633C>A 635T>G), TCG101TCT (638G>T), GTG102GTT (641G>T), CCT103CCA (644T>A), GTG104GTT (647G>T), CTC106TTG (651C>T 653C>G), ACA107ACC (656A>C), AGA108CGA (657A>C), TCG109TGT (661C>G 662G>T), GGT110GGA (665T>A), ATC111ATT (668C>T), CGT113AAG (672C>A 673G>A 674T>G), ATC115ATA (680C>A), CCC116CCT (683C>T), AAA117GCC (684A>G 685A>C 686A>C), TTG119TTA (692G>A), CGT120CGA (695T>A), GCA122CAC (699G>C 700C>A 701A>C), ATA123GTA (702A>G), CGT124AGG (705C>A 707T>G), AGG125GCA (708A>G 709G>C 710G>A), AAA126AAG (713A>G), GAT127TCT (714G>T 715A>C), GAA128GAT (719A>T), CAC129CAT (722C>T), GTT134GTC (737T>C), TAC137TAT (746C>T), TCA139TCT (752A>T), GGG142GGA (761G>A), GCT144TCA (765G>T 767T>A), AAG145AAA (770G>A), GTT146TAG (771G>T 772T>A 773T>G), TTG147ATT (774T>A 776G>T), AAA148GCG (777A>G 778A>C 779A>G), GTT149GTG (782T>G), AGT154AAG (796G>A 797T>G), GCA156GCC (803A>C), TTT158TTC (809T>C), GAA159CAA (810G>C), TCC160TCA (815C>A), ATA161ATT (818A>T), ACT162ACG (821T>G), ACC163ACT (824C>T), CCA164CCT (827A>T), CCT166TCA (831C>T 833T>A), GGG169GAT (841G>A 842G>T), CTA172CGT (850T>G 851A>T), GTG174GTT (857G>T), CTG175CTT (860G>T), GAA176GGA (862A>G), ATT178ATG (869T>G), ACG180ACT (875G>T), TTC182TGA (880T>G 881C>A), CAT184AAG (885C>A 887T>G), CAA186CGA (892A>G), CTA190CTT (905A>T), CCC191CCA (908C>A), TTT192CAT (909T>C 910T>A), CGA194CAA (916G>A), GGC195TCC (918G>T 919G>C), ATT196ATA (923T>A), CCA197CCT (926A>T), ATG198CTT (927A>C 929G>T), ATT200AAG (934T>A 935T>G), GGG201GGT (938G>T), TCT203AAT (942T>A 943C>A), CAA205AAA (948C>A), CCC212CCA (971C>A), CTA213TTA (972C>T), AAT214CTC (975A>C 976A>T 977T>C), AAT216AAC (983T>C), AGG219CGC (990A>C 992G>C), TCA220CGG (993T>C 994C>G 995A>G), TAC221TTT (997A>T 998C>T), GGT222CAA (999G>C 1000G>A 1001T>A), TTA223TAT (1003T>A 1004A>T), GCC224TCG (1005G>T 1007C>G), GTA225GTG (1010A>G), GAC227GAT (1016C>T), AAA228GTT (1017A>G 1018A>T 1019A>T), GTT229GTA (1022T>A), GAT230GAC (1025T>C), CAA231GGA (1026C>G 1027A>G), GAG232GCA (1030A>C 1031G>A), GCT233GCG (1034T>G), AGT234GCT (1035A>G 1036G>C), TTC237GTT (1044T>G 1046C>T), AAG238AAA (1049G>A), TTT239GAA (1050T>G 1051T>A 1052T>A), CCA240CAC (1054C>A 1055A>C), ATT242ATC (1061T>C), GTT244GTG (1067T>G), TTG246TTA (1073G>A), AAA247AAG (1076A>G), CTA250ATA (1083C>A), GCG251GCT (1088G>T), GCC252GCG (1091C>G), TGG255TTG (1099G>T), ACA257GTG (1104A>G 1105C>T 1106A>G), ATT260ATG (1115T>G), TCA262TCC (1121A>C), TTC263ATT (1122T>A 1124C>T), CAG264CAA (1127G>A), GAC265GAT (1130C>T), GGC266GGG (1133C>G), TCA269TCC (1142A>C), CCC270CCT (1145C>T), GGG271GGT (1148G>T), ATG272ATT (1151G>T), TTG273CTT (1152T>C 1154G>T), TCC275TCT (1160C>T), AAA276CAA (1161A>C), TCT277ATG (1164T>A 1165C>T 1166T>G), GTC278GTT (1169C>T), TTC279TTA (1172C>A), TTT280TAC (1174T>A 1175T>C), CCT281CCA (1178T>A), TTA282TTT (1181A>T), GAC283GAT (1184C>T), AGG284TCT (1185A>T 1186G>C 1187G>T), GCT285AAT (1188G>A 1189C>A), GCA286AAT (1191G>A 1192C>A 1193A>T), ACT287ACC (1196T>C), GAA288del (1197_1199delGAA), TTA290TTT (1205A>T), AAC292AAT (1211C>T), TTA293GAA (1212T>G 1213T>A), TCT294TCC (1217T>C), CTC295CTT (1220C>T), GAA296GAT (1223A>T), CAC297TTA (1224C>T 1225A>T 1226C>A), GAG299GAA (1232G>A), AGT301CAA (1236A>C 1237G>A 1238T>A), GTC302GTA (1241C>A), CCA304CCC (1247A>C), TGG305CTT (1248T>C 1249G>T 1250G>T), TTT306TTC (1253T>C), TCA307TCT (1256A>T), ACC308AGC (1258C>G), TTG310TTA (1265G>A), AGT311GGG (1266A>G 1268T>G), CCT312GCC (1269C>G 1271T>C), TAT313TAC (1274T>C), CGA314CAA (1276G>A), GGG315GGA (1280G>A), GTG316GTT (1283G>T), GTT319GTG (1292T>G), AGG322AGA (1301G>A), CTT323TTG (1302C>T 1304T>G), GGT324GCT (1306G>C), GCA325CAG (1308G>C 1309C>A 1310A>G), GTG326GCC (1312T>C 1313G>C), ATA327ATT (1316A>T), GAG328GAA (1319G>A), GGC329GGT (1322C>T), GGA331GGG (1328A>G), AGA333CGG (1332A>C 1334A>G), GTA335ATC (1338G>A 1340A>C), GTA337GCT (1345T>C 1346A>T), ATG338ATA (1349G>A), AAC340AAT (1355C>T), TAC341TCT (1357A>C 1358C>T), AGA345AGG (1370A>G), CTT346TGA (1371C>T 1372T>G 1373T>A), CTC347CTG (1376C>G), CAC348TTT (1377C>T 1378A>T 1379C>T), CCT349CCC (1382T>C), GTT350GTC (1385T>C), GAT352CAA (1389G>C 1391T>A), GCT354GCA (1397T>A), ACA356AAA (1402C>A), CTG358CTA (1409G>A), AAA360CGC (1413A>C 1414A>G 1415A>C), ATA361CTT (1416A>C 1418A>T), AAG362CCG (1419A>C 1420A>C), ACA363GCG (1422A>G 1424A>G), GAC364GAT (1427C>T), GGG365GGA (1430G>A), ACT366GCA (1431A>G 1433T>A), GAG370GAA (1445G>A), AAA371CGT (1446A>C 1447A>G 1448A>T), CCT372CCG (1451T>G), TTG374GTA (1455T>G 1457G>A), GCT375CGA (1458G>C 1459C>G 1460T>A), AAG377CGA (1464A>C 1465A>G 1466G>A), CAA378TCT (1467C>T 1468A>C 1469A>T), AAG379CAA (1470A>C 1472G>A), AAC380AAG (1475C>G), TTT381AAA (1476T>A 1477T>A 1478T>A), AAA382GGT (1479A>G 1480A>G 1481A>T), TGT384TCT (1486G>C), TAT385TTC (1489A>T 1490T>C), TTA389TTC (1502A>C), AAG390AAA (1505G>A), TCA391TCG (1508A>G), CCG397CCA (1526G>A), ATG401ATA (1538G>A), TAT402TAC (1541T>C), CTT404TTC (1545C>T 1547T>C), TCT406TCC (1553T>C), ATG407TGC (1554A>T 1555T>G 1556G>C), ATA408ATG (1559A>G), CCT411AGT (1566C>A 1567C>G), ACC412ACA (1571C>A), TTG413CTG (1572T>C), TCC415TCA (1580C>A), GTT418GTC (1589T>C), AGC420del (1593_1597delAGCTC), TCT421--A (1593_1597delAGCTC 1598T>A), CTT424CTC (1607T>C), ACG426ACT (1613G>T), ATC427TTC (1614A>T), CTT428TTG (1617C>T 1619T>G), GTT429ATC (1620G>A 1622T>C), AAC430GTC (1623A>G 1624A>T), AAA431CCT (1626A>C 1627A>C 1628A>T), TTA433ATG (1632T>A 1634A>G), ACA434GTC (1635A>G 1636C>T 1637A>C), AGA436AGG (1643A>G), TAT438AGT (1647T>A 1648A>G), ATA440ATT (1655A>T), TTT442TTC (1661T>C), CTG443TTA (1662C>T 1664G>A), ACT444ACA (1667T>A), GGG445GGA (1670G>A), CCT447CCG (1676T>G), TTG448TTA (1679G>A), GGC449GGT (1682C>T), TTC450TAC (1684T>A), CAC451TAC (1686C>T), GGT452GGA (1691T>A), TCT453TCA (1694T>A), GCA455TCC (1698G>T 1700A>C), CTT456CTA (1703T>A), TTC457TTT (1706C>T), TCG458GCG (1707T>G), TTG459CTT (1710T>C 1712G>T), TCT460TCC (1715T>C), CAC462CAT (1721C>T), ATA464ATG (1727A>G), TTA467CTA (1734T>C), GCA468GCT (1739A>T), TGT476AGT (1761T>A), AAC477ACT (1765A>C 1766C>T), TCT478ACC (1767T>A 1769T>C), CCA479CCT (1772A>T), GGG481GCT (1777G>C 1778G>T), TAC483TAT (1784C>T), GCT484GCC (1787T>C), TTA485CTG (1788T>C 1790A>G), AGT495ACA (1819G>C 1820T>A), AAA496GAG (1821A>G 1823A>G), GCC498GCG (1829C>G), AAT499CGG (1830A>C 1831A>G 1832T>G), GAG500CAG (1833G>C), AAG502AGG (1840A>G), AGA507AGG (1856A>G), CTC508CTT (1859C>T), AAT509GGT (1860A>G 1861A>G), GTC510GTT (1865C>T), AAA511ACC (1867A>C 1868A>C), TCG513TCT (1874G>T), ATA514TTA (1875A>T), CCA515GCT (1878C>G 1880A>T), AAA516AAG (1883A>G), TCT517TCA (1886T>A), TTG518ATT (1887T>A 1889G>T), ATA519ATT (1892A>T), TCT520TCG (1895T>G), GAT521GAG (1898T>G), GGA523GGT (1904A>T), ACC524ACT (1907C>T), GCG528GCC (1919G>C), AAG529AAA (1922G>A), AGG530CGG (1923A>C), TCA535TCG (1940A>G), ATT538GTT (1947A>G), TCC544TCT (1967C>T), CTT545GTG (1968C>G 1970T>G), AGA546AAG (1972G>A 1973A>G), GCT547GCA (1976T>A), TTA549ACA (1980T>A 1981T>C), TCT550TTC (1984C>T 1985T>C), CGT552CGC (1991T>C), GTT555GTG (2000T>G), GGA556GGT (2003A>T), CTT557CTG (2006T>G), GCC559AAC (2010G>A 2011C>A), CTC560ATT (2013C>A 2015C>T), ATC562ACC (2020T>C), GAT565GAC (2030T>C), AAC567AAT (2036C>T), ATA568ATG (2039A>G), AGA573CGG (2052A>C 2054A>G), GGG575GGT (2060G>T), GGT576GGA (2063T>A), GCC577GCT (2066C>T), TAC579TTC (2071A>T), AAG580AGG (2074A>G), GCG583GCC (2084G>C), CGC584CGT (2087C>T), TTA585CTT (2088T>C 2090A>T), TTC586TTT (2093C>T), CAA589CAG (2102A>G), AGC590TCA (2103A>T 2104G>C 2105C>A), AAG591AAA (2108G>A), CGT592CGA (2111T>A), GAG594GAA (2117G>A), CGT595AGG (2118C>A 2120T>G), CTA596TTA (2121C>T), GCT598GTG (2128C>T 2129T>G), CAT600AGG (2133C>A 2134A>G 2135T>G), CTT601CTC (2138T>C), AAG602AAA (2141G>A), CCA603CTA (2143C>T), GTT604GGT (2146T>G), CGG605AGG (2148C>A), TCA606TTA (2152C>T), TTA608TCA (2158T>C), TAC611TTA (2167A>T 2168C>A), TGG613TTT (2173G>T 2174G>T), TGG614TGT (2177G>T), AGG617AGA (2186G>A), ATG619AAA (2191T>A 2192G>A), CCC620CCT (2195C>T), CCC623CCT (2204C>T), TTG625TTA (2210G>A), ATA629ATG (2222A>G), GTA630GTG (2225A>G), TAC632TTT (2230A>T 2231C>T), CTC633CTT (2234C>T), CTT634CTA (2237T>A), AAA635AGG (2239A>G 2240A>G), GAA636AAG (2241G>A 2243A>G), GTT639CCC (2250G>C 2251T>C 2252T>C), CAG643CAT (2264G>T), CCA646ACT (2271C>A 2273A>T), AAG647GAA (2274A>G 2276G>A), CTG649ATG (2280C>A), GTG650GTA (2285G>A), TTC651TTT (2288C>T), GTT655TGG (2298G>T 2299T>G 2300T>G), GAA656GAG (2303A>G), ATA657ATC (2306A>C), CTA658CTT (2309A>T), CGT660TGC (2313C>T 2315T>C), CTA663TTA (2322C>T), CGC664CTC (2326G>T), CAA665AAT (2328C>A 2330A>T), GTA667GTG (2336A>G), GAA668AAA (2337G>A), TTA671CTT (2346T>C 2348A>T), AAG672CGG (2349A>C 2350A>G), CTC674GTT (2355C>G 2357C>T), AAC675TCT (2358A>T 2359A>C 2360C>T), CAT678CAC (2369T>C), ACG679ACT (2372G>T), TGT681TTG (2377G>T 2378T>G), AAC683TCT (2382A>T 2383A>C 2384C>T), CCA684TTT (2385C>T 2386C>T 2387A>T), GAT685GAA (2390T>A), TCC687TCT (2396C>T), ATT688ATC (2399T>C), AAG689GAA (2400A>G 2402G>A), AGT690CAA (2403A>C 2404G>A 2405T>A), CTC691TTG (2406C>T 2408C>G), ATC692ATG (2411C>G), GAC693GAT (2414C>T), TCC694GCT (2415T>G 2417C>T), CCT695CCC (2420T>C), ATA696ATT (2423A>T), ACA699AAG (2431C>A 2432A>G), TCC700TCA (2435C>A), AAA702AAG (2441A>G), AGG703CGA (2442A>C 2444G>A), CTC704TCG (2445C>T 2446T>C 2447C>G), GAA705AAT (2448G>A 2450A>T), ACT706GAA (2451A>G 2452C>A 2453T>A), GAT707GAC (2456T>C), TTT708CTG (2457T>C 2459T>G), GAT709AAA (2460G>A 2462T>A), TTA710TTG (2465A>G), ATC711GTC (2466A>G), GGC714GGT (2477C>T), CTA715TTA (2478C>T), AAA718AAG (2489A>G), TGT719TGC (2492T>C), TAC720TAT (2495C>T), GGT723GGA (2504T>A), GGT725GGC (2510T>C), AAT727GAT (2514A>G), CCC728CTG (2518C>T 2519C>G), ACC730ACT (2525C>T), ACT731ACA (2528T>A), CAG733AGG (2532C>A 2533A>G), TGG734TAT (2536G>A 2537G>T), TTT736CTG (2541T>C 2543T>G), AAC739AGT (2551A>G 2552C>T), ACT740ACC (2555T>C), GTT741TCT (2556G>T 2557T>C), ATT742ATC (2561T>C), AAT745GAT (2568A>G), AAA746CGA (2571A>C 2572A>G), TTA749TTG (2582A>G), GGG750GGA (2585G>A), GGG754GGA (2597G>A), AAC755GCT (2598A>G 2599A>C 2600C>T), ATT758ATA (2609T>A), TCT760GCA (2613T>G 2615T>A), CCT761CCA (2618T>A), GAA763GAT (2624A>T), TAC764TTA (2626A>T 2627C>A), CAA765CCA (2629A>C), ACT766TCT (2631A>T), ACA767-CT (2634delA 2636A>T), GGA768GGT (2639A>T), AAA769GAA (2640A>G), GAG770AGG (2643G>A 2644A>G), GAA771GTG (2647A>T 2648A>G), GTG772TAG (2649G>T 2650T>A), GGC773GTC (2653G>T), TTA774ATA (2655T>A)

Proteins

RNA-dependent RNA polymerase (YP_010798874.1)	32	777	95.1%	3439	66.5%	737 (99.7%)	512 (69.3%)	0/2/2/2	5

	Begin	End	Coverage	Score	Concordance	Matches	Identities	I/D/M/F*	Stop Codons
**NT**	**428**	**2700**	**81.3%**	**1676**	**37.6%**	**2246 (99.6%)**	**1551 (68.8%)**	**0/9**	
Protein mutations:	I36L (441A>C 443C>T), L38F (447C>T), A44V (466C>T), A46M (471G>A 472C>T 473T>G), L48M (477T>A), T49H (480A>C 481C>A 482A>T), I52V (489A>G), V60I (513G>A), S61K (516T>A 517C>A 518T>A), K62T (520A>C), T64V (525A>G 526C>T 527T>G), T65K (529C>A 530C>A), R67K (534C>A 535G>A 536C>A), K69R (540A>C 541A>G 542G>T), Y72F (550A>T), F74Y (556T>A 557C>T), A83T (582G>A), C85S (589G>C), F94Y (616T>A), R95S (618C>T 619G>C 620A>T), E97N (624G>A 626A>T), E98D (629A>C), P99T (630C>A 632A>C), L100M (633C>A 635T>G), S109C (661C>G 662G>T), R113K (672C>A 673G>A 674T>G), K117A (684A>G 685A>C 686A>C), A122H (699G>C 700C>A 701A>C), I123V (702A>G), R125A (708A>G 709G>C 710G>A), D127S (714G>T 715A>C), E128D (719A>T), A144S (765G>T 767T>A), V146* (771G>T 772T>A 773T>G), L147I (774T>A 776G>T), K148A (777A>G 778A>C 779A>G), S154K (796G>A 797T>G), E159Q (810G>C), P166S (831C>T 833T>A), G169D (841G>A 842G>T), L172R (850T>G 851A>T), E176G (862A>G), I178M (869T>G), F182* (880T>G 881C>A), H184K (885C>A 887T>G), Q186R (892A>G), F192H (909T>C 910T>A), R194Q (916G>A), G195S (918G>T 919G>C), M198L (927A>C 929G>T), I200K (934T>A 935T>G), S203N (942T>A 943C>A), Q205K (948C>A), N214L (975A>C 976A>T 977T>C), S220R (993T>C 994C>G 995A>G), Y221F (997A>T 998C>T), G222Q (999G>C 1000G>A 1001T>A), L223Y (1003T>A 1004A>T), A224S (1005G>T 1007C>G), K228V (1017A>G 1018A>T 1019A>T), Q231G (1026C>G 1027A>G), E232A (1030A>C 1031G>A), S234A (1035A>G 1036G>C), F237V (1044T>G 1046C>T), F239E (1050T>G 1051T>A 1052T>A), P240H (1054C>A 1055A>C), L250I (1083C>A), W255L (1099G>T), T257V (1104A>G 1105C>T 1106A>G), I260M (1115T>G), F263I (1122T>A 1124C>T), M272I (1151G>T), K276Q (1161A>C), S277M (1164T>A 1165C>T 1166T>G), F279L (1172C>A), F280Y (1174T>A 1175T>C), L282F (1181A>T), R284S (1185A>T 1186G>C 1187G>T), A285N (1188G>A 1189C>A), A286N (1191G>A 1192C>A 1193A>T), E288del (1197_1199delGAA), L290F (1205A>T), L293E (1212T>G 1213T>A), E296D (1223A>T), H297L (1224C>T 1225A>T 1226C>A), S301Q (1236A>C 1237G>A 1238T>A), W305L (1248T>C 1249G>T 1250G>T), T308S (1258C>G), S311G (1266A>G 1268T>G), P312A (1269C>G 1271T>C), R314Q (1276G>A), G324A (1306G>C), A325Q (1308G>C 1309C>A 1310A>G), V326A (1312T>C 1313G>C), V335I (1338G>A 1340A>C), V337A (1345T>C 1346A>T), M338I (1349G>A), Y341S (1357A>C 1358C>T), L346* (1371C>T 1372T>G 1373T>A), H348F (1377C>T 1378A>T 1379C>T), D352Q (1389G>C 1391T>A), T356K (1402C>A), K360R (1413A>C 1414A>G 1415A>C), I361L (1416A>C 1418A>T), K362P (1419A>C 1420A>C), T363A (1422A>G 1424A>G), T366A (1431A>G 1433T>A), K371R (1446A>C 1447A>G 1448A>T), L374V (1455T>G 1457G>A), A375R (1458G>C 1459C>G 1460T>A), K377R (1464A>C 1465A>G 1466G>A), Q378S (1467C>T 1468A>C 1469A>T), K379Q (1470A>C 1472G>A), N380K (1475C>G), F381K (1476T>A 1477T>A 1478T>A), K382G (1479A>G 1480A>G 1481A>T), C384S (1486G>C), Y385F (1489A>T 1490T>C), L389F (1502A>C), M401I (1538G>A), L404F (1545C>T 1547T>C), M407C (1554A>T 1555T>G 1556G>C), I408M (1559A>G), P411S (1566C>A 1567C>G), S420del (1593_1597delAGCTC), I427F (1614A>T), V429I (1620G>A 1622T>C), N430V (1623A>G 1624A>T), K431P (1626A>C 1627A>C 1628A>T), L433M (1632T>A 1634A>G), T434V (1635A>G 1636C>T 1637A>C), Y438S (1647T>A 1648A>G), F450Y (1684T>A), H451Y (1686C>T), A455S (1698G>T 1700A>C), S458A (1707T>G), I464M (1727A>G), C476S (1761T>A), N477T (1765A>C 1766C>T), S478T (1767T>A 1769T>C), G481A (1777G>C 1778G>T), S495T (1819G>C 1820T>A), K496E (1821A>G 1823A>G), N499R (1830A>C 1831A>G 1832T>G), E500Q (1833G>C), K502R (1840A>G), N509G (1860A>G 1861A>G), K511T (1867A>C 1868A>C), I514L (1875A>T), P515A (1878C>G 1880A>T), L518I (1887T>A 1889G>T), D521E (1898T>G), I538V (1947A>G), L545V (1968C>G 1970T>G), R546K (1972G>A 1973A>G), L549T (1980T>A 1981T>C), S550F (1984C>T 1985T>C), A559N (2010G>A 2011C>A), L560I (2013C>A 2015C>T), I562T (2020T>C), I568M (2039A>G), Y579F (2071A>T), K580R (2074A>G), A598V (2128C>T 2129T>G), H600R (2133C>A 2134A>G 2135T>G), P603L (2143C>T), V604G (2146T>G), S606L (2152C>T), L608S (2158T>C), Y611L (2167A>T 2168C>A), W613F (2173G>T 2174G>T), W614C (2177G>T), M619K (2191T>A 2192G>A), I629M (2222A>G), Y632F (2230A>T 2231C>T), K635R (2239A>G 2240A>G), E636K (2241G>A 2243A>G), V639P (2250G>C 2251T>C 2252T>C), Q643H (2264G>T), P646T (2271C>A 2273A>T), K647E (2274A>G 2276G>A), L649M (2280C>A), V655W (2298G>T 2299T>G 2300T>G), R660C (2313C>T 2315T>C), R664L (2326G>T), Q665N (2328C>A 2330A>T), E668K (2337G>A), K672R (2349A>C 2350A>G), L674V (2355C>G 2357C>T), N675S (2358A>T 2359A>C 2360C>T), C681L (2377G>T 2378T>G), N683S (2382A>T 2383A>C 2384C>T), P684F (2385C>T 2386C>T 2387A>T), D685E (2390T>A), K689E (2400A>G 2402G>A), S690Q (2403A>C 2404G>A 2405T>A), I692M (2411C>G), S694A (2415T>G 2417C>T), T699K (2431C>A 2432A>G), L704S (2445C>T 2446T>C 2447C>G), E705N (2448G>A 2450A>T), T706E (2451A>G 2452C>A 2453T>A), F708L (2457T>C 2459T>G), D709K (2460G>A 2462T>A), I711V (2466A>G), N727D (2514A>G), P728L (2518C>T 2519C>G), Q733R (2532C>A 2533A>G), W734Y (2536G>A 2537G>T), F736L (2541T>C 2543T>G), N739S (2551A>G 2552C>T), V741S (2556G>T 2557T>C), N745D (2568A>G), K746R (2571A>C 2572A>G), N755A (2598A>G 2599A>C 2600C>T), S760A (2613T>G 2615T>A), E763D (2624A>T), Y764L (2626A>T 2627C>A), Q765P (2629A>C), T766S (2631A>T), K769E (2640A>G), E770R (2643G>A 2644A>G), E771V (2647A>T 2648A>G), V772* (2649G>T 2650T>A), G773V (2653G>T), L774I (2655T>A)								

	Begin	End	Coverage	Score	Concordance	Matches	Identities	I/D/M/F*	Stop Codons
**NT**	**428**	**2700**	**81.3%**	**1676**	**37.6%**	**2246 (99.6%)**	**1551 (68.8%)**	**0/9**	
Codon mutations:	AAA34AAG (437A>G), ATC36CTT (441A>C 443C>T), CTT38TTT (447C>T), CTA42TTA (459C>T), GCC44GTC (466C>T), TCC45TCT (470C>T), GCT46ATG (471G>A 472C>T 473T>G), TTG48ATG (477T>A), ACA49CAT (480A>C 481C>A 482A>T), AAA50AAG (485A>G), ATC52GTC (489A>G), GCT53GCC (494T>C), CTT54CTA (497T>A), GCT55GCG (500T>G), GGA56GGC (503A>C), TGT57TGC (506T>C), GAC58GAT (509C>T), TTT59TTC (512T>C), GTA60ATA (513G>A), TCT61AAA (516T>A 517C>A 518T>A), AAA62ACA (520A>C), GTG63GTT (524G>T), ACT64GTG (525A>G 526C>T 527T>G), ACC65AAA (529C>A 530C>A), TTA66CTG (531T>C 533A>G), CGC67AAA (534C>A 535G>A 536C>A), CGG68CGA (539G>A), AAG69CGT (540A>C 541A>G 542G>T), TCG70TCA (545G>A), GGC71GGT (548C>T), TAT72TTT (550A>T), TTC74TAT (556T>A 557C>T), ACA75ACT (560A>T), TAC78TAT (569C>T), AAG80AAA (575G>A), TGC82TGT (581C>T), GCA83ACA (582G>A), GTA84GTT (587A>T), TGC85TCC (589G>C), TTA86CTT (591T>C 593A>T), CGA88CGT (599A>T), TAC89TAT (602C>T), TCC93AGT (612T>A 613C>G 614C>T), TTT94TAT (616T>A), CGA95TCT (618C>T 619G>C 620A>T), GAA97AAT (624G>A 626A>T), GAA98GAC (629A>C), CCA99ACC (630C>A 632A>C), CTT100ATG (633C>A 635T>G), TCG101TCT (638G>T), GTG102GTT (641G>T), CCT103CCA (644T>A), GTG104GTT (647G>T), CTC106TTG (651C>T 653C>G), ACA107ACC (656A>C), AGA108CGA (657A>C), TCG109TGT (661C>G 662G>T), GGT110GGA (665T>A), ATC111ATT (668C>T), CGT113AAG (672C>A 673G>A 674T>G), ATC115ATA (680C>A), CCC116CCT (683C>T), AAA117GCC (684A>G 685A>C 686A>C), TTG119TTA (692G>A), CGT120CGA (695T>A), GCA122CAC (699G>C 700C>A 701A>C), ATA123GTA (702A>G), CGT124AGG (705C>A 707T>G), AGG125GCA (708A>G 709G>C 710G>A), AAA126AAG (713A>G), GAT127TCT (714G>T 715A>C), GAA128GAT (719A>T), CAC129CAT (722C>T), GTT134GTC (737T>C), TAC137TAT (746C>T), TCA139TCT (752A>T), GGG142GGA (761G>A), GCT144TCA (765G>T 767T>A), AAG145AAA (770G>A), GTT146TAG (771G>T 772T>A 773T>G), TTG147ATT (774T>A 776G>T), AAA148GCG (777A>G 778A>C 779A>G), GTT149GTG (782T>G), AGT154AAG (796G>A 797T>G), GCA156GCC (803A>C), TTT158TTC (809T>C), GAA159CAA (810G>C), TCC160TCA (815C>A), ATA161ATT (818A>T), ACT162ACG (821T>G), ACC163ACT (824C>T), CCA164CCT (827A>T), CCT166TCA (831C>T 833T>A), GGG169GAT (841G>A 842G>T), CTA172CGT (850T>G 851A>T), GTG174GTT (857G>T), CTG175CTT (860G>T), GAA176GGA (862A>G), ATT178ATG (869T>G), ACG180ACT (875G>T), TTC182TGA (880T>G 881C>A), CAT184AAG (885C>A 887T>G), CAA186CGA (892A>G), CTA190CTT (905A>T), CCC191CCA (908C>A), TTT192CAT (909T>C 910T>A), CGA194CAA (916G>A), GGC195TCC (918G>T 919G>C), ATT196ATA (923T>A), CCA197CCT (926A>T), ATG198CTT (927A>C 929G>T), ATT200AAG (934T>A 935T>G), GGG201GGT (938G>T), TCT203AAT (942T>A 943C>A), CAA205AAA (948C>A), CCC212CCA (971C>A), CTA213TTA (972C>T), AAT214CTC (975A>C 976A>T 977T>C), AAT216AAC (983T>C), AGG219CGC (990A>C 992G>C), TCA220CGG (993T>C 994C>G 995A>G), TAC221TTT (997A>T 998C>T), GGT222CAA (999G>C 1000G>A 1001T>A), TTA223TAT (1003T>A 1004A>T), GCC224TCG (1005G>T 1007C>G), GTA225GTG (1010A>G), GAC227GAT (1016C>T), AAA228GTT (1017A>G 1018A>T 1019A>T), GTT229GTA (1022T>A), GAT230GAC (1025T>C), CAA231GGA (1026C>G 1027A>G), GAG232GCA (1030A>C 1031G>A), GCT233GCG (1034T>G), AGT234GCT (1035A>G 1036G>C), TTC237GTT (1044T>G 1046C>T), AAG238AAA (1049G>A), TTT239GAA (1050T>G 1051T>A 1052T>A), CCA240CAC (1054C>A 1055A>C), ATT242ATC (1061T>C), GTT244GTG (1067T>G), TTG246TTA (1073G>A), AAA247AAG (1076A>G), CTA250ATA (1083C>A), GCG251GCT (1088G>T), GCC252GCG (1091C>G), TGG255TTG (1099G>T), ACA257GTG (1104A>G 1105C>T 1106A>G), ATT260ATG (1115T>G), TCA262TCC (1121A>C), TTC263ATT (1122T>A 1124C>T), CAG264CAA (1127G>A), GAC265GAT (1130C>T), GGC266GGG (1133C>G), TCA269TCC (1142A>C), CCC270CCT (1145C>T), GGG271GGT (1148G>T), ATG272ATT (1151G>T), TTG273CTT (1152T>C 1154G>T), TCC275TCT (1160C>T), AAA276CAA (1161A>C), TCT277ATG (1164T>A 1165C>T 1166T>G), GTC278GTT (1169C>T), TTC279TTA (1172C>A), TTT280TAC (1174T>A 1175T>C), CCT281CCA (1178T>A), TTA282TTT (1181A>T), GAC283GAT (1184C>T), AGG284TCT (1185A>T 1186G>C 1187G>T), GCT285AAT (1188G>A 1189C>A), GCA286AAT (1191G>A 1192C>A 1193A>T), ACT287ACC (1196T>C), GAA288del (1197_1199delGAA), TTA290TTT (1205A>T), AAC292AAT (1211C>T), TTA293GAA (1212T>G 1213T>A), TCT294TCC (1217T>C), CTC295CTT (1220C>T), GAA296GAT (1223A>T), CAC297TTA (1224C>T 1225A>T 1226C>A), GAG299GAA (1232G>A), AGT301CAA (1236A>C 1237G>A 1238T>A), GTC302GTA (1241C>A), CCA304CCC (1247A>C), TGG305CTT (1248T>C 1249G>T 1250G>T), TTT306TTC (1253T>C), TCA307TCT (1256A>T), ACC308AGC (1258C>G), TTG310TTA (1265G>A), AGT311GGG (1266A>G 1268T>G), CCT312GCC (1269C>G 1271T>C), TAT313TAC (1274T>C), CGA314CAA (1276G>A), GGG315GGA (1280G>A), GTG316GTT (1283G>T), GTT319GTG (1292T>G), AGG322AGA (1301G>A), CTT323TTG (1302C>T 1304T>G), GGT324GCT (1306G>C), GCA325CAG (1308G>C 1309C>A 1310A>G), GTG326GCC (1312T>C 1313G>C), ATA327ATT (1316A>T), GAG328GAA (1319G>A), GGC329GGT (1322C>T), GGA331GGG (1328A>G), AGA333CGG (1332A>C 1334A>G), GTA335ATC (1338G>A 1340A>C), GTA337GCT (1345T>C 1346A>T), ATG338ATA (1349G>A), AAC340AAT (1355C>T), TAC341TCT (1357A>C 1358C>T), AGA345AGG (1370A>G), CTT346TGA (1371C>T 1372T>G 1373T>A), CTC347CTG (1376C>G), CAC348TTT (1377C>T 1378A>T 1379C>T), CCT349CCC (1382T>C), GTT350GTC (1385T>C), GAT352CAA (1389G>C 1391T>A), GCT354GCA (1397T>A), ACA356AAA (1402C>A), CTG358CTA (1409G>A), AAA360CGC (1413A>C 1414A>G 1415A>C), ATA361CTT (1416A>C 1418A>T), AAG362CCG (1419A>C 1420A>C), ACA363GCG (1422A>G 1424A>G), GAC364GAT (1427C>T), GGG365GGA (1430G>A), ACT366GCA (1431A>G 1433T>A), GAG370GAA (1445G>A), AAA371CGT (1446A>C 1447A>G 1448A>T), CCT372CCG (1451T>G), TTG374GTA (1455T>G 1457G>A), GCT375CGA (1458G>C 1459C>G 1460T>A), AAG377CGA (1464A>C 1465A>G 1466G>A), CAA378TCT (1467C>T 1468A>C 1469A>T), AAG379CAA (1470A>C 1472G>A), AAC380AAG (1475C>G), TTT381AAA (1476T>A 1477T>A 1478T>A), AAA382GGT (1479A>G 1480A>G 1481A>T), TGT384TCT (1486G>C), TAT385TTC (1489A>T 1490T>C), TTA389TTC (1502A>C), AAG390AAA (1505G>A), TCA391TCG (1508A>G), CCG397CCA (1526G>A), ATG401ATA (1538G>A), TAT402TAC (1541T>C), CTT404TTC (1545C>T 1547T>C), TCT406TCC (1553T>C), ATG407TGC (1554A>T 1555T>G 1556G>C), ATA408ATG (1559A>G), CCT411AGT (1566C>A 1567C>G), ACC412ACA (1571C>A), TTG413CTG (1572T>C), TCC415TCA (1580C>A), GTT418GTC (1589T>C), AGC420del (1593_1597delAGCTC), TCT421--A (1593_1597delAGCTC 1598T>A), CTT424CTC (1607T>C), ACG426ACT (1613G>T), ATC427TTC (1614A>T), CTT428TTG (1617C>T 1619T>G), GTT429ATC (1620G>A 1622T>C), AAC430GTC (1623A>G 1624A>T), AAA431CCT (1626A>C 1627A>C 1628A>T), TTA433ATG (1632T>A 1634A>G), ACA434GTC (1635A>G 1636C>T 1637A>C), AGA436AGG (1643A>G), TAT438AGT (1647T>A 1648A>G), ATA440ATT (1655A>T), TTT442TTC (1661T>C), CTG443TTA (1662C>T 1664G>A), ACT444ACA (1667T>A), GGG445GGA (1670G>A), CCT447CCG (1676T>G), TTG448TTA (1679G>A), GGC449GGT (1682C>T), TTC450TAC (1684T>A), CAC451TAC (1686C>T), GGT452GGA (1691T>A), TCT453TCA (1694T>A), GCA455TCC (1698G>T 1700A>C), CTT456CTA (1703T>A), TTC457TTT (1706C>T), TCG458GCG (1707T>G), TTG459CTT (1710T>C 1712G>T), TCT460TCC (1715T>C), CAC462CAT (1721C>T), ATA464ATG (1727A>G), TTA467CTA (1734T>C), GCA468GCT (1739A>T), TGT476AGT (1761T>A), AAC477ACT (1765A>C 1766C>T), TCT478ACC (1767T>A 1769T>C), CCA479CCT (1772A>T), GGG481GCT (1777G>C 1778G>T), TAC483TAT (1784C>T), GCT484GCC (1787T>C), TTA485CTG (1788T>C 1790A>G), AGT495ACA (1819G>C 1820T>A), AAA496GAG (1821A>G 1823A>G), GCC498GCG (1829C>G), AAT499CGG (1830A>C 1831A>G 1832T>G), GAG500CAG (1833G>C), AAG502AGG (1840A>G), AGA507AGG (1856A>G), CTC508CTT (1859C>T), AAT509GGT (1860A>G 1861A>G), GTC510GTT (1865C>T), AAA511ACC (1867A>C 1868A>C), TCG513TCT (1874G>T), ATA514TTA (1875A>T), CCA515GCT (1878C>G 1880A>T), AAA516AAG (1883A>G), TCT517TCA (1886T>A), TTG518ATT (1887T>A 1889G>T), ATA519ATT (1892A>T), TCT520TCG (1895T>G), GAT521GAG (1898T>G), GGA523GGT (1904A>T), ACC524ACT (1907C>T), GCG528GCC (1919G>C), AAG529AAA (1922G>A), AGG530CGG (1923A>C), TCA535TCG (1940A>G), ATT538GTT (1947A>G), TCC544TCT (1967C>T), CTT545GTG (1968C>G 1970T>G), AGA546AAG (1972G>A 1973A>G), GCT547GCA (1976T>A), TTA549ACA (1980T>A 1981T>C), TCT550TTC (1984C>T 1985T>C), CGT552CGC (1991T>C), GTT555GTG (2000T>G), GGA556GGT (2003A>T), CTT557CTG (2006T>G), GCC559AAC (2010G>A 2011C>A), CTC560ATT (2013C>A 2015C>T), ATC562ACC (2020T>C), GAT565GAC (2030T>C), AAC567AAT (2036C>T), ATA568ATG (2039A>G), AGA573CGG (2052A>C 2054A>G), GGG575GGT (2060G>T), GGT576GGA (2063T>A), GCC577GCT (2066C>T), TAC579TTC (2071A>T), AAG580AGG (2074A>G), GCG583GCC (2084G>C), CGC584CGT (2087C>T), TTA585CTT (2088T>C 2090A>T), TTC586TTT (2093C>T), CAA589CAG (2102A>G), AGC590TCA (2103A>T 2104G>C 2105C>A), AAG591AAA (2108G>A), CGT592CGA (2111T>A), GAG594GAA (2117G>A), CGT595AGG (2118C>A 2120T>G), CTA596TTA (2121C>T), GCT598GTG (2128C>T 2129T>G), CAT600AGG (2133C>A 2134A>G 2135T>G), CTT601CTC (2138T>C), AAG602AAA (2141G>A), CCA603CTA (2143C>T), GTT604GGT (2146T>G), CGG605AGG (2148C>A), TCA606TTA (2152C>T), TTA608TCA (2158T>C), TAC611TTA (2167A>T 2168C>A), TGG613TTT (2173G>T 2174G>T), TGG614TGT (2177G>T), AGG617AGA (2186G>A), ATG619AAA (2191T>A 2192G>A), CCC620CCT (2195C>T), CCC623CCT (2204C>T), TTG625TTA (2210G>A), ATA629ATG (2222A>G), GTA630GTG (2225A>G), TAC632TTT (2230A>T 2231C>T), CTC633CTT (2234C>T), CTT634CTA (2237T>A), AAA635AGG (2239A>G 2240A>G), GAA636AAG (2241G>A 2243A>G), GTT639CCC (2250G>C 2251T>C 2252T>C), CAG643CAT (2264G>T), CCA646ACT (2271C>A 2273A>T), AAG647GAA (2274A>G 2276G>A), CTG649ATG (2280C>A), GTG650GTA (2285G>A), TTC651TTT (2288C>T), GTT655TGG (2298G>T 2299T>G 2300T>G), GAA656GAG (2303A>G), ATA657ATC (2306A>C), CTA658CTT (2309A>T), CGT660TGC (2313C>T 2315T>C), CTA663TTA (2322C>T), CGC664CTC (2326G>T), CAA665AAT (2328C>A 2330A>T), GTA667GTG (2336A>G), GAA668AAA (2337G>A), TTA671CTT (2346T>C 2348A>T), AAG672CGG (2349A>C 2350A>G), CTC674GTT (2355C>G 2357C>T), AAC675TCT (2358A>T 2359A>C 2360C>T), CAT678CAC (2369T>C), ACG679ACT (2372G>T), TGT681TTG (2377G>T 2378T>G), AAC683TCT (2382A>T 2383A>C 2384C>T), CCA684TTT (2385C>T 2386C>T 2387A>T), GAT685GAA (2390T>A), TCC687TCT (2396C>T), ATT688ATC (2399T>C), AAG689GAA (2400A>G 2402G>A), AGT690CAA (2403A>C 2404G>A 2405T>A), CTC691TTG (2406C>T 2408C>G), ATC692ATG (2411C>G), GAC693GAT (2414C>T), TCC694GCT (2415T>G 2417C>T), CCT695CCC (2420T>C), ATA696ATT (2423A>T), ACA699AAG (2431C>A 2432A>G), TCC700TCA (2435C>A), AAA702AAG (2441A>G), AGG703CGA (2442A>C 2444G>A), CTC704TCG (2445C>T 2446T>C 2447C>G), GAA705AAT (2448G>A 2450A>T), ACT706GAA (2451A>G 2452C>A 2453T>A), GAT707GAC (2456T>C), TTT708CTG (2457T>C 2459T>G), GAT709AAA (2460G>A 2462T>A), TTA710TTG (2465A>G), ATC711GTC (2466A>G), GGC714GGT (2477C>T), CTA715TTA (2478C>T), AAA718AAG (2489A>G), TGT719TGC (2492T>C), TAC720TAT (2495C>T), GGT723GGA (2504T>A), GGT725GGC (2510T>C), AAT727GAT (2514A>G), CCC728CTG (2518C>T 2519C>G), ACC730ACT (2525C>T), ACT731ACA (2528T>A), CAG733AGG (2532C>A 2533A>G), TGG734TAT (2536G>A 2537G>T), TTT736CTG (2541T>C 2543T>G), AAC739AGT (2551A>G 2552C>T), ACT740ACC (2555T>C), GTT741TCT (2556G>T 2557T>C), ATT742ATC (2561T>C), AAT745GAT (2568A>G), AAA746CGA (2571A>C 2572A>G), TTA749TTG (2582A>G), GGG750GGA (2585G>A), GGG754GGA (2597G>A), AAC755GCT (2598A>G 2599A>C 2600C>T), ATT758ATA (2609T>A), TCT760GCA (2613T>G 2615T>A), CCT761CCA (2618T>A), GAA763GAT (2624A>T), TAC764TTA (2626A>T 2627C>A), CAA765CCA (2629A>C), ACT766TCT (2631A>T), ACA767-CT (2634delA 2636A>T), GGA768GGT (2639A>T), AAA769GAA (2640A>G), GAG770AGG (2643G>A 2644A>G), GAA771GTG (2647A>T 2648A>G), GTG772TAG (2649G>T 2650T>A), GGC773GTC (2653G>T), TTA774ATA (2655T>A)								

*: Inserts / Deletes / Misaligned / Frameshifts

Analysis details

This analysis was performed with panviral2.64

NGS Details (UN8): Solendovirus venanicotianae

Assembly

**Coverage Length**	2332 (4 contig(s))
**Depth Of Coverage**	21.1
**Number Of Reads**	430
**Reads Per Million**	7.19 rpm (after QC)
**Ambiguities**	7
**Assembly Method**	de novo + reference guided assembly
**Consensus Caller**	Bcf Tools

Coverage Map

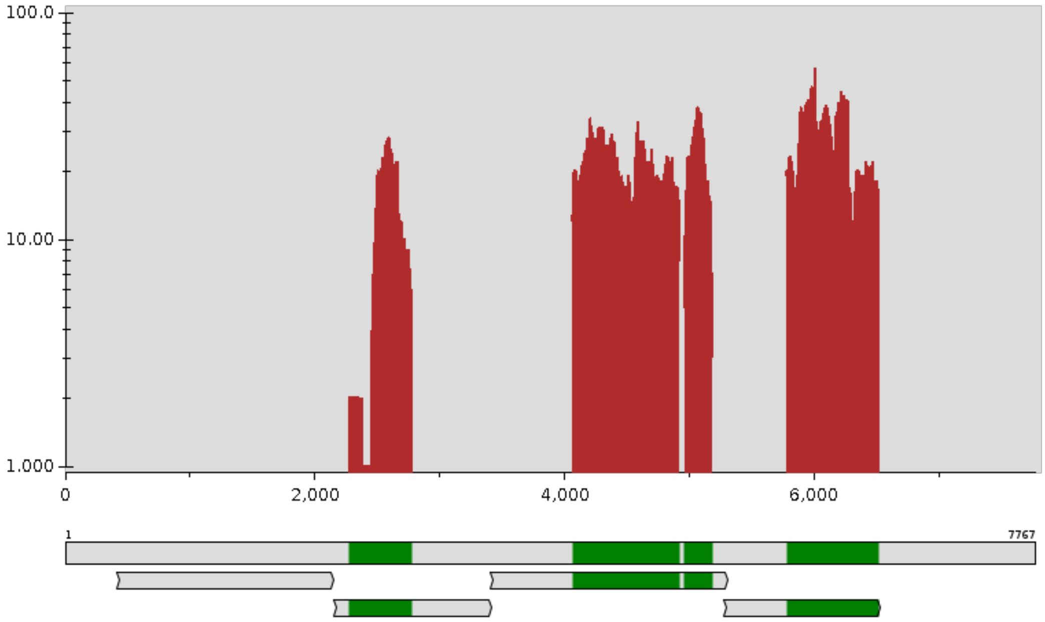

Assignment

**Type**	Solendovirus venanicotianae (Taxonomy ID: 3048371)
**Reference Genome**	NC_003378.1
**NT Identity (%)**	79.7655
**AA Identity (%)**	76.7927
**Number Of Stop Codons**	4
**Number Of CDS**	4

Alignment

**Alignment Score**	2691.0 (NT) + 3770.0 (AA) = 6461.0
**Concordance (%)**	67.8676

**Alignment Method**	Global, seeded, nucleotide + amino acids (AGA)

Genome Region

Sequence starts at position 2271 and ends at position 6519 relative to NC_003378.1 reference sequence.

Alignment Detailed Statistics

	Begin	End	Coverage	Score	Concordance	Matches	Identities	I/D/M/F*	Stop Codons
**NT**	**2271**	**6519**	**30.0%**	**2691**	**59.3%**	**2303 (98.8%)**	**1837 (78.8%)**	**0/29**	
Mutations:	2274C>T, 2280T>C, 2281G>A, 2283C>A, 2294T>A, 2295T>C, 2299G>A, 2301A>G, 2304T>G, 2306C>A, 2307G>A, 2308A>T, 2313A>C, 2314A>T, 2316T>C, 2328A>G, 2330A>C, 2332A>G, 2333A>T, 2343C>T, 2347G>A, 2350G>A, 2352T>A, 2355C>T, 2368A>G, 2370A>G, 2373T>A, 2376T>G, 2388A>C, 2389G>A, 2390G>A, 2393G>A, 2394T>A, 2397G>A, 2398G>A, 2400C>T, 2402A>G, 2412A>C, 2418C>T, 2422A>T, 2424A>G, 2437G>A, 2443C>T, 2448T>A, 2452A>C, 2454G>A, 2461A>G, 2463C>T, 2472G>A, 2475T>C, 2481C>T, 2482T>C, 2493T>A, 2511A>T, 2535C>A, 2551C>T, 2568A>T, 2571C>A, 2572C>G, 2589C>T, 2602A>G, 2622T>C, 2628G>A, 2637T>C, 2638A>G, 2643A>C, 2646C>T, 2652C>T, 2665C>A, 2668A>G, 2670T>A, 2676A>T, 2687A>G, 2692T>C, 2709G>A, 2717G>R, 2718A>R, 2724G>A, 2725T>C, 2728G>A, 2729C>M, 2730A>M, 2733C>A, 2737A>K, 2743G>A, 2754C>A, 2756G>R, 2759G>S, 2763G>A, 2766C>T, 2767C>T, 2772C>T, 2775A>T, 2776T>C, 4079A>G, 4086A>G, 4091T>A, 4098G>A, 4100T>A, 4103T>A, 4104A>T, 4105A>G, 4124T>C, 4127C>T, 4181A>T, 4190G>A, 4196C>T, 4199A>T, 4202C>T, 4235T>C, 4241T>C, 4250C>T, 4253T>C, 4260T>C, 4265G>A, 4284T>A, 4292C>T, 4298T>C, 4307C>T, 4313G>A, 4325T>C, 4328T>C, 4329T>C, 4331G>A, 4334A>G, 4340G>A, 4343T>A, 4355A>G, 4362G>T, 4364T>A, 4400T>A, 4430A>T, 4436C>T, 4451C>T, 4463T>C, 4470G>C, 4475T>C, 4478T>C, 4484A>T, 4500C>A, 4503C>T, 4508T>C, 4513G>A, 4523T>A, 4530A>T, 4550C>T, 4551G>A, 4552C>T, 4554C>A, 4559T>A, 4568C>T, 4571T>A, 4574T>A, 4581T>C, 4591C>A, 4592C>G, 4598A>T, 4601A>G, 4604T>A, 4605A>T, 4607G>T, 4626T>C, 4643T>C, 4653A>G, 4660T>C, 4665A>C, 4666C>A, 4667T>A, 4679A>G, 4687T>C, 4688C>A, 4691T>C, 4692C>T, 4693T>C, 4694T>A, 4697T>A, 4701A>G, 4703C>A, 4704A>T, 4706T>A, 4709T>C, 4721A>G, 4734C>T, 4740A>T, 4755A>C, 4757G>A, 4769T>A, 4773T>C, 4784C>T, 4785T>C, 4793T>A, 4806C>T, 4811A>T, 4814G>A, 4817T>C, 4828G>A, 4831T>A, 4836G>A, 4839A>G, 4847G>A, 4848G>A, 4850A>C, 4854A>G, 4857A>C, 4859G>A, 4865T>A, 4868G>A, 4869A>T, 4872T>C, 4874G>T, 4877T>C, 4896T>C, 4913C>T, 4920T>A, 4970T>A, 4971C>T, 4973T>A, 4976G>A, 4979T>C, 4982A>G, 4986A>G, 4988T>A, 5011G>A, 5012C>T, 5018C>T, 5021T>C, 5027A>T, 5028T>A, 5030T>A, 5032A>T, 5033T>C, 5034A>T, 5037G>A, 5039A>C, 5040C>T, 5043C>G, 5046G>A, 5047A>T, 5053G>A, 5055G>A, 5076T>C, 5077T>A, 5079G>T, 5094C>T, 5117A>T, 5120T>G, 5123C>T, 5125C>A, 5128G>A, 5135T>A, 5136G>A, 5147C>T, 5150T>C, 5156A>G, 5174T>A, 5781A>C, 5784G>A, 5786A>C, 5787T>A, 5789T>A, 5796T>C, 5797A>T, 5798A>T, 5802T>A, 5805A>C, 5807G>A, 5811C>A, 5812C>A, 5814A>T, 5815T>A, 5816C>T, 5817T>C, 5820C>T, 5821C>A, 5824A>G, 5826T>A, 5831A>C, 5833G>C, 5838C>T, 5844T>A, 5850C>A, 5851C>A, 5853T>A, 5857A>C, 5858C>A, 5865C>T, 5872T>C, 5874G>T, 5877A>T, 5880T>A, 5881T>C, 5882T>A, 5886A>C, 5889T>A, 5892A>T, 5894G>A, 5897C>T, 5898A>C, 5901A>T, 5902T>C, 5907A>T, 5908G>A, 5909C>G, 5910C>A, 5913C>A, 5916C>T, 5919C>T, 5921A>G, 5922C>T, 5925C>T, 5935G>A, 5936A>G, 5940T>C, 5950C>T, 5952G>C, 5953A>G, 5954C>A, 5955A>C, 5959C>G, 5964G>A, 5968G>T, 5970C>A, 5972A>C, 5979T>A, 5991G>A, 5997C>T, 5999T>C, 6000G>A, 6001C>A, 6002A>C, 6009G>A, 6011G>A, 6012G>A, 6014T>A, 6015A>T, 6018T>A, 6020A>G, 6024G>A, 6026C>A, 6027A>T, 6031T>G, 6033C>T, 6035A>T, 6036C>T, 6037G>A, 6040C>A, 6041G>A, 6044T>G, 6045C>T, 6049T>A, 6051G>A, 6054T>C, 6055A>C, 6060A>G, 6067C>T, 6071A>T, 6075A>T, 6084G>A, 6087T>A, 6088A>G, 6090C>T, 6094C>T, 6097G>A, 6099T>A, 6108T>A, 6112C>T, 6114C>A, 6117A>C, 6120G>A, 6123A>T, 6129C>T, 6132A>T, 6139A>G, 6141G>T, 6144A>T, 6145G>A, 6146A>C, 6148G>C, 6150A>G, 6153A>G, 6154G>C, 6155A>C, 6156A>G, 6163G>C, 6164A>C, 6170T>A, 6172A>G, 6174T>G, 6177T>A, 6179C>T, 6181G>A, 6183A>T, 6186C>T, 6192T>C, 6201G>A, 6204A>T, 6211A>C, 6219T>A, 6223C>A, 6225A>T, 6232C>T, 6236C>G, 6240C>T, 6247C>A, 6253C>G, 6254A>G, 6258T>C, 6259G>C, 6264T>G, 6269G>A, 6270C>T, 6271T>C, 6277_6283delTCAAGGG, 6285A>C, 6290C>T, 6298_6315delTATTCAAATTGCAGAGAT, 6318A>C, 6319_6321delAGA, 6325G>A, 6326C>G, 6340C>G, 6341T>C, 6344G>T, 6345A>G, 6352G>A, 6354C>A, 6355T>A, 6357A>G, 6359G>C, 6360T>A, 6361C>T, 6363T>G, 6369A>T, 6375A>G, 6376C>A, 6380C>A, 6381T>A, 6382A>C, 6384A>T, 6386C>T, 6387C>T, 6401G>A, 6402G>A, 6404G>A, 6406delA, 6407A>G, 6408T>A, 6411C>T, 6414C>T, 6417C>G, 6418C>G, 6419C>A, 6420A>T, 6423T>A, 6424C>T, 6426G>A, 6427T>C, 6446T>A, 6451A>G, 6453T>G, 6456C>A, 6462T>C, 6471C>T, 6477C>T, 6480A>G, 6489A>C, 6502A>G, 6504T>A, 6505G>A, 6507T>C								

CDS

	Begin	End	Coverage	Score	Concordance	Matches	Identities	I/D/M/F*	Stop Codons
Tvvgp2	40	209	40.5%	821	71.7%	170 (100%)	133 (78.2%)	0/0/5/0	1
Protein mutations:	V43I (2281G>A 2283C>A), F47Y (2294T>A 2295T>C), V49M (2299G>A 2301A>G), T51K (2306C>A 2307G>A), I52L (2308A>T), K53N (2313A>C), N54Y (2314A>T 2316T>C), Q59P (2330A>C), K60V (2332A>G 2333A>T), V65I (2347G>A), V66I (2350G>A 2352T>A), I72V (2368A>G 2370A>G), D74E (2376T>G), G79K (2389G>A 2390G>A), G80E (2393G>A 2394T>A), A82T (2398G>A 2400C>T), K83R (2402A>G), R90W (2422A>T 2424A>G), E95K (2437G>A), Q97* (2443C>T), D98E (2448T>A), K100Q (2452A>C 2454G>A), N103D (2461A>G 2463C>T), Q140E (2572C>G), I150V (2602A>G), I162V (2638A>G), L171I (2665C>A), N172E (2668A>G 2670T>A), R174S (2676A>T), K178R (2687A>G), V197I (2743G>A)								
Codon mutations:	GAC40GAT (2274C>T), AGT42AGC (2280T>C), GTC43ATA (2281G>A 2283C>A), TTT47TAC (2294T>A 2295T>C), GTA49ATG (2299G>A 2301A>G), CCT50CCG (2304T>G), ACG51AAA (2306C>A 2307G>A), ATA52TTA (2308A>T), AAA53AAC (2313A>C), AAT54TAC (2314A>T 2316T>C), AGA58AGG (2328A>G), CAA59CCA (2330A>C), AAA60GTA (2332A>G 2333A>T), TAC63TAT (2343C>T), GTA65ATA (2347G>A), GTT66ATA (2350G>A 2352T>A), AGC67AGT (2355C>T), ATA72GTG (2368A>G 2370A>G), ATT73ATA (2373T>A), GAT74GAG (2376T>G), ACA78ACC (2388A>C), GGA79AAA (2389G>A 2390G>A), GGT80GAA (2393G>A 2394T>A), AAG81AAA (2397G>A), GCC82ACT (2398G>A 2400C>T), AAA83AGA (2402A>G), ATA86ATC (2412A>C), AAC88AAT (2418C>T), AGA90TGG (2422A>T 2424A>G), GAA95AAA (2437G>A), CAA97TAA (2443C>T), GAT98GAA (2448T>A), AAG100CAA (2452A>C 2454G>A), AAC103GAT (2461A>G 2463C>T), AAG106AAA (2472G>A), TAT107TAC (2475T>C), CAC109CAT (2481C>T), TTA110CTA (2482T>C), ACT113ACA (2493T>A), GCA119GCT (2511A>T), ACC127ACA (2535C>A), CTA133TTA (2551C>T), ATA138ATT (2568A>T), ATC139ATA (2571C>A), CAA140GAA (2572C>G), AGC145AGT (2589C>T), ATA150GTA (2602A>G), TAT156TAC (2622T>C), AAG158AAA (2628G>A), TTT161TTC (2637T>C), ATA162GTA (2638A>G), ATA163ATC (2643A>C), AGC164AGT (2646C>T), AAC166AAT (2652C>T), CTA171ATA (2665C>A), AAT172GAA (2668A>G 2670T>A), AGA174AGT (2676A>T), AAA178AGA (2687A>G), TTA180CTA (2692T>C), AAG185AAA (2709G>A), GGA188GRR (2717G>R 2718A>R), GAG190GAA (2724G>A), TTA191CTA (2725T>C), GCA192AMM (2728G>A 2729C>M 2730A>M), CCC193CCA (2733C>A), AGT195KGT (2737A>K), GTA197ATA (2743G>A), GCC200GCA (2754C>A), AGA201ARA (2756G>R), TGT202TST (2759G>S), AAG203AAA (2763G>A), AAC204AAT (2766C>T), CTA205TTA (2767C>T), TAC206TAT (2772C>T), GTA207GTT (2775A>T), TTA208CTA (2776T>C)								
Tvvgp3	219	591	56.6%	2134	85.0%	360 (100%)	306 (85.0%)	0/0/0/0	2
Protein mutations:	I223M (4079A>G), K226E (4086A>G), D227E (4091T>A), D230K (4098G>A 4100T>A), N231K (4103T>A), K232* (4104A>T 4105A>G), Y292N (4284T>A), D311E (4343T>A), A318S (4362G>T 4364T>A), E354Q (4470G>C), L364I (4500C>A), R368K (4513G>A), D371E (4523T>A), I374L (4530A>T), A381I (4551G>A 4552C>T), H382N (4554C>A), T394K (4591C>A 4592C>G), M399F (4605A>T 4607G>T), I415V (4653A>G), M417T (4660T>C), T419Q (4665A>C 4666C>A 4667T>A), I426T (4687T>C 4688C>A), L428S (4692C>T 4693T>C 4694T>A), D429E (4697T>A), N431E (4701A>G 4703C>A), I432L (4704A>T 4706T>A), I444L (4740A>T), K467N (4811A>T), S473N (4828G>A), F474Y (4831T>A), E476K (4836G>A), K477E (4839A>G), E480N (4848G>A 4850A>C), I482V (4854A>G), K483Q (4857A>C 4859G>A), I487L (4869A>T), Y496H (4896T>C), N526E (4986A>G 4988T>A), C534Y (5011G>A 5012C>T), S540T (5028T>A 5030T>A), Y541F (5032A>T 5033T>C), T542S (5034A>T), E543N (5037G>A 5039A>C), P544S (5040C>T), Q545E (5043C>G), E546I (5046G>A 5047A>T), W548* (5053G>A), E549K (5055G>A), F556H (5076T>C 5077T>A), A557S (5079G>T), T572N (5125C>A), R573K (5128G>A), V576I (5136G>A)								
Codon mutations:	ATA223ATG (4079A>G), AAA226GAA (4086A>G), GAT227GAA (4091T>A), GAT230AAA (4098G>A 4100T>A), AAT231AAA (4103T>A), AAA232TGA (4104A>T 4105A>G), AAT238AAC (4124T>C), AGC239AGT (4127C>T), GGA257GGT (4181A>T), AGG260AGA (4190G>A), GTC262GTT (4196C>T), ATA263ATT (4199A>T), GAC264GAT (4202C>T), TAT275TAC (4235T>C), TAT277TAC (4241T>C), CCC280CCT (4250C>T), AAT281AAC (4253T>C), TTA284CTA (4260T>C), AAG285AAA (4265G>A), TAT292AAT (4284T>A), TAC294TAT (4292C>T), AGT296AGC (4298T>C), GAC299GAT (4307C>T), AAG301AAA (4313G>A), TAT305TAC (4325T>C), CAT306CAC (4328T>C), TTG307CTA (4329T>C 4331G>A), AAA308AAG (4334A>G), GAG310GAA (4340G>A), GAT311GAA (4343T>A), AAA315AAG (4355A>G), GCT318TCA (4362G>T 4364T>A), GTT330GTA (4400T>A), GGA340GGT (4430A>T), TAC342TAT (4436C>T), GAC347GAT (4451C>T), AAT351AAC (4463T>C), GAA354CAA (4470G>C), AAT355AAC (4475T>C), TGT356TGC (4478T>C), GTA358GTT (4484A>T), CTA364ATA (4500C>A), CTA365TTA (4503C>T), TAT366TAC (4508T>C), AGA368AAA (4513G>A), GAT371GAA (4523T>A), ATA374TTA (4530A>T), TTC380TTT (4550C>T), GCA381ATA (4551G>A 4552C>T), CAT382AAT (4554C>A), ATT383ATA (4559T>A), AAC386AAT (4568C>T), TCT387TCA (4571T>A), GGT388GGA (4574T>A), TTA391CTA (4581T>C), ACC394AAG (4591C>A 4592C>G), GCA396GCT (4598A>T), GAA397GAG (4601A>G), ATT398ATA (4604T>A), ATG399TTT (4605A>T 4607G>T), TTA406CTA (4626T>C), GAT411GAC (4643T>C), ATA415GTA (4653A>G), ATG417ACG (4660T>C), ACT419CAA (4665A>C 4666C>A 4667T>A), CAA423CAG (4679A>G), ATC426ACA (4687T>C 4688C>A), AAT427AAC (4691T>C), CTT428TCA (4692C>T 4693T>C 4694T>A), GAT429GAA (4697T>A), AAC431GAA (4701A>G 4703C>A), ATT432TTA (4704A>T 4706T>A), GAT433GAC (4709T>C), AAA437AAG (4721A>G), CTA442TTA (4734C>T), ATA444TTA (4740A>T), AGG449CGA (4755A>C 4757G>A), CCT453CCA (4769T>A), TTA455CTA (4773T>C), AAC458AAT (4784C>T), TTA459CTA (4785T>C), CCT461CCA (4793T>A), CTA466TTA (4806C>T), AAA467AAT (4811A>T), AAG468AAA (4814G>A), GAT469GAC (4817T>C), AGT473AAT (4828G>A), TTT474TAT (4831T>A), GAA476AAA (4836G>A), AAA477GAA (4839A>G), AAG479AAA (4847G>A), GAA480AAC (4848G>A 4850A>C), ATA482GTA (4854A>G), AAG483CAA (4857A>C 4859G>A), ATT485ATA (4865T>A), AAG486AAA (4868G>A), ATA487TTA (4869A>T), TTG488CTT (4872T>C 4874G>T), TGT489TGC (4877T>C), TAC496CAC (4896T>C), AAC501AAT (4913C>T), TTT504AT. (4920T>A), GTT520GTA (4970T>A), CTT521TTA (4971C>T 4973T>A), AAG522AAA (4976G>A), TAT523TAC (4979T>C), AGA524AGG (4982A>G), AAT526GAA (4986A>G 4988T>A), TGC534TAT (5011G>A 5012C>T), TAC536TAT (5018C>T), TAT537TAC (5021T>C), GGA539GGT (5027A>T), TCT540ACA (5028T>A 5030T>A), TAT541TTC (5032A>T 5033T>C), ACA542TCA (5034A>T), GAA543AAC (5037G>A 5039A>C), CCA544TCA (5040C>T), CAA545GAA (5043C>G), GAA546ATA (5046G>A 5047A>T), TGG548TAG (5053G>A), GAA549AAA (5055G>A), TTT556CAT (5076T>C 5077T>A), GCA557TCA (5079G>T), CTA562TTA (5094C>T), ATA569ATT (5117A>T), GTT570GTG (5120T>G), TAC571TAT (5123C>T), ACC572AAC (5125C>A), AGA573AAA (5128G>A), ATT575ATA (5135T>A), GTA576ATA (5136G>A), GAC579GAT (5147C>T), AAT580AAC (5150T>C), CAA582CAG (5156A>G), ACT588ACA (5174T>A)								
Tvvgp4	169	415	58.9%	815	50.7%	237 (96.3%)	150 (61.0%)	0/9/2/2	1

	Begin	End	Coverage	Score	Concordance	Matches	Identities	I/D/M/F*	Stop Codons
**NT**	**2271**	**6519**	**30.0%**	**2691**	**59.3%**	**2303 (98.8%)**	**1837 (78.8%)**	**0/29**	
Protein mutations:	N171T (5786A>C 5787T>A), F172Y (5789T>A), K175L (5797A>T 5798A>T), N176K (5802T>A), R178K (5807G>A), Q180N (5812C>A 5814A>T), S181I (5815T>A 5816C>T 5817T>C), Q183K (5821C>A), N184E (5824A>G 5826T>A), N186T (5831A>C), E187Q (5833G>C), H192Q (5850C>A), H193K (5851C>A 5853T>A), T195Q (5857A>C 5858C>A), L203Q (5881T>C 5882T>A), N205K (5889T>A), S207N (5894G>A), A208V (5897C>T 5898A>C), K209N (5901A>T), A212R (5908G>A 5909C>G 5910C>A), N216S (5921A>G 5922C>T), D221S (5935G>A 5936A>G), Q226Y (5950C>T 5952G>C), T227D (5953A>G 5954C>A 5955A>C), Q229E (5959C>G), A232S (5968G>T 5970C>A), N233T (5972A>C), M242T (5999T>C 6000G>A), Q243T (6001C>A 6002A>C), R246K (6011G>A 6012G>A), I247N (6014T>A 6015A>T), K249R (6020A>G), T251N (6026C>A 6027A>T), F253V (6031T>G 6033C>T), Y254F (6035A>T 6036C>T), V255I (6037G>A), R256K (6040C>A 6041G>A), F257C (6044T>G 6045C>T), S259T (6049T>A 6051G>A), T261P (6055A>C), Y266F (6071A>T), E267D (6075A>T), I272V (6088A>G 6090C>T), Q274* (6094C>T), V275I (6097G>A 6099T>A), E283D (6123A>T), K289D (6139A>G 6141G>T), E291T (6145G>A 6146A>C), E292Q (6148G>C 6150A>G), E294P (6154G>C 6155A>C 6156A>G), E297P (6163G>C 6164A>C), V299E (6170T>A), N300E (6172A>G 6174T>G), P302L (6179C>T), E303N (6181G>A 6183A>T), I313L (6211A>C), L317I (6223C>A 6225A>T), A321G (6236C>G), L325I (6247C>A), Q327G (6253C>G 6254A>G), A329P (6259G>C), I330M (6264T>G), S332N (6269G>A 6270C>T), Y333H (6271T>C), S335_R336del (6277_6283delTCAAGGG), T339I (6290C>T), Y342_D347del (6298_6315delTATTCAAATTGCAGAGAT), R349del (6319_6321delAGA), A351R (6325G>A 6326C>G), L356A (6340C>G 6341T>C), R357M (6344G>T 6345A>G), V360I (6352G>A 6354C>A), L361M (6355T>A 6357A>G), S362T (6359G>C 6360T>A), K365N (6369A>T), Q368K (6376C>A), P369Q (6380C>A 6381T>A), T370P (6382A>C 6384A>T), T371I (6386C>T 6387C>T), R376K (6401G>A 6402G>A), R377K (6404G>A), P382D (6418C>G 6419C>A 6420A>T), D383E (6423T>A), L391Q (6446T>A), S393G (6451A>G 6453T>G), H394Q (6456C>A), K405N (6489A>C), I410V (6502A>G 6504T>A), V411I (6505G>A 6507T>C)								
Codon mutations:	ATA169ATC (5781A>C), CAG170CAA (5784G>A), AAT171ACA (5786A>C 5787T>A), TTT172TAT (5789T>A), AAT174AAC (5796T>C), AAA175TTA (5797A>T 5798A>T), AAT176AAA (5802T>A), CCA177CCC (5805A>C), AGA178AAA (5807G>A), TCC179TCA (5811C>A), CAA180AAT (5812C>A 5814A>T), TCT181ATC (5815T>A 5816C>T 5817T>C), ACC182ACT (5820C>T), CAA183AAA (5821C>A), AAT184GAA (5824A>G 5826T>A), AAC186ACC (5831A>C), GAA187CAA (5833G>C), GAC188GAT (5838C>T), ATT190ATA (5844T>A), CAC192CAA (5850C>A), CAT193AAA (5851C>A 5853T>A), ACA195CAA (5857A>C 5858C>A), TAC197TAT (5865C>T), TTG200CTT (5872T>C 5874G>T), ATA201ATT (5877A>T), GCT202GCA (5880T>A), TTA203CAA (5881T>C 5882T>A), CCA204CCC (5886A>C), AAT205AAA (5889T>A), ACA206ACT (5892A>T), AGT207AAT (5894G>A), GCA208GTC (5897C>T 5898A>C), AAA209AAT (5901A>T), TTA210CTA (5902T>C), GTA211GTT (5907A>T), GCC212AGA (5908G>A 5909C>G 5910C>A), ACC213ACA (5913C>A), TGC214TGT (5916C>T), TAC215TAT (5919C>T), AAC216AGT (5921A>G 5922C>T), TAC217TAT (5925C>T), GAT221AGT (5935G>A 5936A>G), ACT222ACC (5940T>C), CAG226TAC (5950C>T 5952G>C), ACA227GAC (5953A>G 5954C>A 5955A>C), CAA229GAA (5959C>G), GAG230GAA (5964G>A), GCC232TCA (5968G>T 5970C>A), AAT233ACT (5972A>C), CCT235CCA (5979T>A), AAG239AAA (5991G>A), TTC241TTT (5997C>T), ATG242ACA (5999T>C 6000G>A), CAA243ACA (6001C>A 6002A>C), AAG245AAA (6009G>A), AGG246AAA (6011G>A 6012G>A), ATA247AAT (6014T>A 6015A>T), ACT248ACA (6018T>A), AAA249AGA (6020A>G), GGG250GGA (6024G>A), ACA251AAT (6026C>A 6027A>T), TTC253GTT (6031T>G 6033C>T), TAC254TTT (6035A>T 6036C>T), GTA255ATA (6037G>A), CGA256AAA (6040C>A 6041G>A), TTC257TGT (6044T>G 6045C>T), TCG259ACA (6049T>A 6051G>A), GCT260GCC (6054T>C), ACA261CCA (6055A>C), GCA262GCG (6060A>G), CTA265TTA (6067C>T), TAT266TTT (6071A>T), GAA267GAT (6075A>T), AAG270AAA (6084G>A), CCT271CCA (6087T>A), ATC272GTT (6088A>G 6090C>T), CAA274TAA (6094C>T), GTT275ATA (6097G>A 6099T>A), ATT278ATA (6108T>A), CTC280TTA (6112C>T 6114C>A), ACA281ACC (6117A>C), AGG282AGA (6120G>A), GAA283GAT (6123A>T), ATC285ATT (6129C>T), ATA286ATT (6132A>T), AAG289GAT (6139A>G 6141G>T), ATA290ATT (6144A>T), GAA291ACA (6145G>A 6146A>C), GAA292CAG (6148G>C 6150A>G), CAA293CAG (6153A>G), GAA294CCG (6154G>C 6155A>C 6156A>G), GAA297CCA (6163G>C 6164A>C), GTA299GAA (6170T>A), AAT300GAG (6172A>G 6174T>G), ATT301ATA (6177T>A), CCA302CTA (6179C>T), GAA303AAT (6181G>A 6183A>T), TTC304TTT (6186C>T), GCT306GCC (6192T>C), AGG309AGA (6201G>A), ATA310ATT (6204A>T), ATA313CTA (6211A>C), ACT315ACA (6219T>A), CTA317ATT (6223C>A 6225A>T), CTA320TTA (6232C>T), GCA321GGA (6236C>G), AAC322AAT (6240C>T), CTA325ATA (6247C>A), CAG327GGG (6253C>G 6254A>G), AAT328AAC (6258T>C), GCA329CCA (6259G>C), ATT330ATG (6264T>G), AGC332AAT (6269G>A 6270C>T), TAT333CAT (6271T>C), TCA335_AGG336del (6277_6283delTCAAGGG), GAA337-AC (6277_6283delTCAAGGG 6285A>C), ACA339ATA (6290C>T), TAT342_GAT347del (6298_6315delTATTCAAATTGCAGAGAT), ATA348ATC (6318A>C), AGA349del (6319_6321delAGA), GCA351AGA (6325G>A 6326C>G), CTA356GCA (6340C>G 6341T>C), AGA357ATG (6344G>T 6345A>G), GTC360ATA (6352G>A 6354C>A), TTA361ATG (6355T>A 6357A>G), AGT362ACA (6359G>C 6360T>A), CTT363TTG (6361C>T 6363T>G), AAA365AAT (6369A>T), GAA367GAG (6375A>G), CAA368AAA (6376C>A), CCT369CAA (6380C>A 6381T>A), ACA370CCT (6382A>C 6384A>T), ACC371ATT (6386C>T 6387C>T), AGG376AAA (6401G>A 6402G>A), AGG377AAG (6404G>A), AAT378-GA (6406delA 6407A>G 6408T>A), TTC379TTT (6411C>T), ATC380ATT (6414C>T), TCC381TCG (6417C>G), CCA382GAT (6418C>G 6419C>A 6420A>T), GAT383GAA (6423T>A), CTG384TTA (6424C>T 6426G>A), TTA385CTA (6427T>C), CTA391CAA (6446T>A), AGT393GGG (6451A>G 6453T>G), CAC394CAA (6456C>A), TAT396TAC (6462T>C), CAC399CAT (6471C>T), TGC401TGT (6477C>T), TCA402TCG (6480A>G), AAA405AAC (6489A>C), ATT410GTA (6502A>G 6504T>A), GTT411ATC (6505G>A 6507T>C)								

Proteins

	Begin	End	Coverage	Score	Concordance	Matches	Identities	I/D/M/F*	Stop Codons
putative cell-to-cell movement protein (NP_569140.1)	40	209	40.5%	821	71.7%	170 (100%)	133 (78.2%)	0/0/5/0	1
Protein mutations:	V43I (2281G>A 2283C>A), F47Y (2294T>A 2295T>C), V49M (2299G>A 2301A>G), T51K (2306C>A 2307G>A), I52L (2308A>T), K53N (2313A>C), N54Y (2314A>T 2316T>C), Q59P (2330A>C), K60V (2332A>G 2333A>T), V65I (2347G>A), V66I (2350G>A 2352T>A), I72V (2368A>G 2370A>G), D74E (2376T>G), G79K (2389G>A 2390G>A), G80E (2393G>A 2394T>A), A82T (2398G>A 2400C>T), K83R (2402A>G), R90W (2422A>T 2424A>G), E95K (2437G>A), Q97* (2443C>T), D98E (2448T>A), K100Q (2452A>C 2454G>A), N103D (2461A>G 2463C>T), Q140E (2572C>G), I150V (2602A>G), I162V (2638A>G), L171I (2665C>A), N172E (2668A>G 2670T>A), R174S (2676A>T), K178R (2687A>G), V197I (2743G>A)								
Codon mutations:	GAC40GAT (2274C>T), AGT42AGC (2280T>C), GTC43ATA (2281G>A 2283C>A), TTT47TAC (2294T>A 2295T>C), GTA49ATG (2299G>A 2301A>G), CCT50CCG (2304T>G), ACG51AAA (2306C>A 2307G>A), ATA52TTA (2308A>T), AAA53AAC (2313A>C), AAT54TAC (2314A>T 2316T>C), AGA58AGG (2328A>G), CAA59CCA (2330A>C), AAA60GTA (2332A>G 2333A>T), TAC63TAT (2343C>T), GTA65ATA (2347G>A), GTT66ATA (2350G>A 2352T>A), AGC67AGT (2355C>T), ATA72GTG (2368A>G 2370A>G), ATT73ATA (2373T>A), GAT74GAG (2376T>G), ACA78ACC (2388A>C), GGA79AAA (2389G>A 2390G>A), GGT80GAA (2393G>A 2394T>A), AAG81AAA (2397G>A), GCC82ACT (2398G>A 2400C>T), AAA83AGA (2402A>G), ATA86ATC (2412A>C), AAC88AAT (2418C>T), AGA90TGG (2422A>T 2424A>G), GAA95AAA (2437G>A), CAA97TAA (2443C>T), GAT98GAA (2448T>A), AAG100CAA (2452A>C 2454G>A), AAC103GAT (2461A>G 2463C>T), AAG106AAA (2472G>A), TAT107TAC (2475T>C), CAC109CAT (2481C>T), TTA110CTA (2482T>C), ACT113ACA (2493T>A), GCA119GCT (2511A>T), ACC127ACA (2535C>A), CTA133TTA (2551C>T), ATA138ATT (2568A>T), ATC139ATA (2571C>A), CAA140GAA (2572C>G), AGC145AGT (2589C>T), ATA150GTA (2602A>G), TAT156TAC (2622T>C), AAG158AAA (2628G>A), TTT161TTC (2637T>C), ATA162GTA (2638A>G), ATA163ATC (2643A>C), AGC164AGT (2646C>T), AAC166AAT (2652C>T), CTA171ATA (2665C>A), AAT172GAA (2668A>G 2670T>A), AGA174AGT (2676A>T), AAA178AGA (2687A>G), TTA180CTA (2692T>C), AAG185AAA (2709G>A), GGA188GRR (2717G>R 2718A>R), GAG190GAA (2724G>A), TTA191CTA (2725T>C), GCA192AMM (2728G>A 2729C>M 2730A>M), CCC193CCA (2733C>A), AGT195KGT (2737A>K), GTA197ATA (2743G>A), GCC200GCA (2754C>A), AGA201ARA (2756G>R), TGT202TST (2759G>S), AAG203AAA (2763G>A), AAC204AAT (2766C>T), CTA205TTA (2767C>T), TAC206TAT (2772C>T), GTA207GTT (2775A>T), TTA208CTA (2776T>C)								
polyprotein (NP_569141.1)	219	591	56.6%	2134	85.0%	360 (100%)	306 (85.0%)	0/0/0/0	2
Protein mutations:	I223M (4079A>G), K226E (4086A>G), D227E (4091T>A), D230K (4098G>A 4100T>A), N231K (4103T>A), K232* (4104A>T 4105A>G), Y292N (4284T>A), D311E (4343T>A), A318S (4362G>T 4364T>A), E354Q (4470G>C), L364I (4500C>A), R368K (4513G>A), D371E (4523T>A), I374L (4530A>T), A381I (4551G>A 4552C>T), H382N (4554C>A), T394K (4591C>A 4592C>G), M399F (4605A>T 4607G>T), I415V (4653A>G), M417T (4660T>C), T419Q (4665A>C 4666C>A 4667T>A), I426T (4687T>C 4688C>A), L428S (4692C>T 4693T>C 4694T>A), D429E (4697T>A), N431E (4701A>G 4703C>A), I432L (4704A>T 4706T>A), I444L (4740A>T), K467N (4811A>T), S473N (4828G>A), F474Y (4831T>A), E476K (4836G>A), K477E (4839A>G), E480N (4848G>A 4850A>C), I482V (4854A>G), K483Q (4857A>C 4859G>A), I487L (4869A>T), Y496H (4896T>C), N526E (4986A>G 4988T>A), C534Y (5011G>A 5012C>T), S540T (5028T>A 5030T>A), Y541F (5032A>T 5033T>C), T542S (5034A>T), E543N (5037G>A 5039A>C), P544S (5040C>T), Q545E (5043C>G), E546I (5046G>A 5047A>T), W548* (5053G>A), E549K (5055G>A), F556H (5076T>C 5077T>A), A557S (5079G>T), T572N (5125C>A), R573K (5128G>A), V576I (5136G>A)								
Codon mutations:	ATA223ATG (4079A>G), AAA226GAA (4086A>G), GAT227GAA (4091T>A), GAT230AAA (4098G>A 4100T>A), AAT231AAA (4103T>A), AAA232TGA (4104A>T 4105A>G), AAT238AAC (4124T>C), AGC239AGT (4127C>T), GGA257GGT (4181A>T), AGG260AGA (4190G>A), GTC262GTT (4196C>T), ATA263ATT (4199A>T), GAC264GAT (4202C>T), TAT275TAC (4235T>C), TAT277TAC (4241T>C), CCC280CCT (4250C>T), AAT281AAC (4253T>C), TTA284CTA (4260T>C), AAG285AAA (4265G>A), TAT292AAT (4284T>A), TAC294TAT (4292C>T), AGT296AGC (4298T>C), GAC299GAT (4307C>T), AAG301AAA (4313G>A), TAT305TAC (4325T>C), CAT306CAC (4328T>C), TTG307CTA (4329T>C 4331G>A), AAA308AAG (4334A>G), GAG310GAA (4340G>A), GAT311GAA (4343T>A), AAA315AAG (4355A>G), GCT318TCA (4362G>T 4364T>A), GTT330GTA (4400T>A), GGA340GGT (4430A>T), TAC342TAT (4436C>T), GAC347GAT (4451C>T), AAT351AAC (4463T>C), GAA354CAA (4470G>C), AAT355AAC (4475T>C), TGT356TGC (4478T>C), GTA358GTT (4484A>T), CTA364ATA (4500C>A), CTA365TTA (4503C>T), TAT366TAC (4508T>C), AGA368AAA (4513G>A), GAT371GAA (4523T>A), ATA374TTA (4530A>T), TTC380TTT (4550C>T), GCA381ATA (4551G>A 4552C>T), CAT382AAT (4554C>A), ATT383ATA (4559T>A), AAC386AAT (4568C>T), TCT387TCA (4571T>A), GGT388GGA (4574T>A), TTA391CTA (4581T>C), ACC394AAG (4591C>A 4592C>G), GCA396GCT (4598A>T), GAA397GAG (4601A>G), ATT398ATA (4604T>A), ATG399TTT (4605A>T 4607G>T), TTA406CTA (4626T>C), GAT411GAC (4643T>C), ATA415GTA (4653A>G), ATG417ACG (4660T>C), ACT419CAA (4665A>C 4666C>A 4667T>A), CAA423CAG (4679A>G), ATC426ACA (4687T>C 4688C>A), AAT427AAC (4691T>C), CTT428TCA (4692C>T 4693T>C 4694T>A), GAT429GAA (4697T>A), AAC431GAA (4701A>G 4703C>A), ATT432TTA (4704A>T 4706T>A), GAT433GAC (4709T>C), AAA437AAG (4721A>G), CTA442TTA (4734C>T), ATA444TTA (4740A>T), AGG449CGA (4755A>C 4757G>A), CCT453CCA (4769T>A), TTA455CTA (4773T>C), AAC458AAT (4784C>T), TTA459CTA (4785T>C), CCT461CCA (4793T>A), CTA466TTA (4806C>T), AAA467AAT (4811A>T), AAG468AAA (4814G>A), GAT469GAC (4817T>C), AGT473AAT (4828G>A), TTT474TAT (4831T>A), GAA476AAA (4836G>A), AAA477GAA (4839A>G), AAG479AAA (4847G>A), GAA480AAC (4848G>A 4850A>C), ATA482GTA (4854A>G), AAG483CAA (4857A>C 4859G>A), ATT485ATA (4865T>A), AAG486AAA (4868G>A), ATA487TTA (4869A>T), TTG488CTT (4872T>C 4874G>T), TGT489TGC (4877T>C), TAC496CAC (4896T>C), AAC501AAT (4913C>T), TTT504AT. (4920T>A), GTT520GTA (4970T>A), CTT521TTA (4971C>T 4973T>A), AAG522AAA (4976G>A), TAT523TAC (4979T>C), AGA524AGG (4982A>G), AAT526GAA (4986A>G 4988T>A), TGC534TAT (5011G>A 5012C>T), TAC536TAT (5018C>T), TAT537TAC (5021T>C), GGA539GGT (5027A>T), TCT540ACA (5028T>A 5030T>A), TAT541TTC (5032A>T 5033T>C), ACA542TCA (5034A>T), GAA543AAC (5037G>A 5039A>C), CCA544TCA (5040C>T), CAA545GAA (5043C>G), GAA546ATA (5046G>A 5047A>T), TGG548TAG (5053G>A), GAA549AAA (5055G>A), TTT556CAT (5076T>C 5077T>A), GCA557TCA (5079G>T), CTA562TTA (5094C>T), ATA569ATT (5117A>T), GTT570GTG (5120T>G), TAC571TAT (5123C>T), ACC572AAC (5125C>A), AGA573AAA (5128G>A), ATT575ATA (5135T>A), GTA576ATA (5136G>A), GAC579GAT (5147C>T), AAT580AAC (5150T>C), CAA582CAG (5156A>G), ACT588ACA (5174T>A)								
putative transactivator factor (NP_569142.1)	169	415	58.9%	815	50.7%	237 (96.3%)	150 (61.0%)	0/9/2/2	1

	Begin	End	Coverage	Score	Concordance	Matches	Identities	I/D/M/F*	Stop Codons
**NT**	**2271**	**6519**	**30.0%**	**2691**	**59.3%**	**2303 (98.8%)**	**1837 (78.8%)**	**0/29**	
Protein mutations:	N171T (5786A>C 5787T>A), F172Y (5789T>A), K175L (5797A>T 5798A>T), N176K (5802T>A), R178K (5807G>A), Q180N (5812C>A 5814A>T), S181I (5815T>A 5816C>T 5817T>C), Q183K (5821C>A), N184E (5824A>G 5826T>A), N186T (5831A>C), E187Q (5833G>C), H192Q (5850C>A), H193K (5851C>A 5853T>A), T195Q (5857A>C 5858C>A), L203Q (5881T>C 5882T>A), N205K (5889T>A), S207N (5894G>A), A208V (5897C>T 5898A>C), K209N (5901A>T), A212R (5908G>A 5909C>G 5910C>A), N216S (5921A>G 5922C>T), D221S (5935G>A 5936A>G), Q226Y (5950C>T 5952G>C), T227D (5953A>G 5954C>A 5955A>C), Q229E (5959C>G), A232S (5968G>T 5970C>A), N233T (5972A>C), M242T (5999T>C 6000G>A), Q243T (6001C>A 6002A>C), R246K (6011G>A 6012G>A), I247N (6014T>A 6015A>T), K249R (6020A>G), T251N (6026C>A 6027A>T), F253V (6031T>G 6033C>T), Y254F (6035A>T 6036C>T), V255I (6037G>A), R256K (6040C>A 6041G>A), F257C (6044T>G 6045C>T), S259T (6049T>A 6051G>A), T261P (6055A>C), Y266F (6071A>T), E267D (6075A>T), I272V (6088A>G 6090C>T), Q274* (6094C>T), V275I (6097G>A 6099T>A), E283D (6123A>T), K289D (6139A>G 6141G>T), E291T (6145G>A 6146A>C), E292Q (6148G>C 6150A>G), E294P (6154G>C 6155A>C 6156A>G), E297P (6163G>C 6164A>C), V299E (6170T>A), N300E (6172A>G 6174T>G), P302L (6179C>T), E303N (6181G>A 6183A>T), I313L (6211A>C), L317I (6223C>A 6225A>T), A321G (6236C>G), L325I (6247C>A), Q327G (6253C>G 6254A>G), A329P (6259G>C), I330M (6264T>G), S332N (6269G>A 6270C>T), Y333H (6271T>C), S335_R336del (6277_6283delTCAAGGG), T339I (6290C>T), Y342_D347del (6298_6315delTATTCAAATTGCAGAGAT), R349del (6319_6321delAGA), A351R (6325G>A 6326C>G), L356A (6340C>G 6341T>C), R357M (6344G>T 6345A>G), V360I (6352G>A 6354C>A), L361M (6355T>A 6357A>G), S362T (6359G>C 6360T>A), K365N (6369A>T), Q368K (6376C>A), P369Q (6380C>A 6381T>A), T370P (6382A>C 6384A>T), T371I (6386C>T 6387C>T), R376K (6401G>A 6402G>A), R377K (6404G>A), P382D (6418C>G 6419C>A 6420A>T), D383E (6423T>A), L391Q (6446T>A), S393G (6451A>G 6453T>G), H394Q (6456C>A), K405N (6489A>C), I410V (6502A>G 6504T>A), V411I (6505G>A 6507T>C)								
Codon mutations:	ATA169ATC (5781A>C), CAG170CAA (5784G>A), AAT171ACA (5786A>C 5787T>A), TTT172TAT (5789T>A), AAT174AAC (5796T>C), AAA175TTA (5797A>T 5798A>T), AAT176AAA (5802T>A), CCA177CCC (5805A>C), AGA178AAA (5807G>A), TCC179TCA (5811C>A), CAA180AAT (5812C>A 5814A>T), TCT181ATC (5815T>A 5816C>T 5817T>C), ACC182ACT (5820C>T), CAA183AAA (5821C>A), AAT184GAA (5824A>G 5826T>A), AAC186ACC (5831A>C), GAA187CAA (5833G>C), GAC188GAT (5838C>T), ATT190ATA (5844T>A), CAC192CAA (5850C>A), CAT193AAA (5851C>A 5853T>A), ACA195CAA (5857A>C 5858C>A), TAC197TAT (5865C>T), TTG200CTT (5872T>C 5874G>T), ATA201ATT (5877A>T), GCT202GCA (5880T>A), TTA203CAA (5881T>C 5882T>A), CCA204CCC (5886A>C), AAT205AAA (5889T>A), ACA206ACT (5892A>T), AGT207AAT (5894G>A), GCA208GTC (5897C>T 5898A>C), AAA209AAT (5901A>T), TTA210CTA (5902T>C), GTA211GTT (5907A>T), GCC212AGA (5908G>A 5909C>G 5910C>A), ACC213ACA (5913C>A), TGC214TGT (5916C>T), TAC215TAT (5919C>T), AAC216AGT (5921A>G 5922C>T), TAC217TAT (5925C>T), GAT221AGT (5935G>A 5936A>G), ACT222ACC (5940T>C), CAG226TAC (5950C>T 5952G>C), ACA227GAC (5953A>G 5954C>A 5955A>C), CAA229GAA (5959C>G), GAG230GAA (5964G>A), GCC232TCA (5968G>T 5970C>A), AAT233ACT (5972A>C), CCT235CCA (5979T>A), AAG239AAA (5991G>A), TTC241TTT (5997C>T), ATG242ACA (5999T>C 6000G>A), CAA243ACA (6001C>A 6002A>C), AAG245AAA (6009G>A), AGG246AAA (6011G>A 6012G>A), ATA247AAT (6014T>A 6015A>T), ACT248ACA (6018T>A), AAA249AGA (6020A>G), GGG250GGA (6024G>A), ACA251AAT (6026C>A 6027A>T), TTC253GTT (6031T>G 6033C>T), TAC254TTT (6035A>T 6036C>T), GTA255ATA (6037G>A), CGA256AAA (6040C>A 6041G>A), TTC257TGT (6044T>G 6045C>T), TCG259ACA (6049T>A 6051G>A), GCT260GCC (6054T>C), ACA261CCA (6055A>C), GCA262GCG (6060A>G), CTA265TTA (6067C>T), TAT266TTT (6071A>T), GAA267GAT (6075A>T), AAG270AAA (6084G>A), CCT271CCA (6087T>A), ATC272GTT (6088A>G 6090C>T), CAA274TAA (6094C>T), GTT275ATA (6097G>A 6099T>A), ATT278ATA (6108T>A), CTC280TTA (6112C>T 6114C>A), ACA281ACC (6117A>C), AGG282AGA (6120G>A), GAA283GAT (6123A>T), ATC285ATT (6129C>T), ATA286ATT (6132A>T), AAG289GAT (6139A>G 6141G>T), ATA290ATT (6144A>T), GAA291ACA (6145G>A 6146A>C), GAA292CAG (6148G>C 6150A>G), CAA293CAG (6153A>G), GAA294CCG (6154G>C 6155A>C 6156A>G), GAA297CCA (6163G>C 6164A>C), GTA299GAA (6170T>A), AAT300GAG (6172A>G 6174T>G), ATT301ATA (6177T>A), CCA302CTA (6179C>T), GAA303AAT (6181G>A 6183A>T), TTC304TTT (6186C>T), GCT306GCC (6192T>C), AGG309AGA (6201G>A), ATA310ATT (6204A>T), ATA313CTA (6211A>C), ACT315ACA (6219T>A), CTA317ATT (6223C>A 6225A>T), CTA320TTA (6232C>T), GCA321GGA (6236C>G), AAC322AAT (6240C>T), CTA325ATA (6247C>A), CAG327GGG (6253C>G 6254A>G), AAT328AAC (6258T>C), GCA329CCA (6259G>C), ATT330ATG (6264T>G), AGC332AAT (6269G>A 6270C>T), TAT333CAT (6271T>C), TCA335_AGG336del (6277_6283delTCAAGGG), GAA337-AC (6277_6283delTCAAGGG 6285A>C), ACA339ATA (6290C>T), TAT342_GAT347del (6298_6315delTATTCAAATTGCAGAGAT), ATA348ATC (6318A>C), AGA349del (6319_6321delAGA), GCA351AGA (6325G>A 6326C>G), CTA356GCA (6340C>G 6341T>C), AGA357ATG (6344G>T 6345A>G), GTC360ATA (6352G>A 6354C>A), TTA361ATG (6355T>A 6357A>G), AGT362ACA (6359G>C 6360T>A), CTT363TTG (6361C>T 6363T>G), AAA365AAT (6369A>T), GAA367GAG (6375A>G), CAA368AAA (6376C>A), CCT369CAA (6380C>A 6381T>A), ACA370CCT (6382A>C 6384A>T), ACC371ATT (6386C>T 6387C>T), AGG376AAA (6401G>A 6402G>A), AGG377AAG (6404G>A), AAT378-GA (6406delA 6407A>G 6408T>A), TTC379TTT (6411C>T), ATC380ATT (6414C>T), TCC381TCG (6417C>G), CCA382GAT (6418C>G 6419C>A 6420A>T), GAT383GAA (6423T>A), CTG384TTA (6424C>T 6426G>A), TTA385CTA (6427T>C), CTA391CAA (6446T>A), AGT393GGG (6451A>G 6453T>G), CAC394CAA (6456C>A), TAT396TAC (6462T>C), CAC399CAT (6471C>T), TGC401TGT (6477C>T), TCA402TCG (6480A>G), AAA405AAC (6489A>C), ATT410GTA (6502A>G 6504T>A), GTT411ATC (6505G>A 6507T>C)								

*: Inserts / Deletes / Misaligned / Frameshifts

Analysis details

This analysis was performed with panviral2.64

NGS Details (UN8): Harvey murine sarcoma virus

Assembly

**Coverage Length**	279 (1 contig(s))
**Depth Of Coverage**	3.9
**Number Of Reads**	8
**Reads Per Million**	0.13 rpm (after QC)
**Ambiguities**	0
**Assembly Method**	de novo + reference guided assembly
**Consensus Caller**	Bcf Tools

Coverage Map

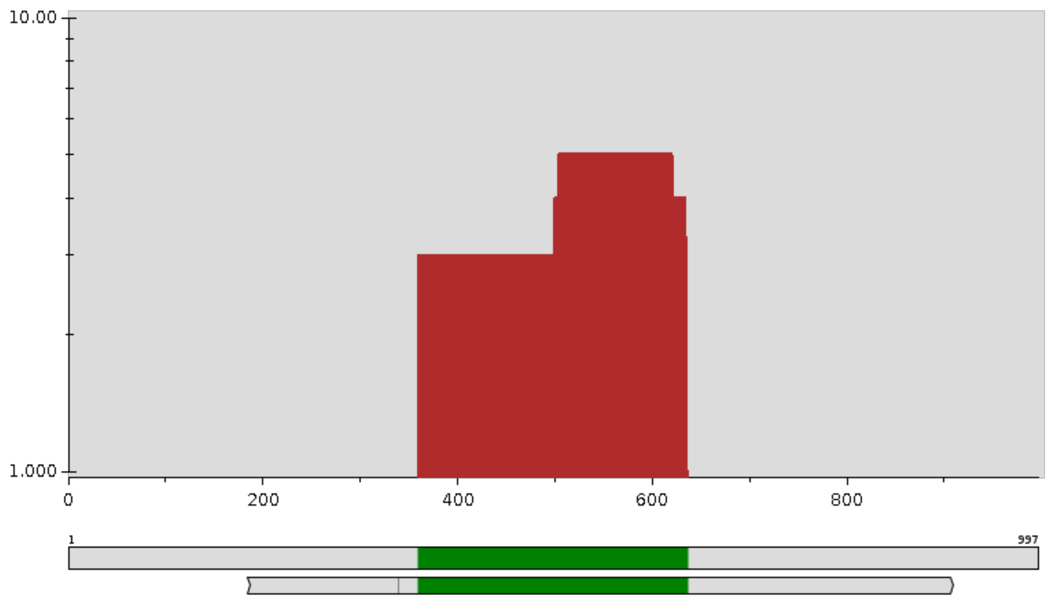

Assignment

**Type**	Harvey murine sarcoma virus (Taxonomy ID: 11807)
**Reference Genome**	NC_038668.1
**NT Identity (%)**	73.3333
**AA Identity (%)**	90.0
**Number Of Stop Codons**	0
**Number Of CDS**	2

Alignment

**Alignment Score**	234.0 (NT) + 1076.0 (AA) = 1310.0
**Concordance (%)**	75.1147

Alignment Method	Global, seeded, nucleotide + amino acids (AGA)

Genome Region

Sequence starts at position 360 and ends at position 638 relative to NC_038668.1 reference sequence.

Alignment Detailed Statistics

	Begin	End	Coverage	Score	Concordance	Matches	Identities	I/D/M/F*	Stop Codons
**NT**	**360**	**638**	**28.0%**	**234**	**44.8%**	**270 (96.8%)**	**198 (71.0%)**	**0/9**	
Mutations:	367G>C, 370C>T, 373T>C, 374A>G, 376A>T, 379C>T, 382G>T, 385A>T, 388G>A, 394C>T, 397G>C, 403C>T, 407C>T, 409G>A, 412C>T, 415G>A, 418C>T, 427G>T, 430C>T, 433G>A, 442C>A, 448A>T, 451G>A, 454C>T, 460C>T, 463G>T, 466A>G, 469G>A, 472A>C, 475C>G, 481T>C, 484G>A, 487G>A, 490G>A, 491_499delTGTTTACTG, 500G>T, 502C>T, 505C>T, 508A>G, 514A>T, 515A>G, 517A>C, 520T>A, 532T>C, 542C>A, 544G>A, 547C>T, 550G>A, 553C>T, 562A>T, 568G>A, 571C>T, 574C>T, 575C>T, 577C>G, 578T>C, 579G>T, 590A>C, 592C>T, 595C>T, 598C>T, 599A>G, 601C>G, 604G>A, 607C>A, 619C>T, 620C>T, 621A>C, 622T>A, 623C>A, 624A>T, 631G>A, 634G>A								
**CDS**									
D1R95_gp1	60	152	38.4%	538	86.5%	90 (96.8%)	81 (87.1%)	0/3/0/0	0
Protein mutations:	R64G (374A>G 376A>T), C103_L105del (491_499delTGTTTACTG), D106Y (500G>T 502C>T), T111A (515A>G 517A>C), C132L (578T>C 579G>T), I136L (590A>C 592C>T), T139A (599A>G 601C>G), H146S (620C>T 621A>C 622T>A), Q147M (623C>A 624A>T)								
Codon mutations:	GTG61GTC (367G>C), GGC62GGT (370C>T), GCT63GCC (373T>C), AGA64GGT (374A>G 376A>T), GGC65GGT (379C>T), GTG66GTT (382G>T), GGA67GGT (385A>T), AAG68AAA (388G>A), GCC70GCT (394C>T), CTG71CTC (397G>C), ATC73ATT (403C>T), CTG75TTA (407C>T 409G>A), ATC76ATT (412C>T), CAG77CAA (415G>A), AAC78AAT (418C>T), GTG81GTT (427G>T), GAC82GAT (430C>T), GAG83GAA (433G>A), CCC86CCA (442C>A), ATA88ATT (448A>T), GAG89GAA (451G>A), GAC90GAT (454C>T), TAC92TAT (460C>T), CGG93CGT (463G>T), AAA94AAG (466A>G), CAG95CAA (469G>A), GTA96GTC (472A>C), GTC97GTG (475C>G), GAT99GAC (481T>C), GGG100GGA (484G>A), GAG101GAA (487G>A), ACG102ACA (490G>A), TGT103_CTG105del (491_499delTGTTTACTG), GAC106TAT (500G>T 502C>T), ATC107ATT (505C>T), TTA108TTG (508A>G), ACA110ACT (514A>T), ACA111GCC (515A>G 517A>C), GGT112GGA (520T>A), TAT116TAC (532T>C), CGG120AGA (542C>A 544G>A), GAC121GAT (547C>T), CAG122CAA (550G>A), TAC123TAT (553C>T), ACA126ACT (562A>T), GAG128GAA (568G>A), GGC129GGT (571C>T), TTC130TTT (574C>T), CTC131TTG (575C>T 577C>G), TGT132CTT (578T>C 579G>T), ATC136CTT (590A>C 592C>T), AAC137AAT (595C>T), AAC138AAT (598C>T), ACC139GCG (599A>G 601C>G), AAG140AAA (604G>A), TCC141TCA (607C>A), ATC145ATT (619C>T), CAT146TCA (620C>T 621A>C 622T>A), CAG147ATG (623C>A 624A>T), AGG149AGA (631G>A), GAG150GAA (634G>A)								
D1R95_gp2	8	100	48.9%	538	86.5%	90 (96.8%)	81 (87.1%)	0/3/0/0	0
Protein mutations:	R12G (374A>G 376A>T), C51_L53del (491_499delTGTTTACTG), D54Y (500G>T 502C>T), T59A (515A>G 517A>C), C80L (578T>C 579G>T), I84L (590A>C 592C>T), T87A (599A>G 601C>G), H94S (620C>T 621A>C 622T>A), Q95M (623C>A 624A>T)								
Codon mutations:	GTG9GTC (367G>C), GGC10GGT (370C>T), GCT11GCC (373T>C), AGA12GGT (374A>G 376A>T), GGC13GGT (379C>T), GTG14GTT (382G>T), GGA15GGT (385A>T), AAG16AAA (388G>A), GCC18GCT (394C>T), CTG19CTC (397G>C), ATC21ATT (403C>T), CTG23TTA (407C>T 409G>A), ATC24ATT (412C>T), CAG25CAA (415G>A), AAC26AAT (418C>T), GTG29GTT (427G>T), GAC30GAT (430C>T), GAG31GAA (433G>A), CCC34CCA (442C>A), ATA36ATT (448A>T), GAG37GAA (451G>A), GAC38GAT (454C>T), TAC40TAT (460C>T), CGG41CGT (463G>T), AAA42AAG (466A>G), CAG43CAA (469G>A), GTA44GTC (472A>C), GTC45GTG (475C>G), GAT47GAC (481T>C), GGG48GGA (484G>A), GAG49GAA (487G>A), ACG50ACA (490G>A), TGT51_CTG53del (491_499delTGTTTACTG), GAC54TAT (500G>T 502C>T), ATC55ATT (505C>T), TTA56TTG (508A>G), ACA58ACT (514A>T), ACA59GCC (515A>G 517A>C), GGT60GGA (520T>A), TAT64TAC (532T>C), CGG68AGA (542C>A 544G>A), GAC69GAT (547C>T), CAG70CAA (550G>A), TAC71TAT (553C>T), ACA74ACT (562A>T), GAG76GAA (568G>A), GGC77GGT (571C>T), TTC78TTT (574C>T), CTC79TTG (575C>T 577C>G), TGT80CTT (578T>C 579G>T), ATC84CTT (590A>C 592C>T), AAC85AAT (595C>T), AAC86AAT (598C>T), ACC87GCG (599A>G 601C>G), AAG88AAA (604G>A), TCC89TCA (607C>A), ATC93ATT (619C>T), CAT94TCA (620C>T 621A>C 622T>A), CAG95ATG (623C>A 624A>T), AGG97AGA (631G>A), GAG98GAA (634G>A)								
**Proteins**									
hypothetical protein (YP_009507788.1)	60	152	38.4%	538	86.5%	90 (96.8%)	81 (87.1%)	0/3/0/0	0
Protein mutations:	R64G (374A>G 376A>T), C103_L105del (491_499delTGTTTACTG), D106Y (500G>T 502C>T), T111A (515A>G 517A>C), C132L (578T>C 579G>T), I136L (590A>C 592C>T), T139A (599A>G 601C>G), H146S (620C>T 621A>C 622T>A), Q147M (623C>A 624A>T)								
Codon mutations:	GTG61GTC (367G>C), GGC62GGT (370C>T), GCT63GCC (373T>C), AGA64GGT (374A>G 376A>T), GGC65GGT (379C>T), GTG66GTT (382G>T), GGA67GGT (385A>T), AAG68AAA (388G>A), GCC70GCT (394C>T), CTG71CTC (397G>C), ATC73ATT (403C>T), CTG75TTA (407C>T 409G>A), ATC76ATT (412C>T), CAG77CAA (415G>A), AAC78AAT (418C>T), GTG81GTT (427G>T), GAC82GAT (430C>T), GAG83GAA (433G>A), CCC86CCA (442C>A), ATA88ATT (448A>T), GAG89GAA (451G>A), GAC90GAT (454C>T), TAC92TAT (460C>T), CGG93CGT (463G>T), AAA94AAG (466A>G), CAG95CAA (469G>A), GTA96GTC (472A>C), GTC97GTG (475C>G), GAT99GAC (481T>C), GGG100GGA (484G>A), GAG101GAA (487G>A), ACG102ACA (490G>A), TGT103_CTG105del (491_499delTGTTTACTG), GAC106TAT (500G>T 502C>T), ATC107ATT (505C>T), TTA108TTG (508A>G), ACA110ACT (514A>T), ACA111GCC (515A>G 517A>C), GGT112GGA (520T>A), TAT116TAC (532T>C), CGG120AGA (542C>A 544G>A), GAC121GAT (547C>T), CAG122CAA (550G>A), TAC123TAT (553C>T), ACA126ACT (562A>T), GAG128GAA (568G>A), GGC129GGT (571C>T), TTC130TTT (574C>T), CTC131TTG (575C>T 577C>G), TGT132CTT (578T>C 579G>T), ATC136CTT (590A>C 592C>T), AAC137AAT (595C>T), AAC138AAT (598C>T), ACC139GCG (599A>G 601C>G), AAG140AAA (604G>A), TCC141TCA (607C>A), ATC145ATT (619C>T), CAT146TCA (620C>T 621A>C 622T>A), CAG147ATG (623C>A 624A>T), AGG149AGA (631G>A), GAG150GAA (634G>A)								
hypothetical protein (YP_009507789.1)	8	100	48.9%	538	86.5%	90 (96.8%)	81 (87.1%)	0/3/0/0	0
Protein mutations:	R12G (374A>G 376A>T), C51_L53del (491_499delTGTTTACTG), D54Y (500G>T 502C>T), T59A (515A>G 517A>C), C80L (578T>C 579G>T), I84L (590A>C 592C>T), T87A (599A>G 601C>G), H94S (620C>T 621A>C 622T>A), Q95M (623C>A 624A>T)								
Codon mutations:	GTG9GTC (367G>C), GGC10GGT (370C>T), GCT11GCC (373T>C), AGA12GGT (374A>G 376A>T), GGC13GGT (379C>T), GTG14GTT (382G>T), GGA15GGT (385A>T), AAG16AAA (388G>A), GCC18GCT (394C>T), CTG19CTC (397G>C), ATC21ATT (403C>T), CTG23TTA (407C>T 409G>A), ATC24ATT (412C>T), CAG25CAA (415G>A), AAC26AAT (418C>T), GTG29GTT (427G>T), GAC30GAT (430C>T), GAG31GAA (433G>A), CCC34CCA (442C>A), ATA36ATT (448A>T), GAG37GAA (451G>A), GAC38GAT (454C>T), TAC40TAT (460C>T), CGG41CGT (463G>T), AAA42AAG (466A>G), CAG43CAA (469G>A), GTA44GTC (472A>C), GTC45GTG (475C>G), GAT47GAC (481T>C), GGG48GGA (484G>A), GAG49GAA (487G>A), ACG50ACA (490G>A), TGT51_CTG53del (491_499delTGTTTACTG), GAC54TAT (500G>T 502C>T), ATC55ATT (505C>T), TTA56TTG (508A>G), ACA58ACT (514A>T), ACA59GCC (515A>G 517A>C), GGT60GGA (520T>A), TAT64TAC (532T>C), CGG68AGA (542C>A 544G>A), GAC69GAT (547C>T), CAG70CAA (550G>A), TAC71TAT (553C>T), ACA74ACT (562A>T), GAG76GAA (568G>A), GGC77GGT (571C>T), TTC78TTT (574C>T), CTC79TTG (575C>T 577C>G), TGT80CTT (578T>C 579G>T), ATC84CTT (590A>C 592C>T), AAC85AAT (595C>T), AAC86AAT (598C>T), ACC87GCG (599A>G 601C>G), AAG88AAA (604G>A), TCC89TCA (607C>A), ATC93ATT (619C>T), CAT94TCA (620C>T 621A>C 622T>A), CAG95ATG (623C>A 624A>T), AGG97AGA (631G>A), GAG98GAA (634G>A)								

*: Inserts / Deletes / Misaligned / Frameshifts

Analysis details

This analysis was performed with panviral2.64

NGS Details (UN8): Tomato chocolate spot virus (segment RNA2)

Assembly

**Coverage Length**	393 (1 contig(s))
**Depth Of Coverage**	4272.8
**Number Of Reads**	14918
**Reads Per Million**	249.32 rpm (after QC)
**Ambiguities**	0
**Assembly Method**	de novo + reference guided assembly
**Consensus Caller**	Bcf Tools

Coverage Map

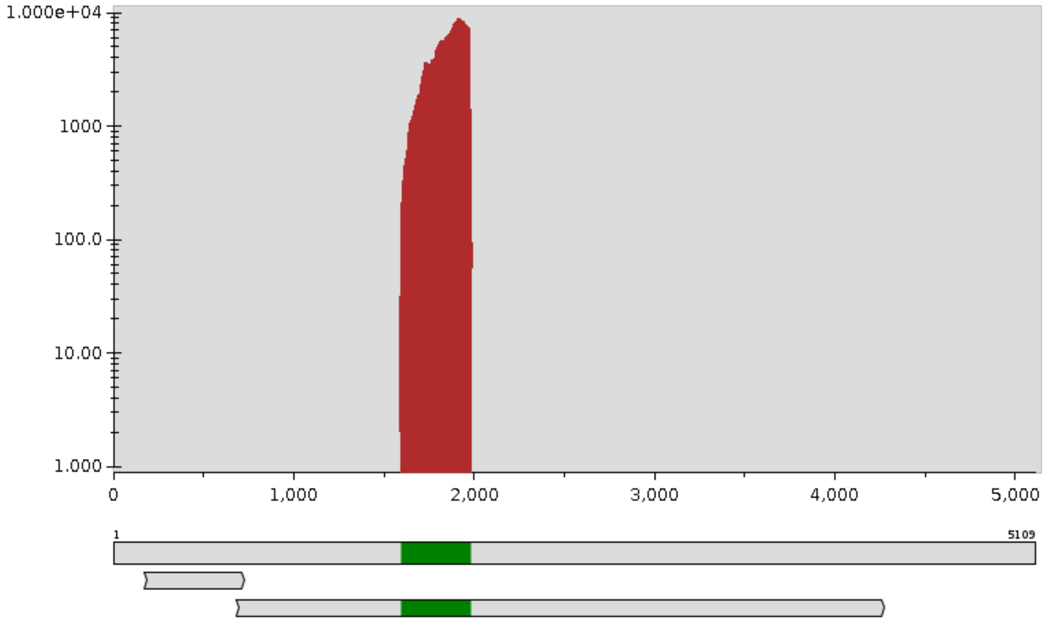

Assignment

**Type**	Tomato chocolate spot virus (Taxonomy ID: 661101)
**Reference Genome**	NC_013076.1
**NT Identity (%)**	56.2341
**AA Identity (%)**	51.9084
**Number Of Stop Codons**	0
**Number Of CDS**	2

Alignment

**Alignment Score**	98.0 (NT) + 555.0 (AA) = 653.0
**Concordance (%)**	37.4427

Alignment Method	Global, seeded, nucleotide + amino acids (AGA)

Genome Region

Sequence starts at position 1593 and ends at position 1985 relative to NC_013076.1 reference sequence.

Alignment Detailed Statistics

	Begin	End	Coverage	Score	Concordance	Matches	Identities	I/D/M/F*	Stop Codons
**NT**	**1593**	**1985**	**7.7%**	**98**	**12.5%**	**393 (100%)**	**221 (56.2%)**	**0/0**	
Mutations:	1603A>C, 1606T>C, 1610A>G, 1618A>G, 1620G>C, 1622T>A, 1624C>T, 1625A>G, 1627C>T, 1628A>T, 1630G>T, 1633T>C, 1634G>C, 1635T>A, 1636T>G, 1645T>C, 1649C>T, 1650T>A, 1651G>T, 1657A>C, 1658G>A, 1659A>C, 1660C>T, 1661T>G, 1662C>A, 1663C>T, 1668C>A, 1669A>T, 1670C>T, 1671A>C, 1672C>T, 1673A>T, 1678C>T, 1690T>G, 1696T>A, 1697G>A, 1698A>C, 1699G>C, 1700C>T, 1702C>G, 1703A>C, 1704C>A, 1705G>T, 1708T>A, 1709G>T, 1711G>A, 1712C>A, 1713A>G, 1714A>G, 1717G>A, 1718G>A, 1721C>G, 1723A>T, 1725A>T, 1726C>T, 1727A>G, 1729T>G, 1730C>T, 1734T>C, 1736G>A, 1737A>C, 1738T>C, 1739G>C, 1740A>T, 1741G>C, 1742G>C, 1743C>T, 1744T>C, 1745T>C, 1746C>G, 1747A>C, 1749G>A, 1750A>G, 1753C>G, 1755A>G, 1756T>A, 1757G>T, 1758T>G, 1759G>T, 1760A>C, 1761T>A, 1762C>T, 1763G>C, 1768G>C, 1771A>G, 1774T>C, 1775G>A, 1777A>T, 1778T>G, 1779C>A, 1780A>G, 1782A>G, 1783G>A, 1784A>C, 1786G>A, 1789G>C, 1790C>T, 1791A>C, 1792G>C, 1798T>C, 1801T>A, 1802T>A, 1805A>C, 1806A>T, 1807G>A, 1811C>T, 1816C>T, 1817A>G, 1818C>A, 1823G>A, 1825C>T, 1832G>C, 1834T>A, 1835G>C, 1837T>G, 1838A>G, 1840T>G, 1843T>C, 1844G>A, 1845C>A, 1846T>G, 1848C>A, 1861C>A, 1864G>T, 1867A>G, 1870A>T, 1871A>T, 1872A>C, 1873T>C, 1876C>A, 1877T>G, 1878C>A, 1879T>G, 1883T>A, 1885A>T, 1887T>G, 1888G>C, 1889G>A, 1897T>C, 1898G>T, 1899A>C, 1901A>T, 1903G>A, 1904T>A, 1906G>T, 1909T>C, 1910C>A, 1911A>G, 1912T>G, 1915G>A, 1918A>G, 1921C>G, 1924T>A, 1926C>A, 1927C>A, 1928C>T, 1930A>G, 1936C>T, 1939A>G, 1945T>C, 1948C>T, 1952G>T, 1953T>A, 1954G>C, 1959T>G, 1960A>G, 1961A>T, 1962T>A, 1963G>T, 1970T>G, 1971T>A, 1972T>G								

CDS

	Begin	End	Coverage	Score	Concordance	Matches	Identities	I/D/M/F*	Stop Codons
ToChV_s2_gp2	304	434	10.9%	555	56.7%	131 (100%)	68 (51.9%)	0/0/0/0	0
Protein mutations:	K309E (1610A>G), G312A (1620G>C), F313I (1622T>A 1624C>T), N314D (1625A>G 1627C>T), M315F (1628A>T 1630G>T), V317Q (1634G>C 1635T>A 1636T>G), L322Y (1649C>T 1650T>A 1651G>T), D325T (1658G>A 1659A>C 1660C>T), S326D (1661T>G 1662C>A 1663C>T), T328N (1668C>A 1669A>T), H329S (1670C>T 1671A>C 1672C>T), I330F (1673A>T), D335E (1690T>G), E338T (1697G>A 1698A>C 1699G>C), R339W (1700C>T 1702C>G), T340H (1703A>C 1704C>A 1705G>T), A342S (1709G>T 1711G>A), Q343R (1712C>A 1713A>G 1714A>G), V345I (1718G>A), Q346D (1721C>G 1723A>T), Y347F (1725A>T 1726C>T), I348V (1727A>G 1729T>G), F350S (1734T>C), D351T (1736G>A 1737A>C 1738T>C), E352L (1739G>C 1740A>T 1741G>C), A353L (1742G>C 1743C>T 1744T>C), S354R (1745T>C 1746C>G 1747A>C), R355Q (1749G>A 1750A>G), N357R (1755A>G 1756T>A), V358C (1757G>T 1758T>G 1759G>T), I359H (1760A>C 1761T>A 1762C>T), V360L (1763G>C), V364I (1775G>A 1777A>T), S365E (1778T>G 1779C>A 1780A>G), K366R (1782A>G 1783G>A), E368D (1789G>C), Q369S (1790C>T 1791A>C 1792G>C), Y373N (1802T>A), K374L (1805A>C 1806A>T 1807G>A), H376Y (1811C>T), T378D (1817A>G 1818C>A), D380N (1823G>A 1825C>T), V383L (1832G>C 1834T>A), D384Q (1835G>C 1837T>G), I385V (1838A>G 1840T>G), A387K (1844G>A 1845C>A 1846T>G), A388E (1848C>A), K393N (1864G>T), N396S (1871A>T 1872A>C 1873T>C), S398E (1877T>G 1878C>A 1879T>G), S400T (1883T>A 1885A>T), L401C (1887T>G 1888G>C), D402N (1889G>A), D405S (1898G>T 1899A>C), M406L (1901A>T 1903G>A), S407T (1904T>A 1906G>T), H409R (1910C>A 1911A>G 1912T>G), A414E (1926C>A 1927C>A), V423Y (1952G>T 1953T>A 1954G>C), I425R (1959T>G 1960A>G), M426Y (1961A>T 1962T>A 1963G>T), F429E (1970T>G 1971T>A 1972T>G)								
Codon mutations:	CCA306CCC (1603A>C), GTT307GTC (1606T>C), AAG309GAG (1610A>G), CAA311CAG (1618A>G), GGC312GCC (1620G>C), TTC313ATT (1622T>A 1624C>T), AAC314GAT (1625A>G 1627C>T), ATG315TTT (1628A>T 1630G>T), GGT316GGC (1633T>C), GTT317CAG (1634G>C 1635T>A 1636T>G), GAT320GAC (1645T>C), CTG322TAT (1649C>T 1650T>A 1651G>T), ATA324ATC (1657A>C), GAC325ACT (1658G>A 1659A>C 1660C>T), TCC326GAT (1661T>G 1662C>A 1663C>T), ACA328AAT (1668C>A 1669A>T), CAC329TCT (1670C>T 1671A>C 1672C>T), ATT330TTT (1673A>T), GAC331GAT (1678C>T), GAT335GAG (1690T>G), ACT337ACA (1696T>A), GAG338ACC (1697G>A 1698A>C 1699G>C), CGC339TGG (1700C>T 1702C>G), ACG340CAT (1703A>C 1704C>A 1705G>T), ATT341ATA (1708T>A), GCG342TCA (1709G>T 1711G>A), CAA343AGG (1712C>A 1713A>G 1714A>G), CAG344CAA (1717G>A), GTT345ATT (1718G>A), CAA346GAT (1721C>G 1723A>T), TAC347TTT (1725A>T 1726C>T), ATT348GTG (1727A>G 1729T>G), CTG349TTG (1730C>T), TTT350TCT (1734T>C), GAT351ACC (1736G>A 1737A>C 1738T>C), GAG352CTC (1739G>C 1740A>T 1741G>C), GCT353CTC (1742G>C 1743C>T 1744T>C), TCA354CGC (1745T>C 1746C>G 1747A>C), CGA355CAG (1749G>A 1750A>G), GGC356GGG (1753C>G), AAT357AGA (1755A>G 1756T>A), GTG358TGT (1757G>T 1758T>G 1759G>T), ATC359CAT (1760A>C 1761T>A 1762C>T), GTG360CTG (1763G>C), CCG361CCC (1768G>C), AAA362AAG (1771A>G), CAT363CAC (1774T>C), GTA364ATT (1775G>A 1777A>T), TCA365GAG (1778T>G 1779C>A 1780A>G), AAG366AGA (1782A>G 1783G>A), AGG367CGA (1784A>C 1786G>A), GAG368GAC (1789G>C), CAG369TCC (1790C>T 1791A>C 1792G>C), AAT371AAC (1798T>C), ATT372ATA (1801T>A), TAC373AAC (1802T>A), AAG374CTA (1805A>C 1806A>T 1807G>A), CAC376TAC (1811C>T), ATC377ATT (1816C>T), ACT378GAT (1817A>G 1818C>A), GAC380AAT (1823G>A 1825C>T), GTT383CTA (1832G>C 1834T>A), GAT384CAG (1835G>C 1837T>G), ATT385GTG (1838A>G 1840T>G), CTT386CTC (1843T>C), GCT387AAG (1844G>A 1845C>A 1846T>G), GCA388GAA (1848C>A), ACC392ACA (1861C>A), AAG393AAT (1864G>T), GTA394GTG (1867A>G), GGA395GGT (1870A>T), AAT396TCC (1871A>T 1872A>C 1873T>C), CTC397CTA (1876C>A), TCT398GAG (1877T>G 1878C>A 1879T>G), TCA400ACT (1883T>A 1885A>T), TTG401TGC (1887T>G 1888G>C), GAT402AAT (1889G>A), TAT404TAC (1897T>C), GAT405TCT (1898G>T 1899A>C), ATG406TTA (1901A>T 1903G>A), TCG407ACT (1904T>A 1906G>T), CTT408CTC (1909T>C), CAT409AGG (1910C>A 1911A>G 1912T>G), GAG410GAA (1915G>A), AGA411AGG (1918A>G), GTC412GTG (1921C>G), ATT413ATA (1924T>A), GCC414GAA (1926C>A 1927C>A), CTA415TTG (1928C>T 1930A>G), CAC417CAT (1936C>T), CAA418CAG (1939A>G), GAT420GAC (1945T>C), CAC421CAT (1948C>T), GTG423TAC (1952G>T 1953T>A 1954G>C), ATA425AGG (1959T>G 1960A>G), ATG426TAT (1961A>T 1962T>A 1963G>T), TTT429GAG (1970T>G 1971T>A 1972T>G)								

Proteins

	Begin	End	Coverage	Score	Concordance	Matches	Identities	I/D/M/F*	Stop Codons
polyprotein (YP_003097231.1)	304	434	10.9%	555	56.7%	131 (100%)	68 (51.9%)	0/0/0/0	0
Protein mutations:	K309E (1610A>G), G312A (1620G>C), F313I (1622T>A 1624C>T), N314D (1625A>G 1627C>T), M315F (1628A>T 1630G>T), V317Q (1634G>C 1635T>A 1636T>G), L322Y (1649C>T 1650T>A 1651G>T), D325T (1658G>A 1659A>C 1660C>T), S326D (1661T>G 1662C>A 1663C>T), T328N (1668C>A 1669A>T), H329S (1670C>T 1671A>C 1672C>T), I330F (1673A>T), D335E (1690T>G), E338T (1697G>A 1698A>C 1699G>C), R339W (1700C>T 1702C>G), T340H (1703A>C 1704C>A 1705G>T), A342S (1709G>T 1711G>A), Q343R (1712C>A 1713A>G 1714A>G), V345I (1718G>A), Q346D (1721C>G 1723A>T), Y347F (1725A>T 1726C>T), I348V (1727A>G 1729T>G), F350S (1734T>C), D351T (1736G>A 1737A>C 1738T>C), E352L (1739G>C 1740A>T 1741G>C), A353L (1742G>C 1743C>T 1744T>C), S354R (1745T>C 1746C>G 1747A>C), R355Q (1749G>A 1750A>G), N357R (1755A>G 1756T>A), V358C (1757G>T 1758T>G 1759G>T), I359H (1760A>C 1761T>A 1762C>T), V360L (1763G>C), V364I (1775G>A 1777A>T), S365E (1778T>G 1779C>A 1780A>G), K366R (1782A>G 1783G>A), E368D (1789G>C), Q369S (1790C>T 1791A>C 1792G>C), Y373N (1802T>A), K374L (1805A>C 1806A>T 1807G>A), H376Y (1811C>T), T378D (1817A>G 1818C>A), D380N (1823G>A 1825C>T), V383L (1832G>C 1834T>A), D384Q (1835G>C 1837T>G), I385V (1838A>G 1840T>G), A387K (1844G>A 1845C>A 1846T>G), A388E (1848C>A), K393N (1864G>T), N396S (1871A>T 1872A>C 1873T>C), S398E (1877T>G 1878C>A 1879T>G), S400T (1883T>A 1885A>T), L401C (1887T>G 1888G>C), D402N (1889G>A), D405S (1898G>T 1899A>C), M406L (1901A>T 1903G>A), S407T (1904T>A 1906G>T), H409R (1910C>A 1911A>G 1912T>G), A414E (1926C>A 1927C>A), V423Y (1952G>T 1953T>A 1954G>C), I425R (1959T>G 1960A>G), M426Y (1961A>T 1962T>A 1963G>T), F429E (1970T>G 1971T>A 1972T>G)								
Codon mutations:	CCA306CCC (1603A>C), GTT307GTC (1606T>C), AAG309GAG (1610A>G), CAA311CAG (1618A>G), GGC312GCC (1620G>C), TTC313ATT (1622T>A 1624C>T), AAC314GAT (1625A>G 1627C>T), ATG315TTT (1628A>T 1630G>T), GGT316GGC (1633T>C), GTT317CAG (1634G>C 1635T>A 1636T>G), GAT320GAC (1645T>C), CTG322TAT (1649C>T 1650T>A 1651G>T), ATA324ATC (1657A>C), GAC325ACT (1658G>A 1659A>C 1660C>T), TCC326GAT (1661T>G 1662C>A 1663C>T), ACA328AAT (1668C>A 1669A>T), CAC329TCT (1670C>T 1671A>C 1672C>T), ATT330TTT (1673A>T), GAC331GAT (1678C>T), GAT335GAG (1690T>G), ACT337ACA (1696T>A), GAG338ACC (1697G>A 1698A>C 1699G>C), CGC339TGG (1700C>T 1702C>G), ACG340CAT (1703A>C 1704C>A 1705G>T), ATT341ATA (1708T>A), GCG342TCA (1709G>T 1711G>A), CAA343AGG (1712C>A 1713A>G 1714A>G), CAG344CAA (1717G>A), GTT345ATT (1718G>A), CAA346GAT (1721C>G 1723A>T), TAC347TTT (1725A>T 1726C>T), ATT348GTG (1727A>G 1729T>G), CTG349TTG (1730C>T), TTT350TCT (1734T>C), GAT351ACC (1736G>A 1737A>C 1738T>C), GAG352CTC (1739G>C 1740A>T 1741G>C), GCT353CTC (1742G>C 1743C>T 1744T>C), TCA354CGC (1745T>C 1746C>G 1747A>C), CGA355CAG (1749G>A 1750A>G), GGC356GGG (1753C>G), AAT357AGA (1755A>G 1756T>A), GTG358TGT (1757G>T 1758T>G 1759G>T), ATC359CAT (1760A>C 1761T>A 1762C>T), GTG360CTG (1763G>C), CCG361CCC (1768G>C), AAA362AAG (1771A>G), CAT363CAC (1774T>C), GTA364ATT (1775G>A 1777A>T), TCA365GAG (1778T>G 1779C>A 1780A>G), AAG366AGA (1782A>G 1783G>A), AGG367CGA (1784A>C 1786G>A), GAG368GAC (1789G>C), CAG369TCC (1790C>T 1791A>C 1792G>C), AAT371AAC (1798T>C), ATT372ATA (1801T>A), TAC373AAC (1802T>A), AAG374CTA (1805A>C 1806A>T 1807G>A), CAC376TAC (1811C>T), ATC377ATT (1816C>T), ACT378GAT (1817A>G 1818C>A), GAC380AAT (1823G>A 1825C>T), GTT383CTA (1832G>C 1834T>A), GAT384CAG (1835G>C 1837T>G), ATT385GTG (1838A>G 1840T>G), CTT386CTC (1843T>C), GCT387AAG (1844G>A 1845C>A 1846T>G), GCA388GAA (1848C>A), ACC392ACA (1861C>A), AAG393AAT (1864G>T), GTA394GTG (1867A>G), GGA395GGT (1870A>T), AAT396TCC (1871A>T 1872A>C 1873T>C), CTC397CTA (1876C>A), TCT398GAG (1877T>G 1878C>A 1879T>G), TCA400ACT (1883T>A 1885A>T), TTG401TGC (1887T>G 1888G>C), GAT402AAT (1889G>A), TAT404TAC (1897T>C), GAT405TCT (1898G>T 1899A>C), ATG406TTA (1901A>T 1903G>A), TCG407ACT (1904T>A 1906G>T), CTT408CTC (1909T>C), CAT409AGG (1910C>A 1911A>G 1912T>G), GAG410GAA (1915G>A), AGA411AGG (1918A>G), GTC412GTG (1921C>G), ATT413ATA (1924T>A), GCC414GAA (1926C>A 1927C>A), CTA415TTG (1928C>T 1930A>G), CAC417CAT (1936C>T), CAA418CAG (1939A>G), GAT420GAC (1945T>C), CAC421CAT (1948C>T), GTG423TAC (1952G>T 1953T>A 1954G>C), ATA425AGG (1959T>G 1960A>G), ATG426TAT (1961A>T 1962T>A 1963G>T), TTT429GAG (1970T>G 1971T>A 1972T>G)								

*: Inserts / Deletes / Misaligned / Frameshifts

Analysis details

This analysis was performed with panviral2.64

NGS Details (UN8): Tomato chocolate spot virus (segment RNA 1)

Assembly

**Coverage Length**	277 (1 contig(s))
**Depth Of Coverage**	15605.2
**Number Of Reads**	36615
**Reads Per Million**	611.93 rpm (after QC)
**Ambiguities**	0
**Assembly Method**	de novo + reference guided assembly
**Consensus Caller**	Bcf Tools

Coverage Map

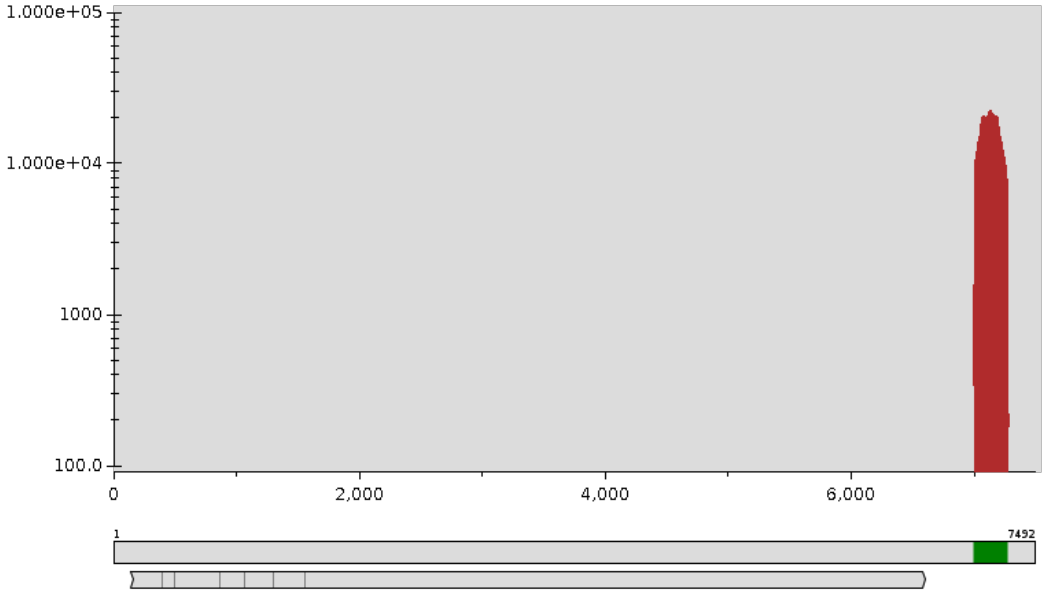

Assignment

**Type**	Tomato chocolate spot virus (Taxonomy ID: 661101)
**Reference Genome**	NC_013075.1
**NT Identity (%)**	82.3741
**AA Identity (%)**	0.0
**Number Of Stop Codons**	0
**Number Of CDS**	1

Alignment

**Alignment Score**	352.0 (NT) + 0.0 (AA) = 352.0
**Concordance (%)**	63.5379

**Alignment Method**	Global, seeded, nucleotide + amino acids (AGA)

Genome Region

Sequence starts at position 6999 and ends at position 7275 relative to NC_013075.1 reference sequence.

Alignment Detailed Statistics

	Begin	End	Coverage	Score	Concordance	Matches	Identities	I/D/M/F*	Stop Codons
**NT**	**6999**	**7275**	**3.7%**	**352**	**63.5%**	**277 (99.6%)**	**229 (82.4%)**	**1/0**	
Mutations:	7012T>G, 7018C>T, 7019A>G, 7020A>G, 7020_7021insT, 7023C>A, 7042G>T, 7044A>T, 7047G>A, 7048C>G, 7066G>A, 7082G>A, 7083T>C, 7098C>T, 7099T>C, 7104T>A, 7107G>A, 7116C>G, 7121G>A, 7127A>C, 7140C>T, 7151A>T, 7152T>C, 7183C>T, 7184T>C, 7185G>T, 7186T>G, 7187T>C, 7188G>A, 7196C>T, 7197A>G, 7198A>C, 7199C>A, 7200A>G, 7201G>A, 7207A>T, 7208A>T, 7209T>G, 7210C>T, 7212T>A, 7219T>A, 7224T>C, 7242A>T, 7255A>T, 7257G>A, 7258G>C, 7260T>C, 7262C>G, 7264T>C								

CDS

Proteins

*: Inserts / Deletes / Misaligned / Frameshifts

Analysis details

This analysis was performed with panviral2.64

NGS Details (UN8): Tomato chocolate spot virus (segment RNA2)

Assembly

**Coverage Length**	1253 (2 contig(s))
**Depth Of Coverage**	5116.5
**Number Of Reads**	50187
**Reads Per Million**	838.75 rpm (after QC)
**Ambiguities**	0
**Assembly Method**	de novo + reference guided assembly
**Consensus Caller**	Bcf Tools

Coverage Map

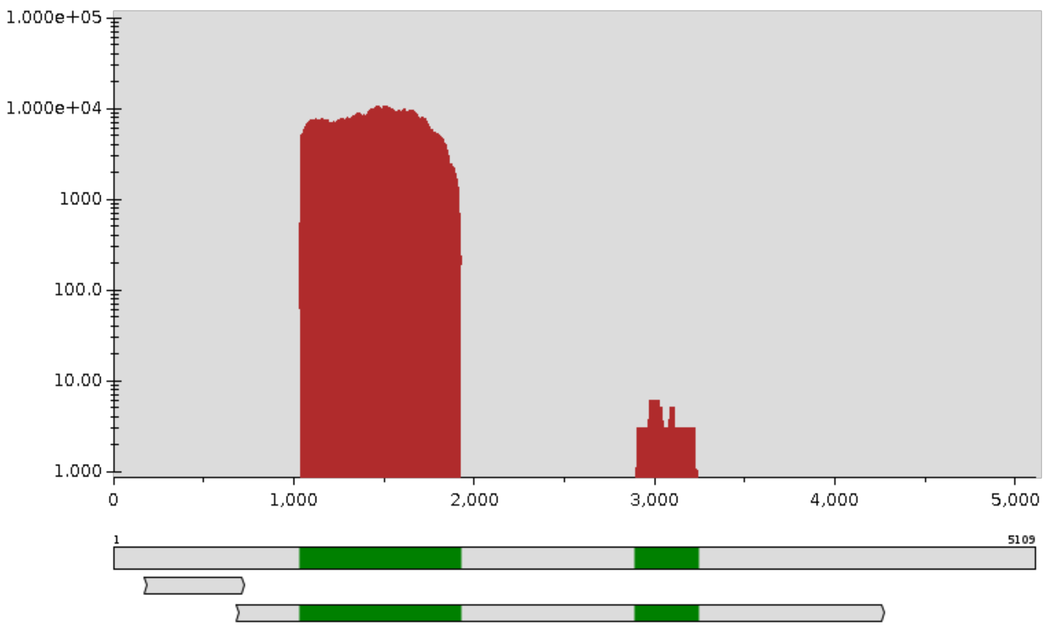

Assignment

**Type**	Tomato chocolate spot virus (Taxonomy ID: 661101)
**Reference Genome**	NC_013076.1
**NT Identity (%)**	65.2105
**AA Identity (%)**	70.4762
**Number Of Stop Codons**	0
**Number Of CDS**	2

Alignment

**Alignment Score**	763.0 (NT) + 2195.0 (AA) = 2958.0
**Concordance (%)**	54.3051

Alignment Method	Global, seeded, nucleotide + amino acids (AGA)

Genome Region

Sequence starts at position 1033 and ends at position 3248 relative to NC_013076.1 reference sequence.

Alignment Detailed Statistics

	Begin	End	Coverage	Score	Concordance	Matches	Identities	I/D/M/F*	Stop Codons
**NT**	**1033**	**3248**	**24.5%**	**763**	**30.4%**	**1253 (99.5%)**	**821 (65.2%)**	**6/0**	
Mutations:	1042G>C, 1054C>A, 1056T>C, 1057C>T, 1058A>G, 1059G>A, 1063G>T, 1066G>T, 1069T>A, 1072A>G, 1075G>A, 1079T>A, 1081C>G, 1084G>A, 1086C>T, 1087A>C, 1088C>T, 1090A>T, 1091G>T, 1093T>A, 1095A>G, 1096T>G, 1099T>A, 1102A>T, 1105A>G, 1108C>T, 1111G>C, 1112C>T, 1114C>G, 1124A>G, 1125G>A, 1126T>A, 1129C>T, 1138C>T, 1142C>G, 1144C>T, 1153A>G, 1156A>C, 1162C>T, 1181G>C, 1182A>T, 1183G>T, 1186T>C, 1192T>G, 1195A>T, 1197A>T, 1198G>A, 1201A>T, 1204T>A, 1210C>T, 1213G>T, 1216A>G, 1219G>A, 1222A>T, 1228A>G, 1231A>G, 1238G>A, 1239A>G, 1240T>C, 1241A>C, 1246G>A, 1252C>T, 1255A>T, 1258C>T, 1259T>A, 1261T>G, 1262C>G, 1263A>C, 1267G>A, 1273T>A, 1277T>A, 1278C>G, 1285C>A, 1291A>G, 1294A>T, 1295T>C, 1297G>C, 1300C>T, 1306T>A, 1309A>G, 1312T>C, 1315C>T, 1316C>T, 1317A>C, 1318A>T, 1327G>A, 1333T>A, 1336C>A, 1351A>T, 1354A>C, 1360T>G, 1366G>A, 1367C>G, 1369A>G, 1372T>G, 1373T>G, 1375C>G, 1376C>T, 1377A>C, 1378G>T, 1379A>C, 1380T>A, 1381G>A, 1385T>C, 1387G>A, 1394C>A, 1396T>A, 1397G>C, 1405T>A, 1408A>T, 1410A>C, 1411G>A, 1412A>C, 1413G>T, 1414G>T, 1423T>C, 1426A>T, 1428G>A, 1429A>C, 1433G>A, 1438A>T, 1440T>C, 1441G>C, 1442A>C, 1443G>A, 1444T>G, 1448A>T, 1449G>C, 1450C>T, 1453C>A, 1454A>T, 1456G>A, 1458C>T, 1459T>G, 1463A>G, 1464G>C, 1465C>G, 1468T>A, 1471C>G, 1474G>T, 1477A>C, 1483G>A, 1489C>A, 1493G>A, 1495G>C, 1501T>C, 1503G>A, 1505C>A, 1513A>T, 1516C>G, 1522A>G, 1525A>G, 1531C>T, 1532G>T, 1534G>T, 1535C>T, 1537T>A, 1539A>G, 1540C>G, 1541A>G, 1547C>T, 1548G>C, 1551T>C, 1552A>G, 1555_1556insGTAGGT, 1556A>G, 1559C>A, 1572C>G, 1573T>G, 1574C>G, 1575A>T, 1576C>A, 1582A>T, 1585C>T, 1586A>T, 1588T>C, 1589G>A, 1590G>A, 1592G>T, 1593G>T, 1595T>C, 1603A>C, 1606T>C, 1610A>G, 1618A>G, 1620G>C, 1622T>A, 1624C>T, 1625A>G, 1627C>T, 1628A>T, 1630G>T, 1633T>C, 1634G>C, 1635T>A, 1636T>G, 1645T>C, 1649C>T, 1650T>A, 1651G>T, 1657A>C, 1658G>A, 1659A>C, 1660C>T, 1661T>G, 1662C>A, 1663C>T, 1668C>A, 1669A>T, 1670C>T, 1671A>C, 1672C>T, 1673A>T, 1678C>T, 1690T>G, 1696T>A, 1697G>A, 1698A>C, 1699G>C, 1700C>T, 1702C>G, 1703A>C, 1704C>A, 1705G>T, 1708T>A, 1709G>T, 1711G>A, 1712C>A, 1713A>G, 1714A>G, 1717G>A, 1718G>A, 1721C>G, 1723A>T, 1725A>T, 1726C>T, 1727A>G, 1729T>G, 1730C>T, 1734T>C, 1736G>A, 1737A>C, 1738T>C, 1739G>C, 1740A>T, 1741G>C, 1742G>C, 1743C>T, 1744T>C, 1745T>C, 1746C>G, 1747A>C, 1749G>A, 1750A>G, 1753C>G, 1755A>G, 1756T>A, 1757G>T, 1758T>G, 1759G>T, 1760A>C, 1761T>A, 1762C>T, 1763G>C, 1768G>C, 1771A>G, 1774T>C, 1775G>A, 1777A>T, 1778T>G, 1779C>A, 1780A>G, 1782A>G, 1783G>A, 1784A>C, 1786G>A, 1789G>C, 1790C>T, 1791A>C, 1792G>C, 1798T>C, 1801T>A, 1802T>A, 1805A>C, 1806A>T, 1807G>A, 1811C>T, 1816C>T, 1817A>G, 1818C>A, 1823G>A, 1825C>T, 1832G>C, 1834T>A, 1835G>C, 1837T>G, 1838A>G, 1840T>G, 1843T>C, 1844G>A, 1845C>A, 1846T>G, 1848C>A, 1861C>A, 1864G>T, 1867A>G, 1870A>T, 1871A>T, 1872A>C, 1873T>C, 1876C>A, 1877T>G, 1878C>A, 1879T>G, 1883T>A, 1885A>T, 1887T>G, 1888G>C, 1889G>A, 1897T>C, 1898G>T, 1899A>C, 1901A>T, 1903G>A, 1904T>A, 1906G>T, 1909T>C, 1910C>A, 1911A>G, 1912T>G, 1915G>A, 1918A>G, 2890G>A, 2894C>A, 2895A>C, 2899A>C, 2901A>T, 2902T>C, 2905C>T, 2906C>A, 2907C>G, 2908G>T, 2911G>C, 2917C>T, 2920C>A, 2926A>G, 2929T>G, 2930T>C, 2932G>T, 2933T>C, 2935A>G, 2938T>C, 2941T>C, 2948G>A, 2950A>T, 2951A>G, 2952C>T, 2953C>A, 2962A>C, 2968C>A, 2975C>T, 2977T>G, 2980G>A, 2983C>T, 2984A>G, 2986A>C, 2992G>C, 2995T>A, 2998G>A, 3001T>A, 3004C>T, 3010G>A, 3020G>A, 3022A>C, 3023C>T, 3025G>A, 3028A>G, 3034C>A, 3035T>C, 3037A>G, 3043T>A, 3046T>C, 3047G>A, 3049A>T, 3052G>A, 3053C>A, 3055G>A, 3072C>G, 3073T>A, 3076G>C, 3079G>C, 3085C>T, 3091G>A, 3092A>G, 3094T>G, 3098A>G, 3109G>T, 3112C>G, 3115C>T, 3118C>T, 3121A>T, 3124A>G, 3128T>C, 3130G>T, 3133C>T, 3139A>G, 3140C>T, 3142C>G, 3145G>C, 3148A>G, 3152A>G, 3153A>C, 3154C>A, 3158A>G, 3160A>G, 3166A>C, 3168A>C, 3169T>A, 3172A>G, 3178T>C, 3181T>A, 3184T>G, 3193T>C, 3199A>G, 3205C>T, 3209C>T, 3211T>G, 3214G>A, 3220C>G, 3223C>A, 3225A>G, 3231G>A, 3232C>A								

CDS

ToChV_s2_gp2	117	855	34.9%	2195	74.0%	418 (99.5%)	296 (70.5%)	2/0/0/0	0
Protein mutations:	I124T (1056T>C 1057C>T), S125D (1058A>G 1059G>A), S132T (1079T>A 1081C>G), S134F (1086C>T 1087A>C), L135F (1088C>T 1090A>T), A136S (1091G>T 1093T>A), D137G (1095A>G 1096T>G), S147E (1124A>G 1125G>A 1126T>A), L153V (1142C>G 1144C>T), E166L (1181G>C 1182A>T 1183G>T), Q171L (1197A>T 1198G>A), D185S (1238G>A 1239A>G 1240T>C), T186P (1241A>C), S192T (1259T>A 1261T>G), Q193A (1262C>G 1263A>C), Q211S (1316C>T 1317A>C 1318A>T), Q228E (1367C>G 1369A>G), S229R (1372T>G), S230A (1373T>G 1375C>G), Q231S (1376C>T 1377A>C 1378G>T), M232Q (1379A>C 1380T>A 1381G>A), A238P (1397G>C), E242A (1410A>C 1411G>A), R243L (1412A>C 1413G>T 1414G>T), R248H (1428G>A 1429A>C), D250N (1433G>A), L252P (1440T>C 1441G>C), S253Q (1442A>C 1443G>A 1444T>G), M257L (1454A>T 1456G>A), T258M (1458C>T 1459T>G), S260A (1463A>G 1464G>C 1465C>G), V270I (1493G>A 1495G>C), R273K (1503G>A), V283F (1532G>T 1534G>T), P284S (1535C>T 1537T>A), N285R (1539A>G 1540C>G), T286A (1541A>G), R288S (1547C>T 1548G>C), L289S (1551T>C 1552A>G), T290_M291insVG (1555_1556insGTAGGT), M291V (1556A>G), A296G (1572C>G 1573T>G), H297V (1574C>G 1575A>T 1576C>A), E299D (1582A>T), T301S (1586A>T 1588T>C), G302N (1589G>A 1590G>A), G303L (1592G>T 1593G>T), K309E (1610A>G), G312A (1620G>C), F313I (1622T>A 1624C>T), N314D (1625A>G 1627C>T), M315F (1628A>T 1630G>T), V317Q (1634G>C 1635T>A 1636T>G), L322Y (1649C>T 1650T>A 1651G>T), D325T (1658G>A 1659A>C 1660C>T), S326D (1661T>G 1662C>A 1663C>T), T328N (1668C>A 1669A>T), H329S (1670C>T 1671A>C 1672C>T), I330F (1673A>T), D335E (1690T>G), E338T (1697G>A 1698A>C 1699G>C), R339W (1700C>T 1702C>G), T340H (1703A>C 1704C>A 1705G>T), A342S (1709G>T 1711G>A), Q343R (1712C>A 1713A>G 1714A>G), V345I (1718G>A), Q346D (1721C>G 1723A>T), Y347F (1725A>T 1726C>T), I348V (1727A>G 1729T>G), F350S (1734T>C), D351T (1736G>A 1737A>C 1738T>C), E352L (1739G>C 1740A>T 1741G>C), A353L (1742G>C 1743C>T 1744T>C), S354R (1745T>C 1746C>G 1747A>C), R355Q (1749G>A 1750A>G), N357R (1755A>G 1756T>A), V358C (1757G>T 1758T>G 1759G>T), I359H (1760A>C 1761T>A 1762C>T), V360L (1763G>C), V364I (1775G>A 1777A>T), S365E (1778T>G 1779C>A 1780A>G), K366R (1782A>G 1783G>A), E368D (1789G>C), Q369S (1790C>T 1791A>C 1792G>C), Y373N (1802T>A), K374L (1805A>C 1806A>T 1807G>A), H376Y (1811C>T), T378D (1817A>G 1818C>A), D380N (1823G>A 1825C>T), V383L (1832G>C 1834T>A), D384Q (1835G>C 1837T>G), I385V (1838A>G 1840T>G), A387K (1844G>A 1845C>A 1846T>G), A388E (1848C>A), K393N (1864G>T), N396S (1871A>T 1872A>C 1873T>C), S398E (1877T>G 1878C>A 1879T>G), S400T (1883T>A 1885A>T), L401C (1887T>G 1888G>C), D402N (1889G>A), D405S (1898G>T 1899A>C), M406L (1901A>T 1903G>A), S407T (1904T>A 1906G>T), H409R (1910C>A 1911A>G 1912T>G), Q737T (2894C>A 2895A>C), Y739F (2901A>T 2902T>C), P741S (2906C>A 2907C>G 2908G>T), G755S (2948G>A 2950A>T), T756V (2951A>G 2952C>T 2953C>A), I767V (2984A>G 2986A>C), V779I (3020G>A 3022A>C), V788I (3047G>A 3049A>T), T796R (3072C>G 3073T>A), I803V (3092A>G 3094T>G), I805V (3098A>G), N823A (3152A>G 3153A>C 3154C>A), K825E (3158A>G 3160A>G), N828T (3168A>C 3169T>A), F833L (3184T>G), K847R (3225A>G), R849Q (3231G>A 3232C>A)								

	Begin	End	Coverage	Score	Concordance	Matches	Identities	I/D/M/F*	Stop Codons
**NT**	**1033**	**3248**	**24.5%**	**763**	**30.4%**	**1253 (99.5%)**	**821 (65.2%)**	**6/0**	
Codon mutations:	TCG119TCC (1042G>C), CCC123CCA (1054C>A), ATC124ACT (1056T>C 1057C>T), AGC125GAC (1058A>G 1059G>A), CCG126CCT (1063G>T), GTG127GTT (1066G>T), ATT128ATA (1069T>A), CAA129CAG (1072A>G), GAG130GAA (1075G>A), TCC132ACG (1079T>A 1081C>G), TTG133TTA (1084G>A), TCA134TTC (1086C>T 1087A>C), CTA135TTT (1088C>T 1090A>T), GCT136TCA (1091G>T 1093T>A), GAT137GGG (1095A>G 1096T>G), GGT138GGA (1099T>A), GCA139GCT (1102A>T), CAA140CAG (1105A>G), GCC141GCT (1108C>T), GTG142GTC (1111G>C), CTC143TTG (1112C>T 1114C>G), AGT147GAA (1124A>G 1125G>A 1126T>A), ATC148ATT (1129C>T), CCC151CCT (1138C>T), CTC153GTT (1142C>G 1144C>T), GGA156GGG (1153A>G), CCA157CCC (1156A>C), TTC159TTT (1162C>T), GAG166CTT (1181G>C 1182A>T 1183G>T), AAT167AAC (1186T>C), CGT169CGG (1192T>G), GCA170GCT (1195A>T), CAG171CTA (1197A>T 1198G>A), ATA172ATT (1201A>T), CCT173CCA (1204T>A), TCC175TCT (1210C>T), GTG176GTT (1213G>T), GTA177GTG (1216A>G), GCG178GCA (1219G>A), ATA179ATT (1222A>T), AAA181AAG (1228A>G), CAA182CAG (1231A>G), GAT185AGC (1238G>A 1239A>G 1240T>C), ACA186CCA (1241A>C), AGG187AGA (1246G>A), GCC189GCT (1252C>T), GTA190GTT (1255A>T), TAC191TAT (1258C>T), TCT192ACG (1259T>A 1261T>G), CAA193GCA (1262C>G 1263A>C), CAG194CAA (1267G>A), ACT196ACA (1273T>A), TCT198AGT (1277T>A 1278C>G), GCC200GCA (1285C>A), GAA202GAG (1291A>G), GCA203GCT (1294A>T), TTG204CTC (1295T>C 1297G>C), AAC205AAT (1300C>T), CCT207CCA (1306T>A), CAA208CAG (1309A>G), TTT209TTC (1312T>C), TTC210TTT (1315C>T), CAA211TCT (1316C>T 1317A>C 1318A>T), CAG214CAA (1327G>A), GCT216GCA (1333T>A), GCC217GCA (1336C>A), TCA222TCT (1351A>T), GTA223GTC (1354A>C), TCT225TCG (1360T>G), GGG227GGA (1366G>A), CAA228GAG (1367C>G 1369A>G), AGT229AGG (1372T>G), TCC230GCG (1373T>G 1375C>G), CAG231TCT (1376C>T 1377A>C 1378G>T), ATG232CAA (1379A>C 1380T>A 1381G>A), TTG234CTA (1385T>C 1387G>A), CGT237AGA (1394C>A 1396T>A), GCT238CCT (1397G>C), GTT240GTA (1405T>A), GGA241GGT (1408A>T), GAG242GCA (1410A>C 1411G>A), AGG243CTT (1412A>C 1413G>T 1414G>T), AAT246AAC (1423T>C), ACA247ACT (1426A>T), CGA248CAC (1428G>A 1429A>C), GAT250AAT (1433G>A), GTA251GTT (1438A>T), CTG252CCC (1440T>C 1441G>C), AGT253CAG (1442A>C 1443G>A 1444T>G), AGC255TCT (1448A>T 1449G>C 1450C>T), TCC256TCA (1453C>A), ATG257TTA (1454A>T 1456G>A), ACT258ATG (1458C>T 1459T>G), AGC260GCG (1463A>G 1464G>C 1465C>G), TCT261TCA (1468T>A), TCC262TCG (1471C>G), CTG263CTT (1474G>T), CGA264CGC (1477A>C), CCG266CCA (1483G>A), CTC268CTA (1489C>A), GTG270ATC (1493G>A 1495G>C), AGT272AGC (1501T>C), AGG273AAG (1503G>A), CGG274AGG (1505C>A), CCA276CCT (1513A>T), TCC277TCG (1516C>G), GAA279GAG (1522A>G), CTA280CTG (1525A>G), GAC282GAT (1531C>T), GTG283TTT (1532G>T 1534G>T), CCT284TCA (1535C>T 1537T>A), AAC285AGG (1539A>G 1540C>G), ACC286GCC (1541A>G), CGA288TCA (1547C>T 1548G>C), TTA289TCG (1551T>C 1552A>G), ACA290_ATG291insGTAGGT (1555_1556insGTAGGT), ATG291GTG (1556A>G), CGA292AGA (1559C>A), GCT296GGG (1572C>G 1573T>G), CAC297GTA (1574C>G 1575A>T 1576C>A), GAA299GAT (1582A>T), GAC300GAT (1585C>T), ACT301TCC (1586A>T 1588T>C), GGT302AAT (1589G>A 1590G>A), GGA303TTA (1592G>T 1593G>T), TTG304CTG (1595T>C), CCA306CCC (1603A>C), GTT307GTC (1606T>C), AAG309GAG (1610A>G), CAA311CAG (1618A>G), GGC312GCC (1620G>C), TTC313ATT (1622T>A 1624C>T), AAC314GAT (1625A>G 1627C>T), ATG315TTT (1628A>T 1630G>T), GGT316GGC (1633T>C), GTT317CAG (1634G>C 1635T>A 1636T>G), GAT320GAC (1645T>C), CTG322TAT (1649C>T 1650T>A 1651G>T), ATA324ATC (1657A>C), GAC325ACT (1658G>A 1659A>C 1660C>T), TCC326GAT (1661T>G 1662C>A 1663C>T), ACA328AAT (1668C>A 1669A>T), CAC329TCT (1670C>T 1671A>C 1672C>T), ATT330TTT (1673A>T), GAC331GAT (1678C>T), GAT335GAG (1690T>G), ACT337ACA (1696T>A), GAG338ACC (1697G>A 1698A>C 1699G>C), CGC339TGG (1700C>T 1702C>G), ACG340CAT (1703A>C 1704C>A 1705G>T), ATT341ATA (1708T>A), GCG342TCA (1709G>T 1711G>A), CAA343AGG (1712C>A 1713A>G 1714A>G), CAG344CAA (1717G>A), GTT345ATT (1718G>A), CAA346GAT (1721C>G 1723A>T), TAC347TTT (1725A>T 1726C>T), ATT348GTG (1727A>G 1729T>G), CTG349TTG (1730C>T), TTT350TCT (1734T>C), GAT351ACC (1736G>A 1737A>C 1738T>C), GAG352CTC (1739G>C 1740A>T 1741G>C), GCT353CTC (1742G>C 1743C>T 1744T>C), TCA354CGC (1745T>C 1746C>G 1747A>C), CGA355CAG (1749G>A 1750A>G), GGC356GGG (1753C>G), AAT357AGA (1755A>G 1756T>A), GTG358TGT (1757G>T 1758T>G 1759G>T), ATC359CAT (1760A>C 1761T>A 1762C>T), GTG360CTG (1763G>C), CCG361CCC (1768G>C), AAA362AAG (1771A>G), CAT363CAC (1774T>C), GTA364ATT (1775G>A 1777A>T), TCA365GAG (1778T>G 1779C>A 1780A>G), AAG366AGA (1782A>G 1783G>A), AGG367CGA (1784A>C 1786G>A), GAG368GAC (1789G>C), CAG369TCC (1790C>T 1791A>C 1792G>C), AAT371AAC (1798T>C), ATT372ATA (1801T>A), TAC373AAC (1802T>A), AAG374CTA (1805A>C 1806A>T 1807G>A), CAC376TAC (1811C>T), ATC377ATT (1816C>T), ACT378GAT (1817A>G 1818C>A), GAC380AAT (1823G>A 1825C>T), GTT383CTA (1832G>C 1834T>A), GAT384CAG (1835G>C 1837T>G), ATT385GTG (1838A>G 1840T>G), CTT386CTC (1843T>C), GCT387AAG (1844G>A 1845C>A 1846T>G), GCA388GAA (1848C>A), ACC392ACA (1861C>A), AAG393AAT (1864G>T), GTA394GTG (1867A>G), GGA395GGT (1870A>T), AAT396TCC (1871A>T 1872A>C 1873T>C), CTC397CTA (1876C>A), TCT398GAG (1877T>G 1878C>A 1879T>G), TCA400ACT (1883T>A 1885A>T), TTG401TGC (1887T>G 1888G>C), GAT402AAT (1889G>A), TAT404TAC (1897T>C), GAT405TCT (1898G>T 1899A>C), ATG406TTA (1901A>T 1903G>A), TCG407ACT (1904T>A 1906G>T), CTT408CTC (1909T>C), CAT409AGG (1910C>A 1911A>G 1912T>G), GAG410GAA (1915G>A), AGA411AGG (1918A>G), CCG735..A (2890G>A), CAA737ACA (2894C>A 2895A>C), ACA738ACC (2899A>C), TAT739TTC (2901A>T 2902T>C), TTC740TTT (2905C>T), CCG741AGT (2906C>A 2907C>G 2908G>T), CGG742CGC (2911G>C), CCC744CCT (2917C>T), ACC745ACA (2920C>A), CAA747CAG (2926A>G), GTT748GTG (2929T>G), TTG749CTT (2930T>C 2932G>T), TTA750CTG (2933T>C 2935A>G), CAT751CAC (2938T>C), TAT752TAC (2941T>C), GGA755AGT (2948G>A 2950A>T), ACC756GTA (2951A>G 2952C>T 2953C>A), GCA759GCC (2962A>C), GGC761GGA (2968C>A), CTT764TTG (2975C>T 2977T>G), GTG765GTA (2980G>A), AGC766AGT (2983C>T), ATA767GTC (2984A>G 2986A>C), TCG769TCC (2992G>C), CCT770CCA (2995T>A), TCG771TCA (2998G>A), GGT772GGA (3001T>A), ATC773ATT (3004C>T), GAG775GAA (3010G>A), GTA779ATC (3020G>A 3022A>C), CTG780TTA (3023C>T 3025G>A), CAA781CAG (3028A>G), TCC783TCA (3034C>A), TTA784CTG (3035T>C 3037A>G), GGT786GGA (3043T>A), AAT787AAC (3046T>C), GTA788ATT (3047G>A 3049A>T), GCG789GCA (3052G>A), CGG790AGA (3053C>A 3055G>A), ACT796AGA (3072C>G 3073T>A), GGG797GGC (3076G>C), ACG798ACC (3079G>C), GTC800GTT (3085C>T), GAG802GAA (3091G>A), ATT803GTG (3092A>G 3094T>G), ATT805GTT (3098A>G), ACG808ACT (3109G>T), CTC809CTG (3112C>G), TTC810TTT (3115C>T), CAC811CAT (3118C>T), TCA812TCT (3121A>T), GGA813GGG (3124A>G), TTG815CTT (3128T>C 3130G>T), GCC816GCT (3133C>T), GGA818GGG (3139A>G), CTC819TTG (3140C>T 3142C>G), GGG820GGC (3145G>C), ACA821ACG (3148A>G), AAC823GCA (3152A>G 3153A>C 3154C>A), AAA825GAG (3158A>G 3160A>G), TCA827TCC (3166A>C), AAT828ACA (3168A>C 3169T>A), GCA829GCG (3172A>G), GAT831GAC (3178T>C), ATT832ATA (3181T>A), TTT833TTG (3184T>G), CCT836CCC (3193T>C), GTA838GTG (3199A>G), TGC840TGT (3205C>T), CTT842TTG (3209C>T 3211T>G), GAG843GAA (3214G>A), GGC845GGG (3220C>G), CGC846CGA (3223C>A), AAG847AGG (3225A>G), CGC849CAA (3231G>A 3232C>A)								

Proteins

	Begin	End	Coverage	Score	Concordance	Matches	Identities	I/D/M/F*	Stop Codons
polyprotein (YP_003097231.1)	117	855	34.9%	2195	74.0%	418 (99.5%)	296 (70.5%)	2/0/0/0	0
Protein mutations:	I124T (1056T>C 1057C>T), S125D (1058A>G 1059G>A), S132T (1079T>A 1081C>G), S134F (1086C>T 1087A>C), L135F (1088C>T 1090A>T), A136S (1091G>T 1093T>A), D137G (1095A>G 1096T>G), S147E (1124A>G 1125G>A 1126T>A), L153V (1142C>G 1144C>T), E166L (1181G>C 1182A>T 1183G>T), Q171L (1197A>T 1198G>A), D185S (1238G>A 1239A>G 1240T>C), T186P (1241A>C), S192T (1259T>A 1261T>G), Q193A (1262C>G 1263A>C), Q211S (1316C>T 1317A>C 1318A>T), Q228E (1367C>G 1369A>G), S229R (1372T>G), S230A (1373T>G 1375C>G), Q231S (1376C>T 1377A>C 1378G>T), M232Q (1379A>C 1380T>A 1381G>A), A238P (1397G>C), E242A (1410A>C 1411G>A), R243L (1412A>C 1413G>T 1414G>T), R248H (1428G>A 1429A>C), D250N (1433G>A), L252P (1440T>C 1441G>C), S253Q (1442A>C 1443G>A 1444T>G), M257L (1454A>T 1456G>A), T258M (1458C>T 1459T>G), S260A (1463A>G 1464G>C 1465C>G), V270I (1493G>A 1495G>C), R273K (1503G>A), V283F (1532G>T 1534G>T), P284S (1535C>T 1537T>A), N285R (1539A>G 1540C>G), T286A (1541A>G), R288S (1547C>T 1548G>C), L289S (1551T>C 1552A>G), T290_M291insVG (1555_1556insGTAGGT), M291V (1556A>G), A296G (1572C>G 1573T>G), H297V (1574C>G 1575A>T 1576C>A), E299D (1582A>T), T301S (1586A>T 1588T>C), G302N (1589G>A 1590G>A), G303L (1592G>T 1593G>T), K309E (1610A>G), G312A (1620G>C), F313I (1622T>A 1624C>T), N314D (1625A>G 1627C>T), M315F (1628A>T 1630G>T), V317Q (1634G>C 1635T>A 1636T>G), L322Y (1649C>T 1650T>A 1651G>T), D325T (1658G>A 1659A>C 1660C>T), S326D (1661T>G 1662C>A 1663C>T), T328N (1668C>A 1669A>T), H329S (1670C>T 1671A>C 1672C>T), I330F (1673A>T), D335E (1690T>G), E338T (1697G>A 1698A>C 1699G>C), R339W (1700C>T 1702C>G), T340H (1703A>C 1704C>A 1705G>T), A342S (1709G>T 1711G>A), Q343R (1712C>A 1713A>G 1714A>G), V345I (1718G>A), Q346D (1721C>G 1723A>T), Y347F (1725A>T 1726C>T), I348V (1727A>G 1729T>G), F350S (1734T>C), D351T (1736G>A 1737A>C 1738T>C), E352L (1739G>C 1740A>T 1741G>C), A353L (1742G>C 1743C>T 1744T>C), S354R (1745T>C 1746C>G 1747A>C), R355Q (1749G>A 1750A>G), N357R (1755A>G 1756T>A), V358C (1757G>T 1758T>G 1759G>T), I359H (1760A>C 1761T>A 1762C>T), V360L (1763G>C), V364I (1775G>A 1777A>T), S365E (1778T>G 1779C>A 1780A>G), K366R (1782A>G 1783G>A), E368D (1789G>C), Q369S (1790C>T 1791A>C 1792G>C), Y373N (1802T>A), K374L (1805A>C 1806A>T 1807G>A), H376Y (1811C>T), T378D (1817A>G 1818C>A), D380N (1823G>A 1825C>T), V383L (1832G>C 1834T>A), D384Q (1835G>C 1837T>G), I385V (1838A>G 1840T>G), A387K (1844G>A 1845C>A 1846T>G), A388E (1848C>A), K393N (1864G>T), N396S (1871A>T 1872A>C 1873T>C), S398E (1877T>G 1878C>A 1879T>G), S400T (1883T>A 1885A>T), L401C (1887T>G 1888G>C), D402N (1889G>A), D405S (1898G>T 1899A>C), M406L (1901A>T 1903G>A), S407T (1904T>A 1906G>T), H409R (1910C>A 1911A>G 1912T>G), Q737T (2894C>A 2895A>C), Y739F (2901A>T 2902T>C), P741S (2906C>A 2907C>G 2908G>T), G755S (2948G>A 2950A>T), T756V (2951A>G 2952C>T 2953C>A), I767V (2984A>G 2986A>C), V779I (3020G>A 3022A>C), V788I (3047G>A 3049A>T), T796R (3072C>G 3073T>A), I803V (3092A>G 3094T>G), I805V (3098A>G), N823A (3152A>G 3153A>C 3154C>A), K825E (3158A>G 3160A>G), N828T (3168A>C 3169T>A), F833L (3184T>G), K847R (3225A>G), R849Q (3231G>A 3232C>A)								

	Begin	End	Coverage	Score	Concordance	Matches	Identities	I/D/M/F*	Stop Codons
**NT**	**1033**	**3248**	**24.5%**	**763**	**30.4%**	**1253 (99.5%)**	**821 (65.2%)**	**6/0**	
Codon mutations:	TCG119TCC (1042G>C), CCC123CCA (1054C>A), ATC124ACT (1056T>C 1057C>T), AGC125GAC (1058A>G 1059G>A), CCG126CCT (1063G>T), GTG127GTT (1066G>T), ATT128ATA (1069T>A), CAA129CAG (1072A>G), GAG130GAA (1075G>A), TCC132ACG (1079T>A 1081C>G), TTG133TTA (1084G>A), TCA134TTC (1086C>T 1087A>C), CTA135TTT (1088C>T 1090A>T), GCT136TCA (1091G>T 1093T>A), GAT137GGG (1095A>G 1096T>G), GGT138GGA (1099T>A), GCA139GCT (1102A>T), CAA140CAG (1105A>G), GCC141GCT (1108C>T), GTG142GTC (1111G>C), CTC143TTG (1112C>T 1114C>G), AGT147GAA (1124A>G 1125G>A 1126T>A), ATC148ATT (1129C>T), CCC151CCT (1138C>T), CTC153GTT (1142C>G 1144C>T), GGA156GGG (1153A>G), CCA157CCC (1156A>C), TTC159TTT (1162C>T), GAG166CTT (1181G>C 1182A>T 1183G>T), AAT167AAC (1186T>C), CGT169CGG (1192T>G), GCA170GCT (1195A>T), CAG171CTA (1197A>T 1198G>A), ATA172ATT (1201A>T), CCT173CCA (1204T>A), TCC175TCT (1210C>T), GTG176GTT (1213G>T), GTA177GTG (1216A>G), GCG178GCA (1219G>A), ATA179ATT (1222A>T), AAA181AAG (1228A>G), CAA182CAG (1231A>G), GAT185AGC (1238G>A 1239A>G 1240T>C), ACA186CCA (1241A>C), AGG187AGA (1246G>A), GCC189GCT (1252C>T), GTA190GTT (1255A>T), TAC191TAT (1258C>T), TCT192ACG (1259T>A 1261T>G), CAA193GCA (1262C>G 1263A>C), CAG194CAA (1267G>A), ACT196ACA (1273T>A), TCT198AGT (1277T>A 1278C>G), GCC200GCA (1285C>A), GAA202GAG (1291A>G), GCA203GCT (1294A>T), TTG204CTC (1295T>C 1297G>C), AAC205AAT (1300C>T), CCT207CCA (1306T>A), CAA208CAG (1309A>G), TTT209TTC (1312T>C), TTC210TTT (1315C>T), CAA211TCT (1316C>T 1317A>C 1318A>T), CAG214CAA (1327G>A), GCT216GCA (1333T>A), GCC217GCA (1336C>A), TCA222TCT (1351A>T), GTA223GTC (1354A>C), TCT225TCG (1360T>G), GGG227GGA (1366G>A), CAA228GAG (1367C>G 1369A>G), AGT229AGG (1372T>G), TCC230GCG (1373T>G 1375C>G), CAG231TCT (1376C>T 1377A>C 1378G>T), ATG232CAA (1379A>C 1380T>A 1381G>A), TTG234CTA (1385T>C 1387G>A), CGT237AGA (1394C>A 1396T>A), GCT238CCT (1397G>C), GTT240GTA (1405T>A), GGA241GGT (1408A>T), GAG242GCA (1410A>C 1411G>A), AGG243CTT (1412A>C 1413G>T 1414G>T), AAT246AAC (1423T>C), ACA247ACT (1426A>T), CGA248CAC (1428G>A 1429A>C), GAT250AAT (1433G>A), GTA251GTT (1438A>T), CTG252CCC (1440T>C 1441G>C), AGT253CAG (1442A>C 1443G>A 1444T>G), AGC255TCT (1448A>T 1449G>C 1450C>T), TCC256TCA (1453C>A), ATG257TTA (1454A>T 1456G>A), ACT258ATG (1458C>T 1459T>G), AGC260GCG (1463A>G 1464G>C 1465C>G), TCT261TCA (1468T>A), TCC262TCG (1471C>G), CTG263CTT (1474G>T), CGA264CGC (1477A>C), CCG266CCA (1483G>A), CTC268CTA (1489C>A), GTG270ATC (1493G>A 1495G>C), AGT272AGC (1501T>C), AGG273AAG (1503G>A), CGG274AGG (1505C>A), CCA276CCT (1513A>T), TCC277TCG (1516C>G), GAA279GAG (1522A>G), CTA280CTG (1525A>G), GAC282GAT (1531C>T), GTG283TTT (1532G>T 1534G>T), CCT284TCA (1535C>T 1537T>A), AAC285AGG (1539A>G 1540C>G), ACC286GCC (1541A>G), CGA288TCA (1547C>T 1548G>C), TTA289TCG (1551T>C 1552A>G), ACA290_ATG291insGTAGGT (1555_1556insGTAGGT), ATG291GTG (1556A>G), CGA292AGA (1559C>A), GCT296GGG (1572C>G 1573T>G), CAC297GTA (1574C>G 1575A>T 1576C>A), GAA299GAT (1582A>T), GAC300GAT (1585C>T), ACT301TCC (1586A>T 1588T>C), GGT302AAT (1589G>A 1590G>A), GGA303TTA (1592G>T 1593G>T), TTG304CTG (1595T>C), CCA306CCC (1603A>C), GTT307GTC (1606T>C), AAG309GAG (1610A>G), CAA311CAG (1618A>G), GGC312GCC (1620G>C), TTC313ATT (1622T>A 1624C>T), AAC314GAT (1625A>G 1627C>T), ATG315TTT (1628A>T 1630G>T), GGT316GGC (1633T>C), GTT317CAG (1634G>C 1635T>A 1636T>G), GAT320GAC (1645T>C), CTG322TAT (1649C>T 1650T>A 1651G>T), ATA324ATC (1657A>C), GAC325ACT (1658G>A 1659A>C 1660C>T), TCC326GAT (1661T>G 1662C>A 1663C>T), ACA328AAT (1668C>A 1669A>T), CAC329TCT (1670C>T 1671A>C 1672C>T), ATT330TTT (1673A>T), GAC331GAT (1678C>T), GAT335GAG (1690T>G), ACT337ACA (1696T>A), GAG338ACC (1697G>A 1698A>C 1699G>C), CGC339TGG (1700C>T 1702C>G), ACG340CAT (1703A>C 1704C>A 1705G>T), ATT341ATA (1708T>A), GCG342TCA (1709G>T 1711G>A), CAA343AGG (1712C>A 1713A>G 1714A>G), CAG344CAA (1717G>A), GTT345ATT (1718G>A), CAA346GAT (1721C>G 1723A>T), TAC347TTT (1725A>T 1726C>T), ATT348GTG (1727A>G 1729T>G), CTG349TTG (1730C>T), TTT350TCT (1734T>C), GAT351ACC (1736G>A 1737A>C 1738T>C), GAG352CTC (1739G>C 1740A>T 1741G>C), GCT353CTC (1742G>C 1743C>T 1744T>C), TCA354CGC (1745T>C 1746C>G 1747A>C), CGA355CAG (1749G>A 1750A>G), GGC356GGG (1753C>G), AAT357AGA (1755A>G 1756T>A), GTG358TGT (1757G>T 1758T>G 1759G>T), ATC359CAT (1760A>C 1761T>A 1762C>T), GTG360CTG (1763G>C), CCG361CCC (1768G>C), AAA362AAG (1771A>G), CAT363CAC (1774T>C), GTA364ATT (1775G>A 1777A>T), TCA365GAG (1778T>G 1779C>A 1780A>G), AAG366AGA (1782A>G 1783G>A), AGG367CGA (1784A>C 1786G>A), GAG368GAC (1789G>C), CAG369TCC (1790C>T 1791A>C 1792G>C), AAT371AAC (1798T>C), ATT372ATA (1801T>A), TAC373AAC (1802T>A), AAG374CTA (1805A>C 1806A>T 1807G>A), CAC376TAC (1811C>T), ATC377ATT (1816C>T), ACT378GAT (1817A>G 1818C>A), GAC380AAT (1823G>A 1825C>T), GTT383CTA (1832G>C 1834T>A), GAT384CAG (1835G>C 1837T>G), ATT385GTG (1838A>G 1840T>G), CTT386CTC (1843T>C), GCT387AAG (1844G>A 1845C>A 1846T>G), GCA388GAA (1848C>A), ACC392ACA (1861C>A), AAG393AAT (1864G>T), GTA394GTG (1867A>G), GGA395GGT (1870A>T), AAT396TCC (1871A>T 1872A>C 1873T>C), CTC397CTA (1876C>A), TCT398GAG (1877T>G 1878C>A 1879T>G), TCA400ACT (1883T>A 1885A>T), TTG401TGC (1887T>G 1888G>C), GAT402AAT (1889G>A), TAT404TAC (1897T>C), GAT405TCT (1898G>T 1899A>C), ATG406TTA (1901A>T 1903G>A), TCG407ACT (1904T>A 1906G>T), CTT408CTC (1909T>C), CAT409AGG (1910C>A 1911A>G 1912T>G), GAG410GAA (1915G>A), AGA411AGG (1918A>G), CCG735..A (2890G>A), CAA737ACA (2894C>A 2895A>C), ACA738ACC (2899A>C), TAT739TTC (2901A>T 2902T>C), TTC740TTT (2905C>T), CCG741AGT (2906C>A 2907C>G 2908G>T), CGG742CGC (2911G>C), CCC744CCT (2917C>T), ACC745ACA (2920C>A), CAA747CAG (2926A>G), GTT748GTG (2929T>G), TTG749CTT (2930T>C 2932G>T), TTA750CTG (2933T>C 2935A>G), CAT751CAC (2938T>C), TAT752TAC (2941T>C), GGA755AGT (2948G>A 2950A>T), ACC756GTA (2951A>G 2952C>T 2953C>A), GCA759GCC (2962A>C), GGC761GGA (2968C>A), CTT764TTG (2975C>T 2977T>G), GTG765GTA (2980G>A), AGC766AGT (2983C>T), ATA767GTC (2984A>G 2986A>C), TCG769TCC (2992G>C), CCT770CCA (2995T>A), TCG771TCA (2998G>A), GGT772GGA (3001T>A), ATC773ATT (3004C>T), GAG775GAA (3010G>A), GTA779ATC (3020G>A 3022A>C), CTG780TTA (3023C>T 3025G>A), CAA781CAG (3028A>G), TCC783TCA (3034C>A), TTA784CTG (3035T>C 3037A>G), GGT786GGA (3043T>A), AAT787AAC (3046T>C), GTA788ATT (3047G>A 3049A>T), GCG789GCA (3052G>A), CGG790AGA (3053C>A 3055G>A), ACT796AGA (3072C>G 3073T>A), GGG797GGC (3076G>C), ACG798ACC (3079G>C), GTC800GTT (3085C>T), GAG802GAA (3091G>A), ATT803GTG (3092A>G 3094T>G), ATT805GTT (3098A>G), ACG808ACT (3109G>T), CTC809CTG (3112C>G), TTC810TTT (3115C>T), CAC811CAT (3118C>T), TCA812TCT (3121A>T), GGA813GGG (3124A>G), TTG815CTT (3128T>C 3130G>T), GCC816GCT (3133C>T), GGA818GGG (3139A>G), CTC819TTG (3140C>T 3142C>G), GGG820GGC (3145G>C), ACA821ACG (3148A>G), AAC823GCA (3152A>G 3153A>C 3154C>A), AAA825GAG (3158A>G 3160A>G), TCA827TCC (3166A>C), AAT828ACA (3168A>C 3169T>A), GCA829GCG (3172A>G), GAT831GAC (3178T>C), ATT832ATA (3181T>A), TTT833TTG (3184T>G), CCT836CCC (3193T>C), GTA838GTG (3199A>G), TGC840TGT (3205C>T), CTT842TTG (3209C>T 3211T>G), GAG843GAA (3214G>A), GGC845GGG (3220C>G), CGC846CGA (3223C>A), AAG847AGG (3225A>G), CGC849CAA (3231G>A 3232C>A)								

*: Inserts / Deletes / Misaligned / Frameshifts

Analysis details

This analysis was performed with panviral2.64

NGS Details (UN8): Lausannevirus

Assembly

**Coverage Length**	137 (1 contig(s))
**Depth Of Coverage**	23002.5
**Number Of Reads**	34961
**Reads Per Million**	584.29 rpm (after QC)
**Ambiguities**	0
**Assembly Method**	de novo + reference guided assembly
**Consensus Caller**	Bcf Tools

Coverage Map

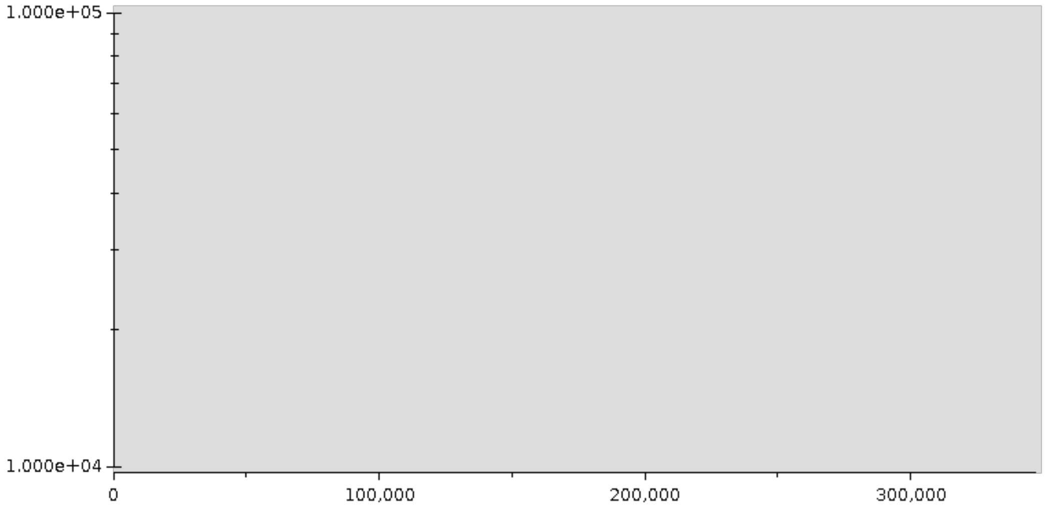

Assignment

**Type**	Lausannevirus (Taxonomy ID: 999883)
**Reference Genome**	NC_015326.1
**NT Identity (%)**	80.292
**AA Identity (%)**	93.4783
**Number Of Stop Codons**	0
**Number Of CDS**	444

Alignment

**Alignment Score**	166.0 (NT) + 291.0 (AA) = 457.0
**Concordance (%)**	79.2028
**Alignment Method**	Local, heuristic, nucleotide (BLASTN)

Genome Region

Sequence starts at position 194953 and ends at position 195089 relative to NC_015326.1 reference sequence.

Alignment Detailed Statistics

	Begin	End	Coverage	Score	Concordance	Matches	Identities	I/D/M/F*	Stop Codons
**NT**	**194953**	**195089**	**0.1%**	**166**	**60.6%**	**137 (100%)**	**110 (80.3%)**	**0/0**	

Mutations: 194967A>G, 194970G>A, 194973A>T, 194976A>G, 194979T>G, 194981T>G, 194982C>G, 194991G>C, 194993G>A, 195000A>T, 195006G>A, 195012A>C, 195014G>A, 195015T>C, 195017G>T, 195018T>C, 195021T>C, 195030A>G, 195042T>C, 195045G>A, 195048T>C, 195051A>G, 195054T>C, 195057T>A, 195058T>G, 195059G>C, 195069C>G

*: Inserts / Deletes / Misaligned / Frameshifts

Analysis details

This analysis was performed with panviral2.64

NGS Details (UN8): Yellowstone lake phycodnavirus 1

Assembly

**Coverage Length**	173 (1 contig(s))
**Depth Of Coverage**	15316.6
**Number Of Reads**	29336
**Reads Per Million**	490.28 rpm (after QC)
**Ambiguities**	0
**Assembly Method**	de novo + reference guided assembly
**Consensus Caller**	Bcf Tools

Coverage Map

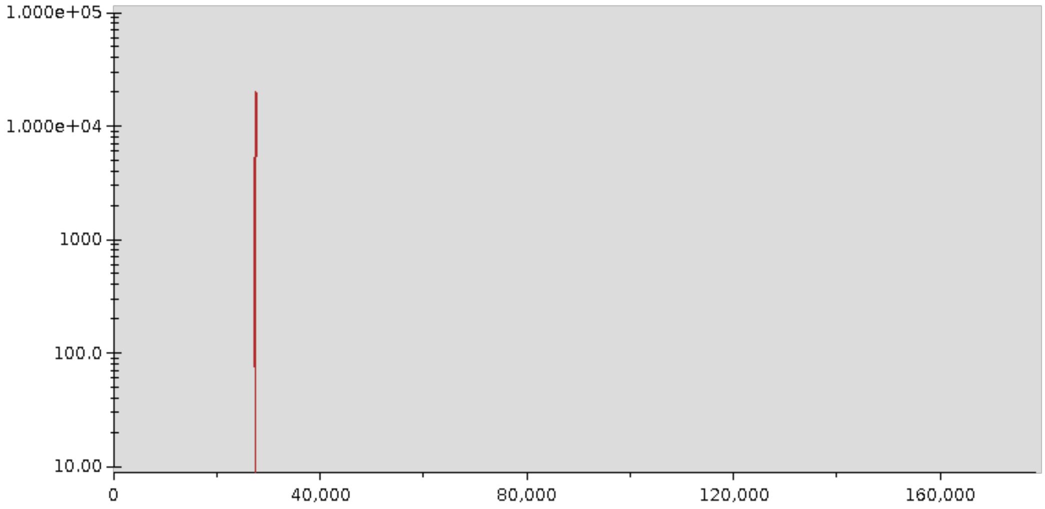

Assignment

**Type**	Yellowstone lake phycodnavirus 1 (Taxonomy ID: 1586713)
**Reference Genome**	NC_028112.1
**NT Identity (%)**	82.0809
**AA Identity (%)**	93.1034
**Number Of Stop Codons**	0
**Number Of CDS**	248

Alignment

**Alignment Score**	222.0 (NT) + 350.0 (AA) = 572.0
**Concordance (%)**	80.5634
**Alignment Method**	Local, heuristic, nucleotide (BLASTN)

Genome Region

Sequence starts at position 27469 and ends at position 27641 relative to NC_028112.1 reference sequence.

Alignment Detailed Statistics

	Begin	End	Coverage	Score	Concordance	Matches	Identities	I/D/M/F*	Stop Codons
**NT**	**27469**	**27641**	**0.1%**	**222**	**64.2%**	**173 (100%)**	**142 (82.1%)**	**0/0**	

Mutations: 27481T>G, 27486G>A, 27492A>G, 27510A>G, 27513T>C, 27516T>A, 27529C>A, 27531T>G, 27534A>G, 27537T>A, 27540T>C, 27543T>A, 27552A>G, 27558G>A, 27563A>G, 27564T>C, 27567T>C, 27573A>T, 27576A>G, 27582T>C, 27586G>A, 27591A>G, 27597A>G, 27600C>A, 27606T>G, 27609T>C, 27612A>T, 27616T>C, 27618G>C, 27624T>C, 27627C>T

*: Inserts / Deletes / Misaligned / Frameshifts

Analysis details

This analysis was performed with panviral2.64

NGS Details (UN8): Euproctis digramma nucleopolyhedrovirus

Assembly

**Coverage Length**	151 (1 contig(s))
**Depth Of Coverage**	15809.8
**Number Of Reads**	26156
**Reads Per Million**	437.13 rpm (after QC)
**Ambiguities**	0
**Assembly Method**	de novo + reference guided assembly
**Consensus Caller**	Bcf Tools

Coverage Map

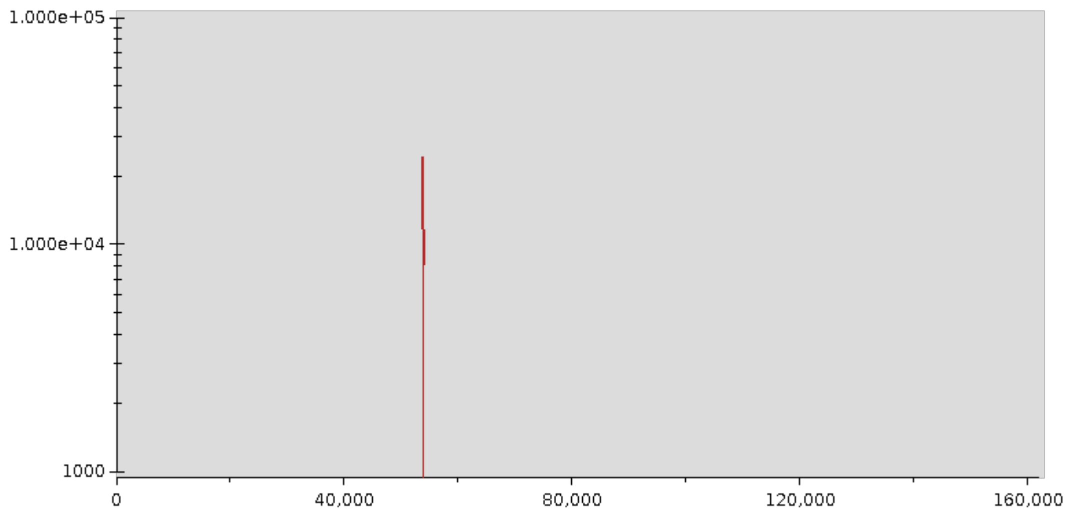

Assignment

**Type**	Euproctis digramma nucleopolyhedrovirus (Taxonomy ID: 307466)
**Reference Genome**	NC_076728.1
**NT Identity (%)**	79.4702
**AA Identity (%)**	92.0
**Number Of Stop Codons**	1
**Number Of CDS**	149

Alignment

**Alignment Score**	178.0 (NT) + 311.0 (AA) = 489.0
**Concordance (%)**	77.619
**Alignment Method**	Local, heuristic, nucleotide (BLASTN)

Genome Region

Sequence starts at position 53764 and ends at position 53914 relative to NC_076728.1 reference sequence.

Alignment Detailed Statistics

	Begin	End	Coverage	Score	Concordance	Matches	Identities	I/D/M/F*	Stop Codons
NT	53764	53914	0.1%	178	58.9%	151 (100%)	120 (79.5%)	0/0	

Mutations: 53777C>A, 53780C>G, 53782A>G, 53783T>A, 53785T>G, 53786C>A, 53804G>A, 53807T>C, 53810T>C, 53813T>C, 53816A>G, 53819A>G, 53825A>G, 53828G>A, 53829C>G, 53830T>C, 53831A>T, 53833T>G, 53834C>A, 53840C>G, 53841G>C, 53842A>C, 53843G>A, 53855T>C, 53858G>T, 53861G>T, 53865T>A, 53875G>T, 53882A>G, 53885G>T, 53900T>C

*: Inserts / Deletes / Misaligned / Frameshifts

Analysis details

This analysis was performed with panviral2.64

NGS Details (UN8): Betabaculovirus clanastomosis

Assembly

**Coverage Length**	134 (1 contig(s))
**Depth Of Coverage**	13042.5
**Number Of Reads**	22579
**Reads Per Million**	377.35 rpm (after QC)
**Ambiguities**	0
**Assembly Method**	de novo + reference guided assembly
**Consensus Caller**	Bcf Tools

Coverage Map

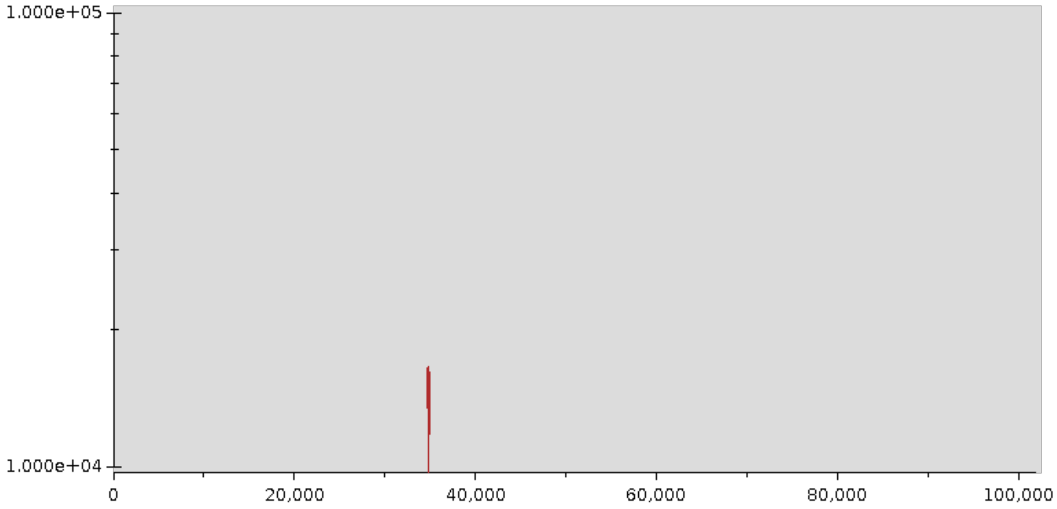

Assignment

**Type**	Betabaculovirus clanastomosis (Taxonomy ID: 3047763)
**Reference Genome**	NC_022646.1
**NT Identity (%)**	83.5821
**AA Identity (%)**	86.6667
**Number Of Stop Codons**	0
**Number Of CDS**	122

Alignment

**Alignment Score**	180.0 (NT) + 289.0 (AA) = 469.0
**Concordance (%)**	80.5842
**Alignment Method**	Local, heuristic, nucleotide (BLASTN)

Genome Region

Sequence starts at position 34740 and ends at position 34873 relative to NC_022646.1 reference sequence.

Alignment Detailed Statistics

	Begin	End	Coverage	Score	Concordance	Matches	Identities	I/D/M/F*	Stop Codons
**NT**	**34740**	**34873**	**0.1%**	**180**	**67.2%**	**134 (100%)**	**112 (83.6%)**	**0/0**	

Mutations: 34753T>A, 34761A>G, 34764T>G, 34768G>A, 34773G>A, 34779T>G, 34784C>G, 34785T>A, 34801A>G, 34804G>A, 34809A>C, 34810T>A, 34814C>G, 34819C>G, 34820T>C, 34825G>C, 34828C>A, 34831T>C, 34837T>A, 34840G>A, 34851G>A, 34852T>C

*: Inserts / Deletes / Misaligned / Frameshifts

Analysis details

This analysis was performed with panviral2.64

NGS Details (UN8): Lausannevirus

Assembly

**Coverage Length**	146 (1 contig(s))
**Depth Of Coverage**	7006.5
**Number Of Reads**	11975
**Reads Per Million**	200.13 rpm (after QC)
**Ambiguities**	0
**Assembly Method**	de novo + reference guided assembly
**Consensus Caller**	Bcf Tools

Coverage Map

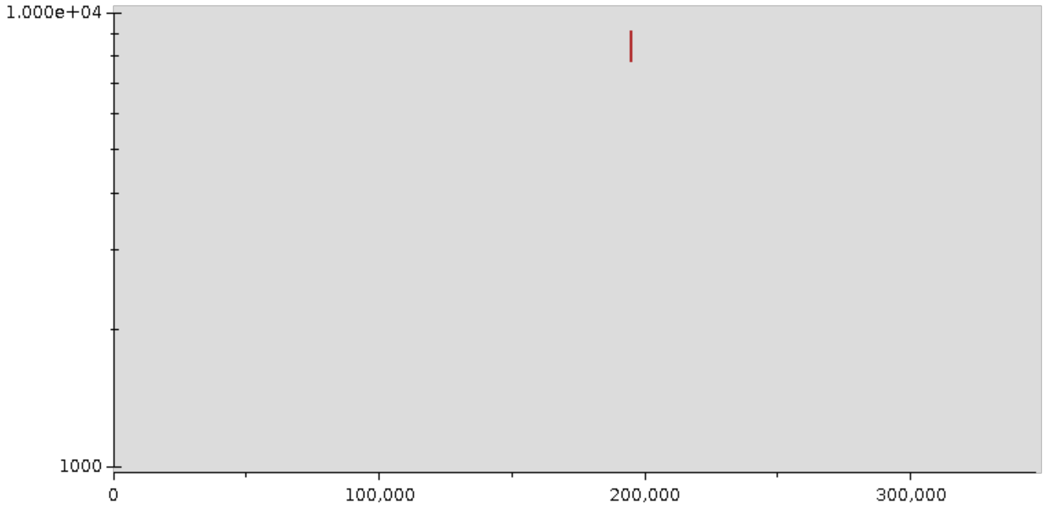

Assignment

**Type**	Lausannevirus (Taxonomy ID: 999883)
**Reference Genome**	NC_015326.1
**NT Identity (%)**	84.2466
**AA Identity (%)**	100.0
**Number Of Stop Codons**	1
**Number Of CDS**	444

Alignment

**Alignment Score**	200.0 (NT) + 309.0 (AA) = 509.0
**Concordance (%)**	84.6922
**Alignment Method**	Local, heuristic, nucleotide (BLASTN)

Genome Region

Sequence starts at position 194911 and ends at position 195056 relative to NC_015326.1 reference sequence.

Alignment Detailed Statistics

	Begin	End	Coverage	Score	Concordance	Matches	Identities	I/D/M/F*	Stop Codons
NT	194911	195056	0.1%	200	68.5%	146 (100%)	123 (84.2%)	0/0	

Mutations: 194925T>C, 194931A>C, 194937G>A, 194943A>G, 194946G>C, 194949C>T, 194955T>C, 194967A>G, 194970G>A, 194979T>C, 194982C>T, 194991G>C, 194993G>A, 195003T>A, 195006G>A, 195009G>A, 195012A>C, 195015T>C, 195017G>T, 195021T>C, 195030A>G, 195039T>C, 195042T>C

*: Inserts / Deletes / Misaligned / Frameshifts

Analysis details

This analysis was performed with panviral2.64

NGS Details (UN8): Yellowstone lake phycodnavirus 1

Assembly

**Coverage Length**	153 (1 contig(s))
**Depth Of Coverage**	6804.1
**Number Of Reads**	11161
**Reads Per Million**	186.53 rpm (after QC)
**Ambiguities**	0
**Assembly Method**	de novo + reference guided assembly
**Consensus Caller**	Bcf Tools

Coverage Map

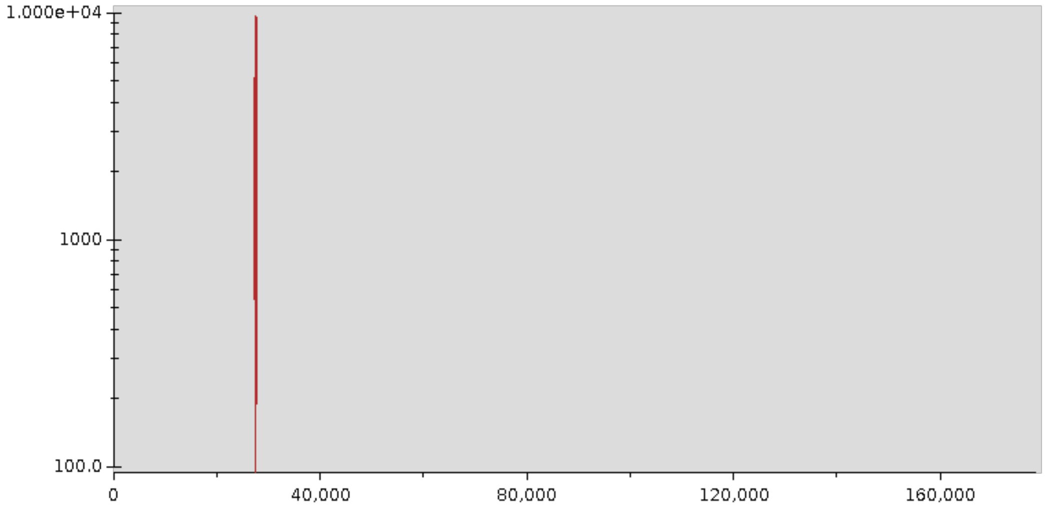

Assignment

**Type**	Yellowstone lake phycodnavirus 1 (Taxonomy ID: 1586713)
**Reference Genome**	NC_028112.1
**NT Identity (%)**	83.0065
**AA Identity (%)**	90.1961
**Number Of Stop Codons**	0
**Number Of CDS**	248

Alignment

**Alignment Score**	202.0 (NT) + 312.0 (AA) = 514.0
**Concordance (%)**	80.8176
**Alignment Method**	Local, heuristic, nucleotide (BLASTN)

Genome Region

Sequence starts at position 27462 and ends at position 27614 relative to NC_028112.1 reference sequence.

Alignment Detailed Statistics

	Begin	End	Coverage	Score	Concordance	Matches	Identities	I/D/M/F*	Stop Codons
**NT**	**27462**	**27614**	**0.1%**	**202**	**66.0%**	**153 (100%)**	**127 (83.0%)**	**0/0**	

Mutations: 27474T>C, 27476C>A, 27477A>C, 27481T>G, 27486G>A, 27492A>G, 27507G>A, 27510A>G, 27513T>C, 27529C>A, 27531T>G, 27534A>G, 27537T>C, 27540T>C, 27552A>G, 27563A>G, 27564T>C, 27567T>C, 27570C>T, 27573A>T, 27576A>G, 27582T>C, 27586G>A, 27591A>G, 27597A>G, 27600C>A

*: Inserts / Deletes / Misaligned / Frameshifts

Analysis details

This analysis was performed with panviral2.64

NGS Details (UN8): Lausannevirus

Assembly

**Coverage Length**	151 (1 contig(s))
**Depth Of Coverage**	3607.8
**Number Of Reads**	6209
**Reads Per Million**	103.77 rpm (after QC)
**Ambiguities**	0
**Assembly Method**	de novo + reference guided assembly
**Consensus Caller**	Bcf Tools

Coverage Map

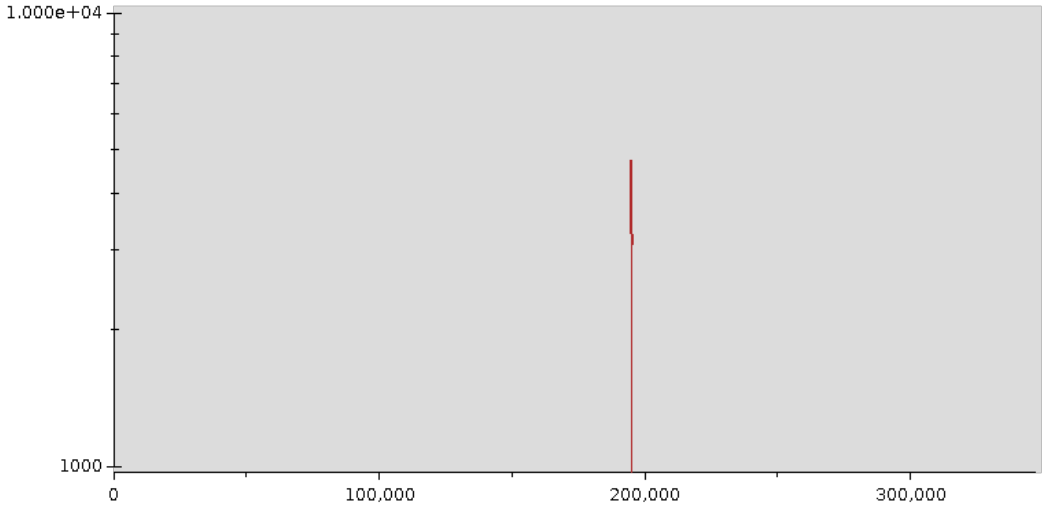

Assignment

**Type**	Lausannevirus (Taxonomy ID: 999883)
**Reference Genome**	NC_015326.1
**NT Identity (%)**	82.1192
**AA Identity (%)**	94.1176
**Number Of Stop Codons**	0
**Number Of CDS**	444

Alignment

**Alignment Score**	194.0 (NT) + 316.0 (AA) = 510.0
**Concordance (%)**	80.9524
**Alignment Method**	Local, heuristic, nucleotide (BLASTN)

Genome Region

Sequence starts at position 194954 and ends at position 195104 relative to NC_015326.1 reference sequence.

Alignment Detailed Statistics

	Begin	End	Coverage	Score	Concordance	Matches	Identities	I/D/M/F*	Stop Codons
**NT**	**194954**	**195104**	**0.1%**	**194**	**64.2%**	**151 (100%)**	**124 (82.1%)**	**0/0**	

Mutations: 194967A>G, 194970G>T, 194973A>C, 194979T>C, 194982C>T, 194991G>C, 195000A>C, 195006G>A, 195012A>C, 195015T>C, 195017G>T, 195018T>C, 195021T>C, 195030A>G, 195042T>C, 195048T>C, 195054T>C, 195057T>A, 195058T>G, 195059G>C, 195063G>C, 195066G>A, 195069C>G, 195075G>A, 195078G>A, 195081C>A, 195093T>C

*: Inserts / Deletes / Misaligned / Frameshifts

Analysis details

This analysis was performed with panviral2.64

NGS Details (UN8): Synechococcus phage S-CRM01

Assembly

**Coverage Length**	151 (1 contig(s))
**Depth Of Coverage**	3685.1
**Number Of Reads**	6209
**Reads Per Million**	103.77 rpm (after QC)
**Ambiguities**	0
**Assembly Method**	de novo + reference guided assembly
**Consensus Caller**	Bcf Tools

Coverage Map

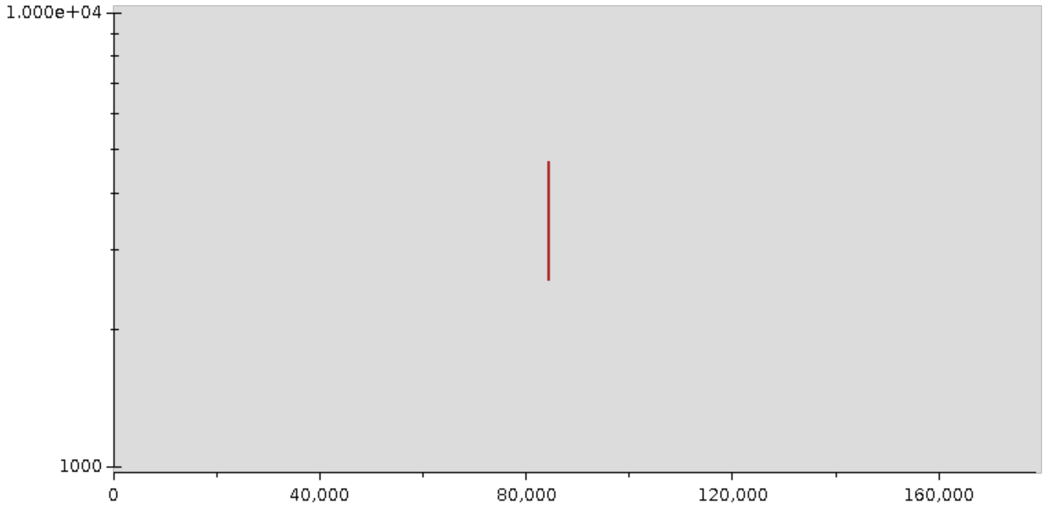

Assignment

**Type**	Synechococcus phage S-CRM01 (Taxonomy ID: 1026955)
**Reference Genome**	NC_015569.1
**NT Identity (%)**	78.8079
**AA Identity (%)**	92.0
**Number Of Stop Codons**	0
**Number Of CDS**	297

Alignment

**Alignment Score**	174.0 (NT) + 311.0 (AA) = 485.0
**Concordance (%)**	75.5452
**Alignment Method**	Local, heuristic, nucleotide (BLASTN)

Genome Region

Sequence starts at position 84284 and ends at position 84434 relative to NC_015569.1 reference sequence.

Alignment Detailed Statistics

	Begin	End	Coverage	Score	Concordance	Matches	Identities	I/D/M/F*	Stop Codons
**NT**	**84284**	**84434**	**0.1%**	**174**	**57.6%**	**151 (100%)**	**119 (78.8%)**	**0/0**	

Mutations: 84299A>T, 84301A>C, 84302G>A, 84305T>A, 84306T>G, 84307G>C, 84308T>A, 84311G>T, 84312A>T, 84323A>G, 84329T>C, 84331G>T, 84335T>C, 84337G>A, 84339G>C, 84340A>T, 84347G>T, 84350A>C, 84352G>A, 84353T>G, 84365T>A, 84369G>C, 84370A>T, 84377A>G, 84379C>A, 84380T>G, 84383A>G, 84398A>C, 84401T>G, 84404C>T, 84416G>T, 84419A>G

*: Inserts / Deletes / Misaligned / Frameshifts

Analysis details

This analysis was performed with panviral2.64

NGS Details (UN8): Marseillevirus marseillevirus

Assembly

**Coverage Length**	126 (1 contig(s))
**Depth Of Coverage**	3706.7
**Number Of Reads**	5547
**Reads Per Million**	92.70 rpm (after QC)
**Ambiguities**	0
**Assembly Method**	de novo + reference guided assembly
**Consensus Caller**	Bcf Tools

Coverage Map

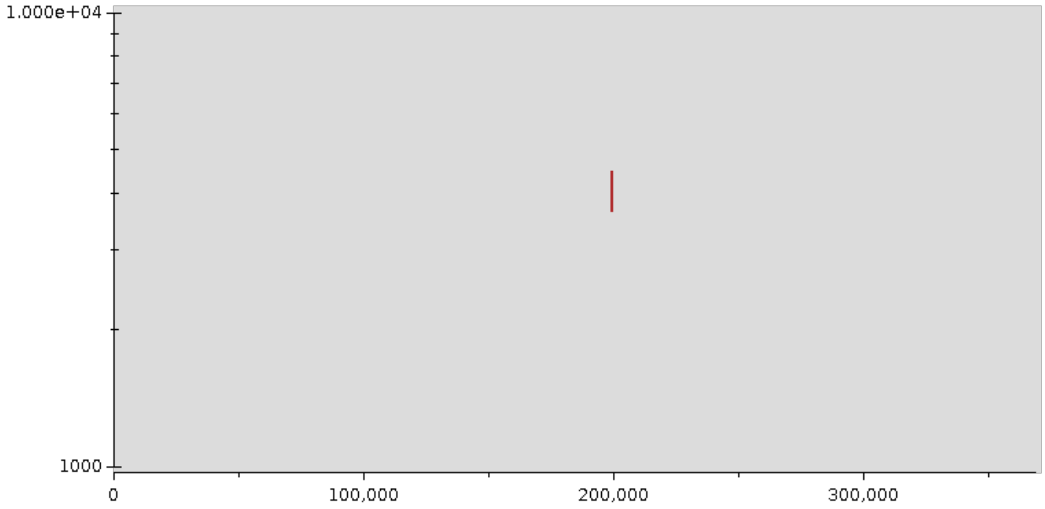

Assignment

**Type**	Marseillevirus marseillevirus (Taxonomy ID: 694581)
**Reference Genome**	NC_013756.1
**NT Identity (%)**	85.7143
**AA Identity (%)**	97.5
**Number Of Stop Codons**	1
**Number Of CDS**	428

Alignment

**Alignment Score**	180.0 (NT) + 261.0 (AA) = 441.0
**Concordance (%)**	85.7977
**Alignment Method**	Local, heuristic, nucleotide (BLASTN)

Genome Region

Sequence starts at position 199400 and ends at position 199525 relative to NC_013756.1 reference sequence.

Alignment Detailed Statistics

	Begin	End	Coverage	Score	Concordance	Matches	Identities	I/D/M/F*	Stop Codons
**NT**	**199400**	**199525**	**0.1%**	**180**	**71.4%**	**126 (100%)**	**108 (85.7%)**	**0/0**	

Mutations: 199411A>G, 199414T>A, 199416T>G, 199417C>G, 199429G>C, 199431G>A, 199435C>G, 199449C>T, 199459A>C, 199464A>G, 199471T>A, 199489T>C, 199492G>T, 199495G>A, 199498G>A, 199501G>C, 199504A>C, 199506G>T

*: Inserts / Deletes / Misaligned / Frameshifts

Analysis details

This analysis was performed with panviral2.64

NGS Details (UN8): Lausannevirus

Assembly

**Coverage Length**	125 (1 contig(s))
**Depth Of Coverage**	3324.6
**Number Of Reads**	4798
**Reads Per Million**	80.19 rpm (after QC)
**Ambiguities**	0
**Assembly Method**	de novo + reference guided assembly
**Consensus Caller**	Bcf Tools

Coverage Map

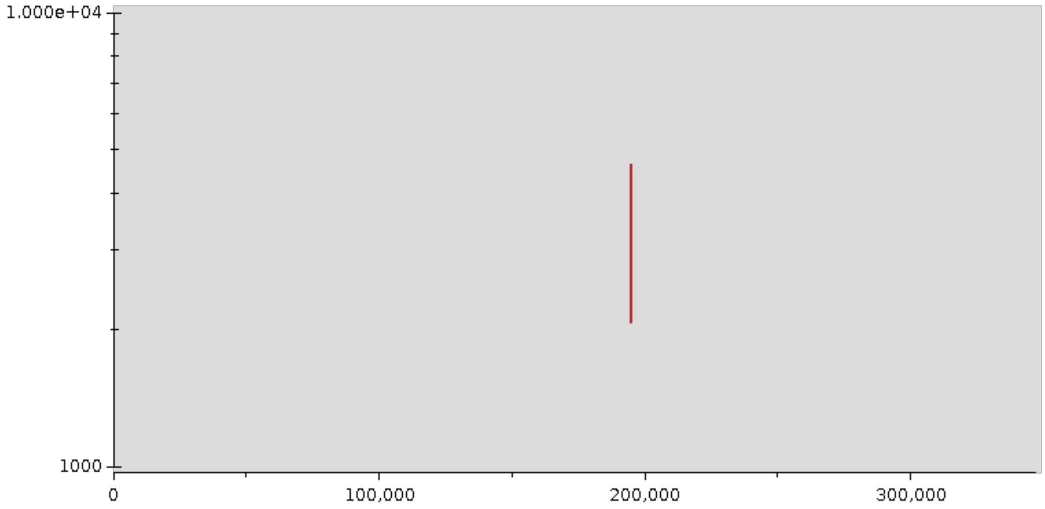

Assignment

**Type**	Lausannevirus (Taxonomy ID: 999883)
**Reference Genome**	NC_015326.1
**NT Identity (%)**	80.0
**AA Identity (%)**	95.2381
**Number Of Stop Codons**	0
**Number Of CDS**	444

Alignment

**Alignment Score**	150.0 (NT) + 275.0 (AA) = 425.0
**Concordance (%)**	79.7373
**Alignment Method**	Local, heuristic, nucleotide (BLASTN)

Genome Region

Sequence starts at position 194935 and ends at position 195059 relative to NC_015326.1 reference sequence.

Alignment Detailed Statistics

	Begin	End	Coverage	Score	Concordance	Matches	Identities	I/D/M/F*	Stop Codons
**NT**	**194935**	**195059**	**0.1%**	**150**	**60.0%**	**125 (100%)**	**100 (80.0%)**	**0/0**	

Mutations: 194940A>C, 194946G>T, 194952T>C, 194955T>C, 194958A>G, 194967A>G, 194970G>C, 194976A>G, 194979T>G, 194981T>G, 194982C>G, 195009G>T, 195012A>C, 195015T>C, 195017G>T, 195018T>C, 195021T>C, 195030A>T, 195033A>G, 195036G>C, 195039T>C, 195042T>C, 195048T>C, 195054T>C, 195057T>A

*: Inserts / Deletes / Misaligned / Frameshifts

Analysis details

This analysis was performed with panviral2.64

NGS Details (UN8): Betabaculovirus disaccharalis

Assembly

**Coverage Length**	125 (1 contig(s))
**Depth Of Coverage**	1794.9
**Number Of Reads**	2967
**Reads Per Million**	49.59 rpm (after QC)
**Ambiguities**	0
**Assembly Method**	de novo + reference guided assembly
**Consensus Caller**	Bcf Tools

Coverage Map

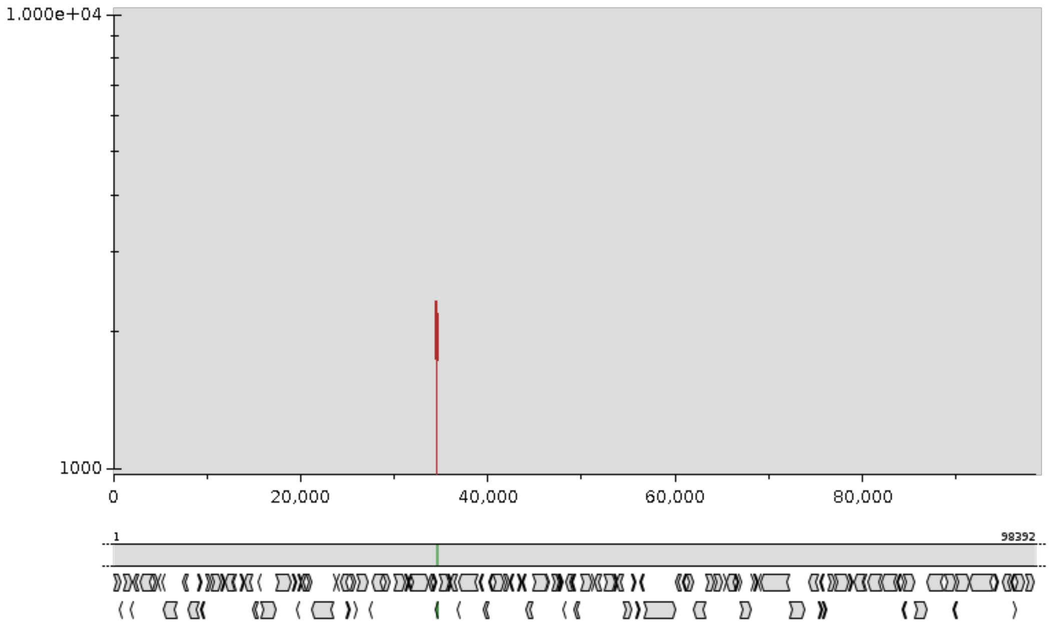

Assignment

**Type**	Betabaculovirus disaccharalis (Taxonomy ID: 3047795)
**Reference Genome**	NC_028491.1
**NT Identity (%)**	82.4
**AA Identity (%)**	92.8571
**Number Of Stop Codons**	0
**Number Of CDS**	125

Alignment

**Alignment Score**	162.0 (NT) + 265.0 (AA) = 427.0
**Concordance (%)**	81.1787

**Alignment Method**	Local, heuristic, nucleotide (BLASTN)

Genome Region

Sequence starts at position 34471 and ends at position 34595 relative to NC_028491.1 reference sequence.

Alignment Detailed Statistics

	Begin	End	Coverage	Score	Concordance	Matches	Identities	I/D/M/F*	Stop Codons
**NT**	**34471**	**34595**	**0.1%**	**162**	**64.8%**	**125 (100%)**	**103 (82.4%)**	**0/0**	
Mutations:	34482G>T, 34485T>C, 34488A>C, 34490G>A, 34491A>C, 34512G>T, 34513C>G, 34514T>A, 34521T>C, 34524A>G, 34533A>G, 34538A>C, 34545T>C, 34547G>T, 34548C>G, 34549T>C, 34551G>A, 34554T>C, 34569G>A, 34575T>A, 34578T>C, 34580A>G								
**CDS**									
v-ubq	61	102	35.3%	265	88.9%	42 (100%)	39 (92.9%)	0/0/0/0	0
Protein mutations:	E76G (34548C>G 34549T>C), S80A (34538A>C)								
Codon mutations:	TTA66CTG (34578T>C 34580A>G), ATA67ATT (34575T>A), GCC69GCT (34569G>A), GAA74GAG (34554T>C), GAC75GAT (34551G>A), GAG76GGC (34548C>G 34549T>C), CGA77AGG (34545T>C 34547G>T), TCA80GCA (34538A>C), GAT81GAC (34533A>G), ATT84ATC (34524A>G), CAA85CAG (34521T>C), AGC88TCA (34512G>T 34513C>G 34514T>A), CGT95CGG (34491A>C), CTT96TTG (34488A>C 34490G>A), AGA97AGG (34485T>C), GGC98GGA (34482G>T)								
**Proteins**									
ubiquitin-like protein (YP_009182246.1)	61	102	35.3%	265	88.9%	42 (100%)	39 (92.9%)	0/0/0/0	0
Protein mutations:	E76G (34548C>G 34549T>C), S80A (34538A>C)								
Codon mutations:	TTA66CTG (34578T>C 34580A>G), ATA67ATT (34575T>A), GCC69GCT (34569G>A), GAA74GAG (34554T>C), GAC75GAT (34551G>A), GAG76GGC (34548C>G 34549T>C), CGA77AGG (34545T>C 34547G>T), TCA80GCA (34538A>C), GAT81GAC (34533A>G), ATT84ATC (34524A>G), CAA85CAG (34521T>C), AGC88TCA (34512G>T 34513C>G 34514T>A), CGT95CGG (34491A>C), CTT96TTG (34488A>C 34490G>A), AGA97AGG (34485T>C), GGC98GGA (34482G>T)								

*: Inserts / Deletes / Misaligned / Frameshifts

Analysis details

This analysis was performed with panviral2.64

NGS Details (UN8): Lausannevirus

Assembly

**Coverage Length**	151 (1 contig(s))
**Depth Of Coverage**	1791.9
**Number Of Reads**	2902
**Reads Per Million**	48.50 rpm (after QC)
**Ambiguities**	0
**Assembly Method**	de novo + reference guided assembly
**Consensus Caller**	Bcf Tools

Coverage Map

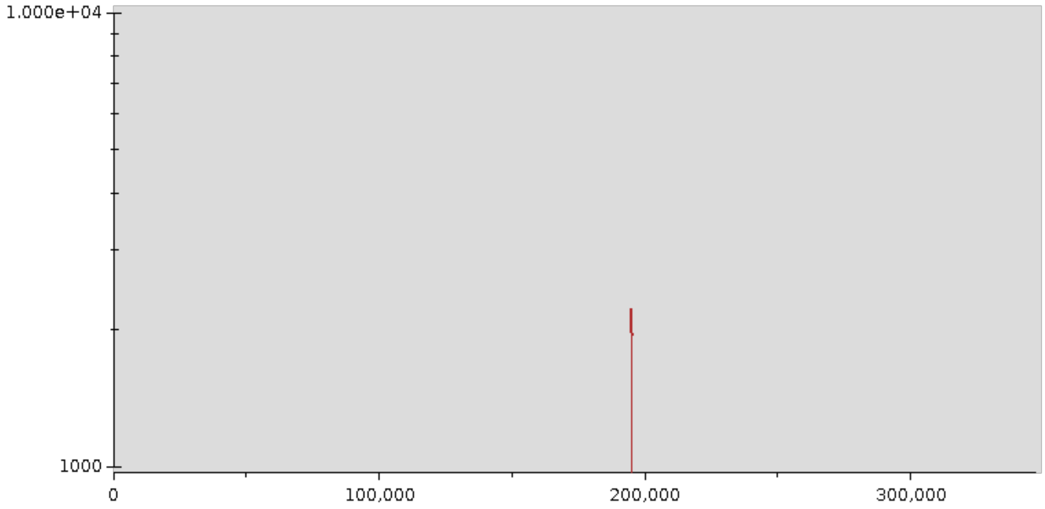

Assignment

**Type**	Lausannevirus (Taxonomy ID: 999883)
**Reference Genome**	NC_015326.1
**NT Identity (%)**	80.7947
**AA Identity (%)**	94.1176
**Number Of Stop Codons**	0
**Number Of CDS**	444

Alignment

**Alignment Score**	186.0 (NT) + 316.0 (AA) = 502.0
**Concordance (%)**	79.6825
**Alignment Method**	Local, heuristic, nucleotide (BLASTN)

Genome Region

Sequence starts at position 194954 and ends at position 195104 relative to NC_015326.1 reference sequence.

Alignment Detailed Statistics

	Begin	End	Coverage	Score	Concordance	Matches	Identities	I/D/M/F*	Stop Codons
**NT**	**194954**	**195104**	**0.1%**	**186**	**61.6%**	**151 (100%)**	**122 (80.8%)**	**0/0**	

Mutations: 194970G>T, 194973A>T, 194979T>C, 194988T>C, 194991G>A, 195000A>G, 195003T>A, 195006G>A, 195009G>A, 195012A>T, 195014G>A, 195015T>A, 195018T>C, 195021T>C, 195030A>T, 195033A>G, 195036G>C, 195045G>A, 195051A>G, 195054T>C, 195058T>G, 195059G>C, 195060C>T, 195063G>C, 195066G>A, 195069C>A, 195081C>T, 195090C>T, 195093T>C

*: Inserts / Deletes / Misaligned / Frameshifts

Analysis details

This analysis was performed with panviral2.64

NGS Details (UN8): Lausannevirus

Assembly

**Coverage Length**	130 (1 contig(s))
**Depth Of Coverage**	631.3
**Number Of Reads**	1319
**Reads Per Million**	22.04 rpm (after QC)
**Ambiguities**	0
**Assembly Method**	de novo + reference guided assembly
**Consensus Caller**	Bcf Tools

Coverage Map

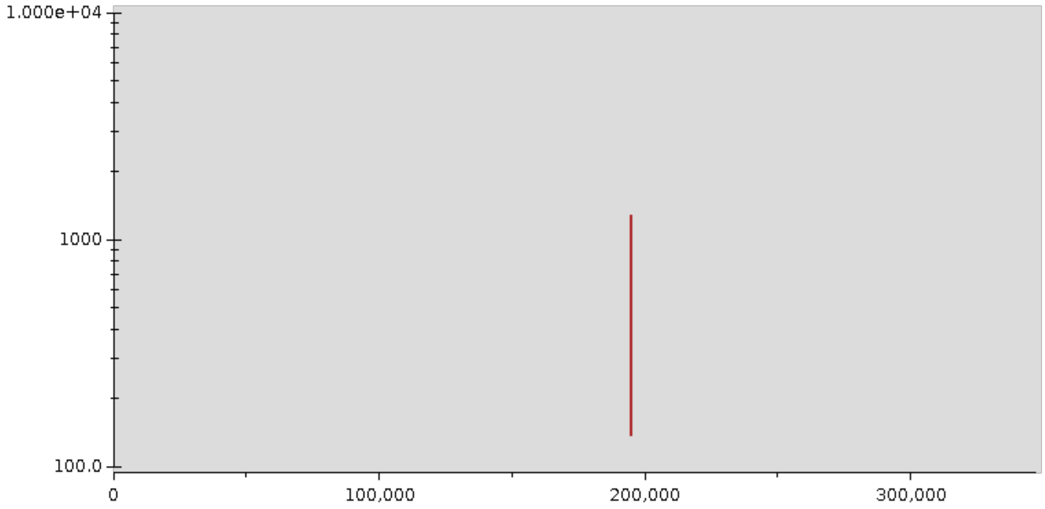

Assignment

**Type**	Lausannevirus (Taxonomy ID: 999883)
**Reference Genome**	NC_015326.1
**NT Identity (%)**	83.8462
**AA Identity (%)**	95.3488
**Number Of Stop Codons**	1
**Number Of CDS**	444

Alignment

**Alignment Score**	176.0 (NT) + 264.0 (AA) = 440.0
**Concordance (%)**	81.1808
**Alignment Method**	Local, heuristic, nucleotide (BLASTN)

Genome Region

Sequence starts at position 194915 and ends at position 195044 relative to NC_015326.1 reference sequence.

Alignment Detailed Statistics

	Begin	End	Coverage	Score	Concordance	Matches	Identities	I/D/M/F*	Stop Codons
NT	194915	195044	0.1%	176	67.7%	130 (100%)	109 (83.8%)	0/0	

Mutations: 194925T>C, 194931A>C, 194937G>A, 194943A>G, 194946G>C, 194949C>T, 194955T>C, 194967A>T, 194970G>A, 194979T>C, 194982C>T, 194987C>A, 194991G>C, 194993G>A, 195003T>A, 195006G>A, 195009G>A, 195015T>C, 195017G>T, 195021T>C, 195030A>G

*: Inserts / Deletes / Misaligned / Frameshifts

Analysis details

This analysis was performed with panviral2.64

NGS Details (UN8): Cladosporium fulvum T-1 virus

Assembly

**Coverage Length**	1429 (6 contig(s))
**Depth Of Coverage**	75.0
**Number Of Reads**	1081
**Reads Per Million**	18.07 rpm (after QC)
**Ambiguities**	0
**Assembly Method**	de novo + reference guided assembly
**Consensus Caller**	Bcf Tools

Coverage Map

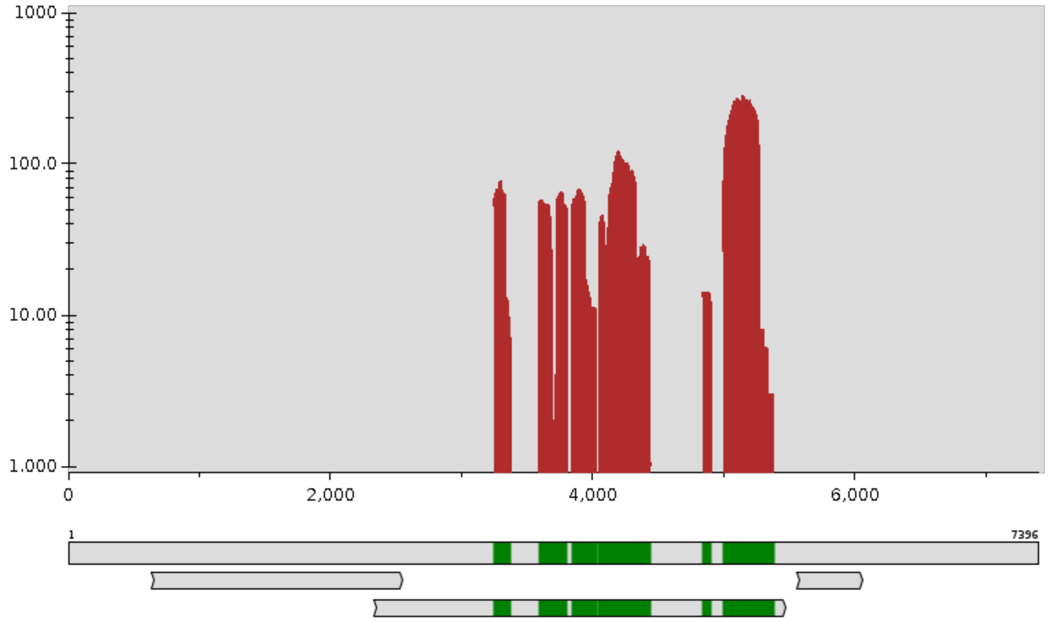

Assignment

**Type**	Cladosporium fulvum T-1 virus (Taxonomy ID: 2052899)
**Reference Genome**	NC_043491.1
**NT Identity (%)**	53.1825
**AA Identity (%)**	48.0932
**Number Of Stop Codons**	0
**Number Of CDS**	3

Alignment

**Alignment Score**	138.0 (NT) + 1641.0 (AA) = 1779.0
**Concordance (%)**	29.2647

Alignment Method	Global, seeded, nucleotide + amino acids (AGA)

Genome Region

Sequence starts at position 3246 and ends at position 5386 relative to NC_043491.1 reference sequence.

Alignment Detailed Statistics

	Begin	End	Coverage	Score	Concordance	Matches	Identities	I/D/M/F*	Stop Codons
**NT**	**3246**	**5386**	**19.3%**	**138**	**5.0%**	**1414 (99.0%)**	**752 (52.6%)**	**0/15**	
Mutations:	3247A>G, 3248C>G, 3249C>A, 3250A>G, 3251T>A, 3252G>T, 3253G>A, 3257C>T, 3258A>T, 3259C>T, 3260A>G, 3261A>G, 3262G>C, 3265A>T, 3266A>G, 3269A>T, 3271T>G, 3272C>T, 3273A>T, 3277A>T, 3279G>A, 3280G>C, 3282A>C, 3283A>C, 3284G>C, 3286G>A, 3289T>A, 3290C>A, 3291C>T, 3292A>C, 3294G>C, 3295G>C, 3296G>A, 3297G>T, 3298A>T, 3301C>T, 3303T>C, 3304A>G, 3309A>G, 3310A>T, 3314T>G, 3317G>C, 3318A>C, 3320A>G, 3321A>C, 3325G>A, 3326C>T, 3328A>G, 3329C>A, 3332A>G, 3333C>A, 3334C>G, 3338C>A, 3339G>A, 3343A>G, 3344T>C, 3345G>A, 3347C>T, 3349G>A, 3355G>T, 3356A>T, 3357A>T, 3359C>T, 3361A>G, 3363C>A, 3367A>G, 3370A>T, 3372G>T, 3373G>T, 3376A>T, 3381G>C, 3584C>A, 3586A>G, 3589G>A, 3598A>G, 3601A>T, 3604A>G, 3605T>C, 3606G>C, 3607G>T, 3610A>G, 3613C>T, 3614G>A, 3618T>G, 3619C>T, 3620A>G, 3621G>T, 3622G>A, 3625A>T, 3628A>G, 3631C>T, 3634A>G, 3636T>A, 3637C>A, 3640C>T, 3646C>T, 3647T>C, 3649G>T, 3652C>A, 3658A>T, 3659A>T, 3661G>T, 3664A>G, 3667G>A, 3670C>T, 3673C>T, 3676A>C, 3679C>G, 3682A>T, 3683T>A, 3687G>T, 3688C>T, 3689C>A, 3690A>T, 3692G>A, 3695C>T, 3697T>A, 3698G>A, 3700C>G, 3703C>T, 3704G>A, 3706A>G, 3707A>G, 3708C>T, 3710C>T, 3712T>C, 3714G>A, 3715A>T, 3716G>C, 3717A>C, 3719C>T, 3720T>A, 3721A>T, 3724C>T, 3728G>A, 3729T>A, 3732G>T, 3733C>T, 3736C>G, 3739T>A, 3741C>T, 3742T>C, 3748G>A, 3751C>T, 3757A>T, 3758C>G, 3760G>C, 3761G>A, 3763C>A, 3766C>T, 3768C>G, 3769A>T, 3770A>C, 3771A>G, 3774G>A, 3775A>T, 3776T>A, 3778C>G, 3779C>T, 3781C>G, 3782C>G, 3791A>G, 3792C>T, 3793C>G, 3796G>C, 3799A>T, 3800G>T, 3802T>G, 3803C>A, 3804A>G, 3805A>G, 3806G>A, 3807A>T, 3808T>G, 3838C>G, 3839A>T, 3841G>T, 3842A>G, 3843C>T, 3844A>T, 3845G>A, 3846C>A, 3848C>A, 3849C>T, 3850C>G, 3856A>G, 3859C>T, 3862A>G, 3865C>T, 3866C>T, 3867A>C, 3868C>G, 3869A>C, 3870A>T, 3871G>A, 3874A>G, 3877A>G, 3880C>G, 3886T>C, 3887T>C, 3893T>C, 3894T>A, 3898C>A, 3899A>G, 3901C>T, 3905A>G, 3906C>A, 3908A>G, 3909C>A, 3910A>C, 3913G>A, 3918C>G, 3922C>G, 3925C>T, 3929G>A, 3930C>A, 3931A>G, 3934G>A, 3935A>G, 3936C>T, 3937A>G, 3938C>G, 3940G>A, 3941T>G, 3943A>G, 3946C>T, 3948G>T, 3949A>G, 3952A>T, 3959G>A, 3960A>G, 3964G>C, 3970A>T, 3973C>G, 3975A>C, 3980G>C, 3982A>T, 3983C>A, 3984A>G, 3986T>A, 3987C>G, 3988A>T, 3992C>T, 3994T>A, 3997A>G, 3998C>T, 4000C>G, 4003C>A, 4009C>T, 4010A>T, 4025A>G, 4027G>T, 4029A>G, 4030C>A, 4033T>C, 4045A>T, 4052A>T, 4057G>T, 4058A>G, 4059T>A, 4060G>T, 4061C>T, 4063T>G, 4064A>C, 4065C>T, 4066A>G, 4068G>A, 4069A>G, 4072A>G, 4075C>G, 4081C>G, 4085A>G, 4086A>T, 4091G>T, 4092G>C, 4093A>C, 4094A>G, 4096A>G, 4100C>T, 4101A>G, 4102G>C, 4103A>G, 4104C>A, 4105C>A, 4106G>A, 4111G>T, 4115A>C, 4119G>A, 4120A>G, 4123C>G, 4129A>C, 4130C>G, 4131A>C, 4132G>T, 4133T>A, 4134G>T, 4135C>T, 4141A>T, 4143C>A, 4144C>A, 4149C>T, 4150G>A, 4153T>G, 4154C>A, 4155G>A, 4156A>G, 4159A>G, 4162C>T, 4165T>G, 4166G>C, 4167G>T, 4168T>A, 4169A>C, 4170G>C, 4171C>T, 4172A>T, 4173A>T, 4174G>T, 4177A>G, 4180C>A, 4184_4189delATCGAG, 4192C>T, 4195C>T, 4201T>G, 4204T>C, 4206T>A, 4207G>A, 4210A>T, 4211A>G, 4213A>G, 4216C>A, 4218C>G, 4219A>C, 4220T>G, 4221G>T, 4222T>A, 4223C>T, 4225A>G, 4226A>G, 4227C>T, 4228A>G, 4231G>A, 4233C>A, 4234A>T, 4235C>G, 4240T>A, 4243G>T, 4244_4249delAAAAGA, 4252C>T, 4255A>G, 4263A>T, 4265T>G, 4267T>A, 4268T>A, 4269C>G, 4271C>A, 4277A>T, 4279G>A, 4281C>A, 4282C>T, 4283A>G, 4284C>A, 4285A>T, 4288G>T, 4291A>G, 4296A>G, 4297C>G, 4301G>T, 4302A>C, 4303C>T, 4304A>G, 4305T>C, 4306C>A, 4312C>A, 4319C>A, 4321T>G, 4322C>G, 4324A>G, 4327C>A, 4328A>T, 4330T>G, 4331G>C, 4333T>C, 4334G>C, 4335C>A, 4336C>T, 4337G>T, 4338C>G, 4339C>T, 4340A>C, 4342G>T, 4346C>A, 4347A>C, 4348T>A, 4354A>G, 4360C>T, 4361G>C, 4364G>T, 4365A>T, 4366G>A, 4369C>A, 4370C>A, 4373_4375delCCG, 4381A>C, 4382A>G, 4383C>T, 4384G>A, 4385A>G, 4387T>A, 4388C>A, 4389T>A, 4390T>G, 4391T>A, 4393A>G, 4397C>A, 4399C>T, 4400A>G, 4401A>T, 4403A>G, 4404A>C, 4406C>A, 4407T>A, 4408C>T, 4411G>T, 4419C>A, 4420G>A, 4423G>T, 4424A>C, 4425C>A, 4426G>A, 4427A>C, 4430G>A, 4433C>T, 4435T>G, 4838A>G, 4840C>G, 4841A>C, 4845A>T, 4846A>T, 4849C>T, 4850G>C, 4852A>T, 4853C>G, 4855C>G, 4865A>G, 4870A>G, 4872C>T, 4873T>A, 4875C>A, 4876A>G, 4878G>A, 4881A>G, 4882C>A, 4883G>C, 4884C>A, 4885G>A, 4886A>C, 4887A>C, 4888A>C, 4889T>G, 4891C>A, 4894T>A, 4896A>T, 4897C>T, 4898C>T, 4901C>G, 4904T>C, 4995C>G, 4996C>G, 5002C>T, 5003A>T, 5004T>C, 5005G>C, 5008A>T, 5009C>T, 5010T>G, 5011A>G, 5014C>G, 5017G>T, 5020C>G, 5027C>T, 5029C>G, 5032A>T, 5035A>G, 5037A>C, 5038T>A, 5041A>G, 5047C>T, 5048A>C, 5055C>T, 5056A>G, 5057T>A, 5058C>G, 5059A>C, 5060G>A, 5061A>C, 5062A>T, 5064T>G, 5065A>C, 5070C>G, 5079A>G, 5080G>A, 5081C>T, 5083C>G, 5085G>C, 5086A>T, 5087T>A, 5088A>G, 5089C>A, 5090C>T, 5092C>G, 5093G>T, 5094T>A, 5095A>T, 5096C>G, 5099G>C, 5100A>G, 5101C>T, 5102A>G, 5103G>A, 5104A>G, 5105T>A, 5107G>T, 5108A>G, 5111C>A, 5115A>T, 5116T>G, 5119C>T, 5123T>G, 5125C>G, 5128G>T, 5130A>T, 5131A>T, 5132G>T, 5133T>C, 5134A>T, 5135T>A, 5137C>T, 5138A>G, 5140T>G, 5141A>T, 5143A>T, 5146C>T, 5147A>C, 5151A>G, 5154A>C, 5156C>G, 5158C>G, 5161C>T, 5164A>C, 5167A>G, 5168A>C, 5169A>G, 5170C>T, 5172A>T, 5178A>G, 5179G>A, 5180A>G, 5182G>C, 5183C>T, 5185C>A, 5186A>C, 5187T>A, 5189G>A, 5190G>A, 5191A>G, 5192A>G, 5193C>A, 5194G>T, 5195A>T, 5197T>G, 5200A>T, 5202T>C, 5205A>G, 5206G>A, 5207C>T, 5208A>T, 5209C>G, 5210A>G, 5212G>T, 5213T>A, 5216T>A, 5217C>G, 5218A>T, 5219A>T, 5226A>T, 5227C>T, 5233A>T, 5234G>C, 5239G>C, 5245G>C, 5248A>G, 5249A>T, 5251G>T, 5254A>G, 5257A>G, 5260G>C, 5262A>T, 5263C>T, 5266G>A, 5269A>T, 5272C>T, 5276C>G, 5278A>C, 5279T>A, 5280A>T, 5281C>G, 5282C>T, 5284A>G, 5285C>A, 5287G>A, 5288C>G, 5289A>C, 5290C>T, 5292A>G, 5294A>G, 5296C>G, 5298A>T, 5299C>G, 5300T>G, 5302C>A, 5303G>T, 5304C>T, 5305A>G, 5306C>A, 5310A>G, 5311C>G, 5313A>G, 5314C>T, 5319T>A, 5321T>G, 5322C>A, 5323A>T, 5324T>C, 5325T>A, 5326A>T, 5332A>C, 5338G>A, 5341G>A, 5342A>T, 5344C>T, 5345G>T, 5346C>G, 5347A>C, 5348C>T, 5349T>A, 5350G>C, 5352A>G, 5353C>T, 5354A>C, 5355A>T, 5356C>G, 5359C>T, 5362A>G, 5366G>T, 5367A>C, 5369A>G, 5374T>A, 5375T>A, 5376C>A, 5379C>T, 5382C>G, 5383G>T, 5384C>T, 5386A>G								
**CDS**									
homologue_of_retroviral_POL_genes	308	1020	45.6%	1641	49.0%	472 (99.0%)	227 (47.6%)	0/5/0/0	0

	Begin	End	Coverage	Score	Concordance	Matches	Identities	I/D/M/F*	Stop Codons
**NT**	**3246**	**5386**	**19.3%**	**138**	**5.0%**	**1414 (99.0%)**	**752 (52.6%)**	**0/15**	
Protein mutations:	P308E (3248C>G 3249C>A 3250A>G), W309I (3251T>A 3252G>T 3253G>A), H311F (3257C>T 3258A>T 3259C>T), K312G (3260A>G 3261A>G 3262G>C), N314D (3266A>G), I315L (3269A>T 3271T>G), Q316L (3272C>T 3273A>T), G318D (3279G>A 3280G>C), K319T (3282A>C 3283A>C), E320Q (3284G>C 3286G>A), P322I (3290C>A 3291C>T 3292A>C), W323S (3294G>C 3295G>C), G324I (3296G>A 3297G>T 3298A>T), L326P (3303T>C 3304A>G), Q328R (3309A>G 3310A>T), S330A (3314T>G), E331P (3317G>C 3318A>C), K332A (3320A>G 3321A>C), Q335K (3329C>A), T336E (3332A>G 3333C>A 3334C>G), R338K (3338C>A 3339G>A), W340Q (3344T>C 3345G>A), E343D (3355G>T), K344L (3356A>T 3357A>T), A346D (3363C>A), W349F (3372G>T 3373G>T), M421I (3589G>A), E425D (3601A>T), W427P (3605T>C 3606G>C 3607G>T), A430T (3614G>A), F431C (3618T>G 3619C>T), R432V (3620A>G 3621G>T 3622G>A), L437Q (3636T>A 3637C>A), M445F (3659A>T 3661G>T), S453T (3683T>A), C454F (3687G>T 3688C>T), Q455M (3689C>A 3690A>T), D456N (3692G>A), V458M (3698G>A 3700C>G), E460K (3704G>A 3706A>G), T461V (3707A>G 3708C>T), L462F (3710C>T 3712T>C), R463N (3714G>A 3715A>T), D464P (3716G>C 3717A>C), L465Y (3719C>T 3720T>A 3721A>T), V468K (3728G>A 3729T>A), C469F (3732G>T 3733C>T), A472V (3741C>T 3742T>C), M474I (3748G>A), L478V (3758C>G 3760G>C), V479I (3761G>A 3763C>A), T481S (3768C>G 3769A>T), K482R (3770A>C 3771A>G), G483D (3774G>A 3775A>T), S484T (3776T>A 3778C>G), Q486E (3782C>G), T489V (3791A>G 3792C>T 3793C>G), K490N (3796G>C), Q491H (3799A>T), V492L (3800G>T 3802T>G), Q493R (3803C>A 3804A>G 3805A>G), D494M (3806G>A 3807A>T 3808T>G), K505Y (3839A>T 3841G>T), T506V (3842A>G 3843C>T 3844A>T), A507K (3845G>A 3846C>A), P508M (3848C>A 3849C>T 3850C>G), H514S (3866C>T 3867A>C 3868C>G), K515L (3869A>C 3870A>T 3871G>A), F523H (3893T>C 3894T>A), I525V (3899A>G 3901C>T), T527E (3905A>G 3906C>A), T528D (3908A>G 3909C>A 3910A>C), T531R (3918C>G), I532M (3922C>G), A535K (3929G>A 3930C>A 3931A>G), T537V (3935A>G 3936C>T 3937A>G), Q538E (3938C>G 3940G>A), S539A (3941T>G 3943A>G), R541M (3948G>T 3949A>G), E542D (3952A>T), E545R (3959G>A 3960A>G), K550T (3975A>C), V552L (3980G>C 3982A>T), Q553R (3983C>A 3984A>G), N562Y (4010A>T), K567D (4025A>G 4027G>T), D568G (4029A>G 4030C>A), M576L (4052A>T), M578D (4058A>G 4059T>A 4060G>T), T580L (4064A>C 4065C>T 4066A>G), R581K (4068G>A 4069A>G), D583E (4075C>G), N585K (4081C>G), K587V (4085A>G 4086A>T), G589S (4091G>T 4092G>C 4093A>C), K590E (4094A>G 4096A>G), Q592C (4100C>T 4101A>G 4102G>C), T593E (4103A>G 4104C>A 4105C>A), E594K (4106G>A), K597Q (4115A>C), R598K (4119G>A 4120A>G), E601D (4129A>C), Q602A (4130C>G 4131A>C 4132G>T), C603I (4133T>A 4134G>T 4135C>T), A606E (4143C>A 4144C>A), T608I (4149C>T 4150G>A), R610K (4154C>A 4155G>A 4156A>G), D613E (4165T>G), G614L (4166G>C 4167G>T 4168T>A), S615P (4169A>C 4170G>C 4171C>T), K616F (4172A>T 4173A>T 4174G>T), I620_E621del (4184_4189delATCGAG), M627K (4206T>A 4207G>A), I629V (4211A>G 4213A>G), A631G (4218C>G 4219A>C), C632V (4220T>G 4221G>T 4222T>A), T634V (4226A>G 4227C>T 4228A>G), T636N (4233C>A 4234A>T), H637D (4235C>G), D638E (4240T>A), K640_R641del (4244_4249delAAAAGA), Y646F (4263A>T), Y647E (4265T>G 4267T>A), M651L (4277A>T 4279G>A), T652N (4281C>A 4282C>T), T653D (4283A>G 4284C>A 4285A>T), N657R (4296A>G 4297C>G), D659S (4301G>T 4302A>C 4303C>T), I660A (4304A>G 4305T>C 4306C>A), D662E (4312C>A), L665M (4319C>A 4321T>G), L666V (4322C>G 4324A>G), I668L (4328A>T 4330T>G), V669L (4331G>C 4333T>C), A670H (4334G>C 4335C>A 4336C>T), A671C (4337G>T 4338C>G 4339C>T), M672L (4340A>C 4342G>T), H674T (4346C>A 4347A>C 4348T>A), V679L (4361G>C), E680L (4364G>T 4365A>T 4366G>A), P682T (4370C>A), P683del (4373_4375delCCG), L685F (4381A>C), T686V (4382A>G 4383C>T 4384G>A), I687V (4385A>G 4387T>A), L688K (4388C>A 4389T>A 4390T>G), S689T (4391T>A 4393A>G), H691N (4397C>A 4399C>T), K692V (4400A>G 4401A>T), N693A (4403A>G 4404A>C), L694N (4406C>A 4407T>A 4408C>T), T698K (4419C>A 4420G>A), T700Q (4424A>C 4425C>A 4426G>A), K701Q (4427A>C), E702K (4430G>A), I838V (4838A>G 4840C>G), K839Q (4841A>C), K840I (4845A>T 4846A>T), V842L (4850G>C 4852A>T), H843E (4853C>G 4855C>G), N847D (4865A>G), A849V (4872C>T 4873T>A), A850E (4875C>A 4876A>G), R851Q (4878G>A), H852R (4881A>G 4882C>A), A853Q (4883G>C 4884C>A 4885G>A), K854P (4886A>C 4887A>C 4888A>C), Y855E (4889T>G 4891C>A), H857L (4896A>T 4897C>T), Q859E (4901C>G), F860L (4904T>C), A890G (4995C>G 4996C>G), M893S (5003A>T 5004T>C 5005G>C), L895W (5009C>T 5010T>G 5011A>G), M897I (5017G>T), Y904S (5037A>C 5038T>A), I908L (5048A>C), A910V (5055C>T 5056A>G), E912T (5060G>A 5061A>C 5062A>T), I913S (5064T>G 5065A>C), T915S (5070C>G), Q918R (5079A>G 5080G>A), G920A (5085G>C 5086A>T), Y921R (5087T>A 5088A>G 5089C>A), V923Y (5093G>T 5094T>A 5095A>T), L924V (5096C>G), D925R (5099G>C 5100A>G 5101C>T), R926E (5102A>G 5103G>A 5104A>G), L927I (5105T>A 5107G>T), I928V (5108A>G), Y930L (5115A>T 5116T>G), F933V (5123T>G 5125C>G), E935V (5130A>T 5131A>T), V936S (5132G>T 5133T>C 5134A>T), F937I (5135T>A 5137C>T), I938V (5138A>G 5140T>G), T939S (5141A>T 5143A>T), D942G (5151A>G), K943T (5154A>C), L944V (5156C>G 5158C>G), N948R (5168A>C 5169A>G 5170C>T), Y949F (5172A>T), K951R (5178A>G 5179G>A), T952A (5180A>G 5182G>C), M954Q (5186A>C 5187T>A), G955K (5189G>A 5190G>A 5191A>G), T956D (5192A>G 5193C>A 5194G>T), I957L (5195A>T 5197T>G), I959T (5202T>C), K960R (5205A>G 5206G>A), H961L (5207C>T 5208A>T 5209C>G), K962D (5210A>G 5212G>T), L963M (5213T>A), T965S (5219A>T), Y967F (5226A>T 5227C>T), E970Q (5234G>C), T975S (5249A>T 5251G>T), N979I (5262A>T 5263C>T), Q984D (5276C>G 5278A>C), Y985M (5279T>A 5280A>T 5281C>G), H988A (5288C>G 5289A>C 5290C>T), Y989C (5292A>G), I990V (5294A>G 5296C>G), N991M (5298A>T 5299C>G), Y992E (5300T>G 5302C>A), A993L (5303G>T 5304C>T 5305A>G), Q994K (5306C>A), D995G (5310A>G 5311C>G), N996S (5313A>G 5314C>T), V998D (5319T>A), S999D (5321T>G 5322C>A 5323A>T), L1000H (5324T>C 5325T>A 5326A>T), I1006F (5342A>T 5344C>T), A1007C (5345G>T 5346C>G 5347A>C), L1008Y (5348C>T 5349T>A 5350G>C), N1009S (5352A>G 5353C>T), N1010L (5354A>C 5355A>T 5356C>G), E1014S (5366G>T 5367A>C), T1015A (5369A>G), S1017K (5375T>A 5376C>A), T1018M (5379C>T), T1019S (5382C>G 5383G>T), P1020S (5384C>T 5386A>G)								

	Begin	End	Coverage	Score	Concordance	Matches	Identities	I/D/M/F*	Stop Codons
**NT**	**3246**	**5386**	**19.3%**	**138**	**5.0%**	**1414 (99.0%)**	**752 (52.6%)**	**0/15**	
Codon mutations:	CAA307.AG (3247A>G), CCA308GAG (3248C>G 3249C>A 3250A>G), TGG309ATA (3251T>A 3252G>T 3253G>A), CAC311TTT (3257C>T 3258A>T 3259C>T), AAG312GGC (3260A>G 3261A>G 3262G>C), ATA313ATT (3265A>T), AAC314GAC (3266A>G), ATT315TTG (3269A>T 3271T>G), CAG316TTG (3272C>T 3273A>T), CCA317CCT (3277A>T), GGG318GAC (3279G>A 3280G>C), AAA319ACC (3282A>C 3283A>C), GAG320CAA (3284G>C 3286G>A), CCT321CCA (3289T>A), CCA322ATC (3290C>A 3291C>T 3292A>C), TGG323TCC (3294G>C 3295G>C), GGA324ATT (3296G>A 3297G>T 3298A>T), CCC325CCT (3301C>T), CTA326CCG (3303T>C 3304A>G), CAA328CGT (3309A>G 3310A>T), TCT330GCT (3314T>G), GAG331CCG (3317G>C 3318A>C), AAA332GCA (3320A>G 3321A>C), GAG333GAA (3325G>A), CTA334TTG (3326C>T 3328A>G), CAG335AAG (3329C>A), ACC336GAG (3332A>G 3333C>A 3334C>G), CGA338AAA (3338C>A 3339G>A), GAA339GAG (3343A>G), TGG340CAG (3344T>C 3345G>A), CTG341TTA (3347C>T 3349G>A), GAG343GAT (3355G>T), AAG344TTG (3356A>T 3357A>T), CTA345TTG (3359C>T 3361A>G), GCC346GAC (3363C>A), AAA347AAG (3367A>G), GGA348GGT (3370A>T), TGG349TTT (3372G>T 3373G>T), ATA350ATT (3376A>T), CGA352CC. (3381G>C), CGA420AGG (3584C>A 3586A>G), ATG421ATA (3589G>A), GGA424GGG (3598A>G), GAA425GAT (3601A>T), GAA426GAG (3604A>G), TGG427CCT (3605T>C 3606G>C 3607G>T), AAA428AAG (3610A>G), ACC429ACT (3613C>T), GCT430ACT (3614G>A), TTC431TGT (3618T>G 3619C>T), AGG432GTA (3620A>G 3621G>T 3622G>A), ACA433ACT (3625A>T), AGA434AGG (3628A>G), TAC435TAT (3631C>T), GGA436GGG (3634A>G), CTC437CAA (3636T>A 3637C>A), TAC438TAT (3640C>T), TTC440TTT (3646C>T), TTG441CTT (3647T>C 3649G>T), GTC442GTA (3652C>A), CCA444CCT (3658A>T), ATG445TTT (3659A>T 3661G>T), GGA446GGG (3664A>G), TTG447TTA (3667G>A), ACC448ACT (3670C>T), AAC449AAT (3673C>T), GCA450GCC (3676A>C), CCC451CCG (3679C>G), GCA452GCT (3682A>T), TCC453ACC (3683T>A), TGC454TTT (3687G>T 3688C>T), CAG455ATG (3689C>A 3690A>T), GAC456AAC (3692G>A), CTT457TTA (3695C>T 3697T>A), GTC458ATG (3698G>A 3700C>G), AAC459AAT (3703C>T), GAA460AAG (3704G>A 3706A>G), ACA461GTA (3707A>G 3708C>T), CTT462TTC (3710C>T 3712T>C), AGA463AAT (3714G>A 3715A>T), GAC464CCC (3716G>C 3717A>C), CTA465TAT (3719C>T 3720T>A 3721A>T), CTC466CTT (3724C>T), GTG468AAG (3728G>A 3729T>A), TGC469TTT (3732G>T 3733C>T), GTC470GTG (3736C>G), GTT471GTA (3739T>A), GCT472GTC (3741C>T 3742T>C), ATG474ATA (3748G>A), GAC475GAT (3751C>T), ATA477ATT (3757A>T), CTG478GTC (3758C>G 3760G>C), GTC479ATA (3761G>A 3763C>A), TAC480TAT (3766C>T), ACA481AGT (3768C>G 3769A>T), AAA482CGA (3770A>C 3771A>G), GGA483GAT (3774G>A 3775A>T), TCC484ACG (3776T>A 3778C>G), CTC485TTG (3779C>T 3781C>G), CAG486GAG (3782C>G), ACC489GTG (3791A>G 3792C>T 3793C>G), AAG490AAC (3796G>C), CAA491CAT (3799A>T), GTT492TTG (3800G>T 3802T>G), CAA493AGG (3803C>A 3804A>G 3805A>G), GAT494ATG (3806G>A 3807A>T 3808T>G), TTC504..G (3838C>G), AAG505TAT (3839A>T 3841G>T), ACA506GTT (3842A>G 3843C>T 3844A>T), GCA507AAA (3845G>A 3846C>A), CCC508ATG (3848C>A 3849C>T 3850C>G), AAA510AAG (3856A>G), TGC511TGT (3859C>T), GAA512GAG (3862A>G), TTC513TTT (3865C>T), CAC514TCG (3866C>T 3867A>C 3868C>G), AAG515CTA (3869A>C 3870A>T 3871G>A), AAA516AAG (3874A>G), GAA517GAG (3877A>G), GTC518GTG (3880C>G), TTT520TTC (3886T>C), TTA521CTA (3887T>C), TTT523CAT (3893T>C 3894T>A), ATC524ATA (3898C>A), ATC525GTT (3899A>G 3901C>T), ACA527GAA (3905A>G 3906C>A), ACA528GAC (3908A>G 3909C>A 3910A>C), GGG529GGA (3913G>A), ACG531AGG (3918C>G), ATC532ATG (3922C>G), GAC533GAT (3925C>T), GCA535AAG (3929G>A 3930C>A 3931A>G), AAG536AAA (3934G>A), ACA537GTG (3935A>G 3936C>T 3937A>G), CAG538GAA (3938C>G 3940G>A), TCA539GCG (3941T>G 3943A>G), ATC540ATT (3946C>T), AGA541ATG (3948G>T 3949A>G), GAA542GAT (3952A>T), GAA545AGA (3959G>A 3960A>G), CCG546CCC (3964G>C), ACA548ACT (3970A>T), GTC549GTG (3973C>G), AAG550ACG (3975A>C), GTA552CTT (3980G>C 3982A>T), CAG553AGG (3983C>A 3984A>G), TCA554AGT (3986T>A 3987C>G 3988A>T), CTT556TTA (3992C>T 3994T>A), GGA557GGG (3997A>G), CTC558TTG (3998C>T 4000C>G), GCC559GCA (4003C>A), TAC561TAT (4009C>T), AAC562TAC (4010A>T), AAG567GAT (4025A>G 4027G>T), GAC568GGA (4029A>G 4030C>A), TAT569TAC (4033T>C), GCA573.CT (4045A>T), ATG576TTG (4052A>T), ACG577ACT (4057G>T), ATG578GAT (4058A>G 4059T>A 4060G>T), CTT579TTG (4061C>T 4063T>G), ACA580CTG (4064A>C 4065C>T 4066A>G), AGA581AAG (4068G>A 4069A>G), AAA582AAG (4072A>G), GAC583GAG (4075C>G), AAC585AAG (4081C>G), AAA587GTA (4085A>G 4086A>T), GGA589TCC (4091G>T 4092G>C 4093A>C), AAA590GAG (4094A>G 4096A>G), CAG592TGC (4100C>T 4101A>G 4102G>C), ACC593GAA (4103A>G 4104C>A 4105C>A), GAA594AAA (4106G>A), GCG595GCT (4111G>T), AAA597CAA (4115A>C), AGA598AAG (4119G>A 4120A>G), CTC599CTG (4123C>G), GAA601GAC (4129A>C), CAG602GCT (4130C>G 4131A>C 4132G>T), TGC603ATT (4133T>A 4134G>T 4135C>T), TCA605TCT (4141A>T), GCC606GAA (4143C>A 4144C>A), ACG608ATA (4149C>T 4150G>A), CTT609CTG (4153T>G), CGA610AAG (4154C>A 4155G>A 4156A>G), CTA611CTG (4159A>G), TTC612TTT (4162C>T), GAT613GAG (4165T>G), GGT614CTA (4166G>C 4167G>T 4168T>A), AGC615CCT (4169A>C 4170G>C 4171C>T), AAG616TTT (4172A>T 4173A>T 4174G>T), GAA617GAG (4177A>G), GTC618GTA (4180C>A), ATC620_GAG621del (4184_4189delATCGAG), ACC622ACT (4192C>T), GAC623GAT (4195C>T), TCT625TCG (4201T>G), GAT626GAC (4204T>C), ATG627AAA (4206T>A 4207G>A), GCA628GCT (4210A>T), ATA629GTG (4211A>G 4213A>G), GGC630GGA (4216C>A), GCA631GGC (4218C>G 4219A>C), TGT632GTA (4220T>G 4221G>T 4222T>A), CTA633TTG (4223C>T 4225A>G), ACA634GTG (4226A>G 4227C>T 4228A>G), CAG635CAA (4231G>A), ACA636AAT (4233C>A 4234A>T), CAC637GAC (4235C>G), GAT638GAA (4240T>A), GGG639GGT (4243G>T), AAA640_AGA641del (4244_4249delAAAAGA), CAC642CAT (4252C>T), CCA643CCG (4255A>G), TAT646TTT (4263A>T), TAT647GAA (4265T>G 4267T>A), TCC648AGC (4268T>A 4269C>G), CGG649AGG (4271C>A), ATG651TTA (4277A>T 4279G>A), ACC652AAT (4281C>A 4282C>T), ACA653GAT (4283A>G 4284C>A 4285A>T), GCG654GCT (4288G>T), GAA655GAG (4291A>G), AAC657AGG (4296A>G 4297C>G), GAC659TCT (4301G>T 4302A>C 4303C>T), ATC660GCA (4304A>G 4305T>C 4306C>A), GAC662GAA (4312C>A), CTT665ATG (4319C>A 4321T>G), CTA666GTG (4322C>G 4324A>G), GCC667GCA (4327C>A), ATT668TTG (4328A>T 4330T>G), GTT669CTC (4331G>C 4333T>C), GCC670CAT (4334G>C 4335C>A 4336C>T), GCC671TGT (4337G>T 4338C>G 4339C>T), ATG672CTT (4340A>C 4342G>T), CAT674ACA (4346C>A 4347A>C 4348T>A), AGA676AGG (4354A>G), TAC678TAT (4360C>T), GTC679CTC (4361G>C), GAG680TTA (4364G>T 4365A>T 4366G>A), GGC681GGA (4369C>A), CCA682ACA (4370C>A), CCG683del (4373_4375delCCG), TTA685TTC (4381A>C), ACG686GTA (4382A>G 4383C>T 4384G>A), ATT687GTA (4385A>G 4387T>A), CTT688AAG (4388C>A 4389T>A 4390T>G), TCA689ACG (4391T>A 4393A>G), CAC691AAT (4397C>A 4399C>T), AAG692GTG (4400A>G 4401A>T), AAT693GCT (4403A>G 4404A>C), CTC694AAT (4406C>A 4407T>A 4408C>T), ACG695ACT (4411G>T), ACG698AAA (4419C>A 4420G>A), ACG699ACT (4423G>T), ACG700CAA (4424A>C 4425C>A 4426G>A), AAG701CAG (4427A>C), GAA702AAA (4430G>A), CTT703TTG (4433C>T 4435T>G), ATC838GTG (4838A>G 4840C>G), AAG839CAG (4841A>C), AAA840ATT (4845A>T 4846A>T), TGC841TGT (4849C>T), GTA842CTT (4850G>C 4852A>T), CAC843GAG (4853C>G 4855C>G), AAC847GAC (4865A>G), AAA848AAG (4870A>G), GCT849GTA (4872C>T 4873T>A), GCA850GAG (4875C>A 4876A>G), CGG851CAG (4878G>A), CAC852CGA (4881A>G 4882C>A), GCG853CAA (4883G>C 4884C>A 4885G>A), AAA854CCC (4886A>C 4887A>C 4888A>C), TAC855GAA (4889T>G 4891C>A), GGT856GGA (4894T>A), CAC857CTT (4896A>T 4897C>T), CTA858TTA (4898C>T), CAG859GAG (4901C>G), TTC860CTC (4904T>C), GCC890GGG (4995C>G 4996C>G), GAC892GAT (5002C>T), ATG893TCC (5003A>T 5004T>C 5005G>C), ATA894ATT (5008A>T), CTA895TGG (5009C>T 5010T>G 5011A>G), GTC896GTG (5014C>G), ATG897ATT (5017G>T), GTC898GTG (5020C>G), CTC901TTG (5027C>T 5029C>G), ACA902ACT (5032A>T), AAA903AAG (5035A>G), TAT904TCA (5037A>C 5038T>A), GCA905GCG (5041A>G), TTC907TTT (5047C>T), ATT908CTT (5048A>C), GCA910GTG (5055C>T 5056A>G), TCA911AGC (5057T>A 5058C>G 5059A>C), GAA912ACT (5060G>A 5061A>C 5062A>T), ATA913AGC (5064T>G 5065A>C), ACT915AGT (5070C>G), CAG918CGA (5079A>G 5080G>A), CTC919TTG (5081C>T 5083C>G), GGA920GCT (5085G>C 5086A>T), TAC921AGA (5087T>A 5088A>G 5089C>A), CTC922TTG (5090C>T 5092C>G), GTA923TAT (5093G>T 5094T>A 5095A>T), CTG924GTG (5096C>G), GAC925CGT (5099G>C 5100A>G 5101C>T), AGA926GAG (5102A>G 5103G>A 5104A>G), TTG927ATT (5105T>A 5107G>T), ATC928GTC (5108A>G), CGA929AGA (5111C>A), TAT930TTG (5115A>T 5116T>G), CAC931CAT (5119C>T), TTC933GTG (5123T>G 5125C>G), CCG934CCT (5128G>T), GAA935GTT (5130A>T 5131A>T), GTA936TCT (5132G>T 5133T>C 5134A>T), TTC937ATT (5135T>A 5137C>T), ATT938GTG (5138A>G 5140T>G), ACA939TCT (5141A>T 5143A>T), GAC940GAT (5146C>T), AGA941CGA (5147A>C), GAC942GGC (5151A>G), AAG943ACG (5154A>C), CTC944GTG (5156C>G 5158C>G), TTC945TTT (5161C>T), ACA946ACC (5164A>C), TCA947TCG (5167A>G), AAC948CGT (5168A>C 5169A>G 5170C>T), TAC949TTC (5172A>T), AAG951AGA (5178A>G 5179G>A), ACG952GCC (5180A>G 5182G>C), CTC953TTA (5183C>T 5185C>A), ATG954CAG (5186A>C 5187T>A), GGA955AAG (5189G>A 5190G>A 5191A>G), ACG956GAT (5192A>G 5193C>A 5194G>T), ATT957TTG (5195A>T 5197T>G), GGA958GGT (5200A>T), ATC959ACC (5202T>C), AAG960AGA (5205A>G 5206G>A), CAC961TTG (5207C>T 5208A>T 5209C>G), AAG962GAT (5210A>G 5212G>T), TTG963ATG (5213T>A), TCA964AGT (5216T>A 5217C>G 5218A>T), ACA965TCA (5219A>T), TAC967TTT (5226A>T 5227C>T), CCA969CCT (5233A>T), GAG970CAG (5234G>C), ACG971ACC (5239G>C), GGG973GGC (5245G>C), CAA974CAG (5248A>G), ACG975TCT (5249A>T 5251G>T), GAA976GAG (5254A>G), AGA977AGG (5257A>G), ACG978ACC (5260G>C), AAC979ATT (5262A>T 5263C>T), CAG980CAA (5266G>A), ACA981ACT (5269A>T), CTC982CTT (5272C>T), CAA984GAC (5276C>G 5278A>C), TAC985ATG (5279T>A 5280A>T 5281C>G), CTA986TTG (5282C>T 5284A>G), CGG987AGA (5285C>A 5287G>A), CAC988GCT (5288C>G 5289A>C 5290C>T), TAC989TGC (5292A>G), ATC990GTG (5294A>G 5296C>G), AAC991ATG (5298A>T 5299C>G), TAC992GAA (5300T>G 5302C>A), GCA993TTG (5303G>T 5304C>T 5305A>G), CAA994AAA (5306C>A), GAC995GGG (5310A>G 5311C>G), AAC996AGT (5313A>G 5314C>T), GTT998GAT (5319T>A), TCA999GAT (5321T>G 5322C>A 5323A>T), TTA1000CAT (5324T>C 5325T>A 5326A>T), CCA1002CCC (5332A>C), GCG1004GCA (5338G>A), CAG1005CAA (5341G>A), ATC1006TTT (5342A>T 5344C>T), GCA1007TGC (5345G>T 5346C>G 5347A>C), CTG1008TAC (5348C>T 5349T>A 5350G>C), AAC1009AGT (5352A>G 5353C>T), AAC1010CTG (5354A>C 5355A>T 5356C>G), CAC1011CAT (5359C>T), AAA1012AAG (5362A>G), GAG1014TCG (5366G>T 5367A>C), ACA1015GCA (5369A>G), ACT1016ACA (5374T>A), TCG1017AAG (5375T>A 5376C>A), ACG1018ATG (5379C>T), ACG1019AGT (5382C>G 5383G>T), CCA1020TCG (5384C>T 5386A>G)								
**Proteins**									
Reverse Transcriptase (YP_009666308.1)	308	1020	45.6%	1641	49.0%	472 (99.0%)	227 (47.6%)	0/5/0/0	0

	Begin	End	Coverage	Score	Concordance	Matches	Identities	I/D/M/F*	Stop Codons
**NT**	**3246**	**5386**	**19.3%**	**138**	**5.0%**	**1414 (99.0%)**	**752 (52.6%)**	**0/15**	
Protein mutations:	P308E (3248C>G 3249C>A 3250A>G), W309I (3251T>A 3252G>T 3253G>A), H311F (3257C>T 3258A>T 3259C>T), K312G (3260A>G 3261A>G 3262G>C), N314D (3266A>G), I315L (3269A>T 3271T>G), Q316L (3272C>T 3273A>T), G318D (3279G>A 3280G>C), K319T (3282A>C 3283A>C), E320Q (3284G>C 3286G>A), P322I (3290C>A 3291C>T 3292A>C), W323S (3294G>C 3295G>C), G324I (3296G>A 3297G>T 3298A>T), L326P (3303T>C 3304A>G), Q328R (3309A>G 3310A>T), S330A (3314T>G), E331P (3317G>C 3318A>C), K332A (3320A>G 3321A>C), Q335K (3329C>A), T336E (3332A>G 3333C>A 3334C>G), R338K (3338C>A 3339G>A), W340Q (3344T>C 3345G>A), E343D (3355G>T), K344L (3356A>T 3357A>T), A346D (3363C>A), W349F (3372G>T 3373G>T), M421I (3589G>A), E425D (3601A>T), W427P (3605T>C 3606G>C 3607G>T), A430T (3614G>A), F431C (3618T>G 3619C>T), R432V (3620A>G 3621G>T 3622G>A), L437Q (3636T>A 3637C>A), M445F (3659A>T 3661G>T), S453T (3683T>A), C454F (3687G>T 3688C>T), Q455M (3689C>A 3690A>T), D456N (3692G>A), V458M (3698G>A 3700C>G), E460K (3704G>A 3706A>G), T461V (3707A>G 3708C>T), L462F (3710C>T 3712T>C), R463N (3714G>A 3715A>T), D464P (3716G>C 3717A>C), L465Y (3719C>T 3720T>A 3721A>T), V468K (3728G>A 3729T>A), C469F (3732G>T 3733C>T), A472V (3741C>T 3742T>C), M474I (3748G>A), L478V (3758C>G 3760G>C), V479I (3761G>A 3763C>A), T481S (3768C>G 3769A>T), K482R (3770A>C 3771A>G), G483D (3774G>A 3775A>T), S484T (3776T>A 3778C>G), Q486E (3782C>G), T489V (3791A>G 3792C>T 3793C>G), K490N (3796G>C), Q491H (3799A>T), V492L (3800G>T 3802T>G), Q493R (3803C>A 3804A>G 3805A>G), D494M (3806G>A 3807A>T 3808T>G), K505Y (3839A>T 3841G>T), T506V (3842A>G 3843C>T 3844A>T), A507K (3845G>A 3846C>A), P508M (3848C>A 3849C>T 3850C>G), H514S (3866C>T 3867A>C 3868C>G), K515L (3869A>C 3870A>T 3871G>A), F523H (3893T>C 3894T>A), I525V (3899A>G 3901C>T), T527E (3905A>G 3906C>A), T528D (3908A>G 3909C>A 3910A>C), T531R (3918C>G), I532M (3922C>G), A535K (3929G>A 3930C>A 3931A>G), T537V (3935A>G 3936C>T 3937A>G), Q538E (3938C>G 3940G>A), S539A (3941T>G 3943A>G), R541M (3948G>T 3949A>G), E542D (3952A>T), E545R (3959G>A 3960A>G), K550T (3975A>C), V552L (3980G>C 3982A>T), Q553R (3983C>A 3984A>G), N562Y (4010A>T), K567D (4025A>G 4027G>T), D568G (4029A>G 4030C>A), M576L (4052A>T), M578D (4058A>G 4059T>A 4060G>T), T580L (4064A>C 4065C>T 4066A>G), R581K (4068G>A 4069A>G), D583E (4075C>G), N585K (4081C>G), K587V (4085A>G 4086A>T), G589S (4091G>T 4092G>C 4093A>C), K590E (4094A>G 4096A>G), Q592C (4100C>T 4101A>G 4102G>C), T593E (4103A>G 4104C>A 4105C>A), E594K (4106G>A), K597Q (4115A>C), R598K (4119G>A 4120A>G), E601D (4129A>C), Q602A (4130C>G 4131A>C 4132G>T), C603I (4133T>A 4134G>T 4135C>T), A606E (4143C>A 4144C>A), T608I (4149C>T 4150G>A), R610K (4154C>A 4155G>A 4156A>G), D613E (4165T>G), G614L (4166G>C 4167G>T 4168T>A), S615P (4169A>C 4170G>C 4171C>T), K616F (4172A>T 4173A>T 4174G>T), I620_E621del (4184_4189delATCGAG), M627K (4206T>A 4207G>A), I629V (4211A>G 4213A>G), A631G (4218C>G 4219A>C), C632V (4220T>G 4221G>T 4222T>A), T634V (4226A>G 4227C>T 4228A>G), T636N (4233C>A 4234A>T), H637D (4235C>G), D638E (4240T>A), K640_R641del (4244_4249delAAAAGA), Y646F (4263A>T), Y647E (4265T>G 4267T>A), M651L (4277A>T 4279G>A), T652N (4281C>A 4282C>T), T653D (4283A>G 4284C>A 4285A>T), N657R (4296A>G 4297C>G), D659S (4301G>T 4302A>C 4303C>T), I660A (4304A>G 4305T>C 4306C>A), D662E (4312C>A), L665M (4319C>A 4321T>G), L666V (4322C>G 4324A>G), I668L (4328A>T 4330T>G), V669L (4331G>C 4333T>C), A670H (4334G>C 4335C>A 4336C>T), A671C (4337G>T 4338C>G 4339C>T), M672L (4340A>C 4342G>T), H674T (4346C>A 4347A>C 4348T>A), V679L (4361G>C), E680L (4364G>T 4365A>T 4366G>A), P682T (4370C>A), P683del (4373_4375delCCG), L685F (4381A>C), T686V (4382A>G 4383C>T 4384G>A), I687V (4385A>G 4387T>A), L688K (4388C>A 4389T>A 4390T>G), S689T (4391T>A 4393A>G), H691N (4397C>A 4399C>T), K692V (4400A>G 4401A>T), N693A (4403A>G 4404A>C), L694N (4406C>A 4407T>A 4408C>T), T698K (4419C>A 4420G>A), T700Q (4424A>C 4425C>A 4426G>A), K701Q (4427A>C), E702K (4430G>A), I838V (4838A>G 4840C>G), K839Q (4841A>C), K840I (4845A>T 4846A>T), V842L (4850G>C 4852A>T), H843E (4853C>G 4855C>G), N847D (4865A>G), A849V (4872C>T 4873T>A), A850E (4875C>A 4876A>G), R851Q (4878G>A), H852R (4881A>G 4882C>A), A853Q (4883G>C 4884C>A 4885G>A), K854P (4886A>C 4887A>C 4888A>C), Y855E (4889T>G 4891C>A), H857L (4896A>T 4897C>T), Q859E (4901C>G), F860L (4904T>C), A890G (4995C>G 4996C>G), M893S (5003A>T 5004T>C 5005G>C), L895W (5009C>T 5010T>G 5011A>G), M897I (5017G>T), Y904S (5037A>C 5038T>A), I908L (5048A>C), A910V (5055C>T 5056A>G), E912T (5060G>A 5061A>C 5062A>T), I913S (5064T>G 5065A>C), T915S (5070C>G), Q918R (5079A>G 5080G>A), G920A (5085G>C 5086A>T), Y921R (5087T>A 5088A>G 5089C>A), V923Y (5093G>T 5094T>A 5095A>T), L924V (5096C>G), D925R (5099G>C 5100A>G 5101C>T), R926E (5102A>G 5103G>A 5104A>G), L927I (5105T>A 5107G>T), I928V (5108A>G), Y930L (5115A>T 5116T>G), F933V (5123T>G 5125C>G), E935V (5130A>T 5131A>T), V936S (5132G>T 5133T>C 5134A>T), F937I (5135T>A 5137C>T), I938V (5138A>G 5140T>G), T939S (5141A>T 5143A>T), D942G (5151A>G), K943T (5154A>C), L944V (5156C>G 5158C>G), N948R (5168A>C 5169A>G 5170C>T), Y949F (5172A>T), K951R (5178A>G 5179G>A), T952A (5180A>G 5182G>C), M954Q (5186A>C 5187T>A), G955K (5189G>A 5190G>A 5191A>G), T956D (5192A>G 5193C>A 5194G>T), I957L (5195A>T 5197T>G), I959T (5202T>C), K960R (5205A>G 5206G>A), H961L (5207C>T 5208A>T 5209C>G), K962D (5210A>G 5212G>T), L963M (5213T>A), T965S (5219A>T), Y967F (5226A>T 5227C>T), E970Q (5234G>C), T975S (5249A>T 5251G>T), N979I (5262A>T 5263C>T), Q984D (5276C>G 5278A>C), Y985M (5279T>A 5280A>T 5281C>G), H988A (5288C>G 5289A>C 5290C>T), Y989C (5292A>G), I990V (5294A>G 5296C>G), N991M (5298A>T 5299C>G), Y992E (5300T>G 5302C>A), A993L (5303G>T 5304C>T 5305A>G), Q994K (5306C>A), D995G (5310A>G 5311C>G), N996S (5313A>G 5314C>T), V998D (5319T>A), S999D (5321T>G 5322C>A 5323A>T), L1000H (5324T>C 5325T>A 5326A>T), I1006F (5342A>T 5344C>T), A1007C (5345G>T 5346C>G 5347A>C), L1008Y (5348C>T 5349T>A 5350G>C), N1009S (5352A>G 5353C>T), N1010L (5354A>C 5355A>T 5356C>G), E1014S (5366G>T 5367A>C), T1015A (5369A>G), S1017K (5375T>A 5376C>A), T1018M (5379C>T), T1019S (5382C>G 5383G>T), P1020S (5384C>T 5386A>G)								

	Begin	End	Coverage	Score	Concordance	Matches	Identities	I/D/M/F*	Stop Codons
**NT**	**3246**	**5386**	**19.3%**	**138**	**5.0%**	**1414 (99.0%)**	**752 (52.6%)**	**0/15**	

Codon mutations:

CAA307.AG (3247A>G), CCA308GAG (3248C>G 3249C>A 3250A>G), TGG309ATA (3251T>A 3252G>T 3253G>A), CAC311TTT (3257C>T 3258A>T 3259C>T), AAG312GGC (3260A>G 3261A>G 3262G>C), ATA313ATT (3265A>T), AAC314GAC (3266A>G), ATT315TTG (3269A>T 3271T>G), CAG316TTG (3272C>T 3273A>T), CCA317CCT (3277A>T), GGG318GAC (3279G>A 3280G>C), AAA319ACC (3282A>C 3283A>C), GAG320CAA (3284G>C 3286G>A), CCT321CCA (3289T>A), CCA322ATC (3290C>A 3291C>T 3292A>C), TGG323TCC (3294G>C 3295G>C), GGA324ATT (3296G>A 3297G>T 3298A>T), CCC325CCT (3301C>T), CTA326CCG (3303T>C 3304A>G), CAA328CGT (3309A>G 3310A>T), TCT330GCT (3314T>G), GAG331CCG (3317G>C 3318A>C), AAA332GCA (3320A>G 3321A>C), GAG333GAA (3325G>A), CTA334TTG (3326C>T 3328A>G), CAG335AAG (3329C>A), ACC336GAG (3332A>G 3333C>A 3334C>G), CGA338AAA (3338C>A 3339G>A), GAA339GAG (3343A>G), TGG340CAG (3344T>C 3345G>A), CTG341TTA (3347C>T 3349G>A), GAG343GAT (3355G>T), AAG344TTG (3356A>T 3357A>T), CTA345TTG (3359C>T 3361A>G), GCC346GAC (3363C>A), AAA347AAG (3367A>G), GGA348GGT (3370A>T), TGG349TTT (3372G>T 3373G>T), ATA350ATT (3376A>T), CGA352CC. (3381G>C), CGA420AGG (3584C>A 3586A>G), ATG421ATA (3589G>A), GGA424GGG (3598A>G), GAA425GAT (3601A>T), GAA426GAG (3604A>G), TGG427CCT (3605T>C 3606G>C 3607G>T), AAA428AAG (3610A>G), ACC429ACT (3613C>T), GCT430ACT (3614G>A), TTC431TGT (3618T>G 3619C>T), AGG432GTA (3620A>G 3621G>T 3622G>A), ACA433ACT (3625A>T), AGA434AGG (3628A>G), TAC435TAT (3631C>T), GGA436GGG (3634A>G), CTC437CAA (3636T>A 3637C>A), TAC438TAT (3640C>T), TTC440TTT (3646C>T), TTG441CTT (3647T>C 3649G>T), GTC442GTA (3652C>A), CCA444CCT (3658A>T), ATG445TTT (3659A>T 3661G>T), GGA446GGG (3664A>G), TTG447TTA (3667G>A), ACC448ACT (3670C>T), AAC449AAT (3673C>T), GCA450GCC (3676A>C), CCC451CCG (3679C>G), GCA452GCT (3682A>T), TCC453ACC (3683T>A), TGC454TTT (3687G>T 3688C>T), CAG455ATG (3689C>A 3690A>T), GAC456AAC (3692G>A), CTT457TTA (3695C>T 3697T>A), GTC458ATG (3698G>A 3700C>G), AAC459AAT (3703C>T), GAA460AAG (3704G>A 3706A>G), ACA461GTA (3707A>G 3708C>T), CTT462TTC (3710C>T 3712T>C), AGA463AAT (3714G>A 3715A>T), GAC464CCC (3716G>C 3717A>C), CTA465TAT (3719C>T 3720T>A 3721A>T), CTC466CTT (3724C>T), GTG468AAG (3728G>A 3729T>A), TGC469TTT (3732G>T 3733C>T), GTC470GTG (3736C>G), GTT471GTA (3739T>A), GCT472GTC (3741C>T 3742T>C), ATG474ATA (3748G>A), GAC475GAT (3751C>T), ATA477ATT (3757A>T), CTG478GTC (3758C>G 3760G>C), GTC479ATA (3761G>A 3763C>A), TAC480TAT (3766C>T), ACA481AGT (3768C>G 3769A>T), AAA482CGA (3770A>C 3771A>G), GGA483GAT (3774G>A 3775A>T), TCC484ACG (3776T>A 3778C>G), CTC485TTG (3779C>T 3781C>G), CAG486GAG (3782C>G), ACC489GTG (3791A>G 3792C>T 3793C>G), AAG490AAC (3796G>C), CAA491CAT (3799A>T), GTT492TTG (3800G>T 3802T>G), CAA493AGG (3803C>A 3804A>G 3805A>G), GAT494ATG (3806G>A 3807A>T 3808T>G), TTC504..G (3838C>G), AAG505TAT (3839A>T 3841G>T), ACA506GTT (3842A>G 3843C>T 3844A>T), GCA507AAA (3845G>A 3846C>A), CCC508ATG (3848C>A 3849C>T 3850C>G), AAA510AAG (3856A>G), TGC511TGT (3859C>T), GAA512GAG (3862A>G), TTC513TTT (3865C>T), CAC514TCG (3866C>T 3867A>C 3868C>G), AAG515CTA (3869A>C 3870A>T 3871G>A), AAA516AAG (3874A>G), GAA517GAG (3877A>G), GTC518GTG (3880C>G), TTT520TTC (3886T>C), TTA521CTA (3887T>C), TTT523CAT (3893T>C 3894T>A), ATC524ATA (3898C>A), ATC525GTT (3899A>G 3901C>T), ACA527GAA (3905A>G 3906C>A), ACA528GAC (3908A>G 3909C>A 3910A>C), GGG529GGA (3913G>A), ACG531AGG (3918C>G), ATC532ATG (3922C>G), GAC533GAT (3925C>T), GCA535AAG (3929G>A 3930C>A 3931A>G), AAG536AAA (3934G>A), ACA537GTG (3935A>G 3936C>T 3937A>G), CAG538GAA (3938C>G 3940G>A), TCA539GCG (3941T>G 3943A>G), ATC540ATT (3946C>T), AGA541ATG (3948G>T 3949A>G), GAA542GAT (3952A>T), GAA545AGA (3959G>A 3960A>G), CCG546CCC (3964G>C), ACA548ACT (3970A>T), GTC549GTG (3973C>G), AAG550ACG (3975A>C), GTA552CTT (3980G>C 3982A>T), CAG553AGG (3983C>A 3984A>G), TCA554AGT (3986T>A 3987C>G 3988A>T), CTT556TTA (3992C>T 3994T>A), GGA557GGG (3997A>G), CTC558TTG (3998C>T 4000C>G), GCC559GCA (4003C>A), TAC561TAT (4009C>T), AAC562TAC (4010A>T), AAG567GAT (4025A>G 4027G>T), GAC568GGA (4029A>G 4030C>A), TAT569TAC (4033T>C), GCA573.CT (4045A>T), ATG576TTG (4052A>T), ACG577ACT (4057G>T), ATG578GAT (4058A>G 4059T>A 4060G>T), CTT579TTG (4061C>T 4063T>G), ACA580CTG (4064A>C 4065C>T 4066A>G), AGA581AAG (4068G>A 4069A>G), AAA582AAG (4072A>G), GAC583GAG (4075C>G), AAC585AAG (4081C>G), AAA587GTA (4085A>G 4086A>T), GGA589TCC (4091G>T 4092G>C 4093A>C), AAA590GAG (4094A>G 4096A>G), CAG592TGC (4100C>T 4101A>G 4102G>C), ACC593GAA (4103A>G 4104C>A 4105C>A), GAA594AAA (4106G>A), GCG595GCT (4111G>T), AAA597CAA (4115A>C), AGA598AAG (4119G>A 4120A>G), CTC599CTG (4123C>G), GAA601GAC (4129A>C), CAG602GCT (4130C>G 4131A>C 4132G>T), TGC603ATT (4133T>A 4134G>T 4135C>T), TCA605TCT (4141A>T), GCC606GAA (4143C>A 4144C>A), ACG608ATA (4149C>T 4150G>A), CTT609CTG (4153T>G), CGA610AAG (4154C>A 4155G>A 4156A>G), CTA611CTG (4159A>G), TTC612TTT (4162C>T), GAT613GAG (4165T>G), GGT614CTA (4166G>C 4167G>T 4168T>A), AGC615CCT (4169A>C 4170G>C 4171C>T), AAG616TTT (4172A>T 4173A>T 4174G>T), GAA617GAG (4177A>G), GTC618GTA (4180C>A), ATC620_GAG621del (4184_4189delATCGAG), ACC622ACT (4192C>T), GAC623GAT (4195C>T), TCT625TCG (4201T>G), GAT626GAC (4204T>C), ATG627AAA (4206T>A 4207G>A), GCA628GCT (4210A>T), ATA629GTG (4211A>G 4213A>G), GGC630GGA (4216C>A), GCA631GGC (4218C>G 4219A>C), TGT632GTA (4220T>G 4221G>T 4222T>A), CTA633TTG (4223C>T 4225A>G), ACA634GTG (4226A>G 4227C>T 4228A>G), CAG635CAA (4231G>A), ACA636AAT (4233C>A 4234A>T), CAC637GAC (4235C>G), GAT638GAA (4240T>A), GGG639GGT (4243G>T), AAA640_AGA641del (4244_4249delAAAAGA), CAC642CAT (4252C>T), CCA643CCG (4255A>G), TAT646TTT (4263A>T), TAT647GAA (4265T>G 4267T>A), TCC648AGC (4268T>A 4269C>G), CGG649AGG (4271C>A), ATG651TTA (4277A>T 4279G>A), ACC652AAT (4281C>A 4282C>T), ACA653GAT (4283A>G 4284C>A 4285A>T), GCG654GCT (4288G>T), GAA655GAG (4291A>G), AAC657AGG (4296A>G 4297C>G), GAC659TCT (4301G>T 4302A>C 4303C>T), ATC660GCA (4304A>G 4305T>C 4306C>A), GAC662GAA (4312C>A), CTT665ATG (4319C>A 4321T>G), CTA666GTG (4322C>G 4324A>G), GCC667GCA (4327C>A), ATT668TTG (4328A>T 4330T>G), GTT669CTC (4331G>C 4333T>C), GCC670CAT (4334G>C 4335C>A 4336C>T), GCC671TGT (4337G>T 4338C>G 4339C>T), ATG672CTT (4340A>C 4342G>T), CAT674ACA (4346C>A 4347A>C 4348T>A), AGA676AGG (4354A>G), TAC678TAT (4360C>T), GTC679CTC (4361G>C), GAG680TTA (4364G>T 4365A>T 4366G>A), GGC681GGA (4369C>A), CCA682ACA (4370C>A), CCG683del (4373_4375delCCG), TTA685TTC (4381A>C), ACG686GTA (4382A>G 4383C>T 4384G>A), ATT687GTA (4385A>G 4387T>A), CTT688AAG (4388C>A 4389T>A 4390T>G), TCA689ACG (4391T>A 4393A>G), CAC691AAT (4397C>A 4399C>T), AAG692GTG (4400A>G 4401A>T), AAT693GCT (4403A>G 4404A>C), CTC694AAT (4406C>A 4407T>A 4408C>T), ACG695ACT (4411G>T), ACG698AAA (4419C>A 4420G>A), ACG699ACT (4423G>T), ACG700CAA (4424A>C 4425C>A 4426G>A), AAG701CAG (4427A>C), GAA702AAA (4430G>A), CTT703TTG (4433C>T 4435T>G), ATC838GTG (4838A>G 4840C>G), AAG839CAG (4841A>C), AAA840ATT (4845A>T 4846A>T), TGC841TGT (4849C>T), GTA842CTT (4850G>C 4852A>T), CAC843GAG (4853C>G 4855C>G), AAC847GAC (4865A>G), AAA848AAG (4870A>G), GCT849GTA (4872C>T 4873T>A), GCA850GAG (4875C>A 4876A>G), CGG851CAG (4878G>A), CAC852CGA (4881A>G 4882C>A), GCG853CAA (4883G>C 4884C>A 4885G>A), AAA854CCC (4886A>C 4887A>C 4888A>C), TAC855GAA (4889T>G 4891C>A), GGT856GGA (4894T>A), CAC857CTT (4896A>T 4897C>T), CTA858TTA (4898C>T), CAG859GAG (4901C>G), TTC860CTC (4904T>C), GCC890GGG (4995C>G 4996C>G), GAC892GAT (5002C>T), ATG893TCC (5003A>T 5004T>C 5005G>C), ATA894ATT (5008A>T), CTA895TGG (5009C>T 5010T>G 5011A>G), GTC896GTG (5014C>G), ATG897ATT (5017G>T), GTC898GTG (5020C>G), CTC901TTG (5027C>T 5029C>G), ACA902ACT (5032A>T), AAA903AAG (5035A>G), TAT904TCA (5037A>C 5038T>A), GCA905GCG (5041A>G), TTC907TTT (5047C>T), ATT908CTT (5048A>C), GCA910GTG (5055C>T 5056A>G), TCA911AGC (5057T>A 5058C>G 5059A>C), GAA912ACT (5060G>A 5061A>C 5062A>T), ATA913AGC (5064T>G 5065A>C), ACT915AGT (5070C>G), CAG918CGA (5079A>G 5080G>A), CTC919TTG (5081C>T 5083C>G), GGA920GCT (5085G>C 5086A>T), TAC921AGA (5087T>A 5088A>G 5089C>A), CTC922TTG (5090C>T 5092C>G), GTA923TAT (5093G>T 5094T>A 5095A>T), CTG924GTG (5096C>G), GAC925CGT (5099G>C 5100A>G 5101C>T), AGA926GAG (5102A>G 5103G>A 5104A>G), TTG927ATT (5105T>A 5107G>T), ATC928GTC (5108A>G), CGA929AGA (5111C>A), TAT930TTG (5115A>T 5116T>G), CAC931CAT (5119C>T), TTC933GTG (5123T>G 5125C>G), CCG934CCT (5128G>T), GAA935GTT (5130A>T 5131A>T), GTA936TCT (5132G>T 5133T>C 5134A>T), TTC937ATT (5135T>A 5137C>T), ATT938GTG (5138A>G 5140T>G), ACA939TCT (5141A>T 5143A>T), GAC940GAT (5146C>T), AGA941CGA (5147A>C), GAC942GGC (5151A>G), AAG943ACG (5154A>C), CTC944GTG (5156C>G 5158C>G), TTC945TTT (5161C>T), ACA946ACC (5164A>C), TCA947TCG (5167A>G), AAC948CGT (5168A>C 5169A>G 5170C>T), TAC949TTC (5172A>T), AAG951AGA (5178A>G 5179G>A), ACG952GCC (5180A>G 5182G>C), CTC953TTA (5183C>T 5185C>A), ATG954CAG (5186A>C 5187T>A), GGA955AAG (5189G>A 5190G>A 5191A>G), ACG956GAT (5192A>G 5193C>A 5194G>T), ATT957TTG (5195A>T 5197T>G), GGA958GGT (5200A>T), ATC959ACC (5202T>C), AAG960AGA (5205A>G 5206G>A), CAC961TTG (5207C>T 5208A>T 5209C>G), AAG962GAT (5210A>G 5212G>T), TTG963ATG (5213T>A), TCA964AGT (5216T>A 5217C>G 5218A>T), ACA965TCA (5219A>T), TAC967TTT (5226A>T 5227C>T), CCA969CCT (5233A>T), GAG970CAG (5234G>C), ACG971ACC (5239G>C), GGG973GGC (5245G>C), CAA974CAG (5248A>G), ACG975TCT (5249A>T 5251G>T), GAA976GAG (5254A>G), AGA977AGG (5257A>G), ACG978ACC (5260G>C), AAC979ATT (5262A>T 5263C>T), CAG980CAA (5266G>A), ACA981ACT (5269A>T), CTC982CTT (5272C>T), CAA984GAC (5276C>G 5278A>C), TAC985ATG (5279T>A 5280A>T 5281C>G), CTA986TTG (5282C>T 5284A>G), CGG987AGA (5285C>A 5287G>A), CAC988GCT (5288C>G 5289A>C 5290C>T), TAC989TGC (5292A>G), ATC990GTG (5294A>G 5296C>G), AAC991ATG (5298A>T 5299C>G), TAC992GAA (5300T>G 5302C>A), GCA993TTG (5303G>T 5304C>T 5305A>G), CAA994AAA (5306C>A), GAC995GGG (5310A>G 5311C>G), AAC996AGT (5313A>G 5314C>T), GTT998GAT (5319T>A), TCA999GAT (5321T>G 5322C>A 5323A>T), TTA1000CAT (5324T>C 5325T>A 5326A>T), CCA1002CCC (5332A>C), GCG1004GCA (5338G>A), CAG1005CAA (5341G>A), ATC1006TTT (5342A>T 5344C>T), GCA1007TGC (5345G>T 5346C>G 5347A>C), CTG1008TAC (5348C>T 5349T>A 5350G>C), AAC1009AGT (5352A>G 5353C>T), AAC1010CTG (5354A>C 5355A>T 5356C>G), CAC1011CAT (5359C>T), AAA1012AAG (5362A>G), GAG1014TCG (5366G>T 5367A>C), ACA1015GCA (5369A>G), ACT1016ACA (5374T>A), TCG1017AAG (5375T>A 5376C>A), ACG1018ATG (5379C>T), ACG1019AGT (5382C>G 5383G>T), CCA1020TCG (5384C>T 5386A>G)

*: Inserts / Deletes / Misaligned / Frameshifts

Analysis details

This analysis was performed with panviral2.64

NGS Details (UN8): Errantivirus

Assembly

**Coverage Length**	698 (1 contig(s))
**Depth Of Coverage**	139.9
**Number Of Reads**	891
**Reads Per Million**	14.89 rpm (after QC)
**Ambiguities**	0
**Assembly Method**	de novo + reference guided assembly
**Consensus Caller**	Bcf Tools

Coverage Map

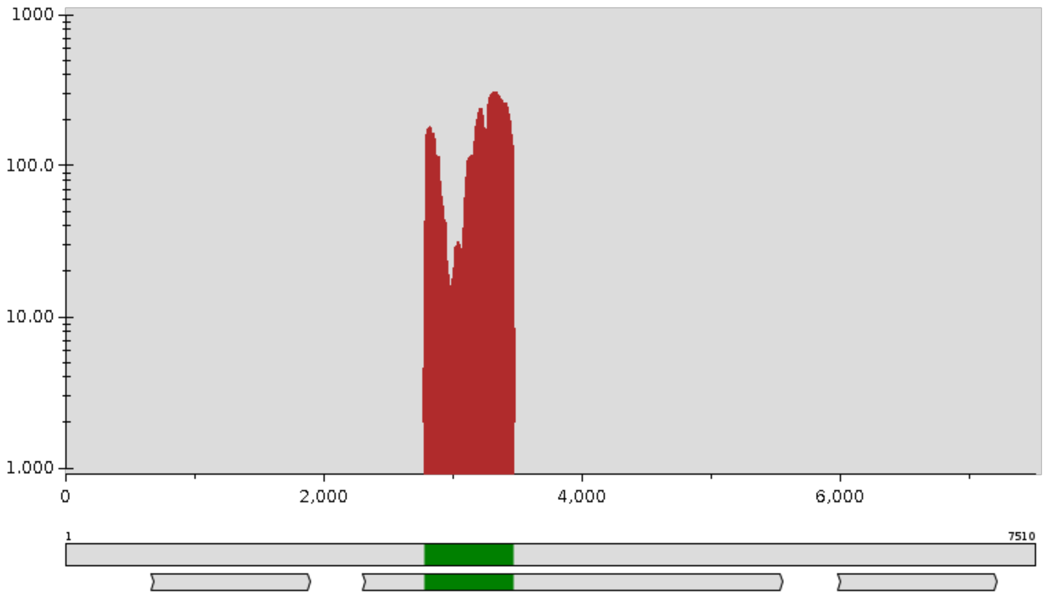

Assignment

**Type**	Errantivirus (Taxonomy ID: 186666)
**Reference Genome**	NC_038512.1
**NT Identity (%)**	53.1479
**AA Identity (%)**	40.7895
**Number Of Stop Codons**	0
**Number Of CDS**	3

Alignment

**Alignment Score**	44.0 (NT) + 709.0 (AA) = 753.0
**Concordance (%)**	26.0734

Alignment Method	Global, seeded, nucleotide + amino acids (AGA)

Genome Region

Sequence starts at position 2779 and ends at position 3476 relative to NC_038512.1 reference sequence.

Alignment Detailed Statistics

	Begin	End	Coverage	Score	Concordance	Matches	Identities	I/D/M/F*	Stop Codons
**NT**	**2779**	**3476**	**9.3%**	**44**	**3.3%**	**683 (97.9%)**	**363 (52.0%)**	**0/15**	

Mutations: 2786A>T, 2791G>A, 2792G>A, 2794C>G, 2795C>A, 2797A>G, 2801A>T, 2806A>C, 2809A>G, 2812A>T, 2813T>A, 2814C>G, 2815A>T, 2817A>T, 2818C>G, 2821T>A, 2822G>C, 2824A>T, 2828A>G, 2831T>G, 2836C>T, 2839A>G, 2841G>T, 2843G>T, 2849C>A, 2850C>A, 2851C>A, 2856A>G, 2858_2860delATC, 2863C>T, 2865C>G, 2870_2872delGGG, 2874A>T, 2875A>G, 2878A>C, 2880A>T, 2884G>T, 2885_2893delCGTCTCGTA, 2894G>A, 2899C>T, 2901T>A, 2902C>T, 2905T>C, 2906A>C, 2915G>A, 2918A>G, 2919A>T, 2927G>A, 2929T>G, 2930G>A, 2935A>G, 2938C>T, 2941G>C, 2942A>T, 2944A>G, 2947A>C, 2948A>C, 2949A>G, 2953A>T, 2954A>G, 2955G>A, 2960G>T, 2963C>T, 2969A>C, 2972T>C, 2975G>C, 2976G>A, 2977T>G, 2978A>G, 2980G>A, 2981T>G, 2982G>C, 2984C>T, 2985A>C, 2986A>C, 2987T>G, 2989C>G, 2992C>T, 2993A>T, 2995C>T, 2997C>A, 2998C>G, 2999T>A, 3001A>C, 3003A>G, 3004T>C, 3008G>A, 3009C>G, 3010A>G, 3011A>T, 3012G>C, 3013T>G, 3016G>C, 3018T>A, 3019T>C, 3020T>C, 3022T>C, 3026G>T, 3029G>A, 3031G>A, 3032A>G, 3034G>T, 3035G>C, 3036A>G, 3037C>G, 3038C>G, 3039C>A, 3040T>A, 3041C>T, 3042A>C, 3047A>G, 3049A>C, 3050T>C, 3055A>G, 3059G>A, 3061G>C, 3065A>C, 3066A>G, 3067C>G, 3068G>A, 3069T>C, 3071G>C, 3072A>G, 3073A>T, 3074C>T, 3076C>T, 3082T>C, 3084T>A, 3085T>C, 3088A>G, 3092C>G, 3094T>G, 3095C>G, 3096G>T, 3097A>C, 3101C>T, 3104A>T, 3106G>C, 3109A>G, 3110T>C, 3112A>T, 3114A>C, 3115A>T, 3118C>T, 3119T>G, 3121A>C, 3124A>T, 3125T>G, 3128A>G, 3134C>A, 3135A>T, 3136A>G, 3137A>G, 3138G>A, 3139A>G, 3140G>T, 3142T>G, 3146G>A, 3148C>T, 3149A>C, 3151T>G, 3154C>G, 3155C>T, 3157A>T, 3159G>A, 3161G>C, 3162G>C, 3163T>C, 3164C>T, 3165T>A, 3167C>T, 3168A>T, 3170A>G, 3173A>C, 3175C>G, 3176A>T, 3179T>G, 3180G>T, 3181T>G, 3182C>A, 3184C>T, 3187C>G, 3189A>T, 3190C>T, 3191C>A, 3193T>A, 3196C>T, 3202T>C, 3203A>T, 3205T>G, 3208C>T, 3212A>T, 3213G>C, 3214T>C, 3216C>A, 3217T>G, 3218T>A, 3219C>G, 3220C>T, 3221C>G, 3222T>A, 3223A>G, 3224C>A, 3225A>G, 3226G>T, 3229A>C, 3232C>T, 3233C>G, 3236G>A, 3237A>G, 3238G>A, 3239A>C, 3245G>A, 3246A>G, 3247A>G, 3248C>A, 3249G>T, 3250A>T, 3253T>G, 3256C>G, 3259A>G, 3263C>T, 3265T>G, 3272A>G, 3273G>A, 3274T>A, 3277C>G, 3280C>A, 3281A>T, 3283A>T, 3284A>G, 3285T>C, 3286T>C, 3287C>A, 3289A>G, 3290A>T, 3292G>C, 3293G>T, 3294A>C, 3295C>A, 3300C>G, 3301C>T, 3304A>G, 3307C>T, 3309T>G, 3311A>C, 3312A>T, 3313G>A, 3315T>A, 3316C>G, 3317G>T, 3318A>C, 3320A>G, 3321C>T, 3322T>A, 3323G>T, 3327A>T, 3331T>A, 3337C>T, 3338A>G, 3340C>G, 3341A>G, 3343A>G, 3345G>C, 3346C>T, 3348G>A, 3352C>T, 3355T>C, 3358C>T, 3360A>T, 3362C>G, 3363C>T, 3365A>G, 3367C>T, 3370T>G, 3372A>C, 3373T>C, 3376G>A, 3377A>G, 3379T>G, 3380T>G, 3382C>T, 3385T>G, 3386A>G, 3388T>G, 3389C>A, 3390A>G, 3392A>G, 3396A>G, 3397T>G, 3398C>G, 3399T>C, 3400G>C, 3402T>G, 3403T>G, 3406A>T, 3408A>C, 3410A>T, 3413C>G, 3414C>T, 3415T>G, 3417A>C, 3418G>T, 3421A>G, 3424A>T, 3425A>C, 3426A>G, 3427A>T, 3428C>A, 3429A>G, 3430A>T, 3433T>C, 3434T>C, 3436A>T, 3440C>T, 3442T>G, 3443C>G, 3444T>C, 3445C>A, 3457A>G, 3459A>G, 3463C>T, 3464A>G

CDS

	Begin	End	Coverage	Score	Concordance	Matches	Identities	I/D/M/F*	Stop Codons
D1R33_gp2	159	391	21.5%	709	45.0%	228 (97.9%)	93 (39.9%)	0/5/0/0	0

Protein mutations: M161L (2786A>T), D163K (2792G>A 2794C>G), Q164K (2795C>A 2797A>G), I166F (2801A>T), D171V (2817A>T 2818C>G), A173P (2822G>C 2824A>T), S175G (2828A>G), S176A (2831T>G), I178M (2839A>G), W179L (2841G>T), V180F (2843G>T), P182K (2849C>A 2850C>A 2851C>A), K184R (2856A>G), I185del (2858_2860delATC), A187G (2865C>G), G189del (2870_2872delGGG), K190M (2874A>T 2875A>G), Q191H (2878A>C), K192M (2880A>T), W193C (2884G>T), R194_V196del (2885_2893delCGTCTCGTA), V197I (2894G>A), F199Y (2901T>A 2902C>T), K201Q (2906A>C), E204K (2915G>A), K205V (2918A>G 2919A>T), D208K (2927G>A 2929T>G), D209N (2930G>A), I213L (2942A>T 2944A>G), N215R (2948A>C 2949A>G), S217D (2954A>G 2955G>A), V219L (2960G>T), L220F (2963C>T), K222Q (2969A>C), G224Q (2975G>C 2976G>A 2977T>G), K225E (2978A>G 2980G>A), C226A (2981T>G 2982G>C), Q227S (2984C>T 2985A>C 2986A>C), Y228E (2987T>G 2989C>G), T230S (2993A>T 2995C>T), T231K (2997C>A 2998C>G), L232I (2999T>A 3001A>C), D233G (3003A>G 3004T>C), A235R (3008G>A 3009C>G 3010A>G), F238Y (3018T>A 3019T>C), Y239H (3020T>C 3022T>C), V241L (3026G>T), E242K (3029G>A 3031G>A), M243V (3032A>G 3034G>T), D244R (3035G>C 3036A>G 3037C>G), P245E (3038C>G 3039C>A 3040T>A), Q246S (3041C>T 3042A>C), I248V (3047A>G 3049A>C), S249P (3050T>C), A252T (3059G>A 3061G>C), N254R (3065A>C 3066A>G 3067C>G), V255T (3068G>A 3069T>C), E256R (3071G>C 3072A>G 3073A>T), H257Y (3074C>T 3076C>T), F260Y (3084T>A 3085T>C), L263V (3092C>G 3094T>G), R264V (3095C>G 3096G>T 3097A>C), P266S (3101C>T), M267F (3104A>T 3106G>C), K270T (3114A>C 3115A>T), S272A (3119T>G 3121A>C), S274A (3125T>G), T275A (3128A>G), Q277M (3134C>A 3135A>T 3136A>G), R278E (3137A>G 3138G>A 3139A>G), V279L (3140G>T 3142T>G), D281N (3146G>A 3148C>T), N282Q (3149A>C 3151T>G), L284F (3155C>T 3157A>T), R285K (3159G>A), G286P (3161G>C 3162G>C 3163T>C), L287Y (3164C>T 3165T>A), Q288L (3167C>T 3168A>T), N289D (3170A>G), N290Q (3173A>C 3175C>G), I291F (3176A>T), C292V (3179T>G 3180G>T 3181T>G), L293I (3182C>A 3184C>T), Y295F (3189A>T 3190C>T), L296I (3191C>A 3193T>A), I300L (3203A>T 3205T>G), T304K (3216C>A 3217T>G), L306E (3221C>G 3222T>A 3223A>G), Q307S (3224C>A 3225A>G 3226G>T), E308D (3229A>C), L310V (3233C>G), E311R (3236G>A 3237A>G 3238G>A), N312H (3239A>C), E314R (3245G>A 3246A>G 3247A>G), R315I (3248C>A 3249G>T 3250A>T), F317L (3256C>G), S323E (3272A>G 3273G>A 3274T>A), N324K (3277C>G), F325L (3280C>A), K326Y (3281A>T 3283A>T), I327A (3284A>G 3285T>C 3286T>C), Q328K (3287C>A 3289A>G), M329F (3290A>T 3292G>C), D330S (3293G>T 3294A>C 3295C>A), S332C (3300C>G 3301C>T), L335W (3309T>G), K336L (3311A>C 3312A>T 3313G>A), L337Q (3315T>A 3316C>G), E338S (3317G>T 3318A>C), T339V (3320A>G 3321C>T 3322T>A), A340S (3323G>T), Y341F (3327A>T), I345V (3338A>G 3340C>G), I346V (3341A>G 3343A>G), S347T (3345G>C 3346C>T), R348K (3348G>A), K352M (3360A>T), P353V (3362C>G 3363C>T), N354D (3365A>G 3367C>T), D356A (3372A>C 3373T>C), I358V (3377A>G 3379T>G), S359A (3380T>G 3382C>T), I361V (3386A>G 3388T>G), Q362R (3389C>A 3390A>G), K363E (3392A>G), Y364W (3396A>G 3397T>G), L365A (3398C>G 3399T>C 3400G>C), I366R (3402T>G 3403T>G), K368T (3408A>C), T369S (3410A>T), P370V (3413C>G 3414C>T 3415T>G), K371T (3417A>C 3418G>T), K374R (3425A>C 3426A>G 3427A>T), Q375S (3428C>A 3429A>G 3430A>T), L380A (3443C>G 3444T>C 3445C>A), K385R (3459A>G), I387V (3464A>G)

Codon mutations: ATG161TTG (2786A>T), TTG162TTA (2791G>A), GAC163AAG (2792G>A 2794C>G), CAA164AAG (2795C>A 2797A>G), ATT166TTT (2801A>T), ATA167ATC (2806A>C), AGA168AGG (2809A>G), CCA169CCT (2812A>T), TCA170AGT (2813T>A 2814C>G 2815A>T), GAC171GTG (2817A>T 2818C>G), TCT172TCA (2821T>A), GCA173CCT (2822G>C 2824A>T), AGC175GGC (2828A>G), TCA176GCA (2831T>G), CCC177CCT (2836C>T), ATA178ATG (2839A>G), TGG179TTG (2841G>T), GTT180TTT (2843G>T), CCC182AAA (2849C>A 2850C>A 2851C>A), AAA184AGA (2856A>G), ATC185del (2858_2860delATC), GAC186GAT (2863C>T), GCT187GGT (2865C>G), GGG189del (2870_2872delGGG), AAA190ATG (2874A>T 2875A>G), CAA191CAC (2878A>C), AAG192ATG (2880A>T), TGG193TGT (2884G>T), CGT194_GTA196del (2885_2893delCGTCTCGTA), GTT197ATT (2894G>A), GAC198GAT (2899C>T), TTC199TAT (2901T>A 2902C>T), CGT200CGC (2905T>C), AAG201CAG (2906A>C), GAG204AAG (2915G>A), AAG205GTG (2918A>G 2919A>T), GAT208AAG (2927G>A 2929T>G), GAC209AAC (2930G>A), AAA210AAG (2935A>G), TAC211TAT (2938C>T), CCG212CCC (2941G>C), ATA213TTG (2942A>T 2944A>G), CCA214CCC (2947A>C), AAC215CGC (2948A>C 2949A>G), ATA216ATT (2953A>T), AGT217GAT (2954A>G 2955G>A), GTA219TTA (2960G>T), CTT220TTT (2963C>T), AAG222CAG (2969A>C), TTA223CTA (2972T>C), GGT224CAG (2975G>C 2976G>A 2977T>G), AAG225GAA (2978A>G 2980G>A), TGC226GCC (2981T>G 2982G>C), CAA227TCC (2984C>T 2985A>C 2986A>C), TAC228GAG (2987T>G 2989C>G), TTC229TTT (2992C>T), ACC230TCT (2993A>T 2995C>T), ACC231AAG (2997C>A 2998C>G), TTA232ATC (2999T>A 3001A>C), GAT233GGC (3003A>G 3004T>C), GCA235AGG (3008G>A 3009C>G 3010A>G), AGT236TCG (3011A>T 3012G>C 3013T>G), GGG237GGC (3016G>C), TTT238TAC (3018T>A 3019T>C), TAT239CAC (3020T>C 3022T>C), GTG241TTG (3026G>T), GAG242AAA (3029G>A 3031G>A), ATG243GTT (3032A>G 3034G>T), GAC244CGG (3035G>C 3036A>G 3037C>G), CCT245GAA (3038C>G 3039C>A 3040T>A), CAA246TCA (3041C>T 3042A>C), ATA248GTC (3047A>G 3049A>C), TCG249CCG (3050T>C), AAA250AAG (3055A>G), GCG252ACC (3059G>A 3061G>C), AAC254CGG (3065A>C 3066A>G 3067C>G), GTA255ACA (3068G>A 3069T>C), GAA256CGT (3071G>C 3072A>G 3073A>T), CAC257TAT (3074C>T 3076C>T), CAT259CAC (3082T>C), TTT260TAC (3084T>A 3085T>C), GAA261GAG (3088A>G), CTT263GTG (3092C>G 3094T>G), CGA264GTC (3095C>G 3096G>T 3097A>C), CCT266TCT (3101C>T), ATG267TTC (3104A>T 3106G>C), GGA268GGG (3109A>G), TTA269CTT (3110T>C 3112A>T), AAA270ACT (3114A>C 3115A>T), AAC271AAT (3118C>T), TCA272GCC (3119T>G 3121A>C), CCA273CCT (3124A>T), TCT274GCT (3125T>G), ACT275GCT (3128A>G), CAA277ATG (3134C>A 3135A>T 3136A>G), AGA278GAG (3137A>G 3138G>A 3139A>G), GTT279TTG (3140G>T 3142T>G), GAC281AAT (3146G>A 3148C>T), AAT282CAG (3149A>C 3151T>G), GTC283GTG (3154C>G), CTA284TTT (3155C>T 3157A>T), AGA285AAA (3159G>A), GGT286CCC (3161G>C 3162G>C 3163T>C), CTC287TAC (3164C>T 3165T>A), CAA288TTA (3167C>T 3168A>T), AAT289GAT (3170A>G), AAC290CAG (3173A>C 3175C>G), ATC291TTC (3176A>T), TGT292GTG (3179T>G 3180G>T 3181T>G), CTC293ATT (3182C>A 3184C>T), GTC294GTG (3187C>G), TAC295TTT (3189A>T 3190C>T), CTT296ATA (3191C>A 3193T>A), GAC297GAT (3196C>T), ATT299ATC (3202T>C), ATT300TTG (3203A>T 3205T>G), GTC301GTT (3208C>T), AGT303TCC (3212A>T 3213G>C 3214T>C), ACT304AAG (3216C>A 3217T>G), TCC305AGT (3218T>A 3219C>G 3220C>T), CTA306GAG (3221C>G 3222T>A 3223A>G), CAG307AGT (3224C>A 3225A>G 3226G>T), GAA308GAC (3229A>C), CAC309CAT (3232C>T), CTG310GTG (3233C>G), GAG311AGA (3236G>A 3237A>G 3238G>A), AAC312CAC (3239A>C), GAA314AGG (3245G>A 3246A>G 3247A>G), CGA315ATT (3248C>A 3249G>T 3250A>T), GTT316GTG (3253T>G), TTC317TTG (3256C>G), CAA318CAG (3259A>G), CTT320TTG (3263C>T 3265T>G), AGT323GAA (3272A>G 3273G>A 3274T>A), AAC324AAG (3277C>G), TTC325TTA (3280C>A), AAA326TAT (3281A>T 3283A>T), ATT327GCC (3284A>G 3285T>C 3286T>C), CAA328AAG (3287C>A 3289A>G), ATG329TTC (3290A>T 3292G>C), GAC330TCA (3293G>T 3294A>C 3295C>A), TCC332TGT (3300C>G 3301C>T), GAA333GAG (3304A>G), TTC334TTT (3307C>T), TTG335TGG (3309T>G), AAG336CTA (3311A>C 3312A>T 3313G>A), CTC337CAG (3315T>A 3316C>G), GAA338TCA (3317G>T 3318A>C), ACT339GTA (3320A>G 3321C>T 3322T>A), GCT340TCT (3323G>T), TAT341TTT (3327A>T), CTT342CTA (3331T>A), CAC344CAT (3337C>T), ATC345GTG (3338A>G 3340C>G), ATA346GTG (3341A>G 3343A>G), AGC347ACT (3345G>C 3346C>T), AGG348AAG (3348G>A), GAC349GAT (3352C>T), GGT350GGC (3355T>C), ATC351ATT (3358C>T), AAG352ATG (3360A>T), CCT353GTT (3362C>G 3363C>T), AAC354GAT (3365A>G 3367C>T), CCT355CCG (3370T>G), GAT356GCC (3372A>C 3373T>C), AAG357AAA (3376G>A), ATT358GTG (3377A>G 3379T>G), TCC359GCT (3380T>G 3382C>T), GCT360GCG (3385T>G), ATT361GTG (3386A>G 3388T>G), CAA362AGA (3389C>A 3390A>G), AAA363GAA (3392A>G), TAT364TGG (3396A>G 3397T>G), CTG365GCC (3398C>G 3399T>C 3400G>C), ATT366AGG (3402T>G 3403T>G), CCA367CCT (3406A>T), AAG368ACG (3408A>C), ACC369TCC (3410A>T), CCT370GTG (3413C>G 3414C>T 3415T>G), AAG371ACT (3417A>C 3418G>T), GAA372GAG (3421A>G), ATA373ATT (3424A>T), AAA374CGT (3425A>C 3426A>G 3427A>T), CAA375AGT (3428C>A 3429A>G 3430A>T), TTT376TTC (3433T>C), TTA377CTT (3434T>C 3436A>T), CTT379TTG (3440C>T 3442T>G), CTC380GCA (3443C>G 3444T>C 3445C>A), CGA384CGG (3457A>G), AAA385AGA (3459A>G), TTC386TTT (3463C>T), ATT387GTT (3464A>G)

Proteins

	Begin	End	Coverage	Score	Concordance	Matches	Identities	I/D/M/F*	Stop Codons
ORF B (YP_009507248.1)	159	391	21.5%	709	45.0%	228 (97.9%)	93 (39.9%)	0/5/0/0	0

	Begin	End	Coverage	Score	Concordance	Matches	Identities	I/D/M/F*	Stop Codons
**NT**	**2779**	**3476**	**9.3%**	**44**	**3.3%**	**683 (97.9%)**	**363 (52.0%)**	**0/15**	

Protein mutations:

M161L (2786A>T), D163K (2792G>A 2794C>G), Q164K (2795C>A 2797A>G), I166F (2801A>T), D171V (2817A>T 2818C>G), A173P (2822G>C 2824A>T), S175G (2828A>G), S176A (2831T>G), I178M (2839A>G), W179L (2841G>T), V180F (2843G>T), P182K (2849C>A 2850C>A 2851C>A), K184R (2856A>G), I185del (2858_2860delATC), A187G (2865C>G), G189del (2870_2872delGGG), K190M (2874A>T 2875A>G), Q191H (2878A>C), K192M (2880A>T), W193C (2884G>T), R194_V196del (2885_2893delCGTCTCGTA), V197I (2894G>A), F199Y (2901T>A 2902C>T), K201Q (2906A>C), E204K (2915G>A), K205V (2918A>G 2919A>T), D208K (2927G>A 2929T>G), D209N (2930G>A), I213L (2942A>T 2944A>G), N215R (2948A>C 2949A>G), S217D (2954A>G 2955G>A), V219L (2960G>T), L220F (2963C>T), K222Q (2969A>C), G224Q (2975G>C 2976G>A 2977T>G), K225E (2978A>G 2980G>A), C226A (2981T>G 2982G>C), Q227S (2984C>T 2985A>C 2986A>C), Y228E (2987T>G 2989C>G), T230S (2993A>T 2995C>T), T231K (2997C>A 2998C>G), L232I (2999T>A 3001A>C), D233G (3003A>G 3004T>C), A235R (3008G>A 3009C>G 3010A>G), F238Y (3018T>A 3019T>C), Y239H (3020T>C 3022T>C), V241L (3026G>T), E242K (3029G>A 3031G>A), M243V (3032A>G 3034G>T), D244R (3035G>C 3036A>G 3037C>G), P245E (3038C>G 3039C>A 3040T>A), Q246S (3041C>T 3042A>C), I248V (3047A>G 3049A>C), S249P (3050T>C), A252T (3059G>A 3061G>C), N254R (3065A>C 3066A>G 3067C>G), V255T (3068G>A 3069T>C), E256R (3071G>C 3072A>G 3073A>T), H257Y (3074C>T 3076C>T), F260Y (3084T>A 3085T>C), L263V (3092C>G 3094T>G), R264V (3095C>G 3096G>T 3097A>C), P266S (3101C>T), M267F (3104A>T 3106G>C), K270T (3114A>C 3115A>T), S272A (3119T>G 3121A>C), S274A (3125T>G), T275A (3128A>G), Q277M (3134C>A 3135A>T 3136A>G), R278E (3137A>G 3138G>A 3139A>G), V279L (3140G>T 3142T>G), D281N (3146G>A 3148C>T), N282Q (3149A>C 3151T>G), L284F (3155C>T 3157A>T), R285K (3159G>A), G286P (3161G>C 3162G>C 3163T>C), L287Y (3164C>T 3165T>A), Q288L (3167C>T 3168A>T), N289D (3170A>G), N290Q (3173A>C 3175C>G), I291F (3176A>T), C292V (3179T>G 3180G>T 3181T>G), L293I (3182C>A 3184C>T), Y295F (3189A>T 3190C>T), L296I (3191C>A 3193T>A), I300L (3203A>T 3205T>G), T304K (3216C>A 3217T>G), L306E (3221C>G 3222T>A 3223A>G), Q307S (3224C>A 3225A>G 3226G>T), E308D (3229A>C), L310V (3233C>G), E311R (3236G>A 3237A>G 3238G>A), N312H (3239A>C), E314R (3245G>A 3246A>G 3247A>G), R315I (3248C>A 3249G>T 3250A>T), F317L (3256C>G), S323E (3272A>G 3273G>A 3274T>A), N324K (3277C>G), F325L (3280C>A), K326Y (3281A>T 3283A>T), I327A (3284A>G 3285T>C 3286T>C), Q328K (3287C>A 3289A>G), M329F (3290A>T 3292G>C), D330S (3293G>T 3294A>C 3295C>A), S332C (3300C>G 3301C>T), L335W (3309T>G), K336L (3311A>C 3312A>T 3313G>A), L337Q (3315T>A 3316C>G), E338S (3317G>T 3318A>C), T339V (3320A>G 3321C>T 3322T>A), A340S (3323G>T), Y341F (3327A>T), I345V (3338A>G 3340C>G), I346V (3341A>G 3343A>G), S347T (3345G>C 3346C>T), R348K (3348G>A), K352M (3360A>T), P353V (3362C>G 3363C>T), N354D (3365A>G 3367C>T), D356A (3372A>C 3373T>C), I358V (3377A>G 3379T>G), S359A (3380T>G 3382C>T), I361V (3386A>G 3388T>G), Q362R (3389C>A 3390A>G), K363E (3392A>G), Y364W (3396A>G 3397T>G), L365A (3398C>G 3399T>C 3400G>C), I366R (3402T>G 3403T>G), K368T (3408A>C), T369S (3410A>T), P370V (3413C>G 3414C>T 3415T>G), K371T (3417A>C 3418G>T), K374R (3425A>C 3426A>G 3427A>T), Q375S (3428C>A 3429A>G 3430A>T), L380A (3443C>G 3444T>C 3445C>A), K385R (3459A>G), I387V (3464A>G)

Codon mutations:

ATG161TTG (2786A>T), TTG162TTA (2791G>A), GAC163AAG (2792G>A 2794C>G), CAA164AAG (2795C>A 2797A>G), ATT166TTT (2801A>T), ATA167ATC (2806A>C), AGA168AGG (2809A>G), CCA169CCT (2812A>T), TCA170AGT (2813T>A 2814C>G 2815A>T), GAC171GTG (2817A>T 2818C>G), TCT172TCA (2821T>A), GCA173CCT (2822G>C 2824A>T), AGC175GGC (2828A>G), TCA176GCA (2831T>G), CCC177CCT (2836C>T), ATA178ATG (2839A>G), TGG179TTG (2841G>T), GTT180TTT (2843G>T), CCC182AAA (2849C>A 2850C>A 2851C>A), AAA184AGA (2856A>G), ATC185del (2858_2860delATC), GAC186GAT (2863C>T), GCT187GGT (2865C>G), GGG189del (2870_2872delGGG), AAA190ATG (2874A>T 2875A>G), CAA191CAC (2878A>C), AAG192ATG (2880A>T), TGG193TGT (2884G>T), CGT194_GTA196del (2885_2893delCGTCTCGTA), GTT197ATT (2894G>A), GAC198GAT (2899C>T), TTC199TAT (2901T>A 2902C>T), CGT200CGC (2905T>C), AAG201CAG (2906A>C), GAG204AAG (2915G>A), AAG205GTG (2918A>G 2919A>T), GAT208AAG (2927G>A 2929T>G), GAC209AAC (2930G>A), AAA210AAG (2935A>G), TAC211TAT (2938C>T), CCG212CCC (2941G>C), ATA213TTG (2942A>T 2944A>G), CCA214CCC (2947A>C), AAC215CGC (2948A>C 2949A>G), ATA216ATT (2953A>T), AGT217GAT (2954A>G 2955G>A), GTA219TTA (2960G>T), CTT220TTT (2963C>T), AAG222CAG (2969A>C), TTA223CTA (2972T>C), GGT224CAG (2975G>C 2976G>A 2977T>G), AAG225GAA (2978A>G 2980G>A), TGC226GCC (2981T>G 2982G>C), CAA227TCC (2984C>T 2985A>C 2986A>C), TAC228GAG (2987T>G 2989C>G), TTC229TTT (2992C>T), ACC230TCT (2993A>T 2995C>T), ACC231AAG (2997C>A 2998C>G), TTA232ATC (2999T>A 3001A>C), GAT233GGC (3003A>G 3004T>C), GCA235AGG (3008G>A 3009C>G 3010A>G), AGT236TCG (3011A>T 3012G>C 3013T>G), GGG237GGC (3016G>C), TTT238TAC (3018T>A 3019T>C), TAT239CAC (3020T>C 3022T>C), GTG241TTG (3026G>T), GAG242AAA (3029G>A 3031G>A), ATG243GTT (3032A>G 3034G>T), GAC244CGG (3035G>C 3036A>G 3037C>G), CCT245GAA (3038C>G 3039C>A 3040T>A), CAA246TCA (3041C>T 3042A>C), ATA248GTC (3047A>G 3049A>C), TCG249CCG (3050T>C), AAA250AAG (3055A>G), GCG252ACC (3059G>A 3061G>C), AAC254CGG (3065A>C 3066A>G 3067C>G), GTA255ACA (3068G>A 3069T>C), GAA256CGT (3071G>C 3072A>G 3073A>T), CAC257TAT (3074C>T 3076C>T), CAT259CAC (3082T>C), TTT260TAC (3084T>A 3085T>C), GAA261GAG (3088A>G), CTT263GTG (3092C>G 3094T>G), CGA264GTC (3095C>G 3096G>T 3097A>C), CCT266TCT (3101C>T), ATG267TTC (3104A>T 3106G>C), GGA268GGG (3109A>G), TTA269CTT (3110T>C 3112A>T), AAA270ACT (3114A>C 3115A>T), AAC271AAT (3118C>T), TCA272GCC (3119T>G 3121A>C), CCA273CCT (3124A>T), TCT274GCT (3125T>G), ACT275GCT (3128A>G), CAA277ATG (3134C>A 3135A>T 3136A>G), AGA278GAG (3137A>G 3138G>A 3139A>G), GTT279TTG (3140G>T 3142T>G), GAC281AAT (3146G>A 3148C>T), AAT282CAG (3149A>C 3151T>G), GTC283GTG (3154C>G), CTA284TTT (3155C>T 3157A>T), AGA285AAA (3159G>A), GGT286CCC (3161G>C 3162G>C 3163T>C), CTC287TAC (3164C>T 3165T>A), CAA288TTA (3167C>T 3168A>T), AAT289GAT (3170A>G), AAC290CAG (3173A>C 3175C>G), ATC291TTC (3176A>T), TGT292GTG (3179T>G 3180G>T 3181T>G), CTC293ATT (3182C>A 3184C>T), GTC294GTG (3187C>G), TAC295TTT (3189A>T 3190C>T), CTT296ATA (3191C>A 3193T>A), GAC297GAT (3196C>T), ATT299ATC (3202T>C), ATT300TTG (3203A>T 3205T>G), GTC301GTT (3208C>T), AGT303TCC (3212A>T 3213G>C 3214T>C), ACT304AAG (3216C>A 3217T>G), TCC305AGT (3218T>A 3219C>G 3220C>T), CTA306GAG (3221C>G 3222T>A 3223A>G), CAG307AGT (3224C>A 3225A>G 3226G>T), GAA308GAC (3229A>C), CAC309CAT (3232C>T), CTG310GTG (3233C>G), GAG311AGA (3236G>A 3237A>G 3238G>A), AAC312CAC (3239A>C), GAA314AGG (3245G>A 3246A>G 3247A>G), CGA315ATT (3248C>A 3249G>T 3250A>T), GTT316GTG (3253T>G), TTC317TTG (3256C>G), CAA318CAG (3259A>G), CTT320TTG (3263C>T 3265T>G), AGT323GAA (3272A>G 3273G>A 3274T>A), AAC324AAG (3277C>G), TTC325TTA (3280C>A), AAA326TAT (3281A>T 3283A>T), ATT327GCC (3284A>G 3285T>C 3286T>C), CAA328AAG (3287C>A 3289A>G), ATG329TTC (3290A>T 3292G>C), GAC330TCA (3293G>T 3294A>C 3295C>A), TCC332TGT (3300C>G 3301C>T), GAA333GAG (3304A>G), TTC334TTT (3307C>T), TTG335TGG (3309T>G), AAG336CTA (3311A>C 3312A>T 3313G>A), CTC337CAG (3315T>A 3316C>G), GAA338TCA (3317G>T 3318A>C), ACT339GTA (3320A>G 3321C>T 3322T>A), GCT340TCT (3323G>T), TAT341TTT (3327A>T), CTT342CTA (3331T>A), CAC344CAT (3337C>T), ATC345GTG (3338A>G 3340C>G), ATA346GTG (3341A>G 3343A>G), AGC347ACT (3345G>C 3346C>T), AGG348AAG (3348G>A), GAC349GAT (3352C>T), GGT350GGC (3355T>C), ATC351ATT (3358C>T), AAG352ATG (3360A>T), CCT353GTT (3362C>G 3363C>T), AAC354GAT (3365A>G 3367C>T), CCT355CCG (3370T>G), GAT356GCC (3372A>C 3373T>C), AAG357AAA (3376G>A), ATT358GTG (3377A>G 3379T>G), TCC359GCT (3380T>G 3382C>T), GCT360GCG (3385T>G), ATT361GTG (3386A>G 3388T>G), CAA362AGA (3389C>A 3390A>G), AAA363GAA (3392A>G), TAT364TGG (3396A>G 3397T>G), CTG365GCC (3398C>G 3399T>C 3400G>C), ATT366AGG (3402T>G 3403T>G), CCA367CCT (3406A>T), AAG368ACG (3408A>C), ACC369TCC (3410A>T), CCT370GTG (3413C>G 3414C>T 3415T>G), AAG371ACT (3417A>C 3418G>T), GAA372GAG (3421A>G), ATA373ATT (3424A>T), AAA374CGT (3425A>C 3426A>G 3427A>T), CAA375AGT (3428C>A 3429A>G 3430A>T), TTT376TTC (3433T>C), TTA377CTT (3434T>C 3436A>T), CTT379TTG (3440C>T 3442T>G), CTC380GCA (3443C>G 3444T>C 3445C>A), CGA384CGG (3457A>G), AAA385AGA (3459A>G), TTC386TTT (3463C>T), ATT387GTT (3464A>G)

*: Inserts / Deletes / Misaligned / Frameshifts

Analysis details

This analysis was performed with panviral2.64

NGS Details (UN8): Human gammaherpesvirus 8

Assembly

**Coverage Length**	135 (1 contig(s))
**Depth Of Coverage**	228.3
**Number Of Reads**	368
**Reads Per Million**	6.15 rpm (after QC)
**Ambiguities**	0
**Assembly Method**	de novo + reference guided assembly
**Consensus Caller**	Bcf Tools

Coverage Map

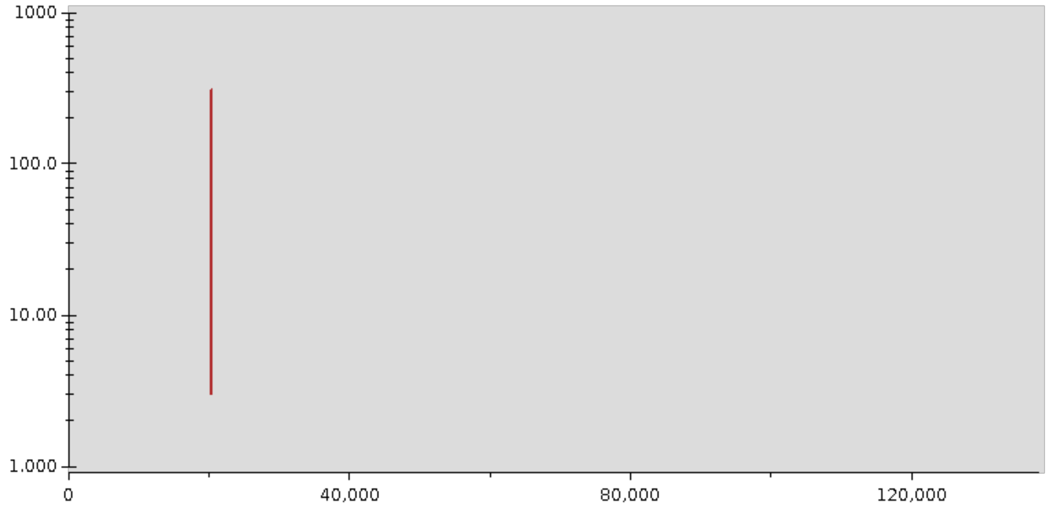

Assignment

**Type**	Human gammaherpesvirus 8 (Taxonomy ID: 37296)
**Subtype**	Could not assign
**Reference Genome**	NC_009333.1
**NT Identity (%)**	83.7037
**AA Identity (%)**	84.4444
**Number Of Stop Codons**	0
**Number Of CDS**	86

Alignment

**Alignment Score**	182.0 (NT) + 287.0 (AA) = 469.0
**Concordance (%)**	78.2972
**Alignment Method**	Local, heuristic, nucleotide (BLASTN)

Genome Region

Sequence starts at position 20273 and ends at position 20407 relative to NC_009333.1 reference sequence.

Alignment Detailed Statistics

	Begin	End	Coverage	Score	Concordance	Matches	Identities	I/D/M/F*	Stop Codons
**NT**	**20273**	**20407**	**0.1%**	**182**	**67.4%**	**135 (100%)**	**113 (83.7%)**	**0/0**	

Mutations: 20293A>T, 20296A>C, 20311G>A, 20314T>G, 20315C>G, 20317C>A, 20320C>T, 20322T>G, 20331G>T, 20341A>C, 20347A>C, 20350G>A, 20352C>T, 20353A>G, 20369C>G, 20370A>C, 20371T>A, 20376A>T, 20383G>A, 20386C>T, 20389A>G, 20392C>T

*: Inserts / Deletes / Misaligned / Frameshifts

Analysis details

This analysis was performed with panviral2.64

NGS Details (UN8): Epiphyllum badnavirus 1

Assembly

**Coverage Length**	256 (1 contig(s))
**Depth Of Coverage**	148.9
**Number Of Reads**	360
**Reads Per Million**	6.02 rpm (after QC)
**Ambiguities**	0
**Assembly Method**	de novo + reference guided assembly
**Consensus Caller**	Bcf Tools

Coverage Map

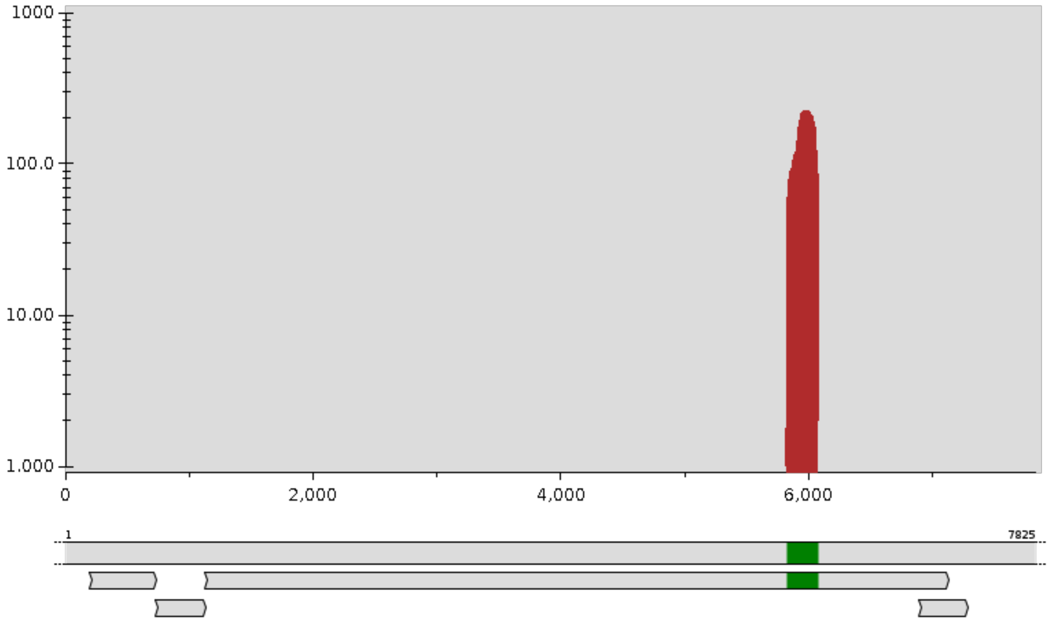

Assignment

**Type**	Epiphyllum badnavirus 1 (Taxonomy ID: 2518008)
**Reference Genome**	NC_076247.1
**NT Identity (%)**	57.529
**AA Identity (%)**	56.9767
**Number Of Stop Codons**	0
**Number Of CDS**	4

Alignment

**Alignment Score**	72.0 (NT) + 324.0 (AA) = 396.0
**Concordance (%)**	34.7673

**Alignment Method**	Global, seeded, nucleotide + amino acids (AGA)

Genome Region

Sequence starts at position 5823 and ends at position 6078 relative to NC_076247.1 reference sequence.

Alignment Detailed Statistics

	Begin	End	Coverage	Score	Concordance	Matches	Identities	I/D/M/F*	Stop Codons
**NT**	**5823**	**6078**	**3.3%**	**72**	**14.1%**	**256 (98.8%)**	**149 (57.5%)**	**3/0**	
Mutations:	5832T>G, 5835C>T, 5839G>T, 5840T>G, 5847C>A, 5851T>A, 5853C>A, 5859G>T, 5860A>C, 5861A>G, 5862G>T, 5863A>T, 5864G>C, 5865C>G, 5868A>T, 5870T>A, 5874C>T, 5878A>T, 5880T>G, 5882T>A, 5884A>G, 5886G>C, 5887G>A, 5888A>G, 5889C>G, 5892A>G, 5893G>A, 5894A>G, 5895A>T, 5896T>G, 5897C>A, 5898C>T, 5905T>A, 5906G>A, 5910C>A, 5913C>G, 5918T>G, 5922C>A, 5923C>A, 5924C>G, 5925A>G, 5926G>T, 5933T>A, 5936A>T, 5937C>T, 5940A>G, 5942G>T, 5943G>T, 5944T>C, 5946A>T, 5949T>C, 5953C>T, 5955A>C, 5958C>T, 5961A>T, 5964G>A, 5966A>C, 5967G>T, 5970T>C, 5973C>T, 5976T>G, 5982G>C, 5985C>T, 5986C>A, 5987A>T, 5988A>G, 5989C>G, 5990G>A, 5991A>T, 5992A>C, 5993A>T, 5994G>T, 5998G>A, 6000C>T, 6002A>G, 6003C>G, 6004T>A, 6005G>T, 6006T>A, 6011G>A, 6012A>G, 6012_6013insCCT, 6013G>T, 6018G>T, 6019T>G, 6020C>A, 6021A>C, 6022G>A, 6023G>T, 6024C>G, 6026T>A, 6030A>G, 6032C>T, 6033A>G, 6036T>G, 6038A>T, 6039C>T, 6054G>A, 6059T>A, 6060C>T, 6061A>T, 6062G>C, 6063T>G, 6065A>G, 6066C>A, 6067A>G, 6068C>A, 6069T>C								
**CDS**									
QKM20_gp3	1566	1650	4.2%	324	51.3%	85 (98.8%)	49 (57.0%)	1/0/0/0	0
Protein mutations:	N1568K (5832T>G), V1571C (5839G>T 5840T>G), F1575I (5851T>A 5853C>A), K1578R (5860A>C 5861A>G 5862G>T), F1581Y (5870T>A), I1584L (5878A>T 5880T>G), M1585K (5882T>A), M1586V (5884A>G 5886G>C), D1587R (5887G>A 5888A>G 5889C>G), E1589S (5893G>A 5894A>G 5895A>T), S1590D (5896T>G 5897C>A 5898C>T), W1593K (5905T>A 5906G>A), L1597R (5918T>G), P1599R (5923C>A 5924C>G 5925A>G), D1600Y (5926G>T), L1602H (5933T>A), Y1603F (5936A>T 5937C>T), W1605F (5942G>T 5943G>T), P1609S (5953C>T 5955A>C), K1613T (5966A>C 5967G>T), Q1620M (5986C>A 5987A>T 5988A>G), R1621D (5989C>G 5990G>A 5991A>T), K1622L (5992A>C 5993A>T 5994G>T), D1624N (5998G>A 6000C>T), N1625R (6002A>G 6003C>G), C1626I (6004T>A 6005G>T 6006T>A), R1628K (6011G>A 6012A>G), R1628_D1629insP (6012_6013insCCT), D1629Y (6013G>T), S1631D (6019T>G 6020C>A 6021A>C), G1632M (6022G>A 6023G>T 6024C>G), F1633Y (6026T>A), A1635V (6032C>T 6033A>G), Y1637F (6038A>T 6039C>T), F1644Y (6059T>A 6060C>T), N1646R (6065A>G 6066C>A), T1647D (6067A>G 6068C>A 6069T>C)								
Codon mutations:	AAT1568AAG (5832T>G), GCC1569GCT (5835C>T), GTC1571TGC (5839G>T 5840T>G), TCC1573TCA (5847C>A), TTC1575ATA (5851T>A 5853C>A), CTG1577CTT (5859G>T), AAG1578CGT (5860A>C 5861A>G 5862G>T), AGC1579TCG (5863A>T 5864G>C 5865C>G), GGA1580GGT (5868A>T), TTC1581TAC (5870T>A), CAC1582CAT (5874C>T), ATT1584TTG (5878A>T 5880T>G), ATG1585AAG (5882T>A), ATG1586GTC (5884A>G 5886G>C), GAC1587AGG (5887G>A 5888A>G 5889C>G), GAA1588GAG (5892A>G), GAA1589AGT (5893G>A 5894A>G 5895A>T), TCC1590GAT (5896T>G 5897C>A 5898C>T), TGG1593AAG (5905T>A 5906G>A), ACC1594ACA (5910C>A), GCC1595GCG (5913C>G), CTG1597CGG (5918T>G), ACC1598ACA (5922C>A), CCA1599AGG (5923C>A 5924C>G 5925A>G), GAT1600TAT (5926G>T), CTT1602CAT (5933T>A), TAC1603TTT (5936A>T 5937C>T), GAA1604GAG (5940A>G), TGG1605TTT (5942G>T 5943G>T), TTA1606CTT (5944T>C 5946A>T), GTT1607GTC (5949T>C), CCA1609TCC (5953C>T 5955A>C), TTC1610TTT (5958C>T), GGA1611GGT (5961A>T), TTG1612TTA (5964G>A), AAG1613ACT (5966A>C 5967G>T), AAT1614AAC (5970T>C), GCC1615GCT (5973C>T), CCT1616CCG (5976T>G), GTG1618GTC (5982G>C), TTC1619TTT (5985C>T), CAA1620ATG (5986C>A 5987A>T 5988A>G), CGA1621GAT (5989C>G 5990G>A 5991A>T), AAG1622CTT (5992A>C 5993A>T 5994G>T), GAC1624AAT (5998G>A 6000C>T), AAC1625AGG (6002A>G 6003C>G), TGT1626ATA (6004T>A 6005G>T 6006T>A), AGA1628AAG (6011G>A 6012A>G), AGA1628_GAC1629insCCT (6012_6013insCCT), GAC1629TAC (6013G>T), CTG1630CTT (6018G>T), TCA1631GAC (6019T>G 6020C>A 6021A>C), GGC1632ATG (6022G>A 6023G>T 6024C>G), TTT1633TAT (6026T>A), GTA1634GTG (6030A>G), GCA1635GTG (6032C>T 6033A>G), GTT1636GTG (6036T>G), TAC1637TTT (6038A>T 6039C>T), CTG1642CTA (6054G>A), TTC1644TAT (6059T>A 6060C>T), AGT1645TCG (6061A>T 6062G>C 6063T>G), AAC1646AGA (6065A>G 6066C>A), ACT1647GAC (6067A>G 6068C>A 6069T>C)								
**Proteins**									
polyprotein (YP_010797894.1)	1566	1650	4.2%	324	51.3%	85 (98.8%)	49 (57.0%)	1/0/0/0	0
Protein mutations:	N1568K (5832T>G), V1571C (5839G>T 5840T>G), F1575I (5851T>A 5853C>A), K1578R (5860A>C 5861A>G 5862G>T), F1581Y (5870T>A), I1584L (5878A>T 5880T>G), M1585K (5882T>A), M1586V (5884A>G 5886G>C), D1587R (5887G>A 5888A>G 5889C>G), E1589S (5893G>A 5894A>G 5895A>T), S1590D (5896T>G 5897C>A 5898C>T), W1593K (5905T>A 5906G>A), L1597R (5918T>G), P1599R (5923C>A 5924C>G 5925A>G), D1600Y (5926G>T), L1602H (5933T>A), Y1603F (5936A>T 5937C>T), W1605F (5942G>T 5943G>T), P1609S (5953C>T 5955A>C), K1613T (5966A>C 5967G>T), Q1620M (5986C>A 5987A>T 5988A>G), R1621D (5989C>G 5990G>A 5991A>T), K1622L (5992A>C 5993A>T 5994G>T), D1624N (5998G>A 6000C>T), N1625R (6002A>G 6003C>G), C1626I (6004T>A 6005G>T 6006T>A), R1628K (6011G>A 6012A>G), R1628_D1629insP (6012_6013insCCT), D1629Y (6013G>T), S1631D (6019T>G 6020C>A 6021A>C), G1632M (6022G>A 6023G>T 6024C>G), F1633Y (6026T>A), A1635V (6032C>T 6033A>G), Y1637F (6038A>T 6039C>T), F1644Y (6059T>A 6060C>T), N1646R (6065A>G 6066C>A), T1647D (6067A>G 6068C>A 6069T>C)								
Codon mutations:	AAT1568AAG (5832T>G), GCC1569GCT (5835C>T), GTC1571TGC (5839G>T 5840T>G), TCC1573TCA (5847C>A), TTC1575ATA (5851T>A 5853C>A), CTG1577CTT (5859G>T), AAG1578CGT (5860A>C 5861A>G 5862G>T), AGC1579TCG (5863A>T 5864G>C 5865C>G), GGA1580GGT (5868A>T), TTC1581TAC (5870T>A), CAC1582CAT (5874C>T), ATT1584TTG (5878A>T 5880T>G), ATG1585AAG (5882T>A), ATG1586GTC (5884A>G 5886G>C), GAC1587AGG (5887G>A 5888A>G 5889C>G), GAA1588GAG (5892A>G), GAA1589AGT (5893G>A 5894A>G 5895A>T), TCC1590GAT (5896T>G 5897C>A 5898C>T), TGG1593AAG (5905T>A 5906G>A), ACC1594ACA (5910C>A), GCC1595GCG (5913C>G), CTG1597CGG (5918T>G), ACC1598ACA (5922C>A), CCA1599AGG (5923C>A 5924C>G 5925A>G), GAT1600TAT (5926G>T), CTT1602CAT (5933T>A), TAC1603TTT (5936A>T 5937C>T), GAA1604GAG (5940A>G), TGG1605TTT (5942G>T 5943G>T), TTA1606CTT (5944T>C 5946A>T), GTT1607GTC (5949T>C), CCA1609TCC (5953C>T 5955A>C), TTC1610TTT (5958C>T), GGA1611GGT (5961A>T), TTG1612TTA (5964G>A), AAG1613ACT (5966A>C 5967G>T), AAT1614AAC (5970T>C), GCC1615GCT (5973C>T), CCT1616CCG (5976T>G), GTG1618GTC (5982G>C), TTC1619TTT (5985C>T), CAA1620ATG (5986C>A 5987A>T 5988A>G), CGA1621GAT (5989C>G 5990G>A 5991A>T), AAG1622CTT (5992A>C 5993A>T 5994G>T), GAC1624AAT (5998G>A 6000C>T), AAC1625AGG (6002A>G 6003C>G), TGT1626ATA (6004T>A 6005G>T 6006T>A), AGA1628AAG (6011G>A 6012A>G), AGA1628_GAC1629insCCT (6012_6013insCCT), GAC1629TAC (6013G>T), CTG1630CTT (6018G>T), TCA1631GAC (6019T>G 6020C>A 6021A>C), GGC1632ATG (6022G>A 6023G>T 6024C>G), TTT1633TAT (6026T>A), GTA1634GTG (6030A>G), GCA1635GTG (6032C>T 6033A>G), GTT1636GTG (6036T>G), TAC1637TTT (6038A>T 6039C>T), CTG1642CTA (6054G>A), TTC1644TAT (6059T>A 6060C>T), AGT1645TCG (6061A>T 6062G>C 6063T>G), AAC1646AGA (6065A>G 6066C>A), ACT1647GAC (6067A>G 6068C>A 6069T>C)								

*: Inserts / Deletes / Misaligned / Frameshifts

Analysis details

This analysis was performed with panviral2.64

NGS Details (UN8): Cassava brown streak virus

Assembly

**Coverage Length**	527 (1 contig(s))
**Depth Of Coverage**	70.8
**Number Of Reads**	308
**Reads Per Million**	5.15 rpm (after QC)
**Ambiguities**	0
**Assembly Method**	de novo + reference guided assembly
**Consensus Caller**	Bcf Tools

Coverage Map

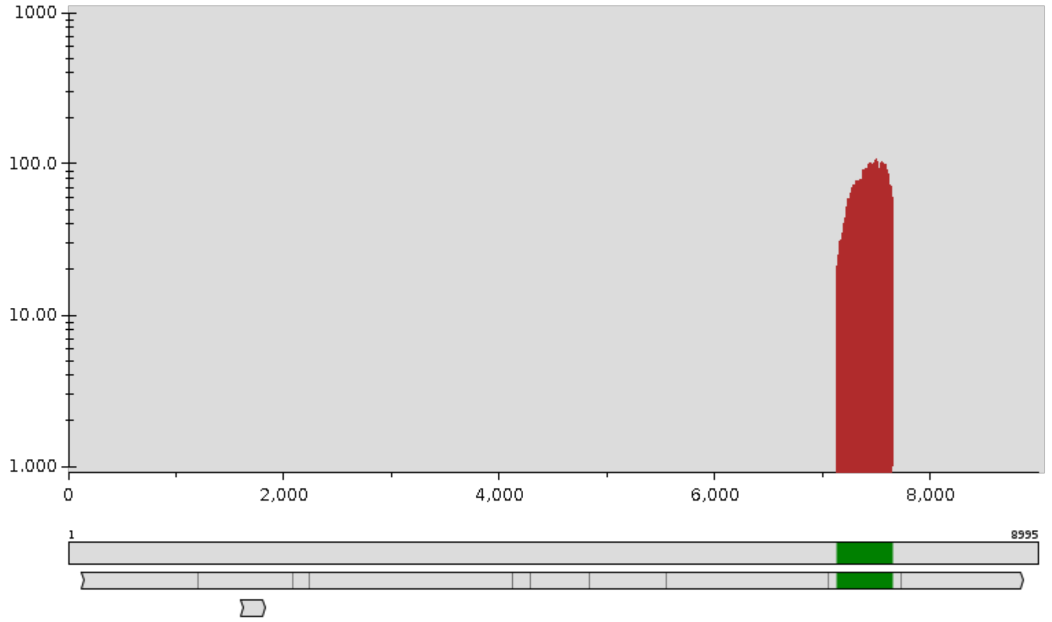

Assignment

**Type**	Cassava brown streak virus (Taxonomy ID: 137758)
**Reference Genome**	NC_012698.2
**NT Identity (%)**	60.9108
**AA Identity (%)**	57.9545
**Number Of Stop Codons**	0
**Number Of CDS**	2

Alignment

**Alignment Score**	230.0 (NT) + 742.0 (AA) = 972.0
**Concordance (%)**	42.7253

Alignment Method	Global, seeded, nucleotide + amino acids (AGA)

Genome Region

Sequence starts at position 7124 and ends at position 7650 relative to NC_012698.2 reference sequence.

Alignment Detailed Statistics

	Begin	End	Coverage	Score	Concordance	Matches	Identities	I/D/M/F*	Stop Codons
**NT**	**7124**	**7650**	**5.9%**	**230**	**21.8%**	**527 (100%)**	**321 (60.9%)**	**0/0**	
Mutations:	7130A>G, 7133A>G, 7134A>G, 7136T>G, 7142C>T, 7145A>T, 7148A>C, 7151G>A, 7157A>T, 7158C>A, 7163A>G, 7167A>G, 7175A>T, 7177A>G, 7178A>G, 7179C>G, 7180A>C, 7184A>T, 7185T>C, 7190T>C, 7192C>A, 7193C>G, 7194A>T, 7202A>C, 7203A>T, 7205C>T, 7206A>C, 7207T>A, 7208C>G, 7211T>C, 7213G>T, 7214G>T, 7217A>G, 7218G>C, 7229A>T, 7234C>T, 7235A>T, 7241A>G, 7242A>G, 7243C>A, 7244A>G, 7245G>C, 7246T>C, 7250G>A, 7253A>T, 7256T>A, 7257A>T, 7258T>C, 7270T>C, 7271A>T, 7272C>A, 7273G>A, 7274T>A, 7275G>A, 7277A>T, 7280G>T, 7281T>G, 7283T>C, 7284G>A, 7286G>A, 7287T>G, 7288T>A, 7290A>G, 7292T>G, 7293G>A, 7294G>A, 7295A>T, 7298G>A, 7304T>G, 7307C>A, 7310A>T, 7313A>G, 7319A>T, 7320A>T, 7323T>C, 7325G>C, 7331T>C, 7332G>A, 7334T>C, 7337T>C, 7340C>T, 7343T>G, 7355A>G, 7358A>T, 7361C>T, 7364T>C, 7374A>T, 7377G>C, 7380G>A, 7381G>A, 7385T>C, 7388A>T, 7390T>A, 7391A>T, 7397A>T, 7398T>C, 7400A>T, 7401T>A, 7403T>C, 7406G>C, 7409G>A, 7410G>T, 7413G>A, 7414A>T, 7416C>G, 7418G>T, 7422C>G, 7424A>G, 7425A>G, 7432T>C, 7433G>A, 7436T>A, 7437A>T, 7438G>A, 7442T>C, 7443C>A, 7445C>G, 7448C>T, 7449G>A, 7451G>C, 7455G>T, 7457T>A, 7458T>C, 7462T>C, 7463A>T, 7464A>C, 7465A>T, 7467A>G, 7468A>G, 7469A>G, 7470G>C, 7471T>C, 7472T>A, 7473G>A, 7474G>A, 7476G>A, 7477A>C, 7481T>G, 7484T>G, 7487A>G, 7489T>C, 7490C>T, 7494A>G, 7495A>T, 7496G>T, 7499T>A, 7500G>A, 7501T>A, 7502G>A, 7503C>A, 7504T>C, 7505A>G, 7506A>C, 7507G>T, 7508A>G, 7511T>A, 7512G>A, 7517T>A, 7520A>G, 7521A>C, 7522T>C, 7523G>A, 7524C>G, 7526A>T, 7527C>A, 7529C>G, 7535A>C, 7539T>A, 7540C>A, 7541A>T, 7544T>C, 7547G>A, 7553C>T, 7556A>G, 7559T>A, 7562C>T, 7568A>T, 7570T>A, 7572A>G, 7573A>G, 7576G>A, 7577G>T, 7578A>G, 7579G>A, 7580A>C, 7581A>C, 7586A>T, 7588T>A, 7589T>C, 7599A>C, 7600T>C, 7601G>C, 7602G>A, 7603C>A, 7604C>G, 7607G>A, 7610G>A, 7616T>C, 7618T>A, 7622A>T, 7628T>C, 7631A>C, 7632T>G, 7633T>G, 7635C>A, 7636G>A								

CDS

	Begin	End	Coverage	Score	Concordance	Matches	Identities	I/D/M/F*	Stop Codons
CBSV_gp1	2334	2509	6.0%	742	60.2%	176 (100%)	102 (58.0%)	0/0/0/0	0
Protein mutations:	I2337V (7134A>G 7136T>G), Q2345K (7158C>A), K2348E (7167A>G), K2351R (7177A>G 7178A>G), Q2352A (7179C>G 7180A>C), F2354L (7185T>C), P2356Q (7192C>A 7193C>G), T2357S (7194A>T), I2360F (7203A>T 7205C>T), I2361Q (7206A>C 7207T>A 7208C>G), R2363L (7213G>T 7214G>T), V2365L (7218G>C), P2370L (7234C>T 7235A>T), T2373E (7242A>G 7243C>A 7244A>G), V2374P (7245G>C 7246T>C), E2376D (7253A>T), I2378S (7257A>T 7258T>C), V2382A (7270T>C 7271A>T), R2383K (7272C>A 7273G>A 7274T>A), V2384I (7275G>A 7277A>T), S2386A (7281T>G 7283T>C), E2387K (7284G>A 7286G>A), L2388E (7287T>G 7288T>A), I2389V (7290A>G 7292T>G), G2390N (7293G>A 7294G>A 7295A>T), S2399C (7320A>T), D2403N (7332G>A 7334T>C), N2406K (7343T>G), M2417L (7374A>T), E2418Q (7377G>C), G2419K (7380G>A 7381G>A), L2422H (7390T>A 7391A>T), Y2426N (7401T>A 7403T>C), K2427N (7406G>C), V2429L (7410G>T), E2430M (7413G>A 7414A>T), P2431A (7416C>G 7418G>T), Q2433E (7422C>G 7424A>G), N2434D (7425A>G), M2436T (7432T>C 7433G>A), S2438Y (7437A>T 7438G>A), L2440M (7443C>A 7445C>G), V2442I (7449G>A 7451G>C), A2444S (7455G>T 7457T>A), F2445L (7458T>C), V2446A (7462T>C 7463A>T), N2447L (7464A>C 7465A>T), K2448G (7467A>G 7468A>G 7469A>G), V2449P (7470G>C 7471T>C 7472T>A), G2450N (7473G>A 7474G>A), D2451T (7476G>A 7477A>C), D2452E (7481T>G), I2454M (7487A>G), I2455T (7489T>C 7490C>T), K2457V (7494A>G 7495A>T 7496G>T), V2459K (7500G>A 7501T>A 7502G>A), L2460T (7503C>A 7504T>C 7505A>G), R2461L (7506A>C 7507G>T 7508A>G), E2463K (7512G>A), M2466P (7521A>C 7522T>C 7523G>A), P2467A (7524C>G 7526A>T), S2472N (7539T>A 7540C>A 7541A>T), L2482H (7570T>A), N2483G (7572A>G 7573A>G), W2484Y (7576G>A 7577G>T), R2485D (7578A>G 7579G>A 7580A>C), K2486Q (7581A>C), F2488Y (7588T>A 7589T>C), M2492P (7599A>C 7600T>C 7601G>C), A2493K (7602G>A 7603C>A 7604C>G), M2498K (7618T>A), F2503G (7632T>G 7633T>G), R2504K (7635C>A 7636G>A)								
Codon mutations:	GCA2335GCG (7130A>G), CCA2336CCG (7133A>G), ATT2337GTG (7134A>G 7136T>G), TTC2339TTT (7142C>T), GTA2340GTT (7145A>T), ACA2341ACC (7148A>C), GGG2342GGA (7151G>A), GCA2344GCT (7157A>T), CAA2345AAA (7158C>A), AAA2346AAG (7163A>G), AAG2348GAG (7167A>G), GTA2350GTT (7175A>T), AAA2351AGG (7177A>G 7178A>G), CAA2352GCA (7179C>G 7180A>C), ATA2353ATT (7184A>T), TTT2354CTT (7185T>C), GGT2355GGC (7190T>C), CCC2356CAG (7192C>A 7193C>G), ACT2357TCT (7194A>T), CCA2359CCC (7202A>C), ATC2360TTT (7203A>T 7205C>T), ATC2361CAG (7206A>C 7207T>A 7208C>G), TCT2362TCC (7211T>C), CGG2363CTT (7213G>T 7214G>T), AAA2364AAG (7217A>G), GTT2365CTT (7218G>C), CCA2368CCT (7229A>T), CCA2370CTT (7234C>T 7235A>T), GGA2372GGG (7241A>G), ACA2373GAG (7242A>G 7243C>A 7244A>G), GTT2374CCT (7245G>C 7246T>C), GAG2375GAA (7250G>A), GAA2376GAT (7253A>T), ATT2377ATA (7256T>A), ATC2378TCC (7257A>T 7258T>C), GTA2382GCT (7270T>C 7271A>T), CGT2383AAA (7272C>A 7273G>A 7274T>A), GTA2384ATT (7275G>A 7277A>T), GCG2385GCT (7280G>T), TCT2386GCC (7281T>G 7283T>C), GAG2387AAA (7284G>A 7286G>A), TTG2388GAG (7287T>G 7288T>A), ATT2389GTG (7290A>G 7292T>G), GGA2390AAT (7293G>A 7294G>A 7295A>T), GGG2391GGA (7298G>A), GTT2393GTG (7304T>G), CTC2394CTA (7307C>A), GTA2395GTT (7310A>T), GAA2396GAG (7313A>G), ACA2398ACT (7319A>T), AGT2399TGT (7320A>T), TTG2400CTC (7323T>C 7325G>C), TTT2402TTC (7331T>C), GAT2403AAC (7332G>A 7334T>C), GCT2404GCC (7337T>C), CTC2405CTT (7340C>T), AAT2406AAG (7343T>G), GGA2410GGG (7355A>G), CCA2411CCT (7358A>T), TAC2412TAT (7361C>T), ATT2413ATC (7364T>C), ATG2417TTG (7374A>T), GAA2418CAA (7377G>C), GGG2419AAG (7380G>A 7381G>A), ATT2420ATC (7385T>C), GGA2421GGT (7388A>T), CTA2422CAT (7390T>A 7391A>T), GGA2424GGT (7397A>T), TTA2425CTT (7398T>C 7400A>T), TAT2426AAC (7401T>A 7403T>C), AAG2427AAC (7406G>C), TTG2428TTA (7409G>A), GTG2429TTG (7410G>T), GAG2430ATG (7413G>A 7414A>T), CCG2431GCT (7416C>G 7418G>T), CAA2433GAG (7422C>G 7424A>G), AAT2434GAT (7425A>G), ATG2436ACA (7432T>C 7433G>A), GCT2437GCA (7436T>A), AGT2438TAT (7437A>T 7438G>A), GCT2439GCC (7442T>C), CTC2440ATG (7443C>A 7445C>G), TGC2441TGT (7448C>T), GTG2442ATC (7449G>A 7451G>C), GCT2444TCA (7455G>T 7457T>A), TTT2445CTT (7458T>C), GTA2446GCT (7462T>C 7463A>T), AAT2447CTT (7464A>C 7465A>T), AAA2448GGG (7467A>G 7468A>G 7469A>G), GTT2449CCA (7470G>C 7471T>C 7472T>A), GGT2450AAT (7473G>A 7474G>A), GAT2451ACT (7476G>A 7477A>C), GAT2452GAG (7481T>G), CCT2453CCG (7484T>G), ATA2454ATG (7487A>G), ATC2455ACT (7489T>C 7490C>T), AAG2457GTT (7494A>G 7495A>T 7496G>T), GGT2458GGA (7499T>A), GTG2459AAA (7500G>A 7501T>A 7502G>A), CTA2460ACG (7503C>A 7504T>C 7505A>G), AGA2461CTG (7506A>C 7507G>T 7508A>G), GGT2462GGA (7511T>A), GAG2463AAG (7512G>A), ATT2464ATA (7517T>A), GTA2465GTG (7520A>G), ATG2466CCA (7521A>C 7522T>C 7523G>A), CCA2467GCT (7524C>G 7526A>T), CGC2468AGG (7527C>A 7529C>G), CCA2470CCC (7535A>C), TCA2472AAT (7539T>A 7540C>A 7541A>T), TTT2473TTC (7544T>C), GGG2474GGA (7547G>A), GAC2476GAT (7553C>T), CCA2477CCG (7556A>G), ATT2478ATA (7559T>A), TTC2479TTT (7562C>T), CCA2481CCT (7568A>T), CTT2482CAT (7570T>A), AAC2483GGC (7572A>G 7573A>G), TGG2484TAT (7576G>A 7577G>T), AGA2485GAC (7578A>G 7579G>A 7580A>C), AAG2486CAG (7581A>C), ACA2487ACT (7586A>T), TTT2488TAC (7588T>A 7589T>C), ATG2492CCC (7599A>C 7600T>C 7601G>C), GCC2493AAG (7602G>A 7603C>A 7604C>G), GAG2494GAA (7607G>A), GAG2495GAA (7610G>A), AAT2497AAC (7616T>C), ATG2498AAG (7618T>A), ATA2499ATT (7622A>T), CAT2501CAC (7628T>C), CGA2502CGC (7631A>C), TTT2503GGT (7632T>G 7633T>G), CGA2504AAA (7635C>A 7636G>A)								

Proteins

	Begin	End	Coverage	Score	Concordance	Matches	Identities	I/D/M/F*	Stop Codons
polyprotein (YP_007027011.1)	2334	2509	6.0%	742	60.2%	176 (100%)	102 (58.0%)	0/0/0/0	0
Protein mutations:	I2337V (7134A>G 7136T>G), Q2345K (7158C>A), K2348E (7167A>G), K2351R (7177A>G 7178A>G), Q2352A (7179C>G 7180A>C), F2354L (7185T>C), P2356Q (7192C>A 7193C>G), T2357S (7194A>T), I2360F (7203A>T 7205C>T), I2361Q (7206A>C 7207T>A 7208C>G), R2363L (7213G>T 7214G>T), V2365L (7218G>C), P2370L (7234C>T 7235A>T), T2373E (7242A>G 7243C>A 7244A>G), V2374P (7245G>C 7246T>C), E2376D (7253A>T), I2378S (7257A>T 7258T>C), V2382A (7270T>C 7271A>T), R2383K (7272C>A 7273G>A 7274T>A), V2384I (7275G>A 7277A>T), S2386A (7281T>G 7283T>C), E2387K (7284G>A 7286G>A), L2388E (7287T>G 7288T>A), I2389V (7290A>G 7292T>G), G2390N (7293G>A 7294G>A 7295A>T), S2399C (7320A>T), D2403N (7332G>A 7334T>C), N2406K (7343T>G), M2417L (7374A>T), E2418Q (7377G>C), G2419K (7380G>A 7381G>A), L2422H (7390T>A 7391A>T), Y2426N (7401T>A 7403T>C), K2427N (7406G>C), V2429L (7410G>T), E2430M (7413G>A 7414A>T), P2431A (7416C>G 7418G>T), Q2433E (7422C>G 7424A>G), N2434D (7425A>G), M2436T (7432T>C 7433G>A), S2438Y (7437A>T 7438G>A), L2440M (7443C>A 7445C>G), V2442I (7449G>A 7451G>C), A2444S (7455G>T 7457T>A), F2445L (7458T>C), V2446A (7462T>C 7463A>T), N2447L (7464A>C 7465A>T), K2448G (7467A>G 7468A>G 7469A>G), V2449P (7470G>C 7471T>C 7472T>A), G2450N (7473G>A 7474G>A), D2451T (7476G>A 7477A>C), D2452E (7481T>G), I2454M (7487A>G), I2455T (7489T>C 7490C>T), K2457V (7494A>G 7495A>T 7496G>T), V2459K (7500G>A 7501T>A 7502G>A), L2460T (7503C>A 7504T>C 7505A>G), R2461L (7506A>C 7507G>T 7508A>G), E2463K (7512G>A), M2466P (7521A>C 7522T>C 7523G>A), P2467A (7524C>G 7526A>T), S2472N (7539T>A 7540C>A 7541A>T), L2482H (7570T>A), N2483G (7572A>G 7573A>G), W2484Y (7576G>A 7577G>T), R2485D (7578A>G 7579G>A 7580A>C), K2486Q (7581A>C), F2488Y (7588T>A 7589T>C), M2492P (7599A>C 7600T>C 7601G>C), A2493K (7602G>A 7603C>A 7604C>G), M2498K (7618T>A), F2503G (7632T>G 7633T>G), R2504K (7635C>A 7636G>A)								

	Begin	End	Coverage	Score	Concordance	Matches	Identities	I/D/M/F*	Stop Codons
**NT**	**7124**	**7650**	**5.9%**	**230**	**21.8%**	**527 (100%)**	**321 (60.9%)**	**0/0**	
Codon mutations:	GCA2335GCG (7130A>G), CCA2336CCG (7133A>G), ATT2337GTG (7134A>G 7136T>G), TTC2339TTT (7142C>T), GTA2340GTT (7145A>T), ACA2341ACC (7148A>C), GGG2342GGA (7151G>A), GCA2344GCT (7157A>T), CAA2345AAA (7158C>A), AAA2346AAG (7163A>G), AAG2348GAG (7167A>G), GTA2350GTT (7175A>T), AAA2351AGG (7177A>G 7178A>G), CAA2352GCA (7179C>G 7180A>C), ATA2353ATT (7184A>T), TTT2354CTT (7185T>C), GGT2355GGC (7190T>C), CCC2356CAG (7192C>A 7193C>G), ACT2357TCT (7194A>T), CCA2359CCC (7202A>C), ATC2360TTT (7203A>T 7205C>T), ATC2361CAG (7206A>C 7207T>A 7208C>G), TCT2362TCC (7211T>C), CGG2363CTT (7213G>T 7214G>T), AAA2364AAG (7217A>G), GTT2365CTT (7218G>C), CCA2368CCT (7229A>T), CCA2370CTT (7234C>T 7235A>T), GGA2372GGG (7241A>G), ACA2373GAG (7242A>G 7243C>A 7244A>G), GTT2374CCT (7245G>C 7246T>C), GAG2375GAA (7250G>A), GAA2376GAT (7253A>T), ATT2377ATA (7256T>A), ATC2378TCC (7257A>T 7258T>C), GTA2382GCT (7270T>C 7271A>T), CGT2383AAA (7272C>A 7273G>A 7274T>A), GTA2384ATT (7275G>A 7277A>T), GCG2385GCT (7280G>T), TCT2386GCC (7281T>G 7283T>C), GAG2387AAA (7284G>A 7286G>A), TTG2388GAG (7287T>G 7288T>A), ATT2389GTG (7290A>G 7292T>G), GGA2390AAT (7293G>A 7294G>A 7295A>T), GGG2391GGA (7298G>A), GTT2393GTG (7304T>G), CTC2394CTA (7307C>A), GTA2395GTT (7310A>T), GAA2396GAG (7313A>G), ACA2398ACT (7319A>T), AGT2399TGT (7320A>T), TTG2400CTC (7323T>C 7325G>C), TTT2402TTC (7331T>C), GAT2403AAC (7332G>A 7334T>C), GCT2404GCC (7337T>C), CTC2405CTT (7340C>T), AAT2406AAG (7343T>G), GGA2410GGG (7355A>G), CCA2411CCT (7358A>T), TAC2412TAT (7361C>T), ATT2413ATC (7364T>C), ATG2417TTG (7374A>T), GAA2418CAA (7377G>C), GGG2419AAG (7380G>A 7381G>A), ATT2420ATC (7385T>C), GGA2421GGT (7388A>T), CTA2422CAT (7390T>A 7391A>T), GGA2424GGT (7397A>T), TTA2425CTT (7398T>C 7400A>T), TAT2426AAC (7401T>A 7403T>C), AAG2427AAC (7406G>C), TTG2428TTA (7409G>A), GTG2429TTG (7410G>T), GAG2430ATG (7413G>A 7414A>T), CCG2431GCT (7416C>G 7418G>T), CAA2433GAG (7422C>G 7424A>G), AAT2434GAT (7425A>G), ATG2436ACA (7432T>C 7433G>A), GCT2437GCA (7436T>A), AGT2438TAT (7437A>T 7438G>A), GCT2439GCC (7442T>C), CTC2440ATG (7443C>A 7445C>G), TGC2441TGT (7448C>T), GTG2442ATC (7449G>A 7451G>C), GCT2444TCA (7455G>T 7457T>A), TTT2445CTT (7458T>C), GTA2446GCT (7462T>C 7463A>T), AAT2447CTT (7464A>C 7465A>T), AAA2448GGG (7467A>G 7468A>G 7469A>G), GTT2449CCA (7470G>C 7471T>C 7472T>A), GGT2450AAT (7473G>A 7474G>A), GAT2451ACT (7476G>A 7477A>C), GAT2452GAG (7481T>G), CCT2453CCG (7484T>G), ATA2454ATG (7487A>G), ATC2455ACT (7489T>C 7490C>T), AAG2457GTT (7494A>G 7495A>T 7496G>T), GGT2458GGA (7499T>A), GTG2459AAA (7500G>A 7501T>A 7502G>A), CTA2460ACG (7503C>A 7504T>C 7505A>G), AGA2461CTG (7506A>C 7507G>T 7508A>G), GGT2462GGA (7511T>A), GAG2463AAG (7512G>A), ATT2464ATA (7517T>A), GTA2465GTG (7520A>G), ATG2466CCA (7521A>C 7522T>C 7523G>A), CCA2467GCT (7524C>G 7526A>T), CGC2468AGG (7527C>A 7529C>G), CCA2470CCC (7535A>C), TCA2472AAT (7539T>A 7540C>A 7541A>T), TTT2473TTC (7544T>C), GGG2474GGA (7547G>A), GAC2476GAT (7553C>T), CCA2477CCG (7556A>G), ATT2478ATA (7559T>A), TTC2479TTT (7562C>T), CCA2481CCT (7568A>T), CTT2482CAT (7570T>A), AAC2483GGC (7572A>G 7573A>G), TGG2484TAT (7576G>A 7577G>T), AGA2485GAC (7578A>G 7579G>A 7580A>C), AAG2486CAG (7581A>C), ACA2487ACT (7586A>T), TTT2488TAC (7588T>A 7589T>C), ATG2492CCC (7599A>C 7600T>C 7601G>C), GCC2493AAG (7602G>A 7603C>A 7604C>G), GAG2494GAA (7607G>A), GAG2495GAA (7610G>A), AAT2497AAC (7616T>C), ATG2498AAG (7618T>A), ATA2499ATT (7622A>T), CAT2501CAC (7628T>C), CGA2502CGC (7631A>C), TTT2503GGT (7632T>G 7633T>G), CGA2504AAA (7635C>A 7636G>A)								
HAM1-like protein (YP_007032446.1)	26	201	77.9%	742	60.2%	176 (100%)	102 (58.0%)	0/0/0/0	0
Protein mutations:	I29V (7134A>G 7136T>G), Q37K (7158C>A), K40E (7167A>G), K43R (7177A>G 7178A>G), Q44A (7179C>G 7180A>C), F46L (7185T>C), P48Q (7192C>A 7193C>G), T49S (7194A>T), I52F (7203A>T 7205C>T), I53Q (7206A>C 7207T>A 7208C>G), R55L (7213G>T 7214G>T), V57L (7218G>C), P62L (7234C>T 7235A>T), T65E (7242A>G 7243C>A 7244A>G), V66P (7245G>C 7246T>C), E68D (7253A>T), I70S (7257A>T 7258T>C), V74A (7270T>C 7271A>T), R75K (7272C>A 7273G>A 7274T>A), V76I (7275G>A 7277A>T), S78A (7281T>G 7283T>C), E79K (7284G>A 7286G>A), L80E (7287T>G 7288T>A), I81V (7290A>G 7292T>G), G82N (7293G>A 7294G>A 7295A>T), S91C (7320A>T), D95N (7332G>A 7334T>C), N98K (7343T>G), M109L (7374A>T), E110Q (7377G>C), G111K (7380G>A 7381G>A), L114H (7390T>A 7391A>T), Y118N (7401T>A 7403T>C), K119N (7406G>C), V121L (7410G>T), E122M (7413G>A 7414A>T), P123A (7416C>G 7418G>T), Q125E (7422C>G 7424A>G), N126D (7425A>G), M128T (7432T>C 7433G>A), S130Y (7437A>T 7438G>A), L132M (7443C>A 7445C>G), V134I (7449G>A 7451G>C), A136S (7455G>T 7457T>A), F137L (7458T>C), V138A (7462T>C 7463A>T), N139L (7464A>C 7465A>T), K140G (7467A>G 7468A>G 7469A>G), V141P (7470G>C 7471T>C 7472T>A), G142N (7473G>A 7474G>A), D143T (7476G>A 7477A>C), D144E (7481T>G), I146M (7487A>G), I147T (7489T>C 7490C>T), K149V (7494A>G 7495A>T 7496G>T), V151K (7500G>A 7501T>A 7502G>A), L152T (7503C>A 7504T>C 7505A>G), R153L (7506A>C 7507G>T 7508A>G), E155K (7512G>A), M158P (7521A>C 7522T>C 7523G>A), P159A (7524C>G 7526A>T), S164N (7539T>A 7540C>A 7541A>T), L174H (7570T>A), N175G (7572A>G 7573A>G), W176Y (7576G>A 7577G>T), R177D (7578A>G 7579G>A 7580A>C), K178Q (7581A>C), F180Y (7588T>A 7589T>C), M184P (7599A>C 7600T>C 7601G>C), A185K (7602G>A 7603C>A 7604C>G), M190K (7618T>A), F195G (7632T>G 7633T>G), R196K (7635C>A 7636G>A)								
Codon mutations:	GCA27GCG (7130A>G), CCA28CCG (7133A>G), ATT29GTG (7134A>G 7136T>G), TTC31TTT (7142C>T), GTA32GTT (7145A>T), ACA33ACC (7148A>C), GGG34GGA (7151G>A), GCA36GCT (7157A>T), CAA37AAA (7158C>A), AAA38AAG (7163A>G), AAG40GAG (7167A>G), GTA42GTT (7175A>T), AAA43AGG (7177A>G 7178A>G), CAA44GCA (7179C>G 7180A>C), ATA45ATT (7184A>T), TTT46CTT (7185T>C), GGT47GGC (7190T>C), CCC48CAG (7192C>A 7193C>G), ACT49TCT (7194A>T), CCA51CCC (7202A>C), ATC52TTT (7203A>T 7205C>T), ATC53CAG (7206A>C 7207T>A 7208C>G), TCT54TCC (7211T>C), CGG55CTT (7213G>T 7214G>T), AAA56AAG (7217A>G), GTT57CTT (7218G>C), CCA60CCT (7229A>T), CCA62CTT (7234C>T 7235A>T), GGA64GGG (7241A>G), ACA65GAG (7242A>G 7243C>A 7244A>G), GTT66CCT (7245G>C 7246T>C), GAG67GAA (7250G>A), GAA68GAT (7253A>T), ATT69ATA (7256T>A), ATC70TCC (7257A>T 7258T>C), GTA74GCT (7270T>C 7271A>T), CGT75AAA (7272C>A 7273G>A 7274T>A), GTA76ATT (7275G>A 7277A>T), GCG77GCT (7280G>T), TCT78GCC (7281T>G 7283T>C), GAG79AAA (7284G>A 7286G>A), TTG80GAG (7287T>G 7288T>A), ATT81GTG (7290A>G 7292T>G), GGA82AAT (7293G>A 7294G>A 7295A>T), GGG83GGA (7298G>A), GTT85GTG (7304T>G), CTC86CTA (7307C>A), GTA87GTT (7310A>T), GAA88GAG (7313A>G), ACA90ACT (7319A>T), AGT91TGT (7320A>T), TTG92CTC (7323T>C 7325G>C), TTT94TTC (7331T>C), GAT95AAC (7332G>A 7334T>C), GCT96GCC (7337T>C), CTC97CTT (7340C>T), AAT98AAG (7343T>G), GGA102GGG (7355A>G), CCA103CCT (7358A>T), TAC104TAT (7361C>T), ATT105ATC (7364T>C), ATG109TTG (7374A>T), GAA110CAA (7377G>C), GGG111AAG (7380G>A 7381G>A), ATT112ATC (7385T>C), GGA113GGT (7388A>T), CTA114CAT (7390T>A 7391A>T), GGA116GGT (7397A>T), TTA117CTT (7398T>C 7400A>T), TAT118AAC (7401T>A 7403T>C), AAG119AAC (7406G>C), TTG120TTA (7409G>A), GTG121TTG (7410G>T), GAG122ATG (7413G>A 7414A>T), CCG123GCT (7416C>G 7418G>T), CAA125GAG (7422C>G 7424A>G), AAT126GAT (7425A>G), ATG128ACA (7432T>C 7433G>A), GCT129GCA (7436T>A), AGT130TAT (7437A>T 7438G>A), GCT131GCC (7442T>C), CTC132ATG (7443C>A 7445C>G), TGC133TGT (7448C>T), GTG134ATC (7449G>A 7451G>C), GCT136TCA (7455G>T 7457T>A), TTT137CTT (7458T>C), GTA138GCT (7462T>C 7463A>T), AAT139CTT (7464A>C 7465A>T), AAA140GGG (7467A>G 7468A>G 7469A>G), GTT141CCA (7470G>C 7471T>C 7472T>A), GGT142AAT (7473G>A 7474G>A), GAT143ACT (7476G>A 7477A>C), GAT144GAG (7481T>G), CCT145CCG (7484T>G), ATA146ATG (7487A>G), ATC147ACT (7489T>C 7490C>T), AAG149GTT (7494A>G 7495A>T 7496G>T), GGT150GGA (7499T>A), GTG151AAA (7500G>A 7501T>A 7502G>A), CTA152ACG (7503C>A 7504T>C 7505A>G), AGA153CTG (7506A>C 7507G>T 7508A>G), GGT154GGA (7511T>A), GAG155AAG (7512G>A), ATT156ATA (7517T>A), GTA157GTG (7520A>G), ATG158CCA (7521A>C 7522T>C 7523G>A), CCA159GCT (7524C>G 7526A>T), CGC160AGG (7527C>A 7529C>G), CCA162CCC (7535A>C), TCA164AAT (7539T>A 7540C>A 7541A>T), TTT165TTC (7544T>C), GGG166GGA (7547G>A), GAC168GAT (7553C>T), CCA169CCG (7556A>G), ATT170ATA (7559T>A), TTC171TTT (7562C>T), CCA173CCT (7568A>T), CTT174CAT (7570T>A), AAC175GGC (7572A>G 7573A>G), TGG176TAT (7576G>A 7577G>T), AGA177GAC (7578A>G 7579G>A 7580A>C), AAG178CAG (7581A>C), ACA179ACT (7586A>T), TTT180TAC (7588T>A 7589T>C), ATG184CCC (7599A>C 7600T>C 7601G>C), GCC185AAG (7602G>A 7603C>A 7604C>G), GAG186GAA (7607G>A), GAG187GAA (7610G>A), AAT189AAC (7616T>C), ATG190AAG (7618T>A), ATA191ATT (7622A>T), CAT193CAC (7628T>C), CGA194CGC (7631A>C), TTT195GGT (7632T>G 7633T>G), CGA196AAA (7635C>A 7636G>A)								

*: Inserts / Deletes / Misaligned / Frameshifts

Analysis details

This analysis was performed with panviral2.64

NGS Details (UN8): Epiphyllum badnavirus 1

Assembly

**Coverage Length**	275 (1 contig(s))
**Depth Of Coverage**	50.7
**Number Of Reads**	137
**Reads Per Million**	2.29 rpm (after QC)
**Ambiguities**	0
**Assembly Method**	de novo + reference guided assembly
**Consensus Caller**	Bcf Tools

Coverage Map

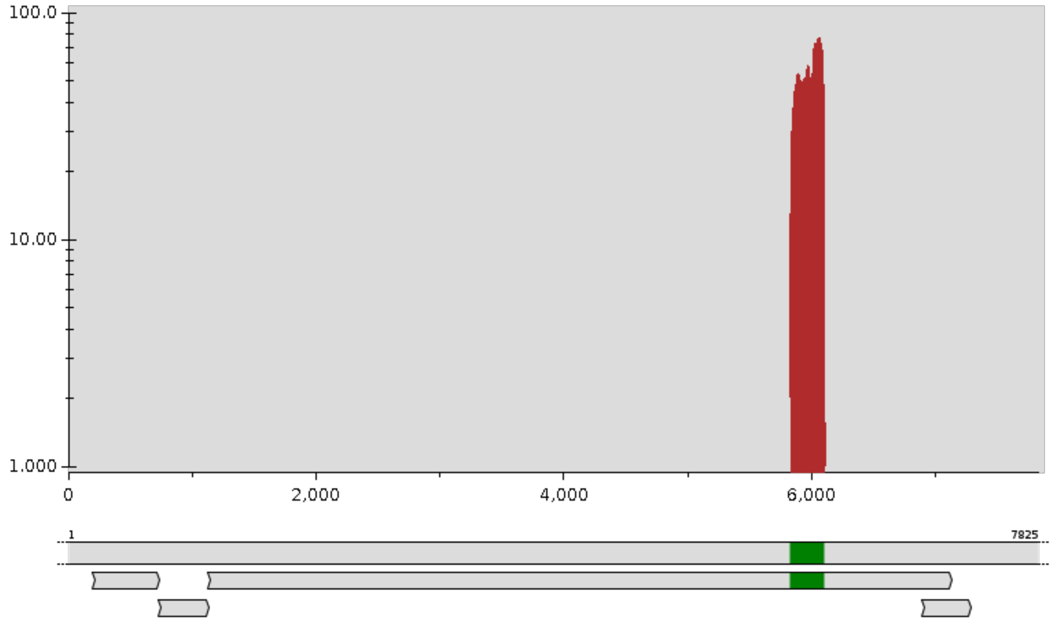

Assignment

**Type**	Epiphyllum badnavirus 1 (Taxonomy ID: 2518008)
**Reference Genome**	NC_076247.1
**NT Identity (%)**	57.554
**AA Identity (%)**	52.6882
**Number Of Stop Codons**	0
**Number Of CDS**	4

Alignment

**Alignment Score**	78.0 (NT) + 337.0 (AA) = 415.0
**Concordance (%)**	34.2127

Alignment Method	Global, seeded, nucleotide + amino acids (AGA)

Genome Region

Sequence starts at position 5829 and ends at position 6103 relative to NC_076247.1 reference sequence.

Alignment Detailed Statistics

	Begin	End	Coverage	Score	Concordance	Matches	Identities	I/D/M/F*	Stop Codons
**NT**	**5829**	**6103**	**3.5%**	**78**	**14.2%**	**275 (98.9%)**	**160 (57.6%)**	**3/0**	
Mutations:	5832T>A, 5835C>T, 5837G>C, 5841C>G, 5844C>T, 5847C>T, 5851T>A, 5853C>T, 5856T>A, 5857C>T, 5861A>G, 5863A>T, 5864G>C, 5868A>T, 5870T>A, 5871C>T, 5874C>T, 5878A>T, 5880T>C, 5882T>G, 5886G>C, 5887G>A, 5888A>G, 5889C>G, 5891A>C, 5895A>G, 5896T>G, 5897C>A, 5898C>T, 5901C>A, 5905T>A, 5906G>A, 5910C>T, 5916C>T, 5918T>G, 5924C>A, 5925A>T, 5926G>T, 5931A>C, 5933T>A, 5937C>T, 5940A>G, 5942G>T, 5943G>T, 5944T>C, 5949T>G, 5953C>T, 5958C>T, 5961A>G, 5962T>C, 5964G>T, 5966A>C, 5967G>T, 5976T>A, 5979T>A, 5981T>C, 5982G>T, 5983T>C, 5985C>T, 5986C>A, 5987A>T, 5988A>G, 5989C>G, 5990G>A, 5991A>C, 5992A>T, 5993A>T, 5998G>A, 5999A>C, 6000C>A, 6001A>T, 6002A>G, 6003C>G, 6004T>G, 6005G>T, 6006T>A, 6009C>T, 6012A>G, 6013G>C, 6014A>C, 6015_6016insTAT, 6016C>A, 6018G>T, 6019T>G, 6020C>A, 6021A>C, 6022G>T, 6023G>C, 6027T>C, 6030A>G, 6032C>T, 6033A>T, 6038A>T, 6039C>T, 6042T>C, 6048C>T, 6051C>A, 6054G>T, 6057C>G, 6059T>A, 6060C>T, 6061A>T, 6062G>C, 6063T>A, 6064A>C, 6065A>G, 6066C>G, 6068C>G, 6070G>A, 6071A>G, 6078C>G, 6083C>T, 6094A>C, 6095A>G, 6096G>T, 6097T>A								

CDS

	Begin	End	Coverage	Score	Concordance	Matches	Identities	I/D/M/F*	Stop Codons
QKM20_gp3	1568	1659	4.6%	337	49.3%	92 (98.9%)	49 (52.7%)	1/0/0/0	0
Protein mutations:	N1568K (5832T>A), S1570T (5837G>C), F1575I (5851T>A 5853C>T), D1576E (5856T>A), K1578R (5861A>G), F1581Y (5870T>A 5871C>T), I1584F (5878A>T 5880T>C), M1585R (5882T>G), M1586I (5886G>C), D1587R (5887G>A 5888A>G 5889C>G), E1588A (5891A>C), S1590D (5896T>G 5897C>A 5898C>T), W1593K (5905T>A 5906G>A), L1597R (5918T>G), P1599H (5924C>A 5925A>T), D1600Y (5926G>T), L1602H (5933T>A), W1605F (5942G>T 5943G>T), P1609S (5953C>T), K1613T (5966A>C 5967G>T), V1618A (5981T>C 5982G>T), F1619L (5983T>C 5985C>T), Q1620M (5986C>A 5987A>T 5988A>G), R1621D (5989C>G 5990G>A 5991A>C), K1622L (5992A>T 5993A>T), D1624T (5998G>A 5999A>C 6000C>A), N1625W (6001A>T 6002A>G 6003C>G), C1626V (6004T>G 6005G>T 6006T>A), D1629P (6013G>C 6014A>C), D1629_L1630insY (6015_6016insTAT), L1630I (6016C>A 6018G>T), S1631D (6019T>G 6020C>A 6021A>C), G1632S (6022G>T 6023G>C), A1635V (6032C>T 6033A>T), Y1637F (6038A>T 6039C>T), F1644Y (6059T>A 6060C>T), N1646R (6064A>C 6065A>G 6066C>G), T1647S (6068C>G), E1648R (6070G>A 6071A>G), D1650E (6078C>G), A1652V (6083C>T), K1656R (6094A>C 6095A>G 6096G>T), S1657T (6097T>A)								
Codon mutations:	AAT1568AAA (5832T>A), GCC1569GCT (5835C>T), AGT1570ACT (5837G>C), GTC1571GTG (5841C>G), TTC1572TTT (5844C>T), TCC1573TCT (5847C>T), TTC1575ATT (5851T>A 5853C>T), GAT1576GAA (5856T>A), CTG1577TTG (5857C>T), AAG1578AGG (5861A>G), AGC1579TCC (5863A>T 5864G>C), GGA1580GGT (5868A>T), TTC1581TAT (5870T>A 5871C>T), CAC1582CAT (5874C>T), ATT1584TTC (5878A>T 5880T>C), ATG1585AGG (5882T>G), ATG1586ATC (5886G>C), GAC1587AGG (5887G>A 5888A>G 5889C>G), GAA1588GCA (5891A>C), GAA1589GAG (5895A>G), TCC1590GAT (5896T>G 5897C>A 5898C>T), ATC1591ATA (5901C>A), TGG1593AAG (5905T>A 5906G>A), ACC1594ACT (5910C>T), TTC1596TTT (5916C>T), CTG1597CGG (5918T>G), CCA1599CAT (5924C>A 5925A>T), GAT1600TAT (5926G>T), GGA1601GGC (5931A>C), CTT1602CAT (5933T>A), TAC1603TAT (5937C>T), GAA1604GAG (5940A>G), TGG1605TTT (5942G>T 5943G>T), TTA1606CTA (5944T>C), GTT1607GTG (5949T>G), CCA1609TCA (5953C>T), TTC1610TTT (5958C>T), GGA1611GGG (5961A>G), TTG1612CTT (5962T>C 5964G>T), AAG1613ACT (5966A>C 5967G>T), CCT1616CCA (5976T>A), GCT1617GCA (5979T>A), GTG1618GCT (5981T>C 5982G>T), TTC1619CTT (5983T>C 5985C>T), CAA1620ATG (5986C>A 5987A>T 5988A>G), CGA1621GAC (5989C>G 5990G>A 5991A>C), AAG1622TTG (5992A>T 5993A>T), GAC1624ACA (5998G>A 5999A>C 6000C>A), AAC1625TGG (6001A>T 6002A>G 6003C>G), TGT1626GTA (6004T>G 6005G>T 6006T>A), TTC1627TTT (6009C>T), AGA1628AGG (6012A>G), GAC1629CCC (6013G>C 6014A>C), GAC1629_CTG1630insTAT (6015_6016insTAT), CTG1630ATT (6016C>A 6018G>T), TCA1631GAC (6019T>G 6020C>A 6021A>C), GGC1632TCC (6022G>T 6023G>C), TTT1633TTC (6027T>C), GTA1634GTG (6030A>G), GCA1635GTT (6032C>T 6033A>T), TAC1637TTT (6038A>T 6039C>T), ATT1638ATC (6042T>C), GAC1640GAT (6048C>T), ATC1641ATA (6051C>A), CTG1642CTT (6054G>T), GTC1643GTG (6057C>G), TTC1644TAT (6059T>A 6060C>T), AGT1645TCA (6061A>T 6062G>C 6063T>A), AAC1646CGG (6064A>C 6065A>G 6066C>G), ACT1647AGT (6068C>G), GAA1648AGA (6070G>A 6071A>G), GAC1650GAG (6078C>G), GCA1652GTA (6083C>T), AAG1656CGT (6094A>C 6095A>G 6096G>T), TCC1657ACC (6097T>A)								

Proteins

	Begin	End	Coverage	Score	Concordance	Matches	Identities	I/D/M/F*	Stop Codons
polyprotein (YP_010797894.1)	1568	1659	4.6%	337	49.3%	92 (98.9%)	49 (52.7%)	1/0/0/0	0
Protein mutations:	N1568K (5832T>A), S1570T (5837G>C), F1575I (5851T>A 5853C>T), D1576E (5856T>A), K1578R (5861A>G), F1581Y (5870T>A 5871C>T), I1584F (5878A>T 5880T>C), M1585R (5882T>G), M1586I (5886G>C), D1587R (5887G>A 5888A>G 5889C>G), E1588A (5891A>C), S1590D (5896T>G 5897C>A 5898C>T), W1593K (5905T>A 5906G>A), L1597R (5918T>G), P1599H (5924C>A 5925A>T), D1600Y (5926G>T), L1602H (5933T>A), W1605F (5942G>T 5943G>T), P1609S (5953C>T), K1613T (5966A>C 5967G>T), V1618A (5981T>C 5982G>T), F1619L (5983T>C 5985C>T), Q1620M (5986C>A 5987A>T 5988A>G), R1621D (5989C>G 5990G>A 5991A>C), K1622L (5992A>T 5993A>T), D1624T (5998G>A 5999A>C 6000C>A), N1625W (6001A>T 6002A>G 6003C>G), C1626V (6004T>G 6005G>T 6006T>A), D1629P (6013G>C 6014A>C), D1629_L1630insY (6015_6016insTAT), L1630I (6016C>A 6018G>T), S1631D (6019T>G 6020C>A 6021A>C), G1632S (6022G>T 6023G>C), A1635V (6032C>T 6033A>T), Y1637F (6038A>T 6039C>T), F1644Y (6059T>A 6060C>T), N1646R (6064A>C 6065A>G 6066C>G), T1647S (6068C>G), E1648R (6070G>A 6071A>G), D1650E (6078C>G), A1652V (6083C>T), K1656R (6094A>C 6095A>G 6096G>T), S1657T (6097T>A)								
Codon mutations:	AAT1568AAA (5832T>A), GCC1569GCT (5835C>T), AGT1570ACT (5837G>C), GTC1571GTG (5841C>G), TTC1572TTT (5844C>T), TCC1573TCT (5847C>T), TTC1575ATT (5851T>A 5853C>T), GAT1576GAA (5856T>A), CTG1577TTG (5857C>T), AAG1578AGG (5861A>G), AGC1579TCC (5863A>T 5864G>C), GGA1580GGT (5868A>T), TTC1581TAT (5870T>A 5871C>T), CAC1582CAT (5874C>T), ATT1584TTC (5878A>T 5880T>C), ATG1585AGG (5882T>G), ATG1586ATC (5886G>C), GAC1587AGG (5887G>A 5888A>G 5889C>G), GAA1588GCA (5891A>C), GAA1589GAG (5895A>G), TCC1590GAT (5896T>G 5897C>A 5898C>T), ATC1591ATA (5901C>A), TGG1593AAG (5905T>A 5906G>A), ACC1594ACT (5910C>T), TTC1596TTT (5916C>T), CTG1597CGG (5918T>G), CCA1599CAT (5924C>A 5925A>T), GAT1600TAT (5926G>T), GGA1601GGC (5931A>C), CTT1602CAT (5933T>A), TAC1603TAT (5937C>T), GAA1604GAG (5940A>G), TGG1605TTT (5942G>T 5943G>T), TTA1606CTA (5944T>C), GTT1607GTG (5949T>G), CCA1609TCA (5953C>T), TTC1610TTT (5958C>T), GGA1611GGG (5961A>G), TTG1612CTT (5962T>C 5964G>T), AAG1613ACT (5966A>C 5967G>T), CCT1616CCA (5976T>A), GCT1617GCA (5979T>A), GTG1618GCT (5981T>C 5982G>T), TTC1619CTT (5983T>C 5985C>T), CAA1620ATG (5986C>A 5987A>T 5988A>G), CGA1621GAC (5989C>G 5990G>A 5991A>C), AAG1622TTG (5992A>T 5993A>T), GAC1624ACA (5998G>A 5999A>C 6000C>A), AAC1625TGG (6001A>T 6002A>G 6003C>G), TGT1626GTA (6004T>G 6005G>T 6006T>A), TTC1627TTT (6009C>T), AGA1628AGG (6012A>G), GAC1629CCC (6013G>C 6014A>C), GAC1629_CTG1630insTAT (6015_6016insTAT), CTG1630ATT (6016C>A 6018G>T), TCA1631GAC (6019T>G 6020C>A 6021A>C), GGC1632TCC (6022G>T 6023G>C), TTT1633TTC (6027T>C), GTA1634GTG (6030A>G), GCA1635GTT (6032C>T 6033A>T), TAC1637TTT (6038A>T 6039C>T), ATT1638ATC (6042T>C), GAC1640GAT (6048C>T), ATC1641ATA (6051C>A), CTG1642CTT (6054G>T), GTC1643GTG (6057C>G), TTC1644TAT (6059T>A 6060C>T), AGT1645TCA (6061A>T 6062G>C 6063T>A), AAC1646CGG (6064A>C 6065A>G 6066C>G), ACT1647AGT (6068C>G), GAA1648AGA (6070G>A 6071A>G), GAC1650GAG (6078C>G), GCA1652GTA (6083C>T), AAG1656CGT (6094A>C 6095A>G 6096G>T), TCC1657ACC (6097T>A)								

*: Inserts / Deletes / Misaligned / Frameshifts

Analysis details

This analysis was performed with panviral2.64

NGS Details (UN8): Errantivirus

Assembly

**Coverage Length**	716 (1 contig(s))
**Depth Of Coverage**	22.2
**Number Of Reads**	133
**Reads Per Million**	2.22 rpm (after QC)
**Ambiguities**	0
**Assembly Method**	de novo + reference guided assembly
**Consensus Caller**	Bcf Tools

Coverage Map

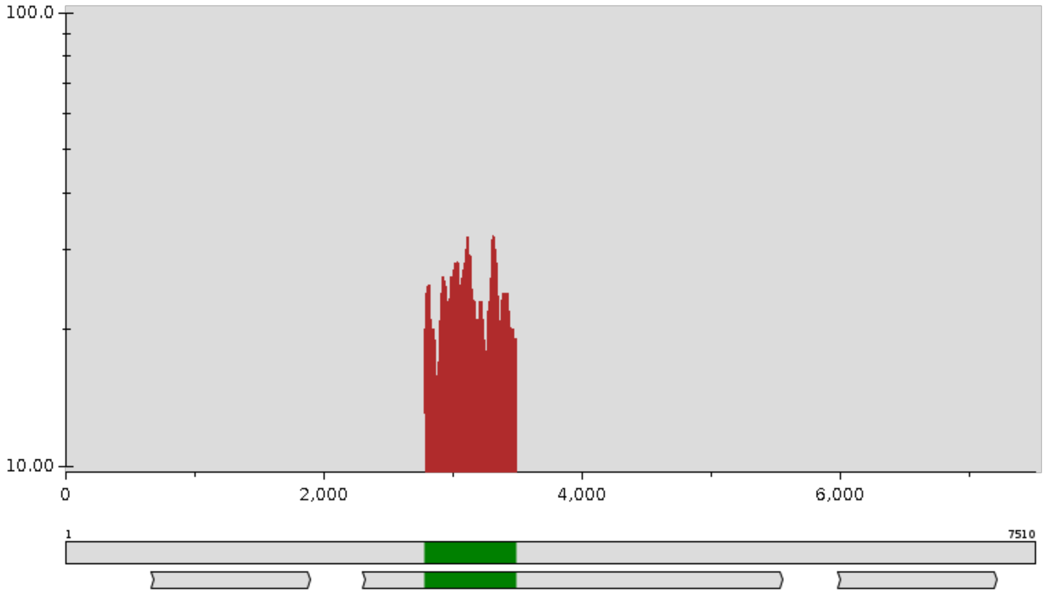

Assignment

**Type**	Errantivirus (Taxonomy ID: 186666)
**Reference Genome**	NC_038512.1
**NT Identity (%)**	52.3605
**AA Identity (%)**	46.1538
**Number Of Stop Codons**	1
**Number Of CDS**	3

Alignment

**Alignment Score**	22.0 (NT) + 632.0 (AA) = 654.0
**Concordance (%)**	23.0769

Alignment Method	Global, seeded, nucleotide + amino acids (AGA)

Genome Region

Sequence starts at position 2783 and ends at position 3498 relative to NC_038512.1 reference sequence.

Alignment Detailed Statistics

	Begin	End	Coverage	Score	Concordance	Matches	Identities	I/D/M/F*	Stop Codons
**NT**	**2783**	**3498**	**9.5%**	**22**	**1.6%**	**699 (97.6%)**	**366 (51.1%)**	**0/17**	
Mutations:	2783A>G, 2785A>T, 2789T>C, 2791G>T, 2794C>T, 2800A>C, 2803T>C, 2806A>C, 2807A>C, 2808G>A, 2812A>C, 2813T>A, 2814C>G, 2815A>C, 2816G>T, 2817A>G, 2819T>A, 2820C>A, 2821T>C, 2822G>C, 2824A>T, 2826G>A, 2827G>T, 2828A>G, 2829G>C, 2832C>T, 2836C>T, 2837A>G, 2840T>G, 2841G>T, 2842G>C, 2843G>C, 2845T>C, 2849C>G, 2850C>G, 2851C>A, 2857A>G, 2858_2860delATC, 2863C>T, 2864G>A, 2865C>G, 2869T>A, 2870_2881delGGGAAACAAAAG, 2885C>A, 2887T>G, 2888C>T, 2890C>A, 2891G>T, 2892T>G, 2893A>T, 2896T>A, 2901T>A, 2902C>T, 2903C>A, 2905T>G, 2906A>G, 2907A>C, 2908G>T, 2914C>T, 2915G>A, 2918A>T, 2919A>T, 2927G>A, 2929T>A, 2932C>T, 2937A>T, 2938C>T, 2939C>A, 2941G>A, 2944A>T, 2947A>C, 2949A>T, 2953A>T, 2954A>G, 2955G>A, 2956T>G, 2959C>G, 2960G>T, 2965T>G, 2968C>T, 2969A>G, 2971G>A, 2974A>G, 2977T>A, 2978A>G, 2979A>G, 2980G>T, 2982G>C, 2986A>G, 2987T>A, 2988A>T, 2997C>A, 2998C>G, 2999T>C, 3001A>T, 3005T>C, 3008G>A, 3009C>G, 3011A>T, 3012G>C, 3013T>A, 3016G>T, 3018T>A, 3020T>C, 3025G>A, 3026G>A, 3028G>T, 3029G>A, 3030A>G, 3035G>A, 3036A>C, 3037C>A, 3038C>A, 3039C>A, 3040T>G, 3041C>G, 3043A>G, 3047A>G, 3049A>C, 3050T>C, 3051C>A, 3058C>T, 3059G>A, 3061G>A, 3066A>G, 3067C>G, 3068G>A, 3069T>C, 3070A>T, 3071G>C, 3073A>T, 3074C>T, 3075A>C, 3076C>T, 3079G>C, 3082T>C, 3084T>A, 3088A>G, 3090T>A, 3094T>A, 3095C>G, 3096G>T, 3103T>C, 3104A>T, 3106G>T, 3109A>C, 3110T>C, 3112A>C, 3114A>C, 3115A>T, 3118C>T, 3119T>G, 3124A>T, 3127T>A, 3129C>G, 3137A>G, 3139A>G, 3140G>C, 3142T>G, 3146G>A, 3148C>T, 3149A>C, 3154C>T, 3155C>T, 3157A>T, 3159G>A, 3160A>G, 3162G>A, 3163T>A, 3165T>A, 3168A>T, 3169A>T, 3171A>G, 3172T>G, 3175C>G, 3176A>T, 3179T>A, 3180G>T, 3182C>T, 3184C>G, 3187C>T, 3189A>T, 3190C>T, 3191_3192delCT, 3196C>T, 3203A>C, 3205T>G, 3206G>A, 3211T>C, 3214T>C, 3216C>A, 3217T>G, 3218T>A, 3219C>G, 3220C>T, 3224C>G, 3226G>A, 3229A>T, 3233C>T, 3236G>C, 3238G>A, 3239A>C, 3245G>A, 3246A>G, 3247A>G, 3248C>G, 3249G>T, 3250A>T, 3251G>A, 3252T>C, 3256C>T, 3257C>A, 3259A>C, 3260A>T, 3261G>T, 3263C>T, 3265T>G, 3267G>T, 3268A>C, 3269G>C, 3272A>C, 3273G>A, 3276A>G, 3277C>A, 3278T>C, 3280C>G, 3281A>T, 3282A>T, 3283A>G, 3286T>C, 3287C>A, 3289A>T, 3291T>A, 3293G>T, 3294A>C, 3295C>A, 3300C>G, 3301C>T, 3302G>T, 3303A>C, 3304A>T, 3307C>T, 3308T>G, 3309T>G, 3310G>A, 3311A>G, 3312A>C, 3313G>A, 3314C>T, 3315T>C, 3316C>A, 3317G>A, 3318A>G, 3319A>G, 3320A>G, 3321C>T, 3324C>A, 3325T>A, 3328T>C, 3333G>A, 3338A>T, 3340C>T, 3344A>T, 3345G>C, 3346C>T, 3347A>G, 3348G>C, 3349G>T, 3352C>G, 3355T>A, 3356A>G, 3358C>G, 3359A>G, 3360A>C, 3361G>T, 3362C>A, 3364T>A, 3365A>G, 3367C>T, 3371G>A, 3373T>G, 3380T>G, 3382C>A, 3385T>A, 3386A>G, 3388T>A, 3389C>T, 3392A>G, 3396A>G, 3397T>G, 3399T>C, 3400G>A, 3406A>T, 3407A>C, 3409G>T, 3412C>T, 3413C>A, 3414C>T, 3415T>A, 3421A>G, 3422A>T, 3424A>G, 3426A>G, 3427A>G, 3428C>G, 3429A>G, 3434T>C, 3436A>T, 3439C>G, 3440C>T, 3442T>G, 3443C>A, 3444T>C, 3445C>A, 3454C>T, 3455C>A, 3457A>G, 3459A>G, 3460A>G, 3467C>A, 3468C>G, 3470G>A, 3471A>G, 3472T>A, 3474T>A, 3477C>G, 3478A>G, 3479C>A, 3480G>T, 3481A>C, 3482C>A, 3484C>T, 3486C>G, 3487A>T, 3493C>T, 3498C>A								

CDS

	Begin	End	Coverage	Score	Concordance	Matches	Identities	I/D/M/F*	Stop Codons
D1R33_gp2	160	398	22.0%	632	39.3%	234 (97.9%)	108 (45.2%)	0/5/1/1	1
Protein mutations:	K160D (2783A>G 2785A>T), R168Q (2807A>C 2808G>A), D171C (2816G>T 2817A>G), S172N (2819T>A 2820C>A 2821T>C), A173P (2822G>C 2824A>T), W174Y (2826G>A 2827G>T), S175A (2828A>G 2829G>C), S176L (2832C>T), I178V (2837A>G), W179V (2840T>G 2841G>T 2842G>C), V180L (2843G>C 2845T>C), P182G (2849C>G 2850C>G 2851C>A), I185del (2858_2860delATC), A187S (2864G>A 2865C>G), G189_K192del (2870_2881delGGGAAACAAAAG), V196C (2891G>T 2892T>G 2893A>T), F199Y (2901T>A 2902C>T), K201A (2906A>G 2907A>C 2908G>T), E204K (2915G>A), K205L (2918A>T 2919A>T), D208K (2927G>A 2929T>A), Y211F (2937A>T 2938C>T), P212T (2939C>A 2941G>A), N215I (2949A>T), S217E (2954A>G 2955G>A 2956T>G), D218E (2959C>G), V219L (2960G>T), K222E (2969A>G 2971G>A), K225G (2978A>G 2979A>G 2980G>T), C226S (2982G>C), Y228I (2987T>A 2988A>T), T231K (2997C>A 2998C>G), A235R (3008G>A 3009C>G), F238Y (3018T>A), Y239H (3020T>C), V241I (3026G>A 3028G>T), E242R (3029G>A 3030A>G), D244T (3035G>A 3036A>C 3037C>A), P245K (3038C>A 3039C>A 3040T>G), Q246E (3041C>G 3043A>G), I248V (3047A>G 3049A>C), S249Q (3050T>C 3051C>A), A252T (3059G>A 3061G>A), N254R (3066A>G 3067C>G), V255T (3068G>A 3069T>C 3070A>T), E256H (3071G>C 3073A>T), H257S (3074C>T 3075A>C 3076C>T), F260Y (3084T>A), F262Y (3090T>A), R264V (3095C>G 3096G>T), M267F (3104A>T 3106G>T), K270T (3114A>C 3115A>T), S272A (3119T>G), T275S (3129C>G), R278G (3137A>G 3139A>G), V279L (3140G>C 3142T>G), D281N (3146G>A 3148C>T), N282H (3149A>C), L284F (3155C>T 3157A>T), R285K (3159G>A 3160A>G), G286E (3162G>A 3163T>A), L287H (3165T>A), Q288L (3168A>T 3169A>T), N289R (3171A>G 3172T>G), N290K (3175C>G), I291F (3176A>T), C292I (3179T>A 3180G>T), Y295F (3189A>T 3190C>T), I300L (3203A>C 3205T>G), V301I (3206G>A), T304K (3216C>A 3217T>G), Q307E (3224C>G 3226G>A), E308D (3229A>T), E311Q (3236G>C 3238G>A), N312H (3239A>C), E314R (3245G>A 3246A>G 3247A>G), R315V (3248C>G 3249G>T 3250A>T), V316T (3251G>A 3252T>C), Q318N (3257C>A 3259A>C), R319L (3260A>T 3261G>T), R321I (3267G>T 3268A>C), E322Q (3269G>C), S323H (3272A>C 3273G>A), N324R (3276A>G 3277C>A), F325L (3278T>C 3280C>G), K326L (3281A>T 3282A>T 3283A>G), Q328N (3287C>A 3289A>T), M329K (3291T>A), D330S (3293G>T 3294A>C 3295C>A), S332C (3300C>G 3301C>T), E333S (3302G>T 3303A>C 3304A>T), L335G (3308T>G 3309T>G 3310G>A), K336A (3311A>G 3312A>C 3313G>A), L337S (3314C>T 3315T>C 3316C>A), E338R (3317G>A 3318A>G 3319A>G), T339V (3320A>G 3321C>T), A340E (3324C>A 3325T>A), G343D (3333G>A), I345F (3338A>T 3340C>T), R348A (3347A>G 3348G>C 3349G>T), D349E (3352C>G), I351V (3356A>G 3358C>G), K352A (3359A>G 3360A>C 3361G>T), P353T (3362C>A 3364T>A), N354D (3365A>G 3367C>T), D356K (3371G>A 3373T>G), S359A (3380T>G 3382C>A), I361V (3386A>G 3388T>A), Q362* (3389C>T), K363E (3392A>G), Y364W (3396A>G 3397T>G), L365P (3399T>C 3400G>A), K368H (3407A>C 3409G>T), P370I (3413C>A 3414C>T 3415T>A), I373L (3422A>T 3424A>G), K374R (3426A>G 3427A>G), Q375G (3428C>G 3429A>G), L380T (3443C>A 3444T>C 3445C>A), K385R (3459A>G 3460A>G), P388R (3467C>A 3468C>G), D389R (3470G>A 3471A>G 3472T>A), F390Y (3474T>A), A391G (3477C>G 3478A>G), R392I (3479C>A 3480G>T 3481A>C), L393I (3482C>A 3484C>T), T394S (3486C>G 3487A>T)								
Codon mutations:	AAA160GAT (2783A>G 2785A>T), TTG162CTT (2789T>C 2791G>T), GAC163GAT (2794C>T), GGA165GGC (2800A>C), ATT166ATC (2803T>C), ATA167ATC (2806A>C), AGA168CAA (2807A>C 2808G>A), CCA169CCC (2812A>C), TCA170AGC (2813T>A 2814C>G 2815A>C), GAC171TGC (2816G>T 2817A>G), TCT172AAC (2819T>A 2820C>A 2821T>C), GCA173CCT (2822G>C 2824A>T), TGG174TAT (2826G>A 2827G>T), AGC175GCC (2828A>G 2829G>C), TCA176TTA (2832C>T), CCC177CCT (2836C>T), ATA178GTA (2837A>G), TGG179GTC (2840T>G 2841G>T 2842G>C), GTT180CTC (2843G>C 2845T>C), CCC182GGA (2849C>G 2850C>G 2851C>A), AAA184AAG (2857A>G), ATC185del (2858_2860delATC), GAC186GAT (2863C>T), GCT187AGT (2864G>A 2865C>G), TCT188TCA (2869T>A), GGG189_AAG192del (2870_2881delGGGAAACAAAAG), CGT194AGG (2885C>A 2887T>G), CTC195TTA (2888C>T 2890C>A), GTA196TGT (2891G>T 2892T>G 2893A>T), GTT197GTA (2896T>A), TTC199TAT (2901T>A 2902C>T), CGT200AGG (2903C>A 2905T>G), AAG201GCT (2906A>G 2907A>C 2908G>T), AAC203AAT (2914C>T), GAG204AAG (2915G>A), AAG205TTG (2918A>T 2919A>T), GAT208AAA (2927G>A 2929T>A), GAC209GAT (2932C>T), TAC211TTT (2937A>T 2938C>T), CCG212ACA (2939C>A 2941G>A), ATA213ATT (2944A>T), CCA214CCC (2947A>C), AAC215ATC (2949A>T), ATA216ATT (2953A>T), AGT217GAG (2954A>G 2955G>A 2956T>G), GAC218GAG (2959C>G), GTA219TTA (2960G>T), CTT220CTG (2965T>G), GAC221GAT (2968C>T), AAG222GAA (2969A>G 2971G>A), TTA223TTG (2974A>G), GGT224GGA (2977T>A), AAG225GGT (2978A>G 2979A>G 2980G>T), TGC226TCC (2982G>C), CAA227CAG (2986A>G), TAC228ATC (2987T>A 2988A>T), ACC231AAG (2997C>A 2998C>G), TTA232CTT (2999T>C 3001A>T), TTG234CTG (3005T>C), GCA235AGA (3008G>A 3009C>G), AGT236TCA (3011A>T 3012G>C 3013T>A), GGG237GGT (3016G>T), TTT238TAT (3018T>A), TAT239CAT (3020T>C), CAG240CAA (3025G>A), GTG241ATT (3026G>A 3028G>T), GAG242AGG (3029G>A 3030A>G), GAC244ACA (3035G>A 3036A>C 3037C>A), CCT245AAG (3038C>A 3039C>A 3040T>G), CAA246GAG (3041C>G 3043A>G), ATA248GTC (3047A>G 3049A>C), TCG249CAG (3050T>C 3051C>A), ACC251ACT (3058C>T), GCG252ACA (3059G>A 3061G>A), AAC254AGG (3066A>G 3067C>G), GTA255ACT (3068G>A 3069T>C 3070A>T), GAA256CAT (3071G>C 3073A>T), CAC257TCT (3074C>T 3075A>C 3076C>T), GGG258GGC (3079G>C), CAT259CAC (3082T>C), TTT260TAT (3084T>A), GAA261GAG (3088A>G), TTC262TAC (3090T>A), CTT263CTA (3094T>A), CGA264GTA (3095C>G 3096G>T), CCT266CCC (3103T>C), ATG267TTT (3104A>T 3106G>T), GGA268GGC (3109A>C), TTA269CTC (3110T>C 3112A>C), AAA270ACT (3114A>C 3115A>T), AAC271AAT (3118C>T), TCA272GCA (3119T>G), CCA273CCT (3124A>T), TCT274TCA (3127T>A), ACT275AGT (3129C>G), AGA278GGG (3137A>G 3139A>G), GTT279CTG (3140G>C 3142T>G), GAC281AAT (3146G>A 3148C>T), AAT282CAT (3149A>C), GTC283GTT (3154C>T), CTA284TTT (3155C>T 3157A>T), AGA285AAG (3159G>A 3160A>G), GGT286GAA (3162G>A 3163T>A), CTC287CAC (3165T>A), CAA288CTT (3168A>T 3169A>T), AAT289AGG (3171A>G 3172T>G), AAC290AAG (3175C>G), ATC291TTC (3176A>T), TGT292ATT (3179T>A 3180G>T), CTC293TTG (3182C>T 3184C>G), GTC294GTT (3187C>T), TAC295TTT (3189A>T 3190C>T), CTT296--T (3191_3192delCT), GAC297GAT (3196C>T), ATT300CTG (3203A>C 3205T>G), GTC301ATC (3206G>A), TAT302TAC (3211T>C), AGT303AGC (3214T>C), ACT304AAG (3216C>A 3217T>G), TCC305AGT (3218T>A 3219C>G 3220C>T), CAG307GAA (3224C>G 3226G>A), GAA308GAT (3229A>T), CTG310TTG (3233C>T), GAG311CAA (3236G>C 3238G>A), AAC312CAC (3239A>C), GAA314AGG (3245G>A 3246A>G 3247A>G), CGA315GTT (3248C>G 3249G>T 3250A>T), GTT316ACT (3251G>A 3252T>C), TTC317TTT (3256C>T), CAA318AAC (3257C>A 3259A>C), AGA319TTA (3260A>T 3261G>T), CTT320TTG (3263C>T 3265T>G), AGA321ATC (3267G>T 3268A>C), GAA322CAA (3269G>C), AGT323CAT (3272A>C 3273G>A), AAC324AGA (3276A>G 3277C>A), TTC325CTG (3278T>C 3280C>G), AAA326TTG (3281A>T 3282A>T 3283A>G), ATT327ATC (3286T>C), CAA328AAT (3287C>A 3289A>T), ATG329AAG (3291T>A), GAC330TCA (3293G>T 3294A>C 3295C>A), TCC332TGT (3300C>G 3301C>T), GAA333TCT (3302G>T 3303A>C 3304A>T), TTC334TTT (3307C>T), TTG335GGA (3308T>G 3309T>G 3310G>A), AAG336GCA (3311A>G 3312A>C 3313G>A), CTC337TCA (3314C>T 3315T>C 3316C>A), GAA338AGG (3317G>A 3318A>G 3319A>G), ACT339GTT (3320A>G 3321C>T), GCT340GAA (3324C>A 3325T>A), TAT341TAC (3328T>C), GGT343GAT (3333G>A), ATC345TTT (3338A>T 3340C>T), AGC347TCT (3344A>T 3345G>C 3346C>T), AGG348GCT (3347A>G 3348G>C 3349G>T), GAC349GAG (3352C>G), GGT350GGA (3355T>A), ATC351GTG (3356A>G 3358C>G), AAG352GCT (3359A>G 3360A>C 3361G>T), CCT353ACA (3362C>A 3364T>A), AAC354GAT (3365A>G 3367C>T), GAT356AAG (3371G>A 3373T>G), TCC359GCA (3380T>G 3382C>A), GCT360GCA (3385T>A), ATT361GTA (3386A>G 3388T>A), CAA362TAA (3389C>T), AAA363GAA (3392A>G), TAT364TGG (3396A>G 3397T>G), CTG365CCA (3399T>C 3400G>A), CCA367CCT (3406A>T), AAG368CAT (3407A>C 3409G>T), ACC369ACT (3412C>T), CCT370ATA (3413C>A 3414C>T 3415T>A), GAA372GAG (3421A>G), ATA373TTG (3422A>T 3424A>G), AAA374AGG (3426A>G 3427A>G), CAA375GGA (3428C>G 3429A>G), TTA377CTT (3434T>C 3436A>T), GGC378GGG (3439C>G), CTT379TTG (3440C>T 3442T>G), CTC380ACA (3443C>A 3444T>C 3445C>A), TAC383TAT (3454C>T), CGA384AGG (3455C>A 3457A>G), AAA385AGG (3459A>G 3460A>G), CCA388AGA (3467C>A 3468C>G), GAT389AGA (3470G>A 3471A>G 3472T>A), TTT390TAT (3474T>A), GCA391GGG (3477C>G 3478A>G), CGA392ATC (3479C>A 3480G>T 3481A>C), CTC393ATT (3482C>A 3484C>T), ACA394AGT (3486C>G 3487A>T), CCC396CCT (3493C>T), ACA398AA. (3498C>A)								

Proteins

	Begin	End	Coverage	Score	Concordance	Matches	Identities	I/D/M/F*	Stop Codons
ORF B (YP_009507248.1)	160	398	22.0%	632	39.3%	234 (97.9%)	108 (45.2%)	0/5/1/1	1

	Begin	End	Coverage	Score	Concordance	Matches	Identities	I/D/M/F*	Stop Codons
**NT**	**2783**	**3498**	**9.5%**	**22**	**1.6%**	**699 (97.6%)**	**366 (51.1%)**	**0/17**	
Protein mutations:	K160D (2783A>G 2785A>T), R168Q (2807A>C 2808G>A), D171C (2816G>T 2817A>G), S172N (2819T>A 2820C>A 2821T>C), A173P (2822G>C 2824A>T), W174Y (2826G>A 2827G>T), S175A (2828A>G 2829G>C), S176L (2832C>T), I178V (2837A>G), W179V (2840T>G 2841G>T 2842G>C), V180L (2843G>C 2845T>C), P182G (2849C>G 2850C>G 2851C>A), I185del (2858_2860delATC), A187S (2864G>A 2865C>G), G189_K192del (2870_2881delGGGAAACAAAAG), V196C (2891G>T 2892T>G 2893A>T), F199Y (2901T>A 2902C>T), K201A (2906A>G 2907A>C 2908G>T), E204K (2915G>A), K205L (2918A>T 2919A>T), D208K (2927G>A 2929T>A), Y211F (2937A>T 2938C>T), P212T (2939C>A 2941G>A), N215I (2949A>T), S217E (2954A>G 2955G>A 2956T>G), D218E (2959C>G), V219L (2960G>T), K222E (2969A>G 2971G>A), K225G (2978A>G 2979A>G 2980G>T), C226S (2982G>C), Y228I (2987T>A 2988A>T), T231K (2997C>A 2998C>G), A235R (3008G>A 3009C>G), F238Y (3018T>A), Y239H (3020T>C), V241I (3026G>A 3028G>T), E242R (3029G>A 3030A>G), D244T (3035G>A 3036A>C 3037C>A), P245K (3038C>A 3039C>A 3040T>G), Q246E (3041C>G 3043A>G), I248V (3047A>G 3049A>C), S249Q (3050T>C 3051C>A), A252T (3059G>A 3061G>A), N254R (3066A>G 3067C>G), V255T (3068G>A 3069T>C 3070A>T), E256H (3071G>C 3073A>T), H257S (3074C>T 3075A>C 3076C>T), F260Y (3084T>A), F262Y (3090T>A), R264V (3095C>G 3096G>T), M267F (3104A>T 3106G>T), K270T (3114A>C 3115A>T), S272A (3119T>G), T275S (3129C>G), R278G (3137A>G 3139A>G), V279L (3140G>C 3142T>G), D281N (3146G>A 3148C>T), N282H (3149A>C), L284F (3155C>T 3157A>T), R285K (3159G>A 3160A>G), G286E (3162G>A 3163T>A), L287H (3165T>A), Q288L (3168A>T 3169A>T), N289R (3171A>G 3172T>G), N290K (3175C>G), I291F (3176A>T), C292I (3179T>A 3180G>T), Y295F (3189A>T 3190C>T), I300L (3203A>C 3205T>G), V301I (3206G>A), T304K (3216C>A 3217T>G), Q307E (3224C>G 3226G>A), E308D (3229A>T), E311Q (3236G>C 3238G>A), N312H (3239A>C), E314R (3245G>A 3246A>G 3247A>G), R315V (3248C>G 3249G>T 3250A>T), V316T (3251G>A 3252T>C), Q318N (3257C>A 3259A>C), R319L (3260A>T 3261G>T), R321I (3267G>T 3268A>C), E322Q (3269G>C), S323H (3272A>C 3273G>A), N324R (3276A>G 3277C>A), F325L (3278T>C 3280C>G), K326L (3281A>T 3282A>T 3283A>G), Q328N (3287C>A 3289A>T), M329K (3291T>A), D330S (3293G>T 3294A>C 3295C>A), S332C (3300C>G 3301C>T), E333S (3302G>T 3303A>C 3304A>T), L335G (3308T>G 3309T>G 3310G>A), K336A (3311A>G 3312A>C 3313G>A), L337S (3314C>T 3315T>C 3316C>A), E338R (3317G>A 3318A>G 3319A>G), T339V (3320A>G 3321C>T), A340E (3324C>A 3325T>A), G343D (3333G>A), I345F (3338A>T 3340C>T), R348A (3347A>G 3348G>C 3349G>T), D349E (3352C>G), I351V (3356A>G 3358C>G), K352A (3359A>G 3360A>C 3361G>T), P353T (3362C>A 3364T>A), N354D (3365A>G 3367C>T), D356K (3371G>A 3373T>G), S359A (3380T>G 3382C>A), I361V (3386A>G 3388T>A), Q362* (3389C>T), K363E (3392A>G), Y364W (3396A>G 3397T>G), L365P (3399T>C 3400G>A), K368H (3407A>C 3409G>T), P370I (3413C>A 3414C>T 3415T>A), I373L (3422A>T 3424A>G), K374R (3426A>G 3427A>G), Q375G (3428C>G 3429A>G), L380T (3443C>A 3444T>C 3445C>A), K385R (3459A>G 3460A>G), P388R (3467C>A 3468C>G), D389R (3470G>A 3471A>G 3472T>A), F390Y (3474T>A), A391G (3477C>G 3478A>G), R392I (3479C>A 3480G>T 3481A>C), L393I (3482C>A 3484C>T), T394S (3486C>G 3487A>T)								
Codon mutations:	AAA160GAT (2783A>G 2785A>T), TTG162CTT (2789T>C 2791G>T), GAC163GAT (2794C>T), GGA165GGC (2800A>C), ATT166ATC (2803T>C), ATA167ATC (2806A>C), AGA168CAA (2807A>C 2808G>A), CCA169CCC (2812A>C), TCA170AGC (2813T>A 2814C>G 2815A>C), GAC171TGC (2816G>T 2817A>G), TCT172AAC (2819T>A 2820C>A 2821T>C), GCA173CCT (2822G>C 2824A>T), TGG174TAT (2826G>A 2827G>T), AGC175GCC (2828A>G 2829G>C), TCA176TTA (2832C>T), CCC177CCT (2836C>T), ATA178GTA (2837A>G), TGG179GTC (2840T>G 2841G>T 2842G>C), GTT180CTC (2843G>C 2845T>C), CCC182GGA (2849C>G 2850C>G 2851C>A), AAA184AAG (2857A>G), ATC185del (2858_2860delATC), GAC186GAT (2863C>T), GCT187AGT (2864G>A 2865C>G), TCT188TCA (2869T>A), GGG189_AAG192del (2870_2881delGGGAAACAAAAG), CGT194AGG (2885C>A 2887T>G), CTC195TTA (2888C>T 2890C>A), GTA196TGT (2891G>T 2892T>G 2893A>T), GTT197GTA (2896T>A), TTC199TAT (2901T>A 2902C>T), CGT200AGG (2903C>A 2905T>G), AAG201GCT (2906A>G 2907A>C 2908G>T), AAC203AAT (2914C>T), GAG204AAG (2915G>A), AAG205TTG (2918A>T 2919A>T), GAT208AAA (2927G>A 2929T>A), GAC209GAT (2932C>T), TAC211TTT (2937A>T 2938C>T), CCG212ACA (2939C>A 2941G>A), ATA213ATT (2944A>T), CCA214CCC (2947A>C), AAC215ATC (2949A>T), ATA216ATT (2953A>T), AGT217GAG (2954A>G 2955G>A 2956T>G), GAC218GAG (2959C>G), GTA219TTA (2960G>T), CTT220CTG (2965T>G), GAC221GAT (2968C>T), AAG222GAA (2969A>G 2971G>A), TTA223TTG (2974A>G), GGT224GGA (2977T>A), AAG225GGT (2978A>G 2979A>G 2980G>T), TGC226TCC (2982G>C), CAA227CAG (2986A>G), TAC228ATC (2987T>A 2988A>T), ACC231AAG (2997C>A 2998C>G), TTA232CTT (2999T>C 3001A>T), TTG234CTG (3005T>C), GCA235AGA (3008G>A 3009C>G), AGT236TCA (3011A>T 3012G>C 3013T>A), GGG237GGT (3016G>T), TTT238TAT (3018T>A), TAT239CAT (3020T>C), CAG240CAA (3025G>A), GTG241ATT (3026G>A 3028G>T), GAG242AGG (3029G>A 3030A>G), GAC244ACA (3035G>A 3036A>C 3037C>A), CCT245AAG (3038C>A 3039C>A 3040T>G), CAA246GAG (3041C>G 3043A>G), ATA248GTC (3047A>G 3049A>C), TCG249CAG (3050T>C 3051C>A), ACC251ACT (3058C>T), GCG252ACA (3059G>A 3061G>A), AAC254AGG (3066A>G 3067C>G), GTA255ACT (3068G>A 3069T>C 3070A>T), GAA256CAT (3071G>C 3073A>T), CAC257TCT (3074C>T 3075A>C 3076C>T), GGG258GGC (3079G>C), CAT259CAC (3082T>C), TTT260TAT (3084T>A), GAA261GAG (3088A>G), TTC262TAC (3090T>A), CTT263CTA (3094T>A), CGA264GTA (3095C>G 3096G>T), CCT266CCC (3103T>C), ATG267TTT (3104A>T 3106G>T), GGA268GGC (3109A>C), TTA269CTC (3110T>C 3112A>C), AAA270ACT (3114A>C 3115A>T), AAC271AAT (3118C>T), TCA272GCA (3119T>G), CCA273CCT (3124A>T), TCT274TCA (3127T>A), ACT275AGT (3129C>G), AGA278GGG (3137A>G 3139A>G), GTT279CTG (3140G>C 3142T>G), GAC281AAT (3146G>A 3148C>T), AAT282CAT (3149A>C), GTC283GTT (3154C>T), CTA284TTT (3155C>T 3157A>T), AGA285AAG (3159G>A 3160A>G), GGT286GAA (3162G>A 3163T>A), CTC287CAC (3165T>A), CAA288CTT (3168A>T 3169A>T), AAT289AGG (3171A>G 3172T>G), AAC290AAG (3175C>G), ATC291TTC (3176A>T), TGT292ATT (3179T>A 3180G>T), CTC293TTG (3182C>T 3184C>G), GTC294GTT (3187C>T), TAC295TTT (3189A>T 3190C>T), CTT296--T (3191_3192delCT), GAC297GAT (3196C>T), ATT300CTG (3203A>C 3205T>G), GTC301ATC (3206G>A), TAT302TAC (3211T>C), AGT303AGC (3214T>C), ACT304AAG (3216C>A 3217T>G), TCC305AGT (3218T>A 3219C>G 3220C>T), CAG307GAA (3224C>G 3226G>A), GAA308GAT (3229A>T), CTG310TTG (3233C>T), GAG311CAA (3236G>C 3238G>A), AAC312CAC (3239A>C), GAA314AGG (3245G>A 3246A>G 3247A>G), CGA315GTT (3248C>G 3249G>T 3250A>T), GTT316ACT (3251G>A 3252T>C), TTC317TTT (3256C>T), CAA318AAC (3257C>A 3259A>C), AGA319TTA (3260A>T 3261G>T), CTT320TTG (3263C>T 3265T>G), AGA321ATC (3267G>T 3268A>C), GAA322CAA (3269G>C), AGT323CAT (3272A>C 3273G>A), AAC324AGA (3276A>G 3277C>A), TTC325CTG (3278T>C 3280C>G), AAA326TTG (3281A>T 3282A>T 3283A>G), ATT327ATC (3286T>C), CAA328AAT (3287C>A 3289A>T), ATG329AAG (3291T>A), GAC330TCA (3293G>T 3294A>C 3295C>A), TCC332TGT (3300C>G 3301C>T), GAA333TCT (3302G>T 3303A>C 3304A>T), TTC334TTT (3307C>T), TTG335GGA (3308T>G 3309T>G 3310G>A), AAG336GCA (3311A>G 3312A>C 3313G>A), CTC337TCA (3314C>T 3315T>C 3316C>A), GAA338AGG (3317G>A 3318A>G 3319A>G), ACT339GTT (3320A>G 3321C>T), GCT340GAA (3324C>A 3325T>A), TAT341TAC (3328T>C), GGT343GAT (3333G>A), ATC345TTT (3338A>T 3340C>T), AGC347TCT (3344A>T 3345G>C 3346C>T), AGG348GCT (3347A>G 3348G>C 3349G>T), GAC349GAG (3352C>G), GGT350GGA (3355T>A), ATC351GTG (3356A>G 3358C>G), AAG352GCT (3359A>G 3360A>C 3361G>T), CCT353ACA (3362C>A 3364T>A), AAC354GAT (3365A>G 3367C>T), GAT356AAG (3371G>A 3373T>G), TCC359GCA (3380T>G 3382C>A), GCT360GCA (3385T>A), ATT361GTA (3386A>G 3388T>A), CAA362TAA (3389C>T), AAA363GAA (3392A>G), TAT364TGG (3396A>G 3397T>G), CTG365CCA (3399T>C 3400G>A), CCA367CCT (3406A>T), AAG368CAT (3407A>C 3409G>T), ACC369ACT (3412C>T), CCT370ATA (3413C>A 3414C>T 3415T>A), GAA372GAG (3421A>G), ATA373TTG (3422A>T 3424A>G), AAA374AGG (3426A>G 3427A>G), CAA375GGA (3428C>G 3429A>G), TTA377CTT (3434T>C 3436A>T), GGC378GGG (3439C>G), CTT379TTG (3440C>T 3442T>G), CTC380ACA (3443C>A 3444T>C 3445C>A), TAC383TAT (3454C>T), CGA384AGG (3455C>A 3457A>G), AAA385AGG (3459A>G 3460A>G), CCA388AGA (3467C>A 3468C>G), GAT389AGA (3470G>A 3471A>G 3472T>A), TTT390TAT (3474T>A), GCA391GGG (3477C>G 3478A>G), CGA392ATC (3479C>A 3480G>T 3481A>C), CTC393ATT (3482C>A 3484C>T), ACA394AGT (3486C>G 3487A>T), CCC396CCT (3493C>T), ACA398AA. (3498C>A)								

*: Inserts / Deletes / Misaligned / Frameshifts

Analysis details

This analysis was performed with panviral2.64

NGS Details (UN8): Dioscovirus dioscoreae

Assembly

**Coverage Length**	1064 (2 contig(s))
**Depth Of Coverage**	10.3
**Number Of Reads**	87
**Reads Per Million**	1.45 rpm (after QC)
**Ambiguities**	0
**Assembly Method**	de novo + reference guided assembly
**Consensus Caller**	Bcf Tools

Coverage Map

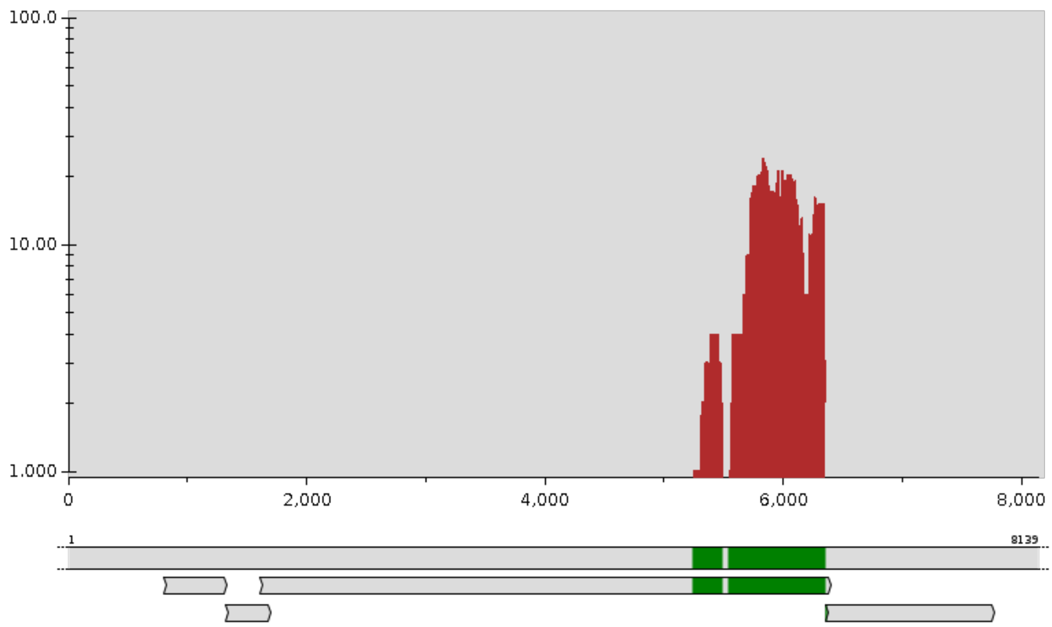

Assignment

**Type**	Dioscovirus dioscoreae (Taxonomy ID: 3052184)
**Reference Genome**	NC_040712.1
**NT Identity (%)**	61.7121
**AA Identity (%)**	49.8592
**Number Of Stop Codons**	1
**Number Of CDS**	4

Alignment

**Alignment Score**	461.0 (NT) + 1175.0 (AA) = 1636.0
**Concordance (%)**	37.5933

Alignment Method	Global, seeded, nucleotide + amino acids (AGA)

Genome Region

Sequence starts at position 5241 and ends at position 6355 relative to NC_040712.1 reference sequence.

Alignment Detailed Statistics

	Begin	End	Coverage	Score	Concordance	Matches	Identities	I/D/M/F*	Stop Codons
**NT**	**5241**	**6355**	**13.1%**	**461**	**22.2%**	**1057 (98.8%)**	**656 (61.3%)**	**6/7**	
Mutations:	5248G>C, 5250C>A, 5252T>A, 5255G>A, 5259T>A, 5260A>T, 5261C>A, 5270G>A, 5271C>A, 5276A>T, 5280G>A, 5288A>T, 5289T>A, 5290A>G, 5291C>A, 5293A>C, 5294A>G, 5297C>T, 5298C>G, 5299A>C, 5303C>T, 5305C>A, 5307C>T, 5309C>A, 5313A>G, 5314G>A, 5317T>A, 5318T>A, 5319G>A, 5320A>G, 5321C>T, 5322T>G, 5325C>T, 5327C>A, 5328C>A, 5330A>T, 5331C>A, 5332T>A, 5335A>G, 5339T>G, 5340A>T, 5345C>T, 5348G>A, 5350T>A, 5351T>A, 5352G>T, 5354C>A, 5356A>T, 5360C>T, 5370C>T, 5371T>G, 5372A>T, 5375G>A, 5379G>C, 5380G>T, 5381A>C, 5383T>A, 5384C>T, 5385C>T, 5386A>G, 5387C>G, 5390G>A, 5391A>G, 5395T>A, 5396G>A, 5400G>C, 5406C>G, 5408G>C, 5412delA, 5424G>A, 5429T>C, 5430G>A, 5431T>C, 5432C>A, 5435C>T, 5438A>T, 5439C>G, 5444T>A, 5457A>C, 5461T>C, 5462A>C, 5471C>T, 5474A>T, 5475C>T, 5477C>A, 5482A>C, 5483T>A, 5486A>G, 5549T>C, 5553A>G, 5558C>T, 5561T>C, 5564T>C, 5565T>C, 5567G>A, 5568A>G, 5573T>C, 5574T>A, 5575C>G, 5577G>A, 5578A>G, 5580A>G, 5581G>A, 5585A>G, 5589C>G, 5595G>C, 5598C>G, 5607T>C, 5608T>A, 5610C>A, 5611A>C, 5612G>A, 5613T>A, 5615C>T, 5618C>T, 5619C>A, 5620A>G, 5621A>G, 5622G>T, 5626G>T, 5627T>C, 5628A>G, 5629A>T, 5630G>A, 5631G>A, 5632A>G, 5633A>T, 5634G>A, 5639G>A, 5640C>A, 5642T>A, 5645T>A, 5646T>A, 5648G>A, 5649T>A, 5651A>C, 5656C>A, 5657A>G, 5661T>A, 5664A>G, 5667A>T, 5670G>T, 5672A>T, 5673G>A, 5674T>A, 5676G>A, 5679A>C, 5685G>A, 5687A>C, 5691C>T, 5693T>A, 5696C>A, 5697C>G, 5700G>A, 5701A>C, 5702A>T, 5703A>T, 5711A>T, 5714A>G, 5717A>G, 5718G>A, 5720T>A, 5721C>A, 5726C>A, 5739T>G, 5740T>C, 5741A>T, 5748A>G, 5756A>T, 5757T>A, 5759T>G, 5762T>A, 5763_5765delGAA, 5768C>T, 5769C>A, 5777A>T, 5779C>A, 5781T>A, 5782T>C, 5786G>A, 5788G>A, 5790C>T, 5795G>A, 5798G>A, 5807G>A, 5808A>T, 5811T>G, 5813A>G, 5816C>T, 5822C>T, 5828T>C, 5830A>T, 5834C>A, 5836C>A, 5843G>A, 5844A>G, 5846T>A, 5850T>A, 5851A>T, 5853A>G, 5855T>A, 5856A>G, 5859A>C, 5860T>C, 5864T>A, 5868A>G, 5869A>C, 5870T>A, 5873G>A, 5874T>A, 5875G>T, 5876T>A, 5877T>G, 5878C>G, 5879C>A, 5882C>T, 5889G>C, 5893G>A, 5895A>C, 5897A>C, 5903T>C, 5904T>A, 5906T>A, 5907C>G, 5910G>A, 5912C>T, 5913T>A, 5914G>T, 5915G>T, 5918G>A, 5919A>C, 5924T>A, 5925A>C, 5926G>A, 5928A>C, 5933C>T, 5940G>A, 5941T>A, 5945T>A, 5947C>A, 5948C>A, 5951A>C, 5952T>A, 5957C>A, 5958C>G, 5959C>A, 5960T>C, 5963G>A, 5965T>G, 5966T>C, 5967T>A, 5973A>C, 5974A>T, 5976C>G, 5977C>A, 5979C>T, 5980A>C, 5982A>G, 5983G>A, 5988A>C, 5990T>A, 5999G>A, 6005T>C, 6009T>A, 6010C>G, 6011T>C, 6014A>C, 6015G>C, 6026G>A, 6028G>C, 6029A>T, 6030A>G, 6032T>A, 6039T>G, 6040G>C, 6041G>A, 6047C>A, 6048T>A, 6049C>A, 6050T>C, 6051C>A, 6054C>T, 6056A>C, 6057G>A, 6058A>G, 6060T>C, 6062A>T, 6063C>A, 6064C>A, 6065T>A, 6066T>A, 6067C>A, 6069A>G, 6070C>A, 6082C>G, 6083A>T, 6084C>G, 6085G>C, 6086G>T, 6089T>C, 6090T>C, 6091G>A, 6092T>A, 6100G>A, 6101C>A, 6104T>C, 6107_6108insGAAAAG, 6108T>G, 6109C>G, 6111G>A, 6112C>A, 6116C>G, 6117T>A, 6118C>G, 6120G>A, 6121C>G, 6122A>T, 6130C>T, 6131T>C, 6135A>G, 6138A>T, 6140G>A, 6144T>A, 6145G>T, 6146T>A, 6154T>G, 6156T>C, 6159G>A, 6163A>G, 6164G>T, 6167T>C, 6171A>T, 6175T>A, 6177T>A, 6179A>T, 6181A>T, 6182T>A, 6183G>A, 6185G>C, 6190A>G, 6191A>T, 6195T>A, 6197T>A, 6198A>G, 6199C>T, 6200C>A, 6201C>G, 6203T>A, 6206G>A, 6212C>T, 6215T>C, 6216A>G, 6217T>A, 6218T>A, 6221A>T, 6224T>A, 6230T>A, 6234T>C, 6237A>C, 6239C>A, 6243C>A, 6245T>A, 6246A>G, 6248T>C, 6249A>C, 6251T>G, 6257A>T, 6258T>A, 6259T>G, 6260G>T, 6262A>G, 6265T>A, 6268A>G, 6269T>A, 6270C>A, 6272T>A, 6276G>C, 6284C>T, 6285T>A, 6286G>T, 6287T>A, 6290T>C, 6291T>A, 6292T>C, 6293A>C, 6297A>T, 6299A>T, 6300G>A, 6301G>T, 6304T>A, 6305A>C, 6306G>C, 6307G>C, 6309_6311delTTA, 6313C>A, 6318A>G, 6319A>T, 6320A>T, 6321A>T, 6323A>C, 6327C>T, 6332C>A, 6333C>A, 6335G>A, 6338T>A								

CDS

	Begin	End	Coverage	Score	Concordance	Matches	Identities	I/D/M/F*	Stop Codons
EXK67_gp3	1211	1582	22.2%	1175	47.7%	353 (98.9%)	177 (49.6%)	2/2/1/1	1
Protein mutations:	S1213T (5248G>C), M1215I (5255G>A), Y1217I (5259T>A 5260A>T 5261C>A), D1224N (5280G>A), Y1227R (5289T>A 5290A>G 5291C>A), K1228T (5293A>C 5294A>G), Q1230A (5298C>G 5299A>C), T1232K (5305C>A), S1235D (5313A>G 5314G>A), I1236K (5317T>A 5318T>A), D1237S (5319G>A 5320A>G 5321C>T), Y1238D (5322T>G), L1240I (5328C>A 5330A>T), L1241N (5331C>A 5332T>A), K1242R (5335A>G), I1243M (5339T>G), K1244* (5340A>T), I1247K (5350T>A 5351T>A), V1248L (5352G>T 5354C>A), Y1249F (5356A>T), L1254C (5370C>T 5371T>G 5372A>T), G1257L (5379G>C 5380G>T 5381A>C), F1258Y (5383T>A 5384C>T), H1259W (5385C>T 5386A>G 5387C>G), I1261V (5391A>G), M1262K (5395T>A 5396G>A), D1264H (5400G>C), Q1266D (5406C>G 5408G>C), A1272T (5424G>A), V1274T (5430G>A 5431T>C 5432C>A), Q1277E (5439C>G), I1283L (5457A>C), V1284A (5461T>C 5462A>C), N1291T (5482A>C 5483T>A), I1315V (5553A>G), I1320V (5568A>G), E1323R (5577G>A 5578A>G), S1324D (5580A>G 5581G>A), I1325M (5585A>G), Q1327E (5589C>G), V1329L (5595G>C), Q1330E (5598C>G), L1333Q (5607T>C 5608T>A), Q1334T (5610C>A 5611A>C 5612G>A), F1335I (5613T>A 5615C>T), Q1337R (5619C>A 5620A>G 5621A>G), V1338L (5622G>T), C1339F (5626G>T 5627T>C), K1340V (5628A>G 5629A>T 5630G>A), E1341S (5631G>A 5632A>G 5633A>T), E1342K (5634G>A), L1344I (5640C>A 5642T>A), L1346I (5646T>A 5648G>A), S1347T (5649T>A 5651A>C), T1349K (5656C>A 5657A>G), L1351M (5661T>A), K1352E (5664A>G), I1353L (5667A>T), G1354C (5670G>T 5672A>T), V1355K (5673G>A 5674T>A), A1356T (5676G>A), N1357H (5679A>C), E1359N (5685G>A 5687A>C), L1363V (5697C>G), E1364T (5700G>A 5701A>C 5702A>T), I1365L (5703A>T), E1367D (5711A>T), V1370I (5718G>A 5720T>A), Q1371K (5721C>A), L1377A (5739T>G 5740T>C 5741A>T), I1380V (5748A>G), E1382D (5756A>T), F1383M (5757T>A 5759T>G), E1385del (5763_5765delGAA), Q1387K (5769C>A), E1389D (5777A>T), T1390K (5779C>A), L1391T (5781T>A 5782T>C), G1393D (5788G>A), I1400L (5808A>T), L1401V (5811T>G 5813A>G), Y1407F (5830A>T), P1409Q (5836C>A), S1412G (5844A>G 5846T>A), Y1414I (5850T>A 5851A>T), T1415A (5853A>G 5855T>A), R1416G (5856A>G), I1417P (5859A>C 5860T>C), F1418L (5864T>A), N1420A (5868A>G 5869A>C 5870T>A), C1422I (5874T>A 5875G>T 5876T>A), S1423G (5877T>G 5878C>G 5879C>A), E1427Q (5889G>C), R1428K (5893G>A), K1429H (5895A>C 5897A>C), S1432T (5904T>A 5906T>A), Q1433E (5907C>G), D1434N (5910G>A 5912C>T), W1435I (5913T>A 5914G>T 5915G>T), M1437L (5919A>C), R1439Q (5925A>C 5926G>A), K1440Q (5928A>C), V1444K (5940G>A 5941T>A), T1446K (5947C>A 5948C>A), K1447N (5951A>C), L1448I (5952T>A), P1450D (5958C>G 5959C>A 5960T>C), I1452S (5965T>G 5966T>C), L1453I (5967T>A), K1455L (5973A>C 5974A>T), P1456E (5976C>G 5977C>A), Q1457S (5979C>T 5980A>C), S1458D (5982A>G 5983G>A), I1460L (5988A>C 5990T>A), E1469Q (6015G>C), G1473A (6028G>C 6029A>T), I1474V (6030A>G 6032T>A), W1477A (6039T>G 6040G>C 6041G>A), S1480N (6048T>A 6049C>A 6050T>C), Q1481K (6051C>A), Q1482Y (6054C>T 6056A>C), D1483S (6057G>A 6058A>G), S1484P (6060T>C 6062A>T), P1485K (6063C>A 6064C>A 6065T>A), S1486K (6066T>A 6067C>A), T1487E (6069A>G 6070C>A), S1491C (6082C>G 6083A>T), R1492A (6084C>G 6085G>C 6086G>T), C1494Q (6090T>C 6091G>A 6092T>A), S1497K (6100G>A 6101C>A), K1499_S1500insEK (6107_6108insGAAAAG), S1500G (6108T>G 6109C>G), A1501N (6111G>A 6112C>A), I1502M (6116C>G), A1504S (6120G>A 6121C>G 6122A>T), A1507V (6130C>T 6131T>C), I1509V (6135A>G), M1510L (6138A>T 6140G>A), C1512I (6144T>A 6145G>T 6146T>A), V1515G (6154T>G), D1517N (6159G>A), K1518S (6163A>G 6164G>T), I1521L (6171A>T), F1522Y (6175T>A), L1523I (6177T>A 6179A>T), Y1524L (6181A>T 6182T>A), E1525N (6183G>A 6185G>C), K1527S (6190A>G 6191A>T), F1529I (6195T>A 6197T>A), T1530V (6198A>G 6199C>T 6200C>A), L1531V (6201C>G 6203T>A), I1536E (6216A>G 6217T>A 6218T>A), N1540K (6230T>A), Y1542H (6234T>C), N1543Q (6237A>C 6239C>A), L1545I (6243C>A 6245T>A), N1546D (6246A>G 6248T>C), N1547Q (6249A>C 6251T>G), K1549N (6257A>T), L1550S (6258T>A 6259T>G 6260G>T), N1551S (6262A>G), I1552K (6265T>A), N1553R (6268A>G 6269T>A), V1556L (6276G>C), C1559I (6285T>A 6286G>T 6287T>A), L1561T (6291T>A 6292T>C 6293A>C), T1563S (6297A>T 6299A>T), G1564I (6300G>A 6301G>T), L1565Y (6304T>A 6305A>C), G1566P (6306G>C 6307G>C), L1567del (6309_6311delTTA), T1568N (6313C>A), K1570V (6318A>G 6319A>T 6320A>T), I1571F (6321A>T 6323A>C), H1573Y (6327C>T), Q1575K (6333C>A 6335G>A)								

	Begin	End	Coverage	Score	Concordance	Matches	Identities	I/D/M/F*	Stop Codons
**NT**	**5241**	**6355**	**13.1%**	**461**	**22.2%**	**1057 (98.8%)**	**656 (61.3%)**	**6/7**	

Codon mutations:

AGT1213ACT (5248G>C), CGT1214AGA (5250C>A 5252T>A), ATG1215ATA (5255G>A), TAC1217ATA (5259T>A 5260A>T 5261C>A), AAG1220AAA (5270G>A), CGA1221AGA (5271C>A), CTA1222CTT (5276A>T), GAT1224AAT (5280G>A), ACA1226ACT (5288A>T), TAC1227AGA (5289T>A 5290A>G 5291C>A), AAA1228ACG (5293A>C 5294A>G), GAC1229GAT (5297C>T), CAA1230GCA (5298C>G 5299A>C), TAC1231TAT (5303C>T), ACA1232AAA (5305C>A), CTC1233TTA (5307C>T 5309C>A), AGT1235GAT (5313A>G 5314G>A), ATT1236AAA (5317T>A 5318T>A), GAC1237AGT (5319G>A 5320A>G 5321C>T), TAT1238GAT (5322T>G), CTC1239TTA (5325C>T 5327C>A), CTA1240ATT (5328C>A 5330A>T), CTT1241AAT (5331C>A 5332T>A), AAA1242AGA (5335A>G), ATT1243ATG (5339T>G), AAA1244TAA (5340A>T), GAC1245GAT (5345C>T), AAG1246AAA (5348G>A), ATT1247AAA (5350T>A 5351T>A), GTC1248TTA (5352G>T 5354C>A), TAC1249TTC (5356A>T), AGC1250AGT (5360C>T), CTA1254TGT (5370C>T 5371T>G 5372A>T), AAG1255AAA (5375G>A), GGA1257CTC (5379G>C 5380G>T 5381A>C), TTC1258TAT (5383T>A 5384C>T), CAC1259TGG (5385C>T 5386A>G 5387C>G), CAG1260CAA (5390G>A), ATT1261GTT (5391A>G), ATG1262AAA (5395T>A 5396G>A), GAT1264CAT (5400G>C), CAG1266GAC (5406C>G 5408G>C), ATA1268-TA (5412delA), GCA1272ACA (5424G>A), TTT1273TTC (5429T>C), GTC1274ACA (5430G>A 5431T>C 5432C>A), TGC1275TGT (5435C>T), CCA1276CCT (5438A>T), CAG1277GAG (5439C>G), GGT1278GGA (5444T>A), ATA1283CTA (5457A>C), GTA1284GCC (5461T>C 5462A>C), TTC1287TTT (5471C>T), GGA1288GGT (5474A>T), CTC1289TTA (5475C>T 5477C>A), AAT1291ACA (5482A>C 5483T>A), GCA1292GCG (5486A>G), GTT1313.TC (5549T>C), ATA1315GTA (5553A>G), GAC1316GAT (5558C>T), GAT1317GAC (5561T>C), ATT1318ATC (5564T>C), TTG1319CTA (5565T>C 5567G>A), ATA1320GTA (5568A>G), TTT1321TTC (5573T>C), TCT1322AGT (5574T>A 5575C>G), GAA1323AGA (5577G>A 5578A>G), AGT1324GAT (5580A>G 5581G>A), ATA1325ATG (5585A>G), CAA1327GAA (5589C>G), GTA1329CTA (5595G>C), CAA1330GAA (5598C>G), TTA1333CAA (5607T>C 5608T>A), CAG1334ACA (5610C>A 5611A>C 5612G>A), TTC1335ATT (5613T>A 5615C>T), TTC1336TTT (5618C>T), CAA1337AGG (5619C>A 5620A>G 5621A>G), GTA1338TTA (5622G>T), TGT1339TTC (5626G>T 5627T>C), AAG1340GTA (5628A>G 5629A>T 5630G>A), GAA1341AGT (5631G>A 5632A>G 5633A>T), GAA1342AAA (5634G>A), GGG1343GGA (5639G>A), CTT1344ATA (5640C>A 5642T>A), ATT1345ATA (5645T>A), TTG1346ATA (5646T>A 5648G>A), TCA1347ACC (5649T>A 5651A>C), ACA1349AAG (5656C>A 5657A>G), TTG1351ATG (5661T>A), AAA1352GAA (5664A>G), ATA1353TTA (5667A>T), GGA1354TGT (5670G>T 5672A>T), GTA1355AAA (5673G>A 5674T>A), GCA1356ACA (5676G>A), AAT1357CAT (5679A>C), GAA1359AAC (5685G>A 5687A>C), CTT1361TTA (5691C>T 5693T>A), GGC1362GGA (5696C>A), CTA1363GTA (5697C>G), GAA1364ACT (5700G>A 5701A>C 5702A>T), ATA1365TTA (5703A>T), GAA1367GAT (5711A>T), GGA1368GGG (5714A>G), AAA1369AAG (5717A>G), GTT1370ATA (5718G>A 5720T>A), CAA1371AAA (5721C>A), CTC1372CTA (5726C>A), TTA1377GCT (5739T>G 5740T>C 5741A>T), ATA1380GTA (5748A>G), GAA1382GAT (5756A>T), TTT1383ATG (5757T>A 5759T>G), CCT1384CCA (5762T>A), GAA1385del (5763_5765delGAA), GAC1386GAT (5768C>T), CAA1387AAA (5769C>A), GAA1389GAT (5777A>T), ACA1390AAA (5779C>A), TTA1391ACA (5781T>A 5782T>C), AAG1392AAA (5786G>A), GGT1393GAT (5788G>A), CTA1394TTA (5790C>T), CAG1395CAA (5795G>A), AAG1396AAA (5798G>A), GGG1399GGA (5807G>A), ATA1400TTA (5808A>T), TTA1401GTG (5811T>G 5813A>G), AAC1402AAT (5816C>T), GCC1404GCT (5822C>T), AAT1406AAC (5828T>C), TAT1407TTT (5830A>T), ATC1408ATA (5834C>A), CCA1409CAA (5836C>A), TTG1411TTA (5843G>A), AGT1412GGA (5844A>G 5846T>A), TAT1414ATT (5850T>A 5851A>T), ACT1415GCA (5853A>G 5855T>A), AGA1416GGA (5856A>G), ATA1417CCA (5859A>C 5860T>C), TTT1418TTA (5864T>A), AAT1420GCA (5868A>G 5869A>C 5870T>A), AAG1421AAA (5873G>A), TGT1422ATA (5874T>A 5875G>T 5876T>A), TCC1423GGA (5877T>G 5878C>G 5879C>A), AGC1424AGT (5882C>T), GAA1427CAA (5889G>C), AGA1428AAA (5893G>A), AAA1429CAC (5895A>C 5897A>C), AAT1431AAC (5903T>C), TCT1432ACA (5904T>A 5906T>A), CAA1433GAA (5907C>G), GAC1434AAT (5910G>A 5912C>T), TGG1435ATT (5913T>A 5914G>T 5915G>T), AAG1436AAA (5918G>A), ATG1437CTG (5919A>C), GTT1438GTA (5924T>A), AGA1439CAA (5925A>C 5926G>A), AAA1440CAA (5928A>C), ATC1441ATT (5933C>T), GTA1444AAA (5940G>A 5941T>A), ATT1445ATA (5945T>A), ACC1446AAA (5947C>A 5948C>A), AAA1447AAC (5951A>C), TTA1448ATA (5952T>A), CCC1449CCA (5957C>A), CCT1450GAC (5958C>G 5959C>A 5960T>C), TTG1451TTA (5963G>A), ATT1452AGC (5965T>G 5966T>C), TTA1453ATA (5967T>A), AAA1455CTA (5973A>C 5974A>T), CCA1456GAA (5976C>G 5977C>A), CAA1457TCA (5979C>T 5980A>C), AGT1458GAT (5982A>G 5983G>A), ATT1460CTA (5988A>C 5990T>A), GAG1463GAA (5999G>A), GAT1465GAC (6005T>C), TCT1467AGC (6009T>A 6010C>G 6011T>C), CTA1468CTC (6014A>C), GAA1469CAA (6015G>C), GGG1472GGA (6026G>A), GGA1473GCT (6028G>C 6029A>T), ATT1474GTA (6030A>G 6032T>A), TGG1477GCA (6039T>G 6040G>C 6041G>A), CCC1479CCA (6047C>A), TCT1480AAC (6048T>A 6049C>A 6050T>C), CAA1481AAA (6051C>A), CAA1482TAC (6054C>T 6056A>C), GAT1483AGT (6057G>A 6058A>G), TCA1484CCT (6060T>C 6062A>T), CCT1485AAA (6063C>A 6064C>A 6065T>A), TCA1486AAA (6066T>A 6067C>A), ACA1487GAA (6069A>G 6070C>A), TCA1491TGT (6082C>G 6083A>T), CGG1492GCT (6084C>G 6085G>C 6086G>T), TAT1493TAC (6089T>C), TGT1494CAA (6090T>C 6091G>A 6092T>A), AGC1497AAA (6100G>A 6101C>A), TAT1498TAC (6104T>C), AAA1499_TCT1500insGAAAAG (6107_6108insGAAAAG), TCT1500GGT (6108T>G 6109C>G), GCC1501AAC (6111G>A 6112C>A), ATC1502ATG (6116C>G), TCT1503AGT (6117T>A 6118C>G), GCA1504AGT (6120G>A 6121C>G 6122A>T), GCT1507GTC (6130C>T 6131T>C), ATA1509GTA (6135A>G), ATG1510TTA (6138A>T 6140G>A), TGT1512ATA (6144T>A 6145G>T 6146T>A), GTA1515GGA (6154T>G), TTA1516CTA (6156T>C), GAT1517AAT (6159G>A), AAG1518AGT (6163A>G 6164G>T), TTT1519TTC (6167T>C), ATA1521TTA (6171A>T), TTT1522TAT (6175T>A), TTA1523ATT (6177T>A 6179A>T), TAT1524TTA (6181A>T 6182T>A), GAG1525AAC (6183G>A 6185G>C), AAA1527AGT (6190A>G 6191A>T), TTT1529ATA (6195T>A 6197T>A), ACC1530GTA (6198A>G 6199C>T 6200C>A), CTT1531GTA (6201C>G 6203T>A), AGG1532AGA (6206G>A), GAC1534GAT (6212C>T), TGT1535TGC (6215T>C), ATT1536GAA (6216A>G 6217T>A 6218T>A), GCA1537GCT (6221A>T), ATT1538ATA (6224T>A), AAT1540AAA (6230T>A), TAT1542CAT (6234T>C), AAC1543CAA (6237A>C 6239C>A), CTT1545ATA (6243C>A 6245T>A), AAT1546GAC (6246A>G 6248T>C), AAT1547CAG (6249A>C 6251T>G), AAA1549AAT (6257A>T), TTG1550AGT (6258T>A 6259T>G 6260G>T), AAT1551AGT (6262A>G), ATA1552AAA (6265T>A), AAT1553AGA (6268A>G 6269T>A), CGT1554AGA (6270C>A 6272T>A), GTA1556CTA (6276G>C), TTC1558TTT (6284C>T), TGT1559ATA (6285T>A 6286G>T 6287T>A), GAT1560GAC (6290T>C), TTA1561ACC (6291T>A 6292T>C 6293A>C), ACA1563TCT (6297A>T 6299A>T), GGA1564ATA (6300G>A 6301G>T), TTA1565TAC (6304T>A 6305A>C), GGT1566CCT (6306G>C 6307G>C), TTA1567del (6309_6311delTTA), ACT1568AAT (6313C>A), AAA1570GTT (6318A>G 6319A>T 6320A>T), ATA1571TTC (6321A>T 6323A>C), CAT1573TAT (6327C>T), ATC1574ATA (6332C>A), CAG1575AAA (6333C>A 6335G>A), GGT1576GGA (6338T>A)

Proteins

	Begin	End	Coverage	Score	Concordance	Matches	Identities	I/D/M/F*	Stop Codons
ORF3 (YP_009553219.1)	1211	1582	22.2%	1175	47.7%	353 (98.9%)	177 (49.6%)	2/2/1/1	1

Protein mutations:

S1213T (5248G>C), M1215I (5255G>A), Y1217I (5259T>A 5260A>T 5261C>A), D1224N (5280G>A), Y1227R (5289T>A 5290A>G 5291C>A), K1228T (5293A>C 5294A>G), Q1230A (5298C>G 5299A>C), T1232K (5305C>A), S1235D (5313A>G 5314G>A), I1236K (5317T>A 5318T>A), D1237S (5319G>A 5320A>G 5321C>T), Y1238D (5322T>G), L1240I (5328C>A 5330A>T), L1241N (5331C>A 5332T>A), K1242R (5335A>G), I1243M (5339T>G), K1244* (5340A>T), I1247K (5350T>A 5351T>A), V1248L (5352G>T 5354C>A), Y1249F (5356A>T), L1254C (5370C>T 5371T>G 5372A>T), G1257L (5379G>C 5380G>T 5381A>C), F1258Y (5383T>A 5384C>T), H1259W (5385C>T 5386A>G 5387C>G), I1261V (5391A>G), M1262K (5395T>A 5396G>A), D1264H (5400G>C), Q1266D (5406C>G 5408G>C), A1272T (5424G>A), V1274T (5430G>A 5431T>C 5432C>A), Q1277E (5439C>G), I1283L (5457A>C), V1284A (5461T>C 5462A>C), N1291T (5482A>C 5483T>A), I1315V (5553A>G), I1320V (5568A>G), E1323R (5577G>A 5578A>G), S1324D (5580A>G 5581G>A), I1325M (5585A>G), Q1327E (5589C>G), V1329L (5595G>C), Q1330E (5598C>G), L1333Q (5607T>C 5608T>A), Q1334T (5610C>A 5611A>C 5612G>A), F1335I (5613T>A 5615C>T), Q1337R (5619C>A 5620A>G 5621A>G), V1338L (5622G>T), C1339F (5626G>T 5627T>C), K1340V (5628A>G 5629A>T 5630G>A), E1341S (5631G>A 5632A>G 5633A>T), E1342K (5634G>A), L1344I (5640C>A 5642T>A), L1346I (5646T>A 5648G>A), S1347T (5649T>A 5651A>C), T1349K (5656C>A 5657A>G), L1351M (5661T>A), K1352E (5664A>G), I1353L (5667A>T), G1354C (5670G>T 5672A>T), V1355K (5673G>A 5674T>A), A1356T (5676G>A), N1357H (5679A>C), E1359N (5685G>A 5687A>C), L1363V (5697C>G), E1364T (5700G>A 5701A>C 5702A>T), I1365L (5703A>T), E1367D (5711A>T), V1370I (5718G>A 5720T>A), Q1371K (5721C>A), L1377A (5739T>G 5740T>C 5741A>T), I1380V (5748A>G), E1382D (5756A>T), F1383M (5757T>A 5759T>G), E1385del (5763_5765delGAA), Q1387K (5769C>A), E1389D (5777A>T), T1390K (5779C>A), L1391T (5781T>A 5782T>C), G1393D (5788G>A), I1400L (5808A>T), L1401V (5811T>G 5813A>G), Y1407F (5830A>T), P1409Q (5836C>A), S1412G (5844A>G 5846T>A), Y1414I (5850T>A 5851A>T), T1415A (5853A>G 5855T>A), R1416G (5856A>G), I1417P (5859A>C 5860T>C), F1418L (5864T>A), N1420A (5868A>G 5869A>C 5870T>A), C1422I (5874T>A 5875G>T 5876T>A), S1423G (5877T>G 5878C>G 5879C>A), E1427Q (5889G>C), R1428K (5893G>A), K1429H (5895A>C 5897A>C), S1432T (5904T>A 5906T>A), Q1433E (5907C>G), D1434N (5910G>A 5912C>T), W1435I (5913T>A 5914G>T 5915G>T), M1437L (5919A>C), R1439Q (5925A>C 5926G>A), K1440Q (5928A>C), V1444K (5940G>A 5941T>A), T1446K (5947C>A 5948C>A), K1447N (5951A>C), L1448I (5952T>A), P1450D (5958C>G 5959C>A 5960T>C), I1452S (5965T>G 5966T>C), L1453I (5967T>A), K1455L (5973A>C 5974A>T), P1456E (5976C>G 5977C>A), Q1457S (5979C>T 5980A>C), S1458D (5982A>G 5983G>A), I1460L (5988A>C 5990T>A), E1469Q (6015G>C), G1473A (6028G>C 6029A>T), I1474V (6030A>G 6032T>A), W1477A (6039T>G 6040G>C 6041G>A), S1480N (6048T>A 6049C>A 6050T>C), Q1481K (6051C>A), Q1482Y (6054C>T 6056A>C), D1483S (6057G>A 6058A>G), S1484P (6060T>C 6062A>T), P1485K (6063C>A 6064C>A 6065T>A), S1486K (6066T>A 6067C>A), T1487E (6069A>G 6070C>A), S1491C (6082C>G 6083A>T), R1492A (6084C>G 6085G>C 6086G>T), C1494Q (6090T>C 6091G>A 6092T>A), S1497K (6100G>A 6101C>A), K1499_S1500insEK (6107_6108insGAAAAG), S1500G (6108T>G 6109C>G), A1501N (6111G>A 6112C>A), I1502M (6116C>G), A1504S (6120G>A 6121C>G 6122A>T), A1507V (6130C>T 6131T>C), I1509V (6135A>G), M1510L (6138A>T 6140G>A), C1512I (6144T>A 6145G>T 6146T>A), V1515G (6154T>G), D1517N (6159G>A), K1518S (6163A>G 6164G>T), I1521L (6171A>T), F1522Y (6175T>A), L1523I (6177T>A 6179A>T), Y1524L (6181A>T 6182T>A), E1525N (6183G>A 6185G>C), K1527S (6190A>G 6191A>T), F1529I (6195T>A 6197T>A), T1530V (6198A>G 6199C>T 6200C>A), L1531V (6201C>G 6203T>A), I1536E (6216A>G 6217T>A 6218T>A), N1540K (6230T>A), Y1542H (6234T>C), N1543Q (6237A>C 6239C>A), L1545I (6243C>A 6245T>A), N1546D (6246A>G 6248T>C), N1547Q (6249A>C 6251T>G), K1549N (6257A>T), L1550S (6258T>A 6259T>G 6260G>T), N1551S (6262A>G), I1552K (6265T>A), N1553R (6268A>G 6269T>A), V1556L (6276G>C), C1559I (6285T>A 6286G>T 6287T>A), L1561T (6291T>A 6292T>C 6293A>C), T1563S (6297A>T 6299A>T), G1564I (6300G>A 6301G>T), L1565Y (6304T>A 6305A>C), G1566P (6306G>C 6307G>C), L1567del (6309_6311delTTA), T1568N (6313C>A), K1570V (6318A>G 6319A>T 6320A>T), I1571F (6321A>T 6323A>C), H1573Y (6327C>T), Q1575K (6333C>A 6335G>A)

	Begin	End	Coverage	Score	Concordance	Matches	Identities	I/D/M/F*	Stop Codons
**NT**	**5241**	**6355**	**13.1%**	**461**	**22.2%**	**1057 (98.8%)**	**656 (61.3%)**	**6/7**	
Codon mutations:	AGT1213ACT (5248G>C), CGT1214AGA (5250C>A 5252T>A), ATG1215ATA (5255G>A), TAC1217ATA (5259T>A 5260A>T 5261C>A), AAG1220AAA (5270G>A), CGA1221AGA (5271C>A), CTA1222CTT (5276A>T), GAT1224AAT (5280G>A), ACA1226ACT (5288A>T), TAC1227AGA (5289T>A 5290A>G 5291C>A), AAA1228ACG (5293A>C 5294A>G), GAC1229GAT (5297C>T), CAA1230GCA (5298C>G 5299A>C), TAC1231TAT (5303C>T), ACA1232AAA (5305C>A), CTC1233TTA (5307C>T 5309C>A), AGT1235GAT (5313A>G 5314G>A), ATT1236AAA (5317T>A 5318T>A), GAC1237AGT (5319G>A 5320A>G 5321C>T), TAT1238GAT (5322T>G), CTC1239TTA (5325C>T 5327C>A), CTA1240ATT (5328C>A 5330A>T), CTT1241AAT (5331C>A 5332T>A), AAA1242AGA (5335A>G), ATT1243ATG (5339T>G), AAA1244TAA (5340A>T), GAC1245GAT (5345C>T), AAG1246AAA (5348G>A), ATT1247AAA (5350T>A 5351T>A), GTC1248TTA (5352G>T 5354C>A), TAC1249TTC (5356A>T), AGC1250AGT (5360C>T), CTA1254TGT (5370C>T 5371T>G 5372A>T), AAG1255AAA (5375G>A), GGA1257CTC (5379G>C 5380G>T 5381A>C), TTC1258TAT (5383T>A 5384C>T), CAC1259TGG (5385C>T 5386A>G 5387C>G), CAG1260CAA (5390G>A), ATT1261GTT (5391A>G), ATG1262AAA (5395T>A 5396G>A), GAT1264CAT (5400G>C), CAG1266GAC (5406C>G 5408G>C), ATA1268-TA (5412delA), GCA1272ACA (5424G>A), TTT1273TTC (5429T>C), GTC1274ACA (5430G>A 5431T>C 5432C>A), TGC1275TGT (5435C>T), CCA1276CCT (5438A>T), CAG1277GAG (5439C>G), GGT1278GGA (5444T>A), ATA1283CTA (5457A>C), GTA1284GCC (5461T>C 5462A>C), TTC1287TTT (5471C>T), GGA1288GGT (5474A>T), CTC1289TTA (5475C>T 5477C>A), AAT1291ACA (5482A>C 5483T>A), GCA1292GCG (5486A>G), GTT1313.TC (5549T>C), ATA1315GTA (5553A>G), GAC1316GAT (5558C>T), GAT1317GAC (5561T>C), ATT1318ATC (5564T>C), TTG1319CTA (5565T>C 5567G>A), ATA1320GTA (5568A>G), TTT1321TTC (5573T>C), TCT1322AGT (5574T>A 5575C>G), GAA1323AGA (5577G>A 5578A>G), AGT1324GAT (5580A>G 5581G>A), ATA1325ATG (5585A>G), CAA1327GAA (5589C>G), GTA1329CTA (5595G>C), CAA1330GAA (5598C>G), TTA1333CAA (5607T>C 5608T>A), CAG1334ACA (5610C>A 5611A>C 5612G>A), TTC1335ATT (5613T>A 5615C>T), TTC1336TTT (5618C>T), CAA1337AGG (5619C>A 5620A>G 5621A>G), GTA1338TTA (5622G>T), TGT1339TTC (5626G>T 5627T>C), AAG1340GTA (5628A>G 5629A>T 5630G>A), GAA1341AGT (5631G>A 5632A>G 5633A>T), GAA1342AAA (5634G>A), GGG1343GGA (5639G>A), CTT1344ATA (5640C>A 5642T>A), ATT1345ATA (5645T>A), TTG1346ATA (5646T>A 5648G>A), TCA1347ACC (5649T>A 5651A>C), ACA1349AAG (5656C>A 5657A>G), TTG1351ATG (5661T>A), AAA1352GAA (5664A>G), ATA1353TTA (5667A>T), GGA1354TGT (5670G>T 5672A>T), GTA1355AAA (5673G>A 5674T>A), GCA1356ACA (5676G>A), AAT1357CAT (5679A>C), GAA1359AAC (5685G>A 5687A>C), CTT1361TTA (5691C>T 5693T>A), GGC1362GGA (5696C>A), CTA1363GTA (5697C>G), GAA1364ACT (5700G>A 5701A>C 5702A>T), ATA1365TTA (5703A>T), GAA1367GAT (5711A>T), GGA1368GGG (5714A>G), AAA1369AAG (5717A>G), GTT1370ATA (5718G>A 5720T>A), CAA1371AAA (5721C>A), CTC1372CTA (5726C>A), TTA1377GCT (5739T>G 5740T>C 5741A>T), ATA1380GTA (5748A>G), GAA1382GAT (5756A>T), TTT1383ATG (5757T>A 5759T>G), CCT1384CCA (5762T>A), GAA1385del (5763_5765delGAA), GAC1386GAT (5768C>T), CAA1387AAA (5769C>A), GAA1389GAT (5777A>T), ACA1390AAA (5779C>A), TTA1391ACA (5781T>A 5782T>C), AAG1392AAA (5786G>A), GGT1393GAT (5788G>A), CTA1394TTA (5790C>T), CAG1395CAA (5795G>A), AAG1396AAA (5798G>A), GGG1399GGA (5807G>A), ATA1400TTA (5808A>T), TTA1401GTG (5811T>G 5813A>G), AAC1402AAT (5816C>T), GCC1404GCT (5822C>T), AAT1406AAC (5828T>C), TAT1407TTT (5830A>T), ATC1408ATA (5834C>A), CCA1409CAA (5836C>A), TTG1411TTA (5843G>A), AGT1412GGA (5844A>G 5846T>A), TAT1414ATT (5850T>A 5851A>T), ACT1415GCA (5853A>G 5855T>A), AGA1416GGA (5856A>G), ATA1417CCA (5859A>C 5860T>C), TTT1418TTA (5864T>A), AAT1420GCA (5868A>G 5869A>C 5870T>A), AAG1421AAA (5873G>A), TGT1422ATA (5874T>A 5875G>T 5876T>A), TCC1423GGA (5877T>G 5878C>G 5879C>A), AGC1424AGT (5882C>T), GAA1427CAA (5889G>C), AGA1428AAA (5893G>A), AAA1429CAC (5895A>C 5897A>C), AAT1431AAC (5903T>C), TCT1432ACA (5904T>A 5906T>A), CAA1433GAA (5907C>G), GAC1434AAT (5910G>A 5912C>T), TGG1435ATT (5913T>A 5914G>T 5915G>T), AAG1436AAA (5918G>A), ATG1437CTG (5919A>C), GTT1438GTA (5924T>A), AGA1439CAA (5925A>C 5926G>A), AAA1440CAA (5928A>C), ATC1441ATT (5933C>T), GTA1444AAA (5940G>A 5941T>A), ATT1445ATA (5945T>A), ACC1446AAA (5947C>A 5948C>A), AAA1447AAC (5951A>C), TTA1448ATA (5952T>A), CCC1449CCA (5957C>A), CCT1450GAC (5958C>G 5959C>A 5960T>C), TTG1451TTA (5963G>A), ATT1452AGC (5965T>G 5966T>C), TTA1453ATA (5967T>A), AAA1455CTA (5973A>C 5974A>T), CCA1456GAA (5976C>G 5977C>A), CAA1457TCA (5979C>T 5980A>C), AGT1458GAT (5982A>G 5983G>A), ATT1460CTA (5988A>C 5990T>A), GAG1463GAA (5999G>A), GAT1465GAC (6005T>C), TCT1467AGC (6009T>A 6010C>G 6011T>C), CTA1468CTC (6014A>C), GAA1469CAA (6015G>C), GGG1472GGA (6026G>A), GGA1473GCT (6028G>C 6029A>T), ATT1474GTA (6030A>G 6032T>A), TGG1477GCA (6039T>G 6040G>C 6041G>A), CCC1479CCA (6047C>A), TCT1480AAC (6048T>A 6049C>A 6050T>C), CAA1481AAA (6051C>A), CAA1482TAC (6054C>T 6056A>C), GAT1483AGT (6057G>A 6058A>G), TCA1484CCT (6060T>C 6062A>T), CCT1485AAA (6063C>A 6064C>A 6065T>A), TCA1486AAA (6066T>A 6067C>A), ACA1487GAA (6069A>G 6070C>A), TCA1491TGT (6082C>G 6083A>T), CGG1492GCT (6084C>G 6085G>C 6086G>T), TAT1493TAC (6089T>C), TGT1494CAA (6090T>C 6091G>A 6092T>A), AGC1497AAA (6100G>A 6101C>A), TAT1498TAC (6104T>C), AAA1499_TCT1500insGAAAAG (6107_6108insGAAAAG), TCT1500GGT (6108T>G 6109C>G), GCC1501AAC (6111G>A 6112C>A), ATC1502ATG (6116C>G), TCT1503AGT (6117T>A 6118C>G), GCA1504AGT (6120G>A 6121C>G 6122A>T), GCT1507GTC (6130C>T 6131T>C), ATA1509GTA (6135A>G), ATG1510TTA (6138A>T 6140G>A), TGT1512ATA (6144T>A 6145G>T 6146T>A), GTA1515GGA (6154T>G), TTA1516CTA (6156T>C), GAT1517AAT (6159G>A), AAG1518AGT (6163A>G 6164G>T), TTT1519TTC (6167T>C), ATA1521TTA (6171A>T), TTT1522TAT (6175T>A), TTA1523ATT (6177T>A 6179A>T), TAT1524TTA (6181A>T 6182T>A), GAG1525AAC (6183G>A 6185G>C), AAA1527AGT (6190A>G 6191A>T), TTT1529ATA (6195T>A 6197T>A), ACC1530GTA (6198A>G 6199C>T 6200C>A), CTT1531GTA (6201C>G 6203T>A), AGG1532AGA (6206G>A), GAC1534GAT (6212C>T), TGT1535TGC (6215T>C), ATT1536GAA (6216A>G 6217T>A 6218T>A), GCA1537GCT (6221A>T), ATT1538ATA (6224T>A), AAT1540AAA (6230T>A), TAT1542CAT (6234T>C), AAC1543CAA (6237A>C 6239C>A), CTT1545ATA (6243C>A 6245T>A), AAT1546GAC (6246A>G 6248T>C), AAT1547CAG (6249A>C 6251T>G), AAA1549AAT (6257A>T), TTG1550AGT (6258T>A 6259T>G 6260G>T), AAT1551AGT (6262A>G), ATA1552AAA (6265T>A), AAT1553AGA (6268A>G 6269T>A), CGT1554AGA (6270C>A 6272T>A), GTA1556CTA (6276G>C), TTC1558TTT (6284C>T), TGT1559ATA (6285T>A 6286G>T 6287T>A), GAT1560GAC (6290T>C), TTA1561ACC (6291T>A 6292T>C 6293A>C), ACA1563TCT (6297A>T 6299A>T), GGA1564ATA (6300G>A 6301G>T), TTA1565TAC (6304T>A 6305A>C), GGT1566CCT (6306G>C 6307G>C), TTA1567del (6309_6311delTTA), ACT1568AAT (6313C>A), AAA1570GTT (6318A>G 6319A>T 6320A>T), ATA1571TTC (6321A>T 6323A>C), CAT1573TAT (6327C>T), ATC1574ATA (6332C>A), CAG1575AAA (6333C>A 6335G>A), GGT1576GGA (6338T>A)								

*: Inserts / Deletes / Misaligned / Frameshifts

Analysis details

This analysis was performed with panviral2.64

NGS Details (UN8): Badnavirus occultipomeae

Assembly

**Coverage Length**	322 (1 contig(s))
**Depth Of Coverage**	29.0
**Number Of Reads**	84
**Reads Per Million**	1.40 rpm (after QC)
**Ambiguities**	0
**Assembly Method**	de novo + reference guided assembly
**Consensus Caller**	Bcf Tools

Coverage Map

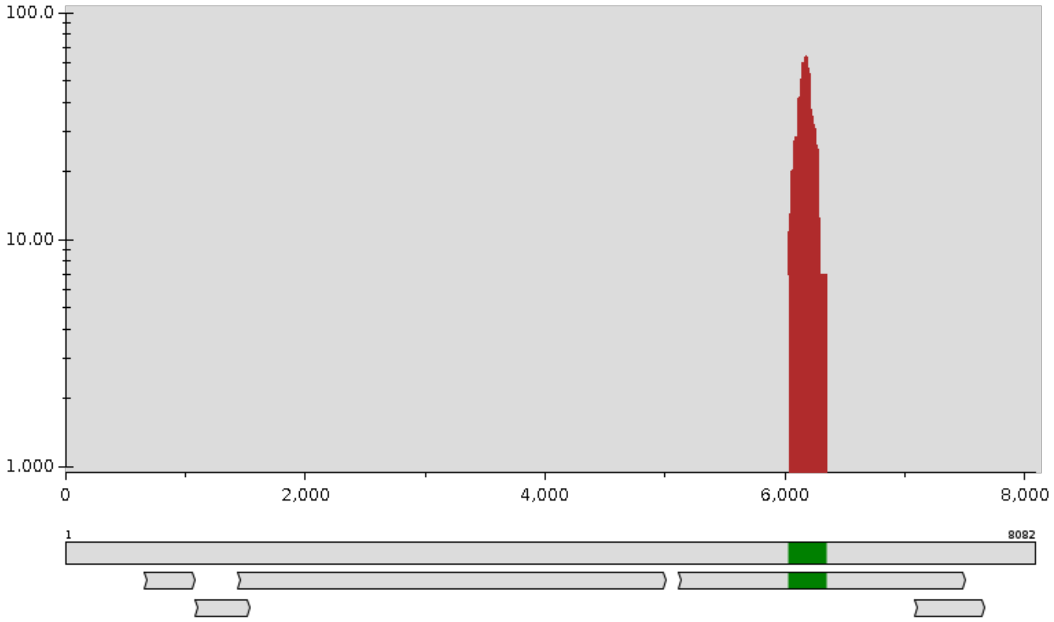

Assignment

**Type**	Badnavirus occultipomeae (Taxonomy ID: 3048353)
**Reference Genome**	NC_015655.1
**NT Identity (%)**	51.0769
**AA Identity (%)**	41.2844
**Number Of Stop Codons**	2
**Number Of CDS**	5

Alignment

**Alignment Score**	8.0 (NT) + 331.0 (AA) = 339.0
**Concordance (%)**	24.2663

**Alignment Method**	Global, seeded, nucleotide + amino acids (AGA)

Genome Region

Sequence starts at position 6028 and ends at position 6349 relative to NC_015655.1 reference sequence.

Alignment Detailed Statistics

	Begin	End	Coverage	Score	Concordance	Matches	Identities	I/D/M/F*	Stop Codons
**NT**	**6028**	**6349**	**4.0%**	**8**	**1.2%**	**322 (99.1%)**	**166 (51.1%)**	**3/0**	
Mutations:	6029A>G, 6030G>T, 6031A>T, 6032T>G, 6033A>T, 6036C>T, 6042A>G, 6043T>A, 6045T>C, 6051C>T, 6052A>C, 6053A>G, 6054A>T, 6055G>T, 6057A>T, 6060C>G, 6062T>A, 6066T>C, 6069G>A, 6070A>C, 6072A>T, 6075A>G, 6076A>G, 6078G>A, 6079G>A, 6084A>G, 6086A>G, 6087G>T, 6088T>G, 6089C>A, 6090C>T, 6092A>T, 6093A>T, 6097T>A, 6098G>A, 6099G>A, 6101C>T, 6102T>A, 6114T>G, 6115C>A, 6116C>G, 6117A>G, 6118G>T, 6120A>T, 6124C>A, 6125T>A, 6126C>T, 6128A>T, 6129T>C, 6130G>A, 6135T>C, 6136G>C, 6137A>T, 6138A>T, 6139G>T, 6145C>T, 6147C>T, 6153A>T, 6154C>T, 6156G>A, 6158T>C, 6159G>T, 6165A>C, 6168A>G, 6169G>A, 6173A>T, 6174T>G, 6178C>A, 6179A>T, 6181A>G, 6182G>A, 6183A>T, 6184A>T, 6185A>T, 6186G>A, 6190G>A, 6192T>C, 6194A>G, 6195T>G, 6197C>T, 6198A>T, 6201T>C, 6205G>C, 6206G>C, 6207A>T, 6207_6208insTTT, 6208A>C, 6209C>T, 6210A>C, 6211G>A, 6213T>C, 6214G>A, 6216A>G, 6220A>T, 6222C>G, 6224C>T, 6225A>G, 6228A>C, 6230A>T, 6234T>C, 6240C>T, 6241A>G, 6243A>G, 6244T>C, 6246G>A, 6247G>A, 6251T>A, 6255T>C, 6256G>C, 6257A>G, 6260A>G, 6262G>A, 6263A>G, 6264A>G, 6270G>A, 6279T>G, 6282T>C, 6283C>T, 6285C>A, 6286T>C, 6287T>G, 6290A>T, 6291T>G, 6292T>G, 6295G>T, 6296C>T, 6297C>A, 6298C>T, 6300A>G, 6303T>A, 6304G>T, 6307A>T, 6311G>A, 6312A>T, 6315A>G, 6316G>A, 6317G>A, 6319C>T, 6321T>G, 6322A>T, 6323T>A, 6325C>G, 6327A>G, 6329G>A, 6330C>G, 6331C>T, 6332C>T, 6333A>T, 6335C>A, 6336A>C, 6340A>T, 6341T>G, 6342G>T, 6343A>G, 6345A>G, 6346A>T, 6349G>T								
**CDS**									
SPBVa_gp4	305	412	13.6%	331	43.4%	108 (99.1%)	45 (41.3%)	1/0/0/0	2
Protein mutations:	K305S (6029A>G 6030G>T), I306C (6031A>T 6032T>G 6033A>T), F310I (6043T>A 6045T>C), K313R (6052A>C 6053A>G 6054A>T), A314S (6055G>T 6057A>T), F316Y (6062T>A), I319L (6070A>C 6072A>T), M321V (6076A>G 6078G>A), E322K (6079G>A), K324S (6086A>G 6087G>T), S325D (6088T>G 6089C>A 6090C>T), K326I (6092A>T 6093A>T), W328K (6097T>A 6098G>A 6099G>A), T329I (6101C>T 6102T>A), P334R (6115C>A 6116C>G 6117A>G), E335Y (6118G>T 6120A>T), L337N (6124C>A 6125T>A 6126C>T), Y338F (6128A>T 6129T>C), E339K (6130G>A), E341L (6136G>C 6137A>T 6138A>T), V342F (6139G>T), P344S (6145C>T 6147C>T), M348T (6158T>C 6159G>T), A352T (6169G>A), D353V (6173A>T 6174T>G), Q355M (6178C>A 6179A>T), R356D (6181A>G 6182G>A 6183A>T), K357L (6184A>T 6185A>T 6186G>A), D359N (6190G>A 6192T>C), N360R (6194A>G 6195T>G), A361V (6197C>T 6198A>T), G364P (6205G>C 6206G>C 6207A>T), G364_T365insF (6207_6208insTTT), T365L (6208A>C 6209C>T 6210A>C), D366N (6211G>A 6213T>C), A367T (6214G>A 6216A>G), I369L (6220A>T 6222C>G), A370V (6224C>T 6225A>G), Y372F (6230A>T), I376V (6241A>G 6243A>G), V378I (6247G>A), F379Y (6251T>A), E381R (6256G>C 6257A>G), N382S (6260A>G), E383R (6262G>A 6263A>G 6264A>G), D388E (6279T>G), L391R (6286T>C 6287T>G), N392M (6290A>T 6291T>G), L393V (6292T>G), A394L (6295G>T 6296C>T 6297C>A), Q395* (6298C>T 6300A>G), V397L (6304G>T), R398* (6307A>T), R399N (6311G>A 6312A>T), G401K (6316G>A 6317G>A), I403Y (6322A>T 6323T>A), L404V (6325C>G 6327A>G), S405K (6329G>A 6330C>G), P406F (6331C>T 6332C>T 6333A>T), T407N (6335C>A 6336A>C), M409C (6340A>T 6341T>G 6342G>T), K410E (6343A>G 6345A>G), I411F (6346A>T)								
Codon mutations:	AAG305AGT (6029A>G 6030G>T), ATA306TGT (6031A>T 6032T>G 6033A>T), TTC307TTT (6036C>T), AAA309AAG (6042A>G), TTT310ATC (6043T>A 6045T>C), CTC312CTT (6051C>T), AAA313CGT (6052A>C 6053A>G 6054A>T), GCA314TCT (6055G>T 6057A>T), GGC315GGG (6060C>G), TTT316TAT (6062T>A), CAT317CAC (6066T>C), CAG318CAA (6069G>A), ATA319CTT (6070A>C 6072A>T), AGA320AGG (6075A>G), ATG321GTA (6076A>G 6078G>A), GAG322AAG (6079G>A), GAA323GAG (6084A>G), AAG324AGT (6086A>G 6087G>T), TCC325GAT (6088T>G 6089C>A 6090C>T), AAA326ATT (6092A>T 6093A>T), TGG328AAA (6097T>A 6098G>A 6099G>A), ACT329ATA (6101C>T 6102T>A), ACT333ACG (6114T>G), CCA334AGG (6115C>A 6116C>G 6117A>G), GAA335TAT (6118G>T 6120A>T), CTC337AAT (6124C>A 6125T>A 6126C>T), TAT338TTC (6128A>T 6129T>C), GAA339AAA (6130G>A), TTT340TTC (6135T>C), GAA341CTT (6136G>C 6137A>T 6138A>T), GTC342TTC (6139G>T), CCC344TCT (6145C>T 6147C>T), GGA346GGT (6153A>T), CTG347TTA (6154C>T 6156G>A), ATG348ACT (6158T>C 6159G>T), GCA350GCC (6165A>C), CCA351CCG (6168A>G), GCA352ACA (6169G>A), GAT353GTG (6173A>T 6174T>G), CAG355ATG (6178C>A 6179A>T), AGA356GAT (6181A>G 6182G>A 6183A>T), AAG357TTA (6184A>T 6185A>T 6186G>A), GAT359AAC (6190G>A 6192T>C), AAT360AGG (6194A>G 6195T>G), GCA361GTT (6197C>T 6198A>T), TTT362TTC (6201T>C), GGA364CCT (6205G>C 6206G>C 6207A>T), GGA364_ACA365insTTT (6207_6208insTTT), ACA365CTC (6208A>C 6209C>T 6210A>C), GAT366AAC (6211G>A 6213T>C), GCA367ACG (6214G>A 6216A>G), ATC369TTG (6220A>T 6222C>G), GCA370GTG (6224C>T 6225A>G), GTA371GTC (6228A>C), TAT372TTT (6230A>T), ATT373ATC (6234T>C), GAC375GAT (6240C>T), ATA376GTG (6241A>G 6243A>G), TTG377CTA (6244T>C 6246G>A), GTA378ATA (6247G>A), TTC379TAC (6251T>A), TCT380TCC (6255T>C), GAA381CGA (6256G>C 6257A>G), AAT382AGT (6260A>G), GAA383AGG (6262G>A 6263A>G 6264A>G), GAG385GAA (6270G>A), GAT388GAG (6279T>G), CAT389CAC (6282T>C), CTC390TTA (6283C>T 6285C>A), TTA391CGA (6286T>C 6287T>G), AAT392ATG (6290A>T 6291T>G), TTG393GTG (6292T>G), GCC394TTA (6295G>T 6296C>T 6297C>A), CAA395TAG (6298C>T 6300A>G), ATT396ATA (6303T>A), GTG397TTG (6304G>T), AGA398TGA (6307A>T), AGA399AAT (6311G>A 6312A>T), GAA400GAG (6315A>G), GGG401AAG (6316G>A 6317G>A), CTT402TTG (6319C>T 6321T>G), ATT403TAT (6322A>T 6323T>A), CTA404GTG (6325C>G 6327A>G), AGC405AAG (6329G>A 6330C>G), CCA406TTT (6331C>T 6332C>T 6333A>T), ACA407AAC (6335C>A 6336A>C), ATG409TGT (6340A>T 6341T>G 6342G>T), AAA410GAG (6343A>G 6345A>G), ATT411TTT (6346A>T), GGA412T.. (6349G>T)								
**Proteins**									
RNaseH/reverse transcriptase (YP_004581513.1)	305	412	13.6%	331	43.4%	108 (99.1%)	45 (41.3%)	1/0/0/0	2
Protein mutations:	K305S (6029A>G 6030G>T), I306C (6031A>T 6032T>G 6033A>T), F310I (6043T>A 6045T>C), K313R (6052A>C 6053A>G 6054A>T), A314S (6055G>T 6057A>T), F316Y (6062T>A), I319L (6070A>C 6072A>T), M321V (6076A>G 6078G>A), E322K (6079G>A), K324S (6086A>G 6087G>T), S325D (6088T>G 6089C>A 6090C>T), K326I (6092A>T 6093A>T), W328K (6097T>A 6098G>A 6099G>A), T329I (6101C>T 6102T>A), P334R (6115C>A 6116C>G 6117A>G), E335Y (6118G>T 6120A>T), L337N (6124C>A 6125T>A 6126C>T), Y338F (6128A>T 6129T>C), E339K (6130G>A), E341L (6136G>C 6137A>T 6138A>T), V342F (6139G>T), P344S (6145C>T 6147C>T), M348T (6158T>C 6159G>T), A352T (6169G>A), D353V (6173A>T 6174T>G), Q355M (6178C>A 6179A>T), R356D (6181A>G 6182G>A 6183A>T), K357L (6184A>T 6185A>T 6186G>A), D359N (6190G>A 6192T>C), N360R (6194A>G 6195T>G), A361V (6197C>T 6198A>T), G364P (6205G>C 6206G>C 6207A>T), G364_T365insF (6207_6208insTTT), T365L (6208A>C 6209C>T 6210A>C), D366N (6211G>A 6213T>C), A367T (6214G>A 6216A>G), I369L (6220A>T 6222C>G), A370V (6224C>T 6225A>G), Y372F (6230A>T), I376V (6241A>G 6243A>G), V378I (6247G>A), F379Y (6251T>A), E381R (6256G>C 6257A>G), N382S (6260A>G), E383R (6262G>A 6263A>G 6264A>G), D388E (6279T>G), L391R (6286T>C 6287T>G), N392M (6290A>T 6291T>G), L393V (6292T>G), A394L (6295G>T 6296C>T 6297C>A), Q395* (6298C>T 6300A>G), V397L (6304G>T), R398* (6307A>T), R399N (6311G>A 6312A>T), G401K (6316G>A 6317G>A), I403Y (6322A>T 6323T>A), L404V (6325C>G 6327A>G), S405K (6329G>A 6330C>G), P406F (6331C>T 6332C>T 6333A>T), T407N (6335C>A 6336A>C), M409C (6340A>T 6341T>G 6342G>T), K410E (6343A>G 6345A>G), I411F (6346A>T)								
Codon mutations:	AAG305AGT (6029A>G 6030G>T), ATA306TGT (6031A>T 6032T>G 6033A>T), TTC307TTT (6036C>T), AAA309AAG (6042A>G), TTT310ATC (6043T>A 6045T>C), CTC312CTT (6051C>T), AAA313CGT (6052A>C 6053A>G 6054A>T), GCA314TCT (6055G>T 6057A>T), GGC315GGG (6060C>G), TTT316TAT (6062T>A), CAT317CAC (6066T>C), CAG318CAA (6069G>A), ATA319CTT (6070A>C 6072A>T), AGA320AGG (6075A>G), ATG321GTA (6076A>G 6078G>A), GAG322AAG (6079G>A), GAA323GAG (6084A>G), AAG324AGT (6086A>G 6087G>T), TCC325GAT (6088T>G 6089C>A 6090C>T), AAA326ATT (6092A>T 6093A>T), TGG328AAA (6097T>A 6098G>A 6099G>A), ACT329ATA (6101C>T 6102T>A), ACT333ACG (6114T>G), CCA334AGG (6115C>A 6116C>G 6117A>G), GAA335TAT (6118G>T 6120A>T), CTC337AAT (6124C>A 6125T>A 6126C>T), TAT338TTC (6128A>T 6129T>C), GAA339AAA (6130G>A), TTT340TTC (6135T>C), GAA341CTT (6136G>C 6137A>T 6138A>T), GTC342TTC (6139G>T), CCC344TCT (6145C>T 6147C>T), GGA346GGT (6153A>T), CTG347TTA (6154C>T 6156G>A), ATG348ACT (6158T>C 6159G>T), GCA350GCC (6165A>C), CCA351CCG (6168A>G), GCA352ACA (6169G>A), GAT353GTG (6173A>T 6174T>G), CAG355ATG (6178C>A 6179A>T), AGA356GAT (6181A>G 6182G>A 6183A>T), AAG357TTA (6184A>T 6185A>T 6186G>A), GAT359AAC (6190G>A 6192T>C), AAT360AGG (6194A>G 6195T>G), GCA361GTT (6197C>T 6198A>T), TTT362TTC (6201T>C), GGA364CCT (6205G>C 6206G>C 6207A>T), GGA364_ACA365insTTT (6207_6208insTTT), ACA365CTC (6208A>C 6209C>T 6210A>C), GAT366AAC (6211G>A 6213T>C), GCA367ACG (6214G>A 6216A>G), ATC369TTG (6220A>T 6222C>G), GCA370GTG (6224C>T 6225A>G), GTA371GTC (6228A>C), TAT372TTT (6230A>T), ATT373ATC (6234T>C), GAC375GAT (6240C>T), ATA376GTG (6241A>G 6243A>G), TTG377CTA (6244T>C 6246G>A), GTA378ATA (6247G>A), TTC379TAC (6251T>A), TCT380TCC (6255T>C), GAA381CGA (6256G>C 6257A>G), AAT382AGT (6260A>G), GAA383AGG (6262G>A 6263A>G 6264A>G), GAG385GAA (6270G>A), GAT388GAG (6279T>G), CAT389CAC (6282T>C), CTC390TTA (6283C>T 6285C>A), TTA391CGA (6286T>C 6287T>G), AAT392ATG (6290A>T 6291T>G), TTG393GTG (6292T>G), GCC394TTA (6295G>T 6296C>T 6297C>A), CAA395TAG (6298C>T 6300A>G), ATT396ATA (6303T>A), GTG397TTG (6304G>T), AGA398TGA (6307A>T), AGA399AAT (6311G>A 6312A>T), GAA400GAG (6315A>G), GGG401AAG (6316G>A 6317G>A), CTT402TTG (6319C>T 6321T>G), ATT403TAT (6322A>T 6323T>A), CTA404GTG (6325C>G 6327A>G), AGC405AAG (6329G>A 6330C>G), CCA406TTT (6331C>T 6332C>T 6333A>T), ACA407AAC (6335C>A 6336A>C), ATG409TGT (6340A>T 6341T>G 6342G>T), AAA410GAG (6343A>G 6345A>G), ATT411TTT (6346A>T), GGA412T.. (6349G>T)								

*: Inserts / Deletes / Misaligned / Frameshifts

Analysis details

This analysis was performed with panviral2.64

NGS Details (UN8): Errantivirus

Assembly

**Coverage Length**	380 (1 contig(s))
**Depth Of Coverage**	22.1
**Number Of Reads**	73
**Reads Per Million**	1.22 rpm (after QC)
**Ambiguities**	0
**Assembly Method**	de novo + reference guided assembly
**Consensus Caller**	Bcf Tools

Coverage Map

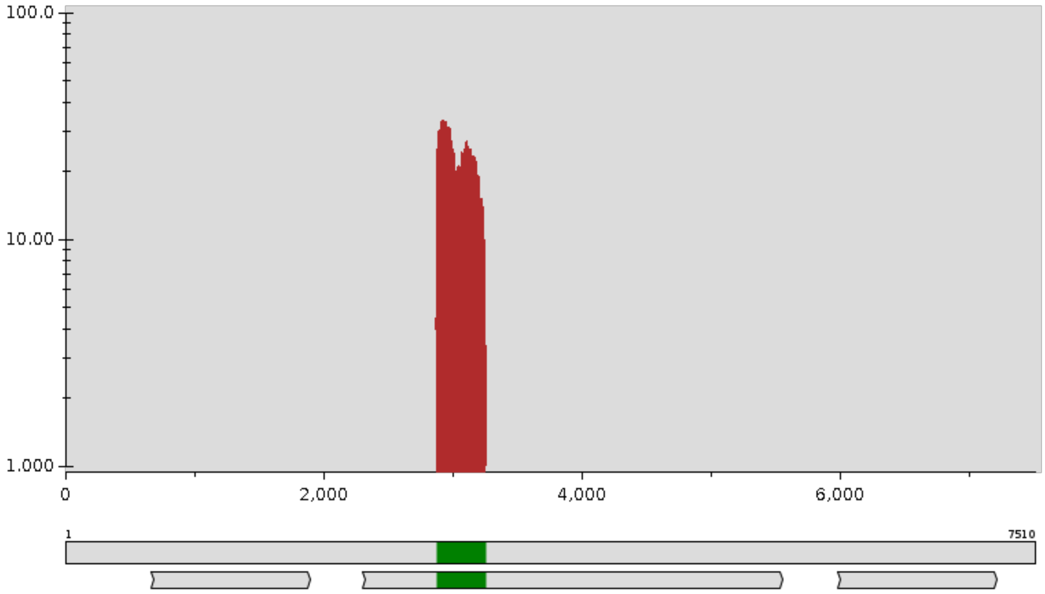

Assignment

**Type**	Errantivirus (Taxonomy ID: 186666)
**Reference Genome**	NC_038512.1
**NT Identity (%)**	54.5932
**AA Identity (%)**	45.3125
**Number Of Stop Codons**	1
**Number Of CDS**	3

Alignment

**Alignment Score**	62.0 (NT) + 310.0 (AA) = 372.0
**Concordance (%)**	24.1505

Alignment Method	Global, seeded, nucleotide + amino acids (AGA)

Genome Region

Sequence starts at position 2876 and ends at position 3255 relative to NC_038512.1 reference sequence.

Alignment Detailed Statistics

	Begin	End	Coverage	Score	Concordance	Matches	Identities	I/D/M/F*	Stop Codons
**NT**	**2876**	**3255**	**5.1%**	**62**	**8.2%**	**380 (99.7%)**	**208 (54.6%)**	**1/0**	
Mutations:	2885C>A, 2887T>G, 2888C>T, 2890C>G, 2891G>T, 2892T>G, 2893A>T, 2896T>A, 2901T>A, 2902C>T, 2903C>A, 2905T>G, 2906A>G, 2908G>T, 2909T>C, 2911G>T, 2914C>T, 2915G>A, 2918A>C, 2920G>C, 2924A>G, 2926C>T, 2927G>A, 2929T>G, 2930G>A, 2937A>T, 2941G>T, 2947A>T, 2948A>C, 2949A>T, 2950C>A, 2951A>G, 2954A>G, 2955G>A, 2956T>A, 2959C>T, 2960G>T, 2962A>G, 2963C>T, 2965T>G, 2968C>T, 2969A>G, 2971G>A, 2972T>C, 2974A>T, 2977T>G, 2978A>G, 2979A>G, 2980G>T, 2982G>C, 2983C>A, 2984C>G, 2987T>A, 2988A>T, 2989C>T, 2992C>T, 2993A>T, 2997C>A, 2998C>A, 2999T>A, 3004T>C, 3005T>C, 3007G>T, 3008G>A, 3009C>G, 3010A>G, 3011A>G, 3012G>C, 3014G>A, 3018T>A, 3019T>C, 3020T>C, 3026G>T, 3029G>A, 3030A>G, 3031G>A, 3036A>C, 3038C>A, 3039C>A, 3040T>A, 3041C>G, 3046T>C, 3050T>C, 3052G>A, 3058C>A, 3061G>A, 3064T>C, 3066A>G, 3067C>A, 3068G>A, 3069T>C, 3070A>T, 3071G>C, 3073A>C, 3074C>T, 3075A>C, 3076C>A, 3090T>A, 3091C>T, 3092C>T, 3094T>G, 3095C>G, 3096G>T, 3103T>A, 3104A>T, 3106G>T, 3110T>C, 3113A>T, 3114A>C, 3115A>T, 3118C>T, 3119T>G, 3125T>G, 3127T>A, 3130T>C, 3137A>G, 3139A>G, 3140G>T, 3142T>G, 3146G>A, 3148C>T, 3150A>C, 3154C>G, 3155C>T, 3157A>T, 3158A>T, 3159G>A, 3161G>A, 3162G>A, 3163T>G, 3164C>T, 3166C>T, 3167C>T, 3168A>T, 3169A>G, 3171A>G, 3172T>G, 3175C>G, 3176A>T, 3177T>C, 3178C>A, 3179T>G, 3180G>T, 3182C>T, 3184C>A, 3187C>T, 3189A>T, 3190C>T, 3191C>T, 3193_3194insT, 3196C>T, 3203A>C, 3205T>G, 3206G>A, 3209T>G, 3215A>C, 3216C>A, 3217T>G, 3220C>T, 3221C>A, 3223A>T, 3224C>G, 3226G>A, 3229A>C, 3232C>T, 3235G>A, 3236G>T, 3237A>T, 3239A>C, 3245G>A, 3246A>G, 3248C>A, 3249G>A								

CDS

	Begin	End	Coverage	Score	Concordance	Matches	Identities	I/D/M/F*	Stop Codons
D1R33_gp2	191	317	11.7%	310	34.5%	127 (99.2%)	58 (45.3%)	1/0/1/1	1
Protein mutations:	V196C (2891G>T 2892T>G 2893A>T), F199Y (2901T>A 2902C>T), K201D (2906A>G 2908G>T), E204K (2915G>A), K205H (2918A>C 2920G>C), I207V (2924A>G 2926C>T), D208K (2927G>A 2929T>G), D209N (2930G>A), Y211F (2937A>T), N215L (2948A>C 2949A>T 2950C>A), I216V (2951A>G), S217E (2954A>G 2955G>A 2956T>A), V219L (2960G>T 2962A>G), K222E (2969A>G 2971G>A), K225G (2978A>G 2979A>G 2980G>T), C226S (2982G>C 2983C>A), Q227E (2984C>G), Y228I (2987T>A 2988A>T 2989C>T), T230S (2993A>T), T231K (2997C>A 2998C>A), L232I (2999T>A), A235R (3008G>A 3009C>G 3010A>G), S236A (3011A>G 3012G>C), G237R (3014G>A), F238Y (3018T>A 3019T>C), Y239H (3020T>C), V241L (3026G>T), E242R (3029G>A 3030A>G 3031G>A), D244A (3036A>C), P245K (3038C>A 3039C>A 3040T>A), Q246E (3041C>G), S249P (3050T>C 3052G>A), N254R (3066A>G 3067C>A), V255T (3068G>A 3069T>C 3070A>T), E256H (3071G>C 3073A>C), H257S (3074C>T 3075A>C 3076C>A), F262Y (3090T>A 3091C>T), R264V (3095C>G 3096G>T), M267F (3104A>T 3106G>T), K270S (3113A>T 3114A>C 3115A>T), S272A (3119T>G), S274A (3125T>G 3127T>A), R278G (3137A>G 3139A>G), V279L (3140G>T 3142T>G), D281N (3146G>A 3148C>T), N282T (3150A>C), L284F (3155C>T 3157A>T), R285* (3158A>T 3159G>A), G286K (3161G>A 3162G>A 3163T>G), L287F (3164C>T 3166C>T), Q288L (3167C>T 3168A>T 3169A>G), N289R (3171A>G 3172T>G), N290K (3175C>G), I291S (3176A>T 3177T>C 3178C>A), C292V (3179T>G 3180G>T), Y295F (3189A>T 3190C>T), L296F (3191C>T), L296_D297insX (3193_3194insT), I300L (3203A>C 3205T>G), V301I (3206G>A), Y302D (3209T>G), T304Q (3215A>C 3216C>A 3217T>G), L306I (3221C>A 3223A>T), Q307E (3224C>G 3226G>A), E308D (3229A>C), E311L (3236G>T 3237A>T), N312H (3239A>C), E314R (3245G>A 3246A>G), R315K (3248C>A 3249G>A)								
Codon mutations:	CGT194AGG (2885C>A 2887T>G), CTC195TTG (2888C>T 2890C>G), GTA196TGT (2891G>T 2892T>G 2893A>T), GTT197GTA (2896T>A), TTC199TAT (2901T>A 2902C>T), CGT200AGG (2903C>A 2905T>G), AAG201GAT (2906A>G 2908G>T), TTG202CTT (2909T>C 2911G>T), AAC203AAT (2914C>T), GAG204AAG (2915G>A), AAG205CAC (2918A>C 2920G>C), ATC207GTT (2924A>G 2926C>T), GAT208AAG (2927G>A 2929T>G), GAC209AAC (2930G>A), TAC211TTC (2937A>T), CCG212CCT (2941G>T), CCA214CCT (2947A>T), AAC215CTA (2948A>C 2949A>T 2950C>A), ATA216GTA (2951A>G), AGT217GAA (2954A>G 2955G>A 2956T>A), GAC218GAT (2959C>T), GTA219TTG (2960G>T 2962A>G), CTT220TTG (2963C>T 2965T>G), GAC221GAT (2968C>T), AAG222GAA (2969A>G 2971G>A), TTA223CTT (2972T>C 2974A>T), GGT224GGG (2977T>G), AAG225GGT (2978A>G 2979A>G 2980G>T), TGC226TCA (2982G>C 2983C>A), CAA227GAA (2984C>G), TAC228ATT (2987T>A 2988A>T 2989C>T), TTC229TTT (2992C>T), ACC230TCC (2993A>T), ACC231AAA (2997C>A 2998C>A), TTA232ATA (2999T>A), GAT233GAC (3004T>C), TTG234CTT (3005T>C 3007G>T), GCA235AGG (3008G>A 3009C>G 3010A>G), AGT236GCT (3011A>G 3012G>C), GGG237AGG (3014G>A), TTT238TAC (3018T>A 3019T>C), TAT239CAT (3020T>C), GTG241TTG (3026G>T), GAG242AGA (3029G>A 3030A>G 3031G>A), GAC244GCC (3036A>C), CCT245AAA (3038C>A 3039C>A 3040T>A), CAA246GAA (3041C>G), GAT247GAC (3046T>C), TCG249CCA (3050T>C 3052G>A), ACC251ACA (3058C>A), GCG252GCA (3061G>A), TTT253TTC (3064T>C), AAC254AGA (3066A>G 3067C>A), GTA255ACT (3068G>A 3069T>C 3070A>T), GAA256CAC (3071G>C 3073A>C), CAC257TCA (3074C>T 3075A>C 3076C>A), TTC262TAT (3090T>A 3091C>T), CTT263TTG (3092C>T 3094T>G), CGA264GTA (3095C>G 3096G>T), CCT266CCA (3103T>A), ATG267TTT (3104A>T 3106G>T), TTA269CTA (3110T>C), AAA270TCT (3113A>T 3114A>C 3115A>T), AAC271AAT (3118C>T), TCA272GCA (3119T>G), TCT274GCA (3125T>G 3127T>A), ACT275ACC (3130T>C), AGA278GGG (3137A>G 3139A>G), GTT279TTG (3140G>T 3142T>G), GAC281AAT (3146G>A 3148C>T), AAT282ACT (3150A>C), GTC283GTG (3154C>G), CTA284TTT (3155C>T 3157A>T), AGA285TAA (3158A>T 3159G>A), GGT286AAG (3161G>A 3162G>A 3163T>G), CTC287TTT (3164C>T 3166C>T), CAA288TTG (3167C>T 3168A>T 3169A>G), AAT289AGG (3171A>G 3172T>G), AAC290AAG (3175C>G), ATC291TCA (3176A>T 3177T>C 3178C>A), TGT292GTT (3179T>G 3180G>T), CTC293TTA (3182C>T 3184C>A), GTC294GTT (3187C>T), TAC295TTT (3189A>T 3190C>T), CTT296TTT (3191C>T), CTT296_GAC297insT-- (3193_3194insT), GAC297GAT (3196C>T), ATT300CTG (3203A>C 3205T>G), GTC301ATC (3206G>A), TAT302GAT (3209T>G), ACT304CAG (3215A>C 3216C>A 3217T>G), TCC305TCT (3220C>T), CTA306ATT (3221C>A 3223A>T), CAG307GAA (3224C>G 3226G>A), GAA308GAC (3229A>C), CAC309CAT (3232C>T), CTG310CTA (3235G>A), GAG311TTG (3236G>T 3237A>T), AAC312CAC (3239A>C), GAA314AGA (3245G>A 3246A>G), CGA315AAA (3248C>A 3249G>A)								

Proteins

	Begin	End	Coverage	Score	Concordance	Matches	Identities	I/D/M/F*	Stop Codons
ORF B (YP_009507248.1)	191	317	11.7%	310	34.5%	127 (99.2%)	58 (45.3%)	1/0/1/1	1
Protein mutations:	V196C (2891G>T 2892T>G 2893A>T), F199Y (2901T>A 2902C>T), K201D (2906A>G 2908G>T), E204K (2915G>A), K205H (2918A>C 2920G>C), I207V (2924A>G 2926C>T), D208K (2927G>A 2929T>G), D209N (2930G>A), Y211F (2937A>T), N215L (2948A>C 2949A>T 2950C>A), I216V (2951A>G), S217E (2954A>G 2955G>A 2956T>A), V219L (2960G>T 2962A>G), K222E (2969A>G 2971G>A), K225G (2978A>G 2979A>G 2980G>T), C226S (2982G>C 2983C>A), Q227E (2984C>G), Y228I (2987T>A 2988A>T 2989C>T), T230S (2993A>T), T231K (2997C>A 2998C>A), L232I (2999T>A), A235R (3008G>A 3009C>G 3010A>G), S236A (3011A>G 3012G>C), G237R (3014G>A), F238Y (3018T>A 3019T>C), Y239H (3020T>C), V241L (3026G>T), E242R (3029G>A 3030A>G 3031G>A), D244A (3036A>C), P245K (3038C>A 3039C>A 3040T>A), Q246E (3041C>G), S249P (3050T>C 3052G>A), N254R (3066A>G 3067C>A), V255T (3068G>A 3069T>C 3070A>T), E256H (3071G>C 3073A>C), H257S (3074C>T 3075A>C 3076C>A), F262Y (3090T>A 3091C>T), R264V (3095C>G 3096G>T), M267F (3104A>T 3106G>T), K270S (3113A>T 3114A>C 3115A>T), S272A (3119T>G), S274A (3125T>G 3127T>A), R278G (3137A>G 3139A>G), V279L (3140G>T 3142T>G), D281N (3146G>A 3148C>T), N282T (3150A>C), L284F (3155C>T 3157A>T), R285* (3158A>T 3159G>A), G286K (3161G>A 3162G>A 3163T>G), L287F (3164C>T 3166C>T), Q288L (3167C>T 3168A>T 3169A>G), N289R (3171A>G 3172T>G), N290K (3175C>G), I291S (3176A>T 3177T>C 3178C>A), C292V (3179T>G 3180G>T), Y295F (3189A>T 3190C>T), L296F (3191C>T), L296_D297insX (3193_3194insT), I300L (3203A>C 3205T>G), V301I (3206G>A), Y302D (3209T>G), T304Q (3215A>C 3216C>A 3217T>G), L306I (3221C>A 3223A>T), Q307E (3224C>G 3226G>A), E308D (3229A>C), E311L (3236G>T 3237A>T), N312H (3239A>C), E314R (3245G>A 3246A>G), R315K (3248C>A 3249G>A)								
Codon mutations:	CGT194AGG (2885C>A 2887T>G), CTC195TTG (2888C>T 2890C>G), GTA196TGT (2891G>T 2892T>G 2893A>T), GTT197GTA (2896T>A), TTC199TAT (2901T>A 2902C>T), CGT200AGG (2903C>A 2905T>G), AAG201GAT (2906A>G 2908G>T), TTG202CTT (2909T>C 2911G>T), AAC203AAT (2914C>T), GAG204AAG (2915G>A), AAG205CAC (2918A>C 2920G>C), ATC207GTT (2924A>G 2926C>T), GAT208AAG (2927G>A 2929T>G), GAC209AAC (2930G>A), TAC211TTC (2937A>T), CCG212CCT (2941G>T), CCA214CCT (2947A>T), AAC215CTA (2948A>C 2949A>T 2950C>A), ATA216GTA (2951A>G), AGT217GAA (2954A>G 2955G>A 2956T>A), GAC218GAT (2959C>T), GTA219TTG (2960G>T 2962A>G), CTT220TTG (2963C>T 2965T>G), GAC221GAT (2968C>T), AAG222GAA (2969A>G 2971G>A), TTA223CTT (2972T>C 2974A>T), GGT224GGG (2977T>G), AAG225GGT (2978A>G 2979A>G 2980G>T), TGC226TCA (2982G>C 2983C>A), CAA227GAA (2984C>G), TAC228ATT (2987T>A 2988A>T 2989C>T), TTC229TTT (2992C>T), ACC230TCC (2993A>T), ACC231AAA (2997C>A 2998C>A), TTA232ATA (2999T>A), GAT233GAC (3004T>C), TTG234CTT (3005T>C 3007G>T), GCA235AGG (3008G>A 3009C>G 3010A>G), AGT236GCT (3011A>G 3012G>C), GGG237AGG (3014G>A), TTT238TAC (3018T>A 3019T>C), TAT239CAT (3020T>C), GTG241TTG (3026G>T), GAG242AGA (3029G>A 3030A>G 3031G>A), GAC244GCC (3036A>C), CCT245AAA (3038C>A 3039C>A 3040T>A), CAA246GAA (3041C>G), GAT247GAC (3046T>C), TCG249CCA (3050T>C 3052G>A), ACC251ACA (3058C>A), GCG252GCA (3061G>A), TTT253TTC (3064T>C), AAC254AGA (3066A>G 3067C>A), GTA255ACT (3068G>A 3069T>C 3070A>T), GAA256CAC (3071G>C 3073A>C), CAC257TCA (3074C>T 3075A>C 3076C>A), TTC262TAT (3090T>A 3091C>T), CTT263TTG (3092C>T 3094T>G), CGA264GTA (3095C>G 3096G>T), CCT266CCA (3103T>A), ATG267TTT (3104A>T 3106G>T), TTA269CTA (3110T>C), AAA270TCT (3113A>T 3114A>C 3115A>T), AAC271AAT (3118C>T), TCA272GCA (3119T>G), TCT274GCA (3125T>G 3127T>A), ACT275ACC (3130T>C), AGA278GGG (3137A>G 3139A>G), GTT279TTG (3140G>T 3142T>G), GAC281AAT (3146G>A 3148C>T), AAT282ACT (3150A>C), GTC283GTG (3154C>G), CTA284TTT (3155C>T 3157A>T), AGA285TAA (3158A>T 3159G>A), GGT286AAG (3161G>A 3162G>A 3163T>G), CTC287TTT (3164C>T 3166C>T), CAA288TTG (3167C>T 3168A>T 3169A>G), AAT289AGG (3171A>G 3172T>G), AAC290AAG (3175C>G), ATC291TCA (3176A>T 3177T>C 3178C>A), TGT292GTT (3179T>G 3180G>T), CTC293TTA (3182C>T 3184C>A), GTC294GTT (3187C>T), TAC295TTT (3189A>T 3190C>T), CTT296TTT (3191C>T), CTT296_GAC297insT-- (3193_3194insT), GAC297GAT (3196C>T), ATT300CTG (3203A>C 3205T>G), GTC301ATC (3206G>A), TAT302GAT (3209T>G), ACT304CAG (3215A>C 3216C>A 3217T>G), TCC305TCT (3220C>T), CTA306ATT (3221C>A 3223A>T), CAG307GAA (3224C>G 3226G>A), GAA308GAC (3229A>C), CAC309CAT (3232C>T), CTG310CTA (3235G>A), GAG311TTG (3236G>T 3237A>T), AAC312CAC (3239A>C), GAA314AGA (3245G>A 3246A>G), CGA315AAA (3248C>A 3249G>A)								

*: Inserts / Deletes / Misaligned / Frameshifts

Analysis details

This analysis was performed with panviral2.64

NGS Details (UN8): Errantivirus

Assembly

**Coverage Length**	353 (1 contig(s))
**Depth Of Coverage**	20.6
**Number Of Reads**	64
**Reads Per Million**	1.07 rpm (after QC)
**Ambiguities**	0
**Assembly Method**	de novo + reference guided assembly
**Consensus Caller**	Bcf Tools

Coverage Map

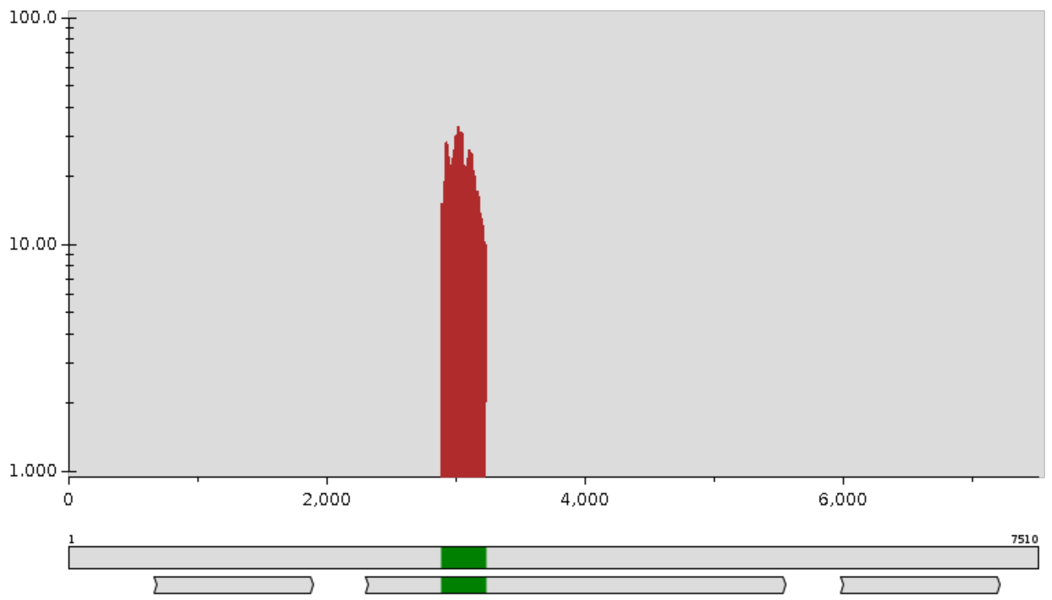

Assignment

**Type**	Errantivirus (Taxonomy ID: 186666)
**Reference Genome**	NC_038512.1
**NT Identity (%)**	56.9405
**AA Identity (%)**	47.4576
**Number Of Stop Codons**	0
**Number Of CDS**	3

Alignment

**Alignment Score**	98.0 (NT) + 431.0 (AA) = 529.0
**Concordance (%)**	34.7113

**Alignment Method**	Global, seeded, nucleotide + amino acids (AGA)

Genome Region

Sequence starts at position 2887 and ends at position 3239 relative to NC_038512.1 reference sequence.

Alignment Detailed Statistics

	Begin	End	Coverage	Score	Concordance	Matches	Identities	I/D/M/F*	Stop Codons
**NT**	**2887**	**3239**	**4.7%**	**98**	**13.9%**	**353 (100%)**	**201 (56.9%)**	**0/0**	
Mutations:	2893A>T, 2896T>A, 2901T>A, 2902C>T, 2903C>A, 2905T>G, 2906A>G, 2908G>C, 2911G>A, 2914C>T, 2915G>A, 2918A>T, 2919A>C, 2920G>C, 2927G>A, 2929T>G, 2930G>A, 2935A>G, 2937A>T, 2938C>T, 2941G>A, 2944A>T, 2947A>T, 2949A>T, 2950C>A, 2951A>G, 2953A>G, 2954A>G, 2955G>A, 2956T>G, 2960G>C, 2963C>T, 2965T>G, 2969A>G, 2971G>A, 2977T>A, 2978A>G, 2979A>G, 2982G>C, 2984C>A, 2987T>A, 2988A>T, 2989C>A, 2993A>T, 2995C>T, 2997C>A, 2998C>A, 2999T>A, 3004T>C, 3007G>A, 3008G>A, 3009C>G, 3011A>T, 3012G>C, 3016G>A, 3018T>A, 3019T>C, 3020T>C, 3025G>A, 3026G>C, 3028G>T, 3029G>A, 3030A>G, 3031G>A, 3036A>C, 3038C>A, 3040T>A, 3041C>G, 3043A>G, 3047A>G, 3049A>G, 3050T>C, 3052G>A, 3058C>T, 3061G>C, 3066A>G, 3067C>A, 3068G>A, 3069T>C, 3070A>T, 3071G>C, 3073A>C, 3074C>T, 3075A>C, 3076C>T, 3088A>G, 3090T>A, 3092C>T, 3094T>A, 3095C>G, 3096G>T, 3097A>C, 3103T>C, 3104A>T, 3106G>T, 3109A>T, 3110T>C, 3112A>G, 3113A>T, 3114A>C, 3118C>T, 3119T>G, 3121A>C, 3125T>G, 3127T>C, 3130T>A, 3137A>G, 3140G>T, 3142T>A, 3146G>A, 3148C>T, 3149A>T, 3150A>C, 3154C>G, 3155C>T, 3157A>C, 3158A>C, 3159G>A, 3161G>C, 3162G>A, 3163T>A, 3164C>T, 3166C>T, 3168A>T, 3171A>G, 3172T>G, 3175C>G, 3176A>C, 3177T>A, 3178C>T, 3179T>A, 3180G>T, 3181T>G, 3182C>T, 3184C>G, 3187C>A, 3189A>T, 3190C>T, 3191C>T, 3196C>T, 3202T>A, 3203A>C, 3206G>A, 3208C>A, 3214T>C, 3216C>A, 3217T>A, 3218T>A, 3221C>A, 3224C>G, 3226G>A, 3229A>T								
**CDS**									
D1R33_gp2	195	312	10.9%	431	51.7%	118 (100%)	56 (47.5%)	0/0/0/0	0
Protein mutations:	F199Y (2901T>A 2902C>T), K201D (2906A>G 2908G>C), E204K (2915G>A), K205S (2918A>T 2919A>C 2920G>C), D208K (2927G>A 2929T>G), D209N (2930G>A), Y211F (2937A>T 2938C>T), N215I (2949A>T 2950C>A), I216V (2951A>G 2953A>G), S217E (2954A>G 2955G>A 2956T>G), V219L (2960G>C), K222E (2969A>G 2971G>A), K225G (2978A>G 2979A>G), C226S (2982G>C), Q227K (2984C>A), Y228I (2987T>A 2988A>T 2989C>A), T230S (2993A>T 2995C>T), T231K (2997C>A 2998C>A), L232I (2999T>A), A235R (3008G>A 3009C>G), F238Y (3018T>A 3019T>C), Y239H (3020T>C), V241L (3026G>C 3028G>T), E242R (3029G>A 3030A>G 3031G>A), D244A (3036A>C), P245T (3038C>A 3040T>A), Q246E (3041C>G 3043A>G), I248V (3047A>G 3049A>G), S249P (3050T>C 3052G>A), N254R (3066A>G 3067C>A), V255T (3068G>A 3069T>C 3070A>T), E256H (3071G>C 3073A>C), H257S (3074C>T 3075A>C 3076C>T), F262Y (3090T>A), R264V (3095C>G 3096G>T 3097A>C), M267F (3104A>T 3106G>T), K270S (3113A>T 3114A>C), S272A (3119T>G 3121A>C), S274A (3125T>G 3127T>C), R278G (3137A>G), V279L (3140G>T 3142T>A), D281N (3146G>A 3148C>T), N282S (3149A>T 3150A>C), L284F (3155C>T 3157A>C), R285Q (3158A>C 3159G>A), G286Q (3161G>C 3162G>A 3163T>A), L287F (3164C>T 3166C>T), Q288L (3168A>T), N289R (3171A>G 3172T>G), N290K (3175C>G), I291H (3176A>C 3177T>A 3178C>T), C292M (3179T>A 3180G>T 3181T>G), Y295F (3189A>T 3190C>T), L296F (3191C>T), I300L (3203A>C), V301I (3206G>A 3208C>A), T304K (3216C>A 3217T>A), S305T (3218T>A), L306I (3221C>A), Q307E (3224C>G 3226G>A), E308D (3229A>T)								
Codon mutations:	GTA196GTT (2893A>T), GTT197GTA (2896T>A), TTC199TAT (2901T>A 2902C>T), CGT200AGG (2903C>A 2905T>G), AAG201GAC (2906A>G 2908G>C), TTG202TTA (2911G>A), AAC203AAT (2914C>T), GAG204AAG (2915G>A), AAG205TCC (2918A>T 2919A>C 2920G>C), GAT208AAG (2927G>A 2929T>G), GAC209AAC (2930G>A), AAA210AAG (2935A>G), TAC211TTT (2937A>T 2938C>T), CCG212CCA (2941G>A), ATA213ATT (2944A>T), CCA214CCT (2947A>T), AAC215ATA (2949A>T 2950C>A), ATA216GTG (2951A>G 2953A>G), AGT217GAG (2954A>G 2955G>A 2956T>G), GTA219CTA (2960G>C), CTT220TTG (2963C>T 2965T>G), AAG222GAA (2969A>G 2971G>A), GGT224GGA (2977T>A), AAG225GGG (2978A>G 2979A>G), TGC226TCC (2982G>C), CAA227AAA (2984C>A), TAC228ATA (2987T>A 2988A>T 2989C>A), ACC230TCT (2993A>T 2995C>T), ACC231AAA (2997C>A 2998C>A), TTA232ATA (2999T>A), GAT233GAC (3004T>C), TTG234TTA (3007G>A), GCA235AGA (3008G>A 3009C>G), AGT236TCT (3011A>T 3012G>C), GGG237GGA (3016G>A), TTT238TAC (3018T>A 3019T>C), TAT239CAT (3020T>C), CAG240CAA (3025G>A), GTG241CTT (3026G>C 3028G>T), GAG242AGA (3029G>A 3030A>G 3031G>A), GAC244GCC (3036A>C), CCT245ACA (3038C>A 3040T>A), CAA246GAG (3041C>G 3043A>G), ATA248GTG (3047A>G 3049A>G), TCG249CCA (3050T>C 3052G>A), ACC251ACT (3058C>T), GCG252GCC (3061G>C), AAC254AGA (3066A>G 3067C>A), GTA255ACT (3068G>A 3069T>C 3070A>T), GAA256CAC (3071G>C 3073A>C), CAC257TCT (3074C>T 3075A>C 3076C>T), GAA261GAG (3088A>G), TTC262TAC (3090T>A), CTT263TTA (3092C>T 3094T>A), CGA264GTC (3095C>G 3096G>T 3097A>C), CCT266CCC (3103T>C), ATG267TTT (3104A>T 3106G>T), GGA268GGT (3109A>T), TTA269CTG (3110T>C 3112A>G), AAA270TCA (3113A>T 3114A>C), AAC271AAT (3118C>T), TCA272GCC (3119T>G 3121A>C), TCT274GCC (3125T>G 3127T>C), ACT275ACA (3130T>A), AGA278GGA (3137A>G), GTT279TTA (3140G>T 3142T>A), GAC281AAT (3146G>A 3148C>T), AAT282TCT (3149A>T 3150A>C), GTC283GTG (3154C>G), CTA284TTC (3155C>T 3157A>C), AGA285CAA (3158A>C 3159G>A), GGT286CAA (3161G>C 3162G>A 3163T>A), CTC287TTT (3164C>T 3166C>T), CAA288CTA (3168A>T), AAT289AGG (3171A>G 3172T>G), AAC290AAG (3175C>G), ATC291CAT (3176A>C 3177T>A 3178C>T), TGT292ATG (3179T>A 3180G>T 3181T>G), CTC293TTG (3182C>T 3184C>G), GTC294GTA (3187C>A), TAC295TTT (3189A>T 3190C>T), CTT296TTT (3191C>T), GAC297GAT (3196C>T), ATT299ATA (3202T>A), ATT300CTT (3203A>C), GTC301ATA (3206G>A 3208C>A), AGT303AGC (3214T>C), ACT304AAA (3216C>A 3217T>A), TCC305ACC (3218T>A), CTA306ATA (3221C>A), CAG307GAA (3224C>G 3226G>A), GAA308GAT (3229A>T)								
**Proteins**									
ORF B (YP_009507248.1)	195	312	10.9%	431	51.7%	118 (100%)	56 (47.5%)	0/0/0/0	0
Protein mutations:	F199Y (2901T>A 2902C>T), K201D (2906A>G 2908G>C), E204K (2915G>A), K205S (2918A>T 2919A>C 2920G>C), D208K (2927G>A 2929T>G), D209N (2930G>A), Y211F (2937A>T 2938C>T), N215I (2949A>T 2950C>A), I216V (2951A>G 2953A>G), S217E (2954A>G 2955G>A 2956T>G), V219L (2960G>C), K222E (2969A>G 2971G>A), K225G (2978A>G 2979A>G), C226S (2982G>C), Q227K (2984C>A), Y228I (2987T>A 2988A>T 2989C>A), T230S (2993A>T 2995C>T), T231K (2997C>A 2998C>A), L232I (2999T>A), A235R (3008G>A 3009C>G), F238Y (3018T>A 3019T>C), Y239H (3020T>C), V241L (3026G>C 3028G>T), E242R (3029G>A 3030A>G 3031G>A), D244A (3036A>C), P245T (3038C>A 3040T>A), Q246E (3041C>G 3043A>G), I248V (3047A>G 3049A>G), S249P (3050T>C 3052G>A), N254R (3066A>G 3067C>A), V255T (3068G>A 3069T>C 3070A>T), E256H (3071G>C 3073A>C), H257S (3074C>T 3075A>C 3076C>T), F262Y (3090T>A), R264V (3095C>G 3096G>T 3097A>C), M267F (3104A>T 3106G>T), K270S (3113A>T 3114A>C), S272A (3119T>G 3121A>C), S274A (3125T>G 3127T>C), R278G (3137A>G), V279L (3140G>T 3142T>A), D281N (3146G>A 3148C>T), N282S (3149A>T 3150A>C), L284F (3155C>T 3157A>C), R285Q (3158A>C 3159G>A), G286Q (3161G>C 3162G>A 3163T>A), L287F (3164C>T 3166C>T), Q288L (3168A>T), N289R (3171A>G 3172T>G), N290K (3175C>G), I291H (3176A>C 3177T>A 3178C>T), C292M (3179T>A 3180G>T 3181T>G), Y295F (3189A>T 3190C>T), L296F (3191C>T), I300L (3203A>C), V301I (3206G>A 3208C>A), T304K (3216C>A 3217T>A), S305T (3218T>A), L306I (3221C>A), Q307E (3224C>G 3226G>A), E308D (3229A>T)								
Codon mutations:	GTA196GTT (2893A>T), GTT197GTA (2896T>A), TTC199TAT (2901T>A 2902C>T), CGT200AGG (2903C>A 2905T>G), AAG201GAC (2906A>G 2908G>C), TTG202TTA (2911G>A), AAC203AAT (2914C>T), GAG204AAG (2915G>A), AAG205TCC (2918A>T 2919A>C 2920G>C), GAT208AAG (2927G>A 2929T>G), GAC209AAC (2930G>A), AAA210AAG (2935A>G), TAC211TTT (2937A>T 2938C>T), CCG212CCA (2941G>A), ATA213ATT (2944A>T), CCA214CCT (2947A>T), AAC215ATA (2949A>T 2950C>A), ATA216GTG (2951A>G 2953A>G), AGT217GAG (2954A>G 2955G>A 2956T>G), GTA219CTA (2960G>C), CTT220TTG (2963C>T 2965T>G), AAG222GAA (2969A>G 2971G>A), GGT224GGA (2977T>A), AAG225GGG (2978A>G 2979A>G), TGC226TCC (2982G>C), CAA227AAA (2984C>A), TAC228ATA (2987T>A 2988A>T 2989C>A), ACC230TCT (2993A>T 2995C>T), ACC231AAA (2997C>A 2998C>A), TTA232ATA (2999T>A), GAT233GAC (3004T>C), TTG234TTA (3007G>A), GCA235AGA (3008G>A 3009C>G), AGT236TCT (3011A>T 3012G>C), GGG237GGA (3016G>A), TTT238TAC (3018T>A 3019T>C), TAT239CAT (3020T>C), CAG240CAA (3025G>A), GTG241CTT (3026G>C 3028G>T), GAG242AGA (3029G>A 3030A>G 3031G>A), GAC244GCC (3036A>C), CCT245ACA (3038C>A 3040T>A), CAA246GAG (3041C>G 3043A>G), ATA248GTG (3047A>G 3049A>G), TCG249CCA (3050T>C 3052G>A), ACC251ACT (3058C>T), GCG252GCC (3061G>C), AAC254AGA (3066A>G 3067C>A), GTA255ACT (3068G>A 3069T>C 3070A>T), GAA256CAC (3071G>C 3073A>C), CAC257TCT (3074C>T 3075A>C 3076C>T), GAA261GAG (3088A>G), TTC262TAC (3090T>A), CTT263TTA (3092C>T 3094T>A), CGA264GTC (3095C>G 3096G>T 3097A>C), CCT266CCC (3103T>C), ATG267TTT (3104A>T 3106G>T), GGA268GGT (3109A>T), TTA269CTG (3110T>C 3112A>G), AAA270TCA (3113A>T 3114A>C), AAC271AAT (3118C>T), TCA272GCC (3119T>G 3121A>C), TCT274GCC (3125T>G 3127T>C), ACT275ACA (3130T>A), AGA278GGA (3137A>G), GTT279TTA (3140G>T 3142T>A), GAC281AAT (3146G>A 3148C>T), AAT282TCT (3149A>T 3150A>C), GTC283GTG (3154C>G), CTA284TTC (3155C>T 3157A>C), AGA285CAA (3158A>C 3159G>A), GGT286CAA (3161G>C 3162G>A 3163T>A), CTC287TTT (3164C>T 3166C>T), CAA288CTA (3168A>T), AAT289AGG (3171A>G 3172T>G), AAC290AAG (3175C>G), ATC291CAT (3176A>C 3177T>A 3178C>T), TGT292ATG (3179T>A 3180G>T 3181T>G), CTC293TTG (3182C>T 3184C>G), GTC294GTA (3187C>A), TAC295TTT (3189A>T 3190C>T), CTT296TTT (3191C>T), GAC297GAT (3196C>T), ATT299ATA (3202T>A), ATT300CTT (3203A>C), GTC301ATA (3206G>A 3208C>A), AGT303AGC (3214T>C), ACT304AAA (3216C>A 3217T>A), TCC305ACC (3218T>A), CTA306ATA (3221C>A), CAG307GAA (3224C>G 3226G>A), GAA308GAT (3229A>T)								

*: Inserts / Deletes / Misaligned / Frameshifts

Analysis details

This analysis was performed with panviral2.64

NGS Details (UN8): Errantivirus

Assembly

**Coverage Length**	372 (1 contig(s))
**Depth Of Coverage**	18.4
**Number Of Reads**	59
**Reads Per Million**	0.99 rpm (after QC)
**Ambiguities**	0
**Assembly Method**	de novo + reference guided assembly
**Consensus Caller**	Bcf Tools

Coverage Map

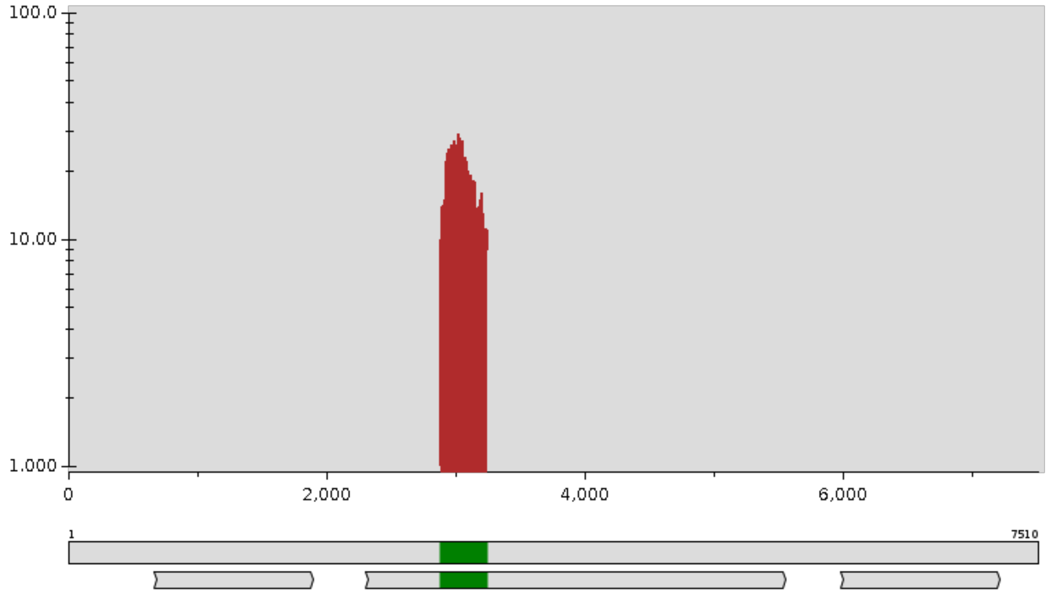

Assignment

**Type**	Errantivirus (Taxonomy ID: 186666)
**Reference Genome**	NC_038512.1
**NT Identity (%)**	53.0667
**AA Identity (%)**	42.4
**Number Of Stop Codons**	0
**Number Of CDS**	3

Alignment

**Alignment Score**	40.0 (NT) + 399.0 (AA) = 439.0
**Concordance (%)**	27.2502

Alignment Method	Global, seeded, nucleotide + amino acids (AGA)

Genome Region

Sequence starts at position 2878 and ends at position 3249 relative to NC_038512.1 reference sequence.

Alignment Detailed Statistics

	Begin	End	Coverage	Score	Concordance	Matches	Identities	I/D/M/F*	Stop Codons
**NT**	**2878**	**3249**	**5.0%**	**40**	**5.4%**	**372 (99.2%)**	**199 (53.1%)**	**3/0**	
Mutations:	2885C>A, 2887T>G, 2888C>T, 2890C>G, 2891G>T, 2892T>G, 2893A>T, 2896T>A, 2901T>A, 2902C>T, 2903C>A, 2905T>G, 2906A>G, 2908G>T, 2909T>C, 2911G>T, 2914C>T, 2915G>A, 2918A>C, 2920G>C, 2924A>G, 2926C>T, 2927G>A, 2929T>G, 2930G>A, 2937A>T, 2939C>T, 2941G>T, 2947A>T, 2948A>C, 2949A>T, 2950C>A, 2951A>G, 2954A>G, 2955G>A, 2956T>A, 2959C>T, 2960G>C, 2963C>T, 2965T>G, 2968C>T, 2969A>G, 2971G>A, 2972T>C, 2974A>T, 2977T>G, 2978A>G, 2979A>G, 2980G>T, 2982G>C, 2983C>A, 2984C>G, 2985A>T, 2987T>A, 2988A>T, 2991T>G, 2992C>T, 2993A>T, 2994C>T, 2997C>A, 2998C>A, 2999T>A, 3004T>C, 3005T>C, 3007G>T, 3008G>A, 3009C>G, 3010A>G, 3011A>G, 3012G>C, 3018T>A, 3019T>C, 3020T>C, 3026G>T, 3029G>A, 3030A>G, 3031G>A, 3036A>C, 3038C>A, 3039C>A, 3040T>A, 3041C>G, 3046T>C, 3050T>C, 3052G>A, 3058C>A, 3061G>A, 3064T>G, 3066A>G, 3067C>A, 3068G>A, 3069T>C, 3070A>T, 3071G>C, 3073A>C, 3074C>T, 3075A>T, 3076C>A, 3088A>G, 3090T>A, 3091C>T, 3092C>T, 3094T>G, 3095C>G, 3096G>T, 3103T>A, 3104A>T, 3106G>T, 3113A>T, 3114A>C, 3115A>T, 3118C>T, 3119T>G, 3125T>A, 3127T>A, 3130T>C, 3137A>G, 3139A>G, 3140G>T, 3142T>G, 3146G>A, 3148C>T, 3150A>C, 3154C>G, 3155C>T, 3157A>T, 3158A>C, 3159G>A, 3160_3161insAAG, 3161G>T, 3162G>T, 3164C>T, 3166C>G, 3167C>A, 3168A>G, 3169A>G, 3172T>G, 3173A>C, 3174A>C, 3175C>T, 3176A>G, 3178C>T, 3180G>T, 3181T>A, 3182C>G, 3184C>T, 3185G>T, 3187C>T, 3189A>T, 3190C>T, 3191C>T, 3194G>T, 3196C>T, 3203A>C, 3205T>G, 3206G>A, 3215A>C, 3216C>A, 3217T>G, 3220C>T, 3221C>A, 3223A>T, 3224C>G, 3226G>A, 3229A>C, 3232C>T, 3235G>A, 3236G>T, 3237A>T, 3239A>C, 3245G>A, 3246A>G, 3248C>A, 3249G>A								

CDS

	Begin	End	Coverage	Score	Concordance	Matches	Identities	I/D/M/F*	Stop Codons
D1R33_gp2	192	315	11.4%	399	45.1%	124 (99.2%)	53 (42.4%)	1/0/0/0	0
Protein mutations:	V196C (2891G>T 2892T>G 2893A>T), F199Y (2901T>A 2902C>T), K201D (2906A>G 2908G>T), E204K (2915G>A), K205H (2918A>C 2920G>C), I207V (2924A>G 2926C>T), D208K (2927G>A 2929T>G), D209N (2930G>A), Y211F (2937A>T), P212S (2939C>T 2941G>T), N215L (2948A>C 2949A>T 2950C>A), I216V (2951A>G), S217E (2954A>G 2955G>A 2956T>A), V219L (2960G>C), K222E (2969A>G 2971G>A), K225G (2978A>G 2979A>G 2980G>T), C226S (2982G>C 2983C>A), Q227V (2984C>G 2985A>T), Y228I (2987T>A 2988A>T), F229C (2991T>G 2992C>T), T230F (2993A>T 2994C>T), T231K (2997C>A 2998C>A), L232I (2999T>A), A235R (3008G>A 3009C>G 3010A>G), S236A (3011A>G 3012G>C), F238Y (3018T>A 3019T>C), Y239H (3020T>C), V241L (3026G>T), E242R (3029G>A 3030A>G 3031G>A), D244A (3036A>C), P245K (3038C>A 3039C>A 3040T>A), Q246E (3041C>G), S249P (3050T>C 3052G>A), F253L (3064T>G), N254R (3066A>G 3067C>A), V255T (3068G>A 3069T>C 3070A>T), E256H (3071G>C 3073A>C), H257L (3074C>T 3075A>T 3076C>A), F262Y (3090T>A 3091C>T), R264V (3095C>G 3096G>T), M267F (3104A>T 3106G>T), K270S (3113A>T 3114A>C 3115A>T), S272A (3119T>G), S274T (3125T>A 3127T>A), R278G (3137A>G 3139A>G), V279L (3140G>T 3142T>G), D281N (3146G>A 3148C>T), N282T (3150A>C), L284F (3155C>T 3157A>T), R285Q (3158A>C 3159G>A), R285_G286insK (3160_3161insAAG), G286F (3161G>T 3162G>T), Q288R (3167C>A 3168A>G 3169A>G), N289K (3172T>G), N290P (3173A>C 3174A>C 3175C>T), I291V (3176A>G 3178C>T), C292L (3180G>T 3181T>A), L293V (3182C>G 3184C>T), V294F (3185G>T 3187C>T), Y295F (3189A>T 3190C>T), L296F (3191C>T), D297Y (3194G>T 3196C>T), I300L (3203A>C 3205T>G), V301I (3206G>A), T304Q (3215A>C 3216C>A 3217T>G), L306I (3221C>A 3223A>T), Q307E (3224C>G 3226G>A), E308D (3229A>C), E311L (3236G>T 3237A>T), N312H (3239A>C), E314R (3245G>A 3246A>G)								
Codon mutations:	CGT194AGG (2885C>A 2887T>G), CTC195TTG (2888C>T 2890C>G), GTA196TGT (2891G>T 2892T>G 2893A>T), GTT197GTA (2896T>A), TTC199TAT (2901T>A 2902C>T), CGT200AGG (2903C>A 2905T>G), AAG201GAT (2906A>G 2908G>T), TTG202CTT (2909T>C 2911G>T), AAC203AAT (2914C>T), GAG204AAG (2915G>A), AAG205CAC (2918A>C 2920G>C), ATC207GTT (2924A>G 2926C>T), GAT208AAG (2927G>A 2929T>G), GAC209AAC (2930G>A), TAC211TTC (2937A>T), CCG212TCT (2939C>T 2941G>T), CCA214CCT (2947A>T), AAC215CTA (2948A>C 2949A>T 2950C>A), ATA216GTA (2951A>G), AGT217GAA (2954A>G 2955G>A 2956T>A), GAC218GAT (2959C>T), GTA219CTA (2960G>C), CTT220TTG (2963C>T 2965T>G), GAC221GAT (2968C>T), AAG222GAA (2969A>G 2971G>A), TTA223CTT (2972T>C 2974A>T), GGT224GGG (2977T>G), AAG225GGT (2978A>G 2979A>G 2980G>T), TGC226TCA (2982G>C 2983C>A), CAA227GTA (2984C>G 2985A>T), TAC228ATC (2987T>A 2988A>T), TTC229TGT (2991T>G 2992C>T), ACC230TTC (2993A>T 2994C>T), ACC231AAA (2997C>A 2998C>A), TTA232ATA (2999T>A), GAT233GAC (3004T>C), TTG234CTT (3005T>C 3007G>T), GCA235AGG (3008G>A 3009C>G 3010A>G), AGT236GCT (3011A>G 3012G>C), TTT238TAC (3018T>A 3019T>C), TAT239CAT (3020T>C), GTG241TTG (3026G>T), GAG242AGA (3029G>A 3030A>G 3031G>A), GAC244GCC (3036A>C), CCT245AAA (3038C>A 3039C>A 3040T>A), CAA246GAA (3041C>G), GAT247GAC (3046T>C), TCG249CCA (3050T>C 3052G>A), ACC251ACA (3058C>A), GCG252GCA (3061G>A), TTT253TTG (3064T>G), AAC254AGA (3066A>G 3067C>A), GTA255ACT (3068G>A 3069T>C 3070A>T), GAA256CAC (3071G>C 3073A>C), CAC257TTA (3074C>T 3075A>T 3076C>A), GAA261GAG (3088A>G), TTC262TAT (3090T>A 3091C>T), CTT263TTG (3092C>T 3094T>G), CGA264GTA (3095C>G 3096G>T), CCT266CCA (3103T>A), ATG267TTT (3104A>T 3106G>T), AAA270TCT (3113A>T 3114A>C 3115A>T), AAC271AAT (3118C>T), TCA272GCA (3119T>G), TCT274ACA (3125T>A 3127T>A), ACT275ACC (3130T>C), AGA278GGG (3137A>G 3139A>G), GTT279TTG (3140G>T 3142T>G), GAC281AAT (3146G>A 3148C>T), AAT282ACT (3150A>C), GTC283GTG (3154C>G), CTA284TTT (3155C>T 3157A>T), AGA285CAA (3158A>C 3159G>A), AGA285_GGT286insAAG (3160_3161insAAG), GGT286TTT (3161G>T 3162G>T), CTC287TTG (3164C>T 3166C>G), CAA288AGG (3167C>A 3168A>G 3169A>G), AAT289AAG (3172T>G), AAC290CCT (3173A>C 3174A>C 3175C>T), ATC291GTT (3176A>G 3178C>T), TGT292TTA (3180G>T 3181T>A), CTC293GTT (3182C>G 3184C>T), GTC294TTT (3185G>T 3187C>T), TAC295TTT (3189A>T 3190C>T), CTT296TTT (3191C>T), GAC297TAT (3194G>T 3196C>T), ATT300CTG (3203A>C 3205T>G), GTC301ATC (3206G>A), ACT304CAG (3215A>C 3216C>A 3217T>G), TCC305TCT (3220C>T), CTA306ATT (3221C>A 3223A>T), CAG307GAA (3224C>G 3226G>A), GAA308GAC (3229A>C), CAC309CAT (3232C>T), CTG310CTA (3235G>A), GAG311TTG (3236G>T 3237A>T), AAC312CAC (3239A>C), GAA314AGA (3245G>A 3246A>G), CGA315AA. (3248C>A 3249G>A)								

Proteins

	Begin	End	Coverage	Score	Concordance	Matches	Identities	I/D/M/F*	Stop Codons
ORF B (YP_009507248.1)	192	315	11.4%	399	45.1%	124 (99.2%)	53 (42.4%)	1/0/0/0	0
Protein mutations:	V196C (2891G>T 2892T>G 2893A>T), F199Y (2901T>A 2902C>T), K201D (2906A>G 2908G>T), E204K (2915G>A), K205H (2918A>C 2920G>C), I207V (2924A>G 2926C>T), D208K (2927G>A 2929T>G), D209N (2930G>A), Y211F (2937A>T), P212S (2939C>T 2941G>T), N215L (2948A>C 2949A>T 2950C>A), I216V (2951A>G), S217E (2954A>G 2955G>A 2956T>A), V219L (2960G>C), K222E (2969A>G 2971G>A), K225G (2978A>G 2979A>G 2980G>T), C226S (2982G>C 2983C>A), Q227V (2984C>G 2985A>T), Y228I (2987T>A 2988A>T), F229C (2991T>G 2992C>T), T230F (2993A>T 2994C>T), T231K (2997C>A 2998C>A), L232I (2999T>A), A235R (3008G>A 3009C>G 3010A>G), S236A (3011A>G 3012G>C), F238Y (3018T>A 3019T>C), Y239H (3020T>C), V241L (3026G>T), E242R (3029G>A 3030A>G 3031G>A), D244A (3036A>C), P245K (3038C>A 3039C>A 3040T>A), Q246E (3041C>G), S249P (3050T>C 3052G>A), F253L (3064T>G), N254R (3066A>G 3067C>A), V255T (3068G>A 3069T>C 3070A>T), E256H (3071G>C 3073A>C), H257L (3074C>T 3075A>T 3076C>A), F262Y (3090T>A 3091C>T), R264V (3095C>G 3096G>T), M267F (3104A>T 3106G>T), K270S (3113A>T 3114A>C 3115A>T), S272A (3119T>G), S274T (3125T>A 3127T>A), R278G (3137A>G 3139A>G), V279L (3140G>T 3142T>G), D281N (3146G>A 3148C>T), N282T (3150A>C), L284F (3155C>T 3157A>T), R285Q (3158A>C 3159G>A), R285_G286insK (3160_3161insAAG), G286F (3161G>T 3162G>T), Q288R (3167C>A 3168A>G 3169A>G), N289K (3172T>G), N290P (3173A>C 3174A>C 3175C>T), I291V (3176A>G 3178C>T), C292L (3180G>T 3181T>A), L293V (3182C>G 3184C>T), V294F (3185G>T 3187C>T), Y295F (3189A>T 3190C>T), L296F (3191C>T), D297Y (3194G>T 3196C>T), I300L (3203A>C 3205T>G), V301I (3206G>A), T304Q (3215A>C 3216C>A 3217T>G), L306I (3221C>A 3223A>T), Q307E (3224C>G 3226G>A), E308D (3229A>C), E311L (3236G>T 3237A>T), N312H (3239A>C), E314R (3245G>A 3246A>G)								
Codon mutations:	CGT194AGG (2885C>A 2887T>G), CTC195TTG (2888C>T 2890C>G), GTA196TGT (2891G>T 2892T>G 2893A>T), GTT197GTA (2896T>A), TTC199TAT (2901T>A 2902C>T), CGT200AGG (2903C>A 2905T>G), AAG201GAT (2906A>G 2908G>T), TTG202CTT (2909T>C 2911G>T), AAC203AAT (2914C>T), GAG204AAG (2915G>A), AAG205CAC (2918A>C 2920G>C), ATC207GTT (2924A>G 2926C>T), GAT208AAG (2927G>A 2929T>G), GAC209AAC (2930G>A), TAC211TTC (2937A>T), CCG212TCT (2939C>T 2941G>T), CCA214CCT (2947A>T), AAC215CTA (2948A>C 2949A>T 2950C>A), ATA216GTA (2951A>G), AGT217GAA (2954A>G 2955G>A 2956T>A), GAC218GAT (2959C>T), GTA219CTA (2960G>C), CTT220TTG (2963C>T 2965T>G), GAC221GAT (2968C>T), AAG222GAA (2969A>G 2971G>A), TTA223CTT (2972T>C 2974A>T), GGT224GGG (2977T>G), AAG225GGT (2978A>G 2979A>G 2980G>T), TGC226TCA (2982G>C 2983C>A), CAA227GTA (2984C>G 2985A>T), TAC228ATC (2987T>A 2988A>T), TTC229TGT (2991T>G 2992C>T), ACC230TTC (2993A>T 2994C>T), ACC231AAA (2997C>A 2998C>A), TTA232ATA (2999T>A), GAT233GAC (3004T>C), TTG234CTT (3005T>C 3007G>T), GCA235AGG (3008G>A 3009C>G 3010A>G), AGT236GCT (3011A>G 3012G>C), TTT238TAC (3018T>A 3019T>C), TAT239CAT (3020T>C), GTG241TTG (3026G>T), GAG242AGA (3029G>A 3030A>G 3031G>A), GAC244GCC (3036A>C), CCT245AAA (3038C>A 3039C>A 3040T>A), CAA246GAA (3041C>G), GAT247GAC (3046T>C), TCG249CCA (3050T>C 3052G>A), ACC251ACA (3058C>A), GCG252GCA (3061G>A), TTT253TTG (3064T>G), AAC254AGA (3066A>G 3067C>A), GTA255ACT (3068G>A 3069T>C 3070A>T), GAA256CAC (3071G>C 3073A>C), CAC257TTA (3074C>T 3075A>T 3076C>A), GAA261GAG (3088A>G), TTC262TAT (3090T>A 3091C>T), CTT263TTG (3092C>T 3094T>G), CGA264GTA (3095C>G 3096G>T), CCT266CCA (3103T>A), ATG267TTT (3104A>T 3106G>T), AAA270TCT (3113A>T 3114A>C 3115A>T), AAC271AAT (3118C>T), TCA272GCA (3119T>G), TCT274ACA (3125T>A 3127T>A), ACT275ACC (3130T>C), AGA278GGG (3137A>G 3139A>G), GTT279TTG (3140G>T 3142T>G), GAC281AAT (3146G>A 3148C>T), AAT282ACT (3150A>C), GTC283GTG (3154C>G), CTA284TTT (3155C>T 3157A>T), AGA285CAA (3158A>C 3159G>A), AGA285_GGT286insAAG (3160_3161insAAG), GGT286TTT (3161G>T 3162G>T), CTC287TTG (3164C>T 3166C>G), CAA288AGG (3167C>A 3168A>G 3169A>G), AAT289AAG (3172T>G), AAC290CCT (3173A>C 3174A>C 3175C>T), ATC291GTT (3176A>G 3178C>T), TGT292TTA (3180G>T 3181T>A), CTC293GTT (3182C>G 3184C>T), GTC294TTT (3185G>T 3187C>T), TAC295TTT (3189A>T 3190C>T), CTT296TTT (3191C>T), GAC297TAT (3194G>T 3196C>T), ATT300CTG (3203A>C 3205T>G), GTC301ATC (3206G>A), ACT304CAG (3215A>C 3216C>A 3217T>G), TCC305TCT (3220C>T), CTA306ATT (3221C>A 3223A>T), CAG307GAA (3224C>G 3226G>A), GAA308GAC (3229A>C), CAC309CAT (3232C>T), CTG310CTA (3235G>A), GAG311TTG (3236G>T 3237A>T), AAC312CAC (3239A>C), GAA314AGA (3245G>A 3246A>G), CGA315AA. (3248C>A 3249G>A)								

*: Inserts / Deletes / Misaligned / Frameshifts

Analysis details

This analysis was performed with panviral2.64

NGS Details (UN8): Badnavirus maculacommelinae

Assembly

**Coverage Length**	909 (1 contig(s))
**Depth Of Coverage**	7.8
**Number Of Reads**	55
**Reads Per Million**	0.92 rpm (after QC)
**Ambiguities**	0
**Assembly Method**	de novo + reference guided assembly
**Consensus Caller**	Bcf Tools

Coverage Map

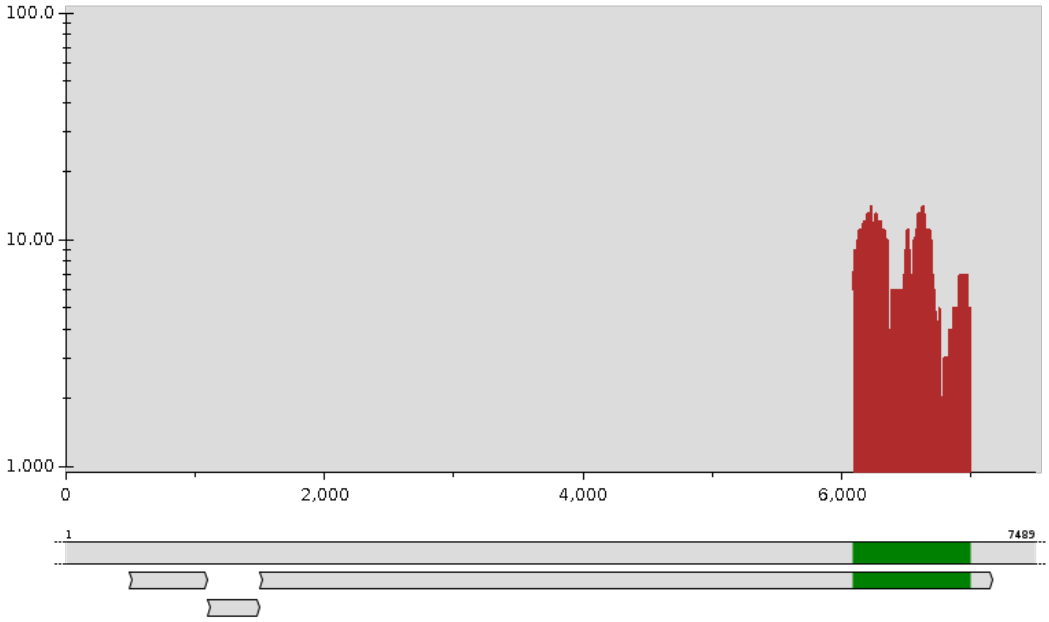

Assignment

**Type**	Badnavirus maculacommelinae (Taxonomy ID: 3047769)
**Reference Genome**	NC_001343.1
**NT Identity (%)**	54.6858
**AA Identity (%)**	47.8548
**Number Of Stop Codons**	3
**Number Of CDS**	3

Alignment

**Alignment Score**	123.0 (NT) + 750.0 (AA) = 873.0
**Concordance (%)**	24.9451

**Alignment Method**	Global, seeded, nucleotide + amino acids (AGA)

Genome Region

Sequence starts at position 6090 and ends at position 6998 relative to NC_001343.1 reference sequence.

Alignment Detailed Statistics

	Begin	End	Coverage	Score	Concordance	Matches	Identities	I/D/M/F*	Stop Codons
**NT**	**6090**	**6998**	**12.1%**	**123**	**7.0%**	**901 (98.5%)**	**496 (54.2%)**	**6/8**	
Mutations:	6091T>A, 6094A>T, 6102C>T, 6104T>A, 6111C>T, 6119T>A, 6120C>T, 6122C>G, 6125A>G, 6127A>C, 6128T>A, 6131G>A, 6134C>A, 6135G>C, 6143T>C, 6149G>A, 6161C>T, 6162G>A, 6164C>T, 6167C>T, 6168A>G, 6169A>G, 6172G>A, 6173G>T, 6174A>T, 6175C>A, 6176A>C, 6177G>A, 6181A>G, 6182G>A, 6185C>T, 6186A>G, 6188A>C, 6189G>C, 6190C>T, 6191T>A, 6194T>A, 6197C>T, 6198A>G, 6200T>A, 6206T>C, 6209A>T, 6212T>A, 6215T>A, 6218C>T, 6219T>A, 6220C>A, 6221A>T, 6222G>A, 6225A>G, 6226C>A, 6227A>C, 6228G>A, 6229C>T, 6231G>A, 6232A>G, 6233A>G, 6234C>G, 6236A>G, 6240T>C, 6241C>T, 6242G>A, 6243C>G, 6244A>G, 6245G>T, 6251C>A, 6252T>C, 6254T>G, 6258A>G, 6260G>A, 6261C>T, 6263G>T, 6264C>A, 6265A>G, 6266G>A, 6269T>A, 6271G>T, 6272C>T, 6273A>G, 6274A>T, 6275G>A, 6276G>A, 6277A>G, 6278A>T, 6281T>C, 6284G>T, 6285T>A, 6290C>A, 6291C>G, 6293T>A, 6297C>A, 6298C>A, 6299T>A, 6301C>A, 6302C>G, 6311G>A, 6312A>T, 6315G>T, 6319C>A, 6320T>A, 6321C>A, 6323G>C, 6324G>C, 6326G>T, 6329C>A, 6330G>A, 6340G>A, 6342G>A, 6343C>T, 6345T>A, 6346C>T, 6347T>A, 6348C>T, 6350C>A, 6354T>G, 6355G>A, 6356T>A, 6357A>T, 6358C>G, 6362A>G, 6365C>A, 6368G>A, 6369C>T, 6371T>A, 6374G>A, 6378_6380delCAC, 6383C>A, 6384A>T, 6385T>A, 6386T>G, 6387T>C, 6388C>T, 6394T>G, 6395A>G, 6397G>C, 6399G>T, 6401C>G, 6402T>A, 6403T>G, 6404C>A, 6406C>G, 6407A>C, 6408G>C, 6411G>A, 6414A>T, 6415A>T, 6417C>G, 6418T>A, 6420G>A, 6421C>A, 6422A>C, 6426C>A, 6427C>A, 6428T>A, 6432delG, 6433G>T, 6436T>C, 6437G>A, 6439G>A, 6442G>T, 6443C>T, 6444T>C, 6445G>T, 6446G>T, 6447T>A, 6449G>A, 6453A>T, 6455C>A, 6456C>A, 6458C>A, 6459T>A, 6460C>A, 6461A>T, 6475A>T, 6479T>A, 6480C>A, 6482G>A, 6485T>C, 6486A>T, 6488C>A, 6491C>A, 6495T>A, 6497G>A, 6500G>A, 6501C>G, 6502A>G, 6510A>T, 6511G>A, 6512A>C, 6513C>T, 6514A>C, 6515A>T, 6520T>C, 6521G>A, 6523C>G, 6525C>A, 6526C>G, 6527A>T, 6529C>A, 6534G>C, 6536C>A, 6540A>T, 6541G>A, 6542A>T, 6543A>T, 6545G>T, 6549C>A, 6550C>A, 6551A>T, 6555A>G, 6556C>A, 6557A>T, 6558T>A, 6559G>T, 6560G>C, 6563G>A, 6564A>T, 6566G>A, 6569A>C, 6570A>C, 6571G>A, 6573C>A, 6578A>C, 6584A>T, 6587G>A, 6590G>T, 6596T>C, 6597C>A, 6602T>A, 6606C>T, 6608T>G, 6609C>G, 6610A>C, 6611G>T, 6612T>A, 6614A>T, 6617A>T, 6619C>T, 6620T>A, 6621A>G, 6624G>T, 6625A>C, 6626T>C, 6627T>G, 6628C>A, 6629A>T, 6631T>A, 6632C>T, 6633A>T, 6635C>A, 6644G>A, 6647G>A, 6653T>G, 6654T>A, 6656T>C, 6657A>T, 6658T>C, 6659G>C, 6662T>A, 6665C>A, 6674C>A, 6675G>A, 6677C>T, 6679G>T, 6680C>G, 6683A>G, 6684T>G, 6685G>C, 6686G>C, 6690A>C, 6691T>C, 6692G>A, 6693T>A, 6694C>G, 6695A>T, 6698G>A, 6699C>T, 6702G>A, 6703A>G, 6705C>G, 6706C>A, 6707A>T, 6709G>A, 6712G>A, 6713C>T, 6716C>A, 6721G>A, 6722A>G, 6725T>A, 6730C>G, 6731C>T, 6735G>C, 6736C>A, 6737T>A, 6740T>C, 6743A>T, 6744T>A, 6745C>A, 6748T>A, 6752T>A, 6753C>G, 6754C>A, 6757T>A, 6758_6759insGGAAAT, 6760A>T, 6761A>G, 6762T>A, 6763C>G, 6764A>T, 6766C>G, 6767C>T, 6770C>A, 6771G>A, 6774G>T, 6775C>T, 6776A>T, 6779G>A, 6782T>A, 6784A>T, 6785G>T, 6788G>A, 6790C>T, 6791A>T, 6793T>A, 6794C>T, 6795delC, 6800C>T, 6801C>T, 6803G>A, 6804G>A, 6808A>G, 6809A>T, 6812C>T, 6814A>G, 6816A>C, 6818T>A, 6821T>C, 6822T>A, 6823A>T, 6825C>A, 6827T>A, 6828G>A, 6830T>C, 6836G>A, 6839G>A, 6840C>A, 6842C>A, 6843A>C, 6845A>G, 6846A>G, 6848T>G, 6849C>A, 6851C>A, 6852T>A, 6869T>A, 6870A>G, 6872C>A, 6879T>C, 6881C>T, 6882A>C, 6884C>A, 6889C>T, 6890G>A, 6893C>T, 6899T>A, 6901A>C, 6902G>T, 6903C>A, 6904C>G, 6905G>T, 6906T>A, 6907C>G, 6912G>A, 6913T>G, 6914T>A, 6915A>C, 6921T>C, 6923A>T, 6925C>A, 6926A>T, 6930T>C, 6931C>T, 6936T>A, 6937T>C, 6938C>A, 6939T>A, 6942A>T, 6944A>T, 6945G>A, 6946G>C, 6948C>T, 6949T>A, 6950T>C, 6952G>A, 6953A>G, 6954_6956delATC, 6958C>A, 6959A>T, 6960G>A, 6964C>T, 6965A>T, 6968C>T, 6974C>T, 6978G>A, 6980T>A, 6986G>A, 6987C>A, 6989T>A, 6993G>A, 6994G>A								
**CDS**									
ComYMVgp3	1529	1831	16.1%	750	35.5%	301 (98.7%)	145 (47.5%)	2/2/2/2	3
Protein mutations:	L1529H (6091T>A), Y1530F (6094A>T), P1536S (6111C>T), N1541T (6127A>C 6128T>A), A1544P (6135G>C), V1553I (6162G>A 6164C>T), K1555G (6168A>G 6169A>G), G1556D (6172G>A 6173G>T), T1557Y (6174A>T 6175C>A 6176A>C), E1558K (6177G>A), K1559R (6181A>G 6182G>A), I1561V (6186A>G 6188A>C), A1562L (6189G>C 6190C>T 6191T>A), I1565V (6198A>G 6200T>A), S1572N (6219T>A 6220C>A 6221A>T), E1573K (6222G>A), T1574D (6225A>G 6226C>A 6227A>C), A1575I (6228G>A 6229C>T), E1576R (6231G>A 6232A>G 6233A>G), Q1577E (6234C>G 6236A>G), S1579L (6240T>C 6241C>T 6242G>A), Q1580G (6243C>G 6244A>G 6245G>T), Y1583Q (6252T>C 6254T>G), M1585V (6258A>G 6260G>A), L1586F (6261C>T 6263G>T), Q1587R (6264C>A 6265A>G 6266G>A), C1589F (6271G>T 6272C>T), K1590V (6273A>G 6274A>T 6275G>A), E1591S (6276G>A 6277A>G 6278A>T), L1594I (6285T>A), L1596V (6291C>G 6293T>A), P1598K (6297C>A 6298C>A 6299T>A), T1599K (6301C>A 6302C>G), I1603L (6312A>T), G1604C (6315G>T), T1605K (6319C>A 6320T>A), P1606T (6321C>A 6323G>C), E1607H (6324G>C 6326G>T), D1609N (6330G>A), G1612E (6340G>A), A1613I (6342G>A 6343C>T), S1614I (6345T>A 6346C>T 6347T>A), C1617E (6354T>G 6355G>A 6356T>A), T1618* (6357A>T 6358C>G), H1625del (6378_6380delCAC), I1627* (6384A>T 6385T>A 6386T>G), S1628L (6387T>C 6388C>T), I1630R (6394T>G 6395A>G), C1631S (6397G>C), D1632* (6399G>T 6401C>G), F1633R (6402T>A 6403T>G 6404C>A), S1634C (6406C>G 6407A>C), D1635H (6408G>C), E1636K (6411G>A), K1637L (6414A>T 6415A>T), L1638E (6417C>G 6418T>A), A1639N (6420G>A 6421C>A 6422A>C), P1641K (6426C>A 6427C>A 6428T>A), M1644T (6436T>C 6437G>A), R1645K (6439G>A), S1646I (6442G>T 6443C>T), W1647L (6444T>C 6445G>T 6446G>T), L1648I (6447T>A 6449G>A), I1650L (6453A>T 6455C>A), L1651I (6456C>A 6458C>A), S1652N (6459T>A 6460C>A 6461A>T), Y1657F (6475A>T), Q1659K (6480C>A 6482G>A), I1661L (6486A>T 6488C>A), L1664I (6495T>A 6497G>A), Q1666G (6501C>G 6502A>G), R1669Y (6510A>T 6511G>A 6512A>C), Q1670S (6513C>T 6514A>C 6515A>T), M1672T (6520T>C 6521G>A), A1673G (6523C>G), P1674S (6525C>A 6526C>G 6527A>T), T1675K (6529C>A), D1677Q (6534G>C 6536C>A), R1679Y (6540A>T 6541G>A 6542A>T), M1680F (6543A>T 6545G>T), P1682N (6549C>A 6550C>A 6551A>T), T1684D (6555A>G 6556C>A 6557A>T), W1685I (6558T>A 6559G>T 6560G>C), M1687L (6564A>T 6566G>A), R1689Q (6570A>C 6571G>A), Q1690K (6573C>A), E1693D (6584A>T), L1698I (6597C>A), Q1702A (6609C>G 6610A>C 6611G>T), L1703I (6612T>A 6614A>T), P1705L (6619C>T 6620T>A), K1706E (6621A>G), D1707S (6624G>T 6625A>C 6626T>C), S1708D (6627T>G 6628C>A 6629A>T), F1709Y (6631T>A 6632C>T), I1710L (6633A>T 6635C>A), C1717S (6654T>A 6656T>C), M1718S (6657A>T 6658T>C 6659G>C), V1724I (6675G>A 6677C>T), C1725L (6679G>T 6680C>G), W1727A (6684T>G 6685G>C 6686G>C), M1729P (6690A>C 6691T>C 6692G>A), H1732Y (6699C>T), D1733S (6702G>A 6703A>G), P1734D (6705C>G 6706C>A 6707A>T), R1735K (6709G>A), S1736N (6712G>A 6713C>T), R1739K (6721G>A 6722A>G), A1742G (6730C>G 6731C>T), A1744Q (6735G>C 6736C>A 6737T>A), S1747K (6744T>A 6745C>A), F1748Y (6748T>A), N1749K (6752T>A), P1750E (6753C>G 6754C>A), I1751K (6757T>A), I1751_K1752insGN (6758_6759insGGAAAT), K1752M (6760A>T 6761A>G), T1754S (6766C>G 6767C>T), D1756N (6771G>A), A1757F (6774G>T 6775C>T 6776A>T), Q1760L (6784A>T 6785G>T), A1762V (6790C>T 6791A>T), I1763N (6793T>A 6794C>T), D1767N (6804G>A), K1768S (6808A>G 6809A>T), K1770R (6814A>G), I1771L (6816A>C 6818T>A), Y1773I (6822T>A 6823A>T), L1774I (6825C>A 6827T>A), D1775N (6828G>A 6830T>C), L1779I (6840C>A 6842C>A), I1780L (6843A>C 6845A>G), I1781V (6846A>G 6848T>G), S1783T (6852T>A), I1789V (6870A>G 6872C>A), Y1792H (6879T>C 6881C>T), N1793Q (6882A>C 6884C>A), T1795I (6889C>T 6890G>A), N1798K (6899T>A), K1799T (6901A>C 6902G>T), P1800S (6903C>A 6904C>G 6905G>T), V1803R (6912G>A 6913T>G 6914T>A), T1807N (6925C>A 6926A>T), S1809L (6930T>C 6931C>T), F1811T (6936T>A 6937T>C 6938C>A), L1812I (6939T>A), T1813S (6942A>T 6944A>T), G1814T (6945G>A 6946G>C), L1815Y (6948C>T 6949T>A 6950T>C), G1816E (6952G>A 6953A>G), I1817del (6954_6956delATC), T1818N (6958C>A 6959A>T), V1819I (6960G>A), T1820I (6964C>T 6965A>T), D1825K (6978G>A 6980T>A), H1828K (6987C>A 6989T>A), G1830N (6993G>A 6994G>A)								

	Begin	End	Coverage	Score	Concordance	Matches	Identities	I/D/M/F*	Stop Codons
**NT**	**6090**	**6998**	**12.1%**	**123**	**7.0%**	**901 (98.5%)**	**496 (54.2%)**	**6/8**	
Codon mutations:	CTT1529CAT (6091T>A), TAT1530TTT (6094A>T), CTT1533TTA (6102C>T 6104T>A), CCA1536TCA (6111C>T), GGT1538GGA (6119T>A), CTC1539TTG (6120C>T 6122C>G), AAA1540AAG (6125A>G), AAT1541ACA (6127A>C 6128T>A), GCG1542GCA (6131G>A), CCC1543CCA (6134C>A), GCT1544CCT (6135G>C), TTT1546TTC (6143T>C), AGG1548AGA (6149G>A), AAC1552AAT (6161C>T), GTC1553ATT (6162G>A 6164C>T), TTC1554TTT (6167C>T), AAA1555GGA (6168A>G 6169A>G), GGG1556GAT (6172G>A 6173G>T), ACA1557TAC (6174A>T 6175C>A 6176A>C), GAA1558AAA (6177G>A), AAG1559AGA (6181A>G 6182G>A), TTC1560TTT (6185C>T), ATA1561GTC (6186A>G 6188A>C), GCT1562CTA (6189G>C 6190C>T 6191T>A), GTT1563GTA (6194T>A), TAC1564TAT (6197C>T), ATT1565GTA (6198A>G 6200T>A), GAT1567GAC (6206T>C), ATA1568ATT (6209A>T), CTT1569CTA (6212T>A), GTT1570GTA (6215T>A), TTC1571TTT (6218C>T), TCA1572AAT (6219T>A 6220C>A 6221A>T), GAA1573AAA (6222G>A), ACA1574GAC (6225A>G 6226C>A 6227A>C), GCA1575ATA (6228G>A 6229C>T), GAA1576AGG (6231G>A 6232A>G 6233A>G), CAA1577GAG (6234C>G 6236A>G), TCG1579CTA (6240T>C 6241C>T 6242G>A), CAG1580GGT (6243C>G 6244A>G 6245G>T), CTC1582CTA (6251C>A), TAT1583CAG (6252T>C 6254T>G), ATG1585GTA (6258A>G 6260G>A), CTG1586TTT (6261C>T 6263G>T), CAG1587AGA (6264C>A 6265A>G 6266G>A), CTT1588CTA (6269T>A), TGC1589TTT (6271G>T 6272C>T), AAG1590GTA (6273A>G 6274A>T 6275G>A), GAA1591AGT (6276G>A 6277A>G 6278A>T), AAT1592AAC (6281T>C), GGG1593GGT (6284G>T), TTA1594ATA (6285T>A), ATC1595ATA (6290C>A), CTT1596GTA (6291C>G 6293T>A), CCT1598AAA (6297C>A 6298C>A 6299T>A), ACC1599AAG (6301C>A 6302C>G), AAG1602AAA (6311G>A), ATA1603TTA (6312A>T), GGT1604TGT (6315G>T), ACT1605AAA (6319C>A 6320T>A), CCG1606ACC (6321C>A 6323G>C), GAG1607CAT (6324G>C 6326G>T), ATC1608ATA (6329C>A), GAT1609AAT (6330G>A), GGG1612GAG (6340G>A), GCT1613ATT (6342G>A 6343C>T), TCT1614ATA (6345T>A 6346C>T 6347T>A), CTC1615TTA (6348C>T 6350C>A), TGT1617GAA (6354T>G 6355G>A 6356T>A), ACA1618TGA (6357A>T 6358C>G), AAA1619AAG (6362A>G), ATC1620ATA (6365C>A), AAG1621AAA (6368G>A), CTT1622TTA (6369C>T 6371T>A), CAG1623CAA (6374G>A), CAC1625del (6378_6380delCAC), ATC1626ATA (6383C>A), ATT1627TAG (6384A>T 6385T>A 6386T>G), TCA1628CTA (6387T>C 6388C>T), ATA1630AGG (6394T>G 6395A>G), TGT1631TCT (6397G>C), GAC1632TAG (6399G>T 6401C>G), TTC1633AGA (6402T>A 6403T>G 6404C>A), TCA1634TGC (6406C>G 6407A>C), GAT1635CAT (6408G>C), GAA1636AAA (6411G>A), AAA1637TTA (6414A>T 6415A>T), CTA1638GAA (6417C>G 6418T>A), GCA1639AAC (6420G>A 6421C>A 6422A>C), CCT1641AAA (6426C>A 6427C>A 6428T>A), GGT1643-TT (6432delG 6433G>T), ATG1644ACA (6436T>C 6437G>A), AGA1645AAA (6439G>A), AGC1646ATT (6442G>T 6443C>T), TGG1647CTT (6444T>C 6445G>T 6446G>T), TTG1648ATA (6447T>A 6449G>A), ATC1650TTA (6453A>T 6455C>A), CTC1651ATA (6456C>A 6458C>A), TCA1652AAT (6459T>A 6460C>A 6461A>T), TAT1657TTT (6475A>T), ATT1658ATA (6479T>A), CAG1659AAA (6480C>A 6482G>A), GAT1660GAC (6485T>C), ATC1661TTA (6486A>T 6488C>A), GGC1662GGA (6491C>A), TTG1664ATA (6495T>A 6497G>A), GTG1665GTA (6500G>A), CAA1666GGA (6501C>G 6502A>G), AGA1669TAC (6510A>T 6511G>A 6512A>C), CAA1670TCT (6513C>T 6514A>C 6515A>T), ATG1672ACA (6520T>C 6521G>A), GCA1673GGA (6523C>G), CCA1674AGT (6525C>A 6526C>G 6527A>T), ACA1675AAA (6529C>A), GAC1677CAA (6534G>C 6536C>A), AGA1679TAT (6540A>T 6541G>A 6542A>T), ATG1680TTT (6543A>T 6545G>T), CCA1682AAT (6549C>A 6550C>A 6551A>T), ACA1684GAT (6555A>G 6556C>A 6557A>T), TGG1685ATC (6558T>A 6559G>T 6560G>C), AAG1686AAA (6563G>A), ATG1687TTA (6564A>T 6566G>A), GTA1688GTC (6569A>C), AGA1689CAA (6570A>C 6571G>A), CAG1690AAG (6573C>A), ATA1691ATC (6578A>C), GAA1693GAT (6584A>T), AAG1694AAA (6587G>A), GTG1695GTT (6590G>T), AAT1697AAC (6596T>C), CTC1698ATC (6597C>A), CCT1699CCA (6602T>A), CTT1701TTG (6606C>T 6608T>G), CAG1702GCT (6609C>G 6610A>C 6611G>T), TTA1703ATT (6612T>A 6614A>T), CCA1704CCT (6617A>T), CCT1705CTA (6619C>T 6620T>A), AAA1706GAA (6621A>G), GAT1707TCC (6624G>T 6625A>C 6626T>C), TCA1708GAT (6627T>G 6628C>A 6629A>T), TTC1709TAT (6631T>A 6632C>T), ATC1710TTA (6633A>T 6635C>A), GAG1713GAA (6644G>A), ACG1714ACA (6647G>A), GGT1716GGG (6653T>G), TGT1717AGC (6654T>A 6656T>C), ATG1718TCC (6657A>T 6658T>C 6659G>C), ACT1719ACA (6662T>A), GGC1720GGA (6665C>A), GCC1723GCA (6674C>A), GTC1724ATT (6675G>A 6677C>T), TGC1725TTG (6679G>T 6680C>G), AAA1726AAG (6683A>G), TGG1727GCC (6684T>G 6685G>C 6686G>C), ATG1729CCA (6690A>C 6691T>C 6692G>A), TCA1730AGT (6693T>A 6694C>G 6695A>T), AAG1731AAA (6698G>A), CAT1732TAT (6699C>T), GAT1733AGT (6702G>A 6703A>G), CCA1734GAT (6705C>G 6706C>A 6707A>T), AGA1735AAA (6709G>A), AGC1736AAT (6712G>A 6713C>T), ACC1737ACA (6716C>A), AGA1739AAG (6721G>A 6722A>G), ATT1740ATA (6725T>A), GCC1742GGT (6730C>G 6731C>T), GCT1744CAA (6735G>C 6736C>A 6737T>A), AGT1745AGC (6740T>C), GGA1746GGT (6743A>T), TCA1747AAA (6744T>A 6745C>A), TTC1748TAC (6748T>A), AAT1749AAA (6752T>A), CCA1750GAA (6753C>G 6754C>A), ATA1751AAA (6757T>A), ATA1751_AAA1752insGGAAAT (6758_6759insGGAAAT), AAA1752ATG (6760A>T 6761A>G), TCA1753AGT (6762T>A 6763C>G 6764A>T), ACC1754AGT (6766C>G 6767C>T), ATC1755ATA (6770C>A), GAT1756AAT (6771G>A), GCA1757TTT (6774G>T 6775C>T 6776A>T), GAG1758GAA (6779G>A), ATT1759ATA (6782T>A), CAG1760CTT (6784A>T 6785G>T), GCG1761GCA (6788G>A), GCA1762GTT (6790C>T 6791A>T), ATC1763AAT (6793T>A 6794C>T), CAT1764-AT (6795delC), GGC1765GGT (6800C>T), CTG1766TTA (6801C>T 6803G>A), GAT1767AAT (6804G>A), AAA1768AGT (6808A>G 6809A>T), TTC1769TTT (6812C>T), AAA1770AGA (6814A>G), ATT1771CTA (6816A>C 6818T>A), TAT1772TAC (6821T>C), TAT1773ATT (6822T>A 6823A>T), CTT1774ATA (6825C>A 6827T>A), GAT1775AAC (6828G>A 6830T>C), AAG1777AAA (6836G>A), GAG1778GAA (6839G>A), CTC1779ATA (6840C>A 6842C>A), ATA1780CTG (6843A>C 6845A>G), ATT1781GTG (6846A>G 6848T>G), CGC1782AGA (6849C>A 6851C>A), TCA1783ACA (6852T>A), ATT1788ATA (6869T>A), ATC1789GTA (6870A>G 6872C>A), TAC1792CAT (6879T>C 6881C>T), AAC1793CAA (6882A>C 6884C>A), ACG1795ATA (6889C>T 6890G>A), AAC1796AAT (6893C>T), AAT1798AAA (6899T>A), AAG1799ACT (6901A>C 6902G>T), CCG1800AGT (6903C>A 6904C>G 6905G>T), TCT1801AGT (6906T>A 6907C>G), GTT1803AGA (6912G>A 6913T>G 6914T>A), AGA1804CGA (6915A>C), TTA1806CTT (6921T>C 6923A>T), ACA1807AAT (6925C>A 6926A>T), TCA1809CTA (6930T>C 6931C>T), TTC1811ACA (6936T>A 6937T>C 6938C>A), TTA1812ATA (6939T>A), ACA1813TCT (6942A>T 6944A>T), GGT1814ACT (6945G>A 6946G>C), CTT1815TAC (6948C>T 6949T>A 6950T>C), GGA1816GAG (6952G>A 6953A>G), ATC1817del (6954_6956delATC), ACA1818AAT (6958C>A 6959A>T), GTT1819ATT (6960G>A), ACA1820ATT (6964C>T 6965A>T), TTC1821TTT (6968C>T), CAC1823CAT (6974C>T), GAT1825AAA (6978G>A 6980T>A), AAG1827AAA (6986G>A), CAT1828AAA (6987C>A 6989T>A), GGC1830AAC (6993G>A 6994G>A)								

Proteins

	Begin	End	Coverage	Score	Concordance	Matches	Identities	I/D/M/F*	Stop Codons
polyprotein (NP_039820.1)	1529	1831	16.1%	750	35.5%	301 (98.7%)	145 (47.5%)	2/2/2/2	3
Protein mutations:	L1529H (6091T>A), Y1530F (6094A>T), P1536S (6111C>T), N1541T (6127A>C 6128T>A), A1544P (6135G>C), V1553I (6162G>A 6164C>T), K1555G (6168A>G 6169A>G), G1556D (6172G>A 6173G>T), T1557Y (6174A>T 6175C>A 6176A>C), E1558K (6177G>A), K1559R (6181A>G 6182G>A), I1561V (6186A>G 6188A>C), A1562L (6189G>C 6190C>T 6191T>A), I1565V (6198A>G 6200T>A), S1572N (6219T>A 6220C>A 6221A>T), E1573K (6222G>A), T1574D (6225A>G 6226C>A 6227A>C), A1575I (6228G>A 6229C>T), E1576R (6231G>A 6232A>G 6233A>G), Q1577E (6234C>G 6236A>G), S1579L (6240T>C 6241C>T 6242G>A), Q1580G (6243C>G 6244A>G 6245G>T), Y1583Q (6252T>C 6254T>G), M1585V (6258A>G 6260G>A), L1586F (6261C>T 6263G>T), Q1587R (6264C>A 6265A>G 6266G>A), C1589F (6271G>T 6272C>T), K1590V (6273A>G 6274A>T 6275G>A), E1591S (6276G>A 6277A>G 6278A>T), L1594I (6285T>A), L1596V (6291C>G 6293T>A), P1598K (6297C>A 6298C>A 6299T>A), T1599K (6301C>A 6302C>G), I1603L (6312A>T), G1604C (6315G>T), T1605K (6319C>A 6320T>A), P1606T (6321C>A 6323G>C), E1607H (6324G>C 6326G>T), D1609N (6330G>A), G1612E (6340G>A), A1613I (6342G>A 6343C>T), S1614I (6345T>A 6346C>T 6347T>A), C1617E (6354T>G 6355G>A 6356T>A), T1618* (6357A>T 6358C>G), H1625del (6378_6380delCAC), I1627* (6384A>T 6385T>A 6386T>G), S1628L (6387T>C 6388C>T), I1630R (6394T>G 6395A>G), C1631S (6397G>C), D1632* (6399G>T 6401C>G), F1633R (6402T>A 6403T>G 6404C>A), S1634C (6406C>G 6407A>C), D1635H (6408G>C), E1636K (6411G>A), K1637L (6414A>T 6415A>T), L1638E (6417C>G 6418T>A), A1639N (6420G>A 6421C>A 6422A>C), P1641K (6426C>A 6427C>A 6428T>A), M1644T (6436T>C 6437G>A), R1645K (6439G>A), S1646I (6442G>T 6443C>T), W1647L (6444T>C 6445G>T 6446G>T), L1648I (6447T>A 6449G>A), I1650L (6453A>T 6455C>A), L1651I (6456C>A 6458C>A), S1652N (6459T>A 6460C>A 6461A>T), Y1657F (6475A>T), Q1659K (6480C>A 6482G>A), I1661L (6486A>T 6488C>A), L1664I (6495T>A 6497G>A), Q1666G (6501C>G 6502A>G), R1669Y (6510A>T 6511G>A 6512A>C), Q1670S (6513C>T 6514A>C 6515A>T), M1672T (6520T>C 6521G>A), A1673G (6523C>G), P1674S (6525C>A 6526C>G 6527A>T), T1675K (6529C>A), D1677Q (6534G>C 6536C>A), R1679Y (6540A>T 6541G>A 6542A>T), M1680F (6543A>T 6545G>T), P1682N (6549C>A 6550C>A 6551A>T), T1684D (6555A>G 6556C>A 6557A>T), W1685I (6558T>A 6559G>T 6560G>C), M1687L (6564A>T 6566G>A), R1689Q (6570A>C 6571G>A), Q1690K (6573C>A), E1693D (6584A>T), L1698I (6597C>A), Q1702A (6609C>G 6610A>C 6611G>T), L1703I (6612T>A 6614A>T), P1705L (6619C>T 6620T>A), K1706E (6621A>G), D1707S (6624G>T 6625A>C 6626T>C), S1708D (6627T>G 6628C>A 6629A>T), F1709Y (6631T>A 6632C>T), I1710L (6633A>T 6635C>A), C1717S (6654T>A 6656T>C), M1718S (6657A>T 6658T>C 6659G>C), V1724I (6675G>A 6677C>T), C1725L (6679G>T 6680C>G), W1727A (6684T>G 6685G>C 6686G>C), M1729P (6690A>C 6691T>C 6692G>A), H1732Y (6699C>T), D1733S (6702G>A 6703A>G), P1734D (6705C>G 6706C>A 6707A>T), R1735K (6709G>A), S1736N (6712G>A 6713C>T), R1739K (6721G>A 6722A>G), A1742G (6730C>G 6731C>T), A1744Q (6735G>C 6736C>A 6737T>A), S1747K (6744T>A 6745C>A), F1748Y (6748T>A), N1749K (6752T>A), P1750E (6753C>G 6754C>A), I1751K (6757T>A), I1751_K1752insGN (6758_6759insGGAAAT), K1752M (6760A>T 6761A>G), T1754S (6766C>G 6767C>T), D1756N (6771G>A), A1757F (6774G>T 6775C>T 6776A>T), Q1760L (6784A>T 6785G>T), A1762V (6790C>T 6791A>T), I1763N (6793T>A 6794C>T), D1767N (6804G>A), K1768S (6808A>G 6809A>T), K1770R (6814A>G), I1771L (6816A>C 6818T>A), Y1773I (6822T>A 6823A>T), L1774I (6825C>A 6827T>A), D1775N (6828G>A 6830T>C), L1779I (6840C>A 6842C>A), I1780L (6843A>C 6845A>G), I1781V (6846A>G 6848T>G), S1783T (6852T>A), I1789V (6870A>G 6872C>A), Y1792H (6879T>C 6881C>T), N1793Q (6882A>C 6884C>A), T1795I (6889C>T 6890G>A), N1798K (6899T>A), K1799T (6901A>C 6902G>T), P1800S (6903C>A 6904C>G 6905G>T), V1803R (6912G>A 6913T>G 6914T>A), T1807N (6925C>A 6926A>T), S1809L (6930T>C 6931C>T), F1811T (6936T>A 6937T>C 6938C>A), L1812I (6939T>A), T1813S (6942A>T 6944A>T), G1814T (6945G>A 6946G>C), L1815Y (6948C>T 6949T>A 6950T>C), G1816E (6952G>A 6953A>G), I1817del (6954_6956delATC), T1818N (6958C>A 6959A>T), V1819I (6960G>A), T1820I (6964C>T 6965A>T), D1825K (6978G>A 6980T>A), H1828K (6987C>A 6989T>A), G1830N (6993G>A 6994G>A)								

	Begin	End	Coverage	Score	Concordance	Matches	Identities	I/D/M/F*	Stop Codons
**NT**	**6090**	**6998**	**12.1%**	**123**	**7.0%**	**901 (98.5%)**	**496 (54.2%)**	**6/8**	
Codon mutations:	CTT1529CAT (6091T>A), TAT1530TTT (6094A>T), CTT1533TTA (6102C>T 6104T>A), CCA1536TCA (6111C>T), GGT1538GGA (6119T>A), CTC1539TTG (6120C>T 6122C>G), AAA1540AAG (6125A>G), AAT1541ACA (6127A>C 6128T>A), GCG1542GCA (6131G>A), CCC1543CCA (6134C>A), GCT1544CCT (6135G>C), TTT1546TTC (6143T>C), AGG1548AGA (6149G>A), AAC1552AAT (6161C>T), GTC1553ATT (6162G>A 6164C>T), TTC1554TTT (6167C>T), AAA1555GGA (6168A>G 6169A>G), GGG1556GAT (6172G>A 6173G>T), ACA1557TAC (6174A>T 6175C>A 6176A>C), GAA1558AAA (6177G>A), AAG1559AGA (6181A>G 6182G>A), TTC1560TTT (6185C>T), ATA1561GTC (6186A>G 6188A>C), GCT1562CTA (6189G>C 6190C>T 6191T>A), GTT1563GTA (6194T>A), TAC1564TAT (6197C>T), ATT1565GTA (6198A>G 6200T>A), GAT1567GAC (6206T>C), ATA1568ATT (6209A>T), CTT1569CTA (6212T>A), GTT1570GTA (6215T>A), TTC1571TTT (6218C>T), TCA1572AAT (6219T>A 6220C>A 6221A>T), GAA1573AAA (6222G>A), ACA1574GAC (6225A>G 6226C>A 6227A>C), GCA1575ATA (6228G>A 6229C>T), GAA1576AGG (6231G>A 6232A>G 6233A>G), CAA1577GAG (6234C>G 6236A>G), TCG1579CTA (6240T>C 6241C>T 6242G>A), CAG1580GGT (6243C>G 6244A>G 6245G>T), CTC1582CTA (6251C>A), TAT1583CAG (6252T>C 6254T>G), ATG1585GTA (6258A>G 6260G>A), CTG1586TTT (6261C>T 6263G>T), CAG1587AGA (6264C>A 6265A>G 6266G>A), CTT1588CTA (6269T>A), TGC1589TTT (6271G>T 6272C>T), AAG1590GTA (6273A>G 6274A>T 6275G>A), GAA1591AGT (6276G>A 6277A>G 6278A>T), AAT1592AAC (6281T>C), GGG1593GGT (6284G>T), TTA1594ATA (6285T>A), ATC1595ATA (6290C>A), CTT1596GTA (6291C>G 6293T>A), CCT1598AAA (6297C>A 6298C>A 6299T>A), ACC1599AAG (6301C>A 6302C>G), AAG1602AAA (6311G>A), ATA1603TTA (6312A>T), GGT1604TGT (6315G>T), ACT1605AAA (6319C>A 6320T>A), CCG1606ACC (6321C>A 6323G>C), GAG1607CAT (6324G>C 6326G>T), ATC1608ATA (6329C>A), GAT1609AAT (6330G>A), GGG1612GAG (6340G>A), GCT1613ATT (6342G>A 6343C>T), TCT1614ATA (6345T>A 6346C>T 6347T>A), CTC1615TTA (6348C>T 6350C>A), TGT1617GAA (6354T>G 6355G>A 6356T>A), ACA1618TGA (6357A>T 6358C>G), AAA1619AAG (6362A>G), ATC1620ATA (6365C>A), AAG1621AAA (6368G>A), CTT1622TTA (6369C>T 6371T>A), CAG1623CAA (6374G>A), CAC1625del (6378_6380delCAC), ATC1626ATA (6383C>A), ATT1627TAG (6384A>T 6385T>A 6386T>G), TCA1628CTA (6387T>C 6388C>T), ATA1630AGG (6394T>G 6395A>G), TGT1631TCT (6397G>C), GAC1632TAG (6399G>T 6401C>G), TTC1633AGA (6402T>A 6403T>G 6404C>A), TCA1634TGC (6406C>G 6407A>C), GAT1635CAT (6408G>C), GAA1636AAA (6411G>A), AAA1637TTA (6414A>T 6415A>T), CTA1638GAA (6417C>G 6418T>A), GCA1639AAC (6420G>A 6421C>A 6422A>C), CCT1641AAA (6426C>A 6427C>A 6428T>A), GGT1643-TT (6432delG 6433G>T), ATG1644ACA (6436T>C 6437G>A), AGA1645AAA (6439G>A), AGC1646ATT (6442G>T 6443C>T), TGG1647CTT (6444T>C 6445G>T 6446G>T), TTG1648ATA (6447T>A 6449G>A), ATC1650TTA (6453A>T 6455C>A), CTC1651ATA (6456C>A 6458C>A), TCA1652AAT (6459T>A 6460C>A 6461A>T), TAT1657TTT (6475A>T), ATT1658ATA (6479T>A), CAG1659AAA (6480C>A 6482G>A), GAT1660GAC (6485T>C), ATC1661TTA (6486A>T 6488C>A), GGC1662GGA (6491C>A), TTG1664ATA (6495T>A 6497G>A), GTG1665GTA (6500G>A), CAA1666GGA (6501C>G 6502A>G), AGA1669TAC (6510A>T 6511G>A 6512A>C), CAA1670TCT (6513C>T 6514A>C 6515A>T), ATG1672ACA (6520T>C 6521G>A), GCA1673GGA (6523C>G), CCA1674AGT (6525C>A 6526C>G 6527A>T), ACA1675AAA (6529C>A), GAC1677CAA (6534G>C 6536C>A), AGA1679TAT (6540A>T 6541G>A 6542A>T), ATG1680TTT (6543A>T 6545G>T), CCA1682AAT (6549C>A 6550C>A 6551A>T), ACA1684GAT (6555A>G 6556C>A 6557A>T), TGG1685ATC (6558T>A 6559G>T 6560G>C), AAG1686AAA (6563G>A), ATG1687TTA (6564A>T 6566G>A), GTA1688GTC (6569A>C), AGA1689CAA (6570A>C 6571G>A), CAG1690AAG (6573C>A), ATA1691ATC (6578A>C), GAA1693GAT (6584A>T), AAG1694AAA (6587G>A), GTG1695GTT (6590G>T), AAT1697AAC (6596T>C), CTC1698ATC (6597C>A), CCT1699CCA (6602T>A), CTT1701TTG (6606C>T 6608T>G), CAG1702GCT (6609C>G 6610A>C 6611G>T), TTA1703ATT (6612T>A 6614A>T), CCA1704CCT (6617A>T), CCT1705CTA (6619C>T 6620T>A), AAA1706GAA (6621A>G), GAT1707TCC (6624G>T 6625A>C 6626T>C), TCA1708GAT (6627T>G 6628C>A 6629A>T), TTC1709TAT (6631T>A 6632C>T), ATC1710TTA (6633A>T 6635C>A), GAG1713GAA (6644G>A), ACG1714ACA (6647G>A), GGT1716GGG (6653T>G), TGT1717AGC (6654T>A 6656T>C), ATG1718TCC (6657A>T 6658T>C 6659G>C), ACT1719ACA (6662T>A), GGC1720GGA (6665C>A), GCC1723GCA (6674C>A), GTC1724ATT (6675G>A 6677C>T), TGC1725TTG (6679G>T 6680C>G), AAA1726AAG (6683A>G), TGG1727GCC (6684T>G 6685G>C 6686G>C), ATG1729CCA (6690A>C 6691T>C 6692G>A), TCA1730AGT (6693T>A 6694C>G 6695A>T), AAG1731AAA (6698G>A), CAT1732TAT (6699C>T), GAT1733AGT (6702G>A 6703A>G), CCA1734GAT (6705C>G 6706C>A 6707A>T), AGA1735AAA (6709G>A), AGC1736AAT (6712G>A 6713C>T), ACC1737ACA (6716C>A), AGA1739AAG (6721G>A 6722A>G), ATT1740ATA (6725T>A), GCC1742GGT (6730C>G 6731C>T), GCT1744CAA (6735G>C 6736C>A 6737T>A), AGT1745AGC (6740T>C), GGA1746GGT (6743A>T), TCA1747AAA (6744T>A 6745C>A), TTC1748TAC (6748T>A), AAT1749AAA (6752T>A), CCA1750GAA (6753C>G 6754C>A), ATA1751AAA (6757T>A), ATA1751_AAA1752insGGAAAT (6758_6759insGGAAAT), AAA1752ATG (6760A>T 6761A>G), TCA1753AGT (6762T>A 6763C>G 6764A>T), ACC1754AGT (6766C>G 6767C>T), ATC1755ATA (6770C>A), GAT1756AAT (6771G>A), GCA1757TTT (6774G>T 6775C>T 6776A>T), GAG1758GAA (6779G>A), ATT1759ATA (6782T>A), CAG1760CTT (6784A>T 6785G>T), GCG1761GCA (6788G>A), GCA1762GTT (6790C>T 6791A>T), ATC1763AAT (6793T>A 6794C>T), CAT1764-AT (6795delC), GGC1765GGT (6800C>T), CTG1766TTA (6801C>T 6803G>A), GAT1767AAT (6804G>A), AAA1768AGT (6808A>G 6809A>T), TTC1769TTT (6812C>T), AAA1770AGA (6814A>G), ATT1771CTA (6816A>C 6818T>A), TAT1772TAC (6821T>C), TAT1773ATT (6822T>A 6823A>T), CTT1774ATA (6825C>A 6827T>A), GAT1775AAC (6828G>A 6830T>C), AAG1777AAA (6836G>A), GAG1778GAA (6839G>A), CTC1779ATA (6840C>A 6842C>A), ATA1780CTG (6843A>C 6845A>G), ATT1781GTG (6846A>G 6848T>G), CGC1782AGA (6849C>A 6851C>A), TCA1783ACA (6852T>A), ATT1788ATA (6869T>A), ATC1789GTA (6870A>G 6872C>A), TAC1792CAT (6879T>C 6881C>T), AAC1793CAA (6882A>C 6884C>A), ACG1795ATA (6889C>T 6890G>A), AAC1796AAT (6893C>T), AAT1798AAA (6899T>A), AAG1799ACT (6901A>C 6902G>T), CCG1800AGT (6903C>A 6904C>G 6905G>T), TCT1801AGT (6906T>A 6907C>G), GTT1803AGA (6912G>A 6913T>G 6914T>A), AGA1804CGA (6915A>C), TTA1806CTT (6921T>C 6923A>T), ACA1807AAT (6925C>A 6926A>T), TCA1809CTA (6930T>C 6931C>T), TTC1811ACA (6936T>A 6937T>C 6938C>A), TTA1812ATA (6939T>A), ACA1813TCT (6942A>T 6944A>T), GGT1814ACT (6945G>A 6946G>C), CTT1815TAC (6948C>T 6949T>A 6950T>C), GGA1816GAG (6952G>A 6953A>G), ATC1817del (6954_6956delATC), ACA1818AAT (6958C>A 6959A>T), GTT1819ATT (6960G>A), ACA1820ATT (6964C>T 6965A>T), TTC1821TTT (6968C>T), CAC1823CAT (6974C>T), GAT1825AAA (6978G>A 6980T>A), AAG1827AAA (6986G>A), CAT1828AAA (6987C>A 6989T>A), GGC1830AAC (6993G>A 6994G>A)								

*: Inserts / Deletes / Misaligned / Frameshifts

Analysis details

This analysis was performed with panviral2.64

NGS Details (UN8): Epiphyllum badnavirus 1

Assembly

**Coverage Length**	405 (1 contig(s))
**Depth Of Coverage**	16.6
**Number Of Reads**	55
**Reads Per Million**	0.92 rpm (after QC)
**Ambiguities**	0
**Assembly Method**	de novo + reference guided assembly
**Consensus Caller**	Bcf Tools

Coverage Map

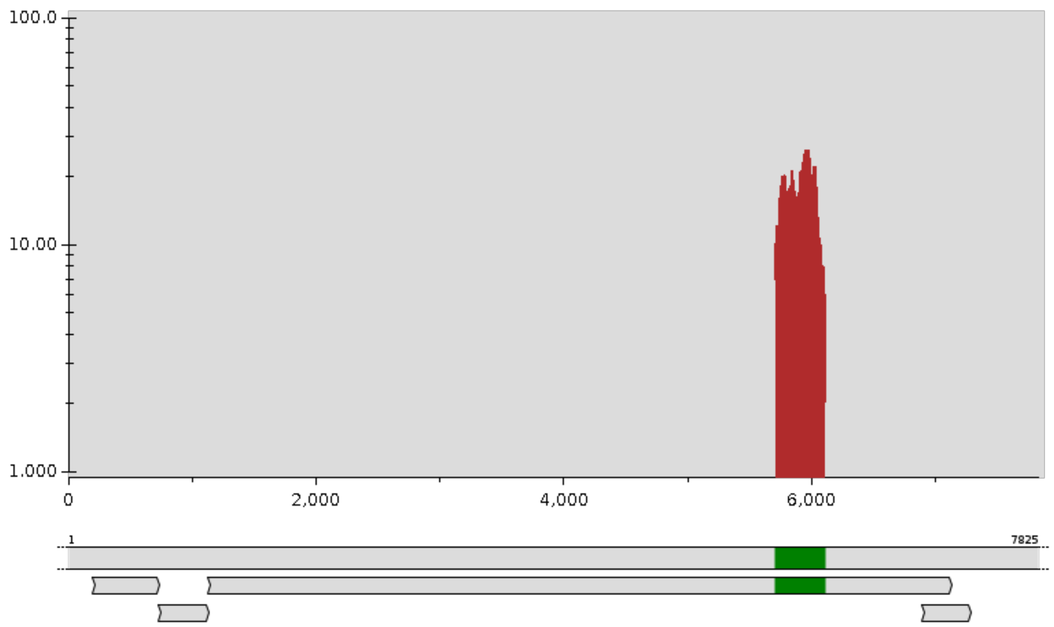

Assignment

**Type**	Epiphyllum badnavirus 1 (Taxonomy ID: 2518008)
**Reference Genome**	NC_076247.1
**NT Identity (%)**	52.3573
**AA Identity (%)**	41.4815
**Number Of Stop Codons**	1
**Number Of CDS**	4

Alignment

**Alignment Score**	9.0 (NT) + 220.0 (AA) = 229.0
**Concordance (%)**	15.6984

Alignment Method	Global, seeded, nucleotide + amino acids (AGA)

Genome Region

Sequence starts at position 5706 and ends at position 6110 relative to NC_076247.1 reference sequence.

Alignment Detailed Statistics

	Begin	End	Coverage	Score	Concordance	Matches	Identities	I/D/M/F*	Stop Codons
**NT**	**5706**	**6110**	**5.2%**	**9**	**1.2%**	**400 (98.0%)**	**211 (51.7%)**	**3/5**	
Mutations:	5706T>C, 5707G>T, 5708C>T, 5709A>G, 5710A>T, 5711C>T, 5714G>T, 5715A>T, 5716G>A, 5718A>G, 5719G>A, 5725A>G, 5727A>C, 5730A>G, 5732A>G, 5733G>C, 5734G>A, 5735A>T, 5736G>A, 5737A>T, 5739A>G, 5742G>A, 5743G>T, 5744T>G, 5745C>T, 5746T>A, 5748C>T, 5749A>G, 5756A>G, 5758A>C, 5759C>A, 5760C>G, 5767G>A, 5769C>G, 5770A>G, 5771A>T, 5772C>A, 5776T>G, 5778C>G, 5781A>G, 5782G>A, 5787G>T, 5791T>C, 5792C>T, 5793T>C, 5794C>A, 5796A>G, 5800G>C, 5806A>G, 5808C>T, 5809_5810delAC, 5811A>G, 5812A>T, 5816T>A, 5817A>C, 5821_5823delAAG, 5824G>C, 5826C>A, 5828G>A, 5829T>G, 5830A>G, 5831A>G, 5835C>T, 5836A>G, 5837G>T, 5838T>C, 5841C>G, 5844C>T, 5847C>T, 5851T>A, 5853C>T, 5857C>G, 5861A>G, 5862G>T, 5863A>T, 5864G>C, 5868A>C, 5870T>A, 5871C>T, 5872C>A, 5874C>T, 5878A>T, 5880T>G, 5882T>G, 5886G>T, 5887G>A, 5889C>G, 5890G>A, 5891A>C, 5892A>T, 5894A>T, 5895A>T, 5896T>G, 5897C>A, 5898C>T, 5901C>T, 5905T>A, 5906G>A, 5908A>G, 5909C>A, 5910C>T, 5913C>T, 5916C>T, 5918T>G, 5919G>A, 5922C>T, 5924C>G, 5925A>C, 5926G>T, 5931A>C, 5933T>A, 5934T>C, 5938G>A, 5940A>G, 5942G>T, 5943G>C, 5944T>C, 5946A>T, 5949T>G, 5953C>T, 5955A>T, 5958C>T, 5961A>G, 5962T>C, 5964G>T, 5966A>C, 5967G>T, 5975C>T, 5976T>C, 5981T>C, 5982G>C, 5985C>T, 5986C>A, 5987A>T, 5989C>G, 5990G>A, 5991A>T, 5992A>C, 5993A>G, 5998G>A, 5999A>C, 6000C>T, 6001A>C, 6002A>G, 6003C>A, 6004T>G, 6005G>T, 6006T>A, 6009C>T, 6012A>G, 6012_6013insCCT, 6013G>T, 6015C>T, 6016C>A, 6018G>T, 6019T>G, 6020C>A, 6021A>T, 6022G>T, 6023G>C, 6030A>G, 6031G>A, 6032C>T, 6033A>T, 6036T>G, 6038A>T, 6051C>T, 6052C>T, 6054G>A, 6057C>A, 6059T>A, 6061A>T, 6062G>C, 6063T>G, 6064A>T, 6065A>G, 6066C>G, 6068C>G, 6070G>A, 6071A>G, 6075T>G, 6078C>G, 6079C>A, 6087G>A, 6090C>T, 6091C>T, 6095A>G, 6097T>G, 6098C>T, 6099C>T, 6100A>G, 6105G>C								

CDS

	Begin	End	Coverage	Score	Concordance	Matches	Identities	I/D/M/F*	Stop Codons
QKM20_gp3	1527	1661	6.7%	220	23.0%	134 (98.5%)	56 (41.2%)	1/1/1/1	1
Protein mutations:	A1527L (5707G>T 5708C>T 5709A>G), T1528F (5710A>T 5711C>T), G1529V (5714G>T 5715A>T), E1530K (5716G>A 5718A>G), E1531K (5719G>A), K1533D (5725A>G 5727A>C), K1535S (5732A>G 5733G>C), E1536I (5734G>A 5735A>T 5736G>A), R1537W (5737A>T 5739A>G), M1538I (5742G>A), V1539C (5743G>T 5744T>G 5745C>T), F1540I (5746T>A 5748C>T), N1541D (5749A>G), K1543R (5756A>G), T1544Q (5758A>C 5759C>A 5760C>G), D1547K (5767G>A 5769C>G), N1548V (5770A>G 5771A>T 5772C>A), F1550V (5776T>G 5778C>G), D1552N (5782G>A), Q1553H (5787G>T), S1555L (5791T>C 5792C>T 5793T>C), L1556M (5794C>A 5796A>G), G1558R (5800G>C), N1560D (5806A>G 5808C>T), I1562F (5812A>T), I1563N (5816T>A 5817A>C), K1565del (5821_5823delAAG), V1566L (5824G>C 5826C>A), C1567* (5828G>A 5829T>G), N1568G (5830A>G 5831A>G), S1570V (5836A>G 5837G>T 5838T>C), F1575I (5851T>A 5853C>T), L1577V (5857C>G), K1578S (5861A>G 5862G>T), F1581Y (5870T>A 5871C>T), H1582N (5872C>A 5874C>T), I1584L (5878A>T 5880T>G), M1585R (5882T>G), M1586I (5886G>T), D1587K (5887G>A 5889C>G), E1588T (5890G>A 5891A>C 5892A>T), E1589V (5894A>T 5895A>T), S1590D (5896T>G 5897C>A 5898C>T), W1593K (5905T>A 5906G>A), T1594D (5908A>G 5909C>A 5910C>T), L1597R (5918T>G 5919G>A), P1599R (5924C>G 5925A>C), D1600Y (5926G>T), L1602H (5933T>A 5934T>C), E1604K (5938G>A 5940A>G), W1605F (5942G>T 5943G>C), P1609S (5953C>T 5955A>T), K1613T (5966A>C 5967G>T), P1616L (5975C>T 5976T>C), V1618A (5981T>C 5982G>C), Q1620I (5986C>A 5987A>T), R1621D (5989C>G 5990G>A 5991A>T), K1622R (5992A>C 5993A>G), D1624T (5998G>A 5999A>C 6000C>T), N1625R (6001A>C 6002A>G 6003C>A), C1626V (6004T>G 6005G>T 6006T>A), R1628_D1629insP (6012_6013insCCT), D1629Y (6013G>T 6015C>T), L1630I (6016C>A 6018G>T), S1631D (6019T>G 6020C>A 6021A>T), G1632S (6022G>T 6023G>C), A1635I (6031G>A 6032C>T 6033A>T), Y1637F (6038A>T), F1644Y (6059T>A), N1646W (6064A>T 6065A>G 6066C>G), T1647S (6068C>G), E1648R (6070G>A 6071A>G), D1649E (6075T>G), D1650E (6078C>G), H1651N (6079C>A), K1656R (6095A>G), S1657V (6097T>G 6098C>T 6099C>T), M1658V (6100A>G)								
Codon mutations:	CCT1526..C (5706T>C), GCA1527TTG (5707G>T 5708C>T 5709A>G), ACT1528TTT (5710A>T 5711C>T), GGA1529GTT (5714G>T 5715A>T), GAA1530AAG (5716G>A 5718A>G), GAG1531AAG (5719G>A), AAA1533GAC (5725A>G 5727A>C), GGA1534GGG (5730A>G), AAG1535AGC (5732A>G 5733G>C), GAG1536ATA (5734G>A 5735A>T 5736G>A), AGA1537TGG (5737A>T 5739A>G), ATG1538ATA (5742G>A), GTC1539TGT (5743G>T 5744T>G 5745C>T), TTC1540ATT (5746T>A 5748C>T), AAC1541GAC (5749A>G), AAA1543AGA (5756A>G), ACC1544CAG (5758A>C 5759C>A 5760C>G), GAC1547AAG (5767G>A 5769C>G), AAC1548GTA (5770A>G 5771A>T 5772C>A), TTC1550GTG (5776T>G 5778C>G), AAA1551AAG (5781A>G), GAT1552AAT (5782G>A), CAG1553CAT (5787G>T), TCT1555CTC (5791T>C 5792C>T 5793T>C), CTA1556ATG (5794C>A 5796A>G), GGC1558CGC (5800G>C), AAC1560GAT (5806A>G 5808C>T), ACA1561--G (5809_5810delAC 5811A>G), ATC1562TTC (5812A>T), ATA1563AAC (5816T>A 5817A>C), AAG1565del (5821_5823delAAG), GTC1566CTA (5824G>C 5826C>A), TGT1567TAG (5828G>A 5829T>G), AAT1568GGT (5830A>G 5831A>G), GCC1569GCT (5835C>T), AGT1570GTC (5836A>G 5837G>T 5838T>C), GTC1571GTG (5841C>G), TTC1572TTT (5844C>T), TCC1573TCT (5847C>T), TTC1575ATT (5851T>A 5853C>T), CTG1577GTG (5857C>G), AAG1578AGT (5861A>G 5862G>T), AGC1579TCC (5863A>T 5864G>C), GGA1580GGC (5868A>C), TTC1581TAT (5870T>A 5871C>T), CAC1582AAT (5872C>A 5874C>T), ATT1584TTG (5878A>T 5880T>G), ATG1585AGG (5882T>G), ATG1586ATT (5886G>T), GAC1587AAG (5887G>A 5889C>G), GAA1588ACT (5890G>A 5891A>C 5892A>T), GAA1589GTT (5894A>T 5895A>T), TCC1590GAT (5896T>G 5897C>A 5898C>T), ATC1591ATT (5901C>T), TGG1593AAG (5905T>A 5906G>A), ACC1594GAT (5908A>G 5909C>A 5910C>T), GCC1595GCT (5913C>T), TTC1596TTT (5916C>T), CTG1597CGA (5918T>G 5919G>A), ACC1598ACT (5922C>T), CCA1599CGC (5924C>G 5925A>C), GAT1600TAT (5926G>T), GGA1601GGC (5931A>C), CTT1602CAC (5933T>A 5934T>C), GAA1604AAG (5938G>A 5940A>G), TGG1605TTC (5942G>T 5943G>C), TTA1606CTT (5944T>C 5946A>T), GTT1607GTG (5949T>G), CCA1609TCT (5953C>T 5955A>T), TTC1610TTT (5958C>T), GGA1611GGG (5961A>G), TTG1612CTT (5962T>C 5964G>T), AAG1613ACT (5966A>C 5967G>T), CCT1616CTC (5975C>T 5976T>C), GTG1618GCC (5981T>C 5982G>C), TTC1619TTT (5985C>T), CAA1620ATA (5986C>A 5987A>T), CGA1621GAT (5989C>G 5990G>A 5991A>T), AAG1622CGG (5992A>C 5993A>G), GAC1624ACT (5998G>A 5999A>C 6000C>T), AAC1625CGA (6001A>C 6002A>G 6003C>A), TGT1626GTA (6004T>G 6005G>T 6006T>A), TTC1627TTT (6009C>T), AGA1628AGG (6012A>G), AGA1628_GAC1629insCCT (6012_6013insCCT), GAC1629TAT (6013G>T 6015C>T), CTG1630ATT (6016C>A 6018G>T), TCA1631GAT (6019T>G 6020C>A 6021A>T), GGC1632TCC (6022G>T 6023G>C), GTA1634GTG (6030A>G), GCA1635ATT (6031G>A 6032C>T 6033A>T), GTT1636GTG (6036T>G), TAC1637TTC (6038A>T), ATC1641ATT (6051C>T), CTG1642TTA (6052C>T 6054G>A), GTC1643GTA (6057C>A), TTC1644TAC (6059T>A), AGT1645TCG (6061A>T 6062G>C 6063T>G), AAC1646TGG (6064A>T 6065A>G 6066C>G), ACT1647AGT (6068C>G), GAA1648AGA (6070G>A 6071A>G), GAT1649GAG (6075T>G), GAC1650GAG (6078C>G), CAT1651AAT (6079C>A), CAG1653CAA (6087G>A), CAC1654CAT (6090C>T), CTG1655TTG (6091C>T), AAG1656AGG (6095A>G), TCC1657GTT (6097T>G 6098C>T 6099C>T), ATG1658GTG (6100A>G), CTG1659CTC (6105G>C)								

Proteins

	Begin	End	Coverage	Score	Concordance	Matches	Identities	I/D/M/F*	Stop Codons
polyprotein (YP_010797894.1)	1527	1661	6.7%	220	23.0%	134 (98.5%)	56 (41.2%)	1/1/1/1	1
Protein mutations:	A1527L (5707G>T 5708C>T 5709A>G), T1528F (5710A>T 5711C>T), G1529V (5714G>T 5715A>T), E1530K (5716G>A 5718A>G), E1531K (5719G>A), K1533D (5725A>G 5727A>C), K1535S (5732A>G 5733G>C), E1536I (5734G>A 5735A>T 5736G>A), R1537W (5737A>T 5739A>G), M1538I (5742G>A), V1539C (5743G>T 5744T>G 5745C>T), F1540I (5746T>A 5748C>T), N1541D (5749A>G), K1543R (5756A>G), T1544Q (5758A>C 5759C>A 5760C>G), D1547K (5767G>A 5769C>G), N1548V (5770A>G 5771A>T 5772C>A), F1550V (5776T>G 5778C>G), D1552N (5782G>A), Q1553H (5787G>T), S1555L (5791T>C 5792C>T 5793T>C), L1556M (5794C>A 5796A>G), G1558R (5800G>C), N1560D (5806A>G 5808C>T), I1562F (5812A>T), I1563N (5816T>A 5817A>C), K1565del (5821_5823delAAG), V1566L (5824G>C 5826C>A), C1567* (5828G>A 5829T>G), N1568G (5830A>G 5831A>G), S1570V (5836A>G 5837G>T 5838T>C), F1575I (5851T>A 5853C>T), L1577V (5857C>G), K1578S (5861A>G 5862G>T), F1581Y (5870T>A 5871C>T), H1582N (5872C>A 5874C>T), I1584L (5878A>T 5880T>G), M1585R (5882T>G), M1586I (5886G>T), D1587K (5887G>A 5889C>G), E1588T (5890G>A 5891A>C 5892A>T), E1589V (5894A>T 5895A>T), S1590D (5896T>G 5897C>A 5898C>T), W1593K (5905T>A 5906G>A), T1594D (5908A>G 5909C>A 5910C>T), L1597R (5918T>G 5919G>A), P1599R (5924C>G 5925A>C), D1600Y (5926G>T), L1602H (5933T>A 5934T>C), E1604K (5938G>A 5940A>G), W1605F (5942G>T 5943G>C), P1609S (5953C>T 5955A>T), K1613T (5966A>C 5967G>T), P1616L (5975C>T 5976T>C), V1618A (5981T>C 5982G>C), Q1620I (5986C>A 5987A>T), R1621D (5989C>G 5990G>A 5991A>T), K1622R (5992A>C 5993A>G), D1624T (5998G>A 5999A>C 6000C>T), N1625R (6001A>C 6002A>G 6003C>A), C1626V (6004T>G 6005G>T 6006T>A), R1628_D1629insP (6012_6013insCCT), D1629Y (6013G>T 6015C>T), L1630I (6016C>A 6018G>T), S1631D (6019T>G 6020C>A 6021A>T), G1632S (6022G>T 6023G>C), A1635I (6031G>A 6032C>T 6033A>T), Y1637F (6038A>T), F1644Y (6059T>A), N1646W (6064A>T 6065A>G 6066C>G), T1647S (6068C>G), E1648R (6070G>A 6071A>G), D1649E (6075T>G), D1650E (6078C>G), H1651N (6079C>A), K1656R (6095A>G), S1657V (6097T>G 6098C>T 6099C>T), M1658V (6100A>G)								

	Begin	End	Coverage	Score	Concordance	Matches	Identities	I/D/M/F*	Stop Codons
**NT**	**5706**	**6110**	**5.2%**	**9**	**1.2%**	**400 (98.0%)**	**211 (51.7%)**	**3/5**	
Codon mutations:	CCT1526..C (5706T>C), GCA1527TTG (5707G>T 5708C>T 5709A>G), ACT1528TTT (5710A>T 5711C>T), GGA1529GTT (5714G>T 5715A>T), GAA1530AAG (5716G>A 5718A>G), GAG1531AAG (5719G>A), AAA1533GAC (5725A>G 5727A>C), GGA1534GGG (5730A>G), AAG1535AGC (5732A>G 5733G>C), GAG1536ATA (5734G>A 5735A>T 5736G>A), AGA1537TGG (5737A>T 5739A>G), ATG1538ATA (5742G>A), GTC1539TGT (5743G>T 5744T>G 5745C>T), TTC1540ATT (5746T>A 5748C>T), AAC1541GAC (5749A>G), AAA1543AGA (5756A>G), ACC1544CAG (5758A>C 5759C>A 5760C>G), GAC1547AAG (5767G>A 5769C>G), AAC1548GTA (5770A>G 5771A>T 5772C>A), TTC1550GTG (5776T>G 5778C>G), AAA1551AAG (5781A>G), GAT1552AAT (5782G>A), CAG1553CAT (5787G>T), TCT1555CTC (5791T>C 5792C>T 5793T>C), CTA1556ATG (5794C>A 5796A>G), GGC1558CGC (5800G>C), AAC1560GAT (5806A>G 5808C>T), ACA1561--G (5809_5810delAC 5811A>G), ATC1562TTC (5812A>T), ATA1563AAC (5816T>A 5817A>C), AAG1565del (5821_5823delAAG), GTC1566CTA (5824G>C 5826C>A), TGT1567TAG (5828G>A 5829T>G), AAT1568GGT (5830A>G 5831A>G), GCC1569GCT (5835C>T), AGT1570GTC (5836A>G 5837G>T 5838T>C), GTC1571GTG (5841C>G), TTC1572TTT (5844C>T), TCC1573TCT (5847C>T), TTC1575ATT (5851T>A 5853C>T), CTG1577GTG (5857C>G), AAG1578AGT (5861A>G 5862G>T), AGC1579TCC (5863A>T 5864G>C), GGA1580GGC (5868A>C), TTC1581TAT (5870T>A 5871C>T), CAC1582AAT (5872C>A 5874C>T), ATT1584TTG (5878A>T 5880T>G), ATG1585AGG (5882T>G), ATG1586ATT (5886G>T), GAC1587AAG (5887G>A 5889C>G), GAA1588ACT (5890G>A 5891A>C 5892A>T), GAA1589GTT (5894A>T 5895A>T), TCC1590GAT (5896T>G 5897C>A 5898C>T), ATC1591ATT (5901C>T), TGG1593AAG (5905T>A 5906G>A), ACC1594GAT (5908A>G 5909C>A 5910C>T), GCC1595GCT (5913C>T), TTC1596TTT (5916C>T), CTG1597CGA (5918T>G 5919G>A), ACC1598ACT (5922C>T), CCA1599CGC (5924C>G 5925A>C), GAT1600TAT (5926G>T), GGA1601GGC (5931A>C), CTT1602CAC (5933T>A 5934T>C), GAA1604AAG (5938G>A 5940A>G), TGG1605TTC (5942G>T 5943G>C), TTA1606CTT (5944T>C 5946A>T), GTT1607GTG (5949T>G), CCA1609TCT (5953C>T 5955A>T), TTC1610TTT (5958C>T), GGA1611GGG (5961A>G), TTG1612CTT (5962T>C 5964G>T), AAG1613ACT (5966A>C 5967G>T), CCT1616CTC (5975C>T 5976T>C), GTG1618GCC (5981T>C 5982G>C), TTC1619TTT (5985C>T), CAA1620ATA (5986C>A 5987A>T), CGA1621GAT (5989C>G 5990G>A 5991A>T), AAG1622CGG (5992A>C 5993A>G), GAC1624ACT (5998G>A 5999A>C 6000C>T), AAC1625CGA (6001A>C 6002A>G 6003C>A), TGT1626GTA (6004T>G 6005G>T 6006T>A), TTC1627TTT (6009C>T), AGA1628AGG (6012A>G), AGA1628_GAC1629insCCT (6012_6013insCCT), GAC1629TAT (6013G>T 6015C>T), CTG1630ATT (6016C>A 6018G>T), TCA1631GAT (6019T>G 6020C>A 6021A>T), GGC1632TCC (6022G>T 6023G>C), GTA1634GTG (6030A>G), GCA1635ATT (6031G>A 6032C>T 6033A>T), GTT1636GTG (6036T>G), TAC1637TTC (6038A>T), ATC1641ATT (6051C>T), CTG1642TTA (6052C>T 6054G>A), GTC1643GTA (6057C>A), TTC1644TAC (6059T>A), AGT1645TCG (6061A>T 6062G>C 6063T>G), AAC1646TGG (6064A>T 6065A>G 6066C>G), ACT1647AGT (6068C>G), GAA1648AGA (6070G>A 6071A>G), GAT1649GAG (6075T>G), GAC1650GAG (6078C>G), CAT1651AAT (6079C>A), CAG1653CAA (6087G>A), CAC1654CAT (6090C>T), CTG1655TTG (6091C>T), AAG1656AGG (6095A>G), TCC1657GTT (6097T>G 6098C>T 6099C>T), ATG1658GTG (6100A>G), CTG1659CTC (6105G>C)								

*: Inserts / Deletes / Misaligned / Frameshifts

Analysis details

This analysis was performed with panviral2.64

NGS Details (UN8): Petuvirus venapetuniae

Assembly

**Coverage Length**	822 (1 contig(s))
**Depth Of Coverage**	7.5
**Number Of Reads**	50
**Reads Per Million**	0.84 rpm (after QC)
**Ambiguities**	0
**Assembly Method**	de novo + reference guided assembly
**Consensus Caller**	Bcf Tools

Coverage Map

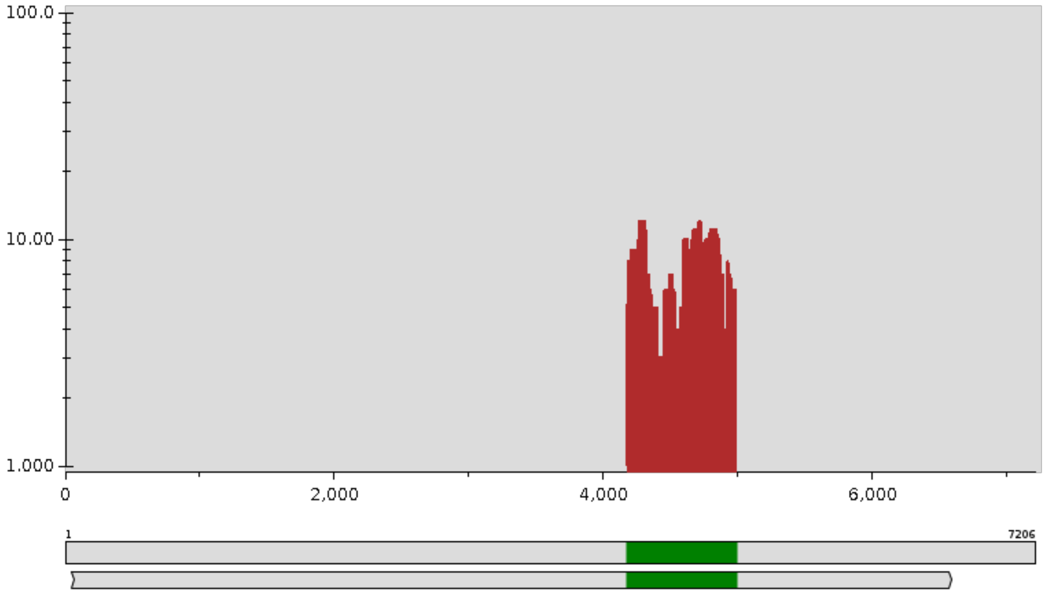

Assignment

**Type**	Petuvirus venapetuniae (Taxonomy ID: 3048265)
**Reference Genome**	NC_001839.2
**NT Identity (%)**	51.7032
**AA Identity (%)**	43.0657
**Number Of Stop Codons**	1
**Number Of CDS**	1

Alignment

**Alignment Score**	56.0 (NT) + 808.0 (AA) = 864.0
**Concordance (%)**	24.2356

Alignment Method	Global, seeded, nucleotide + amino acids (AGA)

Genome Region

Sequence starts at position 4174 and ends at position 4995 relative to NC_001839.2 reference sequence.

Alignment Detailed Statistics

	Begin	End	Coverage	Score	Concordance	Matches	Identities	I/D/M/F*	Stop Codons
**NT**	**4174**	**4995**	**11.4%**	**56**	**3.4%**	**822 (100%)**	**425 (51.7%)**	**0/0**	
Mutations:	4180T>A, 4183A>G, 4185A>T, 4186A>T, 4187G>C, 4193A>G, 4196C>G, 4197A>G, 4198T>C, 4199C>T, 4200A>T, 4201A>T, 4203C>T, 4205C>T, 4208A>T, 4211G>A, 4214A>C, 4217C>G, 4219A>C, 4220T>C, 4221T>A, 4222C>T, 4223T>C, 4224G>C, 4225G>A, 4232T>C, 4234C>A, 4235A>T, 4239C>T, 4240A>T, 4241T>A, 4242C>A, 4244C>G, 4245C>G, 4247G>A, 4248T>A, 4250A>C, 4251G>T, 4252C>G, 4253A>T, 4255T>A, 4259G>A, 4263T>A, 4264G>T, 4265T>C, 4268T>C, 4269G>A, 4270A>C, 4271G>T, 4275C>T, 4276A>T, 4277A>G, 4278C>G, 4280A>T, 4281T>A, 4282T>G, 4284G>A, 4286T>A, 4287C>A, 4289A>C, 4292C>T, 4293G>A, 4294A>G, 4298C>T, 4300C>T, 4302G>A, 4304T>A, 4307A>T, 4309A>C, 4310A>T, 4314G>A, 4315C>G, 4316A>C, 4320G>T, 4321A>C, 4322A>C, 4328T>C, 4334C>T, 4337C>T, 4340G>T, 4341A>G, 4342G>T, 4343G>T, 4344T>G, 4346A>T, 4349G>A, 4350C>A, 4354T>A, 4355G>A, 4356A>T, 4358A>C, 4360G>A, 4361A>G, 4362A>T, 4363A>G, 4364A>T, 4365T>C, 4366T>C, 4370G>A, 4371T>C, 4373A>T, 4376C>T, 4379A>C, 4386C>A, 4391A>T, 4394A>G, 4397C>T, 4398C>A, 4399A>C, 4401T>G, 4402T>C, 4403C>T, 4404C>T, 4406C>A, 4407C>A, 4410G>T, 4411A>G, 4412T>G, 4413G>A, 4414A>T, 4415C>T, 4417A>G, 4418G>A, 4420T>A, 4421T>C, 4424C>T, 4430A>T, 4433T>C, 4436G>A, 4437C>A, 4438T>A, 4439C>G, 4440A>G, 4441C>A, 4442A>T, 4445C>T, 4446T>C, 4448C>A, 4449T>A, 4450C>A, 4451T>A, 4452C>A, 4453A>G, 4454C>A, 4455T>A, 4456T>C, 4457A>C, 4459C>T, 4460C>T, 4469G>C, 4470C>A, 4472C>T, 4474T>A, 4475T>C, 4476T>A, 4477C>G, 4478A>T, 4481G>A, 4488C>A, 4490T>G, 4493G>A, 4499A>G, 4509T>A, 4511A>T, 4512G>C, 4513G>A, 4514A>G, 4517C>T, 4518C>T, 4519A>C, 4521C>G, 4522C>A, 4523T>A, 4526T>A, 4529G>C, 4531G>A, 4533C>T, 4534C>A, 4535A>G, 4538A>G, 4539A>T, 4541A>C, 4543G>C, 4544G>T, 4548T>A, 4549G>A, 4550C>T, 4551A>G, 4556T>G, 4557G>T, 4558A>T, 4560A>G, 4562A>T, 4565T>A, 4566T>C, 4567T>A, 4569C>G, 4577G>C, 4580C>T, 4585C>T, 4586A>T, 4589C>T, 4592T>G, 4600C>A, 4601A>T, 4607T>C, 4610T>A, 4611T>G, 4612T>A, 4613G>A, 4616C>T, 4623G>A, 4624C>T, 4625C>T, 4630T>A, 4631C>T, 4632A>G, 4634A>T, 4638T>A, 4640C>T, 4641C>A, 4643G>T, 4645C>A, 4646C>T, 4647A>T, 4649C>G, 4650C>A, 4651T>G, 4652T>C, 4653T>C, 4654T>A, 4656T>A, 4657C>T, 4658A>G, 4659G>T, 4661T>A, 4662T>A, 4664G>A, 4667C>T, 4670C>T, 4677G>A, 4680A>G, 4682C>T, 4683C>T, 4685T>A, 4686C>A, 4688T>C, 4691T>C, 4697A>G, 4698A>G, 4699C>A, 4700A>C, 4701C>A, 4703G>C, 4706A>T, 4707G>T, 4708A>C, 4712C>T, 4713A>T, 4715A>G, 4719T>C, 4720T>A, 4721G>C, 4724T>A, 4727C>T, 4728C>A, 4729A>T, 4730G>T, 4733C>A, 4734A>T, 4736A>T, 4738G>A, 4739T>C, 4740T>A, 4741T>C, 4742G>C, 4745T>C, 4750A>G, 4752T>A, 4753T>A, 4757A>T, 4758G>T, 4760C>G, 4761A>G, 4763G>A, 4764T>G, 4767T>A, 4768C>G, 4769G>C, 4770G>A, 4772A>T, 4781G>T, 4783T>A, 4784C>A, 4788G>T, 4789C>T, 4795A>C, 4796T>A, 4803C>A, 4804A>G, 4808C>T, 4811A>G, 4814A>T, 4815A>C, 4816T>A, 4817G>T, 4823T>A, 4824G>T, 4825C>A, 4826T>C, 4827G>C, 4829T>A, 4832A>T, 4833A>T, 4837T>G, 4838T>C, 4839T>A, 4840C>G, 4844T>A, 4845G>A, 4846C>T, 4848G>T, 4850T>C, 4851C>A, 4852A>G, 4853C>A, 4854A>G, 4855T>C, 4857T>A, 4858C>T, 4860T>G, 4861T>A, 4863G>T, 4864A>T, 4865A>T, 4866C>T, 4867T>C, 4868C>T, 4869C>A, 4871A>T, 4874G>A, 4880T>C, 4881G>A, 4884A>G, 4885C>T, 4886A>T, 4888A>T, 4890C>A, 4891T>C, 4892C>T, 4893T>G, 4894C>A, 4895G>C, 4896G>A, 4897T>A, 4900A>C, 4901G>C, 4904G>A, 4905A>C, 4907A>G, 4911C>A, 4912A>G, 4913A>G, 4917C>T, 4919T>G, 4922G>A, 4924T>G, 4926G>C, 4931T>C, 4935A>G, 4938A>G, 4939G>C, 4940A>T, 4943T>C, 4946T>C, 4952A>T, 4953G>A, 4960C>G, 4961T>A, 4962G>C, 4964G>A, 4965C>G, 4966A>T, 4968A>T, 4969T>G, 4971T>A, 4972C>A, 4973C>A, 4974C>T, 4977C>T, 4979T>G, 4980A>T, 4981G>T, 4985T>C, 4987T>G, 4989C>T, 4994G>A									
**CDS**									
PVCVgp1	1376	1649	12.6%	808	41.9%	274 (100%)	118 (43.1%)	0/0/0/0	1
Protein mutations:	F1377Y (4180T>A), K1378R (4183A>G), K1379F (4185A>T 4186A>T 4187G>C), N1382K (4196C>G), I1383A (4197A>G 4198T>C 4199C>T), N1384F (4200A>T 4201A>T), P1385S (4203C>T 4205C>T), S1389R (4217C>G), H1390P (4219A>C 4220T>C), S1391I (4221T>A 4222C>T 4223T>C), G1392Q (4224G>C 4225G>A), P1395H (4234C>A 4235A>T), H1397L (4239C>T 4240A>T 4241T>A), L1398M (4242C>A 4244C>G), Q1399E (4245C>G 4247G>A), L1400I (4248T>A 4250A>C), A1401C (4251G>T 4252C>G 4253A>T), I1402K (4255T>A), C1405I (4263T>A 4264G>T 4265T>C), E1407T (4269G>A 4270A>C 4271G>T), Q1409L (4275C>T 4276A>T 4277A>G), Q1410D (4278C>G 4280A>T), F1411S (4281T>A 4282T>G), D1412K (4284G>A 4286T>A), L1413I (4287C>A 4289A>C), E1415R (4293G>A 4294A>G), S1417F (4300C>T), D1418K (4302G>A 4304T>A), Q1420P (4309A>C 4310A>T), A1422S (4314G>A 4315C>G 4316A>C), E1424S (4320G>T 4321A>C 4322A>C), K1430N (4340G>T), R1431V (4341A>G 4342G>T 4343G>T), S1432A (4344T>G 4346A>T), Q1434K (4350C>A), V1435E (4354T>A 4355G>A), R1436C (4356A>T 4358A>C), G1437E (4360G>A 4361A>G), K1438C (4362A>T 4363A>G 4364A>T), L1439P (4365T>C 4366T>C), Q1446K (4386C>A), H1450T (4398C>A 4399A>C), F1451A (4401T>G 4402T>C 4403C>T), Q1453K (4407C>A), D1454W (4410G>T 4411A>G 4412T>G), D1455I (4413G>A 4414A>T 4415C>T), K1456R (4417A>G 4418G>A), F1457Y (4420T>A 4421T>C), L1463K (4437C>A 4438T>A 4439C>G), T1464D (4440A>G 4441C>A 4442A>T), F1466L (4446T>C 4448C>A), S1467K (4449T>A 4450C>A 4451T>A), H1468R (4452C>A 4453A>G 4454C>A), L1469T (4455T>A 4456T>C 4457A>C), S1470F (4459C>T 4460C>T), K1473N (4469G>C), L1474I (4470C>A 4472C>T), F1475Y (4474T>A 4475T>C), L1480M (4488C>A 4490T>G), L1487I (4509T>A 4511A>T), G1488Q (4512G>C 4513G>A 4514A>G), H1490S (4518C>T 4519A>C), P1491E (4521C>G 4522C>A 4523T>A), N1492K (4526T>A), E1493D (4529G>C), R1494K (4531G>A), P1495* (4533C>T 4534C>A 4535A>G), T1497S (4539A>T 4541A>C), G1498A (4543G>C 4544G>T), C1500N (4548T>A 4549G>A 4550C>T), I1501V (4551A>G), D1503F (4557G>T 4558A>T), R1504G (4560A>G 4562A>T), H1505Q (4565T>A), F1506H (4566T>C 4567T>A), Q1507E (4569C>G), K1509N (4577G>C), P1512L (4585C>T 4586A>T), T1517N (4600C>A 4601A>T), L1521E (4611T>G 4612T>A 4613G>A), A1525I (4623G>A 4624C>T 4625C>T), I1527N (4630T>A 4631C>T), K1528D (4632A>G 4634A>T), F1530I (4638T>A 4640C>T), Q1531N (4641C>A 4643G>T), P1532H (4645C>A 4646C>T), I1533L (4647A>T 4649C>G), L1534S (4650C>A 4651T>G 4652T>C), F1535H (4653T>C 4654T>A), S1536M (4656T>A 4657C>T 4658A>G), A1537S (4659G>T 4661T>A), L1538I (4662T>A 4664G>A), D1543N (4677G>A), I1544V (4680A>G 4682C>T), L1546I (4686C>A 4688T>C), T1550D (4698A>G 4699C>A 4700A>C), L1551I (4701C>A 4703G>C), E1552D (4706A>T), D1553S (4707G>T 4708A>C), I1555L (4713A>T 4715A>G), L1557H (4719T>C 4720T>A 4721G>C), Q1560I (4728C>A 4729A>T 4730G>T), F1561L (4733C>A), I1562F (4734A>T 4736A>T), S1563N (4738G>A 4739T>C), L1564T (4740T>A 4741T>C 4742G>C), K1567R (4750A>G), F1568N (4752T>A 4753T>A), V1570L (4758G>T 4760C>G), M1571V (4761A>G 4763G>A), L1572V (4764T>G), A1574T (4770G>A 4772A>T), M1577I (4781G>T), I1578K (4783T>A 4784C>A), A1580F (4788G>T 4789C>T), N1582T (4795A>C 4796T>A), Q1585R (4803C>A 4804A>G), M1589H (4815A>C 4816T>A 4817G>T), F1591L (4823T>A), A1592Y (4824G>T 4825C>A 4826T>C), D1593Q (4827G>C 4829T>A), T1595S (4833A>T), F1596C (4837T>G 4838T>C), S1597R (4839T>A 4840C>G), A1599I (4845G>A 4846C>T), G1600C (4848G>T 4850T>C), H1601R (4851C>A 4852A>G 4853C>A), I1602A (4854A>G 4855T>C), S1603I (4857T>A 4858C>T), L1604E (4860T>G 4861T>A), E1605F (4863G>T 4864A>T 4865A>T), L1606S (4866C>T 4867T>C 4868C>T), Q1607N (4869C>A 4871A>T), D1611N (4881G>A), T1612V (4884A>G 4885C>T 4886A>T), N1613I (4888A>T), L1614T (4890C>A 4891T>C 4892C>T), S1615D (4893T>G 4894C>A 4895G>C), V1616K (4896G>A 4897T>A), K1617T (4900A>C 4901G>C), I1619L (4905A>C 4907A>G), Q1621R (4911C>A 4912A>G 4913A>G), I1625S (4924T>G), V1626L (4926G>C), I1629V (4935A>G), R1630A (4938A>G 4939G>C 4940A>T), E1635K (4953G>A), T1637R (4960C>G 4961T>A), E1638Q (4962G>C 4964G>A), H1639V (4965C>G 4966A>T), I1640C (4968A>T 4969T>G), S1641K (4971T>A 4972C>A 4973C>A), P1642S (4974C>T), S1644F (4980A>T 4981G>T), M1646R (4987T>G)									

	Begin	End	Coverage	Score	Concordance	Matches	Identities	I/D/M/F*	Stop Codons
**NT**	**4174**	**4995**	**11.4%**	**56**	**3.4%**	**822 (100%)**	**425 (51.7%)**	**0/0**	
Codon mutations:	TTT1377TAT (4180T>A), AAA1378AGA (4183A>G), AAG1379TTC (4185A>T 4186A>T 4187G>C), GAA1381GAG (4193A>G), AAC1382AAG (4196C>G), ATC1383GCT (4197A>G 4198T>C 4199C>T), AAT1384TTT (4200A>T 4201A>T), CCC1385TCT (4203C>T 4205C>T), ACA1386ACT (4208A>T), AAG1387AAA (4211G>A), GCA1388GCC (4214A>C), AGC1389AGG (4217C>G), CAT1390CCC (4219A>C 4220T>C), TCT1391ATC (4221T>A 4222C>T 4223T>C), GGA1392CAA (4224G>C 4225G>A), AAT1394AAC (4232T>C), CCA1395CAT (4234C>A 4235A>T), CAT1397TTA (4239C>T 4240A>T 4241T>A), CTC1398ATG (4242C>A 4244C>G), CAG1399GAA (4245C>G 4247G>A), TTA1400ATC (4248T>A 4250A>C), GCA1401TGT (4251G>T 4252C>G 4253A>T), ATA1402AAA (4255T>A), AAG1403AAA (4259G>A), TGT1405ATC (4263T>A 4264G>T 4265T>C), GAT1406GAC (4268T>C), GAG1407ACT (4269G>A 4270A>C 4271G>T), CAA1409TTG (4275C>T 4276A>T 4277A>G), CAA1410GAT (4278C>G 4280A>T), TTT1411AGT (4281T>A 4282T>G), GAT1412AAA (4284G>A 4286T>A), CTA1413ATC (4287C>A 4289A>C), ATC1414ATT (4292C>T), GAA1415AGA (4293G>A 4294A>G), CCC1416CCT (4298C>T), TCT1417TTT (4300C>T), GAT1418AAA (4302G>A 4304T>A), TCA1419TCT (4307A>T), CAA1420CCT (4309A>C 4310A>T), GCA1422AGC (4314G>A 4315C>G 4316A>C), GAA1424TCC (4320G>T 4321A>C 4322A>C), TTT1426TTC (4328T>C), GTC1428GTT (4334C>T), AAC1429AAT (4337C>T), AAG1430AAT (4340G>T), AGG1431GTT (4341A>G 4342G>T 4343G>T), TCA1432GCT (4344T>G 4346A>T), GAG1433GAA (4349G>A), CAA1434AAA (4350C>A), GTG1435GAA (4354T>A 4355G>A), AGA1436TGC (4356A>T 4358A>C), GGA1437GAG (4360G>A 4361A>G), AAA1438TGT (4362A>T 4363A>G 4364A>T), TTA1439CCA (4365T>C 4366T>C), AGG1440AGA (4370G>A), TTA1441CTT (4371T>C 4373A>T), GTC1442GTT (4376C>T), ATA1443ATC (4379A>C), CAA1446AAA (4386C>A), CCA1447CCT (4391A>T), TTA1448TTG (4394A>G), AAC1449AAT (4397C>T), CAT1450ACT (4398C>A 4399A>C), TTC1451GCT (4401T>G 4402T>C 4403C>T), CTC1452TTA (4404C>T 4406C>A), CAA1453AAA (4407C>A), GAT1454TGG (4410G>T 4411A>G 4412T>G), GAC1455ATT (4413G>A 4414A>T 4415C>T), AAG1456AGA (4417A>G 4418G>A), TTT1457TAC (4420T>A 4421T>C), CCC1458CCT (4424C>T), CCA1460CCT (4430A>T), AAT1461AAC (4433T>C), AAG1462AAA (4436G>A), CTC1463AAG (4437C>A 4438T>A 4439C>G), ACA1464GAT (4440A>G 4441C>A 4442A>T), CTC1465CTT (4445C>T), TTC1466CTA (4446T>C 4448C>A), TCT1467AAA (4449T>A 4450C>A 4451T>A), CAC1468AGA (4452C>A 4453A>G 4454C>A), TTA1469ACC (4455T>A 4456T>C 4457A>C), TCC1470TTT (4459C>T 4460C>T), AAG1473AAC (4469G>C), CTC1474ATT (4470C>A 4472C>T), TTT1475TAC (4474T>A 4475T>C), TCA1476AGT (4476T>A 4477C>G 4478A>T), AAG1477AAA (4481G>A), CTT1480ATG (4488C>A 4490T>G), AAG1481AAA (4493G>A), GGA1483GGG (4499A>G), TTA1487ATT (4509T>A 4511A>T), GGA1488CAG (4512G>C 4513G>A 4514A>G), ATC1489ATT (4517C>T), CAT1490TCT (4518C>T 4519A>C), CCT1491GAA (4521C>G 4522C>A 4523T>A), AAT1492AAA (4526T>A), GAG1493GAC (4529G>C), AGA1494AAA (4531G>A), CCA1495TAG (4533C>T 4534C>A 4535A>G), AAA1496AAG (4538A>G), ACA1497TCC (4539A>T 4541A>C), GGG1498GCT (4543G>C 4544G>T), TGC1500AAT (4548T>A 4549G>A 4550C>T), ATT1501GTT (4551A>G), CCT1502CCG (4556T>G), GAT1503TTT (4557G>T 4558A>T), AGA1504GGT (4560A>G 4562A>T), CAT1505CAA (4565T>A), TTT1506CAT (4566T>C 4567T>A), CAA1507GAA (4569C>G), AAG1509AAC (4577G>C), GTC1510GTT (4580C>T), CCA1512CTT (4585C>T 4586A>T), TTC1513TTT (4589C>T), GGT1514GGG (4592T>G), ACA1517AAT (4600C>A 4601A>T), CCT1519CCC (4607T>C), TCT1520TCA (4610T>A), TTG1521GAA (4611T>G 4612T>A 4613G>A), TTC1522TTT (4616C>T), GCC1525ATT (4623G>A 4624C>T 4625C>T), ATC1527AAT (4630T>A 4631C>T), AAA1528GAT (4632A>G 4634A>T), TTC1530ATT (4638T>A 4640C>T), CAG1531AAT (4641C>A 4643G>T), CCC1532CAT (4645C>A 4646C>T), ATC1533TTG (4647A>T 4649C>G), CTT1534AGC (4650C>A 4651T>G 4652T>C), TTT1535CAT (4653T>C 4654T>A), TCA1536ATG (4656T>A 4657C>T 4658A>G), GCT1537TCA (4659G>T 4661T>A), TTG1538ATA (4662T>A 4664G>A), GTC1539GTT (4667C>T), TAC1540TAT (4670C>T), GAT1543AAT (4677G>A), ATC1544GTT (4680A>G 4682C>T), CTT1545TTA (4683C>T 4685T>A), CTT1546ATC (4686C>A 4688T>C), TTT1547TTC (4691T>C), GAA1549GAG (4697A>G), ACA1550GAC (4698A>G 4699C>A 4700A>C), CTG1551ATC (4701C>A 4703G>C), GAA1552GAT (4706A>T), GAT1553TCT (4707G>T 4708A>C), CAC1554CAT (4712C>T), ATA1555TTG (4713A>T 4715A>G), TTG1557CAC (4719T>C 4720T>A 4721G>C), CTT1558CTA (4724T>A), AAC1559AAT (4727C>T), CAG1560ATT (4728C>A 4729A>T 4730G>T), TTC1561TTA (4733C>A), ATA1562TTT (4734A>T 4736A>T), AGT1563AAC (4738G>A 4739T>C), TTG1564ACC (4740T>A 4741T>C 4742G>C), GTT1565GTC (4745T>C), AAA1567AGA (4750A>G), TTT1568AAT (4752T>A 4753T>A), GGA1569GGT (4757A>T), GTC1570TTG (4758G>T 4760C>G), ATG1571GTA (4761A>G 4763G>A), TTA1572GTA (4764T>G), TCG1573AGC (4767T>A 4768C>G 4769G>C), GCA1574ACT (4770G>A 4772A>T), ATG1577ATT (4781G>T), ATC1578AAA (4783T>A 4784C>A), GCT1580TTT (4788G>T 4789C>T), AAT1582ACA (4795A>C 4796T>A), CAA1585AGA (4803C>A 4804A>G), TTC1586TTT (4808C>T), TTA1587TTG (4811A>G), GGA1588GGT (4814A>T), ATG1589CAT (4815A>C 4816T>A 4817G>T), TTT1591TTA (4823T>A), GCT1592TAC (4824G>T 4825C>A 4826T>C), GAT1593CAA (4827G>C 4829T>A), GGA1594GGT (4832A>T), ACT1595TCT (4833A>T), TTT1596TGC (4837T>G 4838T>C), TCA1597AGA (4839T>A 4840C>G), CCT1598CCA (4844T>A), GCT1599ATT (4845G>A 4846C>T), GGT1600TGC (4848G>T 4850T>C), CAC1601AGA (4851C>A 4852A>G 4853C>A), ATC1602GCC (4854A>G 4855T>C), TCT1603ATT (4857T>A 4858C>T), TTG1604GAG (4860T>G 4861T>A), GAA1605TTT (4863G>T 4864A>T 4865A>T), CTC1606TCT (4866C>T 4867T>C 4868C>T), CAA1607AAT (4869C>A 4871A>T), AAG1608AAA (4874G>A), CCT1610CCC (4880T>C), GAT1611AAT (4881G>A), ACA1612GTT (4884A>G 4885C>T 4886A>T), AAT1613ATT (4888A>T), CTC1614ACT (4890C>A 4891T>C 4892C>T), TCG1615GAC (4893T>G 4894C>A 4895G>C), GTA1616AAA (4896G>A 4897T>A), AAG1617ACC (4900A>C 4901G>C), CAG1618CAA (4904G>A), ATA1619CTG (4905A>C 4907A>G), CAA1621AGG (4911C>A 4912A>G 4913A>G), CTT1623TTG (4917C>T 4919T>G), GGG1624GGA (4922G>A), ATT1625AGT (4924T>G), GTT1626CTT (4926G>C), AAT1627AAC (4931T>C), ATC1629GTC (4935A>G), AGA1630GCT (4938A>G 4939G>C 4940A>T), GAT1631GAC (4943T>C), TTT1632TTC (4946T>C), CCA1634CCT (4952A>T), GAG1635AAG (4953G>A), ACT1637AGA (4960C>G 4961T>A), GAG1638CAA (4962G>C 4964G>A), CAT1639GTT (4965C>G 4966A>T), ATT1640TGT (4968A>T 4969T>G), TCC1641AAA (4971T>A 4972C>A 4973C>A), CCT1642TCT (4974C>T), CTT1643TTG (4977C>T 4979T>G), AGT1644TTT (4980A>T 4981G>T), GAT1645GAC (4985T>C), ATG1646AGG (4987T>G), CTA1647TTA (4989C>T), AAG1648AAA (4994G>A)								

Proteins

	Begin	End	Coverage	Score	Concordance	Matches	Identities	I/D/M/F*	Stop Codons
ORF I polyprotein (NP_127504.1)	1376	1649	12.6%	808	41.9%	274 (100%)	118 (43.1%)	0/0/0/0	1
Protein mutations:	F1377Y (4180T>A), K1378R (4183A>G), K1379F (4185A>T 4186A>T 4187G>C), N1382K (4196C>G), I1383A (4197A>G 4198T>C 4199C>T), N1384F (4200A>T 4201A>T), P1385S (4203C>T 4205C>T), S1389R (4217C>G), H1390P (4219A>C 4220T>C), S1391I (4221T>A 4222C>T 4223T>C), G1392Q (4224G>C 4225G>A), P1395H (4234C>A 4235A>T), H1397L (4239C>T 4240A>T 4241T>A), L1398M (4242C>A 4244C>G), Q1399E (4245C>G 4247G>A), L1400I (4248T>A 4250A>C), A1401C (4251G>T 4252C>G 4253A>T), I1402K (4255T>A), C1405I (4263T>A 4264G>T 4265T>C), E1407T (4269G>A 4270A>C 4271G>T), Q1409L (4275C>T 4276A>T 4277A>G), Q1410D (4278C>G 4280A>T), F1411S (4281T>A 4282T>G), D1412K (4284G>A 4286T>A), L1413I (4287C>A 4289A>C), E1415R (4293G>A 4294A>G), S1417F (4300C>T), D1418K (4302G>A 4304T>A), Q1420P (4309A>C 4310A>T), A1422S (4314G>A 4315C>G 4316A>C), E1424S (4320G>T 4321A>C 4322A>C), K1430N (4340G>T), R1431V (4341A>G 4342G>T 4343G>T), S1432A (4344T>G 4346A>T), Q1434K (4350C>A), V1435E (4354T>A 4355G>A), R1436C (4356A>T 4358A>C), G1437E (4360G>A 4361A>G), K1438C (4362A>T 4363A>G 4364A>T), L1439P (4365T>C 4366T>C), Q1446K (4386C>A), H1450T (4398C>A 4399A>C), F1451A (4401T>G 4402T>C 4403C>T), Q1453K (4407C>A), D1454W (4410G>T 4411A>G 4412T>G), D1455I (4413G>A 4414A>T 4415C>T), K1456R (4417A>G 4418G>A), F1457Y (4420T>A 4421T>C), L1463K (4437C>A 4438T>A 4439C>G), T1464D (4440A>G 4441C>A 4442A>T), F1466L (4446T>C 4448C>A), S1467K (4449T>A 4450C>A 4451T>A), H1468R (4452C>A 4453A>G 4454C>A), L1469T (4455T>A 4456T>C 4457A>C), S1470F (4459C>T 4460C>T), K1473N (4469G>C), L1474I (4470C>A 4472C>T), F1475Y (4474T>A 4475T>C), L1480M (4488C>A 4490T>G), L1487I (4509T>A 4511A>T), G1488Q (4512G>C 4513G>A 4514A>G), H1490S (4518C>T 4519A>C), P1491E (4521C>G 4522C>A 4523T>A), N1492K (4526T>A), E1493D (4529G>C), R1494K (4531G>A), P1495* (4533C>T 4534C>A 4535A>G), T1497S (4539A>T 4541A>C), G1498A (4543G>C 4544G>T), C1500N (4548T>A 4549G>A 4550C>T), I1501V (4551A>G), D1503F (4557G>T 4558A>T), R1504G (4560A>G 4562A>T), H1505Q (4565T>A), F1506H (4566T>C 4567T>A), Q1507E (4569C>G), K1509N (4577G>C), P1512L (4585C>T 4586A>T), T1517N (4600C>A 4601A>T), L1521E (4611T>G 4612T>A 4613G>A), A1525I (4623G>A 4624C>T 4625C>T), I1527N (4630T>A 4631C>T), K1528D (4632A>G 4634A>T), F1530I (4638T>A 4640C>T), Q1531N (4641C>A 4643G>T), P1532H (4645C>A 4646C>T), I1533L (4647A>T 4649C>G), L1534S (4650C>A 4651T>G 4652T>C), F1535H (4653T>C 4654T>A), S1536M (4656T>A 4657C>T 4658A>G), A1537S (4659G>T 4661T>A), L1538I (4662T>A 4664G>A), D1543N (4677G>A), I1544V (4680A>G 4682C>T), L1546I (4686C>A 4688T>C), T1550D (4698A>G 4699C>A 4700A>C), L1551I (4701C>A 4703G>C), E1552D (4706A>T), D1553S (4707G>T 4708A>C), I1555L (4713A>T 4715A>G), L1557H (4719T>C 4720T>A 4721G>C), Q1560I (4728C>A 4729A>T 4730G>T), F1561L (4733C>A), I1562F (4734A>T 4736A>T), S1563N (4738G>A 4739T>C), L1564T (4740T>A 4741T>C 4742G>C), K1567R (4750A>G), F1568N (4752T>A 4753T>A), V1570L (4758G>T 4760C>G), M1571V (4761A>G 4763G>A), L1572V (4764T>G), A1574T (4770G>A 4772A>T), M1577I (4781G>T), I1578K (4783T>A 4784C>A), A1580F (4788G>T 4789C>T), N1582T (4795A>C 4796T>A), Q1585R (4803C>A 4804A>G), M1589H (4815A>C 4816T>A 4817G>T), F1591L (4823T>A), A1592Y (4824G>T 4825C>A 4826T>C), D1593Q (4827G>C 4829T>A), T1595S (4833A>T), F1596C (4837T>G 4838T>C), S1597R (4839T>A 4840C>G), A1599I (4845G>A 4846C>T), G1600C (4848G>T 4850T>C), H1601R (4851C>A 4852A>G 4853C>A), I1602A (4854A>G 4855T>C), S1603I (4857T>A 4858C>T), L1604E (4860T>G 4861T>A), E1605F (4863G>T 4864A>T 4865A>T), L1606S (4866C>T 4867T>C 4868C>T), Q1607N (4869C>A 4871A>T), D1611N (4881G>A), T1612V (4884A>G 4885C>T 4886A>T), N1613I (4888A>T), L1614T (4890C>A 4891T>C 4892C>T), S1615D (4893T>G 4894C>A 4895G>C), V1616K (4896G>A 4897T>A), K1617T (4900A>C 4901G>C), I1619L (4905A>C 4907A>G), Q1621R (4911C>A 4912A>G 4913A>G), I1625S (4924T>G), V1626L (4926G>C), I1629V (4935A>G), R1630A (4938A>G 4939G>C 4940A>T), E1635K (4953G>A), T1637R (4960C>G 4961T>A), E1638Q (4962G>C 4964G>A), H1639V (4965C>G 4966A>T), I1640C (4968A>T 4969T>G), S1641K (4971T>A 4972C>A 4973C>A), P1642S (4974C>T), S1644F (4980A>T 4981G>T), M1646R (4987T>G)								

	Begin	End	Coverage	Score	Concordance	Matches	Identities	I/D/M/F*	Stop Codons
**NT**	**4174**	**4995**	**11.4%**	**56**	**3.4%**	**822 (100%)**	**425 (51.7%)**	**0/0**	
Codon mutations:	TTT1377TAT (4180T>A), AAA1378AGA (4183A>G), AAG1379TTC (4185A>T 4186A>T 4187G>C), GAA1381GAG (4193A>G), AAC1382AAG (4196C>G), ATC1383GCT (4197A>G 4198T>C 4199C>T), AAT1384TTT (4200A>T 4201A>T), CCC1385TCT (4203C>T 4205C>T), ACA1386ACT (4208A>T), AAG1387AAA (4211G>A), GCA1388GCC (4214A>C), AGC1389AGG (4217C>G), CAT1390CCC (4219A>C 4220T>C), TCT1391ATC (4221T>A 4222C>T 4223T>C), GGA1392CAA (4224G>C 4225G>A), AAT1394AAC (4232T>C), CCA1395CAT (4234C>A 4235A>T), CAT1397TTA (4239C>T 4240A>T 4241T>A), CTC1398ATG (4242C>A 4244C>G), CAG1399GAA (4245C>G 4247G>A), TTA1400ATC (4248T>A 4250A>C), GCA1401TGT (4251G>T 4252C>G 4253A>T), ATA1402AAA (4255T>A), AAG1403AAA (4259G>A), TGT1405ATC (4263T>A 4264G>T 4265T>C), GAT1406GAC (4268T>C), GAG1407ACT (4269G>A 4270A>C 4271G>T), CAA1409TTG (4275C>T 4276A>T 4277A>G), CAA1410GAT (4278C>G 4280A>T), TTT1411AGT (4281T>A 4282T>G), GAT1412AAA (4284G>A 4286T>A), CTA1413ATC (4287C>A 4289A>C), ATC1414ATT (4292C>T), GAA1415AGA (4293G>A 4294A>G), CCC1416CCT (4298C>T), TCT1417TTT (4300C>T), GAT1418AAA (4302G>A 4304T>A), TCA1419TCT (4307A>T), CAA1420CCT (4309A>C 4310A>T), GCA1422AGC (4314G>A 4315C>G 4316A>C), GAA1424TCC (4320G>T 4321A>C 4322A>C), TTT1426TTC (4328T>C), GTC1428GTT (4334C>T), AAC1429AAT (4337C>T), AAG1430AAT (4340G>T), AGG1431GTT (4341A>G 4342G>T 4343G>T), TCA1432GCT (4344T>G 4346A>T), GAG1433GAA (4349G>A), CAA1434AAA (4350C>A), GTG1435GAA (4354T>A 4355G>A), AGA1436TGC (4356A>T 4358A>C), GGA1437GAG (4360G>A 4361A>G), AAA1438TGT (4362A>T 4363A>G 4364A>T), TTA1439CCA (4365T>C 4366T>C), AGG1440AGA (4370G>A), TTA1441CTT (4371T>C 4373A>T), GTC1442GTT (4376C>T), ATA1443ATC (4379A>C), CAA1446AAA (4386C>A), CCA1447CCT (4391A>T), TTA1448TTG (4394A>G), AAC1449AAT (4397C>T), CAT1450ACT (4398C>A 4399A>C), TTC1451GCT (4401T>G 4402T>C 4403C>T), CTC1452TTA (4404C>T 4406C>A), CAA1453AAA (4407C>A), GAT1454TGG (4410G>T 4411A>G 4412T>G), GAC1455ATT (4413G>A 4414A>T 4415C>T), AAG1456AGA (4417A>G 4418G>A), TTT1457TAC (4420T>A 4421T>C), CCC1458CCT (4424C>T), CCA1460CCT (4430A>T), AAT1461AAC (4433T>C), AAG1462AAA (4436G>A), CTC1463AAG (4437C>A 4438T>A 4439C>G), ACA1464GAT (4440A>G 4441C>A 4442A>T), CTC1465CTT (4445C>T), TTC1466CTA (4446T>C 4448C>A), TCT1467AAA (4449T>A 4450C>A 4451T>A), CAC1468AGA (4452C>A 4453A>G 4454C>A), TTA1469ACC (4455T>A 4456T>C 4457A>C), TCC1470TTT (4459C>T 4460C>T), AAG1473AAC (4469G>C), CTC1474ATT (4470C>A 4472C>T), TTT1475TAC (4474T>A 4475T>C), TCA1476AGT (4476T>A 4477C>G 4478A>T), AAG1477AAA (4481G>A), CTT1480ATG (4488C>A 4490T>G), AAG1481AAA (4493G>A), GGA1483GGG (4499A>G), TTA1487ATT (4509T>A 4511A>T), GGA1488CAG (4512G>C 4513G>A 4514A>G), ATC1489ATT (4517C>T), CAT1490TCT (4518C>T 4519A>C), CCT1491GAA (4521C>G 4522C>A 4523T>A), AAT1492AAA (4526T>A), GAG1493GAC (4529G>C), AGA1494AAA (4531G>A), CCA1495TAG (4533C>T 4534C>A 4535A>G), AAA1496AAG (4538A>G), ACA1497TCC (4539A>T 4541A>C), GGG1498GCT (4543G>C 4544G>T), TGC1500AAT (4548T>A 4549G>A 4550C>T), ATT1501GTT (4551A>G), CCT1502CCG (4556T>G), GAT1503TTT (4557G>T 4558A>T), AGA1504GGT (4560A>G 4562A>T), CAT1505CAA (4565T>A), TTT1506CAT (4566T>C 4567T>A), CAA1507GAA (4569C>G), AAG1509AAC (4577G>C), GTC1510GTT (4580C>T), CCA1512CTT (4585C>T 4586A>T), TTC1513TTT (4589C>T), GGT1514GGG (4592T>G), ACA1517AAT (4600C>A 4601A>T), CCT1519CCC (4607T>C), TCT1520TCA (4610T>A), TTG1521GAA (4611T>G 4612T>A 4613G>A), TTC1522TTT (4616C>T), GCC1525ATT (4623G>A 4624C>T 4625C>T), ATC1527AAT (4630T>A 4631C>T), AAA1528GAT (4632A>G 4634A>T), TTC1530ATT (4638T>A 4640C>T), CAG1531AAT (4641C>A 4643G>T), CCC1532CAT (4645C>A 4646C>T), ATC1533TTG (4647A>T 4649C>G), CTT1534AGC (4650C>A 4651T>G 4652T>C), TTT1535CAT (4653T>C 4654T>A), TCA1536ATG (4656T>A 4657C>T 4658A>G), GCT1537TCA (4659G>T 4661T>A), TTG1538ATA (4662T>A 4664G>A), GTC1539GTT (4667C>T), TAC1540TAT (4670C>T), GAT1543AAT (4677G>A), ATC1544GTT (4680A>G 4682C>T), CTT1545TTA (4683C>T 4685T>A), CTT1546ATC (4686C>A 4688T>C), TTT1547TTC (4691T>C), GAA1549GAG (4697A>G), ACA1550GAC (4698A>G 4699C>A 4700A>C), CTG1551ATC (4701C>A 4703G>C), GAA1552GAT (4706A>T), GAT1553TCT (4707G>T 4708A>C), CAC1554CAT (4712C>T), ATA1555TTG (4713A>T 4715A>G), TTG1557CAC (4719T>C 4720T>A 4721G>C), CTT1558CTA (4724T>A), AAC1559AAT (4727C>T), CAG1560ATT (4728C>A 4729A>T 4730G>T), TTC1561TTA (4733C>A), ATA1562TTT (4734A>T 4736A>T), AGT1563AAC (4738G>A 4739T>C), TTG1564ACC (4740T>A 4741T>C 4742G>C), GTT1565GTC (4745T>C), AAA1567AGA (4750A>G), TTT1568AAT (4752T>A 4753T>A), GGA1569GGT (4757A>T), GTC1570TTG (4758G>T 4760C>G), ATG1571GTA (4761A>G 4763G>A), TTA1572GTA (4764T>G), TCG1573AGC (4767T>A 4768C>G 4769G>C), GCA1574ACT (4770G>A 4772A>T), ATG1577ATT (4781G>T), ATC1578AAA (4783T>A 4784C>A), GCT1580TTT (4788G>T 4789C>T), AAT1582ACA (4795A>C 4796T>A), CAA1585AGA (4803C>A 4804A>G), TTC1586TTT (4808C>T), TTA1587TTG (4811A>G), GGA1588GGT (4814A>T), ATG1589CAT (4815A>C 4816T>A 4817G>T), TTT1591TTA (4823T>A), GCT1592TAC (4824G>T 4825C>A 4826T>C), GAT1593CAA (4827G>C 4829T>A), GGA1594GGT (4832A>T), ACT1595TCT (4833A>T), TTT1596TGC (4837T>G 4838T>C), TCA1597AGA (4839T>A 4840C>G), CCT1598CCA (4844T>A), GCT1599ATT (4845G>A 4846C>T), GGT1600TGC (4848G>T 4850T>C), CAC1601AGA (4851C>A 4852A>G 4853C>A), ATC1602GCC (4854A>G 4855T>C), TCT1603ATT (4857T>A 4858C>T), TTG1604GAG (4860T>G 4861T>A), GAA1605TTT (4863G>T 4864A>T 4865A>T), CTC1606TCT (4866C>T 4867T>C 4868C>T), CAA1607AAT (4869C>A 4871A>T), AAG1608AAA (4874G>A), CCT1610CCC (4880T>C), GAT1611AAT (4881G>A), ACA1612GTT (4884A>G 4885C>T 4886A>T), AAT1613ATT (4888A>T), CTC1614ACT (4890C>A 4891T>C 4892C>T), TCG1615GAC (4893T>G 4894C>A 4895G>C), GTA1616AAA (4896G>A 4897T>A), AAG1617ACC (4900A>C 4901G>C), CAG1618CAA (4904G>A), ATA1619CTG (4905A>C 4907A>G), CAA1621AGG (4911C>A 4912A>G 4913A>G), CTT1623TTG (4917C>T 4919T>G), GGG1624GGA (4922G>A), ATT1625AGT (4924T>G), GTT1626CTT (4926G>C), AAT1627AAC (4931T>C), ATC1629GTC (4935A>G), AGA1630GCT (4938A>G 4939G>C 4940A>T), GAT1631GAC (4943T>C), TTT1632TTC (4946T>C), CCA1634CCT (4952A>T), GAG1635AAG (4953G>A), ACT1637AGA (4960C>G 4961T>A), GAG1638CAA (4962G>C 4964G>A), CAT1639GTT (4965C>G 4966A>T), ATT1640TGT (4968A>T 4969T>G), TCC1641AAA (4971T>A 4972C>A 4973C>A), CCT1642TCT (4974C>T), CTT1643TTG (4977C>T 4979T>G), AGT1644TTT (4980A>T 4981G>T), GAT1645GAC (4985T>C), ATG1646AGG (4987T>G), CTA1647TTA (4989C>T), AAG1648AAA (4994G>A)								

*: Inserts / Deletes / Misaligned / Frameshifts

Analysis details

This analysis was performed with panviral2.64

NGS Details (UN8): Badnavirus occultipomeae

Assembly

**Coverage Length**	366 (1 contig(s))
**Depth Of Coverage**	11.3
**Number Of Reads**	40
**Reads Per Million**	0.67 rpm (after QC)
**Ambiguities**	0
**Assembly Method**	de novo + reference guided assembly
**Consensus Caller**	Bcf Tools

Coverage Map

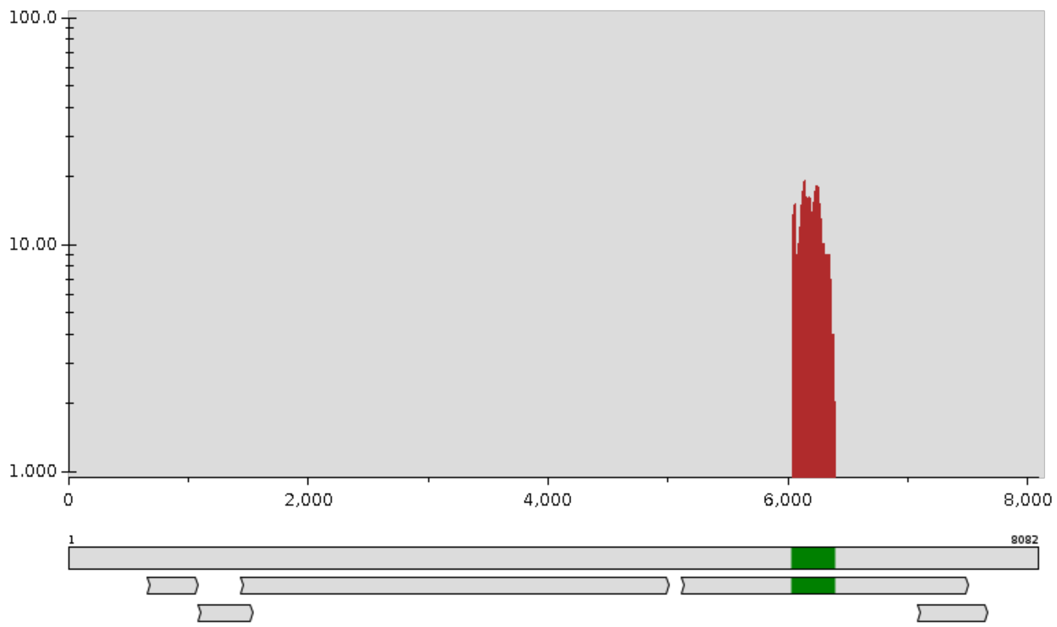

Assignment

**Type**	Badnavirus occultipomeae (Taxonomy ID: 3048353)
**Reference Genome**	NC_015655.1
**NT Identity (%)**	55.0136
**AA Identity (%)**	40.6504
**Number Of Stop Codons**	2
**Number Of CDS**	5

Alignment

**Alignment Score**	68.0 (NT) + 320.0 (AA) = 388.0
**Concordance (%)**	24.5725

Alignment Method	Global, seeded, nucleotide + amino acids (AGA)

Genome Region

Sequence starts at position 6033 and ends at position 6398 relative to NC_015655.1 reference sequence.

Alignment Detailed Statistics

	Begin	End	Coverage	Score	Concordance	Matches	Identities	I/D/M/F*	Stop Codons
**NT**	**6033**	**6398**	**4.5%**	**68**	**9.3%**	**366 (99.2%)**	**203 (55.0%)**	**3/0**	
Mutations:	6033A>T, 6042A>G, 6043T>A, 6045T>A, 6048T>C, 6051C>A, 6053A>G, 6055G>T, 6060C>T, 6062T>A, 6063T>C, 6067C>T, 6070A>T, 6072A>G, 6074G>A, 6075A>G, 6076A>G, 6080A>G, 6081G>A, 6085A>T, 6087G>T, 6088T>G, 6089C>A, 6090C>T, 6092A>T, 6093A>C, 6095C>T, 6096A>G, 6097T>A, 6098G>A, 6099G>A, 6101C>T, 6102T>A, 6103G>A, 6105T>C, 6109T>C, 6114T>C, 6116C>G, 6117A>T, 6118G>T, 6120A>T, 6123G>C, 6125T>A, 6126C>T, 6128A>T, 6130G>A, 6135T>C, 6136G>C, 6137A>T, 6138A>G, 6141C>T, 6145C>T, 6154C>T, 6158T>C, 6159G>T, 6162T>C, 6167C>T, 6168A>T, 6173A>C, 6178C>A, 6179A>T, 6181A>G, 6182G>A, 6183A>T, 6184A>C, 6185A>T, 6186G>T, 6190G>A, 6193A>T, 6194A>G, 6195T>A, 6196G>C, 6197C>T, 6198A>G, 6201T>C, 6203G>A, 6205G>C, 6206G>C, 6207_6208insTGC, 6210A>G, 6214G>A, 6215C>T, 6216A>G, 6219T>C, 6222C>A, 6223G>A, 6224C>T, 6225A>T, 6230A>T, 6243A>T, 6249A>C, 6251T>A, 6255T>C, 6256G>A, 6258A>G, 6264A>G, 6275A>C, 6276G>C, 6277G>A, 6282T>C, 6285C>T, 6286T>A, 6287T>G, 6289A>T, 6290A>T, 6292T>G, 6294G>T, 6295G>C, 6296C>T, 6298C>A, 6302T>C, 6304G>C, 6306G>T, 6308G>A, 6309A>G, 6310A>G, 6311G>A, 6312A>T, 6313G>A, 6314A>G, 6315A>G, 6317G>A, 6319C>T, 6321T>G, 6322A>T, 6323T>A, 6325C>G, 6326T>C, 6329G>A, 6330C>A, 6331C>T, 6332C>T, 6333A>C, 6334A>T, 6339A>G, 6340A>T, 6341T>G, 6342G>T, 6343A>G, 6345A>T, 6346A>T, 6349G>T, 6351A>G, 6352G>A, 6354C>T, 6355A>G, 6356A>C, 6358G>T, 6359A>C, 6365A>T, 6366T>A, 6376A>C, 6377T>A, 6378A>T, 6380A>T, 6381G>T, 6384C>A, 6385C>T, 6386A>C, 6387A>T, 6391A>G, 6394A>G, 6395A>G, 6396G>T								

CDS

	Begin	End	Coverage	Score	Concordance	Matches	Identities	I/D/M/F*	Stop Codons
SPBVa_gp4	307	428	15.3%	320	37.3%	122 (99.2%)	50 (40.7%)	1/0/0/0	2
Protein mutations:	F310I (6043T>A 6045T>A), K313R (6053A>G), A314S (6055G>T), F316Y (6062T>A 6063T>C), Q318* (6067C>T), I319L (6070A>T 6072A>G), R320K (6074G>A 6075A>G), M321V (6076A>G), E322G (6080A>G 6081G>A), K324Y (6085A>T 6087G>T), S325D (6088T>G 6089C>A 6090C>T), K326I (6092A>T 6093A>C), P327L (6095C>T 6096A>G), W328K (6097T>A 6098G>A 6099G>A), T329I (6101C>T 6102T>A), A330T (6103G>A 6105T>C), W332R (6109T>C), P334R (6116C>G 6117A>T), E335Y (6118G>T 6120A>T), L337H (6125T>A 6126C>T), Y338F (6128A>T), E339K (6130G>A), E341L (6136G>C 6137A>T 6138A>G), P344S (6145C>T), M348T (6158T>C 6159G>T), P351L (6167C>T 6168A>T), D353A (6173A>C), Q355M (6178C>A 6179A>T), R356D (6181A>G 6182G>A 6183A>T), K357L (6184A>C 6185A>T 6186G>T), D359N (6190G>A), N360* (6193A>T 6194A>G 6195T>A), A361L (6196G>C 6197C>T 6198A>G), R363K (6203G>A), G364P (6205G>C 6206G>C), G364_T365insC (6207_6208insTGC), A367M (6214G>A 6215C>T 6216A>G), A370I (6223G>A 6224C>T 6225A>T), Y372F (6230A>T), F379Y (6251T>A), E381K (6256G>A 6258A>G), E387A (6275A>C 6276G>C), D388N (6277G>A), L391R (6286T>A 6287T>G), N392F (6289A>T 6290A>T), L393V (6292T>G 6294G>T), A394L (6295G>C 6296C>T), Q395K (6298C>A), I396T (6302T>C), V397L (6304G>C 6306G>T), R398K (6308G>A 6309A>G), R399D (6310A>G 6311G>A 6312A>T), E400R (6313G>A 6314A>G 6315A>G), G401E (6317G>A), I403Y (6322A>T 6323T>A), L404A (6325C>G 6326T>C), S405K (6329G>A 6330C>A), P406F (6331C>T 6332C>T 6333A>C), T407S (6334A>T), M409C (6340A>T 6341T>G 6342G>T), K410D (6343A>G 6345A>T), I411F (6346A>T), G412W (6349G>T 6351A>G), V413I (6352G>A 6354C>T), K414A (6355A>G 6356A>C), E415S (6358G>T 6359A>C), D417V (6365A>T 6366T>A), I421H (6376A>C 6377T>A 6378A>T), K422I (6380A>T 6381G>T), Q424S (6385C>T 6386A>C 6387A>T), N426D (6391A>G), K427G (6394A>G 6395A>G 6396G>T)								
Codon mutations:	ATA306..T (6033A>T), AAA309AAG (6042A>G), TTT310ATA (6043T>A 6045T>A), GAT311GAC (6048T>C), CTC312CTA (6051C>A), AAA313AGA (6053A>G), GCA314TCA (6055G>T), GGC315GGT (6060C>T), TTT316TAC (6062T>A 6063T>C), CAG318TAG (6067C>T), ATA319TTG (6070A>T 6072A>G), AGA320AAG (6074G>A 6075A>G), ATG321GTG (6076A>G), GAG322GGA (6080A>G 6081G>A), AAG324TAT (6085A>T 6087G>T), TCC325GAT (6088T>G 6089C>A 6090C>T), AAA326ATC (6092A>T 6093A>C), CCA327CTG (6095C>T 6096A>G), TGG328AAA (6097T>A 6098G>A 6099G>A), ACT329ATA (6101C>T 6102T>A), GCT330ACC (6103G>A 6105T>C), TGG332CGG (6109T>C), ACT333ACC (6114T>C), CCA334CGT (6116C>G 6117A>T), GAA335TAT (6118G>T 6120A>T), GGG336GGC (6123G>C), CTC337CAT (6125T>A 6126C>T), TAT338TTT (6128A>T), GAA339AAA (6130G>A), TTT340TTC (6135T>C), GAA341CTG (6136G>C 6137A>T 6138A>G), GTC342GTT (6141C>T), CCC344TCC (6145C>T), CTG347TTG (6154C>T), ATG348ACT (6158T>C 6159G>T), AAT349AAC (6162T>C), CCA351CTT (6167C>T 6168A>T), GAT353GCT (6173A>C), CAG355ATG (6178C>A 6179A>T), AGA356GAT (6181A>G 6182G>A 6183A>T), AAG357CTT (6184A>C 6185A>T 6186G>T), GAT359AAT (6190G>A), AAT360TGA (6193A>T 6194A>G 6195T>A), GCA361CTG (6196G>C 6197C>T 6198A>G), TTT362TTC (6201T>C), AGG363AAG (6203G>A), GGA364CCA (6205G>C 6206G>C), GGA364_ACA365insTGC (6207_6208insTGC), ACA365ACG (6210A>G), GCA367ATG (6214G>A 6215C>T 6216A>G), TTT368TTC (6219T>C), ATC369ATA (6222C>A), GCA370ATT (6223G>A 6224C>T 6225A>T), TAT372TTT (6230A>T), ATA376ATT (6243A>T), GTA378GTC (6249A>C), TTC379TAC (6251T>A), TCT380TCC (6255T>C), GAA381AAG (6256G>A 6258A>G), GAA383GAG (6264A>G), GAG387GCC (6275A>C 6276G>C), GAT388AAT (6277G>A), CAT389CAC (6282T>C), CTC390CTT (6285C>T), TTA391AGA (6286T>A 6287T>G), AAT392TTT (6289A>T 6290A>T), TTG393GTT (6292T>G 6294G>T), GCC394CTC (6295G>C 6296C>T), CAA395AAA (6298C>A), ATT396ACT (6302T>C), GTG397CTT (6304G>C 6306G>T), AGA398AAG (6308G>A 6309A>G), AGA399GAT (6310A>G 6311G>A 6312A>T), GAA400AGG (6313G>A 6314A>G 6315A>G), GGG401GAG (6317G>A), CTT402TTG (6319C>T 6321T>G), ATT403TAT (6322A>T 6323T>A), CTA404GCA (6325C>G 6326T>C), AGC405AAA (6329G>A 6330C>A), CCA406TTC (6331C>T 6332C>T 6333A>C), ACA407TCA (6334A>T), AAA408AAG (6339A>G), ATG409TGT (6340A>T 6341T>G 6342G>T), AAA410GAT (6343A>G 6345A>T), ATT411TTT (6346A>T), GGA412TGG (6349G>T 6351A>G), GTC413ATT (6352G>A 6354C>T), AAA414GCA (6355A>G 6356A>C), GAA415TCA (6358G>T 6359A>C), GAT417GTA (6365A>T 6366T>A), ATA421CAT (6376A>C 6377T>A 6378A>T), AAG422ATT (6380A>T 6381G>T), ATC423ATA (6384C>A), CAA424TCT (6385C>T 6386A>C 6387A>T), AAT426GAT (6391A>G), AAG427GGT (6394A>G 6395A>G 6396G>T)								

Proteins

	Begin	End	Coverage	Score	Concordance	Matches	Identities	I/D/M/F*	Stop Codons
RNaseH/reverse transcriptase (YP_004581513.1)	307	428	15.3%	320	37.3%	122 (99.2%)	50 (40.7%)	1/0/0/0	2
Protein mutations:	F310I (6043T>A 6045T>A), K313R (6053A>G), A314S (6055G>T), F316Y (6062T>A 6063T>C), Q318* (6067C>T), I319L (6070A>T 6072A>G), R320K (6074G>A 6075A>G), M321V (6076A>G), E322G (6080A>G 6081G>A), K324Y (6085A>T 6087G>T), S325D (6088T>G 6089C>A 6090C>T), K326I (6092A>T 6093A>C), P327L (6095C>T 6096A>G), W328K (6097T>A 6098G>A 6099G>A), T329I (6101C>T 6102T>A), A330T (6103G>A 6105T>C), W332R (6109T>C), P334R (6116C>G 6117A>T), E335Y (6118G>T 6120A>T), L337H (6125T>A 6126C>T), Y338F (6128A>T), E339K (6130G>A), E341L (6136G>C 6137A>T 6138A>G), P344S (6145C>T), M348T (6158T>C 6159G>T), P351L (6167C>T 6168A>T), D353A (6173A>C), Q355M (6178C>A 6179A>T), R356D (6181A>G 6182G>A 6183A>T), K357L (6184A>C 6185A>T 6186G>T), D359N (6190G>A), N360* (6193A>T 6194A>G 6195T>A), A361L (6196G>C 6197C>T 6198A>G), R363K (6203G>A), G364P (6205G>C 6206G>C), G364_T365insC (6207_6208insTGC), A367M (6214G>A 6215C>T 6216A>G), A370I (6223G>A 6224C>T 6225A>T), Y372F (6230A>T), F379Y (6251T>A), E381K (6256G>A 6258A>G), E387A (6275A>C 6276G>C), D388N (6277G>A), L391R (6286T>A 6287T>G), N392F (6289A>T 6290A>T), L393V (6292T>G 6294G>T), A394L (6295G>C 6296C>T), Q395K (6298C>A), I396T (6302T>C), V397L (6304G>C 6306G>T), R398K (6308G>A 6309A>G), R399D (6310A>G 6311G>A 6312A>T), E400R (6313G>A 6314A>G 6315A>G), G401E (6317G>A), I403Y (6322A>T 6323T>A), L404A (6325C>G 6326T>C), S405K (6329G>A 6330C>A), P406F (6331C>T 6332C>T 6333A>C), T407S (6334A>T), M409C (6340A>T 6341T>G 6342G>T), K410D (6343A>G 6345A>T), I411F (6346A>T), G412W (6349G>T 6351A>G), V413I (6352G>A 6354C>T), K414A (6355A>G 6356A>C), E415S (6358G>T 6359A>C), D417V (6365A>T 6366T>A), I421H (6376A>C 6377T>A 6378A>T), K422I (6380A>T 6381G>T), Q424S (6385C>T 6386A>C 6387A>T), N426D (6391A>G), K427G (6394A>G 6395A>G 6396G>T)								
Codon mutations:	ATA306..T (6033A>T), AAA309AAG (6042A>G), TTT310ATA (6043T>A 6045T>A), GAT311GAC (6048T>C), CTC312CTA (6051C>A), AAA313AGA (6053A>G), GCA314TCA (6055G>T), GGC315GGT (6060C>T), TTT316TAC (6062T>A 6063T>C), CAG318TAG (6067C>T), ATA319TTG (6070A>T 6072A>G), AGA320AAG (6074G>A 6075A>G), ATG321GTG (6076A>G), GAG322GGA (6080A>G 6081G>A), AAG324TAT (6085A>T 6087G>T), TCC325GAT (6088T>G 6089C>A 6090C>T), AAA326ATC (6092A>T 6093A>C), CCA327CTG (6095C>T 6096A>G), TGG328AAA (6097T>A 6098G>A 6099G>A), ACT329ATA (6101C>T 6102T>A), GCT330ACC (6103G>A 6105T>C), TGG332CGG (6109T>C), ACT333ACC (6114T>C), CCA334CGT (6116C>G 6117A>T), GAA335TAT (6118G>T 6120A>T), GGG336GGC (6123G>C), CTC337CAT (6125T>A 6126C>T), TAT338TTT (6128A>T), GAA339AAA (6130G>A), TTT340TTC (6135T>C), GAA341CTG (6136G>C 6137A>T 6138A>G), GTC342GTT (6141C>T), CCC344TCC (6145C>T), CTG347TTG (6154C>T), ATG348ACT (6158T>C 6159G>T), AAT349AAC (6162T>C), CCA351CTT (6167C>T 6168A>T), GAT353GCT (6173A>C), CAG355ATG (6178C>A 6179A>T), AGA356GAT (6181A>G 6182G>A 6183A>T), AAG357CTT (6184A>C 6185A>T 6186G>T), GAT359AAT (6190G>A), AAT360TGA (6193A>T 6194A>G 6195T>A), GCA361CTG (6196G>C 6197C>T 6198A>G), TTT362TTC (6201T>C), AGG363AAG (6203G>A), GGA364CCA (6205G>C 6206G>C), GGA364_ACA365insTGC (6207_6208insTGC), ACA365ACG (6210A>G), GCA367ATG (6214G>A 6215C>T 6216A>G), TTT368TTC (6219T>C), ATC369ATA (6222C>A), GCA370ATT (6223G>A 6224C>T 6225A>T), TAT372TTT (6230A>T), ATA376ATT (6243A>T), GTA378GTC (6249A>C), TTC379TAC (6251T>A), TCT380TCC (6255T>C), GAA381AAG (6256G>A 6258A>G), GAA383GAG (6264A>G), GAG387GCC (6275A>C 6276G>C), GAT388AAT (6277G>A), CAT389CAC (6282T>C), CTC390CTT (6285C>T), TTA391AGA (6286T>A 6287T>G), AAT392TTT (6289A>T 6290A>T), TTG393GTT (6292T>G 6294G>T), GCC394CTC (6295G>C 6296C>T), CAA395AAA (6298C>A), ATT396ACT (6302T>C), GTG397CTT (6304G>C 6306G>T), AGA398AAG (6308G>A 6309A>G), AGA399GAT (6310A>G 6311G>A 6312A>T), GAA400AGG (6313G>A 6314A>G 6315A>G), GGG401GAG (6317G>A), CTT402TTG (6319C>T 6321T>G), ATT403TAT (6322A>T 6323T>A), CTA404GCA (6325C>G 6326T>C), AGC405AAA (6329G>A 6330C>A), CCA406TTC (6331C>T 6332C>T 6333A>C), ACA407TCA (6334A>T), AAA408AAG (6339A>G), ATG409TGT (6340A>T 6341T>G 6342G>T), AAA410GAT (6343A>G 6345A>T), ATT411TTT (6346A>T), GGA412TGG (6349G>T 6351A>G), GTC413ATT (6352G>A 6354C>T), AAA414GCA (6355A>G 6356A>C), GAA415TCA (6358G>T 6359A>C), GAT417GTA (6365A>T 6366T>A), ATA421CAT (6376A>C 6377T>A 6378A>T), AAG422ATT (6380A>T 6381G>T), ATC423ATA (6384C>A), CAA424TCT (6385C>T 6386A>C 6387A>T), AAT426GAT (6391A>G), AAG427GGT (6394A>G 6395A>G 6396G>T)								

*: Inserts / Deletes / Misaligned / Frameshifts

Analysis details

This analysis was performed with panviral2.64

NGS Details (UN8): Caulimovirus tessellobrassicae

Assembly

**Coverage Length**	725 (2 contig(s))
**Depth Of Coverage**	6.6
**Number Of Reads**	39
**Reads Per Million**	0.65 rpm (after QC)
**Ambiguities**	0
**Assembly Method**	de novo + reference guided assembly
**Consensus Caller**	Bcf Tools

Coverage Map

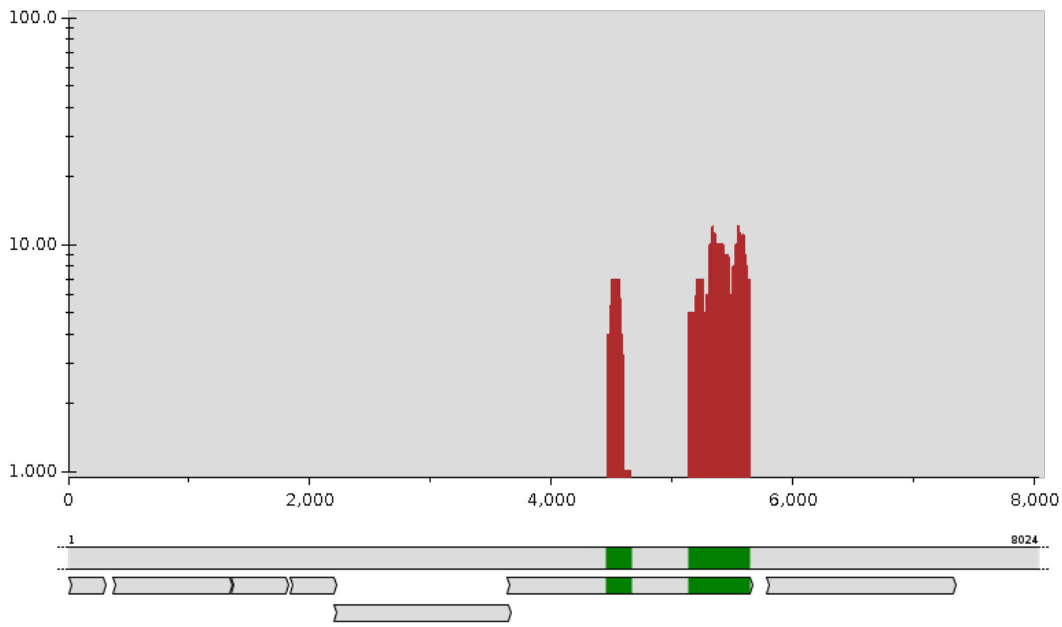

Assignment

**Type**	Caulimovirus tessellobrassicae (Taxonomy ID: 3047738)
**Reference Genome**	NC_001497.2
**NT Identity (%)**	56.6761
**AA Identity (%)**	46.383
**Number Of Stop Codons**	0
**Number Of CDS**	7

Alignment

**Alignment Score**	149.0 (NT) + 792.0 (AA) = 941.0
**Concordance (%)**	31.5772

Alignment Method	Global, seeded, nucleotide + amino acids (AGA)

Genome Region

Sequence starts at position 4456 and ends at position 5647 relative to NC_001497.2 reference sequence.

Alignment Detailed Statistics

	Begin	End	Coverage	Score	Concordance	Matches	Identities	I/D/M/F*	Stop Codons
**NT**	**4456**	**5647**	**9.0%**	**149**	**10.9%**	**704 (97.1%)**	**399 (55.0%)**	**0/21**	
Mutations:	4465A>T, 4467T>C, 4469A>G, 4473C>T, 4476T>A, 4481T>G, 4482G>C, 4483G>A, 4484C>G, 4485A>T, 4488A>C, 4491C>G, 4495T>A, 4500C>A, 4502A>G, 4503C>A, 4506T>C, 4507G>C, 4509A>T, 4512C>G, 4515G>A, 4516A>C, 4518G>A, 4519C>A, 4520G>A, 4522A>C, 4527A>T, 4531A>G, 4532A>C, 4533A>T, 4535G>A, 4536T>A, 4542A>C, 4543G>A, 4545C>A, 4548C>T, 4551C>T, 4556C>A, 4557T>G, 4558A>C, 4560G>T, 4563C>T, 4567G>A, 4568C>A, 4570A>T, 4571C>G, 4575A>T, 4576G>T, 4577G>T, 4578A>T, 4581T>C, 4583C>G, 4584C>A, 4588A>T, 4589A>T, 4591C>A, 4596C>T, 4603G>A, 4606G>A, 4608G>T, 4609T>C, 4611A>T, 4612C>A, 4613T>C, 4616C>A, 4617A>T, 4620C>A, 4621A>G, 4622T>C, 4623T>A, 4624C>A, 4625G>A, 4627G>A, 4632G>A, 4634A>T, 4635G>T, 4636A>T, 4637T>A, 4641C>T, 4644T>A, 4645T>A, 4646C>A, 4647C>A, 4650C>T, 4653C>T, 4660T>A, 4661C>G, 5132T>A, 5133C>G, 5139G>T, 5141C>T, 5143C>T, 5145G>A, 5149G>A, 5150C>G, 5151C>G, 5152A>C, 5153A>T, 5154G>T, 5155C>A, 5158A>C, 5159A>G, 5161G>A, 5164A>C, 5165A>C, 5167G>A, 5168T>A, 5169T>C, 5171C>G, 5173T>A, 5174G>T, 5176A>G, 5185A>G, 5187A>T, 5190G>A, 5191G>C, 5196C>T, 5197C>A, 5198T>G, 5199C>A, 5201A>G, 5202C>T, 5203A>G, 5205G>T, 5210A>G, 5211G>C, 5212G>C, 5214G>A, 5217G>A, 5218A>C, 5221A>G, 5223T>A, 5224C>T, 5225T>G, 5226G>T, 5227C>T, 5228A>C, 5229A>C, 5230G>A, 5231G>A, 5235T>A, 5238T>A, 5239C>A, 5240C>A, 5241A>G, 5245C>A, 5246A>G, 5247T>A, 5249A>T, 5253C>G, 5254T>G, 5255T>A, 5262G>A, 5265G>C, 5268G>C, 5269C>T, 5271G>A, 5272A>G, 5274C>T, 5275A>T, 5277C>A, 5278G>C, 5280G>A, 5283C>A, 5289A>T, 5295C>T, 5296G>T, 5299T>C, 5301C>T, 5309G>C, 5310T>A, 5311A>T, 5313G>A, 5317A>C, 5320G>A, 5323_5325delATC, 5326A>G, 5328A>C, 5329A>C, 5334C>T, 5338_5355delGGTACTAATACTGAGTTA, 5361C>T, 5364A>G, 5367C>T, 5368G>A, 5371T>A, 5372C>G, 5376A>T, 5378G>C, 5379C>A, 5385A>T, 5387C>A, 5388T>A, 5389G>A, 5391A>C, 5396A>C, 5397G>T, 5400T>A, 5404C>T, 5405A>C, 5406C>A, 5408G>C, 5409C>A, 5415C>A, 5421G>A, 5422A>T, 5423C>T, 5430G>C, 5431G>A, 5434A>G, 5438A>G, 5439T>A, 5440A>G, 5441C>G, 5442T>C, 5446A>C, 5447A>G, 5448G>A, 5455A>T, 5456G>C, 5458A>G, 5459T>C, 5462A>T, 5463T>C, 5467A>C, 5468C>T, 5469T>A, 5472T>A, 5473G>A, 5474T>A, 5475T>G, 5478T>A, 5481T>C, 5482C>A, 5484G>C, 5487T>C, 5490G>A, 5491A>T, 5496T>C, 5502T>A, 5505T>G, 5506T>G, 5508C>A, 5509A>T, 5510A>C, 5511G>A, 5512A>G, 5517C>G, 5518G>A, 5520T>A, 5521A>T, 5522A>T, 5524C>A, 5525T>A, 5526C>T, 5529T>A, 5530T>C, 5531A>T, 5532C>T, 5533A>C, 5534A>C, 5536G>T, 5537G>C, 5538A>T, 5541T>A, 5542T>C, 5544G>T, 5545A>C, 5548C>T, 5549T>A, 5551G>A, 5557A>T, 5558A>T, 5559C>A, 5560A>C, 5561T>A, 5562C>T, 5573C>T, 5576G>T, 5578C>T, 5580T>A, 5581A>T, 5582G>C, 5583C>T, 5584C>T, 5585A>T, 5586C>T, 5590T>A, 5592A>C, 5595T>C, 5596G>A, 5598T>A, 5599G>A, 5605C>A, 5607C>T, 5616A>T, 5617A>T, 5619C>T, 5622C>T, 5625C>T, 5626C>G, 5627A>T, 5628C>T, 5634G>A, 5637C>T, 5641C>T, 5643T>A								

CDS

	Begin	End	Coverage	Score	Concordance	Matches	Identities	I/D/M/F*	Stop Codons
CaMVgp6	275	672	35.6%	792	48.5%	235 (97.1%)	109 (45.0%)	0/7/0/0	0
Protein mutations:	S278C (4465A>T 4467T>C), K279R (4469A>G), M283S (4481T>G 4482G>C), A284S (4483G>A 4484C>G 4485A>T), L288M (4495T>A), N290R (4502A>G 4503C>A), E292H (4507G>C 4509A>T), K295Q (4516A>C 4518G>A), R296K (4519C>A 4520G>A), K300A (4531A>G 4532A>C 4533A>T), R301Q (4535G>A 4536T>A), V304I (4543G>A 4545C>A), A308E (4556C>A 4557T>G), M309L (4558A>C 4560G>T), A312N (4567G>A 4568C>A), T313C (4570A>T 4571C>G), G315F (4576G>T 4577G>T 4578A>T), A317G (4583C>G 4584C>A), N319F (4588A>T 4589A>T), L320I (4591C>A), D324N (4603G>A), E325N (4606G>A 4608G>T), L327T (4612C>A 4613T>C), T328N (4616C>A 4617A>T), I330A (4621A>G 4622T>C 4623T>A), R331K (4624C>A 4625G>A), G332R (4627G>A), K334I (4634A>T 4635G>T), I335Y (4636A>T 4637T>A), S338K (4645T>A 4646C>A 4647C>A), I500K (5132T>A 5133C>G), K502N (5139G>T), P503L (5141C>T), A506R (5149G>A 5150C>G 5151C>G), K507L (5152A>C 5153A>T 5154G>T), L508I (5155C>A), K509R (5158A>C 5159A>G), E510K (5161G>A), N511P (5164A>C 5165A>C), V512N (5167G>A 5168T>A 5169T>C), P513R (5171C>G), W514M (5173T>A 5174G>T), R515G (5176A>G), K518D (5185A>G 5187A>T), D520H (5191G>C), L522R (5197C>A 5198T>G 5199C>A), Y523C (5201A>G 5202C>T), M524V (5203A>G 5205G>T), K526S (5210A>G 5211G>C), V527L (5212G>C 5214G>A), K529Q (5218A>C), N530E (5221A>G 5223T>A), L531C (5224C>T 5225T>G 5226G>T), Q532S (5227C>T 5228A>C 5229A>C), G533K (5230G>A 5231G>A), F534L (5235T>A), P536K (5239C>A 5240C>A 5241A>G), H538R (5245C>A 5246A>G 5247T>A), H539L (5249A>T), L541E (5254T>G 5255T>A), E544D (5265G>C), K545N (5268G>C), I547V (5272A>G 5274C>T), I548L (5275A>T 5277C>A), E549Q (5278G>C 5280G>A), D555Y (5296G>T), Y556H (5299T>C 5301C>T), G559A (5309G>C 5310T>A), M560L (5311A>T 5313G>A), K562Q (5317A>C), A563T (5320G>A), I564del (5323_5325delATC), K565D (5326A>G 5328A>C), I566L (5329A>C), G569_L574del (5338_5355delGGTACTAATACTGAGTTA), A579T (5368G>A), S582T (5378G>C 5379C>A), K584N (5385A>T), A585E (5387C>A 5388T>A), A586T (5389G>A 5391A>C), K588T (5396A>C 5397G>T), N589K (5400T>A), H591S (5404C>T 5405A>C 5406C>A), S592T (5408G>C 5409C>A), D594E (5415C>A), T597L (5422A>T 5423C>T), V600I (5431G>A), I601V (5434A>G), N602R (5438A>G 5439T>A), T603G (5440A>G 5441C>G 5442T>C), K605R (5446A>C 5447A>G 5448G>A), I609A (5458A>G 5459T>C), Y610F (5462A>T 5463T>C), T612L (5467A>C 5468C>T 5469T>A), V614K (5473G>A 5474T>A 5475T>G), H615Q (5478T>A), L617I (5482C>A 5484G>C), T620S (5491A>T), H624Q (5505T>G), F625V (5506T>G 5508C>A), K626S (5509A>T 5510A>C 5511G>A), S627G (5512A>G), F628L (5517C>G), V629I (5518G>A 5520T>A), N630F (5521A>T 5522A>T), L631N (5524C>A 5525T>A 5526C>T), N632K (5529T>A), Y633L (5530T>C 5531A>T 5532C>T), K634P (5533A>C 5534A>C), G635S (5536G>T 5537G>C 5538A>T), D636E (5541T>A), S637P (5542T>C 5544G>T), K638Q (5545A>C), L639Y (5548C>T 5549T>A), G640R (5551G>A), N642L (5557A>T 5558A>T 5559C>A), I643H (5560A>C 5561T>A 5562C>T), A647V (5573C>T), W648L (5576G>T), H651F (5584C>T 5585A>T 5586C>T), S653T (5590T>A 5592A>C), D655K (5596G>A 5598T>A), V656I (5599G>A), H658N (5605C>A 5607C>T), T662S (5617A>T 5619C>T), H665V (5626C>G 5627A>T 5628C>T)								
Codon mutations:	AGT278TGC (4465A>T 4467T>C), AAA279AGA (4469A>G), AGC280AGT (4473C>T), CCT281CCA (4476T>A), ATG283AGC (4481T>G 4482G>C), GCA284AGT (4483G>A 4484C>G 4485A>T), CCA285CCC (4488A>C), GCC286GCG (4491C>G), TTG288ATG (4495T>A), GTC289GTA (4500C>A), AAC290AGA (4502A>G 4503C>A), AAT291AAC (4506T>C), GAA292CAT (4507G>C 4509A>T), GCC293GCG (4512C>G), GAG294GAA (4515G>A), AAG295CAA (4516A>C 4518G>A), CGA296AAA (4519C>A 4520G>A), AGA297CGA (4522A>C), GGA298GGT (4527A>T), AAA300GCT (4531A>G 4532A>C 4533A>T), CGT301CAA (4535G>A 4536T>A), GTA303GTC (4542A>C), GTC304ATA (4543G>A 4545C>A), AAC305AAT (4548C>T), TAC306TAT (4551C>T), GCT308GAG (4556C>A 4557T>G), ATG309CTT (4558A>C 4560G>T), AAC310AAT (4563C>T), GCT312AAT (4567G>A 4568C>A), ACT313TGT (4570A>T 4571C>G), GTA314GTT (4575A>T), GGA315TTT (4576G>T 4577G>T 4578A>T), GAT316GAC (4581T>C), GCC317GGA (4583C>G 4584C>A), AAT319TTT (4588A>T 4589A>T), CTT320ATT (4591C>A), CCC321CCT (4596C>T), GAC324AAC (4603G>A), GAG325AAT (4606G>A 4608G>T), TTA326CTT (4609T>C 4611A>T), CTT327ACT (4612C>A 4613T>C), ACA328AAT (4616C>A 4617A>T), CTC329CTA (4620C>A), ATT330GCA (4621A>G 4622T>C 4623T>A), CGA331AAA (4624C>A 4625G>A), GGA332AGA (4627G>A), AAG333AAA (4632G>A), AAG334ATT (4634A>T 4635G>T), ATC335TAC (4636A>T 4637T>A), TTC336TTT (4641C>T), TCT337TCA (4644T>A), TCC338AAA (4645T>A 4646C>A 4647C>A), TTC339TTT (4650C>T), GAC340GAT (4653C>T), TCA343AG. (4660T>A 4661C>G), ATC500AAG (5132T>A 5133C>G), AAG502AAT (5139G>T), CCT503CTT (5141C>T), CTG504TTA (5143C>T 5145G>A), GCC506AGG (5149G>A 5150C>G 5151C>G), AAG507CTT (5152A>C 5153A>T 5154G>T), CTT508ATT (5155C>A), AAA509CGA (5158A>C 5159A>G), GAA510AAA (5161G>A), AAC511CCC (5164A>C 5165A>C), GTT512AAC (5167G>A 5168T>A 5169T>C), CCA513CGA (5171C>G), TGG514ATG (5173T>A 5174G>T), AGA515GGA (5176A>G), AAA518GAT (5185A>G 5187A>T), GAG519GAA (5190G>A), GAT520CAT (5191G>C), ACC521ACT (5196C>T), CTC522AGA (5197C>A 5198T>G 5199C>A), TAC523TGT (5201A>G 5202C>T), ATG524GTT (5203A>G 5205G>T), AAG526AGC (5210A>G 5211G>C), GTG527CTA (5212G>C 5214G>A), AAG528AAA (5217G>A), AAA529CAA (5218A>C), AAT530GAA (5221A>G 5223T>A), CTG531TGT (5224C>T 5225T>G 5226G>T), CAA532TCC (5227C>T 5228A>C 5229A>C), GGA533AAA (5230G>A 5231G>A), TTT534TTA (5235T>A), CCT535CCA (5238T>A), CCA536AAG (5239C>A 5240C>A 5241A>G), CAT538AGA (5245C>A 5246A>G 5247T>A), CAT539CTT (5249A>T), CCC540CCG (5253C>G), TTA541GAA (5254T>G 5255T>A), GAG543GAA (5262G>A), GAG544GAC (5265G>C), AAG545AAC (5268G>C), CTG546TTA (5269C>T 5271G>A), ATC547GTT (5272A>G 5274C>T), ATC548TTA (5275A>T 5277C>A), GAG549CAA (5278G>C 5280G>A), ACC550ACA (5283C>A), GCA552GCT (5289A>T), GAC554GAT (5295C>T), GAC555TAC (5296G>T), TAC556CAT (5299T>C 5301C>T), GGT559GCA (5309G>C 5310T>A), ATG560TTA (5311A>T 5313G>A), AAA562CAA (5317A>C), GCT563ACT (5320G>A), ATC564del (5323_5325delATC), AAA565GAC (5326A>G 5328A>C), ATT566CTT (5329A>C), AAC567AAT (5334C>T), GGT569_TTA574del (5338_5355delGGTACTAATACTGAGTTA), TGC576TGT (5361C>T), AGA577AGG (5364A>G), TAC578TAT (5367C>T), GCA579ACA (5368G>A), TCT580AGT (5371T>A 5372C>G), GGA581GGT (5376A>T), AGC582ACA (5378G>C 5379C>A), AAA584AAT (5385A>T), GCT585GAA (5387C>A 5388T>A), GCA586ACC (5389G>A 5391A>C), AAG588ACT (5396A>C 5397G>T), AAT589AAA (5400T>A), CAC591TCA (5404C>T 5405A>C 5406C>A), AGC592ACA (5408G>C 5409C>A), GAC594GAA (5415C>A), GAG596GAA (5421G>A), ACA597TTA (5422A>T 5423C>T), GCG599GCC (5430G>C), GTA600ATA (5431G>A), ATA601GTA (5434A>G), AAT602AGA (5438A>G 5439T>A), ACT603GGC (5440A>G 5441C>G 5442T>C), AAG605CGA (5446A>C 5447A>G 5448G>A), AGT608TCT (5455A>T 5456G>C), ATT609GCT (5458A>G 5459T>C), TAT610TTC (5462A>T 5463T>C), ACT612CTA (5467A>C 5468C>T 5469T>A), CCT613CCA (5472T>A), GTT614AAG (5473G>A 5474T>A 5475T>G), CAT615CAA (5478T>A), TTT616TTC (5481T>C), CTG617ATC (5482C>A 5484G>C), ATT618ATC (5487T>C), AGG619AGA (5490G>A), ACA620TCA (5491A>T), GAT621GAC (5496T>C), ACT623ACA (5502T>A), CAT624CAG (5505T>G), TTC625GTA (5506T>G 5508C>A), AAG626TCA (5509A>T 5510A>C 5511G>A), AGT627GGT (5512A>G), TTC628TTG (5517C>G), GTT629ATA (5518G>A 5520T>A), AAT630TTT (5521A>T 5522A>T), CTC631AAT (5524C>A 5525T>A 5526C>T), AAT632AAA (5529T>A), TAC633CTT (5530T>C 5531A>T 5532C>T), AAA634CCA (5533A>C 5534A>C), GGA635TCT (5536G>T 5537G>C 5538A>T), GAT636GAA (5541T>A), TCG637CCT (5542T>C 5544G>T), AAA638CAA (5545A>C), CTT639TAT (5548C>T 5549T>A), GGA640AGA (5551G>A), AAC642TTA (5557A>T 5558A>T 5559C>A), ATC643CAT (5560A>C 5561T>A 5562C>T), GCA647GTA (5573C>T), TGG648TTG (5576G>T), CTT649TTA (5578C>T 5580T>A), AGC650TCT (5581A>T 5582G>C 5583C>T), CAC651TTT (5584C>T 5585A>T 5586C>T), TCA653ACC (5590T>A 5592A>C), TTT654TTC (5595T>C), GAT655AAA (5596G>A 5598T>A), GTT656ATT (5599G>A), CAC658AAT (5605C>A 5607C>T), GGA661GGT (5616A>T), ACC662TCT (5617A>T 5619C>T), GAC663GAT (5622C>T), AAC664AAT (5625C>T), CAC665GTT (5626C>G 5627A>T 5628C>T), GCG667GCA (5634G>A), GAC668GAT (5637C>T), CTT670TTA (5641C>T 5643T>A)								

Proteins

	Begin	End	Coverage	Score	Concordance	Matches	Identities	I/D/M/F*	Stop Codons
reverse transcriptase (NP_056728.1)	275	672	35.6%	792	48.5%	235 (97.1%)	109 (45.0%)	0/7/0/0	0

	Begin	End	Coverage	Score	Concordance	Matches	Identities	I/D/M/F*	Stop Codons
**NT**	**4456**	**5647**	**9.0%**	**149**	**10.9%**	**704 (97.1%)**	**399 (55.0%)**	**0/21**	
Protein mutations:	S278C (4465A>T 4467T>C), K279R (4469A>G), M283S (4481T>G 4482G>C), A284S (4483G>A 4484C>G 4485A>T), L288M (4495T>A), N290R (4502A>G 4503C>A), E292H (4507G>C 4509A>T), K295Q (4516A>C 4518G>A), R296K (4519C>A 4520G>A), K300A (4531A>G 4532A>C 4533A>T), R301Q (4535G>A 4536T>A), V304I (4543G>A 4545C>A), A308E (4556C>A 4557T>G), M309L (4558A>C 4560G>T), A312N (4567G>A 4568C>A), T313C (4570A>T 4571C>G), G315F (4576G>T 4577G>T 4578A>T), A317G (4583C>G 4584C>A), N319F (4588A>T 4589A>T), L320I (4591C>A), D324N (4603G>A), E325N (4606G>A 4608G>T), L327T (4612C>A 4613T>C), T328N (4616C>A 4617A>T), I330A (4621A>G 4622T>C 4623T>A), R331K (4624C>A 4625G>A), G332R (4627G>A), K334I (4634A>T 4635G>T), I335Y (4636A>T 4637T>A), S338K (4645T>A 4646C>A 4647C>A), I500K (5132T>A 5133C>G), K502N (5139G>T), P503L (5141C>T), A506R (5149G>A 5150C>G 5151C>G), K507L (5152A>C 5153A>T 5154G>T), L508I (5155C>A), K509R (5158A>C 5159A>G), E510K (5161G>A), N511P (5164A>C 5165A>C), V512N (5167G>A 5168T>A 5169T>C), P513R (5171C>G), W514M (5173T>A 5174G>T), R515G (5176A>G), K518D (5185A>G 5187A>T), D520H (5191G>C), L522R (5197C>A 5198T>G 5199C>A), Y523C (5201A>G 5202C>T), M524V (5203A>G 5205G>T), K526S (5210A>G 5211G>C), V527L (5212G>C 5214G>A), K529Q (5218A>C), N530E (5221A>G 5223T>A), L531C (5224C>T 5225T>G 5226G>T), Q532S (5227C>T 5228A>C 5229A>C), G533K (5230G>A 5231G>A), F534L (5235T>A), P536K (5239C>A 5240C>A 5241A>G), H538R (5245C>A 5246A>G 5247T>A), H539L (5249A>T), L541E (5254T>G 5255T>A), E544D (5265G>C), K545N (5268G>C), I547V (5272A>G 5274C>T), I548L (5275A>T 5277C>A), E549Q (5278G>C 5280G>A), D555Y (5296G>T), Y556H (5299T>C 5301C>T), G559A (5309G>C 5310T>A), M560L (5311A>T 5313G>A), K562Q (5317A>C), A563T (5320G>A), I564del (5323_5325delATC), K565D (5326A>G 5328A>C), I566L (5329A>C), G569_L574del (5338_5355delGGTACTAATACTGAGTTA), A579T (5368G>A), S582T (5378G>C 5379C>A), K584N (5385A>T), A585E (5387C>A 5388T>A), A586T (5389G>A 5391A>C), K588T (5396A>C 5397G>T), N589K (5400T>A), H591S (5404C>T 5405A>C 5406C>A), S592T (5408G>C 5409C>A), D594E (5415C>A), T597L (5422A>T 5423C>T), V600I (5431G>A), I601V (5434A>G), N602R (5438A>G 5439T>A), T603G (5440A>G 5441C>G 5442T>C), K605R (5446A>C 5447A>G 5448G>A), I609A (5458A>G 5459T>C), Y610F (5462A>T 5463T>C), T612L (5467A>C 5468C>T 5469T>A), V614K (5473G>A 5474T>A 5475T>G), H615Q (5478T>A), L617I (5482C>A 5484G>C), T620S (5491A>T), H624Q (5505T>G), F625V (5506T>G 5508C>A), K626S (5509A>T 5510A>C 5511G>A), S627G (5512A>G), F628L (5517C>G), V629I (5518G>A 5520T>A), N630F (5521A>T 5522A>T), L631N (5524C>A 5525T>A 5526C>T), N632K (5529T>A), Y633L (5530T>C 5531A>T 5532C>T), K634P (5533A>C 5534A>C), G635S (5536G>T 5537G>C 5538A>T), D636E (5541T>A), S637P (5542T>C 5544G>T), K638Q (5545A>C), L639Y (5548C>T 5549T>A), G640R (5551G>A), N642L (5557A>T 5558A>T 5559C>A), I643H (5560A>C 5561T>A 5562C>T), A647V (5573C>T), W648L (5576G>T), H651F (5584C>T 5585A>T 5586C>T), S653T (5590T>A 5592A>C), D655K (5596G>A 5598T>A), V656I (5599G>A), H658N (5605C>A 5607C>T), T662S (5617A>T 5619C>T), H665V (5626C>G 5627A>T 5628C>T)								
Codon mutations:	AGT278TGC (4465A>T 4467T>C), AAA279AGA (4469A>G), AGC280AGT (4473C>T), CCT281CCA (4476T>A), ATG283AGC (4481T>G 4482G>C), GCA284AGT (4483G>A 4484C>G 4485A>T), CCA285CCC (4488A>C), GCC286GCG (4491C>G), TTG288ATG (4495T>A), GTC289GTA (4500C>A), AAC290AGA (4502A>G 4503C>A), AAT291AAC (4506T>C), GAA292CAT (4507G>C 4509A>T), GCC293GCG (4512C>G), GAG294GAA (4515G>A), AAG295CAA (4516A>C 4518G>A), CGA296AAA (4519C>A 4520G>A), AGA297CGA (4522A>C), GGA298GGT (4527A>T), AAA300GCT (4531A>G 4532A>C 4533A>T), CGT301CAA (4535G>A 4536T>A), GTA303GTC (4542A>C), GTC304ATA (4543G>A 4545C>A), AAC305AAT (4548C>T), TAC306TAT (4551C>T), GCT308GAG (4556C>A 4557T>G), ATG309CTT (4558A>C 4560G>T), AAC310AAT (4563C>T), GCT312AAT (4567G>A 4568C>A), ACT313TGT (4570A>T 4571C>G), GTA314GTT (4575A>T), GGA315TTT (4576G>T 4577G>T 4578A>T), GAT316GAC (4581T>C), GCC317GGA (4583C>G 4584C>A), AAT319TTT (4588A>T 4589A>T), CTT320ATT (4591C>A), CCC321CCT (4596C>T), GAC324AAC (4603G>A), GAG325AAT (4606G>A 4608G>T), TTA326CTT (4609T>C 4611A>T), CTT327ACT (4612C>A 4613T>C), ACA328AAT (4616C>A 4617A>T), CTC329CTA (4620C>A), ATT330GCA (4621A>G 4622T>C 4623T>A), CGA331AAA (4624C>A 4625G>A), GGA332AGA (4627G>A), AAG333AAA (4632G>A), AAG334ATT (4634A>T 4635G>T), ATC335TAC (4636A>T 4637T>A), TTC336TTT (4641C>T), TCT337TCA (4644T>A), TCC338AAA (4645T>A 4646C>A 4647C>A), TTC339TTT (4650C>T), GAC340GAT (4653C>T), TCA343AG. (4660T>A 4661C>G), ATC500AAG (5132T>A 5133C>G), AAG502AAT (5139G>T), CCT503CTT (5141C>T), CTG504TTA (5143C>T 5145G>A), GCC506AGG (5149G>A 5150C>G 5151C>G), AAG507CTT (5152A>C 5153A>T 5154G>T), CTT508ATT (5155C>A), AAA509CGA (5158A>C 5159A>G), GAA510AAA (5161G>A), AAC511CCC (5164A>C 5165A>C), GTT512AAC (5167G>A 5168T>A 5169T>C), CCA513CGA (5171C>G), TGG514ATG (5173T>A 5174G>T), AGA515GGA (5176A>G), AAA518GAT (5185A>G 5187A>T), GAG519GAA (5190G>A), GAT520CAT (5191G>C), ACC521ACT (5196C>T), CTC522AGA (5197C>A 5198T>G 5199C>A), TAC523TGT (5201A>G 5202C>T), ATG524GTT (5203A>G 5205G>T), AAG526AGC (5210A>G 5211G>C), GTG527CTA (5212G>C 5214G>A), AAG528AAA (5217G>A), AAA529CAA (5218A>C), AAT530GAA (5221A>G 5223T>A), CTG531TGT (5224C>T 5225T>G 5226G>T), CAA532TCC (5227C>T 5228A>C 5229A>C), GGA533AAA (5230G>A 5231G>A), TTT534TTA (5235T>A), CCT535CCA (5238T>A), CCA536AAG (5239C>A 5240C>A 5241A>G), CAT538AGA (5245C>A 5246A>G 5247T>A), CAT539CTT (5249A>T), CCC540CCG (5253C>G), TTA541GAA (5254T>G 5255T>A), GAG543GAA (5262G>A), GAG544GAC (5265G>C), AAG545AAC (5268G>C), CTG546TTA (5269C>T 5271G>A), ATC547GTT (5272A>G 5274C>T), ATC548TTA (5275A>T 5277C>A), GAG549CAA (5278G>C 5280G>A), ACC550ACA (5283C>A), GCA552GCT (5289A>T), GAC554GAT (5295C>T), GAC555TAC (5296G>T), TAC556CAT (5299T>C 5301C>T), GGT559GCA (5309G>C 5310T>A), ATG560TTA (5311A>T 5313G>A), AAA562CAA (5317A>C), GCT563ACT (5320G>A), ATC564del (5323_5325delATC), AAA565GAC (5326A>G 5328A>C), ATT566CTT (5329A>C), AAC567AAT (5334C>T), GGT569_TTA574del (5338_5355delGGTACTAATACTGAGTTA), TGC576TGT (5361C>T), AGA577AGG (5364A>G), TAC578TAT (5367C>T), GCA579ACA (5368G>A), TCT580AGT (5371T>A 5372C>G), GGA581GGT (5376A>T), AGC582ACA (5378G>C 5379C>A), AAA584AAT (5385A>T), GCT585GAA (5387C>A 5388T>A), GCA586ACC (5389G>A 5391A>C), AAG588ACT (5396A>C 5397G>T), AAT589AAA (5400T>A), CAC591TCA (5404C>T 5405A>C 5406C>A), AGC592ACA (5408G>C 5409C>A), GAC594GAA (5415C>A), GAG596GAA (5421G>A), ACA597TTA (5422A>T 5423C>T), GCG599GCC (5430G>C), GTA600ATA (5431G>A), ATA601GTA (5434A>G), AAT602AGA (5438A>G 5439T>A), ACT603GGC (5440A>G 5441C>G 5442T>C), AAG605CGA (5446A>C 5447A>G 5448G>A), AGT608TCT (5455A>T 5456G>C), ATT609GCT (5458A>G 5459T>C), TAT610TTC (5462A>T 5463T>C), ACT612CTA (5467A>C 5468C>T 5469T>A), CCT613CCA (5472T>A), GTT614AAG (5473G>A 5474T>A 5475T>G), CAT615CAA (5478T>A), TTT616TTC (5481T>C), CTG617ATC (5482C>A 5484G>C), ATT618ATC (5487T>C), AGG619AGA (5490G>A), ACA620TCA (5491A>T), GAT621GAC (5496T>C), ACT623ACA (5502T>A), CAT624CAG (5505T>G), TTC625GTA (5506T>G 5508C>A), AAG626TCA (5509A>T 5510A>C 5511G>A), AGT627GGT (5512A>G), TTC628TTG (5517C>G), GTT629ATA (5518G>A 5520T>A), AAT630TTT (5521A>T 5522A>T), CTC631AAT (5524C>A 5525T>A 5526C>T), AAT632AAA (5529T>A), TAC633CTT (5530T>C 5531A>T 5532C>T), AAA634CCA (5533A>C 5534A>C), GGA635TCT (5536G>T 5537G>C 5538A>T), GAT636GAA (5541T>A), TCG637CCT (5542T>C 5544G>T), AAA638CAA (5545A>C), CTT639TAT (5548C>T 5549T>A), GGA640AGA (5551G>A), AAC642TTA (5557A>T 5558A>T 5559C>A), ATC643CAT (5560A>C 5561T>A 5562C>T), GCA647GTA (5573C>T), TGG648TTG (5576G>T), CTT649TTA (5578C>T 5580T>A), AGC650TCT (5581A>T 5582G>C 5583C>T), CAC651TTT (5584C>T 5585A>T 5586C>T), TCA653ACC (5590T>A 5592A>C), TTT654TTC (5595T>C), GAT655AAA (5596G>A 5598T>A), GTT656ATT (5599G>A), CAC658AAT (5605C>A 5607C>T), GGA661GGT (5616A>T), ACC662TCT (5617A>T 5619C>T), GAC663GAT (5622C>T), AAC664AAT (5625C>T), CAC665GTT (5626C>G 5627A>T 5628C>T), GCG667GCA (5634G>A), GAC668GAT (5637C>T), CTT670TTA (5641C>T 5643T>A)								

*: Inserts / Deletes / Misaligned / Frameshifts

Analysis details

This analysis was performed with panviral2.64

NGS Details (UN8): Caulimovirus venafragariae

Assembly

**Coverage Length**	425 (1 contig(s))
**Depth Of Coverage**	11.4
**Number Of Reads**	39
**Reads Per Million**	0.65 rpm (after QC)
**Ambiguities**	0
**Assembly Method**	de novo + reference guided assembly
**Consensus Caller**	Bcf Tools

Coverage Map

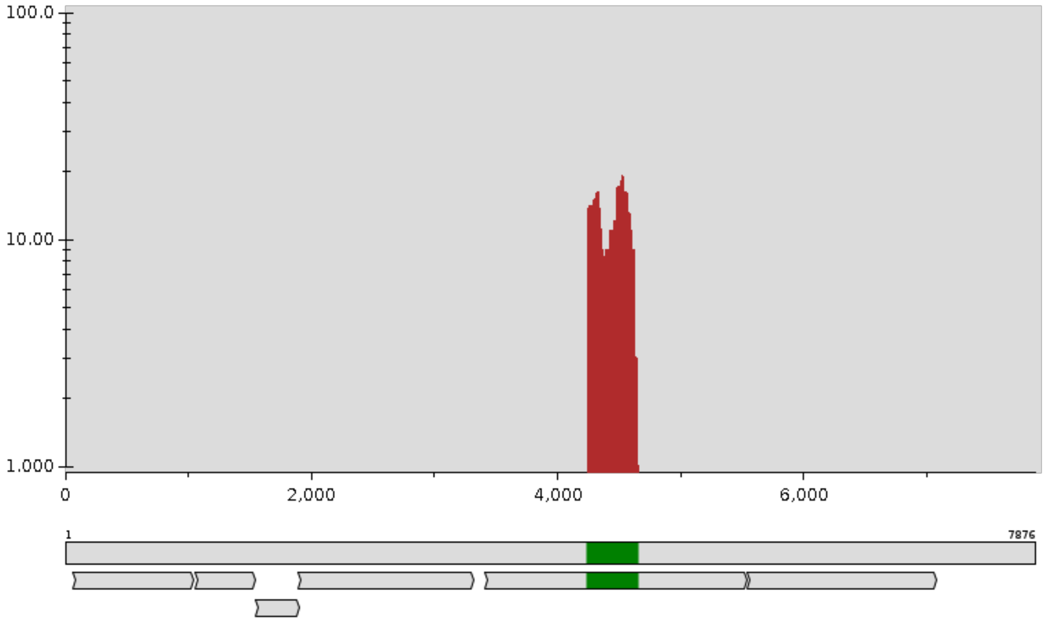

Assignment

**Type**	Caulimovirus venafragariae (Taxonomy ID: 3048344)
**Reference Genome**	NC_001725.1
**NT Identity (%)**	64.0662
**AA Identity (%)**	67.6056
**Number Of Stop Codons**	0
**Number Of CDS**	6

Alignment

**Alignment Score**	227.0 (NT) + 590.0 (AA) = 817.0
**Concordance (%)**	48.2386

Alignment Method	Global, seeded, nucleotide + amino acids (AGA)

Genome Region

Sequence starts at position 4236 and ends at position 4660 relative to NC_001725.1 reference sequence.

Alignment Detailed Statistics

	Begin	End	Coverage	Score	Concordance	Matches	Identities	I/D/M/F*	Stop Codons
**NT**	**4236**	**4660**	**5.4%**	**227**	**27.2%**	**423 (99.5%)**	**271 (63.8%)**	**0/2**	
Mutations:	4236A>C, 4242T>A, 4244A>G, 4245G>A, 4246T>G, 4247G>T, 4259A>T, 4260G>A, 4264T>A, 4265C>A, 4266A>T, 4267C>G, 4268C>A, 4269T>G, 4275C>T, 4276C>A, 4277G>A, 4279G>A, 4280A>C, 4281A>T, 4284G>A, 4292C>T, 4299C>T, 4300G>C, 4306C>T, 4309C>T, 4311C>A, 4314G>T, 4315C>A, 4317T>G, 4318G>A, 4319G>A, 4320T>A, 4321A>T, 4323C>A, 4326C>A, 4330_4331delCC, 4332T>C, 4335C>T, 4338G>A, 4341T>C, 4344A>G, 4348T>A, 4349C>G, 4350C>T, 4351T>A, 4352C>G, 4353A>T, 4359C>A, 4371G>A, 4374T>C, 4380C>G, 4381G>A, 4383G>A, 4384A>C, 4385T>A, 4386C>A, 4395C>A, 4401A>T, 4402A>C, 4413T>A, 4423A>G, 4426T>C, 4428A>T, 4432G>A, 4434C>A, 4435C>A, 4438A>T, 4439C>G, 4440A>T, 4441A>G, 4442A>T, 4443G>C, 4444G>T, 4445G>T, 4446A>C, 4449T>C, 4452C>A, 4455C>T, 4456C>T, 4458A>C, 4459C>A, 4461T>C, 4464T>A, 4467C>T, 4470G>A, 4471G>A, 4473G>T, 4474C>A, 4476A>C, 4479G>T, 4480C>A, 4482T>A, 4483C>A, 4485A>T, 4486A>C, 4487G>T, 4488A>T, 4489A>G, 4490T>C, 4491C>A, 4492G>A, 4493G>A, 4497T>A, 4500G>A, 4505T>A, 4506T>C, 4508A>T, 4509C>T, 4512T>A, 4513T>A, 4514C>A, 4515C>A, 4521C>T, 4524T>C, 4528T>A, 4529C>G, 4542G>A, 4543G>A, 4546C>A, 4547G>A, 4548C>A, 4551T>A, 4553C>A, 4554T>A, 4555C>G, 4556C>A, 4558G>A, 4560A>T, 4561A>T, 4563A>T, 4566T>A, 4567C>G, 4568A>C, 4569G>T, 4570C>T, 4575C>A, 4578T>A, 4584C>T, 4585T>A, 4586G>C, 4587T>A, 4590C>A, 4591C>A, 4593A>T, 4614C>G, 4633C>A, 4635A>T, 4642G>C, 4643C>A, 4644T>A, 4647T>C, 4650C>T								

CDS

	Begin	End	Coverage	Score	Concordance	Matches	Identities	I/D/M/F*	Stop Codons
ORF_V	278	419	20.0%	590	57.0%	142 (100%)	96 (67.6%)	0/0/1/1	0
Protein mutations:	K280R (4244A>G 4245G>A), C281V (4246T>G 4247G>T), Q285L (4259A>T 4260G>A), S287N (4264T>A 4265C>A 4266A>T), P288E (4267C>G 4268C>A 4269T>G), R291K (4276C>A 4277G>A), E292T (4279G>A 4280A>C 4281A>T), T296I (4292C>T), E299Q (4300G>C), K303N (4314G>T), L304M (4315C>A 4317T>G), G305K (4318G>A 4319G>A 4320T>A), I306L (4321A>T 4323C>A), E326K (4381G>A 4383G>A), I327Q (4384A>C 4385T>A 4386C>A), K340E (4423A>G), D343K (4432G>A 4434C>A), H344N (4435C>A), T345C (4438A>T 4439C>G 4440A>T), K346V (4441A>G 4442A>T 4443G>C), G347F (4444G>T 4445G>T 4446A>C), L351F (4456C>T 4458A>C), L352I (4459C>A 4461T>C), E356N (4471G>A 4473G>T), Q357N (4474C>A 4476A>C), L359I (4480C>A 4482T>A), Q360N (4483C>A 4485A>T), R361L (4486A>C 4487G>T 4488A>T), I362A (4489A>G 4490T>C 4491C>A), G363K (4492G>A 4493G>A), F367Y (4505T>A 4506T>C), Y368F (4508A>T 4509C>T), S370K (4513T>A 4514C>A 4515C>A), V380I (4543G>A), R381K (4546C>A 4547G>A 4548C>A), A383E (4553C>A 4554T>A), P384E (4555C>G 4556C>A), E385N (4558G>A 4560A>T), T386S (4561A>T 4563A>T), Q388A (4567C>G 4568A>C 4569G>T), C394T (4585T>A 4586G>C 4587T>A), Q396N (4591C>A 4593A>T), Q410N (4633C>A 4635A>T), A413Q (4642G>C 4643C>A 4644T>A)								
Codon mutations:	AAA277..C (4236A>C), ATT279ATA (4242T>A), AAG280AGA (4244A>G 4245G>A), TGT281GTT (4246T>G 4247G>T), CAG285CTA (4259A>T 4260G>A), TCA287AAT (4264T>A 4265C>A 4266A>T), CCT288GAG (4267C>G 4268C>A 4269T>G), GAC290GAT (4275C>T), CGA291AAA (4276C>A 4277G>A), GAA292ACT (4279G>A 4280A>C 4281A>T), GAG293GAA (4284G>A), ACT296ATT (4292C>T), ATC298ATT (4299C>T), GAA299CAA (4300G>C), CTA301TTA (4306C>T), CTC302TTA (4309C>T 4311C>A), AAG303AAT (4314G>T), CTT304ATG (4315C>A 4317T>G), GGT305AAA (4318G>A 4319G>A 4320T>A), ATC306TTA (4321A>T 4323C>A), ATC307ATA (4326C>A), CCT309--C (4330_4331delCC 4332T>C), AGC310AGT (4335C>T), AAG311AAA (4338G>A), AGT312AGC (4341T>C), CCA313CCG (4344A>G), TCC315AGT (4348T>A 4349C>G 4350C>T), TCA316AGT (4351T>A 4352C>G 4353A>T), GCC318GCA (4359C>A), AGG322AGA (4371G>A), AAT323AAC (4374T>C), GCC325GCG (4380C>G), GAG326AAA (4381G>A 4383G>A), ATC327CAA (4384A>C 4385T>A 4386C>A), GGC330GGA (4395C>A), GCA332GCT (4401A>T), AGA333CGA (4402A>C), ATT336ATA (4413T>A), AAG340GAG (4423A>G), TTA341CTT (4426T>C 4428A>T), GAC343AAA (4432G>A 4434C>A), CAT344AAT (4435C>A), ACA345TGT (4438A>T 4439C>G 4440A>T), AAG346GTC (4441A>G 4442A>T 4443G>C), GGA347TTC (4444G>T 4445G>T 4446A>C), GAT348GAC (4449T>C), GGC349GGA (4452C>A), TAC350TAT (4455C>T), CTA351TTC (4456C>T 4458A>C), CTT352ATC (4459C>A 4461T>C), CCT353CCA (4464T>A), AAC354AAT (4467C>T), AAG355AAA (4470G>A), GAG356AAT (4471G>A 4473G>T), CAA357AAC (4474C>A 4476A>C), CTG358CTT (4479G>T), CTT359ATA (4480C>A 4482T>A), CAA360AAT (4483C>A 4485A>T), AGA361CTT (4486A>C 4487G>T 4488A>T), ATC362GCA (4489A>G 4490T>C 4491C>A), GGA363AAA (4492G>A 4493G>A), GGT364GGA (4497T>A), AAG365AAA (4500G>A), TTT367TAC (4505T>A 4506T>C), TAC368TTT (4508A>T 4509C>T), TCT369TCA (4512T>A), TCC370AAA (4513T>A 4514C>A 4515C>A), GAC372GAT (4521C>T), TGT373TGC (4524T>C), TCT375AGT (4528T>A 4529C>G), CAG379CAA (4542G>A), GTA380ATA (4543G>A), CGC381AAA (4546C>A 4547G>A 4548C>A), CTT382CTA (4551T>A), GCT383GAA (4553C>A 4554T>A), CCA384GAA (4555C>G 4556C>A), GAA385AAT (4558G>A 4560A>T), ACA386TCT (4561A>T 4563A>T), ATT387ATA (4566T>A), CAG388GCT (4567C>G 4568A>C 4569G>T), CTA389TTA (4570C>T), ACC390ACA (4575C>A), GCT391GCA (4578T>A), AGC393AGT (4584C>T), TGT394ACA (4585T>A 4586G>C 4587T>A), CCC395CCA (4590C>A), CAA396AAT (4591C>A 4593A>T), GTC403GTG (4614C>G), CAA410AAT (4633C>A 4635A>T), GCT413CAA (4642G>C 4643C>A 4644T>A), ATT414ATC (4647T>C), TTC415TTT (4650C>T)								

Proteins

	Begin	End	Coverage	Score	Concordance	Matches	Identities	I/D/M/F*	Stop Codons
hypothetical protein (NP_043933.1)	278	419	20.0%	590	57.0%	142 (100%)	96 (67.6%)	0/0/1/1	0
Protein mutations:	K280R (4244A>G 4245G>A), C281V (4246T>G 4247G>T), Q285L (4259A>T 4260G>A), S287N (4264T>A 4265C>A 4266A>T), P288E (4267C>G 4268C>A 4269T>G), R291K (4276C>A 4277G>A), E292T (4279G>A 4280A>C 4281A>T), T296I (4292C>T), E299Q (4300G>C), K303N (4314G>T), L304M (4315C>A 4317T>G), G305K (4318G>A 4319G>A 4320T>A), I306L (4321A>T 4323C>A), E326K (4381G>A 4383G>A), I327Q (4384A>C 4385T>A 4386C>A), K340E (4423A>G), D343K (4432G>A 4434C>A), H344N (4435C>A), T345C (4438A>T 4439C>G 4440A>T), K346V (4441A>G 4442A>T 4443G>C), G347F (4444G>T 4445G>T 4446A>C), L351F (4456C>T 4458A>C), L352I (4459C>A 4461T>C), E356N (4471G>A 4473G>T), Q357N (4474C>A 4476A>C), L359I (4480C>A 4482T>A), Q360N (4483C>A 4485A>T), R361L (4486A>C 4487G>T 4488A>T), I362A (4489A>G 4490T>C 4491C>A), G363K (4492G>A 4493G>A), F367Y (4505T>A 4506T>C), Y368F (4508A>T 4509C>T), S370K (4513T>A 4514C>A 4515C>A), V380I (4543G>A), R381K (4546C>A 4547G>A 4548C>A), A383E (4553C>A 4554T>A), P384E (4555C>G 4556C>A), E385N (4558G>A 4560A>T), T386S (4561A>T 4563A>T), Q388A (4567C>G 4568A>C 4569G>T), C394T (4585T>A 4586G>C 4587T>A), Q396N (4591C>A 4593A>T), Q410N (4633C>A 4635A>T), A413Q (4642G>C 4643C>A 4644T>A)								
Codon mutations:	AAA277..C (4236A>C), ATT279ATA (4242T>A), AAG280AGA (4244A>G 4245G>A), TGT281GTT (4246T>G 4247G>T), CAG285CTA (4259A>T 4260G>A), TCA287AAT (4264T>A 4265C>A 4266A>T), CCT288GAG (4267C>G 4268C>A 4269T>G), GAC290GAT (4275C>T), CGA291AAA (4276C>A 4277G>A), GAA292ACT (4279G>A 4280A>C 4281A>T), GAG293GAA (4284G>A), ACT296ATT (4292C>T), ATC298ATT (4299C>T), GAA299CAA (4300G>C), CTA301TTA (4306C>T), CTC302TTA (4309C>T 4311C>A), AAG303AAT (4314G>T), CTT304ATG (4315C>A 4317T>G), GGT305AAA (4318G>A 4319G>A 4320T>A), ATC306TTA (4321A>T 4323C>A), ATC307ATA (4326C>A), CCT309--C (4330_4331delCC 4332T>C), AGC310AGT (4335C>T), AAG311AAA (4338G>A), AGT312AGC (4341T>C), CCA313CCG (4344A>G), TCC315AGT (4348T>A 4349C>G 4350C>T), TCA316AGT (4351T>A 4352C>G 4353A>T), GCC318GCA (4359C>A), AGG322AGA (4371G>A), AAT323AAC (4374T>C), GCC325GCG (4380C>G), GAG326AAA (4381G>A 4383G>A), ATC327CAA (4384A>C 4385T>A 4386C>A), GGC330GGA (4395C>A), GCA332GCT (4401A>T), AGA333CGA (4402A>C), ATT336ATA (4413T>A), AAG340GAG (4423A>G), TTA341CTT (4426T>C 4428A>T), GAC343AAA (4432G>A 4434C>A), CAT344AAT (4435C>A), ACA345TGT (4438A>T 4439C>G 4440A>T), AAG346GTC (4441A>G 4442A>T 4443G>C), GGA347TTC (4444G>T 4445G>T 4446A>C), GAT348GAC (4449T>C), GGC349GGA (4452C>A), TAC350TAT (4455C>T), CTA351TTC (4456C>T 4458A>C), CTT352ATC (4459C>A 4461T>C), CCT353CCA (4464T>A), AAC354AAT (4467C>T), AAG355AAA (4470G>A), GAG356AAT (4471G>A 4473G>T), CAA357AAC (4474C>A 4476A>C), CTG358CTT (4479G>T), CTT359ATA (4480C>A 4482T>A), CAA360AAT (4483C>A 4485A>T), AGA361CTT (4486A>C 4487G>T 4488A>T), ATC362GCA (4489A>G 4490T>C 4491C>A), GGA363AAA (4492G>A 4493G>A), GGT364GGA (4497T>A), AAG365AAA (4500G>A), TTT367TAC (4505T>A 4506T>C), TAC368TTT (4508A>T 4509C>T), TCT369TCA (4512T>A), TCC370AAA (4513T>A 4514C>A 4515C>A), GAC372GAT (4521C>T), TGT373TGC (4524T>C), TCT375AGT (4528T>A 4529C>G), CAG379CAA (4542G>A), GTA380ATA (4543G>A), CGC381AAA (4546C>A 4547G>A 4548C>A), CTT382CTA (4551T>A), GCT383GAA (4553C>A 4554T>A), CCA384GAA (4555C>G 4556C>A), GAA385AAT (4558G>A 4560A>T), ACA386TCT (4561A>T 4563A>T), ATT387ATA (4566T>A), CAG388GCT (4567C>G 4568A>C 4569G>T), CTA389TTA (4570C>T), ACC390ACA (4575C>A), GCT391GCA (4578T>A), AGC393AGT (4584C>T), TGT394ACA (4585T>A 4586G>C 4587T>A), CCC395CCA (4590C>A), CAA396AAT (4591C>A 4593A>T), GTC403GTG (4614C>G), CAA410AAT (4633C>A 4635A>T), GCT413CAA (4642G>C 4643C>A 4644T>A), ATT414ATC (4647T>C), TTC415TTT (4650C>T)								

*: Inserts / Deletes / Misaligned / Frameshifts

Analysis details

This analysis was performed with panviral2.64

NGS Details (UN8): Caulimovirus deformatiolamii

Assembly

**Coverage Length**	576 (2 contig(s))
**Depth Of Coverage**	5.8
**Number Of Reads**	27
**Reads Per Million**	0.45 rpm (after QC)
**Ambiguities**	0
**Assembly Method**	de novo + reference guided assembly
**Consensus Caller**	Bcf Tools

Coverage Map

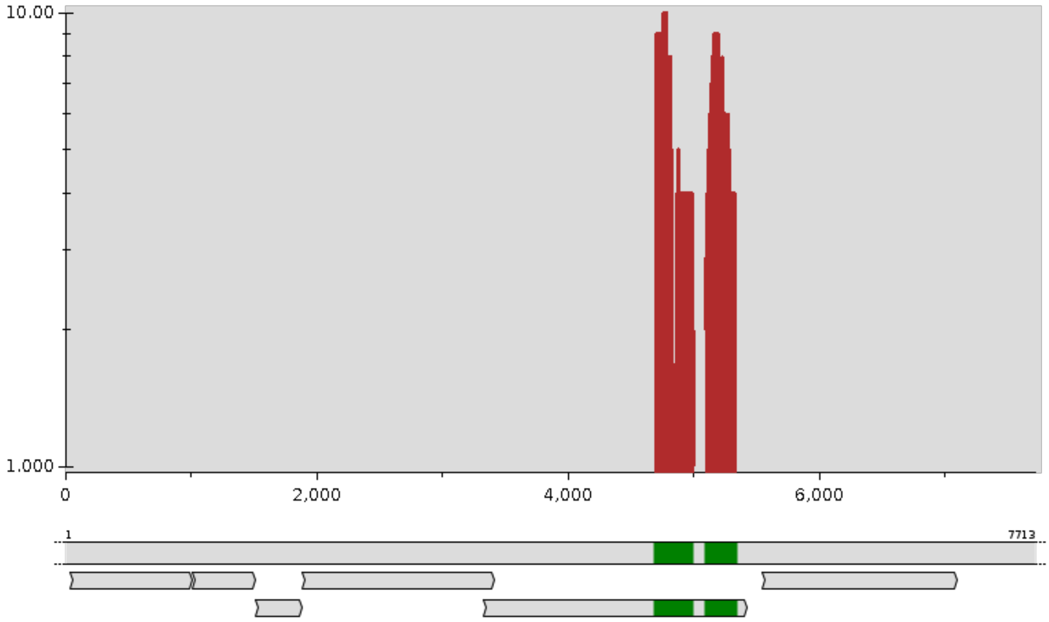

Assignment

**Type**	Caulimovirus deformatiolamii (Taxonomy ID: 3048173)
**Reference Genome**	NC_010737.1
**NT Identity (%)**	54.4503
**AA Identity (%)**	45.0262
**Number Of Stop Codons**	0
**Number Of CDS**	6

Alignment

**Alignment Score**	81.0 (NT) + 632.0 (AA) = 713.0
**Concordance (%)**	28.7964

Alignment Method	Global, seeded, nucleotide + amino acids (AGA)

Genome Region

Sequence starts at position 4686 and ends at position 5347 relative to NC_010737.1 reference sequence.

Alignment Detailed Statistics

	Begin	End	Coverage	Score	Concordance	Matches	Identities	I/D/M/F*	Stop Codons
**NT**	**4686**	**5347**	**7.5%**	**81**	**7.2%**	**570 (98.4%)**	**312 (53.9%)**	**3/6**	
Mutations:	4690G>A, 4691C>T, 4692A>G, 4693C>A, 4696C>T, 4698A>T, 4702A>C, 4704G>A, 4705T>A, 4706G>C, 4707C>A, 4709G>A, 4713T>C, 4715A>G, 4716C>A, 4725C>G, 4727T>A, 4732A>T, 4734T>A, 4735G>T, 4737C>T, 4738G>C, 4743G>A, 4746T>A, 4747C>T, 4749C>T, 4750C>A, 4751G>A, 4752T>A, 4755T>C, 4756C>A, 4757A>T, 4758G>C, 4759A>T, 4760A>G, 4761T>C, 4762C>A, 4763A>G, 4764T>A, 4765A>G, 4766T>C, 4767T>C, 4768C>A, 4774C>T, 4775A>T, 4777A>T, 4778T>C, 4780C>G, 4781A>G, 4785G>A, 4791A>T, 4795A>G, 4800T>C, 4801G>A, 4802A>C, 4803A>T, 4811A>C, 4812A>T, 4816C>T, 4818T>A, 4821A>G, 4827T>C, 4828C>T, 4830T>G, 4833T>A, 4835T>G, 4836T>C, 4837T>C, 4839A>C, 4841C>A, 4847C>T, 4848C>T, 4849A>G, 4850G>C, 4851T>A, 4854T>C, 4856A>T, 4857T>C, 4864C>A, 4867T>G, 4870G>A, 4871C>G, 4873T>C, 4874C>A, 4875T>G, 4876A>G, 4878G>T, 4879C>T, 4881T>C, 4882G>A, 4883C>A, 4884T>A, 4887T>C, 4888T>C, 4890G>C, 4891C>T, 4893G>T, 4896A>T, 4898A>G, 4899G>A, 4902C>T, 4906G>C, 4908A>G, 4908_4909insACC, 4909G>C, 4910A>C, 4917A>C, 4923T>A, 4924T>G, 4925G>A, 4927A>G, 4929G>A, 4932T>A, 4933T>A, 4936G>C, 4937A>T, 4938C>A, 4939A>G, 4941T>G, 4942_4947delGAGTAT, 4951G>C, 4953A>G, 4954G>A, 4962G>A, 4965A>G, 4967G>T, 4968T>G, 4969C>G, 4973C>T, 4974A>G, 4975G>A, 4978T>C, 4980T>C, 4983T>A, 4984A>T, 4985A>G, 4986G>T, 4987C>T, 4990C>G, 4991A>G, 4992T>A, 5088T>A, 5091C>T, 5094C>T, 5097G>A, 5103C>T, 5104T>A, 5105C>G, 5106A>T, 5109T>A, 5111G>C, 5112T>C, 5118A>T, 5119A>G, 5121G>A, 5122G>A, 5128T>A, 5129T>C, 5130A>T, 5132A>G, 5133T>G, 5136T>C, 5137C>T, 5138A>T, 5139T>A, 5140T>A, 5142T>A, 5155A>T, 5157T>A, 5160T>A, 5163A>T, 5164G>A, 5166T>A, 5167A>G, 5169C>A, 5174T>G, 5175T>C, 5178C>A, 5179G>C, 5180C>G, 5181T>A, 5187C>T, 5188A>T, 5191A>G, 5192T>C, 5193T>A, 5195A>T, 5196T>C, 5197C>T, 5200A>C, 5201C>T, 5202T>A, 5205T>A, 5206C>A, 5207T>A, 5208T>G, 5209G>C, 5211G>A, 5214T>C, 5215C>A, 5217G>C, 5220A>C, 5224A>T, 5226A>G, 5229T>C, 5232C>T, 5234A>C, 5236A>C, 5238C>G, 5239T>G, 5241T>A, 5242A>T, 5244C>A, 5245T>G, 5246T>G, 5248T>C, 5250T>A, 5253G>A, 5254A>T, 5255A>T, 5258C>A, 5259C>T, 5262T>A, 5263G>C, 5265G>T, 5266A>T, 5267A>C, 5269G>T, 5270G>C, 5274T>A, 5275T>C, 5276A>C, 5278A>C, 5281C>T, 5283G>T, 5284G>A, 5286A>G, 5290C>T, 5292T>A, 5293G>C, 5294T>A, 5295C>T, 5298G>A, 5304A>G, 5305C>G, 5306A>T, 5309G>T, 5311C>T, 5316C>T, 5317A>T, 5318G>T, 5319A>T, 5322C>T, 5323A>T, 5324G>C, 5331G>A, 5332G>A, 5338C>A, 5340C>T, 5345C>A								

CDS

	Begin	End	Coverage	Score	Concordance	Matches	Identities	I/D/M/F*	Stop Codons
LLDV_gp5	455	675	27.5%	632	46.1%	190 (98.4%)	86 (44.6%)	1/2/0/0	0
Protein mutations:	A456M (4690G>A 4691C>T 4692A>G), Q457K (4693C>A), L458F (4696C>T 4698A>T), K460Q (4702A>C 4704G>A), C461T (4705T>A 4706G>C 4707C>A), R462K (4709G>A), N464R (4715A>G 4716C>A), L468H (4727T>A), I470L (4732A>T 4734T>A), D471Y (4735G>T 4737C>T), E472Q (4738G>C), H475Y (4747C>T 4749C>T), R476K (4750C>A 4751G>A 4752T>A), Q478I (4756C>A 4757A>T 4758G>C), N479C (4759A>T 4760A>G 4761T>C), H480R (4762C>A 4763A>G 4764T>A), I481A (4765A>G 4766T>C 4767T>C), L482I (4768C>A), H484F (4774C>T 4775A>T), I485S (4777A>T 4778T>C), H486G (4780C>G 4781A>G), K491E (4795A>G), E493T (4801G>A 4802A>C 4803A>T), K496T (4811A>C 4812A>T), I504S (4835T>G 4836T>C), T506N (4841C>A), A508V (4847C>T 4848C>T), S509A (4849A>G 4850G>C 4851T>A), Y511F (4856A>T 4857T>C), Q514K (4864C>A), L515V (4867T>G), A516R (4870G>A 4871C>G), S517Q (4873T>C 4874C>A 4875T>G), M518V (4876A>G 4878G>T), R519C (4879C>T 4881T>C), A520K (4882G>A 4883C>A 4884T>A), Q523Y (4891C>T 4893G>T), E524D (4896A>T), K525R (4898A>G 4899G>A), E528Q (4906G>C 4908A>G), E528_D529insT (4908_4909insACC), D529P (4909G>C 4910A>C), N533K (4923T>A), W534E (4924T>G 4925G>A), K535E (4927A>G 4929G>A), H536Q (4932T>A), S537T (4933T>A), D538L (4936G>C 4937A>T 4938C>A), T539A (4939A>G 4941T>G), E540_Y541del (4942_4947delGAGTAT), E543Q (4951G>C 4953A>G), E544K (4954G>A), S548M (4967G>T 4968T>G), L549V (4969C>G), T550M (4973C>T 4974A>G), D551N (4975G>A), F552L (4978T>C 4980T>C), K554C (4984A>T 4985A>G 4986G>T), H556G (4990C>G 4991A>G 4992T>A), S596T (5111G>C 5112T>C), K598N (5118A>T), K599E (5119A>G 5121G>A), A600T (5122G>A), L602T (5128T>A 5129T>C 5130A>T), N603R (5132A>G 5133T>G), H605L (5137C>T 5138A>T 5139T>A), S606T (5140T>A 5142T>A), I611L (5155A>T 5157T>A), V614I (5164G>A 5166T>A), I615V (5167A>G 5169C>A), V617G (5174T>G 5175T>C), A619R (5179G>C 5180C>G 5181T>A), T622S (5188A>T), I623A (5191A>G 5192T>C 5193T>A), Y624F (5195A>T 5196T>C), T626L (5200A>C 5201C>T 5202T>A), L628K (5206C>A 5207T>A 5208T>G), E629Q (5209G>C 5211G>A), L631I (5215C>A 5217G>C), T634S (5224A>T 5226A>G), K637T (5234A>C), N638Q (5236A>C 5238C>G), F639V (5239T>G 5241T>A), T640S (5242A>T 5244C>A), F641G (5245T>G 5246T>G), F642L (5248T>C 5250T>A), M643I (5253G>A), N644F (5254A>T 5255A>T), T645N (5258C>A 5259C>T), N646K (5262T>A), V647L (5263G>C 5265G>T), K648S (5266A>T 5267A>C), G649S (5269G>T 5270G>C), D650E (5274T>A), Y651P (5275T>C 5276A>C), K652Q (5278A>C), Q653Y (5281C>T 5283G>T), G654R (5284G>A 5286A>G), V657H (5293G>C 5294T>A 5295C>T), Q661V (5305C>G 5306A>T), W662L (5309G>T), R665F (5317A>T 5318G>T 5319A>T), V670I (5332G>A), H672N (5338C>A 5340C>T), T674K (5345C>A)								
Codon mutations:	GCA456ATG (4690G>A 4691C>T 4692A>G), CAA457AAA (4693C>A), CTA458TTT (4696C>T 4698A>T), AAG460CAA (4702A>C 4704G>A), TGC461ACA (4705T>A 4706G>C 4707C>A), AGA462AAA (4709G>A), ATT463ATC (4713T>C), AAC464AGA (4715A>G 4716C>A), GGC467GGG (4725C>G), CTT468CAT (4727T>A), ATT470TTA (4732A>T 4734T>A), GAC471TAT (4735G>T 4737C>T), GAA472CAA (4738G>C), GGG473GGA (4743G>A), ACT474ACA (4746T>A), CAC475TAT (4747C>T 4749C>T), CGT476AAA (4750C>A 4751G>A 4752T>A), CCT477CCC (4755T>C), CAG478ATC (4756C>A 4757A>T 4758G>C), AAT479TGC (4759A>T 4760A>G 4761T>C), CAT480AGA (4762C>A 4763A>G 4764T>A), ATT481GCC (4765A>G 4766T>C 4767T>C), CTC482ATC (4768C>A), CAT484TTT (4774C>T 4775A>T), ATT485TCT (4777A>T 4778T>C), CAT486GGT (4780C>G 4781A>G), AAG487AAA (4785G>A), CCA489CCT (4791A>T), AAA491GAA (4795A>G), ATT492ATC (4800T>C), GAA493ACT (4801G>A 4802A>C 4803A>T), AAA496ACT (4811A>C 4812A>T), CTT498TTA (4816C>T 4818T>A), CAA499CAG (4821A>G), TTT501TTC (4827T>C), CTT502TTG (4828C>T 4830T>G), GGT503GGA (4833T>A), ATT504AGC (4835T>G 4836T>C), TTA505CTC (4837T>C 4839A>C), ACT506AAT (4841C>A), GCC508GTT (4847C>T 4848C>T), AGT509GCA (4849A>G 4850G>C 4851T>A), GAT510GAC (4854T>C), TAT511TTC (4856A>T 4857T>C), CAA514AAA (4864C>A), TTA515GTA (4867T>G), GCA516AGA (4870G>A 4871C>G), TCT517CAG (4873T>C 4874C>A 4875T>G), ATG518GTT (4876A>G 4878G>T), CGT519TGC (4879C>T 4881T>C), GCT520AAA (4882G>A 4883C>A 4884T>A), CCT521CCC (4887T>C), TTG522CTC (4888T>C 4890G>C), CAG523TAT (4891C>T 4893G>T), GAA524GAT (4896A>T), AAG525AGA (4898A>G 4899G>A), CTC526CTT (4902C>T), GAA528CAG (4906G>C 4908A>G), GAA528_GAT529insACC (4908_4909insACC), GAT529CCT (4909G>C 4910A>C), CCA531CCC (4917A>C), AAT533AAA (4923T>A), TGG534GAG (4924T>G 4925G>A), AAG535GAA (4927A>G 4929G>A), CAT536CAA (4932T>A), TCT537ACT (4933T>A), GAC538CTA (4936G>C 4937A>T 4938C>A), ACT539GCG (4939A>G 4941T>G), GAG540_TAT541del (4942_4947delGAGTAT), GAA543CAG (4951G>C 4953A>G), GAA544AAA (4954G>A), AAG546AAA (4962G>A), AAA547AAG (4965A>G), AGT548ATG (4967G>T 4968T>G), CTC549GTC (4969C>G), ACA550ATG (4973C>T 4974A>G), GAC551AAC (4975G>A), TTT552CTC (4978T>C 4980T>C), CCT553CCA (4983T>A), AAG554TGT (4984A>T 4985A>G 4986G>T), CTA555TTA (4987C>T), CAT556GGA (4990C>G 4991A>G 4992T>A), CGT588..A (5088T>A), ATC589ATT (5091C>T), TGC590TGT (5094C>T), AGG591AGA (5097G>A), ACC593ACT (5103C>T), TCA594AGT (5104T>A 5105C>G 5106A>T), GGT595GGA (5109T>A), AGT596ACC (5111G>C 5112T>C), AAA598AAT (5118A>T), AAG599GAA (5119A>G 5121G>A), GCA600ACA (5122G>A), TTA602ACT (5128T>A 5129T>C 5130A>T), AAT603AGG (5132A>G 5133T>G), TAT604TAC (5136T>C), CAT605TTA (5137C>T 5138A>T 5139T>A), TCT606ACA (5140T>A 5142T>A), ATT611TTA (5155A>T 5157T>A), CTT612CTA (5160T>A), GCA613GCT (5163A>T), GTT614ATA (5164G>A 5166T>A), ATC615GTA (5167A>G 5169C>A), GTT617GGC (5174T>G 5175T>C), ATC618ATA (5178C>A), GCT619CGA (5179G>C 5180C>G 5181T>A), TTC621TTT (5187C>T), ACT622TCT (5188A>T), ATT623GCA (5191A>G 5192T>C 5193T>A), TAT624TTC (5195A>T 5196T>C), CTA625TTA (5197C>T), ACT626CTA (5200A>C 5201C>T 5202T>A), CCT627CCA (5205T>A), CTT628AAG (5206C>A 5207T>A 5208T>G), GAG629CAA (5209G>C 5211G>A), TTT630TTC (5214T>C), CTG631ATC (5215C>A 5217G>C), ATA632ATC (5220A>C), ACA634TCG (5224A>T 5226A>G), GAT635GAC (5229T>C), AAC636AAT (5232C>T), AAG637ACG (5234A>C), AAC638CAG (5236A>C 5238C>G), TTT639GTA (5239T>G 5241T>A), ACC640TCA (5242A>T 5244C>A), TTT641GGT (5245T>G 5246T>G), TTT642CTA (5248T>C 5250T>A), ATG643ATA (5253G>A), AAC644TTC (5254A>T 5255A>T), ACC645AAT (5258C>A 5259C>T), AAT646AAA (5262T>A), GTG647CTT (5263G>C 5265G>T), AAA648TCA (5266A>T 5267A>C), GGT649TCT (5269G>T 5270G>C), GAT650GAA (5274T>A), TAT651CCT (5275T>C 5276A>C), AAA652CAA (5278A>C), CAG653TAT (5281C>T 5283G>T), GGA654AGG (5284G>A 5286A>G), CTT656TTA (5290C>T 5292T>A), GTC657CAT (5293G>C 5294T>A 5295C>T), AGG658AGA (5298G>A), CAA660CAG (5304A>G), CAA661GTA (5305C>G 5306A>T), TGG662TTG (5309G>T), CTA663TTA (5311C>T), TCC664TCT (5316C>T), AGA665TTT (5317A>T 5318G>T 5319A>T), TAC666TAT (5322C>T), AGT667TCT (5323A>T 5324G>C), AAG669AAA (5331G>A), GTT670ATT (5332G>A), CAC672AAT (5338C>A 5340C>T), ACA674AAA (5345C>A)								

Proteins

	Begin	End	Coverage	Score	Concordance	Matches	Identities	I/D/M/F*	Stop Codons
replicase (YP_001931961.1)	455	675	27.5%	632	46.1%	190 (98.4%)	86 (44.6%)	1/2/0/0	0
Protein mutations:	A456M (4690G>A 4691C>T 4692A>G), Q457K (4693C>A), L458F (4696C>T 4698A>T), K460Q (4702A>C 4704G>A), C461T (4705T>A 4706G>C 4707C>A), R462K (4709G>A), N464R (4715A>G 4716C>A), L468H (4727T>A), I470L (4732A>T 4734T>A), D471Y (4735G>T 4737C>T), E472Q (4738G>C), H475Y (4747C>T 4749C>T), R476K (4750C>A 4751G>A 4752T>A), Q478I (4756C>A 4757A>T 4758G>C), N479C (4759A>T 4760A>G 4761T>C), H480R (4762C>A 4763A>G 4764T>A), I481A (4765A>G 4766T>C 4767T>C), L482I (4768C>A), H484F (4774C>T 4775A>T), I485S (4777A>T 4778T>C), H486G (4780C>G 4781A>G), K491E (4795A>G), E493T (4801G>A 4802A>C 4803A>T), K496T (4811A>C 4812A>T), I504S (4835T>G 4836T>C), T506N (4841C>A), A508V (4847C>T 4848C>T), S509A (4849A>G 4850G>C 4851T>A), Y511F (4856A>T 4857T>C), Q514K (4864C>A), L515V (4867T>G), A516R (4870G>A 4871C>G), S517Q (4873T>C 4874C>A 4875T>G), M518V (4876A>G 4878G>T), R519C (4879C>T 4881T>C), A520K (4882G>A 4883C>A 4884T>A), Q523Y (4891C>T 4893G>T), E524D (4896A>T), K525R (4898A>G 4899G>A), E528Q (4906G>C 4908A>G), E528_D529insT (4908_4909insACC), D529P (4909G>C 4910A>C), N533K (4923T>A), W534E (4924T>G 4925G>A), K535E (4927A>G 4929G>A), H536Q (4932T>A), S537T (4933T>A), D538L (4936G>C 4937A>T 4938C>A), T539A (4939A>G 4941T>G), E540_Y541del (4942_4947delGAGTAT), E543Q (4951G>C 4953A>G), E544K (4954G>A), S548M (4967G>T 4968T>G), L549V (4969C>G), T550M (4973C>T 4974A>G), D551N (4975G>A), F552L (4978T>C 4980T>C), K554C (4984A>T 4985A>G 4986G>T), H556G (4990C>G 4991A>G 4992T>A), S596T (5111G>C 5112T>C), K598N (5118A>T), K599E (5119A>G 5121G>A), A600T (5122G>A), L602T (5128T>A 5129T>C 5130A>T), N603R (5132A>G 5133T>G), H605L (5137C>T 5138A>T 5139T>A), S606T (5140T>A 5142T>A), I611L (5155A>T 5157T>A), V614I (5164G>A 5166T>A), I615V (5167A>G 5169C>A), V617G (5174T>G 5175T>C), A619R (5179G>C 5180C>G 5181T>A), T622S (5188A>T), I623A (5191A>G 5192T>C 5193T>A), Y624F (5195A>T 5196T>C), T626L (5200A>C 5201C>T 5202T>A), L628K (5206C>A 5207T>A 5208T>G), E629Q (5209G>C 5211G>A), L631I (5215C>A 5217G>C), T634S (5224A>T 5226A>G), K637T (5234A>C), N638Q (5236A>C 5238C>G), F639V (5239T>G 5241T>A), T640S (5242A>T 5244C>A), F641G (5245T>G 5246T>G), F642L (5248T>C 5250T>A), M643I (5253G>A), N644F (5254A>T 5255A>T), T645N (5258C>A 5259C>T), N646K (5262T>A), V647L (5263G>C 5265G>T), K648S (5266A>T 5267A>C), G649S (5269G>T 5270G>C), D650E (5274T>A), Y651P (5275T>C 5276A>C), K652Q (5278A>C), Q653Y (5281C>T 5283G>T), G654R (5284G>A 5286A>G), V657H (5293G>C 5294T>A 5295C>T), Q661V (5305C>G 5306A>T), W662L (5309G>T), R665F (5317A>T 5318G>T 5319A>T), V670I (5332G>A), H672N (5338C>A 5340C>T), T674K (5345C>A)								

	Begin	End	Coverage	Score	Concordance	Matches	Identities	I/D/M/F*	Stop Codons
**NT**	**4686**	**5347**	**7.5%**	**81**	**7.2%**	**570 (98.4%)**	**312 (53.9%)**	**3/6**	
Codon mutations:	GCA456ATG (4690G>A 4691C>T 4692A>G), CAA457AAA (4693C>A), CTA458TTT (4696C>T 4698A>T), AAG460CAA (4702A>C 4704G>A), TGC461ACA (4705T>A 4706G>C 4707C>A), AGA462AAA (4709G>A), ATT463ATC (4713T>C), AAC464AGA (4715A>G 4716C>A), GGC467GGG (4725C>G), CTT468CAT (4727T>A), ATT470TTA (4732A>T 4734T>A), GAC471TAT (4735G>T 4737C>T), GAA472CAA (4738G>C), GGG473GGA (4743G>A), ACT474ACA (4746T>A), CAC475TAT (4747C>T 4749C>T), CGT476AAA (4750C>A 4751G>A 4752T>A), CCT477CCC (4755T>C), CAG478ATC (4756C>A 4757A>T 4758G>C), AAT479TGC (4759A>T 4760A>G 4761T>C), CAT480AGA (4762C>A 4763A>G 4764T>A), ATT481GCC (4765A>G 4766T>C 4767T>C), CTC482ATC (4768C>A), CAT484TTT (4774C>T 4775A>T), ATT485TCT (4777A>T 4778T>C), CAT486GGT (4780C>G 4781A>G), AAG487AAA (4785G>A), CCA489CCT (4791A>T), AAA491GAA (4795A>G), ATT492ATC (4800T>C), GAA493ACT (4801G>A 4802A>C 4803A>T), AAA496ACT (4811A>C 4812A>T), CTT498TTA (4816C>T 4818T>A), CAA499CAG (4821A>G), TTT501TTC (4827T>C), CTT502TTG (4828C>T 4830T>G), GGT503GGA (4833T>A), ATT504AGC (4835T>G 4836T>C), TTA505CTC (4837T>C 4839A>C), ACT506AAT (4841C>A), GCC508GTT (4847C>T 4848C>T), AGT509GCA (4849A>G 4850G>C 4851T>A), GAT510GAC (4854T>C), TAT511TTC (4856A>T 4857T>C), CAA514AAA (4864C>A), TTA515GTA (4867T>G), GCA516AGA (4870G>A 4871C>G), TCT517CAG (4873T>C 4874C>A 4875T>G), ATG518GTT (4876A>G 4878G>T), CGT519TGC (4879C>T 4881T>C), GCT520AAA (4882G>A 4883C>A 4884T>A), CCT521CCC (4887T>C), TTG522CTC (4888T>C 4890G>C), CAG523TAT (4891C>T 4893G>T), GAA524GAT (4896A>T), AAG525AGA (4898A>G 4899G>A), CTC526CTT (4902C>T), GAA528CAG (4906G>C 4908A>G), GAA528_GAT529insACC (4908_4909insACC), GAT529CCT (4909G>C 4910A>C), CCA531CCC (4917A>C), AAT533AAA (4923T>A), TGG534GAG (4924T>G 4925G>A), AAG535GAA (4927A>G 4929G>A), CAT536CAA (4932T>A), TCT537ACT (4933T>A), GAC538CTA (4936G>C 4937A>T 4938C>A), ACT539GCG (4939A>G 4941T>G), GAG540_TAT541del (4942_4947delGAGTAT), GAA543CAG (4951G>C 4953A>G), GAA544AAA (4954G>A), AAG546AAA (4962G>A), AAA547AAG (4965A>G), AGT548ATG (4967G>T 4968T>G), CTC549GTC (4969C>G), ACA550ATG (4973C>T 4974A>G), GAC551AAC (4975G>A), TTT552CTC (4978T>C 4980T>C), CCT553CCA (4983T>A), AAG554TGT (4984A>T 4985A>G 4986G>T), CTA555TTA (4987C>T), CAT556GGA (4990C>G 4991A>G 4992T>A), CGT588..A (5088T>A), ATC589ATT (5091C>T), TGC590TGT (5094C>T), AGG591AGA (5097G>A), ACC593ACT (5103C>T), TCA594AGT (5104T>A 5105C>G 5106A>T), GGT595GGA (5109T>A), AGT596ACC (5111G>C 5112T>C), AAA598AAT (5118A>T), AAG599GAA (5119A>G 5121G>A), GCA600ACA (5122G>A), TTA602ACT (5128T>A 5129T>C 5130A>T), AAT603AGG (5132A>G 5133T>G), TAT604TAC (5136T>C), CAT605TTA (5137C>T 5138A>T 5139T>A), TCT606ACA (5140T>A 5142T>A), ATT611TTA (5155A>T 5157T>A), CTT612CTA (5160T>A), GCA613GCT (5163A>T), GTT614ATA (5164G>A 5166T>A), ATC615GTA (5167A>G 5169C>A), GTT617GGC (5174T>G 5175T>C), ATC618ATA (5178C>A), GCT619CGA (5179G>C 5180C>G 5181T>A), TTC621TTT (5187C>T), ACT622TCT (5188A>T), ATT623GCA (5191A>G 5192T>C 5193T>A), TAT624TTC (5195A>T 5196T>C), CTA625TTA (5197C>T), ACT626CTA (5200A>C 5201C>T 5202T>A), CCT627CCA (5205T>A), CTT628AAG (5206C>A 5207T>A 5208T>G), GAG629CAA (5209G>C 5211G>A), TTT630TTC (5214T>C), CTG631ATC (5215C>A 5217G>C), ATA632ATC (5220A>C), ACA634TCG (5224A>T 5226A>G), GAT635GAC (5229T>C), AAC636AAT (5232C>T), AAG637ACG (5234A>C), AAC638CAG (5236A>C 5238C>G), TTT639GTA (5239T>G 5241T>A), ACC640TCA (5242A>T 5244C>A), TTT641GGT (5245T>G 5246T>G), TTT642CTA (5248T>C 5250T>A), ATG643ATA (5253G>A), AAC644TTC (5254A>T 5255A>T), ACC645AAT (5258C>A 5259C>T), AAT646AAA (5262T>A), GTG647CTT (5263G>C 5265G>T), AAA648TCA (5266A>T 5267A>C), GGT649TCT (5269G>T 5270G>C), GAT650GAA (5274T>A), TAT651CCT (5275T>C 5276A>C), AAA652CAA (5278A>C), CAG653TAT (5281C>T 5283G>T), GGA654AGG (5284G>A 5286A>G), CTT656TTA (5290C>T 5292T>A), GTC657CAT (5293G>C 5294T>A 5295C>T), AGG658AGA (5298G>A), CAA660CAG (5304A>G), CAA661GTA (5305C>G 5306A>T), TGG662TTG (5309G>T), CTA663TTA (5311C>T), TCC664TCT (5316C>T), AGA665TTT (5317A>T 5318G>T 5319A>T), TAC666TAT (5322C>T), AGT667TCT (5323A>T 5324G>C), AAG669AAA (5331G>A), GTT670ATT (5332G>A), CAC672AAT (5338C>A 5340C>T), ACA674AAA (5345C>A)								

*: Inserts / Deletes / Misaligned / Frameshifts

Analysis details

This analysis was performed with panviral2.64

NGS Details (UN8): Petuvirus venapetuniae

Assembly

**Coverage Length**	551 (1 contig(s))
**Depth Of Coverage**	5.8
**Number Of Reads**	25
**Reads Per Million**	0.42 rpm (after QC)
**Ambiguities**	0
**Assembly Method**	de novo + reference guided assembly
**Consensus Caller**	Bcf Tools

Coverage Map

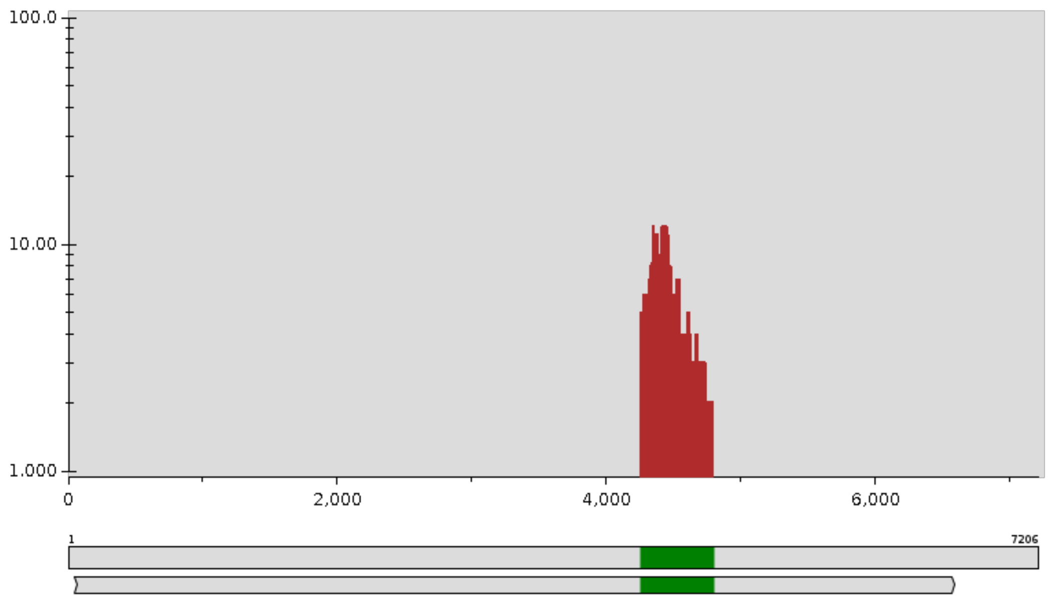

Assignment

**Type**	Petuvirus venapetuniae (Taxonomy ID: 3048265)
**Reference Genome**	NC_001839.2
**NT Identity (%)**	55.1724
**AA Identity (%)**	49.4565
**Number Of Stop Codons**	0
**Number Of CDS**	1

Alignment

**Alignment Score**	114.0 (NT) + 660.0 (AA) = 774.0
**Concordance (%)**	32.3714

Alignment Method	Global, seeded, nucleotide + amino acids (AGA)

Genome Region

Sequence starts at position 4248 and ends at position 4798 relative to NC_001839.2 reference sequence.

Alignment Detailed Statistics

	Begin	End	Coverage	Score	Concordance	Matches	Identities	I/D/M/F*	Stop Codons
**NT**	**4248**	**4798**	**7.6%**	**114**	**10.3%**	**551 (100%)**	**304 (55.2%)**	**0/0**	
Mutations:	4250A>T, 4251G>T, 4252C>G, 4253A>T, 4255T>A, 4259G>A, 4263T>A, 4264G>T, 4265T>C, 4266G>A, 4269G>T, 4270A>C, 4271G>T, 4275C>T, 4276A>T, 4278C>G, 4281T>A, 4282T>A, 4283T>C, 4284G>A, 4286T>A, 4287C>A, 4289A>T, 4293G>A, 4294A>G, 4298C>T, 4302G>A, 4304T>A, 4307A>T, 4309A>C, 4310A>T, 4314G>A, 4315C>G, 4316A>C, 4320G>T, 4321A>C, 4325T>C, 4328T>C, 4334C>T, 4337C>T, 4340G>A, 4342G>C, 4343G>T, 4344T>G, 4346A>T, 4349G>A, 4350C>A, 4354T>A, 4355G>A, 4361A>C, 4362A>T, 4363A>C, 4364A>C, 4365T>C, 4366T>C, 4367A>T, 4376C>T, 4379A>T, 4386C>A, 4388A>G, 4391A>T, 4397C>T, 4398C>T, 4399A>C, 4401T>G, 4403C>T, 4404C>T, 4406C>A, 4407C>A, 4410G>T, 4411A>G, 4412T>G, 4413G>A, 4414A>T, 4415C>T, 4417A>G, 4418G>A, 4420T>A, 4421T>C, 4424C>G, 4430A>T, 4436G>A, 4437C>A, 4439C>G, 4440A>G, 4441C>A, 4442A>T, 4445C>T, 4448C>G, 4449T>A, 4450C>A, 4451T>A, 4452C>A, 4453A>G, 4454C>A, 4455T>A, 4456T>C, 4457A>T, 4459C>T, 4460C>T, 4464G>A, 4466A>C, 4469G>T, 4470C>A, 4472C>T, 4474T>A, 4475T>C, 4476T>A, 4477C>G, 4478A>T, 4481G>A, 4488C>A, 4490T>G, 4493G>A, 4499A>T, 4502T>C, 4509T>A, 4511A>T, 4512G>C, 4513G>A, 4517C>T, 4518C>T, 4519A>C, 4520T>G, 4521C>A, 4522C>A, 4523T>A, 4526T>A, 4529G>T, 4531G>A, 4533C>T, 4534C>A, 4535A>T, 4541A>T, 4543G>C, 4544G>T, 4547T>C, 4548T>A, 4549G>A, 4551A>G, 4553T>C, 4556T>C, 4557G>T, 4558A>T, 4559T>C, 4560A>G, 4565T>A, 4567T>A, 4569C>G, 4577G>T, 4580C>T, 4586A>C, 4589C>T, 4592T>A, 4593C>T, 4595T>A, 4600C>A, 4601A>T, 4611T>G, 4612T>A, 4613G>A, 4616C>T, 4622A>C, 4624C>T, 4625C>T, 4630T>A, 4631C>T, 4632A>T, 4633A>C, 4634A>T, 4637C>T, 4640C>T, 4641C>A, 4643G>T, 4645C>A, 4650C>A, 4651T>G, 4652T>C, 4653T>A, 4654T>A, 4655T>C, 4656T>A, 4657C>T, 4658A>C, 4659G>T, 4662T>A, 4664G>T, 4667C>G, 4670C>T, 4680A>G, 4682C>T, 4683C>T, 4685T>G, 4686C>A, 4688T>A, 4697A>G, 4698A>G, 4699C>A, 4700A>T, 4701C>A, 4703G>T, 4706A>T, 4707G>T, 4708A>C, 4712C>T, 4713A>T, 4715A>T, 4719T>C, 4720T>A, 4721G>T, 4722C>T, 4724T>A, 4727C>T, 4728C>A, 4729A>C, 4730G>C, 4734A>T, 4736A>C, 4738G>A, 4739T>A, 4740T>A, 4742G>T, 4745T>C, 4750A>G, 4752T>A, 4753T>A, 4757A>T, 4758G>C, 4760C>T, 4761A>G, 4763G>C, 4764T>A, 4766A>C, 4767T>A, 4768C>G, 4769G>T, 4772A>T, 4775A>G, 4778G>A, 4783T>A, 4784C>A, 4785C>T, 4787T>G, 4788G>T, 4789C>T, 4795A>C, 4796T>A								

CDS

	Begin	End	Coverage	Score	Concordance	Matches	Identities	I/D/M/F*	Stop Codons
PVCVgp1	1400	1583	8.4%	660	51.0%	184 (100%)	91 (49.5%)	0/0/0/0	0
Protein mutations:	L1400F (4250A>T), A1401C (4251G>T 4252C>G 4253A>T), I1402K (4255T>A), C1405I (4263T>A 4264G>T 4265T>C), D1406N (4266G>A), E1407S (4269G>T 4270A>C 4271G>T), Q1409L (4275C>T 4276A>T), Q1410E (4278C>G), F1411N (4281T>A 4282T>A 4283T>C), D1412K (4284G>A 4286T>A), L1413I (4287C>A 4289A>T), E1415R (4293G>A 4294A>G), D1418K (4302G>A 4304T>A), Q1420P (4309A>C 4310A>T), A1422S (4314G>A 4315C>G 4316A>C), E1424S (4320G>T 4321A>C), R1431T (4342G>C 4343G>T), S1432A (4344T>G 4346A>T), Q1434K (4350C>A), V1435E (4354T>A 4355G>A), K1438S (4362A>T 4363A>C 4364A>C), L1439P (4365T>C 4366T>C 4367A>T), Q1446K (4386C>A 4388A>G), H1450S (4398C>T 4399A>C), F1451V (4401T>G 4403C>T), Q1453K (4407C>A), D1454W (4410G>T 4411A>G 4412T>G), D1455I (4413G>A 4414A>T 4415C>T), K1456R (4417A>G 4418G>A), F1457Y (4420T>A 4421T>C), L1463M (4437C>A 4439C>G), T1464D (4440A>G 4441C>A 4442A>T), F1466L (4448C>G), S1467K (4449T>A 4450C>A 4451T>A), H1468R (4452C>A 4453A>G 4454C>A), L1469T (4455T>A 4456T>C 4457A>T), S1470F (4459C>T 4460C>T), A1472T (4464G>A 4466A>C), K1473N (4469G>T), L1474I (4470C>A 4472C>T), F1475Y (4474T>A 4475T>C), L1480M (4488C>A 4490T>G), L1487I (4509T>A 4511A>T), G1488Q (4512G>C 4513G>A), H1490S (4518C>T 4519A>C 4520T>G), P1491K (4521C>A 4522C>A 4523T>A), N1492K (4526T>A), E1493D (4529G>T), R1494K (4531G>A), P1495Y (4533C>T 4534C>A 4535A>T), G1498A (4543G>C 4544G>T), C1500N (4548T>A 4549G>A), I1501V (4551A>G 4553T>C), D1503F (4557G>T 4558A>T 4559T>C), R1504G (4560A>G), H1505Q (4565T>A), F1506Y (4567T>A), Q1507E (4569C>G), K1509N (4577G>T), T1517N (4600C>A 4601A>T), L1521E (4611T>G 4612T>A 4613G>A), K1524N (4622A>C), A1525V (4624C>T 4625C>T), I1527N (4630T>A 4631C>T), K1528S (4632A>T 4633A>C 4634A>T), Q1531N (4641C>A 4643G>T), P1532H (4645C>A), L1534S (4650C>A 4651T>G 4652T>C), F1535N (4653T>A 4654T>A 4655T>C), S1536I (4656T>A 4657C>T 4658A>C), A1537S (4659G>T), L1538I (4662T>A 4664G>T), I1544V (4680A>G 4682C>T), L1546I (4686C>A 4688T>A), T1550D (4698A>G 4699C>A 4700A>T), L1551I (4701C>A 4703G>T), E1552D (4706A>T), D1553S (4707G>T 4708A>C), I1555F (4713A>T 4715A>T), L1557H (4719T>C 4720T>A 4721G>T), Q1560T (4728C>A 4729A>C 4730G>C), I1562F (4734A>T 4736A>C), S1563K (4738G>A 4739T>A), L1564I (4740T>A 4742G>T), K1567R (4750A>G), F1568N (4752T>A 4753T>A), V1570L (4758G>C 4760C>T), M1571V (4761A>G 4763G>C), L1572I (4764T>A 4766A>C), I1578K (4783T>A 4784C>A), A1580F (4788G>T 4789C>T), N1582T (4795A>C 4796T>A)								
Codon mutations:	TTA1400TTT (4250A>T), GCA1401TGT (4251G>T 4252C>G 4253A>T), ATA1402AAA (4255T>A), AAG1403AAA (4259G>A), TGT1405ATC (4263T>A 4264G>T 4265T>C), GAT1406AAT (4266G>A), GAG1407TCT (4269G>T 4270A>C 4271G>T), CAA1409TTA (4275C>T 4276A>T), CAA1410GAA (4278C>G), TTT1411AAC (4281T>A 4282T>A 4283T>C), GAT1412AAA (4284G>A 4286T>A), CTA1413ATT (4287C>A 4289A>T), GAA1415AGA (4293G>A 4294A>G), CCC1416CCT (4298C>T), GAT1418AAA (4302G>A 4304T>A), TCA1419TCT (4307A>T), CAA1420CCT (4309A>C 4310A>T), GCA1422AGC (4314G>A 4315C>G 4316A>C), GAA1424TCA (4320G>T 4321A>C), GCT1425GCC (4325T>C), TTT1426TTC (4328T>C), GTC1428GTT (4334C>T), AAC1429AAT (4337C>T), AAG1430AAA (4340G>A), AGG1431ACT (4342G>C 4343G>T), TCA1432GCT (4344T>G 4346A>T), GAG1433GAA (4349G>A), CAA1434AAA (4350C>A), GTG1435GAA (4354T>A 4355G>A), GGA1437GGC (4361A>C), AAA1438TCC (4362A>T 4363A>C 4364A>C), TTA1439CCT (4365T>C 4366T>C 4367A>T), GTC1442GTT (4376C>T), ATA1443ATT (4379A>T), CAA1446AAG (4386C>A 4388A>G), CCA1447CCT (4391A>T), AAC1449AAT (4397C>T), CAT1450TCT (4398C>T 4399A>C), TTC1451GTT (4401T>G 4403C>T), CTC1452TTA (4404C>T 4406C>A), CAA1453AAA (4407C>A), GAT1454TGG (4410G>T 4411A>G 4412T>G), GAC1455ATT (4413G>A 4414A>T 4415C>T), AAG1456AGA (4417A>G 4418G>A), TTT1457TAC (4420T>A 4421T>C), CCC1458CCG (4424C>G), CCA1460CCT (4430A>T), AAG1462AAA (4436G>A), CTC1463ATG (4437C>A 4439C>G), ACA1464GAT (4440A>G 4441C>A 4442A>T), CTC1465CTT (4445C>T), TTC1466TTG (4448C>G), TCT1467AAA (4449T>A 4450C>A 4451T>A), CAC1468AGA (4452C>A 4453A>G 4454C>A), TTA1469ACT (4455T>A 4456T>C 4457A>T), TCC1470TTT (4459C>T 4460C>T), GCA1472ACC (4464G>A 4466A>C), AAG1473AAT (4469G>T), CTC1474ATT (4470C>A 4472C>T), TTT1475TAC (4474T>A 4475T>C), TCA1476AGT (4476T>A 4477C>G 4478A>T), AAG1477AAA (4481G>A), CTT1480ATG (4488C>A 4490T>G), AAG1481AAA (4493G>A), GGA1483GGT (4499A>T), TTT1484TTC (4502T>C), TTA1487ATT (4509T>A 4511A>T), GGA1488CAA (4512G>C 4513G>A), ATC1489ATT (4517C>T), CAT1490TCG (4518C>T 4519A>C 4520T>G), CCT1491AAA (4521C>A 4522C>A 4523T>A), AAT1492AAA (4526T>A), GAG1493GAT (4529G>T), AGA1494AAA (4531G>A), CCA1495TAT (4533C>T 4534C>A 4535A>T), ACA1497ACT (4541A>T), GGG1498GCT (4543G>C 4544G>T), TTT1499TTC (4547T>C), TGC1500AAC (4548T>A 4549G>A), ATT1501GTC (4551A>G 4553T>C), CCT1502CCC (4556T>C), GAT1503TTC (4557G>T 4558A>T 4559T>C), AGA1504GGA (4560A>G), CAT1505CAA (4565T>A), TTT1506TAT (4567T>A), CAA1507GAA (4569C>G), AAG1509AAT (4577G>T), GTC1510GTT (4580C>T), CCA1512CCC (4586A>C), TTC1513TTT (4589C>T), GGT1514GGA (4592T>A), CTT1515TTA (4593C>T 4595T>A), ACA1517AAT (4600C>A 4601A>T), TTG1521GAA (4611T>G 4612T>A 4613G>A), TTC1522TTT (4616C>T), AAA1524AAC (4622A>C), GCC1525GTT (4624C>T 4625C>T), ATC1527AAT (4630T>A 4631C>T), AAA1528TCT (4632A>T 4633A>C 4634A>T), ATC1529ATT (4637C>T), TTC1530TTT (4640C>T), CAG1531AAT (4641C>A 4643G>T), CCC1532CAC (4645C>A), CTT1534AGC (4650C>A 4651T>G 4652T>C), TTT1535AAC (4653T>A 4654T>A 4655T>C), TCA1536ATC (4656T>A 4657C>T 4658A>C), GCT1537TCT (4659G>T), TTG1538ATT (4662T>A 4664G>T), GTC1539GTG (4667C>G), TAC1540TAT (4670C>T), ATC1544GTT (4680A>G 4682C>T), CTT1545TTG (4683C>T 4685T>G), CTT1546ATA (4686C>A 4688T>A), GAA1549GAG (4697A>G), ACA1550GAT (4698A>G 4699C>A 4700A>T), CTG1551ATT (4701C>A 4703G>T), GAA1552GAT (4706A>T), GAT1553TCT (4707G>T 4708A>C), CAC1554CAT (4712C>T), ATA1555TTT (4713A>T 4715A>T), TTG1557CAT (4719T>C 4720T>A 4721G>T), CTT1558TTA (4722C>T 4724T>A), AAC1559AAT (4727C>T), CAG1560ACC (4728C>A 4729A>C 4730G>C), ATA1562TTC (4734A>T 4736A>C), AGT1563AAA (4738G>A 4739T>A), TTG1564ATT (4740T>A 4742G>T), GTT1565GTC (4745T>C), AAA1567AGA (4750A>G), TTT1568AAT (4752T>A 4753T>A), GGA1569GGT (4757A>T), GTC1570CTT (4758G>C 4760C>T), ATG1571GTC (4761A>G 4763G>C), TTA1572ATC (4764T>A 4766A>C), TCG1573AGT (4767T>A 4768C>G 4769G>T), GCA1574GCT (4772A>T), AAA1575AAG (4775A>G), AAG1576AAA (4778G>A), ATC1578AAA (4783T>A 4784C>A), CTT1579TTG (4785C>T 4787T>G), GCT1580TTT (4788G>T 4789C>T), AAT1582ACA (4795A>C 4796T>A)								

Proteins

	Begin	End	Coverage	Score	Concordance	Matches	Identities	I/D/M/F*	Stop Codons
ORF I polyprotein (NP_127504.1)	1400	1583	8.4%	660	51.0%	184 (100%)	91 (49.5%)	0/0/0/0	0
Protein mutations:	L1400F (4250A>T), A1401C (4251G>T 4252C>G 4253A>T), I1402K (4255T>A), C1405I (4263T>A 4264G>T 4265T>C), D1406N (4266G>A), E1407S (4269G>T 4270A>C 4271G>T), Q1409L (4275C>T 4276A>T), Q1410E (4278C>G), F1411N (4281T>A 4282T>A 4283T>C), D1412K (4284G>A 4286T>A), L1413I (4287C>A 4289A>T), E1415R (4293G>A 4294A>G), D1418K (4302G>A 4304T>A), Q1420P (4309A>C 4310A>T), A1422S (4314G>A 4315C>G 4316A>C), E1424S (4320G>T 4321A>C), R1431T (4342G>C 4343G>T), S1432A (4344T>G 4346A>T), Q1434K (4350C>A), V1435E (4354T>A 4355G>A), K1438S (4362A>T 4363A>C 4364A>C), L1439P (4365T>C 4366T>C 4367A>T), Q1446K (4386C>A 4388A>G), H1450S (4398C>T 4399A>C), F1451V (4401T>G 4403C>T), Q1453K (4407C>A), D1454W (4410G>T 4411A>G 4412T>G), D1455I (4413G>A 4414A>T 4415C>T), K1456R (4417A>G 4418G>A), F1457Y (4420T>A 4421T>C), L1463M (4437C>A 4439C>G), T1464D (4440A>G 4441C>A 4442A>T), F1466L (4448C>G), S1467K (4449T>A 4450C>A 4451T>A), H1468R (4452C>A 4453A>G 4454C>A), L1469T (4455T>A 4456T>C 4457A>T), S1470F (4459C>T 4460C>T), A1472T (4464G>A 4466A>C), K1473N (4469G>T), L1474I (4470C>A 4472C>T), F1475Y (4474T>A 4475T>C), L1480M (4488C>A 4490T>G), L1487I (4509T>A 4511A>T), G1488Q (4512G>C 4513G>A), H1490S (4518C>T 4519A>C 4520T>G), P1491K (4521C>A 4522C>A 4523T>A), N1492K (4526T>A), E1493D (4529G>T), R1494K (4531G>A), P1495Y (4533C>T 4534C>A 4535A>T), G1498A (4543G>C 4544G>T), C1500N (4548T>A 4549G>A), I1501V (4551A>G 4553T>C), D1503F (4557G>T 4558A>T 4559T>C), R1504G (4560A>G), H1505Q (4565T>A), F1506Y (4567T>A), Q1507E (4569C>G), K1509N (4577G>T), T1517N (4600C>A 4601A>T), L1521E (4611T>G 4612T>A 4613G>A), K1524N (4622A>C), A1525V (4624C>T 4625C>T), I1527N (4630T>A 4631C>T), K1528S (4632A>T 4633A>C 4634A>T), Q1531N (4641C>A 4643G>T), P1532H (4645C>A), L1534S (4650C>A 4651T>G 4652T>C), F1535N (4653T>A 4654T>A 4655T>C), S1536I (4656T>A 4657C>T 4658A>C), A1537S (4659G>T), L1538I (4662T>A 4664G>T), I1544V (4680A>G 4682C>T), L1546I (4686C>A 4688T>A), T1550D (4698A>G 4699C>A 4700A>T), L1551I (4701C>A 4703G>T), E1552D (4706A>T), D1553S (4707G>T 4708A>C), I1555F (4713A>T 4715A>T), L1557H (4719T>C 4720T>A 4721G>T), Q1560T (4728C>A 4729A>C 4730G>C), I1562F (4734A>T 4736A>C), S1563K (4738G>A 4739T>A), L1564I (4740T>A 4742G>T), K1567R (4750A>G), F1568N (4752T>A 4753T>A), V1570L (4758G>C 4760C>T), M1571V (4761A>G 4763G>C), L1572I (4764T>A 4766A>C), I1578K (4783T>A 4784C>A), A1580F (4788G>T 4789C>T), N1582T (4795A>C 4796T>A)								

	Begin	End	Coverage	Score	Concordance	Matches	Identities	I/D/M/F*	Stop Codons
**NT**	**4248**	**4798**	**7.6%**	**114**	**10.3%**	**551 (100%)**	**304 (55.2%)**	**0/0**	
Codon mutations:	TTA1400TTT (4250A>T), GCA1401TGT (4251G>T 4252C>G 4253A>T), ATA1402AAA (4255T>A), AAG1403AAA (4259G>A), TGT1405ATC (4263T>A 4264G>T 4265T>C), GAT1406AAT (4266G>A), GAG1407TCT (4269G>T 4270A>C 4271G>T), CAA1409TTA (4275C>T 4276A>T), CAA1410GAA (4278C>G), TTT1411AAC (4281T>A 4282T>A 4283T>C), GAT1412AAA (4284G>A 4286T>A), CTA1413ATT (4287C>A 4289A>T), GAA1415AGA (4293G>A 4294A>G), CCC1416CCT (4298C>T), GAT1418AAA (4302G>A 4304T>A), TCA1419TCT (4307A>T), CAA1420CCT (4309A>C 4310A>T), GCA1422AGC (4314G>A 4315C>G 4316A>C), GAA1424TCA (4320G>T 4321A>C), GCT1425GCC (4325T>C), TTT1426TTC (4328T>C), GTC1428GTT (4334C>T), AAC1429AAT (4337C>T), AAG1430AAA (4340G>A), AGG1431ACT (4342G>C 4343G>T), TCA1432GCT (4344T>G 4346A>T), GAG1433GAA (4349G>A), CAA1434AAA (4350C>A), GTG1435GAA (4354T>A 4355G>A), GGA1437GGC (4361A>C), AAA1438TCC (4362A>T 4363A>C 4364A>C), TTA1439CCT (4365T>C 4366T>C 4367A>T), GTC1442GTT (4376C>T), ATA1443ATT (4379A>T), CAA1446AAG (4386C>A 4388A>G), CCA1447CCT (4391A>T), AAC1449AAT (4397C>T), CAT1450TCT (4398C>T 4399A>C), TTC1451GTT (4401T>G 4403C>T), CTC1452TTA (4404C>T 4406C>A), CAA1453AAA (4407C>A), GAT1454TGG (4410G>T 4411A>G 4412T>G), GAC1455ATT (4413G>A 4414A>T 4415C>T), AAG1456AGA (4417A>G 4418G>A), TTT1457TAC (4420T>A 4421T>C), CCC1458CCG (4424C>G), CCA1460CCT (4430A>T), AAG1462AAA (4436G>A), CTC1463ATG (4437C>A 4439C>G), ACA1464GAT (4440A>G 4441C>A 4442A>T), CTC1465CTT (4445C>T), TTC1466TTG (4448C>G), TCT1467AAA (4449T>A 4450C>A 4451T>A), CAC1468AGA (4452C>A 4453A>G 4454C>A), TTA1469ACT (4455T>A 4456T>C 4457A>T), TCC1470TTT (4459C>T 4460C>T), GCA1472ACC (4464G>A 4466A>C), AAG1473AAT (4469G>T), CTC1474ATT (4470C>A 4472C>T), TTT1475TAC (4474T>A 4475T>C), TCA1476AGT (4476T>A 4477C>G 4478A>T), AAG1477AAA (4481G>A), CTT1480ATG (4488C>A 4490T>G), AAG1481AAA (4493G>A), GGA1483GGT (4499A>T), TTT1484TTC (4502T>C), TTA1487ATT (4509T>A 4511A>T), GGA1488CAA (4512G>C 4513G>A), ATC1489ATT (4517C>T), CAT1490TCG (4518C>T 4519A>C 4520T>G), CCT1491AAA (4521C>A 4522C>A 4523T>A), AAT1492AAA (4526T>A), GAG1493GAT (4529G>T), AGA1494AAA (4531G>A), CCA1495TAT (4533C>T 4534C>A 4535A>T), ACA1497ACT (4541A>T), GGG1498GCT (4543G>C 4544G>T), TTT1499TTC (4547T>C), TGC1500AAC (4548T>A 4549G>A), ATT1501GTC (4551A>G 4553T>C), CCT1502CCC (4556T>C), GAT1503TTC (4557G>T 4558A>T 4559T>C), AGA1504GGA (4560A>G), CAT1505CAA (4565T>A), TTT1506TAT (4567T>A), CAA1507GAA (4569C>G), AAG1509AAT (4577G>T), GTC1510GTT (4580C>T), CCA1512CCC (4586A>C), TTC1513TTT (4589C>T), GGT1514GGA (4592T>A), CTT1515TTA (4593C>T 4595T>A), ACA1517AAT (4600C>A 4601A>T), TTG1521GAA (4611T>G 4612T>A 4613G>A), TTC1522TTT (4616C>T), AAA1524AAC (4622A>C), GCC1525GTT (4624C>T 4625C>T), ATC1527AAT (4630T>A 4631C>T), AAA1528TCT (4632A>T 4633A>C 4634A>T), ATC1529ATT (4637C>T), TTC1530TTT (4640C>T), CAG1531AAT (4641C>A 4643G>T), CCC1532CAC (4645C>A), CTT1534AGC (4650C>A 4651T>G 4652T>C), TTT1535AAC (4653T>A 4654T>A 4655T>C), TCA1536ATC (4656T>A 4657C>T 4658A>C), GCT1537TCT (4659G>T), TTG1538ATT (4662T>A 4664G>T), GTC1539GTG (4667C>G), TAC1540TAT (4670C>T), ATC1544GTT (4680A>G 4682C>T), CTT1545TTG (4683C>T 4685T>G), CTT1546ATA (4686C>A 4688T>A), GAA1549GAG (4697A>G), ACA1550GAT (4698A>G 4699C>A 4700A>T), CTG1551ATT (4701C>A 4703G>T), GAA1552GAT (4706A>T), GAT1553TCT (4707G>T 4708A>C), CAC1554CAT (4712C>T), ATA1555TTT (4713A>T 4715A>T), TTG1557CAT (4719T>C 4720T>A 4721G>T), CTT1558TTA (4722C>T 4724T>A), AAC1559AAT (4727C>T), CAG1560ACC (4728C>A 4729A>C 4730G>C), ATA1562TTC (4734A>T 4736A>C), AGT1563AAA (4738G>A 4739T>A), TTG1564ATT (4740T>A 4742G>T), GTT1565GTC (4745T>C), AAA1567AGA (4750A>G), TTT1568AAT (4752T>A 4753T>A), GGA1569GGT (4757A>T), GTC1570CTT (4758G>C 4760C>T), ATG1571GTC (4761A>G 4763G>C), TTA1572ATC (4764T>A 4766A>C), TCG1573AGT (4767T>A 4768C>G 4769G>T), GCA1574GCT (4772A>T), AAA1575AAG (4775A>G), AAG1576AAA (4778G>A), ATC1578AAA (4783T>A 4784C>A), CTT1579TTG (4785C>T 4787T>G), GCT1580TTT (4788G>T 4789C>T), AAT1582ACA (4795A>C 4796T>A)								

*: Inserts / Deletes / Misaligned / Frameshifts

Analysis details

This analysis was performed with panviral2.64

NGS Details (UN8): Errantivirus

Assembly

**Coverage Length**	361 (1 contig(s))
**Depth Of Coverage**	7.6
**Number Of Reads**	23
**Reads Per Million**	0.38 rpm (after QC)
**Ambiguities**	0
**Assembly Method**	de novo + reference guided assembly
**Consensus Caller**	Bcf Tools

Coverage Map

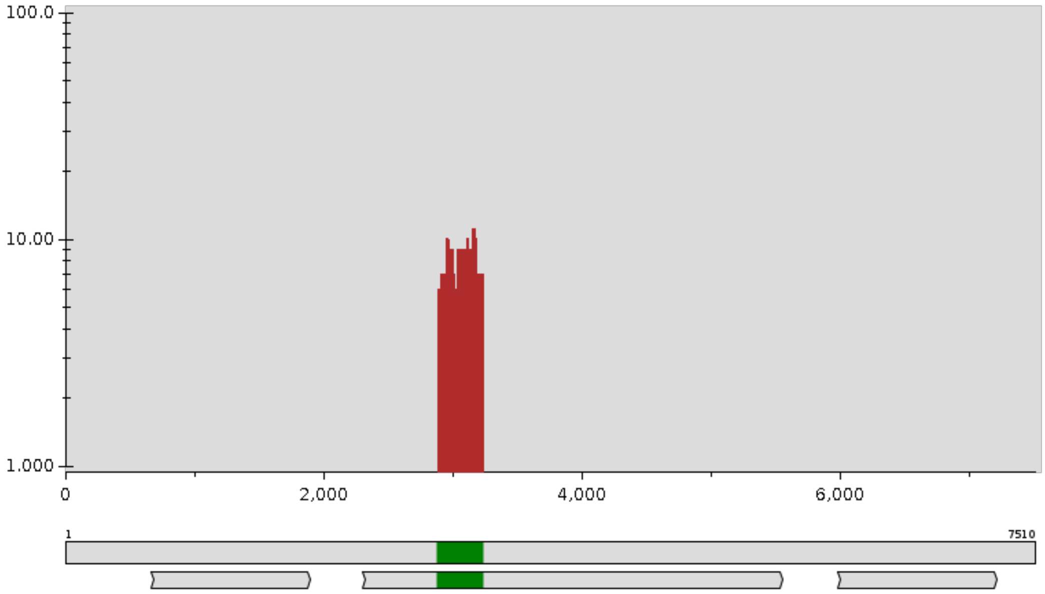

Assignment

**Type**	Errantivirus (Taxonomy ID: 186666)
**Reference Genome**	NC_038512.1
**NT Identity (%)**	55.4017
**AA Identity (%)**	47.5
**Number Of Stop Codons**	1
**Number Of CDS**	3

Alignment

**Alignment Score**	78.0 (NT) + 430.0 (AA) = 508.0
**Concordance (%)**	32.2335

Alignment Method	Global, seeded, nucleotide + amino acids (AGA)

Genome Region

Sequence starts at position 2881 and ends at position 3241 relative to NC_038512.1 reference sequence.

Alignment Detailed Statistics

	Begin	End	Coverage	Score	Concordance	Matches	Identities	I/D/M/F*	Stop Codons
**NT**	**2881**	**3241**	**4.8%**	**78**	**10.8%**	**361 (100%)**	**200 (55.4%)**	**0/0**	
Mutations:	2881G>T, 2887T>G, 2888C>T, 2890C>T, 2891G>T, 2892T>G, 2893A>T, 2896T>G, 2899C>T, 2901T>A, 2907A>G, 2908G>C, 2909T>C, 2911G>T, 2915G>A, 2917G>A, 2919A>T, 2920G>C, 2924A>G, 2927G>C, 2928A>C, 2929T>G, 2930G>A, 2938C>T, 2941G>C, 2944A>T, 2947A>C, 2953A>T, 2954A>G, 2955G>A, 2959C>G, 2960G>T, 2962A>G, 2963C>T, 2965T>A, 2968C>T, 2969A>G, 2972T>C, 2975G>C, 2976G>A, 2978A>G, 2979A>G, 2980G>A, 2981T>G, 2982G>C, 2984C>A, 2985A>T, 2987T>A, 2988A>T, 2989C>T, 2992C>T, 2993A>C, 2995C>A, 2997C>A, 2998C>A, 2999T>A, 3001A>T, 3005T>C, 3007G>T, 3008G>T, 3009C>G, 3010A>T, 3011A>T, 3012G>T, 3013T>G, 3016G>A, 3018T>A, 3019T>C, 3020T>C, 3025G>A, 3026G>A, 3028G>T, 3029G>C, 3030A>G, 3031G>T, 3032A>G, 3034G>C, 3035G>C, 3037C>A, 3040T>A, 3041C>A, 3043A>T, 3047A>G, 3049A>C, 3051C>A, 3052G>T, 3058C>T, 3061G>C, 3065A>C, 3066A>G, 3068G>A, 3069T>C, 3070A>T, 3071G>C, 3073A>T, 3074C>T, 3075A>C, 3076C>A, 3079G>C, 3084T>A, 3088A>G, 3091C>T, 3094T>C, 3095C>G, 3096G>T, 3104A>T, 3106G>T, 3110T>C, 3112A>C, 3114A>C, 3115A>C, 3118C>T, 3119T>G, 3127T>C, 3130T>G, 3134C>T, 3136A>G, 3137A>G, 3139A>C, 3141T>C, 3142T>C, 3146G>A, 3148C>T, 3149A>G, 3152G>C, 3154C>T, 3155C>T, 3157A>C, 3160A>G, 3161G>C, 3162G>C, 3163T>A, 3164C>T, 3165T>A, 3168A>T, 3169A>T, 3171A>G, 3172T>A, 3175C>A, 3176A>T, 3178C>T, 3179T>G, 3180G>T, 3181T>C, 3184C>A, 3187C>G, 3189A>T, 3191C>T, 3196C>T, 3203A>C, 3205T>A, 3206G>A, 3215A>C, 3216C>T, 3218T>G, 3219C>A, 3222T>C, 3223A>T, 3226G>A, 3227G>A, 3228A>C								

CDS

	Begin	End	Coverage	Score	Concordance	Matches	Identities	I/D/M/F*	Stop Codons
D1R33_gp2	193	312	11.1%	430	50.1%	120 (100%)	57 (47.5%)	0/0/0/0	1
Protein mutations:	L195F (2888C>T 2890C>T), V196C (2891G>T 2892T>G 2893A>T), F199Y (2901T>A), K201S (2907A>G 2908G>C), E204K (2915G>A 2917G>A), K205I (2919A>T 2920G>C), I207V (2924A>G), D208P (2927G>C 2928A>C 2929T>G), D209N (2930G>A), S217D (2954A>G 2955G>A), D218E (2959C>G), V219L (2960G>T 2962A>G), K222E (2969A>G), G224H (2975G>C 2976G>A), K225G (2978A>G 2979A>G 2980G>A), C226A (2981T>G 2982G>C), Q227I (2984C>A 2985A>T), Y228I (2987T>A 2988A>T 2989C>T), T230P (2993A>C 2995C>A), T231K (2997C>A 2998C>A), L232I (2999T>A 3001A>T), A235C (3008G>T 3009C>G 3010A>T), S236L (3011A>T 3012G>T 3013T>G), F238Y (3018T>A 3019T>C), Y239H (3020T>C), V241I (3026G>A 3028G>T), E242R (3029G>C 3030A>G 3031G>T), M243V (3032A>G 3034G>C), D244Q (3035G>C 3037C>A), Q246N (3041C>A 3043A>T), I248V (3047A>G 3049A>C), S249Y (3051C>A 3052G>T), N254R (3065A>C 3066A>G), V255T (3068G>A 3069T>C 3070A>T), E256H (3071G>C 3073A>T), H257S (3074C>T 3075A>C 3076C>A), F260Y (3084T>A), R264V (3095C>G 3096G>T), M267F (3104A>T 3106G>T), K270T (3114A>C 3115A>C), S272A (3119T>G), Q277* (3134C>T 3136A>G), R278G (3137A>G 3139A>C), V279A (3141T>C 3142T>C), D281N (3146G>A 3148C>T), N282D (3149A>G), V283L (3152G>C 3154C>T), L284F (3155C>T 3157A>C), G286P (3161G>C 3162G>C 3163T>A), L287Y (3164C>T 3165T>A), Q288L (3168A>T 3169A>T), N289R (3171A>G 3172T>A), N290K (3175C>A), I291F (3176A>T 3178C>T), C292V (3179T>G 3180G>T 3181T>C), Y295F (3189A>T), L296F (3191C>T), I300L (3203A>C 3205T>A), V301I (3206G>A), T304L (3215A>C 3216C>T), S305D (3218T>G 3219C>A), L306P (3222T>C 3223A>T), E308T (3227G>A 3228A>C)								
Codon mutations:	AAG192..T (2881G>T), CGT194CGG (2887T>G), CTC195TTT (2888C>T 2890C>T), GTA196TGT (2891G>T 2892T>G 2893A>T), GTT197GTG (2896T>G), GAC198GAT (2899C>T), TTC199TAC (2901T>A), AAG201AGC (2907A>G 2908G>C), TTG202CTT (2909T>C 2911G>T), GAG204AAA (2915G>A 2917G>A), AAG205ATC (2919A>T 2920G>C), ATC207GTC (2924A>G), GAT208CCG (2927G>C 2928A>C 2929T>G), GAC209AAC (2930G>A), TAC211TAT (2938C>T), CCG212CCC (2941G>C), ATA213ATT (2944A>T), CCA214CCC (2947A>C), ATA216ATT (2953A>T), AGT217GAT (2954A>G 2955G>A), GAC218GAG (2959C>G), GTA219TTG (2960G>T 2962A>G), CTT220TTA (2963C>T 2965T>A), GAC221GAT (2968C>T), AAG222GAG (2969A>G), TTA223CTA (2972T>C), GGT224CAT (2975G>C 2976G>A), AAG225GGA (2978A>G 2979A>G 2980G>A), TGC226GCC (2981T>G 2982G>C), CAA227ATA (2984C>A 2985A>T), TAC228ATT (2987T>A 2988A>T 2989C>T), TTC229TTT (2992C>T), ACC230CCA (2993A>C 2995C>A), ACC231AAA (2997C>A 2998C>A), TTA232ATT (2999T>A 3001A>T), TTG234CTT (3005T>C 3007G>T), GCA235TGT (3008G>T 3009C>G 3010A>T), AGT236TTG (3011A>T 3012G>T 3013T>G), GGG237GGA (3016G>A), TTT238TAC (3018T>A 3019T>C), TAT239CAT (3020T>C), CAG240CAA (3025G>A), GTG241ATT (3026G>A 3028G>T), GAG242CGT (3029G>C 3030A>G 3031G>T), ATG243GTC (3032A>G 3034G>C), GAC244CAA (3035G>C 3037C>A), CCT245CCA (3040T>A), CAA246AAT (3041C>A 3043A>T), ATA248GTC (3047A>G 3049A>C), TCG249TAT (3051C>A 3052G>T), ACC251ACT (3058C>T), GCG252GCC (3061G>C), AAC254CGC (3065A>C 3066A>G), GTA255ACT (3068G>A 3069T>C 3070A>T), GAA256CAT (3071G>C 3073A>T), CAC257TCA (3074C>T 3075A>C 3076C>A), GGG258GGC (3079G>C), TTT260TAT (3084T>A), GAA261GAG (3088A>G), TTC262TTT (3091C>T), CTT263CTC (3094T>C), CGA264GTA (3095C>G 3096G>T), ATG267TTT (3104A>T 3106G>T), TTA269CTC (3110T>C 3112A>C), AAA270ACC (3114A>C 3115A>C), AAC271AAT (3118C>T), TCA272GCA (3119T>G), TCT274TCC (3127T>C), ACT275ACG (3130T>G), CAA277TAG (3134C>T 3136A>G), AGA278GGC (3137A>G 3139A>C), GTT279GCC (3141T>C 3142T>C), GAC281AAT (3146G>A 3148C>T), AAT282GAT (3149A>G), GTC283CTT (3152G>C 3154C>T), CTA284TTC (3155C>T 3157A>C), AGA285AGG (3160A>G), GGT286CCA (3161G>C 3162G>C 3163T>A), CTC287TAC (3164C>T 3165T>A), CAA288CTT (3168A>T 3169A>T), AAT289AGA (3171A>G 3172T>A), AAC290AAA (3175C>A), ATC291TTT (3176A>T 3178C>T), TGT292GTC (3179T>G 3180G>T 3181T>C), CTC293CTA (3184C>A), GTC294GTG (3187C>G), TAC295TTC (3189A>T), CTT296TTT (3191C>T), GAC297GAT (3196C>T), ATT300CTA (3203A>C 3205T>A), GTC301ATC (3206G>A), ACT304CTT (3215A>C 3216C>T), TCC305GAC (3218T>G 3219C>A), CTA306CCT (3222T>C 3223A>T), CAG307CAA (3226G>A), GAA308ACA (3227G>A 3228A>C)								

Proteins

	Begin	End	Coverage	Score	Concordance	Matches	Identities	I/D/M/F*	Stop Codons
ORF B (YP_009507248.1)	193	312	11.1%	430	50.1%	120 (100%)	57 (47.5%)	0/0/0/0	1
Protein mutations:	L195F (2888C>T 2890C>T), V196C (2891G>T 2892T>G 2893A>T), F199Y (2901T>A), K201S (2907A>G 2908G>C), E204K (2915G>A 2917G>A), K205I (2919A>T 2920G>C), I207V (2924A>G), D208P (2927G>C 2928A>C 2929T>G), D209N (2930G>A), S217D (2954A>G 2955G>A), D218E (2959C>G), V219L (2960G>T 2962A>G), K222E (2969A>G), G224H (2975G>C 2976G>A), K225G (2978A>G 2979A>G 2980G>A), C226A (2981T>G 2982G>C), Q227I (2984C>A 2985A>T), Y228I (2987T>A 2988A>T 2989C>T), T230P (2993A>C 2995C>A), T231K (2997C>A 2998C>A), L232I (2999T>A 3001A>T), A235C (3008G>T 3009C>G 3010A>T), S236L (3011A>T 3012G>T 3013T>G), F238Y (3018T>A 3019T>C), Y239H (3020T>C), V241I (3026G>A 3028G>T), E242R (3029G>C 3030A>G 3031G>T), M243V (3032A>G 3034G>C), D244Q (3035G>C 3037C>A), Q246N (3041C>A 3043A>T), I248V (3047A>G 3049A>C), S249Y (3051C>A 3052G>T), N254R (3065A>C 3066A>G), V255T (3068G>A 3069T>C 3070A>T), E256H (3071G>C 3073A>T), H257S (3074C>T 3075A>C 3076C>A), F260Y (3084T>A), R264V (3095C>G 3096G>T), M267F (3104A>T 3106G>T), K270T (3114A>C 3115A>C), S272A (3119T>G), Q277* (3134C>T 3136A>G), R278G (3137A>G 3139A>C), V279A (3141T>C 3142T>C), D281N (3146G>A 3148C>T), N282D (3149A>G), V283L (3152G>C 3154C>T), L284F (3155C>T 3157A>C), G286P (3161G>C 3162G>C 3163T>A), L287Y (3164C>T 3165T>A), Q288L (3168A>T 3169A>T), N289R (3171A>G 3172T>A), N290K (3175C>A), I291F (3176A>T 3178C>T), C292V (3179T>G 3180G>T 3181T>C), Y295F (3189A>T), L296F (3191C>T), I300L (3203A>C 3205T>A), V301I (3206G>A), T304L (3215A>C 3216C>T), S305D (3218T>G 3219C>A), L306P (3222T>C 3223A>T), E308T (3227G>A 3228A>C)								
Codon mutations:	AAG192..T (2881G>T), CGT194CGG (2887T>G), CTC195TTT (2888C>T 2890C>T), GTA196TGT (2891G>T 2892T>G 2893A>T), GTT197GTG (2896T>G), GAC198GAT (2899C>T), TTC199TAC (2901T>A), AAG201AGC (2907A>G 2908G>C), TTG202CTT (2909T>C 2911G>T), GAG204AAA (2915G>A 2917G>A), AAG205ATC (2919A>T 2920G>C), ATC207GTC (2924A>G), GAT208CCG (2927G>C 2928A>C 2929T>G), GAC209AAC (2930G>A), TAC211TAT (2938C>T), CCG212CCC (2941G>C), ATA213ATT (2944A>T), CCA214CCC (2947A>C), ATA216ATT (2953A>T), AGT217GAT (2954A>G 2955G>A), GAC218GAG (2959C>G), GTA219TTG (2960G>T 2962A>G), CTT220TTA (2963C>T 2965T>A), GAC221GAT (2968C>T), AAG222GAG (2969A>G), TTA223CTA (2972T>C), GGT224CAT (2975G>C 2976G>A), AAG225GGA (2978A>G 2979A>G 2980G>A), TGC226GCC (2981T>G 2982G>C), CAA227ATA (2984C>A 2985A>T), TAC228ATT (2987T>A 2988A>T 2989C>T), TTC229TTT (2992C>T), ACC230CCA (2993A>C 2995C>A), ACC231AAA (2997C>A 2998C>A), TTA232ATT (2999T>A 3001A>T), TTG234CTT (3005T>C 3007G>T), GCA235TGT (3008G>T 3009C>G 3010A>T), AGT236TTG (3011A>T 3012G>T 3013T>G), GGG237GGA (3016G>A), TTT238TAC (3018T>A 3019T>C), TAT239CAT (3020T>C), CAG240CAA (3025G>A), GTG241ATT (3026G>A 3028G>T), GAG242CGT (3029G>C 3030A>G 3031G>T), ATG243GTC (3032A>G 3034G>C), GAC244CAA (3035G>C 3037C>A), CCT245CCA (3040T>A), CAA246AAT (3041C>A 3043A>T), ATA248GTC (3047A>G 3049A>C), TCG249TAT (3051C>A 3052G>T), ACC251ACT (3058C>T), GCG252GCC (3061G>C), AAC254CGC (3065A>C 3066A>G), GTA255ACT (3068G>A 3069T>C 3070A>T), GAA256CAT (3071G>C 3073A>T), CAC257TCA (3074C>T 3075A>C 3076C>A), GGG258GGC (3079G>C), TTT260TAT (3084T>A), GAA261GAG (3088A>G), TTC262TTT (3091C>T), CTT263CTC (3094T>C), CGA264GTA (3095C>G 3096G>T), ATG267TTT (3104A>T 3106G>T), TTA269CTC (3110T>C 3112A>C), AAA270ACC (3114A>C 3115A>C), AAC271AAT (3118C>T), TCA272GCA (3119T>G), TCT274TCC (3127T>C), ACT275ACG (3130T>G), CAA277TAG (3134C>T 3136A>G), AGA278GGC (3137A>G 3139A>C), GTT279GCC (3141T>C 3142T>C), GAC281AAT (3146G>A 3148C>T), AAT282GAT (3149A>G), GTC283CTT (3152G>C 3154C>T), CTA284TTC (3155C>T 3157A>C), AGA285AGG (3160A>G), GGT286CCA (3161G>C 3162G>C 3163T>A), CTC287TAC (3164C>T 3165T>A), CAA288CTT (3168A>T 3169A>T), AAT289AGA (3171A>G 3172T>A), AAC290AAA (3175C>A), ATC291TTT (3176A>T 3178C>T), TGT292GTC (3179T>G 3180G>T 3181T>C), CTC293CTA (3184C>A), GTC294GTG (3187C>G), TAC295TTC (3189A>T), CTT296TTT (3191C>T), GAC297GAT (3196C>T), ATT300CTA (3203A>C 3205T>A), GTC301ATC (3206G>A), ACT304CTT (3215A>C 3216C>T), TCC305GAC (3218T>G 3219C>A), CTA306CCT (3222T>C 3223A>T), CAG307CAA (3226G>A), GAA308ACA (3227G>A 3228A>C)								

*: Inserts / Deletes / Misaligned / Frameshifts

Analysis details

This analysis was performed with panviral2.64

NGS Details (UN8): Badnavirus maculaucubae

Assembly

**Coverage Length**	340 (1 contig(s))
**Depth Of Coverage**	8.4
**Number Of Reads**	20
**Reads Per Million**	0.33 rpm (after QC)
**Ambiguities**	0
**Assembly Method**	de novo + reference guided assembly
**Consensus Caller**	Bcf Tools

Coverage Map

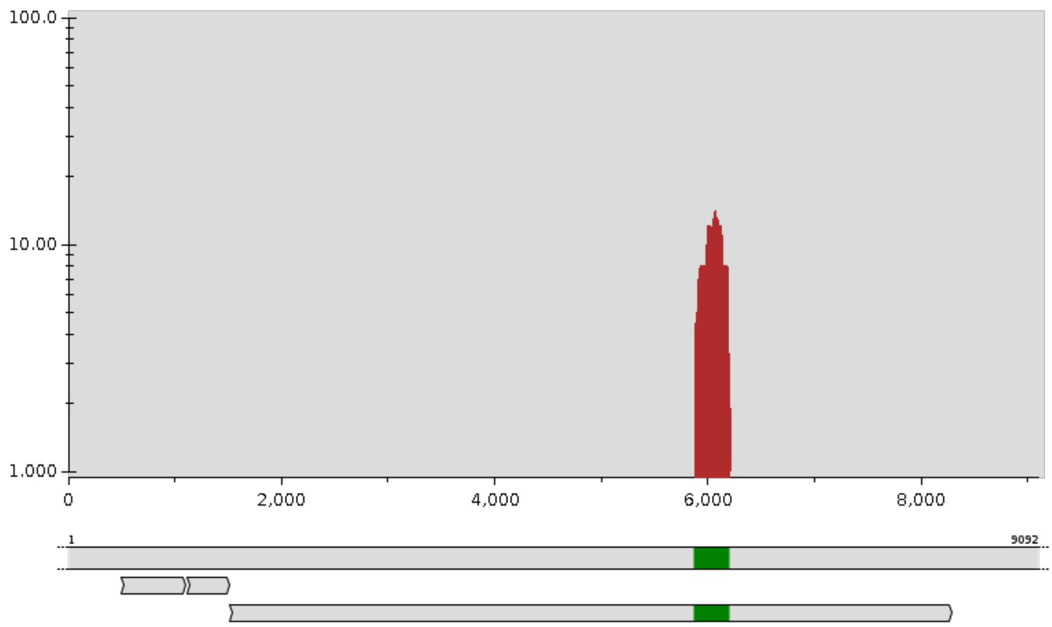

Assignment

**Type**	Badnavirus maculaucubae (Taxonomy ID: 3051986)
**Reference Genome**	NC_076606.1
**NT Identity (%)**	57.2327
**AA Identity (%)**	57.5472
**Number Of Stop Codons**	0
**Number Of CDS**	3

Alignment

**Alignment Score**	52.0 (NT) + 297.0 (AA) = 349.0
**Concordance (%)**	30.7435

Alignment Method	Global, seeded, nucleotide + amino acids (AGA)

Genome Region

Sequence starts at position 5868 and ends at position 6207 relative to NC_076606.1 reference sequence.

Alignment Detailed Statistics

	Begin	End	Coverage	Score	Concordance	Matches	Identities	I/D/M/F*	Stop Codons
**NT**	**5868**	**6207**	**3.7%**	**52**	**8.7%**	**318 (93.5%)**	**182 (53.5%)**	**0/22**	
Mutations:	5868C>A, 5871T>A, 5872A>G, 5873T>A, 5874T>A, 5875C>T, 5877G>A, 5878C>T, 5880G>A, 5884C>T, 5886T>A, 5889T>A, 5892T>A, 5895C>A, 5896C>A, 5899C>A, 5900C>G, 5901C>A, 5902A>T, 5903G>C, 5904T>A, 5906A>C, 5907G>A, 5908A>T, 5909G>C, 5910C>T, 5916T>C, 5920A>T, 5922G>A, 5928T>A, 5934T>G, 5937C>A, 5941T>A, 5944G>A, 5945G>T, 5949T>G, 5950delA, 5951C>A, 5955T>A, 5957A>T, 5958T>C, 5959_5979delCCAGAGACGGGAAAGGAGAAG, 5981A>G, 5985C>A, 5990A>C, 5991A>C, 5992C>A, 5994T>A, 6000C>A, 6001T>A, 6003T>A, 6009C>T, 6012A>G, 6014T>G, 6015G>A, 6018G>C, 6031C>A, 6032A>G, 6033T>A, 6035A>T, 6036A>G, 6040C>G, 6041A>C, 6042A>T, 6045C>T, 6047G>A, 6048C>A, 6049C>T, 6051T>A, 6054C>A, 6056G>A, 6057G>C, 6059T>A, 6060C>A, 6063C>T, 6064T>G, 6065C>A, 6066C>A, 6067A>C, 6069T>A, 6070A>C, 6075A>T, 6076G>A, 6077C>G, 6078C>A, 6081T>A, 6082G>C, 6083G>A, 6085A>G, 6086A>G, 6087T>A, 6088G>A, 6089C>A, 6092A>G, 6094G>A, 6096C>T, 6102C>T, 6111T>C, 6113T>G, 6114G>T, 6120T>A, 6123T>G, 6125T>A, 6127C>T, 6128A>G, 6129C>G, 6132G>A, 6135G>A, 6136G>A, 6137C>A, 6142G>C, 6144A>C, 6145G>C, 6146A>C, 6147A>T, 6148T>G, 6149C>A, 6150A>T, 6153C>T, 6156T>A, 6157C>G, 6158C>A, 6165G>A, 6166G>A, 6168T>A, 6171C>T, 6173T>C, 6174A>T, 6175A>T, 6176C>G, 6183T>A, 6188T>A, 6189A>T, 6191A>T, 6192C>T, 6195A>G, 6199C>T, 6203T>C								

CDS

QKP79_gp3	1454	1566	5.0%	297	42.0%	106 (93.8%)	61 (54.0%)	0/7/1/1	0
Protein mutations:	N1454K (5871T>A), I1455E (5872A>G 5873T>A 5874T>A), P1464R (5899C>A 5900C>G 5901C>A), K1466T (5906A>C 5907G>A), T1471S (5920A>T 5922G>A), I1475M (5934T>G), S1478T (5941T>A), G1479I (5944G>A 5945G>T), D1483V (5957A>T 5958T>C), P1484_K1490del (5959_5979delCCAGAGACGGGAAAGGAGAAG), K1491R (5981A>G), E1494A (5990A>C 5991A>C), F1498I (6001T>A 6003T>A), M1502R (6014T>G 6015G>A), H1508R (6031C>A 6032A>G 6033T>A), K1509M (6035A>T 6036A>G), Q1511A (6040C>G 6041A>C 6042A>T), S1513K (6047G>A 6048C>A), G1516D (6056G>A 6057G>C), I1517K (6059T>A 6060C>A), S1519E (6064T>G 6065C>A 6066C>A), I1520L (6067A>C 6069T>A), I1521L (6070A>C), K1522N (6075A>T), A1523R (6076G>A 6077C>G 6078C>A), G1525Q (6082G>C 6083G>A), N1526G (6085A>G 6086A>G 6087T>A), A1527K (6088G>A 6089C>A), K1528R (6092A>G), V1529I (6094G>A 6096C>T), L1535C (6113T>G 6114G>T), F1539Y (6125T>A), H1540W (6127C>T 6128A>G 6129C>G), A1543K (6136G>A 6137C>A), E1545H (6142G>C 6144A>C), E1546P (6145G>C 6146A>C 6147A>T), S1547D (6148T>G 6149C>A 6150A>T), P1550E (6157C>G 6158C>A), A1553T (6166G>A 6168T>A), I1555T (6173T>C 6174A>T), T1556C (6175A>T 6176C>G), D1558E (6183T>A), L1560H (6188T>A 6189A>T), Y1561F (6191A>T 6192C>T), V1565A (6203T>C)								
Codon mutations:	ATC1453..A (5868C>A), AAT1454AAA (5871T>A), ATT1455GAA (5872A>G 5873T>A 5874T>A), CTG1456TTA (5875C>T 5877G>A), CTG1457TTA (5878C>T 5880G>A), CTT1459TTA (5884C>T 5886T>A), GGT1460GGA (5889T>A), GTT1461GTA (5892T>A), ATC1462ATA (5895C>A), CGA1463AGA (5896C>A), CCC1464AGA (5899C>A 5900C>G 5901C>A), AGT1465TCA (5902A>T 5903G>C 5904T>A), AAG1466ACA (5906A>C 5907G>A), AGC1467TCT (5908A>T 5909G>C 5910C>T), CAT1469CAC (5916T>C), ACG1471TCA (5920A>T 5922G>A), GCT1473GCA (5928T>A), ATT1475ATG (5934T>G), GTC1476GTA (5937C>A), TCC1478ACC (5941T>A), GGA1479ATA (5944G>A 5945G>T), ACT1480ACG (5949T>G), ACA1481-AA (5950delA 5951C>A), ATT1482ATA (5955T>A), GAT1483GTC (5957A>T 5958T>C), CCA1484_AAG1490del (5959_5979delCCAGAGACGGGAAAGGAGAAG), AAA1491AGA (5981A>G), GGC1492GGA (5985C>A), GAA1494GCC (5990A>C 5991A>C), CGT1495AGA (5992C>A 5994T>A), GTC1497GTA (6000C>A), TTT1498ATA (6001T>A 6003T>A), TAC1500TAT (6009C>T), AAA1501AAG (6012A>G), ATG1502AGA (6014T>G 6015G>A), CTG1503CTC (6018G>C), CAT1508AGA (6031C>A 6032A>G 6033T>A), AAA1509ATG (6035A>T 6036A>G), CAA1511GCT (6040C>G 6041A>C 6042A>T), TAC1512TAT (6045C>T), AGC1513AAA (6047G>A 6048C>A), CTT1514TTA (6049C>T 6051T>A), CCC1515CCA (6054C>A), GGG1516GAC (6056G>A 6057G>C), ATC1517AAA (6059T>A 6060C>A), AAC1518AAT (6063C>T), TCC1519GAA (6064T>G 6065C>A 6066C>A), ATT1520CTA (6067A>C 6069T>A), ATA1521CTA (6070A>C), AAA1522AAT (6075A>T), GCC1523AGA (6076G>A 6077C>G 6078C>A), ATT1524ATA (6081T>A), GGA1525CAA (6082G>C 6083G>A), AAT1526GGA (6085A>G 6086A>G 6087T>A), GCA1527AAA (6088G>A 6089C>A), AAA1528AGA (6092A>G), GTC1529ATT (6094G>A 6096C>T), AGC1531AGT (6102C>T), GAT1534GAC (6111T>C), TTG1535TGT (6113T>G 6114G>T), TCT1537TCA (6120T>A), GGT1538GGG (6123T>G), TTT1539TAT (6125T>A), CAC1540TGG (6127C>T 6128A>G 6129C>G), CAG1541CAA (6132G>A), GTG1542GTA (6135G>A), GCA1543AAA (6136G>A 6137C>A), GAA1545CAC (6142G>C 6144A>C), GAA1546CCT (6145G>C 6146A>C 6147A>T), TCA1547GAT (6148T>G 6149C>A 6150A>T), AGC1548AGT (6153C>T), ATT1549ATA (6156T>A), CCA1550GAA (6157C>G 6158C>A), ACG1552ACA (6165G>A), GCT1553ACA (6166G>A 6168T>A), TTC1554TTT (6171C>T), ATA1555ACT (6173T>C 6174A>T), ACT1556TGT (6175A>T 6176C>G), GAT1558GAA (6183T>A), CTA1560CAT (6188T>A 6189A>T), TAC1561TTT (6191A>T 6192C>T), GAA1562GAG (6195A>G), CTA1564TTA (6199C>T), GTA1565GCA (6203T>C)								

Proteins

hypothetical protein (YP_010799265.1)	1454	1566	5.0%	297	42.0%	106 (93.8%)	61 (54.0%)	0/7/1/1	0
Protein mutations:	N1454K (5871T>A), I1455E (5872A>G 5873T>A 5874T>A), P1464R (5899C>A 5900C>G 5901C>A), K1466T (5906A>C 5907G>A), T1471S (5920A>T 5922G>A), I1475M (5934T>G), S1478T (5941T>A), G1479I (5944G>A 5945G>T), D1483V (5957A>T 5958T>C), P1484_K1490del (5959_5979delCCAGAGACGGGAAAGGAGAAG), K1491R (5981A>G), E1494A (5990A>C 5991A>C), F1498I (6001T>A 6003T>A), M1502R (6014T>G 6015G>A), H1508R (6031C>A 6032A>G 6033T>A), K1509M (6035A>T 6036A>G), Q1511A (6040C>G 6041A>C 6042A>T), S1513K (6047G>A 6048C>A), G1516D (6056G>A 6057G>C), I1517K (6059T>A 6060C>A), S1519E (6064T>G 6065C>A 6066C>A), I1520L (6067A>C 6069T>A), I1521L (6070A>C), K1522N (6075A>T), A1523R (6076G>A 6077C>G 6078C>A), G1525Q (6082G>C 6083G>A), N1526G (6085A>G 6086A>G 6087T>A), A1527K (6088G>A 6089C>A), K1528R (6092A>G), V1529I (6094G>A 6096C>T), L1535C (6113T>G 6114G>T), F1539Y (6125T>A), H1540W (6127C>T 6128A>G 6129C>G), A1543K (6136G>A 6137C>A), E1545H (6142G>C 6144A>C), E1546P (6145G>C 6146A>C 6147A>T), S1547D (6148T>G 6149C>A 6150A>T), P1550E (6157C>G 6158C>A), A1553T (6166G>A 6168T>A), I1555T (6173T>C 6174A>T), T1556C (6175A>T 6176C>G), D1558E (6183T>A), L1560H (6188T>A 6189A>T), Y1561F (6191A>T 6192C>T), V1565A (6203T>C)								
Codon mutations:	ATC1453..A (5868C>A), AAT1454AAA (5871T>A), ATT1455GAA (5872A>G 5873T>A 5874T>A), CTG1456TTA (5875C>T 5877G>A), CTG1457TTA (5878C>T 5880G>A), CTT1459TTA (5884C>T 5886T>A), GGT1460GGA (5889T>A), GTT1461GTA (5892T>A), ATC1462ATA (5895C>A), CGA1463AGA (5896C>A), CCC1464AGA (5899C>A 5900C>G 5901C>A), AGT1465TCA (5902A>T 5903G>C 5904T>A), AAG1466ACA (5906A>C 5907G>A), AGC1467TCT (5908A>T 5909G>C 5910C>T), CAT1469CAC (5916T>C), ACG1471TCA (5920A>T 5922G>A), GCT1473GCA (5928T>A), ATT1475ATG (5934T>G), GTC1476GTA (5937C>A), TCC1478ACC (5941T>A), GGA1479ATA (5944G>A 5945G>T), ACT1480ACG (5949T>G), ACA1481-AA (5950delA 5951C>A), ATT1482ATA (5955T>A), GAT1483GTC (5957A>T 5958T>C), CCA1484_AAG1490del (5959_5979delCCAGAGACGGGAAAGGAGAAG), AAA1491AGA (5981A>G), GGC1492GGA (5985C>A), GAA1494GCC (5990A>C 5991A>C), CGT1495AGA (5992C>A 5994T>A), GTC1497GTA (6000C>A), TTT1498ATA (6001T>A 6003T>A), TAC1500TAT (6009C>T), AAA1501AAG (6012A>G), ATG1502AGA (6014T>G 6015G>A), CTG1503CTC (6018G>C), CAT1508AGA (6031C>A 6032A>G 6033T>A), AAA1509ATG (6035A>T 6036A>G), CAA1511GCT (6040C>G 6041A>C 6042A>T), TAC1512TAT (6045C>T), AGC1513AAA (6047G>A 6048C>A), CTT1514TTA (6049C>T 6051T>A), CCC1515CCA (6054C>A), GGG1516GAC (6056G>A 6057G>C), ATC1517AAA (6059T>A 6060C>A), AAC1518AAT (6063C>T), TCC1519GAA (6064T>G 6065C>A 6066C>A), ATT1520CTA (6067A>C 6069T>A), ATA1521CTA (6070A>C), AAA1522AAT (6075A>T), GCC1523AGA (6076G>A 6077C>G 6078C>A), ATT1524ATA (6081T>A), GGA1525CAA (6082G>C 6083G>A), AAT1526GGA (6085A>G 6086A>G 6087T>A), GCA1527AAA (6088G>A 6089C>A), AAA1528AGA (6092A>G), GTC1529ATT (6094G>A 6096C>T), AGC1531AGT (6102C>T), GAT1534GAC (6111T>C), TTG1535TGT (6113T>G 6114G>T), TCT1537TCA (6120T>A), GGT1538GGG (6123T>G), TTT1539TAT (6125T>A), CAC1540TGG (6127C>T 6128A>G 6129C>G), CAG1541CAA (6132G>A), GTG1542GTA (6135G>A), GCA1543AAA (6136G>A 6137C>A), GAA1545CAC (6142G>C 6144A>C), GAA1546CCT (6145G>C 6146A>C 6147A>T), TCA1547GAT (6148T>G 6149C>A 6150A>T), AGC1548AGT (6153C>T), ATT1549ATA (6156T>A), CCA1550GAA (6157C>G 6158C>A), ACG1552ACA (6165G>A), GCT1553ACA (6166G>A 6168T>A), TTC1554TTT (6171C>T), ATA1555ACT (6173T>C 6174A>T), ACT1556TGT (6175A>T 6176C>G), GAT1558GAA (6183T>A), CTA1560CAT (6188T>A 6189A>T), TAC1561TTT (6191A>T 6192C>T), GAA1562GAG (6195A>G), CTA1564TTA (6199C>T), GTA1565GCA (6203T>C)								

*: Inserts / Deletes / Misaligned / Frameshifts

Analysis details

This analysis was performed with panviral2.64

NGS Details (UN8): Caulimovirus maculatractylodei

Assembly

**Coverage Length**	509 (1 contig(s))
**Depth Of Coverage**	4.7
**Number Of Reads**	18
**Reads Per Million**	0.30 rpm (after QC)
**Ambiguities**	0
**Assembly Method**	de novo + reference guided assembly
**Consensus Caller**	Bcf Tools

Coverage Map

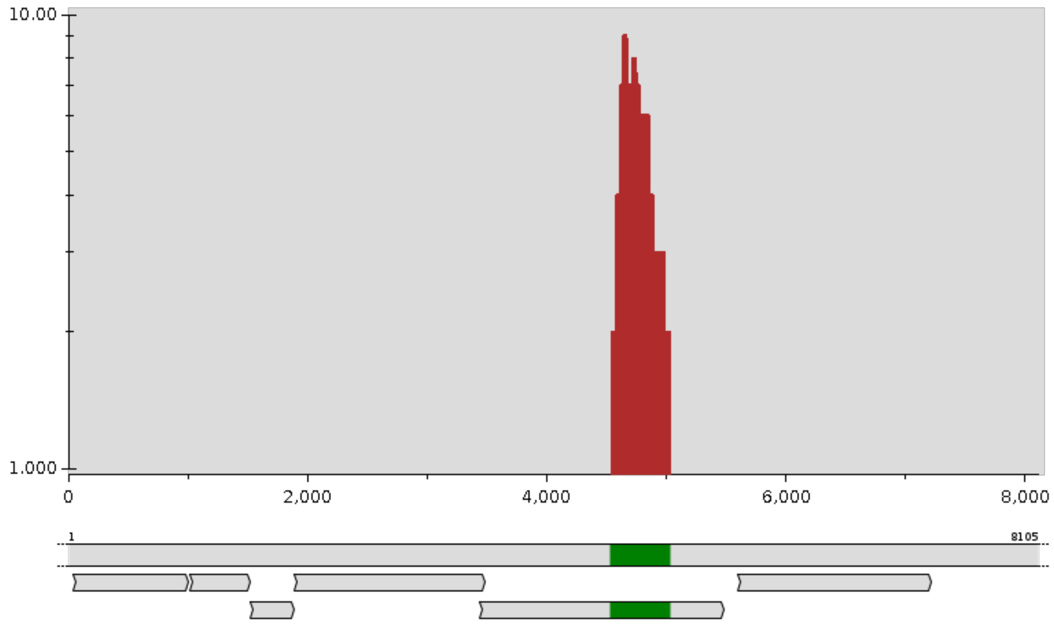

Assignment

**Type**	Caulimovirus maculatractylodei (Taxonomy ID: 3047382)
**Reference Genome**	NC_027924.1
**NT Identity (%)**	56.66
**AA Identity (%)**	47.0238
**Number Of Stop Codons**	0
**Number Of CDS**	6

Alignment

**Alignment Score**	110.0 (NT) + 569.0 (AA) = 679.0
**Concordance (%)**	31.0329

**Alignment Method**	Global, seeded, nucleotide + amino acids (AGA)

Genome Region

Sequence starts at position 4531 and ends at position 5039 relative to NC_027924.1 reference sequence.

Alignment Detailed Statistics

	Begin	End	Coverage	Score	Concordance	Matches	Identities	I/D/M/F*	Stop Codons
**NT**	**4531**	**5039**	**6.3%**	**110**	**11.2%**	**503 (98.8%)**	**285 (56.0%)**	**0/6**	
Mutations:	4533G>T, 4538C>A, 4539C>T, 4540T>G, 4541G>T, 4542T>C, 4545T>C, 4546A>T, 4547C>T, 4548G>T, 4551T>A, 4554T>A, 4558C>G, 4560G>A, 4566G>T, 4569A>T, 4570G>A, 4572C>G, 4575A>T, 4581A>G, 4584C>T, 4588C>A, 4590G>T, 4592C>T, 4593T>C, 4596G>A, 4599T>A, 4600A>G, 4601T>A, 4605C>T, 4610G>A, 4611A>T, 4612C>A, 4613A>T, 4614C>T, 4620C>T, 4621A>T, 4622A>C, 4623T>A, 4624G>A, 4625C>T, 4626G>T, 4629C>T, 4630_4632delAGG, 4633G>A, 4635G>T, 4636T>C, 4639G>A, 4640A>G, 4641A>C, 4642G>C, 4644A>T, 4649G>C, 4651T>A, 4652G>T, 4656T>C, 4659T>C, 4660G>A, 4669A>G, 4671C>T, 4672C>T, 4674C>A, 4675G>A, 4677C>A, 4680C>T, 4681A>T, 4682G>C, 4683C>A, 4684A>G, 4687A>G, 4690G>A, 4691A>T, 4692A>T, 4693A>G, 4694C>A, 4695G>T, 4696G>T, 4697A>C, 4698A>T, 4701C>T, 4702A>T, 4703G>T, 4704G>C, 4711G>C, 4715T>A, 4720A>T, 4723C>T, 4725A>C, 4726G>A, 4729C>A, 4730A>C, 4731G>T, 4732T>A, 4733G>T, 4739A>T, 4741T>A, 4742T>A, 4743G>C, 4746A>C, 4747A>T, 4749C>T, 4750A>G, 4753C>A, 4756T>A, 4757C>G, 4758C>T, 4759A>G, 4760A>C, 4761G>A, 4768G>A, 4769C>T, 4771C>A, 4773T>G, 4774T>C, 4776G>T, 4779C>T, 4780A>C, 4785A>T, 4786A>C, 4791C>A, 4793A>G, 4794T>A, 4797T>C, 4800A>T, 4803C>A, 4804T>C, 4805T>A, 4806A>T, 4809A>T, 4810A>T, 4812C>A, 4813G>T, 4821C>G, 4822T>A, 4825C>T, 4827C>T, 4834C>A, 4835A>T, 4836G>T, 4837A>T, 4838A>G, 4839T>C, 4840C>A, 4841A>G, 4842T>A, 4844T>C, 4846C>A, 4848G>T, 4852C>T, 4853A>T, 4855A>G, 4856T>C, 4858C>G, 4863G>A, 4869A>T, 4874G>C, 4876T>A, 4878A>C, 4879G>A, 4880A>C, 4881A>T, 4884T>C, 4887G>A, 4889A>C, 4890G>A, 4905C>T, 4908C>T, 4911G>A, 4913T>G, 4919C>A, 4923C>T, 4925C>T, 4927T>G, 4934A>T, 4941T>C, 4944G>A, 4945T>G, 4947A>C, 4948G>A, 4949C>G, 4950T>G, 4953G>C, 4956A>T, 4957A>T, 4959G>C, 4965T>C, 4968T>G, 4969C>T, 4970A>T, 4971A>T, 4973C>A, 4974T>C, 4976A>G, 4977A>G, 4980T>C, 4983G>A, 4986A>G, 4990_4992delGTG, 4993A>C, 4995T>C, 4996T>C, 4997G>A, 4998G>A, 4999T>C, 5007T>C, 5008G>A, 5010T>G, 5011A>G, 5012G>A, 5014G>C, 5016T>C, 5020A>G, 5021G>A, 5023C>G, 5024T>C, 5026A>G, 5028G>A, 5031C>T, 5034G>T, 5037C>T								

CDS

APL28_gp5	364	533	25.0%	569	47.0%	168 (98.8%)	79 (46.5%)	0/2/0/0	0
Protein mutations:	T366N (4538C>A 4539C>T), C367V (4540T>G 4541G>T 4542T>C), T369F (4546A>T 4547C>T 4548G>T), H371Q (4554T>A), Q373E (4558C>G 4560G>A), K375N (4566G>T), V377M (4570G>A 4572C>G), Q383N (4588C>A 4590G>T), A384V (4592C>T 4593T>C), I387E (4600A>G 4601T>A), R390N (4610G>A 4611A>T), H391I (4612C>A 4613A>T 4614C>T), N394S (4621A>T 4622A>C 4623T>A), A395I (4624G>A 4625C>T 4626G>T), R397del (4630_4632delAGG), E398N (4633G>A 4635G>T), F399L (4636T>C), E400S (4639G>A 4640A>G 4641A>C), E401H (4642G>C 4644A>T), C403S (4649G>C), C404I (4651T>A 4652G>T), V407I (4660G>A), I410V (4669A>G 4671C>T), V412I (4675G>A 4677C>A), K415E (4684A>G), N416D (4687A>G), E417I (4690G>A 4691A>T 4692A>T), T418D (4693A>G 4694C>A 4695G>T), E419S (4696G>T 4697A>C 4698A>T), R421F (4702A>T 4703G>T 4704G>C), V424L (4711G>C), I425N (4715T>A), I427F (4720A>T), L428F (4723C>T 4725A>C), E429K (4726G>A), Q430T (4729C>A 4730A>C 4731G>T), C431I (4732T>A 4733G>T), N433I (4739A>T), L434N (4741T>A 4742T>A 4743G>C), I436F (4747A>T 4749C>T), I437V (4750A>G), L438I (4753C>A), K440A (4759A>G 4760A>C 4761G>A), A443I (4768G>A 4769C>T), H444K (4771C>A 4773T>G), K447Q (4780A>C), K449Q (4786A>C), N451R (4793A>G 4794T>A), L455H (4804T>C 4805T>A 4806A>T), E456D (4809A>T), I457L (4810A>T 4812C>A), D458Y (4813G>T), S461T (4822T>A), H462Y (4825C>T 4827C>T), Q465I (4834C>A 4835A>T 4836G>T), N466C (4837A>T 4838A>G 4839T>C), H467R (4840C>A 4841A>G 4842T>A), I468T (4844T>C), L469I (4846C>A 4848G>T), H471F (4852C>T 4853A>T), I472A (4855A>G 4856T>C), H473D (4858C>G), R478T (4874G>C), L479I (4876T>A 4878A>C), E480T (4879G>A 4880A>C 4881A>T), K483T (4889A>C 4890G>A), I491S (4913T>G), T493N (4919C>A), A495V (4925C>T), S496A (4927T>G), Y498F (4934A>T), L502V (4945T>G 4947A>C), A503R (4948G>A 4949C>G 4950T>G), Q504H (4953G>C), R506C (4957A>T 4959G>C), F509L (4968T>G), Q510F (4969C>T 4970A>T 4971A>T), A511D (4973C>A 4974T>C), K512R (4976A>G 4977A>G), V517del (4990_4992delGTG), T518P (4993A>C 4995T>C), W519Q (4996T>C 4997G>A 4998G>A), S520P (4999T>C), D523K (5008G>A 5010T>G), S524D (5011A>G 5012G>A), D525H (5014G>C 5016T>C), S527D (5020A>G 5021G>A), L528A (5023C>G 5024T>C), M529V (5026A>G 5028G>A), K531N (5034G>T)								
Codon mutations:	GCG364GCT (4533G>T), ACC366AAT (4538C>A 4539C>T), TGT367GTC (4540T>G 4541G>T 4542T>C), CCT368CCC (4545T>C), ACG369TTT (4546A>T 4547C>T 4548G>T), GGT370GGA (4551T>A), CAT371CAA (4554T>A), CAG373GAA (4558C>G 4560G>A), AAG375AAT (4566G>T), GTA376GTT (4569A>T), GTC377ATG (4570G>A 4572C>G), CCA378CCT (4575A>T), GGA380GGG (4581A>G), CTC381CTT (4584C>T), CAG383AAT (4588C>A 4590G>T), GCT384GTC (4592C>T 4593T>C), CCG385CCA (4596G>A), TCT386TCA (4599T>A), ATA387GAA (4600A>G 4601T>A), TTC388TTT (4605C>T), AGA390AAT (4610G>A 4611A>T), CAC391ATT (4612C>A 4613A>T 4614C>T), AAC393AAT (4620C>T), AAT394TCA (4621A>T 4622A>C 4623T>A), GCG395ATT (4624G>A 4625C>T 4626G>T), TTC396TTT (4629C>T), AGG397del (4630_4632delAGG), GAG398AAT (4633G>A 4635G>T), TTT399CTT (4636T>C), GAA400AGC (4639G>A 4640A>G 4641A>C), GAA401CAT (4642G>C 4644A>T), TGT403TCT (4649G>C), TGT404ATT (4651T>A 4652G>T), GTT405GTC (4656T>C), TAT406TAC (4659T>C), GTT407ATT (4660G>A), ATC410GTT (4669A>G 4671C>T), CTC411TTA (4672C>T 4674C>A), GTC412ATA (4675G>A 4677C>A), TTC413TTT (4680C>T), AGC414TCA (4681A>T 4682G>C 4683C>A), AAA415GAA (4684A>G), AAT416GAT (4687A>G), GAA417ATT (4690G>A 4691A>T 4692A>T), ACG418GAT (4693A>G 4694C>A 4695G>T), GAA419TCT (4696G>T 4697A>C 4698A>T), CAC420CAT (4701C>T), AGG421TTC (4702A>T 4703G>T 4704G>C), GTC424CTC (4711G>C), ATT425AAT (4715T>A), ATC427TTC (4720A>T), CTA428TTC (4723C>T 4725A>C), GAA429AAA (4726G>A), CAG430ACT (4729C>A 4730A>C 4731G>T), TGT431ATT (4732T>A 4733G>T), AAC433ATC (4739A>T), TTG434AAC (4741T>A 4742T>A 4743G>C), GGA435GGC (4746A>C), ATC436TTT (4747A>T 4749C>T), ATC437GTC (4750A>G), CTT438ATT (4753C>A), TCC439AGT (4756T>A 4757C>G 4758C>T), AAG440GCA (4759A>G 4760A>C 4761G>A), GCA443ATA (4768G>A 4769C>T), CAT444AAG (4771C>A 4773T>G), TTG445CTT (4774T>C 4776G>T), TTC446TTT (4779C>T), AAA447CAA (4780A>C), ACA448ACT (4785A>T), AAG449CAG (4786A>C), ATC450ATA (4791C>A), AAT451AGA (4793A>G 4794T>A), TTT452TTC (4797T>C), CTA453CTT (4800A>T), GGC454GGA (4803C>A), TTA455CAT (4804T>C 4805T>A 4806A>T), GAA456GAT (4809A>T), ATC457TTA (4810A>T 4812C>A), GAT458TAT (4813G>T), GGC460GGG (4821C>G), TCT461ACT (4822T>A), CAC462TAT (4825C>T 4827C>T), CAG465ATT (4834C>A 4835A>T 4836G>T), AAT466TGC (4837A>T 4838A>G 4839T>C), CAT467AGA (4840C>A 4841A>G 4842T>A), ATT468ACT (4844T>C), CTG469ATT (4846C>A 4848G>T), CAT471TTT (4852C>T 4853A>T), ATT472GCT (4855A>G 4856T>C), CAT473GAT (4858C>G), AAG474AAA (4863G>A), CCA476CCT (4869A>T), AGA478ACA (4874G>C), TTA479ATC (4876T>A 4878A>C), GAA480ACT (4879G>A 4880A>C 4881A>T), GAT481GAC (4884T>C), AAG482AAA (4887G>A), AAG483ACA (4889A>C 4890G>A), TTC488TTT (4905C>T), CTC489CTT (4908C>T), GGG490GGA (4911G>A), ATC491AGC (4913T>G), ACT493AAT (4919C>A), TAC494TAT (4923C>T), GCT495GTT (4925C>T), TCT496GCT (4927T>G), TAC498TTC (4934A>T), CCT500CCC (4941T>C), AAG501AAA (4944G>A), TTA502GTC (4945T>G 4947A>C), GCT503AGG (4948G>A 4949C>G 4950T>G), CAG504CAC (4953G>C), ATA505ATT (4956A>T), AGG506TGC (4957A>T 4959G>C), CCT508CCC (4965T>C), TTT509TTG (4968T>G), CAA510TTT (4969C>T 4970A>T 4971A>T), GCT511GAC (4973C>A 4974T>C), AAA512AGG (4976A>G 4977A>G), CTT513CTC (4980T>C), AAG514AAA (4983G>A), AAA515AAG (4986A>G), GTG517del (4990_4992delGTG), ACT518CCC (4993A>C 4995T>C), TGG519CAA (4996T>C 4997G>A 4998G>A), TCT520CCT (4999T>C), ACT522ACC (5007T>C), GAT523AAG (5008G>A 5010T>G), AGT524GAT (5011A>G 5012G>A), GAT525CAC (5014G>C 5016T>C), AGT527GAT (5020A>G 5021G>A), CTT528GCT (5023C>G 5024T>C), ATG529GTA (5026A>G 5028G>A), ATC530ATT (5031C>T), AAG531AAT (5034G>T), ATC532ATT (5037C>T)								

Proteins

ORF5 (YP_009165750.1)	364	533	25.0%	569	47.0%	168 (98.8%)	79 (46.5%)	0/2/0/0	0
Protein mutations:	T366N (4538C>A 4539C>T), C367V (4540T>G 4541G>T 4542T>C), T369F (4546A>T 4547C>T 4548G>T), H371Q (4554T>A), Q373E (4558C>G 4560G>A), K375N (4566G>T), V377M (4570G>A 4572C>G), Q383N (4588C>A 4590G>T), A384V (4592C>T 4593T>C), I387E (4600A>G 4601T>A), R390N (4610G>A 4611A>T), H391I (4612C>A 4613A>T 4614C>T), N394S (4621A>T 4622A>C 4623T>A), A395I (4624G>A 4625C>T 4626G>T), R397del (4630_4632delAGG), E398N (4633G>A 4635G>T), F399L (4636T>C), E400S (4639G>A 4640A>G 4641A>C), E401H (4642G>C 4644A>T), C403S (4649G>C), C404I (4651T>A 4652G>T), V407I (4660G>A), I410V (4669A>G 4671C>T), V412I (4675G>A 4677C>A), K415E (4684A>G), N416D (4687A>G), E417I (4690G>A 4691A>T 4692A>T), T418D (4693A>G 4694C>A 4695G>T), E419S (4696G>T 4697A>C 4698A>T), R421F (4702A>T 4703G>T 4704G>C), V424L (4711G>C), I425N (4715T>A), I427F (4720A>T), L428F (4723C>T 4725A>C), E429K (4726G>A), Q430T (4729C>A 4730A>C 4731G>T), C431I (4732T>A 4733G>T), N433I (4739A>T), L434N (4741T>A 4742T>A 4743G>C), I436F (4747A>T 4749C>T), I437V (4750A>G), L438I (4753C>A), K440A (4759A>G 4760A>C 4761G>A), A443I (4768G>A 4769C>T), H444K (4771C>A 4773T>G), K447Q (4780A>C), K449Q (4786A>C), N451R (4793A>G 4794T>A), L455H (4804T>C 4805T>A 4806A>T), E456D (4809A>T), I457L (4810A>T 4812C>A), D458Y (4813G>T), S461T (4822T>A), H462Y (4825C>T 4827C>T), Q465I (4834C>A 4835A>T 4836G>T), N466C (4837A>T 4838A>G 4839T>C), H467R (4840C>A 4841A>G 4842T>A), I468T (4844T>C), L469I (4846C>A 4848G>T), H471F (4852C>T 4853A>T), I472A (4855A>G 4856T>C), H473D (4858C>G), R478T (4874G>C), L479I (4876T>A 4878A>C), E480T (4879G>A 4880A>C 4881A>T), K483T (4889A>C 4890G>A), I491S (4913T>G), T493N (4919C>A), A495V (4925C>T), S496A (4927T>G), Y498F (4934A>T), L502V (4945T>G 4947A>C), A503R (4948G>A 4949C>G 4950T>G), Q504H (4953G>C), R506C (4957A>T 4959G>C), F509L (4968T>G), Q510F (4969C>T 4970A>T 4971A>T), A511D (4973C>A 4974T>C), K512R (4976A>G 4977A>G), V517del (4990_4992delGTG), T518P (4993A>C 4995T>C), W519Q (4996T>C 4997G>A 4998G>A), S520P (4999T>C), D523K (5008G>A 5010T>G), S524D (5011A>G 5012G>A), D525H (5014G>C 5016T>C), S527D (5020A>G 5021G>A), L528A (5023C>G 5024T>C), M529V (5026A>G 5028G>A), K531N (5034G>T)								

	Begin	End	Coverage	Score	Concordance	Matches	Identities	I/D/M/F*	Stop Codons
**NT**	**4531**	**5039**	**6.3%**	**110**	**11.2%**	**503 (98.8%)**	**285 (56.0%)**	**0/6**	
Codon mutations:	GCG364GCT (4533G>T), ACC366AAT (4538C>A 4539C>T), TGT367GTC (4540T>G 4541G>T 4542T>C), CCT368CCC (4545T>C), ACG369TTT (4546A>T 4547C>T 4548G>T), GGT370GGA (4551T>A), CAT371CAA (4554T>A), CAG373GAA (4558C>G 4560G>A), AAG375AAT (4566G>T), GTA376GTT (4569A>T), GTC377ATG (4570G>A 4572C>G), CCA378CCT (4575A>T), GGA380GGG (4581A>G), CTC381CTT (4584C>T), CAG383AAT (4588C>A 4590G>T), GCT384GTC (4592C>T 4593T>C), CCG385CCA (4596G>A), TCT386TCA (4599T>A), ATA387GAA (4600A>G 4601T>A), TTC388TTT (4605C>T), AGA390AAT (4610G>A 4611A>T), CAC391ATT (4612C>A 4613A>T 4614C>T), AAC393AAT (4620C>T), AAT394TCA (4621A>T 4622A>C 4623T>A), GCG395ATT (4624G>A 4625C>T 4626G>T), TTC396TTT (4629C>T), AGG397del (4630_4632delAGG), GAG398AAT (4633G>A 4635G>T), TTT399CTT (4636T>C), GAA400AGC (4639G>A 4640A>G 4641A>C), GAA401CAT (4642G>C 4644A>T), TGT403TCT (4649G>C), TGT404ATT (4651T>A 4652G>T), GTT405GTC (4656T>C), TAT406TAC (4659T>C), GTT407ATT (4660G>A), ATC410GTT (4669A>G 4671C>T), CTC411TTA (4672C>T 4674C>A), GTC412ATA (4675G>A 4677C>A), TTC413TTT (4680C>T), AGC414TCA (4681A>T 4682G>C 4683C>A), AAA415GAA (4684A>G), AAT416GAT (4687A>G), GAA417ATT (4690G>A 4691A>T 4692A>T), ACG418GAT (4693A>G 4694C>A 4695G>T), GAA419TCT (4696G>T 4697A>C 4698A>T), CAC420CAT (4701C>T), AGG421TTC (4702A>T 4703G>T 4704G>C), GTC424CTC (4711G>C), ATT425AAT (4715T>A), ATC427TTC (4720A>T), CTA428TTC (4723C>T 4725A>C), GAA429AAA (4726G>A), CAG430ACT (4729C>A 4730A>C 4731G>T), TGT431ATT (4732T>A 4733G>T), AAC433ATC (4739A>T), TTG434AAC (4741T>A 4742T>A 4743G>C), GGA435GGC (4746A>C), ATC436TTT (4747A>T 4749C>T), ATC437GTC (4750A>G), CTT438ATT (4753C>A), TCC439AGT (4756T>A 4757C>G 4758C>T), AAG440GCA (4759A>G 4760A>C 4761G>A), GCA443ATA (4768G>A 4769C>T), CAT444AAG (4771C>A 4773T>G), TTG445CTT (4774T>C 4776G>T), TTC446TTT (4779C>T), AAA447CAA (4780A>C), ACA448ACT (4785A>T), AAG449CAG (4786A>C), ATC450ATA (4791C>A), AAT451AGA (4793A>G 4794T>A), TTT452TTC (4797T>C), CTA453CTT (4800A>T), GGC454GGA (4803C>A), TTA455CAT (4804T>C 4805T>A 4806A>T), GAA456GAT (4809A>T), ATC457TTA (4810A>T 4812C>A), GAT458TAT (4813G>T), GGC460GGG (4821C>G), TCT461ACT (4822T>A), CAC462TAT (4825C>T 4827C>T), CAG465ATT (4834C>A 4835A>T 4836G>T), AAT466TGC (4837A>T 4838A>G 4839T>C), CAT467AGA (4840C>A 4841A>G 4842T>A), ATT468ACT (4844T>C), CTG469ATT (4846C>A 4848G>T), CAT471TTT (4852C>T 4853A>T), ATT472GCT (4855A>G 4856T>C), CAT473GAT (4858C>G), AAG474AAA (4863G>A), CCA476CCT (4869A>T), AGA478ACA (4874G>C), TTA479ATC (4876T>A 4878A>C), GAA480ACT (4879G>A 4880A>C 4881A>T), GAT481GAC (4884T>C), AAG482AAA (4887G>A), AAG483ACA (4889A>C 4890G>A), TTC488TTT (4905C>T), CTC489CTT (4908C>T), GGG490GGA (4911G>A), ATC491AGC (4913T>G), ACT493AAT (4919C>A), TAC494TAT (4923C>T), GCT495GTT (4925C>T), TCT496GCT (4927T>G), TAC498TTC (4934A>T), CCT500CCC (4941T>C), AAG501AAA (4944G>A), TTA502GTC (4945T>G 4947A>C), GCT503AGG (4948G>A 4949C>G 4950T>G), CAG504CAC (4953G>C), ATA505ATT (4956A>T), AGG506TGC (4957A>T 4959G>C), CCT508CCC (4965T>C), TTT509TTG (4968T>G), CAA510TTT (4969C>T 4970A>T 4971A>T), GCT511GAC (4973C>A 4974T>C), AAA512AGG (4976A>G 4977A>G), CTT513CTC (4980T>C), AAG514AAA (4983G>A), AAA515AAG (4986A>G), GTG517del (4990_4992delGTG), ACT518CCC (4993A>C 4995T>C), TGG519CAA (4996T>C 4997G>A 4998G>A), TCT520CCT (4999T>C), ACT522ACC (5007T>C), GAT523AAG (5008G>A 5010T>G), AGT524GAT (5011A>G 5012G>A), GAT525CAC (5014G>C 5016T>C), AGT527GAT (5020A>G 5021G>A), CTT528GCT (5023C>G 5024T>C), ATG529GTA (5026A>G 5028G>A), ATC530ATT (5031C>T), AAG531AAT (5034G>T), ATC532ATT (5037C>T)								

*: Inserts / Deletes / Misaligned / Frameshifts

Analysis details

This analysis was performed with panviral2.64

NGS Details (UN8): Cavemovirus venamanihotis

Assembly

**Coverage Length**	379 (1 contig(s))
**Depth Of Coverage**	4.8
**Number Of Reads**	15
**Reads Per Million**	0.25 rpm (after QC)
**Ambiguities**	0
**Assembly Method**	de novo + reference guided assembly
**Consensus Caller**	Bcf Tools

Coverage Map

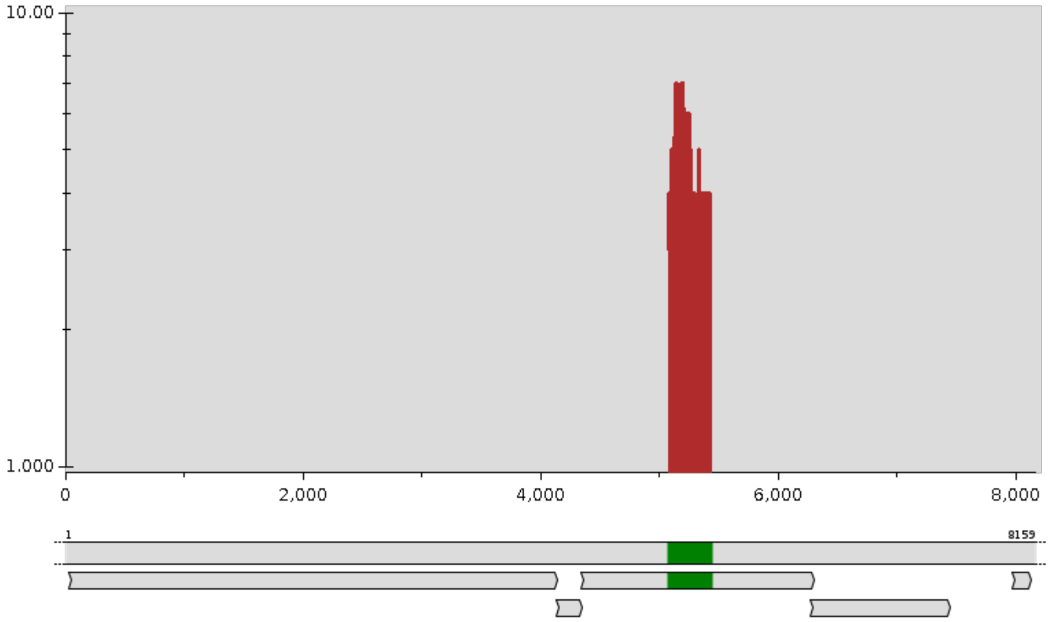

Assignment

**Type**	Cavemovirus venamanihotis (Taxonomy ID: 3047736)
**Reference Genome**	NC_001648.1
**NT Identity (%)**	60.1583
**AA Identity (%)**	50.0
**Number Of Stop Codons**	2
**Number Of CDS**	5

Alignment

**Alignment Score**	154.0 (NT) + 452.0 (AA) = 606.0
**Concordance (%)**	36.2657

**Alignment Method**	Global, seeded, nucleotide + amino acids (AGA)

Genome Region

Sequence starts at position 5072 and ends at position 5450 relative to NC_001648.1 reference sequence.

Alignment Detailed Statistics

	Begin	End	Coverage	Score	Concordance	Matches	Identities	I/D/M/F*	Stop Codons
**NT**	**5072**	**5450**	**4.6%**	**154**	**20.3%**	**379 (100%)**	**228 (60.2%)**	**0/0**	

Mutations: 5075T>G, 5076A>T, 5079C>A, 5081A>T, 5088A>T, 5089T>A, 5090A>T, 5093A>C, 5099A>T, 5100C>A, 5101A>C, 5102T>G, 5103T>G, 5109C>A, 5112A>G, 5115A>C, 5118G>A, 5119G>C, 5121A>G, 5122A>T, 5123A>T, 5124A>C, 5127A>C, 5130A>T, 5132G>A, 5135A>T, 5139G>A, 5141T>C, 5148G>T, 5149A>C, 5160A>T, 5161A>T, 5162A>T, 5163G>T, 5164C>T, 5165T>A, 5168A>G, 5169G>T, 5170T>G, 5172G>A, 5174A>T, 5176A>G, 5177A>G, 5186C>T, 5196G>A, 5197A>G, 5198T>G, 5199A>G, 5200C>A, 5201A>C, 5205A>T, 5208C>T, 5209A>C, 5214A>T, 5215G>T, 5216T>A, 5217A>T, 5218T>A, 5219A>T, 5220C>G, 5222G>T, 5227G>A, 5228A>C, 5229T>A, 5230A>T, 5231C>T, 5233A>T, 5235A>T, 5236G>C, 5237T>A, 5248G>T, 5249T>A, 5260T>A, 5263A>G, 5264T>G, 5267T>A, 5270A>T, 5271A>T, 5274T>A, 5276A>T, 5277G>T, 5278A>T, 5279A>T, 5280G>A, 5285T>G, 5286A>C, 5287G>A, 5290A>C, 5291A>T, 5292A>T, 5294A>T, 5295T>A, 5296A>G, 5297T>A, 5300A>T, 5302C>T, 5308C>A, 5309A>T, 5315G>T, 5316C>T, 5317A>T, 5318G>T, 5322T>C, 5323A>C, 5324T>A, 5328C>G, 5330G>A, 5336A>C, 5337G>T, 5339A>T, 5340T>A, 5342A>G, 5345A>T, 5349G>T, 5351A>T, 5353A>T, 5354T>G, 5355C>A, 5357T>A, 5360C>T, 5361T>G, 5362C>T, 5363A>C, 5366A>T, 5367A>T, 5368G>C, 5370A>G, 5371T>A, 5372T>A, 5375C>T, 5378A>G, 5379C>A, 5382T>A, 5384T>C, 5388G>A, 5391A>G, 5392G>A, 5393A>T, 5396A>T, 5401G>A, 5402A>T, 5410A>T, 5411T>A, 5420T>C, 5426G>T, 5438T>C, 5441A>T, 5444A>G, 5445G>A, 5447C>T, 5449T>A

CDS

	Begin	End	Coverage	Score	Concordance	Matches	Identities	I/D/M/F*	Stop Codons
CsVMVgp3	244	369	19.3%	452	49.0%	126 (100%)	63 (50.0%)	0/0/0/0	2

Protein mutations: S245C (5076A>T), P246T (5079C>A 5081A>T), I249Y (5088A>T 5089T>A 5090A>T), K252N (5099A>T), H253T (5100C>A 5101A>C 5102T>G), S254A (5103T>G), Q256K (5109C>A), K257E (5112A>G), G259T (5118G>A 5119G>C), K260V (5121A>G 5122A>T 5123A>T), T261P (5124A>C), M263L (5130A>T 5132G>A), D266N (5139G>A 5141T>C), D269S (5148G>T 5149A>C), K273F (5160A>T 5161A>T 5162A>T), A274L (5163G>T 5164C>T 5165T>A), V276* (5169G>T 5170T>G), V277I (5172G>A 5174A>T), K278R (5176A>G 5177A>G), D285R (5196G>A 5197A>G 5198T>G), T286D (5199A>G 5200C>A 5201A>C), I288L (5205A>T), H289S (5208C>T 5209A>C), S291L (5214A>T 5215G>T 5216T>A), I292Y (5217A>T 5218T>A 5219A>T), Q293D (5220C>G 5222G>T), R295N (5227G>A 5228A>C), Y296I (5229T>A 5230A>T 5231C>T), Y297F (5233A>T), C302L (5248G>T 5249T>A), F306Y (5260T>A), Y307W (5263A>G 5264T>G), H308Q (5267T>A), K310* (5271A>T), L311I (5274T>A 5276A>T), E312F (5277G>T 5278A>T 5279A>T), E313K (5280G>A), D314E (5285T>G), S315H (5286A>C 5287G>A), K316T (5290A>C 5291A>T), K317Y (5292A>T 5294A>T), Y318R (5295T>A 5296A>G 5297T>A), A320V (5302C>T), T322N (5308C>A 5309A>T), Q325F (5316C>T 5317A>T 5318G>T), Y327P (5322T>C 5323A>C 5324T>A), Q329E (5328C>G 5330G>A), K331N (5336A>C), V332F (5337G>T 5339A>T), L333M (5340T>A 5342A>G), G336C (5349G>T 5351A>T), Y337L (5353A>T 5354T>G), H338K (5355C>A 5357T>A), S340V (5361T>G 5362C>T 5363A>C), I343E (5370A>G 5371T>A 5372T>A), Q346K (5379C>A), F347I (5382T>A 5384T>C), D349N (5388G>A), R350D (5391A>G 5392G>A 5393A>T), R353N (5401G>A 5402A>T), Y356L (5410A>T 5411T>A), V368I (5445G>A 5447C>T), F369Y (5449T>A)

Codon mutations: TCT244TCG (5075T>G), AGC245TGC (5076A>T), CCA246ACT (5079C>A 5081A>T), ATA249TAT (5088A>T 5089T>A 5090A>T), GTA250GTC (5093A>C), AAA252AAT (5099A>T), CAT253ACG (5100C>A 5101A>C 5102T>G), TCA254GCA (5103T>G), CAA256AAA (5109C>A), AAA257GAA (5112A>G), AGA258CGA (5115A>C), GGA259ACA (5118G>A 5119G>C), AAA260GTT (5121A>G 5122A>T 5123A>T), ACT261CCT (5124A>C), AGA262CGA (5127A>C), ATG263TTA (5130A>T 5132G>A), GTA264GTT (5135A>T), GAT266AAC (5139G>A 5141T>C), GAT269TCT (5148G>T 5149A>C), AAA273TTT (5160A>T 5161A>T 5162A>T), GCT274TTA (5163G>T 5164C>T 5165T>A), AAA275AAG (5168A>G), GTA276TGA (5169G>T 5170T>G), GTA277ATT (5172G>A 5174A>T), AAA278AGG (5176A>G 5177A>G), ATC281ATT (5186C>T), GAT285AGG (5196G>A 5197A>G 5198T>G), ACA286GAC (5199A>G 5200C>A 5201A>C), ATA288TTA (5205A>T), CAT289TCT (5208C>T 5209A>C), AGT291TTA (5214A>T 5215G>T 5216T>A), ATA292TAT (5217A>T 5218T>A 5219A>T), CAG293GAT (5220C>G 5222G>T), AGA295AAC (5227G>A 5228A>C), TAC296ATT (5229T>A 5230A>T 5231C>T), TAT297TTT (5233A>T), AGT298TCA (5235A>T 5236G>C 5237T>A), TGT302TTA (5248G>T 5249T>A), TTT306TAT (5260T>A), TAT307TGG (5263A>G 5264T>G), CAT308CAA (5267T>A), ATA309ATT (5270A>T), AAG310TAG (5271A>T), TTA311ATT (5274T>A 5276A>T), GAA312TTT (5277G>T 5278A>T 5279A>T), GAA313AAA (5280G>A), GAT314GAG (5285T>G), AGT315CAT (5286A>C 5287G>A), AAA316ACT (5290A>C 5291A>T), AAA317TAT (5292A>T 5294A>T), TAT318AGA (5295T>A 5296A>G 5297T>A), ACA319ACT (5300A>T), GCT320GTT (5302C>T), ACA322AAT (5308C>A 5309A>T), CCG324CCT (5315G>T), CAG325TTT (5316C>T 5317A>T 5318G>T), TAT327CCA (5322T>C 5323A>C 5324T>A), CAG329GAA (5328C>G 5330G>A), AAA331AAC (5336A>C), GTA332TTT (5337G>T 5339A>T), TTA333ATG (5340T>A 5342A>G), CCA334CCT (5345A>T), GGA336TGT (5349G>T 5351A>T), TAT337TTG (5353A>T 5354T>G), CAT338AAA (5355C>A 5357T>A), AAC339AAT (5360C>T), TCA340GTC (5361T>G 5362C>T 5363A>C), CCA341CCT (5366A>T), AGT342TCT (5367A>T 5368G>C), ATT343GAA (5370A>G 5371T>A 5372T>A), TTC344TTT (5375C>T), CAA345CAG (5378A>G), CAA346AAA (5379C>A), TTT347ATC (5382T>A 5384T>C), GAT349AAT (5388G>A), AGA350GAT (5391A>G 5392G>A 5393A>T), ATA351ATT (5396A>T), AGA353AAT (5401G>A 5402A>T), TAT356TTA (5410A>T 5411T>A), ATT359ATC (5420T>C), GTG361GTT (5426G>T), GAT365GAC (5438T>C), ATA366ATT (5441A>T), TTA367TTG (5444A>G), GTC368ATT (5445G>A 5447C>T), TTT369TAT (5449T>A)

Proteins

	Begin	End	Coverage	Score	Concordance	Matches	Identities	I/D/M/F*	Stop Codons
aspartic protease/reverse transcriptase (NP_056848.1)	244	369	19.3%	452	49.0%	126 (100%)	63 (50.0%)	0/0/0/0	2

Protein mutations: S245C (5076A>T), P246T (5079C>A 5081A>T), I249Y (5088A>T 5089T>A 5090A>T), K252N (5099A>T), H253T (5100C>A 5101A>C 5102T>G), S254A (5103T>G), Q256K (5109C>A), K257E (5112A>G), G259T (5118G>A 5119G>C), K260V (5121A>G 5122A>T 5123A>T), T261P (5124A>C), M263L (5130A>T 5132G>A), D266N (5139G>A 5141T>C), D269S (5148G>T 5149A>C), K273F (5160A>T 5161A>T 5162A>T), A274L (5163G>T 5164C>T 5165T>A), V276* (5169G>T 5170T>G), V277I (5172G>A 5174A>T), K278R (5176A>G 5177A>G), D285R (5196G>A 5197A>G 5198T>G), T286D (5199A>G 5200C>A 5201A>C), I288L (5205A>T), H289S (5208C>T 5209A>C), S291L (5214A>T 5215G>T 5216T>A), I292Y (5217A>T 5218T>A 5219A>T), Q293D (5220C>G 5222G>T), R295N (5227G>A 5228A>C), Y296I (5229T>A 5230A>T 5231C>T), Y297F (5233A>T), C302L (5248G>T 5249T>A), F306Y (5260T>A), Y307W (5263A>G 5264T>G), H308Q (5267T>A), K310* (5271A>T), L311I (5274T>A 5276A>T), E312F (5277G>T 5278A>T 5279A>T), E313K (5280G>A), D314E (5285T>G), S315H (5286A>C 5287G>A), K316T (5290A>C 5291A>T), K317Y (5292A>T 5294A>T), Y318R (5295T>A 5296A>G 5297T>A), A320V (5302C>T), T322N (5308C>A 5309A>T), Q325F (5316C>T 5317A>T 5318G>T), Y327P (5322T>C 5323A>C 5324T>A), Q329E (5328C>G 5330G>A), K331N (5336A>C), V332F (5337G>T 5339A>T), L333M (5340T>A 5342A>G), G336C (5349G>T 5351A>T), Y337L (5353A>T 5354T>G), H338K (5355C>A 5357T>A), S340V (5361T>G 5362C>T 5363A>C), I343E (5370A>G 5371T>A 5372T>A), Q346K (5379C>A), F347I (5382T>A 5384T>C), D349N (5388G>A), R350D (5391A>G 5392G>A 5393A>T), R353N (5401G>A 5402A>T), Y356L (5410A>T 5411T>A), V368I (5445G>A 5447C>T), F369Y (5449T>A)

Codon mutations: TCT244TCG (5075T>G), AGC245TGC (5076A>T), CCA246ACT (5079C>A 5081A>T), ATA249TAT (5088A>T 5089T>A 5090A>T), GTA250GTC (5093A>C), AAA252AAT (5099A>T), CAT253ACG (5100C>A 5101A>C 5102T>G), TCA254GCA (5103T>G), CAA256AAA (5109C>A), AAA257GAA (5112A>G), AGA258CGA (5115A>C), GGA259ACA (5118G>A 5119G>C), AAA260GTT (5121A>G 5122A>T 5123A>T), ACT261CCT (5124A>C), AGA262CGA (5127A>C), ATG263TTA (5130A>T 5132G>A), GTA264GTT (5135A>T), GAT266AAC (5139G>A 5141T>C), GAT269TCT (5148G>T 5149A>C), AAA273TTT (5160A>T 5161A>T 5162A>T), GCT274TTA (5163G>T 5164C>T 5165T>A), AAA275AAG (5168A>G), GTA276TGA (5169G>T 5170T>G), GTA277ATT (5172G>A 5174A>T), AAA278AGG (5176A>G 5177A>G), ATC281ATT (5186C>T), GAT285AGG (5196G>A 5197A>G 5198T>G), ACA286GAC (5199A>G 5200C>A 5201A>C), ATA288TTA (5205A>T), CAT289TCT (5208C>T 5209A>C), AGT291TTA (5214A>T 5215G>T 5216T>A), ATA292TAT (5217A>T 5218T>A 5219A>T), CAG293GAT (5220C>G 5222G>T), AGA295AAC (5227G>A 5228A>C), TAC296ATT (5229T>A 5230A>T 5231C>T), TAT297TTT (5233A>T), AGT298TCA (5235A>T 5236G>C 5237T>A), TGT302TTA (5248G>T 5249T>A), TTT306TAT (5260T>A), TAT307TGG (5263A>G 5264T>G), CAT308CAA (5267T>A), ATA309ATT (5270A>T), AAG310TAG (5271A>T), TTA311ATT (5274T>A 5276A>T), GAA312TTT (5277G>T 5278A>T 5279A>T), GAA313AAA (5280G>A), GAT314GAG (5285T>G), AGT315CAT (5286A>C 5287G>A), AAA316ACT (5290A>C 5291A>T), AAA317TAT (5292A>T 5294A>T), TAT318AGA (5295T>A 5296A>G 5297T>A), ACA319ACT (5300A>T), GCT320GTT (5302C>T), ACA322AAT (5308C>A 5309A>T), CCG324CCT (5315G>T), CAG325TTT (5316C>T 5317A>T 5318G>T), TAT327CCA (5322T>C 5323A>C 5324T>A), CAG329GAA (5328C>G 5330G>A), AAA331AAC (5336A>C), GTA332TTT (5337G>T 5339A>T), TTA333ATG (5340T>A 5342A>G), CCA334CCT (5345A>T), GGA336TGT (5349G>T 5351A>T), TAT337TTG (5353A>T 5354T>G), CAT338AAA (5355C>A 5357T>A), AAC339AAT (5360C>T), TCA340GTC (5361T>G 5362C>T 5363A>C), CCA341CCT (5366A>T), AGT342TCT (5367A>T 5368G>C), ATT343GAA (5370A>G 5371T>A 5372T>A), TTC344TTT (5375C>T), CAA345CAG (5378A>G), CAA346AAA (5379C>A), TTT347ATC (5382T>A 5384T>C), GAT349AAT (5388G>A), AGA350GAT (5391A>G 5392G>A 5393A>T), ATA351ATT (5396A>T), AGA353AAT (5401G>A 5402A>T), TAT356TTA (5410A>T 5411T>A), ATT359ATC (5420T>C), GTG361GTT (5426G>T), GAT365GAC (5438T>C), ATA366ATT (5441A>T), TTA367TTG (5444A>G), GTC368ATT (5445G>A 5447C>T), TTT369TAT (5449T>A)

*: Inserts / Deletes / Misaligned / Frameshifts

Analysis details

This analysis was performed with panviral2.64

NGS Details (UN8): Errantivirus

Assembly

**Coverage Length**	314 (1 contig(s))
**Depth Of Coverage**	5.4
**Number Of Reads**	14
**Reads Per Million**	0.23 rpm (after QC)
**Ambiguities**	0
**Assembly Method**	de novo + reference guided assembly
**Consensus Caller**	Bcf Tools

Coverage Map

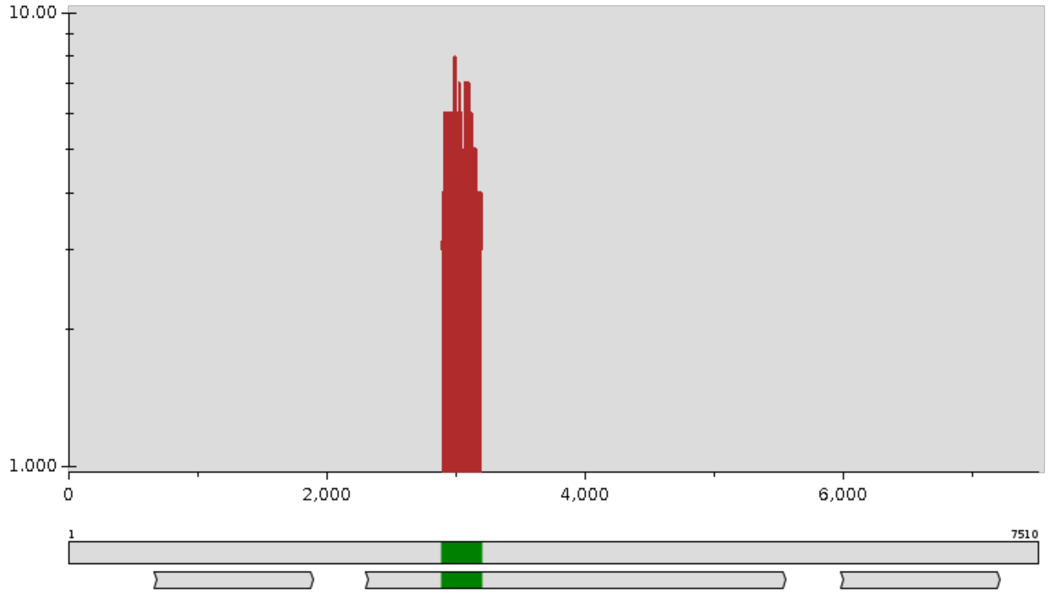

Assignment

**Type**	Errantivirus (Taxonomy ID: 186666)
**Reference Genome**	NC_038512.1
**NT Identity (%)**	56.3694
**AA Identity (%)**	48.5714
**Number Of Stop Codons**	1
**Number Of CDS**	3

Alignment

**Alignment Score**	80.0 (NT) + 360.0 (AA) = 440.0
**Concordance (%)**	32.1637

Alignment Method	Global, seeded, nucleotide + amino acids (AGA)

Genome Region

Sequence starts at position 2893 and ends at position 3206 relative to NC_038512.1 reference sequence.

Alignment Detailed Statistics

	Begin	End	Coverage	Score	Concordance	Matches	Identities	I/D/M/F*	Stop Codons
**NT**	**2893**	**3206**	**4.2%**	**80**	**12.7%**	**314 (100%)**	**177 (56.4%)**	**0/0**	
Mutations:	2893A>T, 2899C>T, 2901T>A, 2902C>T, 2905T>C, 2906A>G, 2914C>T, 2916A>C, 2917G>A, 2920G>A, 2923T>A, 2927G>A, 2929T>A, 2932C>T, 2937A>T, 2938C>T, 2939C>T, 2941G>T, 2944A>T, 2948A>G, 2949A>T, 2950C>A, 2953A>T, 2954A>G, 2956T>A, 2959C>A, 2960G>T, 2962A>G, 2963C>T, 2964T>A, 2965T>G, 2968C>G, 2969A>G, 2971G>A, 2972T>C, 2974A>G, 2975G>T, 2976G>T, 2978A>G, 2979A>G, 2981T>G, 2982G>C, 2983C>T, 2984C>A, 2989C>T, 2993A>T, 2995C>T, 2997C>A, 2998C>A, 3004T>C, 3007G>A, 3008G>C, 3009C>G, 3010A>C, 3011A>C, 3012G>A, 3013T>A, 3016G>T, 3018T>A, 3025G>A, 3028G>T, 3029G>C, 3035G>A, 3037C>A, 3038C>G, 3039C>A, 3040T>G, 3041C>G, 3043A>T, 3047A>G, 3049A>G, 3050T>G, 3051C>A, 3058C>T, 3061G>T, 3065A>C, 3068G>A, 3069T>C, 3070A>T, 3071G>C, 3073A>T, 3076C>T, 3088A>G, 3091C>T, 3092C>T, 3094T>G, 3095C>G, 3096G>T, 3097A>T, 3103T>C, 3104A>T, 3106G>T, 3109A>C, 3114A>C, 3115A>C, 3118C>T, 3119T>G, 3121A>T, 3122C>T, 3130T>C, 3135A>T, 3137A>T, 3138G>C, 3140G>T, 3142T>A, 3146G>A, 3148C>T, 3149A>G, 3151T>G, 3154C>A, 3155C>T, 3157A>T, 3158A>C, 3159G>A, 3160A>G, 3161G>C, 3162G>T, 3165T>A, 3167C>T, 3168A>T, 3169A>G, 3171A>G, 3172T>A, 3175C>A, 3176A>T, 3178C>T, 3179T>A, 3180G>T, 3182C>T, 3184C>G, 3187C>G, 3188T>C, 3189A>T, 3190C>T, 3191C>T, 3196C>T, 3203A>C								
**CDS**									
D1R33_gp2	197	301	9.7%	360	48.0%	105 (100%)	51 (48.6%)	0/0/0/0	1
Protein mutations:	F199Y (2901T>A 2902C>T), K201E (2906A>G), E204A (2916A>C 2917G>A), D208K (2927G>A 2929T>A), Y211F (2937A>T 2938C>T), P212S (2939C>T 2941G>T), N215V (2948A>G 2949A>T 2950C>A), S217G (2954A>G 2956T>A), D218E (2959C>A), V219L (2960G>T 2962A>G), L220* (2963C>T 2964T>A 2965T>G), D221E (2968C>G), K222E (2969A>G 2971G>A), G224F (2975G>T 2976G>T), K225G (2978A>G 2979A>G), C226A (2981T>G 2982G>C 2983C>T), Q227K (2984C>A), T230S (2993A>T 2995C>T), T231K (2997C>A 2998C>A), A235R (3008G>C 3009C>G 3010A>C), S236Q (3011A>C 3012G>A 3013T>A), F238Y (3018T>A), E242Q (3029G>C), D244K (3035G>A 3037C>A), P245E (3038C>G 3039C>A 3040T>G), Q246D (3041C>G 3043A>T), I248V (3047A>G 3049A>G), S249E (3050T>G 3051C>A), N254H (3065A>C), V255T (3068G>A 3069T>C 3070A>T), E256H (3071G>C 3073A>T), R264V (3095C>G 3096G>T 3097A>T), M267F (3104A>T 3106G>T), K270T (3114A>C 3115A>C), S272A (3119T>G 3121A>T), P273S (3122C>T), Q277L (3135A>T), R278S (3137A>T 3138G>C), V279L (3140G>T 3142T>A), D281N (3146G>A 3148C>T), N282E (3149A>G 3151T>G), L284F (3155C>T 3157A>T), R285Q (3158A>C 3159G>A 3160A>G), G286L (3161G>C 3162G>T), L287H (3165T>A), Q288L (3167C>T 3168A>T 3169A>G), N289R (3171A>G 3172T>A), N290K (3175C>A), I291F (3176A>T 3178C>T), C292I (3179T>A 3180G>T), Y295L (3188T>C 3189A>T 3190C>T), L296F (3191C>T), I300L (3203A>C)								
Codon mutations:	GTA196..T (2893A>T), GAC198GAT (2899C>T), TTC199TAT (2901T>A 2902C>T), CGT200CGC (2905T>C), AAG201GAG (2906A>G), AAC203AAT (2914C>T), GAG204GCA (2916A>C 2917G>A), AAG205AAA (2920G>A), ACT206ACA (2923T>A), GAT208AAA (2927G>A 2929T>A), GAC209GAT (2932C>T), TAC211TTT (2937A>T 2938C>T), CCG212TCT (2939C>T 2941G>T), ATA213ATT (2944A>T), AAC215GTA (2948A>G 2949A>T 2950C>A), ATA216ATT (2953A>T), AGT217GGA (2954A>G 2956T>A), GAC218GAA (2959C>A), GTA219TTG (2960G>T 2962A>G), CTT220TAG (2963C>T 2964T>A 2965T>G), GAC221GAG (2968C>G), AAG222GAA (2969A>G 2971G>A), TTA223CTG (2972T>C 2974A>G), GGT224TTT (2975G>T 2976G>T), AAG225GGG (2978A>G 2979A>G), TGC226GCT (2981T>G 2982G>C 2983C>T), CAA227AAA (2984C>A), TAC228TAT (2989C>T), ACC230TCT (2993A>T 2995C>T), ACC231AAA (2997C>A 2998C>A), GAT233GAC (3004T>C), TTG234TTA (3007G>A), GCA235CGC (3008G>C 3009C>G 3010A>C), AGT236CAA (3011A>C 3012G>A 3013T>A), GGG237GGT (3016G>T), TTT238TAT (3018T>A), CAG240CAA (3025G>A), GTG241GTT (3028G>T), GAG242CAG (3029G>C), GAC244AAA (3035G>A 3037C>A), CCT245GAG (3038C>G 3039C>A 3040T>G), CAA246GAT (3041C>G 3043A>T), ATA248GTG (3047A>G 3049A>G), TCG249GAG (3050T>G 3051C>A), ACC251ACT (3058C>T), GCG252GCT (3061G>T), AAC254CAC (3065A>C), GTA255ACT (3068G>A 3069T>C 3070A>T), GAA256CAT (3071G>C 3073A>T), CAC257CAT (3076C>T), GAA261GAG (3088A>G), TTC262TTT (3091C>T), CTT263TTG (3092C>T 3094T>G), CGA264GTT (3095C>G 3096G>T 3097A>T), CCT266CCC (3103T>C), ATG267TTT (3104A>T 3106G>T), GGA268GGC (3109A>C), AAA270ACC (3114A>C 3115A>C), AAC271AAT (3118C>T), TCA272GCT (3119T>G 3121A>T), CCA273TCA (3122C>T), ACT275ACC (3130T>C), CAA277CTA (3135A>T), AGA278TCA (3137A>T 3138G>C), GTT279TTA (3140G>T 3142T>A), GAC281AAT (3146G>A 3148C>T), AAT282GAG (3149A>G 3151T>G), GTC283GTA (3154C>A), CTA284TTT (3155C>T 3157A>T), AGA285CAG (3158A>C 3159G>A 3160A>G), GGT286CTT (3161G>C 3162G>T), CTC287CAC (3165T>A), CAA288TTG (3167C>T 3168A>T 3169A>G), AAT289AGA (3171A>G 3172T>A), AAC290AAA (3175C>A), ATC291TTT (3176A>T 3178C>T), TGT292ATT (3179T>A 3180G>T), CTC293TTG (3182C>T 3184C>G), GTC294GTG (3187C>G), TAC295CTT (3188T>C 3189A>T 3190C>T), CTT296TTT (3191C>T), GAC297GAT (3196C>T), ATT300CTT (3203A>C)								
**Proteins**									
ORF B (YP_009507248.1)	197	301	9.7%	360	48.0%	105 (100%)	51 (48.6%)	0/0/0/0	1
Protein mutations:	F199Y (2901T>A 2902C>T), K201E (2906A>G), E204A (2916A>C 2917G>A), D208K (2927G>A 2929T>A), Y211F (2937A>T 2938C>T), P212S (2939C>T 2941G>T), N215V (2948A>G 2949A>T 2950C>A), S217G (2954A>G 2956T>A), D218E (2959C>A), V219L (2960G>T 2962A>G), L220* (2963C>T 2964T>A 2965T>G), D221E (2968C>G), K222E (2969A>G 2971G>A), G224F (2975G>T 2976G>T), K225G (2978A>G 2979A>G), C226A (2981T>G 2982G>C 2983C>T), Q227K (2984C>A), T230S (2993A>T 2995C>T), T231K (2997C>A 2998C>A), A235R (3008G>C 3009C>G 3010A>C), S236Q (3011A>C 3012G>A 3013T>A), F238Y (3018T>A), E242Q (3029G>C), D244K (3035G>A 3037C>A), P245E (3038C>G 3039C>A 3040T>G), Q246D (3041C>G 3043A>T), I248V (3047A>G 3049A>G), S249E (3050T>G 3051C>A), N254H (3065A>C), V255T (3068G>A 3069T>C 3070A>T), E256H (3071G>C 3073A>T), R264V (3095C>G 3096G>T 3097A>T), M267F (3104A>T 3106G>T), K270T (3114A>C 3115A>C), S272A (3119T>G 3121A>T), P273S (3122C>T), Q277L (3135A>T), R278S (3137A>T 3138G>C), V279L (3140G>T 3142T>A), D281N (3146G>A 3148C>T), N282E (3149A>G 3151T>G), L284F (3155C>T 3157A>T), R285Q (3158A>C 3159G>A 3160A>G), G286L (3161G>C 3162G>T), L287H (3165T>A), Q288L (3167C>T 3168A>T 3169A>G), N289R (3171A>G 3172T>A), N290K (3175C>A), I291F (3176A>T 3178C>T), C292I (3179T>A 3180G>T), Y295L (3188T>C 3189A>T 3190C>T), L296F (3191C>T), I300L (3203A>C)								
Codon mutations:	GTA196..T (2893A>T), GAC198GAT (2899C>T), TTC199TAT (2901T>A 2902C>T), CGT200CGC (2905T>C), AAG201GAG (2906A>G), AAC203AAT (2914C>T), GAG204GCA (2916A>C 2917G>A), AAG205AAA (2920G>A), ACT206ACA (2923T>A), GAT208AAA (2927G>A 2929T>A), GAC209GAT (2932C>T), TAC211TTT (2937A>T 2938C>T), CCG212TCT (2939C>T 2941G>T), ATA213ATT (2944A>T), AAC215GTA (2948A>G 2949A>T 2950C>A), ATA216ATT (2953A>T), AGT217GGA (2954A>G 2956T>A), GAC218GAA (2959C>A), GTA219TTG (2960G>T 2962A>G), CTT220TAG (2963C>T 2964T>A 2965T>G), GAC221GAG (2968C>G), AAG222GAA (2969A>G 2971G>A), TTA223CTG (2972T>C 2974A>G), GGT224TTT (2975G>T 2976G>T), AAG225GGG (2978A>G 2979A>G), TGC226GCT (2981T>G 2982G>C 2983C>T), CAA227AAA (2984C>A), TAC228TAT (2989C>T), ACC230TCT (2993A>T 2995C>T), ACC231AAA (2997C>A 2998C>A), GAT233GAC (3004T>C), TTG234TTA (3007G>A), GCA235CGC (3008G>C 3009C>G 3010A>C), AGT236CAA (3011A>C 3012G>A 3013T>A), GGG237GGT (3016G>T), TTT238TAT (3018T>A), CAG240CAA (3025G>A), GTG241GTT (3028G>T), GAG242CAG (3029G>C), GAC244AAA (3035G>A 3037C>A), CCT245GAG (3038C>G 3039C>A 3040T>G), CAA246GAT (3041C>G 3043A>T), ATA248GTG (3047A>G 3049A>G), TCG249GAG (3050T>G 3051C>A), ACC251ACT (3058C>T), GCG252GCT (3061G>T), AAC254CAC (3065A>C), GTA255ACT (3068G>A 3069T>C 3070A>T), GAA256CAT (3071G>C 3073A>T), CAC257CAT (3076C>T), GAA261GAG (3088A>G), TTC262TTT (3091C>T), CTT263TTG (3092C>T 3094T>G), CGA264GTT (3095C>G 3096G>T 3097A>T), CCT266CCC (3103T>C), ATG267TTT (3104A>T 3106G>T), GGA268GGC (3109A>C), AAA270ACC (3114A>C 3115A>C), AAC271AAT (3118C>T), TCA272GCT (3119T>G 3121A>T), CCA273TCA (3122C>T), ACT275ACC (3130T>C), CAA277CTA (3135A>T), AGA278TCA (3137A>T 3138G>C), GTT279TTA (3140G>T 3142T>A), GAC281AAT (3146G>A 3148C>T), AAT282GAG (3149A>G 3151T>G), GTC283GTA (3154C>A), CTA284TTT (3155C>T 3157A>T), AGA285CAG (3158A>C 3159G>A 3160A>G), GGT286CTT (3161G>C 3162G>T), CTC287CAC (3165T>A), CAA288TTG (3167C>T 3168A>T 3169A>G), AAT289AGA (3171A>G 3172T>A), AAC290AAA (3175C>A), ATC291TTT (3176A>T 3178C>T), TGT292ATT (3179T>A 3180G>T), CTC293TTG (3182C>T 3184C>G), GTC294GTG (3187C>G), TAC295CTT (3188T>C 3189A>T 3190C>T), CTT296TTT (3191C>T), GAC297GAT (3196C>T), ATT300CTT (3203A>C)								

*: Inserts / Deletes / Misaligned / Frameshifts

Analysis details

This analysis was performed with panviral2.64

NGS Details (UN8): Caulimovirus venafragariae

Assembly

**Coverage Length**	254 (1 contig(s))
**Depth Of Coverage**	6.4
**Number Of Reads**	14
**Reads Per Million**	0.23 rpm (after QC)
**Ambiguities**	0
**Assembly Method**	de novo + reference guided assembly
**Consensus Caller**	Bcf Tools

Coverage Map

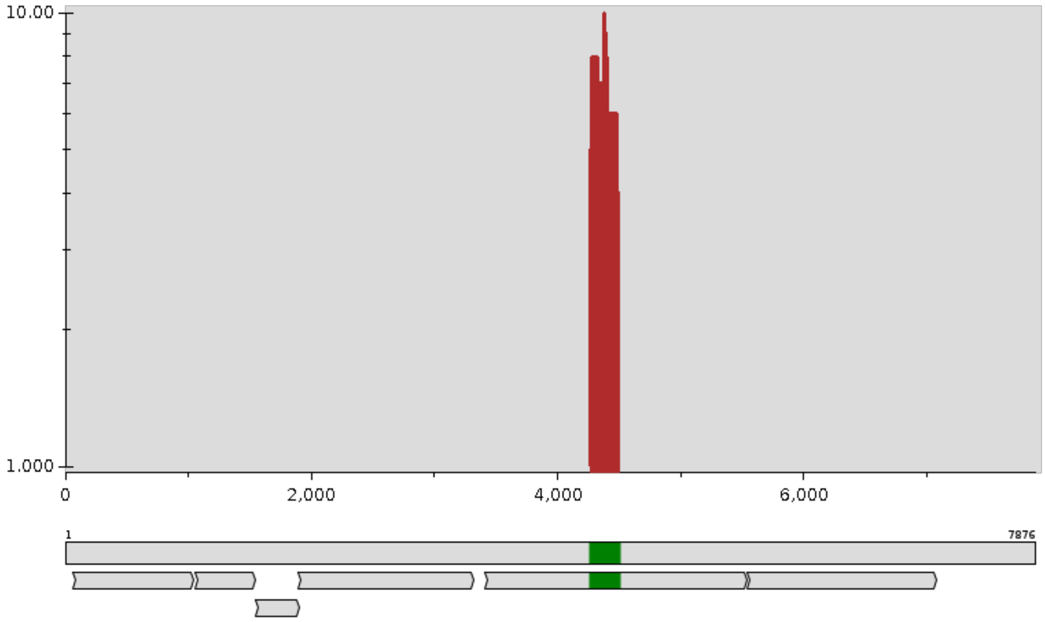

Assignment

**Type**	Caulimovirus venafragariae (Taxonomy ID: 3048344)
**Reference Genome**	NC_001725.1
**NT Identity (%)**	60.8696
**AA Identity (%)**	61.9048
**Number Of Stop Codons**	0
**Number Of CDS**	6

Alignment

**Alignment Score**	100.0 (NT) + 212.0 (AA) = 312.0
**Concordance (%)**	34.6833

**Alignment Method**	Global, seeded, nucleotide + amino acids (AGA)

Genome Region

Sequence starts at position 4259 and ends at position 4512 relative to NC_001725.1 reference sequence.

Alignment Detailed Statistics

	Begin	End	Coverage	Score	Concordance	Matches	Identities	I/D/M/F*	Stop Codons
**NT**	**4259**	**4512**	**3.2%**	**100**	**20.2%**	**253 (99.6%)**	**154 (60.6%)**	**0/1**	
Mutations:	4262A>C, 4264T>A, 4267C>A, 4268C>A, 4276C>A, 4277G>T, 4284G>T, 4288A>C, 4290A>G, 4292C>T, 4293T>A, 4296G>A, 4299C>A, 4300G>A, 4306C>T, 4309C>T, 4311C>A, 4312A>G, 4314G>T, 4315C>T, 4317T>A, 4320T>A, 4321A>G, 4323C>A, 4326C>A, 4330C>A, 4331C>G, 4332T>A, 4333A>T, 4334G>C, 4335C>A, 4337A>C, 4338G>A, 4339A>T, 4340G>C, 4342C>A, 4343C>G, 4347T>C, 4348T>A, 4349C>G, 4350C>A, 4354C>G, 4359C>A, 4369delA, 4370G>A, 4371G>A, 4374T>C, 4378G>A, 4379C>A, 4383G>A, 4386C>A, 4387A>G, 4388A>T, 4389A>C, 4390C>A, 4395C>A, 4401A>C, 4413T>A, 4416C>T, 4422A>G, 4424A>G, 4425G>A, 4426T>C, 4428A>C, 4434C>T, 4435C>A, 4440A>T, 4442A>G, 4443G>A, 4444G>A, 4445G>T, 4446A>G, 4451G>C, 4452C>T, 4455C>T, 4456C>A, 4457T>A, 4459C>T, 4461T>A, 4464T>A, 4465A>G, 4470G>A, 4471G>A, 4473G>T, 4474C>G, 4479G>A, 4482T>A, 4483C>A, 4485A>T, 4491C>A, 4492G>C, 4493G>A, 4497T>A, 4500G>A, 4502C>G, 4503C>A, 4504T>A, 4508A>T, 4510T>A, 4511C>G								

CDS

	Begin	End	Coverage	Score	Concordance	Matches	Identities	I/D/M/F*	Stop Codons
ORF_V	286	369	11.8%	212	36.9%	84 (100%)	52 (61.9%)	0/0/1/1	0
Protein mutations:	Y286S (4262A>C), S287T (4264T>A), P288N (4267C>A 4268C>A), R291I (4276C>A 4277G>T), E293D (4284G>T), K295Q (4288A>C 4290A>G), T296I (4292C>T 4293T>A), E299K (4300G>A), K303D (4312A>G 4314G>T), I306V (4321A>G 4323C>A), P309R (4330C>A 4331C>G 4332T>A), K311T (4337A>C 4338G>A), P313R (4342C>A 4343C>G), S315R (4348T>A 4349C>G 4350C>A), P317A (4354C>G), A325N (4378G>A 4379C>A), K328V (4387A>G 4388A>T 4389A>C), K340R (4424A>G 4425G>A), H344N (4435C>A), K346R (4442A>G 4443G>A), G347M (4444G>A 4445G>T 4446A>G), G349A (4451G>C 4452C>T), L351K (4456C>A 4457T>A), N354D (4465A>G), E356N (4471G>A 4473G>T), Q357E (4474C>G), Q360N (4483C>A 4485A>T), G363Q (4492G>C 4493G>A), T366R (4502C>G 4503C>A), F367I (4504T>A), Y368F (4508A>T)								
Codon mutations:	TAT286TCT (4262A>C), TCA287ACA (4264T>A), CCT288AAT (4267C>A 4268C>A), CGA291ATA (4276C>A 4277G>T), GAG293GAT (4284G>T), AAA295CAG (4288A>C 4290A>G), ACT296ATA (4292C>T 4293T>A), CAG297CAA (4296G>A), ATC298ATA (4299C>A), GAA299AAA (4300G>A), CTA301TTA (4306C>T), CTC302TTA (4309C>T 4311C>A), AAG303GAT (4312A>G 4314G>T), CTT304TTA (4315C>T 4317T>A), GGT305GGA (4320T>A), ATC306GTA (4321A>G 4323C>A), ATC307ATA (4326C>A), CCT309AGA (4330C>A 4331C>G 4332T>A), AGC310TCA (4333A>T 4334G>C 4335C>A), AAG311ACA (4337A>C 4338G>A), AGT312TCT (4339A>T 4340G>C), CCA313AGA (4342C>A 4343C>G), CAT314CAC (4347T>C), TCC315AGA (4348T>A 4349C>G 4350C>A), CCA317GCA (4354C>G), GCC318GCA (4359C>A), AGG322-AA (4369delA 4370G>A 4371G>A), AAT323AAC (4374T>C), GCC325AAC (4378G>A 4379C>A), GAG326GAA (4383G>A), ATC327ATA (4386C>A), AAA328GTC (4387A>G 4388A>T 4389A>C), CGA329AGA (4390C>A), GGC330GGA (4395C>A), GCA332GCC (4401A>C), ATT336ATA (4413T>A), AAC337AAT (4416C>T), AAA339AAG (4422A>G), AAG340AGA (4424A>G 4425G>A), TTA341CTC (4426T>C 4428A>C), GAC343GAT (4434C>T), CAT344AAT (4435C>A), ACA345ACT (4440A>T), AAG346AGA (4442A>G 4443G>A), GGA347ATG (4444G>A 4445G>T 4446A>G), GGC349GCT (4451G>C 4452C>T), TAC350TAT (4455C>T), CTA351AAA (4456C>A 4457T>A), CTT352TTA (4459C>T 4461T>A), CCT353CCA (4464T>A), AAC354GAC (4465A>G), AAG355AAA (4470G>A), GAG356AAT (4471G>A 4473G>T), CAA357GAA (4474C>G), CTG358CTA (4479G>A), CTT359CTA (4482T>A), CAA360AAT (4483C>A 4485A>T), ATC362ATA (4491C>A), GGA363CAA (4492G>C 4493G>A), GGT364GGA (4497T>A), AAG365AAA (4500G>A), ACC366AGA (4502C>G 4503C>A), TTT367ATT (4504T>A), TAC368TTC (4508A>T), TCT369AGT (4510T>A 4511C>G)								

Proteins

	Begin	End	Coverage	Score	Concordance	Matches	Identities	I/D/M/F*	Stop Codons
hypothetical protein (NP_043933.1)	286	369	11.8%	212	36.9%	84 (100%)	52 (61.9%)	0/0/1/1	0
Protein mutations:	Y286S (4262A>C), S287T (4264T>A), P288N (4267C>A 4268C>A), R291I (4276C>A 4277G>T), E293D (4284G>T), K295Q (4288A>C 4290A>G), T296I (4292C>T 4293T>A), E299K (4300G>A), K303D (4312A>G 4314G>T), I306V (4321A>G 4323C>A), P309R (4330C>A 4331C>G 4332T>A), K311T (4337A>C 4338G>A), P313R (4342C>A 4343C>G), S315R (4348T>A 4349C>G 4350C>A), P317A (4354C>G), A325N (4378G>A 4379C>A), K328V (4387A>G 4388A>T 4389A>C), K340R (4424A>G 4425G>A), H344N (4435C>A), K346R (4442A>G 4443G>A), G347M (4444G>A 4445G>T 4446A>G), G349A (4451G>C 4452C>T), L351K (4456C>A 4457T>A), N354D (4465A>G), E356N (4471G>A 4473G>T), Q357E (4474C>G), Q360N (4483C>A 4485A>T), G363Q (4492G>C 4493G>A), T366R (4502C>G 4503C>A), F367I (4504T>A), Y368F (4508A>T)								
Codon mutations:	TAT286TCT (4262A>C), TCA287ACA (4264T>A), CCT288AAT (4267C>A 4268C>A), CGA291ATA (4276C>A 4277G>T), GAG293GAT (4284G>T), AAA295CAG (4288A>C 4290A>G), ACT296ATA (4292C>T 4293T>A), CAG297CAA (4296G>A), ATC298ATA (4299C>A), GAA299AAA (4300G>A), CTA301TTA (4306C>T), CTC302TTA (4309C>T 4311C>A), AAG303GAT (4312A>G 4314G>T), CTT304TTA (4315C>T 4317T>A), GGT305GGA (4320T>A), ATC306GTA (4321A>G 4323C>A), ATC307ATA (4326C>A), CCT309AGA (4330C>A 4331C>G 4332T>A), AGC310TCA (4333A>T 4334G>C 4335C>A), AAG311ACA (4337A>C 4338G>A), AGT312TCT (4339A>T 4340G>C), CCA313AGA (4342C>A 4343C>G), CAT314CAC (4347T>C), TCC315AGA (4348T>A 4349C>G 4350C>A), CCA317GCA (4354C>G), GCC318GCA (4359C>A), AGG322-AA (4369delA 4370G>A 4371G>A), AAT323AAC (4374T>C), GCC325AAC (4378G>A 4379C>A), GAG326GAA (4383G>A), ATC327ATA (4386C>A), AAA328GTC (4387A>G 4388A>T 4389A>C), CGA329AGA (4390C>A), GGC330GGA (4395C>A), GCA332GCC (4401A>C), ATT336ATA (4413T>A), AAC337AAT (4416C>T), AAA339AAG (4422A>G), AAG340AGA (4424A>G 4425G>A), TTA341CTC (4426T>C 4428A>C), GAC343GAT (4434C>T), CAT344AAT (4435C>A), ACA345ACT (4440A>T), AAG346AGA (4442A>G 4443G>A), GGA347ATG (4444G>A 4445G>T 4446A>G), GGC349GCT (4451G>C 4452C>T), TAC350TAT (4455C>T), CTA351AAA (4456C>A 4457T>A), CTT352TTA (4459C>T 4461T>A), CCT353CCA (4464T>A), AAC354GAC (4465A>G), AAG355AAA (4470G>A), GAG356AAT (4471G>A 4473G>T), CAA357GAA (4474C>G), CTG358CTA (4479G>A), CTT359CTA (4482T>A), CAA360AAT (4483C>A 4485A>T), ATC362ATA (4491C>A), GGA363CAA (4492G>C 4493G>A), GGT364GGA (4497T>A), AAG365AAA (4500G>A), ACC366AGA (4502C>G 4503C>A), TTT367ATT (4504T>A), TAC368TTC (4508A>T), TCT369AGT (4510T>A 4511C>G)								

*: Inserts / Deletes / Misaligned / Frameshifts

Analysis details

This analysis was performed with panviral2.64

NGS Details (UN8): Badnavirus maculasmallanthi

Assembly

**Coverage Length**	304 (1 contig(s))
**Depth Of Coverage**	4.7
**Number Of Reads**	13
**Reads Per Million**	0.22 rpm (after QC)
**Ambiguities**	0
**Assembly Method**	de novo + reference guided assembly
**Consensus Caller**	Bcf Tools

Coverage Map

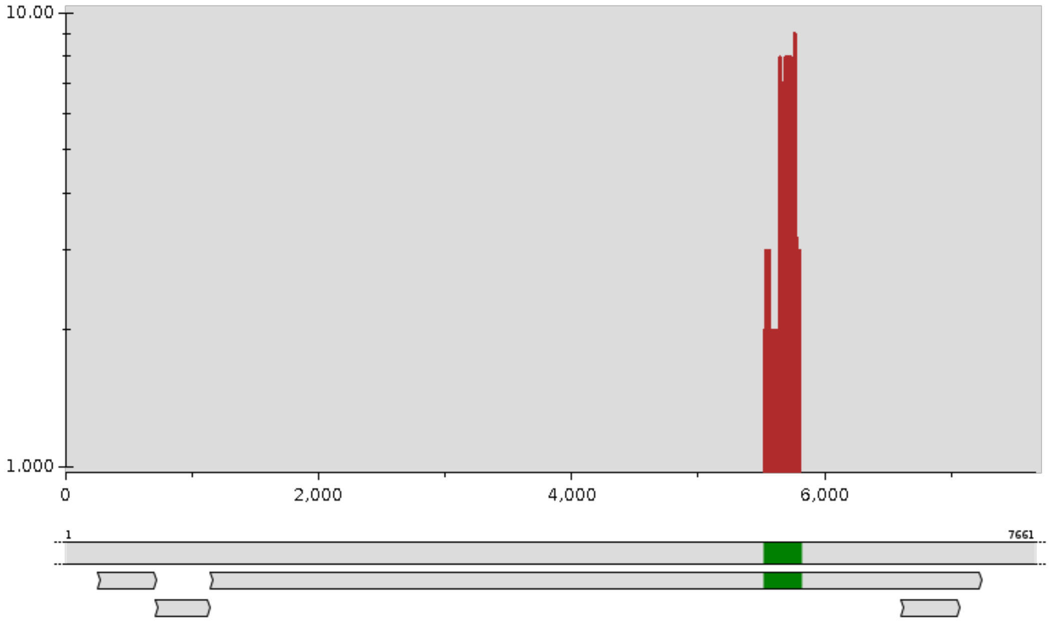

Assignment

**Type**	Badnavirus maculasmallanthi (Taxonomy ID: 3048453)
**Reference Genome**	NC_026472.1
**NT Identity (%)**	51.1401
**AA Identity (%)**	41.1765
**Number Of Stop Codons**	0
**Number Of CDS**	4

Alignment

**Alignment Score**	8.0 (NT) + 290.0 (AA) = 298.0
**Concordance (%)**	22.61

Alignment Method	Global, seeded, nucleotide + amino acids (AGA)

Genome Region

Sequence starts at position 5516 and ends at position 5819 relative to NC_026472.1 reference sequence.

Alignment Detailed Statistics

	Begin	End	Coverage	Score	Concordance	Matches	Identities	I/D/M/F*	Stop Codons
**NT**	**5516**	**5819**	**4.0%**	**8**	**1.3%**	**304 (99.0%)**	**157 (51.1%)**	**3/0**	
Mutations:	5517T>C, 5518T>C, 5527G>C, 5529G>T, 5532A>C, 5535C>T, 5536A>G, 5537C>A, 5542C>T, 5544A>T, 5545A>G, 5547A>T, 5548A>C, 5549G>A, 5550A>G, 5551A>C, 5553A>T, 5554G>C, 5555G>A, 5556G>A, 5558A>G, 5559T>G, 5560G>A, 5561C>T, 5562A>G, 5563A>T, 5564A>C, 5565G>C, 5566A>T, 5568A>G, 5574A>T, 5578T>A, 5584C>T, 5588A>G, 5589G>A, 5592T>C, 5595A>G, 5597T>A, 5599C>T, 5604A>G, 5605G>T, 5607T>G, 5608G>A, 5609C>G, 5610C>G, 5613G>T, 5614G>T, 5615A>G, 5616A>G, 5618A>C, 5620G>T, 5621A>C, 5622A>T, 5623T>G, 5624C>A, 5628A>T, 5631C>T, 5632T>A, 5633G>A, 5637G>A, 5638G>A, 5643T>C, 5644T>A, 5645T>A, 5646A>G, 5648T>C, 5649C>T, 5650C>A, 5651C>A, 5653G>T, 5654G>A, 5655A>C, 5657G>A, 5658A>C, 5659T>C, 5660T>A, 5661A>T, 5664C>T, 5667A>G, 5669G>T, 5670G>C, 5674G>A, 5676C>G, 5680C>T, 5685C>T, 5688A>G, 5691C>A, 5693A>C, 5694A>C, 5700A>C, 5702C>T, 5703C>T, 5705C>T, 5706T>A, 5707A>G, 5708T>C, 5709T>A, 5712T>C, 5713C>A, 5714A>T, 5715G>A, 5716A>G, 5717G>A, 5718A>G, 5719A>T, 5720A>T, 5721A>G, 5725G>A, 5727T>C, 5728A>G, 5729A>G, 5731T>G, 5732G>T, 5733C>G, 5736C>T, 5736_5737insCAG, 5737A>C, 5738A>C, 5740G>T, 5743A>T, 5744C>T, 5748A>T, 5749G>T, 5750A>T, 5757C>G, 5758G>A, 5759C>T, 5760C>T, 5761G>A, 5763A>T, 5765A>T, 5766C>T, 5769C>T, 5772C>T, 5779C>T, 5784A>T, 5793C>G, 5795G>C, 5796T>A, 5797G>A, 5799A>G, 5802G>C, 5805T>C, 5808T>C, 5810A>T, 5811A>T, 5814G>A, 5815C>T								

CDS

	Begin	End	Coverage	Score	Concordance	Matches	Identities	I/D/M/F*	Stop Codons
UF61_gp3	1459	1559	5.0%	290	40.1%	101 (99.0%)	42 (41.2%)	1/0/0/0	0
Protein mutations:	S1459P (5518T>C), G1462R (5527G>C 5529G>T), T1465D (5536A>G 5537C>A), L1467F (5542C>T 5544A>T), K1468D (5545A>G 5547A>T), R1469Q (5548A>C 5549G>A 5550A>G), I1470L (5551A>C 5553A>T), G1471Q (5554G>C 5555G>A 5556G>A), N1472R (5558A>G 5559T>G), A1473M (5560G>A 5561C>T 5562A>G), K1474S (5563A>T 5564A>C 5565G>C), I1475L (5566A>T 5568A>G), F1479I (5578T>A), K1482R (5588A>G 5589G>A), F1485Y (5597T>A), H1486Y (5599C>T), V1488L (5605G>T 5607T>G), A1489R (5608G>A 5609C>G 5610C>G), M1490I (5613G>T), E1491W (5614G>T 5615A>G 5616A>G), E1492A (5618A>C), E1493S (5620G>T 5621A>C 5622A>T), S1494D (5623T>G 5624C>A), W1497K (5632T>A 5633G>A), A1499T (5638G>A), L1501K (5644T>A 5645T>A 5646A>G), I1502T (5648T>C 5649C>T), P1503K (5650C>A 5651C>A), G1504Y (5653G>T 5654G>A 5655A>C), G1505D (5657G>A 5658A>C), L1506H (5659T>C 5660T>A 5661A>T), W1509F (5669G>T 5670G>C), V1511M (5674G>A 5676C>G), P1513S (5680C>T), K1517T (5693A>C 5694A>C), P1520L (5702C>T 5703C>T), A1521V (5705C>T 5706T>A), I1522A (5707A>G 5708T>C 5709T>A), Q1524I (5713C>A 5714A>T 5715G>A), R1525E (5716A>G 5717G>A 5718A>G), K1526L (5719A>T 5720A>T 5721A>G), D1528N (5725G>A 5727T>C), K1529G (5728A>G 5729A>G), C1530V (5731T>G 5732G>T 5733C>G), F1531_K1532insQ (5736_5737insCAG), K1532P (5737A>C 5738A>C), D1533Y (5740G>T), T1534L (5743A>T 5744C>T), E1535D (5748A>T), E1536L (5749G>T 5750A>T), I1538M (5757C>G), A1539I (5758G>A 5759C>T 5760C>T), V1540I (5761G>A 5763A>T), Y1541F (5765A>T 5766C>T), N1550K (5793C>G), S1551T (5795G>C 5796T>A), E1552K (5797G>A 5799A>G), E1556V (5810A>T 5811A>T), H1558Y (5815C>T)								
Codon mutations:	TAT1458.AC (5517T>C), TCC1459CCC (5518T>C), GGG1462CGT (5527G>C 5529G>T), ATA1463ATC (5532A>C), AAC1464AAT (5535C>T), ACC1465GAC (5536A>G 5537C>A), CTA1467TTT (5542C>T 5544A>T), AAA1468GAT (5545A>G 5547A>T), AGA1469CAG (5548A>C 5549G>A 5550A>G), ATA1470CTT (5551A>C 5553A>T), GGG1471CAA (5554G>C 5555G>A 5556G>A), AAT1472AGG (5558A>G 5559T>G), GCA1473ATG (5560G>A 5561C>T 5562A>G), AAG1474TCC (5563A>T 5564A>C 5565G>C), ATA1475TTG (5566A>T 5568A>G), TCA1477TCT (5574A>T), TTT1479ATT (5578T>A), CTG1481TTG (5584C>T), AAG1482AGA (5588A>G 5589G>A), TCT1483TCC (5592T>C), GGA1484GGG (5595A>G), TTT1485TAT (5597T>A), CAT1486TAT (5599C>T), CAA1487CAG (5604A>G), GTT1488TTG (5605G>T 5607T>G), GCC1489AGG (5608G>A 5609C>G 5610C>G), ATG1490ATT (5613G>T), GAA1491TGG (5614G>T 5615A>G 5616A>G), GAA1492GCA (5618A>C), GAA1493TCT (5620G>T 5621A>C 5622A>T), TCC1494GAC (5623T>G 5624C>A), ATA1495ATT (5628A>T), CCC1496CCT (5631C>T), TGG1497AAG (5632T>A 5633G>A), ACG1498ACA (5637G>A), GCC1499ACC (5638G>A), TTT1500TTC (5643T>C), TTA1501AAG (5644T>A 5645T>A 5646A>G), ATC1502ACT (5648T>C 5649C>T), CCA1503AAA (5650C>A 5651C>A), GGA1504TAC (5653G>T 5654G>A 5655A>C), GGA1505GAC (5657G>A 5658A>C), TTA1506CAT (5659T>C 5660T>A 5661A>T), TAC1507TAT (5664C>T), GAA1508GAG (5667A>G), TGG1509TTC (5669G>T 5670G>C), GTC1511ATG (5674G>A 5676C>G), CCA1513TCA (5680C>T), TTC1514TTT (5685C>T), GGA1515GGG (5688A>G), CTC1516CTA (5691C>A), AAA1517ACC (5693A>C 5694A>C), GCA1519GCC (5700A>C), CCC1520CTT (5702C>T 5703C>T), GCT1521GTA (5705C>T 5706T>A), ATT1522GCA (5707A>G 5708T>C 5709T>A), TTT1523TTC (5712T>C), CAG1524ATA (5713C>A 5714A>T 5715G>A), AGA1525GAG (5716A>G 5717G>A 5718A>G), AAA1526TTG (5719A>T 5720A>T 5721A>G), GAT1528AAC (5725G>A 5727T>C), AAA1529GGA (5728A>G 5729A>G), TGC1530GTG (5731T>G 5732G>T 5733C>G), TTC1531TTT (5736C>T), TTC1531_AAA1532insCAG (5736_5737insCAG), AAA1532CCA (5737A>C 5738A>C), GAC1533TAC (5740G>T), ACA1534TTA (5743A>T 5744C>T), GAA1535GAT (5748A>T), GAG1536TTG (5749G>T 5750A>T), ATC1538ATG (5757C>G), GCC1539ATT (5758G>A 5759C>T 5760C>T), GTA1540ATT (5761G>A 5763A>T), TAC1541TTT (5765A>T 5766C>T), ATC1542ATT (5769C>T), GAC1543GAT (5772C>T), CTG1546TTG (5779C>T), GTA1547GTT (5784A>T), AAC1550AAG (5793C>G), AGT1551ACA (5795G>C 5796T>A), GAA1552AAG (5797G>A 5799A>G), GCG1553GCC (5802G>C), GAT1554GAC (5805T>C), CAT1555CAC (5808T>C), GAA1556GTT (5810A>T 5811A>T), AGG1557AGA (5814G>A), CAT1558TAT (5815C>T)								

Proteins

	Begin	End	Coverage	Score	Concordance	Matches	Identities	I/D/M/F*	Stop Codons
ORF3 (YP_009121747.1)	1459	1559	5.0%	290	40.1%	101 (99.0%)	42 (41.2%)	1/0/0/0	0
Protein mutations:	S1459P (5518T>C), G1462R (5527G>C 5529G>T), T1465D (5536A>G 5537C>A), L1467F (5542C>T 5544A>T), K1468D (5545A>G 5547A>T), R1469Q (5548A>C 5549G>A 5550A>G), I1470L (5551A>C 5553A>T), G1471Q (5554G>C 5555G>A 5556G>A), N1472R (5558A>G 5559T>G), A1473M (5560G>A 5561C>T 5562A>G), K1474S (5563A>T 5564A>C 5565G>C), I1475L (5566A>T 5568A>G), F1479I (5578T>A), K1482R (5588A>G 5589G>A), F1485Y (5597T>A), H1486Y (5599C>T), V1488L (5605G>T 5607T>G), A1489R (5608G>A 5609C>G 5610C>G), M1490I (5613G>T), E1491W (5614G>T 5615A>G 5616A>G), E1492A (5618A>C), E1493S (5620G>T 5621A>C 5622A>T), S1494D (5623T>G 5624C>A), W1497K (5632T>A 5633G>A), A1499T (5638G>A), L1501K (5644T>A 5645T>A 5646A>G), I1502T (5648T>C 5649C>T), P1503K (5650C>A 5651C>A), G1504Y (5653G>T 5654G>A 5655A>C), G1505D (5657G>A 5658A>C), L1506H (5659T>C 5660T>A 5661A>T), W1509F (5669G>T 5670G>C), V1511M (5674G>A 5676C>G), P1513S (5680C>T), K1517T (5693A>C 5694A>C), P1520L (5702C>T 5703C>T), A1521V (5705C>T 5706T>A), I1522A (5707A>G 5708T>C 5709T>A), Q1524I (5713C>A 5714A>T 5715G>A), R1525E (5716A>G 5717G>A 5718A>G), K1526L (5719A>T 5720A>T 5721A>G), D1528N (5725G>A 5727T>C), K1529G (5728A>G 5729A>G), C1530V (5731T>G 5732G>T 5733C>G), F1531_K1532insQ (5736_5737insCAG), K1532P (5737A>C 5738A>C), D1533Y (5740G>T), T1534L (5743A>T 5744C>T), E1535D (5748A>T), E1536L (5749G>T 5750A>T), I1538M (5757C>G), A1539I (5758G>A 5759C>T 5760C>T), V1540I (5761G>A 5763A>T), Y1541F (5765A>T 5766C>T), N1550K (5793C>G), S1551T (5795G>C 5796T>A), E1552K (5797G>A 5799A>G), E1556V (5810A>T 5811A>T), H1558Y (5815C>T)								
Codon mutations:	TAT1458.AC (5517T>C), TCC1459CCC (5518T>C), GGG1462CGT (5527G>C 5529G>T), ATA1463ATC (5532A>C), AAC1464AAT (5535C>T), ACC1465GAC (5536A>G 5537C>A), CTA1467TTT (5542C>T 5544A>T), AAA1468GAT (5545A>G 5547A>T), AGA1469CAG (5548A>C 5549G>A 5550A>G), ATA1470CTT (5551A>C 5553A>T), GGG1471CAA (5554G>C 5555G>A 5556G>A), AAT1472AGG (5558A>G 5559T>G), GCA1473ATG (5560G>A 5561C>T 5562A>G), AAG1474TCC (5563A>T 5564A>C 5565G>C), ATA1475TTG (5566A>T 5568A>G), TCA1477TCT (5574A>T), TTT1479ATT (5578T>A), CTG1481TTG (5584C>T), AAG1482AGA (5588A>G 5589G>A), TCT1483TCC (5592T>C), GGA1484GGG (5595A>G), TTT1485TAT (5597T>A), CAT1486TAT (5599C>T), CAA1487CAG (5604A>G), GTT1488TTG (5605G>T 5607T>G), GCC1489AGG (5608G>A 5609C>G 5610C>G), ATG1490ATT (5613G>T), GAA1491TGG (5614G>T 5615A>G 5616A>G), GAA1492GCA (5618A>C), GAA1493TCT (5620G>T 5621A>C 5622A>T), TCC1494GAC (5623T>G 5624C>A), ATA1495ATT (5628A>T), CCC1496CCT (5631C>T), TGG1497AAG (5632T>A 5633G>A), ACG1498ACA (5637G>A), GCC1499ACC (5638G>A), TTT1500TTC (5643T>C), TTA1501AAG (5644T>A 5645T>A 5646A>G), ATC1502ACT (5648T>C 5649C>T), CCA1503AAA (5650C>A 5651C>A), GGA1504TAC (5653G>T 5654G>A 5655A>C), GGA1505GAC (5657G>A 5658A>C), TTA1506CAT (5659T>C 5660T>A 5661A>T), TAC1507TAT (5664C>T), GAA1508GAG (5667A>G), TGG1509TTC (5669G>T 5670G>C), GTC1511ATG (5674G>A 5676C>G), CCA1513TCA (5680C>T), TTC1514TTT (5685C>T), GGA1515GGG (5688A>G), CTC1516CTA (5691C>A), AAA1517ACC (5693A>C 5694A>C), GCA1519GCC (5700A>C), CCC1520CTT (5702C>T 5703C>T), GCT1521GTA (5705C>T 5706T>A), ATT1522GCA (5707A>G 5708T>C 5709T>A), TTT1523TTC (5712T>C), CAG1524ATA (5713C>A 5714A>T 5715G>A), AGA1525GAG (5716A>G 5717G>A 5718A>G), AAA1526TTG (5719A>T 5720A>T 5721A>G), GAT1528AAC (5725G>A 5727T>C), AAA1529GGA (5728A>G 5729A>G), TGC1530GTG (5731T>G 5732G>T 5733C>G), TTC1531TTT (5736C>T), TTC1531_AAA1532insCAG (5736_5737insCAG), AAA1532CCA (5737A>C 5738A>C), GAC1533TAC (5740G>T), ACA1534TTA (5743A>T 5744C>T), GAA1535GAT (5748A>T), GAG1536TTG (5749G>T 5750A>T), ATC1538ATG (5757C>G), GCC1539ATT (5758G>A 5759C>T 5760C>T), GTA1540ATT (5761G>A 5763A>T), TAC1541TTT (5765A>T 5766C>T), ATC1542ATT (5769C>T), GAC1543GAT (5772C>T), CTG1546TTG (5779C>T), GTA1547GTT (5784A>T), AAC1550AAG (5793C>G), AGT1551ACA (5795G>C 5796T>A), GAA1552AAG (5797G>A 5799A>G), GCG1553GCC (5802G>C), GAT1554GAC (5805T>C), CAT1555CAC (5808T>C), GAA1556GTT (5810A>T 5811A>T), AGG1557AGA (5814G>A), CAT1558TAT (5815C>T)								

*: Inserts / Deletes / Misaligned / Frameshifts

Analysis details

This analysis was performed with panviral2.64

NGS Details (UN8): Hubei noda-like virus 9 (segment 2)

Assembly

**Coverage Length**	328 (2 contig(s))
**Depth Of Coverage**	2.7
**Number Of Reads**	9
**Reads Per Million**	0.15 rpm (after QC)
**Ambiguities**	0
**Assembly Method**	de novo + reference guided assembly
**Consensus Caller**	Bcf Tools

Coverage Map

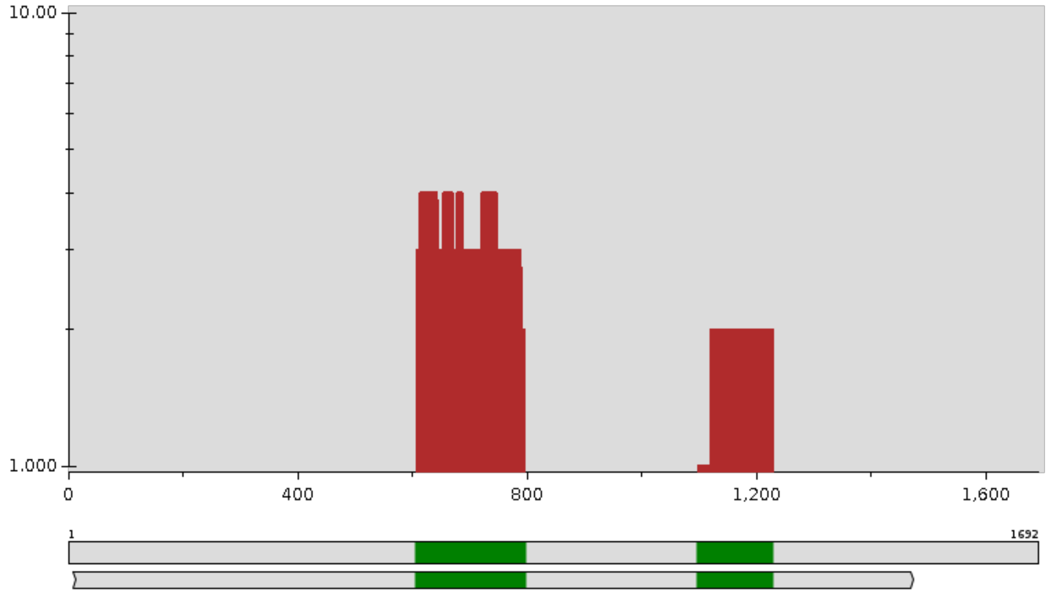

Assignment

**Type**	Hubei noda-like virus 9 (Taxonomy ID: 1922989)
**Reference Genome**	NC_033308.1
**NT Identity (%)**	66.4634
**AA Identity (%)**	69.0909
**Number Of Stop Codons**	0
**Number Of CDS**	1

Alignment

**Alignment Score**	216.0 (NT) + 562.0 (AA) = 778.0
**Concordance (%)**	55.8908

Alignment Method	Global, seeded, nucleotide + amino acids (AGA)

Genome Region

Sequence starts at position 607 and ends at position 1231 relative to NC_033308.1 reference sequence.

Alignment Detailed Statistics

	Begin	End	Coverage	Score	Concordance	Matches	Identities	I/D/M/F*	Stop Codons
**NT**	**607**	**1231**	**19.4%**	**216**	**32.9%**	**328 (100%)**	**218 (66.5%)**	**0/0**	
Mutations:	607G>C, 613T>A, 614C>G, 616C>A, 618T>C, 621A>G, 622G>A, 624A>T, 628T>G, 631T>A, 636A>T, 642A>G, 645T>A, 646T>C, 648A>C, 652A>G, 653C>T, 654T>C, 655G>A, 656C>A, 657A>T, 658C>G, 659C>A, 661G>A, 662G>C, 664A>G, 665A>C, 667T>C, 668T>A, 669C>T, 673A>T, 674A>C, 675T>C, 676C>T, 678T>G, 680T>A, 688A>G, 690C>T, 691C>A, 694A>C, 696A>G, 697A>G, 699C>T, 703G>T, 704C>G, 708T>A, 714A>T, 721T>C, 723G>T, 734C>A, 735C>T, 738T>G, 744C>T, 747G>A, 750T>A, 753T>G, 756A>T, 757G>A, 777C>T, 780T>C, 784T>A, 787G>C, 788A>T, 796A>T, 798T>C, 1098T>A, 1105T>A, 1106C>G, 1107C>T, 1113T>A, 1114G>A, 1115T>C, 1116T>C, 1122T>G, 1126A>T, 1128G>A, 1132C>T, 1134T>A, 1137C>T, 1140G>T, 1146C>T, 1147G>C, 1149G>A, 1152T>A, 1156C>A, 1158C>T, 1167C>T, 1171A>G, 1173T>C, 1174C>T, 1176T>A, 1179A>G, 1188A>C, 1191T>A, 1197T>A, 1200C>G, 1201G>A, 1202T>C, 1203C>A, 1205A>T, 1209T>A, 1210G>C, 1211A>C, 1212T>A, 1219A>T, 1221G>T, 1222C>G, 1223C>T, 1227C>T, 1230C>T								

CDS

	Begin	End	Coverage	Score	Concordance	Matches	Identities	I/D/M/F*	Stop Codons
BWW32_s2gp1	200	408	22.5%	562	73.4%	110 (100%)	76 (69.1%)	0/0/0/0	0
Protein mutations:	E200Q (607G>C), L203I (616C>A 618T>C), A205T (622G>A 624A>T), S207A (628T>G), S208T (631T>A), L209F (636A>T), T215V (652A>G 653C>T 654T>C), A216N (655G>A 656C>A 657A>T), P217D (658C>G 659C>A), G218T (661G>A 662G>C), N219A (664A>G 665A>C), F220H (667T>C 668T>A 669C>T), N222S (673A>T 674A>C 675T>C), F224Y (680T>A), I227V (688A>G 690C>T), T230A (697A>G 699C>T), A232C (703G>T 704C>G), A242D (734C>A 735C>T), V250I (757G>A), S259T (784T>A), E260L (787G>C 788A>T), I263F (796A>T 798T>C), V369T (1114G>A 1115T>C 1116T>C), M373L (1126A>T 1128G>A), E380Q (1147G>C 1149G>A), L383I (1156C>A 1158C>T), N388D (1171A>G 1173T>C), V398T (1201G>A 1202T>C 1203C>A), Y399F (1205A>T), D401P (1210G>C 1211A>C 1212T>A), T404S (1219A>T 1221G>T), P405V (1222C>G 1223C>T)								
Codon mutations:	GAA200CAA (607G>C), TCT202AGT (613T>A 614C>G), CTT203ATC (616C>A 618T>C), TTA204TTG (621A>G), GCA205ACT (622G>A 624A>T), TCC207GCC (628T>G), TCT208ACT (631T>A), TTA209TTT (636A>T), CCA211CCG (642A>G), GCT212GCA (645T>A), TTA213CTC (646T>C 648A>C), ACT215GTC (652A>G 653C>T 654T>C), GCA216AAT (655G>A 656C>A 657A>T), CCT217GAT (658C>G 659C>A), GGT218ACT (661G>A 662G>C), AAC219GCC (664A>G 665A>C), TTC220CAT (667T>C 668T>A 669C>T), AAT222TCC (673A>T 674A>C 675T>C), CTT223TTG (676C>T 678T>G), TTT224TAT (680T>A), ATC227GTT (688A>G 690C>T), CGA228AGA (691C>A), AGA229CGG (694A>C 696A>G), ACC230GCT (697A>G 699C>T), GCT232TGT (703G>T 704C>G), GGT233GGA (708T>A), ACA235ACT (714A>T), TTG238CTT (721T>C 723G>T), GCC242GAT (734C>A 735C>T), CTT243CTG (738T>G), AAC245AAT (744C>T), CAG246CAA (747G>A), GGT247GGA (750T>A), CGT248CGG (753T>G), GTA249GTT (756A>T), GTT250ATT (757G>A), CCC256CCT (777C>T), TCT257TCC (780T>C), TCT259ACT (784T>A), GAG260CTG (787G>C 788A>T), ATT263TTC (796A>T 798T>C), CGT363.GA (1098T>A), TCC366AGT (1105T>A 1106C>G 1107C>T), GGT368GGA (1113T>A), GTT369ACC (1114G>A 1115T>C 1116T>C), CTT371CTG (1122T>G), ATG373TTA (1126A>T 1128G>A), CTT375TTA (1132C>T 1134T>A), GAC376GAT (1137C>T), CCG377CCT (1140G>T), GCC379GCT (1146C>T), GAG380CAA (1147G>C 1149G>A), CTT381CTA (1152T>A), CTC383ATT (1156C>A 1158C>T), GTC386GTT (1167C>T), AAT388GAC (1171A>G 1173T>C), CTT389TTA (1174C>T 1176T>A), GAA390GAG (1179A>G), CCA393CCC (1188A>C), TCT394TCA (1191T>A), TCT396TCA (1197T>A), TCC397TCG (1200C>G), GTC398ACA (1201G>A 1202T>C 1203C>A), TAT399TTT (1205A>T), TCT400TCA (1209T>A), GAT401CCA (1210G>C 1211A>C 1212T>A), ACG404TCT (1219A>T 1221G>T), CCT405GTT (1222C>G 1223C>T), GGC406GGT (1227C>T), TAC407TAT (1230C>T)								

Proteins

	Begin	End	Coverage	Score	Concordance	Matches	Identities	I/D/M/F*	Stop Codons
capsid protein (YP_009337880.1)	200	408	22.5%	562	73.4%	110 (100%)	76 (69.1%)	0/0/0/0	0
Protein mutations:	E200Q (607G>C), L203I (616C>A 618T>C), A205T (622G>A 624A>T), S207A (628T>G), S208T (631T>A), L209F (636A>T), T215V (652A>G 653C>T 654T>C), A216N (655G>A 656C>A 657A>T), P217D (658C>G 659C>A), G218T (661G>A 662G>C), N219A (664A>G 665A>C), F220H (667T>C 668T>A 669C>T), N222S (673A>T 674A>C 675T>C), F224Y (680T>A), I227V (688A>G 690C>T), T230A (697A>G 699C>T), A232C (703G>T 704C>G), A242D (734C>A 735C>T), V250I (757G>A), S259T (784T>A), E260L (787G>C 788A>T), I263F (796A>T 798T>C), V369T (1114G>A 1115T>C 1116T>C), M373L (1126A>T 1128G>A), E380Q (1147G>C 1149G>A), L383I (1156C>A 1158C>T), N388D (1171A>G 1173T>C), V398T (1201G>A 1202T>C 1203C>A), Y399F (1205A>T), D401P (1210G>C 1211A>C 1212T>A), T404S (1219A>T 1221G>T), P405V (1222C>G 1223C>T)								
Codon mutations:	GAA200CAA (607G>C), TCT202AGT (613T>A 614C>G), CTT203ATC (616C>A 618T>C), TTA204TTG (621A>G), GCA205ACT (622G>A 624A>T), TCC207GCC (628T>G), TCT208ACT (631T>A), TTA209TTT (636A>T), CCA211CCG (642A>G), GCT212GCA (645T>A), TTA213CTC (646T>C 648A>C), ACT215GTC (652A>G 653C>T 654T>C), GCA216AAT (655G>A 656C>A 657A>T), CCT217GAT (658C>G 659C>A), GGT218ACT (661G>A 662G>C), AAC219GCC (664A>G 665A>C), TTC220CAT (667T>C 668T>A 669C>T), AAT222TCC (673A>T 674A>C 675T>C), CTT223TTG (676C>T 678T>G), TTT224TAT (680T>A), ATC227GTT (688A>G 690C>T), CGA228AGA (691C>A), AGA229CGG (694A>C 696A>G), ACC230GCT (697A>G 699C>T), GCT232TGT (703G>T 704C>G), GGT233GGA (708T>A), ACA235ACT (714A>T), TTG238CTT (721T>C 723G>T), GCC242GAT (734C>A 735C>T), CTT243CTG (738T>G), AAC245AAT (744C>T), CAG246CAA (747G>A), GGT247GGA (750T>A), CGT248CGG (753T>G), GTA249GTT (756A>T), GTT250ATT (757G>A), CCC256CCT (777C>T), TCT257TCC (780T>C), TCT259ACT (784T>A), GAG260CTG (787G>C 788A>T), ATT263TTC (796A>T 798T>C), CGT363.GA (1098T>A), TCC366AGT (1105T>A 1106C>G 1107C>T), GGT368GGA (1113T>A), GTT369ACC (1114G>A 1115T>C 1116T>C), CTT371CTG (1122T>G), ATG373TTA (1126A>T 1128G>A), CTT375TTA (1132C>T 1134T>A), GAC376GAT (1137C>T), CCG377CCT (1140G>T), GCC379GCT (1146C>T), GAG380CAA (1147G>C 1149G>A), CTT381CTA (1152T>A), CTC383ATT (1156C>A 1158C>T), GTC386GTT (1167C>T), AAT388GAC (1171A>G 1173T>C), CTT389TTA (1174C>T 1176T>A), GAA390GAG (1179A>G), CCA393CCC (1188A>C), TCT394TCA (1191T>A), TCT396TCA (1197T>A), TCC397TCG (1200C>G), GTC398ACA (1201G>A 1202T>C 1203C>A), TAT399TTT (1205A>T), TCT400TCA (1209T>A), GAT401CCA (1210G>C 1211A>C 1212T>A), ACG404TCT (1219A>T 1221G>T), CCT405GTT (1222C>G 1223C>T), GGC406GGT (1227C>T), TAC407TAT (1230C>T)								

*: Inserts / Deletes / Misaligned / Frameshifts

Analysis details

This analysis was performed with panviral2.64

NGS Details (UN8): Hubei noda-like virus 9 (segment 1)

Assembly

**Coverage Length**	706 (3 contig(s))
**Depth Of Coverage**	1.9
**Number Of Reads**	11
**Reads Per Million**	0.18 rpm (after QC)
**Ambiguities**	0
**Assembly Method**	de novo + reference guided assembly
**Consensus Caller**	Bcf Tools

Coverage Map

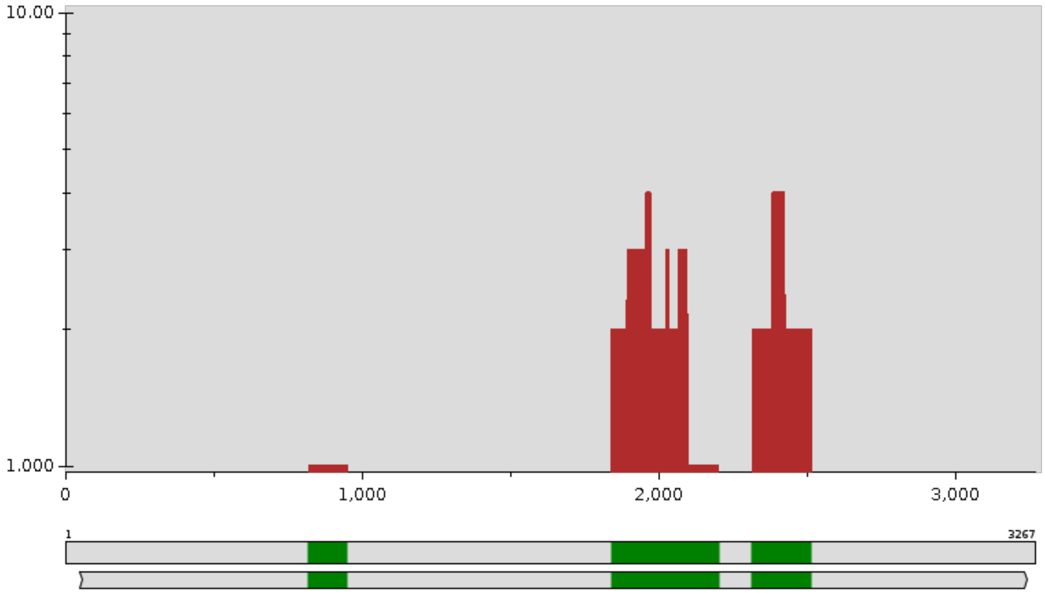

Assignment

**Type**	Hubei noda-like virus 9 (Taxonomy ID: 1922989)
**Reference Genome**	NC_033307.1
**NT Identity (%)**	66.4306
**AA Identity (%)**	69.9153
**Number Of Stop Codons**	0
**Number Of CDS**	1

Alignment

**Alignment Score**	464.0 (NT) + 1227.0 (AA) = 1691.0
**Concordance (%)**	55.479

Alignment Method	Global, seeded, nucleotide + amino acids (AGA)

Genome Region

Sequence starts at position 818 and ends at position 2517 relative to NC_033307.1 reference sequence.

Alignment Detailed Statistics

	Begin	End	Coverage	Score	Concordance	Matches	Identities	I/D/M/F*	Stop Codons
**NT**	**818**	**2517**	**21.6%**	**464**	**32.9%**	**706 (100%)**	**469 (66.4%)**	**0/0**	
Mutations:	824C>G, 833C>T, 836C>T, 839A>C, 840C>A, 842G>A, 843A>G, 848C>T, 866A>G, 878G>C, 880A>G, 881A>G, 885A>G, 886G>A, 887T>A, 896C>T, 902T>C, 908C>T, 909A>G, 910G>C, 917C>T, 918A>T, 920T>A, 921T>C, 922C>A, 923T>A, 924A>G, 927G>A, 928T>C, 932T>C, 933T>A, 934C>A, 935A>G, 936T>A, 937T>A, 942T>A, 943T>A, 944T>C, 1841A>T, 1842C>T, 1847G>T, 1848G>A, 1849C>G, 1852G>A, 1853G>T, 1859T>C, 1862T>A, 1865C>T, 1868G>A, 1871A>C, 1877T>C, 1880A>G, 1885A>G, 1886T>C, 1889C>T, 1894C>G, 1895A>T, 1898C>T, 1901A>G, 1907C>G, 1910A>G, 1913A>T, 1917C>T, 1919G>A, 1920C>G, 1923T>A, 1925T>C, 1927C>T, 1928T>G, 1931A>G, 1932C>T, 1934C>G, 1937G>A, 1941G>A, 1942A>G, 1946T>C, 1949T>C, 1950C>G, 1951A>C, 1952A>T, 1954A>G, 1959G>T, 1960C>G, 1962G>A, 1965C>T, 1970C>T, 1979C>G, 1985C>T, 1988T>C, 1991T>A, 1997A>T, 2007C>T, 2009A>G, 2013C>A, 2014G>A, 2015A>G, 2016T>A, 2021T>A, 2028C>A, 2032T>G, 2033C>T, 2036C>T, 2037C>A, 2039A>G, 2040C>T, 2041G>T, 2046A>T, 2047G>C, 2048T>G, 2050G>A, 2054C>T, 2057A>C, 2060C>T, 2061C>A, 2062T>C, 2063G>T, 2065C>T, 2066A>C, 2067A>T, 2068A>C, 2069C>A, 2070T>A, 2072C>T, 2076T>A, 2083A>G, 2084A>T, 2087C>A, 2090C>T, 2093A>G, 2096A>G, 2097T>A, 2103G>A, 2104T>C, 2108C>T, 2109C>A, 2110G>A, 2111T>A, 2114A>G, 2118A>G, 2119A>T, 2121C>A, 2127T>A, 2128C>G, 2129A>T, 2130G>A, 2132C>A, 2134T>A, 2135T>C, 2144A>T, 2145A>C, 2147A>T, 2153C>T, 2156C>T, 2159T>A, 2171T>C, 2174C>T, 2186C>T, 2189C>T, 2313C>A, 2319A>G, 2320T>A, 2321T>A, 2324T>G, 2326T>C, 2327G>T, 2328G>A, 2329T>A, 2333T>C, 2334T>C, 2339A>C, 2343T>A, 2348C>T, 2357G>T, 2360G>T, 2362C>G, 2363T>G, 2364G>A, 2365A>T, 2366T>G, 2368C>A, 2369C>A, 2372T>G, 2374C>A, 2375G>A, 2376T>C, 2378A>T, 2379A>G, 2381C>T, 2382A>T, 2383A>C, 2388T>C, 2390A>T, 2394C>A, 2396C>G, 2399T>C, 2402C>T, 2405T>A, 2409A>C, 2411C>T, 2412C>G, 2413T>C, 2414T>A, 2415G>C, 2416C>G, 2418T>A, 2420T>A, 2421C>A, 2422T>C, 2423T>A, 2425G>C, 2426T>A, 2429A>C, 2432C>T, 2438C>T, 2443C>A, 2444A>G, 2450A>T, 2456A>C, 2459A>G, 2460T>G, 2472A>G, 2473C>T, 2474A>G, 2477T>C, 2480G>A, 2481A>C, 2482T>A, 2483G>A, 2486A>T, 2489C>T, 2490C>A, 2492T>A, 2495C>G, 2501T>A, 2504A>T, 2506A>T, 2510C>T								

CDS

	Begin	End	Coverage	Score	Concordance	Matches	Identities	I/D/M/F*	Stop Codons
BWW32_s1gp1	257	823	22.2%	1227	74.4%	236 (100%)	165 (69.9%)	0/0/0/0	0
Protein mutations:	D258E (824C>G), Q264K (840C>A 842G>A), N265D (843A>G), K277R (880A>G 881A>G), S279E (885A>G 886G>A 887T>A), S287A (909A>G 910G>C), I290L (918A>T 920T>A), S291Q (921T>C 922C>A 923T>A), I292V (924A>G), V293T (927G>A 928T>C), S295K (933T>A 934C>A 935A>G), F296N (936T>A 937T>A), F298N (942T>A 943T>A 944T>C), A600R (1848G>A 1849C>G), W601Y (1852G>A 1853G>T), E607D (1871A>C), H612R (1885A>G 1886T>C), S615C (1894C>G 1895A>T), Q624E (1920C>G), S625T (1923T>A 1925T>C), A626V (1927C>T 1928T>G), D631S (1941G>A 1942A>G), Q634A (1950C>G 1951A>C 1952A>T), N635S (1954A>G), A637C (1959G>T 1960C>G), V638I (1962G>A), R655K (2013C>A 2014G>A 2015A>G), Y656N (2016T>A), I661S (2032T>G 2033C>T), Q663K (2037C>A 2039A>G), R664L (2040C>T 2041G>T), S667N (2050G>A), L671T (2061C>A 2062T>C 2063G>T), T672I (2065C>T 2066A>C), N673S (2067A>T 2068A>C 2069C>A), S674T (2070T>A 2072C>T), F676I (2076T>A), K678S (2083A>G 2084A>T), S683T (2097T>A), V685T (2103G>A 2104T>C), R687K (2109C>A 2110G>A 2111T>A), K690V (2118A>G 2119A>T), A694T (2130G>A 2132C>A), F695Y (2134T>A 2135T>C), L755I (2313C>A), I757E (2319A>G 2320T>A 2321T>A), V759A (2326T>C 2327G>T), V760N (2328G>A 2329T>A), S765T (2343T>A), E769D (2357G>T), T771R (2362C>G 2363T>G), D772M (2364G>A 2365A>T 2366T>G), P773Q (2368C>A 2369C>A), A775E (2374C>A 2375G>A), S776P (2376T>C 2378A>T), I777V (2379A>G 2381C>T), N778S (2382A>T 2383A>C), I787L (2409A>C 2411C>T), L788A (2412C>G 2413T>C 2414T>A), A789R (2415G>C 2416C>G), S790T (2418T>A 2420T>A), L791T (2421C>A 2422T>C 2423T>A), S792T (2425G>C 2426T>A), T798K (2443C>A 2444A>G), L804V (2460T>G), T808V (2472A>G 2473C>T 2474A>G), M811Q (2481A>C 2482T>A 2483G>A), D815E (2495C>G), Y819F (2506A>T)								
Codon mutations:	GAC258GAG (824C>G), TAC261TAT (833C>T), TAC262TAT (836C>T), ATA263ATC (839A>C), CAG264AAA (840C>A 842G>A), AAT265GAT (843A>G), AAC266AAT (848C>T), GTA272GTG (866A>G), GGG276GGC (878G>C), AAA277AGG (880A>G 881A>G), AGT279GAA (885A>G 886G>A 887T>A), GTC282GTT (896C>T), GAT284GAC (902T>C), GAC286GAT (908C>T), AGT287GCT (909A>G 910G>C), ACC289ACT (917C>T), ATT290TTA (918A>T 920T>A), TCT291CAA (921T>C 922C>A 923T>A), ATC292GTC (924A>G), GTT293ACT (927G>A 928T>C), GAT294GAC (932T>C), TCA295AAG (933T>A 934C>A 935A>G), TTT296AAT (936T>A 937T>A), TTT298AAC (942T>A 943T>A 944T>C), GCA597GCT (1841A>T), CTA598TTA (1842C>T), TCG599TCT (1847G>T), GCA600AGA (1848G>A 1849C>G), TGG601TAT (1852G>A 1853G>T), TAT603TAC (1859T>C), GCT604GCA (1862T>A), TTC605TTT (1865C>T), AAG606AAA (1868G>A), GAA607GAC (1871A>C), TGT609TGC (1877T>C), TTA610TTG (1880A>G), CAT612CGC (1885A>G 1886T>C), CTC613CTT (1889C>T), TCA615TGT (1894C>G 1895A>T), TAC616TAT (1898C>T), GGA617GGG (1901A>G), GGC619GGG (1907C>G), AAA620AAG (1910A>G), ACA621ACT (1913A>T), CTG623TTA (1917C>T 1919G>A), CAG624GAG (1920C>G), TCT625ACC (1923T>A 1925T>C), GCT626GTG (1927C>T 1928T>G), AAA627AAG (1931A>G), CTC628TTG (1932C>T 1934C>G), CTG629CTA (1937G>A), GAT631AGT (1941G>A 1942A>G), ATT632ATC (1946T>C), TGT633TGC (1949T>C), CAA634GCT (1950C>G 1951A>C 1952A>T), AAT635AGT (1954A>G), GCT637TGT (1959G>T 1960C>G), GTC638ATC (1962G>A), CTA639TTA (1965C>T), ACC640ACT (1970C>T), ACC643ACG (1979C>G), TTC645TTT (1985C>T), GAT646GAC (1988T>C), GGT647GGA (1991T>A), ATA649ATT (1997A>T), CTA653TTG (2007C>T 2009A>G), CGA655AAG (2013C>A 2014G>A 2015A>G), TAT656AAT (2016T>A), GTT657GTA (2021T>A), CGG660AGG (2028C>A), ATC661AGT (2032T>G 2033C>T), TAC662TAT (2036C>T), CAA663AAG (2037C>A 2039A>G), CGA664TTA (2040C>T 2041G>T), AGT666TCG (2046A>T 2047G>C 2048T>G), AGT667AAT (2050G>A), GAC668GAT (2054C>T), CCA669CCC (2057A>C), GCC670GCT (2060C>T), CTG671ACT (2061C>A 2062T>C 2063G>T), ACA672ATC (2065C>T 2066A>C), AAC673TCA (2067A>T 2068A>C 2069C>A), TCC674ACT (2070T>A 2072C>T), TTT676ATT (2076T>A), AAA678AGT (2083A>G 2084A>T), ATC679ATA (2087C>A), TTC680TTT (2090C>T), AAA681AAG (2093A>G), CAA682CAG (2096A>G), TCA683ACA (2097T>A), GTA685ACA (2103G>A 2104T>C), ACC686ACT (2108C>T), CGT687AAA (2109C>A 2110G>A 2111T>A), GAA688GAG (2114A>G), AAA690GTA (2118A>G 2119A>T), CGA691AGA (2121C>A), TCA693AGT (2127T>A 2128C>G 2129A>T), GCC694ACA (2130G>A 2132C>A), TTT695TAC (2134T>A 2135T>C), ACA698ACT (2144A>T), AGA699CGT (2145A>C 2147A>T), GGC701GGT (2153C>T), AGC702AGT (2156C>T), CCT703CCA (2159T>A), GAT707GAC (2171T>C), GGC708GGT (2174C>T), ATC712ATT (2186C>T), AAC713AAT (2189C>T), CTT755ATT (2313C>A), ATT757GAA (2319A>G 2320T>A 2321T>A), GTT758GTG (2324T>G), GTG759GCT (2326T>C 2327G>T), GTT760AAT (2328G>A 2329T>A), GAT761GAC (2333T>C), TTA762CTA (2334T>C), GGA763GGC (2339A>C), TCA765ACA (2343T>A), GTC766GTT (2348C>T), GAG769GAT (2357G>T), GTG770GTT (2360G>T), ACT771AGG (2362C>G 2363T>G), GAT772ATG (2364G>A 2365A>T 2366T>G), CCC773CAA (2368C>A 2369C>A), GGT774GGG (2372T>G), GCG775GAA (2374C>A 2375G>A), TCA776CCT (2376T>C 2378A>T), ATC777GTT (2379A>G 2381C>T), AAC778TCC (2382A>T 2383A>C), TTA780CTT (2388T>C 2390A>T), CGC782AGG (2394C>A 2396C>G), ATT783ATC (2399T>C), TTC784TTT (2402C>T), CCT785CCA (2405T>A), ATC787CTT (2409A>C 2411C>T), CTT788GCA (2412C>G 2413T>C 2414T>A), GCA789CGA (2415G>C 2416C>G), TCT790ACA (2418T>A 2420T>A), CTT791ACA (2421C>A 2422T>C 2423T>A), AGT792ACA (2425G>C 2426T>A), TCA793TCC (2429A>C), CAC794CAT (2432C>T), GAC796GAT (2438C>T), ACA798AAG (2443C>A 2444A>G), ACA800ACT (2450A>T), CCA802CCC (2456A>C), AAA803AAG (2459A>G), TTA804GTA (2460T>G), ACA808GTG (2472A>G 2473C>T 2474A>G), AAT809AAC (2477T>C), CAG810CAA (2480G>A), ATG811CAA (2481A>C 2482T>A 2483G>A), GTA812GTT (2486A>T), ACC813ACT (2489C>T), CGT814AGA (2490C>A 2492T>A), GAC815GAG (2495C>G), GCT817GCA (2501T>A), GCA818GCT (2504A>T), TAT819TTT (2506A>T), AAC820AAT (2510C>T)								

Proteins

	Begin	End	Coverage	Score	Concordance	Matches	Identities	I/D/M/F*	Stop Codons
RNA-dependent RNA polymerase (YP_009337879.1)	257	823	22.2%	1227	74.4%	236 (100%)	165 (69.9%)	0/0/0/0	0
Protein mutations:	D258E (824C>G), Q264K (840C>A 842G>A), N265D (843A>G), K277R (880A>G 881A>G), S279E (885A>G 886G>A 887T>A), S287A (909A>G 910G>C), I290L (918A>T 920T>A), S291Q (921T>C 922C>A 923T>A), I292V (924A>G), V293T (927G>A 928T>C), S295K (933T>A 934C>A 935A>G), F296N (936T>A 937T>A), F298N (942T>A 943T>A 944T>C), A600R (1848G>A 1849C>G), W601Y (1852G>A 1853G>T), E607D (1871A>C), H612R (1885A>G 1886T>C), S615C (1894C>G 1895A>T), Q624E (1920C>G), S625T (1923T>A 1925T>C), A626V (1927C>T 1928T>G), D631S (1941G>A 1942A>G), Q634A (1950C>G 1951A>C 1952A>T), N635S (1954A>G), A637C (1959G>T 1960C>G), V638I (1962G>A), R655K (2013C>A 2014G>A 2015A>G), Y656N (2016T>A), I661S (2032T>G 2033C>T), Q663K (2037C>A 2039A>G), R664L (2040C>T 2041G>T), S667N (2050G>A), L671T (2061C>A 2062T>C 2063G>T), T672I (2065C>T 2066A>C), N673S (2067A>T 2068A>C 2069C>A), S674T (2070T>A 2072C>T), F676I (2076T>A), K678S (2083A>G 2084A>T), S683T (2097T>A), V685T (2103G>A 2104T>C), R687K (2109C>A 2110G>A 2111T>A), K690V (2118A>G 2119A>T), A694T (2130G>A 2132C>A), F695Y (2134T>A 2135T>C), L755I (2313C>A), I757E (2319A>G 2320T>A 2321T>A), V759A (2326T>C 2327G>T), V760N (2328G>A 2329T>A), S765T (2343T>A), E769D (2357G>T), T771R (2362C>G 2363T>G), D772M (2364G>A 2365A>T 2366T>G), P773Q (2368C>A 2369C>A), A775E (2374C>A 2375G>A), S776P (2376T>C 2378A>T), I777V (2379A>G 2381C>T), N778S (2382A>T 2383A>C), I787L (2409A>C 2411C>T), L788A (2412C>G 2413T>C 2414T>A), A789R (2415G>C 2416C>G), S790T (2418T>A 2420T>A), L791T (2421C>A 2422T>C 2423T>A), S792T (2425G>C 2426T>A), T798K (2443C>A 2444A>G), L804V (2460T>G), T808V (2472A>G 2473C>T 2474A>G), M811Q (2481A>C 2482T>A 2483G>A), D815E (2495C>G), Y819F (2506A>T)								

	Begin	End	Coverage	Score	Concordance	Matches	Identities	I/D/M/F*	Stop Codons
**NT**	**818**	**2517**	**21.6%**	**464**	**32.9%**	**706 (100%)**	**469 (66.4%)**	**0/0**	
Codon mutations:	GAC258GAG (824C>G), TAC261TAT (833C>T), TAC262TAT (836C>T), ATA263ATC (839A>C), CAG264AAA (840C>A 842G>A), AAT265GAT (843A>G), AAC266AAT (848C>T), GTA272GTG (866A>G), GGG276GGC (878G>C), AAA277AGG (880A>G 881A>G), AGT279GAA (885A>G 886G>A 887T>A), GTC282GTT (896C>T), GAT284GAC (902T>C), GAC286GAT (908C>T), AGT287GCT (909A>G 910G>C), ACC289ACT (917C>T), ATT290TTA (918A>T 920T>A), TCT291CAA (921T>C 922C>A 923T>A), ATC292GTC (924A>G), GTT293ACT (927G>A 928T>C), GAT294GAC (932T>C), TCA295AAG (933T>A 934C>A 935A>G), TTT296AAT (936T>A 937T>A), TTT298AAC (942T>A 943T>A 944T>C), GCA597GCT (1841A>T), CTA598TTA (1842C>T), TCG599TCT (1847G>T), GCA600AGA (1848G>A 1849C>G), TGG601TAT (1852G>A 1853G>T), TAT603TAC (1859T>C), GCT604GCA (1862T>A), TTC605TTT (1865C>T), AAG606AAA (1868G>A), GAA607GAC (1871A>C), TGT609TGC (1877T>C), TTA610TTG (1880A>G), CAT612CGC (1885A>G 1886T>C), CTC613CTT (1889C>T), TCA615TGT (1894C>G 1895A>T), TAC616TAT (1898C>T), GGA617GGG (1901A>G), GGC619GGG (1907C>G), AAA620AAG (1910A>G), ACA621ACT (1913A>T), CTG623TTA (1917C>T 1919G>A), CAG624GAG (1920C>G), TCT625ACC (1923T>A 1925T>C), GCT626GTG (1927C>T 1928T>G), AAA627AAG (1931A>G), CTC628TTG (1932C>T 1934C>G), CTG629CTA (1937G>A), GAT631AGT (1941G>A 1942A>G), ATT632ATC (1946T>C), TGT633TGC (1949T>C), CAA634GCT (1950C>G 1951A>C 1952A>T), AAT635AGT (1954A>G), GCT637TGT (1959G>T 1960C>G), GTC638ATC (1962G>A), CTA639TTA (1965C>T), ACC640ACT (1970C>T), ACC643ACG (1979C>G), TTC645TTT (1985C>T), GAT646GAC (1988T>C), GGT647GGA (1991T>A), ATA649ATT (1997A>T), CTA653TTG (2007C>T 2009A>G), CGA655AAG (2013C>A 2014G>A 2015A>G), TAT656AAT (2016T>A), GTT657GTA (2021T>A), CGG660AGG (2028C>A), ATC661AGT (2032T>G 2033C>T), TAC662TAT (2036C>T), CAA663AAG (2037C>A 2039A>G), CGA664TTA (2040C>T 2041G>T), AGT666TCG (2046A>T 2047G>C 2048T>G), AGT667AAT (2050G>A), GAC668GAT (2054C>T), CCA669CCC (2057A>C), GCC670GCT (2060C>T), CTG671ACT (2061C>A 2062T>C 2063G>T), ACA672ATC (2065C>T 2066A>C), AAC673TCA (2067A>T 2068A>C 2069C>A), TCC674ACT (2070T>A 2072C>T), TTT676ATT (2076T>A), AAA678AGT (2083A>G 2084A>T), ATC679ATA (2087C>A), TTC680TTT (2090C>T), AAA681AAG (2093A>G), CAA682CAG (2096A>G), TCA683ACA (2097T>A), GTA685ACA (2103G>A 2104T>C), ACC686ACT (2108C>T), CGT687AAA (2109C>A 2110G>A 2111T>A), GAA688GAG (2114A>G), AAA690GTA (2118A>G 2119A>T), CGA691AGA (2121C>A), TCA693AGT (2127T>A 2128C>G 2129A>T), GCC694ACA (2130G>A 2132C>A), TTT695TAC (2134T>A 2135T>C), ACA698ACT (2144A>T), AGA699CGT (2145A>C 2147A>T), GGC701GGT (2153C>T), AGC702AGT (2156C>T), CCT703CCA (2159T>A), GAT707GAC (2171T>C), GGC708GGT (2174C>T), ATC712ATT (2186C>T), AAC713AAT (2189C>T), CTT755ATT (2313C>A), ATT757GAA (2319A>G 2320T>A 2321T>A), GTT758GTG (2324T>G), GTG759GCT (2326T>C 2327G>T), GTT760AAT (2328G>A 2329T>A), GAT761GAC (2333T>C), TTA762CTA (2334T>C), GGA763GGC (2339A>C), TCA765ACA (2343T>A), GTC766GTT (2348C>T), GAG769GAT (2357G>T), GTG770GTT (2360G>T), ACT771AGG (2362C>G 2363T>G), GAT772ATG (2364G>A 2365A>T 2366T>G), CCC773CAA (2368C>A 2369C>A), GGT774GGG (2372T>G), GCG775GAA (2374C>A 2375G>A), TCA776CCT (2376T>C 2378A>T), ATC777GTT (2379A>G 2381C>T), AAC778TCC (2382A>T 2383A>C), TTA780CTT (2388T>C 2390A>T), CGC782AGG (2394C>A 2396C>G), ATT783ATC (2399T>C), TTC784TTT (2402C>T), CCT785CCA (2405T>A), ATC787CTT (2409A>C 2411C>T), CTT788GCA (2412C>G 2413T>C 2414T>A), GCA789CGA (2415G>C 2416C>G), TCT790ACA (2418T>A 2420T>A), CTT791ACA (2421C>A 2422T>C 2423T>A), AGT792ACA (2425G>C 2426T>A), TCA793TCC (2429A>C), CAC794CAT (2432C>T), GAC796GAT (2438C>T), ACA798AAG (2443C>A 2444A>G), ACA800ACT (2450A>T), CCA802CCC (2456A>C), AAA803AAG (2459A>G), TTA804GTA (2460T>G), ACA808GTG (2472A>G 2473C>T 2474A>G), AAT809AAC (2477T>C), CAG810CAA (2480G>A), ATG811CAA (2481A>C 2482T>A 2483G>A), GTA812GTT (2486A>T), ACC813ACT (2489C>T), CGT814AGA (2490C>A 2492T>A), GAC815GAG (2495C>G), GCT817GCA (2501T>A), GCA818GCT (2504A>T), TAT819TTT (2506A>T), AAC820AAT (2510C>T)								

*: Inserts / Deletes / Misaligned / Frameshifts

Analysis details

This analysis was performed with panviral2.64

NGS Details (UN8): Caulimovirus venafragariae

Assembly

**Coverage Length**	253 (1 contig(s))
**Depth Of Coverage**	4.2
**Number Of Reads**	9
**Reads Per Million**	0.15 rpm (after QC)
**Ambiguities**	0
**Assembly Method**	de novo + reference guided assembly
**Consensus Caller**	Bcf Tools

Coverage Map

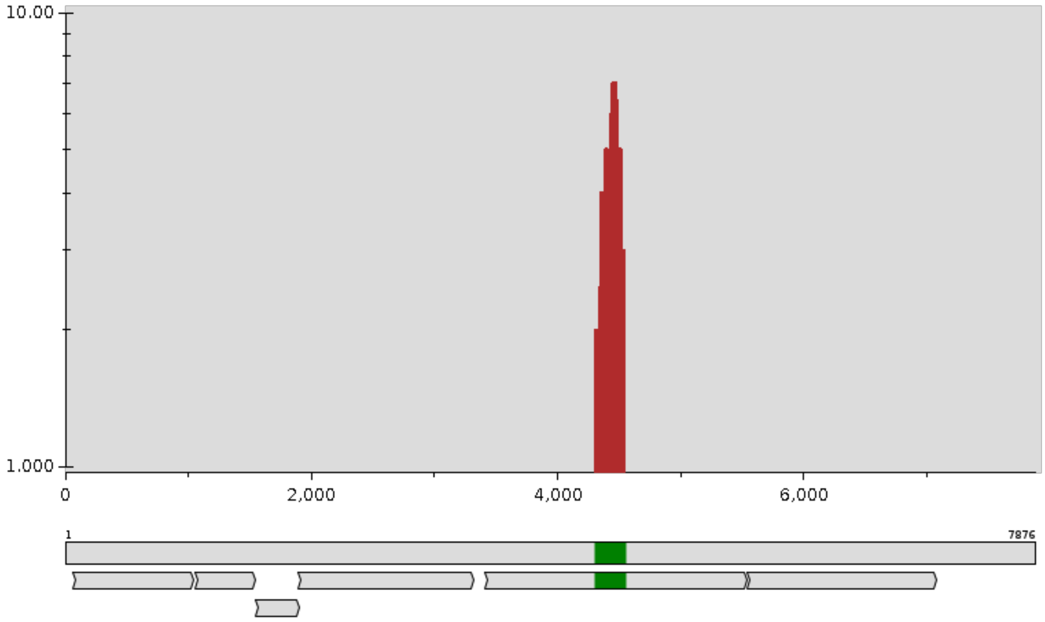

Assignment

**Type**	Caulimovirus venafragariae (Taxonomy ID: 3048344)
**Reference Genome**	NC_001725.1
**NT Identity (%)**	63.6364
**AA Identity (%)**	63.5294
**Number Of Stop Codons**	0
**Number Of CDS**	6

Alignment

**Alignment Score**	138.0 (NT) + 390.0 (AA) = 528.0
**Concordance (%)**	48.4404

Alignment Method	Global, seeded, nucleotide + amino acids (AGA)

Genome Region

Sequence starts at position 4300 and ends at position 4552 relative to NC_001725.1 reference sequence.

Alignment Detailed Statistics

	Begin	End	Coverage	Score	Concordance	Matches	Identities	I/D/M/F*	Stop Codons
**NT**	**4300**	**4552**	**3.2%**	**138**	**27.3%**	**253 (100%)**	**161 (63.6%)**	**0/0**	
Mutations:	4300G>A, 4311C>A, 4314G>C, 4315C>T, 4317T>A, 4320T>A, 4321A>G, 4323C>T, 4326C>A, 4330C>A, 4331C>G, 4332T>A, 4333A>T, 4334G>C, 4335C>T, 4337A>C, 4338G>A, 4339A>T, 4340G>C, 4341T>A, 4342C>A, 4343C>A, 4348T>A, 4349C>G, 4350C>A, 4354C>G, 4362C>T, 4371G>A, 4378G>A, 4379C>G, 4383G>A, 4386C>A, 4387A>G, 4388A>T, 4390C>A, 4395C>T, 4402A>C, 4403G>A, 4408G>A, 4413T>A, 4419T>C, 4424A>G, 4425G>A, 4426T>C, 4428A>T, 4434C>T, 4435C>A, 4437T>C, 4440A>T, 4442A>G, 4443G>A, 4444G>A, 4445G>C, 4446A>G, 4447G>T, 4451G>C, 4452C>T, 4455C>T, 4456C>A, 4457T>A, 4458A>G, 4461T>A, 4464T>A, 4465A>G, 4467C>T, 4470G>A, 4471G>A, 4472A>G, 4473G>T, 4474C>G, 4477C>T, 4479G>A, 4480C>A, 4482T>A, 4483C>A, 4485A>T, 4491C>A, 4492G>C, 4493G>A, 4503C>A, 4504T>G, 4506T>A, 4508A>T, 4510T>A, 4511C>A, 4513T>A, 4514C>A, 4515C>A, 4521C>T, 4530T>A, 4535T>A, 4542G>A								
**CDS**									
ORF_V	299	383	12.0%	390	66.3%	85 (100%)	54 (63.5%)	0/0/0/0	0
Protein mutations:	E299K (4300G>A), K303N (4314G>C), I306V (4321A>G 4323C>T), P309R (4330C>A 4331C>G 4332T>A), K311T (4337A>C 4338G>A), P313K (4342C>A 4343C>A), S315R (4348T>A 4349C>G 4350C>A), P317A (4354C>G), A325S (4378G>A 4379C>G), K328V (4387A>G 4388A>T), R333Q (4402A>C 4403G>A), V335I (4408G>A), K340R (4424A>G 4425G>A), H344N (4435C>A 4437T>C), K346R (4442A>G 4443G>A), G347T (4444G>A 4445G>C 4446A>G), D348Y (4447G>T), G349A (4451G>C 4452C>T), L351K (4456C>A 4457T>A 4458A>G), N354D (4465A>G 4467C>T), E356S (4471G>A 4472A>G 4473G>T), Q357E (4474C>G), L359I (4480C>A 4482T>A), Q360N (4483C>A 4485A>T), G363Q (4492G>C 4493G>A), F367V (4504T>G 4506T>A), Y368F (4508A>T), S369N (4510T>A 4511C>A), S370K (4513T>A 4514C>A 4515C>A), F377Y (4535T>A)								
Codon mutations:	GAA299AAA (4300G>A), CTC302CTA (4311C>A), AAG303AAC (4314G>C), CTT304TTA (4315C>T 4317T>A), GGT305GGA (4320T>A), ATC306GTT (4321A>G 4323C>T), ATC307ATA (4326C>A), CCT309AGA (4330C>A 4331C>G 4332T>A), AGC310TCT (4333A>T 4334G>C 4335C>T), AAG311ACA (4337A>C 4338G>A), AGT312TCA (4339A>T 4340G>C 4341T>A), CCA313AAA (4342C>A 4343C>A), TCC315AGA (4348T>A 4349C>G 4350C>A), CCA317GCA (4354C>G), TTC319TTT (4362C>T), AGG322AGA (4371G>A), GCC325AGC (4378G>A 4379C>G), GAG326GAA (4383G>A), ATC327ATA (4386C>A), AAA328GTA (4387A>G 4388A>T), CGA329AGA (4390C>A), GGC330GGT (4395C>T), AGA333CAA (4402A>C 4403G>A), GTA335ATA (4408G>A), ATT336ATA (4413T>A), TAT338TAC (4419T>C), AAG340AGA (4424A>G 4425G>A), TTA341CTT (4426T>C 4428A>T), GAC343GAT (4434C>T), CAT344AAC (4435C>A 4437T>C), ACA345ACT (4440A>T), AAG346AGA (4442A>G 4443G>A), GGA347ACG (4444G>A 4445G>C 4446A>G), GAT348TAT (4447G>T), GGC349GCT (4451G>C 4452C>T), TAC350TAT (4455C>T), CTA351AAG (4456C>A 4457T>A 4458A>G), CTT352CTA (4461T>A), CCT353CCA (4464T>A), AAC354GAT (4465A>G 4467C>T), AAG355AAA (4470G>A), GAG356AGT (4471G>A 4472A>G 4473G>T), CAA357GAA (4474C>G), CTG358TTA (4477C>T 4479G>A), CTT359ATA (4480C>A 4482T>A), CAA360AAT (4483C>A 4485A>T), ATC362ATA (4491C>A), GGA363CAA (4492G>C 4493G>A), ACC366ACA (4503C>A), TTT367GTA (4504T>G 4506T>A), TAC368TTC (4508A>T), TCT369AAT (4510T>A 4511C>A), TCC370AAA (4513T>A 4514C>A 4515C>A), GAC372GAT (4521C>T), TCT375TCA (4530T>A), TTT377TAT (4535T>A), CAG379CAA (4542G>A)								
**Proteins**									
hypothetical protein (NP_043933.1)	299	383	12.0%	390	66.3%	85 (100%)	54 (63.5%)	0/0/0/0	0
Protein mutations:	E299K (4300G>A), K303N (4314G>C), I306V (4321A>G 4323C>T), P309R (4330C>A 4331C>G 4332T>A), K311T (4337A>C 4338G>A), P313K (4342C>A 4343C>A), S315R (4348T>A 4349C>G 4350C>A), P317A (4354C>G), A325S (4378G>A 4379C>G), K328V (4387A>G 4388A>T), R333Q (4402A>C 4403G>A), V335I (4408G>A), K340R (4424A>G 4425G>A), H344N (4435C>A 4437T>C), K346R (4442A>G 4443G>A), G347T (4444G>A 4445G>C 4446A>G), D348Y (4447G>T), G349A (4451G>C 4452C>T), L351K (4456C>A 4457T>A 4458A>G), N354D (4465A>G 4467C>T), E356S (4471G>A 4472A>G 4473G>T), Q357E (4474C>G), L359I (4480C>A 4482T>A), Q360N (4483C>A 4485A>T), G363Q (4492G>C 4493G>A), F367V (4504T>G 4506T>A), Y368F (4508A>T), S369N (4510T>A 4511C>A), S370K (4513T>A 4514C>A 4515C>A), F377Y (4535T>A)								
Codon mutations:	GAA299AAA (4300G>A), CTC302CTA (4311C>A), AAG303AAC (4314G>C), CTT304TTA (4315C>T 4317T>A), GGT305GGA (4320T>A), ATC306GTT (4321A>G 4323C>T), ATC307ATA (4326C>A), CCT309AGA (4330C>A 4331C>G 4332T>A), AGC310TCT (4333A>T 4334G>C 4335C>T), AAG311ACA (4337A>C 4338G>A), AGT312TCA (4339A>T 4340G>C 4341T>A), CCA313AAA (4342C>A 4343C>A), TCC315AGA (4348T>A 4349C>G 4350C>A), CCA317GCA (4354C>G), TTC319TTT (4362C>T), AGG322AGA (4371G>A), GCC325AGC (4378G>A 4379C>G), GAG326GAA (4383G>A), ATC327ATA (4386C>A), AAA328GTA (4387A>G 4388A>T), CGA329AGA (4390C>A), GGC330GGT (4395C>T), AGA333CAA (4402A>C 4403G>A), GTA335ATA (4408G>A), ATT336ATA (4413T>A), TAT338TAC (4419T>C), AAG340AGA (4424A>G 4425G>A), TTA341CTT (4426T>C 4428A>T), GAC343GAT (4434C>T), CAT344AAC (4435C>A 4437T>C), ACA345ACT (4440A>T), AAG346AGA (4442A>G 4443G>A), GGA347ACG (4444G>A 4445G>C 4446A>G), GAT348TAT (4447G>T), GGC349GCT (4451G>C 4452C>T), TAC350TAT (4455C>T), CTA351AAG (4456C>A 4457T>A 4458A>G), CTT352CTA (4461T>A), CCT353CCA (4464T>A), AAC354GAT (4465A>G 4467C>T), AAG355AAA (4470G>A), GAG356AGT (4471G>A 4472A>G 4473G>T), CAA357GAA (4474C>G), CTG358TTA (4477C>T 4479G>A), CTT359ATA (4480C>A 4482T>A), CAA360AAT (4483C>A 4485A>T), ATC362ATA (4491C>A), GGA363CAA (4492G>C 4493G>A), ACC366ACA (4503C>A), TTT367GTA (4504T>G 4506T>A), TAC368TTC (4508A>T), TCT369AAT (4510T>A 4511C>A), TCC370AAA (4513T>A 4514C>A 4515C>A), GAC372GAT (4521C>T), TCT375TCA (4530T>A), TTT377TAT (4535T>A), CAG379CAA (4542G>A)								

*: Inserts / Deletes / Misaligned / Frameshifts

Analysis details

This analysis was performed with panviral2.64

NGS Details (UN8): Cavemovirus venamanihotis

Assembly

**Coverage Length**	273 (1 contig(s))
**Depth Of Coverage**	3.2
**Number Of Reads**	8
**Reads Per Million**	0.13 rpm (after QC)
**Ambiguities**	0
**Assembly Method**	de novo + reference guided assembly
**Consensus Caller**	Bcf Tools

Coverage Map

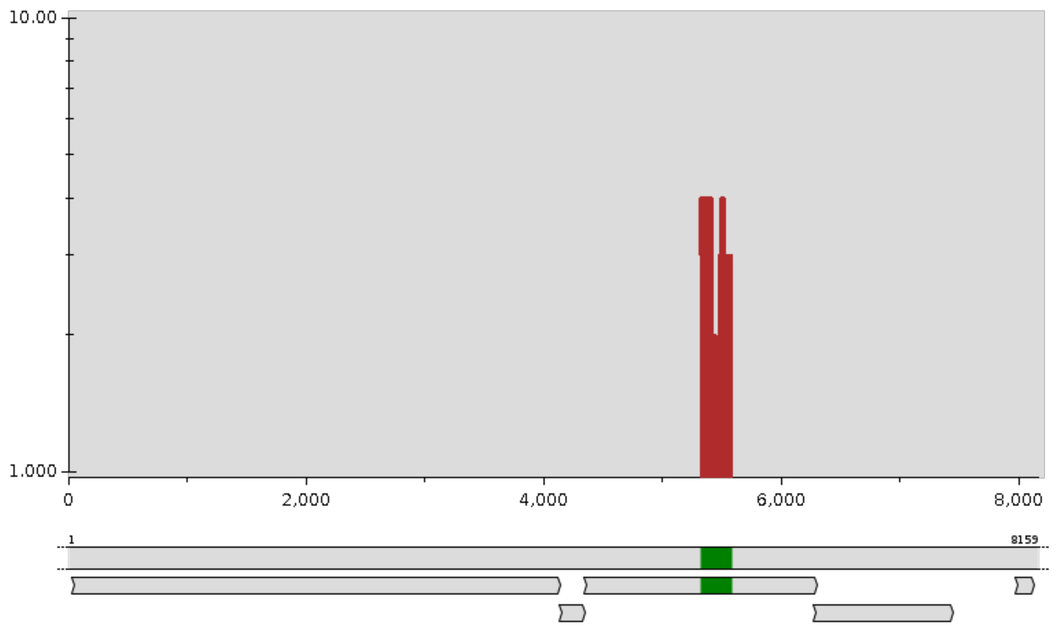

Assignment

**Type**	Cavemovirus venamanihotis (Taxonomy ID: 3047736)
**Reference Genome**	NC_001648.1
**NT Identity (%)**	60.0733
**AA Identity (%)**	51.6484
**Number Of Stop Codons**	0
**Number Of CDS**	5

Alignment

**Alignment Score**	110.0 (NT) + 360.0 (AA) = 470.0
**Concordance (%)**	39.2977

Alignment Method	Global, seeded, nucleotide + amino acids (AGA)

Genome Region

Sequence starts at position 5318 and ends at position 5590 relative to NC_001648.1 reference sequence.

Alignment Detailed Statistics

	Begin	End	Coverage	Score	Concordance	Matches	Identities	I/D/M/F*	Stop Codons
**NT**	**5318**	**5590**	**3.3%**	**110**	**20.1%**	**273 (100%)**	**164 (60.1%)**	**0/0**	
Mutations:	5318G>T, 5321A>G, 5322T>C, 5324T>A, 5328C>G, 5330G>A, 5336A>T, 5339A>T, 5340T>A, 5342A>G, 5345A>T, 5351A>C, 5353A>T, 5354T>A, 5355C>A, 5357T>A, 5360C>T, 5361T>G, 5363A>C, 5366A>T, 5367A>T, 5368G>C, 5370A>G, 5371T>A, 5372T>A, 5375C>T, 5379C>A, 5382T>A, 5388G>A, 5391A>G, 5392G>A, 5393A>T, 5396A>T, 5401G>A, 5402A>T, 5410A>T, 5411T>G, 5426G>C, 5428A>G, 5429T>C, 5438T>C, 5444A>G, 5447C>A, 5449T>A, 5452C>T, 5453T>A, 5456G>A, 5458C>T, 5459T>A, 5460A>T, 5466G>A, 5467A>T, 5473A>T, 5474A>T, 5476T>A, 5477A>G, 5481A>C, 5485C>A, 5486A>C, 5488A>T, 5489A>T, 5494G>A, 5495A>G, 5496G>A, 5498T>A, 5501A>T, 5502A>G, 5503C>T, 5504A>G, 5505C>A, 5507T>A, 5508G>C, 5509C>A, 5516A>T, 5519A>G, 5520A>G, 5522T>G, 5525T>A, 5526A>T, 5527G>C, 5531G>A, 5532A>C, 5533A>C, 5539C>T, 5541G>A, 5542A>G, 5543A>T, 5546A>G, 5548G>T, 5550A>C, 5553G>A, 5554A>C, 5556A>G, 5559A>G, 5561A>C, 5562G>C, 5563A>G, 5564T>A, 5567C>T, 5568C>T, 5570T>A, 5574G>C, 5575T>A, 5576C>T, 5577C>A, 5579A>T, 5583G>T, 5584A>G, 5585A>T								
**CDS**									
CsVMVgp3	326	416	13.9%	360	54.0%	91 (100%)	47 (51.6%)	0/0/0/0	0
Protein mutations:	Y327Q (5322T>C 5324T>A), Q329E (5328C>G 5330G>A), K331N (5336A>T), L333M (5340T>A 5342A>G), Y337L (5353A>T 5354T>A), H338K (5355C>A 5357T>A), S340A (5361T>G 5363A>C), I343E (5370A>G 5371T>A 5372T>A), Q346K (5379C>A), F347I (5382T>A), D349N (5388G>A), R350D (5391A>G 5392G>A 5393A>T), R353N (5401G>A 5402A>T), Y356L (5410A>T 5411T>G), Y362C (5428A>G 5429T>C), F369Y (5449T>A), S370L (5452C>T 5453T>A), T372I (5458C>T 5459T>A), I373F (5460A>T), E375M (5466G>A 5467A>T), K377I (5473A>T 5474A>T), I378K (5476T>A 5477A>G), I380L (5481A>C), A381D (5485C>A 5486A>C), K382I (5488A>T 5489A>T), R384K (5494G>A 5495A>G), D385K (5496G>A 5498T>A), T387V (5502A>G 5503C>T 5504A>G), L388I (5505C>A 5507T>A), A389Q (5508G>C 5509C>A), I393V (5520A>G 5522T>G), K397P (5532A>C 5533A>C), T399I (5539C>T), E400S (5541G>A 5542A>G 5543A>T), C402F (5548G>T), K403Q (5550A>C), E404T (5553G>A 5554A>C), K405E (5556A>G), I406V (5559A>G 5561A>C), D407R (5562G>C 5563A>G 5564T>A), V411H (5574G>C 5575T>A 5576C>T), Q412N (5577C>A 5579A>T), E414C (5583G>T 5584A>G 5585A>T)								
Codon mutations:	CAG325..T (5318G>T), GGA326GGG (5321A>G), TAT327CAA (5322T>C 5324T>A), CAG329GAA (5328C>G 5330G>A), AAA331AAT (5336A>T), GTA332GTT (5339A>T), TTA333ATG (5340T>A 5342A>G), CCA334CCT (5345A>T), GGA336GGC (5351A>C), TAT337TTA (5353A>T 5354T>A), CAT338AAA (5355C>A 5357T>A), AAC339AAT (5360C>T), TCA340GCC (5361T>G 5363A>C), CCA341CCT (5366A>T), AGT342TCT (5367A>T 5368G>C), ATT343GAA (5370A>G 5371T>A 5372T>A), TTC344TTT (5375C>T), CAA346AAA (5379C>A), TTT347ATT (5382T>A), GAT349AAT (5388G>A), AGA350GAT (5391A>G 5392G>A 5393A>T), ATA351ATT (5396A>T), AGA353AAT (5401G>A 5402A>T), TAT356TTG (5410A>T 5411T>G), GTG361GTC (5426G>C), TAT362TGC (5428A>G 5429T>C), GAT365GAC (5438T>C), TTA367TTG (5444A>G), GTC368GTA (5447C>A), TTT369TAT (5449T>A), TCT370TTA (5452C>T 5453T>A), AAG371AAA (5456G>A), ACT372ATA (5458C>T 5459T>A), ATT373TTT (5460A>T), GAG375ATG (5466G>A 5467A>T), AAA377ATT (5473A>T 5474A>T), ATA378AAG (5476T>A 5477A>G), ATT380CTT (5481A>C), GCA381GAC (5485C>A 5486A>C), AAA382ATT (5488A>T 5489A>T), AGA384AAG (5494G>A 5495A>G), GAT385AAA (5496G>A 5498T>A), ATA386ATT (5501A>T), ACA387GTG (5502A>G 5503C>T 5504A>G), CTT388ATA (5505C>A 5507T>A), GCA389CAA (5508G>C 5509C>A), GGA391GGT (5516A>T), TTA392TTG (5519A>G), ATT393GTG (5520A>G 5522T>G), ATT394ATA (5525T>A), AGT395TCT (5526A>T 5527G>C), AAG396AAA (5531G>A), AAA397CCA (5532A>C 5533A>C), ACA399ATA (5539C>T), GAA400AGT (5541G>A 5542A>G 5543A>T), TTA401TTG (5546A>G), TGT402TTT (5548G>T), AAA403CAA (5550A>C), GAA404ACA (5553G>A 5554A>C), AAA405GAA (5556A>G), ATA406GTC (5559A>G 5561A>C), GAT407CGA (5562G>C 5563A>G 5564T>A), TTC408TTT (5567C>T), CTT409TTA (5568C>T 5570T>A), GTC411CAT (5574G>C 5575T>A 5576C>T), CAA412AAT (5577C>A 5579A>T), GAA414TGT (5583G>T 5584A>G 5585A>T)								
**Proteins**									
aspartic protease/reverse transcriptase (NP_056848.1)	326	416	13.9%	360	54.0%	91 (100%)	47 (51.6%)	0/0/0/0	0
Protein mutations:	Y327Q (5322T>C 5324T>A), Q329E (5328C>G 5330G>A), K331N (5336A>T), L333M (5340T>A 5342A>G), Y337L (5353A>T 5354T>A), H338K (5355C>A 5357T>A), S340A (5361T>G 5363A>C), I343E (5370A>G 5371T>A 5372T>A), Q346K (5379C>A), F347I (5382T>A), D349N (5388G>A), R350D (5391A>G 5392G>A 5393A>T), R353N (5401G>A 5402A>T), Y356L (5410A>T 5411T>G), Y362C (5428A>G 5429T>C), F369Y (5449T>A), S370L (5452C>T 5453T>A), T372I (5458C>T 5459T>A), I373F (5460A>T), E375M (5466G>A 5467A>T), K377I (5473A>T 5474A>T), I378K (5476T>A 5477A>G), I380L (5481A>C), A381D (5485C>A 5486A>C), K382I (5488A>T 5489A>T), R384K (5494G>A 5495A>G), D385K (5496G>A 5498T>A), T387V (5502A>G 5503C>T 5504A>G), L388I (5505C>A 5507T>A), A389Q (5508G>C 5509C>A), I393V (5520A>G 5522T>G), K397P (5532A>C 5533A>C), T399I (5539C>T), E400S (5541G>A 5542A>G 5543A>T), C402F (5548G>T), K403Q (5550A>C), E404T (5553G>A 5554A>C), K405E (5556A>G), I406V (5559A>G 5561A>C), D407R (5562G>C 5563A>G 5564T>A), V411H (5574G>C 5575T>A 5576C>T), Q412N (5577C>A 5579A>T), E414C (5583G>T 5584A>G 5585A>T)								
Codon mutations:	CAG325..T (5318G>T), GGA326GGG (5321A>G), TAT327CAA (5322T>C 5324T>A), CAG329GAA (5328C>G 5330G>A), AAA331AAT (5336A>T), GTA332GTT (5339A>T), TTA333ATG (5340T>A 5342A>G), CCA334CCT (5345A>T), GGA336GGC (5351A>C), TAT337TTA (5353A>T 5354T>A), CAT338AAA (5355C>A 5357T>A), AAC339AAT (5360C>T), TCA340GCC (5361T>G 5363A>C), CCA341CCT (5366A>T), AGT342TCT (5367A>T 5368G>C), ATT343GAA (5370A>G 5371T>A 5372T>A), TTC344TTT (5375C>T), CAA346AAA (5379C>A), TTT347ATT (5382T>A), GAT349AAT (5388G>A), AGA350GAT (5391A>G 5392G>A 5393A>T), ATA351ATT (5396A>T), AGA353AAT (5401G>A 5402A>T), TAT356TTG (5410A>T 5411T>G), GTG361GTC (5426G>C), TAT362TGC (5428A>G 5429T>C), GAT365GAC (5438T>C), TTA367TTG (5444A>G), GTC368GTA (5447C>A), TTT369TAT (5449T>A), TCT370TTA (5452C>T 5453T>A), AAG371AAA (5456G>A), ACT372ATA (5458C>T 5459T>A), ATT373TTT (5460A>T), GAG375ATG (5466G>A 5467A>T), AAA377ATT (5473A>T 5474A>T), ATA378AAG (5476T>A 5477A>G), ATT380CTT (5481A>C), GCA381GAC (5485C>A 5486A>C), AAA382ATT (5488A>T 5489A>T), AGA384AAG (5494G>A 5495A>G), GAT385AAA (5496G>A 5498T>A), ATA386ATT (5501A>T), ACA387GTG (5502A>G 5503C>T 5504A>G), CTT388ATA (5505C>A 5507T>A), GCA389CAA (5508G>C 5509C>A), GGA391GGT (5516A>T), TTA392TTG (5519A>G), ATT393GTG (5520A>G 5522T>G), ATT394ATA (5525T>A), AGT395TCT (5526A>T 5527G>C), AAG396AAA (5531G>A), AAA397CCA (5532A>C 5533A>C), ACA399ATA (5539C>T), GAA400AGT (5541G>A 5542A>G 5543A>T), TTA401TTG (5546A>G), TGT402TTT (5548G>T), AAA403CAA (5550A>C), GAA404ACA (5553G>A 5554A>C), AAA405GAA (5556A>G), ATA406GTC (5559A>G 5561A>C), GAT407CGA (5562G>C 5563A>G 5564T>A), TTC408TTT (5567C>T), CTT409TTA (5568C>T 5570T>A), GTC411CAT (5574G>C 5575T>A 5576C>T), CAA412AAT (5577C>A 5579A>T), GAA414TGT (5583G>T 5584A>G 5585A>T)								

*: Inserts / Deletes / Misaligned / Frameshifts

Analysis details

This analysis was performed with panviral2.64

NGS Details (UN8): Alphabaculovirus chofumiferanae

Assembly

**Coverage Length**	136 (1 contig(s))
**Depth Of Coverage**	4.5
**Number Of Reads**	7
**Reads Per Million**	0.12 rpm (after QC)
**Ambiguities**	0
**Assembly Method**	de novo + reference guided assembly
**Consensus Caller**	Bcf Tools

Coverage Map

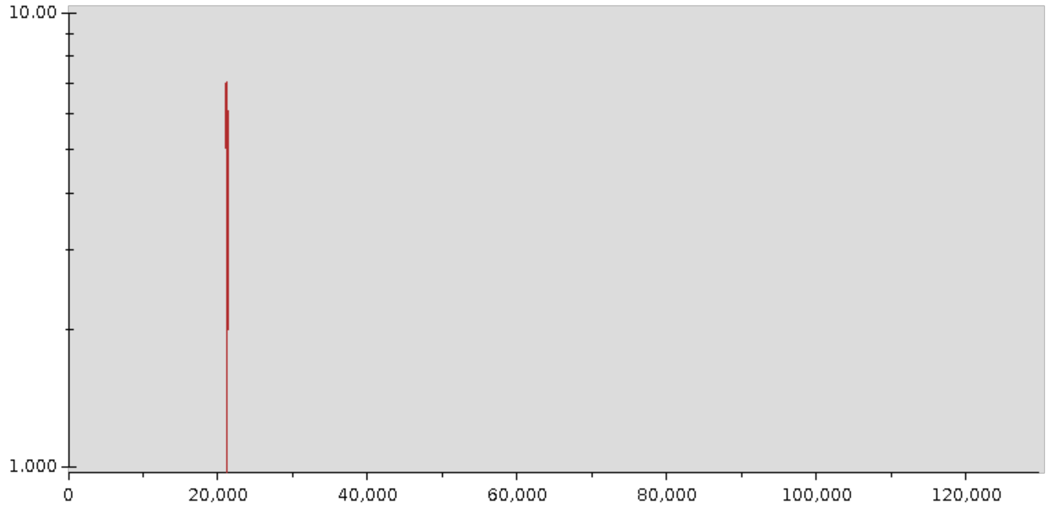

Assignment

**Type**	Alphabaculovirus chofumiferanae (Taxonomy ID: 3047750)
**Reference Genome**	NC_004778.3
**NT Identity (%)**	78.6765
**AA Identity (%)**	80.0
**Number Of Stop Codons**	0
**Number Of CDS**	145

Alignment

**Alignment Score**	156.0 (NT) + 309.0 (AA) = 465.0
**Concordance (%)**	71.9814
**Alignment Method**	Local, heuristic, nucleotide (BLASTN)

Genome Region

Sequence starts at position 21138 and ends at position 21273 relative to NC_004778.3 reference sequence.

Alignment Detailed Statistics

	Begin	End	Coverage	Score	Concordance	Matches	Identities	I/D/M/F*	Stop Codons
**NT**	**21138**	**21273**	**0.1%**	**156**	**57.4%**	**136 (100%)**	**107 (78.7%)**	**0/0**	

Mutations: 21147C>G, 21153G>A, 21162C>T, 21168T>A, 21189A>C, 21192T>G, 21193T>G, 21194G>C, 21195G>T, 21196T>C, 21197G>C, 21198C>G, 21200T>C, 21201A>G, 21207A>G, 21213C>G, 21214T>G, 21215C>G, 21216G>T, 21219G>A, 21222G>A, 21225C>T, 21231G>C, 21241G>T, 21246G>A, 21254C>G, 21260C>T, 21264A>G, 21271T>C

*: Inserts / Deletes / Misaligned / Frameshifts

Analysis details

This analysis was performed with panviral2.64

NGS Details (UN8): Badnavirus volubetulae

Assembly

**Coverage Length**	305 (1 contig(s))
**Depth Of Coverage**	3.2
**Number Of Reads**	7
**Reads Per Million**	0.12 rpm (after QC)
**Ambiguities**	0
**Assembly Method**	de novo + reference guided assembly
**Consensus Caller**	Bcf Tools

Coverage Map

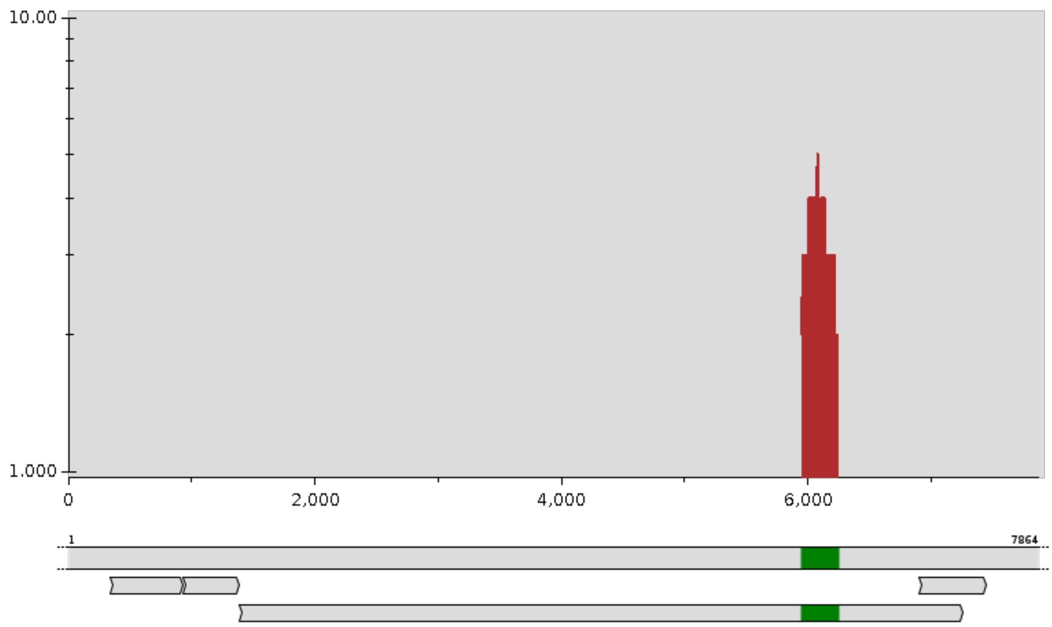

Assignment

**Type**	Badnavirus volubetulae (Taxonomy ID: 3047683)
**Reference Genome**	NC_040635.1
**NT Identity (%)**	61.0738
**AA Identity (%)**	47.4747
**Number Of Stop Codons**	0
**Number Of CDS**	4

Alignment

**Alignment Score**	107.0 (NT) + 244.0 (AA) = 351.0
**Concordance (%)**	30.7895

Alignment Method	Global, seeded, nucleotide + amino acids (AGA)

Genome Region

Sequence starts at position 5945 and ends at position 6249 relative to NC_040635.1 reference sequence.

Alignment Detailed Statistics

	Begin	End	Coverage	Score	Concordance	Matches	Identities	I/D/M/F*	Stop Codons
**NT**	**5945**	**6249**	**3.9%**	**107**	**18.7%**	**298 (97.7%)**	**182 (59.7%)**	**0/7**	
Mutations:	5949C>T, 5956C>T, 5961T>C, 5965C>T, 5973T>G, 5976T>A, 5981A>T, 5982T>G, 5985C>A, 5989G>T, 5992G>A, 5997C>T, 6000G>A, 6009G>T, 6014A>G, 6016T>A, 6017G>T, 6018C>A, 6021T>C, 6023A>G, 6026G>A, 6028A>T, 6029C>A, 6030G>T, 6031G>A, 6034G>A, 6035A>G, 6039C>T, 6042A>T, 6043G>T, 6044C>T, 6045T>A, 6051C>T, 6052A>G, 6054C>A, 6060T>C, 6066G>A, 6069C>A, 6072C>T, 6073T>A, 6074C>G, 6076G>A, 6079A>G, 6082C>A, 6083A>T, 6084T>A, 6087A>G, 6088C>G, 6090G>A, 6092A>G, 6094G>T, 6095A>T, 6099G>A, 6107G>A, 6110A>T, 6111G>A, 6114G>A, 6117G>T, 6118G>A, 6119A>G, 6120G>A, 6121A>C, 6124G>T, 6126C>T, 6127C>G, 6128G>T, 6129C>A, 6130C>A, 6132G>A, 6136G>A, 6137G>A, 6139C>A, 6142G>A, 6144C>A, 6145T>G, 6148_6153delTCACCC, 6155C>G, 6161T>A, 6162G>A, 6165G>T, 6166A>T, 6167T>A, 6168A>T, 6170C>T, 6172T>A, 6174C>A, 6175delT, 6177A>T, 6178G>C, 6180A>T, 6183T>A, 6184G>A, 6190C>T, 6192T>A, 6196G>A, 6197C>T, 6200C>T, 6201T>A, 6202A>T, 6204T>G, 6207C>A, 6208A>G, 6211A>G, 6212G>A, 6213C>A, 6215G>A, 6219T>A, 6220C>A, 6222G>A, 6225G>A, 6231C>T, 6237C>A, 6239T>C, 6240G>T, 6241C>A, 6243G>A, 6246G>A, 6248T>A								
**CDS**									
BLRaV_gp3	1520	1620	5.2%	244	35.0%	99 (98.0%)	47 (46.5%)	0/2/1/1	0
Protein mutations:	P1526S (5965C>T), N1531M (5981A>T 5982T>G), A1534S (5989G>T), V1535I (5992G>A), M1540I (6009G>T), N1542S (6014A>G), C1543I (6016T>A 6017G>T 6018C>A), K1545R (6023A>G), G1546D (6026G>A), T1547Y (6028A>T 6029C>A 6030G>T), E1548K (6031G>A), E1549R (6034G>A 6035A>G), A1552L (6043G>T 6044C>T 6045T>A), I1555V (6052A>G 6054C>A), E1563K (6076G>A), N1564D (6079A>G), H1565I (6082C>A 6083A>T 6084T>A), Q1567E (6088C>G 6090G>A), H1568R (6092A>G), E1569L (6094G>T 6095A>T), R1573Q (6107G>A), K1574I (6110A>T 6111G>A), M1575I (6114G>A), L1576F (6117G>T), E1577R (6118G>A 6119A>G 6120G>A), I1578L (6121A>C), V1579F (6124G>T 6126C>T), R1580V (6127C>G 6128G>T 6129C>A), Q1581K (6130C>A 6132G>A), G1583K (6136G>A 6137G>A), L1584I (6139C>A), V1585I (6142G>A 6144C>A), L1586V (6145T>G), S1587_P1588del (6148_6153delTCACCC), T1589S (6155C>G), M1591K (6161T>A 6162G>A), K1592N (6165G>T), I1593Y (6166A>T 6167T>A 6168A>T), A1594V (6170C>T), C1595R (6172T>A 6174C>A), E1597H (6178G>C 6180A>T), D1599N (6184G>A), A1603I (6196G>A 6197C>T), T1604I (6200C>T 6201T>A), I1605L (6202A>T 6204T>G), N1607D (6208A>G), S1608E (6211A>G 6212G>A 6213C>A), R1609K (6215G>A), Q1611K (6220C>A 6222G>A), V1617A (6239T>C 6240G>T), Q1618K (6241C>A 6243G>A), I1620K (6248T>A)								
Codon mutations:	TAC1520TAT (5949C>T), CTA1523TTA (5956C>T), GTT1524GTC (5961T>C), CCA1526TCA (5965C>T), GGT1528GGG (5973T>G), CTT1529CTA (5976T>A), AAT1531ATG (5981A>T 5982T>G), GCC1532GCA (5985C>A), GCA1534TCA (5989G>T), GTA1535ATA (5992G>A), TTC1536TTT (5997C>T), CAG1537CAA (6000G>A), ATG1540ATT (6009G>T), AAT1542AGT (6014A>G), TGC1543ATA (6016T>A 6017G>T 6018C>A), TTT1544TTC (6021T>C), AAA1545AGA (6023A>G), GGT1546GAT (6026G>A), ACG1547TAT (6028A>T 6029C>A 6030G>T), GAA1548AAA (6031G>A), GAA1549AGA (6034G>A 6035A>G), TTC1550TTT (6039C>T), ATA1551ATT (6042A>T), GCT1552TTA (6043G>T 6044C>T 6045T>A), TAC1554TAT (6051C>T), ATC1555GTA (6052A>G 6054C>A), GAT1557GAC (6060T>C), TTG1559TTA (6066G>A), GTC1560GTA (6069C>A), TTC1561TTT (6072C>T), TCC1562AGC (6073T>A 6074C>G), GAA1563AAA (6076G>A), AAT1564GAT (6079A>G), CAT1565ATA (6082C>A 6083A>T 6084T>A), AAA1566AAG (6087A>G), CAG1567GAA (6088C>G 6090G>A), CAT1568CGT (6092A>G), GAA1569TTA (6094G>T 6095A>T), GGG1570GGA (6099G>A), CGG1573CAG (6107G>A), AAG1574ATA (6110A>T 6111G>A), ATG1575ATA (6114G>A), TTG1576TTT (6117G>T), GAG1577AGA (6118G>A 6119A>G 6120G>A), ATA1578CTA (6121A>C), GTC1579TTT (6124G>T 6126C>T), CGC1580GTA (6127C>G 6128G>T 6129C>A), CAG1581AAA (6130C>A 6132G>A), GGA1583AAA (6136G>A 6137G>A), CTA1584ATA (6139C>A), GTC1585ATA (6142G>A 6144C>A), TTA1586GTA (6145T>G), TCA1587_CCC1588del (6148_6153delTCACCC), ACT1589AGT (6155C>G), ATG1591AAA (6161T>A 6162G>A), AAG1592AAT (6165G>T), ATA1593TAT (6166A>T 6167T>A 6168A>T), GCA1594GTA (6170C>T), TGC1595AGA (6172T>A 6174C>A), TCA1596-CT (6175delT 6177A>T), GAA1597CAT (6178G>C 6180A>T), ATT1598ATA (6183T>A), GAT1599AAT (6184G>A), CTT1601TTA (6190C>T 6192T>A), GCA1603ATA (6196G>A 6197C>T), ACT1604ATA (6200C>T 6201T>A), ATT1605TTG (6202A>T 6204T>G), GGC1606GGA (6207C>A), AAC1607GAC (6208A>G), AGC1608GAA (6211A>G 6212G>A 6213C>A), AGA1609AAA (6215G>A), ATT1610ATA (6219T>A), CAG1611AAA (6220C>A 6222G>A), TTG1612TTA (6225G>A), CCC1614CCT (6231C>T), ATC1616ATA (6237C>A), GTG1617GCT (6239T>C 6240G>T), CAG1618AAA (6241C>A 6243G>A), AAG1619AAA (6246G>A), ATA1620AAA (6248T>A)								
**Proteins**									
polyprotein (YP_009552737.1)	1520	1620	5.2%	244	35.0%	99 (98.0%)	47 (46.5%)	0/2/1/1	0
Protein mutations:	P1526S (5965C>T), N1531M (5981A>T 5982T>G), A1534S (5989G>T), V1535I (5992G>A), M1540I (6009G>T), N1542S (6014A>G), C1543I (6016T>A 6017G>T 6018C>A), K1545R (6023A>G), G1546D (6026G>A), T1547Y (6028A>T 6029C>A 6030G>T), E1548K (6031G>A), E1549R (6034G>A 6035A>G), A1552L (6043G>T 6044C>T 6045T>A), I1555V (6052A>G 6054C>A), E1563K (6076G>A), N1564D (6079A>G), H1565I (6082C>A 6083A>T 6084T>A), Q1567E (6088C>G 6090G>A), H1568R (6092A>G), E1569L (6094G>T 6095A>T), R1573Q (6107G>A), K1574I (6110A>T 6111G>A), M1575I (6114G>A), L1576F (6117G>T), E1577R (6118G>A 6119A>G 6120G>A), I1578L (6121A>C), V1579F (6124G>T 6126C>T), R1580V (6127C>G 6128G>T 6129C>A), Q1581K (6130C>A 6132G>A), G1583K (6136G>A 6137G>A), L1584I (6139C>A), V1585I (6142G>A 6144C>A), L1586V (6145T>G), S1587_P1588del (6148_6153delTCACCC), T1589S (6155C>G), M1591K (6161T>A 6162G>A), K1592N (6165G>T), I1593Y (6166A>T 6167T>A 6168A>T), A1594V (6170C>T), C1595R (6172T>A 6174C>A), E1597H (6178G>C 6180A>T), D1599N (6184G>A), A1603I (6196G>A 6197C>T), T1604I (6200C>T 6201T>A), I1605L (6202A>T 6204T>G), N1607D (6208A>G), S1608E (6211A>G 6212G>A 6213C>A), R1609K (6215G>A), Q1611K (6220C>A 6222G>A), V1617A (6239T>C 6240G>T), Q1618K (6241C>A 6243G>A), I1620K (6248T>A)								
Codon mutations:	TAC1520TAT (5949C>T), CTA1523TTA (5956C>T), GTT1524GTC (5961T>C), CCA1526TCA (5965C>T), GGT1528GGG (5973T>G), CTT1529CTA (5976T>A), AAT1531ATG (5981A>T 5982T>G), GCC1532GCA (5985C>A), GCA1534TCA (5989G>T), GTA1535ATA (5992G>A), TTC1536TTT (5997C>T), CAG1537CAA (6000G>A), ATG1540ATT (6009G>T), AAT1542AGT (6014A>G), TGC1543ATA (6016T>A 6017G>T 6018C>A), TTT1544TTC (6021T>C), AAA1545AGA (6023A>G), GGT1546GAT (6026G>A), ACG1547TAT (6028A>T 6029C>A 6030G>T), GAA1548AAA (6031G>A), GAA1549AGA (6034G>A 6035A>G), TTC1550TTT (6039C>T), ATA1551ATT (6042A>T), GCT1552TTA (6043G>T 6044C>T 6045T>A), TAC1554TAT (6051C>T), ATC1555GTA (6052A>G 6054C>A), GAT1557GAC (6060T>C), TTG1559TTA (6066G>A), GTC1560GTA (6069C>A), TTC1561TTT (6072C>T), TCC1562AGC (6073T>A 6074C>G), GAA1563AAA (6076G>A), AAT1564GAT (6079A>G), CAT1565ATA (6082C>A 6083A>T 6084T>A), AAA1566AAG (6087A>G), CAG1567GAA (6088C>G 6090G>A), CAT1568CGT (6092A>G), GAA1569TTA (6094G>T 6095A>T), GGG1570GGA (6099G>A), CGG1573CAG (6107G>A), AAG1574ATA (6110A>T 6111G>A), ATG1575ATA (6114G>A), TTG1576TTT (6117G>T), GAG1577AGA (6118G>A 6119A>G 6120G>A), ATA1578CTA (6121A>C), GTC1579TTT (6124G>T 6126C>T), CGC1580GTA (6127C>G 6128G>T 6129C>A), CAG1581AAA (6130C>A 6132G>A), GGA1583AAA (6136G>A 6137G>A), CTA1584ATA (6139C>A), GTC1585ATA (6142G>A 6144C>A), TTA1586GTA (6145T>G), TCA1587_CCC1588del (6148_6153delTCACCC), ACT1589AGT (6155C>G), ATG1591AAA (6161T>A 6162G>A), AAG1592AAT (6165G>T), ATA1593TAT (6166A>T 6167T>A 6168A>T), GCA1594GTA (6170C>T), TGC1595AGA (6172T>A 6174C>A), TCA1596-CT (6175delT 6177A>T), GAA1597CAT (6178G>C 6180A>T), ATT1598ATA (6183T>A), GAT1599AAT (6184G>A), CTT1601TTA (6190C>T 6192T>A), GCA1603ATA (6196G>A 6197C>T), ACT1604ATA (6200C>T 6201T>A), ATT1605TTG (6202A>T 6204T>G), GGC1606GGA (6207C>A), AAC1607GAC (6208A>G), AGC1608GAA (6211A>G 6212G>A 6213C>A), AGA1609AAA (6215G>A), ATT1610ATA (6219T>A), CAG1611AAA (6220C>A 6222G>A), TTG1612TTA (6225G>A), CCC1614CCT (6231C>T), ATC1616ATA (6237C>A), GTG1617GCT (6239T>C 6240G>T), CAG1618AAA (6241C>A 6243G>A), AAG1619AAA (6246G>A), ATA1620AAA (6248T>A)								

*: Inserts / Deletes / Misaligned / Frameshifts

Analysis details

This analysis was performed with panviral2.64

NGS Details (UN8): Epiphyllum badnavirus 1

Assembly

**Coverage Length**	307 (1 contig(s))
**Depth Of Coverage**	2.2
**Number Of Reads**	6
**Reads Per Million**	0.10 rpm (after QC)
**Ambiguities**	0
**Assembly Method**	de novo + reference guided assembly
**Consensus Caller**	Bcf Tools

Coverage Map

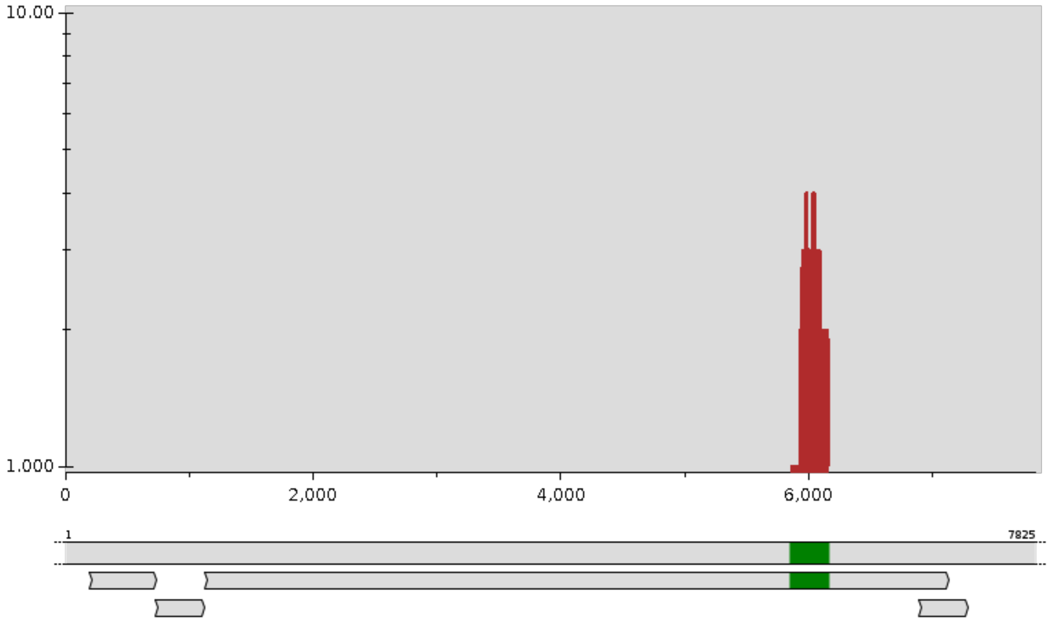

Assignment

**Type**	Epiphyllum badnavirus 1 (Taxonomy ID: 2518008)
**Reference Genome**	NC_076247.1
**NT Identity (%)**	52.8481
**AA Identity (%)**	43.8095
**Number Of Stop Codons**	2
**Number Of CDS**	4

Alignment

**Alignment Score**	18.0 (NT) + 299.0 (AA) = 317.0
**Concordance (%)**	23.6744

Alignment Method	Global, seeded, nucleotide + amino acids (AGA)

Genome Region

Sequence starts at position 5855 and ends at position 6161 relative to NC_076247.1 reference sequence.

Alignment Detailed Statistics

	Begin	End	Coverage	Score	Concordance	Matches	Identities	I/D/M/F*	Stop Codons
**NT**	**5855**	**6161**	**3.9%**	**18**	**2.9%**	**307 (97.2%)**	**167 (52.8%)**	**9/0**	

Mutations: 5857C>T, 5861A>G, 5863A>T, 5864G>C, 5865C>A, 5867G>C, 5870T>A, 5871C>T, 5877G>A, 5880T>G, 5882T>G, 5884A>G, 5887G>A, 5889C>G, 5892A>G, 5893G>A, 5896T>G, 5897C>T, 5901C>A, 5904T>C, 5905T>A, 5906G>A, 5910C>A, 5913C>T, 5916C>T, 5917C>A, 5918T>G, 5919G>A, 5923C>T, 5924C>A, 5926G>T, 5931A>T, 5933T>A, 5936A>T, 5937C>T, 5942G>T, 5943G>T, 5944T>C, 5953C>T, 5958C>T, 5961A>C, 5962T>C, 5964G>A, 5966A>C, 5967G>A, 5973C>A, 5976T>G, 5977G>A, 5979T>A, 5982G>T, 5985C>T, 5986C>A, 5987A>T, 5988A>G, 5989C>G, 5990G>A, 5991A>T, 5992A>T, 5993A>T, 5998G>A, 6000C>T, 6002A>G, 6003C>G, 6004T>G, 6005G>T, 6006T>G, 6011G>A, 6012A>G, 6012_6013insATA, 6013G>T, 6014A>T, 6016C>T, 6018G>A, 6020C>A, 6021A>T, 6023G>T, 6024C>A, 6030A>G, 6031G>A, 6032C>T, 6036T>A, 6038A>T, 6039C>T, 6042T>A, 6043G>A, 6048C>T, 6051C>T, 6052C>T, 6057C>G, 6059T>A, 6060C>T, 6061A>T, 6062G>C, 6063T>A, 6065A>G, 6066C>A, 6067A>T, 6069T>A, 6070G>A, 6075T>G, 6083C>A, 6085C>A, 6087G>T, 6094A>C, 6095A>G, 6097T>C, 6098C>A, 6099C>G, 6100A>G, 6102G>A, 6103C>T, 6105G>A, 6108G>A, 6111_6112insCTT, 6117G>T, 6118A>C, 6121G>C, 6123G>A, 6124G>T, 6125G>T, 6127C>T, 6128T>A, 6129G>T, 6130A>G, 6132_6133insAAG, 6133C>T, 6139C>A, 6140C>A, 6141T>A, 6142A>T, 6143C>G, 6145A>G, 6147G>A, 6148A>T, 6150G>T, 6151A>T, 6152A>G, 6153G>A, 6154A>T, 6157G>A, 6158C>A, 6159T>A, 6160G>T

CDS

	Begin	End	Coverage	Score	Concordance	Matches	Identities	I/D/M/F*	Stop Codons
QKM20_gp3	1577	1678	5.1%	299	40.5%	102 (97.1%)	46 (43.8%)	3/0/0/0	2

Protein mutations: K1578R (5861A>G), G1580A (5867G>C), F1581Y (5870T>A 5871C>T), I1584M (5880T>G), M1585R (5882T>G), M1586V (5884A>G), D1587K (5887G>A 5889C>G), E1589K (5893G>A), S1590V (5896T>G 5897C>T), W1593K (5905T>A 5906G>A), L1597R (5917C>A 5918T>G 5919G>A), P1599* (5923C>T 5924C>A), D1600Y (5926G>T), L1602H (5933T>A), Y1603F (5936A>T 5937C>T), W1605F (5942G>T 5943G>T), P1609S (5953C>T), K1613T (5966A>C 5967G>A), A1617T (5977G>A 5979T>A), Q1620M (5986C>A 5987A>T 5988A>G), R1621D (5989C>G 5990G>A 5991A>T), K1622L (5992A>T 5993A>T), D1624N (5998G>A 6000C>T), N1625R (6002A>G 6003C>G), C1626V (6004T>G 6005G>T 6006T>G), R1628K (6011G>A 6012A>G), R1628_D1629insI (6012_6013insATA), D1629F (6013G>T 6014A>T), S1631Y (6020C>A 6021A>T), G1632V (6023G>T 6024C>A), A1635I (6031G>A 6032C>T), Y1637F (6038A>T 6039C>T), D1639N (6043G>A), F1644Y (6059T>A 6060C>T), N1646R (6065A>G 6066C>A), T1647S (6067A>T 6069T>A), E1648K (6070G>A), D1649E (6075T>G), A1652E (6083C>A), Q1653N (6085C>A 6087G>T), K1656R (6094A>C 6095A>G), S1657Q (6097T>C 6098C>A 6099C>G), M1658V (6100A>G 6102G>A), V1661_C1662insL (6111_6112insCTT), E1663D (6117G>T), E1665Q (6121G>C 6123G>A), G1666L (6124G>T 6125G>T), L1667Y (6127C>T 6128T>A 6129G>T), I1668V (6130A>G), I1668_L1669insK (6132_6133insAAG), L1669F (6133C>T), P1671K (6139C>A 6140C>A 6141T>A), T1672W (6142A>T 6143C>G), K1673E (6145A>G 6147G>A), M1674F (6148A>T 6150G>T), K1675* (6151A>T 6152A>G 6153G>A), I1676L (6154A>T), A1677K (6157G>A 6158C>A 6159T>A)

Codon mutations: CTG1577TTG (5857C>T), AAG1578AGG (5861A>G), AGC1579TCA (5863A>T 5864G>C 5865C>A), GGA1580GCA (5867G>C), TTC1581TAT (5870T>A 5871C>T), CAG1583CAA (5877G>A), ATT1584ATG (5880T>G), ATG1585AGG (5882T>G), ATG1586GTG (5884A>G), GAC1587AAG (5887G>A 5889C>G), GAA1588GAG (5892A>G), GAA1589AAA (5893G>A), TCC1590GTC (5896T>G 5897C>T), ATC1591ATA (5901C>A), CCT1592CCC (5904T>C), TGG1593AAG (5905T>A 5906G>A), ACC1594ACA (5910C>A), GCC1595GCT (5913C>T), TTC1596TTT (5916C>T), CTG1597AGA (5917C>A 5918T>G 5919G>A), CCA1599TAA (5923C>T 5924C>A), GAT1600TAT (5926G>T), GGA1601GGT (5931A>T), CTT1602CAT (5933T>A), TAC1603TTT (5936A>T 5937C>T), TGG1605TTT (5942G>T 5943G>T), TTA1606CTA (5944T>C), CCA1609TCA (5953C>T), TTC1610TTT (5958C>T), GGA1611GGC (5961A>C), TTG1612CTA (5962T>C 5964G>A), AAG1613ACA (5966A>C 5967G>A), GCC1615GCA (5973C>A), CCT1616CCG (5976T>G), GCT1617ACA (5977G>A 5979T>A), GTG1618GTT (5982G>T), TTC1619TTT (5985C>T), CAA1620ATG (5986C>A 5987A>T 5988A>G), CGA1621GAT (5989C>G 5990G>A 5991A>T), AAG1622TTG (5992A>T 5993A>T), GAC1624AAT (5998G>A 6000C>T), AAC1625AGG (6002A>G 6003C>G), TGT1626GTG (6004T>G 6005G>T 6006T>G), AGA1628AAG (6011G>A 6012A>G), AGA1628_GAC1629insATA (6012_6013insATA), GAC1629TTC (6013G>T 6014A>T), CTG1630TTA (6016C>T 6018G>A), TCA1631TAT (6020C>A 6021A>T), GGC1632GTA (6023G>T 6024C>A), GTA1634GTG (6030A>G), GCA1635ATA (6031G>A 6032C>T), GTT1636GTA (6036T>A), TAC1637TTT (6038A>T 6039C>T), ATT1638ATA (6042T>A), GAT1639AAT (6043G>A), GAC1640GAT (6048C>T), ATC1641ATT (6051C>T), CTG1642TTG (6052C>T), GTC1643GTG (6057C>G), TTC1644TAT (6059T>A 6060C>T), AGT1645TCA (6061A>T 6062G>C 6063T>A), AAC1646AGA (6065A>G 6066C>A), ACT1647TCA (6067A>T 6069T>A), GAA1648AAA (6070G>A), GAT1649GAG (6075T>G), GCA1652GAA (6083C>A), CAG1653AAT (6085C>A 6087G>T), AAG1656CGG (6094A>C 6095A>G), TCC1657CAG (6097T>C 6098C>A 6099C>G), ATG1658GTA (6100A>G 6102G>A), CTG1659TTA (6103C>T 6105G>A), CAG1660CAA (6108G>A), GTT1661_TGT1662insCTT (6111_6112insCTT), GAG1663GAT (6117G>T), AGA1664CGA (6118A>C), GAG1665CAA (6121G>C 6123G>A), GGG1666TTG (6124G>T 6125G>T), CTG1667TAT (6127C>T 6128T>A 6129G>T), ATC1668GTC (6130A>G), ATC1668_CTC1669insAAG (6132_6133insAAG), CTC1669TTC (6133C>T), CCT1671AAA (6139C>A 6140C>A 6141T>A), ACG1672TGG (6142A>T 6143C>G), AAG1673GAA (6145A>G 6147G>A), ATG1674TTT (6148A>T 6150G>T), AAG1675TGA (6151A>T 6152A>G 6153G>A), ATA1676TTA (6154A>T), GCT1677AAA (6157G>A 6158C>A 6159T>A), GCA1678TC. (6160G>T)

Proteins

	Begin	End	Coverage	Score	Concordance	Matches	Identities	I/D/M/F*	Stop Codons
polyprotein (YP_010797894.1)	1577	1678	5.1%	299	40.5%	102 (97.1%)	46 (43.8%)	3/0/0/0	2

Protein mutations: K1578R (5861A>G), G1580A (5867G>C), F1581Y (5870T>A 5871C>T), I1584M (5880T>G), M1585R (5882T>G), M1586V (5884A>G), D1587K (5887G>A 5889C>G), E1589K (5893G>A), S1590V (5896T>G 5897C>T), W1593K (5905T>A 5906G>A), L1597R (5917C>A 5918T>G 5919G>A), P1599* (5923C>T 5924C>A), D1600Y (5926G>T), L1602H (5933T>A), Y1603F (5936A>T 5937C>T), W1605F (5942G>T 5943G>T), P1609S (5953C>T), K1613T (5966A>C 5967G>A), A1617T (5977G>A 5979T>A), Q1620M (5986C>A 5987A>T 5988A>G), R1621D (5989C>G 5990G>A 5991A>T), K1622L (5992A>T 5993A>T), D1624N (5998G>A 6000C>T), N1625R (6002A>G 6003C>G), C1626V (6004T>G 6005G>T 6006T>G), R1628K (6011G>A 6012A>G), R1628_D1629insI (6012_6013insATA), D1629F (6013G>T 6014A>T), S1631Y (6020C>A 6021A>T), G1632V (6023G>T 6024C>A), A1635I (6031G>A 6032C>T), Y1637F (6038A>T 6039C>T), D1639N (6043G>A), F1644Y (6059T>A 6060C>T), N1646R (6065A>G 6066C>A), T1647S (6067A>T 6069T>A), E1648K (6070G>A), D1649E (6075T>G), A1652E (6083C>A), Q1653N (6085C>A 6087G>T), K1656R (6094A>C 6095A>G), S1657Q (6097T>C 6098C>A 6099C>G), M1658V (6100A>G 6102G>A), V1661_C1662insL (6111_6112insCTT), E1663D (6117G>T), E1665Q (6121G>C 6123G>A), G1666L (6124G>T 6125G>T), L1667Y (6127C>T 6128T>A 6129G>T), I1668V (6130A>G), I1668_L1669insK (6132_6133insAAG), L1669F (6133C>T), P1671K (6139C>A 6140C>A 6141T>A), T1672W (6142A>T 6143C>G), K1673E (6145A>G 6147G>A), M1674F (6148A>T 6150G>T), K1675* (6151A>T 6152A>G 6153G>A), I1676L (6154A>T), A1677K (6157G>A 6158C>A 6159T>A)

Codon mutations: CTG1577TTG (5857C>T), AAG1578AGG (5861A>G), AGC1579TCA (5863A>T 5864G>C 5865C>A), GGA1580GCA (5867G>C), TTC1581TAT (5870T>A 5871C>T), CAG1583CAA (5877G>A), ATT1584ATG (5880T>G), ATG1585AGG (5882T>G), ATG1586GTG (5884A>G), GAC1587AAG (5887G>A 5889C>G), GAA1588GAG (5892A>G), GAA1589AAA (5893G>A), TCC1590GTC (5896T>G 5897C>T), ATC1591ATA (5901C>A), CCT1592CCC (5904T>C), TGG1593AAG (5905T>A 5906G>A), ACC1594ACA (5910C>A), GCC1595GCT (5913C>T), TTC1596TTT (5916C>T), CTG1597AGA (5917C>A 5918T>G 5919G>A), CCA1599TAA (5923C>T 5924C>A), GAT1600TAT (5926G>T), GGA1601GGT (5931A>T), CTT1602CAT (5933T>A), TAC1603TTT (5936A>T 5937C>T), TGG1605TTT (5942G>T 5943G>T), TTA1606CTA (5944T>C), CCA1609TCA (5953C>T), TTC1610TTT (5958C>T), GGA1611GGC (5961A>C), TTG1612CTA (5962T>C 5964G>A), AAG1613ACA (5966A>C 5967G>A), GCC1615GCA (5973C>A), CCT1616CCG (5976T>G), GCT1617ACA (5977G>A 5979T>A), GTG1618GTT (5982G>T), TTC1619TTT (5985C>T), CAA1620ATG (5986C>A 5987A>T 5988A>G), CGA1621GAT (5989C>G 5990G>A 5991A>T), AAG1622TTG (5992A>T 5993A>T), GAC1624AAT (5998G>A 6000C>T), AAC1625AGG (6002A>G 6003C>G), TGT1626GTG (6004T>G 6005G>T 6006T>G), AGA1628AAG (6011G>A 6012A>G), AGA1628_GAC1629insATA (6012_6013insATA), GAC1629TTC (6013G>T 6014A>T), CTG1630TTA (6016C>T 6018G>A), TCA1631TAT (6020C>A 6021A>T), GGC1632GTA (6023G>T 6024C>A), GTA1634GTG (6030A>G), GCA1635ATA (6031G>A 6032C>T), GTT1636GTA (6036T>A), TAC1637TTT (6038A>T 6039C>T), ATT1638ATA (6042T>A), GAT1639AAT (6043G>A), GAC1640GAT (6048C>T), ATC1641ATT (6051C>T), CTG1642TTG (6052C>T), GTC1643GTG (6057C>G), TTC1644TAT (6059T>A 6060C>T), AGT1645TCA (6061A>T 6062G>C 6063T>A), AAC1646AGA (6065A>G 6066C>A), ACT1647TCA (6067A>T 6069T>A), GAA1648AAA (6070G>A), GAT1649GAG (6075T>G), GCA1652GAA (6083C>A), CAG1653AAT (6085C>A 6087G>T), AAG1656CGG (6094A>C 6095A>G), TCC1657CAG (6097T>C 6098C>A 6099C>G), ATG1658GTA (6100A>G 6102G>A), CTG1659TTA (6103C>T 6105G>A), CAG1660CAA (6108G>A), GTT1661_TGT1662insCTT (6111_6112insCTT), GAG1663GAT (6117G>T), AGA1664CGA (6118A>C), GAG1665CAA (6121G>C 6123G>A), GGG1666TTG (6124G>T 6125G>T), CTG1667TAT (6127C>T 6128T>A 6129G>T), ATC1668GTC (6130A>G), ATC1668_CTC1669insAAG (6132_6133insAAG), CTC1669TTC (6133C>T), CCT1671AAA (6139C>A 6140C>A 6141T>A), ACG1672TGG (6142A>T 6143C>G), AAG1673GAA (6145A>G 6147G>A), ATG1674TTT (6148A>T 6150G>T), AAG1675TGA (6151A>T 6152A>G 6153G>A), ATA1676TTA (6154A>T), GCT1677AAA (6157G>A 6158C>A 6159T>A), GCA1678TC. (6160G>T)

*: Inserts / Deletes / Misaligned / Frameshifts

Analysis details

This analysis was performed with panviral2.64

NGS Details (UN8): Potato virus X

Assembly

**Coverage Length**	236 (1 contig(s))
**Depth Of Coverage**	2.3
**Number Of Reads**	4
**Reads Per Million**	0.07 rpm (after QC)
**Ambiguities**	0
**Assembly Method**	de novo + reference guided assembly
**Consensus Caller**	Bcf Tools

Coverage Map

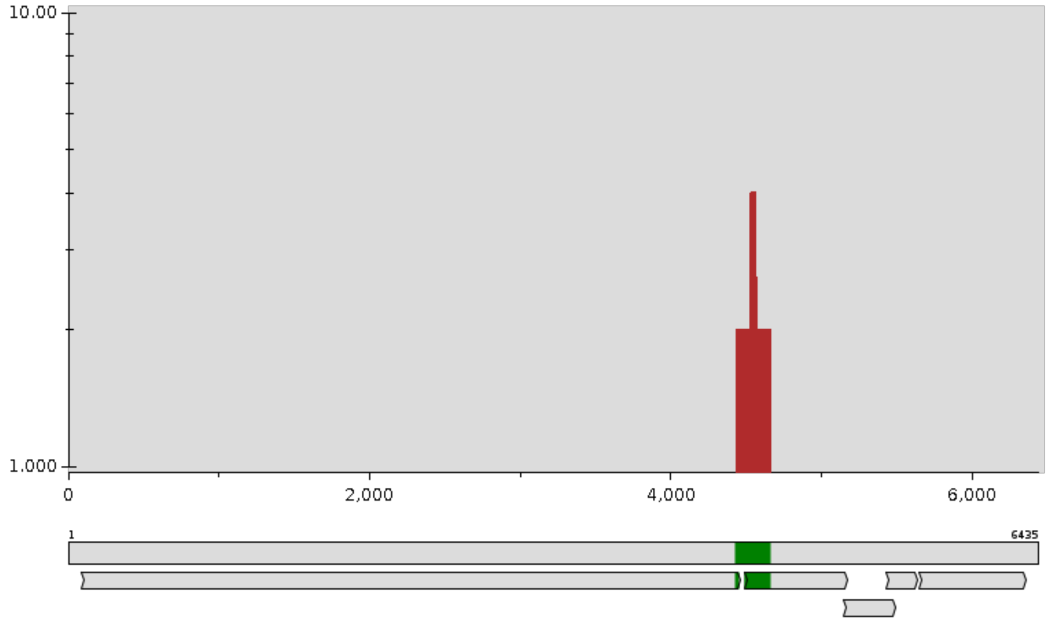

Assignment

**Type**	Potato virus X (Taxonomy ID: 12183)
**Reference Genome**	NC_011620.1
**NT Identity (%)**	96.6102
**AA Identity (%)**	95.5882
**Number Of Stop Codons**	1
**Number Of CDS**	5

Alignment

**Alignment Score**	440.0 (NT) + 407.0 (AA) = 847.0
**Concordance (%)**	94.7427

Alignment Method	Global, seeded, nucleotide + amino acids (AGA)

Genome Region

Sequence starts at position 4430 and ends at position 4665 relative to NC_011620.1 reference sequence.

Alignment Detailed Statistics

	Begin	End	Coverage	Score	Concordance	Matches	Identities	I/D/M/F*	Stop Codons
**NT**	**4430**	**4665**	**3.7%**	**440**	**93.2%**	**236 (100%)**	**228 (96.6%)**	**0/0**	
Mutations:	4439T>A, 4463T>G, 4472T>G, 4502G>T, 4534T>C, 4535C>A, 4584A>C, 4638G>A								
**CDS**									
PVX_gp1	1450	1457	0.5%	49	84.5%	8 (100%)	7 (87.5%)	0/0/0/0	1
Protein mutations:	L1452H (4439T>A)								
Codon mutations:	CTC1452CAC (4439T>A)								
PVX_gp2	1	60	26.4%	358	97.3%	60 (100%)	58 (96.7%)	0/0/0/0	0
Protein mutations:	S6I (4502G>T), S17H (4534T>C 4535C>A)								
Codon mutations:	AGT6ATT (4502G>T), TCT17CAT (4534T>C 4535C>A), GCA33GCC (4584A>C), GTG51GTA (4638G>A)								
**Proteins**									
replicase (YP_002332929.1)	1450	1457	0.5%	49	84.5%	8 (100%)	7 (87.5%)	0/0/0/0	1
Protein mutations:	L1452H (4439T>A)								
Codon mutations:	CTC1452CAC (4439T>A)								
25K protein (YP_002332930.1)	1	60	26.4%	358	97.3%	60 (100%)	58 (96.7%)	0/0/0/0	0
Protein mutations:	S6I (4502G>T), S17H (4534T>C 4535C>A)								
Codon mutations:	AGT6ATT (4502G>T), TCT17CAT (4534T>C 4535C>A), GCA33GCC (4584A>C), GTG51GTA (4638G>A)								

*: Inserts / Deletes / Misaligned / Frameshifts

Analysis details

This analysis was performed with panviral2.64

NGS Details (UN8): Petuvirus venapetuniae

Assembly

**Coverage Length**	225 (1 contig(s))
**Depth Of Coverage**	2.4
**Number Of Reads**	4
**Reads Per Million**	0.07 rpm (after QC)
**Ambiguities**	0
**Assembly Method**	de novo + reference guided assembly
**Consensus Caller**	Bcf Tools

Coverage Map

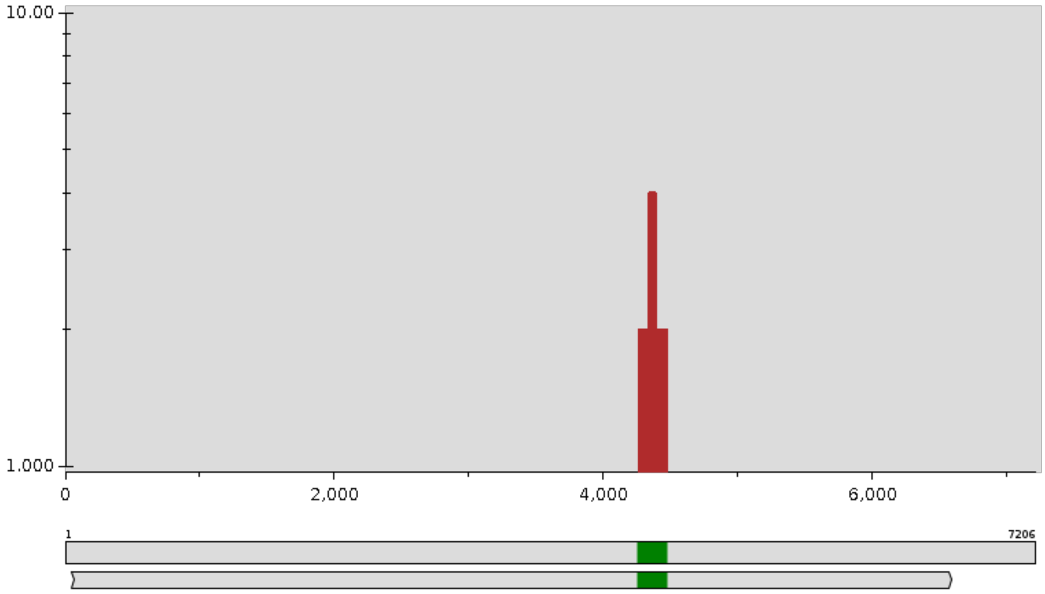

Assignment

**Type**	Petuvirus venapetuniae (Taxonomy ID: 3048265)
**Reference Genome**	NC_001839.2
**NT Identity (%)**	68.4444
**AA Identity (%)**	69.3333
**Number Of Stop Codons**	0
**Number Of CDS**	1

Alignment

**Alignment Score**	166.0 (NT) + 387.0 (AA) = 553.0
**Concordance (%)**	56.1421

Alignment Method	Global, seeded, nucleotide + amino acids (AGA)

Genome Region

Sequence starts at position 4253 and ends at position 4477 relative to NC_001839.2 reference sequence.

Alignment Detailed Statistics

	Begin	End	Coverage	Score	Concordance	Matches	Identities	I/D/M/F*	Stop Codons
**NT**	**4253**	**4477**	**3.1%**	**166**	**36.9%**	**225 (100%)**	**154 (68.4%)**	**0/0**	
Mutations:	4256A>G, 4264G>A, 4269G>A, 4271G>A, 4272C>A, 4275C>T, 4276A>T, 4277A>G, 4278C>A, 4280A>G, 4284G>A, 4285A>G, 4286T>A, 4287C>T, 4292C>T, 4295A>G, 4298C>A, 4307A>C, 4314G>T, 4316A>T, 4320G>A, 4334C>T, 4343G>A, 4344T>G, 4346A>T, 4349G>A, 4354T>C, 4355G>A, 4361A>T, 4365T>C, 4367A>T, 4368A>C, 4370G>A, 4379A>C, 4385T>C, 4388A>G, 4391A>C, 4392T>C, 4394A>C, 4400T>C, 4406C>T, 4407C>T, 4408A>T, 4409A>G, 4413G>A, 4414A>G, 4415C>T, 4418G>A, 4421T>C, 4422C>A, 4424C>T, 4427T>C, 4429C>T, 4436G>T, 4439C>A, 4441C>T, 4442A>T, 4443C>T, 4445C>A, 4450C>T, 4451T>A, 4458T>G, 4459C>T, 4460C>A, 4461A>G, 4466A>C, 4468A>C, 4469G>A, 4470C>T, 4471T>A, 4475T>C								

CDS

PVCVgp1	1402	1476	3.4%	387	71.3%	75 (100%)	52 (69.3%)	0/0/0/0	0
Protein mutations:	I1402M (4256A>G), C1405Y (4264G>A), E1407K (4269G>A 4271G>A), L1408I (4272C>A), Q1409L (4275C>T 4276A>T 4277A>G), Q1410K (4278C>A 4280A>G), D1412R (4284G>A 4285A>G 4286T>A), A1422S (4314G>T 4316A>T), E1424K (4320G>A), S1432A (4344T>G 4346A>T), V1435A (4354T>C 4355G>A), Q1453L (4407C>T 4408A>T 4409A>G), D1455S (4413G>A 4414A>G 4415C>T), P1458T (4422C>A 4424C>T), P1460L (4429C>T), K1462N (4436G>T), T1464I (4441C>T 4442A>T), S1467L (4450C>T 4451T>A), S1470V (4458T>G 4459C>T 4460C>A), K1471E (4461A>G), K1473T (4468A>C 4469G>A), L1474Y (4470C>T 4471T>A)								
Codon mutations:	ATA1402ATG (4256A>G), TGT1405TAT (4264G>A), GAG1407AAA (4269G>A 4271G>A), CTT1408ATT (4272C>A), CAA1409TTG (4275C>T 4276A>T 4277A>G), CAA1410AAG (4278C>A 4280A>G), GAT1412AGA (4284G>A 4285A>G 4286T>A), CTA1413TTA (4287C>T), ATC1414ATT (4292C>T), GAA1415GAG (4295A>G), CCC1416CCA (4298C>A), TCA1419TCC (4307A>C), GCA1422TCT (4314G>T 4316A>T), GAA1424AAA (4320G>A), GTC1428GTT (4334C>T), AGG1431AGA (4343G>A), TCA1432GCT (4344T>G 4346A>T), GAG1433GAA (4349G>A), GTG1435GCA (4354T>C 4355G>A), GGA1437GGT (4361A>T), TTA1439CTT (4365T>C 4367A>T), AGG1440CGA (4368A>C 4370G>A), ATA1443ATC (4379A>C), TAT1445TAC (4385T>C), CAA1446CAG (4388A>G), CCA1447CCC (4391A>C), TTA1448CTC (4392T>C 4394A>C), CAT1450CAC (4400T>C), CTC1452CTT (4406C>T), CAA1453TTG (4407C>T 4408A>T 4409A>G), GAC1455AGT (4413G>A 4414A>G 4415C>T), AAG1456AAA (4418G>A), TTT1457TTC (4421T>C), CCC1458ACT (4422C>A 4424C>T), ATT1459ATC (4427T>C), CCA1460CTA (4429C>T), AAG1462AAT (4436G>T), CTC1463CTA (4439C>A), ACA1464ATT (4441C>T 4442A>T), CTC1465TTA (4443C>T 4445C>A), TCT1467TTA (4450C>T 4451T>A), TCC1470GTA (4458T>G 4459C>T 4460C>A), AAA1471GAA (4461A>G), GCA1472GCC (4466A>C), AAG1473ACA (4468A>C 4469G>A), CTC1474TAC (4470C>T 4471T>A), TTT1475TTC (4475T>C)								

Proteins

ORF I polyprotein (NP_127504.1)	1402	1476	3.4%	387	71.3%	75 (100%)	52 (69.3%)	0/0/0/0	0
Protein mutations:	I1402M (4256A>G), C1405Y (4264G>A), E1407K (4269G>A 4271G>A), L1408I (4272C>A), Q1409L (4275C>T 4276A>T 4277A>G), Q1410K (4278C>A 4280A>G), D1412R (4284G>A 4285A>G 4286T>A), A1422S (4314G>T 4316A>T), E1424K (4320G>A), S1432A (4344T>G 4346A>T), V1435A (4354T>C 4355G>A), Q1453L (4407C>T 4408A>T 4409A>G), D1455S (4413G>A 4414A>G 4415C>T), P1458T (4422C>A 4424C>T), P1460L (4429C>T), K1462N (4436G>T), T1464I (4441C>T 4442A>T), S1467L (4450C>T 4451T>A), S1470V (4458T>G 4459C>T 4460C>A), K1471E (4461A>G), K1473T (4468A>C 4469G>A), L1474Y (4470C>T 4471T>A)								
Codon mutations:	ATA1402ATG (4256A>G), TGT1405TAT (4264G>A), GAG1407AAA (4269G>A 4271G>A), CTT1408ATT (4272C>A), CAA1409TTG (4275C>T 4276A>T 4277A>G), CAA1410AAG (4278C>A 4280A>G), GAT1412AGA (4284G>A 4285A>G 4286T>A), CTA1413TTA (4287C>T), ATC1414ATT (4292C>T), GAA1415GAG (4295A>G), CCC1416CCA (4298C>A), TCA1419TCC (4307A>C), GCA1422TCT (4314G>T 4316A>T), GAA1424AAA (4320G>A), GTC1428GTT (4334C>T), AGG1431AGA (4343G>A), TCA1432GCT (4344T>G 4346A>T), GAG1433GAA (4349G>A), GTG1435GCA (4354T>C 4355G>A), GGA1437GGT (4361A>T), TTA1439CTT (4365T>C 4367A>T), AGG1440CGA (4368A>C 4370G>A), ATA1443ATC (4379A>C), TAT1445TAC (4385T>C), CAA1446CAG (4388A>G), CCA1447CCC (4391A>C), TTA1448CTC (4392T>C 4394A>C), CAT1450CAC (4400T>C), CTC1452CTT (4406C>T), CAA1453TTG (4407C>T 4408A>T 4409A>G), GAC1455AGT (4413G>A 4414A>G 4415C>T), AAG1456AAA (4418G>A), TTT1457TTC (4421T>C), CCC1458ACT (4422C>A 4424C>T), ATT1459ATC (4427T>C), CCA1460CTA (4429C>T), AAG1462AAT (4436G>T), CTC1463CTA (4439C>A), ACA1464ATT (4441C>T 4442A>T), CTC1465TTA (4443C>T 4445C>A), TCT1467TTA (4450C>T 4451T>A), TCC1470GTA (4458T>G 4459C>T 4460C>A), AAA1471GAA (4461A>G), GCA1472GCC (4466A>C), AAG1473ACA (4468A>C 4469G>A), CTC1474TAC (4470C>T 4471T>A), TTT1475TTC (4475T>C)								

*: Inserts / Deletes / Misaligned / Frameshifts

Analysis details

This analysis was performed with panviral2.64

NGS Details (UN8): Tomato associated geminivirus 1

Assembly

**Coverage Length**	236 (1 contig(s))
**Depth Of Coverage**	1.8
**Number Of Reads**	4
**Reads Per Million**	0.07 rpm (after QC)
**Ambiguities**	0
**Assembly Method**	de novo + reference guided assembly
**Consensus Caller**	Bcf Tools

Coverage Map

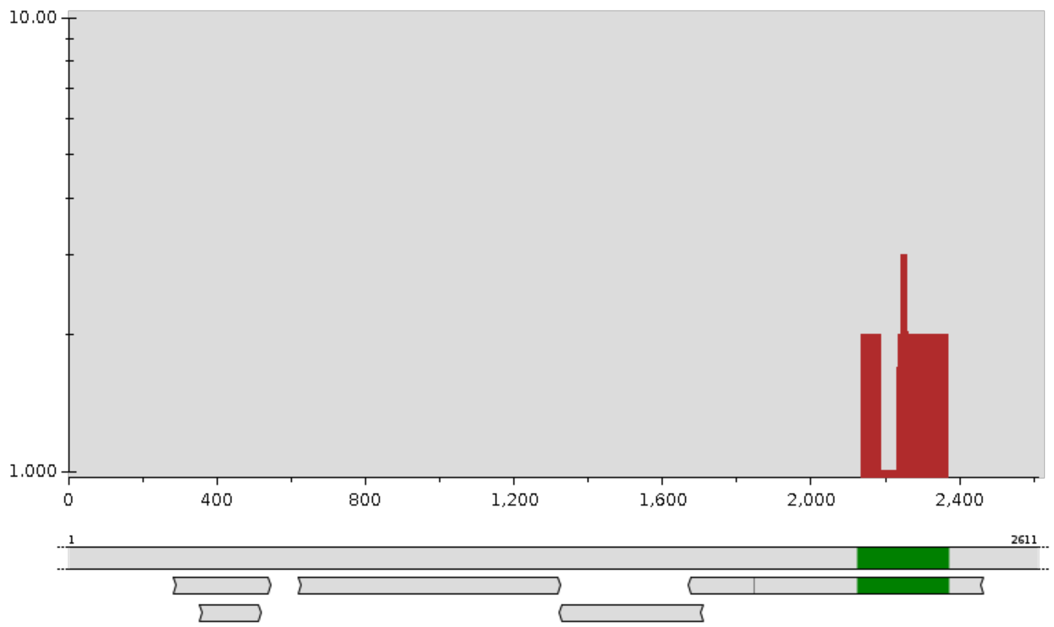

Assignment

**Type**	Tomato associated geminivirus 1 (Taxonomy ID: 2027645)
**Reference Genome**	NC_035676.1
**NT Identity (%)**	63.7097
**AA Identity (%)**	59.0361
**Number Of Stop Codons**	2
**Number Of CDS**	5

Alignment

**Alignment Score**	139.0 (NT) + 778.0 (AA) = 917.0
**Concordance (%)**	54.4537

**Alignment Method**	Global, seeded, nucleotide + amino acids (AGA)

Genome Region

Sequence starts at position 2135 and ends at position 2370 relative to NC_035676.1 reference sequence.

Alignment Detailed Statistics

	Begin	End	Coverage	Score	Concordance	Matches	Identities	I/D/M/F*	Stop Codons
**NT**	**2135**	**2370**	**9.0%**	**139**	**29.4%**	**236 (95.2%)**	**158 (63.7%)**	**12/0**	
Mutations:	2137G>A, 2139A>T, 2142C>T, 2148G>T, 2151A>G, 2154G>A, 2157G>A, 2160C>G, 2162C>A, 2163A>G, 2166A>G, 2168C>G, 2170G>T, 2171C>T, 2172A>G, 2176C>G, 2177T>A, 2181T>C, 2184G>T, 2190G>A, 2191A>G, 2196T>G, 2199A>G, 2203T>A, 2205A>T, 2210C>T, 2213A>G, 2213_2214insATTTGTTGAGCT, 2218T>A, 2219T>C, 2220A>G, 2223G>C, 2226G>A, 2228T>G, 2229G>A, 2234C>T, 2238G>C, 2239A>T, 2244C>G, 2245T>A, 2250C>T, 2251T>C, 2252G>T, 2253C>A, 2254G>A, 2255T>A, 2256C>A, 2259A>G, 2262C>T, 2263C>T, 2271A>G, 2277T>A, 2278A>C, 2279C>A, 2280C>T, 2283A>G, 2289G>A, 2291T>G, 2292G>A, 2298C>T, 2301T>C, 2313G>T, 2314T>G, 2319C>T, 2322A>G, 2323C>G, 2324A>C, 2326G>A, 2333C>T, 2334G>A, 2337T>A, 2338G>T, 2340G>A, 2341A>G, 2342C>G, 2346A>G, 2354T>G, 2358T>A, 2360A>T								
**CDS**									
CK833_gp4	33	111	23.7%	389	62.1%	79 (95.2%)	49 (59.0%)	4/0/0/0	1
Protein mutations:	L36I (2358T>A 2360A>T), K38Q (2354T>G), V42P (2340G>A 2341A>G 2342C>G), T43N (2337T>A 2338G>T), V45I (2333C>T), A47V (2326G>A), C48A (2322A>G 2323C>G 2324A>C), N51T (2313G>T 2314T>G), I59L (2289G>A 2291T>G), V63C (2277T>A 2278A>C 2279C>A), R68K (2262C>T 2263C>T), Q70H (2256C>A), T71F (2253C>A 2254G>A 2255T>A), Q72R (2250C>T 2251T>C 2252G>T), Q74L (2244C>G 2245T>A), F76* (2238G>C 2239A>T), D78N (2234C>T), T80P (2226G>A 2228T>G), D81E (2223G>C), N83V (2218T>A 2219T>C), G84_S85insSSTN (2213_2214insATTTGTTGAGCT), S85P (2213A>G), G86R (2210C>T), Y88F (2203T>A), I92T (2190G>A 2191A>G), R97S (2176C>G 2177T>A), A99K (2170G>T 2171C>T), D100H (2166A>G 2168C>G), A102S (2160C>G 2162C>A), T110I (2137G>A)								
Codon mutations:	TTA36ATT (2358T>A 2360A>T), AAA38CAA (2354T>G), TAT40TAC (2346A>G), GTC42CCT (2340G>A 2341A>G 2342C>G), ACA43AAT (2337T>A 2338G>T), TAC44TAT (2334G>A), GTT45ATT (2333C>T), GCT47GTT (2326G>A), TGT48GCC (2322A>G 2323C>G 2324A>C), ACG49ACA (2319C>T), AAC51ACA (2313G>T 2314T>G), GGA55GGG (2301T>C), GAG56GAA (2298C>T), CAC58CAT (2292G>A), ATC59CTT (2289G>A 2291T>G), TGT61TGC (2283A>G), CTG62CTA (2280C>T), GTA63TGT (2277T>A 2278A>C 2279C>A), ACT65ACC (2271A>G), AGG68AAA (2262C>T 2263C>T), TTT69TTC (2259A>G), CAG70CAT (2256C>A), ACG71TTT (2253C>A 2254G>A 2255T>A), CAG72AGA (2250C>T 2251T>C 2252G>T), CAG74CTC (2244C>G 2245T>A), TTC76TAG (2238G>C 2239A>T), GAC78AAC (2234C>T), ATC79ATT (2229G>A), ACC80CCT (2226G>A 2228T>G), GAC81GAG (2223G>C), CCT82CCC (2220A>G), AAC83GTC (2218T>A 2219T>C), GGA84_TCG85insAGCTCAACAAAT (2213_2214insATTTGTTGAGCT), TCG85CCG (2213A>G), GGA86AGA (2210C>T), ATT87ATA (2205A>T), TAT88TTT (2203T>A), CAT89CAC (2199A>G), CCA90CCC (2196T>G), ATC92ACT (2190G>A 2191A>G), ATC94ATA (2184G>T), CCA95CCG (2181T>C), AGG97TCG (2176C>G 2177T>A), GAT98GAC (2172A>G), GCA99AAA (2170G>T 2171C>T), GAT100CAC (2166A>G 2168C>G), GTT101GTC (2163A>G), GCG102TCC (2160C>G 2162C>A), GAC103GAT (2157G>A), TAC104TAT (2154G>A), ATT105ATC (2151A>G), TCC106TCA (2148G>T), GGG108GGA (2142C>T), GGT109GGA (2139A>T), ACC110ATC (2137G>A)								
CK833_gp5	33	111	30.2%	389	62.1%	79 (95.2%)	49 (59.0%)	4/0/0/0	1
Protein mutations:	L36I (2358T>A 2360A>T), K38Q (2354T>G), V42P (2340G>A 2341A>G 2342C>G), T43N (2337T>A 2338G>T), V45I (2333C>T), A47V (2326G>A), C48A (2322A>G 2323C>G 2324A>C), N51T (2313G>T 2314T>G), I59L (2289G>A 2291T>G), V63C (2277T>A 2278A>C 2279C>A), R68K (2262C>T 2263C>T), Q70H (2256C>A), T71F (2253C>A 2254G>A 2255T>A), Q72R (2250C>T 2251T>C 2252G>T), Q74L (2244C>G 2245T>A), F76* (2238G>C 2239A>T), D78N (2234C>T), T80P (2226G>A 2228T>G), D81E (2223G>C), N83V (2218T>A 2219T>C), G84_S85insSSTN (2213_2214insATTTGTTGAGCT), S85P (2213A>G), G86R (2210C>T), Y88F (2203T>A), I92T (2190G>A 2191A>G), R97S (2176C>G 2177T>A), A99K (2170G>T 2171C>T), D100H (2166A>G 2168C>G), A102S (2160C>G 2162C>A), T110I (2137G>A)								
Codon mutations:	TTA36ATT (2358T>A 2360A>T), AAA38CAA (2354T>G), TAT40TAC (2346A>G), GTC42CCT (2340G>A 2341A>G 2342C>G), ACA43AAT (2337T>A 2338G>T), TAC44TAT (2334G>A), GTT45ATT (2333C>T), GCT47GTT (2326G>A), TGT48GCC (2322A>G 2323C>G 2324A>C), ACG49ACA (2319C>T), AAC51ACA (2313G>T 2314T>G), GGA55GGG (2301T>C), GAG56GAA (2298C>T), CAC58CAT (2292G>A), ATC59CTT (2289G>A 2291T>G), TGT61TGC (2283A>G), CTG62CTA (2280C>T), GTA63TGT (2277T>A 2278A>C 2279C>A), ACT65ACC (2271A>G), AGG68AAA (2262C>T 2263C>T), TTT69TTC (2259A>G), CAG70CAT (2256C>A), ACG71TTT (2253C>A 2254G>A 2255T>A), CAG72AGA (2250C>T 2251T>C 2252G>T), CAG74CTC (2244C>G 2245T>A), TTC76TAG (2238G>C 2239A>T), GAC78AAC (2234C>T), ATC79ATT (2229G>A), ACC80CCT (2226G>A 2228T>G), GAC81GAG (2223G>C), CCT82CCC (2220A>G), AAC83GTC (2218T>A 2219T>C), GGA84_TCG85insAGCTCAACAAAT (2213_2214insATTTGTTGAGCT), TCG85CCG (2213A>G), GGA86AGA (2210C>T), ATT87ATA (2205A>T), TAT88TTT (2203T>A), CAT89CAC (2199A>G), CCA90CCC (2196T>G), ATC92ACT (2190G>A 2191A>G), ATC94ATA (2184G>T), CCA95CCG (2181T>C), AGG97TCG (2176C>G 2177T>A), GAT98GAC (2172A>G), GCA99AAA (2170G>T 2171C>T), GAT100CAC (2166A>G 2168C>G), GTT101GTC (2163A>G), GCG102TCC (2160C>G 2162C>A), GAC103GAT (2157G>A), TAC104TAT (2154G>A), ATT105ATC (2151A>G), TCC106TCA (2148G>T), GGG108GGA (2142C>T), GGT109GGA (2139A>T), ACC110ATC (2137G>A)								
**Proteins**									
replication-associated protein (YP_009417307.1)	33	111	23.7%	389	62.1%	79 (95.2%)	49 (59.0%)	4/0/0/0	1
Protein mutations:	L36I (2358T>A 2360A>T), K38Q (2354T>G), V42P (2340G>A 2341A>G 2342C>G), T43N (2337T>A 2338G>T), V45I (2333C>T), A47V (2326G>A), C48A (2322A>G 2323C>G 2324A>C), N51T (2313G>T 2314T>G), I59L (2289G>A 2291T>G), V63C (2277T>A 2278A>C 2279C>A), R68K (2262C>T 2263C>T), Q70H (2256C>A), T71F (2253C>A 2254G>A 2255T>A), Q72R (2250C>T 2251T>C 2252G>T), Q74L (2244C>G 2245T>A), F76* (2238G>C 2239A>T), D78N (2234C>T), T80P (2226G>A 2228T>G), D81E (2223G>C), N83V (2218T>A 2219T>C), G84_S85insSSTN (2213_2214insATTTGTTGAGCT), S85P (2213A>G), G86R (2210C>T), Y88F (2203T>A), I92T (2190G>A 2191A>G), R97S (2176C>G 2177T>A), A99K (2170G>T 2171C>T), D100H (2166A>G 2168C>G), A102S (2160C>G 2162C>A), T110I (2137G>A)								
Codon mutations:	TTA36ATT (2358T>A 2360A>T), AAA38CAA (2354T>G), TAT40TAC (2346A>G), GTC42CCT (2340G>A 2341A>G 2342C>G), ACA43AAT (2337T>A 2338G>T), TAC44TAT (2334G>A), GTT45ATT (2333C>T), GCT47GTT (2326G>A), TGT48GCC (2322A>G 2323C>G 2324A>C), ACG49ACA (2319C>T), AAC51ACA (2313G>T 2314T>G), GGA55GGG (2301T>C), GAG56GAA (2298C>T), CAC58CAT (2292G>A), ATC59CTT (2289G>A 2291T>G), TGT61TGC (2283A>G), CTG62CTA (2280C>T), GTA63TGT (2277T>A 2278A>C 2279C>A), ACT65ACC (2271A>G), AGG68AAA (2262C>T 2263C>T), TTT69TTC (2259A>G), CAG70CAT (2256C>A), ACG71TTT (2253C>A 2254G>A 2255T>A), CAG72AGA (2250C>T 2251T>C 2252G>T), CAG74CTC (2244C>G 2245T>A), TTC76TAG (2238G>C 2239A>T), GAC78AAC (2234C>T), ATC79ATT (2229G>A), ACC80CCT (2226G>A 2228T>G), GAC81GAG (2223G>C), CCT82CCC (2220A>G), AAC83GTC (2218T>A 2219T>C), GGA84_TCG85insAGCTCAACAAAT (2213_2214insATTTGTTGAGCT), TCG85CCG (2213A>G), GGA86AGA (2210C>T), ATT87ATA (2205A>T), TAT88TTT (2203T>A), CAT89CAC (2199A>G), CCA90CCC (2196T>G), ATC92ACT (2190G>A 2191A>G), ATC94ATA (2184G>T), CCA95CCG (2181T>C), AGG97TCG (2176C>G 2177T>A), GAT98GAC (2172A>G), GCA99AAA (2170G>T 2171C>T), GAT100CAC (2166A>G 2168C>G), GTT101GTC (2163A>G), GCG102TCC (2160C>G 2162C>A), GAC103GAT (2157G>A), TAC104TAT (2154G>A), ATT105ATC (2151A>G), TCC106TCA (2148G>T), GGG108GGA (2142C>T), GGT109GGA (2139A>T), ACC110ATC (2137G>A)								
RepA (YP_009417308.1)	33	111	30.2%	389	62.1%	79 (95.2%)	49 (59.0%)	4/0/0/0	1
Protein mutations:	L36I (2358T>A 2360A>T), K38Q (2354T>G), V42P (2340G>A 2341A>G 2342C>G), T43N (2337T>A 2338G>T), V45I (2333C>T), A47V (2326G>A), C48A (2322A>G 2323C>G 2324A>C), N51T (2313G>T 2314T>G), I59L (2289G>A 2291T>G), V63C (2277T>A 2278A>C 2279C>A), R68K (2262C>T 2263C>T), Q70H (2256C>A), T71F (2253C>A 2254G>A 2255T>A), Q72R (2250C>T 2251T>C 2252G>T), Q74L (2244C>G 2245T>A), F76* (2238G>C 2239A>T), D78N (2234C>T), T80P (2226G>A 2228T>G), D81E (2223G>C), N83V (2218T>A 2219T>C), G84_S85insSSTN (2213_2214insATTTGTTGAGCT), S85P (2213A>G), G86R (2210C>T), Y88F (2203T>A), I92T (2190G>A 2191A>G), R97S (2176C>G 2177T>A), A99K (2170G>T 2171C>T), D100H (2166A>G 2168C>G), A102S (2160C>G 2162C>A), T110I (2137G>A)								
Codon mutations:	TTA36ATT (2358T>A 2360A>T), AAA38CAA (2354T>G), TAT40TAC (2346A>G), GTC42CCT (2340G>A 2341A>G 2342C>G), ACA43AAT (2337T>A 2338G>T), TAC44TAT (2334G>A), GTT45ATT (2333C>T), GCT47GTT (2326G>A), TGT48GCC (2322A>G 2323C>G 2324A>C), ACG49ACA (2319C>T), AAC51ACA (2313G>T 2314T>G), GGA55GGG (2301T>C), GAG56GAA (2298C>T), CAC58CAT (2292G>A), ATC59CTT (2289G>A 2291T>G), TGT61TGC (2283A>G), CTG62CTA (2280C>T), GTA63TGT (2277T>A 2278A>C 2279C>A), ACT65ACC (2271A>G), AGG68AAA (2262C>T 2263C>T), TTT69TTC (2259A>G), CAG70CAT (2256C>A), ACG71TTT (2253C>A 2254G>A 2255T>A), CAG72AGA (2250C>T 2251T>C 2252G>T), CAG74CTC (2244C>G 2245T>A), TTC76TAG (2238G>C 2239A>T), GAC78AAC (2234C>T), ATC79ATT (2229G>A), ACC80CCT (2226G>A 2228T>G), GAC81GAG (2223G>C), CCT82CCC (2220A>G), AAC83GTC (2218T>A 2219T>C), GGA84_TCG85insAGCTCAACAAAT (2213_2214insATTTGTTGAGCT), TCG85CCG (2213A>G), GGA86AGA (2210C>T), ATT87ATA (2205A>T), TAT88TTT (2203T>A), CAT89CAC (2199A>G), CCA90CCC (2196T>G), ATC92ACT (2190G>A 2191A>G), ATC94ATA (2184G>T), CCA95CCG (2181T>C), AGG97TCG (2176C>G 2177T>A), GAT98GAC (2172A>G), GCA99AAA (2170G>T 2171C>T), GAT100CAC (2166A>G 2168C>G), GTT101GTC (2163A>G), GCG102TCC (2160C>G 2162C>A), GAC103GAT (2157G>A), TAC104TAT (2154G>A), ATT105ATC (2151A>G), TCC106TCA (2148G>T), GGG108GGA (2142C>T), GGT109GGA (2139A>T), ACC110ATC (2137G>A)								

*: Inserts / Deletes / Misaligned / Frameshifts

Analysis details

This analysis was performed with panviral2.64

NGS Details (UN8): Bracoviriform congregatae (segment Circle 7)

Assembly

**Coverage Length**	135 (1 contig(s))
**Depth Of Coverage**	2.5
**Number Of Reads**	3
**Reads Per Million**	0.05 rpm (after QC)
**Ambiguities**	0
**Assembly Method**	de novo + reference guided assembly
**Consensus Caller**	Bcf Tools

Coverage Map

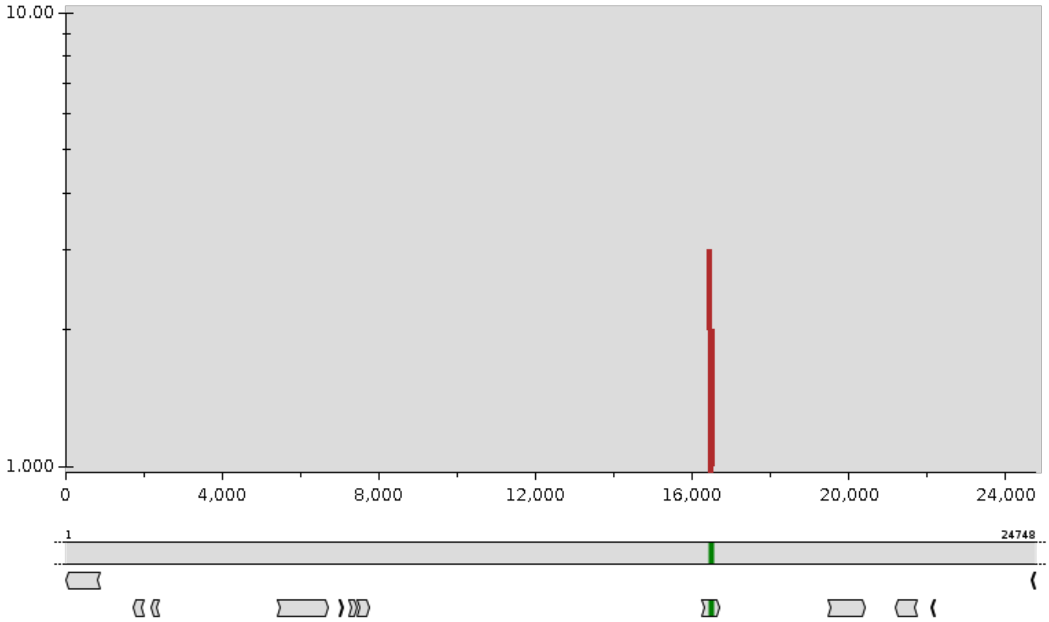

Assignment

**Type**	Bracoviriform congregatae (Taxonomy ID: 39640)
**Reference Genome**	NC_006639.1
**NT Identity (%)**	75.5556
**AA Identity (%)**	86.6667
**Number Of Stop Codons**	0
**Number Of CDS**	5

Alignment

**Alignment Score**	138.0 (NT) + 273.0 (AA) = 411.0
**Concordance (%)**	72.4868

Alignment Method	Global, seeded, nucleotide + amino acids (AGA)

Genome Region

Sequence starts at position 16415 and ends at position 16549 relative to NC_006639.1 reference sequence.

Alignment Detailed Statistics

	Begin	End	Coverage	Score	Concordance	Matches	Identities	I/D/M/F*	Stop Codons
**NT**	**16415**	**16549**	**0.5%**	**138**	**51.1%**	**135 (100%)**	**102 (75.6%)**	**0/0**	
Mutations:	16417A>T, 16418T>C, 16420G>C, 16423A>C, 16426A>G, 16429G>C, 16432C>A, 16435T>C, 16444T>C, 16449G>A, 16450A>G, 16451T>A, 16452C>T, 16454C>T, 16456T>G, 16459A>T, 16462T>C, 16471A>G, 16474A>T, 16486A>C, 16489T>G, 16492C>T, 16501G>C, 16504T>G, 16516A>T, 16526G>A, 16531T>A, 16533G>C, 16534A>C, 16535T>A, 16538G>A, 16543T>C, 16546A>G								
**CDS**									
Histone	62	106	28.8%	273	91.9%	45 (100%)	39 (86.7%)	0/0/0/0	0
Protein mutations:	R73K (16449G>A 16450A>G), S74I (16451T>A 16452C>T), V99I (16526G>A), G101A (16533G>C 16534A>C), L102M (16535T>A), V103I (16538G>A)								
Codon mutations:	GGA62GGT (16417A>T), TTG63CTC (16418T>C 16420G>C), GGA64GGC (16423A>C), AAA65AAG (16426A>G), GGG66GGC (16429G>C), GGC67GGA (16432C>A), GCT68GCC (16435T>C), CAT71CAC (16444T>C), AGA73AAG (16449G>A 16450A>G), TCT74ATT (16451T>A 16452C>T), CTT75TTG (16454C>T 16456T>G), CGA76CGT (16459A>T), GAT77GAC (16462T>C), CAA80CAG (16471A>G), GGA81GGT (16474A>T), CCA85CCC (16486A>C), GCT86GCG (16489T>G), ATC87ATT (16492C>T), CTG90CTC (16501G>C), GCT91GCG (16504T>G), GGA95GGT (16516A>T), GTC99ATC (16526G>A), TCT100TCA (16531T>A), GGA101GCC (16533G>C 16534A>C), TTG102ATG (16535T>A), GTC103ATC (16538G>A), TAT104TAC (16543T>C), GAA105GAG (16546A>G)								
**Proteins**									
hypothetical protein (YP_184795.1)	62	106	28.8%	273	91.9%	45 (100%)	39 (86.7%)	0/0/0/0	0
Protein mutations:	R73K (16449G>A 16450A>G), S74I (16451T>A 16452C>T), V99I (16526G>A), G101A (16533G>C 16534A>C), L102M (16535T>A), V103I (16538G>A)								
Codon mutations:	GGA62GGT (16417A>T), TTG63CTC (16418T>C 16420G>C), GGA64GGC (16423A>C), AAA65AAG (16426A>G), GGG66GGC (16429G>C), GGC67GGA (16432C>A), GCT68GCC (16435T>C), CAT71CAC (16444T>C), AGA73AAG (16449G>A 16450A>G), TCT74ATT (16451T>A 16452C>T), CTT75TTG (16454C>T 16456T>G), CGA76CGT (16459A>T), GAT77GAC (16462T>C), CAA80CAG (16471A>G), GGA81GGT (16474A>T), CCA85CCC (16486A>C), GCT86GCG (16489T>G), ATC87ATT (16492C>T), CTG90CTC (16501G>C), GCT91GCG (16504T>G), GGA95GGT (16516A>T), GTC99ATC (16526G>A), TCT100TCA (16531T>A), GGA101GCC (16533G>C 16534A>C), TTG102ATG (16535T>A), GTC103ATC (16538G>A), TAT104TAC (16543T>C), GAA105GAG (16546A>G)								

*: Inserts / Deletes / Misaligned / Frameshifts

Analysis details

This analysis was performed with panviral2.64

NGS Details (UN8): Tobacco streak virus (segment RNA 1)

Assembly

**Coverage Length**	269 (2 contig(s))
**Depth Of Coverage**	1.0
**Number Of Reads**	2
**Reads Per Million**	0.03 rpm (after QC)
**Ambiguities**	0
**Assembly Method**	de novo + reference guided assembly
**Consensus Caller**	Bcf Tools

Coverage Map

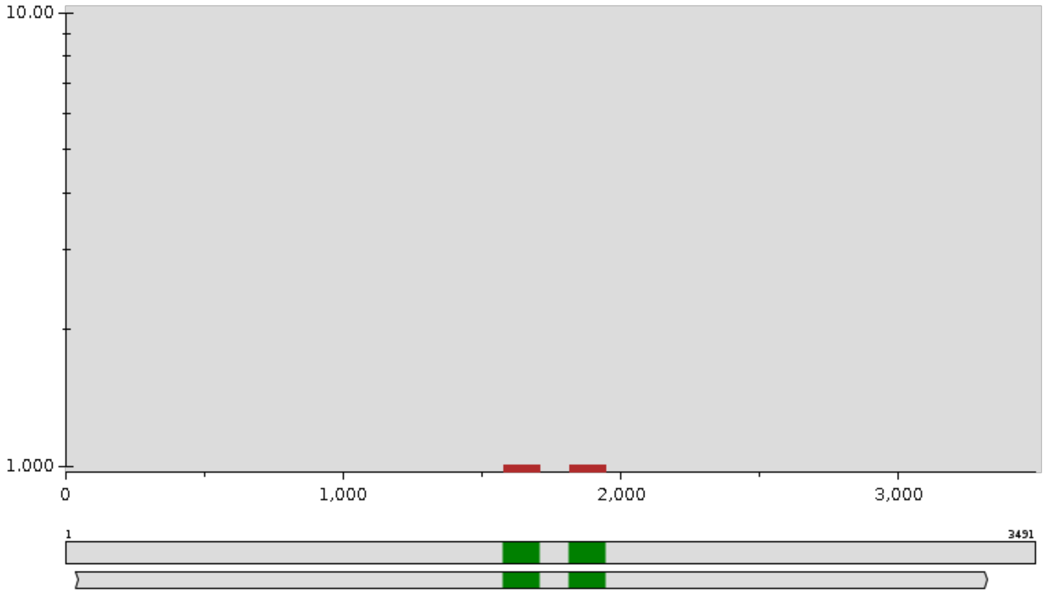

Assignment

**Type**	Tobacco streak virus (Taxonomy ID: 12317)
**Reference Genome**	NC_003844.1
**NT Identity (%)**	74.3494
**AA Identity (%)**	80.0
**Number Of Stop Codons**	0
**Number Of CDS**	1

Alignment

**Alignment Score**	262.0 (NT) + 508.0 (AA) = 770.0
**Concordance (%)**	69.3069

Alignment Method	Global, seeded, nucleotide + amino acids (AGA)

Genome Region

Sequence starts at position 1576 and ends at position 1947 relative to NC_003844.1 reference sequence.

Alignment Detailed Statistics

	Begin	End	Coverage	Score	Concordance	Matches	Identities	I/D/M/F*	Stop Codons
**NT**	**1576**	**1947**	**7.7%**	**262**	**48.7%**	**269 (100%)**	**200 (74.3%)**	**0/0**	
Mutations:	1577A>G, 1579A>G, 1585A>T, 1588G>A, 1590G>A, 1591C>T, 1592C>T, 1595T>C, 1597G>T, 1599G>T, 1603A>G, 1606G>T, 1609T>A, 1612C>T, 1613A>T, 1615G>T, 1618C>G, 1619C>A, 1621A>G, 1623G>A, 1624T>G, 1625G>A, 1626T>C, 1633G>A, 1634C>T, 1636C>G, 1639G>C, 1648C>T, 1651G>T, 1657C>T, 1666T>C, 1669C>T, 1673G>A, 1678T>C, 1681T>C, 1690G>A, 1693G>T, 1694A>G, 1695C>T, 1696T>C, 1699T>C, 1702G>T, 1822T>G, 1825A>G, 1826C>A, 1830C>A, 1831C>T, 1834T>C, 1867A>C, 1870G>C, 1873A>G, 1876T>G, 1879T>C, 1890A>C, 1892A>T, 1894T>C, 1895A>G, 1897C>G, 1899A>G, 1903T>C, 1906T>G, 1912A>G, 1915G>A, 1918T>C, 1930T>C, 1934A>G, 1936T>C, 1938A>G, 1939A>G								
**CDS**									
TbSVs1gp1	514	637	8.2%	508	86.7%	90 (100%)	72 (80.0%)	0/0/0/0	0
Protein mutations:	I514V (1577A>G 1579A>G), S518N (1590G>A 1591C>T), R521L (1599G>T), T526S (1613A>T 1615G>T), S529K (1623G>A 1624T>G), V530T (1625G>A 1626T>C), V546I (1673G>A), T553V (1694A>G 1695C>T 1696T>C), Q597K (1826C>A), A598D (1830C>A 1831C>T), Q618P (1890A>C), T619S (1892A>T 1894T>C), I620V (1895A>G 1897C>G), K621R (1899A>G), I633V (1934A>G 1936T>C), K634R (1938A>G 1939A>G)								
Codon mutations:	ATA514GTG (1577A>G 1579A>G), CCA516CCT (1585A>T), AGG517AGA (1588G>A), AGC518AAT (1590G>A 1591C>T), CTG519TTG (1592C>T), TTG520CTT (1595T>C 1597G>T), CGT521CTT (1599G>T), GAA522GAG (1603A>G), GTG523GTT (1606G>T), ATT524ATA (1609T>A), TAC525TAT (1612C>T), ACG526TCT (1613A>T 1615G>T), GTC527GTG (1618C>G), CGA528AGG (1619C>A 1621A>G), AGT529AAG (1623G>A 1624T>G), GTT530ACT (1625G>A 1626T>C), GGG532GGA (1633G>A), CTC533TTG (1634C>T 1636C>G), ACG534ACC (1639G>C), GAC537GAT (1648C>T), TCG538TCT (1651G>T), TAC540TAT (1657C>T), GTT543GTC (1666T>C), TCC544TCT (1669C>T), GTT546ATT (1673G>A), CCT547CCC (1678T>C), TCT548TCC (1681T>C), AAG551AAA (1690G>A), GCG552GCT (1693G>T), ACT553GTC (1694A>G 1695C>T 1696T>C), CAT554CAC (1699T>C), GTG555GTT (1702G>T), CGT595CGG (1822T>G), CAA596CAG (1825A>G), CAG597AAG (1826C>A), GCC598GAT (1830C>A 1831C>T), TTT599TTC (1834T>C), ATA610ATC (1867A>C), GCG611GCC (1870G>C), AAA612AAG (1873A>G), GCT613GCG (1876T>G), ATT614ATC (1879T>C), CAA618CCA (1890A>C), ACT619TCC (1892A>T 1894T>C), ATC620GTG (1895A>G 1897C>G), AAA621AGA (1899A>G), GAT622GAC (1903T>C), GGT623GGG (1906T>G), TTA625TTG (1912A>G), CCG626CCA (1915G>A), ATT627ATC (1918T>C), TGT631TGC (1930T>C), ATT633GTC (1934A>G 1936T>C), AAA634AGG (1938A>G 1939A>G)								
**Proteins**									
putative viral replicase (NP_620772.1)	514	637	8.2%	508	86.7%	90 (100%)	72 (80.0%)	0/0/0/0	0
Protein mutations:	I514V (1577A>G 1579A>G), S518N (1590G>A 1591C>T), R521L (1599G>T), T526S (1613A>T 1615G>T), S529K (1623G>A 1624T>G), V530T (1625G>A 1626T>C), V546I (1673G>A), T553V (1694A>G 1695C>T 1696T>C), Q597K (1826C>A), A598D (1830C>A 1831C>T), Q618P (1890A>C), T619S (1892A>T 1894T>C), I620V (1895A>G 1897C>G), K621R (1899A>G), I633V (1934A>G 1936T>C), K634R (1938A>G 1939A>G)								
Codon mutations:	ATA514GTG (1577A>G 1579A>G), CCA516CCT (1585A>T), AGG517AGA (1588G>A), AGC518AAT (1590G>A 1591C>T), CTG519TTG (1592C>T), TTG520CTT (1595T>C 1597G>T), CGT521CTT (1599G>T), GAA522GAG (1603A>G), GTG523GTT (1606G>T), ATT524ATA (1609T>A), TAC525TAT (1612C>T), ACG526TCT (1613A>T 1615G>T), GTC527GTG (1618C>G), CGA528AGG (1619C>A 1621A>G), AGT529AAG (1623G>A 1624T>G), GTT530ACT (1625G>A 1626T>C), GGG532GGA (1633G>A), CTC533TTG (1634C>T 1636C>G), ACG534ACC (1639G>C), GAC537GAT (1648C>T), TCG538TCT (1651G>T), TAC540TAT (1657C>T), GTT543GTC (1666T>C), TCC544TCT (1669C>T), GTT546ATT (1673G>A), CCT547CCC (1678T>C), TCT548TCC (1681T>C), AAG551AAA (1690G>A), GCG552GCT (1693G>T), ACT553GTC (1694A>G 1695C>T 1696T>C), CAT554CAC (1699T>C), GTG555GTT (1702G>T), CGT595CGG (1822T>G), CAA596CAG (1825A>G), CAG597AAG (1826C>A), GCC598GAT (1830C>A 1831C>T), TTT599TTC (1834T>C), ATA610ATC (1867A>C), GCG611GCC (1870G>C), AAA612AAG (1873A>G), GCT613GCG (1876T>G), ATT614ATC (1879T>C), CAA618CCA (1890A>C), ACT619TCC (1892A>T 1894T>C), ATC620GTG (1895A>G 1897C>G), AAA621AGA (1899A>G), GAT622GAC (1903T>C), GGT623GGG (1906T>G), TTA625TTG (1912A>G), CCG626CCA (1915G>A), ATT627ATC (1918T>C), TGT631TGC (1930T>C), ATT633GTC (1934A>G 1936T>C), AAA634AGG (1938A>G 1939A>G)								

*: Inserts / Deletes / Misaligned / Frameshifts

Analysis details

This analysis was performed with panviral2.64

NGS Details (UN8): Nepovirus aeonii (segment RNA2)

Assembly

**Coverage Length**	242 (1 contig(s))
**Depth Of Coverage**	1.1
**Number Of Reads**	2
**Reads Per Million**	0.03 rpm (after QC)
**Ambiguities**	0
**Assembly Method**	de novo + reference guided assembly
**Consensus Caller**	Bcf Tools

Coverage Map

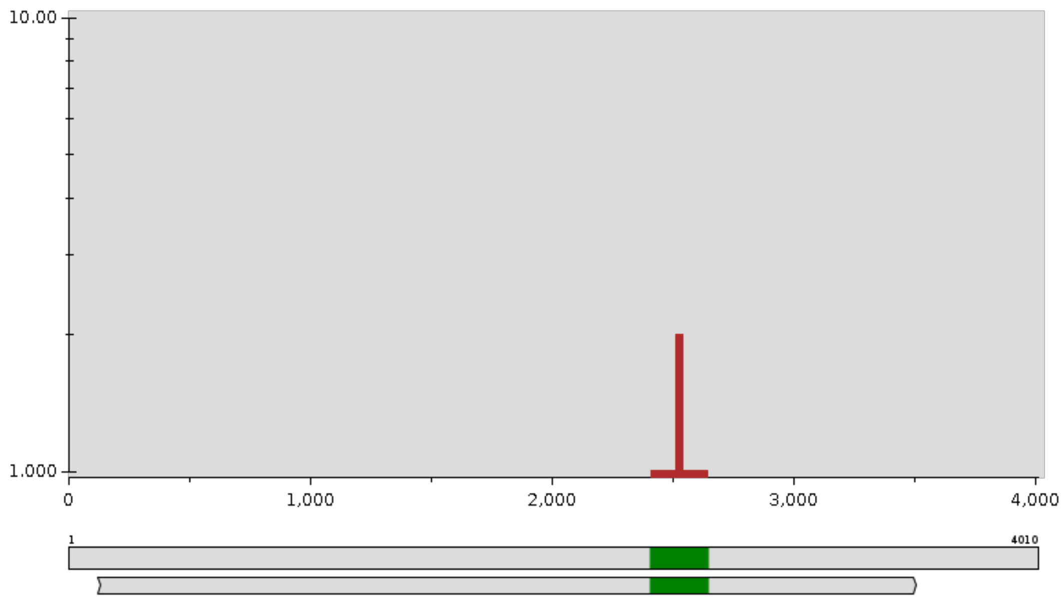

Assignment

**Type**	Nepovirus aeonii (Taxonomy ID: 3047350)
**Reference Genome**	NC_038761.1
**NT Identity (%)**	69.0083
**AA Identity (%)**	66.6667
**Number Of Stop Codons**	0
**Number Of CDS**	1

Alignment

**Alignment Score**	184.0 (NT) + 410.0 (AA) = 594.0
**Concordance (%)**	57.7259

Alignment Method	Global, seeded, nucleotide + amino acids (AGA)

Genome Region

Sequence starts at position 2406 and ends at position 2647 relative to NC_038761.1 reference sequence.

Alignment Detailed Statistics

	Begin	End	Coverage	Score	Concordance	Matches	Identities	I/D/M/F*	Stop Codons
**NT**	**2406**	**2647**	**6.0%**	**184**	**38.0%**	**242 (100%)**	**167 (69.0%)**	**0/0**	
Mutations:	2409T>C, 2412T>C, 2415A>G, 2421G>T, 2422A>C, 2424G>T, 2427G>A, 2428G>T, 2433T>G, 2437A>C, 2439T>G, 2445G>T, 2446G>A, 2447T>C, 2452G>T, 2457T>C, 2460A>G, 2461G>T, 2462A>C, 2463T>A, 2466C>T, 2468C>T, 2469C>T, 2470C>G, 2472G>A, 2474A>C, 2481G>A, 2482T>C, 2483C>A, 2484T>G, 2491C>T, 2493T>G, 2496C>T, 2499C>A, 2502G>A, 2503G>A, 2505T>G, 2514G>T, 2515C>A, 2517G>A, 2519G>C, 2520T>A, 2521G>C, 2522T>A, 2524G>A, 2526G>T, 2529T>C, 2530T>A, 2531C>G, 2532A>T, 2533C>T, 2535C>T, 2536A>G, 2537G>A, 2541T>G, 2542G>A, 2562A>G, 2565G>A, 2571G>A, 2580G>C, 2583T>C, 2592A>G, 2593A>T, 2607T>C, 2608A>G, 2622T>C, 2623A>G, 2629C>T, 2631C>T, 2632A>T, 2634T>A, 2635C>A, 2637A>G, 2643C>T, 2646G>A								

CDS

	Begin	End	Coverage	Score	Concordance	Matches	Identities	I/D/M/F*	Stop Codons
D1S54_s2gp1	762	842	7.2%	410	73.7%	81 (100%)	54 (66.7%)	0/0/0/0	0
Protein mutations:	M767L (2422A>C 2424G>T), A769S (2428G>T), D770E (2433T>G), N772Q (2437A>C 2439T>G), V775T (2446G>A 2447T>C), V777L (2452G>T), I779M (2460A>G), D780S (2461G>T 2462A>C 2463T>A), T782I (2468C>T 2469C>T), Q783E (2470C>G 2472G>A), Q784P (2474A>C), S787Q (2482T>C 2483C>A 2484T>G), V794M (2503G>A 2505T>G), L798I (2515C>A 2517G>A), S799T (2519G>C 2520T>A), V800H (2521G>C 2522T>A), G801S (2524G>A 2526G>T), L804F (2533C>T 2535C>T), S805D (2536A>G 2537G>A), D807N (2542G>A), M824L (2593A>T), N829D (2608A>G), I834V (2623A>G), L836F (2629C>T 2631C>T), T837S (2632A>T 2634T>A), Q838K (2635C>A 2637A>G)								
Codon mutations:	ACT762ACC (2409T>C), AAT763AAC (2412T>C), CAA764CAG (2415A>G), GTG766GTT (2421G>T), ATG767CTT (2422A>C 2424G>T), GCG768GCA (2427G>A), GCA769TCA (2428G>T), GAT770GAG (2433T>G), AAT772CAG (2437A>C 2439T>G), GTG774GTT (2445G>T), GTA775ACA (2446G>A 2447T>C), GTG777TTG (2452G>T), CAT778CAC (2457T>C), ATA779ATG (2460A>G), GAT780TCA (2461G>T 2462A>C 2463T>A), CGC781CGT (2466C>T), ACC782ATT (2468C>T 2469C>T), CAG783GAA (2470C>G 2472G>A), CAA784CCA (2474A>C), GAG786GAA (2481G>A), TCT787CAG (2482T>C 2483C>A 2484T>G), CTT790TTG (2491C>T 2493T>G), GCC791GCT (2496C>T), CCC792CCA (2499C>A), ACG793ACA (2502G>A), GTT794ATG (2503G>A 2505T>G), CCG797CCT (2514G>T), CTG798ATA (2515C>A 2517G>A), AGT799ACA (2519G>C 2520T>A), GTT800CAT (2521G>C 2522T>A), GGG801AGT (2524G>A 2526G>T), GAT802GAC (2529T>C), TCA803AGT (2530T>A 2531C>G 2532A>T), CTC804TTT (2533C>T 2535C>T), AGT805GAT (2536A>G 2537G>A), GTT806GTG (2541T>G), GAT807AAT (2542G>A), AAA813AAG (2562A>G), GAG814GAA (2565G>A), AAG816AAA (2571G>A), GGG819GGC (2580G>C), ACT820ACC (2583T>C), TTA823TTG (2592A>G), ATG824TTG (2593A>T), TAT828TAC (2607T>C), AAT829GAT (2608A>G), CCT833CCC (2622T>C), ATC834GTC (2623A>G), CTC836TTT (2629C>T 2631C>T), ACT837TCA (2632A>T 2634T>A), CAA838AAG (2635C>A 2637A>G), AGC840AGT (2643C>T), TCG841TCA (2646G>A)								

Proteins

	Begin	End	Coverage	Score	Concordance	Matches	Identities	I/D/M/F*	Stop Codons
polyprotein P2 (YP_009507917.1)	762	842	7.2%	410	73.7%	81 (100%)	54 (66.7%)	0/0/0/0	0
Protein mutations:	M767L (2422A>C 2424G>T), A769S (2428G>T), D770E (2433T>G), N772Q (2437A>C 2439T>G), V775T (2446G>A 2447T>C), V777L (2452G>T), I779M (2460A>G), D780S (2461G>T 2462A>C 2463T>A), T782I (2468C>T 2469C>T), Q783E (2470C>G 2472G>A), Q784P (2474A>C), S787Q (2482T>C 2483C>A 2484T>G), V794M (2503G>A 2505T>G), L798I (2515C>A 2517G>A), S799T (2519G>C 2520T>A), V800H (2521G>C 2522T>A), G801S (2524G>A 2526G>T), L804F (2533C>T 2535C>T), S805D (2536A>G 2537G>A), D807N (2542G>A), M824L (2593A>T), N829D (2608A>G), I834V (2623A>G), L836F (2629C>T 2631C>T), T837S (2632A>T 2634T>A), Q838K (2635C>A 2637A>G)								
Codon mutations:	ACT762ACC (2409T>C), AAT763AAC (2412T>C), CAA764CAG (2415A>G), GTG766GTT (2421G>T), ATG767CTT (2422A>C 2424G>T), GCG768GCA (2427G>A), GCA769TCA (2428G>T), GAT770GAG (2433T>G), AAT772CAG (2437A>C 2439T>G), GTG774GTT (2445G>T), GTA775ACA (2446G>A 2447T>C), GTG777TTG (2452G>T), CAT778CAC (2457T>C), ATA779ATG (2460A>G), GAT780TCA (2461G>T 2462A>C 2463T>A), CGC781CGT (2466C>T), ACC782ATT (2468C>T 2469C>T), CAG783GAA (2470C>G 2472G>A), CAA784CCA (2474A>C), GAG786GAA (2481G>A), TCT787CAG (2482T>C 2483C>A 2484T>G), CTT790TTG (2491C>T 2493T>G), GCC791GCT (2496C>T), CCC792CCA (2499C>A), ACG793ACA (2502G>A), GTT794ATG (2503G>A 2505T>G), CCG797CCT (2514G>T), CTG798ATA (2515C>A 2517G>A), AGT799ACA (2519G>C 2520T>A), GTT800CAT (2521G>C 2522T>A), GGG801AGT (2524G>A 2526G>T), GAT802GAC (2529T>C), TCA803AGT (2530T>A 2531C>G 2532A>T), CTC804TTT (2533C>T 2535C>T), AGT805GAT (2536A>G 2537G>A), GTT806GTG (2541T>G), GAT807AAT (2542G>A), AAA813AAG (2562A>G), GAG814GAA (2565G>A), AAG816AAA (2571G>A), GGG819GGC (2580G>C), ACT820ACC (2583T>C), TTA823TTG (2592A>G), ATG824TTG (2593A>T), TAT828TAC (2607T>C), AAT829GAT (2608A>G), CCT833CCC (2622T>C), ATC834GTC (2623A>G), CTC836TTT (2629C>T 2631C>T), ACT837TCA (2632A>T 2634T>A), CAA838AAG (2635C>A 2637A>G), AGC840AGT (2643C>T), TCG841TCA (2646G>A)								

*: Inserts / Deletes / Misaligned / Frameshifts

Analysis details

This analysis was performed with panviral2.64

NGS Details (UN8): Duamitovirus peex1

Assembly

**Coverage Length**	135 (1 contig(s))
**Depth Of Coverage**	1.5
**Number Of Reads**	2
**Reads Per Million**	0.03 rpm (after QC)
**Ambiguities**	0
**Assembly Method**	de novo + reference guided assembly
**Consensus Caller**	Bcf Tools

Coverage Map

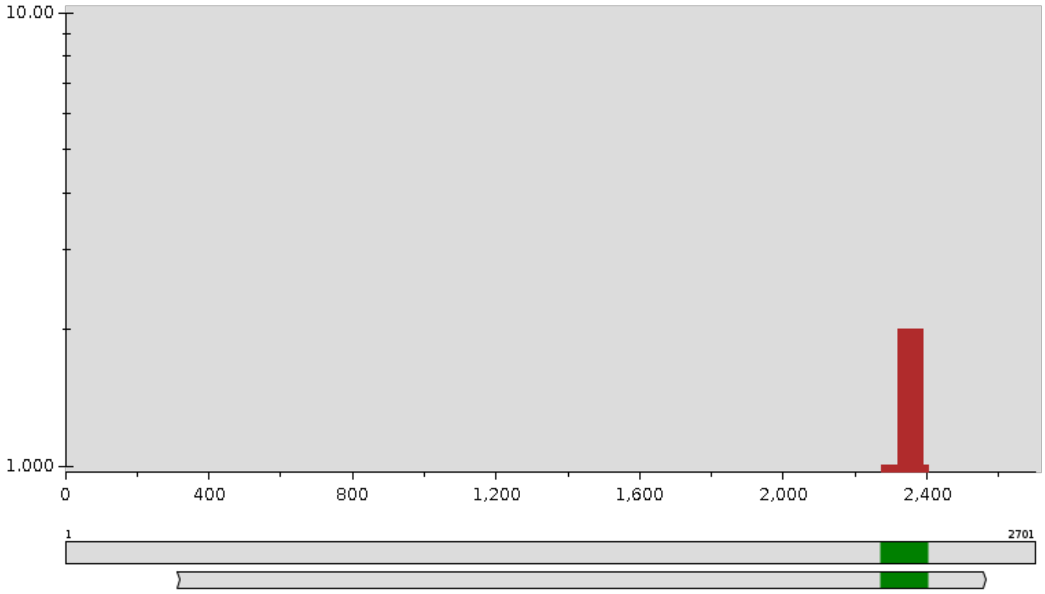

Assignment

**Type**	Duamitovirus peex1 (Taxonomy ID: 2955799)
**Reference Genome**	NC_076525.1
**NT Identity (%)**	70.3704
**AA Identity (%)**	73.3333
**Number Of Stop Codons**	1
**Number Of CDS**	1

Alignment

**Alignment Score**	110.0 (NT) + 266.0 (AA) = 376.0
**Concordance (%)**	58.567

**Alignment Method**	Global, seeded, nucleotide + amino acids (AGA)

Genome Region

Sequence starts at position 2271 and ends at position 2405 relative to NC_076525.1 reference sequence.

Alignment Detailed Statistics

	Begin	End	Coverage	Score	Concordance	Matches	Identities	I/D/M/F*	Stop Codons
**NT**	**2271**	**2405**	**5.0%**	**110**	**40.7%**	**135 (100%)**	**95 (70.4%)**	**0/0**	
Mutations:	2276T>A, 2280A>G, 2283G>A, 2294T>G, 2295C>A, 2296G>A, 2301C>G, 2303T>C, 2306A>T, 2312T>C, 2315C>T, 2321G>T, 2325A>G, 2326A>C, 2327T>A, 2330C>A, 2331C>G, 2333G>A, 2339G>A, 2345A>T, 2348C>T, 2351A>G, 2352C>T, 2354T>G, 2363G>T, 2373G>A, 2374C>G, 2375G>T, 2377C>A, 2378T>A, 2381T>C, 2384G>A, 2388C>A, 2390T>A, 2393T>A, 2394C>A, 2396G>C, 2397A>G, 2398C>A, 2399T>A								
**CDS**									
RdRp	654	698	6.0%	266	71.5%	45 (100%)	33 (73.3%)	0/0/0/0	1
Protein mutations:	H655Q (2276T>A), M657V (2280A>G), E658K (2283G>A), R662K (2295C>A 2296G>A), L664V (2301C>G 2303T>C), N672A (2325A>G 2326A>C 2327T>A), P674A (2331C>G 2333G>A), A688S (2373G>A 2374C>G 2375G>T), T689K (2377C>A 2378T>A), W691* (2384G>A), Q695N (2394C>A 2396G>C), T696E (2397A>G 2398C>A 2399T>A)								
Codon mutations:	CAT655CAA (2276T>A), ATG657GTG (2280A>G), GAA658AAA (2283G>A), CTT661CTG (2294T>G), CGA662AAA (2295C>A 2296G>A), CTT664GTC (2301C>G 2303T>C), TCA665TCT (2306A>T), TAT667TAC (2312T>C), CAC668CAT (2315C>T), GTG670GTT (2321G>T), AAT672GCA (2325A>G 2326A>C 2327T>A), TCC673TCA (2330C>A), CCG674GCA (2331C>G 2333G>A), GTG676GTA (2339G>A), ATA678ATT (2345A>T), GAC679GAT (2348C>T), CAA680CAG (2351A>G), CTT681TTG (2352C>T 2354T>G), GTG684GTT (2363G>T), GCG688AGT (2373G>A 2374C>G 2375G>T), ACT689AAA (2377C>A 2378T>A), TCT690TCC (2381T>C), TGG691TGA (2384G>A), CGT693AGA (2388C>A 2390T>A), ACT694ACA (2393T>A), CAG695AAC (2394C>A 2396G>C), ACT696GAA (2397A>G 2398C>A 2399T>A)								
**Proteins**									
RNA-dependent RNA polymerase (YP_010798875.1)	654	698	6.0%	266	71.5%	45 (100%)	33 (73.3%)	0/0/0/0	1
Protein mutations:	H655Q (2276T>A), M657V (2280A>G), E658K (2283G>A), R662K (2295C>A 2296G>A), L664V (2301C>G 2303T>C), N672A (2325A>G 2326A>C 2327T>A), P674A (2331C>G 2333G>A), A688S (2373G>A 2374C>G 2375G>T), T689K (2377C>A 2378T>A), W691* (2384G>A), Q695N (2394C>A 2396G>C), T696E (2397A>G 2398C>A 2399T>A)								
Codon mutations:	CAT655CAA (2276T>A), ATG657GTG (2280A>G), GAA658AAA (2283G>A), CTT661CTG (2294T>G), CGA662AAA (2295C>A 2296G>A), CTT664GTC (2301C>G 2303T>C), TCA665TCT (2306A>T), TAT667TAC (2312T>C), CAC668CAT (2315C>T), GTG670GTT (2321G>T), AAT672GCA (2325A>G 2326A>C 2327T>A), TCC673TCA (2330C>A), CCG674GCA (2331C>G 2333G>A), GTG676GTA (2339G>A), ATA678ATT (2345A>T), GAC679GAT (2348C>T), CAA680CAG (2351A>G), CTT681TTG (2352C>T 2354T>G), GTG684GTT (2363G>T), GCG688AGT (2373G>A 2374C>G 2375G>T), ACT689AAA (2377C>A 2378T>A), TCT690TCC (2381T>C), TGG691TGA (2384G>A), CGT693AGA (2388C>A 2390T>A), ACT694ACA (2393T>A), CAG695AAC (2394C>A 2396G>C), ACT696GAA (2397A>G 2398C>A 2399T>A)								

*: Inserts / Deletes / Misaligned / Frameshifts

Analysis details

This analysis was performed with panviral2.64

NGS Details (UN8): Moloney murine leukemia virus

Assembly

**Coverage Length**	222 (1 contig(s))
**Depth Of Coverage**	1.3
**Number Of Reads**	2
**Reads Per Million**	0.03 rpm (after QC)
**Ambiguities**	0
**Assembly Method**	de novo + reference guided assembly
**Consensus Caller**	Bcf Tools

Coverage Map

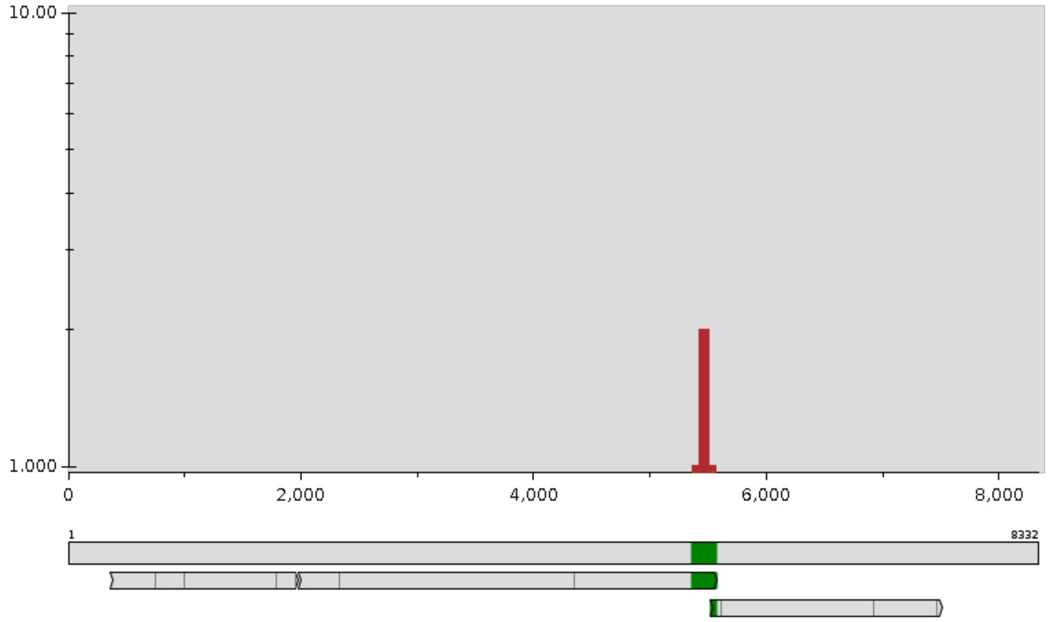

Assignment

**Type**	Moloney murine leukemia virus (Taxonomy ID: 11801)
**Reference Genome**	NC_001501.1
**NT Identity (%)**	81.2207
**AA Identity (%)**	78.2609
**Number Of Stop Codons**	1
**Number Of CDS**	3

Alignment

**Alignment Score**	248.0 (NT) + 498.0 (AA) = 746.0
**Concordance (%)**	72.8516

**Alignment Method**	Global, seeded, nucleotide + amino acids (AGA)

Genome Region

Sequence starts at position 5355 and ends at position 5576 relative to NC_001501.1 reference sequence.

Alignment Detailed Statistics

	Begin	End	Coverage	Score	Concordance	Matches	Identities	I/D/M/F*	Stop Codons
**NT**	**5355**	**5576**	**2.7%**	**248**	**60.8%**	**213 (95.9%)**	**173 (77.9%)**	**0/9**	
Mutations:	5357A>G, 5373C>T, 5381T>C, 5396T>C, 5402A>C, 5426C>T, 5445G>T, 5447A>T, 5450T>G, 5465C>T, 5471G>A, 5477T>G, 5480C>G, 5481G>A, 5482A>C, 5483C>A, 5484C>A, 5486C>T, 5487_5495delGGGGGTGGA, 5498A>T, 5499T>C, 5501C>A, 5502T>G, 5504T>C, 5505A>G, 5508C>A, 5509T>C, 5510G>A, 5511A>G, 5517C>A, 5518G>A, 5519C>G, 5522T>C, 5525A>G, 5528C>T, 5537C>T, 5546A>G, 5552G>A, 5561C>T, 5563A>G, 5572A>G								
**CDS**									
gag-pol	1666	1738	4.2%	421	86.6%	70 (95.9%)	60 (82.2%)	0/3/0/0	1
Protein mutations:	A1696S (5445G>T 5447A>T), D1708T (5481G>A 5482A>C 5483C>A), P1709T (5484C>A 5486C>T), G1710_G1712del (5487_5495delGGGGGTGGA), S1714P (5499T>C 5501C>A), S1715A (5502T>G 5504T>C), R1716G (5505A>G), L1717T (5508C>A 5509T>C 5510G>A), T1718A (5511A>G), R1720K (5517C>A 5518G>A 5519C>G), E1735G (5563A>G)								
Codon mutations:	CGA1666CGG (5357A>G), CTA1672TTA (5373C>T), CCT1674CCC (5381T>C), CCT1679CCC (5396T>C), ACA1681ACC (5402A>C), GCC1689GCT (5426C>T), GCA1696TCT (5445G>T 5447A>T), GCT1697GCG (5450T>G), GCC1702GCT (5465C>T), GTG1704GTA (5471G>A), GCT1706GCG (5477T>G), GCC1707GCG (5480C>G), GAC1708ACA (5481G>A 5482A>C 5483C>A), CCC1709ACT (5484C>A 5486C>T), GGG1710_GGA1712del (5487_5495delGGGGGTGGA), CCA1713CCT (5498A>T), TCC1714CCA (5499T>C 5501C>A), TCT1715GCC (5502T>G 5504T>C), AGA1716GGA (5505A>G), CTG1717ACA (5508C>A 5509T>C 5510G>A), ACA1718GCA (5511A>G), CGC1720AAG (5517C>A 5518G>A 5519C>G), GTT1721GTC (5522T>C), CAA1722CAG (5525A>G), CGC1723CGT (5528C>T), AAC1726AAT (5537C>T), AAA1729AAG (5546A>G), AGG1731AGA (5552G>A), CGC1734CGT (5561C>T), GAG1735GGG (5563A>G), TAA1738TGA (5572A>G)								
env	1	22	3.3%	77	54.2%	22 (100%)	12 (54.5%)	0/0/0/0	0
Protein mutations:	A2E (5517C>A 5518G>A), R3G (5519C>G), S4P (5522T>C), T5A (5525A>G), L6F (5528C>T), P9S (5537C>T), N12D (5546A>G), V14I (5552G>A), R17W (5561C>T 5563A>G)								
Codon mutations:	GCG2GAA (5517C>A 5518G>A), CGT3GGT (5519C>G), TCA4CCA (5522T>C), ACG5GCG (5525A>G), CTC6TTC (5528C>T), CCC9TCC (5537C>T), AAT12GAT (5546A>G), GTT14ATT (5552G>A), CGA17TGG (5561C>T 5563A>G), CTA20CTG (5572A>G)								
**Proteins**									
Pr180 (NP_057933.2)	1666	1738	4.2%	421	86.6%	70 (95.9%)	60 (82.2%)	0/3/0/0	1
Protein mutations:	A1696S (5445G>T 5447A>T), D1708T (5481G>A 5482A>C 5483C>A), P1709T (5484C>A 5486C>T), G1710_G1712del (5487_5495delGGGGGTGGA), S1714P (5499T>C 5501C>A), S1715A (5502T>G 5504T>C), R1716G (5505A>G), L1717T (5508C>A 5509T>C 5510G>A), T1718A (5511A>G), R1720K (5517C>A 5518G>A 5519C>G), E1735G (5563A>G)								
Codon mutations:	CGA1666CGG (5357A>G), CTA1672TTA (5373C>T), CCT1674CCC (5381T>C), CCT1679CCC (5396T>C), ACA1681ACC (5402A>C), GCC1689GCT (5426C>T), GCA1696TCT (5445G>T 5447A>T), GCT1697GCG (5450T>G), GCC1702GCT (5465C>T), GTG1704GTA (5471G>A), GCT1706GCG (5477T>G), GCC1707GCG (5480C>G), GAC1708ACA (5481G>A 5482A>C 5483C>A), CCC1709ACT (5484C>A 5486C>T), GGG1710_GGA1712del (5487_5495delGGGGGTGGA), CCA1713CCT (5498A>T), TCC1714CCA (5499T>C 5501C>A), TCT1715GCC (5502T>G 5504T>C), AGA1716GGA (5505A>G), CTG1717ACA (5508C>A 5509T>C 5510G>A), ACA1718GCA (5511A>G), CGC1720AAG (5517C>A 5518G>A 5519C>G), GTT1721GTC (5522T>C), CAA1722CAG (5525A>G), CGC1723CGT (5528C>T), AAC1726AAT (5537C>T), AAA1729AAG (5546A>G), AGG1731AGA (5552G>A), CGC1734CGT (5561C>T), GAG1735GGG (5563A>G), TAA1738TGA (5572A>G)								
p46 IN (NP_955592.1)	336	407	17.7%	420	86.6%	69 (95.8%)	59 (81.9%)	0/3/0/0	0
Protein mutations:	A366S (5445G>T 5447A>T), D378T (5481G>A 5482A>C 5483C>A), P379T (5484C>A 5486C>T), G380_G382del (5487_5495delGGGGGTGGA), S384P (5499T>C 5501C>A), S385A (5502T>G 5504T>C), R386G (5505A>G), L387T (5508C>A 5509T>C 5510G>A), T388A (5511A>G), R390K (5517C>A 5518G>A 5519C>G), E405G (5563A>G)								
Codon mutations:	CGA336CGG (5357A>G), CTA342TTA (5373C>T), CCT344CCC (5381T>C), CCT349CCC (5396T>C), ACA351ACC (5402A>C), GCC359GCT (5426C>T), GCA366TCT (5445G>T 5447A>T), GCT367GCG (5450T>G), GCC372GCT (5465C>T), GTG374GTA (5471G>A), GCT376GCG (5477T>G), GCC377GCG (5480C>G), GAC378ACA (5481G>A 5482A>C 5483C>A), CCC379ACT (5484C>A 5486C>T), GGG380_GGA382del (5487_5495delGGGGGTGGA), CCA383CCT (5498A>T), TCC384CCA (5499T>C 5501C>A), TCT385GCC (5502T>G 5504T>C), AGA386GGA (5505A>G), CTG387ACA (5508C>A 5509T>C 5510G>A), ACA388GCA (5511A>G), CGC390AAG (5517C>A 5518G>A 5519C>G), GTT391GTC (5522T>C), CAA392CAG (5525A>G), CGC393CGT (5528C>T), AAC396AAT (5537C>T), AAA399AAG (5546A>G), AGG401AGA (5552G>A), CGC404CGT (5561C>T), GAG405GGG (5563A>G)								
gPr80 (NP_057935.1)	1	22	3.3%	77	54.2%	22 (100%)	12 (54.5%)	0/0/0/0	0
Protein mutations:	A2E (5517C>A 5518G>A), R3G (5519C>G), S4P (5522T>C), T5A (5525A>G), L6F (5528C>T), P9S (5537C>T), N12D (5546A>G), V14I (5552G>A), R17W (5561C>T 5563A>G)								
Codon mutations:	GCG2GAA (5517C>A 5518G>A), CGT3GGT (5519C>G), TCA4CCA (5522T>C), ACG5GCG (5525A>G), CTC6TTC (5528C>T), CCC9TCC (5537C>T), AAT12GAT (5546A>G), GTT14ATT (5552G>A), CGA17TGG (5561C>T 5563A>G), CTA20CTG (5572A>G)								

*: Inserts / Deletes / Misaligned / Frameshifts

Analysis details

This analysis was performed with panviral2.64

NGS Details (UN8): Teseptimavirus EG1

Assembly

**Coverage Length**	209 (1 contig(s))
**Depth Of Coverage**	1.2
**Number Of Reads**	2
**Reads Per Million**	0.03 rpm (after QC)
**Ambiguities**	0
**Assembly Method**	de novo + reference guided assembly
**Consensus Caller**	Bcf Tools

Coverage Map

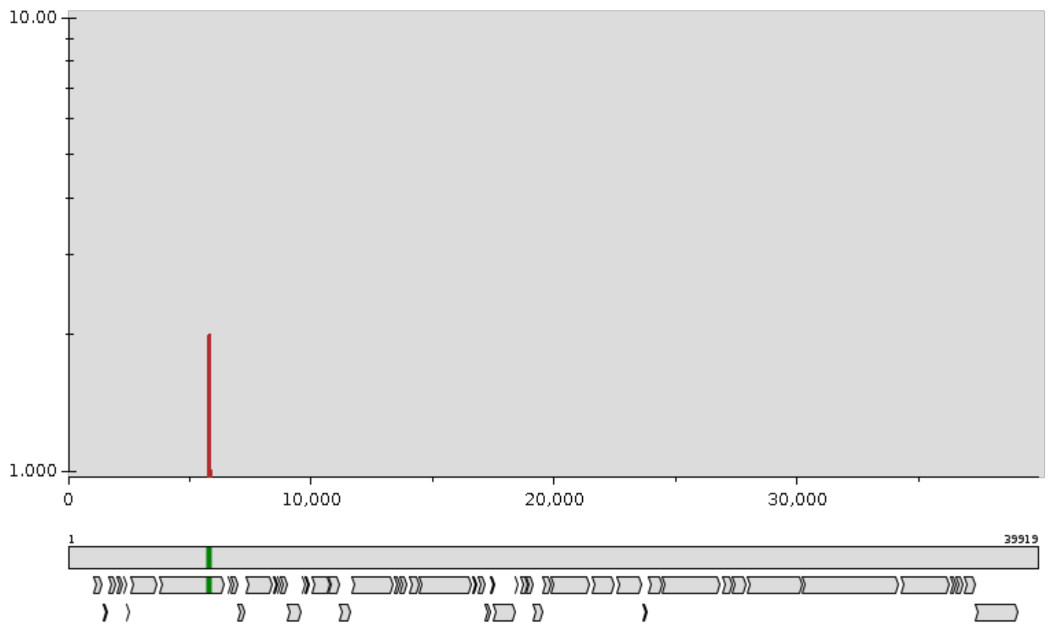

Assignment

**Type**	Teseptimavirus EG1 (Taxonomy ID: 2733876)
**Reference Genome**	NC_047895.1
**NT Identity (%)**	75.1196
**AA Identity (%)**	98.5714
**Number Of Stop Codons**	0
**Number Of CDS**	51

Alignment

**Alignment Score**	210.0 (NT) + 425.0 (AA) = 635.0
**Concordance (%)**	75.3262

Alignment Method	Local, heuristic, nucleotide (BLASTN)

Genome Region

Sequence starts at position 5710 and ends at position 5918 relative to NC_047895.1 reference sequence.

Alignment Detailed Statistics

	Begin	End	Coverage	Score	Concordance	Matches	Identities	I/D/M/F*	Stop Codons
**NT**	**5710**	**5918**	**0.5%**	**210**	**50.2%**	**209 (100%)**	**157 (75.1%)**	**0/0**	
Mutations:	5712A>C, 5715T>G, 5718T>C, 5721T>C, 5722T>A, 5723C>G, 5724A>C, 5727C>T, 5733T>A, 5734C>T, 5745C>G, 5751T>C, 5754T>C, 5757G>A, 5760C>A, 5763T>A, 5766G>T, 5775T>G, 5778G>A, 5779C>T, 5791T>A, 5792C>G, 5793A>T, 5796G>T, 5797A>T, 5798G>C, 5799C>G, 5808A>C, 5814T>C, 5820A>T, 5826T>C, 5832C>T, 5838T>G, 5841G>A, 5842T>A, 5843C>G, 5847T>G, 5853G>A, 5854C>T, 5856G>A, 5859G>C, 5862T>A, 5865T>C, 5868G>A, 5871C>T, 5874A>G, 5883G>A, 5886T>G, 5889T>C, 5892G>A, 5895T>C, 5898C>G								
**CDS**									
HOS48_gp08	657	726	7.9%	425	97.7%	70 (100%)	69 (98.6%)	0/0/0/0	0
**Proteins**									
DNA-dependent RNA polymerase (YP_009795804.1)	657	726	7.9%	425	97.7%	70 (100%)	69 (98.6%)	0/0/0/0	0
Protein mutations:	*none*								
Codon mutations:	CCA657CCC (5712A>C), GCT658GCG (5715T>G), ATT659ATC (5718T>C), GAT660GAC (5721T>C), TCA661AGC (5722T>A 5723C>G 5724A>C), GGC662GGT (5727C>T), GGT664GGA (5733T>A), CTG665TTG (5734C>T), ACC668ACG (5745C>G), CCT670CCC (5751T>C), AAT671AAC (5754T>C), CAG672CAA (5757G>A), GCC673GCA (5760C>A), GCT674GCA (5763T>A), GGG675GGT (5766G>T), GCT678GCG (5775T>G), AAG679AAA (5778G>A), CTG680TTG (5779C>T), TCA684AGT (5791T>A 5792C>G 5793A>T), GTG685GTT (5796G>T), AGC686TCG (5797A>T 5798G>C 5799C>G), GTA689GTC (5808A>C), GCT691GCC (5814T>C), GTA693GTT (5820A>T), GCT695GCC (5826T>C), AAC697AAT (5832C>T), CTT699CTG (5838T>G), AAG700AAA (5841G>A), TCT701AGT (5842T>A 5843C>G), GCT702GCG (5847T>G), AAG704AAA (5853G>A), CTG705TTA (5854C>T 5856G>A), CTG706CTC (5859G>C), GCT707GCA (5862T>A), GCT708GCC (5865T>C), GAG709GAA (5868G>A), GTC710GTT (5871C>T), AAA711AAG (5874A>G), AAG714AAA (5883G>A), ACT715ACG (5886T>G), GGT716GGC (5889T>C), GAG717GAA (5892G>A), ATT718ATC (5895T>C), CTC719CTG (5898C>G)								

*: Inserts / Deletes / Misaligned / Frameshifts

Analysis details

This analysis was performed with panviral2.64

NGS Details (UN8): Duamitovirus peex1

Assembly

**Coverage Length**	135 (1 contig(s))
**Depth Of Coverage**	1.0
**Number Of Reads**	1
**Reads Per Million**	0.02 rpm (after QC)
**Ambiguities**	0
**Assembly Method**	de novo + reference guided assembly
**Consensus Caller**	Bcf Tools

Coverage Map

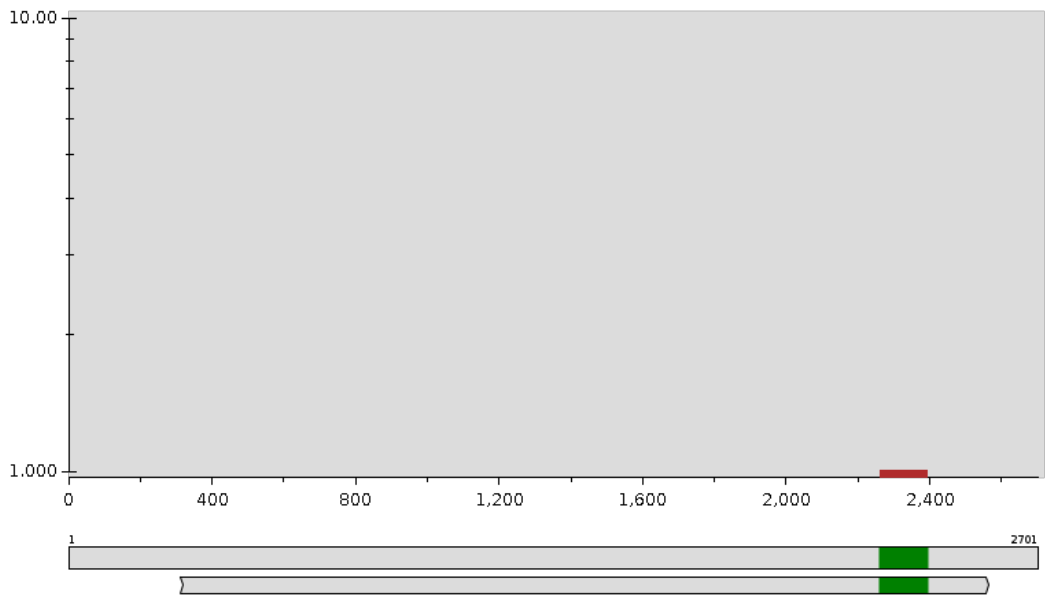

Assignment

**Type**	Duamitovirus peex1 (Taxonomy ID: 2955799)
**Reference Genome**	NC_076525.1
**NT Identity (%)**	80.0
**AA Identity (%)**	80.0
**Number Of Stop Codons**	0
**Number Of CDS**	1

Alignment

**Alignment Score**	162.0 (NT) + 300.0 (AA) = 462.0
**Concordance (%)**	72.3005

Alignment Method	Global, seeded, nucleotide + amino acids (AGA)

Genome Region

Sequence starts at position 2262 and ends at position 2396 relative to NC_076525.1 reference sequence.

Alignment Detailed Statistics

	Begin	End	Coverage	Score	Concordance	Matches	Identities	I/D/M/F*	Stop Codons
**NT**	**2262**	**2396**	**5.0%**	**162**	**60.0%**	**135 (100%)**	**108 (80.0%)**	**0/0**	
Mutations:	2268G>A, 2270A>C, 2272G>T, 2274C>A, 2283G>A, 2292C>T, 2294T>G, 2295C>A, 2296G>C, 2297A>T, 2306A>T, 2315C>T, 2321G>A, 2325A>T, 2326A>T, 2327T>A, 2330C>A, 2333G>A, 2336A>T, 2345A>T, 2348C>T, 2354T>A, 2355A>T, 2357G>T, 2360A>T, 2363G>T, 2375G>C								
**CDS**									
RdRp	651	695	6.0%	300	81.3%	45 (100%)	36 (80.0%)	0/0/0/0	0
Protein mutations:	V653I (2268G>A 2270A>C), R654L (2272G>T), H655N (2274C>A), E658K (2283G>A), R662T (2295C>A 2296G>C 2297A>T), N672L (2325A>T 2326A>T 2327T>A), E675D (2336A>T), M682F (2355A>T 2357G>T), E683D (2360A>T)								
Codon mutations:	GTA653ATC (2268G>A 2270A>C), CGT654CTT (2272G>T), CAT655AAT (2274C>A), GAA658AAA (2283G>A), CTT661TTG (2292C>T 2294T>G), CGA662ACT (2295C>A 2296G>C 2297A>T), TCA665TCT (2306A>T), CAC668CAT (2315C>T), GTG670GTA (2321G>A), AAT672TTA (2325A>T 2326A>T 2327T>A), TCC673TCA (2330C>A), CCG674CCA (2333G>A), GAA675GAT (2336A>T), ATA678ATT (2345A>T), GAC679GAT (2348C>T), CTT681CTA (2354T>A), ATG682TTT (2355A>T 2357G>T), GAA683GAT (2360A>T), GTG684GTT (2363G>T), GCG688GCC (2375G>C)								
**Proteins**									
RNA-dependent RNA polymerase (YP_010798875.1)	651	695	6.0%	300	81.3%	45 (100%)	36 (80.0%)	0/0/0/0	0
Protein mutations:	V653I (2268G>A 2270A>C), R654L (2272G>T), H655N (2274C>A), E658K (2283G>A), R662T (2295C>A 2296G>C 2297A>T), N672L (2325A>T 2326A>T 2327T>A), E675D (2336A>T), M682F (2355A>T 2357G>T), E683D (2360A>T)								
Codon mutations:	GTA653ATC (2268G>A 2270A>C), CGT654CTT (2272G>T), CAT655AAT (2274C>A), GAA658AAA (2283G>A), CTT661TTG (2292C>T 2294T>G), CGA662ACT (2295C>A 2296G>C 2297A>T), TCA665TCT (2306A>T), CAC668CAT (2315C>T), GTG670GTA (2321G>A), AAT672TTA (2325A>T 2326A>T 2327T>A), TCC673TCA (2330C>A), CCG674CCA (2333G>A), GAA675GAT (2336A>T), ATA678ATT (2345A>T), GAC679GAT (2348C>T), CTT681CTA (2354T>A), ATG682TTT (2355A>T 2357G>T), GAA683GAT (2360A>T), GTG684GTT (2363G>T), GCG688GCC (2375G>C)								

*: Inserts / Deletes / Misaligned / Frameshifts

Analysis details

This analysis was performed with panviral2.64

NGS Details (UN8): Cyvirus cyprinidallo3

Assembly

**Coverage Length**	90 (1 contig(s))
**Depth Of Coverage**	1.0
**Number Of Reads**	1
**Reads Per Million**	0.02 rpm (after QC)
**Ambiguities**	0
**Assembly Method**	de novo + reference guided assembly
**Consensus Caller**	Bcf Tools

Coverage Map

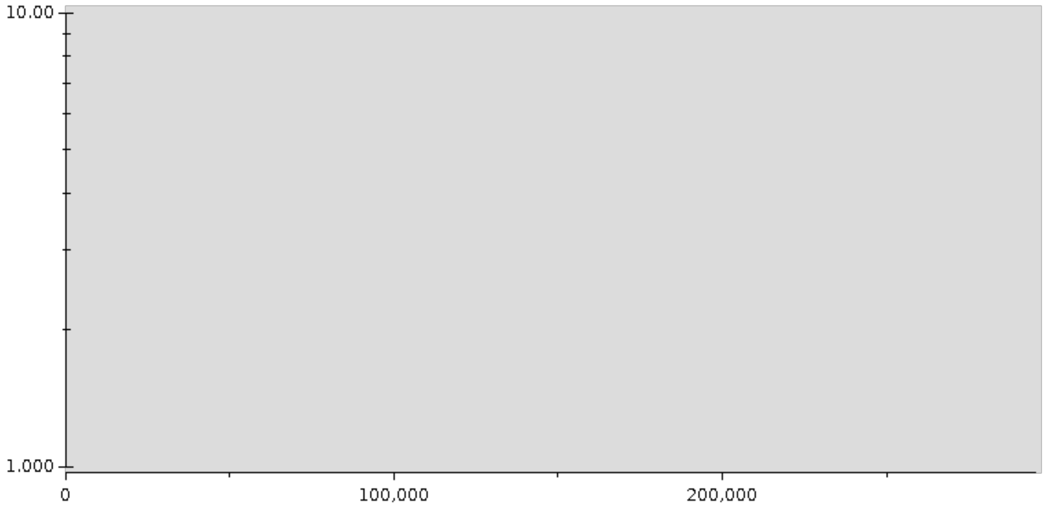

Assignment

**Type**	Cyvirus cyprinidallo3 (Taxonomy ID: 3050271)
**Reference Genome**	NC_009127.1
**NT Identity (%)**	83.3333
**AA Identity (%)**	80.0
**Number Of Stop Codons**	0
**Number Of CDS**	163

Alignment

**Alignment Score**	120.0 (NT) + 177.0 (AA) = 297.0
**Concordance (%)**	74.25
**Alignment Method**	Local, heuristic, nucleotide (BLASTN)

Genome Region

Sequence starts at position 43055 and ends at position 43144 relative to NC_009127.1 reference sequence.

Alignment Detailed Statistics

	Begin	End	Coverage	Score	Concordance	Matches	Identities	I/D/M/F*	Stop Codons
**NT**	**43055**	**43144**	**0.1%**	**120**	**66.7%**	**90 (100%)**	**75 (83.3%)**	**0/0**	

Mutations: 43062A>G, 43065C>A, 43067G>C, 43068T>A, 43069G>A, 43082T>C, 43088G>A, 43097G>C, 43098T>C, 43100G>T, 43102A>C, 43103G>T, 43118T>C, 43133C>T, 43135C>G

*: Inserts / Deletes / Misaligned / Frameshifts

Analysis details

This analysis was performed with panviral2.64

NGS Details (UN8): Diplodia scrobiculata RNA virus 1

Assembly

**Coverage Length**	120 (1 contig(s))
**Depth Of Coverage**	1.0
**Number Of Reads**	1
**Reads Per Million**	0.02 rpm (after QC)
**Ambiguities**	0
**Assembly Method**	de novo + reference guided assembly
**Consensus Caller**	Bcf Tools

Coverage Map

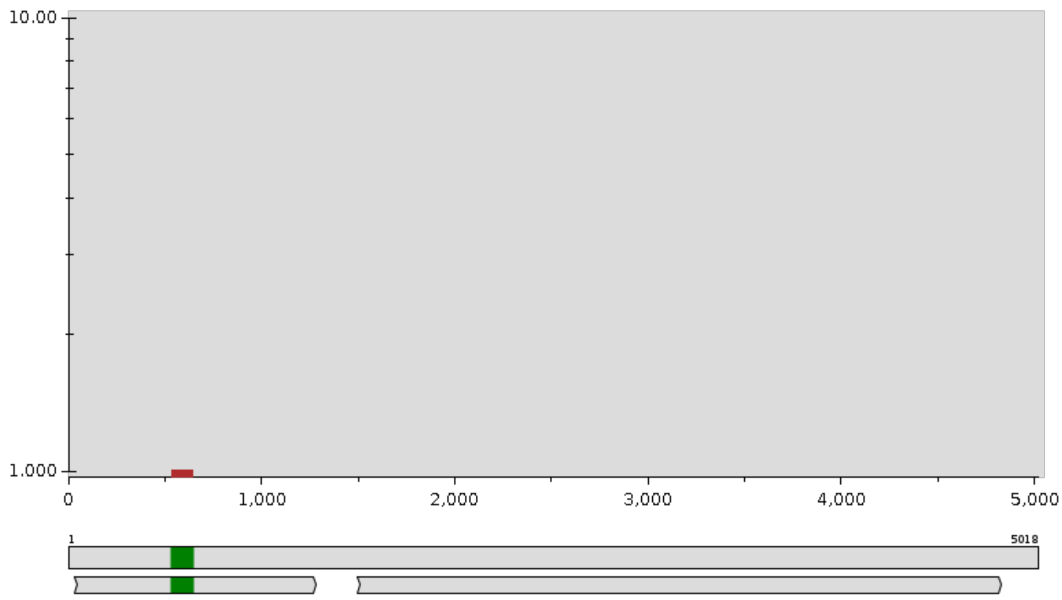

Assignment

**Type**	Diplodia scrobiculata RNA virus 1 (Taxonomy ID: 459770)
**Reference Genome**	NC_013699.1
**NT Identity (%)**	80.8333
**AA Identity (%)**	80.0
**Number Of Stop Codons**	0
**Number Of CDS**	2

Alignment

**Alignment Score**	148.0 (NT) + 195.0 (AA) = 343.0
**Concordance (%)**	72.6695

**Alignment Method**	Global, seeded, nucleotide + amino acids (AGA)

Genome Region

Sequence starts at position 532 and ends at position 651 relative to NC_013699.1 reference sequence.

Alignment Detailed Statistics

	Begin	End	Coverage	Score	Concordance	Matches	Identities	I/D/M/F*	Stop Codons
**NT**	**532**	**651**	**2.4%**	**148**	**61.7%**	**120 (100%)**	**97 (80.8%)**	**0/0**	
Mutations:	536C>G, 560G>A, 564C>G, 566T>G, 581C>T, 585A>C, 587A>C, 590A>T, 603G>T, 608A>G, 616C>G, 617G>C, 621A>G, 623C>G, 624A>C, 625A>G, 626G>C, 628A>G, 629C>T, 632G>T, 641G>A, 644A>C, 648C>T								
**CDS**									
DSRV-1_gp1	169	208	9.6%	195	79.3%	40 (100%)	32 (80.0%)	0/0/0/0	0
Protein mutations:	P179A (564C>G 566T>G), E187D (590A>T), A192S (603G>T), A196G (616C>G 617G>C), I198V (621A>G 623C>G), K199R (624A>C 625A>G 626G>C), N200S (628A>G 629C>T)								
Codon mutations:	CCC169CCG (536C>G), CAG177CAA (560G>A), CCT179GCG (564C>G 566T>G), CTC184CTT (581C>T), AGA186CGC (585A>C 587A>C), GAA187GAT (590A>T), GCG192TCG (603G>T), GAA193GAG (608A>G), GCG196GGC (616C>G 617G>C), ATC198GTG (621A>G 623C>G), AAG199CGC (624A>C 625A>G 626G>C), AAC200AGT (628A>G 629C>T), GTG201GTT (632G>T), CTG204CTA (641G>A), CTA205CTC (644A>C), CTG207TTG (648C>T)								
**Proteins**									
hypothetical protein (YP_003359177.1)	169	208	9.6%	195	79.3%	40 (100%)	32 (80.0%)	0/0/0/0	0
Protein mutations:	P179A (564C>G 566T>G), E187D (590A>T), A192S (603G>T), A196G (616C>G 617G>C), I198V (621A>G 623C>G), K199R (624A>C 625A>G 626G>C), N200S (628A>G 629C>T)								
Codon mutations:	CCC169CCG (536C>G), CAG177CAA (560G>A), CCT179GCG (564C>G 566T>G), CTC184CTT (581C>T), AGA186CGC (585A>C 587A>C), GAA187GAT (590A>T), GCG192TCG (603G>T), GAA193GAG (608A>G), GCG196GGC (616C>G 617G>C), ATC198GTG (621A>G 623C>G), AAG199CGC (624A>C 625A>G 626G>C), AAC200AGT (628A>G 629C>T), GTG201GTT (632G>T), CTG204CTA (641G>A), CTA205CTC (644A>C), CTG207TTG (648C>T)								

*: Inserts / Deletes / Misaligned / Frameshifts

Analysis details

This analysis was performed with panviral2.64
