# Supplementary material for: Nucleotide sequence analysis reveals the presence of PVY-Tam isolates affecting tamarillo in Colombia
Source: Virol J. 2026 Apr 20;23:145. doi: 10.1186/s12985-026-03166-6 (PMC13234967; doi:10.1186/s12985-026-03166-6)
Supplement: Supplementary file 3 — Additional file 3. [file 12985_2026_3166_MOESM3_ESM.pdf]

Analysis of UN18_val

**Technology**	Paired-end short reads
**Input Files**	UN18_val_R1.fq.gz (1.71 GB), UN18_val_R2.fq.gz (1.73 GB)
**Submitted On**	2023-09-26 06:46:26 UTC
**Duration**	3h 31m 58s
**Tool Version**	panviral2.64
**Location**	https://www.genomedetective.com/db/ui/analysis/01461f33-a0b9-4228-b7b0-a45adba24853

Statistics

**Original Read Length**	20 - 150
**Trimmed Read Length**	50 - 135

	# Reads	% of Reads
**Input file**	53669874	100.0%
**After QC**	53280916	99.3%
**After filtering**	2405890	4.5%
**Mapped back to contigs**	232586	0.4%

Assignments

Assignment	No. of Reads	Depth of Coverage	Identity		Genome Coverage	
			NT	AA		
Torradovirus lycopersici (2 segments out of 2)	51427	582.9	86.8%	91.7%	81.0%	
Torradovirus lycopersici (segment RNA 2)	38768	1183.7	83.8%	86.2%	72.7%	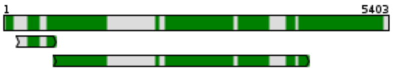
Torradovirus lycopersici (segment RNA 1)	12659	234.6	88.5%	94.1%	86.7%	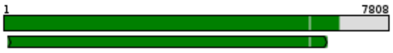
Torradovirus marchitezum (2 segments out of 2)	31334	381.8	65.1%	68.6%	85.5%	
Torradovirus marchitezum (segment RNA 2)	26259	709.3	64.3%	68.0%	94.7%	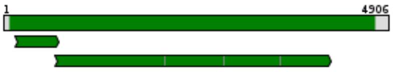
Torradovirus marchitezum (segment RNA 1)	5075	116.5	65.8%	69.0%	79.3%	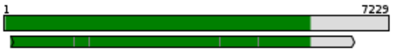
Tomato necrotic dwarf virus (segment RNA1)	21581	468.2	64.6%	66.1%	84.2%	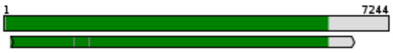
Diachasmimorpha longicaudata entomopoxvirus (2 segments out of 13)	18634	1786.1	61.3%	59.4%	N/A	

Assignment	No. of Reads	Depth of Coverage	Identity		Genome Coverage	
			NT	AA		
Diachasmimorpha longicaudata entomopoxvirus	581	361.0	76.9%	90.9%	0.1%	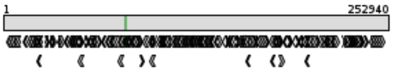
Diachasmimorpha longicaudata entomopoxvirus (segment NC_043455.1)	18053	1953.9	59.5%	55.8%	84.9%	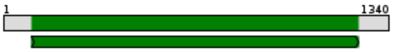
Bracoviriform glomeratae (segment NC_043292.1)	11330	4199.5	72.8%	84.1%	75.8%	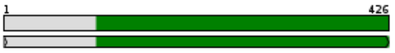
Potato leafroll virus	4303	98.0	98.1%	97.3%	97.4%	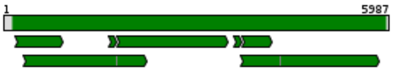
Duamitovirus soch1	853	45.1	68.9%	68.4%	82.0%	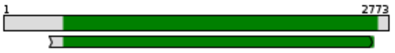
Solendovirus venanicotianae	218	15.1	78.0%	74.0%	21.4%	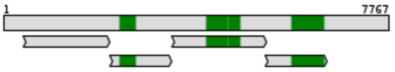
Potato virus Y	45	3.1	85.2%	91.1%	19.3%	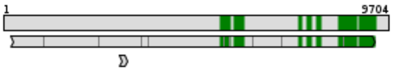
Duamitovirus peex1	32	5.3	69.7%	67.5%	26.1%	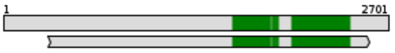
Harvey murine sarcoma virus	6	2.2	73.4%	85.0%	22.3%	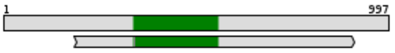

Discoveries

Similar to	No. of Reads	Depth of Coverage	Identity		Genome Coverage	
			NT	AA		
Brazilian marseillevirus	31766	13067.1	81.7%	94.7%	0.1%	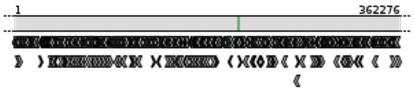
Tomato chocolate spot virus (3 segments out of 2)	23700	1253.2	72.1%	71.7%	18.2%	
Tomato chocolate spot virus (segment RNA2)	14899	1610.5	67.0%	74.8%	21.8%	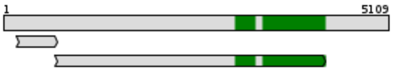
Tomato chocolate spot virus (segment RNA 1)	6736	1872.0	78.3%	74.4%	5.4%	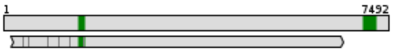
Tomato chocolate spot virus (segment RNA 1)	2065	416.7	76.2%	13.6%	10.3%	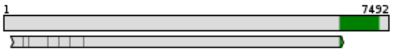
Tokyovirus A1	12764	4800.1	76.8%	90.7%	0.0%	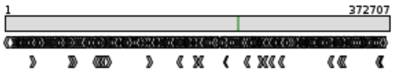
Leucotheavirus sp4	6064	880.3	75.9%	85.4%	0.5%	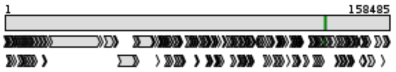

Similar to	No. of Reads	Depth of Coverage	Identity		Genome Coverage	
			NT	AA		
Noumeavirus	4184	2766.6	81.7%	92.2%	0.0%	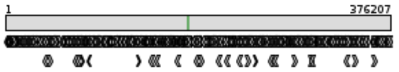
Betabaculovirus disaccharalis	2638	1580.3	82.4%	92.9%	0.1%	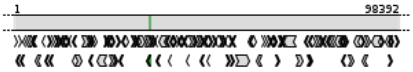
Lausannevirus	2565	1524.8	83.3%	100.0%	0.0%	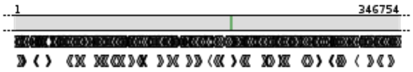
Brazilian marseillevirus	2433	558.0	81.6%	95.9%	0.1%	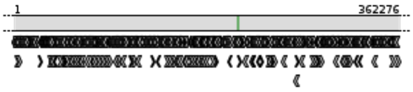
Betabaculovirus disaccharalis	2060	1390.5	83.7%	88.4%	0.1%	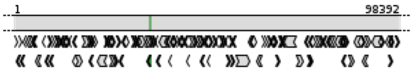
Bracoviriform congregatae (segment Circle 7)	1498	965.9	77.3%	91.2%	0.7%	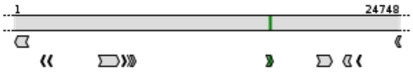
Tunisvirus fontaine2	895	465.1	80.0%	91.4%	0.0%	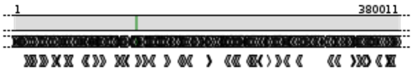
Lowelvirus tuscon4d	634	106.0	76.8%	88.2%	0.3%	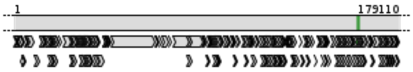
Cassava brown streak virus	450	103.8	60.5%	57.5%	5.8%	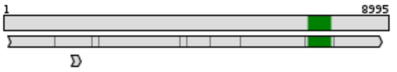
Human gammaherpesvirus 8 (subtype: Could not assign)	294	183.0	83.2%	84.6%	0.1%	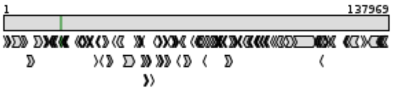
Cladosporium fulvum T-1 virus	179	15.0	53.1%	42.6%	16.3%	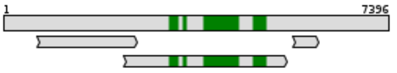
Errantivirus	149	16.0	56.1%	44.8%	9.6%	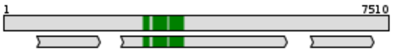
Marseillevirus marseillevirus	75	52.2	81.7%	97.4%	0.0%	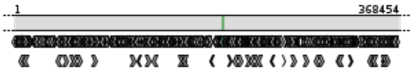
Badnavirus occultipomeae	66	25.5	58.8%	49.5%	3.2%	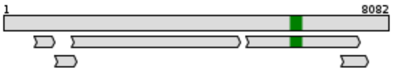
Caulimovirus venafragariae	48	9.9	58.3%	54.9%	7.2%	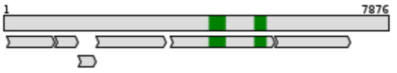
Badnavirus occultipomeae	38	14.4	60.5%	51.8%	3.1%	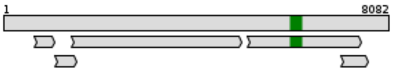

Similar to	No. of Reads	Depth of Coverage	Identity NT	Identity AA	Genome Coverage	
Brazilian marseillevirus	33	15.2	78.5%	91.2%	0.0%	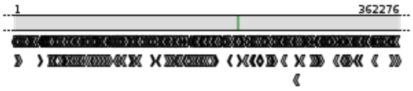
Rahariannevirus raharianne	31	2.4	69.8%	65.3%	3.5%	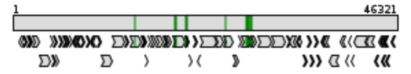
Caulimovirus venafragariae	30	4.8	58.1%	52.9%	10.6%	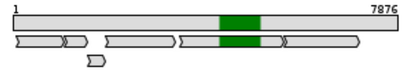
Badnavirus maculapiperis	30	13.7	57.9%	53.2%	3.1%	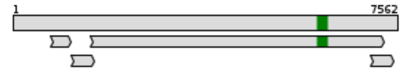
Epiphyllum badnavirus 1	30	12.9	57.1%	49.4%	3.1%	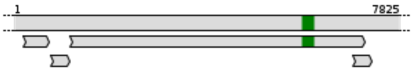
Badnavirus epsiloninflatheobromae	23	3.9	57.4%	48.6%	8.7%	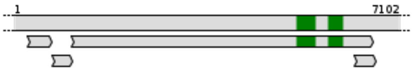
Dioscovirus dioscoreae	23	3.2	61.8%	52.6%	11.2%	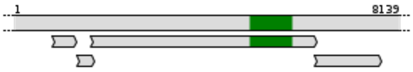
Caulimovirus minutangelicae	17	6.0	54.9%	46.5%	4.7%	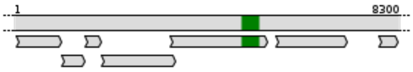
Caulimovirus venafragariae	17	3.8	56.1%	51.2%	7.7%	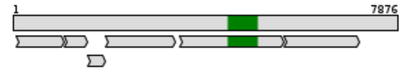
Badnavirus maculasmallanthi	13	5.8	56.3%	48.9%	3.7%	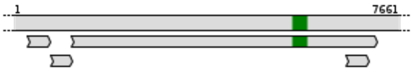
Pinus nigra virus 1	13	3.8	58.0%	48.3%	6.1%	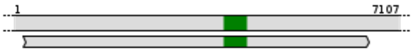
Hibiscus bacilliform virus GD1	12	6.1	56.3%	47.5%	3.2%	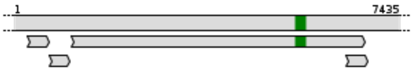
Caulimovirus tessellomirabilis	11	3.1	58.2%	47.7%	5.9%	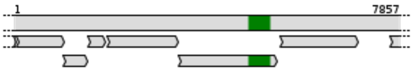
Badnavirus tessellocastaneae	6	2.8	55.9%	54.5%	3.7%	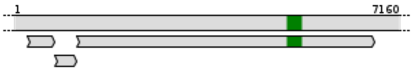
Betabaculovirus erellonis	6	5.9	82.4%	93.3%	0.1%	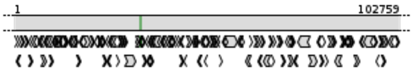
Gihfavirus pelohabitans	4	2.3	97.4%	0.0%	3.5%	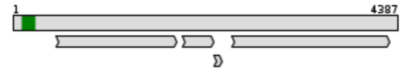
Duamitovirus dapi1	4	3.8	69.4%	66.7%	4.8%	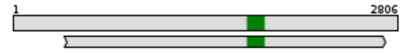

Similar to	No. of Reads	Depth of Coverage	Identity		Genome Coverage	
			NT	AA		
Fusarium graminearum mycotymovirus 1	4	2.0	69.5%	76.7%	3.4%	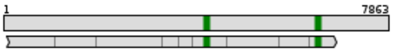
Tokyovirus A1	4	2.9	77.0%	93.3%	0.0%	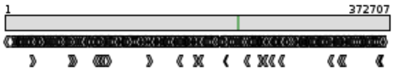
Botryotinia fuckeliana partitivirus 1 (segment RNA 1)	4	3.6	68.1%	71.1%	7.5%	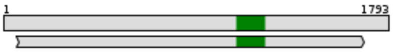
Gammaflexivirus PaGFV-1	2	2.0	80.0%	86.7%	1.4%	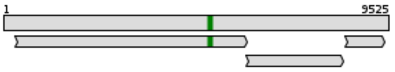
Sugarcane chlorotic streak virus	2	1.9	62.8%	55.8%	4.4%	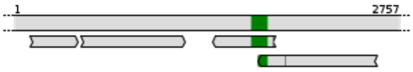
Seussvirus seuss	2	1.1	76.3%	79.5%	0.3%	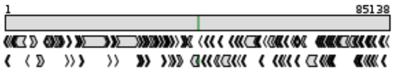
Fusarium poae narnavirus 2	2	1.0	74.8%	74.4%	12.0%	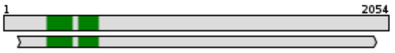

NGS Details (UN18_val): Diachasmimorpha longicaudata entomopoxvirus

Assembly

**Coverage Length**	134 (1 contig(s))
**Depth Of Coverage**	361.0
**Number Of Reads**	581
**Reads Per Million**	10.90 rpm (after QC)
**Ambiguities**	0
**Assembly Method**	de novo + reference guided assembly
**Consensus Caller**	Bcf Tools

Coverage Map

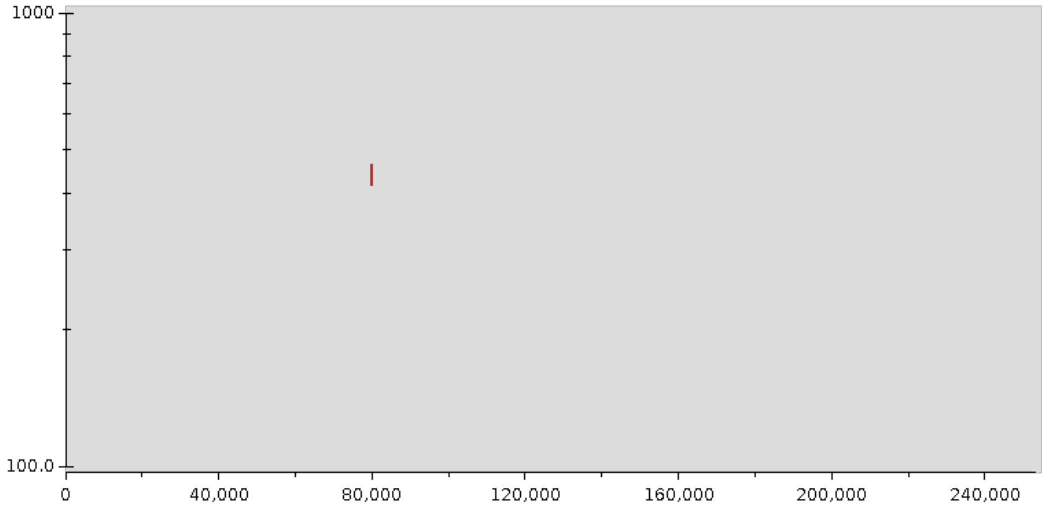

Assignment

**Type**	Diachasmimorpha longicaudata entomopoxvirus (Taxonomy ID: 109981)
**Reference Genome**	NC_076102.1
**NT Identity (%)**	76.8657
**AA Identity (%)**	90.9091
**Number Of Stop Codons**	0
**Number Of CDS**	193

Alignment

**Alignment Score**	144.0 (NT) + 293.0 (AA) = 437.0
**Concordance (%)**	74.0678
**Alignment Method**	Local, heuristic, nucleotide (BLASTN)

Genome Region

Sequence starts at position 79871 and ends at position 80004 relative to NC_076102.1 reference sequence.

Alignment Detailed Statistics

	Begin	End	Coverage	Score	Concordance	Matches	Identities	I/D/M/F*	Stop Codons
**NT**	**79871**	**80004**	**0.1%**	**144**	**53.7%**	**134 (100%)**	**103 (76.9%)**	**0/0**	

Mutations: 79886G>A, 79894T>C, 79895C>A, 79898T>C, 79904G>A, 79910T>A, 79912T>G, 79917C>G, 79918T>A, 79919T>G, 79921T>G, 79928A>G, 79934T>A, 79937A>G, 79940A>G, 79941T>A, 79942T>G, 79943T>C, 79949A>G, 79952A>G, 79954G>T, 79958C>A, 79960A>G, 79961A>G, 79964G>A, 79970G>A, 79973A>C, 79976T>C, 79982T>C, 79984T>C, 79985T>C

*: Inserts / Deletes / Misaligned / Frameshifts

Analysis details

This analysis was performed with panviral2.64

NGS Details (UN18_val): Diachasmimorpha longicaudata entomopoxvirus (segment NC_043455.1)

Assembly

**Coverage Length**	1138 (1 contig(s))
**Depth Of Coverage**	1953.9
**Number Of Reads**	18053
**Reads Per Million**	338.83 rpm (after QC)
**Ambiguities**	0
**Assembly Method**	de novo + reference guided assembly
**Consensus Caller**	Bcf Tools

Coverage Map

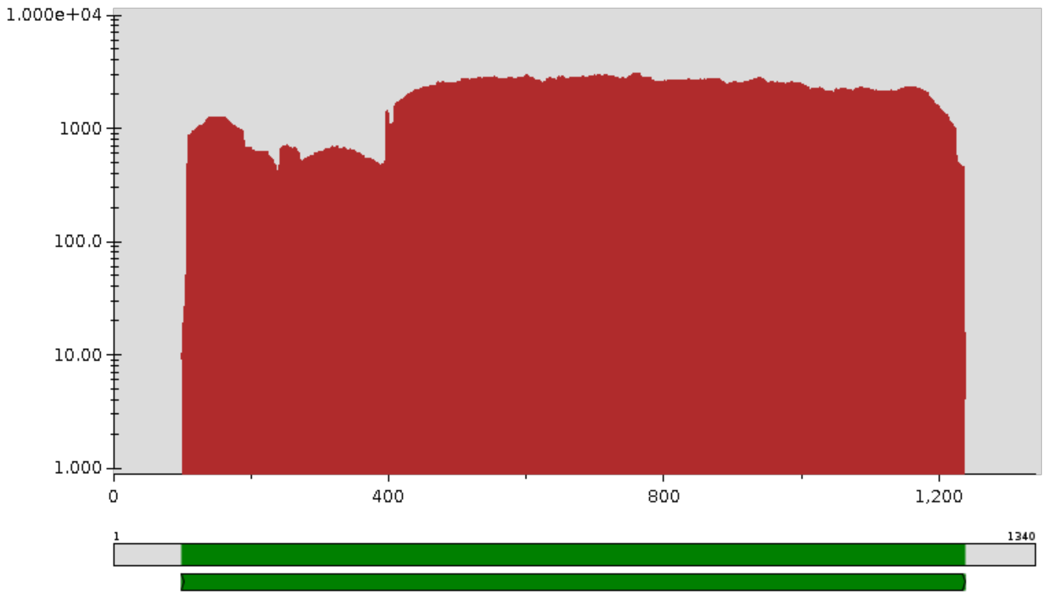

Assignment

**Type**	Diachasmimorpha longicaudata entomopoxvirus (Taxonomy ID: 109981)
**Reference Genome**	NC_043455.1
**NT Identity (%)**	59.4903
**AA Identity (%)**	55.7895
**Number Of Stop Codons**	0
**Number Of CDS**	1

Alignment

**Alignment Score**	414.0 (NT) + 1484.0 (AA) = 1898.0
**Concordance (%)**	39.9159

Alignment Method	Global, seeded, nucleotide + amino acids (AGA)

Genome Region

Sequence starts at position 101 and ends at position 1238 relative to NC_043455.1 reference sequence.

Alignment Detailed Statistics

	Begin	End	Coverage	Score	Concordance	Matches	Identities	I/D/M/F*	Stop Codons
**NT**	**101**	**1238**	**84.9%**	**414**	**18.3%**	**1135 (99.5%)**	**677 (59.3%)**	**3/3**	
Mutations:	110A>G, 112G>T, 117C>A, 119G>A, 120A>G, 121A>T, 127C>T, 128C>A, 129A>C, 130T>C, 136C>T, 137C>T, 139C>G, 140A>C, 142G>A, 143A>G, 148T>C, 149A>C, 151C>T, 154A>G, 156A>G, 157G>A, 160T>C, 163A>C, 167T>G, 169T>C, 171G>A, 175A>T, 181A>G, 187T>A, 190A>T, 191A>G, 193T>C, 199A>G, 204A>G, 207C>G, 208C>T, 211T>A, 212T>G, 214T>G, 217A>T, 219G>T, 221A>T, 222T>G, 223T>C, 224T>A, 225C>A, 226T>G, 230A>C, 231A>T, 232A>T, 235C>T, 238G>C, 241A>T, 242G>C, 243T>A, 244C>T, 247A>G, 256G>T, 262A>C, 268A>G, 271A>C, 274A>C, 276C>T, 277A>G, 278T>A, 279A>T, 283A>T, 284A>C, 286C>T, 288G>C, 289T>C, 294T>G, 295A>C, 298G>A, 299C>G, 300A>T, 301A>T, 302A>G, 304A>G, 310T>C, 311T>A, 312C>A, 313T>A, 320A>G, 322T>G, 323A>G, 325C>T, 335A>G, 337C>A, 341A>G, 343C>T, 346A>T, 352T>G, 355A>G, 356T>C, 358G>T, 361T>A, 362C>G, 363T>C, 364C>T, 368G>A, 370T>G, 371C>G, 374C>A, 375G>A, 376T>G, 379A>G, 380T>A, 382G>T, 383C>T, 384A>T, 385A>G, 386A>G, 394G>A, 395A>G, 397T>C, 403A>G, 404_406delTAT, 407A>G, 408A>G, 409T>C, 410T>G, 412T>G, 415A>G, 416T>G, 417G>T, 421A>T, 423T>C, 428A>G, 433A>G, 436C>T, 439A>C, 443A>G, 446A>C, 447A>G, 448G>T, 451A>G, 452A>G, 453G>A, 454C>T, 458G>C, 459A>G, 460A>T, 462C>T, 463T>C, 466A>T, 467A>C, 471A>G, 472A>T, 472_473insGGT, 474C>T, 475C>T, 478G>T, 481T>A, 482T>G, 485A>G, 490A>T, 496T>A, 499A>C, 502A>T, 503A>G, 505G>C, 506A>T, 511T>C, 512C>A, 514A>G, 517C>G, 518A>C, 519C>G, 521C>A, 523A>G, 524A>C, 526A>G, 527A>T, 528G>C, 530A>C, 532C>T, 533G>C, 534A>G, 535T>C, 536A>C, 538C>T, 539A>G, 541A>C, 542G>C, 543C>A, 544A>C, 547T>C, 550A>G, 553A>G, 554G>T, 559A>T, 560A>T, 562A>G, 574T>C, 577G>A, 581T>C, 583G>C, 584A>T, 585T>C, 586A>T, 587G>A, 588A>G, 589T>A, 590A>G, 591A>G, 595T>C, 596T>A, 597T>A, 602A>C, 604A>G, 607A>T, 608C>T, 610A>T, 616T>A, 620G>C, 622A>G, 623T>C, 628T>G, 629G>C, 630A>C, 632A>C, 633G>C, 634T>A, 635C>A, 637T>A, 638G>A, 646T>G, 647A>G, 648T>G, 650T>G, 652A>T, 655A>C, 658A>T, 661A>C, 664C>T, 665G>A, 667T>G, 671T>C, 674A>G, 675G>A, 678T>C, 680A>C, 682C>T, 683A>G, 685T>G, 687C>T, 688A>T, 689T>A, 692C>A, 693A>G, 694G>A, 695G>A, 696T>A, 697C>G, 700T>C, 705G>A, 706A>C, 707G>A, 709T>A, 712C>T, 715A>G, 717A>G, 718A>G, 724G>T, 727A>G, 730A>G, 731A>C, 732A>G, 735C>A, 739A>G, 740T>C, 742G>C, 745A>T, 748C>T, 757T>C, 759G>A, 765A>T, 770A>G, 772A>C, 778A>T, 779A>G, 781G>C, 784A>G, 785A>G, 787T>A, 790T>G, 792T>G, 793T>G, 796A>G, 797G>C, 798C>T, 799A>T, 805A>T, 810T>G, 811T>C, 820T>C, 823C>G, 824C>A, 825A>C, 830A>G, 831G>C, 833C>A, 838T>C, 841A>G, 843C>G, 844A>T, 845C>G, 847A>C, 850A>C, 854T>G, 855G>T, 856T>G, 859T>C, 862A>C, 863C>A, 865T>G, 866A>C, 868A>T, 869C>A, 878G>T, 879T>G, 880A>G, 885T>C, 886G>T, 889A>T, 890T>A, 891G>A, 892T>G, 893T>A, 895A>G, 896A>C, 897C>G, 898A>C, 900A>G, 901T>C, 902A>C, 904A>T, 905A>G, 908T>C, 909T>A, 915C>T, 919A>T, 920A>G, 921G>C, 922C>A, 924T>C, 925C>T, 934T>C, 938T>G, 939C>A, 943A>G, 944C>A, 946A>C, 947G>A, 948A>C, 949A>T, 950C>A, 952T>A, 956T>A, 958T>C, 965A>C, 966A>G, 970A>G, 973T>C, 974A>C, 977G>T, 978A>C, 982T>G, 983A>T, 984A>C, 986A>T, 989A>C, 991A>T, 992A>G, 994A>G, 997T>C, 998C>A, 1000T>C, 1001T>A, 1007A>G, 1012A>C, 1013T>C, 1015A>T, 1018A>T, 1019A>C, 1021A>T, 1024C>T, 1033T>A, 1036A>G, 1040A>G, 1042A>C, 1045T>C, 1046T>C, 1048A>T, 1051T>C, 1054C>T, 1057T>C, 1063T>C, 1064T>C, 1070C>A, 1071A>C, 1073A>C, 1075T>A, 1076A>C, 1077G>C, 1087T>C, 1088A>C, 1090A>G, 1103A>C, 1105A>T, 1111T>A, 1112A>C, 1120C>A, 1121C>A, 1123A>G, 1126A>G, 1129G>T, 1130A>G, 1141T>C, 1145A>G, 1147A>C, 1150T>C, 1151G>A, 1153A>G, 1156C>T, 1159A>T, 1160T>G, 1161T>A, 1163C>A, 1164C>G, 1165A>G, 1167A>T, 1172G>T, 1173C>T, 1177A>C, 1178T>A, 1181G>C, 1183A>G, 1185C>A, 1186G>A, 1192T>C, 1195T>C, 1196A>G, 1197C>T, 1199A>G, 1200A>T, 1204A>C, 1207T>G, 1210A>G, 1211A>C, 1218A>C, 1219G>C, 1223A>G, 1227T>C, 1232T>C, 1234G>C, 1235A>C, 1237A>G									

CDS

	Begin	End	Coverage	Score	Concordance	Matches	Identities	I/D/M/F*	Stop Codons
FLA14_p101	1	380	100%	1484	59.2%	379 (99.5%)	212 (55.6%)	1/1/0/0	0
Protein mutations:	M4V (110A>G 112G>T), A6D (117C>A), E7S (119G>A 120A>G 121A>T), H10T (128C>A 129A>C 130T>C), K14Q (140A>C 142G>A), K15E (143A>G), I17L (149A>C 151C>T), K19R (156A>G 157G>A), S23A (167T>G 169T>C), C24Y (171G>A), T31A (191A>G 193T>C), K35R (204A>G), A36G (207C>G 208C>T), F38V (212T>G 214T>G), C40F (219G>T), I41C (221A>T 222T>G 223T>C), S42K (224T>A 225C>A 226T>G), K44L (230A>C 231A>T 232A>T), V48H (242G>C 243T>A 244C>T), T59M (276C>T 277A>G), Y60I (278T>A 279A>T), I62L (284A>C 286C>T), S63T (288G>C 289T>C), L65C (294T>G 295A>C), Q67V (299C>G 300A>T 301A>T), I68V (302A>G 304A>G), S71K (311T>A 312C>A 313T>A), N74E (320A>G 322T>G), I75V (323A>G 325C>T), I79V (335A>G 337C>A), T81A (341A>G 343C>T), L88A (362C>G 363T>C 364C>T), A90T (368G>A 370T>G), Q91E (371C>G), R92K (374C>A 375G>A 376T>G), L94I (380T>A 382G>T), Q95L (383C>T 384A>T 385A>G), T96A (386A>G), N99D (395A>G 397T>C), Y102del (404_406delTAT), N103G (407A>G 408A>G 409T>C), F104V (410T>G 412T>G), C106V (416T>G 417G>T), Q107H (421A>T), V108A (423T>C), I110V (428A>G), I115V (443A>G), K116R (446A>C 447A>G 448G>T), S118D (452A>G 453G>A 454C>T), E120R (458G>C 459A>G 460A>T), T121I (462C>T 463T>C), K123Q (467A>C), K124S (471A>G 472A>T), K124_A125insG (472_473insGGT), A125V (474C>T 475C>T), Q126H (478G>T), L128V (482T>G), I129V (485A>G), M135V (503A>G 505G>C), I136F (506A>T), L138M (512C>A 514A>G), T140R (518A>C 519C>G), K142Q (524A>C 526A>G), I144L (530A>C 532C>T), D145R (533G>C 534A>G 535T>C), T146P (536A>C 538C>T), K147D (539A>G 541A>C), A148H (542G>C 543C>A 544A>C), I151M (553A>G), V152F (554G>T), I154L (560A>T 562A>G), I162S (584A>T 585T>C 586A>T), D163R (587G>A 588A>G 589T>A), N164G (590A>G 591A>G), L166K (596T>A 597T>A), K168Q (602A>C 604A>G), Q170Y (608C>T 610A>T), E174Q (620G>C 622A>G), F175L (623T>C), F176L (628T>G), E177P (629G>C 630A>C), S178P (632A>C 633G>C 634T>A), H179K (635C>A 637T>A), V180I (638G>A), I183G (647A>G 648T>G), L184V (650T>G 652A>T), L185F (655A>C), V189M (665G>A 667T>G), S191P (671T>C), R192E (674A>G 675G>A), V193A (678T>C), I194L (680A>C 682C>T), N195E (683A>G 685T>G), T196I (687C>T 688A>T), S197T (689T>A), Q198R (692C>A 693A>G 694G>A), V199K (695G>A 696T>A 697C>G), R202N (705G>A 706A>C), D203K (707G>A 709T>A), K206R (717A>G 718A>G), N211R (731A>C 732A>G), A212D (735C>A), R220K (759G>A), Y222F (765A>T), I224V (770A>G 772A>C), K227D (779A>G 781G>C), N229E (785A>G 787T>A), D230E (790T>G), F231W (792T>G 793T>G), A233L (797G>C 798C>T 799A>T), F237C (810T>G 811T>C), D241E (823C>G), H242T (824C>A 825A>C), S244A (830A>G 831G>C), L245I (833C>A), T248S (843C>G 844A>T), L249V (845C>G 847A>C), C252V (854T>G 855G>T 856T>G), Q257K (869C>A), V260W (878G>T 879T>G 880A>G), M262T (885T>C 886G>T), E263D (889A>T), C264K (890T>A 891G>A 892T>G), L265M (893T>A 895A>G), T266R (896A>C 897C>G 898A>C), N267S (900A>G 901T>C), N269D (905A>G), F270H (908T>C 909T>A), A272V (915C>T), S274A (920A>G 921G>C 922C>A), I275T (924T>C 925C>T), S280D (938T>G 939C>A), Q282N (944C>A 946A>C), E283T (947G>A 948A>C 949A>T), F286I (956T>A 958T>C), K289R (965A>C 966A>G), D293S (977G>T 978A>C), K295S (983A>T 984A>C), T296S (986A>T), I298V (992A>G 994A>G), L300I (998C>A 1000T>C), S301T (1001T>A), N303D (1007A>G), I314V (1040A>G 1042A>C), H324T (1070C>A 1071A>C), N325Q (1073A>C 1075T>A), R326P (1076A>C 1077G>C), I330L (1088A>C 1090A>G), I344V (1130A>G), I349V (1145A>G 1147A>C), E351K (1151G>A 1153A>G), E353D (1159A>T), L354E (1160T>G 1161T>A), P355R (1163C>A 1164C>G 1165A>G), K356M (1167A>T), A358F (1172G>T 1173C>T), E359D (1177A>C), L360I (1178T>A), E361Q (1181G>C 1183A>G), T362K (1185C>A 1186G>A), T366V (1196A>G 1197C>T), K367V (1199A>G 1200A>T), D369E (1207T>G), M371L (1211A>C), E373A (1218A>C 1219G>C), I375V (1223A>G), V376A (1227T>C), I379L (1235A>C 1237A>G)									

	Begin	End	Coverage	Score	Concordance	Matches	Identities	I/D/M/F*	Stop Codons
**NT**	**101**	**1238**	**84.9%**	**414**	**18.3%**	**1135 (99.5%)**	**677 (59.3%)**	**3/3**	
Codon mutations:	ATG4GTT (110A>G 112G>T), GCT6GAT (117C>A), GAA7AGT (119G>A 120A>G 121A>T), GAC9GAT (127C>T), CAT10ACC (128C>A 129A>C 130T>C), GGC12GGT (136C>T), CTC13TTG (137C>T 139C>G), AAG14CAA (140A>C 142G>A), AAA15GAA (143A>G), AAT16AAC (148T>C), ATC17CTT (149A>C 151C>T), CTA18CTG (154A>G), AAG19AGA (156A>G 157G>A), GGT20GGC (160T>C), ATA21ATC (163A>C), TCT23GCC (167T>G 169T>C), TGT24TAT (171G>A), GGA25GGT (175A>T), GAA27GAG (181A>G), CCT29CCA (187T>A), TCA30TCT (190A>T), ACT31GCC (191A>G 193T>C), CAA33CAG (199A>G), AAA35AGA (204A>G), GCC36GGT (207C>G 208C>T), ATT37ATA (211T>A), TTT38GTG (212T>G 214T>G), CCA39CCT (217A>T), TGT40TTT (219G>T), ATT41TGC (221A>T 222T>G 223T>C), TCT42AAG (224T>A 225C>A 226T>G), AAA44CTT (230A>C 231A>T 232A>T), GAC45GAT (235C>T), GTG46GTC (238G>C), ATA47ATT (241A>T), GTC48CAT (242G>C 243T>A 244C>T), CAA49CAG (247A>G), TCG52TCT (256G>T), ACA54ACC (262A>C), AAA56AAG (268A>G), ACA57ACC (271A>C), GCA58GCC (274A>C), ACA59ATG (276C>T 277A>G), TAT60ATT (278T>A 279A>T), GCA61GCT (283A>T), ATC62CTT (284A>C 286C>T), AGT63ACC (288G>C 289T>C), TTA65TGC (294T>G 295A>C), CAG66CAA (298G>A), CAA67GTT (299C>G 300A>T 301A>T), ATA68GTG (302A>G 304A>G), ACT70ACC (310T>C), TCT71AAA (311T>A 312C>A 313T>A), AAT74GAG (320A>G 322T>G), ATC75GTT (323A>G 325C>T), ATC79GTA (335A>G 337C>A), ACC81GCT (341A>G 343C>T), CCA82CCT (346A>T), CGT84CGG (352T>G), GAA85GAG (355A>G), TTG86CTT (356T>C 358G>T), GCT87GCA (361T>A), CTC88GCT (362C>G 363T>C 364C>T), GCT90ACG (368G>A 370T>G), CAA91GAA (371C>G), CGT92AAG (374C>A 375G>A 376T>G), GTA93GTG (379A>G), TTG94ATT (380T>A 382G>T), CAA95TTG (383C>T 384A>T 385A>G), ACA96GCA (386A>G), GGG98GGA (394G>A), AAT99GAC (395A>G 397T>C), CTA101CTG (403A>G), TAT102del (404_406delTAT), AAT103GGC (407A>G 408A>G 409T>C), TTT104GTG (410T>G 412T>G), AAA105AAG (415A>G), TGT106GTT (416T>G 417G>T), CAA107CAT (421A>T), GTT108GCT (423T>C), ATT110GTT (428A>G), GGA111GGG (433A>G), GGC112GGT (436C>T), ACA113ACC (439A>C), ATC115GTC (443A>G), AAG116CGT (446A>C 447A>G 448G>T), GAA117GAG (451A>G), AGC118GAT (452A>G 453G>A 454C>T), GAA120CGT (458G>C 459A>G 460A>T), ACT121ATC (462C>T 463T>C), CTA122CTT (466A>T), AAG123CAG (467A>C), AAA124AGT (471A>G 472A>T), AAA124_GCC125insGGT (472_473insGGT), GCC125GTT (474C>T 475C>T), CAG126CAT (478G>T), GTT127GTA (481T>A), TTG128GTG (482T>G), ATT129GTT (485A>G), GGA130GGT (490A>T), CCT132CCA (496T>A), GGA133GGC (499A>C), CGA134CGT (502A>T), ATG135GTC (503A>G 505G>C), ATT136TTT (506A>T), GAT137GAC (511T>C), CTA138ATG (512C>A 514A>G), CTC139CTG (517C>G), ACC140CGC (518A>C 519C>G), CGA141AGG (521C>A 523A>G), AAA142CAG (524A>C 526A>G), AGT143TCT (527A>T 528G>C), ATC144CTT (530A>C 532C>T), GAT145CGC (533G>C 534A>G 535T>C), ACC146CCT (536A>C 538C>T), AAA147GAC (539A>G 541A>C), GCA148CAC (542G>C 543C>A 544A>C), ATT149ATC (547T>C), AAA150AAG (550A>G), ATA151ATG (553A>G), GTT152TTT (554G>T), GTA153GTT (559A>T), ATA154TTG (560A>T 562A>G), GAT158GAC (574T>C), GAG159GAA (577G>A), TTG161CTC (581T>C 583G>C), ATA162TCT (584A>T 585T>C 586A>T), GAT163AGA (587G>A 588A>G 589T>A), AAT164GGT (590A>G 591A>G), TTT165TTC (595T>C), TTG166AAG (596T>A 597T>A), AAA168CAG (602A>C 604A>G), ATA169ATT (607A>T), CAA170TAT (608C>T 610A>T), ATT172ATA (616T>A), GAA174CAG (620G>C 622A>G), TTT175CTT (623T>C), TTT176TTG (628T>G), GAA177CCA (629G>C 630A>C), AGT178CCA (632A>C 633G>C 634T>A), CAT179AAA (635C>A 637T>A), GTC180ATC (638G>A), GTT182GTG (646T>G), ATT183GGT (647A>G 648T>G), TTA184GTT (650T>G 652A>T), TTA185TTC (655A>C), TCA186TCT (658A>T), GCA187GCC (661A>C), ACC188ACT (664C>T), GTT189ATG (665G>A 667T>G), TCA191CCA (671T>C), AGG192GAG (674A>G 675G>A), GTT193GCT (678T>C), ATC194CTT (680A>C 682C>T), AAT195GAG (683A>G 685T>G), ACA196ATT (687C>T 688A>T), TCT197ACT (689T>A), CAG198AGA (692C>A 693A>G 694G>A), GTC199AAG (695G>A 696T>A 697C>G), TTT200TTC (700T>C), AGA202AAC (705G>A 706A>C), GAT203AAA (707G>A 709T>A), CCC204CCT (712C>T), GTA205GTG (715A>G), AAA206AGG (717A>G 718A>G), CTG208CTT (724G>T), GTA209GTG (727A>G), AAA210AAG (730A>G), AAT211CGT (731A>C 732A>G), GCT212GAT (735C>A), GAA213GAG (739A>G), TTG214CTC (740T>C 742G>C), ACA215ACT (745A>T), CTC216CTT (748C>T), ATT219ATC (757T>C), AGG220AAG (759G>A), TAT222TTT (765A>T), ATA224GTC (770A>G 772A>C), GTA226GTT (778A>T), AAG227GAC (779A>G 781G>C), AAA228AAG (784A>G), AAT229GAA (785A>G 787T>A), GAT230GAG (790T>G), TTT231TGG (792T>G 793T>G), AAA232AAG (796A>G), GCA233CTT (797G>C 798C>T 799A>T), ACA235ACT (805A>T), TTT237TGC (810T>G 811T>C), TAT240TAC (820T>C), GAC241GAG (823C>G), CAC242ACC (824C>A 825A>C), AGC244GCC (830A>G 831G>C), CTC245ATC (833C>A), ACT246ACC (838T>C), CAA247CAG (841A>G), ACA248AGT (843C>G 844A>T), CTA249GTC (845C>G 847A>C), ATA250ATC (850A>C), TGT252GTG (854T>G 855G>T 856T>G), AAT253AAC (859T>C), ACA254ACC (862A>C), CGT255AGG (863C>A 865T>G), AGA256CGT (866A>C 868A>T), CAA257AAA (869C>A), GTA260TGG (878G>T 879T>G 880A>G), ATG262ACT (885T>C 886G>T), GAA263GAT (889A>T), TGT264AAG (890T>A 891G>A 892T>G), TTA265ATG (893T>A 895A>G), ACA266CGC (896A>C 897C>G 898A>C), AAT267AGC (900A>G 901T>C), AGA268CGT (902A>C 904A>T), AAT269GAT (905A>G), TTC270CAC (908T>C 909T>A), GCA272GTA (915C>T), TCA273TCT (919A>T), AGC274GCA (920A>G 921G>C 922C>A), ATC275ACT (924T>C 925C>T), GAT278GAC (934T>C), TCC280GAC (938T>G 939C>A), CAA281CAG (943A>G), CAA282AAC (944C>A 946A>C), GAA283ACT (947G>A 948A>C 949A>T), CGT284AGA (950C>A 952T>A), TTT286ATC (956T>A 958T>C), AAA289CGA (965A>C 966A>G), GAA290GAG (970A>G), TTT291TTC (973T>C), AGA292CGA (974A>C), GAT293TCT (977G>T 978A>C), GGT294GGG (982T>G), AAA295TCA (983A>T 984A>C), ACT296TCT (986A>T), AGA297CGT (989A>C 991A>T), ATA298GTG (992A>G 994A>G), CTT299CTC (997T>C), CTT300ATC (998C>A 1000T>C), TCA301ACA (1001T>A), AAT303GAT (1007A>G), CTA304CTC (1012A>C), TTA305CTT (1013T>C 1015A>T), GCA306GCT (1018A>T), AGA307CGT (1019A>C 1021A>T), GGC308GGT (1024C>T), GTT311GTA (1033T>A), CAA312CAG (1036A>G), ATA314GTC (1040A>G 1042A>C), TCT315TCC (1045T>C), TTA316CTT (1046T>C 1048A>T), GTT317GTC (1051T>C), ATC318ATT (1054C>T), AAT319AAC (1057T>C), GAT321GAC (1063T>C), TTG322CTG (1064T>C), CAT324ACT (1070C>A 1071A>C), AAT325CAA (1073A>C 1075T>A), AGA326CCA (1076A>C 1077G>C), TAT329TAC (1087T>C), ATA330CTG (1088A>C 1090A>G), AGA335CGT (1103A>C 1105A>T), GGT337GGA (1111T>A), AGA338CGA (1112A>C), GGC340GGA (1120C>A), CGA341AGG (1121C>A 1123A>G), AAA342AAG (1126A>G), GGG343GGT (1129G>T), ATT344GTT (1130A>G), AAT347AAC (1141T>C), ATA349GTC (1145A>G 1147A>C), ACT350ACC (1150T>C), GAA351AAG (1151G>A 1153A>G), GAC352GAT (1156C>T), GAA353GAT (1159A>T), TTA354GAA (1160T>G 1161T>A), CCA355AGG (1163C>A 1164C>G 1165A>G), AAG356ATG (1167A>T), GCT358TTT (1172G>T 1173C>T), GAA359GAC (1177A>C), TTA360ATA (1178T>A), GAA361CAG (1181G>C 1183A>G), ACG362AAA (1185C>A 1186G>A), TAT364TAC (1192T>C), AAT365AAC (1195T>C), ACC366GTC (1196A>G 1197C>T), AAA367GTA (1199A>G 1200A>T), ATA368ATC (1204A>C), GAT369GAG (1207T>G), GAA370GAG (1210A>G), ATG371CTG (1211A>C), GAG373GCC (1218A>C 1219G>C), ATT375GTT (1223A>G), GTT376GCT (1227T>C), TTG378CTC (1232T>C 1234G>C), ATA379CTG (1235A>C 1237A>G)								

Proteins

hypothetical protein (YP_009666148.1)	1	380	100%	1484	59.2%	379 (99.5%)	212 (55.6%)	1/1/0/0	0
Protein mutations:	M4V (110A>G 112G>T), A6D (117C>A), E7S (119G>A 120A>G 121A>T), H10T (128C>A 129A>C 130T>C), K14Q (140A>C 142G>A), K15E (143A>G), I17L (149A>C 151C>T), K19R (156A>G 157G>A), S23A (167T>G 169T>C), C24Y (171G>A), T31A (191A>G 193T>C), K35R (204A>G), A36G (207C>G 208C>T), F38V (212T>G 214T>G), C40F (219G>T), I41C (221A>T 222T>G 223T>C), S42K (224T>A 225C>A 226T>G), K44L (230A>C 231A>T 232A>T), V48H (242G>C 243T>A 244C>T), T59M (276C>T 277A>G), Y60I (278T>A 279A>T), I62L (284A>C 286C>T), S63T (288G>C 289T>C), L65C (294T>G 295A>C), Q67V (299C>G 300A>T 301A>T), I68V (302A>G 304A>G), S71K (311T>A 312C>A 313T>A), N74E (320A>G 322T>G), I75V (323A>G 325C>T), I79V (335A>G 337C>A), T81A (341A>G 343C>T), L88A (362C>G 363T>C 364C>T), A90T (368G>A 370T>G), Q91E (371C>G), R92K (374C>A 375G>A 376T>G), L94I (380T>A 382G>T), Q95L (383C>T 384A>T 385A>G), T96A (386A>G), N99D (395A>G 397T>C), Y102del (404_406delTAT), N103G (407A>G 408A>G 409T>C), F104V (410T>G 412T>G), C106V (416T>G 417G>T), Q107H (421A>T), V108A (423T>C), I110V (428A>G), I115V (443A>G), K116R (446A>C 447A>G 448G>T), S118D (452A>G 453G>A 454C>T), E120R (458G>C 459A>G 460A>T), T121I (462C>T 463T>C), K123Q (467A>C), K124S (471A>G 472A>T), K124_A125insG (472_473insGGT), A125V (474C>T 475C>T), Q126H (478G>T), L128V (482T>G), I129V (485A>G), M135V (503A>G 505G>C), I136F (506A>T), L138M (512C>A 514A>G), T140R (518A>C 519C>G), K142Q (524A>C 526A>G), I144L (530A>C 532C>T), D145R (533G>C 534A>G 535T>C), T146P (536A>C 538C>T), K147D (539A>G 541A>C), A148H (542G>C 543C>A 544A>C), I151M (553A>G), V152F (554G>T), I154L (560A>T 562A>G), I162S (584A>T 585T>C 586A>T), D163R (587G>A 588A>G 589T>A), N164G (590A>G 591A>G), L166K (596T>A 597T>A), K168Q (602A>C 604A>G), Q170Y (608C>T 610A>T), E174Q (620G>C 622A>G), F175L (623T>C), F176L (628T>G), E177P (629G>C 630A>C), S178P (632A>C 633G>C 634T>A), H179K (635C>A 637T>A), V180I (638G>A), I183G (647A>G 648T>G), L184V (650T>G 652A>T), L185F (655A>C), V189M (665G>A 667T>G), S191P (671T>C), R192E (674A>G 675G>A), V193A (678T>C), I194L (680A>C 682C>T), N195E (683A>G 685T>G), T196I (687C>T 688A>T), S197T (689T>A), Q198R (692C>A 693A>G 694G>A), V199K (695G>A 696T>A 697C>G), R202N (705G>A 706A>C), D203K (707G>A 709T>A), K206R (717A>G 718A>G), N211R (731A>C 732A>G), A212D (735C>A), R220K (759G>A), Y222F (765A>T), I224V (770A>G 772A>C), K227D (779A>G 781G>C), N229E (785A>G 787T>A), D230E (790T>G), F231W (792T>G 793T>G), A233L (797G>C 798C>T 799A>T), F237C (810T>G 811T>C), D241E (823C>G), H242T (824C>A 825A>C), S244A (830A>G 831G>C), L245I (833C>A), T248S (843C>G 844A>T), L249V (845C>G 847A>C), C252V (854T>G 855G>T 856T>G), Q257K (869C>A), V260W (878G>T 879T>G 880A>G), M262T (885T>C 886G>T), E263D (889A>T), C264K (890T>A 891G>A 892T>G), L265M (893T>A 895A>G), T266R (896A>C 897C>G 898A>C), N267S (900A>G 901T>C), N269D (905A>G), F270H (908T>C 909T>A), A272V (915C>T), S274A (920A>G 921G>C 922C>A), I275T (924T>C 925C>T), S280D (938T>G 939C>A), Q282N (944C>A 946A>C), E283T (947G>A 948A>C 949A>T), F286I (956T>A 958T>C), K289R (965A>C 966A>G), D293S (977G>T 978A>C), K295S (983A>T 984A>C), T296S (986A>T), I298V (992A>G 994A>G), L300I (998C>A 1000T>C), S301T (1001T>A), N303D (1007A>G), I314V (1040A>G 1042A>C), H324T (1070C>A 1071A>C), N325Q (1073A>C 1075T>A), R326P (1076A>C 1077G>C), I330L (1088A>C 1090A>G), I344V (1130A>G), I349V (1145A>G 1147A>C), E351K (1151G>A 1153A>G), E353D (1159A>T), L354E (1160T>G 1161T>A), P355R (1163C>A 1164C>G 1165A>G), K356M (1167A>T), A358F (1172G>T 1173C>T), E359D (1177A>C), L360I (1178T>A), E361Q (1181G>C 1183A>G), T362K (1185C>A 1186G>A), T366V (1196A>G 1197C>T), K367V (1199A>G 1200A>T), D369E (1207T>G), M371L (1211A>C), E373A (1218A>C 1219G>C), I375V (1223A>G), V376A (1227T>C), I379L (1235A>C 1237A>G)								

	Begin	End	Coverage	Score	Concordance	Matches	Identities	I/D/M/F*	Stop Codons
**NT**	**101**	**1238**	**84.9%**	**414**	**18.3%**	**1135 (99.5%)**	**677 (59.3%)**	**3/3**	
Codon mutations:	ATG4GTT (110A>G 112G>T), GCT6GAT (117C>A), GAA7AGT (119G>A 120A>G 121A>T), GAC9GAT (127C>T), CAT10ACC (128C>A 129A>C 130T>C), GGC12GGT (136C>T), CTC13TTG (137C>T 139C>G), AAG14CAA (140A>C 142G>A), AAA15GAA (143A>G), AAT16AAC (148T>C), ATC17CTT (149A>C 151C>T), CTA18CTG (154A>G), AAG19AGA (156A>G 157G>A), GGT20GGC (160T>C), ATA21ATC (163A>C), TCT23GCC (167T>G 169T>C), TGT24TAT (171G>A), GGA25GGT (175A>T), GAA27GAG (181A>G), CCT29CCA (187T>A), TCA30TCT (190A>T), ACT31GCC (191A>G 193T>C), CAA33CAG (199A>G), AAA35AGA (204A>G), GCC36GGT (207C>G 208C>T), ATT37ATA (211T>A), TTT38GTG (212T>G 214T>G), CCA39CCT (217A>T), TGT40TTT (219G>T), ATT41TGC (221A>T 222T>G 223T>C), TCT42AAG (224T>A 225C>A 226T>G), AAA44CTT (230A>C 231A>T 232A>T), GAC45GAT (235C>T), GTG46GTC (238G>C), ATA47ATT (241A>T), GTC48CAT (242G>C 243T>A 244C>T), CAA49CAG (247A>G), TCG52TCT (256G>T), ACA54ACC (262A>C), AAA56AAG (268A>G), ACA57ACC (271A>C), GCA58GCC (274A>C), ACA59ATG (276C>T 277A>G), TAT60ATT (278T>A 279A>T), GCA61GCT (283A>T), ATC62CTT (284A>C 286C>T), AGT63ACC (288G>C 289T>C), TTA65TGC (294T>G 295A>C), CAG66CAA (298G>A), CAA67GTT (299C>G 300A>T 301A>T), ATA68GTG (302A>G 304A>G), ACT70ACC (310T>C), TCT71AAA (311T>A 312C>A 313T>A), AAT74GAG (320A>G 322T>G), ATC75GTT (323A>G 325C>T), ATC79GTA (335A>G 337C>A), ACC81GCT (341A>G 343C>T), CCA82CCT (346A>T), CGT84CGG (352T>G), GAA85GAG (355A>G), TTG86CTT (356T>C 358G>T), GCT87GCA (361T>A), CTC88GCT (362C>G 363T>C 364C>T), GCT90ACG (368G>A 370T>G), CAA91GAA (371C>G), CGT92AAG (374C>A 375G>A 376T>G), GTA93GTG (379A>G), TTG94ATT (380T>A 382G>T), CAA95TTG (383C>T 384A>T 385A>G), ACA96GCA (386A>G), GGG98GGA (394G>A), AAT99GAC (395A>G 397T>C), CTA101CTG (403A>G), TAT102del (404_406delTAT), AAT103GGC (407A>G 408A>G 409T>C), TTT104GTG (410T>G 412T>G), AAA105AAG (415A>G), TGT106GTT (416T>G 417G>T), CAA107CAT (421A>T), GTT108GCT (423T>C), ATT110GTT (428A>G), GGA111GGG (433A>G), GGC112GGT (436C>T), ACA113ACC (439A>C), ATC115GTC (443A>G), AAG116CGT (446A>C 447A>G 448G>T), GAA117GAG (451A>G), AGC118GAT (452A>G 453G>A 454C>T), GAA120CGT (458G>C 459A>G 460A>T), ACT121ATC (462C>T 463T>C), CTA122CTT (466A>T), AAG123CAG (467A>C), AAA124AGT (471A>G 472A>T), AAA124_GCC125insGGT (472_473insGGT), GCC125GTT (474C>T 475C>T), CAG126CAT (478G>T), GTT127GTA (481T>A), TTG128GTG (482T>G), ATT129GTT (485A>G), GGA130GGT (490A>T), CCT132CCA (496T>A), GGA133GGC (499A>C), CGA134CGT (502A>T), ATG135GTC (503A>G 505G>C), ATT136TTT (506A>T), GAT137GAC (511T>C), CTA138ATG (512C>A 514A>G), CTC139CTG (517C>G), ACC140CGC (518A>C 519C>G), CGA141AGG (521C>A 523A>G), AAA142CAG (524A>C 526A>G), AGT143TCT (527A>T 528G>C), ATC144CTT (530A>C 532C>T), GAT145CGC (533G>C 534A>G 535T>C), ACC146CCT (536A>C 538C>T), AAA147GAC (539A>G 541A>C), GCA148CAC (542G>C 543C>A 544A>C), ATT149ATC (547T>C), AAA150AAG (550A>G), ATA151ATG (553A>G), GTT152TTT (554G>T), GTA153GTT (559A>T), ATA154TTG (560A>T 562A>G), GAT158GAC (574T>C), GAG159GAA (577G>A), TTG161CTC (581T>C 583G>C), ATA162TCT (584A>T 585T>C 586A>T), GAT163AGA (587G>A 588A>G 589T>A), AAT164GGT (590A>G 591A>G), TTT165TTC (595T>C), TTG166AAG (596T>A 597T>A), AAA168CAG (602A>C 604A>G), ATA169ATT (607A>T), CAA170TAT (608C>T 610A>T), ATT172ATA (616T>A), GAA174CAG (620G>C 622A>G), TTT175CTT (623T>C), TTT176TTG (628T>G), GAA177CCA (629G>C 630A>C), AGT178CCA (632A>C 633G>C 634T>A), CAT179AAA (635C>A 637T>A), GTC180ATC (638G>A), GTT182GTG (646T>G), ATT183GGT (647A>G 648T>G), TTA184GTT (650T>G 652A>T), TTA185TTC (655A>C), TCA186TCT (658A>T), GCA187GCC (661A>C), ACC188ACT (664C>T), GTT189ATG (665G>A 667T>G), TCA191CCA (671T>C), AGG192GAG (674A>G 675G>A), GTT193GCT (678T>C), ATC194CTT (680A>C 682C>T), AAT195GAG (683A>G 685T>G), ACA196ATT (687C>T 688A>T), TCT197ACT (689T>A), CAG198AGA (692C>A 693A>G 694G>A), GTC199AAG (695G>A 696T>A 697C>G), TTT200TTC (700T>C), AGA202AAC (705G>A 706A>C), GAT203AAA (707G>A 709T>A), CCC204CCT (712C>T), GTA205GTG (715A>G), AAA206AGG (717A>G 718A>G), CTG208CTT (724G>T), GTA209GTG (727A>G), AAA210AAG (730A>G), AAT211CGT (731A>C 732A>G), GCT212GAT (735C>A), GAA213GAG (739A>G), TTG214CTC (740T>C 742G>C), ACA215ACT (745A>T), CTC216CTT (748C>T), ATT219ATC (757T>C), AGG220AAG (759G>A), TAT222TTT (765A>T), ATA224GTC (770A>G 772A>C), GTA226GTT (778A>T), AAG227GAC (779A>G 781G>C), AAA228AAG (784A>G), AAT229GAA (785A>G 787T>A), GAT230GAG (790T>G), TTT231TGG (792T>G 793T>G), AAA232AAG (796A>G), GCA233CTT (797G>C 798C>T 799A>T), ACA235ACT (805A>T), TTT237TGC (810T>G 811T>C), TAT240TAC (820T>C), GAC241GAG (823C>G), CAC242ACC (824C>A 825A>C), AGC244GCC (830A>G 831G>C), CTC245ATC (833C>A), ACT246ACC (838T>C), CAA247CAG (841A>G), ACA248AGT (843C>G 844A>T), CTA249GTC (845C>G 847A>C), ATA250ATC (850A>C), TGT252GTG (854T>G 855G>T 856T>G), AAT253AAC (859T>C), ACA254ACC (862A>C), CGT255AGG (863C>A 865T>G), AGA256CGT (866A>C 868A>T), CAA257AAA (869C>A), GTA260TGG (878G>T 879T>G 880A>G), ATG262ACT (885T>C 886G>T), GAA263GAT (889A>T), TGT264AAG (890T>A 891G>A 892T>G), TTA265ATG (893T>A 895A>G), ACA266CGC (896A>C 897C>G 898A>C), AAT267AGC (900A>G 901T>C), AGA268CGT (902A>C 904A>T), AAT269GAT (905A>G), TTC270CAC (908T>C 909T>A), GCA272GTA (915C>T), TCA273TCT (919A>T), AGC274GCA (920A>G 921G>C 922C>A), ATC275ACT (924T>C 925C>T), GAT278GAC (934T>C), TCC280GAC (938T>G 939C>A), CAA281CAG (943A>G), CAA282AAC (944C>A 946A>C), GAA283ACT (947G>A 948A>C 949A>T), CGT284AGA (950C>A 952T>A), TTT286ATC (956T>A 958T>C), AAA289CGA (965A>C 966A>G), GAA290GAG (970A>G), TTT291TTC (973T>C), AGA292CGA (974A>C), GAT293TCT (977G>T 978A>C), GGT294GGG (982T>G), AAA295TCA (983A>T 984A>C), ACT296TCT (986A>T), AGA297CGT (989A>C 991A>T), ATA298GTG (992A>G 994A>G), CTT299CTC (997T>C), CTT300ATC (998C>A 1000T>C), TCA301ACA (1001T>A), AAT303GAT (1007A>G), CTA304CTC (1012A>C), TTA305CTT (1013T>C 1015A>T), GCA306GCT (1018A>T), AGA307CGT (1019A>C 1021A>T), GGC308GGT (1024C>T), GTT311GTA (1033T>A), CAA312CAG (1036A>G), ATA314GTC (1040A>G 1042A>C), TCT315TCC (1045T>C), TTA316CTT (1046T>C 1048A>T), GTT317GTC (1051T>C), ATC318ATT (1054C>T), AAT319AAC (1057T>C), GAT321GAC (1063T>C), TTG322CTG (1064T>C), CAT324ACT (1070C>A 1071A>C), AAT325CAA (1073A>C 1075T>A), AGA326CCA (1076A>C 1077G>C), TAT329TAC (1087T>C), ATA330CTG (1088A>C 1090A>G), AGA335CGT (1103A>C 1105A>T), GGT337GGA (1111T>A), AGA338CGA (1112A>C), GGC340GGA (1120C>A), CGA341AGG (1121C>A 1123A>G), AAA342AAG (1126A>G), GGG343GGT (1129G>T), ATT344GTT (1130A>G), AAT347AAC (1141T>C), ATA349GTC (1145A>G 1147A>C), ACT350ACC (1150T>C), GAA351AAG (1151G>A 1153A>G), GAC352GAT (1156C>T), GAA353GAT (1159A>T), TTA354GAA (1160T>G 1161T>A), CCA355AGG (1163C>A 1164C>G 1165A>G), AAG356ATG (1167A>T), GCT358TTT (1172G>T 1173C>T), GAA359GAC (1177A>C), TTA360ATA (1178T>A), GAA361CAG (1181G>C 1183A>G), ACG362AAA (1185C>A 1186G>A), TAT364TAC (1192T>C), AAT365AAC (1195T>C), ACC366GTC (1196A>G 1197C>T), AAA367GTA (1199A>G 1200A>T), ATA368ATC (1204A>C), GAT369GAG (1207T>G), GAA370GAG (1210A>G), ATG371CTG (1211A>C), GAG373GCC (1218A>C 1219G>C), ATT375GTT (1223A>G), GTT376GCT (1227T>C), TTG378CTC (1232T>C 1234G>C), ATA379CTG (1235A>C 1237A>G)								

*: Inserts / Deletes / Misaligned / Frameshifts

Analysis details

This analysis was performed with panviral2.64

NGS Details (UN18_val): Torradovirus lycopersici (segment RNA 1)

Assembly

**Coverage Length**	6773 (2 contig(s))
**Depth Of Coverage**	234.6
**Number Of Reads**	12659
**Reads Per Million**	237.59 rpm (after QC)
**Ambiguities**	0
**Assembly Method**	de novo + reference guided assembly
**Consensus Caller**	Bcf Tools

Coverage Map

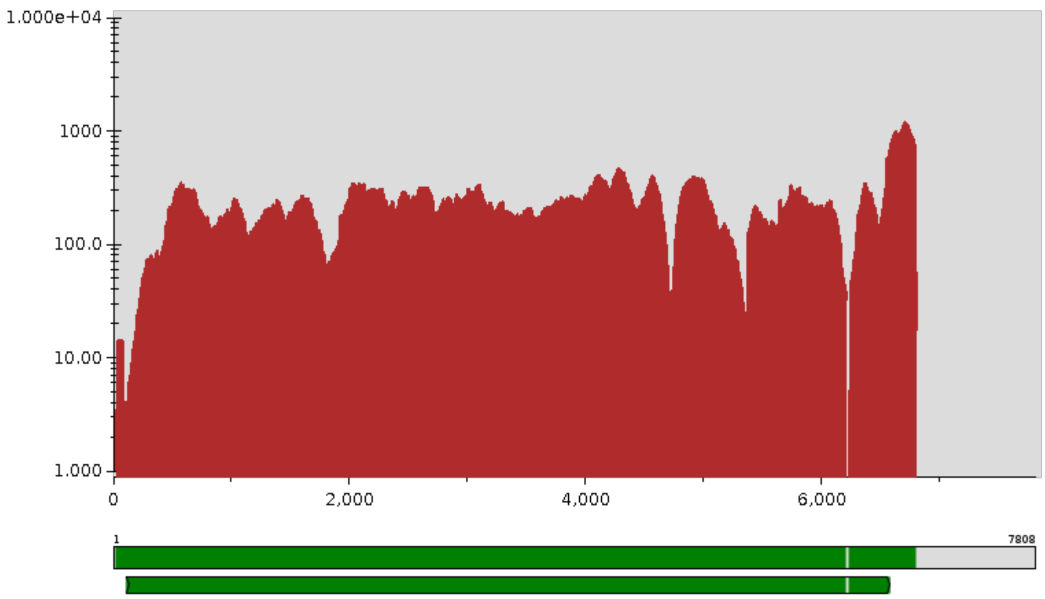

Assignment

**Type**	Torradovirus lycopersici (Taxonomy ID: 3048378)
**Reference Genome**	NC_009013.1
**NT Identity (%)**	88.5183
**AA Identity (%)**	94.1067
**Number Of Stop Codons**	1
**Number Of CDS**	1

Alignment

**Alignment Score**	10416.0 (NT) + 14340.0 (AA) = 24756.0
**Concordance (%)**	86.6321

Alignment Method	Global, seeded, nucleotide + amino acids (AGA)

Genome Region

Sequence starts at position 21 and ends at position 6806 relative to NC_009013.1 reference sequence.

Alignment Detailed Statistics

	Begin	End	Coverage	Score	Concordance	Matches	Identities	I/D/M/F*	Stop Codons
**NT**	**21**	**6806**	**86.7%**	**10416**	**77.0%**	**6772 (99.9%)**	**5998 (88.5%)**	**4/1**	
Mutations:	22T>G, 69C>T, 72C>T, 77A>T, 83C>T, 83_84insT, 88T>C, 90C>G, 93T>A, 101G>A, 102G>C, 115T>C, 122A>T, 128C>T, 130C>T, 134T>C, 135T>C, 138A>G, 140T>C, 142A>C, 144T>C, 145T>A, 146A>G, 147C>T, 148T>G, 155T>A, 157C>T, 159C>T, 161A>G, 178T>A, 187A>T, 190G>A, 196T>A, 197G>C, 198C>A, 199T>A, 220C>T, 223T>A, 226G>T, 241G>A, 247A>T, 259T>C, 262T>A, 265C>T, 271A>G, 280G>A, 289T>G, 292A>T, 301T>A, 302T>G, 307C>T, 310T>C, 311A>T, 313C>T, 315G>A, 322T>C, 324A>G, 325C>T, 328T>A, 331T>C, 334T>A, 340G>A, 343A>G, 349G>A, 358T>A, 359T>C, 361A>T, 364T>A, 367A>G, 370C>T, 376C>T, 382T>A, 385C>T, 388T>C, 394C>T, 397A>C, 409A>G, 412A>C, 427T>G, 433G>A, 439T>C, 443A>G, 451A>G, 454T>C, 473A>G, 485G>A, 487C>T, 490A>G, 493T>C, 496G>A, 502G>T, 541T>C, 559C>T, 578C>T, 586A>T, 599A>T, 604C>T, 606T>C, 615A>G, 618C>T, 619A>T, 655C>T, 661A>G, 676A>G, 694A>G, 707C>T, 718A>G, 719T>G, 748G>T, 751A>T, 760C>T, 778T>A, 781C>T, 790C>T, 799A>T, 805T>C, 806A>C, 820A>C, 821T>A, 832A>T, 835A>C, 841T>C, 842C>T, 847C>T, 883G>A, 886T>C, 892G>A, 901T>C, 913T>C, 925C>T, 938C>T, 952A>G, 955C>T, 964T>G, 970G>A, 973T>C, 977G>A, 997C>T, 1001T>C, 1006T>C, 1018C>T, 1019A>G, 1020C>T, 1033G>A, 1060T>C, 1063C>T, 1081C>T, 1093T>C, 1096C>A, 1109C>T, 1111A>G, 1129A>G, 1135T>A, 1165C>A, 1180G>A, 1186G>A, 1195A>T, 1196A>T, 1198C>T, 1204T>G, 1207T>G, 1231G>A, 1240C>T, 1255G>A, 1267T>C, 1300G>A, 1303A>G, 1319C>T, 1324C>T, 1342C>T, 1357A>C, 1361T>C, 1390A>T, 1408T>C, 1426T>A, 1429C>T, 1441C>T, 1451C>T, 1462G>T, 1480C>T, 1481A>G, 1489T>C, 1495A>T, 1522C>A, 1552C>T, 1553C>A, 1555C>G, 1568C>A, 1570A>G, 1573T>C, 1582C>T, 1585A>C, 1591C>T, 1607G>T, 1612G>A, 1618T>G, 1619C>T, 1624C>A, 1630C>T, 1643A>C, 1645A>G, 1669C>T, 1681T>A, 1684G>A, 1692C>T, 1699T>G, 1706T>G, 1714T>G, 1737A>G, 1738A>G, 1739A>C, 1750A>T, 1755C>T, 1756A>C, 1771C>T, 1781T>C, 1783G>A, 1789A>G, 1810G>A, 1813T>G, 1825C>A, 1834A>G, 1839A>T, 1844G>A, 1849A>C, 1850T>A, 1858G>A, 1867G>A, 1877T>A, 1879A>T, 1885G>A, 1889A>G, 1894A>T, 1906T>A, 1907A>C, 1908T>C, 1909G>A, 1911T>G, 1927C>A, 1942G>A, 1960A>G, 1966A>G, 1984C>T, 1990G>A, 1993G>A, 2009T>C, 2023T>C, 2056T>C, 2062C>T, 2077C>A, 2082T>A, 2092A>G, 2120A>C, 2128T>C, 2130A>C, 2138C>T, 2141T>C, 2143T>A, 2146A>G, 2149T>C, 2155T>C, 2174C>T, 2179A>C, 2182G>A, 2185T>C, 2194T>A, 2200T>C, 2212T>C, 2224C>T, 2227C>T, 2240A>T, 2241A>G, 2250G>A, 2251C>A, 2257C>T, 2263G>A, 2287G>A, 2290C>T, 2299A>T, 2314C>T, 2318C>T, 2323A>T, 2347C>T, 2356C>T, 2359G>A, 2365A>T, 2375A>C, 2377G>T, 2380T>C, 2387C>A, 2392A>G, 2407T>C, 2409G>A, 2443T>C, 2446G>A, 2479G>A, 2485C>T, 2494T>G, 2497G>A, 2500T>C, 2503G>A, 2506T>C, 2509C>T, 2512T>C, 2542G>A, 2548C>T, 2557C>T, 2620G>A, 2626T>C, 2641G>A, 2650G>A, 2662C>T, 2692G>A, 2695T>A, 2710A>G, 2716A>C, 2726G>A, 2731C>T, 2734C>T, 2743A>G, 2744T>C, 2765T>C, 2767G>C, 2768A>C, 2773A>G, 2788C>T, 2809C>T, 2821G>A, 2827A>G, 2842T>C, 2848C>T, 2852C>A, 2854T>A, 2860C>G, 2866T>A, 2869C>T, 2881T>C, 2882G>T, 2926G>T, 2929C>T, 2930T>C, 2941A>G, 2947T>A, 2950C>T, 2974T>C, 2980C>G, 2998A>G, 3031T>C, 3034G>A, 3040T>C, 3049A>C, 3064A>T, 3079T>C, 3083C>A, 3085A>G, 3101T>C, 3103G>T, 3109T>C, 3136T>G, 3146A>T, 3151C>T, 3163T>C, 3169C>T, 3172T>C, 3202T>C, 3241T>C, 3253C>T, 3256G>A, 3262C>T, 3283T>C, 3289T>C, 3298T>C, 3307A>G, 3358A>T, 3376T>G, 3382T>A, 3400A>C, 3406A>G, 3413C>G, 3418C>A, 3421T>C, 3431C>A, 3445G>T, 3463A>G, 3466G>A, 3481C>T, 3484A>T, 3508G>T, 3530C>A, 3532A>G, 3534G>A, 3553T>A, 3559G>A, 3574T>C, 3583C>A, 3586T>C, 3589A>G, 3591C>T, 3593T>C, 3594C>A, 3610A>G, 3625C>T, 3628G>A, 3629G>T, 3646C>T, 3658A>C, 3661A>G, 3664T>C, 3676C>T, 3688A>G, 3691C>T, 3693A>T, 3701C>T, 3706T>C, 3710G>T, 3713A>G, 3716T>C, 3739A>T, 3754G>A, 3758G>A, 3766G>A, 3778G>T, 3781G>A, 3808T>C, 3814C>T, 3817G>A, 3824T>C, 3868C>T, 3871T>C, 3883G>A, 3895A>C, 3907A>T, 3911C>A, 3925A>G, 3928G>A, 3949C>T, 3955G>A, 3961G>A, 3970A>G, 3973T>C, 4034T>C, 4055C>A, 4066T>C, 4084C>T, 4093C>T, 4118C>T, 4129C>T, 4147C>A, 4157C>T, 4159G>A, 4162A>T, 4189T>A, 4192T>G, 4195A>G, 4207G>A, 4210T>C, 4225A>T, 4228C>T, 4234T>C, 4252C>T, 4282C>T, 4288C>A, 4294A>G, 4309T>G, 4318C>T, 4339T>C, 4348C>T, 4354T>C, 4358A>C, 4360A>G, 4396T>C, 4414T>G, 4417T>C, 4426G>T, 4429A>G, 4441A>T, 4459T>C, 4468C>T, 4471T>C, 4474T>C, 4501A>T, 4510T>A, 4514T>C, 4537T>A, 4570C>T, 4576A>T, 4588C>T, 4591T>C, 4625T>C, 4636T>C, 4648T>A, 4651T>A, 4660A>G, 4666G>A, 4678T>C, 4684A>T, 4687G>A, 4696C>T, 4699C>T, 4702G>A, 4705C>T, 4714T>C, 4716G>A, 4719G>A, 4735C>T, 4736T>C, 4747A>G, 4753T>C, 4756A>C, 4759C>T, 4765T>C, 4768T>C, 4774G>A, 4780T>C, 4783A>G, 4786A>G, 4789C>T, 4802C>T, 4813A>G, 4816T>C, 4834C>T, 4843G>A, 4897A>C, 4898G>A, 4903A>G, 4958A>C, 4960C>T, 4969G>A, 4975G>A, 4981C>T, 4999T>C, 5005T>C, 5012T>C, 5017G>A, 5023C>T, 5030G>A, 5041A>T, 5044A>C, 5059G>T, 5074T>C, 5077T>C, 5079T>C, 5083A>T, 5090G>T, 5102T>C, 5113C>T, 5119T>C, 5120G>A, 5122T>C, 5123C>T, 5131T>C, 5132T>A, 5143G>T, 5146T>C, 5167T>C, 5176C>T, 5200G>A, 5206C>T, 5209C>A, 5218T>G, 5269C>A, 5278G>A, 5290G>T, 5293T>C, 5302T>A, 5326C>T, 5347C>A, 5353G>A, 5357A>T, 5360A>C, 5369G>A, 5371G>T, 5386C>G, 5392G>A, 5399A>G, 5407A>T, 5410G>T, 5413C>T, 5419A>C, 5429C>T, 5437T>C, 5459G>A, 5462G>A, 5486G>A, 5491T>G, 5492A>T, 5498C>T, 5500C>G, 5515A>T, 5531C>T, 5533T>G, 5537A>C, 5558G>C, 5578T>C, 5582A>G, 5591G>T, 5602G>A, 5604C>T, 5608A>G, 5611A>T, 5613A>T, 5617A>T, 5626A>T, 5638T>G, 5641T>C, 5656G>A, 5674A>T, 5677G>T, 5686G>A, 5689C>A, 5690T>C, 5710T>A, 5711T>C, 5716G>T, 5719A>C, 5731C>T, 5734T>C, 5737T>C, 5739G>A, 5746T>C, 5774A>G, 5779T>C, 5786T>C, 5797A>G, 5804T>A, 5805T>C, 5806C>T, 5826A>T, 5836A>T, 5849T>C, 5850T>A, 5851G>A, 5869G>A, 5878G>T, 5881T>C, 5884T>C, 5887C>T, 5890C>T, 5894A>C, 5907A>T, 5914T>C, 5923T>C, 5927C>T, 5942C>T, 5944C>T, 5955A>G, 5965G>A, 5974T>C, 5975A>C, 5980G>A, 5986T>C, 5998A>G, 6002A>G, 6003C>T, 6004C>T, 6009G>A, 6016A>G, 6019A>G, 6028C>T, 6031A>T, 6033C>T, 6035G>T, 6055T>A, 6067C>A, 6070T>C, 6079C>G, 6089C>T, 6092C>T, 6100T>G, 6103G>A, 6106G>A, 6112A>G, 6121A>T, 6130G>T, 6139T>C, 6145T>C, 6154C>T, 6163C>G, 6166C>T, 6184A>G, 6185A>G, 6186C>A, 6187T>C, 6189A>G, 6193C>T, 6196A>G, 6199C>T, 6202G>C, 6208A>G, 6211A>G, 6214T>G, 6215C>G, 6230A>G, 6232A>G, 6235G>A, 6239G>A, 6240T>C, 6253A>G, 6256C>A, 6262A>T, 6263C>T, 6265G>A, 6268G>T, 6274C>G, 6291T>A, 6292T>A, 6295T>C, 6296G>C, 6298A>G, 6301T>A, 6304A>G, 6308T>C, 6317A>G, 6319A>T, 6322G>A, 6323A>G, 6344C>T, 6358T>C, 6371G>A, 6382C>T, 6385C>T, 6404T>C, 6405G>A, 6406T>C, 6412C>T, 6414G>T, 6415A>G, 6436C>T, 6437T>A, 6445C>T, 6448G>A, 6457C>A, 6458T>A, 6461C>T, 6481T>A, 6485C>A, 6490A>C, 6493T>C, 6496G>A, 6499A>T, 6500A>G, 6501T>A, 6517C>A, 6532C>T, 6545C>T, 6548T>C, 6556A>C, 6562A>T, 6566G>A, 6567G>A, 6568C>T, 6571C>T, 6574A>G, 6587G>A, 6591T>A, 6592A>T, 6593A>T, 6595T>C, 6602G>A, 6603C>T, 6647_6648insGTT, 6669G>A, 6670C>T, 6675C>T, 6685A>G, 6692A>C, 6715C>T, 6716G>A, 6721A>T, 6727T>C, 6742A>T, 6752T>C, 6759G>A, 6766delC, 6776C>A, 6786T>C, 6787G>A								

CDS

	Begin	End	Coverage	Score	Concordance	Matches	Identities	I/D/M/F*	Stop Codons
ToTV_sRNA1gp1	1	2159	99.8%	14340	95.3%	2155 (100%)	2028 (94.1%)	0/0/0/0	1
Protein mutations:	M6L (122A>T), P8S (128C>T 130C>T), F10P (134T>C 135T>C), N11S (138A>G), S12P (140T>C 142A>C), V13A (144T>C 145T>A), T14V (146A>G 147C>T 148T>G), C17S (155T>A 157C>T), A18V (159C>T), T19A (161A>G), A31Q (197G>C 198C>A 199T>A), S66A (302T>G), T69S (311A>T 313C>T), S70N (315G>A), N73S (324A>G 325C>T), S113G (443A>G), N123D (473A>G), D127N (485G>A 487C>T), T165S (599A>T), I167T (606T>C), N170S (615A>G), T171I (618C>T 619A>T), S205A (719T>G), S239T (821T>A), V291I (977G>A), T305V (1019A>G 1020C>T), T364S (1196A>T 1198C>T), D367E (1207T>G), I459V (1481A>G), A501S (1607G>T), T529I (1692C>T), S534A (1706T>G), K544R (1737A>G 1738A>G), T550I (1755C>T 1756A>C), Y578F (1839A>T), A580T (1844G>A), S582T (1850T>A), S591T (1877T>A 1879A>T), I595V (1889A>G), D600E (1906T>A), M601P (1907A>C 1908T>C 1909G>A), L602W (1911T>G), F659Y (2082T>A), N675T (2130A>C), Y679Q (2141T>C 2143T>A), N712C (2240A>T 2241A>G), S715K (2250G>A 2251C>A), R761S (2387C>A), R768K (2409G>A), G874S (2726G>A), A926S (2882G>T), Q993K (3083C>A 3085A>G), T1014S (3146A>T), Q1103E (3413C>G), R1143K (3534G>A), A1162V (3591C>T), S1163Q (3593T>C 3594C>A), A1175S (3629G>T), Y1196F (3693A>T), A1202S (3710G>T), I1203V (3713A>G), V1218I (3758G>A), I1362M (4192T>G), S1537N (4716G>A), S1538N (4719G>A), V1598I (4898G>A), N1618H (4958A>C 4960C>T), V1642M (5030G>A), I1658T (5079T>C), A1662S (5090G>T), V1672I (5120G>A 5122T>C), S1676T (5132T>A), T1751S (5357A>T), K1752Q (5360A>C), V1755I (5369G>A 5371G>T), I1765V (5399A>G), V1785I (5459G>A), V1786I (5462G>A), D1794N (5486G>A), T1796S (5492A>T), K1811Q (5537A>C), E1818Q (5558G>C), I1826V (5582A>G), A1829S (5591G>T), T1833I (5604C>T), Y1836F (5613A>T), Q1857H (5677G>T), Y1869H (5711T>C), R1878K (5739G>A), I1890V (5774A>G), F1900T (5804T>A 5805T>C 5806C>T), Y1907F (5826A>T), L1915Q (5849T>C 5850T>A 5851G>A), N1930H (5894A>C), Y1934F (5907A>T), H1946Y (5942C>T 5944C>T), N1950S (5955A>G), T1966V (6002A>G 6003C>T 6004C>T), G1968E (6009G>A), T1976I (6033C>T), A1977S (6035G>T), L1996F (6092C>T), I2002M (6112A>G), E2008D (6130G>T), T2027D (6185A>G 6186C>A 6187T>C), H2028R (6189A>G), M2032I (6202G>C), D2036E (6214T>G), I2042V (6230A>G 6232A>G), V2045T (6239G>A 6240T>C), S2056R (6274C>G), V2062E (6291T>A 6292T>A), V2064L (6296G>C 6298A>G), I2071V (6317A>G 6319A>T), I2073V (6323A>G), A2089T (6371G>A), C2100H (6404T>C 6405G>A 6406T>C), R2103L (6414G>T 6415A>G), C2111S (6437T>A), S2118T (6458T>A), L2119F (6461C>T), H2127N (6485C>A), R2128S (6490A>C), I2132D (6500A>G 6501T>A), L2147F (6545C>T), G2154N (6566G>A 6567G>A 6568C>T)								

	Begin	End	Coverage	Score	Concordance	Matches	Identities	I/D/M/F*	Stop Codons
**NT**	**21**	**6806**	**86.7%**	**10416**	**77.0%**	**6772 (99.9%)**	**5998 (88.5%)**	**4/1**	
Codon mutations:	TTT3TTC (115T>C), ATG6TTG (122A>T), CCC8TCT (128C>T 130C>T), TTC10CCC (134T>C 135T>C), AAC11AGC (138A>G), TCA12CCC (140T>C 142A>C), GTT13GCA (144T>C 145T>A), ACT14GTG (146A>G 147C>T 148T>G), TGC17AGT (155T>A 157C>T), GCT18GTT (159C>T), ACC19GCC (161A>G), TCT24TCA (178T>A), TCA27TCT (187A>T), GAG28GAA (190G>A), ACT30ACA (196T>A), GCT31CAA (197G>C 198C>A 199T>A), CGC38CGT (220C>T), ACT39ACA (223T>A), CTG40CTT (226G>T), AAG45AAA (241G>A), TCA47TCT (247A>T), TCT51TCC (259T>C), TCT52TCA (262T>A), CAC53CAT (265C>T), GAA55GAG (271A>G), AAG58AAA (280G>A), GTT61GTG (289T>G), ACA62ACT (292A>T), GCT65GCA (301T>A), TCT66GCT (302T>G), TCC67TCT (307C>T), TTT68TTC (310T>C), ACC69TCT (311A>T 313C>T), AGC70AAC (315G>A), TGT72TGC (322T>C), AAC73AGT (324A>G 325C>T), TCT74TCA (328T>A), GTT75GTC (331T>C), ACT76ACA (334T>A), TTG78TTA (340G>A), TTA79TTG (343A>G), AAG81AAA (349G>A), ACT84ACA (358T>A), TTA85CTT (359T>C 361A>T), ATT86ATA (364T>A), GAA87GAG (367A>G), CCC88CCT (370C>T), ATC90ATT (376C>T), GCT92GCA (382T>A), TAC93TAT (385C>T), TCT94TCC (388T>C), GTC96GTT (394C>T), GCA97GCC (397A>C), AAA101AAG (409A>G), TCA102TCC (412A>C), GTT107GTG (427T>G), AAG109AAA (433G>A), GTT111GTC (439T>C), AGC113GGC (443A>G), AAA115AAG (451A>G), GAT116GAC (454T>C), AAC123GAC (473A>G), GAC127AAT (485G>A 487C>T), AAA128AAG (490A>G), TCT129TCC (493T>C), GAG130GAA (496G>A), GTG132GTT (502G>T), TCT145TCC (541T>C), TTC151TTT (559C>T), CTA158TTA (578C>T), GGA160GGT (586A>T), ACT165TCT (599A>T), CAC166CAT (604C>T), ATA167ACA (606T>C), AAT170AGT (615A>G), ACA171ATT (618C>T 619A>T), GAC183GAT (655C>T), TTA185TTG (661A>G), CAA190CAG (676A>G), GCA196GCG (694A>G), CTA201TTA (707C>T), GAA204GAG (718A>G), TCC205GCC (719T>G), GTG214GTT (748G>T), GTA215GTT (751A>T), CTC218CTT (760C>T), TCT224TCA (778T>A), ACC225ACT (781C>T), TTC228TTT (790C>T), GTA231GTT (799A>T), GGT233GGC (805T>C), AGA234CGA (806A>C), CTA238CTC (820A>C), TCC239ACC (821T>A), CTA242CTT (832A>T), TCA243TCC (835A>C), ATT245ATC (841T>C), CTG246TTG (842C>T), TAC247TAT (847C>T), TTG259TTA (883G>A), TTT260TTC (886T>C), CTG262CTA (892G>A), AAT265AAC (901T>C), TGT269TGC (913T>C), GCC273GCT (925C>T), CTG278TTG (938C>T), GTA282GTG (952A>G), GAC283GAT (955C>T), GGT286GGG (964T>G), AAG288AAA (970G>A), AAT289AAC (973T>C), GTT291ATT (977G>A), GCC297GCT (997C>T), TTG299CTG (1001T>C), TCT300TCC (1006T>C), TTC304TTT (1018C>T), ACG305GTG (1019A>G 1020C>T), GAG309GAA (1033G>A), CAT318CAC (1060T>C), CGC319CGT (1063C>T), AAC325AAT (1081C>T), ACT329ACC (1093T>C), ATC330ATA (1096C>A), CTA335TTG (1109C>T 1111A>G), AAA341AAG (1129A>G), TCT343TCA (1135T>A), GCC353GCA (1165C>A), AGG358AGA (1180G>A), ACG360ACA (1186G>A), ATA363ATT (1195A>T), ACC364TCT (1196A>T 1198C>T), CTT366CTG (1204T>G), GAT367GAG (1207T>G), CAG375CAA (1231G>A), GGC378GGT (1240C>T), AGG383AGA (1255G>A), TTT387TTC (1267T>C), GGG398GGA (1300G>A), AAA399AAG (1303A>G), CTG405TTG (1319C>T), TTC406TTT (1324C>T), GAC412GAT (1342C>T), CCA417CCC (1357A>C), TTG419CTG (1361T>C), CGA428CGT (1390A>T), AAT434AAC (1408T>C), GCT440GCA (1426T>A), ATC441ATT (1429C>T), GAC445GAT (1441C>T), CTG449TTG (1451C>T), GGG452GGT (1462G>T), GAC458GAT (1480C>T), ATT459GTT (1481A>G), GAT461GAC (1489T>C), ATA463ATT (1495A>T), CCC472CCA (1522C>A), GGC482GGT (1552C>T), CGC483AGG (1553C>A 1555C>G), CGA488AGG (1568C>A 1570A>G), TAT489TAC (1573T>C), GCC492GCT (1582C>T), ACA493ACC (1585A>C), AAC495AAT (1591C>T), GCT501TCT (1607G>T), CAG502CAA (1612G>A), GGT504GGG (1618T>G), CTA505TTA (1619C>T), ACC506ACA (1624C>A), CCC508CCT (1630C>T), AGA513CGG (1643A>C 1645A>G), TGC521TGT (1669C>T), GGT525GGA (1681T>A), GAG526GAA (1684G>A), ACT529ATT (1692C>T), GCT531GCG (1699T>G), TCT534GCT (1706T>G), CTT536CTG (1714T>G), AAA544AGG (1737A>G 1738A>G), AGA545CGA (1739A>C), GCA548GCT (1750A>T), ACA550ATC (1755C>T 1756A>C), AGC555AGT (1771C>T), TTG559CTA (1781T>C 1783G>A), GAA561GAG (1789A>G), AAG568AAA (1810G>A), GTT569GTG (1813T>G), ATC573ATA (1825C>A), AAA576AAG (1834A>G), TAT578TTT (1839A>T), GCC580ACC (1844G>A), ACA581ACC (1849A>C), TCA582ACA (1850T>A), AAG584AAA (1858G>A), CAG587CAA (1867G>A), TCA591ACT (1877T>A 1879A>T), GAG593GAA (1885G>A), ATA595GTA (1889A>G), ATA596ATT (1894A>T), GAT600GAA (1906T>A), ATG601CCA (1907A>C 1908T>C 1909G>A), TTG602TGG (1911T>G), ACC607ACA (1927C>A), AAG612AAA (1942G>A), GTA618GTG (1960A>G), GAA620GAG (1966A>G), TAC626TAT (1984C>T), CAG628CAA (1990G>A), GAG629GAA (1993G>A), TTG635CTG (2009T>C), TAT639TAC (2023T>C), CCT650CCC (2056T>C), GAC652GAT (2062C>T), GCC657GCA (2077C>A), TTT659TAT (2082T>A), CTA662CTG (2092A>G), AGG672CGG (2120A>C), GTT674GTC (2128T>C), AAT675ACT (2130A>C), CTG678TTG (2138C>T), TAT679CAA (2141T>C 2143T>A), CAA680CAG (2146A>G), GGT681GGC (2149T>C), TGT683TGC (2155T>C), CTG690TTG (2174C>T), ATA691ATC (2179A>C), AAG692AAA (2182G>A), AAT693AAC (2185T>C), GCT696GCA (2194T>A), TAT698TAC (2200T>C), TTT702TTC (2212T>C), ATC706ATT (2224C>T), TAC707TAT (2227C>T), AAT712TGT (2240A>T 2241A>G), AGC715AAA (2250G>A 2251C>A), GAC717GAT (2257C>T), CAG719CAA (2263G>A), GCG727GCA (2287G>A), CAC728CAT (2290C>T), ATA731ATT (2299A>T), GAC736GAT (2314C>T), CTG738TTG (2318C>T), ATA739ATT (2323A>T), CAC747CAT (2347C>T), TTC750TTT (2356C>T), TTG751TTA (2359G>A), ACA753ACT (2365A>T), AGG757CGT (2375A>C 2377G>T), TAT758TAC (2380T>C), CGC761AGC (2387C>A), AGA762AGG (2392A>G), CCT767CCC (2407T>C), AGG768AAG (2409G>A), ATT779ATC (2443T>C), GTG780GTA (2446G>A), CCG791CCA (2479G>A), ATC793ATT (2485C>T), GTT796GTG (2494T>G), CTG797CTA (2497G>A), ATT798ATC (2500T>C), AAG799AAA (2503G>A), GCT800GCC (2506T>C), GGC801GGT (2509C>T), CTT802CTC (2512T>C), GGG812GGA (2542G>A), GTC814GTT (2548C>T), TTC817TTT (2557C>T), GAG838GAA (2620G>A), AAT840AAC (2626T>C), CGG845CGA (2641G>A), AAG848AAA (2650G>A), ATC852ATT (2662C>T), AAG862AAA (2692G>A), GGT863GGA (2695T>A), AAA868AAG (2710A>G), GCA870GCC (2716A>C), GGC874AGC (2726G>A), TTC875TTT (2731C>T), ATC876ATT (2734C>T), GGA879GGG (2743A>G), TTG880CTG (2744T>C), TTG887CTC (2765T>C 2767G>C), AGA888CGA (2768A>C), CTA889CTG (2773A>G), TAC894TAT (2788C>T), TGC901TGT (2809C>T), GTG905GTA (2821G>A), ACA907ACG (2827A>G), TTT912TTC (2842T>C), AGC914AGT (2848C>T), CGT916AGA (2852C>A 2854T>A), GGC918GGG (2860C>G), ACT920ACA (2866T>A), TTC921TTT (2869C>T), CAT925CAC (2881T>C), GCC926TCC (2882G>T), CGG940CGT (2926G>T), TTC941TTT (2929C>T), TTG942CTG (2930T>C), CAA945CAG (2941A>G), ATT947ATA (2947T>A), GTC948GTT (2950C>T), GAT956GAC (2974T>C), CCC958CCG (2980C>G), AAA964AAG (2998A>G), TCT975TCC (3031T>C), AAG976AAA (3034G>A), ACT978ACC (3040T>C), GTA981GTC (3049A>C), GTA986GTT (3064A>T), GCT991GCC (3079T>C), CAA993AAG (3083C>A 3085A>G), TTG999CTT (3101T>C 3103G>T), TTT1001TTC (3109T>C), CTT1010CTG (3136T>G), ACG1014TCG (3146A>T), TAC1015TAT (3151C>T), TCT1019TCC (3163T>C), TCC1021TCT (3169C>T), TTT1022TTC (3172T>C), TAT1032TAC (3202T>C), CTT1045CTC (3241T>C), AAC1049AAT (3253C>T), GTG1050GTA (3256G>A), GAC1052GAT (3262C>T), GGT1059GGC (3283T>C), CAT1061CAC (3289T>C), TCT1064TCC (3298T>C), GAA1067GAG (3307A>G), CGA1084CGT (3358A>T), CTT1090CTG (3376T>G), ACT1092ACA (3382T>A), GCA1098GCC (3400A>C), GTA1100GTG (3406A>G), CAG1103GAG (3413C>G), CTC1104CTA (3418C>A), CTT1105CTC (3421T>C), CGA1109AGA (3431C>A), GCG1113GCT (3445G>T), AAA1119AAG (3463A>G), CAG1120CAA (3466G>A), GGC1125GGT (3481C>T), ACA1126ACT (3484A>T), ACG1134ACT (3508G>T), CGA1142AGG (3530C>A 3532A>G), AGG1143AAG (3534G>A), GCT1149GCA (3553T>A), GGG1151GGA (3559G>A), GCT1156GCC (3574T>C), ATC1159ATA (3583C>A), ATT1160ATC (3586T>C), TCA1161TCG (3589A>G), GCT1162GTT (3591C>T), TCA1163CAA (3593T>C 3594C>A), GAA1168GAG (3610A>G), TAC1173TAT (3625C>T), GTG1174GTA (3628G>A), GCT1175TCT (3629G>T), TAC1180TAT (3646C>T), GCA1184GCC (3658A>C), CAA1185CAG (3661A>G), AAT1186AAC (3664T>C), GAC1190GAT (3676C>T), CTA1194CTG (3688A>G), GCC1195GCT (3691C>T), TAT1196TTT (3693A>T), CTA1199TTA (3701C>T), CGT1200CGC (3706T>C), GCA1202TCA (3710G>T), ATT1203GTT (3713A>G), TTG1204CTG (3716T>C), CGA1211CGT (3739A>T), AAG1216AAA (3754G>A), GTC1218ATC (3758G>A), CAG1220CAA (3766G>A), GTG1224GTT (3778G>T), GTG1225GTA (3781G>A), TAT1234TAC (3808T>C), GAC1236GAT (3814C>T), GGG1237GGA (3817G>A), TTG1240CTG (3824T>C), GGC1254GGT (3868C>T), ACT1255ACC (3871T>C), GAG1259GAA (3883G>A), CCA1263CCC (3895A>C), GGA1267GGT (3907A>T), CGA1269AGA (3911C>A), AAA1273AAG (3925A>G), AGG1274AGA (3928G>A), GAC1281GAT (3949C>T), GAG1283GAA (3955G>A), TTG1285TTA (3961G>A), GAA1288GAG (3970A>G), ATT1289ATC (3973T>C), TTG1310CTG (4034T>C), CGG1317AGG (4055C>A), CGT1320CGC (4066T>C), CCC1326CCT (4084C>T), TAC1329TAT (4093C>T), CTG1338TTG (4118C>T), TCC1341TCT (4129C>T), TCC1347TCA (4147C>A), CTG1351TTA (4157C>T 4159G>A), CCA1352CCT (4162A>T), GGT1361GGA (4189T>A), ATT1362ATG (4192T>G), GAA1363GAG (4195A>G), TTG1367TTA (4207G>A), GCT1368GCC (4210T>C), GCA1373GCT (4225A>T), GTC1374GTT (4228C>T), GAT1376GAC (4234T>C), GAC1382GAT (4252C>T), ATC1392ATT (4282C>T), GCC1394GCA (4288C>A), CAA1396CAG (4294A>G), CTT1401CTG (4309T>G), GAC1404GAT (4318C>T), GGT1411GGC (4339T>C), GAC1414GAT (4348C>T), AAT1416AAC (4354T>C), AGA1418CGG (4358A>C 4360A>G), ACT1430ACC (4396T>C), GTT1436GTG (4414T>G), TTT1437TTC (4417T>C), GTG1440GTT (4426G>T), GGA1441GGG (4429A>G), TCA1445TCT (4441A>T), GTT1451GTC (4459T>C), AAC1454AAT (4468C>T), TCT1455TCC (4471T>C), CTT1456CTC (4474T>C), GCA1465GCT (4501A>T), TCT1468TCA (4510T>A), TTG1470CTG (4514T>C), GCT1477GCA (4537T>A), AAC1488AAT (4570C>T), CTA1490CTT (4576A>T), GTC1494GTT (4588C>T), TAT1495TAC (4591T>C), TTG1507CTG (4625T>C), TAT1510TAC (4636T>C), GCT1514GCA (4648T>A), ATT1515ATA (4651T>A), GAA1518GAG (4660A>G), GAG1520GAA (4666G>A), GTT1524GTC (4678T>C), ATA1526ATT (4684A>T), AAG1527AAA (4687G>A), ATC1530ATT (4696C>T), GAC1531GAT (4699C>T), AAG1532AAA (4702G>A), AAC1533AAT (4705C>T), GTT1536GTC (4714T>C), AGC1537AAC (4716G>A), AGT1538AAT (4719G>A), GAC1543GAT (4735C>T), TTG1544CTG (4736T>C), CTA1547CTG (4747A>G), TTT1549TTC (4753T>C), CTA1550CTC (4756A>C), AGC1551AGT (4759C>T), GGT1553GGC (4765T>C), TTT1554TTC (4768T>C), AGG1556AGA (4774G>A), CGT1558CGC (4780T>C), CTA1559CTG (4783A>G), GGA1560GGG (4786A>G), TAC1561TAT (4789C>T), CTG1566TTG (4802C>T), GTA1569GTG (4813A>G), TCT1570TCC (4816T>C), TAC1576TAT (4834C>T), AGG1579AGA (4843G>A), GGA1597GGC (4897A>C), GTT1598ATT (4898G>A), AGA1599AGG (4903A>G), AAC1618CAT (4958A>C 4960C>T), AAG1621AAA (4969G>A), CGG1623CGA (4975G>A), TTC1625TTT (4981C>T), CCT1631CCC (4999T>C), TAT1633TAC (5005T>C), TTG1636CTG (5012T>C), GAG1637GAA (5017G>A), GAC1639GAT (5023C>T), GTG1642ATG (5030G>A), ACA1645ACT (5041A>T), GGA1646GGC (5044A>C), GGG1651GGT (5059G>T), CTT1656CTC (5074T>C), CAT1657CAC (5077T>C), ATT1658ACT (5079T>C), GCA1659GCT (5083A>T), GCT1662TCT (5090G>T), TTG1666CTG (5102T>C), GGC1669GGT (5113C>T), ACT1671ACC (5119T>C), GTT1672ATC (5120G>A 5122T>C), CTG1673TTG (5123C>T), TTT1675TTC (5131T>C), TCT1676ACT (5132T>A), GTG1679GTT (5143G>T), CAT1680CAC (5146T>C), AGT1687AGC (5167T>C), TCC1690TCT (5176C>T), CAG1698CAA (5200G>A), GAC1700GAT (5206C>T), ACC1701ACA (5209C>A), GTT1704GTG (5218T>G), ACC1721ACA (5269C>A), AGG1724AGA (5278G>A), GGG1728GGT (5290G>T), CTT1729CTC (5293T>C), CCT1732CCA (5302T>A), GAC1740GAT (5326C>T), ACC1747ACA (5347C>A), GGG1749GGA (5353G>A), ACC1751TCC (5357A>T), AAA1752CAA (5360A>C), GTG1755ATT (5369G>A 5371G>T), GGC1760GGG (5386C>G), GGG1762GGA (5392G>A), ATT1765GTT (5399A>G), CCA1767CCT (5407A>T), GTG1768GTT (5410G>T), TGC1769TGT (5413C>T), GCA1771GCC (5419A>C), CTG1775TTG (5429C>T), ACT1777ACC (5437T>C), GTT1785ATT (5459G>A), GTT1786ATT (5462G>A), GAT1794AAT (5486G>A), GTT1795GTG (5491T>G), ACA1796TCA (5492A>T), CTC1798TTG (5498C>T 5500C>G), GGA1803GGT (5515A>T), CTT1809TTG (5531C>T 5533T>G), AAA1811CAA (5537A>C), GAA1818CAA (5558G>C), TGT1824TGC (5578T>C), ATT1826GTT (5582A>G), GCC1829TCC (5591G>T), GAG1832GAA (5602G>A), ACA1833ATA (5604C>T), CCA1834CCG (5608A>G), GCA1835GCT (5611A>T), TAC1836TTC (5613A>T), ATA1837ATT (5617A>T), GGA1840GGT (5626A>T), CTT1844CTG (5638T>G), GGT1845GGC (5641T>C), GTG1850GTA (5656G>A), CTA1856CTT (5674A>T), CAG1857CAT (5677G>T), CAG1860CAA (5686G>A), CGC1861CGA (5689C>A), TTG1862CTG (5690T>C), CCT1868CCA (5710T>A), TAT1869CAT (5711T>C), GGG1870GGT (5716G>T), GGA1871GGC (5719A>C), CAC1875CAT (5731C>T), ACT1876ACC (5734T>C), ACT1877ACC (5737T>C), AGA1878AAA (5739G>A), CTT1880CTC (5746T>C), ATA1890GTA (5774A>G), TAT1891TAC (5779T>C), TTA1894CTA (5786T>C), AGA1897AGG (5797A>G), TTC1900ACT (5804T>A 5805T>C 5806C>T), TAT1907TTT (5826A>T), TCA1910TCT (5836A>T), TTG1915CAA (5849T>C 5850T>A 5851G>A), AGG1921AGA (5869G>A), GGG1924GGT (5878G>T), TTT1925TTC (5881T>C), TTT1926TTC (5884T>C), CCC1927CCT (5887C>T), CGC1928CGT (5890C>T), AAT1930CAT (5894A>C), TAC1934TTC (5907A>T), CTT1936CTC (5914T>C), TTT1939TTC (5923T>C), CTG1941TTG (5927C>T), CAC1946TAT (5942C>T 5944C>T), AAC1950AGC (5955A>G), ACG1953ACA (5965G>A), TTT1956TTC (5974T>C), AGA1957CGA (5975A>C), AAG1958AAA (5980G>A), TTT1960TTC (5986T>C), AAA1964AAG (5998A>G), ACC1966GTT (6002A>G 6003C>T 6004C>T), GGG1968GAG (6009G>A), AAA1970AAG (6016A>G), GAA1971GAG (6019A>G), TCC1974TCT (6028C>T), GCA1975GCT (6031A>T), ACA1976ATA (6033C>T), GCT1977TCT (6035G>T), GGT1983GGA (6055T>A), TCC1987TCA (6067C>A), GCT1988GCC (6070T>C), CTC1991CTG (6079C>G), CTA1995TTA (6089C>T), CTT1996TTT (6092C>T), CTT1998CTG (6100T>G), CAG1999CAA (6103G>A), AAG2000AAA (6106G>A), ATA2002ATG (6112A>G), TCA2005TCT (6121A>T), GAG2008GAT (6130G>T), TTT2011TTC (6139T>C), ATT2013ATC (6145T>C), GTC2016GTT (6154C>T), GGC2019GGG (6163C>G), CAC2020CAT (6166C>T), GTA2026GTG (6184A>G), ACT2027GAC (6185A>G 6186C>A 6187T>C), CAC2028CGC (6189A>G), TTC2029TTT (6193C>T), CAA2030CAG (6196A>G), CGC2031CGT (6199C>T), ATG2032ATC (6202G>C), CAA2034CAG (6208A>G), CAA2035CAG (6211A>G), GAT2036GAG (6214T>G), CTG2037GT. (6215C>G), ATA2042GTG (6230A>G 6232A>G), GAG2043GAA (6235G>A), GTG2045ACG (6239G>A 6240T>C), TTA2049TTG (6253A>G), ACC2050ACA (6256C>A), ACA2052ACT (6262A>T), CTG2053TTA (6263C>T 6265G>A), TCG2054TCT (6268G>T), AGC2056AGG (6274C>G), GTT2062GAA (6291T>A 6292T>A), GAT2063GAC (6295T>C), GTA2064CTG (6296G>C 6298A>G), CCT2065CCA (6301T>A), TTA2066TTG (6304A>G), TTG2068CTG (6308T>C), ATA2071GTT (6317A>G 6319A>T), GAG2072GAA (6322G>A), ATC2073GTC (6323A>G), CTA2080TTA (6344C>T), AAT2084AAC (6358T>C), GCA2089ACA (6371G>A), GCC2092GCT (6382C>T), ATC2093ATT (6385C>T), TGT2100CAC (6404T>C 6405G>A 6406T>C), AAC2102AAT (6412C>T), CGA2103CTG (6414G>T 6415A>G), TTC2110TTT (6436C>T), TGT2111AGT (6437T>A), TAC2113TAT (6445C>T), ACG2114ACA (6448G>A), TCC2117TCA (6457C>A), TCT2118ACT (6458T>A), CTT2119TTT (6461C>T), CTT2125CTA (6481T>A), CAT2127AAT (6485C>A), AGA2128AGC (6490A>C), CAT2129CAC (6493T>C), CAG2130CAA (6496G>A), ACA2131ACT (6499A>T), ATT2132GAT (6500A>G 6501T>A), TCC2137TCA (6517C>A), CAC2142CAT (6532C>T), CTT2147TTT (6545C>T), TTG2148CTG (6548T>C), CCA2150CCC (6556A>C), CTA2152CTT (6562A>T), GGC2154AAT (6566G>A 6567G>A 6568C>T), TAC2155TAT (6571C>T), AAA2156AAG (6574A>G)								

Proteins

	Begin	End	Coverage	Score	Concordance	Matches	Identities	I/D/M/F*	Stop Codons
polyprotein (YP_001039627.1)	1	2159	99.8%	14340	95.3%	2155 (100%)	2028 (94.1%)	0/0/0/0	1

	Begin	End	Coverage	Score	Concordance	Matches	Identities	I/D/M/F*	Stop Codons
**NT**	**21**	**6806**	**86.7%**	**10416**	**77.0%**	**6772 (99.9%)**	**5998 (88.5%)**	**4/1**	

Protein mutations:	M6L (122A>T), P8S (128C>T 130C>T), F10P (134T>C 135T>C), N11S (138A>G), S12P (140T>C 142A>C), V13A (144T>C 145T>A), T14V (146A>G 147C>T 148T>G), C17S (155T>A 157C>T), A18V (159C>T), T19A (161A>G), A31Q (197G>C 198C>A 199T>A), S66A (302T>G), T69S (311A>T 313C>T), S70N (315G>A), N73S (324A>G 325C>T), S113G (443A>G), N123D (473A>G), D127N (485G>A 487C>T), T165S (599A>T), I167T (606T>C), N170S (615A>G), T171I (618C>T 619A>T), S205A (719T>G), S239T (821T>A), V291I (977G>A), T305V (1019A>G 1020C>T), T364S (1196A>T 1198C>T), D367E (1207T>G), I459V (1481A>G), A501S (1607G>T), T529I (1692C>T), S534A (1706T>G), K544R (1737A>G 1738A>G), T550I (1755C>T 1756A>C), Y578F (1839A>T), A580T (1844G>A), S582T (1850T>A), S591T (1877T>A 1879A>T), I595V (1889A>G), D600E (1906T>A), M601P (1907A>C 1908T>C 1909G>A), L602W (1911T>G), F659Y (2082T>A), N675T (2130A>C), Y679Q (2141T>C 2143T>A), N712C (2240A>T 2241A>G), S715K (2250G>A 2251C>A), R761S (2387C>A), R768K (2409G>A), G874S (2726G>A), A926S (2882G>T), Q993K (3083C>A 3085A>G), T1014S (3146A>T), Q1103E (3413C>G), R1143K (3534G>A), A1162V (3591C>T), S1163Q (3593T>C 3594C>A), A1175S (3629G>T), Y1196F (3693A>T), A1202S (3710G>T), I1203V (3713A>G), V1218I (3758G>A), I1362M (4192T>G), S1537N (4716G>A), S1538N (4719G>A), V1598I (4898G>A), N1618H (4958A>C 4960C>T), V1642M (5030G>A), I1658T (5079T>C), A1662S (5090G>T), V1672I (5120G>A 5122T>C), S1676T (5132T>A), T1751S (5357A>T), K1752Q (5360A>C), V1755I (5369G>A 5371G>T), I1765V (5399A>G), V1785I (5459G>A), V1786I (5462G>A), D1794N (5486G>A), T1796S (5492A>T), K1811Q (5537A>C), E1818Q (5558G>C), I1826V (5582A>G), A1829S (5591G>T), T1833I (5604C>T), Y1836F (5613A>T), Q1857H (5677G>T), Y1869H (5711T>C), R1878K (5739G>A), I1890V (5774A>G), F1900T (5804T>A 5805T>C 5806C>T), Y1907F (5826A>T), L1915Q (5849T>C 5850T>A 5851G>A), N1930H (5894A>C), Y1934F (5907A>T), H1946Y (5942C>T 5944C>T), N1950S (5955A>G), T1966V (6002A>G 6003C>T 6004C>T), G1968E (6009G>A), T1976I (6033C>T), A1977S (6035G>T), L1996F (6092C>T), I2002M (6112A>G), E2008D (6130G>T), T2027D (6185A>G 6186C>A 6187T>C), H2028R (6189A>G), M2032I (6202G>C), D2036E (6214T>G), I2042V (6230A>G 6232A>G), V2045T (6239G>A 6240T>C), S2056R (6274C>G), V2062E (6291T>A 6292T>A), V2064L (6296G>C 6298A>G), I2071V (6317A>G 6319A>T), I2073V (6323A>G), A2089T (6371G>A), C2100H (6404T>C 6405G>A 6406T>C), R2103L (6414G>T 6415A>G), C2111S (6437T>A), S2118T (6458T>A), L2119F (6461C>T), H2127N (6485C>A), R2128S (6490A>C), I2132D (6500A>G 6501T>A), L2147F (6545C>T), G2154N (6566G>A 6567G>A 6568C>T)

	Begin	End	Coverage	Score	Concordance	Matches	Identities	I/D/M/F*	Stop Codons
NT	21	6806	86.7%	10416	77.0%	6772 (99.9%)	5998 (88.5%)	4/1	

Codon mutations:

TTT3TTC (115T>C), ATG6TTG (122A>T), CCC8TCT (128C>T 130C>T), TTC10CCC (134T>C 135T>C), AAC11AGC (138A>G), TCA12CCC (140T>C 142A>C), GTT13GCA (144T>C 145T>A), ACT14GTG (146A>G 147C>T 148T>G), TGC17AGT (155T>A 157C>T), GCT18GTT (159C>T), ACC19GCC (161A>G), TCT24TCA (178T>A), TCA27TCT (187A>T), GAG28GAA (190G>A), ACT30ACA (196T>A), GCT31CAA (197G>C 198C>A 199T>A), CGC38CGT (220C>T), ACT39ACA (223T>A), CTG40CTT (226G>T), AAG45AAA (241G>A), TCA47TCT (247A>T), TCT51TCC (259T>C), TCT52TCA (262T>A), CAC53CAT (265C>T), GAA55GAG (271A>G), AAG58AAA (280G>A), GTT61GTG (289T>G), ACA62ACT (292A>T), GCT65GCA (301T>A), TCT66GCT (302T>G), TCC67TCT (307C>T), TTT68TTC (310T>C), ACC69TCT (311A>T 313C>T), AGC70AAC (315G>A), TGT72TGC (322T>C), AAC73AGT (324A>G 325C>T), TCT74TCA (328T>A), GTT75GTC (331T>C), ACT76ACA (334T>A), TTG78TTA (340G>A), TTA79TTG (343A>G), AAG81AAA (349G>A), ACT84ACA (358T>A), TTA85CTT (359T>C 361A>T), ATT86ATA (364T>A), GAA87GAG (367A>G), CCC88CCT (370C>T), ATC90ATT (376C>T), GCT92GCA (382T>A), TAC93TAT (385C>T), TCT94TCC (388T>C), GTC96GTT (394C>T), GCA97GCC (397A>C), AAA101AAG (409A>G), TCA102TCC (412A>C), GTT107GTG (427T>G), AAG109AAA (433G>A), GTT111GTC (439T>C), AGC113GGC (443A>G), AAA115AAG (451A>G), GAT116GAC (454T>C), AAC123GAC (473A>G), GAC127AAT (485G>A 487C>T), AAA128AAG (490A>G), TCT129TCC (493T>C), GAG130GAA (496G>A), GTG132GTT (502G>T), TCT145TCC (541T>C), TTC151TTT (559C>T), CTA158TTA (578C>T), GGA160GGT (586A>T), ACT165TCT (599A>T), CAC166CAT (604C>T), ATA167ACA (606T>C), AAT170AGT (615A>G), ACA171ATT (618C>T 619A>T), GAC183GAT (655C>T), TTA185TTG (661A>G), CAA190CAG (676A>G), GCA196GCG (694A>G), CTA201TTA (707C>T), GAA204GAG (718A>G), TCC205GCC (719T>G), GTG214GTT (748G>T), GTA215GTT (751A>T), CTC218CTT (760C>T), TCT224TCA (778T>A), ACC225ACT (781C>T), TTC228TTT (790C>T), GTA231GTT (799A>T), GGT233GGC (805T>C), AGA234CGA (806A>C), CTA238CTC (820A>C), TCC239ACC (821T>A), CTA242CTT (832A>T), TCA243TCC (835A>C), ATT245ATC (841T>C), CTG246TTG (842C>T), TAC247TAT (847C>T), TTG259TTA (883G>A), TTT260TTC (886T>C), CTG262CTA (892G>A), AAT265AAC (901T>C), TGT269TGC (913T>C), GCC273GCT (925C>T), CTG278TTG (938C>T), GTA282GTG (952A>G), GAC283GAT (955C>T), GGT286GGG (964T>G), AAG288AAA (970G>A), AAT289AAC (973T>C), GTT291ATT (977G>A), GCC297GCT (997C>T), TTG299CTG (1001T>C), TCT300TCC (1006T>C), TTC304TTT (1018C>T), ACG305GTG (1019A>G 1020C>T), GAG309GAA (1033G>A), CAT318CAC (1060T>C), CGC319CGT (1063C>T), AAC325AAT (1081C>T), ACT329ACC (1093T>C), ATC330ATA (1096C>A), CTA335TTG (1109C>T 1111A>G), AAA341AAG (1129A>G), TCT343TCA (1135T>A), GCC353GCA (1165C>A), AGG358AGA (1180G>A), ACG360ACA (1186G>A), ATA363ATT (1195A>T), ACC364TCT (1196A>T 1198C>T), CTT366CTG (1204T>G), GAT367GAG (1207T>G), CAG375CAA (1231G>A), GGC378GGT (1240C>T), AGG383AGA (1255G>A), TTT387TTC (1267T>C), GGG398GGA (1300G>A), AAA399AAG (1303A>G), CTG405TTG (1319C>T), TTC406TTT (1324C>T), GAC412GAT (1342C>T), CCA417CCC (1357A>C), TTG419CTG (1361T>C), CGA428CGT (1390A>T), AAT434AAC (1408T>C), GCT440GCA (1426T>A), ATC441ATT (1429C>T), GAC445GAT (1441C>T), CTG449TTG (1451C>T), GGG452GGT (1462G>T), GAC458GAT (1480C>T), ATT459GTT (1481A>G), GAT461GAC (1489T>C), ATA463ATT (1495A>T), CCC472CCA (1522C>A), GGC482GGT (1552C>T), CGC483AGG (1553C>A 1555C>G), CGA488AGG (1568C>A 1570A>G), TAT489TAC (1573T>C), GCC492GCT (1582C>T), ACA493ACC (1585A>C), AAC495AAT (1591C>T), GCT501TCT (1607G>T), CAG502CAA (1612G>A), GGT504GGG (1618T>G), CTA505TTA (1619C>T), ACC506ACA (1624C>A), CCC508CCT (1630C>T), AGA513CGG (1643A>C 1645A>G), TGC521TGT (1669C>T), GGT525GGA (1681T>A), GAG526GAA (1684G>A), ACT529ATT (1692C>T), GCT531GCG (1699T>G), TCT534GCT (1706T>G), CTT536CTG (1714T>G), AAA544AGG (1737A>G 1738A>G), AGA545CGA (1739A>C), GCA548GCT (1750A>T), ACA550ATC (1755C>T 1756A>C), AGC555AGT (1771C>T), TTG559CTA (1781T>C 1783G>A), GAA561GAG (1789A>G), AAG568AAA (1810G>A), GTT569GTG (1813T>G), ATC573ATA (1825C>A), AAA576AAG (1834A>G), TAT578TTT (1839A>T), GCC580ACC (1844G>A), ACA581ACC (1849A>C), TCA582ACA (1850T>A), AAG584AAA (1858G>A), CAG587CAA (1867G>A), TCA591ACT (1877T>A 1879A>T), GAG593GAA (1885G>A), ATA595GTA (1889A>G), ATA596ATT (1894A>T), GAT600GAA (1906T>A), ATG601CCA (1907A>C 1908T>C 1909G>A), TTG602TGG (1911T>G), ACC607ACA (1927C>A), AAG612AAA (1942G>A), GTA618GTG (1960A>G), GAA620GAG (1966A>G), TAC626TAT (1984C>T), CAG628CAA (1990G>A), GAG629GAA (1993G>A), TTG635CTG (2009T>C), TAT639TAC (2023T>C), CCT650CCC (2056T>C), GAC652GAT (2062C>T), GCC657GCA (2077C>A), TTT659TAT (2082T>A), CTA662CTG (2092A>G), AGG672CGG (2120A>C), GTT674GTC (2128T>C), AAT675ACT (2130A>C), CTG678TTG (2138C>T), TAT679CAA (2141T>C 2143T>A), CAA680CAG (2146A>G), GGT681GGC (2149T>C), TGT683TGC (2155T>C), CTG690TTG (2174C>T), ATA691ATC (2179A>C), AAG692AAA (2182G>A), AAT693AAC (2185T>C), GCT696GCA (2194T>A), TAT698TAC (2200T>C), TTT702TTC (2212T>C), ATC706ATT (2224C>T), TAC707TAT (2227C>T), AAT712TGT (2240A>T 2241A>G), AGC715AAA (2250G>A 2251C>A), GAC717GAT (2257C>T), CAG719CAA (2263G>A), GCG727GCA (2287G>A), CAC728CAT (2290C>T), ATA731ATT (2299A>T), GAC736GAT (2314C>T), CTG738TTG (2318C>T), ATA739ATT (2323A>T), CAC747CAT (2347C>T), TTC750TTT (2356C>T), TTG751TTA (2359G>A), ACA753ACT (2365A>T), AGG757CGT (2375A>C 2377G>T), TAT758TAC (2380T>C), CGC761AGC (2387C>A), AGA762AGG (2392A>G), CCT767CCC (2407T>C), AGG768AAG (2409G>A), ATT779ATC (2443T>C), GTG780GTA (2446G>A), CCG791CCA (2479G>A), ATC793ATT (2485C>T), GTT796GTG (2494T>G), CTG797CTA (2497G>A), ATT798ATC (2500T>C), AAG799AAA (2503G>A), GCT800GCC (2506T>C), GGC801GGT (2509C>T), CTT802CTC (2512T>C), GGG812GGA (2542G>A), GTC814GTT (2548C>T), TTC817TTT (2557C>T), GAG838GAA (2620G>A), AAT840AAC (2626T>C), CGG845CGA (2641G>A), AAG848AAA (2650G>A), ATC852ATT (2662C>T), AAG862AAA (2692G>A), GGT863GGA (2695T>A), AAA868AAG (2710A>G), GCA870GCC (2716A>C), GGC874AGC (2726G>A), TTC875TTT (2731C>T), ATC876ATT (2734C>T), GGA879GGG (2743A>G), TTG880CTG (2744T>C), TTG887CTC (2765T>C 2767G>C), AGA888CGA (2768A>C), CTA889CTG (2773A>G), TAC894TAT (2788C>T), TGC901TGT (2809C>T), GTG905GTA (2821G>A), ACA907ACG (2827A>G), TTT912TTC (2842T>C), AGC914AGT (2848C>T), CGT916AGA (2852C>A 2854T>A), GGC918GGG (2860C>G), ACT920ACA (2866T>A), TTC921TTT (2869C>T), CAT925CAC (2881T>C), GCC926TCC (2882G>T), CGG940CGT (2926G>T), TTC941TTT (2929C>T), TTG942CTG (2930T>C), CAA945CAG (2941A>G), ATT947ATA (2947T>A), GTC948GTT (2950C>T), GAT956GAC (2974T>C), CCC958CCG (2980C>G), AAA964AAG (2998A>G), TCT975TCC (3031T>C), AAG976AAA (3034G>A), ACT978ACC (3040T>C), GTA981GTC (3049A>C), GTA986GTT (3064A>T), GCT991GCC (3079T>C), CAA993AAG (3083C>A 3085A>G), TTG999CTT (3101T>C 3103G>T), TTT1001TTC (3109T>C), CTT1010CTG (3136T>G), ACG1014TCG (3146A>T), TAC1015TAT (3151C>T), TCT1019TCC (3163T>C), TCC1021TCT (3169C>T), TTT1022TTC (3172T>C), TAT1032TAC (3202T>C), CTT1045CTC (3241T>C), AAC1049AAT (3253C>T), GTG1050GTA (3256G>A), GAC1052GAT (3262C>T), GGT1059GGC (3283T>C), CAT1061CAC (3289T>C), TCT1064TCC (3298T>C), GAA1067GAG (3307A>G), CGA1084CGT (3358A>T), CTT1090CTG (3376T>G), ACT1092ACA (3382T>A), GCA1098GCC (3400A>C), GTA1100GTG (3406A>G), CAG1103GAG (3413C>G), CTC1104CTA (3418C>A), CTT1105CTC (3421T>C), CGA1109AGA (3431C>A), GCG1113GCT (3445G>T), AAA1119AAG (3463A>G), CAG1120CAA (3466G>A), GGC1125GGT (3481C>T), ACA1126ACT (3484A>T), ACG1134ACT (3508G>T), CGA1142AGG (3530C>A 3532A>G), AGG1143AAG (3534G>A), GCT1149GCA (3553T>A), GGG1151GGA (3559G>A), GCT1156GCC (3574T>C), ATC1159ATA (3583C>A), ATT1160ATC (3586T>C), TCA1161TCG (3589A>G), GCT1162GTT (3591C>T), TCA1163CAA (3593T>C 3594C>A), GAA1168GAG (3610A>G), TAC1173TAT (3625C>T), GTG1174GTA (3628G>A), GCT1175TCT (3629G>T), TAC1180TAT (3646C>T), GCA1184GCC (3658A>C), CAA1185CAG (3661A>G), AAT1186AAC (3664T>C), GAC1190GAT (3676C>T), CTA1194CTG (3688A>G), GCC1195GCT (3691C>T), TAT1196TTT (3693A>T), CTA1199TTA (3701C>T), CGT1200CGC (3706T>C), GCA1202TCA (3710G>T), ATT1203GTT (3713A>G), TTG1204CTG (3716T>C), CGA1211CGT (3739A>T), AAG1216AAA (3754G>A), GTC1218ATC (3758G>A), CAG1220CAA (3766G>A), GTG1224GTT (3778G>T), GTG1225GTA (3781G>A), TAT1234TAC (3808T>C), GAC1236GAT (3814C>T), GGG1237GGA (3817G>A), TTG1240CTG (3824T>C), GGC1254GGT (3868C>T), ACT1255ACC (3871T>C), GAG1259GAA (3883G>A), CCA1263CCC (3895A>C), GGA1267GGT (3907A>T), CGA1269AGA (3911C>A), AAA1273AAG (3925A>G), AGG1274AGA (3928G>A), GAC1281GAT (3949C>T), GAG1283GAA (3955G>A), TTG1285TTA (3961G>A), GAA1288GAG (3970A>G), ATT1289ATC (3973T>C), TTG1310CTG (4034T>C), CGG1317AGG (4055C>A), CGT1320CGC (4066T>C), CCC1326CCT (4084C>T), TAC1329TAT (4093C>T), CTG1338TTG (4118C>T), TCC1341TCT (4129C>T), TCC1347TCA (4147C>A), CTG1351TTA (4157C>T 4159G>A), CCA1352CCT (4162A>T), GGT1361GGA (4189T>A), ATT1362ATG (4192T>G), GAA1363GAG (4195A>G), TTG1367TTA (4207G>A), GCT1368GCC (4210T>C), GCA1373GCT (4225A>T), GTC1374GTT (4228C>T), GAT1376GAC (4234T>C), GAC1382GAT (4252C>T), ATC1392ATT (4282C>T), GCC1394GCA (4288C>A), CAA1396CAG (4294A>G), CTT1401CTG (4309T>G), GAC1404GAT (4318C>T), GGT1411GGC (4339T>C), GAC1414GAT (4348C>T), AAT1416AAC (4354T>C), AGA1418CGG (4358A>C 4360A>G), ACT1430ACC (4396T>C), GTT1436GTG (4414T>G), TTT1437TTC (4417T>C), GTG1440GTT (4426G>T), GGA1441GGG (4429A>G), TCA1445TCT (4441A>T), GTT1451GTC (4459T>C), AAC1454AAT (4468C>T), TCT1455TCC (4471T>C), CTT1456CTC (4474T>C), GCA1465GCT (4501A>T), TCT1468TCA (4510T>A), TTG1470CTG (4514T>C), GCT1477GCA (4537T>A), AAC1488AAT (4570C>T), CTA1490CTT (4576A>T), GTC1494GTT (4588C>T), TAT1495TAC (4591T>C), TTG1507CTG (4625T>C), TAT1510TAC (4636T>C), GCT1514GCA (4648T>A), ATT1515ATA (4651T>A), GAA1518GAG (4660A>G), GAG1520GAA (4666G>A), GTT1524GTC (4678T>C), ATA1526ATT (4684A>T), AAG1527AAA (4687G>A), ATC1530ATT (4696C>T), GAC1531GAT (4699C>T), AAG1532AAA (4702G>A), AAC1533AAT (4705C>T), GTT1536GTC (4714T>C), AGC1537AAC (4716G>A), AGT1538AAT (4719G>A), GAC1543GAT (4735C>T), TTG1544CTG (4736T>C), CTA1547CTG (4747A>G), TTT1549TTC (4753T>C), CTA1550CTC (4756A>C), AGC1551AGT (4759C>T), GGT1553GGC (4765T>C), TTT1554TTC (4768T>C), AGG1556AGA (4774G>A), CGT1558CGC (4780T>C), CTA1559CTG (4783A>G), GGA1560GGG (4786A>G), TAC1561TAT (4789C>T), CTG1566TTG (4802C>T), GTA1569GTG (4813A>G), TCT1570TCC (4816T>C), TAC1576TAT (4834C>T), AGG1579AGA (4843G>A), GGA1597GGC (4897A>C), GTT1598ATT (4898G>A), AGA1599AGG (4903A>G), AAC1618CAT (4958A>C 4960C>T), AAG1621AAA (4969G>A), CGG1623CGA (4975G>A), TTC1625TTT (4981C>T), CCT1631CCC (4999T>C), TAT1633TAC (5005T>C), TTG1636CTG (5012T>C), GAG1637GAA (5017G>A), GAC1639GAT (5023C>T), GTG1642ATG (5030G>A), ACA1645ACT (5041A>T), GGA1646GGC (5044A>C), GGG1651GGT (5059G>T), CTT1656CTC (5074T>C), CAT1657CAC (5077T>C), ATT1658ACT (5079T>C), GCA1659GCT (5083A>T), GCT1662TCT (5090G>T), TTG1666CTG (5102T>C), GGC1669GGT (5113C>T), ACT1671ACC (5119T>C), GTT1672ATC (5120G>A 5122T>C), CTG1673TTG (5123C>T), TTT1675TTC (5131T>C), TCT1676ACT (5132T>A), GTG1679GTT (5143G>T), CAT1680CAC (5146T>C), AGT1687AGC (5167T>C), TCC1690TCT (5176C>T), CAG1698CAA (5200G>A), GAC1700GAT (5206C>T), ACC1701ACA (5209C>A), GTT1704GTG (5218T>G), ACC1721ACA (5269C>A), AGG1724AGA (5278G>A), GGG1728GGT (5290G>T), CTT1729CTC (5293T>C), CCT1732CCA (5302T>A), GAC1740GAT (5326C>T), ACC1747ACA (5347C>A), GGG1749GGA (5353G>A), ACC1751TCC (5357A>T), AAA1752CAA (5360A>C), GTG1755ATT (5369G>A 5371G>T), GGC1760GGG (5386C>G), GGG1762GGA (5392G>A), ATT1765GTT (5399A>G), CCA1767CCT (5407A>T), GTG1768GTT (5410G>T), TGC1769TGT (5413C>T), GCA1771GCC (5419A>C), CTG1775TTG (5429C>T), ACT1777ACC (5437T>C), GTT1785ATT (5459G>A), GTT1786ATT (5462G>A), GAT1794AAT (5486G>A), GTT1795GTG (5491T>G), ACA1796TCA (5492A>T), CTC1798TTG (5498C>T 5500C>G), GGA1803GGT (5515A>T), CTT1809TTG (5531C>T 5533T>G), AAA1811CAA (5537A>C), GAA1818CAA (5558G>C), TGT1824TGC (5578T>C), ATT1826GTT (5582A>G), GCC1829TCC (5591G>T), GAG1832GAA (5602G>A), ACA1833ATA (5604C>T), CCA1834CCG (5608A>G), GCA1835GCT (5611A>T), TAC1836TTC (5613A>T), ATA1837ATT (5617A>T), GGA1840GGT (5626A>T), CTT1844CTG (5638T>G), GGT1845GGC (5641T>C), GTG1850GTA (5656G>A), CTA1856CTT (5674A>T), CAG1857CAT (5677G>T), CAG1860CAA (5686G>A), CGC1861CGA (5689C>A), TTG1862CTG (5690T>C), CCT1868CCA (5710T>A), TAT1869CAT (5711T>C), GGG1870GGT (5716G>T), GGA1871GGC (5719A>C), CAC1875CAT (5731C>T), ACT1876ACC (5734T>C), ACT1877ACC (5737T>C), AGA1878AAA (5739G>A), CTT1880CTC (5746T>C), ATA1890GTA (5774A>G), TAT1891TAC (5779T>C), TTA1894CTA (5786T>C), AGA1897AGG (5797A>G), TTC1900ACT (5804T>A 5805T>C 5806C>T), TAT1907TTT (5826A>T), TCA1910TCT (5836A>T), TTG1915CAA (5849T>C 5850T>A 5851G>A), AGG1921AGA (5869G>A), GGG1924GGT (5878G>T), TTT1925TTC (5881T>C), TTT1926TTC (5884T>C), CCC1927CCT (5887C>T), CGC1928CGT (5890C>T), AAT1930CAT (5894A>C), TAC1934TTC (5907A>T), CTT1936CTC (5914T>C), TTT1939TTC (5923T>C), CTG1941TTG (5927C>T), CAC1946TAT (5942C>T 5944C>T), AAC1950AGC (5955A>G), ACG1953ACA (5965G>A), TTT1956TTC (5974T>C), AGA1957CGA (5975A>C), AAG1958AAA (5980G>A), TTT1960TTC (5986T>C), AAA1964AAG (5998A>G), ACC1966GTT (6002A>G 6003C>T 6004C>T), GGG1968GAG (6009G>A), AAA1970AAG (6016A>G), GAA1971GAG (6019A>G), TCC1974TCT (6028C>T), GCA1975GCT (6031A>T), ACA1976ATA (6033C>T), GCT1977TCT (6035G>T), GGT1983GGA (6055T>A), TCC1987TCA (6067C>A), GCT1988GCC (6070T>C), CTC1991CTG (6079C>G), CTA1995TTA (6089C>T), CTT1996TTT (6092C>T), CTT1998CTG (6100T>G), CAG1999CAA (6103G>A), AAG2000AAA (6106G>A), ATA2002ATG (6112A>G), TCA2005TCT (6121A>T), GAG2008GAT (6130G>T), TTT2011TTC (6139T>C), ATT2013ATC (6145T>C), GTC2016GTT (6154C>T), GGC2019GGG (6163C>G), CAC2020CAT (6166C>T), GTA2026GTG (6184A>G), ACT2027GAC (6185A>G 6186C>A 6187T>C), CAC2028CGC (6189A>G), TTC2029TTT (6193C>T), CAA2030CAG (6196A>G), CGC2031CGT (6199C>T), ATG2032ATC (6202G>C), CAA2034CAG (6208A>G), CAA2035CAG (6211A>G), GAT2036GAG (6214T>G), CTG2037GT. (6215C>G), ATA2042GTG (6230A>G 6232A>G), GAG2043GAA (6235G>A), GTG2045ACG (6239G>A 6240T>C), TTA2049TTG (6253A>G), ACC2050ACA (6256C>A), ACA2052ACT (6262A>T), CTG2053TTA (6263C>T 6265G>A), TCG2054TCT (6268G>T), AGC2056AGG (6274C>G), GTT2062GAA (6291T>A 6292T>A), GAT2063GAC (6295T>C), GTA2064CTG (6296G>C 6298A>G), CCT2065CCA (6301T>A), TTA2066TTG (6304A>G), TTG2068CTG (6308T>C), ATA2071GTT (6317A>G 6319A>T), GAG2072GAA (6322G>A), ATC2073GTC (6323A>G), CTA2080TTA (6344C>T), AAT2084AAC (6358T>C), GCA2089ACA (6371G>A), GCC2092GCT (6382C>T), ATC2093ATT (6385C>T), TGT2100CAC (6404T>C 6405G>A 6406T>C), AAC2102AAT (6412C>T), CGA2103CTG (6414G>T 6415A>G), TTC2110TTT (6436C>T), TGT2111AGT (6437T>A), TAC2113TAT (6445C>T), ACG2114ACA (6448G>A), TCC2117TCA (6457C>A), TCT2118ACT (6458T>A), CTT2119TTT (6461C>T), CTT2125CTA (6481T>A), CAT2127AAT (6485C>A), AGA2128AGC (6490A>C), CAT2129CAC (6493T>C), CAG2130CAA (6496G>A), ACA2131ACT (6499A>T), ATT2132GAT (6500A>G 6501T>A), TCC2137TCA (6517C>A), CAC2142CAT (6532C>T), CTT2147TTT (6545C>T), TTG2148CTG (6548T>C), CCA2150CCC (6556A>C), CTA2152CTT (6562A>T), GGC2154AAT (6566G>A 6567G>A 6568C>T), TAC2155TAT (6571C>T), AAA2156AAG (6574A>G)

*: Inserts / Deletes / Misaligned / Frameshifts

Analysis details

This analysis was performed with panviral2.64

NGS Details (UN18_val): Torradovirus lycopersici (segment RNA 2)

Assembly

**Coverage Length**	3928 (8 contig(s))
**Depth Of Coverage**	1183.7
**Number Of Reads**	38768
**Reads Per Million**	727.62 rpm (after QC)
**Ambiguities**	0
**Assembly Method**	de novo + reference guided assembly
**Consensus Caller**	Bcf Tools

Coverage Map

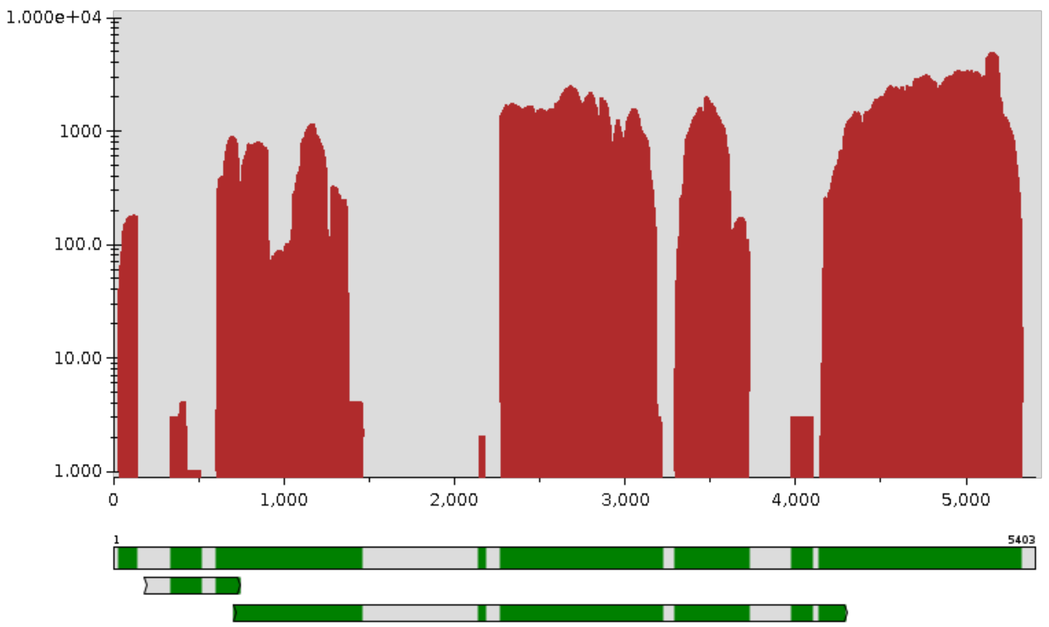

Assignment

**Type**	Torradovirus lycopersici (Taxonomy ID: 3048378)
**Reference Genome**	NC_009032.1
**NT Identity (%)**	83.834
**AA Identity (%)**	86.1702
**Number Of Stop Codons**	4
**Number Of CDS**	2

Alignment

**Alignment Score**	5298.0 (NT) + 5645.0 (AA) = 10943.0
**Concordance (%)**	76.9442

**Alignment Method**	Global, seeded, nucleotide + amino acids (AGA)

Genome Region

Sequence starts at position 30 and ends at position 5329 relative to NC_009032.1 reference sequence.

Alignment Detailed Statistics

	Begin	End	Coverage	Score	Concordance	Matches	Identities	I/D/M/F*	Stop Codons
**NT**	**30**	**5329**	**72.7%**	**5298**	**67.5%**	**3927 (99.9%)**	**3293 (83.8%)**	**1/1**	
Mutations:	52C>T, 58T>C, 60G>A, 75T>C, 77T>G, 78C>T, 80A>T, 90C>T, 96C>T, 104A>G, 105G>T, 107G>A, 110C>T, 111C>A, 112G>A, 117C>T, 121A>C, 137T>C, 139C>T, 144A>G, 340A>T, 350C>A, 352A>C, 355T>C, 356G>A, 370C>T, 372A>C, 376C>T, 388T>A, 400T>C, 401T>C, 403A>G, 406G>A, 418T>G, 424A>C, 430T>A, 432G>C, 433C>T, 436T>C, 439G>A, 442A>C, 445T>A, 448C>A, 451A>G, 460T>G, 461A>C, 472T>A, 484C>G, 487A>G, 496A>T, 502T>A, 505G>T, 507G>A, 508G>A, 509A>G, 511T>A, 514T>C, 515G>A, 520C>A, 604C>A, 607G>A, 610C>A, 613A>G, 618G>C, 619C>T, 622A>G, 626C>G, 628C>A, 631A>T, 632G>A, 637A>G, 644C>G, 645T>C, 646T>C, 649A>G, 654T>A, 658C>T, 664A>C, 665T>A, 670A>G, 671T>C, 673A>T, 677T>C, 679G>A, 683T>A, 685G>C, 686T>C, 687C>A, 688C>G, 689A>G, 690A>C, 691C>A, 696T>C, 703T>C, 706T>A, 718T>G, 731G>A, 733A>G, 750A>G, 751G>C, 755A>C, 758T>A, 761A>C, 788A>T, 797T>G, 806C>G, 818G>C, 821T>G, 827A>G, 828C>T, 833C>A, 844A>T, 848G>C, 854C>G, 860C>A, 863C>T, 866A>G, 869G>A, 884A>T, 887G>T, 911T>G, 929T>A, 932A>G, 935T>C, 956G>C, 986G>A, 995T>C, 1010T>G, 1031A>G, 1034T>G, 1040A>C, 1052A>G, 1061T>C, 1073C>T, 1083C>A, 1095G>A, 1097T>G, 1100G>A, 1109C>T, 1115G>A, 1118A>T, 1130A>G, 1139G>T, 1142C>A, 1149C>T, 1151A>G, 1163A>G, 1169C>G, 1178C>T, 1187A>T, 1205T>A, 1220T>A, 1226A>T, 1235C>A, 1247A>G, 1262T>A, 1277A>G, 1313T>C, 1316C>T, 1317C>A, 1319A>G, 1322T>A, 1325A>G, 1328T>C, 1331C>T, 1334G>T, 1343G>A, 1355G>A, 1361A>T, 1364A>T, 1367A>T, 1370A>C, 1376T>G, 1383A>G, 1385C>G, 1388C>T, 1390C>A, 1394A>C, 1395A>C, 1397T>A, 1400T>G, 1403A>T, 1406G>A, 1407C>T, 1409T>A, 1412G>T, 1418G>A, 1421A>T, 1426A>T, 1427G>T, 1433C>G, 1434G>A, 1439A>T, 1451G>T, 1454A>C, 1455A>C, 1456G>A, 1457C>G, 1460A>G, 1461G>T, 2140A>G, 2142A>G, 2144C>G, 2159T>A, 2162T>A, 2164T>G, 2167A>G, 2169G>A, 2170A>G, 2171A>C, 2173T>G, 2174A>G, 2175C>T, 2177A>C, 2178T>A, 2183C>T, 2271A>T, 2272G>C, 2273G>A, 2276C>T, 2277A>G, 2279T>C, 2282A>T, 2285T>C, 2286A>T, 2288G>C, 2294A>C, 2301C>G, 2303A>G, 2311A>T, 2312T>G, 2313A>G, 2315T>A, 2318A>T, 2320G>C, 2321T>C, 2322G>C, 2324G>C, 2326C>T, 2327A>T, 2328G>C, 2330G>A, 2331C>T, 2339A>G, 2345A>G, 2357G>T, 2363T>C, 2366C>G, 2367T>G, 2368C>A, 2369C>G, 2375C>T, 2376A>C, 2381T>C, 2384A>C, 2387T>C, 2393G>A, 2397G>A, 2398C>A, 2399A>C, 2409A>T, 2410G>C, 2411C>T, 2412A>G, 2413G>C, 2414T>C, 2415T>A, 2416C>A, 2417T>C, 2420T>G, 2424G>C, 2425C>A, 2426T>A, 2430G>C, 2432G>A, 2436T>G, 2438A>T, 2444G>C, 2445C>T, 2447C>A, 2453G>A, 2456T>A, 2459C>G, 2462T>C, 2463G>A, 2469T>A, 2470C>A, 2471C>G, 2474G>A, 2477G>A, 2479T>C, 2480G>T, 2483A>C, 2489A>C, 2492G>A, 2495A>G, 2501A>G, 2503G>A, 2507T>C, 2516C>T, 2521T>C, 2522A>C, 2523A>C, 2525T>G, 2528G>T, 2530A>T, 2537C>A, 2540C>T, 2546T>C, 2549T>A, 2552C>T, 2555C>T, 2561T>G, 2565A>G, 2567T>A, 2570G>A, 2573T>A, 2582C>A, 2583C>T, 2588T>A, 2597G>A, 2605T>C, 2609C>A, 2613A>C, 2615A>T, 2617A>C, 2618G>C, 2627C>T, 2628A>G, 2630C>G, 2633C>T, 2639C>A, 2640T>A, 2643C>G, 2645G>C, 2660T>C, 2661A>C, 2662G>A, 2663G>T, 2672A>T, 2678T>A, 2681A>G, 2685A>T, 2686G>C, 2687T>A, 2693G>T, 2696A>T, 2700T>G, 2701G>T, 2702T>A, 2708A>T, 2711T>G, 2712A>C, 2713C>A, 2714G>A, 2717T>C, 2720C>A, 2725A>C, 2726A>C, 2729C>T, 2730G>A, 2735A>T, 2736C>T, 2738T>G, 2747C>T, 2748T>G, 2750G>T, 2753G>C, 2755C>T, 2756T>G, 2792C>T, 2795C>T, 2798G>T, 2801T>C, 2819G>A, 2820A>T, 2828T>C, 2835A>G, 2837C>T, 2841C>T, 2843G>A, 2846G>T, 2852A>G, 2861G>C, 2864C>T, 2870C>A, 2879T>A, 2882T>G, 2885T>G, 2886T>C, 2894A>T, 2900A>T, 2909T>A, 2915A>G, 2921C>G, 2936A>T, 2939A>G, 2957A>T, 2958C>T, 2966C>T, 2972T>C, 2978A>G, 2981G>A, 2982G>A, 2987T>A, 2989G>C, 2996T>C, 2999T>A, 3003T>C, 3011C>T, 3012A>G, 3014C>A, 3020T>C, 3023G>T, 3032A>T, 3044C>T, 3047T>C, 3050G>A, 3062C>A, 3063T>C, 3077A>T, 3080A>T, 3083C>A, 3089C>T, 3101T>A, 3107T>A, 3113G>T, 3122T>A, 3126A>G, 3128T>C, 3131G>A, 3137T>C, 3143C>T, 3146T>C, 3152T>A, 3156T>C, 3158G>T, 3167A>T, 3176A>T, 3179G>A, 3182C>A, 3187G>A, 3197C>A, 3206C>T, 3210T>C, 3221G>C, 3293A>C, 3299T>G, 3303A>T, 3305C>T, 3308T>A, 3311T>G, 3323T>A, 3324C>T, 3329G>A, 3335C>A, 3350C>T, 3354A>C, 3357T>C, 3359G>A, 3368A>G, 3371C>G, 3372T>A, 3386A>T, 3398A>T, 3404A>G, 3410G>T, 3413C>T, 3419A>C, 3422A>G, 3428G>A, 3437C>A, 3443A>G, 3449G>A, 3452C>G, 3455T>A, 3458G>C, 3461C>A, 3473T>G, 3479C>T, 3482T>C, 3485C>T, 3488A>T, 3491C>T, 3503A>G, 3508A>G, 3512T>C, 3515G>T, 3521C>T, 3522C>T, 3524A>G, 3527C>T, 3533G>A, 3543T>A, 3551C>T, 3563G>A, 3567T>C, 3569A>G, 3572C>A, 3575G>A, 3581C>T, 3590A>C, 3617A>C, 3623A>T, 3624C>G, 3625A>C, 3627G>C, 3629G>T, 3630C>G, 3631C>G, 3635C>T, 3638A>G, 3641C>A, 3650A>T, 3656G>A, 3662A>T, 3674G>T, 3689C>T, 3698A>C, 3702T>G, 3708T>C, 3716C>T, 3722C>G, 3728C>T, 3983T>A, 3984A>G, 3986G>T, 3988G>C, 3990G>A, 3991A>C, 3995C>T, 3998C>T, 4016T>A, 4025G>A, 4031T>C, 4035A>C, 4037A>G, 4040C>G, 4046C>T, 4049A>G, 4052C>A, 4059A>G, 4061A>T, 4067A>T, 4069A>G, 4070T>G, 4072C>G, 4076A>T, 4077A>C, 4079A>T, 4082A>T, 4094A>G, 4103A>G, 4106A>T, 4139G>A, 4142A>T, 4148T>C, 4149C>A, 4151G>T, 4158T>A, 4163C>T, 4172A>G, 4181C>T, 4187G>A, 4188C>G, 4196T>C, 4208A>G, 4211A>T, 4223G>A, 4224C>T, 4227T>C, 4229G>T, 4250C>T, 4256T>A, 4262T>C, 4271T>C, 4280A>G, 4284A>G, 4286T>C, 4289T>C, 4300T>C, 4354G>C, 4355T>A, 4381A>G, 4382A>T, 4383C>G, 4388A>T, 4393A>G, 4424C>T, 4429T>C, 4457A>G, 4468T>C, 4496T>A, 4506A>T, 4525T>C, 4605T>C, 4610delT, 4633T>A, 4634G>A, 4643G>A, 4648G>A, 4650T>C, 4651G>A, 4659A>G, 4675A>T, 4812C>T, 4813C>T, 4860T>C, 4915G>A, 5020C>T, 5079C>T, 5089T>C, 5109A>G, 5123C>T, 5206C>T, 5213A>G, 5214C>T, 5222C>T, 5224G>A, 5225C>A, 5226T>G, 5231A>T, 5233G>T, 5235T>A, 5236G>A, 5239_5240insA, 5242T>G, 5249A>G, 5258A>G, 5259T>A, 5262A>T, 5282T>C, 5313A>T, 5315T>C, 5325G>A, 5327A>C								

CDS

ToTV_sRNA2gp1	52	188	58.0%	561	81.1%	109 (100%)	86 (78.9%)	0/0/0/0	1
Protein mutations:	L57I (350C>A 352A>C), V59I (356G>A), N64T (372A>C), G84A (432G>C 433C>T), H93Q (460T>G), Q108H (505G>T), R109K (507G>A 508G>A), I110V (509A>G 511T>A), V112I (515G>A), R146P (618G>C 619C>T), L149V (626C>G 628C>A), K150N (631A>T), E151K (632G>A), L155A (644C>G 645T>C 646T>C), L158Q (654T>A), S162T (665T>A), L168I (683T>A 685G>C), S169Q (686T>C 687C>A 688C>G), N170A (689A>G 690A>C 691C>A), L172P (696T>C), D175E (706T>A), S179R (718T>G), E184K (731G>A 733A>G)								
Codon mutations:	GCA53GCT (340A>T), CTA57ATC (350C>A 352A>C), TCT58TCC (355T>C), GTT59ATT (356G>A), AAC63AAT (370C>T), AAT64ACT (372A>C), AGC65AGT (376C>T), GTT69GTA (388T>A), TAT73TAC (400T>C), TTA74CTG (401T>C 403A>G), CTG75CTA (406G>A), ACT79ACG (418T>G), CCA81CCC (424A>C), ACT83ACA (430T>A), GGC84GCT (432G>C 433C>T), GTT85GTC (436T>C), AAG86AAA (439G>A), CTA87CTC (442A>C), TCT88TCA (445T>A), GGC89GGA (448C>A), TTA90TTG (451A>G), CAT93CAG (460T>G), AGA94CGA (461A>C), CGT97CGA (472T>A), ACC101ACG (484C>G), AGA102AGG (487A>G), CTA105CTT (496A>T), GCT107GCA (502T>A), CAG108CAT (505G>T), AGG109AAA (507G>A 508G>A), ATT110GTA (509A>G 511T>A), TCT111TCC (514T>C), GTA112ATA (515G>A), TCC113TCA (520C>A), GAC141..A (604C>A), GAG142GAA (607G>A), GCC143GCA (610C>A), AAA144AAG (613A>G), CGC146CCT (618G>C 619C>T), TTA147TTG (622A>G), CTC149GTA (626C>G 628C>A), AAA150AAT (631A>T), GAG151AAG (632G>A), AAA152AAG (637A>G), CTT155GCC (644C>G 645T>C 646T>C), GAA156GAG (649A>G), CTA158CAA (654T>A), TCC159TCT (658C>T), ATA161ATC (664A>C), TCC162ACC (665T>A), AAA163AAG (670A>G), TTA164CTT (671T>C 673A>T), TTG166CTA (677T>C 679G>A), TTG168ATC (683T>A 685G>C), TCC169CAG (686T>C 687C>A 688C>G), AAC170GCA (689A>G 690A>C 691C>A), CTA172CCA (696T>C), AAT174AAC (703T>C), GAT175GAA (706T>A), AGT179AGG (718T>G), GAA184AAG (731G>A 733A>G)								
ToTV_sRNA2gp2	1	1199	69.3%	5084	88.7%	831 (100%)	724 (87.1%)	0/0/0/0	3
Protein mutations:	M1T (703T>C), M2K (706T>A), V6G (718T>G), K11R (733A>G), S17A (750A>G 751G>C), Y48F (844A>T), E61D (884A>T), N70K (911T>G), L128M (1083C>A), V132M (1095G>A 1097T>G), Q211H (1334G>T), T228A (1383A>G 1385C>G), S230* (1390C>A), T232P (1395A>C 1397T>A), D233E (1400T>G), Q242L (1426A>T 1427G>T), D245N (1434G>A), S252Q (1455A>C 1456G>A 1457C>G), A254S (1461G>T), N481E (2142A>G 2144C>G), S487R (2162T>A), V488G (2164T>G), E489G (2167A>G), E490S (2169G>A 2170A>G 2171A>C), I491R (2173T>G 2174A>G), P492S (2175C>T 2177A>C), S493T (2178T>A), R524S (2271A>T 2272G>C 2273G>A), N526D (2277A>G 2279T>C), E527D (2282A>T), M529F (2286A>T 2288G>C), E531D (2294A>C), Q534E (2301C>G 2303A>G), N537M (2311A>T 2312T>G), N538E (2313A>G 2315T>A), E539D (2318A>T), G540A (2320G>C 2321T>C), A541P (2322G>C 2324G>C), T542I (2326C>T 2327A>T), E543Q (2328G>C 2330G>A), N555K (2366C>G), S556E (2367T>G 2368C>A 2369C>G), N559H (2376A>C), A566N (2397G>A 2398C>A 2399A>C), S571A (2412A>G 2413G>C 2414T>C), S572N (2415T>A 2416C>A 2417T>C), A575Q (2424G>C 2425C>A 2426T>A), V577L (2430G>C 2432G>A), S579A (2436T>G 2438A>T), G588S (2463G>A), S590K (2469T>A 2470C>A 2471C>G), M593T (2479T>C 2480G>T), R601K (2503G>A), L607S (2521T>C 2522A>C), I608L (2523A>C 2525T>G), M609I (2528G>T), Y610F (2530A>T), I622V (2565A>G 2567T>A), V635A (2605T>C), K639T (2617A>C 2618G>C), I643V (2628A>G 2630C>G), S647T (2640T>A), L648V (2643C>G 2645G>C), R654H (2661A>C 2662G>A 2663G>T), E665D (2696A>T), C667V (2700T>G 2701G>T 2702T>A), T671Q (2712A>C 2713C>A 2714G>A), K675T (2725A>C 2726A>C), V677I (2730G>A), S683A (2748T>G 2750G>T), A685V (2755C>T 2756T>G), L699F (2798G>T), S707C (2820A>T), I712V (2835A>G 2837C>T), Q715H (2846G>T), D740E (2921C>G), A761T (2982G>A), S763T (2989G>C), I771V (3012A>G 3014C>A), I809V (3126A>G 3128T>C), N827K (3182C>A), S829N (3187G>A), K864N (3293A>C), T868S (3303A>T 3305C>T), L891M (3372T>A), C912* (3437C>A), N936S (3508A>G), S948T (3543T>A), Q975A (3624C>G 3625A>C), V976L (3627G>C 3629G>T), P977G (3630C>G 3631C>G), D980E (3641C>A), L1001V (3702T>G), M1095V (3984A>G 3986G>T), S1096T (3988G>C), D1097T (3990G>A 3991A>C), H1105Q (4016T>A), I1120V (4059A>G 4061A>T), N1123R (4069A>G 4070T>G), A1124G (4072C>G), L1150I (4149C>A 4151G>T), L1153M (4158T>A), Q1163E (4188C>G), H1175Y (4224C>T), S1195G (4284A>G 4286T>C)								

	Begin	End	Coverage	Score	Concordance	Matches	Identities	I/D/M/F*	Stop Codons
**NT**	**30**	**5329**	**72.7%**	**5298**	**67.5%**	**3927 (99.9%)**	**3293 (83.8%)**	**1/1**	

Codon mutations: ATG1ACG (703T>C), ATG2AAG (706T>A), GTG6GGG (718T>G), AGG10AGA (731G>A), AAG11AGG (733A>G), AGT17GCT (750A>G 751G>C), GCA18GCC (755A>C), GCT19GCA (758T>A), GTA20GTC (761A>C), ACA29ACT (788A>T), GTT32GTG (797T>G), GGC35GGG (806C>G), CTG39CTC (818G>C), CTT40CTG (821T>G), AAA42AAG (827A>G), CTG43TTG (828C>T), GGC44GGA (833C>A), TAT48TTT (844A>T), GGG49GGC (848G>C), CTC51CTG (854C>G), GGC53GGA (860C>A), GCC54GCT (863C>T), CAA55CAG (866A>G), GAG56GAA (869G>A), GAA61GAT (884A>T), CTG62CTT (887G>T), AAT70AAG (911T>G), TCT76TCA (929T>A), TCA77TCG (932A>G), TTT78TTC (935T>C), TCG85TCC (956G>C), TTG95TTA (986G>A), GTT98GTC (995T>C), GTT103GTG (1010T>G), GAA110GAG (1031A>G), GGT111GGG (1034T>G), GTA113GTC (1040A>C), TTA117TTG (1052A>G), AGT120AGC (1061T>C), ACC124ACT (1073C>T), CTG128ATG (1083C>A), GTT132ATG (1095G>A 1097T>G), TTG133TTA (1100G>A), TCC136TCT (1109C>T), GGG138GGA (1115G>A), GCA139GCT (1118A>T), TTA143TTG (1130A>G), CCG146CCT (1139G>T), ACC147ACA (1142C>A), CTA150TTG (1149C>T 1151A>G), AAA154AAG (1163A>G), GGC156GGG (1169C>G), TTC159TTT (1178C>T), ACA162ACT (1187A>T), ACT168ACA (1205T>A), CCT173CCA (1220T>A), TCA175TCT (1226A>T), GCC178GCA (1235C>A), CAA182CAG (1247A>G), CGT187CGA (1262T>A), TCA192TCG (1277A>G), CTT204CTC (1313T>C), CAC205CAT (1316C>T), CGA206AGG (1317C>A 1319A>G), CCT207CCA (1322T>A), CAA208CAG (1325A>G), TTT209TTC (1328T>C), TTC210TTT (1331C>T), CAG211CAT (1334G>T), CAG214CAA (1343G>A), CAG218CAA (1355G>A), ATA220ATT (1361A>T), TCA221TCT (1364A>T), TCA222TCT (1367A>T), GTA223GTC (1370A>C), TCT225TCG (1376T>G), ACC228GCG (1383A>G 1385C>G), TCC229TCT (1388C>T), TCA230TAA (1390C>A), GCA231GCC (1394A>C), ACT232CCA (1395A>C 1397T>A), GAT233GAG (1400T>G), TCA234TCT (1403A>T), GTG235GTA (1406G>A), CTT236TTA (1407C>T 1409T>A), CCG237CCT (1412G>T), GTG239GTA (1418G>A), GGA240GGT (1421A>T), CAG242CTT (1426A>T 1427G>T), GGC244GGG (1433C>G), GAC245AAC (1434G>A), ACA246ACT (1439A>T), GTG250GTT (1451G>T), CCA251CCC (1454A>C), AGC252CAG (1455A>C 1456G>A 1457C>G), GAA253GAG (1460A>G), GCA254TCA (1461G>T), AAA480.GA (2140A>G), AAC481GAG (2142A>G 2144C>G), ACT486ACA (2159T>A), AGT487AGA (2162T>A), GTG488GGG (2164T>G), GAA489GGA (2167A>G), GAA490AGC (2169G>A 2170A>G 2171A>C), ATA491AGG (2173T>G 2174A>G), CCA492TCC (2175C>T 2177A>C), TCC493ACC (2178T>A), ACC494ACT (2183C>T), AGG524TCA (2271A>T 2272G>C 2273G>A), CCC525CCT (2276C>T), AAT526GAC (2277A>G 2279T>C), GAA527GAT (2282A>T), GTT528GTC (2285T>C), ATG529TTC (2286A>T 2288G>C), GAA531GAC (2294A>C), CAA534GAG (2301C>G 2303A>G), AAT537ATG (2311A>T 2312T>G), AAT538GAA (2313A>G 2315T>A), GAA539GAT (2318A>T), GGT540GCC (2320G>C 2321T>C), GCG541CCC (2322G>C 2324G>C), ACA542ATT (2326C>T 2327A>T), GAG543CAA (2328G>C 2330G>A), CTG544TTG (2331C>T), TTA546TTG (2339A>G), CAA548CAG (2345A>G), GTG552GTT (2357G>T), AGT554AGC (2363T>C), AAC555AAG (2366C>G), TCC556GAG (2367T>G 2368C>A 2369C>G), TTC558TTT (2375C>T), AAT559CAT (2376A>C), GTT560GTC (2381T>C), GGA561GGC (2384A>C), GTT562GTC (2387T>C), GAG564GAA (2393G>A), GCA566AAC (2397G>A 2398C>A 2399A>C), AGC570TCT (2409A>T 2410G>C 2411C>T), AGT571GCC (2412A>G 2413G>C 2414T>C), TCT572AAC (2415T>A 2416C>A 2417T>C), GTT573GTG (2420T>G), GCT575CAA (2424G>C 2425C>A 2426T>A), GTG577CTA (2430G>C 2432G>A), TCA579GCT (2436T>G 2438A>T), GCG581GCC (2444G>C), CTC582TTA (2445C>T 2447C>A), GCG584GCA (2453G>A), GCT585GCA (2456T>A), CTC586CTG (2459C>G), TTT587TTC (2462T>C), GGT588AGT (2463G>A), TCC590AAG (2469T>A 2470C>A 2471C>G), AAG591AAA (2474G>A), GAG592GAA (2477G>A), ATG593ACT (2479T>C 2480G>T), TCA594TCC (2483A>C), GGA596GGC (2489A>C), TCG597TCA (2492G>A), CAA598CAG (2495A>G), CTA600CTG (2501A>G), AGG601AAG (2503G>A), TAT602TAC (2507T>C), GCC605GCT (2516C>T), TTA607TCC (2521T>C 2522A>C), ATT608CTG (2523A>C 2525T>G), ATG609ATT (2528G>T), TAC610TTC (2530A>T), GTC612GTA (2537C>A), ATC613ATT (2540C>T), TAT615TAC (2546T>C), ATT616ATA (2549T>A), TCC617TCT (2552C>T), GGC618GGT (2555C>T), GGT620GGG (2561T>G), ATT622GTA (2565A>G 2567T>A), TCG623TCA (2570G>A), GGT624GGA (2573T>A), GCC627GCA (2582C>A), CTG628TTG (2583C>T), GTT629GTA (2588T>A), GAG632GAA (2597G>A), GTG635GCG (2605T>C), CTC636CTA (2609C>A), AGA638CGT (2613A>C 2615A>T), AAG639ACC (2617A>C 2618G>C), TTC642TTT (2627C>T), ATC643GTG (2628A>G 2630C>G), AAC644AAT (2633C>T), GCC646GCA (2639C>A), TCT647ACT (2640T>A), CTG648GTC (2643C>G 2645G>C), CAT653CAC (2660T>C), AGG654CAT (2661A>C 2662G>A 2663G>T), TCA657TCT (2672A>T), TCT659TCA (2678T>A), GAA660GAG (2681A>G), AGT662TCA (2685A>T 2686G>C 2687T>A), GGG664GGT (2693G>T), GAA665GAT (2696A>T), TGT667GTA (2700T>G 2701G>T 2702T>A), ACA669ACT (2708A>T), CCT670CCG (2711T>G), ACG671CAA (2712A>C 2713C>A 2714G>A), GGT672GGC (2717T>C), ATC673ATA (2720C>A), AAA675ACC (2725A>C 2726A>C), TTC676TTT (2729C>T), GTT677ATT (2730G>A), CCA678CCT (2735A>T), CTT679TTG (2736C>T 2738T>G), GCC682GCT (2747C>T), TCG683GCT (2748T>G 2750G>T), GGG684GGC (2753G>C), GCT685GTG (2755C>T 2756T>G), CAC697CAT (2792C>T), CCC698CCT (2795C>T), TTG699TTT (2798G>T), GCT700GCC (2801T>C), GAG706GAA (2819G>A), AGC707TGC (2820A>T), CCT709CCC (2828T>C), ATC712GTT (2835A>G 2837C>T), CTG714TTA (2841C>T 2843G>A), CAG715CAT (2846G>T), AAA717AAG (2852A>G), TCG720TCC (2861G>C), ACC721ACT (2864C>T), ATC723ATA (2870C>A), CCT726CCA (2879T>A), CCT727CCG (2882T>G), CGT728CGG (2885T>G), TTG729CTG (2886T>C), GCA731GCT (2894A>T), GCA733GCT (2900A>T), GGT736GGA (2909T>A), AAA738AAG (2915A>G), GAC740GAG (2921C>G), CCA745CCT (2936A>T), CGA746CGG (2939A>G), GTA752GTT (2957A>T), CTG753TTG (2958C>T), CAC755CAT (2966C>T), AAT757AAC (2972T>C), GGA759GGG (2978A>G), GTG760GTA (2981G>A), GCT761ACT (2982G>A), GCT762GCA (2987T>A), AGC763ACC (2989G>C), GGT765GGC (2996T>C), ACT766ACA (2999T>A), TTA768CTA (3003T>C), AGC770AGT (3011C>T), ATC771GTA (3012A>G 3014C>A), TCT773TCC (3020T>C), CCG774CCT (3023G>T), ATA777ATT (3032A>T), GAC781GAT (3044C>T), GGT782GGC (3047T>C), ACG783ACA (3050G>A), TCC787TCA (3062C>A), TTG788CTG (3063T>C), ATA792ATT (3077A>T), GCA793GCT (3080A>T), CGC794CGA (3083C>A), TGC796TGT (3089C>T), ACT800ACA (3101T>A), ACT802ACA (3107T>A), GTG804GTT (3113G>T), ATT807ATA (3122T>A), ATT809GTC (3126A>G 3128T>C), GAG810GAA (3131G>A), ACT812ACC (3137T>C), TTC814TTT (3143C>T), CAT815CAC (3146T>C), GGT817GGA (3152T>A), TTG819CTT (3156T>C 3158G>T), GGA822GGT (3167A>T), ACA825ACT (3176A>T), CTG826CTA (3179G>A), AAC827AAA (3182C>A), AGC829AAC (3187G>A), ACC832ACA (3197C>A), GAC835GAT (3206C>T), TTA837CTA (3210T>C), CCG840CCC (3221G>C), AAA864AAC (3293A>C), CTT866CTG (3299T>G), ACC868TCT (3303A>T 3305C>T), ACT869ACA (3308T>A), GGT870GGG (3311T>G), TCT874TCA (3323T>A), CTA875TTA (3324C>T), CCG876CCA (3329G>A), CCC878CCA (3335C>A), CAC883CAT (3350C>T), AGG885CGG (3354A>C), TTG886CTA (3357T>C 3359G>A), ACA889ACG (3368A>G), GTC890GTG (3371C>G), TTG891ATG (3372T>A), GTA895GTT (3386A>T), ATA899ATT (3398A>T), CTA901CTG (3404A>G), ACG903ACT (3410G>T), GTC904GTT (3413C>T), GTA906GTC (3419A>C), ACA907ACG (3422A>G), CAG909CAA (3428G>A), TGC912TGA (3437C>A), GAA914GAG (3443A>G), TTG916TTA (3449G>A), GTC917GTG (3452C>G), CTT918CTA (3455T>A), GGG919GGC (3458G>C), GGC920GGA (3461C>A), GTT924GTG (3473T>G), CCC926CCT (3479C>T), ATT927ATC (3482T>C), TAC928TAT (3485C>T), GGA929GGT (3488A>T), CAC930CAT (3491C>T), GGA934GGG (3503A>G), AAT936AGT (3508A>G), GCT937GCC (3512T>C), GTG938GTT (3515G>T), TTC940TTT (3521C>T), CTA941TTG (3522C>T 3524A>G), TTC942TTT (3527C>T), GAG944GAA (3533G>A), TCT948ACT (3543T>A), CGC950CGT (3551C>T), GAG954GAA (3563G>A), TTA956CTG (3567T>C 3569A>G), CGC957CGA (3572C>A), AAG958AAA (3575G>A), AAC960AAT (3581C>T), ACA963ACC (3590A>C), CCA972CCC (3617A>C), GTA974GTT (3623A>T), CAA975GCA (3624C>G 3625A>C), GTG976CTT (3627G>C 3629G>T), CCG977GGG (3630C>G 3631C>G), CTC978CTT (3635C>T), AGA979AGG (3638A>G), GAC980GAA (3641C>A), TCA983TCT (3650A>T), GGG985GGA (3656G>A), GTA987GTT (3662A>T), GTG991GTT (3674G>T), GAC996GAT (3689C>T), CGA999CGC (3698A>C), TTG1001GTG (3702T>G), TTA1003CTA (3708T>C), TGC1005TGT (3716C>T), CCC1007CCG (3722C>G), TCC1009TCT (3728C>T), GGT1094GGA (3983T>A), ATG1095GTT (3984A>G 3986G>T), AGC1096ACC (3988G>C), GAC1097ACC (3990G>A 3991A>C), AAC1098AAT (3995C>T), ATC1099ATT (3998C>T), CAT1105CAA (4016T>A), GAG1108GAA (4025G>A), TTT1110TTC (4031T>C), AGA1112CGG (4035A>C 4037A>G), CGC1113CGG (4040C>G), ACC1115ACT (4046C>T), AAA1116AAG (4049A>G), GCC1117GCA (4052C>A), ATA1120GTT (4059A>G 4061A>T), CCA1122CCT (4067A>T), AAT1123AGG (4069A>G 4070T>G), GCT1124GGT (4072C>G), TCA1125TCT (4076A>T), AGA1126CGT (4077A>C 4079A>T), ATA1127ATT (4082A>T), CAA1131CAG (4094A>G), CTA1134CTG (4103A>G), GGA1135GGT (4106A>T), GTG1146.TA (4139G>A), ATA1147ATT (4142A>T), TCT1149TCC (4148T>C), CTG1150ATT (4149C>A 4151G>T), TTG1153ATG (4158T>A), GTC1154GTT (4163C>T), GGA1157GGG (4172A>G), TTC1160TTT (4181C>T), TTG1162TTA (4187G>A), CAA1163GAA (4188C>G), GCT1165GCC (4196T>C), TCA1169TCG (4208A>G), GCA1170GCT (4211A>T), AAG1174AAA (4223G>A), CAT1175TAT (4224C>T), TTG1176CTT (4227T>C 4229G>T), ACC1183ACT (4250C>T), CTT1185CTA (4256T>A), ACT1187ACC (4262T>C), TTT1190TTC (4271T>C), CTA1193CTG (4280A>G), AGT1195GGC (4284A>G 4286T>C), TTT1196TTC (4289T>C)

Proteins

	Begin	End	Coverage	Score	Concordance	Matches	Identities	I/D/M/F*	Stop Codons
Orf1 (YP_001040017.1)	52	188	58.0%	561	81.1%	109 (100%)	86 (78.9%)	0/0/0/0	1

Protein mutations: L57I (350C>A 352A>C), V59I (356G>A), N64T (372A>C), G84A (432G>C 433C>T), H93Q (460T>G), Q108H (505G>T), R109K (507G>A 508G>A), I110V (509A>G 511T>A), V112I (515G>A), R146P (618G>C 619C>T), L149V (626C>G 628C>A), K150N (631A>T), E151K (632G>A), L155A (644C>G 645T>C 646T>C), L158Q (654T>A), S162T (665T>A), L168I (683T>A 685G>C), S169Q (686T>C 687C>A 688C>G), N170A (689A>G 690A>C 691C>A), L172P (696T>C), D175E (706T>A), S179R (718T>G), E184K (731G>A 733A>G)

Codon mutations: GCA53GCT (340A>T), CTA57ATC (350C>A 352A>C), TCT58TCC (355T>C), GTT59ATT (356G>A), AAC63AAT (370C>T), AAT64ACT (372A>C), AGC65AGT (376C>T), GTT69GTA (388T>A), TAT73TAC (400T>C), TTA74CTG (401T>C 403A>G), CTG75CTA (406G>A), ACT79ACG (418T>G), CCA81CCC (424A>C), ACT83ACA (430T>A), GGC84GCT (432G>C 433C>T), GTT85GTC (436T>C), AAG86AAA (439G>A), CTA87CTC (442A>C), TCT88TCA (445T>A), GGC89GGA (448C>A), TTA90TTG (451A>G), CAT93CAG (460T>G), AGA94CGA (461A>C), CGT97CGA (472T>A), ACC101ACG (484C>G), AGA102AGG (487A>G), CTA105CTT (496A>T), GCT107GCA (502T>A), CAG108CAT (505G>T), AGG109AAA (507G>A 508G>A), ATT110GTA (509A>G 511T>A), TCT111TCC (514T>C), GTA112ATA (515G>A), TCC113TCA (520C>A), GAC141..A (604C>A), GAG142GAA (607G>A), GCC143GCA (610C>A), AAA144AAG (613A>G), CGC146CCT (618G>C 619C>T), TTA147TTG (622A>G), CTC149GTA (626C>G 628C>A), AAA150AAT (631A>T), GAG151AAG (632G>A), AAA152AAG (637A>G), CTT155GCC (644C>G 645T>C 646T>C), GAA156GAG (649A>G), CTA158CAA (654T>A), TCC159TCT (658C>T), ATA161ATC (664A>C), TCC162ACC (665T>A), AAA163AAG (670A>G), TTA164CTT (671T>C 673A>T), TTG166CTA (677T>C 679G>A), TTG168ATC (683T>A 685G>C), TCC169CAG (686T>C 687C>A 688C>G), AAC170GCA (689A>G 690A>C 691C>A), CTA172CCA (696T>C), AAT174AAC (703T>C), GAT175GAA (706T>A), AGT179AGG (718T>G), GAA184AAG (731G>A 733A>G)

	Begin	End	Coverage	Score	Concordance	Matches	Identities	I/D/M/F*	Stop Codons
polyprotein (YP_001040018.1)	1	1199	69.3%	5084	88.7%	831 (100%)	724 (87.1%)	0/0/0/0	3

Protein mutations: M1T (703T>C), M2K (706T>A), V6G (718T>G), K11R (733A>G), S17A (750A>G 751G>C), Y48F (844A>T), E61D (884A>T), N70K (911T>G), L128M (1083C>A), V132M (1095G>A 1097T>G), Q211H (1334G>T), T228A (1383A>G 1385C>G), S230* (1390C>A), T232P (1395A>C 1397T>A), D233E (1400T>G), Q242L (1426A>T 1427G>T), D245N (1434G>A), S252Q (1455A>C 1456G>A 1457C>G), A254S (1461G>T), N481E (2142A>G 2144C>G), S487R (2162T>A), V488G (2164T>G), E489G (2167A>G), E490S (2169G>A 2170A>G 2171A>C), I491R (2173T>G 2174A>G), P492S (2175C>T 2177A>C), S493T (2178T>A), R524S (2271A>T 2272G>C 2273G>A), N526D (2277A>G 2279T>C), E527D (2282A>T), M529F (2286A>T 2288G>C), E531D (2294A>C), Q534E (2301C>G 2303A>G), N537M (2311A>T 2312T>G), N538E (2313A>G 2315T>A), E539D (2318A>T), G540A (2320G>C 2321T>C), A541P (2322G>C 2324G>C), T542I (2326C>T 2327A>T), E543Q (2328G>C 2330G>A), N555K (2366C>G), S556E (2367T>G 2368C>A 2369C>G), N559H (2376A>C), A566N (2397G>A 2398C>A 2399A>C), S571A (2412A>G 2413G>C 2414T>C), S572N (2415T>A 2416C>A 2417T>C), A575Q (2424G>C 2425C>A 2426T>A), V577L (2430G>C 2432G>A), S579A (2436T>G 2438A>T), G588S (2463G>A), S590K (2469T>A 2470C>A 2471C>G), M593T (2479T>C 2480G>T), R601K (2503G>A), L607S (2521T>C 2522A>C), I608L (2523A>C 2525T>G), M609I (2528G>T), Y610F (2530A>T), I622V (2565A>G 2567T>A), V635A (2605T>C), K639T (2617A>C 2618G>C), I643V (2628A>G 2630C>G), S647T (2640T>A), L648V (2643C>G 2645G>C), R654H (2661A>C 2662G>A 2663G>T), E665D (2696A>T), C667V (2700T>G 2701G>T 2702T>A), T671Q (2712A>C 2713C>A 2714G>A), K675T (2725A>C 2726A>C), V677I (2730G>A), S683A (2748T>G 2750G>T), A685V (2755C>T 2756T>G), L699F (2798G>T), S707C (2820A>T), I712V (2835A>G 2837C>T), Q715H (2846G>T), D740E (2921C>G), A761T (2982G>A), S763T (2989G>C), I771V (3012A>G 3014C>A), I809V (3126A>G 3128T>C), N827K (3182C>A), S829N (3187G>A), K864N (3293A>C), T868S (3303A>T 3305C>T), L891M (3372T>A), C912* (3437C>A), N936S (3508A>G), S948T (3543T>A), Q975A (3624C>G 3625A>C), V976L (3627G>C 3629G>T), P977G (3630C>G 3631C>G), D980E (3641C>A), L1001V (3702T>G), M1095V (3984A>G 3986G>T), S1096T (3988G>C), D1097T (3990G>A 3991A>C), H1105Q (4016T>A), I1120V (4059A>G 4061A>T), N1123R (4069A>G 4070T>G), A1124G (4072C>G), L1150I (4149C>A 4151G>T), L1153M (4158T>A), Q1163E (4188C>G), H1175Y (4224C>T), S1195G (4284A>G 4286T>C)

	Begin	End	Coverage	Score	Concordance	Matches	Identities	I/D/M/F*	Stop Codons
**NT**	**30**	**5329**	**72.7%**	**5298**	**67.5%**	**3927 (99.9%)**	**3293 (83.8%)**	**1/1**	
Codon mutations:	ATG1ACG (703T>C), ATG2AAG (706T>A), GTG6GGG (718T>G), AGG10AGA (731G>A), AAG11AGG (733A>G), AGT17GCT (750A>G 751G>C), GCA18GCC (755A>C), GCT19GCA (758T>A), GTA20GTC (761A>C), ACA29ACT (788A>T), GTT32GTG (797T>G), GGC35GGG (806C>G), CTG39CTC (818G>C), CTT40CTG (821T>G), AAA42AAG (827A>G), CTG43TTG (828C>T), GGC44GGA (833C>A), TAT48TTT (844A>T), GGG49GGC (848G>C), CTC51CTG (854C>G), GGC53GGA (860C>A), GCC54GCT (863C>T), CAA55CAG (866A>G), GAG56GAA (869G>A), GAA61GAT (884A>T), CTG62CTT (887G>T), AAT70AAG (911T>G), TCT76TCA (929T>A), TCA77TCG (932A>G), TTT78TTC (935T>C), TCG85TCC (956G>C), TTG95TTA (986G>A), GTT98GTC (995T>C), GTT103GTG (1010T>G), GAA110GAG (1031A>G), GGT111GGG (1034T>G), GTA113GTC (1040A>C), TTA117TTG (1052A>G), AGT120AGC (1061T>C), ACC124ACT (1073C>T), CTG128ATG (1083C>A), GTT132ATG (1095G>A 1097T>G), TTG133TTA (1100G>A), TCC136TCT (1109C>T), GGG138GGA (1115G>A), GCA139GCT (1118A>T), TTA143TTG (1130A>G), CCG146CCT (1139G>T), ACC147ACA (1142C>A), CTA150TTG (1149C>T 1151A>G), AAA154AAG (1163A>G), GGC156GGG (1169C>G), TTC159TTT (1178C>T), ACA162ACT (1187A>T), ACT168ACA (1205T>A), CCT173CCA (1220T>A), TCA175TCT (1226A>T), GCC178GCA (1235C>A), CAA182CAG (1247A>G), CGT187CGA (1262T>A), TCA192TCG (1277A>G), CTT204CTC (1313T>C), CAC205CAT (1316C>T), CGA206AGG (1317C>A 1319A>G), CCT207CCA (1322T>A), CAA208CAG (1325A>G), TTT209TTC (1328T>C), TTC210TTT (1331C>T), CAG211CAT (1334G>T), CAG214CAA (1343G>A), CAG218CAA (1355G>A), ATA220ATT (1361A>T), TCA221TCT (1364A>T), TCA222TCT (1367A>T), GTA223GTC (1370A>C), TCT225TCG (1376T>G), ACC228GCG (1383A>G 1385C>G), TCC229TCT (1388C>T), TCA230TAA (1390C>A), GCA231GCC (1394A>C), ACT232CCA (1395A>C 1397T>A), GAT233GAG (1400T>G), TCA234TCT (1403A>T), GTG235GTA (1406G>A), CTT236TTA (1407C>T 1409T>A), CCG237CCT (1412G>T), GTG239GTA (1418G>A), GGA240GGT (1421A>T), CAG242CTT (1426A>T 1427G>T), GGC244GGG (1433C>G), GAC245AAC (1434G>A), ACA246ACT (1439A>T), GTG250GTT (1451G>T), CCA251CCC (1454A>C), AGC252CAG (1455A>C 1456G>A 1457C>G), GAA253GAG (1460A>G), GCA254TCA (1461G>T), AAA480.GA (2140A>G), AAC481GAG (2142A>G 2144C>G), ACT486ACA (2159T>A), AGT487AGA (2162T>A), GTG488GGG (2164T>G), GAA489GGA (2167A>G), GAA490AGC (2169G>A 2170A>G 2171A>C), ATA491AGG (2173T>G 2174A>G), CCA492TCC (2175C>T 2177A>C), TCC493ACC (2178T>A), ACC494ACT (2183C>T), AGG524TCA (2271A>T 2272G>C 2273G>A), CCC525CCT (2276C>T), AAT526GAC (2277A>G 2279T>C), GAA527GAT (2282A>T), GTT528GTC (2285T>C), ATG529TTC (2286A>T 2288G>C), GAA531GAC (2294A>C), CAA534GAG (2301C>G 2303A>G), AAT537ATG (2311A>T 2312T>G), AAT538GAA (2313A>G 2315T>A), GAA539GAT (2318A>T), GGT540GCC (2320G>C 2321T>C), GCG541CCC (2322G>C 2324G>C), ACA542ATT (2326C>T 2327A>T), GAG543CAA (2328G>C 2330G>A), CTG544TTG (2331C>T), TTA546TTG (2339A>G), CAA548CAG (2345A>G), GTG552GTT (2357G>T), AGT554AGC (2363T>C), AAC555AAG (2366C>G), TCC556GAG (2367T>G 2368C>A 2369C>G), TTC558TTT (2375C>T), AAT559CAT (2376A>C), GTT560GTC (2381T>C), GGA561GGC (2384A>C), GTT562GTC (2387T>C), GAG564GAA (2393G>A), GCA566AAC (2397G>A 2398C>A 2399A>C), AGC570TCT (2409A>T 2410G>C 2411C>T), AGT571GCC (2412A>G 2413G>C 2414T>C), TCT572AAC (2415T>A 2416C>A 2417T>C), GTT573GTG (2420T>G), GCT575CAA (2424G>C 2425C>A 2426T>A), GTG577CTA (2430G>C 2432G>A), TCA579GCT (2436T>G 2438A>T), GCG581GCC (2444G>C), CTC582TTA (2445C>T 2447C>A), GCG584GCA (2453G>A), GCT585GCA (2456T>A), CTC586CTG (2459C>G), TTT587TTC (2462T>C), GGT588AGT (2463G>A), TCC590AAG (2469T>A 2470C>A 2471C>G), AAG591AAA (2474G>A), GAG592GAA (2477G>A), ATG593ACT (2479T>C 2480G>T), TCA594TCC (2483A>C), GGA596GGC (2489A>C), TCG597TCA (2492G>A), CAA598CAG (2495A>G), CTA600CTG (2501A>G), AGG601AAG (2503G>A), TAT602TAC (2507T>C), GCC605GCT (2516C>T), TTA607TCC (2521T>C 2522A>C), ATT608CTG (2523A>C 2525T>G), ATG609ATT (2528G>T), TAC610TTC (2530A>T), GTC612GTA (2537C>A), ATC613ATT (2540C>T), TAT615TAC (2546T>C), ATT616ATA (2549T>A), TCC617TCT (2552C>T), GGC618GGT (2555C>T), GGT620GGG (2561T>G), ATT622GTA (2565A>G 2567T>A), TCG623TCA (2570G>A), GGT624GGA (2573T>A), GCC627GCA (2582C>A), CTG628TTG (2583C>T), GTT629GTA (2588T>A), GAG632GAA (2597G>A), GTG635GCG (2605T>C), CTC636CTA (2609C>A), AGA638CGT (2613A>C 2615A>T), AAG639ACC (2617A>C 2618G>C), TTC642TTT (2627C>T), ATC643GTG (2628A>G 2630C>G), AAC644AAT (2633C>T), GCC646GCA (2639C>A), TCT647ACT (2640T>A), CTG648GTC (2643C>G 2645G>C), CAT653CAC (2660T>C), AGG654CAT (2661A>C 2662G>A 2663G>T), TCA657TCT (2672A>T), TCT659TCA (2678T>A), GAA660GAG (2681A>G), AGT662TCA (2685A>T 2686G>C 2687T>A), GGG664GGT (2693G>T), GAA665GAT (2696A>T), TGT667GTA (2700T>G 2701G>T 2702T>A), ACA669ACT (2708A>T), CCT670CCG (2711T>G), ACG671CAA (2712A>C 2713C>A 2714G>A), GGT672GGC (2717T>C), ATC673ATA (2720C>A), AAA675ACC (2725A>C 2726A>C), TTC676TTT (2729C>T), GTT677ATT (2730G>A), CCA678CCT (2735A>T), CTT679TTG (2736C>T 2738T>G), GCC682GCT (2747C>T), TCG683GCT (2748T>G 2750G>T), GGG684GGC (2753G>C), GCT685GTG (2755C>T 2756T>G), CAC697CAT (2792C>T), CCC698CCT (2795C>T), TTG699TTT (2798G>T), GCT700GCC (2801T>C), GAG706GAA (2819G>A), AGC707TGC (2820A>T), CCT709CCC (2828T>C), ATC712GTT (2835A>G 2837C>T), CTG714TTA (2841C>T 2843G>A), CAG715CAT (2846G>T), AAA717AAG (2852A>G), TCG720TCC (2861G>C), ACC721ACT (2864C>T), ATC723ATA (2870C>A), CCT726CCA (2879T>A), CCT727CCG (2882T>G), CGT728CGG (2885T>G), TTG729CTG (2886T>C), GCA731GCT (2894A>T), GCA733GCT (2900A>T), GGT736GGA (2909T>A), AAA738AAG (2915A>G), GAC740GAG (2921C>G), CCA745CCT (2936A>T), CGA746CGG (2939A>G), GTA752GTT (2957A>T), CTG753TTG (2958C>T), CAC755CAT (2966C>T), AAT757AAC (2972T>C), GGA759GGG (2978A>G), GTG760GTA (2981G>A), GCT761ACT (2982G>A), GCT762GCA (2987T>A), AGC763ACC (2989G>C), GGT765GGC (2996T>C), ACT766ACA (2999T>A), TTA768CTA (3003T>C), AGC770AGT (3011C>T), ATC771GTA (3012A>G 3014C>A), TCT773TCC (3020T>C), CCG774CCT (3023G>T), ATA777ATT (3032A>T), GAC781GAT (3044C>T), GGT782GGC (3047T>C), ACG783ACA (3050G>A), TCC787TCA (3062C>A), TTG788CTG (3063T>C), ATA792ATT (3077A>T), GCA793GCT (3080A>T), CGC794CGA (3083C>A), TGC796TGT (3089C>T), ACT800ACA (3101T>A), ACT802ACA (3107T>A), GTG804GTT (3113G>T), ATT807ATA (3122T>A), ATT809GTC (3126A>G 3128T>C), GAG810GAA (3131G>A), ACT812ACC (3137T>C), TTC814TTT (3143C>T), CAT815CAC (3146T>C), GGT817GGA (3152T>A), TTG819CTT (3156T>C 3158G>T), GGA822GGT (3167A>T), ACA825ACT (3176A>T), CTG826CTA (3179G>A), AAC827AAA (3182C>A), AGC829AAC (3187G>A), ACC832ACA (3197C>A), GAC835GAT (3206C>T), TTA837CTA (3210T>C), CCG840CCC (3221G>C), AAA864AAC (3293A>C), CTT866CTG (3299T>G), ACC868TCT (3303A>T 3305C>T), ACT869ACA (3308T>A), GGT870GGG (3311T>G), TCT874TCA (3323T>A), CTA875TTA (3324C>T), CCG876CCA (3329G>A), CCC878CCA (3335C>A), CAC883CAT (3350C>T), AGG885CGG (3354A>C), TTG886CTA (3357T>C 3359G>A), ACA889ACG (3368A>G), GTC890GTG (3371C>G), TTG891ATG (3372T>A), GTA895GTT (3386A>T), ATA899ATT (3398A>T), CTA901CTG (3404A>G), ACG903ACT (3410G>T), GTC904GTT (3413C>T), GTA906GTC (3419A>C), ACA907ACG (3422A>G), CAG909CAA (3428G>A), TGC912TGA (3437C>A), GAA914GAG (3443A>G), TTG916TTA (3449G>A), GTC917GTG (3452C>G), CTT918CTA (3455T>A), GGG919GGC (3458G>C), GGC920GGA (3461C>A), GTT924GTG (3473T>G), CCC926CCT (3479C>T), ATT927ATC (3482T>C), TAC928TAT (3485C>T), GGA929GGT (3488A>T), CAC930CAT (3491C>T), GGA934GGG (3503A>G), AAT936AGT (3508A>G), GCT937GCC (3512T>C), GTG938GTT (3515G>T), TTC940TTT (3521C>T), CTA941TTG (3522C>T 3524A>G), TTC942TTT (3527C>T), GAG944GAA (3533G>A), TCT948ACT (3543T>A), CGC950CGT (3551C>T), GAG954GAA (3563G>A), TTA956CTG (3567T>C 3569A>G), CGC957CGA (3572C>A), AAG958AAA (3575G>A), AAC960AAT (3581C>T), ACA963ACC (3590A>C), CCA972CCC (3617A>C), GTA974GTT (3623A>T), CAA975GCA (3624C>G 3625A>C), GTG976CTT (3627G>C 3629G>T), CCG977GGG (3630C>G 3631C>G), CTC978CTT (3635C>T), AGA979AGG (3638A>G), GAC980GAA (3641C>A), TCA983TCT (3650A>T), GGG985GGA (3656G>A), GTA987GTT (3662A>T), GTG991GTT (3674G>T), GAC996GAT (3689C>T), CGA999CGC (3698A>C), TTG1001GTG (3702T>G), TTA1003CTA (3708T>C), TGC1005TGT (3716C>T), CCC1007CCG (3722C>G), TCC1009TCT (3728C>T), GGT1094GGA (3983T>A), ATG1095GTT (3984A>G 3986G>T), AGC1096ACC (3988G>C), GAC1097ACC (3990G>A 3991A>C), AAC1098AAT (3995C>T), ATC1099ATT (3998C>T), CAT1105CAA (4016T>A), GAG1108GAA (4025G>A), TTT1110TTC (4031T>C), AGA1112CGG (4035A>C 4037A>G), CGC1113CGG (4040C>G), ACC1115ACT (4046C>T), AAA1116AAG (4049A>G), GCC1117GCA (4052C>A), ATA1120GTT (4059A>G 4061A>T), CCA1122CCT (4067A>T), AAT1123AGG (4069A>G 4070T>G), GCT1124GGT (4072C>G), TCA1125TCT (4076A>T), AGA1126CGT (4077A>C 4079A>T), ATA1127ATT (4082A>T), CAA1131CAG (4094A>G), CTA1134CTG (4103A>G), GGA1135GGT (4106A>T), GTG1146.TA (4139G>A), ATA1147ATT (4142A>T), TCT1149TCC (4148T>C), CTG1150ATT (4149C>A 4151G>T), TTG1153ATG (4158T>A), GTC1154GTT (4163C>T), GGA1157GGG (4172A>G), TTC1160TTT (4181C>T), TTG1162TTA (4187G>A), CAA1163GAA (4188C>G), GCT1165GCC (4196T>C), TCA1169TCG (4208A>G), GCA1170GCT (4211A>T), AAG1174AAA (4223G>A), CAT1175TAT (4224C>T), TTG1176CTT (4227T>C 4229G>T), ACC1183ACT (4250C>T), CTT1185CTA (4256T>A), ACT1187ACC (4262T>C), TTT1190TTC (4271T>C), CTA1193CTG (4280A>G), AGT1195GGC (4284A>G 4286T>C), TTT1196TTC (4289T>C)								

*: Inserts / Deletes / Misaligned / Frameshifts

Analysis details

This analysis was performed with panviral2.64

NGS Details (UN18_val): Torradovirus marchitezum (segment RNA 1)

Assembly

**Coverage Length**	5735 (1 contig(s))
**Depth Of Coverage**	116.5
**Number Of Reads**	5075
**Reads Per Million**	95.25 rpm (after QC)
**Ambiguities**	0
**Assembly Method**	de novo + reference guided assembly
**Consensus Caller**	Bcf Tools

Coverage Map

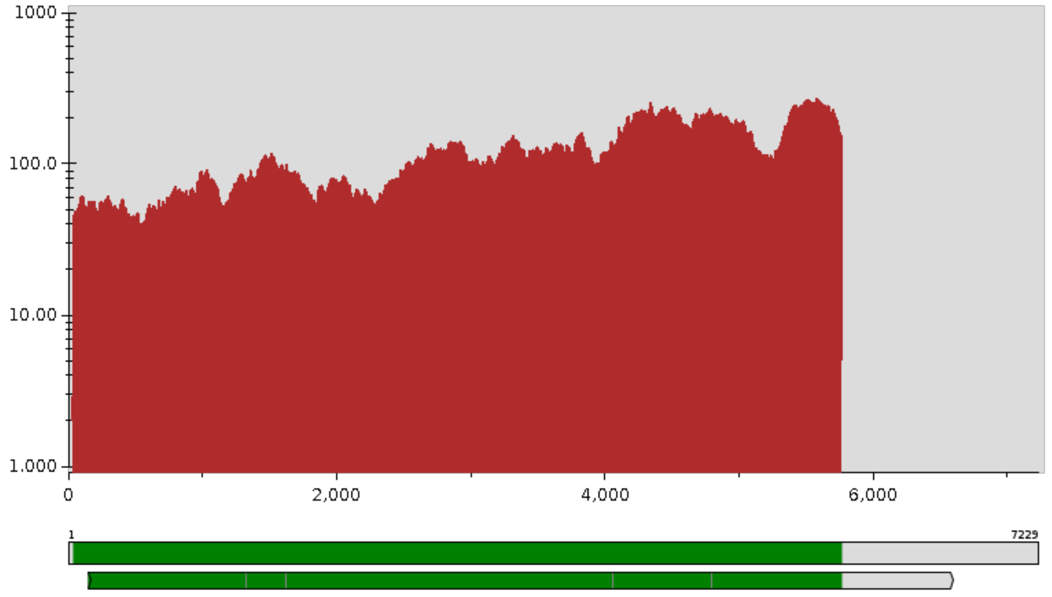

Assignment

**Type**	Torradovirus marchitezum (Taxonomy ID: 3048376)
**Reference Genome**	NC_010987.1
**NT Identity (%)**	65.7968
**AA Identity (%)**	68.984
**Number Of Stop Codons**	0
**Number Of CDS**	1

Alignment

**Alignment Score**	3466.0 (NT) + 9403.0 (AA) = 12869.0
**Concordance (%)**	52.9392

Alignment Method	Global, seeded, nucleotide + amino acids (AGA)

Genome Region

Sequence starts at position 35 and ends at position 5769 relative to NC_010987.1 reference sequence.

Alignment Detailed Statistics

	Begin	End	Coverage	Score	Concordance	Matches	Identities	I/D/M/F*	Stop Codons
NT	35	5769	79.3%	3466	30.8%	5682 (98.6%)	3757 (65.2%)	28/53	

	Begin	End	Coverage	Score	Concordance	Matches	Identities	I/D/M/F*	Stop Codons
**NT**	**35**	**5769**	**79.3%**	**3466**	**30.8%**	**5682 (98.6%)**	**3757 (65.2%)**	**28/53**	

Mutations:

41A>C, 44G>T, 45A>T, 50G>C, 51C>T, 52C>T, 55T>A, 57T>A, 58T>A, 62T>C, 63G>C, 66A>T, 66_67insGTT, 70G>A, 74C>T, 75T>G, 79A>T, 80A>C, 81A>C, 82C>A, 88C>T, 88_89insG, 91C>T, 92A>T, 95T>C, 96T>C, 97C>A, 105C>T, 106T>C, 108A>G, 109C>G, 111C>T, 112G>T, 113A>T, 117T>C, 118T>A, 121T>A, 122T>C, 123A>C, 125A>C, 127T>A, 128_135delCCTCAATT, 139C>A, 146T>A, 155G>A, 158G>C, 159T>G, 161C>T, 162_191delTCCAAGGTTGCTGGTGTTGATGACGCCAGT, 198T>G, 200C>T, 203C>T, 204A>C, 209A>T, 212C>T, 213T>C, 216G>T, 217G>C, 221A>G, 222C>T, 224T>G, 227A>T, 228A>C, 230T>G, 231T>A, 233T>A, 234A>G, 236T>G, 239C>A, 240A>T, 241A>C, 243T>C, 245T>G, 248A>C, 251A>G, 252G>A, 254G>A, 255G>A, 257T>G, 259G>A, 260T>G, 262A>G, 266T>G, 268C>G, 269C>T, 271C>A, 272T>A, 275G>A, 278A>T, 279C>G, 284G>A, 285A>T, 287T>C, 292A>G, 293T>C, 297C>A, 302G>A, 305T>A, 306C>T, 308G>A, 314A>T, 317T>A, 318G>A, 320G>T, 321A>G, 322C>T, 324G>A, 326T>A, 327G>A, 328T>C, 329G>T, 330T>G, 332C>A, 333A>G, 334C>A, 335A>T, 338T>C, 342G>A, 343T>A, 344G>T, 345G>A, 346C>G, 347C>T, 348A>G, 349C>T, 359A>T, 360C>G, 362T>A, 363A>G, 365T>A, 369G>T, 371T>A, 374G>A, 376A>G, 377C>G, 385G>C, 386T>C, 387G>A, 389A>G, 390C>G, 395A>T, 398T>A, 401A>T, 405C>G, 412C>G, 413G>T, 414T>A, 415A>T, 417T>A, 418C>A, 425G>C, 426T>A, 427C>A, 428C>A, 437C>T, 439A>G, 441T>A, 443T>C, 446C>A, 448A>T, 449G>T, 459T>C, 461A>G, 465T>G, 467G>T, 468T>G, 471G>T, 472A>T, 474A>G, 476T>C, 477T>A, 479G>C, 480T>A, 481C>A, 482C>A, 488A>T, 489G>T, 493A>T, 494A>T, 495T>A, 496T>G, 502T>A, 504G>T, 506T>A, 510G>C, 515A>G, 516A>G, 518T>A, 520A>C, 523G>C, 524T>A, 526A>G, 527A>T, 530T>A, 531G>T, 532T>A, 533A>T, 537A>G, 539A>T, 542T>A, 545A>T, 546T>A, 547A>T, 548T>A, 550G>C, 557T>A, 560A>C, 564G>T, 566T>C, 567A>C, 569A>T, 570A>T, 573T>C, 575G>A, 578A>G, 579G>T, 581G>A, 582C>T, 584T>A, 586T>A, 588T>A, 590T>A, 591G>A, 593T>C, 600A>G, 601G>T, 602T>A, 611T>A, 614G>A, 615G>A, 616G>T, 617T>G, 621T>A, 622C>G, 623T>A, 625A>G, 626C>T, 627A>G, 628C>T, 629T>A, 632C>T, 633A>T, 634A>G, 635A>T, 641C>A, 645T>C, 647G>A, 648A>G, 650A>T, 653C>A, 656A>T, 660A>T, 662G>C, 665T>C, 668A>T, 669A>T, 670G>C, 674A>G, 677C>T, 681T>A, 683A>T, 684G>A, 685A>G, 695A>C, 696A>C, 697A>T, 698A>C, 702G>T, 704C>T, 707A>G, 708C>A, 709G>A, 713C>T, 714A>T, 716C>T, 717A>C, 718T>A, 720G>A, 725C>A, 728A>G, 732A>T, 733G>C, 735A>G, 740T>G, 746G>T, 749A>G, 750T>A, 751C>G, 752A>C, 753G>T, 755T>A, 756T>A, 757C>A, 758A>T, 770G>C, 771T>A, 772C>G, 776G>A, 780A>C, 785C>T, 786A>T, 788A>T, 789A>T, 791A>G, 792T>A, 794G>A, 797T>A, 803G>C, 806T>C, 809G>A, 818G>A, 821C>T, 822A>G, 824T>G, 830G>A, 839A>G, 849T>C, 851G>T, 853G>C, 854C>A, 860A>G, 861T>C, 863G>A, 873C>T, 875C>G, 881G>A, 882A>G, 884A>G, 887A>C, 893T>A, 899G>A, 914A>C, 918A>C, 919C>A, 920T>A, 921C>T, 923T>G, 926C>T, 927C>A, 928G>A, 929T>G, 932T>C, 938A>G, 947C>G, 962A>T, 968T>G, 969C>T, 971T>G, 977A>T, 978A>G, 980A>T, 982T>C, 983C>T, 985G>A, 986C>T, 988C>T, 989C>A, 992T>C, 995A>G, 998C>T, 1004T>C, 1010G>T, 1011T>C, 1016A>T, 1019T>A, 1022A>T, 1031G>A, 1032C>A, 1034G>T, 1043T>C, 1046G>C, 1049C>T, 1059C>A, 1060A>C, 1061G>T, 1064G>A, 1068G>T, 1069A>C, 1077T>G, 1078G>C, 1091G>A, 1092C>A, 1094C>G, 1100G>A, 1109C>T, 1113A>C, 1114A>C, 1115G>T, 1121C>T, 1122C>T, 1124T>A, 1126C>T, 1142G>T, 1144G>A, 1145G>A, 1148A>G, 1149G>C, 1151A>G, 1154T>A, 1163C>A, 1169T>C, 1172C>T, 1178A>T, 1187A>T, 1190T>G, 1199C>T, 1200A>T, 1205C>T, 1211G>A, 1215A>T, 1216G>C, 1221G>A, 1223C>A, 1228C>T, 1232G>A, 1235A>G, 1238A>T, 1242G>C, 1244T>C, 1245T>A, 1246A>T, 1247T>A, 1250T>A, 1255G>A, 1256T>C, 1260A>C, 1261G>A, 1262G>A, 1265C>T, 1267T>C, 1268G>A, 1271C>A, 1277C>T, 1283T>C, 1284C>A, 1286T>A, 1295T>C, 1304A>T, 1313C>T, 1316G>T, 1317T>G, 1318C>A, 1319C>G, 1325G>A, 1328T>C, 1331T>C, 1337T>C, 1340A>G, 1341T>A, 1342C>G, 1349C>A, 1350T>C, 1352A>G, 1355T>C, 1358T>C, 1367G>A, 1373C>T, 1374A>G, 1375G>C, 1376A>C, 1380G>C, 1381G>A, 1382T>A, 1388A>C, 1391A>G, 1394C>G, 1397T>C, 1398C>A, 1401C>T, 1403A>G, 1412A>T, 1419A>C, 1421G>C, 1427A>T, 1430T>C, 1436C>A, 1439C>T, 1463G>C, 1472C>T, 1478A>T, 1482T>C, 1487A>G, 1493G>A, 1499G>C, 1502G>A, 1508A>G, 1511T>C, 1517A>G, 1520T>C, 1523G>C, 1529T>C, 1538A>C, 1553C>A, 1556A>G, 1559A>C, 1565C>T, 1571T>A, 1574G>A, 1583C>G, 1584A>C, 1595C>A, 1598C>A, 1604T>C, 1616A>C, 1622T>C, 1623C>T, 1626A>G, 1628C>T, 1634T>A, 1637T>A, 1640C>A, 1646T>C, 1649T>G, 1652T>G, 1661A>T, 1673G>A, 1674C>A, 1679A>G, 1680A>C, 1688C>T, 1689C>T, 1697G>A, 1700T>C, 1701A>C, 1702T>G, 1706G>A, 1708A>T, 1712C>G, 1715G>A, 1718T>C, 1721C>A, 1724T>A, 1727A>G, 1728G>A, 1730T>C, 1733T>A, 1736G>A, 1738G>C, 1748A>C, 1751T>C, 1757A>C, 1758G>A, 1760G>T, 1762A>G, 1763G>A, 1769A>G, 1772A>G, 1781T>C, 1784C>A, 1787T>A, 1793G>A, 1794A>G, 1796T>C, 1797T>C, 1799G>C, 1802C>T, 1806A>T, 1807G>C, 1811T>C, 1812C>T, 1814T>G, 1820G>A, 1829A>G, 1835A>G, 1838T>C, 1839C>A, 1844A>C, 1847T>C, 1848G>C, 1849C>A, 1850A>C, 1854G>A, 1856A>T, 1857T>G, 1861A>C, 1862G>C, 1866T>C, 1868G>C, 1870T>A, 1871T>C, 1872G>A, 1874A>C, 1875G>C, 1876C>A, 1877T>G, 1878G>T, 1879A>C, 1881T>A, 1883T>A, 1886C>T, 1887A>T, 1888T>G, 1889A>T, 1890A>G, 1891G>A, 1892T>A, 1893C>G, 1894C>A, 1895T>G, 1898G>A, 1899A>G, 1901A>T, 1904T>C, 1910T>G, 1911C>G, 1921C>T, 1922A>T, 1925T>A, 1928T>A, 1934A>G, 1935G>A, 1937G>A, 1940T>C, 1941G>T, 1943G>T, 1944G>C, 1948G>A, 1949G>T, 1953A>C, 1954G>A, 1956T>A, 1958G>A, 1959C>A, 1962A>T, 1963C>T, 1964C>T, 1965A>G, 1967G>C, 1968C>G, 1972A>T, 1974G>A, 1975A>G, 1976A>C, 1979A>C, 1981T>G, 1982A>T, 1983T>G, 1985G>T, 1986_1991delGTGCCC, 1994T>A, 1998G>C, 2000G>C, 2001A>T, 2002G>C, 2006A>G, 2007A>G, 2019A>G, 2020T>G, 2021C>A, 2025G>T, 2028A>C, 2029G>A, 2030A>G, 2033G>A, 2036C>A, 2039G>T, 2042A>G, 2045G>A, 2049T>C, 2054A>G, 2060A>T, 2063C>T, 2066G>A, 2069C>A, 2070A>G, 2071G>C, 2072T>A, 2075G>A, 2076G>A, 2081T>A, 2082G>T, 2083A>T, 2084G>T, 2088C>T, 2089T>A, 2096T>C, 2102C>T, 2103C>G, 2104A>C, 2106C>T, 2107A>C, 2108T>A, 2117C>A, 2120G>T, 2122A>T, 2127T>G, 2128C>T, 2129T>C, 2132T>G, 2133G>T, 2134A>C, 2135T>A, 2136G>A, 2137A>G, 2142C>G, 2143A>C, 2144T>C, 2152T>G, 2153T>C, 2156C>T, 2158A>C, 2159C>T, 2160A>C, 2161A>G, 2162A>C, 2166A>G, 2167G>T, 2169G>A, 2170T>A, 2171C>T, 2172A>G, 2174C>G, 2178T>A, 2180G>T, 2183T>C, 2184A>G, 2185G>A, 2186A>G, 2189A>G, 2192A>T, 2193G>T, 2194A>C, 2195T>G, 2196G>A, 2200A>C, 2202C>G, 2203A>G, 2204G>A, 2208C>G, 2209G>C, 2210A>C, 2217G>A, 2219A>C, 2220C>A, 2222A>G, 2223A>G, 2224A>C, 2228T>C, 2231T>A, 2241A>G, 2243A>C, 2255G>A, 2258T>C, 2268G>A, 2269C>G, 2270A>T, 2271T>A, 2277A>G, 2279C>G, 2282T>A, 2285G>A, 2286A>G, 2287A>C, 2288G>T, 2289A>C, 2291A>C, 2292C>A, 2294A>G, 2296C>A, 2297A>T, 2300G>A, 2303G>A, 2305C>G, 2306A>G, 2307G>A, 2308A>T, 2310A>T, 2313A>T, 2314G>A, 2315G>C, 2317C>A, 2321A>C, 2322T>A, 2323T>G, 2324T>A, 2331T>A, 2332C>G, 2333T>A, 2334T>G, 2335G>C, 2336C>A, 2337A>C, 2339T>G, 2342T>A, 2345A>T, 2350T>C, 2357C>T, 2358C>T, 2360C>A, 2363A>G, 2369C>T, 2372G>A, 2375G>A, 2381A>T, 2385T>C, 2386T>A, 2390T>C, 2393G>A, 2396T>C, 2397T>C, 2402T>C, 2415A>C, 2417A>G, 2426_2427insAGAAGG, 2429C>G, 2431A>T, 2432A>G, 2435A>C, 2436T>A, 2438A>T, 2441A>G, 2444G>A, 2445C>A, 2448_2453delAAGGAA, 2456A>T, 2460A>G, 2462C>A, 2463A>C, 2466A>T, 2467T>C, 2468T>G, 2469T>C, 2470G>T, 2474C>T, 2475C>G, 2477T>C, 2478A>T, 2479A>C, 2480A>C, 2482T>C, 2483C>A, 2484G>A, 2488A>G, 2489A>G, 2490T>A, 2491A>G, 2492T>A, 2495C>G, 2496A>C, 2498G>A, 2501T>A, 2503C>T, 2504A>G, 2508T>A, 2509G>A, 2510G>T, 2511A>G, 2512G>A, 2513C>T, 2516T>G, 2522T>C, 2523G>A, 2524C>T, 2525A>T, 2526C>T, 2530G>A, 2537C>T, 2549T>G, 2556A>C, 2561G>A, 2567T>C, 2573A>G, 2574G>A, 2575T>C, 2577T>A, 2579T>G, 2582A>T, 2584G>C, 2586A>G, 2588T>A, 2591A>T, 2597T>C, 2598T>A, 2600G>T, 2601G>T, 2603G>T, 2610C>A, 2612G>A, 2615T>C, 2617A>G, 2620C>T, 2621T>G, 2622T>A, 2624C>T, 2629A>C, 2631G>T, 2633A>C, 2634T>C, 2635C>A, 2641G>A, 2651G>T, 2663C>G, 2669T>C, 2675C>A, 2678T>C, 2693T>A, 2695A>G, 2696T>A, 2696_2697insTCC, 2697A>C, 2699G>T, 2700C>G, 2702C>A, 2703A>T, 2704G>C, 2708T>G, 2709C>A, 2711T>G, 2714C>G, 2715G>T, 2718G>A, 2720T>C, 2729T>A, 2733T>C, 2736T>A, 2737T>A, 2738G>T, 2740C>A, 2741A>T, 2744A>G, 2744_2745insGGA, 2745G>A, 2747T>A, 2754G>T, 2756T>C, 2757G>A, 2758A>G, 2759A>G, 2765A>T, 2771A>G, 2774T>C, 2775C>G, 2777G>A, 2778A>T, 2779G>C, 2780C>T, 2783C>T, 2784A>G, 2785A>C, 2786G>C, 2798G>A, 2802T>C, 2807A>G, 2808C>G, 2810T>G, 2812G>C, 2813T>A, 2822G>T, 2829A>C, 2831A>T, 2834C>A, 2837C>T, 2846T>C, 2847T>A, 2848C>G, 2849T>C, 2851G>A, 2852T>A, 2864C>T, 2870T>A, 2873T>C, 2876A>T, 2880C>A, 2881A>C, 2882C>G, 2883A>C, 2885T>G, 2886G>C, 2887C>T, 2888T>C, 2891A>T, 2897T>A, 2900T>C, 2907A>T, 2915C>T, 2920C>A, 2921C>A, 2922A>C, 2928C>G, 2929G>T, 2930A>C, 2932C>G, 2933T>C, 2934A>C, 2936A>G, 2939T>G, 2948C>T, 2953G>A, 2955G>C, 2957A>T, 2958T>G, 2960A>C, 2962G>A, 2964A>T, 2967A>T, 2969T>G, 2970G>C, 2971A>C, 2972G>T, 2974G>A, 2975C>A, 2981C>T, 2982A>G, 2984C>T, 2985A>G, 2987T>A, 2988C>T, 2990G>A, 2991T>C, 2994C>A, 2996C>G, 3002T>C, 3003G>T, 3004A>G, 3008A>T, 3011T>C, 3014T>G, 3017A>T, 3020G>A, 3021C>G, 3022C>T, 3023T>C, 3030C>A, 3032T>A, 3035A>G, 3041C>T, 3042C>G, 3044A>T, 3045A>G, 3046G>A, 3055A>C, 3056G>A, 3071G>A, 3074C>A, 3077A>T, 3078C>G, 3083A>C, 3089C>T, 3090T>G, 3091G>T, 3092T>C, 3098T>A, 3101T>G, 3104G>A, 3107T>A, 3109C>A, 3116A>T, 3117C>G, 3118G>A, 3119T>G, 3120C>A, 3121T>A, 3122T>A, 3123G>C, 3124G>A, 3125C>G, 3128T>C, 3131A>G, 3134G>C, 3144A>T, 3145T>A, 3146G>T, 3147C>T, 3161A>C, 3164G>A, 3170C>T, 3175T>A, 3176T>G, 3182G>C, 3183G>A, 3184A>C, 3188T>C, 3189G>A, 3190A>G, 3191A>G, 3192C>A, 3196A>G, 3198T>A, 3200C>A, 3201T>A, 3202C>A, 3203C>T, 3204A>T, 3205G>C, 3206T>A, 3210A>C, 3212G>C, 3212_3213insTCA, 3215T>C, 3216T>A, 3218C>T, 3219T>G, 3225C>G, 3228T>G, 3230T>C, 3242C>T, 3243A>G, 3245A>C, 3249G>A, 3251G>C, 3263C>T, 3269C>A, 3272G>T, 3279C>T, 3281G>A, 3284A>G, 3288A>T, 3289G>C, 3291G>A, 3293T>C, 3296G>A, 3299T>C, 3308T>A, 3309G>T, 3310T>G, 3311G>C, 3314C>T, 3320C>T, 3323C>T, 3326C>T, 3332A>T, 3335A>C, 3343G>A, 3344C>A, 3346T>C, 3347A>T, 3351G>A, 3353T>A, 3356A>G, 3357C>T, 3358G>T, 3359T>G, 3366A>G, 3368C>A, 3369T>G, 3371T>A, 3380T>C, 3383A>T, 3387A>G, 3389C>A, 3398A>C, 3401A>G, 3404A>G, 3405G>C, 3407G>A, 3414C>G, 3416A>T, 3417A>C, 3418C>A, 3419T>A, 3425G>A, 3428C>T, 3432G>A, 3433C>T, 3434A>G, 3437A>C, 3443A>G, 3444C>A, 3446C>A, 3452T>C, 3453A>T, 3455T>G, 3458G>C, 3461G>A, 3462T>C, 3463C>A, 3464A>T, 3465A>G, 3468A>C, 3471A>T, 3472G>C, 3473C>A, 3482A>T, 3483C>G, 3484A>C, 3485G>T, 3488T>C, 3494G>C, 3500C>G, 3506G>T, 3507T>G, 3508A>C, 3509C>T, 3511A>T, 3512C>T, 3513C>T, 3515T>G, 3518A>C, 3521A>T, 3527A>T, 3530G>A, 3534T>A, 3539C>A, 3542C>A, 3545A>G, 3548T>G, 3551C>A, 3563T>C, 3566G>C, 3567C>A, 3577A>C, 3579T>C, 3587A>G, 3593C>T, 3596A>T, 3597C>G, 3598C>A, 3599T>A, 3603G>C, 3605G>A, 3608A>T, 3611A>T, 3614A>T, 3617C>A, 3618A>C, 3620T>G, 3623A>C, 3624A>T, 3626A>T, 3629T>G, 3630C>A, 3632T>A, 3635C>T, 3644T>G, 3650T>A, 3656C>T, 3659C>T, 3665G>T, 3671G>C, 3672G>A, 3674A>T, 3675A>G, 3676T>A, 3695T>C, 3697G>C, 3698T>A, 3699G>A, 3700G>A, 3701A>T, 3707T>G, 3710T>C, 3711G>A, 3712A>G, 3719C>A, 3720A>C, 3721A>G, 3722A>G, 3723C>G, 3724T>C, 3725A>T, 3731C>T, 3735A>G, 3736A>G, 3745G>C, 3746C>T, 3747T>G, 3749G>C, 3750C>A, 3752G>T, 3753C>T, 3758T>A, 3759A>G, 3761C>T, 3762A>C, 3764G>C, 3765C>A, 3766G>A, 3767T>G, 3770C>T, 3773G>A, 3775A>G, 3777A>G, 3779C>G, 3782T>A, 3785G>T, 3788A>T, 3789C>A, 3790G>T, 3791T>C, 3794A>C, 3795T>A, 3796G>C, 3797T>A, 3798G>A, 3799A>C, 3801A>C, 3803G>T, 3804G>T, 3805A>G, 3812T>C, 3813G>A, 3815A>C, 3818G>C, 3821C>G, 3827A>T, 3828A>G, 3830A>T, 3836A>T, 3838G>T, 3842T>C, 3844A>T, 3847A>T, 3852G>T, 3853G>C, 3860G>A, 3863G>C, 3872T>A, 3875C>T, 3881T>C, 3884C>T, 3890C>T, 3893G>A, 3894A>C, 3899G>T, 3900A>T, 3905G>T, 3907T>C, 3908G>C, 3911C>T, 3914G>A, 3917A>G, 3923T>C, 3926G>T, 3927C>G, 3930T>G, 3932C>A, 3933A>C, 3934C>A, 3935T>G, 3937G>A, 3938C>G, 3939G>A, 3940A>G, 3941A>T, 3942G>A, 3943G>C, 3944A>C, 3945G>C, 3947G>A, 3950A>G, 3953G>A, 3954G>C, 3955A>T, 3962A>G, 3966G>A, 3968C>T, 3969A>T, 3977A>T, 3981G>C, 3992A>G, 3995G>A, 3996C>T, 4005C>G, 4007G>A, 4010T>A, 4016A>G, 4019C>T, 4021T>A, 4023C>T, 4031T>A, 4032C>A, 4037C>T, 4043C>T, 4049C>T, 4052C>T, 4055A>G, 4058T>C, 4062C>A, 4064C>G, 4067C>A, 4070A>T, 4073T>G, 4076G>C, 4079A>G, 4082C>A, 4085T>C, 4088C>G, 4091G>A, 4093G>A, 4094A>G, 4097G>A, 4101A>C, 4103G>T, 4106C>T, 4115A>T, 4116C>T, 4118T>G, 4121T>A, 4130C>T, 4137T>C, 4142C>G, 4143A>C, 4145G>C, 4148G>A, 4154T>C, 4163C>T, 4166A>T, 4169C>A, 4170A>T, 4171C>G, 4172A>T, 4175G>A, 4182T>G, 4183T>C, 4184G>A, 4187C>T, 4192C>T, 4194A>C, 4196A>G, 4199A>C, 4208T>G, 4214T>C, 4215G>A, 4220T>C, 4222C>T, 4223A>T, 4224A>T, 4225G>C, 4226T>A, 4227T>C, 4228C>A, 4238A>C, 4239T>A, 4240T>C, 4241G>C, 4242T>C, 4250T>C, 4251G>C, 4252G>A, 4256T>C, 4257A>C, 4259A>G, 4260G>A, 4261C>G, 4268A>T, 4271C>T, 4274G>T, 4277A>T, 4283A>T, 4286T>A, 4289T>C, 4292T>C, 4310A>G, 4313A>G, 4316T>A, 4317G>A, 4319T>A, 4325T>A, 4331G>A, 4337T>A, 4354A>C, 4361C>T, 4364A>G, 4368A>G, 4369A>C, 4370G>T, 4373T>A, 4376C>A, 4379T>C, 4380C>G, 4381C>A, 4382T>G, 4388C>A, 4394T>A, 4395C>A, 4403A>T, 4406T>G, 4409A>G, 4418A>T, 4424T>C, 4433A>T, 4436G>A, 4449G>C, 4451G>C, 4454T>C, 4460G>A, 4466G>T, 4469C>A, 4475A>T, 4478A>T, 4487A>T, 4490A>C, 4493G>C, 4499C>A, 4502C>T, 4505T>C, 4508T>C, 4511C>T, 4512C>T, 4514C>A, 4517T>C, 4532A>G, 4538C>T, 4541T>C, 4547A>G, 4548T>C, 4550G>C, 4551T>C, 4553A>C, 4559A>G, 4565T>C, 4568T>C, 4569G>A, 4570C>A, 4571T>A, 4574C>A, 4576G>A, 4580T>A, 4581A>T, 4583A>G, 4586A>T, 4589T>A, 4592A>G, 4593C>T, 4604C>T, 4608C>G, 4610G>C, 4611C>T, 4616C>T, 4619A>T, 4622T>A, 4625T>G, 4631A>G, 4646T>G, 4652A>G, 4656T>A, 4658A>G, 4661T>C, 4664C>T, 4667G>A, 4673T>C, 4674T>G, 4675C>A, 4676T>G, 4677C>T, 4679G>A, 4682A>T, 4683A>G, 4684A>C, 4685T>A, 4694T>A, 4698T>C, 4705T>A, 4706G>A, 4709C>G, 4712T>C, 4715A>T, 4718C>T, 4721T>C, 4730T>C, 4736C>T, 4740T>A, 4741C>A, 4742G>C, 4746G>A, 4751T>C, 4757G>T, 4760A>C, 4761C>A, 4766T>C, 4769A>G, 4782C>T, 4784A>G, 4787T>G, 4791C>T, 4796T>C, 4802T>C, 4805T>C, 4808A>G, 4811A>C, 4818C>T, 4820T>G, 4823A>T, 4829C>A, 4830A>C, 4832G>A, 4835T>C, 4838T>C, 4853A>C, 4856C>T, 4857A>G, 4859T>G, 4862G>A, 4865C>A, 4877A>C, 4883T>A, 4886C>A, 4887C>G, 4888C>T, 4889A>T, 4892T>G, 4898T>A, 4901C>T, 4903C>T, 4904T>C, 4905C>A, 4906A>C, 4907G>T, 4914A>T, 4915T>A, 4916G>T, 4931A>G, 4934A>T, 4935A>G, 4938C>A, 4940T>A, 4943A>G, 4952T>G, 4955C>T, 4958T>A, 4960G>A, 4961T>G, 4964G>C, 4966C>T, 4975A>C, 4976G>A, 4977C>T, 4979T>G, 4980G>A, 4981T>C, 4982C>A, 4983T>G, 4984T>A, 4985A>C, 4986G>A, 4991T>C, 4994G>A, 4996C>A, 4997G>A, 5000G>A, 5001T>A, 5003G>A, 5004G>A, 5005A>T, 5006T>G, 5007G>C, 5009A>G, 5010A>C, 5012G>A, 5015T>C, 5018C>G, 5019A>C, 5020G>C, 5022G>A, 5023C>T, 5024T>C, 5028A>C, 5029T>A, 5030C>T, 5036T>A, 5039C>T, 5043T>G, 5044T>C, 5045G>T, 5049G>C, 5050T>A, 5051G>A, 5055C>A, 5056A>G, 5057G>T, 5060A>G, 5064A>C, 5066T>A, 5067G>C, 5068C>A, 5070A>G, 5071A>C, 5072G>C, 5075A>T, 5078A>T, 5081G>A, 5084T>C, 5085T>A, 5086C>G, 5087A>T, 5089G>A, 5090T>C, 5091G>A, 5092C>T, 5093C>A, 5094C>G, 5095T>G, 5097A>G, 5099A>T, 5100A>C, 5102T>G, 5103T>C, 5104G>T, 5105T>G, 5109A>C, 5112G>A, 5113C>G, 5114A>C, 5117A>G, 5119C>A, 5120A>T, 5127T>A, 5129C>T, 5132T>C, 5133A>G, 5134C>T, 5138A>T, 5139T>C, 5141G>T, 5142G>C, 5143A>C, 5144C>A, 5146C>A, 5150T>A, 5151G>A, 5153G>A, 5156G>A, 5159C>T, 5160A>G, 5161C>A, 5163A>G, 5169G>T, 5170A>C, 5171G>A, 5173G>A, 5174T>A, 5176A>G, 5178C>G, 5180G>C, 5181A>C, 5184T>G, 5185G>A, 5186G>A, 5190G>T, 5191C>G, 5192A>T, 5193C>T, 5195A>T, 5196A>G, 5197A>G, 5198C>A, 5199A>G, 5202G>T, 5203G>T, 5204T>C, 5207T>C, 5214G>T, 5216A>G, 5219T>A, 5222T>A, 5224C>A, 5227C>T, 5228C>T, 5229C>A, 5231C>T, 5232T>C, 5234C>T, 5236C>A, 5237G>A, 5246A>G, 5248G>A, 5249G>C, 5250T>A, 5251A>C, 5252C>A, 5256A>C, 5264C>T, 5267T>C, 5279G>A, 5282G>A, 5283T>A, 5285C>A, 5287A>C, 5288T>A, 5294T>G, 5339G>C, 5342C>T, 5345T>A, 5349T>G, 5354T>C, 5357A>C, 5359T>A, 5363T>C, 5364G>A, 5365C>A, 5366C>T, 5370A>C, 5372G>A, 5374A>G, 5376G>C, 5377T>G, 5378G>C, 5382G>T, 5385G>A, 5386G>T, 5387A>G, 5390A>G, 5391C>A, 5392A>T, 5395T>G, 5397G>A, 5399A>C, 5400A>C, 5401C>A, 5402C>A, 5405T>A, 5408C>T, 5411T>C, 5415G>A, 5417G>T, 5423A>G, 5429A>T, 5438C>G, 5439G>A, 5441C>T, 5444A>C, 5453A>T, 5456G>T, 5460A>T, 5462G>T, 5465C>A, 5469G>T, 5474A>C, 5476G>A, 5477G>T, 5480A>G, 5484T>A, 5486T>A, 5488T>C, 5489G>T, 5493G>C, 5495C>A, 5496A>C, 5498A>T, 5504T>C, 5511C>A, 5513C>A, 5517A>T, 5519T>G, 5521G>C, 5522A>G, 5523A>G, 5524A>G, 5525T>C, 5526G>T, 5531T>G, 5534C>T, 5535C>T, 5537C>G, 5541A>C, 5542C>A, 5543C>G, 5555C>T, 5559G>A, 5561G>T, 5564G>A, 5570T>C, 5573T>C, 5574C>A, 5575A>C, 5579_5580insGTC, 5584C>A, 5585T>G, 5586C>A, 5588T>A, 5589G>A, 5591A>T, 5592G>T, 5593A>G, 5597A>G, 5598G>A, 5599T>C, 5600G>A, 5607A>G, 5608G>C, 5610G>A, 5614C>G, 5615A>G, 5616_5618delTCG, 5620T>G, 5622C>G, 5624C>T, 5625A>G, 5627T>A, 5629G>T, 5630T>G, 5633T>G, 5635T>A, 5636G>C, 5642A>T, 5642_5643insCATGCT, 5644G>C, 5645G>T, 5647T>A, 5650A>C, 5651A>C, 5652T>C, 5654G>T, 5657C>A, 5663C>T, 5666C>A, 5669T>A, 5670A>G, 5672A>G, 5673C>G, 5674A>G, 5675T>C, 5676G>T, 5679G>T, 5680A>C, 5681T>A, 5682T>A, 5684A>T, 5685G>T, 5689A>G,

	5691C>A, 5695C>T, 5696A>G, 5700A>G, 5703C>A, 5705G>A, 5706A>T, 5711A>C, 5714T>C, 5715G>A, 5716T>C, 5717G>T, 5720A>T, 5723A>G, 5727C>A, 5728A>T, 5729T>G, 5732A>T, 5735A>T, 5740G>C, 5742A>G, 5746T>G, 5747T>C, 5750C>A, 5754C>A, 5755A>G, 5756G>A, 5757G>A

CDS

ToMarV_RNA1gp1	1	1877	87.2%	9403	72.1%	1862 (98.8%)	1290 (68.4%)	8/15/0/0	0

Protein mutations:	M6I (158G>C), F7V (159T>G 161C>T), S8_S17del (162_191delTCCAAGGTTGCTGGTGTTGATGACGCCAGT), S20A (198T>G 200C>T), T22P (204A>C), F25L (213T>C), G26S (216G>T 217G>C), N30Q (228A>C 230T>G), S31T (231T>A 233T>A), I32V (234A>G 236T>G), K34S (240A>T 241A>C), F35L (243T>C 245T>G), A38T (252G>A 254G>A), V39M (255G>A 257T>G), S40K (259G>A 260T>G), N41S (262A>G), I42M (266T>G), T43S (268C>G 269C>T), T44K (271C>A 272T>A), M45I (275G>A), Q47E (279C>G), I49F (285A>T 287T>C), D51G (292A>G 293T>C), L53I (297C>A), D55E (305T>A), V60I (318G>A 320G>T), T61V (321A>G 322C>T), D62K (324G>A 326T>A), V63T (327G>A 328T>C 329G>T), S64A (330T>G 332C>A), T65D (333A>G 334C>A 335A>T), V68N (342G>A 343T>A 344G>T), A69S (345G>A 346C>G 347C>T), T70V (348A>G 349C>T), L74V (360C>G 362T>A), N75E (363A>G 365T>A), V77L (369G>T 371T>A), D79G (376A>G 377C>G), G82A (385G>C 386T>C), A83T (387G>A 389A>G), L84V (390C>G), D86E (398T>A), L89V (405C>G), A91G (412C>G 413G>T), Y92I (414T>A 415A>T), S93N (417T>A 418C>A), L95F (425G>C), S96K (426T>A 427C>A 428C>A), K100R (439A>G), S101T (441T>A 443T>C), K103I (448A>T 449G>T), L109V (465T>G 467G>T), F110V (468T>G), E111L (471G>T 472A>T), S112G (474A>G 476T>C), L113I (477T>A 479G>C), S114K (480T>A 481C>A 482C>A), K116N (488A>T), V117F (489G>T), K118I (493A>T 494A>T), L119R (495T>A 496T>G), F121Y (502T>A), A122S (504G>T 506T>A), V124L (510G>C), N126E (516A>G 518T>A), K127T (520A>C), S128T (523G>C 524T>A), E129G (526A>G 527A>T), D130E (530T>A), V131Y (531G>T 532T>A 533A>T), I133V (537A>G 539A>T), Y136I (546T>A 547A>T 548T>A), G137A (550G>C), A142S (564G>T 566T>C), I143L (567A>C 569A>T), T144S (570A>T), V147L (579G>T 581G>A), L149Q (586T>A), F150I (588T>A 590T>A), V151I (591G>A 593T>C), S154V (600A>G 601G>T 602T>A), G159M (615G>A 616G>T 617T>G), S161R (621T>A 622C>G 623T>A), N162S (625A>G 626C>T), T163V (627A>G 628C>T 629T>A), K165C (633A>T 634A>G 635A>T), I170V (648A>G 650A>T), M174F (660A>T 662G>C), L181I (681T>A 683A>T), D182S (684G>A 685A>G), K186L (696A>C 697A>T 698A>C), A188S (702G>T 704C>T), R190K (708C>A 709G>A), T192S (714A>T 716C>T), M193Q (717A>C 718T>A), V194M (720G>A), M199V (735A>G), V205L (753G>T 755T>A), S206N (756T>A 757C>A 758A>T), M214L (780A>C), T216S (786A>T 788A>T), I217L (789A>T 791A>G), L218I (792T>A 794G>A), I228V (822A>G 824T>G), S238T (853G>C 854C>A), T248A (882A>G 884A>G), T260Q (918A>C 919C>A 920T>A), R263K (927C>A 928G>A 929T>G), I280V (978A>G 980A>T), V281A (982T>C 983C>T), G282D (985G>A 986C>T), T283I (988C>T 989C>A), L298I (1032C>A 1034G>T), Q307T (1059C>A 1060A>C 1061G>T), D310S (1068G>T 1069A>C), C313A (1077T>G 1078G>C), K325P (1113A>C 1114A>C 1115G>T), P328S (1122C>T 1124T>A), T329I (1126C>T), R335K (1144G>A 1145G>A), E337Q (1149G>C 1151A>G), M354L (1200A>T), A361T (1221G>A 1223C>A), A363V (1228C>T), E366D (1238A>T), V368L (1242G>C 1244T>C), Y369I (1245T>A 1246A>T 1247T>A), S372N (1255G>A 1256T>C), R374Q (1260A>C 1261G>A 1262G>A), M376T (1267T>C 1268G>A), S393E (1317T>G 1318C>A 1319C>G), R412A (1374A>G 1375G>C 1376A>C), G414Q (1380G>C 1381G>A 1382T>A), I496V (1626A>G 1628C>T), M521R (1701A>C 1702T>G), E523V (1708A>T), D530N (1728G>A 1730T>C), G533A (1738G>C), R536S (1748A>C), V540I (1758G>A 1760G>T), E541G (1762A>G 1763G>A), N552D (1794A>G 1796T>C), Q567K (1839C>A), A570H (1848G>C 1849C>A 1850A>C), V572I (1854G>A 1856A>T), S573A (1857T>G), K574T (1861A>C 1862G>C), F577Y (1870T>A 1871T>C), E578N (1872G>A 1874A>C), A579Q (1875G>C 1876C>A 1877T>G), E580S (1878G>T 1879A>C), S581T (1881T>A 1883T>A), I583C (1887A>T 1888T>G 1889A>T), S584E (1890A>G 1891G>A 1892T>A), P585E (1893C>G 1894C>A 1895T>G), I587V (1899A>G 1901A>T), Q591E (1911C>G), A594V (1921C>T 1922A>T), D595E (1925T>A), D596E (1928T>A), I598M (1934A>G), A599T (1935G>A 1937G>A), V601F (1941G>T 1943G>T), D602H (1944G>C), G603D (1948G>A 1949G>T), R605Q (1953A>C 1954G>A), L606I (1956T>A 1958G>A), R607S (1959C>A), T608F (1962A>T 1963C>T 1964C>T), K609D (1965A>G 1967G>C), Q610E (1968C>G), D611V (1972A>T), E612S (1974G>A 1975A>G 1976A>C), I614S (1981T>G 1982A>T), L615V (1983T>G 1985G>T), V616_P617del (1986_1991delGTGCCC), V620L (1998G>C 2000G>C), N623D (2007A>G), I627G (2019A>G 2020T>G 2021C>A), A629S (2025G>T), R630Q (2028A>C 2029G>A 2030A>G), S644A (2070A>G 2071G>C 2072T>A), D646N (2076G>A), E648F (2082G>T 2083A>T 2084G>T), L650Y (2088C>T 2089T>A), H655A (2103C>G 2104A>C), H656S (2106C>T 2107A>C 2108T>A), M660I (2120G>T), Y661F (2122A>T), S663V (2127T>G 2128C>T 2129T>C), D665S (2133G>T 2134A>C 2135T>A), D666S (2136G>A 2137A>G), H668A (2142C>G 2143A>C 2144T>C), I671S (2152T>G 2153T>C), N673T (2158A>C 2159C>T), K674R (2160A>C 2161A>G 2162A>C), R676V (2166A>G 2167G>T), V677N (2169G>A 2170T>A 2171C>T), N678E (2172A>G 2174C>G), L680I (2178T>A 2180G>T), R682E (2184A>G 2185G>A 2186A>G), D685S (2193G>T 2194A>C 2195T>G), A686T (2196G>A), E687A (2200A>C), Q688G (2202C>G 2203A>G 2204G>A), R690A (2208C>G 2209G>C 2210A>C), V693I (2217G>A 2219A>C), Q694K (2220C>A 2222A>G), N695A (2223A>G 2224A>C), I701V (2241A>G 2243A>C), A710S (2268G>A 2269C>G 2270A>T), S711T (2271T>A), I713V (2277A>G 2279C>G), K716A (2286A>G 2287A>C 2288G>T), K717H (2289A>C 2291A>C), L718M (2292C>A 2294A>G), A719D (2296C>A 2297A>T), T722R (2305C>G 2306A>G), E723M (2307G>A 2308A>T), M724L (2310A>T), R725Y (2313A>T 2314G>A 2315G>C), T726N (2317C>A), F728R (2322T>A 2323T>G 2324T>A), S731R (2331T>A 2332C>G 2333T>A), C732A (2334T>G 2335G>C 2336C>A), I733L (2337A>C 2339T>G), D734E (2342T>A), V737A (2350T>C), I741M (2363A>G), F749H (2385T>C 2386T>A), C762_N763insRR (2426_2427insAGAAGG), N763K (2429C>G), K764M (2431A>T 2432A>G), S766T (2436T>A 2438A>T), P769T (2445C>A), K770_E771del (2448_2453delAAGGAA), I774V (2460A>G 2462C>A), K775Q (2463A>C), I776S (2466A>T 2467T>C 2468T>G), C777L (2469T>C 2470G>T), H779D (2475C>G 2477T>C), K780S (2478A>T 2479A>C 2480A>C), I781T (2482T>C 2483C>A), V782I (2484G>A), E783G (2488A>G 2489A>G), Y784R (2490T>A 2491A>G 2492T>A), I785M (2495C>G), K786Q (2496A>C 2498G>A), D787E (2501T>A), T788M (2503C>T 2504A>G), W790N (2508T>A 2509G>A 2510G>T), S791D (2511A>G 2512G>A 2513C>T), A795I (2523G>A 2524C>T 2525A>T), R797K (2530G>A), I806L (2556A>C), M807I (2561G>A), V812T (2574G>A 2575T>C), F813M (2577T>A 2579T>G), G815A (2584G>C), I816V (2586A>G 2588T>A), L820I (2598T>A 2600G>T), A821S (2601G>T 2603G>T), Q824K (2610C>A 2612G>A), N826S (2617A>G), S827L (2620C>T 2621T>G), F828I (2622T>A 2624C>T), N830T (2629A>C), A831S (2631G>T 2633A>C), S832Q (2634T>C 2635C>A), R834K (2641G>A), N852R (2695A>G 2696T>A), N852_K853insS (2696_2697insTCC), K853H (2697A>C 2699G>T), L854V (2700C>G 2702C>A), R855S (2703A>T 2704G>C), N856K (2708T>G), L857M (2709C>A 2711T>G), V859F (2715G>T), G860S (2718G>A 2720T>C), S865P (2733T>C), L866N (2736T>A 2737T>A 2738G>T), A867D (2740C>A 2741A>T), Q868_D869insG (2744_2745insGGA), D869K (2745G>A 2747T>A), A872S (2754G>T 2756T>C), E873R (2757G>A 2758A>G 2759A>G), Q879E (2775C>G 2777G>A), K882A (2784A>G 2785A>C 2786G>C), L890V (2808C>G 2810T>G), G891A (2812G>C 2813T>A), G904E (2851G>A 2852T>A), H914T (2880C>A 2881A>C 2882C>G), N915Q (2883A>C 2885T>G), A916L (2886G>C 2887C>T 2888T>C), T923S (2907A>T), A927E (2920C>A 2921C>A), N928H (2922A>C), R930V (2928C>G 2929G>T 2930A>C), S931C (2932C>G 2933T>C), K932Q (2934A>C 2936A>G), R938K (2953G>A), E939H (2955G>C 2957A>T), S940A (2958T>G 2960A>C), R941K (2962G>A), I942F (2964A>T), I943L (2967A>T 2969T>G), E944P (2970G>C 2971A>C 2972G>T), G945E (2974G>A 2975C>A), I948V (2982A>G 2984C>T), I949V (2985A>G 2987T>A), D955C (3003G>T 3004A>G), P961V (3021C>G 3022C>T 3023T>C), P968A (3042C>G 3044A>T), R969E (3045A>G 3046G>A), E972A (3055A>C 3056G>A), Q980E (3078C>G), C984V (3090T>G 3091G>T 3092T>C), T990K (3109C>A), R993E (3117C>G 3118G>A 3119T>G), L994K (3120C>A 3121T>A 3122T>A), G995Q (3123G>C 3124G>A 3125C>G), E998D (3134G>C), M1002Y (3144A>T 3145T>A 3146G>T), P1003S (3147C>T), V1012E (3175T>A 3176T>G), E1015T (3183G>A 3184A>C), E1017R (3189G>A 3190A>G 3191A>G), L1018M (3192C>A), N1019S (3196A>G), S1020T (3198T>A 3200C>A), S1021N (3201T>A 3202C>A 3203C>T), K1024H (3210A>C 3212G>C), K1024_I1025insS (3212_3213insTCA), C1026S (3216T>A 3218C>T), S1027A (3219T>G), Q1029E (3225C>G), S1030A (3228T>G 3230T>C), I1035V (3243A>G 3245A>C), G1037S (3249G>A 3251G>C), V1051I (3291G>A 3293T>C), V1057C (3309G>T 3310T>G 3311G>C), G1068E (3343G>A 3344C>A), V1069A (3346T>C 3347A>T), A1071T (3351G>A 3353T>A), R1073L (3357C>T 3358G>T 3359T>G), I1076V (3366A>G 3368C>A), S1077A (3369T>G 3371T>A), I1083V (3387A>G 3389C>A), E1086D (3398A>C), E1089Q (3405G>C 3407G>A), P1092A (3414C>G 3416A>T), T1093Q (3417A>C 3418C>A 3419T>A), A1098M (3432G>A 3433C>T 3434A>G), I1105L (3453A>T 3455T>G), S1108H (3462T>C 3463C>A 3464A>T), I1109V (3465A>G), Q1115A (3483C>G 3484A>C 3485G>T), E1118D (3494G>C), N1120K (3500C>G), Y1123A (3507T>G 3508A>C 3509C>T), Y1124F (3511A>T 3512C>T), L1132M (3534T>A), Q1146P (3577A>C), P1153E (3597C>G 3598C>A 3599T>A), E1155Q (3603G>C 3605G>A), I1160L (3618A>C 3620T>G), T1162S (3624A>T 3626A>T), H1164K (3630C>A 3632T>A), V1178I (3672G>A 3674A>T), M1179E (3675A>G 3676T>A), C1186S (3697G>C 3698T>A), G1187N (3699G>A 3700G>A 3701A>T), D1189E (3707T>G), D1191S (3711G>A 3712A>G), K1194R (3720A>C 3721A>G 3722A>G), L1195A (3723C>G 3724T>C 3725A>T), N1199G (3735A>G 3736A>G), C1202S (3745G>C 3746C>T), S1203A (3747T>G 3749G>C), L1204I (3750C>A 3752G>T), I1207V (3759A>G 3761C>T), M1208L (3762A>C 3764G>C), R1209K (3765C>A 3766G>A 3767T>G), K1212R (3775A>G), I1213V (3777A>G 3779C>G), R1217I (3789C>A 3790G>T 3791T>C), C1219T (3795T>A 3796G>C 3797T>A), E1220T (3798G>A 3799A>C), D1222C (3804G>T 3805A>G), V1225I (3813G>A 3815A>C), I1230V (3828A>G 3830A>T), C1233F (3838G>T), Y1235F (3844A>T), Y1236F (3847A>T), G1238S (3852G>T 3853G>C), M1252L (3894A>C), M1254L (3900A>T), M1256T (3907T>C 3908G>C), Q1263E (3927C>G), S1264A (3930T>G 3932C>A), T1265Q (3933A>C 3934C>A 3935T>G), G1266E (3937G>A 3938C>G), E1267S (3939G>A 3940A>G 3941A>T), G1268T (3942G>A 3943G>C 3944A>C), E1269Q (3945G>C 3947G>A), E1272L (3954G>C 3955A>T), D1276N (3966G>A 3968C>T), T1277S (3969A>T), E1281Q (3981G>C), Q1289E (4005C>G 4007G>A), I1294N (4021T>A), H1295Y (4023C>T), D1297E (4031T>A), L1298M (4032C>A), E1312D (4076G>C), R1318K (4093G>A 4094A>G), T1344C (4170A>T 4171C>G 4172A>T), L1348A (4182T>G 4183T>C 4184G>A), A1351V (4192C>T), I1352L (4194A>C 4196A>G), D1359N (4215G>A), T1361I (4222C>T 4223A>T), S1363H (4227T>C 4228C>A), L1367T (4239T>A 4240T>C 4241G>C), G1371Q (4251G>C 4252G>A), A1374S (4260G>A 4261C>G), V1393I (4317G>A 4319T>A), F1399L (4337T>A), E1405A (4354A>C), K1410A (4368A>G 4369A>C 4370G>T), P1414E (4380C>G 4381C>A 4382T>G), V1437L (4449G>C 4451G>C), A1477K (4569G>A 4570C>A 4571T>A), R1479K (4576G>A), I1481L (4581A>T 4583A>G), P1485S (4593C>T), Q1490D (4608C>G 4610G>C), L1506M (4656T>A 4658A>G), S1512E (4674T>G 4675C>A 4676T>G), N1515A (4683A>G 4684A>C 4685T>A), H1518Q (4694T>A), L1522Q (4705T>A 4706G>A), S1534N (4740T>A 4741C>A 4742G>C), D1536N (4746G>A), E1539D (4757G>T), Q1541K (4761C>A), H1563Q (4829C>A), M1564L (4830A>C 4832G>A), I1573V (4857A>G 4859T>G), P1583V (4887C>G 4888C>T 4889A>T), T1588I (4903C>T 4904T>C), Q1589T (4905C>A 4906A>C 4907G>T), M1592Y (4914A>T 4915T>A 4916G>T), I1599V (4935A>G), H1604Q (4952T>G), R1607Q (4960G>A 4961T>G), A1609V (4966C>T), K1612T (4975A>C 4976G>A), V1614T (4980G>A 4981T>C 4982C>A), L1615D (4983T>G 4984T>A 4985A>C), D1616N (4986G>A), T1619K (4996C>A 4997G>A), L1621I (5001T>A 5003G>A), D1622M (5004G>A 5005A>T 5006T>G), E1623Q (5007G>C 5009A>G), F1626L (5018C>G), R1627P (5019A>C 5020G>C), A1628I (5022G>A 5023C>T 5024T>C), I1630H (5028A>C 5029T>A 5030C>T), L1635A (5043T>G 5044T>C 5045G>T), V1637Q (5049G>C 5050T>A 5051G>A), Q1639S (5055C>A 5056A>G 5057G>T), I1642L (5064A>C 5066T>A), A1643Q (5067G>C 5068C>A), K1644A (5070A>G 5071A>C 5072G>C), S1650N (5089G>A 5090T>C), A1651I (5091G>A 5092C>T 5093C>A), L1652G (5094C>G 5095T>G), T1653A (5097A>G 5099A>T), I1654L (5100A>C 5102T>G), C1655L (5103T>C 5104G>T 5105T>G), M1657L (5109A>C), A1658S (5112G>A 5113C>G 5114A>C), A1660D (5119C>A 5120A>T), S1663T (5127T>A 5129C>T), T1665V (5133A>G 5134C>T), D1668P (5142G>C 5143A>C 5144C>A), A1669E (5146C>A), E1671K (5151G>A 5153G>A), T1674E (5160A>G 5161C>A), N1675D (5163A>G), E1677S (5169G>T 5170A>C 5171G>A), R1678Q (5173G>A 5174T>A), D1679G (5176A>G), L1680V (5178C>G 5180G>C), N1681H (5181A>C), W1682E (5184T>G 5185G>A 5186G>A), A1684C (5190G>T 5191C>G 5192A>T), P1685S (5193C>T 5195A>T), N1686G (5196A>G 5197A>G 5198C>A), I1687V (5199A>G), G1688F (5202G>T 5203G>T 5204T>C), A1692S (5214G>T 5216A>G), A1695E (5224C>A), A1696V (5227C>T 5228C>T), H1697N (5229C>A 5231C>T), Y1698H (5232T>C 5234C>T), T1699K (5236C>A 5237G>A), G1703D (5248G>A 5249G>C), Y1704T (5250T>A 5251A>C 5252C>A), I1706L (5256A>C), S1715T (5283T>A 5285C>A), N1716T (5287A>C 5288T>A), S1737A (5349T>G), M1740K (5359T>A), A1742N (5364G>A 5365C>A 5366C>T), K1744Q (5370A>C 5372G>A), N1745S (5374A>G), V1746R (5376G>C 5377T>G 5378G>C), A1748S (5382G>T), G1749M (5385G>A 5386G>T 5387A>G), H1751I (5391C>A 5392A>T), V1752G (5395T>G), A1753T (5397G>A 5399A>C), T1754Q (5400A>C 5401C>A 5402C>A), N1755K (5405T>A), V1759I (5415G>A 5417G>T), G1767S (5439G>A 5441C>T), M1774F (5460A>T 5462G>T), A1777S (5469G>T), R1779N (5476G>A 5477G>T), C1782R (5484T>A 5486T>A), V1783A (5488T>C 5489G>T), D1785Q (5493G>C 5495C>A), I1786L (5496A>C 5498A>T), I1793L (5517A>T 5519T>G), G1794A (5521G>C 5522A>G), N1795G (5523A>G 5524A>G 5525T>C), V1796L (5526G>T), T1801Q (5541A>C 5542C>A 5543C>G), V1807I (5559G>A 5561G>T), Q1812T (5574C>A 5575A>C), C1813_V1814insV (5579_5580insGTC), P1815Q (5584C>A 5585T>G), H1816K (5586C>A 5588T>A), G1817S (5589G>A 5591A>T), D1818C (5592G>T 5593A>G), V1820T (5598G>A 5599T>C 5600G>A), S1823A (5607A>G 5608G>C), G1824S (5610G>A), A1825G (5614C>G 5615A>G), S1826del (5616_5618delTCG), L1827R (5620T>G), H1828D (5622C>G 5624C>T), S1829G (5625A>G 5627T>A), S1830M (5629G>T 5630T>G), F1831L (5633T>G), M1832N (5635T>A 5636G>C), Q1834H (5642A>T), Q1834_G1835insHA (5642_5643insCATGCT), G1835A (5644G>C 5645G>T), F1836Y (5647T>A), E1837A (5650A>C 5651A>C), I1844V (5670A>G 5672A>G), H1845G (5673C>G 5674A>G 5675T>C), G1846C (5676G>T), D1847S (5679G>T 5680A>C 5681T>A), L1848I (5682T>A 5684A>T), A1849S (5685G>T), K1850R (5689A>G), Q1851K (5691C>A), T1852M (5695C>T 5696A>G), M1854V (5700A>G), M1856L (5706A>T), V1859T (5715G>A 5716T>C 5717G>T), H1863M (5727C>A 5728A>T 5729T>G), G1867A (5740G>C), N1868D (5742A>G), F1869C (5746T>G 5747T>C), Q1872R (5754C>A 5755A>G 5756G>A), D1873N (5757G>A)

	Begin	End	Coverage	Score	Concordance	Matches	Identities	I/D/M/F*	Stop Codons
**NT**	**35**	**5769**	**79.3%**	**3466**	**30.8%**	**5682 (98.6%)**	**3757 (65.2%)**	**28/53**	

Codon mutations:

GCT2GCA (146T>A), AAG5AAA (155G>A), ATG6ATC (158G>C), TTC7GTT (159T>G 161C>T), TCC8_AGT17del (162_191delTCCAAGGTTGCTGGTGTTGATGACGCCAGT), TCC20GCT (198T>G 200C>T), ACC21ACT (203C>T), ACT22CCT (204A>C), TCA23TCT (209A>T), TTC24TTT (212C>T), TTT25CTT (213T>C), GGT26TCT (216G>T 217G>C), GAA27GAG (221A>G), CTT28TTG (222C>T 224T>G), TCA29TCT (227A>T), AAT30CAG (228A>C 230T>G), TCT31ACA (231T>A 233T>A), ATT32GTG (234A>G 236T>G), TCC33TCA (239C>A), AAA34TCA (240A>T 241A>C), TTT35CTG (243T>C 245T>G), TCA36TCC (248A>C), AAA37AAG (251A>G), GCG38ACA (252G>A 254G>A), GTT39ATG (255G>A 257T>G), AGT40AAG (259G>A 260T>G), AAT41AGT (262A>G), ATT42ATG (266T>G), ACC43AGT (268C>G 269C>T), ACT44AAA (271C>A 272T>A), ATG45ATA (275G>A), TCA46TCT (278A>T), CAG47GAG (279C>G), AAG48AAA (284G>A), ATT49TTC (285A>T 287T>C), GAT51GGC (292A>G 293T>C), CTT53ATT (297C>A), GAG54GAA (302G>A), GAT55GAA (305T>A), CTG56TTA (306C>T 308G>A), CCA58CCT (314A>T), TCT59TCA (317T>A), GTG60ATT (318G>A 320G>T), ACT61GTT (321A>G 322C>T), GAT62AAA (324G>A 326T>A), GTG63ACT (327G>A 328T>C 329G>T), TCC64GCA (330T>G 332C>A), ACA65GAT (333A>G 334C>A 335A>T), TCT66TCC (338T>C), GTG68AAT (342G>A 343T>A 344G>T), GCC69AGT (345G>A 346C>G 347C>T), ACT70GTT (348A>G 349C>T), TCA73TCT (359A>T), CTT74GTA (360C>G 362T>A), AAT75GAA (363A>G 365T>A), GTT77TTA (369G>T 371T>A), TTG78TTA (374G>A), GAC79GGG (376A>G 377C>G), GGT82GCC (385G>C 386T>C), GCA83ACG (387G>A 389A>G), CTT84GTT (390C>G), ATA85ATT (395A>T), GAT86GAA (398T>A), CCA87CCT (401A>T), CTT89GTT (405C>G), GCG91GGT (412C>G 413G>T), TAC92ATC (414T>A 415A>T), TCT93AAT (417T>A 418C>A), TTG95TTC (425G>C), TCC96AAA (426T>A 427C>A 428C>A), TAC99TAT (437C>T), AAA100AGA (439A>G), TCT101ACC (441T>A 443T>C), ATC102ATA (446C>A), AAG103ATT (448A>T 449G>T), TTA107CTG (459T>C 461A>G), TTG109GTT (465T>G 467G>T), TTT110GTT (468T>G), GAG111TTG (471G>T 472A>T), AGT112GGC (474A>G 476T>C), TTG113ATC (477T>A 479G>C), TCC114AAA (480T>A 481C>A 482C>A), AAA116AAT (488A>T), GTT117TTT (489G>T), AAA118ATT (493A>T 494A>T), TTA119AGA (495T>A 496T>G), TTT121TAT (502T>A), GCT122TCA (504G>T 506T>A), GTG124CTG (510G>C), TTA125TTG (515A>G), AAT126GAA (516A>G 518T>A), AAA127ACA (520A>C), AGT128ACA (523G>C 524T>A), GAA129GGT (526A>G 527A>T), GAT130GAA (530T>A), GTA131TAT (531G>T 532T>A 533A>T), ATA133GTT (537A>G 539A>T), GTT134GTA (542T>A), GTA135GTT (545A>T), TAT136ATA (546T>A 547A>T 548T>A), GGT137GCT (550G>C), CTT139CTA (557T>A), GTA140GTC (560A>C), GCT142TCC (564G>T 566T>C), ATA143CTT (567A>C 569A>T), ACA144TCA (570A>T), TTG145CTA (573T>C 575G>A), TTA146TTG (578A>G), GTG147TTA (579G>T 581G>A), CTT148TTA (582C>T 584T>A), CTA149CAA (586T>A), TTT150ATA (588T>A 590T>A), GTT151ATC (591G>A 593T>C), AGT154GTA (600A>G 601G>T 602T>A), GTT157GTA (611T>A), GAG158GAA (614G>A), GGT159ATG (615G>A 616G>T 617T>G), TCT161AGA (621T>A 622C>G 623T>A), AAC162AGT (625A>G 626C>T), ACT163GTA (627A>G 628C>T 629T>A), GTC164GTT (632C>T), AAA165TGT (633A>T 634A>G 635A>T), ATC167ATA (641C>A), TTG169CTA (645T>C 647G>A), ATA170GTT (648A>G 650A>T), GTC171GTA (653C>A), GGA172GGT (656A>T), ATG174TTC (660A>T 662G>C), TTT175TTC (665T>C), TCA176TCT (668A>T), AGT177TCT (669A>T 670G>C), CTA178CTG (674A>G), TAC179TAT (677C>T), TTA181ATT (681T>A 683A>T), GAT182AGT (684G>A 685A>G), CCA185CCC (695A>C), AAA186CTC (696A>C 697A>T 698A>C), GCC188TCT (702G>T 704C>T), GAA189GAG (707A>G), CGA190AAA (708C>A 709G>A), TTC191TTT (713C>T), ACC192TCT (714A>T 716C>T), ATG193CAG (717A>C 718T>A), GTG194ATG (720G>A), GCC195GCA (725C>A), CAA196CAG (728A>G), AGT198TCT (732A>T 733G>C), ATG199GTG (735A>G), CTT200CTG (740T>G), GGG202GGT (746G>T), GAA203GAG (749A>G), TCA204AGC (750T>A 751C>G 752A>C), GTT205TTA (753G>T 755T>A), TCA206AAT (756T>A 757C>A 758A>T), ACG210ACC (770G>C), TCT211AGT (771T>A 772C>G), CAG212CAA (776G>A), ATG214CTG (780A>C), TCC215TCT (785C>T), ACA216TCT (786A>T 788A>T), ATA217TTG (789A>T 791A>G), TTG218ATA (792T>A 794G>A), GCT219GCA (797T>A), GGG221GGC (803G>C), ATT222ATC (806T>C), TCG223TCA (809G>A), GTG226GTA (818G>A), TTC227TTT (821C>T), ATT228GTG (822A>G 824T>G), GTG230GTA (830G>A), AGA233AGG (839A>G), TTG237CTT (849T>C 851G>T), AGC238ACA (853G>C 854C>A), CCA240CCG (860A>G), TTG241CTA (861T>C 863G>A), CTC245TTG (873C>T 875C>G), TCG247TCA (881G>A), ACA248GCG (882A>G 884A>G), GGA249GGC (887A>C), GGT251GGA (893T>A), CAG253CAA (899G>A), CTA258CTC (914A>C), ACT260CAA (918A>C 919C>A 920T>A), CTT261TTG (921C>T 923T>G), TAC262TAT (926C>T), CGT263AAG (927C>A 928G>A 929T>G), AAT264AAC (932T>C), AAA266AAG (938A>G), ACC269ACG (947C>G), TCA274TCT (962A>T), GTT276GTG (968T>G), CTT277TTG (969C>T 971T>G), ATA279ATT (977A>T), ATA280GTT (978A>G 980A>T), GTC281GCT (982T>C 983C>T), GGC282GAT (985G>A 986C>T), ACC283ATA (988C>T 989C>A), TTT284TTC (992T>C), GGA285GGG (995A>G), TTC286TTT (998C>T), AAT288AAC (1004T>C), GTG290GTT (1010G>T), TTG291CTG (1011T>C), TCA292TCT (1016A>T), GCT293GCA (1019T>A), ATA294ATT (1022A>T), ACG297ACA (1031G>A), CTG298ATT (1032C>A 1034G>T), GAT301GAC (1043T>C), CTG302CTC (1046G>C), TTC303TTT (1049C>T), CAG307ACT (1059C>A 1060A>C 1061G>T), GAG308GAA (1064G>A), GAT310TCT (1068G>T 1069A>C), TGT313GCT (1077T>G 1078G>C), ACG317ACA (1091G>A), CGC318AGG (1092C>A 1094C>G), GAG320GAA (1100G>A), GCC323GCT (1109C>T), AAG325CCT (1113A>C 1114A>C 1115G>T), TTC327TTT (1121C>T), CCT328TCA (1122C>T 1124T>A), ACC329ATC (1126C>T), CTG334CTT (1142G>T), AGG335AAA (1144G>A 1145G>A), GAA336GAG (1148A>G), GAA337CAG (1149G>C 1151A>G), GCT338GCA (1154T>A), ATC341ATA (1163C>A), GCT343GCC (1169T>C), TAC344TAT (1172C>T), GCA346GCT (1178A>T), CCA349CCT (1187A>T), GTT350GTG (1190T>G), TTC353TTT (1199C>T), ATG354TTG (1200A>T), AGC355AGT (1205C>T), AGG357AGA (1211G>A), AGT359TCT (1215A>T 1216G>C), GCC361ACA (1221G>A 1223C>A), GCG363GTG (1228C>T), CAG364CAA (1232G>A), TTA365TTG (1235A>G), GAA366GAT (1238A>T), GTT368CTC (1242G>C 1244T>C), TAT369ATA (1245T>A 1246A>T 1247T>A), GCT370GCA (1250T>A), AGT372AAC (1255G>A 1256T>C), AGG374CAA (1260A>C 1261G>A 1262G>A), CAC375CAT (1265C>T), ATG376ACA (1267T>C 1268G>A), GGC377GGA (1271C>A), GGC379GGT (1277C>T), TAT381TAC (1283T>C), CGT382AGA (1284C>A 1286T>A), CCT385CCC (1295T>C), GTA388GTT (1304A>T), TTC391TTT (1313C>T), GGG392GGT (1316G>T), TCC393GAG (1317T>G 1318C>A 1319C>G), GGG395GGA (1325G>A), TGT396TGC (1328T>C), GGT397GGC (1331T>C), TCT399TCC (1337T>C), ACA400ACG (1340A>G), TCC401AGC (1341T>A 1342C>G), CGC403CGA (1349C>A), TTA404CTG (1350T>C 1352A>G), TTT405TTC (1355T>C), ATT406ATC (1358T>C), GTG409GTA (1367G>A), GAC411GAT (1373C>T), AGA412GCC (1374A>G 1375G>C 1376A>C), GGT414CAA (1380G>C 1381G>A 1382T>A), CCA416CCC (1388A>C), AAA417AAG (1391A>G), CTC418CTG (1394C>G), AAT419AAC (1397T>C), CGG420AGG (1398C>A), CTA421TTG (1401C>T 1403A>G), GTA424GTT (1412A>T), AGG427CGC (1419A>C 1421G>C), GCA429GCT (1427A>T), TAT430TAC (1430T>C), TCC432TCA (1436C>A), AAC433AAT (1439C>T), CTG441CTC (1463G>C), GAC444GAT (1472C>T), GGA446GGT (1478A>T), TTG448CTG (1482T>C), CGA449CGG (1487A>G), GGG451GGA (1493G>A), GGG453GGC (1499G>C), CAG454CAA (1502G>A), CAA456CAG (1508A>G), GAT457GAC (1511T>C), AAA459AAG (1517A>G), GAT460GAC (1520T>C), CTG461CTC (1523G>C), GAT463GAC (1529T>C), TCA466TCC (1538A>C), CCC471CCA (1553C>A), TTA472TTG (1556A>G), CCA473CCC (1559A>C), GCC475GCT (1565C>T), GTT477GTA (1571T>A), GAG478GAA (1574G>A), GGC481GGG (1583C>G), AGG482CGG (1584A>C), ACC485ACA (1595C>A), TCC486TCA (1598C>A), TAT488TAC (1604T>C), ACA492ACC (1616A>C), AAT494AAC (1622T>C), CTG495TTG (1623C>T), ATC496GTT (1626A>G 1628C>T), GCT498GCA (1634T>A), CCT499CCA (1637T>A), GCC500GCA (1640C>A), TGT502TGC (1646T>C), GGT503GGG (1649T>G), CTT504CTG (1652T>G), CCA507CCT (1661A>T), GAG511GAA (1673G>A), CGG512AGG (1674C>A), AGA513AGG (1679A>G), AGG514CGG (1680A>C), ATC516ATT (1688C>T), CTG517TTG (1689C>T), GAG519GAA (1697G>A), TGT520TGC (1700T>C), ATG521CGG (1701A>C 1702T>G), AAG522AAA (1706G>A), GAG523GTG (1708A>T), GGC524GGG (1712C>G), GAG525GAA (1715G>A), TTT526TTC (1718T>C), TCC527TCA (1721C>A), ACT528ACA (1724T>A), GAA529GAG (1727A>G), GAT530AAC (1728G>A 1730T>C), CCT531CCA (1733T>A), ACG532ACA (1736G>A), GGA533GCA (1738G>C), AGA536AGC (1748A>C), TTT537TTC (1751T>C), ATA539ATC (1757A>C), GTG540ATT (1758G>A 1760G>T), GAG541GGA (1762A>G 1763G>A), AGA543AGG (1769A>G), AGA544AGG (1772A>G), GCT547GCC (1781T>C), ATC548ATA (1784C>A), ACT549ACA (1787T>A), AGG551AGA (1793G>A), AAT552GAC (1794A>G 1796T>C), TTG553CTC (1797T>C 1799G>C), ACC554ACT (1802C>T), AGT556TCT (1806A>T 1807G>C), GAT557GAC (1811T>C), CTT558TTG (1812C>T 1814T>G), GAG560GAA (1820G>A), GTA563GTG (1829A>G), AAA565AAG (1835A>G), TGT566TGC (1838T>C), CAG567AAG (1839C>A), GTA568GTC (1844A>C), CAT569CAC (1847T>C), GCA570CAC (1848G>C 1849C>A 1850A>C), GTA572ATT (1854G>A 1856A>T), TCA573GCA (1857T>G), AAG574ACC (1861A>C 1862G>C), TTG576CTC (1866T>C 1868G>C), TTT577TAC (1870T>A 1871T>C), GAA578AAC (1872G>A 1874A>C), GCT579CAG (1875G>C 1876C>A 1877T>G), GAA580TCA (1878G>T 1879A>C), TCT581ACA (1881T>A 1883T>A), GGC582GGT (1886C>T), ATA583TGT (1887A>T 1888T>G 1889A>T), AGT584GAA (1890A>G 1891G>A 1892T>A), CCT585GAG (1893C>G 1894C>A 1895T>G), AAG586AAA (1898G>A), ATA587GTT (1899A>G 1901A>T), GCT588GCC (1904T>C), GTT590GTG (1910T>G), CAA591GAA (1911C>G), GCA594GTT (1921C>T 1922A>T), GAT595GAA (1925T>A), GAT596GAA (1928T>A), ATA598ATG (1934A>G), GCG599ACA (1935G>A 1937G>A), TCT600TCC (1940T>C), GTG601TTT (1941G>T 1943G>T), GAT602CAT (1944G>C), GGG603GAT (1948G>A 1949G>T), AGA605CAA (1953A>C 1954G>A), TTG606ATA (1956T>A 1958G>A), CGC607AGC (1959C>A), ACC608TTT (1962A>T 1963C>T 1964C>T), AAG609GAC (1965A>G 1967G>C), CAA610GAA (1968C>G), GAT611GTT (1972A>T), GAA612AGC (1974G>A 1975A>G 1976A>C), CCA613CCC (1979A>C), ATA614AGT (1981T>G 1982A>T), TTG615GTT (1983T>G 1985G>T), GTG616_CCC617del (1986_1991delGTGCCC), ACT618ACA (1994T>A), GTG620CTC (1998G>C 2000G>C), AGT621TCT (2001A>T 2002G>C), GAA622GAG (2006A>G), AAT623GAT (2007A>G), ATC627GGA (2019A>G 2020T>G 2021C>A), GCA629TCA (2025G>T), AGA630CAG (2028A>C 2029G>A 2030A>G), GAG631GAA (2033G>A), CTC632CTA (2036C>A), ACG633ACT (2039G>T), GTA634GTG (2042A>G), GAG635GAA (2045G>A), TTG637CTG (2049T>C), AAA638AAG (2054A>G), GCA640GCT (2060A>T), TAC641TAT (2063C>T), CAG642CAA (2066G>A), GGC643GGA (2069C>A), AGT644GCA (2070A>G 2071G>C 2072T>A), TTG645TTA (2075G>A), GAC646AAC (2076G>A), CCT647CCA (2081T>A), GAG648TTT (2082G>T 2083A>T 2084G>T), CTT650TAT (2088C>T 2089T>A), CCT652CCC (2096T>C), GAC654GAT (2102C>T), CAT655GCT (2103C>G 2104A>C), CAT656TCA (2106C>T 2107A>C 2108T>A), GCC659GCA (2117C>A), ATG660ATT (2120G>T), TAT661TTT (2122A>T), TCT663GTC (2127T>G 2128C>T 2129T>C), CTT664CTG (2132T>G), GAT665TCA (2133G>T 2134A>C 2135T>A), GAT666AGT (2136G>A 2137A>G), CAT668GCC (2142C>G 2143A>C 2144T>C), ATT671AGC (2152T>G 2153T>C), TTC672TTT (2156C>T), AAC673ACT (2158A>C 2159C>T), AAA674CGC (2160A>C 2161A>G 2162A>C), AGA676GTA (2166A>G 2167G>T), GTC677AAT (2169G>A 2170T>A 2171C>T), AAC678GAG (2172A>G 2174C>G), TTG680ATT (2178T>A 2180G>T), TAT681TAC (2183T>C), AGA682GAG (2184A>G 2185G>A 2186A>G), GGA683GGG (2189A>G), GCA684GCT (2192A>T), GAT685TCG (2193G>T 2194A>C 2195T>G), GCT686ACT (2196G>A), GAA687GCA (2200A>C), CAG688GGA (2202C>G 2203A>G 2204G>A), CGA690GCC (2208C>G 2209G>C 2210A>C), GTA693ATC (2217G>A 2219A>C), CAA694AAG (2220C>A 2222A>G), AAC695GCC (2223A>G 2224A>C), ATT696ATC (2228T>C), CCT697CCA (2231T>A), ATA701GTC (2241A>G 2243A>C), AAG705AAA (2255G>A), AGT706AGC (2258T>C), GCA710AGT (2268G>A 2269C>G 2270A>T), TCA711ACA (2271T>A), ATC713GTG (2277A>G 2279C>G), TCT714TCA (2282T>A), GAG715GAA (2285G>A), AAG716GCT (2286A>G 2287A>C 2288G>T), AAA717CAC (2289A>C 2291A>C), CTA718ATG (2292C>A 2294A>G), GCA719GAT (2296C>A 2297A>T), GTG720GTA (2300G>A), CAG721CAA (2303G>A), ACA722AGG (2305C>G 2306A>G), GAG723ATG (2307G>A 2308A>T), ATG724TTG (2310A>T), AGG725TAC (2313A>T 2314G>A 2315G>C), ACT726AAT (2317C>A), GGA727GGC (2321A>C), TTT728AGA (2322T>A 2323T>G 2324T>A), TCT731AGA (2331T>A 2332C>G 2333T>A), TGC732GCA (2334T>G 2335G>C 2336C>A), ATT733CTG (2337A>C 2339T>G), GAT734GAA (2342T>A), GCA735GCT (2345A>T), GTT737GCT (2350T>C), ACC739ACT (2357C>T), CTC740TTA (2358C>T 2360C>A), ATA741ATG (2363A>G), ATC743ATT (2369C>T), GAG744GAA (2372G>A), CAG745CAA (2375G>A), CCA747CCT (2381A>T), TTT749CAT (2385T>C 2386T>A), GTT750GTC (2390T>C), CAG751CAA (2393G>A), TTT752TTC (2396T>C), TTA753CTA (2397T>C), TAT754TAC (2402T>C), AGA759CGG (2415A>C 2417A>G), TGC762_AAC763insAGAAGG (2426_2427insAGAAGG), AAC763AAG (2429C>G), AAA764ATG (2431A>T 2432A>G), GTA765GTC (2435A>C), TCA766ACT (2436T>A 2438A>T), AAA767AAG (2441A>G), GAG768GAA (2444G>A), CCA769ACA (2445C>A), AAG770_GAA771del (2448_2453delAAGGAA), TCA772TCT (2456A>T), ATC774GTA (2460A>G 2462C>A), AAA775CAA (2463A>C), ATT776TCG (2466A>T 2467T>C 2468T>G), TGC777CTC (2469T>C 2470G>T), TAC778TAT (2474C>T), CAT779GAC (2475C>G 2477T>C), AAA780TCC (2478A>T 2479A>C 2480A>C), ATC781ACA (2482T>C 2483C>A), GTT782ATT (2484G>A), GAA783GGG (2488A>G 2489A>G), TAT784AGA (2490T>A 2491A>G 2492T>A), ATC785ATG (2495C>G), AAG786CAA (2496A>C 2498G>A), GAT787GAA (2501T>A), ACA788ATG (2503C>T 2504A>G), TGG790AAT (2508T>A 2509G>A 2510G>T), AGC791GAT (2511A>G 2512G>A 2513C>T), CTT792CTG (2516T>G), TAT794TAC (2522T>C), GCA795ATT (2523G>A 2524C>T 2525A>T), CTG796TTG (2526C>T), AGA797AAA (2530G>A), CTC799CTT (2537C>T), GGT803GGG (2549T>G), ATA806CTA (2556A>C), ATG807ATA (2561G>A), CTT809CTC (2567T>C), GGA811GGG (2573A>G), GTT812ACT (2574G>A 2575T>C), TTT813ATG (2577T>A 2579T>G), GGA814GGT (2582A>T), GGC815GCC (2584G>C), ATT816GTA (2586A>G 2588T>A), ACA817ACT (2591A>T), TTT819TTC (2597T>C), TTG820ATT (2598T>A 2600G>T), GCG821TCT (2601G>T 2603G>T), CAG824AAA (2610C>A 2612G>A), AGT825AGC (2615T>C), AAC826AGC (2617A>G), TCT827TTG (2620C>T 2621T>G), TTC828ATT (2622T>A 2624C>T), AAT830ACT (2629A>C), GCA831TCC (2631G>T 2633A>C), TCG832CAG (2634T>C 2635C>A), AGA834AAA (2641G>A), GTG837GTT (2651G>T), TCC841TCG (2663C>G), AGT843AGC (2669T>C), TCC845TCA (2675C>A), AGT846AGC (2678T>C), GGT851GGA (2693T>A), AAT852AGA (2695A>G 2696T>A), AAT852_AAG853insTCC (2696_2697insTCC), AAG853CAT (2697A>C 2699G>T), CTC854GTA (2700C>G 2702C>A), AGA855TCA (2703A>T 2704G>C), AAT856AAG (2708T>G), CTT857ATG (2709C>A 2711T>G), CTC858CTG (2714C>G), GTT859TTT (2715G>T), GGT860AGC (2718G>A 2720T>C), TCT863TCA (2729T>A), TCA865CCA (2733T>C), TTG866AAT (2736T>A 2737T>A 2738G>T), GCA867GAT (2740C>A 2741A>T), CAA868CAG (2744A>G), CAA868_GAT869insGGA (2744_2745insGGA), GAT869AAA (2745G>A 2747T>A), GCT872TCC (2754G>T 2756T>C), GAA873AGG (2757G>A 2758A>G 2759A>G), GGA875GGT (2765A>T), GTA877GTG (2771A>G), AAT878AAC (2774T>C), CAG879GAA (2775C>G 2777G>A), AGC880TCT (2778A>T 2779G>C 2780C>T), CTC881CTT (2783C>T), AAG882GCC (2784A>G 2785A>C 2786G>C), GTG886GTA (2798G>A), TTA888CTA (2802T>C), AGA889AGG (2807A>G), CTT890GTG (2808C>G 2810T>G), GGT891GCA (2812G>C 2813T>A), GTG894GTT (2822G>T), AGA897CGT (2829A>C 2831A>T), GGC898GGA (2834C>A), ACC899ACT (2837C>T), TGT902TGC (2846T>C), TCT903AGC (2847T>A 2848C>G 2849T>C), GGT904GAA (2851G>A 2852T>A), ACC908ACT (2864C>T), GCT910GCA (2870T>A), CAT911CAC (2873T>C), GCA912GCT (2876A>T), CAC914ACG (2880C>A 2881A>C 2882C>G), AAT915CAG (2883A>C 2885T>G), GCT916CTC (2886G>C 2887C>T 2888T>C), CGA917CGT (2891A>T), GGT919GGA (2897T>A), ACT920ACC (2900T>C), ACA923TCA (2907A>T), ATC925ATT (2915C>T), GCC927GAA (2920C>A 2921C>A), AAT928CAT (2922A>C), CGA930GTC (2928C>G 2929G>T 2930A>C), TCT931TGC (2932C>G 2933T>C), AAA932CAG (2934A>C 2936A>G), GTT933GTG (2939T>G), AAC936AAT (2948C>T), AGA938AAA (2953G>A), GAA939CAT (2955G>C 2957A>T), TCA940GCC (2958T>G 2960A>C), AGG941AAG (2962G>A), ATT942TTT (2964A>T), ATT943TTG (2967A>T 2969T>G), GAG944CCT (2970G>C 2971A>C 2972G>T), GGC945GAA (2974G>A 2975C>A), GAC947GAT (2981C>T), ATC948GTT (2982A>G 2984C>T), ATT949GTA (2985A>G 2987T>A), CTG950TTA (2988C>T 2990G>A), TTG951CTG (2991T>C), CGC952AGG (2994C>A 2996C>G), GGT954GGC (3002T>C), GAT955TGT (3003G>T 3004A>G), CCA956CCT (3008A>T), GAT957GAC (3011T>C), GGT958GGG (3014T>G), CCA959CCT (3017A>T), AAG960AAA (3020G>A), CCT961GTC (3021C>G 3022C>T 3023T>C), CGT964AGA (3030C>A 3032T>A), AAA965AAG (3035A>G), TTC967TTT (3041C>T), CCA968GCT (3042C>G 3044A>T), AGA969GAA (3045A>G 3046G>A), GAG972GCA (3055A>C 3056G>A), AAG977AAA

(3071G>A), GGC978GGA (3074C>A), TCA979TCT (3077A>T), CAA980GAA (3078C>G), GGA981GGC (3083A>C), TGC983TGT (3089C>T), TGT984GTC (3090T>G 3091G>T 3092T>C), GCT986GCA (3098T>A), GTT987GTG (3101T>G), GCG988GCA (3104G>A), TCT989TCA (3107T>A), ACA990AAA (3109C>A), CCA992CCT (3116A>T), CGT993GAG (3117C>G 3118G>A 3119T>G), CTT994AAA (3120C>A 3121T>A 3122T>A), GGC995CAG (3123G>C 3124G>A 3125C>G), AAT996AAC (3128T>C), TTA997TTG (3131A>G), GAG998GAC (3134G>C), ATG1002TAT (3144A>T 3145T>A 3146G>T), CCA1003TCA (3147C>T), TCA1007TCC (3161A>C), AAG1008AAA (3164G>A), TAC1010TAT (3170C>T), GTT1012GAG (3175T>A 3176T>G), GTG1014GTC (3182G>C), GAA1015ACA (3183G>A 3184A>C), TAT1016TAC (3188T>C), GAA1017AGG (3189G>A 3190A>G 3191A>G), CTG1018ATG (3192C>A), AAC1019AGC (3196A>G), TCC1020ACA (3198T>A 3200C>A), TCC1021AAT (3201T>A 3202C>A 3203C>T), AGT1022TCA (3204A>T 3205G>C 3206T>A), AAG1024CAC (3210A>C 3212G>C), AAG1024_ATT1025insTCA (3212_3213insTCA), ATT1025ATC (3215T>C), TGC1026AGT (3216T>A 3218C>T), TCT1027GCT (3219T>G), CAA1029GAA (3225C>G), TCT1030GCC (3228T>G 3230T>C), CAC1034CAT (3242C>T), ATA1035GTC (3243A>G 3245A>C), GGG1037AGC (3249G>A 3251G>C), GAC1041GAT (3263C>T), GGC1043GGA (3269C>A), ACG1044ACT (3272G>T), CTG1047TTA (3279C>T 3281G>A), TTA1048TTG (3284A>G), AGT1050TCT (3288A>T 3289G>C), GTT1051ATC (3291G>A 3293T>C), CAG1052CAA (3296G>A), AAT1053AAC (3299T>C), CCT1056CCA (3308T>A), GTG1057TGC (3309G>T 3310T>G 3311G>C), ATC1058ATT (3314C>T), GGC1060GGT (3320C>T), ATC1061ATT (3323C>T), CAC1062CAT (3326C>T), GCA1064GCT (3332A>T), TCA1065TCC (3335A>C), GGC1068GAA (3343G>A 3344C>A), GTA1069GCT (3346T>C 3347A>T), GCT1071ACA (3351G>A 3353T>A), GAA1072GAG (3356A>G), CGT1073TTG (3357C>T 3358G>T 3359T>G), ATC1076GTA (3366A>G 3368C>A), TCT1077GCA (3369T>G 3371T>A), GCT1080GCC (3380T>C), ACA1081ACT (3383A>T), ATC1083GTA (3387A>G 3389C>A), GAA1086GAC (3398A>C), CAA1087CAG (3401A>G), CTA1088CTG (3404A>G), GAG1089CAA (3405G>C 3407G>A), CCA1092GCT (3414C>G 3416A>T), ACT1093CAA (3417A>C 3418C>A 3419T>A), CCG1095CCA (3425G>A), GTC1096GTT (3428C>T), GCA1098ATG (3432G>A 3433C>T 3434A>G), GCA1099GCC (3437A>C), GTA1101GTG (3443A>G), CGC1102AGA (3444C>A 3446C>A), GAT1104GAC (3452T>C), ATT1105TTG (3453A>T 3455T>G), CTG1106CTC (3458G>C), AAG1107AAA (3461G>A), TCA1108CAT (3462T>C 3463C>A 3464A>T), ATT1109GTT (3465A>G), AGA1110CGA (3468A>C), AGC1111TCA (3471A>T 3472G>C 3473C>A), ACA1114ACT (3482A>T), CAG1115GCT (3483C>G 3484A>C 3485G>T), CTT1116CTC (3488T>C), GAG1118GAC (3494G>C), AAC1120AAG (3500C>G), GTG1122GTT (3506G>T), TAC1123GCT (3507T>G 3508A>C 3509C>T), TAC1124TTT (3511A>T 3512C>T), CTT1125TTG (3513C>T 3515T>G), GGA1126GGC (3518A>C), ACA1127ACT (3521A>T), CCA1129CCT (3527A>T), CAG1130CAA (3530G>A), TTG1132ATG (3534T>A), GCC1133GCA (3539C>A), GCC1134GCA (3542C>A), ACA1135ACG (3545A>G), GTT1136GTG (3548T>G), CCC1137CCA (3551C>A), ACT1141ACC (3563T>C), CTG1142CTC (3566G>C), CGG1143AGG (3567C>A), CAA1146CCA (3577A>C), TTG1147CTG (3579T>C), GAA1149GAG (3587A>G), TTC1151TTT (3593C>T), GGA1152GGT (3596A>T), CCT1153GAA (3597C>G 3598C>A 3599T>A), GAG1155CAA (3603G>C 3605G>A), ACA1156ACT (3608A>T), GCA1157GCT (3611A>T), CCA1158CCT (3614A>T), TCC1159TCA (3617C>A), ATT1160CTG (3618A>C 3620T>G), CTA1161CTC (3623A>C), ACA1162TCT (3624A>T 3626A>T), GTT1163GTG (3629T>G), CAT1164AAA (3630C>A 3632T>A), GAC1165GAT (3635C>T), GGT1168GGG (3644T>G), GGT1170GGA (3650T>A), GAC1172GAT (3656C>T), CCC1173CCT (3659C>T), GTG1175GTT (3665G>T), GGG1177GGC (3671G>C), GTA1178ATT (3672G>A 3674A>T), ATG1179GAG (3675A>G 3676T>A), GCT1185GCC (3695T>C), TGT1186TCA (3697G>C 3698T>A), GGA1187AAT (3699G>A 3700G>A 3701A>T), GAT1189GAG (3707T>G), GAT1190GAC (3710T>C), GAC1191AGC (3711G>A 3712A>G), GCC1193GCA (3719C>A), AAA1194CGG (3720A>C 3721A>G 3722A>G), CTA1195GCT (3723C>G 3724T>C 3725A>T), TTC1197TTT (3731C>T), AAT1199GGT (3735A>G 3736A>G), TGC1202TCT (3745G>C 3746C>T), TCG1203GCC (3747T>G 3749G>C), CTG1204ATT (3750C>A 3752G>T), CTA1205TTA (3753C>T), CCT1206CCA (3758T>A), ATC1207GTT (3759A>G 3761C>T), ATG1208CTC (3762A>C 3764G>C), CGT1209AAG (3765C>A 3766G>A 3767T>G), AGC1210AGT (3770C>T), CAG1211CAA (3773G>A), AAG1212AGG (3775A>G), ATC1213GTG (3777A>G 3779C>G), CCT1214CCA (3782T>A), GGG1215GGT (3785G>T), GGA1216GGT (3788A>T), CGT1217ATC (3789C>A 3790G>T 3791T>C), CCA1218CCC (3794A>C), TGT1219ACA (3795T>A 3796G>C 3797T>A), GAA1220ACA (3798G>A 3799A>C), AGG1221CGT (3801A>C 3803G>T), GAT1222TGT (3804G>T 3805A>G), GAT1224GAC (3812T>C), GTA1225ATC (3813G>A 3815A>C), GTG1226GTC (3818G>C), CTC1227CTG (3821C>G), GGA1229GGT (3827A>T), ATA1230GTT (3828A>G 3830A>T), GGA1232GGT (3836A>T), TGT1233TTT (3838G>T), GAT1234GAC (3842T>C), TAC1235TTC (3844A>T), TAT1236TTT (3847A>T), GGC1238TCC (3852G>T 3853G>C), GAG1240GAA (3860G>A), CTG1241CTC (3863G>C), TCT1244TCA (3872T>A), TGC1245TGT (3875C>T), TAT1247TAC (3881T>C), CCC1248CCT (3884C>T), AAC1250AAT (3890C>T), AAG1251AAA (3893G>A), ATG1252CTG (3894A>C), GGG1253GGT (3899G>T), ATG1254TTG (3900A>T), GGG1255GGT (3905G>T), ATG1256ACC (3907T>C 3908G>C), AAC1257AAT (3911C>T), AAG1258AAA (3914G>A), AGA1259AGG (3917A>G), TTT1261TTC (3923T>C), GTG1262GTT (3926G>T), CAA1263GAA (3927C>G), TCC1264GCA (3930T>G 3932C>A), ACT1265CAG (3933A>C 3934C>A 3935T>G), GGC1266GAG (3937G>A 3938C>G), GAA1267AGT (3939G>A 3940A>G 3941A>T), GGA1268ACC (3942G>A 3943G>C 3944A>C), GAG1269CAA (3945G>C 3947G>A), AGA1270AGG (3950A>G), GTG1271GTA (3953G>A), GAA1272CTA (3954G>C 3955A>T), AAA1274AAG (3962A>G), GAC1276AAT (3966G>A 3968C>T), ACT1277TCT (3969A>T), GTA1279GTT (3977A>T), GAA1281CAA (3981G>C), GAA1284GAG (3992A>G), GAG1285GAA (3995G>A), CTA1286TTA (3996C>T), CAG1289GAA (4005C>G 4007G>A), ATT1290ATA (4010T>A), AAA1292AAG (4016A>G), GGC1293GGT (4019C>T), ATC1294AAC (4021T>A), CAT1295TAT (4023C>T), GAT1297GAA (4031T>A), CTG1298ATG (4032C>A), GTC1299GTT (4037C>T), ACC1301ACT (4043C>T), TGC1303TGT (4049C>T), GCC1304GCT (4052C>T), AAA1305AAG (4055A>G), GAT1306GAC (4058T>C), CGC1308AGG (4062C>A 4064C>G), CTC1309CTA (4067C>A), CCA1310CCT (4070A>T), CTT1311CTG (4073T>G), GAG1312GAC (4076G>C), AAA1313AAG (4079A>G), ATC1314ATA (4082C>A), TAT1315TAC (4085T>C), GGC1316GGG (4088C>G), AAG1317AAA (4091G>A), AGA1318AAG (4093G>A 4094A>G), AAG1319AAA (4097G>A), AGG1321CGT (4101A>C 4103G>T), CTC1322CTT (4106C>T), ATA1325ATT (4115A>T), CTT1326TTG (4116C>T 4118T>G), CCT1327CCA (4121T>A), TAC1330TAT (4130C>T), TTG1333CTG (4137T>C), GTC1334GTG (4142C>G), AGG1335CGC (4143A>C 4145G>C), AAG1336AAA (4148G>A), TTT1338TTC (4154T>C), TTC1341TTT (4163C>T), TCA1342TCT (4166A>T), GCC1343GCA (4169C>A), ACA1344TGT (4170A>T 4171C>G 4172A>T), TTG1345TTA (4175G>A), TTG1348GCA (4182T>G 4183T>C 4184G>A), CAC1349CAT (4187C>T), GCT1351GTT (4192C>T), ATA1352CTG (4194A>C 4196A>G), CCA1353CCC (4199A>C), GTT1356GTG (4208T>G), ATT1358ATC (4214T>C), GAT1359AAT (4215G>A), CCT1360CCC (4220T>C), ACA1361ATT (4222C>T 4223A>T), AGT1362TCA (4224A>T 4225G>C 4226T>A), TCT1363CAT (4227T>C 4228C>A), ACA1366ACC (4238A>C), TTG1367ACC (4239T>A 4240T>C 4241G>C), TTG1368CTG (4242T>C), AAT1370AAC (4250T>C), GGG1371CAG (4251G>C 4252G>A), TTT1372TTC (4256T>C), AGA1373CGG (4257A>C 4259A>G), GCT1374AGT (4260G>A 4261C>G), TCA1376TCT (4268A>T), GAC1377GAT (4271C>T), GTG1378GTT (4274G>T), GGA1379GGT (4277A>T), TCA1381TCT (4283A>T), GCT1382GCA (4286T>A), GAT1383GAC (4289T>C), TAT1384TAC (4292T>C), AGA1390AGG (4310A>G), GCA1391GCG (4313A>G), CCT1392CCA (4316T>A), GTT1393ATA (4317G>A 4319T>A), GCT1395GCA (4325T>A), CAG1397CAA (4331G>A), TTT1399TTA (4337T>A), GAA1405GCA (4354A>C), TAC1407TAT (4361C>T), GGA1408GGG (4364A>G), AAG1410GCT (4368A>G 4369A>C 4370G>T), CCT1411CCA (4373T>A), GGC1412GGA (4376C>A), AGT1413AGC (4379T>C), CCT1414GAG (4380C>G 4381C>A 4382T>G), TCC1416TCA (4388C>A), GCT1418GCA (4394T>A), CGA1419AGA (4395C>A), GCA1421GCT (4403A>T), CTT1422CTG (4406T>G), TTA1423TTG (4409A>G), GCA1426GCT (4418A>T), TGT1428TGC (4424T>C), ACA1431ACT (4433A>T), CTG1432CTA (4436G>A), GTG1437CTC (4449G>C 4451G>C), TTT1438TTC (4454T>C), TTG1440TTA (4460G>A), GGG1442GGT (4466G>T), GGC1443GGA (4469C>A), CCA1445CCT (4475A>T), TCA1446TCT (4478A>T), GCA1449GCT (4487A>T), CTA1450CTC (4490A>C), ACG1451ACC (4493G>C), ATC1453ATA (4499C>A), TTC1454TTT (4502C>T), AAT1455AAC (4505T>C), TCT1456TCC (4508T>C), CTC1457CTT (4511C>T), CTC1458TTA (4512C>T 4514C>A), AAT1459AAC (4517T>C), CGA1464CGG (4532A>G), GCC1466GCT (4538C>T), TTT1467TTC (4541T>C), TCA1469TCG (4547A>G), TTG1470CTC (4548T>C 4550G>C), TTA1471CTC (4551T>C 4553A>C), AGA1473AGG (4559A>G), CAT1475CAC (4565T>C), ATT1476ATC (4568T>C), GCT1477AAA (4569G>A 4570C>A 4571T>A), GCC1478GCA (4574C>A), AGG1479AAG (4576G>A), GCT1480GCA (4580T>A), ATA1481TTG (4581A>T 4583A>G), GGA1482GGT (4586A>T), GTT1483GTA (4589T>A), AAA1484AAG (4592A>G), CCA1485TCA (4593C>T), TTC1488TTT (4604C>T), CAG1490GAC (4608C>G 4610G>C), CTA1491TTA (4611C>T), TTC1492TTT (4616C>T), ATA1493ATT (4619A>T), GCT1494GCA (4622T>A), GTT1495GTG (4625T>G), GGA1497GGG (4631A>G), GTT1502GTG (4646T>G), GTA1504GTG (4652A>G), TTA1506ATG (4656T>A 4658A>G), CAT1507CAC (4661T>C), CTC1508CTT (4664C>T), CAG1509CAA (4667G>A), TAT1511TAC (4673T>C), TCT1512GAG (4674T>G 4675C>A 4676T>G), CTG1513TTA (4677C>T 4679G>A), CCA1514CCT (4682A>T), AAT1515GCA (4683A>G 4684A>C 4685T>A), CAT1518CAA (4694T>A), TTA1520CTA (4698T>C), CTG1522CAA (4705T>A 4706G>A), GTC1523GTG (4709C>G), AAT1524AAC (4712T>C), GTA1525GTT (4715A>T), ATC1526ATT (4718C>T), ATT1527ATC (4721T>C), GGT1530GGC (4730T>C), GAC1532GAT (4736C>T), TCG1534AAC (4740T>A 4741C>A 4742G>C), GAT1536AAT (4746G>A), GTT1537GTC (4751T>C), GAG1539GAT (4757G>T), GTA1540GTC (4760A>C), CAA1541AAA (4761C>A), TTT1542TTC (4766T>C), CAA1543CAG (4769A>G), CTA1548TTG (4782C>T 4784A>G), ACT1549ACG (4787T>G), CTG1551TTG (4791C>T), AGT1552AGC (4796T>C), GGT1554GGC (4802T>C), TTT1555TTC (4805T>C), AAA1556AAG (4808A>G), CGA1557CGC (4811A>C), CTT1560TTG (4818C>T 4820T>G), GGA1561GGT (4823A>T), CAC1563CAA (4829C>A), ATG1564CTA (4830A>C 4832G>A), GCT1565GCC (4835T>C), CCT1566CCC (4838T>C), TCA1571TCC (4853A>C), ATC1572ATT (4856C>T), ATT1573GTG (4857A>G 4859T>G), GAG1574GAA (4862G>A), CCC1575CCA (4865C>A), ATA1579ATC (4877A>C), CCT1581CCA (4883T>A), GCC1582GCA (4886C>A), CCA1583GTT (4887C>G 4888C>T 4889A>T), GGT1584GGG (4892T>G), CCT1586CCA (4898T>A), GAC1587GAT (4901C>T), ACT1588ATC (4903C>T 4904T>C), CAG1589ACT (4905C>A 4906A>C 4907G>T), ATG1592TAT (4914A>T 4915T>A 4916G>T), ACA1597ACG (4931A>G), GGA1598GGT (4934A>T), ATA1599GTA (4935A>G), CGT1600AGA (4938C>A 4940T>A), GAA1601GAG (4943A>G), CAT1604CAG (4952T>G), CAC1605CAT (4955C>T), GGT1606GGA (4958T>A), CGT1607CAG (4960G>A 4961T>G), GTG1608GTC (4964G>C), GCT1609GTT (4966C>T), AAG1612ACA (4975A>C 4976G>A), CTT1613TTG (4977C>T 4979T>G), GTC1614ACA (4980G>A 4981T>C 4982C>A), TTA1615GAC (4983T>G 4984T>A 4985A>C), GAT1616AAT (4986G>A), GTT1617GTC (4991T>C), CAG1618CAA (4994G>A), ACG1619AAA (4996C>A 4997G>A), GCG1620GCA (5000G>A), TTG1621ATA (5001T>A 5003G>A), GAT1622ATG (5004G>A 5005A>T 5006T>G), GAA1623CAG (5007G>C 5009A>G), AGG1624CGA (5010A>C 5012G>A), GGT1625GGC (5015T>C), TTC1626TTG (5018C>G), AGA1627CCA (5019A>C 5020G>C), GCT1628ATC (5022G>A 5023C>T 5024T>C), ATC1630CAT (5028A>C 5029T>A 5030C>T), CCT1632CCA (5036T>A), TCC1633TCT (5039C>T), TTG1635GCT (5043T>G 5044T>C 5045G>T), GTG1637CAA (5049G>C 5050T>A 5051G>A), CAG1639AGT (5055C>A 5056A>G 5057G>T), GAA1640GAG (5060A>G), ATT1642CTA (5064A>C 5066T>A), GCA1643CAA (5067G>C 5068C>A), AAG1644GCC (5070A>G 5071A>C 5072G>C), GTA1645GTT (5075A>T), ACA1646ACT (5078A>T), GGG1647GGA (5081G>A), GAT1648GAC (5084T>C), TCA1649AGT (5085T>A 5086C>G 5087A>T), AGT1650AAC (5089G>A 5090T>C), GCC1651ATA (5091G>A 5092C>T 5093C>A), CTG1652GGG (5094C>G 5095T>G), ACA1653GCT (5097A>G 5099A>T), ATT1654CTG (5100A>C 5102T>G), TGT1655CTG (5103T>C 5104G>T 5105T>G), ATG1657CTG (5109A>C), GCA1658AGC (5112G>A 5113C>G 5114A>C), AAA1659AAG (5117A>G), GCA1660GAT (5119C>A 5120A>T), TCC1663ACT (5127T>A 5129C>T), TAT1664TAC (5132T>C), ACG1665GTG (5133A>G 5134C>T), CCA1666CCT (5138A>T), TTG1667CTT (5139T>C 5141G>T), GAC1668CCA (5142G>C 5143A>C 5144C>A), GCA1669GAA (5146C>A), GGT1670GGA (5150T>A), GAG1671AAA (5151G>A 5153G>A), AAG1672AAA (5156G>A), ATC1673ATT (5159C>T), ACA1674GAA (5160A>G 5161C>A), AAT1675GAT (5163A>G), GAG1677TCA (5169G>T 5170A>C 5171G>A), CGT1678CAA (5173G>A 5174T>A), GAT1679GGT (5176A>G), CTG1680GTC (5178C>G 5180G>C), AAT1681CAT (5181A>C), TGG1682GAA (5184T>G 5185G>A 5186G>A), GCA1684TGT (5190G>T 5191C>G 5192A>T), CCA1685TCT (5193C>T 5195A>T), AAC1686GGA (5196A>G 5197A>G 5198C>A), ATT1687GTT (5199A>G), GGT1688TTC (5202G>T 5203G>T 5204T>C), TTT1689TTC (5207T>C), GCA1692TCG (5214G>T 5216A>G), CGT1693CGA (5219T>A), ACT1694ACA (5222T>A), GCA1695GAA (5224C>A), GCC1696GTT (5227C>T 5228C>T), CAC1697AAT (5229C>A 5231C>T), TAC1698CAT (5232T>C 5234C>T), ACG1699AAA (5236C>A 5237G>A), GAA1702GAG (5246A>G), GGG1703GAC (5248G>A 5249G>C), TAC1704ACA (5250T>A 5251A>C 5252C>A), ATT1706CTT (5256A>C), AAC1708AAT (5264C>T), TGT1709TGC (5267T>C), AAG1713AAA (5279G>A), AAG1714AAA (5282G>A), TCC1715ACA (5283T>A 5285C>A), AAT1716ACA (5287A>C 5288T>A), GTT1718GTG (5294T>G), CCG1733CCC (5339G>C), TAC1734TAT (5342C>T), ACT1735ACA (5345T>A), TCA1737GCA (5349T>G), GCT1738GCC (5354T>C), ATA1739ATC (5357A>C), ATG1740AAG (5359T>A), GAT1741GAC (5363T>C), GCC1742AAT (5364G>A 5365C>A 5366C>T), AAG1744CAA (5370A>C 5372G>A), AAT1745AGT (5374A>G), GTG1746CGC (5376G>C 5377T>G 5378G>C), GCA1748TCA (5382G>T), GGA1749ATG (5385G>A 5386G>T 5387A>G), GGA1750GGG (5390A>G), CAT1751ATT (5391C>A 5392A>T), GTA1752GGA (5395T>G), GCA1753ACC (5397G>A 5399A>C), ACC1754CAA (5400A>C 5401C>A 5402C>A), AAT1755AAA (5405T>A), GTC1756GTT (5408C>T), GTT1757GTC (5411T>C), GTG1759ATT (5415G>A 5417G>T), GGA1761GGG (5423A>G), GGA1763GGT (5429A>T), GTC1766GTG (5438C>G), GGC1767AGT (5439G>A 5441C>T), CCA1768CCC (5444A>C), GCA1771GCT (5453A>T), GCG1772GCT (5456G>T), ATG1774TTT (5460A>T 5462G>T), GCC1775GCA (5465C>A), GCA1777TCA (5469G>T), ACA1778ACC (5474A>C), AGG1779AAT (5476G>A 5477G>T), CAA1780CAG (5480A>G), TGT1782AGA (5484T>A 5486T>A), GTG1783GCT (5488T>C 5489G>T), GAC1785CAA (5493G>C 5495C>A), ATA1786CTT (5496A>C 5498A>T), GTT1788GTC (5504T>C), CGC1791AGA (5511C>A 5513C>A), ATT1793TTG (5517A>T 5519T>G), GGA1794GCG (5521G>C 5522A>G), AAT1795GGC (5523A>G 5524A>G 5525T>C), GTG1796TTG (5526G>T), CTT1797CTG (5531T>G), GAC1798GAT (5534C>T), CTC1799TTG (5535C>T 5537C>G), ACC1801CAG (5541A>C 5542C>A 5543C>G), GGC1805GGT (5555C>T), GTG1807ATT (5559G>A 5561G>T), CAG1808CAA (5564G>A), TTT1810TTC (5570T>C), CTT1811CTC (5573T>C), CAA1812ACA (5574C>A 5575A>C), TGT1813_GTG1814insGTC (5579_5580insGTC), CCT1815CAG (5584C>A 5585T>G), CAT1816AAA (5586C>A 5588T>A), GGA1817AGT (5589G>A 5591A>T), GAC1818TGC (5592G>T 5593A>G), AAA1819AAG (5597A>G), GTG1820ACA (5598G>A 5599T>C 5600G>A), AGT1823GCT (5607A>G 5608G>C), GGT1824AGT (5610G>A), GCA1825GGG (5614C>G 5615A>G), TCG1826del (5616_5618delTCG), CTC1827CGC (5620T>G), CAC1828GAT (5622C>G 5624C>T), AGT1829GGA (5625A>G 5627T>A), AGT1830ATG (5629G>T 5630T>G), TTT1831TTG (5633T>G), ATG1832AAC (5635T>A 5636G>C), CAA1834CAT (5642A>T), CAA1834_GGG1835insCATGCT (5642_5643insCATGCT), GGG1835GCT (5644G>C 5645G>T), TTT1836TAT (5647T>A), GAA1837GCC (5650A>C 5651A>C), TTG1838CTT (5652T>C 5654G>T), GGC1839GGA (5657C>A), CTC1841CTT (5663C>T), CGC1842CGA (5666C>A), ATT1843ATA (5669T>A), ATA1844GTG (5670A>G 5672A>G), CAT1845GGC (5673C>G 5674A>G 5675T>C), GGT1846TGT (5676G>T), GAT1847TCA (5679G>T 5680A>C 5681T>A), TTA1848ATT (5682T>A 5684A>T), GCA1849TCA (5685G>T), AAA1850AGA (5689A>G), CAG1851AAG (5691C>A), ACA1852ATG (5695C>T 5696A>G), ATG1854GTG (5700A>G), CGG1855AGA (5703C>A 5705G>A), ATG1856TTG (5706A>T), CCA1857CCC (5711A>C), TAT1858TAC (5714T>C), GTG1859ACT (5715G>A 5716T>C 5717G>T), GTA1860GTT (5720A>T), GGA1861GGG (5723A>G), CAT1863ATG (5727C>A 5728A>T 5729T>G), GGA1864GGT (5732A>T), GGA1865GGT (5735A>T), GGA1867GCA (5740G>C), AAT1868GAT (5742A>G), TTT1869TGC (5746T>G 5747T>C), TCC1870TCA (5750C>A), CAG1872AGA (5754C>A 5755A>G 5756G>A), GAT1873AAT (5757G>A)

Proteins

polyprotein (YP_001976147.1)	1	1877	87.2%	9403	72.1%	1862 (98.8%)	1290 (68.4%)	8/15/0/0	0

	Begin	End	Coverage	Score	Concordance	Matches	Identities	I/D/M/F*	Stop Codons
**NT**	**35**	**5769**	**79.3%**	**3466**	**30.8%**	**5682 (98.6%)**	**3757 (65.2%)**	**28/53**	
Protein mutations:	M6I (158G>C), F7V (159T>G 161C>T), S8_S17del (162_191delTCCAAGGTTGCTGGTGTTGATGACGCCAGT), S20A (198T>G 200C>T), T22P (204A>C), F25L (213T>C), G26S (216G>T 217G>C), N30Q (228A>C 230T>G), S31T (231T>A 233T>A), I32V (234A>G 236T>G), K34S (240A>T 241A>C), F35L (243T>C 245T>G), A38T (252G>A 254G>A), V39M (255G>A 257T>G), S40K (259G>A 260T>G), N41S (262A>G), I42M (266T>G), T43S (268C>G 269C>T), T44K (271C>A 272T>A), M45I (275G>A), Q47E (279C>G), I49F (285A>T 287T>C), D51G (292A>G 293T>C), L53I (297C>A), D55E (305T>A), V60I (318G>A 320G>T), T61V (321A>G 322C>T), D62K (324G>A 326T>A), V63T (327G>A 328T>C 329G>T), S64A (330T>G 332C>A), T65D (333A>G 334C>A 335A>T), V68N (342G>A 343T>A 344G>T), A69S (345G>A 346C>G 347C>T), T70V (348A>G 349C>T), L74V (360C>G 362T>A), N75E (363A>G 365T>A), V77L (369G>T 371T>A), D79G (376A>G 377C>G), G82A (385G>C 386T>C), A83T (387G>A 389A>G), L84V (390C>G), D86E (398T>A), L89V (405C>G), A91G (412C>G 413G>T), Y92I (414T>A 415A>T), S93N (417T>A 418C>A), L95F (425G>C), S96K (426T>A 427C>A 428C>A), K100R (439A>G), S101T (441T>A 443T>C), K103I (448A>T 449G>T), L109V (465T>G 467G>T), F110V (468T>G), E111L (471G>T 472A>T), S112G (474A>G 476T>C), L113I (477T>A 479G>C), S114K (480T>A 481C>A 482C>A), K116N (488A>T), V117F (489G>T), K118I (493A>T 494A>T), L119R (495T>A 496T>G), F121Y (502T>A), A122S (504G>T 506T>A), V124L (510G>C), N126E (516A>G 518T>A), K127T (520A>C), S128T (523G>C 524T>A), E129G (526A>G 527A>T), D130E (530T>A), V131Y (531G>T 532T>A 533A>T), I133V (537A>G 539A>T), Y136I (546T>A 547A>T 548T>A), G137A (550G>C), A142S (564G>T 566T>C), I143L (567A>C 569A>T), T144S (570A>T), V147L (579G>T 581G>A), L149Q (586T>A), F150I (588T>A 590T>A), V151I (591G>A 593T>C), S154V (600A>G 601G>T 602T>A), G159M (615G>A 616G>T 617T>G), S161R (621T>A 622C>G 623T>A), N162S (625A>G 626C>T), T163V (627A>G 628C>T 629T>A), K165C (633A>T 634A>G 635A>T), I170V (648A>G 650A>T), M174F (660A>T 662G>C), L181I (681T>A 683A>T), D182S (684G>A 685A>G), K186L (696A>C 697A>T 698A>C), A188S (702G>T 704C>T), R190K (708C>A 709G>A), T192S (714A>T 716C>T), M193Q (717A>C 718T>A), V194M (720G>A), M199V (735A>G), V205L (753G>T 755T>A), S206N (756T>A 757C>A 758A>T), M214L (780A>C), T216S (786A>T 788A>T), I217L (789A>T 791A>G), L218I (792T>A 794G>A), I228V (822A>G 824T>G), S238T (853G>C 854C>A), T248A (882A>G 884A>G), T260Q (918A>C 919C>A 920T>A), R263K (927C>A 928G>A 929T>G), I280V (978A>G 980A>T), V281A (982T>C 983C>T), G282D (985G>A 986C>T), T283I (988C>T 989C>A), L298I (1032C>A 1034G>T), Q307T (1059C>A 1060A>C 1061G>T), D310S (1068G>T 1069A>C), C313A (1077T>G 1078G>C), K325P (1113A>C 1114A>C 1115G>T), P328S (1122C>T 1124T>A), T329I (1126C>T), R335K (1144G>A 1145G>A), E337Q (1149G>C 1151A>G), M354L (1200A>T), A361T (1221G>A 1223C>A), A363V (1228C>T), E366D (1238A>T), V368L (1242G>C 1244T>C), Y369I (1245T>A 1246A>T 1247T>A), S372N (1255G>A 1256T>C), R374Q (1260A>C 1261G>A 1262G>A), M376T (1267T>C 1268G>A), S393E (1317T>G 1318C>A 1319C>G), R412A (1374A>G 1375G>C 1376A>C), G414Q (1380G>C 1381G>A 1382T>A), I496V (1626A>G 1628C>T), M521R (1701A>C 1702T>G), E523V (1708A>T), D530N (1728G>A 1730T>C), G533A (1738G>C), R536S (1748A>C), V540I (1758G>A 1760G>T), E541G (1762A>G 1763G>A), N552D (1794A>G 1796T>C), Q567K (1839C>A), A570H (1848G>C 1849C>A 1850A>C), V572I (1854G>A 1856A>T), S573A (1857T>G), K574T (1861A>C 1862G>C), F577Y (1870T>A 1871T>C), E578N (1872G>A 1874A>C), A579Q (1875G>C 1876C>A 1877T>G), E580S (1878G>T 1879A>C), S581T (1881T>A 1883T>A), I583C (1887A>T 1888T>G 1889A>T), S584E (1890A>G 1891G>A 1892T>A), P585E (1893C>G 1894C>A 1895T>G), I587V (1899A>G 1901A>T), Q591E (1911C>G), A594V (1921C>T 1922A>T), D595E (1925T>A), D596E (1928T>A), I598M (1934A>G), A599T (1935G>A 1937G>A), V601F (1941G>T 1943G>T), D602H (1944G>C), G603D (1948G>A 1949G>T), R605Q (1953A>C 1954G>A), L606I (1956T>A 1958G>A), R607S (1959C>A), T608F (1962A>T 1963C>T 1964C>T), K609D (1965A>G 1967G>C), Q610E (1968C>G), D611V (1972A>T), E612S (1974G>A 1975A>G 1976A>C), I614S (1981T>G 1982A>T), L615V (1983T>G 1985G>T), V616_P617del (1986_1991delGTGCCC), V620L (1998G>C 2000G>C), N623D (2007A>G), I627G (2019A>G 2020T>G 2021C>A), A629S (2025G>T), R630Q (2028A>C 2029G>A 2030A>G), S644A (2070A>G 2071G>C 2072T>A), D646N (2076G>A), E648F (2082G>T 2083A>T 2084G>T), L650Y (2088C>T 2089T>A), H655A (2103C>G 2104A>C), H656S (2106C>T 2107A>C 2108T>A), M660I (2120G>T), Y661F (2122A>T), S663V (2127T>G 2128C>T 2129T>C), D665S (2133G>T 2134A>C 2135T>A), D666S (2136G>A 2137A>G), H668A (2142C>G 2143A>C 2144T>C), I671S (2152T>G 2153T>C), N673T (2158A>C 2159C>T), K674R (2160A>C 2161A>G 2162A>C), R676V (2166A>G 2167G>T), V677N (2169G>A 2170T>A 2171C>T), N678E (2172A>G 2174C>G), L680I (2178T>A 2180G>T), R682E (2184A>G 2185G>A 2186A>G), D685S (2193G>T 2194A>C 2195T>G), A686T (2196G>A), E687A (2200A>C), Q688G (2202C>G 2203A>G 2204G>A), R690A (2208C>G 2209G>C 2210A>C), V693I (2217G>A 2219A>C), Q694K (2220C>A 2222A>G), N695A (2223A>G 2224A>C), I701V (2241A>G 2243A>C), A710S (2268G>A 2269C>G 2270A>T), S711T (2271T>A), I713V (2277A>G 2279C>G), K716A (2286A>G 2287A>C 2288G>T), K717H (2289A>C 2291A>C), L718M (2292C>A 2294A>G), A719D (2296C>A 2297A>T), T722R (2305C>G 2306A>G), E723M (2307G>A 2308A>T), M724L (2310A>T), R725Y (2313A>T 2314G>A 2315G>C), T726N (2317C>A), F728R (2322T>A 2323T>G 2324T>A), S731R (2331T>A 2332C>G 2333T>A), C732A (2334T>G 2335G>C 2336C>A), I733L (2337A>C 2339T>G), D734E (2342T>A), V737A (2350T>C), I741M (2363A>G), F749H (2385T>C 2386T>A), C762_N763insRR (2426_2427insAGAAGG), N763K (2429C>G), K764M (2431A>T 2432A>G), S766T (2436T>A 2438A>T), P769T (2445C>A), K770_E771del (2448_2453delAAGGAA), I774V (2460A>G 2462C>A), K775Q (2463A>C), I776S (2466A>T 2467T>C 2468T>G), C777L (2469T>C 2470G>T), H779D (2475C>G 2477T>C), K780S (2478A>T 2479A>C 2480A>C), I781T (2482T>C 2483C>A), V782I (2484G>A), E783G (2488A>G 2489A>G), Y784R (2490T>A 2491A>G 2492T>A), I785M (2495C>G), K786Q (2496A>C 2498G>A), D787E (2501T>A), T788M (2503C>T 2504A>G), W790N (2508T>A 2509G>A 2510G>T), S791D (2511A>G 2512G>A 2513C>T), A795I (2523G>A 2524C>T 2525A>T), R797K (2530G>A), I806L (2556A>C), M807I (2561G>A), V812T (2574G>A 2575T>C), F813M (2577T>A 2579T>G), G815A (2584G>C), I816V (2586A>G 2588T>A), L820I (2598T>A 2600G>T), A821S (2601G>T 2603G>T), Q824K (2610C>A 2612G>A), N826S (2617A>G), S827L (2620C>T 2621T>G), F828I (2622T>A 2624C>T), N830T (2629A>C), A831S (2631G>T 2633A>C), S832Q (2634T>C 2635C>A), R834K (2641G>A), N852R (2695A>G 2696T>A), N852_K853insS (2696_2697insTCC), K853H (2697A>C 2699G>T), L854V (2700C>G 2702C>A), R855S (2703A>T 2704G>C), N856K (2708T>G), L857M (2709C>A 2711T>G), V859F (2715G>T), G860S (2718G>A 2720T>C), S865P (2733T>C), L866N (2736T>A 2737T>A 2738G>T), A867D (2740C>A 2741A>T), Q868_D869insG (2744_2745insGGA), D869K (2745G>A 2747T>A), A872S (2754G>T 2756T>C), E873R (2757G>A 2758A>G 2759A>G), Q879E (2775C>G 2777G>A), K882A (2784A>G 2785A>C 2786G>C), L890V (2808C>G 2810T>G), G891A (2812G>C 2813T>A), G904E (2851G>A 2852T>A), H914T (2880C>A 2881A>C 2882C>G), N915Q (2883A>C 2885T>G), A916L (2886G>C 2887C>T 2888T>C), T923S (2907A>T), A927E (2920C>A 2921C>A), N928H (2922A>C), R930V (2928C>G 2929G>T 2930A>C), S931C (2932C>G 2933T>C), K932Q (2934A>C 2936A>G), R938K (2953G>A), E939H (2955G>C 2957A>T), S940A (2958T>G 2960A>C), R941K (2962G>A), I942F (2964A>T), I943L (2967A>T 2969T>G), E944P (2970G>C 2971A>C 2972G>T), G945E (2974G>A 2975C>A), I948V (2982A>G 2984C>T), I949V (2985A>G 2987T>A), D955C (3003G>T 3004A>G), P961V (3021C>G 3022C>T 3023T>C), P968A (3042C>G 3044A>T), R969E (3045A>G 3046G>A), E972A (3055A>C 3056G>A), Q980E (3078C>G), C984V (3090T>G 3091G>T 3092T>C), T990K (3109C>A), R993E (3117C>G 3118G>A 3119T>G), L994K (3120C>A 3121T>A 3122T>A), G995Q (3123G>C 3124G>A 3125C>G), E998D (3134G>C), M1002Y (3144A>T 3145T>A 3146G>T), P1003S (3147C>T), V1012E (3175T>A 3176T>G), E1015T (3183G>A 3184A>C), E1017R (3189G>A 3190A>G 3191A>G), L1018M (3192C>A), N1019S (3196A>G), S1020T (3198T>A 3200C>A), S1021N (3201T>A 3202C>A 3203C>T), K1024H (3210A>C 3212G>C), K1024_I1025insS (3212_3213insTCA), C1026S (3216T>A 3218C>T), S1027A (3219T>G), Q1029E (3225C>G), S1030A (3228T>G 3230T>C), I1035V (3243A>G 3245A>C), G1037S (3249G>A 3251G>C), V1051I (3291G>A 3293T>C), V1057C (3309G>T 3310T>G 3311G>C), G1068E (3343G>A 3344C>A), V1069A (3346T>C 3347A>T), A1071T (3351G>A 3353T>A), R1073L (3357C>T 3358G>T 3359T>G), I1076V (3366A>G 3368C>A), S1077A (3369T>G 3371T>A), I1083V (3387A>G 3389C>A), E1086D (3398A>C), E1089Q (3405G>C 3407G>A), P1092A (3414C>G 3416A>T), T1093Q (3417A>C 3418C>A 3419T>A), A1098M (3432G>A 3433C>T 3434A>G), I1105L (3453A>T 3455T>G), S1108H (3462T>C 3463C>A 3464A>T), I1109V (3465A>G), Q1115A (3483C>G 3484A>C 3485G>T), E1118D (3494G>C), N1120K (3500C>G), Y1123A (3507T>G 3508A>C 3509C>T), Y1124F (3511A>T 3512C>T), L1132M (3534T>A), Q1146P (3577A>C), P1153E (3597C>G 3598C>A 3599T>A), E1155Q (3603G>C 3605G>A), I1160L (3618A>C 3620T>G), T1162S (3624A>T 3626A>T), H1164K (3630C>A 3632T>A), V1178I (3672G>A 3674A>T), M1179E (3675A>G 3676T>A), C1186S (3697G>C 3698T>A), G1187N (3699G>A 3700G>A 3701A>T), D1189E (3707T>G), D1191S (3711G>A 3712A>G), K1194R (3720A>C 3721A>G 3722A>G), L1195A (3723C>G 3724T>C 3725A>T), N1199G (3735A>G 3736A>G), C1202S (3745G>C 3746C>T), S1203A (3747T>G 3749G>C), L1204I (3750C>A 3752G>T), I1207V (3759A>G 3761C>T), M1208L (3762A>C 3764G>C), R1209K (3765C>A 3766G>A 3767T>G), K1212R (3775A>G), I1213V (3777A>G 3779C>G), R1217I (3789C>A 3790G>T 3791T>C), C1219T (3795T>A 3796G>C 3797T>A), E1220T (3798G>A 3799A>C), D1222C (3804G>T 3805A>G), V1225I (3813G>A 3815A>C), I1230V (3828A>G 3830A>T), C1233F (3838G>T), Y1235F (3844A>T), Y1236F (3847A>T), G1238S (3852G>T 3853G>C), M1252L (3894A>C), M1254L (3900A>T), M1256T (3907T>C 3908G>C), Q1263E (3927C>G), S1264A (3930T>G 3932C>A), T1265Q (3933A>C 3934C>A 3935T>G), G1266E (3937G>A 3938C>G), E1267S (3939G>A 3940A>G 3941A>T), G1268T (3942G>A 3943G>C 3944A>C), E1269Q (3945G>C 3947G>A), E1272L (3954G>C 3955A>T), D1276N (3966G>A 3968C>T), T1277S (3969A>T), E1281Q (3981G>C), Q1289E (4005C>G 4007G>A), I1294N (4021T>A), H1295Y (4023C>T), D1297E (4031T>A), L1298M (4032C>A), E1312D (4076G>C), R1318K (4093G>A 4094A>G), T1344C (4170A>T 4171C>G 4172A>T), L1348A (4182T>G 4183T>C 4184G>A), A1351V (4192C>T), I1352L (4194A>C 4196A>G), D1359N (4215G>A), T1361I (4222C>T 4223A>T), S1363H (4227T>C 4228C>A), L1367T (4239T>A 4240T>C 4241G>C), G1371Q (4251G>C 4252G>A), A1374S (4260G>A 4261C>G), V1393I (4317G>A 4319T>A), F1399L (4337T>A), E1405A (4354A>C), K1410A (4368A>G 4369A>C 4370G>T), P1414E (4380C>G 4381C>A 4382T>G), V1437L (4449G>C 4451G>C), A1477K (4569G>A 4570C>A 4571T>A), R1479K (4576G>A), I1481L (4581A>T 4583A>G), P1485S (4593C>T), Q1490D (4608C>G 4610G>C), L1506M (4656T>A 4658A>G), S1512E (4674T>G 4675C>A 4676T>G), N1515A (4683A>G 4684A>C 4685T>A), H1518Q (4694T>A), L1522Q (4705T>A 4706G>A), S1534N (4740T>A 4741C>A 4742G>C), D1536N (4746G>A), E1539D (4757G>T), Q1541K (4761C>A), H1563Q (4829C>A), M1564L (4830A>C 4832G>A), I1573V (4857A>G 4859T>G), P1583V (4887C>G 4888C>T 4889A>T), T1588I (4903C>T 4904T>C), Q1589T (4905C>A 4906A>C 4907G>T), M1592Y (4914A>T 4915T>A 4916G>T), I1599V (4935A>G), H1604Q (4952T>G), R1607Q (4960G>A 4961T>G), A1609V (4966C>T), K1612T (4975A>C 4976G>A), V1614T (4980G>A 4981T>C 4982C>A), L1615D (4983T>G 4984T>A 4985A>C), D1616N (4986G>A), T1619K (4996C>A 4997G>A), L1621I (5001T>A 5003G>A), D1622M (5004G>A 5005A>T 5006T>G), E1623Q (5007G>C 5009A>G), F1626L (5018C>G), R1627P (5019A>C 5020G>C), A1628I (5022G>A 5023C>T 5024T>C), I1630H (5028A>C 5029T>A 5030C>T), L1635A (5043T>G 5044T>C 5045G>T), V1637Q (5049G>C 5050T>A 5051G>A), Q1639S (5055C>A 5056A>G 5057G>T), I1642L (5064A>C 5066T>A), A1643Q (5067G>C 5068C>A), K1644A (5070A>G 5071A>C 5072G>C), S1650N (5089G>A 5090T>C), A1651I (5091G>A 5092C>T 5093C>A), L1652G (5094C>G 5095T>G), T1653A (5097A>G 5099A>T), I1654L (5100A>C 5102T>G), C1655L (5103T>C 5104G>T 5105T>G), M1657L (5109A>C), A1658S (5112G>A 5113C>G 5114A>C), A1660D (5119C>A 5120A>T), S1663T (5127T>A 5129C>T), T1665V (5133A>G 5134C>T), D1668P (5142G>C 5143A>C 5144C>A), A1669E (5146C>A), E1671K (5151G>A 5153G>A), T1674E (5160A>G 5161C>A), N1675D (5163A>G), E1677S (5169G>T 5170A>C 5171G>A), R1678Q (5173G>A 5174T>A), D1679G (5176A>G), L1680V (5178C>G 5180G>C), N1681H (5181A>C), W1682E (5184T>G 5185G>A 5186G>A), A1684C (5190G>T 5191C>G 5192A>T), P1685S (5193C>T 5195A>T), N1686G (5196A>G 5197A>G 5198C>A), I1687V (5199A>G), G1688F (5202G>T 5203G>T 5204T>C), A1692S (5214G>T 5216A>G), A1695E (5224C>A), A1696V (5227C>T 5228C>T), H1697N (5229C>A 5231C>T), Y1698H (5232T>C 5234C>T), T1699K (5236C>A 5237G>A), G1703D (5248G>A 5249G>C), Y1704T (5250T>A 5251A>C 5252C>A), I1706L (5256A>C), S1715T (5283T>A 5285C>A), N1716T (5287A>C 5288T>A), S1737A (5349T>G), M1740K (5359T>A), A1742N (5364G>A 5365C>A 5366C>T), K1744Q (5370A>C 5372G>A), N1745S (5374A>G), V1746R (5376G>C 5377T>G 5378G>C), A1748S (5382G>T), G1749M (5385G>A 5386G>T 5387A>G), H1751I (5391C>A 5392A>T), V1752G (5395T>G), A1753T (5397G>A 5399A>C), T1754Q (5400A>C 5401C>A 5402C>A), N1755K (5405T>A), V1759I (5415G>A 5417G>T), G1767S (5439G>A 5441C>T), M1774F (5460A>T 5462G>T), A1777S (5469G>T), R1779N (5476G>A 5477G>T), C1782R (5484T>A 5486T>A), V1783A (5488T>C 5489G>T), D1785Q (5493G>C 5495C>A), I1786L (5496A>C 5498A>T), I1793L (5517A>T 5519T>G), G1794A (5521G>C 5522A>G), N1795G (5523A>G 5524A>G 5525T>C), V1796L (5526G>T), T1801Q (5541A>C 5542C>A 5543C>G), V1807I (5559G>A 5561G>T), Q1812T (5574C>A 5575A>C), C1813_V1814insV (5579_5580insGTC), P1815Q (5584C>A 5585T>G), H1816K (5586C>A 5588T>A), G1817S (5589G>A 5591A>T), D1818C (5592G>T 5593A>G), V1820T (5598G>A 5599T>C 5600G>A), S1823A (5607A>G 5608G>C), G1824S (5610G>A), A1825G (5614C>G 5615A>G), S1826del (5616_5618delTCG), L1827R (5620T>G), H1828D (5622C>G 5624C>T), S1829G (5625A>G 5627T>A), S1830M (5629G>T 5630T>G), F1831L (5633T>G), M1832N (5635T>A 5636G>C), Q1834H (5642A>T), Q1834_G1835insHA (5642_5643insCATGCT), G1835A (5644G>C 5645G>T), F1836Y (5647T>A), E1837A (5650A>C 5651A>C), I1844V (5670A>G 5672A>G), H1845G (5673C>G 5674A>G 5675T>C), G1846C (5676G>T), D1847S (5679G>T 5680A>C 5681T>A), L1848I (5682T>A 5684A>T), A1849S (5685G>T), K1850R (5689A>G), Q1851K (5691C>A), T1852M (5695C>T 5696A>G), M1854V (5700A>G), M1856L (5706A>T), V1859T (5715G>A 5716T>C 5717G>T), H1863M (5727C>A 5728A>T 5729T>G), G1867A (5740G>C), N1868D (5742A>G), F1869C (5746T>G 5747T>C), Q1872R (5754C>A 5755A>G 5756G>A), D1873N (5757G>A)									

	Begin	End	Coverage	Score	Concordance	Matches	Identities	I/D/M/F*	Stop Codons
**NT**	**35**	**5769**	**79.3%**	**3466**	**30.8%**	**5682 (98.6%)**	**3757 (65.2%)**	**28/53**	

Codon mutations:

GCT2GCA (146T>A), AAG5AAA (155G>A), ATG6ATC (158G>C), TTC7GTT (159T>G 161C>T), TCC8_AGT17del (162_191delTCCAAGGTTGCTGGTGTTGATGACGCCAGT), TCC20GCT (198T>G 200C>T), ACC21ACT (203C>T), ACT22CCT (204A>C), TCA23TCT (209A>T), TTC24TTT (212C>T), TTT25CTT (213T>C), GGT26TCT (216G>T 217G>C), GAA27GAG (221A>G), CTT28TTG (222C>T 224T>G), TCA29TCT (227A>T), AAT30CAG (228A>C 230T>G), TCT31ACA (231T>A 233T>A), ATT32GTG (234A>G 236T>G), TCC33TCA (239C>A), AAA34TCA (240A>T 241A>C), TTT35CTG (243T>C 245T>G), TCA36TCC (248A>C), AAA37AAG (251A>G), GCG38ACA (252G>A 254G>A), GTT39ATG (255G>A 257T>G), AGT40AAG (259G>A 260T>G), AAT41AGT (262A>G), ATT42ATG (266T>G), ACC43AGT (268C>G 269C>T), ACT44AAA (271C>A 272T>A), ATG45ATA (275G>A), TCA46TCT (278A>T), CAG47GAG (279C>G), AAG48AAA (284G>A), ATT49TTC (285A>T 287T>C), GAT51GGC (292A>G 293T>C), CTT53ATT (297C>A), GAG54GAA (302G>A), GAT55GAA (305T>A), CTG56TTA (306C>T 308G>A), CCA58CCT (314A>T), TCT59TCA (317T>A), GTG60ATT (318G>A 320G>T), ACT61GTT (321A>G 322C>T), GAT62AAA (324G>A 326T>A), GTG63ACT (327G>A 328T>C 329G>T), TCC64GCA (330T>G 332C>A), ACA65GAT (333A>G 334C>A 335A>T), TCT66TCC (338T>C), GTG68AAT (342G>A 343T>A 344G>T), GCC69AGT (345G>A 346C>G 347C>T), ACT70GTT (348A>G 349C>T), TCA73TCT (359A>T), CTT74GTA (360C>G 362T>A), AAT75GAA (363A>G 365T>A), GTT77TTA (369G>T 371T>A), TTG78TTA (374G>A), GAC79GGG (376A>G 377C>G), GGT82GCC (385G>C 386T>C), GCA83ACG (387G>A 389A>G), CTT84GTT (390C>G), ATA85ATT (395A>T), GAT86GAA (398T>A), CCA87CCT (401A>T), CTT89GTT (405C>G), GCG91GGT (412C>G 413G>T), TAC92ATC (414T>A 415A>T), TCT93AAT (417T>A 418C>A), TTG95TTC (425G>C), TCC96AAA (426T>A 427C>A 428C>A), TAC99TAT (437C>T), AAA100AGA (439A>G), TCT101ACC (441T>A 443T>C), ATC102ATA (446C>A), AAG103ATT (448A>T 449G>T), TTA107CTG (459T>C 461A>G), TTG109GTT (465T>G 467G>T), TTT110GTT (468T>G), GAG111TTG (471G>T 472A>T), AGT112GGC (474A>G 476T>C), TTG113ATC (477T>A 479G>C), TCC114AAA (480T>A 481C>A 482C>A), AAA116AAT (488A>T), GTT117TTT (489G>T), AAA118ATT (493A>T 494A>T), TTA119AGA (495T>A 496T>G), TTT121TAT (502T>A), GCT122TCA (504G>T 506T>A), GTG124CTG (510G>C), TTA125TTG (515A>G), AAT126GAA (516A>G 518T>A), AAA127ACA (520A>C), AGT128ACA (523G>C 524T>A), GAA129GGT (526A>G 527A>T), GAT130GAA (530T>A), GTA131TAT (531G>T 532T>A 533A>T), ATA133GTT (537A>G 539A>T), GTT134GTA (542T>A), GTA135GTT (545A>T), TAT136ATA (546T>A 547A>T 548T>A), GGT137GCT (550G>C), CTT139CTA (557T>A), GTA140GTC (560A>C), GCT142TCC (564G>T 566T>C), ATA143CTT (567A>C 569A>T), ACA144TCA (570A>T), TTG145CTA (573T>C 575G>A), TTA146TTG (578A>G), GTG147TTA (579G>T 581G>A), CTT148TTA (582C>T 584T>A), CTA149CAA (586T>A), TTT150ATA (588T>A 590T>A), GTT151ATC (591G>A 593T>C), AGT154GTA (600A>G 601G>T 602T>A), GTT157GTA (611T>A), GAG158GAA (614G>A), GGT159ATG (615G>A 616G>T 617T>G), TCT161AGA (621T>A 622C>G 623T>A), AAC162AGT (625A>G 626C>T), ACT163GTA (627A>G 628C>T 629T>A), GTC164GTT (632C>T), AAA165TGT (633A>T 634A>G 635A>T), ATC167ATA (641C>A), TTG169CTA (645T>C 647G>A), ATA170GTT (648A>G 650A>T), GTC171GTA (653C>A), GGA172GGT (656A>T), ATG174TTC (660A>T 662G>C), TTT175TTC (665T>C), TCA176TCT (668A>T), AGT177TCT (669A>T 670G>C), CTA178CTG (674A>G), TAC179TAT (677C>T), TTA181ATT (681T>A 683A>T), GAT182AGT (684G>A 685A>G), CCA185CCC (695A>C), AAA186CTC (696A>C 697A>T 698A>C), GCC188TCT (702G>T 704C>T), GAA189GAG (707A>G), CGA190AAA (708C>A 709G>A), TTC191TTT (713C>T), ACC192TCT (714A>T 716C>T), ATG193CAG (717A>C 718T>A), GTG194ATG (720G>A), GCC195GCA (725C>A), CAA196CAG (728A>G), AGT198TCT (732A>T 733G>C), ATG199GTG (735A>G), CTT200CTG (740T>G), GGG202GGT (746G>T), GAA203GAG (749A>G), TCA204AGC (750T>A 751C>G 752A>C), GTT205TTA (753G>T 755T>A), TCA206AAT (756T>A 757C>A 758A>T), ACG210ACC (770G>C), TCT211AGT (771T>A 772C>G), CAG212CAA (776G>A), ATG214CTG (780A>C), TCC215TCT (785C>T), ACA216TCT (786A>T 788A>T), ATA217TTG (789A>T 791A>G), TTG218ATA (792T>A 794G>A), GCT219GCA (797T>A), GGG221GGC (803G>C), ATT222ATC (806T>C), TCG223TCA (809G>A), GTG226GTA (818G>A), TTC227TTT (821C>T), ATT228GTG (822A>G 824T>G), GTG230GTA (830G>A), AGA233AGG (839A>G), TTG237CTT (849T>C 851G>T), AGC238ACA (853G>C 854C>A), CCA240CCG (860A>G), TTG241CTA (861T>C 863G>A), CTC245TTG (873C>T 875C>G), TCG247TCA (881G>A), ACA248GCG (882A>G 884A>G), GGA249GGC (887A>C), GGT251GGA (893T>A), CAG253CAA (899G>A), CTA258CTC (914A>C), ACT260CAA (918A>C 919C>A 920T>A), CTT261TTG (921C>T 923T>G), TAC262TAT (926C>T), CGT263AAG (927C>A 928G>A 929T>G), AAT264AAC (932T>C), AAA266AAG (938A>G), ACC269ACG (947C>G), TCA274TCT (962A>T), GTT276GTG (968T>G), CTT277TTG (969C>T 971T>G), ATA279ATT (977A>T), ATA280GTT (978A>G 980A>T), GTC281GCT (982T>C 983C>T), GGC282GAT (985G>A 986C>T), ACC283ATA (988C>T 989C>A), TTT284TTC (992T>C), GGA285GGG (995A>G), TTC286TTT (998C>T), AAT288AAC (1004T>C), GTG290GTT (1010G>T), TTG291CTG (1011T>C), TCA292TCT (1016A>T), GCT293GCA (1019T>A), ATA294ATT (1022A>T), ACG297ACA (1031G>A), CTG298ATT (1032C>A 1034G>T), GAT301GAC (1043T>C), CTG302CTC (1046G>C), TTC303TTT (1049C>T), CAG307ACT (1059C>A 1060A>C 1061G>T), GAG308GAA (1064G>A), GAT310TCT (1068G>T 1069A>C), TGT313GCT (1077T>G 1078G>C), ACG317ACA (1091G>A), CGC318AGG (1092C>A 1094C>G), GAG320GAA (1100G>A), GCC323GCT (1109C>T), AAG325CCT (1113A>C 1114A>C 1115G>T), TTC327TTT (1121C>T), CCT328TCA (1122C>T 1124T>A), ACC329ATC (1126C>T), CTG334CTT (1142G>T), AGG335AAA (1144G>A 1145G>A), GAA336GAG (1148A>G), GAA337CAG (1149G>C 1151A>G), GCT338GCA (1154T>A), ATC341ATA (1163C>A), GCT343GCC (1169T>C), TAC344TAT (1172C>T), GCA346GCT (1178A>T), CCA349CCT (1187A>T), GTT350GTG (1190T>G), TTC353TTT (1199C>T), ATG354TTG (1200A>T), AGC355AGT (1205C>T), AGG357AGA (1211G>A), AGT359TCT (1215A>T 1216G>C), GCC361ACA (1221G>A 1223C>A), GCG363GTG (1228C>T), CAG364CAA (1232G>A), TTA365TTG (1235A>G), GAA366GAT (1238A>T), GTT368CTC (1242G>C 1244T>C), TAT369ATA (1245T>A 1246A>T 1247T>A), GCT370GCA (1250T>A), AGT372AAC (1255G>A 1256T>C), AGG374CAA (1260A>C 1261G>A 1262G>A), CAC375CAT (1265C>T), ATG376ACA (1267T>C 1268G>A), GGC377GGA (1271C>A), GGC379GGT (1277C>T), TAT381TAC (1283T>C), CGT382AGA (1284C>A 1286T>A), CCT385CCC (1295T>C), GTA388GTT (1304A>T), TTC391TTT (1313C>T), GGG392GGT (1316G>T), TCC393GAG (1317T>G 1318C>A 1319C>G), GGG395GGA (1325G>A), TGT396TGC (1328T>C), GGT397GGC (1331T>C), TCT399TCC (1337T>C), ACA400ACG (1340A>G), TCC401AGC (1341T>A 1342C>G), CGC403CGA (1349C>A), TTA404CTG (1350T>C 1352A>G), TTT405TTC (1355T>C), ATT406ATC (1358T>C), GTG409GTA (1367G>A), GAC411GAT (1373C>T), AGA412GCC (1374A>G 1375G>C 1376A>C), GGT414CAA (1380G>C 1381G>A 1382T>A), CCA416CCC (1388A>C), AAA417AAG (1391A>G), CTC418CTG (1394C>G), AAT419AAC (1397T>C), CGG420AGG (1398C>A), CTA421TTG (1401C>T 1403A>G), GTA424GTT (1412A>T), AGG427CGC (1419A>C 1421G>C), GCA429GCT (1427A>T), TAT430TAC (1430T>C), TCC432TCA (1436C>A), AAC433AAT (1439C>T), CTG441CTC (1463G>C), GAC444GAT (1472C>T), GGA446GGT (1478A>T), TTG448CTG (1482T>C), CGA449CGG (1487A>G), GGG451GGA (1493G>A), GGG453GGC (1499G>C), CAG454CAA (1502G>A), CAA456CAG (1508A>G), GAT457GAC (1511T>C), AAA459AAG (1517A>G), GAT460GAC (1520T>C), CTG461CTC (1523G>C), GAT463GAC (1529T>C), TCA466TCC (1538A>C), CCC471CCA (1553C>A), TTA472TTG (1556A>G), CCA473CCC (1559A>C), GCC475GCT (1565C>T), GTT477GTA (1571T>A), GAG478GAA (1574G>A), GGC481GGG (1583C>G), AGG482CGG (1584A>C), ACC485ACA (1595C>A), TCC486TCA (1598C>A), TAT488TAC (1604T>C), ACA492ACC (1616A>C), AAT494AAC (1622T>C), CTG495TTG (1623C>T), ATC496GTT (1626A>G 1628C>T), GCT498GCA (1634T>A), CCT499CCA (1637T>A), GCC500GCA (1640C>A), TGT502TGC (1646T>C), GGT503GGG (1649T>G), CTT504CTG (1652T>G), CCA507CCT (1661A>T), GAG511GAA (1673G>A), CGG512AGG (1674C>A), AGA513AGG (1679A>G), AGG514CGG (1680A>C), ATC516ATT (1688C>T), CTG517TTG (1689C>T), GAG519GAA (1697G>A), TGT520TGC (1700T>C), ATG521CGG (1701A>C 1702T>G), AAG522AAA (1706G>A), GAG523GTG (1708A>T), GGC524GGG (1712C>G), GAG525GAA (1715G>A), TTT526TTC (1718T>C), TCC527TCA (1721C>A), ACT528ACA (1724T>A), GAA529GAG (1727A>G), GAT530AAC (1728G>A 1730T>C), CCT531CCA (1733T>A), ACG532ACA (1736G>A), GGA533GCA (1738G>C), AGA536AGC (1748A>C), TTT537TTC (1751T>C), ATA539ATC (1757A>C), GTG540ATT (1758G>A 1760G>T), GAG541GGA (1762A>G 1763G>A), AGA543AGG (1769A>G), AGA544AGG (1772A>G), GCT547GCC (1781T>C), ATC548ATA (1784C>A), ACT549ACA (1787T>A), AGG551AGA (1793G>A), AAT552GAC (1794A>G 1796T>C), TTG553CTC (1797T>C 1799G>C), ACC554ACT (1802C>T), AGT556TCT (1806A>T 1807G>C), GAT557GAC (1811T>C), CTT558TTG (1812C>T 1814T>G), GAG560GAA (1820G>A), GTA563GTG (1829A>G), AAA565AAG (1835A>G), TGT566TGC (1838T>C), CAG567AAG (1839C>A), GTA568GTC (1844A>C), CAT569CAC (1847T>C), GCA570CAC (1848G>C 1849C>A 1850A>C), GTA572ATT (1854G>A 1856A>T), TCA573GCA (1857T>G), AAG574ACC (1861A>C 1862G>C), TTG576CTC (1866T>C 1868G>C), TTT577TAC (1870T>A 1871T>C), GAA578AAC (1872G>A 1874A>C), GCT579CAG (1875G>C 1876C>A 1877T>G), GAA580TCA (1878G>T 1879A>C), TCT581ACA (1881T>A 1883T>A), GGC582GGT (1886C>T), ATA583TGT (1887A>T 1888T>G 1889A>T), AGT584GAA (1890A>G 1891G>A 1892T>A), CCT585GAG (1893C>G 1894C>A 1895T>G), AAG586AAA (1898G>A), ATA587GTT (1899A>G 1901A>T), GCT588GCC (1904T>C), GTT590GTG (1910T>G), CAA591GAA (1911C>G), GCA594GTT (1921C>T 1922A>T), GAT595GAA (1925T>A), GAT596GAA (1928T>A), ATA598ATG (1934A>G), GCG599ACA (1935G>A 1937G>A), TCT600TCC (1940T>C), GTG601TTT (1941G>T 1943G>T), GAT602CAT (1944G>C), GGG603GAT (1948G>A 1949G>T), AGA605CAA (1953A>C 1954G>A), TTG606ATA (1956T>A 1958G>A), CGC607AGC (1959C>A), ACC608TTT (1962A>T 1963C>T 1964C>T), AAG609GAC (1965A>G 1967G>C), CAA610GAA (1968C>G), GAT611GTT (1972A>T), GAA612AGC (1974G>A 1975A>G 1976A>C), CCA613CCC (1979A>C), ATA614AGT (1981T>G 1982A>T), TTG615GTT (1983T>G 1985G>T), GTG616_CCC617del (1986_1991delGTGCCC), ACT618ACA (1994T>A), GTG620CTC (1998G>C 2000G>C), AGT621TCT (2001A>T 2002G>C), GAA622GAG (2006A>G), AAT623GAT (2007A>G), ATC627GGA (2019A>G 2020T>G 2021C>A), GCA629TCA (2025G>T), AGA630CAG (2028A>C 2029G>A 2030A>G), GAG631GAA (2033G>A), CTC632CTA (2036C>A), ACG633ACT (2039G>T), GTA634GTG (2042A>G), GAG635GAA (2045G>A), TTG637CTG (2049T>C), AAA638AAG (2054A>G), GCA640GCT (2060A>T), TAC641TAT (2063C>T), CAG642CAA (2066G>A), GGC643GGA (2069C>A), AGT644GCA (2070A>G 2071G>C 2072T>A), TTG645TTA (2075G>A), GAC646AAC (2076G>A), CCT647CCA (2081T>A), GAG648TTT (2082G>T 2083A>T 2084G>T), CTT650TAT (2088C>T 2089T>A), CCT652CCC (2096T>C), GAC654GAT (2102C>T), CAT655GCT (2103C>G 2104A>C), CAT656TCA (2106C>T 2107A>C 2108T>A), GCC659GCA (2117C>A), ATG660ATT (2120G>T), TAT661TTT (2122A>T), TCT663GTC (2127T>G 2128C>T 2129T>C), CTT664CTG (2132T>G), GAT665TCA (2133G>T 2134A>C 2135T>A), GAT666AGT (2136G>A 2137A>G), CAT668GCC (2142C>G 2143A>C 2144T>C), ATT671AGC (2152T>G 2153T>C), TTC672TTT (2156C>T), AAC673ACT (2158A>C 2159C>T), AAA674CGC (2160A>C 2161A>G 2162A>C), AGA676GTA (2166A>G 2167G>T), GTC677AAT (2169G>A 2170T>A 2171C>T), AAC678GAG (2172A>G 2174C>G), TTG680ATT (2178T>A 2180G>T), TAT681TAC (2183T>C), AGA682GAG (2184A>G 2185G>A 2186A>G), GGA683GGG (2189A>G), GCA684GCT (2192A>T), GAT685TCG (2193G>T 2194A>C 2195T>G), GCT686ACT (2196G>A), GAA687GCA (2200A>C), CAG688GGA (2202C>G 2203A>G 2204G>A), CGA690GCC (2208C>G 2209G>C 2210A>C), GTA693ATC (2217G>A 2219A>C), CAA694AAG (2220C>A 2222A>G), AAC695GCC (2223A>G 2224A>C), ATT696ATC (2228T>C), CCT697CCA (2231T>A), ATA701GTC (2241A>G 2243A>C), AAG705AAA (2255G>A), AGT706AGC (2258T>C), GCA710AGT (2268G>A 2269C>G 2270A>T), TCA711ACA (2271T>A), ATC713GTG (2277A>G 2279C>G), TCT714TCA (2282T>A), GAG715GAA (2285G>A), AAG716GCT (2286A>G 2287A>C 2288G>T), AAA717CAC (2289A>C 2291A>C), CTA718ATG (2292C>A 2294A>G), GCA719GAT (2296C>A 2297A>T), GTG720GTA (2300G>A), CAG721CAA (2303G>A), ACA722AGG (2305C>G 2306A>G), GAG723ATG (2307G>A 2308A>T), ATG724TTG (2310A>T), AGG725TAC (2313A>T 2314G>A 2315G>C), ACT726AAT (2317C>A), GGA727GGC (2321A>C), TTT728AGA (2322T>A 2323T>G 2324T>A), TCT731AGA (2331T>A 2332C>G 2333T>A), TGC732GCA (2334T>G 2335G>C 2336C>A), ATT733CTG (2337A>C 2339T>G), GAT734GAA (2342T>A), GCA735GCT (2345A>T), GTT737GCT (2350T>C), ACC739ACT (2357C>T), CTC740TTA (2358C>T 2360C>A), ATA741ATG (2363A>G), ATC743ATT (2369C>T), GAG744GAA (2372G>A), CAG745CAA (2375G>A), CCA747CCT (2381A>T), TTT749CAT (2385T>C 2386T>A), GTT750GTC (2390T>C), CAG751CAA (2393G>A), TTT752TTC (2396T>C), TTA753CTA (2397T>C), TAT754TAC (2402T>C), AGA759CGG (2415A>C 2417A>G), TGC762_AAC763insAGAAGG (2426_2427insAGAAGG), AAC763AAG (2429C>G), AAA764ATG (2431A>T 2432A>G), GTA765GTC (2435A>C), TCA766ACT (2436T>A 2438A>T), AAA767AAG (2441A>G), GAG768GAA (2444G>A), CCA769ACA (2445C>A), AAG770_GAA771del (2448_2453delAAGGAA), TCA772TCT (2456A>T), ATC774GTA (2460A>G 2462C>A), AAA775CAA (2463A>C), ATT776TCG (2466A>T 2467T>C 2468T>G), TGC777CTC (2469T>C 2470G>T), TAC778TAT (2474C>T), CAT779GAC (2475C>G 2477T>C), AAA780TCC (2478A>T 2479A>C 2480A>C), ATC781ACA (2482T>C 2483C>A), GTT782ATT (2484G>A), GAA783GGG (2488A>G 2489A>G), TAT784AGA (2490T>A 2491A>G 2492T>A), ATC785ATG (2495C>G), AAG786CAA (2496A>C 2498G>A), GAT787GAA (2501T>A), ACA788ATG (2503C>T 2504A>G), TGG790AAT (2508T>A 2509G>A 2510G>T), AGC791GAT (2511A>G 2512G>A 2513C>T), CTT792CTG (2516T>G), TAT794TAC (2522T>C), GCA795ATT (2523G>A 2524C>T 2525A>T), CTG796TTG (2526C>T), AGA797AAA (2530G>A), CTC799CTT (2537C>T), GGT803GGG (2549T>G), ATA806CTA (2556A>C), ATG807ATA (2561G>A), CTT809CTC (2567T>C), GGA811GGG (2573A>G), GTT812ACT (2574G>A 2575T>C), TTT813ATG (2577T>A 2579T>G), GGA814GGT (2582A>T), GGC815GCC (2584G>C), ATT816GTA (2586A>G 2588T>A), ACA817ACT (2591A>T), TTT819TTC (2597T>C), TTG820ATT (2598T>A 2600G>T), GCG821TCT (2601G>T 2603G>T), CAG824AAA (2610C>A 2612G>A), AGT825AGC (2615T>C), AAC826AGC (2617A>G), TCT827TTG (2620C>T 2621T>G), TTC828ATT (2622T>A 2624C>T), AAT830ACT (2629A>C), GCA831TCC (2631G>T 2633A>C), TCG832CAG (2634T>C 2635C>A), AGA834AAA (2641G>A), GTG837GTT (2651G>T), TCC841TCG (2663C>G), AGT843AGC (2669T>C), TCC845TCA (2675C>A), AGT846AGC (2678T>C), GGT851GGA (2693T>A), AAT852AGA (2695A>G 2696T>A), AAT852_AAG853insTCC (2696_2697insTCC), AAG853CAT (2697A>C 2699G>T), CTC854GTA (2700C>G 2702C>A), AGA855TCA (2703A>T 2704G>C), AAT856AAG (2708T>G), CTT857ATG (2709C>A 2711T>G), CTC858CTG (2714C>G), GTT859TTT (2715G>T), GGT860AGC (2718G>A 2720T>C), TCT863TCA (2729T>A), TCA865CCA (2733T>C), TTG866AAT (2736T>A 2737T>A 2738G>T), GCA867GAT (2740C>A 2741A>T), CAA868CAG (2744A>G), CAA868_GAT869insGGA (2744_2745insGGA), GAT869AAA (2745G>A 2747T>A), GCT872TCC (2754G>T 2756T>C), GAA873AGG (2757G>A 2758A>G 2759A>G), GGA875GGT (2765A>T), GTA877GTG (2771A>G), AAT878AAC (2774T>C), CAG879GAA (2775C>G 2777G>A), AGC880TCT (2778A>T 2779G>C 2780C>T), CTC881CTT (2783C>T), AAG882GCC (2784A>G 2785A>C 2786G>C), GTG886GTA (2798G>A), TTA888CTA (2802T>C), AGA889AGG (2807A>G), CTT890GTG (2808C>G 2810T>G), GGT891GCA (2812G>C 2813T>A), GTG894GTT (2822G>T), AGA897CGT (2829A>C 2831A>T), GGC898GGA (2834C>A), ACC899ACT (2837C>T), TGT902TGC (2846T>C), TCT903AGC (2847T>A 2848C>G 2849T>C), GGT904GAA (2851G>A 2852T>A), ACC908ACT (2864C>T), GCT910GCA (2870T>A), CAT911CAC (2873T>C), GCA912GCT (2876A>T), CAC914ACG (2880C>A 2881A>C 2882C>G), AAT915CAG (2883A>C 2885T>G), GCT916CTC (2886G>C 2887C>T 2888T>C), CGA917CGT (2891A>T), GGT919GGA (2897T>A), ACT920ACC (2900T>C), ACA923TCA (2907A>T), ATC925ATT (2915C>T), GCC927GAA (2920C>A 2921C>A), AAT928CAT (2922A>C), CGA930GTC (2928C>G 2929G>T 2930A>C), TCT931TGC (2932C>G 2933T>C), AAA932CAG (2934A>C 2936A>G), GTT933GTG (2939T>G), AAC936AAT (2948C>T), AGA938AAA (2953G>A), GAA939CAT (2955G>C 2957A>T), TCA940GCC (2958T>G 2960A>C), AGG941AAG (2962G>A), ATT942TTT (2964A>T), ATT943TTG (2967A>T 2969T>G), GAG944CCT (2970G>C 2971A>C 2972G>T), GGC945GAA (2974G>A 2975C>A), GAC947GAT (2981C>T), ATC948GTT (2982A>G 2984C>T), ATT949GTA (2985A>G 2987T>A), CTG950TTA (2988C>T 2990G>A), TTG951CTG (2991T>C), CGC952AGG (2994C>A 2996C>G), GGT954GGC (3002T>C), GAT955TGT (3003G>T 3004A>G), CCA956CCT (3008A>T), GAT957GAC (3011T>C), GGT958GGG (3014T>G), CCA959CCT (3017A>T), AAG960AAA (3020G>A), CCT961GTC (3021C>G 3022C>T 3023T>C), CGT964AGA (3030C>A 3032T>A), AAA965AAG (3035A>G), TTC967TTT (3041C>T), CCA968GCT (3042C>G 3044A>T), AGA969GAA (3045A>G 3046G>A), GAG972GCA (3055A>C 3056G>A), AAG977AAA

	(3071G>A), GGC978GGA (3074C>A), TCA979TCT (3077A>T), CAA980GAA (3078C>G), GGA981GGC (3083A>C), TGC983TGT (3089C>T), TGT984GTC (3090T>G 3091G>T 3092T>C), GCT986GCA (3098T>A), GTT987GTG (3101T>G), GCG988GCA (3104G>A), TCT989TCA (3107T>A), ACA990AAA (3109C>A), CCA992CCT (3116A>T), CGT993GAG (3117C>G 3118G>A 3119T>G), CTT994AAA (3120C>A 3121T>A 3122T>A), GGC995CAG (3123G>C 3124G>A 3125C>G), AAT996AAC (3128T>C), TTA997TTG (3131A>G), GAG998GAC (3134G>C), ATG1002TAT (3144A>T 3145T>A 3146G>T), CCA1003TCA (3147C>T), TCA1007TCC (3161A>C), AAG1008AAA (3164G>A), TAC1010TAT (3170C>T), GTT1012GAG (3175T>A 3176T>G), GTG1014GTC (3182G>C), GAA1015ACA (3183G>A 3184A>C), TAT1016TAC (3188T>C), GAA1017AGG (3189G>A 3190A>G 3191A>G), CTG1018ATG (3192C>A), AAC1019AGC (3196A>G), TCC1020ACA (3198T>A 3200C>A), TCC1021AAT (3201T>A 3202C>A 3203C>T), AGT1022TCA (3204A>T 3205G>C 3206T>A), AAG1024CAC (3210A>C 3212G>C), AAG1024_ATT1025insTCA (3212_3213insTCA), ATT1025ATC (3215T>C), TGC1026AGT (3216T>A 3218C>T), TCT1027GCT (3219T>G), CAA1029GAA (3225C>G), TCT1030GCC (3228T>G 3230T>C), CAC1034CAT (3242C>T), ATA1035GTC (3243A>G 3245A>C), GGG1037AGC (3249G>A 3251G>C), GAC1041GAT (3263C>T), GGC1043GGA (3269C>A), ACG1044ACT (3272G>T), CTG1047TTA (3279C>T 3281G>A), TTA1048TTG (3284A>G), AGT1050TCT (3288A>T 3289G>C), GTT1051ATC (3291G>A 3293T>C), CAG1052CAA (3296G>A), AAT1053AAC (3299T>C), CCT1056CCA (3308T>A), GTG1057TGC (3309G>T 3310T>G 3311G>C), ATC1058ATT (3314C>T), GGC1060GGT (3320C>T), ATC1061ATT (3323C>T), CAC1062CAT (3326C>T), GCA1064GCT (3332A>T), TCA1065TCC (3335A>C), GGC1068GAA (3343G>A 3344C>A), GTA1069GCT (3346T>C 3347A>T), GCT1071ACA (3351G>A 3353T>A), GAA1072GAG (3356A>G), CGT1073TTG (3357C>T 3358G>T 3359T>G), ATC1076GTA (3366A>G 3368C>A), TCT1077GCA (3369T>G 3371T>A), GCT1080GCC (3380T>C), ACA1081ACT (3383A>T), ATC1083GTA (3387A>G 3389C>A), GAA1086GAC (3398A>C), CAA1087CAG (3401A>G), CTA1088CTG (3404A>G), GAG1089CAA (3405G>C 3407G>A), CCA1092GCT (3414C>G 3416A>T), ACT1093CAA (3417A>C 3418C>A 3419T>A), CCG1095CCA (3425G>A), GTC1096GTT (3428C>T), GCA1098ATG (3432G>A 3433C>T 3434A>G), GCA1099GCC (3437A>C), GTA1101GTG (3443A>G), CGC1102AGA (3444C>A 3446C>A), GAT1104GAC (3452T>C), ATT1105TTG (3453A>T 3455T>G), CTG1106CTC (3458G>C), AAG1107AAA (3461G>A), TCA1108CAT (3462T>C 3463C>A 3464A>T), ATT1109GTT (3465A>G), AGA1110CGA (3468A>C), AGC1111TCA (3471A>T 3472G>C 3473C>A), ACA1114ACT (3482A>T), CAG1115GCT (3483C>G 3484A>C 3485G>T), CTT1116CTC (3488T>C), GAG1118GAC (3494G>C), AAC1120AAG (3500C>G), GTG1122GTT (3506G>T), TAC1123GCT (3507T>G 3508A>C 3509C>T), TAC1124TTT (3511A>T 3512C>T), CTT1125TTG (3513C>T 3515T>G), GGA1126GGC (3518A>C), ACA1127ACT (3521A>T), CCA1129CCT (3527A>T), CAG1130CAA (3530G>A), TTG1132ATG (3534T>A), GCC1133GCA (3539C>A), GCC1134GCA (3542C>A), ACA1135ACG (3545A>G), GTT1136GTG (3548T>G), CCC1137CCA (3551C>A), ACT1141ACC (3563T>C), CTG1142CTC (3566G>C), CGG1143AGG (3567C>A), CAA1146CCA (3577A>C), TTG1147CTG (3579T>C), GAA1149GAG (3587A>G), TTC1151TTT (3593C>T), GGA1152GGT (3596A>T), CCT1153GAA (3597C>G 3598C>A 3599T>A), GAG1155CAA (3603G>C 3605G>A), ACA1156ACT (3608A>T), GCA1157GCT (3611A>T), CCA1158CCT (3614A>T), TCC1159TCA (3617C>A), ATT1160CTG (3618A>C 3620T>G), CTA1161CTC (3623A>C), ACA1162TCT (3624A>T 3626A>T), GTT1163GTG (3629T>G), CAT1164AAA (3630C>A 3632T>A), GAC1165GAT (3635C>T), GGT1168GGG (3644T>G), GGT1170GGA (3650T>A), GAC1172GAT (3656C>T), CCC1173CCT (3659C>T), GTG1175GTT (3665G>T), GGG1177GGC (3671G>C), GTA1178ATT (3672G>A 3674A>T), ATG1179GAG (3675A>G 3676T>A), GCT1185GCC (3695T>C), TGT1186TCA (3697G>C 3698T>A), GGA1187AAT (3699G>A 3700G>A 3701A>T), GAT1189GAG (3707T>G), GAT1190GAC (3710T>C), GAC1191AGC (3711G>A 3712A>G), GCC1193GCA (3719C>A), AAA1194CGG (3720A>C 3721A>G 3722A>G), CTA1195GCT (3723C>G 3724T>C 3725A>T), TTC1197TTT (3731C>T), AAT1199GGT (3735A>G 3736A>G), TGC1202TCT (3745G>C 3746C>T), TCG1203GCC (3747T>G 3749G>C), CTG1204ATT (3750C>A 3752G>T), CTA1205TTA (3753C>T), CCT1206CCA (3758T>A), ATC1207GTT (3759A>G 3761C>T), ATG1208CTC (3762A>C 3764G>C), CGT1209AAG (3765C>A 3766G>A 3767T>G), AGC1210AGT (3770C>T), CAG1211CAA (3773G>A), AAG1212AGG (3775A>G), ATC1213GTG (3777A>G 3779C>G), CCT1214CCA (3782T>A), GGG1215GGT (3785G>T), GGA1216GGT (3788A>T), CGT1217ATC (3789C>A 3790G>T 3791T>C), CCA1218CCC (3794A>C), TGT1219ACA (3795T>A 3796G>C 3797T>A), GAA1220ACA (3798G>A 3799A>C), AGG1221CGT (3801A>C 3803G>T), GAT1222TGT (3804G>T 3805A>G), GAT1224GAC (3812T>C), GTA1225ATC (3813G>A 3815A>C), GTG1226GTC (3818G>C), CTC1227CTG (3821C>G), GGA1229GGT (3827A>T), ATA1230GTT (3828A>G 3830A>T), GGA1232GGT (3836A>T), TGT1233TTT (3838G>T), GAT1234GAC (3842T>C), TAC1235TTC (3844A>T), TAT1236TTT (3847A>T), GGC1238TCC (3852G>T 3853G>C), GAG1240GAA (3860G>A), CTG1241CTC (3863G>C), TCT1244TCA (3872T>A), TGC1245TGT (3875C>T), TAT1247TAC (3881T>C), CCC1248CCT (3884C>T), AAC1250AAT (3890C>T), AAG1251AAA (3893G>A), ATG1252CTG (3894A>C), GGG1253GGT (3899G>T), ATG1254TTG (3900A>T), GGG1255GGT (3905G>T), ATG1256ACC (3907T>C 3908G>C), AAC1257AAT (3911C>T), AAG1258AAA (3914G>A), AGA1259AGG (3917A>G), TTT1261TTC (3923T>C), GTG1262GTT (3926G>T), CAA1263GAA (3927C>G), TCC1264GCA (3930T>G 3932C>A), ACT1265CAG (3933A>C 3934C>A 3935T>G), GGC1266GAG (3937G>A 3938C>G), GAA1267AGT (3939G>A 3940A>G 3941A>T), GGA1268ACC (3942G>A 3943G>C 3944A>C), GAG1269CAA (3945G>C 3947G>A), AGA1270AGG (3950A>G), GTG1271GTA (3953G>A), GAA1272CTA (3954G>C 3955A>T), AAA1274AAG (3962A>G), GAC1276AAT (3966G>A 3968C>T), ACT1277TCT (3969A>T), GTA1279GTT (3977A>T), GAA1281CAA (3981G>C), GAA1284GAG (3992A>G), GAG1285GAA (3995G>A), CTA1286TTA (3996C>T), CAG1289GAA (4005C>G 4007G>A), ATT1290ATA (4010T>A), AAA1292AAG (4016A>G), GGC1293GGT (4019C>T), ATC1294AAC (4021T>A), CAT1295TAT (4023C>T), GAT1297GAA (4031T>A), CTG1298ATG (4032C>A), GTC1299GTT (4037C>T), ACC1301ACT (4043C>T), TGC1303TGT (4049C>T), GCC1304GCT (4052C>T), AAA1305AAG (4055A>G), GAT1306GAC (4058T>C), CGC1308AGG (4062C>A 4064C>G), CTC1309CTA (4067C>A), CCA1310CCT (4070A>T), CTT1311CTG (4073T>G), GAG1312GAC (4076G>C), AAA1313AAG (4079A>G), ATC1314ATA (4082C>A), TAT1315TAC (4085T>C), GGC1316GGG (4088C>G), AAG1317AAA (4091G>A), AGA1318AAG (4093G>A 4094A>G), AAG1319AAA (4097G>A), AGG1321CGT (4101A>C 4103G>T), CTC1322CTT (4106C>T), ATA1325ATT (4115A>T), CTT1326TTG (4116C>T 4118T>G), CCT1327CCA (4121T>A), TAC1330TAT (4130C>T), TTG1333CTG (4137T>C), GTC1334GTG (4142C>G), AGG1335CGC (4143A>C 4145G>C), AAG1336AAA (4148G>A), TTT1338TTC (4154T>C), TTC1341TTT (4163C>T), TCA1342TCT (4166A>T), GCC1343GCA (4169C>A), ACA1344TGT (4170A>T 4171C>G 4172A>T), TTG1345TTA (4175G>A), TTG1348GCA (4182T>G 4183T>C 4184G>A), CAC1349CAT (4187C>T), GCT1351GTT (4192C>T), ATA1352CTG (4194A>C 4196A>G), CCA1353CCC (4199A>C), GTT1356GTG (4208T>G), ATT1358ATC (4214T>C), GAT1359AAT (4215G>A), CCT1360CCC (4220T>C), ACA1361ATT (4222C>T 4223A>T), AGT1362TCA (4224A>T 4225G>C 4226T>A), TCT1363CAT (4227T>C 4228C>A), ACA1366ACC (4238A>C), TTG1367ACC (4239T>A 4240T>C 4241G>C), TTG1368CTG (4242T>C), AAT1370AAC (4250T>C), GGG1371CAG (4251G>C 4252G>A), TTT1372TTC (4256T>C), AGA1373CGG (4257A>C 4259A>G), GCT1374AGT (4260G>A 4261C>G), TCA1376TCT (4268A>T), GAC1377GAT (4271C>T), GTG1378GTT (4274G>T), GGA1379GGT (4277A>T), TCA1381TCT (4283A>T), GCT1382GCA (4286T>A), GAT1383GAC (4289T>C), TAT1384TAC (4292T>C), AGA1390AGG (4310A>G), GCA1391GCG (4313A>G), CCT1392CCA (4316T>A), GTT1393ATA (4317G>A 4319T>A), GCT1395GCA (4325T>A), CAG1397CAA (4331G>A), TTT1399TTA (4337T>A), GAA1405GCA (4354A>C), TAC1407TAT (4361C>T), GGA1408GGG (4364A>G), AAG1410GCT (4368A>G 4369A>C 4370G>T), CCT1411CCA (4373T>A), GGC1412GGA (4376C>A), AGT1413AGC (4379T>C), CCT1414GAG (4380C>G 4381C>A 4382T>G), TCC1416TCA (4388C>A), GCT1418GCA (4394T>A), CGA1419AGA (4395C>A), GCA1421GCT (4403A>T), CTT1422CTG (4406T>G), TTA1423TTG (4409A>G), GCA1426GCT (4418A>T), TGT1428TGC (4424T>C), ACA1431ACT (4433A>T), CTG1432CTA (4436G>A), GTG1437CTC (4449G>C 4451G>C), TTT1438TTC (4454T>C), TTG1440TTA (4460G>A), GGG1442GGT (4466G>T), GGC1443GGA (4469C>A), CCA1445CCT (4475A>T), TCA1446TCT (4478A>T), GCA1449GCT (4487A>T), CTA1450CTC (4490A>C), ACG1451ACC (4493G>C), ATC1453ATA (4499C>A), TTC1454TTT (4502C>T), AAT1455AAC (4505T>C), TCT1456TCC (4508T>C), CTC1457CTT (4511C>T), CTC1458TTA (4512C>T 4514C>A), AAT1459AAC (4517T>C), CGA1464CGG (4532A>G), GCC1466GCT (4538C>T), TTT1467TTC (4541T>C), TCA1469TCG (4547A>G), TTG1470CTC (4548T>C 4550G>C), TTA1471CTC (4551T>C 4553A>C), AGA1473AGG (4559A>G), CAT1475CAC (4565T>C), ATT1476ATC (4568T>C), GCT1477AAA (4569G>A 4570C>A 4571T>A), GCC1478GCA (4574C>A), AGG1479AAG (4576G>A), GCT1480GCA (4580T>A), ATA1481TTG (4581A>T 4583A>G), GGA1482GGT (4586A>T), GTT1483GTA (4589T>A), AAA1484AAG (4592A>G), CCA1485TCA (4593C>T), TTC1488TTT (4604C>T), CAG1490GAC (4608C>G 4610G>C), CTA1491TTA (4611C>T), TTC1492TTT (4616C>T), ATA1493ATT (4619A>T), GCT1494GCA (4622T>A), GTT1495GTG (4625T>G), GGA1497GGG (4631A>G), GTT1502GTG (4646T>G), GTA1504GTG (4652A>G), TTA1506ATG (4656T>A 4658A>G), CAT1507CAC (4661T>C), CTC1508CTT (4664C>T), CAG1509CAA (4667G>A), TAT1511TAC (4673T>C), TCT1512GAG (4674T>G 4675C>A 4676T>G), CTG1513TTA (4677C>T 4679G>A), CCA1514CCT (4682A>T), AAT1515GCA (4683A>G 4684A>C 4685T>A), CAT1518CAA (4694T>A), TTA1520CTA (4698T>C), CTG1522CAA (4705T>A 4706G>A), GTC1523GTG (4709C>G), AAT1524AAC (4712T>C), GTA1525GTT (4715A>T), ATC1526ATT (4718C>T), ATT1527ATC (4721T>C), GGT1530GGC (4730T>C), GAC1532GAT (4736C>T), TCG1534AAC (4740T>A 4741C>A 4742G>C), GAT1536AAT (4746G>A), GTT1537GTC (4751T>C), GAG1539GAT (4757G>T), GTA1540GTC (4760A>C), CAA1541AAA (4761C>A), TTT1542TTC (4766T>C), CAA1543CAG (4769A>G), CTA1548TTG (4782C>T 4784A>G), ACT1549ACG (4787T>G), CTG1551TTG (4791C>T), AGT1552AGC (4796T>C), GGT1554GGC (4802T>C), TTT1555TTC (4805T>C), AAA1556AAG (4808A>G), CGA1557CGC (4811A>C), CTT1560TTG (4818C>T 4820T>G), GGA1561GGT (4823A>T), CAC1563CAA (4829C>A), ATG1564CTA (4830A>C 4832G>A), GCT1565GCC (4835T>C), CCT1566CCC (4838T>C), TCA1571TCC (4853A>C), ATC1572ATT (4856C>T), ATT1573GTG (4857A>G 4859T>G), GAG1574GAA (4862G>A), CCC1575CCA (4865C>A), ATA1579ATC (4877A>C), CCT1581CCA (4883T>A), GCC1582GCA (4886C>A), CCA1583GTT (4887C>G 4888C>T 4889A>T), GGT1584GGG (4892T>G), CCT1586CCA (4898T>A), GAC1587GAT (4901C>T), ACT1588ATC (4903C>T 4904T>C), CAG1589ACT (4905C>A 4906A>C 4907G>T), ATG1592TAT (4914A>T 4915T>A 4916G>T), ACA1597ACG (4931A>G), GGA1598GGT (4934A>T), ATA1599GTA (4935A>G), CGT1600AGA (4938C>A 4940T>A), GAA1601GAG (4943A>G), CAT1604CAG (4952T>G), CAC1605CAT (4955C>T), GGT1606GGA (4958T>A), CGT1607CAG (4960G>A 4961T>G), GTG1608GTC (4964G>C), GCT1609GTT (4966C>T), AAG1612ACA (4975A>C 4976G>A), CTT1613TTG (4977C>T 4979T>G), GTC1614ACA (4980G>A 4981T>C 4982C>A), TTA1615GAC (4983T>G 4984T>A 4985A>C), GAT1616AAT (4986G>A), GTT1617GTC (4991T>C), CAG1618CAA (4994G>A), ACG1619AAA (4996C>A 4997G>A), GCG1620GCA (5000G>A), TTG1621ATA (5001T>A 5003G>A), GAT1622ATG (5004G>A 5005A>T 5006T>G), GAA1623CAG (5007G>C 5009A>G), AGG1624CGA (5010A>C 5012G>A), GGT1625GGC (5015T>C), TTC1626TTG (5018C>G), AGA1627CCA (5019A>C 5020G>C), GCT1628ATC (5022G>A 5023C>T 5024T>C), ATC1630CAT (5028A>C 5029T>A 5030C>T), CCT1632CCA (5036T>A), TCC1633TCT (5039C>T), TTG1635GCT (5043T>G 5044T>C 5045G>T), GTG1637CAA (5049G>C 5050T>A 5051G>A), CAG1639AGT (5055C>A 5056A>G 5057G>T), GAA1640GAG (5060A>G), ATT1642CTA (5064A>C 5066T>A), GCA1643CAA (5067G>C 5068C>A), AAG1644GCC (5070A>G 5071A>C 5072G>C), GTA1645GTT (5075A>T), ACA1646ACT (5078A>T), GGG1647GGA (5081G>A), GAT1648GAC (5084T>C), TCA1649AGT (5085T>A 5086C>G 5087A>T), AGT1650AAC (5089G>A 5090T>C), GCC1651ATA (5091G>A 5092C>T 5093C>A), CTG1652GGG (5094C>G 5095T>G), ACA1653GCT (5097A>G 5099A>T), ATT1654CTG (5100A>C 5102T>G), TGT1655CTG (5103T>C 5104G>T 5105T>G), ATG1657CTG (5109A>C), GCA1658AGC (5112G>A 5113C>G 5114A>C), AAA1659AAG (5117A>G), GCA1660GAT (5119C>A 5120A>T), TCC1663ACT (5127T>A 5129C>T), TAT1664TAC (5132T>C), ACG1665GTG (5133A>G 5134C>T), CCA1666CCT (5138A>T), TTG1667CTT (5139T>C 5141G>T), GAC1668CCA (5142G>C 5143A>C 5144C>A), GCA1669GAA (5146C>A), GGT1670GGA (5150T>A), GAG1671AAA (5151G>A 5153G>A), AAG1672AAA (5156G>A), ATC1673ATT (5159C>T), ACA1674GAA (5160A>G 5161C>A), AAT1675GAT (5163A>G), GAG1677TCA (5169G>T 5170A>C 5171G>A), CGT1678CAA (5173G>A 5174T>A), GAT1679GGT (5176A>G), CTG1680GTC (5178C>G 5180G>C), AAT1681CAT (5181A>C), TGG1682GAA (5184T>G 5185G>A 5186G>A), GCA1684TGT (5190G>T 5191C>G 5192A>T), CCA1685TCT (5193C>T 5195A>T), AAC1686GGA (5196A>G 5197A>G 5198C>A), ATT1687GTT (5199A>G), GGT1688TTC (5202G>T 5203G>T 5204T>C), TTT1689TTC (5207T>C), GCA1692TCG (5214G>T 5216A>G), CGT1693CGA (5219T>A), ACT1694ACA (5222T>A), GCA1695GAA (5224C>A), GCC1696GTT (5227C>T 5228C>T), CAC1697AAT (5229C>A 5231C>T), TAC1698CAT (5232T>C 5234C>T), ACG1699AAA (5236C>A 5237G>A), GAA1702GAG (5246A>G), GGG1703GAC (5248G>A 5249G>C), TAC1704ACA (5250T>A 5251A>C 5252C>A), ATT1706CTT (5256A>C), AAC1708AAT (5264C>T), TGT1709TGC (5267T>C), AAG1713AAA (5279G>A), AAG1714AAA (5282G>A), TCC1715ACA (5283T>A 5285C>A), AAT1716ACA (5287A>C 5288T>A), GTT1718GTG (5294T>G), CCG1733CCC (5339G>C), TAC1734TAT (5342C>T), ACT1735ACA (5345T>A), TCA1737GCA (5349T>G), GCT1738GCC (5354T>C), ATA1739ATC (5357A>C), ATG1740AAG (5359T>A), GAT1741GAC (5363T>C), GCC1742AAT (5364G>A 5365C>A 5366C>T), AAG1744CAA (5370A>C 5372G>A), AAT1745AGT (5374A>G), GTG1746CGC (5376G>C 5377T>G 5378G>C), GCA1748TCA (5382G>T), GGA1749ATG (5385G>A 5386G>T 5387A>G), GGA1750GGG (5390A>G), CAT1751ATT (5391C>A 5392A>T), GTA1752GGA (5395T>G), GCA1753ACC (5397G>A 5399A>C), ACC1754CAA (5400A>C 5401C>A 5402C>A), AAT1755AAA (5405T>A), GTC1756GTT (5408C>T), GTT1757GTC (5411T>C), GTG1759ATT (5415G>A 5417G>T), GGA1761GGG (5423A>G), GGA1763GGT (5429A>T), GTC1766GTG (5438C>G), GGC1767AGT (5439G>A 5441C>T), CCA1768CCC (5444A>C), GCA1771GCT (5453A>T), GCG1772GCT (5456G>T), ATG1774TTT (5460A>T 5462G>T), GCC1775GCA (5465C>A), GCA1777TCA (5469G>T), ACA1778ACC (5474A>C), AGG1779AAT (5476G>A 5477G>T), CAA1780CAG (5480A>G), TGT1782AGA (5484T>A 5486T>A), GTG1783GCT (5488T>C 5489G>T), GAC1785CAA (5493G>C 5495C>A), ATA1786CTT (5496A>C 5498A>T), GTT1788GTC (5504T>C), CGC1791AGA (5511C>A 5513C>A), ATT1793TTG (5517A>T 5519T>G), GGA1794GCG (5521G>C 5522A>G), AAT1795GGC (5523A>G 5524A>G 5525T>C), GTG1796TTG (5526G>T), CTT1797CTG (5531T>G), GAC1798GAT (5534C>T), CTC1799TTG (5535C>T 5537C>G), ACC1801CAG (5541A>C 5542C>A 5543C>G), GGC1805GGT (5555C>T), GTG1807ATT (5559G>A 5561G>T), CAG1808CAA (5564G>A), TTT1810TTC (5570T>C), CTT1811CTC (5573T>C), CAA1812ACA (5574C>A 5575A>C), TGT1813_GTG1814insGTC (5579_5580insGTC), CCT1815CAG (5584C>A 5585T>G), CAT1816AAA (5586C>A 5588T>A), GGA1817AGT (5589G>A 5591A>T), GAC1818TGC (5592G>T 5593A>G), AAA1819AAG (5597A>G), GTG1820ACA (5598G>A 5599T>C 5600G>A), AGT1823GCT (5607A>G 5608G>C), GGT1824AGT (5610G>A), GCA1825GGG (5614C>G 5615A>G), TCG1826del (5616_5618delTCG), CTC1827CGC (5620T>G), CAC1828GAT (5622C>G 5624C>T), AGT1829GGA (5625A>G 5627T>A), AGT1830ATG (5629G>T 5630T>G), TTT1831TTG (5633T>G), ATG1832AAC (5635T>A 5636G>C), CAA1834CAT (5642A>T), CAA1834_GGG1835insCATGCT (5642_5643insCATGCT), GGG1835GCT (5644G>C 5645G>T), TTT1836TAT (5647T>A), GAA1837GCC (5650A>C 5651A>C), TTG1838CTT (5652T>C 5654G>T), GGC1839GGA (5657C>A), CTC1841CTT (5663C>T), CGC1842CGA (5666C>A), ATT1843ATA (5669T>A), ATA1844GTG (5670A>G 5672A>G), CAT1845GGC (5673C>G 5674A>G 5675T>C), GGT1846TGT (5676G>T), GAT1847TCA (5679G>T 5680A>C 5681T>A), TTA1848ATT (5682T>A 5684A>T), GCA1849TCA (5685G>T), AAA1850AGA (5689A>G), CAG1851AAG (5691C>A), ACA1852ATG (5695C>T 5696A>G), ATG1854GTG (5700A>G), CGG1855AGA (5703C>A 5705G>A), ATG1856TTG (5706A>T), CCA1857CCC (5711A>C), TAT1858TAC (5714T>C), GTG1859ACT (5715G>A 5716T>C 5717G>T), GTA1860GTT (5720A>T), GGA1861GGG (5723A>G), CAT1863ATG (5727C>A 5728A>T 5729T>G), GGA1864GGT (5732A>T), GGA1865GGT (5735A>T), GGA1867GCA (5740G>C), AAT1868GAT (5742A>G), TTT1869TGC (5746T>G 5747T>C), TCC1870TCA (5750C>A), CAG1872AGA (5754C>A 5755A>G 5756G>A), GAT1873AAT (5757G>A)								
helicase (YP_001976154.1)	1	98	100%	652	97.2%	98 (100%)	96 (98.0%)	0/0/0/0	0
Protein mutations:	R16A (1374A>G 1375G>C 1376A>C), G18Q (1380G>C 1381G>A 1382T>A)								

	Begin	End	Coverage	Score	Concordance	Matches	Identities	I/D/M/F*	Stop Codons
**NT**	**35**	**5769**	**79.3%**	**3466**	**30.8%**	**5682 (98.6%)**	**3757 (65.2%)**	**28/53**	
Codon mutations:	GGT1GGC (1331T>C), TCT3TCC (1337T>C), ACA4ACG (1340A>G), TCC5AGC (1341T>A 1342C>G), CGC7CGA (1349C>A), TTA8CTG (1350T>C 1352A>G), TTT9TTC (1355T>C), ATT10ATC (1358T>C), GTG13GTA (1367G>A), GAC15GAT (1373C>T), AGA16GCC (1374A>G 1375G>C 1376A>C), GGT18CAA (1380G>C 1381G>A 1382T>A), CCA20CCC (1388A>C), AAA21AAG (1391A>G), CTC22CTG (1394C>G), AAT23AAC (1397T>C), CGG24AGG (1398C>A), CTA25TTG (1401C>T 1403A>G), GTA28GTT (1412A>T), AGG31CGC (1419A>C 1421G>C), GCA33GCT (1427A>T), TAT34TAC (1430T>C), TCC36TCA (1436C>A), AAC37AAT (1439C>T), CTG45CTC (1463G>C), GAC48GAT (1472C>T), GGA50GGT (1478A>T), TTG52CTG (1482T>C), CGA53CGG (1487A>G), GGG55GGA (1493G>A), GGG57GGC (1499G>C), CAG58CAA (1502G>A), CAA60CAG (1508A>G), GAT61GAC (1511T>C), AAA63AAG (1517A>G), GAT64GAC (1520T>C), CTG65CTC (1523G>C), GAT67GAC (1529T>C), TCA70TCC (1538A>C), CCC75CCA (1553C>A), TTA76TTG (1556A>G), CCA77CCC (1559A>C), GCC79GCT (1565C>T), GTT81GTA (1571T>A), GAG82GAA (1574G>A), GGC85GGG (1583C>G), AGG86CGG (1584A>C), ACC89ACA (1595C>A), TCC90TCA (1598C>A), TAT92TAC (1604T>C), ACA96ACC (1616A>C), AAT98AAC (1622T>C)								
RNA-dependent RNA polymerase (YP_001976153.1)	1	249	100%	1561	88.9%	249 (100%)	217 (87.1%)	0/0/0/0	0
Protein mutations:	E8D (4076G>C), R14K (4093G>A 4094A>G), T40C (4170A>T 4171C>G 4172A>T), L44A (4182T>G 4183T>C 4184G>A), A47V (4192C>T), I48L (4194A>C 4196A>G), D55N (4215G>A), T57I (4222C>T 4223A>T), S59H (4227T>C 4228C>A), L63T (4239T>A 4240T>C 4241G>C), G67Q (4251G>C 4252G>A), A70S (4260G>A 4261C>G), V89I (4317G>A 4319T>A), F95L (4337T>A), E101A (4354A>C), K106A (4368A>G 4369A>C 4370G>T), P110E (4380C>G 4381C>A 4382T>G), V133L (4449G>C 4451G>C), A173K (4569G>A 4570C>A 4571T>A), R175K (4576G>A), I177L (4581A>T 4583A>G), P181S (4593C>T), Q186D (4608C>G 4610G>C), L202M (4656T>A 4658A>G), S208E (4674T>G 4675C>A 4676T>G), N211A (4683A>G 4684A>C 4685T>A), H214Q (4694T>A), L218Q (4705T>A 4706G>A), S230N (4740T>A 4741C>A 4742G>C), D232N (4746G>A), E235D (4757G>T), Q237K (4761C>A)								
Codon mutations:	AAA1AAG (4055A>G), GAT2GAC (4058T>C), CGC4AGG (4062C>A 4064C>G), CTC5CTA (4067C>A), CCA6CCT (4070A>T), CTT7CTG (4073T>G), GAG8GAC (4076G>C), AAA9AAG (4079A>G), ATC10ATA (4082C>A), TAT11TAC (4085T>C), GGC12GGG (4088C>G), AAG13AAA (4091G>A), AGA14AAG (4093G>A 4094A>G), AAG15AAA (4097G>A), AGG17CGT (4101A>C 4103G>T), CTC18CTT (4106C>T), ATA21ATT (4115A>T), CTT22TTG (4116C>T 4118T>G), CCT23CCA (4121T>A), TAC26TAT (4130C>T), TTG29CTG (4137T>C), GTC30GTG (4142C>G), AGG31CGC (4143A>C 4145G>C), AAG32AAA (4148G>A), TTT34TTC (4154T>C), TTC37TTT (4163C>T), TCA38TCT (4166A>T), GCC39GCA (4169C>A), ACA40TGT (4170A>T 4171C>G 4172A>T), TTG41TTA (4175G>A), TTG44GCA (4182T>G 4183T>C 4184G>A), CAC45CAT (4187C>T), GCT47GTT (4192C>T), ATA48CTG (4194A>C 4196A>G), CCA49CCC (4199A>C), GTT52GTG (4208T>G), ATT54ATC (4214T>C), GAT55AAT (4215G>A), CCT56CCC (4220T>C), ACA57ATT (4222C>T 4223A>T), AGT58TCA (4224A>T 4225G>C 4226T>A), TCT59CAT (4227T>C 4228C>A), ACA62ACC (4238A>C), TTG63ACC (4239T>A 4240T>C 4241G>C), TTG64CTG (4242T>C), AAT66AAC (4250T>C), GGG67CAG (4251G>C 4252G>A), TTT68TTC (4256T>C), AGA69CGG (4257A>C 4259A>G), GCT70AGT (4260G>A 4261C>G), TCA72TCT (4268A>T), GAC73GAT (4271C>T), GTG74GTT (4274G>T), GGA75GGT (4277A>T), TCA77TCT (4283A>T), GCT78GCA (4286T>A), GAT79GAC (4289T>C), TAT80TAC (4292T>C), AGA86AGG (4310A>G), GCA87GCG (4313A>G), CCT88CCA (4316T>A), GTT89ATA (4317G>A 4319T>A), GCT91GCA (4325T>A), CAG93CAA (4331G>A), TTT95TTA (4337T>A), GAA101GCA (4354A>C), TAC103TAT (4361C>T), GGA104GGG (4364A>G), AAG106GCT (4368A>G 4369A>C 4370G>T), CCT107CCA (4373T>A), GGC108GGA (4376C>A), AGT109AGC (4379T>C), CCT110GAG (4380C>G 4381C>A 4382T>G), TCC112TCA (4388C>A), GCT114GCA (4394T>A), CGA115AGA (4395C>A), GCA117GCT (4403A>T), CTT118CTG (4406T>G), TTA119TTG (4409A>G), GCA122GCT (4418A>T), TGT124TGC (4424T>C), ACA127ACT (4433A>T), CTG128CTA (4436G>A), GTG133CTC (4449G>C 4451G>C), TTT134TTC (4454T>C), TTG136TTA (4460G>A), GGG138GGT (4466G>T), GGC139GGA (4469C>A), CCA141CCT (4475A>T), TCA142TCT (4478A>T), GCA145GCT (4487A>T), CTA146CTC (4490A>C), ACG147ACC (4493G>C), ATC149ATA (4499C>A), TTC150TTT (4502C>T), AAT151AAC (4505T>C), TCT152TCC (4508T>C), CTC153CTT (4511C>T), CTC154TTA (4512C>T 4514C>A), AAT155AAC (4517T>C), CGA160CGG (4532A>G), GCC162GCT (4538C>T), TTT163TTC (4541T>C), TCA165TCG (4547A>G), TTG166CTC (4548T>C 4550G>C), TTA167CTC (4551T>C 4553A>C), AGA169AGG (4559A>G), CAT171CAC (4565T>C), ATT172ATC (4568T>C), GCT173AAA (4569G>A 4570C>A 4571T>A), GCC174GCA (4574C>A), AGG175AAG (4576G>A), GCT176GCA (4580T>A), ATA177TTG (4581A>T 4583A>G), GGA178GGT (4586A>T), GTT179GTA (4589T>A), AAA180AAG (4592A>G), CCA181TCA (4593C>T), TTC184TTT (4604C>T), CAG186GAC (4608C>G 4610G>C), CTA187TTA (4611C>T), TTC188TTT (4616C>T), ATA189ATT (4619A>T), GCT190GCA (4622T>A), GTT191GTG (4625T>G), GGA193GGG (4631A>G), GTT198GTG (4646T>G), GTA200GTG (4652A>G), TTA202ATG (4656T>A 4658A>G), CAT203CAC (4661T>C), CTC204CTT (4664C>T), CAG205CAA (4667G>A), TAT207TAC (4673T>C), TCT208GAG (4674T>G 4675C>A 4676T>G), CTG209TTA (4677C>T 4679G>A), CCA210CCT (4682A>T), AAT211GCA (4683A>G 4684A>C 4685T>A), CAT214CAA (4694T>A), TTA216CTA (4698T>C), CTG218CAA (4705T>A 4706G>A), GTC219GTG (4709C>G), AAT220AAC (4712T>C), GTA221GTT (4715A>T), ATC222ATT (4718C>T), ATT223ATC (4721T>C), GGT226GGC (4730T>C), GAC228GAT (4736C>T), TCG230AAC (4740T>A 4741C>A 4742G>C), GAT232AAT (4746G>A), GTT233GTC (4751T>C), GAG235GAT (4757G>T), GTA236GTC (4760A>C), CAA237AAA (4761C>A), TTT238TTC (4766T>C), CAA239CAG (4769A>G), CTA244TTG (4782C>T 4784A>G), ACT245ACG (4787T>G), CTG247TTG (4791C>T), AGT248AGC (4796T>C)								

*: Inserts / Deletes / Misaligned / Frameshifts

Analysis details

This analysis was performed with panviral2.64

NGS Details (UN18_val): Torradovirus marchitezum (segment RNA 2)

Assembly

**Coverage Length**	4645 (1 contig(s))
**Depth Of Coverage**	709.3
**Number Of Reads**	26259
**Reads Per Million**	492.84 rpm (after QC)
**Ambiguities**	0
**Assembly Method**	de novo + reference guided assembly
**Consensus Caller**	Bcf Tools

Coverage Map

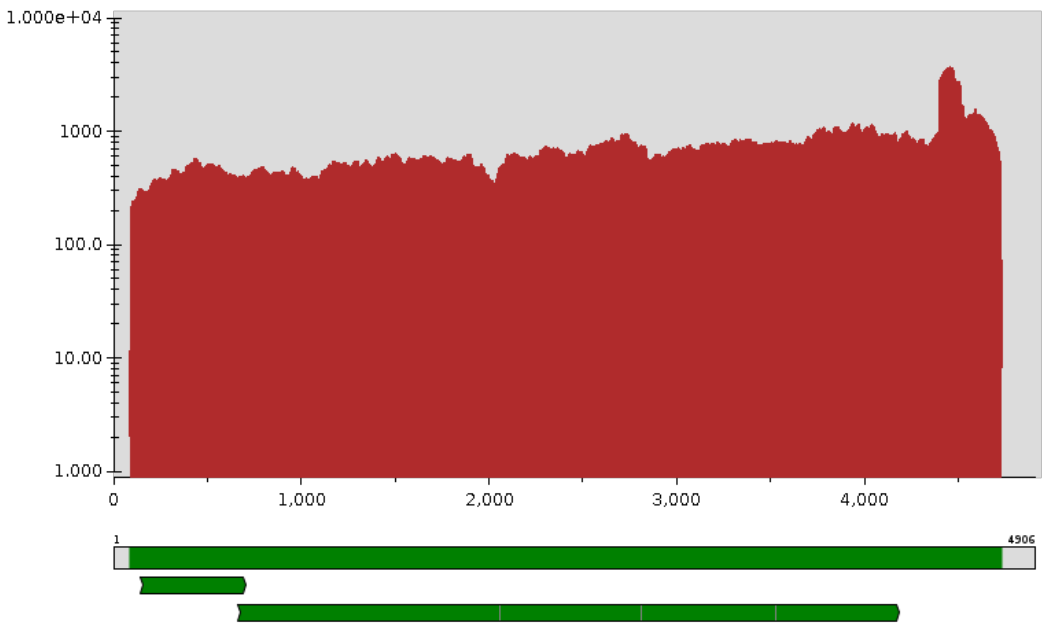

Assignment

**Type**	Torradovirus marchitezum (Taxonomy ID: 3048376)
**Reference Genome**	NC_010988.1
**NT Identity (%)**	64.3495
**AA Identity (%)**	67.9739
**Number Of Stop Codons**	2
**Number Of CDS**	2

Alignment

**Alignment Score**	2570.0 (NT) + 6793.0 (AA) = 9363.0
**Concordance (%)**	50.7067

**Alignment Method**	Global, seeded, nucleotide + amino acids (AGA)

Genome Region

Sequence starts at position 89 and ends at position 4733 relative to NC_010988.1 reference sequence.

Alignment Detailed Statistics

	Begin	End	Coverage	Score	Concordance	Matches	Identities	I/D/M/F*	Stop Codons
**NT**	**89**	**4733**	**94.7%**	**2570**	**28.4%**	**4597 (96.7%)**	**3027 (63.7%)**	**107/48**	

Mutations:

101G>T, 106C>A, 109A>C, 110T>C, 111T>C, 118T>C, 120C>A, 121T>A, 122T>A, 124G>C, 128T>A, 129G>A, 130T>A, 132G>T, 133_134insCACTCT, 135C>T, 137T>G, 144A>T, 147C>T, 150T>C, 151G>A, 153G>T, 154C>A, 155G>A, 156T>G, 157T>C, 159G>C, 162T>C, 165A>C, 166T>C, 167C>A, 168T>A, 169G>A, 174C>G, 177A>T, 182C>A, 183T>A, 186T>C, 190C>A, 198T>A, 201C>A, 202A>G, 204C>A, 207G>T, 209A>C, 210T>C, 214A>G, 220T>A, 221C>G, 229G>A, 230T>C, 234C>T, 235A>G, 236C>T, 237C>T, 241T>A, 243G>T, 246A>T, 261C>T, 262C>T, 264T>A, 267C>T, 273A>G, 274G>A, 277G>T, 278T>G, 282T>C, 291T>A, 298G>A, 300C>T, 303A>G, 306T>G, 307T>C, 309A>T, 310A>T, 312C>T, 315T>G, 316T>A, 317C>G, 324G>A, 333G>T, 336A>C, 340T>G, 342A>T, 348T>A, 351A>C, 354C>T, 357T>C, 358T>C, 360G>A, 361T>C, 363G>A, 374A>C, 375T>G, 379C>G, 380C>A, 381C>T, 384T>C, 387G>A, 388A>G, 389A>C, 390C>A, 402T>A, 405T>C, 408A>G, 409C>A, 414C>G, 416A>G, 417T>A, 423A>C, 424G>A, 426G>T, 427C>A, 428G>A, 429T>A, 430C>T, 432T>G, 441C>A, 442A>C, 444A>G, 448G>T, 450C>G, 451C>T, 453A>G, 456G>A, 458A>C, 459A>C, 462C>T, 465G>A, 468T>G, 469T>C, 470C>A, 477A>T, 478G>T, 480A>T, 481T>A, 483A>C, 489A>T, 492T>A, 495T>C, 496C>A, 497C>A, 498T>C, 499T>C, 501T>C, 508_525delGACAGTAAAGGCAAGAGT, 527A>C, 529G>T, 532G>T, 533A>C, 534G>A, 535C>G, 538C>A, 540C>T, 541A>G, 544_549delCGAGAG, 550G>A, 551A>G, 553C>T, 555C>A, 557T>G, 558T>C, 559A>G, 560A>G, 564A>G, 565G>A, 566T>A, 567T>C, 568G>A, 572T>C, 573T>A, 576C>T, 579A>G, 580C>G, 581G>A, 582T>A, 584A>C, 585G>C, 586T>C, 588A>T, 591G>A, 592A>G, 593G>T, 594A>T, 595T>G, 596T>C, 597T>C, 598C>A, 599A>G, 600G>A, 606G>A, 607T>C, 610G>C, 611C>A, 613A>G, 614G>A, 615T>A, 616C>G, 617A>C, 619A>C, 620A>G, 621A>G, 629A>T, 631C>G, 632A>T, 633G>C, 639A>C, 640C>A, 641A>G, 642A>G, 643C>T, 645T>G, 649C>A, 651C>T, 652T>C, 653C>A, 654C>A, 655A>G, 656A>G, 661G>C, 662T>C, 663T>A, 666T>C, 669T>C, 670A>G, 672T>G, 675C>T, 676A>G, 681C>T, 682T>G, 687T>C, 693T>G, 694G>A, 695A>G, 696A>T, 697A>G, 698G>A, 702G>C, 706C>A, 710A>G, 712T>C, 714A>G, 716C>T, 717A>C, 718G>A, 719_721delCAG, 722G>A, 724G>A, 729C>T, 730C>A, 733C>T, 736G>C, 738G>A, 739G>A, 745A>T, 748A>T, 751A>G, 754A>C, 760T>G, 766G>T, 769G>A, 771C>A, 772C>A, 775A>T, 784T>C, 785T>C, 787G>T, 790C>T, 792G>A, 796G>A, 797C>T, 799T>G, 802C>A, 811C>A, 814C>T, 817G>C, 818G>A, 820T>A, 821C>T, 823T>G, 828G>C, 829T>A, 835A>G, 841G>A, 844A>G, 853G>T, 854T>C, 856G>T, 858T>A, 862C>T, 874A>T, 875T>A, 876C>G, 877A>T, 880G>A, 881A>G, 883C>G, 884A>G, 892T>A, 899A>G, 901A>T, 904C>T, 908C>A, 909A>G, 910T>G, 912A>C, 913G>C, 914T>A, 916T>A, 917G>A, 918T>C, 919T>A, 922G>A, 925T>A, 926A>C, 927A>G, 928G>T, 931C>T, 936C>G, 937A>G, 940C>T, 943G>A, 949T>C, 953C>T, 955G>A, 956T>C, 958T>C, 961T>G, 965C>T, 967T>G, 970C>T, 973C>T, 979C>T, 982A>C, 987T>C, 988T>A, 993G>A, 997G>A, 1003A>T, 1004A>G, 1005G>C, 1006G>C, 1009T>C, 1012G>A, 1015A>C, 1018T>C, 1019C>T, 1021T>G, 1024T>C, 1027A>T, 1030T>C, 1033T>C, 1040C>A, 1041T>C, 1042C>T, 1043T>G, 1044C>A, 1045A>T, 1048G>T, 1057A>G, 1063G>A, 1065C>T, 1066A>T, 1067C>T, 1071C>A, 1075G>T, 1076G>T, 1078C>T, 1080A>G, 1081T>A, 1087G>A, 1090G>A, 1093A>C, 1099C>A, 1105C>T, 1108C>T, 1111C>T, 1127C>T, 1129C>G, 1132A>G, 1135A>G, 1138G>C, 1156C>A, 1162G>A, 1165C>T, 1167A>T, 1168A>C, 1171T>C, 1174C>G, 1177G>A, 1180A>T, 1183G>A, 1189G>T, 1195C>T, 1198G>A, 1204C>A, 1207A>T, 1223G>A, 1224A>G, 1225C>T, 1226A>C, 1228A>T, 1229A>C, 1231G>T, 1237A>T, 1240T>A, 1244T>A, 1247C>G, 1249G>C, 1252A>T, 1255A>C, 1258A>T, 1262T>A, 1263C>G, 1270C>T, 1273A>T, 1276A>G, 1279A>T, 1282C>T, 1285T>C, 1286C>A, 1288T>A, 1291G>T, 1294A>G, 1306T>C, 1309A>G, 1315G>A, 1318T>A, 1321T>A, 1327T>C, 1330A>T, 1333T>A, 1339T>G, 1342T>C, 1345G>C, 1347G>A, 1351_1352insGTA, 1354A>G, 1357T>A, 1359C>G, 1363A>G, 1364A>C, 1367_1369delGCT, 1370C>T, 1372T>G, 1375A>G, 1380A>G, 1382G>C, 1384C>T, 1390T>G, 1393A>T, 1395A>C, 1396A>C, 1397C>G, 1398A>C, 1399G>A, 1405G>T, 1407G>A, 1408C>T, 1411A>C, 1418G>A, 1420C>T, 1423C>G, 1424A>C, 1425T>C, 1427A>C, 1429T>G, 1432A>G, 1433A>G, 1434G>C, 1435C>A, 1437C>A, 1442A>G, 1444C>G, 1447G>C, 1448A>T, 1449G>C, 1456T>A, 1462A>G, 1471T>C, 1474C>A, 1477A>G, 1480G>A, 1488A>G, 1492C>A, 1496C>G, 1498G>A, 1501C>A, 1508C>T, 1513G>T, 1513_1514insGACTTT, 1517G>T, 1518T>C, 1521G>C, 1523A>T, 1525A>T, 1526T>A, 1527C>G, 1529T>G, 1531C>G, 1533T>A, 1534A>T, 1536T>A, 1537C>G, 1538A>G, 1540C>A, 1541A>G, 1542C>T, 1543A>G, 1544C>A, 1546T>A, 1549A>G, 1555A>T, 1557T>G, 1559C>A, 1560A>T, 1565_1567delGAG, 1574T>A, 1575G>C, 1576T>G, 1577G>T, 1578G>C, 1579G>T, 1580G>C, 1581G>T, 1583T>C, 1591A>T, 1594A>C, 1595_1600delCCCAAG, 1603A>G, 1605A>T, 1606G>A, 1607G>T, 1608G>C, 1609C>A, 1615C>A, 1616T>G, 1617T>G, 1618C>A, 1620A>C, 1621C>A, 1622A>C, 1624G>C, 1625G>C, 1626G>A, 1627T>A, 1629C>A, 1630C>A, 1631A>G, 1633T>C, 1639T>C, 1644T>A, 1645G>T, 1651G>T, 1652G>C, 1654G>A, 1655T>A, 1656C>G, 1657G>T, 1660C>T, 1662C>A, 1664C>G, 1666C>T, 1667A>T, 1671A>C, 1672T>C, 1679G>A, 1680A>G, 1682G>A, 1684T>C, 1692A>C, 1693A>C, 1695G>T, 1696A>T, 1700A>G, 1702A>G, 1703G>C, 1705T>C, 1706C>G, 1711G>A, 1712G>A, 1714A>T, 1715C>G, 1717A>T, 1719T>A, 1721A>G, 1723C>T, 1726C>T, 1727T>G, 1729T>G, 1730A>C, 1731G>A, 1732T>G, 1733G>A, 1740A>G, 1742A>G, 1743G>C, 1744A>T, 1747A>T, 1750C>T, 1751G>C, 1753T>C, 1754A>T, 1755T>G, 1759G>C, 1764G>A, 1769G>A, 1773C>A, 1774A>G, 1776A>T, 1778C>A, 1781C>G, 1783T>C, 1784C>T, 1789T>C, 1790A>T, 1791A>G, 1795C>A, 1799A>T, 1800G>T, 1801G>T, 1804G>A, 1807C>T, 1810A>C, 1813C>T, 1818A>C, 1828A>T, 1831A>T, 1834A>T, 1835C>T, 1837T>G, 1839A>C, 1840A>G, 1843T>A, 1849T>A, 1858T>G, 1859A>G, 1861C>T, 1862C>G, 1863A>G, 1864A>G, 1865G>A, 1866G>T, 1867C>G, 1871A>G, 1872C>A, 1873T>A, 1874C>A, 1876C>A, 1879T>A, 1880T>C, 1881T>C, 1882C>A, 1891T>C, 1892G>T, 1895A>T, 1898T>A, 1899C>G, 1900T>C, 1903C>G, 1907G>C, 1915T>G, 1918G>T, 1924C>A, 1930T>C, 1931C>A, 1932A>T, 1933A>G, 1936A>G, 1939T>C, 1943T>C, 1944C>A, 1945C>A, 1948C>T, 1953T>G, 1954T>G, 1955C>T, 1957A>T, 1958G>T, 1961C>G, 1963G>T, 1964A>T, 1965C>T, 1966T>A, 1968A>C, 1969C>A, 1972T>C, 1975A>G, 1981G>T, 1982A>G, 1984T>C, 1991T>G, 1992G>T, 1993C>G, 2002A>T, 2003T>C, 2005A>T, 2008C>A, 2009T>C, 2012A>G, 2013A>C, 2017G>T, 2018C>A, 2020T>G, 2020_2021insGTGAAA, 2023G>T, 2024T>C, 2025T>C, 2026G>C, 2027A>T, 2029T>G, 2030G>A, 2033G>A, 2034A>G, 2035G>T, 2038G>T, 2040A>T, 2043T>C, 2044G>A, 2045G>T, 2046C>G, 2050_2051insTTGAAT, 2051A>C, 2053A>C, 2054A>T, 2055G>T, 2057T>G, 2058C>G, 2059T>A, 2060G>A, 2063A>C, 2064G>C, 2066C>A, 2067A>G, 2068A>C, 2069G>A, 2070C>T, 2071A>G, 2072C>A, 2073A>G, 2074G>T, 2078A>G, 2081G>C, 2082C>A, 2085G>C, 2086G>T, 2089C>A, 2091T>A, 2092A>T, 2095T>C, 2095_2096insAGC, 2096G>A, 2098A>G, 2099A>T, 2101T>G, 2104G>A, 2105C>A, 2112A>G, 2113C>A, 2115G>A, 2116A>G, 2120A>C, 2121C>T, 2122T>G, 2128C>A, 2134G>T, 2135T>A, 2140T>C, 2143C>A, 2144A>G, 2146G>A, 2147A>C, 2149G>T, 2152A>G, 2152_2153insAGT, 2155T>G, 2156C>G, 2157C>G, 2158C>T, 2160C>T, 2162A>C, 2164G>A, 2165C>A, 2166C>G, 2168G>A, 2170T>G, 2171A>G, 2174A>G, 2176A>G, 2177G>T, 2178A>C, 2184T>A, 2185T>G, 2188A>T, 2191G>A, 2193G>A, 2194A>T, 2195G>C, 2197A>G, 2202A>G, 2203A>G, 2204G>A, 2209T>A, 2210C>G, 2212A>G, 2216G>A, 2217G>A, 2221T>C, 2222G>A, 2225A>T, 2227T>C, 2233C>T, 2239A>G, 2240C>G, 2241C>A, 2242A>G, 2249G>A, 2250A>T, 2251C>G, 2252A>G, 2253C>G, 2254A>T, 2255T>G, 2256C>A, 2257C>T, 2258A>G, 2259T>C, 2260G>C, 2261G>C, 2263A>G, 2264G>T, 2266T>A, 2267G>A, 2269A>T, 2275T>G, 2276A>T, 2281G>A, 2284A>G, 2287G>A, 2289T>C, 2290G>A, 2296A>G, 2302C>T, 2308T>G, 2311C>T, 2323A>G, 2324G>A, 2335C>T, 2336G>C, 2338T>G, 2342G>A, 2344G>A, 2348T>A, 2349C>G, 2351G>A, 2352C>A, 2353T>C, 2365G>A, 2368A>G, 2371A>T, 2372A>C, 2374G>T, 2375A>G, 2376G>C, 2377C>A, 2378T>C, 2380G>A, 2381G>T, 2383T>C, 2384T>C, 2386G>C, 2389C>A, 2390G>A, 2391C>T, 2392T>G, 2395C>A, 2396C>T, 2398A>G, 2401C>T, 2402T>A, 2403C>G, 2404A>C, 2407A>G, 2409G>A, 2410C>G, 2413A>G, 2417A>G, 2422A>C, 2428T>G, 2429G>T, 2431G>C, 2440T>C, 2446T>C, 2449C>T, 2452T>C, 2455A>T, 2458T>A, 2459C>A, 2460T>A, 2461A>C, 2462A>C, 2464C>T, 2469A>T, 2470C>T, 2474A>G, 2476A>C, 2482T>G, 2485T>C, 2486G>A, 2495G>A, 2497T>G, 2503T>G, 2504A>G, 2506C>G, 2509T>G, 2512T>A, 2515A>G, 2516C>T, 2518G>A, 2521T>C, 2536A>G, 2539T>C, 2544T>C, 2545G>A, 2552C>A, 2556A>C, 2557G>C, 2572C>T, 2575C>A, 2581A>C, 2582T>A, 2584G>T, 2596G>A, 2600A>C, 2601C>A, 2602A>T, 2603T>C, 2608T>G, 2618C>G, 2624A>T, 2625A>C, 2631A>G, 2633G>A, 2639T>G, 2640G>T, 2641C>G, 2644T>C, 2645A>T, 2647C>A, 2650A>T, 2651A>C, 2652C>A, 2656G>T, 2662C>T, 2664A>C, 2665G>C, 2667A>T, 2668C>T, 2671G>A, 2674T>A, 2675C>T, 2681G>A, 2682A>C, 2683A>T, 2684G>T, 2685G>C, 2686T>A, 2687A>C, 2688C>T, 2689A>G, 2692A>T, 2694C>T, 2695C>T, 2696A>C, 2701T>A, 2702T>C, 2704A>T, 2707T>C, 2713G>T, 2728A>C, 2731C>T, 2737A>G, 2738T>G, 2740T>A, 2746A>C, 2752G>A, 2756A>G, 2758T>G, 2759A>T, 2760G>C, 2761T>A, 2762G>A, 2767A>T, 2770C>T, 2773T>C, 2774T>A, 2776G>A, 2780T>C, 2783C>T, 2784A>C, 2785G>C, 2791A>G, 2795T>C, 2797A>C, 2803C>A, 2810C>A, 2812T>G, 2822C>A, 2824A>G, 2825A>T, 2829T>G, 2832C>G, 2833A>G, 2836G>A, 2842A>G, 2844A>T, 2848C>A, 2852A>C, 2853A>G, 2855G>C, 2857G>A, 2860G>C, 2861C>A, 2862A>C, 2863G>A, 2866A>C, 2868A>T, 2869T>C, 2873C>A, 2874C>G, 2875A>T, 2876A>C, 2878G>C, 2881T>C, 2884A>T, 2887T>A, 2896T>G, 2897T>C, 2899G>T, 2900T>C, 2902A>G, 2905T>C, 2908T>C, 2915G>A, 2917G>T, 2918A>G, 2919C>T, 2921T>G, 2923A>T, 2924T>G, 2926C>A, 2927C>G, 2929A>C, 2941A>T, 2950C>T, 2951A>G, 2953A>C, 2956T>C, 2959A>C, 2971A>T, 2977G>A, 2986C>T, 2988C>T, 2992G>A, 2998G>A, 3001T>A, 3002T>C, 3010T>A, 3016A>T, 3019C>A, 3022G>A, 3031G>A, 3039C>G, 3040T>A, 3043A>C, 3046T>C, 3059A>G, 3061T>G, 3064C>T, 3065A>G, 3076C>T, 3077T>C, 3088A>T, 3091T>G, 3097G>T, 3100A>T, 3103T>A, 3106A>G, 3107C>T, 3109T>G, 3112A>C, 3115T>G, 3119A>G, 3120A>C, 3121C>A, 3124C>A, 3125A>G, 3127A>G, 3131A>T, 3135A>C, 3136T>A, 3139T>G, 3151T>G, 3160A>C, 3169A>G, 3172C>T, 3176C>T, 3178T>G, 3187T>G, 3192A>G, 3193A>G, 3198G>A, 3199G>A, 3203C>A, 3204G>A, 3205G>A, 3208T>C, 3211T>A, 3212A>C, 3214T>G, 3217A>C, 3229A>G, 3230A>T, 3231A>G, 3232A>C, 3235T>C, 3236T>C, 3238G>C, 3241T>G, 3244C>T, 3247A>C, 3250G>A, 3253A>C, 3256G>A, 3262C>A, 3265G>A, 3275C>A, 3280C>T, 3281T>C, 3282T>A, 3283T>A, 3286C>T, 3290T>A, 3295C>T, 3296T>C, 3301T>C, 3307G>T, 3310A>T, 3322C>G, 3331G>A, 3335A>T, 3337A>G, 3340T>C, 3347T>A, 3349C>T, 3355T>A, 3358C>A, 3367G>A, 3373G>A, 3374T>C, 3377C>A, 3383A>G, 3388T>C, 3391T>G, 3392T>C, 3394G>T, 3397T>G, 3400C>T, 3401A>T, 3403A>T, 3406A>G, 3409T>A, 3412G>T, 3415A>G, 3421A>T, 3427G>T, 3436A>C, 3439A>G, 3442C>G, 3444A>G, 3448C>T, 3449T>G, 3451A>T, 3454T>G, 3460C>T, 3463T>G, 3466C>T, 3476G>A, 3478T>C, 3479T>C, 3481A>G, 3482T>A, 3483C>A, 3484A>T, 3490T>C, 3497A>C, 3502A>G, 3505G>A, 3506T>C, 3514G>A, 3517C>A, 3520C>T, 3521A>G, 3526G>T, 3527G>A, 3528A>T, 3529G>C, 3531A>T, 3532C>T, 3533C>G, 3535G>C, 3536G>C, 3544C>G, 3548G>A, 3550T>C, 3551_3556delCCGCCC, 3561A>C, 3562G>A, 3563G>T, 3569A>G, 3570G>T, 3572A>T, 3573T>C, 3574T>C, 3575C>T, 3577G>C, 3578T>C, 3579C>A, 3580C>G, 3583A>C, 3586A>G, 3589G>A, 3592A>G, 3595T>C, 3596T>A, 3598C>A, 3604T>A, 3607T>A, 3610A>G, 3619C>T, 3622C>T, 3623A>G, 3624T>C, 3625G>T, 3627G>C, 3628T>A, 3638G>A, 3639A>C, 3640C>A, 3643A>C, 3650T>G, 3652G>A, 3655A>T, 3656A>T, 3658T>G, 3661T>A, 3667A>C, 3670A>T, 3676C>A, 3677A>G, 3679A>G, 3680A>C, 3682G>C, 3688A>T, 3690G>C, 3691G>A, 3692C>T, 3693A>C, 3700T>A, 3701G>A, 3706G>A, 3715C>A, 3721T>C, 3727T>C, 3733T>C, 3740A>T, 3742A>C, 3745A>T, 3760A>T, 3763T>A, 3770A>G, 3772T>A, 3776T>A, 3779A>T, 3784G>A, 3787A>G, 3791A>C, 3794G>A, 3795T>C, 3796A>T, 3799A>G, 3800T>A, 3801C>G, 3803A>T, 3804G>C, 3805T>C, 3806C>A, 3807C>A, 3808T>C, 3810A>G, 3812G>A, 3814T>A, 3817A>T, 3821G>A, 3823A>C, 3824C>T, 3826A>G, 3829T>C, 3830G>A, 3835T>A, 3836T>C, 3841T>C, 3842G>A, 3844C>A, 3850C>G, 3859T>C, 3861C>A, 3863G>T, 3864C>T, 3868G>T, 3869C>G, 3871T>A, 3872A>T, 3873A>C, 3875A>G, 3876A>C, 3877A>G, 3881A>G, 3882A>C, 3883T>A, 3884T>C, 3890G>T, 3901T>C, 3904A>T, 3907C>A, 3910A>T, 3925A>C, 3928C>T, 3931T>G, 3934G>T, 3935G>A, 3937A>C, 3940A>C, 3943T>C, 3945C>T, 3946G>C, 3952A>T, 3955C>T, 3956A>G, 3957C>T, 3958T>A, 3971G>C, 3976T>C, 3979C>T, 3982A>T, 3983A>C, 3985G>C, 3988G>T, 3989C>T, 3990A>T, 3991T>C, 3996G>A, 3998A>G, 3999T>C, 4000G>C, 4002G>A, 4003G>A, 4004G>T, 4005A>C, 4006A>C, 4008T>A, 4009C>T, 4010T>G, 4011C>A, 4012A>T, 4014G>A, 4015C>G, 4016A>G, 4018G>A, 4019C>A, 4020A>G, 4021A>T, 4024C>A, 4025C>A, 4027C>A, 4030C>T, 4031A>G, 4033G>T, 4036A>T, 4037C>T, 4039A>G, 4045T>C, 4047G>A, 4051T>A, 4054A>C, 4055A>T, 4056A>T, 4057T>G, 4058C>T, 4061A>C, 4063C>A, 4066A>C, 4070T>C, 4072G>T, 4075T>A, 4078T>A, 4079T>G, 4080C>T, 4081C>T, 4083C>A, 4084C>A, 4085A>C, 4087A>T, 4088G>A, 4090G>T, 4096C>A, 4102G>A, 4105A>C, 4106C>A, 4108T>A, 4109A>G, 4111A>C, 4112T>A, 4113C>A, 4114T>G, 4117T>A, 4120A>T, 4122T>C, 4129C>T, 4132G>A, 4144C>T, 4150T>C, 4153T>A, 4154T>C, 4155C>T, 4159C>A, 4163C>A, 4168A>G, 4171A>G, 4177T>C, 4180C>A, 4181A>G, 4189A>G, 4192T>G, 4195C>T, 4198C>T, 4210C>A, 4213A>T, 4228A>G, 4242T>A, 4247A>G, 4248A>C, 4259C>T, 4261A>G, 4267T>A, 4274T>C, 4276G>A, 4279A>T, 4280A>T, 4281A>T, 4283A>C, 4284C>T, 4285A>T, 4286C>T, 4287T>G, 4289A>T, 4291C>A, 4291_4292insCGTA, 4294C>T, 4304T>G, 4306G>A, 4309G>A, 4312G>T, 4312_4313insAGTAAATAAATAAAGT, 4317G>T, 4320G>T, 4324T>G, 4325T>G, 4330G>C, 4331C>A, 4333A>G, 4336A>T, 4339A>G, 4340G>T, 4342G>T, 4343A>T, 4345A>T, 4346G>C, 4349A>G, 4351G>A, 4363T>C, 4364C>T, 4365T>C, 4370A>T, 4372A>G, 4373G>A, 4374A>G, 4384C>T, 4385C>G, 4386T>A, 4391T>G, 4393C>T, 4394A>G, 4398A>T, 4399G>T, 4400A>G, 4404A>G, 4415T>C, 4420delA, 4422A>G, 4426G>A, 4437C>T, 4441T>C, 4449A>T, 4451T>G, 4472A>G, 4477_4478insTCCTCCACA, 4479A>C, 4480T>A, 4482T>A, 4486G>T, 4488T>A, 4489T>A, 4490A>C, 4491A>G, 4492C>G, 4493A>T, 4497C>G, 4499C>G, 4500G>T, 4501T>G, 4502T>A, 4506A>T, 4510T>A, 4510_4511insTCACTTA, 4518A>G, 4519G>A, 4520A>C, 4521C>A, 4523T>C, 4525A>G, 4526T>G, 4532C>G, 4534A>T, 4535A>T, 4539A>C, 4540_4541insAGTTAACGCGAACACAGGTTGTTTCCCTATAGAAACTA, 4542C>A, 4544T>C, 4548T>G, 4552C>G, 4553A>C, 4554T>A, 4556T>C, 4557A>T, 4559G>A, 4562A>G, 4563_4564delTA, 4566A>G, 4568G>A, 4569A>G, 4570G>C, 4581C>T, 4594T>C, 4595T>A, 4602C>G, 4606A>T, 4613T>C, 4614C>T, 4616C>T, 4618A>G, 4626T>C, 4628G>A, 4630G>A, 4632C>T, 4637T>A, 4639G>A, 4642A>T, 4643G>T, 4646C>A, 4654C>T, 4675G>C, 4680A>G, 4684T>A, 4685T>A, 4688C>T, 4689C>G, 4704G>C, 4706G>A, 4708T>A, 4722T>G

CDS

	Begin	End	Coverage	Score	Concordance	Matches	Identities	I/D/M/F*	Stop Codons
**NT**	**89**	**4733**	**94.7%**	**2570**	**28.4%**	**4597 (96.7%)**	**3027 (63.7%)**	**107/48**	
ToMarV_RNA2gp1	1	191	100%	784	66.7%	183 (95.8%)	116 (60.7%)	0/8/0/0	1
Protein mutations:	G5S (151G>A 153G>T), R6K (154C>A 155G>A 156T>G), S10Q (166T>C 167C>A 168T>A), V11I (169G>A), D12E (174C>G), E13D (177A>T), A15E (182C>A 183T>A), H18N (190C>A), T22A (202A>G 204C>A), N24T (209A>C 210T>C), I26V (214A>G), V31T (229G>A 230T>C), T33V (235A>G 236C>T 237C>T), L35I (241T>A 243G>T), V46I (274G>A), V47C (277G>T 278T>G), V54I (298G>A 300C>T), T58S (310A>T 312C>T), L68V (340T>G 342A>T), N79T (374A>C 375T>G), P81D (379C>G 380C>A 381C>T), N84A (388A>G 389A>C 390C>A), L91I (409C>A), H93R (416A>G 417T>A), V96I (424G>A 426G>T), R97K (427C>A 428G>A 429T>A), V104L (448G>T 450C>G), E107A (458A>C 459A>C), S111H (469T>C 470C>A), A114S (478G>T 480A>T), S115T (481T>A 483A>C), P120N (496C>A 497C>A 498T>C), S121P (499T>C 501T>C), D124_S129del (508_525delGACAGTAAAGGCAAGAGT), K130T (527A>C), V131L (529G>T), E132S (532G>T 533A>C 534G>A), Q133E (535C>G), P134T (538C>A 540C>T), T135A (541A>G), R136_E137del (544_549delCGAGAG), D138S (550G>A 551A>G), I140S (557T>G 558T>C), K141G (559A>G 560A>G), V143N (565G>A 566T>A 567T>C), E144K (568G>A), V145A (572T>C 573T>A), R148E (580C>G 581G>A 582T>A), E149A (584A>C 585G>C), R152V (592A>G 593G>T 594A>T), F153A (595T>G 596T>C 597T>C), Q154R (598C>A 599A>G 600G>A), A158Q (610G>C 611C>A), S159E (613A>G 614G>A 615T>A), Q160A (616C>G 617A>C), K161R (619A>C 620A>G 621A>G), N164I (629A>T), Q165V (631C>G 632A>T 633G>C), Q168R (640C>A 641A>G 642A>G), L171I (649C>A 651C>T), S172Q (652T>C 653C>A 654C>A), N173G (655A>G 656A>G), V175P (661G>C 662T>C 663T>A), N178E (670A>G 672T>G), I180V (676A>G), S182A (682T>G), E186S (694G>A 695A>G 696A>T), S187D (697A>G 698G>A), Q190K (706C>A)								
Codon mutations:	TCA2TCT (144A>T), TTC3TTT (147C>T), ATT4ATC (150T>C), GGG5AGT (151G>A 153G>T), CGT6AAG (154C>A 155G>A 156T>G), TTG7CTC (157T>C 159G>C), AAT8AAC (162T>C), ACA9ACC (165A>C), TCT10CAA (166T>C 167C>A 168T>A), GTA11ATA (169G>A), GAC12GAG (174C>G), GAA13GAT (177A>T), GCT15GAA (182C>A 183T>A), TTT16TTC (186T>C), CAT18AAT (190C>A), GTT20GTA (198T>A), GCC21GCA (201C>A), ACC22GCA (202A>G 204C>A), TCG23TCT (207G>T), AAT24ACC (209A>C 210T>C), ATT26GTT (214A>G), TCT28AGT (220T>A 221C>G), GTT31ACT (229G>A 230T>C), GGC32GGT (234C>T), ACC33GTT (235A>G 236C>T 237C>T), TTG35ATT (241T>A 243G>T), ATA36ATT (246A>T), ACC41ACT (261C>T), CTT42TTA (262C>T 264T>A), GAC43GAT (267C>T), AAA45AAG (273A>G), GTT46ATT (274G>A), GTT47TGT (277G>T 278T>G), CCT48CCC (282T>C), GGT51GGA (291T>A), GTC54ATT (298G>A 300C>T), TCA55TCG (303A>G), GTT56GTG (306T>G), TTA57CTT (307T>C 309A>T), ACC58TCT (310A>T 312C>T), GTT59GTG (315T>G), TCT60AGT (316T>A 317C>G), GAG62GAA (324G>A), ACG65ACT (333G>T), CCA66CCC (336A>C), TTA68GTT (340T>G 342A>T), CCT70CCA (348T>A), GGA71GGC (351A>C), CAC72CAT (354C>T), TAT73TAC (357T>C), TTG74CTA (358T>C 360G>A), TTG75CTA (361T>C 363G>A), AAT79ACG (374A>C 375T>G), CCC81GAT (379C>G 380C>A 381C>T), ATT82ATC (384T>C), AAG83AAA (387G>A), AAC84GCA (388A>G 389A>C 390C>A), TCT88TCA (402T>A), GGT89GGC (405T>C), TTA90TTG (408A>G), CTT91ATT (409C>A), GTC92GTG (414C>G), CAT93CGA (416A>G 417T>A), TCA95TCC (423A>C), GTG96ATT (424G>A 426G>T), CGT97AAA (427C>A 428G>A 429T>A), CTT98TTG (430C>T 432T>G), ACC101ACA (441C>A), AGA102CGG (442A>C 444A>G), GTC104TTG (448G>T 450C>G), CTA105TTG (451C>T 453A>G), GAG106GAA (456G>A), GAA107GCC (458A>C 459A>C), AAC108AAT (462C>T), AAG109AAA (465G>A), GTT110GTG (468T>G), TCT111CAT (469T>C 470C>A), TCA113TCT (477A>T), GCA114TCT (478G>T 480A>T), TCA115ACC (481T>A 483A>C), TCA117TCT (489A>T), TCT118TCA (492T>A), TCT119TCC (495T>C), CCT120AAC (496C>A 497C>A 498T>C), TCT121CCC (499T>C 501T>C), GAC124_AGT129del (508_525delGACAGTAAAGGCAAGAGT), AAA130ACA (527A>C), GTA131TTA (529G>T), GAG132TCA (532G>T 533A>C 534G>A), CAA133GAA (535C>G), CCC134ACT (538C>A 540C>T), ACA135GCA (541A>G), CGA136_GAG137del (544_549delCGAGAG), GAT138AGT (550G>A 551A>G), CTC139TTA (553C>T 555C>A), ATT140AGC (557T>G 558T>C), AAA141GGA (559A>G 560A>G), GAA142GAG (564A>G), GTT143AAC (565G>A 566T>A 567T>C), GAG144AAG (568G>A), GTT145GCA (572T>C 573T>A), CTC146CTT (576C>T), AAA147AAG (579A>G), CGT148GAA (580C>G 581G>A 582T>A), GAG149GCC (584A>C 585G>C), TTA150CTT (586T>C 588A>T), GAG151GAA (591G>A), AGA152GTT (592A>G 593G>T 594A>T), TTT153GCC (595T>G 596T>C 597T>C), CAG154AGA (598C>A 599A>G 600G>A), GAG156GAA (606G>A), TTG157CTG (607T>C), GCA158CAA (610G>C 611C>A), AGT159GAA (613A>G 614G>A 615T>A), CAA160GCA (616C>G 617A>C), AAA161CGG (619A>C 620A>G 621A>G), AAT164ATT (629A>T), CAG165GTC (631C>G 632A>T 633G>C), CTA167CTC (639A>C), CAA168AGG (640C>A 641A>G 642A>G), CTT169TTG (643C>T 645T>G), CTC171ATT (649C>A 651C>T), TCC172CAA (652T>C 653C>A 654C>A), AAT173GGT (655A>G 656A>G), GTT175CCA (661G>C 662T>C 663T>A), AGT176AGC (666T>C), AAT177AAC (669T>C), AAT178GAG (670A>G 672T>G), GAC179GAT (675C>T), ATC180GTC (676A>G), TTC181TTT (681C>T), TCA182GCA (682T>G), GGT183GGC (687T>C), ACT185ACG (693T>G), GAA186AGT (694G>A 695A>G 696A>T), AGT187GAT (697A>G 698G>A), GGG188GGC (702G>C), CAG190AAG (706C>A), TAA191TGA (710A>G)								
ToMarV_RNA2gp2	1	1192	100%	6009	73.1%	1185 (98.7%)	820 (68.3%)	9/7/0/0	1
Protein mutations:	M1R (672T>G), T2M (675C>T 676A>G), S4L (681C>T 682T>G), V6A (687T>C), L8R (693T>G 694G>A), K9V (695A>G 696A>T 697A>G), V10M (698G>A), G11A (702G>C), N14D (710A>G 712T>C), H15R (714A>G), Q16S (716C>T 717A>C 718G>A), Q17del (719_721delCAG), E18K (722G>A 724G>A), A20V (729C>T 730C>A), E22D (736G>C), R23K (738G>A 739G>A), A34E (771C>A 772C>A), R41K (792G>A), V50I (818G>A 820T>A), G53A (828G>C 829T>A), E61D (853G>T), F63Y (858T>A), I71V (881A>G 883C>G), T72A (884A>G), T77A (899A>G 901A>T), H80R (908C>A 909A>G 910T>G), K81T (912A>C 913G>C), F82I (914T>A 916T>A), V83T (917G>A 918T>C 919T>A), K86R (926A>C 927A>G 928G>T), A89G (936C>G 937A>G), Y96H (956T>C 958T>C), V106A (987T>C 988T>A), S108N (993G>A), R112A (1004A>G 1005G>C 1006G>C), L124T (1040C>A 1041T>C 1042C>T), S125D (1043T>G 1044C>A 1045A>T), T132I (1065C>T 1066A>T), S134Y (1071C>A), L135F (1075G>T), A136S (1076G>T 1078C>T), D137G (1080A>G 1081T>A), Q166L (1167A>T 1168A>C), D185S (1223G>A 1224A>G 1225C>T), T186P (1226A>C 1228A>T), S192T (1244T>A), Q193D (1247C>G 1249G>C), Q194H (1252A>T), E195D (1255A>C), S226N (1347G>A), G227_Q228insV (1351_1352insGTA), S229R (1357T>A), T230S (1359C>G), M232L (1364A>C), A233del (1367_1369delGCT), K237R (1380A>G), A238P (1382G>C 1384C>T), E242A (1395A>C 1396A>C), Q243A (1397C>G 1398A>C 1399G>A), G246D (1407G>A 1408C>T), D250N (1418G>A 1420C>T), M252P (1424A>C 1425T>C), N253Q (1427A>C 1429T>G), S255A (1433A>G 1434G>C 1435C>A), T256N (1437C>A), I258V (1442A>G 1444C>G), K273R (1488A>G), P276A (1496C>G 1498G>A), P281_A282insDF (1513_1514insGACTTT), V283S (1517G>T 1518T>C), S284T (1521G>C), T285S (1523A>T 1525A>T), S286R (1526T>A 1527C>G), S287A (1529T>G 1531C>G), L288Y (1533T>A 1534A>T), L289Q (1536T>A 1537C>G), S290G (1538A>G 1540C>A), T291V (1541A>G 1542C>T 1543A>G), V296G (1557T>G), H297I (1559C>A 1560A>T), E299del (1565_1567delGAG), C302T (1574T>A 1575G>C 1576T>G), G303S (1577G>T 1578G>C 1579G>T), G304L (1580G>C 1581G>T), P309_K310del (1595_1600delCCCAAG), K312I (1605A>T 1606G>A), G313S (1607G>T 1608G>C 1609C>A), F316G (1616T>G 1617T>G 1618C>A), N317T (1620A>C 1621C>A), M318L (1622A>C 1624G>C), G319Q (1625G>C 1626G>A 1627T>A), A320E (1629C>A 1630C>A), I321V (1631A>G 1633T>C), L325Y (1644T>A 1645G>T), M327I (1651G>T), E328Q (1652G>C 1654G>A), A331E (1662C>A), H332D (1664C>G 1666C>T), I333F (1667A>T), D334A (1671A>C 1672T>C), D337S (1679G>A 1680A>G), D338N (1682G>A 1684T>C), E341A (1692A>C 1693A>C), R342L (1695G>T 1696A>T), I344V (1700A>G 1702A>G), A345P (1703G>C 1705T>C), Q346E (1706C>G), V348I (1712G>A 1714A>T), Q349D (1715C>G 1717A>T), F350Y (1719T>A), I351V (1721A>G 1723C>T), F353V (1727T>G 1729T>G), S354Q (1730A>C 1731G>A 1732T>G), E355K (1733G>A), K357R (1740A>G), R358A (1742A>G 1743G>C 1744A>T), V361L (1751G>C 1753T>C), I362C (1754A>T 1755T>G), R365K (1764G>A), V367I (1769G>A), A368E (1773C>A 1774A>G), K369M (1776A>T), H371D (1781C>G 1783T>C), N374C (1790A>T 1791A>G), R377F (1799A>T 1800G>T 1801G>T), D383A (1818A>C), E387D (1831A>T), E390A (1839A>C 1840A>G), N396K (1858T>G), I397V (1859A>G 1861C>T), Q398G (1862C>G 1863A>G 1864A>G), G399M (1865G>A 1866G>T 1867C>G), T401E (1871A>G 1872C>A 1873T>A), F404P (1880T>C 1881T>C 1882C>A), A408S (1892G>T), M409L (1895A>T), E413Q (1907G>C), Q421M (1931C>A 1932A>T 1933A>G), S425Q (1943T>C 1944C>A 1945C>A), I428R (1953T>G 1954T>G), Q429Y (1955C>T 1957A>T), G430W (1958G>T), Q431D (1961C>G 1963G>T), T432L (1964A>T 1965C>T 1966T>A), N433T (1968A>C 1969C>A), N438D (1982A>G 1984T>C), C441V (1991T>G 1992G>T 1993C>G), K448A (2012A>G 2013A>C), E449D (2017G>T), R450_Q451insVK (2020_2021insGTGAAA), Q451H (2023G>T), L452P (2024T>C 2025T>C 2026G>C), I453L (2027A>T 2029T>G), V454M (2030G>A), E455S (2033G>A 2034A>G 2035G>T), E456D (2038G>T), Q457L (2040A>T), V458A (2043T>C 2044G>A), A459C (2045G>T 2046C>G), Q460_R461insLN (2050_2051insTTGAAT), R462L (2054A>T 2055G>T), S463G (2057T>G 2058C>G 2059T>A), E464K (2060G>A), R465P (2063A>C 2064G>C), Q466S (2066C>A 2067A>G 2068A>C), A467M (2069G>A 2070C>T 2071A>G), Q468S (2072C>A 2073A>G 2074G>T), T470A (2078A>G), A471Q (2081G>C 2082C>A), R472T (2085G>C 2086G>T), I474N (2091T>A 2092A>T), A475_E476insS (2095_2096insAGC), E476K (2096G>A 2098A>G), S477W (2099A>T 2101T>G), P479T (2105C>A), D481G (2112A>G 2113C>A), R482K (2115G>A 2116A>G), T484L (2120A>C 2121C>T 2122T>G), S489T (2135T>A), T492A (2144A>G 2146G>A), M493L (2147A>C 2149G>T), E494_D495insS (2152_2153insAGT), D495E (2155T>G), P496G (2156C>G 2157C>G 2158C>T), T497I (2160C>T), K498Q (2162A>C 2164G>A), P499R (2165C>A 2166C>G), D500K (2168G>A 2170T>G), K501E (2171A>G), I502V (2174A>G 2176A>G), E503S (2177G>T 2178A>C), V505E (2184T>A 2185T>G), G508D (2193G>A 2194A>T), A509P (2195G>C 2197A>G), E511G (2202A>G 2203A>G), E512K (2204G>A), Q514E (2210C>G 2212A>G), G516N (2216G>A 2217G>A), V518I (2222G>A), I519F (2225A>T 2227T>C), P524E (2240C>G 2241C>A 2242A>G), D527M (2249G>A 2250A>T 2251C>G), T528G (2252A>G 2253C>G 2254A>T), S529D (2255T>G 2256C>A 2257C>T), M530A (2258A>G 2259T>C 2260G>C), A531P (2261G>C 2263A>G), V532L (2264G>T 2266T>A), E533N (2267G>A 2269A>T), D535E (2275T>G), M536L (2276A>T), V540A (2289T>C 2290G>A), D546E (2308T>G), V552I (2324G>A), V556L (2336G>C 2338T>G), E558K (2342G>A 2344G>A), A561N (2351G>A 2352C>A 2353T>C), M568L (2372A>C 2374G>T), S569A (2375A>G 2376G>C 2377C>A), A571S (2381G>T 2383T>C), A574M (2390G>A 2391C>T 2392T>G), S580K (2409G>A 2410C>G), T583A (2417A>G), A587S (2429G>T 2431G>C), L597N (2459C>A 2460T>A 2461A>C), I598L (2462A>C 2464C>T), Y600F (2469A>T 2470C>T), I602V (2474A>G 2476A>C), V606I (2486G>A), V609M (2495G>A 2497T>G), I612V (2504A>G 2506C>G), V625A (2544T>C 2545G>A), K629T (2556A>C 2557G>C), L638I (2582T>A 2584G>T), T644H (2600A>C 2601C>A 2602A>T), Q650E (2618C>G), N652S (2624A>T 2625A>C), E654G (2631A>G), E655K (2633G>A), C657V (2639T>G 2640G>T 2641C>G), T659S (2645A>T 2647C>A), T661Q (2651A>C 2652C>A), K665T (2664A>C 2665G>C), Y666F (2667A>T 2668C>T), E671T (2681G>A 2682A>C 2683A>T), G672S (2684G>T 2685G>C 2686T>A), T673L (2687A>C 2688C>T 2689A>G), A675V (2694C>T 2695C>T), T676P (2696A>C), D677E (2701T>A), S690A (2738T>G 2740T>A), N696E (2756A>G 2758T>G), V698I (2762G>A), L702I (2774T>A 2776G>A), Q705S (2783C>T 2784A>C 2785G>C), L714M (2810C>A 2812T>G), M719L (2825A>T), I720R (2829T>G), A721G (2832C>G 2833A>G), Y725F (2844A>T), K728R (2852A>C 2853A>G), A729P (2855G>C 2857G>A), Q731T (2861C>A 2862A>C 2863G>A), Y733F (2868A>T 2869T>C), P735S (2873C>A 2874C>G 2875A>T), G749S (2915G>A 2917G>T), T750V (2918A>G 2919C>T), S751A (2921T>G 2923A>T), S752A (2924T>G 2926C>A), P753A (2927C>G 2929A>C), I761V (2951A>G 2953A>C), T773I (2988C>T), T790R (3039C>G 3040T>A), I797V (3059A>G 3061T>G), I799V (3065A>G), N817A (3119A>G 3120A>C 3121C>A), K819E (3125A>G 3127A>G), T821S (3131A>T), N822T (3135A>C 3136T>A), F827L (3151T>G), K841R (3192A>G 3193A>G), R843Q (3198G>A 3199G>A), R845K (3203C>A 3204G>A 3205G>A), I848L (3212A>C 3214T>G), K854C (3230A>T 3231A>G 3232A>C), Q869K (3275C>A), F871Q (3281T>C 3282T>A 3283T>A), L874M (3290T>A), I889L (3335A>T 3337A>G), S893T (3347T>A 3349C>T), C902R (3374T>C), L903I (3377C>A), N905D (3383A>G), T911S (3401A>T 3403A>T), K925R (3444A>G), S927A (3449T>G 3451A>T), D936N (3476G>A 3478T>C), S938N (3482T>A 3483C>A 3484A>T), I943L (3497A>C), D949E (3517C>A), I951V (3521A>G), E952D (3526G>T), E953I (3527G>A 3528A>T 3529G>C), Y954F (3531A>T 3532C>T), Q955D (3533C>G 3535G>C), E956Q (3536G>C), D960N (3548G>A 3550T>C), P961_P962del (3551_3556delCCGCCC), K964T (3561A>C 3562G>A), A965S (3563G>T), S967V (3569A>G 3570G>T), I968S (3572A>T 3573T>C 3574T>C), L969F (3575C>T 3577G>C), S970Q (3578T>C 3579C>A 3580C>G), S976T (3596T>A 3598C>A), M985A (3623A>G 3624T>C 3625G>T), G986A (3627G>C 3628T>A), D990T (3638G>A 3639A>C 3640C>A), E991D (3643A>C), L994V (3650T>G 3652G>A), I996L (3656A>T 3658T>G), I1003V (3677A>G 3679A>G), E1006D (3688A>T), G1007A (3690G>C 3691G>A), H1008S (3692C>T 3693A>C), V1011I (3701G>A), T1024S (3740A>T 3742A>C), N1034E (3770A>G 3772T>A), S1036T (3776T>A), I1037F (3779A>T), I1039M (3787A>G), V1042T (3794G>A 3795T>C 3796A>T), P1046N (3806C>A 3807C>A 3808T>C), N1047S (3810A>G), V1048I (3812G>A 3814T>A), V1051I (3821G>A 3823A>C), V1054I (3830G>A), F1056L (3836T>C), A1058T (3842G>A 3844C>A), P1064Q (3861C>A), A1065F (3863G>T 3864C>T), L1067V (3869C>G 3871T>A), N1068S (3872A>T 3873A>C), K1069A (3875A>G 3876A>C 3877A>G), N1071A (3881A>G 3882A>C 3883T>A), Y1072H (3884T>C), V1074L (3890G>T), A1089T (3935G>A 3937A>C), T1092I (3945C>T 3946G>C), T1096V (3956A>G 3957C>T 3958T>A), E1101Q (3971G>C), H1107F (3989C>T 3990A>T 3991T>C), R1109K (3996G>A), M1110A (3998A>G 3999T>C 4000G>C), R1111K (4002G>A 4003G>A), E1112S (4004G>T 4005A>C 4006A>C), F1113Y (4008T>A 4009C>T), S1114D (4010T>G 4011C>A 4012A>T), S1115K (4014G>A 4015C>G), K1116E (4016A>G 4018G>A), Q1117S (4019C>A 4020A>G 4021A>T), M1121V (4031A>G 4033G>T), R1126K (4047G>A), N1129L (4055A>T 4056A>T 4057T>G), I1131L (4061A>C 4063C>A), S1137V (4079T>G 4080C>T 4081C>T), A1138E (4083C>A 4084C>A), I1139L (4085A>C 4087A>T), V1140I (4088G>A 4090G>T), L1146I (4106C>A 4108T>A), I1147V (4109A>G 4111A>C), S1148K (4112T>A 4113C>A 4114T>G), L1151P (4122T>C), S1162L (4154T>C 4155C>T), H1165N (4163C>A), N1171D (4181A>G), I1191N (4242T>A)								

	Begin	End	Coverage	Score	Concordance	Matches	Identities	I/D/M/F*	Stop Codons
**NT**	**89**	**4733**	**94.7%**	**2570**	**28.4%**	**4597 (96.7%)**	**3027 (63.7%)**	**107/48**	

Codon mutations:

ATG1AGG (672T>G), ACA2ATG (675C>T 676A>G), TCT4TTG (681C>T 682T>G), GTT6GCT (687T>C), CTG8CGA (693T>G 694G>A), AAA9GTG (695A>G 696A>T 697A>G), GTG10ATG (698G>A), GGC11GCC (702G>C), CCC12CCA (706C>A), AAT14GAC (710A>G 712T>C), CAC15CGC (714A>G), CAG16TCA (716C>T 717A>C 718G>A), CAG17del (719_721delCAG), GAG18AAA (722G>A 724G>A), GCC20GTA (729C>T 730C>A), TCC21TCT (733C>T), GAG22GAC (736G>C), AGG23AAA (738G>A 739G>A), ATA25ATT (745A>T), TCA26TCT (748A>T), CAA27CAG (751A>G), ATA28ATC (754A>C), GCT30GCG (760T>G), GTG32GTT (766G>T), GAG33GAA (769G>A), GCC34GAA (771C>A 772C>A), GGA35GGT (775A>T), AAT38AAC (784T>C), TTG39CTT (785T>C 787G>T), CTC40CTT (790C>T), AGA41AAA (792G>A), AAG42AAA (796G>A), CTT43TTG (797C>T 799T>G), GGC44GGA (802C>A), TCC47TCA (811C>A), TAC48TAT (814C>T), GGG49GGC (817G>C), GTT50ATA (818G>A 820T>A), CTT51TTG (821C>T 823T>G), GGT53GCA (828G>C 829T>A), CAA55CAG (835A>G), AAG57AAA (841G>A), AGA58AGG (844A>G), GAG61GAT (853G>T), TTG62CTT (854T>C 856G>T), TTT63TAT (858T>A), GAC64GAT (862C>T), GTA68GTT (874A>T), TCA69AGT (875T>A 876C>G 877A>T), AAG70AAA (880G>A), ATC71GTG (881A>G 883C>G), ACA72GCA (884A>G), CTT74CTA (892T>A), ACA77GCT (899A>G 901A>T), TTC78TTT (904C>T), CAT80AGG (908C>A 909A>G 910T>G), AAG81ACC (912A>C 913G>C), TTT82ATA (914T>A 916T>A), GTT83ACA (917G>A 918T>C 919T>A), GAG84GAA (922G>A), TCT85TCA (925T>A), AAG86CGT (926A>C 927A>G 928G>T), GAC87GAT (931C>T), GCA89GGG (936C>G 937A>G), AAC90AAT (940C>T), TTG91TTA (943G>A), TTT93TTC (949T>C), CTG95TTA (953C>T 955G>A), TAT96CAC (956T>C 958T>C), GGT97GGG (961T>G), CTT99TTG (965C>T 967T>G), TTC100TTT (970C>T), TTC101TTT (973C>T), GTC103GTT (979C>T), CCA104CCC (982A>C), GTT106GCA (987T>C 988T>A), AGT108AAT (993G>A), GGG109GGA (997G>A), GGA111GGT (1003A>T), AGG112GCC (1004A>G 1005G>C 1006G>C), GTT113GTC (1009T>C), AAG114AAA (1012G>A), ATA115ATC (1015A>C), AGT116AGC (1018T>C), CTT117TTG (1019C>T 1021T>G), TGT118TGC (1024T>C), TCA119TCT (1027A>T), AGT120AGC (1030T>C), AAT121AAC (1033T>C), CTC124ACT (1040C>A 1041T>C 1042C>T), TCA125GAT (1043T>G 1044C>A 1045A>T), CCG126CCT (1048G>T), CAA129CAG (1057A>G), AAG131AAA (1063G>A), ACA132ATT (1065C>T 1066A>T), CTG133TTG (1067C>T), TCC134TAC (1071C>A), TTG135TTT (1075G>T), GCC136TCT (1076G>T 1078C>T), GAT137GGA (1080A>G 1081T>A), GCG139GCA (1087G>A), CAG140CAA (1090G>A), GCA141GCC (1093A>C), CTC143CTA (1099C>A), AGC145AGT (1105C>T), CCC146CCT (1108C>T), AGC147AGT (1111C>T), CTC153TTG (1127C>T 1129C>G), AAA154AAG (1132A>G), AGA155AGG (1135A>G), GGG156GGC (1138G>C), ACC162ACA (1156C>A), GAG164GAA (1162G>A), TGC165TGT (1165C>T), CAA166CTC (1167A>T 1168A>C), AAT167AAC (1171T>C), ACC168ACG (1174C>G), AGG169AGA (1177G>A), GCA170GCT (1180A>T), CAG171CAA (1183G>A), CCG173CCT (1189G>T), TCC175TCT (1195C>T), GTG176GTA (1198G>A), GCC178GCA (1204C>A), ATA179ATT (1207A>T), GAC185AGT (1223G>A 1224A>G 1225C>T), ACA186CCT (1226A>C 1228A>T), AGG187CGT (1229A>C 1231G>T), GCA189GCT (1237A>T), GTT190GTA (1240T>A), TCA192ACA (1244T>A), CAG193GAC (1247C>G 1249G>C), CAA194CAT (1252A>T), GAA195GAC (1255A>C), ACA196ACT (1258A>T), TCT198AGT (1262T>A 1263C>G), GCC200GCT (1270C>T), ATA201ATT (1273A>T), GAA202GAG (1276A>G), GCA203GCT (1279A>T), CTC204CTT (1282C>T), AAT205AAC (1285T>C), CGT206AGA (1286C>A 1288T>A), CCG207CCT (1291G>T), CAA208CAG (1294A>G), GAT212GAC (1306T>C), AGA213AGG (1309A>G), GAG215GAA (1315G>A), GCT216GCA (1318T>A), GCT217GCA (1321T>A), TAT219TAC (1327T>C), ATA220ATT (1330A>T), GCT221GCA (1333T>A), GTT223GTG (1339T>G), TAT224TAC (1342T>C), TCG225TCC (1345G>C), AGT226AAT (1347G>A), GGG227_CAA228insGTA (1351_1352insGTA), CAA228CAG (1354A>G), AGT229AGA (1357T>A), ACT230AGT (1359C>G), CAA231CAG (1363A>G), ATG232CTG (1364A>C), GCT233del (1367_1369delGCT), CTT234TTG (1370C>T 1372T>G), GAA235GAG (1375A>G), AAA237AGA (1380A>G), GCC238CCT (1382G>C 1384C>T), GTT240GTG (1390T>G), GGA241GGT (1393A>T), GAA242GCC (1395A>C 1396A>C), CAG243GCA (1397C>G 1398A>C 1399G>A), GGG245GGT (1405G>T), GGC246GAT (1407G>A 1408C>T), ACA247ACC (1411A>C), GAC250AAT (1418G>A 1420C>T), GTC251GTG (1423C>G), ATG252CCG (1424A>C 1425T>C), AAT253CAG (1427A>C 1429T>G), GAA254GAG (1432A>G), AGC255GCA (1433A>G 1434G>C 1435C>A), ACT256AAT (1437C>A), ATC258GTG (1442A>G 1444C>G), CGG259CGC (1447G>C), AGT260TCT (1448A>T 1449G>C), TCT262TCA (1456T>A), AGA264AGG (1462A>G), ACT267ACC (1471T>C), CTC268CTA (1474C>A), AAA269AAG (1477A>G), GTG270GTA (1480G>A), AAG273AGG (1488A>G), CGC274CGA (1492C>A), CCG276GCA (1496C>G 1498G>A), TCC277TCA (1501C>A), CTG280TTG (1508C>T), CCG281CCT (1513G>T), CCG281_GCT282insGACTTT (1513_1514insGACTTT), GTA283TCA (1517G>T 1518T>C), AGT284ACT (1521G>C), ACA285TCT (1523A>T 1525A>T), TCA286AGA (1526T>A 1527C>G), TCC287GCG (1529T>G 1531C>G), TTA288TAT (1533T>A 1534A>T), CTC289CAG (1536T>A 1537C>G), AGC290GGA (1538A>G 1540C>A), ACA291GTG (1541A>G 1542C>T 1543A>G), CGT292AGA (1544C>A 1546T>A), GAA293GAG (1549A>G), ACA295ACT (1555A>T), GTT296GGT (1557T>G), CAT297ATT (1559C>A 1560A>T), GAG299del (1565_1567delGAG), TGT302ACG (1574T>A 1575G>C 1576T>G), GGG303TCT (1577G>T 1578G>C 1579G>T), GGA304CTA (1580G>C 1581G>T), TTG305CTG (1583T>C), CCA307CCT (1591A>T), GCA308GCC (1594A>C), CCC309_AAG310del (1595_1600delCCCAAG), AAA311AAG (1603A>G), AAG312ATA (1605A>T 1606G>A), GGC313TCA (1607G>T 1608G>C 1609C>A), GCC315GCA (1615C>A), TTC316GGA (1616T>G 1617T>G 1618C>A), AAC317ACA (1620A>C 1621C>A), ATG318CTC (1622A>C 1624G>C), GGT319CAA (1625G>C 1626G>A 1627T>A), GCC320GAA (1629C>A 1630C>A), ATT321GTC (1631A>G 1633T>C), GAT323GAC (1639T>C), TTG325TAT (1644T>A 1645G>T), ATG327ATT (1651G>T), GAG328CAA (1652G>C 1654G>A), TCG329AGT (1655T>A 1656C>G 1657G>T), TTC330TTT (1660C>T), GCA331GAA (1662C>A), CAC332GAT (1664C>G 1666C>T), ATT333TTT (1667A>T), GAT334GCC (1671A>C 1672T>C), GAT337AGT (1679G>A 1680A>G), GAT338AAC (1682G>A 1684T>C), GAA341GCC (1692A>C 1693A>C), CGA342CTT (1695G>T 1696A>T), ATA344GTG (1700A>G 1702A>G), GCT345CCC (1703G>C 1705T>C), CAG346GAG (1706C>G), CAG347CAA (1711G>A), GTA348ATT (1712G>A 1714A>T), CAA349GAT (1715C>G 1717A>T), TTT350TAT (1719T>A), ATC351GTT (1721A>G 1723C>T), CTC352CTT (1726C>T), TTT353GTG (1727T>G 1729T>G), AGT354CAG (1730A>C 1731G>A 1732T>G), GAG355AAG (1733G>A), AAA357AGA (1740A>G), AGA358GCT (1742A>G 1743G>C 1744A>T), GGA359GGT (1747A>T), AAC360AAT (1750C>T), GTT361CTC (1751G>C 1753T>C), ATT362TGT (1754A>T 1755T>G), GTG363GTC (1759G>C), AGA365AAA (1764G>A), GTT367ATT (1769G>A), GCA368GAG (1773C>A 1774A>G), AAG369ATG (1776A>T), CGG370AGG (1778C>A), CAT371GAC (1781C>G 1783T>C), CTG372TTG (1784C>T), CAT373CAC (1789T>C), AAT374TGT (1790A>T 1791A>G), ATC375ATA (1795C>A), AGG377TTT (1799A>T 1800G>T 1801G>T), GAG378GAA (1804G>A), CAC379CAT (1807C>T), ATA380ATC (1810A>C), ACC381ACT (1813C>T), GAC383GCC (1818A>C), GTA386GTT (1828A>T), GAA387GAT (1831A>T), ATA388ATT (1834A>T), CTT389TTG (1835C>T 1837T>G), GAA390GCG (1839A>C 1840A>G), GGT391GGA (1843T>A), GGT393GGA (1849T>A), AAT396AAG (1858T>G), ATC397GTT (1859A>G 1861C>T), CAA398GGG (1862C>G 1863A>G 1864A>G), GGC399ATG (1865G>A 1866G>T 1867C>G), ACT401GAA (1871A>G 1872C>A 1873T>A), CGC402AGA (1874C>A 1876C>A), ACT403ACA (1879T>A), TTC404CCA (1880T>C 1881T>C 1882C>A), TAT407TAC (1891T>C), GCT408TCT (1892G>T), ATG409TTG (1895A>T), TCT410AGC (1898T>A 1899C>G 1900T>C), CTC411CTG (1903C>G), GAG413CAG (1907G>C), GTT415GTG (1915T>G), GTG416GTT (1918G>T), CTC418CTA (1924C>A), CAT420CAC (1930T>C), CAA421ATG (1931C>A 1932A>T 1933A>G), AGA422AGG (1936A>G), GAT423GAC (1939T>C), TCC425CAA (1943T>C 1944C>A 1945C>A), TTC426TTT (1948C>T), ATT428AGG (1953T>G 1954T>G), CAA429TAT (1955C>T 1957A>T), GGG430TGG (1958G>T), CAG431GAT (1961C>G 1963G>T), ACT432TTA (1964A>T 1965C>T 1966T>A), AAC433ACA (1968A>C 1969C>A), AAT434AAC (1972T>C), CCA435CCG (1975A>G), CCG437CCT (1981G>T), AAT438GAC (1982A>G 1984T>C), TGC441GTG (1991T>G 1992G>T 1993C>G), GGA444GGT (2002A>T), TTA445CTT (2003T>C 2005A>T), ACC446ACA (2008C>A), TTG447CTG (2009T>C), AAA448GCA (2012A>G 2013A>C), GAG449GAT (2017G>T), CGT450AGG (2018C>A 2020T>G), CGT450_CAG451insGTGAAA (2020_2021insGTGAAA), CAG451CAT (2023G>T), TTG452CCC (2024T>C 2025T>C 2026G>C), ATT453TTG (2027A>T 2029T>G), GTG454ATG (2030G>A), GAG455AGT (2033G>A 2034A>G 2035G>T), GAG456GAT (2038G>T), CAA457CTA (2040A>T), GTG458GCA (2043T>C 2044G>A), GCC459TGC (2045G>T 2046C>G), CAA460_AGA461insTTGAAT (2050_2051insTTGAAT), AGA461CGC (2051A>C 2053A>C), AGG462TTG (2054A>T 2055G>T), TCT463GGA (2057T>G 2058C>G 2059T>A), GAA464AAA (2060G>A), AGA465CCA (2063A>C 2064G>C), CAA466AGC (2066C>A 2067A>G 2068A>C), GCA467ATG (2069G>A 2070C>T 2071A>G), CAG468AGT (2072C>A 2073A>G 2074G>T), ACA470GCA (2078A>G), GCA471CAA (2081G>C 2082C>A), AGG472ACT (2085G>C 2086G>T), GGC473GGA (2089C>A), ATA474AAT (2091T>A 2092A>T), GCT475GCC (2095T>C), GCT475_GAA476insAGC (2095_2096insAGC), GAA476AAG (2096G>A 2098A>G), AGT477TGG (2099A>T 2101T>G), CAG478CAA (2104G>A), CCT479ACT (2105C>A), GAC481GGA (2112A>G 2113C>A), AGA482AAG (2115G>A 2116A>G), ACT484CTG (2120A>C 2121C>T 2122T>G), TCC486TCA (2128C>A), GTG488GTT (2134G>T), TCA489ACA (2135T>A), ACT490ACC (2140T>C), ACC491ACA (2143C>A), ACG492GCA (2144A>G 2146G>A), ATG493CTT (2147A>C 2149G>T), GAA494GAG (2152A>G), GAA494_GAT495insAGT (2152_2153insAGT), GAT495GAG (2155T>G), CCC496GGT (2156C>G 2157C>G 2158C>T), ACC497ATC (2160C>T), AAG498CAA (2162A>C 2164G>A), CCA499AGA (2165C>A 2166C>G), GAT500AAG (2168G>A 2170T>G), AAG501GAG (2171A>G), ATA502GTG (2174A>G 2176A>G), GAA503TCA (2177G>T 2178A>C), GTT505GAG (2184T>A 2185T>G), GCA506GCT (2188A>T), GAG507GAA (2191G>A), GGA508GAT (2193G>A 2194A>T), GCA509CCG (2195G>C 2197A>G), GAA511GGG (2202A>G 2203A>G), GAA512AAA (2204G>A), ACT513ACA (2209T>A), CAA514GAG (2210C>G 2212A>G), GGT516AAT (2216G>A 2217G>A), GAT517GAC (2221T>C), GTT518ATT (2222G>A), ATT519TTC (2225A>T 2227T>C), GAC521GAT (2233C>T), GGA523GGG (2239A>G), CCA524GAG (2240C>G 2241C>A 2242A>G), GAC527ATG (2249G>A 2250A>T 2251C>G), ACA528GGT (2252A>G 2253C>G 2254A>T), TCC529GAT (2255T>G 2256C>A 2257C>T), ATG530GCC (2258A>G 2259T>C 2260G>C), GCA531CCG (2261G>C 2263A>G), GTT532TTA (2264G>T 2266T>A), GAA533AAT (2267G>A 2269A>T), GAT535GAG (2275T>G), ATG536TTG (2276A>T), CAG537CAA (2281G>A), CAA538CAG (2284A>G), CCG539CCA (2287G>A), GTG540GCA (2289T>C 2290G>A), GTA542GTG (2296A>G), AGC544AGT (2302C>T), GAT546GAG (2308T>G), TTC547TTT (2311C>T), GGA551GGG (2323A>G), GTT552ATT (2324G>A), TTC555TTT (2335C>T), GTT556CTG (2336G>C 2338T>G), GAG558AAA (2342G>A 2344G>A), TCC560AGC (2348T>A 2349C>G), GCT561AAC (2351G>A 2352C>A 2353T>C), GAG565GAA (2365G>A), CAA566CAG (2368A>G), GTA567GTT (2371A>T), ATG568CTT (2372A>C 2374G>T), AGC569GCA (2375A>G 2376G>C 2377C>A), TTG570CTA (2378T>C 2380G>A), GCT571TCC (2381G>T 2383T>C), TTG572CTC (2384T>C 2386G>C), CCC573CCA (2389C>A), GCT574ATG (2390G>A 2391C>T 2392T>G), GCC575GCA (2395C>A), CTA576TTG (2396C>T 2398A>G), TTC577TTT (2401C>T), TCA578AGC (2402T>A 2403C>G 2404A>C), AAA579AAG (2407A>G), AGC580AAG (2409G>A 2410C>G), AAA581AAG (2413A>G), ACT583GCT (2417A>G), TCA584TCC (2422A>C), GGT586GGG (2428T>G), GCG587TCC (2429G>T 2431G>C), CTT590CTC (2440T>C), TAT592TAC (2446T>C), TAC593TAT (2449C>T), GAT594GAC (2452T>C), GCA595GCT (2455A>T), GCT596GCA (2458T>A), CTA597AAC (2459C>A 2460T>A 2461A>C), ATC598CTT (2462A>C 2464C>T), TAC600TTT (2469A>T 2470C>T), ATA602GTC (2474A>G 2476A>C), CTT604CTG (2482T>G), TAT605TAC (2485T>C), GTT606ATT (2486G>A), GTT609ATG (2495G>A 2497T>G), GCT611GCG (2503T>G), ATC612GTG (2504A>G 2506C>G), TCT613TCG (2509T>G), GGT614GGA (2512T>A), CAA615CAG (2515A>G), CTG616TTA (2516C>T 2518G>A), GCT617GCC (2521T>C), GAA622GAG (2536A>G), TGT623TGC (2539T>C), GTG625GCA (2544T>C 2545G>A), CGA628AGA (2552C>A), AAG629ACC (2556A>C 2557G>C), AAC634AAT (2572C>T), ATC635ATA (2575C>A), ACA637ACC (2581A>C), TTG638ATT (2582T>A 2584G>T), AAG642AAA (2596G>A), ACA644CAT (2600A>C 2601C>A 2602A>T), TTG645CTG (2603T>C), GTT646GTG (2608T>G), CAA650GAA (2618C>G), AAC652TCC (2624A>T 2625A>C), GAG654GGG (2631A>G), GAG655AAG (2633G>A), TGC657GTG (2639T>G 2640G>T 2641C>G), TTT658TTC (2644T>C), ACC659TCA (2645A>T 2647C>A), CCA660CCT (2650A>T), ACA661CAA (2651A>C 2652C>A), GGG662GGT (2656G>T), GGC664GGT (2662C>T), AAG665ACC (2664A>C 2665G>C), TAC666TTT (2667A>T 2668C>T), GTG667GTA (2671G>A), CCT668CCA (2674T>A), CTG669TTG (2675C>T), GAA671ACT (2681G>A 2682A>C 2683A>T), GGT672TCA (2684G>T 2685G>C 2686T>A), ACA673CTG (2687A>C 2688C>T 2689A>G), GGA674GGT (2692A>T), GCC675GTT (2694C>T 2695C>T), ACT676CCT (2696A>C), GAT677GAA (2701T>A), TTA678CTT (2702T>C 2704A>T), GGT679GGC (2707T>C), GTG681GTT (2713G>T), ACA686ACC (2728A>C), CAC687CAT (2731C>T), TTA689TTG (2737A>G), TCT690GCA (2738T>G 2740T>A), GCA692GCC (2746A>C), GAG694GAA (2752G>A), AAT696GAG (2756A>G 2758T>G), AGT697TCA (2759A>T 2760G>C 2761T>A), GTA698ATA (2762G>A), CCA699CCT (2767A>T), TGC700TGT (2770C>T), CAT701CAC (2773T>C), TTG702ATA (2774T>A 2776G>A), TTA704CTA (2780T>C), CAG705TCC (2783C>T 2784A>C 2785G>C), AAA707AAG (2791A>G), TTA709CTC (2795T>C 2797A>C), ACC711ACA (2803C>A), CTT714ATG (2810C>A 2812T>G), CGA718AGG (2822C>A 2824A>G), ATG719TTG (2825A>T), ATA720AGA (2829T>G), GCA721GGG (2832C>G 2833A>G), CAG722CAA (2836G>A), CAA724CAG (2842A>G), TAT725TTT (2844A>T), GGC726GGA (2848C>A), AAG728CGG (2852A>C 2853A>G), GCG729CCA (2855G>C 2857G>A), GGG730GGC (2860G>C), CAG731ACA (2861C>A 2862A>C 2863G>A), ACA732ACC (2866A>C), TAT733TTC (2868A>T 2869T>C), CCA735AGT (2873C>A 2874C>G 2875A>T), AGG736CGC (2876A>C 2878G>C), TTT737TTC (2881T>C), CCA738CCT (2884A>T), ACT739ACA (2887T>A), GTT742GTG (2896T>G), TTG743CTT (2897T>C 2899G>T), TTA744CTG (2900T>C 2902A>G), CAT745CAC (2905T>C), TAT746TAC (2908T>C), GGG749AGT (2915G>A 2917G>T), ACA750GTA (2918A>G 2919C>T), TCA751GCT (2921T>G 2923A>T), TCC752GCA (2924T>G 2926C>A), CCA753GCC (2927C>G 2929A>C), ACA757ACT (2941A>T), AGC760AGT (2950C>T), ATA761GTC (2951A>G 2953A>C), TTT762TTC (2956T>C), TCA763TCC (2959A>C), ATA767ATT (2971A>T), GAG769GAA (2977G>A), GGC772GGT (2986C>T), ACC773ATC (2988C>T), TTG774TTA (2992G>A), CCG776CCA (2998G>A), TCT777TCA (3001T>A), TTG778CTG (3002T>C), GGT780GGA (3010T>A), ATA782ATT (3016A>T), GCC783GCA (3019C>A), AGG784AGA (3022G>A), AAG787AAA (3031G>A), ACT790AGA (3039C>G 3040T>A), GGA791GGC (3043A>C), ACT792ACC (3046T>C), ATT797GTG (3059A>G 3061T>G), TGC798TGT (3064C>T), ATT799GTT (3065A>G), ACC802ACT (3076C>T), TTG803CTG (3077T>C), TCA806TCT (3088A>T), GGT807GGG (3091T>G), CTG809CTT (3097G>T), GCA810GCT (3100A>T), ATT811ATA (3103T>A), GGA812GGG (3106A>G), CTT813TTG (3107C>T 3109T>G), GGA814GGC (3112A>C), ACT815ACG (3115T>G), AAC817GCA (3119A>G 3120A>C 3121C>A), ACC818ACA (3124C>A), AAA819GAG (3125A>G 3127A>G), ACC821TCC (3131A>T), AAT822ACA (3135A>C 3136T>A), GCT823GCG (3139T>G), TTT827TTG (3151T>G), CCA830CCC (3160A>C), GTA833GTG (3169A>G), TGC834TGT (3172C>T), CTT836TTG (3176C>T 3178T>G), GGT839GGG (3187T>G), AAA841AGG (3192A>G 3193A>G), CGG843CAA (3198G>A 3199G>A), CGG845AAA (3203C>A 3204G>A 3205G>A), TGT846TGC (3208T>C), TCT847TCA (3211T>A), ATT848CTG (3212A>C 3214T>G), ACA849ACC (3217A>C), GGA853GGG (3229A>G), AAA854TGC (3230A>T 3231A>G 3232A>C), AAT855AAC (3235T>C), TTG856CTC (3236T>C 3238G>C), CTT857CTG (3241T>G), TCC858TCT (3244C>T), ACA859ACC (3247A>C), GGG860GGA (3250G>A), CGA861CGC (3253A>C), AAG862AAA (3256G>A), TCC864TCA (3262C>A), TTG865TTA (3265G>A), CAG869AAG (3275C>A), CAC870CAT (3280C>T), TTT871CAA (3281T>C 3282T>A 3283T>A), TCC872TCT (3286C>T), TTG874ATG (3290T>A), CGC875CGT (3295C>T), TTG876CTG (3296T>C), TTT877TTC (3301T>C), ACG879ACT (3307G>T), GTA880GTT (3310A>T), CTC884CTG (3322C>G), ACG887ACA (3331G>A), ATA889TTG (3335A>T 3337A>G), CAT890CAC (3340T>C), TCC893ACT (3347T>A 3349C>T), GGT895GGA (3355T>A), GTC896GTA (3358C>A), CAG899CAA (3367G>A), AAG901AAA (3373G>A), TGC902CGC (3374T>C), CTT903ATT (3377C>A), AAT905GAT (3383A>G), CTT906CTC (3388T>C), ACT907ACG (3391T>G), TTG908CTT (3392T>C 3394G>T), GGT909GGG (3397T>G), GGC910GGT (3400C>T), ACA911TCT (3401A>T 3403A>T), GTA912GTG (3406A>G), TCT913TCA (3409T>A), GTG914GTT (3412G>T), AAA915AAG (3415A>G), ATA917ATT (3421A>T), GGG919GGT (3427G>T), ACA922ACC (3436A>C), AAA923AAG (3439A>G), GGC924GGG (3442C>G), AAA925AGA (3444A>G), AGC926AGT (3448C>T), TCA927GCT (3449T>G 3451A>T), GTT928GTG (3454T>G),

	TTC930TTT (3460C>T), CTT931CTG (3463T>G), TTC932TTT (3466C>T), GAT936AAC (3476G>A 3478T>C), TTA937CTG (3479T>C 3481A>G), TCA938AAT (3482T>A 3483C>A 3484A>T), CGT940CGC (3490T>C), ATT943CTT (3497A>C), GAA944GAG (3502A>G), AAG945AAA (3505G>A), TTG946CTG (3506T>C), AAG948AAA (3514G>A), GAC949GAA (3517C>A), AAC950AAT (3520C>T), ATT951GTT (3521A>G), GAG952GAT (3526G>T), GAG953ATC (3527G>A 3528A>T 3529G>C), TAC954TTT (3531A>T 3532C>T), CAG955GAC (3533C>G 3535G>C), GAG956CAG (3536G>C), GGC958GGG (3544C>G), GAT960AAC (3548G>A 3550T>C), CCG961_CCC962del (3551_3556delCCGCCC), AAG964ACA (3561A>C 3562G>A), GCT965TCT (3563G>T), AGT967GTT (3569A>G 3570G>T), ATT968TCC (3572A>T 3573T>C 3574T>C), CTG969TTC (3575C>T 3577G>C), TCC970CAG (3578T>C 3579C>A 3580C>G), ATA971ATC (3583A>C), AGA972AGG (3586A>G), GAG973GAA (3589G>A), AAA974AAG (3592A>G), TTT975TTC (3595T>C), TCC976ACA (3596T>A 3598C>A), GGT978GGA (3604T>A), GCT979GCA (3607T>A), GTA980GTG (3610A>G), TTC983TTT (3619C>T), TGC984TGT (3622C>T), ATG985GCT (3623A>G 3624T>C 3625G>T), GGT986GCA (3627G>C 3628T>A), GAC990ACA (3638G>A 3639A>C 3640C>A), GAA991GAC (3643A>C), TTG994GTA (3650T>G 3652G>A), GTA995GTT (3655A>T), ATT996TTG (3656A>T 3658T>G), CCT997CCA (3661T>A), GCA999GCC (3667A>C), CCA1000CCT (3670A>T), TCC1002TCA (3676C>A), ATA1003GTG (3677A>G 3679A>G), AGG1004CGC (3680A>C 3682G>C), GAA1006GAT (3688A>T), GGG1007GCA (3690G>C 3691G>A), CAC1008TCC (3692C>T 3693A>C), CCT1010CCA (3700T>A), GTT1011ATT (3701G>A), AAG1012AAA (3706G>A), ATC1015ATA (3715C>A), TGT1017TGC (3721T>C), TTT1019TTC (3727T>C), GAT1021GAC (3733T>C), ACA1024TCC (3740A>T 3742A>C), TCA1025TCT (3745A>T), TCA1030TCT (3760A>T), GGT1031GGA (3763T>A), AAT1034GAA (3770A>G 3772T>A), TCA1036ACA (3776T>A), ATT1037TTT (3779A>T), GTG1038GTA (3784G>A), ATA1039ATG (3787A>G), AGA1041CGA (3791A>C), GTA1042ACT (3794G>A 3795T>C 3796A>T), CAA1043CAG (3799A>G), TCC1044AGC (3800T>A 3801C>G), AGT1045TCC (3803A>T 3804G>C 3805T>C), CCT1046AAC (3806C>A 3807C>A 3808T>C), AAT1047AGT (3810A>G), GTT1048ATA (3812G>A 3814T>A), GGA1049GGT (3817A>T), GTA1051ATC (3821G>A 3823A>C), CTA1052TTG (3824C>T 3826A>G), AAT1053AAC (3829T>C), GTT1054ATT (3830G>A), GCT1055GCA (3835T>A), TTT1056CTT (3836T>C), GAT1057GAC (3841T>C), GCC1058ACA (3842G>A 3844C>A), GGC1060GGG (3850C>G), TTT1063TTC (3859T>C), CCA1064CAA (3861C>A), GCT1065TTT (3863G>T 3864C>T), GGG1066GGT (3868G>T), CTT1067GTA (3869C>G 3871T>A), AAT1068TCT (3872A>T 3873A>C), AAA1069GCG (3875A>G 3876A>C 3877A>G), AAT1071GCA (3881A>G 3882A>C 3883T>A), TAT1072CAT (3884T>C), GTA1074TTA (3890G>T), GGT1077GGC (3901T>C), GGA1078GGT (3904A>T), GGC1079GGA (3907C>A), ACA1080ACT (3910A>T), TCA1085TCC (3925A>C), TAC1086TAT (3928C>T), GGT1087GGG (3931T>G), GTG1088GTT (3934G>T), GCA1089ACC (3935G>A 3937A>C), ACA1090ACC (3940A>C), AAT1091AAC (3943T>C), ACG1092ATC (3945C>T 3946G>C), TCA1094TCT (3952A>T), TTC1095TTT (3955C>T), ACT1096GTA (3956A>G 3957C>T 3958T>A), GAG1101CAG (3971G>C), TTT1102TTC (3976T>C), TTC1103TTT (3979C>T), CCA1104CCT (3982A>T), AGG1105CGC (3983A>C 3985G>C), CGG1106CGT (3988G>T), CAT1107TTC (3989C>T 3990A>T 3991T>C), AGG1109AAG (3996G>A), ATG1110GCC (3998A>G 3999T>C 4000G>C), AGG1111AAA (4002G>A 4003G>A), GAA1112TCC (4004G>T 4005A>C 4006A>C), TTC1113TAT (4008T>A 4009C>T), TCA1114GAT (4010T>G 4011C>A 4012A>T), AGC1115AAG (4014G>A 4015C>G), AAG1116GAA (4016A>G 4018G>A), CAA1117AGT (4019C>A 4020A>G 4021A>T), TCC1118TCA (4024C>A), CGC1119AGA (4025C>A 4027C>A), ATC1120ATT (4030C>T), ATG1121GTT (4031A>G 4033G>T), TCA1122TCT (4036A>T), CTA1123TTG (4037C>T 4039A>G), GAT1125GAC (4045T>C), AGG1126AAG (4047G>A), CTT1127CTA (4051T>A), GGA1128GGC (4054A>C), AAT1129TTG (4055A>T 4056A>T 4057T>G), CTG1130TTG (4058C>T), ATC1131CTA (4061A>C 4063C>A), ATA1132ATC (4066A>C), TTG1134CTT (4070T>C 4072G>T), CCT1135CCA (4075T>A), CCT1136CCA (4078T>A), TCC1137GTT (4079T>G 4080C>T 4081C>T), GCC1138GAA (4083C>A 4084C>A), ATA1139CTT (4085A>C 4087A>T), GTG1140ATT (4088G>A 4090G>T), TCC1142TCA (4096C>A), GAG1144GAA (4102G>A), ATA1145ATC (4105A>C), CTT1146ATA (4106C>A 4108T>A), ATA1147GTC (4109A>G 4111A>C), TCT1148AAG (4112T>A 4113C>A 4114T>G), CCT1149CCA (4117T>A), GGA1150GGT (4120A>T), CTT1151CCT (4122T>C), TTC1153TTT (4129C>T), AAG1154AAA (4132G>A), GCC1158GCT (4144C>T), CCT1160CCC (4150T>C), CCT1161CCA (4153T>A), TCT1162CTT (4154T>C 4155C>T), GCC1163GCA (4159C>A), CAT1165AAT (4163C>A), GAA1166GAG (4168A>G), AAA1167AAG (4171A>G), CTT1169CTC (4177T>C), GGC1170GGA (4180C>A), AAT1171GAT (4181A>G), CAA1173CAG (4189A>G), ACT1174ACG (4192T>G), CAC1175CAT (4195C>T), ACC1176ACT (4198C>T), ACC1180ACA (4210C>A), TCA1181TCT (4213A>T), CTA1186CTG (4228A>G), ATT1191AAT (4242T>A)

Proteins

hypothetical protein (YP_001976148.1)	1	191	100%	784	66.7%	183 (95.8%)	116 (60.7%)	0/8/0/0	1
Protein mutations:	G5S (151G>A 153G>T), R6K (154C>A 155G>A 156T>G), S10Q (166T>C 167C>A 168T>A), V11I (169G>A), D12E (174C>G), E13D (177A>T), A15E (182C>A 183T>A), H18N (190C>A), T22A (202A>G 204C>A), N24T (209A>C 210T>C), I26V (214A>G), V31T (229G>A 230T>C), T33V (235A>G 236C>T 237C>T), L35I (241T>A 243G>T), V46I (274G>A), V47C (277G>T 278T>G), V54I (298G>A 300C>T), T58S (310A>T 312C>T), L68V (340T>G 342A>T), N79T (374A>C 375T>G), P81D (379C>G 380C>A 381C>T), N84A (388A>G 389A>C 390C>A), L91I (409C>A), H93R (416A>G 417T>A), V96I (424G>A 426G>T), R97K (427C>A 428G>A 429T>A), V104L (448G>T 450C>G), E107A (458A>C 459A>C), S111H (469T>C 470C>A), A114S (478G>T 480A>T), S115T (481T>A 483A>C), P120N (496C>A 497C>A 498T>C), S121P (499T>C 501T>C), D124_S129del (508_525delGACAGTAAAGGCAAGAGT), K130T (527A>C), V131L (529G>T), E132S (532G>T 533A>C 534G>A), Q133E (535C>G), P134T (538C>A 540C>T), T135A (541A>G), R136_E137del (544_549delCGAGAG), D138S (550G>A 551A>G), I140S (557T>G 558T>C), K141G (559A>G 560A>G), V143N (565G>A 566T>A 567T>C), E144K (568G>A), V145A (572T>C 573T>A), R148E (580C>G 581G>A 582T>A), E149A (584A>C 585G>C), R152V (592A>G 593G>T 594A>T), F153A (595T>G 596T>C 597T>C), Q154R (598C>A 599A>G 600G>A), A158Q (610G>C 611C>A), S159E (613A>G 614G>A 615T>A), Q160A (616C>G 617A>C), K161R (619A>C 620A>G 621A>G), N164I (629A>T), Q165V (631C>G 632A>T 633G>C), Q168R (640C>A 641A>G 642A>G), L171I (649C>A 651C>T), S172Q (652T>C 653C>A 654C>A), N173G (655A>G 656A>G), V175P (661G>C 662T>C 663T>A), N178E (670A>G 672T>G), I180V (676A>G), S182A (682T>G), E186S (694G>A 695A>G 696A>T), S187D (697A>G 698G>A), Q190K (706C>A)								
Codon mutations:	TCA2TCT (144A>T), TTC3TTT (147C>T), ATT4ATC (150T>C), GGG5AGT (151G>A 153G>T), CGT6AAG (154C>A 155G>A 156T>G), TTG7CTC (157T>C 159G>C), AAT8AAC (162T>C), ACA9ACC (165A>C), TCT10CAA (166T>C 167C>A 168T>A), GTA11ATA (169G>A), GAC12GAG (174C>G), GAA13GAT (177A>T), GCT15GAA (182C>A 183T>A), TTT16TTC (186T>C), CAT18AAT (190C>A), GTT20GTA (198T>A), GCC21GCA (201C>A), ACC22GCA (202A>G 204C>A), TCG23TCT (207G>T), AAT24ACC (209A>C 210T>C), ATT26GTT (214A>G), TCT28AGT (220T>A 221C>G), GTT31ACT (229G>A 230T>C), GGC32GGT (234C>T), ACC33GTT (235A>G 236C>T 237C>T), TTG35ATT (241T>A 243G>T), ATA36ATT (246A>T), ACC41ACT (261C>T), CTT42TTA (262C>T 264T>A), GAC43GAT (267C>T), AAA45AAG (273A>G), GTT46ATT (274G>A), GTT47TGT (277G>T 278T>G), CCT48CCC (282T>C), GGT51GGA (291T>A), GTC54ATT (298G>A 300C>T), TCA55TCG (303A>G), GTT56GTG (306T>G), TTA57CTT (307T>C 309A>T), ACC58TCT (310A>T 312C>T), GTT59GTG (315T>G), TCT60AGT (316T>A 317C>G), GAG62GAA (324G>A), ACG65ACT (333G>T), CCA66CCC (336A>C), TTA68GTT (340T>G 342A>T), CCT70CCA (348T>A), GGA71GGC (351A>C), CAC72CAT (354C>T), TAT73TAC (357T>C), TTG74CTA (358T>C 360G>A), TTG75CTA (361T>C 363G>A), AAT79ACG (374A>C 375T>G), CCC81GAT (379C>G 380C>A 381C>T), ATT82ATC (384T>C), AAG83AAA (387G>A), AAC84GCA (388A>G 389A>C 390C>A), TCT88TCA (402T>A), GGT89GGC (405T>C), TTA90TTG (408A>G), CTT91ATT (409C>A), GTC92GTG (414C>G), CAT93CGA (416A>G 417T>A), TCA95TCC (423A>C), GTG96ATT (424G>A 426G>T), CGT97AAA (427C>A 428G>A 429T>A), CTT98TTG (430C>T 432T>G), ACC101ACA (441C>A), AGA102CGG (442A>C 444A>G), GTC104TTG (448G>T 450C>G), CTA105TTG (451C>T 453A>G), GAG106GAA (456G>A), GAA107GCC (458A>C 459A>C), AAC108AAT (462C>T), AAG109AAA (465G>A), GTT110GTG (468T>G), TCT111CAT (469T>C 470C>A), TCA113TCT (477A>T), GCA114TCT (478G>T 480A>T), TCA115ACC (481T>A 483A>C), TCA117TCT (489A>T), TCT118TCA (492T>A), TCT119TCC (495T>C), CCT120AAC (496C>A 497C>A 498T>C), TCT121CCC (499T>C 501T>C), GAC124_AGT129del (508_525delGACAGTAAAGGCAAGAGT), AAA130ACA (527A>C), GTA131TTA (529G>T), GAG132TCA (532G>T 533A>C 534G>A), CAA133GAA (535C>G), CCC134ACT (538C>A 540C>T), ACA135GCA (541A>G), CGA136_GAG137del (544_549delCGAGAG), GAT138AGT (550G>A 551A>G), CTC139TTA (553C>T 555C>A), ATT140AGC (557T>G 558T>C), AAA141GGA (559A>G 560A>G), GAA142GAG (564A>G), GTT143AAC (565G>A 566T>A 567T>C), GAG144AAG (568G>A), GTT145GCA (572T>C 573T>A), CTC146CTT (576C>T), AAA147AAG (579A>G), CGT148GAA (580C>G 581G>A 582T>A), GAG149GCC (584A>C 585G>C), TTA150CTT (586T>C 588A>T), GAG151GAA (591G>A), AGA152GTT (592A>G 593G>T 594A>T), TTT153GCC (595T>G 596T>C 597T>C), CAG154AGA (598C>A 599A>G 600G>A), GAG156GAA (606G>A), TTG157CTG (607T>C), GCA158CAA (610G>C 611C>A), AGT159GAA (613A>G 614G>A 615T>A), CAA160GCA (616C>G 617A>C), AAA161CGG (619A>C 620A>G 621A>G), AAT164ATT (629A>T), CAG165GTC (631C>G 632A>T 633G>C), CTA167CTC (639A>C), CAA168AGG (640C>A 641A>G 642A>G), CTT169TTG (643C>T 645T>G), CTC171ATT (649C>A 651C>T), TCC172CAA (652T>C 653C>A 654C>A), AAT173GGT (655A>G 656A>G), GTT175CCA (661G>C 662T>C 663T>A), AGT176AGC (666T>C), AAT177AAC (669T>C), AAT178GAG (670A>G 672T>G), GAC179GAT (675C>T), ATC180GTC (676A>G), TTC181TTT (681C>T), TCA182GCA (682T>G), GGT183GGC (687T>C), ACT185ACG (693T>G), GAA186AGT (694G>A 695A>G 696A>T), AGT187GAT (697A>G 698G>A), GGG188GGC (702G>C), CAG190AAG (706C>A), TAA191TGA (710A>G)								
polyprotein (YP_001976149.1)	1	1192	100%	6009	73.1%	1185 (98.7%)	820 (68.3%)	9/7/0/0	1

	Begin	End	Coverage	Score	Concordance	Matches	Identities	I/D/M/F*	Stop Codons
**NT**	**89**	**4733**	**94.7%**	**2570**	**28.4%**	**4597 (96.7%)**	**3027 (63.7%)**	**107/48**	
Protein mutations:	M1R (672T>G), T2M (675C>T 676A>G), S4L (681C>T 682T>G), V6A (687T>C), L8R (693T>G 694G>A), K9V (695A>G 696A>T 697A>G), V10M (698G>A), G11A (702G>C), N14D (710A>G 712T>C), H15R (714A>G), Q16S (716C>T 717A>C 718G>A), Q17del (719_721delCAG), E18K (722G>A 724G>A), A20V (729C>T 730C>A), E22D (736G>C), R23K (738G>A 739G>A), A34E (771C>A 772C>A), R41K (792G>A), V50I (818G>A 820T>A), G53A (828G>C 829T>A), E61D (853G>T), F63Y (858T>A), I71V (881A>G 883C>G), T72A (884A>G), T77A (899A>G 901A>T), H80R (908C>A 909A>G 910T>G), K81T (912A>C 913G>C), F82I (914T>A 916T>A), V83T (917G>A 918T>C 919T>A), K86R (926A>C 927A>G 928G>T), A89G (936C>G 937A>G), Y96H (956T>C 958T>C), V106A (987T>C 988T>A), S108N (993G>A), R112A (1004A>G 1005G>C 1006G>C), L124T (1040C>A 1041T>C 1042C>T), S125D (1043T>G 1044C>A 1045A>T), T132I (1065C>T 1066A>T), S134Y (1071C>A), L135F (1075G>T), A136S (1076G>T 1078C>T), D137G (1080A>G 1081T>A), Q166L (1167A>T 1168A>C), D185S (1223G>A 1224A>G 1225C>T), T186P (1226A>C 1228A>T), S192T (1244T>A), Q193D (1247C>G 1249G>C), Q194H (1252A>T), E195D (1255A>C), S226N (1347G>A), G227_Q228insV (1351_1352insGTA), S229R (1357T>A), T230S (1359C>G), M232L (1364A>C), A233del (1367_1369delGCT), K237R (1380A>G), A238P (1382G>C 1384C>T), E242A (1395A>C 1396A>C), Q243A (1397C>G 1398A>C 1399G>A), G246D (1407G>A 1408C>T), D250N (1418G>A 1420C>T), M252P (1424A>C 1425T>C), N253Q (1427A>C 1429T>G), S255A (1433A>G 1434G>C 1435C>A), T256N (1437C>A), I258V (1442A>G 1444C>G), K273R (1488A>G), P276A (1496C>G 1498G>A), P281_A282insDF (1513_1514insGACTTT), V283S (1517G>T 1518T>C), S284T (1521G>C), T285S (1523A>T 1525A>T), S286R (1526T>A 1527C>G), S287A (1529T>G 1531C>G), L288Y (1533T>A 1534A>T), L289Q (1536T>A 1537C>G), S290G (1538A>G 1540C>A), T291V (1541A>G 1542C>T 1543A>G), V296G (1557T>G), H297I (1559C>A 1560A>T), E299del (1565_1567delGAG), C302T (1574T>A 1575G>C 1576T>G), G303S (1577G>T 1578G>C 1579G>T), G304L (1580G>C 1581G>T), P309_K310del (1595_1600delCCCAAG), K312I (1605A>T 1606G>A), G313S (1607G>T 1608G>C 1609C>A), F316G (1616T>G 1617T>G 1618C>A), N317T (1620A>C 1621C>A), M318L (1622A>C 1624G>C), G319Q (1625G>C 1626G>A 1627T>A), A320E (1629C>A 1630C>A), I321V (1631A>G 1633T>C), L325Y (1644T>A 1645G>T), M327I (1651G>T), E328Q (1652G>C 1654G>A), A331E (1662C>A), H332D (1664C>G 1666C>T), I333F (1667A>T), D334A (1671A>C 1672T>C), D337S (1679G>A 1680A>G), D338N (1682G>A 1684T>C), E341A (1692A>C 1693A>C), R342L (1695G>T 1696A>T), I344V (1700A>G 1702A>G), A345P (1703G>C 1705T>C), Q346E (1706C>G), V348I (1712G>A 1714A>T), Q349D (1715C>G 1717A>T), F350Y (1719T>A), I351V (1721A>G 1723C>T), F353V (1727T>G 1729T>G), S354Q (1730A>C 1731G>A 1732T>G), E355K (1733G>A), K357R (1740A>G), R358A (1742A>G 1743G>C 1744A>T), V361L (1751G>C 1753T>C), I362C (1754A>T 1755T>G), R365K (1764G>A), V367I (1769G>A), A368E (1773C>A 1774A>G), K369M (1776A>T), H371D (1781C>G 1783T>C), N374C (1790A>T 1791A>G), R377F (1799A>T 1800G>T 1801G>T), D383A (1818A>C), E387D (1831A>T), E390A (1839A>C 1840A>G), N396K (1858T>G), I397V (1859A>G 1861C>T), Q398G (1862C>G 1863A>G 1864A>G), G399M (1865G>A 1866G>T 1867C>G), T401E (1871A>G 1872C>A 1873T>A), F404P (1880T>C 1881T>C 1882C>A), A408S (1892G>T), M409L (1895A>T), E413Q (1907G>C), Q421M (1931C>A 1932A>T 1933A>G), S425Q (1943T>C 1944C>A 1945C>A), I428R (1953T>G 1954T>G), Q429Y (1955C>T 1957A>T), G430W (1958G>T), Q431D (1961C>G 1963G>T), T432L (1964A>T 1965C>T 1966T>A), N433T (1968A>C 1969C>A), N438D (1982A>G 1984T>C), C441V (1991T>G 1992G>T 1993C>G), K448A (2012A>G 2013A>C), E449D (2017G>T), R450_Q451insVK (2020_2021insGTGAAA), Q451H (2023G>T), L452P (2024T>C 2025T>C 2026G>C), I453L (2027A>T 2029T>G), V454M (2030G>A), E455S (2033G>A 2034A>G 2035G>T), E456D (2038G>T), Q457L (2040A>T), V458A (2043T>C 2044G>A), A459C (2045G>T 2046C>G), Q460_R461insLN (2050_2051insTTGAAT), R462L (2054A>T 2055G>T), S463G (2057T>G 2058C>G 2059T>A), E464K (2060G>A), R465P (2063A>C 2064G>C), Q466S (2066C>A 2067A>G 2068A>C), A467M (2069G>A 2070C>T 2071A>G), Q468S (2072C>A 2073A>G 2074G>T), T470A (2078A>G), A471Q (2081G>C 2082C>A), R472T (2085G>C 2086G>T), I474N (2091T>A 2092A>T), A475_E476insS (2095_2096insAGC), E476K (2096G>A 2098A>G), S477W (2099A>T 2101T>G), P479T (2105C>A), D481G (2112A>G 2113C>A), R482K (2115G>A 2116A>G), T484L (2120A>C 2121C>T 2122T>G), S489T (2135T>A), T492A (2144A>G 2146G>A), M493L (2147A>C 2149G>T), E494_D495insS (2152_2153insAGT), D495E (2155T>G), P496G (2156C>G 2157C>G 2158C>T), T497I (2160C>T), K498Q (2162A>C 2164G>A), P499R (2165C>A 2166C>G), D500K (2168G>A 2170T>G), K501E (2171A>G), I502V (2174A>G 2176A>G), E503S (2177G>T 2178A>C), V505E (2184T>A 2185T>G), G508D (2193G>A 2194A>T), A509P (2195G>C 2197A>G), E511G (2202A>G 2203A>G), E512K (2204G>A), Q514E (2210C>G 2212A>G), G516N (2216G>A 2217G>A), V518I (2222G>A), I519F (2225A>T 2227T>C), P524E (2240C>G 2241C>A 2242A>G), D527M (2249G>A 2250A>T 2251C>G), T528G (2252A>G 2253C>G 2254A>T), S529D (2255T>G 2256C>A 2257C>T), M530A (2258A>G 2259T>C 2260G>C), A531P (2261G>C 2263A>G), V532L (2264G>T 2266T>A), E533N (2267G>A 2269A>T), D535E (2275T>G), M536L (2276A>T), V540A (2289T>C 2290G>A), D546E (2308T>G), V552I (2324G>A), V556L (2336G>C 2338T>G), E558K (2342G>A 2344G>A), A561N (2351G>A 2352C>A 2353T>C), M568L (2372A>C 2374G>T), S569A (2375A>G 2376G>C 2377C>A), A571S (2381G>T 2383T>C), A574M (2390G>A 2391C>T 2392T>G), S580K (2409G>A 2410C>G), T583A (2417A>G), A587S (2429G>T 2431G>C), L597N (2459C>A 2460T>A 2461A>C), I598L (2462A>C 2464C>T), Y600F (2469A>T 2470C>T), I602V (2474A>G 2476A>C), V606I (2486G>A), V609M (2495G>A 2497T>G), I612V (2504A>G 2506C>G), V625A (2544T>C 2545G>A), K629T (2556A>C 2557G>C), L638I (2582T>A 2584G>T), T644H (2600A>C 2601C>A 2602A>T), Q650E (2618C>G), N652S (2624A>T 2625A>C), E654G (2631A>G), E655K (2633G>A), C657V (2639T>G 2640G>T 2641C>G), T659S (2645A>T 2647C>A), T661Q (2651A>C 2652C>A), K665T (2664A>C 2665G>C), Y666F (2667A>T 2668C>T), E671T (2681G>A 2682A>C 2683A>T), G672S (2684G>T 2685G>C 2686T>A), T673L (2687A>C 2688C>T 2689A>G), A675V (2694C>T 2695C>T), T676P (2696A>C), D677E (2701T>A), S690A (2738T>G 2740T>A), N696E (2756A>G 2758T>G), V698I (2762G>A), L702I (2774T>A 2776G>A), Q705S (2783C>T 2784A>C 2785G>C), L714M (2810C>A 2812T>G), M719L (2825A>T), I720R (2829T>G), A721G (2832C>G 2833A>G), Y725F (2844A>T), K728R (2852A>C 2853A>G), A729P (2855G>C 2857G>A), Q731T (2861C>A 2862A>C 2863G>A), Y733F (2868A>T 2869T>C), P735S (2873C>A 2874C>G 2875A>T), G749S (2915G>A 2917G>T), T750V (2918A>G 2919C>T), S751A (2921T>G 2923A>T), S752A (2924T>G 2926C>A), P753A (2927C>G 2929A>C), I761V (2951A>G 2953A>C), T773I (2988C>T), T790R (3039C>G 3040T>A), I797V (3059A>G 3061T>G), I799V (3065A>G), N817A (3119A>G 3120A>C 3121C>A), K819E (3125A>G 3127A>G), T821S (3131A>T), N822T (3135A>C 3136T>A), F827L (3151T>G), K841R (3192A>G 3193A>G), R843Q (3198G>A 3199G>A), R845K (3203C>A 3204G>A 3205G>A), I848L (3212A>C 3214T>G), K854C (3230A>T 3231A>G 3232A>C), Q869K (3275C>A), F871Q (3281T>C 3282T>A 3283T>A), L874M (3290T>A), I889L (3335A>T 3337A>G), S893T (3347T>A 3349C>T), C902R (3374T>C), L903I (3377C>A), N905D (3383A>G), T911S (3401A>T 3403A>T), K925R (3444A>G), S927A (3449T>G 3451A>T), D936N (3476G>A 3478T>C), S938N (3482T>A 3483C>A 3484A>T), I943L (3497A>C), D949E (3517C>A), I951V (3521A>G), E952D (3526G>T), E953I (3527G>A 3528A>T 3529G>C), Y954F (3531A>T 3532C>T), Q955D (3533C>G 3535G>C), E956Q (3536G>C), D960N (3548G>A 3550T>C), P961_P962del (3551_3556delCCGCCC), K964T (3561A>C 3562G>A), A965S (3563G>T), S967V (3569A>G 3570G>T), I968S (3572A>T 3573T>C 3574T>C), L969F (3575C>T 3577G>C), S970Q (3578T>C 3579C>A 3580C>G), S976T (3596T>A 3598C>A), M985A (3623A>G 3624T>C 3625G>T), G986A (3627G>C 3628T>A), D990T (3638G>A 3639A>C 3640C>A), E991D (3643A>C), L994V (3650T>G 3652G>A), I996L (3656A>T 3658T>G), I1003V (3677A>G 3679A>G), E1006D (3688A>T), G1007A (3690G>C 3691G>A), H1008S (3692C>T 3693A>C), V1011I (3701G>A), T1024S (3740A>T 3742A>C), N1034E (3770A>G 3772T>A), S1036T (3776T>A), I1037F (3779A>T), I1039M (3787A>G), V1042T (3794G>A 3795T>C 3796A>T), P1046N (3806C>A 3807C>A 3808T>C), N1047S (3810A>G), V1048I (3812G>A 3814T>A), V1051I (3821G>A 3823A>C), V1054I (3830G>A), F1056L (3836T>C), A1058T (3842G>A 3844C>A), P1064Q (3861C>A), A1065F (3863G>T 3864C>T), L1067V (3869C>G 3871T>A), N1068S (3872A>T 3873A>C), K1069A (3875A>G 3876A>C 3877A>G), N1071A (3881A>G 3882A>C 3883T>A), Y1072H (3884T>C), V1074L (3890G>T), A1089T (3935G>A 3937A>C), T1092I (3945C>T 3946G>C), T1096V (3956A>G 3957C>T 3958T>A), E1101Q (3971G>C), H1107F (3989C>T 3990A>T 3991T>C), R1109K (3996G>A), M1110A (3998A>G 3999T>C 4000G>C), R1111K (4002G>A 4003G>A), E1112S (4004G>T 4005A>C 4006A>C), F1113Y (4008T>A 4009C>T), S1114D (4010T>G 4011C>A 4012A>T), S1115K (4014G>A 4015C>G), K1116E (4016A>G 4018G>A), Q1117S (4019C>A 4020A>G 4021A>T), M1121V (4031A>G 4033G>T), R1126K (4047G>A), N1129L (4055A>T 4056A>T 4057T>G), I1131L (4061A>C 4063C>A), S1137V (4079T>G 4080C>T 4081C>T), A1138E (4083C>A 4084C>A), I1139L (4085A>C 4087A>T), V1140I (4088G>A 4090G>T), L1146I (4106C>A 4108T>A), I1147V (4109A>G 4111A>C), S1148K (4112T>A 4113C>A 4114T>G), L1151P (4122T>C), S1162L (4154T>C 4155C>T), H1165N (4163C>A), N1171D (4181A>G), I1191N (4242T>A)								

	Begin	End	Coverage	Score	Concordance	Matches	Identities	I/D/M/F*	Stop Codons
**NT**	**89**	**4733**	**94.7%**	**2570**	**28.4%**	**4597 (96.7%)**	**3027 (63.7%)**	**107/48**	

Codon mutations:

ATG1AGG (672T>G), ACA2ATG (675C>T 676A>G), TCT4TTG (681C>T 682T>G), GTT6GCT (687T>C), CTG8CGA (693T>G 694G>A), AAA9GTG (695A>G 696A>T 697A>G), GTG10ATG (698G>A), GGC11GCC (702G>C), CCC12CCA (706C>A), AAT14GAC (710A>G 712T>C), CAC15CGC (714A>G), CAG16TCA (716C>T 717A>C 718G>A), CAG17del (719_721delCAG), GAG18AAA (722G>A 724G>A), GCC20GTA (729C>T 730C>A), TCC21TCT (733C>T), GAG22GAC (736G>C), AGG23AAA (738G>A 739G>A), ATA25ATT (745A>T), TCA26TCT (748A>T), CAA27CAG (751A>G), ATA28ATC (754A>C), GCT30GCG (760T>G), GTG32GTT (766G>T), GAG33GAA (769G>A), GCC34GAA (771C>A 772C>A), GGA35GGT (775A>T), AAT38AAC (784T>C), TTG39CTT (785T>C 787G>T), CTC40CTT (790C>T), AGA41AAA (792G>A), AAG42AAA (796G>A), CTT43TTG (797C>T 799T>G), GGC44GGA (802C>A), TCC47TCA (811C>A), TAC48TAT (814C>T), GGG49GGC (817G>C), GTT50ATA (818G>A 820T>A), CTT51TTG (821C>T 823T>G), GGT53GCA (828G>C 829T>A), CAA55CAG (835A>G), AAG57AAA (841G>A), AGA58AGG (844A>G), GAG61GAT (853G>T), TTG62CTT (854T>C 856G>T), TTT63TAT (858T>A), GAC64GAT (862C>T), GTA68GTT (874A>T), TCA69AGT (875T>A 876C>G 877A>T), AAG70AAA (880G>A), ATC71GTG (881A>G 883C>G), ACA72GCA (884A>G), CTT74CTA (892T>A), ACA77GCT (899A>G 901A>T), TTC78TTT (904C>T), CAT80AGG (908C>A 909A>G 910T>G), AAG81ACC (912A>C 913G>C), TTT82ATA (914T>A 916T>A), GTT83ACA (917G>A 918T>C 919T>A), GAG84GAA (922G>A), TCT85TCA (925T>A), AAG86CGT (926A>C 927A>G 928G>T), GAC87GAT (931C>T), GCA89GGG (936C>G 937A>G), AAC90AAT (940C>T), TTG91TTA (943G>A), TTT93TTC (949T>C), CTG95TTA (953C>T 955G>A), TAT96CAC (956T>C 958T>C), GGT97GGG (961T>G), CTT99TTG (965C>T 967T>G), TTC100TTT (970C>T), TTC101TTT (973C>T), GTC103GTT (979C>T), CCA104CCC (982A>C), GTT106GCA (987T>C 988T>A), AGT108AAT (993G>A), GGG109GGA (997G>A), GGA111GGT (1003A>T), AGG112GCC (1004A>G 1005G>C 1006G>C), GTT113GTC (1009T>C), AAG114AAA (1012G>A), ATA115ATC (1015A>C), AGT116AGC (1018T>C), CTT117TTG (1019C>T 1021T>G), TGT118TGC (1024T>C), TCA119TCT (1027A>T), AGT120AGC (1030T>C), AAT121AAC (1033T>C), CTC124ACT (1040C>A 1041T>C 1042C>T), TCA125GAT (1043T>G 1044C>A 1045A>T), CCG126CCT (1048G>T), CAA129CAG (1057A>G), AAG131AAA (1063G>A), ACA132ATT (1065C>T 1066A>T), CTG133TTG (1067C>T), TCC134TAC (1071C>A), TTG135TTT (1075G>T), GCC136TCT (1076G>T 1078C>T), GAT137GGA (1080A>G 1081T>A), GCG139GCA (1087G>A), CAG140CAA (1090G>A), GCA141GCC (1093A>C), CTC143CTA (1099C>A), AGC145AGT (1105C>T), CCC146CCT (1108C>T), AGC147AGT (1111C>T), CTC153TTG (1127C>T 1129C>G), AAA154AAG (1132A>G), AGA155AGG (1135A>G), GGG156GGC (1138G>C), ACC162ACA (1156C>A), GAG164GAA (1162G>A), TGC165TGT (1165C>T), CAA166CTC (1167A>T 1168A>C), AAT167AAC (1171T>C), ACC168ACG (1174C>G), AGG169AGA (1177G>A), GCA170GCT (1180A>T), CAG171CAA (1183G>A), CCG173CCT (1189G>T), TCC175TCT (1195C>T), GTG176GTA (1198G>A), GCC178GCA (1204C>A), ATA179ATT (1207A>T), GAC185AGT (1223G>A 1224A>G 1225C>T), ACA186CCT (1226A>C 1228A>T), AGG187CGT (1229A>C 1231G>T), GCA189GCT (1237A>T), GTT190GTA (1240T>A), TCA192ACA (1244T>A), CAG193GAC (1247C>G 1249G>C), CAA194CAT (1252A>T), GAA195GAC (1255A>C), ACA196ACT (1258A>T), TCT198AGT (1262T>A 1263C>G), GCC200GCT (1270C>T), ATA201ATT (1273A>T), GAA202GAG (1276A>G), GCA203GCT (1279A>T), CTC204CTT (1282C>T), AAT205AAC (1285T>C), CGT206AGA (1286C>A 1288T>A), CCG207CCT (1291G>T), CAA208CAG (1294A>G), GAT212GAC (1306T>C), AGA213AGG (1309A>G), GAG215GAA (1315G>A), GCT216GCA (1318T>A), GCT217GCA (1321T>A), TAT219TAC (1327T>C), ATA220ATT (1330A>T), GCT221GCA (1333T>A), GTT223GTG (1339T>G), TAT224TAC (1342T>C), TCG225TCC (1345G>C), AGT226AAT (1347G>A), GGG227_CAA228insGTA (1351_1352insGTA), CAA228CAG (1354A>G), AGT229AGA (1357T>A), ACT230AGT (1359C>G), CAA231CAG (1363A>G), ATG232CTG (1364A>C), GCT233del (1367_1369delGCT), CTT234TTG (1370C>T 1372T>G), GAA235GAG (1375A>G), AAA237AGA (1380A>G), GCC238CCT (1382G>C 1384C>T), GTT240GTG (1390T>G), GGA241GGT (1393A>T), GAA242GCC (1395A>C 1396A>C), CAG243GCA (1397C>G 1398A>C 1399G>A), GGG245GGT (1405G>T), GGC246GAT (1407G>A 1408C>T), ACA247ACC (1411A>C), GAC250AAT (1418G>A 1420C>T), GTC251GTG (1423C>G), ATG252CCG (1424A>C 1425T>C), AAT253CAG (1427A>C 1429T>G), GAA254GAG (1432A>G), AGC255GCA (1433A>G 1434G>C 1435C>A), ACT256AAT (1437C>A), ATC258GTG (1442A>G 1444C>G), CGG259CGC (1447G>C), AGT260TCT (1448A>T 1449G>C), TCT262TCA (1456T>A), AGA264AGG (1462A>G), ACT267ACC (1471T>C), CTC268CTA (1474C>A), AAA269AAG (1477A>G), GTG270GTA (1480G>A), AAG273AGG (1488A>G), CGC274CGA (1492C>A), CCG276GCA (1496C>G 1498G>A), TCC277TCA (1501C>A), CTG280TTG (1508C>T), CCG281CCT (1513G>T), CCG281_GCT282insGACTTT (1513_1514insGACTTT), GTA283TCA (1517G>T 1518T>C), AGT284ACT (1521G>C), ACA285TCT (1523A>T 1525A>T), TCA286AGA (1526T>A 1527C>G), TCC287GCG (1529T>G 1531C>G), TTA288TAT (1533T>A 1534A>T), CTC289CAG (1536T>A 1537C>G), AGC290GGA (1538A>G 1540C>A), ACA291GTG (1541A>G 1542C>T 1543A>G), CGT292AGA (1544C>A 1546T>A), GAA293GAG (1549A>G), ACA295ACT (1555A>T), GTT296GGT (1557T>G), CAT297ATT (1559C>A 1560A>T), GAG299del (1565_1567delGAG), TGT302ACG (1574T>A 1575G>C 1576T>G), GGG303TCT (1577G>T 1578G>C 1579G>T), GGA304CTA (1580G>C 1581G>T), TTG305CTG (1583T>C), CCA307CCT (1591A>T), GCA308GCC (1594A>C), CCC309_AAG310del (1595_1600delCCCAAG), AAA311AAG (1603A>G), AAG312ATA (1605A>T 1606G>A), GGC313TCA (1607G>T 1608G>C 1609C>A), GCC315GCA (1615C>A), TTC316GGA (1616T>G 1617T>G 1618C>A), AAC317ACA (1620A>C 1621C>A), ATG318CTC (1622A>C 1624G>C), GGT319CAA (1625G>C 1626G>A 1627T>A), GCC320GAA (1629C>A 1630C>A), ATT321GTC (1631A>G 1633T>C), GAT323GAC (1639T>C), TTG325TAT (1644T>A 1645G>T), ATG327ATT (1651G>T), GAG328CAA (1652G>C 1654G>A), TCG329AGT (1655T>A 1656C>G 1657G>T), TTC330TTT (1660C>T), GCA331GAA (1662C>A), CAC332GAT (1664C>G 1666C>T), ATT333TTT (1667A>T), GAT334GCC (1671A>C 1672T>C), GAT337AGT (1679G>A 1680A>G), GAT338AAC (1682G>A 1684T>C), GAA341GCC (1692A>C 1693A>C), CGA342CTT (1695G>T 1696A>T), ATA344GTG (1700A>G 1702A>G), GCT345CCC (1703G>C 1705T>C), CAG346GAG (1706C>G), CAG347CAA (1711G>A), GTA348ATT (1712G>A 1714A>T), CAA349GAT (1715C>G 1717A>T), TTT350TAT (1719T>A), ATC351GTT (1721A>G 1723C>T), CTC352CTT (1726C>T), TTT353GTG (1727T>G 1729T>G), AGT354CAG (1730A>C 1731G>A 1732T>G), GAG355AAG (1733G>A), AAA357AGA (1740A>G), AGA358GCT (1742A>G 1743G>C 1744A>T), GGA359GGT (1747A>T), AAC360AAT (1750C>T), GTT361CTC (1751G>C 1753T>C), ATT362TGT (1754A>T 1755T>G), GTG363GTC (1759G>C), AGA365AAA (1764G>A), GTT367ATT (1769G>A), GCA368GAG (1773C>A 1774A>G), AAG369ATG (1776A>T), CGG370AGG (1778C>A), CAT371GAC (1781C>G 1783T>C), CTG372TTG (1784C>T), CAT373CAC (1789T>C), AAT374TGT (1790A>T 1791A>G), ATC375ATA (1795C>A), AGG377TTT (1799A>T 1800G>T 1801G>T), GAG378GAA (1804G>A), CAC379CAT (1807C>T), ATA380ATC (1810A>C), ACC381ACT (1813C>T), GAC383GCC (1818A>C), GTA386GTT (1828A>T), GAA387GAT (1831A>T), ATA388ATT (1834A>T), CTT389TTG (1835C>T 1837T>G), GAA390GCG (1839A>C 1840A>G), GGT391GGA (1843T>A), GGT393GGA (1849T>A), AAT396AAG (1858T>G), ATC397GTT (1859A>G 1861C>T), CAA398GGG (1862C>G 1863A>G 1864A>G), GGC399ATG (1865G>A 1866G>T 1867C>G), ACT401GAA (1871A>G 1872C>A 1873T>A), CGC402AGA (1874C>A 1876C>A), ACT403ACA (1879T>A), TTC404CCA (1880T>C 1881T>C 1882C>A), TAT407TAC (1891T>C), GCT408TCT (1892G>T), ATG409TTG (1895A>T), TCT410AGC (1898T>A 1899C>G 1900T>C), CTC411CTG (1903C>G), GAG413CAG (1907G>C), GTT415GTG (1915T>G), GTG416GTT (1918G>T), CTC418CTA (1924C>A), CAT420CAC (1930T>C), CAA421ATG (1931C>A 1932A>T 1933A>G), AGA422AGG (1936A>G), GAT423GAC (1939T>C), TCC425CAA (1943T>C 1944C>A 1945C>A), TTC426TTT (1948C>T), ATT428AGG (1953T>G 1954T>G), CAA429TAT (1955C>T 1957A>T), GGG430TGG (1958G>T), CAG431GAT (1961C>G 1963G>T), ACT432TTA (1964A>T 1965C>T 1966T>A), AAC433ACA (1968A>C 1969C>A), AAT434AAC (1972T>C), CCA435CCG (1975A>G), CCG437CCT (1981G>T), AAT438GAC (1982A>G 1984T>C), TGC441GTG (1991T>G 1992G>T 1993C>G), GGA444GGT (2002A>T), TTA445CTT (2003T>C 2005A>T), ACC446ACA (2008C>A), TTG447CTG (2009T>C), AAA448GCA (2012A>G 2013A>C), GAG449GAT (2017G>T), CGT450AGG (2018C>A 2020T>G), CGT450_CAG451insGTGAAA (2020_2021insGTGAAA), CAG451CAT (2023G>T), TTG452CCC (2024T>C 2025T>C 2026G>C), ATT453TTG (2027A>T 2029T>G), GTG454ATG (2030G>A), GAG455AGT (2033G>A 2034A>G 2035G>T), GAG456GAT (2038G>T), CAA457CTA (2040A>T), GTG458GCA (2043T>C 2044G>A), GCC459TGC (2045G>T 2046C>G), CAA460_AGA461insTTGAAT (2050_2051insTTGAAT), AGA461CGC (2051A>C 2053A>C), AGG462TTG (2054A>T 2055G>T), TCT463GGA (2057T>G 2058C>G 2059T>A), GAA464AAA (2060G>A), AGA465CCA (2063A>C 2064G>C), CAA466AGC (2066C>A 2067A>G 2068A>C), GCA467ATG (2069G>A 2070C>T 2071A>G), CAG468AGT (2072C>A 2073A>G 2074G>T), ACA470GCA (2078A>G), GCA471CAA (2081G>C 2082C>A), AGG472ACT (2085G>C 2086G>T), GGC473GGA (2089C>A), ATA474AAT (2091T>A 2092A>T), GCT475GCC (2095T>C), GCT475_GAA476insAGC (2095_2096insAGC), GAA476AAG (2096G>A 2098A>G), AGT477TGG (2099A>T 2101T>G), CAG478CAA (2104G>A), CCT479ACT (2105C>A), GAC481GGA (2112A>G 2113C>A), AGA482AAG (2115G>A 2116A>G), ACT484CTG (2120A>C 2121C>T 2122T>G), TCC486TCA (2128C>A), GTG488GTT (2134G>T), TCA489ACA (2135T>A), ACT490ACC (2140T>C), ACC491ACA (2143C>A), ACG492GCA (2144A>G 2146G>A), ATG493CTT (2147A>C 2149G>T), GAA494GAG (2152A>G), GAA494_GAT495insAGT (2152_2153insAGT), GAT495GAG (2155T>G), CCC496GGT (2156C>G 2157C>G 2158C>T), ACC497ATC (2160C>T), AAG498CAA (2162A>C 2164G>A), CCA499AGA (2165C>A 2166C>G), GAT500AAG (2168G>A 2170T>G), AAG501GAG (2171A>G), ATA502GTG (2174A>G 2176A>G), GAA503TCA (2177G>T 2178A>C), GTT505GAG (2184T>A 2185T>G), GCA506GCT (2188A>T), GAG507GAA (2191G>A), GGA508GAT (2193G>A 2194A>T), GCA509CCG (2195G>C 2197A>G), GAA511GGG (2202A>G 2203A>G), GAA512AAA (2204G>A), ACT513ACA (2209T>A), CAA514GAG (2210C>G 2212A>G), GGT516AAT (2216G>A 2217G>A), GAT517GAC (2221T>C), GTT518ATT (2222G>A), ATT519TTC (2225A>T 2227T>C), GAC521GAT (2233C>T), GGA523GGG (2239A>G), CCA524GAG (2240C>G 2241C>A 2242A>G), GAC527ATG (2249G>A 2250A>T 2251C>G), ACA528GGT (2252A>G 2253C>G 2254A>T), TCC529GAT (2255T>G 2256C>A 2257C>T), ATG530GCC (2258A>G 2259T>C 2260G>C), GCA531CCG (2261G>C 2263A>G), GTT532TTA (2264G>T 2266T>A), GAA533AAT (2267G>A 2269A>T), GAT535GAG (2275T>G), ATG536TTG (2276A>T), CAG537CAA (2281G>A), CAA538CAG (2284A>G), CCG539CCA (2287G>A), GTG540GCA (2289T>C 2290G>A), GTA542GTG (2296A>G), AGC544AGT (2302C>T), GAT546GAG (2308T>G), TTC547TTT (2311C>T), GGA551GGG (2323A>G), GTT552ATT (2324G>A), TTC555TTT (2335C>T), GTT556CTG (2336G>C 2338T>G), GAG558AAA (2342G>A 2344G>A), TCC560AGC (2348T>A 2349C>G), GCT561AAC (2351G>A 2352C>A 2353T>C), GAG565GAA (2365G>A), CAA566CAG (2368A>G), GTA567GTT (2371A>T), ATG568CTT (2372A>C 2374G>T), AGC569GCA (2375A>G 2376G>C 2377C>A), TTG570CTA (2378T>C 2380G>A), GCT571TCC (2381G>T 2383T>C), TTG572CTC (2384T>C 2386G>C), CCC573CCA (2389C>A), GCT574ATG (2390G>A 2391C>T 2392T>G), GCC575GCA (2395C>A), CTA576TTG (2396C>T 2398A>G), TTC577TTT (2401C>T), TCA578AGC (2402T>A 2403C>G 2404A>C), AAA579AAG (2407A>G), AGC580AAG (2409G>A 2410C>G), AAA581AAG (2413A>G), ACT583GCT (2417A>G), TCA584TCC (2422A>C), GGT586GGG (2428T>G), GCG587TCC (2429G>T 2431G>C), CTT590CTC (2440T>C), TAT592TAC (2446T>C), TAC593TAT (2449C>T), GAT594GAC (2452T>C), GCA595GCT (2455A>T), GCT596GCA (2458T>A), CTA597AAC (2459C>A 2460T>A 2461A>C), ATC598CTT (2462A>C 2464C>T), TAC600TTT (2469A>T 2470C>T), ATA602GTC (2474A>G 2476A>C), CTT604CTG (2482T>G), TAT605TAC (2485T>C), GTT606ATT (2486G>A), GTT609ATG (2495G>A 2497T>G), GCT611GCG (2503T>G), ATC612GTG (2504A>G 2506C>G), TCT613TCG (2509T>G), GGT614GGA (2512T>A), CAA615CAG (2515A>G), CTG616TTA (2516C>T 2518G>A), GCT617GCC (2521T>C), GAA622GAG (2536A>G), TGT623TGC (2539T>C), GTG625GCA (2544T>C 2545G>A), CGA628AGA (2552C>A), AAG629ACC (2556A>C 2557G>C), AAC634AAT (2572C>T), ATC635ATA (2575C>A), ACA637ACC (2581A>C), TTG638ATT (2582T>A 2584G>T), AAG642AAA (2596G>A), ACA644CAT (2600A>C 2601C>A 2602A>T), TTG645CTG (2603T>C), GTT646GTG (2608T>G), CAA650GAA (2618C>G), AAC652TCC (2624A>T 2625A>C), GAG654GGG (2631A>G), GAG655AAG (2633G>A), TGC657GTG (2639T>G 2640G>T 2641C>G), TTT658TTC (2644T>C), ACC659TCA (2645A>T 2647C>A), CCA660CCT (2650A>T), ACA661CAA (2651A>C 2652C>A), GGG662GGT (2656G>T), GGC664GGT (2662C>T), AAG665ACC (2664A>C 2665G>C), TAC666TTT (2667A>T 2668C>T), GTG667GTA (2671G>A), CCT668CCA (2674T>A), CTG669TTG (2675C>T), GAA671ACT (2681G>A 2682A>C 2683A>T), GGT672TCA (2684G>T 2685G>C 2686T>A), ACA673CTG (2687A>C 2688C>T 2689A>G), GGA674GGT (2692A>T), GCC675GTT (2694C>T 2695C>T), ACT676CCT (2696A>C), GAT677GAA (2701T>A), TTA678CTT (2702T>C 2704A>T), GGT679GGC (2707T>C), GTG681GTT (2713G>T), ACA686ACC (2728A>C), CAC687CAT (2731C>T), TTA689TTG (2737A>G), TCT690GCA (2738T>G 2740T>A), GCA692GCC (2746A>C), GAG694GAA (2752G>A), AAT696GAG (2756A>G 2758T>G), AGT697TCA (2759A>T 2760G>C 2761T>A), GTA698ATA (2762G>A), CCA699CCT (2767A>T), TGC700TGT (2770C>T), CAT701CAC (2773T>C), TTG702ATA (2774T>A 2776G>A), TTA704CTA (2780T>C), CAG705TCC (2783C>T 2784A>C 2785G>C), AAA707AAG (2791A>G), TTA709CTC (2795T>C 2797A>C), ACC711ACA (2803C>A), CTT714ATG (2810C>A 2812T>G), CGA718AGG (2822C>A 2824A>G), ATG719TTG (2825A>T), ATA720AGA (2829T>G), GCA721GGG (2832C>G 2833A>G), CAG722CAA (2836G>A), CAA724CAG (2842A>G), TAT725TTT (2844A>T), GGC726GGA (2848C>A), AAG728CGG (2852A>C 2853A>G), GCG729CCA (2855G>C 2857G>A), GGG730GGC (2860G>C), CAG731ACA (2861C>A 2862A>C 2863G>A), ACA732ACC (2866A>C), TAT733TTC (2868A>T 2869T>C), CCA735AGT (2873C>A 2874C>G 2875A>T), AGG736CGC (2876A>C 2878G>C), TTT737TTC (2881T>C), CCA738CCT (2884A>T), ACT739ACA (2887T>A), GTT742GTG (2896T>G), TTG743CTT (2897T>C 2899G>T), TTA744CTG (2900T>C 2902A>G), CAT745CAC (2905T>C), TAT746TAC (2908T>C), GGG749AGT (2915G>A 2917G>T), ACA750GTA (2918A>G 2919C>T), TCA751GCT (2921T>G 2923A>T), TCC752GCA (2924T>G 2926C>A), CCA753GCC (2927C>G 2929A>C), ACA757ACT (2941A>T), AGC760AGT (2950C>T), ATA761GTC (2951A>G 2953A>C), TTT762TTC (2956T>C), TCA763TCC (2959A>C), ATA767ATT (2971A>T), GAG769GAA (2977G>A), GGC772GGT (2986C>T), ACC773ATC (2988C>T), TTG774TTA (2992G>A), CCG776CCA (2998G>A), TCT777TCA (3001T>A), TTG778CTG (3002T>C), GGT780GGA (3010T>A), ATA782ATT (3016A>T), GCC783GCA (3019C>A), AGG784AGA (3022G>A), AAG787AAA (3031G>A), ACT790AGA (3039C>G 3040T>A), GGA791GGC (3043A>C), ACT792ACC (3046T>C), ATT797GTG (3059A>G 3061T>G), TGC798TGT (3064C>T), ATT799GTT (3065A>G), ACC802ACT (3076C>T), TTG803CTG (3077T>C), TCA806TCT (3088A>T), GGT807GGG (3091T>G), CTG809CTT (3097G>T), GCA810GCT (3100A>T), ATT811ATA (3103T>A), GGA812GGG (3106A>G), CTT813TTG (3107C>T 3109T>G), GGA814GGC (3112A>C), ACT815ACG (3115T>G), AAC817GCA (3119A>G 3120A>C 3121C>A), ACC818ACA (3124C>A), AAA819GAG (3125A>G 3127A>G), ACC821TCC (3131A>T), AAT822ACA (3135A>C 3136T>A), GCT823GCG (3139T>G), TTT827TTG (3151T>G), CCA830CCC (3160A>C), GTA833GTG (3169A>G), TGC834TGT (3172C>T), CTT836TTG (3176C>T 3178T>G), GGT839GGG (3187T>G), AAA841AGG (3192A>G 3193A>G), CGG843CAA (3198G>A 3199G>A), CGG845AAA (3203C>A 3204G>A 3205G>A), TGT846TGC (3208T>C), TCT847TCA (3211T>A), ATT848CTG (3212A>C 3214T>G), ACA849ACC (3217A>C), GGA853GGG (3229A>G), AAA854TGC (3230A>T 3231A>G 3232A>C), AAT855AAC (3235T>C), TTG856CTC (3236T>C 3238G>C), CTT857CTG (3241T>G), TCC858TCT (3244C>T), ACA859ACC (3247A>C), GGG860GGA (3250G>A), CGA861CGC (3253A>C), AAG862AAA (3256G>A), TCC864TCA (3262C>A), TTG865TTA (3265G>A), CAG869AAG (3275C>A), CAC870CAT (3280C>T), TTT871CAA (3281T>C 3282T>A 3283T>A), TCC872TCT (3286C>T), TTG874ATG (3290T>A), CGC875CGT (3295C>T), TTG876CTG (3296T>C), TTT877TTC (3301T>C), ACG879ACT (3307G>T), GTA880GTT (3310A>T), CTC884CTG (3322C>G), ACG887ACA (3331G>A), ATA889TTG (3335A>T 3337A>G), CAT890CAC (3340T>C), TCC893ACT (3347T>A 3349C>T), GGT895GGA (3355T>A), GTC896GTA (3358C>A), CAG899CAA (3367G>A), AAG901AAA (3373G>A), TGC902CGC (3374T>C), CTT903ATT (3377C>A), AAT905GAT (3383A>G), CTT906CTC (3388T>C), ACT907ACG (3391T>G), TTG908CTT (3392T>C 3394G>T), GGT909GGG (3397T>G), GGC910GGT (3400C>T), ACA911TCT (3401A>T 3403A>T), GTA912GTG (3406A>G), TCT913TCA (3409T>A), GTG914GTT (3412G>T), AAA915AAG (3415A>G), ATA917ATT (3421A>T), GGG919GGT (3427G>T), ACA922ACC (3436A>C), AAA923AAG (3439A>G), GGC924GGG (3442C>G), AAA925AGA (3444A>G), AGC926AGT (3448C>T), TCA927GCT (3449T>G 3451A>T), GTT928GTG (3454T>G),

	TTC930TTT (3460C>T), CTT931CTG (3463T>G), TTC932TTT (3466C>T), GAT936AAC (3476G>A 3478T>C), TTA937CTG (3479T>C 3481A>G), TCA938AAT (3482T>A 3483C>A 3484A>T), CGT940CGC (3490T>C), ATT943CTT (3497A>C), GAA944GAG (3502A>G), AAG945AAA (3505G>A), TTG946CTG (3506T>C), AAG948AAA (3514G>A), GAC949GAA (3517C>A), AAC950AAT (3520C>T), ATT951GTT (3521A>G), GAG952GAT (3526G>T), GAG953ATC (3527G>A 3528A>T 3529G>C), TAC954TTT (3531A>T 3532C>T), CAG955GAC (3533C>G 3535G>C), GAG956CAG (3536G>C), GGC958GGG (3544C>G), GAT960AAC (3548G>A 3550T>C), CCG961_CCC962del (3551_3556delCCGCCC), AAG964ACA (3561A>C 3562G>A), GCT965TCT (3563G>T), AGT967GTT (3569A>G 3570G>T), ATT968TCC (3572A>T 3573T>C 3574T>C), CTG969TTC (3575C>T 3577G>C), TCC970CAG (3578T>C 3579C>A 3580C>G), ATA971ATC (3583A>C), AGA972AGG (3586A>G), GAG973GAA (3589G>A), AAA974AAG (3592A>G), TTT975TTC (3595T>C), TCC976ACA (3596T>A 3598C>A), GGT978GGA (3604T>A), GCT979GCA (3607T>A), GTA980GTG (3610A>G), TTC983TTT (3619C>T), TGC984TGT (3622C>T), ATG985GCT (3623A>G 3624T>C 3625G>T), GGT986GCA (3627G>C 3628T>A), GAC990ACA (3638G>A 3639A>C 3640C>A), GAA991GAC (3643A>C), TTG994GTA (3650T>G 3652G>A), GTA995GTT (3655A>T), ATT996TTG (3656A>T 3658T>G), CCT997CCA (3661T>A), GCA999GCC (3667A>C), CCA1000CCT (3670A>T), TCC1002TCA (3676C>A), ATA1003GTG (3677A>G 3679A>G), AGG1004CGC (3680A>C 3682G>C), GAA1006GAT (3688A>T), GGG1007GCA (3690G>C 3691G>A), CAC1008TCC (3692C>T 3693A>C), CCT1010CCA (3700T>A), GTT1011ATT (3701G>A), AAG1012AAA (3706G>A), ATC1015ATA (3715C>A), TGT1017TGC (3721T>C), TTT1019TTC (3727T>C), GAT1021GAC (3733T>C), ACA1024TCC (3740A>T 3742A>C), TCA1025TCT (3745A>T), TCA1030TCT (3760A>T), GGT1031GGA (3763T>A), AAT1034GAA (3770A>G 3772T>A), TCA1036ACA (3776T>A), ATT1037TTT (3779A>T), GTG1038GTA (3784G>A), ATA1039ATG (3787A>G), AGA1041CGA (3791A>C), GTA1042ACT (3794G>A 3795T>C 3796A>T), CAA1043CAG (3799A>G), TCC1044AGC (3800T>A 3801C>G), AGT1045TCC (3803A>T 3804G>C 3805T>C), CCT1046AAC (3806C>A 3807C>A 3808T>C), AAT1047AGT (3810A>G), GTT1048ATA (3812G>A 3814T>A), GGA1049GGT (3817A>T), GTA1051ATC (3821G>A 3823A>C), CTA1052TTG (3824C>T 3826A>G), AAT1053AAC (3829T>C), GTT1054ATT (3830G>A), GCT1055GCA (3835T>A), TTT1056CTT (3836T>C), GAT1057GAC (3841T>C), GCC1058ACA (3842G>A 3844C>A), GGC1060GGG (3850C>G), TTT1063TTC (3859T>C), CCA1064CAA (3861C>A), GCT1065TTT (3863G>T 3864C>T), GGG1066GGT (3868G>T), CTT1067GTA (3869C>G 3871T>A), AAT1068TCT (3872A>T 3873A>C), AAA1069GCG (3875A>G 3876A>C 3877A>G), AAT1071GCA (3881A>G 3882A>C 3883T>A), TAT1072CAT (3884T>C), GTA1074TTA (3890G>T), GGT1077GGC (3901T>C), GGA1078GGT (3904A>T), GGC1079GGA (3907C>A), ACA1080ACT (3910A>T), TCA1085TCC (3925A>C), TAC1086TAT (3928C>T), GGT1087GGG (3931T>G), GTG1088GTT (3934G>T), GCA1089ACC (3935G>A 3937A>C), ACA1090ACC (3940A>C), AAT1091AAC (3943T>C), ACG1092ATC (3945C>T 3946G>C), TCA1094TCT (3952A>T), TTC1095TTT (3955C>T), ACT1096GTA (3956A>G 3957C>T 3958T>A), GAG1101CAG (3971G>C), TTT1102TTC (3976T>C), TTC1103TTT (3979C>T), CCA1104CCT (3982A>T), AGG1105CGC (3983A>C 3985G>C), CGG1106CGT (3988G>T), CAT1107TTC (3989C>T 3990A>T 3991T>C), AGG1109AAG (3996G>A), ATG1110GCC (3998A>G 3999T>C 4000G>C), AGG1111AAA (4002G>A 4003G>A), GAA1112TCC (4004G>T 4005A>C 4006A>C), TTC1113TAT (4008T>A 4009C>T), TCA1114GAT (4010T>G 4011C>A 4012A>T), AGC1115AAG (4014G>A 4015C>G), AAG1116GAA (4016A>G 4018G>A), CAA1117AGT (4019C>A 4020A>G 4021A>T), TCC1118TCA (4024C>A), CGC1119AGA (4025C>A 4027C>A), ATC1120ATT (4030C>T), ATG1121GTT (4031A>G 4033G>T), TCA1122TCT (4036A>T), CTA1123TTG (4037C>T 4039A>G), GAT1125GAC (4045T>C), AGG1126AAG (4047G>A), CTT1127CTA (4051T>A), GGA1128GGC (4054A>C), AAT1129TTG (4055A>T 4056A>T 4057T>G), CTG1130TTG (4058C>T), ATC1131CTA (4061A>C 4063C>A), ATA1132ATC (4066A>C), TTG1134CTT (4070T>C 4072G>T), CCT1135CCA (4075T>A), CCT1136CCA (4078T>A), TCC1137GTT (4079T>G 4080C>T 4081C>T), GCC1138GAA (4083C>A 4084C>A), ATA1139CTT (4085A>C 4087A>T), GTG1140ATT (4088G>A 4090G>T), TCC1142TCA (4096C>A), GAG1144GAA (4102G>A), ATA1145ATC (4105A>C), CTT1146ATA (4106C>A 4108T>A), ATA1147GTC (4109A>G 4111A>C), TCT1148AAG (4112T>A 4113C>A 4114T>G), CCT1149CCA (4117T>A), GGA1150GGT (4120A>T), CTT1151CCT (4122T>C), TTC1153TTT (4129C>T), AAG1154AAA (4132G>A), GCC1158GCT (4144C>T), CCT1160CCC (4150T>C), CCT1161CCA (4153T>A), TCT1162CTT (4154T>C 4155C>T), GCC1163GCA (4159C>A), CAT1165AAT (4163C>A), GAA1166GAG (4168A>G), AAA1167AAG (4171A>G), CTT1169CTC (4177T>C), GGC1170GGA (4180C>A), AAT1171GAT (4181A>G), CAA1173CAG (4189A>G), ACT1174ACG (4192T>G), CAC1175CAT (4195C>T), ACC1176ACT (4198C>T), ACC1180ACA (4210C>A), TCA1181TCT (4213A>T), CTA1186CTG (4228A>G), ATT1191AAT (4242T>A)								
Coat protein A (YP_001976155.1)	1	254	100%	1189	70.1%	254 (99.2%)	165 (64.5%)	2/0/0/0	0
Protein mutations:	T2A (2078A>G), A3Q (2081G>C 2082C>A), R4T (2085G>C 2086G>T), I6N (2091T>A 2092A>T), A7_E8insS (2095_2096insAGC), E8K (2096G>A 2098A>G), S9W (2099A>T 2101T>G), P11T (2105C>A), D13G (2112A>G 2113C>A), R14K (2115G>A 2116A>G), T16L (2120A>C 2121C>T 2122T>G), S21T (2135T>A), T24A (2144A>G 2146G>A), M25L (2147A>C 2149G>T), E26_D27insS (2152_2153insAGT), D27E (2155T>G), P28G (2156C>G 2157C>G 2158C>T), T29I (2160C>T), K30Q (2162A>C 2164G>A), P31R (2165C>A 2166C>G), D32K (2168G>A 2170T>G), K33E (2171A>G), I34V (2174A>G 2176A>G), E35S (2177G>T 2178A>C), V37E (2184T>A 2185T>G), G40D (2193G>A 2194A>T), A41P (2195G>C 2197A>G), E43G (2202A>G 2203A>G), E44K (2204G>A), Q46E (2210C>G 2212A>G), G48N (2216G>A 2217G>A), V50I (2222G>A), I51F (2225A>T 2227T>C), P56E (2240C>G 2241C>A 2242A>G), D59M (2249G>A 2250A>T 2251C>G), T60G (2252A>G 2253C>G 2254A>T), S61D (2255T>G 2256C>A 2257C>T), M62A (2258A>G 2259T>C 2260G>C), A63P (2261G>C 2263A>G), V64L (2264G>T 2266T>A), E65N (2267G>A 2269A>T), D67E (2275T>G), M68L (2276A>T), V72A (2289T>C 2290G>A), D78E (2308T>G), V84I (2324G>A), V88L (2336G>C 2338T>G), E90K (2342G>A 2344G>A), A93N (2351G>A 2352C>A 2353T>C), M100L (2372A>C 2374G>T), S101A (2375A>G 2376G>C 2377C>A), A103S (2381G>T 2383T>C), A106M (2390G>A 2391C>T 2392T>G), S112K (2409G>A 2410C>G), T115A (2417A>G), A119S (2429G>T 2431G>C), L129N (2459C>A 2460T>A 2461A>C), I130L (2462A>C 2464C>T), Y132F (2469A>T 2470C>T), I134V (2474A>G 2476A>C), V138I (2486G>A), V141M (2495G>A 2497T>G), I144V (2504A>G 2506C>G), V157A (2544T>C 2545G>A), K161T (2556A>C 2557G>C), L170I (2582T>A 2584G>T), T176H (2600A>C 2601C>A 2602A>T), Q182E (2618C>G), N184S (2624A>T 2625A>C), E186G (2631A>G), E187K (2633G>A), C189V (2639T>G 2640G>T 2641C>G), T191S (2645A>T 2647C>A), T193Q (2651A>C 2652C>A), K197T (2664A>C 2665G>C), Y198F (2667A>T 2668C>T), E203T (2681G>A 2682A>C 2683A>T), G204S (2684G>T 2685G>C 2686T>A), T205L (2687A>C 2688C>T 2689A>G), A207V (2694C>T 2695C>T), T208P (2696A>C), D209E (2701T>A), S222A (2738T>G 2740T>A), N228E (2756A>G 2758T>G), V230I (2762G>A), L234I (2774T>A 2776G>A), Q237S (2783C>T 2784A>C 2785G>C), L246M (2810C>A 2812T>G), M251L (2825A>T), I252R (2829T>G), A253G (2832C>G 2833A>G)								
Codon mutations:	ACA2GCA (2078A>G), GCA3CAA (2081G>C 2082C>A), AGG4ACT (2085G>C 2086G>T), GGC5GGA (2089C>A), ATA6AAT (2091T>A 2092A>T), GCT7GCC (2095T>C), GCT7_GAA8insAGC (2095_2096insAGC), GAA8AAG (2096G>A 2098A>G), AGT9TGG (2099A>T 2101T>G), CAG10CAA (2104G>A), CCT11ACT (2105C>A), GAC13GGA (2112A>G 2113C>A), AGA14AAG (2115G>A 2116A>G), ACT16CTG (2120A>C 2121C>T 2122T>G), TCC18TCA (2128C>A), GTG20GTT (2134G>T), TCA21ACA (2135T>A), ACT22ACC (2140T>C), ACC23ACA (2143C>A), ACG24GCA (2144A>G 2146G>A), ATG25CTT (2147A>C 2149G>T), GAA26GAG (2152A>G), GAA26_GAT27insAGT (2152_2153insAGT), GAT27GAG (2155T>G), CCC28GGT (2156C>G 2157C>G 2158C>T), ACC29ATC (2160C>T), AAG30CAA (2162A>C 2164G>A), CCA31AGA (2165C>A 2166C>G), GAT32AAG (2168G>A 2170T>G), AAG33GAG (2171A>G), ATA34GTG (2174A>G 2176A>G), GAA35TCA (2177G>T 2178A>C), GTT37GAG (2184T>A 2185T>G), GCA38GCT (2188A>T), GAG39GAA (2191G>A), GGA40GAT (2193G>A 2194A>T), GCA41CCG (2195G>C 2197A>G), GAA43GGG (2202A>G 2203A>G), GAA44AAA (2204G>A), ACT45ACA (2209T>A), CAA46GAG (2210C>G 2212A>G), GGT48AAT (2216G>A 2217G>A), GAT49GAC (2221T>C), GTT50ATT (2222G>A), ATT51TTC (2225A>T 2227T>C), GAC53GAT (2233C>T), GGA55GGG (2239A>G), CCA56GAG (2240C>G 2241C>A 2242A>G), GAC59ATG (2249G>A 2250A>T 2251C>G), ACA60GGT (2252A>G 2253C>G 2254A>T), TCC61GAT (2255T>G 2256C>A 2257C>T), ATG62GCC (2258A>G 2259T>C 2260G>C), GCA63CCG (2261G>C 2263A>G), GTT64TTA (2264G>T 2266T>A), GAA65AAT (2267G>A 2269A>T), GAT67GAG (2275T>G), ATG68TTG (2276A>T), CAG69CAA (2281G>A), CAA70CAG (2284A>G), CCG71CCA (2287G>A), GTG72GCA (2289T>C 2290G>A), GTA74GTG (2296A>G), AGC76AGT (2302C>T), GAT78GAG (2308T>G), TTC79TTT (2311C>T), GGA83GGG (2323A>G), GTT84ATT (2324G>A), TTC87TTT (2335C>T), GTT88CTG (2336G>C 2338T>G), GAG90AAA (2342G>A 2344G>A), TCC92AGC (2348T>A 2349C>G), GCT93AAC (2351G>A 2352C>A 2353T>C), GAG97GAA (2365G>A), CAA98CAG (2368A>G), GTA99GTT (2371A>T), ATG100CTT (2372A>C 2374G>T), AGC101GCA (2375A>G 2376G>C 2377C>A), TTG102CTA (2378T>C 2380G>A), GCT103TCC (2381G>T 2383T>C), TTG104CTC (2384T>C 2386G>C), CCC105CCA (2389C>A), GCT106ATG (2390G>A 2391C>T 2392T>G), GCC107GCA (2395C>A), CTA108TTG (2396C>T 2398A>G), TTC109TTT (2401C>T), TCA110AGC (2402T>A 2403C>G 2404A>C), AAA111AAG (2407A>G), AGC112AAG (2409G>A 2410C>G), AAA113AAG (2413A>G), ACT115GCT (2417A>G), TCA116TCC (2422A>C), GGT118GGG (2428T>G), GCG119TCC (2429G>T 2431G>C), CTT122CTC (2440T>C), TAT124TAC (2446T>C), TAC125TAT (2449C>T), GAT126GAC (2452T>C), GCA127GCT (2455A>T), GCT128GCA (2458T>A), CTA129AAC (2459C>A 2460T>A 2461A>C), ATC130CTT (2462A>C 2464C>T), TAC132TTT (2469A>T 2470C>T), ATA134GTC (2474A>G 2476A>C), CTT136CTG (2482T>G), TAT137TAC (2485T>C), GTT138ATT (2486G>A), GTT141ATG (2495G>A 2497T>G), GCT143GCG (2503T>G), ATC144GTG (2504A>G 2506C>G), TCT145TCG (2509T>G), GGT146GGA (2512T>A), CAA147CAG (2515A>G), CTG148TTA (2516C>T 2518G>A), GCT149GCC (2521T>C), GAA154GAG (2536A>G), TGT155TGC (2539T>C), GTG157GCA (2544T>C 2545G>A), CGA160AGA (2552C>A), AAG161ACC (2556A>C 2557G>C), AAC166AAT (2572C>T), ATC167ATA (2575C>A), ACA169ACC (2581A>C), TTG170ATT (2582T>A 2584G>T), AAG174AAA (2596G>A), ACA176CAT (2600A>C 2601C>A 2602A>T), TTG177CTG (2603T>C), GTT178GTG (2608T>G), CAA182GAA (2618C>G), AAC184TCC (2624A>T 2625A>C), GAG186GGG (2631A>G), GAG187AAG (2633G>A), TGC189GTG (2639T>G 2640G>T 2641C>G), TTT190TTC (2644T>C), ACC191TCA (2645A>T 2647C>A), CCA192CCT (2650A>T), ACA193CAA (2651A>C 2652C>A), GGG194GGT (2656G>T), GGC196GGT (2662C>T), AAG197ACC (2664A>C 2665G>C), TAC198TTT (2667A>T 2668C>T), GTG199GTA (2671G>A), CCT200CCA (2674T>A), CTG201TTG (2675C>T), GAA203ACT (2681G>A 2682A>C 2683A>T), GGT204TCA (2684G>T 2685G>C 2686T>A), ACA205CTG (2687A>C 2688C>T 2689A>G), GGA206GGT (2692A>T), GCC207GTT (2694C>T 2695C>T), ACT208CCT (2696A>C), GAT209GAA (2701T>A), TTA210CTT (2702T>C 2704A>T), GGT211GGC (2707T>C), GTG213GTT (2713G>T), ACA218ACC (2728A>C), CAC219CAT (2731C>T), TTA221TTG (2737A>G), TCT222GCA (2738T>G 2740T>A), GCA224GCC (2746A>C), GAG226GAA (2752G>A), AAT228GAG (2756A>G 2758T>G), AGT229TCA (2759A>T 2760G>C 2761T>A), GTA230ATA (2762G>A), CCA231CCT (2767A>T), TGC232TGT (2770C>T), CAT233CAC (2773T>C), TTG234ATA (2774T>A 2776G>A), TTA236CTA (2780T>C), CAG237TCC (2783C>T 2784A>C 2785G>C), AAA239AAG (2791A>G), TTA241CTC (2795T>C 2797A>C), ACC243ACA (2803C>A), CTT246ATG (2810C>A 2812T>G), CGA250AGG (2822C>A 2824A>G), ATG251TTG (2825A>T), ATA252AGA (2829T>G), GCA253GGG (2832C>G 2833A>G), CAG254CAA (2836G>A)								
Coat protein B (YP_001976156.1)	1	244	100%	1422	83.4%	242 (99.2%)	192 (78.7%)	0/2/0/0	0
Protein mutations:	Y3F (2844A>T), K6R (2852A>C 2853A>G), A7P (2855G>C 2857G>A), Q9T (2861C>A 2862A>C 2863G>A), Y11F (2868A>T 2869T>C), P13S (2873C>A 2874C>G 2875A>T), G27S (2915G>A 2917G>T), T28V (2918A>G 2919C>T), S29A (2921T>G 2923A>T), S30A (2924T>G 2926C>A), P31A (2927C>G 2929A>C), I39V (2951A>G 2953A>C), T51I (2988C>T), T68R (3039C>G 3040T>A), I75V (3059A>G 3061T>G), I77V (3065A>G), N95A (3119A>G 3120A>C 3121C>A), K97E (3125A>G 3127A>G), T99S (3131A>T), N100T (3135A>C 3136T>A), F105L (3151T>G), K119R (3192A>G 3193A>G), R121Q (3198G>A 3199G>A), R123K (3203C>A 3204G>A 3205G>A), I126L (3212A>C 3214T>G), K132C (3230A>T 3231A>G 3232A>C), Q147K (3275C>A), F149Q (3281T>C 3282T>A 3283T>A), L152M (3290T>A), I167L (3335A>T 3337A>G), S171T (3347T>A 3349C>T), C180R (3374T>C), L181I (3377C>A), N183D (3383A>G), T189S (3401A>T 3403A>T), K203R (3444A>G), S205A (3449T>G 3451A>T), D214N (3476G>A 3478T>C), S216N (3482T>A 3483C>A 3484A>T), I221L (3497A>C), D227E (3517C>A), I229V (3521A>G), E230D (3526G>T), E231I (3527G>A 3528A>T 3529G>C), Y232F (3531A>T 3532C>T), Q233D (3533C>G 3535G>C), E234Q (3536G>C), D238N (3548G>A 3550T>C), P239_P240del (3551_3556delCCGCCC), K242T (3561A>C 3562G>A), A243S (3563G>T)								

	Begin	End	Coverage	Score	Concordance	Matches	Identities	I/D/M/F*	Stop Codons
**NT**	**89**	**4733**	**94.7%**	**2570**	**28.4%**	**4597 (96.7%)**	**3027 (63.7%)**	**107/48**	
Codon mutations:	CAA2CAG (2842A>G), TAT3TTT (2844A>T), GGC4GGA (2848C>A), AAG6CGG (2852A>C 2853A>G), GCG7CCA (2855G>C 2857G>A), GGG8GGC (2860G>C), CAG9ACA (2861C>A 2862A>C 2863G>A), ACA10ACC (2866A>C), TAT11TTC (2868A>T 2869T>C), CCA13AGT (2873C>A 2874C>G 2875A>T), AGG14CGC (2876A>C 2878G>C), TTT15TTC (2881T>C), CCA16CCT (2884A>T), ACT17ACA (2887T>A), GTT20GTG (2896T>G), TTG21CTT (2897T>C 2899G>T), TTA22CTG (2900T>C 2902A>G), CAT23CAC (2905T>C), TAT24TAC (2908T>C), GGG27AGT (2915G>A 2917G>T), ACA28GTA (2918A>G 2919C>T), TCA29GCT (2921T>G 2923A>T), TCC30GCA (2924T>G 2926C>A), CCA31GCC (2927C>G 2929A>C), ACA35ACT (2941A>T), AGC38AGT (2950C>T), ATA39GTC (2951A>G 2953A>C), TTT40TTC (2956T>C), TCA41TCC (2959A>C), ATA45ATT (2971A>T), GAG47GAA (2977G>A), GGC50GGT (2986C>T), ACC51ATC (2988C>T), TTG52TTA (2992G>A), CCG54CCA (2998G>A), TCT55TCA (3001T>A), TTG56CTG (3002T>C), GGT58GGA (3010T>A), ATA60ATT (3016A>T), GCC61GCA (3019C>A), AGG62AGA (3022G>A), AAG65AAA (3031G>A), ACT68AGA (3039C>G 3040T>A), GGA69GGC (3043A>C), ACT70ACC (3046T>C), ATT75GTG (3059A>G 3061T>G), TGC76TGT (3064C>T), ATT77GTT (3065A>G), ACC80ACT (3076C>T), TTG81CTG (3077T>C), TCA84TCT (3088A>T), GGT85GGG (3091T>G), CTG87CTT (3097G>T), GCA88GCT (3100A>T), ATT89ATA (3103T>A), GGA90GGG (3106A>G), CTT91TTG (3107C>T 3109T>G), GGA92GGC (3112A>C), ACT93ACG (3115T>G), AAC95GCA (3119A>G 3120A>C 3121C>A), ACC96ACA (3124C>A), AAA97GAG (3125A>G 3127A>G), ACC99TCC (3131A>T), AAT100ACA (3135A>C 3136T>A), GCT101GCG (3139T>G), TTT105TTG (3151T>G), CCA108CCC (3160A>C), GTA111GTG (3169A>G), TGC112TGT (3172C>T), CTT114TTG (3176C>T 3178T>G), GGT117GGG (3187T>G), AAA119AGG (3192A>G 3193A>G), CGG121CAA (3198G>A 3199G>A), CGG123AAA (3203C>A 3204G>A 3205G>A), TGT124TGC (3208T>C), TCT125TCA (3211T>A), ATT126CTG (3212A>C 3214T>G), ACA127ACC (3217A>C), GGA131GGG (3229A>G), AAA132TGC (3230A>T 3231A>G 3232A>C), AAT133AAC (3235T>C), TTG134CTC (3236T>C 3238G>C), CTT135CTG (3241T>G), TCC136TCT (3244C>T), ACA137ACC (3247A>C), GGG138GGA (3250G>A), CGA139CGC (3253A>C), AAG140AAA (3256G>A), TCC142TCA (3262C>A), TTG143TTA (3265G>A), CAG147AAG (3275C>A), CAC148CAT (3280C>T), TTT149CAA (3281T>C 3282T>A 3283T>A), TCC150TCT (3286C>T), TTG152ATG (3290T>A), CGC153CGT (3295C>T), TTG154CTG (3296T>C), TTT155TTC (3301T>C), ACG157ACT (3307G>T), GTA158GTT (3310A>T), CTC162CTG (3322C>G), ACG165ACA (3331G>A), ATA167TTG (3335A>T 3337A>G), CAT168CAC (3340T>C), TCC171ACT (3347T>A 3349C>T), GGT173GGA (3355T>A), GTC174GTA (3358C>A), CAG177CAA (3367G>A), AAG179AAA (3373G>A), TGC180CGC (3374T>C), CTT181ATT (3377C>A), AAT183GAT (3383A>G), CTT184CTC (3388T>C), ACT185ACG (3391T>G), TTG186CTT (3392T>C 3394G>T), GGT187GGG (3397T>G), GGC188GGT (3400C>T), ACA189TCT (3401A>T 3403A>T), GTA190GTG (3406A>G), TCT191TCA (3409T>A), GTG192GTT (3412G>T), AAA193AAG (3415A>G), ATA195ATT (3421A>T), GGG197GGT (3427G>T), ACA200ACC (3436A>C), AAA201AAG (3439A>G), GGC202GGG (3442C>G), AAA203AGA (3444A>G), AGC204AGT (3448C>T), TCA205GCT (3449T>G 3451A>T), GTT206GTG (3454T>G), TTC208TTT (3460C>T), CTT209CTG (3463T>G), TTC210TTT (3466C>T), GAT214AAC (3476G>A 3478T>C), TTA215CTG (3479T>C 3481A>G), TCA216AAT (3482T>A 3483C>A 3484A>T), CGT218CGC (3490T>C), ATT221CTT (3497A>C), GAA222GAG (3502A>G), AAG223AAA (3505G>A), TTG224CTG (3506T>C), AAG226AAA (3514G>A), GAC227GAA (3517C>A), AAC228AAT (3520C>T), ATT229GTT (3521A>G), GAG230GAT (3526G>T), GAG231ATC (3527G>A 3528A>T 3529G>C), TAC232TTT (3531A>T 3532C>T), CAG233GAC (3533C>G 3535G>C), GAG234CAG (3536G>C), GGC236GGG (3544C>G), GAT238AAC (3548G>A 3550T>C), CCG239_CCC240del (3551_3556delCCGCCC), AAG242ACA (3561A>C 3562G>A), GCT243TCT (3563G>T)								
Coat protein C (YP_001976157.1)	1	225	100%	1271	77.0%	225 (100%)	158 (70.2%)	0/0/0/0	0
Protein mutations:	S1V (3569A>G 3570G>T), I2S (3572A>T 3573T>C 3574T>C), L3F (3575C>T 3577G>C), S4Q (3578T>C 3579C>A 3580C>G), S10T (3596T>A 3598C>A), M19A (3623A>G 3624T>C 3625G>T), G20A (3627G>C 3628T>A), D24T (3638G>A 3639A>C 3640C>A), E25D (3643A>C), L28V (3650T>G 3652G>A), I30L (3656A>T 3658T>G), I37V (3677A>G 3679A>G), E40D (3688A>T), G41A (3690G>C 3691G>A), H42S (3692C>T 3693A>C), V45I (3701G>A), T58S (3740A>T 3742A>C), N68E (3770A>G 3772T>A), S70T (3776T>A), I71F (3779A>T), I73M (3787A>G), V76T (3794G>A 3795T>C 3796A>T), P80N (3806C>A 3807C>A 3808T>C), N81S (3810A>G), V82I (3812G>A 3814T>A), V85I (3821G>A 3823A>C), V88I (3830G>A), F90L (3836T>C), A92T (3842G>A 3844C>A), P98Q (3861C>A), A99F (3863G>T 3864C>T), L101V (3869C>G 3871T>A), N102S (3872A>T 3873A>C), K103A (3875A>G 3876A>C 3877A>G), N105A (3881A>G 3882A>C 3883T>A), Y106H (3884T>C), V108L (3890G>T), A123T (3935G>A 3937A>C), T126I (3945C>T 3946G>C), T130V (3956A>G 3957C>T 3958T>A), E135Q (3971G>C), H141F (3989C>T 3990A>T 3991T>C), R143K (3996G>A), M144A (3998A>G 3999T>C 4000G>C), R145K (4002G>A 4003G>A), E146S (4004G>T 4005A>C 4006A>C), F147Y (4008T>A 4009C>T), S148D (4010T>G 4011C>A 4012A>T), S149K (4014G>A 4015C>G), K150E (4016A>G 4018G>A), Q151S (4019C>A 4020A>G 4021A>T), M155V (4031A>G 4033G>T), R160K (4047G>A), N163L (4055A>T 4056A>T 4057T>G), I165L (4061A>C 4063C>A), S171V (4079T>G 4080C>T 4081C>T), A172E (4083C>A 4084C>A), I173L (4085A>C 4087A>T), V174I (4088G>A 4090G>T), L180I (4106C>A 4108T>A), I181V (4109A>G 4111A>C), S182K (4112T>A 4113C>A 4114T>G), L185P (4122T>C), S196L (4154T>C 4155C>T), H199N (4163C>A), N205D (4181A>G), I225N (4242T>A)								
Codon mutations:	AGT1GTT (3569A>G 3570G>T), ATT2TCC (3572A>T 3573T>C 3574T>C), CTG3TTC (3575C>T 3577G>C), TCC4CAG (3578T>C 3579C>A 3580C>G), ATA5ATC (3583A>C), AGA6AGG (3586A>G), GAG7GAA (3589G>A), AAA8AAG (3592A>G), TTT9TTC (3595T>C), TCC10ACA (3596T>A 3598C>A), GGT12GGA (3604T>A), GCT13GCA (3607T>A), GTA14GTG (3610A>G), TTC17TTT (3619C>T), TGC18TGT (3622C>T), ATG19GCT (3623A>G 3624T>C 3625G>T), GGT20GCA (3627G>C 3628T>A), GAC24ACA (3638G>A 3639A>C 3640C>A), GAA25GAC (3643A>C), TTG28GTA (3650T>G 3652G>A), GTA29GTT (3655A>T), ATT30TTG (3656A>T 3658T>G), CCT31CCA (3661T>A), GCA33GCC (3667A>C), CCA34CCT (3670A>T), TCC36TCA (3676C>A), ATA37GTG (3677A>G 3679A>G), AGG38CGC (3680A>C 3682G>C), GAA40GAT (3688A>T), GGG41GCA (3690G>C 3691G>A), CAC42TCC (3692C>T 3693A>C), CCT44CCA (3700T>A), GTT45ATT (3701G>A), AAG46AAA (3706G>A), ATC49ATA (3715C>A), TGT51TGC (3721T>C), TTT53TTC (3727T>C), GAT55GAC (3733T>C), ACA58TCC (3740A>T 3742A>C), TCA59TCT (3745A>T), TCA64TCT (3760A>T), GGT65GGA (3763T>A), AAT68GAA (3770A>G 3772T>A), TCA70ACA (3776T>A), ATT71TTT (3779A>T), GTG72GTA (3784G>A), ATA73ATG (3787A>G), AGA75CGA (3791A>C), GTA76ACT (3794G>A 3795T>C 3796A>T), CAA77CAG (3799A>G), TCC78AGC (3800T>A 3801C>G), AGT79TCC (3803A>T 3804G>C 3805T>C), CCT80AAC (3806C>A 3807C>A 3808T>C), AAT81AGT (3810A>G), GTT82ATA (3812G>A 3814T>A), GGA83GGT (3817A>T), GTA85ATC (3821G>A 3823A>C), CTA86TTG (3824C>T 3826A>G), AAT87AAC (3829T>C), GTT88ATT (3830G>A), GCT89GCA (3835T>A), TTT90CTT (3836T>C), GAT91GAC (3841T>C), GCC92ACA (3842G>A 3844C>A), GGC94GGG (3850C>G), TTT97TTC (3859T>C), CCA98CAA (3861C>A), GCT99TTT (3863G>T 3864C>T), GGG100GGT (3868G>T), CTT101GTA (3869C>G 3871T>A), AAT102TCT (3872A>T 3873A>C), AAA103GCG (3875A>G 3876A>C 3877A>G), AAT105GCA (3881A>G 3882A>C 3883T>A), TAT106CAT (3884T>C), GTA108TTA (3890G>T), GGT111GGC (3901T>C), GGA112GGT (3904A>T), GGC113GGA (3907C>A), ACA114ACT (3910A>T), TCA119TCC (3925A>C), TAC120TAT (3928C>T), GGT121GGG (3931T>G), GTG122GTT (3934G>T), GCA123ACC (3935G>A 3937A>C), ACA124ACC (3940A>C), AAT125AAC (3943T>C), ACG126ATC (3945C>T 3946G>C), TCA128TCT (3952A>T), TTC129TTT (3955C>T), ACT130GTA (3956A>G 3957C>T 3958T>A), GAG135CAG (3971G>C), TTT136TTC (3976T>C), TTC137TTT (3979C>T), CCA138CCT (3982A>T), AGG139CGC (3983A>C 3985G>C), CGG140CGT (3988G>T), CAT141TTC (3989C>T 3990A>T 3991T>C), AGG143AAG (3996G>A), ATG144GCC (3998A>G 3999T>C 4000G>C), AGG145AAA (4002G>A 4003G>A), GAA146TCC (4004G>T 4005A>C 4006A>C), TTC147TAT (4008T>A 4009C>T), TCA148GAT (4010T>G 4011C>A 4012A>T), AGC149AAG (4014G>A 4015C>G), AAG150GAA (4016A>G 4018G>A), CAA151AGT (4019C>A 4020A>G 4021A>T), TCC152TCA (4024C>A), CGC153AGA (4025C>A 4027C>A), ATC154ATT (4030C>T), ATG155GTT (4031A>G 4033G>T), TCA156TCT (4036A>T), CTA157TTG (4037C>T 4039A>G), GAT159GAC (4045T>C), AGG160AAG (4047G>A), CTT161CTA (4051T>A), GGA162GGC (4054A>C), AAT163TTG (4055A>T 4056A>T 4057T>G), CTG164TTG (4058C>T), ATC165CTA (4061A>C 4063C>A), ATA166ATC (4066A>C), TTG168CTT (4070T>C 4072G>T), CCT169CCA (4075T>A), CCT170CCA (4078T>A), TCC171GTT (4079T>G 4080C>T 4081C>T), GCC172GAA (4083C>A 4084C>A), ATA173CTT (4085A>C 4087A>T), GTG174ATT (4088G>A 4090G>T), TCC176TCA (4096C>A), GAG178GAA (4102G>A), ATA179ATC (4105A>C), CTT180ATA (4106C>A 4108T>A), ATA181GTC (4109A>G 4111A>C), TCT182AAG (4112T>A 4113C>A 4114T>G), CCT183CCA (4117T>A), GGA184GGT (4120A>T), CTT185CCT (4122T>C), TTC187TTT (4129C>T), AAG188AAA (4132G>A), GCC192GCT (4144C>T), CCT194CCC (4150T>C), CCT195CCA (4153T>A), TCT196CTT (4154T>C 4155C>T), GCC197GCA (4159C>A), CAT199AAT (4163C>A), GAA200GAG (4168A>G), AAA201AAG (4171A>G), CTT203CTC (4177T>C), GGC204GGA (4180C>A), AAT205GAT (4181A>G), CAA207CAG (4189A>G), ACT208ACG (4192T>G), CAC209CAT (4195C>T), ACC210ACT (4198C>T), ACC214ACA (4210C>A), TCA215TCT (4213A>T), CTA220CTG (4228A>G), ATT225AAT (4242T>A)								

*: Inserts / Deletes / Misaligned / Frameshifts

Analysis details

This analysis was performed with panviral2.64

NGS Details (UN18_val): Tomato necrotic dwarf virus (segment RNA1)

Assembly

**Coverage Length**	6096 (1 contig(s))
**Depth Of Coverage**	468.2
**Number Of Reads**	21581
**Reads Per Million**	405.04 rpm (after QC)
**Ambiguities**	0
**Assembly Method**	de novo + reference guided assembly
**Consensus Caller**	Bcf Tools

Coverage Map

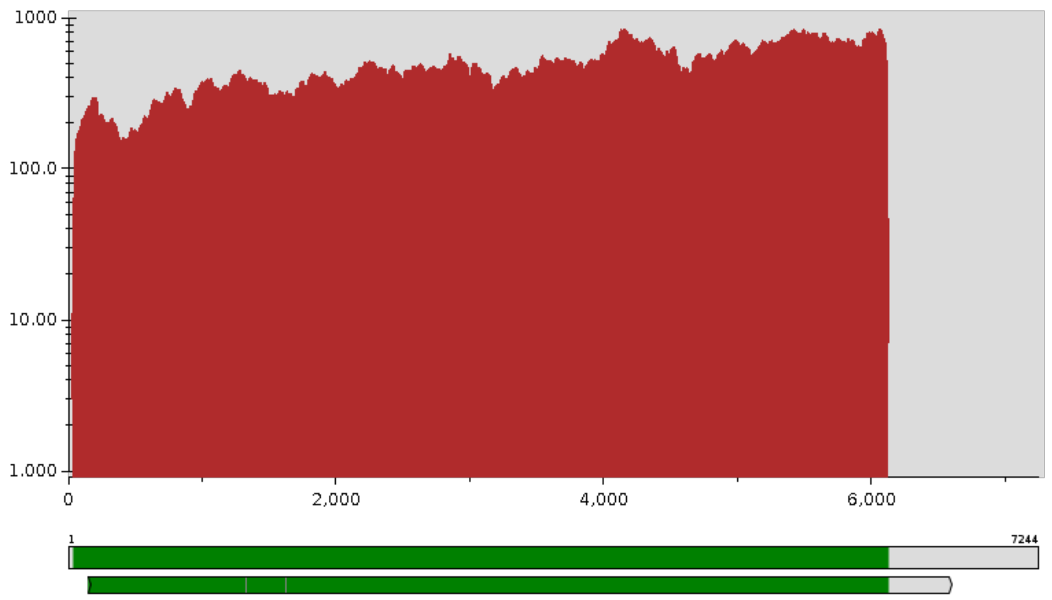

Assignment

**Type**	Tomato necrotic dwarf virus (Taxonomy ID: 1481465)
**Reference Genome**	NC_027926.1
**NT Identity (%)**	64.5613
**AA Identity (%)**	66.0633
**Number Of Stop Codons**	0
**Number Of CDS**	1

Alignment

**Alignment Score**	3374.0 (NT) + 9636.0 (AA) = 13010.0
**Concordance (%)**	50.6383

Alignment Method	Global, seeded, nucleotide + amino acids (AGA)

Genome Region

Sequence starts at position 33 and ends at position 6128 relative to NC_027926.1 reference sequence.

Alignment Detailed Statistics

	Begin	End	Coverage	Score	Concordance	Matches	Identities	I/D/M/F*	Stop Codons
NT	33	6128	84.2%	3374	28.2%	6043 (98.8%)	3915 (64.0%)	21/53	

	Begin	End	Coverage	Score	Concordance	Matches	Identities	I/D/M/F*	Stop Codons
**NT**	**33**	**6128**	**84.2%**	**3374**	**28.2%**	**6043 (98.8%)**	**3915 (64.0%)**	**21/53**	

Mutations:

35T>C, 40G>A, 42C>T, 43C>T, 49T>C, 50C>T, 52A>C, 54T>C, 62A>C, 64T>C, 65T>A, 66G>T, 67G>A, 69A>C, 70T>A, 71T>A, 74A>C, 77G>T, 79T>C, 82A>T, 84G>T,
85_93delCTAAACTTC, 99C>A, 101T>C, 109_119delGAAACTTTTAA, 121A>C, 122C>T, 123A>C, 125T>A, 129T>C, 130T>C, 131C>T, 134C>G, 135T>A, 136T>A, 137A>T, 138T>C,
142G>A, 143T>C, 144G>A, 145A>T, 146T>A, 148A>C, 156T>A, 157C>T, 165A>G, 170T>G, 171T>G, 172G>A, 175T>C, 178T>C, 180A>T, 181T>G,
184_201delGAGAAGACTGGATCAGTT, 202C>T, 204C>T, 207T>A, 208A>C, 213T>A, 221G>C, 222T>A, 226C>G, 228G>T, 232G>T, 233G>C, 238A>T, 240C>T, 243A>T, 244A>T,
245A>C, 246A>T, 247T>C, 249C>T, 255A>G, 256G>A, 259A>C, 261T>C, 262A>T, 263G>C, 267C>T, 268A>G, 270C>T, 272C>A, 273A>T, 274T>A, 275C>A, 279A>T, 287A>C,
288A>C, 292T>G, 293C>T, 294T>A, 300T>C, 301T>A, 306T>G, 310T>G, 312G>C, 315A>G, 318A>G, 319T>C, 321T>A, 324G>T, 326C>T, 327T>G, 329A>C, 330T>A, 334T>G,
336T>A, 337T>A, 339T>A, 342T>A, 347T>C, 348T>C, 357T>C, 358T>A, 359C>A, 360C>T, 363A>T, 364T>G, 366G>A, 367A>G, 369T>A, 371A>G, 375G>T, 376C>T, 378T>A,
387A>T, 389G>C, 390T>A, 391T>A, 392C>A, 393C>A, 394T>A, 396G>A, 399T>G, 405T>C, 408T>C, 409C>A, 411G>C, 414A>G, 416C>G, 417A>T, 420C>T, 421T>G, 422C>A,
427T>G, 429G>A, 430G>A, 431C>A, 435C>T, 445T>A, 450C>A, 451A>G, 452A>T, 453G>A, 457A>C, 459G>T, 462T>A, 463T>C, 465A>T, 466C>A, 467G>A, 469T>A, 472T>G,
474C>T, 475G>T, 476A>T, 481T>G, 483G>C, 484T>A, 485C>A, 486C>G, 487A>G, 493G>T, 495T>C, 497G>T, 498A>C, 499A>C, 506T>A, 507T>C, 508C>G, 510G>A, 514G>T,
516T>A, 522G>A, 525G>A, 526A>G, 527G>T, 532G>T, 533A>C, 534T>A, 535G>T, 543T>A, 546A>G, 547G>T, 550T>A, 551A>T, 552T>A, 554G>C, 561G>A, 570A>T, 571A>T,
573A>G, 574A>G, 577C>A, 580C>T, 582T>G, 583G>C, 585T>A, 586T>C, 588G>A, 589T>C, 593G>A, 597T>A, 604T>G, 605C>T, 609C>T, 612A>T, 613A>G, 615T>A, 616G>A,
617A>C, 618A>G, 619G>C, 620G>C, 621A>T, 622G>T, 624A>T, 625T>A, 626C>A, 629G>A, 631A>G, 632C>T, 633C>A, 634A>G, 637A>G, 638A>T, 639A>T, 641A>C, 643A>T,
645T>G, 648C>T, 650T>C, 652A>G, 654A>T, 657A>T, 660G>A, 664A>T, 666G>T, 669T>C, 673A>T, 674G>C, 675T>A, 684C>T, 687G>A, 690C>T, 699C>A, 701A>C, 702G>A,
706G>T, 710A>G, 717C>T, 718A>T, 720A>C, 721A>C, 722T>A, 724G>C, 726A>G, 729A>G, 736A>G, 737G>C, 738T>A, 739A>G, 741G>A, 744A>T, 753G>A, 754T>A, 755C>G,
756T>C, 757G>T, 759C>G, 760T>A, 762A>G, 765C>T, 768T>A, 774A>T, 775T>A, 776C>G, 780G>A, 781T>C, 783G>T, 784A>T, 786G>A, 789A>C, 790A>T, 792T>A, 793A>T,
795T>G, 796A>T, 801A>T, 810T>C, 813C>A, 816G>T, 817C>T, 819C>A, 822G>A, 826A>G, 828A>T, 831A>T, 834G>T, 837A>T, 840A>G, 846C>A, 853T>C, 855G>T, 856A>T,
857G>C, 858C>A, 861T>C, 864C>A, 867C>G, 876T>C, 885A>T, 886A>G, 891G>A, 897G>T, 900T>G, 915A>G, 916T>C, 921T>C, 922A>C, 923C>A, 924A>G, 933A>G, 936T>C,
942A>G, 948C>T, 951T>A, 954A>T, 960A>C, 963T>C, 966T>A, 972G>T, 978A>G, 981T>A, 982A>G, 984A>G, 988G>A, 990T>C, 991G>A, 992C>T, 1002C>T, 1005G>A, 1014G>A,
1015C>T, 1017T>G, 1020T>A, 1023A>G, 1026T>A, 1028G>A, 1029C>T, 1032A>T, 1035A>C, 1038G>A, 1041T>C, 1044T>C, 1048T>C, 1050G>T, 1054G>C, 1056A>G, 1063C>A,
1064A>C, 1071G>T, 1072G>T, 1073A>C, 1074T>G, 1076C>A, 1077T>G, 1081T>G, 1082G>C, 1086T>A, 1090G>T, 1094C>T, 1095C>G, 1096A>C, 1098G>C, 1099T>C, 1101G>T,
1104G>A, 1107C>T, 1113C>A, 1117A>C, 1118A>C, 1119A>T, 1122C>A, 1125C>T, 1128T>A, 1129G>A, 1130A>T, 1131C>T, 1134A>G, 1138A>C, 1140T>A, 1143C>T, 1144C>T,
1146T>A, 1148G>A, 1149G>A, 1158A>C, 1159C>G, 1161G>C, 1164G>A, 1167T>A, 1173A>T, 1185A>C, 1188C>T, 1194T>C, 1197C>A, 1204A>T, 1212T>C, 1213C>A, 1215A>G,
1218G>T, 1219A>T, 1220G>C, 1224A>T, 1227T>C, 1230C>A, 1233T>G, 1239T>A, 1242A>T, 1245G>A, 1246G>C, 1248C>G, 1249T>G, 1250A>T, 1254G>T, 1258A>G, 1259G>C,
1260T>C, 1261T>A, 1264A>C, 1265G>A, 1270G>T, 1271T>C, 1278T>G, 1281T>C, 1284G>A, 1290A>G, 1296A>G, 1299C>T, 1308T>G, 1316A>T, 1320T>A, 1323C>T, 1329G>A,
1344A>G, 1345G>T, 1346C>G, 1347A>T, 1353A>C, 1354T>C, 1356G>T, 1372T>C, 1374G>C, 1377T>C, 1378C>G, 1379G>C, 1380C>A, 1386G>T, 1389G>A, 1392T>A, 1398T>G,
1402A>C, 1404A>C, 1405C>T, 1407T>G, 1413T>G, 1416T>A, 1419C>T, 1422G>A, 1423C>A, 1425C>A, 1431C>A, 1440T>C, 1443T>C, 1449C>A, 1452C>T, 1455G>A, 1461T>A,
1465T>C, 1467G>T, 1476C>T, 1482A>T, 1485A>T, 1486T>C, 1488G>T, 1489A>C, 1491A>G, 1503A>G, 1506A>G, 1512A>G, 1518T>A, 1536T>C, 1542C>T, 1545A>C, 1551A>T,
1557A>T, 1560G>T, 1563C>A, 1569A>T, 1572T>A, 1575G>C, 1578A>G, 1581C>T, 1584G>A, 1587G>A, 1593T>C, 1599T>A, 1608T>C, 1617A>C, 1626T>C, 1627C>T, 1629T>G,
1630G>A, 1635A>T, 1638A>T, 1641T>A, 1644C>T, 1656T>A, 1659C>G, 1671T>A, 1677G>A, 1678C>A, 1680T>G, 1683A>G, 1686G>A, 1692C>T, 1704T>C, 1706T>G, 1707C>A,
1712A>T, 1713A>G, 1716T>C, 1719A>T, 1723T>G, 1724C>A, 1726A>C, 1729G>A, 1731G>T, 1733A>C, 1734C>T, 1740C>A, 1742G>C, 1743A>C, 1749G>A, 1750A>G, 1752G>T,
1756A>G, 1758C>T, 1759A>G, 1761T>G, 1771C>A, 1773G>A, 1776G>A, 1788A>C, 1798C>G, 1799A>C, 1800C>T, 1804A>T, 1805G>C, 1806T>C, 1809C>T, 1810T>G, 1811C>A,
1812G>T, 1821A>G, 1822G>C, 1824C>A, 1836C>A, 1842T>C, 1844A>G, 1848T>G, 1851T>C, 1852G>T, 1853C>A, 1858A>G, 1860A>G, 1865A>G, 1866G>C, 1869G>A, 1872G>T,
1874T>A, 1875T>C, 1876G>A, 1878A>T, 1879G>A, 1881A>T, 1882G>A, 1883A>C, 1885A>T, 1887T>G, 1890C>A, 1891A>T, 1893T>G, 1894T>G, 1896A>T, 1897A>G, 1903A>G,
1904C>T, 1905A>T, 1908C>T, 1914T>C, 1915G>A, 1916C>A, 1920A>T, 1921T>G, 1924T>G, 1925C>T, 1929A>T, 1932G>A, 1935T>G, 1936G>C, 1940C>A, 1941A>T, 1945G>T,
1947T>C, 1948G>C, 1950T>A, 1952G>A, 1953G>T, 1956T>C, 1957_1959delCGC, 1962C>A, 1963A>C, 1966A>G, 1967C>T, 1968A>T, 1969A>T, 1970A>T, 1971A>G, 1973A>C,
1974A>T, 1975G>C, 1976A>C, 1979T>A, 1984A>G, 1985T>C, 1988T>C, 1989G>T, 1990A>T, 1991C>A, 1993C>G, 1994C>A, 1995C>G, 1996A>C, 1997C>A, 1998A>G, 1999G>A,
2000T>C, 2001T>A, 2004A>T, 2010G>A, 2012A>C, 2013A>T, 2016T>A, 2018G>A, 2020G>A, 2022G>T, 2023A>G, 2031T>C, 2032A>C, 2034A>G, 2038T>C, 2040A>C, 2046G>T,
2049A>G, 2055T>C, 2060T>A, 2067T>C, 2070G>A, 2073C>G, 2077T>A, 2080G>A, 2083C>A, 2084C>A, 2085G>A, 2086G>T, 2087A>T, 2088G>T, 2089A>G, 2090G>A, 2091T>G,
2094G>T, 2097T>C, 2100T>A, 2107T>C, 2108A>T, 2109T>C, 2110C>T, 2111A>C, 2112A>C, 2115A>G, 2118A>G, 2122C>A, 2126T>A, 2131T>G, 2132C>T, 2133C>G, 2137G>T,
2138A>C, 2140G>T, 2142A>T, 2145G>T, 2146C>G, 2147A>C, 2149C>A, 2151G>A, 2154A>G, 2155G>A, 2156T>A, 2157C>T, 2160C>T, 2161A>G, 2162T>A, 2163G>A, 2165G>T,
2166A>G, 2170A>G, 2172G>A, 2174C>A, 2178C>T, 2179A>T, 2182A>T, 2184C>G, 2185T>G, 2186A>T, 2193T>A, 2196C>A, 2198A>G, 2200G>C, 2203G>C, 2205A>G, 2208A>G,
2211C>T, 2212A>G, 2213G>C, 2214G>T, 2225A>G, 2226A>G, 2229T>C, 2245C>A, 2256T>C, 2259A>G, 2262C>T, 2268C>T, 2272G>A, 2273C>G, 2274T>C, 2275T>A, 2277C>A,
2283C>T, 2285G>C, 2290A>G, 2293A>C, 2294A>T, 2300C>A, 2304A>C, 2308A>C, 2309C>G, 2310A>C, 2311G>A, 2313G>T, 2314A>C, 2316G>T, 2317C>G, 2318G>A, 2319C>G,
2320A>C, 2321C>A, 2325A>G, 2326T>A, 2327T>A, 2328T>A, 2331A>G, 2332C>T, 2337T>G, 2338T>G, 2339G>C, 2340T>G, 2341A>C, 2344G>C, 2346T>G, 2354T>C, 2355T>A,
2362T>C, 2364G>T, 2366T>C, 2367T>G, 2370C>T, 2373C>T, 2374C>G, 2376G>A, 2389T>A, 2390T>G, 2391T>A, 2394G>A, 2400T>C, 2401T>C, 2409A>T, 2412C>T, 2421T>C,
2424T>C, 2432C>G, 2433C>G, 2440T>G, 2442C>A, 2442_2443insGGT, 2444A>C, 2445A>T, 2448G>C, 2449G>A, 2450C>A, 2451C>G, 2454A>G, 2455G>A, 2456A>G, 2457C>A,
2458T>A, 2459C>A, 2464G>A, 2466C>T, 2468A>T, 2469G>C, 2470A>G, 2471T>A, 2475T>C, 2476T>A, 2477A>T, 2478C>G, 2479C>A, 2480A>T, 2481C>T, 2482A>G, 2483A>G,
2484A>T, 2487T>C, 2488G>A, 2489T>A, 2493T>C, 2494T>A, 2496C>G, 2501A>T, 2502A>C, 2503G>C, 2505A>G, 2507C>T, 2508T>G, 2512T>A, 2513G>A, 2514G>T, 2515A>G,
2520A>G, 2523A>C, 2525A>T, 2526T>C, 2527G>A, 2528C>T, 2529A>C, 2530C>T, 2534G>A, 2553C>A, 2556A>G, 2560A>T, 2562C>G, 2565G>A, 2568G>A, 2577A>C, 2578T>A,
2579T>C, 2580G>C, 2581T>A, 2583T>G, 2584G>A, 2585G>A, 2586G>T, 2588G>C, 2589A>C, 2590A>G, 2592A>T, 2595A>T, 2601T>C, 2602C>A, 2605G>A, 2610T>C, 2611T>C,
2612G>T, 2613G>C, 2614C>A, 2616A>G, 2617A>G, 2618G>C, 2619C>A, 2620A>G, 2621A>C, 2622T>A, 2623T>A, 2627T>C, 2628T>C, 2631A>T, 2632A>T, 2633G>T, 2634T>A,
2638A>G, 2639G>A, 2643A>C, 2649T>G, 2652T>C, 2655C>A, 2658A>C, 2673C>T, 2679T>C, 2685A>G, 2688G>A, 2691C>T, 2694G>A, 2697C>G, 2698C>A, 2699C>A, 2700C>G,
2701T>A, 2705G>A, 2707C>T, 2709G>C, 2710A>G, 2711A>C, 2712G>A, 2715T>A, 2717T>C, 2718G>C, 2722G>C, 2724G>A, 2730G>A, 2731A>G, 2732G>C, 2733T>G, 2734A>G,
2735G>C, 2736C>A, 2738C>A, 2744T>A, 2745T>C, 2745_2746insCAT, 2746A>G, 2747C>T, 2748A>G, 2749C>G, 2751A>C, 2752G>C, 2753A>G, 2762C>A, 2763T>A, 2764G>C,
2766A>G, 2772G>A, 2778C>T, 2781C>T, 2782C>G, 2785G>A, 2786C>G, 2787A>T, 2790C>G, 2791A>C, 2793A>C, 2796G>A, 2799T>C, 2800A>T, 2803A>G, 2808A>T, 2809C>T,
2812C>A, 2814G>A, 2815C>A, 2819G>C, 2820A>T, 2823G>A, 2829G>C, 2832C>T, 2844A>G, 2850G>T, 2856A>T, 2859G>T, 2865A>T, 2866A>C, 2868G>C, 2883A>C, 2886C>T,
2887C>A, 2888A>G, 2889C>A, 2892T>C, 2893G>A, 2898A>G, 2904A>C, 2907A>T, 2914A>T, 2919T>C, 2922C>T, 2926C>G, 2927C>A, 2928T>G, 2929A>C, 2931C>T, 2934A>C,
2936G>T, 2937G>T, 2939C>G, 2940A>T, 2943G>T, 2951A>T, 2955C>T, 2959C>A, 2960G>C, 2961G>C, 2962G>C, 2967C>A, 2969A>G, 2971G>T, 2973C>G, 2976T>A, 2982A>T,
2989A>G, 2991C>G, 2995C>T, 2998C>T, 3000A>G, 3001C>A, 3003T>G, 3006G>A, 3009T>G, 3012C>T, 3015T>A, 3028C>T, 3030T>A, 3036T>A, 3037C>A, 3039C>A, 3042G>A,
3051A>T, 3063G>T, 3066C>G, 3069C>T, 3071T>A, 3073A>T, 3075C>T, 3078A>G, 3081T>C, 3084T>A, 3085C>A, 3086A>T, 3093A>G, 3096T>C, 3097T>G, 3098G>T, 3099T>G,
3102A>G, 3105C>T, 3108G>A, 3111G>T, 3116C>A, 3117C>G, 3123A>C, 3125G>A, 3126A>G, 3127C>A, 3128T>A, 3129G>A, 3130G>T, 3131G>C, 3135C>T, 3136C>T, 3138A>G,
3141A>G, 3142T>G, 3145T>C, 3147G>T, 3152T>A, 3168C>A, 3171A>G, 3177T>C, 3180A>C, 3182T>A, 3183C>A, 3186G>A, 3189G>C, 3190G>A, 3191A>C, 3192G>A, 3197A>G,
3198G>A, 3204C>T, 3207T>G, 3208G>A, 3209C>A, 3210C>T, 3211A>G, 3212G>C, 3213T>A, 3217A>G, 3218A>C, 3219A>T, 3220T>C, 3222G>T, 3225T>C, 3228G>A, 3231G>A,
3232C>G, 3233A>T, 3234A>T, 3235T>G, 3243A>G, 3246T>C, 3249T>C, 3252G>T, 3256G>A, 3258A>T, 3267T>A, 3276A>T, 3283T>C, 3285A>C, 3286C>T, 3288T>A, 3294C>A,
3296G>C, 3300T>G, 3309A>G, 3312A>G, 3315T>G, 3316G>T, 3317T>G, 3318T>C, 3324G>T, 3327C>A, 3330T>A, 3339T>A, 3342A>T, 3350G>A, 3353T>C, 3354T>C, 3357A>T,
3363A>G, 3369A>T, 3371T>A, 3373A>G, 3375T>G, 3376T>G, 3381C>A, 3384T>C, 3387C>A, 3390G>A, 3393C>A, 3394A>G, 3396C>G, 3399C>T, 3400C>A, 3402G>A, 3405C>T,
3408G>A, 3409C>T, 3411T>G, 3415A>G, 3417C>T, 3429G>A, 3432T>C, 3433G>A, 3440C>T, 3444A>T, 3453A>C, 3459C>A, 3460A>C, 3462T>G, 3463C>T, 3465C>G, 3466A>G,
3468A>G, 3469G>C, 3470C>A, 3472A>C, 3474T>G, 3477G>A, 3478A>T, 3479G>C, 3480T>C, 3482G>A, 3483A>G, 3486A>G, 3487A>G, 3488C>A, 3489C>A, 3491C>G, 3492T>A,
3493C>T, 3495T>C, 3501A>G, 3507C>A, 3510A>G, 3513C>G, 3514T>G, 3516C>T, 3519T>C, 3520C>T, 3522T>G, 3531T>A, 3534T>G, 3535C>T, 3536A>T, 3537A>G, 3541T>A,
3546C>T, 3549A>T, 3555A>G, 3558C>G, 3561T>C, 3567T>A, 3570A>C, 3573C>T, 3575G>A, 3577A>C, 3578A>G, 3579G>C, 3584A>C, 3585A>C, 3586T>C, 3597A>T, 3603T>G,
3604C>G, 3605C>A, 3606T>C, 3610G>C, 3612A>G, 3615C>T, 3618T>C, 3621C>A, 3624A>C, 3627C>T, 3630C>A, 3631A>T, 3633C>T, 3635T>C, 3639T>A, 3646C>A, 3654G>A,
3657A>G, 3660C>T, 3666A>C, 3675A>T, 3681G>C, 3682A>G, 3683T>A, 3687A>G, 3690C>T, 3699T>A, 3703C>T, 3704A>C, 3705T>C, 3706G>A, 3707G>A, 3708T>C, 3714T>G,
3717T>G, 3718G>C, 3719A>C, 3720T>A, 3726T>A, 3729A>G, 3730T>C, 3733T>G, 3741A>G, 3742A>G, 3743C>G, 3747C>T, 3751A>G, 3752G>C, 3753T>A, 3756A>C, 3757C>A,
3759G>T, 3762G>T, 3765A>T, 3766A>G, 3767C>T, 3768A>G, 3769A>C, 3771G>T, 3774A>G, 3776A>T, 3777C>G, 3780A>G, 3781A>C, 3782A>G, 3795C>G, 3801A>T, 3802C>G,
3803A>C, 3804G>A, 3805G>A, 3806A>T, 3807G>C, 3811G>A, 3822G>A, 3828G>C, 3837T>A, 3845G>T, 3854A>T, 3860G>C, 3861C>A, 3868C>T, 3870C>G, 3876A>G, 3879C>A,
3888C>T, 3894C>T, 3901A>T, 3906C>A, 3907A>C, 3909G>T, 3912A>T, 3913A>T, 3914T>C, 3915G>T, 3921G>A, 3922C>A, 3927G>A, 3933C>G, 3935T>A, 3937C>G, 3939A>C,
3940A>C, 3941C>A, 3943G>C, 3944G>C, 3946G>A, 3948A>C, 3949G>T, 3950G>C, 3951T>G, 3952G>C, 3954T>G, 3956A>G, 3957A>G, 3960A>T, 3961G>C, 3962A>T, 3963G>T,
3966T>C, 3968G>A, 3973G>A, 3981A>T, 3984G>C, 3990A>T, 3993T>A, 3999G>A, 4002A>G, 4003C>T, 4008C>T, 4009A>G, 4010T>C, 4011T>G, 4014A>G, 4015A>G, 4017T>A,
4023G>A, 4028T>C, 4030C>T, 4031A>T, 4035G>T, 4038T>C, 4041A>C, 4042A>G, 4047C>A, 4050A>C, 4053A>G, 4059C>G, 4062G>A, 4068G>A, 4071C>G, 4072C>T, 4074T>G,
4077T>A, 4083A>G, 4104A>G, 4107A>C, 4108A>C, 4110A>C, 4111T>C, 4113A>G, 4116T>C, 4119G>A, 4122A>C, 4137C>T, 4146G>A, 4149C>G, 4150A>C, 4161T>C, 4173C>T,
4176C>T, 4177A>T, 4178C>G, 4179C>T, 4188A>T, 4189T>G, 4190T>C, 4197T>C, 4200C>A, 4201A>C, 4206A>T, 4209T>C, 4215T>A, 4218A>T, 4221A>C, 4222G>A, 4230C>G,
4233T>C, 4234T>C, 4235C>A, 4243T>A, 4245T>A, 4246C>G, 4248T>G, 4251T>G, 4254C>T, 4256A>G, 4257C>T, 4259A>G, 4263C>T, 4267G>T, 4269T>A, 4279A>G, 4280C>A,
4281T>G, 4284G>A, 4287T>C, 4293T>C, 4302A>G, 4305T>C, 4311C>T, 4314G>A, 4315C>A, 4317C>A, 4323A>C, 4324G>A, 4326G>C, 4329C>T, 4332T>A, 4335T>C, 4338G>A,
4351T>C, 4356T>G, 4361A>C, 4362G>T, 4364A>T, 4370A>G, 4372A>G, 4373C>A, 4374C>T, 4375A>G, 4383A>G, 4384G>A, 4385A>G, 4386G>T, 4391C>A, 4392T>G, 4395C>A,
4401A>T, 4404A>G, 4407T>C, 4408G>A, 4410T>A, 4411C>T, 4413T>A, 4414C>T, 4416C>A, 4425A>C, 4428A>T, 4437C>T, 4441T>C, 4443A>C, 4450G>A, 4452T>C, 4455A>G,
4456G>T, 4458T>G, 4461C>T, 4464T>C, 4465T>C, 4467G>A, 4470T>G, 4476C>A, 4482G>C, 4494A>T, 4495C>T, 4497T>G, 4500C>T, 4509C>T, 4512C>T, 4516C>T, 4519T>C,
4521G>A, 4537C>A, 4542T>C, 4545T>G, 4548C>T, 4551C>A, 4557T>G, 4560A>G, 4563A>G, 4566G>A, 4569T>A, 4572C>T, 4573A>G, 4575T>C, 4576G>A, 4577C>A, 4578C>A,
4581T>C, 4583G>A, 4585G>T, 4588A>G, 4590A>G, 4593T>C, 4594G>A, 4596A>T, 4602T>A, 4603G>A, 4604G>A, 4608T>C, 4614C>T, 4618C>T, 4620T>G, 4638A>T, 4647T>C,
4648C>T, 4653G>T, 4656T>G, 4659G>T, 4662T>A, 4663C>A, 4669C>T, 4681T>G, 4682C>A, 4683A>T, 4686G>A, 4689T>A, 4690A>T, 4691A>C, 4692T>C, 4698C>A, 4699C>A,
4700A>G, 4701G>A, 4707T>G, 4710C>A, 4711A>G, 4712T>C, 4713G>T, 4716A>T, 4719T>C, 4728C>T, 4731A>G, 4737G>T, 4738C>T, 4743T>C, 4749T>A, 4759A>G, 4761C>G,
4762C>A, 4765G>A, 4766T>C, 4767G>A, 4768C>A, 4770G>A, 4773C>T, 4775A>T, 4779C>T, 4785A>T, 4788A>G, 4794T>C, 4803C>T, 4809A>T, 4812T>C, 4815A>G, 4818T>A,
4824C>A, 4825C>T, 4827T>G, 4830G>A, 4836C>A, 4857G>T, 4861A>G, 4863A>C, 4866T>C, 4872T>C, 4875G>T, 4878C>T, 4884C>T, 4887A>G, 4890T>A, 4894C>G, 4895C>T,
4899T>A, 4909A>G, 4910C>T, 4911T>A, 4912G>C, 4913T>A, 4914G>A, 4917A>T, 4921A>T, 4922T>A, 4923G>T, 4926G>A, 4930G>A, 4932G>T, 4938T>A, 4942C>A, 4944T>C,
4945C>A, 4947T>A, 4950G>A, 4956T>C, 4966A>C, 4967G>A, 4968A>G, 4970T>C, 4971T>C, 4972G>A, 4974A>T, 4980G>A, 4982A>G, 4983G>T, 4986A>T, 4987A>G, 4988C>T,
4989T>A, 4990T>A, 4999C>T, 5000A>T, 5001G>T, 5002T>G, 5004T>A, 5007C>T, 5012G>A, 5013A>G, 5014G>C, 5017C>A, 5019T>A, 5020G>A, 5021G>A, 5022A>T, 5026G>C,
5027G>C, 5029G>A, 5030C>T, 5031A>T, 5033C>T, 5034A>G, 5038T>A, 5040T>G, 5043T>C, 5044T>A, 5049T>C, 5050A>T, 5051T>A, 5052G>T, 5056G>C, 5058G>T, 5061G>A,
5062C>A, 5063A>G, 5064G>T, 5065G>C, 5071A>T, 5073C>G, 5074G>A, 5077A>G, 5093C>A, 5094A>G, 5095T>A, 5096C>G, 5097A>C, 5098G>A, 5099C>T, 5100G>T, 5101T>G,
5102T>G, 5104A>G, 5105C>T, 5106A>T, 5107A>T, 5109C>G, 5110T>A, 5111G>T, 5112T>G, 5115A>G, 5116A>C, 5118G>A, 5119G>T, 5121C>A, 5122A>G, 5124A>T, 5128T>A,
5129C>A, 5130T>A, 5132T>A, 5133C>T, 5136T>A, 5138T>A, 5142A>C, 5148G>A, 5151A>T, 5152C>G, 5158A>G, 5160T>A, 5164G>A, 5167_5169delGAA, 5178A>G, 5179A>G,
5180G>A, 5181A>G, 5183A>G, 5185C>G, 5188A>C, 5191T>G, 5192G>A, 5193G>A, 5198C>T, 5199C>G, 5202A>T, 5203A>G, 5205C>T, 5206A>G, 5208T>A, 5209G>T, 5210G>T,
5217C>T, 5218T>A, 5219C>G, 5220A>T, 5223C>T, 5226T>G, 5233G>T, 5235C>A, 5238G>C, 5239A>C, 5240C>A, 5241T>A, 5242G>C, 5248G>T, 5249A>C, 5250G>A, 5251C>T,
5253T>A, 5256G>T, 5257T>A, 5258G>A, 5262A>C, 5263G>C, 5265A>G, 5271C>T, 5274T>C, 5277A>T, 5280G>A, 5283G>C, 5284A>C, 5285A>G, 5286G>C, 5288A>G, 5289G>A,
5291G>C, 5292C>A, 5294A>C, 5301T>G, 5302C>A, 5304C>A, 5307A>G, 5313T>C, 5315G>A, 5325T>C, 5340G>A, 5346A>C, 5349T>C, 5352C>T, 5353A>T, 5356T>G, 5364T>C,
5366T>A, 5371G>C, 5372C>A, 5377A>C, 5379A>C, 5380G>A, 5381G>A, 5382C>G, 5384A>G, 5385A>T, 5386A>C, 5389T>G, 5390C>A, 5393A>T, 5394G>T, 5396A>G, 5397C>T,
5398A>G, 5399T>A, 5402C>G, 5403T>G, 5410G>T, 5411A>C, 5415T>C, 5416G>A, 5417T>C, 5418G>C, 5424G>T, 5430G>T, 5442T>A, 5443G>A, 5445G>A, 5446A>G, 5447G>C,
5448C>T, 5463T>A, 5466A>G, 5467A>T, 5469G>T, 5472T>A, 5473G>T, 5483G>A, 5487G>A, 5490T>C, 5491T>A, 5493C>G, 5494A>G, 5495T>C, 5496T>C, 5497G>A, 5498A>C,
5499G>A, 5502T>C, 5503A>C, 5505C>T, 5512A>C, 5514G>C, 5515T>C, 5517G>A, 5520C>A, 5523T>C, 5526T>G, 5528G>C, 5529C>T, 5530A>G, 5531A>G, 5532T>G, 5536T>C,
5550A>C, 5553T>C, 5556C>T, 5567T>C, 5568T>G, 5571G>A, 5577T>G, 5581C>A, 5589G>T, 5590C>G, 5591C>A, 5592T>A, 5593C>G, 5595G>C, 5595_5596insACC, 5596G>T,
5597A>C, 5599G>C, 5600G>A, 5601G>C, 5604A>G, 5605A>T, 5609C>G, 5614G>A, 5615T>G, 5620T>A, 5622G>A, 5623_5625delTCT, 5627T>A, 5628G>T, 5629C>G, 5630A>G,
5631A>C, 5633G>C, 5634T>C, 5636G>C, 5642T>A, 5643G>C, 5649A>T, 5649_5650insCACAGT, 5651G>C, 5652T>A, 5656G>T, 5657A>C, 5658G>A, 5659C>G, 5664C>A,

	5666A>T, 5667A>T, 5668A>C, 5670T>A, 5671C>A, 5673T>G, 5674G>A, 5676C>A, 5677A>G, 5679C>T, 5681A>C, 5682C>T, 5683G>T, 5684G>C, 5685T>C, 5688T>C, 5689T>C, 5691A>C, 5694A>T, 5695A>G, 5696T>A, 5697G>A, 5698G>A, 5700A>G, 5707T>G, 5709G>T, 5712C>T, 5713A>T, 5721T>C, 5722G>A, 5723T>C, 5724T>A, 5725G>A, 5727G>T, 5730C>T, 5733A>T, 5734C>G, 5736T>A, 5739T>A, 5742A>T, 5747G>C, 5749A>C, 5751C>G, 5753T>G, 5754T>C, 5755A>T, 5757C>A, 5760A>G, 5763A>G, 5766T>C, 5769T>G, 5770G>A, 5772T>G, 5775A>T, 5776T>G, 5777T>C, 5778G>T, 5781T>G, 5783C>A, 5784A>G, 5786G>C, 5787T>A, 5788C>T, 5790T>G, 5791G>T, 5792A>C, 5793A>T, 5794A>G, 5795A>T, 5796G>C, 5797T>A, 5806G>A, 5807A>C, 5808A>T, 5809T>C, 5811G>T, 5812C>G, 5813T>C, 5814T>C, 5819A>G, 5820G>A, 5821A>G, 5822A>G, 5823C>A, 5824A>G, 5825C>T, 5826G>C, 5830C>G, 5835C>A, 5845G>A, 5847G>A, 5848A>G, 5851A>T, 5853G>C, 5854A>T, 5856T>C, 5860A>C, 5862A>C, 5866G>T, 5867T>G, 5868G>T, 5870C>A, 5872C>A, 5873A>T, 5874A>G, 5878G>C, 5880C>T, 5881A>G, 5882G>A, 5883T>G, 5887G>C, 5889A>G, 5890A>G, 5893C>A, 5894A>G, 5895G>T, 5902T>G, 5904C>A, 5905T>G, 5906A>T, 5907T>A, 5908C>T, 5909C>T, 5912A>T, 5915C>G, 5916C>G, 5918C>G, 5919A>C, 5920A>G, 5921C>T, 5922C>T, 5923G>A, 5924T>A, 5926G>A, 5931G>A, 5932C>A, 5934C>G, 5935A>T, 5936A>T, 5938G>T, 5939T>C, 5940A>C, 5943T>C, 5944G>A, 5945C>T, 5946T>G, 5947G>A, 5950G>C, 5951C>A, 5952T>A, 5955T>G, 5955_5956insATG, 5956A>T, 5958C>A, 5960T>A, 5961T>A, 5962G>A, 5963T>C, 5965A>C, 5968A>G, 5969C>T, 5973T>C, 5975A>G, 5976A>C, 5979T>A, 5981T>G, 5982C>T, 5983C>T, 5985G>T, 5986_5988delGGA, 5991A>C, 5992G>A, 5993T>C, 5994T>A, 5997C>T, 5998C>A, 6000G>T, 6004A>G, 6005A>C, 6006T>A, 6008T>G, 6009T>G, 6012C>A, 6014G>C, 6016C>A, 6017C>A, 6018T>G, 6021G>C, 6022T>A, 6023G>T, 6024G>T, 6024_6025insGTC, 6026A>T, 6027A>G, 6029T>C, 6030T>A, 6031G>A, 6032G>A, 6037G>A, 6042C>G, 6043A>C, 6046_6048delATG, 6050C>G, 6052A>T, 6054A>T, 6055C>A, 6057C>T, 6058T>C, 6060A>T, 6066G>A, 6068G>C, 6069T>A, 6070C>G, 6072T>G, 6073C>T, 6077C>T, 6078A>T, 6086T>C, 6087G>T, 6090G>A, 6094T>C, 6095C>T, 6098C>T, 6099C>T, 6100A>G, 6104A>G, 6105G>A, 6109T>A, 6112C>T, 6114C>G, 6115A>C, 6119G>A

CDS

APL33_sRA11gp1	1	1993	92.7%	9636	70.1%	1982 (99.1%)	1314 (65.7%)	7/11/0/0	0

	Begin	End	Coverage	Score	Concordance	Matches	Identities	I/D/M/F*	Stop Codons
**NT**	**33**	**6128**	**84.2%**	**3374**	**28.2%**	**6043 (98.8%)**	**3915 (64.0%)**	**21/53**	
Protein mutations:	L3F (157C>T), F7W (170T>G 171T>G), G8S (172G>A), S9P (175T>C), S10P (178T>C 180A>T), S11A (181T>G), E12_V17del (184_201delGAGAAGACTGGATCAGTT), P18S (202C>T 204C>T), T20P (208A>C), G24A (221G>C 222T>A), L26V (226C>G 228G>T), G28S (232G>T 233G>C), I30F (238A>T 240C>T), K32S (244A>T 245A>C 246A>T), F33L (247T>C 249C>T), A36T (256G>A), I37L (259A>C 261T>C), I40V (268A>G 270C>T), T41N (272C>A 273A>T), S42K (274T>A 275C>A), K46T (287A>C 288A>C), S48V (292T>G 293C>T 294T>A), L51I (301T>A), D52E (306T>G), L54V (310T>G 312G>C), S57P (319T>C 321T>A), T59M (326C>T 327T>G), D60A (329A>C 330T>A), S62A (334T>G 336T>A), S63T (337T>A 339T>A), V66A (347T>C 348T>C), S70N (358T>A 359C>A 360C>T), L72V (364T>G 366G>A), N73E (367A>G 369T>A), K74R (371A>G), G80A (389G>C 390T>A), S81K (391T>A 392C>A 393C>A), L82I (394T>A 396G>A), I83M (399T>G), L87I (409C>A 411G>C), A89G (416C>G 417A>T), S91D (421T>G 422C>A), L93V (427T>G 429G>A), A94K (430G>A 431C>A), S99T (445T>A), K101V (451A>G 452A>T 453G>A), M103L (457A>C 459G>T), R106K (466C>A 467G>A), L107M (469T>A), F108V (472T>G 474C>T), E109L (475G>T 476A>T), L111V (481T>G 483G>C), S112K (484T>A 485C>A 486C>G), N113D (487A>G), V115F (493G>T 495T>C), R116I (497G>T 498A>C), I117L (499A>C), F119Y (506T>A 507T>C), Q120E (508C>G 510G>A), V122L (514G>T 516T>A), S126V (526A>G 527G>T), D128S (532G>T 533A>C 534T>A), V129L (535G>T), V133F (547G>T), Y134I (550T>A 551A>T 552T>A), G135A (554G>C), I141L (571A>T 573A>G), T142A (574A>G), L143I (577C>A), V145L (583G>C 585T>A), C148Y (593G>A), S152V (604T>G 605C>T), I155V (613A>G 615T>A), E156T (616G>A 617A>C 618A>G), G157P (619G>C 620G>C 621A>T), V158F (622G>T 624A>T), S159N (625T>A 626C>A), S160N (629G>A), T161V (631A>G 632C>T 633C>A), I162V (634A>G), K163V (637A>G 638A>T 639A>T), N164T (641A>C), I165L (643A>T 645T>G), L167S (650T>C), I168V (652A>G 654A>T), M172F (664A>T 666G>T), K184T (701A>C 702G>A), A186S (706G>T), E187G (710A>G), T190S (718A>T 720A>C), M191Q (721A>C 722T>A), V192L (724G>C 726A>G), S196A (736A>G 737G>C 738T>A), M197V (739A>G 741G>A), V203L (757G>T 759C>G), S204T (760T>A 762A>G), M212L (784A>T 786G>A), T214S (790A>T 792T>A), I215L (793A>T 795T>G), M216L (796A>T), I226V (826A>G 828A>T), T246A (886A>G), T258Q (922A>C 923C>A 924A>G), I278V (982A>G 984A>G), G280S (988G>A 990T>C), A281I (991G>A 992C>T), S293N (1028G>A 1029C>T), E302Q (1054G>C 1056A>G), Q305T (1063C>A 1064A>C), D308S (1072G>T 1073A>C 1074T>G), A309E (1076C>A 1077T>G), C311A (1081T>G 1082G>C), D312E (1086T>A), A314S (1090G>T), T315M (1094C>T 1095C>G), K323P (1117A>C 1118A>C 1119A>T), D327I (1129G>A 1130A>T 1131C>T), N330Q (1138A>C 1140T>A), R333K (1148G>A 1149G>A), L337V (1159C>G 1161G>C), M352L (1204A>T), E364D (1242A>T), V366L (1246G>C 1248C>G), Y367V (1249T>G 1250A>T), S370A (1258A>G 1259G>C 1260T>C), C371S (1261T>A), R372Q (1264A>C 1265G>A), V374S (1270G>T 1271T>C), Y389F (1316A>T), A399C (1345G>T 1346C>G 1347A>T), R410A (1378C>G 1379G>C 1380C>A), V494I (1630G>A), I519R (1706T>G 1707C>A), E521V (1712A>T 1713A>G), E523D (1719A>T), S525D (1723T>G 1724C>A), T526P (1726A>C), E527N (1729G>A 1731G>T), D528A (1733A>C 1734C>T), G531A (1742G>C 1743A>C), M534V (1750A>G 1752G>T), N536D (1756A>G 1758C>T), I537V (1759A>G 1761T>G), H550A (1798C>G 1799A>C 1800C>T), S554D (1810T>G 1811C>A 1812G>T), D558Q (1822G>C 1824C>A), Q565R (1844A>G), A568Y (1852G>T 1853C>A), I570V (1858A>G 1860A>G), K572S (1865A>G 1866G>C), F575Y (1874T>A 1875T>C), E576N (1876G>A 1878A>T), A577T (1879G>A 1881A>T), E578T (1882G>A 1883A>C), T579S (1885A>T 1887T>G), I581L (1891A>T 1893T>G), S582A (1894T>G 1896A>T), N583D (1897A>G), T585V (1903A>G 1904C>T 1905A>T), A589N (1915G>A 1916C>A), S591A (1921T>G), S592V (1924T>G 1925C>T), E593D (1929A>T), V596L (1936G>C), A597D (1940C>A 1941A>T), V599F (1945G>T 1947T>C), D600Q (1948G>C 1950T>A), G601D (1952G>A 1953G>T), R603del (1957_1959delCGC), F604L (1962C>A), K605Q (1963A>C), T606V (1966A>G 1967C>T 1968A>T), K607L (1969A>T 1970A>T 1971A>G), Q608P (1973A>C 1974A>T), D609P (1975G>C 1976A>C), V610E (1979T>A), I612A (1984A>G 1985T>C), V613A (1988T>C 1989G>T), T614Y (1990A>T 1991C>A), P615E (1993C>G 1994C>A 1995C>G), T616Q (1996A>C 1997C>A 1998A>G), V617T (1999G>A 2000T>C 2001T>A), E621A (2012A>C 2013A>T), D622E (2016T>A), R623K (2018G>A), V624I (2020G>A 2022G>T), I625V (2023A>G), K628Q (2032A>C 2034A>G), F637Y (2060T>A), L643M (2077T>A), D644N (2080G>A), P645K (2083C>A 2084C>A 2085G>A), E646F (2086G>T 2087A>T 2088G>T), S647E (2089A>G 2090G>A 2091T>G), L648F (2094G>T), Y653L (2107T>C 2108A>T 2109T>C), Q654S (2110C>T 2111A>C 2112A>C), L658I (2122C>A), F659Y (2126T>A), S661V (2131T>G 2132C>T 2133C>G), D663S (2137G>T 2138A>C), A664S (2140G>T 2142A>T), E665D (2145G>T), H666A (2146C>G 2147A>C), Q667K (2149C>A 2151G>A), V669N (2155G>A 2156T>A 2157C>T), M671E (2161A>G 2162T>A 2163G>A), R672M (2165G>T 2166A>G), M674V (2170A>G 2172G>A), T675N (2174C>A), M677L (2179A>T), I678L (2182A>T 2184C>G), Y679V (2185T>G 2186A>T), N683S (2198A>G), A684P (2200G>C), E685Q (2203G>C 2205A>G), R688A (2212A>G 2213G>C 2214G>T), Q692R (2225A>G 2226A>G), L699I (2245C>A), A708S (2272G>A 2273C>G 2274T>C), S709T (2275T>A 2277C>A), C712S (2285G>C), K714E (2290A>G), K715L (2293A>C 2294A>T), A717D (2300C>A), T720R (2308A>C 2309C>G 2310A>C), E721N (2311G>A 2313G>T), M722L (2314A>C 2316G>T), R723E (2317C>G 2318G>A 2319C>G), T724Q (2320A>C 2321C>A), F726K (2326T>A 2327T>A 2328T>A), H728Y (2332C>T), N729K (2337T>G), C730A (2338T>G 2339G>C 2340T>G), I731L (2341A>C), D732Q (2344G>C 2346T>G), V735A (2354T>C 2355T>A), I739T (2366T>C 2367T>G), Q742E (2374C>G 2376G>A), F747R (2389T>A 2390T>G 2391T>A), T761R (2432C>G 2433C>G), S764A (2440T>G 2442C>A), S764_K765insG (2442_2443insGGT), K765T (2444A>C 2445A>T), E766D (2448G>C), A767K (2449G>A 2450C>A 2451C>G), D769R (2455G>A 2456A>G 2457C>A), S770N (2458T>A 2459C>A), V772I (2464G>A 2466C>T), K773I (2468A>T 2469G>C), I774E (2470A>G 2471T>A), Y776M (2476T>A 2477A>T 2478C>G), H777I (2479C>A 2480A>T 2481C>T), K778G (2482A>G 2483A>G 2484A>T), V780K (2488G>A 2489T>A), Y782K (2494T>A 2496C>G), K784I (2501A>T 2502A>C), E785Q (2503G>C 2505A>G), T786M (2507C>T 2508T>G), W788N (2512T>A 2513G>A 2514G>T), N789D (2515A>G), Y792F (2525A>T 2526T>C), A793I (2527G>A 2528C>T 2529A>C), R795K (2534G>A), I804L (2560A>T 2562C>G), M805I (2565G>A), L810T (2578T>A 2579T>C 2580G>C), F811M (2581T>A 2583T>G), G812N (2584G>A 2585G>A 2586G>T), G813A (2588G>C 2589A>C), I814V (2590A>G 2592A>T), L818I (2602C>A), A819T (2605G>A), W821L (2611T>C 2612G>T 2613G>C), Q822K (2614C>A 2616A>G), S823A (2617A>G 2618G>C 2619C>A), N824A (2620A>G 2621A>C 2622T>A), S825T (2623T>A), F826S (2627T>C 2628T>C), S828L (2632A>T 2633G>T 2634T>A), S830D (2638A>G 2639G>A), P850K (2698C>A 2699C>A 2700C>G), S851T (2701T>A), R852H (2705G>A), L853F (2707C>T 2709G>C), K854A (2710A>G 2711A>C 2712G>A), N855K (2715T>A), L856S (2717T>C 2718G>C), V858L (2722G>C 2724G>A), S861A (2731A>G 2732G>C 2733T>G), S862A (2734A>G 2735G>C 2736C>A), A863D (2738C>A), I865N (2744T>A 2745T>C), I865_T866insH (2745_2746insCAT), T866V (2746A>G 2747C>T 2748A>G), Q867D (2749C>G 2751A>C), E868R (2752G>C 2753A>G), A871E (2762C>A 2763T>A), E872Q (2764G>C 2766A>G), Q878E (2782C>G), A879S (2785G>A 2786C>G 2787A>T), K881H (2791A>C 2793A>C), M884L (2800A>T), I885V (2803A>G), L889I (2815C>A), G890A (2819G>C 2820A>T), M906L (2866A>C 2868G>C), H913R (2887C>A 2888A>G 2889C>A), V915M (2893G>A), T922S (2914A>T), P926E (2926C>G 2927C>A 2928T>G), N927H (2929A>C 2931C>T), R929I (2936G>T 2937G>T), S930C (2939C>G 2940A>T), K931N (2943G>T), Y934F (2951A>T), R937T (2959C>A 2960G>C 2961G>C), E938Q (2962G>C), K940R (2969A>G), V941L (2971G>T 2973C>G), I947V (2989A>G 2991C>G), P960S (3028C>T 3030T>A), E971D (3063G>T), F974Y (3071T>A), T975S (3073A>T 3075C>T), Q979M (3085C>A 3086A>T), C983V (3097T>G 3098G>T 3099T>G), T989K (3116C>A 3117C>G), R992K (3125G>A 3126A>G), L993K (3127C>A 3128T>A 3129G>A), G994S (3130G>T 3131G>C), F998V (3142T>G), F1001Y (3152T>A), V1011E (3182T>A 3183C>A), E1014T (3190G>A 3191A>C 3192G>A), E1016G (3197A>G 3198G>A), A1020N (3208G>A 3209C>A 3210C>T), S1021A (3211A>G 3212G>C 3213T>A), K1023A (3217A>G 3218A>C 3219A>T), Q1028V (3232C>G 3233A>T 3234A>T), S1029A (3235T>G), M1034I (3252G>T), G1036S (3256G>A 3258A>T), S1049T (3296G>C), V1056C (3316G>T 3317T>G 3318T>C), G1067D (3350G>A), I1068T (3353T>C 3354T>C), F1074Y (3371T>A), I1075V (3373A>G 3375T>G), S1076A (3376T>G), I1082V (3394A>G 3396C>G), N1089D (3415A>G 3417C>T), V1095I (3433G>A), A1097V (3440C>T), D1103E (3459C>A), I1104L (3460A>C 3462T>G), K1106E (3466A>G 3468A>G), A1107H (3469G>C 3470C>A), I1108L (3472A>C 3474T>G), R1111K (3482G>A 3483A>G), T1113E (3487A>G 3488C>A 3489C>A), T1114R (3491C>G 3492T>A), L1115F (3493C>T 3495T>C), N1119K (3507C>A), S1122A (3514T>G 3516C>T), Q1129L (3535C>T 3536A>T 3537A>G), L1131M (3541T>A), R1142K (3575G>A), K1143R (3577A>C 3578A>G 3579G>C), Q1145P (3584A>C 3585A>C), P1152D (3604C>G 3605C>A 3606T>C), E1154Q (3610G>C 3612A>G), T1161S (3631A>T 3633C>T), V1162A (3635T>C), N1163K (3639T>A), M1178E (3682A>G 3683T>A), H1185S (3703C>T 3704A>C 3705T>C), G1186N (3706G>A 3707G>A 3708T>C), D1188E (3714T>G), D1189E (3717T>G), D1190P (3718G>C 3719A>C 3720T>A), S1195A (3733T>G), T1198G (3742A>G 3743C>G), S1201A (3751A>G 3752G>C 3753T>A), L1203I (3757C>A 3759G>T), T1206V (3766A>G 3767C>T 3768A>G), M1207L (3769A>C 3771G>T), N1209M (3776A>T 3777C>G), K1211R (3781A>C 3782A>G), Q1218A (3802C>G 3803A>C 3804G>A), E1219I (3805G>A 3806A>T 3807G>C), D1221N (3811G>A), C1232F (3845G>T), Y1235F (3854A>T), G1237A (3860G>C 3861C>A), M1251L (3901A>T), M1253L (3907A>C 3909G>T), M1255S (3913A>T 3914T>C 3915G>T), V1262E (3935T>A), P1263A (3937C>G 3939A>C), T1264Q (3940A>C 3941C>A), G1265P (3943G>C 3944G>C), E1266N (3946G>A 3948A>C), G1267S (3949G>T 3950G>C 3951T>G), D1268Q (3952G>C 3954T>G), K1269R (3956A>G 3957A>G), E1271L (3961G>C 3962A>T 3963G>T), R1273K (3968G>A), D1275N (3973G>A), E1280D (3990A>T), I1287A (4009A>G 4010T>C 4011T>G), I1289V (4015A>G 4017T>A), I1293T (4028T>C), H1294F (4030C>T 4031A>T), I1298V (4042A>G), T1343C (4177A>T 4178C>G 4179C>T), L1347A (4189T>G 4190T>C), I1351L (4201A>C), D1358N (4222G>A), S1362H (4234T>C 4235C>A), S1365T (4243T>A 4245T>A), L1366V (4246C>G 4248T>G), N1369S (4256A>G 4257C>T), N1370S (4259A>G), A1373S (4267G>T 4269T>A), T1377E (4279A>G 4280C>A 4281T>G), V1392I (4324G>A 4326G>C), E1404A (4361A>C 4362G>T), Y1405F (4364A>T), D1407G (4370A>G), T1408D (4372A>G 4373C>A 4374C>T), K1409E (4375A>G), E1412S (4384G>A 4385A>G 4386G>T), A1414E (4391C>A 4392T>G), A1420T (4408G>A 4410T>A), D1434N (4450G>A 4452T>C), V1436L (4456G>T 4458T>G), I1475V (4573A>G 4575T>C), A1476K (4576G>A 4577C>A 4578C>A), R1478K (4583G>A), A1479S (4585G>T), I1480V (4588A>G 4590A>G), V1482I (4594G>A 4596A>T), G1485N (4603G>A 4604G>A), L1505M (4663C>A), S1511D (4681T>G 4682C>A 4683A>T), N1514S (4690A>T 4691A>C 4692T>C), Q1517R (4699C>A 4700A>G 4701G>A), D1520E (4710C>A), M1521A (4711A>G 4712T>C 4713G>T), N1537E (4759A>G 4761C>G), Q1538K (4762C>A), V1539T (4765G>A 4766T>C 4767G>A), Q1540K (4768C>A 4770G>A), Q1542L (4775A>T), H1562Q (4836C>A), I1571V (4861A>G 4863A>C), M1575I (4875G>T), P1582V (4894C>G 4895C>T), T1587V (4909A>G 4910C>T 4911T>A), V1588Q (4912G>C 4913T>A 4914G>A), M1591Y (4921A>T 4922T>A 4923G>T), V1594I (4930G>A 4932G>T), L1598I (4942C>A 4944T>C), R1606Q (4966A>C 4967G>A 4968A>G), V1607A (4970T>C 4971T>C), A1608T (4972G>A 4974A>T), K1611S (4982A>G 4983G>T), L1612F (4986A>T), T1613V (4987A>G 4988C>T 4989T>A), Y1614N (4990T>A), Q1617F (4999C>T 5000A>T 5001G>T), C1618G (5002T>G 5004T>A), R1621K (5012G>A 5013A>G), E1622Q (5014G>C), G1624N (5020G>A 5021G>A 5022A>T), G1626P (5026G>C 5027G>C), A1627I (5029G>A 5030C>T 5031A>T), T1628M (5033C>T 5034A>G), F1630M (5038T>A 5040T>G), S1632T (5044T>A), M1634Y (5050A>T 5051T>A 5052G>T), V1636L (5056G>C 5058G>T), Q1638S (5062C>A 5063A>G 5064G>T), E1639Q (5065G>C), I1641L (5071A>T 5073C>G), A1642T (5074G>A), K1643E (5077A>G), A1648E (5093C>A 5094A>G), A1650I (5098G>A 5099C>T 5100G>T), L1651G (5101T>G 5102T>G), T1652V (5104A>G 5105C>T 5106A>T), I1653L (5107A>T 5109C>G), C1654M (5110T>A 5111G>T 5112T>G), M1656L (5116A>C 5118G>A), A1657S (5119G>T 5121C>A), K1658D (5122A>G 5124A>T), S1660K (5128T>A 5129C>A 5130T>A), I1661N (5132T>A 5133C>T), F1663Y (5138T>A), P1668A (5152C>G), N1670E (5158A>G 5160T>A), V1672I (5164G>A), E1673del (5167_5169delGAA), R1677E (5179A>G 5180G>A 5181A>G), D1678G (5183A>G), L1679V (5185C>G), N1680H (5188A>C), W1681E (5191T>G 5192G>A 5193G>A), A1683V (5198C>T 5199C>G), N1685D (5203A>G 5205C>T), I1686V (5206A>G 5208T>A), G1687F (5209G>T 5210G>T), A1695S (5233G>T 5235C>A), K1696N (5238G>C), T1697Q (5239A>C 5240C>A 5241T>A), E1698Q (5242G>C), E1700S (5248G>T 5249A>C 5250G>A), P1701S (5251C>T 5253T>A), C1703N (5257T>A 5258G>A), V1705L (5263G>C 5265A>G), K1712R (5284A>C 5285A>G 5286G>C), K1713R (5288A>G 5289G>A), S1714T (5291G>C 5292C>A), K1715T (5294A>C), S1722N (5315G>A), M1730I (5340G>A), M1735L (5353A>T), S1736A (5356T>G), L1739Q (5366T>A), A1741H (5371G>C 5372C>A), G1744K (5380G>A 5381G>A 5382C>G), K1745S (5384A>G 5385A>T), M1746L (5386A>C), S1747E (5389T>G 5390C>A), E1748V (5393A>T 5394G>T), D1749G (5396A>G 5397C>T), I1750D (5398A>G 5399T>A), A1751G (5402C>G 5403T>G), D1754S (5410G>T 5411A>C), V1756T (5416G>A 5417T>C 5418G>C), V1765I (5443G>A 5445G>A), S1766A (5446A>G 5447G>C 5448C>T), M1773F (5467A>T 5469G>T), V1775L (5473G>T), R1778H (5483G>A), C1781R (5491T>A 5493C>G), I1782A (5494A>G 5495T>C 5496T>C), E1783T (5497G>A 5498A>C 5499G>A), I1785L (5503A>C 5505C>T), I1792M (5526T>G), G1793A (5528G>C 5529C>T), N1794G (5530A>G 5531A>G 5532T>G), V1806A (5567T>C 5568T>G), F1809L (5577T>G), Q1811K (5581C>A), P1814E (5590C>G 5591C>A 5592T>A), Q1815D (5593C>G 5595G>C), Q1815_E1816insT (5595_5596insACC), E1816S (5596G>T 5597A>C), G1817H (5599G>C 5600G>A 5601G>C), M1819L (5605A>T), A1820G (5609C>G), V1822S (5614G>A 5615T>G), S1824T (5620T>A 5622G>A), S1825del (5623_5625delTCT), L1826Y (5627T>A 5628G>T), Q1827G (5629C>G 5630A>G 5631A>C), S1828T (5633G>C 5634T>C), S1829T (5636G>C), M1831N (5642T>A 5643G>C), Q1833H (5649A>T), Q1833_G1834insHS (5649_5650insCACAGT), G1834A (5651G>C 5652T>A), E1836S (5656G>T 5657A>C 5658G>A), L1837V (5659C>G), Q1839L (5666A>T 5667A>T), I1840L (5668A>C 5670T>A), V1842I (5674G>A 5676C>A), I1843V (5677A>G 5679C>T), H1844P (5681A>C 5682C>T), G1845S (5683G>T 5684G>C 5685T>C), M1849E (5695A>G 5696T>A 5697G>A), E1850K (5698G>A 5700A>G), L1853V (5707T>G 5709G>T), M1855L (5713A>T), V1858T (5722G>A 5723T>C 5724T>A), V1859I (5725G>A 5727G>T), H1862E (5734C>G 5736T>A), G1866A (5747G>C), N1867Q (5749A>C 5751C>G), F1868C (5753T>G 5754T>C), T1869S (5755A>T 5757C>A), D1874K (5770G>A 5772T>G), Q1875H (5775A>T), L1876A (5776T>G 5777T>C 5778G>T), F1877L (5781T>G), A1878E (5783C>A 5784A>G), S1879T (5786G>C 5787T>A), E1881S (5791G>T 5792A>C 5793A>T), K1882V (5794A>G 5795A>T 5796G>C), C1883S (5797T>A), E1886T (5806G>A 5807A>C 5808A>T), L1888A (5812C>G 5813T>C 5814T>C), K1890R (5819A>G 5820G>A), N1891G (5821A>G 5822A>G 5823C>A), T1892V (5824A>G 5825C>T 5826G>C), L1894V (5830C>G), E1899K (5845G>A 5847G>A), K1900E (5848A>G), M1901F (5851A>T 5853G>C), T1902S (5854A>T 5856T>C), K1904H (5860A>C 5862A>C), V1906C (5866G>T 5867T>G 5868G>T), A1907E (5870C>A), Q1908M (5872C>A 5873A>T 5874A>G), V1910L (5878G>C 5880C>T), S1911E (5881A>G 5882G>A 5883T>G), V1913L (5887G>C 5889A>G), K1914E (5890A>G), Q1915S (5893C>A 5894A>G 5895G>T), F1918V (5902T>G 5904C>A), Y1919V (5905T>G 5906A>T 5907T>A), P1920F (5908C>T 5909C>T), K1921M (5912A>T), A1922G (5915C>G 5916C>G), T1923S (5918C>G 5919A>C), T1924V (5920A>G 5921C>T 5922C>T), V1925K (5923G>A 5924T>A), E1926K (5926G>A), L1928M (5932C>A 5934C>G), K1929L (5935A>T 5936A>T), V1930S (5938G>T 5939T>C 5940A>C), A1932M (5944G>A 5945C>T 5946T>G), D1933N (5947G>A), A1934Q (5950G>C 5951C>A 5952T>A), H1935Q (5955T>G), H1935_T1936insM (5955_5956insATG), T1936S (5956A>T 5958C>A), I1937K (5960T>A 5961T>A), V1938T (5962G>A 5963T>C), K1939Q (5965A>C), T1940V (5968A>G 5969C>T), K1942S (5975A>G 5976A>C), F1944C (5981T>G 5982C>T), R1945C (5983C>T 5985G>T), G1946del (5986_5988delGGA), V1948T (5992G>A 5993T>C 5994T>A), R1950S (5998C>A 6000G>T), N1952A (6004A>G 6005A>C 6006T>A), F1953W (6008T>G 6009T>G), S1955T (6014G>C), P1956K (6016C>A 6017C>A 6018T>G), E1957D (6021G>C), W1958I (6022T>A 6023G>T 6024G>T), W1958_K1959insV (6024_6025insGTC), K1959M (6026A>T 6027A>G), I1960T (6029T>C 6030T>A), G1961K (6031G>A 6032G>A), E1963K (6037G>A), N1964K (6042C>G), T1965P (6043A>C), M1966del (6046_6048delATG), A1967G (6050C>G), T1968S (6052A>T 6054A>T), L1969I (6055C>A 6057C>T), S1970P (6058T>C 6060A>T), S1973T (6068G>C 6069T>A), L1974V (6070C>G 6072T>G), L1975F (6073C>T), P1976L (6077C>T 6078A>T), L1979P (6086T>C 6087G>T), S1982L (6094T>C 6095C>T), A1983V (6098C>T 6099C>T), M1984V (6100A>G), K1985R (6104A>G 6105G>A), L1987M (6109T>A), S1989R (6115A>C), R1990K (6119G>A)								

	Begin	End	Coverage	Score	Concordance	Matches	Identities	I/D/M/F*	Stop Codons
**NT**	**33**	**6128**	**84.2%**	**3374**	**28.2%**	**6043 (98.8%)**	**3915 (64.0%)**	**21/53**	

GCT2GCA (156T>A), CTT3TTT (157C>T), AAA5AAG (165A>G), TTT7TGG (170T>G 171T>G), GGT8AGT (172G>A), TCT9CCT (175T>C), TCA10CCT (178T>C 180A>T), TCT11GCT (181T>G), GAG12_GTT17del (184_201delGAGAAGACTGGATCAGTT), CCC18TCT (202C>T 204C>T), TCT19TCA (207T>A), ACT20CCT (208A>C), TCT21TCA (213T>A), GGT24GCA (221G>C 222T>A), CTG26GTT (226C>G 228G>T), GGT28TCT (232G>T 233G>C), ATC30TTT (238A>T 240C>T), TCA31TCT (243A>T), AAA32TCT (244A>T 245A>C 246A>T), TTC33CTT (247T>C 249C>T), AAA35AAG (255A>G), GCT36ACT (256G>A), ATT37CTC (259A>C 261T>C), AGC38TCC (262A>T 263G>C), AAC39AAT (267C>T), ATC40GTT (268A>G 270C>T), ACA41AAT (272C>A 273A>T), TCA42AAA (274T>A 275C>A), GTA43GTT (279A>T), AAA46ACC (287A>C 288A>C), TCT48GTA (292T>G 293C>T 294T>A), CAT50CAC (300T>C), TTA51ATA (301T>A), GAT52GAG (306T>G), TTG54GTC (310T>G 312G>C), AAA55AAG (315A>G), CCA56CCG (318A>G), TCT57CCA (319T>C 321T>A), GTG58GTT (324G>T), ACT59ATG (326C>T 327T>G), GAT60GCA (329A>C 330T>A), TCT62GCA (334T>G 336T>A), TCT63ACA (337T>A 339T>A), TCT64TCA (342T>A), GTT66GCC (347T>C 348T>C), TGT69TGC (357T>C), TCC70AAT (358T>A 359C>A 360C>T), GCA71GCT (363A>T), TTG72GTA (364T>G 366G>A), AAT73GAA (367A>G 369T>A), AAA74AGA (371A>G), GTG75GTT (375G>T), CTT76TTA (376C>T 378T>A), ATA79ATT (387A>T), GGT80GCA (389G>C 390T>A), TCC81AAA (391T>A 392C>A 393C>A), TTG82ATA (394T>A 396G>A), ATT83ATG (399T>G), CCT85CCC (405T>C), TTT86TTC (408T>C), CTG87ATC (409C>A 411G>C), AAA88AAG (414A>G), GCA89GGT (416C>G 417A>T), TAC90TAT (420C>T), TCT91GAT (421T>G 422C>A), TTG93GTA (427T>G 429G>A), GCA94AAA (430G>A 431C>A), ACC95ACT (435C>T), TCT99ACT (445T>A), ATC100ATA (450C>A), AAG101GTA (451A>G 452A>T 453G>A), ATG103CTT (457A>C 459G>T), GTT104GTA (462T>A), TTA105CTT (463T>C 465A>T), CGG106AAG (466C>A 467G>A), TTG107ATG (469T>A), TTC108GTT (472T>G 474C>T), GAA109TTA (475G>T 476A>T), TTG111GTC (481T>G 483G>C), TCC112AAG (484T>A 485C>A 486C>G), AAT113GAT (487A>G), GTT115TTC (493G>T 495T>C), AGA116ATC (497G>T 498A>C), ATA117CTA (499A>C), TTT119TAC (506T>A 507T>C), CAG120GAA (508C>G 510G>A), GTT122TTA (514G>T 516T>A), GAG124GAA (522G>A), AAG125AAA (525G>A), AGT126GTT (526A>G 527G>T), GAT128TCA (532G>T 533A>C 534T>A), GTA129TTA (535G>T), ATT131ATA (543T>A), GTA132GTG (546A>G), GTT133TTT (547G>T), TAT134ATA (550T>A 551A>T 552T>A), GGT135GCT (554G>C), TTG137TTA (561G>A), GCA140GCT (570A>T), ATA141TTG (571A>T 573A>G), ACT142GCT (574A>G), CTT143ATT (577C>A), CTT144TTG (580C>T 582T>G), GTT145CTA (583G>C 585T>A), TTG146CTA (586T>C 588G>A), TTG147CTG (589T>C), TGT148TAT (593G>A), GTT149GTA (597T>A), TCA152GTA (604T>G 605C>T), GAC153GAT (609C>T), ATA154ATT (612A>T), ATT155GTA (613A>G 615T>A), GAA156ACG (616G>A 617A>C 618A>G), GGA157CCT (619G>C 620G>C 621A>T), GTA158TTT (622G>T 624A>T), TCT159AAT (625T>A 626C>A), AGT160AAT (629G>A), ACC161GTA (631A>G 632C>T 633C>A), ATT162GTT (634A>G), AAA163GTT (637A>G 638A>T 639A>T), AAT164ACT (641A>C), ATT165TTG (643A>T 645T>G), TTC166TTT (648C>T), TTA167TCA (650T>C), ATA168GTT (652A>G 654A>T), GTA169GTT (657A>T), GGG170GGA (660G>A), ATG172TTT (664A>T 666G>T), TTT173TTC (669T>C), AGT175TCA (673A>T 674G>C 675T>A), AAC178AAT (684C>T), TTG179TTA (687G>A), GAC180GAT (690C>T), CCC183CCA (699C>A), AAG184ACA (701A>C 702G>A), GCT186TCT (706G>T), GAG187GGG (710A>G), TTC189TTT (717C>T), ACA190TCC (718A>T 720A>C), ATG191CAG (721A>C 722T>A), GTA192CTG (724G>C 726A>G), GCA193GCG (729A>G), AGT196GCA (736A>G 737G>C 738T>A), ATG197GTA (739A>G 741G>A), CTA198CTT (744A>T), GAG201GAA (753G>A), TCT202AGC (754T>A 755C>G 756T>C), GTC203TTG (757G>T 759C>G), TCA204ACG (760T>A 762A>G), CAC205CAT (765C>T), ACT206ACA (768T>A), ACA208ACT (774A>T), TCC209AGC (775T>A 776C>G), CAG210CAA (780G>A), TTG211CTT (781T>C 783G>T), ATG212TTA (784A>T 786G>A), TCA213TCC (789A>C), ACT214TCA (790A>T 792T>A), ATT215TTG (793A>T 795T>G), ATG216TTG (796A>T), GCA217GCT (801A>T), ATT220ATC (810T>C), TCC221TCA (813C>A), ACG222ACT (816G>T), CTC223TTA (817C>T 819C>A), GTG224GTA (822G>A), ATA226GTT (826A>G 828A>T), GCA227GCT (831A>T), GTG228GTT (834G>T), CCA229CCT (837A>T), GGA230GGG (840A>G), CCC232CCA (846C>A), TTG235CTT (853T>C 855G>T), AGC236TCA (856A>T 857G>C 858C>A), AAT237AAC (861T>C), CCC238CCA (864C>A), CTC239CTG (867C>G), ATT242ATC (876T>C), TCA245TCT (885A>T), ACT246GCT (886A>G), GGG247GGA (891G>A), GGG249GGT (897G>T), GCT250GCG (900T>G), CAA255CAG (915A>G), TTG256CTG (916T>C), TTT257TTC (921T>C), ACA258CAG (922A>C 923C>A 924A>G), AGA261AGG (933A>G), AAT262AAC (936T>C), AAA264AAG (942A>G), TGC266TGT (948C>T), ACT267ACA (951T>A), TCA268TCT (954A>T), GCA270GCC (960A>C), TTT271TTC (963T>C), TCT272TCA (966T>A), GTG274GTT (972G>T), GAA276GAG (978A>G), ATT277ATA (981T>A), ATA278GTG (982A>G 984A>G), GGT280AGC (988G>A 990T>C), GCT281ATT (991G>A 992C>T), TTC284TTT (1002C>T), AAG285AAA (1005G>A), GTG288GTA (1014G>A), CTT289TTG (1015C>T 1017T>G), TCT290TCA (1020T>A), GCA291GCG (1023A>G), ATT292ATA (1026T>A), AGC293AAT (1028G>A 1029C>T), GCA294GCT (1032A>T), ACA295ACC (1035A>C), TTG296TTA (1038G>A), TCT297TCC (1041T>C), ACT298ACC (1044T>C), TTG300CTT (1048T>C 1050G>T), GAA302CAG (1054G>C 1056A>G), CAG305ACG (1063C>A 1064A>C), GTG307GTT (1071G>T), GAT308TCG (1072G>T 1073A>C 1074T>G), GCT309GAG (1076C>A 1077T>G), TGT311GCT (1081T>G 1082G>C), GAT312GAA (1086T>A), GCT314TCT (1090G>T), ACC315ATG (1094C>T 1095C>G), AGG316CGC (1096A>C 1098G>C), TTG317CTT (1099T>C 1101G>T), GAG318GAA (1104G>A), AAC319AAT (1107C>T), GCC321GCA (1113C>A), AAA323CCT (1117A>C 1118A>C 1119A>T), GCC324GCA (1122C>A), TTC325TTT (1125C>T), CCT326CCA (1128T>A), GAC327ATT (1129G>A 1130A>T 1131C>T), AAA328AAG (1134A>G), AAT330CAA (1138A>C 1140T>A), CAC331CAT (1143C>T), CTT332TTA (1144C>T 1146T>A), AGG333AAA (1148G>A 1149G>A), GCA336GCC (1158A>C), CTG337GTC (1159C>G 1161G>C), AAG338AAA (1164G>A), ATT339ATA (1167T>A), GCA341GCT (1173A>T), ACA345ACC (1185A>C), CAC346CAT (1188C>T), GTT348GTC (1194T>C), GCC349GCA (1197C>A), ATG352TTG (1204A>T), CAT354CAC (1212T>C), CGA355AGG (1213C>A 1215A>G), GTG356GTT (1218G>T), AGT357TCT (1219A>T 1220G>C), GCA358GCT (1224A>T), GCT359GCC (1227T>C), ATC360ATA (1230C>A), ACT361ACG (1233T>G), CTT363CTA (1239T>A), GAA364GAT (1242A>T), AAG365AAA (1245G>A), GTC366CTG (1246G>C 1248C>G), TAT367GTT (1249T>G 1250A>T), GCG368GCT (1254G>T), AGT370GCC (1258A>G 1259G>C 1260T>C), TGC371AGC (1261T>A), AGA372CAA (1264A>C 1265G>A), GTT374TCT (1270G>T 1271T>C), GTT376GTG (1278T>G), GGT377GGC (1281T>C), CAG378CAA (1284G>A), AGA380AGG (1290A>G), GAA382GAG (1296A>G), CCC383CCT (1299C>T), GTT386GTG (1308T>G), TAT389TTT (1316A>T), GGT390GGA (1320T>A), GCC391GCT (1323C>T), GGG393GGA (1329G>A), ACA398ACG (1344A>G), GCA399TGT (1345G>T 1346C>G 1347A>T), CGA401CGC (1353A>C), TTG402CTT (1354T>C 1356G>T), TTG408CTC (1372T>C 1374G>C), GAT409GAC (1377T>C), CGC410GCA (1378C>G 1379G>C 1380C>A), GGG412GGT (1386G>T), GAG413GAA (1389G>A), CCT414CCA (1392T>A), CTT416CTG (1398T>G), AGA418CGC (1402A>C 1404A>C), CTT419TTG (1405C>T 1407T>G), GCT421GCG (1413T>G), GTT422GTA (1416T>A), AGC423AGT (1419C>T), AAG424AAA (1422G>A), CGC425AGA (1423C>A 1425C>A), GCC427GCA (1431C>A), TCT430TCC (1440T>C), AAT431AAC (1443T>C), GCC433GCA (1449C>A), CAC434CAT (1452C>T), CAG435CAA (1455G>A), GCT437GCA (1461T>A), TTG439CTT (1465T>C 1467G>T), GAC442GAT (1476C>T), GGA444GGT (1482A>T), GCA445GCT (1485A>T), TTG446CTT (1486T>C 1488G>T), AGA447CGG (1489A>C 1491A>G), GGA451GGG (1503A>G), CAA452CAG (1506A>G), CAA454CAG (1512A>G), ATT456ATA (1518T>A), ATT462ATC (1536T>C), TCC464TCT (1542C>T), ACA465ACC (1545A>C), CCA467CCT (1551A>T), CCA469CCT (1557A>T), CTG470CTT (1560G>T), CCC471CCA (1563C>A), GCA473GCT (1569A>T), GCT474GCA (1572T>A), GTG475GTC (1575G>C), GAA476GAG (1578A>G), GAC477GAT (1581C>T), AAG478AAA (1584G>A), GGG479GGA (1587G>A), CAT481CAC (1593T>C), ACT483ACA (1599T>A), TAT486TAC (1608T>C), GCA489GCC (1617A>C), AAT492AAC (1626T>C), CTT493TTG (1627C>T 1629T>G), GTA494ATA (1630G>A), TCA495TCT (1635A>T), GCA496GCT (1638A>T), CCT497CCA (1641T>A), GCC498GCT (1644C>T), CTT502CTA (1656T>A), ACC503ACG (1659C>G), GCT507GCA (1671T>A), GAG509GAA (1677G>A), CGT510AGG (1678C>A 1680T>G), AGA511AGG (1683A>G), AGG512AGA (1686G>A), ATC514ATT (1692C>T), TGT518TGC (1704T>C), ATC519AGA (1706T>G 1707C>A), GAA521GTG (1712A>T 1713A>G), GGT522GGC (1716T>C), GAA523GAT (1719A>T), TCT525GAT (1723T>G 1724C>A), ACA526CCA (1726A>C), GAG527AAT (1729G>A 1731G>T), GAC528GCT (1733A>C 1734C>T), ACC530ACA (1740C>A), GGA531GCC (1742G>C 1743A>C), CTG533CTA (1749G>A), ATG534GTT (1750A>G 1752G>T), AAC536GAT (1756A>G 1758C>T), ATT537GTG (1759A>G 1761T>G), CGG541AGA (1771C>A 1773G>A), AGG542AGA (1776G>A), ATA546ATC (1788A>C), CAC550GCT (1798C>G 1799A>C 1800C>T), AGT552TCC (1804A>T 1805G>C 1806T>C), TAC553TAT (1809C>T), TCG554GAT (1810T>G 1811C>A 1812G>T), TTA557TTG (1821A>G), GAC558CAA (1822G>C 1824C>A), GCC562GCA (1836C>A), TGT564TGC (1842T>C), CAG565CGG (1844A>G), GTT566GTG (1848T>G), CAT567CAC (1851T>C), GCT568TAT (1852G>T 1853C>A), ATA570GTG (1858A>G 1860A>G), AAG572AGC (1865A>G 1866G>C), CAG573CAA (1869G>A), CTG574CTT (1872G>T), TTT575TAC (1874T>A 1875T>C), GAA576AAT (1876G>A 1878A>T), GCA577ACT (1879G>A 1881A>T), GAA578ACA (1882G>A 1883A>C), ACT579TCG (1885A>T 1887T>G), GGC580GGA (1890C>A), ATT581TTG (1891A>T 1893T>G), TCA582GCT (1894T>G 1896A>T), AAT583GAT (1897A>G), ACA585GTT (1903A>G 1904C>T 1905A>T), GCC586GCT (1908C>T), GTT588GTC (1914T>C), GCT589AAT (1915G>A 1916C>A), GTA590GTT (1920A>T), TCT591GCT (1921T>G), TCT592GTT (1924T>G 1925C>T), GAA593GAT (1929A>T), GAG594GAA (1932G>A), GTT595GTG (1935T>G), GTG596CTG (1936G>C), GCA597GAT (1940C>A 1941A>T), GTT599TTC (1945G>T 1947T>C), GAT600CAA (1948G>C 1950T>A), GGG601GAT (1952G>A 1953G>T), GCT602GCC (1956T>C), CGC603del (1957_1959delCGC), TTC604TTA (1962C>A), AAA605CAA (1963A>C), ACA606GTT (1966A>G 1967C>T 1968A>T), AAA607TTG (1969A>T 1970A>T 1971A>G), CAA608CCT (1973A>C 1974A>T), GAT609CCT (1975G>C 1976A>C), GTG610GAG (1979T>A), ATA612GCA (1984A>G 1985T>C), GTG613GCT (1988T>C 1989G>T), ACT614TAT (1990A>T 1991C>A), CCC615GAG (1993C>G 1994C>A 1995C>G), ACA616CAG (1996A>C 1997C>A 1998A>G), GTT617ACA (1999G>A 2000T>C 2001T>A), ATA618ATT (2004A>T), GAG620GAA (2010G>A), GAA621GCT (2012A>C 2013A>T), GAT622GAA (2016T>A), AGA623AAA (2018G>A), GTG624ATT (2020G>A 2022G>T), ATT625GTT (2023A>G), TCT627TCC (2031T>C), AAA628CAG (2032A>C 2034A>G), TTA630CTC (2038T>C 2040A>C), GTG632GTT (2046G>T), GAA633GAG (2049A>G), CTT635CTC (2055T>C), TTT637TAT (2060T>A), TAT639TAC (2067T>C), CAG640CAA (2070G>A), GGC641GGG (2073C>G), TTG643ATG (2077T>A), GAC644AAC (2080G>A), CCG645AAA (2083C>A 2084C>A 2085G>A), GAG646TTT (2086G>T 2087A>T 2088G>T), AGT647GAG (2089A>G 2090G>A 2091T>G), TTG648TTT (2094G>T), TTT649TTC (2097T>C), CCT650CCA (2100T>A), TAT653CTC (2107T>C 2108A>T 2109T>C), CAA654TCC (2110C>T 2111A>C 2112A>C), AAA655AAG (2115A>G), CAA656CAG (2118A>G), CTC658ATC (2122C>A), TTT659TAT (2126T>A), TCC661GTG (2131T>G 2132C>T 2133C>G), GAT663TCT (2137G>T 2138A>C), GCA664TCT (2140G>T 2142A>T), GAG665GAT (2145G>T), CAC666GCC (2146C>G 2147A>C), CAG667AAA (2149C>A 2151G>A), CAA668CAG (2154A>G), GTC669AAT (2155G>A 2156T>A 2157C>T), TTC670TTT (2160C>T), ATG671GAA (2161A>G 2162T>A 2163G>A), AGA672ATG (2165G>T 2166A>G), ATG674GTA (2170A>G 2172G>A), ACT675AAT (2174C>A), GAC676GAT (2178C>T), ATG677TTG (2179A>T), ATC678TTG (2182A>T 2184C>G), TAC679GTC (2185T>G 2186A>T), GGT681GGA (2193T>A), GCC682GCA (2196C>A), AAT683AGT (2198A>G), GCT684CCT (2200G>C), GAA685CAG (2203G>C 2205A>G), CAA686CAG (2208A>G), TAC687TAT (2211C>T), AGG688GCT (2212A>G 2213G>C 2214G>T), CAA692CGG (2225A>G 2226A>G), AAT693AAC (2229T>C), CTC699ATC (2245C>A), TTT702TTC (2256T>C), AAA703AAG (2259A>G), AGC704AGT (2262C>T), ATC706ATT (2268C>T), GCT708AGC (2272G>A 2273C>G 2274T>C), TCC709ACA (2275T>A 2277C>A), ATC711ATT (2283C>T), TGT712TCT (2285G>C), AAG714GAG (2290A>G), AAG715CTG (2293A>C 2294A>T), GCT717GAT (2300C>A), GTA718GTC (2304A>C), ACA720CGC (2308A>C 2309C>G 2310A>C), GAG721AAT (2311G>A 2313G>T), ATG722CTT (2314A>C 2316G>T), CGC723GAG (2317C>G 2318G>A 2319C>G), ACA724CAA (2320A>C 2321C>A), GGA725GGG (2325A>G), TTT726AAA (2326T>A 2327T>A 2328T>A), GCA727GCG (2331A>G), CAT728TAT (2332C>T), AAT729AAG (2337T>G), TGT730GCG (2338T>G 2339G>C 2340T>G), ATT731CTT (2341A>C), GAT732CAG (2344G>C 2346T>G), GTT735GCA (2354T>C 2355T>A), TTG738CTT (2362T>C 2364G>T), ATT739ACG (2366T>C 2367T>G), TGC740TGT (2370C>T), ATC741ATT (2373C>T), CAG742GAA (2374C>G 2376G>A), TTT747AGA (2389T>A 2390T>G 2391T>A), GTG748GTA (2394G>A), TTT750TTC (2400T>C), TTG751CTG (2401T>C), ACA753ACT (2409A>T), GCC754GCT (2412C>T), CGT757CGC (2421T>C), TAT758TAC (2424T>C), ACC761AGG (2432C>G 2433C>G), TCC764GCA (2440T>G 2442C>A), TCC764_AAA765insGGT (2442_2443insGGT), AAA765ACT (2444A>C 2445A>T), GAG766GAC (2448G>C), GCC767AAG (2449G>A 2450C>A 2451C>G), AAA768AAG (2454A>G), GAC769AGA (2455G>A 2456A>G 2457C>A), TCT770AAT (2458T>A 2459C>A), GTC772ATT (2464G>A 2466C>T), AAG773ATC (2468A>T 2469G>C), ATA774GAA (2470A>G 2471T>A), TGT775TGC (2475T>C), TAC776ATG (2476T>A 2477A>T 2478C>G), CAC777ATT (2479C>A 2480A>T 2481C>T), AAA778GGT (2482A>G 2483A>G 2484A>T), ATT779ATC (2487T>C), GTG780AAG (2488G>A 2489T>A), GAT781GAC (2493T>C), TAC782AAG (2494T>A 2496C>G), AAA784ATC (2501A>T 2502A>C), GAA785CAG (2503G>C 2505A>G), ACT786ATG (2507C>T 2508T>G), TGG788AAT (2512T>A 2513G>A 2514G>T), AAC789GAC (2515A>G), TTA790TTG (2520A>G), CCA791CCC (2523A>C), TAT792TTC (2525A>T 2526T>C), GCA793ATC (2527G>A 2528C>T 2529A>C), CTG794TTG (2530C>T), AGG795AAG (2534G>A), GGC801GGA (2553C>A), CTA802CTG (2556A>G), ATC804TTG (2560A>T 2562C>G), ATG805ATA (2565G>A), GCG806GCA (2568G>A), GGA809GGC (2577A>C), TTG810ACC (2578T>A 2579T>C 2580G>C), TTT811ATG (2581T>A 2583T>G), GGG812AAT (2584G>A 2585G>A 2586G>T), GGA813GCC (2588G>C 2589A>C), ATA814GTT (2590A>G 2592A>T), ACA815ACT (2595A>T), TTT817TTC (2601T>C), CTT818ATT (2602C>A), GCT819ACT (2605G>A), TGT820TGC (2610T>C), TGG821CTC (2611T>C 2612G>T 2613G>C), CAA822AAG (2614C>A 2616A>G), AGC823GCA (2617A>G 2618G>C 2619C>A), AAT824GCA (2620A>G 2621A>C 2622T>A), TCT825ACT (2623T>A), TTT826TCC (2627T>C 2628T>C), CCA827CCT (2631A>T), AGT828TTA (2632A>T 2633G>T 2634T>A), AGT830GAT (2638A>G 2639G>A), GGA831GGC (2643A>C), GGT833GGG (2649T>G), GGT834GGC (2652T>C), GTC835GTA (2655C>A), ACA836ACC (2658A>C), AGC841AGT (2673C>T), TCT843TCC (2679T>C), AAA845AAG (2685A>G), AAG846AAA (2688G>A), AAC847AAT (2691C>T), AAG848AAA (2694G>A), GGC849GGG (2697C>G), CCC850AAG (2698C>A 2699C>A 2700C>G), TCT851ACT (2701T>A), CGT852CAT (2705G>A), CTG853TTC (2707C>T 2709G>C), AAG854GCA (2710A>G 2711A>C 2712G>A), AAT855AAA (2715T>A), TTG856TCC (2717T>C 2718G>C), GTG858CTA (2722G>C 2724G>A), CAG860CAA (2730G>A), AGT861GCG (2731A>G 2732G>C 2733T>G), AGC862GCA (2734A>G 2735G>C 2736C>A), GCT863GAT (2738C>A), ATT865AAC (2744T>A 2745T>C), ATT865_ACA866insCAT (2745_2746insCAT), ACA866GTG (2746A>G 2747C>T 2748A>G), CAA867GAC (2749C>G 2751A>C), GAA868CGA (2752G>C 2753A>G), GCT871GAA (2762C>A 2763T>A), GAA872CAG (2764G>C 2766A>G), GGG874GGA (2772G>A), GTC876GTT (2778C>T), AAC877AAT (2781C>T), CAA878GAA (2782C>G), GCA879AGT (2785G>A 2786C>G 2787A>T), CTC880CTG (2790C>G), AAA881CAC (2791A>C 2793A>C), AAG882AAA (2796G>A), AAT883AAC (2799T>C), ATG884TTG (2800A>T), ATT885GTT (2803A>G), GTA886GTT (2808A>T), CTG887TTG (2809C>T), CGG888AGA (2812C>A 2814G>A), CTT889ATT (2815C>A), GGA890GCT (2819G>C 2820A>T), GAG891GAA (2823G>A), GTG893GTC (2829G>C), TAC894TAT (2832C>T), ACA898ACG (2844A>G), GTG900GTT (2850G>T), TCA902TCT (2856A>T), GGG903GGT (2859G>T), ATA905ATT (2865A>T), ATG906CTC (2866A>C 2868G>C), GCA911GCC (2883A>C), TTC912TTT (2886C>T), CAC913AGA (2887C>A 2888A>G 2889C>A), AAT914AAC (2892T>C), GTG915ATG (2893G>A), AGA916AGG (2898A>G), GGA918GGC (2904A>C), ACA919ACT (2907A>T), ACC922TCC (2914A>T), ATT923ATC (2919T>C), ATC924ATT (2922C>T), CCT926GAG (2926C>G 2927C>A 2928T>G), AAC927CAT (2929A>C 2931C>T), TCA928TCC (2934A>C), AGG929ATT (2936G>T 2937G>T), TCA930TGT (2939C>G 2940A>T), AAG931AAT (2943G>T), TAC934TTC (2951A>T), AAC935AAT (2955C>T), CGG937ACC (2959C>A 2960G>C 2961G>C), GAA938CAA (2962G>C), TCC939TCA (2967C>A), AAG940AGG (2969A>G), GTC941TTG (2971G>T 2973C>G), ATT942ATA (2976T>A), GGA944GGT (2982A>T), ATC947GTG (2989A>G 2991C>G), CTG949TTG (2995C>T), CTA950TTG

Codon mutations:

(2998C>T 3000A>G), CGT951AGG (3001C>A 3003T>G), GTG952GTA (3006G>A), GGT953GGG (3009T>G), GAC954GAT (3012C>T), CCT955CCA (3015T>A), CCT960TCA (3028C>T 3030T>A), ATT962ATA (3036T>A), CGC963AGA (3037C>A 3039C>A), AAG964AAA (3042G>A), CCA967CCT (3051A>T), GAG971GAT (3063G>T), GTC972GTG (3066C>G), TGC973TGT (3069C>T), TTC974TAC (3071T>A), ACC975TCT (3073A>T 3075C>T), AAA976AAG (3078A>G), GGT977GGC (3081T>C), TCT978TCA (3084T>A), CAG979ATG (3085C>A 3086A>T), TTA981TTG (3093A>G), TGT982TGC (3096T>C), TGT983GTG (3097T>G 3098G>T 3099T>G), AGA984AGG (3102A>G), GCC985GCT (3105C>T), GTG986GTA (3108G>A), GCG987GCT (3111G>T), ACC989AAG (3116C>A 3117C>G), CCA991CCC (3123A>C), AGA992AAG (3125G>A 3126A>G), CTG993AAA (3127C>A 3128T>A 3129G>A), GGC994TCC (3130G>T 3131G>C), AAC995AAT (3135C>T), CTA996TTG (3136C>T 3138A>G), GAA997GAG (3141A>G), TTT998GTT (3142T>G), TTG999CTT (3145T>C 3147G>T), TTT1001TAT (3152T>A), TCC1006TCA (3168C>A), AAA1007AAG (3171A>G), TAT1009TAC (3177T>C), ACA1010ACC (3180A>C), GTC1011GAA (3182T>A 3183C>A), AAG1012AAA (3186G>A), GTG1013GTC (3189G>C), GAG1014ACA (3190G>A 3191A>C 3192G>A), GAG1016GGA (3197A>G 3198G>A), GAC1018GAT (3204C>T), TCT1019TCG (3207T>G), GCC1020AAT (3208G>A 3209C>A 3210C>T), AGT1021GCA (3211A>G 3212G>C 3213T>A), AAA1023GCT (3217A>G 3218A>C 3219A>T), TTG1024CTT (3220T>C 3222G>T), AGT1025AGC (3225T>C), TCG1026TCA (3228G>A), CAG1027CAA (3231G>A), CAA1028GTT (3232C>G 3233A>T 3234A>T), TCA1029GCA (3235T>G), GAA1031GAG (3243A>G), TAT1032TAC (3246T>C), CAT1033CAC (3249T>C), ATG1034ATT (3252G>T), GGA1036AGT (3256G>A 3258A>T), GGT1039GGA (3267T>A), GGA1042GGT (3276A>T), TTA1045CTC (3283T>C 3285A>C), CTT1046TTA (3286C>T 3288T>A), CCC1048CCA (3294C>A), AGT1049ACT (3296G>C), GTT1050GTG (3300T>G), AAA1053AAG (3309A>G), CAA1054CAG (3312A>G), CCT1055CCG (3315T>G), GTT1056TGC (3316G>T 3317T>G 3318T>C), GTG1058GTT (3324G>T), GGC1059GGA (3327C>A), ATT1060ATA (3330T>A), GCT1063GCA (3339T>A), TCA1064TCT (3342A>T), GGT1067GAT (3350G>A), ATT1068ACC (3353T>C 3354T>C), GCA1069GCT (3357A>T), GAA1071GAG (3363A>G), GGA1073GGT (3369A>T), TTC1074TAC (3371T>A), ATT1075GTG (3373A>G 3375T>G), TCT1076GCT (3376T>G), TCC1077TCA (3381C>A), AAT1078AAC (3384T>C), GCC1079GCA (3387C>A), ACG1080ACA (3390G>A), GCC1081GCA (3393C>A), ATC1082GTG (3394A>G 3396C>G), TTC1083TTT (3399C>T), CGG1084AGA (3400C>A 3402G>A), GAC1085GAT (3405C>T), CAG1086CAA (3408G>A), CTT1087TTG (3409C>T 3411T>G), AAC1089GAT (3415A>G 3417C>T), GGG1093GGA (3429G>A), CCT1094CCC (3432T>C), GTT1095ATT (3433G>A), GCG1097GTG (3440C>T), GCA1098GCT (3444A>T), CGA1101CGC (3453A>C), GAC1103GAA (3459C>A), ATT1104CTG (3460A>C 3462T>G), CTC1105TTG (3463C>T 3465C>G), AAA1106GAG (3466A>G 3468A>G), GCC1107CAC (3469G>C 3470C>A), ATT1108CTG (3472A>C 3474T>G), AGG1109AGA (3477G>A), AGT1110TCC (3478A>T 3479G>C 3480T>C), AGA1111AAG (3482G>A 3483A>G), GAA1112GAG (3486A>G), ACC1113GAA (3487A>G 3488C>A 3489C>A), ACT1114AGA (3491C>G 3492T>A), CTT1115TTC (3493C>T 3495T>C), GAA1117GAG (3501A>G), AAC1119AAA (3507C>A), CAA1120CAG (3510A>G), GTC1121GTG (3513C>G), TCC1122GCT (3514T>G 3516C>T), TTT1123TTC (3519T>C), CTT1124TTG (3520C>T 3522T>G), GTT1127GTA (3531T>A), CCT1128CCG (3534T>G), CAA1129TTG (3535C>T 3536A>T 3537A>G), TTG1131ATG (3541T>A), GCC1132GCT (3546C>T), GCA1133GCT (3549A>T), GTA1135GTG (3555A>G), CCC1136CCG (3558C>G), CAT1137CAC (3561T>C), ACT1139ACA (3567T>A), ACA1140ACC (3570A>C), CTC1141CTT (3573C>T), AGA1142AAA (3575G>A), AAG1143CGC (3577A>C 3578A>G 3579G>C), CAA1145CCC (3584A>C 3585A>C), TTG1146CTG (3586T>C), GCA1149GCT (3597A>T), GGT1151GGG (3603T>G), CCT1152GAC (3604C>G 3605C>A 3606T>C), GAA1154CAG (3610G>C 3612A>G), ACC1155ACT (3615C>T), GCT1156GCC (3618T>C), CCC1157CCA (3621C>A), TCA1158TCC (3624A>C), ATC1159ATT (3627C>T), CTC1160CTA (3630C>A), ACC1161TCT (3631A>T 3633C>T), GTC1162GCC (3635T>C), AAT1163AAA (3639T>A), CGG1166AGG (3646C>A), GAG1168GAA (3654G>A), GGA1169GGG (3657A>G), TTC1170TTT (3660C>T), CCA1172CCC (3666A>C), GCA1175GCT (3675A>T), GTG1177GTC (3681G>C), ATG1178GAG (3682A>G 3683T>A), AAA1179AAG (3687A>G), TAC1180TAT (3690C>T), ACT1183ACA (3699T>A), CAT1185TCC (3703C>T 3704A>C 3705T>C), GGT1186AAC (3706G>A 3707G>A 3708T>C), GAT1188GAG (3714T>G), GAT1189GAG (3717T>G), GAT1190CCA (3718G>C 3719A>C 3720T>A), GCT1192GCA (3726T>A), AAA1193AAG (3729A>G), TTG1194CTG (3730T>C), TCC1195GCC (3733T>G), GAA1197GAG (3741A>G), ACT1198GGT (3742A>G 3743C>G), CTC1199CTT (3747C>T), AGT1201GCA (3751A>G 3752G>C 3753T>A), TCA1202TCC (3756A>C), CTG1203ATT (3757C>A 3759G>T), CTG1204CTT (3762G>T), CCA1205CCT (3765A>T), ACA1206GTG (3766A>G 3767C>T 3768A>G), ATG1207CTT (3769A>C 3771G>T), AAA1208AAG (3774A>G), AAC1209ATG (3776A>T 3777C>G), CAA1210CAG (3780A>G), AAA1211CGA (3781A>C 3782A>G), GGC1215GGG (3795C>G), CCA1217CCT (3801A>T), CAG1218GCA (3802C>G 3803A>C 3804G>A), GAG1219ATC (3805G>A 3806A>T 3807G>C), GAT1221AAT (3811G>A), GTG1224GTA (3822G>A), CTG1226CTC (3828G>C), ATT1229ATA (3837T>A), TGT1232TTT (3845G>T), TAT1235TTT (3854A>T), GGC1237GCA (3860G>C 3861C>A), CTC1240TTG (3868C>T 3870C>G), ACA1242ACG (3876A>G), TCC1243TCA (3879C>A), TAC1246TAT (3888C>T), TTC1248TTT (3894C>T), ATG1251TTG (3901A>T), GGC1252GGA (3906C>A), ATG1253CTT (3907A>C 3909G>T), GGA1254GGT (3912A>T), ATG1255TCT (3913A>T 3914T>C 3915G>T), AAG1257AAA (3921G>A), CGG1258AGG (3922C>A), GAG1259GAA (3927G>A), GTC1261GTG (3933C>G), GTA1262GAA (3935T>A), CCA1263GCC (3937C>G 3939A>C), ACA1264CAA (3940A>C 3941C>A), GGA1265CCA (3943G>C 3944G>C), GAA1266AAC (3946G>A 3948A>C), GGT1267TCG (3949G>T 3950G>C 3951T>G), GAT1268CAG (3952G>C 3954T>G), AAA1269AGG (3956A>G 3957A>G), GTA1270GTT (3960A>T), GAG1271CTT (3961G>C 3962A>T 3963G>T), CTT1272CTC (3966T>C), AGA1273AAA (3968G>A), GAC1275AAC (3973G>A), CCA1277CCT (3981A>T), GTG1278GTC (3984G>C), GAA1280GAT (3990A>T), GCT1281GCA (3993T>A), GAG1283GAA (3999G>A), GAA1284GAG (4002A>G), CTG1285TTG (4003C>T), GAC1286GAT (4008C>T), ATT1287GCG (4009A>G 4010T>C 4011T>G), CAA1288CAG (4014A>G), ATT1289GTA (4015A>G 4017T>A), CAG1291CAA (4023G>A), ATA1293ACA (4028T>C), CAT1294TTT (4030C>T 4031A>T), GTG1295GTT (4035G>T), GAT1296GAC (4038T>C), CTA1297CTC (4041A>C), ATC1298GTC (4042A>G), ACC1299ACA (4047C>A), ACA1300ACC (4050A>C), CAA1301CAG (4053A>G), GCC1303GCG (4059C>G), AAG1304AAA (4062G>A), GAG1306GAA (4068G>A), CGC1307CGG (4071C>G), CTT1308TTG (4072C>T 4074T>G), CCT1309CCA (4077T>A), GAA1311GAG (4083A>G), AAA1318AAG (4104A>G), ACA1319ACC (4107A>C), AGA1320CGC (4108A>C 4110A>C), TTA1321CTG (4111T>C 4113A>G), TTT1322TTC (4116T>C), GAG1323GAA (4119G>A), ATA1324ATC (4122A>C), TAC1329TAT (4137C>T), CTG1332CTA (4146G>A), GTC1333GTG (4149C>G), AGG1334CGG (4150A>C), TTT1337TTC (4161T>C), TCC1341TCT (4173C>T), GCC1342GCT (4176C>T), ACC1343TGT (4177A>T 4178C>G 4179C>T), GCA1346GCT (4188A>T), TTA1347GCA (4189T>G 4190T>C), AAT1349AAC (4197T>C), GTC1350GTA (4200C>A), ATC1351CTC (4201A>C), CCA1352CCT (4206A>T), TGT1353TGC (4209T>C), GTT1355GTA (4215T>A), GGA1356GGT (4218A>T), ATA1357ATC (4221A>C), GAT1358AAT (4222G>A), ACC1360ACG (4230C>G), AGT1361AGC (4233T>C), TCT1362CAT (4234T>C 4235C>A), TCT1365ACA (4243T>A 4245T>A), CTT1366GTG (4246C>G 4248T>G), CTT1367CTG (4251T>G), GCC1368GCT (4254C>T), AAC1369AGT (4256A>G 4257C>T), AAC1370AGC (4259A>G), TTC1371TTT (4263C>T), GCT1373TCA (4267G>T 4269T>A), ACT1377GAG (4279A>G 4280C>A 4281T>G), GGG1378GGA (4284G>A), TTT1379TTC (4287T>C), GCT1381GCC (4293T>C), TCA1384TCG (4302A>G), AGT1385AGC (4305T>C), GAC1387GAT (4311C>T), GGG1388GGA (4314G>A), CGC1389AGA (4315C>A 4317C>A), CCA1391CCC (4323A>C), GTG1392ATC (4324G>A 4326G>C), TTC1393TTT (4329C>T), GCT1394GCA (4332T>A), TTT1395TTC (4335T>C), CAG1396CAA (4338G>A), TTG1401CTG (4351T>C), GTT1402GTG (4356T>G), GAG1404GCT (4361A>C 4362G>T), TAT1405TTT (4364A>T), GAT1407GGT (4370A>G), ACC1408GAT (4372A>G 4373C>A 4374C>T), AAG1409GAG (4375A>G), GGA1411GGG (4383A>G), GAG1412AGT (4384G>A 4385A>G 4386G>T), GCT1414GAG (4391C>A 4392T>G), TCC1415TCA (4395C>A), GCA1417GCT (4401A>T), AGA1418AGG (4404A>G), CAT1419CAC (4407T>C), GCT1420ACA (4408G>A 4410T>A), CTT1421TTA (4411C>T 4413T>A), CTC1422TTA (4414C>T 4416C>A), GCA1425GCC (4425A>C), TCA1426TCT (4428A>T), TAC1429TAT (4437C>T), TTA1431CTC (4441T>C 4443A>C), GAT1434AAC (4450G>A 4452T>C), AAA1435AAG (4455A>G), GTT1436TTG (4456G>T 4458T>G), TTC1437TTT (4461C>T), CGT1438CGC (4464T>C), TTG1439CTA (4465T>C 4467G>A), GTT1440GTG (4470T>G), GGC1442GGA (4476C>A), CCG1444CCC (4482G>C), GCA1448GCT (4494A>T), CTT1449TTG (4495C>T 4497T>G), ACC1450ACT (4500C>T), TTC1453TTT (4509C>T), AAC1454AAT (4512C>T), CTG1456TTG (4516C>T), TTG1457CTA (4519T>C 4521G>A), CGG1463AGG (4537C>A), TAT1464TAC (4542T>C), GCT1465GCG (4545T>G), TTC1466TTT (4548C>T), ATC1467ATA (4551C>A), CTT1469CTG (4557T>G), TTA1470TTG (4560A>G), AGA1471AGG (4563A>G), AGG1472AGA (4566G>A), CCT1473CCA (4569T>A), CAC1474CAT (4572C>T), ATT1475GTC (4573A>G 4575T>C), GCC1476AAA (4576G>A 4577C>A 4578C>A), GCT1477GCC (4581T>C), AGA1478AAA (4583G>A), GCT1479TCT (4585G>T), ATA1480GTG (4588A>G 4590A>G), GGT1481GGC (4593T>C), GTA1482ATT (4594G>A 4596A>T), CCT1484CCA (4602T>A), GGT1485AAT (4603G>A 4604G>A), GAT1486GAC (4608T>C), AAC1488AAT (4614C>T), CTT1490TTG (4618C>T 4620T>G), GGA1496GGT (4638A>T), AAT1499AAC (4647T>C), CTG1500TTG (4648C>T), GTG1501GTT (4653G>T), GCT1502GCG (4656T>G), GTG1503GTT (4659G>T), CCT1504CCA (4662T>A), CTG1505ATG (4663C>A), CTG1507TTG (4669C>T), TCA1511GAT (4681T>G 4682C>A 4683A>T), CTG1512CTA (4686G>A), CCT1513CCA (4689T>A), AAT1514TCC (4690A>T 4691A>C 4692T>C), GCC1516GCA (4698C>A), CAG1517AGA (4699C>A 4700A>G 4701G>A), CTT1519CTG (4707T>G), GAC1520GAA (4710C>A), ATG1521GCT (4711A>G 4712T>C 4713G>T), GTA1522GTT (4716A>T), AAT1523AAC (4719T>C), ATC1526ATT (4728C>T), AAA1527AAG (4731A>G), GGG1529GGT (4737G>T), CTG1530TTG (4738C>T), GAT1531GAC (4743T>C), TCT1533TCA (4749T>A), AAC1537GAG (4759A>G 4761C>G), CAG1538AAG (4762C>A), GTG1539ACA (4765G>A 4766T>C 4767G>A), CAG1540AAA (4768C>A 4770G>A), TTC1541TTT (4773C>T), CAG1542CTG (4775A>T), GAC1543GAT (4779C>T), TCA1545TCT (4785A>T), GAA1546GAG (4788A>G), ACT1548ACC (4794T>C), AGC1551AGT (4803C>T), GGA1553GGT (4809A>T), TTT1554TTC (4812T>C), AAA1555AAG (4815A>G), CGT1556CGA (4818T>A), GCC1558GCA (4824C>A), CTT1559TTG (4825C>T 4827T>G), GGG1560GGA (4830G>A), CAC1562CAA (4836C>A), GTG1569GTT (4857G>T), ATA1571GTC (4861A>G 4863A>C), ATT1572ATC (4866T>C), CCT1574CCC (4872T>C), ATG1575ATT (4875G>T), TAC1576TAT (4878C>T), ATC1578ATT (4884C>T), AGA1579AGG (4887A>G), CCT1580CCA (4890T>A), CCG1582GTG (4894C>G 4895C>T), GGT1583GGA (4899T>A), ACT1587GTA (4909A>G 4910C>T 4911T>A), GTG1588CAA (4912G>C 4913T>A 4914G>A), GCA1589GCT (4917A>T), ATG1591TAT (4921A>T 4922T>A 4923G>T), GAG1592GAA (4926G>A), GTG1594ATT (4930G>A 4932G>T), ACT1596ACA (4938T>A), CTT1598ATC (4942C>A 4944T>C), CGT1599AGA (4945C>A 4947T>A), GAG1600GAA (4950G>A), TTT1602TTC (4956T>C), AGA1606CAG (4966A>C 4967G>A 4968A>G), GTT1607GCC (4970T>C 4971T>C), GCA1608ACT (4972G>A 4974A>T), GAG1610GAA (4980G>A), AAG1611AGT (4982A>G 4983G>T), TTA1612TTT (4986A>T), ACT1613GTA (4987A>G 4988C>T 4989T>A), TAT1614AAT (4990T>A), CAG1617TTT (4999C>T 5000A>T 5001G>T), TGT1618GGA (5002T>G 5004T>A), GCC1619GCT (5007C>T), AGA1621AAG (5012G>A 5013A>G), GAG1622CAG (5014G>C), CGT1623AGA (5017C>A 5019T>A), GGA1624AAT (5020G>A 5021G>A 5022A>T), GGC1626CCC (5026G>C 5027G>C), GCA1627ATT (5029G>A 5030C>T 5031A>T), ACA1628ATG (5033C>T 5034A>G), TTT1630ATG (5038T>A 5040T>G), CCT1631CCC (5043T>C), TCT1632ACT (5044T>A), TAT1633TAC (5049T>C), ATG1634TAT (5050A>T 5051T>A 5052G>T), GTG1636CTT (5056G>C 5058G>T), GAG1637GAA (5061G>A), CAG1638AGT (5062C>A 5063A>G 5064G>T), GAA1639CAA (5065G>C), ATC1641TTG (5071A>T 5073C>G), GCA1642ACA (5074G>A), AAG1643GAG (5077A>G), GCA1648GAG (5093C>A 5094A>G), TCA1649AGC (5095T>A 5096C>G 5097A>C), GCG1650ATT (5098G>A 5099C>T 5100G>T), TTG1651GGG (5101T>G 5102T>G), ACA1652GTT (5104A>G 5105C>T 5106A>T), ATC1653TTG (5107A>T 5109C>G), TGT1654ATG (5110T>A 5111G>T 5112T>G), GAA1655GAG (5115A>G), ATG1656CTA (5116A>C 5118G>A), GCC1657TCA (5119G>T 5121C>A), AAA1658GAT (5122A>G 5124A>T), TCT1660AAA (5128T>A 5129C>A 5130T>A), ATC1661AAT (5132T>A 5133C>T), ACT1662ACA (5136T>A), TTC1663TAC (5138T>A), ACA1664ACC (5142A>C), TTG1666TTA (5148G>A), CCA1667CCT (5151A>T), CCT1668GCT (5152C>G), AAT1670GAA (5158A>G 5160T>A), GTT1672ATT (5164G>A), GAA1673del (5167_5169delGAA), GAA1676GAG (5178A>G), AGA1677GAG (5179A>G 5180G>A 5181A>G), GAT1678GGT (5183A>G), CTC1679GTC (5185C>G), AAT1680CAT (5188A>C), TGG1681GAA (5191T>G 5192G>A 5193G>A), GCC1683GTG (5198C>T 5199C>G), CCA1684CCT (5202A>T), AAC1685GAT (5203A>G 5205C>T), ATT1686GTA (5206A>G 5208T>A), GGT1687TTT (5209G>T 5210G>T), TGC1689TGT (5217C>T), TCA1690AGT (5218T>A 5219C>G 5220A>T), GCC1691GCT (5223C>T), CGT1692CGG (5226T>G), GCC1695TCA (5233G>T 5235C>A), AAG1696AAC (5238G>C), ACT1697CAA (5239A>C 5240C>A 5241T>A), GAA1698CAA (5242G>C), GAG1700TCA (5248G>T 5249A>C 5250G>A), CCT1701TCA (5251C>T 5253T>A), GGG1702GGT (5256G>T), TGC1703AAC (5257T>A 5258G>A), ATA1704ATC (5262A>C), GTA1705CTG (5263G>C 5265A>G), AAC1707AAT (5271C>T), TGT1708TGC (5274T>C), ACA1709ACT (5277A>T), GGG1710GGA (5280G>A), GCG1711GCC (5283G>C), AAG1712CGC (5284A>C 5285A>G 5286G>C), AAG1713AGA (5288A>G 5289G>A), AGC1714ACA (5291G>C 5292C>A), AAA1715ACA (5294A>C), GTT1717GTG (5301T>G), CGC1718AGA (5302C>A 5304C>A), GGA1719GGG (5307A>G), GCT1721GCC (5313T>C), AGT1722AAT (5315G>A), GAT1725GAC (5325T>C), ATG1730ATA (5340G>A), CCA1732CCC (5346A>C), TAT1733TAC (5349T>C), ACC1734ACT (5352C>T), ATG1735TTG (5353A>T), TCA1736GCA (5356T>G), ATT1738ATC (5364T>C), CTG1739CAG (5366T>A), GCT1741CAT (5371G>C 5372C>A), AGA1743CGC (5377A>C 5379A>C), GGC1744AAG (5380G>A 5381G>A 5382C>G), AAA1745AGT (5384A>G 5385A>T), ATG1746CTG (5386A>C), TCA1747GAA (5389T>G 5390C>A), GAG1748GTT (5393A>T 5394G>T), GAC1749GGT (5396A>G 5397C>T), ATT1750GAT (5398A>G 5399T>A), GCT1751GGG (5402C>G 5403T>G), GAT1754TCT (5410G>T 5411A>C), GTT1755GTC (5415T>C), GTG1756ACC (5416G>A 5417T>C 5418G>C), GTG1758GTT (5424G>T), GGG1760GGT (5430G>T), GCT1764GCA (5442T>A), GTG1765ATA (5443G>A 5445G>A), AGC1766GCT (5446A>G 5447G>C 5448C>T), GCT1771GCA (5463T>A), TTA1772TTG (5466A>G), ATG1773TTT (5467A>T 5469G>T), GCT1774GCA (5472T>A), GTG1775TTG (5473G>T), CGT1778CAT (5483G>A), CAG1779CAA (5487G>A), TAT1780TAC (5490T>C), TGC1781AGG (5491T>A 5493C>G), ATT1782GCC (5494A>G 5495T>C 5496T>C), GAG1783ACA (5497G>A 5498A>C 5499G>A), GAT1784GAC (5502T>C), ATC1785CTT (5503A>C 5505C>T), AGG1788CGC (5512A>C 5514G>C), TTG1789CTA (5515T>C 5517G>A), CGC1790CGA (5520C>A), ACT1791ACC (5523T>C), ATT1792ATG (5526T>G), GGC1793GCT (5528G>C 5529C>T), AAT1794GGG (5530A>G 5531A>G 5532T>G), TTG1796CTG (5536T>C), ACA1800ACC (5550A>C), TAT1801TAC (5553T>C), CCC1802CCT (5556C>T), GTT1806GCG (5567T>C 5568T>G), CAG1807CAA (5571G>A), TTT1809TTG (5577T>G), CAA1811AAA (5581C>A), GTG1813GTT (5589G>T), CCT1814GAA (5590C>G 5591C>A 5592T>A), CAG1815GAC (5593C>G 5595G>C), CAG1815_GAA1816insACC (5595_5596insACC), GAA1816TCA (5596G>T 5597A>C), GGG1817CAC (5599G>C 5600G>A 5601G>C), AAA1818AAG (5604A>G), ATG1819TTG (5605A>T), GCA1820GGA (5609C>G), GTT1822AGT (5614G>A 5615T>G), TCG1824ACA (5620T>A 5622G>A), TCT1825del (5623_5625delTCT), TTG1826TAT (5627T>A 5628G>T), CAA1827GGC (5629C>G 5630A>G 5631A>C), AGT1828ACC (5633G>C 5634T>C), AGT1829ACT (5636G>C), ATG1831AAC (5642T>A 5643G>C), CAA1833CAT (5649A>T), CAA1833_GGT1834insCACAGT (5649_5650insCACAGT), GGT1834GCA (5651G>C 5652T>A), GAG1836TCA (5656G>T 5657A>C 5658G>A), CTT1837GTT (5659C>G), GGC1838GGA (5664C>A), CAA1839CTT (5666A>T 5667A>T), ATT1840CTA (5668A>C 5670T>A), CGT1841AGG (5671C>A 5673T>G), GTC1842ATA (5674G>A 5676C>A), ATC1843GTT (5677A>G 5679C>T), CAC1844CCT (5681A>C 5682C>T), GGT1845TCC (5683G>T 5684G>C 5685T>C), GAT1846GAC (5688T>C), TTA1847CTC (5689T>C 5691A>C), TCA1848TCT (5694A>T), ATG1849GAA (5695A>G 5696T>A 5697G>A), GAA1850AAG (5698G>A 5700A>G), TTG1853GTT (5707T>G 5709G>T), CGC1854CGT (5712C>T), ATG1855TTG (5713A>T), TAT1857TAC (5721T>C), GTT1858ACA (5722G>A 5723T>C 5724T>A), GTG1859ATT (5725G>A 5727G>T), GGC1860GGT (5730C>T), CCA1861CCT (5733A>T), CAT1862GAA (5734C>G 5736T>A), GGT1863GGA (5739T>A), GGA1864GGT (5742A>T), GGC1866GCC (5747G>C), AAC1867CAG (5749A>C 5751C>G), TTT1868TGC (5753T>G 5754T>C), ACC1869TCA (5755A>T 5757C>A), ACA1870ACG (5760A>G), AAA1871AAG (5763A>G), AAT1872AAC (5766T>C), CTT1873CTG (5769T>G), GAT1874AAG (5770G>A 5772T>G), CAA1875CAT (5775A>T), TTG1876GCT (5776T>G 5777T>C 5778G>T), TTT1877TTG (5781T>G), GCA1878GAG (5783C>A 5784A>G), AGT1879ACA (5786G>C 5787T>A), CTT1880TTG (5788C>T 5790T>G), GAA1881TCT (5791G>T 5792A>C 5793A>T), AAG1882GTC (5794A>G 5795A>T 5796G>C), TGT1883AGT (5797T>A), GAA1886ACT (5806G>A 5807A>C 5808A>T), TTG1887CTT (5809T>C 5811G>T), CTT1888GCC (5812C>G 5813T>C 5814T>C), AAG1890AGA (5819A>G 5820G>A), AAC1891GGA (5821A>G 5822A>G 5823C>A), ACG1892GTC (5824A>G 5825C>T 5826G>C), CTG1894GTG (5830C>G), ACC1895ACA (5835C>A), GAG1899AAA (5845G>A 5847G>A), AAA1900GAA (5848A>G), ATG1901TTC (5851A>T 5853G>C), ACT1902TCC (5854A>T 5856T>C), AAA1904CAC (5860A>C 5862A>C), GTG1906TGT (5866G>T 5867T>G 5868G>T), GCA1907GAA (5870C>A), CAA1908ATG (5872C>A 5873A>T 5874A>G), GTC1910CTT (5878G>C 5880C>T), AGT1911GAG (5881A>G 5882G>A 5883T>G), GTA1913CTG (5887G>C 5889A>G), AAA1914GAA (5890A>G), CAG1915AGT (5893C>A 5894A>G 5895G>T), TTC1918GTA

	(5902T>G 5904C>A), TAT1919GTA (5905T>G 5906A>T 5907T>A), CCC1920TTC (5908C>T 5909C>T), AAG1921ATG (5912A>T), GCC1922GGG (5915C>G 5916C>G), ACA1923AGC (5918C>G 5919A>C), ACC1924GTT (5920A>G 5921C>T 5922C>T), GTG1925AAG (5923G>A 5924T>A), GAA1926AAA (5926G>A), CAG1927CAA (5931G>A), CTC1928ATG (5932C>A 5934C>G), AAG1929TTG (5935A>T 5936A>T), GTA1930TCC (5938G>T 5939T>C 5940A>C), TTT1931TTC (5943T>C), GCT1932ATG (5944G>A 5945C>T 5946T>G), GAT1933AAT (5947G>A), GCT1934CAA (5950G>C 5951C>A 5952T>A), CAT1935CAG (5955T>G), CAT1935_ACC1936insATG (5955_5956insATG), ACC1936TCA (5956A>T 5958C>A), ATT1937AAA (5960T>A 5961T>A), GTC1938ACC (5962G>A 5963T>C), AAA1939CAA (5965A>C), ACT1940GTT (5968A>G 5969C>T), GCT1941GCC (5973T>C), AAA1942AGC (5975A>G 5976A>C), CCT1943CCA (5979T>A), TTC1944TGT (5981T>G 5982C>T), CGG1945TGT (5983C>T 5985G>T), GGA1946del (5986_5988delGGA), GTA1947GTC (5991A>C), GTT1948ACA (5992G>A 5993T>C 5994T>A), TTC1949TTT (5997C>T), CGG1950AGT (5998C>A 6000G>T), AAT1952GCA (6004A>G 6005A>C 6006T>A), TTT1953TGG (6008T>G 6009T>G), CTC1954CTA (6012C>A), AGT1955ACT (6014G>C), CCT1956AAG (6016C>A 6017C>A 6018T>G), GAG1957GAC (6021G>C), TGG1958ATT (6022T>A 6023G>T 6024G>T), TGG1958_AAA1959insGTC (6024_6025insGTC), AAA1959ATG (6026A>T 6027A>G), ATT1960ACA (6029T>C 6030T>A), GGA1961AAA (6031G>A 6032G>A), GAG1963AAG (6037G>A), AAC1964AAG (6042C>G), ACA1965CCA (6043A>C), ATG1966del (6046_6048delATG), GCA1967GGA (6050C>G), ACA1968TCT (6052A>T 6054A>T), CTC1969ATT (6055C>A 6057C>T), TCA1970CCT (6058T>C 6060A>T), GAG1972GAA (6066G>A), AGT1973ACA (6068G>C 6069T>A), CTT1974GTG (6070C>G 6072T>G), CTT1975TTT (6073C>T), CCA1976CTT (6077C>T 6078A>T), CTG1979CCT (6086T>C 6087G>T), TCG1980TCA (6090G>A), TCA1982CTA (6094T>C 6095C>T), GCC1983GTT (6098C>T 6099C>T), ATG1984GTG (6100A>G), AAG1985AGA (6104A>G 6105G>A), TTG1987ATG (6109T>A), CTC1988TTG (6112C>T 6114C>G), AGC1989CGC (6115A>C), AGA1990AAA (6119G>A)

Proteins

polyprotein (YP_009165993.1)	1	1993	92.7%	9636	70.1%	1982 (99.1%)	1314 (65.7%)	7/11/0/0	0

	Begin	End	Coverage	Score	Concordance	Matches	Identities	I/D/M/F*	Stop Codons
**NT**	**33**	**6128**	**84.2%**	**3374**	**28.2%**	**6043 (98.8%)**	**3915 (64.0%)**	**21/53**	
Protein mutations:	L3F (157C>T), F7W (170T>G 171T>G), G8S (172G>A), S9P (175T>C), S10P (178T>C 180A>T), S11A (181T>G), E12_V17del (184_201delGAGAAGACTGGATCAGTT), P18S (202C>T 204C>T), T20P (208A>C), G24A (221G>C 222T>A), L26V (226C>G 228G>T), G28S (232G>T 233G>C), I30F (238A>T 240C>T), K32S (244A>T 245A>C 246A>T), F33L (247T>C 249C>T), A36T (256G>A), I37L (259A>C 261T>C), I40V (268A>G 270C>T), T41N (272C>A 273A>T), S42K (274T>A 275C>A), K46T (287A>C 288A>C), S48V (292T>G 293C>T 294T>A), L51I (301T>A), D52E (306T>G), L54V (310T>G 312G>C), S57P (319T>C 321T>A), T59M (326C>T 327T>G), D60A (329A>C 330T>A), S62A (334T>G 336T>A), S63T (337T>A 339T>A), V66A (347T>C 348T>C), S70N (358T>A 359C>A 360C>T), L72V (364T>G 366G>A), N73E (367A>G 369T>A), K74R (371A>G), G80A (389G>C 390T>A), S81K (391T>A 392C>A 393C>A), L82I (394T>A 396G>A), I83M (399T>G), L87I (409C>A 411G>C), A89G (416C>G 417A>T), S91D (421T>G 422C>A), L93V (427T>G 429G>A), A94K (430G>A 431C>A), S99T (445T>A), K101V (451A>G 452A>T 453G>A), M103L (457A>C 459G>T), R106K (466C>A 467G>A), L107M (469T>A), F108V (472T>G 474C>T), E109L (475G>T 476A>T), L111V (481T>G 483G>C), S112K (484T>A 485C>A 486C>G), N113D (487A>G), V115F (493G>T 495T>C), R116I (497G>T 498A>C), I117L (499A>C), F119Y (506T>A 507T>C), Q120E (508C>G 510G>A), V122L (514G>T 516T>A), S126V (526A>G 527G>T), D128S (532G>T 533A>C 534T>A), V129L (535G>T), V133F (547G>T), Y134I (550T>A 551A>T 552T>A), G135A (554G>C), I141L (571A>T 573A>G), T142A (574A>G), L143I (577C>A), V145L (583G>C 585T>A), C148Y (593G>A), S152V (604T>G 605C>T), I155V (613A>G 615T>A), E156T (616G>A 617A>C 618A>G), G157P (619G>C 620G>C 621A>T), V158F (622G>T 624A>T), S159N (625T>A 626C>A), S160N (629G>A), T161V (631A>G 632C>T 633C>A), I162V (634A>G), K163V (637A>G 638A>T 639A>T), N164T (641A>C), I165L (643A>T 645T>G), L167S (650T>C), I168V (652A>G 654A>T), M172F (664A>T 666G>T), K184T (701A>C 702G>A), A186S (706G>T), E187G (710A>G), T190S (718A>T 720A>C), M191Q (721A>C 722T>A), V192L (724G>C 726A>G), S196A (736A>G 737G>C 738T>A), M197V (739A>G 741G>A), V203L (757G>T 759C>G), S204T (760T>A 762A>G), M212L (784A>T 786G>A), T214S (790A>T 792T>A), I215L (793A>T 795T>G), M216L (796A>T), I226V (826A>G 828A>T), T246A (886A>G), T258Q (922A>C 923C>A 924A>G), I278V (982A>G 984A>G), G280S (988G>A 990T>C), A281I (991G>A 992C>T), S293N (1028G>A 1029C>T), E302Q (1054G>C 1056A>G), Q305T (1063C>A 1064A>C), D308S (1072G>T 1073A>C 1074T>G), A309E (1076C>A 1077T>G), C311A (1081T>G 1082G>C), D312E (1086T>A), A314S (1090G>T), T315M (1094C>T 1095C>G), K323P (1117A>C 1118A>C 1119A>T), D327I (1129G>A 1130A>T 1131C>T), N330Q (1138A>C 1140T>A), R333K (1148G>A 1149G>A), L337V (1159C>G 1161G>C), M352L (1204A>T), E364D (1242A>T), V366L (1246G>C 1248C>G), Y367V (1249T>G 1250A>T), S370A (1258A>G 1259G>C 1260T>C), C371S (1261T>A), R372Q (1264A>C 1265G>A), V374S (1270G>T 1271T>C), Y389F (1316A>T), A399C (1345G>T 1346C>G 1347A>T), R410A (1378C>G 1379G>C 1380C>A), V494I (1630G>A), I519R (1706T>G 1707C>A), E521V (1712A>T 1713A>G), E523D (1719A>T), S525D (1723T>G 1724C>A), T526P (1726A>C), E527N (1729G>A 1731G>T), D528A (1733A>C 1734C>T), G531A (1742G>C 1743A>C), M534V (1750A>G 1752G>T), N536D (1756A>G 1758C>T), I537V (1759A>G 1761T>G), H550A (1798C>G 1799A>C 1800C>T), S554D (1810T>G 1811C>A 1812G>T), D558Q (1822G>C 1824C>A), Q565R (1844A>G), A568Y (1852G>T 1853C>A), I570V (1858A>G 1860A>G), K572S (1865A>G 1866G>C), F575Y (1874T>A 1875T>C), E576N (1876G>A 1878A>T), A577T (1879G>A 1881A>T), E578T (1882G>A 1883A>C), T579S (1885A>T 1887T>G), I581L (1891A>T 1893T>G), S582A (1894T>G 1896A>T), N583D (1897A>G), T585V (1903A>G 1904C>T 1905A>T), A589N (1915G>A 1916C>A), S591A (1921T>G), S592V (1924T>G 1925C>T), E593D (1929A>T), V596L (1936G>C), A597D (1940C>A 1941A>T), V599F (1945G>T 1947T>C), D600Q (1948G>C 1950T>A), G601D (1952G>A 1953G>T), R603del (1957_1959delCGC), F604L (1962C>A), K605Q (1963A>C), T606V (1966A>G 1967C>T 1968A>T), K607L (1969A>T 1970A>T 1971A>G), Q608P (1973A>C 1974A>T), D609P (1975G>C 1976A>C), V610E (1979T>A), I612A (1984A>G 1985T>C), V613A (1988T>C 1989G>T), T614Y (1990A>T 1991C>A), P615E (1993C>G 1994C>A 1995C>G), T616Q (1996A>C 1997C>A 1998A>G), V617T (1999G>A 2000T>C 2001T>A), E621A (2012A>C 2013A>T), D622E (2016T>A), R623K (2018G>A), V624I (2020G>A 2022G>T), I625V (2023A>G), K628Q (2032A>C 2034A>G), F637Y (2060T>A), L643M (2077T>A), D644N (2080G>A), P645K (2083C>A 2084C>A 2085G>A), E646F (2086G>T 2087A>T 2088G>T), S647E (2089A>G 2090G>A 2091T>G), L648F (2094G>T), Y653L (2107T>C 2108A>T 2109T>C), Q654S (2110C>T 2111A>C 2112A>C), L658I (2122C>A), F659Y (2126T>A), S661V (2131T>G 2132C>T 2133C>G), D663S (2137G>T 2138A>C), A664S (2140G>T 2142A>T), E665D (2145G>T), H666A (2146C>G 2147A>C), Q667K (2149C>A 2151G>A), V669N (2155G>A 2156T>A 2157C>T), M671E (2161A>G 2162T>A 2163G>A), R672M (2165G>T 2166A>G), M674V (2170A>G 2172G>A), T675N (2174C>A), M677L (2179A>T), I678L (2182A>T 2184C>G), Y679V (2185T>G 2186A>T), N683S (2198A>G), A684P (2200G>C), E685Q (2203G>C 2205A>G), R688A (2212A>G 2213G>C 2214G>T), Q692R (2225A>G 2226A>G), L699I (2245C>A), A708S (2272G>A 2273C>G 2274T>C), S709T (2275T>A 2277C>A), C712S (2285G>C), K714E (2290A>G), K715L (2293A>C 2294A>T), A717D (2300C>A), T720R (2308A>C 2309C>G 2310A>C), E721N (2311G>A 2313G>T), M722L (2314A>C 2316G>T), R723E (2317C>G 2318G>A 2319C>G), T724Q (2320A>C 2321C>A), F726K (2326T>A 2327T>A 2328T>A), H728Y (2332C>T), N729K (2337T>G), C730A (2338T>G 2339G>C 2340T>G), I731L (2341A>C), D732Q (2344G>C 2346T>G), V735A (2354T>C 2355T>A), I739T (2366T>C 2367T>G), Q742E (2374C>G 2376G>A), F747R (2389T>A 2390T>G 2391T>A), T761R (2432C>G 2433C>G), S764A (2440T>G 2442C>A), S764_K765insG (2442_2443insGGT), K765T (2444A>C 2445A>T), E766D (2448G>C), A767K (2449G>A 2450C>A 2451C>G), D769R (2455G>A 2456A>G 2457C>A), S770N (2458T>A 2459C>A), V772I (2464G>A 2466C>T), K773I (2468A>T 2469G>C), I774E (2470A>G 2471T>A), Y776M (2476T>A 2477A>T 2478C>G), H777I (2479C>A 2480A>T 2481C>T), K778G (2482A>G 2483A>G 2484A>T), V780K (2488G>A 2489T>A), Y782K (2494T>A 2496C>G), K784I (2501A>T 2502A>C), E785Q (2503G>C 2505A>G), T786M (2507C>T 2508T>G), W788N (2512T>A 2513G>A 2514G>T), N789D (2515A>G), Y792F (2525A>T 2526T>C), A793I (2527G>A 2528C>T 2529A>C), R795K (2534G>A), I804L (2560A>T 2562C>G), M805I (2565G>A), L810T (2578T>A 2579T>C 2580G>C), F811M (2581T>A 2583T>G), G812N (2584G>A 2585G>A 2586G>T), G813A (2588G>C 2589A>C), I814V (2590A>G 2592A>T), L818I (2602C>A), A819T (2605G>A), W821L (2611T>C 2612G>T 2613G>C), Q822K (2614C>A 2616A>G), S823A (2617A>G 2618G>C 2619C>A), N824A (2620A>G 2621A>C 2622T>A), S825T (2623T>A), F826S (2627T>C 2628T>C), S828L (2632A>T 2633G>T 2634T>A), S830D (2638A>G 2639G>A), P850K (2698C>A 2699C>A 2700C>G), S851T (2701T>A), R852H (2705G>A), L853F (2707C>T 2709G>C), K854A (2710A>G 2711A>C 2712G>A), N855K (2715T>A), L856S (2717T>C 2718G>C), V858L (2722G>C 2724G>A), S861A (2731A>G 2732G>C 2733T>G), S862A (2734A>G 2735G>C 2736C>A), A863D (2738C>A), I865N (2744T>A 2745T>C), I865_T866insH (2745_2746insCAT), T866V (2746A>G 2747C>T 2748A>G), Q867D (2749C>G 2751A>C), E868R (2752G>C 2753A>G), A871E (2762C>A 2763T>A), E872Q (2764G>C 2766A>G), Q878E (2782C>G), A879S (2785G>A 2786C>G 2787A>T), K881H (2791A>C 2793A>C), M884L (2800A>T), I885V (2803A>G), L889I (2815C>A), G890A (2819G>C 2820A>T), M906L (2866A>C 2868G>C), H913R (2887C>A 2888A>G 2889C>A), V915M (2893G>A), T922S (2914A>T), P926E (2926C>G 2927C>A 2928T>G), N927H (2929A>C 2931C>T), R929I (2936G>T 2937G>T), S930C (2939C>G 2940A>T), K931N (2943G>T), Y934F (2951A>T), R937T (2959C>A 2960G>C 2961G>C), E938Q (2962G>C), K940R (2969A>G), V941L (2971G>T 2973C>G), I947V (2989A>G 2991C>G), P960S (3028C>T 3030T>A), E971D (3063G>T), F974Y (3071T>A), T975S (3073A>T 3075C>T), Q979M (3085C>A 3086A>T), C983V (3097T>G 3098G>T 3099T>G), T989K (3116C>A 3117C>G), R992K (3125G>A 3126A>G), L993K (3127C>A 3128T>A 3129G>A), G994S (3130G>T 3131G>C), F998V (3142T>G), F1001Y (3152T>A), V1011E (3182T>A 3183C>A), E1014T (3190G>A 3191A>C 3192G>A), E1016G (3197A>G 3198G>A), A1020N (3208G>A 3209C>A 3210C>T), S1021A (3211A>G 3212G>C 3213T>A), K1023A (3217A>G 3218A>C 3219A>T), Q1028V (3232C>G 3233A>T 3234A>T), S1029A (3235T>G), M1034I (3252G>T), G1036S (3256G>A 3258A>T), S1049T (3296G>C), V1056C (3316G>T 3317T>G 3318T>C), G1067D (3350G>A), I1068T (3353T>C 3354T>C), F1074Y (3371T>A), I1075V (3373A>G 3375T>G), S1076A (3376T>G), I1082V (3394A>G 3396C>G), N1089D (3415A>G 3417C>T), V1095I (3433G>A), A1097V (3440C>T), D1103E (3459C>A), I1104L (3460A>C 3462T>G), K1106E (3466A>G 3468A>G), A1107H (3469G>C 3470C>A), I1108L (3472A>C 3474T>G), R1111K (3482G>A 3483A>G), T1113E (3487A>G 3488C>A 3489C>A), T1114R (3491C>G 3492T>A), L1115F (3493C>T 3495T>C), N1119K (3507C>A), S1122A (3514T>G 3516C>T), Q1129L (3535C>T 3536A>T 3537A>G), L1131M (3541T>A), R1142K (3575G>A), K1143R (3577A>C 3578A>G 3579G>C), Q1145P (3584A>C 3585A>C), P1152D (3604C>G 3605C>A 3606T>C), E1154Q (3610G>C 3612A>G), T1161S (3631A>T 3633C>T), V1162A (3635T>C), N1163K (3639T>A), M1178E (3682A>G 3683T>A), H1185S (3703C>T 3704A>C 3705T>C), G1186N (3706G>A 3707G>A 3708T>C), D1188E (3714T>G), D1189E (3717T>G), D1190P (3718G>C 3719A>C 3720T>A), S1195A (3733T>G), T1198G (3742A>G 3743C>G), S1201A (3751A>G 3752G>C 3753T>A), L1203I (3757C>A 3759G>T), T1206V (3766A>G 3767C>T 3768A>G), M1207L (3769A>C 3771G>T), N1209M (3776A>T 3777C>G), K1211R (3781A>C 3782A>G), Q1218A (3802C>G 3803A>C 3804G>A), E1219I (3805G>A 3806A>T 3807G>C), D1221N (3811G>A), C1232F (3845G>T), Y1235F (3854A>T), G1237A (3860G>C 3861C>A), M1251L (3901A>T), M1253L (3907A>C 3909G>T), M1255S (3913A>T 3914T>C 3915G>T), V1262E (3935T>A), P1263A (3937C>G 3939A>C), T1264Q (3940A>C 3941C>A), G1265P (3943G>C 3944G>C), E1266N (3946G>A 3948A>C), G1267S (3949G>T 3950G>C 3951T>G), D1268Q (3952G>C 3954T>G), K1269R (3956A>G 3957A>G), E1271L (3961G>C 3962A>T 3963G>T), R1273K (3968G>A), D1275N (3973G>A), E1280D (3990A>T), I1287A (4009A>G 4010T>C 4011T>G), I1289V (4015A>G 4017T>A), I1293T (4028T>C), H1294F (4030C>T 4031A>T), I1298V (4042A>G), T1343C (4177A>T 4178C>G 4179C>T), L1347A (4189T>G 4190T>C), I1351L (4201A>C), D1358N (4222G>A), S1362H (4234T>C 4235C>A), S1365T (4243T>A 4245T>A), L1366V (4246C>G 4248T>G), N1369S (4256A>G 4257C>T), N1370S (4259A>G), A1373S (4267G>T 4269T>A), T1377E (4279A>G 4280C>A 4281T>G), V1392I (4324G>A 4326G>C), E1404A (4361A>C 4362G>T), Y1405F (4364A>T), D1407G (4370A>G), T1408D (4372A>G 4373C>A 4374C>T), K1409E (4375A>G), E1412S (4384G>A 4385A>G 4386G>T), A1414E (4391C>A 4392T>G), A1420T (4408G>A 4410T>A), D1434N (4450G>A 4452T>C), V1436L (4456G>T 4458T>G), I1475V (4573A>G 4575T>C), A1476K (4576G>A 4577C>A 4578C>A), R1478K (4583G>A), A1479S (4585G>T), I1480V (4588A>G 4590A>G), V1482I (4594G>A 4596A>T), G1485N (4603G>A 4604G>A), L1505M (4663C>A), S1511D (4681T>G 4682C>A 4683A>T), N1514S (4690A>T 4691A>C 4692T>C), Q1517R (4699C>A 4700A>G 4701G>A), D1520E (4710C>A), M1521A (4711A>G 4712T>C 4713G>T), N1537E (4759A>G 4761C>G), Q1538K (4762C>A), V1539T (4765G>A 4766T>C 4767G>A), Q1540K (4768C>A 4770G>A), Q1542L (4775A>T), H1562Q (4836C>A), I1571V (4861A>G 4863A>C), M1575I (4875G>T), P1582V (4894C>G 4895C>T), T1587V (4909A>G 4910C>T 4911T>A), V1588Q (4912G>C 4913T>A 4914G>A), M1591Y (4921A>T 4922T>A 4923G>T), V1594I (4930G>A 4932G>T), L1598I (4942C>A 4944T>C), R1606Q (4966A>C 4967G>A 4968A>G), V1607A (4970T>C 4971T>C), A1608T (4972G>A 4974A>T), K1611S (4982A>G 4983G>T), L1612F (4986A>T), T1613V (4987A>G 4988C>T 4989T>A), Y1614N (4990T>A), Q1617F (4999C>T 5000A>T 5001G>T), C1618G (5002T>G 5004T>A), R1621K (5012G>A 5013A>G), E1622Q (5014G>C), G1624N (5020G>A 5021G>A 5022A>T), G1626P (5026G>C 5027G>C), A1627I (5029G>A 5030C>T 5031A>T), T1628M (5033C>T 5034A>G), F1630M (5038T>A 5040T>G), S1632T (5044T>A), M1634Y (5050A>T 5051T>A 5052G>T), V1636L (5056G>C 5058G>T), Q1638S (5062C>A 5063A>G 5064G>T), E1639Q (5065G>C), I1641L (5071A>T 5073C>G), A1642T (5074G>A), K1643E (5077A>G), A1648E (5093C>A 5094A>G), A1650I (5098G>A 5099C>T 5100G>T), L1651G (5101T>G 5102T>G), T1652V (5104A>G 5105C>T 5106A>T), I1653L (5107A>T 5109C>G), C1654M (5110T>A 5111G>T 5112T>G), M1656L (5116A>C 5118G>A), A1657S (5119G>T 5121C>A), K1658D (5122A>G 5124A>T), S1660K (5128T>A 5129C>A 5130T>A), I1661N (5132T>A 5133C>T), F1663Y (5138T>A), P1668A (5152C>G), N1670E (5158A>G 5160T>A), V1672I (5164G>A), E1673del (5167_5169delGAA), R1677E (5179A>G 5180G>A 5181A>G), D1678G (5183A>G), L1679V (5185C>G), N1680H (5188A>C), W1681E (5191T>G 5192G>A 5193G>A), A1683V (5198C>T 5199C>G), N1685D (5203A>G 5205C>T), I1686V (5206A>G 5208T>A), G1687F (5209G>T 5210G>T), A1695S (5233G>T 5235C>A), K1696N (5238G>C), T1697Q (5239A>C 5240C>A 5241T>A), E1698Q (5242G>C), E1700S (5248G>T 5249A>C 5250G>A), P1701S (5251C>T 5253T>A), C1703N (5257T>A 5258G>A), V1705L (5263G>C 5265A>G), K1712R (5284A>C 5285A>G 5286G>C), K1713R (5288A>G 5289G>A), S1714T (5291G>C 5292C>A), K1715T (5294A>C), S1722N (5315G>A), M1730I (5340G>A), M1735L (5353A>T), S1736A (5356T>G), L1739Q (5366T>A), A1741H (5371G>C 5372C>A), G1744K (5380G>A 5381G>A 5382C>G), K1745S (5384A>G 5385A>T), M1746L (5386A>C), S1747E (5389T>G 5390C>A), E1748V (5393A>T 5394G>T), D1749G (5396A>G 5397C>T), I1750D (5398A>G 5399T>A), A1751G (5402C>G 5403T>G), D1754S (5410G>T 5411A>C), V1756T (5416G>A 5417T>C 5418G>C), V1765I (5443G>A 5445G>A), S1766A (5446A>G 5447G>C 5448C>T), M1773F (5467A>T 5469G>T), V1775L (5473G>T), R1778H (5483G>A), C1781R (5491T>A 5493C>G), I1782A (5494A>G 5495T>C 5496T>C), E1783T (5497G>A 5498A>C 5499G>A), I1785L (5503A>C 5505C>T), I1792M (5526T>G), G1793A (5528G>C 5529C>T), N1794G (5530A>G 5531A>G 5532T>G), V1806A (5567T>C 5568T>G), F1809L (5577T>G), Q1811K (5581C>A), P1814E (5590C>G 5591C>A 5592T>A), Q1815D (5593C>G 5595G>C), Q1815_E1816insT (5595_5596insACC), E1816S (5596G>T 5597A>C), G1817H (5599G>C 5600G>A 5601G>C), M1819L (5605A>T), A1820G (5609C>G), V1822S (5614G>A 5615T>G), S1824T (5620T>A 5622G>A), S1825del (5623_5625delTCT), L1826Y (5627T>A 5628G>T), Q1827G (5629C>G 5630A>G 5631A>C), S1828T (5633G>C 5634T>C), S1829T (5636G>C), M1831N (5642T>A 5643G>C), Q1833H (5649A>T), Q1833_G1834insHS (5649_5650insCACAGT), G1834A (5651G>C 5652T>A), E1836S (5656G>T 5657A>C 5658G>A), L1837V (5659C>G), Q1839L (5666A>T 5667A>T), I1840L (5668A>C 5670T>A), V1842I (5674G>A 5676C>A), I1843V (5677A>G 5679C>T), H1844P (5681A>C 5682C>T), G1845S (5683G>T 5684G>C 5685T>C), M1849E (5695A>G 5696T>A 5697G>A), E1850K (5698G>A 5700A>G), L1853V (5707T>G 5709G>T), M1855L (5713A>T), V1858T (5722G>A 5723T>C 5724T>A), V1859I (5725G>A 5727G>T), H1862E (5734C>G 5736T>A), G1866A (5747G>C), N1867Q (5749A>C 5751C>G), F1868C (5753T>G 5754T>C), T1869S (5755A>T 5757C>A), D1874K (5770G>A 5772T>G), Q1875H (5775A>T), L1876A (5776T>G 5777T>C 5778G>T), F1877L (5781T>G), A1878E (5783C>A 5784A>G), S1879T (5786G>C 5787T>A), E1881S (5791G>T 5792A>C 5793A>T), K1882V (5794A>G 5795A>T 5796G>C), C1883S (5797T>A), E1886T (5806G>A 5807A>C 5808A>T), L1888A (5812C>G 5813T>C 5814T>C), K1890R (5819A>G 5820G>A), N1891G (5821A>G 5822A>G 5823C>A), T1892V (5824A>G 5825C>T 5826G>C), L1894V (5830C>G), E1899K (5845G>A 5847G>A), K1900E (5848A>G), M1901F (5851A>T 5853G>C), T1902S (5854A>T 5856T>C), K1904H (5860A>C 5862A>C), V1906C (5866G>T 5867T>G 5868G>T), A1907E (5870C>A), Q1908M (5872C>A 5873A>T 5874A>G), V1910L (5878G>C 5880C>T), S1911E (5881A>G 5882G>A 5883T>G), V1913L (5887G>C 5889A>G), K1914E (5890A>G), Q1915S (5893C>A 5894A>G 5895G>T), F1918V (5902T>G 5904C>A), Y1919V (5905T>G 5906A>T 5907T>A), P1920F (5908C>T 5909C>T), K1921M (5912A>T), A1922G (5915C>G 5916C>G), T1923S (5918C>G 5919A>C), T1924V (5920A>G 5921C>T 5922C>T), V1925K (5923G>A 5924T>A), E1926K (5926G>A), L1928M (5932C>A 5934C>G), K1929L (5935A>T 5936A>T), V1930S (5938G>T 5939T>C 5940A>C), A1932M (5944G>A 5945C>T 5946T>G), D1933N (5947G>A), A1934Q (5950G>C 5951C>A 5952T>A), H1935Q (5955T>G), H1935_T1936insM (5955_5956insATG), T1936S (5956A>T 5958C>A), I1937K (5960T>A 5961T>A), V1938T (5962G>A 5963T>C), K1939Q (5965A>C), T1940V (5968A>G 5969C>T), K1942S (5975A>G 5976A>C), F1944C (5981T>G 5982C>T), R1945C (5983C>T 5985G>T), G1946del (5986_5988delGGA), V1948T (5992G>A 5993T>C 5994T>A), R1950S (5998C>A 6000G>T), N1952A (6004A>G 6005A>C 6006T>A), F1953W (6008T>G 6009T>G), S1955T (6014G>C), P1956K (6016C>A 6017C>A 6018T>G), E1957D (6021G>C), W1958I (6022T>A 6023G>T 6024G>T), W1958_K1959insV (6024_6025insGTC), K1959M (6026A>T 6027A>G), I1960T (6029T>C 6030T>A), G1961K (6031G>A 6032G>A), E1963K (6037G>A), N1964K (6042C>G), T1965P (6043A>C), M1966del (6046_6048delATG), A1967G (6050C>G), T1968S (6052A>T 6054A>T), L1969I (6055C>A 6057C>T), S1970P (6058T>C 6060A>T), S1973T (6068G>C 6069T>A), L1974V (6070C>G 6072T>G), L1975F (6073C>T), P1976L (6077C>T 6078A>T), L1979P (6086T>C 6087G>T), S1982L (6094T>C 6095C>T), A1983V (6098C>T 6099C>T), M1984V (6100A>G), K1985R (6104A>G 6105G>A), L1987M (6109T>A), S1989R (6115A>C), R1990K (6119G>A)								

	Begin	End	Coverage	Score	Concordance	Matches	Identities	I/D/M/F*	Stop Codons
**NT**	**33**	**6128**	**84.2%**	**3374**	**28.2%**	**6043 (98.8%)**	**3915 (64.0%)**	**21/53**	

GCT2GCA (156T>A), CTT3TTT (157C>T), AAA5AAG (165A>G), TTT7TGG (170T>G 171T>G), GGT8AGT (172G>A), TCT9CCT (175T>C), TCA10CCT (178T>C 180A>T), TCT11GCT
(181T>G), GAG12_GTT17del (184_201delGAGAAGACTGGATCAGTT), CCC18TCT (202C>T 204C>T), TCT19TCA (207T>A), ACT20CCT (208A>C), TCT21TCA (213T>A),
GGT24GCA (221G>C 222T>A), CTG26GTT (226C>G 228G>T), GGT28TCT (232G>T 233G>C), ATC30TTT (238A>T 240C>T), TCA31TCT (243A>T), AAA32TCT (244A>T 245A>C
246A>T), TTC33CTT (247T>C 249C>T), AAA35AAG (255A>G), GCT36ACT (256G>A), ATT37CTC (259A>C 261T>C), AGC38TCC (262A>T 263G>C), AAC39AAT (267C>T),
ATC40GTT (268A>G 270C>T), ACA41AAT (272C>A 273A>T), TCA42AAA (274T>A 275C>A), GTA43GTT (279A>T), AAA46ACC (287A>C 288A>C), TCT48GTA (292T>G 293C>T
294T>A), CAT50CAC (300T>C), TTA51ATA (301T>A), GAT52GAG (306T>G), TTG54GTC (310T>G 312G>C), AAA55AAG (315A>G), CCA56CCG (318A>G), TCT57CCA (319T>C
321T>A), GTG58GTT (324G>T), ACT59ATG (326C>T 327T>G), GAT60GCA (329A>C 330T>A), TCT62GCA (334T>G 336T>A), TCT63ACA (337T>A 339T>A), TCT64TCA (342T>A),
GTT66GCC (347T>C 348T>C), TGT69TGC (357T>C), TCC70AAT (358T>A 359C>A 360C>T), GCA71GCT (363A>T), TTG72GTA (364T>G 366G>A), AAT73GAA (367A>G 369T>A),
AAA74AGA (371A>G), GTG75GTT (375G>T), CTT76TTA (376C>T 378T>A), ATA79ATT (387A>T), GGT80GCA (389G>C 390T>A), TCC81AAA (391T>A 392C>A 393C>A),
TTG82ATA (394T>A 396G>A), ATT83ATG (399T>G), CCT85CCC (405T>C), TTT86TTC (408T>C), CTG87ATC (409C>A 411G>C), AAA88AAG (414A>G), GCA89GGT (416C>G
417A>T), TAC90TAT (420C>T), TCT91GAT (421T>G 422C>A), TTG93GTA (427T>G 429G>A), GCA94AAA (430G>A 431C>A), ACC95ACT (435C>T), TCT99ACT (445T>A),
ATC100ATA (450C>A), AAG101GTA (451A>G 452A>T 453G>A), ATG103CTT (457A>C 459G>T), GTT104GTA (462T>A), TTA105CTT (463T>C 465A>T), CGG106AAG (466C>A
467G>A), TTG107ATG (469T>A), TTC108GTT (472T>G 474C>T), GAA109TTA (475G>T 476A>T), TTG111GTC (481T>G 483G>C), TCC112AAG (484T>A 485C>A 486C>G),
AAT113GAT (487A>G), GTT115TTC (493G>T 495T>C), AGA116ATC (497G>T 498A>C), ATA117CTA (499A>C), TTT119TAC (506T>A 507T>C), CAG120GAA (508C>G 510G>A),
GTT122TTA (514G>T 516T>A), GAG124GAA (522G>A), AAG125AAA (525G>A), AGT126GTT (526A>G 527G>T), GAT128TCA (532G>T 533A>C 534T>A), GTA129TTA (535G>T),
ATT131ATA (543T>A), GTA132GTG (546A>G), GTT133TTT (547G>T), TAT134ATA (550T>A 551A>T 552T>A), GGT135GCT (554G>C), TTG137TTA (561G>A), GCA140GCT
(570A>T), ATA141TTG (571A>T 573A>G), ACT142GCT (574A>G), CTT143ATT (577C>A), CTT144TTG (580C>T 582T>G), GTT145CTA (583G>C 585T>A), TTG146CTA (586T>C
588G>A), TTG147CTG (589T>C), TGT148TAT (593G>A), GTT149GTA (597T>A), TCA152GTA (604T>G 605C>T), GAC153GAT (609C>T), ATA154ATT (612A>T), ATT155GTA
(613A>G 615T>A), GAA156ACG (616G>A 617A>C 618A>G), GGA157CCT (619G>C 620G>C 621A>T), GTA158TTT (622G>T 624A>T), TCT159AAT (625T>A 626C>A), AGT160AAT
(629G>A), ACC161GTA (631A>G 632C>T 633C>A), ATT162GTT (634A>G), AAA163GTT (637A>G 638A>T 639A>T), AAT164ACT (641A>C), ATT165TTG (643A>T 645T>G),
TTC166TTT (648C>T), TTA167TCA (650T>C), ATA168GTT (652A>G 654A>T), GTA169GTT (657A>T), GGG170GGA (660G>A), ATG172TTT (664A>T 666G>T), TTT173TTC
(669T>C), AGT175TCA (673A>T 674G>C 675T>A), AAC178AAT (684C>T), TTG179TTA (687G>A), GAC180GAT (690C>T), CCC183CCA (699C>A), AAG184ACA (701A>C 702G>A),
GCT186TCT (706G>T), GAG187GGG (710A>G), TTC189TTT (717C>T), ACA190TCC (718A>T 720A>C), ATG191CAG (721A>C 722T>A), GTA192CTG (724G>C 726A>G),
GCA193GCG (729A>G), AGT196GCA (736A>G 737G>C 738T>A), ATG197GTA (739A>G 741G>A), CTA198CTT (744A>T), GAG201GAA (753G>A), TCT202AGC (754T>A 755C>G
756T>C), GTC203TTG (757G>T 759C>G), TCA204ACG (760T>A 762A>G), CAC205CAT (765C>T), ACT206ACA (768T>A), ACA208ACT (774A>T), TCC209AGC (775T>A 776C>G),
CAG210CAA (780G>A), TTG211CTT (781T>C 783G>T), ATG212TTA (784A>T 786G>A), TCA213TCC (789A>C), ACT214TCA (790A>T 792T>A), ATT215TTG (793A>T 795T>G),
ATG216TTG (796A>T), GCA217GCT (801A>T), ATT220ATC (810T>C), TCC221TCA (813C>A), ACG222ACT (816G>T), CTC223TTA (817C>T 819C>A), GTG224GTA (822G>A),
ATA226GTT (826A>G 828A>T), GCA227GCT (831A>T), GTG228GTT (834G>T), CCA229CCT (837A>T), GGA230GGG (840A>G), CCC232CCA (846C>A), TTG235CTT (853T>C
855G>T), AGC236TCA (856A>T 857G>C 858C>A), AAT237AAC (861T>C), CCC238CCA (864C>A), CTC239CTG (867C>G), ATT242ATC (876T>C), TCA245TCT (885A>T),
ACT246GCT (886A>G), GGG247GGA (891G>A), GGG249GGT (897G>T), GCT250GCG (900T>G), CAA255CAG (915A>G), TTG256CTG (916T>C), TTT257TTC (921T>C),
ACA258CAG (922A>C 923C>A 924A>G), AGA261AGG (933A>G), AAT262AAC (936T>C), AAA264AAG (942A>G), TGC266TGT (948C>T), ACT267ACA (951T>A), TCA268TCT
(954A>T), GCA270GCC (960A>C), TTT271TTC (963T>C), TCT272TCA (966T>A), GTG274GTT (972G>T), GAA276GAG (978A>G), ATT277ATA (981T>A), ATA278GTG (982A>G
984A>G), GGT280AGC (988G>A 990T>C), GCT281ATT (991G>A 992C>T), TTC284TTT (1002C>T), AAG285AAA (1005G>A), GTG288GTA (1014G>A), CTT289TTG (1015C>T
1017T>G), TCT290TCA (1020T>A), GCA291GCG (1023A>G), ATT292ATA (1026T>A), AGC293AAT (1028G>A 1029C>T), GCA294GCT (1032A>T), ACA295ACC (1035A>C),
TTG296TTA (1038G>A), TCT297TCC (1041T>C), ACT298ACC (1044T>C), TTG300CTT (1048T>C 1050G>T), GAA302CAG (1054G>C 1056A>G), CAG305ACG (1063C>A
1064A>C), GTG307GTT (1071G>T), GAT308TCG (1072G>T 1073A>C 1074T>G), GCT309GAG (1076C>A 1077T>G), TGT311GCT (1081T>G 1082G>C), GAT312GAA (1086T>A),
GCT314TCT (1090G>T), ACC315ATG (1094C>T 1095C>G), AGG316CGC (1096A>C 1098G>C), TTG317CTT (1099T>C 1101G>T), GAG318GAA (1104G>A), AAC319AAT
(1107C>T), GCC321GCA (1113C>A), AAA323CCT (1117A>C 1118A>C 1119A>T), GCC324GCA (1122C>A), TTC325TTT (1125C>T), CCT326CCA (1128T>A), GAC327ATT
(1129G>A 1130A>T 1131C>T), AAA328AAG (1134A>G), AAT330CAA (1138A>C 1140T>A), CAC331CAT (1143C>T), CTT332TTA (1144C>T 1146T>A), AGG333AAA (1148G>A
1149G>A), GCA336GCC (1158A>C), CTG337GTC (1159C>G 1161G>C), AAG338AAA (1164G>A), ATT339ATA (1167T>A), GCA341GCT (1173A>T), ACA345ACC (1185A>C),
CAC346CAT (1188C>T), GTT348GTC (1194T>C), GCC349GCA (1197C>A), ATG352TTG (1204A>T), CAT354CAC (1212T>C), CGA355AGG (1213C>A 1215A>G), GTG356GTT
(1218G>T), AGT357TCT (1219A>T 1220G>C), GCA358GCT (1224A>T), GCT359GCC (1227T>C), ATC360ATA (1230C>A), ACT361ACG (1233T>G), CTT363CTA (1239T>A),
GAA364GAT (1242A>T), AAG365AAA (1245G>A), GTC366CTG (1246G>C 1248C>G), TAT367GTT (1249T>G 1250A>T), GCG368GCT (1254G>T), AGT370GCC (1258A>G
1259G>C 1260T>C), TGC371AGC (1261T>A), AGA372CAA (1264A>C 1265G>A), GTT374TCT (1270G>T 1271T>C), GTT376GTG (1278T>G), GGT377GGC (1281T>C),
CAG378CAA (1284G>A), AGA380AGG (1290A>G), GAA382GAG (1296A>G), CCC383CCT (1299C>T), GTT386GTG (1308T>G), TAT389TTT (1316A>T), GGT390GGA (1320T>A),
GCC391GCT (1323C>T), GGG393GGA (1329G>A), ACA398ACG (1344A>G), GCA399TGT (1345G>T 1346C>G 1347A>T), CGA401CGC (1353A>C), TTG402CTT (1354T>C
1356G>T), TTG408CTC (1372T>C 1374G>C), GAT409GAC (1377T>C), CGC410GCA (1378C>G 1379G>C 1380C>A), GGG412GGT (1386G>T), GAG413GAA (1389G>A),
CCT414CCA (1392T>A), CTT416CTG (1398T>G), AGA418CGC (1402A>C 1404A>C), CTT419TTG (1405C>T 1407T>G), GCT421GCG (1413T>G), GTT422GTA (1416T>A),
AGC423AGT (1419C>T), AAG424AAA (1422G>A), CGC425AGA (1423C>A 1425C>A), GCC427GCA (1431C>A), TCT430TCC (1440T>C), AAT431AAC (1443T>C), GCC433GCA
(1449C>A), CAC434CAT (1452C>T), CAG435CAA (1455G>A), GCT437GCA (1461T>A), TTG439CTT (1465T>C 1467G>T), GAC442GAT (1476C>T), GGA444GGT (1482A>T),
GCA445GCT (1485A>T), TTG446CTT (1486T>C 1488G>T), AGA447CGG (1489A>C 1491A>G), GGA451GGG (1503A>G), CAA452CAG (1506A>G), CAA454CAG (1512A>G),
ATT456ATA (1518T>A), ATT462ATC (1536T>C), TCC464TCT (1542C>T), ACA465ACC (1545A>C), CCA467CCT (1551A>T), CCA469CCT (1557A>T), CTG470CTT (1560G>T),
CCC471CCA (1563C>A), GCA473GCT (1569A>T), GCT474GCA (1572T>A), GTG475GTC (1575G>C), GAA476GAG (1578A>G), GAC477GAT (1581C>T), AAG478AAA (1584G>A),
GGG479GGA (1587G>A), CAT481CAC (1593T>C), ACT483ACA (1599T>A), TAT486TAC (1608T>C), GCA489GCC (1617A>C), AAT492AAC (1626T>C), CTT493TTG (1627C>T
1629T>G), GTA494ATA (1630G>A), TCA495TCT (1635A>T), GCA496GCT (1638A>T), CCT497CCA (1641T>A), GCC498GCT (1644C>T), CTT502CTA (1656T>A), ACC503ACG
(1659C>G), GCT507GCA (1671T>A), GAG509GAA (1677G>A), CGT510AGG (1678C>A 1680T>G), AGA511AGG (1683A>G), AGG512AGA (1686G>A), ATC514ATT (1692C>T),
TGT518TGC (1704T>C), ATC519AGA (1706T>G 1707C>A), GAA521GTG (1712A>T 1713A>G), GGT522GGC (1716T>C), GAA523GAT (1719A>T), TCT525GAT (1723T>G
1724C>A), ACA526CCA (1726A>C), GAG527AAT (1729G>A 1731G>T), GAC528GCT (1733A>C 1734C>T), ACC530ACA (1740C>A), GGA531GCC (1742G>C 1743A>C),
CTG533CTA (1749G>A), ATG534GTT (1750A>G 1752G>T), AAC536GAT (1756A>G 1758C>T), ATT537GTG (1759A>G 1761T>G), CGG541AGA (1771C>A 1773G>A), AGG542AGA
(1776G>A), ATA546ATC (1788A>C), CAC550GCT (1798C>G 1799A>C 1800C>T), AGT552TCC (1804A>T 1805G>C 1806T>C), TAC553TAT (1809C>T), TCG554GAT (1810T>G
1811C>A 1812G>T), TTA557TTG (1821A>G), GAC558CAA (1822G>C 1824C>A), GCC562GCA (1836C>A), TGT564TGC (1842T>C), CAG565CGG (1844A>G), GTT566GTG
(1848T>G), CAT567CAC (1851T>C), GCT568TAT (1852G>T 1853C>A), ATA570GTG (1858A>G 1860A>G), AAG572AGC (1865A>G 1866G>C), CAG573CAA (1869G>A),
CTG574CTT (1872G>T), TTT575TAC (1874T>A 1875T>C), GAA576AAT (1876G>A 1878A>T), GCA577ACT (1879G>A 1881A>T), GAA578ACA (1882G>A 1883A>C), ACT579TCG
(1885A>T 1887T>G), GGC580GGA (1890C>A), ATT581TTG (1891A>T 1893T>G), TCA582GCT (1894T>G 1896A>T), AAT583GAT (1897A>G), ACA585GTT (1903A>G 1904C>T
1905A>T), GCC586GCT (1908C>T), GTT588GTC (1914T>C), GCT589AAT (1915G>A 1916C>A), GTA590GTT (1920A>T), TCT591GCT (1921T>G), TCT592GTT (1924T>G
1925C>T), GAA593GAT (1929A>T), GAG594GAA (1932G>A), GTT595GTG (1935T>G), GTG596CTG (1936G>C), GCA597GAT (1940C>A 1941A>T), GTT599TTC (1945G>T
1947T>C), GAT600CAA (1948G>C 1950T>A), GGG601GAT (1952G>A 1953G>T), GCT602GCC (1956T>C), CGC603del (1957_1959delCGC), TTC604TTA (1962C>A), AAA605CAA
(1963A>C), ACA606GTT (1966A>G 1967C>T 1968A>T), AAA607TTG (1969A>T 1970A>T 1971A>G), CAA608CCT (1973A>C 1974A>T), GAT609CCT (1975G>C 1976A>C),
GTG610GAG (1979T>A), ATA612GCA (1984A>G 1985T>C), GTG613GCT (1988T>C 1989G>T), ACT614TAT (1990A>T 1991C>A), CCC615GAG (1993C>G 1994C>A 1995C>G),
ACA616CAG (1996A>C 1997C>A 1998A>G), GTT617ACA (1999G>A 2000T>C 2001T>A), ATA618ATT (2004A>T), GAG620GAA (2010G>A), GAA621GCT (2012A>C 2013A>T),
GAT622GAA (2016T>A), AGA623AAA (2018G>A), GTG624ATT (2020G>A 2022G>T), ATT625GTT (2023A>G), TCT627TCC (2031T>C), AAA628CAG (2032A>C 2034A>G),
TTA630CTC (2038T>C 2040A>C), GTG632GTT (2046G>T), GAA633GAG (2049A>G), CTT635CTC (2055T>C), TTT637TAT (2060T>A), TAT639TAC (2067T>C), CAG640CAA
(2070G>A), GGC641GGG (2073C>G), TTG643ATG (2077T>A), GAC644AAC (2080G>A), CCG645AAA (2083C>A 2084C>A 2085G>A), GAG646TTT (2086G>T 2087A>T 2088G>T),
AGT647GAG (2089A>G 2090G>A 2091T>G), TTG648TTT (2094G>T), TTT649TTC (2097T>C), CCT650CCA (2100T>A), TAT653CTC (2107T>C 2108A>T 2109T>C), CAA654TCC
(2110C>T 2111A>C 2112A>C), AAA655AAG (2115A>G), CAA656CAG (2118A>G), CTC658ATC (2122C>A), TTT659TAT (2126T>A), TCC661GTG (2131T>G 2132C>T 2133C>G),
GAT663TCT (2137G>T 2138A>C), GCA664TCT (2140G>T 2142A>T), GAG665GAT (2145G>T), CAC666GCC (2146C>G 2147A>C), CAG667AAA (2149C>A 2151G>A), CAA668CAG
(2154A>G), GTC669AAT (2155G>A 2156T>A 2157C>T), TTC670TTT (2160C>T), ATG671GAA (2161A>G 2162T>A 2163G>A), AGA672ATG (2165G>T 2166A>G), ATG674GTA
(2170A>G 2172G>A), ACT675AAT (2174C>A), GAC676GAT (2178C>T), ATG677TTG (2179A>T), ATC678TTG (2182A>T 2184C>G), TAC679GTC (2185T>G 2186A>T),
GGT681GGA (2193T>A), GCC682GCA (2196C>A), AAT683AGT (2198A>G), GCT684CCT (2200G>C), GAA685CAG (2203G>C 2205A>G), CAA686CAG (2208A>G), TAC687TAT
(2211C>T), AGG688GCT (2212A>G 2213G>C 2214G>T), CAA692CGG (2225A>G 2226A>G), AAT693AAC (2229T>C), CTC699ATC (2245C>A), TTT702TTC (2256T>C),
AAA703AAG (2259A>G), AGC704AGT (2262C>T), ATC706ATT (2268C>T), GCT708AGC (2272G>A 2273C>G 2274T>C), TCC709ACA (2275T>A 2277C>A), ATC711ATT (2283C>T),
TGT712TCT (2285G>C), AAG714GAG (2290A>G), AAG715CTG (2293A>C 2294A>T), GCT717GAT (2300C>A), GTA718GTC (2304A>C), ACA720CGC (2308A>C 2309C>G
2310A>C), GAG721AAT (2311G>A 2313G>T), ATG722CTT (2314A>C 2316G>T), CGC723GAG (2317C>G 2318G>A 2319C>G), ACA724CAA (2320A>C 2321C>A), GGA725GGG
(2325A>G), TTT726AAA (2326T>A 2327T>A 2328T>A), GCA727GCG (2331A>G), CAT728TAT (2332C>T), AAT729AAG (2337T>G), TGT730GCG (2338T>G 2339G>C 2340T>G),
ATT731CTT (2341A>C), GAT732CAG (2344G>C 2346T>G), GTT735GCA (2354T>C 2355T>A), TTG738CTT (2362T>C 2364G>T), ATT739ACG (2366T>C 2367T>G), TGC740TGT
(2370C>T), ATC741ATT (2373C>T), CAG742GAA (2374C>G 2376G>A), TTT747AGA (2389T>A 2390T>G 2391T>A), GTG748GTA (2394G>A), TTT750TTC (2400T>C), TTG751CTG
(2401T>C), ACA753ACT (2409A>T), GCC754GCT (2412C>T), CGT757CGC (2421T>C), TAT758TAC (2424T>C), ACC761AGG (2432C>G 2433C>G), TCC764GCA (2440T>G
2442C>A), TCC764_AAA765insGGT (2442_2443insGGT), AAA765ACT (2444A>C 2445A>T), GAG766GAC (2448G>C), GCC767AAG (2449G>A 2450C>A 2451C>G), AAA768AAG
(2454A>G), GAC769AGA (2455G>A 2456A>G 2457C>A), TCT770AAT (2458T>A 2459C>A), GTC772ATT (2464G>A 2466C>T), AAG773ATC (2468A>T 2469G>C), ATA774GAA
(2470A>G 2471T>A), TGT775TGC (2475T>C), TAC776ATG (2476T>A 2477A>T 2478C>G), CAC777ATT (2479C>A 2480A>T 2481C>T), AAA778GGT (2482A>G 2483A>G
2484A>T), ATT779ATC (2487T>C), GTG780AAG (2488G>A 2489T>A), GAT781GAC (2493T>C), TAC782AAG (2494T>A 2496C>G), AAA784ATC (2501A>T 2502A>C), GAA785CAG
(2503G>C 2505A>G), ACT786ATG (2507C>T 2508T>G), TGG788AAT (2512T>A 2513G>A 2514G>T), AAC789GAC (2515A>G), TTA790TTG (2520A>G), CCA791CCC (2523A>C),
TAT792TTC (2525A>T 2526T>C), GCA793ATC (2527G>A 2528C>T 2529A>C), CTG794TTG (2530C>T), AGG795AAG (2534G>A), GGC801GGA (2553C>A), CTA802CTG
(2556A>G), ATC804TTG (2560A>T 2562C>G), ATG805ATA (2565G>A), GCG806GCA (2568G>A), GGA809GGC (2577A>C), TTG810ACC (2578T>A 2579T>C 2580G>C),
TTT811ATG (2581T>A 2583T>G), GGG812AAT (2584G>A 2585G>A 2586G>T), GGA813GCC (2588G>C 2589A>C), ATA814GTT (2590A>G 2592A>T), ACA815ACT (2595A>T),
TTT817TTC (2601T>C), CTT818ATT (2602C>A), GCT819ACT (2605G>A), TGT820TGC (2610T>C), TGG821CTC (2611T>C 2612G>T 2613G>C), CAA822AAG (2614C>A 2616A>G),
AGC823GCA (2617A>G 2618G>C 2619C>A), AAT824GCA (2620A>G 2621A>C 2622T>A), TCT825ACT (2623T>A), TTT826TCC (2627T>C 2628T>C), CCA827CCT (2631A>T),
AGT828TTA (2632A>T 2633G>T 2634T>A), AGT830GAT (2638A>G 2639G>A), GGA831GGC (2643A>C), GGT833GGG (2649T>G), GGT834GGC (2652T>C), GTC835GTA
(2655C>A), ACA836ACC (2658A>C), AGC841AGT (2673C>T), TCT843TCC (2679T>C), AAA845AAG (2685A>G), AAG846AAA (2688G>A), AAC847AAT (2691C>T), AAG848AAA
(2694G>A), GGC849GGG (2697C>G), CCC850AAG (2698C>A 2699C>A 2700C>G), TCT851ACT (2701T>A), CGT852CAT (2705G>A), CTG853TTC (2707C>T 2709G>C),
AAG854GCA (2710A>G 2711A>C 2712G>A), AAT855AAA (2715T>A), TTG856TCC (2717T>C 2718G>C), GTG858CTA (2722G>C 2724G>A), CAG860CAA (2730G>A), AGT861GCG
(2731A>G 2732G>C 2733T>G), AGC862GCA (2734A>G 2735G>C 2736C>A), GCT863GAT (2738C>A), ATT865AAC (2744T>A 2745T>C), ATT865_ACA866insCAT
(2745_2746insCAT), ACA866GTG (2746A>G 2747C>T 2748A>G), CAA867GAC (2749C>G 2751A>C), GAA868CGA (2752G>C 2753A>G), GCT871GAA (2762C>A 2763T>A),
GAA872CAG (2764G>C 2766A>G), GGG874GGA (2772G>A), GTC876GTT (2778C>T), AAC877AAT (2781C>T), CAA878GAA (2782C>G), GCA879AGT (2785G>A 2786C>G
2787A>T), CTC880CTG (2790C>G), AAA881CAC (2791A>C 2793A>C), AAG882AAA (2796G>A), AAT883AAC (2799T>C), ATG884TTG (2800A>T), ATT885GTT (2803A>G),
GTA886GTT (2808A>T), CTG887TTG (2809C>T), CGG888AGA (2812C>A 2814G>A), CTT889ATT (2815C>A), GGA890GCT (2819G>C 2820A>T), GAG891GAA (2823G>A),
GTG893GTC (2829G>C), TAC894TAT (2832C>T), ACA898ACG (2844A>G), GTG900GTT (2850G>T), TCA902TCT (2856A>T), GGG903GGT (2859G>T), ATA905ATT (2865A>T),
ATG906CTC (2866A>C 2868G>C), GCA911GCC (2883A>C), TTC912TTT (2886C>T), CAC913AGA (2887C>A 2888A>G 2889C>A), AAT914AAC (2892T>C), GTG915ATG
(2893G>A), AGA916AGG (2898A>G), GGA918GGC (2904A>C), ACA919ACT (2907A>T), ACC922TCC (2914A>T), ATT923ATC (2919T>C), ATC924ATT (2922C>T), CCT926GAG
(2926C>G 2927C>A 2928T>G), AAC927CAT (2929A>C 2931C>T), TCA928TCC (2934A>C), AGG929ATT (2936G>T 2937G>T), TCA930TGT (2939C>G 2940A>T), AAG931AAT
(2943G>T), TAC934TTC (2951A>T), AAC935AAT (2955C>T), CGG937ACC (2959C>A 2960G>C 2961G>C), GAA938CAA (2962G>C), TCC939TCA (2967C>A), AAG940AGG
(2969A>G), GTC941TTG (2971G>T 2973C>G), ATT942ATA (2976T>A), GGA944GGT (2982A>T), ATC947GTG (2989A>G 2991C>G), CTG949TTG (2995C>T), CTA950TTG

Codon mutations:

(2998C>T 3000A>G), CGT951AGG (3001C>A 3003T>G), GTG952GTA (3006G>A), GGT953GGG (3009T>G), GAC954GAT (3012C>T), CCT955CCA (3015T>A), CCT960TCA (3028C>T 3030T>A), ATT962ATA (3036T>A), CGC963AGA (3037C>A 3039C>A), AAG964AAA (3042G>A), CCA967CCT (3051A>T), GAG971GAT (3063G>T), GTC972GTG (3066C>G), TGC973TGT (3069C>T), TTC974TAC (3071T>A), ACC975TCT (3073A>T 3075C>T), AAA976AAG (3078A>G), GGT977GGC (3081T>C), TCT978TCA (3084T>A), CAG979ATG (3085C>A 3086A>T), TTA981TTG (3093A>G), TGT982TGC (3096T>C), TGT983GTG (3097T>G 3098G>T 3099T>G), AGA984AGG (3102A>G), GCC985GCT (3105C>T), GTG986GTA (3108G>A), GCG987GCT (3111G>T), ACC989AAG (3116C>A 3117C>G), CCA991CCC (3123A>C), AGA992AAG (3125G>A 3126A>G), CTG993AAA (3127C>A 3128T>A 3129G>A), GGC994TCC (3130G>T 3131G>C), AAC995AAT (3135C>T), CTA996TTG (3136C>T 3138A>G), GAA997GAG (3141A>G), TTT998GTT (3142T>G), TTG999CTT (3145T>C 3147G>T), TTT1001TAT (3152T>A), TCC1006TCA (3168C>A), AAA1007AAG (3171A>G), TAT1009TAC (3177T>C), ACA1010ACC (3180A>C), GTC1011GAA (3182T>A 3183C>A), AAG1012AAA (3186G>A), GTG1013GTC (3189G>C), GAG1014ACA (3190G>A 3191A>C 3192G>A), GAG1016GGA (3197A>G 3198G>A), GAC1018GAT (3204C>T), TCT1019TCG (3207T>G), GCC1020AAT (3208G>A 3209C>A 3210C>T), AGT1021GCA (3211A>G 3212G>C 3213T>A), AAA1023GCT (3217A>G 3218A>C 3219A>T), TTG1024CTT (3220T>C 3222G>T), AGT1025AGC (3225T>C), TCG1026TCA (3228G>A), CAG1027CAA (3231G>A), CAA1028GTT (3232C>G 3233A>T 3234A>T), TCA1029GCA (3235T>G), GAA1031GAG (3243A>G), TAT1032TAC (3246T>C), CAT1033CAC (3249T>C), ATG1034ATT (3252G>T), GGA1036AGT (3256G>A 3258A>T), GGT1039GGA (3267T>A), GGA1042GGT (3276A>T), TTA1045CTC (3283T>C 3285A>C), CTT1046TTA (3286C>T 3288T>A), CCC1048CCA (3294C>A), AGT1049ACT (3296G>C), GTT1050GTG (3300T>G), AAA1053AAG (3309A>G), CAA1054CAG (3312A>G), CCT1055CCG (3315T>G), GTT1056TGC (3316G>T 3317T>G 3318T>C), GTG1058GTT (3324G>T), GGC1059GGA (3327C>A), ATT1060ATA (3330T>A), GCT1063GCA (3339T>A), TCA1064TCT (3342A>T), GGT1067GAT (3350G>A), ATT1068ACC (3353T>C 3354T>C), GCA1069GCT (3357A>T), GAA1071GAG (3363A>G), GGA1073GGT (3369A>T), TTC1074TAC (3371T>A), ATT1075GTG (3373A>G 3375T>G), TCT1076GCT (3376T>G), TCC1077TCA (3381C>A), AAT1078AAC (3384T>C), GCC1079GCA (3387C>A), ACG1080ACA (3390G>A), GCC1081GCA (3393C>A), ATC1082GTG (3394A>G 3396C>G), TTC1083TTT (3399C>T), CGG1084AGA (3400C>A 3402G>A), GAC1085GAT (3405C>T), CAG1086CAA (3408G>A), CTT1087TTG (3409C>T 3411T>G), AAC1089GAT (3415A>G 3417C>T), GGG1093GGA (3429G>A), CCT1094CCC (3432T>C), GTT1095ATT (3433G>A), GCG1097GTG (3440C>T), GCA1098GCT (3444A>T), CGA1101CGC (3453A>C), GAC1103GAA (3459C>A), ATT1104CTG (3460A>C 3462T>G), CTC1105TTG (3463C>T 3465C>G), AAA1106GAG (3466A>G 3468A>G), GCC1107CAC (3469G>C 3470C>A), ATT1108CTG (3472A>C 3474T>G), AGG1109AGA (3477G>A), AGT1110TCC (3478A>T 3479G>C 3480T>C), AGA1111AAG (3482G>A 3483A>G), GAA1112GAG (3486A>G), ACC1113GAA (3487A>G 3488C>A 3489C>A), ACT1114AGA (3491C>G 3492T>A), CTT1115TTC (3493C>T 3495T>C), GAA1117GAG (3501A>G), AAC1119AAA (3507C>A), CAA1120CAG (3510A>G), GTC1121GTG (3513C>G), TCC1122GCT (3514T>G 3516C>T), TTT1123TTC (3519T>C), CTT1124TTG (3520C>T 3522T>G), GTT1127GTA (3531T>A), CCT1128CCG (3534T>G), CAA1129TTG (3535C>T 3536A>T 3537A>G), TTG1131ATG (3541T>A), GCC1132GCT (3546C>T), GCA1133GCT (3549A>T), GTA1135GTG (3555A>G), CCC1136CCG (3558C>G), CAT1137CAC (3561T>C), ACT1139ACA (3567T>A), ACA1140ACC (3570A>C), CTC1141CTT (3573C>T), AGA1142AAA (3575G>A), AAG1143CGC (3577A>C 3578A>G 3579G>C), CAA1145CCC (3584A>C 3585A>C), TTG1146CTG (3586T>C), GCA1149GCT (3597A>T), GGT1151GGG (3603T>G), CCT1152GAC (3604C>G 3605C>A 3606T>C), GAA1154CAG (3610G>C 3612A>G), ACC1155ACT (3615C>T), GCT1156GCC (3618T>C), CCC1157CCA (3621C>A), TCA1158TCC (3624A>C), ATC1159ATT (3627C>T), CTC1160CTA (3630C>A), ACC1161TCT (3631A>T 3633C>T), GTC1162GCC (3635T>C), AAT1163AAA (3639T>A), CGG1166AGG (3646C>A), GAG1168GAA (3654G>A), GGA1169GGG (3657A>G), TTC1170TTT (3660C>T), CCA1172CCC (3666A>C), GCA1175GCT (3675A>T), GTG1177GTC (3681G>C), ATG1178GAG (3682A>G 3683T>A), AAA1179AAG (3687A>G), TAC1180TAT (3690C>T), ACT1183ACA (3699T>A), CAT1185TCC (3703C>T 3704A>C 3705T>C), GGT1186AAC (3706G>A 3707G>A 3708T>C), GAT1188GAG (3714T>G), GAT1189GAG (3717T>G), GAT1190CCA (3718G>C 3719A>C 3720T>A), GCT1192GCA (3726T>A), AAA1193AAG (3729A>G), TTG1194CTG (3730T>C), TCC1195GCC (3733T>G), GAA1197GAG (3741A>G), ACT1198GGT (3742A>G 3743C>G), CTC1199CTT (3747C>T), AGT1201GCA (3751A>G 3752G>C 3753T>A), TCA1202TCC (3756A>C), CTG1203ATT (3757C>A 3759G>T), CTG1204CTT (3762G>T), CCA1205CCT (3765A>T), ACA1206GTG (3766A>G 3767C>T 3768A>G), ATG1207CTT (3769A>C 3771G>T), AAA1208AAG (3774A>G), AAC1209ATG (3776A>T 3777C>G), CAA1210CAG (3780A>G), AAA1211CGA (3781A>C 3782A>G), GGC1215GGG (3795C>G), CCA1217CCT (3801A>T), CAG1218GCA (3802C>G 3803A>C 3804G>A), GAG1219ATC (3805G>A 3806A>T 3807G>C), GAT1221AAT (3811G>A), GTG1224GTA (3822G>A), CTG1226CTC (3828G>C), ATT1229ATA (3837T>A), TGT1232TTT (3845G>T), TAT1235TTT (3854A>T), GGC1237GCA (3860G>C 3861C>A), CTC1240TTG (3868C>T 3870C>G), ACA1242ACG (3876A>G), TCC1243TCA (3879C>A), TAC1246TAT (3888C>T), TTC1248TTT (3894C>T), ATG1251TTG (3901A>T), GGC1252GGA (3906C>A), ATG1253CTT (3907A>C 3909G>T), GGA1254GGT (3912A>T), ATG1255TCT (3913A>T 3914T>C 3915G>T), AAG1257AAA (3921G>A), CGG1258AGG (3922C>A), GAG1259GAA (3927G>A), GTC1261GTG (3933C>G), GTA1262GAA (3935T>A), CCA1263GCC (3937C>G 3939A>C), ACA1264CAA (3940A>C 3941C>A), GGA1265CCA (3943G>C 3944G>C), GAA1266AAC (3946G>A 3948A>C), GGT1267TCG (3949G>T 3950G>C 3951T>G), GAT1268CAG (3952G>C 3954T>G), AAA1269AGG (3956A>G 3957A>G), GTA1270GTT (3960A>T), GAG1271CTT (3961G>C 3962A>T 3963G>T), CTT1272CTC (3966T>C), AGA1273AAA (3968G>A), GAC1275AAC (3973G>A), CCA1277CCT (3981A>T), GTG1278GTC (3984G>C), GAA1280GAT (3990A>T), GCT1281GCA (3993T>A), GAG1283GAA (3999G>A), GAA1284GAG (4002A>G), CTG1285TTG (4003C>T), GAC1286GAT (4008C>T), ATT1287GCG (4009A>G 4010T>C 4011T>G), CAA1288CAG (4014A>G), ATT1289GTA (4015A>G 4017T>A), CAG1291CAA (4023G>A), ATA1293ACA (4028T>C), CAT1294TTT (4030C>T 4031A>T), GTG1295GTT (4035G>T), GAT1296GAC (4038T>C), CTA1297CTC (4041A>C), ATC1298GTC (4042A>G), ACC1299ACA (4047C>A), ACA1300ACC (4050A>C), CAA1301CAG (4053A>G), GCC1303GCG (4059C>G), AAG1304AAA (4062G>A), GAG1306GAA (4068G>A), CGC1307CGG (4071C>G), CTT1308TTG (4072C>T 4074T>G), CCT1309CCA (4077T>A), GAA1311GAG (4083A>G), AAA1318AAG (4104A>G), ACA1319ACC (4107A>C), AGA1320CGC (4108A>C 4110A>C), TTA1321CTG (4111T>C 4113A>G), TTT1322TTC (4116T>C), GAG1323GAA (4119G>A), ATA1324ATC (4122A>C), TAC1329TAT (4137C>T), CTG1332CTA (4146G>A), GTC1333GTG (4149C>G), AGG1334CGG (4150A>C), TTT1337TTC (4161T>C), TCC1341TCT (4173C>T), GCC1342GCT (4176C>T), ACC1343TGT (4177A>T 4178C>G 4179C>T), GCA1346GCT (4188A>T), TTA1347GCA (4189T>G 4190T>C), AAT1349AAC (4197T>C), GTC1350GTA (4200C>A), ATC1351CTC (4201A>C), CCA1352CCT (4206A>T), TGT1353TGC (4209T>C), GTT1355GTA (4215T>A), GGA1356GGT (4218A>T), ATA1357ATC (4221A>C), GAT1358AAT (4222G>A), ACC1360ACG (4230C>G), AGT1361AGC (4233T>C), TCT1362CAT (4234T>C 4235C>A), TCT1365ACA (4243T>A 4245T>A), CTT1366GTG (4246C>G 4248T>G), CTT1367CTG (4251T>G), GCC1368GCT (4254C>T), AAC1369AGT (4256A>G 4257C>T), AAC1370AGC (4259A>G), TTC1371TTT (4263C>T), GCT1373TCA (4267G>T 4269T>A), ACT1377GAG (4279A>G 4280C>A 4281T>G), GGG1378GGA (4284G>A), TTT1379TTC (4287T>C), GCT1381GCC (4293T>C), TCA1384TCG (4302A>G), AGT1385AGC (4305T>C), GAC1387GAT (4311C>T), GGG1388GGA (4314G>A), CGC1389AGA (4315C>A 4317C>A), CCA1391CCC (4323A>C), GTG1392ATC (4324G>A 4326G>C), TTC1393TTT (4329C>T), GCT1394GCA (4332T>A), TTT1395TTC (4335T>C), CAG1396CAA (4338G>A), TTG1401CTG (4351T>C), GTT1402GTG (4356T>G), GAG1404GCT (4361A>C 4362G>T), TAT1405TTT (4364A>T), GAT1407GGT (4370A>G), ACC1408GAT (4372A>G 4373C>A 4374C>T), AAG1409GAG (4375A>G), GGA1411GGG (4383A>G), GAG1412AGT (4384G>A 4385A>G 4386G>T), GCT1414GAG (4391C>A 4392T>G), TCC1415TCA (4395C>A), GCA1417GCT (4401A>T), AGA1418AGG (4404A>G), CAT1419CAC (4407T>C), GCT1420ACA (4408G>A 4410T>A), CTT1421TTA (4411C>T 4413T>A), CTC1422TTA (4414C>T 4416C>A), GCA1425GCC (4425A>C), TCA1426TCT (4428A>T), TAC1429TAT (4437C>T), TTA1431CTC (4441T>C 4443A>C), GAT1434AAC (4450G>A 4452T>C), AAA1435AAG (4455A>G), GTT1436TTG (4456G>T 4458T>G), TTC1437TTT (4461C>T), CGT1438CGC (4464T>C), TTG1439CTA (4465T>C 4467G>A), GTT1440GTG (4470T>G), GGC1442GGA (4476C>A), CCG1444CCC (4482G>C), GCA1448GCT (4494A>T), CTT1449TTG (4495C>T 4497T>G), ACC1450ACT (4500C>T), TTC1453TTT (4509C>T), AAC1454AAT (4512C>T), CTG1456TTG (4516C>T), TTG1457CTA (4519T>C 4521G>A), CGG1463AGG (4537C>A), TAT1464TAC (4542T>C), GCT1465GCG (4545T>G), TTC1466TTT (4548C>T), ATC1467ATA (4551C>A), CTT1469CTG (4557T>G), TTA1470TTG (4560A>G), AGA1471AGG (4563A>G), AGG1472AGA (4566G>A), CCT1473CCA (4569T>A), CAC1474CAT (4572C>T), ATT1475GTC (4573A>G 4575T>C), GCC1476AAA (4576G>A 4577C>A 4578C>A), GCT1477GCC (4581T>C), AGA1478AAA (4583G>A), GCT1479TCT (4585G>T), ATA1480GTG (4588A>G 4590A>G), GGT1481GGC (4593T>C), GTA1482ATT (4594G>A 4596A>T), CCT1484CCA (4602T>A), GGT1485AAT (4603G>A 4604G>A), GAT1486GAC (4608T>C), AAC1488AAT (4614C>T), CTT1490TTG (4618C>T 4620T>G), GGA1496GGT (4638A>T), AAT1499AAC (4647T>C), CTG1500TTG (4648C>T), GTG1501GTT (4653G>T), GCT1502GCG (4656T>G), GTG1503GTT (4659G>T), CCT1504CCA (4662T>A), CTG1505ATG (4663C>A), CTG1507TTG (4669C>T), TCA1511GAT (4681T>G 4682C>A 4683A>T), CTG1512CTA (4686G>A), CCT1513CCA (4689T>A), AAT1514TCC (4690A>T 4691A>C 4692T>C), GCC1516GCA (4698C>A), CAG1517AGA (4699C>A 4700A>G 4701G>A), CTT1519CTG (4707T>G), GAC1520GAA (4710C>A), ATG1521GCT (4711A>G 4712T>C 4713G>T), GTA1522GTT (4716A>T), AAT1523AAC (4719T>C), ATC1526ATT (4728C>T), AAA1527AAG (4731A>G), GGG1529GGT (4737G>T), CTG1530TTG (4738C>T), GAT1531GAC (4743T>C), TCT1533TCA (4749T>A), AAC1537GAG (4759A>G 4761C>G), CAG1538AAG (4762C>A), GTG1539ACA (4765G>A 4766T>C 4767G>A), CAG1540AAA (4768C>A 4770G>A), TTC1541TTT (4773C>T), CAG1542CTG (4775A>T), GAC1543GAT (4779C>T), TCA1545TCT (4785A>T), GAA1546GAG (4788A>G), ACT1548ACC (4794T>C), AGC1551AGT (4803C>T), GGA1553GGT (4809A>T), TTT1554TTC (4812T>C), AAA1555AAG (4815A>G), CGT1556CGA (4818T>A), GCC1558GCA (4824C>A), CTT1559TTG (4825C>T 4827T>G), GGG1560GGA (4830G>A), CAC1562CAA (4836C>A), GTG1569GTT (4857G>T), ATA1571GTC (4861A>G 4863A>C), ATT1572ATC (4866T>C), CCT1574CCC (4872T>C), ATG1575ATT (4875G>T), TAC1576TAT (4878C>T), ATC1578ATT (4884C>T), AGA1579AGG (4887A>G), CCT1580CCA (4890T>A), CCG1582GTG (4894C>G 4895C>T), GGT1583GGA (4899T>A), ACT1587GTA (4909A>G 4910C>T 4911T>A), GTG1588CAA (4912G>C 4913T>A 4914G>A), GCA1589GCT (4917A>T), ATG1591TAT (4921A>T 4922T>A 4923G>T), GAG1592GAA (4926G>A), GTG1594ATT (4930G>A 4932G>T), ACT1596ACA (4938T>A), CTT1598ATC (4942C>A 4944T>C), CGT1599AGA (4945C>A 4947T>A), GAG1600GAA (4950G>A), TTT1602TTC (4956T>C), AGA1606CAG (4966A>C 4967G>A 4968A>G), GTT1607GCC (4970T>C 4971T>C), GCA1608ACT (4972G>A 4974A>T), GAG1610GAA (4980G>A), AAG1611AGT (4982A>G 4983G>T), TTA1612TTT (4986A>T), ACT1613GTA (4987A>G 4988C>T 4989T>A), TAT1614AAT (4990T>A), CAG1617TTT (4999C>T 5000A>T 5001G>T), TGT1618GGA (5002T>G 5004T>A), GCC1619GCT (5007C>T), AGA1621AAG (5012G>A 5013A>G), GAG1622CAG (5014G>C), CGT1623AGA (5017C>A 5019T>A), GGA1624AAT (5020G>A 5021G>A 5022A>T), GGC1626CCC (5026G>C 5027G>C), GCA1627ATT (5029G>A 5030C>T 5031A>T), ACA1628ATG (5033C>T 5034A>G), TTT1630ATG (5038T>A 5040T>G), CCT1631CCC (5043T>C), TCT1632ACT (5044T>A), TAT1633TAC (5049T>C), ATG1634TAT (5050A>T 5051T>A 5052G>T), GTG1636CTT (5056G>C 5058G>T), GAG1637GAA (5061G>A), CAG1638AGT (5062C>A 5063A>G 5064G>T), GAA1639CAA (5065G>C), ATC1641TTG (5071A>T 5073C>G), GCA1642ACA (5074G>A), AAG1643GAG (5077A>G), GCA1648GAG (5093C>A 5094A>G), TCA1649AGC (5095T>A 5096C>G 5097A>C), GCG1650ATT (5098G>A 5099C>T 5100G>T), TTG1651GGG (5101T>G 5102T>G), ACA1652GTT (5104A>G 5105C>T 5106A>T), ATC1653TTG (5107A>T 5109C>G), TGT1654ATG (5110T>A 5111G>T 5112T>G), GAA1655GAG (5115A>G), ATG1656CTA (5116A>C 5118G>A), GCC1657TCA (5119G>T 5121C>A), AAA1658GAT (5122A>G 5124A>T), TCT1660AAA (5128T>A 5129C>A 5130T>A), ATC1661AAT (5132T>A 5133C>T), ACT1662ACA (5136T>A), TTC1663TAC (5138T>A), ACA1664ACC (5142A>C), TTG1666TTA (5148G>A), CCA1667CCT (5151A>T), CCT1668GCT (5152C>G), AAT1670GAA (5158A>G 5160T>A), GTT1672ATT (5164G>A), GAA1673del (5167_5169delGAA), GAA1676GAG (5178A>G), AGA1677GAG (5179A>G 5180G>A 5181A>G), GAT1678GGT (5183A>G), CTC1679GTC (5185C>G), AAT1680CAT (5188A>C), TGG1681GAA (5191T>G 5192G>A 5193G>A), GCC1683GTG (5198C>T 5199C>G), CCA1684CCT (5202A>T), AAC1685GAT (5203A>G 5205C>T), ATT1686GTA (5206A>G 5208T>A), GGT1687TTT (5209G>T 5210G>T), TGC1689TGT (5217C>T), TCA1690AGT (5218T>A 5219C>G 5220A>T), GCC1691GCT (5223C>T), CGT1692CGG (5226T>G), GCC1695TCA (5233G>T 5235C>A), AAG1696AAC (5238G>C), ACT1697CAA (5239A>C 5240C>A 5241T>A), GAA1698CAA (5242G>C), GAG1700TCA (5248G>T 5249A>C 5250G>A), CCT1701TCA (5251C>T 5253T>A), GGG1702GGT (5256G>T), TGC1703AAC (5257T>A 5258G>A), ATA1704ATC (5262A>C), GTA1705CTG (5263G>C 5265A>G), AAC1707AAT (5271C>T), TGT1708TGC (5274T>C), ACA1709ACT (5277A>T), GGG1710GGA (5280G>A), GCG1711GCC (5283G>C), AAG1712CGC (5284A>C 5285A>G 5286G>C), AAG1713AGA (5288A>G 5289G>A), AGC1714ACA (5291G>C 5292C>A), AAA1715ACA (5294A>C), GTT1717GTG (5301T>G), CGC1718AGA (5302C>A 5304C>A), GGA1719GGG (5307A>G), GCT1721GCC (5313T>C), AGT1722AAT (5315G>A), GAT1725GAC (5325T>C), ATG1730ATA (5340G>A), CCA1732CCC (5346A>C), TAT1733TAC (5349T>C), ACC1734ACT (5352C>T), ATG1735TTG (5353A>T), TCA1736GCA (5356T>G), ATT1738ATC (5364T>C), CTG1739CAG (5366T>A), GCT1741CAT (5371G>C 5372C>A), AGA1743CGC (5377A>C 5379A>C), GGC1744AAG (5380G>A 5381G>A 5382C>G), AAA1745AGT (5384A>G 5385A>T), ATG1746CTG (5386A>C), TCA1747GAA (5389T>G 5390C>A), GAG1748GTT (5393A>T 5394G>T), GAC1749GGT (5396A>G 5397C>T), ATT1750GAT (5398A>G 5399T>A), GCT1751GGG (5402C>G 5403T>G), GAT1754TCT (5410G>T 5411A>C), GTT1755GTC (5415T>C), GTG1756ACC (5416G>A 5417T>C 5418G>C), GTG1758GTT (5424G>T), GGG1760GGT (5430G>T), GCT1764GCA (5442T>A), GTG1765ATA (5443G>A 5445G>A), AGC1766GCT (5446A>G 5447G>C 5448C>T), GCT1771GCA (5463T>A), TTA1772TTG (5466A>G), ATG1773TTT (5467A>T 5469G>T), GCT1774GCA (5472T>A), GTG1775TTG (5473G>T), CGT1778CAT (5483G>A), CAG1779CAA (5487G>A), TAT1780TAC (5490T>C), TGC1781AGG (5491T>A 5493C>G), ATT1782GCC (5494A>G 5495T>C 5496T>C), GAG1783ACA (5497G>A 5498A>C 5499G>A), GAT1784GAC (5502T>C), ATC1785CTT (5503A>C 5505C>T), AGG1788CGC (5512A>C 5514G>C), TTG1789CTA (5515T>C 5517G>A), CGC1790CGA (5520C>A), ACT1791ACC (5523T>C), ATT1792ATG (5526T>G), GGC1793GCT (5528G>C 5529C>T), AAT1794GGG (5530A>G 5531A>G 5532T>G), TTG1796CTG (5536T>C), ACA1800ACC (5550A>C), TAT1801TAC (5553T>C), CCC1802CCT (5556C>T), GTT1806GCG (5567T>C 5568T>G), CAG1807CAA (5571G>A), TTT1809TTG (5577T>G), CAA1811AAA (5581C>A), GTG1813GTT (5589G>T), CCT1814GAA (5590C>G 5591C>A 5592T>A), CAG1815GAC (5593C>G 5595G>C), CAG1815_GAA1816insACC (5595_5596insACC), GAA1816TCA (5596G>T 5597A>C), GGG1817CAC (5599G>C 5600G>A 5601G>C), AAA1818AAG (5604A>G), ATG1819TTG (5605A>T), GCA1820GGA (5609C>G), GTT1822AGT (5614G>A 5615T>G), TCG1824ACA (5620T>A 5622G>A), TCT1825del (5623_5625delTCT), TTG1826TAT (5627T>A 5628G>T), CAA1827GGC (5629C>G 5630A>G 5631A>C), AGT1828ACC (5633G>C 5634T>C), AGT1829ACT (5636G>C), ATG1831AAC (5642T>A 5643G>C), CAA1833CAT (5649A>T), CAA1833_GGT1834insCACAGT (5649_5650insCACAGT), GGT1834GCA (5651G>C 5652T>A), GAG1836TCA (5656G>T 5657A>C 5658G>A), CTT1837GTT (5659C>G), GGC1838GGA (5664C>A), CAA1839CTT (5666A>T 5667A>T), ATT1840CTA (5668A>C 5670T>A), CGT1841AGG (5671C>A 5673T>G), GTC1842ATA (5674G>A 5676C>A), ATC1843GTT (5677A>G 5679C>T), CAC1844CCT (5681A>C 5682C>T), GGT1845TCC (5683G>T 5684G>C 5685T>C), GAT1846GAC (5688T>C), TTA1847CTC (5689T>C 5691A>C), TCA1848TCT (5694A>T), ATG1849GAA (5695A>G 5696T>A 5697G>A), GAA1850AAG (5698G>A 5700A>G), TTG1853GTT (5707T>G 5709G>T), CGC1854CGT (5712C>T), ATG1855TTG (5713A>T), TAT1857TAC (5721T>C), GTT1858ACA (5722G>A 5723T>C 5724T>A), GTG1859ATT (5725G>A 5727G>T), GGC1860GGT (5730C>T), CCA1861CCT (5733A>T), CAT1862GAA (5734C>G 5736T>A), GGT1863GGA (5739T>A), GGA1864GGT (5742A>T), GGC1866GCC (5747G>C), AAC1867CAG (5749A>C 5751C>G), TTT1868TGC (5753T>G 5754T>C), ACC1869TCA (5755A>T 5757C>A), ACA1870ACG (5760A>G), AAA1871AAG (5763A>G), AAT1872AAC (5766T>C), CTT1873CTG (5769T>G), GAT1874AAG (5770G>A 5772T>G), CAA1875CAT (5775A>T), TTG1876GCT (5776T>G 5777T>C 5778G>T), TTT1877TTG (5781T>G), GCA1878GAG (5783C>A 5784A>G), AGT1879ACA (5786G>C 5787T>A), CTT1880TTG (5788C>T 5790T>G), GAA1881TCT (5791G>T 5792A>C 5793A>T), AAG1882GTC (5794A>G 5795A>T 5796G>C), TGT1883AGT (5797T>A), GAA1886ACT (5806G>A 5807A>C 5808A>T), TTG1887CTT (5809T>C 5811G>T), CTT1888GCC (5812C>G 5813T>C 5814T>C), AAG1890AGA (5819A>G 5820G>A), AAC1891GGA (5821A>G 5822A>G 5823C>A), ACG1892GTC (5824A>G 5825C>T 5826G>C), CTG1894GTG (5830C>G), ACC1895ACA (5835C>A), GAG1899AAA (5845G>A 5847G>A), AAA1900GAA (5848A>G), ATG1901TTC (5851A>T 5853G>C), ACT1902TCC (5854A>T 5856T>C), AAA1904CAC (5860A>C 5862A>C), GTG1906TGT (5866G>T 5867T>G 5868G>T), GCA1907GAA (5870C>A), CAA1908ATG (5872C>A 5873A>T 5874A>G), GTC1910CTT (5878G>C 5880C>T), AGT1911GAG (5881A>G 5882G>A 5883T>G), GTA1913CTG (5887G>C 5889A>G), AAA1914GAA (5890A>G), CAG1915AGT (5893C>A 5894A>G 5895G>T), TTC1918GTA

	(5902T>G 5904C>A), TAT1919GTA (5905T>G 5906A>T 5907T>A), CCC1920TTC (5908C>T 5909C>T), AAG1921ATG (5912A>T), GCC1922GGG (5915C>G 5916C>G), ACA1923AGC (5918C>G 5919A>C), ACC1924GTT (5920A>G 5921C>T 5922C>T), GTG1925AAG (5923G>A 5924T>A), GAA1926AAA (5926G>A), CAG1927CAA (5931G>A), CTC1928ATG (5932C>A 5934C>G), AAG1929TTG (5935A>T 5936A>T), GTA1930TCC (5938G>T 5939T>C 5940A>C), TTT1931TTC (5943T>C), GCT1932ATG (5944G>A 5945C>T 5946T>G), GAT1933AAT (5947G>A), GCT1934CAA (5950G>C 5951C>A 5952T>A), CAT1935CAG (5955T>G), CAT1935_ACC1936insATG (5955_5956insATG), ACC1936TCA (5956A>T 5958C>A), ATT1937AAA (5960T>A 5961T>A), GTC1938ACC (5962G>A 5963T>C), AAA1939CAA (5965A>C), ACT1940GTT (5968A>G 5969C>T), GCT1941GCC (5973T>C), AAA1942AGC (5975A>G 5976A>C), CCT1943CCA (5979T>A), TTC1944TGT (5981T>G 5982C>T), CGG1945TGT (5983C>T 5985G>T), GGA1946del (5986_5988delGGA), GTA1947GTC (5991A>C), GTT1948ACA (5992G>A 5993T>C 5994T>A), TTC1949TTT (5997C>T), CGG1950AGT (5998C>A 6000G>T), AAT1952GCA (6004A>G 6005A>C 6006T>A), TTT1953TGG (6008T>G 6009T>G), CTC1954CTA (6012C>A), AGT1955ACT (6014G>C), CCT1956AAG (6016C>A 6017C>A 6018T>G), GAG1957GAC (6021G>C), TGG1958ATT (6022T>A 6023G>T 6024G>T), TGG1958_AAA1959insGTC (6024_6025insGTC), AAA1959ATG (6026A>T 6027A>G), ATT1960ACA (6029T>C 6030T>A), GGA1961AAA (6031G>A 6032G>A), GAG1963AAG (6037G>A), AAC1964AAG (6042C>G), ACA1965CCA (6043A>C), ATG1966del (6046_6048delATG), GCA1967GGA (6050C>G), ACA1968TCT (6052A>T 6054A>T), CTC1969ATT (6055C>A 6057C>T), TCA1970CCT (6058T>C 6060A>T), GAG1972GAA (6066G>A), AGT1973ACA (6068G>C 6069T>A), CTT1974GTG (6070C>G 6072T>G), CTT1975TTT (6073C>T), CCA1976CTT (6077C>T 6078A>T), CTG1979CCT (6086T>C 6087G>T), TCG1980TCA (6090G>A), TCA1982CTA (6094T>C 6095C>T), GCC1983GTT (6098C>T 6099C>T), ATG1984GTG (6100A>G), AAG1985AGA (6104A>G 6105G>A), TTG1987ATG (6109T>A), CTC1988TTG (6112C>T 6114C>G), AGC1989CGC (6115A>C), AGA1990AAA (6119G>A)								
helicase motif A-C (YP_009167369.1)	1	98	100%	654	97.6%	98 (100%)	96 (98.0%)	0/0/0/0	0
Protein mutations:	A5C (1345G>T 1346C>G 1347A>T), R16A (1378C>G 1379G>C 1380C>A)								
Codon mutations:	ACA4ACG (1344A>G), GCA5TGT (1345G>T 1346C>G 1347A>T), CGA7CGC (1353A>C), TTG8CTT (1354T>C 1356G>T), TTG14CTC (1372T>C 1374G>C), GAT15GAC (1377T>C), CGC16GCA (1378C>G 1379G>C 1380C>A), GGG18GGT (1386G>T), GAG19GAA (1389G>A), CCT20CCA (1392T>A), CTT22CTG (1398T>G), AGA24CGC (1402A>C 1404A>C), CTT25TTG (1405C>T 1407T>G), GCT27GCG (1413T>G), GTT28GTA (1416T>A), AGC29AGT (1419C>T), AAG30AAA (1422G>A), CGC31AGA (1423C>A 1425C>A), GCC33GCA (1431C>A), TCT36TCC (1440T>C), AAT37AAC (1443T>C), GCC39GCA (1449C>A), CAC40CAT (1452C>T), CAG41CAA (1455G>A), GCT43GCA (1461T>A), TTG45CTT (1465T>C 1467G>T), GAC48GAT (1476C>T), GGA50GGT (1482A>T), GCA51GCT (1485A>T), TTG52CTT (1486T>C 1488G>T), AGA53CGG (1489A>C 1491A>G), GGA57GGG (1503A>G), CAA58CAG (1506A>G), CAA60CAG (1512A>G), ATT62ATA (1518T>A), ATT68ATC (1536T>C), TCC70TCT (1542C>T), ACA71ACC (1545A>C), CCA73CCT (1551A>T), CCA75CCT (1557A>T), CTG76CTT (1560G>T), CCC77CCA (1563C>A), GCA79GCT (1569A>T), GCT80GCA (1572T>A), GTG81GTC (1575G>C), GAA82GAG (1578A>G), GAC83GAT (1581C>T), AAG84AAA (1584G>A), GGG85GGA (1587G>A), CAT87CAC (1593T>C), ACT89ACA (1599T>A), TAT92TAC (1608T>C), GCA95GCC (1617A>C), AAT98AAC (1626T>C)								

*: Inserts / Deletes / Misaligned / Frameshifts

Analysis details

This analysis was performed with panviral2.64

NGS Details (UN18_val): Bracoviriform glomeratae (segment NC_043292.1)

Assembly

**Coverage Length**	323 (1 contig(s))
**Depth Of Coverage**	4199.5
**Number Of Reads**	11330
**Reads Per Million**	212.65 rpm (after QC)
**Ambiguities**	0
**Assembly Method**	de novo + reference guided assembly
**Consensus Caller**	Bcf Tools

Coverage Map

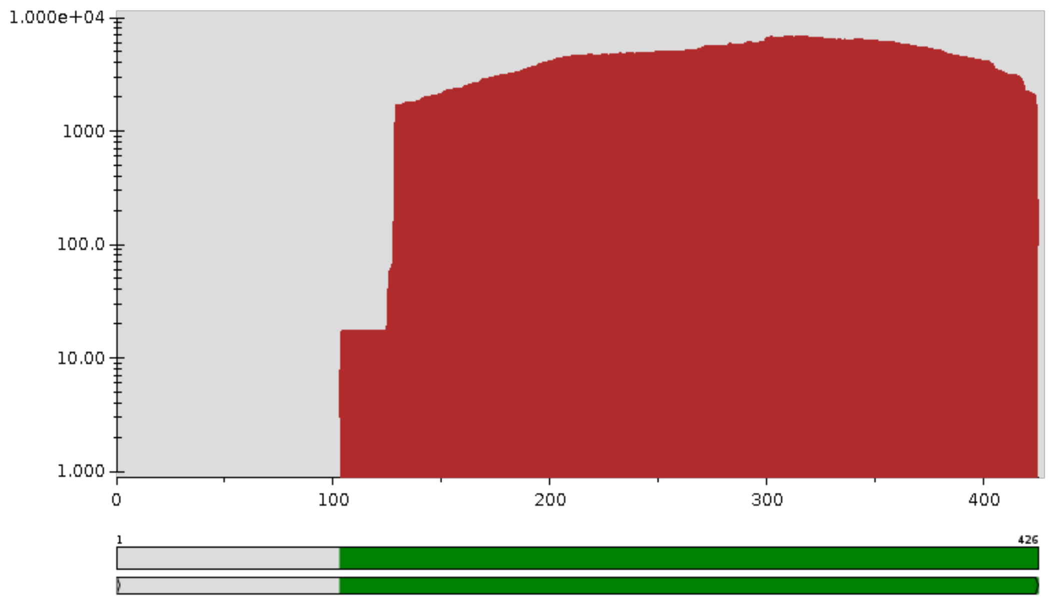

Assignment

**Type**	Bracoviriform glomeratae (Taxonomy ID: 257816)
**Reference Genome**	NC_043292.1
**NT Identity (%)**	72.7554
**AA Identity (%)**	84.1121
**Number Of Stop Codons**	1
**Number Of CDS**	1

Alignment

**Alignment Score**	294.0 (NT) + 580.0 (AA) = 874.0
**Concordance (%)**	65.7637

Alignment Method	Global, seeded, nucleotide + amino acids (AGA)

Genome Region

Sequence starts at position 104 and ends at position 426 relative to NC_043292.1 reference sequence.

Alignment Detailed Statistics

	Begin	End	Coverage	Score	Concordance	Matches	Identities	I/D/M/F*	Stop Codons
**NT**	**104**	**426**	**75.8%**	**294**	**45.5%**	**323 (100%)**	**235 (72.8%)**	**0/0**	
Mutations:	105G>A, 108C>T, 111A>G, 113A>G, 117A>C, 119G>A, 129C>A, 132A>G, 136A>G, 137C>G, 138G>C, 150A>C, 153A>G, 155C>G, 156G>A, 159C>A, 166C>A, 168T>G, 169T>C, 172C>A, 174C>G, 177A>G, 183T>A, 184C>A, 189T>C, 198A>G, 201A>C, 207C>T, 214A>G, 219C>T, 222T>C, 223C>A, 225T>G, 228G>T, 235C>A, 237T>G, 243A>T, 246C>G, 255C>T, 261A>G, 264A>G, 265G>A, 273A>G, 278T>C, 279T>C, 284A>G, 285T>A, 288T>G, 289T>C, 300C>T, 301C>T, 303C>G, 309C>T, 312T>G, 315C>T, 322G>T, 329T>C, 333T>C, 336C>T, 339A>G, 345A>C, 347A>G, 348A>G, 349C>A, 351T>A, 360C>T, 363C>T, 366C>T, 375C>T, 378C>T, 384A>C, 385T>C, 387G>C, 390A>G, 391C>A, 392A>G, 393T>A, 394A>C, 396A>G, 399C>A, 400C>A, 402T>G, 404T>C, 405G>T, 406A>C, 414T>A, 416A>T, 420A>G								
**CDS**									
FK954_p501	36	142	75.4%	580	84.4%	107 (100%)	90 (84.1%)	0/0/0/0	1
Protein mutations:	E38G (113A>G), R40K (119G>A), T46G (136A>G 137C>G 138G>C), A52G (155C>G 156G>A), Y57H (169T>C), T72A (214A>G), V89I (265G>A), I93T (278T>C 279T>C), D95G (284A>G 285T>A), A108S (322G>T), I110T (329T>C), K116R (347A>G 348A>G), H131R (391C>A 392A>G 393T>A), K132Q (394A>C 396A>G), M135T (404T>C 405G>T), I136L (406A>C), Y139F (416A>T)								
Codon mutations:	TTG35.TA (105G>A), GGC36GGT (108C>T), AAA37AAG (111A>G), GAA38GGA (113A>G), GGA39GGC (117A>C), AGA40AAA (119G>A), GGC43GGA (129C>A), AAA44AAG (132A>G), ACG46GGC (136A>G 137C>G 138G>C), GGA50GGC (150A>C), AAA51AAG (153A>G), GCG52GGA (155C>G 156G>A), GGC53GGA (159C>A), CGT56AGG (166C>A 168T>G), TAT57CAT (169T>C), CGC58AGG (172C>A 174C>G), AAA59AAG (177A>G), CTT61CTA (183T>A), CGA62AGA (184C>A), GAT63GAC (189T>C), CAA66CAG (198A>G), GGA67GGC (201A>C), ACC69ACT (207C>T), ACT72GCT (214A>G), ATC73ATT (219C>T), CGT74CGC (222T>C), CGT75AGG (223C>A 225T>G), CTG76CTT (228G>T), CGT79AGG (235C>A 237T>G), GGA81GGT (243A>T), GTC82GTG (246C>G), ATC85ATT (255C>T), GGA87GGG (261A>G), TTA88TTG (264A>G), GTC89ATC (265G>A), GAA91GAG (273A>G), ATT93ACC (278T>C 279T>C), GAT95GGA (284A>G 285T>A), GTT96GTG (288T>G), TTG97CTG (289T>C), TTC100TTT (300C>T), CTC101TTG (301C>T 303C>G), AAC103AAT (309C>T), GTT104GTG (312T>G), ATC105ATT (315C>T), GCT108TCT (322G>T), ATC110ACC (329T>C), TAT111TAC (333T>C), ACC112ACT (336C>T), GAA113GAG (339A>G), GCA115GCC (345A>C), AAA116AGG (347A>G 348A>G), CGT117AGA (349C>A 351T>A), GTC120GTT (360C>T), ACC121ACT (363C>T), GCC122GCT (366C>T), GTC125GTT (375C>T), GTC126GTT (378C>T), GCA128GCC (384A>C), TTG129CTC (385T>C 387G>C), AAA130AAG (390A>G), CAT131AGA (391C>A 392A>G 393T>A), AAA132CAG (394A>C 396A>G), GGC133GGA (399C>A), CGT134AGG (400C>A 402T>G), ATG135ACT (404T>C 405G>T), ATC136CTC (406A>C), GGT138GGA (414T>A), TAT139TTT (416A>T), GGA140GGG (420A>G)								
**Proteins**									
putative histone 4 (YP_009665791.1)	36	142	75.4%	580	84.4%	107 (100%)	90 (84.1%)	0/0/0/0	1
Protein mutations:	E38G (113A>G), R40K (119G>A), T46G (136A>G 137C>G 138G>C), A52G (155C>G 156G>A), Y57H (169T>C), T72A (214A>G), V89I (265G>A), I93T (278T>C 279T>C), D95G (284A>G 285T>A), A108S (322G>T), I110T (329T>C), K116R (347A>G 348A>G), H131R (391C>A 392A>G 393T>A), K132Q (394A>C 396A>G), M135T (404T>C 405G>T), I136L (406A>C), Y139F (416A>T)								
Codon mutations:	TTG35.TA (105G>A), GGC36GGT (108C>T), AAA37AAG (111A>G), GAA38GGA (113A>G), GGA39GGC (117A>C), AGA40AAA (119G>A), GGC43GGA (129C>A), AAA44AAG (132A>G), ACG46GGC (136A>G 137C>G 138G>C), GGA50GGC (150A>C), AAA51AAG (153A>G), GCG52GGA (155C>G 156G>A), GGC53GGA (159C>A), CGT56AGG (166C>A 168T>G), TAT57CAT (169T>C), CGC58AGG (172C>A 174C>G), AAA59AAG (177A>G), CTT61CTA (183T>A), CGA62AGA (184C>A), GAT63GAC (189T>C), CAA66CAG (198A>G), GGA67GGC (201A>C), ACC69ACT (207C>T), ACT72GCT (214A>G), ATC73ATT (219C>T), CGT74CGC (222T>C), CGT75AGG (223C>A 225T>G), CTG76CTT (228G>T), CGT79AGG (235C>A 237T>G), GGA81GGT (243A>T), GTC82GTG (246C>G), ATC85ATT (255C>T), GGA87GGG (261A>G), TTA88TTG (264A>G), GTC89ATC (265G>A), GAA91GAG (273A>G), ATT93ACC (278T>C 279T>C), GAT95GGA (284A>G 285T>A), GTT96GTG (288T>G), TTG97CTG (289T>C), TTC100TTT (300C>T), CTC101TTG (301C>T 303C>G), AAC103AAT (309C>T), GTT104GTG (312T>G), ATC105ATT (315C>T), GCT108TCT (322G>T), ATC110ACC (329T>C), TAT111TAC (333T>C), ACC112ACT (336C>T), GAA113GAG (339A>G), GCA115GCC (345A>C), AAA116AGG (347A>G 348A>G), CGT117AGA (349C>A 351T>A), GTC120GTT (360C>T), ACC121ACT (363C>T), GCC122GCT (366C>T), GTC125GTT (375C>T), GTC126GTT (378C>T), GCA128GCC (384A>C), TTG129CTC (385T>C 387G>C), AAA130AAG (390A>G), CAT131AGA (391C>A 392A>G 393T>A), AAA132CAG (394A>C 396A>G), GGC133GGA (399C>A), CGT134AGG (400C>A 402T>G), ATG135ACT (404T>C 405G>T), ATC136CTC (406A>C), GGT138GGA (414T>A), TAT139TTT (416A>T), GGA140GGG (420A>G)								

*: Inserts / Deletes / Misaligned / Frameshifts

Analysis details

This analysis was performed with panviral2.64

NGS Details (UN18_val): Potato leafroll virus

Assembly

**Coverage Length**	5830 (1 contig(s))
**Depth Of Coverage**	98.0
**Number Of Reads**	4303
**Reads Per Million**	80.76 rpm (after QC)
**Ambiguities**	0
**Assembly Method**	read mapping against reference + variant calling
**Consensus Caller**	Bcf Tools

Coverage Map

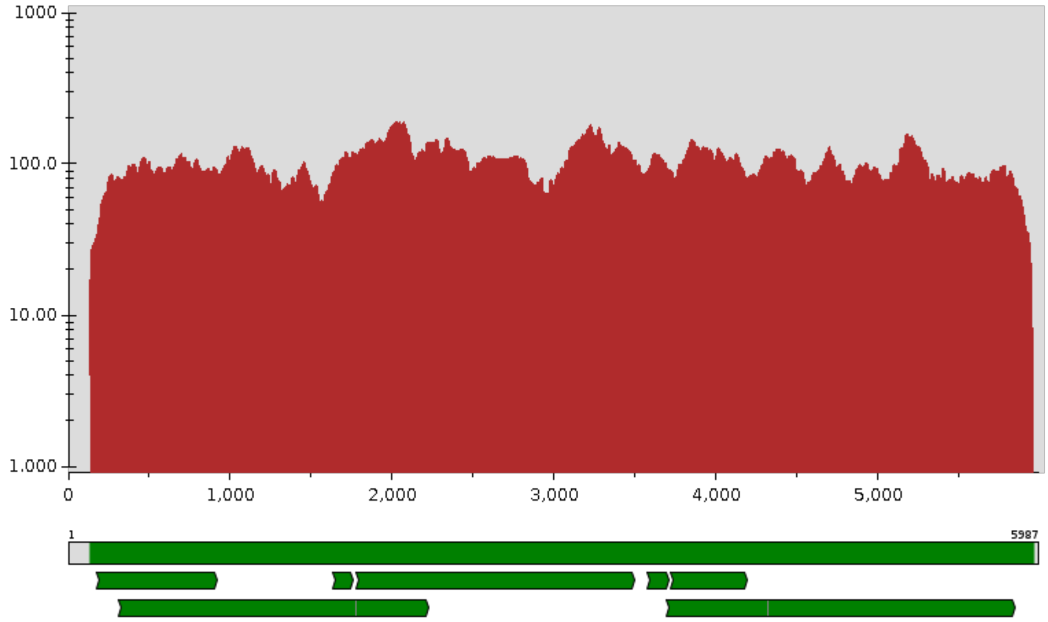

Assignment

**Type**	Potato leafroll virus (Taxonomy ID: 12045)
**Reference Genome**	NC_001747.1
**NT Identity (%)**	98.0614
**AA Identity (%)**	97.3103
**Number Of Stop Codons**	9
**Number Of CDS**	8

Alignment

**Alignment Score**	11196.0 (NT) + 21113.0 (AA) = 32309.0
**Concordance (%)**	97.0444

Alignment Method	Global, seeded, nucleotide + amino acids (AGA)

Genome Region

Sequence starts at position 135 and ends at position 5964 relative to NC_001747.1 reference sequence.

Alignment Detailed Statistics

	Begin	End	Coverage	Score	Concordance	Matches	Identities	I/D/M/F*	Stop Codons
**NT**	**135**	**5964**	**97.4%**	**11196**	**96.1%**	**5829 (99.9%)**	**5716 (98.0%)**	**0/1**	
Mutations:	243C>T, 249C>T, 279G>T, 293T>C, 343C>T, 513C>T, 525C>T, 561C>T, 600T>C, 648T>C, 699A>C, 715C>T, 745G>A, 822C>T, 825T>G, 864C>T, 922G>A, 931A>T, 934A>G, 1079G>A, 1093A>G, 1117G>A, 1664G>A, 1674C>T, 1678T>A, 1755T>C, 1795C>A, 1815A>T, 1823G>A, 2007T>C, 2051G>A, 2079C>T, 2154C>T, 2265A>G, 2283G>A, 2313A>G, 2316T>G, 2325C>T, 2470G>A, 2580A>G, 2586A>G, 2613C>T, 2622T>C, 2637C>T, 2649C>T, 2697G>A, 2729A>G, 2811T>A, 2817C>T, 2856G>A, 2877C>T, 2898T>C, 2931T>C, 2982C>T, 3000T>C, 3033C>T, 3036C>T, 3069C>T, 3075T>A, 3102T>C, 3117C>T, 3321C>T, 3468T>C, 3510delA, 3525A>G, 3548C>T, 3585A>G, 3604T>C, 3605T>A, 3652T>A, 3721T>G, 3748T>G, 3750C>A, 3788T>G, 3876G>A, 3878T>A, 3911G>C, 3935C>T, 3939A>T, 4031A>G, 4034C>T, 4113G>A, 4166C>T, 4172T>C, 4214T>G, 4601A>G, 4608A>G, 4610C>T, 4616C>T, 4817T>C, 4883C>T, 4886T>C, 4895T>C, 4898C>T, 4979C>T, 5038C>T, 5042G>A, 5048C>T, 5062C>T, 5071A>G, 5072C>T, 5129G>A, 5199C>T, 5214C>A, 5215G>A, 5229G>A, 5385G>A, 5501C>T, 5508T>C, 5522C>T, 5781G>A, 5816A>G, 5824A>G, 5925T>C								
**CDS**									
PLRVgp1	1	248	100%	1695	97.9%	248 (100%)	243 (98.0%)	0/0/0/0	1
Protein mutations:	Q35H (279G>T), I40T (293T>C), H57Y (343C>T), Q175H (699A>C), V191I (745G>A)								
Codon mutations:	TTC23TTT (243C>T), GTC25GTT (249C>T), CAG35CAT (279G>T), ATT40ACT (293T>C), CAT57TAT (343C>T), ACC113ACT (513C>T), TAC117TAT (525C>T), TAC129TAT (561C>T), CAT142CAC (600T>C), CGT158CGC (648T>C), CAA175CAC (699A>C), CTA181TTA (715C>T), GTT191ATT (745G>A), CGC216CGT (822C>T), GCT217GCG (825T>G), GTC230GTT (864C>T)								
PLRVgp2	1	1063	100%	7338	98.4%	1063 (100%)	1043 (98.1%)	0/0/0/0	1
Protein mutations:	P69L (513C>T), T73I (525C>T), T85I (561C>T), M98T (600T>C), V114A (648T>C), N131T (699A>C), A172V (822C>T), L173R (825T>G), S186F (864C>T), E208D (931A>T), E258K (1079G>A), E453K (1664G>A), T456I (1674C>T), N457K (1678T>A), L483P (1755T>C), Q497K (1795C>A), R506Q (1823G>A), G582E (2051G>A), A722T (2470G>A), N808S (2729A>G)								
Codon mutations:	TTC12TTT (343C>T), CCG69CTG (513C>T), ACA73ATA (525C>T), ACA85ATA (561C>T), ATG98ACG (600T>C), GTG114GCG (648T>C), AAC131ACC (699A>C), CTC136CTT (715C>T), TTG146TTA (745G>A), GCG172GTG (822C>T), CTA173CGA (825T>G), TCC186TTC (864C>T), AGG205AGA (922G>A), GAA208GAT (931A>T), GGA209GGG (934A>G), GAA258AAA (1079G>A), GCA262GCG (1093A>G), AGG270AGA (1117G>A), GAG453AAG (1664G>A), ACA456ATA (1674C>T), AAT457AAA (1678T>A), CTA483CCA (1755T>C), CAA497AAA (1795C>A), CGA503CGT (1815A>T), CGA506CAA (1823G>A), TTT567TTC (2007T>C), GGA582GAA (2051G>A), TGC591TGT (2079C>T), CAC616CAT (2154C>T), TCA653TCG (2265A>G), GCG659GCA (2283G>A), ACA669ACG (2313A>G), GTT670GTG (2316T>G), TAC673TAT (2325C>T), GCA722ACA (2470G>A), GAA758GAG (2580A>G), CTA760CTG (2586A>G), ATC769ATT (2613C>T), TTT772TTC (2622T>C), CCC777CCT (2637C>T), AGC781AGT (2649C>T), GTG797GTA (2697G>A), AAT808AGT (2729A>G), GCT835GCA (2811T>A), TTC837TTT (2817C>T), GTG850GTA (2856G>A), CAC857CAT (2877C>T), ACT864ACC (2898T>C), TAT875TAC (2931T>C), AAC892AAT (2982C>T), CGT898CGC (3000T>C), AAC909AAT (3033C>T), TCC910TCT (3036C>T), GCC921GCT (3069C>T), ACT923ACA (3075T>A), AGT932AGC (3102T>C), TCC937TCT (3117C>T), AAC1005AAT (3321C>T), GTT1054GTC (3468T>C)								
PLRVgp3	1	640	100%	4167	97.1%	640 (100%)	619 (96.7%)	0/0/0/0	1
Protein mutations:	P69L (513C>T), T73I (525C>T), T85I (561C>T), M98T (600T>C), V114A (648T>C), N131T (699A>C), A172V (822C>T), L173R (825T>G), S186F (864C>T), E208D (931A>T), E258K (1079G>A), E453K (1664G>A), T456I (1674C>T), N457K (1678T>A), L483P (1755T>C), D503V (1815A>T), D506N (1823G>A), L567S (2007T>C), E582K (2051G>A), A591V (2079C>T), T616I (2154C>T)								
Codon mutations:	TTC12TTT (343C>T), CCG69CTG (513C>T), ACA73ATA (525C>T), ACA85ATA (561C>T), ATG98ACG (600T>C), GTG114GCG (648T>C), AAC131ACC (699A>C), CTC136CTT (715C>T), TTG146TTA (745G>A), GCG172GTG (822C>T), CTA173CGA (825T>G), TCC186TTC (864C>T), AGG205AGA (922G>A), GAA208GAT (931A>T), GGA209GGG (934A>G), GAA258AAA (1079G>A), GCA262GCG (1093A>G), AGG270AGA (1117G>A), GAG453AAG (1664G>A), ACA456ATA (1674C>T), AAT457AAA (1678T>A), CTA483CCA (1755T>C), GCC496GCA (1795C>A), GAC503GTC (1815A>T), GAC506AAC (1823G>A), TTA567TCA (2007T>C), GAG582AAG (2051G>A), GCG591GTG (2079C>T), ACC616ATC (2154C>T)								
Rap1	1	42	100%	260	90.6%	42 (100%)	39 (92.9%)	0/0/0/0	1
Protein mutations:	Q15* (1674C>T), M16K (1678T>A), *42Q (1755T>C)								
Codon mutations:	GAG11GAA (1664G>A), CAA15TAA (1674C>T), ATG16AAG (1678T>A), TAA42CAA (1755T>C)								
ORF3a	1	46	100%	286	95.7%	46 (100%)	43 (93.5%)	0/0/0/0	1
Protein mutations:	K4R (3585A>G), L11I (3605T>A), F26L (3652T>A)								
Codon mutations:	AAA4AGA (3585A>G), GCT10GCC (3604T>C), TTA11ATA (3605T>A), TTT26TTA (3652T>A)								
PLRVgp4	1	718	100%	4944	98.1%	718 (100%)	701 (97.6%)	0/0/0/0	2
Protein mutations:	V10G (3721T>G), M19R (3748T>G), V62I (3876G>A 3878T>A), T83S (3939A>T), V141I (4113G>A), I306V (4608A>G 4610C>T), P449L (5038C>T), A457V (5062C>T), H460R (5071A>G 5072C>T), P503S (5199C>T), R508K (5214C>A 5215G>A), V513I (5229G>A), E565K (5385G>A), S606P (5508T>C), E697K (5781G>A), I708M (5816A>G), K711R (5824A>G)								
Codon mutations:	GTC10GGC (3721T>G), ATG19AGG (3748T>G), CGA20AGA (3750C>A), GTT32GTG (3788T>G), GTT62ATA (3876G>A 3878T>A), GTG73GTC (3911G>C), GGC81GGT (3935C>T), ACC83TCC (3939A>T), ACA113ACG (4031A>G), AGC114AGT (4034C>T), GTA141ATA (4113G>A), GCC158GCT (4166C>T), ACT160ACC (4172T>C), TCT174TCG (4214T>G), TCA303TCG (4601A>G), ATC306GTT (4608A>G 4610C>T), TGC308TGT (4616C>T), GGT375GGC (4817T>C), TTC397TTT (4883C>T), CTT398CTC (4886T>C), CCT401CCC (4895T>C), GCC402GCT (4898C>T), ACC429ACT (4979C>T), CCA449CTA (5038C>T), CGG450CGA (5042G>A), GGC452GGT (5048C>T), GCC457GTC (5062C>T), CAC460CGT (5071A>G 5072C>T), ACG479ACA (5129G>A), CCA503TCA (5199C>T), CGA508AAA (5214C>A 5215G>A), GTA513ATA (5229G>A), GAA565AAA (5385G>A), AAC603AAT (5501C>T), TCC606CCC (5508T>C), CCC610CCT (5522C>T), GAA697AAA (5781G>A), ATA708ATG (5816A>G), AAG711AGG (5824A>G)								
PLRVgp5	1	209	100%	1416	98.7%	209 (100%)	204 (97.6%)	0/0/0/0	1
Protein mutations:	V10G (3721T>G), M19R (3748T>G), V62I (3876G>A 3878T>A), T83S (3939A>T), V141I (4113G>A)								
Codon mutations:	GTC10GGC (3721T>G), ATG19AGG (3748T>G), CGA20AGA (3750C>A), GTT32GTG (3788T>G), GTT62ATA (3876G>A 3878T>A), GTG73GTC (3911G>C), GGC81GGT (3935C>T), ACC83TCC (3939A>T), ACA113ACG (4031A>G), AGC114AGT (4034C>T), GTA141ATA (4113G>A), GCC158GCT (4166C>T), ACT160ACC (4172T>C), TCT174TCG (4214T>G)								
PLRVgp6	1	157	100%	1007	91.4%	157 (100%)	147 (93.6%)	0/0/0/0	1
Protein mutations:	S2A (3721T>G), C11G (3748T>G 3750C>A), F24C (3788T>G), F54Y (3878T>A), C65S (3911G>C), A73V (3935C>T), Q105R (4031A>G), A106V (4034C>T), P150L (4166C>T), L152P (4172T>C)								
Codon mutations:	TCA2GCA (3721T>G), TGC11GGA (3748T>G 3750C>A), TTC24TGC (3788T>G), GAG53GAA (3876G>A), TTC54TAC (3878T>A), TGT65TCT (3911G>C), GCA73GTA (3935C>T), ACA74ACT (3939A>T), CAA105CGA (4031A>G), GCA106GTA (4034C>T), AAG132AAA (4113G>A), CCA150CTA (4166C>T), CTT152CCT (4172T>C)								
**Proteins**									
P0 protein (NP_056746.1)	1	248	100%	1695	97.9%	248 (100%)	243 (98.0%)	0/0/0/0	1
Protein mutations:	Q35H (279G>T), I40T (293T>C), H57Y (343C>T), Q175H (699A>C), V191I (745G>A)								

	Begin	End	Coverage	Score	Concordance	Matches	Identities	I/D/M/F*	Stop Codons
**NT**	**135**	**5964**	**97.4%**	**11196**	**96.1%**	**5829 (99.9%)**	**5716 (98.0%)**	**0/1**	
Codon mutations:	TTC23TTT (243C>T), GTC25GTT (249C>T), CAG35CAT (279G>T), ATT40ACT (293T>C), CAT57TAT (343C>T), ACC113ACT (513C>T), TAC117TAT (525C>T), TAC129TAT (561C>T), CAT142CAC (600T>C), CGT158CGC (648T>C), CAA175CAC (699A>C), CTA181TTA (715C>T), GTT191ATT (745G>A), CGC216CGT (822C>T), GCT217GCG (825T>G), GTC230GTT (864C>T)								
RNA-dependent RNA polymerase (NP_056748.3)	1	1063	100%	7338	98.4%	1063 (100%)	1043 (98.1%)	0/0/0/0	1
Protein mutations:	P69L (513C>T), T73I (525C>T), T85I (561C>T), M98T (600T>C), V114A (648T>C), N131T (699A>C), A172V (822C>T), L173R (825T>G), S186F (864C>T), E208D (931A>T), E258K (1079G>A), E453K (1664G>A), T456I (1674C>T), N457K (1678T>A), L483P (1755T>C), Q497K (1795C>A), R506Q (1823G>A), G582E (2051G>A), A722T (2470G>A), N808S (2729A>G)								
Codon mutations:	TTC12TTT (343C>T), CCG69CTG (513C>T), ACA73ATA (525C>T), ACA85ATA (561C>T), ATG98ACG (600T>C), GTG114GCG (648T>C), AAC131ACC (699A>C), CTC136CTT (715C>T), TTG146TTA (745G>A), GCG172GTG (822C>T), CTA173CGA (825T>G), TCC186TTC (864C>T), AGG205AGA (922G>A), GAA208GAT (931A>T), GGA209GGG (934A>G), GAA258AAA (1079G>A), GCA262GCG (1093A>G), AGG270AGA (1117G>A), GAG453AAG (1664G>A), ACA456ATA (1674C>T), AAT457AAA (1678T>A), CTA483CCA (1755T>C), CAA497AAA (1795C>A), CGA503CGT (1815A>T), CGA506CAA (1823G>A), TTT567TTC (2007T>C), GGA582GAA (2051G>A), TGC591TGT (2079C>T), CAC616CAT (2154C>T), TCA653TCG (2265A>G), GCG659GCA (2283G>A), ACA669ACG (2313A>G), GTT670GTG (2316T>G), TAC673TAT (2325C>T), GCA722ACA (2470G>A), GAA758GAG (2580A>G), CTA760CTG (2586A>G), ATC769ATT (2613C>T), TTT772TTC (2622T>C), CCC777CCT (2637C>T), AGC781AGT (2649C>T), GTG797GTA (2697G>A), AAT808AGT (2729A>G), GCT835GCA (2811T>A), TTC837TTT (2817C>T), GTG850GTA (2856G>A), CAC857CAT (2877C>T), ACT864ACC (2898T>C), TAT875TAC (2931T>C), AAC892AAT (2982C>T), CGT898CGC (3000T>C), AAC909AAT (3033C>T), TCC910TCT (3036C>T), GCC921GCT (3069C>T), ACT923ACA (3075T>A), AGT932AGC (3102T>C), TCC937TCT (3117C>T), AAC1005AAT (3321C>T), GTT1054GTC (3468T>C)								
P1 protein (NP_056747.1)	1	640	100%	4167	97.1%	640 (100%)	619 (96.7%)	0/0/0/0	1
Protein mutations:	P69L (513C>T), T73I (525C>T), T85I (561C>T), M98T (600T>C), V114A (648T>C), N131T (699A>C), A172V (822C>T), L173R (825T>G), S186F (864C>T), E208D (931A>T), E258K (1079G>A), E453K (1664G>A), T456I (1674C>T), N457K (1678T>A), L483P (1755T>C), D503V (1815A>T), D506N (1823G>A), L567S (2007T>C), E582K (2051G>A), A591V (2079C>T), T616I (2154C>T)								
Codon mutations:	TTC12TTT (343C>T), CCG69CTG (513C>T), ACA73ATA (525C>T), ACA85ATA (561C>T), ATG98ACG (600T>C), GTG114GCG (648T>C), AAC131ACC (699A>C), CTC136CTT (715C>T), TTG146TTA (745G>A), GCG172GTG (822C>T), CTA173CGA (825T>G), TCC186TTC (864C>T), AGG205AGA (922G>A), GAA208GAT (931A>T), GGA209GGG (934A>G), GAA258AAA (1079G>A), GCA262GCG (1093A>G), AGG270AGA (1117G>A), GAG453AAG (1664G>A), ACA456ATA (1674C>T), AAT457AAA (1678T>A), CTA483CCA (1755T>C), GCC496GCA (1795C>A), GAC503GTC (1815A>T), GAC506AAC (1823G>A), TTA567TCA (2007T>C), GAG582AAG (2051G>A), GCG591GTG (2079C>T), ACC616ATC (2154C>T)								
Replication-associated protein (YP_006355442.1)	1	42	100%	260	90.6%	42 (100%)	39 (92.9%)	0/0/0/0	1
Protein mutations:	Q15* (1674C>T), M16K (1678T>A), *42Q (1755T>C)								
Codon mutations:	GAG11GAA (1664G>A), CAA15TAA (1674C>T), ATG16AAG (1678T>A), TAA42CAA (1755T>C)								
protein 3a (YP_009179365.2)	1	46	100%	286	95.7%	46 (100%)	43 (93.5%)	0/0/0/0	1
Protein mutations:	K4R (3585A>G), L11I (3605T>A), F26L (3652T>A)								
Codon mutations:	AAA4AGA (3585A>G), GCT10GCC (3604T>C), TTA11ATA (3605T>A), TTT26TTA (3652T>A)								
CP read-through protein (NP_056751.2)	1	718	100%	4944	98.1%	718 (100%)	701 (97.6%)	0/0/0/0	2
Protein mutations:	V10G (3721T>G), M19R (3748T>G), V62I (3876G>A 3878T>A), T83S (3939A>T), V141I (4113G>A), I306V (4608A>G 4610C>T), P449L (5038C>T), A457V (5062C>T), H460R (5071A>G 5072C>T), P503S (5199C>T), R508K (5214C>A 5215G>A), V513I (5229G>A), E565K (5385G>A), S606P (5508T>C), E697K (5781G>A), I708M (5816A>G), K711R (5824A>G)								
Codon mutations:	GTC10GGC (3721T>G), ATG19AGG (3748T>G), CGA20AGA (3750C>A), GTT32GTG (3788T>G), GTT62ATA (3876G>A 3878T>A), GTG73GTC (3911G>C), GGC81GGT (3935C>T), ACC83TCC (3939A>T), ACA113ACG (4031A>G), AGC114AGT (4034C>T), GTA141ATA (4113G>A), GCC158GCT (4166C>T), ACT160ACC (4172T>C), TCT174TCG (4214T>G), TCA303TCG (4601A>G), ATC306GTT (4608A>G 4610C>T), TGC308TGT (4616C>T), GGT375GGC (4817T>C), TTC397TTT (4883C>T), CTT398CTC (4886T>C), CCT401CCC (4895T>C), GCC402GCT (4898C>T), ACC429ACT (4979C>T), CCA449CTA (5038C>T), CGG450CGA (5042G>A), GGC452GGT (5048C>T), GCC457GTC (5062C>T), CAC460CGT (5071A>G 5072C>T), ACG479ACA (5129G>A), CCA503TCA (5199C>T), CGA508AAA (5214C>A 5215G>A), GTA513ATA (5229G>A), GAA565AAA (5385G>A), AAC603AAT (5501C>T), TCC606CCC (5508T>C), CCC610CCT (5522C>T), GAA697AAA (5781G>A), ATA708ATG (5816A>G), AAG711AGG (5824A>G)								
coat protein (NP_056749.1)	1	209	100%	1416	98.7%	209 (100%)	204 (97.6%)	0/0/0/0	1
Protein mutations:	V10G (3721T>G), M19R (3748T>G), V62I (3876G>A 3878T>A), T83S (3939A>T), V141I (4113G>A)								
Codon mutations:	GTC10GGC (3721T>G), ATG19AGG (3748T>G), CGA20AGA (3750C>A), GTT32GTG (3788T>G), GTT62ATA (3876G>A 3878T>A), GTG73GTC (3911G>C), GGC81GGT (3935C>T), ACC83TCC (3939A>T), ACA113ACG (4031A>G), AGC114AGT (4034C>T), GTA141ATA (4113G>A), GCC158GCT (4166C>T), ACT160ACC (4172T>C), TCT174TCG (4214T>G)								
movement protein (NP_056750.1)	1	157	100%	1007	91.4%	157 (100%)	147 (93.6%)	0/0/0/0	1
Protein mutations:	S2A (3721T>G), C11G (3748T>G 3750C>A), F24C (3788T>G), F54Y (3878T>A), C65S (3911G>C), A73V (3935C>T), Q105R (4031A>G), A106V (4034C>T), P150L (4166C>T), L152P (4172T>C)								
Codon mutations:	TCA2GCA (3721T>G), TGC11GGA (3748T>G 3750C>A), TTC24TGC (3788T>G), GAG53GAA (3876G>A), TTC54TAC (3878T>A), TGT65TCT (3911G>C), GCA73GTA (3935C>T), ACA74ACT (3939A>T), CAA105CGA (4031A>G), GCA106GTA (4034C>T), AAG132AAA (4113G>A), CCA150CTA (4166C>T), CTT152CCT (4172T>C)								

*: Inserts / Deletes / Misaligned / Frameshifts

Analysis details

This analysis was performed with panviral2.64

NGS Details (UN18_val): Duamitovirus soch1

Assembly

**Coverage Length**	2273 (1 contig(s))
**Depth Of Coverage**	45.1
**Number Of Reads**	853
**Reads Per Million**	16.01 rpm (after QC)
**Ambiguities**	0
**Assembly Method**	de novo + reference guided assembly
**Consensus Caller**	Bcf Tools

Coverage Map

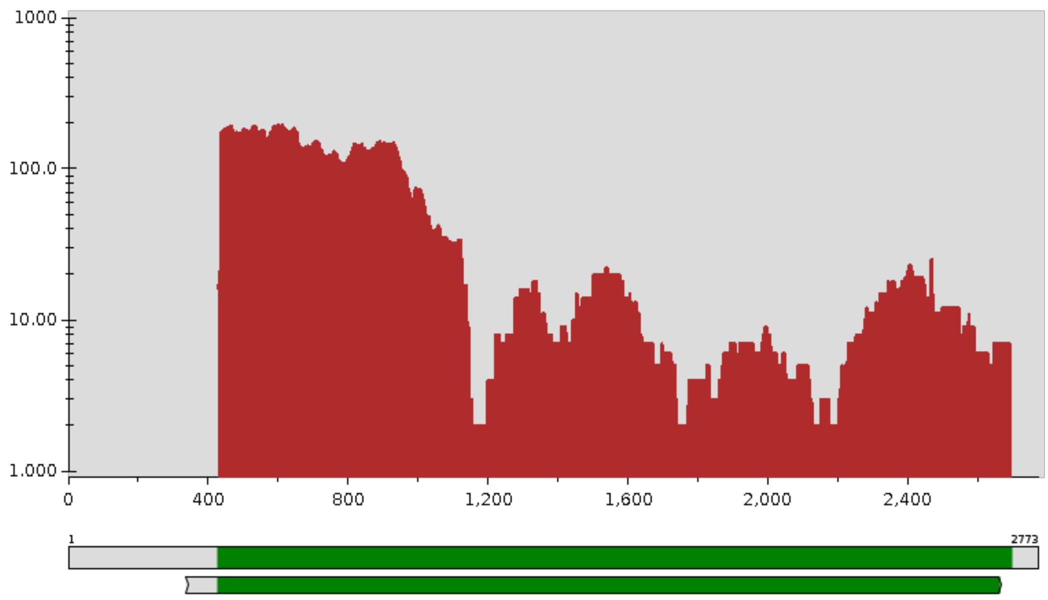

Assignment

**Type**	Duamitovirus soch1 (Taxonomy ID: 2955838)
**Reference Genome**	NC_076524.1
**NT Identity (%)**	68.9124
**AA Identity (%)**	68.3646
**Number Of Stop Codons**	4
**Number Of CDS**	1

Alignment

**Alignment Score**	1680.0 (NT) + 3316.0 (AA) = 4996.0
**Concordance (%)**	52.8854

Alignment Method	Global, seeded, nucleotide + amino acids (AGA)

Genome Region

Sequence starts at position 428 and ends at position 2700 relative to NC_076524.1 reference sequence.

Alignment Detailed Statistics

	Begin	End	Coverage	Score	Concordance	Matches	Identities	I/D/M/F*	Stop Codons
**NT**	**428**	**2700**	**82.0%**	**1680**	**37.3%**	**2266 (99.5%)**	**1565 (68.7%)**	**5/7**	
Mutations:	437A>G, 441A>C, 443C>T, 447C>T, 459C>T, 466C>T, 470C>T, 471G>A, 472C>T, 473T>G, 477T>A, 480A>C, 481C>A, 482A>T, 485A>G, 489A>G, 494T>C, 497T>A, 500T>G, 503A>C, 506T>C, 509C>T, 512T>C, 513G>A, 516T>A, 517C>A, 518T>A, 520A>C, 524G>T, 525A>G, 526C>T, 527T>G, 529C>A, 530C>A, 531T>C, 533A>G, 534C>A, 535G>A, 536C>A, 539G>A, 540A>C, 541A>G, 542G>T, 545G>A, 548C>T, 550A>T, 556T>A, 557C>T, 560A>T, 569C>T, 575G>A, 581C>T, 582G>A, 587A>T, 589G>C, 591T>C, 593A>T, 599A>T, 602C>T, 612T>A, 613C>G, 614C>T, 616T>A, 618C>T, 619G>C, 620A>T, 624G>A, 626A>T, 629A>C, 630C>A, 632A>C, 633C>A, 635T>G, 638G>T, 641G>T, 644T>A, 647G>T, 651C>T, 653C>G, 656A>C, 657A>C, 661C>G, 662G>T, 665T>A, 668C>T, 672C>A, 673G>A, 674T>G, 680C>A, 683C>T, 684A>G, 685A>C, 686A>C, 692G>A, 695T>A, 699G>C, 700C>A, 701A>C, 702A>G, 705C>A, 707T>G, 708A>G, 709G>C, 710G>A, 713A>G, 714G>T, 715A>C, 719A>T, 722C>T, 737T>C, 746C>T, 752A>T, 761G>A, 765G>T, 767T>A, 770G>A, 771G>T, 772T>A, 773T>G, 774T>A, 776G>T, 777A>G, 778A>C, 779A>G, 782T>G, 796G>A, 797T>G, 803A>C, 809T>C, 810G>C, 815C>A, 818A>T, 821T>G, 824C>T, 827A>T, 831C>T, 833T>A, 841G>A, 842G>T, 850T>G, 851A>T, 857G>T, 860G>T, 862A>G, 869T>G, 875G>T, 880T>G, 881C>A, 885C>A, 887T>G, 892A>G, 905A>T, 908C>A, 909T>C, 910T>A, 916G>A, 918G>T, 919G>C, 923T>A, 926A>T, 927A>C, 929G>T, 934T>A, 935T>G, 938G>T, 942T>A, 943C>A, 948C>A, 971C>A, 972C>T, 975A>C, 976A>T, 977T>C, 983T>C, 990A>C, 992G>C, 993T>C, 994C>G, 995A>G, 997A>T, 998C>T, 999G>C, 1000G>A, 1001T>A, 1003T>A, 1004A>T, 1005G>T, 1007C>G, 1010A>G, 1016C>T, 1017A>G, 1018A>T, 1019A>T, 1022T>A, 1025T>C, 1026C>G, 1027A>G, 1030A>C, 1031G>A, 1034T>G, 1035A>G, 1036G>C, 1044T>G, 1046C>T, 1049G>A, 1050T>G, 1051T>A, 1052T>A, 1054C>A, 1055A>C, 1061T>C, 1067T>G, 1073G>A, 1076A>G, 1083C>A, 1088G>T, 1091C>G, 1099G>T, 1104A>G, 1105C>T, 1106A>G, 1115T>G, 1121A>C, 1122T>A, 1124C>T, 1127G>A, 1130C>T, 1133C>G, 1142A>C, 1145C>T, 1205A>T, 1211C>T, 1212T>G, 1213T>A, 1217T>C, 1220C>T, 1223A>T, 1224C>T, 1225A>T, 1226C>A, 1232G>A, 1235A>G, 1236A>T, 1237G>C, 1241C>A, 1244A>C, 1247A>C, 1249G>A, 1250G>C, 1254T>A, 1255C>G, 1256A>T, 1257A>T, 1259C>T, 1260T>C, 1262A>T, 1266A>G, 1268T>G, 1269C>G, 1274T>C, 1275C>A, 1276G>A, 1277A>G, 1280G>T, 1283G>T, 1286A>G, 1291T>C, 1292T>A, 1295T>G, 1298T>A, 1302C>T, 1304T>A, 1306G>C, 1308G>C, 1309C>A, 1322C>A, 1328A>T, 1335A>C, 1338G>A, 1340A>C, 1345T>C, 1349G>A, 1355C>T, 1359A>G, 1364_1365insAAAAA, 1369G>A, 1370A>G, 1371C>T, 1372T>G, 1373T>A, 1376C>G, 1377C>T, 1378A>T, 1379C>T, 1385T>C, 1389G>C, 1391T>A, 1397T>A, 1402C>A, 1409G>A, 1413A>C, 1414A>G, 1415A>C, 1416A>C, 1418A>T, 1419A>C, 1420A>C, 1422A>G, 1424A>G, 1427C>T, 1430G>A, 1431A>G, 1433T>A, 1445G>A, 1446A>C, 1447A>G, 1448A>T, 1451T>G, 1455T>G, 1457G>A, 1458G>C, 1459C>G, 1460T>A, 1464A>C, 1465A>G, 1466G>A, 1467C>T, 1468A>C, 1469A>T, 1470A>C, 1472G>A, 1475C>G, 1476T>A, 1477T>A, 1478T>A, 1479A>G, 1480A>G, 1481A>T, 1486G>C, 1489A>T, 1490T>C, 1502A>C, 1505G>A, 1508A>G, 1526G>A, 1538G>A, 1541T>C, 1545C>T, 1547T>C, 1553T>C, 1554A>T, 1555T>G, 1556G>C, 1559A>G, 1566C>A, 1567C>G, 1571C>A, 1572T>C, 1580C>A, 1589T>C, 1593_1597delAGCTC, 1598T>A, 1607T>C, 1613G>T, 1614A>T, 1617C>T, 1619T>G, 1620G>A, 1622T>C, 1623A>G, 1624A>T, 1626A>C, 1627A>C, 1628A>T, 1632T>A, 1634A>G, 1635A>G, 1636C>T, 1637A>C, 1643A>G, 1644G>A, 1647T>A, 1648A>G, 1664G>A, 1670G>A, 1676T>G, 1679G>A, 1682C>T, 1684T>A, 1686C>T, 1691T>A, 1694T>A, 1698G>T, 1700A>C, 1703T>A, 1707T>G, 1710T>C, 1712G>T, 1715T>C, 1736A>G, 1739A>T, 1744G>A, 1745C>A, 1746A>T, 1748A>T, 1754T>C, 1758G>A, 1759G>C, 1760A>G, 1761T>A, 1765A>C, 1766C>T, 1767T>A, 1769T>C, 1772A>T, 1777G>C, 1778G>T, 1787T>C, 1788T>C, 1790A>G, 1791C>T, 1819G>C, 1820T>A, 1821A>G, 1823A>G, 1829C>G, 1830A>C, 1831A>G, 1832T>G, 1833G>C, 1840A>G, 1847A>G, 1848T>C, 1856A>G, 1859C>T, 1860A>G, 1861A>G, 1865C>T, 1867A>C, 1871T>C, 1874G>T, 1875delA, 1878C>G, 1880A>T, 1886T>G, 1887T>A, 1888T>G, 1889G>A, 1895T>C, 1898T>G, 1901T>C, 1910T>C, 1919G>T, 1925G>A, 1934T>C, 1937A>G, 1938T>G, 1939C>A, 1940A>C, 1950G>T, 1952T>C, 1953T>C, 1955A>T, 1964T>C, 1967C>T, 1976T>A, 1980T>A, 1981T>C, 1982A>T, 1985T>C, 1989C>A, 1991T>G, 1994T>A, 2000T>G, 2003A>T, 2006T>G, 2009C>T, 2010G>C, 2011C>A, 2012C>A, 2020T>C, 2028G>T, 2029A>C, 2030T>G, 2034A>T, 2035A>C, 2036C>A, 2038T>C, 2042A>G, 2043G>A, 2050A>C, 2051A>C, 2053G>T, 2054A>T, 2057G>T, 2058G>T, 2063T>G, 2069G>A, 2071A>T, 2074A>G, 2075G>A, 2078A>T, 2082G>T, 2084G>T, 2087C>T, 2090A>G, 2091T>A, 2093C>G, 2094A>T, 2099C>T, 2103A>T, 2104G>C, 2105C>T, 2108G>A, 2117G>A, 2118C>A, 2120T>A, 2123A>G, 2129T>G, 2133C>G, 2134A>C, 2135T>A, 2136C>A, 2137T>G, 2138T>C, 2141G>A, 2145G>C, 2146T>A, 2147T>C, 2148C>A, 2156C>T, 2157T>C, 2160C>T, 2166T>C, 2167A>T, 2173G>A, 2174G>T, 2183A>G, 2186G>A, 2187G>T, 2188G>T, 2191T>A, 2192G>A, 2195C>T, 2204C>T, 2206A>T, 2213A>G, 2216G>A, 2223G>A, 2226A>G, 2227G>A, 2231C>T, 2236T>G, 2237T>G, 2240A>G, 2248A>C, 2250G>C, 2251T>C, 2259T>A, 2263A>G, 2264G>A, 2265A>C, 2276G>T, 2279T>G, 2280C>T, 2288C>T, 2297A>G, 2298G>A, 2299T>G, 2300T>A, 2306A>C, 2309A>T, 2315T>C, 2322C>T, 2326G>T, 2328C>A, 2329A>G, 2336A>G, 2337G>A, 2346T>C, 2348A>G, 2351G>A, 2355C>G, 2358A>T, 2359A>C, 2360C>T, 2372G>T, 2373A>G, 2375C>T, 2376T>G, 2377G>C, 2378T>A, 2379A>G, 2380T>C, 2381G>A, 2382A>T, 2383A>C, 2384C>A, 2385C>G, 2390T>A, 2394T>A, 2400A>G, 2402G>T, 2403A>C, 2404G>A, 2405T>G, 2406C>T, 2408C>G, 2411C>G, 2414C>A, 2415T>G, 2416C>T, 2417C>T, 2423A>G, 2426C>T, 2427G>A, 2428C>G, 2429A>T, 2431C>A, 2438G>A, 2444G>A, 2445C>A, 2446T>C, 2447C>A, 2448G>A, 2450A>C, 2451A>G, 2452C>A, 2453T>A, 2457T>C, 2465A>G, 2477C>T, 2478C>T, 2489A>G, 2492T>C, 2495C>T, 2504T>A, 2510T>C, 2514A>G, 2518C>T, 2519C>G, 2524C>T, 2525C>T, 2528T>A, 2532C>A, 2533A>G, 2536G>A, 2537G>T, 2543T>G, 2551A>G, 2552C>T, 2555T>C, 2556G>T, 2557T>C, 2561T>C, 2568A>G, 2571A>C, 2572A>G, 2582A>T, 2585G>A, 2597G>A, 2598A>G, 2599A>C, 2600C>T, 2601G>A, 2609T>A, 2613T>G, 2615T>A, 2618T>A, 2622G>T, 2624A>T, 2626A>T, 2627C>A, 2629A>C, 2631A>T, 2634A>C, 2635C>T, 2638G>T, 2639A>G, 2644A>G, 2646G>T, 2647A>G, 2648A>T, 2649G>A, 2650T>G, 2652G>T, 2653G>C, 2654C>A, 2656T>A, 2657A>T, 2658A>G, 2659G>A, 2660A>C, 2661C>T, 2664T>A, 2665A>G, 2667delG, 2674T>A, 2678C>T, 2681G>A, 2686T>A, 2689T>C, 2690G>C, 2694A>G, 2695T>A								

CDS

	Begin	End	Coverage	Score	Concordance	Matches	Identities	I/D/M/F*	Stop Codons
RdRp	32	777	95.9%	3316	63.5%	744 (99.6%)	510 (68.3%)	2/1/3/3	4
Protein mutations:	I36L (441A>C 443C>T), L38F (447C>T), A44V (466C>T), A46M (471G>A 472C>T 473T>G), L48M (477T>A), T49H (480A>C 481C>A 482A>T), I52V (489A>G), V60I (513G>A), S61K (516T>A 517C>A 518T>A), K62T (520A>C), T64V (525A>G 526C>T 527T>G), T65K (529C>A 530C>A), R67K (534C>A 535G>A 536C>A), K69R (540A>C 541A>G 542G>T), Y72F (550A>T), F74Y (556T>A 557C>T), A83T (582G>A), C85S (589G>C), F94Y (616T>A), R95S (618C>T 619G>C 620A>T), E97N (624G>A 626A>T), E98D (629A>C), P99T (630C>A 632A>C), L100M (633C>A 635T>G), S109C (661C>G 662G>T), R113K (672C>A 673G>A 674T>G), K117A (684A>G 685A>C 686A>C), A122H (699G>C 700C>A 701A>C), I123V (702A>G), R125A (708A>G 709G>C 710G>A), D127S (714G>T 715A>C), E128D (719A>T), A144S (765G>T 767T>A), V146* (771G>T 772T>A 773T>G), L147I (774T>A 776G>T), K148A (777A>G 778A>C 779A>G), S154K (796G>A 797T>G), E159Q (810G>C), P166S (831C>T 833T>A), G169D (841G>A 842G>T), L172R (850T>G 851A>T), E176G (862A>G), I178M (869T>G), F182* (880T>G 881C>A), H184K (885C>A 887T>G), Q186R (892A>G), F192H (909T>C 910T>A), R194Q (916G>A), G195S (918G>T 919G>C), M198L (927A>C 929G>T), I200K (934T>A 935T>G), S203N (942T>A 943C>A), Q205K (948C>A), N214L (975A>C 976A>T 977T>C), S220R (993T>C 994C>G 995A>G), Y221F (997A>T 998C>T), G222Q (999G>C 1000G>A 1001T>A), L223Y (1003T>A 1004A>T), A224S (1005G>T 1007C>G), K228V (1017A>G 1018A>T 1019A>T), Q231G (1026C>G 1027A>G), E232A (1030A>C 1031G>A), S234A (1035A>G 1036G>C), F237V (1044T>G 1046C>T), F239E (1050T>G 1051T>A 1052T>A), P240H (1054C>A 1055A>C), L250I (1083C>A), W255L (1099G>T), T257V (1104A>G 1105C>T 1106A>G), I260M (1115T>G), F263I (1122T>A 1124C>T), L290F (1205A>T), L293E (1212T>G 1213T>A), E296D (1223A>T), H297L (1224C>T 1225A>T 1226C>A), W305Y (1249G>A 1250G>C), T308S (1257A>T 1259C>T), S311G (1266A>G 1268T>G), P312A (1269C>G), R314K (1275C>A 1276G>A 1277A>G), V319A (1291T>C 1292T>A), G324A (1306G>C), A325Q (1308G>C 1309C>A), V335I (1338G>A 1340A>C), V337A (1345T>C), M338I (1349G>A), I342V (1359A>G), K343_Q344insKX (1364_1365insAAAAA), R345K (1369G>A 1370A>G), L346* (1371C>T 1372T>G 1373T>A), H348F (1377C>T 1378A>T 1379C>T), D352Q (1389G>C 1391T>A), T356K (1402C>A), K360R (1413A>C 1414A>G 1415A>C), I361L (1416A>C 1418A>T), K362P (1419A>C 1420A>C), T363A (1422A>G 1424A>G), T366A (1431A>G 1433T>A), K371R (1446A>C 1447A>G 1448A>T), L374V (1455T>G 1457G>A), A375R (1458G>C 1459C>G 1460T>A), K377R (1464A>C 1465A>G 1466G>A), Q378S (1467C>T 1468A>C 1469A>T), K379Q (1470A>C 1472G>A), N380K (1475C>G), F381K (1476T>A 1477T>A 1478T>A), K382G (1479A>G 1480A>G 1481A>T), C384S (1486G>C), Y385F (1489A>T 1490T>C), L389F (1502A>C), M401I (1538G>A), L404F (1545C>T 1547T>C), M407C (1554A>T 1555T>G 1556G>C), I408M (1559A>G), P411S (1566C>A 1567C>G), S420del (1593_1597delAGCTC), I427F (1614A>T), V429I (1620G>A 1622T>C), N430V (1623A>G 1624A>T), K431P (1626A>C 1627A>C 1628A>T), L433M (1632T>A 1634A>G), T434V (1635A>G 1636C>T 1637A>C), V437M (1644G>A), Y438S (1647T>A 1648A>G), F450Y (1684T>A), H451Y (1686C>T), A455S (1698G>T 1700A>C), S458A (1707T>G), S470K (1744G>A 1745C>A), K471Y (1746A>T 1748A>T), G475T (1758G>A 1759G>C 1760A>G), C476S (1761T>A), N477T (1765A>C 1766C>T), S478T (1767T>A 1769T>C), G481A (1777G>C 1778G>T), S495T (1819G>C 1820T>A), K496E (1821A>G 1823A>G), N499R (1830A>C 1831A>G 1832T>G), E500Q (1833G>C), K502R (1840A>G), N509G (1860A>G 1861A>G), K511T (1867A>C), P515A (1878C>G 1880A>T), L518R (1887T>A 1888T>G 1889G>A), D521E (1898T>G), S535D (1938T>G 1939C>A 1940A>C), D539Y (1950G>T 1952T>C), L549T (1980T>A 1981T>C 1982A>T), A559Q (2010G>C 2011C>A 2012C>A), I562T (2020T>C), D565S (2028G>T 2029A>C 2030T>G), N567S (2034A>T 2035A>C 2036C>A), I568T (2038T>C), V570I (2043G>A), Q572P (2050A>C 2051A>C), R573I (2053G>T 2054A>T), L574F (2057G>T), G575W (2058G>T), Y579F (2071A>T), K580R (2074A>G 2075G>A), A583S (2082G>T 2084G>T), F586M (2091T>A 2093C>G), T587S (2094A>T), H600A (2133C>G 2134A>C 2135T>A), L601S (2136C>A 2137T>G 2138T>C), V604H (2145G>C 2146T>A 2147T>C), Y611L (2166T>C 2167A>T), W613Y (2173G>A 2174G>T), G618F (2187G>T 2188G>T), M619K (2191T>A 2192G>A), Y624F (2206A>T), V630I (2223G>A), S631D (2226A>G 2227G>A), L634R (2236T>G 2237T>G), K638T (2248A>C), V639P (2250G>C 2251T>C), L642I (2259T>A), Q643R (2263A>G 2264G>A), I644L (2265A>C), K647N (2276G>T), D648E (2279T>G), V655R (2298G>A 2299T>G 2300T>A), R664L (2326G>T), Q665R (2328C>A 2329A>G), E668K (2337G>A), L674V (2355C>G), N675S (2358A>T 2359A>C 2360C>T), I680V (2373A>G 2375C>T), C681A (2376T>G 2377G>C 2378T>A), M682A (2379A>G 2380T>C 2381G>A), N683S (2382A>T 2383A>C 2384C>A), P684A (2385C>G), D685E (2390T>A), S687T (2394T>A), K689D (2400A>G 2402G>T), S690Q (2403A>C 2404G>A 2405T>G), I692M (2411C>G), D693E (2414C>A), S694V (2415T>G 2416C>T 2417C>T), I696M (2423A>G), A698S (2427G>A 2428C>G 2429A>T), T699K (2431C>A), W701* (2438G>A), L704T (2445C>A 2446T>C 2447C>A), E705N (2448G>A 2450A>C), T706E (2451A>G 2452C>A 2453T>A), F708L (2457T>C), N727D (2514A>G), P728L (2518C>T 2519C>G), T730I (2524C>T 2525C>T), Q733R (2532C>A 2533A>G), W734Y (2536G>A 2537G>T), F736L (2543T>G), N739S (2551A>G 2552C>T), V741S (2556G>T 2557T>C), N745D (2568A>G), K746R (2571A>C 2572A>G), L749F (2582A>T), N755A (2598A>G 2599A>C 2600C>T), D756N (2601G>A), S760A (2613T>G 2615T>A), E763Y (2622G>T 2624A>T), Y764L (2626A>T 2627C>A), Q765P (2629A>C), T766S (2631A>T), T767L (2634A>C 2635C>T), G768V (2638G>T 2639A>G), E770G (2644A>G), E771C (2646G>T 2647A>G 2648A>T), V772R (2649G>A 2650T>G), G773S (2652G>T 2653G>C 2654C>A), L774Y (2656T>A 2657A>T), R775D (2658A>G 2659G>A 2660A>C), L776F (2661C>T), *777R (2664T>A 2665A>G)								

	Begin	End	Coverage	Score	Concordance	Matches	Identities	I/D/M/F*	Stop Codons
**NT**	**428**	**2700**	**82.0%**	**1680**	**37.3%**	**2266 (99.5%)**	**1565 (68.7%)**	**5/7**	

Codon mutations: AAA34AAG (437A>G), ATC36CTT (441A>C 443C>T), CTT38TTT (447C>T), CTA42TTA (459C>T), GCC44GTC (466C>T), TCC45TCT (470C>T), GCT46ATG (471G>A 472C>T 473T>G), TTG48ATG (477T>A), ACA49CAT (480A>C 481C>A 482A>T), AAA50AAG (485A>G), ATC52GTC (489A>G), GCT53GCC (494T>C), CTT54CTA (497T>A), GCT55GCG (500T>G), GGA56GGC (503A>C), TGT57TGC (506T>C), GAC58GAT (509C>T), TTT59TTC (512T>C), GTA60ATA (513G>A), TCT61AAA (516T>A 517C>A 518T>A), AAA62ACA (520A>C), GTG63GTT (524G>T), ACT64GTG (525A>G 526C>T 527T>G), ACC65AAA (529C>A 530C>A), TTA66CTG (531T>C 533A>G), CGC67AAA (534C>A 535G>A 536C>A), CGG68CGA (539G>A), AAG69CGT (540A>C 541A>G 542G>T), TCG70TCA (545G>A), GGC71GGT (548C>T), TAT72TTT (550A>T), TTC74TAT (556T>A 557C>T), ACA75ACT (560A>T), TAC78TAT (569C>T), AAG80AAA (575G>A), TGC82TGT (581C>T), GCA83ACA (582G>A), GTA84GTT (587A>T), TGC85TCC (589G>C), TTA86CTT (591T>C 593A>T), CGA88CGT (599A>T), TAC89TAT (602C>T), TCC93AGT (612T>A 613C>G 614C>T), TTT94TAT (616T>A), CGA95TCT (618C>T 619G>C 620A>T), GAA97AAT (624G>A 626A>T), GAA98GAC (629A>C), CCA99ACC (630C>A 632A>C), CTT100ATG (633C>A 635T>G), TCG101TCT (638G>T), GTG102GTT (641G>T), CCT103CCA (644T>A), GTG104GTT (647G>T), CTC106TTG (651C>T 653C>G), ACA107ACC (656A>C), AGA108CGA (657A>C), TCG109TGT (661C>G 662G>T), GGT110GGA (665T>A), ATC111ATT (668C>T), CGT113AAG (672C>A 673G>A 674T>G), ATC115ATA (680C>A), CCC116CCT (683C>T), AAA117GCC (684A>G 685A>C 686A>C), TTG119TTA (692G>A), CGT120CGA (695T>A), GCA122CAC (699G>C 700C>A 701A>C), ATA123GTA (702A>G), CGT124AGG (705C>A 707T>G), AGG125GCA (708A>G 709G>C 710G>A), AAA126AAG (713A>G), GAT127TCT (714G>T 715A>C), GAA128GAT (719A>T), CAC129CAT (722C>T), GTT134GTC (737T>C), TAC137TAT (746C>T), TCA139TCT (752A>T), GGG142GGA (761G>A), GCT144TCA (765G>T 767T>A), AAG145AAA (770G>A), GTT146TAG (771G>T 772T>A 773T>G), TTG147ATT (774T>A 776G>T), AAA148GCG (777A>G 778A>C 779A>G), GTT149GTG (782T>G), AGT154AAG (796G>A 797T>G), GCA156GCC (803A>C), TTT158TTC (809T>C), GAA159CAA (810G>C), TCC160TCA (815C>A), ATA161ATT (818A>T), ACT162ACG (821T>G), ACC163ACT (824C>T), CCA164CCT (827A>T), CCT166TCA (831C>T 833T>A), GGG169GAT (841G>A 842G>T), CTA172CGT (850T>G 851A>T), GTG174GTT (857G>T), CTG175CTT (860G>T), GAA176GGA (862A>G), ATT178ATG (869T>G), ACG180ACT (875G>T), TTC182TGA (880T>G 881C>A), CAT184AAG (885C>A 887T>G), CAA186CGA (892A>G), CTA190CTT (905A>T), CCC191CCA (908C>A), TTT192CAT (909T>C 910T>A), CGA194CAA (916G>A), GGC195TCC (918G>T 919G>C), ATT196ATA (923T>A), CCA197CCT (926A>T), ATG198CTT (927A>C 929G>T), ATT200AAG (934T>A 935T>G), GGG201GGT (938G>T), TCT203AAT (942T>A 943C>A), CAA205AAA (948C>A), CCC212CCA (971C>A), CTA213TTA (972C>T), AAT214CTC (975A>C 976A>T 977T>C), AAT216AAC (983T>C), AGG219CGC (990A>C 992G>C), TCA220CGG (993T>C 994C>G 995A>G), TAC221TTT (997A>T 998C>T), GGT222CAA (999G>C 1000G>A 1001T>A), TTA223TAT (1003T>A 1004A>T), GCC224TCG (1005G>T 1007C>G), GTA225GTG (1010A>G), GAC227GAT (1016C>T), AAA228GTT (1017A>G 1018A>T 1019A>T), GTT229GTA (1022T>A), GAT230GAC (1025T>C), CAA231GGA (1026C>G 1027A>G), GAG232GCA (1030A>C 1031G>A), GCT233GCG (1034T>G), AGT234GCT (1035A>G 1036G>C), TTC237GTT (1044T>G 1046C>T), AAG238AAA (1049G>A), TTT239GAA (1050T>G 1051T>A 1052T>A), CCA240CAC (1054C>A 1055A>C), ATT242ATC (1061T>C), GTT244GTG (1067T>G), TTG246TTA (1073G>A), AAA247AAG (1076A>G), CTA250ATA (1083C>A), GCG251GCT (1088G>T), GCC252GCG (1091C>G), TGG255TTG (1099G>T), ACA257GTG (1104A>G 1105C>T 1106A>G), ATT260ATG (1115T>G), TCA262TCC (1121A>C), TTC263ATT (1122T>A 1124C>T), CAG264CAA (1127G>A), GAC265GAT (1130C>T), GGC266GGG (1133C>G), TCA269TCC (1142A>C), CCC270CCT (1145C>T), TTA290TTT (1205A>T), AAC292AAT (1211C>T), TTA293GAA (1212T>G 1213T>A), TCT294TCC (1217T>C), CTC295CTT (1220C>T), GAA296GAT (1223A>T), CAC297TTA (1224C>T 1225A>T 1226C>A), GAG299GAA (1232G>A), AAA300AAG (1235A>G), AGT301TCT (1236A>T 1237G>C), GTC302GTA (1241C>A), GGA303GGC (1244A>C), CCA304CCC (1247A>C), TGG305TAC (1249G>A 1250G>C), TCA307AGT (1254T>A 1255C>G 1256A>T), ACC308TCT (1257A>T 1259C>T), TTA309CTT (1260T>C 1262A>T), AGT311GGG (1266A>G 1268T>G), CCT312GCT (1269C>G), TAT313TAC (1274T>C), CGA314AAG (1275C>A 1276G>A 1277A>G), GGG315GGT (1280G>T), GTG316GTT (1283G>T), CCA317CCG (1286A>G), GTT319GCA (1291T>C 1292T>A), ACT320ACG (1295T>G), GGT321GGA (1298T>A), CTT323TTA (1302C>T 1304T>A), GGT324GCT (1306G>C), GCA325CAA (1308G>C 1309C>A), GGC329GGA (1322C>A), GGA331GGT (1328A>T), AGG334CGG (1335A>C), GTA335ATC (1338G>A 1340A>C), GTA337GCA (1345T>C), ATG338ATA (1349G>A), AAC340AAT (1355C>T), ATA342GTA (1359A>G), AAA343_CAA344insAAAAA- (1364_1365insAAAAA), AGA345AAG (1369G>A 1370A>G), CTT346TGA (1371C>T 1372T>G 1373T>A), CTC347CTG (1376C>G), CAC348TTT (1377C>T 1378A>T 1379C>T), GTT350GTC (1385T>C), GAT352CAA (1389G>C 1391T>A), GCT354GCA (1397T>A), ACA356AAA (1402C>A), CTG358CTA (1409G>A), AAA360CGC (1413A>C 1414A>G 1415A>C), ATA361CTT (1416A>C 1418A>T), AAG362CCG (1419A>C 1420A>C), ACA363GCG (1422A>G 1424A>G), GAC364GAT (1427C>T), GGG365GGA (1430G>A), ACT366GCA (1431A>G 1433T>A), GAG370GAA (1445G>A), AAA371CGT (1446A>C 1447A>G 1448A>T), CCT372CCG (1451T>G), TTG374GTA (1455T>G 1457G>A), GCT375CGA (1458G>C 1459C>G 1460T>A), AAG377CGA (1464A>C 1465A>G 1466G>A), CAA378TCT (1467C>T 1468A>C 1469A>T), AAG379CAA (1470A>C 1472G>A), AAC380AAG (1475C>G), TTT381AAA (1476T>A 1477T>A 1478T>A), AAA382GGT (1479A>G 1480A>G 1481A>T), TGT384TCT (1486G>C), TAT385TTC (1489A>T 1490T>C), TTA389TTC (1502A>C), AAG390AAA (1505G>A), TCA391TCG (1508A>G), CCG397CCA (1526G>A), ATG401ATA (1538G>A), TAT402TAC (1541T>C), CTT404TTC (1545C>T 1547T>C), TCT406TCC (1553T>C), ATG407TGC (1554A>T 1555T>G 1556G>C), ATA408ATG (1559A>G), CCT411AGT (1566C>A 1567C>G), ACC412ACA (1571C>A), TTG413CTG (1572T>C), TCC415TCA (1580C>A), GTT418GTC (1589T>C), AGC420del (1593_1597delAGCTC), TCT421--A (1593_1597delAGCTC 1598T>A), CTT424CTC (1607T>C), ACG426ACT (1613G>T), ATC427TTC (1614A>T), CTT428TTG (1617C>T 1619T>G), GTT429ATC (1620G>A 1622T>C), AAC430GTC (1623A>G 1624A>T), AAA431CCT (1626A>C 1627A>C 1628A>T), TTA433ATG (1632T>A 1634A>G), ACA434GTC (1635A>G 1636C>T 1637A>C), AGA436AGG (1643A>G), GTG437ATG (1644G>A), TAT438AGT (1647T>A 1648A>G), CTG443CTA (1664G>A), GGG445GGA (1670G>A), CCT447CCG (1676T>G), TTG448TTA (1679G>A), GGC449GGT (1682C>T), TTC450TAC (1684T>A), CAC451TAC (1686C>T), GGT452GGA (1691T>A), TCT453TCA (1694T>A), GCA455TCC (1698G>T 1700A>C), CTT456CTA (1703T>A), TCG458GCG (1707T>G), TTG459CTT (1710T>C 1712G>T), TCT460TCC (1715T>C), TTA467TTG (1736A>G), GCA468GCT (1739A>T), AGC470AAA (1744G>A 1745C>A), AAA471TAT (1746A>T 1748A>T), TAT473TAC (1754T>C), GGA475ACG (1758G>A 1759G>C 1760A>G), TGT476AGT (1761T>A), AAC477ACT (1765A>C 1766C>T), TCT478ACC (1767T>A 1769T>C), CCA479CCT (1772A>T), GGG481GCT (1777G>C 1778G>T), GCT484GCC (1787T>C), TTA485CTG (1788T>C 1790A>G), CTA486TTA (1791C>T), AGT495ACA (1819G>C 1820T>A), AAA496GAG (1821A>G 1823A>G), GCC498GCG (1829C>G), AAT499CGG (1830A>C 1831A>G 1832T>G), GAG500CAG (1833G>C), AAG502AGG (1840A>G), TTA504TTG (1847A>G), TTG505CTG (1848T>C), AGA507AGG (1856A>G), CTC508CTT (1859C>T), AAT509GGT (1860A>G 1861A>G), GTC510GTT (1865C>T), AAA511ACA (1867A>C), ATT512ATC (1871T>C), TCG513TCT (1874G>T), ATA514-TA (1875delA), CCA515GCT (1878C>G 1880A>T), TCT517TCG (1886T>G), TTG518AGA (1887T>A 1888T>G 1889G>A), TCT520TCC (1895T>C), GAT521GAG (1898T>G), AAT522AAC (1901T>C), ATT525ATC (1910T>C), GCG528GCT (1919G>T), AGG530AGA (1925G>A), ACT533ACC (1934T>C), AAA534AAG (1937A>G), TCA535GAC (1938T>G 1939C>A 1940A>C), GAT539TAC (1950G>T 1952T>C), TTA540CTT (1953T>C 1955A>T), ATT543ATC (1964T>C), TCC544TCT (1967C>T), GCT547GCA (1976T>A), TTA549ACT (1980T>A 1981T>C 1982A>T), TCT550TCC (1985T>C), CGT552AGG (1989C>A 1991T>G), ACT553ACA (1994T>A), GTT555GTG (2000T>G), GGA556GGT (2003A>T), CTT557CTG (2006T>G), TGC558TGT (2009C>T), GCC559CAA (2010G>C 2011C>A 2012C>A), ATC562ACC (2020T>C), GAT565TCG (2028G>T 2029A>C 2030T>G), AAC567TCA (2034A>T 2035A>C 2036C>A), ATA568ACA (2038T>C), TCA569TCG (2042A>G), GTT570ATT (2043G>A), CAA572CCC (2050A>C 2051A>C), AGA573ATT (2053G>T 2054A>T), TTG574TTT (2057G>T), GGG575TGG (2058G>T), GGT576GGG (2063T>G), GGG578GGA (2069G>A), TAC579TTC (2071A>T), AAG580AGA (2074A>G 2075G>A), GTA581GTT (2078A>T), GCG583TCT (2082G>T 2084G>T), CGC584CGT (2087C>T), TTA585TTG (2090A>G), TTC586ATG (2091T>A 2093C>G), ACT587TCT (2094A>T), ACC588ACT (2099C>T), AGC590TCT (2103A>T 2104G>C 2105C>T), AAG591AAA (2108G>A), GAG594GAA (2117G>A), CGT595AGA (2118C>A 2120T>A), CTA596CTG (2123A>G), GCT598GCG (2129T>G), CAT600GCA (2133C>G 2134A>C 2135T>A), CTT601AGC (2136C>A 2137T>G 2138T>C), AAG602AAA (2141G>A), GTT604CAC (2145G>C 2146T>A 2147T>C), CGG605AGG (2148C>A), CAC607CAT (2156C>T), TTA608CTA (2157T>C), CTA609TTA (2160C>T), TAC611CTC (2166T>C 2167A>T), TGG613TAT (2173G>A 2174G>T), GGA616GGG (2183A>G), AGG617AGA (2186G>A), GGT618TTT (2187G>T 2188G>T), ATG619AAA (2191T>A 2192G>A), CCC620CCT (2195C>T), CCC623CCT (2204C>T), TAT624TTT (2206A>T), AAA626AAG (2213A>G), GGG627GGA (2216G>A), GTA630ATA (2223G>A), AGT631GAT (2226A>G 2227G>A), TAC632TAT (2231C>T), CTT634CGG (2236T>G 2237T>G), AAA635AAG (2240A>G), AAA638ACA (2248A>C), GTT639CCT (2250G>C 2251T>C), TTA642ATA (2259T>A), CAG643CGA (2263A>G 2264G>A), ATC644CTC (2265A>C), AAG647AAT (2276G>T), GAT648GAG (2279T>G), CTG649TTG (2280C>T), TTC651TTT (2288C>T), GAA654GAG (2297A>G), GTT655AGA (2298G>A 2299T>G 2300T>A), ATA657ATC (2306A>C), CTA658CTT (2309A>T), CGT660CGC (2315T>C), CTA663TTA (2322C>T), CGC664CTC (2326G>T), CAA665AGA (2328C>A 2329A>G), GTA667GTG (2336A>G), GAA668AAA (2337G>A), TTA671CTG (2346T>C 2348A>G), AAG672AAA (2351G>A), CTC674GTC (2355C>G), AAC675TCT (2358A>T 2359A>C 2360C>T), ACG679ACT (2372G>T), ATC680GTT (2373A>G 2375C>T), TGT681GCA (2376T>G 2377G>C 2378T>A), ATG682GCA (2379A>G 2380T>C 2381G>A), AAC683TCA (2382A>T 2383A>C 2384C>A), CCA684GCA (2385C>G), GAT685GAA (2390T>A), TCC687ACC (2394T>A), AAG689GAT (2400A>G 2402G>T), AGT690CAG (2403A>C 2404G>A 2405T>G), CTC691TTG (2406C>T 2408C>G), ATC692ATG (2411C>G), GAC693GAA (2414C>A), TCC694GTT (2415T>G 2416C>T 2417C>T), ATA696ATG (2423A>G), TGC697TGT (2426C>T), GCA698AGT (2427G>A 2428C>G 2429A>T), ACA699AAA (2431C>A), TGG701TGA (2438G>A), AGG703AGA (2444G>A), CTC704ACA (2445C>A 2446T>C 2447C>A), GAA705AAC (2448G>A 2450A>C), ACT706GAA (2451A>G 2452C>A 2453T>A), TTT708CTT (2457T>C), TTA710TTG (2465A>G), GGC714GGT (2477C>T), CTA715TTA (2478C>T), AAA718AAG (2489A>G), TGT719TGC (2492T>C), TAC720TAT (2495C>T), GGT723GGA (2504T>A), GGT725GGC (2510T>C), AAT727GAT (2514A>G), CCC728CTG (2518C>T 2519C>G), ACC730ATT (2524C>T 2525C>T), ACT731ACA (2528T>A), CAG733AGG (2532C>A 2533A>G), TGG734TAT (2536G>A 2537G>T), TTT736TTG (2543T>G), AAC739AGT (2551A>G 2552C>T), ACT740ACC (2555T>C), GTT741TCT (2556G>T 2557T>C), ATT742ATC (2561T>C), AAT745GAT (2568A>G), AAA746CGA (2571A>C 2572A>G), TTA749TTT (2582A>T), GGG750GGA (2585G>A), GGG754GGA (2597G>A), AAC755GCT (2598A>G 2599A>C 2600C>T), GAT756AAT (2601G>A), ATT758ATA (2609T>A), TCT760GCA (2613T>G 2615T>A), CCT761CCA (2618T>A), GAA763TAT (2622G>T 2624A>T), TAC764TTA (2626A>T 2627C>A), CAA765CCA (2629A>C), ACT766TCT (2631A>T), ACA767CTA (2634A>C 2635C>T), GGA768GTG (2638G>T 2639A>G), GAG770GGG (2644A>G), GAA771TGT (2646G>T 2647A>G 2648A>T), GTG772AGG (2649G>A 2650T>G), GGC773TCA (2652G>T 2653G>C 2654C>A), TTA774TAT (2656T>A 2657A>T), AGA775GAC (2658A>G 2659G>A 2660A>C), CTT776TTT (2661C>T), TAG777AGG (2664T>A 2665A>G)

Proteins

	Begin	End	Coverage	Score	Concordance	Matches	Identities	I/D/M/F*	Stop Codons
RNA-dependent RNA polymerase (YP_010798874.1)	32	777	95.9%	3316	63.5%	744 (99.6%)	510 (68.3%)	2/1/3/3	4

	Begin	End	Coverage	Score	Concordance	Matches	Identities	I/D/M/F*	Stop Codons
**NT**	**428**	**2700**	**82.0%**	**1680**	**37.3%**	**2266 (99.5%)**	**1565 (68.7%)**	**5/7**	

Protein mutations:

I36L (441A>C 443C>T), L38F (447C>T), A44V (466C>T), A46M (471G>A 472C>T 473T>G), L48M (477T>A), T49H (480A>C 481C>A 482A>T), I52V (489A>G), V60I (513G>A), S61K (516T>A 517C>A 518T>A), K62T (520A>C), T64V (525A>G 526C>T 527T>G), T65K (529C>A 530C>A), R67K (534C>A 535G>A 536C>A), K69R (540A>C 541A>G 542G>T), Y72F (550A>T), F74Y (556T>A 557C>T), A83T (582G>A), C85S (589G>C), F94Y (616T>A), R95S (618C>T 619G>C 620A>T), E97N (624G>A 626A>T), E98D (629A>C), P99T (630C>A 632A>C), L100M (633C>A 635T>G), S109C (661C>G 662G>T), R113K (672C>A 673G>A 674T>G), K117A (684A>G 685A>C 686A>C), A122H (699G>C 700C>A 701A>C), I123V (702A>G), R125A (708A>G 709G>C 710G>A), D127S (714G>T 715A>C), E128D (719A>T), A144S (765G>T 767T>A), V146* (771G>T 772T>A 773T>G), L147I (774T>A 776G>T), K148A (777A>G 778A>C 779A>G), S154K (796G>A 797T>G), E159Q (810G>C), P166S (831C>T 833T>A), G169D (841G>A 842G>T), L172R (850T>G 851A>T), E176G (862A>G), I178M (869T>G), F182* (880T>G 881C>A), H184K (885C>A 887T>G), Q186R (892A>G), F192H (909T>C 910T>A), R194Q (916G>A), G195S (918G>T 919G>C), M198L (927A>C 929G>T), I200K (934T>A 935T>G), S203N (942T>A 943C>A), Q205K (948C>A), N214L (975A>C 976A>T 977T>C), S220R (993T>C 994C>G 995A>G), Y221F (997A>T 998C>T), G222Q (999G>C 1000G>A 1001T>A), L223Y (1003T>A 1004A>T), A224S (1005G>T 1007C>G), K228V (1017A>G 1018A>T 1019A>T), Q231G (1026C>G 1027A>G), E232A (1030A>C 1031G>A), S234A (1035A>G 1036G>C), F237V (1044T>G 1046C>T), F239E (1050T>G 1051T>A 1052T>A), P240H (1054C>A 1055A>C), L250I (1083C>A), W255L (1099G>T), T257V (1104A>G 1105C>T 1106A>G), I260M (1115T>G), F263I (1122T>A 1124C>T), L290F (1205A>T), L293E (1212T>G 1213T>A), E296D (1223A>T), H297L (1224C>T 1225A>T 1226C>A), W305Y (1249G>A 1250G>C), T308S (1257A>T 1259C>T), S311G (1266A>G 1268T>G), P312A (1269C>G), R314K (1275C>A 1276G>A 1277A>G), V319A (1291T>C 1292T>A), G324A (1306G>C), A325Q (1308G>C 1309C>A), V335I (1338G>A 1340A>C), V337A (1345T>C), M338I (1349G>A), I342V (1359A>G), K343_Q344insKX (1364_1365insAAAAA), R345K (1369G>A 1370A>G), L346* (1371C>T 1372T>G 1373T>A), H348F (1377C>T 1378A>T 1379C>T), D352Q (1389G>C 1391T>A), T356K (1402C>A), K360R (1413A>C 1414A>G 1415A>C), I361L (1416A>C 1418A>T), K362P (1419A>C 1420A>C), T363A (1422A>G 1424A>G), T366A (1431A>G 1433T>A), K371R (1446A>C 1447A>G 1448A>T), L374V (1455T>G 1457G>A), A375R (1458G>C 1459C>G 1460T>A), K377R (1464A>C 1465A>G 1466G>A), Q378S (1467C>T 1468A>C 1469A>T), K379Q (1470A>C 1472G>A), N380K (1475C>G), F381K (1476T>A 1477T>A 1478T>A), K382G (1479A>G 1480A>G 1481A>T), C384S (1486G>C), Y385F (1489A>T 1490T>C), L389F (1502A>C), M401I (1538G>A), L404F (1545C>T 1547T>C), M407C (1554A>T 1555T>G 1556G>C), I408M (1559A>G), P411S (1566C>A 1567C>G), S420del (1593_1597delAGCTC), I427F (1614A>T), V429I (1620G>A 1622T>C), N430V (1623A>G 1624A>T), K431P (1626A>C 1627A>C 1628A>T), L433M (1632T>A 1634A>G), T434V (1635A>G 1636C>T 1637A>C), V437M (1644G>A), Y438S (1647T>A 1648A>G), F450Y (1684T>A), H451Y (1686C>T), A455S (1698G>T 1700A>C), S458A (1707T>G), S470K (1744G>A 1745C>A), K471Y (1746A>T 1748A>T), G475T (1758G>A 1759G>C 1760A>G), C476S (1761T>A), N477T (1765A>C 1766C>T), S478T (1767T>A 1769T>C), G481A (1777G>C 1778G>T), S495T (1819G>C 1820T>A), K496E (1821A>G 1823A>G), N499R (1830A>C 1831A>G 1832T>G), E500Q (1833G>C), K502R (1840A>G), N509G (1860A>G 1861A>G), K511T (1867A>C), P515A (1878C>G 1880A>T), L518R (1887T>A 1888T>G 1889G>A), D521E (1898T>G), S535D (1938T>G 1939C>A 1940A>C), D539Y (1950G>T 1952T>C), L549T (1980T>A 1981T>C 1982A>T), A559Q (2010G>C 2011C>A 2012C>A), I562T (2020T>C), D565S (2028G>T 2029A>C 2030T>G), N567S (2034A>T 2035A>C 2036C>A), I568T (2038T>C), V570I (2043G>A), Q572P (2050A>C 2051A>C), R573I (2053G>T 2054A>T), L574F (2057G>T), G575W (2058G>T), Y579F (2071A>T), K580R (2074A>G 2075G>A), A583S (2082G>T 2084G>T), F586M (2091T>A 2093C>G), T587S (2094A>T), H600A (2133C>G 2134A>C 2135T>A), L601S (2136C>A 2137T>G 2138T>C), V604H (2145G>C 2146T>A 2147T>C), Y611L (2166T>C 2167A>T), W613Y (2173G>A 2174G>T), G618F (2187G>T 2188G>T), M619K (2191T>A 2192G>A), Y624F (2206A>T), V630I (2223G>A), S631D (2226A>G 2227G>A), L634R (2236T>G 2237T>G), K638T (2248A>C), V639P (2250G>C 2251T>C), L642I (2259T>A), Q643R (2263A>G 2264G>A), I644L (2265A>C), K647N (2276G>T), D648E (2279T>G), V655R (2298G>A 2299T>G 2300T>A), R664L (2326G>T), Q665R (2328C>A 2329A>G), E668K (2337G>A), L674V (2355C>G), N675S (2358A>T 2359A>C 2360C>T), I680V (2373A>G 2375C>T), C681A (2376T>G 2377G>C 2378T>A), M682A (2379A>G 2380T>C 2381G>A), N683S (2382A>T 2383A>C 2384C>A), P684A (2385C>G), D685E (2390T>A), S687T (2394T>A), K689D (2400A>G 2402G>T), S690Q (2403A>C 2404G>A 2405T>G), I692M (2411C>G), D693E (2414C>A), S694V (2415T>G 2416C>T 2417C>T), I696M (2423A>G), A698S (2427G>A 2428C>G 2429A>T), T699K (2431C>A), W701* (2438G>A), L704T (2445C>A 2446T>C 2447C>A), E705N (2448G>A 2450A>C), T706E (2451A>G 2452C>A 2453T>A), F708L (2457T>C), N727D (2514A>G), P728L (2518C>T 2519C>G), T730I (2524C>T 2525C>T), Q733R (2532C>A 2533A>G), W734Y (2536G>A 2537G>T), F736L (2543T>G), N739S (2551A>G 2552C>T), V741S (2556G>T 2557T>C), N745D (2568A>G), K746R (2571A>C 2572A>G), L749F (2582A>T), N755A (2598A>G 2599A>C 2600C>T), D756N (2601G>A), S760A (2613T>G 2615T>A), E763Y (2622G>T 2624A>T), Y764L (2626A>T 2627C>A), Q765P (2629A>C), T766S (2631A>T), T767L (2634A>C 2635C>T), G768V (2638G>T 2639A>G), E770G (2644A>G), E771C (2646G>T 2647A>G 2648A>T), V772R (2649G>A 2650T>G), G773S (2652G>T 2653G>C 2654C>A), L774Y (2656T>A 2657A>T), R775D (2658A>G 2659G>A 2660A>C), L776F (2661C>T), *777R (2664T>A 2665A>G)

	Begin	End	Coverage	Score	Concordance	Matches	Identities	I/D/M/F*	Stop Codons
NT	428	2700	82.0%	1680	37.3%	2266 (99.5%)	1565 (68.7%)	5/7	
Codon mutations:	AAA34AAG (437A>G), ATC36CTT (441A>C 443C>T), CTT38TTT (447C>T), CTA42TTA (459C>T), GCC44GTC (466C>T), TCC45TCT (470C>T), GCT46ATG (471G>A 472C>T 473T>G), TTG48ATG (477T>A), ACA49CAT (480A>C 481C>A 482A>T), AAA50AAG (485A>G), ATC52GTC (489A>G), GCT53GCC (494T>C), CTT54CTA (497T>A), GCT55GCG (500T>G), GGA56GGC (503A>C), TGT57TGC (506T>C), GAC58GAT (509C>T), TTT59TTC (512T>C), GTA60ATA (513G>A), TCT61AAA (516T>A 517C>A 518T>A), AAA62ACA (520A>C), GTG63GTT (524G>T), ACT64GTG (525A>G 526C>T 527T>G), ACC65AAA (529C>A 530C>A), TTA66CTG (531T>C 533A>G), CGC67AAA (534C>A 535G>A 536C>A), CGG68CGA (539G>A), AAG69CGT (540A>C 541A>G 542G>T), TCG70TCA (545G>A), GGC71GGT (548C>T), TAT72TTT (550A>T), TTC74TAT (556T>A 557C>T), ACA75ACT (560A>T), TAC78TAT (569C>T), AAG80AAA (575G>A), TGC82TGT (581C>T), GCA83ACA (582G>A), GTA84GTT (587A>T), TGC85TCC (589G>C), TTA86CTT (591T>C 593A>T), CGA88CGT (599A>T), TAC89TAT (602C>T), TCC93AGT (612T>A 613C>G 614C>T), TTT94TAT (616T>A), CGA95TCT (618C>T 619G>C 620A>T), GAA97AAT (624G>A 626A>T), GAA98GAC (629A>C), CCA99ACC (630C>A 632A>C), CTT100ATG (633C>A 635T>G), TCG101TCT (638G>T), GTG102GTT (641G>T), CCT103CCA (644T>A), GTG104GTT (647G>T), CTC106TTG (651C>T 653C>G), ACA107ACC (656A>C), AGA108CGA (657A>C), TCG109TGT (661C>G 662G>T), GGT110GGA (665T>A), ATC111ATT (668C>T), CGT113AAG (672C>A 673G>A 674T>G), ATC115ATA (680C>A), CCC116CCT (683C>T), AAA117GCC (684A>G 685A>C 686A>C), TTG119TTA (692G>A), CGT120CGA (695T>A), GCA122CAC (699G>C 700C>A 701A>C), ATA123GTA (702A>G), CGT124AGG (705C>A 707T>G), AGG125GCA (708A>G 709G>C 710G>A), AAA126AAG (713A>G), GAT127TCT (714G>T 715A>C), GAA128GAT (719A>T), CAC129CAT (722C>T), GTT134GTC (737T>C), TAC137TAT (746C>T), TCA139TCT (752A>T), GGG142GGA (761G>A), GCT144TCA (765G>T 767T>A), AAG145AAA (770G>A), GTT146TAG (771G>T 772T>A 773T>G), TTG147ATT (774T>A 776G>T), AAA148GCG (777A>G 778A>C 779A>G), GTT149GTG (782T>G), AGT154AAG (796G>A 797T>G), GCA156GCC (803A>C), TTT158TTC (809T>C), GAA159CAA (810G>C), TCC160TCA (815C>A), ATA161ATT (818A>T), ACT162ACG (821T>G), ACC163ACT (824C>T), CCA164CCT (827A>T), CCT166TCA (831C>T 833T>A), GGG169GAT (841G>A 842G>T), CTA172CGT (850T>G 851A>T), GTG174GTT (857G>T), CTG175CTT (860G>T), GAA176GGA (862A>G), ATT178ATG (869T>G), ACG180ACT (875G>T), TTC182TGA (880T>G 881C>A), CAT184AAG (885C>A 887T>G), CAA186CGA (892A>G), CTA190CTT (905A>T), CCC191CCA (908C>A), TTT192CAT (909T>C 910T>A), CGA194CAA (916G>A), GGC195TCC (918G>T 919G>C), ATT196ATA (923T>A), CCA197CCT (926A>T), ATG198CTT (927A>C 929G>T), ATT200AAG (934T>A 935T>G), GGG201GGT (938G>T), TCT203AAT (942T>A 943C>A), CAA205AAA (948C>A), CCC212CCA (971C>A), CTA213TTA (972C>T), AAT214CTC (975A>C 976A>T 977T>C), AAT216AAC (983T>C), AGG219CGC (990A>C 992G>C), TCA220CGG (993T>C 994C>G 995A>G), TAC221TTT (997A>T 998C>T), GGT222CAA (999G>C 1000G>A 1001T>A), TTA223TAT (1003T>A 1004A>T), GCC224TCG (1005G>T 1007C>G), GTA225GTG (1010A>G), GAC227GAT (1016C>T), AAA228GTT (1017A>G 1018A>T 1019A>T), GTT229GTA (1022T>A), GAT230GAC (1025T>C), CAA231GGA (1026C>G 1027A>G), GAG232GCA (1030A>C 1031G>A), GCT233GCG (1034T>G), AGT234GCT (1035A>G 1036G>C), TTC237GTT (1044T>G 1046C>T), AAG238AAA (1049G>A), TTT239GAA (1050T>G 1051T>A 1052T>A), CCA240CAC (1054C>A 1055A>C), ATT242ATC (1061T>C), GTT244GTG (1067T>G), TTG246TTA (1073G>A), AAA247AAG (1076A>G), CTA250ATA (1083C>A), GCG251GCT (1088G>T), GCC252GCG (1091C>G), TGG255TTG (1099G>T), ACA257GTG (1104A>G 1105C>T 1106A>G), ATT260ATG (1115T>G), TCA262TCC (1121A>C), TTC263ATT (1122T>A 1124C>T), CAG264CAA (1127G>A), GAC265GAT (1130C>T), GGC266GGG (1133C>G), TCA269TCC (1142A>C), CCC270CCT (1145C>T), TTA290TTT (1205A>T), AAC292AAT (1211C>T), TTA293GAA (1212T>G 1213T>A), TCT294TCC (1217T>C), CTC295CTT (1220C>T), GAA296GAT (1223A>T), CAC297TTA (1224C>T 1225A>T 1226C>A), GAG299GAA (1232G>A), AAA300AAG (1235A>G), AGT301TCT (1236A>T 1237G>C), GTC302GTA (1241C>A), GGA303GGC (1244A>C), CCA304CCC (1247A>C), TGG305TAC (1249G>A 1250G>C), TCA307AGT (1254T>A 1255C>G 1256A>T), ACC308TCT (1257A>T 1259C>T), TTA309CTT (1260T>C 1262A>T), AGT311GGG (1266A>G 1268T>G), CCT312GCT (1269C>G), TAT313TAC (1274T>C), CGA314AAG (1275C>A 1276G>A 1277A>G), GGG315GGT (1280G>T), GTG316GTT (1283G>T), CCA317CCG (1286A>G), GTT319GCA (1291T>C 1292T>A), ACT320ACG (1295T>G), GGT321GGA (1298T>A), CTT323TTA (1302C>T 1304T>A), GGT324GCT (1306G>C), GCA325CAA (1308G>C 1309C>A), GGC329GGA (1322C>A), GGA331GGT (1328A>T), AGG334CGG (1335A>C), GTA335ATC (1338G>A 1340A>C), GTA337GCA (1345T>C), ATG338ATA (1349G>A), AAC340AAT (1355C>T), ATA342GTA (1359A>G), AAA343_CAA344insAAAAA- (1364_1365insAAAAA), AGA345AAG (1369G>A 1370A>G), CTT346TGA (1371C>T 1372T>G 1373T>A), CTC347CTG (1376C>G), CAC348TTT (1377C>T 1378A>T 1379C>T), GTT350GTC (1385T>C), GAT352CAA (1389G>C 1391T>A), GCT354GCA (1397T>A), ACA356AAA (1402C>A), CTG358CTA (1409G>A), AAA360CGC (1413A>C 1414A>G 1415A>C), ATA361CTT (1416A>C 1418A>T), AAG362CCG (1419A>C 1420A>C), ACA363GCG (1422A>G 1424A>G), GAC364GAT (1427C>T), GGG365GGA (1430G>A), ACT366GCA (1431A>G 1433T>A), GAG370GAA (1445G>A), AAA371CGT (1446A>C 1447A>G 1448A>T), CCT372CCG (1451T>G), TTG374GTA (1455T>G 1457G>A), GCT375CGA (1458G>C 1459C>G 1460T>A), AAG377CGA (1464A>C 1465A>G 1466G>A), CAA378TCT (1467C>T 1468A>C 1469A>T), AAG379CAA (1470A>C 1472G>A), AAC380AAG (1475C>G), TTT381AAA (1476T>A 1477T>A 1478T>A), AAA382GGT (1479A>G 1480A>G 1481A>T), TGT384TCT (1486G>C), TAT385TTC (1489A>T 1490T>C), TTA389TTC (1502A>C), AAG390AAA (1505G>A), TCA391TCG (1508A>G), CCG397CCA (1526G>A), ATG401ATA (1538G>A), TAT402TAC (1541T>C), CTT404TTC (1545C>T 1547T>C), TCT406TCC (1553T>C), ATG407TGC (1554A>T 1555T>G 1556G>C), ATA408ATG (1559A>G), CCT411AGT (1566C>A 1567C>G), ACC412ACA (1571C>A), TTG413CTG (1572T>C), TCC415TCA (1580C>A), GTT418GTC (1589T>C), AGC420del (1593_1597delAGCTC), TCT421--A (1593_1597delAGCTC 1598T>A), CTT424CTC (1607T>C), ACG426ACT (1613G>T), ATC427TTC (1614A>T), CTT428TTG (1617C>T 1619T>G), GTT429ATC (1620G>A 1622T>C), AAC430GTC (1623A>G 1624A>T), AAA431CCT (1626A>C 1627A>C 1628A>T), TTA433ATG (1632T>A 1634A>G), ACA434GTC (1635A>G 1636C>T 1637A>C), AGA436AGG (1643A>G), GTG437ATG (1644G>A), TAT438AGT (1647T>A 1648A>G), CTG443CTA (1664G>A), GGG445GGA (1670G>A), CCT447CCG (1676T>G), TTG448TTA (1679G>A), GGC449GGT (1682C>T), TTC450TAC (1684T>A), CAC451TAC (1686C>T), GGT452GGA (1691T>A), TCT453TCA (1694T>A), GCA455TCC (1698G>T 1700A>C), CTT456CTA (1703T>A), TCG458GCG (1707T>G), TTG459CTT (1710T>C 1712G>T), TCT460TCC (1715T>C), TTA467TTG (1736A>G), GCA468GCT (1739A>T), AGC470AAA (1744G>A 1745C>A), AAA471TAT (1746A>T 1748A>T), TAT473TAC (1754T>C), GGA475ACG (1758G>A 1759G>C 1760A>G), TGT476AGT (1761T>A), AAC477ACT (1765A>C 1766C>T), TCT478ACC (1767T>A 1769T>C), CCA479CCT (1772A>T), GGG481GCT (1777G>C 1778G>T), GCT484GCC (1787T>C), TTA485CTG (1788T>C 1790A>G), CTA486TTA (1791C>T), AGT495ACA (1819G>C 1820T>A), AAA496GAG (1821A>G 1823A>G), GCC498GCG (1829C>G), AAT499CGG (1830A>C 1831A>G 1832T>G), GAG500CAG (1833G>C), AAG502AGG (1840A>G), TTA504TTG (1847A>G), TTG505CTG (1848T>C), AGA507AGG (1856A>G), CTC508CTT (1859C>T), AAT509GGT (1860A>G 1861A>G), GTC510GTT (1865C>T), AAA511ACA (1867A>C), ATT512ATC (1871T>C), TCG513TCT (1874G>T), ATA514-TA (1875delA), CCA515GCT (1878C>G 1880A>T), TCT517TCG (1886T>G), TTG518AGA (1887T>A 1888T>G 1889G>A), TCT520TCC (1895T>C), GAT521GAG (1898T>G), AAT522AAC (1901T>C), ATT525ATC (1910T>C), GCG528GCT (1919G>T), AGG530AGA (1925G>A), ACT533ACC (1934T>C), AAA534AAG (1937A>G), TCA535GAC (1938T>G 1939C>A 1940A>C), GAT539TAC (1950G>T 1952T>C), TTA540CTT (1953T>C 1955A>T), ATT543ATC (1964T>C), TCC544TCT (1967C>T), GCT547GCA (1976T>A), TTA549ACT (1980T>A 1981T>C 1982A>T), TCT550TCC (1985T>C), CGT552AGG (1989C>A 1991T>G), ACT553ACA (1994T>A), GTT555GTG (2000T>G), GGA556GGT (2003A>T), CTT557CTG (2006T>G), TGC558TGT (2009C>T), GCC559CAA (2010G>C 2011C>A 2012C>A), ATC562ACC (2020T>C), GAT565TCG (2028G>T 2029A>C 2030T>G), AAC567TCA (2034A>T 2035A>C 2036C>A), ATA568ACA (2038T>C), TCA569TCG (2042A>G), GTT570ATT (2043G>A), CAA572CCC (2050A>C 2051A>C), AGA573ATT (2053G>T 2054A>T), TTG574TTT (2057G>T), GGG575TGG (2058G>T), GGT576GGG (2063T>G), GGG578GGA (2069G>A), TAC579TTC (2071A>T), AAG580AGA (2074A>G 2075G>A), GTA581GTT (2078A>T), GCG583TCT (2082G>T 2084G>T), CGC584CGT (2087C>T), TTA585TTG (2090A>G), TTC586ATG (2091T>A 2093C>G), ACT587TCT (2094A>T), ACC588ACT (2099C>T), AGC590TCT (2103A>T 2104G>C 2105C>T), AAG591AAA (2108G>A), GAG594GAA (2117G>A), CGT595AGA (2118C>A 2120T>A), CTA596CTG (2123A>G), GCT598GCG (2129T>G), CAT600GCA (2133C>G 2134A>C 2135T>A), CTT601AGC (2136C>A 2137T>G 2138T>C), AAG602AAA (2141G>A), GTT604CAC (2145G>C 2146T>A 2147T>C), CGG605AGG (2148C>A), CAC607CAT (2156C>T), TTA608CTA (2157T>C), CTA609TTA (2160C>T), TAC611CTC (2166T>C 2167A>T), TGG613TAT (2173G>A 2174G>T), GGA616GGG (2183A>G), AGG617AGA (2186G>A), GGT618TTT (2187G>T 2188G>T), ATG619AAA (2191T>A 2192G>A), CCC620CCT (2195C>T), CCC623CCT (2204C>T), TAT624TTT (2206A>T), AAA626AAG (2213A>G), GGG627GGA (2216G>A), GTA630ATA (2223G>A), AGT631GAT (2226A>G 2227G>A), TAC632TAT (2231C>T), CTT634CGG (2236T>G 2237T>G), AAA635AAG (2240A>G), AAA638ACA (2248A>C), GTT639CCT (2250G>C 2251T>C), TTA642ATA (2259T>A), CAG643CGA (2263A>G 2264G>A), ATC644CTC (2265A>C), AAG647AAT (2276G>T), GAT648GAG (2279T>G), CTG649TTG (2280C>T), TTC651TTT (2288C>T), GAA654GAG (2297A>G), GTT655AGA (2298G>A 2299T>G 2300T>A), ATA657ATC (2306A>C), CTA658CTT (2309A>T), CGT660CGC (2315T>C), CTA663TTA (2322C>T), CGC664CTC (2326G>T), CAA665AGA (2328C>A 2329A>G), GTA667GTG (2336A>G), GAA668AAA (2337G>A), TTA671CTG (2346T>C 2348A>G), AAG672AAA (2351G>A), CTC674GTC (2355C>G), AAC675TCT (2358A>T 2359A>C 2360C>T), ACG679ACT (2372G>T), ATC680GTT (2373A>G 2375C>T), TGT681GCA (2376T>G 2377G>C 2378T>A), ATG682GCA (2379A>G 2380T>C 2381G>A), AAC683TCA (2382A>T 2383A>C 2384C>A), CCA684GCA (2385C>G), GAT685GAA (2390T>A), TCC687ACC (2394T>A), AAG689GAT (2400A>G 2402G>T), AGT690CAG (2403A>C 2404G>A 2405T>G), CTC691TTG (2406C>T 2408C>G), ATC692ATG (2411C>G), GAC693GAA (2414C>A), TCC694GTT (2415T>G 2416C>T 2417C>T), ATA696ATG (2423A>G), TGC697TGT (2426C>T), GCA698AGT (2427G>A 2428C>G 2429A>T), ACA699AAA (2431C>A), TGG701TGA (2438G>A), AGG703AGA (2444G>A), CTC704ACA (2445C>A 2446T>C 2447C>A), GAA705AAC (2448G>A 2450A>C), ACT706GAA (2451A>G 2452C>A 2453T>A), TTT708CTT (2457T>C), TTA710TTG (2465A>G), GGC714GGT (2477C>T), CTA715TTA (2478C>T), AAA718AAG (2489A>G), TGT719TGC (2492T>C), TAC720TAT (2495C>T), GGT723GGA (2504T>A), GGT725GGC (2510T>C), AAT727GAT (2514A>G), CCC728CTG (2518C>T 2519C>G), ACC730ATT (2524C>T 2525C>T), ACT731ACA (2528T>A), CAG733AGG (2532C>A 2533A>G), TGG734TAT (2536G>A 2537G>T), TTT736TTG (2543T>G), AAC739AGT (2551A>G 2552C>T), ACT740ACC (2555T>C), GTT741TCT (2556G>T 2557T>C), ATT742ATC (2561T>C), AAT745GAT (2568A>G), AAA746CGA (2571A>C 2572A>G), TTA749TTT (2582A>T), GGG750GGA (2585G>A), GGG754GGA (2597G>A), AAC755GCT (2598A>G 2599A>C 2600C>T), GAT756AAT (2601G>A), ATT758ATA (2609T>A), TCT760GCA (2613T>G 2615T>A), CCT761CCA (2618T>A), GAA763TAT (2622G>T 2624A>T), TAC764TTA (2626A>T 2627C>A), CAA765CCA (2629A>C), ACT766TCT (2631A>T), ACA767CTA (2634A>C 2635C>T), GGA768GTG (2638G>T 2639A>G), GAG770GGG (2644A>G), GAA771TGT (2646G>T 2647A>G 2648A>T), GTG772AGG (2649G>A 2650T>G), GGC773TCA (2652G>T 2653G>C 2654C>A), TTA774TAT (2656T>A 2657A>T), AGA775GAC (2658A>G 2659G>A 2660A>C), CTT776TTT (2661C>T), TAG777AGG (2664T>A 2665A>G)								

*: Inserts / Deletes / Misaligned / Frameshifts

Analysis details

This analysis was performed with panviral2.64

NGS Details (UN18_val): Solendovirus venanicotianae

Assembly

**Coverage Length**	1662 (4 contig(s))
**Depth Of Coverage**	15.1
**Number Of Reads**	218
**Reads Per Million**	4.09 rpm (after QC)
**Ambiguities**	0
**Assembly Method**	de novo + reference guided assembly
**Consensus Caller**	Bcf Tools

Coverage Map

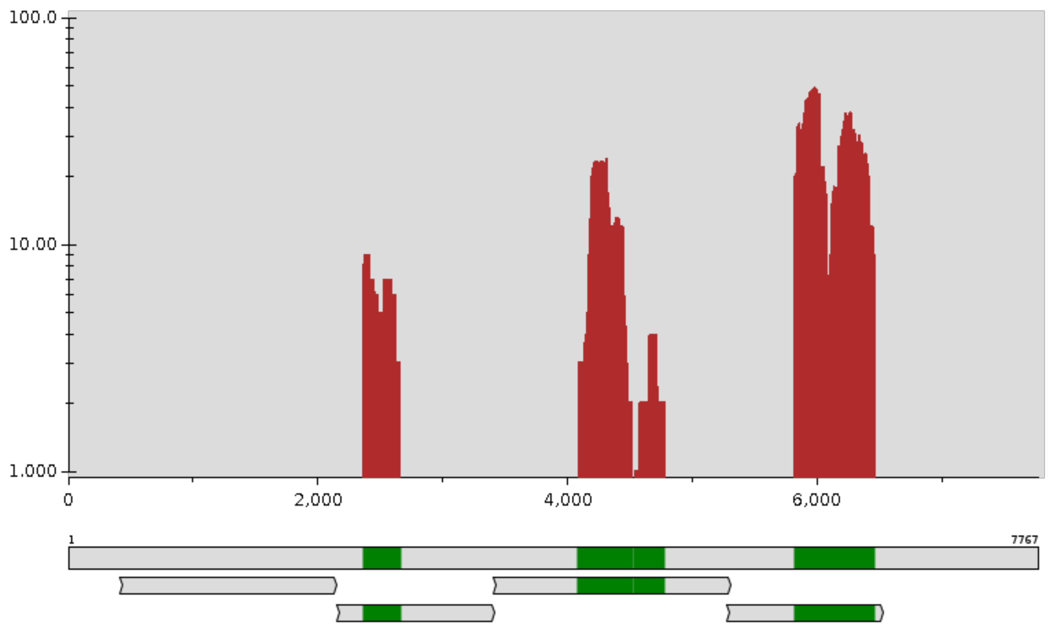

Assignment

**Type**	Solendovirus venanicotianae (Taxonomy ID: 3048371)
**Reference Genome**	NC_003378.1
**NT Identity (%)**	77.9855
**AA Identity (%)**	73.9602
**Number Of Stop Codons**	2
**Number Of CDS**	4

Alignment

**Alignment Score**	1834.0 (NT) + 2746.0 (AA) = 4580.0
**Concordance (%)**	66.3971

**Alignment Method**	Global, seeded, nucleotide + amino acids (AGA)

Genome Region

Sequence starts at position 2358 and ends at position 6465 relative to NC_003378.1 reference sequence.

Alignment Detailed Statistics

	Begin	End	Coverage	Score	Concordance	Matches	Identities	I/D/M/F*	Stop Codons
**NT**	**2358**	**6465**	**21.4%**	**1834**	**55.7%**	**1658 (99.8%)**	**1293 (77.8%)**	**0/4**	
Mutations:	2358A>G, 2373T>A, 2376T>C, 2379C>T, 2388A>T, 2389G>A, 2390G>A, 2394T>A, 2397G>A, 2406A>T, 2426T>C, 2442A>T, 2454G>A, 2464C>T, 2469T>A, 2472G>A, 2482T>C, 2493T>A, 2503_2505delATA, 2535C>A, 2538T>A, 2545A>C, 2551C>T, 2555C>T, 2559T>C, 2562T>C, 2565A>G, 2568A>T, 2569A>T, 2571C>A, 2572C>G, 2589C>T, 2590A>G, 2595A>C, 2597G>C, 2598T>A, 2602A>G, 2606A>G, 2609G>A, 2613T>C, 2622T>C, 2628G>A, 2637T>C, 2638A>G, 2643A>C, 2646C>T, 2647G>C, 2649C>A, 2665C>T, 4086A>G, 4091T>A, 4097G>A, 4100T>G, 4103T>A, 4116G>A, 4118A>C, 4127C>T, 4139T>C, 4143G>A, 4161C>T, 4181A>T, 4187C>T, 4190G>A, 4196C>T, 4199A>T, 4241T>C, 4250C>A, 4256A>G, 4265G>A, 4292C>T, 4307C>T, 4313G>A, 4325T>C, 4328T>C, 4329T>C, 4331G>T, 4334A>G, 4340G>A, 4343T>A, 4357T>A, 4358A>T, 4362G>T, 4364T>A, 4367T>C, 4370A>T, 4380G>A, 4400T>A, 4411G>A, 4430A>T, 4436C>T, 4451C>T, 4454C>T, 4457T>C, 4467C>T, 4472A>T, 4482G>A, 4496T>C, 4503C>T, 4508T>C, 4513G>A, 4516C>T, 4550C>T, 4551G>A, 4552C>T, 4554C>G, 4568C>T, 4571T>A, 4574T>A, 4580C>G, 4587A>C, 4589A>G, 4591C>A, 4592C>A, 4595A>G, 4601A>C, 4604T>A, 4606T>A, 4607G>T, 4614C>G, 4616G>A, 4619A>T, 4626T>C, 4628A>C, 4634A>G, 4635C>A, 4636A>T, 4640A>T, 4641G>T, 4642A>C, 4643T>A, 4652A>C, 4655A>T, 4656A>G, 4659A>T, 4661G>A, 4670T>C, 4674G>T, 4675T>C, 4677C>A, 4682A>G, 4685A>C, 4686A>T, 4687T>G, 4688C>T, 4689A>G, 4691T>A, 4692C>T, 4695G>T, 4696A>C, 4697T>A, 4700A>T, 4703C>A, 4704A>C, 4706T>A, 4707G>A, 4709T>C, 4716A>C, 4718G>A, 4719A>G, 4722T>A, 4724A>C, 4728T>A, 4729C>G, 4730A>G, 4736A>T, 4740A>T, 4741T>G, 4742A>T, 4743G>T, 4748C>T, 4749C>T, 4751A>C, 4753T>C, 4757G>T, 4762A>T, 4769T>A, 4770A>G, 4772A>C, 4773T>C, 4779G>A, 5817T>C, 5820C>T, 5821C>A, 5824A>G, 5826T>A, 5831A>C, 5832C>A, 5833G>C, 5835A>G, 5838C>T, 5850C>A, 5851C>A, 5853T>A, 5857A>C, 5858C>A, 5859A>G, 5865C>T, 5868T>C, 5871A>G, 5872T>C, 5874G>A, 5877A>T, 5880T>A, 5881T>C, 5882T>A, 5889T>A, 5892A>T, 5894G>A, 5898A>T, 5901A>C, 5902T>C, 5907A>T, 5908G>A, 5909C>A, 5910C>A, 5913C>A, 5916C>T, 5921A>G, 5922C>T, 5925C>T, 5935G>A, 5936A>G, 5937T>C, 5940T>A, 5941G>A, 5950C>T, 5952G>T, 5953A>G, 5954C>A, 5955A>C, 5956G>T, 5959C>G, 5964G>A, 5968G>T, 5970C>A, 5972A>C, 5979T>A, 5991G>A, 5997C>T, 6001C>A, 6009G>A, 6012G>A, 6015A>T, 6018T>A, 6023G>A, 6024G>A, 6026C>A, 6027A>T, 6033C>T, 6036C>T, 6037G>A, 6040C>A, 6041G>A, 6045C>T, 6049T>A, 6051G>A, 6054T>A, 6055A>C, 6067C>T, 6075A>T, 6084G>A, 6087T>A, 6088A>G, 6099T>C, 6108T>A, 6112C>T, 6114C>G, 6117A>T, 6120G>A, 6123A>T, 6129C>T, 6132A>T, 6136G>T, 6139A>G, 6141G>T, 6144A>T, 6145G>A, 6146A>C, 6148G>C, 6150A>G, 6153A>G, 6154G>C, 6155A>C, 6156A>G, 6163G>C, 6164A>C, 6170T>A, 6172A>G, 6174T>G, 6177T>A, 6179C>T, 6181G>A, 6183A>T, 6186C>T, 6192T>C, 6201G>A, 6204A>T, 6207T>C, 6211A>C, 6214T>G, 6219T>A, 6223C>A, 6225A>T, 6232C>T, 6236C>G, 6240C>T, 6247C>A, 6253C>G, 6255G>A, 6258T>C, 6259G>C, 6264T>G, 6269G>T, 6270C>T, 6277T>A, 6282G>A, 6285A>T, 6288G>A, 6290C>T, 6308G>C, 6309C>A, 6315T>A, 6318A>G, 6319A>C, 6322G>A, 6325G>C, 6326C>A, 6334G>A, 6340C>G, 6341T>C, 6345A>G, 6352G>A, 6354C>A, 6355T>A, 6357A>G, 6359G>C, 6360T>A, 6363T>G, 6369A>T, 6371C>T, 6376C>A, 6377A>G, 6380C>A, 6381T>A, 6382A>C, 6384A>T, 6401G>A, 6402G>A, 6404G>A, 6406delA, 6407A>G, 6408T>A, 6411C>T, 6414C>T, 6417C>G, 6418C>G, 6419C>A, 6420A>T, 6423T>A, 6424C>T, 6426G>A, 6427T>C, 6446T>A, 6451A>G, 6453T>G, 6456C>A, 6462T>C								

CDS

	Begin	End	Coverage	Score	Concordance	Matches	Identities	I/D/M/F*	Stop Codons
Tvvgp2	69	172	24.8%	605	87.6%	103 (99.0%)	88 (84.6%)	0/1/0/0	0
Protein mutations:	G79K (2389G>A 2390G>A), I91T (2426T>C), P104S (2464C>T), I117del (2503_2505delATA), I131L (2545A>C), A134V (2555C>T), I139L (2569A>T 2571C>A), Q140E (2572C>G), I146V (2590A>G), S148T (2597G>C 2598T>A), I150V (2602A>G), K151R (2606A>G), G152E (2609G>A), I162V (2638A>G), A165P (2647G>C 2649C>A)								
Codon mutations:	CAA68..G (2358A>G), ATT73ATA (2373T>A), GAT74GAC (2376T>C), TGC75TGT (2379C>T), ACA78ACT (2388A>T), GGA79AAA (2389G>A 2390G>A), GGT80GGA (2394T>A), AAG81AAA (2397G>A), ATA84ATT (2406A>T), ATA91ACA (2426T>C), ATA96ATT (2442A>T), AAG100AAA (2454G>A), CCA104TCA (2464C>T), ATT105ATA (2469T>A), AAG106AAA (2472G>A), TTA110CTA (2482T>C), ACT113ACA (2493T>A), ATA117del (2503_2505delATA), ACC127ACA (2535C>A), CCT128CCA (2538T>A), ATA131CTA (2545A>C), CTA133TTA (2551C>T), GCA134GTA (2555C>T), GAT135GAC (2559T>C), GAT136GAC (2562T>C), AGA137AGG (2565A>G), ATA138ATT (2568A>T), ATC139TTA (2569A>T 2571C>A), CAA140GAA (2572C>G), AGC145AGT (2589C>T), ATA146GTA (2590A>G), ATA147ATC (2595A>C), AGT148ACA (2597G>C 2598T>A), ATA150GTA (2602A>G), AAA151AGA (2606A>G), GGA152GAA (2609G>A), AAT153AAC (2613T>C), TAT156TAC (2622T>C), AAG158AAA (2628G>A), TTT161TTC (2637T>C), ATA162GTA (2638A>G), ATA163ATC (2643A>C), AGC164AGT (2646C>T), GCC165CCA (2647G>C 2649C>A), CTA171TTA (2665C>T)								
Tvvgp3	225	458	36.5%	1263	78.5%	232 (100%)	179 (77.2%)	0/0/0/0	0
Protein mutations:	K226E (4086A>G), D227E (4091T>A), D230E (4100T>G), N231K (4103T>A), E236N (4116G>A 4118A>C), A245T (4143G>A), H251Y (4161C>T), D311E (4343T>A), L316Y (4357T>A 4358A>T), A318S (4362G>T 4364T>A), G324R (4380G>A), G334E (4411G>A), E354D (4472A>T), V358I (4482G>A), R368K (4513G>A), T369I (4516C>T), A381I (4551G>A 4552C>T), H382D (4554C>G), S390R (4580C>G), K393Q (4587A>C 4589A>G), T394K (4591C>A 4592C>A), E397D (4601A>C), M399N (4606T>A 4607G>T), Q402E (4614C>G 4616G>A), I408M (4634A>G), Q409I (4635C>A 4636A>T), D411S (4641G>T 4642A>C 4643T>A), K416E (4656A>G), M417L (4659A>T 4661G>A), V422S (4674G>T 4675T>C), Q423K (4677C>A), I426C (4686A>T 4687T>G 4688C>T), N427E (4689A>G 4691T>A), L428F (4692C>T), D429S (4695G>T 4696A>C 4697T>A), E430D (4700A>T), N431K (4703C>A), I432L (4704A>C 4706T>A), D433N (4707G>A 4709T>C), K436Q (4716A>C 4718G>A), K437E (4719A>G), L438I (4722T>A 4724A>C), S440R (4728T>A 4729C>G 4730A>G), I444C (4740A>T 4741T>G 4742A>T), V445L (4743G>T), Q447Y (4749C>T 4751A>C), V448A (4753T>C), R449S (4757G>T), Y451F (4762A>T), K454D (4770A>G 4772A>C), E457K (4779G>A)								
Codon mutations:	AAA226GAA (4086A>G), GAT227GAA (4091T>A), TTG229TTA (4097G>A), GAT230GAG (4100T>G), AAT231AAA (4103T>A), GAA236AAC (4116G>A 4118A>C), AGC239AGT (4127C>T), AGT243AGC (4139T>C), GCA245ACA (4143G>A), CAT251TAT (4161C>T), GGA257GGT (4181A>T), AGC259AGT (4187C>T), AGG260AGA (4190G>A), GTC262GTT (4196C>T), ATA263ATT (4199A>T), TAT277TAC (4241T>C), CCC280CCA (4250C>A), AAA282AAG (4256A>G), AAG285AAA (4265G>A), TAC294TAT (4292C>T), GAC299GAT (4307C>T), AAG301AAA (4313G>A), TAT305TAC (4325T>C), CAT306CAC (4328T>C), TTG307CTT (4329T>C 4331G>T), AAA308AAG (4334A>G), GAG310GAA (4340G>A), GAT311GAA (4343T>A), TTA316TAT (4357T>A 4358A>T), GCT318TCA (4362G>T 4364T>A), TTT319TTC (4367T>C), ACA320ACT (4370A>T), GGA324AGA (4380G>A), GTT330GTA (4400T>A), GGA334GAA (4411G>A), GGA340GGT (4430A>T), TAC342TAT (4436C>T), GAC347GAT (4451C>T), AAC348AAT (4454C>T), TAT349TAC (4457T>C), CTA353TTA (4467C>T), GAA354GAT (4472A>T), GTA358ATA (4482G>A), GAT362GAC (4496T>C), CTA365TTA (4503C>T), TAT366TAC (4508T>C), AGA368AAA (4513G>A), ACA369ATA (4516C>T), TTC380TTT (4550C>T), GCA381ATA (4551G>A 4552C>T), CAT382GAT (4554C>G), AAC386AAT (4568C>T), TCT387TCA (4571T>A), GGT388GGA (4574T>A), AGC390AGG (4580C>G), AAA393CAG (4587A>C 4589A>G), ACC394AAA (4591C>A 4592C>A), AAA395AAG (4595A>G), GAA397GAC (4601A>C), ATT398ATA (4604T>A), ATG399AAT (4606T>A 4607G>T), CAG402GAA (4614C>G 4616G>A), ATA403ATT (4619A>T), TTA406CTC (4626T>C 4628A>C), ATA408ATG (4634A>G), CAA409ATA (4635C>A 4636A>T), ATA410ATT (4640A>T), GAT411TCA (4641G>T 4642A>C 4643T>A), GGA414GGC (4652A>C), ATA415ATT (4655A>T), AAA416GAA (4656A>G), ATG417TTA (4659A>T 4661G>A), CAT420CAC (4670T>C), GTA422TCA (4674G>T 4675T>C), CAA423AAA (4677C>A), AAA424AAG (4682A>G), ATA425ATC (4685A>C), ATC426TGT (4686A>T 4687T>G 4688C>T), AAT427GAA (4689A>G 4691T>A), CTT428TTT (4692C>T), GAT429TCA (4695G>T 4696A>C 4697T>A), GAA430GAT (4700A>T), AAC431AAA (4703C>A), ATT432CTA (4704A>C 4706T>A), GAT433AAC (4707G>A 4709T>C), AAG436CAA (4716A>C 4718G>A), AAA437GAA (4719A>G), TTA438ATC (4722T>A 4724A>C), TCA440AGG (4728T>A 4729C>G 4730A>G), CTA442CTT (4736A>T), ATA444TGT (4740A>T 4741T>G 4742A>T), GTA445TTA (4743G>T), AAC446AAT (4748C>T), CAA447TAC (4749C>T 4751A>C), GTA448GCA (4753T>C), AGG449AGT (4757G>T), TAT451TTT (4762A>T), CCT453CCA (4769T>A), AAA454GAC (4770A>G 4772A>C), TTA455CTA (4773T>C), GAA457AAA (4779G>A)								
Tvvgp4	180	397	52.2%	878	59.0%	218 (100%)	142 (65.1%)	0/0/1/1	2
Protein mutations:	Q183K (5821C>A), N184E (5824A>G 5826T>A), N186T (5831A>C 5832C>A), E187Q (5833G>C 5835A>G), H192Q (5850C>A), H193K (5851C>A 5853T>A), T195Q (5857A>C 5858C>A 5859A>G), L203Q (5881T>C 5882T>A), N205K (5889T>A), S207N (5894G>A), K209N (5901A>C), A212K (5908G>A 5909C>A 5910C>A), N216S (5921A>G 5922C>T), D221S (5935G>A 5936A>G 5937T>C), V223I (5941G>A), Q226Y (5950C>T 5952G>T), T227D (5953A>G 5954C>A 5955A>C), G228* (5956G>T), Q229E (5959C>G), A232S (5968G>T 5970C>A), N233T (5972A>C), Q243K (6001C>A), G250E (6023G>A 6024G>A), T251N (6026C>A 6027A>T), V255I (6037G>A), R256K (6040C>A 6041G>A), S259T (6049T>A 6051G>A), T261P (6055A>C), E267D (6075A>T), I272V (6088A>G), E283D (6123A>T), E288* (6136G>T), K289D (6139A>G 6141G>T), E291T (6145G>A 6146A>C), E292Q (6148G>C 6150A>G), E294P (6154G>C 6155A>C 6156A>G), E297P (6163G>C 6164A>C), V299E (6170T>A), N300E (6172A>G 6174T>G), P302L (6179C>T), E303N (6181G>A 6183A>T), I313L (6211A>C), S314A (6214T>G), L317I (6223C>A 6225A>T), A321G (6236C>G), L325I (6247C>A), Q327E (6253C>G 6255G>A), A329P (6259G>C), I330M (6264T>G), S332I (6269G>T 6270C>T), S335T (6277T>A), E337D (6285A>T), T339I (6290C>T), C345S (6308G>C 6309C>A), D347E (6315T>A), I348M (6318A>G), E350K (6322G>A), A351Q (6325G>C 6326C>A), E354K (6334G>A), L356A (6340C>G 6341T>C), V360I (6352G>A 6354C>A), L361M (6355T>A 6357A>G), S362T (6359G>C 6360T>A), K365N (6369A>T), P366L (6371C>T), Q368R (6376C>A 6377A>G), P369Q (6380C>A 6381T>A), T370P (6382A>C 6384A>T), R376K (6401G>A 6402G>A), R377K (6404G>A), P382D (6418C>G 6419C>A 6420A>T), D383E (6423T>A), L391Q (6446T>A), S393G (6451A>G 6453T>G), H394Q (6456C>A)								

	Begin	End	Coverage	Score	Concordance	Matches	Identities	I/D/M/F*	Stop Codons
**NT**	**2358**	**6465**	**21.4%**	**1834**	**55.7%**	**1658 (99.8%)**	**1293 (77.8%)**	**0/4**	
Codon mutations:	TCT181TCC (5817T>C), ACC182ACT (5820C>T), CAA183AAA (5821C>A), AAT184GAA (5824A>G 5826T>A), AAC186ACA (5831A>C 5832C>A), GAA187CAG (5833G>C 5835A>G), GAC188GAT (5838C>T), CAC192CAA (5850C>A), CAT193AAA (5851C>A 5853T>A), ACA195CAG (5857A>C 5858C>A 5859A>G), TAC197TAT (5865C>T), AAT198AAC (5868T>C), AAA199AAG (5871A>G), TTG200CTA (5872T>C 5874G>A), ATA201ATT (5877A>T), GCT202GCA (5880T>A), TTA203CAA (5881T>C 5882T>A), AAT205AAA (5889T>A), ACA206ACT (5892A>T), AGT207AAT (5894G>A), GCA208GCT (5898A>T), AAA209AAC (5901A>C), TTA210CTA (5902T>C), GTA211GTT (5907A>T), GCC212AAA (5908G>A 5909C>A 5910C>A), ACC213ACA (5913C>A), TGC214TGT (5916C>T), AAC216AGT (5921A>G 5922C>T), TAC217TAT (5925C>T), GAT221AGC (5935G>A 5936A>G 5937T>C), ACT222ACA (5940T>A), GTA223ATA (5941G>A), CAG226TAT (5950C>T 5952G>T), ACA227GAC (5953A>G 5954C>A 5955A>C), GGA228TGA (5956G>T), CAA229GAA (5959C>G), GAG230GAA (5964G>A), GCC232TCA (5968G>T 5970C>A), AAT233ACT (5972A>C), CCT235CCA (5979T>A), AAG239AAA (5991G>A), TTC241TTT (5997C>T), CAA243AAA (6001C>A), AAG245AAA (6009G>A), AGG246AGA (6012G>A), ATA247ATT (6015A>T), ACT248ACA (6018T>A), GGG250GAA (6023G>A 6024G>A), ACA251AAT (6026C>A 6027A>T), TTC253TTT (6033C>T), TAC254TAT (6036C>T), GTA255ATA (6037G>A), CGA256AAA (6040C>A 6041G>A), TTC257TTT (6045C>T), TCG259ACA (6049T>A 6051G>A), GCT260GCA (6054T>A), ACA261CCA (6055A>C), CTA265TTA (6067C>T), GAA267GAT (6075A>T), AAG270AAA (6084G>A), CCT271CCA (6087T>A), ATC272GTC (6088A>G), GTT275GTC (6099T>C), ATT278ATA (6108T>A), CTC280TTG (6112C>T 6114C>G), ACA281ACT (6117A>T), AGG282AGA (6120G>A), GAA283GAT (6123A>T), ATC285ATT (6129C>T), ATA286ATT (6132A>T), GAA288TAA (6136G>T), AAG289GAT (6139A>G 6141G>T), ATA290ATT (6144A>T), GAA291ACA (6145G>A 6146A>C), GAA292CAG (6148G>C 6150A>G), CAA293CAG (6153A>G), GAA294CCG (6154G>C 6155A>C 6156A>G), GAA297CCA (6163G>C 6164A>C), GTA299GAA (6170T>A), AAT300GAG (6172A>G 6174T>G), ATT301ATA (6177T>A), CCA302CTA (6179C>T), GAA303AAT (6181G>A 6183A>T), TTC304TTT (6186C>T), GCT306GCC (6192T>C), AGG309AGA (6201G>A), ATA310ATT (6204A>T), ATT311ATC (6207T>C), ATA313CTA (6211A>C), TCT314GCT (6214T>G), ACT315ACA (6219T>A), CTA317ATT (6223C>A 6225A>T), CTA320TTA (6232C>T), GCA321GGA (6236C>G), AAC322AAT (6240C>T), CTA325ATA (6247C>A), CAG327GAA (6253C>G 6255G>A), AAT328AAC (6258T>C), GCA329CCA (6259G>C), ATT330ATG (6264T>G), AGC332ATT (6269G>T 6270C>T), TCA335ACA (6277T>A), AGG336AGA (6282G>A), GAA337GAT (6285A>T), CAG338CAA (6288G>A), ACA339ATA (6290C>T), TGC345TCA (6308G>C 6309C>A), GAT347GAA (6315T>A), ATA348ATG (6318A>G), AGA349CGA (6319A>C), GAA350AAA (6322G>A), GCA351CAA (6325G>C 6326C>A), GAA354AAA (6334G>A), CTA356GCA (6340C>G 6341T>C), AGA357AGG (6345A>G), GTC360ATA (6352G>A 6354C>A), TTA361ATG (6355T>A 6357A>G), AGT362ACA (6359G>C 6360T>A), CTT363CTG (6363T>G), AAA365AAT (6369A>T), CCA366CTA (6371C>T), CAA368AGA (6376C>A 6377A>G), CCT369CAA (6380C>A 6381T>A), ACA370CCT (6382A>C 6384A>T), AGG376AAA (6401G>A 6402G>A), AGG377AAG (6404G>A), AAT378-GA (6406delA 6407A>G 6408T>A), TTC379TTT (6411C>T), ATC380ATT (6414C>T), TCC381TCG (6417C>G), CCA382GAT (6418C>G 6419C>A 6420A>T), GAT383GAA (6423T>A), CTG384TTA (6424C>T 6426G>A), TTA385CTA (6427T>C), CTA391CAA (6446T>A), AGT393GGG (6451A>G 6453T>G), CAC394CAA (6456C>A), TAT396TAC (6462T>C)								

Proteins

	Begin	End	Coverage	Score	Concordance	Matches	Identities	I/D/M/F*	Stop Codons
putative cell-to-cell movement protein (NP_569140.1)	69	172	24.8%	605	87.6%	103 (99.0%)	88 (84.6%)	0/1/0/0	0
Protein mutations:	G79K (2389G>A 2390G>A), I91T (2426T>C), P104S (2464C>T), I117del (2503_2505delATA), I131L (2545A>C), A134V (2555C>T), I139L (2569A>T 2571C>A), Q140E (2572C>G), I146V (2590A>G), S148T (2597G>C 2598T>A), I150V (2602A>G), K151R (2606A>G), G152E (2609G>A), I162V (2638A>G), A165P (2647G>C 2649C>A)								
Codon mutations:	CAA68..G (2358A>G), ATT73ATA (2373T>A), GAT74GAC (2376T>C), TGC75TGT (2379C>T), ACA78ACT (2388A>T), GGA79AAA (2389G>A 2390G>A), GGT80GGA (2394T>A), AAG81AAA (2397G>A), ATA84ATT (2406A>T), ATA91ACA (2426T>C), ATA96ATT (2442A>T), AAG100AAA (2454G>A), CCA104TCA (2464C>T), ATT105ATA (2469T>A), AAG106AAA (2472G>A), TTA110CTA (2482T>C), ACT113ACA (2493T>A), ATA117del (2503_2505delATA), ACC127ACA (2535C>A), CCT128CCA (2538T>A), ATA131CTA (2545A>C), CTA133TTA (2551C>T), GCA134GTA (2555C>T), GAT135GAC (2559T>C), GAT136GAC (2562T>C), AGA137AGG (2565A>G), ATA138ATT (2568A>T), ATC139TTA (2569A>T 2571C>A), CAA140GAA (2572C>G), AGC145AGT (2589C>T), ATA146GTA (2590A>G), ATA147ATC (2595A>C), AGT148ACA (2597G>C 2598T>A), ATA150GTA (2602A>G), AAA151AGA (2606A>G), GGA152GAA (2609G>A), AAT153AAC (2613T>C), TAT156TAC (2622T>C), AAG158AAA (2628G>A), TTT161TTC (2637T>C), ATA162GTA (2638A>G), ATA163ATC (2643A>C), AGC164AGT (2646C>T), GCC165CCA (2647G>C 2649C>A), CTA171TTA (2665C>T)								
polyprotein (NP_569141.1)	225	458	36.5%	1263	78.5%	232 (100%)	179 (77.2%)	0/0/0/0	0
Protein mutations:	K226E (4086A>G), D227E (4091T>A), D230E (4100T>G), N231K (4103T>A), E236N (4116G>A 4118A>C), A245T (4143G>A), H251Y (4161C>T), D311E (4343T>A), L316Y (4357T>A 4358A>T), A318S (4362G>T 4364T>A), G324R (4380G>A), G334E (4411G>A), E354D (4472A>T), V358I (4482G>A), R368K (4513G>A), T369I (4516C>T), A381I (4551G>A 4552C>T), H382D (4554C>G), S390R (4580C>G), K393Q (4587A>C 4589A>G), T394K (4591C>A 4592C>A), E397D (4601A>C), M399N (4606T>A 4607G>T), Q402E (4614C>G 4616G>A), I408M (4634A>G), Q409I (4635C>A 4636A>T), D411S (4641G>T 4642A>C 4643T>A), K416E (4656A>G), M417L (4659A>T 4661G>A), V422S (4674G>T 4675T>C), Q423K (4677C>A), I426C (4686A>T 4687T>G 4688C>T), N427E (4689A>G 4691T>A), L428F (4692C>T), D429S (4695G>T 4696A>C 4697T>A), E430D (4700A>T), N431K (4703C>A), I432L (4704A>C 4706T>A), D433N (4707G>A 4709T>C), K436Q (4716A>C 4718G>A), K437E (4719A>G), L438I (4722T>A 4724A>C), S440R (4728T>A 4729C>G 4730A>G), I444C (4740A>T 4741T>G 4742A>T), V445L (4743G>T), Q447Y (4749C>T 4751A>C), V448A (4753T>C), R449S (4757G>T), Y451F (4762A>T), K454D (4770A>G 4772A>C), E457K (4779G>A)								
Codon mutations:	AAA226GAA (4086A>G), GAT227GAA (4091T>A), TTG229TTA (4097G>A), GAT230GAG (4100T>G), AAT231AAA (4103T>A), GAA236AAC (4116G>A 4118A>C), AGC239AGT (4127C>T), AGT243AGC (4139T>C), GCA245ACA (4143G>A), CAT251TAT (4161C>T), GGA257GGT (4181A>T), AGC259AGT (4187C>T), AGG260AGA (4190G>A), GTC262GTT (4196C>T), ATA263ATT (4199A>T), TAT277TAC (4241T>C), CCC280CCA (4250C>A), AAA282AAG (4256A>G), AAG285AAA (4265G>A), TAC294TAT (4292C>T), GAC299GAT (4307C>T), AAG301AAA (4313G>A), TAT305TAC (4325T>C), CAT306CAC (4328T>C), TTG307CTT (4329T>C 4331G>T), AAA308AAG (4334A>G), GAG310GAA (4340G>A), GAT311GAA (4343T>A), TTA316TAT (4357T>A 4358A>T), GCT318TCA (4362G>T 4364T>A), TTT319TTC (4367T>C), ACA320ACT (4370A>T), GGA324AGA (4380G>A), GTT330GTA (4400T>A), GGA334GAA (4411G>A), GGA340GGT (4430A>T), TAC342TAT (4436C>T), GAC347GAT (4451C>T), AAC348AAT (4454C>T), TAT349TAC (4457T>C), CTA353TTA (4467C>T), GAA354GAT (4472A>T), GTA358ATA (4482G>A), GAT362GAC (4496T>C), CTA365TTA (4503C>T), TAT366TAC (4508T>C), AGA368AAA (4513G>A), ACA369ATA (4516C>T), TTC380TTT (4550C>T), GCA381ATA (4551G>A 4552C>T), CAT382GAT (4554C>G), AAC386AAT (4568C>T), TCT387TCA (4571T>A), GGT388GGA (4574T>A), AGC390AGG (4580C>G), AAA393CAG (4587A>C 4589A>G), ACC394AAA (4591C>A 4592C>A), AAA395AAG (4595A>G), GAA397GAC (4601A>C), ATT398ATA (4604T>A), ATG399AAT (4606T>A 4607G>T), CAG402GAA (4614C>G 4616G>A), ATA403ATT (4619A>T), TTA406CTC (4626T>C 4628A>C), ATA408ATG (4634A>G), CAA409ATA (4635C>A 4636A>T), ATA410ATT (4640A>T), GAT411TCA (4641G>T 4642A>C 4643T>A), GGA414GGC (4652A>C), ATA415ATT (4655A>T), AAA416GAA (4656A>G), ATG417TTA (4659A>T 4661G>A), CAT420CAC (4670T>C), GTA422TCA (4674G>T 4675T>C), CAA423AAA (4677C>A), AAA424AAG (4682A>G), ATA425ATC (4685A>C), ATC426TGT (4686A>T 4687T>G 4688C>T), AAT427GAA (4689A>G 4691T>A), CTT428TTT (4692C>T), GAT429TCA (4695G>T 4696A>C 4697T>A), GAA430GAT (4700A>T), AAC431AAA (4703C>A), ATT432CTA (4704A>C 4706T>A), GAT433AAC (4707G>A 4709T>C), AAG436CAA (4716A>C 4718G>A), AAA437GAA (4719A>G), TTA438ATC (4722T>A 4724A>C), TCA440AGG (4728T>A 4729C>G 4730A>G), CTA442CTT (4736A>T), ATA444TGT (4740A>T 4741T>G 4742A>T), GTA445TTA (4743G>T), AAC446AAT (4748C>T), CAA447TAC (4749C>T 4751A>C), GTA448GCA (4753T>C), AGG449AGT (4757G>T), TAT451TTT (4762A>T), CCT453CCA (4769T>A), AAA454GAC (4770A>G 4772A>C), TTA455CTA (4773T>C), GAA457AAA (4779G>A)								
putative transactivator factor (NP_569142.1)	180	397	52.2%	878	59.0%	218 (100%)	142 (65.1%)	0/0/1/1	2
Protein mutations:	Q183K (5821C>A), N184E (5824A>G 5826T>A), N186T (5831A>C 5832C>A), E187Q (5833G>C 5835A>G), H192Q (5850C>A), H193K (5851C>A 5853T>A), T195Q (5857A>C 5858C>A 5859A>G), L203Q (5881T>C 5882T>A), N205K (5889T>A), S207N (5894G>A), K209N (5901A>C), A212K (5908G>A 5909C>A 5910C>A), N216S (5921A>G 5922C>T), D221S (5935G>A 5936A>G 5937T>C), V223I (5941G>A), Q226Y (5950C>T 5952G>T), T227D (5953A>G 5954C>A 5955A>C), G228* (5956G>T), Q229E (5959C>G), A232S (5968G>T 5970C>A), N233T (5972A>C), Q243K (6001C>A), G250E (6023G>A 6024G>A), T251N (6026C>A 6027A>T), V255I (6037G>A), R256K (6040C>A 6041G>A), S259T (6049T>A 6051G>A), T261P (6055A>C), E267D (6075A>T), I272V (6088A>G), E283D (6123A>T), E288* (6136G>T), K289D (6139A>G 6141G>T), E291T (6145G>A 6146A>C), E292Q (6148G>C 6150A>G), E294P (6154G>C 6155A>C 6156A>G), E297P (6163G>C 6164A>C), V299E (6170T>A), N300E (6172A>G 6174T>G), P302L (6179C>T), E303N (6181G>A 6183A>T), I313L (6211A>C), S314A (6214T>G), L317I (6223C>A 6225A>T), A321G (6236C>G), L325I (6247C>A), Q327E (6253C>G 6255G>A), A329P (6259G>C), I330M (6264T>G), S332I (6269G>T 6270C>T), S335T (6277T>A), E337D (6285A>T), T339I (6290C>T), C345S (6308G>C 6309C>A), D347E (6315T>A), I348M (6318A>G), E350K (6322G>A), A351Q (6325G>C 6326C>A), E354K (6334G>A), L356A (6340C>G 6341T>C), V360I (6352G>A 6354C>A), L361M (6355T>A 6357A>G), S362T (6359G>C 6360T>A), K365N (6369A>T), P366L (6371C>T), Q368R (6376C>A 6377A>G), P369Q (6380C>A 6381T>A), T370P (6382A>C 6384A>T), R376K (6401G>A 6402G>A), R377K (6404G>A), P382D (6418C>G 6419C>A 6420A>T), D383E (6423T>A), L391Q (6446T>A), S393G (6451A>G 6453T>G), H394Q (6456C>A)								

	Begin	End	Coverage	Score	Concordance	Matches	Identities	I/D/M/F*	Stop Codons
**NT**	**2358**	**6465**	**21.4%**	**1834**	**55.7%**	**1658 (99.8%)**	**1293 (77.8%)**	**0/4**	
Codon mutations:	TCT181TCC (5817T>C), ACC182ACT (5820C>T), CAA183AAA (5821C>A), AAT184GAA (5824A>G 5826T>A), AAC186ACA (5831A>C 5832C>A), GAA187CAG (5833G>C 5835A>G), GAC188GAT (5838C>T), CAC192CAA (5850C>A), CAT193AAA (5851C>A 5853T>A), ACA195CAG (5857A>C 5858C>A 5859A>G), TAC197TAT (5865C>T), AAT198AAC (5868T>C), AAA199AAG (5871A>G), TTG200CTA (5872T>C 5874G>A), ATA201ATT (5877A>T), GCT202GCA (5880T>A), TTA203CAA (5881T>C 5882T>A), AAT205AAA (5889T>A), ACA206ACT (5892A>T), AGT207AAT (5894G>A), GCA208GCT (5898A>T), AAA209AAC (5901A>C), TTA210CTA (5902T>C), GTA211GTT (5907A>T), GCC212AAA (5908G>A 5909C>A 5910C>A), ACC213ACA (5913C>A), TGC214TGT (5916C>T), AAC216AGT (5921A>G 5922C>T), TAC217TAT (5925C>T), GAT221AGC (5935G>A 5936A>G 5937T>C), ACT222ACA (5940T>A), GTA223ATA (5941G>A), CAG226TAT (5950C>T 5952G>T), ACA227GAC (5953A>G 5954C>A 5955A>C), GGA228TGA (5956G>T), CAA229GAA (5959C>G), GAG230GAA (5964G>A), GCC232TCA (5968G>T 5970C>A), AAT233ACT (5972A>C), CCT235CCA (5979T>A), AAG239AAA (5991G>A), TTC241TTT (5997C>T), CAA243AAA (6001C>A), AAG245AAA (6009G>A), AGG246AGA (6012G>A), ATA247ATT (6015A>T), ACT248ACA (6018T>A), GGG250GAA (6023G>A 6024G>A), ACA251AAT (6026C>A 6027A>T), TTC253TTT (6033C>T), TAC254TAT (6036C>T), GTA255ATA (6037G>A), CGA256AAA (6040C>A 6041G>A), TTC257TTT (6045C>T), TCG259ACA (6049T>A 6051G>A), GCT260GCA (6054T>A), ACA261CCA (6055A>C), CTA265TTA (6067C>T), GAA267GAT (6075A>T), AAG270AAA (6084G>A), CCT271CCA (6087T>A), ATC272GTC (6088A>G), GTT275GTC (6099T>C), ATT278ATA (6108T>A), CTC280TTG (6112C>T 6114C>G), ACA281ACT (6117A>T), AGG282AGA (6120G>A), GAA283GAT (6123A>T), ATC285ATT (6129C>T), ATA286ATT (6132A>T), GAA288TAA (6136G>T), AAG289GAT (6139A>G 6141G>T), ATA290ATT (6144A>T), GAA291ACA (6145G>A 6146A>C), GAA292CAG (6148G>C 6150A>G), CAA293CAG (6153A>G), GAA294CCG (6154G>C 6155A>C 6156A>G), GAA297CCA (6163G>C 6164A>C), GTA299GAA (6170T>A), AAT300GAG (6172A>G 6174T>G), ATT301ATA (6177T>A), CCA302CTA (6179C>T), GAA303AAT (6181G>A 6183A>T), TTC304TTT (6186C>T), GCT306GCC (6192T>C), AGG309AGA (6201G>A), ATA310ATT (6204A>T), ATT311ATC (6207T>C), ATA313CTA (6211A>C), TCT314GCT (6214T>G), ACT315ACA (6219T>A), CTA317ATT (6223C>A 6225A>T), CTA320TTA (6232C>T), GCA321GGA (6236C>G), AAC322AAT (6240C>T), CTA325ATA (6247C>A), CAG327GAA (6253C>G 6255G>A), AAT328AAC (6258T>C), GCA329CCA (6259G>C), ATT330ATG (6264T>G), AGC332ATT (6269G>T 6270C>T), TCA335ACA (6277T>A), AGG336AGA (6282G>A), GAA337GAT (6285A>T), CAG338CAA (6288G>A), ACA339ATA (6290C>T), TGC345TCA (6308G>C 6309C>A), GAT347GAA (6315T>A), ATA348ATG (6318A>G), AGA349CGA (6319A>C), GAA350AAA (6322G>A), GCA351CAA (6325G>C 6326C>A), GAA354AAA (6334G>A), CTA356GCA (6340C>G 6341T>C), AGA357AGG (6345A>G), GTC360ATA (6352G>A 6354C>A), TTA361ATG (6355T>A 6357A>G), AGT362ACA (6359G>C 6360T>A), CTT363CTG (6363T>G), AAA365AAT (6369A>T), CCA366CTA (6371C>T), CAA368AGA (6376C>A 6377A>G), CCT369CAA (6380C>A 6381T>A), ACA370CCT (6382A>C 6384A>T), AGG376AAA (6401G>A 6402G>A), AGG377AAG (6404G>A), AAT378-GA (6406delA 6407A>G 6408T>A), TTC379TTT (6411C>T), ATC380ATT (6414C>T), TCC381TCG (6417C>G), CCA382GAT (6418C>G 6419C>A 6420A>T), GAT383GAA (6423T>A), CTG384TTA (6424C>T 6426G>A), TTA385CTA (6427T>C), CTA391CAA (6446T>A), AGT393GGG (6451A>G 6453T>G), CAC394CAA (6456C>A), TAT396TAC (6462T>C)								

*: Inserts / Deletes / Misaligned / Frameshifts

Analysis details

This analysis was performed with panviral2.64

NGS Details (UN18_val): Potato virus Y

Assembly

**Coverage Length**	1875 (7 contig(s))
**Depth Of Coverage**	3.1
**Number Of Reads**	45
**Reads Per Million**	0.84 rpm (after QC)
**Ambiguities**	0
**Assembly Method**	de novo + reference guided assembly
**Consensus Caller**	Bcf Tools

Coverage Map

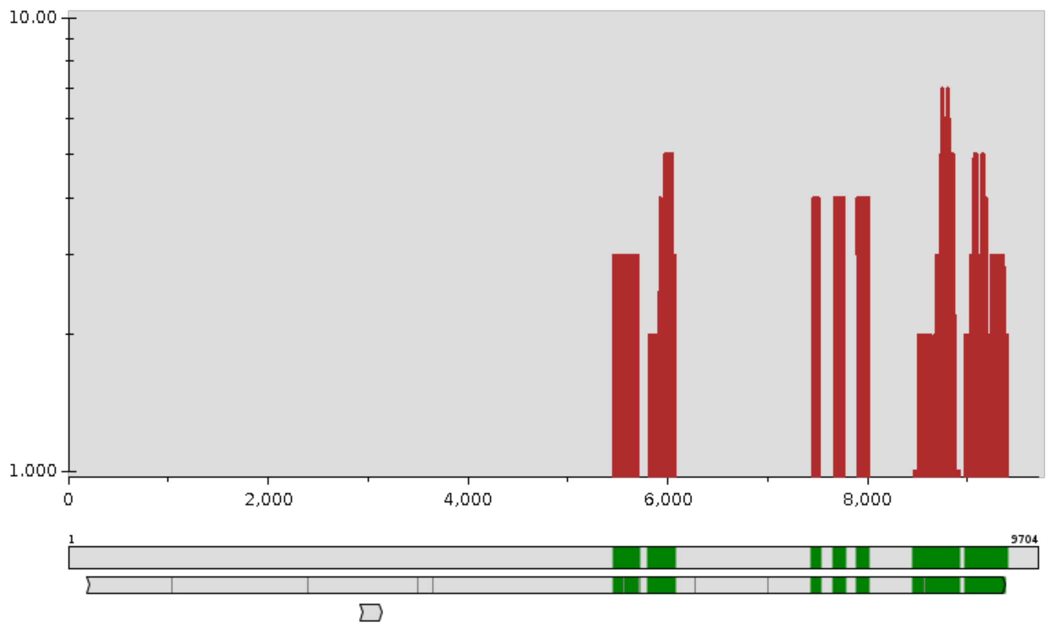

Assignment

**Type**	Potato virus Y (Taxonomy ID: 12216)
**Reference Genome**	NC_001616.1
**NT Identity (%)**	85.1634
**AA Identity (%)**	91.1475
**Number Of Stop Codons**	1
**Number Of CDS**	2

Alignment

**Alignment Score**	2600.0 (NT) + 3847.0 (AA) = 6447.0
**Concordance (%)**	82.2321

**Alignment Method**	Global, seeded, nucleotide + amino acids (AGA)

Genome Region

Sequence starts at position 5448 and ends at position 9414 relative to NC_001616.1 reference sequence.

Alignment Detailed Statistics

	Begin	End	Coverage	Score	Concordance	Matches	Identities	I/D/M/F*	Stop Codons
**NT**	**5448**	**9414**	**19.3%**	**2600**	**70.0%**	**1866 (99.5%)**	**1590 (84.8%)**	**1/9**	
Mutations:	5452T>C, 5458G>A, 5467T>C, 5470A>G, 5471C>T, 5473A>G, 5482C>T, 5485A>C, 5488T>C, 5489T>C, 5491G>A, 5497T>C, 5506T>C, 5509G>A, 5512G>A, 5516T>C, 5530T>C, 5533G>A, 5536G>T, 5539G>A, 5548T>G, 5554C>T, 5560T>A, 5561G>A, 5563G>A, 5566G>C, 5569A>G, 5582_5590delCTCAAGTTG, 5591A>C, 5593G>A, 5596G>A, 5597A>G, 5605C>A, 5617G>T, 5619C>T, 5620T>G, 5629G>A, 5630A>C, 5641C>T, 5650A>T, 5662A>T, 5665A>G, 5689A>G, 5693G>A, 5698G>A, 5713A>G, 5716G>C, 5719A>G, 5803C>T, 5836G>A, 5839A>G, 5845A>G, 5848A>G, 5857T>C, 5860C>T, 5863A>T, 5869T>A, 5875T>C, 5884C>T, 5893C>T, 5894A>G, 5896C>G, 5899C>T, 5908G>A, 5911T>C, 5923G>A, 5938A>G, 5941C>T, 5944T>C, 5947T>C, 5959G>A, 5960C>G, 5961G>C, 5962G>C, 5974A>G, 5986T>A, 5989C>T, 5990A>G, 5998T>C, 6008A>C, 6010A>T, 6019A>T, 6022G>T, 6025A>T, 6030A>C, 6049C>A, 6058G>A, 6061A>G, 6064C>A, 6070T>C, 6072G>C, 6073T>C, 6079G>A, 6081C>A, 6085A>T, 7432T>C, 7441T>C, 7459G>A, 7462A>G, 7468A>T, 7471C>A, 7477A>G, 7480C>T, 7486G>T, 7495T>C, 7498A>G, 7501T>C, 7503T>C, 7504G>A, 7513T>A, 7519C>T, 7522A>G, 7531G>A, 7666A>G, 7672T>C, 7678A>T, 7684G>A, 7690T>C, 7696T>C, 7702T>G, 7709G>A, 7713A>G, 7714A>G, 7715C>T, 7726T>C, 7735G>A, 7744A>G, 7747C>T, 7762C>T, 7768G>A, 7771T>C, 7780A>G, 7783A>C, 7894A>T, 7897T>C, 7921C>T, 7927G>A, 7936T>C, 7939T>C, 7942T>C, 7966G>A, 7969T>C, 7975T>C, 7987C>T, 7990T>C, 8005T>C, 8458A>G, 8460G>A, 8464A>G, 8467G>A, 8480G>A, 8482T>A, 8497G>A, 8498C>T, 8500A>G, 8502G>A, 8503A>G, 8506C>T, 8515A>T, 8524C>T, 8527A>C, 8528T>C, 8530A>G, 8539G>C, 8546C>T, 8547T>G, 8552T>A, 8563A>G, 8574C>G, 8585A>G, 8598G>A, 8602C>T, 8603A>T, 8604A>C, 8605C>T, 8620A>G, 8622C>A, 8625A>G, 8626G>A, 8629G>A, 8632C>T, 8638C>T, 8641G>A, 8647C>T, 8648C>T, 8649C>T, 8650G>C, 8653C>T, 8656A>G, 8658G>A, 8664A>C, 8665T>G, 8674T>G, 8679C>T, 8680A>T, 8683C>A, 8692G>A, 8695A>T, 8698T>C, 8707G>A, 8708A>C, 8716G>A, 8722C>T, 8725G>A, 8731A>G, 8737A>G, 8743C>T, 8745C>A, 8746A>G, 8749C>T, 8752G>A, 8755A>T, 8770C>T, 8771T>C, 8776A>G, 8779T>C, 8782G>A, 8791T>C, 8797A>G, 8800A>G, 8803A>G, 8809T>C, 8812T>C, 8818T>C, 8824G>A, 8827A>G, 8848G>A, 8854T>C, 8856A>G, 8857G>A, 8863G>A, 8865G>A, 8867A>C, 8869G>T, 8872A>G, 8883G>A, 8888A>T, 8929C>T, 8983G>A, 8984A>G, 8986T>C, 8988A>T, 8998G>A, 9004G>A, 9007G>A, 9016C>T, 9037C>A, 9085A>T, 9097C>T, 9131A>G, 9136A>T, 9145G>T, 9149A>G, 9150T>G, 9151G>A, 9152G>A, 9157A>G, 9160G>T, 9163T>C, 9184G>A, 9196A>G, 9214G>A, 9217A>G, 9220G>A, 9244A>T, 9247G>A, 9256C>T, 9260C>T, 9262T>C, 9274G>A, 9280C>T, 9289C>T, 9304G>A, 9334C>T, 9364C>G, 9377_9378insT, 9387C>T, 9404G>A								

CDS

	Begin	End	Coverage	Score	Concordance	Matches	Identities	I/D/M/F*	Stop Codons
PVYgp1	1756	3064	20.0%	3847	91.5%	610 (99.5%)	556 (90.7%)	0/3/0/0	1
Protein mutations:	A1793T (5561G>A 5563G>A), L1800_L1802del (5582_5590delCTCAAGTTG), K1803Q (5591A>C 5593G>A), I1805V (5597A>G), N1807K (5605C>A), A1812V (5619C>T 5620T>G), I1816L (5630A>C), V1837I (5693G>A), I1904V (5894A>G 5896C>G), R1926A (5960C>G 5961G>C 5962G>C), I1936V (5990A>G), E1945D (6019A>T), K1949T (6030A>C), D1955E (6049C>A), M1958I (6058G>A), S1963T (6072G>C 6073T>C), T1966N (6081C>A), L2440S (7503T>C 7504G>A), D2509N (7709G>A), K2510R (7713A>G 7714A>G), R2759K (8460G>A), A2766T (8480G>A 8482T>A), R2773K (8502G>A 8503A>G), E2777D (8515A>T), E2785D (8539G>C), L2788C (8546C>T 8547T>G), S2790T (8552T>A), A2797G (8574C>G), I2801V (8585A>G), G2805E (8598G>A), N2807S (8603A>T 8604A>C 8605C>T), P2813Q (8622C>A), E2814G (8625A>G 8626G>A), P2822F (8648C>T 8649C>T 8650G>C), G2825E (8658G>A), D2827A (8664A>C 8665T>G), A2832V (8679C>T 8680A>T), T2854K (8745C>A 8746A>G), E2891G (8856A>G 8857G>A), R2894Q (8865G>A), M2895L (8867A>C 8869G>T), G2900E (8883G>A), T2902S (8888A>T), N2934D (8984A>G 8986T>C), E2935V (8988A>T), I2983V (9131A>G), M2989G (9149A>G 9150T>G 9151G>A), G2990S (9152G>A), P3026S (9260C>T 9262T>C)								
Codon mutations:	CTT1756CTC (5452T>C), AAG1758AAA (5458G>A), AGT1761AGC (5467T>C), CAA1762CAG (5470A>G), CTA1763TTG (5471C>T 5473A>G), TTC1766TTT (5482C>T), TCA1767TCC (5485A>C), AAT1768AAC (5488T>C), TTG1769CTA (5489T>C 5491G>A), GGT1771GGC (5497T>C), TGT1774TGC (5506T>C), GAG1775GAA (5509G>A), GAG1776GAA (5512G>A), TTA1778CTA (5516T>C), TAT1782TAC (5530T>C), GAG1783GAA (5533G>A), TCG1784TCT (5536G>T), TTG1785TTA (5539G>A), GTT1788GTG (5548T>G), CAC1790CAT (5554C>T), GCT1792GCA (5560T>A), GCG1793ACA (5561G>A 5563G>A), ACG1794ACC (5566G>C), TCA1795TCG (5569A>G), CTC1800_TTG1802del (5582_5590delCTCAAGTTG), AAG1803CAA (5591A>C 5593G>A), GGG1804GGA (5596G>A), ATT1805GTT (5597A>G), AAC1807AAA (5605C>A), GTG1811GTT (5617G>T), GCT1812GTG (5619C>T 5620T>G), TTG1815TTA (5629G>A), ATC1816CTC (5630A>C), GGC1819GGT (5641C>T), GCA1822GCT (5650A>T), ATA1826ATT (5662A>T), GGA1827GGG (5665A>G), CAA1835CAG (5689A>G), GTT1837ATT (5693G>A), GAG1838GAA (5698G>A), CAA1843CAG (5713A>G), GGG1844GGC (5716G>C), AAA1845AAG (5719A>G), GAC1873GAT (5803C>T), AGG1884AGA (5836G>A), AAA1885AAG (5839A>G), GGA1887GGG (5845A>G), AAA1888AAG (5848A>G), GGT1891GGC (5857T>C), ACC1892ACT (5860C>T), ACA1893ACT (5863A>T), GGT1895GGA (5869T>A), GGT1897GGC (5875T>C), AGC1900AGT (5884C>T), TTC1903TTT (5893C>T), ATC1904GTG (5894A>G 5896C>G), AAC1905AAT (5899C>T), GGG1908GGA (5908G>A), TTT1909TTC (5911T>C), GAG1913GAA (5923G>A), CAA1918CAG (5938A>G), TTC1919TTT (5941C>T), GTT1920GTC (5944T>C), GAT1921GAC (5947T>C), GGG1925GGA (5959G>A), CGG1926GCC (5960C>G 5961G>C 5962G>C), GAA1930GAG (5974A>G), GCT1934GCA (5986T>A), GAC1935GAT (5989C>T), ATT1936GTT (5990A>G), GAT1938GAC (5998T>C), AGA1942CGT (6008A>C 6010A>T), GAA1945GAT (6019A>T), GTG1946GTT (6022G>T), CGA1947CGT (6025A>T), AAA1949ACA (6030A>C), GAC1955GAA (6049C>A), ATG1958ATA (6058G>A), CAA1959CAG (6061A>G), GCC1960GCA (6064C>A), GGT1962GGC (6070T>C), AGT1963ACC (6072G>C 6073T>C), ACG1965ACA (6079G>A), ACC1966AAC (6081C>A), ATA1967ATT (6085A>T), TAT2416..C (7432T>C), CAT2419CAC (7441T>C), AAG2425AAA (7459G>A), GAA2426GAG (7462A>G), ATA2428ATT (7468A>T), GTC2429GTA (7471C>A), CAA2431CAG (7477A>G), AGC2432AGT (7480C>T), CTG2434CTT (7486G>T), TAT2437TAC (7495T>C), AAA2438AAG (7498A>G), GGT2439GGC (7501T>C), TTG2440TCA (7503T>C 7504G>A), ATT2443ATA (7513T>A), AAC2445AAT (7519C>T), GGA2446GGG (7522A>G), AAG2449AAA (7531G>A), GAA2494GAG (7666A>G), TGT2496TGC (7672T>C), ACA2498ACT (7678A>T), GGG2500GGA (7684G>A), ACT2502ACC (7690T>C), TTT2504TTC (7696T>C), GGT2506GGG (7702T>G), GAT2509AAT (7709G>A), AAA2510AGG (7713A>G 7714A>G), CTG2511TTG (7715C>T), CGT2514CGC (7726T>C), GAG2517GAA (7735G>A), GTA2520GTG (7744A>G), TAC2521TAT (7747C>T), GGC2526GGT (7762C>T), CAG2528CAA (7768G>A), TTT2529TTC (7771T>C), TCA2532TCG (7780A>G), CTA2533CTC (7783A>C), CCA2570CCT (7894A>T), ATT2571ATC (7897T>C), GTC2579GTT (7921C>T), AAG2581AAA (7927G>A), GGT2584GGC (7936T>C), AAT2585AAC (7939T>C), AAT2586AAC (7942T>C), GTG2594GTA (7966G>A), GAT2595GAC (7969T>C), TCT2597TCC (7975T>C), GTC2601GTT (7987C>T), CTT2602CTC (7990T>C), GCT2607GCC (8005T>C), CTA2758CTG (8458A>G), AGG2759AAG (8460G>A), AAA2760AAG (8464A>G), CTG2761CTA (8467G>A), GCT2766ACA (8480G>A 8482T>A), GAG2771GAA (8497G>A), CTA2772TTG (8498C>T 8500A>G), AGA2773AAG (8502G>A 8503A>G), GCC2774GCT (8506C>T), GAA2777GAT (8515A>T), GTC2780GTT (8524C>T), GCA2781GCC (8527A>C), TTA2782CTG (8528T>C 8530A>G), GAG2785GAC (8539G>C), CTT2788TGT (8546C>T 8547T>G), TCT2790ACT (8552T>A), GTA2793GTG (8563A>G), GCA2797GGA (8574C>G), ATT2801GTT (8585A>G), GGA2805GAA (8598G>A), AGC2806AGT (8602C>T), AAC2807TCT (8603A>T 8604A>C 8605C>T), AAA2812AAG (8620A>G), CCA2813CAA (8622C>A), GAG2814GGA (8625A>G 8626G>A), CAG2815CAA (8629G>A), GGC2816GGT (8632C>T), ATC2818ATT (8638C>T), CAG2819CAA (8641G>A), AAC2821AAT (8647C>T), CCG2822TTC (8648C>T 8649C>T 8650G>C), AAC2823AAT (8653C>T), AAA2824AAG (8656A>G), GGA2825GAA (8658G>A), GAT2827GCG (8664A>C 8665T>G), GTT2830GTG (8674T>G), GCA2832GTT (8679C>T 8680A>T), GGC2833GGA (8683C>A), GGG2836GGA (8692G>A), ACA2837ACT (8695A>T), CAT2838CAC (8698T>C), CCG2841CCA (8707G>A), AGA2842CGA (8708A>C), AAG2844AAA (8716G>A), ATC2846ATT (8722C>T), ACG2847ACA (8725G>A), AAA2849AAG (8731A>G), AGA2851AGG (8737A>G), CCC2853CCT (8743C>T), ACA2854AAG (8745C>A 8746A>G), AGC2855AGT (8749C>T), AAG2856AAA (8752G>A), GGA2857GGT (8755A>T), AAC2862AAT (8770C>T), TTA2863CTA (8771T>C), GAA2864GAG (8776A>G), CAT2865CAC (8779T>C), TTG2866TTA (8782G>A), TAT2869TAC (8791T>C), CCA2871CCG (8797A>G), CAA2872CAG (8800A>G), CAA2873CAG (8803A>G), GAT2875GAC (8809T>C), ATT2876ATC (8812T>C), AAT2878AAC (8818T>C), CGG2880CGA (8824G>A), GCA2881GCG (8827A>G), ACG2888ACA (8848G>A), TAT2890TAC (8854T>C), GAG2891GGA (8856A>G 8857G>A), GTG2893GTA (8863G>A), CGG2894CAG (8865G>A), ATG2895CTT (8867A>C 8869G>T), GCA2896GCG (8872A>G), GGA2900GAA (8883G>A), ACT2902TCT (8888A>T), TGC2915TGT (8929C>T), GGG2933GGA (8983G>A), AAT2934GAC (8984A>G 8986T>C), GAA2935GTA (8988A>T), GAG2938GAA (8998G>A), CCG2940CCA (9004G>A), TTG2941TTA (9007G>A), ATC2944ATT (9016C>T), ACC2951ACA (9037C>A), ATA2967ATT (9085A>T), AAC2971AAT (9097C>T), ATT2983GTT (9131A>G), CGA2984CGT (9136A>T), CGG2987CGT (9145G>T), ATG2989GGA (9149A>G 9150T>G 9151G>A), GGT2990AGT (9152G>A), TTA2991TTG (9157A>G), GCG2992GCT (9160G>T), CGT2993CGC (9163T>C), GAG3000GAA (9184G>A), CGA3004CGG (9196A>G), AGG3010AGA (9214G>A), GAA3011GAG (9217A>G), GCG3012GCA (9220G>A), GCA3020GCT (9244A>T), TTG3021TTA (9247G>A), GCC3024GCT (9256C>T), CCT3026TCC (9260C>T 9262T>C), GGG3030GGA (9274G>A), GAC3032GAT (9280C>T), ATC3035ATT (9289C>T), GAG3040GAA (9304G>A), GTC3050GTT (9334C>T), GTC3060GTG (9364C>G)								

Proteins

	Begin	End	Coverage	Score	Concordance	Matches	Identities	I/D/M/F*	Stop Codons
polyprotein (NP_056759.1)	1756	3064	20.0%	3847	91.5%	610 (99.5%)	556 (90.7%)	0/3/0/0	1
Protein mutations:	A1793T (5561G>A 5563G>A), L1800_L1802del (5582_5590delCTCAAGTTG), K1803Q (5591A>C 5593G>A), I1805V (5597A>G), N1807K (5605C>A), A1812V (5619C>T 5620T>G), I1816L (5630A>C), V1837I (5693G>A), I1904V (5894A>G 5896C>G), R1926A (5960C>G 5961G>C 5962G>C), I1936V (5990A>G), E1945D (6019A>T), K1949T (6030A>C), D1955E (6049C>A), M1958I (6058G>A), S1963T (6072G>C 6073T>C), T1966N (6081C>A), L2440S (7503T>C 7504G>A), D2509N (7709G>A), K2510R (7713A>G 7714A>G), R2759K (8460G>A), A2766T (8480G>A 8482T>A), R2773K (8502G>A 8503A>G), E2777D (8515A>T), E2785D (8539G>C), L2788C (8546C>T 8547T>G), S2790T (8552T>A), A2797G (8574C>G), I2801V (8585A>G), G2805E (8598G>A), N2807S (8603A>T 8604A>C 8605C>T), P2813Q (8622C>A), E2814G (8625A>G 8626G>A), P2822F (8648C>T 8649C>T 8650G>C), G2825E (8658G>A), D2827A (8664A>C 8665T>G), A2832V (8679C>T 8680A>T), T2854K (8745C>A 8746A>G), E2891G (8856A>G 8857G>A), R2894Q (8865G>A), M2895L (8867A>C 8869G>T), G2900E (8883G>A), T2902S (8888A>T), N2934D (8984A>G 8986T>C), E2935V (8988A>T), I2983V (9131A>G), M2989G (9149A>G 9150T>G 9151G>A), G2990S (9152G>A), P3026S (9260C>T 9262T>C)								

	Begin	End	Coverage	Score	Concordance	Matches	Identities	I/D/M/F*	Stop Codons
**NT**	**5448**	**9414**	**19.3%**	**2600**	**70.0%**	**1866 (99.5%)**	**1590 (84.8%)**	**1/9**	
Codon mutations:	CTT1756CTC (5452T>C), AAG1758AAA (5458G>A), AGT1761AGC (5467T>C), CAA1762CAG (5470A>G), CTA1763TTG (5471C>T 5473A>G), TTC1766TTT (5482C>T), TCA1767TCC (5485A>C), AAT1768AAC (5488T>C), TTG1769CTA (5489T>C 5491G>A), GGT1771GGC (5497T>C), TGT1774TGC (5506T>C), GAG1775GAA (5509G>A), GAG1776GAA (5512G>A), TTA1778CTA (5516T>C), TAT1782TAC (5530T>C), GAG1783GAA (5533G>A), TCG1784TCT (5536G>T), TTG1785TTA (5539G>A), GTT1788GTG (5548T>G), CAC1790CAT (5554C>T), GCT1792GCA (5560T>A), GCG1793ACA (5561G>A 5563G>A), ACG1794ACC (5566G>C), TCA1795TCG (5569A>G), CTC1800_TTG1802del (5582_5590delCTCAAGTTG), AAG1803CAA (5591A>C 5593G>A), GGG1804GGA (5596G>A), ATT1805GTT (5597A>G), AAC1807AAA (5605C>A), GTG1811GTT (5617G>T), GCT1812GTG (5619C>T 5620T>G), TTG1815TTA (5629G>A), ATC1816CTC (5630A>C), GGC1819GGT (5641C>T), GCA1822GCT (5650A>T), ATA1826ATT (5662A>T), GGA1827GGG (5665A>G), CAA1835CAG (5689A>G), GTT1837ATT (5693G>A), GAG1838GAA (5698G>A), CAA1843CAG (5713A>G), GGG1844GGC (5716G>C), AAA1845AAG (5719A>G), GAC1873GAT (5803C>T), AGG1884AGA (5836G>A), AAA1885AAG (5839A>G), GGA1887GGG (5845A>G), AAA1888AAG (5848A>G), GGT1891GGC (5857T>C), ACC1892ACT (5860C>T), ACA1893ACT (5863A>T), GGT1895GGA (5869T>A), GGT1897GGC (5875T>C), AGC1900AGT (5884C>T), TTC1903TTT (5893C>T), ATC1904GTG (5894A>G 5896C>G), AAC1905AAT (5899C>T), GGG1908GGA (5908G>A), TTT1909TTC (5911T>C), GAG1913GAA (5923G>A), CAA1918CAG (5938A>G), TTC1919TTT (5941C>T), GTT1920GTC (5944T>C), GAT1921GAC (5947T>C), GGG1925GGA (5959G>A), CGG1926GCC (5960C>G 5961G>C 5962G>C), GAA1930GAG (5974A>G), GCT1934GCA (5986T>A), GAC1935GAT (5989C>T), ATT1936GTT (5990A>G), GAT1938GAC (5998T>C), AGA1942CGT (6008A>C 6010A>T), GAA1945GAT (6019A>T), GTG1946GTT (6022G>T), CGA1947CGT (6025A>T), AAA1949ACA (6030A>C), GAC1955GAA (6049C>A), ATG1958ATA (6058G>A), CAA1959CAG (6061A>G), GCC1960GCA (6064C>A), GGT1962GGC (6070T>C), AGT1963ACC (6072G>C 6073T>C), ACG1965ACA (6079G>A), ACC1966AAC (6081C>A), ATA1967ATT (6085A>T), TAT2416..C (7432T>C), CAT2419CAC (7441T>C), AAG2425AAA (7459G>A), GAA2426GAG (7462A>G), ATA2428ATT (7468A>T), GTC2429GTA (7471C>A), CAA2431CAG (7477A>G), AGC2432AGT (7480C>T), CTG2434CTT (7486G>T), TAT2437TAC (7495T>C), AAA2438AAG (7498A>G), GGT2439GGC (7501T>C), TTG2440TCA (7503T>C 7504G>A), ATT2443ATA (7513T>A), AAC2445AAT (7519C>T), GGA2446GGG (7522A>G), AAG2449AAA (7531G>A), GAA2494GAG (7666A>G), TGT2496TGC (7672T>C), ACA2498ACT (7678A>T), GGG2500GGA (7684G>A), ACT2502ACC (7690T>C), TTT2504TTC (7696T>C), GGT2506GGG (7702T>G), GAT2509AAT (7709G>A), AAA2510AGG (7713A>G 7714A>G), CTG2511TTG (7715C>T), CGT2514CGC (7726T>C), GAG2517GAA (7735G>A), GTA2520GTG (7744A>G), TAC2521TAT (7747C>T), GGC2526GGT (7762C>T), CAG2528CAA (7768G>A), TTT2529TTC (7771T>C), TCA2532TCG (7780A>G), CTA2533CTC (7783A>C), CCA2570CCT (7894A>T), ATT2571ATC (7897T>C), GTC2579GTT (7921C>T), AAG2581AAA (7927G>A), GGT2584GGC (7936T>C), AAT2585AAC (7939T>C), AAT2586AAC (7942T>C), GTG2594GTA (7966G>A), GAT2595GAC (7969T>C), TCT2597TCC (7975T>C), GTC2601GTT (7987C>T), CTT2602CTC (7990T>C), GCT2607GCC (8005T>C), CTA2758CTG (8458A>G), AGG2759AAG (8460G>A), AAA2760AAG (8464A>G), CTG2761CTA (8467G>A), GCT2766ACA (8480G>A 8482T>A), GAG2771GAA (8497G>A), CTA2772TTG (8498C>T 8500A>G), AGA2773AAG (8502G>A 8503A>G), GCC2774GCT (8506C>T), GAA2777GAT (8515A>T), GTC2780GTT (8524C>T), GCA2781GCC (8527A>C), TTA2782CTG (8528T>C 8530A>G), GAG2785GAC (8539G>C), CTT2788TGT (8546C>T 8547T>G), TCT2790ACT (8552T>A), GTA2793GTG (8563A>G), GCA2797GGA (8574C>G), ATT2801GTT (8585A>G), GGA2805GAA (8598G>A), AGC2806AGT (8602C>T), AAC2807TCT (8603A>T 8604A>C 8605C>T), AAA2812AAG (8620A>G), CCA2813CAA (8622C>A), GAG2814GGA (8625A>G 8626G>A), CAG2815CAA (8629G>A), GGC2816GGT (8632C>T), ATC2818ATT (8638C>T), CAG2819CAA (8641G>A), AAC2821AAT (8647C>T), CCG2822TTC (8648C>T 8649C>T 8650G>C), AAC2823AAT (8653C>T), AAA2824AAG (8656A>G), GGA2825GAA (8658G>A), GAT2827GCG (8664A>C 8665T>G), GTT2830GTG (8674T>G), GCA2832GTT (8679C>T 8680A>T), GGC2833GGA (8683C>A), GGG2836GGA (8692G>A), ACA2837ACT (8695A>T), CAT2838CAC (8698T>C), CCG2841CCA (8707G>A), AGA2842CGA (8708A>C), AAG2844AAA (8716G>A), ATC2846ATT (8722C>T), ACG2847ACA (8725G>A), AAA2849AAG (8731A>G), AGA2851AGG (8737A>G), CCC2853CCT (8743C>T), ACA2854AAG (8745C>A 8746A>G), AGC2855AGT (8749C>T), AAG2856AAA (8752G>A), GGA2857GGT (8755A>T), AAC2862AAT (8770C>T), TTA2863CTA (8771T>C), GAA2864GAG (8776A>G), CAT2865CAC (8779T>C), TTG2866TTA (8782G>A), TAT2869TAC (8791T>C), CCA2871CCG (8797A>G), CAA2872CAG (8800A>G), CAA2873CAG (8803A>G), GAT2875GAC (8809T>C), ATT2876ATC (8812T>C), AAT2878AAC (8818T>C), CGG2880CGA (8824G>A), GCA2881GCG (8827A>G), ACG2888ACA (8848G>A), TAT2890TAC (8854T>C), GAG2891GGA (8856A>G 8857G>A), GTG2893GTA (8863G>A), CGG2894CAG (8865G>A), ATG2895CTT (8867A>C 8869G>T), GCA2896GCG (8872A>G), GGA2900GAA (8883G>A), ACT2902TCT (8888A>T), TGC2915TGT (8929C>T), GGG2933GGA (8983G>A), AAT2934GAC (8984A>G 8986T>C), GAA2935GTA (8988A>T), GAG2938GAA (8998G>A), CCG2940CCA (9004G>A), TTG2941TTA (9007G>A), ATC2944ATT (9016C>T), ACC2951ACA (9037C>A), ATA2967ATT (9085A>T), AAC2971AAT (9097C>T), ATT2983GTT (9131A>G), CGA2984CGT (9136A>T), CGG2987CGT (9145G>T), ATG2989GGA (9149A>G 9150T>G 9151G>A), GGT2990AGT (9152G>A), TTA2991TTG (9157A>G), GCG2992GCT (9160G>T), CGT2993CGC (9163T>C), GAG3000GAA (9184G>A), CGA3004CGG (9196A>G), AGG3010AGA (9214G>A), GAA3011GAG (9217A>G), GCG3012GCA (9220G>A), GCA3020GCT (9244A>T), TTG3021TTA (9247G>A), GCC3024GCT (9256C>T), CCT3026TCC (9260C>T 9262T>C), GGG3030GGA (9274G>A), GAC3032GAT (9280C>T), ATC3035ATT (9289C>T), GAG3040GAA (9304G>A), GTC3050GTT (9334C>T), GTC3060GTG (9364C>G)								
CI protein (NP_734246.1)	599	634	5.7%	245	98.4%	36 (100%)	36 (100%)	0/0/0/0	0
Protein mutations:	*none*								
Codon mutations:	CTT599CTC (5452T>C), AAG601AAA (5458G>A), AGT604AGC (5467T>C), CAA605CAG (5470A>G), CTA606TTG (5471C>T 5473A>G), TTC609TTT (5482C>T), TCA610TCC (5485A>C), AAT611AAC (5488T>C), TTG612CTA (5489T>C 5491G>A), GGT614GGC (5497T>C), TGT617TGC (5506T>C), GAG618GAA (5509G>A), GAG619GAA (5512G>A), TTA621CTA (5516T>C), TAT625TAC (5530T>C), GAG626GAA (5533G>A), TCG627TCT (5536G>T), TTG628TTA (5539G>A), GTT631GTG (5548T>G), CAC633CAT (5554C>T)								
6K2 protein (NP_734247.1)	1	52	100%	277	91.7%	49 (94.2%)	42 (80.8%)	0/3/0/0	0
Protein mutations:	A2T (5561G>A 5563G>A), L9_L11del (5582_5590delCTCAAGTTG), K12Q (5591A>C 5593G>A), I14V (5597A>G), N16K (5605C>A), A21V (5619C>T 5620T>G), I25L (5630A>C), V46I (5693G>A)								
Codon mutations:	GCT1GCA (5560T>A), GCG2ACA (5561G>A 5563G>A), ACG3ACC (5566G>C), TCA4TCG (5569A>G), CTC9_TTG11del (5582_5590delCTCAAGTTG), AAG12CAA (5591A>C 5593G>A), GGG13GGA (5596G>A), ATT14GTT (5597A>G), AAC16AAA (5605C>A), GTG20GTT (5617G>T), GCT21GTG (5619C>T 5620T>G), TTG24TTA (5629G>A), ATC25CTC (5630A>C), GGC28GGT (5641C>T), GCA31GCT (5650A>T), ATA35ATT (5662A>T), GGA36GGG (5665A>G), CAA44CAG (5689A>G), GTT46ATT (5693G>A), GAG47GAA (5698G>A), CAA52CAG (5713A>G)								
NIa-VPg protein (NP_734252.1)	1	125	54.3%	631	91.2%	102 (100%)	91 (89.2%)	0/0/0/0	0
Protein mutations:	I61V (5894A>G 5896C>G), R83A (5960C>G 5961G>C 5962G>C), I93V (5990A>G), E102D (6019A>T), K106T (6030A>C), D112E (6049C>A), M115I (6058G>A), S120T (6072G>C 6073T>C), T123N (6081C>A)								
Codon mutations:	GGG1GGC (5716G>C), AAA2AAG (5719A>G), GAC30GAT (5803C>T), AGG41AGA (5836G>A), AAA42AAG (5839A>G), GGA44GGG (5845A>G), AAA45AAG (5848A>G), GGT48GGC (5857T>C), ACC49ACT (5860C>T), ACA50ACT (5863A>T), GGT52GGA (5869T>A), GGT54GGC (5875T>C), AGC57AGT (5884C>T), TTC60TTT (5893C>T), ATC61GTG (5894A>G 5896C>G), AAC62AAT (5899C>T), GGG65GGA (5908G>A), TTT66TTC (5911T>C), GAG70GAA (5923G>A), CAA75CAG (5938A>G), TTC76TTT (5941C>T), GTT77GTC (5944T>C), GAT78GAC (5947T>C), GGG82GGA (5959G>A), CGG83GCC (5960C>G 5961G>C 5962G>C), GAA87GAG (5974A>G), GCT91GCA (5986T>A), GAC92GAT (5989C>T), ATT93GTT (5990A>G), GAT95GAC (5998T>C), AGA99CGT (6008A>C 6010A>T), GAA102GAT (6019A>T), GTG103GTT (6022G>T), CGA104CGT (6025A>T), AAA106ACA (6030A>C), GAC112GAA (6049C>A), ATG115ATA (6058G>A), CAA116CAG (6061A>G), GCC117GCA (6064C>A), GGT119GGC (6070T>C), AGT120ACC (6072G>C 6073T>C), ACG122ACA (6079G>A), ACC123AAC (6081C>A), ATA124ATT (6085A>T)								
NIb protein (NP_734249.1)	142	521	31.7%	1109	92.3%	165 (100%)	152 (92.1%)	0/0/0/0	0
Protein mutations:	L165S (7503T>C 7504G>A), D234N (7709G>A), K235R (7713A>G 7714A>G), R484K (8460G>A), A491T (8480G>A 8482T>A), R498K (8502G>A 8503A>G), E502D (8515A>T), E510D (8539G>C), L513C (8546C>T 8547T>G), S515T (8552T>A)								
Codon mutations:	TAT141..C (7432T>C), CAT144CAC (7441T>C), AAG150AAA (7459G>A), GAA151GAG (7462A>G), ATA153ATT (7468A>T), GTC154GTA (7471C>A), CAA156CAG (7477A>G), AGC157AGT (7480C>T), CTG159CTT (7486G>T), TAT162TAC (7495T>C), AAA163AAG (7498A>G), GGT164GGC (7501T>C), TTG165TCA (7503T>C 7504G>A), ATT168ATA (7513T>A), AAC170AAT (7519C>T), GGA171GGG (7522A>G), AAG174AAA (7531G>A), GAA219GAG (7666A>G), TGT221TGC (7672T>C), ACA223ACT (7678A>T), GGG225GGA (7684G>A), ACT227ACC (7690T>C), TTT229TTC (7696T>C), GGT231GGG (7702T>G), GAT234AAT (7709G>A), AAA235AGG (7713A>G 7714A>G), CTG236TTG (7715C>T), CGT239CGC (7726T>C), GAG242GAA (7735G>A), GTA245GTG (7744A>G), TAC246TAT (7747C>T), GGC251GGT (7762C>T), CAG253CAA (7768G>A), TTT254TTC (7771T>C), TCA257TCG (7780A>G), CTA258CTC (7783A>C), CCA295CCT (7894A>T), ATT296ATC (7897T>C), GTC304GTT (7921C>T), AAG306AAA (7927G>A), GGT309GGC (7936T>C), AAT310AAC (7939T>C), AAT311AAC (7942T>C), GTG319GTA (7966G>A), GAT320GAC (7969T>C), TCT322TCC (7975T>C), GTC326GTT (7987C>T), CTT327CTC (7990T>C), GCT332GCC (8005T>C), CTA483CTG (8458A>G), AGG484AAG (8460G>A), AAA485AAG (8464A>G), CTG486CTA (8467G>A), GCT491ACA (8480G>A 8482T>A), GAG496GAA (8497G>A), CTA497TTG (8498C>T 8500A>G), AGA498AAG (8502G>A 8503A>G), GCC499GCT (8506C>T), GAA502GAT (8515A>T), GTC505GTT (8524C>T), GCA506GCC (8527A>C), TTA507CTG (8528T>C 8530A>G), GAG510GAC (8539G>C), CTT513TGT (8546C>T 8547T>G), TCT515ACT (8552T>A), GTA518GTG (8563A>G)								
coat protein (NP_734250.1)	1	267	96.3%	1584	90.1%	257 (100%)	234 (91.1%)	0/0/0/0	0
Protein mutations:	A1G (8574C>G), I5V (8585A>G), G9E (8598G>A), N11S (8603A>T 8604A>C 8605C>T), P17Q (8622C>A), E18G (8625A>G 8626G>A), P26F (8648C>T 8649C>T 8650G>C), G29E (8658G>A), D31A (8664A>C 8665T>G), A36V (8679C>T 8680A>T), T58K (8745C>A 8746A>G), E95G (8856A>G 8857G>A), R98Q (8865G>A), M99L (8867A>C 8869G>T), G104E (8883G>A), T106S (8888A>T), N138D (8984A>G 8986T>C), E139V (8988A>T), I187V (9131A>G), M193G (9149A>G 9150T>G 9151G>A), G194S (9152G>A), P230S (9260C>T 9262T>C)								

	Begin	End	Coverage	Score	Concordance	Matches	Identities	I/D/M/F*	Stop Codons
**NT**	**5448**	**9414**	**19.3%**	**2600**	**70.0%**	**1866 (99.5%)**	**1590 (84.8%)**	**1/9**	
Codon mutations:	GCA1GGA (8574C>G), ATT5GTT (8585A>G), GGA9GAA (8598G>A), AGC10AGT (8602C>T), AAC11TCT (8603A>T 8604A>C 8605C>T), AAA16AAG (8620A>G), CCA17CAA (8622C>A), GAG18GGA (8625A>G 8626G>A), CAG19CAA (8629G>A), GGC20GGT (8632C>T), ATC22ATT (8638C>T), CAG23CAA (8641G>A), AAC25AAT (8647C>T), CCG26TTC (8648C>T 8649C>T 8650G>C), AAC27AAT (8653C>T), AAA28AAG (8656A>G), GGA29GAA (8658G>A), GAT31GCG (8664A>C 8665T>G), GTT34GTG (8674T>G), GCA36GTT (8679C>T 8680A>T), GGC37GGA (8683C>A), GGG40GGA (8692G>A), ACA41ACT (8695A>T), CAT42CAC (8698T>C), CCG45CCA (8707G>A), AGA46CGA (8708A>C), AAG48AAA (8716G>A), ATC50ATT (8722C>T), ACG51ACA (8725G>A), AAA53AAG (8731A>G), AGA55AGG (8737A>G), CCC57CCT (8743C>T), ACA58AAG (8745C>A 8746A>G), AGC59AGT (8749C>T), AAG60AAA (8752G>A), GGA61GGT (8755A>T), AAC66AAT (8770C>T), TTA67CTA (8771T>C), GAA68GAG (8776A>G), CAT69CAC (8779T>C), TTG70TTA (8782G>A), TAT73TAC (8791T>C), CCA75CCG (8797A>G), CAA76CAG (8800A>G), CAA77CAG (8803A>G), GAT79GAC (8809T>C), ATT80ATC (8812T>C), AAT82AAC (8818T>C), CGG84CGA (8824G>A), GCA85GCG (8827A>G), ACG92ACA (8848G>A), TAT94TAC (8854T>C), GAG95GGA (8856A>G 8857G>A), GTG97GTA (8863G>A), CGG98CAG (8865G>A), ATG99CTT (8867A>C 8869G>T), GCA100GCG (8872A>G), GGA104GAA (8883G>A), ACT106TCT (8888A>T), TGC119TGT (8929C>T), GGG137GGA (8983G>A), AAT138GAC (8984A>G 8986T>C), GAA139GTA (8988A>T), GAG142GAA (8998G>A), CCG144CCA (9004G>A), TTG145TTA (9007G>A), ATC148ATT (9016C>T), ACC155ACA (9037C>A), ATA171ATT (9085A>T), AAC175AAT (9097C>T), ATT187GTT (9131A>G), CGA188CGT (9136A>T), CGG191CGT (9145G>T), ATG193GGA (9149A>G 9150T>G 9151G>A), GGT194AGT (9152G>A), TTA195TTG (9157A>G), GCG196GCT (9160G>T), CGT197CGC (9163T>C), GAG204GAA (9184G>A), CGA208CGG (9196A>G), AGG214AGA (9214G>A), GAA215GAG (9217A>G), GCG216GCA (9220G>A), GCA224GCT (9244A>T), TTG225TTA (9247G>A), GCC228GCT (9256C>T), CCT230TCC (9260C>T 9262T>C), GGG234GGA (9274G>A), GAC236GAT (9280C>T), ATC239ATT (9289C>T), GAG244GAA (9304G>A), GTC254GTT (9334C>T), GTC264GTG (9364C>G)								

*: Inserts / Deletes / Misaligned / Frameshifts

Analysis details

This analysis was performed with panviral2.64

NGS Details (UN18_val): Duamitovirus peex1

Assembly

**Coverage Length**	705 (3 contig(s))
**Depth Of Coverage**	5.3
**Number Of Reads**	32
**Reads Per Million**	0.60 rpm (after QC)
**Ambiguities**	0
**Assembly Method**	de novo + reference guided assembly
**Consensus Caller**	Bcf Tools

Coverage Map

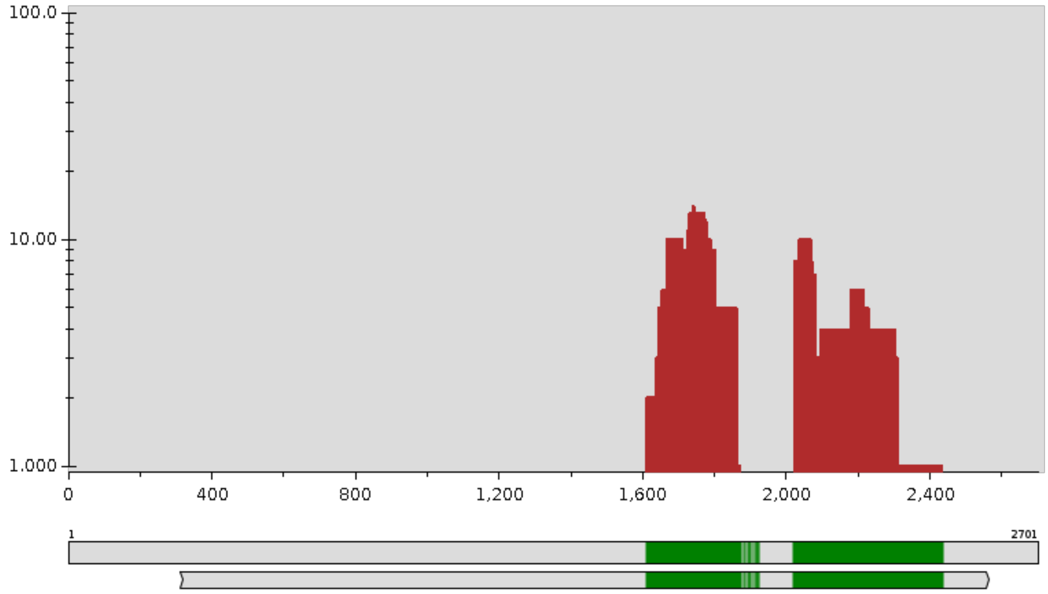

Assignment

**Type**	Duamitovirus peex1 (Taxonomy ID: 2955799)
**Reference Genome**	NC_076525.1
**NT Identity (%)**	69.7443
**AA Identity (%)**	67.5325
**Number Of Stop Codons**	0
**Number Of CDS**	1

Alignment

**Alignment Score**	546.0 (NT) + 1021.0 (AA) = 1567.0
**Concordance (%)**	54.6484

Alignment Method	Global, seeded, nucleotide + amino acids (AGA)

Genome Region

Sequence starts at position 1609 and ends at position 2439 relative to NC_076525.1 reference sequence.

Alignment Detailed Statistics

	Begin	End	Coverage	Score	Concordance	Matches	Identities	I/D/M/F*	Stop Codons
**NT**	**1609**	**2439**	**26.1%**	**546**	**39.1%**	**704 (99.9%)**	**491 (69.6%)**	**0/1**	
Mutations:	1611G>A, 1613T>A, 1616G>A, 1622T>G, 1623C>T, 1628C>T, 1632C>T, 1633T>A, 1634G>C, 1655C>G, 1658G>T, 1661A>C, 1671T>A, 1676A>G, 1680T>C, 1682G>A, 1685G>T, 1689G>A, 1691G>A, 1692A>T, 1694G>T, 1696T>C, 1697C>A, 1703G>A, 1704G>A, 1705G>C, 1706C>G, 1707C>A, 1711G>C, 1713C>A, 1714G>C, 1715G>C, 1721C>T, 1722T>G, 1723G>C, 1724G>T, 1727A>T, 1730T>C, 1736T>G, 1737C>T, 1739C>A, 1751T>C, 1754C>A, 1757T>C, 1758G>A, 1764G>A, 1765A>C, 1766G>A, 1772C>G, 1774G>C, 1775C>G, 1776A>C, 1777A>G, 1781A>G, 1784T>C, 1785G>A, 1786C>G, 1788G>A, 1789A>T, 1790A>T, 1792A>T, 1793C>G, 1799G>T, 1800C>A, 1808A>T, 1814C>A, 1817T>C, 1820G>T, 1821delA, 1824C>G, 1825A>C, 1834T>G, 1841G>C, 1842A>G, 1844T>G, 1847T>C, 1850T>A, 1851T>A, 1853T>C, 1854C>A, 1856T>C, 1862C>T, 1865A>T, 1871G>A, 1880T>C, 1884T>G, 1885T>A, 1886A>C, 1893G>A, 1896G>T, 1898T>C, 1899T>C, 1901A>T, 1902A>T, 1903G>C, 1908G>A, 1914T>C, 1916A>T, 1918A>G, 1920T>G, 1922C>A, 2020G>A, 2024T>C, 2027A>T, 2033C>T, 2036T>G, 2040A>T, 2042G>T, 2051T>A, 2052A>C, 2053A>C, 2057C>A, 2066G>A, 2067A>C, 2069T>A, 2075T>G, 2077T>C, 2088T>C, 2090T>A, 2091T>A, 2095T>G, 2096A>G, 2098C>T, 2102G>T, 2104G>T, 2105G>A, 2109T>C, 2119G>T, 2120G>T, 2133G>T, 2134G>T, 2135A>T, 2136A>G, 2137A>G, 2138G>A, 2139C>A, 2145G>A, 2156A>G, 2158G>A, 2159A>G, 2161G>C, 2162T>G, 2165G>A, 2171C>A, 2173C>A, 2180T>G, 2184A>G, 2188A>C, 2189G>T, 2190T>G, 2195A>G, 2202G>C, 2210G>A, 2211T>A, 2213G>C, 2214A>T, 2216A>T, 2223C>G, 2225A>T, 2226C>T, 2231G>A, 2234A>T, 2240T>A, 2245A>G, 2246T>G, 2249G>A, 2255A>T, 2258A>G, 2261C>G, 2268G>A, 2270A>C, 2272G>T, 2274C>A, 2283G>A, 2292C>T, 2294T>G, 2295C>A, 2296G>C, 2297A>T, 2306A>T, 2315C>T, 2321G>A, 2325A>T, 2326A>T, 2327T>A, 2330C>A, 2333G>A, 2336A>T, 2345A>T, 2348C>T, 2354T>A, 2355A>T, 2357G>T, 2360A>T, 2363G>T, 2375G>C, 2390T>A, 2391A>T, 2393T>A, 2394C>A, 2396G>C, 2397A>G, 2398C>A, 2399T>A, 2403C>T, 2404T>A, 2406C>G, 2408T>A, 2412G>A, 2414G>C, 2425T>A, 2426A>C, 2427A>G, 2429C>T								

CDS

	Begin	End	Coverage	Score	Concordance	Matches	Identities	I/D/M/F*	Stop Codons
RdRp	434	710	30.8%	1021	59.2%	231 (100%)	156 (67.5%)	0/0/2/1	0
Protein mutations:	A434T (1611G>A 1613T>A), L441Y (1632C>T 1633T>A 1634G>C), L454M (1671T>A), E460K (1689G>A 1691G>A), K461Y (1692A>T 1694G>T), V462A (1696T>C 1697C>A), G465T (1704G>A 1705G>C 1706C>G), R466S (1707C>A), S467T (1711G>C), R468T (1713C>A 1714G>C 1715G>C), W471A (1722T>G 1723G>C 1724G>T), E472D (1727A>T), A483T (1758G>A), E485T (1764G>A 1765A>C 1766G>A), G488A (1774G>C 1775C>G), K489R (1776A>C 1777A>G), A492R (1785G>A 1786C>G), E493I (1788G>A 1789A>T 1790A>T), Y494L (1792A>T 1793C>G), E496D (1799G>T), H505A (1824C>G 1825A>C), I508R (1834T>G), N511E (1842A>G 1844T>G), S514T (1851T>A 1853T>C), L515I (1854C>A 1856T>C), L525D (1884T>G 1885T>A 1886A>C), T577S (2040A>T 2042G>T), K581P (2052A>C 2053A>C), I586L (2067A>C 2069T>A), V589A (2077T>C), S593P (2088T>C 2090T>A), Y594N (2091T>A), I595R (2095T>G 2096A>G), S596L (2098C>T), Q597H (2102G>T), R598L (2104G>T 2105G>A), S600P (2109T>C), W603F (2119G>T 2120G>T), G608F (2133G>T 2134G>T 2135A>T), K609G (2136A>G 2137A>G 2138G>A), P610T (2139C>A), D612N (2145G>A), R616K (2158G>A 2159A>G), G617A (2161G>C 2162T>G), A621D (2173C>A), K625E (2184A>G), E626A (2188A>C 2189G>T), L627V (2190T>G), E631Q (2202G>C), L634I (2211T>A 2213G>C), I635F (2214A>T 2216A>T), Q638D (2223C>G 2225A>T), L641F (2234A>T), H645R (2245A>G 2246T>G), V653I (2268G>A 2270A>C), R654L (2272G>T), H655N (2274C>A), E658K (2283G>A), R662T (2295C>A 2296G>C 2297A>T), N672L (2325A>T 2326A>T 2327T>A), E675D (2336A>T), M682F (2355A>T 2357G>T), E683D (2360A>T), T694S (2391A>T 2393T>A), Q695N (2394C>A 2396G>C), T696E (2397A>G 2398C>A 2399T>A), L698Y (2403C>T 2404T>A), H699E (2406C>G 2408T>A), V701I (2412G>A 2414G>C), L705Y (2425T>A 2426A>C), I706V (2427A>G 2429C>T)								
Codon mutations:	GCT434ACA (1611G>A 1613T>A), GGG435GGA (1616G>A), CCT437CCG (1622T>G), CTA438TTA (1623C>T), GGC439GGT (1628C>T), CTG441TAC (1632C>T 1633T>A 1634G>C), GCC448GCG (1655C>G), CTG449CTT (1658G>T), TCA450TCC (1661A>C), TTG454ATG (1671T>A), GTA455GTG (1676A>G), TTG457CTA (1680T>C 1682G>A), GCG458GCT (1685G>T), GAG460AAA (1689G>A 1691G>A), AAG461TAT (1692A>T 1694G>T), GTC462GCA (1696T>C 1697C>A), CCG464CCA (1703G>A), GGC465ACG (1704G>A 1705G>C 1706C>G), CGT466AGT (1707C>A), AGT467ACT (1711G>C), CGG468ACC (1713C>A 1714G>C 1715G>C), TTC470TTT (1721C>T), TGG471GCT (1722T>G 1723G>C 1724G>T), GAA472GAT (1727A>T), TAT473TAC (1730T>C), CTT475CTG (1736T>G), CTC476TTA (1737C>T 1739C>A), ATT480ATC (1751T>C), CTC481CTA (1754C>A), ATT482ATC (1757T>C), GCC483ACC (1758G>A), GAG485ACA (1764G>A 1765A>C 1766G>A), GTC487GTG (1772C>G), GGC488GCG (1774G>C 1775C>G), AAG489CGG (1776A>C 1777A>G), CAA490CAG (1781A>G), TAT491TAC (1784T>C), GCG492AGG (1785G>A 1786C>G), GAA493ATT (1788G>A 1789A>T 1790A>T), TAC494TTG (1792A>T 1793C>G), GAG496GAT (1799G>T), CGG497AGG (1800C>A), GGA499GGT (1808A>T), ACC501ACA (1814C>A), ATT502ATC (1817T>C), TCG503TCT (1820G>T), ATA504-TA (1821delA), CAT505GCT (1824C>G 1825A>C), ATA508AGA (1834T>G), TCG510TCC (1841G>C), AAT511GAG (1842A>G 1844T>G), AAT512AAC (1847T>C), GGT513GGA (1850T>A), TCT514ACC (1851T>A 1853T>C), CTT515ATC (1854C>A 1856T>C), TTC517TTT (1862C>T), GCA518GCT (1865A>T), AGG520AGA (1871G>A), ACT523..C (1880T>C), TTA525GAC (1884T>G 1885T>A 1886A>C), GTT528A.. (1893G>A), GAT529T.C (1896G>T 1898T>C), TTA530C.T (1899T>C 1901A>T), AGT531TC. (1902A>T 1903G>C), GTC533A.. (1908G>A), TTA535C.T (1914T>C 1916A>T), AAA536.G. (1918A>G), TCC537G.A (1920T>G 1922C>A), AGG570.AG (2020G>A), GTT571GTC (2024T>C), CGA572CGT (2027A>T), CGC574CGT (2033C>T), CTT575CTG (2036T>G), ACG577TCT (2040A>T 2042G>T), TCT580TCA (2051T>A), AAA581CCA (2052A>C 2053A>C), CGC582CGA (2057C>A), AGG585AGA (2066G>A), ATT586CTA (2067A>C 2069T>A), GCT588GCG (2075T>G), GTT589GCT (2077T>C), TCT593CCA (2088T>C 2090T>A), TAC594AAC (2091T>A), ATA595AGG (2095T>G 2096A>G), TCA596TTA (2098C>T), CAG597CAT (2102G>T), CGG598CTA (2104G>T 2105G>A), TCT600CCT (2109T>C), TGG603TTT (2119G>T 2120G>T), GGA608TTT (2133G>T 2134G>T 2135A>T), AAG609GGA (2136A>G 2137A>G 2138G>A), CCC610ACC (2139C>A), GAT612AAT (2145G>A), TTA615TTG (2156A>G), AGA616AAG (2158G>A 2159A>G), GGT617GCG (2161G>C 2162T>G), AAG618AAA (2165G>A), ATC620ATA (2171C>A), GCT621GAT (2173C>A), CTT623CTG (2180T>G), AAG625GAG (2184A>G), GAG626GCT (2188A>C 2189G>T), TTA627GTA (2190T>G), AAA628AAG (2195A>G), GAG631CAG (2202G>C), CAG633CAA (2210G>A), TTG634ATC (2211T>A 2213G>C), ATA635TTT (2214A>T 2216A>T), CAA638GAT (2223C>G 2225A>T), CTG639TTG (2226C>T), GTG640GTA (2231G>A), TTA641TTT (2234A>T), GGT643GGA (2240T>A), CAT645CGG (2245A>G 2246T>G), GAG646GAA (2249G>A), CTA648CTT (2255A>T), GAA649GAG (2258A>G), CGC650CGG (2261C>G), GTA653ATC (2268G>A 2270A>C), CGT654CTT (2272G>T), CAT655AAT (2274C>A), GAA658AAA (2283G>A), CTT661TTG (2292C>T 2294T>G), CGA662ACT (2295C>A 2296G>C 2297A>T), TCA665TCT (2306A>T), CAC668CAT (2315C>T), GTG670GTA (2321G>A), AAT672TTA (2325A>T 2326A>T 2327T>A), TCC673TCA (2330C>A), CCG674CCA (2333G>A), GAA675GAT (2336A>T), ATA678ATT (2345A>T), GAC679GAT (2348C>T), CTT681CTA (2354T>A), ATG682TTT (2355A>T 2357G>T), GAA683GAT (2360A>T), GTG684GTT (2363G>T), GCG688GCC (2375G>C), CGT693CGA (2390T>A), ACT694TCA (2391A>T 2393T>A), CAG695AAC (2394C>A 2396G>C), ACT696GAA (2397A>G 2398C>A 2399T>A), CTT698TAT (2403C>T 2404T>A), CAT699GAA (2406C>G 2408T>A), GTG701ATC (2412G>A 2414G>C), TTA705TAC (2425T>A 2426A>C), ATC706GTT (2427A>G 2429C>T)								

Proteins

	Begin	End	Coverage	Score	Concordance	Matches	Identities	I/D/M/F*	Stop Codons
RNA-dependent RNA polymerase (YP_010798875.1)	434	710	30.8%	1021	59.2%	231 (100%)	156 (67.5%)	0/0/2/1	0
Protein mutations:	A434T (1611G>A 1613T>A), L441Y (1632C>T 1633T>A 1634G>C), L454M (1671T>A), E460K (1689G>A 1691G>A), K461Y (1692A>T 1694G>T), V462A (1696T>C 1697C>A), G465T (1704G>A 1705G>C 1706C>G), R466S (1707C>A), S467T (1711G>C), R468T (1713C>A 1714G>C 1715G>C), W471A (1722T>G 1723G>C 1724G>T), E472D (1727A>T), A483T (1758G>A), E485T (1764G>A 1765A>C 1766G>A), G488A (1774G>C 1775C>G), K489R (1776A>C 1777A>G), A492R (1785G>A 1786C>G), E493I (1788G>A 1789A>T 1790A>T), Y494L (1792A>T 1793C>G), E496D (1799G>T), H505A (1824C>G 1825A>C), I508R (1834T>G), N511E (1842A>G 1844T>G), S514T (1851T>A 1853T>C), L515I (1854C>A 1856T>C), L525D (1884T>G 1885T>A 1886A>C), T577S (2040A>T 2042G>T), K581P (2052A>C 2053A>C), I586L (2067A>C 2069T>A), V589A (2077T>C), S593P (2088T>C 2090T>A), Y594N (2091T>A), I595R (2095T>G 2096A>G), S596L (2098C>T), Q597H (2102G>T), R598L (2104G>T 2105G>A), S600P (2109T>C), W603F (2119G>T 2120G>T), G608F (2133G>T 2134G>T 2135A>T), K609G (2136A>G 2137A>G 2138G>A), P610T (2139C>A), D612N (2145G>A), R616K (2158G>A 2159A>G), G617A (2161G>C 2162T>G), A621D (2173C>A), K625E (2184A>G), E626A (2188A>C 2189G>T), L627V (2190T>G), E631Q (2202G>C), L634I (2211T>A 2213G>C), I635F (2214A>T 2216A>T), Q638D (2223C>G 2225A>T), L641F (2234A>T), H645R (2245A>G 2246T>G), V653I (2268G>A 2270A>C), R654L (2272G>T), H655N (2274C>A), E658K (2283G>A), R662T (2295C>A 2296G>C 2297A>T), N672L (2325A>T 2326A>T 2327T>A), E675D (2336A>T), M682F (2355A>T 2357G>T), E683D (2360A>T), T694S (2391A>T 2393T>A), Q695N (2394C>A 2396G>C), T696E (2397A>G 2398C>A 2399T>A), L698Y (2403C>T 2404T>A), H699E (2406C>G 2408T>A), V701I (2412G>A 2414G>C), L705Y (2425T>A 2426A>C), I706V (2427A>G 2429C>T)								

	Begin	End	Coverage	Score	Concordance	Matches	Identities	I/D/M/F*	Stop Codons
**NT**	**1609**	**2439**	**26.1%**	**546**	**39.1%**	**704 (99.9%)**	**491 (69.6%)**	**0/1**	
Codon mutations:	GCT434ACA (1611G>A 1613T>A), GGG435GGA (1616G>A), CCT437CCG (1622T>G), CTA438TTA (1623C>T), GGC439GGT (1628C>T), CTG441TAC (1632C>T 1633T>A 1634G>C), GCC448GCG (1655C>G), CTG449CTT (1658G>T), TCA450TCC (1661A>C), TTG454ATG (1671T>A), GTA455GTG (1676A>G), TTG457CTA (1680T>C 1682G>A), GCG458GCT (1685G>T), GAG460AAA (1689G>A 1691G>A), AAG461TAT (1692A>T 1694G>T), GTC462GCA (1696T>C 1697C>A), CCG464CCA (1703G>A), GGC465ACG (1704G>A 1705G>C 1706C>G), CGT466AGT (1707C>A), AGT467ACT (1711G>C), CGG468ACC (1713C>A 1714G>C 1715G>C), TTC470TTT (1721C>T), TGG471GCT (1722T>G 1723G>C 1724G>T), GAA472GAT (1727A>T), TAT473TAC (1730T>C), CTT475CTG (1736T>G), CTC476TTA (1737C>T 1739C>A), ATT480ATC (1751T>C), CTC481CTA (1754C>A), ATT482ATC (1757T>C), GCC483ACC (1758G>A), GAG485ACA (1764G>A 1765A>C 1766G>A), GTC487GTG (1772C>G), GGC488GCG (1774G>C 1775C>G), AAG489CGG (1776A>C 1777A>G), CAA490CAG (1781A>G), TAT491TAC (1784T>C), GCG492AGG (1785G>A 1786C>G), GAA493ATT (1788G>A 1789A>T 1790A>T), TAC494TTG (1792A>T 1793C>G), GAG496GAT (1799G>T), CGG497AGG (1800C>A), GGA499GGT (1808A>T), ACC501ACA (1814C>A), ATT502ATC (1817T>C), TCG503TCT (1820G>T), ATA504-TA (1821delA), CAT505GCT (1824C>G 1825A>C), ATA508AGA (1834T>G), TCG510TCC (1841G>C), AAT511GAG (1842A>G 1844T>G), AAT512AAC (1847T>C), GGT513GGA (1850T>A), TCT514ACC (1851T>A 1853T>C), CTT515ATC (1854C>A 1856T>C), TTC517TTT (1862C>T), GCA518GCT (1865A>T), AGG520AGA (1871G>A), ACT523..C (1880T>C), TTA525GAC (1884T>G 1885T>A 1886A>C), GTT528A.. (1893G>A), GAT529T.C (1896G>T 1898T>C), TTA530C.T (1899T>C 1901A>T), AGT531TC. (1902A>T 1903G>C), GTC533A.. (1908G>A), TTA535C.T (1914T>C 1916A>T), AAA536.G. (1918A>G), TCC537G.A (1920T>G 1922C>A), AGG570.AG (2020G>A), GTT571GTC (2024T>C), CGA572CGT (2027A>T), CGC574CGT (2033C>T), CTT575CTG (2036T>G), ACG577TCT (2040A>T 2042G>T), TCT580TCA (2051T>A), AAA581CCA (2052A>C 2053A>C), CGC582CGA (2057C>A), AGG585AGA (2066G>A), ATT586CTA (2067A>C 2069T>A), GCT588GCG (2075T>G), GTT589GCT (2077T>C), TCT593CCA (2088T>C 2090T>A), TAC594AAC (2091T>A), ATA595AGG (2095T>G 2096A>G), TCA596TTA (2098C>T), CAG597CAT (2102G>T), CGG598CTA (2104G>T 2105G>A), TCT600CCT (2109T>C), TGG603TTT (2119G>T 2120G>T), GGA608TTT (2133G>T 2134G>T 2135A>T), AAG609GGA (2136A>G 2137A>G 2138G>A), CCC610ACC (2139C>A), GAT612AAT (2145G>A), TTA615TTG (2156A>G), AGA616AAG (2158G>A 2159A>G), GGT617GCG (2161G>C 2162T>G), AAG618AAA (2165G>A), ATC620ATA (2171C>A), GCT621GAT (2173C>A), CTT623CTG (2180T>G), AAG625GAG (2184A>G), GAG626GCT (2188A>C 2189G>T), TTA627GTA (2190T>G), AAA628AAG (2195A>G), GAG631CAG (2202G>C), CAG633CAA (2210G>A), TTG634ATC (2211T>A 2213G>C), ATA635TTT (2214A>T 2216A>T), CAA638GAT (2223C>G 2225A>T), CTG639TTG (2226C>T), GTG640GTA (2231G>A), TTA641TTT (2234A>T), GGT643GGA (2240T>A), CAT645CGG (2245A>G 2246T>G), GAG646GAA (2249G>A), CTA648CTT (2255A>T), GAA649GAG (2258A>G), CGC650CGG (2261C>G), GTA653ATC (2268G>A 2270A>C), CGT654CTT (2272G>T), CAT655AAT (2274C>A), GAA658AAA (2283G>A), CTT661TTG (2292C>T 2294T>G), CGA662ACT (2295C>A 2296G>C 2297A>T), TCA665TCT (2306A>T), CAC668CAT (2315C>T), GTG670GTA (2321G>A), AAT672TTA (2325A>T 2326A>T 2327T>A), TCC673TCA (2330C>A), CCG674CCA (2333G>A), GAA675GAT (2336A>T), ATA678ATT (2345A>T), GAC679GAT (2348C>T), CTT681CTA (2354T>A), ATG682TTT (2355A>T 2357G>T), GAA683GAT (2360A>T), GTG684GTT (2363G>T), GCG688GCC (2375G>C), CGT693CGA (2390T>A), ACT694TCA (2391A>T 2393T>A), CAG695AAC (2394C>A 2396G>C), ACT696GAA (2397A>G 2398C>A 2399T>A), CTT698TAT (2403C>T 2404T>A), CAT699GAA (2406C>G 2408T>A), GTG701ATC (2412G>A 2414G>C), TTA705TAC (2425T>A 2426A>C), ATC706GTT (2427A>G 2429C>T)								

*: Inserts / Deletes / Misaligned / Frameshifts

Analysis details

This analysis was performed with panviral2.64

NGS Details (UN18_val): Harvey murine sarcoma virus

Assembly

**Coverage Length**	222 (1 contig(s))
**Depth Of Coverage**	2.2
**Number Of Reads**	6
**Reads Per Million**	0.11 rpm (after QC)
**Ambiguities**	0
**Assembly Method**	de novo + reference guided assembly
**Consensus Caller**	Bcf Tools

Coverage Map

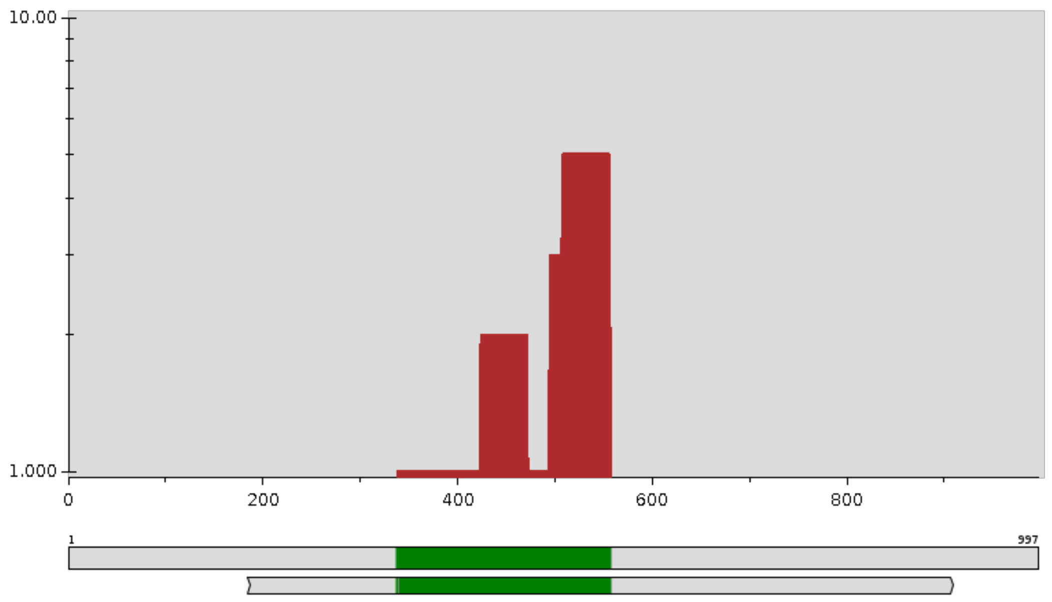

Assignment

**Type**	Harvey murine sarcoma virus (Taxonomy ID: 11807)
**Reference Genome**	NC_038668.1
**NT Identity (%)**	73.4234
**AA Identity (%)**	85.034
**Number Of Stop Codons**	0
**Number Of CDS**	2

Alignment

**Alignment Score**	208.0 (NT) + 814.0 (AA) = 1022.0
**Concordance (%)**	71.669

Alignment Method	Global, seeded, nucleotide + amino acids (AGA)

Genome Region

Sequence starts at position 338 and ends at position 559 relative to NC_038668.1 reference sequence.

Alignment Detailed Statistics

	Begin	End	Coverage	Score	Concordance	Matches	Identities	I/D/M/F*	Stop Codons
**NT**	**338**	**559**	**22.3%**	**208**	**46.8%**	**222 (100%)**	**163 (73.4%)**	**0/0**	
Mutations:	346A>G, 358T>C, 364G>A, 367G>C, 370C>T, 372C>G, 374A>G, 376A>T, 379C>A, 382G>C, 392G>T, 393C>G, 394C>T, 395C>T, 409G>C, 417A>G, 424T>C, 427G>C, 433G>A, 439T>C, 442C>T, 445T>C, 448A>C, 451G>A, 457C>T, 461C>A, 466A>G, 469G>A, 470G>T, 471T>G, 472A>C, 475C>G, 478T>C, 483G>A, 488A>G, 489C>T, 490G>C, 491T>G, 492G>C, 493T>G, 494T>C, 496A>G, 499G>A, 503A>G, 505C>G, 506T>C, 508A>C, 514A>G, 515A>G, 517A>C, 520T>C, 523A>G, 526A>G, 532T>C, 533A>T, 534G>C, 544G>C, 547C>G, 550G>A								
**CDS**									
D1R95_gp1	52	125	30.6%	409	83.0%	74 (100%)	63 (85.1%)	0/0/0/0	0
Protein mutations:	A63G (372C>G), R64G (374A>G 376A>T), A70C (392G>T 393C>G 394C>T), N78S (417A>G), V96C (470G>T 471T>G 472A>C), G100E (483G>A), T102V (488A>G 489C>T 490G>C), C103A (491T>G 492G>C 493T>G), I107V (503A>G 505C>G), T111A (515A>G 517A>C), D121E (547C>G)								
Codon mutations:	ACA54ACG (346A>G), CTT58CTC (358T>C), GTG60GTA (364G>A), GTG61GTC (367G>C), GGC62GGT (370C>T), GCT63GGT (372C>G), AGA64GGT (374A>G 376A>T), GGC65GGA (379C>A), GTG66GTC (382G>C), GCC70TGT (392G>T 393C>G 394C>T), CTG71TTG (395C>T), CTG75CTC (409G>C), AAC78AGC (417A>G), TTT80TTC (424T>C), GTG81GTC (427G>C), GAG83GAA (433G>A), GAT85GAC (439T>C), CCC86CCT (442C>T), ACT87ACC (445T>C), ATA88ATC (448A>C), GAG89GAA (451G>A), TCC91TCT (457C>T), CGG93AGG (461C>A), AAA94AAG (466A>G), CAG95CAA (469G>A), GTA96TGC (470G>T 471T>G 472A>C), GTC97GTG (475C>G), ATT98ATC (478T>C), GGG100GAG (483G>A), ACG102GTC (488A>G 489C>T 490G>C), TGT103GCG (491T>G 492G>C 493T>G), TTA104CTG (494T>C 496A>G), CTG105CTA (499G>A), ATC107GTG (503A>G 505C>G), TTA108CTC (506T>C 508A>C), ACA110ACG (514A>G), ACA111GCC (515A>G 517A>C), GGT112GGC (520T>C), CAA113CAG (523A>G), GAA114GAG (526A>G), TAT116TAC (532T>C), AGT117TCT (533A>T 534G>C), CGG120CGC (544G>C), GAC121GAG (547C>G), CAG122CAA (550G>A)								
D1R95_gp2	1	73	38.4%	405	82.8%	73 (100%)	62 (84.9%)	0/0/0/0	0
Protein mutations:	A11G (372C>G), R12G (374A>G 376A>T), A18C (392G>T 393C>G 394C>T), N26S (417A>G), V44C (470G>T 471T>G 472A>C), G48E (483G>A), T50V (488A>G 489C>T 490G>C), C51A (491T>G 492G>C 493T>G), I55V (503A>G 505C>G), T59A (515A>G 517A>C), D69E (547C>G)								
Codon mutations:	ACA2ACG (346A>G), CTT6CTC (358T>C), GTG8GTA (364G>A), GTG9GTC (367G>C), GGC10GGT (370C>T), GCT11GGT (372C>G), AGA12GGT (374A>G 376A>T), GGC13GGA (379C>A), GTG14GTC (382G>C), GCC18TGT (392G>T 393C>G 394C>T), CTG19TTG (395C>T), CTG23CTC (409G>C), AAC26AGC (417A>G), TTT28TTC (424T>C), GTG29GTC (427G>C), GAG31GAA (433G>A), GAT33GAC (439T>C), CCC34CCT (442C>T), ACT35ACC (445T>C), ATA36ATC (448A>C), GAG37GAA (451G>A), TCC39TCT (457C>T), CGG41AGG (461C>A), AAA42AAG (466A>G), CAG43CAA (469G>A), GTA44TGC (470G>T 471T>G 472A>C), GTC45GTG (475C>G), ATT46ATC (478T>C), GGG48GAG (483G>A), ACG50GTC (488A>G 489C>T 490G>C), TGT51GCG (491T>G 492G>C 493T>G), TTA52CTG (494T>C 496A>G), CTG53CTA (499G>A), ATC55GTG (503A>G 505C>G), TTA56CTC (506T>C 508A>C), ACA58ACG (514A>G), ACA59GCC (515A>G 517A>C), GGT60GGC (520T>C), CAA61CAG (523A>G), GAA62GAG (526A>G), TAT64TAC (532T>C), AGT65TCT (533A>T 534G>C), CGG68CGC (544G>C), GAC69GAG (547C>G), CAG70CAA (550G>A)								
**Proteins**									
hypothetical protein (YP_009507788.1)	52	125	30.6%	409	83.0%	74 (100%)	63 (85.1%)	0/0/0/0	0
Protein mutations:	A63G (372C>G), R64G (374A>G 376A>T), A70C (392G>T 393C>G 394C>T), N78S (417A>G), V96C (470G>T 471T>G 472A>C), G100E (483G>A), T102V (488A>G 489C>T 490G>C), C103A (491T>G 492G>C 493T>G), I107V (503A>G 505C>G), T111A (515A>G 517A>C), D121E (547C>G)								
Codon mutations:	ACA54ACG (346A>G), CTT58CTC (358T>C), GTG60GTA (364G>A), GTG61GTC (367G>C), GGC62GGT (370C>T), GCT63GGT (372C>G), AGA64GGT (374A>G 376A>T), GGC65GGA (379C>A), GTG66GTC (382G>C), GCC70TGT (392G>T 393C>G 394C>T), CTG71TTG (395C>T), CTG75CTC (409G>C), AAC78AGC (417A>G), TTT80TTC (424T>C), GTG81GTC (427G>C), GAG83GAA (433G>A), GAT85GAC (439T>C), CCC86CCT (442C>T), ACT87ACC (445T>C), ATA88ATC (448A>C), GAG89GAA (451G>A), TCC91TCT (457C>T), CGG93AGG (461C>A), AAA94AAG (466A>G), CAG95CAA (469G>A), GTA96TGC (470G>T 471T>G 472A>C), GTC97GTG (475C>G), ATT98ATC (478T>C), GGG100GAG (483G>A), ACG102GTC (488A>G 489C>T 490G>C), TGT103GCG (491T>G 492G>C 493T>G), TTA104CTG (494T>C 496A>G), CTG105CTA (499G>A), ATC107GTG (503A>G 505C>G), TTA108CTC (506T>C 508A>C), ACA110ACG (514A>G), ACA111GCC (515A>G 517A>C), GGT112GGC (520T>C), CAA113CAG (523A>G), GAA114GAG (526A>G), TAT116TAC (532T>C), AGT117TCT (533A>T 534G>C), CGG120CGC (544G>C), GAC121GAG (547C>G), CAG122CAA (550G>A)								
hypothetical protein (YP_009507789.1)	1	73	38.4%	405	82.8%	73 (100%)	62 (84.9%)	0/0/0/0	0
Protein mutations:	A11G (372C>G), R12G (374A>G 376A>T), A18C (392G>T 393C>G 394C>T), N26S (417A>G), V44C (470G>T 471T>G 472A>C), G48E (483G>A), T50V (488A>G 489C>T 490G>C), C51A (491T>G 492G>C 493T>G), I55V (503A>G 505C>G), T59A (515A>G 517A>C), D69E (547C>G)								
Codon mutations:	ACA2ACG (346A>G), CTT6CTC (358T>C), GTG8GTA (364G>A), GTG9GTC (367G>C), GGC10GGT (370C>T), GCT11GGT (372C>G), AGA12GGT (374A>G 376A>T), GGC13GGA (379C>A), GTG14GTC (382G>C), GCC18TGT (392G>T 393C>G 394C>T), CTG19TTG (395C>T), CTG23CTC (409G>C), AAC26AGC (417A>G), TTT28TTC (424T>C), GTG29GTC (427G>C), GAG31GAA (433G>A), GAT33GAC (439T>C), CCC34CCT (442C>T), ACT35ACC (445T>C), ATA36ATC (448A>C), GAG37GAA (451G>A), TCC39TCT (457C>T), CGG41AGG (461C>A), AAA42AAG (466A>G), CAG43CAA (469G>A), GTA44TGC (470G>T 471T>G 472A>C), GTC45GTG (475C>G), ATT46ATC (478T>C), GGG48GAG (483G>A), ACG50GTC (488A>G 489C>T 490G>C), TGT51GCG (491T>G 492G>C 493T>G), TTA52CTG (494T>C 496A>G), CTG53CTA (499G>A), ATC55GTG (503A>G 505C>G), TTA56CTC (506T>C 508A>C), ACA58ACG (514A>G), ACA59GCC (515A>G 517A>C), GGT60GGC (520T>C), CAA61CAG (523A>G), GAA62GAG (526A>G), TAT64TAC (532T>C), AGT65TCT (533A>T 534G>C), CGG68CGC (544G>C), GAC69GAG (547C>G), CAG70CAA (550G>A)								

*: Inserts / Deletes / Misaligned / Frameshifts

Analysis details

This analysis was performed with panviral2.64

NGS Details (UN18_val): Brazilian marseillevirus

Assembly

**Coverage Length**	240 (1 contig(s))
**Depth Of Coverage**	13067.1
**Number Of Reads**	31766
**Reads Per Million**	596.20 rpm (after QC)
**Ambiguities**	0
**Assembly Method**	de novo + reference guided assembly
**Consensus Caller**	Bcf Tools

Coverage Map

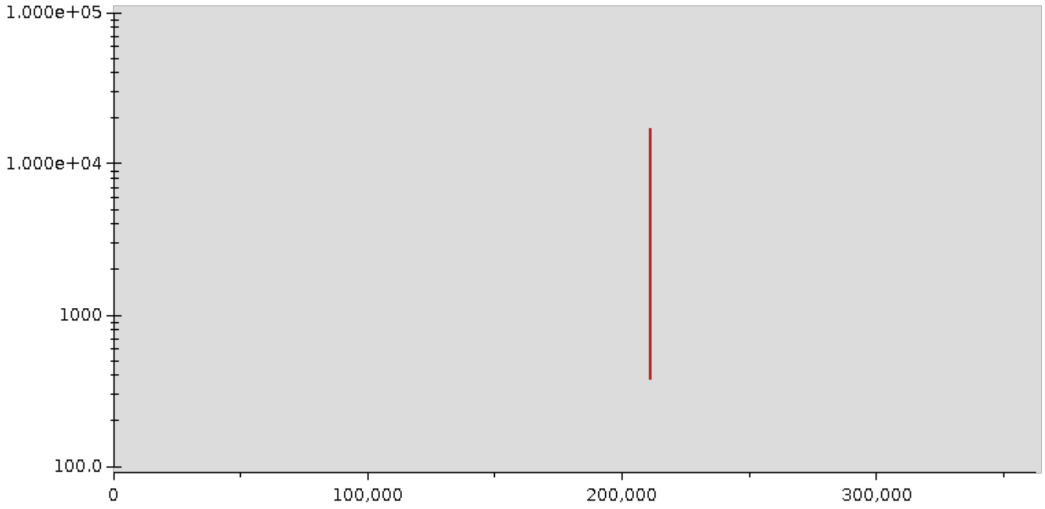

Assignment

**Type**	Brazilian marseillevirus (Taxonomy ID: 1813599)
**Reference Genome**	NC_029692.1
**NT Identity (%)**	81.6667
**AA Identity (%)**	94.6667
**Number Of Stop Codons**	1
**Number Of CDS**	491

Alignment

**Alignment Score**	304.0 (NT) + 454.0 (AA) = 758.0
**Concordance (%)**	79.9578
**Alignment Method**	Local, heuristic, nucleotide (BLASTN)

Genome Region

Sequence starts at position 210884 and ends at position 211123 relative to NC_029692.1 reference sequence.

Alignment Detailed Statistics

	Begin	End	Coverage	Score	Concordance	Matches	Identities	I/D/M/F*	Stop Codons
**NT**	**210884**	**211123**	**0.1%**	**304**	**63.3%**	**240 (100%)**	**196 (81.7%)**	**0/0**	

Mutations: 210899T>A, 210901T>G, 210902T>G, 210905G>C, 210908A>C, 210910G>A, 210917A>G, 210926T>C, 210929T>C, 210932G>A, 210938A>G, 210953C>G, 210955T>G, 210956T>G, 210962T>C, 210965C>A, 210974G>A, 210977T>A, 210986A>C, 210988G>A, 210989G>C, 210995T>C, 211001A>T, 211004A>T, 211007A>G, 211009C>T, 211013T>C, 211031T>G, 211032T>G, 211033G>C, 211034T>C, 211037C>A, 211040G>A, 211043T>G, 211046G>A, 211061T>C, 211070G>C, 211079C>G, 211088C>G, 211091G>A, 211100T>A, 211103A>G, 211106C>G, 211108C>T

*: Inserts / Deletes / Misaligned / Frameshifts

Analysis details

This analysis was performed with panviral2.64

NGS Details (UN18_val): Tomato chocolate spot virus (segment RNA 1)

Assembly

**Coverage Length**	775 (1 contig(s))
**Depth Of Coverage**	416.7
**Number Of Reads**	2065
**Reads Per Million**	38.76 rpm (after QC)
**Ambiguities**	0
**Assembly Method**	de novo + reference guided assembly
**Consensus Caller**	Bcf Tools

Coverage Map

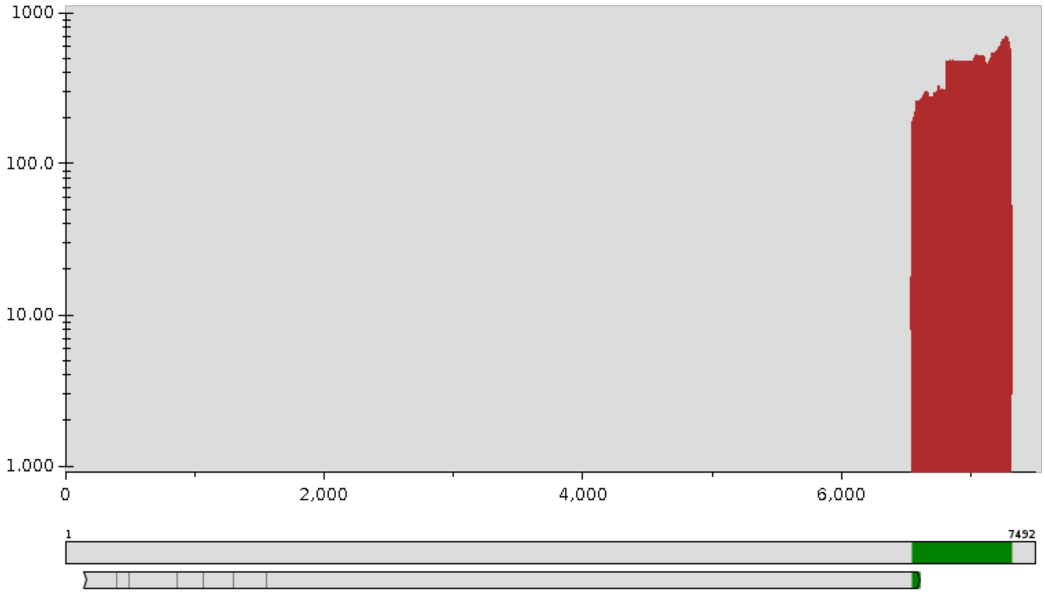

Assignment

**Type**	Tomato chocolate spot virus (Taxonomy ID: 661101)
**Reference Genome**	NC_013075.1
**NT Identity (%)**	76.2393
**AA Identity (%)**	13.6364
**Number Of Stop Codons**	0
**Number Of CDS**	1

Alignment

**Alignment Score**	352.0 (NT) + 12.0 (AA) = 364.0
**Concordance (%)**	33.9869

Alignment Method	Global, seeded, nucleotide + amino acids (AGA)

Genome Region

Sequence starts at position 6539 and ends at position 7313 relative to NC_013075.1 reference sequence.

Alignment Detailed Statistics

	Begin	End	Coverage	Score	Concordance	Matches	Identities	I/D/M/F*	Stop Codons
**NT**	**6539**	**7313**	**10.3%**	**352**	**38.5%**	**584 (75.3%)**	**446 (57.5%)**	**1/191**	
Mutations:	6548T>A, 6550C>A, 6552G>A, 6554C>G, 6555C>T, 6556A>C, 6557C>T, 6558T>G, 6559T>G, 6560T>A, 6562G>A, 6564T>G, 6566G>C, 6569A>G, 6570C>A, 6571T>A, 6572G>T, 6574C>G, 6577T>A, 6579C>A, 6582C>G, 6583C>A, 6585T>G, 6586A>T, 6587C>T, 6589A>G, 6592T>G, 6593A>T, 6595C>A, 6598G>T, 6600G>C, 6604T>G, 6607G>T, 6608T>C, 6609C>G, 6610A>G, 6612G>C, 6615T>A, 6618delT, 6621C>T, 6622T>C, 6625G>T, 6626T>C, 6628T>A, 6629G>A, 6630T>C, 6633_6634insT, 6636T>A, 6637T>A, 6638A>G, 6639C>T, 6651A>G, 6653T>A, 6658_6660delGTG, 6662G>C, 6667G>A, 6668G>T, 6670A>G, 6671G>T, 6674T>A, 6675T>A, 6676G>T, 6677C>T, 6678A>C, 6683T>A, 6684_6688delATAAC, 6700T>C, 6711A>G, 6721G>T, 6723T>A, 6729T>A, 6735G>T, 6737A>G, 6740C>A, 6741G>A, 6753G>T, 6756T>C, 6758A>G, 6768A>G, 6777T>C, 6779T>A, 6783T>C, 6788T>C, 6795A>G, 6800G>A, 6806C>T, 6814C>T, 6815_6840delAATTGTTTGGTGTTGTCAACGCGCCG, 6846_6855delGGCCAAGAAA, 6861_6917delCCTTAGGCTTTATTGGTAAAGTCAAGATGTGGAGAAGGAATGCCAGCAAGCATAAGC, 6922_7010delTCTCTTAGGGTCGGGCTTAAGATGGCAGCCAGAATTAGAGCCTTTGAGTATGCTTGTGGGCATCTGGTTTTCACATCAACTCGTTTGGA, 7013A>T, 7014T>A, 7018C>G, 7019A>G, 7020A>T, 7023C>A, 7042G>T, 7044A>T, 7062G>A, 7070T>C, 7071C>T, 7084A>G, 7087T>C, 7098C>T, 7099T>C, 7107G>A, 7116C>T, 7118T>A, 7121G>A, 7122T>C, 7123C>T, 7124T>G, 7137A>T, 7138G>A, 7139A>G, 7140C>T, 7148A>G, 7151A>T, 7159A>T, 7162T>C, 7165G>A, 7171T>G, 7173C>T, 7177A>C, 7185G>A, 7199C>T, 7208A>T, 7209T>A, 7210C>T, 7211A>G, 7213C>T, 7214A>T, 7229T>C, 7245T>C, 7250G>A, 7253T>A, 7254G>A, 7256T>C, 7257G>A, 7258G>T, 7259C>G, 7274G>C, 7275T>C, 7292T>G, 7303T>A								

CDS

ToChSV_s1_gp1	2135	2156	1.0%	12	7.5%	22 (100%)	3 (13.6%)	0/0/0/0	0
Protein mutations:	V2137D (6548T>A), Q2138K (6550C>A 6552G>A), S2139C (6554C>G 6555C>T), T2140L (6556A>C 6557C>T 6558T>G), F2141D (6559T>G 6560T>A), V2142M (6562G>A 6564T>G), R2143T (6566G>C), N2144R (6569A>G 6570C>A), C2145I (6571T>A 6572G>T), Q2146E (6574C>G), S2147T (6577T>A 6579C>A), I2148M (6582C>G), L2149M (6583C>A 6585T>G), T2150L (6586A>T 6587C>T), N2151D (6589A>G), Y2152V (6592T>G 6593A>T), Q2153K (6595C>A), E2154Y (6598G>T 6600G>C), *2156G (6604T>G)								
Codon mutations:	GTC2137GAC (6548T>A), CAG2138AAA (6550C>A 6552G>A), TCC2139TGT (6554C>G 6555C>T), ACT2140CTG (6556A>C 6557C>T 6558T>G), TTT2141GAT (6559T>G 6560T>A), GTT2142ATG (6562G>A 6564T>G), AGA2143ACA (6566G>C), AAC2144AGA (6569A>G 6570C>A), TGT2145ATT (6571T>A 6572G>T), CAA2146GAA (6574C>G), TCC2147ACA (6577T>A 6579C>A), ATC2148ATG (6582C>G), CTT2149ATG (6583C>A 6585T>G), ACA2150TTA (6586A>T 6587C>T), AAT2151GAT (6589A>G), TAT2152GTT (6592T>G 6593A>T), CAG2153AAG (6595C>A), GAG2154TAC (6598G>T 6600G>C), TGA2156GGA (6604T>G)								

Proteins

polyprotein (YP_003097229.1)	2135	2156	1.0%	12	7.5%	22 (100%)	3 (13.6%)	0/0/0/0	0
Protein mutations:	V2137D (6548T>A), Q2138K (6550C>A 6552G>A), S2139C (6554C>G 6555C>T), T2140L (6556A>C 6557C>T 6558T>G), F2141D (6559T>G 6560T>A), V2142M (6562G>A 6564T>G), R2143T (6566G>C), N2144R (6569A>G 6570C>A), C2145I (6571T>A 6572G>T), Q2146E (6574C>G), S2147T (6577T>A 6579C>A), I2148M (6582C>G), L2149M (6583C>A 6585T>G), T2150L (6586A>T 6587C>T), N2151D (6589A>G), Y2152V (6592T>G 6593A>T), Q2153K (6595C>A), E2154Y (6598G>T 6600G>C), *2156G (6604T>G)								
Codon mutations:	GTC2137GAC (6548T>A), CAG2138AAA (6550C>A 6552G>A), TCC2139TGT (6554C>G 6555C>T), ACT2140CTG (6556A>C 6557C>T 6558T>G), TTT2141GAT (6559T>G 6560T>A), GTT2142ATG (6562G>A 6564T>G), AGA2143ACA (6566G>C), AAC2144AGA (6569A>G 6570C>A), TGT2145ATT (6571T>A 6572G>T), CAA2146GAA (6574C>G), TCC2147ACA (6577T>A 6579C>A), ATC2148ATG (6582C>G), CTT2149ATG (6583C>A 6585T>G), ACA2150TTA (6586A>T 6587C>T), AAT2151GAT (6589A>G), TAT2152GTT (6592T>G 6593A>T), CAG2153AAG (6595C>A), GAG2154TAC (6598G>T 6600G>C), TGA2156GGA (6604T>G)								

*: Inserts / Deletes / Misaligned / Frameshifts

Analysis details

This analysis was performed with panviral2.64

NGS Details (UN18_val): Tomato chocolate spot virus (segment RNA 1)

Assembly

**Coverage Length**	405 (2 contig(s))
**Depth Of Coverage**	1872.0
**Number Of Reads**	6736
**Reads Per Million**	126.42 rpm (after QC)
**Ambiguities**	0
**Assembly Method**	de novo + reference guided assembly
**Consensus Caller**	Bcf Tools

Coverage Map

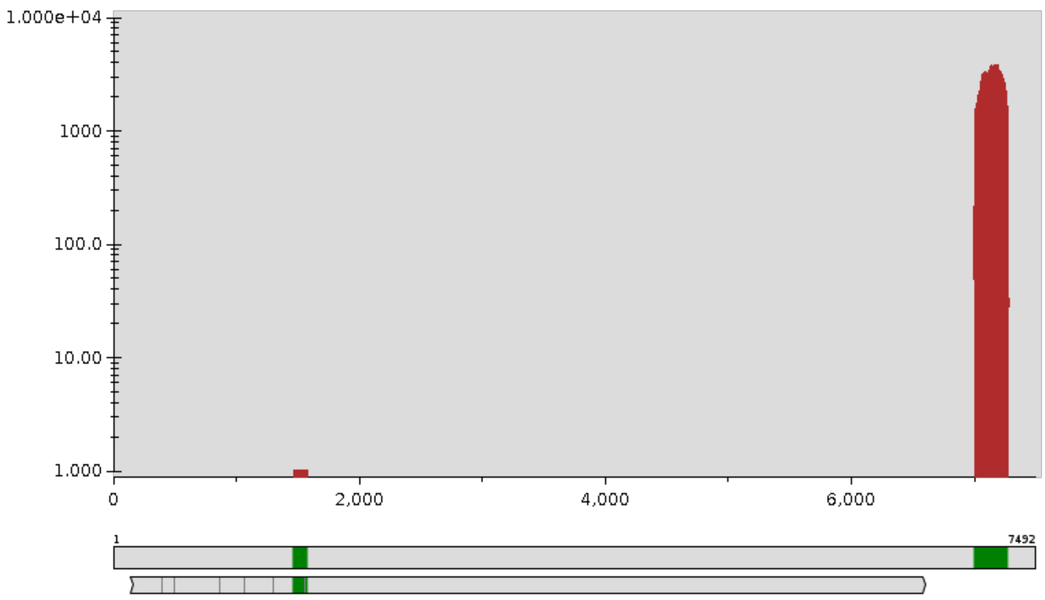

Assignment

**Type**	Tomato chocolate spot virus (Taxonomy ID: 661101)
**Reference Genome**	NC_013075.1
**NT Identity (%)**	78.3251
**AA Identity (%)**	74.4186
**Number Of Stop Codons**	1
**Number Of CDS**	1

Alignment

**Alignment Score**	452.0 (NT) + 207.0 (AA) = 659.0
**Concordance (%)**	60.1277

Alignment Method	Global, seeded, nucleotide + amino acids (AGA)

Genome Region

Sequence starts at position 1458 and ends at position 7275 relative to NC_013075.1 reference sequence.

Alignment Detailed Statistics

	Begin	End	Coverage	Score	Concordance	Matches	Identities	I/D/M/F*	Stop Codons
**NT**	**1458**	**7275**	**5.4%**	**452**	**55.8%**	**405 (99.8%)**	**318 (78.3%)**	**1/0**	
Mutations:	1459A>G, 1461T>G, 1473C>T, 1479A>T, 1483T>C, 1485G>A, 1486C>A, 1488C>G, 1494T>A, 1497G>A, 1500T>G, 1503A>G, 1504T>A, 1506T>A, 1509A>G, 1512T>C, 1515C>A, 1518A>G, 1530C>A, 1536G>A, 1539A>T, 1542G>C, 1554G>T, 1555T>C, 1557A>T, 1559C>A, 1560T>A, 1562T>A, 1563G>C, 1564G>T, 1566T>A, 1567G>C, 1569T>A, 1570G>T, 1571T>A, 1572G>C, 1573G>T, 1576G>A, 1584G>A, 7012T>G, 7018C>T, 7019A>G, 7020A>G, 7020_7021insT, 7023C>A, 7042G>T, 7044A>T, 7047G>A, 7048C>G, 7066G>A, 7082G>A, 7083T>C, 7098C>T, 7099T>C, 7104T>A, 7107G>A, 7116C>G, 7121G>A, 7127A>C, 7140C>T, 7151A>T, 7152T>C, 7183C>T, 7184T>C, 7185G>T, 7186T>G, 7187T>C, 7188G>A, 7196C>T, 7197A>G, 7198A>C, 7199C>A, 7200A>G, 7201G>A, 7207A>T, 7208A>T, 7209T>G, 7210C>T, 7212T>A, 7219T>A, 7224T>C, 7242A>T, 7255A>T, 7257G>A, 7258G>C, 7260T>C, 7262C>G, 7264T>C								
**CDS**									
ToChSV_s1_gp1	441	483	2.0%	207	69.2%	43 (100%)	32 (74.4%)	0/0/0/0	1
Protein mutations:	I441V (1459A>G 1461T>G), C456R (1504T>A 1506T>A), D464E (1530C>A), P474Q (1559C>A 1560T>A), M475N (1562T>A 1563G>C), A476S (1564G>T 1566T>A), A477P (1567G>C 1569T>A), V478Y (1570G>T 1571T>A 1572G>C), E479* (1573G>T), D480N (1576G>A)								
Codon mutations:	ATT441GTG (1459A>G 1461T>G), GAC445GAT (1473C>T), GGA447GGT (1479A>T), TTG449CTA (1483T>C 1485G>A), CGC450AGG (1486C>A 1488C>G), GGT452GGA (1494T>A), GCG453GCA (1497G>A), GGT454GGG (1500T>G), CAA455CAG (1503A>G), TGT456AGA (1504T>A 1506T>A), CAA457CAG (1509A>G), GAT458GAC (1512T>C), ATC459ATA (1515C>A), AAA460AAG (1518A>G), GAC464GAA (1530C>A), AAG466AAA (1536G>A), TCA467TCT (1539A>T), ACG468ACC (1542G>C), CCG472CCT (1554G>T), TTA473CTT (1555T>C 1557A>T), CCT474CAA (1559C>A 1560T>A), ATG475AAC (1562T>A 1563G>C), GCT476TCA (1564G>T 1566T>A), GCT477CCA (1567G>C 1569T>A), GTG478TAC (1570G>T 1571T>A 1572G>C), GAG479TAG (1573G>T), GAT480AAT (1576G>A), GGG482GGA (1584G>A)								
**Proteins**									
polyprotein (YP_003097229.1)	441	483	2.0%	207	69.2%	43 (100%)	32 (74.4%)	0/0/0/0	1
Protein mutations:	I441V (1459A>G 1461T>G), C456R (1504T>A 1506T>A), D464E (1530C>A), P474Q (1559C>A 1560T>A), M475N (1562T>A 1563G>C), A476S (1564G>T 1566T>A), A477P (1567G>C 1569T>A), V478Y (1570G>T 1571T>A 1572G>C), E479* (1573G>T), D480N (1576G>A)								
Codon mutations:	ATT441GTG (1459A>G 1461T>G), GAC445GAT (1473C>T), GGA447GGT (1479A>T), TTG449CTA (1483T>C 1485G>A), CGC450AGG (1486C>A 1488C>G), GGT452GGA (1494T>A), GCG453GCA (1497G>A), GGT454GGG (1500T>G), CAA455CAG (1503A>G), TGT456AGA (1504T>A 1506T>A), CAA457CAG (1509A>G), GAT458GAC (1512T>C), ATC459ATA (1515C>A), AAA460AAG (1518A>G), GAC464GAA (1530C>A), AAG466AAA (1536G>A), TCA467TCT (1539A>T), ACG468ACC (1542G>C), CCG472CCT (1554G>T), TTA473CTT (1555T>C 1557A>T), CCT474CAA (1559C>A 1560T>A), ATG475AAC (1562T>A 1563G>C), GCT476TCA (1564G>T 1566T>A), GCT477CCA (1567G>C 1569T>A), GTG478TAC (1570G>T 1571T>A 1572G>C), GAG479TAG (1573G>T), GAT480AAT (1576G>A), GGG482GGA (1584G>A)								
RNA-dependent RNA polymerase (YP_003097234.1)	52	84	39.3%	201	85.9%	33 (100%)	30 (90.9%)	0/0/0/0	0
Protein mutations:	I52V (1459A>G 1461T>G), C67R (1504T>A 1506T>A), D75E (1530C>A)								
Codon mutations:	ATT52GTG (1459A>G 1461T>G), GAC56GAT (1473C>T), GGA58GGT (1479A>T), TTG60CTA (1483T>C 1485G>A), CGC61AGG (1486C>A 1488C>G), GGT63GGA (1494T>A), GCG64GCA (1497G>A), GGT65GGG (1500T>G), CAA66CAG (1503A>G), TGT67AGA (1504T>A 1506T>A), CAA68CAG (1509A>G), GAT69GAC (1512T>C), ATC70ATA (1515C>A), AAA71AAG (1518A>G), GAC75GAA (1530C>A), AAG77AAA (1536G>A), TCA78TCT (1539A>T), ACG79ACC (1542G>C), CCG83CCT (1554G>T), TTA84CTT (1555T>C 1557A>T)								

*: Inserts / Deletes / Misaligned / Frameshifts

Analysis details

This analysis was performed with panviral2.64

NGS Details (UN18_val): Tomato chocolate spot virus (segment RNA2)

Assembly

**Coverage Length**	1113 (2 contig(s))
**Depth Of Coverage**	1610.5
**Number Of Reads**	14899
**Reads Per Million**	279.63 rpm (after QC)
**Ambiguities**	0
**Assembly Method**	de novo + reference guided assembly
**Consensus Caller**	Bcf Tools

Coverage Map

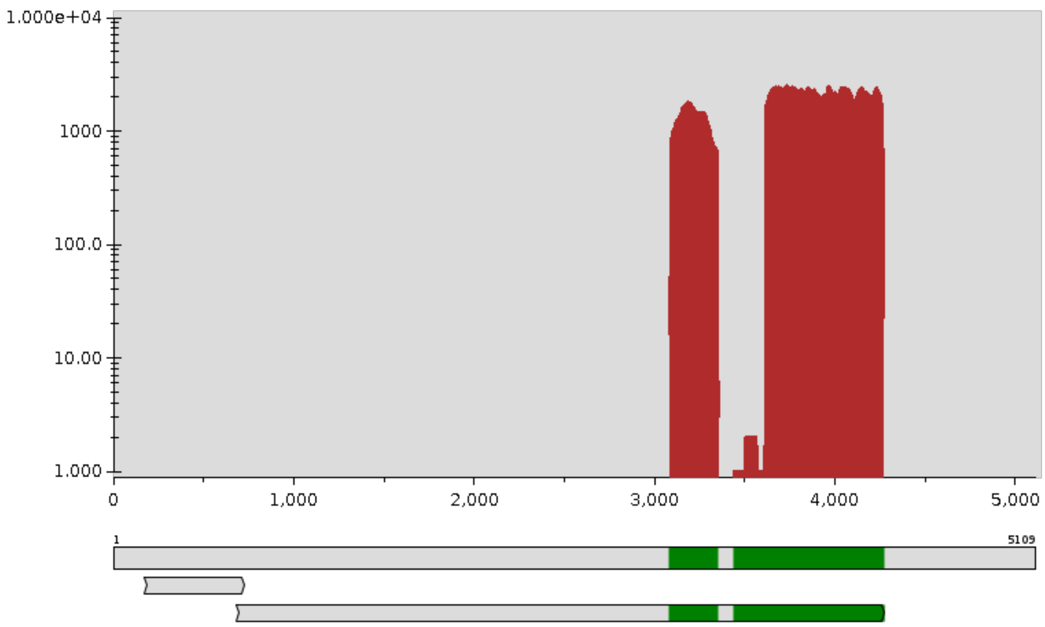

Assignment

**Type**	Tomato chocolate spot virus (Taxonomy ID: 661101)
**Reference Genome**	NC_013076.1
**NT Identity (%)**	67.028
**AA Identity (%)**	74.7967
**Number Of Stop Codons**	1
**Number Of CDS**	2

Alignment

**Alignment Score**	739.0 (NT) + 2112.0 (AA) = 2851.0
**Concordance (%)**	58.9659

Alignment Method	Global, seeded, nucleotide + amino acids (AGA)

Genome Region

Sequence starts at position 3084 and ends at position 4276 relative to NC_013076.1 reference sequence.

Alignment Detailed Statistics

	Begin	End	Coverage	Score	Concordance	Matches	Identities	I/D/M/F*	Stop Codons
**NT**	**3084**	**4276**	**21.8%**	**739**	**33.6%**	**1107 (99.5%)**	**742 (66.7%)**	**0/6**	
Mutations:	3094T>A, 3098A>G, 3100T>C, 3103G>A, 3106A>G, 3109G>C, 3110C>T, 3112C>A, 3115C>T, 3121A>T, 3128T>C, 3130G>T, 3136A>T, 3139A>T, 3140C>T, 3142C>G, 3145G>T, 3148A>T, 3151G>A, 3152A>G, 3153A>C, 3154C>A, 3158A>G, 3164T>G, 3166A>C, 3168A>C, 3169T>A, 3172A>T, 3182T>C, 3184T>A, 3187C>T, 3193T>C, 3196T>C, 3199A>G, 3202G>A, 3211T>A, 3214G>A, 3215A>G, 3224A>C, 3225A>G, 3226G>T, 3231G>A, 3232C>G, 3238A>G, 3242A>T, 3244T>C, 3245G>C, 3247C>A, 3250G>A, 3253C>T, 3263A>T, 3264A>G, 3265G>C, 3268C>T, 3269T>C, 3272T>C, 3274G>C, 3277C>T, 3280T>A, 3283A>G, 3284A>C, 3289G>A, 3292C>T, 3296C>T, 3298G>A, 3301G>A, 3307G>A, 3308G>A, 3309G>A, 3310A>G, 3314T>C, 3315T>A, 3316T>A, 3317T>G, 3319C>T, 3323T>A, 3325A>G, 3328G>C, 3331T>A, 3337T>A, 3343T>G, 3442T>A, 3445G>T, 3454C>T, 3460A>T, 3463C>T, 3468C>A, 3469A>C, 3472A>T, 3475A>T, 3477A>G, 3478G>A, 3482G>T, 3484G>T, 3490C>T, 3499C>T, 3505G>A, 3509G>A, 3511T>C, 3512A>C, 3516C>A, 3517C>T, 3520G>T, 3522G>T, 3526G>T, 3530A>C, 3533G>T, 3535A>G, 3538G>A, 3541C>G, 3544G>A, 3547G>A, 3550T>A, 3553C>T, 3556G>T, 3559A>T, 3560C>A, 3561A>T, 3562A>C, 3564A>T, 3568T>C, 3569G>C, 3570C>A, 3571C>G, 3574G>A, 3577T>G, 3581C>A, 3583G>C, 3584_3589delCCAGTC, 3593C>A, 3594A>C, 3596A>T, 3597G>C, 3598C>T, 3601G>A, 3604G>T, 3605A>T, 3606A>C, 3607T>C, 3610G>C, 3611T>C, 3612C>A, 3613C>G, 3614A>C, 3617A>C, 3622G>A, 3628T>C, 3631A>T, 3637T>A, 3640A>T, 3643C>T, 3646G>A, 3652C>T, 3655C>T, 3656A>T, 3657T>C, 3658G>T, 3660G>C, 3671G>A, 3672A>C, 3673A>C, 3674G>A, 3675A>G, 3676A>C, 3677A>C, 3679G>C, 3680T>C, 3683C>G, 3685T>G, 3688A>T, 3689A>C, 3691A>T, 3703C>G, 3709A>T, 3712G>A, 3715C>A, 3718T>C, 3719G>T, 3720A>C, 3722G>C, 3724T>C, 3725C>A, 3726A>C, 3727G>C, 3731G>C, 3733T>C, 3745A>C, 3751C>G, 3757T>C, 3760C>T, 3772T>C, 3773A>T, 3775A>T, 3778C>A, 3784T>C, 3787C>T, 3793A>T, 3799C>T, 3802C>T, 3803A>G, 3805T>G, 3808T>C, 3809T>A, 3812A>C, 3814A>C, 3815G>A, 3817T>C, 3818G>A, 3820G>C, 3823C>T, 3824C>A, 3826A>G, 3829T>C, 3832G>A, 3833T>G, 3834C>G, 3836A>T, 3837G>C, 3838C>T, 3839C>A, 3840C>A, 3844T>C, 3845G>A, 3847C>T, 3850A>T, 3853C>T, 3854A>G, 3856T>A, 3859G>T, 3865A>G, 3869C>T, 3871T>G, 3875T>A, 3880T>A, 3883T>C, 3889A>T, 3893C>T, 3895A>T, 3896G>T, 3897C>A, 3898A>T, 3901C>T, 3902C>G, 3905A>G, 3906G>C, 3907T>A, 3908A>G, 3913A>G, 3914A>G, 3915A>C, 3916T>A, 3917T>C, 3922A>G, 3923G>C, 3934A>C, 3937A>T, 3940A>T, 3946G>A, 3952C>T, 3955T>C, 3961T>C, 3964T>A, 3970A>C, 3973A>T, 3979A>T, 3985T>C, 3989A>G, 3990C>T, 3994T>G, 4000T>C, 4004G>A, 4013T>C, 4014G>C, 4016A>C, 4021A>G, 4022C>T, 4024C>T, 4028C>A, 4029G>A, 4030C>G, 4031A>G, 4032T>C, 4033G>A, 4034A>C, 4039G>A, 4042C>T, 4043T>G, 4044C>A, 4045A>C, 4048A>T, 4050C>A, 4051T>G, 4052C>A, 4053A>G, 4054A>T, 4058A>C, 4060A>T, 4063A>T, 4064A>G, 4066G>T, 4070C>T, 4072T>G, 4078T>C, 4080G>A, 4081G>A, 4082C>T, 4084C>G, 4088A>C, 4089A>T, 4090C>T, 4091T>C, 4093A>C, 4094A>T, 4096A>G, 4099A>C, 4105T>A, 4108C>A, 4116G>A, 4117G>C, 4118C>T, 4120T>A, 4121G>A, 4123G>T, 4126T>C, 4129A>C, 4132A>T, 4135G>A, 4138T>C, 4139C>A, 4141C>G, 4142A>G, 4144C>T, 4145T>A, 4146C>A, 4147C>G, 4150G>A, 4153T>G, 4156T>A, 4159C>T, 4162C>T, 4164A>G, 4165A>G, 4166C>T, 4168G>A, 4172G>C, 4174G>T, 4177A>C, 4187T>C, 4188C>T, 4189T>G, 4192C>T, 4195C>T, 4198C>T, 4201G>A, 4204G>A, 4206T>A, 4213G>T, 4214A>G, 4216C>T, 4225A>G, 4228C>T, 4234T>C, 4255T>A, 4258G>A, 4261C>G, 4267T>C									

CDS

	Begin	End	Coverage	Score	Concordance	Matches	Identities	I/D/M/F*	Stop Codons
ToChV_s2_gp2	801	1197	31.0%	2112	79.8%	369 (99.5%)	276 (74.4%)	0/2/0/0	1
Protein mutations:	I805V (3098A>G 3100T>C), N823A (3152A>G 3153A>C 3154C>A), K825E (3158A>G), S827A (3164T>G 3166A>C), N828T (3168A>C 3169T>A), F833L (3182T>C 3184T>A), M844V (3215A>G), K847R (3224A>C 3225A>G 3226G>T), R849Q (3231G>A 3232C>G), T853S (3242A>T 3244T>C), V854L (3245G>C 3247C>A), K860C (3263A>T 3264A>G 3265G>C), G875K (3308G>A 3309G>A 3310A>G), F877Q (3314T>C 3315T>A 3316T>A), S878A (3317T>G 3319C>T), L880M (3323T>A 3325A>G), T928N (3468C>A 3469A>C), K929N (3472A>T), K931R (3477A>G 3478G>A), A933S (3482G>T 3484G>T), D942N (3509G>A 3511T>C), M943L (3512A>C), T944N (3516C>A 3517C>T), Q945H (3520G>T), R946L (3522G>T), K947N (3526G>T), I949L (3530A>C), E950* (3533G>T 3535A>G), D955E (3550T>A), E958D (3559A>T), Q959I (3560C>A 3561A>T 3562A>C), Y960F (3564A>T), A962Q (3569G>C 3570C>A 3571C>G), Q966N (3581C>A 3583G>C), P967_V968del (3584_3589delCCAGTC), Q970T (3593C>A 3594A>C), N974S (3605A>T 3606A>C 3607T>C), L975F (3610G>C), S976Q (3611T>C 3612C>A 3613C>G), I977L (3614A>C), M991S (3656A>T 3657T>C 3658G>T), G992A (3660G>C), E996T (3671G>A 3672A>C 3673A>C), E997S (3674G>A 3675A>G 3676A>C), L1000V (3683C>G 3685T>G), I1002L (3689A>C 3691A>T), E1012S (3719G>T 3720A>C), A1013P (3722G>C 3724T>C), Q1014T (3725C>A 3726A>C 3727G>C), A1016P (3731G>C 3733T>C), T1030S (3773A>T 3775A>T), N1040E (3803A>G 3805T>G), S1042T (3809T>A), I1043L (3812A>C 3814A>C), V1044I (3815G>A 3817T>C), V1045I (3818G>A 3820G>C), S1050G (3833T>G 3834C>G), P1052N (3839C>A 3840C>A), V1054I (3845G>A 3847C>T), I1057V (3854A>G 3856T>A), S1064T (3875T>A), P1070S (3893C>T 3895A>T), A1071Y (3896G>T 3897C>A 3898A>T), L1073V (3902C>G), S1074A (3905A>G 3906G>C 3907T>A), K1075E (3908A>G), N1077A (3914A>G 3915A>C 3916T>A), Y1078H (3917T>C), V1080L (3923G>C), T1102V (3989A>G 3990C>T), E1107K (4004G>A), C1110P (4013T>C 4014G>C), H1113Y (4022C>T 4024C>T), R1115K (4028C>A 4029G>A 4030C>G), M1116A (4031A>G 4032T>C 4033G>A), S1120D (4043T>G 4044C>A 4045A>C), K1121N (4048A>T), T1122K (4050C>A 4051T>G), Q1123S (4052C>A 4053A>G 4054A>T), M1127V (4064A>G 4066G>T), R1132K (4080G>A 4081G>A), N1135L (4088A>C 4089A>T 4090C>T), I1137L (4094A>T 4096A>G), G1144D (4116G>A 4117G>C), V1146I (4121G>A 4123G>T), L1152M (4139C>A 4141C>G), I1153V (4142A>G 4144C>T), S1154K (4145T>A 4146C>A 4147C>G), K1160R (4164A>G 4165A>G), V1163L (4172G>C 4174G>T), S1168L (4187T>C 4188C>T 4189T>G), F1174Y (4206T>A), N1177D (4214A>G 4216C>T)									
Codon mutations:	ATT803ATA (3094T>A), ATT805GTC (3098A>G 3100T>C), GAG806GAA (3103G>A), AAA807AAG (3106A>G), ACG808ACC (3109G>C), CTC809TTA (3110C>T 3112C>A), TTC810TTT (3115C>T), TCA812TCT (3121A>T), TTG815CTT (3128T>C 3130G>T), ATA817ATT (3136A>T), GGA818GGT (3139A>T), CTC819TTG (3140C>T 3142C>G), GGG820GGT (3145G>T), ACA821ACT (3148A>T), CTG822CTA (3151G>A), AAC823GCA (3152A>G 3153A>C 3154C>A), AAA825GAA (3158A>G), TCA827GCC (3164T>G 3166A>C), AAT828ACA (3168A>C 3169T>A), GCA829GCT (3172A>T), TTT833CTA (3182T>C 3184T>A), AAC834AAT (3187C>T), CCT836CCC (3193T>C), CAT837CAC (3196T>C), GTA838GTG (3199A>G), GTG839GTA (3202G>A), CTT842CTA (3211T>A), GAG843GAA (3214G>A), ATG844GTG (3215A>G), AAG847CGT (3224A>C 3225A>G 3226G>T), CGC849CAG (3231G>A 3232C>G), AAA851AAG (3238A>G), ACT853TCC (3242A>T 3244T>C), GTC854CTA (3245G>C 3247C>A), ACG855ACA (3250G>A), AAC856AAT (3253C>T), AAG860TGC (3263A>T 3264A>G 3265G>C), AAC861AAT (3268C>T), TTG862CTG (3269T>C), TTG863CTC (3272T>C 3274G>C), TCC864TCT (3277C>T), ACT865ACA (3280T>A), GGA866GGG (3283A>G), AGG867CGG (3284A>C), AAG868AAA (3289G>A), AGC869AGT (3292C>T), CTG871TTA (3296C>T 3298G>A), CCG872CCA (3301G>A), CCG874CCA (3307G>A), GGA875AAG (3308G>A 3309G>A 3310A>G), TTT877CAA (3314T>C 3315T>A 3316T>A), TCC878GCT (3317T>G 3319C>T), TTA880ATG (3323T>A 3325A>G), CGG881CGC (3328G>C), CTT882CTA (3331T>A), GCT884GCA (3337T>A), GTT886GTG (3343T>G), TCT919TCA (3442T>A), GTG920GTT (3445G>T), ATC923ATT (3454C>T), GGA925GGT (3460A>T), CAC926CAT (3463C>T), ACA928AAC (3468C>A 3469A>C), AAA929AAT (3472A>T), GGA930GGT (3475A>T), AAG931AGA (3477A>G 3478G>A), GCG933TCT (3482G>T 3484G>T), GAC935GAT (3490C>T), TTC938TTT (3499C>T), GAG940GAA (3505G>A), GAT942AAC (3509G>A 3511T>C), ATG943CTG (3512A>C), ACC944AAT (3516C>A 3517C>T), CAG945CAT (3520G>T), CGC946CTC (3522G>T), AAG947AAT (3526G>T), ATT949CTT (3530A>C), GAA950TAG (3533G>T 3535A>G), AAG951AAA (3538G>A), CTC952CTG (3541C>G), AGG953AGA (3544G>A), AAG954AAA (3547G>A), GAT955GAA (3550T>A), AAC956AAT (3553C>T), GTG957GTT (3556G>T), GAA958GAT (3559A>T), CAA959ATC (3560C>A 3561A>T 3562A>C), TAT960TTT (3564A>T), GAT961GAC (3568T>C), GCC962CAG (3569G>C 3570C>A 3571C>G), AAG963AAA (3574G>A), GGT964GGG (3577T>G), CAG966AAC (3581C>A 3583G>C), CCA967_GTC968del (3584_3589delCCAGTC), CAA970ACA (3593C>A 3594A>C), AGC971TCT (3596A>T 3597G>C 3598C>T), CAG972CAA (3601G>A), GTG973GTT (3604G>T), AAT974TCC (3605A>T 3606A>C 3607T>C), TTG975TTC (3610G>C), TCC976CAG (3611T>C 3612C>A 3613C>G), ATT977CTT (3614A>C), AGG978CGG (3617A>C), GAG979GAA (3622G>A), TTT981TTC (3628T>C), TCA982TCT (3631A>T), GGT984GGA (3637T>A), GCA985GCT (3640A>T), GTC986GTT (3643C>T), CAG987CAA (3646G>A), TTC989TTT (3652C>T), TGC990TGT (3655C>T), ATG991TCT (3656A>T 3657T>C 3658G>T), GGC992GCC (3660G>C), GAA996ACC (3671G>A 3672A>C 3673A>C), GAA997AGC (3674G>A 3675A>G 3676A>C), AGG998CGC (3677A>C 3679G>C), TTG999CTG (3680T>C), CTT1000GTG (3683C>G 3685T>G), GTA1001GTT (3688A>T), ATA1002CTT (3689A>C 3691A>T), CCC1006CCG (3703C>G), TCA1008TCT (3709A>T), GTG1009GTA (3712G>A), CGC1010CGA (3715C>A), TTT1011TTC (3718T>C), GAA1012TCA (3719G>T 3720A>C), GCT1013CCC (3722G>C 3724T>C), CAG1014ACC (3725C>A 3726A>C 3727G>C), GCT1016CCC (3731G>C 3733T>C), GCA1020GCC (3745A>C), ACC1022ACG (3751C>G), CCT1024CCC (3757T>C), TTC1025TTT (3760C>T), TGT1029TGC (3772T>C), ACA1030TCT (3773A>T 3775A>T), TCC1031TCA (3778C>A), TGT1033TGC (3784T>C), TAC1034TAT (3787C>T), TCA1036TCT (3793A>T), AGC1038AGT (3799C>T), CTC1039CTT (3802C>T), AAT1040GAG (3803A>G 3805T>G), TAT1041TAC (3808T>C), TCC1042ACC (3809T>A), ATA1043CTC (3812A>C 3814A>C), GTT1044ATC (3815G>A 3817T>C), GTG1045ATC (3818G>A 3820G>C), CAC1046CAT (3823C>T), CGA1047AGG (3824C>A 3826A>G), GTT1048GTC (3829T>C), CAG1049CAA (3832G>A), TCC1050GGC (3833T>G 3834C>G), AGC1051TCT (3836A>T 3837G>C 3838C>T), CCT1052AAT (3839C>A 3840C>A), AAT1053AAC (3844T>C), GTC1054ATT (3845G>A 3847C>T), GGA1055GGT (3850A>T), GGC1056GGT (3853C>T), ATT1057GTA (3854A>G 3856T>A), CTG1058CTT (3859G>T), GTA1060GTG (3865A>G), CTT1062TTG (3869C>T 3871T>G), TCC1064ACC (3875T>A), TCT1065TCA (3880T>A), GGT1066GGC (3883T>C), CCA1068CCT (3889A>T), CCA1070TCT (3893C>T 3895A>T), GCA1071TAT (3896G>T 3897C>A 3898A>T), GGC1072GGT (3901C>T), CTG1073GTG (3902C>G), AGT1074GCA (3905A>G 3906G>C 3907T>A), AAG1075GAG (3908A>G), GGA1076GGG (3913A>G), AAT1077GCA (3914A>G 3915A>C 3916T>A), TAT1078CAT (3917T>C), GTA1079GTG (3922A>G), GTT1080CTT (3923G>C), GGA1083GGC (3934A>C), GGA1084GGT (3937A>T), GGA1085GGT (3940A>T), AAG1087AAA (3946G>A), AAC1089AAT (3952C>T), TTT1090TTC (3955T>C), TAT1092TAC (3961T>C), GGT1093GGA (3964T>A), ACA1095ACC (3970A>C), ACA1096ACT (3973A>T), ATA1098ATT (3979A>T), TCT1100TCC (3985T>C), ACG1102GTG (3989A>G 3990C>T), GTT1103GTG (3994T>G), GAT1105GAC (4000T>C), GAA1107AAA (4004G>A), TGT1110CCT (4013T>C 4014G>C), AGG1111CGG (4016A>C), AGA1112AGG (4021A>G), CAC1113TAT (4022C>T 4024C>T), CGC1115AAG (4028C>A 4029G>A 4030C>G), ATG1116GCA (4031A>G 4032T>C 4033G>A), AGA1117CGA (4034A>C), GAG1118GAA (4039G>A), TTC1119TTT (4042C>T), TCA1120GAC (4043T>G 4044C>A 4045A>C), AAA1121AAT (4048A>T), ACT1122AAG (4050C>A 4051T>G), CAA1123AGT (4052C>A 4053A>G 4054A>T), AGA1125CGT (4058A>C 4060A>T), ATA1126ATT (4063A>T), ATG1127GTT (4064A>G 4066G>T), CTT1129TTG (4070C>T 4072T>G), GAT1131GAC (4078T>C), AGG1132AAA (4080G>A 4081G>A), CTC1133TTG (4082C>T 4084C>G), AAC1135CTT (4088A>C 4089A>T 4090C>T), TTA1136CTC (4091T>C 4093A>C), ATA1137TTG (4094A>T 4096A>G), ATA1138ATC (4099A>C), CTT1140CTA (4105T>A), CCC1141CCA (4108C>A), GGG1144GAC (4116G>A 4117G>C), CTT1145TTA (4118C>T 4120T>A), GTG1146ATT (4121G>A 4123G>T), AGT1147AGC (4126T>C), TCA1148TCC (4129A>C), ATA1149ATT (4132A>T), GAG1150GAA (4135G>A), ATT1151ATC (4138T>C), CTC1152ATG (4139C>A 4141C>G), ATC1153GTT (4142A>G 4144C>T), TCC1154AAG (4145T>A 4146C>A 4147C>G), CCG1155CCA (4150G>A), GGT1156GGG (4153T>G), CCT1157CCA (4156T>A), GAC1158GAT (4159C>T), TTC1159TTT (4162C>T), AAA1160AGG (4164A>G 4165A>G), CTG1161TTA (4166C>T 4168G>A), GTG1163CTT (4172G>C 4174G>T), GCA1164GCC (4177A>C), TCT1168CTG (4187T>C 4188C>T 4189T>G), GCC1169GCT (4192C>T), AAC1170AAT (4195C>T), CAC1171CAT (4198C>T), GAG1172GAA (4201G>A), AAG1173AAA (4204G>A), TTT1174TAT (4206T>A), GGG1176GGT (4213G>T), AAC1177GAT (4214A>G 4216C>T), ACA1180ACG (4225A>G), CAC1181CAT (4228C>T), TAT1183TAC (4234T>C), TCT1190TCA (4255T>A), GAG1191GAA (4258G>A), CTC1192CTG (4261C>G), GAT1194GAC (4267T>C)									

Proteins

	Begin	End	Coverage	Score	Concordance	Matches	Identities	I/D/M/F*	Stop Codons
polyprotein (YP_003097231.1)	801	1197	31.0%	2112	79.8%	369 (99.5%)	276 (74.4%)	0/2/0/0	1

	Begin	End	Coverage	Score	Concordance	Matches	Identities	I/D/M/F*	Stop Codons
**NT**	**3084**	**4276**	**21.8%**	**739**	**33.6%**	**1107 (99.5%)**	**742 (66.7%)**	**0/6**	
Protein mutations:	I805V (3098A>G 3100T>C), N823A (3152A>G 3153A>C 3154C>A), K825E (3158A>G), S827A (3164T>G 3166A>C), N828T (3168A>C 3169T>A), F833L (3182T>C 3184T>A), M844V (3215A>G), K847R (3224A>C 3225A>G 3226G>T), R849Q (3231G>A 3232C>G), T853S (3242A>T 3244T>C), V854L (3245G>C 3247C>A), K860C (3263A>T 3264A>G 3265G>C), G875K (3308G>A 3309G>A 3310A>G), F877Q (3314T>C 3315T>A 3316T>A), S878A (3317T>G 3319C>T), L880M (3323T>A 3325A>G), T928N (3468C>A 3469A>C), K929N (3472A>T), K931R (3477A>G 3478G>A), A933S (3482G>T 3484G>T), D942N (3509G>A 3511T>C), M943L (3512A>C), T944N (3516C>A 3517C>T), Q945H (3520G>T), R946L (3522G>T), K947N (3526G>T), I949L (3530A>C), E950* (3533G>T 3535A>G), D955E (3550T>A), E958D (3559A>T), Q959I (3560C>A 3561A>T 3562A>C), Y960F (3564A>T), A962Q (3569G>C 3570C>A 3571C>G), Q966N (3581C>A 3583G>C), P967_V968del (3584_3589delCCAGTC), Q970T (3593C>A 3594A>C), N974S (3605A>T 3606A>C 3607T>C), L975F (3610G>C), S976Q (3611T>C 3612C>A 3613C>G), I977L (3614A>C), M991S (3656A>T 3657T>C 3658G>T), G992A (3660G>C), E996T (3671G>A 3672A>C 3673A>C), E997S (3674G>A 3675A>G 3676A>C), L1000V (3683C>G 3685T>G), I1002L (3689A>C 3691A>T), E1012S (3719G>T 3720A>C), A1013P (3722G>C 3724T>C), Q1014T (3725C>A 3726A>C 3727G>C), A1016P (3731G>C 3733T>C), T1030S (3773A>T 3775A>T), N1040E (3803A>G 3805T>G), S1042T (3809T>A), I1043L (3812A>C 3814A>C), V1044I (3815G>A 3817T>C), V1045I (3818G>A 3820G>C), S1050G (3833T>G 3834C>G), P1052N (3839C>A 3840C>A), V1054I (3845G>A 3847C>T), I1057V (3854A>G 3856T>A), S1064T (3875T>A), P1070S (3893C>T 3895A>T), A1071Y (3896G>T 3897C>A 3898A>T), L1073V (3902C>G), S1074A (3905A>G 3906G>C 3907T>A), K1075E (3908A>G), N1077A (3914A>G 3915A>C 3916T>A), Y1078H (3917T>C), V1080L (3923G>C), T1102V (3989A>G 3990C>T), E1107K (4004G>A), C1110P (4013T>C 4014G>C), H1113Y (4022C>T 4024C>T), R1115K (4028C>A 4029G>A 4030C>G), M1116A (4031A>G 4032T>C 4033G>A), S1120D (4043T>G 4044C>A 4045A>C), K1121N (4048A>T), T1122K (4050C>A 4051T>G), Q1123S (4052C>A 4053A>G 4054A>T), M1127V (4064A>G 4066G>T), R1132K (4080G>A 4081G>A), N1135L (4088A>C 4089A>T 4090C>T), I1137L (4094A>T 4096A>G), G1144D (4116G>A 4117G>C), V1146I (4121G>A 4123G>T), L1152M (4139C>A 4141C>G), I1153V (4142A>G 4144C>T), S1154K (4145T>A 4146C>A 4147C>G), K1160R (4164A>G 4165A>G), V1163L (4172G>C 4174G>T), S1168L (4187T>C 4188C>T 4189T>G), F1174Y (4206T>A), N1177D (4214A>G 4216C>T)								
Codon mutations:	ATT803ATA (3094T>A), ATT805GTC (3098A>G 3100T>C), GAG806GAA (3103G>A), AAA807AAG (3106A>G), ACG808ACC (3109G>C), CTC809TTA (3110C>T 3112C>A), TTC810TTT (3115C>T), TCA812TCT (3121A>T), TTG815CTT (3128T>C 3130G>T), ATA817ATT (3136A>T), GGA818GGT (3139A>T), CTC819TTG (3140C>T 3142C>G), GGG820GGT (3145G>T), ACA821ACT (3148A>T), CTG822CTA (3151G>A), AAC823GCA (3152A>G 3153A>C 3154C>A), AAA825GAA (3158A>G), TCA827GCC (3164T>G 3166A>C), AAT828ACA (3168A>C 3169T>A), GCA829GCT (3172A>T), TTT833CTA (3182T>C 3184T>A), AAC834AAT (3187C>T), CCT836CCC (3193T>C), CAT837CAC (3196T>C), GTA838GTG (3199A>G), GTG839GTA (3202G>A), CTT842CTA (3211T>A), GAG843GAA (3214G>A), ATG844GTG (3215A>G), AAG847CGT (3224A>C 3225A>G 3226G>T), CGC849CAG (3231G>A 3232C>G), AAA851AAG (3238A>G), ACT853TCC (3242A>T 3244T>C), GTC854CTA (3245G>C 3247C>A), ACG855ACA (3250G>A), AAC856AAT (3253C>T), AAG860TGC (3263A>T 3264A>G 3265G>C), AAC861AAT (3268C>T), TTG862CTG (3269T>C), TTG863CTC (3272T>C 3274G>C), TCC864TCT (3277C>T), ACT865ACA (3280T>A), GGA866GGG (3283A>G), AGG867CGG (3284A>C), AAG868AAA (3289G>A), AGC869AGT (3292C>T), CTG871TTA (3296C>T 3298G>A), CCG872CCA (3301G>A), CCG874CCA (3307G>A), GGA875AAG (3308G>A 3309G>A 3310A>G), TTT877CAA (3314T>C 3315T>A 3316T>A), TCC878GCT (3317T>G 3319C>T), TTA880ATG (3323T>A 3325A>G), CGG881CGC (3328G>C), CTT882CTA (3331T>A), GCT884GCA (3337T>A), GTT886GTG (3343T>G), TCT919TCA (3442T>A), GTG920GTT (3445G>T), ATC923ATT (3454C>T), GGA925GGT (3460A>T), CAC926CAT (3463C>T), ACA928AAC (3468C>A 3469A>C), AAA929AAT (3472A>T), GGA930GGT (3475A>T), AAG931AGA (3477A>G 3478G>A), GCG933TCT (3482G>T 3484G>T), GAC935GAT (3490C>T), TTC938TTT (3499C>T), GAG940GAA (3505G>A), GAT942AAC (3509G>A 3511T>C), ATG943CTG (3512A>C), ACC944AAT (3516C>A 3517C>T), CAG945CAT (3520G>T), CGC946CTC (3522G>T), AAG947AAT (3526G>T), ATT949CTT (3530A>C), GAA950TAG (3533G>T 3535A>G), AAG951AAA (3538G>A), CTC952CTG (3541C>G), AGG953AGA (3544G>A), AAG954AAA (3547G>A), GAT955GAA (3550T>A), AAC956AAT (3553C>T), GTG957GTT (3556G>T), GAA958GAT (3559A>T), CAA959ATC (3560C>A 3561A>T 3562A>C), TAT960TTT (3564A>T), GAT961GAC (3568T>C), GCC962CAG (3569G>C 3570C>A 3571C>G), AAG963AAA (3574G>A), GGT964GGG (3577T>G), CAG966AAC (3581C>A 3583G>C), CCA967_GTC968del (3584_3589delCCAGTC), CAA970ACA (3593C>A 3594A>C), AGC971TCT (3596A>T 3597G>C 3598C>T), CAG972CAA (3601G>A), GTG973GTT (3604G>T), AAT974TCC (3605A>T 3606A>C 3607T>C), TTG975TTC (3610G>C), TCC976CAG (3611T>C 3612C>A 3613C>G), ATT977CTT (3614A>C), AGG978CGG (3617A>C), GAG979GAA (3622G>A), TTT981TTC (3628T>C), TCA982TCT (3631A>T), GGT984GGA (3637T>A), GCA985GCT (3640A>T), GTC986GTT (3643C>T), CAG987CAA (3646G>A), TTC989TTT (3652C>T), TGC990TGT (3655C>T), ATG991TCT (3656A>T 3657T>C 3658G>T), GGC992GCC (3660G>C), GAA996ACC (3671G>A 3672A>C 3673A>C), GAA997AGC (3674G>A 3675A>G 3676A>C), AGG998CGC (3677A>C 3679G>C), TTG999CTG (3680T>C), CTT1000GTG (3683C>G 3685T>G), GTA1001GTT (3688A>T), ATA1002CTT (3689A>C 3691A>T), CCC1006CCG (3703C>G), TCA1008TCT (3709A>T), GTG1009GTA (3712G>A), CGC1010CGA (3715C>A), TTT1011TTC (3718T>C), GAA1012TCA (3719G>T 3720A>C), GCT1013CCC (3722G>C 3724T>C), CAG1014ACC (3725C>A 3726A>C 3727G>C), GCT1016CCC (3731G>C 3733T>C), GCA1020GCC (3745A>C), ACC1022ACG (3751C>G), CCT1024CCC (3757T>C), TTC1025TTT (3760C>T), TGT1029TGC (3772T>C), ACA1030TCT (3773A>T 3775A>T), TCC1031TCA (3778C>A), TGT1033TGC (3784T>C), TAC1034TAT (3787C>T), TCA1036TCT (3793A>T), AGC1038AGT (3799C>T), CTC1039CTT (3802C>T), AAT1040GAG (3803A>G 3805T>G), TAT1041TAC (3808T>C), TCC1042ACC (3809T>A), ATA1043CTC (3812A>C 3814A>C), GTT1044ATC (3815G>A 3817T>C), GTG1045ATC (3818G>A 3820G>C), CAC1046CAT (3823C>T), CGA1047AGG (3824C>A 3826A>G), GTT1048GTC (3829T>C), CAG1049CAA (3832G>A), TCC1050GGC (3833T>G 3834C>G), AGC1051TCT (3836A>T 3837G>C 3838C>T), CCT1052AAT (3839C>A 3840C>A), AAT1053AAC (3844T>C), GTC1054ATT (3845G>A 3847C>T), GGA1055GGT (3850A>T), GGC1056GGT (3853C>T), ATT1057GTA (3854A>G 3856T>A), CTG1058CTT (3859G>T), GTA1060GTG (3865A>G), CTT1062TTG (3869C>T 3871T>G), TCC1064ACC (3875T>A), TCT1065TCA (3880T>A), GGT1066GGC (3883T>C), CCA1068CCT (3889A>T), CCA1070TCT (3893C>T 3895A>T), GCA1071TAT (3896G>T 3897C>A 3898A>T), GGC1072GGT (3901C>T), CTG1073GTG (3902C>G), AGT1074GCA (3905A>G 3906G>C 3907T>A), AAG1075GAG (3908A>G), GGA1076GGG (3913A>G), AAT1077GCA (3914A>G 3915A>C 3916T>A), TAT1078CAT (3917T>C), GTA1079GTG (3922A>G), GTT1080CTT (3923G>C), GGA1083GGC (3934A>C), GGA1084GGT (3937A>T), GGA1085GGT (3940A>T), AAG1087AAA (3946G>A), AAC1089AAT (3952C>T), TTT1090TTC (3955T>C), TAT1092TAC (3961T>C), GGT1093GGA (3964T>A), ACA1095ACC (3970A>C), ACA1096ACT (3973A>T), ATA1098ATT (3979A>T), TCT1100TCC (3985T>C), ACG1102GTG (3989A>G 3990C>T), GTT1103GTG (3994T>G), GAT1105GAC (4000T>C), GAA1107AAA (4004G>A), TGT1110CCT (4013T>C 4014G>C), AGG1111CGG (4016A>C), AGA1112AGG (4021A>G), CAC1113TAT (4022C>T 4024C>T), CGC1115AAG (4028C>A 4029G>A 4030C>G), ATG1116GCA (4031A>G 4032T>C 4033G>A), AGA1117CGA (4034A>C), GAG1118GAA (4039G>A), TTC1119TTT (4042C>T), TCA1120GAC (4043T>G 4044C>A 4045A>C), AAA1121AAT (4048A>T), ACT1122AAG (4050C>A 4051T>G), CAA1123AGT (4052C>A 4053A>G 4054A>T), AGA1125CGT (4058A>C 4060A>T), ATA1126ATT (4063A>T), ATG1127GTT (4064A>G 4066G>T), CTT1129TTG (4070C>T 4072T>G), GAT1131GAC (4078T>C), AGG1132AAA (4080G>A 4081G>A), CTC1133TTG (4082C>T 4084C>G), AAC1135CTT (4088A>C 4089A>T 4090C>T), TTA1136CTC (4091T>C 4093A>C), ATA1137TTG (4094A>T 4096A>G), ATA1138ATC (4099A>C), CTT1140CTA (4105T>A), CCC1141CCA (4108C>A), GGG1144GAC (4116G>A 4117G>C), CTT1145TTA (4118C>T 4120T>A), GTG1146ATT (4121G>A 4123G>T), AGT1147AGC (4126T>C), TCA1148TCC (4129A>C), ATA1149ATT (4132A>T), GAG1150GAA (4135G>A), ATT1151ATC (4138T>C), CTC1152ATG (4139C>A 4141C>G), ATC1153GTT (4142A>G 4144C>T), TCC1154AAG (4145T>A 4146C>A 4147C>G), CCG1155CCA (4150G>A), GGT1156GGG (4153T>G), CCT1157CCA (4156T>A), GAC1158GAT (4159C>T), TTC1159TTT (4162C>T), AAA1160AGG (4164A>G 4165A>G), CTG1161TTA (4166C>T 4168G>A), GTG1163CTT (4172G>C 4174G>T), GCA1164GCC (4177A>C), TCT1168CTG (4187T>C 4188C>T 4189T>G), GCC1169GCT (4192C>T), AAC1170AAT (4195C>T), CAC1171CAT (4198C>T), GAG1172GAA (4201G>A), AAG1173AAA (4204G>A), TTT1174TAT (4206T>A), GGG1176GGT (4213G>T), AAC1177GAT (4214A>G 4216C>T), ACA1180ACG (4225A>G), CAC1181CAT (4228C>T), TAT1183TAC (4234T>C), TCT1190TCA (4255T>A), GAG1191GAA (4258G>A), CTC1192CTG (4261C>G), GAT1194GAC (4267T>C)								

*: Inserts / Deletes / Misaligned / Frameshifts

Analysis details

This analysis was performed with panviral2.64

NGS Details (UN18_val): Tokyovirus A1

Assembly

**Coverage Length**	164 (1 contig(s))
**Depth Of Coverage**	4800.1
**Number Of Reads**	12764
**Reads Per Million**	239.56 rpm (after QC)
**Ambiguities**	0
**Assembly Method**	de novo + reference guided assembly
**Consensus Caller**	Bcf Tools

Coverage Map

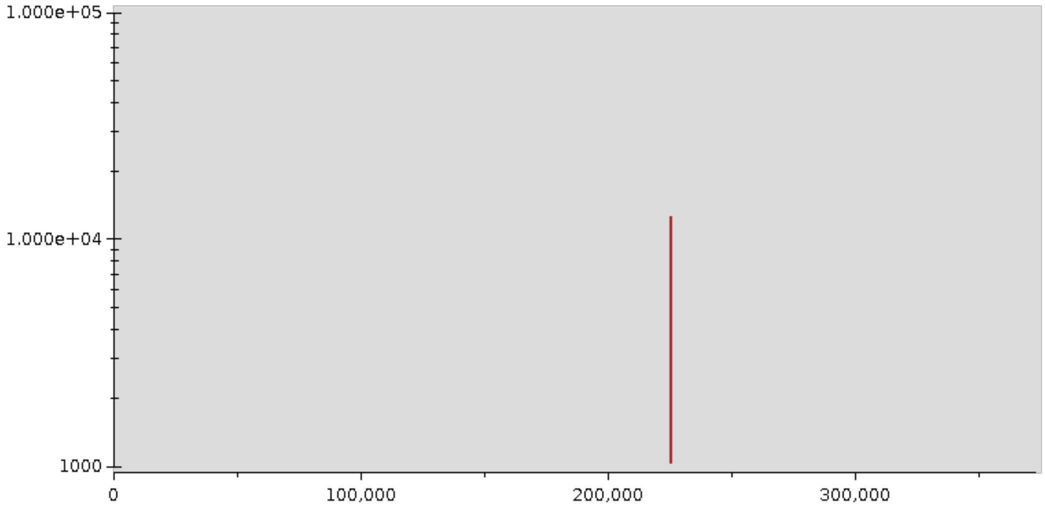

Assignment

**Type**	Tokyovirus A1 (Taxonomy ID: 1826170)
**Reference Genome**	NC_030230.1
**NT Identity (%)**	76.8293
**AA Identity (%)**	90.7407
**Number Of Stop Codons**	0
**Number Of CDS**	470

Alignment

**Alignment Score**	176.0 (NT) + 332.0 (AA) = 508.0
**Concordance (%)**	74.4868
**Alignment Method**	Local, heuristic, nucleotide (BLASTN)

Genome Region

Sequence starts at position 225492 and ends at position 225655 relative to NC_030230.1 reference sequence.

Alignment Detailed Statistics

	Begin	End	Coverage	Score	Concordance	Matches	Identities	I/D/M/F*	Stop Codons
**NT**	**225492**	**225655**	**0.1%**	**176**	**53.7%**	**164 (100%)**	**126 (76.8%)**	**0/0**	

Mutations: 225498T>G, 225500T>G, 225507A>G, 225510G>A, 225513G>C, 225516A>T, 225522T>C, 225525T>C, 225528T>C, 225531G>A, 225533C>T, 225534A>G, 225540A>G, 225545C>A, 225552T>G, 225554T>G, 225555C>G, 225561T>C, 225564T>A, 225573C>G, 225585A>C, 225587G>A, 225588T>G, 225591T>C, 225594T>C, 225600G>T, 225603C>G, 225609A>G, 225618G>A, 225621T>C, 225630T>G, 225631T>C, 225632G>T, 225636C>G, 225640G>T, 225641C>T, 225642T>G, 225651G>A

*: Inserts / Deletes / Misaligned / Frameshifts

Analysis details

This analysis was performed with panviral2.64

NGS Details (UN18_val): Leucotheavirus sp4

Assembly

**Coverage Length**	863 (1 contig(s))
**Depth Of Coverage**	880.3
**Number Of Reads**	6064
**Reads Per Million**	113.81 rpm (after QC)
**Ambiguities**	0
**Assembly Method**	de novo + reference guided assembly
**Consensus Caller**	Bcf Tools

Coverage Map

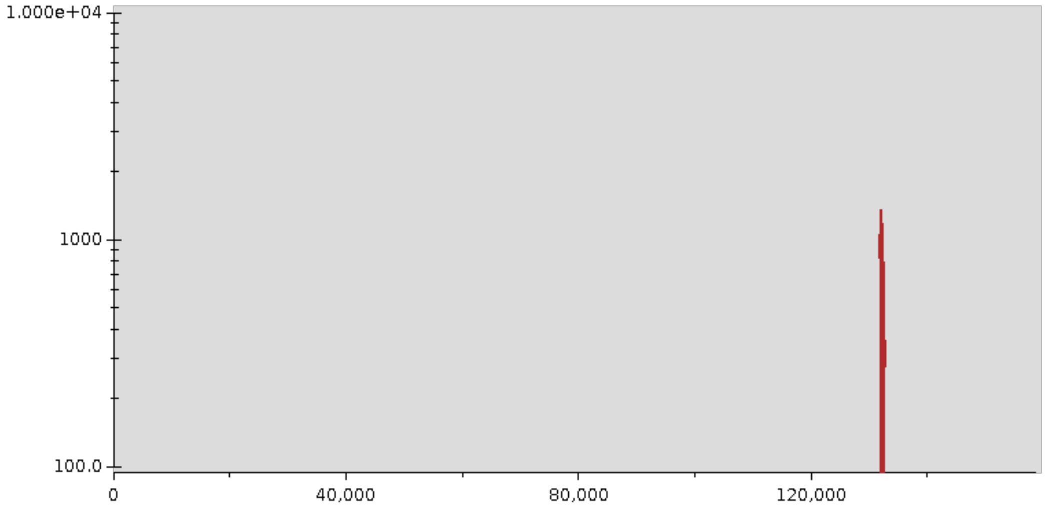

Assignment

**Type**	Leucotheavirus sp4 (Taxonomy ID: 2734113)
**Reference Genome**	NC_048102.1
**NT Identity (%)**	75.898
**AA Identity (%)**	85.4167
**Number Of Stop Codons**	0
**Number Of CDS**	194

Alignment

**Alignment Score**	894.0 (NT) + 1851.0 (AA) = 2745.0
**Concordance (%)**	72.7731
**Alignment Method**	Local, heuristic, nucleotide (BLASTN)

Genome Region

Sequence starts at position 131800 and ends at position 132662 relative to NC_048102.1 reference sequence.

Alignment Detailed Statistics

	Begin	End	Coverage	Score	Concordance	Matches	Identities	I/D/M/F*	Stop Codons
NT	131800	132662	0.5%	894	51.8%	863 (100%)	655 (75.9%)	0/0	
Mutations:	131802T>A, 131816G>T, 131818T>A, 131821T>G, 131824A>T, 131827T>A, 131830G>T, 131833T>C, 131836T>A, 131842C>T, 131845C>T, 131851T>C, 131857T>C, 131858G>A, 131861G>A, 131868G>C, 131872A>T, 131873A>G, 131874A>C, 131875T>A, 131878A>T, 131881T>A, 131885C>T, 131887T>A, 131890C>T, 131896T>C, 131899C>A, 131902T>C, 131911A>G, 131915A>T, 131917C>T, 131918C>G, 131920C>T, 131926G>A, 131932G>A, 131938T>C, 131949T>A, 131950C>T, 131951C>G, 131956C>A, 131957G>A, 131959A>T, 131960A>G, 131962C>T, 131963T>C, 131965T>A, 131972C>T, 131974G>A, 131975A>C, 131980T>C, 131981A>G, 131983C>A, 131984T>G, 131985T>C, 131987G>T, 131988C>G, 131989A>T, 131998A>T, 132004A>G, 132010A>G, 132014T>A, 132015C>G, 132018A>T, 132019T>C, 132025T>G, 132034T>A, 132043C>T, 132044T>G, 132045G>C, 132055C>T, 132058T>A, 132061A>T, 132073A>T, 132076A>T, 132077T>A, 132085C>T, 132089C>T, 132092G>A, 132097T>C, 132100T>A, 132101T>A, 132112T>A, 132113T>A, 132114C>G, 132115G>T, 132118C>T, 132124C>T, 132126C>G, 132127A>T, 132136C>A, 132139C>A, 132142T>C, 132157C>T, 132159A>T, 132164C>A, 132166G>T, 132169G>A, 132175A>G, 132178A>T, 132193G>T, 132202C>A, 132205C>T, 132208T>C, 132212C>T, 132214C>A, 132220T>G, 132226C>T, 132235T>C, 132238A>C, 132241T>C, 132244G>A, 132253A>T, 132266C>T, 132271T>A, 132278T>A, 132279C>G, 132280A>T, 132281C>T, 132284G>A, 132286T>C, 132287C>A, 132289T>G, 132298T>A, 132303C>A, 132307G>A, 132310C>T, 132311C>G, 132312A>C, 132313G>T, 132316C>T, 132317T>G, 132319T>A, 132327A>G, 132328G>A, 132340A>G, 132346G>A, 132352C>T, 132355C>T, 132358T>C, 132361T>A, 132364T>C, 132367A>T, 132373A>T, 132376C>T, 132379C>T, 132382T>C, 132385T>A, 132386C>T, 132397G>A, 132403A>T, 132404T>A, 132405C>G, 132406G>T, 132418C>T, 132425C>T, 132427G>A, 132439T>A, 132445A>T, 132454C>A, 132457T>A, 132469C>T, 132472T>C, 132475A>T, 132476C>T, 132478T>A, 132482G>A, 132484T>C, 132490C>T, 132496A>T, 132505G>A, 132517C>T, 132526G>A, 132530A>G, 132532C>A, 132533C>G, 132538T>C, 132542T>C, 132543C>A, 132544T>G, 132548A>C, 132549A>G, 132550G>T, 132553T>A, 132554C>A, 132556G>T, 132557C>A, 132558C>A, 132559T>C, 132562C>T, 132568A>T, 132571C>T, 132572G>A, 132574T>C, 132575C>A, 132583C>T, 132587G>A, 132588G>A, 132589T>C, 132590T>C, 132592G>T, 132613G>A, 132619C>T, 132622A>T, 132625C>T, 132637C>A, 132643T>A, 132650G>A, 132652T>C								

*: Inserts / Deletes / Misaligned / Frameshifts

Analysis details

This analysis was performed with panviral2.64

NGS Details (UN18_val): Noumeavirus

Assembly

**Coverage Length**	153 (1 contig(s))
**Depth Of Coverage**	2766.6
**Number Of Reads**	4184
**Reads Per Million**	78.53 rpm (after QC)
**Ambiguities**	0
**Assembly Method**	de novo + reference guided assembly
**Consensus Caller**	Bcf Tools

Coverage Map

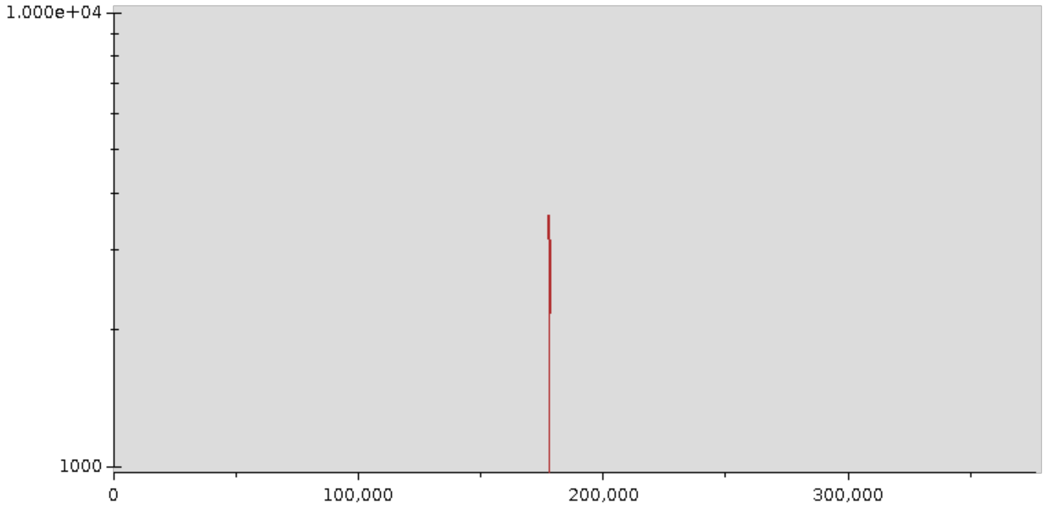

Assignment

**Type**	Noumeavirus (Taxonomy ID: 1955558)
**Reference Genome**	NC_033775.1
**NT Identity (%)**	81.6993
**AA Identity (%)**	92.1569
**Number Of Stop Codons**	0
**Number Of CDS**	452

Alignment

**Alignment Score**	194.0 (NT) + 300.0 (AA) = 494.0
**Concordance (%)**	79.6774
**Alignment Method**	Local, heuristic, nucleotide (BLASTN)

Genome Region

Sequence starts at position 177941 and ends at position 178093 relative to NC_033775.1 reference sequence.

Alignment Detailed Statistics

	Begin	End	Coverage	Score	Concordance	Matches	Identities	I/D/M/F*	Stop Codons
**NT**	**177941**	**178093**	**0.1%**	**194**	**63.4%**	**153 (100%)**	**125 (81.7%)**	**0/0**	

Mutations: 177953A>G, 177956G>C, 177977C>T, 177980A>T, 177989C>T, 177992G>C, 177996G>A, 178001A>G, 178002C>A, 178003A>C, 178004A>C, 178010T>C, 178019A>G, 178022A>G, 178025A>G, 178028C>T, 178034G>A, 178037C>T, 178043A>G, 178044C>A, 178046A>G, 178049T>G, 178055C>T, 178058C>T, 178061A>G, 178064A>G, 178079A>T, 178080A>C

*: Inserts / Deletes / Misaligned / Frameshifts

Analysis details

This analysis was performed with panviral2.64

NGS Details (UN18_val): Betabaculovirus disaccharalis

Assembly

**Coverage Length**	125 (1 contig(s))
**Depth Of Coverage**	1580.3
**Number Of Reads**	2638
**Reads Per Million**	49.51 rpm (after QC)
**Ambiguities**	0
**Assembly Method**	de novo + reference guided assembly
**Consensus Caller**	Bcf Tools

Coverage Map

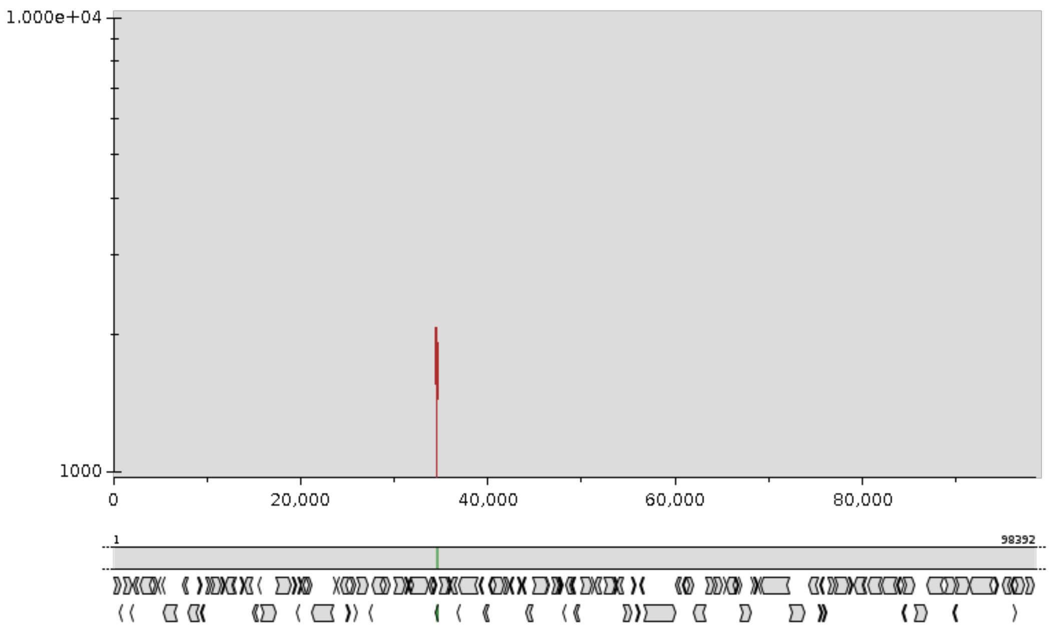

Assignment

**Type**	Betabaculovirus disaccharalis (Taxonomy ID: 3047795)
**Reference Genome**	NC_028491.1
**NT Identity (%)**	82.4
**AA Identity (%)**	92.8571
**Number Of Stop Codons**	0
**Number Of CDS**	125

Alignment

**Alignment Score**	162.0 (NT) + 265.0 (AA) = 427.0
**Concordance (%)**	81.1787

Alignment Method	Local, heuristic, nucleotide (BLASTN)

Genome Region

Sequence starts at position 34471 and ends at position 34595 relative to NC_028491.1 reference sequence.

Alignment Detailed Statistics

	Begin	End	Coverage	Score	Concordance	Matches	Identities	I/D/M/F*	Stop Codons
**NT**	**34471**	**34595**	**0.1%**	**162**	**64.8%**	**125 (100%)**	**103 (82.4%)**	**0/0**	
Mutations:	34482G>T, 34485T>C, 34488A>C, 34490G>A, 34491A>C, 34512G>T, 34513C>G, 34514T>A, 34521T>C, 34524A>G, 34533A>G, 34538A>C, 34545T>C, 34547G>T, 34548C>G, 34549T>C, 34551G>A, 34554T>C, 34569G>A, 34575T>A, 34578T>C, 34580A>G								
**CDS**									
v-ubq	61	102	35.3%	265	88.9%	42 (100%)	39 (92.9%)	0/0/0/0	0
Protein mutations:	E76G (34548C>G 34549T>C), S80A (34538A>C)								
Codon mutations:	TTA66CTG (34578T>C 34580A>G), ATA67ATT (34575T>A), GCC69GCT (34569G>A), GAA74GAG (34554T>C), GAC75GAT (34551G>A), GAG76GGC (34548C>G 34549T>C), CGA77AGG (34545T>C 34547G>T), TCA80GCA (34538A>C), GAT81GAC (34533A>G), ATT84ATC (34524A>G), CAA85CAG (34521T>C), AGC88TCA (34512G>T 34513C>G 34514T>A), CGT95CGG (34491A>C), CTT96TTG (34488A>C 34490G>A), AGA97AGG (34485T>C), GGC98GGA (34482G>T)								
**Proteins**									
ubiquitin-like protein (YP_009182246.1)	61	102	35.3%	265	88.9%	42 (100%)	39 (92.9%)	0/0/0/0	0
Protein mutations:	E76G (34548C>G 34549T>C), S80A (34538A>C)								
Codon mutations:	TTA66CTG (34578T>C 34580A>G), ATA67ATT (34575T>A), GCC69GCT (34569G>A), GAA74GAG (34554T>C), GAC75GAT (34551G>A), GAG76GGC (34548C>G 34549T>C), CGA77AGG (34545T>C 34547G>T), TCA80GCA (34538A>C), GAT81GAC (34533A>G), ATT84ATC (34524A>G), CAA85CAG (34521T>C), AGC88TCA (34512G>T 34513C>G 34514T>A), CGT95CGG (34491A>C), CTT96TTG (34488A>C 34490G>A), AGA97AGG (34485T>C), GGC98GGA (34482G>T)								

*: Inserts / Deletes / Misaligned / Frameshifts

Analysis details

This analysis was performed with panviral2.64

NGS Details (UN18_val): Lausannevirus

Assembly

**Coverage Length**	132 (1 contig(s))
**Depth Of Coverage**	1524.8
**Number Of Reads**	2565
**Reads Per Million**	48.14 rpm (after QC)
**Ambiguities**	0
**Assembly Method**	de novo + reference guided assembly
**Consensus Caller**	Bcf Tools

Coverage Map

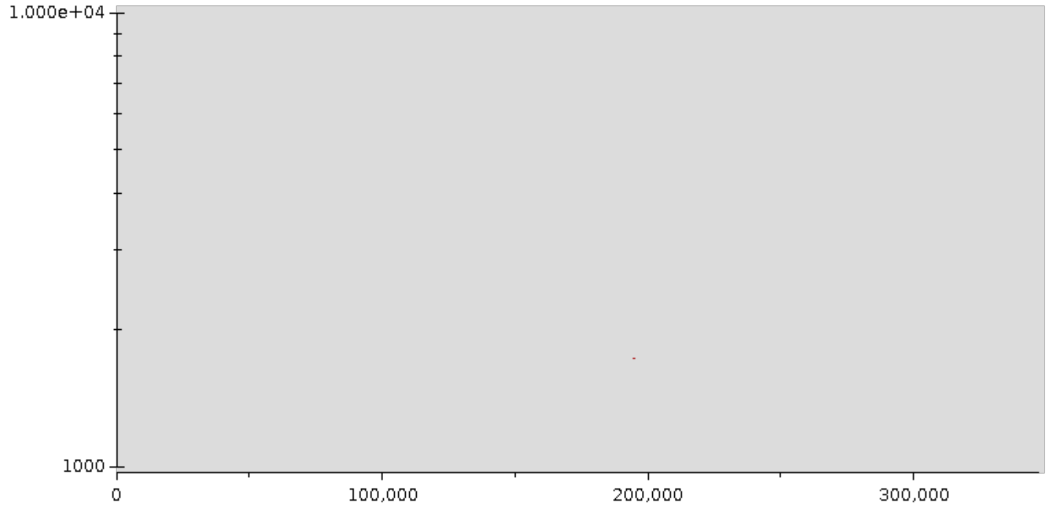

Assignment

**Type**	Lausannevirus (Taxonomy ID: 999883)
**Reference Genome**	NC_015326.1
**NT Identity (%)**	83.3333
**AA Identity (%)**	100.0
**Number Of Stop Codons**	1
**Number Of CDS**	444

Alignment

**Alignment Score**	176.0 (NT) + 282.0 (AA) = 458.0
**Concordance (%)**	83.8828
**Alignment Method**	Local, heuristic, nucleotide (BLASTN)

Genome Region

Sequence starts at position 194913 and ends at position 195044 relative to NC_015326.1 reference sequence.

Alignment Detailed Statistics

	Begin	End	Coverage	Score	Concordance	Matches	Identities	I/D/M/F*	Stop Codons
**NT**	**194913**	**195044**	**0.1%**	**176**	**66.7%**	**132 (100%)**	**110 (83.3%)**	**0/0**	

Mutations: 194925T>C, 194930A>G, 194931A>G, 194937G>A, 194946G>A, 194955T>C, 194958A>G, 194967A>G, 194970G>T, 194976A>G, 194979T>G, 194981T>G, 194982C>G, 194988T>C, 194991G>A, 195003T>A, 195012A>G, 195015T>C, 195017G>T, 195018T>C, 195024G>A, 195030A>T

*: Inserts / Deletes / Misaligned / Frameshifts

Analysis details

This analysis was performed with panviral2.64

NGS Details (UN18_val): Brazilian marseillevirus

Assembly

**Coverage Length**	228 (1 contig(s))
**Depth Of Coverage**	558.0
**Number Of Reads**	2433
**Reads Per Million**	45.66 rpm (after QC)
**Ambiguities**	0
**Assembly Method**	de novo + reference guided assembly
**Consensus Caller**	Bcf Tools

Coverage Map

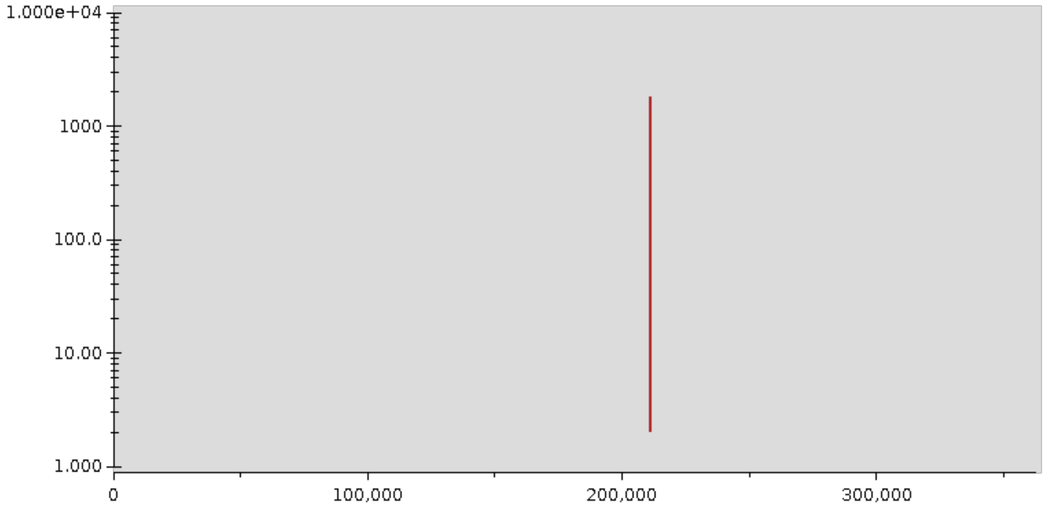

Assignment

**Type**	Brazilian marseillevirus (Taxonomy ID: 1813599)
**Reference Genome**	NC_029692.1
**NT Identity (%)**	81.5789
**AA Identity (%)**	95.8904
**Number Of Stop Codons**	0
**Number Of CDS**	491

Alignment

**Alignment Score**	288.0 (NT) + 452.0 (AA) = 740.0
**Concordance (%)**	80.8743
**Alignment Method**	Local, heuristic, nucleotide (BLASTN)

Genome Region

Sequence starts at position 210896 and ends at position 211123 relative to NC_029692.1 reference sequence.

Alignment Detailed Statistics

	Begin	End	Coverage	Score	Concordance	Matches	Identities	I/D/M/F*	Stop Codons
NT	210896	211123	0.1%	288	63.2%	228 (100%)	186 (81.6%)	0/0	

Mutations: 210905G>C, 210908A>G, 210914A>G, 210917A>G, 210920G>C, 210926T>C, 210929T>C, 210938A>G, 210941A>G, 210944C>G, 210947A>G, 210953C>A, 210955T>G, 210956T>G, 210959A>G, 210962T>C, 210965C>G, 210977T>A, 210986A>G, 210989G>A, 210992T>C, 210995T>C, 211001A>G, 211004A>G, 211009C>T, 211010T>G, 211013T>C, 211031T>C, 211034T>C, 211043T>C, 211055A>G, 211056G>A, 211061T>C, 211064A>G, 211073A>G, 211079C>G, 211088C>G, 211094A>G, 211100T>G, 211103A>G, 211106C>G, 211108C>T

*: Inserts / Deletes / Misaligned / Frameshifts

Analysis details

This analysis was performed with panviral2.64

NGS Details (UN18_val): Betabaculovirus disaccharalis

Assembly

**Coverage Length**	129 (1 contig(s))
**Depth Of Coverage**	1390.5
**Number Of Reads**	2060
**Reads Per Million**	38.66 rpm (after QC)
**Ambiguities**	0
**Assembly Method**	de novo + reference guided assembly
**Consensus Caller**	Bcf Tools

Coverage Map

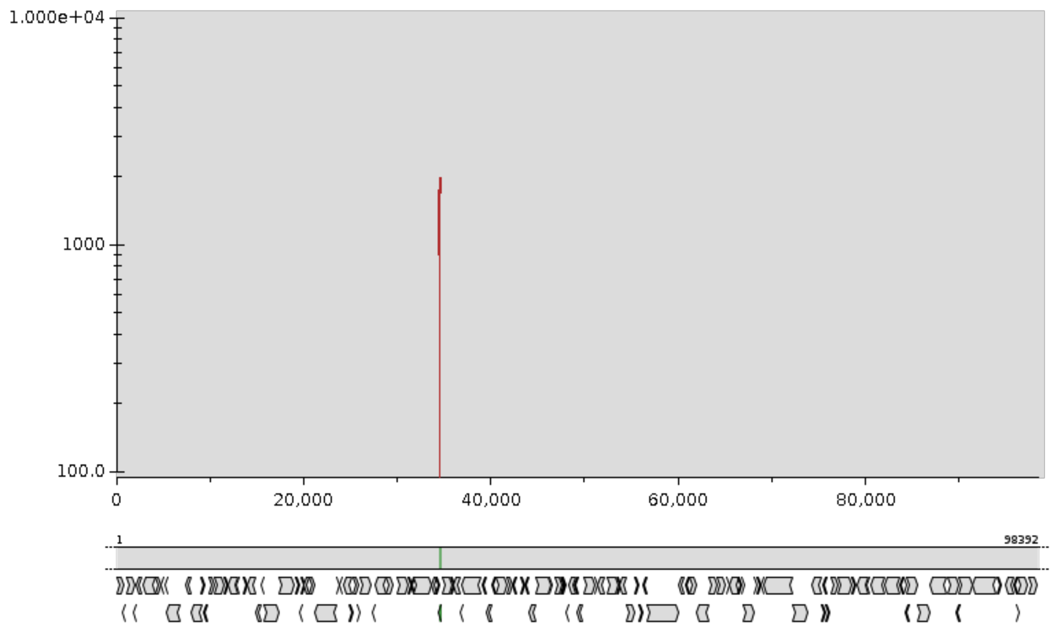

Assignment

**Type**	Betabaculovirus disaccharalis (Taxonomy ID: 3047795)
**Reference Genome**	NC_028491.1
**NT Identity (%)**	83.7209
**AA Identity (%)**	88.3721
**Number Of Stop Codons**	0
**Number Of CDS**	125

Alignment

**Alignment Score**	174.0 (NT) + 257.0 (AA) = 431.0
**Concordance (%)**	79.0826

Alignment Method	Local, heuristic, nucleotide (BLASTN)

Genome Region

Sequence starts at position 34471 and ends at position 34599 relative to NC_028491.1 reference sequence.

Alignment Detailed Statistics

	Begin	End	Coverage	Score	Concordance	Matches	Identities	I/D/M/F*	Stop Codons
**NT**	**34471**	**34599**	**0.1%**	**174**	**67.4%**	**129 (100%)**	**108 (83.7%)**	**0/0**	
Mutations:	34482G>T, 34485T>C, 34488A>C, 34490G>A, 34503A>C, 34511T>G, 34513C>G, 34514T>A, 34521T>C, 34524A>G, 34533A>G, 34538A>C, 34542T>A, 34545T>C, 34547G>T, 34549T>C, 34554T>C, 34569G>A, 34575T>G, 34578T>C, 34587T>C								
**CDS**									
v-ubq	60	102	36.1%	257	81.8%	43 (100%)	38 (88.4%)	0/0/0/0	0
Protein mutations:	E76G (34549T>C), S80A (34538A>C), T89P (34511T>G), H91Q (34503A>C)								
Codon mutations:	CAA63CAG (34587T>C), TTA66TTG (34578T>C), ATA67ATC (34575T>G), GCC69GCT (34569G>A), GAA74GAG (34554T>C), GAG76GGG (34549T>C), CGA77AGG (34545T>C 34547G>T), ACA78ACT (34542T>A), TCA80GCA (34538A>C), GAT81GAC (34533A>G), ATT84ATC (34524A>G), CAA85CAG (34521T>C), AGC88TCC (34513C>G 34514T>A), ACT89CCT (34511T>G), CAT91CAG (34503A>C), CTT96TTG (34488A>C 34490G>A), AGA97AGG (34485T>C), GGC98GGA (34482G>T)								
**Proteins**									
ubiquitin-like protein (YP_009182246.1)	60	102	36.1%	257	81.8%	43 (100%)	38 (88.4%)	0/0/0/0	0
Protein mutations:	E76G (34549T>C), S80A (34538A>C), T89P (34511T>G), H91Q (34503A>C)								
Codon mutations:	CAA63CAG (34587T>C), TTA66TTG (34578T>C), ATA67ATC (34575T>G), GCC69GCT (34569G>A), GAA74GAG (34554T>C), GAG76GGG (34549T>C), CGA77AGG (34545T>C 34547G>T), ACA78ACT (34542T>A), TCA80GCA (34538A>C), GAT81GAC (34533A>G), ATT84ATC (34524A>G), CAA85CAG (34521T>C), AGC88TCC (34513C>G 34514T>A), ACT89CCT (34511T>G), CAT91CAG (34503A>C), CTT96TTG (34488A>C 34490G>A), AGA97AGG (34485T>C), GGC98GGA (34482G>T)								

*: Inserts / Deletes / Misaligned / Frameshifts

Analysis details

This analysis was performed with panviral2.64

NGS Details (UN18_val): Bracoviriform congregatae (segment Circle 7)

Assembly

**Coverage Length**	172 (1 contig(s))
**Depth Of Coverage**	965.9
**Number Of Reads**	1498
**Reads Per Million**	28.12 rpm (after QC)
**Ambiguities**	0
**Assembly Method**	de novo + reference guided assembly
**Consensus Caller**	Bcf Tools

Coverage Map

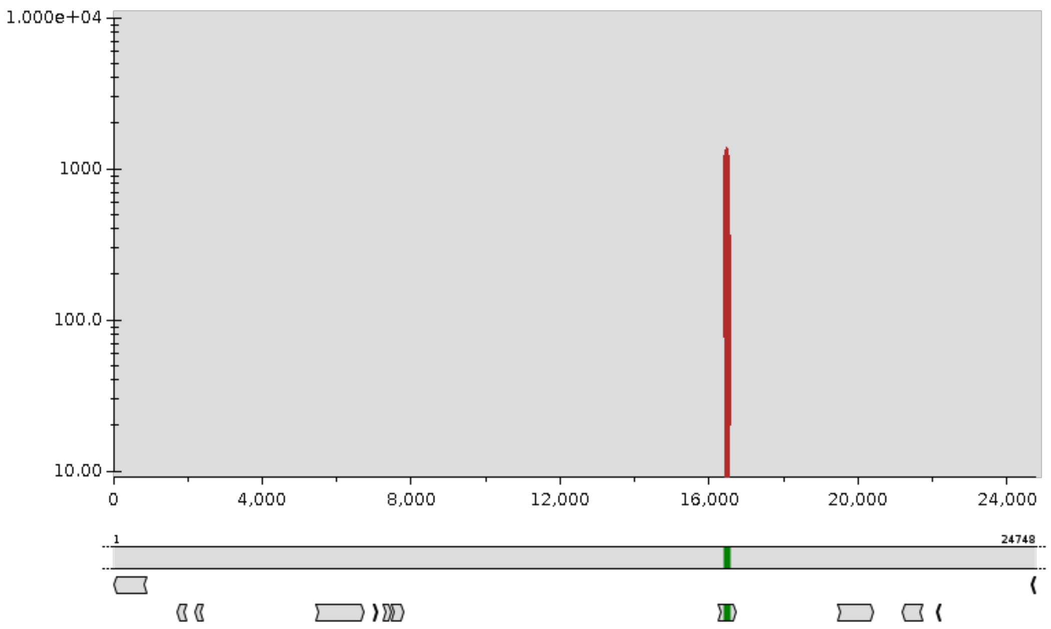

Assignment

**Type**	Bracoviriform congregatae (Taxonomy ID: 39640)
**Reference Genome**	NC_006639.1
**NT Identity (%)**	77.3256
**AA Identity (%)**	91.2281
**Number Of Stop Codons**	0
**Number Of CDS**	5

Alignment

**Alignment Score**	188.0 (NT) + 350.0 (AA) = 538.0
**Concordance (%)**	75.3501

Alignment Method	Global, seeded, nucleotide + amino acids (AGA)

Genome Region

Sequence starts at position 16393 and ends at position 16564 relative to NC_006639.1 reference sequence.

Alignment Detailed Statistics

	Begin	End	Coverage	Score	Concordance	Matches	Identities	I/D/M/F*	Stop Codons
**NT**	**16393**	**16564**	**0.7%**	**188**	**54.7%**	**172 (100%)**	**133 (77.3%)**	**0/0**	
Mutations:	16402A>T, 16405A>G, 16411G>C, 16414A>G, 16423A>C, 16429G>A, 16432C>A, 16435T>C, 16439C>A, 16441T>G, 16444T>C, 16445C>A, 16447C>G, 16449G>A, 16450A>G, 16451T>G, 16452C>T, 16453T>G, 16456T>A, 16457C>A, 16459A>G, 16471A>G, 16474A>T, 16477C>T, 16480C>T, 16492C>T, 16496C>A, 16498T>G, 16501G>T, 16508C>A, 16510T>A, 16516A>T, 16519C>G, 16526G>A, 16528C>T, 16538G>A, 16540C>T, 16546A>G, 16551T>C								

CDS

	Begin	End	Coverage	Score	Concordance	Matches	Identities	I/D/M/F*	Stop Codons
Histone	55	111	36.5%	350	93.6%	57 (100%)	52 (91.2%)	0/0/0/0	0
Protein mutations:	R73K (16449G>A 16450A>G), S74V (16451T>G 16452C>T 16453T>G), V99I (16526G>A 16528C>T), V103I (16538G>A 16540C>T), I107T (16551T>C)								
Codon mutations:	GTA57GTT (16402A>T), AAA58AAG (16405A>G), GGG60GGC (16411G>C), AAA61AAG (16414A>G), GGA64GGC (16423A>C), GGG66GGA (16429G>A), GGC67GGA (16432C>A), GCT68GCC (16435T>C), CGT70AGG (16439C>A 16441T>G), CAT71CAC (16444T>C), CGC72AGG (16445C>A 16447C>G), AGA73AAG (16449G>A 16450A>G), TCT74GTG (16451T>G 16452C>T 16453T>G), CTT75CTA (16456T>A), CGA76AGG (16457C>A 16459A>G), CAA80CAG (16471A>G), GGA81GGT (16474A>T), ATC82ATT (16477C>T), ACC83ACT (16480C>T), ATC87ATT (16492C>T), CGT89AGG (16496C>A 16498T>G), CTG90CTT (16501G>T), CGT93AGA (16508C>A 16510T>A), GGA95GGT (16516A>T), GTC96GTG (16519C>G), GTC99ATT (16526G>A 16528C>T), GTC103ATT (16538G>A 16540C>T), GAA105GAG (16546A>G), ATT107ACT (16551T>C)								

Proteins

	Begin	End	Coverage	Score	Concordance	Matches	Identities	I/D/M/F*	Stop Codons
hypothetical protein (YP_184795.1)	55	111	36.5%	350	93.6%	57 (100%)	52 (91.2%)	0/0/0/0	0
Protein mutations:	R73K (16449G>A 16450A>G), S74V (16451T>G 16452C>T 16453T>G), V99I (16526G>A 16528C>T), V103I (16538G>A 16540C>T), I107T (16551T>C)								
Codon mutations:	GTA57GTT (16402A>T), AAA58AAG (16405A>G), GGG60GGC (16411G>C), AAA61AAG (16414A>G), GGA64GGC (16423A>C), GGG66GGA (16429G>A), GGC67GGA (16432C>A), GCT68GCC (16435T>C), CGT70AGG (16439C>A 16441T>G), CAT71CAC (16444T>C), CGC72AGG (16445C>A 16447C>G), AGA73AAG (16449G>A 16450A>G), TCT74GTG (16451T>G 16452C>T 16453T>G), CTT75CTA (16456T>A), CGA76AGG (16457C>A 16459A>G), CAA80CAG (16471A>G), GGA81GGT (16474A>T), ATC82ATT (16477C>T), ACC83ACT (16480C>T), ATC87ATT (16492C>T), CGT89AGG (16496C>A 16498T>G), CTG90CTT (16501G>T), CGT93AGA (16508C>A 16510T>A), GGA95GGT (16516A>T), GTC96GTG (16519C>G), GTC99ATT (16526G>A 16528C>T), GTC103ATT (16538G>A 16540C>T), GAA105GAG (16546A>G), ATT107ACT (16551T>C)								

*: Inserts / Deletes / Misaligned / Frameshifts

Analysis details

This analysis was performed with panviral2.64

NGS Details (UN18_val): Tunisvirus fontaine2

Assembly

**Coverage Length**	175 (1 contig(s))
**Depth Of Coverage**	465.1
**Number Of Reads**	895
**Reads Per Million**	16.80 rpm (after QC)
**Ambiguities**	0
**Assembly Method**	de novo + reference guided assembly
**Consensus Caller**	Bcf Tools

Coverage Map

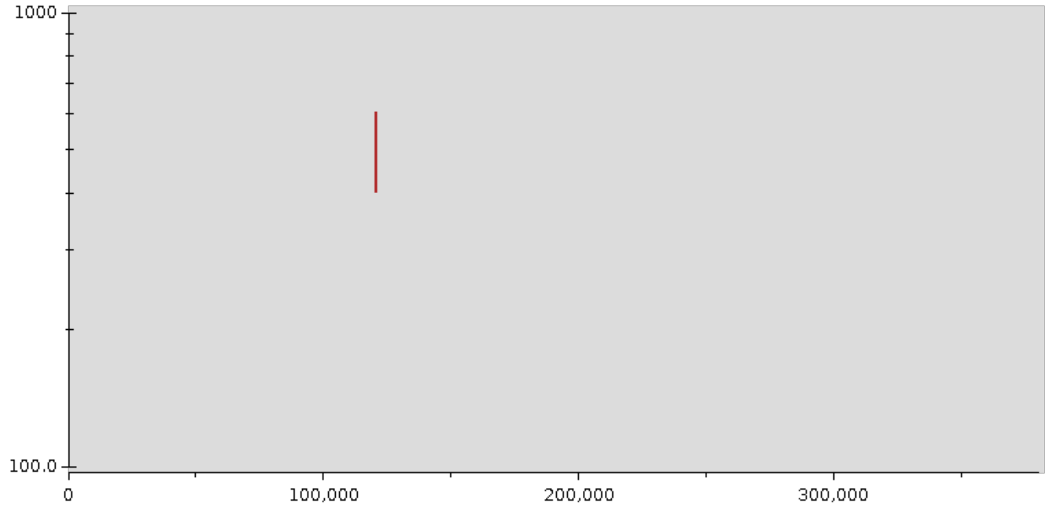

Assignment

**Type**	Tunisvirus fontaine2 (Taxonomy ID: 1421067)
**Reference Genome**	NC_038511.1
**NT Identity (%)**	80.0
**AA Identity (%)**	91.3793
**Number Of Stop Codons**	1
**Number Of CDS**	484

Alignment

**Alignment Score**	210.0 (NT) + 342.0 (AA) = 552.0
**Concordance (%)**	77.2028
**Alignment Method**	Local, heuristic, nucleotide (BLASTN)

Genome Region

Sequence starts at position 120430 and ends at position 120604 relative to NC_038511.1 reference sequence.

Alignment Detailed Statistics

	Begin	End	Coverage	Score	Concordance	Matches	Identities	I/D/M/F*	Stop Codons
**NT**	**120430**	**120604**	**0.1%**	**210**	**60.0%**	**175 (100%)**	**140 (80.0%)**	**0/0**	

Mutations: 120440T>C, 120449A>C, 120458A>T, 120461G>T, 120470T>C, 120475C>T, 120476G>A, 120479A>G, 120485G>A, 120488A>C, 120494C>T, 120496T>G, 120497C>A, 120508G>A, 120509A>C, 120510C>T, 120511T>G, 120512C>T, 120515C>T, 120518G>A, 120521G>A, 120524G>A, 120527A>T, 120532G>T, 120536T>C, 120542C>A, 120548A>T, 120551G>C, 120560A>G, 120563T>C, 120572T>G, 120573T>G, 120574G>T, 120582C>T, 120584C>G

*: Inserts / Deletes / Misaligned / Frameshifts

Analysis details

This analysis was performed with panviral2.64

NGS Details (UN18_val): Lowelvirus tuscon4d

Assembly

**Coverage Length**	619 (2 contig(s))
**Depth Of Coverage**	106.0
**Number Of Reads**	634
**Reads Per Million**	11.90 rpm (after QC)
**Ambiguities**	0
**Assembly Method**	de novo + reference guided assembly
**Consensus Caller**	Bcf Tools

Coverage Map

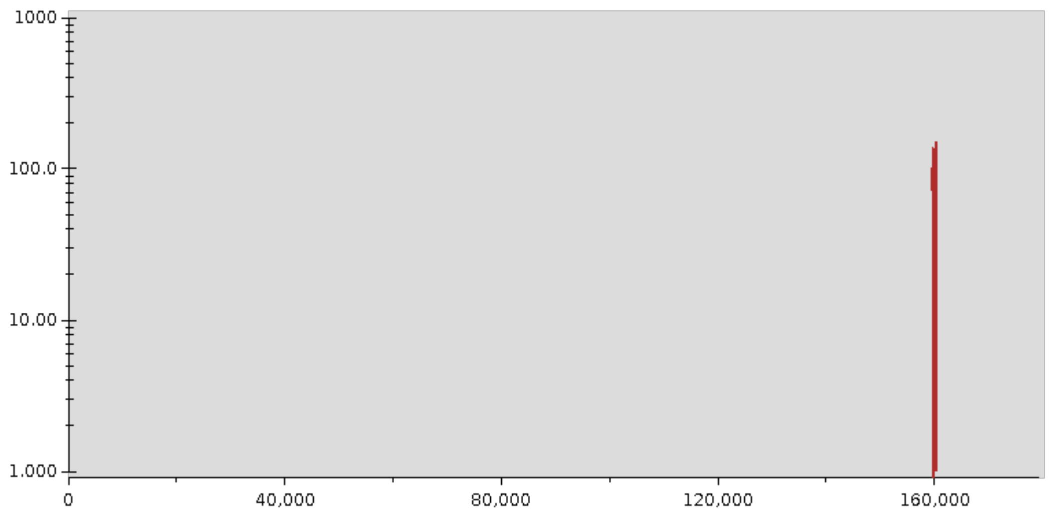

Assignment

**Type**	Lowelvirus tuscon4d (Taxonomy ID: 2956131)
**Reference Genome**	NC_026923.1
**NT Identity (%)**	76.8233
**AA Identity (%)**	88.2353
**Number Of Stop Codons**	1
**Number Of CDS**	218

Alignment

**Alignment Score**	651.0 (NT) + 1264.0 (AA) = 1915.0
**Concordance (%)**	74.0275
**Alignment Method**	Local, heuristic, nucleotide (BLASTN)

Genome Region

Sequence starts at position 159602 and ends at position 160401 relative to NC_026923.1 reference sequence.

Alignment Detailed Statistics

	Begin	End	Coverage	Score	Concordance	Matches	Identities	I/D/M/F*	Stop Codons
NT	159602	160401	0.3%	651	53.2%	617 (99.7%)	474 (76.6%)	0/2	

Mutations: 159612T>A, 159624G>A, 159625T>G, 159627T>A, 159633C>A, 159636C>T, 159639C>T, 159640C>A, 159641A>C, 159642A>T, 159645C>T, 159651C>T, 159655C>T, 159657T>G, 159660A>G, 159663G>T, 159664A>C, 159666T>G, 159670T>A, 159672C>T, 159687C>T, 159690T>A, 159693A>T, 159696C>T, 159698C>G, 159699T>C, 159702C>A, 159711T>C, 159715C>T, 159720G>T, 159726T>C, 159729A>G, 159730A>C, 159738T>A, 159739C>T, 159740T>C, 159741A>T, 159745G>C, 159746G>A, 159747T>A, 159748A>T, 159750C>G, 159751C>A, 159753T>A, 159759C>T, 159765T>A, 159768T>C, 159771G>A, 159783T>A, 159798C>T, 159799A>T, 159800G>C, 159801C>T, 159807C>T, 159810T>G, 159813C>T, 159822G>A, 159832T>G, 159833C>G, 159834A>T, 159855C>T, 159858G>T, 159867A>T, 159870C>A, 159874A>C, 159880C>A, 159882G>C, 159883T>C, 159885G>A, 159888C>T, 159889C>T, 159894G>A, 159897T>G, 159900C>T, 159903C>T, 159906C>T, 159913C>T, 159915C>G, 159924C>T, 159942T>C, 159946A>G, 159949C>T, 159951A>G, 159954T>C, 159956G>C, 159957A>T, 159960A>T, 159961C>T, 159964T>C, 159966G>A, 159969T>C, 159972A>C, 159975C>T, 159987T>C, 159993G>A, 159996C>T, 159999A>T, 160002G>A, 160011T>C, 160014A>T, 160017A>T, 160018C>G, 160019A>G, 160020A>T, 160026C>T, 160032T>C, 160033A>C, 160034A>G, 160035G>T, 160038G>T, 160042G>A, 160044G>C, 160246A>C, 160248A>C, 160254A>G, 160270G>A, 160275C>T, 160287G>A, 160290C>T, 160291_160292delAT, 160293C>A, 160296A>C, 160299G>A, 160302T>C, 160308A>T, 160309C>A, 160326A>G, 160327C>G, 160330G>C, 160331C>A, 160332T>G, 160338A>G, 160341A>T, 160350C>T, 160356C>A, 160359C>T, 160368A>G, 160371A>T, 160377T>A, 160378A>C, 160380A>T, 160383T>A, 160389A>T, 160392G>T

*: Inserts / Deletes / Misaligned / Frameshifts

Analysis details

This analysis was performed with panviral2.64

NGS Details (UN18_val): Cassava brown streak virus

Assembly

**Coverage Length**	521 (1 contig(s))
**Depth Of Coverage**	103.8
**Number Of Reads**	450
**Reads Per Million**	8.45 rpm (after QC)
**Ambiguities**	0
**Assembly Method**	de novo + reference guided assembly
**Consensus Caller**	Bcf Tools

Coverage Map

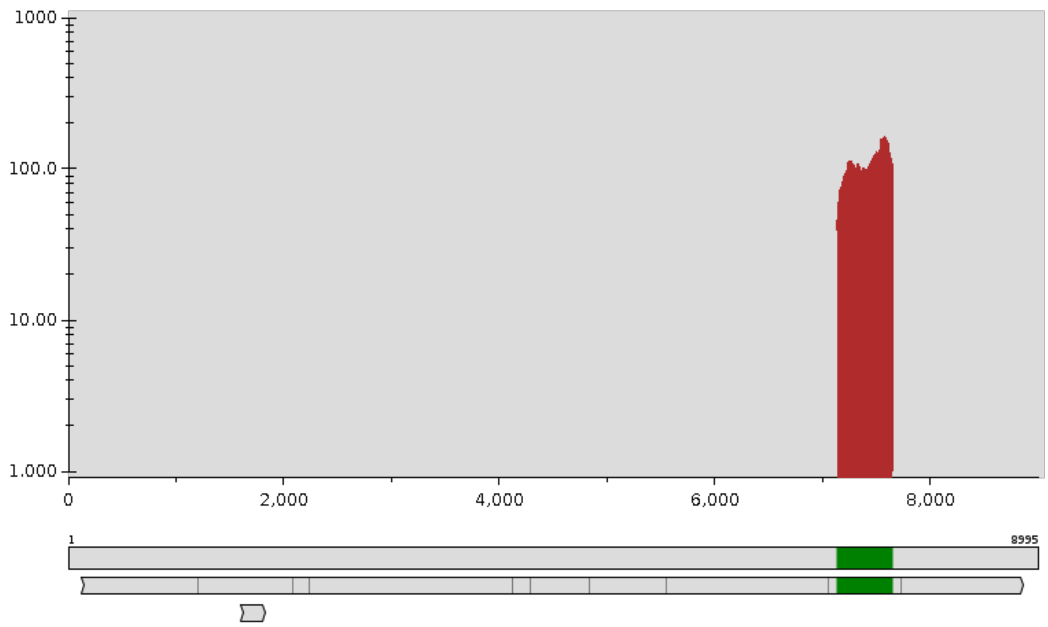

Assignment

**Type**	Cassava brown streak virus (Taxonomy ID: 137758)
**Reference Genome**	NC_012698.2
**NT Identity (%)**	60.4607
**AA Identity (%)**	57.4713
**Number Of Stop Codons**	0
**Number Of CDS**	2

Alignment

**Alignment Score**	218.0 (NT) + 732.0 (AA) = 950.0
**Concordance (%)**	42.166

Alignment Method	Global, seeded, nucleotide + amino acids (AGA)

Genome Region

Sequence starts at position 7130 and ends at position 7650 relative to NC_012698.2 reference sequence.

Alignment Detailed Statistics

	Begin	End	Coverage	Score	Concordance	Matches	Identities	I/D/M/F*	Stop Codons
**NT**	**7130**	**7650**	**5.8%**	**218**	**20.9%**	**521 (100%)**	**315 (60.5%)**	**0/0**	
Mutations:	7130A>G, 7133A>G, 7134A>G, 7136T>G, 7142C>T, 7145A>T, 7148A>C, 7151G>A, 7157A>T, 7158C>A, 7163A>G, 7167A>G, 7175A>T, 7177A>G, 7178A>G, 7179C>G, 7180A>C, 7184A>T, 7185T>C, 7190T>C, 7192C>A, 7193C>G, 7194A>T, 7202A>C, 7203A>T, 7205C>T, 7206A>C, 7207T>A, 7208C>G, 7211T>C, 7213G>T, 7214G>T, 7217A>G, 7218G>C, 7229A>T, 7234C>T, 7235A>T, 7241A>G, 7242A>G, 7243C>A, 7244A>G, 7245G>C, 7246T>C, 7250G>A, 7253A>T, 7256T>A, 7257A>T, 7258T>C, 7270T>C, 7271A>T, 7272C>A, 7273G>A, 7274T>A, 7275G>A, 7277A>T, 7280G>T, 7281T>G, 7283T>C, 7284G>A, 7286G>A, 7287T>G, 7288T>A, 7290A>G, 7292T>G, 7293G>A, 7294G>A, 7295A>T, 7298G>A, 7304T>G, 7307C>A, 7310A>T, 7313A>G, 7319A>T, 7320A>T, 7323T>C, 7325G>C, 7331T>C, 7332G>A, 7334T>C, 7337T>C, 7340C>T, 7343T>G, 7355A>G, 7358A>T, 7361C>T, 7364T>C, 7374A>T, 7377G>C, 7380G>A, 7381G>A, 7385T>C, 7388A>T, 7390T>A, 7391A>T, 7397A>T, 7398T>C, 7400A>T, 7401T>A, 7403T>C, 7406G>C, 7409G>A, 7410G>T, 7413G>A, 7414A>T, 7416C>G, 7418G>T, 7422C>G, 7424A>G, 7425A>G, 7432T>C, 7433G>A, 7436T>A, 7437A>T, 7438G>A, 7442T>C, 7443C>A, 7445C>G, 7448C>T, 7449G>A, 7451G>C, 7455G>T, 7457T>A, 7458T>C, 7462T>C, 7463A>T, 7464A>C, 7465A>T, 7467A>G, 7468A>G, 7469A>G, 7470G>C, 7471T>C, 7472T>A, 7473G>A, 7474G>A, 7476G>A, 7477A>C, 7481T>G, 7484T>G, 7487A>G, 7489T>C, 7490C>T, 7494A>G, 7495A>T, 7496G>T, 7499T>A, 7500G>A, 7501T>A, 7502G>A, 7503C>A, 7504T>C, 7505A>G, 7506A>C, 7507G>T, 7508A>G, 7511T>A, 7512G>A, 7517T>A, 7520A>G, 7521A>C, 7522T>C, 7523G>A, 7524C>G, 7526A>T, 7527C>A, 7529C>G, 7535A>C, 7539T>A, 7540C>A, 7541A>T, 7544T>C, 7547G>A, 7553C>T, 7556A>G, 7559T>A, 7562C>T, 7568A>T, 7570T>A, 7572A>G, 7573A>G, 7576G>A, 7577G>T, 7578A>G, 7579G>A, 7580A>C, 7581A>C, 7586A>T, 7588T>A, 7589T>C, 7599A>C, 7600T>C, 7601G>C, 7602G>A, 7603C>A, 7604C>G, 7607G>A, 7610G>A, 7616T>C, 7618T>A, 7622A>T, 7628T>C, 7631A>C, 7632T>G, 7633T>G, 7635C>A, 7636G>A								

CDS

	Begin	End	Coverage	Score	Concordance	Matches	Identities	I/D/M/F*	Stop Codons
CBSV_gp1	2336	2509	6.0%	732	60.0%	174 (100%)	100 (57.5%)	0/0/0/0	0
Protein mutations:	I2337V (7134A>G 7136T>G), Q2345K (7158C>A), K2348E (7167A>G), K2351R (7177A>G 7178A>G), Q2352A (7179C>G 7180A>C), F2354L (7185T>C), P2356Q (7192C>A 7193C>G), T2357S (7194A>T), I2360F (7203A>T 7205C>T), I2361Q (7206A>C 7207T>A 7208C>G), R2363L (7213G>T 7214G>T), V2365L (7218G>C), P2370L (7234C>T 7235A>T), T2373E (7242A>G 7243C>A 7244A>G), V2374P (7245G>C 7246T>C), E2376D (7253A>T), I2378S (7257A>T 7258T>C), V2382A (7270T>C 7271A>T), R2383K (7272C>A 7273G>A 7274T>A), V2384I (7275G>A 7277A>T), S2386A (7281T>G 7283T>C), E2387K (7284G>A 7286G>A), L2388E (7287T>G 7288T>A), I2389V (7290A>G 7292T>G), G2390N (7293G>A 7294G>A 7295A>T), S2399C (7320A>T), D2403N (7332G>A 7334T>C), N2406K (7343T>G), M2417L (7374A>T), E2418Q (7377G>C), G2419K (7380G>A 7381G>A), L2422H (7390T>A 7391A>T), Y2426N (7401T>A 7403T>C), K2427N (7406G>C), V2429L (7410G>T), E2430M (7413G>A 7414A>T), P2431A (7416C>G 7418G>T), Q2433E (7422C>G 7424A>G), N2434D (7425A>G), M2436T (7432T>C 7433G>A), S2438Y (7437A>T 7438G>A), L2440M (7443C>A 7445C>G), V2442I (7449G>A 7451G>C), A2444S (7455G>T 7457T>A), F2445L (7458T>C), V2446A (7462T>C 7463A>T), N2447L (7464A>C 7465A>T), K2448G (7467A>G 7468A>G 7469A>G), V2449P (7470G>C 7471T>C 7472T>A), G2450N (7473G>A 7474G>A), D2451T (7476G>A 7477A>C), D2452E (7481T>G), I2454M (7487A>G), I2455T (7489T>C 7490C>T), K2457V (7494A>G 7495A>T 7496G>T), V2459K (7500G>A 7501T>A 7502G>A), L2460T (7503C>A 7504T>C 7505A>G), R2461L (7506A>C 7507G>T 7508A>G), E2463K (7512G>A), M2466P (7521A>C 7522T>C 7523G>A), P2467A (7524C>G 7526A>T), S2472N (7539T>A 7540C>A 7541A>T), L2482H (7570T>A), N2483G (7572A>G 7573A>G), W2484Y (7576G>A 7577G>T), R2485D (7578A>G 7579G>A 7580A>C), K2486Q (7581A>C), F2488Y (7588T>A 7589T>C), M2492P (7599A>C 7600T>C 7601G>C), A2493K (7602G>A 7603C>A 7604C>G), M2498K (7618T>A), F2503G (7632T>G 7633T>G), R2504K (7635C>A 7636G>A)								
Codon mutations:	GCA2335..G (7130A>G), CCA2336CCG (7133A>G), ATT2337GTG (7134A>G 7136T>G), TTC2339TTT (7142C>T), GTA2340GTT (7145A>T), ACA2341ACC (7148A>C), GGG2342GGA (7151G>A), GCA2344GCT (7157A>T), CAA2345AAA (7158C>A), AAA2346AAG (7163A>G), AAG2348GAG (7167A>G), GTA2350GTT (7175A>T), AAA2351AGG (7177A>G 7178A>G), CAA2352GCA (7179C>G 7180A>C), ATA2353ATT (7184A>T), TTT2354CTT (7185T>C), GGT2355GGC (7190T>C), CCC2356CAG (7192C>A 7193C>G), ACT2357TCT (7194A>T), CCA2359CCC (7202A>C), ATC2360TTT (7203A>T 7205C>T), ATC2361CAG (7206A>C 7207T>A 7208C>G), TCT2362TCC (7211T>C), CGG2363CTT (7213G>T 7214G>T), AAA2364AAG (7217A>G), GTT2365CTT (7218G>C), CCA2368CCT (7229A>T), CCA2370CTT (7234C>T 7235A>T), GGA2372GGG (7241A>G), ACA2373GAG (7242A>G 7243C>A 7244A>G), GTT2374CCT (7245G>C 7246T>C), GAG2375GAA (7250G>A), GAA2376GAT (7253A>T), ATT2377ATA (7256T>A), ATC2378TCC (7257A>T 7258T>C), GTA2382GCT (7270T>C 7271A>T), CGT2383AAA (7272C>A 7273G>A 7274T>A), GTA2384ATT (7275G>A 7277A>T), GCG2385GCT (7280G>T), TCT2386GCC (7281T>G 7283T>C), GAG2387AAA (7284G>A 7286G>A), TTG2388GAG (7287T>G 7288T>A), ATT2389GTG (7290A>G 7292T>G), GGA2390AAT (7293G>A 7294G>A 7295A>T), GGG2391GGA (7298G>A), GTT2393GTG (7304T>G), CTC2394CTA (7307C>A), GTA2395GTT (7310A>T), GAA2396GAG (7313A>G), ACA2398ACT (7319A>T), AGT2399TGT (7320A>T), TTG2400CTC (7323T>C 7325G>C), TTT2402TTC (7331T>C), GAT2403AAC (7332G>A 7334T>C), GCT2404GCC (7337T>C), CTC2405CTT (7340C>T), AAT2406AAG (7343T>G), GGA2410GGG (7355A>G), CCA2411CCT (7358A>T), TAC2412TAT (7361C>T), ATT2413ATC (7364T>C), ATG2417TTG (7374A>T), GAA2418CAA (7377G>C), GGG2419AAG (7380G>A 7381G>A), ATT2420ATC (7385T>C), GGA2421GGT (7388A>T), CTA2422CAT (7390T>A 7391A>T), GGA2424GGT (7397A>T), TTA2425CTT (7398T>C 7400A>T), TAT2426AAC (7401T>A 7403T>C), AAG2427AAC (7406G>C), TTG2428TTA (7409G>A), GTG2429TTG (7410G>T), GAG2430ATG (7413G>A 7414A>T), CCG2431GCT (7416C>G 7418G>T), CAA2433GAG (7422C>G 7424A>G), AAT2434GAT (7425A>G), ATG2436ACA (7432T>C 7433G>A), GCT2437GCA (7436T>A), AGT2438TAT (7437A>T 7438G>A), GCT2439GCC (7442T>C), CTC2440ATG (7443C>A 7445C>G), TGC2441TGT (7448C>T), GTG2442ATC (7449G>A 7451G>C), GCT2444TCA (7455G>T 7457T>A), TTT2445CTT (7458T>C), GTA2446GCT (7462T>C 7463A>T), AAT2447CTT (7464A>C 7465A>T), AAA2448GGG (7467A>G 7468A>G 7469A>G), GTT2449CCA (7470G>C 7471T>C 7472T>A), GGT2450AAT (7473G>A 7474G>A), GAT2451ACT (7476G>A 7477A>C), GAT2452GAG (7481T>G), CCT2453CCG (7484T>G), ATA2454ATG (7487A>G), ATC2455ACT (7489T>C 7490C>T), AAG2457GTT (7494A>G 7495A>T 7496G>T), GGT2458GGA (7499T>A), GTG2459AAA (7500G>A 7501T>A 7502G>A), CTA2460ACG (7503C>A 7504T>C 7505A>G), AGA2461CTG (7506A>C 7507G>T 7508A>G), GGT2462GGA (7511T>A), GAG2463AAG (7512G>A), ATT2464ATA (7517T>A), GTA2465GTG (7520A>G), ATG2466CCA (7521A>C 7522T>C 7523G>A), CCA2467GCT (7524C>G 7526A>T), CGC2468AGG (7527C>A 7529C>G), CCA2470CCC (7535A>C), TCA2472AAT (7539T>A 7540C>A 7541A>T), TTT2473TTC (7544T>C), GGG2474GGA (7547G>A), GAC2476GAT (7553C>T), CCA2477CCG (7556A>G), ATT2478ATA (7559T>A), TTC2479TTT (7562C>T), CCA2481CCT (7568A>T), CTT2482CAT (7570T>A), AAC2483GGC (7572A>G 7573A>G), TGG2484TAT (7576G>A 7577G>T), AGA2485GAC (7578A>G 7579G>A 7580A>C), AAG2486CAG (7581A>C), ACA2487ACT (7586A>T), TTT2488TAC (7588T>A 7589T>C), ATG2492CCC (7599A>C 7600T>C 7601G>C), GCC2493AAG (7602G>A 7603C>A 7604C>G), GAG2494GAA (7607G>A), GAG2495GAA (7610G>A), AAT2497AAC (7616T>C), ATG2498AAG (7618T>A), ATA2499ATT (7622A>T), CAT2501CAC (7628T>C), CGA2502CGC (7631A>C), TTT2503GGT (7632T>G 7633T>G), CGA2504AAA (7635C>A 7636G>A)								

Proteins

	Begin	End	Coverage	Score	Concordance	Matches	Identities	I/D/M/F*	Stop Codons
polyprotein (YP_007027011.1)	2336	2509	6.0%	732	60.0%	174 (100%)	100 (57.5%)	0/0/0/0	0
Protein mutations:	I2337V (7134A>G 7136T>G), Q2345K (7158C>A), K2348E (7167A>G), K2351R (7177A>G 7178A>G), Q2352A (7179C>G 7180A>C), F2354L (7185T>C), P2356Q (7192C>A 7193C>G), T2357S (7194A>T), I2360F (7203A>T 7205C>T), I2361Q (7206A>C 7207T>A 7208C>G), R2363L (7213G>T 7214G>T), V2365L (7218G>C), P2370L (7234C>T 7235A>T), T2373E (7242A>G 7243C>A 7244A>G), V2374P (7245G>C 7246T>C), E2376D (7253A>T), I2378S (7257A>T 7258T>C), V2382A (7270T>C 7271A>T), R2383K (7272C>A 7273G>A 7274T>A), V2384I (7275G>A 7277A>T), S2386A (7281T>G 7283T>C), E2387K (7284G>A 7286G>A), L2388E (7287T>G 7288T>A), I2389V (7290A>G 7292T>G), G2390N (7293G>A 7294G>A 7295A>T), S2399C (7320A>T), D2403N (7332G>A 7334T>C), N2406K (7343T>G), M2417L (7374A>T), E2418Q (7377G>C), G2419K (7380G>A 7381G>A), L2422H (7390T>A 7391A>T), Y2426N (7401T>A 7403T>C), K2427N (7406G>C), V2429L (7410G>T), E2430M (7413G>A 7414A>T), P2431A (7416C>G 7418G>T), Q2433E (7422C>G 7424A>G), N2434D (7425A>G), M2436T (7432T>C 7433G>A), S2438Y (7437A>T 7438G>A), L2440M (7443C>A 7445C>G), V2442I (7449G>A 7451G>C), A2444S (7455G>T 7457T>A), F2445L (7458T>C), V2446A (7462T>C 7463A>T), N2447L (7464A>C 7465A>T), K2448G (7467A>G 7468A>G 7469A>G), V2449P (7470G>C 7471T>C 7472T>A), G2450N (7473G>A 7474G>A), D2451T (7476G>A 7477A>C), D2452E (7481T>G), I2454M (7487A>G), I2455T (7489T>C 7490C>T), K2457V (7494A>G 7495A>T 7496G>T), V2459K (7500G>A 7501T>A 7502G>A), L2460T (7503C>A 7504T>C 7505A>G), R2461L (7506A>C 7507G>T 7508A>G), E2463K (7512G>A), M2466P (7521A>C 7522T>C 7523G>A), P2467A (7524C>G 7526A>T), S2472N (7539T>A 7540C>A 7541A>T), L2482H (7570T>A), N2483G (7572A>G 7573A>G), W2484Y (7576G>A 7577G>T), R2485D (7578A>G 7579G>A 7580A>C), K2486Q (7581A>C), F2488Y (7588T>A 7589T>C), M2492P (7599A>C 7600T>C 7601G>C), A2493K (7602G>A 7603C>A 7604C>G), M2498K (7618T>A), F2503G (7632T>G 7633T>G), R2504K (7635C>A 7636G>A)								

	Begin	End	Coverage	Score	Concordance	Matches	Identities	I/D/M/F*	Stop Codons
**NT**	**7130**	**7650**	**5.8%**	**218**	**20.9%**	**521 (100%)**	**315 (60.5%)**	**0/0**	
Codon mutations:	GCA2335..G (7130A>G), CCA2336CCG (7133A>G), ATT2337GTG (7134A>G 7136T>G), TTC2339TTT (7142C>T), GTA2340GTT (7145A>T), ACA2341ACC (7148A>C), GGG2342GGA (7151G>A), GCA2344GCT (7157A>T), CAA2345AAA (7158C>A), AAA2346AAG (7163A>G), AAG2348GAG (7167A>G), GTA2350GTT (7175A>T), AAA2351AGG (7177A>G 7178A>G), CAA2352GCA (7179C>G 7180A>C), ATA2353ATT (7184A>T), TTT2354CTT (7185T>C), GGT2355GGC (7190T>C), CCC2356CAG (7192C>A 7193C>G), ACT2357TCT (7194A>T), CCA2359CCC (7202A>C), ATC2360TTT (7203A>T 7205C>T), ATC2361CAG (7206A>C 7207T>A 7208C>G), TCT2362TCC (7211T>C), CGG2363CTT (7213G>T 7214G>T), AAA2364AAG (7217A>G), GTT2365CTT (7218G>C), CCA2368CCT (7229A>T), CCA2370CTT (7234C>T 7235A>T), GGA2372GGG (7241A>G), ACA2373GAG (7242A>G 7243C>A 7244A>G), GTT2374CCT (7245G>C 7246T>C), GAG2375GAA (7250G>A), GAA2376GAT (7253A>T), ATT2377ATA (7256T>A), ATC2378TCC (7257A>T 7258T>C), GTA2382GCT (7270T>C 7271A>T), CGT2383AAA (7272C>A 7273G>A 7274T>A), GTA2384ATT (7275G>A 7277A>T), GCG2385GCT (7280G>T), TCT2386GCC (7281T>G 7283T>C), GAG2387AAA (7284G>A 7286G>A), TTG2388GAG (7287T>G 7288T>A), ATT2389GTG (7290A>G 7292T>G), GGA2390AAT (7293G>A 7294G>A 7295A>T), GGG2391GGA (7298G>A), GTT2393GTG (7304T>G), CTC2394CTA (7307C>A), GTA2395GTT (7310A>T), GAA2396GAG (7313A>G), ACA2398ACT (7319A>T), AGT2399TGT (7320A>T), TTG2400CTC (7323T>C 7325G>C), TTT2402TTC (7331T>C), GAT2403AAC (7332G>A 7334T>C), GCT2404GCC (7337T>C), CTC2405CTT (7340C>T), AAT2406AAG (7343T>G), GGA2410GGG (7355A>G), CCA2411CCT (7358A>T), TAC2412TAT (7361C>T), ATT2413ATC (7364T>C), ATG2417TTG (7374A>T), GAA2418CAA (7377G>C), GGG2419AAG (7380G>A 7381G>A), ATT2420ATC (7385T>C), GGA2421GGT (7388A>T), CTA2422CAT (7390T>A 7391A>T), GGA2424GGT (7397A>T), TTA2425CTT (7398T>C 7400A>T), TAT2426AAC (7401T>A 7403T>C), AAG2427AAC (7406G>C), TTG2428TTA (7409G>A), GTG2429TTG (7410G>T), GAG2430ATG (7413G>A 7414A>T), CCG2431GCT (7416C>G 7418G>T), CAA2433GAG (7422C>G 7424A>G), AAT2434GAT (7425A>G), ATG2436ACA (7432T>C 7433G>A), GCT2437GCA (7436T>A), AGT2438TAT (7437A>T 7438G>A), GCT2439GCC (7442T>C), CTC2440ATG (7443C>A 7445C>G), TGC2441TGT (7448C>T), GTG2442ATC (7449G>A 7451G>C), GCT2444TCA (7455G>T 7457T>A), TTT2445CTT (7458T>C), GTA2446GCT (7462T>C 7463A>T), AAT2447CTT (7464A>C 7465A>T), AAA2448GGG (7467A>G 7468A>G 7469A>G), GTT2449CCA (7470G>C 7471T>C 7472T>A), GGT2450AAT (7473G>A 7474G>A), GAT2451ACT (7476G>A 7477A>C), GAT2452GAG (7481T>G), CCT2453CCG (7484T>G), ATA2454ATG (7487A>G), ATC2455ACT (7489T>C 7490C>T), AAG2457GTT (7494A>G 7495A>T 7496G>T), GGT2458GGA (7499T>A), GTG2459AAA (7500G>A 7501T>A 7502G>A), CTA2460ACG (7503C>A 7504T>C 7505A>G), AGA2461CTG (7506A>C 7507G>T 7508A>G), GGT2462GGA (7511T>A), GAG2463AAG (7512G>A), ATT2464ATA (7517T>A), GTA2465GTG (7520A>G), ATG2466CCA (7521A>C 7522T>C 7523G>A), CCA2467GCT (7524C>G 7526A>T), CGC2468AGG (7527C>A 7529C>G), CCA2470CCC (7535A>C), TCA2472AAT (7539T>A 7540C>A 7541A>T), TTT2473TTC (7544T>C), GGG2474GGA (7547G>A), GAC2476GAT (7553C>T), CCA2477CCG (7556A>G), ATT2478ATA (7559T>A), TTC2479TTT (7562C>T), CCA2481CCT (7568A>T), CTT2482CAT (7570T>A), AAC2483GGC (7572A>G 7573A>G), TGG2484TAT (7576G>A 7577G>T), AGA2485GAC (7578A>G 7579G>A 7580A>C), AAG2486CAG (7581A>C), ACA2487ACT (7586A>T), TTT2488TAC (7588T>A 7589T>C), ATG2492CCC (7599A>C 7600T>C 7601G>C), GCC2493AAG (7602G>A 7603C>A 7604C>G), GAG2494GAA (7607G>A), GAG2495GAA (7610G>A), AAT2497AAC (7616T>C), ATG2498AAG (7618T>A), ATA2499ATT (7622A>T), CAT2501CAC (7628T>C), CGA2502CGC (7631A>C), TTT2503GGT (7632T>G 7633T>G), CGA2504AAA (7635C>A 7636G>A)								
HAM1-like protein (YP_007032446.1)	28	201	77.0%	732	60.0%	174 (100%)	100 (57.5%)	0/0/0/0	0
Protein mutations:	I29V (7134A>G 7136T>G), Q37K (7158C>A), K40E (7167A>G), K43R (7177A>G 7178A>G), Q44A (7179C>G 7180A>C), F46L (7185T>C), P48Q (7192C>A 7193C>G), T49S (7194A>T), I52F (7203A>T 7205C>T), I53Q (7206A>C 7207T>A 7208C>G), R55L (7213G>T 7214G>T), V57L (7218G>C), P62L (7234C>T 7235A>T), T65E (7242A>G 7243C>A 7244A>G), V66P (7245G>C 7246T>C), E68D (7253A>T), I70S (7257A>T 7258T>C), V74A (7270T>C 7271A>T), R75K (7272C>A 7273G>A 7274T>A), V76I (7275G>A 7277A>T), S78A (7281T>G 7283T>C), E79K (7284G>A 7286G>A), L80E (7287T>G 7288T>A), I81V (7290A>G 7292T>G), G82N (7293G>A 7294G>A 7295A>T), S91C (7320A>T), D95N (7332G>A 7334T>C), N98K (7343T>G), M109L (7374A>T), E110Q (7377G>C), G111K (7380G>A 7381G>A), L114H (7390T>A 7391A>T), Y118N (7401T>A 7403T>C), K119N (7406G>C), V121L (7410G>T), E122M (7413G>A 7414A>T), P123A (7416C>G 7418G>T), Q125E (7422C>G 7424A>G), N126D (7425A>G), M128T (7432T>C 7433G>A), S130Y (7437A>T 7438G>A), L132M (7443C>A 7445C>G), V134I (7449G>A 7451G>C), A136S (7455G>T 7457T>A), F137L (7458T>C), V138A (7462T>C 7463A>T), N139L (7464A>C 7465A>T), K140G (7467A>G 7468A>G 7469A>G), V141P (7470G>C 7471T>C 7472T>A), G142N (7473G>A 7474G>A), D143T (7476G>A 7477A>C), D144E (7481T>G), I146M (7487A>G), I147T (7489T>C 7490C>T), K149V (7494A>G 7495A>T 7496G>T), V151K (7500G>A 7501T>A 7502G>A), L152T (7503C>A 7504T>C 7505A>G), R153L (7506A>C 7507G>T 7508A>G), E155K (7512G>A), M158P (7521A>C 7522T>C 7523G>A), P159A (7524C>G 7526A>T), S164N (7539T>A 7540C>A 7541A>T), L174H (7570T>A), N175G (7572A>G 7573A>G), W176Y (7576G>A 7577G>T), R177D (7578A>G 7579G>A 7580A>C), K178Q (7581A>C), F180Y (7588T>A 7589T>C), M184P (7599A>C 7600T>C 7601G>C), A185K (7602G>A 7603C>A 7604C>G), M190K (7618T>A), F195G (7632T>G 7633T>G), R196K (7635C>A 7636G>A)								
Codon mutations:	GCA27..G (7130A>G), CCA28CCG (7133A>G), ATT29GTG (7134A>G 7136T>G), TTC31TTT (7142C>T), GTA32GTT (7145A>T), ACA33ACC (7148A>C), GGG34GGA (7151G>A), GCA36GCT (7157A>T), CAA37AAA (7158C>A), AAA38AAG (7163A>G), AAG40GAG (7167A>G), GTA42GTT (7175A>T), AAA43AGG (7177A>G 7178A>G), CAA44GCA (7179C>G 7180A>C), ATA45ATT (7184A>T), TTT46CTT (7185T>C), GGT47GGC (7190T>C), CCC48CAG (7192C>A 7193C>G), ACT49TCT (7194A>T), CCA51CCC (7202A>C), ATC52TTT (7203A>T 7205C>T), ATC53CAG (7206A>C 7207T>A 7208C>G), TCT54TCC (7211T>C), CGG55CTT (7213G>T 7214G>T), AAA56AAG (7217A>G), GTT57CTT (7218G>C), CCA60CCT (7229A>T), CCA62CTT (7234C>T 7235A>T), GGA64GGG (7241A>G), ACA65GAG (7242A>G 7243C>A 7244A>G), GTT66CCT (7245G>C 7246T>C), GAG67GAA (7250G>A), GAA68GAT (7253A>T), ATT69ATA (7256T>A), ATC70TCC (7257A>T 7258T>C), GTA74GCT (7270T>C 7271A>T), CGT75AAA (7272C>A 7273G>A 7274T>A), GTA76ATT (7275G>A 7277A>T), GCG77GCT (7280G>T), TCT78GCC (7281T>G 7283T>C), GAG79AAA (7284G>A 7286G>A), TTG80GAG (7287T>G 7288T>A), ATT81GTG (7290A>G 7292T>G), GGA82AAT (7293G>A 7294G>A 7295A>T), GGG83GGA (7298G>A), GTT85GTG (7304T>G), CTC86CTA (7307C>A), GTA87GTT (7310A>T), GAA88GAG (7313A>G), ACA90ACT (7319A>T), AGT91TGT (7320A>T), TTG92CTC (7323T>C 7325G>C), TTT94TTC (7331T>C), GAT95AAC (7332G>A 7334T>C), GCT96GCC (7337T>C), CTC97CTT (7340C>T), AAT98AAG (7343T>G), GGA102GGG (7355A>G), CCA103CCT (7358A>T), TAC104TAT (7361C>T), ATT105ATC (7364T>C), ATG109TTG (7374A>T), GAA110CAA (7377G>C), GGG111AAG (7380G>A 7381G>A), ATT112ATC (7385T>C), GGA113GGT (7388A>T), CTA114CAT (7390T>A 7391A>T), GGA116GGT (7397A>T), TTA117CTT (7398T>C 7400A>T), TAT118AAC (7401T>A 7403T>C), AAG119AAC (7406G>C), TTG120TTA (7409G>A), GTG121TTG (7410G>T), GAG122ATG (7413G>A 7414A>T), CCG123GCT (7416C>G 7418G>T), CAA125GAG (7422C>G 7424A>G), AAT126GAT (7425A>G), ATG128ACA (7432T>C 7433G>A), GCT129GCA (7436T>A), AGT130TAT (7437A>T 7438G>A), GCT131GCC (7442T>C), CTC132ATG (7443C>A 7445C>G), TGC133TGT (7448C>T), GTG134ATC (7449G>A 7451G>C), GCT136TCA (7455G>T 7457T>A), TTT137CTT (7458T>C), GTA138GCT (7462T>C 7463A>T), AAT139CTT (7464A>C 7465A>T), AAA140GGG (7467A>G 7468A>G 7469A>G), GTT141CCA (7470G>C 7471T>C 7472T>A), GGT142AAT (7473G>A 7474G>A), GAT143ACT (7476G>A 7477A>C), GAT144GAG (7481T>G), CCT145CCG (7484T>G), ATA146ATG (7487A>G), ATC147ACT (7489T>C 7490C>T), AAG149GTT (7494A>G 7495A>T 7496G>T), GGT150GGA (7499T>A), GTG151AAA (7500G>A 7501T>A 7502G>A), CTA152ACG (7503C>A 7504T>C 7505A>G), AGA153CTG (7506A>C 7507G>T 7508A>G), GGT154GGA (7511T>A), GAG155AAG (7512G>A), ATT156ATA (7517T>A), GTA157GTG (7520A>G), ATG158CCA (7521A>C 7522T>C 7523G>A), CCA159GCT (7524C>G 7526A>T), CGC160AGG (7527C>A 7529C>G), CCA162CCC (7535A>C), TCA164AAT (7539T>A 7540C>A 7541A>T), TTT165TTC (7544T>C), GGG166GGA (7547G>A), GAC168GAT (7553C>T), CCA169CCG (7556A>G), ATT170ATA (7559T>A), TTC171TTT (7562C>T), CCA173CCT (7568A>T), CTT174CAT (7570T>A), AAC175GGC (7572A>G 7573A>G), TGG176TAT (7576G>A 7577G>T), AGA177GAC (7578A>G 7579G>A 7580A>C), AAG178CAG (7581A>C), ACA179ACT (7586A>T), TTT180TAC (7588T>A 7589T>C), ATG184CCC (7599A>C 7600T>C 7601G>C), GCC185AAG (7602G>A 7603C>A 7604C>G), GAG186GAA (7607G>A), GAG187GAA (7610G>A), AAT189AAC (7616T>C), ATG190AAG (7618T>A), ATA191ATT (7622A>T), CAT193CAC (7628T>C), CGA194CGC (7631A>C), TTT195GGT (7632T>G 7633T>G), CGA196AAA (7635C>A 7636G>A)								

*: Inserts / Deletes / Misaligned / Frameshifts

Analysis details

This analysis was performed with panviral2.64

NGS Details (UN18_val): Human gammaherpesvirus 8

Assembly

**Coverage Length**	119 (1 contig(s))
**Depth Of Coverage**	183.0
**Number Of Reads**	294
**Reads Per Million**	5.52 rpm (after QC)
**Ambiguities**	0
**Assembly Method**	de novo + reference guided assembly
**Consensus Caller**	Bcf Tools

Coverage Map

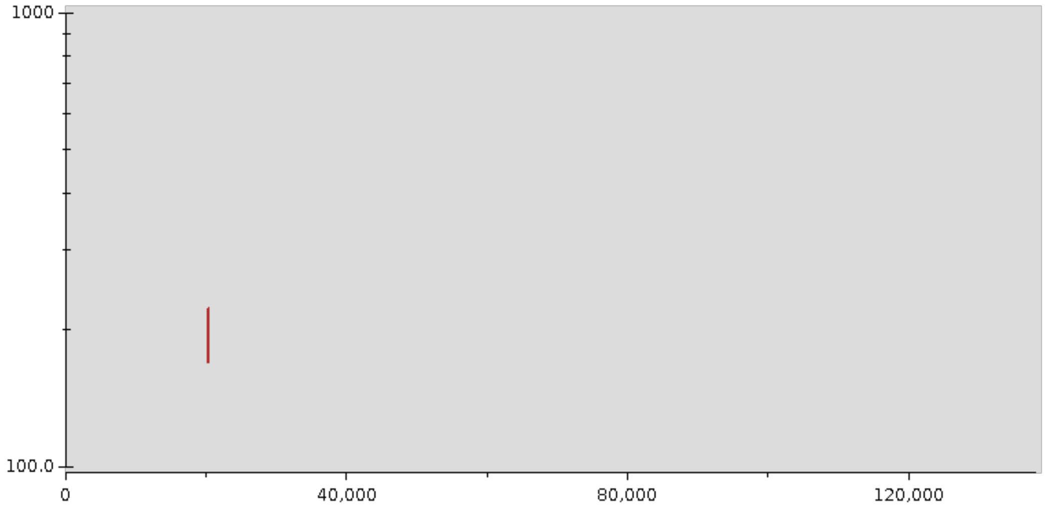

Assignment

**Type**	Human gammaherpesvirus 8 (Taxonomy ID: 37296)
**Subtype**	Could not assign
**Reference Genome**	NC_009333.1
**NT Identity (%)**	83.1933
**AA Identity (%)**	84.6154
**Number Of Stop Codons**	0
**Number Of CDS**	86

Alignment

**Alignment Score**	158.0 (NT) + 260.0 (AA) = 418.0
**Concordance (%)**	77.4074
**Alignment Method**	Local, heuristic, nucleotide (BLASTN)

Genome Region

Sequence starts at position 20278 and ends at position 20396 relative to NC_009333.1 reference sequence.

Alignment Detailed Statistics

	Begin	End	Coverage	Score	Concordance	Matches	Identities	I/D/M/F*	Stop Codons
**NT**	**20278**	**20396**	**0.1%**	**158**	**66.4%**	**119 (100%)**	**99 (83.2%)**	**0/0**	

Mutations: 20293A>T, 20296A>C, 20311G>A, 20314T>G, 20315C>G, 20317C>A, 20320C>T, 20322T>G, 20331G>T, 20341A>C, 20347A>C, 20350G>A, 20352C>T, 20353A>G, 20369C>G, 20370A>C, 20371T>A, 20376A>T, 20383G>A, 20386C>T

*: Inserts / Deletes / Misaligned / Frameshifts

Analysis details

This analysis was performed with panviral2.64

NGS Details (UN18_val): Cladosporium fulvum T-1 virus

Assembly

**Coverage Length**	1209 (4 contig(s))
**Depth Of Coverage**	15.0
**Number Of Reads**	179
**Reads Per Million**	3.36 rpm (after QC)
**Ambiguities**	0
**Assembly Method**	de novo + reference guided assembly
**Consensus Caller**	Bcf Tools

Coverage Map

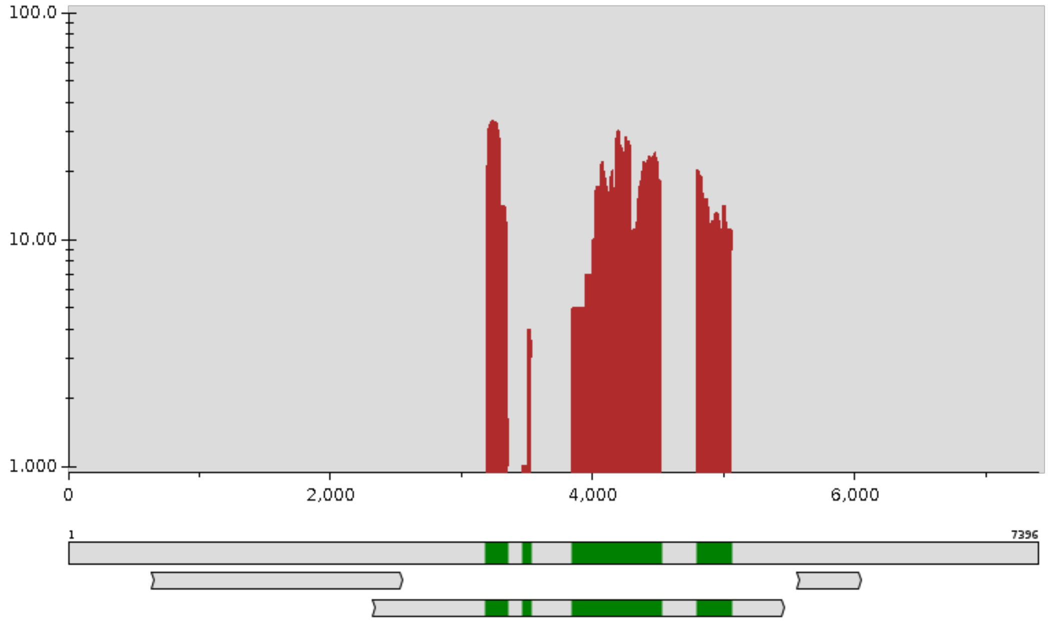

Assignment

**Type**	Cladosporium fulvum T-1 virus (Taxonomy ID: 2052899)
**Reference Genome**	NC_043491.1
**NT Identity (%)**	53.1486
**AA Identity (%)**	42.5693
**Number Of Stop Codons**	0
**Number Of CDS**	3

Alignment

**Alignment Score**	96.0 (NT) + 1234.0 (AA) = 1330.0
**Concordance (%)**	26.2586

Alignment Method	Global, seeded, nucleotide + amino acids (AGA)

Genome Region

Sequence starts at position 3189 and ends at position 5061 relative to NC_043491.1 reference sequence.

Alignment Detailed Statistics

	Begin	End	Coverage	Score	Concordance	Matches	Identities	I/D/M/F*	Stop Codons
**NT**	**3189**	**5061**	**16.3%**	**96**	**4.2%**	**1182 (97.0%)**	**633 (52.0%)**	**9/27**	
Mutations:	3189G>C, 3190C>A, 3191A>G, 3194T>C, 3195G>T, 3197T>C, 3198C>T, 3200C>A, 3201G>A, 3204T>A, 3205C>G, 3207T>A, 3208T>C, 3210A>C, 3211A>T, 3214G>T, 3216A>T, 3217A>G, 3219G>T, 3220A>G, 3221G>C, 3222G>C, 3223C>A, 3224A>C, 3225A>C, 3226G>T, 3229C>G, 3230G>T, 3231C>T, 3232C>G, 3233T>C, 3234T>C, 3236C>A, 3237C>A, 3238T>G, 3243A>T, 3244C>G, 3246A>C, 3251T>A, 3253_3254insAGGGATATT, 3261A>G, 3262G>A, 3265A>T, 3266A>G, 3268C>G, 3269A>T, 3271T>G, 3272C>A, 3273A>T, 3280G>T, 3281A>T, 3282A>C, 3283A>T, 3284G>A, 3285A>C, 3289T>G, 3292A>C, 3294G>C, 3295G>T, 3296G>A, 3297G>T, 3298A>C, 3301C>T, 3303T>C, 3304A>T, 3307T>C, 3308C>A, 3309A>G, 3314T>G, 3317G>C, 3318A>C, 3319G>T, 3320A>G, 3321A>C, 3326C>T, 3328A>G, 3329C>G, 3330A>C, 3331G>T, 3332A>G, 3333C>A, 3334C>A, 3337A>G, 3338C>A, 3339G>A, 3340A>G, 3341G>A, 3343A>G, 3344T>C, 3345G>A, 3347C>T, 3457C>T, 3460A>G, 3461A>C, 3464T>C, 3469C>T, 3470G>A, 3473A>G, 3475C>A, 3478G>T, 3479A>G, 3481C>G, 3488C>A, 3489G>A, 3490A>G, 3496G>C, 3497C>A, 3499A>G, 3502C>T, 3504A>G, 3505C>A, 3508C>T, 3511A>T, 3514A>T, 3515G>T, 3516C>T, 3517A>G, 3518C>T, 3519A>T, 3520A>T, 3524A>C, 3525G>A, 3527T>C, 3529A>T, 3530A>C, 3531C>A, 3532C>G, 3839A>T, 3841G>T, 3842A>G, 3843C>T, 3844A>C, 3845G>A, 3846C>A, 3847A>G, 3848C>A, 3849C>T, 3850C>G, 3856A>G, 3859C>T, 3862A>G, 3865C>T, 3866C>G, 3867A>C, 3869A>C, 3871G>A, 3874A>G, 3880C>A, 3882A>C, 3883G>C, 3886T>C, 3887T>C, 3892C>G, 3893T>C, 3894T>A, 3898C>A, 3899A>G, 3901C>T, 3905A>G, 3906C>A, 3908A>G, 3909C>A, 3910A>C, 3912G>A, 3913G>A, 3914A>G, 3918C>G, 3922C>G, 3929G>A, 3930C>A, 3931A>G, 3934G>A, 3935A>G, 3936C>T, 3937A>G, 3940G>A, 3941T>G, 3943A>G, 3946C>T, 3948G>T, 3952A>T, 3957C>A, 3959G>A, 3960A>C, 3964G>C, 3970A>T, 3973C>G, 3975A>C, 3979T>A, 3980G>C, 3982A>T, 3983C>A, 3984A>G, 3985G>A, 3988A>T, 3992C>T, 3997A>G, 4000C>A, 4003C>T, 4009C>T, 4010A>T, 4025A>G, 4027G>T, 4029A>G, 4030C>A, 4033T>C, 4038A>C, 4041C>A, 4045A>C, 4048A>T, 4052A>C, 4057G>T, 4058A>G, 4059T>A, 4060G>T, 4061C>T, 4063T>G, 4064A>C, 4065C>T, 4066A>G, 4068G>A, 4069A>G, 4072A>G, 4075C>A, 4080A>C, 4081C>G, 4085A>G, 4091G>T, 4092G>C, 4093A>C, 4094A>G, 4097G>A, 4100C>T, 4101A>G, 4102G>C, 4103A>G, 4104C>A, 4105C>A, 4111G>T, 4115A>C, 4117A>G, 4119G>A, 4120A>T, 4129A>C, 4130C>G, 4131A>C, 4132G>A, 4133T>A, 4134G>T, 4135C>T, 4138T>A, 4143C>A, 4144C>A, 4149C>T, 4150G>A, 4153T>G, 4154C>A, 4155G>A, 4156A>G, 4159A>G, 4160T>C, 4161T>C, 4162C>A, 4165T>C, 4166G>T, 4167G>T, 4169A>G, 4170G>A, 4171C>G, 4172A>C, 4173A>T, 4174G>A, 4175G>C, 4176A>C, 4177A>T, 4178G>T, 4180C>T, 4181C>G, 4183C>G, 4184A>G, 4186C>A, 4187G>C, 4189G>C, 4192C>T, 4195C>T, 4201T>G, 4204T>C, 4206T>A, 4207G>A, 4210A>T, 4211A>G, 4213A>G, 4216C>A, 4218C>G, 4219A>C, 4220T>G, 4221G>T, 4222T>A, 4223C>T, 4225A>G, 4226A>G, 4227C>T, 4228A>G, 4231G>A, 4232_4237delACACAC, 4240T>A, 4243G>T, 4244A>G, 4248G>A, 4252C>T, 4255A>G, 4263A>T, 4265T>G, 4267T>A, 4268T>A, 4269C>G, 4271C>A, 4277A>T, 4279G>A, 4281C>A, 4283A>G, 4284C>A, 4285A>G, 4286G>A, 4288G>A, 4293A>G, 4294G>A, 4295A>C, 4296A>G, 4297C>G, 4301G>A, 4302A>C, 4303C>A, 4304A>G, 4309T>A, 4312C>G, 4319C>A, 4321T>G, 4322C>A, 4323T>C, 4330T>C, 4333T>C, 4334G>C, 4335C>A, 4337G>T, 4338C>G, 4340A>C, 4344A>G, 4345A>C, 4346C>A, 4347A>C, 4348T>C, 4355G>C, 4356T>A, 4357G>C, 4361G>T, 4363C>G, 4364G>C, 4365A>T, 4366G>T, 4369C>T, 4370C>T, 4372A>T, 4373_4375delCCG, 4381A>C, 4382A>T, 4383C>T, 4384G>A, 4385A>G, 4387T>C, 4388C>A, 4389T>A, 4390T>A, 4391T>A, 4393A>C, 4397C>A, 4400A>G, 4401A>T, 4403A>G, 4404A>C, 4405T>C, 4406C>A, 4407T>C, 4410C>G, 4411G>C, 4418A>C, 4419C>A, 4420G>A, 4421A>T, 4423G>T, 4424A>C, 4425C>A, 4426G>A, 4430G>A, 4438C>G, 4440G>C, 4441A>C, 4443G>A, 4444A>G, 4450C>A, 4451C>A, 4453C>G, 4457T>C, 4458C>A, 4459G>A, 4462G>T, 4463C>T, 4465G>C, 4466C>T, 4468T>G, 4470G>C, 4471G>A, 4472C>G, 4474G>A, 4476A>T, 4477C>T, 4478A>G, 4480G>C, 4482T>A, 4486A>G, 4487A>C, 4489C>G, 4490A>G, 4492A>G, 4497C>A, 4498T>G, 4504A>C, 4506C>A, 4507A>G, 4509A>G, 4510G>C, 4513C>T, 4515G>T, 4516C>T, 4517C>G, 4518C>T, 4519G>T, 4522A>C, 4789A>T, 4790C>G, 4795C>A, 4796A>G, 4797G>C, 4798C>T, 4800T>A, 4803C>A, 4804A>T, 4806T>G, 4807C>G, 4810A>C, 4813G>A, 4819A>G, 4820C>G, 4821T>A, 4823A>G, 4825G>T, 4829C>G, 4830T>A, 4831A>G, 4833G>T, 4838A>G, 4845A>C, 4846A>T, 4849C>T, 4850G>C, 4852A>T, 4853C>G, 4854A>T, 4855C>G, 4861A>G, 4865A>G, 4870A>G, 4872C>T, 4873T>G, 4875C>A, 4878G>A, 4879G>A, 4881A>G, 4882C>T, 4883G>C, 4885G>C, 4886A>G, 4887A>C, 4889T>G, 4890A>C, 4891C>T, 4896A>T, 4897C>A, 4901C>G, 4904T>C, 4905T>C, 4906C>A, 4907A>C, 4908G>T, 4910A>C, 4912A>C, 4913C>A, 4914C>T, 4916C>G, 4918A>C, 4919A>G, 4920C>A, 4923A>G, 4933C>A, 4934G>A, 4935A>G, 4936G>T, 4937G>A, 4940A>T, 4942G>C, 4954T>C, 4957G>A, 4958A>T, 4959A>G, 4960A>C, 4961C>T, 4963C>A, 4966G>A, 4968G>A, 4969G>A, 4973_4990delAAGGATCGAGTCACAGGA, 4991C>G, 4995C>G, 4996C>G, 4998A>G, 4999T>C, 5000G>A, 5001A>G, 5004T>A, 5005G>T, 5009C>A, 5011A>G, 5014C>T, 5015A>G, 5017G>C, 5020C>G, 5027C>T, 5029C>T, 5031C>G, 5032A>C, 5038T>C, 5040C>G, 5042C>G, 5043A>T, 5044C>A, 5050T>A, 5053T>A, 5055C>T, 5056A>C, 5057T>C, 5059A>G, 5060G>A, 5061A>C								

CDS

	Begin	End	Coverage	Score	Concordance	Matches	Identities	I/D/M/F*	Stop Codons
homologue_of_retroviral_POL_genes	289	912	38.5%	1234	44.2%	394 (97.0%)	169 (41.6%)	3/9/0/0	0
Protein mutations:	K289E (3191A>G), W290L (3194T>C 3195G>T), S291L (3197T>C 3198C>T), R292K (3200C>A 3201G>A), L293Q (3204T>A 3205C>G), F294Y (3207T>A 3208T>C), E295A (3210A>C 3211A>T), E296D (3214G>T), E297V (3216A>T 3217A>G), R298M (3219G>T 3220A>G), G299P (3221G>C 3222G>C 3223C>A), K300P (3224A>C 3225A>C 3226G>T), D301E (3229C>G), A302L (3230G>T 3231C>T 3232C>G), L303P (3233T>C 3234T>C), P304K (3236C>A 3237C>A 3238T>G), H306L (3243A>T 3244C>G), Q307P (3246A>C), W309R (3251T>A), W309_D310insRDI (3253_3254insAGGGATATT), K312R (3261A>G 3262G>A), N314E (3266A>G 3268C>G), I315L (3269A>T 3271T>G), Q316M (3272C>A 3273A>T), K319S (3281A>T 3282A>C 3283A>T), E320T (3284G>A 3285A>C), W323S (3294G>C 3295G>T), G324I (3296G>A 3297G>T 3298A>C), L326P (3303T>C 3304A>T), Q328R (3308C>A 3309A>G), S330A (3314T>G), E331P (3317G>C 3318A>C 3319G>T), K332A (3320A>G 3321A>C), Q335A (3329C>G 3330A>C 3331G>T), T336E (3332A>G 3333C>A 3334C>A), R338K (3338C>A 3339G>A 3340A>G), E339K (3341G>A 3343A>G), W340Q (3344T>C 3345G>A), K379Q (3461A>C), E382K (3470G>A), I383V (3473A>G 3475C>A), I385V (3479A>G 3481C>G), R388K (3488C>A 3489G>A 3490A>G), L391M (3497C>A 3499A>G), N393R (3504A>G 3505C>A), E395D (3511A>T), E396D (3514A>T), A397L (3515G>T 3516C>T 3517A>G), Q398F (3518C>T 3519A>T 3520A>T), R400Q (3524A>C 3525G>A), T402Q (3530A>C 3531C>A 3532C>G), K505Y (3839A>T 3841G>T), T506V (3842A>G 3843C>T 3844A>C), A507K (3845G>A 3846C>A 3847A>G), P508M (3848C>A 3849C>T 3850C>G), H514A (3866C>G 3867A>C), K515Q (3869A>C 3871G>A), K519T (3882A>C 3883G>C), F523H (3893T>C 3894T>A), I525V (3899A>G 3901C>T), T527E (3905A>G 3906C>A), T528D (3908A>G 3909C>A 3910A>C), G529E (3912G>A 3913G>A), I530V (3914A>G), T531R (3918C>G), I532M (3922C>G), A535K (3929G>A 3930C>A 3931A>G), T537V (3935A>G 3936C>T 3937A>G), S539A (3941T>G 3943A>G), R541I (3948G>T), E542D (3952A>T), P544Q (3957C>A), E545T (3959G>A 3960A>C), K550T (3975A>C), D551E (3979T>A), V552L (3980G>C 3982A>T), Q553R (3983C>A 3984A>G 3985G>A), L556F (3992C>T), N562Y (4010A>T), K567D (4025A>G 4027G>T), D568G (4029A>G 4030C>A), K571T (4038A>C), T572K (4041C>A), M576L (4052A>C), M578D (4058A>G 4059T>A 4060G>T), T580L (4064A>C 4065C>T 4066A>G), R581K (4068G>A 4069A>G), D583E (4075C>A), N585T (4080A>C 4081C>G), K587E (4085A>G), G589S (4091G>T 4092G>C 4093A>C), K590E (4094A>G), E591K (4097G>A), Q592C (4100C>T 4101A>G 4102G>C), T593E (4103A>G 4104C>A 4105C>A), K597Q (4115A>C 4117A>G), R598N (4119G>A 4120A>T), E601D (4129A>C), Q602A (4130C>G 4131A>C 4132G>A), C603I (4133T>A 4134G>T 4135C>T), A606E (4143C>A 4144C>A), T608I (4149C>T 4150G>A), R610K (4154C>A 4155G>A 4156A>G), F612P (4160T>C 4161T>C 4162C>A), G614F (4166G>T 4167G>T), S615E (4169A>G 4170G>A 4171C>G), K616L (4172A>C 4173A>T 4174G>A), E617P (4175G>C 4176A>C 4177A>T), V618F (4178G>T 4180C>T), H619E (4181C>G 4183C>G), I620V (4184A>G 4186C>A), E621H (4187G>C 4189G>C), M627K (4206T>A 4207G>A), I629V (4211A>G 4213A>G), A631G (4218C>G 4219A>C), C632V (4220T>G 4221G>T 4222T>A), T634V (4226A>G 4227C>T 4228A>G), T636_H637del (4232_4237delACACAC), D638E (4240T>A), K640E (4244A>G), R641K (4248G>A), Y646F (4263A>T), Y647E (4265T>G 4267T>A), M651L (4277A>T 4279G>A), T652N (4281C>A), T653E (4283A>G 4284C>A 4285A>G), A654T (4286G>A 4288G>A), Q656R (4293A>G 4294G>A), N657R (4295A>C 4296A>G 4297C>G), D659T (4301G>A 4302A>C 4303C>A), I660V (4304A>G), H661Q (4309T>A), D662E (4312C>G), L665M (4319C>A 4321T>G), L666T (4322C>A 4323T>C), A670H (4334G>C 4335C>A), A671C (4337G>T 4338C>G), M672L (4340A>C), Q673R (4344A>G 4345A>C), H674T (4346C>A 4347A>C 4348T>C), V677H (4355G>C 4356T>A 4357G>C), V679L (4361G>T 4363C>G), E680L (4364G>C 4365A>T 4366G>T), P682S (4370C>T 4372A>T), P683del (4373_4375delCCG), L685F (4381A>C), T686L (4382A>T 4383C>T 4384G>A), I687V (4385A>G 4387T>C), L688K (4388C>A 4389T>A 4390T>A), S689T (4391T>A 4393A>C), H691N (4397C>A), K692V (4400A>G 4401A>T), N693A (4403A>G 4404A>C 4405T>C), L694T (4406C>A 4407T>C), T695S (4410C>G 4411G>C), T698Q (4418A>C 4419C>A 4420G>A), T699S (4421A>T 4423G>T), T700Q (4424A>C 4425C>A 4426G>A), E702K (4430G>A), R705P (4440G>C 4441A>C), R706K (4443G>A 4444A>G), S711Q (4457T>C 4458C>A 4459G>A), E712D (4462G>T), L713F (4463C>T 4465G>C), G715A (4470G>C 4471G>A), Q716E (4472C>G 4474G>A), Y717F (4476A>T 4477C>T), K718D (4478A>G 4480G>C), F719Y (4482T>A), I721L (4487A>C 4489C>G), K722E (4490A>G 4492A>G), T724K (4497C>A 4498T>G), T727K (4506C>A 4507A>G), E728G (4509A>G 4510G>C), G730V (4515G>T 4516C>T), P731V (4517C>G 4518C>T 4519G>T), Q822E (4790C>G), S824A (4796A>G 4797G>C 4798C>T), F825Y (4800T>A), S826Y (4803C>A 4804A>T), F827W (4806T>G 4807C>G), L832D (4820C>G 4821T>A), K833D (4823A>G 4825G>T), L835E (4829C>G 4830T>A 4831A>G), R836L (4833G>T), I838V (4838A>G), K840T (4845A>C 4846A>T), V842L (4850G>C 4852A>T), H843V (4853C>G 4854A>T 4855C>G), N847D (4865A>G), A849V (4872C>T 4873T>G), A850E (4875C>A), R851Q (4878G>A 4879G>A), H852R (4881A>G 4882C>T), A853P (4883G>C 4885G>C), K854A (4886A>G 4887A>C), Y855A (4889T>G 4890A>C 4891C>T), H857L (4896A>T 4897C>A), Q859E (4901C>G), F860P (4904T>C 4905T>C 4906C>A), R861L (4907A>C 4908G>T), T862P (4910A>C 4912A>C), P863I (4913C>A 4914C>T), P864A (4916C>G 4918A>C), T865E (4919A>G 4920C>A), K866R (4923A>G), D869E (4933C>A), E870S (4934G>A 4935A>G 4936G>T), V871I (4937G>A), T872S (4940A>T 4942G>C), K878C (4958A>T 4959A>G 4960A>C), R881K (4968G>A 4969G>A), K883_G888del (4973_4990delAAGGATCGAGTCACAGGA), Q889E (4991C>G), A890G (4995C>G 4996C>G), Y891C (4998A>G 4999T>C), D892S (5000G>A 5001A>G), M893N (5004T>A 5005G>T), L895M (5009C>A 5011A>G), M897V (5015A>G 5017G>C), L901F (5027C>T 5029C>T), T902S (5031C>G 5032A>C), A905G (5040C>G), H906V (5042C>G 5043A>T 5044C>A), A910V (5055C>T 5056A>C), S911P (5057T>C 5059A>G)								

	Begin	End	Coverage	Score	Concordance	Matches	Identities	I/D/M/F*	Stop Codons
**NT**	**3189**	**5061**	**16.3%**	**96**	**4.2%**	**1182 (97.0%)**	**633 (52.0%)**	**9/27**	

Codon mutations: CGC288.CA (3189G>C 3190C>A), AAG289GAG (3191A>G), TGG290CTG (3194T>C 3195G>T), TCG291CTG (3197T>C 3198C>T), CGA292AAA (3200C>A 3201G>A), CTC293CAG (3204T>A 3205C>G), TTT294TAC (3207T>A 3208T>C), GAA295GCT (3210A>C 3211A>T), GAG296GAT (3214G>T), GAA297GTG (3216A>T 3217A>G), AGA298ATG (3219G>T 3220A>G), GGC299CCA (3221G>C 3222G>C 3223C>A), AAG300CCT (3224A>C 3225A>C 3226G>T), GAC301GAG (3229C>G), GCC302TTG (3230G>T 3231C>T 3232C>G), TTA303CCA (3233T>C 3234T>C), CCT304AAG (3236C>A 3237C>A 3238T>G), CAC306CTG (3243A>T 3244C>G), CAA307CCA (3246A>C), TGG309AGG (3251T>A), TGG309_GAT310insAGGGATATT (3253_3254insAGGGATATT), AAG312AGA (3261A>G 3262G>A), ATA313ATT (3265A>T), AAC314GAG (3266A>G 3268C>G), ATT315TTG (3269A>T 3271T>G), CAG316ATG (3272C>A 3273A>T), GGG318GGT (3280G>T), AAA319TCT (3281A>T 3282A>C 3283A>T), GAG320ACG (3284G>A 3285A>C), CCT321CCG (3289T>G), CCA322CCC (3292A>C), TGG323TCT (3294G>C 3295G>T), GGA324ATC (3296G>A 3297G>T 3298A>C), CCC325CCT (3301C>T), CTA326CCT (3303T>C 3304A>T), TAT327TAC (3307T>C), CAA328AGA (3308C>A 3309A>G), TCT330GCT (3314T>G), GAG331CCT (3317G>C 3318A>C 3319G>T), AAA332GCA (3320A>G 3321A>C), CTA334TTG (3326C>T 3328A>G), CAG335GCT (3329C>G 3330A>C 3331G>T), ACC336GAA (3332A>G 3333C>A 3334C>A), CTA337CTG (3337A>G), CGA338AAG (3338C>A 3339G>A 3340A>G), GAA339AAG (3341G>A 3343A>G), TGG340CAG (3344T>C 3345G>A), CTG341TTG (3347C>T), TAC377TAT (3457C>T), CGA378CGG (3460A>G), AAG379CAG (3461A>C), TTG380CTG (3464T>C), AAC381AAT (3469C>T), GAG382AAG (3470G>A), ATC383GTA (3473A>G 3475C>A), ACG384ACT (3478G>T), ATC385GTG (3479A>G 3481C>G), CGA388AAG (3488C>A 3489G>A 3490A>G), CCG390CCC (3496G>C), CTA391ATG (3497C>A 3499A>G), CCC392CCT (3502C>T), AAC393AGA (3504A>G 3505C>A), ATC394ATT (3508C>T), GAA395GAT (3511A>T), GAA396GAT (3514A>T), GCA397TTG (3515G>T 3516C>T 3517A>G), CAA398TTT (3518C>T 3519A>T 3520A>T), AGA400CAA (3524A>C 3525G>A), TTA401CTT (3527T>C 3529A>T), ACC402CAG (3530A>C 3531C>A 3532C>G), AAG505TAT (3839A>T 3841G>T), ACA506GTC (3842A>G 3843C>T 3844A>C), GCA507AAG (3845G>A 3846C>A 3847A>G), CCC508ATG (3848C>A 3849C>T 3850C>G), AAA510AAG (3856A>G), TGC511TGT (3859C>T), GAA512GAG (3862A>G), TTC513TTT (3865C>T), CAC514GCC (3866C>G 3867A>C), AAG515CAA (3869A>C 3871G>A), AAA516AAG (3874A>G), GTC518GTA (3880C>A), AAG519ACC (3882A>C 3883G>C), TTT520TTC (3886T>C), TTA521CTA (3887T>C), GGC522GGG (3892C>G), TTT523CAT (3893T>C 3894T>A), ATC524ATA (3898C>A), ATC525GTT (3899A>G 3901C>T), ACA527GAA (3905A>G 3906C>A), ACA528GAC (3908A>G 3909C>A 3910A>C), GGG529GAA (3912G>A 3913G>A), ATA530GTA (3914A>G), ACG531AGG (3918C>G), ATC532ATG (3922C>G), GCA535AAG (3929G>A 3930C>A 3931A>G), AAG536AAA (3934G>A), ACA537GTG (3935A>G 3936C>T 3937A>G), CAG538CAA (3940G>A), TCA539GCG (3941T>G 3943A>G), ATC540ATT (3946C>T), AGA541ATA (3948G>T), GAA542GAT (3952A>T), CCA544CAA (3957C>A), GAA545ACA (3959G>A 3960A>C), CCG546CCC (3964G>C), ACA548ACT (3970A>T), GTC549GTG (3973C>G), AAG550ACG (3975A>C), GAT551GAA (3979T>A), GTA552CTT (3980G>C 3982A>T), CAG553AGA (3983C>A 3984A>G 3985G>A), TCA554TCT (3988A>T), CTT556TTT (3992C>T), GGA557GGG (3997A>G), CTC558CTA (4000C>A), GCC559GCT (4003C>T), TAC561TAT (4009C>T), AAC562TAC (4010A>T), AAG567GAT (4025A>G 4027G>T), GAC568GGA (4029A>G 4030C>A), TAT569TAC (4033T>C), AAG571ACG (4038A>C), ACA572AAA (4041C>A), GCA573GCC (4045A>C), GCA574GCT (4048A>T), ATG576CTG (4052A>C), ACG577ACT (4057G>T), ATG578GAT (4058A>G 4059T>A 4060G>T), CTT579TTG (4061C>T 4063T>G), ACA580CTG (4064A>C 4065C>T 4066A>G), AGA581AAG (4068G>A 4069A>G), AAA582AAG (4072A>G), GAC583GAA (4075C>A), AAC585ACG (4080A>C 4081C>G), AAA587GAA (4085A>G), GGA589TCC (4091G>T 4092G>C 4093A>C), AAA590GAA (4094A>G), GAA591AAA (4097G>A), CAG592TGC (4100C>T 4101A>G 4102G>C), ACC593GAA (4103A>G 4104C>A 4105C>A), GCG595GCT (4111G>T), AAA597CAG (4115A>C 4117A>G), AGA598AAT (4119G>A 4120A>T), GAA601GAC (4129A>C), CAG602GCA (4130C>G 4131A>C 4132G>A), TGC603ATT (4133T>A 4134G>T 4135C>T), GCT604GCA (4138T>A), GCC606GAA (4143C>A 4144C>A), ACG608ATA (4149C>T 4150G>A), CTT609CTG (4153T>G), CGA610AAG (4154C>A 4155G>A 4156A>G), CTA611CTG (4159A>G), TTC612CCA (4160T>C 4161T>C 4162C>A), GAT613GAC (4165T>C), GGT614TTT (4166G>T 4167G>T), AGC615GAG (4169A>G 4170G>A 4171C>G), AAG616CTA (4172A>C 4173A>T 4174G>A), GAA617CCT (4175G>C 4176A>C 4177A>T), GTC618TTT (4178G>T 4180C>T), CAC619GAG (4181C>G 4183C>G), ATC620GTA (4184A>G 4186C>A), GAG621CAC (4187G>C 4189G>C), ACC622ACT (4192C>T), GAC623GAT (4195C>T), TCT625TCG (4201T>G), GAT626GAC (4204T>C), ATG627AAA (4206T>A 4207G>A), GCA628GCT (4210A>T), ATA629GTG (4211A>G 4213A>G), GGC630GGA (4216C>A), GCA631GGC (4218C>G 4219A>C), TGT632GTA (4220T>G 4221G>T 4222T>A), CTA633TTG (4223C>T 4225A>G), ACA634GTG (4226A>G 4227C>T 4228A>G), CAG635CAA (4231G>A), ACA636_CAC637del (4232_4237delACACAC), GAT638GAA (4240T>A), GGG639GGT (4243G>T), AAA640GAA (4244A>G), AGA641AAA (4248G>A), CAC642CAT (4252C>T), CCA643CCG (4255A>G), TAT646TTT (4263A>T), TAT647GAA (4265T>G 4267T>A), TCC648AGC (4268T>A 4269C>G), CGG649AGG (4271C>A), ATG651TTA (4277A>T 4279G>A), ACC652AAC (4281C>A), ACA653GAG (4283A>G 4284C>A 4285A>G), GCG654ACA (4286G>A 4288G>A), CAG656CGA (4293A>G 4294G>A), AAC657CGG (4295A>C 4296A>G 4297C>G), GAC659ACA (4301G>A 4302A>C 4303C>A), ATC660GTC (4304A>G), CAT661CAA (4309T>A), GAC662GAG (4312C>G), CTT665ATG (4319C>A 4321T>G), CTA666ACA (4322C>A 4323T>C), ATT668ATC (4330T>C), GTT669GTC (4333T>C), GCC670CAC (4334G>C 4335C>A), GCC671TGC (4337G>T 4338C>G), ATG672CTG (4340A>C), CAA673CGC (4344A>G 4345A>C), CAT674ACC (4346C>A 4347A>C 4348T>C), GTG677CAC (4355G>C 4356T>A 4357G>C), GTC679TTG (4361G>T 4363C>G), GAG680CTT (4364G>C 4365A>T 4366G>T), GGC681GGT (4369C>T), CCA682TCT (4370C>T 4372A>T), CCG683del (4373_4375delCCG), TTA685TTC (4381A>C), ACG686TTA (4382A>T 4383C>T 4384G>A), ATT687GTC (4385A>G 4387T>C), CTT688AAA (4388C>A 4389T>A 4390T>A), TCA689ACC (4391T>A 4393A>C), CAC691AAC (4397C>A), AAG692GTG (4400A>G 4401A>T), AAT693GCC (4403A>G 4404A>C 4405T>C), CTC694ACC (4406C>A 4407T>C), ACG695AGC (4410C>G 4411G>C), ACG698CAA (4418A>C 4419C>A 4420G>A), ACG699TCT (4421A>T 4423G>T), ACG700CAA (4424A>C 4425C>A 4426G>A), GAA702AAA (4430G>A), ACC704ACG (4438C>G), CGA705CCC (4440G>C 4441A>C), AGA706AAG (4443G>A 4444A>G), GCC708GCA (4450C>A), CGC709AGG (4451C>A 4453C>G), TCG711CAA (4457T>C 4458C>A 4459G>A), GAG712GAT (4462G>T), CTG713TTC (4463C>T 4465G>C), CTT714TTG (4466C>T 4468T>G), GGG715GCA (4470G>C 4471G>A), CAG716GAA (4472C>G 4474G>A), TAC717TTT (4476A>T 4477C>T), AAG718GAC (4478A>G 4480G>C), TTC719TAC (4482T>A), GAA720GAG (4486A>G), ATC721CTG (4487A>C 4489C>G), AAA722GAG (4490A>G 4492A>G), ACT724AAG (4497C>A 4498T>G), GGA726GGC (4504A>C), ACA727AAG (4506C>A 4507A>G), GAG728GGC (4509A>G 4510G>C), AAC729AAT (4513C>T), GGC730GTT (4515G>T 4516C>T), CCG731GTT (4517C>G 4518C>T 4519G>T), GCA732GCC (4522A>C), ATA821ATT (4789A>T), CAG822GAG (4790C>G), CGC823CGA (4795C>A), AGC824GCT (4796A>G 4797G>C 4798C>T), TTC825TAC (4800T>A), TCA826TAT (4803C>A 4804A>T), TTC827TGG (4806T>G 4807C>G), CCA828CCC (4810A>C), CAG829CAA (4813G>A), AGA831AGG (4819A>G), CTT832GAT (4820C>G 4821T>A), AAG833GAT (4823A>G 4825G>T), CTA835GAG (4829C>G 4830T>A 4831A>G), CGC836CTC (4833G>T), ATC838GTC (4838A>G), AAA840ACT (4845A>C 4846A>T), TGC841TGT (4849C>T), GTA842CTT (4850G>C 4852A>T), CAC843GTG (4853C>G 4854A>T 4855C>G), CAA845CAG (4861A>G), AAC847GAC (4865A>G), AAA848AAG (4870A>G), GCT849GTG (4872C>T 4873T>G), GCA850GAA (4875C>A), CGG851CAA (4878G>A 4879G>A), CAC852CGT (4881A>G 4882C>T), GCG853CCC (4883G>C 4885G>C), AAA854GCA (4886A>G 4887A>C), TAC855GCT (4889T>G 4890A>C 4891C>T), CAC857CTA (4896A>T 4897C>A), CAG859GAG (4901C>G), TTC860CCA (4904T>C 4905T>C 4906C>A), AGG861CTG (4907A>C 4908G>T), ACA862CCC (4910A>C 4912A>C), CCA863ATA (4913C>A 4914C>T), CCA864GCC (4916C>G 4918A>C), ACG865GAG (4919A>G 4920C>A), AAA866AGA (4923A>G), GAC869GAA (4933C>A), GAG870AGT (4934G>A 4935A>G 4936G>T), GTT871ATT (4937G>A), ACG872TCC (4940A>T 4942G>C), ATT876ATC (4954T>C), ACG877ACA (4957G>A), AAA878TGC (4958A>T 4959A>G 4960A>C), CTC879TTA (4961C>T 4963C>A), CCG880CCA (4966G>A), AGG881AAA (4968G>A 4969G>A), AAG883_GGA888del (4973_4990delAAGGATCGAGTCACAGGA), CAA889GAA (4991C>G), GCC890GGG (4995C>G 4996C>G), TAT891TGC (4998A>G 4999T>C), GAC892AGC (5000G>A 5001A>G), ATG893AAT (5004T>A 5005G>T), CTA895ATG (5009C>A 5011A>G), GTC896GTT (5014C>T), ATG897GTC (5015A>G 5017G>C), GTC898GTG (5020C>G), CTC901TTT (5027C>T 5029C>T), ACA902AGC (5031C>G 5032A>C), TAT904TAC (5038T>C), GCA905GGA (5040C>G), CAC906GTA (5042C>G 5043A>T 5044C>A), ATT908ATA (5050T>A), CCT909CCA (5053T>A), GCA910GTC (5055C>T 5056A>C), TCA911CCG (5057T>C 5059A>G), GAA912AC. (5060G>A 5061A>C)

Proteins

	Begin	End	Coverage	Score	Concordance	Matches	Identities	I/D/M/F*	Stop Codons
Reverse Transcriptase (YP_009666308.1)	289	912	38.5%	1234	44.2%	394 (97.0%)	169 (41.6%)	3/9/0/0	0

	Begin	End	Coverage	Score	Concordance	Matches	Identities	I/D/M/F*	Stop Codons
**NT**	**3189**	**5061**	**16.3%**	**96**	**4.2%**	**1182 (97.0%)**	**633 (52.0%)**	**9/27**	
Protein mutations:	K289E (3191A>G), W290L (3194T>C 3195G>T), S291L (3197T>C 3198C>T), R292K (3200C>A 3201G>A), L293Q (3204T>A 3205C>G), F294Y (3207T>A 3208T>C), E295A (3210A>C 3211A>T), E296D (3214G>T), E297V (3216A>T 3217A>G), R298M (3219G>T 3220A>G), G299P (3221G>C 3222G>C 3223C>A), K300P (3224A>C 3225A>C 3226G>T), D301E (3229C>G), A302L (3230G>T 3231C>T 3232C>G), L303P (3233T>C 3234T>C), P304K (3236C>A 3237C>A 3238T>G), H306L (3243A>T 3244C>G), Q307P (3246A>C), W309R (3251T>A), W309_D310insRDI (3253_3254insAGGGATATT), K312R (3261A>G 3262G>A), N314E (3266A>G 3268C>G), I315L (3269A>T 3271T>G), Q316M (3272C>A 3273A>T), K319S (3281A>T 3282A>C 3283A>T), E320T (3284G>A 3285A>C), W323S (3294G>C 3295G>T), G324I (3296G>A 3297G>T 3298A>C), L326P (3303T>C 3304A>T), Q328R (3308C>A 3309A>G), S330A (3314T>G), E331P (3317G>C 3318A>C 3319G>T), K332A (3320A>G 3321A>C), Q335A (3329C>G 3330A>C 3331G>T), T336E (3332A>G 3333C>A 3334C>A), R338K (3338C>A 3339G>A 3340A>G), E339K (3341G>A 3343A>G), W340Q (3344T>C 3345G>A), K379Q (3461A>C), E382K (3470G>A), I383V (3473A>G 3475C>A), I385V (3479A>G 3481C>G), R388K (3488C>A 3489G>A 3490A>G), L391M (3497C>A 3499A>G), N393R (3504A>G 3505C>A), E395D (3511A>T), E396D (3514A>T), A397L (3515G>T 3516C>T 3517A>G), Q398F (3518C>T 3519A>T 3520A>T), R400Q (3524A>C 3525G>A), T402Q (3530A>C 3531C>A 3532C>G), K505Y (3839A>T 3841G>T), T506V (3842A>G 3843C>T 3844A>C), A507K (3845G>A 3846C>A 3847A>G), P508M (3848C>A 3849C>T 3850C>G), H514A (3866C>G 3867A>C), K515Q (3869A>C 3871G>A), K519T (3882A>C 3883G>C), F523H (3893T>C 3894T>A), I525V (3899A>G 3901C>T), T527E (3905A>G 3906C>A), T528D (3908A>G 3909C>A 3910A>C), G529E (3912G>A 3913G>A), I530V (3914A>G), T531R (3918C>G), I532M (3922C>G), A535K (3929G>A 3930C>A 3931A>G), T537V (3935A>G 3936C>T 3937A>G), S539A (3941T>G 3943A>G), R541I (3948G>T), E542D (3952A>T), P544Q (3957C>A), E545T (3959G>A 3960A>C), K550T (3975A>C), D551E (3979T>A), V552L (3980G>C 3982A>T), Q553R (3983C>A 3984A>G 3985G>A), L556F (3992C>T), N562Y (4010A>T), K567D (4025A>G 4027G>T), D568G (4029A>G 4030C>A), K571T (4038A>C), T572K (4041C>A), M576L (4052A>C), M578D (4058A>G 4059T>A 4060G>T), T580L (4064A>C 4065C>T 4066A>G), R581K (4068G>A 4069A>G), D583E (4075C>A), N585T (4080A>C 4081C>G), K587E (4085A>G), G589S (4091G>T 4092G>C 4093A>C), K590E (4094A>G), E591K (4097G>A), Q592C (4100C>T 4101A>G 4102G>C), T593E (4103A>G 4104C>A 4105C>A), K597Q (4115A>C 4117A>G), R598N (4119G>A 4120A>T), E601D (4129A>C), Q602A (4130C>G 4131A>C 4132G>A), C603I (4133T>A 4134G>T 4135C>T), A606E (4143C>A 4144C>A), T608I (4149C>T 4150G>A), R610K (4154C>A 4155G>A 4156A>G), F612P (4160T>C 4161T>C 4162C>A), G614F (4166G>T 4167G>T), S615E (4169A>G 4170G>A 4171C>G), K616L (4172A>C 4173A>T 4174G>A), E617P (4175G>C 4176A>C 4177A>T), V618F (4178G>T 4180C>T), H619E (4181C>G 4183C>G), I620V (4184A>G 4186C>A), E621H (4187G>C 4189G>C), M627K (4206T>A 4207G>A), I629V (4211A>G 4213A>G), A631G (4218C>G 4219A>C), C632V (4220T>G 4221G>T 4222T>A), T634V (4226A>G 4227C>T 4228A>G), T636_H637del (4232_4237delACACAC), D638E (4240T>A), K640E (4244A>G), R641K (4248G>A), Y646F (4263A>T), Y647E (4265T>G 4267T>A), M651L (4277A>T 4279G>A), T652N (4281C>A), T653E (4283A>G 4284C>A 4285A>G), A654T (4286G>A 4288G>A), Q656R (4293A>G 4294G>A), N657R (4295A>C 4296A>G 4297C>G), D659T (4301G>A 4302A>C 4303C>A), I660V (4304A>G), H661Q (4309T>A), D662E (4312C>G), L665M (4319C>A 4321T>G), L666T (4322C>A 4323T>C), A670H (4334G>C 4335C>A), A671C (4337G>T 4338C>G), M672L (4340A>C), Q673R (4344A>G 4345A>C), H674T (4346C>A 4347A>C 4348T>C), V677H (4355G>C 4356T>A 4357G>C), V679L (4361G>T 4363C>G), E680L (4364G>C 4365A>T 4366G>T), P682S (4370C>T 4372A>T), P683del (4373_4375delCCG), L685F (4381A>C), T686L (4382A>T 4383C>T 4384G>A), I687V (4385A>G 4387T>C), L688K (4388C>A 4389T>A 4390T>A), S689T (4391T>A 4393A>C), H691N (4397C>A), K692V (4400A>G 4401A>T), N693A (4403A>G 4404A>C 4405T>C), L694T (4406C>A 4407T>C), T695S (4410C>G 4411G>C), T698Q (4418A>C 4419C>A 4420G>A), T699S (4421A>T 4423G>T), T700Q (4424A>C 4425C>A 4426G>A), E702K (4430G>A), R705P (4440G>C 4441A>C), R706K (4443G>A 4444A>G), S711Q (4457T>C 4458C>A 4459G>A), E712D (4462G>T), L713F (4463C>T 4465G>C), G715A (4470G>C 4471G>A), Q716E (4472C>G 4474G>A), Y717F (4476A>T 4477C>T), K718D (4478A>G 4480G>C), F719Y (4482T>A), I721L (4487A>C 4489C>G), K722E (4490A>G 4492A>G), T724K (4497C>A 4498T>G), T727K (4506C>A 4507A>G), E728G (4509A>G 4510G>C), G730V (4515G>T 4516C>T), P731V (4517C>G 4518C>T 4519G>T), Q822E (4790C>G), S824A (4796A>G 4797G>C 4798C>T), F825Y (4800T>A), S826Y (4803C>A 4804A>T), F827W (4806T>G 4807C>G), L832D (4820C>G 4821T>A), K833D (4823A>G 4825G>T), L835E (4829C>G 4830T>A 4831A>G), R836L (4833G>T), I838V (4838A>G), K840T (4845A>C 4846A>T), V842L (4850G>C 4852A>T), H843V (4853C>G 4854A>T 4855C>G), N847D (4865A>G), A849V (4872C>T 4873T>G), A850E (4875C>A), R851Q (4878G>A 4879G>A), H852R (4881A>G 4882C>T), A853P (4883G>C 4885G>C), K854A (4886A>G 4887A>C), Y855A (4889T>G 4890A>C 4891C>T), H857L (4896A>T 4897C>A), Q859E (4901C>G), F860P (4904T>C 4905T>C 4906C>A), R861L (4907A>C 4908G>T), T862P (4910A>C 4912A>C), P863I (4913C>A 4914C>T), P864A (4916C>G 4918A>C), T865E (4919A>G 4920C>A), K866R (4923A>G), D869E (4933C>A), E870S (4934G>A 4935A>G 4936G>T), V871I (4937G>A), T872S (4940A>T 4942G>C), K878C (4958A>T 4959A>G 4960A>C), R881K (4968G>A 4969G>A), K883_G888del (4973_4990delAAGGATCGAGTCACAGGA), Q889E (4991C>G), A890G (4995C>G 4996C>G), Y891C (4998A>G 4999T>C), D892S (5000G>A 5001A>G), M893N (5004T>A 5005G>T), L895M (5009C>A 5011A>G), M897V (5015A>G 5017G>C), L901F (5027C>T 5029C>T), T902S (5031C>G 5032A>C), A905G (5040C>G), H906V (5042C>G 5043A>T 5044C>A), A910V (5055C>T 5056A>C), S911P (5057T>C 5059A>G)								

	Begin	End	Coverage	Score	Concordance	Matches	Identities	I/D/M/F*	Stop Codons
**NT**	**3189**	**5061**	**16.3%**	**96**	**4.2%**	**1182 (97.0%)**	**633 (52.0%)**	**9/27**	
Codon mutations:	CGC288.CA (3189G>C 3190C>A), AAG289GAG (3191A>G), TGG290CTG (3194T>C 3195G>T), TCG291CTG (3197T>C 3198C>T), CGA292AAA (3200C>A 3201G>A), CTC293CAG (3204T>A 3205C>G), TTT294TAC (3207T>A 3208T>C), GAA295GCT (3210A>C 3211A>T), GAG296GAT (3214G>T), GAA297GTG (3216A>T 3217A>G), AGA298ATG (3219G>T 3220A>G), GGC299CCA (3221G>C 3222G>C 3223C>A), AAG300CCT (3224A>C 3225A>C 3226G>T), GAC301GAG (3229C>G), GCC302TTG (3230G>T 3231C>T 3232C>G), TTA303CCA (3233T>C 3234T>C), CCT304AAG (3236C>A 3237C>A 3238T>G), CAC306CTG (3243A>T 3244C>G), CAA307CCA (3246A>C), TGG309AGG (3251T>A), TGG309_GAT310insAGGGATATT (3253_3254insAGGGATATT), AAG312AGA (3261A>G 3262G>A), ATA313ATT (3265A>T), AAC314GAG (3266A>G 3268C>G), ATT315TTG (3269A>T 3271T>G), CAG316ATG (3272C>A 3273A>T), GGG318GGT (3280G>T), AAA319TCT (3281A>T 3282A>C 3283A>T), GAG320ACG (3284G>A 3285A>C), CCT321CCG (3289T>G), CCA322CCC (3292A>C), TGG323TCT (3294G>C 3295G>T), GGA324ATC (3296G>A 3297G>T 3298A>C), CCC325CCT (3301C>T), CTA326CCT (3303T>C 3304A>T), TAT327TAC (3307T>C), CAA328AGA (3308C>A 3309A>G), TCT330GCT (3314T>G), GAG331CCT (3317G>C 3318A>C 3319G>T), AAA332GCA (3320A>G 3321A>C), CTA334TTG (3326C>T 3328A>G), CAG335GCT (3329C>G 3330A>C 3331G>T), ACC336GAA (3332A>G 3333C>A 3334C>A), CTA337CTG (3337A>G), CGA338AAG (3338C>A 3339G>A 3340A>G), GAA339AAG (3341G>A 3343A>G), TGG340CAG (3344T>C 3345G>A), CTG341TTG (3347C>T), TAC377TAT (3457C>T), CGA378CGG (3460A>G), AAG379CAG (3461A>C), TTG380CTG (3464T>C), AAC381AAT (3469C>T), GAG382AAG (3470G>A), ATC383GTA (3473A>G 3475C>A), ACG384ACT (3478G>T), ATC385GTG (3479A>G 3481C>G), CGA388AAG (3488C>A 3489G>A 3490A>G), CCG390CCC (3496G>C), CTA391ATG (3497C>A 3499A>G), CCC392CCT (3502C>T), AAC393AGA (3504A>G 3505C>A), ATC394ATT (3508C>T), GAA395GAT (3511A>T), GAA396GAT (3514A>T), GCA397TTG (3515G>T 3516C>T 3517A>G), CAA398TTT (3518C>T 3519A>T 3520A>T), AGA400CAA (3524A>C 3525G>A), TTA401CTT (3527T>C 3529A>T), ACC402CAG (3530A>C 3531C>A 3532C>G), AAG505TAT (3839A>T 3841G>T), ACA506GTC (3842A>G 3843C>T 3844A>C), GCA507AAG (3845G>A 3846C>A 3847A>G), CCC508ATG (3848C>A 3849C>T 3850C>G), AAA510AAG (3856A>G), TGC511TGT (3859C>T), GAA512GAG (3862A>G), TTC513TTT (3865C>T), CAC514GCC (3866C>G 3867A>C), AAG515CAA (3869A>C 3871G>A), AAA516AAG (3874A>G), GTC518GTA (3880C>A), AAG519ACC (3882A>C 3883G>C), TTT520TTC (3886T>C), TTA521CTA (3887T>C), GGC522GGG (3892C>G), TTT523CAT (3893T>C 3894T>A), ATC524ATA (3898C>A), ATC525GTT (3899A>G 3901C>T), ACA527GAA (3905A>G 3906C>A), ACA528GAC (3908A>G 3909C>A 3910A>C), GGG529GAA (3912G>A 3913G>A), ATA530GTA (3914A>G), ACG531AGG (3918C>G), ATC532ATG (3922C>G), GCA535AAG (3929G>A 3930C>A 3931A>G), AAG536AAA (3934G>A), ACA537GTG (3935A>G 3936C>T 3937A>G), CAG538CAA (3940G>A), TCA539GCG (3941T>G 3943A>G), ATC540ATT (3946C>T), AGA541ATA (3948G>T), GAA542GAT (3952A>T), CCA544CAA (3957C>A), GAA545ACA (3959G>A 3960A>C), CCG546CCC (3964G>C), ACA548ACT (3970A>T), GTC549GTG (3973C>G), AAG550ACG (3975A>C), GAT551GAA (3979T>A), GTA552CTT (3980G>C 3982A>T), CAG553AGA (3983C>A 3984A>G 3985G>A), TCA554TCT (3988A>T), CTT556TTT (3992C>T), GGA557GGG (3997A>G), CTC558CTA (4000C>A), GCC559GCT (4003C>T), TAC561TAT (4009C>T), AAC562TAC (4010A>T), AAG567GAT (4025A>G 4027G>T), GAC568GGA (4029A>G 4030C>A), TAT569TAC (4033T>C), AAG571ACG (4038A>C), ACA572AAA (4041C>A), GCA573GCC (4045A>C), GCA574GCT (4048A>T), ATG576CTG (4052A>C), ACG577ACT (4057G>T), ATG578GAT (4058A>G 4059T>A 4060G>T), CTT579TTG (4061C>T 4063T>G), ACA580CTG (4064A>C 4065C>T 4066A>G), AGA581AAG (4068G>A 4069A>G), AAA582AAG (4072A>G), GAC583GAA (4075C>A), AAC585ACG (4080A>C 4081C>G), AAA587GAA (4085A>G), GGA589TCC (4091G>T 4092G>C 4093A>C), AAA590GAA (4094A>G), GAA591AAA (4097G>A), CAG592TGC (4100C>T 4101A>G 4102G>C), ACC593GAA (4103A>G 4104C>A 4105C>A), GCG595GCT (4111G>T), AAA597CAG (4115A>C 4117A>G), AGA598AAT (4119G>A 4120A>T), GAA601GAC (4129A>C), CAG602GCA (4130C>G 4131A>C 4132G>A), TGC603ATT (4133T>A 4134G>T 4135C>T), GCT604GCA (4138T>A), GCC606GAA (4143C>A 4144C>A), ACG608ATA (4149C>T 4150G>A), CTT609CTG (4153T>G), CGA610AAG (4154C>A 4155G>A 4156A>G), CTA611CTG (4159A>G), TTC612CCA (4160T>C 4161T>C 4162C>A), GAT613GAC (4165T>C), GGT614TTT (4166G>T 4167G>T), AGC615GAG (4169A>G 4170G>A 4171C>G), AAG616CTA (4172A>C 4173A>T 4174G>A), GAA617CCT (4175G>C 4176A>C 4177A>T), GTC618TTT (4178G>T 4180C>T), CAC619GAG (4181C>G 4183C>G), ATC620GTA (4184A>G 4186C>A), GAG621CAC (4187G>C 4189G>C), ACC622ACT (4192C>T), GAC623GAT (4195C>T), TCT625TCG (4201T>G), GAT626GAC (4204T>C), ATG627AAA (4206T>A 4207G>A), GCA628GCT (4210A>T), ATA629GTG (4211A>G 4213A>G), GGC630GGA (4216C>A), GCA631GGC (4218C>G 4219A>C), TGT632GTA (4220T>G 4221G>T 4222T>A), CTA633TTG (4223C>T 4225A>G), ACA634GTG (4226A>G 4227C>T 4228A>G), CAG635CAA (4231G>A), ACA636_CAC637del (4232_4237delACACAC), GAT638GAA (4240T>A), GGG639GGT (4243G>T), AAA640GAA (4244A>G), AGA641AAA (4248G>A), CAC642CAT (4252C>T), CCA643CCG (4255A>G), TAT646TTT (4263A>T), TAT647GAA (4265T>G 4267T>A), TCC648AGC (4268T>A 4269C>G), CGG649AGG (4271C>A), ATG651TTA (4277A>T 4279G>A), ACC652AAC (4281C>A), ACA653GAG (4283A>G 4284C>A 4285A>G), GCG654ACA (4286G>A 4288G>A), CAG656CGA (4293A>G 4294G>A), AAC657CGG (4295A>C 4296A>G 4297C>G), GAC659ACA (4301G>A 4302A>C 4303C>A), ATC660GTC (4304A>G), CAT661CAA (4309T>A), GAC662GAG (4312C>G), CTT665ATG (4319C>A 4321T>G), CTA666ACA (4322C>A 4323T>C), ATT668ATC (4330T>C), GTT669GTC (4333T>C), GCC670CAC (4334G>C 4335C>A), GCC671TGC (4337G>T 4338C>G), ATG672CTG (4340A>C), CAA673CGC (4344A>G 4345A>C), CAT674ACC (4346C>A 4347A>C 4348T>C), GTG677CAC (4355G>C 4356T>A 4357G>C), GTC679TTG (4361G>T 4363C>G), GAG680CTT (4364G>C 4365A>T 4366G>T), GGC681GGT (4369C>T), CCA682TCT (4370C>T 4372A>T), CCG683del (4373_4375delCCG), TTA685TTC (4381A>C), ACG686TTA (4382A>T 4383C>T 4384G>A), ATT687GTC (4385A>G 4387T>C), CTT688AAA (4388C>A 4389T>A 4390T>A), TCA689ACC (4391T>A 4393A>C), CAC691AAC (4397C>A), AAG692GTG (4400A>G 4401A>T), AAT693GCC (4403A>G 4404A>C 4405T>C), CTC694ACC (4406C>A 4407T>C), ACG695AGC (4410C>G 4411G>C), ACG698CAA (4418A>C 4419C>A 4420G>A), ACG699TCT (4421A>T 4423G>T), ACG700CAA (4424A>C 4425C>A 4426G>A), GAA702AAA (4430G>A), ACC704ACG (4438C>G), CGA705CCC (4440G>C 4441A>C), AGA706AAG (4443G>A 4444A>G), GCC708GCA (4450C>A), CGC709AGG (4451C>A 4453C>G), TCG711CAA (4457T>C 4458C>A 4459G>A), GAG712GAT (4462G>T), CTG713TTC (4463C>T 4465G>C), CTT714TTG (4466C>T 4468T>G), GGG715GCA (4470G>C 4471G>A), CAG716GAA (4472C>G 4474G>A), TAC717TTT (4476A>T 4477C>T), AAG718GAC (4478A>G 4480G>C), TTC719TAC (4482T>A), GAA720GAG (4486A>G), ATC721CTG (4487A>C 4489C>G), AAA722GAG (4490A>G 4492A>G), ACT724AAG (4497C>A 4498T>G), GGA726GGC (4504A>C), ACA727AAG (4506C>A 4507A>G), GAG728GGC (4509A>G 4510G>C), AAC729AAT (4513C>T), GGC730GTT (4515G>T 4516C>T), CCG731GTT (4517C>G 4518C>T 4519G>T), GCA732GCC (4522A>C), ATA821ATT (4789A>T), CAG822GAG (4790C>G), CGC823CGA (4795C>A), AGC824GCT (4796A>G 4797G>C 4798C>T), TTC825TAC (4800T>A), TCA826TAT (4803C>A 4804A>T), TTC827TGG (4806T>G 4807C>G), CCA828CCC (4810A>C), CAG829CAA (4813G>A), AGA831AGG (4819A>G), CTT832GAT (4820C>G 4821T>A), AAG833GAT (4823A>G 4825G>T), CTA835GAG (4829C>G 4830T>A 4831A>G), CGC836CTC (4833G>T), ATC838GTC (4838A>G), AAA840ACT (4845A>C 4846A>T), TGC841TGT (4849C>T), GTA842CTT (4850G>C 4852A>T), CAC843GTG (4853C>G 4854A>T 4855C>G), CAA845CAG (4861A>G), AAC847GAC (4865A>G), AAA848AAG (4870A>G), GCT849GTG (4872C>T 4873T>G), GCA850GAA (4875C>A), CGG851CAA (4878G>A 4879G>A), CAC852CGT (4881A>G 4882C>T), GCG853CCC (4883G>C 4885G>C), AAA854GCA (4886A>G 4887A>C), TAC855GCT (4889T>G 4890A>C 4891C>T), CAC857CTA (4896A>T 4897C>A), CAG859GAG (4901C>G), TTC860CCA (4904T>C 4905T>C 4906C>A), AGG861CTG (4907A>C 4908G>T), ACA862CCC (4910A>C 4912A>C), CCA863ATA (4913C>A 4914C>T), CCA864GCC (4916C>G 4918A>C), ACG865GAG (4919A>G 4920C>A), AAA866AGA (4923A>G), GAC869GAA (4933C>A), GAG870AGT (4934G>A 4935A>G 4936G>T), GTT871ATT (4937G>A), ACG872TCC (4940A>T 4942G>C), ATT876ATC (4954T>C), ACG877ACA (4957G>A), AAA878TGC (4958A>T 4959A>G 4960A>C), CTC879TTA (4961C>T 4963C>A), CCG880CCA (4966G>A), AGG881AAA (4968G>A 4969G>A), AAG883_GGA888del (4973_4990delAAGGATCGAGTCACAGGA), CAA889GAA (4991C>G), GCC890GGG (4995C>G 4996C>G), TAT891TGC (4998A>G 4999T>C), GAC892AGC (5000G>A 5001A>G), ATG893AAT (5004T>A 5005G>T), CTA895ATG (5009C>A 5011A>G), GTC896GTT (5014C>T), ATG897GTC (5015A>G 5017G>C), GTC898GTG (5020C>G), CTC901TTT (5027C>T 5029C>T), ACA902AGC (5031C>G 5032A>C), TAT904TAC (5038T>C), GCA905GGA (5040C>G), CAC906GTA (5042C>G 5043A>T 5044C>A), ATT908ATA (5050T>A), CCT909CCA (5053T>A), GCA910GTC (5055C>T 5056A>C), TCA911CCG (5057T>C 5059A>G), GAA912AC. (5060G>A 5061A>C)								

*: Inserts / Deletes / Misaligned / Frameshifts

Analysis details

This analysis was performed with panviral2.64

NGS Details (UN18_val): Errantivirus

Assembly

**Coverage Length**	724 (3 contig(s))
**Depth Of Coverage**	16.0
**Number Of Reads**	149
**Reads Per Million**	2.80 rpm (after QC)
**Ambiguities**	0
**Assembly Method**	de novo + reference guided assembly
**Consensus Caller**	Bcf Tools

Coverage Map

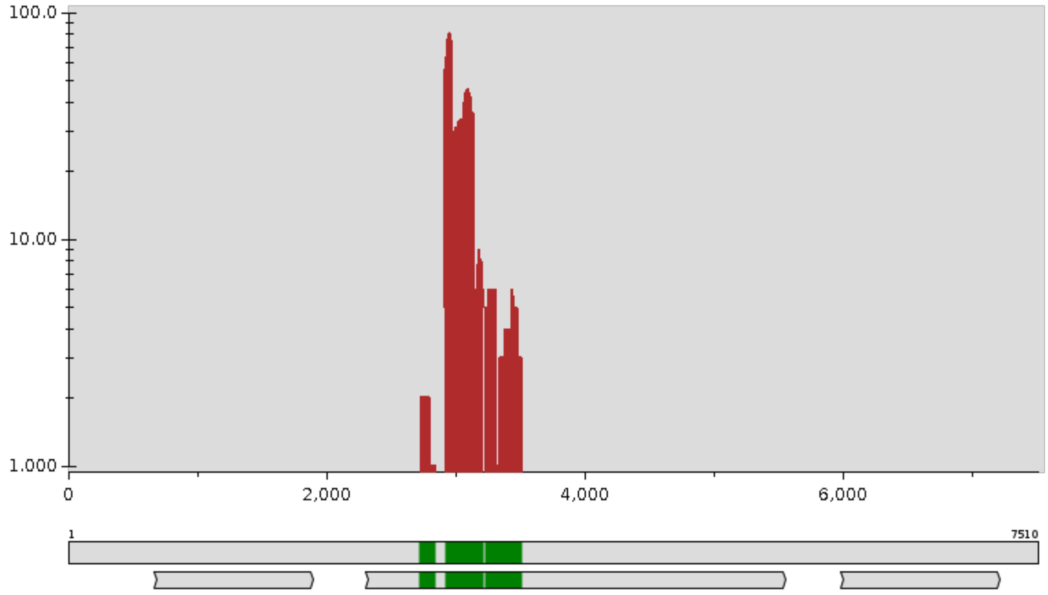

Assignment

**Type**	Errantivirus (Taxonomy ID: 186666)
**Reference Genome**	NC_038512.1
**NT Identity (%)**	56.0773
**AA Identity (%)**	44.8133
**Number Of Stop Codons**	0
**Number Of CDS**	3

Alignment

**Alignment Score**	176.0 (NT) + 804.0 (AA) = 980.0
**Concordance (%)**	31.2799

Alignment Method	Global, seeded, nucleotide + amino acids (AGA)

Genome Region

Sequence starts at position 2720 and ends at position 3520 relative to NC_038512.1 reference sequence.

Alignment Detailed Statistics

	Begin	End	Coverage	Score	Concordance	Matches	Identities	I/D/M/F*	Stop Codons
**NT**	**2720**	**3520**	**9.6%**	**176**	**12.2%**	**724 (100%)**	**406 (56.1%)**	**0/0**	

Mutations: 2720G>A, 2722G>A, 2723T>A, 2727C>T, 2730A>G, 2732A>C, 2733G>C, 2734T>A, 2738C>A, 2746C>T, 2747T>A, 2748T>C, 2749C>T, 2752C>A, 2755T>G, 2756C>A, 2757G>A, 2758C>G, 2759C>G, 2761G>T, 2765G>A, 2768A>G, 2769G>A, 2770G>A, 2771G>A, 2773C>G, 2774C>A, 2775A>T, 2776A>G, 2777A>G, 2780A>G, 2781C>A, 2782G>T, 2783A>G, 2785A>G, 2786A>T, 2789T>C, 2795C>A, 2796A>C, 2801A>G, 2806A>T, 2809A>G, 2811C>A, 2812A>G, 2813T>A, 2814C>G, 2815A>T, 2816G>A, 2817A>T, 2818C>T, 2819T>A, 2820C>G, 2822G>C, 2827G>T, 2828A>T, 2829G>C, 2839A>T, 2917G>C, 2919A>T, 2920G>C, 2924A>G, 2926C>G, 2927G>A, 2928A>G, 2929T>G, 2932C>T, 2934A>G, 2935A>G, 2937A>T, 2941G>A, 2944A>C, 2949A>C, 2950C>T, 2953A>T, 2954A>G, 2955G>A, 2959C>G, 2960G>C, 2963C>T, 2968C>T, 2969A>G, 2972T>G, 2975G>A, 2976G>A, 2978A>G, 2979A>G, 2980G>C, 2981T>G, 2982G>C, 2983C>T, 2984C>G, 2985A>T, 2986A>T, 2989C>T, 2993A>T, 2995C>A, 2997C>A, 2998C>G, 2999T>C, 3001A>T, 3008G>T, 3009C>T, 3011A>T, 3012G>C, 3016G>A, 3018T>A, 3019T>C, 3020T>G, 3025G>A, 3026G>A, 3028G>T, 3029G>C, 3030A>G, 3032A>G, 3034G>T, 3035G>A, 3037C>G, 3038C>G, 3039C>T, 3040T>G, 3041C>G, 3042A>C, 3050T>C, 3052G>C, 3058C>T, 3061G>T, 3066A>G, 3067C>G, 3068G>A, 3069T>C, 3071G>C, 3073A>C, 3074C>G, 3076C>T, 3079G>T, 3084T>A, 3085T>C, 3091C>T, 3092C>T, 3094T>A, 3095C>G, 3096G>T, 3097A>C, 3104A>T, 3106G>T, 3109A>G, 3112A>G, 3113A>T, 3114A>C, 3115A>T, 3119T>G, 3121A>G, 3122C>T, 3124A>G, 3127T>C, 3130T>A, 3139A>C, 3140G>C, 3142T>C, 3146G>A, 3151T>G, 3152G>A, 3155C>T, 3157A>T, 3158A>C, 3159G>A, 3160A>C, 3161G>T, 3162G>C, 3164C>T, 3165T>A, 3168A>T, 3169A>C, 3171A>G, 3172T>A, 3175C>G, 3176A>T, 3179T>A, 3180G>T, 3182C>T, 3184C>G, 3187C>A, 3189A>T, 3190C>T, 3191C>T, 3193T>C, 3196C>T, 3202T>C, 3203A>C, 3205T>C, 3206G>A, 3215A>T, 3226G>A, 3229A>G, 3233C>A, 3235G>A, 3245G>A, 3246A>G, 3248C>A, 3249G>C, 3250A>T, 3253T>G, 3254T>C, 3256C>G, 3259A>G, 3260A>G, 3261G>T, 3262A>T, 3263C>T, 3265T>G, 3272A>G, 3273G>A, 3274T>A, 3275A>C, 3277C>G, 3280C>A, 3281A>T, 3283A>T, 3284A>G, 3285T>C, 3286T>A, 3287C>A, 3290A>T, 3291T>A, 3292G>T, 3295C>T, 3300C>G, 3301C>T, 3309T>G, 3311A>T, 3312A>T, 3313G>T, 3316C>G, 3320A>G, 3321C>T, 3322T>G, 3324C>A, 3325T>G, 3329C>T, 3331T>G, 3338A>T, 3340C>T, 3346C>T, 3347A>G, 3348G>C, 3349G>C, 3351A>C, 3352C>T, 3355T>A, 3356A>G, 3358C>T, 3359A>G, 3360A>C, 3361G>A, 3362C>A, 3365A>G, 3367C>T, 3371G>A, 3373T>G, 3379T>A, 3380T>G, 3381C>A, 3382C>T, 3384C>T, 3386A>G, 3389C>A, 3393A>C, 3396A>G, 3397T>G, 3398C>A, 3399T>C, 3400G>A, 3403T>A, 3408A>C, 3409G>A, 3413C>G, 3414C>T, 3415T>G, 3419G>C, 3422A>T, 3424A>G, 3426A>G, 3428C>G, 3429A>G, 3430A>C, 3433T>C, 3436A>T, 3439C>T, 3443C>G, 3444T>C, 3445C>A, 3451T>C, 3454C>T, 3455C>A, 3459A>G, 3467C>A, 3468C>G, 3471A>G, 3477C>G, 3478A>T, 3479C>G, 3480G>T, 3481A>T, 3482C>A, 3484C>T, 3485A>G, 3489A>G, 3498C>A, 3499A>T, 3500C>G, 3504G>T, 3505C>G, 3511A>G, 3515G>A, 3516G>A, 3517T>G, 3519G>T, 3520T>A

CDS

	Begin	End	Coverage	Score	Concordance	Matches	Identities	I/D/M/F*	Stop Codons
D1R33_gp2	139	405	22.2%	804	47.2%	241 (100%)	108 (44.8%)	0/0/0/0	0

Protein mutations: V139I (2720G>A 2722G>A), Y140N (2723T>A), T141I (2727C>T), K142R (2730A>G), S143P (2732A>C 2733G>C 2734T>A), F148T (2747T>A 2748T>C 2749C>T), H150Q (2755T>G), R151K (2756C>A 2757G>A 2758C>G), Q152D (2759C>G 2761G>T), V154I (2765G>A), R155E (2768A>G 2769G>A 2770G>A), D156K (2771G>A 2773C>G), Q157M (2774C>A 2775A>T 2776A>G), I158V (2777A>G), T159D (2780A>G 2781C>A 2782G>T), K160E (2783A>G 2785A>G), M161L (2786A>T), Q164T (2795C>A 2796A>C), I166V (2801A>G), P169Q (2811C>A 2812A>G), D171I (2816G>A 2817A>T 2818C>T), A173P (2822G>C), W174C (2827G>T), K205I (2919A>T 2920G>C), I207V (2924A>G 2926C>G), D208R (2927G>A 2928A>G 2929T>G), K210R (2934A>G 2935A>G), Y211F (2937A>T), N215T (2949A>C 2950C>T), S217D (2954A>G 2955G>A), D218E (2959C>G), V219L (2960G>C), L220F (2963C>T), K222E (2969A>G), L223V (2972T>G), G224N (2975G>A 2976G>A), K225G (2978A>G 2979A>G 2980G>C), C226A (2981T>G 2982G>C 2983C>T), Q227V (2984C>G 2985A>T 2986A>T), T230S (2993A>T 2995C>A), T231K (2997C>A 2998C>G), A235L (3008G>T 3009C>T), F238Y (3018T>A 3019T>C), Y239D (3020T>G), V241I (3026G>A 3028G>T), E242R (3029G>C 3030A>G), M243V (3032A>G 3034G>T), D244K (3035G>A 3037C>G), P245V (3038C>G 3039C>T 3040T>G), Q246A (3041C>G 3042A>C), S249P (3050T>C 3052G>C), N254R (3066A>G 3067C>G), V255T (3068G>A 3069T>C), E256H (3071G>C 3073A>C), H257D (3074C>G 3076C>T), F260Y (3084T>A 3085T>C), R264V (3095C>G 3096G>T 3097A>C), M267F (3104A>T 3106G>T), K270S (3113A>T 3114A>C 3115A>T), S272A (3119T>G 3121A>G), P273S (3122C>T 3124A>G), R278S (3139A>C), V279L (3140G>C 3142T>C), D281N (3146G>A), N282K (3151T>G), V283I (3152G>A), L284F (3155C>T 3157A>T), R285H (3158A>C 3159G>A 3160A>C), G286S (3161G>T 3162G>C), L287Y (3164C>T 3165T>A), Q288L (3168A>T 3169A>C), N289R (3171A>G 3172T>A), N290K (3175C>G), I291F (3176A>T), C292I (3179T>A 3180G>T), Y295F (3189A>T 3190C>T), L296F (3191C>T 3193T>C), I300L (3203A>C 3205T>C), V301I (3206G>A), L310I (3233C>A 3235G>A), E314R (3245G>A 3246A>G), R315T (3248C>A 3249G>C 3250A>T), F317L (3254T>C 3256C>G), R319V (3260A>G 3261G>T 3262A>T), S323E (3272A>G 3273G>A 3274T>A), N324Q (3275A>C 3277C>G), F325L (3280C>A), K326Y (3281A>T 3283A>T), I327A (3284A>G 3285T>C 3286T>A), Q328K (3287C>A), M329Y (3290A>T 3291T>A 3292G>T), S332C (3300C>G 3301C>T), L335W (3309T>G), K336F (3311A>T 3312A>T 3313G>T), T339V (3320A>G 3321C>T 3322T>G), A340E (3324C>A 3325T>G), I345F (3338A>T 3340C>T), R348A (3347A>G 3348G>C 3349G>C), D349A (3351A>C 3352C>T), I351V (3356A>G 3358C>T), K352A (3359A>G 3360A>C 3361G>A), P353T (3362C>A), N354D (3365A>G 3367C>T), D356K (3371G>A 3373T>G), S359D (3380T>G 3381C>A 3382C>T), A360V (3384C>T), I361V (3386A>G), Q362K (3389C>A), K363T (3393A>C), Y364W (3396A>G 3397T>G), L365T (3398C>A 3399T>C 3400G>A), K368T (3408A>C 3409G>A), P370V (3413C>G 3414C>T 3415T>G), E372Q (3419G>C), I373L (3422A>T 3424A>G), K374R (3426A>G), Q375G (3428C>G 3429A>G 3430A>C), L377F (3436A>T), L380A (3443C>G 3444T>C 3445C>A), K385R (3459A>G), P388R (3467C>A 3468C>G), D389G (3471A>G), A391G (3477C>G 3478A>T), R392V (3479C>G 3480G>T 3481A>T), L393I (3482C>A 3484C>T), T394A (3485A>G), K395R (3489A>G), T398N (3498C>A 3499A>T), Q399E (3500C>G), C400L (3504G>T 3505C>G), G404K (3515G>A 3516G>A 3517T>G), S405I (3519G>T 3520T>A)

Codon mutations: GTG139ATA (2720G>A 2722G>A), TAC140AAC (2723T>A), ACC141ATC (2727C>T), AAA142AGA (2730A>G), AGT143CCA (2732A>C 2733G>C 2734T>A), CGG145AGG (2738C>A), CCC147CCT (2746C>T), TTC148ACT (2747T>A 2748T>C 2749C>T), ATC149ATA (2752C>A), CAT150CAG (2755T>G), CGC151AAG (2756C>A 2757G>A 2758C>G), CAG152GAT (2759C>G 2761G>T), GTT154ATT (2765G>A), AGG155GAA (2768A>G 2769G>A 2770G>A), GAC156AAG (2771G>A 2773C>G), CAA157ATG (2774C>A 2775A>T 2776A>G), ATC158GTC (2777A>G), ACG159GAT (2780A>G 2781C>A 2782G>T), AAA160GAG (2783A>G 2785A>G), ATG161TTG (2786A>T), TTG162CTG (2789T>C), CAA164ACA (2795C>A 2796A>C), ATT166GTT (2801A>G), ATA167ATT (2806A>T), AGA168AGG (2809A>G), CCA169CAG (2811C>A 2812A>G), TCA170AGT (2813T>A 2814C>G 2815A>T), GAC171ATT (2816G>A 2817A>T 2818C>T), TCT172AGT (2819T>A 2820C>G), GCA173CCA (2822G>C), TGG174TGT (2827G>T), AGC175TCC (2828A>T 2829G>C), ATA178ATT (2839A>T), GAG204..C (2917G>C), AAG205ATC (2919A>T 2920G>C), ATC207GTG (2924A>G 2926C>G), GAT208AGG (2927G>A 2928A>G 2929T>G), GAC209GAT (2932C>T), AAA210AGG (2934A>G 2935A>G), TAC211TTC (2937A>T), CCG212CCA (2941G>A), ATA213ATC (2944A>C), AAC215ACT (2949A>C 2950C>T), ATA216ATT (2953A>T), AGT217GAT (2954A>G 2955G>A), GAC218GAG (2959C>G), GTA219CTA (2960G>C), CTT220TTT (2963C>T), GAC221GAT (2968C>T), AAG222GAG (2969A>G), TTA223GTA (2972T>G), GGT224AAT (2975G>A 2976G>A), AAG225GGC (2978A>G 2979A>G 2980G>C), TGC226GCT (2981T>G 2982G>C 2983C>T), CAA227GTT (2984C>G 2985A>T 2986A>T), TAC228TAT (2989C>T), ACC230TCA (2993A>T 2995C>A), ACC231AAG (2997C>A 2998C>G), TTA232CTT (2999T>C 3001A>T), GCA235TTA (3008G>T 3009C>T), AGT236TCT (3011A>T 3012G>C), GGG237GGA (3016G>A), TTT238TAC (3018T>A 3019T>C), TAT239GAT (3020T>G), CAG240CAA (3025G>A), GTG241ATT (3026G>A 3028G>T), GAG242CGG (3029G>C 3030A>G), ATG243GTT (3032A>G 3034G>T), GAC244AAG (3035G>A 3037C>G), CCT245GTG (3038C>G 3039C>T 3040T>G), CAA246GCA (3041C>G 3042A>C), TCG249CCC (3050T>C 3052G>C), ACC251ACT (3058C>T), GCG252GCT (3061G>T), AAC254AGG (3066A>G 3067C>G), GTA255ACA (3068G>A 3069T>C), GAA256CAC (3071G>C 3073A>C), CAC257GAT (3074C>G 3076C>T), GGG258GGT (3079G>T), TTT260TAC (3084T>A 3085T>C), TTC262TTT (3091C>T), CTT263TTA (3092C>T 3094T>A), CGA264GTC (3095C>G 3096G>T 3097A>C), ATG267TTT (3104A>T 3106G>T), GGA268GGG (3109A>G), TTA269TTG (3112A>G), AAA270TCT (3113A>T 3114A>C 3115A>T), TCA272GCG (3119T>G 3121A>G), CCA273TCG (3122C>T 3124A>G), TCT274TCC (3127T>C), ACT275ACA (3130T>A), AGA278AGC (3139A>C), GTT279CTC (3140G>C 3142T>C), GAC281AAC (3146G>A), AAT282AAG (3151T>G), GTC283ATC (3152G>A), CTA284TTT (3155C>T 3157A>T), AGA285CAC (3158A>C 3159G>A 3160A>C), GGT286TCT (3161G>T 3162G>C), CTC287TAC (3164C>T 3165T>A), CAA288CTC (3168A>T 3169A>C), AAT289AGA (3171A>G 3172T>A), AAC290AAG (3175C>G), ATC291TTC (3176A>T), TGT292ATT (3179T>A 3180G>T), CTC293TTG (3182C>T 3184C>G), GTC294GTA (3187C>A), TAC295TTT (3189A>T 3190C>T), CTT296TTC (3191C>T 3193T>C), GAC297GAT (3196C>T), ATT299ATC (3202T>C), ATT300CTC (3203A>C 3205T>C), GTC301ATC (3206G>A), ACT304TC. (3215A>T), CAG307..A (3226G>A), GAA308GAG (3229A>G), CTG310ATA (3233C>A 3235G>A), GAA314AGA (3245G>A 3246A>G), CGA315ACT (3248C>A 3249G>C 3250A>T), GTT316GTG (3253T>G), TTC317CTG (3254T>C 3256C>G), CAA318CAG (3259A>G), AGA319GTT (3260A>G 3261G>T 3262A>T), CTT320TTG (3263C>T 3265T>G), AGT323GAA (3272A>G 3273G>A 3274T>A), AAC324CAG (3275A>C 3277C>G), TTC325TTA (3280C>A), AAA326TAT (3281A>T 3283A>T), ATT327GCA (3284A>G 3285T>C 3286T>A), CAA328AAA (3287C>A), ATG329TAT (3290A>T 3291T>A 3292G>T), GAC330GAT (3295C>T), TCC332TGT (3300C>G 3301C>T), TTG335TGG (3309T>G), AAG336TTT (3311A>T 3312A>T 3313G>T), CTC337CTG (3316C>G), ACT339GTG (3320A>G 3321C>T 3322T>G), GCT340GAG (3324C>A 3325T>G), CTT342TTG (3329C>T 3331T>G), ATC345TTT (3338A>T 3340C>T), AGC347AGT (3346C>T), AGG348GCC (3347A>G 3348G>C 3349G>C), GAC349GCT (3351A>C 3352C>T), GGT350GGA (3355T>A), ATC351GTT (3356A>G 3358C>T), AAG352GCA (3359A>G 3360A>C 3361G>A), CCT353ACT (3362C>A), AAC354GAT (3365A>G 3367C>T), GAT356AAG (3371G>A 3373T>G), ATT358ATA (3379T>A), TCC359GAT (3380T>G 3381C>A 3382C>T), GCT360GTT (3384C>T), ATT361GTT (3386A>G), CAA362AAA (3389C>A), AAA363ACA (3393A>C), TAT364TGG (3396A>G 3397T>G), CTG365ACA (3398C>A 3399T>C 3400G>A), ATT366ATA (3403T>A), AAG368ACA (3408A>C 3409G>A), CCT370GTG (3413C>G 3414C>T 3415T>G), GAA372CAA (3419G>C), ATA373TTG (3422A>T 3424A>G), AAA374AGA (3426A>G), CAA375GGC (3428C>G 3429A>G 3430A>C), TTT376TTC (3433T>C), TTA377TTT (3436A>T), GGC378GGT (3439C>T), CTC380GCA (3443C>G 3444T>C 3445C>A), TAT382TAC (3451T>C), TAC383TAT (3454C>T), CGA384AGA (3455C>A), AAA385AGA (3459A>G), CCA388AGA (3467C>A 3468C>G), GAT389GGT (3471A>G), GCA391GGT (3477C>G 3478A>T), CGA392GTT (3479C>G 3480G>T 3481A>T), CTC393ATT (3482C>A 3484C>T), ACA394GCA (3485A>G), AAA395AGA (3489A>G), ACA398AAT (3498C>A 3499A>T), CAG399GAG (3500C>G), TGC400TTG (3504G>T 3505C>G), AAA402AAG (3511A>G), GGT404AAG (3515G>A 3516G>A 3517T>G), AGT405ATA (3519G>T 3520T>A)

Proteins

	Begin	End	Coverage	Score	Concordance	Matches	Identities	I/D/M/F*	Stop Codons
ORF B (YP_009507248.1)	139	405	22.2%	804	47.2%	241 (100%)	108 (44.8%)	0/0/0/0	0

	Begin	End	Coverage	Score	Concordance	Matches	Identities	I/D/M/F*	Stop Codons
**NT**	**2720**	**3520**	**9.6%**	**176**	**12.2%**	**724 (100%)**	**406 (56.1%)**	**0/0**	
Protein mutations:	V139I (2720G>A 2722G>A), Y140N (2723T>A), T141I (2727C>T), K142R (2730A>G), S143P (2732A>C 2733G>C 2734T>A), F148T (2747T>A 2748T>C 2749C>T), H150Q (2755T>G), R151K (2756C>A 2757G>A 2758C>G), Q152D (2759C>G 2761G>T), V154I (2765G>A), R155E (2768A>G 2769G>A 2770G>A), D156K (2771G>A 2773C>G), Q157M (2774C>A 2775A>T 2776A>G), I158V (2777A>G), T159D (2780A>G 2781C>A 2782G>T), K160E (2783A>G 2785A>G), M161L (2786A>T), Q164T (2795C>A 2796A>C), I166V (2801A>G), P169Q (2811C>A 2812A>G), D171I (2816G>A 2817A>T 2818C>T), A173P (2822G>C), W174C (2827G>T), K205I (2919A>T 2920G>C), I207V (2924A>G 2926C>G), D208R (2927G>A 2928A>G 2929T>G), K210R (2934A>G 2935A>G), Y211F (2937A>T), N215T (2949A>C 2950C>T), S217D (2954A>G 2955G>A), D218E (2959C>G), V219L (2960G>C), L220F (2963C>T), K222E (2969A>G), L223V (2972T>G), G224N (2975G>A 2976G>A), K225G (2978A>G 2979A>G 2980G>C), C226A (2981T>G 2982G>C 2983C>T), Q227V (2984C>G 2985A>T 2986A>T), T230S (2993A>T 2995C>A), T231K (2997C>A 2998C>G), A235L (3008G>T 3009C>T), F238Y (3018T>A 3019T>C), Y239D (3020T>G), V241I (3026G>A 3028G>T), E242R (3029G>C 3030A>G), M243V (3032A>G 3034G>T), D244K (3035G>A 3037C>G), P245V (3038C>G 3039C>T 3040T>G), Q246A (3041C>G 3042A>C), S249P (3050T>C 3052G>C), N254R (3066A>G 3067C>G), V255T (3068G>A 3069T>C), E256H (3071G>C 3073A>C), H257D (3074C>G 3076C>T), F260Y (3084T>A 3085T>C), R264V (3095C>G 3096G>T 3097A>C), M267F (3104A>T 3106G>T), K270S (3113A>T 3114A>C 3115A>T), S272A (3119T>G 3121A>G), P273S (3122C>T 3124A>G), R278S (3139A>C), V279L (3140G>C 3142T>C), D281N (3146G>A), N282K (3151T>G), V283I (3152G>A), L284F (3155C>T 3157A>T), R285H (3158A>C 3159G>A 3160A>C), G286S (3161G>T 3162G>C), L287Y (3164C>T 3165T>A), Q288L (3168A>T 3169A>C), N289R (3171A>G 3172T>A), N290K (3175C>G), I291F (3176A>T), C292I (3179T>A 3180G>T), Y295F (3189A>T 3190C>T), L296F (3191C>T 3193T>C), I300L (3203A>C 3205T>C), V301I (3206G>A), L310I (3233C>A 3235G>A), E314R (3245G>A 3246A>G), R315T (3248C>A 3249G>C 3250A>T), F317L (3254T>C 3256C>G), R319V (3260A>G 3261G>T 3262A>T), S323E (3272A>G 3273G>A 3274T>A), N324Q (3275A>C 3277C>G), F325L (3280C>A), K326Y (3281A>T 3283A>T), I327A (3284A>G 3285T>C 3286T>A), Q328K (3287C>A), M329Y (3290A>T 3291T>A 3292G>T), S332C (3300C>G 3301C>T), L335W (3309T>G), K336F (3311A>T 3312A>T 3313G>T), T339V (3320A>G 3321C>T 3322T>G), A340E (3324C>A 3325T>G), I345F (3338A>T 3340C>T), R348A (3347A>G 3348G>C 3349G>C), D349A (3351A>C 3352C>T), I351V (3356A>G 3358C>T), K352A (3359A>G 3360A>C 3361G>A), P353T (3362C>A), N354D (3365A>G 3367C>T), D356K (3371G>A 3373T>G), S359D (3380T>G 3381C>A 3382C>T), A360V (3384C>T), I361V (3386A>G), Q362K (3389C>A), K363T (3393A>C), Y364W (3396A>G 3397T>G), L365T (3398C>A 3399T>C 3400G>A), K368T (3408A>C 3409G>A), P370V (3413C>G 3414C>T 3415T>G), E372Q (3419G>C), I373L (3422A>T 3424A>G), K374R (3426A>G), Q375G (3428C>G 3429A>G 3430A>C), L377F (3436A>T), L380A (3443C>G 3444T>C 3445C>A), K385R (3459A>G), P388R (3467C>A 3468C>G), D389G (3471A>G), A391G (3477C>G 3478A>T), R392V (3479C>G 3480G>T 3481A>T), L393I (3482C>A 3484C>T), T394A (3485A>G), K395R (3489A>G), T398N (3498C>A 3499A>T), Q399E (3500C>G), C400L (3504G>T 3505C>G), G404K (3515G>A 3516G>A 3517T>G), S405I (3519G>T 3520T>A)								
Codon mutations:	GTG139ATA (2720G>A 2722G>A), TAC140AAC (2723T>A), ACC141ATC (2727C>T), AAA142AGA (2730A>G), AGT143CCA (2732A>C 2733G>C 2734T>A), CGG145AGG (2738C>A), CCC147CCT (2746C>T), TTC148ACT (2747T>A 2748T>C 2749C>T), ATC149ATA (2752C>A), CAT150CAG (2755T>G), CGC151AAG (2756C>A 2757G>A 2758C>G), CAG152GAT (2759C>G 2761G>T), GTT154ATT (2765G>A), AGG155GAA (2768A>G 2769G>A 2770G>A), GAC156AAG (2771G>A 2773C>G), CAA157ATG (2774C>A 2775A>T 2776A>G), ATC158GTC (2777A>G), ACG159GAT (2780A>G 2781C>A 2782G>T), AAA160GAG (2783A>G 2785A>G), ATG161TTG (2786A>T), TTG162CTG (2789T>C), CAA164ACA (2795C>A 2796A>C), ATT166GTT (2801A>G), ATA167ATT (2806A>T), AGA168AGG (2809A>G), CCA169CAG (2811C>A 2812A>G), TCA170AGT (2813T>A 2814C>G 2815A>T), GAC171ATT (2816G>A 2817A>T 2818C>T), TCT172AGT (2819T>A 2820C>G), GCA173CCA (2822G>C), TGG174TGT (2827G>T), AGC175TCC (2828A>T 2829G>C), ATA178ATT (2839A>T), GAG204..C (2917G>C), AAG205ATC (2919A>T 2920G>C), ATC207GTG (2924A>G 2926C>G), GAT208AGG (2927G>A 2928A>G 2929T>G), GAC209GAT (2932C>T), AAA210AGG (2934A>G 2935A>G), TAC211TTC (2937A>T), CCG212CCA (2941G>A), ATA213ATC (2944A>C), AAC215ACT (2949A>C 2950C>T), ATA216ATT (2953A>T), AGT217GAT (2954A>G 2955G>A), GAC218GAG (2959C>G), GTA219CTA (2960G>C), CTT220TTT (2963C>T), GAC221GAT (2968C>T), AAG222GAG (2969A>G), TTA223GTA (2972T>G), GGT224AAT (2975G>A 2976G>A), AAG225GGC (2978A>G 2979A>G 2980G>C), TGC226GCT (2981T>G 2982G>C 2983C>T), CAA227GTT (2984C>G 2985A>T 2986A>T), TAC228TAT (2989C>T), ACC230TCA (2993A>T 2995C>A), ACC231AAG (2997C>A 2998C>G), TTA232CTT (2999T>C 3001A>T), GCA235TTA (3008G>T 3009C>T), AGT236TCT (3011A>T 3012G>C), GGG237GGA (3016G>A), TTT238TAC (3018T>A 3019T>C), TAT239GAT (3020T>G), CAG240CAA (3025G>A), GTG241ATT (3026G>A 3028G>T), GAG242CGG (3029G>C 3030A>G), ATG243GTT (3032A>G 3034G>T), GAC244AAG (3035G>A 3037C>G), CCT245GTG (3038C>G 3039C>T 3040T>G), CAA246GCA (3041C>G 3042A>C), TCG249CCC (3050T>C 3052G>C), ACC251ACT (3058C>T), GCG252GCT (3061G>T), AAC254AGG (3066A>G 3067C>G), GTA255ACA (3068G>A 3069T>C), GAA256CAC (3071G>C 3073A>C), CAC257GAT (3074C>G 3076C>T), GGG258GGT (3079G>T), TTT260TAC (3084T>A 3085T>C), TTC262TTT (3091C>T), CTT263TTA (3092C>T 3094T>A), CGA264GTC (3095C>G 3096G>T 3097A>C), ATG267TTT (3104A>T 3106G>T), GGA268GGG (3109A>G), TTA269TTG (3112A>G), AAA270TCT (3113A>T 3114A>C 3115A>T), TCA272GCG (3119T>G 3121A>G), CCA273TCG (3122C>T 3124A>G), TCT274TCC (3127T>C), ACT275ACA (3130T>A), AGA278AGC (3139A>C), GTT279CTC (3140G>C 3142T>C), GAC281AAC (3146G>A), AAT282AAG (3151T>G), GTC283ATC (3152G>A), CTA284TTT (3155C>T 3157A>T), AGA285CAC (3158A>C 3159G>A 3160A>C), GGT286TCT (3161G>T 3162G>C), CTC287TAC (3164C>T 3165T>A), CAA288CTC (3168A>T 3169A>C), AAT289AGA (3171A>G 3172T>A), AAC290AAG (3175C>G), ATC291TTC (3176A>T), TGT292ATT (3179T>A 3180G>T), CTC293TTG (3182C>T 3184C>G), GTC294GTA (3187C>A), TAC295TTT (3189A>T 3190C>T), CTT296TTC (3191C>T 3193T>C), GAC297GAT (3196C>T), ATT299ATC (3202T>C), ATT300CTC (3203A>C 3205T>C), GTC301ATC (3206G>A), ACT304TC. (3215A>T), CAG307..A (3226G>A), GAA308GAG (3229A>G), CTG310ATA (3233C>A 3235G>A), GAA314AGA (3245G>A 3246A>G), CGA315ACT (3248C>A 3249G>C 3250A>T), GTT316GTG (3253T>G), TTC317CTG (3254T>C 3256C>G), CAA318CAG (3259A>G), AGA319GTT (3260A>G 3261G>T 3262A>T), CTT320TTG (3263C>T 3265T>G), AGT323GAA (3272A>G 3273G>A 3274T>A), AAC324CAG (3275A>C 3277C>G), TTC325TTA (3280C>A), AAA326TAT (3281A>T 3283A>T), ATT327GCA (3284A>G 3285T>C 3286T>A), CAA328AAA (3287C>A), ATG329TAT (3290A>T 3291T>A 3292G>T), GAC330GAT (3295C>T), TCC332TGT (3300C>G 3301C>T), TTG335TGG (3309T>G), AAG336TTT (3311A>T 3312A>T 3313G>T), CTC337CTG (3316C>G), ACT339GTG (3320A>G 3321C>T 3322T>G), GCT340GAG (3324C>A 3325T>G), CTT342TTG (3329C>T 3331T>G), ATC345TTT (3338A>T 3340C>T), AGC347AGT (3346C>T), AGG348GCC (3347A>G 3348G>C 3349G>C), GAC349GCT (3351A>C 3352C>T), GGT350GGA (3355T>A), ATC351GTT (3356A>G 3358C>T), AAG352GCA (3359A>G 3360A>C 3361G>A), CCT353ACT (3362C>A), AAC354GAT (3365A>G 3367C>T), GAT356AAG (3371G>A 3373T>G), ATT358ATA (3379T>A), TCC359GAT (3380T>G 3381C>A 3382C>T), GCT360GTT (3384C>T), ATT361GTT (3386A>G), CAA362AAA (3389C>A), AAA363ACA (3393A>C), TAT364TGG (3396A>G 3397T>G), CTG365ACA (3398C>A 3399T>C 3400G>A), ATT366ATA (3403T>A), AAG368ACA (3408A>C 3409G>A), CCT370GTG (3413C>G 3414C>T 3415T>G), GAA372CAA (3419G>C), ATA373TTG (3422A>T 3424A>G), AAA374AGA (3426A>G), CAA375GGC (3428C>G 3429A>G 3430A>C), TTT376TTC (3433T>C), TTA377TTT (3436A>T), GGC378GGT (3439C>T), CTC380GCA (3443C>G 3444T>C 3445C>A), TAT382TAC (3451T>C), TAC383TAT (3454C>T), CGA384AGA (3455C>A), AAA385AGA (3459A>G), CCA388AGA (3467C>A 3468C>G), GAT389GGT (3471A>G), GCA391GGT (3477C>G 3478A>T), CGA392GTT (3479C>G 3480G>T 3481A>T), CTC393ATT (3482C>A 3484C>T), ACA394GCA (3485A>G), AAA395AGA (3489A>G), ACA398AAT (3498C>A 3499A>T), CAG399GAG (3500C>G), TGC400TTG (3504G>T 3505C>G), AAA402AAG (3511A>G), GGT404AAG (3515G>A 3516G>A 3517T>G), AGT405ATA (3519G>T 3520T>A)								

*: Inserts / Deletes / Misaligned / Frameshifts

Analysis details

This analysis was performed with panviral2.64

NGS Details (UN18_val): Marseillevirus marseillevirus

Assembly

**Coverage Length**	115 (1 contig(s))
**Depth Of Coverage**	52.2
**Number Of Reads**	75
**Reads Per Million**	1.41 rpm (after QC)
**Ambiguities**	0
**Assembly Method**	de novo + reference guided assembly
**Consensus Caller**	Bcf Tools

Coverage Map

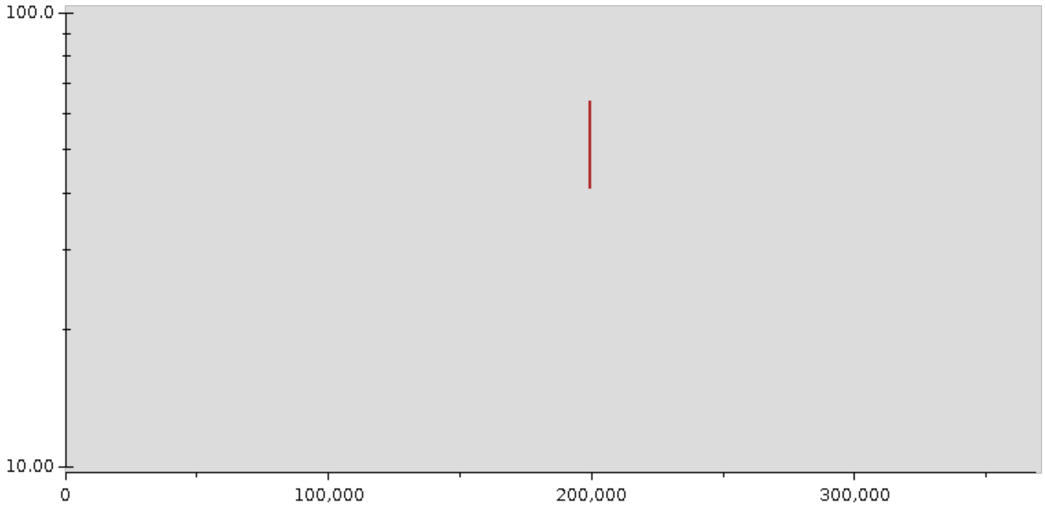

Assignment

**Type**	Marseillevirus marseillevirus (Taxonomy ID: 694581)
**Reference Genome**	NC_013756.1
**NT Identity (%)**	81.7391
**AA Identity (%)**	97.3684
**Number Of Stop Codons**	1
**Number Of CDS**	428

Alignment

**Alignment Score**	146.0 (NT) + 245.0 (AA) = 391.0
**Concordance (%)**	82.1429
**Alignment Method**	Local, heuristic, nucleotide (BLASTN)

Genome Region

Sequence starts at position 199404 and ends at position 199518 relative to NC_013756.1 reference sequence.

Alignment Detailed Statistics

	Begin	End	Coverage	Score	Concordance	Matches	Identities	I/D/M/F*	Stop Codons
**NT**	**199404**	**199518**	**0.1%**	**146**	**63.5%**	**115 (100%)**	**94 (81.7%)**	**0/0**	

Mutations: 199411A>C, 199414T>A, 199416T>G, 199423A>G, 199432A>G, 199444T>C, 199449C>T, 199459A>C, 199462C>A, 199464A>G, 199465A>G, 199470T>G, 199471T>G, 199474A>G, 199477T>C, 199480C>G, 199489T>G, 199495G>A, 199501G>C, 199503G>A, 199516C>T

*: Inserts / Deletes / Misaligned / Frameshifts

Analysis details

This analysis was performed with panviral2.64

NGS Details (UN18_val): Badnavirus occultipomeae

Assembly

**Coverage Length**	260 (1 contig(s))
**Depth Of Coverage**	25.5
**Number Of Reads**	66
**Reads Per Million**	1.24 rpm (after QC)
**Ambiguities**	0
**Assembly Method**	de novo + reference guided assembly
**Consensus Caller**	Bcf Tools

Coverage Map

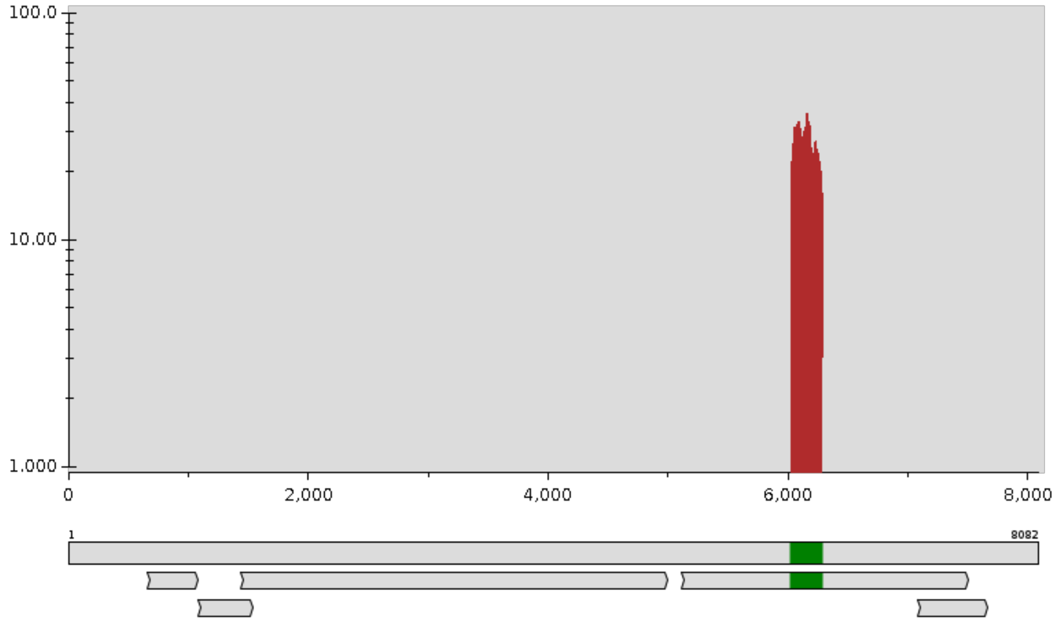

Assignment

**Type**	Badnavirus occultipomeae (Taxonomy ID: 3048353)
**Reference Genome**	NC_015655.1
**NT Identity (%)**	58.7591
**AA Identity (%)**	49.4505
**Number Of Stop Codons**	1
**Number Of CDS**	5

Alignment

**Alignment Score**	101.0 (NT) + 190.0 (AA) = 291.0
**Concordance (%)**	25.2824

Alignment Method	Global, seeded, nucleotide + amino acids (AGA)

Genome Region

Sequence starts at position 6026 and ends at position 6285 relative to NC_015655.1 reference sequence.

Alignment Detailed Statistics

	Begin	End	Coverage	Score	Concordance	Matches	Identities	I/D/M/F*	Stop Codons
**NT**	**6026**	**6285**	**3.2%**	**101**	**19.4%**	**260 (94.9%)**	**161 (58.8%)**	**14/0**	
Mutations:	6033A>T, 6042A>G, 6043T>A, 6045T>A, 6048T>C, 6051C>A, 6053A>G, 6055G>T, 6062T>A, 6067C>T, 6070A>T, 6074G>A, 6075A>G, 6076A>G, 6078G>A, 6079G>A, 6081G>A, 6084A>G, 6085A>T, 6086A>G, 6087G>T, 6088T>G, 6089C>A, 6090C>T, 6092A>T, 6093A>C, 6097T>A, 6098G>A, 6099G>A, 6109T>C, 6111G>A, 6116C>A, 6117A>T, 6118G>T, 6120A>T, 6123G>C, 6124C>T, 6125T>A, 6126C>T, 6128A>T, 6132A>G, 6135T>C, 6136G>T, 6137A>T, 6138A>G, 6145C>T, 6153A>G, 6154C>T, 6158T>C, 6159G>T, 6168A>T, 6173A>C, 6174T>C, 6178C>A, 6179A>T, 6181A>G, 6182G>A, 6183A>T, 6184A>C, 6185A>T, 6186G>C, 6190G>A, 6194A>G, 6195T>A, 6197C>T, 6198A>G, 6201T>C, 6203G>A, 6205G>C, 6206G>C, 6207A>T, 6207_6208insTATTCAAGCCTTAT, 6208A>C, 6209C>T, 6210A>T, 6213T>C, 6214G>A, 6215C>T, 6216A>G, 6219T>A, 6220A>G, 6222C>T, 6223G>A, 6224C>T, 6225A>T, 6228A>C, 6230A>T, 6240C>T, 6243A>C, 6249A>C, 6251T>A, 6255T>C, 6256G>A, 6258A>G, 6264A>G, 6270G>A, 6271C>T, 6275A>C, 6276G>C, 6277G>T								

CDS

	Begin	End	Coverage	Score	Concordance	Matches	Identities	I/D/M/F*	Stop Codons
SPBVa_gp4	305	390	10.8%	190	29.9%	86 (94.5%)	45 (49.5%)	5/0/1/1	1
Protein mutations:	F310I (6043T>A 6045T>A), K313R (6053A>G), A314S (6055G>T), F316Y (6062T>A), Q318* (6067C>T), I319L (6070A>T), R320K (6074G>A 6075A>G), M321V (6076A>G 6078G>A), E322K (6079G>A 6081G>A), K324C (6085A>T 6086A>G 6087G>T), S325D (6088T>G 6089C>A 6090C>T), K326I (6092A>T 6093A>C), W328K (6097T>A 6098G>A 6099G>A), W332R (6109T>C 6111G>A), P334H (6116C>A 6117A>T), E335Y (6118G>T 6120A>T), L337Y (6124C>T 6125T>A 6126C>T), Y338F (6128A>T), E341L (6136G>T 6137A>T 6138A>G), P344S (6145C>T), M348T (6158T>C 6159G>T), D353A (6173A>C 6174T>C), Q355M (6178C>A 6179A>T), R356D (6181A>G 6182G>A 6183A>T), K357L (6184A>C 6185A>T 6186G>C), D359N (6190G>A), N360R (6194A>G 6195T>A), A361V (6197C>T 6198A>G), R363K (6203G>A), G364P (6205G>C 6206G>C 6207A>T), G364_T365insYSSLX (6207_6208insTATTCAAGCCTTAT), T365L (6208A>C 6209C>T 6210A>T), A367M (6214G>A 6215C>T 6216A>G), F368L (6219T>A), I369V (6220A>G 6222C>T), A370I (6223G>A 6224C>T 6225A>T), Y372F (6230A>T), F379Y (6251T>A), E381K (6256G>A 6258A>G), H386Y (6271C>T), E387A (6275A>C 6276G>C), D388Y (6277G>T)								
Codon mutations:	ATA306ATT (6033A>T), AAA309AAG (6042A>G), TTT310ATA (6043T>A 6045T>A), GAT311GAC (6048T>C), CTC312CTA (6051C>A), AAA313AGA (6053A>G), GCA314TCA (6055G>T), TTT316TAT (6062T>A), CAG318TAG (6067C>T), ATA319TTA (6070A>T), AGA320AAG (6074G>A 6075A>G), ATG321GTA (6076A>G 6078G>A), GAG322AAA (6079G>A 6081G>A), GAA323GAG (6084A>G), AAG324TGT (6085A>T 6086A>G 6087G>T), TCC325GAT (6088T>G 6089C>A 6090C>T), AAA326ATC (6092A>T 6093A>C), TGG328AAA (6097T>A 6098G>A 6099G>A), TGG332CGA (6109T>C 6111G>A), CCA334CAT (6116C>A 6117A>T), GAA335TAT (6118G>T 6120A>T), GGG336GGC (6123G>C), CTC337TAT (6124C>T 6125T>A 6126C>T), TAT338TTT (6128A>T), GAA339GAG (6132A>G), TTT340TTC (6135T>C), GAA341TTG (6136G>T 6137A>T 6138A>G), CCC344TCC (6145C>T), GGA346GGG (6153A>G), CTG347TTG (6154C>T), ATG348ACT (6158T>C 6159G>T), CCA351CCT (6168A>T), GAT353GCC (6173A>C 6174T>C), CAG355ATG (6178C>A 6179A>T), AGA356GAT (6181A>G 6182G>A 6183A>T), AAG357CTC (6184A>C 6185A>T 6186G>C), GAT359AAT (6190G>A), AAT360AGA (6194A>G 6195T>A), GCA361GTG (6197C>T 6198A>G), TTT362TTC (6201T>C), AGG363AAG (6203G>A), GGA364CCT (6205G>C 6206G>C 6207A>T), GGA364_ACA365insTATTCAAGCCTTAT-(6207_6208insTATTCAAGCCTTAT), ACA365CTT (6208A>C 6209C>T 6210A>T), GAT366GAC (6213T>C), GCA367ATG (6214G>A 6215C>T 6216A>G), TTT368TTA (6219T>A), ATC369GTT (6220A>G 6222C>T), GCA370ATT (6223G>A 6224C>T 6225A>T), GTA371GTC (6228A>C), TAT372TTT (6230A>T), GAC375GAT (6240C>T), ATA376ATC (6243A>C), GTA378GTC (6249A>C), TTC379TAC (6251T>A), TCT380TCC (6255T>C), GAA381AAG (6256G>A 6258A>G), GAA383GAG (6264A>G), GAG385GAA (6270G>A), CAT386TAT (6271C>T), GAG387GCC (6275A>C 6276G>C), GAT388TAT (6277G>T)								

Proteins

	Begin	End	Coverage	Score	Concordance	Matches	Identities	I/D/M/F*	Stop Codons
RNaseH/reverse transcriptase (YP_004581513.1)	305	390	10.8%	190	29.9%	86 (94.5%)	45 (49.5%)	5/0/1/1	1
Protein mutations:	F310I (6043T>A 6045T>A), K313R (6053A>G), A314S (6055G>T), F316Y (6062T>A), Q318* (6067C>T), I319L (6070A>T), R320K (6074G>A 6075A>G), M321V (6076A>G 6078G>A), E322K (6079G>A 6081G>A), K324C (6085A>T 6086A>G 6087G>T), S325D (6088T>G 6089C>A 6090C>T), K326I (6092A>T 6093A>C), W328K (6097T>A 6098G>A 6099G>A), W332R (6109T>C 6111G>A), P334H (6116C>A 6117A>T), E335Y (6118G>T 6120A>T), L337Y (6124C>T 6125T>A 6126C>T), Y338F (6128A>T), E341L (6136G>T 6137A>T 6138A>G), P344S (6145C>T), M348T (6158T>C 6159G>T), D353A (6173A>C 6174T>C), Q355M (6178C>A 6179A>T), R356D (6181A>G 6182G>A 6183A>T), K357L (6184A>C 6185A>T 6186G>C), D359N (6190G>A), N360R (6194A>G 6195T>A), A361V (6197C>T 6198A>G), R363K (6203G>A), G364P (6205G>C 6206G>C 6207A>T), G364_T365insYSSLX (6207_6208insTATTCAAGCCTTAT), T365L (6208A>C 6209C>T 6210A>T), A367M (6214G>A 6215C>T 6216A>G), F368L (6219T>A), I369V (6220A>G 6222C>T), A370I (6223G>A 6224C>T 6225A>T), Y372F (6230A>T), F379Y (6251T>A), E381K (6256G>A 6258A>G), H386Y (6271C>T), E387A (6275A>C 6276G>C), D388Y (6277G>T)								
Codon mutations:	ATA306ATT (6033A>T), AAA309AAG (6042A>G), TTT310ATA (6043T>A 6045T>A), GAT311GAC (6048T>C), CTC312CTA (6051C>A), AAA313AGA (6053A>G), GCA314TCA (6055G>T), TTT316TAT (6062T>A), CAG318TAG (6067C>T), ATA319TTA (6070A>T), AGA320AAG (6074G>A 6075A>G), ATG321GTA (6076A>G 6078G>A), GAG322AAA (6079G>A 6081G>A), GAA323GAG (6084A>G), AAG324TGT (6085A>T 6086A>G 6087G>T), TCC325GAT (6088T>G 6089C>A 6090C>T), AAA326ATC (6092A>T 6093A>C), TGG328AAA (6097T>A 6098G>A 6099G>A), TGG332CGA (6109T>C 6111G>A), CCA334CAT (6116C>A 6117A>T), GAA335TAT (6118G>T 6120A>T), GGG336GGC (6123G>C), CTC337TAT (6124C>T 6125T>A 6126C>T), TAT338TTT (6128A>T), GAA339GAG (6132A>G), TTT340TTC (6135T>C), GAA341TTG (6136G>T 6137A>T 6138A>G), CCC344TCC (6145C>T), GGA346GGG (6153A>G), CTG347TTG (6154C>T), ATG348ACT (6158T>C 6159G>T), CCA351CCT (6168A>T), GAT353GCC (6173A>C 6174T>C), CAG355ATG (6178C>A 6179A>T), AGA356GAT (6181A>G 6182G>A 6183A>T), AAG357CTC (6184A>C 6185A>T 6186G>C), GAT359AAT (6190G>A), AAT360AGA (6194A>G 6195T>A), GCA361GTG (6197C>T 6198A>G), TTT362TTC (6201T>C), AGG363AAG (6203G>A), GGA364CCT (6205G>C 6206G>C 6207A>T), GGA364_ACA365insTATTCAAGCCTTAT-(6207_6208insTATTCAAGCCTTAT), ACA365CTT (6208A>C 6209C>T 6210A>T), GAT366GAC (6213T>C), GCA367ATG (6214G>A 6215C>T 6216A>G), TTT368TTA (6219T>A), ATC369GTT (6220A>G 6222C>T), GCA370ATT (6223G>A 6224C>T 6225A>T), GTA371GTC (6228A>C), TAT372TTT (6230A>T), GAC375GAT (6240C>T), ATA376ATC (6243A>C), GTA378GTC (6249A>C), TTC379TAC (6251T>A), TCT380TCC (6255T>C), GAA381AAG (6256G>A 6258A>G), GAA383GAG (6264A>G), GAG385GAA (6270G>A), CAT386TAT (6271C>T), GAG387GCC (6275A>C 6276G>C), GAT388TAT (6277G>T)								

*: Inserts / Deletes / Misaligned / Frameshifts

Analysis details

This analysis was performed with panviral2.64

NGS Details (UN18_val): Caulimovirus venafragariae

Assembly

**Coverage Length**	570 (2 contig(s))
**Depth Of Coverage**	9.9
**Number Of Reads**	48
**Reads Per Million**	0.90 rpm (after QC)
**Ambiguities**	0
**Assembly Method**	de novo + reference guided assembly
**Consensus Caller**	Bcf Tools

Coverage Map

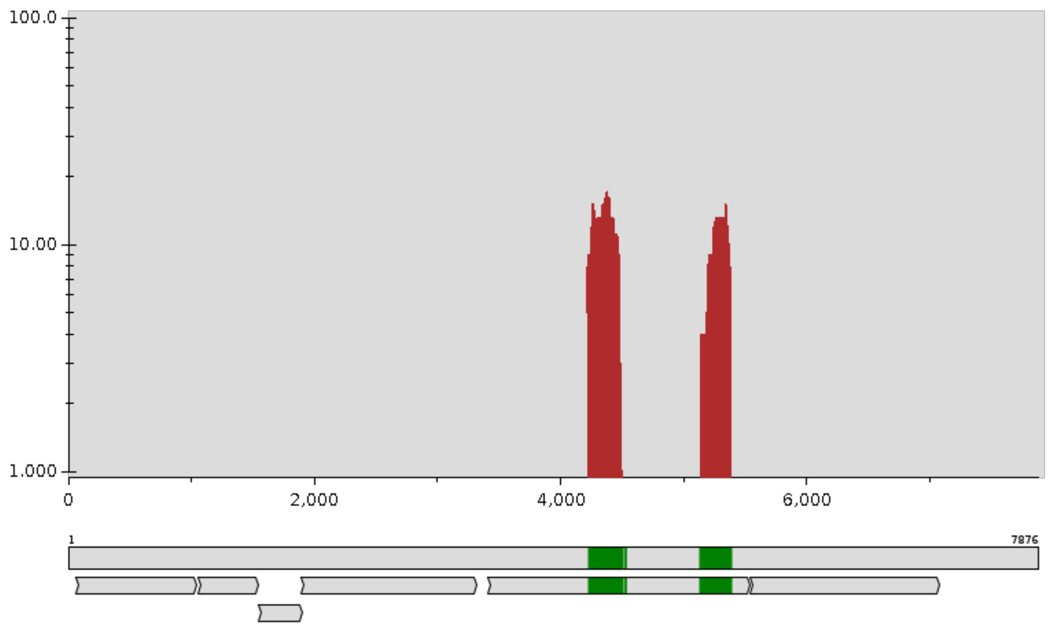

Assignment

**Type**	Caulimovirus venafragariae (Taxonomy ID: 3048344)
**Reference Genome**	NC_001725.1
**NT Identity (%)**	58.2734
**AA Identity (%)**	54.8913
**Number Of Stop Codons**	0
**Number Of CDS**	6

Alignment

**Alignment Score**	152.0 (NT) + 606.0 (AA) = 758.0
**Concordance (%)**	35.4578

Alignment Method	Global, seeded, nucleotide + amino acids (AGA)

Genome Region

Sequence starts at position 4219 and ends at position 5394 relative to NC_001725.1 reference sequence.

Alignment Detailed Statistics

	Begin	End	Coverage	Score	Concordance	Matches	Identities	I/D/M/F*	Stop Codons
**NT**	**4219**	**5394**	**7.2%**	**152**	**14.1%**	**556 (97.5%)**	**324 (56.8%)**	**0/14**	
Mutations:	4219T>C, 4222T>A, 4223T>A, 4228A>C, 4230T>C, 4232A>G, 4235A>T, 4236A>C, 4242T>A, 4244A>G, 4245G>A, 4246T>G, 4247G>T, 4254A>T, 4259A>T, 4260G>A, 4263T>C, 4264T>A, 4265C>A, 4266A>T, 4267C>G, 4268C>A, 4269T>A, 4275C>T, 4276C>A, 4277G>A, 4279G>A, 4280A>C, 4281A>T, 4284G>A, 4292C>T, 4299C>T, 4300G>C, 4306C>T, 4309C>T, 4311C>A, 4314G>T, 4315C>A, 4317T>G, 4318G>A, 4319G>A, 4320T>A, 4321A>T, 4323C>A, 4326C>A, 4330_4331delCC, 4332T>C, 4335C>T, 4338G>A, 4341T>C, 4344A>G, 4348T>A, 4349C>G, 4350C>T, 4351T>A, 4352C>G, 4353A>T, 4359C>A, 4371G>A, 4374T>C, 4380C>G, 4381G>A, 4383G>A, 4384A>C, 4385T>A, 4386C>A, 4395C>A, 4401A>T, 4402A>C, 4413T>A, 4423A>G, 4426T>C, 4428A>T, 4432G>A, 4434C>A, 4435C>A, 4438A>T, 4439C>G, 4440A>T, 4441A>G, 4442A>T, 4443G>C, 4444G>T, 4445G>T, 4446A>T, 4452C>A, 4455C>T, 4456C>T, 4458A>T, 4459C>A, 4464T>A, 4470G>A, 4471G>A, 4473G>T, 4474C>A, 4476A>T, 4479G>T, 4480C>A, 4482T>A, 4483C>A, 4485A>T, 4486A>C, 4487G>T, 4489A>G, 4490T>C, 4492G>A, 4493G>A, 4497T>A, 4500G>A, 4505T>A, 4506T>C, 4508A>T, 4509C>T, 4512T>A, 4513T>A, 4514C>A, 4515C>A, 4521C>T, 4524T>C, 4528T>A, 4529C>G, 5126C>A, 5133G>A, 5139G>C, 5141C>T, 5142T>A, 5143A>G, 5145C>A, 5146A>T, 5148C>A, 5149G>C, 5152T>A, 5153G>C, 5154C>T, 5160A>T, 5163A>T, 5167G>T, 5169C>T, 5182A>T, 5184C>A, 5185C>T, 5187C>A, 5188A>C, 5191G>A, 5194A>G, 5196A>T, 5199T>A, 5200C>A, 5201C>A, 5202A>T, 5206_5217delGGTAAAGAGGTA, 5220A>T, 5223C>T, 5226G>A, 5230G>A, 5233T>A, 5234C>G, 5235A>T, 5247A>T, 5248C>G, 5249C>A, 5251G>A, 5253T>A, 5256G>A, 5258A>C, 5259G>T, 5261A>G, 5262T>G, 5266C>T, 5267A>T, 5268T>A, 5270G>C, 5271T>A, 5280G>A, 5284A>T, 5286C>A, 5289T>A, 5290T>G, 5292C>T, 5295T>A, 5296A>G, 5298T>A, 5300A>G, 5301G>A, 5303C>G, 5304A>C, 5307C>A, 5308A>C, 5309A>G, 5311G>A, 5312C>A, 5313T>G, 5317A>T, 5318G>C, 5319A>T, 5322T>A, 5324A>T, 5325T>C, 5326A>T, 5328C>A, 5331T>A, 5334T>A, 5335T>A, 5337T>A, 5338A>C, 5343T>C, 5344C>A, 5346T>C, 5347G>A, 5349A>C, 5352G>A, 5353A>T, 5355T>G, 5358T>C, 5364T>G, 5365A>C, 5367T>G, 5369C>T, 5370T>A, 5371G>T, 5373T>A, 5374T>G, 5375A>G, 5377T>C, 5379T>A, 5380G>A, 5382G>A, 5383A>T, 5384G>T, 5385A>C, 5387C>A, 5388A>T, 5391T>A, 5392A>C								

CDS

ORF_V	272	663	26.5%	606	47.3%	184 (97.9%)	101 (53.7%)	0/4/1/1	0
Protein mutations:	L273K (4222T>A 4223T>A), T275P (4228A>C 4230T>C), N276S (4232A>G), K277I (4235A>T 4236A>C), K280R (4244A>G 4245G>A), C281V (4246T>G 4247G>T), Q285L (4259A>T 4260G>A), S287N (4264T>A 4265C>A 4266A>T), P288E (4267C>G 4268C>A 4269T>A), R291K (4276C>A 4277G>A), E292T (4279G>A 4280A>C 4281A>T), T296I (4292C>T), E299Q (4300G>C), K303N (4314G>T), L304M (4315C>A 4317T>G), G305K (4318G>A 4319G>A 4320T>A), I306L (4321A>T 4323C>A), E326K (4381G>A 4383G>A), I327Q (4384A>C 4385T>A 4386C>A), K340E (4423A>G), D343K (4432G>A 4434C>A), H344N (4435C>A), T345C (4438A>T 4439C>G 4440A>T), K346V (4441A>G 4442A>T 4443G>C), G347F (4444G>T 4445G>T 4446A>T), L351F (4456C>T 4458A>T), L352I (4459C>A), E356N (4471G>A 4473G>T), Q357N (4474C>A 4476A>T), L359I (4480C>A 4482T>A), Q360N (4483C>A 4485A>T), R361L (4486A>C 4487G>T), I362A (4489A>G 4490T>C), G363K (4492G>A 4493G>A), F367Y (4505T>A 4506T>C), Y368F (4508A>T 4509C>T), S370K (4513T>A 4514C>A 4515C>A), K578N (5139G>C), P579L (5141C>T 5142T>A), I580V (5143A>G 5145C>A), I581L (5146A>T 5148C>A), E582Q (5149G>C), C583T (5152T>A 5153G>C 5154C>T), D588Y (5167G>T 5169C>T), I593L (5182A>T 5184C>A), K595Q (5188A>C), A596T (5191G>A), K597D (5194A>G 5196A>T), P599N (5200C>A 5201C>A 5202A>T), G601_V604del (5206_5217delGGTAAAGAGGTA), A609T (5230G>A), K614N (5247A>T), P615E (5248C>G 5249C>A), A616T (5251G>A 5253T>A), K618T (5258A>C 5259G>T), N619R (5261A>G 5262T>G), H621L (5266C>T 5267A>T 5268T>A), S622T (5270G>C 5271T>A), I627L (5284A>T 5286C>A), S629A (5290T>G 5292C>T), I631V (5296A>G 5298T>A), K632R (5300A>G 5301G>A), A633G (5303C>G 5304A>C), K635R (5308A>C 5309A>G), A636K (5311G>A 5312C>A 5313T>G), R638S (5317A>T 5318G>C 5319A>T), Y640F (5324A>T 5325T>C), I641L (5326A>T 5328C>A), Y644K (5335T>A 5337T>A), K645Q (5338A>C), L647I (5344C>A 5346T>C), V648I (5347G>A 5349A>C), T650S (5353A>T 5355T>G), N654Q (5365A>C 5367T>G), A655V (5369C>T 5370T>A), A656S (5371G>T 5373T>A), Y657G (5374T>G 5375A>G), F658L (5377T>C 5379T>A), V659I (5380G>A 5382G>A), R660F (5383A>T 5384G>T 5385A>C), T661N (5387C>A 5388A>T), N662K (5391T>A), I663L (5392A>C)								
Codon mutations:	TTA272CTA (4219T>C), TTA273AAA (4222T>A 4223T>A), ACT275CCC (4228A>C 4230T>C), AAC276AGC (4232A>G), AAA277ATC (4235A>T 4236A>C), ATT279ATA (4242T>A), AAG280AGA (4244A>G 4245G>A), TGT281GTT (4246T>G 4247G>T), CCA283CCT (4254A>T), CAG285CTA (4259A>T 4260G>A), TAT286TAC (4263T>C), TCA287AAT (4264T>A 4265C>A 4266A>T), CCT288GAA (4267C>G 4268C>A 4269T>A), GAC290GAT (4275C>T), CGA291AAA (4276C>A 4277G>A), GAA292ACT (4279G>A 4280A>C 4281A>T), GAG293GAA (4284G>A), ACT296ATT (4292C>T), ATC298ATT (4299C>T), GAA299CAA (4300G>C), CTA301TTA (4306C>T), CTC302TTA (4309C>T 4311C>A), AAG303AAT (4314G>T), CTT304ATG (4315C>A 4317T>G), GGT305AAA (4318G>A 4319G>A 4320T>A), ATC306TTA (4321A>T 4323C>A), ATC307ATA (4326C>A), CCT309--C (4330_4331delCC 4332T>C), AGC310AGT (4335C>T), AAG311AAA (4338G>A), AGT312AGC (4341T>C), CCA313CCG (4344A>G), TCC315AGT (4348T>A 4349C>G 4350C>T), TCA316AGT (4351T>A 4352C>G 4353A>T), GCC318GCA (4359C>A), AGG322AGA (4371G>A), AAT323AAC (4374T>C), GCC325GCG (4380C>G), GAG326AAA (4381G>A 4383G>A), ATC327CAA (4384A>C 4385T>A 4386C>A), GGC330GGA (4395C>A), GCA332GCT (4401A>T), AGA333CGA (4402A>C), ATT336ATA (4413T>A), AAG340GAG (4423A>G), TTA341CTT (4426T>C 4428A>T), GAC343AAA (4432G>A 4434C>A), CAT344AAT (4435C>A), ACA345TGT (4438A>T 4439C>G 4440A>T), AAG346GTC (4441A>G 4442A>T 4443G>C), GGA347TTT (4444G>T 4445G>T 4446A>T), GGC349GGA (4452C>A), TAC350TAT (4455C>T), CTA351TTT (4456C>T 4458A>T), CTT352ATT (4459C>A), CCT353CCA (4464T>A), AAG355AAA (4470G>A), GAG356AAT (4471G>A 4473G>T), CAA357AAT (4474C>A 4476A>T), CTG358CTT (4479G>T), CTT359ATA (4480C>A 4482T>A), CAA360AAT (4483C>A 4485A>T), AGA361CTA (4486A>C 4487G>T), ATC362GCC (4489A>G 4490T>C), GGA363AAA (4492G>A 4493G>A), GGT364GGA (4497T>A), AAG365AAA (4500G>A), TTT367TAC (4505T>A 4506T>C), TAC368TTT (4508A>T 4509C>T), TCT369TCA (4512T>A), TCC370AAA (4513T>A 4514C>A 4515C>A), GAC372..T (4521C>T), TGT373..C (4524T>C), TCT375AG. (4528T>A 4529C>G), TCA574.AA (5126C>A), GAG576GAA (5133G>A), AAG578AAC (5139G>C), CCT579CTA (5141C>T 5142T>A), ATC580GTA (5143A>G 5145C>A), ATC581TTA (5146A>T 5148C>A), GAA582CAA (5149G>C), TGC583ACT (5152T>A 5153G>C 5154C>T), GCA585GCT (5160A>T), TCA586TCT (5163A>T), GAC588TAT (5167G>T 5169C>T), ATC593TTA (5182A>T 5184C>A), CTC594TTA (5185C>T 5187C>A), AAA595CAA (5188A>C), GCA596ACA (5191G>A), AAA597GAT (5194A>G 5196A>T), CTT598CTA (5199T>A), CCA599AAT (5200C>A 5201C>A 5202A>T), GGT601_GTA604del (5206_5217delGGTAAAGAGGTA), ATA605ATT (5220A>T), TGC606TGT (5223C>T), AGG607AGA (5226G>A), GCT609ACT (5230G>A), TCA610AGT (5233T>A 5234C>G 5235A>T), AAA614AAT (5247A>T), CCA615GAA (5248C>G 5249C>A), GCT616ACA (5251G>A 5253T>A), GAG617GAA (5256G>A), AAG618ACT (5258A>C 5259G>T), AAT619AGG (5261A>G 5262T>G), CAT621TTA (5266C>T 5267A>T 5268T>A), AGT622ACA (5270G>C 5271T>A), AAG625AAA (5280G>A), ATC627TTA (5284A>T 5286C>A), CTT628CTA (5289T>A), TCC629GCT (5290T>G 5292C>T), ATT630ATA (5295T>A), ATT631GTA (5296A>G 5298T>A), AAG632AGA (5300A>G 5301G>A), GCA633GGC (5303C>G 5304A>C), ATC634ATA (5307C>A), AAA635CGA (5308A>C 5309A>G), GCT636AAG (5311G>A 5312C>A 5313T>G), AGA638TCT (5317A>T 5318G>C 5319A>T), GCT639GCA (5322T>A), TAT640TTC (5324A>T 5325T>C), ATC641TTA (5326A>T 5328C>A), CTT642CTA (5331T>A), CCT643CCA (5334T>A), TAT644AAA (5335T>A 5337T>A), AAA645CAA (5338A>C), TTT646TTC (5343T>C), CTT647ATC (5344C>A 5346T>C), GTA648ATC (5347G>A 5349A>C), AGG649AGA (5352G>A), ACT650TCG (5353A>T 5355T>G), GAT651GAC (5358T>C), ACT653ACG (5364T>G), AAT654CAG (5365A>C 5367T>G), GCT655GTA (5369C>T 5370T>A), GCT656TCA (5371G>T 5373T>A), TAT657GGT (5374T>G 5375A>G), TTT658CTA (5377T>C 5379T>A), GTG659ATA (5380G>A 5382G>A), AGA660TTC (5383A>T 5384G>T 5385A>C), ACA661AAT (5387C>A 5388A>T), AAT662AAA (5391T>A), ATT663CTT (5392A>C)								

Proteins

hypothetical protein (NP_043933.1)	272	663	26.5%	606	47.3%	184 (97.9%)	101 (53.7%)	0/4/1/1	0
Protein mutations:	L273K (4222T>A 4223T>A), T275P (4228A>C 4230T>C), N276S (4232A>G), K277I (4235A>T 4236A>C), K280R (4244A>G 4245G>A), C281V (4246T>G 4247G>T), Q285L (4259A>T 4260G>A), S287N (4264T>A 4265C>A 4266A>T), P288E (4267C>G 4268C>A 4269T>A), R291K (4276C>A 4277G>A), E292T (4279G>A 4280A>C 4281A>T), T296I (4292C>T), E299Q (4300G>C), K303N (4314G>T), L304M (4315C>A 4317T>G), G305K (4318G>A 4319G>A 4320T>A), I306L (4321A>T 4323C>A), E326K (4381G>A 4383G>A), I327Q (4384A>C 4385T>A 4386C>A), K340E (4423A>G), D343K (4432G>A 4434C>A), H344N (4435C>A), T345C (4438A>T 4439C>G 4440A>T), K346V (4441A>G 4442A>T 4443G>C), G347F (4444G>T 4445G>T 4446A>T), L351F (4456C>T 4458A>T), L352I (4459C>A), E356N (4471G>A 4473G>T), Q357N (4474C>A 4476A>T), L359I (4480C>A 4482T>A), Q360N (4483C>A 4485A>T), R361L (4486A>C 4487G>T), I362A (4489A>G 4490T>C), G363K (4492G>A 4493G>A), F367Y (4505T>A 4506T>C), Y368F (4508A>T 4509C>T), S370K (4513T>A 4514C>A 4515C>A), K578N (5139G>C), P579L (5141C>T 5142T>A), I580V (5143A>G 5145C>A), I581L (5146A>T 5148C>A), E582Q (5149G>C), C583T (5152T>A 5153G>C 5154C>T), D588Y (5167G>T 5169C>T), I593L (5182A>T 5184C>A), K595Q (5188A>C), A596T (5191G>A), K597D (5194A>G 5196A>T), P599N (5200C>A 5201C>A 5202A>T), G601_V604del (5206_5217delGGTAAAGAGGTA), A609T (5230G>A), K614N (5247A>T), P615E (5248C>G 5249C>A), A616T (5251G>A 5253T>A), K618T (5258A>C 5259G>T), N619R (5261A>G 5262T>G), H621L (5266C>T 5267A>T 5268T>A), S622T (5270G>C 5271T>A), I627L (5284A>T 5286C>A), S629A (5290T>G 5292C>T), I631V (5296A>G 5298T>A), K632R (5300A>G 5301G>A), A633G (5303C>G 5304A>C), K635R (5308A>C 5309A>G), A636K (5311G>A 5312C>A 5313T>G), R638S (5317A>T 5318G>C 5319A>T), Y640F (5324A>T 5325T>C), I641L (5326A>T 5328C>A), Y644K (5335T>A 5337T>A), K645Q (5338A>C), L647I (5344C>A 5346T>C), V648I (5347G>A 5349A>C), T650S (5353A>T 5355T>G), N654Q (5365A>C 5367T>G), A655V (5369C>T 5370T>A), A656S (5371G>T 5373T>A), Y657G (5374T>G 5375A>G), F658L (5377T>C 5379T>A), V659I (5380G>A 5382G>A), R660F (5383A>T 5384G>T 5385A>C), T661N (5387C>A 5388A>T), N662K (5391T>A), I663L (5392A>C)								

	Begin	End	Coverage	Score	Concordance	Matches	Identities	I/D/M/F*	Stop Codons
**NT**	**4219**	**5394**	**7.2%**	**152**	**14.1%**	**556 (97.5%)**	**324 (56.8%)**	**0/14**	
Codon mutations:	TTA272CTA (4219T>C), TTA273AAA (4222T>A 4223T>A), ACT275CCC (4228A>C 4230T>C), AAC276AGC (4232A>G), AAA277ATC (4235A>T 4236A>C), ATT279ATA (4242T>A), AAG280AGA (4244A>G 4245G>A), TGT281GTT (4246T>G 4247G>T), CCA283CCT (4254A>T), CAG285CTA (4259A>T 4260G>A), TAT286TAC (4263T>C), TCA287AAT (4264T>A 4265C>A 4266A>T), CCT288GAA (4267C>G 4268C>A 4269T>A), GAC290GAT (4275C>T), CGA291AAA (4276C>A 4277G>A), GAA292ACT (4279G>A 4280A>C 4281A>T), GAG293GAA (4284G>A), ACT296ATT (4292C>T), ATC298ATT (4299C>T), GAA299CAA (4300G>C), CTA301TTA (4306C>T), CTC302TTA (4309C>T 4311C>A), AAG303AAT (4314G>T), CTT304ATG (4315C>A 4317T>G), GGT305AAA (4318G>A 4319G>A 4320T>A), ATC306TTA (4321A>T 4323C>A), ATC307ATA (4326C>A), CCT309--C (4330_4331delCC 4332T>C), AGC310AGT (4335C>T), AAG311AAA (4338G>A), AGT312AGC (4341T>C), CCA313CCG (4344A>G), TCC315AGT (4348T>A 4349C>G 4350C>T), TCA316AGT (4351T>A 4352C>G 4353A>T), GCC318GCA (4359C>A), AGG322AGA (4371G>A), AAT323AAC (4374T>C), GCC325GCG (4380C>G), GAG326AAA (4381G>A 4383G>A), ATC327CAA (4384A>C 4385T>A 4386C>A), GGC330GGA (4395C>A), GCA332GCT (4401A>T), AGA333CGA (4402A>C), ATT336ATA (4413T>A), AAG340GAG (4423A>G), TTA341CTT (4426T>C 4428A>T), GAC343AAA (4432G>A 4434C>A), CAT344AAT (4435C>A), ACA345TGT (4438A>T 4439C>G 4440A>T), AAG346GTC (4441A>G 4442A>T 4443G>C), GGA347TTT (4444G>T 4445G>T 4446A>T), GGC349GGA (4452C>A), TAC350TAT (4455C>T), CTA351TTT (4456C>T 4458A>T), CTT352ATT (4459C>A), CCT353CCA (4464T>A), AAG355AAA (4470G>A), GAG356AAT (4471G>A 4473G>T), CAA357AAT (4474C>A 4476A>T), CTG358CTT (4479G>T), CTT359ATA (4480C>A 4482T>A), CAA360AAT (4483C>A 4485A>T), AGA361CTA (4486A>C 4487G>T), ATC362GCC (4489A>G 4490T>C), GGA363AAA (4492G>A 4493G>A), GGT364GGA (4497T>A), AAG365AAA (4500G>A), TTT367TAC (4505T>A 4506T>C), TAC368TTT (4508A>T 4509C>T), TCT369TCA (4512T>A), TCC370AAA (4513T>A 4514C>A 4515C>A), GAC372..T (4521C>T), TGT373..C (4524T>C), TCT375AG. (4528T>A 4529C>G), TCA574.AA (5126C>A), GAG576GAA (5133G>A), AAG578AAC (5139G>C), CCT579CTA (5141C>T 5142T>A), ATC580GTA (5143A>G 5145C>A), ATC581TTA (5146A>T 5148C>A), GAA582CAA (5149G>C), TGC583ACT (5152T>A 5153G>C 5154C>T), GCA585GCT (5160A>T), TCA586TCT (5163A>T), GAC588TAT (5167G>T 5169C>T), ATC593TTA (5182A>T 5184C>A), CTC594TTA (5185C>T 5187C>A), AAA595CAA (5188A>C), GCA596ACA (5191G>A), AAA597GAT (5194A>G 5196A>T), CTT598CTA (5199T>A), CCA599AAT (5200C>A 5201C>A 5202A>T), GGT601_GTA604del (5206_5217delGGTAAAGAGGTA), ATA605ATT (5220A>T), TGC606TGT (5223C>T), AGG607AGA (5226G>A), GCT609ACT (5230G>A), TCA610AGT (5233T>A 5234C>G 5235A>T), AAA614AAT (5247A>T), CCA615GAA (5248C>G 5249C>A), GCT616ACA (5251G>A 5253T>A), GAG617GAA (5256G>A), AAG618ACT (5258A>C 5259G>T), AAT619AGG (5261A>G 5262T>G), CAT621TTA (5266C>T 5267A>T 5268T>A), AGT622ACA (5270G>C 5271T>A), AAG625AAA (5280G>A), ATC627TTA (5284A>T 5286C>A), CTT628CTA (5289T>A), TCC629GCT (5290T>G 5292C>T), ATT630ATA (5295T>A), ATT631GTA (5296A>G 5298T>A), AAG632AGA (5300A>G 5301G>A), GCA633GGC (5303C>G 5304A>C), ATC634ATA (5307C>A), AAA635CGA (5308A>C 5309A>G), GCT636AAG (5311G>A 5312C>A 5313T>G), AGA638TCT (5317A>T 5318G>C 5319A>T), GCT639GCA (5322T>A), TAT640TTC (5324A>T 5325T>C), ATC641TTA (5326A>T 5328C>A), CTT642CTA (5331T>A), CCT643CCA (5334T>A), TAT644AAA (5335T>A 5337T>A), AAA645CAA (5338A>C), TTT646TTC (5343T>C), CTT647ATC (5344C>A 5346T>C), GTA648ATC (5347G>A 5349A>C), AGG649AGA (5352G>A), ACT650TCG (5353A>T 5355T>G), GAT651GAC (5358T>C), ACT653ACG (5364T>G), AAT654CAG (5365A>C 5367T>G), GCT655GTA (5369C>T 5370T>A), GCT656TCA (5371G>T 5373T>A), TAT657GGT (5374T>G 5375A>G), TTT658CTA (5377T>C 5379T>A), GTG659ATA (5380G>A 5382G>A), AGA660TTC (5383A>T 5384G>T 5385A>C), ACA661AAT (5387C>A 5388A>T), AAT662AAA (5391T>A), ATT663CTT (5392A>C)								

*: Inserts / Deletes / Misaligned / Frameshifts

Analysis details

This analysis was performed with panviral2.64

NGS Details (UN18_val): Badnavirus occultipomeae

Assembly

**Coverage Length**	253 (1 contig(s))
**Depth Of Coverage**	14.4
**Number Of Reads**	38
**Reads Per Million**	0.71 rpm (after QC)
**Ambiguities**	0
**Assembly Method**	de novo + reference guided assembly
**Consensus Caller**	Bcf Tools

Coverage Map

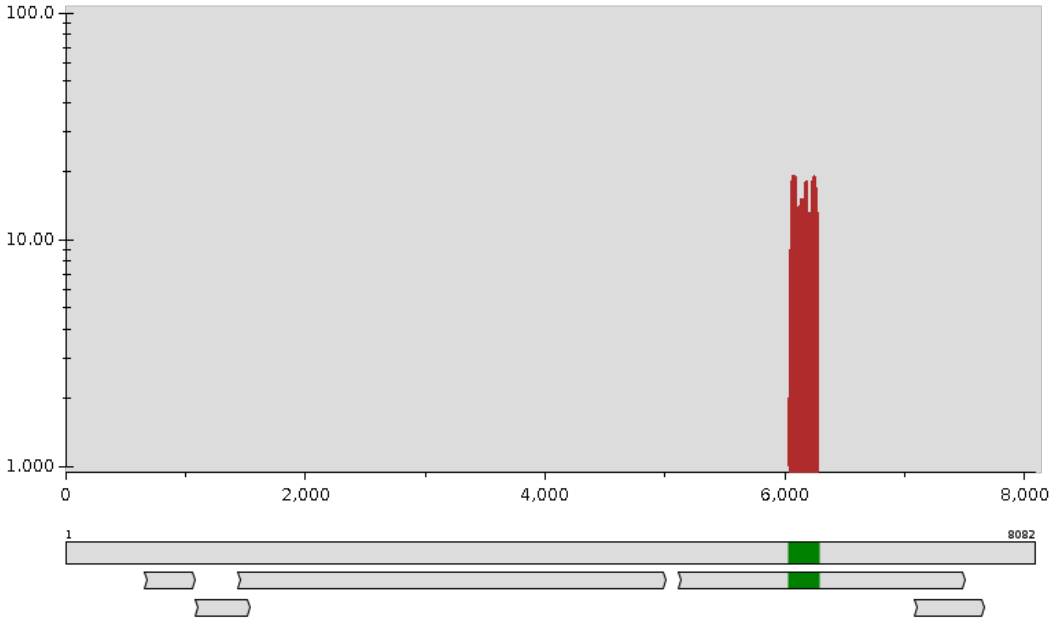

Assignment

**Type**	Badnavirus occultipomeae (Taxonomy ID: 3048353)
**Reference Genome**	NC_015655.1
**NT Identity (%)**	60.5469
**AA Identity (%)**	51.7647
**Number Of Stop Codons**	0
**Number Of CDS**	5

Alignment

**Alignment Score**	102.0 (NT) + 312.0 (AA) = 414.0
**Concordance (%)**	36.7347

Alignment Method	Global, seeded, nucleotide + amino acids (AGA)

Genome Region

Sequence starts at position 6033 and ends at position 6285 relative to NC_015655.1 reference sequence.

Alignment Detailed Statistics

	Begin	End	Coverage	Score	Concordance	Matches	Identities	I/D/M/F*	Stop Codons
**NT**	**6033**	**6285**	**3.1%**	**102**	**20.2%**	**253 (98.8%)**	**155 (60.5%)**	**3/0**	
Mutations:	6033A>C, 6039A>T, 6042A>G, 6043T>A, 6045T>A, 6048T>C, 6053A>G, 6055G>T, 6062T>A, 6070A>T, 6072A>G, 6074G>A, 6076A>G, 6079G>A, 6080A>G, 6081G>A, 6085A>T, 6086A>G, 6087G>T, 6088T>G, 6089C>A, 6092A>T, 6093A>C, 6096A>G, 6097T>A, 6098G>A, 6102T>G, 6109T>C, 6114T>C, 6116C>G, 6117A>G, 6118G>T, 6120A>T, 6123G>T, 6125T>A, 6128A>T, 6134T>G, 6136G>T, 6137A>T, 6138A>G, 6141C>A, 6145C>T, 6150T>C, 6153A>T, 6154C>T, 6155T>C, 6158T>C, 6159G>A, 6168A>T, 6173A>C, 6174T>C, 6178C>A, 6179A>T, 6181A>G, 6182G>A, 6183A>T, 6184A>T, 6185A>T, 6190G>A, 6192T>C, 6194A>G, 6195T>A, 6197C>T, 6201T>C, 6204G>A, 6205G>C, 6206G>A, 6207A>G, 6207_6208insTAT, 6208A>C, 6209C>T, 6210A>T, 6213T>C, 6214G>A, 6215C>T, 6216A>G, 6219T>C, 6220A>G, 6222C>A, 6223G>A, 6224C>T, 6225A>C, 6228A>G, 6230A>T, 6234T>C, 6240C>T, 6243A>C, 6251T>A, 6252C>T, 6255T>C, 6256G>A, 6257A>G, 6259A>G, 6264A>G, 6267A>G, 6275A>T, 6276G>T, 6277G>A, 6282T>C								
**CDS**									
SPBVa_gp4	307	390	10.6%	312	49.8%	84 (98.8%)	44 (51.8%)	1/0/0/0	0
Protein mutations:	F310I (6043T>A 6045T>A), K313R (6053A>G), A314S (6055G>T), F316Y (6062T>A), I319L (6070A>T 6072A>G), R320K (6074G>A), M321V (6076A>G), E322R (6079G>A 6080A>G 6081G>A), K324C (6085A>T 6086A>G 6087G>T), S325D (6088T>G 6089C>A), K326I (6092A>T 6093A>C), W328K (6097T>A 6098G>A), W332R (6109T>C), P334R (6116C>G 6117A>G), E335Y (6118G>T 6120A>T), L337H (6125T>A), Y338F (6128A>T), F340C (6134T>G), E341L (6136G>T 6137A>T 6138A>G), P344S (6145C>T), L347S (6154C>T 6155T>C), M348T (6158T>C 6159G>A), D353A (6173A>C 6174T>C), Q355M (6178C>A 6179A>T), R356D (6181A>G 6182G>A 6183A>T), K357L (6184A>T 6185A>T), D359N (6190G>A 6192T>C), N360R (6194A>G 6195T>A), A361V (6197C>T), G364Q (6205G>C 6206G>A 6207A>G), G364_T365insY (6207_6208insTAT), T365L (6208A>C 6209C>T 6210A>T), A367M (6214G>A 6215C>T 6216A>G), I369V (6220A>G 6222C>A), A370I (6223G>A 6224C>T 6225A>C), Y372F (6230A>T), F379Y (6251T>A 6252C>T), E381R (6256G>A 6257A>G), N382D (6259A>G), E387V (6275A>T 6276G>T), D388N (6277G>A)								
Codon mutations:	ATA306..C (6033A>C), TCA308TCT (6039A>T), AAA309AAG (6042A>G), TTT310ATA (6043T>A 6045T>A), GAT311GAC (6048T>C), AAA313AGA (6053A>G), GCA314TCA (6055G>T), TTT316TAT (6062T>A), ATA319TTG (6070A>T 6072A>G), AGA320AAA (6074G>A), ATG321GTG (6076A>G), GAG322AGA (6079G>A 6080A>G 6081G>A), AAG324TGT (6085A>T 6086A>G 6087G>T), TCC325GAC (6088T>G 6089C>A), AAA326ATC (6092A>T 6093A>C), CCA327CCG (6096A>G), TGG328AAG (6097T>A 6098G>A), ACT329ACG (6102T>G), TGG332CGG (6109T>C), ACT333ACC (6114T>C), CCA334CGG (6116C>G 6117A>G), GAA335TAT (6118G>T 6120A>T), GGG336GGT (6123G>T), CTC337CAC (6125T>A), TAT338TTT (6128A>T), TTT340TGT (6134T>G), GAA341TTG (6136G>T 6137A>T 6138A>G), GTC342GTA (6141C>A), CCC344TCC (6145C>T), TTT345TTC (6150T>C), GGA346GGT (6153A>T), CTG347TCG (6154C>T 6155T>C), ATG348ACA (6158T>C 6159G>A), CCA351CCT (6168A>T), GAT353GCC (6173A>C 6174T>C), CAG355ATG (6178C>A 6179A>T), AGA356GAT (6181A>G 6182G>A 6183A>T), AAG357TTG (6184A>T 6185A>T), GAT359AAC (6190G>A 6192T>C), AAT360AGA (6194A>G 6195T>A), GCA361GTA (6197C>T), TTT362TTC (6201T>C), AGG363AGA (6204G>A), GGA364CAG (6205G>C 6206G>A 6207A>G), GGA364_ACA365insTAT (6207_6208insTAT), ACA365CTT (6208A>C 6209C>T 6210A>T), GAT366GAC (6213T>C), GCA367ATG (6214G>A 6215C>T 6216A>G), TTT368TTC (6219T>C), ATC369GTA (6220A>G 6222C>A), GCA370ATC (6223G>A 6224C>T 6225A>C), GTA371GTG (6228A>G), TAT372TTT (6230A>T), ATT373ATC (6234T>C), GAC375GAT (6240C>T), ATA376ATC (6243A>C), TTC379TAT (6251T>A 6252C>T), TCT380TCC (6255T>C), GAA381AGA (6256G>A 6257A>G), AAT382GAT (6259A>G), GAA383GAG (6264A>G), GAA384GAG (6267A>G), GAG387GTT (6275A>T 6276G>T), GAT388AAT (6277G>A), CAT389CAC (6282T>C)								
**Proteins**									
RNaseH/reverse transcriptase (YP_004581513.1)	307	390	10.6%	312	49.8%	84 (98.8%)	44 (51.8%)	1/0/0/0	0
Protein mutations:	F310I (6043T>A 6045T>A), K313R (6053A>G), A314S (6055G>T), F316Y (6062T>A), I319L (6070A>T 6072A>G), R320K (6074G>A), M321V (6076A>G), E322R (6079G>A 6080A>G 6081G>A), K324C (6085A>T 6086A>G 6087G>T), S325D (6088T>G 6089C>A), K326I (6092A>T 6093A>C), W328K (6097T>A 6098G>A), W332R (6109T>C), P334R (6116C>G 6117A>G), E335Y (6118G>T 6120A>T), L337H (6125T>A), Y338F (6128A>T), F340C (6134T>G), E341L (6136G>T 6137A>T 6138A>G), P344S (6145C>T), L347S (6154C>T 6155T>C), M348T (6158T>C 6159G>A), D353A (6173A>C 6174T>C), Q355M (6178C>A 6179A>T), R356D (6181A>G 6182G>A 6183A>T), K357L (6184A>T 6185A>T), D359N (6190G>A 6192T>C), N360R (6194A>G 6195T>A), A361V (6197C>T), G364Q (6205G>C 6206G>A 6207A>G), G364_T365insY (6207_6208insTAT), T365L (6208A>C 6209C>T 6210A>T), A367M (6214G>A 6215C>T 6216A>G), I369V (6220A>G 6222C>A), A370I (6223G>A 6224C>T 6225A>C), Y372F (6230A>T), F379Y (6251T>A 6252C>T), E381R (6256G>A 6257A>G), N382D (6259A>G), E387V (6275A>T 6276G>T), D388N (6277G>A)								
Codon mutations:	ATA306..C (6033A>C), TCA308TCT (6039A>T), AAA309AAG (6042A>G), TTT310ATA (6043T>A 6045T>A), GAT311GAC (6048T>C), AAA313AGA (6053A>G), GCA314TCA (6055G>T), TTT316TAT (6062T>A), ATA319TTG (6070A>T 6072A>G), AGA320AAA (6074G>A), ATG321GTG (6076A>G), GAG322AGA (6079G>A 6080A>G 6081G>A), AAG324TGT (6085A>T 6086A>G 6087G>T), TCC325GAC (6088T>G 6089C>A), AAA326ATC (6092A>T 6093A>C), CCA327CCG (6096A>G), TGG328AAG (6097T>A 6098G>A), ACT329ACG (6102T>G), TGG332CGG (6109T>C), ACT333ACC (6114T>C), CCA334CGG (6116C>G 6117A>G), GAA335TAT (6118G>T 6120A>T), GGG336GGT (6123G>T), CTC337CAC (6125T>A), TAT338TTT (6128A>T), TTT340TGT (6134T>G), GAA341TTG (6136G>T 6137A>T 6138A>G), GTC342GTA (6141C>A), CCC344TCC (6145C>T), TTT345TTC (6150T>C), GGA346GGT (6153A>T), CTG347TCG (6154C>T 6155T>C), ATG348ACA (6158T>C 6159G>A), CCA351CCT (6168A>T), GAT353GCC (6173A>C 6174T>C), CAG355ATG (6178C>A 6179A>T), AGA356GAT (6181A>G 6182G>A 6183A>T), AAG357TTG (6184A>T 6185A>T), GAT359AAC (6190G>A 6192T>C), AAT360AGA (6194A>G 6195T>A), GCA361GTA (6197C>T), TTT362TTC (6201T>C), AGG363AGA (6204G>A), GGA364CAG (6205G>C 6206G>A 6207A>G), GGA364_ACA365insTAT (6207_6208insTAT), ACA365CTT (6208A>C 6209C>T 6210A>T), GAT366GAC (6213T>C), GCA367ATG (6214G>A 6215C>T 6216A>G), TTT368TTC (6219T>C), ATC369GTA (6220A>G 6222C>A), GCA370ATC (6223G>A 6224C>T 6225A>C), GTA371GTG (6228A>G), TAT372TTT (6230A>T), ATT373ATC (6234T>C), GAC375GAT (6240C>T), ATA376ATC (6243A>C), TTC379TAT (6251T>A 6252C>T), TCT380TCC (6255T>C), GAA381AGA (6256G>A 6257A>G), AAT382GAT (6259A>G), GAA383GAG (6264A>G), GAA384GAG (6267A>G), GAG387GTT (6275A>T 6276G>T), GAT388AAT (6277G>A), CAT389CAC (6282T>C)								

*: Inserts / Deletes / Misaligned / Frameshifts

Analysis details

This analysis was performed with panviral2.64

NGS Details (UN18_val): Brazilian marseillevirus

Assembly

**Coverage Length**	172 (1 contig(s))
**Depth Of Coverage**	15.2
**Number Of Reads**	33
**Reads Per Million**	0.62 rpm (after QC)
**Ambiguities**	0
**Assembly Method**	de novo + reference guided assembly
**Consensus Caller**	Bcf Tools

Coverage Map

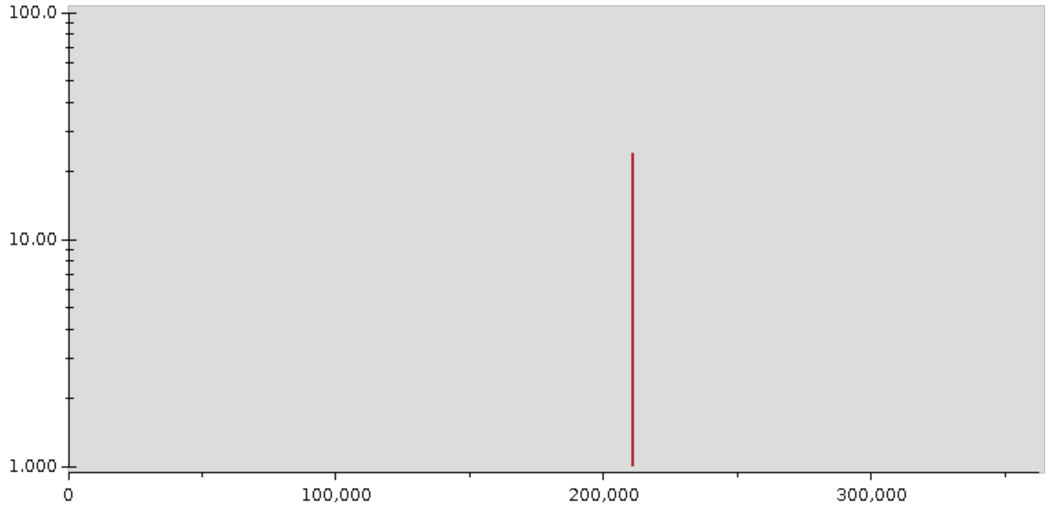

Assignment

**Type**	Brazilian marseillevirus (Taxonomy ID: 1813599)
**Reference Genome**	NC_029692.1
**NT Identity (%)**	78.4884
**AA Identity (%)**	91.2281
**Number Of Stop Codons**	0
**Number Of CDS**	491

Alignment

**Alignment Score**	196.0 (NT) + 345.0 (AA) = 541.0
**Concordance (%)**	76.5205
**Alignment Method**	Local, heuristic, nucleotide (BLASTN)

Genome Region

Sequence starts at position 210894 and ends at position 211065 relative to NC_029692.1 reference sequence.

Alignment Detailed Statistics

	Begin	End	Coverage	Score	Concordance	Matches	Identities	I/D/M/F*	Stop Codons
**NT**	**210894**	**211065**	**0.1%**	**196**	**57.0%**	**172 (100%)**	**135 (78.5%)**	**0/0**	

Mutations: 210896A>G, 210901T>G, 210902T>C, 210904G>A, 210908A>G, 210914A>C, 210923C>T, 210926T>C, 210929T>C, 210932G>A, 210938A>G, 210941A>G, 210946C>A, 210953C>T, 210956T>A, 210959A>G, 210962T>C, 210965C>G, 210968C>T, 210971C>T, 210974G>A, 210977T>G, 210986A>G, 210989G>T, 210992T>C, 210995T>C, 211004A>T, 211007A>G, 211009C>T, 211013T>C, 211031T>G, 211032T>C, 211033G>T, 211034T>C, 211040G>A, 211043T>G, 211055A>C

*: Inserts / Deletes / Misaligned / Frameshifts

Analysis details

This analysis was performed with panviral2.64

NGS Details (UN18_val): Rahariannevirus raharianne

Assembly

**Coverage Length**	1643 (6 contig(s))
**Depth Of Coverage**	2.4
**Number Of Reads**	31
**Reads Per Million**	0.58 rpm (after QC)
**Ambiguities**	0
**Assembly Method**	de novo + reference guided assembly
**Consensus Caller**	Bcf Tools

Coverage Map

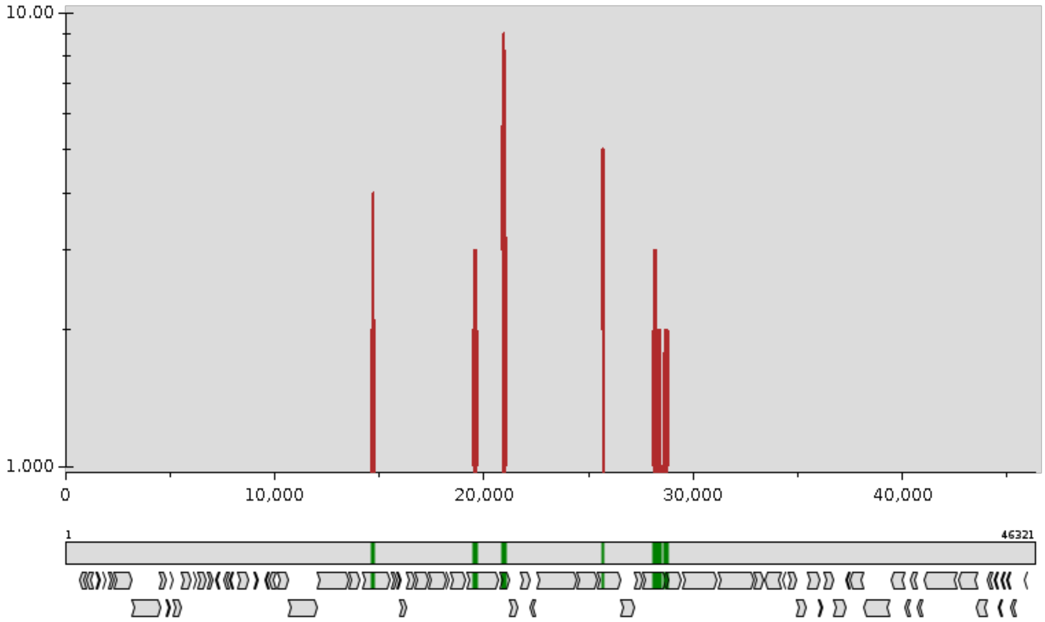

Assignment

**Type**	Rahariannevirus raharianne (Taxonomy ID: 2846050)
**Reference Genome**	NC_054955.1
**NT Identity (%)**	69.7974
**AA Identity (%)**	65.3061
**Number Of Stop Codons**	1
**Number Of CDS**	75

Alignment

**Alignment Score**	1249.0 (NT) + 2380.0 (AA) = 3629.0
**Concordance (%)**	55.1939

Alignment Method	Local, heuristic, nucleotide (BLASTN)

Genome Region

Sequence starts at position 14591 and ends at position 28832 relative to NC_054955.1 reference sequence.

Alignment Detailed Statistics

	Begin	End	Coverage	Score	Concordance	Matches	Identities	I/D/M/F*	Stop Codons
**NT**	**14591**	**28832**	**3.5%**	**1249**	**38.8%**	**1626 (98.8%)**	**1137 (69.1%)**	**3/17**	
Mutations:	14595A>G, 14596A>C, 14597T>C, 14600C>G, 14603G>C, 14609T>C, 14640A>G, 14641C>T, 14642G>C, 14648G>C, 14652T>G, 14661G>C, 14662C>T, 14663C>G, 14666C>G, 14670T>A, 14671C>G, 14672G>C, 14678C>T, 14682A>G, 14684C>G, 14697A>C, 14699C>G, 14715C>G, 14716A>T, 14718T>A, 14723C>T, 14729G>C, 14734G>C, 14753G>C, 14759T>G, 14769A>G, 14780G>C, 14783T>C, 14788C>T, 14789A>G, 14794C>T, 14798C>T, 14802T>G, 14803C>G, 19482C>A, 19483G>A, 19484C>A, 19485T>G, 19486G>C, 19493C>G, 19496C>G, 19512A>G, 19513C>T, 19520G>C, 19521T>A, 19522C>G, 19523G>C, 19525C>T, 19526G>C, 19527A>G, 19528T>C, 19529C>G, 19534A>T, 19542G>T, 19545G>A, 19547G>C, 19550G>C, 19551C>T, 19553G>C, 19557G>T, 19559G>C, 19574G>C, 19575C>T, 19577G>C, 19584T>G, 19585C>G, 19586G>C, 19587G>A, 19589G>C, 19595C>G, 19596G>A, 19601C>G, 19602G>T, 19604C>G, 19607G>C, 19611A>T, 19612G>C, 19613C>G, 19614G>C, 19615C>A, 19616C>G, 19620A>T, 19621A>C, 19622T>G, 19626T>C, 19627G>A, 19633T>C, 19634A>C, 19635A>G, 19636C>T, 19640C>T, 19641G>T, 19650A>G, 19651C>G, 19652G>C, 19653C>T, 19655A>G, 19656A>G, 19657A>T, 19658C>G, 19666T>C, 19667T>C, 19683G>T, 19685T>G, 20839G>C, 20840G>A, 20841T>C, 20846C>G, 20847A>T, 20852T>G, 20854A>C, 20856C>G, 20857T>C, 20861A>C, 20866G>C, 20867C>A, 20869G>C, 20871C>T, 20874A>G, 20876A>C, 20877A>T, 20884C>G, 20885T>C, 20886C>A, 20887T>G, 20891A>C, 20894A>T, 20895G>C, 20896C>G, 20897A>G, 20898A>C, 20901A>C, 20902G>C, 20905T>C, 20906G>A, 20909A>G, 20914T>G, 20915G>A, 20917G>C, 20918C>A, 20919G>A, 20920C>G, 20921G>A, 20927C>G, 20929C>T, 20945G>C, 20946A>G, 20950C>T, 20952T>C, 20959T>C, 20972T>C, 20973C>G, 20974T>C, 20978T>A, 20979C>G, 20980G>C, 20984G>A, 20985C>A, 20986G>C, 20987A>G, 20989T>G, 20992A>G, 20998A>G, 21002G>A, 21003C>G, 21004G>C, 21005G>A, 21007G>A, 21014A>G, 21028A>G, 21034A>G, 21035T>G, 21037G>C, 21062T>C, 21063C>A, 21067C>G, 21071C>A, 21072A>C, 21076T>C, 21077T>A, 21078A>C, 21079T>C, 21085G>C, 25634T>C, 25635T>C, 25637C>G, 25650T>A, 25651C>G, 25653G>A, 25659A>G, 25662A>G, 25665T>C, 25666A>C, 25667C>G, 25672A>C, 25673G>C, 25675C>A, 25676G>C, 25679C>A, 25682C>G, 25683A>T, 25685C>G, 25691G>C, 25697T>C, 25700G>C, 25706G>C, 25709T>C, 25720A>T, 25724C>T, 25727C>G, 25733G>C, 25737C>A, 25744C>G, 25771C>T, 25772G>C, 28051T>G, 28053G>C, 28054C>G, 28062T>C, 28064G>A, 28065C>T, 28066T>C, 28068G>C, 28072C>G, 28073G>A, 28075A>C, 28076T>A, 28077C>A, 28078G>C, 28081C>G, 28082A>C, 28086T>G, 28088G>C, 28089G>A, 28090C>G, 28093A>C, 28096T>C, 28097T>G, 28099C>G, 28108T>C, 28114C>G, 28117G>C, 28123C>G, 28126A>G, 28137A>C, 28138C>G, 28141C>T, 28147T>C, 28154G>A, 28156A>G, 28159C>T, 28162T>C, 28165G>C, 28166C>T, 28168G>C, 28171T>C, 28172A>T, 28177C>G, 28181A>G, 28185C>G, 28190G>A, 28192A>C, 28193G>A, 28194C>A, 28195G>C, 28207G>A, 28213G>C, 28215A>G, 28216A>G, 28217T>G, 28219C>T, 28222T>C, 28223G>A, 28229T>A, 28238A>C, 28239C>T, 28240C>G, 28241T>G, 28243G>C, 28244C>A, 28246G>C, 28254G>A, 28255A>C, 28256C>A, 28258G>C, 28260G>A, 28264T>C, 28270G>C, 28271C>A, 28272T>C, 28273T>C, 28277A>G, 28278A>G, 28280C>T, 28282G>T, 28283A>G, 28285G>C, 28290C>G, 28296T>C, 28298A>G, 28304A>C, 28309T>C, 28310A>T, 28316G>T, 28317T>A, 28318G>C, 28321C>G, 28322A>G, 28324C>G, 28336A>C, 28339G>C, 28342C>T, 28343T>G, 28348A>G, 28351G>C, 28357G>C, 28358C>T, 28360T>C, 28366G>C, 28367T>A, 28369A>C, 28372T>C, 28373T>A, 28374C>G, 28375G>C, 28381T>C, 28390T>C, 28399A>G, 28400C>G, 28402A>C, 28403T>G, 28405C>T, 28406A>T, 28407G>C, 28408C>G, 28410G>C, 28426T>G, 28429G>T, 28433G>C, 28435G>T, 28438T>G, 28440A>C, 28441T>G, 28445T>A, 28446C>A, 28447T>C, 28457A>G, 28459G>C, 28460G>A, 28462G>C, 28465C>T, 28467T>C, 28468C>G, 28472G>A, 28473C>G, 28474G>C, 28480T>C, 28481G>A, 28484A>C, 28485C>A, 28486G>C, 28488T>C, 28489G>C, 28492G>C, 28494C>T, 28495G>C, 28496A>G, 28497A>T, 28498G>C, 28501G>T, 28502T>C, 28505A>G, 28507C>G, 28508G>C, 28509T>A, 28510G>A, 28511A>G, 28516A>C, 28517C>G, 28520A>C, 28523A>T, 28525G>C, 28528T>C, 28553G>A, 28555A>G, 28561G>C, 28564T>C, 28565T>A, 28567G>C, 28568C>A, 28569G>A, 28574G>T, 28576C>G, 28579A>G, 28580G>C, 28582G>C, 28585T>C, 28588T>C, 28590A>G, 28594T>G, 28600T>G, 28602G>C, 28603C>A, 28609G>C, 28613A>G, 28618G>C, 28622G>A, 28624G>C, 28634A>G, 28645G>T, 28648T>C, 28652G>A, 28654T>G, 28657G>C, 28660G>C, 28662G>A, 28665T>A, 28666T>G, 28669G>A, 28675A>C, 28678A>G, 28679T>G, 28680G>C, 28681G>C, 28682T>G, 28683C>G, 28684G>C, 28685G>A, 28687A>G, 28688G>A, 28691A>T, 28692G>C, 28693C>G, 28697G>A, 28699A>C, 28703T>G, 28706_28719delAAACAACTCTGGTG, 28724A>T, 28725A>C, 28729A>G, 28730G>C, 28731C>A, 28732G>A, 28734T>C, 28737C>T, 28740T>C, 28743A>G, 28744C>A, 28745A>G, 28746A>C, 28749T>C, 28750G>A, 28753T>A, 28754A>G, 28756C>A, 28757A>C, 28767C>G, 28768G>C, 28769G>A, 28770C>G, 28771T>C, 28772A>T, 28774C>A, 28776G>C, 28779T>G, 28780C>G, 28782_28783insGGG, 28789_28791delGGC, 28792G>C, 28794T>G, 28795G>C, 28796C>A, 28797G>A, 28799C>G, 28800G>C, 28802A>T, 28803G>C, 28804T>G, 28805C>T, 28809A>G, 28810G>A, 28814T>G, 28815G>C, 28816A>G, 28817C>A, 28830G>A								

CDS

KMC43_gp27	133	207	16.9%	411	85.8%	75 (100%)	62 (82.7%)	0/0/0/0	0
Protein mutations:	N134A (14595A>G 14596A>C 14597T>C), T149V (14640A>G 14641C>T 14642G>C), S153A (14652T>G), A156L (14661G>C 14662C>T 14663C>G), I163V (14682A>G 14684C>G), N168Q (14697A>C 14699C>G), Q174V (14715C>G 14716A>T), L175M (14718T>A), G180A (14734G>C), I192V (14769A>G), A198V (14788C>T 14789A>G), A200V (14794C>T), S203G (14802T>G 14803C>G)								
Codon mutations:	AAT134GCC (14595A>G 14596A>C 14597T>C), GCC135GCG (14600C>G), CTG136CTC (14603G>C), AAT138AAC (14609T>C), ACG149GTC (14640A>G 14641C>T 14642G>C), CTG151CTC (14648G>C), TCG153GCG (14652T>G), GCC156CTG (14661G>C 14662C>T 14663C>G), ACC157ACG (14666C>G), TCG159AGC (14670T>A 14671C>G 14672G>C), GAC161GAT (14678C>T), ATC163GTG (14682A>G 14684C>G), AAC168CAG (14697A>C 14699C>G), CAG174GTG (14715C>G 14716A>T), TTG175ATG (14718T>A), GTC176GTT (14723C>T), GTG178GTC (14729G>C), GGC180GCC (14734G>C), GTG186GTC (14753G>C), GTT188GTG (14759T>G), ATC192GTC (14769A>G), CTG195CTC (14780G>C), ACT196ACC (14783T>C), GCA198GTG (14788C>T 14789A>G), GCC200GTC (14794C>T), GAC201GAT (14798C>T), TCC203GGC (14802T>G 14803C>G)								
KMC43_gp36	89	162	14.5%	317	67.3%	74 (100%)	49 (66.2%)	0/0/0/0	0
Protein mutations:	R92K (19482C>A 19483G>A 19484C>A), C93A (19485T>G 19486G>C), T102V (19512A>G 19513C>T), T106I (19525C>T 19526G>C), I107A (19527A>G 19528T>C 19529C>G), Q109L (19534A>T), G112C (19542G>T), V113I (19545G>A 19547G>C), L115F (19551C>T 19553G>C), A117S (19557G>T 19559G>C), L123F (19575C>T 19577G>C), S126G (19584T>G 19585C>G 19586G>C), V127I (19587G>A 19589G>C), A130T (19596G>A), A132S (19602G>T 19604C>G), A136Q (19614G>C 19615C>A 19616C>G), N138S (19620A>T 19621A>C 19622T>G), W140Q (19626T>C 19627G>A), V142A (19633T>C 19634A>C), T143V (19635A>G 19636C>T), A145S (19641G>T), T148G (19650A>G 19651C>G 19652G>C), N150V (19656A>G 19657A>T 19658C>G), V153A (19666T>C 19667T>C), A159S (19683G>T 19685T>G)								
Codon mutations:	CGC92AAA (19482C>A 19483G>A 19484C>A), TGC93GCC (19485T>G 19486G>C), CGC95CGG (19493C>G), GTC96GTG (19496C>G), ACG102GTG (19512A>G 19513C>T), GCG104GCC (19520G>C), TCG105AGC (19521T>A 19522C>G 19523G>C), ACG106ATC (19525C>T 19526G>C), ATC107GCG (19527A>G 19528T>C 19529C>G), CAG109CTG (19534A>T), GGC112TGC (19542G>T), GTG113ATC (19545G>A 19547G>C), ACG114ACC (19550G>C), CTG115TTC (19551C>T 19553G>C), GCG117TCC (19557G>T 19559G>C), ACG122ACC (19574G>C), CTG123TTC (19575C>T 19577G>C), TCG126GGC (19584T>G 19585C>G 19586G>C), GTG127ATC (19587G>A 19589G>C), GTC129GTG (19595C>G), GCG130ACG (19596G>A), CTC131CTG (19601C>G), GCC132TCG (19602G>T 19604C>G), GCG133GCC (19607G>C), AGC135TCG (19611A>T 19612G>C 19613C>G), GCC136CAG (19614G>C 19615C>A 19616C>G), AAT138TCG (19620A>T 19621A>C 19622T>G), TGG140CAG (19626T>C 19627G>A), GTA142GCC (19633T>C 19634A>C), ACG143GTG (19635A>G 19636C>T), GTC144GTT (19640C>T), GCG145TCG (19641G>T), ACG148GGC (19650A>G 19651C>G 19652G>C), CTA149TTG (19653C>T 19655A>G), AAC150GTG (19656A>G 19657A>T 19658C>G), GTT153GCC (19666T>C 19667T>C), GCT159TCG (19683G>T 19685T>G)								
KMC43_gp37	13	99	60.4%	399	66.6%	87 (100%)	54 (62.1%)	0/0/0/0	0
Protein mutations:	V15T (20840G>A 20841T>C), Q17V (20846C>G 20847A>T), S19A (20852T>G 20854A>C), T20S (20856C>G 20857T>C), T22P (20861A>C), Q24N (20867C>A 20869G>C), P25L (20871C>T), N26S (20874A>G), K27L (20876A>C 20877A>T), S30Q (20885T>C 20886C>A 20887T>G), K32Q (20891A>C), K34A (20897A>G 20898A>C), Q35P (20901A>C 20902G>C), V37M (20906G>A), T38A (20909A>G), D39E (20914T>G), V40I (20915G>A 20917G>C), R41K (20918C>A 20919G>A 20920C>G), V42I (20921G>A), R44G (20927C>G 20929C>T), D50R (20945G>C 20946A>G), V52A (20952T>C), S59R (20972T>C 20973C>G 20974T>C), A63N (20984G>A 20985C>A 20986G>C), I64V (20987A>G 20989T>G), A69S (21002G>A 21003C>G 21004G>C), A70T (21005G>A 21007G>A), N73D (21014A>G), S80A (21035T>G 21037G>C), S89Q (21062T>C 21063C>A), Q92T (21071C>A 21072A>C), Y94T (21077T>A 21078A>C 21079T>C)								
Codon mutations:	CTG14CTC (20839G>C), GTC15ACC (20840G>A 20841T>C), CAG17GTG (20846C>G 20847A>T), TCA19GCC (20852T>G 20854A>C), ACT20AGC (20856C>G 20857T>C), ACG22CCG (20861A>C), CTG23CTC (20866G>C), CAG24AAC (20867C>A 20869G>C), CCC25CTC (20871C>T), AAC26AGC (20874A>G), AAG27CTG (20876A>C 20877A>T), ACC29ACG (20884C>G), TCT30CAG (20885T>C 20886C>A 20887T>G), AAG32CAG (20891A>C), AGC33TCG (20894A>T 20895G>C 20896C>G), AAG34GCG (20897A>G 20898A>C), CAG35CCC (20901A>C 20902G>C), GAT36GAC (20905T>C), GTG37ATG (20906G>A), ACC38GCC (20909A>G), GAT39GAG (20914T>G), GTG40ATC (20915G>A 20917G>C), CGC41AAG (20918C>A 20919G>A 20920C>G), GTC42ATC (20921G>A), CGC44GGT (20927C>G 20929C>T), GAC50CGC (20945G>C 20946A>G), CAC51CAT (20950C>T), GTT52GCT (20952T>C), TTT54TTC (20959T>C), TCT59CGC (20972T>C 20973C>G 20974T>C), TCG61AGC (20978T>A 20979C>G 20980G>C), GCG63AAC (20984G>A 20985C>A 20986G>C), ATT64GTG (20987A>G 20989T>G), GAA65GAG (20992A>G), CAA67CAG (20998A>G), GCG69AGC (21002G>A 21003C>G 21004G>C), GCG70ACA (21005G>A 21007G>A), AAC73GAC (21014A>G), CAA77CAG (21028A>G), GAA79GAG (21034A>G), TCG80GCC (21035T>G 21037G>C), TCG89CAG (21062T>C 21063C>A), CCC90CCG (21067C>G), CAG92ACG (21071C>A 21072A>C), ATT93ATC (21076T>C), TAT94ACC (21077T>A 21078A>C 21079T>C), ACG96ACC (21085G>C)								
KMC43_gp43	71	122	13.9%	262	73.8%	52 (100%)	38 (73.1%)	0/0/0/0	0
Protein mutations:	F74L (25635T>C 25637C>G), D80N (25653G>A), T82A (25659A>G), N83D (25662A>G), Y84P (25665T>C 25666A>C 25667C>G), E86A (25672A>C 25673G>C), T87N (25675C>A 25676G>C), D88E (25679C>A), T90S (25683A>T 25685C>G), Y102F (25720A>T), L108M (25737C>A), T110S (25744C>G), A119V (25771C>T 25772G>C)								
Codon mutations:	ATT73ATC (25634T>C), TTC74CTG (25635T>C 25637C>G), TCC79AGC (25650T>A 25651C>G), GAT80AAT (25653G>A), ACG82GCG (25659A>G), AAC83GAC (25662A>G), TAC84CCG (25665T>C 25666A>C 25667C>G), GAG86GCC (25672A>C 25673G>C), ACG87AAC (25675C>A 25676G>C), GAC88GAA (25679C>A), CTC89CTG (25682C>G), ACC90TCG (25683A>T 25685C>G), CTG92CTC (25691G>C), TAT94TAC (25697T>C), GGG95GGC (25700G>C), GTG97GTC (25706G>C), GAT98GAC (25709T>C), TAC102TTC (25720A>T), GAC103GAT (25724C>T), CCC104CCG (25727C>G), TCG106TCC (25733G>C), CTG108ATG (25737C>A), ACC110AGC (25744C>G), GCG119GTC (25771C>T 25772G>C)								
KMC43_gp46	157	375	57.3%	867	68.1%	215 (100%)	133 (61.9%)	0/0/0/0	1

	Begin	End	Coverage	Score	Concordance	Matches	Identities	I/D/M/F*	Stop Codons
**NT**	**14591**	**28832**	**3.5%**	**1249**	**38.8%**	**1626 (98.8%)**	**1137 (69.1%)**	**3/17**	
Protein mutations:	G159A (28053G>C 28054C>G), V162A (28062T>C), A163I (28064G>A 28065C>T 28066T>C), G164A (28068G>C), V166I (28073G>A 28075A>C), S167N (28076T>A 28077C>A 28078G>C), I169L (28082A>C), V170G (28086T>G), G171Q (28088G>C 28089G>A 28090C>G), S174A (28097T>G 28099C>G), N187T (28137A>C 28138C>G), A193T (28154G>A 28156A>G), L197F (28166C>T 28168G>C), T199S (28172A>T), I202V (28181A>G), A203G (28185C>G), V205I (28190G>A 28192A>C), A206N (28193G>A 28194C>A 28195G>C), K213R (28215A>G 28216A>G), S214A (28217T>G 28219C>T), V216I (28223G>A), Y218N (28229T>A), T221L (28238A>C 28239C>T 28240C>G), S222A (28241T>G 28243G>C), L223I (28244C>A 28246G>C), G226D (28254G>A 28255A>C), L227I (28256C>A 28258G>C), S228N (28260G>A), L232T (28271C>A 28272T>C 28273T>C), N234G (28277A>G 28278A>G), Q235Y (28280C>T 28282G>T), T236A (28283A>G 28285G>C), A238G (28290C>G), V240A (28296T>C), T241A (28298A>G), M243L (28304A>C), N245Y (28310A>T), V247Y (28316G>T 28317T>A 28318G>C), I249V (28322A>G 28324C>G), S256A (28343T>G), L261F (28358C>T 28360T>C), S264T (28367T>A 28369A>C), L275V (28400C>G 28402A>C), C276G (28403T>G 28405C>T), S278T (28410G>C), V286L (28433G>C 28435G>T), N288T (28440A>C 28441T>G), S290N (28445T>A 28446C>A 28447T>C), T294A (28457A>G 28459G>C), A295T (28460G>A 28462G>C), I297T (28467T>C 28468C>G), A299S (28472G>A 28473C>G 28474G>C), D302N (28481G>A), T303H (28484A>C 28485C>A 28486G>C), V304A (28488T>C 28489G>C), T306I (28494C>T 28495G>C), K307V (28496A>G 28497A>T 28498G>C), I310V (28505A>G 28507C>G), V311Q (28508G>C 28509T>A 28510G>A), T312A (28511A>G), L314V (28517C>G), K315Q (28520A>C), T316S (28523A>T 28525G>C), A326T (28553G>A 28555A>G), S330T (28565T>A 28567G>C), R331K (28568C>A 28569G>A), A333S (28574G>T 28576C>G), V335L (28580G>C 28582G>C), D338G (28590A>G), G342A (28602G>C 28603C>A), I346V (28613A>G), V349I (28622G>A 28624G>C), S353G (28634A>G), A359T (28652G>A 28654T>G), S362N (28662G>A), L363Q (28665T>A 28666T>G), W368A (28679T>G 28680G>C 28681G>C), S369G (28682T>G 28683C>G 28684G>C), A370T (28685G>A 28687A>G), V371I (28688G>A), A374T (28697G>A 28699A>C)								
Codon mutations:	GCT158GCG (28051T>G), GGC159GCG (28053G>C 28054C>G), GTG162GCG (28062T>C), GCT163ATC (28064G>A 28065C>T 28066T>C), GGC164GCC (28068G>C), GCC165GCG (28072C>G), GTA166ATC (28073G>A 28075A>C), TCG167AAC (28076T>A 28077C>A 28078G>C), ACC168ACG (28081C>G), ATC169CTC (28082A>C), GTC170GGC (28086T>G), GGC171CAG (28088G>C 28089G>A 28090C>G), GCA172GCC (28093A>C), ATT173ATC (28096T>C), TCC174GCG (28097T>G 28099C>G), GAT177GAC (28108T>C), GTC179GTG (28114C>G), ACG180ACC (28117G>C), GCC182GCG (28123C>G), GCA183GCG (28126A>G), AAC187ACG (28137A>C 28138C>G), GGC188GGT (28141C>T), GAT190GAC (28147T>C), GCA193ACG (28154G>A 28156A>G), GAC194GAT (28159C>T), GCT195GCC (28162T>C), GCG196GCC (28165G>C), CTG197TTC (28166C>T 28168G>C), CGT198CGC (28171T>C), ACG199TCG (28172A>T), CGC200CGG (28177C>G), ATC202GTC (28181A>G), GCC203GGC (28185C>G), GTA205ATC (28190G>A 28192A>C), GCG206AAC (28193G>A 28194C>A 28195G>C), AAG210AAA (28207G>A), ACG212ACC (28213G>C), AAA213AGG (28215A>G 28216A>G), TCC214GCT (28217T>G 28219C>T), GCT215GCC (28222T>C), GTC216ATC (28223G>A), TAT218AAT (28229T>A), ACC221CTG (28238A>C 28239C>T 28240C>G), TCG222GCC (28241T>G 28243G>C), CTG223ATC (28244C>A 28246G>C), GGA226GAC (28254G>A 28255A>C), CTG227ATC (28256C>A 28258G>C), AGC228AAC (28260G>A), TAT229TAC (28264T>C), CTG231CTC (28270G>C), CTT232ACC (28271C>A 28272T>C 28273T>C), AAC234GGC (28277A>G 28278A>G), CAG235TAT (28280C>T 28282G>T), ACG236GCC (28283A>G 28285G>C), GCC238GGC (28290C>G), GTG240GCG (28296T>C), ACG241GCG (28298A>G), ATG243CTG (28304A>C), GGT244GGC (28309T>C), AAC245TAC (28310A>T), GTG247TAC (28316G>T 28317T>A 28318G>C), GTC248GTG (28321C>G), ATC249GTG (28322A>G 28324C>G), GGA253GGC (28336A>C), ACG254ACC (28339G>C), GGC255GGT (28342C>T), TCG256GCG (28343T>G), CCA257CCG (28348A>G), TCG258TCC (28351G>C), ACG260ACC (28357G>C), CTT261TTC (28358C>T 28360T>C), TCG263TCC (28366G>C), TCA264ACC (28367T>A 28369A>C), GTT265GTC (28372T>C), TCG266AGC (28373T>A 28374C>G 28375G>C), GCT268GCC (28381T>C), GCT271GCC (28390T>C), CCA274CCG (28399A>G), CTA275GTC (28400C>G 28402A>C), TGC276GGT (28403T>G 28405C>T), AGC277TCG (28406A>T 28407G>C 28408C>G), AGC278ACC (28410G>C), GCT283GCG (28426T>G), CCG284CCT (28429G>T), GTG286CTT (28433G>C 28435G>T), GTT287GTG (28438T>G), AAT288ACG (28440A>C 28441T>G), TCT290AAC (28445T>A 28446C>A 28447T>C), ACG294GCC (28457A>G 28459G>C), GCG295ACC (28460G>A 28462G>C), ACC296ACT (28465C>T), ATC297ACG (28467T>C 28468C>G), GCG299AGC (28472G>A 28473C>G 28474G>C), TAT301TAC (28480T>C), GAC302AAC (28481G>A), ACG303CAC (28484A>C 28485C>A 28486G>C), GTG304GCC (28488T>C 28489G>C), GCG305GCC (28492G>C), ACG306ATC (28494C>T 28495G>C), AAG307GTC (28496A>G 28497A>T 28498G>C), GCG308GCT (28501G>T), TTG309CTG (28502T>C), ATC310GTG (28505A>G 28507C>G), GTG311CAA (28508G>C 28509T>A 28510G>A), ACC312GCC (28511A>G), GCA313GCC (28516A>C), CTG314GTG (28517C>G), AAG315CAG (28520A>C), ACG316TCC (28523A>T 28525G>C), TAT317TAC (28528T>C), GCA326ACG (28553G>A 28555A>G), CCG328CCC (28561G>C), TAT329TAC (28564T>C), TCG330ACC (28565T>A 28567G>C), CGG331AAG (28568C>A 28569G>A), GCC333TCG (28574G>T 28576C>G), CAA334CAG (28579A>G), GTG335CTC (28580G>C 28582G>C), GCT336GCC (28585T>C), TAT337TAC (28588T>C), GAC338GGC (28590A>G), GCT339GCG (28594T>G), CCT341CCG (28600T>G), GGC342GCA (28602G>C 28603C>A), ACG344ACC (28609G>C), ATC346GTC (28613A>G), ACG347ACC (28618G>C), GTG349ATC (28622G>A 28624G>C), AGC353GGC (28634A>G), GCG356GCT (28645G>T), GAT357GAC (28648T>C), GCT359ACG (28652G>A 28654T>G), GCG360GCC (28657G>C), ACG361ACC (28660G>C), AGT362AAT (28662G>A), CTT363CAG (28665T>A 28666T>G), CAG364CAA (28669G>A), ATA366ATC (28675A>C), AAA367AAG (28678A>G), TGG368GCC (28679T>G 28680G>C 28681G>C), TCG369GGC (28682T>G 28683C>G 28684G>C), GCA370ACG (28685G>A 28687A>G), GTC371ATC (28688G>A), AGC372TCG (28691A>T 28692G>C 28693C>G), GCA374ACC (28697G>A 28699A>C)								
KMC43_gp47	1	36	14.8%	124	50.6%	35 (94.6%)	16 (43.2%)	1/1/0/0	0
Protein mutations:	S2A (28729A>G 28730G>C 28731C>A), V3I (28732G>A 28734T>C), Q7S (28744C>A 28745A>G 28746A>C), V9I (28750G>A), Y10S (28753T>A 28754A>G), Q11T (28756C>A 28757A>C), G15Q (28768G>C 28769G>A 28770C>G), Y16L (28771T>C 28772A>T), L17I (28774C>A 28776G>C), R19G (28780C>G), R19_W20insG (28782_28783insGGG), G22del (28789_28791delGGC), D23Q (28792G>C 28794T>G), A24Q (28795G>C 28796C>A 28797G>A), A25G (28799C>G 28800G>C), Q26L (28802A>T 28803G>C), S27V (28804T>G 28805C>T), V29I (28810G>A), L30R (28814T>G 28815G>C), T31D (28816A>G 28817C>A)								
Codon mutations:	AGC2GCA (28729A>G 28730G>C 28731C>A), GTT3ATC (28732G>A 28734T>C), GGC4GGT (28737C>T), GAT5GAC (28740T>C), CAA6CAG (28743A>G), CAA7AGC (28744C>A 28745A>G 28746A>C), GAT8GAC (28749T>C), GTC9ATC (28750G>A), TAC10AGC (28753T>A 28754A>G), CAG11ACG (28756C>A 28757A>C), CGC14CGG (28767C>G), GGC15CAG (28768G>C 28769G>A 28770C>G), TAC16CTC (28771T>C 28772A>T), CTG17ATC (28774C>A 28776G>C), CCT18CCG (28779T>G), CGC19GGC (28780C>G), CGC19_TGG20insGGG (28782_28783insGGG), GGC22del (28789_28791delGGC), GAT23CAG (28792G>C 28794T>G), GCG24CAA (28795G>C 28796C>A 28797G>A), GCG25GGC (28799C>G 28800G>C), CAG26CTC (28802A>T 28803G>C), TCG27GTG (28804T>G 28805C>T), CCA28CCG (28809A>G), GTC29ATC (28810G>A), CTG30CGC (28814T>G 28815G>C), ACC31GAC (28816A>G 28817C>A), CAG35CAA (28830G>A)								

Proteins

DUF4043 domain-containing protein (YP_010078151.1)	133	207	16.9%	411	85.8%	75 (100%)	62 (82.7%)	0/0/0/0	0
Protein mutations:	N134A (14595A>G 14596A>C 14597T>C), T149V (14640A>G 14641C>T 14642G>C), S153A (14652T>G), A156L (14661G>C 14662C>T 14663C>G), I163V (14682A>G 14684C>G), N168Q (14697A>C 14699C>G), Q174V (14715C>G 14716A>T), L175M (14718T>A), G180A (14734G>C), I192V (14769A>G), A198V (14788C>T 14789A>G), A200V (14794C>T), S203G (14802T>G 14803C>G)								
Codon mutations:	AAT134GCC (14595A>G 14596A>C 14597T>C), GCC135GCG (14600C>G), CTG136CTC (14603G>C), AAT138AAC (14609T>C), ACG149GTC (14640A>G 14641C>T 14642G>C), CTG151CTC (14648G>C), TCG153GCG (14652T>G), GCC156CTG (14661G>C 14662C>T 14663C>G), ACC157ACG (14666C>G), TCG159AGC (14670T>A 14671C>G 14672G>C), GAC161GAT (14678C>T), ATC163GTG (14682A>G 14684C>G), AAC168CAG (14697A>C 14699C>G), CAG174GTG (14715C>G 14716A>T), TTG175ATG (14718T>A), GTC176GTT (14723C>T), GTG178GTC (14729G>C), GGC180GCC (14734G>C), GTG186GTC (14753G>C), GTT188GTG (14759T>G), ATC192GTC (14769A>G), CTG195CTC (14780G>C), ACT196ACC (14783T>C), GCA198GTG (14788C>T 14789A>G), GCC200GTC (14794C>T), GAC201GAT (14798C>T), TCC203GGC (14802T>G 14803C>G)								
tail protein (YP_010078160.1)	89	162	14.5%	317	67.3%	74 (100%)	49 (66.2%)	0/0/0/0	0
Protein mutations:	R92K (19482C>A 19483G>A 19484C>A), C93A (19485T>G 19486G>C), T102V (19512A>G 19513C>T), T106I (19525C>T 19526G>C), I107A (19527A>G 19528T>C 19529C>G), Q109L (19534A>T), G112C (19542G>T), V113I (19545G>A 19547G>C), L115F (19551C>T 19553G>C), A117S (19557G>T 19559G>C), L123F (19575C>T 19577G>C), S126G (19584T>G 19585C>G 19586G>C), V127I (19587G>A 19589G>C), A130T (19596G>A), A132S (19602G>T 19604C>G), A136Q (19614G>C 19615C>A 19616C>G), N138S (19620A>T 19621A>C 19622T>G), W140Q (19626T>C 19627G>A), V142A (19633T>C 19634A>C), T143V (19635A>G 19636C>T), A145S (19641G>T), T148G (19650A>G 19651C>G 19652G>C), N150V (19656A>G 19657A>T 19658C>G), V153A (19666T>C 19667T>C), A159S (19683G>T 19685T>G)								
Codon mutations:	CGC92AAA (19482C>A 19483G>A 19484C>A), TGC93GCC (19485T>G 19486G>C), CGC95CGG (19493C>G), GTC96GTG (19496C>G), ACG102GTG (19512A>G 19513C>T), GCG104GCC (19520G>C), TCG105AGC (19521T>A 19522C>G 19523G>C), ACG106ATC (19525C>T 19526G>C), ATC107GCG (19527A>G 19528T>C 19529C>G), CAG109CTG (19534A>T), GGC112TGC (19542G>T), GTG113ATC (19545G>A 19547G>C), ACG114ACC (19550G>C), CTG115TTC (19551C>T 19553G>C), GCG117TCC (19557G>T 19559G>C), ACG122ACC (19574G>C), CTG123TTC (19575C>T 19577G>C), TCG126GGC (19584T>G 19585C>G 19586G>C), GTG127ATC (19587G>A 19589G>C), GTC129GTG (19595C>G), GCG130ACG (19596G>A), CTC131CTG (19601C>G), GCC132TCG (19602G>T 19604C>G), GCG133GCC (19607G>C), AGC135TCG (19611A>T 19612G>C 19613C>G), GCC136CAG (19614G>C 19615C>A 19616C>G), AAT138TCG (19620A>T 19621A>C 19622T>G), TGG140CAG (19626T>C 19627G>A), GTA142GCC (19633T>C 19634A>C), ACG143GTG (19635A>G 19636C>T), GTC144GTT (19640C>T), GCG145TCG (19641G>T), ACG148GGC (19650A>G 19651C>G 19652G>C), CTA149TTG (19653C>T 19655A>G), AAC150GTG (19656A>G 19657A>T 19658C>G), GTT153GCC (19666T>C 19667T>C), GCT159TCG (19683G>T 19685T>G)								
hypothetical protein (YP_010078161.1)	13	99	60.4%	399	66.6%	87 (100%)	54 (62.1%)	0/0/0/0	0
Protein mutations:	V15T (20840G>A 20841T>C), Q17V (20846C>G 20847A>T), S19A (20852T>G 20854A>C), T20S (20856C>G 20857T>C), T22P (20861A>C), Q24N (20867C>A 20869G>C), P25L (20871C>T), N26S (20874A>G), K27L (20876A>C 20877A>T), S30Q (20885T>C 20886C>A 20887T>G), K32Q (20891A>C), K34A (20897A>G 20898A>C), Q35P (20901A>C 20902G>C), V37M (20906G>A), T38A (20909A>G), D39E (20914T>G), V40I (20915G>A 20917G>C), R41K (20918C>A 20919G>A 20920C>G), V42I (20921G>A), R44G (20927C>G 20929C>T), D50R (20945G>C 20946A>G), V52A (20952T>C), S59R (20972T>C 20973C>G 20974T>C), A63N (20984G>A 20985C>A 20986G>C), I64V (20987A>G 20989T>G), A69S (21002G>A 21003C>G 21004G>C), A70T (21005G>A 21007G>A), N73D (21014A>G), S80A (21035T>G 21037G>C), S89Q (21062T>C 21063C>A), Q92T (21071C>A 21072A>C), Y94T (21077T>A 21078A>C 21079T>C)								

	Begin	End	Coverage	Score	Concordance	Matches	Identities	I/D/M/F*	Stop Codons
**NT**	**14591**	**28832**	**3.5%**	**1249**	**38.8%**	**1626 (98.8%)**	**1137 (69.1%)**	**3/17**	
Codon mutations:	CTG14CTC (20839G>C), GTC15ACC (20840G>A 20841T>C), CAG17GTG (20846C>G 20847A>T), TCA19GCC (20852T>G 20854A>C), ACT20AGC (20856C>G 20857T>C), ACG22CCG (20861A>C), CTG23CTC (20866G>C), CAG24AAC (20867C>A 20869G>C), CCC25CTC (20871C>T), AAC26AGC (20874A>G), AAG27CTG (20876A>C 20877A>T), ACC29ACG (20884C>G), TCT30CAG (20885T>C 20886C>A 20887T>G), AAG32CAG (20891A>C), AGC33TCG (20894A>T 20895G>C 20896C>G), AAG34GCG (20897A>G 20898A>C), CAG35CCC (20901A>C 20902G>C), GAT36GAC (20905T>C), GTG37ATG (20906G>A), ACC38GCC (20909A>G), GAT39GAG (20914T>G), GTG40ATC (20915G>A 20917G>C), CGC41AAG (20918C>A 20919G>A 20920C>G), GTC42ATC (20921G>A), CGC44GGT (20927C>G 20929C>T), GAC50CGC (20945G>C 20946A>G), CAC51CAT (20950C>T), GTT52GCT (20952T>C), TTT54TTC (20959T>C), TCT59CGC (20972T>C 20973C>G 20974T>C), TCG61AGC (20978T>A 20979C>G 20980G>C), GCG63AAC (20984G>A 20985C>A 20986G>C), ATT64GTG (20987A>G 20989T>G), GAA65GAG (20992A>G), CAA67CAG (20998A>G), GCG69AGC (21002G>A 21003C>G 21004G>C), GCG70ACA (21005G>A 21007G>A), AAC73GAC (21014A>G), CAA77CAG (21028A>G), GAA79GAG (21034A>G), TCG80GCC (21035T>G 21037G>C), TCG89CAG (21062T>C 21063C>A), CCC90CCG (21067C>G), CAG92ACG (21071C>A 21072A>C), ATT93ATC (21076T>C), TAT94ACC (21077T>A 21078A>C 21079T>C), ACG96ACC (21085G>C)								
hypothetical protein (YP_010078167.1)	71	122	13.9%	262	73.8%	52 (100%)	38 (73.1%)	0/0/0/0	0
Protein mutations:	F74L (25635T>C 25637C>G), D80N (25653G>A), T82A (25659A>G), N83D (25662A>G), Y84P (25665T>C 25666A>C 25667C>G), E86A (25672A>C 25673G>C), T87N (25675C>A 25676G>C), D88E (25679C>A), T90S (25683A>T 25685C>G), Y102F (25720A>T), L108M (25737C>A), T110S (25744C>G), A119V (25771C>T 25772G>C)								
Codon mutations:	ATT73ATC (25634T>C), TTC74CTG (25635T>C 25637C>G), TCC79AGC (25650T>A 25651C>G), GAT80AAT (25653G>A), ACG82GCG (25659A>G), AAC83GAC (25662A>G), TAC84CCG (25665T>C 25666A>C 25667C>G), GAG86GCC (25672A>C 25673G>C), ACG87AAC (25675C>A 25676G>C), GAC88GAA (25679C>A), CTC89CTG (25682C>G), ACC90TCG (25683A>T 25685C>G), CTG92CTC (25691G>C), TAT94TAC (25697T>C), GGG95GGC (25700G>C), GTG97GTC (25706G>C), GAT98GAC (25709T>C), TAC102TTC (25720A>T), GAC103GAT (25724C>T), CCC104CCG (25727C>G), TCG106TCC (25733G>C), CTG108ATG (25737C>A), ACC110AGC (25744C>G), GCG119GTC (25771C>T 25772G>C)								
hypothetical protein (YP_010078170.1)	157	375	57.3%	867	68.1%	215 (100%)	133 (61.9%)	0/0/0/0	1
Protein mutations:	G159A (28053G>C 28054C>G), V162A (28062T>C), A163I (28064G>A 28065C>T 28066T>C), G164A (28068G>C), V166I (28073G>A 28075A>C), S167N (28076T>A 28077C>A 28078G>C), I169L (28082A>C), V170G (28086T>G), G171Q (28088G>C 28089G>A 28090C>G), S174A (28097T>G 28099C>G), N187T (28137A>C 28138C>G), A193T (28154G>A 28156A>G), L197F (28166C>T 28168G>C), T199S (28172A>T), I202V (28181A>G), A203G (28185C>G), V205I (28190G>A 28192A>C), A206N (28193G>A 28194C>A 28195G>C), K213R (28215A>G 28216A>G), S214A (28217T>G 28219C>T), V216I (28223G>A), Y218N (28229T>A), T221L (28238A>C 28239C>T 28240C>G), S222A (28241T>G 28243G>C), L223I (28244C>A 28246G>C), G226D (28254G>A 28255A>C), L227I (28256C>A 28258G>C), S228N (28260G>A), L232T (28271C>A 28272T>C 28273T>C), N234G (28277A>G 28278A>G), Q235Y (28280C>T 28282G>T), T236A (28283A>G 28285G>C), A238G (28290C>G), V240A (28296T>C), T241A (28298A>G), M243L (28304A>C), N245Y (28310A>T), V247Y (28316G>T 28317T>A 28318G>C), I249V (28322A>G 28324C>G), S256A (28343T>G), L261F (28358C>T 28360T>C), S264T (28367T>A 28369A>C), L275V (28400C>G 28402A>C), C276G (28403T>G 28405C>T), S278T (28410G>C), V286L (28433G>C 28435G>T), N288T (28440A>C 28441T>G), S290N (28445T>A 28446C>A 28447T>C), T294A (28457A>G 28459G>C), A295T (28460G>A 28462G>C), I297T (28467T>C 28468C>G), A299S (28472G>A 28473C>G 28474G>C), D302N (28481G>A), T303H (28484A>C 28485C>A 28486G>C), V304A (28488T>C 28489G>C), T306I (28494C>T 28495G>C), K307V (28496A>G 28497A>T 28498G>C), I310V (28505A>G 28507C>G), V311Q (28508G>C 28509T>A 28510G>A), T312A (28511A>G), L314V (28517C>G), K315Q (28520A>C), T316S (28523A>T 28525G>C), A326T (28553G>A 28555A>G), S330T (28565T>A 28567G>C), R331K (28568C>A 28569G>A), A333S (28574G>T 28576C>G), V335L (28580G>C 28582G>C), D338G (28590A>G), G342A (28602G>C 28603C>A), I346V (28613A>G), V349I (28622G>A 28624G>C), S353G (28634A>G), A359T (28652G>A 28654T>G), S362N (28662G>A), L363Q (28665T>A 28666T>G), W368A (28679T>G 28680G>C 28681G>C), S369G (28682T>G 28683C>G 28684G>C), A370T (28685G>A 28687A>G), V371I (28688G>A), A374T (28697G>A 28699A>C)								
Codon mutations:	GCT158GCG (28051T>G), GGC159GCG (28053G>C 28054C>G), GTG162GCG (28062T>C), GCT163ATC (28064G>A 28065C>T 28066T>C), GGC164GCC (28068G>C), GCC165GCG (28072C>G), GTA166ATC (28073G>A 28075A>C), TCG167AAC (28076T>A 28077C>A 28078G>C), ACC168ACG (28081C>G), ATC169CTC (28082A>C), GTC170GGC (28086T>G), GGC171CAG (28088G>C 28089G>A 28090C>G), GCA172GCC (28093A>C), ATT173ATC (28096T>C), TCC174GCG (28097T>G 28099C>G), GAT177GAC (28108T>C), GTC179GTG (28114C>G), ACG180ACC (28117G>C), GCC182GCG (28123C>G), GCA183GCG (28126A>G), AAC187ACG (28137A>C 28138C>G), GGC188GGT (28141C>T), GAT190GAC (28147T>C), GCA193ACG (28154G>A 28156A>G), GAC194GAT (28159C>T), GCT195GCC (28162T>C), GCG196GCC (28165G>C), CTG197TTC (28166C>T 28168G>C), CGT198CGC (28171T>C), ACG199TCG (28172A>T), CGC200CGG (28177C>G), ATC202GTC (28181A>G), GCC203GGC (28185C>G), GTA205ATC (28190G>A 28192A>C), GCG206AAC (28193G>A 28194C>A 28195G>C), AAG210AAA (28207G>A), ACG212ACC (28213G>C), AAA213AGG (28215A>G 28216A>G), TCC214GCT (28217T>G 28219C>T), GCT215GCC (28222T>C), GTC216ATC (28223G>A), TAT218AAT (28229T>A), ACC221CTG (28238A>C 28239C>T 28240C>G), TCG222GCC (28241T>G 28243G>C), CTG223ATC (28244C>A 28246G>C), GGA226GAC (28254G>A 28255A>C), CTG227ATC (28256C>A 28258G>C), AGC228AAC (28260G>A), TAT229TAC (28264T>C), CTG231CTC (28270G>C), CTT232ACC (28271C>A 28272T>C 28273T>C), AAC234GGC (28277A>G 28278A>G), CAG235TAT (28280C>T 28282G>T), ACG236GCC (28283A>G 28285G>C), GCC238GGC (28290C>G), GTG240GCG (28296T>C), ACG241GCG (28298A>G), ATG243CTG (28304A>C), GGT244GGC (28309T>C), AAC245TAC (28310A>T), GTG247TAC (28316G>T 28317T>A 28318G>C), GTC248GTG (28321C>G), ATC249GTG (28322A>G 28324C>G), GGA253GGC (28336A>C), ACG254ACC (28339G>C), GGC255GGT (28342C>T), TCG256GCG (28343T>G), CCA257CCG (28348A>G), TCG258TCC (28351G>C), ACG260ACC (28357G>C), CTT261TTC (28358C>T 28360T>C), TCG263TCC (28366G>C), TCA264ACC (28367T>A 28369A>C), GTT265GTC (28372T>C), TCG266AGC (28373T>A 28374C>G 28375G>C), GCT268GCC (28381T>C), GCT271GCC (28390T>C), CCA274CCG (28399A>G), CTA275GTC (28400C>G 28402A>C), TGC276GGT (28403T>G 28405C>T), AGC277TCG (28406A>T 28407G>C 28408C>G), AGC278ACC (28410G>C), GCT283GCG (28426T>G), CCG284CCT (28429G>T), GTG286CTT (28433G>C 28435G>T), GTT287GTG (28438T>G), AAT288ACG (28440A>C 28441T>G), TCT290AAC (28445T>A 28446C>A 28447T>C), ACG294GCC (28457A>G 28459G>C), GCG295ACC (28460G>A 28462G>C), ACC296ACT (28465C>T), ATC297ACG (28467T>C 28468C>G), GCG299AGC (28472G>A 28473C>G 28474G>C), TAT301TAC (28480T>C), GAC302AAC (28481G>A), ACG303CAC (28484A>C 28485C>A 28486G>C), GTG304GCC (28488T>C 28489G>C), GCG305GCC (28492G>C), ACG306ATC (28494C>T 28495G>C), AAG307GTC (28496A>G 28497A>T 28498G>C), GCG308GCT (28501G>T), TTG309CTG (28502T>C), ATC310GTG (28505A>G 28507C>G), GTG311CAA (28508G>C 28509T>A 28510G>A), ACC312GCC (28511A>G), GCA313GCC (28516A>C), CTG314GTG (28517C>G), AAG315CAG (28520A>C), ACG316TCC (28523A>T 28525G>C), TAT317TAC (28528T>C), GCA326ACG (28553G>A 28555A>G), CCG328CCC (28561G>C), TAT329TAC (28564T>C), TCG330ACC (28565T>A 28567G>C), CGG331AAG (28568C>A 28569G>A), GCC333TCG (28574G>T 28576C>G), CAA334CAG (28579A>G), GTG335CTC (28580G>C 28582G>C), GCT336GCC (28585T>C), TAT337TAC (28588T>C), GAC338GGC (28590A>G), GCT339GCG (28594T>G), CCT341CCG (28600T>G), GGC342GCA (28602G>C 28603C>A), ACG344ACC (28609G>C), ATC346GTC (28613A>G), ACG347ACC (28618G>C), GTG349ATC (28622G>A 28624G>C), AGC353GGC (28634A>G), GCG356GCT (28645G>T), GAT357GAC (28648T>C), GCT359ACG (28652G>A 28654T>G), GCG360GCC (28657G>C), ACG361ACC (28660G>C), AGT362AAT (28662G>A), CTT363CAG (28665T>A 28666T>G), CAG364CAA (28669G>A), ATA366ATC (28675A>C), AAA367AAG (28678A>G), TGG368GCC (28679T>G 28680G>C 28681G>C), TCG369GGC (28682T>G 28683C>G 28684G>C), GCA370ACG (28685G>A 28687A>G), GTC371ATC (28688G>A), AGC372TCG (28691A>T 28692G>C 28693C>G), GCA374ACC (28697G>A 28699A>C)								
hypothetical protein (YP_010078171.1)	1	36	14.8%	124	50.6%	35 (94.6%)	16 (43.2%)	1/1/0/0	0
Protein mutations:	S2A (28729A>G 28730G>C 28731C>A), V3I (28732G>A 28734T>C), Q7S (28744C>A 28745A>G 28746A>C), V9I (28750G>A), Y10S (28753T>A 28754A>G), Q11T (28756C>A 28757A>C), G15Q (28768G>C 28769G>A 28770C>G), Y16L (28771T>C 28772A>T), L17I (28774C>A 28776G>C), R19G (28780C>G), R19_W20insG (28782_28783insGGG), G22del (28789_28791delGGC), D23Q (28792G>C 28794T>G), A24Q (28795G>C 28796C>A 28797G>A), A25G (28799C>G 28800G>C), Q26L (28802A>T 28803G>C), S27V (28804T>G 28805C>T), V29I (28810G>A), L30R (28814T>G 28815G>C), T31D (28816A>G 28817C>A)								
Codon mutations:	AGC2GCA (28729A>G 28730G>C 28731C>A), GTT3ATC (28732G>A 28734T>C), GGC4GGT (28737C>T), GAT5GAC (28740T>C), CAA6CAG (28743A>G), CAA7AGC (28744C>A 28745A>G 28746A>C), GAT8GAC (28749T>C), GTC9ATC (28750G>A), TAC10AGC (28753T>A 28754A>G), CAG11ACG (28756C>A 28757A>C), CGC14CGG (28767C>G), GGC15CAG (28768G>C 28769G>A 28770C>G), TAC16CTC (28771T>C 28772A>T), CTG17ATC (28774C>A 28776G>C), CCT18CCG (28779T>G), CGC19GGC (28780C>G), CGC19_TGG20insGGG (28782_28783insGGG), GGC22del (28789_28791delGGC), GAT23CAG (28792G>C 28794T>G), GCG24CAA (28795G>C 28796C>A 28797G>A), GCG25GGC (28799C>G 28800G>C), CAG26CTC (28802A>T 28803G>C), TCG27GTG (28804T>G 28805C>T), CCA28CCG (28809A>G), GTC29ATC (28810G>A), CTG30CGC (28814T>G 28815G>C), ACC31GAC (28816A>G 28817C>A), CAG35CAA (28830G>A)								

*: Inserts / Deletes / Misaligned / Frameshifts

Analysis details

This analysis was performed with panviral2.64

NGS Details (UN18_val): Caulimovirus venafragariae

Assembly

**Coverage Length**	838 (1 contig(s))
**Depth Of Coverage**	4.8
**Number Of Reads**	30
**Reads Per Million**	0.56 rpm (after QC)
**Ambiguities**	0
**Assembly Method**	de novo + reference guided assembly
**Consensus Caller**	Bcf Tools

Coverage Map

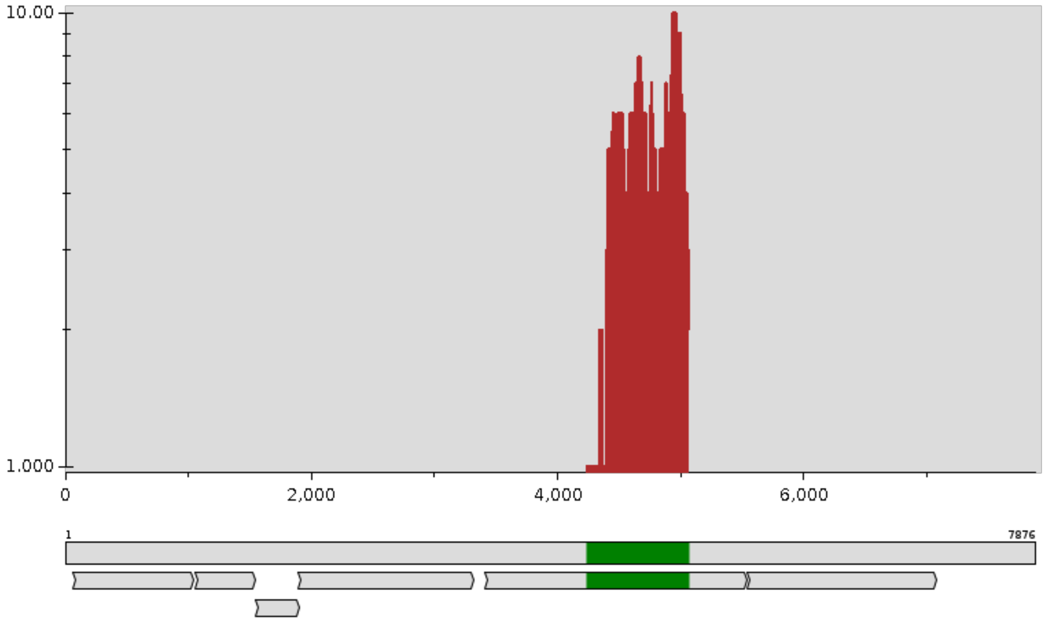

Assignment

**Type**	Caulimovirus venafragariae (Taxonomy ID: 3048344)
**Reference Genome**	NC_001725.1
**NT Identity (%)**	58.0723
**AA Identity (%)**	52.8986
**Number Of Stop Codons**	1
**Number Of CDS**	6

Alignment

**Alignment Score**	242.0 (NT) + 916.0 (AA) = 1158.0
**Concordance (%)**	33.7413

**Alignment Method**	Global, seeded, nucleotide + amino acids (AGA)

Genome Region

Sequence starts at position 4232 and ends at position 5069 relative to NC_001725.1 reference sequence.

Alignment Detailed Statistics

	Begin	End	Coverage	Score	Concordance	Matches	Identities	I/D/M/F*	Stop Codons
**NT**	**4232**	**5069**	**10.6%**	**242**	**14.8%**	**830 (99.0%)**	**482 (57.5%)**	**0/8**	
Mutations:	4233C>T, 4235A>T, 4239T>C, 4242T>A, 4244A>G, 4245G>A, 4246T>G, 4247G>T, 4249A>C, 4254A>C, 4258C>T, 4259A>T, 4260G>T, 4264T>A, 4265C>A, 4266A>C, 4267C>G, 4268C>A, 4270C>G, 4275C>T, 4276C>A, 4277G>A, 4279G>A, 4281A>T, 4284G>A, 4287T>C, 4289A>G, 4292C>T, 4299C>T, 4300G>C, 4302A>T, 4308A>T, 4314G>A, 4315C>A, 4317T>G, 4318G>A, 4319G>A, 4320T>G, 4321A>T, 4323C>A, 4330C>G, 4332T>A, 4337A>G, 4338G>A, 4347T>C, 4348T>A, 4349C>G, 4351T>A, 4352C>G, 4353A>T, 4356A>C, 4359C>G, 4371G>A, 4374T>C, 4380C>G, 4383G>A, 4384A>C, 4385T>A, 4386C>A, 4395C>T, 4398A>G, 4401A>T, 4402A>C, 4410A>T, 4413T>A, 4423A>G, 4426T>C, 4428A>T, 4432G>A, 4434C>A, 4435C>A, 4437T>C, 4438A>T, 4439C>G, 4440A>T, 4442A>T, 4443G>T, 4444G>T, 4445G>T, 4446A>T, 4449T>C, 4452C>A, 4456C>T, 4458A>T, 4459C>A, 4461T>C, 4467C>T, 4470G>A, 4471G>A, 4472A>C, 4473G>T, 4474C>A, 4476A>T, 4479G>T, 4480C>A, 4482T>A, 4483C>A, 4485A>T, 4486A>C, 4487G>T, 4489A>G, 4492G>A, 4493G>A, 4496G>T, 4497T>A, 4500G>A, 4505T>A, 4506T>C, 4508A>T, 4509C>T, 4512T>C, 4513T>A, 4514C>A, 4515C>A, 4521C>T, 4528T>A, 4529C>G, 4543G>A, 4546C>A, 4548C>A, 4551T>C, 4552G>T, 4554T>A, 4555C>G, 4556C>A, 4557A>G, 4561A>T, 4568A>C, 4569G>T, 4575C>A, 4578T>A, 4585T>A, 4586G>C, 4590C>T, 4591C>A, 4593A>T, 4599C>T, 4602C>T, 4614C>A, 4618C>T, 4620T>A, 4633C>A, 4635A>T, 4638C>A, 4641T>A, 4642G>C, 4643C>A, 4644T>A, 4650C>T, 4656G>A, 4657C>A, 4659C>A, 4663G>T, 4665C>T, 4666G>A, 4670G>T, 4671T>A, 4672C>T, 4674C>T, 4675_4679delAGCAA, 4680C>T, 4681A>C, 4684T>A, 4686T>A, 4687C>A, 4688C>A, 4689A>G, 4690C>A, 4692G>A, 4697G>T, 4698T>G, 4699G>A, 4700C>T, 4701T>A, 4707T>C, 4708G>A, 4710T>A, 4719C>T, 4720A>T, 4722C>G, 4725C>A, 4727T>G, 4729A>T, 4730G>C, 4731C>A, 4733A>C, 4737T>A, 4738G>T, 4740A>T, 4741G>C, 4743A>G, 4750T>C, 4752A>T, 4753G>A, 4754G>C, 4759G>C, 4761C>T, 4765A>G, 4766T>A, 4767T>A, 4768G>T, 4771C>T, 4772T>C, 4776C>A, 4777A>C, 4778G>T, 4779A>C, 4782C>T, 4783A>G, 4784A>T, 4785A>C, 4786G>A, 4787C>A, 4788C>T, 4789C>A, 4790T>A, 4791A>T, 4793G>A, 4794A>C, 4800G>A, 4801C>T, 4803T>A, 4806C>T, 4807A>C, 4809A>G, 4812G>A, 4818C>A, 4819C>G, 4821G>A, 4822T>A, 4824G>A, 4825T>A, 4826G>A, 4827C>T, 4832C>A, 4833A>T, 4835C>A, 4836T>A, 4839C>A, 4840A>G, 4842T>A, 4851T>A, 4852C>A, 4854A>G, 4855G>A, 4857A>T, 4860A>T, 4861G>T, 4862A>C, 4865G>A, 4867G>A, 4868G>A, 4869A>T, 4870A>G, 4871A>G, 4872T>A, 4873C>A, 4875C>A, 4876A>G, 4879G>T, 4881C>A, 4884G>A, 4886G>C, 4890T>C, 4893C>A, 4894G>T, 4895G>C, 4896A>C, 4897C>A, 4898T>A, 4900C>A, 4902C>G, 4903T>A, 4906G>T, 4908T>A, 4910C>A, 4911T>G, 4917A>T, 4920T>C, 4921C>A, 4923A>G, 4924C>T, 4926T>A, 4928G>T, 4929T>A, 4930G>A, 4931A>C, 4932T>C, 4934G>A, 4935G>A, 4936A>C, 4938T>A, 4940C>A, 4941C>A, 4942T>A, 4954T>C, 4956A>T, 4959C>A, 4960C>T, 4961T>G, 4963C>T, 4968T>C, 4972A>G, 4973T>C, 4974C>A, 4975T>G, 4976C>G, 4977C>A, 4979C>A, 4980T>A, 4982A>T, 4983T>C, 4984T>A, 4986T>A, 4990A>G, 4993A>T, 5001C>G, 5002C>A, 5003T>A, 5004T>G, 5007G>A, 5008T>A, 5009C>A, 5010A>T, 5012C>T, 5014T>C, 5016A>T, 5019G>A, 5020_5022delGTT, 5028T>C, 5030A>T, 5031G>C, 5033A>G, 5035G>A, 5037G>A, 5038A>T, 5039T>C, 5042C>A, 5043T>C, 5044T>C, 5046G>A, 5048C>T, 5052G>A, 5054C>G, 5056G>T, 5057A>T, 5058G>T, 5059A>T, 5060A>T, 5061A>C								

CDS

	Begin	End	Coverage	Score	Concordance	Matches	Identities	I/D/M/F*	Stop Codons
ORF_V	277	555	39.2%	916	47.2%	276 (99.3%)	146 (52.5%)	0/2/1/1	1
Protein mutations:	K277I (4235A>T), K280R (4244A>G 4245G>A), C281V (4246T>G 4247G>T), K282Q (4249A>C), Q285F (4258C>T 4259A>T 4260G>T), S287N (4264T>A 4265C>A 4266A>C), P288D (4267C>G 4268C>A), Q289E (4270C>G), R291K (4276C>A 4277G>A), E292N (4279G>A 4281A>T), K295R (4289A>G), T296I (4292C>T), E299H (4300G>C 4302A>T), L304M (4315C>A 4317T>G), G305K (4318G>A 4319G>A 4320T>G), I306L (4321A>T 4323C>A), P309A (4330C>G 4332T>A), K311R (4337A>G 4338G>A), I327Q (4384A>C 4385T>A 4386C>A), K340E (4423A>G), D343K (4432G>A 4434C>A), H344N (4435C>A 4437T>C), T345C (4438A>T 4439C>G 4440A>T), K346I (4442A>T 4443G>T), G347F (4444G>T 4445G>T 4446A>T), L351F (4456C>T 4458A>T), L352I (4459C>A 4461T>C), E356T (4471G>A 4472A>C 4473G>T), Q357N (4474C>A 4476A>T), L359I (4480C>A 4482T>A), Q360N (4483C>A 4485A>T), R361L (4486A>C 4487G>T), I362V (4489A>G), G363K (4492G>A 4493G>A), G364V (4496G>T 4497T>A), F367Y (4505T>A 4506T>C), Y368F (4508A>T 4509C>T), S370K (4513T>A 4514C>A 4515C>A), V380I (4543G>A), A383S (4552G>T 4554T>A), P384E (4555C>G 4556C>A 4557A>G), T386S (4561A>T), Q388P (4568A>C 4569G>T), C394T (4585T>A 4586G>C), Q396N (4591C>A 4593A>T), P405S (4618C>T 4620T>A), Q410N (4633C>A 4635A>T), A413Q (4642G>C 4643C>A 4644T>A), H418K (4657C>A 4659C>A), D420Y (4663G>T 4665C>T), E421K (4666G>A), S422I (4670G>T 4671T>A), L423F (4672C>T 4674C>T), S424del (4675_4679delAGCAA), M426L (4681A>C), Y427K (4684T>A 4686T>A), P428K (4687C>A 4688C>A 4689A>G), Q429K (4690C>A 4692G>A), C431L (4697G>T 4698T>G), A432I (4699G>A 4700C>T 4701T>A), V435I (4708G>A 4710T>A), I439L (4720A>T 4722C>G), F441C (4727T>G), K443T (4733A>C), E445Y (4738G>T 4740A>T), E446Q (4741G>C 4743A>G), G450T (4753G>A 4754G>C), V452L (4759G>C 4761C>T), I454E (4765A>G 4766T>A 4767T>A), V455F (4768G>T), L456S (4771C>T 4772T>C), N457K (4776C>A), R458L (4777A>C 4778G>T 4779A>C), K460V (4783A>G 4784A>T 4785A>C), A461N (4786G>A 4787C>A 4788C>T), L462N (4789C>A 4790T>A 4791A>T), G463D (4793G>A 4794A>C), K468Q (4807A>C 4809A>G), Q472E (4819C>G 4821G>A), L473I (4822T>A 4824G>A), C474N (4825T>A 4826G>A 4827C>T), T476N (4832C>A 4833A>T), T477K (4835C>A 4836T>A), N479E (4840A>G 4842T>A), L483M (4852C>A 4854A>G), V484I (4855G>A 4857A>T), E486S (4861G>T 4862A>C), R487K (4865G>A), G488N (4867G>A 4868G>A 4869A>T), N489G (4870A>G 4871A>G 4872T>A), L490I (4873C>A 4875C>A), K491E (4876A>G), V492L (4879G>T 4881C>A), S494T (4886G>C), G497S (4894G>T 4895G>C 4896A>C), L498K (4897C>A 4898T>A), H499K (4900C>A 4902C>G), L500I (4903T>A), V501L (4906G>T 4908T>A), A502E (4910C>A 4911T>G), Q506K (4921C>A 4923A>G), S508I (4928G>T 4929T>A), D509T (4930G>A 4931A>C 4932T>C), R510K (4934G>A 4935G>A), N511Q (4936A>C 4938T>A), A512E (4940C>A 4941C>A), L513I (4942T>A), L519C (4960C>T 4961T>G), I523A (4972A>G 4973T>C 4974C>A), S524G (4975T>G 4976C>G 4977C>A), A525E (4979C>A 4980T>A), Y526F (4982A>T 4983T>C), F527I (4984T>A 4986T>A), K529E (4990A>G), I530L (4993A>T), N532K (5001C>G), L533K (5002C>A 5003T>A 5004T>G), S535N (5008T>A 5009C>A 5010A>T), P536L (5012C>T), V539del (5020_5022delGTT), K542I (5030A>T 5031G>C), K543R (5033A>G), E544K (5035G>A 5037G>A), I545S (5038A>T 5039T>C), T546N (5042C>A 5043T>C), W547R (5044T>C 5046G>A), S548F (5048C>T), W549* (5052G>A), T550S (5054C>G), E551F (5056G>T 5057A>T 5058G>T), K552F (5059A>T 5060A>T 5061A>C)								
Codon mutations:	AAC276.AT (4233C>T), AAA277ATA (4235A>T), ATT278ATC (4239T>C), ATT279ATA (4242T>A), AAG280AGA (4244A>G 4245G>A), TGT281GTT (4246T>G 4247G>T), AAA282CAA (4249A>C), CCA283CCC (4254A>C), CAG285TTT (4258C>T 4259A>T 4260G>T), TCA287AAC (4264T>A 4265C>A 4266A>C), CCT288GAT (4267C>G 4268C>A), CAA289GAA (4270C>G), GAC290GAT (4275C>T), CGA291AAA (4276C>A 4277G>A), GAA292AAT (4279G>A 4281A>T), GAG293GAA (4284G>A), TTT294TTC (4287T>C), AAA295AGA (4289A>G), ACT296ATT (4292C>T), ATC298ATT (4299C>T), GAA299CAT (4300G>C 4302A>T), CTA301CTT (4308A>T), AAG303AAA (4314G>A), CTT304ATG (4315C>A 4317T>G), GGT305AAG (4318G>A 4319G>A 4320T>G), ATC306TTA (4321A>T 4323C>A), CCT309GCA (4330C>G 4332T>A), AAG311AGA (4337A>G 4338G>A), CAT314CAC (4347T>C), TCC315AGC (4348T>A 4349C>G), TCA316AGT (4351T>A 4352C>G 4353A>T), CCA317CCC (4356A>C), GCC318GCG (4359C>G), AGG322AGA (4371G>A), AAT323AAC (4374T>C), GCC325GCG (4380C>G), GAG326GAA (4383G>A), ATC327CAA (4384A>C 4385T>A 4386C>A), GGC330GGT (4395C>T), AAA331AAG (4398A>G), GCA332GCT (4401A>T), AGA333CGA (4402A>C), GTA335GTT (4410A>T), ATT336ATA (4413T>A), AAG340GAG (4423A>G), TTA341CTT (4426T>C 4428A>T), GAC343AAA (4432G>A 4434C>A), CAT344AAC (4435C>A 4437T>C), ACA345TGT (4438A>T 4439C>G 4440A>T), AAG346ATT (4442A>T 4443G>T), GGA347TTT (4444G>T 4445G>T 4446A>T), GAT348GAC (4449T>C), GGC349GGA (4452C>A), CTA351TTT (4456C>T 4458A>T), CTT352ATC (4459C>A 4461T>C), AAC354AAT (4467C>T), AAG355AAA (4470G>A), GAG356ACT (4471G>A 4472A>C 4473G>T), CAA357AAT (4474C>A 4476A>T), CTG358CTT (4479G>T), CTT359ATA (4480C>A 4482T>A), CAA360AAT (4483C>A 4485A>T), AGA361CTA (4486A>C 4487G>T), ATC362GTC (4489A>G), GGA363AAA (4492G>A 4493G>A), GGT364GTA (4496G>T 4497T>A), AAG365AAA (4500G>A), TTT367TAC (4505T>A 4506T>C), TAC368TTT (4508A>T 4509C>T), TCT369TCC (4512T>C), TCC370AAA (4513T>A 4514C>A 4515C>A), GAC372GAT (4521C>T), TCT375AGT (4528T>A 4529C>G), GTA380ATA (4543G>A), CGC381AGA (4546C>A 4548C>A), CTT382CTC (4551T>C), GCT383TCA (4552G>T 4554T>A), CCA384GAG (4555C>G 4556C>A 4557A>G), ACA386TCA (4561A>T), CAG388CCT (4568A>C 4569G>T), ACC390ACA (4575C>A), GCT391GCA (4578T>A), TGT394ACT (4585T>A 4586G>C), CCC395CCT (4590C>T), CAA396AAT (4591C>A 4593A>T), CAC398CAT (4599C>T), TAC399TAT (4602C>T), GTC403GTA (4614C>A), CCT405TCA (4618C>T 4620T>A), CAA410AAT (4633C>A 4635A>T), GCC411GCA (4638C>A), CCT412CCA (4641T>A), GCT413CAA (4642G>C 4643C>A 4644T>A), TTC415TTT (4650C>T), AGG417AGA (4656G>A), CAC418AAA (4657C>A 4659C>A), GAC420TAT (4663G>T 4665C>T), GAA421AAA (4666G>A), AGT422ATA (4670G>T 4671T>A), CTC423TTT (4672C>T 4674C>T), AGC424del (4675_4679delAGCAA), AAC425--T (4675_4679delAGCAA 4680C>T), ATG426CTG (4681A>C), TAT427AAA (4684T>A 4686T>A), CCA428AAG (4687C>A 4688C>A 4689A>G), CAG429AAA (4690C>A 4692G>A), TGT431TTG (4697G>T 4698T>G), GCT432ATA (4699G>A 4700C>T 4701T>A), TAT434TAC (4707T>C), GTT435ATA (4708G>A 4710T>A), ATC438ATT (4719C>T), ATC439TTG (4720A>T 4722C>G), GTC440GTA (4725C>A), TTC441TGC (4727T>G), AGC442TCA (4729A>T 4730G>C 4731C>A), AAA443ACA (4733A>C), ACT444ACA (4737T>A), GAA445TAT (4738G>T 4740A>T), GAA446CAG (4741G>C 4743A>G), TTA449CTT (4750T>C 4752A>T), GGA450ACA (4753G>A 4754G>C), GTC452CTT (4759G>C 4761C>T), ATT454GAA (4765A>G 4766T>A 4767T>A), GTT455TTT (4768G>T), CTA456TCA (4771C>T 4772T>C), AAC457AAA (4776C>A), AGA458CTC (4777A>C 4778G>T 4779A>C), TGC459TGT (4782C>T), AAA460GTC (4783A>G 4784A>T 4785A>C), GCC461AAT (4786G>A 4787C>A 4788C>T), CTA462AAT (4789C>A 4790T>A 4791A>T), GGA463GAC (4793G>A 4794A>C), GTG465GTA (4800G>A), CTT466TTA (4801C>T 4803T>A), AGC467AGT (4806C>T), AAA468CAG (4807A>C 4809A>G), AAG469AAA (4812G>A), GCC471GCA (4818C>A), CAG472GAA (4819C>G 4821G>A), TTG473ATA (4822T>A 4824G>A), TGC474AAT (4825T>A 4826G>A 4827C>T), ACA476AAT (4832C>A 4833A>T), ACT477AAA (4835C>A 4836T>A), ATC478ATA (4839C>A), AAT479GAA (4840A>G 4842T>A), GGT482GGA (4851T>A), CTA483ATG (4852C>A 4854A>G), GTA484ATT (4855G>A 4857A>T), ATA485ATT (4860A>T), GAA486TCA (4861G>T 4862A>C), AGA487AAA (4865G>A), GGA488AAT (4867G>A 4868G>A 4869A>T), AAT489GGA (4870A>G 4871A>G 4872T>A), CTC490ATA (4873C>A 4875C>A), AAA491GAA (4876A>G), GTC492TTA (4879G>T 4881C>A), CAG493CAA (4884G>A), AGT494ACT (4886G>C), CAT495CAC (4890T>C), ATC496ATA (4893C>A), GGA497TCC (4894G>T 4895G>C 4896A>C), CTA498AAA (4897C>A 4898T>A), CAC499AAG (4900C>A 4902C>G), TTA500ATA (4903T>A), GTT501TTA (4906G>T 4908T>A), GCT502GAG (4910C>A 4911T>G), CCA504CCT (4917A>T), GAT505GAC (4920T>C), CAA506AAG (4921C>A 4923A>G), CTT507TTA (4924C>T 4926T>A), AGT508ATA (4928G>T 4929T>A), GAT509ACC (4930G>A 4931A>C 4932T>C), AGG510AAA (4934G>A 4935G>A), AAT511CAA (4936A>C 4938T>A), GCC512GAA (4940C>A 4941C>A), TTA513ATA (4942T>A), TTA517CTT (4954T>C 4956A>T), GGC518GGA (4959C>A), CTC519TGC (4960C>T 4961T>G), CTA520TTA (4963C>T), AAT521AAC (4968T>C), ATC523GCA (4972A>G 4973T>C 4974C>A), TCC524GGA (4975T>G 4976C>G 4977C>A), GCT525GAA (4979C>A 4980T>A), TAT526TTC (4982A>T 4983T>C), TTT527ATA (4984T>A 4986T>A), AAA529GAA (4990A>G), ATA530TTA (4993A>T), AAC532AAG (5001C>G), CTT533AAG (5002C>A 5003T>A 5004T>G), AGG534AGA (5007G>A), TCA535AAT (5008T>A 5009C>A 5010A>T), CCT536CTT (5012C>T), TTA537CTT (5014T>C 5016A>T), CAG538CAA (5019G>A), GTT539del (5020_5022delGTT), CTT541CTC (5028T>C), AAG542ATC (5030A>T 5031G>C), AAA543AGA (5033A>G), GAG544AAA (5035G>A 5037G>A), ATA545TCA (5038A>T 5039T>C), ACT546AAC (5042C>A 5043T>C), TGG547CGA (5044T>C 5046G>A), TCT548TTT (5048C>T), TGG549TGA (5052G>A), ACT550AGT (5054C>G), GAG551TTT (5056G>T 5057A>T 5058G>T), AAA552TTC (5059A>T 5060A>T 5061A>C)								

Proteins

	Begin	End	Coverage	Score	Concordance	Matches	Identities	I/D/M/F*	Stop Codons
hypothetical protein (NP_043933.1)	277	555	39.2%	916	47.2%	276 (99.3%)	146 (52.5%)	0/2/1/1	1

	Begin	End	Coverage	Score	Concordance	Matches	Identities	I/D/M/F*	Stop Codons
**NT**	**4232**	**5069**	**10.6%**	**242**	**14.8%**	**830 (99.0%)**	**482 (57.5%)**	**0/8**	
Protein mutations:	K277I (4235A>T), K280R (4244A>G 4245G>A), C281V (4246T>G 4247G>T), K282Q (4249A>C), Q285F (4258C>T 4259A>T 4260G>T), S287N (4264T>A 4265C>A 4266A>C), P288D (4267C>G 4268C>A), Q289E (4270C>G), R291K (4276C>A 4277G>A), E292N (4279G>A 4281A>T), K295R (4289A>G), T296I (4292C>T), E299H (4300G>C 4302A>T), L304M (4315C>A 4317T>G), G305K (4318G>A 4319G>A 4320T>G), I306L (4321A>T 4323C>A), P309A (4330C>G 4332T>A), K311R (4337A>G 4338G>A), I327Q (4384A>C 4385T>A 4386C>A), K340E (4423A>G), D343K (4432G>A 4434C>A), H344N (4435C>A 4437T>C), T345C (4438A>T 4439C>G 4440A>T), K346I (4442A>T 4443G>T), G347F (4444G>T 4445G>T 4446A>T), L351F (4456C>T 4458A>T), L352I (4459C>A 4461T>C), E356T (4471G>A 4472A>C 4473G>T), Q357N (4474C>A 4476A>T), L359I (4480C>A 4482T>A), Q360N (4483C>A 4485A>T), R361L (4486A>C 4487G>T), I362V (4489A>G), G363K (4492G>A 4493G>A), G364V (4496G>T 4497T>A), F367Y (4505T>A 4506T>C), Y368F (4508A>T 4509C>T), S370K (4513T>A 4514C>A 4515C>A), V380I (4543G>A), A383S (4552G>T 4554T>A), P384E (4555C>G 4556C>A 4557A>G), T386S (4561A>T), Q388P (4568A>C 4569G>T), C394T (4585T>A 4586G>C), Q396N (4591C>A 4593A>T), P405S (4618C>T 4620T>A), Q410N (4633C>A 4635A>T), A413Q (4642G>C 4643C>A 4644T>A), H418K (4657C>A 4659C>A), D420Y (4663G>T 4665C>T), E421K (4666G>A), S422I (4670G>T 4671T>A), L423F (4672C>T 4674C>T), S424del (4675_4679delAGCAA), M426L (4681A>C), Y427K (4684T>A 4686T>A), P428K (4687C>A 4688C>A 4689A>G), Q429K (4690C>A 4692G>A), C431L (4697G>T 4698T>G), A432I (4699G>A 4700C>T 4701T>A), V435I (4708G>A 4710T>A), I439L (4720A>T 4722C>G), F441C (4727T>G), K443T (4733A>C), E445Y (4738G>T 4740A>T), E446Q (4741G>C 4743A>G), G450T (4753G>A 4754G>C), V452L (4759G>C 4761C>T), I454E (4765A>G 4766T>A 4767T>A), V455F (4768G>T), L456S (4771C>T 4772T>C), N457K (4776C>A), R458L (4777A>C 4778G>T 4779A>C), K460V (4783A>G 4784A>T 4785A>C), A461N (4786G>A 4787C>A 4788C>T), L462N (4789C>A 4790T>A 4791A>T), G463D (4793G>A 4794A>C), K468Q (4807A>C 4809A>G), Q472E (4819C>G 4821G>A), L473I (4822T>A 4824G>A), C474N (4825T>A 4826G>A 4827C>T), T476N (4832C>A 4833A>T), T477K (4835C>A 4836T>A), N479E (4840A>G 4842T>A), L483M (4852C>A 4854A>G), V484I (4855G>A 4857A>T), E486S (4861G>T 4862A>C), R487K (4865G>A), G488N (4867G>A 4868G>A 4869A>T), N489G (4870A>G 4871A>G 4872T>A), L490I (4873C>A 4875C>A), K491E (4876A>G), V492L (4879G>T 4881C>A), S494T (4886G>C), G497S (4894G>T 4895G>C 4896A>C), L498K (4897C>A 4898T>A), H499K (4900C>A 4902C>G), L500I (4903T>A), V501L (4906G>T 4908T>A), A502E (4910C>A 4911T>G), Q506K (4921C>A 4923A>G), S508I (4928G>T 4929T>A), D509T (4930G>A 4931A>C 4932T>C), R510K (4934G>A 4935G>A), N511Q (4936A>C 4938T>A), A512E (4940C>A 4941C>A), L513I (4942T>A), L519C (4960C>T 4961T>G), I523A (4972A>G 4973T>C 4974C>A), S524G (4975T>G 4976C>G 4977C>A), A525E (4979C>A 4980T>A), Y526F (4982A>T 4983T>C), F527I (4984T>A 4986T>A), K529E (4990A>G), I530L (4993A>T), N532K (5001C>G), L533K (5002C>A 5003T>A 5004T>G), S535N (5008T>A 5009C>A 5010A>T), P536L (5012C>T), V539del (5020_5022delGTT), K542I (5030A>T 5031G>C), K543R (5033A>G), E544K (5035G>A 5037G>A), I545S (5038A>T 5039T>C), T546N (5042C>A 5043T>C), W547R (5044T>C 5046G>A), S548F (5048C>T), W549* (5052G>A), T550S (5054C>G), E551F (5056G>T 5057A>T 5058G>T), K552F (5059A>T 5060A>T 5061A>C)								
Codon mutations:	AAC276.AT (4233C>T), AAA277ATA (4235A>T), ATT278ATC (4239T>C), ATT279ATA (4242T>A), AAG280AGA (4244A>G 4245G>A), TGT281GTT (4246T>G 4247G>T), AAA282CAA (4249A>C), CCA283CCC (4254A>C), CAG285TTT (4258C>T 4259A>T 4260G>T), TCA287AAC (4264T>A 4265C>A 4266A>C), CCT288GAT (4267C>G 4268C>A), CAA289GAA (4270C>G), GAC290GAT (4275C>T), CGA291AAA (4276C>A 4277G>A), GAA292AAT (4279G>A 4281A>T), GAG293GAA (4284G>A), TTT294TTC (4287T>C), AAA295AGA (4289A>G), ACT296ATT (4292C>T), ATC298ATT (4299C>T), GAA299CAT (4300G>C 4302A>T), CTA301CTT (4308A>T), AAG303AAA (4314G>A), CTT304ATG (4315C>A 4317T>G), GGT305AAG (4318G>A 4319G>A 4320T>G), ATC306TTA (4321A>T 4323C>A), CCT309GCA (4330C>G 4332T>A), AAG311AGA (4337A>G 4338G>A), CAT314CAC (4347T>C), TCC315AGC (4348T>A 4349C>G), TCA316AGT (4351T>A 4352C>G 4353A>T), CCA317CCC (4356A>C), GCC318GCG (4359C>G), AGG322AGA (4371G>A), AAT323AAC (4374T>C), GCC325GCG (4380C>G), GAG326GAA (4383G>A), ATC327CAA (4384A>C 4385T>A 4386C>A), GGC330GGT (4395C>T), AAA331AAG (4398A>G), GCA332GCT (4401A>T), AGA333CGA (4402A>C), GTA335GTT (4410A>T), ATT336ATA (4413T>A), AAG340GAG (4423A>G), TTA341CTT (4426T>C 4428A>T), GAC343AAA (4432G>A 4434C>A), CAT344AAC (4435C>A 4437T>C), ACA345TGT (4438A>T 4439C>G 4440A>T), AAG346ATT (4442A>T 4443G>T), GGA347TTT (4444G>T 4445G>T 4446A>T), GAT348GAC (4449T>C), GGC349GGA (4452C>A), CTA351TTT (4456C>T 4458A>T), CTT352ATC (4459C>A 4461T>C), AAC354AAT (4467C>T), AAG355AAA (4470G>A), GAG356ACT (4471G>A 4472A>C 4473G>T), CAA357AAT (4474C>A 4476A>T), CTG358CTT (4479G>T), CTT359ATA (4480C>A 4482T>A), CAA360AAT (4483C>A 4485A>T), AGA361CTA (4486A>C 4487G>T), ATC362GTC (4489A>G), GGA363AAA (4492G>A 4493G>A), GGT364GTA (4496G>T 4497T>A), AAG365AAA (4500G>A), TTT367TAC (4505T>A 4506T>C), TAC368TTT (4508A>T 4509C>T), TCT369TCC (4512T>C), TCC370AAA (4513T>A 4514C>A 4515C>A), GAC372GAT (4521C>T), TCT375AGT (4528T>A 4529C>G), GTA380ATA (4543G>A), CGC381AGA (4546C>A 4548C>A), CTT382CTC (4551T>C), GCT383TCA (4552G>T 4554T>A), CCA384GAG (4555C>G 4556C>A 4557A>G), ACA386TCA (4561A>T), CAG388CCT (4568A>C 4569G>T), ACC390ACA (4575C>A), GCT391GCA (4578T>A), TGT394ACT (4585T>A 4586G>C), CCC395CCT (4590C>T), CAA396AAT (4591C>A 4593A>T), CAC398CAT (4599C>T), TAC399TAT (4602C>T), GTC403GTA (4614C>A), CCT405TCA (4618C>T 4620T>A), CAA410AAT (4633C>A 4635A>T), GCC411GCA (4638C>A), CCT412CCA (4641T>A), GCT413CAA (4642G>C 4643C>A 4644T>A), TTC415TTT (4650C>T), AGG417AGA (4656G>A), CAC418AAA (4657C>A 4659C>A), GAC420TAT (4663G>T 4665C>T), GAA421AAA (4666G>A), AGT422ATA (4670G>T 4671T>A), CTC423TTT (4672C>T 4674C>T), AGC424del (4675_4679delAGCAA), AAC425--T (4675_4679delAGCAA 4680C>T), ATG426CTG (4681A>C), TAT427AAA (4684T>A 4686T>A), CCA428AAG (4687C>A 4688C>A 4689A>G), CAG429AAA (4690C>A 4692G>A), TGT431TTG (4697G>T 4698T>G), GCT432ATA (4699G>A 4700C>T 4701T>A), TAT434TAC (4707T>C), GTT435ATA (4708G>A 4710T>A), ATC438ATT (4719C>T), ATC439TTG (4720A>T 4722C>G), GTC440GTA (4725C>A), TTC441TGC (4727T>G), AGC442TCA (4729A>T 4730G>C 4731C>A), AAA443ACA (4733A>C), ACT444ACA (4737T>A), GAA445TAT (4738G>T 4740A>T), GAA446CAG (4741G>C 4743A>G), TTA449CTT (4750T>C 4752A>T), GGA450ACA (4753G>A 4754G>C), GTC452CTT (4759G>C 4761C>T), ATT454GAA (4765A>G 4766T>A 4767T>A), GTT455TTT (4768G>T), CTA456TCA (4771C>T 4772T>C), AAC457AAA (4776C>A), AGA458CTC (4777A>C 4778G>T 4779A>C), TGC459TGT (4782C>T), AAA460GTC (4783A>G 4784A>T 4785A>C), GCC461AAT (4786G>A 4787C>A 4788C>T), CTA462AAT (4789C>A 4790T>A 4791A>T), GGA463GAC (4793G>A 4794A>C), GTG465GTA (4800G>A), CTT466TTA (4801C>T 4803T>A), AGC467AGT (4806C>T), AAA468CAG (4807A>C 4809A>G), AAG469AAA (4812G>A), GCC471GCA (4818C>A), CAG472GAA (4819C>G 4821G>A), TTG473ATA (4822T>A 4824G>A), TGC474AAT (4825T>A 4826G>A 4827C>T), ACA476AAT (4832C>A 4833A>T), ACT477AAA (4835C>A 4836T>A), ATC478ATA (4839C>A), AAT479GAA (4840A>G 4842T>A), GGT482GGA (4851T>A), CTA483ATG (4852C>A 4854A>G), GTA484ATT (4855G>A 4857A>T), ATA485ATT (4860A>T), GAA486TCA (4861G>T 4862A>C), AGA487AAA (4865G>A), GGA488AAT (4867G>A 4868G>A 4869A>T), AAT489GGA (4870A>G 4871A>G 4872T>A), CTC490ATA (4873C>A 4875C>A), AAA491GAA (4876A>G), GTC492TTA (4879G>T 4881C>A), CAG493CAA (4884G>A), AGT494ACT (4886G>C), CAT495CAC (4890T>C), ATC496ATA (4893C>A), GGA497TCC (4894G>T 4895G>C 4896A>C), CTA498AAA (4897C>A 4898T>A), CAC499AAG (4900C>A 4902C>G), TTA500ATA (4903T>A), GTT501TTA (4906G>T 4908T>A), GCT502GAG (4910C>A 4911T>G), CCA504CCT (4917A>T), GAT505GAC (4920T>C), CAA506AAG (4921C>A 4923A>G), CTT507TTA (4924C>T 4926T>A), AGT508ATA (4928G>T 4929T>A), GAT509ACC (4930G>A 4931A>C 4932T>C), AGG510AAA (4934G>A 4935G>A), AAT511CAA (4936A>C 4938T>A), GCC512GAA (4940C>A 4941C>A), TTA513ATA (4942T>A), TTA517CTT (4954T>C 4956A>T), GGC518GGA (4959C>A), CTC519TGC (4960C>T 4961T>G), CTA520TTA (4963C>T), AAT521AAC (4968T>C), ATC523GCA (4972A>G 4973T>C 4974C>A), TCC524GGA (4975T>G 4976C>G 4977C>A), GCT525GAA (4979C>A 4980T>A), TAT526TTC (4982A>T 4983T>C), TTT527ATA (4984T>A 4986T>A), AAA529GAA (4990A>G), ATA530TTA (4993A>T), AAC532AAG (5001C>G), CTT533AAG (5002C>A 5003T>A 5004T>G), AGG534AGA (5007G>A), TCA535AAT (5008T>A 5009C>A 5010A>T), CCT536CTT (5012C>T), TTA537CTT (5014T>C 5016A>T), CAG538CAA (5019G>A), GTT539del (5020_5022delGTT), CTT541CTC (5028T>C), AAG542ATC (5030A>T 5031G>C), AAA543AGA (5033A>G), GAG544AAA (5035G>A 5037G>A), ATA545TCA (5038A>T 5039T>C), ACT546AAC (5042C>A 5043T>C), TGG547CGA (5044T>C 5046G>A), TCT548TTT (5048C>T), TGG549TGA (5052G>A), ACT550AGT (5054C>G), GAG551TTT (5056G>T 5057A>T 5058G>T), AAA552TTC (5059A>T 5060A>T 5061A>C)								

*: Inserts / Deletes / Misaligned / Frameshifts

Analysis details

This analysis was performed with panviral2.64

NGS Details (UN18_val): Badnavirus maculapiperis

Assembly

**Coverage Length**	234 (1 contig(s))
**Depth Of Coverage**	13.7
**Number Of Reads**	30
**Reads Per Million**	0.56 rpm (after QC)
**Ambiguities**	0
**Assembly Method**	de novo + reference guided assembly
**Consensus Caller**	Bcf Tools

Coverage Map

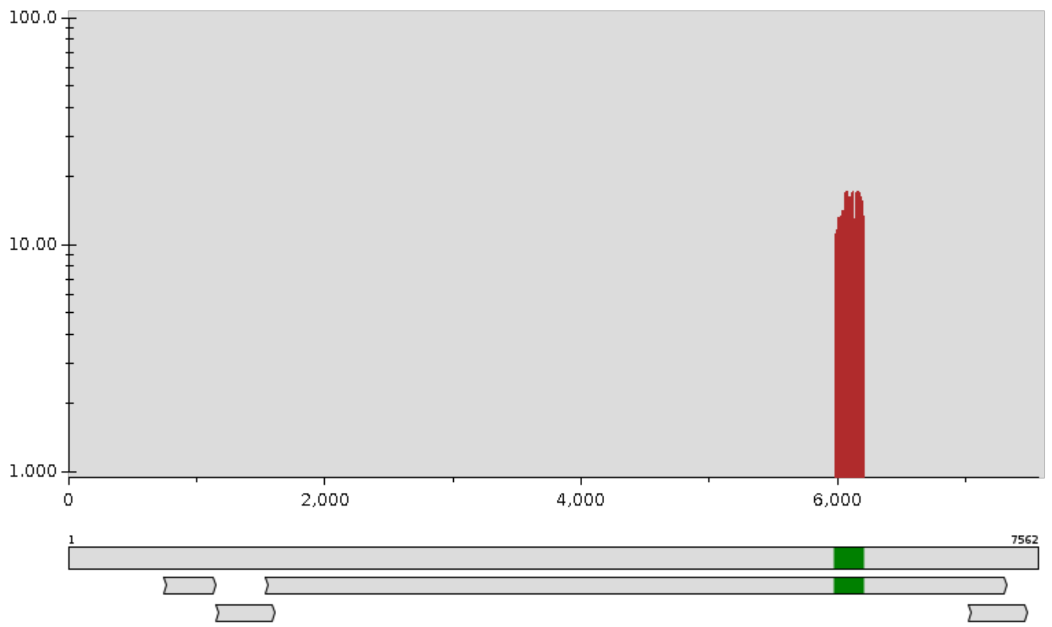

Assignment

**Type**	Badnavirus maculapiperis (Taxonomy ID: 3048272)
**Reference Genome**	NC_022365.1
**NT Identity (%)**	57.8723
**AA Identity (%)**	53.1646
**Number Of Stop Codons**	0
**Number Of CDS**	4

Alignment

**Alignment Score**	57.0 (NT) + 188.0 (AA) = 245.0
**Concordance (%)**	29.5852

Alignment Method	Global, seeded, nucleotide + amino acids (AGA)

Genome Region

Sequence starts at position 5977 and ends at position 6210 relative to NC_022365.1 reference sequence.

Alignment Detailed Statistics

	Begin	End	Coverage	Score	Concordance	Matches	Identities	I/D/M/F*	Stop Codons
**NT**	**5977**	**6210**	**3.1%**	**57**	**12.6%**	**232 (97.9%)**	**136 (57.4%)**	**3/2**	
Mutations:	5989T>A, 5991A>C, 5994A>G, 5995T>C, 5997C>T, 6000C>T, 6003A>G, 6005A>G, 6009T>A, 6014T>A, 6018C>T, 6022G>A, 6024A>T, 6025G>A, 6026C>G, 6031G>A, 6032A>C, 6033C>A, 6034C>A, 6035C>A, 6036T>G, 6039A>G, 6040T>G, 6041C>A, 6043A>G, 6045A>C, 6046G>C, 6049T>A, 6050G>A, 6051G>A, 6055G>A, 6057C>A, 6060C>T, 6063C>G, 6066C>T, 6068C>A, 6070A>T, 6071A>C, 6075G>C, 6077T>A, 6078A>C, 6081C>T, 6084A>G, 6086G>A, 6087G>C, 6090T>A, 6093C>A, 6099A>C, 6105G>C, 6108T>C, 6110A>C, 6111A>T, 6121G>T, 6123T>A, 6124G>A, 6125T>G, 6126G>T, 6129C>T, 6133A>G, 6135A>G, 6136A>C, 6137A>T, 6138A>G, 6142G>A, 6144C>T, 6145A>C, 6147C>T, 6148T>G, 6149G>T, 6153C>T, 6156A>G, 6158G>A, 6159T>A, 6159_6160insCAC, 6160A>C, 6162G>T, 6163G>A, 6164A>G, 6165A>G, 6166G>A, 6168T>G, 6171T>C, 6174A>T, 6175G>T, 6176C>T, 6177T>G, 6180C>T, 6182A>T, 6183C>T, 6184_6185delAT, 6199G>A, 6201G>C, 6203T>A, 6205T>A, 6206C>G, 6207A>C, 6208G>A, 6210A>G								

CDS

	Begin	End	Coverage	Score	Concordance	Matches	Identities	I/D/M/F*	Stop Codons
P878_gp3	1481	1558	4.0%	188	33.4%	78 (98.7%)	42 (53.2%)	1/0/1/1	0
Protein mutations:	S1485T (5989T>A 5991A>C), F1487L (5995T>C 5997C>T), K1490R (6005A>G), F1493Y (6014T>A), V1496I (6022G>A 6024A>T), A1497R (6025G>A 6026C>G), D1499T (6031G>A 6032A>C 6033C>A), P1500K (6034C>A 6035C>A 6036T>G), S1502D (6040T>G 6041C>A), I1503V (6043A>G 6045A>C), E1504Q (6046G>C), W1505K (6049T>A 6050G>A 6051G>A), A1507T (6055G>A 6057C>A), S1509R (6063C>G), P1511H (6068C>A), N1512S (6070A>T 6071A>C), L1514H (6077T>A 6078A>C), W1517Y (6086G>A 6087G>C), K1525T (6110A>C 6111A>T), A1529S (6121G>T 6123T>A), V1530S (6124G>A 6125T>G 6126G>T), R1533G (6133A>G 6135A>G), K1534L (6136A>C 6137A>T 6138A>G), D1536N (6142G>A 6144C>T), N1537H (6145A>C 6147C>T), C1538V (6148T>G 6149G>T), G1541E (6158G>A 6159T>A), G1541_M1542insH (6159_6160insCAC), M1542L (6160A>C 6162G>T), E1543R (6163G>A 6164A>G 6165A>G), D1544K (6166G>A 6168T>G), A1547L (6175G>T 6176C>T 6177T>G), Y1549F (6182A>T 6183C>T), V1555I (6199G>A 6201G>C), F1556Y (6203T>A), E1558K (6208G>A 6210A>G)								
Codon mutations:	TCA1485ACC (5989T>A 5991A>C), AAA1486AAG (5994A>G), TTC1487CTT (5995T>C 5997C>T), GAC1488GAT (6000C>T), CTA1489CTG (6003A>G), AAA1490AGA (6005A>G), TCT1491TCA (6009T>A), TTT1493TAT (6014T>A), CAC1494CAT (6018C>T), GTA1496ATT (6022G>A 6024A>T), GCG1497AGG (6025G>A 6026C>G), GAC1499ACA (6031G>A 6032A>C 6033C>A), CCT1500AAG (6034C>A 6035C>A 6036T>G), GAA1501GAG (6039A>G), TCT1502GAT (6040T>G 6041C>A), ATA1503GTC (6043A>G 6045A>C), GAG1504CAG (6046G>C), TGG1505AAA (6049T>A 6050G>A 6051G>A), GCC1507ACA (6055G>A 6057C>A), TTC1508TTT (6060C>T), AGC1509AGG (6063C>G), ACC1510ACT (6066C>T), CCT1511CAT (6068C>A), AAT1512TCT (6070A>T 6071A>C), GGG1513GGC (6075G>C), CTA1514CAC (6077T>A 6078A>C), TAC1515TAT (6081C>T), GAA1516GAG (6084A>G), TGG1517TAC (6086G>A 6087G>C), CTT1518CTA (6090T>A), GTC1519GTA (6093C>A), CCA1521CCC (6099A>C), GGG1523GGC (6105G>C), CTT1524CTC (6108T>C), AAA1525ACT (6110A>C 6111A>T), GCT1529TCA (6121G>T 6123T>A), GTG1530AGT (6124G>A 6125T>G 6126G>T), TTC1531TTT (6129C>T), AGA1533GGG (6133A>G 6135A>G), AAA1534CTG (6136A>C 6137A>T 6138A>G), GAC1536AAT (6142G>A 6144C>T), AAC1537CAT (6145A>C 6147C>T), TGT1538GTT (6148T>G 6149G>T), TTC1539TTT (6153C>T), AAA1540AAG (6156A>G), GGT1541GAA (6158G>A 6159T>A), GGT1541_ATG1542insCAC (6159_6160insCAC), ATG1542CTT (6160A>C 6162G>T), GAA1543AGG (6163G>A 6164A>G 6165A>G), GAT1544AAG (6166G>A 6168T>G), TTT1545TTC (6171T>C), ATA1546ATT (6174A>T), GCT1547TTG (6175G>T 6176C>T 6177T>G), GTC1548GTT (6180C>T), TAC1549TTT (6182A>T 6183C>T), ATT1550--T (6184_6185delAT), GTG1555ATC (6199G>A 6201G>C), TTC1556TAC (6203T>A), TCA1557AGC (6205T>A 6206C>G 6207A>C), GAA1558AAG (6208G>A 6210A>G)								

Proteins

	Begin	End	Coverage	Score	Concordance	Matches	Identities	I/D/M/F*	Stop Codons
ORF3 (YP_008567619.1)	1481	1558	4.0%	188	33.4%	78 (98.7%)	42 (53.2%)	1/0/1/1	0
Protein mutations:	S1485T (5989T>A 5991A>C), F1487L (5995T>C 5997C>T), K1490R (6005A>G), F1493Y (6014T>A), V1496I (6022G>A 6024A>T), A1497R (6025G>A 6026C>G), D1499T (6031G>A 6032A>C 6033C>A), P1500K (6034C>A 6035C>A 6036T>G), S1502D (6040T>G 6041C>A), I1503V (6043A>G 6045A>C), E1504Q (6046G>C), W1505K (6049T>A 6050G>A 6051G>A), A1507T (6055G>A 6057C>A), S1509R (6063C>G), P1511H (6068C>A), N1512S (6070A>T 6071A>C), L1514H (6077T>A 6078A>C), W1517Y (6086G>A 6087G>C), K1525T (6110A>C 6111A>T), A1529S (6121G>T 6123T>A), V1530S (6124G>A 6125T>G 6126G>T), R1533G (6133A>G 6135A>G), K1534L (6136A>C 6137A>T 6138A>G), D1536N (6142G>A 6144C>T), N1537H (6145A>C 6147C>T), C1538V (6148T>G 6149G>T), G1541E (6158G>A 6159T>A), G1541_M1542insH (6159_6160insCAC), M1542L (6160A>C 6162G>T), E1543R (6163G>A 6164A>G 6165A>G), D1544K (6166G>A 6168T>G), A1547L (6175G>T 6176C>T 6177T>G), Y1549F (6182A>T 6183C>T), V1555I (6199G>A 6201G>C), F1556Y (6203T>A), E1558K (6208G>A 6210A>G)								
Codon mutations:	TCA1485ACC (5989T>A 5991A>C), AAA1486AAG (5994A>G), TTC1487CTT (5995T>C 5997C>T), GAC1488GAT (6000C>T), CTA1489CTG (6003A>G), AAA1490AGA (6005A>G), TCT1491TCA (6009T>A), TTT1493TAT (6014T>A), CAC1494CAT (6018C>T), GTA1496ATT (6022G>A 6024A>T), GCG1497AGG (6025G>A 6026C>G), GAC1499ACA (6031G>A 6032A>C 6033C>A), CCT1500AAG (6034C>A 6035C>A 6036T>G), GAA1501GAG (6039A>G), TCT1502GAT (6040T>G 6041C>A), ATA1503GTC (6043A>G 6045A>C), GAG1504CAG (6046G>C), TGG1505AAA (6049T>A 6050G>A 6051G>A), GCC1507ACA (6055G>A 6057C>A), TTC1508TTT (6060C>T), AGC1509AGG (6063C>G), ACC1510ACT (6066C>T), CCT1511CAT (6068C>A), AAT1512TCT (6070A>T 6071A>C), GGG1513GGC (6075G>C), CTA1514CAC (6077T>A 6078A>C), TAC1515TAT (6081C>T), GAA1516GAG (6084A>G), TGG1517TAC (6086G>A 6087G>C), CTT1518CTA (6090T>A), GTC1519GTA (6093C>A), CCA1521CCC (6099A>C), GGG1523GGC (6105G>C), CTT1524CTC (6108T>C), AAA1525ACT (6110A>C 6111A>T), GCT1529TCA (6121G>T 6123T>A), GTG1530AGT (6124G>A 6125T>G 6126G>T), TTC1531TTT (6129C>T), AGA1533GGG (6133A>G 6135A>G), AAA1534CTG (6136A>C 6137A>T 6138A>G), GAC1536AAT (6142G>A 6144C>T), AAC1537CAT (6145A>C 6147C>T), TGT1538GTT (6148T>G 6149G>T), TTC1539TTT (6153C>T), AAA1540AAG (6156A>G), GGT1541GAA (6158G>A 6159T>A), GGT1541_ATG1542insCAC (6159_6160insCAC), ATG1542CTT (6160A>C 6162G>T), GAA1543AGG (6163G>A 6164A>G 6165A>G), GAT1544AAG (6166G>A 6168T>G), TTT1545TTC (6171T>C), ATA1546ATT (6174A>T), GCT1547TTG (6175G>T 6176C>T 6177T>G), GTC1548GTT (6180C>T), TAC1549TTT (6182A>T 6183C>T), ATT1550--T (6184_6185delAT), GTG1555ATC (6199G>A 6201G>C), TTC1556TAC (6203T>A), TCA1557AGC (6205T>A 6206C>G 6207A>C), GAA1558AAG (6208G>A 6210A>G)								

*: Inserts / Deletes / Misaligned / Frameshifts

Analysis details

This analysis was performed with panviral2.64

NGS Details (UN18_val): Epiphyllum badnavirus 1

Assembly

Coverage Length	244 (1 contig(s))
Depth Of Coverage	12.9
Number Of Reads	30
Reads Per Million	0.56 rpm (after QC)
Ambiguities	0
Assembly Method	de novo + reference guided assembly
Consensus Caller	Bcf Tools

Coverage Map

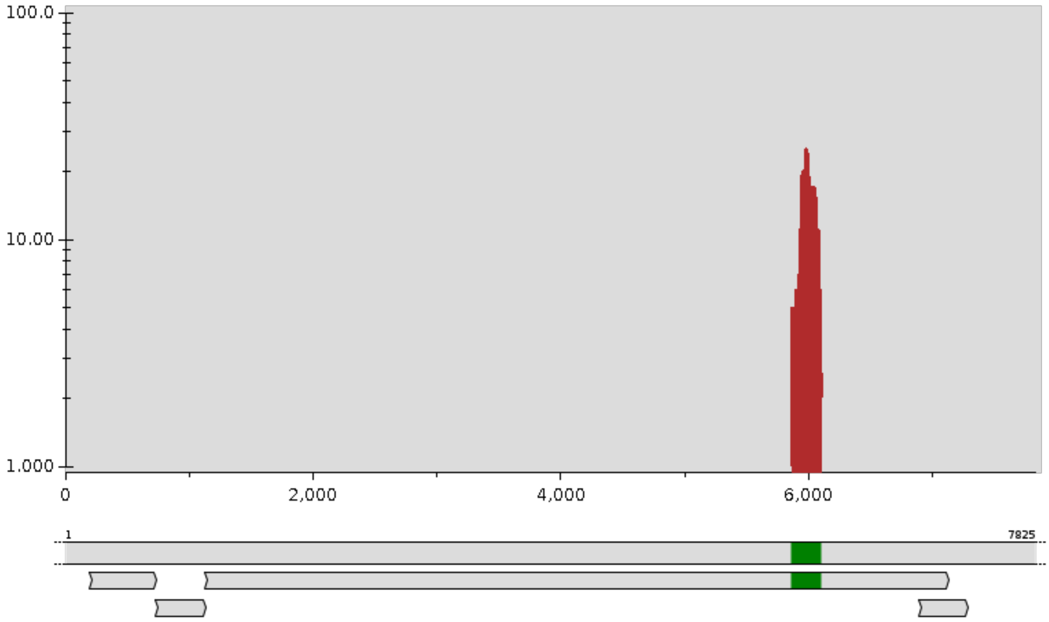

Assignment

Type	Epiphyllum badnavirus 1 (Taxonomy ID: 2518008)
Reference Genome	NC_076247.1
NT Identity (%)	57.085
AA Identity (%)	49.3976
Number Of Stop Codons	0
Number Of CDS	4

Alignment

Alignment Score	64.0 (NT) + 291.0 (AA) = 355.0
Concordance (%)	32.5987

**Alignment Method**	Global, seeded, nucleotide + amino acids (AGA)

Genome Region

Sequence starts at position 5860 and ends at position 6103 relative to NC_076247.1 reference sequence.

Alignment Detailed Statistics

	Begin	End	Coverage	Score	Concordance	Matches	Identities	I/D/M/F*	Stop Codons
**NT**	**5860**	**6103**	**3.1%**	**64**	**13.1%**	**244 (98.8%)**	**141 (57.1%)**	**3/0**	
Mutations:	5865C>G, 5868A>C, 5870T>A, 5878A>T, 5880T>G, 5882T>A, 5883G>A, 5884A>G, 5886G>T, 5887G>C, 5888A>G, 5889C>G, 5893G>T, 5894A>C, 5896T>G, 5897C>A, 5898C>T, 5899A>G, 5904T>G, 5905T>A, 5906G>A, 5911G>A, 5916C>T, 5918T>G, 5922C>A, 5924C>G, 5925A>T, 5926G>T, 5931A>G, 5933T>A, 5934T>C, 5940A>G, 5942G>T, 5943G>C, 5944T>G, 5946A>G, 5949T>C, 5953C>T, 5955A>T, 5961A>G, 5962T>C, 5964G>T, 5966A>C, 5967G>T, 5981T>C, 5982G>T, 5985C>T, 5986C>A, 5987A>T, 5988A>G, 5989C>G, 5990G>A, 5991A>G, 5992A>T, 5993A>T, 5998G>A, 6000C>T, 6001A>C, 6002A>G, 6003C>A, 6004T>G, 6005G>T, 6006T>G, 6009C>T, 6011G>A, 6012A>G, 6012_6013insCCC, 6013G>T, 6016C>T, 6019T>G, 6020C>A, 6021A>C, 6022G>C, 6023G>A, 6024C>G, 6027T>C, 6030A>G, 6031G>A, 6032C>T, 6033A>T, 6036T>G, 6038A>T, 6039C>T, 6043G>A, 6048C>T, 6052C>T, 6057C>T, 6059T>A, 6060C>T, 6061A>T, 6062G>C, 6063T>C, 6066C>G, 6068C>G, 6072A>G, 6073G>A, 6074A>T, 6078C>T, 6084A>G, 6095A>G, 6097T>A, 6098C>T, 6100A>G, 6103C>T								
**CDS**									
QKM20_gp3	1578	1659	4.1%	291	48.1%	82 (98.8%)	41 (49.4%)	1/0/0/0	0
Protein mutations:	S1579R (5865C>G), F1581Y (5870T>A), I1584L (5878A>T 5880T>G), M1585K (5882T>A 5883G>A), M1586V (5884A>G 5886G>T), D1587R (5887G>C 5888A>G 5889C>G), E1589S (5893G>T 5894A>C), S1590D (5896T>G 5897C>A 5898C>T), I1591V (5899A>G), W1593K (5905T>A 5906G>A), A1595T (5911G>A), L1597R (5918T>G), P1599R (5924C>G 5925A>T), D1600Y (5926G>T), L1602H (5933T>A 5934T>C), W1605F (5942G>T 5943G>C), L1606V (5944T>G 5946A>G), P1609S (5953C>T 5955A>T), K1613T (5966A>C 5967G>T), V1618A (5981T>C 5982G>T), Q1620M (5986C>A 5987A>T 5988A>G), R1621E (5989C>G 5990G>A 5991A>G), K1622L (5992A>T 5993A>T), D1624N (5998G>A 6000C>T), N1625R (6001A>C 6002A>G 6003C>A), C1626V (6004T>G 6005G>T 6006T>G), R1628K (6011G>A 6012A>G), R1628_D1629insP (6012_6013insCCC), D1629Y (6013G>T), S1631D (6019T>G 6020C>A 6021A>C), G1632Q (6022G>C 6023G>A 6024C>G), A1635I (6031G>A 6032C>T 6033A>T), Y1637F (6038A>T 6039C>T), D1639N (6043G>A), F1644Y (6059T>A 6060C>T), N1646K (6066C>G), T1647S (6068C>G), D1649I (6073G>A 6074A>T), K1656R (6095A>G), S1657I (6097T>A 6098C>T), M1658V (6100A>G)								
Codon mutations:	AGC1579AGG (5865C>G), GGA1580GGC (5868A>C), TTC1581TAC (5870T>A), ATT1584TTG (5878A>T 5880T>G), ATG1585AAA (5882T>A 5883G>A), ATG1586GTT (5884A>G 5886G>T), GAC1587CGG (5887G>C 5888A>G 5889C>G), GAA1589TCA (5893G>T 5894A>C), TCC1590GAT (5896T>G 5897C>A 5898C>T), ATC1591GTC (5899A>G), CCT1592CCG (5904T>G), TGG1593AAG (5905T>A 5906G>A), GCC1595ACC (5911G>A), TTC1596TTT (5916C>T), CTG1597CGG (5918T>G), ACC1598ACA (5922C>A), CCA1599CGT (5924C>G 5925A>T), GAT1600TAT (5926G>T), GGA1601GGG (5931A>G), CTT1602CAC (5933T>A 5934T>C), GAA1604GAG (5940A>G), TGG1605TTC (5942G>T 5943G>C), TTA1606GTG (5944T>G 5946A>G), GTT1607GTC (5949T>C), CCA1609TCT (5953C>T 5955A>T), GGA1611GGG (5961A>G), TTG1612CTT (5962T>C 5964G>T), AAG1613ACT (5966A>C 5967G>T), GTG1618GCT (5981T>C 5982G>T), TTC1619TTT (5985C>T), CAA1620ATG (5986C>A 5987A>T 5988A>G), CGA1621GAG (5989C>G 5990G>A 5991A>G), AAG1622TTG (5992A>T 5993A>T), GAC1624AAT (5998G>A 6000C>T), AAC1625CGA (6001A>C 6002A>G 6003C>A), TGT1626GTG (6004T>G 6005G>T 6006T>G), TTC1627TTT (6009C>T), AGA1628AAG (6011G>A 6012A>G), AGA1628_GAC1629insCCC (6012_6013insCCC), GAC1629TAC (6013G>T), CTG1630TTG (6016C>T), TCA1631GAC (6019T>G 6020C>A 6021A>C), GGC1632CAG (6022G>C 6023G>A 6024C>G), TTT1633TTC (6027T>C), GTA1634GTG (6030A>G), GCA1635ATT (6031G>A 6032C>T 6033A>T), GTT1636GTG (6036T>G), TAC1637TTT (6038A>T 6039C>T), GAT1639AAT (6043G>A), GAC1640GAT (6048C>T), CTG1642TTG (6052C>T), GTC1643GTT (6057C>T), TTC1644TAT (6059T>A 6060C>T), AGT1645TCC (6061A>T 6062G>C 6063T>C), AAC1646AAG (6066C>G), ACT1647AGT (6068C>G), GAA1648GAG (6072A>G), GAT1649ATT (6073G>A 6074A>T), GAC1650GAT (6078C>T), GCA1652GCG (6084A>G), AAG1656AGG (6095A>G), TCC1657ATC (6097T>A 6098C>T), ATG1658GTG (6100A>G), CTG1659T.. (6103C>T)								
**Proteins**									
polyprotein (YP_010797894.1)	1578	1659	4.1%	291	48.1%	82 (98.8%)	41 (49.4%)	1/0/0/0	0
Protein mutations:	S1579R (5865C>G), F1581Y (5870T>A), I1584L (5878A>T 5880T>G), M1585K (5882T>A 5883G>A), M1586V (5884A>G 5886G>T), D1587R (5887G>C 5888A>G 5889C>G), E1589S (5893G>T 5894A>C), S1590D (5896T>G 5897C>A 5898C>T), I1591V (5899A>G), W1593K (5905T>A 5906G>A), A1595T (5911G>A), L1597R (5918T>G), P1599R (5924C>G 5925A>T), D1600Y (5926G>T), L1602H (5933T>A 5934T>C), W1605F (5942G>T 5943G>C), L1606V (5944T>G 5946A>G), P1609S (5953C>T 5955A>T), K1613T (5966A>C 5967G>T), V1618A (5981T>C 5982G>T), Q1620M (5986C>A 5987A>T 5988A>G), R1621E (5989C>G 5990G>A 5991A>G), K1622L (5992A>T 5993A>T), D1624N (5998G>A 6000C>T), N1625R (6001A>C 6002A>G 6003C>A), C1626V (6004T>G 6005G>T 6006T>G), R1628K (6011G>A 6012A>G), R1628_D1629insP (6012_6013insCCC), D1629Y (6013G>T), S1631D (6019T>G 6020C>A 6021A>C), G1632Q (6022G>C 6023G>A 6024C>G), A1635I (6031G>A 6032C>T 6033A>T), Y1637F (6038A>T 6039C>T), D1639N (6043G>A), F1644Y (6059T>A 6060C>T), N1646K (6066C>G), T1647S (6068C>G), D1649I (6073G>A 6074A>T), K1656R (6095A>G), S1657I (6097T>A 6098C>T), M1658V (6100A>G)								
Codon mutations:	AGC1579AGG (5865C>G), GGA1580GGC (5868A>C), TTC1581TAC (5870T>A), ATT1584TTG (5878A>T 5880T>G), ATG1585AAA (5882T>A 5883G>A), ATG1586GTT (5884A>G 5886G>T), GAC1587CGG (5887G>C 5888A>G 5889C>G), GAA1589TCA (5893G>T 5894A>C), TCC1590GAT (5896T>G 5897C>A 5898C>T), ATC1591GTC (5899A>G), CCT1592CCG (5904T>G), TGG1593AAG (5905T>A 5906G>A), GCC1595ACC (5911G>A), TTC1596TTT (5916C>T), CTG1597CGG (5918T>G), ACC1598ACA (5922C>A), CCA1599CGT (5924C>G 5925A>T), GAT1600TAT (5926G>T), GGA1601GGG (5931A>G), CTT1602CAC (5933T>A 5934T>C), GAA1604GAG (5940A>G), TGG1605TTC (5942G>T 5943G>C), TTA1606GTG (5944T>G 5946A>G), GTT1607GTC (5949T>C), CCA1609TCT (5953C>T 5955A>T), GGA1611GGG (5961A>G), TTG1612CTT (5962T>C 5964G>T), AAG1613ACT (5966A>C 5967G>T), GTG1618GCT (5981T>C 5982G>T), TTC1619TTT (5985C>T), CAA1620ATG (5986C>A 5987A>T 5988A>G), CGA1621GAG (5989C>G 5990G>A 5991A>G), AAG1622TTG (5992A>T 5993A>T), GAC1624AAT (5998G>A 6000C>T), AAC1625CGA (6001A>C 6002A>G 6003C>A), TGT1626GTG (6004T>G 6005G>T 6006T>G), TTC1627TTT (6009C>T), AGA1628AAG (6011G>A 6012A>G), AGA1628_GAC1629insCCC (6012_6013insCCC), GAC1629TAC (6013G>T), CTG1630TTG (6016C>T), TCA1631GAC (6019T>G 6020C>A 6021A>C), GGC1632CAG (6022G>C 6023G>A 6024C>G), TTT1633TTC (6027T>C), GTA1634GTG (6030A>G), GCA1635ATT (6031G>A 6032C>T 6033A>T), GTT1636GTG (6036T>G), TAC1637TTT (6038A>T 6039C>T), GAT1639AAT (6043G>A), GAC1640GAT (6048C>T), CTG1642TTG (6052C>T), GTC1643GTT (6057C>T), TTC1644TAT (6059T>A 6060C>T), AGT1645TCC (6061A>T 6062G>C 6063T>C), AAC1646AAG (6066C>G), ACT1647AGT (6068C>G), GAA1648GAG (6072A>G), GAT1649ATT (6073G>A 6074A>T), GAC1650GAT (6078C>T), GCA1652GCG (6084A>G), AAG1656AGG (6095A>G), TCC1657ATC (6097T>A 6098C>T), ATG1658GTG (6100A>G), CTG1659T.. (6103C>T)								

*: Inserts / Deletes / Misaligned / Frameshifts

Analysis details

This analysis was performed with panviral2.64

NGS Details (UN18_val): Badnavirus epsiloninflatheobromae

Assembly

**Coverage Length**	616 (2 contig(s))
**Depth Of Coverage**	3.9
**Number Of Reads**	23
**Reads Per Million**	0.43 rpm (after QC)
**Ambiguities**	0
**Assembly Method**	de novo + reference guided assembly
**Consensus Caller**	Bcf Tools

Coverage Map

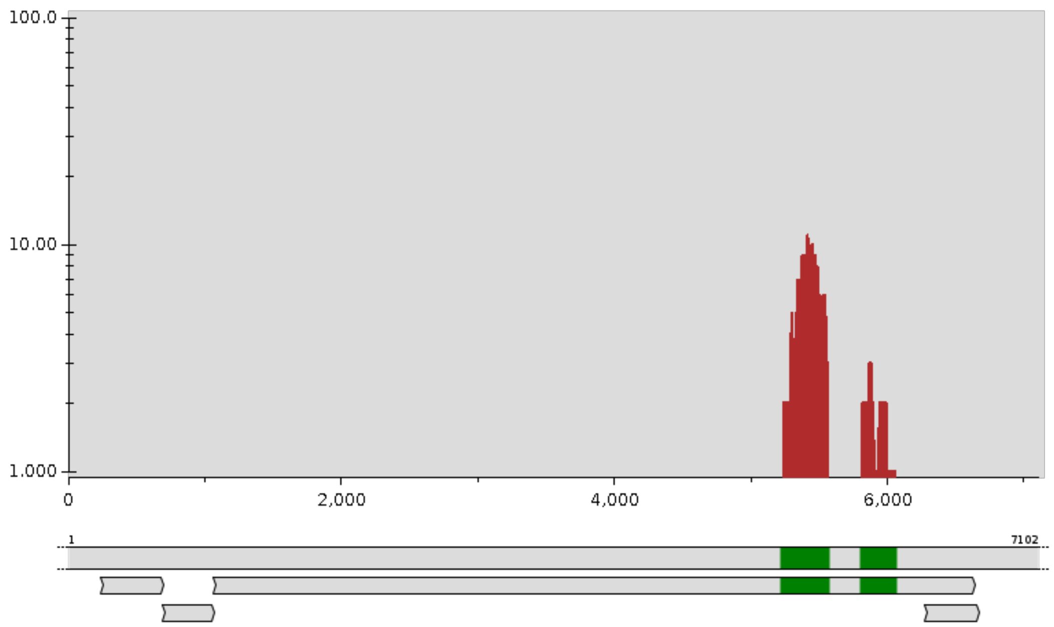

Assignment

**Type**	Badnavirus epsiloninflatheobromae (Taxonomy ID: 3047711)
**Reference Genome**	NC_043535.1
**NT Identity (%)**	57.44
**AA Identity (%)**	48.5577
**Number Of Stop Codons**	0
**Number Of CDS**	4

Alignment

**Alignment Score**	177.0 (NT) + 779.0 (AA) = 956.0
**Concordance (%)**	34.8651

Alignment Method	Global, seeded, nucleotide + amino acids (AGA)

Genome Region

Sequence starts at position 5228 and ends at position 6068 relative to NC_043535.1 reference sequence.

Alignment Detailed Statistics

	Begin	End	Coverage	Score	Concordance	Matches	Identities	I/D/M/F*	Stop Codons
**NT**	**5228**	**6068**	**8.7%**	**177**	**14.4%**	**616 (98.6%)**	**359 (57.4%)**	**9/0**	
Mutations:	5228T>G, 5231A>G, 5233T>A, 5240G>A, 5243C>T, 5244G>A, 5245C>G, 5256G>C, 5257A>C, 5258G>C, 5259T>G, 5260C>A, 5261A>C, 5264C>T, 5267A>C, 5268T>A, 5269G>A, 5270G>A, 5273C>T, 5276G>C, 5280T>A, 5282G>A, 5283G>A, 5284T>C, 5285A>T, 5287C>A, 5288T>C, 5291A>T, 5294A>T, 5296T>C, 5303A>G, 5307C>T, 5312T>G, 5315G>T, 5318A>C, 5321T>C, 5325C>T, 5327C>A, 5329A>C, 5330A>C, 5333T>C, 5344T>C, 5345C>T, 5349C>T, 5350A>G, 5351A>C, 5353G>C, 5354A>G, 5355A>C, 5356A>T, 5361G>A, 5363T>A, 5364C>A, 5366T>G, 5367T>A, 5368G>T, 5369T>A, 5372_5373insCAT, 5373G>C, 5375A>T, 5376G>T, 5377G>A, 5378A>C, 5379A>C, 5380C>T, 5384A>T, 5385G>C, 5387G>A, 5390T>C, 5391A>G, 5393T>G, 5395C>T, 5396T>A, 5403A>T, 5405T>G, 5415A>G, 5420T>C, 5422T>A, 5429T>C, 5432A>C, 5433G>T, 5434A>T, 5439C>G, 5441G>A, 5444T>C, 5445A>G, 5446A>T, 5453T>C, 5454C>T, 5463T>G, 5465C>A, 5466C>T, 5468G>C, 5469G>C, 5471G>A, 5472A>G, 5473G>T, 5474A>C, 5476G>T, 5477C>A, 5478A>C, 5479A>G, 5481C>G, 5484C>A, 5488G>A, 5490T>C, 5492G>A, 5493G>T, 5494T>A, 5495G>C, 5496T>G, 5498A>C, 5500G>A, 5501C>G, 5503C>A, 5504A>G, 5505A>G, 5506C>A, 5507A>G, 5511A>T, 5512T>G, 5513G>C, 5514A>G, 5516A>G, 5517A>T, 5519A>T, 5521G>C, 5525G>A, 5526A>C, 5528A>T, 5529A>G, 5530G>A, 5531G>A, 5532A>G, 5534C>G, 5535G>C, 5537G>C, 5543A>G, 5547G>C, 5548C>A, 5549T>C, 5550G>T, 5552G>C, 5555C>T, 5557A>G, 5559G>C, 5564G>T, 5565A>G, 5568A>C, 5574C>A, 5576A>G, 5577C>G, 5804T>G, 5813G>A, 5814G>A, 5815A>G, 5816G>C, 5819C>A, 5824C>T, 5829G>T, 5830A>C, 5833C>A, 5834G>T, 5837C>T, 5838A>C, 5840C>A, 5846T>A, 5852T>A, 5859T>A, 5862A>C, 5864G>C, 5865G>C, 5878G>C, 5879A>T, 5880A>G, 5882C>A, 5884G>T, 5885T>A, 5888G>A, 5889T>G, 5890G>C, 5891G>A, 5893A>G, 5897T>A, 5898T>A, 5899T>A, 5900T>C, 5904A>T, 5905G>A, 5906G>C, 5907G>A, 5908A>G, 5909C>T, 5912A>T, 5913C>A, 5914G>A, 5921C>A, 5924G>A, 5927G>A, 5930A>T, 5936C>T, 5939T>C, 5940G>C, 5941C>A, 5948A>T, 5953T>A, 5956G>A, 5958C>G, 5959C>A, 5960_5961insAAGGGT, 5962T>A, 5965A>T, 5966A>G, 5967T>A, 5968C>G, 5969C>T, 5971C>G, 5972A>T, 5975C>A, 5980C>T, 5981T>C, 5984G>A, 5985A>G, 5988C>T, 5989A>T, 5990C>A, 5993G>T, 5994G>A, 5999G>A, 6000A>T, 6003A>G, 6005T>A, 6006T>C, 6011G>T, 6013A>G, 6014A>T, 6017T>C, 6020G>A, 6021C>T, 6027T>A, 6028A>T, 6034G>A, 6035T>C, 6039C>A, 6040C>G, 6041A>T, 6043C>A, 6044G>A, 6045G>A, 6047C>A, 6048A>G, 6049C>T, 6050T>A, 6051A>G, 6053C>A, 6056G>A, 6065T>C, 6066C>G, 6068G>A								

CDS

FLA70_gp3	1390	1669	11.0%	779	51.0%	205 (98.6%)	101 (48.6%)	3/0/0/0	0
Protein mutations:	F1391Y (5233T>A), A1395R (5244G>A 5245C>G), E1399P (5256G>C 5257A>C 5258G>C), S1400D (5259T>G 5260C>A 5261A>C), W1403K (5268T>A 5269G>A 5270G>A), W1407R (5280T>A 5282G>A), V1408T (5283G>A 5284T>C 5285A>T), P1409H (5287C>A 5288T>C), Q1410H (5291A>T), L1412P (5296T>C), M1418I (5315G>T), K1423T (5329A>C 5330A>C), I1428T (5344T>C 5345C>T), Q1430C (5349C>T 5350A>G 5351A>C), R1431T (5353G>C 5354A>G), K1432L (5355A>C 5356A>T), D1434K (5361G>A 5363T>A), H1435K (5364C>A 5366T>G), C1436I (5367T>A 5368G>T 5369T>A), F1437_A1438insH (5372_5373insCAT), A1438P (5373G>C 5375A>T), G1439Y (5376G>T 5377G>A 5378A>C), T1440L (5379A>C 5380C>T), E1441D (5384A>T), E1442Q (5385G>C 5387G>A), I1444V (5391A>G 5393T>G), A1445V (5395C>T 5396T>A), I1448L (5403A>T 5405T>G), I1452V (5415A>G), F1454Y (5422T>A), E1458L (5433G>T 5434A>T), Q1460E (5439C>G 5441G>A), K1462V (5445A>G 5446A>T), F1468V (5463T>G 5465C>A), L1469F (5466C>T 5468G>C), E1470Q (5469G>C 5471G>A), R1471V (5472A>G 5473G>T 5474A>C), C1472L (5476G>T 5477C>A), K1473R (5478A>C 5479A>G), Q1474E (5481C>G), H1475N (5484C>A), G1476E (5488G>A), V1478Y (5493G>T 5494T>A 5495G>C), L1479V (5496T>G 5498A>C), S1480K (5500G>A 5501C>G), P1481Q (5503C>A 5504A>G), T1482E (5505A>G 5506C>A 5507A>G), M1484C (5511A>T 5512T>G 5513G>C), K1485E (5514A>G 5516A>G), I1486F (5517A>T 5519A>T), G1487A (5521G>C), K1489H (5526A>C 5528A>T), R1490E (5529A>G 5530G>A 5531G>A), I1491V (5532A>G 5534C>G), E1492H (5535G>C 5537G>C), A1496H (5547G>C 5548C>A 5549T>C), V1497F (5550G>T 5552G>C), N1499S (5557A>G), E1500Q (5559G>C), K1502E (5565A>G), I1503L (5568A>C), L1505M (5574C>A 5576A>G), E1585S (5814G>A 5815A>G 5816G>C), P1588L (5824C>T), E1590S (5829G>T 5830A>C), A1591D (5833C>A 5834G>T), I1593L (5838A>C 5840C>A), C1600S (5859T>A), M1601L (5862A>C 5864G>C), E1602Q (5865G>C), G1606A (5878G>C 5879A>T), I1607V (5880A>G 5882C>A), C1608L (5884G>T 5885T>A), W1610A (5889T>G 5890G>C 5891G>A), K1611R (5893A>G), F1613N (5898T>A 5899T>A 5900T>C), R1615Y (5904A>T 5905G>A 5906G>C), D1616S (5907G>A 5908A>G 5909C>T), R1618K (5913C>A 5914G>A), D1620E (5921C>A), A1627Q (5940G>C 5941C>A), F1631Y (5953T>A), R1632K (5956G>A), P1633E (5958C>G 5959C>A), P1633_I1634insKG (5960_5961insAAGGGT), I1634N (5962T>A), K1635M (5965A>T 5966A>G), T1637S (5971C>G 5972A>T), A1640V (5980C>T 5981T>C), I1642V (5985A>G), H1643L (5988C>T 5989A>T 5990C>A), V1645I (5994G>A), M1646I (5999G>A), N1647Y (6000A>T), S1648G (6003A>G 6005T>A), K1650N (6011G>T), K1651S (6013A>G 6014A>T), Y1656I (6027T>A 6028A>T), S1658N (6034G>A 6035T>C), P1660S (6039C>A 6040C>G 6041A>T), A1661E (6043C>A 6044G>A), V1662I (6045G>A 6047C>A), T1663V (6048A>G 6049C>T 6050T>A), I1664V (6051A>G 6053C>A), Q1669E (6066C>G 6068G>A)								
Codon mutations:	AGT1389..G (5228T>G), GGA1390GGG (5231A>G), TTT1391TAT (5233T>A), CAG1393CAA (5240G>A), ATC1394ATT (5243C>T), GCA1395AGA (5244G>A 5245C>G), GAG1399CCC (5256G>C 5257A>C 5258G>C), TCA1400GAC (5259T>G 5260C>A 5261A>C), ATC1401ATT (5264C>T), CCA1402CCC (5267A>C), TGG1403AAA (5268T>A 5269G>A 5270G>A), ACC1404ACT (5273C>T), GCG1405GCC (5276G>C), TGG1407AGA (5280T>A 5282G>A), GTA1408ACT (5283G>A 5284T>C 5285A>T), CCT1409CAC (5287C>A 5288T>C), CAA1410CAT (5291A>T), GGA1411GGT (5294A>T), CTT1412CCT (5296T>C), GAA1414GAG (5303A>G), CTG1416TTG (5307C>T), GTT1417GTG (5312T>G), ATG1418ATT (5315G>T), CCA1419CCC (5318A>C), TTT1420TTC (5321T>C), CTC1422TTA (5325C>T 5327C>A), AAA1423ACC (5329A>C 5330A>C), AAT1424AAC (5333T>C), ATC1428ACT (5344T>C 5345C>T), CAA1430TGC (5349C>T 5350A>G 5351A>C), AGA1431ACG (5353G>C 5354A>G), AAA1432CTA (5355A>C 5356A>T), GAT1434AAA (5361G>A 5363T>A), CAT1435AAG (5364C>A 5366T>G), TGT1436ATA (5367T>A 5368G>T 5369T>A), TTT1437_GCA1438insCAT (5372_5373insCAT), GCA1438CCT (5373G>C 5375A>T), GGA1439TAC (5376G>T 5377G>A 5378A>C), ACA1440CTA (5379A>C 5380C>T), GAA1441GAT (5384A>T), GAG1442CAA (5385G>C 5387G>A), TTT1443TTC (5390T>C), ATT1444GTG (5391A>G 5393T>G), GCT1445GTA (5395C>T 5396T>A), ATT1448TTG (5403A>T 5405T>G), ATC1452GTC (5415A>G), ATT1453ATC (5420T>C), TTC1454TAC (5422T>A), AAT1456AAC (5429T>C), ACA1457ACC (5432A>C), GAG1458TTG (5433G>T 5434A>T), CAG1460GAA (5439C>G 5441G>A), CAT1461CAC (5444T>C), AAG1462GTG (5445A>G 5446A>T), CAT1464CAC (5453T>C), CTG1465TTG (5454C>T), TTC1468GTA (5463T>G 5465C>A), CTG1469TTC (5466C>T 5468G>C), GAG1470CAA (5469G>C 5471G>A), AGA1471GTC (5472A>G 5473G>T 5474A>C), TGC1472TTA (5476G>T 5477C>A), AAG1473CGG (5478A>C 5479A>G), CAA1474GAA (5481C>G), CAT1475AAT (5484C>A), GGG1476GAG (5488G>A), TTG1477CTA (5490T>C 5492G>A), GTG1478TAC (5493G>T 5494T>A 5495G>C), TTA1479GTC (5496T>G 5498A>C), AGC1480AAG (5500G>A 5501C>G), CCA1481CAG (5503C>A 5504A>G), ACA1482GAG (5505A>G 5506C>A 5507A>G), ATG1484TGC (5511A>T 5512T>G 5513G>C), AAA1485GAG (5514A>G 5516A>G), ATA1486TTT (5517A>T 5519A>T), GGC1487GCC (5521G>C), CAG1488CAA (5525G>A), AAA1489CAT (5526A>C 5528A>T), AGG1490GAA (5529A>G 5530G>A 5531G>A), ATC1491GTG (5532A>G 5534C>G), GAG1492CAC (5535G>C 5537G>C), TTA1494TTG (5543A>G), GCT1496CAC (5547G>C 5548C>A 5549T>C), GTG1497TTC (5550G>T 5552G>C), ATC1498ATT (5555C>T), AAC1499AGC (5557A>G), GAA1500CAA (5559G>C), GGG1501GGT (5564G>T), AAG1502GAG (5565A>G), ATA1503CTA (5568A>C), CTA1505ATG (5574C>A 5576A>G), CAG1506GA. (5577C>G), CTT1581..G (5804T>G), TTG1584TTA (5813G>A), GAG1585AGC (5814G>A 5815A>G 5816G>C), ATC1586ATA (5819C>A), CCA1588CTA (5824C>T), GAA1590TCA (5829G>T 5830A>C), GCG1591GAT (5833C>A 5834G>T), TAC1592TAT (5837C>T), ATC1593CTA (5838A>C 5840C>A), ATT1595ATA (5846T>A), ACT1597ACA (5852T>A), TGC1600AGC (5859T>A), ATG1601CTC (5862A>C 5864G>C), GAA1602CAA (5865G>C), GGA1606GCT (5878G>C 5879A>T), ATC1607GTA (5880A>G 5882C>A), TGT1608TTA (5884G>T 5885T>A), AAG1609AAA (5888G>A), TGG1610GCA (5889T>G 5890G>C 5891G>A), AAA1611AGA (5893A>G), CCT1612CCA (5897T>A), TTT1613AAC (5898T>A 5899T>A 5900T>C), AGG1615TAC (5904A>T 5905G>A 5906G>C), GAC1616AGT (5907G>A 5908A>G 5909C>T), CCA1617CCT (5912A>T), CGA1618AAA (5913C>A 5914G>A), GAC1620GAA (5921C>A), GAG1621GAA (5924G>A), AAG1622AAA (5927G>A), ATA1623ATT (5930A>T), GCC1625GCT (5936C>T), TAT1626TAC (5939T>C), GCA1627CAA (5940G>C 5941C>A), GGA1629GGT (5948A>T), TTC1631TAC (5953T>A), AGA1632AAA (5956G>A), CCA1633GAA (5958C>G 5959C>A), CCA1633_ATC1634insAAGGGT (5960_5961insAAGGGT), ATC1634AAC (5962T>A), AAA1635ATG (5965A>T 5966A>G), TCC1636AGT (5967T>A 5968C>G 5969C>T), ACA1637AGT (5971C>G 5972A>T), ATC1638ATA (5975C>A), GCT1640GTC (5980C>T 5981T>C), GAG1641GAA (5984G>A), ATA1642GTA (5985A>G), CAC1643TTA (5988C>T 5989A>T 5990C>A), GCG1644GCT (5993G>T), GTA1645ATA (5994G>A), ATG1646ATA (5999G>A), AAT1647TAT (6000A>T), AGT1648GGA (6003A>G 6005T>A), TTA1649CTA (6006T>C), AAG1650AAT (6011G>T), AAA1651AGT (6013A>G 6014A>T), TTT1652TTC (6017T>C), AAG1653AAA (6020G>A), CTA1654TTA (6021C>T), TAT1656ATT (6027T>A 6028A>T), AGT1658AAC (6034G>A 6035T>C), CCA1660AGT (6039C>A 6040C>G 6041A>T), GCG1661GAA (6043C>A 6044G>A), GTC1662ATA (6045G>A 6047C>A), ACT1663GTA (6048A>G 6049C>T 6050T>A), ATC1664GTA (6051A>G 6053C>A), AGG1665AGA (6056G>A), TGT1668TGC (6065T>C), CAG1669GAA (6066C>G 6068G>A)								

Proteins

ORF3 polyprotein (YP_009666830.1)	1390	1669	11.0%	779	51.0%	205 (98.6%)	101 (48.6%)	3/0/0/0	0
Protein mutations:	F1391Y (5233T>A), A1395R (5244G>A 5245C>G), E1399P (5256G>C 5257A>C 5258G>C), S1400D (5259T>G 5260C>A 5261A>C), W1403K (5268T>A 5269G>A 5270G>A), W1407R (5280T>A 5282G>A), V1408T (5283G>A 5284T>C 5285A>T), P1409H (5287C>A 5288T>C), Q1410H (5291A>T), L1412P (5296T>C), M1418I (5315G>T), K1423T (5329A>C 5330A>C), I1428T (5344T>C 5345C>T), Q1430C (5349C>T 5350A>G 5351A>C), R1431T (5353G>C 5354A>G), K1432L (5355A>C 5356A>T), D1434K (5361G>A 5363T>A), H1435K (5364C>A 5366T>G), C1436I (5367T>A 5368G>T 5369T>A), F1437_A1438insH (5372_5373insCAT), A1438P (5373G>C 5375A>T), G1439Y (5376G>T 5377G>A 5378A>C), T1440L (5379A>C 5380C>T), E1441D (5384A>T), E1442Q (5385G>C 5387G>A), I1444V (5391A>G 5393T>G), A1445V (5395C>T 5396T>A), I1448L (5403A>T 5405T>G), I1452V (5415A>G), F1454Y (5422T>A), E1458L (5433G>T 5434A>T), Q1460E (5439C>G 5441G>A), K1462V (5445A>G 5446A>T), F1468V (5463T>G 5465C>A), L1469F (5466C>T 5468G>C), E1470Q (5469G>C 5471G>A), R1471V (5472A>G 5473G>T 5474A>C), C1472L (5476G>T 5477C>A), K1473R (5478A>C 5479A>G), Q1474E (5481C>G), H1475N (5484C>A), G1476E (5488G>A), V1478Y (5493G>T 5494T>A 5495G>C), L1479V (5496T>G 5498A>C), S1480K (5500G>A 5501C>G), P1481Q (5503C>A 5504A>G), T1482E (5505A>G 5506C>A 5507A>G), M1484C (5511A>T 5512T>G 5513G>C), K1485E (5514A>G 5516A>G), I1486F (5517A>T 5519A>T), G1487A (5521G>C), K1489H (5526A>C 5528A>T), R1490E (5529A>G 5530G>A 5531G>A), I1491V (5532A>G 5534C>G), E1492H (5535G>C 5537G>C), A1496H (5547G>C 5548C>A 5549T>C), V1497F (5550G>T 5552G>C), N1499S (5557A>G), E1500Q (5559G>C), K1502E (5565A>G), I1503L (5568A>C), L1505M (5574C>A 5576A>G), E1585S (5814G>A 5815A>G 5816G>C), P1588L (5824C>T), E1590S (5829G>T 5830A>C), A1591D (5833C>A 5834G>T), I1593L (5838A>C 5840C>A), C1600S (5859T>A), M1601L (5862A>C 5864G>C), E1602Q (5865G>C), G1606A (5878G>C 5879A>T), I1607V (5880A>G 5882C>A), C1608L (5884G>T 5885T>A), W1610A (5889T>G 5890G>C 5891G>A), K1611R (5893A>G), F1613N (5898T>A 5899T>A 5900T>C), R1615Y (5904A>T 5905G>A 5906G>C), D1616S (5907G>A 5908A>G 5909C>T), R1618K (5913C>A 5914G>A), D1620E (5921C>A), A1627Q (5940G>C 5941C>A), F1631Y (5953T>A), R1632K (5956G>A), P1633E (5958C>G 5959C>A), P1633_I1634insKG (5960_5961insAAGGGT), I1634N (5962T>A), K1635M (5965A>T 5966A>G), T1637S (5971C>G 5972A>T), A1640V (5980C>T 5981T>C), I1642V (5985A>G), H1643L (5988C>T 5989A>T 5990C>A), V1645I (5994G>A), M1646I (5999G>A), N1647Y (6000A>T), S1648G (6003A>G 6005T>A), K1650N (6011G>T), K1651S (6013A>G 6014A>T), Y1656I (6027T>A 6028A>T), S1658N (6034G>A 6035T>C), P1660S (6039C>A 6040C>G 6041A>T), A1661E (6043C>A 6044G>A), V1662I (6045G>A 6047C>A), T1663V (6048A>G 6049C>T 6050T>A), I1664V (6051A>G 6053C>A), Q1669E (6066C>G 6068G>A)								

	Begin	End	Coverage	Score	Concordance	Matches	Identities	I/D/M/F*	Stop Codons
**NT**	**5228**	**6068**	**8.7%**	**177**	**14.4%**	**616 (98.6%)**	**359 (57.4%)**	**9/0**	
Codon mutations:	AGT1389..G (5228T>G), GGA1390GGG (5231A>G), TTT1391TAT (5233T>A), CAG1393CAA (5240G>A), ATC1394ATT (5243C>T), GCA1395AGA (5244G>A 5245C>G), GAG1399CCC (5256G>C 5257A>C 5258G>C), TCA1400GAC (5259T>G 5260C>A 5261A>C), ATC1401ATT (5264C>T), CCA1402CCC (5267A>C), TGG1403AAA (5268T>A 5269G>A 5270G>A), ACC1404ACT (5273C>T), GCG1405GCC (5276G>C), TGG1407AGA (5280T>A 5282G>A), GTA1408ACT (5283G>A 5284T>C 5285A>T), CCT1409CAC (5287C>A 5288T>C), CAA1410CAT (5291A>T), GGA1411GGT (5294A>T), CTT1412CCT (5296T>C), GAA1414GAG (5303A>G), CTG1416TTG (5307C>T), GTT1417GTG (5312T>G), ATG1418ATT (5315G>T), CCA1419CCC (5318A>C), TTT1420TTC (5321T>C), CTC1422TTA (5325C>T 5327C>A), AAA1423ACC (5329A>C 5330A>C), AAT1424AAC (5333T>C), ATC1428ACT (5344T>C 5345C>T), CAA1430TGC (5349C>T 5350A>G 5351A>C), AGA1431ACG (5353G>C 5354A>G), AAA1432CTA (5355A>C 5356A>T), GAT1434AAA (5361G>A 5363T>A), CAT1435AAG (5364C>A 5366T>G), TGT1436ATA (5367T>A 5368G>T 5369T>A), TTT1437_GCA1438insCAT (5372_5373insCAT), GCA1438CCT (5373G>C 5375A>T), GGA1439TAC (5376G>T 5377G>A 5378A>C), ACA1440CTA (5379A>C 5380C>T), GAA1441GAT (5384A>T), GAG1442CAA (5385G>C 5387G>A), TTT1443TTC (5390T>C), ATT1444GTG (5391A>G 5393T>G), GCT1445GTA (5395C>T 5396T>A), ATT1448TTG (5403A>T 5405T>G), ATC1452GTC (5415A>G), ATT1453ATC (5420T>C), TTC1454TAC (5422T>A), AAT1456AAC (5429T>C), ACA1457ACC (5432A>C), GAG1458TTG (5433G>T 5434A>T), CAG1460GAA (5439C>G 5441G>A), CAT1461CAC (5444T>C), AAG1462GTG (5445A>G 5446A>T), CAT1464CAC (5453T>C), CTG1465TTG (5454C>T), TTC1468GTA (5463T>G 5465C>A), CTG1469TTC (5466C>T 5468G>C), GAG1470CAA (5469G>C 5471G>A), AGA1471GTC (5472A>G 5473G>T 5474A>C), TGC1472TTA (5476G>T 5477C>A), AAG1473CGG (5478A>C 5479A>G), CAA1474GAA (5481C>G), CAT1475AAT (5484C>A), GGG1476GAG (5488G>A), TTG1477CTA (5490T>C 5492G>A), GTG1478TAC (5493G>T 5494T>A 5495G>C), TTA1479GTC (5496T>G 5498A>C), AGC1480AAG (5500G>A 5501C>G), CCA1481CAG (5503C>A 5504A>G), ACA1482GAG (5505A>G 5506C>A 5507A>G), ATG1484TGC (5511A>T 5512T>G 5513G>C), AAA1485GAG (5514A>G 5516A>G), ATA1486TTT (5517A>T 5519A>T), GGC1487GCC (5521G>C), CAG1488CAA (5525G>A), AAA1489CAT (5526A>C 5528A>T), AGG1490GAA (5529A>G 5530G>A 5531G>A), ATC1491GTG (5532A>G 5534C>G), GAG1492CAC (5535G>C 5537G>C), TTA1494TTG (5543A>G), GCT1496CAC (5547G>C 5548C>A 5549T>C), GTG1497TTC (5550G>T 5552G>C), ATC1498ATT (5555C>T), AAC1499AGC (5557A>G), GAA1500CAA (5559G>C), GGG1501GGT (5564G>T), AAG1502GAG (5565A>G), ATA1503CTA (5568A>C), CTA1505ATG (5574C>A 5576A>G), CAG1506GA. (5577C>G), CTT1581..G (5804T>G), TTG1584TTA (5813G>A), GAG1585AGC (5814G>A 5815A>G 5816G>C), ATC1586ATA (5819C>A), CCA1588CTA (5824C>T), GAA1590TCA (5829G>T 5830A>C), GCG1591GAT (5833C>A 5834G>T), TAC1592TAT (5837C>T), ATC1593CTA (5838A>C 5840C>A), ATT1595ATA (5846T>A), ACT1597ACA (5852T>A), TGC1600AGC (5859T>A), ATG1601CTC (5862A>C 5864G>C), GAA1602CAA (5865G>C), GGA1606GCT (5878G>C 5879A>T), ATC1607GTA (5880A>G 5882C>A), TGT1608TTA (5884G>T 5885T>A), AAG1609AAA (5888G>A), TGG1610GCA (5889T>G 5890G>C 5891G>A), AAA1611AGA (5893A>G), CCT1612CCA (5897T>A), TTT1613AAC (5898T>A 5899T>A 5900T>C), AGG1615TAC (5904A>T 5905G>A 5906G>C), GAC1616AGT (5907G>A 5908A>G 5909C>T), CCA1617CCT (5912A>T), CGA1618AAA (5913C>A 5914G>A), GAC1620GAA (5921C>A), GAG1621GAA (5924G>A), AAG1622AAA (5927G>A), ATA1623ATT (5930A>T), GCC1625GCT (5936C>T), TAT1626TAC (5939T>C), GCA1627CAA (5940G>C 5941C>A), GGA1629GGT (5948A>T), TTC1631TAC (5953T>A), AGA1632AAA (5956G>A), CCA1633GAA (5958C>G 5959C>A), CCA1633_ATC1634insAAGGGT (5960_5961insAAGGGT), ATC1634AAC (5962T>A), AAA1635ATG (5965A>T 5966A>G), TCC1636AGT (5967T>A 5968C>G 5969C>T), ACA1637AGT (5971C>G 5972A>T), ATC1638ATA (5975C>A), GCT1640GTC (5980C>T 5981T>C), GAG1641GAA (5984G>A), ATA1642GTA (5985A>G), CAC1643TTA (5988C>T 5989A>T 5990C>A), GCG1644GCT (5993G>T), GTA1645ATA (5994G>A), ATG1646ATA (5999G>A), AAT1647TAT (6000A>T), AGT1648GGA (6003A>G 6005T>A), TTA1649CTA (6006T>C), AAG1650AAT (6011G>T), AAA1651AGT (6013A>G 6014A>T), TTT1652TTC (6017T>C), AAG1653AAA (6020G>A), CTA1654TTA (6021C>T), TAT1656ATT (6027T>A 6028A>T), AGT1658AAC (6034G>A 6035T>C), CCA1660AGT (6039C>A 6040C>G 6041A>T), GCG1661GAA (6043C>A 6044G>A), GTC1662ATA (6045G>A 6047C>A), ACT1663GTA (6048A>G 6049C>T 6050T>A), ATC1664GTA (6051A>G 6053C>A), AGG1665AGA (6056G>A), TGT1668TGC (6065T>C), CAG1669GAA (6066C>G 6068G>A)								

*: Inserts / Deletes / Misaligned / Frameshifts

Analysis details

This analysis was performed with panviral2.64

NGS Details (UN18_val): Dioscovirus dioscoreae

Assembly

**Coverage Length**	915 (1 contig(s))
**Depth Of Coverage**	3.2
**Number Of Reads**	23
**Reads Per Million**	0.43 rpm (after QC)
**Ambiguities**	0
**Assembly Method**	de novo + reference guided assembly
**Consensus Caller**	Bcf Tools

Coverage Map

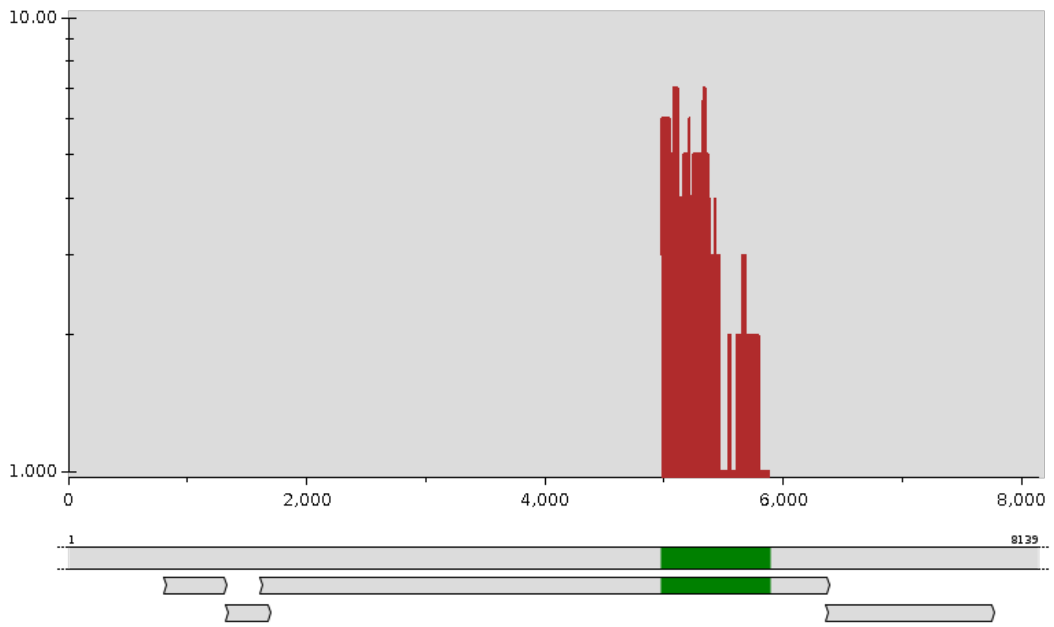

Assignment

**Type**	Dioscovirus dioscoreae (Taxonomy ID: 3052184)
**Reference Genome**	NC_040712.1
**NT Identity (%)**	61.8421
**AA Identity (%)**	52.6316
**Number Of Stop Codons**	0
**Number Of CDS**	4

Alignment

**Alignment Score**	420.0 (NT) + 1186.0 (AA) = 1606.0
**Concordance (%)**	40.8444

**Alignment Method**	Global, seeded, nucleotide + amino acids (AGA)

Genome Region

Sequence starts at position 4980 and ends at position 5894 relative to NC_040712.1 reference sequence.

Alignment Detailed Statistics

	Begin	End	Coverage	Score	Concordance	Matches	Identities	I/D/M/F*	Stop Codons
**NT**	**4980**	**5894**	**11.2%**	**420**	**23.2%**	**912 (99.7%)**	**564 (61.6%)**	**0/3**	
Mutations:	4983C>A, 4986C>A, 4988T>A, 4989A>T, 4990A>T, 4992T>C, 4994G>A, 4997A>T, 5001A>T, 5003C>A, 5004C>G, 5009T>A, 5012T>A, 5015G>A, 5016A>G, 5018T>A, 5019G>A, 5021C>A, 5024A>C, 5025T>C, 5029A>T, 5030G>A, 5031T>C, 5038A>G, 5040A>G, 5041A>C, 5042G>C, 5046C>A, 5047A>G, 5048G>A, 5051T>A, 5053G>T, 5054A>G, 5057C>T, 5063G>A, 5066C>T, 5069C>A, 5078T>A, 5081T>A, 5082C>T, 5083T>A, 5091C>A, 5093G>A, 5094G>A, 5095A>T, 5096C>A, 5097A>G, 5098A>C, 5099G>A, 5100C>G, 5102T>A, 5105T>A, 5106A>G, 5107T>A, 5109C>G, 5112T>A, 5115C>A, 5116C>A, 5117A>T, 5118G>C, 5121A>G, 5122T>A, 5123G>C, 5124G>A, 5125C>T, 5126G>A, 5127A>G, 5132A>T, 5134A>T, 5136G>C, 5138A>G, 5140A>T, 5141G>A, 5144T>A, 5147T>A, 5149C>A, 5157C>T, 5159G>A, 5161C>A, 5162A>T, 5163C>T, 5165C>A, 5166A>G, 5167A>G, 5168G>A, 5171T>A, 5174C>A, 5178C>A, 5179C>G, 5180T>A, 5183C>A, 5184C>A, 5185A>C, 5187A>T, 5188G>C, 5190C>A, 5192C>A, 5195T>C, 5198G>A, 5199A>T, 5207T>A, 5213T>G, 5216C>A, 5219C>A, 5222A>C, 5226T>A, 5227C>A, 5228T>C, 5232C>A, 5233A>T, 5237A>C, 5243T>A, 5247A>G, 5248G>C, 5249T>C, 5250C>A, 5252T>A, 5259T>A, 5260A>T, 5261C>A, 5271C>A, 5276A>C, 5288A>T, 5289T>A, 5290A>G, 5291C>A, 5293A>C, 5294A>G, 5295G>T, 5297C>T, 5298C>G, 5299A>C, 5300A>T, 5303C>T, 5305C>A, 5306A>G, 5309C>A, 5313A>G, 5314G>A, 5317T>A, 5318T>A, 5319G>A, 5320A>G, 5321C>T, 5322T>G, 5324T>A, 5325C>T, 5327C>A, 5328C>A, 5331C>A, 5332T>A, 5335A>G, 5339T>A, 5340A>C, 5344A>G, 5345C>T, 5350T>C, 5351T>A, 5354C>A, 5356A>T, 5359G>A, 5360C>T, 5370C>T, 5371T>G, 5372A>T, 5375G>A, 5383T>A, 5384C>T, 5385C>T, 5386A>G, 5387C>G, 5390G>A, 5391A>G, 5395T>G, 5396G>A, 5400G>C, 5403C>G, 5404C>A, 5406C>G, 5408G>T, 5410G>A, 5424G>A, 5429T>C, 5430G>A, 5431T>C, 5432C>T, 5438A>T, 5439C>G, 5441G>A, 5444T>A, 5457A>T, 5471C>T, 5475C>T, 5477C>G, 5480A>G, 5482A>C, 5489T>A, 5490T>C, 5492A>T, 5493G>A, 5502C>A, 5507A>G, 5513T>C, 5519A>T, 5522C>T, 5523A>G, 5524A>G, 5526A>G, 5528A>T, 5531T>C, 5532T>A, 5533C>A, 5535G>A, 5536A>G, 5544T>C, 5545G>T, 5546T>A, 5549T>A, 5552C>T, 5553A>G, 5556G>T, 5558C>T, 5561T>C, 5564T>C, 5565T>C, 5567G>A, 5568A>G, 5573T>C, 5574T>A, 5575C>G, 5577G>A, 5578A>G, 5580A>G, 5581G>A, 5585A>G, 5589C>G, 5595G>C, 5598C>G, 5607T>C, 5608T>A, 5610C>A, 5611A>C, 5612G>A, 5613T>A, 5615C>T, 5618C>T, 5619C>A, 5620A>G, 5621A>G, 5622G>T, 5626G>T, 5627T>C, 5628A>G, 5629A>T, 5630G>A, 5631G>A, 5632A>G, 5633A>T, 5634G>A, 5639G>A, 5640C>A, 5642T>A, 5645T>A, 5646T>A, 5648G>A, 5649T>A, 5651A>C, 5656C>A, 5657A>G, 5661T>A, 5664A>G, 5667A>T, 5670G>T, 5672A>T, 5673G>A, 5674T>A, 5676G>A, 5679A>C, 5685G>A, 5687A>C, 5691C>T, 5693T>A, 5696C>A, 5697C>G, 5700G>A, 5701A>C, 5702A>T, 5703A>T, 5711A>T, 5714A>G, 5717A>G, 5718G>A, 5720T>A, 5721C>A, 5726C>A, 5739T>G, 5740T>C, 5741A>T, 5748A>G, 5756A>T, 5757T>A, 5759T>G, 5762T>A, 5763_5765delGAA, 5768C>T, 5769C>A, 5777A>T, 5779C>A, 5781T>A, 5782T>C, 5786G>A, 5788G>A, 5790C>T, 5795G>A, 5798G>A, 5807G>A, 5808A>T, 5811T>G, 5813A>G, 5816C>T, 5822C>T, 5828T>C, 5830A>T, 5834C>A, 5836C>A, 5843G>A, 5844A>G, 5846T>A, 5850T>A, 5851A>T, 5853A>G, 5855T>A, 5856A>G, 5859A>C, 5860T>C, 5864T>A, 5868A>G, 5869A>C, 5870T>A, 5873G>A, 5874T>A, 5875G>T, 5876T>A, 5877T>G, 5878C>G, 5879C>A, 5882C>T, 5889G>C, 5893G>A								

CDS

	Begin	End	Coverage	Score	Concordance	Matches	Identities	I/D/M/F*	Stop Codons
EXK67_gp3	1124	1428	19.1%	1186	55.9%	304 (99.7%)	160 (52.5%)	0/1/0/0	0
Protein mutations:	L1125I (4983C>A), H1126K (4986C>A 4988T>A), K1127L (4989A>T 4990A>T), E1129D (4997A>T), I1131L (5001A>T 5003C>A), Q1132E (5004C>G), N1136E (5016A>G 5018T>A), D1137K (5019G>A 5021C>A), K1140I (5029A>T 5030G>A), Y1141H (5031T>C), E1143G (5038A>G), K1144A (5040A>G 5041A>C 5042G>C), Q1146R (5046C>A 5047A>G 5048G>A), R1148M (5053G>T 5054A>G), D1157E (5081T>A), L1158Y (5082C>T 5083T>A), Q1161K (5091C>A 5093G>A), D1162I (5094G>A 5095A>T 5096C>A), K1163A (5097A>G 5098A>C 5099G>A), P1164A (5100C>G 5102T>A), I1166E (5106A>G 5107T>A), P1167A (5109C>G), S1168T (5112T>A), P1169N (5115C>A 5116C>A 5117A>T), E1170Q (5118G>C), M1171D (5121A>G 5122T>A 5123G>C), A1172I (5124G>A 5125C>T 5126G>A), K1173E (5127A>G), E1174D (5132A>T), Y1175F (5134A>T), E1176Q (5136G>C 5138A>G), K1177I (5140A>T 5141G>A), H1178Q (5144T>A), T1180K (5149C>A), A1184D (5161C>A 5162A>T), K1186G (5166A>G 5167A>G 5168G>A), P1190R (5178C>A 5179C>G 5180T>A), Q1192T (5184C>A 5185A>C), T1197S (5199A>T), I1201M (5213T>G), N1203K (5219C>A), K1204N (5222A>C), S1206N (5226T>A 5227C>A 5228T>C), Q1208I (5232C>A 5233A>T), S1213A (5247A>G 5248G>C 5249T>C), Y1217I (5259T>A 5260A>T 5261C>A), Y1227R (5289T>A 5290A>G 5291C>A), K1228T (5293A>C 5294A>G), D1229Y (5295G>T 5297C>T), Q1230A (5298C>G 5299A>C 5300A>T), T1232K (5305C>A 5306A>G), S1235D (5313A>G 5314G>A), I1236K (5317T>A 5318T>A), D1237S (5319G>A 5320A>G 5321C>T), Y1238E (5322T>G 5324T>A), L1240I (5328C>A), L1241N (5331C>A 5332T>A), K1242R (5335A>G), K1244Q (5340A>C), D1245G (5344A>G 5345C>T), I1247T (5350T>C 5351T>A), Y1249F (5356A>T), S1250N (5359G>A 5360C>T), L1254C (5370C>T 5371T>G 5372A>T), F1258Y (5383T>A 5384C>T), H1259W (5385C>T 5386A>G 5387C>G), I1261V (5391A>G), M1262R (5395T>G 5396G>A), D1264H (5400G>C), P1265E (5403C>G 5404C>A), Q1266D (5406C>G 5408G>T), S1267N (5410G>A), A1272T (5424G>A), V1274T (5430G>A 5431T>C 5432C>T), Q1277E (5439C>G 5441G>A), I1283L (5457A>T), N1291T (5482A>C), S1294P (5490T>C 5492A>T), V1295I (5493G>A), K1305G (5523A>G 5524A>G), K1306D (5526A>G 5528A>T), S1308K (5532T>A 5533C>A), E1309R (5535G>A 5536A>G), C1312L (5544T>C 5545G>T 5546T>A), I1315V (5553A>G), D1316Y (5556G>T 5558C>T), I1320V (5568A>G), E1323R (5577G>A 5578A>G), S1324D (5580A>G 5581G>A), I1325M (5585A>G), Q1327E (5589C>G), V1329L (5595G>C), Q1330E (5598C>G), L1333Q (5607T>C 5608T>A), Q1334T (5610C>A 5611A>C 5612G>A), F1335I (5613T>A 5615C>T), Q1337R (5619C>A 5620A>G 5621A>G), V1338L (5622G>T), C1339F (5626G>T 5627T>C), K1340V (5628A>G 5629A>T 5630G>A), E1341S (5631G>A 5632A>G 5633A>T), E1342K (5634G>A), L1344I (5640C>A 5642T>A), L1346I (5646T>A 5648G>A), S1347T (5649T>A 5651A>C), T1349K (5656C>A 5657A>G), L1351M (5661T>A), K1352E (5664A>G), I1353L (5667A>T), G1354C (5670G>T 5672A>T), V1355K (5673G>A 5674T>A), A1356T (5676G>A), N1357H (5679A>C), E1359N (5685G>A 5687A>C), L1363V (5697C>G), E1364T (5700G>A 5701A>C 5702A>T), I1365L (5703A>T), E1367D (5711A>T), V1370I (5718G>A 5720T>A), Q1371K (5721C>A), L1377A (5739T>G 5740T>C 5741A>T), I1380V (5748A>G), E1382D (5756A>T), F1383M (5757T>A 5759T>G), E1385del (5763_5765delGAA), Q1387K (5769C>A), E1389D (5777A>T), T1390K (5779C>A), L1391T (5781T>A 5782T>C), G1393D (5788G>A), I1400L (5808A>T), L1401V (5811T>G 5813A>G), Y1407F (5830A>T), P1409Q (5836C>A), S1412G (5844A>G 5846T>A), Y1414I (5850T>A 5851A>T), T1415A (5853A>G 5855T>A), R1416G (5856A>G), I1417P (5859A>C 5860T>C), F1418L (5864T>A), N1420A (5868A>G 5869A>C 5870T>A), C1422I (5874T>A 5875G>T 5876T>A), S1423G (5877T>G 5878C>G 5879C>A), E1427Q (5889G>C), R1428K (5893G>A)								
Codon mutations:	CTA1125ATA (4983C>A), CAT1126AAA (4986C>A 4988T>A), AAA1127TTA (4989A>T 4990A>T), TTG1128CTA (4992T>C 4994G>A), GAA1129GAT (4997A>T), ATC1131TTA (5001A>T 5003C>A), CAA1132GAA (5004C>G), ATT1133ATA (5009T>A), ATT1134ATA (5012T>A), GGG1135GGA (5015G>A), AAT1136GAA (5016A>G 5018T>A), GAC1137AAA (5019G>A 5021C>A), CCA1138CCC (5024A>C), TTA1139CTA (5025T>C), AAG1140ATA (5029A>T 5030G>A), TAT1141CAT (5031T>C), GAA1143GGA (5038A>G), AAG1144GCC (5040A>G 5041A>C 5042G>C), CAG1146AGA (5046C>A 5047A>G 5048G>A), ATT1147ATA (5051T>A), AGA1148ATG (5053G>T 5054A>G), TGC1149TGT (5057C>T), CTG1151CTA (5063G>A), GAC1152GAT (5066C>T), ATC1153ATA (5069C>A), CCT1156CCA (5078T>A), GAT1157GAA (5081T>A), CTT1158TAT (5082C>T 5083T>A), CAG1161AAA (5091C>A 5093G>A), GAC1162ATA (5094G>A 5095A>T 5096C>A), AAG1163GCA (5097A>G 5098A>C 5099G>A), CCT1164GCA (5100C>G 5102T>A), ATT1165ATA (5105T>A), ATA1166GAA (5106A>G 5107T>A), CCT1167GCT (5109C>G), TCA1168ACA (5112T>A), CCA1169AAT (5115C>A 5116C>A 5117A>T), GAA1170CAA (5118G>C), ATG1171GAC (5121A>G 5122T>A 5123G>C), GCG1172ATA (5124G>A 5125C>T 5126G>A), AAA1173GAA (5127A>G), GAA1174GAT (5132A>T), TAT1175TTT (5134A>T), GAA1176CAG (5136G>C 5138A>G), AAG1177ATA (5140A>T 5141G>A), CAT1178CAA (5144T>A), ATT1179ATA (5147T>A), ACA1180AAA (5149C>A), CTG1183TTA (5157C>T 5159G>A), GCA1184GAT (5161C>A 5162A>T), CTC1185TTA (5163C>T 5165C>A), AAG1186GGA (5166A>G 5167A>G 5168G>A), GTT1187GTA (5171T>A), ATC1188ATA (5174C>A), CCT1190AGA (5178C>A 5179C>G 5180T>A), TCC1191TCA (5183C>A), CAA1192ACA (5184C>A 5185A>C), AGT1193TCT (5187A>T 5188G>C), CGC1194AGA (5190C>A 5192C>A), CAT1195CAC (5195T>C), AGG1196AGA (5198G>A), ACA1197TCA (5199A>T), GCT1199GCA (5207T>A), ATT1201ATG (5213T>G), GTC1202GTA (5216C>A), AAC1203AAA (5219C>A), AAA1204AAC (5222A>C), TCT1206AAC (5226T>A 5227C>A 5228T>C), CAA1208ATA (5232C>A 5233A>T), GTA1209GTC (5237A>C), GGT1211GGA (5243T>A), AGT1213GCC (5247A>G 5248G>C 5249T>C), CGT1214AGA (5250C>A 5252T>A), TAC1217ATA (5259T>A 5260A>T 5261C>A), CGA1221AGA (5271C>A), CTA1222CTC (5276A>C), ACA1226ACT (5288A>T), TAC1227AGA (5289T>A 5290A>G 5291C>A), AAA1228ACG (5293A>C 5294A>G), GAC1229TAT (5295G>T 5297C>T), CAA1230GCT (5298C>G 5299A>C 5300A>T), TAC1231TAT (5303C>T), ACA1232AAG (5305C>A 5306A>G), CTC1233CTA (5309C>A), AGT1235GAT (5313A>G 5314G>A), ATT1236AAA (5317T>A 5318T>A), GAC1237AGT (5319G>A 5320A>G 5321C>T), TAT1238GAA (5322T>G 5324T>A), CTC1239TTA (5325C>T 5327C>A), CTA1240ATA (5328C>A), CTT1241AAT (5331C>A 5332T>A), AAA1242AGA (5335A>G), ATT1243ATA (5339T>A), AAA1244CAA (5340A>C), GAC1245GGT (5344A>G 5345C>T), ATT1247ACA (5350T>C 5351T>A), GTC1248GTA (5354C>A), TAC1249TTC (5356A>T), AGC1250AAT (5359G>A 5360C>T), CTA1254TGT (5370C>T 5371T>G 5372A>T), AAG1255AAA (5375G>A), TTC1258TAT (5383T>A 5384C>T), CAC1259TGG (5385C>T 5386A>G 5387C>G), CAG1260CAA (5390G>A), ATT1261GTT (5391A>G), ATG1262AGA (5395T>G 5396G>A), GAT1264CAT (5400G>C), CCA1265GAA (5403C>G 5404C>A), CAG1266GAT (5406C>G 5408G>T), AGT1267AAT (5410G>A), GCA1272ACA (5424G>A), TTT1273TTC (5429T>C), GTC1274ACT (5430G>A 5431T>C 5432C>T), CCA1276CCT (5438A>T), CAG1277GAA (5439C>G 5441G>A), GGT1278GGA (5444T>A), ATA1283TTA (5457A>T), TTC1287TTT (5471C>T), CTC1289TTG (5475C>T 5477C>G), AAA1290AAG (5480A>G), AAT1291ACT (5482A>C), CCT1293CCA (5489T>A), TCA1294CCT (5490T>C 5492A>T), GTA1295ATA (5493G>A), CGA1298AGA (5502C>A), AAA1299AAG (5507A>G), GAT1301GAC (5513T>C), ATA1303ATT (5519A>T), TTC1304TTT (5522C>T), AAA1305GGA (5523A>G 5524A>G), AAA1306GAT (5526A>G 5528A>T), TAT1307TAC (5531T>C), TCA1308AAA (5532T>A 5533C>A), GAA1309AGA (5535G>A 5536A>G), TGT1312CTA (5544T>C 5545G>T 5546T>A), GTT1313GTA (5549T>A), TAC1314TAT (5552C>T), ATA1315GTA (5553A>G), GAC1316TAT (5556G>T 5558C>T), GAT1317GAC (5561T>C), ATT1318ATC (5564T>C), TTG1319CTA (5565T>C 5567G>A), ATA1320GTA (5568A>G), TTT1321TTC (5573T>C), TCT1322AGT (5574T>A 5575C>G), GAA1323AGA (5577G>A 5578A>G), AGT1324GAT (5580A>G 5581G>A), ATA1325ATG (5585A>G), CAA1327GAA (5589C>G), GTA1329CTA (5595G>C), CAA1330GAA (5598C>G), TTA1333CAA (5607T>C 5608T>A), CAG1334ACA (5610C>A 5611A>C 5612G>A), TTC1335ATT (5613T>A 5615C>T), TTC1336TTT (5618C>T), CAA1337AGG (5619C>A 5620A>G 5621A>G), GTA1338TTA (5622G>T), TGT1339TTC (5626G>T 5627T>C), AAG1340GTA (5628A>G 5629A>T 5630G>A), GAA1341AGT (5631G>A 5632A>G 5633A>T), GAA1342AAA (5634G>A), GGG1343GGA (5639G>A), CTT1344ATA (5640C>A 5642T>A), ATT1345ATA (5645T>A), TTG1346ATA (5646T>A 5648G>A), TCA1347ACC (5649T>A 5651A>C), ACA1349AAG (5656C>A 5657A>G), TTG1351ATG (5661T>A), AAA1352GAA (5664A>G), ATA1353TTA (5667A>T), GGA1354TGT (5670G>T 5672A>T), GTA1355AAA (5673G>A 5674T>A), GCA1356ACA (5676G>A), AAT1357CAT (5679A>C), GAA1359AAC (5685G>A 5687A>C), CTT1361TTA (5691C>T 5693T>A), GGC1362GGA (5696C>A), CTA1363GTA (5697C>G), GAA1364ACT (5700G>A 5701A>C 5702A>T), ATA1365TTA (5703A>T), GAA1367GAT (5711A>T), GGA1368GGG (5714A>G), AAA1369AAG (5717A>G), GTT1370ATA (5718G>A 5720T>A), CAA1371AAA (5721C>A), CTC1372CTA (5726C>A), TTA1377GCT (5739T>G 5740T>C 5741A>T), ATA1380GTA (5748A>G), GAA1382GAT (5756A>T), TTT1383ATG (5757T>A 5759T>G), CCT1384CCA (5762T>A), GAA1385del (5763_5765delGAA), GAC1386GAT (5768C>T), CAA1387AAA (5769C>A), GAA1389GAT (5777A>T), ACA1390AAA (5779C>A), TTA1391ACA (5781T>A 5782T>C), AAG1392AAA (5786G>A), GGT1393GAT (5788G>A), CTA1394TTA (5790C>T), CAG1395CAA (5795G>A), AAG1396AAA (5798G>A), GGG1399GGA (5807G>A), ATA1400TTA (5808A>T), TTA1401GTG (5811T>G 5813A>G), AAC1402AAT (5816C>T), GCC1404GCT (5822C>T), AAT1406AAC (5828T>C), TAT1407TTT (5830A>T), ATC1408ATA (5834C>A), CCA1409CAA (5836C>A), TTG1411TTA (5843G>A), AGT1412GGA (5844A>G 5846T>A), TAT1414ATT (5850T>A 5851A>T), ACT1415GCA (5853A>G 5855T>A), AGA1416GGA (5856A>G), ATA1417CCA (5859A>C 5860T>C), TTT1418TTA (5864T>A), AAT1420GCA (5868A>G 5869A>C 5870T>A), AAG1421AAA (5873G>A), TGT1422ATA (5874T>A 5875G>T 5876T>A), TCC1423GGA (5877T>G 5878C>G 5879C>A), AGC1424AGT (5882C>T), GAA1427CAA (5889G>C), AGA1428AAA (5893G>A)								

Proteins

	Begin	End	Coverage	Score	Concordance	Matches	Identities	I/D/M/F*	Stop Codons
**NT**	**4980**	**5894**	**11.2%**	**420**	**23.2%**	**912 (99.7%)**	**564 (61.6%)**	**0/3**	
ORF3 (YP_009553219.1)	1124	1428	19.1%	1186	55.9%	304 (99.7%)	160 (52.5%)	0/1/0/0	0
Protein mutations:	L1125I (4983C>A), H1126K (4986C>A 4988T>A), K1127L (4989A>T 4990A>T), E1129D (4997A>T), I1131L (5001A>T 5003C>A), Q1132E (5004C>G), N1136E (5016A>G 5018T>A), D1137K (5019G>A 5021C>A), K1140I (5029A>T 5030G>A), Y1141H (5031T>C), E1143G (5038A>G), K1144A (5040A>G 5041A>C 5042G>C), Q1146R (5046C>A 5047A>G 5048G>A), R1148M (5053G>T 5054A>G), D1157E (5081T>A), L1158Y (5082C>T 5083T>A), Q1161K (5091C>A 5093G>A), D1162I (5094G>A 5095A>T 5096C>A), K1163A (5097A>G 5098A>C 5099G>A), P1164A (5100C>G 5102T>A), I1166E (5106A>G 5107T>A), P1167A (5109C>G), S1168T (5112T>A), P1169N (5115C>A 5116C>A 5117A>T), E1170Q (5118G>C), M1171D (5121A>G 5122T>A 5123G>C), A1172I (5124G>A 5125C>T 5126G>A), K1173E (5127A>G), E1174D (5132A>T), Y1175F (5134A>T), E1176Q (5136G>C 5138A>G), K1177I (5140A>T 5141G>A), H1178Q (5144T>A), T1180K (5149C>A), A1184D (5161C>A 5162A>T), K1186G (5166A>G 5167A>G 5168G>A), P1190R (5178C>A 5179C>G 5180T>A), Q1192T (5184C>A 5185A>C), T1197S (5199A>T), I1201M (5213T>G), N1203K (5219C>A), K1204N (5222A>C), S1206N (5226T>A 5227C>A 5228T>C), Q1208I (5232C>A 5233A>T), S1213A (5247A>G 5248G>C 5249T>C), Y1217I (5259T>A 5260A>T 5261C>A), Y1227R (5289T>A 5290A>G 5291C>A), K1228T (5293A>C 5294A>G), D1229Y (5295G>T 5297C>T), Q1230A (5298C>G 5299A>C 5300A>T), T1232K (5305C>A 5306A>G), S1235D (5313A>G 5314G>A), I1236K (5317T>A 5318T>A), D1237S (5319G>A 5320A>G 5321C>T), Y1238E (5322T>G 5324T>A), L1240I (5328C>A), L1241N (5331C>A 5332T>A), K1242R (5335A>G), K1244Q (5340A>C), D1245G (5344A>G 5345C>T), I1247T (5350T>C 5351T>A), Y1249F (5356A>T), S1250N (5359G>A 5360C>T), L1254C (5370C>T 5371T>G 5372A>T), F1258Y (5383T>A 5384C>T), H1259W (5385C>T 5386A>G 5387C>G), I1261V (5391A>G), M1262R (5395T>G 5396G>A), D1264H (5400G>C), P1265E (5403C>G 5404C>A), Q1266D (5406C>G 5408G>T), S1267N (5410G>A), A1272T (5424G>A), V1274T (5430G>A 5431T>C 5432C>T), Q1277E (5439C>G 5441G>A), I1283L (5457A>T), N1291T (5482A>C), S1294P (5490T>C 5492A>T), V1295I (5493G>A), K1305G (5523A>G 5524A>G), K1306D (5526A>G 5528A>T), S1308K (5532T>A 5533C>A), E1309R (5535G>A 5536A>G), C1312L (5544T>C 5545G>T 5546T>A), I1315V (5553A>G), D1316Y (5556G>T 5558C>T), I1320V (5568A>G), E1323R (5577G>A 5578A>G), S1324D (5580A>G 5581G>A), I1325M (5585A>G), Q1327E (5589C>G), V1329L (5595G>C), Q1330E (5598C>G), L1333Q (5607T>C 5608T>A), Q1334T (5610C>A 5611A>C 5612G>A), F1335I (5613T>A 5615C>T), Q1337R (5619C>A 5620A>G 5621A>G), V1338L (5622G>T), C1339F (5626G>T 5627T>C), K1340V (5628A>G 5629A>T 5630G>A), E1341S (5631G>A 5632A>G 5633A>T), E1342K (5634G>A), L1344I (5640C>A 5642T>A), L1346I (5646T>A 5648G>A), S1347T (5649T>A 5651A>C), T1349K (5656C>A 5657A>G), L1351M (5661T>A), K1352E (5664A>G), I1353L (5667A>T), G1354C (5670G>T 5672A>T), V1355K (5673G>A 5674T>A), A1356T (5676G>A), N1357H (5679A>C), E1359N (5685G>A 5687A>C), L1363V (5697C>G), E1364T (5700G>A 5701A>C 5702A>T), I1365L (5703A>T), E1367D (5711A>T), V1370I (5718G>A 5720T>A), Q1371K (5721C>A), L1377A (5739T>G 5740T>C 5741A>T), I1380V (5748A>G), E1382D (5756A>T), F1383M (5757T>A 5759T>G), E1385del (5763_5765delGAA), Q1387K (5769C>A), E1389D (5777A>T), T1390K (5779C>A), L1391T (5781T>A 5782T>C), G1393D (5788G>A), I1400L (5808A>T), L1401V (5811T>G 5813A>G), Y1407F (5830A>T), P1409Q (5836C>A), S1412G (5844A>G 5846T>A), Y1414I (5850T>A 5851A>T), T1415A (5853A>G 5855T>A), R1416G (5856A>G), I1417P (5859A>C 5860T>C), F1418L (5864T>A), N1420A (5868A>G 5869A>C 5870T>A), C1422I (5874T>A 5875G>T 5876T>A), S1423G (5877T>G 5878C>G 5879C>A), E1427Q (5889G>C), R1428K (5893G>A)								
Codon mutations:	CTA1125ATA (4983C>A), CAT1126AAA (4986C>A 4988T>A), AAA1127TTA (4989A>T 4990A>T), TTG1128CTA (4992T>C 4994G>A), GAA1129GAT (4997A>T), ATC1131TTA (5001A>T 5003C>A), CAA1132GAA (5004C>G), ATT1133ATA (5009T>A), ATT1134ATA (5012T>A), GGG1135GGA (5015G>A), AAT1136GAA (5016A>G 5018T>A), GAC1137AAA (5019G>A 5021C>A), CCA1138CCC (5024A>C), TTA1139CTA (5025T>C), AAG1140ATA (5029A>T 5030G>A), TAT1141CAT (5031T>C), GAA1143GGA (5038A>G), AAG1144GCC (5040A>G 5041A>C 5042G>C), CAG1146AGA (5046C>A 5047A>G 5048G>A), ATT1147ATA (5051T>A), AGA1148ATG (5053G>T 5054A>G), TGC1149TGT (5057C>T), CTG1151CTA (5063G>A), GAC1152GAT (5066C>T), ATC1153ATA (5069C>A), CCT1156CCA (5078T>A), GAT1157GAA (5081T>A), CTT1158TAT (5082C>T 5083T>A), CAG1161AAA (5091C>A 5093G>A), GAC1162ATA (5094G>A 5095A>T 5096C>A), AAG1163GCA (5097A>G 5098A>C 5099G>A), CCT1164GCA (5100C>G 5102T>A), ATT1165ATA (5105T>A), ATA1166GAA (5106A>G 5107T>A), CCT1167GCT (5109C>G), TCA1168ACA (5112T>A), CCA1169AAT (5115C>A 5116C>A 5117A>T), GAA1170CAA (5118G>C), ATG1171GAC (5121A>G 5122T>A 5123G>C), GCG1172ATA (5124G>A 5125C>T 5126G>A), AAA1173GAA (5127A>G), GAA1174GAT (5132A>T), TAT1175TTT (5134A>T), GAA1176CAG (5136G>C 5138A>G), AAG1177ATA (5140A>T 5141G>A), CAT1178CAA (5144T>A), ATT1179ATA (5147T>A), ACA1180AAA (5149C>A), CTG1183TTA (5157C>T 5159G>A), GCA1184GAT (5161C>A 5162A>T), CTC1185TTA (5163C>T 5165C>A), AAG1186GGA (5166A>G 5167A>G 5168G>A), GTT1187GTA (5171T>A), ATC1188ATA (5174C>A), CCT1190AGA (5178C>A 5179C>G 5180T>A), TCC1191TCA (5183C>A), CAA1192ACA (5184C>A 5185A>C), AGT1193TCT (5187A>T 5188G>C), CGC1194AGA (5190C>A 5192C>A), CAT1195CAC (5195T>C), AGG1196AGA (5198G>A), ACA1197TCA (5199A>T), GCT1199GCA (5207T>A), ATT1201ATG (5213T>G), GTC1202GTA (5216C>A), AAC1203AAA (5219C>A), AAA1204AAC (5222A>C), TCT1206AAC (5226T>A 5227C>A 5228T>C), CAA1208ATA (5232C>A 5233A>T), GTA1209GTC (5237A>C), GGT1211GGA (5243T>A), AGT1213GCC (5247A>G 5248G>C 5249T>C), CGT1214AGA (5250C>A 5252T>A), TAC1217ATA (5259T>A 5260A>T 5261C>A), CGA1221AGA (5271C>A), CTA1222CTC (5276A>C), ACA1226ACT (5288A>T), TAC1227AGA (5289T>A 5290A>G 5291C>A), AAA1228ACG (5293A>C 5294A>G), GAC1229TAT (5295G>T 5297C>T), CAA1230GCT (5298C>G 5299A>C 5300A>T), TAC1231TAT (5303C>T), ACA1232AAG (5305C>A 5306A>G), CTC1233CTA (5309C>A), AGT1235GAT (5313A>G 5314G>A), ATT1236AAA (5317T>A 5318T>A), GAC1237AGT (5319G>A 5320A>G 5321C>T), TAT1238GAA (5322T>G 5324T>A), CTC1239TTA (5325C>T 5327C>A), CTA1240ATA (5328C>A), CTT1241AAT (5331C>A 5332T>A), AAA1242AGA (5335A>G), ATT1243ATA (5339T>A), AAA1244CAA (5340A>C), GAC1245GGT (5344A>G 5345C>T), ATT1247ACA (5350T>C 5351T>A), GTC1248GTA (5354C>A), TAC1249TTC (5356A>T), AGC1250AAT (5359G>A 5360C>T), CTA1254TGT (5370C>T 5371T>G 5372A>T), AAG1255AAA (5375G>A), TTC1258TAT (5383T>A 5384C>T), CAC1259TGG (5385C>T 5386A>G 5387C>G), CAG1260CAA (5390G>A), ATT1261GTT (5391A>G), ATG1262AGA (5395T>G 5396G>A), GAT1264CAT (5400G>C), CCA1265GAA (5403C>G 5404C>A), CAG1266GAT (5406C>G 5408G>T), AGT1267AAT (5410G>A), GCA1272ACA (5424G>A), TTT1273TTC (5429T>C), GTC1274ACT (5430G>A 5431T>C 5432C>T), CCA1276CCT (5438A>T), CAG1277GAA (5439C>G 5441G>A), GGT1278GGA (5444T>A), ATA1283TTA (5457A>T), TTC1287TTT (5471C>T), CTC1289TTG (5475C>T 5477C>G), AAA1290AAG (5480A>G), AAT1291ACT (5482A>C), CCT1293CCA (5489T>A), TCA1294CCT (5490T>C 5492A>T), GTA1295ATA (5493G>A), CGA1298AGA (5502C>A), AAA1299AAG (5507A>G), GAT1301GAC (5513T>C), ATA1303ATT (5519A>T), TTC1304TTT (5522C>T), AAA1305GGA (5523A>G 5524A>G), AAA1306GAT (5526A>G 5528A>T), TAT1307TAC (5531T>C), TCA1308AAA (5532T>A 5533C>A), GAA1309AGA (5535G>A 5536A>G), TGT1312CTA (5544T>C 5545G>T 5546T>A), GTT1313GTA (5549T>A), TAC1314TAT (5552C>T), ATA1315GTA (5553A>G), GAC1316TAT (5556G>T 5558C>T), GAT1317GAC (5561T>C), ATT1318ATC (5564T>C), TTG1319CTA (5565T>C 5567G>A), ATA1320GTA (5568A>G), TTT1321TTC (5573T>C), TCT1322AGT (5574T>A 5575C>G), GAA1323AGA (5577G>A 5578A>G), AGT1324GAT (5580A>G 5581G>A), ATA1325ATG (5585A>G), CAA1327GAA (5589C>G), GTA1329CTA (5595G>C), CAA1330GAA (5598C>G), TTA1333CAA (5607T>C 5608T>A), CAG1334ACA (5610C>A 5611A>C 5612G>A), TTC1335ATT (5613T>A 5615C>T), TTC1336TTT (5618C>T), CAA1337AGG (5619C>A 5620A>G 5621A>G), GTA1338TTA (5622G>T), TGT1339TTC (5626G>T 5627T>C), AAG1340GTA (5628A>G 5629A>T 5630G>A), GAA1341AGT (5631G>A 5632A>G 5633A>T), GAA1342AAA (5634G>A), GGG1343GGA (5639G>A), CTT1344ATA (5640C>A 5642T>A), ATT1345ATA (5645T>A), TTG1346ATA (5646T>A 5648G>A), TCA1347ACC (5649T>A 5651A>C), ACA1349AAG (5656C>A 5657A>G), TTG1351ATG (5661T>A), AAA1352GAA (5664A>G), ATA1353TTA (5667A>T), GGA1354TGT (5670G>T 5672A>T), GTA1355AAA (5673G>A 5674T>A), GCA1356ACA (5676G>A), AAT1357CAT (5679A>C), GAA1359AAC (5685G>A 5687A>C), CTT1361TTA (5691C>T 5693T>A), GGC1362GGA (5696C>A), CTA1363GTA (5697C>G), GAA1364ACT (5700G>A 5701A>C 5702A>T), ATA1365TTA (5703A>T), GAA1367GAT (5711A>T), GGA1368GGG (5714A>G), AAA1369AAG (5717A>G), GTT1370ATA (5718G>A 5720T>A), CAA1371AAA (5721C>A), CTC1372CTA (5726C>A), TTA1377GCT (5739T>G 5740T>C 5741A>T), ATA1380GTA (5748A>G), GAA1382GAT (5756A>T), TTT1383ATG (5757T>A 5759T>G), CCT1384CCA (5762T>A), GAA1385del (5763_5765delGAA), GAC1386GAT (5768C>T), CAA1387AAA (5769C>A), GAA1389GAT (5777A>T), ACA1390AAA (5779C>A), TTA1391ACA (5781T>A 5782T>C), AAG1392AAA (5786G>A), GGT1393GAT (5788G>A), CTA1394TTA (5790C>T), CAG1395CAA (5795G>A), AAG1396AAA (5798G>A), GGG1399GGA (5807G>A), ATA1400TTA (5808A>T), TTA1401GTG (5811T>G 5813A>G), AAC1402AAT (5816C>T), GCC1404GCT (5822C>T), AAT1406AAC (5828T>C), TAT1407TTT (5830A>T), ATC1408ATA (5834C>A), CCA1409CAA (5836C>A), TTG1411TTA (5843G>A), AGT1412GGA (5844A>G 5846T>A), TAT1414ATT (5850T>A 5851A>T), ACT1415GCA (5853A>G 5855T>A), AGA1416GGA (5856A>G), ATA1417CCA (5859A>C 5860T>C), TTT1418TTA (5864T>A), AAT1420GCA (5868A>G 5869A>C 5870T>A), AAG1421AAA (5873G>A), TGT1422ATA (5874T>A 5875G>T 5876T>A), TCC1423GGA (5877T>G 5878C>G 5879C>A), AGC1424AGT (5882C>T), GAA1427CAA (5889G>C), AGA1428AAA (5893G>A)								

*: Inserts / Deletes / Misaligned / Frameshifts

Analysis details

This analysis was performed with panviral2.64

NGS Details (UN18_val): Caulimovirus minutangelicae

Assembly

**Coverage Length**	391 (1 contig(s))
**Depth Of Coverage**	6.0
**Number Of Reads**	17
**Reads Per Million**	0.32 rpm (after QC)
**Ambiguities**	0
**Assembly Method**	de novo + reference guided assembly
**Consensus Caller**	Bcf Tools

Coverage Map

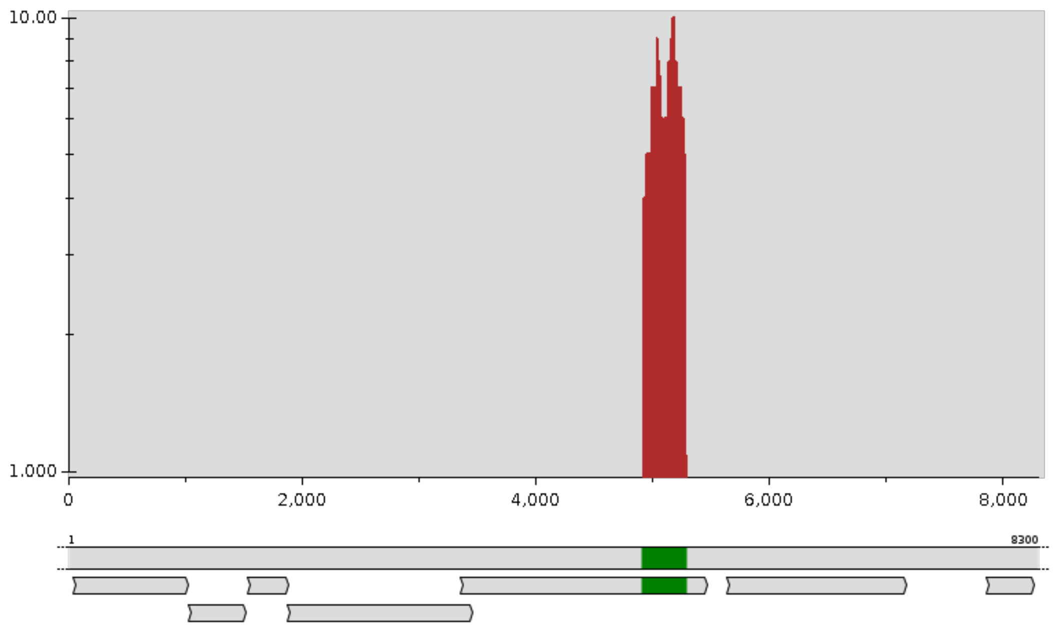

Assignment

**Type**	Caulimovirus minutangelicae (Taxonomy ID: 3047363)
**Reference Genome**	NC_043523.1
**NT Identity (%)**	54.8813
**AA Identity (%)**	46.4567
**Number Of Stop Codons**	0
**Number Of CDS**	7

Alignment

**Alignment Score**	44.0 (NT) + 416.0 (AA) = 460.0
**Concordance (%)**	29.2435

**Alignment Method**	Global, seeded, nucleotide + amino acids (AGA)

Genome Region

Sequence starts at position 4910 and ends at position 5300 relative to NC_043523.1 reference sequence.

Alignment Detailed Statistics

	Begin	End	Coverage	Score	Concordance	Matches	Identities	I/D/M/F*	Stop Codons
**NT**	**4910**	**5300**	**4.7%**	**44**	**6.0%**	**379 (96.9%)**	**208 (53.2%)**	**0/12**	
Mutations:	4912T>C, 4915T>A, 4916T>A, 4917C>G, 4921T>A, 4923A>T, 4924C>T, 4927T>A, 4932G>A, 4933T>C, 4940T>A, 4941C>A, 4942C>A, 4945A>G, 4949G>A, 4950C>A, 4951A>T, 4953C>T, 4955C>T, 4960G>A, 4961G>A, 4962T>G, 4963A>G, 4964A>C, 4965A>T, 4966A>T, 4967C>A, 4969C>T, 4970A>C, 4971A>G, 4975G>A, 4976G>T, 4977A>C, 4978T>C, 4980T>A, 4981A>C, 4982T>C, 4983C>G, 4984T>A, 4985T>A, 4986G>T, 4988A>G, 4989A>G, 4990T>A, 4998C>A, 5000T>G, 5001C>A, 5003G>C, 5005C>T, 5008T>C, 5009T>A, 5010C>A, 5013A>G, 5014C>T, 5017G>T, 5018G>C, 5019C>A, 5020T>A, 5022A>G, 5023G>C, 5024T>C, 5029G>A, 5030A>C, 5034G>A, 5035T>A, 5037T>G, 5038G>T, 5039A>T, 5040A>C, 5041T>C, 5044T>A, 5047C>A, 5050G>A, 5053A>G, 5054T>C, 5057T>A, 5058A>G, 5059T>A, 5061A>T, 5066C>G, 5067T>A, 5068T>A, 5071T>G, 5074T>A, 5077T>C, 5080G>C, 5081C>T, 5084A>G, 5086C>T, 5087A>C, 5089C>A, 5090G>C, 5101A>T, 5104T>A, 5105C>A, 5107A>T, 5108G>T, 5110T>C, 5111T>C, 5121G>C, 5122C>A, 5123A>T, 5125T>A, 5128G>A, 5129A>C, 5131G>A, 5132G>A, 5134A>T, 5136C>A, 5137A>C, 5138A>C, 5139A>T, 5141_5143delACA, 5148G>A, 5150_5158delGAAGAGCTC, 5167A>G, 5170C>T, 5171G>A, 5173T>A, 5174T>A, 5175C>G, 5176A>T, 5179A>T, 5181G>C, 5182C>A, 5188A>T, 5189T>G, 5190C>A, 5192G>A, 5194A>C, 5201C>A, 5202G>A, 5206T>C, 5207C>T, 5208A>C, 5209T>A, 5210T>A, 5212T>A, 5221A>G, 5224G>A, 5226A>T, 5227T>A, 5230A>G, 5233T>C, 5234G>A, 5236T>A, 5237A>G, 5239T>A, 5244T>G, 5245T>C, 5248T>A, 5249A>C, 5250A>G, 5257C>T, 5260G>T, 5261A>G, 5262T>C, 5265A>T, 5270A>T, 5271C>T, 5272T>A, 5276G>A, 5277T>A, 5278A>G, 5281T>A, 5284T>C, 5286C>T, 5287T>C, 5288G>A, 5290T>C, 5294A>T, 5296A>G, 5299T>C								

CDS

	Begin	End	Coverage	Score	Concordance	Matches	Identities	I/D/M/F*	Stop Codons
FLA58_gp5	517	647	18.6%	416	48.8%	127 (96.9%)	59 (45.0%)	0/4/0/0	0
Protein mutations:	D520E (4921T>A), Y521F (4923A>T 4924C>T), G524D (4932G>A 4933T>C), S527K (4940T>A 4941C>A 4942C>A), A530N (4949G>A 4950C>A 4951A>T), P531L (4953C>T), V534R (4961G>A 4962T>G 4963A>G), K535L (4964A>C 4965A>T 4966A>T), L536I (4967C>A 4969C>T), K537R (4970A>C 4971A>G), D539S (4976G>T 4977A>C 4978T>C), I540N (4980T>A 4981A>C), S541R (4982T>C 4983C>G 4984T>A), W542M (4985T>A 4986G>T), N543G (4988A>G 4989A>G 4990T>A), A546D (4998C>A), S547E (5000T>G 5001C>A), D548H (5003G>C 5005C>T), S550K (5009T>A 5010C>A), Y551C (5013A>G 5014C>T), A553Q (5018G>C 5019C>A 5020T>A), K554S (5022A>G 5023G>C), K557Q (5030A>C), G558E (5034G>A 5035T>A), L559C (5037T>G 5038G>T), N560S (5039A>T 5040A>C 5041T>C), N561K (5044T>A), F562L (5047C>A), Y566R (5057T>A 5058A>G 5059T>A), H567L (5061A>T), L569E (5066C>G 5067T>A 5068T>A), D571E (5074T>A), K573N (5080G>C), I575V (5084A>G 5086C>T), I576L (5087A>C 5089C>A), E577Q (5090G>C), Q582N (5105C>A 5107A>T), D583Y (5108G>T 5110T>C), Y584H (5111T>C), G587A (5121G>C 5122C>A), I588L (5123A>T 5125T>A), K590Q (5129A>C 5131G>A), A591T (5132G>A 5134A>T), A592D (5136C>A 5137A>C), N593L (5138A>C 5139A>T), T594del (5141_5143delACA), G596E (5148G>A), E597_L599del (5150_5158delGAAGAGCTC), A604T (5171G>A 5173T>A), S607T (5181G>C 5182C>A), K609N (5188A>T), S610E (5189T>G 5190C>A), A611T (5192G>A 5194A>C), R614K (5201C>A 5202G>A), H616S (5207C>T 5208A>C 5209T>A), S617T (5210T>A 5212T>A), Y622L (5226A>T 5227T>A), V625I (5234G>A 5236T>A), I626V (5237A>G 5239T>A), V628G (5244T>G 5245T>C), K630R (5249A>C 5250A>G), I634A (5261A>G 5262T>C), Y635F (5265A>T), T637L (5270A>T 5271C>T 5272T>A), V639K (5276G>A 5277T>A 5278A>G), H640Q (5281T>A), T642I (5286C>T 5287T>C), V643I (5288G>A 5290T>C), T645S (5294A>T 5296A>G)								
Codon mutations:	TAT517TAC (4912T>C), GCT518GCA (4915T>A), TCT519AGT (4916T>A 4917C>G), GAT520GAA (4921T>A), TAC521TTT (4923A>T 4924C>T), ATT522ATA (4927T>A), GGT524GAC (4932G>A 4933T>C), TCC527AAA (4940T>A 4941C>A 4942C>A), AAA528AAG (4945A>G), GCA530AAT (4949G>A 4950C>A 4951A>T), CCT531CTT (4953C>T), CTA532TTA (4955C>T), CAG533CAA (4960G>A), GTA534AGG (4961G>A 4962T>G 4963A>G), AAA535CTT (4964A>C 4965A>T 4966A>T), CTC536ATT (4967C>A 4969C>T), AAA537CGA (4970A>C 4971A>G), AAG538AAA (4975G>A), GAT539TCC (4976G>T 4977A>C 4978T>C), ATA540AAC (4980T>A 4981A>C), TCT541CGA (4982T>C 4983C>G 4984T>A), TGG542ATG (4985T>A 4986G>T), AAT543GGA (4988A>G 4989A>G 4990T>A), GCT546GAT (4998C>A), TCA547GAA (5000T>G 5001C>A), GAC548CAT (5003G>C 5005C>T), ACT549ACC (5008T>C), TCA550AAA (5009T>A 5010C>A), TAC551TGT (5013A>G 5014C>T), GTG552GTT (5017G>T), GCT553CAA (5018G>C 5019C>A 5020T>A), AAG554AGC (5022A>G 5023G>C), TTA555CTA (5024T>C), AAG556AAA (5029G>A), AAA557CAA (5030A>C), GGT558GAA (5034G>A 5035T>A), TTG559TGT (5037T>G 5038G>T), AAT560TCC (5039A>T 5040A>C 5041T>C), AAT561AAA (5044T>A), TTC562TTA (5047C>A), CCG563CCA (5050G>A), AAA564AAG (5053A>G), TTA565CTA (5054T>C), TAT566AGA (5057T>A 5058A>G 5059T>A), CAT567CTT (5061A>T), CTT569GAA (5066C>G 5067T>A 5068T>A), CCT570CCG (5071T>G), GAT571GAA (5074T>A), GAT572GAC (5077T>C), AAG573AAC (5080G>C), CTA574TTA (5081C>T), ATC575GTT (5084A>G 5086C>T), ATC576CTA (5087A>C 5089C>A), GAA577CAA (5090G>C), GCA580GCT (5101A>T), TCT581TCA (5104T>A), CAA582AAT (5105C>A 5107A>T), GAT583TAC (5108G>T 5110T>C), TAT584CAT (5111T>C), GGC587GCA (5121G>C 5122C>A), ATT588TTA (5123A>T 5125T>A), TTG589TTA (5128G>A), AAG590CAA (5129A>C 5131G>A), GCA591ACT (5132G>A 5134A>T), GCA592GAC (5136C>A 5137A>C), AAT593CTT (5138A>C 5139A>T), ACA594del (5141_5143delACA), GGA596GAA (5148G>A), GAA597_CTC599del (5150_5158delGAAGAGCTC), AGA602AGG (5167A>G), TAC603TAT (5170C>T), GCT604ACA (5171G>A 5173T>A), TCA605AGT (5174T>A 5175C>G 5176A>T), GGA606GGT (5179A>T), AGC607ACA (5181G>C 5182C>A), AAA609AAT (5188A>T), TCA610GAA (5189T>G 5190C>A), GCA611ACC (5192G>A 5194A>C), CGA614AAA (5201C>A 5202G>A), TAT615TAC (5206T>C), CAT616TCA (5207C>T 5208A>C 5209T>A), TCT617ACA (5210T>A 5212T>A), AAA620AAG (5221A>G), GAG621GAA (5224G>A), TAT622TTA (5226A>T 5227T>A), TTA623TTG (5230A>G), GCT624GCC (5233T>C), GTT625ATA (5234G>A 5236T>A), ATT626GTA (5237A>G 5239T>A), GTT628GGC (5244T>G 5245T>C), ATT629ATA (5248T>A), AAA630CGA (5249A>C 5250A>G), TTC632TTT (5257C>T), TCG633TCT (5260G>T), ATT634GCT (5261A>G 5262T>C), TAT635TTT (5265A>T), ACT637TTA (5270A>T 5271C>T 5272T>A), GTA639AAG (5276G>A 5277T>A 5278A>G), CAT640CAA (5281T>A), TTT641TTC (5284T>C), ACT642ATC (5286C>T 5287T>C), GTT643ATC (5288G>A 5290T>C), ACA645TCG (5294A>T 5296A>G), GAT646GAC (5299T>C)								

Proteins

	Begin	End	Coverage	Score	Concordance	Matches	Identities	I/D/M/F*	Stop Codons
reverse transcriptase (YP_009666503.1)	517	647	18.6%	416	48.8%	127 (96.9%)	59 (45.0%)	0/4/0/0	0
Protein mutations:	D520E (4921T>A), Y521F (4923A>T 4924C>T), G524D (4932G>A 4933T>C), S527K (4940T>A 4941C>A 4942C>A), A530N (4949G>A 4950C>A 4951A>T), P531L (4953C>T), V534R (4961G>A 4962T>G 4963A>G), K535L (4964A>C 4965A>T 4966A>T), L536I (4967C>A 4969C>T), K537R (4970A>C 4971A>G), D539S (4976G>T 4977A>C 4978T>C), I540N (4980T>A 4981A>C), S541R (4982T>C 4983C>G 4984T>A), W542M (4985T>A 4986G>T), N543G (4988A>G 4989A>G 4990T>A), A546D (4998C>A), S547E (5000T>G 5001C>A), D548H (5003G>C 5005C>T), S550K (5009T>A 5010C>A), Y551C (5013A>G 5014C>T), A553Q (5018G>C 5019C>A 5020T>A), K554S (5022A>G 5023G>C), K557Q (5030A>C), G558E (5034G>A 5035T>A), L559C (5037T>G 5038G>T), N560S (5039A>T 5040A>C 5041T>C), N561K (5044T>A), F562L (5047C>A), Y566R (5057T>A 5058A>G 5059T>A), H567L (5061A>T), L569E (5066C>G 5067T>A 5068T>A), D571E (5074T>A), K573N (5080G>C), I575V (5084A>G 5086C>T), I576L (5087A>C 5089C>A), E577Q (5090G>C), Q582N (5105C>A 5107A>T), D583Y (5108G>T 5110T>C), Y584H (5111T>C), G587A (5121G>C 5122C>A), I588L (5123A>T 5125T>A), K590Q (5129A>C 5131G>A), A591T (5132G>A 5134A>T), A592D (5136C>A 5137A>C), N593L (5138A>C 5139A>T), T594del (5141_5143delACA), G596E (5148G>A), E597_L599del (5150_5158delGAAGAGCTC), A604T (5171G>A 5173T>A), S607T (5181G>C 5182C>A), K609N (5188A>T), S610E (5189T>G 5190C>A), A611T (5192G>A 5194A>C), R614K (5201C>A 5202G>A), H616S (5207C>T 5208A>C 5209T>A), S617T (5210T>A 5212T>A), Y622L (5226A>T 5227T>A), V625I (5234G>A 5236T>A), I626V (5237A>G 5239T>A), V628G (5244T>G 5245T>C), K630R (5249A>C 5250A>G), I634A (5261A>G 5262T>C), Y635F (5265A>T), T637L (5270A>T 5271C>T 5272T>A), V639K (5276G>A 5277T>A 5278A>G), H640Q (5281T>A), T642I (5286C>T 5287T>C), V643I (5288G>A 5290T>C), T645S (5294A>T 5296A>G)								
Codon mutations:	TAT517TAC (4912T>C), GCT518GCA (4915T>A), TCT519AGT (4916T>A 4917C>G), GAT520GAA (4921T>A), TAC521TTT (4923A>T 4924C>T), ATT522ATA (4927T>A), GGT524GAC (4932G>A 4933T>C), TCC527AAA (4940T>A 4941C>A 4942C>A), AAA528AAG (4945A>G), GCA530AAT (4949G>A 4950C>A 4951A>T), CCT531CTT (4953C>T), CTA532TTA (4955C>T), CAG533CAA (4960G>A), GTA534AGG (4961G>A 4962T>G 4963A>G), AAA535CTT (4964A>C 4965A>T 4966A>T), CTC536ATT (4967C>A 4969C>T), AAA537CGA (4970A>C 4971A>G), AAG538AAA (4975G>A), GAT539TCC (4976G>T 4977A>C 4978T>C), ATA540AAC (4980T>A 4981A>C), TCT541CGA (4982T>C 4983C>G 4984T>A), TGG542ATG (4985T>A 4986G>T), AAT543GGA (4988A>G 4989A>G 4990T>A), GCT546GAT (4998C>A), TCA547GAA (5000T>G 5001C>A), GAC548CAT (5003G>C 5005C>T), ACT549ACC (5008T>C), TCA550AAA (5009T>A 5010C>A), TAC551TGT (5013A>G 5014C>T), GTG552GTT (5017G>T), GCT553CAA (5018G>C 5019C>A 5020T>A), AAG554AGC (5022A>G 5023G>C), TTA555CTA (5024T>C), AAG556AAA (5029G>A), AAA557CAA (5030A>C), GGT558GAA (5034G>A 5035T>A), TTG559TGT (5037T>G 5038G>T), AAT560TCC (5039A>T 5040A>C 5041T>C), AAT561AAA (5044T>A), TTC562TTA (5047C>A), CCG563CCA (5050G>A), AAA564AAG (5053A>G), TTA565CTA (5054T>C), TAT566AGA (5057T>A 5058A>G 5059T>A), CAT567CTT (5061A>T), CTT569GAA (5066C>G 5067T>A 5068T>A), CCT570CCG (5071T>G), GAT571GAA (5074T>A), GAT572GAC (5077T>C), AAG573AAC (5080G>C), CTA574TTA (5081C>T), ATC575GTT (5084A>G 5086C>T), ATC576CTA (5087A>C 5089C>A), GAA577CAA (5090G>C), GCA580GCT (5101A>T), TCT581TCA (5104T>A), CAA582AAT (5105C>A 5107A>T), GAT583TAC (5108G>T 5110T>C), TAT584CAT (5111T>C), GGC587GCA (5121G>C 5122C>A), ATT588TTA (5123A>T 5125T>A), TTG589TTA (5128G>A), AAG590CAA (5129A>C 5131G>A), GCA591ACT (5132G>A 5134A>T), GCA592GAC (5136C>A 5137A>C), AAT593CTT (5138A>C 5139A>T), ACA594del (5141_5143delACA), GGA596GAA (5148G>A), GAA597_CTC599del (5150_5158delGAAGAGCTC), AGA602AGG (5167A>G), TAC603TAT (5170C>T), GCT604ACA (5171G>A 5173T>A), TCA605AGT (5174T>A 5175C>G 5176A>T), GGA606GGT (5179A>T), AGC607ACA (5181G>C 5182C>A), AAA609AAT (5188A>T), TCA610GAA (5189T>G 5190C>A), GCA611ACC (5192G>A 5194A>C), CGA614AAA (5201C>A 5202G>A), TAT615TAC (5206T>C), CAT616TCA (5207C>T 5208A>C 5209T>A), TCT617ACA (5210T>A 5212T>A), AAA620AAG (5221A>G), GAG621GAA (5224G>A), TAT622TTA (5226A>T 5227T>A), TTA623TTG (5230A>G), GCT624GCC (5233T>C), GTT625ATA (5234G>A 5236T>A), ATT626GTA (5237A>G 5239T>A), GTT628GGC (5244T>G 5245T>C), ATT629ATA (5248T>A), AAA630CGA (5249A>C 5250A>G), TTC632TTT (5257C>T), TCG633TCT (5260G>T), ATT634GCT (5261A>G 5262T>C), TAT635TTT (5265A>T), ACT637TTA (5270A>T 5271C>T 5272T>A), GTA639AAG (5276G>A 5277T>A 5278A>G), CAT640CAA (5281T>A), TTT641TTC (5284T>C), ACT642ATC (5286C>T 5287T>C), GTT643ATC (5288G>A 5290T>C), ACA645TCG (5294A>T 5296A>G), GAT646GAC (5299T>C)								

*: Inserts / Deletes / Misaligned / Frameshifts

Analysis details

This analysis was performed with panviral2.64

NGS Details (UN18_val): Caulimovirus venafragariae

Assembly

**Coverage Length**	607 (1 contig(s))
**Depth Of Coverage**	3.8
**Number Of Reads**	17
**Reads Per Million**	0.32 rpm (after QC)
**Ambiguities**	0
**Assembly Method**	de novo + reference guided assembly
**Consensus Caller**	Bcf Tools

Coverage Map

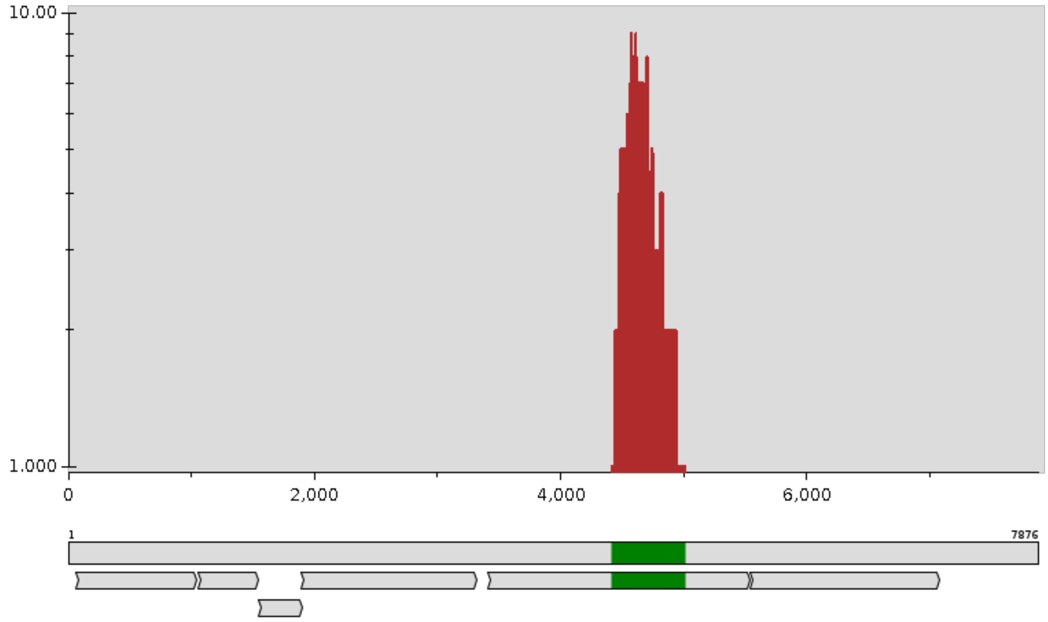

Assignment

**Type**	Caulimovirus venafragariae (Taxonomy ID: 3048344)
**Reference Genome**	NC_001725.1
**NT Identity (%)**	56.0732
**AA Identity (%)**	51.2438
**Number Of Stop Codons**	0
**Number Of CDS**	6

Alignment

**Alignment Score**	131.0 (NT) + 766.0 (AA) = 897.0
**Concordance (%)**	34.66

**Alignment Method**	Global, seeded, nucleotide + amino acids (AGA)

Genome Region

Sequence starts at position 4411 and ends at position 5017 relative to NC_001725.1 reference sequence.

Alignment Detailed Statistics

	Begin	End	Coverage	Score	Concordance	Matches	Identities	I/D/M/F*	Stop Codons
**NT**	**4411**	**5017**	**7.7%**	**131**	**11.0%**	**601 (99.0%)**	**337 (55.5%)**	**0/6**	
Mutations:	4413T>A, 4423A>G, 4426T>C, 4428A>T, 4432G>A, 4434C>A, 4435C>A, 4438A>T, 4439C>G, 4440A>T, 4441A>G, 4442A>T, 4443G>C, 4444G>T, 4445G>T, 4446A>C, 4449T>C, 4452C>A, 4455C>T, 4456C>T, 4458A>C, 4459C>A, 4461T>C, 4464T>A, 4467C>T, 4470G>A, 4471G>A, 4473G>T, 4474C>A, 4476A>C, 4479G>T, 4480C>A, 4482T>A, 4483C>A, 4485A>T, 4486A>C, 4487G>T, 4488A>T, 4489A>G, 4490T>C, 4492G>A, 4493G>A, 4497T>A, 4500G>A, 4505T>A, 4506T>C, 4508A>T, 4509C>T, 4512T>A, 4513T>A, 4514C>A, 4515C>A, 4521C>T, 4524T>C, 4528T>A, 4529C>G, 4542G>A, 4543G>A, 4546C>A, 4547G>A, 4548C>A, 4551T>A, 4553C>A, 4554T>A, 4555C>G, 4556C>A, 4558G>A, 4560A>T, 4561A>T, 4563A>T, 4566T>A, 4567C>G, 4568A>C, 4569G>T, 4570C>T, 4575C>A, 4578T>A, 4584C>T, 4585T>A, 4586G>C, 4587T>A, 4590C>A, 4591C>A, 4593A>T, 4614C>G, 4633C>A, 4635A>T, 4642G>C, 4643C>A, 4644T>A, 4647T>C, 4650C>T, 4653A>G, 4657C>A, 4659C>A, 4665C>T, 4666G>A, 4670G>T, 4671T>A, 4672C>T, 4674C>T, 4675A>T, 4676G>C, 4678A>C, 4680C>A, 4682T>A, 4684T>A, 4687_4692delCCACAG, 4696T>C, 4697G>T, 4698T>A, 4699G>A, 4700C>T, 4701T>C, 4704A>C, 4707T>C, 4708G>A, 4710T>A, 4716C>T, 4719C>A, 4720A>T, 4722C>A, 4723G>A, 4725C>A, 4727T>G, 4728C>T, 4729A>T, 4730G>C, 4734A>C, 4735A>T, 4737T>A, 4738G>T, 4740A>T, 4741G>C, 4753G>A, 4754G>C, 4755A>T, 4759G>C, 4761C>T, 4765A>C, 4766T>A, 4767T>A, 4768G>T, 4771C>T, 4772T>C, 4773A>T, 4774A>C, 4776C>A, 4778G>T, 4782C>T, 4783A>T, 4784A>G, 4785A>C, 4787C>A, 4788C>A, 4789C>A, 4790T>A, 4791A>T, 4797A>T, 4801C>T, 4803T>A, 4807A>C, 4809A>G, 4812G>A, 4815A>G, 4818C>A, 4819C>G, 4821G>C, 4822T>A, 4824G>A, 4825T>A, 4826G>A, 4827C>T, 4832C>A, 4833A>T, 4834A>G, 4835C>A, 4836T>A, 4839C>T, 4840A>G, 4842T>A, 4845C>T, 4848T>C, 4851T>A, 4852C>A, 4854A>G, 4855G>A, 4860A>T, 4861G>T, 4862A>C, 4865G>A, 4866A>G, 4867G>A, 4868G>A, 4869A>T, 4870A>G, 4871A>G, 4872T>C, 4873C>A, 4875C>T, 4876A>G, 4879G>T, 4881C>A, 4884G>A, 4886G>C, 4893C>A, 4894G>T, 4895G>C, 4897C>A, 4898T>A, 4900C>A, 4902C>G, 4903T>A, 4905A>C, 4906G>T, 4907T>G, 4910C>A, 4911T>A, 4921C>A, 4926T>A, 4928G>A, 4930G>A, 4931A>C, 4932T>A, 4934G>A, 4935G>A, 4936A>C, 4938T>A, 4940C>A, 4941C>A, 4942T>A, 4944A>C, 4950A>G, 4954T>C, 4956A>T, 4959C>A, 4960C>T, 4961T>G, 4962C>T, 4963C>T, 4971T>C, 4972A>G, 4973T>C, 4974C>A, 4975T>A, 4976C>G, 4977C>T, 4979C>A, 4980T>A, 4982A>T, 4983T>C, 4984T>A, 4986T>A, 4990A>G, 4992A>C, 4993A>T, 5001C>A, 5002C>A, 5003T>A, 5004T>G, 5007G>A, 5008T>A, 5009C>A, 5010A>T, 5012C>T								

CDS

	Begin	End	Coverage	Score	Concordance	Matches	Identities	I/D/M/F*	Stop Codons
ORF_V	336	538	28.6%	766	54.4%	201 (99.0%)	103 (50.7%)	0/2/0/0	0
Protein mutations:	K340E (4423A>G), D343K (4432G>A 4434C>A), H344N (4435C>A), T345C (4438A>T 4439C>G 4440A>T), K346V (4441A>G 4442A>T 4443G>C), G347F (4444G>T 4445G>T 4446A>C), L351F (4456C>T 4458A>C), L352I (4459C>A 4461T>C), E356N (4471G>A 4473G>T), Q357N (4474C>A 4476A>C), L359I (4480C>A 4482T>A), Q360N (4483C>A 4485A>T), R361L (4486A>C 4487G>T 4488A>T), I362A (4489A>G 4490T>C), G363K (4492G>A 4493G>A), F367Y (4505T>A 4506T>C), Y368F (4508A>T 4509C>T), S370K (4513T>A 4514C>A 4515C>A), V380I (4543G>A), R381K (4546C>A 4547G>A 4548C>A), A383E (4553C>A 4554T>A), P384E (4555C>G 4556C>A), E385N (4558G>A 4560A>T), T386S (4561A>T 4563A>T), Q388A (4567C>G 4568A>C 4569G>T), C394T (4585T>A 4586G>C 4587T>A), Q396N (4591C>A 4593A>T), Q410N (4633C>A 4635A>T), A413Q (4642G>C 4643C>A 4644T>A), H418K (4657C>A 4659C>A), E421K (4666G>A), S422I (4670G>T 4671T>A), L423F (4672C>T 4674C>T), N425Q (4678A>C 4680C>A), M426K (4682T>A), Y427N (4684T>A), P428_Q429del (4687_4692delCCACAG), C431L (4696T>C 4697G>T 4698T>A), A432I (4699G>A 4700C>T 4701T>C), V435I (4708G>A 4710T>A), I439L (4720A>T 4722C>A), V440I (4723G>A 4725C>A), F441C (4727T>G 4728C>T), K443N (4734A>C), T444S (4735A>T 4737T>A), E445Y (4738G>T 4740A>T), E446Q (4741G>C), G450T (4753G>A 4754G>C 4755A>T), V452L (4759G>C 4761C>T), I454Q (4765A>C 4766T>A 4767T>A), V455F (4768G>T), L456S (4771C>T 4772T>C 4773A>T), N457Q (4774A>C 4776C>A), R458I (4778G>T), K460C (4783A>T 4784A>G 4785A>C), A461E (4787C>A 4788C>A), L462N (4789C>A 4790T>A 4791A>T), K468Q (4807A>C 4809A>G), Q472D (4819C>G 4821G>C), L473I (4822T>A 4824G>A), C474N (4825T>A 4826G>A 4827C>T), T476N (4832C>A 4833A>T), T477E (4834A>G 4835C>A 4836T>A), N479E (4840A>G 4842T>A), L483M (4852C>A 4854A>G), V484I (4855G>A), E486S (4861G>T 4862A>C), R487K (4865G>A 4866A>G), G488N (4867G>A 4868G>A 4869A>T), N489G (4870A>G 4871A>G 4872T>C), L490I (4873C>A 4875C>T), K491E (4876A>G), V492L (4879G>T 4881C>A), S494T (4886G>C), G497S (4894G>T 4895G>C), L498K (4897C>A 4898T>A), H499K (4900C>A 4902C>G), L500I (4903T>A 4905A>C), V501C (4906G>T 4907T>G), A502E (4910C>A 4911T>A), Q506K (4921C>A), S508N (4928G>A), D509T (4930G>A 4931A>C 4932T>A), R510K (4934G>A 4935G>A), N511Q (4936A>C 4938T>A), A512E (4940C>A 4941C>A), L513I (4942T>A 4944A>C), L519C (4960C>T 4961T>G 4962C>T), I523A (4972A>G 4973T>C 4974C>A), A525E (4979C>A 4980T>A), Y526F (4982A>T 4983T>C), F527I (4984T>A 4986T>A), K529D (4990A>G 4992A>C), I530L (4993A>T), N532K (5001C>A), L533K (5002C>A 5003T>A 5004T>G), S535N (5008T>A 5009C>A 5010A>T), P536L (5012C>T)								
Codon mutations:	ATT336ATA (4413T>A), AAG340GAG (4423A>G), TTA341CTT (4426T>C 4428A>T), GAC343AAA (4432G>A 4434C>A), CAT344AAT (4435C>A), ACA345TGT (4438A>T 4439C>G 4440A>T), AAG346GTC (4441A>G 4442A>T 4443G>C), GGA347TTC (4444G>T 4445G>T 4446A>C), GAT348GAC (4449T>C), GGC349GGA (4452C>A), TAC350TAT (4455C>T), CTA351TTC (4456C>T 4458A>C), CTT352ATC (4459C>A 4461T>C), CCT353CCA (4464T>A), AAC354AAT (4467C>T), AAG355AAA (4470G>A), GAG356AAT (4471G>A 4473G>T), CAA357AAC (4474C>A 4476A>C), CTG358CTT (4479G>T), CTT359ATA (4480C>A 4482T>A), CAA360AAT (4483C>A 4485A>T), AGA361CTT (4486A>C 4487G>T 4488A>T), ATC362GCC (4489A>G 4490T>C), GGA363AAA (4492G>A 4493G>A), GGT364GGA (4497T>A), AAG365AAA (4500G>A), TTT367TAC (4505T>A 4506T>C), TAC368TTT (4508A>T 4509C>T), TCT369TCA (4512T>A), TCC370AAA (4513T>A 4514C>A 4515C>A), GAC372GAT (4521C>T), TGT373TGC (4524T>C), TCT375AGT (4528T>A 4529C>G), CAG379CAA (4542G>A), GTA380ATA (4543G>A), CGC381AAA (4546C>A 4547G>A 4548C>A), CTT382CTA (4551T>A), GCT383GAA (4553C>A 4554T>A), CCA384GAA (4555C>G 4556C>A), GAA385AAT (4558G>A 4560A>T), ACA386TCT (4561A>T 4563A>T), ATT387ATA (4566T>A), CAG388GCT (4567C>G 4568A>C 4569G>T), CTA389TTA (4570C>T), ACC390ACA (4575C>A), GCT391GCA (4578T>A), AGC393AGT (4584C>T), TGT394ACA (4585T>A 4586G>C 4587T>A), CCC395CCA (4590C>A), CAA396AAT (4591C>A 4593A>T), GTC403GTG (4614C>G), CAA410AAT (4633C>A 4635A>T), GCT413CAA (4642G>C 4643C>A 4644T>A), ATT414ATC (4647T>C), TTC415TTT (4650C>T), CAA416CAG (4653A>G), CAC418AAA (4657C>A 4659C>A), GAC420GAT (4665C>T), GAA421AAA (4666G>A), AGT422ATA (4670G>T 4671T>A), CTC423TTT (4672C>T 4674C>T), AGC424TCC (4675A>T 4676G>C), AAC425CAA (4678A>C 4680C>A), ATG426AAG (4682T>A), TAT427AAT (4684T>A), CCA428_CAG429del (4687_4692delCCACAG), TGT431CTA (4696T>C 4697G>T 4698T>A), GCT432ATC (4699G>A 4700C>T 4701T>C), GTA433GTC (4704A>C), TAT434TAC (4707T>C), GTT435ATA (4708G>A 4710T>A), GAC437GAT (4716C>T), ATC438ATA (4719C>A), ATC439TTA (4720A>T 4722C>A), GTC440ATA (4723G>A 4725C>A), TTC441TGT (4727T>G 4728C>T), AGC442TCC (4729A>T 4730G>C), AAA443AAC (4734A>C), ACT444TCA (4735A>T 4737T>A), GAA445TAT (4738G>T 4740A>T), GAA446CAA (4741G>C), GGA450ACT (4753G>A 4754G>C 4755A>T), GTC452CTT (4759G>C 4761C>T), ATT454CAA (4765A>C 4766T>A 4767T>A), GTT455TTT (4768G>T), CTA456TCT (4771C>T 4772T>C 4773A>T), AAC457CAA (4774A>C 4776C>A), AGA458ATA (4778G>T), TGC459TGT (4782C>T), AAA460TGC (4783A>T 4784A>G 4785A>C), GCC461GAA (4787C>A 4788C>A), CTA462AAT (4789C>A 4790T>A 4791A>T), ATA464ATT (4797A>T), CTT466TTA (4801C>T 4803T>A), AAA468CAG (4807A>C 4809A>G), AAG469AAA (4812G>A), AAA470AAG (4815A>G), GCC471GCA (4818C>A), CAG472GAC (4819C>G 4821G>C), TTG473ATA (4822T>A 4824G>A), TGC474AAT (4825T>A 4826G>A 4827C>T), ACA476AAT (4832C>A 4833A>T), ACT477GAA (4834A>G 4835C>A 4836T>A), ATC478ATT (4839C>T), AAT479GAA (4840A>G 4842T>A), TTC480TTT (4845C>T), CTT481CTC (4848T>C), GGT482GGA (4851T>A), CTA483ATG (4852C>A 4854A>G), GTA484ATA (4855G>A), ATA485ATT (4860A>T), GAA486TCA (4861G>T 4862A>C), AGA487AAG (4865G>A 4866A>G), GGA488AAT (4867G>A 4868G>A 4869A>T), AAT489GGC (4870A>G 4871A>G 4872T>C), CTC490ATT (4873C>A 4875C>T), AAA491GAA (4876A>G), GTC492TTA (4879G>T 4881C>A), CAG493CAA (4884G>A), AGT494ACT (4886G>C), ATC496ATA (4893C>A), GGA497TCA (4894G>T 4895G>C), CTA498AAA (4897C>A 4898T>A), CAC499AAG (4900C>A 4902C>G), TTA500ATC (4903T>A 4905A>C), GTT501TGT (4906G>T 4907T>G), GCT502GAA (4910C>A 4911T>A), CAA506AAA (4921C>A), CTT507CTA (4926T>A), AGT508AAT (4928G>A), GAT509ACA (4930G>A 4931A>C 4932T>A), AGG510AAA (4934G>A 4935G>A), AAT511CAA (4936A>C 4938T>A), GCC512GAA (4940C>A 4941C>A), TTA513ATC (4942T>A 4944A>C), AGA515AGG (4950A>G), TTA517CTT (4954T>C 4956A>T), GGC518GGA (4959C>A), CTC519TGT (4960C>T 4961T>G 4962C>T), CTA520TTA (4963C>T), TAT522TAC (4971T>C), ATC523GCA (4972A>G 4973T>C 4974C>A), TCC524AGT (4975T>A 4976C>G 4977C>T), GCT525GAA (4979C>A 4980T>A), TAT526TTC (4982A>T 4983T>C), TTT527ATA (4984T>A 4986T>A), AAA529GAC (4990A>G 4992A>C), ATA530TTA (4993A>T), AAC532AAA (5001C>A), CTT533AAG (5002C>A 5003T>A 5004T>G), AGG534AGA (5007G>A), TCA535AAT (5008T>A 5009C>A 5010A>T), CCT536CTT (5012C>T)								

Proteins

	Begin	End	Coverage	Score	Concordance	Matches	Identities	I/D/M/F*	Stop Codons
hypothetical protein (NP_043933.1)	336	538	28.6%	766	54.4%	201 (99.0%)	103 (50.7%)	0/2/0/0	0
Protein mutations:	K340E (4423A>G), D343K (4432G>A 4434C>A), H344N (4435C>A), T345C (4438A>T 4439C>G 4440A>T), K346V (4441A>G 4442A>T 4443G>C), G347F (4444G>T 4445G>T 4446A>C), L351F (4456C>T 4458A>C), L352I (4459C>A 4461T>C), E356N (4471G>A 4473G>T), Q357N (4474C>A 4476A>C), L359I (4480C>A 4482T>A), Q360N (4483C>A 4485A>T), R361L (4486A>C 4487G>T 4488A>T), I362A (4489A>G 4490T>C), G363K (4492G>A 4493G>A), F367Y (4505T>A 4506T>C), Y368F (4508A>T 4509C>T), S370K (4513T>A 4514C>A 4515C>A), V380I (4543G>A), R381K (4546C>A 4547G>A 4548C>A), A383E (4553C>A 4554T>A), P384E (4555C>G 4556C>A), E385N (4558G>A 4560A>T), T386S (4561A>T 4563A>T), Q388A (4567C>G 4568A>C 4569G>T), C394T (4585T>A 4586G>C 4587T>A), Q396N (4591C>A 4593A>T), Q410N (4633C>A 4635A>T), A413Q (4642G>C 4643C>A 4644T>A), H418K (4657C>A 4659C>A), E421K (4666G>A), S422I (4670G>T 4671T>A), L423F (4672C>T 4674C>T), N425Q (4678A>C 4680C>A), M426K (4682T>A), Y427N (4684T>A), P428_Q429del (4687_4692delCCACAG), C431L (4696T>C 4697G>T 4698T>A), A432I (4699G>A 4700C>T 4701T>C), V435I (4708G>A 4710T>A), I439L (4720A>T 4722C>A), V440I (4723G>A 4725C>A), F441C (4727T>G 4728C>T), K443N (4734A>C), T444S (4735A>T 4737T>A), E445Y (4738G>T 4740A>T), E446Q (4741G>C), G450T (4753G>A 4754G>C 4755A>T), V452L (4759G>C 4761C>T), I454Q (4765A>C 4766T>A 4767T>A), V455F (4768G>T), L456S (4771C>T 4772T>C 4773A>T), N457Q (4774A>C 4776C>A), R458I (4778G>T), K460C (4783A>T 4784A>G 4785A>C), A461E (4787C>A 4788C>A), L462N (4789C>A 4790T>A 4791A>T), K468Q (4807A>C 4809A>G), Q472D (4819C>G 4821G>C), L473I (4822T>A 4824G>A), C474N (4825T>A 4826G>A 4827C>T), T476N (4832C>A 4833A>T), T477E (4834A>G 4835C>A 4836T>A), N479E (4840A>G 4842T>A), L483M (4852C>A 4854A>G), V484I (4855G>A), E486S (4861G>T 4862A>C), R487K (4865G>A 4866A>G), G488N (4867G>A 4868G>A 4869A>T), N489G (4870A>G 4871A>G 4872T>C), L490I (4873C>A 4875C>T), K491E (4876A>G), V492L (4879G>T 4881C>A), S494T (4886G>C), G497S (4894G>T 4895G>C), L498K (4897C>A 4898T>A), H499K (4900C>A 4902C>G), L500I (4903T>A 4905A>C), V501C (4906G>T 4907T>G), A502E (4910C>A 4911T>A), Q506K (4921C>A), S508N (4928G>A), D509T (4930G>A 4931A>C 4932T>A), R510K (4934G>A 4935G>A), N511Q (4936A>C 4938T>A), A512E (4940C>A 4941C>A), L513I (4942T>A 4944A>C), L519C (4960C>T 4961T>G 4962C>T), I523A (4972A>G 4973T>C 4974C>A), A525E (4979C>A 4980T>A), Y526F (4982A>T 4983T>C), F527I (4984T>A 4986T>A), K529D (4990A>G 4992A>C), I530L (4993A>T), N532K (5001C>A), L533K (5002C>A 5003T>A 5004T>G), S535N (5008T>A 5009C>A 5010A>T), P536L (5012C>T)								

	Begin	End	Coverage	Score	Concordance	Matches	Identities	I/D/M/F*	Stop Codons
**NT**	**4411**	**5017**	**7.7%**	**131**	**11.0%**	**601 (99.0%)**	**337 (55.5%)**	**0/6**	
Codon mutations:	ATT336ATA (4413T>A), AAG340GAG (4423A>G), TTA341CTT (4426T>C 4428A>T), GAC343AAA (4432G>A 4434C>A), CAT344AAT (4435C>A), ACA345TGT (4438A>T 4439C>G 4440A>T), AAG346GTC (4441A>G 4442A>T 4443G>C), GGA347TTC (4444G>T 4445G>T 4446A>C), GAT348GAC (4449T>C), GGC349GGA (4452C>A), TAC350TAT (4455C>T), CTA351TTC (4456C>T 4458A>C), CTT352ATC (4459C>A 4461T>C), CCT353CCA (4464T>A), AAC354AAT (4467C>T), AAG355AAA (4470G>A), GAG356AAT (4471G>A 4473G>T), CAA357AAC (4474C>A 4476A>C), CTG358CTT (4479G>T), CTT359ATA (4480C>A 4482T>A), CAA360AAT (4483C>A 4485A>T), AGA361CTT (4486A>C 4487G>T 4488A>T), ATC362GCC (4489A>G 4490T>C), GGA363AAA (4492G>A 4493G>A), GGT364GGA (4497T>A), AAG365AAA (4500G>A), TTT367TAC (4505T>A 4506T>C), TAC368TTT (4508A>T 4509C>T), TCT369TCA (4512T>A), TCC370AAA (4513T>A 4514C>A 4515C>A), GAC372GAT (4521C>T), TGT373TGC (4524T>C), TCT375AGT (4528T>A 4529C>G), CAG379CAA (4542G>A), GTA380ATA (4543G>A), CGC381AAA (4546C>A 4547G>A 4548C>A), CTT382CTA (4551T>A), GCT383GAA (4553C>A 4554T>A), CCA384GAA (4555C>G 4556C>A), GAA385AAT (4558G>A 4560A>T), ACA386TCT (4561A>T 4563A>T), ATT387ATA (4566T>A), CAG388GCT (4567C>G 4568A>C 4569G>T), CTA389TTA (4570C>T), ACC390ACA (4575C>A), GCT391GCA (4578T>A), AGC393AGT (4584C>T), TGT394ACA (4585T>A 4586G>C 4587T>A), CCC395CCA (4590C>A), CAA396AAT (4591C>A 4593A>T), GTC403GTG (4614C>G), CAA410AAT (4633C>A 4635A>T), GCT413CAA (4642G>C 4643C>A 4644T>A), ATT414ATC (4647T>C), TTC415TTT (4650C>T), CAA416CAG (4653A>G), CAC418AAA (4657C>A 4659C>A), GAC420GAT (4665C>T), GAA421AAA (4666G>A), AGT422ATA (4670G>T 4671T>A), CTC423TTT (4672C>T 4674C>T), AGC424TCC (4675A>T 4676G>C), AAC425CAA (4678A>C 4680C>A), ATG426AAG (4682T>A), TAT427AAT (4684T>A), CCA428_CAG429del (4687_4692delCCACAG), TGT431CTA (4696T>C 4697G>T 4698T>A), GCT432ATC (4699G>A 4700C>T 4701T>C), GTA433GTC (4704A>C), TAT434TAC (4707T>C), GTT435ATA (4708G>A 4710T>A), GAC437GAT (4716C>T), ATC438ATA (4719C>A), ATC439TTA (4720A>T 4722C>A), GTC440ATA (4723G>A 4725C>A), TTC441TGT (4727T>G 4728C>T), AGC442TCC (4729A>T 4730G>C), AAA443AAC (4734A>C), ACT444TCA (4735A>T 4737T>A), GAA445TAT (4738G>T 4740A>T), GAA446CAA (4741G>C), GGA450ACT (4753G>A 4754G>C 4755A>T), GTC452CTT (4759G>C 4761C>T), ATT454CAA (4765A>C 4766T>A 4767T>A), GTT455TTT (4768G>T), CTA456TCT (4771C>T 4772T>C 4773A>T), AAC457CAA (4774A>C 4776C>A), AGA458ATA (4778G>T), TGC459TGT (4782C>T), AAA460TGC (4783A>T 4784A>G 4785A>C), GCC461GAA (4787C>A 4788C>A), CTA462AAT (4789C>A 4790T>A 4791A>T), ATA464ATT (4797A>T), CTT466TTA (4801C>T 4803T>A), AAA468CAG (4807A>C 4809A>G), AAG469AAA (4812G>A), AAA470AAG (4815A>G), GCC471GCA (4818C>A), CAG472GAC (4819C>G 4821G>C), TTG473ATA (4822T>A 4824G>A), TGC474AAT (4825T>A 4826G>A 4827C>T), ACA476AAT (4832C>A 4833A>T), ACT477GAA (4834A>G 4835C>A 4836T>A), ATC478ATT (4839C>T), AAT479GAA (4840A>G 4842T>A), TTC480TTT (4845C>T), CTT481CTC (4848T>C), GGT482GGA (4851T>A), CTA483ATG (4852C>A 4854A>G), GTA484ATA (4855G>A), ATA485ATT (4860A>T), GAA486TCA (4861G>T 4862A>C), AGA487AAG (4865G>A 4866A>G), GGA488AAT (4867G>A 4868G>A 4869A>T), AAT489GGC (4870A>G 4871A>G 4872T>C), CTC490ATT (4873C>A 4875C>T), AAA491GAA (4876A>G), GTC492TTA (4879G>T 4881C>A), CAG493CAA (4884G>A), AGT494ACT (4886G>C), ATC496ATA (4893C>A), GGA497TCA (4894G>T 4895G>C), CTA498AAA (4897C>A 4898T>A), CAC499AAG (4900C>A 4902C>G), TTA500ATC (4903T>A 4905A>C), GTT501TGT (4906G>T 4907T>G), GCT502GAA (4910C>A 4911T>A), CAA506AAA (4921C>A), CTT507CTA (4926T>A), AGT508AAT (4928G>A), GAT509ACA (4930G>A 4931A>C 4932T>A), AGG510AAA (4934G>A 4935G>A), AAT511CAA (4936A>C 4938T>A), GCC512GAA (4940C>A 4941C>A), TTA513ATC (4942T>A 4944A>C), AGA515AGG (4950A>G), TTA517CTT (4954T>C 4956A>T), GGC518GGA (4959C>A), CTC519TGT (4960C>T 4961T>G 4962C>T), CTA520TTA (4963C>T), TAT522TAC (4971T>C), ATC523GCA (4972A>G 4973T>C 4974C>A), TCC524AGT (4975T>A 4976C>G 4977C>T), GCT525GAA (4979C>A 4980T>A), TAT526TTC (4982A>T 4983T>C), TTT527ATA (4984T>A 4986T>A), AAA529GAC (4990A>G 4992A>C), ATA530TTA (4993A>T), AAC532AAA (5001C>A), CTT533AAG (5002C>A 5003T>A 5004T>G), AGG534AGA (5007G>A), TCA535AAT (5008T>A 5009C>A 5010A>T), CCT536CTT (5012C>T)								

*: Inserts / Deletes / Misaligned / Frameshifts

Analysis details

This analysis was performed with panviral2.64

NGS Details (UN18_val): Badnavirus maculasmallanthi

Assembly

**Coverage Length**	281 (1 contig(s))
**Depth Of Coverage**	5.8
**Number Of Reads**	13
**Reads Per Million**	0.24 rpm (after QC)
**Ambiguities**	0
**Assembly Method**	de novo + reference guided assembly
**Consensus Caller**	Bcf Tools

Coverage Map

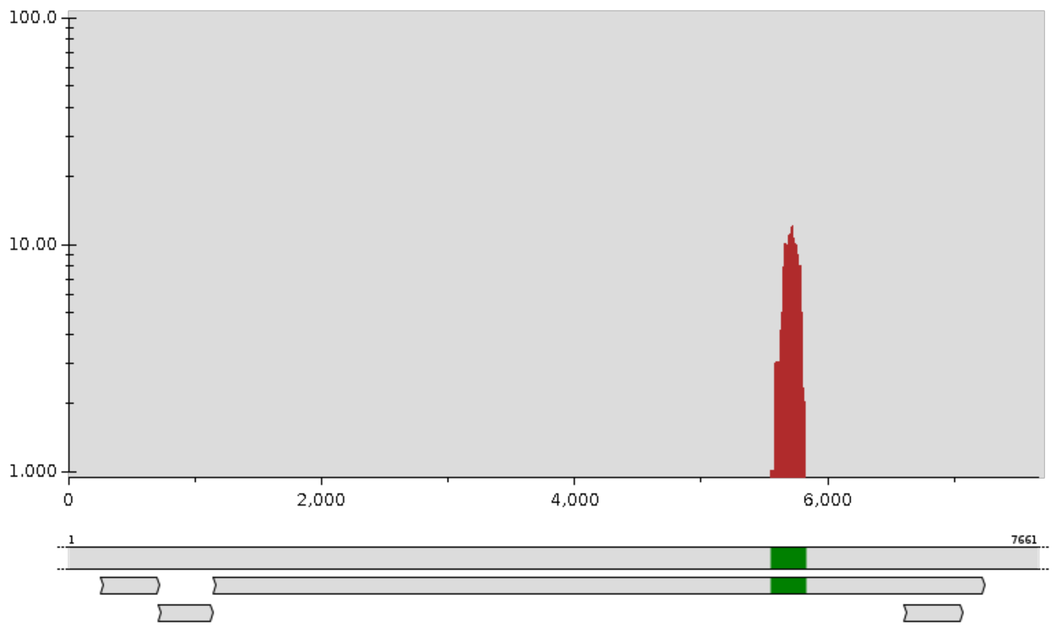

Assignment

**Type**	Badnavirus maculasmallanthi (Taxonomy ID: 3048453)
**Reference Genome**	NC_026472.1
**NT Identity (%)**	56.338
**AA Identity (%)**	48.9362
**Number Of Stop Codons**	1
**Number Of CDS**	4

Alignment

**Alignment Score**	66.0 (NT) + 305.0 (AA) = 371.0
**Concordance (%)**	30.261

Alignment Method	Global, seeded, nucleotide + amino acids (AGA)

Genome Region

Sequence starts at position 5549 and ends at position 5829 relative to NC_026472.1 reference sequence.

Alignment Detailed Statistics

	Begin	End	Coverage	Score	Concordance	Matches	Identities	I/D/M/F*	Stop Codons
**NT**	**5549**	**5829**	**3.7%**	**66**	**11.7%**	**281 (98.9%)**	**160 (56.3%)**	**3/0**	
Mutations:	5549G>A, 5551A>T, 5554G>C, 5557A>G, 5558A>G, 5562A>T, 5563A>T, 5564A>T, 5566A>G, 5568A>G, 5574A>C, 5578T>A, 5587A>C, 5589G>T, 5595A>G, 5597T>A, 5601T>C, 5604A>G, 5605G>T, 5607T>G, 5608G>A, 5609C>G, 5610C>A, 5611A>G, 5613G>T, 5614G>T, 5615A>G, 5619A>G, 5620G>T, 5621A>C, 5623T>G, 5624C>A, 5625C>T, 5628A>C, 5630C>T, 5631C>A, 5632T>A, 5633G>A, 5634G>A, 5637G>T, 5638G>A, 5640C>T, 5644T>A, 5645T>G, 5646A>G, 5648T>C, 5649C>A, 5651C>G, 5652A>G, 5653G>T, 5654G>A, 5655A>C, 5658A>G, 5659T>C, 5660T>A, 5661A>T, 5664C>T, 5667A>G, 5669G>T, 5670G>T, 5671C>G, 5673T>G, 5676C>T, 5680C>T, 5682A>C, 5685C>T, 5686G>A, 5691C>A, 5693A>C, 5694A>T, 5703C>T, 5706T>C, 5707A>G, 5708T>C, 5709T>A, 5713C>A, 5714A>T, 5716A>G, 5717G>A, 5718A>G, 5719A>T, 5720A>T, 5721A>G, 5725G>A, 5727T>C, 5728A>C, 5729A>G, 5731T>G, 5732G>T, 5733C>G, 5736C>T, 5739_5740insCCC, 5740G>T, 5743A>T, 5744C>T, 5745A>G, 5748A>C, 5749G>T, 5750A>C, 5755A>G, 5757C>G, 5758G>A, 5759C>T, 5760C>T, 5763A>G, 5765A>T, 5775C>T, 5779C>T, 5787C>T, 5793C>G, 5799A>G, 5801C>A, 5805T>C, 5810A>T, 5811A>C, 5812A>C, 5814G>T, 5817T>C, 5818T>C, 5822A>G, 5825T>C, 5827A>G								

CDS

	Begin	End	Coverage	Score	Concordance	Matches	Identities	I/D/M/F*	Stop Codons
UF61_gp3	1470	1562	4.6%	305	45.4%	93 (98.9%)	46 (48.9%)	1/0/0/0	1
Protein mutations:	I1470L (5551A>T), G1471R (5554G>C), N1472G (5557A>G 5558A>G), K1474L (5563A>T 5564A>T), I1475V (5566A>G 5568A>G), F1479I (5578T>A), K1482H (5587A>C 5589G>T), F1485Y (5597T>A), V1488L (5605G>T 5607T>G), A1489R (5608G>A 5609C>G 5610C>A), M1490V (5611A>G 5613G>T), E1491* (5614G>T 5615A>G), E1493S (5620G>T 5621A>C), S1494D (5623T>G 5624C>A 5625C>T), P1496L (5630C>T 5631C>A), W1497K (5632T>A 5633G>A 5634G>A), A1499T (5638G>A 5640C>T), L1501R (5644T>A 5645T>G 5646A>G), I1502T (5648T>C 5649C>A), P1503R (5651C>G 5652A>G), G1504Y (5653G>T 5654G>A 5655A>C), L1506H (5659T>C 5660T>A 5661A>T), W1509F (5669G>T 5670G>T), L1510V (5671C>G 5673T>G), P1513S (5680C>T 5682A>C), G1515R (5686G>A), K1517T (5693A>C 5694A>T), I1522A (5707A>G 5708T>C 5709T>A), Q1524M (5713C>A 5714A>T), R1525E (5716A>G 5717G>A 5718A>G), K1526L (5719A>T 5720A>T 5721A>G), D1528N (5725G>A 5727T>C), K1529R (5728A>C 5729A>G), C1530V (5731T>G 5732G>T 5733C>G), K1532_D1533insP (5739_5740insCCC), D1533Y (5740G>T), T1534L (5743A>T 5744C>T 5745A>G), E1535D (5748A>C), E1536S (5749G>T 5750A>C), I1538V (5755A>G 5757C>G), A1539I (5758G>A 5759C>T 5760C>T), Y1541F (5765A>T), N1550K (5793C>G), A1553E (5801C>A), E1556V (5810A>T 5811A>C), K1560R (5822A>G), I1561T (5825T>C), M1562V (5827A>G)								
Codon mutations:	AGA1469.AA (5549G>A), ATA1470TTA (5551A>T), GGG1471CGG (5554G>C), AAT1472GGT (5557A>G 5558A>G), GCA1473GCT (5562A>T), AAG1474TTG (5563A>T 5564A>T), ATA1475GTG (5566A>G 5568A>G), TCA1477TCC (5574A>C), TTT1479ATT (5578T>A), AAG1482CAT (5587A>C 5589G>T), GGA1484GGG (5595A>G), TTT1485TAT (5597T>A), CAT1486CAC (5601T>C), CAA1487CAG (5604A>G), GTT1488TTG (5605G>T 5607T>G), GCC1489AGA (5608G>A 5609C>G 5610C>A), ATG1490GTT (5611A>G 5613G>T), GAA1491TGA (5614G>T 5615A>G), GAA1492GAG (5619A>G), GAA1493TCA (5620G>T 5621A>C), TCC1494GAT (5623T>G 5624C>A 5625C>T), ATA1495ATC (5628A>C), CCC1496CTA (5630C>T 5631C>A), TGG1497AAA (5632T>A 5633G>A 5634G>A), ACG1498ACT (5637G>T), GCC1499ACT (5638G>A 5640C>T), TTA1501AGG (5644T>A 5645T>G 5646A>G), ATC1502ACA (5648T>C 5649C>A), CCA1503CGG (5651C>G 5652A>G), GGA1504TAC (5653G>T 5654G>A 5655A>C), GGA1505GGG (5658A>G), TTA1506CAT (5659T>C 5660T>A 5661A>T), TAC1507TAT (5664C>T), GAA1508GAG (5667A>G), TGG1509TTT (5669G>T 5670G>T), CTT1510GTG (5671C>G 5673T>G), GTC1511GTT (5676C>T), CCA1513TCC (5680C>T 5682A>C), TTC1514TTT (5685C>T), GGA1515AGA (5686G>A), CTC1516CTA (5691C>A), AAA1517ACT (5693A>C 5694A>T), CCC1520CCT (5703C>T), GCT1521GCC (5706T>C), ATT1522GCA (5707A>G 5708T>C 5709T>A), CAG1524ATG (5713C>A 5714A>T), AGA1525GAG (5716A>G 5717G>A 5718A>G), AAA1526TTG (5719A>T 5720A>T 5721A>G), GAT1528AAC (5725G>A 5727T>C), AAA1529CGA (5728A>C 5729A>G), TGC1530GTG (5731T>G 5732G>T 5733C>G), TTC1531TTT (5736C>T), AAA1532_GAC1533insCCC (5739_5740insCCC), GAC1533TAC (5740G>T), ACA1534TTG (5743A>T 5744C>T 5745A>G), GAA1535GAC (5748A>C), GAG1536TCG (5749G>T 5750A>C), ATC1538GTG (5755A>G 5757C>G), GCC1539ATT (5758G>A 5759C>T 5760C>T), GTA1540GTG (5763A>G), TAC1541TTC (5765A>T), GAC1544GAT (5775C>T), CTG1546TTG (5779C>T), TAC1548TAT (5787C>T), AAC1550AAG (5793C>G), GAA1552GAG (5799A>G), GCG1553GAG (5801C>A), GAT1554GAC (5805T>C), GAA1556GTC (5810A>T 5811A>C), AGG1557CGT (5812A>C 5814G>T), CAT1558CAC (5817T>C), TTA1559CTA (5818T>C), AAG1560AGG (5822A>G), ATT1561ACT (5825T>C), ATG1562GTG (5827A>G)								

Proteins

	Begin	End	Coverage	Score	Concordance	Matches	Identities	I/D/M/F*	Stop Codons
ORF3 (YP_009121747.1)	1470	1562	4.6%	305	45.4%	93 (98.9%)	46 (48.9%)	1/0/0/0	1
Protein mutations:	I1470L (5551A>T), G1471R (5554G>C), N1472G (5557A>G 5558A>G), K1474L (5563A>T 5564A>T), I1475V (5566A>G 5568A>G), F1479I (5578T>A), K1482H (5587A>C 5589G>T), F1485Y (5597T>A), V1488L (5605G>T 5607T>G), A1489R (5608G>A 5609C>G 5610C>A), M1490V (5611A>G 5613G>T), E1491* (5614G>T 5615A>G), E1493S (5620G>T 5621A>C), S1494D (5623T>G 5624C>A 5625C>T), P1496L (5630C>T 5631C>A), W1497K (5632T>A 5633G>A 5634G>A), A1499T (5638G>A 5640C>T), L1501R (5644T>A 5645T>G 5646A>G), I1502T (5648T>C 5649C>A), P1503R (5651C>G 5652A>G), G1504Y (5653G>T 5654G>A 5655A>C), L1506H (5659T>C 5660T>A 5661A>T), W1509F (5669G>T 5670G>T), L1510V (5671C>G 5673T>G), P1513S (5680C>T 5682A>C), G1515R (5686G>A), K1517T (5693A>C 5694A>T), I1522A (5707A>G 5708T>C 5709T>A), Q1524M (5713C>A 5714A>T), R1525E (5716A>G 5717G>A 5718A>G), K1526L (5719A>T 5720A>T 5721A>G), D1528N (5725G>A 5727T>C), K1529R (5728A>C 5729A>G), C1530V (5731T>G 5732G>T 5733C>G), K1532_D1533insP (5739_5740insCCC), D1533Y (5740G>T), T1534L (5743A>T 5744C>T 5745A>G), E1535D (5748A>C), E1536S (5749G>T 5750A>C), I1538V (5755A>G 5757C>G), A1539I (5758G>A 5759C>T 5760C>T), Y1541F (5765A>T), N1550K (5793C>G), A1553E (5801C>A), E1556V (5810A>T 5811A>C), K1560R (5822A>G), I1561T (5825T>C), M1562V (5827A>G)								
Codon mutations:	AGA1469.AA (5549G>A), ATA1470TTA (5551A>T), GGG1471CGG (5554G>C), AAT1472GGT (5557A>G 5558A>G), GCA1473GCT (5562A>T), AAG1474TTG (5563A>T 5564A>T), ATA1475GTG (5566A>G 5568A>G), TCA1477TCC (5574A>C), TTT1479ATT (5578T>A), AAG1482CAT (5587A>C 5589G>T), GGA1484GGG (5595A>G), TTT1485TAT (5597T>A), CAT1486CAC (5601T>C), CAA1487CAG (5604A>G), GTT1488TTG (5605G>T 5607T>G), GCC1489AGA (5608G>A 5609C>G 5610C>A), ATG1490GTT (5611A>G 5613G>T), GAA1491TGA (5614G>T 5615A>G), GAA1492GAG (5619A>G), GAA1493TCA (5620G>T 5621A>C), TCC1494GAT (5623T>G 5624C>A 5625C>T), ATA1495ATC (5628A>C), CCC1496CTA (5630C>T 5631C>A), TGG1497AAA (5632T>A 5633G>A 5634G>A), ACG1498ACT (5637G>T), GCC1499ACT (5638G>A 5640C>T), TTA1501AGG (5644T>A 5645T>G 5646A>G), ATC1502ACA (5648T>C 5649C>A), CCA1503CGG (5651C>G 5652A>G), GGA1504TAC (5653G>T 5654G>A 5655A>C), GGA1505GGG (5658A>G), TTA1506CAT (5659T>C 5660T>A 5661A>T), TAC1507TAT (5664C>T), GAA1508GAG (5667A>G), TGG1509TTT (5669G>T 5670G>T), CTT1510GTG (5671C>G 5673T>G), GTC1511GTT (5676C>T), CCA1513TCC (5680C>T 5682A>C), TTC1514TTT (5685C>T), GGA1515AGA (5686G>A), CTC1516CTA (5691C>A), AAA1517ACT (5693A>C 5694A>T), CCC1520CCT (5703C>T), GCT1521GCC (5706T>C), ATT1522GCA (5707A>G 5708T>C 5709T>A), CAG1524ATG (5713C>A 5714A>T), AGA1525GAG (5716A>G 5717G>A 5718A>G), AAA1526TTG (5719A>T 5720A>T 5721A>G), GAT1528AAC (5725G>A 5727T>C), AAA1529CGA (5728A>C 5729A>G), TGC1530GTG (5731T>G 5732G>T 5733C>G), TTC1531TTT (5736C>T), AAA1532_GAC1533insCCC (5739_5740insCCC), GAC1533TAC (5740G>T), ACA1534TTG (5743A>T 5744C>T 5745A>G), GAA1535GAC (5748A>C), GAG1536TCG (5749G>T 5750A>C), ATC1538GTG (5755A>G 5757C>G), GCC1539ATT (5758G>A 5759C>T 5760C>T), GTA1540GTG (5763A>G), TAC1541TTC (5765A>T), GAC1544GAT (5775C>T), CTG1546TTG (5779C>T), TAC1548TAT (5787C>T), AAC1550AAG (5793C>G), GAA1552GAG (5799A>G), GCG1553GAG (5801C>A), GAT1554GAC (5805T>C), GAA1556GTC (5810A>T 5811A>C), AGG1557CGT (5812A>C 5814G>T), CAT1558CAC (5817T>C), TTA1559CTA (5818T>C), AAG1560AGG (5822A>G), ATT1561ACT (5825T>C), ATG1562GTG (5827A>G)								

*: Inserts / Deletes / Misaligned / Frameshifts

Analysis details

This analysis was performed with panviral2.64

NGS Details (UN18_val): Pinus nigra virus 1

Assembly

**Coverage Length**	437 (1 contig(s))
**Depth Of Coverage**	3.8
**Number Of Reads**	13
**Reads Per Million**	0.24 rpm (after QC)
**Ambiguities**	0
**Assembly Method**	de novo + reference guided assembly
**Consensus Caller**	Bcf Tools

Coverage Map

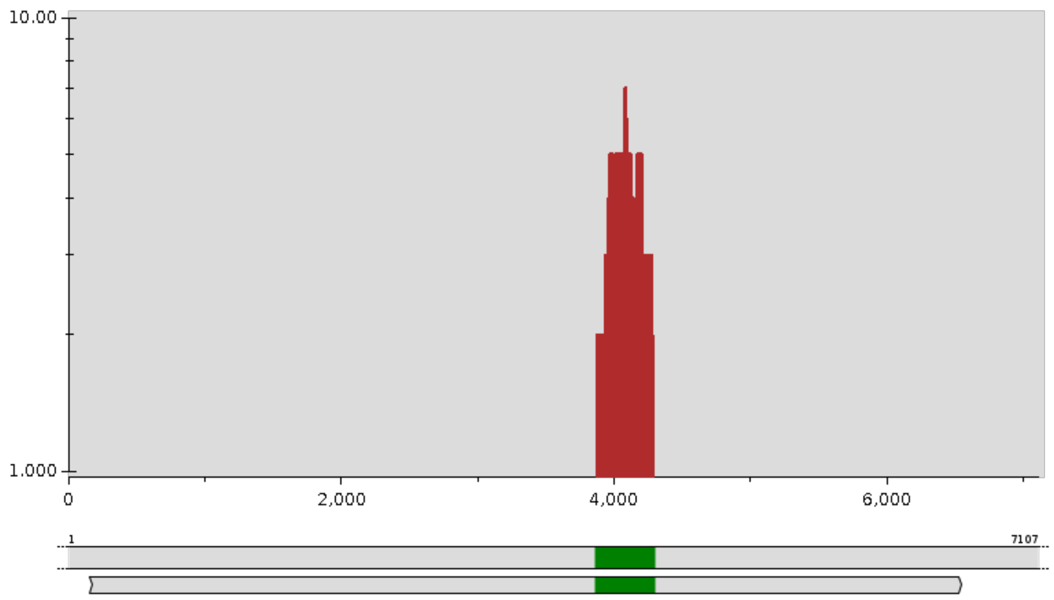

Assignment

**Type**	Pinus nigra virus 1 (Taxonomy ID: 2267679)
**Reference Genome**	NC_040841.1
**NT Identity (%)**	57.9545
**AA Identity (%)**	48.2993
**Number Of Stop Codons**	0
**Number Of CDS**	1

Alignment

**Alignment Score**	134.0 (NT) + 536.0 (AA) = 670.0
**Concordance (%)**	34.4473

Alignment Method	Global, seeded, nucleotide + amino acids (AGA)

Genome Region

Sequence starts at position 3866 and ends at position 4302 relative to NC_040841.1 reference sequence.

Alignment Detailed Statistics

	Begin	End	Coverage	Score	Concordance	Matches	Identities	I/D/M/F*	Stop Codons
**NT**	**3866**	**4302**	**6.1%**	**134**	**15.3%**	**437 (99.3%)**	**255 (58.0%)**	**3/0**	

Mutations: 3868A>C, 3871T>C, 3873T>A, 3874A>T, 3877A>T, 3879G>A, 3880G>T, 3883T>A, 3884C>A, 3885A>C, 3887T>G, 3892G>A, 3894T>A, 3897T>A, 3904T>C, 3905A>T, 3906A>C, 3907A>C, 3910A>T, 3913A>G, 3914A>T, 3916G>A, 3919A>T, 3922A>T, 3931A>G, 3935C>T, 3937T>A, 3941G>T, 3943C>T, 3944A>G, 3946C>T, 3947A>T, 3948C>T, 3949T>A, 3950C>A, 3952G>A, 3953A>T, 3954A>G, 3955T>G, 3956T>A, 3960A>G, 3961T>A, 3967A>G, 3968T>A, 3970G>T, 3973A>T, 3975G>A, 3976A>T, 3977C>A, 3978C>A, 3980G>A, 3981A>T, 3982A>G, 3984T>A, 3985G>T, 3986A>C, 3989A>T, 3992C>A, 3996A>G, 3999T>C, 4001C>T, 4002A>T, 4003G>T, 4004C>A, 4006T>A, 4008G>C, 4009T>C, 4012G>T, 4013G>A, 4015G>T, 4017T>A, 4021C>T, 4024G>A, 4030C>T, 4036G>A, 4042G>T, 4044A>T, 4045T>C, 4047A>G, 4048T>G, 4051G>A, 4054A>T, 4061C>T, 4062A>C, 4064C>A, 4065C>A, 4067G>A, 4074G>A, 4076C>T, 4078C>T, 4084A>T, 4092T>A, 4093A>C, 4094T>G, 4095G>T, 4096T>C, 4099A>C, 4100G>T, 4101C>T, 4102A>C, 4106T>C, 4107T>A, 4108C>A, 4112C>G, 4114G>A, 4120A>T, 4124G>A, 4126T>G, 4129A>C, 4135T>A, 4138G>A, 4141G>A, 4147G>C, 4150A>T, 4151G>T, 4153C>T, 4154T>G, 4155T>A, 4156C>A, 4164G>A, 4165A>C, 4166A>G, 4167G>T, 4168G>T, 4172G>A, 4176A>C, 4180C>T, 4184G>A, 4185C>A, 4187A>C, 4189A>C, 4190T>A, 4191A>T, 4192T>C, 4193G>A, 4194A>G, 4195T>C, 4196T>A, 4197T>A, 4198T>C, 4201A>C, 4201_4202insTCT, 4202G>A, 4205A>G, 4206C>T, 4207A>G, 4210C>T, 4213A>T, 4220A>G, 4225A>G, 4228T>A, 4229C>T, 4230A>T, 4232A>T, 4233G>C, 4235C>G, 4236C>A, 4237A>G, 4241G>A, 4243A>T, 4244C>G, 4246G>T, 4247A>T, 4248A>C, 4255G>T, 4258G>A, 4261C>T, 4265G>A, 4267A>T, 4269T>C, 4270T>C, 4273T>C, 4274C>T, 4276T>C, 4277G>A, 4280G>A, 4281A>T, 4282A>T, 4288G>A, 4290A>G, 4292C>A, 4297A>T, 4298A>C

CDS

	Begin	End	Coverage	Score	Concordance	Matches	Identities	I/D/M/F*	Stop Codons
EXL67_gp1	1238	1383	6.9%	536	49.8%	146 (99.3%)	71 (48.3%)	1/0/0/0	0

Protein mutations: L1240Y (3873T>A 3874A>T), R1242N (3879G>A 3880G>T), N1243K (3883T>A), H1244T (3884C>A 3885A>C), S1245A (3887T>G), I1247K (3894T>A), V1248E (3897T>A), K1251S (3905A>T 3906A>C 3907A>C), M1254L (3914A>T 3916G>A), A1263S (3941G>T 3943C>T), I1264V (3944A>G 3946C>T), T1265L (3947A>T 3948C>T 3949T>A), Q1266K (3950C>A 3952G>A), N1267W (3953A>T 3954A>G 3955T>G), F1268I (3956T>A), N1269R (3960A>G 3961T>A), L1272I (3968T>A 3970G>T), R1274N (3975G>A 3976A>T), P1275K (3977C>A 3978C>A), E1276M (3980G>A 3981A>T 3982A>G), V1277D (3984T>A 3985G>T), I1278L (3986A>C), M1279L (3989A>T), Q1280K (3992C>A), K1281R (3996A>G), I1282T (3999T>C), Q1283F (4001C>T 4002A>T 4003G>T), H1284K (4004C>A 4006T>A), S1285T (4008G>C 4009T>C), K1286N (4012G>T), V1287I (4013G>A 4015G>T), F1288Y (4017T>A), Y1297F (4044A>T 4045T>C), Y1298W (4047A>G 4048T>G), Q1303S (4061C>T 4062A>C), P1304K (4064C>A 4065C>A), E1305K (4067G>A), R1307K (4074G>A), H1308Y (4076C>T 4078C>T), I1313N (4092T>A 4093A>C), C1314V (4094T>G 4095G>T 4096T>C), A1316F (4100G>T 4101C>T 4102A>C), F1318Q (4106T>C 4107T>A 4108C>A), Q1320E (4112C>G 4114G>A), K1322N (4120A>T), V1324M (4124G>A 4126T>G), A1333S (4151G>T 4153C>T), F1334E (4154T>G 4155T>A 4156C>A), R1337N (4164G>A 4165A>C), R1338V (4166A>G 4167G>T 4168G>T), D1340N (4172G>A), Y1341S (4176A>C), A1344N (4184G>A 4185C>A), K1345H (4187A>C 4189A>C), Y1346I (4190T>A 4191A>T 4192T>C), D1347S (4193G>A 4194A>G 4195T>C), F1348N (4196T>A 4197T>A 4198T>C), I1349_V1350insS (4201_4202insTCT), V1350I (4202G>A), T1351V (4205A>G 4206C>T 4207A>G), I1356V (4220A>G), H1359F (4229C>T 4230A>T), P1361E (4235C>G 4236C>A 4237A>G), V1363I (4241G>A 4243A>T), Q1364D (4244C>G 4246G>T), N1365S (4247A>T 4248A>C), L1367F (4255G>T), E1371N (4265G>A 4267A>T), I1372T (4269T>C 4270T>C), L1374F (4274C>T 4276T>C), E1375K (4277G>A), E1376I (4280G>A 4281A>T 4282A>T), K1379R (4290A>G), H1380N (4292C>A), I1382L (4298A>C)

Codon mutations: GCA1238GCC (3868A>C), TTT1239TTC (3871T>C), TTA1240TAT (3873T>A 3874A>T), GTA1241GTT (3877A>T), AGG1242AAT (3879G>A 3880G>T), AAT1243AAA (3883T>A), CAT1244ACT (3884C>A 3885A>C), TCT1245GCT (3887T>G), GAG1246GAA (3892G>A), ATA1247AAA (3894T>A), GTA1248GAA (3897T>A), GGT1250GGC (3904T>C), AAA1251TCC (3905A>T 3906A>C 3907A>C), CCA1252CCT (3910A>T), AGA1253AGG (3913A>G), ATG1254TTA (3914A>T 3916G>A), GTA1255GTT (3919A>T), ATA1256ATT (3922A>T), AAA1259AAG (3931A>G), CTT1261TTA (3935C>T 3937T>A), GCC1263TCT (3941G>T 3943C>T), ATC1264GTT (3944A>G 3946C>T), ACT1265TTA (3947A>T 3948C>T 3949T>A), CAG1266AAA (3950C>A 3952G>A), AAT1267TGG (3953A>T 3954A>G 3955T>G), TTT1268ATT (3956T>A), AAT1269AGA (3960A>G 3961T>A), CCA1271CCG (3967A>G), TTG1272ATT (3968T>A 3970G>T), CCA1273CCT (3973A>T), AGA1274AAT (3975G>A 3976A>T), CCA1275AAA (3977C>A 3978C>A), GAA1276ATG (3980G>A 3981A>T 3982A>G), GTG1277GAT (3984T>A 3985G>T), ATT1278CTT (3986A>C), ATG1279TTG (3989A>T), CAA1280AAA (3992C>A), AAA1281AGA (3996A>G), ATT1282ACT (3999T>C), CAG1283TTT (4001C>T 4002A>T 4003G>T), CAT1284AAA (4004C>A 4006T>A), AGT1285ACC (4008G>C 4009T>C), AAG1286AAT (4012G>T), GTG1287ATT (4013G>A 4015G>T), TTC1288TAC (4017T>A), AGC1289AGT (4021C>T), AAG1290AAA (4024G>A), GAC1292GAT (4030C>T), AAG1294AAA (4036G>A), GGG1296GGT (4042G>T), TAT1297TTC (4044A>T 4045T>C), TAT1298TGG (4047A>G 4048T>G), CAG1299CAA (4051G>A), ATA1300ATT (4054A>T), CAG1303TCG (4061C>T 4062A>C), CCA1304AAA (4064C>A 4065C>A), GAA1305AAA (4067G>A), AGA1307AAA (4074G>A), CAC1308TAT (4076C>T 4078C>T), ACA1310ACT (4084A>T), ATA1313AAC (4092T>A 4093A>C), TGT1314GTC (4094T>G 4095G>T 4096T>C), CCA1315CCC (4099A>C), GCA1316TTC (4100G>T 4101C>T 4102A>C), TTC1318CAA (4106T>C 4107T>A 4108C>A), CAG1320GAA (4112C>G 4114G>A), AAA1322AAT (4120A>T), GTT1324ATG (4124G>A 4126T>G), CCA1325CCC (4129A>C), GGT1327GGA (4135T>A), TTG1328TTA (4138G>A), AAG1329AAA (4141G>A), GCG1331GCC (4147G>C), CCA1332CCT (4150A>T), GCC1333TCT (4151G>T 4153C>T), TTC1334GAA (4154T>G 4155T>A 4156C>A), AGA1337AAC (4164G>A 4165A>C), AGG1338GTT (4166A>G 4167G>T 4168G>T), GAT1340AAT (4172G>A), TAT1341TCT (4176A>C), ATC1342ATT (4180C>T), GCT1344AAT (4184G>A 4185C>A), AAA1345CAC (4187A>C 4189A>C), TAT1346ATC (4190T>A 4191A>T 4192T>C), GAT1347AGC (4193G>A 4194A>G 4195T>C), TTT1348AAC (4196T>A 4197T>A 4198T>C), ATA1349ATC (4201A>C), ATA1349_GTT1350insTCT (4201_4202insTCT), GTT1350ATT (4202G>A), ACA1351GTG (4205A>G 4206C>T 4207A>G), TAC1352TAT (4210C>T), ATA1353ATT (4213A>T), ATT1356GTT (4220A>G), TTA1357TTG (4225A>G), ATT1358ATA (4228T>A), CAT1359TTT (4229C>T 4230A>T), AGT1360TCT (4232A>T 4233G>C), CCA1361GAG (4235C>G 4236C>A 4237A>G), GTA1363ATT (4241G>A 4243A>T), CAG1364GAT (4244C>G 4246G>T), AAT1365TCT (4247A>T 4248A>C), TTG1367TTT (4255G>T), AAG1368AAA (4258G>A), CAC1369CAT (4261C>T), GAA1371AAT (4265G>A 4267A>T), ATT1372ACC (4269T>C 4270T>C), TTT1373TTC (4273T>C), CTT1374TTC (4274C>T 4276T>C), GAA1375AAA (4277G>A), GAA1376ATT (4280G>A 4281A>T 4282A>T), AAG1378AAA (4288G>A), AAA1379AGA (4290A>G), CAT1380AAT (4292C>A), GGA1381GGT (4297A>T), ATT1382CTT (4298A>C)

Proteins

	Begin	End	Coverage	Score	Concordance	Matches	Identities	I/D/M/F*	Stop Codons
polyprotein (YP_009553669.1)	1238	1383	6.9%	536	49.8%	146 (99.3%)	71 (48.3%)	1/0/0/0	0

Protein mutations: L1240Y (3873T>A 3874A>T), R1242N (3879G>A 3880G>T), N1243K (3883T>A), H1244T (3884C>A 3885A>C), S1245A (3887T>G), I1247K (3894T>A), V1248E (3897T>A), K1251S (3905A>T 3906A>C 3907A>C), M1254L (3914A>T 3916G>A), A1263S (3941G>T 3943C>T), I1264V (3944A>G 3946C>T), T1265L (3947A>T 3948C>T 3949T>A), Q1266K (3950C>A 3952G>A), N1267W (3953A>T 3954A>G 3955T>G), F1268I (3956T>A), N1269R (3960A>G 3961T>A), L1272I (3968T>A 3970G>T), R1274N (3975G>A 3976A>T), P1275K (3977C>A 3978C>A), E1276M (3980G>A 3981A>T 3982A>G), V1277D (3984T>A 3985G>T), I1278L (3986A>C), M1279L (3989A>T), Q1280K (3992C>A), K1281R (3996A>G), I1282T (3999T>C), Q1283F (4001C>T 4002A>T 4003G>T), H1284K (4004C>A 4006T>A), S1285T (4008G>C 4009T>C), K1286N (4012G>T), V1287I (4013G>A 4015G>T), F1288Y (4017T>A), Y1297F (4044A>T 4045T>C), Y1298W (4047A>G 4048T>G), Q1303S (4061C>T 4062A>C), P1304K (4064C>A 4065C>A), E1305K (4067G>A), R1307K (4074G>A), H1308Y (4076C>T 4078C>T), I1313N (4092T>A 4093A>C), C1314V (4094T>G 4095G>T 4096T>C), A1316F (4100G>T 4101C>T 4102A>C), F1318Q (4106T>C 4107T>A 4108C>A), Q1320E (4112C>G 4114G>A), K1322N (4120A>T), V1324M (4124G>A 4126T>G), A1333S (4151G>T 4153C>T), F1334E (4154T>G 4155T>A 4156C>A), R1337N (4164G>A 4165A>C), R1338V (4166A>G 4167G>T 4168G>T), D1340N (4172G>A), Y1341S (4176A>C), A1344N (4184G>A 4185C>A), K1345H (4187A>C 4189A>C), Y1346I (4190T>A 4191A>T 4192T>C), D1347S (4193G>A 4194A>G 4195T>C), F1348N (4196T>A 4197T>A 4198T>C), I1349_V1350insS (4201_4202insTCT), V1350I (4202G>A), T1351V (4205A>G 4206C>T 4207A>G), I1356V (4220A>G), H1359F (4229C>T 4230A>T), P1361E (4235C>G 4236C>A 4237A>G), V1363I (4241G>A 4243A>T), Q1364D (4244C>G 4246G>T), N1365S (4247A>T 4248A>C), L1367F (4255G>T), E1371N (4265G>A 4267A>T), I1372T (4269T>C 4270T>C), L1374F (4274C>T 4276T>C), E1375K (4277G>A), E1376I (4280G>A 4281A>T 4282A>T), K1379R (4290A>G), H1380N (4292C>A), I1382L (4298A>C)

Codon mutations: GCA1238GCC (3868A>C), TTT1239TTC (3871T>C), TTA1240TAT (3873T>A 3874A>T), GTA1241GTT (3877A>T), AGG1242AAT (3879G>A 3880G>T), AAT1243AAA (3883T>A), CAT1244ACT (3884C>A 3885A>C), TCT1245GCT (3887T>G), GAG1246GAA (3892G>A), ATA1247AAA (3894T>A), GTA1248GAA (3897T>A), GGT1250GGC (3904T>C), AAA1251TCC (3905A>T 3906A>C 3907A>C), CCA1252CCT (3910A>T), AGA1253AGG (3913A>G), ATG1254TTA (3914A>T 3916G>A), GTA1255GTT (3919A>T), ATA1256ATT (3922A>T), AAA1259AAG (3931A>G), CTT1261TTA (3935C>T 3937T>A), GCC1263TCT (3941G>T 3943C>T), ATC1264GTT (3944A>G 3946C>T), ACT1265TTA (3947A>T 3948C>T 3949T>A), CAG1266AAA (3950C>A 3952G>A), AAT1267TGG (3953A>T 3954A>G 3955T>G), TTT1268ATT (3956T>A), AAT1269AGA (3960A>G 3961T>A), CCA1271CCG (3967A>G), TTG1272ATT (3968T>A 3970G>T), CCA1273CCT (3973A>T), AGA1274AAT (3975G>A 3976A>T), CCA1275AAA (3977C>A 3978C>A), GAA1276ATG (3980G>A 3981A>T 3982A>G), GTG1277GAT (3984T>A 3985G>T), ATT1278CTT (3986A>C), ATG1279TTG (3989A>T), CAA1280AAA (3992C>A), AAA1281AGA (3996A>G), ATT1282ACT (3999T>C), CAG1283TTT (4001C>T 4002A>T 4003G>T), CAT1284AAA (4004C>A 4006T>A), AGT1285ACC (4008G>C 4009T>C), AAG1286AAT (4012G>T), GTG1287ATT (4013G>A 4015G>T), TTC1288TAC (4017T>A), AGC1289AGT (4021C>T), AAG1290AAA (4024G>A), GAC1292GAT (4030C>T), AAG1294AAA (4036G>A), GGG1296GGT (4042G>T), TAT1297TTC (4044A>T 4045T>C), TAT1298TGG (4047A>G 4048T>G), CAG1299CAA (4051G>A), ATA1300ATT (4054A>T), CAG1303TCG (4061C>T 4062A>C), CCA1304AAA (4064C>A 4065C>A), GAA1305AAA (4067G>A), AGA1307AAA (4074G>A), CAC1308TAT (4076C>T 4078C>T), ACA1310ACT (4084A>T), ATA1313AAC (4092T>A 4093A>C), TGT1314GTC (4094T>G 4095G>T 4096T>C), CCA1315CCC (4099A>C), GCA1316TTC (4100G>T 4101C>T 4102A>C), TTC1318CAA (4106T>C 4107T>A 4108C>A), CAG1320GAA (4112C>G 4114G>A), AAA1322AAT (4120A>T), GTT1324ATG (4124G>A 4126T>G), CCA1325CCC (4129A>C), GGT1327GGA (4135T>A), TTG1328TTA (4138G>A), AAG1329AAA (4141G>A), GCG1331GCC (4147G>C), CCA1332CCT (4150A>T), GCC1333TCT (4151G>T 4153C>T), TTC1334GAA (4154T>G 4155T>A 4156C>A), AGA1337AAC (4164G>A 4165A>C), AGG1338GTT (4166A>G 4167G>T 4168G>T), GAT1340AAT (4172G>A), TAT1341TCT (4176A>C), ATC1342ATT (4180C>T), GCT1344AAT (4184G>A 4185C>A), AAA1345CAC (4187A>C 4189A>C), TAT1346ATC (4190T>A 4191A>T 4192T>C), GAT1347AGC (4193G>A 4194A>G 4195T>C), TTT1348AAC (4196T>A 4197T>A 4198T>C), ATA1349ATC (4201A>C), ATA1349_GTT1350insTCT (4201_4202insTCT), GTT1350ATT (4202G>A), ACA1351GTG (4205A>G 4206C>T 4207A>G), TAC1352TAT (4210C>T), ATA1353ATT (4213A>T), ATT1356GTT (4220A>G), TTA1357TTG (4225A>G), ATT1358ATA (4228T>A), CAT1359TTT (4229C>T 4230A>T), AGT1360TCT (4232A>T 4233G>C), CCA1361GAG (4235C>G 4236C>A 4237A>G), GTA1363ATT (4241G>A 4243A>T), CAG1364GAT (4244C>G 4246G>T), AAT1365TCT (4247A>T 4248A>C), TTG1367TTT (4255G>T), AAG1368AAA (4258G>A), CAC1369CAT (4261C>T), GAA1371AAT (4265G>A 4267A>T), ATT1372ACC (4269T>C 4270T>C), TTT1373TTC (4273T>C), CTT1374TTC (4274C>T 4276T>C), GAA1375AAA (4277G>A), GAA1376ATT (4280G>A 4281A>T 4282A>T), AAG1378AAA (4288G>A), AAA1379AGA (4290A>G), CAT1380AAT (4292C>A), GGA1381GGT (4297A>T), ATT1382CTT (4298A>C)

*: Inserts / Deletes / Misaligned / Frameshifts

Analysis details

This analysis was performed with panviral2.64

NGS Details (UN18_val): Hibiscus bacilliform virus GD1

Assembly

**Coverage Length**	235 (1 contig(s))
**Depth Of Coverage**	6.1
**Number Of Reads**	12
**Reads Per Million**	0.23 rpm (after QC)
**Ambiguities**	0
**Assembly Method**	de novo + reference guided assembly
**Consensus Caller**	Bcf Tools

Coverage Map

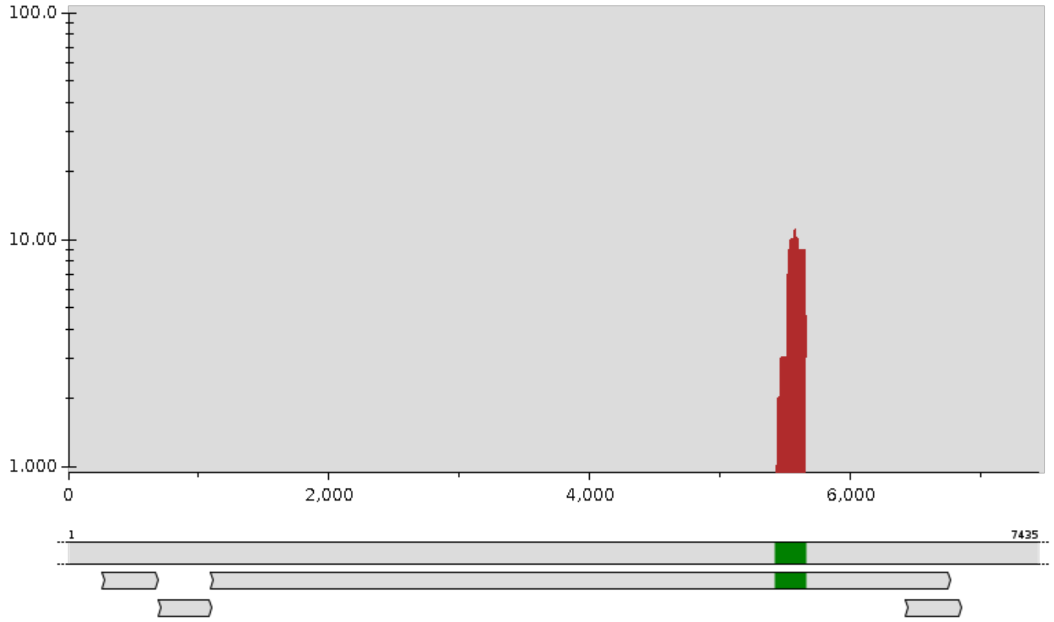

Assignment

**Type**	Hibiscus bacilliform virus GD1 (Taxonomy ID: 1459800)
**Reference Genome**	NC_023485.1
**NT Identity (%)**	56.3025
**AA Identity (%)**	47.5
**Number Of Stop Codons**	0
**Number Of CDS**	4

Alignment

**Alignment Score**	54.0 (NT) + 274.0 (AA) = 328.0
**Concordance (%)**	31.5688

Alignment Method	Global, seeded, nucleotide + amino acids (AGA)

Genome Region

Sequence starts at position 5421 and ends at position 5655 relative to NC_023485.1 reference sequence.

Alignment Detailed Statistics

	Begin	End	Coverage	Score	Concordance	Matches	Identities	I/D/M/F*	Stop Codons
**NT**	**5421**	**5655**	**3.2%**	**54**	**11.5%**	**235 (98.7%)**	**134 (56.3%)**	**3/0**	
Mutations:	5422C>A, 5426C>T, 5427A>C, 5430T>A, 5431G>A, 5435T>A, 5436G>A, 5441C>T, 5442T>C, 5445G>A, 5446T>C, 5447T>C, 5449C>A, 5450A>T, 5451G>T, 5453T>C, 5456A>C, 5458T>A, 5459G>C, 5465A>G, 5467G>T, 5468G>T, 5470T>C, 5480A>T, 5486A>G, 5489T>A, 5491A>C, 5492A>T, 5495C>T, 5502G>A, 5503C>A, 5505G>A, 5506T>C, 5511C>A, 5512A>T, 5514C>G, 5515G>A, 5516A>T, 5517A>C, 5518A>T, 5523G>A, 5524A>C, 5525C>T, 5526A>C, 5527A>G, 5528C>G, 5531C>G, 5534C>T, 5536A>G, 5537A>G, 5537_5538insCCA, 5538G>T, 5539G>A, 5541A>C, 5542C>T, 5546A>T, 5547G>A, 5552T>C, 5555C>T, 5556G>A, 5557C>T, 5561A>G, 5563A>T, 5564C>T, 5573C>T, 5576A>T, 5579C>G, 5584T>A, 5588C>T, 5590A>G, 5591G>C, 5592T>A, 5593C>G, 5595G>C, 5601G>C, 5603A>G, 5607A>G, 5608T>C, 5609C>T, 5610A>C, 5615C>T, 5616A>C, 5618A>T, 5619C>A, 5622G>A, 5624C>T, 5625A>G, 5626T>C, 5627G>A, 5630A>G, 5631G>C, 5633A>G, 5635A>C, 5637T>C, 5638G>T, 5640C>A, 5642G>A, 5646A>C, 5648T>C, 5649G>A, 5654T>A, 5655G>T								

CDS

	Begin	End	Coverage	Score	Concordance	Matches	Identities	I/D/M/F*	Stop Codons
BU35_gp3	1444	1522	4.2%	274	47.7%	79 (98.8%)	38 (47.5%)	1/0/0/0	0
Protein mutations:	S1444Y (5422C>A), K1446Q (5427A>C), W1447K (5430T>A 5431G>A), A1449T (5436G>A), W1451R (5442T>C), V1452T (5445G>A 5446T>C 5447T>C), P1453H (5449C>A 5450A>T), D1454Y (5451G>T 5453T>C), L1456H (5458T>A 5459G>C), W1459F (5467G>T 5468G>T), L1460P (5470T>C), K1467T (5491A>C 5492A>T), A1471K (5502G>A 5503C>A), V1472T (5505G>A 5506T>C), Q1474M (5511C>A 5512A>T), R1475D (5514C>G 5515G>A 5516A>T), K1476L (5517A>C 5518A>T), D1478T (5523G>A 5524A>C 5525C>T), N1479R (5526A>C 5527A>G 5528C>G), K1482R (5536A>G 5537A>G), K1482_G1483insP (5537_5538insCCA), G1483Y (5538G>T 5539G>A), T1484L (5541A>C 5542C>T), E1485D (5546A>T), A1486T (5547G>A), A1489I (5556G>A 5557C>T), Y1491F (5563A>T 5564C>T), F1498Y (5584T>A), Q1500R (5590A>G 5591G>C), E1502Q (5595G>C), E1504Q (5601G>C 5603A>G), I1506A (5607A>G 5608T>C 5609C>T), K1507Q (5610A>C), I1509L (5616A>C 5618A>T), V1511I (5622G>A 5624C>T), M1512A (5625A>G 5626T>C 5627G>A), E1514Q (5631G>C 5633A>G), K1515T (5635A>C), C1516L (5637T>C 5638G>T), N1519H (5646A>C 5648T>C), G1520R (5649G>A)								
Codon mutations:	TCT1444TAT (5422C>A), ATC1445ATT (5426C>T), AAG1446CAG (5427A>C), TGG1447AAG (5430T>A 5431G>A), ACT1448ACA (5435T>A), GCT1449ACT (5436G>A), TTC1450TTT (5441C>T), TGG1451CGG (5442T>C), GTT1452ACC (5445G>A 5446T>C 5447T>C), CCA1453CAT (5449C>A 5450A>T), GAT1454TAC (5451G>T 5453T>C), GGA1455GGC (5456A>C), CTG1456CAC (5458T>A 5459G>C), GAA1458GAG (5465A>G), TGG1459TTT (5467G>T 5468G>T), CTA1460CCA (5470T>C), CCA1463CCT (5480A>T), GGA1465GGG (5486A>G), CTT1466CTA (5489T>A), AAA1467ACT (5491A>C 5492A>T), AAC1468AAT (5495C>T), GCA1471AAA (5502G>A 5503C>A), GTA1472ACA (5505G>A 5506T>C), CAG1474ATG (5511C>A 5512A>T), CGA1475GAT (5514C>G 5515G>A 5516A>T), AAG1476CTG (5517A>C 5518A>T), GAC1478ACT (5523G>A 5524A>C 5525C>T), AAC1479CGG (5526A>C 5527A>G 5528C>G), GTC1480GTG (5531C>G), TTC1481TTT (5534C>T), AAA1482AGG (5536A>G 5537A>G), AAA1482_GGT1483insCCA (5537_5538insCCA), GGT1483TAT (5538G>T 5539G>A), ACT1484CTT (5541A>C 5542C>T), GAA1485GAT (5546A>T), GCC1486ACC (5547G>A), TTT1487TTC (5552T>C), ATC1488ATT (5555C>T), GCT1489ATT (5556G>A 5557C>T), GTA1490GTG (5561A>G), TAC1491TTT (5563A>T 5564C>T), GAC1494GAT (5573C>T), ATA1495ATT (5576A>T), CTC1496CTG (5579C>G), TTT1498TAT (5584T>A), TCC1499TCT (5588C>T), CAG1500CGC (5590A>G 5591G>C), TCT1501AGT (5592T>A 5593C>G), GAG1502CAG (5595G>C), GAA1504CAG (5601G>C 5603A>G), ATC1506GCT (5607A>G 5608T>C 5609C>T), AAG1507CAG (5610A>C), CAC1508CAT (5615C>T), ATA1509CTT (5616A>C 5618A>T), CGG1510AGG (5619C>A), GTC1511ATT (5622G>A 5624C>T), ATG1512GCA (5625A>G 5626T>C 5627G>A), TTA1513TTG (5630A>G), GAA1514CAG (5631G>C 5633A>G), AAA1515ACA (5635A>C), TGC1516CTC (5637T>C 5638G>T), CGG1517AGA (5640C>A 5642G>A), AAT1519CAC (5646A>C 5648T>C), GGG1520AGG (5649G>A), CTT1521CTA (5654T>A), GTT1522T.. (5655G>T)								

Proteins

	Begin	End	Coverage	Score	Concordance	Matches	Identities	I/D/M/F*	Stop Codons
polyprotein (YP_009002585.1)	1444	1522	4.2%	274	47.7%	79 (98.8%)	38 (47.5%)	1/0/0/0	0
Protein mutations:	S1444Y (5422C>A), K1446Q (5427A>C), W1447K (5430T>A 5431G>A), A1449T (5436G>A), W1451R (5442T>C), V1452T (5445G>A 5446T>C 5447T>C), P1453H (5449C>A 5450A>T), D1454Y (5451G>T 5453T>C), L1456H (5458T>A 5459G>C), W1459F (5467G>T 5468G>T), L1460P (5470T>C), K1467T (5491A>C 5492A>T), A1471K (5502G>A 5503C>A), V1472T (5505G>A 5506T>C), Q1474M (5511C>A 5512A>T), R1475D (5514C>G 5515G>A 5516A>T), K1476L (5517A>C 5518A>T), D1478T (5523G>A 5524A>C 5525C>T), N1479R (5526A>C 5527A>G 5528C>G), K1482R (5536A>G 5537A>G), K1482_G1483insP (5537_5538insCCA), G1483Y (5538G>T 5539G>A), T1484L (5541A>C 5542C>T), E1485D (5546A>T), A1486T (5547G>A), A1489I (5556G>A 5557C>T), Y1491F (5563A>T 5564C>T), F1498Y (5584T>A), Q1500R (5590A>G 5591G>C), E1502Q (5595G>C), E1504Q (5601G>C 5603A>G), I1506A (5607A>G 5608T>C 5609C>T), K1507Q (5610A>C), I1509L (5616A>C 5618A>T), V1511I (5622G>A 5624C>T), M1512A (5625A>G 5626T>C 5627G>A), E1514Q (5631G>C 5633A>G), K1515T (5635A>C), C1516L (5637T>C 5638G>T), N1519H (5646A>C 5648T>C), G1520R (5649G>A)								
Codon mutations:	TCT1444TAT (5422C>A), ATC1445ATT (5426C>T), AAG1446CAG (5427A>C), TGG1447AAG (5430T>A 5431G>A), ACT1448ACA (5435T>A), GCT1449ACT (5436G>A), TTC1450TTT (5441C>T), TGG1451CGG (5442T>C), GTT1452ACC (5445G>A 5446T>C 5447T>C), CCA1453CAT (5449C>A 5450A>T), GAT1454TAC (5451G>T 5453T>C), GGA1455GGC (5456A>C), CTG1456CAC (5458T>A 5459G>C), GAA1458GAG (5465A>G), TGG1459TTT (5467G>T 5468G>T), CTA1460CCA (5470T>C), CCA1463CCT (5480A>T), GGA1465GGG (5486A>G), CTT1466CTA (5489T>A), AAA1467ACT (5491A>C 5492A>T), AAC1468AAT (5495C>T), GCA1471AAA (5502G>A 5503C>A), GTA1472ACA (5505G>A 5506T>C), CAG1474ATG (5511C>A 5512A>T), CGA1475GAT (5514C>G 5515G>A 5516A>T), AAG1476CTG (5517A>C 5518A>T), GAC1478ACT (5523G>A 5524A>C 5525C>T), AAC1479CGG (5526A>C 5527A>G 5528C>G), GTC1480GTG (5531C>G), TTC1481TTT (5534C>T), AAA1482AGG (5536A>G 5537A>G), AAA1482_GGT1483insCCA (5537_5538insCCA), GGT1483TAT (5538G>T 5539G>A), ACT1484CTT (5541A>C 5542C>T), GAA1485GAT (5546A>T), GCC1486ACC (5547G>A), TTT1487TTC (5552T>C), ATC1488ATT (5555C>T), GCT1489ATT (5556G>A 5557C>T), GTA1490GTG (5561A>G), TAC1491TTT (5563A>T 5564C>T), GAC1494GAT (5573C>T), ATA1495ATT (5576A>T), CTC1496CTG (5579C>G), TTT1498TAT (5584T>A), TCC1499TCT (5588C>T), CAG1500CGC (5590A>G 5591G>C), TCT1501AGT (5592T>A 5593C>G), GAG1502CAG (5595G>C), GAA1504CAG (5601G>C 5603A>G), ATC1506GCT (5607A>G 5608T>C 5609C>T), AAG1507CAG (5610A>C), CAC1508CAT (5615C>T), ATA1509CTT (5616A>C 5618A>T), CGG1510AGG (5619C>A), GTC1511ATT (5622G>A 5624C>T), ATG1512GCA (5625A>G 5626T>C 5627G>A), TTA1513TTG (5630A>G), GAA1514CAG (5631G>C 5633A>G), AAA1515ACA (5635A>C), TGC1516CTC (5637T>C 5638G>T), CGG1517AGA (5640C>A 5642G>A), AAT1519CAC (5646A>C 5648T>C), GGG1520AGG (5649G>A), CTT1521CTA (5654T>A), GTT1522T.. (5655G>T)								

*: Inserts / Deletes / Misaligned / Frameshifts

Analysis details

This analysis was performed with panviral2.64

NGS Details (UN18_val): Caulimovirus tessellomirabilis

Assembly

**Coverage Length**	467 (1 contig(s))
**Depth Of Coverage**	3.1
**Number Of Reads**	11
**Reads Per Million**	0.21 rpm (after QC)
**Ambiguities**	0
**Assembly Method**	de novo + reference guided assembly
**Consensus Caller**	Bcf Tools

Coverage Map

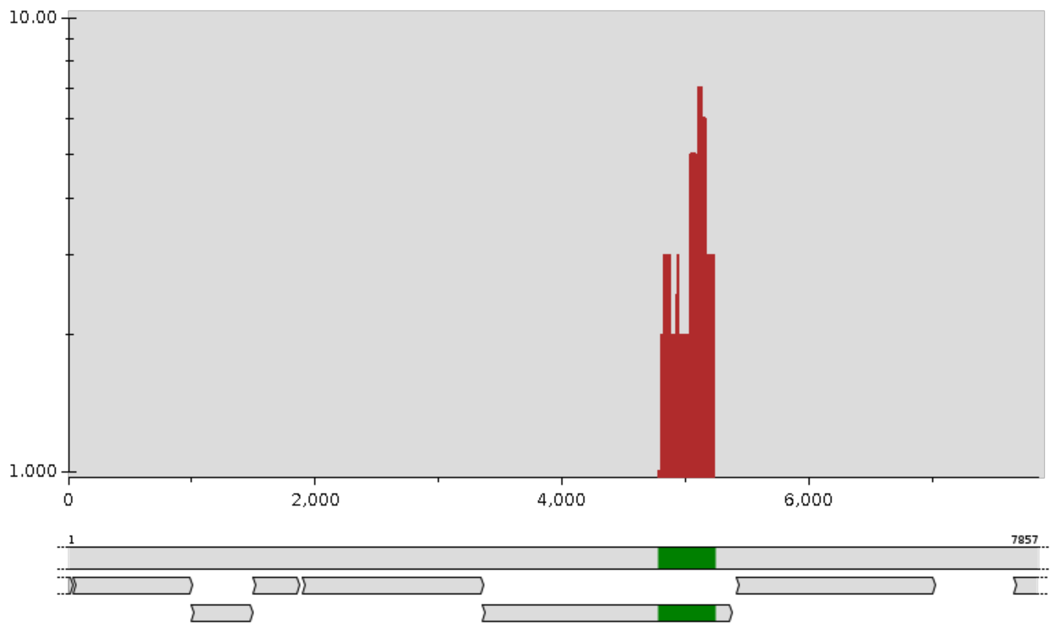

Assignment

**Type**	Caulimovirus tessellomirabilis (Taxonomy ID: 3048203)
**Reference Genome**	NC_004036.1
**NT Identity (%)**	58.1858
**AA Identity (%)**	47.6821
**Number Of Stop Codons**	0
**Number Of CDS**	7

Alignment

**Alignment Score**	106.0 (NT) + 464.0 (AA) = 570.0
**Concordance (%)**	30.8108

Alignment Method	Global, seeded, nucleotide + amino acids (AGA)

Genome Region

Sequence starts at position 4777 and ends at position 5243 relative to NC_004036.1 reference sequence.

Alignment Detailed Statistics

	Begin	End	Coverage	Score	Concordance	Matches	Identities	I/D/M/F*	Stop Codons
**NT**	**4777**	**5243**	**5.9%**	**106**	**12.3%**	**452 (96.8%)**	**263 (56.3%)**	**0/15**	
Mutations:	4780G>A, 4790G>T, 4791T>G, 4792T>C, 4793C>T, 4797C>A, 4798A>C, 4806A>G, 4807T>A, 4808A>G, 4809G>A, 4810C>A, 4812A>T, 4813T>C, 4816C>A, 4820A>G, 4822G>A, 4823C>T, 4825T>A, 4829G>A, 4834A>G, 4835C>A, 4837G>A, 4840A>T, 4842C>T, 4843G>T, 4844T>C, 4849G>A, 4850G>A, 4851T>A, 4853A>C, 4854A>T, 4855G>T, 4856T>A, 4858G>C, 4860A>G, 4861G>A, 4865G>T, 4866A>C, 4867T>A, 4868C>A, 4870G>T, 4871G>C, 4872T>G, 4875G>T, 4877A>G, 4878T>G, 4879C>A, 4885G>A, 4886C>A, 4889T>A, 4890C>A, 4892G>C, 4898G>C, 4899A>T, 4902A>G, 4903C>T, 4907A>C, 4912G>A, 4913A>C, 4915A>C, 4918G>A, 4919A>G, 4923G>A, 4926T>G, 4927A>T, 4928A>G, 4929T>C, 4933T>A, 4934T>C, 4946T>C, 4947A>G, 4948T>A, 4949C>T, 4955A>G, 4957G>A, 4958A>C, 4959A>C, 4966C>T, 4968G>A, 4972A>G, 4976A>T, 4978T>A, 4979G>C, 4990T>C, 4993T>C, 4997C>T, 4999C>T, 5000T>C, 5001T>A, 5002C>T, 5008T>A, 5010G>C, 5012G>T, 5014C>A, 5015C>T, 5017T>A, 5018_5023delAAGGCC, 5029C>A, 5030A>G, 5031C>A, 5033G>C, 5034A>T, 5036G>A, 5037G>A, 5042_5047delGAATTA, 5050C>T, 5053C>T, 5056G>A, 5059T>C, 5060T>A, 5062T>A, 5063T>A, 5064C>G, 5065A>T, 5071A>C, 5074C>T, 5077A>C, 5078C>G, 5079C>A, 5087T>G, 5089G>T, 5095C>T, 5096C>T, 5097A>C, 5098T>C, 5100G>C, 5101T>A, 5107G>A, 5117C>T, 5122G>A, 5126A>G, 5127A>T, 5129C>A, 5130A>G, 5131G>A, 5133T>G, 5134G>A, 5137T>A, 5138A>C, 5139C>G, 5140T>A, 5147A>T, 5148G>C, 5149T>A, 5151T>C, 5152T>A, 5154A>C, 5155T>C, 5156C>T, 5159A>T, 5160C>T, 5161C>A, 5163C>A, 5166T>A, 5167T>A, 5168T>C, 5169G>C, 5170T>A, 5174A>G, 5175C>T, 5176A>T, 5177G>A, 5179C>T, 5181G>A, 5182G>A, 5184C>T, 5192G>A, 5193T>C, 5195A>C, 5197T>A, 5198_5200delCTT, 5201T>A, 5204A>T, 5205A>C, 5212T>G, 5215G>A, 5216A>T, 5217A>T, 5221A>T, 5224G>A, 5225A>C, 5227T>C, 5228A>C, 5231G>T, 5232G>C, 5236C>A, 5237A>C, 5238G>C, 5240A>C, 5241A>C, 5242A>T								

CDS

	Begin	End	Coverage	Score	Concordance	Matches	Identities	I/D/M/F*	Stop Codons
MiMVgp6	475	630	23.1%	464	46.2%	151 (96.8%)	72 (46.2%)	0/5/0/0	0
Protein mutations:	V479C (4790G>T 4791T>G 4792T>C), T481N (4797C>A 4798A>C), D484G (4806A>G 4807T>A), S485E (4808A>G 4809G>A 4810C>A), Y486F (4812A>T 4813T>C), K489E (4820A>G 4822G>A), E492K (4829G>A), K495N (4840A>T), P496L (4842C>T 4843G>T), V499K (4850G>A 4851T>A), K500L (4853A>C 4854A>T 4855G>T), L501I (4856T>A 4858G>C), K502R (4860A>G 4861G>A), D504S (4865G>T 4866A>C 4867T>A), Q505N (4868C>A 4870G>T), V506R (4871G>C 4872T>G), W507L (4875G>T), I508G (4877A>G 4878T>G 4879C>A), Q511K (4886C>A), S512K (4889T>A 4890C>A), D513H (4892G>C), D515L (4898G>C 4899A>T), Y516C (4902A>G 4903C>T), K518Q (4907A>C), I520L (4913A>C 4915A>C), K522E (4919A>G), G523E (4923G>A), L524C (4926T>G 4927A>T), I525A (4928A>G 4929T>C), N526K (4933T>A), F527L (4934T>C), Y531R (4946T>C 4947A>G 4948T>A), K534E (4955A>G 4957G>A), K535P (4958A>C 4959A>C), S538N (4968G>A), I541L (4976A>T 4978T>A), E542Q (4979G>C), H548Y (4997C>T 4999C>T), F549H (5000T>C 5001T>A 5002C>T), G552A (5010G>C), V553L (5012G>T 5014C>A), K555_A556del (5018_5023delAAGGCC), T559D (5030A>G 5031C>A), E560L (5033G>C 5034A>T), G561N (5036G>A 5037G>A), E563_L564del (5042_5047delGAATTA), S569T (5060T>A 5062T>A), K574N (5077A>C), P575E (5078C>G 5079C>A), L578V (5087T>G 5089G>T), H581S (5096C>T 5097A>C 5098T>C), S582T (5100G>C 5101T>A), K591V (5126A>G 5127A>T), Q592R (5129C>A 5130A>G 5131G>A), V593G (5133T>G 5134G>A), T595R (5138A>C 5139C>G 5140T>A), I599T (5151T>C 5152T>A), Y600S (5154A>C 5155T>C), T602L (5159A>T 5160C>T 5161C>A), P603H (5163C>A), V604E (5166T>A 5167T>A), C605P (5168T>C 5169G>C 5170T>A), T607V (5174A>G 5175C>T 5176A>T), V608I (5177G>A 5179C>T), R609K (5181G>A 5182G>A), T610I (5184C>T), V613T (5192G>A 5193T>C), N614Q (5195A>C 5197T>A), L615del (5198_5200delCTT), L616I (5201T>A), K617S (5204A>T 5205A>C), F619L (5212T>G), M620I (5215G>A), N621F (5216A>T 5217A>T), K622N (5221A>T), I624L (5225A>C 5227T>C), T625P (5228A>C), G626S (5231G>T 5232G>C), D627E (5236C>A), S628P (5237A>C 5238G>C), K629P (5240A>C 5241A>C 5242A>T)								
Codon mutations:	AGG475AGA (4780G>A), GTT479TGC (4790G>T 4791T>G 4792T>C), CTA480TTA (4793C>T), ACA481AAC (4797C>A 4798A>C), GAT484GGA (4806A>G 4807T>A), AGC485GAA (4808A>G 4809G>A 4810C>A), TAT486TTC (4812A>T 4813T>C), ATC487ATA (4816C>A), AAG489GAA (4820A>G 4822G>A), CTT490TTA (4823C>T 4825T>A), GAA492AAA (4829G>A), AAA493AAG (4834A>G), CGG494AGA (4835C>A 4837G>A), AAA495AAT (4840A>T), CCG496CTT (4842C>T 4843G>T), TTA497CTA (4844T>C), CAG498CAA (4849G>A), GTA499AAA (4850G>A 4851T>A), AAG500CTT (4853A>C 4854A>T 4855G>T), TTG501ATC (4856T>A 4858G>C), AAG502AGA (4860A>G 4861G>A), GAT504TCA (4865G>T 4866A>C 4867T>A), CAG505AAT (4868C>A 4870G>T), GTA506CGA (4871G>C 4872T>G), TGG507TTG (4875G>T), ATC508GGA (4877A>G 4878T>G 4879C>A), ACG510ACA (4885G>A), CAA511AAA (4886C>A), TCA512AAA (4889T>A 4890C>A), GAT513CAT (4892G>C), GAT515CTT (4898G>C 4899A>T), TAC516TGT (4902A>G 4903C>T), AAA518CAA (4907A>C), AAG519AAA (4912G>A), ATA520CTC (4913A>C 4915A>C), AAG521AAA (4918G>A), AAA522GAA (4919A>G), GGA523GAA (4923G>A), TTA524TGT (4926T>G 4927A>T), ATT525GCT (4928A>G 4929T>C), AAT526AAA (4933T>A), TTT527CTT (4934T>C), TAT531CGA (4946T>C 4947A>G 4948T>A), CTA532TTA (4949C>T), AAG534GAA (4955A>G 4957G>A), AAA535CCA (4958A>C 4959A>C), GAC537GAT (4966C>T), AGT538AAT (4968G>A), TTA539TTG (4972A>G), ATT541TTA (4976A>T 4978T>A), GAA542CAA (4979G>C), GCT545GCC (4990T>C), TCT546TCC (4993T>C), CAC548TAT (4997C>T 4999C>T), TTC549CAT (5000T>C 5001T>A 5002C>T), GGT551GGA (5008T>A), GGA552GCA (5010G>C), GTC553TTA (5012G>T 5014C>A), CTT554TTA (5015C>T 5017T>A), AAG555_GCC556del (5018_5023delAAGGCC), ACC558ACA (5029C>A), ACT559GAT (5030A>G 5031C>A), GAA560CTA (5033G>C 5034A>T), GGT561AAT (5036G>A 5037G>A), GAA563_TTA564del (5042_5047delGAATTA), ATC565ATT (5050C>T), TGC566TGT (5053C>T), AGG567AGA (5056G>A), TAT568TAC (5059T>C), TCT569ACA (5060T>A 5062T>A), TCA570AGT (5063T>A 5064C>G 5065A>T), ACA572ACC (5071A>C), TTC573TTT (5074C>T), AAA574AAC (5077A>C), CCA575GAA (5078C>G 5079C>A), TTG578GTT (5087T>G 5089G>T), TAC580TAT (5095C>T), CAT581TCC (5096C>T 5097A>C 5098T>C), AGT582ACA (5100G>C 5101T>A), GAG584GAA (5107G>A), CTA588TTA (5117C>T), GCG589GCA (5122G>A), AAA591GTA (5126A>G 5127A>T), CAG592AGA (5129C>A 5130A>G 5131G>A), GTG593GGA (5133T>G 5134G>A), ATT594ATA (5137T>A), ACT595CGA (5138A>C 5139C>G 5140T>A), AGT598TCA (5147A>T 5148G>C 5149T>A), ATT599ACA (5151T>C 5152T>A), TAT600TCC (5154A>C 5155T>C), CTA601TTA (5156C>T), ACC602TTA (5159A>T 5160C>T 5161C>A), CCT603CAT (5163C>A), GTT604GAA (5166T>A 5167T>A), TGT605CCA (5168T>C 5169G>C 5170T>A), ACA607GTT (5174A>G 5175C>T 5176A>T), GTC608ATT (5177G>A 5179C>T), AGG609AAA (5181G>A 5182G>A), ACA610ATA (5184C>T), GTA613ACA (5192G>A 5193T>C), AAT614CAA (5195A>C 5197T>A), CTT615del (5198_5200delCTT), TTA616ATA (5201T>A), AAA617TCA (5204A>T 5205A>C), TTT619TTG (5212T>G), ATG620ATA (5215G>A), AAT621TTT (5216A>T 5217A>T), AAA622AAT (5221A>T), AAG623AAA (5224G>A), ATT624CTC (5225A>C 5227T>C), ACT625CCT (5228A>C), GGT626TCT (5231G>T 5232G>C), GAC627GAA (5236C>A), AGT628CCT (5237A>C 5238G>C), AAA629CCT (5240A>C 5241A>C 5242A>T)								

Proteins

	Begin	End	Coverage	Score	Concordance	Matches	Identities	I/D/M/F*	Stop Codons
hypothetical protein (NP_659397.1)	475	630	23.1%	464	46.2%	151 (96.8%)	72 (46.2%)	0/5/0/0	0
Protein mutations:	V479C (4790G>T 4791T>G 4792T>C), T481N (4797C>A 4798A>C), D484G (4806A>G 4807T>A), S485E (4808A>G 4809G>A 4810C>A), Y486F (4812A>T 4813T>C), K489E (4820A>G 4822G>A), E492K (4829G>A), K495N (4840A>T), P496L (4842C>T 4843G>T), V499K (4850G>A 4851T>A), K500L (4853A>C 4854A>T 4855G>T), L501I (4856T>A 4858G>C), K502R (4860A>G 4861G>A), D504S (4865G>T 4866A>C 4867T>A), Q505N (4868C>A 4870G>T), V506R (4871G>C 4872T>G), W507L (4875G>T), I508G (4877A>G 4878T>G 4879C>A), Q511K (4886C>A), S512K (4889T>A 4890C>A), D513H (4892G>C), D515L (4898G>C 4899A>T), Y516C (4902A>G 4903C>T), K518Q (4907A>C), I520L (4913A>C 4915A>C), K522E (4919A>G), G523E (4923G>A), L524C (4926T>G 4927A>T), I525A (4928A>G 4929T>C), N526K (4933T>A), F527L (4934T>C), Y531R (4946T>C 4947A>G 4948T>A), K534E (4955A>G 4957G>A), K535P (4958A>C 4959A>C), S538N (4968G>A), I541L (4976A>T 4978T>A), E542Q (4979G>C), H548Y (4997C>T 4999C>T), F549H (5000T>C 5001T>A 5002C>T), G552A (5010G>C), V553L (5012G>T 5014C>A), K555_A556del (5018_5023delAAGGCC), T559D (5030A>G 5031C>A), E560L (5033G>C 5034A>T), G561N (5036G>A 5037G>A), E563_L564del (5042_5047delGAATTA), S569T (5060T>A 5062T>A), K574N (5077A>C), P575E (5078C>G 5079C>A), L578V (5087T>G 5089G>T), H581S (5096C>T 5097A>C 5098T>C), S582T (5100G>C 5101T>A), K591V (5126A>G 5127A>T), Q592R (5129C>A 5130A>G 5131G>A), V593G (5133T>G 5134G>A), T595R (5138A>C 5139C>G 5140T>A), I599T (5151T>C 5152T>A), Y600S (5154A>C 5155T>C), T602L (5159A>T 5160C>T 5161C>A), P603H (5163C>A), V604E (5166T>A 5167T>A), C605P (5168T>C 5169G>C 5170T>A), T607V (5174A>G 5175C>T 5176A>T), V608I (5177G>A 5179C>T), R609K (5181G>A 5182G>A), T610I (5184C>T), V613T (5192G>A 5193T>C), N614Q (5195A>C 5197T>A), L615del (5198_5200delCTT), L616I (5201T>A), K617S (5204A>T 5205A>C), F619L (5212T>G), M620I (5215G>A), N621F (5216A>T 5217A>T), K622N (5221A>T), I624L (5225A>C 5227T>C), T625P (5228A>C), G626S (5231G>T 5232G>C), D627E (5236C>A), S628P (5237A>C 5238G>C), K629P (5240A>C 5241A>C 5242A>T)								
Codon mutations:	AGG475AGA (4780G>A), GTT479TGC (4790G>T 4791T>G 4792T>C), CTA480TTA (4793C>T), ACA481AAC (4797C>A 4798A>C), GAT484GGA (4806A>G 4807T>A), AGC485GAA (4808A>G 4809G>A 4810C>A), TAT486TTC (4812A>T 4813T>C), ATC487ATA (4816C>A), AAG489GAA (4820A>G 4822G>A), CTT490TTA (4823C>T 4825T>A), GAA492AAA (4829G>A), AAA493AAG (4834A>G), CGG494AGA (4835C>A 4837G>A), AAA495AAT (4840A>T), CCG496CTT (4842C>T 4843G>T), TTA497CTA (4844T>C), CAG498CAA (4849G>A), GTA499AAA (4850G>A 4851T>A), AAG500CTT (4853A>C 4854A>T 4855G>T), TTG501ATC (4856T>A 4858G>C), AAG502AGA (4860A>G 4861G>A), GAT504TCA (4865G>T 4866A>C 4867T>A), CAG505AAT (4868C>A 4870G>T), GTA506CGA (4871G>C 4872T>G), TGG507TTG (4875G>T), ATC508GGA (4877A>G 4878T>G 4879C>A), ACG510ACA (4885G>A), CAA511AAA (4886C>A), TCA512AAA (4889T>A 4890C>A), GAT513CAT (4892G>C), GAT515CTT (4898G>C 4899A>T), TAC516TGT (4902A>G 4903C>T), AAA518CAA (4907A>C), AAG519AAA (4912G>A), ATA520CTC (4913A>C 4915A>C), AAG521AAA (4918G>A), AAA522GAA (4919A>G), GGA523GAA (4923G>A), TTA524TGT (4926T>G 4927A>T), ATT525GCT (4928A>G 4929T>C), AAT526AAA (4933T>A), TTT527CTT (4934T>C), TAT531CGA (4946T>C 4947A>G 4948T>A), CTA532TTA (4949C>T), AAG534GAA (4955A>G 4957G>A), AAA535CCA (4958A>C 4959A>C), GAC537GAT (4966C>T), AGT538AAT (4968G>A), TTA539TTG (4972A>G), ATT541TTA (4976A>T 4978T>A), GAA542CAA (4979G>C), GCT545GCC (4990T>C), TCT546TCC (4993T>C), CAC548TAT (4997C>T 4999C>T), TTC549CAT (5000T>C 5001T>A 5002C>T), GGT551GGA (5008T>A), GGA552GCA (5010G>C), GTC553TTA (5012G>T 5014C>A), CTT554TTA (5015C>T 5017T>A), AAG555_GCC556del (5018_5023delAAGGCC), ACC558ACA (5029C>A), ACT559GAT (5030A>G 5031C>A), GAA560CTA (5033G>C 5034A>T), GGT561AAT (5036G>A 5037G>A), GAA563_TTA564del (5042_5047delGAATTA), ATC565ATT (5050C>T), TGC566TGT (5053C>T), AGG567AGA (5056G>A), TAT568TAC (5059T>C), TCT569ACA (5060T>A 5062T>A), TCA570AGT (5063T>A 5064C>G 5065A>T), ACA572ACC (5071A>C), TTC573TTT (5074C>T), AAA574AAC (5077A>C), CCA575GAA (5078C>G 5079C>A), TTG578GTT (5087T>G 5089G>T), TAC580TAT (5095C>T), CAT581TCC (5096C>T 5097A>C 5098T>C), AGT582ACA (5100G>C 5101T>A), GAG584GAA (5107G>A), CTA588TTA (5117C>T), GCG589GCA (5122G>A), AAA591GTA (5126A>G 5127A>T), CAG592AGA (5129C>A 5130A>G 5131G>A), GTG593GGA (5133T>G 5134G>A), ATT594ATA (5137T>A), ACT595CGA (5138A>C 5139C>G 5140T>A), AGT598TCA (5147A>T 5148G>C 5149T>A), ATT599ACA (5151T>C 5152T>A), TAT600TCC (5154A>C 5155T>C), CTA601TTA (5156C>T), ACC602TTA (5159A>T 5160C>T 5161C>A), CCT603CAT (5163C>A), GTT604GAA (5166T>A 5167T>A), TGT605CCA (5168T>C 5169G>C 5170T>A), ACA607GTT (5174A>G 5175C>T 5176A>T), GTC608ATT (5177G>A 5179C>T), AGG609AAA (5181G>A 5182G>A), ACA610ATA (5184C>T), GTA613ACA (5192G>A 5193T>C), AAT614CAA (5195A>C 5197T>A), CTT615del (5198_5200delCTT), TTA616ATA (5201T>A), AAA617TCA (5204A>T 5205A>C), TTT619TTG (5212T>G), ATG620ATA (5215G>A), AAT621TTT (5216A>T 5217A>T), AAA622AAT (5221A>T), AAG623AAA (5224G>A), ATT624CTC (5225A>C 5227T>C), ACT625CCT (5228A>C), GGT626TCT (5231G>T 5232G>C), GAC627GAA (5236C>A), AGT628CCT (5237A>C 5238G>C), AAA629CCT (5240A>C 5241A>C 5242A>T)								

*: Inserts / Deletes / Misaligned / Frameshifts

Analysis details

This analysis was performed with panviral2.64

NGS Details (UN18_val): Badnavirus tessellocastaneae

Assembly

**Coverage Length**	263 (1 contig(s))
**Depth Of Coverage**	2.8
**Number Of Reads**	6
**Reads Per Million**	0.11 rpm (after QC)
**Ambiguities**	0
**Assembly Method**	de novo + reference guided assembly
**Consensus Caller**	Bcf Tools

Coverage Map

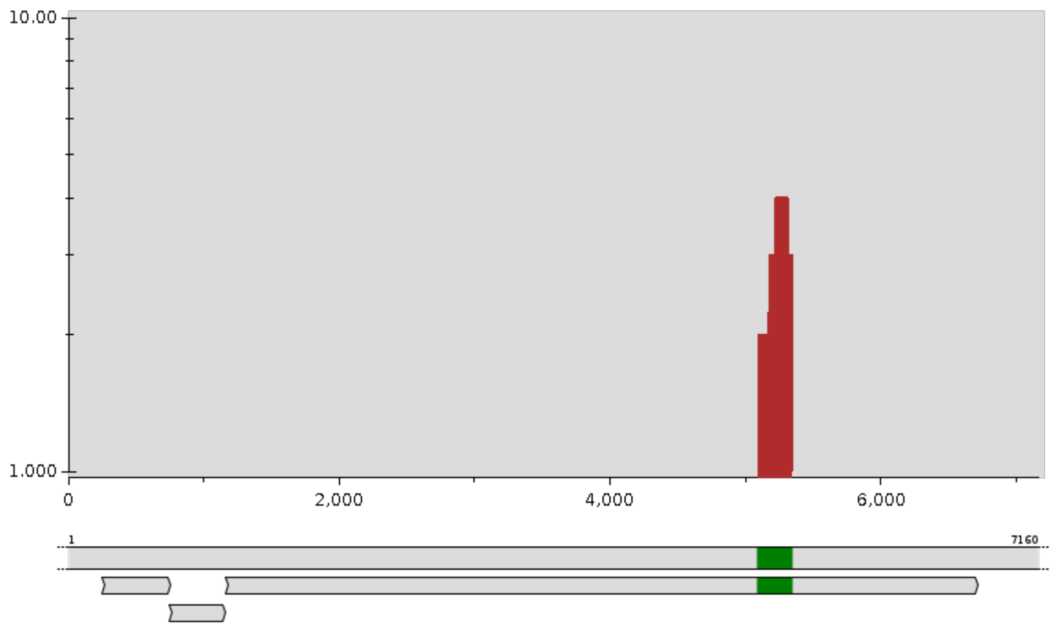

Assignment

**Type**	Badnavirus tessellocastaneae (Taxonomy ID: 3051987)
**Reference Genome**	NC_076885.1
**NT Identity (%)**	55.8935
**AA Identity (%)**	54.5455
**Number Of Stop Codons**	0
**Number Of CDS**	3

Alignment

**Alignment Score**	62.0 (NT) + 353.0 (AA) = 415.0
**Concordance (%)**	36.1813

Alignment Method	Global, seeded, nucleotide + amino acids (AGA)

Genome Region

Sequence starts at position 5087 and ends at position 5349 relative to NC_076885.1 reference sequence.

Alignment Detailed Statistics

	Begin	End	Coverage	Score	Concordance	Matches	Identities	I/D/M/F*	Stop Codons
**NT**	**5087**	**5349**	**3.7%**	**62**	**11.8%**	**263 (100%)**	**147 (55.9%)**	**0/0**	
Mutations:	5087C>G, 5092T>C, 5098A>G, 5100C>A, 5102C>T, 5104C>A, 5109T>C, 5110G>C, 5113T>C, 5116C>T, 5117C>G, 5118A>T, 5122A>G, 5125C>T, 5126C>A, 5130A>T, 5132T>C, 5134C>A, 5135C>A, 5137C>T, 5142G>T, 5143A>T, 5146A>T, 5148C>A, 5149A>G, 5150T>G, 5151C>A, 5152C>A, 5153A>T, 5155C>A, 5156A>T, 5158C>A, 5159A>G, 5161G>T, 5162A>G, 5163G>A, 5164A>G, 5165G>T, 5167T>G, 5168G>C, 5169C>A, 5170A>T, 5174T>G, 5176A>C, 5180G>T, 5181T>A, 5182C>T, 5188C>T, 5192T>C, 5194T>A, 5197T>C, 5200A>G, 5202A>G, 5203G>A, 5204A>T, 5205G>C, 5209G>T, 5211T>A, 5215T>C, 5219G>A, 5221G>A, 5222G>A, 5223T>G, 5228G>T, 5229A>T, 5230A>T, 5234G>T, 5235A>C, 5236G>T, 5237T>A, 5238C>A, 5239A>T, 5245A>T, 5246T>A, 5247G>A, 5248G>A, 5251A>G, 5252G>A, 5254G>A, 5258G>A, 5259T>G, 5260G>A, 5261G>A, 5262T>C, 5263A>C, 5265C>A, 5266A>T, 5267G>C, 5268G>A, 5272A>G, 5273T>C, 5274T>A, 5275A>C, 5278C>T, 5283G>T, 5284G>C, 5285C>T, 5287G>A, 5290T>G, 5296A>C, 5302G>C, 5305A>T, 5307G>C, 5308A>C, 5314A>C, 5317A>C, 5320A>C, 5323C>A, 5326C>T, 5329A>G, 5332G>C, 5333A>C, 5334A>T, 5335G>C, 5339G>A, 5341C>T								

CDS

	Begin	End	Coverage	Score	Concordance	Matches	Identities	I/D/M/F*	Stop Codons
ORF3	1309	1396	4.7%	353	55.9%	88 (100%)	48 (54.5%)	0/0/0/0	0
Protein mutations:	L1309V (5087C>G), A1313E (5100C>A), M1316T (5109T>C 5110G>C), H1319V (5117C>G 5118A>T), Q1322K (5126C>A), Y1323F (5130A>T), S1324P (5132T>C 5134C>A), L1325I (5135C>A 5137C>T), G1327V (5142G>T 5143A>T), T1329K (5148C>A 5149A>G), S1330E (5150T>G 5151C>A 5152C>A), I1331L (5153A>T 5155C>A), I1332L (5156A>T 5158C>A), K1333D (5159A>G 5161G>T), R1334E (5162A>G 5163G>A 5164A>G), V1335L (5165G>T 5167T>G), A1336H (5168G>C 5169C>A 5170A>T), S1338A (5174T>G 5176A>C), V1340Y (5180G>T 5181T>A 5182C>T), F1344L (5192T>C 5194T>A), K1347R (5202A>G 5203G>A), F1350Y (5211T>A), V1353I (5219G>A 5221G>A), V1354R (5222G>A 5223T>G), E1356F (5228G>T 5229A>T 5230A>T), E1358S (5234G>T 5235A>C 5236G>T), S1359N (5237T>A 5238C>A 5239A>T), W1362K (5246T>A 5247G>A 5248G>A), A1364T (5252G>A 5254G>A), V1366R (5258G>A 5259T>G 5260G>A), V1367T (5261G>A 5262T>C 5263A>C), P1368H (5265C>A 5266A>T), G1369Q (5267G>C 5268G>A), L1371H (5273T>C 5274T>A 5275A>C), W1374F (5283G>T 5284G>C), R1382T (5307G>C 5308A>C), R1390S (5332G>C), K1391L (5333A>C 5334A>T 5335G>C), D1393N (5339G>A 5341C>T)								
Codon mutations:	CTA1309GTA (5087C>G), GAT1310GAC (5092T>C), AGA1312AGG (5098A>G), GCA1313GAA (5100C>A), CTC1314TTA (5102C>T 5104C>A), ATG1316ACC (5109T>C 5110G>C), AAT1317AAC (5113T>C), ACC1318ACT (5116C>T), CAC1319GTC (5117C>G 5118A>T), AAA1320AAG (5122A>G), GAC1321GAT (5125C>T), CAG1322AAG (5126C>A), TAT1323TTT (5130A>T), TCC1324CCA (5132T>C 5134C>A), CTC1325ATT (5135C>A 5137C>T), GGA1327GTT (5142G>T 5143A>T), ATA1328ATT (5146A>T), ACA1329AAG (5148C>A 5149A>G), TCC1330GAA (5150T>G 5151C>A 5152C>A), ATC1331TTA (5153A>T 5155C>A), ATC1332TTA (5156A>T 5158C>A), AAG1333GAT (5159A>G 5161G>T), AGA1334GAG (5162A>G 5163G>A 5164A>G), GTT1335TTG (5165G>T 5167T>G), GCA1336CAT (5168G>C 5169C>A 5170A>T), TCA1338GCC (5174T>G 5176A>C), GTC1340TAT (5180G>T 5181T>A 5182C>T), TCC1342TCT (5188C>T), TTT1344CTA (5192T>C 5194T>A), GAT1345GAC (5197T>C), TTA1346TTG (5200A>G), AAG1347AGA (5202A>G 5203G>A), AGT1348TCT (5204A>T 5205G>C), GGG1349GGT (5209G>T), TTC1350TAC (5211T>A), CAT1351CAC (5215T>C), GTG1353ATA (5219G>A 5221G>A), GTA1354AGA (5222G>A 5223T>G), GAA1356TTT (5228G>T 5229A>T 5230A>T), GAG1358TCT (5234G>T 5235A>C 5236G>T), TCA1359AAT (5237T>A 5238C>A 5239A>T), CCA1361CCT (5245A>T), TGG1362AAA (5246T>A 5247G>A 5248G>A), ACA1363ACG (5251A>G), GCG1364ACA (5252G>A 5254G>A), GTG1366AGA (5258G>A 5259T>G 5260G>A), GTA1367ACC (5261G>A 5262T>C 5263A>C), CCA1368CAT (5265C>A 5266A>T), GGA1369CAA (5267G>C 5268G>A), GGA1370GGG (5272A>G), TTA1371CAC (5273T>C 5274T>A 5275A>C), TAC1372TAT (5278C>T), TGG1374TTC (5283G>T 5284G>C), CTG1375TTA (5285C>T 5287G>A), GTT1376GTG (5290T>G), CCA1378CCC (5296A>C), GGG1380GGC (5302G>C), CTA1381CTT (5305A>T), AGA1382ACC (5307G>C 5308A>C), GCA1384GCC (5314A>C), CCA1385CCC (5317A>C), TCA1386TCC (5320A>C), ACC1387ACA (5323C>A), TTC1388TTT (5326C>T), CAA1389CAG (5329A>G), AGG1390AGC (5332G>C), AAG1391CTC (5333A>C 5334A>T 5335G>C), GAC1393AAT (5339G>A 5341C>T)								

Proteins

	Begin	End	Coverage	Score	Concordance	Matches	Identities	I/D/M/F*	Stop Codons
polyprotein (YP_010800602.1)	1309	1396	4.7%	353	55.9%	88 (100%)	48 (54.5%)	0/0/0/0	0
Protein mutations:	L1309V (5087C>G), A1313E (5100C>A), M1316T (5109T>C 5110G>C), H1319V (5117C>G 5118A>T), Q1322K (5126C>A), Y1323F (5130A>T), S1324P (5132T>C 5134C>A), L1325I (5135C>A 5137C>T), G1327V (5142G>T 5143A>T), T1329K (5148C>A 5149A>G), S1330E (5150T>G 5151C>A 5152C>A), I1331L (5153A>T 5155C>A), I1332L (5156A>T 5158C>A), K1333D (5159A>G 5161G>T), R1334E (5162A>G 5163G>A 5164A>G), V1335L (5165G>T 5167T>G), A1336H (5168G>C 5169C>A 5170A>T), S1338A (5174T>G 5176A>C), V1340Y (5180G>T 5181T>A 5182C>T), F1344L (5192T>C 5194T>A), K1347R (5202A>G 5203G>A), F1350Y (5211T>A), V1353I (5219G>A 5221G>A), V1354R (5222G>A 5223T>G), E1356F (5228G>T 5229A>T 5230A>T), E1358S (5234G>T 5235A>C 5236G>T), S1359N (5237T>A 5238C>A 5239A>T), W1362K (5246T>A 5247G>A 5248G>A), A1364T (5252G>A 5254G>A), V1366R (5258G>A 5259T>G 5260G>A), V1367T (5261G>A 5262T>C 5263A>C), P1368H (5265C>A 5266A>T), G1369Q (5267G>C 5268G>A), L1371H (5273T>C 5274T>A 5275A>C), W1374F (5283G>T 5284G>C), R1382T (5307G>C 5308A>C), R1390S (5332G>C), K1391L (5333A>C 5334A>T 5335G>C), D1393N (5339G>A 5341C>T)								
Codon mutations:	CTA1309GTA (5087C>G), GAT1310GAC (5092T>C), AGA1312AGG (5098A>G), GCA1313GAA (5100C>A), CTC1314TTA (5102C>T 5104C>A), ATG1316ACC (5109T>C 5110G>C), AAT1317AAC (5113T>C), ACC1318ACT (5116C>T), CAC1319GTC (5117C>G 5118A>T), AAA1320AAG (5122A>G), GAC1321GAT (5125C>T), CAG1322AAG (5126C>A), TAT1323TTT (5130A>T), TCC1324CCA (5132T>C 5134C>A), CTC1325ATT (5135C>A 5137C>T), GGA1327GTT (5142G>T 5143A>T), ATA1328ATT (5146A>T), ACA1329AAG (5148C>A 5149A>G), TCC1330GAA (5150T>G 5151C>A 5152C>A), ATC1331TTA (5153A>T 5155C>A), ATC1332TTA (5156A>T 5158C>A), AAG1333GAT (5159A>G 5161G>T), AGA1334GAG (5162A>G 5163G>A 5164A>G), GTT1335TTG (5165G>T 5167T>G), GCA1336CAT (5168G>C 5169C>A 5170A>T), TCA1338GCC (5174T>G 5176A>C), GTC1340TAT (5180G>T 5181T>A 5182C>T), TCC1342TCT (5188C>T), TTT1344CTA (5192T>C 5194T>A), GAT1345GAC (5197T>C), TTA1346TTG (5200A>G), AAG1347AGA (5202A>G 5203G>A), AGT1348TCT (5204A>T 5205G>C), GGG1349GGT (5209G>T), TTC1350TAC (5211T>A), CAT1351CAC (5215T>C), GTG1353ATA (5219G>A 5221G>A), GTA1354AGA (5222G>A 5223T>G), GAA1356TTT (5228G>T 5229A>T 5230A>T), GAG1358TCT (5234G>T 5235A>C 5236G>T), TCA1359AAT (5237T>A 5238C>A 5239A>T), CCA1361CCT (5245A>T), TGG1362AAA (5246T>A 5247G>A 5248G>A), ACA1363ACG (5251A>G), GCG1364ACA (5252G>A 5254G>A), GTG1366AGA (5258G>A 5259T>G 5260G>A), GTA1367ACC (5261G>A 5262T>C 5263A>C), CCA1368CAT (5265C>A 5266A>T), GGA1369CAA (5267G>C 5268G>A), GGA1370GGG (5272A>G), TTA1371CAC (5273T>C 5274T>A 5275A>C), TAC1372TAT (5278C>T), TGG1374TTC (5283G>T 5284G>C), CTG1375TTA (5285C>T 5287G>A), GTT1376GTG (5290T>G), CCA1378CCC (5296A>C), GGG1380GGC (5302G>C), CTA1381CTT (5305A>T), AGA1382ACC (5307G>C 5308A>C), GCA1384GCC (5314A>C), CCA1385CCC (5317A>C), TCA1386TCC (5320A>C), ACC1387ACA (5323C>A), TTC1388TTT (5326C>T), CAA1389CAG (5329A>G), AGG1390AGC (5332G>C), AAG1391CTC (5333A>C 5334A>T 5335G>C), GAC1393AAT (5339G>A 5341C>T)								

*: Inserts / Deletes / Misaligned / Frameshifts

Analysis details

This analysis was performed with panviral2.64

NGS Details (UN18_val): Betabaculovirus erellonis

Assembly

**Coverage Length**	91 (1 contig(s))
**Depth Of Coverage**	5.9
**Number Of Reads**	6
**Reads Per Million**	0.11 rpm (after QC)
**Ambiguities**	0
**Assembly Method**	de novo + reference guided assembly
**Consensus Caller**	Bcf Tools

Coverage Map

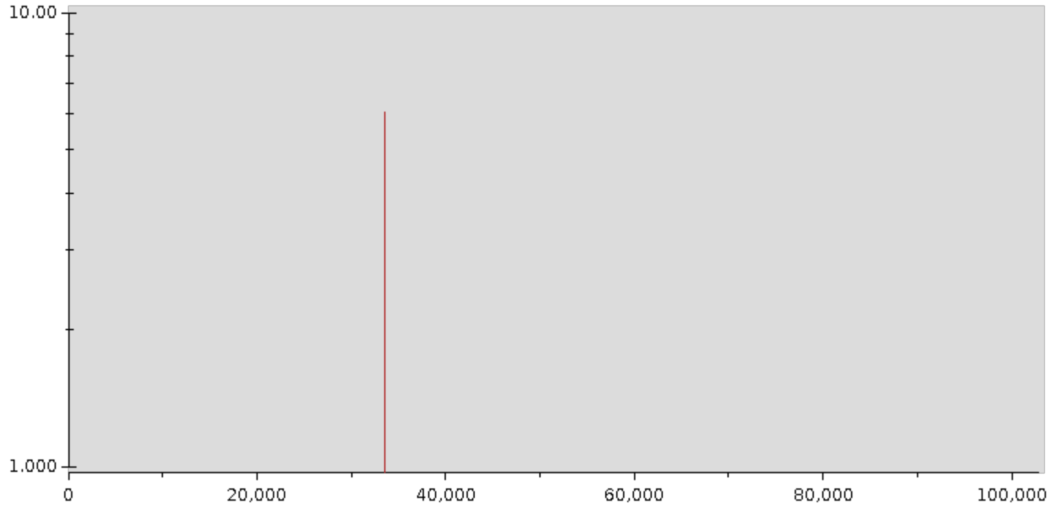

Assignment

**Type**	Betabaculovirus erellonis (Taxonomy ID: 3047828)
**Reference Genome**	NC_025257.1
**NT Identity (%)**	82.4176
**AA Identity (%)**	93.3333
**Number Of Stop Codons**	0
**Number Of CDS**	130

Alignment

**Alignment Score**	118.0 (NT) + 178.0 (AA) = 296.0
**Concordance (%)**	80.4348
**Alignment Method**	Local, heuristic, nucleotide (BLASTN)

Genome Region

Sequence starts at position 33523 and ends at position 33613 relative to NC_025257.1 reference sequence.

Alignment Detailed Statistics

	Begin	End	Coverage	Score	Concordance	Matches	Identities	I/D/M/F*	Stop Codons
**NT**	**33523**	**33613**	**0.1%**	**118**	**64.8%**	**91 (100%)**	**75 (82.4%)**	**0/0**	

Mutations: 33525A>T, 33537T>A, 33539A>G, 33540C>G, 33549T>C, 33556C>G, 33557T>A, 33561T>C, 33567T>G, 33576A>G, 33580G>C, 33581A>T, 33582G>C, 33585T>A, 33592T>C, 33606T>C

*: Inserts / Deletes / Misaligned / Frameshifts

Analysis details

This analysis was performed with panviral2.64

NGS Details (UN18_val): Gihfavirus pelohabitans

Assembly

**Coverage Length**	155 (1 contig(s))
**Depth Of Coverage**	2.3
**Number Of Reads**	4
**Reads Per Million**	0.08 rpm (after QC)
**Ambiguities**	0
**Assembly Method**	de novo + reference guided assembly
**Consensus Caller**	Bcf Tools

Coverage Map

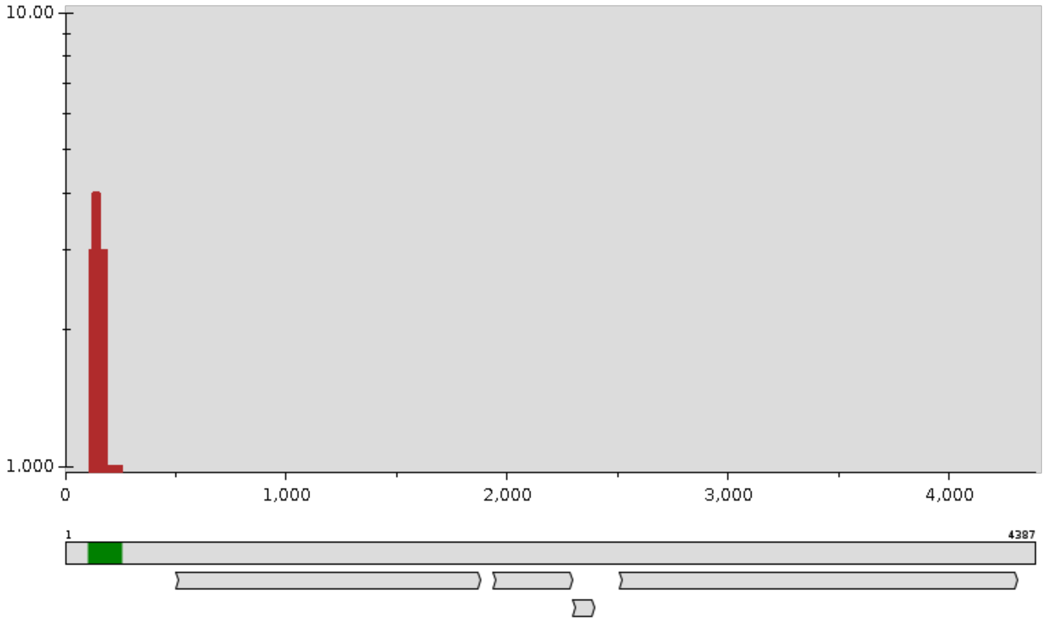

Assignment

**Type**	Gihfavirus pelohabitans (Taxonomy ID: 2844652)
**Reference Genome**	NC_074583.1
**NT Identity (%)**	97.4194
**AA Identity (%)**	0.0
**Number Of Stop Codons**	0
**Number Of CDS**	4

Alignment

**Alignment Score**	294.0 (NT) + 0.0 (AA) = 294.0
**Concordance (%)**	94.8387

**Alignment Method**	Global, seeded, nucleotide + amino acids (AGA)

Genome Region

Sequence starts at position 106 and ends at position 260 relative to NC_074583.1 reference sequence.

Alignment Detailed Statistics

	Begin	End	Coverage	Score	Concordance	Matches	Identities	I/D/M/F*	Stop Codons
**NT**	**106**	**260**	**3.5%**	**294**	**94.8%**	**155 (100%)**	**151 (97.4%)**	**0/0**	
Mutations:	106G>T, 169A>C, 178T>G, 182T>C								

CDS

Proteins

*: Inserts / Deletes / Misaligned / Frameshifts

Analysis details

This analysis was performed with panviral2.64

NGS Details (UN18_val): Duamitovirus dapi1

Assembly

**Coverage Length**	134 (1 contig(s))
**Depth Of Coverage**	3.8
**Number Of Reads**	4
**Reads Per Million**	0.08 rpm (after QC)
**Ambiguities**	0
**Assembly Method**	de novo + reference guided assembly
**Consensus Caller**	Bcf Tools

Coverage Map

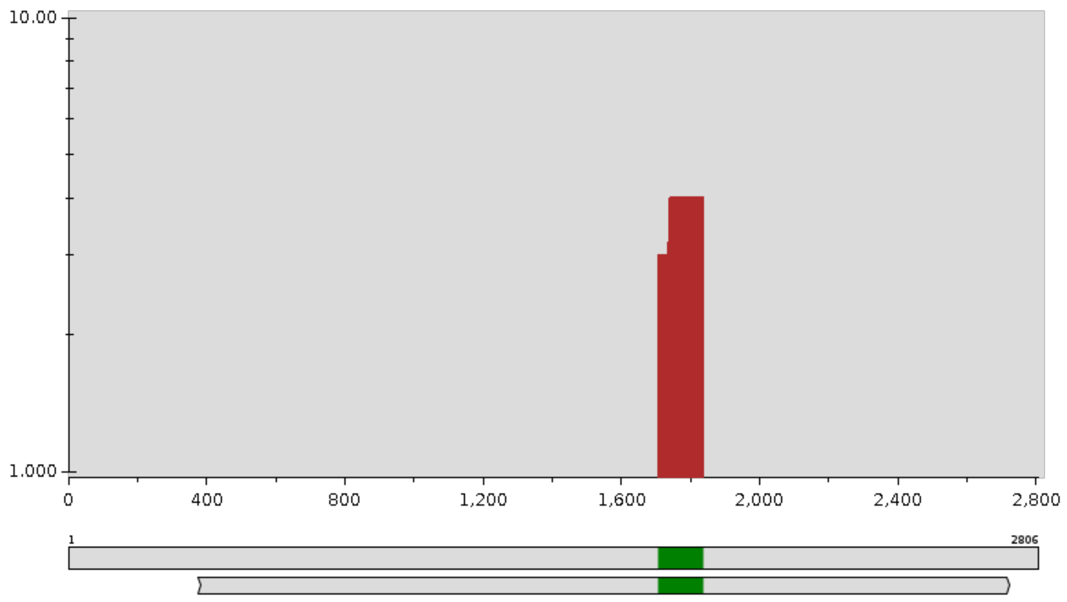

Assignment

**Type**	Duamitovirus dapi1 (Taxonomy ID: 2955778)
**Reference Genome**	NC_076528.1
**NT Identity (%)**	69.403
**AA Identity (%)**	66.6667
**Number Of Stop Codons**	0
**Number Of CDS**	1

Alignment

**Alignment Score**	104.0 (NT) + 249.0 (AA) = 353.0
**Concordance (%)**	57.6797

Alignment Method	Global, seeded, nucleotide + amino acids (AGA)

Genome Region

Sequence starts at position 1707 and ends at position 1840 relative to NC_076528.1 reference sequence.

Alignment Detailed Statistics

	Begin	End	Coverage	Score	Concordance	Matches	Identities	I/D/M/F*	Stop Codons
**NT**	**1707**	**1840**	**4.8%**	**104**	**38.8%**	**134 (100%)**	**93 (69.4%)**	**0/0**	
Mutations:	1710C>T, 1713A>G, 1715G>A, 1725T>C, 1728G>C, 1734C>T, 1736A>T, 1737C>G, 1738T>A, 1739C>G, 1743C>T, 1749G>T, 1750T>C, 1751C>T, 1756G>A, 1763C>A, 1774G>C, 1776A>T, 1783C>A, 1785G>A, 1788G>T, 1789G>A, 1791C>T, 1792T>G, 1793G>A, 1794G>A, 1800A>G, 1804C>G, 1805C>G, 1806T>C, 1809T>G, 1810C>A, 1811A>C, 1816T>C, 1819A>T, 1820A>G, 1821G>C, 1827C>T, 1830T>C, 1831C>A, 1839T>C								
**CDS**									
RdRp	445	489	5.7%	249	68.8%	45 (100%)	30 (66.7%)	0/0/0/0	0
Protein mutations:	R447K (1715G>A), Y454L (1736A>T 1737C>G), S459L (1750T>C 1751C>T), A461T (1756G>A), T463K (1763C>A), V467L (1774G>C 1776A>T), L470I (1783C>A 1785G>A), A472T (1789G>A 1791C>T), W473E (1792T>G 1793G>A 1794G>A), P477G (1804C>G 1805C>G 1806T>C), Q479T (1810C>A 1811A>C), F481L (1816T>C), K482C (1819A>T 1820A>G 1821G>C), L486I (1831C>A)								
Codon mutations:	TTC445TTT (1710C>T), ACA446ACG (1713A>G), AGG447AAG (1715G>A), CCT450CCC (1725T>C), CTG451CTC (1728G>C), TTC453TTT (1734C>T), TAC454TTG (1736A>T 1737C>G), TCT455AGT (1738T>A 1739C>G), TCC456TCT (1743C>T), CCG458CCT (1749G>T), TCA459CTA (1750T>C 1751C>T), GCA461ACA (1756G>A), ACA463AAA (1763C>A), GTA467CTT (1774G>C 1776A>T), CTG470ATA (1783C>A 1785G>A), GCG471GCT (1788G>T), GCC472ACT (1789G>A 1791C>T), TGG473GAA (1792T>G 1793G>A 1794G>A), GTA475GTG (1800A>G), CCT477GGC (1804C>G 1805C>G 1806T>C), ACT478ACG (1809T>G), CAG479ACG (1810C>A 1811A>C), TTT481CTT (1816T>C), AAG482TGC (1819A>T 1820A>G 1821G>C), TAC484TAT (1827C>T), GCT485GCC (1830T>C), CTT486ATT (1831C>A), GGT488GGC (1839T>C)								
**Proteins**									
RNA-dependent RNA polymerase (YP_010798878.1)	445	489	5.7%	249	68.8%	45 (100%)	30 (66.7%)	0/0/0/0	0
Protein mutations:	R447K (1715G>A), Y454L (1736A>T 1737C>G), S459L (1750T>C 1751C>T), A461T (1756G>A), T463K (1763C>A), V467L (1774G>C 1776A>T), L470I (1783C>A 1785G>A), A472T (1789G>A 1791C>T), W473E (1792T>G 1793G>A 1794G>A), P477G (1804C>G 1805C>G 1806T>C), Q479T (1810C>A 1811A>C), F481L (1816T>C), K482C (1819A>T 1820A>G 1821G>C), L486I (1831C>A)								
Codon mutations:	TTC445TTT (1710C>T), ACA446ACG (1713A>G), AGG447AAG (1715G>A), CCT450CCC (1725T>C), CTG451CTC (1728G>C), TTC453TTT (1734C>T), TAC454TTG (1736A>T 1737C>G), TCT455AGT (1738T>A 1739C>G), TCC456TCT (1743C>T), CCG458CCT (1749G>T), TCA459CTA (1750T>C 1751C>T), GCA461ACA (1756G>A), ACA463AAA (1763C>A), GTA467CTT (1774G>C 1776A>T), CTG470ATA (1783C>A 1785G>A), GCG471GCT (1788G>T), GCC472ACT (1789G>A 1791C>T), TGG473GAA (1792T>G 1793G>A 1794G>A), GTA475GTG (1800A>G), CCT477GGC (1804C>G 1805C>G 1806T>C), ACT478ACG (1809T>G), CAG479ACG (1810C>A 1811A>C), TTT481CTT (1816T>C), AAG482TGC (1819A>T 1820A>G 1821G>C), TAC484TAT (1827C>T), GCT485GCC (1830T>C), CTT486ATT (1831C>A), GGT488GGC (1839T>C)								

*: Inserts / Deletes / Misaligned / Frameshifts

Analysis details

This analysis was performed with panviral2.64

NGS Details (UN18_val): Fusarium graminearum mycotymovirus 1

Assembly

**Coverage Length**	269 (2 contig(s))
**Depth Of Coverage**	2.0
**Number Of Reads**	4
**Reads Per Million**	0.08 rpm (after QC)
**Ambiguities**	0
**Assembly Method**	de novo + reference guided assembly
**Consensus Caller**	Bcf Tools

Coverage Map

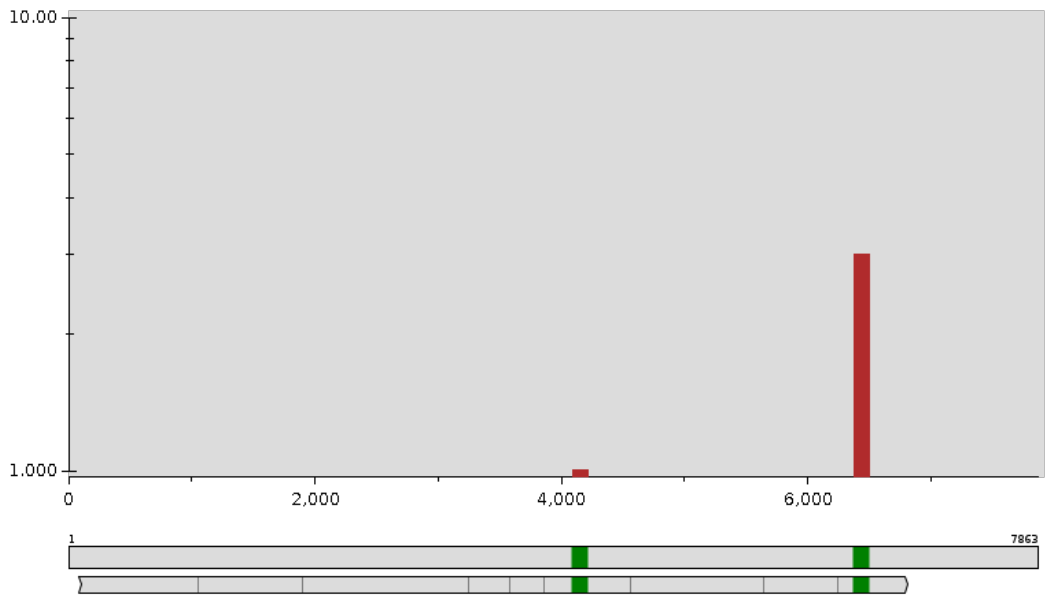

Assignment

**Type**	Fusarium graminearum mycotymovirus 1 (Taxonomy ID: 1809243)
**Reference Genome**	NC_040784.1
**NT Identity (%)**	69.5167
**AA Identity (%)**	76.6667
**Number Of Stop Codons**	0
**Number Of CDS**	1

Alignment

**Alignment Score**	210.0 (NT) + 483.0 (AA) = 693.0
**Concordance (%)**	59.7929

Alignment Method	Global, seeded, nucleotide + amino acids (AGA)

Genome Region

Sequence starts at position 4085 and ends at position 6503 relative to NC_040784.1 reference sequence.

Alignment Detailed Statistics

	Begin	End	Coverage	Score	Concordance	Matches	Identities	I/D/M/F*	Stop Codons
**NT**	**4085**	**6503**	**3.4%**	**210**	**39.0%**	**269 (100%)**	**187 (69.5%)**	**0/0**	
Mutations:	4085T>A, 4087T>G, 4093T>C, 4100A>G, 4105C>T, 4106G>T, 4107C>T, 4108A>C, 4111T>C, 4114G>C, 4123C>T, 4124A>C, 4125G>A, 4126C>G, 4129G>C, 4138A>C, 4141G>T, 4142C>G, 4144G>T, 4145A>G, 4150T>A, 4153C>T, 4156C>T, 4159T>C, 4162A>G, 4165G>A, 4168A>T, 4170G>A, 4171T>G, 4177T>C, 4183A>C, 4184G>A, 4186C>T, 4187T>G, 4188G>C, 4189C>T, 4192T>G, 4193T>A, 4194C>G, 4201A>T, 4205T>A, 4207C>T, 4213C>T, 4215G>C, 6370A>C, 6376C>T, 6379A>G, 6385G>T, 6388A>T, 6397T>C, 6406C>T, 6407A>C, 6409G>C, 6415C>T, 6421C>T, 6424T>C, 6425G>A, 6426G>A, 6427C>T, 6434G>A, 6435C>G, 6436G>C, 6438A>C, 6439A>C, 6440T>A, 6450A>G, 6454G>T, 6455C>T, 6457C>A, 6458A>G, 6459A>C, 6460C>T, 6463A>G, 6466T>C, 6472G>C, 6478C>T, 6481C>T, 6482C>G, 6484C>G, 6493C>T, 6496C>T, 6499C>T								

CDS

	Begin	End	Coverage	Score	Concordance	Matches	Identities	I/D/M/F*	Stop Codons
EXL10_gp1	1335	2141	4.0%	483	75.0%	90 (100%)	69 (76.7%)	0/0/0/0	0
Protein mutations:	F1335M (4085T>A 4087T>G), I1340V (4100A>G), A1342F (4106G>T 4107C>T 4108A>C), S1348Q (4124A>C 4125G>A 4126C>G), L1354V (4142C>G 4144G>T), I1355V (4145A>G), G1363E (4170G>A 4171T>G), D1368N (4184G>A 4186C>T), C1369A (4187T>G 4188G>C 4189C>T), D1370E (4192T>G), S1375T (4205T>A 4207C>T), C1378S (4215G>C), M2109L (6407A>C 6409G>C), G2115N (6425G>A 6426G>A 6427C>T), A2118S (6434G>A 6435C>G 6436G>C), K2119T (6438A>C 6439A>C), S2120T (6440T>A), N2123S (6450A>G), N2126A (6458A>G 6459A>C 6460C>T), L2134V (6482C>G 6484C>G)								
Codon mutations:	TTT1335ATG (4085T>A 4087T>G), CCT1337CCC (4093T>C), ATC1340GTC (4100A>G), GAC1341GAT (4105C>T), GCA1342TTC (4106G>T 4107C>T 4108A>C), TGT1343TGC (4111T>C), CTG1344CTC (4114G>C), GAC1347GAT (4123C>T), AGC1348CAG (4124A>C 4125G>A 4126C>G), ACG1349ACC (4129G>C), TCA1352TCC (4138A>C), GTG1353GTT (4141G>T), CTG1354GTT (4142C>G 4144G>T), ATC1355GTC (4145A>G), ATT1356ATA (4150T>A), GGC1357GGT (4153C>T), GAC1358GAT (4156C>T), GTT1359GTC (4159T>C), ACA1360ACG (4162A>G), CAG1361CAA (4165G>A), GGA1362GGT (4168A>T), GGT1363GAG (4170G>A 4171T>G), CAT1365CAC (4177T>C), TCA1367TCC (4183A>C), GAC1368AAT (4184G>A 4186C>T), TGC1369GCT (4187T>G 4188G>C 4189C>T), GAT1370GAG (4192T>G), TCC1371AGC (4193T>A 4194C>G), CTA1373CTT (4201A>T), TCC1375ACT (4205T>A 4207C>T), GGC1377GGT (4213C>T), TGT1378TCT (4215G>C), GGA2096..C (6370A>C), ATC2098ATT (6376C>T), AAA2099AAG (6379A>G), CCG2101CCT (6385G>T), CGA2102CGT (6388A>T), TTT2105TTC (6397T>C), CTC2108CTT (6406C>T), ATG2109CTC (6407A>C 6409G>C), GCC2111GCT (6415C>T), GAC2113GAT (6421C>T), CGT2114CGC (6424T>C), GGC2115AAT (6425G>A 6426G>A 6427C>T), GCG2118AGC (6434G>A 6435C>G 6436G>C), AAA2119ACC (6438A>C 6439A>C), TCT2120ACT (6440T>A), AAC2123AGC (6450A>G), CTG2124CTT (6454G>T), CTC2125TTA (6455C>T 6457C>A), AAC2126GCT (6458A>G 6459A>C 6460C>T), GAA2127GAG (6463A>G), GTT2128GTC (6466T>C), GTG2130GTC (6472G>C), TAC2132TAT (6478C>T), CAC2133CAT (6481C>T), CTC2134GTG (6482C>G 6484C>G), CAC2137CAT (6493C>T), GTC2138GTT (6496C>T), TAC2139TAT (6499C>T)								

Proteins

	Begin	End	Coverage	Score	Concordance	Matches	Identities	I/D/M/F*	Stop Codons
replication-associated polyprotein (YP_009553357.1)	1335	2141	4.0%	483	75.0%	90 (100%)	69 (76.7%)	0/0/0/0	0
Protein mutations:	F1335M (4085T>A 4087T>G), I1340V (4100A>G), A1342F (4106G>T 4107C>T 4108A>C), S1348Q (4124A>C 4125G>A 4126C>G), L1354V (4142C>G 4144G>T), I1355V (4145A>G), G1363E (4170G>A 4171T>G), D1368N (4184G>A 4186C>T), C1369A (4187T>G 4188G>C 4189C>T), D1370E (4192T>G), S1375T (4205T>A 4207C>T), C1378S (4215G>C), M2109L (6407A>C 6409G>C), G2115N (6425G>A 6426G>A 6427C>T), A2118S (6434G>A 6435C>G 6436G>C), K2119T (6438A>C 6439A>C), S2120T (6440T>A), N2123S (6450A>G), N2126A (6458A>G 6459A>C 6460C>T), L2134V (6482C>G 6484C>G)								
Codon mutations:	TTT1335ATG (4085T>A 4087T>G), CCT1337CCC (4093T>C), ATC1340GTC (4100A>G), GAC1341GAT (4105C>T), GCA1342TTC (4106G>T 4107C>T 4108A>C), TGT1343TGC (4111T>C), CTG1344CTC (4114G>C), GAC1347GAT (4123C>T), AGC1348CAG (4124A>C 4125G>A 4126C>G), ACG1349ACC (4129G>C), TCA1352TCC (4138A>C), GTG1353GTT (4141G>T), CTG1354GTT (4142C>G 4144G>T), ATC1355GTC (4145A>G), ATT1356ATA (4150T>A), GGC1357GGT (4153C>T), GAC1358GAT (4156C>T), GTT1359GTC (4159T>C), ACA1360ACG (4162A>G), CAG1361CAA (4165G>A), GGA1362GGT (4168A>T), GGT1363GAG (4170G>A 4171T>G), CAT1365CAC (4177T>C), TCA1367TCC (4183A>C), GAC1368AAT (4184G>A 4186C>T), TGC1369GCT (4187T>G 4188G>C 4189C>T), GAT1370GAG (4192T>G), TCC1371AGC (4193T>A 4194C>G), CTA1373CTT (4201A>T), TCC1375ACT (4205T>A 4207C>T), GGC1377GGT (4213C>T), TGT1378TCT (4215G>C), GGA2096..C (6370A>C), ATC2098ATT (6376C>T), AAA2099AAG (6379A>G), CCG2101CCT (6385G>T), CGA2102CGT (6388A>T), TTT2105TTC (6397T>C), CTC2108CTT (6406C>T), ATG2109CTC (6407A>C 6409G>C), GCC2111GCT (6415C>T), GAC2113GAT (6421C>T), CGT2114CGC (6424T>C), GGC2115AAT (6425G>A 6426G>A 6427C>T), GCG2118AGC (6434G>A 6435C>G 6436G>C), AAA2119ACC (6438A>C 6439A>C), TCT2120ACT (6440T>A), AAC2123AGC (6450A>G), CTG2124CTT (6454G>T), CTC2125TTA (6455C>T 6457C>A), AAC2126GCT (6458A>G 6459A>C 6460C>T), GAA2127GAG (6463A>G), GTT2128GTC (6466T>C), GTG2130GTC (6472G>C), TAC2132TAT (6478C>T), CAC2133CAT (6481C>T), CTC2134GTG (6482C>G 6484C>G), CAC2137CAT (6493C>T), GTC2138GTT (6496C>T), TAC2139TAT (6499C>T)								
viral RNA helicase (YP_009553722.1)	76	120	19.1%	236	70.9%	45 (100%)	33 (73.3%)	0/0/0/0	0
Protein mutations:	F76M (4085T>A 4087T>G), I81V (4100A>G), A83F (4106G>T 4107C>T 4108A>C), S89Q (4124A>C 4125G>A 4126C>G), L95V (4142C>G 4144G>T), I96V (4145A>G), G104E (4170G>A 4171T>G), D109N (4184G>A 4186C>T), C110A (4187T>G 4188G>C 4189C>T), D111E (4192T>G), S116T (4205T>A 4207C>T), C119S (4215G>C)								
Codon mutations:	TTT76ATG (4085T>A 4087T>G), CCT78CCC (4093T>C), ATC81GTC (4100A>G), GAC82GAT (4105C>T), GCA83TTC (4106G>T 4107C>T 4108A>C), TGT84TGC (4111T>C), CTG85CTC (4114G>C), GAC88GAT (4123C>T), AGC89CAG (4124A>C 4125G>A 4126C>G), ACG90ACC (4129G>C), TCA93TCC (4138A>C), GTG94GTT (4141G>T), CTG95GTT (4142C>G 4144G>T), ATC96GTC (4145A>G), ATT97ATA (4150T>A), GGC98GGT (4153C>T), GAC99GAT (4156C>T), GTT100GTC (4159T>C), ACA101ACG (4162A>G), CAG102CAA (4165G>A), GGA103GGT (4168A>T), GGT104GAG (4170G>A 4171T>G), CAT106CAC (4177T>C), TCA108TCC (4183A>C), GAC109AAT (4184G>A 4186C>T), TGC110GCT (4187T>G 4188G>C 4189C>T), GAT111GAG (4192T>G), TCC112AGC (4193T>A 4194C>G), CTA114CTT (4201A>T), TCC116ACT (4205T>A 4207C>T), GGC118GGT (4213C>T), TGT119TCT (4215G>C)								

*: Inserts / Deletes / Misaligned / Frameshifts

Analysis details

This analysis was performed with panviral2.64

NGS Details (UN18_val): Tokyovirus A1

Assembly

**Coverage Length**	135 (1 contig(s))
**Depth Of Coverage**	2.9
**Number Of Reads**	4
**Reads Per Million**	0.08 rpm (after QC)
**Ambiguities**	0
**Assembly Method**	de novo + reference guided assembly
**Consensus Caller**	Bcf Tools

Coverage Map

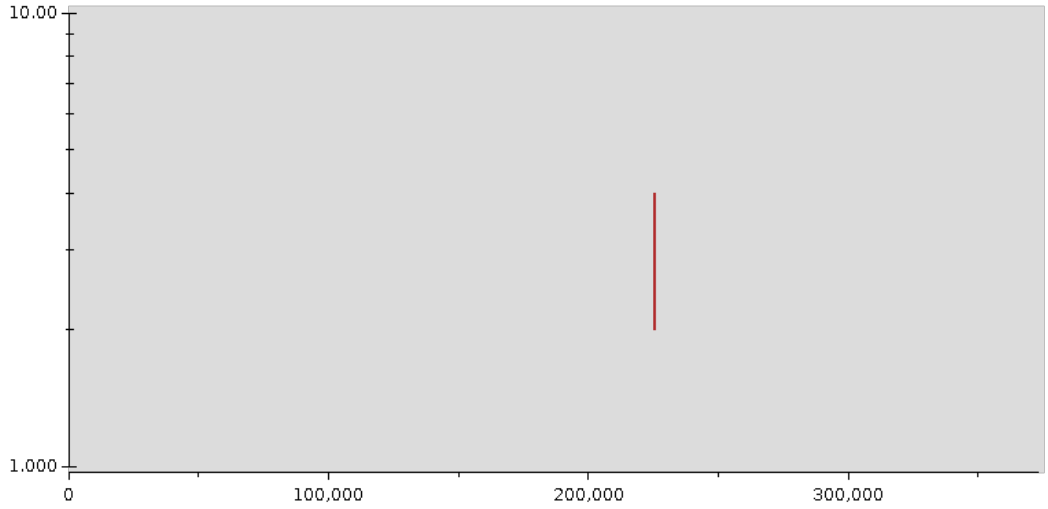

Assignment

**Type**	Tokyovirus A1 (Taxonomy ID: 1826170)
**Reference Genome**	NC_030230.1
**NT Identity (%)**	77.037
**AA Identity (%)**	93.3333
**Number Of Stop Codons**	0
**Number Of CDS**	470

Alignment

**Alignment Score**	146.0 (NT) + 291.0 (AA) = 437.0
**Concordance (%)**	77.3451
**Alignment Method**	Local, heuristic, nucleotide (BLASTN)

Genome Region

Sequence starts at position 225496 and ends at position 225630 relative to NC_030230.1 reference sequence.

Alignment Detailed Statistics

	Begin	End	Coverage	Score	Concordance	Matches	Identities	I/D/M/F*	Stop Codons
**NT**	**225496**	**225630**	**0.1%**	**146**	**54.1%**	**135 (100%)**	**104 (77.0%)**	**0/0**	

Mutations: 225503G>A, 225504G>C, 225507A>C, 225509G>A, 225513G>C, 225515G>A, 225516A>G, 225519G>A, 225520G>C, 225521A>T, 225522T>C, 225525T>C, 225528T>C, 225531G>A, 225533C>T, 225534A>G, 225540A>G, 225544G>C, 225545C>T, 225552T>C, 225555C>T, 225558A>G, 225564T>G, 225567C>T, 225570C>T, 225573C>T, 225585A>G, 225597G>A, 225603C>A, 225606A>G, 225621T>C

*: Inserts / Deletes / Misaligned / Frameshifts

Analysis details

This analysis was performed with panviral2.64

NGS Details (UN18_val): Botryotinia fuckeliana partitivirus 1 (segment RNA 1)

Assembly

**Coverage Length**	135 (1 contig(s))
**Depth Of Coverage**	3.6
**Number Of Reads**	4
**Reads Per Million**	0.08 rpm (after QC)
**Ambiguities**	0
**Assembly Method**	de novo + reference guided assembly
**Consensus Caller**	Bcf Tools

Coverage Map

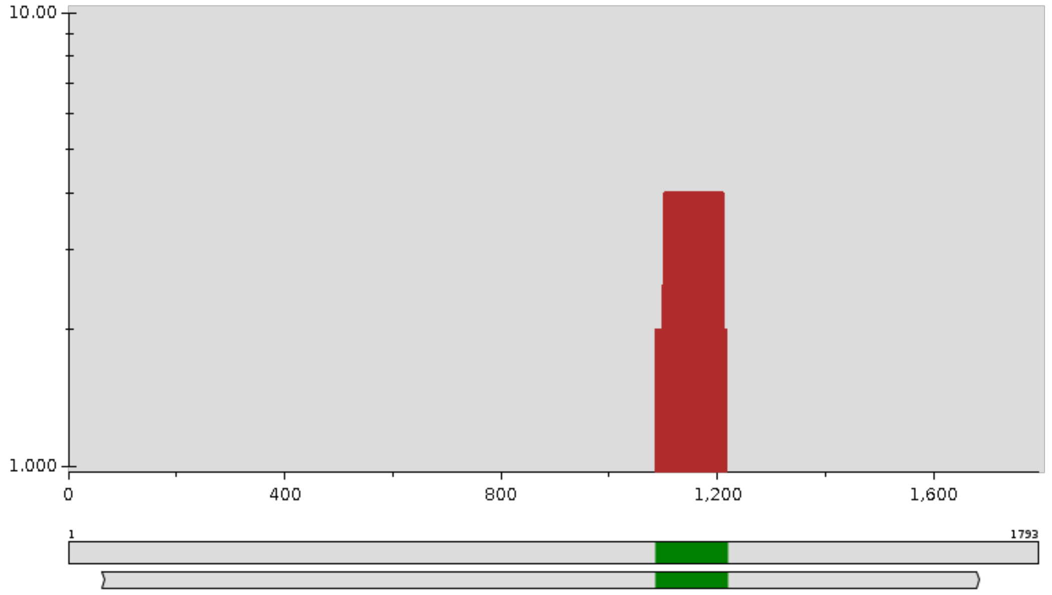

Assignment

**Type**	Botryotinia fuckeliana partitivirus 1 (Taxonomy ID: 425010)
**Reference Genome**	NC_010349.1
**NT Identity (%)**	68.1481
**AA Identity (%)**	71.1111
**Number Of Stop Codons**	0
**Number Of CDS**	1

Alignment

**Alignment Score**	98.0 (NT) + 243.0 (AA) = 341.0
**Concordance (%)**	55.6281

Alignment Method	Global, seeded, nucleotide + amino acids (AGA)

Genome Region

Sequence starts at position 1086 and ends at position 1220 relative to NC_010349.1 reference sequence.

Alignment Detailed Statistics

	Begin	End	Coverage	Score	Concordance	Matches	Identities	I/D/M/F*	Stop Codons
**NT**	**1086**	**1220**	**7.5%**	**98**	**36.3%**	**135 (100%)**	**92 (68.1%)**	**0/0**	
Mutations:	1096A>T, 1102T>C, 1103C>A, 1105T>A, 1110T>A, 1111C>T, 1112A>G, 1114G>A, 1117T>A, 1123T>A, 1129A>T, 1130T>A, 1132G>C, 1134G>A, 1135G>C, 1137G>T, 1138G>C, 1141G>C, 1144G>A, 1145A>C, 1147G>C, 1150T>C, 1156T>C, 1165T>C, 1166C>A, 1167A>C, 1171A>T, 1172C>G, 1174A>T, 1177T>C, 1178G>A, 1183T>C, 1184T>C, 1186G>C, 1187G>A, 1192C>T, 1195C>T, 1201T>C, 1202G>A, 1204A>G, 1205G>A, 1206A>C, 1210C>T								
**CDS**									
RdRp	343	387	8.3%	243	67.9%	45 (100%)	32 (71.1%)	0/0/0/0	0
Protein mutations:	F350Y (1110T>A 1111C>T), R351G (1112A>G 1114G>A), S357T (1130T>A 1132G>C), W358Y (1134G>A 1135G>C), W359F (1137G>T 1138G>C), M362L (1145A>C 1147G>C), H369T (1166C>A 1167A>C), L371V (1172C>G 1174A>T), D373N (1178G>A), A376T (1187G>A), V381M (1202G>A 1204A>G), E382T (1205G>A 1206A>C)								
Codon mutations:	CGA345CGT (1096A>T), TTT347TTC (1102T>C), CGT348AGA (1103C>A 1105T>A), TTC350TAT (1110T>A 1111C>T), AGG351GGA (1112A>G 1114G>A), GGT352GGA (1117T>A), CCT354CCA (1123T>A), GGA356GGT (1129A>T), TCG357ACC (1130T>A 1132G>C), TGG358TAC (1134G>A 1135G>C), TGG359TTC (1137G>T 1138G>C), ACG360ACC (1141G>C), CAG361CAA (1144G>A), ATG362CTC (1145A>C 1147G>C), ATT363ATC (1150T>C), AGT365AGC (1156T>C), AAT368AAC (1165T>C), CAC369ACC (1166C>A 1167A>C), ATA370ATT (1171A>T), CTA371GTT (1172C>G 1174A>T), ATT372ATC (1177T>C), GAT373AAT (1178G>A), TAT374TAC (1183T>C), TTG375CTC (1184T>C 1186G>C), GCA376ACA (1187G>A), GAC377GAT (1192C>T), TGC378TGT (1195C>T), GAT380GAC (1201T>C), GTA381ATG (1202G>A 1204A>G), GAG382ACG (1205G>A 1206A>C), ATC383ATT (1210C>T)								
**Proteins**									
RNA-dependent RNA polymerase (YP_001686789.1)	343	387	8.3%	243	67.9%	45 (100%)	32 (71.1%)	0/0/0/0	0
Protein mutations:	F350Y (1110T>A 1111C>T), R351G (1112A>G 1114G>A), S357T (1130T>A 1132G>C), W358Y (1134G>A 1135G>C), W359F (1137G>T 1138G>C), M362L (1145A>C 1147G>C), H369T (1166C>A 1167A>C), L371V (1172C>G 1174A>T), D373N (1178G>A), A376T (1187G>A), V381M (1202G>A 1204A>G), E382T (1205G>A 1206A>C)								
Codon mutations:	CGA345CGT (1096A>T), TTT347TTC (1102T>C), CGT348AGA (1103C>A 1105T>A), TTC350TAT (1110T>A 1111C>T), AGG351GGA (1112A>G 1114G>A), GGT352GGA (1117T>A), CCT354CCA (1123T>A), GGA356GGT (1129A>T), TCG357ACC (1130T>A 1132G>C), TGG358TAC (1134G>A 1135G>C), TGG359TTC (1137G>T 1138G>C), ACG360ACC (1141G>C), CAG361CAA (1144G>A), ATG362CTC (1145A>C 1147G>C), ATT363ATC (1150T>C), AGT365AGC (1156T>C), AAT368AAC (1165T>C), CAC369ACC (1166C>A 1167A>C), ATA370ATT (1171A>T), CTA371GTT (1172C>G 1174A>T), ATT372ATC (1177T>C), GAT373AAT (1178G>A), TAT374TAC (1183T>C), TTG375CTC (1184T>C 1186G>C), GCA376ACA (1187G>A), GAC377GAT (1192C>T), TGC378TGT (1195C>T), GAT380GAC (1201T>C), GTA381ATG (1202G>A 1204A>G), GAG382ACG (1205G>A 1206A>C), ATC383ATT (1210C>T)								

*: Inserts / Deletes / Misaligned / Frameshifts

Analysis details

This analysis was performed with panviral2.64

NGS Details (UN18_val): Gammaflexivirus PaGFV-1

Assembly

**Coverage Length**	135 (1 contig(s))
**Depth Of Coverage**	2.0
**Number Of Reads**	2
**Reads Per Million**	0.04 rpm (after QC)
**Ambiguities**	0
**Assembly Method**	de novo + reference guided assembly
**Consensus Caller**	Bcf Tools

Coverage Map

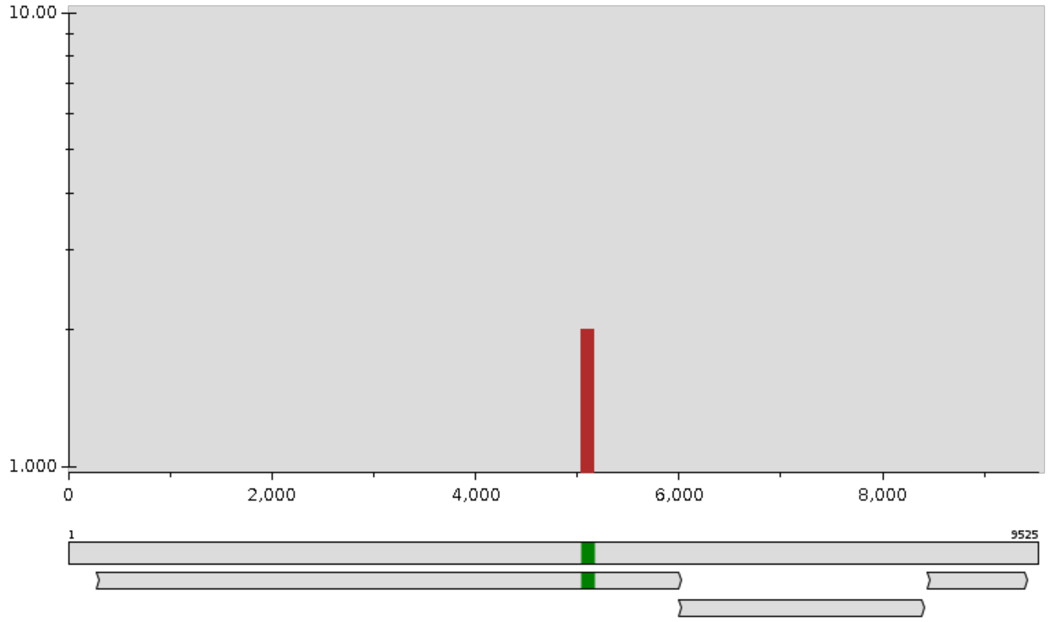

Assignment

**Type**	Gammaflexivirus PaGFV-1 (Taxonomy ID: 2955928)
**Reference Genome**	NC_076696.1
**NT Identity (%)**	80.0
**AA Identity (%)**	86.6667
**Number Of Stop Codons**	0
**Number Of CDS**	3

Alignment

**Alignment Score**	162.0 (NT) + 246.0 (AA) = 408.0
**Concordance (%)**	73.7794

Alignment Method	Global, seeded, nucleotide + amino acids (AGA)

Genome Region

Sequence starts at position 5038 and ends at position 5172 relative to NC_076696.1 reference sequence.

Alignment Detailed Statistics

	Begin	End	Coverage	Score	Concordance	Matches	Identities	I/D/M/F*	Stop Codons
**NT**	**5038**	**5172**	**1.4%**	**162**	**60.0%**	**135 (100%)**	**108 (80.0%)**	**0/0**	
Mutations:	5040T>A, 5042T>A, 5045G>C, 5048C>T, 5052C>G, 5053A>G, 5054C>T, 5057G>A, 5063G>A, 5072T>C, 5078A>T, 5084T>C, 5093G>A, 5096T>C, 5102C>G, 5103C>G, 5111T>G, 5112G>A, 5114T>A, 5126C>T, 5138T>C, 5147G>C, 5150T>G, 5159C>T, 5160T>G, 5163A>C, 5165A>C								
**CDS**									
QKQ70_gp1	1588	1632	2.3%	246	80.4%	45 (100%)	39 (86.7%)	0/0/0/0	0
Protein mutations:	S1588T (5040T>A 5042T>A), H1592G (5052C>G 5053A>G 5054C>T), Q1609E (5103C>G), D1612K (5112G>A 5114T>A), S1628A (5160T>G)								
Codon mutations:	TCT1588ACA (5040T>A 5042T>A), CTG1589CTC (5045G>C), GAC1590GAT (5048C>T), CAC1592GGT (5052C>G 5053A>G 5054C>T), AAG1593AAA (5057G>A), GAG1595GAA (5063G>A), CTT1598CTC (5072T>C), TCA1600TCT (5078A>T), GAT1602GAC (5084T>C), AAG1605AAA (5093G>A), CTT1606CTC (5096T>C), ACC1608ACG (5102C>G), CAG1609GAG (5103C>G), CGT1611CGG (5111T>G), GAT1612AAA (5112G>A 5114T>A), GGC1616GGT (5126C>T), ACT1620ACC (5138T>C), GCG1623GCC (5147G>C), GCT1624GCG (5150T>G), AAC1627AAT (5159C>T), TCT1628GCT (5160T>G), AGA1629CGC (5163A>C 5165A>C)								
**Proteins**									
replicase (YP_010799909.1)	1588	1632	2.3%	246	80.4%	45 (100%)	39 (86.7%)	0/0/0/0	0
Protein mutations:	S1588T (5040T>A 5042T>A), H1592G (5052C>G 5053A>G 5054C>T), Q1609E (5103C>G), D1612K (5112G>A 5114T>A), S1628A (5160T>G)								
Codon mutations:	TCT1588ACA (5040T>A 5042T>A), CTG1589CTC (5045G>C), GAC1590GAT (5048C>T), CAC1592GGT (5052C>G 5053A>G 5054C>T), AAG1593AAA (5057G>A), GAG1595GAA (5063G>A), CTT1598CTC (5072T>C), TCA1600TCT (5078A>T), GAT1602GAC (5084T>C), AAG1605AAA (5093G>A), CTT1606CTC (5096T>C), ACC1608ACG (5102C>G), CAG1609GAG (5103C>G), CGT1611CGG (5111T>G), GAT1612AAA (5112G>A 5114T>A), GGC1616GGT (5126C>T), ACT1620ACC (5138T>C), GCG1623GCC (5147G>C), GCT1624GCG (5150T>G), AAC1627AAT (5159C>T), TCT1628GCT (5160T>G), AGA1629CGC (5163A>C 5165A>C)								

*: Inserts / Deletes / Misaligned / Frameshifts

Analysis details

This analysis was performed with panviral2.64

NGS Details (UN18_val): Sugarcane chlorotic streak virus

Assembly

**Coverage Length**	121 (1 contig(s))
**Depth Of Coverage**	1.9
**Number Of Reads**	2
**Reads Per Million**	0.04 rpm (after QC)
**Ambiguities**	0
**Assembly Method**	de novo + reference guided assembly
**Consensus Caller**	Bcf Tools

Coverage Map

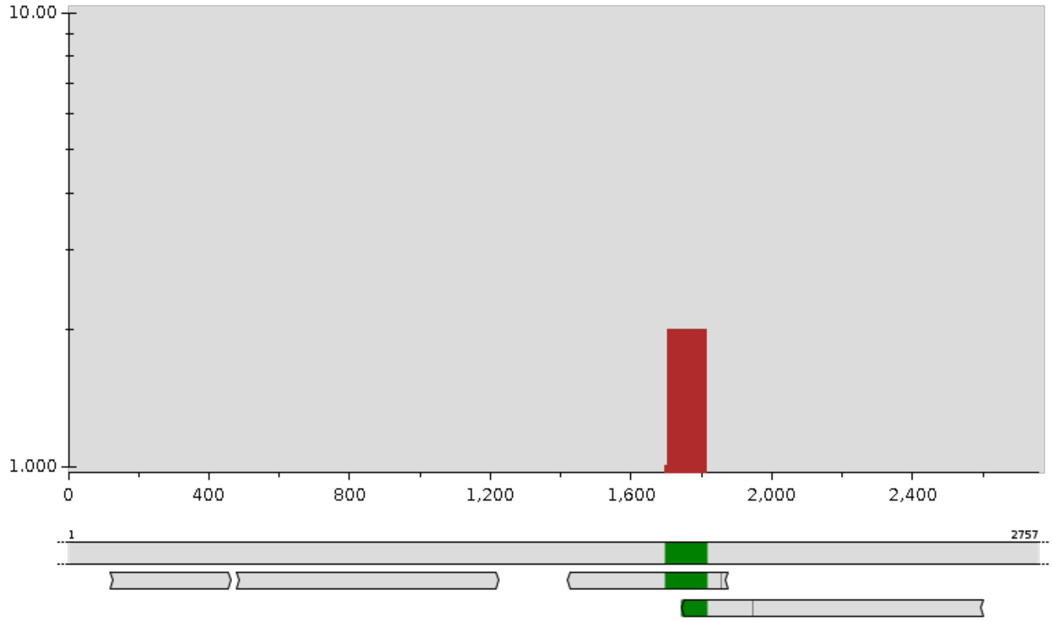

Assignment

**Type**	Sugarcane chlorotic streak virus (Taxonomy ID: 1919062)
**Reference Genome**	NC_032004.1
**NT Identity (%)**	62.8099
**AA Identity (%)**	55.7692
**Number Of Stop Codons**	1
**Number Of CDS**	5

Alignment

**Alignment Score**	62.0 (NT) + 441.0 (AA) = 503.0
**Concordance (%)**	50.1996

Alignment Method	Global, seeded, nucleotide + amino acids (AGA)

Genome Region

Sequence starts at position 1696 and ends at position 1816 relative to NC_032004.1 reference sequence.

Alignment Detailed Statistics

	Begin	End	Coverage	Score	Concordance	Matches	Identities	I/D/M/F*	Stop Codons
**NT**	**1696**	**1816**	**4.4%**	**62**	**25.6%**	**121 (100%)**	**76 (62.8%)**	**0/0**	
Mutations:	1701A>G, 1704A>T, 1710G>A, 1713G>A, 1714A>T, 1715C>A, 1716G>C, 1718C>A, 1720G>C, 1721C>T, 1722C>G, 1723T>A, 1724C>T, 1725T>A, 1734A>G, 1735G>T, 1736A>T, 1737G>A, 1738G>C, 1740C>G, 1741C>A, 1744G>T, 1755T>G, 1756T>C, 1757G>A, 1758C>A, 1759C>A, 1761G>A, 1767A>G, 1776C>A, 1779G>A, 1784T>G, 1785A>T, 1791A>T, 1793A>T, 1794T>C, 1796A>T, 1797C>T, 1800T>A, 1803T>A, 1806C>T, 1809T>A, 1811T>A, 1812T>A, 1815G>C								
**CDS**									
BS327_gp3	234	273	11.0%	206	65.8%	40 (100%)	25 (62.5%)	0/0/0/0	0
Protein mutations:	T235S (1809T>A 1811T>A), S240T (1794T>C 1796A>T), S241T (1791A>T 1793A>T), W252F (1758C>A 1759C>A), Q253C (1755T>G 1756T>C 1757G>A), A257D (1744G>T), W258F (1740C>G 1741C>A), T259S (1737G>A 1738G>C), S260N (1734A>G 1735G>T 1736A>T), E263D (1725T>A), E264I (1722C>G 1723T>A 1724C>T), A265R (1720G>C 1721C>T), V266L (1716G>C 1718C>A), V267Y (1713G>A 1714A>T 1715C>A)								
Codon mutations:	GGC233.GG (1815G>C), CCA234CCT (1812T>A), ACA235TCT (1809T>A 1811T>A), AGG236AGA (1806C>T), ACA237ACT (1803T>A), GGA238GGT (1800T>A), AAG239AAA (1797C>T), TCA240ACG (1794T>C 1796A>T), TCT241ACA (1791A>T 1793A>T), GCT243GCA (1785A>T), AGA244CGA (1784T>G), AGC245AGT (1779G>A), CTG246CTT (1776C>A), CAT249CAC (1767A>G), TAC251TAT (1761G>A), TGG252TTT (1758C>A 1759C>A), CAA253TGC (1755T>G 1756T>C 1757G>A), GCC257GAC (1744G>T), TGG258TTC (1740C>G 1741C>A), ACC259AGT (1737G>A 1738G>C), TCT260AAC (1734A>G 1735G>T 1736A>T), GAA263GAT (1725T>A), GAG264ATC (1722C>G 1723T>A 1724C>T), GCA265AGA (1720G>C 1721C>T), GTC266TTG (1716G>C 1718C>A), GTC267TAT (1713G>A 1714A>T 1715C>A), AAC268AAT (1710G>A), ATT270ATA (1704A>T), GAT271GAC (1701A>G)								
BS327_gp4	22	61	26.3%	206	65.8%	40 (100%)	25 (62.5%)	0/0/0/0	0
Protein mutations:	T23S (1809T>A 1811T>A), S28T (1794T>C 1796A>T), S29T (1791A>T 1793A>T), W40F (1758C>A 1759C>A), Q41C (1755T>G 1756T>C 1757G>A), A45D (1744G>T), W46F (1740C>G 1741C>A), T47S (1737G>A 1738G>C), S48N (1734A>G 1735G>T 1736A>T), E51D (1725T>A), E52I (1722C>G 1723T>A 1724C>T), A53R (1720G>C 1721C>T), V54L (1716G>C 1718C>A), V55Y (1713G>A 1714A>T 1715C>A)								
Codon mutations:	GGC21.GG (1815G>C), CCA22CCT (1812T>A), ACA23TCT (1809T>A 1811T>A), AGG24AGA (1806C>T), ACA25ACT (1803T>A), GGA26GGT (1800T>A), AAG27AAA (1797C>T), TCA28ACG (1794T>C 1796A>T), TCT29ACA (1791A>T 1793A>T), GCT31GCA (1785A>T), AGA32CGA (1784T>G), AGC33AGT (1779G>A), CTG34CTT (1776C>A), CAT37CAC (1767A>G), TAC39TAT (1761G>A), TGG40TTT (1758C>A 1759C>A), CAA41TGC (1755T>G 1756T>C 1757G>A), GCC45GAC (1744G>T), TGG46TTC (1740C>G 1741C>A), ACC47AGT (1737G>A 1738G>C), TCT48AAC (1734A>G 1735G>T 1736A>T), GAA51GAT (1725T>A), GAG52ATC (1722C>G 1723T>A 1724C>T), GCA53AGA (1720G>C 1721C>T), GTC54TTG (1716G>C 1718C>A), GTC55TAT (1713G>A 1714A>T 1715C>A), AAC56AAT (1710G>A), ATT58ATA (1704A>T), GAT59GAC (1701A>G)								
BS327_gp5	264	287	8.4%	29	17.9%	24 (100%)	8 (33.3%)	0/0/0/0	1
Protein mutations:	A264G (1815G>C), Q265L (1811T>A 1812T>A), Q266L (1809T>A), G267E (1806C>T), Q268L (1803T>A), E269V (1800T>A), S270K (1796A>T 1797C>T), H271R (1793A>T 1794T>C), L272H (1791A>T), L274H (1784T>G 1785A>T), A276V (1779G>A), W277L (1776C>A), I280T (1767A>G), T282I (1761G>A), G283F (1757G>A 1758C>A 1759C>A), K284A (1755T>G 1756T>C)								
Codon mutations:	GCC264GGC (1815G>C), CAA265CTT (1811T>A 1812T>A), CAA266CTA (1809T>A), GGA267GAA (1806C>T), CAG268CTG (1803T>A), GAA269GTA (1800T>A), AGT270AAA (1796A>T 1797C>T), CAT271CGA (1793A>T 1794T>C), CTT272CAT (1791A>T), CTA274CAC (1784T>G 1785A>T), GCC276GTC (1779G>A), TGG277TTG (1776C>A), ATA280ACA (1767A>G), ACT282ATT (1761G>A), GGC283TTT (1757G>A 1758C>A 1759C>A), AAA284GCA (1755T>G 1756T>C)								
**Proteins**									
Rep-associated protein (YP_009325925.1)	234	273	11.0%	206	65.8%	40 (100%)	25 (62.5%)	0/0/0/0	0
Protein mutations:	T235S (1809T>A 1811T>A), S240T (1794T>C 1796A>T), S241T (1791A>T 1793A>T), W252F (1758C>A 1759C>A), Q253C (1755T>G 1756T>C 1757G>A), A257D (1744G>T), W258F (1740C>G 1741C>A), T259S (1737G>A 1738G>C), S260N (1734A>G 1735G>T 1736A>T), E263D (1725T>A), E264I (1722C>G 1723T>A 1724C>T), A265R (1720G>C 1721C>T), V266L (1716G>C 1718C>A), V267Y (1713G>A 1714A>T 1715C>A)								
Codon mutations:	GGC233.GG (1815G>C), CCA234CCT (1812T>A), ACA235TCT (1809T>A 1811T>A), AGG236AGA (1806C>T), ACA237ACT (1803T>A), GGA238GGT (1800T>A), AAG239AAA (1797C>T), TCA240ACG (1794T>C 1796A>T), TCT241ACA (1791A>T 1793A>T), GCT243GCA (1785A>T), AGA244CGA (1784T>G), AGC245AGT (1779G>A), CTG246CTT (1776C>A), CAT249CAC (1767A>G), TAC251TAT (1761G>A), TGG252TTT (1758C>A 1759C>A), CAA253TGC (1755T>G 1756T>C 1757G>A), GCC257GAC (1744G>T), TGG258TTC (1740C>G 1741C>A), ACC259AGT (1737G>A 1738G>C), TCT260AAC (1734A>G 1735G>T 1736A>T), GAA263GAT (1725T>A), GAG264ATC (1722C>G 1723T>A 1724C>T), GCA265AGA (1720G>C 1721C>T), GTC266TTG (1716G>C 1718C>A), GTC267TAT (1713G>A 1714A>T 1715C>A), AAC268AAT (1710G>A), ATT270ATA (1704A>T), GAT271GAC (1701A>G)								
RepB (YP_009325926.1)	22	61	26.3%	206	65.8%	40 (100%)	25 (62.5%)	0/0/0/0	0
Protein mutations:	T23S (1809T>A 1811T>A), S28T (1794T>C 1796A>T), S29T (1791A>T 1793A>T), W40F (1758C>A 1759C>A), Q41C (1755T>G 1756T>C 1757G>A), A45D (1744G>T), W46F (1740C>G 1741C>A), T47S (1737G>A 1738G>C), S48N (1734A>G 1735G>T 1736A>T), E51D (1725T>A), E52I (1722C>G 1723T>A 1724C>T), A53R (1720G>C 1721C>T), V54L (1716G>C 1718C>A), V55Y (1713G>A 1714A>T 1715C>A)								
Codon mutations:	GGC21.GG (1815G>C), CCA22CCT (1812T>A), ACA23TCT (1809T>A 1811T>A), AGG24AGA (1806C>T), ACA25ACT (1803T>A), GGA26GGT (1800T>A), AAG27AAA (1797C>T), TCA28ACG (1794T>C 1796A>T), TCT29ACA (1791A>T 1793A>T), GCT31GCA (1785A>T), AGA32CGA (1784T>G), AGC33AGT (1779G>A), CTG34CTT (1776C>A), CAT37CAC (1767A>G), TAC39TAT (1761G>A), TGG40TTT (1758C>A 1759C>A), CAA41TGC (1755T>G 1756T>C 1757G>A), GCC45GAC (1744G>T), TGG46TTC (1740C>G 1741C>A), ACC47AGT (1737G>A 1738G>C), TCT48AAC (1734A>G 1735G>T 1736A>T), GAA51GAT (1725T>A), GAG52ATC (1722C>G 1723T>A 1724C>T), GCA53AGA (1720G>C 1721C>T), GTC54TTG (1716G>C 1718C>A), GTC55TAT (1713G>A 1714A>T 1715C>A), AAC56AAT (1710G>A), ATT58ATA (1704A>T), GAT59GAC (1701A>G)								
RepA (YP_009325927.1)	264	287	8.4%	29	17.9%	24 (100%)	8 (33.3%)	0/0/0/0	1
Protein mutations:	A264G (1815G>C), Q265L (1811T>A 1812T>A), Q266L (1809T>A), G267E (1806C>T), Q268L (1803T>A), E269V (1800T>A), S270K (1796A>T 1797C>T), H271R (1793A>T 1794T>C), L272H (1791A>T), L274H (1784T>G 1785A>T), A276V (1779G>A), W277L (1776C>A), I280T (1767A>G), T282I (1761G>A), G283F (1757G>A 1758C>A 1759C>A), K284A (1755T>G 1756T>C)								
Codon mutations:	GCC264GGC (1815G>C), CAA265CTT (1811T>A 1812T>A), CAA266CTA (1809T>A), GGA267GAA (1806C>T), CAG268CTG (1803T>A), GAA269GTA (1800T>A), AGT270AAA (1796A>T 1797C>T), CAT271CGA (1793A>T 1794T>C), CTT272CAT (1791A>T), CTA274CAC (1784T>G 1785A>T), GCC276GTC (1779G>A), TGG277TTG (1776C>A), ATA280ACA (1767A>G), ACT282ATT (1761G>A), GGC283TTT (1757G>A 1758C>A 1759C>A), AAA284GCA (1755T>G 1756T>C)								

*: Inserts / Deletes / Misaligned / Frameshifts

Analysis details

This analysis was performed with panviral2.64

NGS Details (UN18_val): Seussvirus seuss

Assembly

**Coverage Length**	232 (1 contig(s))
**Depth Of Coverage**	1.1
**Number Of Reads**	2
**Reads Per Million**	0.04 rpm (after QC)
**Ambiguities**	0
**Assembly Method**	de novo + reference guided assembly
**Consensus Caller**	Bcf Tools

Coverage Map

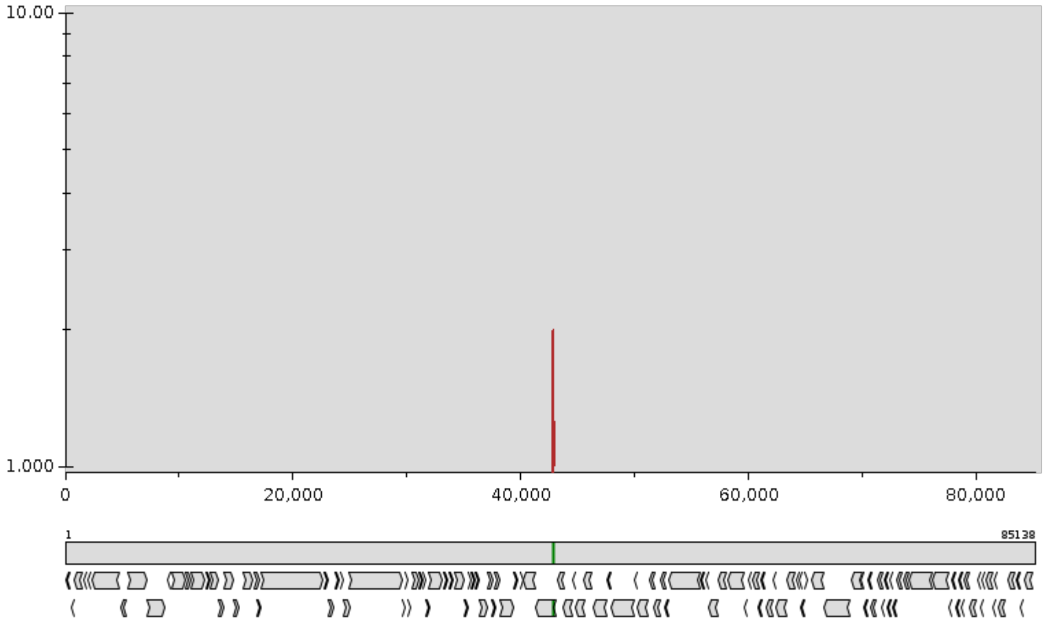

Assignment

**Type**	Seussvirus seuss (Taxonomy ID: 2734278)
**Reference Genome**	NC_047757.1
**NT Identity (%)**	76.2931
**AA Identity (%)**	79.4872
**Number Of Stop Codons**	0
**Number Of CDS**	120

Alignment

**Alignment Score**	244.0 (NT) + 499.0 (AA) = 743.0
**Concordance (%)**	70.4265

**Alignment Method**	Local, heuristic, nucleotide (BLASTN)

Genome Region

Sequence starts at position 42730 and ends at position 42961 relative to NC_047757.1 reference sequence.

Alignment Detailed Statistics

	Begin	End	Coverage	Score	Concordance	Matches	Identities	I/D/M/F*	Stop Codons
**NT**	**42730**	**42961**	**0.3%**	**244**	**52.6%**	**232 (100%)**	**177 (76.3%)**	**0/0**	
Mutations:	42734T>G, 42737C>T, 42747G>C, 42748A>C, 42752G>C, 42756T>C, 42757C>T, 42764G>A, 42782A>G, 42786T>C, 42787T>G, 42788G>C, 42789C>G, 42790T>A, 42791G>T, 42794C>G, 42801G>A, 42802T>C, 42820T>C, 42821C>G, 42822T>A, 42823G>C, 42830C>G, 42831G>T, 42832C>T, 42837T>C, 42842C>G, 42843T>G, 42844C>G, 42845G>C, 42848G>C, 42849C>G, 42850C>A, 42851G>A, 42854C>G, 42858A>T, 42860A>G, 42872A>C, 42886C>T, 42893A>G, 42899G>C, 42902G>C, 42904T>C, 42908G>C, 42928G>A, 42929C>G, 42930T>C, 42933G>C, 42934A>T, 42935C>G, 42938G>A, 42941G>A, 42944A>G, 42956G>C, 42959G>A								

CDS

	Begin	End	Coverage	Score	Concordance	Matches	Identities	I/D/M/F*	Stop Codons
HOR08_gp052	48	125	13.1%	499	83.2%	78 (100%)	62 (79.5%)	0/0/0/0	0
Protein mutations:	M56I (42935C>G), Q58R (42929C>G 42930T>C), I67V (42902G>C 42904T>C), A73T (42886C>T), F82Y (42858A>T), G85S (42848G>C 42849C>G 42850C>A), E87P (42842C>G 42843T>G 42844C>G), N89S (42837T>C), A91N (42830C>G 42831G>T 42832C>T), Q94V (42821C>G 42822T>A 42823G>C), N95D (42820T>C), T101V (42801G>A 42802T>C), N106R (42786T>C 42787T>G), D116S (42756T>C 42757C>T), S119G (42747G>C 42748A>C)								
Codon mutations:	CAC48CAT (42959G>A), GCC49GCG (42956G>C), TAT53TAC (42944A>G), GAC54GAT (42941G>A), TAC55TAT (42938G>A), ATG56ATC (42935C>G), TCC57AGC (42933G>C 42934A>T), CAG58CGC (42929C>G 42930T>C), CTG59TTG (42928G>A), ACC65ACG (42908G>C), ATC67GTG (42902G>C 42904T>C), CTC68CTG (42899G>C), AAT70AAC (42893A>G), GCG73ACG (42886C>T), CTT77CTG (42872A>C), TGT81TGC (42860A>G), TTC82TAC (42858A>T), CTG83CTC (42854C>G), AAC84AAT (42851G>A), GGC85TCG (42848G>C 42849C>G 42850C>A), GTC86GTG (42845G>C), GAG87CCC (42842C>G 42843T>G 42844C>G), AAC89AGC (42837T>C), GCG91AAC (42830C>G 42831G>T 42832C>T), CAG94GTC (42821C>G 42822T>A 42823G>C), AAC95GAC (42820T>C), ACC101GTC (42801G>A 42802T>C), CTG103CTC (42794C>G), GCC104GCA (42791G>T), AGC105TCG (42788G>C 42789C>G 42790T>A), AAC106CGC (42786T>C 42787T>G), GGT107GGC (42782A>G), TAC113TAT (42764G>A), GAC116AGC (42756T>C 42757C>T), GTC117GTG (42752G>C), TCC119GGC (42747G>C 42748A>C), GAG122GAA (42737C>T), CCA123CCC (42734T>G)								

Proteins

	Begin	End	Coverage	Score	Concordance	Matches	Identities	I/D/M/F*	Stop Codons
ribonucleotide reductase large subunit (YP_009785562.1)	48	125	13.1%	499	83.2%	78 (100%)	62 (79.5%)	0/0/0/0	0
Protein mutations:	M56I (42935C>G), Q58R (42929C>G 42930T>C), I67V (42902G>C 42904T>C), A73T (42886C>T), F82Y (42858A>T), G85S (42848G>C 42849C>G 42850C>A), E87P (42842C>G 42843T>G 42844C>G), N89S (42837T>C), A91N (42830C>G 42831G>T 42832C>T), Q94V (42821C>G 42822T>A 42823G>C), N95D (42820T>C), T101V (42801G>A 42802T>C), N106R (42786T>C 42787T>G), D116S (42756T>C 42757C>T), S119G (42747G>C 42748A>C)								
Codon mutations:	CAC48CAT (42959G>A), GCC49GCG (42956G>C), TAT53TAC (42944A>G), GAC54GAT (42941G>A), TAC55TAT (42938G>A), ATG56ATC (42935C>G), TCC57AGC (42933G>C 42934A>T), CAG58CGC (42929C>G 42930T>C), CTG59TTG (42928G>A), ACC65ACG (42908G>C), ATC67GTG (42902G>C 42904T>C), CTC68CTG (42899G>C), AAT70AAC (42893A>G), GCG73ACG (42886C>T), CTT77CTG (42872A>C), TGT81TGC (42860A>G), TTC82TAC (42858A>T), CTG83CTC (42854C>G), AAC84AAT (42851G>A), GGC85TCG (42848G>C 42849C>G 42850C>A), GTC86GTG (42845G>C), GAG87CCC (42842C>G 42843T>G 42844C>G), AAC89AGC (42837T>C), GCG91AAC (42830C>G 42831G>T 42832C>T), CAG94GTC (42821C>G 42822T>A 42823G>C), AAC95GAC (42820T>C), ACC101GTC (42801G>A 42802T>C), CTG103CTC (42794C>G), GCC104GCA (42791G>T), AGC105TCG (42788G>C 42789C>G 42790T>A), AAC106CGC (42786T>C 42787T>G), GGT107GGC (42782A>G), TAC113TAT (42764G>A), GAC116AGC (42756T>C 42757C>T), GTC117GTG (42752G>C), TCC119GGC (42747G>C 42748A>C), GAG122GAA (42737C>T), CCA123CCC (42734T>G)								

*: Inserts / Deletes / Misaligned / Frameshifts

Analysis details

This analysis was performed with panviral2.64

NGS Details (UN18_val): Fusarium poae narnavirus 2

Assembly

**Coverage Length**	246 (2 contig(s))
**Depth Of Coverage**	1.0
**Number Of Reads**	2
**Reads Per Million**	0.04 rpm (after QC)
**Ambiguities**	0
**Assembly Method**	de novo + reference guided assembly
**Consensus Caller**	Bcf Tools

Coverage Map

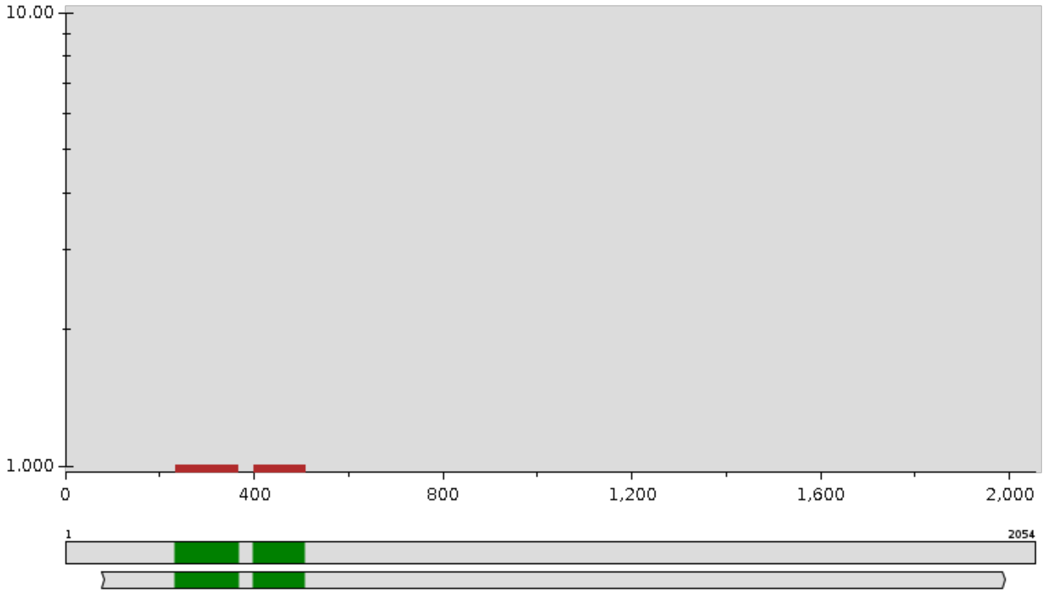

Assignment

**Type**	Fusarium poae narnavirus 2 (Taxonomy ID: 1849532)
**Reference Genome**	NC_030866.1
**NT Identity (%)**	74.7967
**AA Identity (%)**	74.3902
**Number Of Stop Codons**	0
**Number Of CDS**	1

Alignment

**Alignment Score**	244.0 (NT) + 507.0 (AA) = 751.0
**Concordance (%)**	66.7556

**Alignment Method**	Global, seeded, nucleotide + amino acids (AGA)

Genome Region

Sequence starts at position 233 and ends at position 509 relative to NC_030866.1 reference sequence.

Alignment Detailed Statistics

	Begin	End	Coverage	Score	Concordance	Matches	Identities	I/D/M/F*	Stop Codons
**NT**	**233**	**509**	**12.0%**	**244**	**49.6%**	**246 (100%)**	**184 (74.8%)**	**0/0**	
Mutations:	234T>C, 238C>A, 240G>A, 242G>A, 243A>G, 244A>G, 245A>G, 249C>T, 250A>G, 252T>C, 255G>A, 256C>G, 257C>A, 258T>A, 263C>A, 264A>C, 268T>C, 269T>A, 270G>C, 272A>G, 273C>G, 279T>C, 282G>T, 283T>C, 285T>G, 288C>T, 289C>T, 290G>C, 294G>C, 301C>A, 304C>G, 309C>T, 312C>A, 315T>C, 321C>T, 332A>G, 334A>T, 335G>T, 336C>G, 337A>G, 338T>A, 339A>T, 355A>G, 399G>A, 403C>T, 405T>A, 406A>T, 408T>A, 411A>G, 420T>C, 423A>G, 429T>G, 447G>C, 450C>A, 468C>G, 471G>A, 475A>T, 476A>C, 477C>T, 480G>C, 484C>T, 486T>C								
**CDS**									
BHR96_gp1	53	144	12.9%	507	77.2%	82 (100%)	61 (74.4%)	0/0/0/0	0
Protein mutations:	R55K (242G>A 243A>G), K56G (244A>G 245A>G), I58V (250A>G 252T>C), P60E (256C>G 257C>A 258T>A), A62D (263C>A 264A>C), L64H (268T>C 269T>A 270G>C), D65G (272A>G 273C>G), S69P (283T>C 285T>G), R71S (289C>T 290G>C), E72D (294G>C), Q75K (301C>A), L76V (304C>G), D85G (332A>G), S86L (334A>T 335G>T 336C>G), I87D (337A>G 338T>A 339A>T), I93V (355A>G), T110S (406A>T 408T>A), N133S (475A>T 476A>C 477C>T), H136Y (484C>T 486T>C)								
Codon mutations:	TCT52.CC (234T>C), CGG54AGA (238C>A 240G>A), AGA55AAG (242G>A 243A>G), AAA56GGA (244A>G 245A>G), TTC57TTT (249C>T), ATT58GTC (250A>G 252T>C), GCG59GCA (255G>A), CCT60GAA (256C>G 257C>A 258T>A), GCA62GAC (263C>A 264A>C), TTG64CAC (268T>C 269T>A 270G>C), GAC65GGG (272A>G 273C>G), CAT67CAC (279T>C), CGG68CGT (282G>T), TCT69CCG (283T>C 285T>G), TTC70TTT (288C>T), CGC71TCC (289C>T 290G>C), GAG72GAC (294G>C), CAA75AAA (301C>A), CTC76GTC (304C>G), GTC77GTT (309C>T), ATC78ATA (312C>A), GAT79GAC (315T>C), TCC81TCT (321C>T), GAT85GGT (332A>G), AGC86TTG (334A>T 335G>T 336C>G), ATA87GAT (337A>G 338T>A 339A>T), ATC93GTC (355A>G), CGG107..A (399G>A), CTT109TTA (403C>T 405T>A), ACT110TCA (406A>T 408T>A), GGA111GGG (411A>G), AAT114AAC (420T>C), CGA115CGG (423A>G), CTT117CTG (429T>G), TCG123TCC (447G>C), ATC124ATA (450C>A), GTC130GTG (468C>G), CTG131CTA (471G>A), AAC133TCT (475A>T 476A>C 477C>T), GGG134GGC (480G>C), CAT136TAC (484C>T 486T>C)								
**Proteins**									
RNA-dependent RNA polymerase (YP_009272903.1)	53	144	12.9%	507	77.2%	82 (100%)	61 (74.4%)	0/0/0/0	0
Protein mutations:	R55K (242G>A 243A>G), K56G (244A>G 245A>G), I58V (250A>G 252T>C), P60E (256C>G 257C>A 258T>A), A62D (263C>A 264A>C), L64H (268T>C 269T>A 270G>C), D65G (272A>G 273C>G), S69P (283T>C 285T>G), R71S (289C>T 290G>C), E72D (294G>C), Q75K (301C>A), L76V (304C>G), D85G (332A>G), S86L (334A>T 335G>T 336C>G), I87D (337A>G 338T>A 339A>T), I93V (355A>G), T110S (406A>T 408T>A), N133S (475A>T 476A>C 477C>T), H136Y (484C>T 486T>C)								
Codon mutations:	TCT52.CC (234T>C), CGG54AGA (238C>A 240G>A), AGA55AAG (242G>A 243A>G), AAA56GGA (244A>G 245A>G), TTC57TTT (249C>T), ATT58GTC (250A>G 252T>C), GCG59GCA (255G>A), CCT60GAA (256C>G 257C>A 258T>A), GCA62GAC (263C>A 264A>C), TTG64CAC (268T>C 269T>A 270G>C), GAC65GGG (272A>G 273C>G), CAT67CAC (279T>C), CGG68CGT (282G>T), TCT69CCG (283T>C 285T>G), TTC70TTT (288C>T), CGC71TCC (289C>T 290G>C), GAG72GAC (294G>C), CAA75AAA (301C>A), CTC76GTC (304C>G), GTC77GTT (309C>T), ATC78ATA (312C>A), GAT79GAC (315T>C), TCC81TCT (321C>T), GAT85GGT (332A>G), AGC86TTG (334A>T 335G>T 336C>G), ATA87GAT (337A>G 338T>A 339A>T), ATC93GTC (355A>G), CGG107..A (399G>A), CTT109TTA (403C>T 405T>A), ACT110TCA (406A>T 408T>A), GGA111GGG (411A>G), AAT114AAC (420T>C), CGA115CGG (423A>G), CTT117CTG (429T>G), TCG123TCC (447G>C), ATC124ATA (450C>A), GTC130GTG (468C>G), CTG131CTA (471G>A), AAC133TCT (475A>T 476A>C 477C>T), GGG134GGC (480G>C), CAT136TAC (484C>T 486T>C)								

*: Inserts / Deletes / Misaligned / Frameshifts

Analysis details

This analysis was performed with panviral2.64
