# Supplementary material for: Nucleotide sequence analysis reveals the presence of PVY-Tam isolates affecting tamarillo in Colombia
Source: Virol J. 2026 Apr 20;23:145. doi: 10.1186/s12985-026-03166-6 (PMC13234967; doi:10.1186/s12985-026-03166-6)
Supplement: Supplementary file 4 — Additional file 4. [file 12985_2026_3166_MOESM4_ESM.pdf]

Analysis of UN24

**Technology**	Paired-end short reads
**Input Files**	UN24_R1.fq.gz (1.61 GB), UN24_R2.fq.gz (1.67 GB)
**Submitted On**	2023-09-26 13:12:56 UTC
**Duration**	3h 59m 33s
**Tool Version**	panviral2.64
**Location**	https://www.genomedetective.com/db/ui/analysis/d87ef240-8dcb-4afa-b65b-122051e282e6

Statistics

**Original Read Length**	20 - 150
**Trimmed Read Length**	50 - 135

	# Reads	% of Reads
**Input file**	50312498	100.0%
**After QC**	49956410	99.3%
**After filtering**	1799410	3.6%
**Mapped back to contigs**	79354	0.2%

Assignments

Assignment	No. of Reads	Depth of Coverage	Identity		Genome Coverage	
			NT	AA		
Diachasmimorpha longicaudata entomopoxvirus (segment NC_043455.1)	10408	1124.4	60.2%	55.7%	84.0%	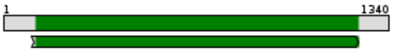
Bracoviriform glomeratae (segment NC_043292.1)	5765	2222.6	72.9%	84.5%	72.8%	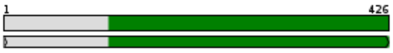
Duamitovirus soch1	434	27.9	68.6%	70.1%	67.7%	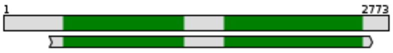
Colombian datura virus	252	5.7	76.3%	85.0%	60.8%	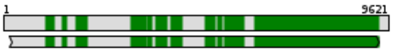
Solendovirus venanicotianae	201	11.2	78.2%	75.3%	26.1%	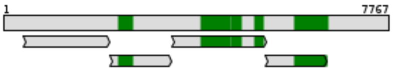
Potato virus Y	77	3.3	84.5%	90.8%	31.6%	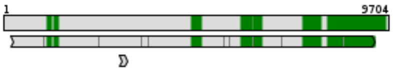
Torradovirus lycopersici (2 segments out of 2)	62	2.5	86.3%	92.5%	24.8%	
Torradovirus lycopersici (segment RNA 1)	16	2.6	87.5%	97.8%	10.6%	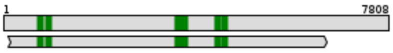

Assignment	No. of Reads	Depth of Coverage	Identity		Genome Coverage	
			NT	AA		
Torradovirus lycopersici (segment RNA 2)	46	2.4	85.9%	89.9%	45.3%	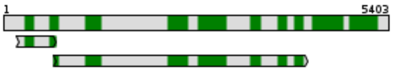

Discoveries

Similar to	No. of Reads	Depth of Coverage	Identity		Genome Coverage	
			NT	AA		
Lausannevirus	20011	11097.3	80.5%	96.6%	0.1%	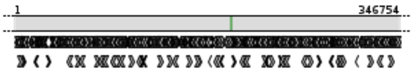
Noumeavirus	14282	5688.3	82.0%	95.8%	0.1%	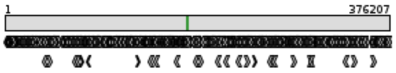
Makelovirus prm1	7006	894.1	76.7%	87.2%	0.7%	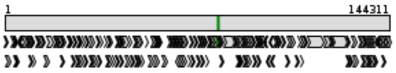
Brazilian marseillevirus	4824	2168.2	82.2%	93.3%	0.1%	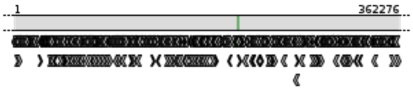
Tokyovirus A1	3678	1769.2	80.6%	93.1%	0.0%	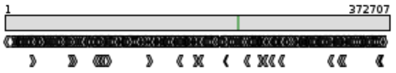
Marseillevirus marseillevirus	2867	2178.8	83.6%	94.3%	0.0%	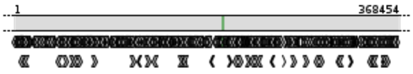
Tunisvirus fontaine2	1907	946.0	78.2%	90.5%	0.1%	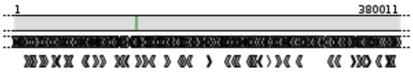
Yellowstone lake phycodnavirus 1	1645	856.7	81.6%	90.3%	0.1%	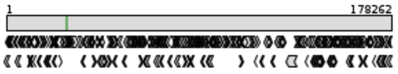
Amsacta moorei entomopoxvirus	1423	860.5	80.2%	81.8%	0.1%	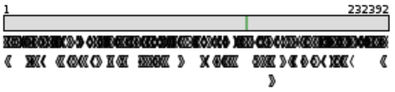
Yellowstone lake phycodnavirus 1	1029	648.7	81.9%	83.3%	0.1%	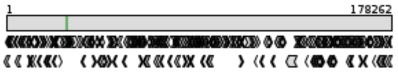
Cladosporium fulvum T-1 virus	749	56.9	53.1%	47.6%	18.9%	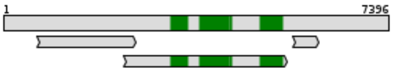
Errantivirus	503	36.2	52.1%	41.3%	14.8%	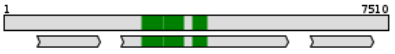
Cassava brown streak virus	421	94.4	60.8%	57.7%	5.8%	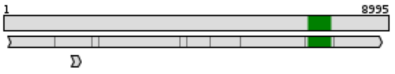
Nodensvirus spm2	374	194.4	81.4%	86.8%	0.1%	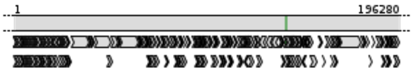

Similar to	No. of Reads	Depth of Coverage	Identity		Genome Coverage	
			NT	AA		
Raharianneviru s raharianne	176	5.0	70.8%	68.1%	9.4%	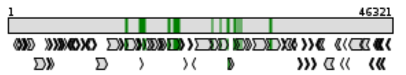
Caulimovirus venafragariae	168	21.5	58.5%	54.4%	10.9%	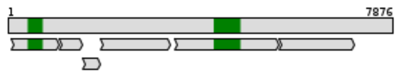
Dioscovirus dioscoreae	161	12.2	59.7%	49.6%	16.9%	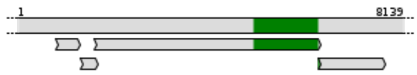
Badnavirus occultipomeae	157	53.6	58.1%	52.3%	3.9%	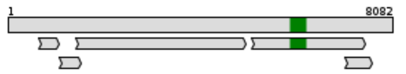
Pseudomonas phage phi297	122	7.3	72.7%	67.2%	4.3%	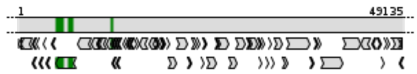
Epiphyllum badnavirus 1	90	35.8	53.9%	51.1%	3.6%	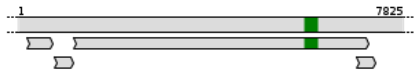
Caulimovirus venafragariae	74	31.5	57.6%	47.1%	3.3%	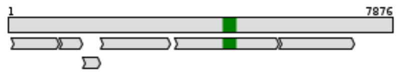
Badnavirus volubetulae	63	27.0	54.1%	49.4%	3.1%	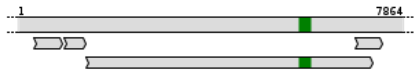
Yellowstone lake phycodnavirus 1	55	35.6	83.7%	93.3%	0.1%	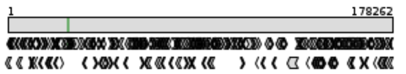
Caulimovirus tessellobrassicae	32	4.8	55.4%	44.9%	10.1%	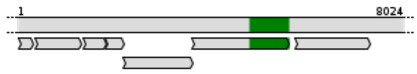
Bracoviriform congregatae (2 segments out of 30)	25	8.2	78.9%	86.2%	N/A	
Bracoviriform congregatae (segment Circle 7)	20	12.2	77.8%	84.4%	0.5%	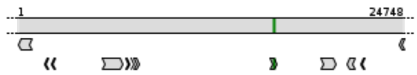
Bracoviriform congregatae (segment Circle 7)	5	4.4	80.0%	87.8%	0.6%	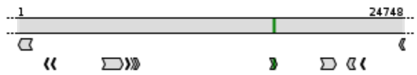
Pinus nigra virus 1	24	10.8	54.4%	40.5%	3.5%	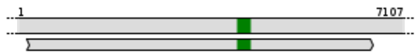
Badnavirus venatheobromae	21	8.9	58.0%	48.9%	3.7%	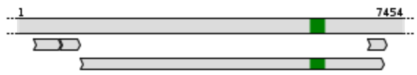
Unknown	21	7.4	58.0%	55.8%	3.2%	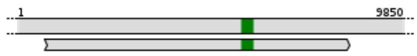
Petuvirus venapetuniae	20	6.3	61.1%	54.3%	4.4%	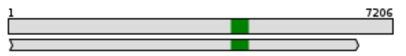

Similar to	No. of Reads	Depth of Coverage	Identity		Genome Coverage	
			NT	AA		
Marseillevirus marseillevirus	18	11.4	82.1%	92.3%	0.0%	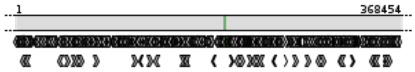
Metaplexis yellow mottle-associated virus	17	6.8	58.7%	45.9%	3.8%	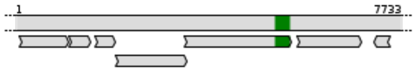
Cavemovirus venamanihotis	15	3.3	57.2%	47.4%	7.1%	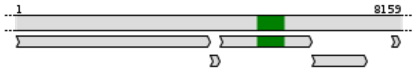
Caulimovirus tessellobrassicae	13	4.5	52.4%	48.4%	4.8%	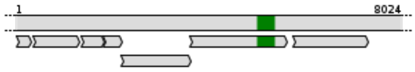
Duamitovirus peex1	12	8.0	70.4%	66.7%	5.0%	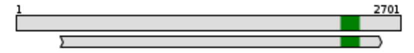
Caulimovirus tessellobrassicae	12	4.9	58.3%	45.6%	4.1%	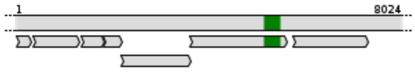
Badnavirus venaribis	12	6.4	53.9%	48.1%	3.1%	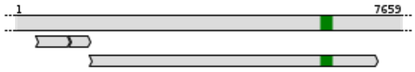
Badnavirus rutilanscamelliae	11	3.5	56.0%	53.7%	4.4%	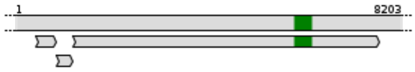
Soymovirus virgarachidis	10	3.9	59.4%	48.1%	4.1%	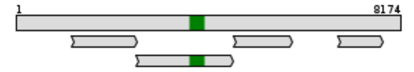
Dioscovirus dioscoreae	10	2.6	63.9%	57.3%	6.3%	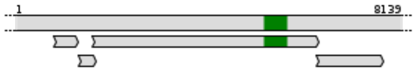
Caulimovirus minutangelicae	9	3.2	56.7%	50.0%	3.3%	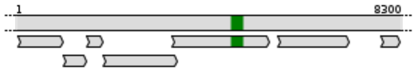
Badnavirus maculaucubae	9	3.6	56.7%	45.7%	3.1%	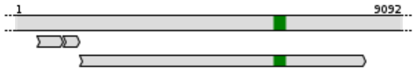
Caulimovirus venafragariae	8	3.8	61.5%	58.0%	3.4%	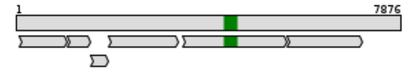
Caulimovirus venafragariae	8	3.5	62.3%	64.7%	3.3%	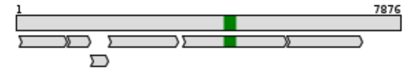
Petuvirus venapetuniae	8	1.9	59.6%	51.5%	5.6%	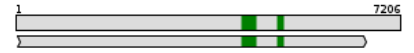
Epiphyllum badnavirus 1	8	3.9	53.8%	46.6%	3.3%	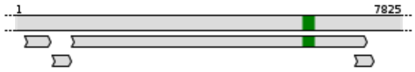
Chinaberry tree badnavirus 1	7	2.7	59.8%	47.7%	3.7%	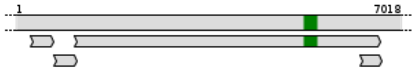

Similar to	No. of Reads	Depth of Coverage	Identity		Genome Coverage	
			NT	AA		
Gihfavirus pelohabitans	6	2.4	98.2%	0.0%	6.3%	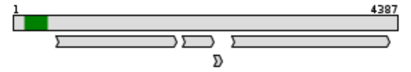
Duamitovirus peex1	6	3.1	72.3%	64.8%	9.8%	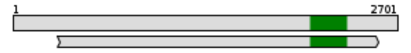
Badnavirus venatheobromae	6	2.3	54.1%	42.9%	4.7%	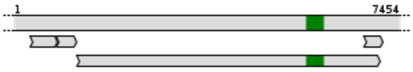
Petuvirus venapetuniae	6	2.5	62.5%	53.2%	3.9%	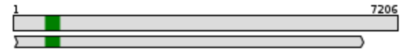
Marseillevirus marseillevirus	6	4.7	80.9%	94.6%	0.0%	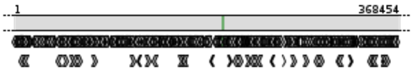
Punavirus P1	4	2.2	100.0%	98.0%	0.2%	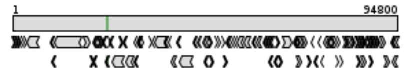
Sugarcane chlorotic streak virus	2	2.0	64.9%	58.4%	4.9%	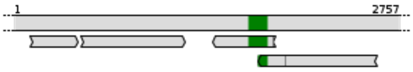
Caulimovirus venafragariae	2	1.1	59.4%	51.9%	3.1%	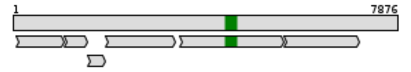
Tomato associated geminivirus 1	2	1.0	76.0%	79.1%	9.9%	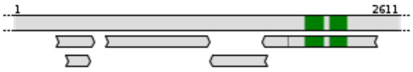

NGS Details (UN24): Torradovirus lycopersici (segment RNA 1)

Assembly

**Coverage Length**	826 (5 contig(s))
**Depth Of Coverage**	2.6
**Number Of Reads**	16
**Reads Per Million**	0.32 rpm (after QC)
**Ambiguities**	0
**Assembly Method**	de novo + reference guided assembly
**Consensus Caller**	Bcf Tools

Coverage Map

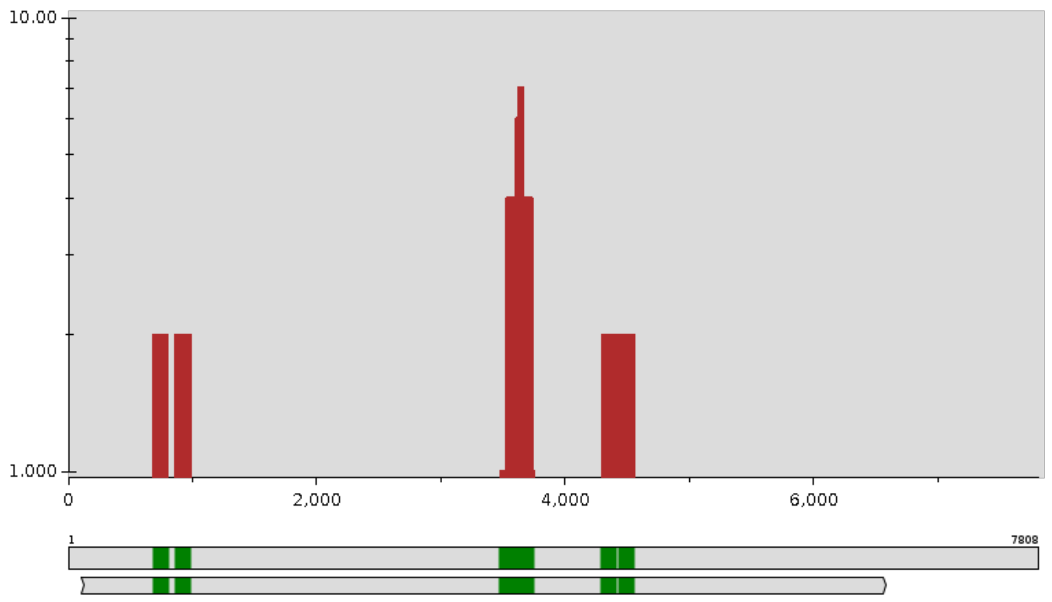

Assignment

**Type**	Torradovirus lycopersici (Taxonomy ID: 3048378)
**Reference Genome**	NC_009013.1
**NT Identity (%)**	87.5303
**AA Identity (%)**	97.8182
**Number Of Stop Codons**	0
**Number Of CDS**	1

Alignment

**Alignment Score**	1240.0 (NT) + 1909.0 (AA) = 3149.0
**Concordance (%)**	87.9363

Alignment Method	Global, seeded, nucleotide + amino acids (AGA)

Genome Region

Sequence starts at position 677 and ends at position 4563 relative to NC_009013.1 reference sequence.

Alignment Detailed Statistics

	Begin	End	Coverage	Score	Concordance	Matches	Identities	I/D/M/F*	Stop Codons
**NT**	**677**	**4563**	**10.6%**	**1240**	**75.1%**	**826 (100%)**	**723 (87.5%)**	**0/0**	
Mutations:	694A>G, 707C>T, 715G>A, 718A>G, 719T>G, 721C>T, 722A>G, 727A>T, 730C>T, 739T>G, 748G>T, 751A>T, 754A>G, 758C>T, 760C>A, 763A>T, 766C>T, 772G>C, 775T>A, 778T>A, 781C>A, 790C>T, 793G>A, 796T>A, 799A>T, 802T>G, 805T>C, 806A>C, 808A>T, 862T>A, 865T>C, 877T>C, 881T>C, 883G>T, 886T>C, 889C>A, 895C>T, 898G>A, 901T>C, 913T>C, 949A>T, 958T>A, 964T>A, 967T>C, 976A>T, 977G>A, 979T>A, 988T>A, 3481C>T, 3484A>T, 3508G>A, 3534G>A, 3538T>C, 3580T>C, 3583C>T, 3610A>G, 3625C>T, 3628G>A, 3643A>G, 3676C>T, 3685G>A, 3701C>T, 3710G>A, 3739A>T, 3751A>G, 3754G>A, 4288C>T, 4291T>C, 4294A>G, 4309T>G, 4312G>T, 4318C>T, 4324T>C, 4327A>T, 4345A>T, 4348C>T, 4357C>G, 4358A>C, 4369T>A, 4381T>A, 4384A>C, 4399T>C, 4414T>G, 4429A>G, 4441A>T, 4444A>G, 4453C>G, 4459T>C, 4471T>C, 4486T>C, 4489T>C, 4493C>A, 4495T>G, 4501A>T, 4507T>C, 4510T>A, 4514T>C, 4525A>C, 4528T>C, 4531A>T, 4537T>A, 4549G>A, 4561T>G								

CDS

	Begin	End	Coverage	Score	Concordance	Matches	Identities	I/D/M/F*	Stop Codons
ToTV_sRNA1gp1	191	1486	12.7%	1909	97.8%	275 (100%)	269 (97.8%)	0/0/0/0	0
Protein mutations:	S205A (719T>G 721C>T), M206V (722A>G), V291I (977G>A 979T>A), R1143K (3534G>A), A1202T (3710G>A)								
Codon mutations:	GCA196GCG (694A>G), CTA201TTA (707C>T), GGG203GGA (715G>A), GAA204GAG (718A>G), TCC205GCT (719T>G 721C>T), ATG206GTG (722A>G), TCA207TCT (727A>T), CAC208CAT (730C>T), CTT211CTG (739T>G), GTG214GTT (748G>T), GTA215GTT (751A>T), GCA216GCG (754A>G), CTC218TTA (758C>T 760C>A), ATA219ATT (763A>T), GCC220GCT (766C>T), GGG222GGC (772G>C), ATT223ATA (775T>A), TCT224TCA (778T>A), ACC225ACA (781C>A), TTC228TTT (790C>T), GTG229GTA (793G>A), GCT230GCA (796T>A), GTA231GTT (799A>T), CCT232CCG (802T>G), GGT233GGC (805T>C), AGA234CGT (806A>C 808A>T), GGT252GGA (862T>A), GCT253GCC (865T>C), AAT257AAC (877T>C), TTG259CTT (881T>C 883G>T), TTT260TTC (886T>C), ACC261ACA (889C>A), TTC263TTT (895C>T), AGG264AGA (898G>A), AAT265AAC (901T>C), TGT269TGC (913T>C), ATA281ATT (949A>T), ATT284ATA (958T>A), GGT286GGA (964T>A), TTT287TTC (967T>C), CCA290CCT (976A>T), GTT291ATA (977G>A 979T>A), GCT294GCA (988T>A), GGC1125GGT (3481C>T), ACA1126ACT (3484A>T), ACG1134ACA (3508G>A), AGG1143AAG (3534G>A), AGT1144AGC (3538T>C), TCT1158TCC (3580T>C), ATC1159ATT (3583C>T), GAA1168GAG (3610A>G), TAC1173TAT (3625C>T), GTG1174GTA (3628G>A), AAA1179AAG (3643A>G), GAC1190GAT (3676C>T), AGG1193AGA (3685G>A), CTA1199TTA (3701C>T), GCA1202ACA (3710G>A), CGA1211CGT (3739A>T), GGA1215GGG (3751A>G), AAG1216AAA (3754G>A), GCC1394GCT (4288C>T), TTT1395TTC (4291T>C), CAA1396CAG (4294A>G), CTT1401CTG (4309T>G), GTG1402GTT (4312G>T), GAC1404GAT (4318C>T), TAT1406TAC (4324T>C), GGA1407GGT (4327A>T), CCA1413CCT (4345A>T), GAC1414GAT (4348C>T), GCC1417GCG (4357C>G), AGA1418CGA (4358A>C), CTT1421CTA (4369T>A), GCT1425GCA (4381T>A), TCA1426TCC (4384A>C), ATT1431ATC (4399T>C), GTT1436GTG (4414T>G), GGA1441..G (4429A>G), TCA1445TCT (4441A>T), GGA1446GGG (4444A>G), CTC1449CTG (4453C>G), GTT1451GTC (4459T>C), TCT1455TCC (4471T>C), TTT1460TTC (4486T>C), TAT1461TAC (4489T>C), CGT1463AGG (4493C>A 4495T>G), GCA1465GCT (4501A>T), ATT1467ATC (4507T>C), TCT1468TCA (4510T>A), TTG1470CTG (4514T>C), CCA1473CCC (4525A>C), CAT1474CAC (4528T>C), ATA1475ATT (4531A>T), GCT1477GCA (4537T>A), GGG1481GGA (4549G>A), TCT1485TCG (4561T>G)								

Proteins

	Begin	End	Coverage	Score	Concordance	Matches	Identities	I/D/M/F*	Stop Codons
polyprotein (YP_001039627.1)	191	1486	12.7%	1909	97.8%	275 (100%)	269 (97.8%)	0/0/0/0	0
Protein mutations:	S205A (719T>G 721C>T), M206V (722A>G), V291I (977G>A 979T>A), R1143K (3534G>A), A1202T (3710G>A)								
Codon mutations:	GCA196GCG (694A>G), CTA201TTA (707C>T), GGG203GGA (715G>A), GAA204GAG (718A>G), TCC205GCT (719T>G 721C>T), ATG206GTG (722A>G), TCA207TCT (727A>T), CAC208CAT (730C>T), CTT211CTG (739T>G), GTG214GTT (748G>T), GTA215GTT (751A>T), GCA216GCG (754A>G), CTC218TTA (758C>T 760C>A), ATA219ATT (763A>T), GCC220GCT (766C>T), GGG222GGC (772G>C), ATT223ATA (775T>A), TCT224TCA (778T>A), ACC225ACA (781C>A), TTC228TTT (790C>T), GTG229GTA (793G>A), GCT230GCA (796T>A), GTA231GTT (799A>T), CCT232CCG (802T>G), GGT233GGC (805T>C), AGA234CGT (806A>C 808A>T), GGT252GGA (862T>A), GCT253GCC (865T>C), AAT257AAC (877T>C), TTG259CTT (881T>C 883G>T), TTT260TTC (886T>C), ACC261ACA (889C>A), TTC263TTT (895C>T), AGG264AGA (898G>A), AAT265AAC (901T>C), TGT269TGC (913T>C), ATA281ATT (949A>T), ATT284ATA (958T>A), GGT286GGA (964T>A), TTT287TTC (967T>C), CCA290CCT (976A>T), GTT291ATA (977G>A 979T>A), GCT294GCA (988T>A), GGC1125GGT (3481C>T), ACA1126ACT (3484A>T), ACG1134ACA (3508G>A), AGG1143AAG (3534G>A), AGT1144AGC (3538T>C), TCT1158TCC (3580T>C), ATC1159ATT (3583C>T), GAA1168GAG (3610A>G), TAC1173TAT (3625C>T), GTG1174GTA (3628G>A), AAA1179AAG (3643A>G), GAC1190GAT (3676C>T), AGG1193AGA (3685G>A), CTA1199TTA (3701C>T), GCA1202ACA (3710G>A), CGA1211CGT (3739A>T), GGA1215GGG (3751A>G), AAG1216AAA (3754G>A), GCC1394GCT (4288C>T), TTT1395TTC (4291T>C), CAA1396CAG (4294A>G), CTT1401CTG (4309T>G), GTG1402GTT (4312G>T), GAC1404GAT (4318C>T), TAT1406TAC (4324T>C), GGA1407GGT (4327A>T), CCA1413CCT (4345A>T), GAC1414GAT (4348C>T), GCC1417GCG (4357C>G), AGA1418CGA (4358A>C), CTT1421CTA (4369T>A), GCT1425GCA (4381T>A), TCA1426TCC (4384A>C), ATT1431ATC (4399T>C), GTT1436GTG (4414T>G), GGA1441..G (4429A>G), TCA1445TCT (4441A>T), GGA1446GGG (4444A>G), CTC1449CTG (4453C>G), GTT1451GTC (4459T>C), TCT1455TCC (4471T>C), TTT1460TTC (4486T>C), TAT1461TAC (4489T>C), CGT1463AGG (4493C>A 4495T>G), GCA1465GCT (4501A>T), ATT1467ATC (4507T>C), TCT1468TCA (4510T>A), TTG1470CTG (4514T>C), CCA1473CCC (4525A>C), CAT1474CAC (4528T>C), ATA1475ATT (4531A>T), GCT1477GCA (4537T>A), GGG1481GGA (4549G>A), TCT1485TCG (4561T>G)								

*: Inserts / Deletes / Misaligned / Frameshifts

Analysis details

This analysis was performed with panviral2.64

NGS Details (UN24): Torradovirus lycopersici (segment RNA 2)

Assembly

**Coverage Length**	2450 (10 contig(s))
**Depth Of Coverage**	2.4
**Number Of Reads**	46
**Reads Per Million**	0.92 rpm (after QC)
**Ambiguities**	0
**Assembly Method**	de novo + reference guided assembly
**Consensus Caller**	Bcf Tools

Coverage Map

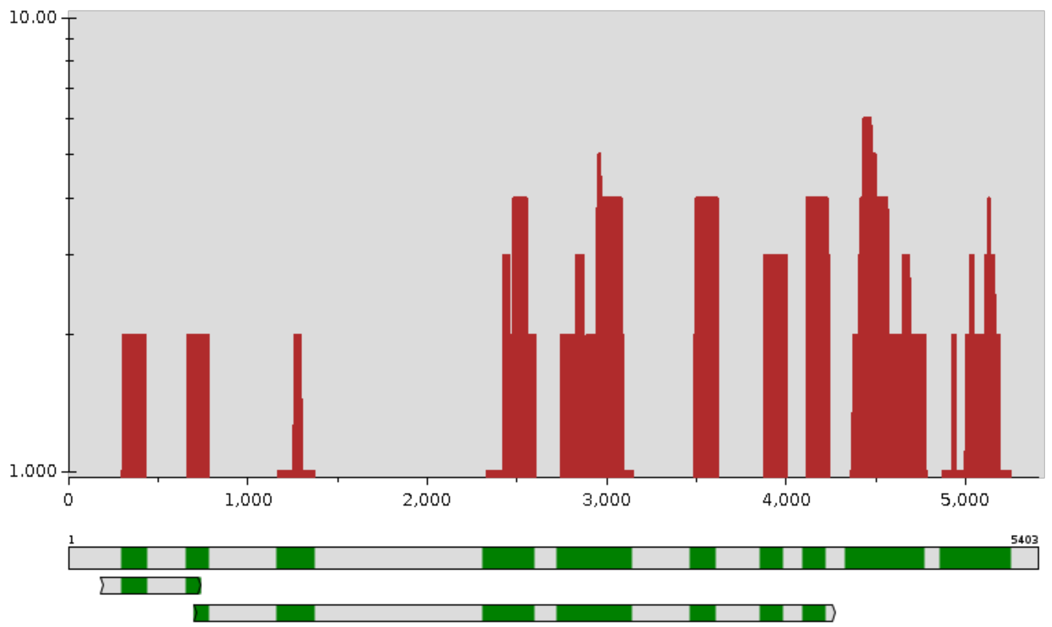

Assignment

**Type**	Torradovirus lycopersici (Taxonomy ID: 3048378)
**Reference Genome**	NC_009032.1
**NT Identity (%)**	85.9457
**AA Identity (%)**	89.9281
**Number Of Stop Codons**	1
**Number Of CDS**	2

Alignment

**Alignment Score**	3470.0 (NT) + 3541.0 (AA) = 7011.0
**Concordance (%)**	80.7998

Alignment Method	Global, seeded, nucleotide + amino acids (AGA)

Genome Region

Sequence starts at position 294 and ends at position 5255 relative to NC_009032.1 reference sequence.

Alignment Detailed Statistics

	Begin	End	Coverage	Score	Concordance	Matches	Identities	I/D/M/F*	Stop Codons
**NT**	**294**	**5255**	**45.3%**	**3470**	**71.6%**	**2439 (98.3%)**	**2122 (85.6%)**	**30/11**	
Mutations:	301A>T, 304T>C, 305C>T, 307A>G, 313C>T, 316G>A, 325C>T, 329A>G, 334T>A, 340A>T, 352A>T, 356G>A, 370C>T, 372A>C, 376C>T, 382A>G, 383C>T, 385G>A, 388T>A, 400T>C, 401T>C, 403A>C, 404C>T, 409A>C, 415A>T, 418T>G, 428A>T, 433C>A, 658C>A, 661A>G, 665T>G, 670A>G, 673A>G, 675G>A, 678T>C, 679G>A, 683T>A, 685G>A, 686T>G, 688C>T, 689A>T, 690A>C, 694A>G, 696T>C, 697A>T, 699G>C, 700T>A, 704G>A, 715C>T, 721C>T, 733A>G, 742G>A, 748A>G, 749A>G, 755A>T, 758T>A, 760T>C, 773A>G, 788A>T, 1166A>G, 1178C>T, 1188A>C, 1196C>T, 1199T>C, 1200G>A, 1201G>A, 1205T>G, 1206C>A, 1208G>A, 1223C>T, 1226A>T, 1232G>T, 1235C>A, 1254G>A, 1255A>G, 1257A>C, 1258T>C, 1259C>T, 1268C>T, 1269A>G, 1271T>A, 1274C>T, 1275T>A, 1278A>G, 1280G>C, 1283G>T, 1286A>C, 1289A>T, 1293T>A, 1294C>G, 1314C>A, 1317C>A, 1325A>G, 1337T>C, 1340A>G, 1346G>A, 1355G>A, 1361A>T, 1362T>G, 1367A>T, 1370A>G, 1373T>C, 1376T>C, 2326C>T, 2336A>T, 2339A>G, 2351C>A, 2354C>T, 2357G>T, 2387T>G, 2393G>A, 2405G>A, 2408A>G, 2414T>C, 2433C>T, 2465T>C, 2501A>C, 2503G>A, 2519A>G, 2573T>C, 2612C>T, 2792C>T, 2819G>A, 2840T>C, 2841C>T, 2885T>C, 2919G>A, 2939A>G, 2968A>C, 2969C>T, 3003T>C, 3050G>A, 3104C>T, 3122T>C, 3128T>C, 3131G>A, 3485C>T, 3488A>G, 3491C>T, 3497T>C, 3503A>G, 3508A>G, 3515G>T, 3522C>T, 3524A>G, 3527C>T, 3530T>A, 3533G>A, 3539C>T, 3540T>C, 3542G>C, 3545T>A, 3555G>A, 3556A>C, 3557A>C, 3560T>C, 3564A>C, 3567T>A, 3569A>G, 3575G>A, 3578A>G, 3581C>T, 3590A>C, 3605A>G, 3611G>A, 3617A>T, 3620G>A, 3623A>T, 3629G>C, 3631C>A, 3878G>C, 3881C>T, 3882A>G, 3884C>A, 3885A>G, 3890A>G, 3893T>C, 3896A>T, 3911T>C, 3914T>A, 3920A>T, 3923C>T, 3926A>C, 3927A>C, 3928A>G, 3929G>A, 3932C>A, 3933A>G, 3935C>T, 3941T>G, 3944C>A, 3947T>C, 3950T>A, 3953T>A, 3960G>A, 3971T>A, 3983T>A, 3984A>G, 3986G>T, 3988G>A, 3989C>T, 3990G>A, 3991A>G, 3995C>T, 3998C>T, 4001T>C, 4112C>G, 4124A>G, 4133A>G, 4142A>C, 4145C>T, 4149C>A, 4151G>A, 4154A>G, 4157C>A, 4166G>A, 4181C>T, 4187G>A, 4188C>G, 4196T>A, 4202T>C, 4205T>A, 4208A>T, 4211A>T, 4214T>C, 4223G>A, 4224C>T, 4225A>T, 4227T>C, 4229G>T, 4232T>C, 4241A>G, 4244G>A, 4354G>A, 4355T>G, 4381A>G, 4382A>C, 4383C>T, 4391A>T, 4392T>C, 4397A>G, 4399T>A, 4410_4411insTAACGGAGA, 4424C>T, 4429T>C, 4447T>A, 4448_4449insT, 4457A>G, 4468T>C, 4496T>A, 4506A>T, 4525T>C, 4567_4568insT, 4605T>C, 4671C>T, 4749T>A, 4888G>T, 4889A>T, 4894A>T, 4896G>A, 4908T>A, 4915G>T, 4916C>T, 4917G>T, 4928A>T, 4930G>A, 4940C>A, 4941C>G, 4943A>T, 4946_4947insC, 4951_4952insGAGCACTGCCATTA, 4955A>C, 4957G>A, 4958A>C, 4959A>C, 4961C>T, 4969A>T, 4972T>G, 4973T>G, 4974T>A, 4976A>T, 4977A>T, 4978G>A, 4993G>A, 4994C>A, 4996A>C, 5000C>T, 5003A>T, 5004G>T, 5019A>T, 5020C>T, 5031T>C, 5032G>A, 5040C>T, 5041A>G, 5053A>G, 5054T>C, 5056G>A, 5099C>T, 5101A>G, 5102T>C, 5107_5108insT, 5109A>C, 5110A>T, 5111A>C, 5121_5122insG, 5122T>A, 5122_5123insG, 5141T>A, 5150A>G, 5185T>A, 5196T>G, 5197T>A, 5198T>G, 5199_5200insC, 5206C>T, 5208G>A, 5211A>G, 5212A>C, 5213A>T, 5214C>T, 5221_5227delACTGCTT, 5233_5236delGTTG, 5241T>A, 5242T>A, 5244G>T, 5246T>A, 5249A>T								

CDS

	Begin	End	Coverage	Score	Concordance	Matches	Identities	I/D/M/F*	Stop Codons
ToTV_sRNA2gp1	39	188	41.5%	490	88.3%	78 (100%)	65 (83.3%)	0/0/0/0	1
Protein mutations:	T50A (329A>G), V59I (356G>A), N64T (372A>C), T83S (428A>T), S162A (665T>G), R165K (675G>A), L166S (678T>C 679G>A), L168I (683T>A 685G>A), S169A (686T>G 688C>T), N170S (689A>T 690A>C), L172P (696T>C 697A>T), S173T (699G>C 700T>A), D175N (704G>A)								
Codon mutations:	CCA40CCT (301A>T), ACT41ACC (304T>C), CTA42TTG (305C>T 307A>G), TTC44TTT (313C>T), AAG45AAA (316G>A), CCC48CCT (325C>T), ACA50GCA (329A>G), GGT51GGA (334T>A), GCA53GCT (340A>T), CTA57CTT (352A>T), GTT59ATT (356G>A), AAC63AAT (370C>T), AAT64ACT (372A>C), AGC65AGT (376C>T), CAA67CAG (382A>G), CTG68TTA (383C>T 385G>A), GTT69GTA (388T>A), TAT73TAC (400T>C), TTA74CTC (401T>C 403A>C), CTG75TTG (404C>T), CGA76CGC (409A>C), GGA78GGT (415A>T), ACT79ACG (418T>G), ACT83TCT (428A>T), GGC84GGA (433C>A), TCC159TCA (658C>A), GAA160GAG (661A>G), TCC162GCC (665T>G), AAA163AAG (670A>G), TTA164TTG (673A>G), AGA165AAA (675G>A), TTG166TCA (678T>C 679G>A), TTG168ATA (683T>A 685G>A), TCC169GCT (686T>G 688C>T), AAC170TCC (689A>T 690A>C), CAA171CAG (694A>G), CTA172CCT (696T>C 697A>T), AGT173ACA (699G>C 700T>A), GAT175AAT (704G>A), TTC178TTT (715C>T), GGC180GGT (721C>T), GAA184GAG (733A>G), AAG187AAA (742G>A)								
ToTV_sRNA2gp2	1	1182	39.9%	3051	91.6%	478 (100%)	435 (91.0%)	0/0/0/0	0
Protein mutations:	M1I (704G>A), S5L (715C>T), A7V (721C>T), K11R (733A>G), S14N (742G>A), K16R (748A>G 749A>G), V20A (760T>C), M163L (1188A>C), G167N (1200G>A 1201G>A), D185S (1254G>A 1255A>G), I186P (1257A>C 1258T>C 1259C>T), I190V (1269A>G 1271T>A), S192T (1275T>A), K193D (1278A>G 1280G>C), Q194H (1283G>T), E195D (1286A>C), H205N (1314C>A), S221A (1362T>G), T542I (2326C>T), E545D (2336A>T), R601K (2503G>A), D740N (2919G>A), Y756S (2968A>C 2969C>T), N936S (3508A>G), E952T (3555G>A 3556A>C 3557A>C), K955Q (3564A>C), L956M (3567T>A 3569A>G), P977Q (3631C>A), I1061V (3882A>G 3884C>A), T1062A (3885A>G), K1076R (3927A>C 3928A>G 3929G>A), T1078A (3933A>G 3935C>T), A1087T (3960G>A), M1095V (3984A>G 3986G>T), S1096N (3988G>A 3989C>T), D1097S (3990G>A 3991A>G), L1150I (4149C>A 4151G>A), Q1163E (4188C>G), H1175F (4224C>T 4225A>T)								
Codon mutations:	ATG1ATA (704G>A), TCA5TTA (715C>T), GCT7GTT (721C>T), AAG11AGG (733A>G), AGT14AAT (742G>A), AAA16AGG (748A>G 749A>G), GCA18GCT (755A>T), GCT19GCA (758T>A), GTA20GCA (760T>C), TTA24TTG (773A>G), ACA29ACT (788A>T), AGA155AGG (1166A>G), TTC159TTT (1178C>T), ATG163CTG (1188A>C), TGC165TGT (1196C>T), CTT166CTC (1199T>C), GGC167AAC (1200G>A 1201G>A), ACT168ACG (1205T>G), CGG169AGA (1206C>A 1208G>A), TGC174TGT (1223C>T), TCA175TCT (1226A>T), GTG177GTT (1232G>T), GCC178GCA (1235C>A), GAT185AGT (1254G>A 1255A>G), ATC186CCT (1257A>C 1258T>C 1259C>T), GCC189GCT (1268C>T), ATT190GTA (1269A>G 1271T>A), TAC191TAT (1274C>T), TCA192ACA (1275T>A), AAG193GAC (1278A>G 1280G>C), CAG194CAT (1283G>T), GAA195GAC (1286A>C), ACA196ACT (1289A>T), TCT198AGT (1293T>A 1294C>G), CAC205AAC (1314C>A), CGA206AGA (1317C>A), CAA208CAG (1325A>G), GAT212GAC (1337T>C), AGA213AGG (1340A>G), GAG215GAA (1346G>A), CAG218CAA (1355G>A), ATA220ATT (1361A>T), TCA221GCA (1362T>G), TCA222TCT (1367A>T), GTA223GTG (1370A>G), TAT224TAC (1373T>C), TCT225TCC (1376T>C), ACA542ATA (2326C>T), GAA545GAT (2336A>T), TTA546TTG (2339A>G), GCC550GCA (2351C>A), TGC551TGT (2354C>T), GTG552GTT (2357G>T), GTT562GTG (2387T>G), GAG564GAA (2393G>A), AAG568AAA (2405G>A), AAA569AAG (2408A>G), AGT571AGC (2414T>C), CTA578TTA (2433C>T), GGT588GGC (2465T>C), CTA600CTC (2501A>C), AGG601AAG (2503G>A), GCA606GCG (2519A>G), GGT624GGC (2573T>C), AAC637AAT (2612C>T), CAC697CAT (2792C>T), GAG706GAA (2819G>A), CAT713CAC (2840T>C), CTG714TTG (2841C>T), CGT728CGC (2885T>C), GAC740AAC (2919G>A), CGA746CGG (2939A>G), TAC756TCT (2968A>C 2969C>T), TTA768CTA (3003T>C), ACG783ACA (3050G>A), GGC801GGT (3104C>T), ATT807ATC (3122T>C), ATT809ATC (3128T>C), GAG810GAA (3131G>A), TAC928..T (3485C>T), GGA929GGG (3488A>G), CAC930CAT (3491C>T), ACT932ACC (3497T>C), GGA934GGG (3503A>G), AAT936AGT (3508A>G), GTG938GTT (3515G>T), CTA941TTG (3522C>T 3524A>G), TTC942TTT (3527C>T), TCT943TCA (3530T>A), GAG944GAA (3533G>A), GAC946GAT (3539C>T), TTG947CTC (3540T>C 3542G>C), TCT948TCA (3545T>A), GAA952ACC (3555G>A 3556A>C 3557A>C), ATT953ATC (3560T>C), AAA955CAA (3564A>C), TTA956ATG (3567T>A 3569A>G), AAG958AAA (3575G>A), GAA959GAG (3578A>G), AAC960AAT (3581C>T), ACA963ACC (3590A>C), GGA968GGG (3605A>G), AAG970AAA (3611G>A), CCA972CCT (3617A>T), CAG973CAA (3620G>A), GTA974GTT (3623A>T), GTG976GTC (3629G>C), CCG977CAG (3631C>A), CTG1059CTC (3878G>C), AAC1060AAT (3881C>T), ATC1061GTA (3882A>G 3884C>A), ACC1062GCC (3885A>G), TTA1063TTG (3890A>G), GAT1064GAC (3893T>C), TCA1065TCT (3896A>T), TTT1070TTC (3911T>C), CCT1071CCA (3914T>A), GGA1073GGT (3920A>T), ATC1074ATT (3923C>T), TCA1075TCC (3926A>C), AAG1076CGA (3927A>C 3928A>G 3929G>A), GGC1077GGA (3932C>A), ACC1078GCT (3933A>G 3935C>T), GTT1080GTG (3941T>G), GTC1081GTA (3944C>A), TCT1082TCC (3947T>C), GCT1083GCA (3950T>A), GGT1084GGA (3953T>A), GCA1087ACA (3960G>A), GCT1090GCA (3971T>A), GGT1094GGA (3983T>A), ATG1095GTT (3984A>G 3986G>T), AGC1096AAT (3988G>A 3989C>T), GAC1097AGC (3990G>A 3991A>G), AAC1098AAT (3995C>T), ATC1099ATT (3998C>T), TTT1100TTC (4001T>C), CTC1137..G (4112C>G), TTA1141TTG (4124A>G), AAA1144AAG (4133A>G), ATA1147ATC (4142A>C), AGC1148AGT (4145C>T), CTG1150ATA (4149C>A 4151G>A), GAA1151GAG (4154A>G), ATC1152ATA (4157C>A), AAG1155AAA (4166G>A), TTC1160TTT (4181C>T), TTG1162TTA (4187G>A), CAA1163GAA (4188C>G), GCT1165GCA (4196T>A), GCT1167GCC (4202T>C), CCT1168CCA (4205T>A), TCA1169TCT (4208A>T), GCA1170GCT (4211A>T), AAT1171AAC (4214T>C), AAG1174AAA (4223G>A), CAT1175TTT (4224C>T 4225A>T), TTG1176CTT (4227T>C 4229G>T), GGT1177GGC (4232T>C), CAA1180CAG (4241A>G), ACG1181ACA (4244G>A)								

Proteins

	Begin	End	Coverage	Score	Concordance	Matches	Identities	I/D/M/F*	Stop Codons
Orf1 (YP_001040017.1)	39	188	41.5%	490	88.3%	78 (100%)	65 (83.3%)	0/0/0/0	1
Protein mutations:	T50A (329A>G), V59I (356G>A), N64T (372A>C), T83S (428A>T), S162A (665T>G), R165K (675G>A), L166S (678T>C 679G>A), L168I (683T>A 685G>A), S169A (686T>G 688C>T), N170S (689A>T 690A>C), L172P (696T>C 697A>T), S173T (699G>C 700T>A), D175N (704G>A)								
Codon mutations:	CCA40CCT (301A>T), ACT41ACC (304T>C), CTA42TTG (305C>T 307A>G), TTC44TTT (313C>T), AAG45AAA (316G>A), CCC48CCT (325C>T), ACA50GCA (329A>G), GGT51GGA (334T>A), GCA53GCT (340A>T), CTA57CTT (352A>T), GTT59ATT (356G>A), AAC63AAT (370C>T), AAT64ACT (372A>C), AGC65AGT (376C>T), CAA67CAG (382A>G), CTG68TTA (383C>T 385G>A), GTT69GTA (388T>A), TAT73TAC (400T>C), TTA74CTC (401T>C 403A>C), CTG75TTG (404C>T), CGA76CGC (409A>C), GGA78GGT (415A>T), ACT79ACG (418T>G), ACT83TCT (428A>T), GGC84GGA (433C>A), TCC159TCA (658C>A), GAA160GAG (661A>G), TCC162GCC (665T>G), AAA163AAG (670A>G), TTA164TTG (673A>G), AGA165AAA (675G>A), TTG166TCA (678T>C 679G>A), TTG168ATA (683T>A 685G>A), TCC169GCT (686T>G 688C>T), AAC170TCC (689A>T 690A>C), CAA171CAG (694A>G), CTA172CCT (696T>C 697A>T), AGT173ACA (699G>C 700T>A), GAT175AAT (704G>A), TTC178TTT (715C>T), GGC180GGT (721C>T), GAA184GAG (733A>G), AAG187AAA (742G>A)								
polyprotein (YP_001040018.1)	1	1182	39.9%	3051	91.6%	478 (100%)	435 (91.0%)	0/0/0/0	0

	Begin	End	Coverage	Score	Concordance	Matches	Identities	I/D/M/F*	Stop Codons
**NT**	**294**	**5255**	**45.3%**	**3470**	**71.6%**	**2439 (98.3%)**	**2122 (85.6%)**	**30/11**	

Protein mutations:	M1I (704G>A), S5L (715C>T), A7V (721C>T), K11R (733A>G), S14N (742G>A), K16R (748A>G 749A>G), V20A (760T>C), M163L (1188A>C), G167N (1200G>A 1201G>A), D185S (1254G>A 1255A>G), I186P (1257A>C 1258T>C 1259C>T), I190V (1269A>G 1271T>A), S192T (1275T>A), K193D (1278A>G 1280G>C), Q194H (1283G>T), E195D (1286A>C), H205N (1314C>A), S221A (1362T>G), T542I (2326C>T), E545D (2336A>T), R601K (2503G>A), D740N (2919G>A), Y756S (2968A>C 2969C>T), N936S (3508A>G), E952T (3555G>A 3556A>C 3557A>C), K955Q (3564A>C), L956M (3567T>A 3569A>G), P977Q (3631C>A), I1061V (3882A>G 3884C>A), T1062A (3885A>G), K1076R (3927A>C 3928A>G 3929G>A), T1078A (3933A>G 3935C>T), A1087T (3960G>A), M1095V (3984A>G 3986G>T), S1096N (3988G>A 3989C>T), D1097S (3990G>A 3991A>G), L1150I (4149C>A 4151G>A), Q1163E (4188C>G), H1175F (4224C>T 4225A>T)
Codon mutations:	ATG1ATA (704G>A), TCA5TTA (715C>T), GCT7GTT (721C>T), AAG11AGG (733A>G), AGT14AAT (742G>A), AAA16AGG (748A>G 749A>G), GCA18GCT (755A>T), GCT19GCA (758T>A), GTA20GCA (760T>C), TTA24TTG (773A>G), ACA29ACT (788A>T), AGA155AGG (1166A>G), TTC159TTT (1178C>T), ATG163CTG (1188A>C), TGC165TGT (1196C>T), CTT166CTC (1199T>C), GGC167AAC (1200G>A 1201G>A), ACT168ACG (1205T>G), CGG169AGA (1206C>A 1208G>A), TGC174TGT (1223C>T), TCA175TCT (1226A>T), GTG177GTT (1232G>T), GCC178GCA (1235C>A), GAT185AGT (1254G>A 1255A>G), ATC186CCT (1257A>C 1258T>C 1259C>T), GCC189GCT (1268C>T), ATT190GTA (1269A>G 1271T>A), TAC191TAT (1274C>T), TCA192ACA (1275T>A), AAG193GAC (1278A>G 1280G>C), CAG194CAT (1283G>T), GAA195GAC (1286A>C), ACA196ACT (1289A>T), TCT198AGT (1293T>A 1294C>G), CAC205AAC (1314C>A), CGA206AGA (1317C>A), CAA208CAG (1325A>G), GAT212GAC (1337T>C), AGA213AGG (1340A>G), GAG215GAA (1346G>A), CAG218CAA (1355G>A), ATA220ATT (1361A>T), TCA221GCA (1362T>G), TCA222TCT (1367A>T), GTA223GTG (1370A>G), TAT224TAC (1373T>C), TCT225TCC (1376T>C), ACA542ATA (2326C>T), GAA545GAT (2336A>T), TTA546TTG (2339A>G), GCC550GCA (2351C>A), TGC551TGT (2354C>T), GTG552GTT (2357G>T), GTT562GTG (2387T>G), GAG564GAA (2393G>A), AAG568AAA (2405G>A), AAA569AAG (2408A>G), AGT571AGC (2414T>C), CTA578TTA (2433C>T), GGT588GGC (2465T>C), CTA600CTC (2501A>C), AGG601AAG (2503G>A), GCA606GCG (2519A>G), GGT624GGC (2573T>C), AAC637AAT (2612C>T), CAC697CAT (2792C>T), GAG706GAA (2819G>A), CAT713CAC (2840T>C), CTG714TTG (2841C>T), CGT728CGC (2885T>C), GAC740AAC (2919G>A), CGA746CGG (2939A>G), TAC756TCT (2968A>C 2969C>T), TTA768CTA (3003T>C), ACG783ACA (3050G>A), GGC801GGT (3104C>T), ATT807ATC (3122T>C), ATT809ATC (3128T>C), GAG810GAA (3131G>A), TAC928..T (3485C>T), GGA929GGG (3488A>G), CAC930CAT (3491C>T), ACT932ACC (3497T>C), GGA934GGG (3503A>G), AAT936AGT (3508A>G), GTG938GTT (3515G>T), CTA941TTG (3522C>T 3524A>G), TTC942TTT (3527C>T), TCT943TCA (3530T>A), GAG944GAA (3533G>A), GAC946GAT (3539C>T), TTG947CTC (3540T>C 3542G>C), TCT948TCA (3545T>A), GAA952ACC (3555G>A 3556A>C 3557A>C), ATT953ATC (3560T>C), AAA955CAA (3564A>C), TTA956ATG (3567T>A 3569A>G), AAG958AAA (3575G>A), GAA959GAG (3578A>G), AAC960AAT (3581C>T), ACA963ACC (3590A>C), GGA968GGG (3605A>G), AAG970AAA (3611G>A), CCA972CCT (3617A>T), CAG973CAA (3620G>A), GTA974GTT (3623A>T), GTG976GTC (3629G>C), CCG977CAG (3631C>A), CTG1059CTC (3878G>C), AAC1060AAT (3881C>T), ATC1061GTA (3882A>G 3884C>A), ACC1062GCC (3885A>G), TTA1063TTG (3890A>G), GAT1064GAC (3893T>C), TCA1065TCT (3896A>T), TTT1070TTC (3911T>C), CCT1071CCA (3914T>A), GGA1073GGT (3920A>T), ATC1074ATT (3923C>T), TCA1075TCC (3926A>C), AAG1076CGA (3927A>C 3928A>G 3929G>A), GGC1077GGA (3932C>A), ACC1078GCT (3933A>G 3935C>T), GTT1080GTG (3941T>G), GTC1081GTA (3944C>A), TCT1082TCC (3947T>C), GCT1083GCA (3950T>A), GGT1084GGA (3953T>A), GCA1087ACA (3960G>A), GCT1090GCA (3971T>A), GGT1094GGA (3983T>A), ATG1095GTT (3984A>G 3986G>T), AGC1096AAT (3988G>A 3989C>T), GAC1097AGC (3990G>A 3991A>G), AAC1098AAT (3995C>T), ATC1099ATT (3998C>T), TTT1100TTC (4001T>C), CTC1137..G (4112C>G), TTA1141TTG (4124A>G), AAA1144AAG (4133A>G), ATA1147ATC (4142A>C), AGC1148AGT (4145C>T), CTG1150ATA (4149C>A 4151G>A), GAA1151GAG (4154A>G), ATC1152ATA (4157C>A), AAG1155AAA (4166G>A), TTC1160TTT (4181C>T), TTG1162TTA (4187G>A), CAA1163GAA (4188C>G), GCT1165GCA (4196T>A), GCT1167GCC (4202T>C), CCT1168CCA (4205T>A), TCA1169TCT (4208A>T), GCA1170GCT (4211A>T), AAT1171AAC (4214T>C), AAG1174AAA (4223G>A), CAT1175TTT (4224C>T 4225A>T), TTG1176CTT (4227T>C 4229G>T), GGT1177GGC (4232T>C), CAA1180CAG (4241A>G), ACG1181ACA (4244G>A)

*: Inserts / Deletes / Misaligned / Frameshifts

Analysis details

This analysis was performed with panviral2.64

NGS Details (UN24): Diachasmimorpha longicaudata entomopoxvirus (segment NC_043455.1)

Assembly

**Coverage Length**	1126 (1 contig(s))
**Depth Of Coverage**	1124.4
**Number Of Reads**	10408
**Reads Per Million**	208.34 rpm (after QC)
**Ambiguities**	0
**Assembly Method**	de novo + reference guided assembly
**Consensus Caller**	Bcf Tools

Coverage Map

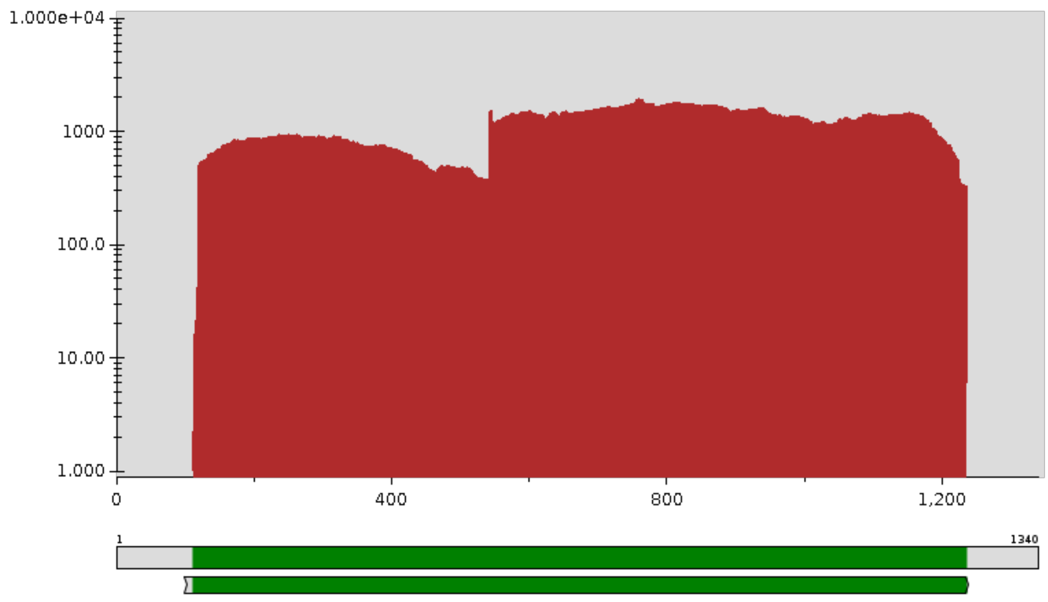

Assignment

**Type**	Diachasmimorpha longicaudata entomopoxvirus (Taxonomy ID: 109981)
**Reference Genome**	NC_043455.1
**NT Identity (%)**	60.2131
**AA Identity (%)**	55.7333
**Number Of Stop Codons**	0
**Number Of CDS**	1

Alignment

**Alignment Score**	442.0 (NT) + 1481.0 (AA) = 1923.0
**Concordance (%)**	40.7934

Alignment Method	Global, seeded, nucleotide + amino acids (AGA)

Genome Region

Sequence starts at position 112 and ends at position 1237 relative to NC_043455.1 reference sequence.

Alignment Detailed Statistics

	Begin	End	Coverage	Score	Concordance	Matches	Identities	I/D/M/F*	Stop Codons
**NT**	**112**	**1237**	**84.0%**	**442**	**19.8%**	**1123 (99.5%)**	**678 (60.1%)**	**3/3**	
Mutations:	121A>T, 126A>C, 127C>T, 128C>G, 130T>G, 136C>G, 137C>A, 139C>T, 142G>A, 143A>G, 145A>T, 146A>G, 148T>C, 149A>C, 151C>T, 154A>T, 156A>G, 157G>A, 160T>G, 163A>T, 166T>C, 167T>C, 168C>A, 169T>G, 171G>A, 172T>C, 175A>G, 178T>C, 187T>A, 190A>T, 191A>G, 193T>C, 203A>C, 204A>G, 208C>T, 209A>G, 211T>A, 214T>G, 217A>T, 218T>A, 219G>T, 223T>A, 226T>G, 230A>C, 231A>G, 232A>T, 238G>C, 241A>T, 243T>C, 244C>T, 247A>G, 256G>C, 259A>T, 262A>C, 271A>C, 272G>T, 274A>C, 276C>T, 277A>G, 278T>A, 279A>T, 283A>T, 284A>C, 286C>T, 288G>C, 289T>C, 294T>G, 295A>C, 298G>A, 299C>G, 300A>T, 301A>T, 302A>G, 304A>G, 310T>C, 311T>A, 312C>A, 313T>A, 320A>G, 322T>G, 323A>G, 325C>A, 331T>A, 337C>A, 341A>T, 343C>T, 346A>T, 352T>G, 355A>G, 356T>C, 358G>T, 361T>A, 362C>G, 363T>C, 364C>T, 368G>A, 370T>G, 371C>G, 374C>A, 375G>A, 376T>G, 379A>G, 380T>A, 382G>T, 383C>T, 384A>T, 385A>G, 386A>G, 394G>A, 395A>G, 400T>C, 401C>A, 403A>T, 404_406delTAT, 409T>C, 410T>A, 412T>A, 413A>C, 415A>G, 416T>G, 417G>C, 418T>A, 421A>T, 423T>C, 424T>G, 438C>A, 442T>C, 443A>G, 445C>G, 446A>G, 447A>G, 448G>T, 452A>G, 453G>A, 454C>T, 455C>A, 456A>T, 457G>C, 458G>A, 459A>G, 462C>A, 463T>G, 467A>G, 469_470insCAT, 470A>G, 471A>G, 474C>T, 475C>A, 478G>A, 482T>G, 484G>C, 485A>T, 486T>C, 500C>A, 503A>G, 505G>C, 506A>T, 507T>G, 511T>C, 512C>A, 514A>G, 515C>A, 517C>T, 519C>A, 520C>A, 521C>A, 523A>G, 525A>G, 528G>C, 530A>T, 532C>A, 533G>C, 534A>G, 535T>A, 540A>G, 541A>G, 544A>C, 547T>C, 550A>G, 553A>G, 554G>T, 559A>T, 560A>T, 562A>G, 574T>C, 577G>A, 581T>C, 583G>C, 584A>T, 585T>C, 586A>T, 587G>A, 588A>G, 589T>A, 590A>G, 591A>G, 595T>C, 596T>A, 597T>A, 602A>C, 604A>G, 607A>T, 608C>T, 610A>T, 616T>A, 620G>C, 622A>G, 623T>C, 628T>G, 629G>C, 630A>C, 632A>C, 633G>C, 634T>A, 635C>A, 637T>A, 638G>A, 646T>G, 647A>G, 648T>G, 650T>G, 652A>T, 655A>C, 658A>T, 661A>C, 664C>T, 665G>A, 667T>G, 671T>C, 674A>G, 675G>A, 678T>C, 680A>C, 682C>T, 683A>G, 685T>G, 687C>T, 688A>T, 689T>A, 692C>A, 693A>G, 694G>A, 695G>A, 696T>A, 697C>G, 700T>C, 705G>A, 706A>C, 707G>A, 709T>G, 715A>G, 717A>G, 718A>G, 724G>T, 727A>G, 730A>G, 731A>C, 732A>G, 735C>A, 739A>G, 740T>C, 742G>C, 745A>T, 748C>T, 754C>T, 759G>A, 765A>T, 770A>G, 772A>C, 778A>C, 779A>G, 781G>T, 784A>G, 785A>G, 787T>A, 790T>G, 792T>G, 793T>G, 796A>G, 797G>C, 798C>T, 799A>T, 810T>G, 820T>C, 823C>G, 824C>A, 825A>C, 826C>T, 829A>G, 830A>G, 831G>C, 833C>A, 838T>C, 841A>G, 843C>G, 844A>T, 845C>G, 847A>C, 850A>C, 854T>G, 855G>T, 859T>C, 862A>T, 863C>A, 865T>G, 866A>C, 869C>A, 878G>T, 879T>G, 880A>G, 885T>C, 886G>T, 889A>T, 890T>A, 891G>A, 892T>G, 893T>A, 895A>G, 896A>C, 897C>G, 898A>C, 900A>G, 901T>C, 902A>C, 904A>T, 905A>G, 908T>C, 909T>A, 915C>T, 919A>T, 920A>G, 921G>C, 922C>A, 924T>C, 925C>T, 934T>C, 938T>G, 939C>A, 943A>G, 944C>A, 946A>C, 947G>A, 948A>C, 949A>T, 950C>A, 952T>A, 956T>A, 958T>C, 965A>C, 966A>G, 967A>G, 970A>G, 973T>C, 974A>C, 977G>T, 978A>C, 982T>C, 983A>T, 984A>C, 986A>T, 989A>C, 991A>T, 992A>G, 994A>G, 998C>A, 1000T>C, 1001T>A, 1007A>G, 1012A>C, 1013T>C, 1015A>T, 1018A>T, 1019A>C, 1021A>T, 1024C>T, 1033T>A, 1036A>G, 1040A>G, 1042A>C, 1045T>C, 1046T>C, 1048A>T, 1051T>C, 1054C>T, 1057T>C, 1063T>C, 1064T>C, 1070C>A, 1071A>C, 1073A>C, 1075T>A, 1076A>C, 1077G>C, 1087T>C, 1088A>C, 1090A>G, 1103A>C, 1105A>T, 1111T>A, 1112A>C, 1120C>A, 1121C>A, 1123A>G, 1126A>G, 1129G>T, 1130A>G, 1141T>C, 1145A>G, 1147A>C, 1150T>C, 1151G>A, 1153A>G, 1156C>T, 1159A>T, 1160T>G, 1161T>A, 1163C>A, 1164C>G, 1165A>G, 1167A>T, 1172G>T, 1173C>T, 1177A>C, 1178T>A, 1181G>C, 1183A>G, 1185C>A, 1186G>A, 1192T>C, 1195T>C, 1196A>G, 1197C>T, 1199A>G, 1200A>T, 1204A>T, 1207T>G, 1210A>G, 1211A>C, 1213G>C, 1218A>C, 1219G>C, 1223A>G, 1227T>C, 1232T>C, 1234G>C, 1235A>C, 1237A>G								
**CDS**									
FLA14_p101	5	379	98.7%	1481	59.6%	374 (99.5%)	209 (55.6%)	1/1/0/0	0
Protein mutations:	E7D (121A>T), D9A (126A>C 127C>T), H10E (128C>G 130T>G), L13I (137C>A 139C>T), K15D (143A>G 145A>T), N16D (146A>G 148T>C), I17L (149A>C 151C>T), K19R (156A>G 157G>A), S23Q (167T>C 168C>A 169T>G), C24Y (171G>A 172T>C), T31A (191A>G 193T>C), K35R (203A>C 204A>G), I37V (209A>G 211T>A), F38L (214T>G), C40I (218T>A 219G>T), K44R (230A>C 231A>G 232A>T), V48A (243T>C 244C>T), A58S (272G>T 274A>C), T59M (276C>T 277A>G), Y60I (278T>A 279A>T), I62L (284A>C 286C>T), S63T (288G>C 289T>C), L65C (294T>G 295A>C), Q67V (299C>G 300A>T 301A>T), I68V (302A>G 304A>G), S71K (311T>A 312C>A 313T>A), N74E (320A>G 322T>G), I75V (323A>G 325C>A), T81S (341A>T 343C>T), L88A (362C>G 363T>C 364C>T), A90T (368G>A 370T>G), Q91E (371C>G), R92K (374C>A 375G>A 376T>G), L94I (380T>A 382G>T), Q95L (383C>T 384A>T 385A>G), T96A (386A>G), N99D (395A>G), L101I (401C>A 403A>T), Y102del (404_406delTAT), F104I (410T>A 412T>A), K105Q (413A>C 415A>G), C106A (416T>G 417G>C 418T>A), Q107H (421A>T), V108A (423T>C 424T>G), T113K (438C>A), I115V (443A>G 445C>G), K116G (446A>G 447A>G 448G>T), S118D (452A>G 453G>A 454C>T), Q119I (455C>A 456A>T 457G>C), E120R (458G>A 459A>G), T121K (462C>A 463T>G), K123E (467A>G), K123_K124insH (469_470insCAT), K124G (470A>G 471A>G), A125V (474C>T 475C>A), L128V (482T>G 484G>C), I129S (485A>T 486T>C), M135V (503A>G 505G>C), I136C (506A>T 507T>G), L138M (512C>A 514A>G), L139I (515C>A 517C>T), T140K (519C>A 520C>A), K142R (525A>G), S143T (528G>C), I144L (530A>T 532C>A), D145R (533G>C 534A>G 535T>A), K147R (540A>G 541A>G), I151M (553A>G), V152F (554G>T), I154L (560A>T 562A>G), I162S (584A>T 585T>C 586A>T), D163R (587G>A 588A>G 589T>A), N164G (590A>G 591A>G), L166K (596T>A 597T>A), K168Q (602A>C 604A>G), Q170Y (608C>T 610A>T), E174Q (620G>C 622A>G), F175L (623T>C), F176L (628T>G), E177P (629G>C 630A>C), S178P (632A>C 633G>C 634T>A), H179K (635C>A 637T>A), V180I (638G>A), I183G (647A>G 648T>G), L184V (650T>G 652A>T), L185F (655A>C), V189M (665G>A 667T>G), S191P (671T>C), R192E (674A>G 675G>A), V193A (678T>C), I194L (680A>C 682C>T), N195E (683A>G 685T>G), T196I (687C>T 688A>T), S197T (689T>A), Q198R (692C>A 693A>G 694G>A), V199K (695G>A 696T>A 697C>G), R202N (705G>A 706A>C), D203K (707G>A 709T>G), K206R (717A>G 718A>G), N211R (731A>C 732A>G), A212D (735C>A), R220K (759G>A), Y222F (765A>T), I224V (770A>G 772A>C), K227D (779A>G 781G>T), N229E (785A>G 787T>A), D230E (790T>G), F231W (792T>G 793T>G), A233L (797G>C 798C>T 799A>T), F237C (810T>G), D241E (823C>G), H242T (824C>A 825A>C 826C>T), S244A (830A>G 831G>C), L245I (833C>A), T248S (843C>G 844A>T), L249V (845C>G 847A>C), C252V (854T>G 855G>T), Q257K (869C>A), V260W (878G>T 879T>G 880A>G), M262T (885T>C 886G>T), E263D (889A>T), C264K (890T>A 891G>A 892T>G), L265M (893T>A 895A>G), T266R (896A>C 897C>G 898A>C), N267S (900A>G 901T>C), N269D (905A>G), F270H (908T>C 909T>A), A272V (915C>T), S274A (920A>G 921G>C 922C>A), I275T (924T>C 925C>T), S280D (938T>G 939C>A), Q282N (944C>A 946A>C), E283T (947G>A 948A>C 949A>T), F286I (956T>A 958T>C), K289R (965A>C 966A>G 967A>G), D293S (977G>T 978A>C), K295S (983A>T 984A>C), T296S (986A>T), I298V (992A>G 994A>G), L300I (998C>A 1000T>C), S301T (1001T>A), N303D (1007A>G), I314V (1040A>G 1042A>C), H324T (1070C>A 1071A>C), N325Q (1073A>C 1075T>A), R326P (1076A>C 1077G>C), I330L (1088A>C 1090A>G), I344V (1130A>G), I349V (1145A>G 1147A>C), E351K (1151G>A 1153A>G), E353D (1159A>T), L354E (1160T>G 1161T>A), P355R (1163C>A 1164C>G 1165A>G), K356M (1167A>T), A358F (1172G>T 1173C>T), E359D (1177A>C), L360I (1178T>A), E361Q (1181G>C 1183A>G), T362K (1185C>A 1186G>A), T366V (1196A>G 1197C>T), K367V (1199A>G 1200A>T), D369E (1207T>G), M371L (1211A>C 1213G>C), E373A (1218A>C 1219G>C), I375V (1223A>G), V376A (1227T>C), I379L (1235A>C 1237A>G)								

	Begin	End	Coverage	Score	Concordance	Matches	Identities	I/D/M/F*	Stop Codons
**NT**	**112**	**1237**	**84.0%**	**442**	**19.8%**	**1123 (99.5%)**	**678 (60.1%)**	**3/3**	

Codon mutations: GAA7GAT (121A>T), GAC9GCT (126A>C 127C>T), CAT10GAG (128C>G 130T>G), GGC12GGG (136C>G), CTC13ATT (137C>A 139C>T), AAG14AAA (142G>A), AAA15GAT (143A>G 145A>T), AAT16GAC (146A>G 148T>C), ATC17CTT (149A>C 151C>T), CTA18CTT (154A>T), AAG19AGA (156A>G 157G>A), GGT20GGG (160T>G), ATA21ATT (163A>T), TAT22TAC (166T>C), TCT23CAG (167T>C 168C>A 169T>G), TGT24TAC (171G>A 172T>C), GGA25GGG (175A>G), TTT26TTC (178T>C), CCT29CCA (187T>A), TCA30TCT (190A>T), ACT31GCC (191A>G 193T>C), AAA35CGA (203A>C 204A>G), GCC36GCT (208C>T), ATT37GTA (209A>G 211T>A), TTT38TTG (214T>G), CCA39CCT (217A>T), TGT40ATT (218T>A 219G>T), ATT41ATA (223T>A), TCT42TCG (226T>G), AAA44CGT (230A>C 231A>G 232A>T), GTG46GTC (238G>C), ATA47ATT (241A>T), GTC48GCT (243T>C 244C>T), CAA49CAG (247A>G), TCG52TCC (256G>C), GGA53GGT (259A>T), ACA54ACC (262A>C), ACA57ACC (271A>C), GCA58TCC (272G>T 274A>C), ACA59ATG (276C>T 277A>G), TAT60ATT (278T>A 279A>T), GCA61GCT (283A>T), ATC62CTT (284A>C 286C>T), AGT63ACC (288G>C 289T>C), TTA65TGC (294T>G 295A>C), CAG66CAA (298G>A), CAA67GTT (299C>G 300A>T 301A>T), ATA68GTG (302A>G 304A>G), ACT70ACC (310T>C), TCT71AAA (311T>A 312C>A 313T>A), AAT74GAG (320A>G 322T>G), ATC75GTA (323A>G 325C>A), GCT77GCA (331T>A), ATC79ATA (337C>A), ACC81TCT (341A>T 343C>T), CCA82CCT (346A>T), CGT84CGG (352T>G), GAA85GAG (355A>G), TTG86CTT (356T>C 358G>T), GCT87GCA (361T>A), CTC88GCT (362C>G 363T>C 364C>T), GCT90ACG (368G>A 370T>G), CAA91GAA (371C>G), CGT92AAG (374C>A 375G>A 376T>G), GTA93GTG (379A>G), TTG94ATT (380T>A 382G>T), CAA95TTG (383C>T 384A>T 385A>G), ACA96GCA (386A>G), GGG98GGA (394G>A), AAT99GAT (395A>G), TAT100TAC (400T>C), CTA101ATT (401C>A 403A>T), TAT102del (404_406delTAT), AAT103AAC (409T>C), TTT104ATA (410T>A 412T>A), AAA105CAG (413A>C 415A>G), TGT106GCA (416T>G 417G>C 418T>A), CAA107CAT (421A>T), GTT108GCG (423T>C 424T>G), ACA113AAA (438C>A), AGT114AGC (442T>C), ATC115GTG (443A>G 445C>G), AAG116GGT (446A>G 447A>G 448G>T), AGC118GAT (452A>G 453G>A 454C>T), CAG119ATC (455C>A 456A>T 457G>C), GAA120AGA (458G>A 459A>G), ACT121AAG (462C>A 463T>G), AAG123GAG (467A>G), AAG123_AAA124insCAT (469_470insCAT), AAA124GGA (470A>G 471A>G), GCC125GTA (474C>T 475C>A), CAG126CAA (478G>A), TTG128GTC (482T>G 484G>C), ATT129TCT (485A>T 486T>C), CGA134AGA (500C>A), ATG135GTC (503A>G 505G>C), ATT136TGT (506A>T 507T>G), GAT137GAC (511T>C), CTA138ATG (512C>A 514A>G), CTC139ATT (515C>A 517C>T), ACC140AAA (519C>A 520C>A), CGA141AGG (521C>A 523A>G), AAA142AGA (525A>G), AGT143ACT (528G>C), ATC144TTA (530A>T 532C>A), GAT145CGA (533G>C 534A>G 535T>A), AAA147AGG (540A>G 541A>G), GCA148GCC (544A>C), ATT149ATC (547T>C), AAA150AAG (550A>G), ATA151ATG (553A>G), GTT152TTT (554G>T), GTA153GTT (559A>T), ATA154TTG (560A>T 562A>G), GAT158GAC (574T>C), GAG159GAA (577G>A), TTG161CTC (581T>C 583G>C), ATA162TCT (584A>T 585T>C 586A>T), GAT163AGA (587G>A 588A>G 589T>A), AAT164GGT (590A>G 591A>G), TTT165TTC (595T>C), TTG166AAG (596T>A 597T>A), AAA168CAG (602A>C 604A>G), ATA169ATT (607A>T), CAA170TAT (608C>T 610A>T), ATT172ATA (616T>A), GAA174CAG (620G>C 622A>G), TTT175CTT (623T>C), TTT176TTG (628T>G), GAA177CCA (629G>C 630A>C), AGT178CCA (632A>C 633G>C 634T>A), CAT179AAA (635C>A 637T>A), GTC180ATC (638G>A), GTT182GTG (646T>G), ATT183GGT (647A>G 648T>G), TTA184GTT (650T>G 652A>T), TTA185TTC (655A>C), TCA186TCT (658A>T), GCA187GCC (661A>C), ACC188ACT (664C>T), GTT189ATG (665G>A 667T>G), TCA191CCA (671T>C), AGG192GAG (674A>G 675G>A), GTT193GCT (678T>C), ATC194CTT (680A>C 682C>T), AAT195GAG (683A>G 685T>G), ACA196ATT (687C>T 688A>T), TCT197ACT (689T>A), CAG198AGA (692C>A 693A>G 694G>A), GTC199AAG (695G>A 696T>A 697C>G), TTT200TTC (700T>C), AGA202AAC (705G>A 706A>C), GAT203AAG (707G>A 709T>G), GTA205GTG (715A>G), AAA206AGG (717A>G 718A>G), CTG208CTT (724G>T), GTA209GTG (727A>G), AAA210AAG (730A>G), AAT211CGT (731A>C 732A>G), GCT212GAT (735C>A), GAA213GAG (739A>G), TTG214CTC (740T>C 742G>C), ACA215ACT (745A>T), CTC216CTT (748C>T), GGC218GGT (754C>T), AGG220AAG (759G>A), TAT222TTT (765A>T), ATA224GTC (770A>G 772A>C), GTA226GTC (778A>C), AAG227GAT (779A>G 781G>T), AAA228AAG (784A>G), AAT229GAA (785A>G 787T>A), GAT230GAG (790T>G), TTT231TGG (792T>G 793T>G), AAA232AAG (796A>G), GCA233CTT (797G>C 798C>T 799A>T), TTT237TGT (810T>G), TAT240TAC (820T>C), GAC241GAG (823C>G), CAC242ACT (824C>A 825A>C 826C>T), TTA243TTG (829A>G), AGC244GCC (830A>G 831G>C), CTC245ATC (833C>A), ACT246ACC (838T>C), CAA247CAG (841A>G), ACA248AGT (843C>G 844A>T), CTA249GTC (845C>G 847A>C), ATA250ATC (850A>C), TGT252GTT (854T>G 855G>T), AAT253AAC (859T>C), ACA254ACT (862A>T), CGT255AGG (863C>A 865T>G), AGA256CGA (866A>C), CAA257AAA (869C>A), GTA260TGG (878G>T 879T>G 880A>G), ATG262ACT (885T>C 886G>T), GAA263GAT (889A>T), TGT264AAG (890T>A 891G>A 892T>G), TTA265ATG (893T>A 895A>G), ACA266CGC (896A>C 897C>G 898A>C), AAT267AGC (900A>G 901T>C), AGA268CGT (902A>C 904A>T), AAT269GAT (905A>G), TTC270CAC (908T>C 909T>A), GCA272GTA (915C>T), TCA273TCT (919A>T), AGC274GCA (920A>G 921G>C 922C>A), ATC275ACT (924T>C 925C>T), GAT278GAC (934T>C), TCC280GAC (938T>G 939C>A), CAA281CAG (943A>G), CAA282AAC (944C>A 946A>C), GAA283ACT (947G>A 948A>C 949A>T), CGT284AGA (950C>A 952T>A), TTT286ATC (956T>A 958T>C), AAA289CGG (965A>C 966A>G 967A>G), GAA290GAG (970A>G), TTT291TTC (973T>C), AGA292CGA (974A>C), GAT293TCT (977G>T 978A>C), GGT294GGC (982T>C), AAA295TCA (983A>T 984A>C), ACT296TCT (986A>T), AGA297CGT (989A>C 991A>T), ATA298GTG (992A>G 994A>G), CTT300ATC (998C>A 1000T>C), TCA301ACA (1001T>A), AAT303GAT (1007A>G), CTA304CTC (1012A>C), TTA305CTT (1013T>C 1015A>T), GCA306GCT (1018A>T), AGA307CGT (1019A>C 1021A>T), GGC308GGT (1024C>T), GTT311GTA (1033T>A), CAA312CAG (1036A>G), ATA314GTC (1040A>G 1042A>C), TCT315TCC (1045T>C), TTA316CTT (1046T>C 1048A>T), GTT317GTC (1051T>C), ATC318ATT (1054C>T), AAT319AAC (1057T>C), GAT321GAC (1063T>C), TTG322CTG (1064T>C), CAT324ACT (1070C>A 1071A>C), AAT325CAA (1073A>C 1075T>A), AGA326CCA (1076A>C 1077G>C), TAT329TAC (1087T>C), ATA330CTG (1088A>C 1090A>G), AGA335CGT (1103A>C 1105A>T), GGT337GGA (1111T>A), AGA338CGA (1112A>C), GGC340GGA (1120C>A), CGA341AGG (1121C>A 1123A>G), AAA342AAG (1126A>G), GGG343GGT (1129G>T), ATT344GTT (1130A>G), AAT347AAC (1141T>C), ATA349GTC (1145A>G 1147A>C), ACT350ACC (1150T>C), GAA351AAG (1151G>A 1153A>G), GAC352GAT (1156C>T), GAA353GAT (1159A>T), TTA354GAA (1160T>G 1161T>A), CCA355AGG (1163C>A 1164C>G 1165A>G), AAG356ATG (1167A>T), GCT358TTT (1172G>T 1173C>T), GAA359GAC (1177A>C), TTA360ATA (1178T>A), GAA361CAG (1181G>C 1183A>G), ACG362AAA (1185C>A 1186G>A), TAT364TAC (1192T>C), AAT365AAC (1195T>C), ACC366GTC (1196A>G 1197C>T), AAA367GTA (1199A>G 1200A>T), ATA368ATT (1204A>T), GAT369GAG (1207T>G), GAA370GAG (1210A>G), ATG371CTC (1211A>C 1213G>C), GAG373GCC (1218A>C 1219G>C), ATT375GTT (1223A>G), GTT376GCT (1227T>C), TTG378CTC (1232T>C 1234G>C), ATA379CTG (1235A>C 1237A>G)

Proteins

	Begin	End	Coverage	Score	Concordance	Matches	Identities	I/D/M/F*	Stop Codons
hypothetical protein (YP_009666148.1)	5	379	98.7%	1481	59.6%	374 (99.5%)	209 (55.6%)	1/1/0/0	0

Protein mutations: E7D (121A>T), D9A (126A>C 127C>T), H10E (128C>G 130T>G), L13I (137C>A 139C>T), K15D (143A>G 145A>T), N16D (146A>G 148T>C), I17L (149A>C 151C>T), K19R (156A>G 157G>A), S23Q (167T>C 168C>A 169T>G), C24Y (171G>A 172T>C), T31A (191A>G 193T>C), K35R (203A>C 204A>G), I37V (209A>G 211T>A), F38L (214T>G), C40I (218T>A 219G>T), K44R (230A>C 231A>G 232A>T), V48A (243T>C 244C>T), A58S (272G>T 274A>C), T59M (276C>T 277A>G), Y60I (278T>A 279A>T), I62L (284A>C 286C>T), S63T (288G>C 289T>C), L65C (294T>G 295A>C), Q67V (299C>G 300A>T 301A>T), I68V (302A>G 304A>G), S71K (311T>A 312C>A 313T>A), N74E (320A>G 322T>G), I75V (323A>G 325C>A), T81S (341A>T 343C>T), L88A (362C>G 363T>C 364C>T), A90T (368G>A 370T>G), Q91E (371C>G), R92K (374C>A 375G>A 376T>G), L94I (380T>A 382G>T), Q95L (383C>T 384A>T 385A>G), T96A (386A>G), N99D (395A>G), L101I (401C>A 403A>T), Y102del (404_406delTAT), F104I (410T>A 412T>A), K105Q (413A>C 415A>G), C106A (416T>G 417G>C 418T>A), Q107H (421A>T), V108A (423T>C 424T>G), T113K (438C>A), I115V (443A>G 445C>G), K116G (446A>G 447A>G 448G>T), S118D (452A>G 453G>A 454C>T), Q119I (455C>A 456A>T 457G>C), E120R (458G>A 459A>G), T121K (462C>A 463T>G), K123E (467A>G), K123_K124insH (469_470insCAT), K124G (470A>G 471A>G), A125V (474C>T 475C>A), L128V (482T>G 484G>C), I129S (485A>T 486T>C), M135V (503A>G 505G>C), I136C (506A>T 507T>G), L138M (512C>A 514A>G), L139I (515C>A 517C>T), T140K (519C>A 520C>A), K142R (525A>G), S143T (528G>C), I144L (530A>T 532C>A), D145R (533G>C 534A>G 535T>A), K147R (540A>G 541A>G), I151M (553A>G), V152F (554G>T), I154L (560A>T 562A>G), I162S (584A>T 585T>C 586A>T), D163R (587G>A 588A>G 589T>A), N164G (590A>G 591A>G), L166K (596T>A 597T>A), K168Q (602A>C 604A>G), Q170Y (608C>T 610A>T), E174Q (620G>C 622A>G), F175L (623T>C), F176L (628T>G), E177P (629G>C 630A>C), S178P (632A>C 633G>C 634T>A), H179K (635C>A 637T>A), V180I (638G>A), I183G (647A>G 648T>G), L184V (650T>G 652A>T), L185F (655A>C), V189M (665G>A 667T>G), S191P (671T>C), R192E (674A>G 675G>A), V193A (678T>C), I194L (680A>C 682C>T), N195E (683A>G 685T>G), T196I (687C>T 688A>T), S197T (689T>A), Q198R (692C>A 693A>G 694G>A), V199K (695G>A 696T>A 697C>G), R202N (705G>A 706A>C), D203K (707G>A 709T>G), K206R (717A>G 718A>G), N211R (731A>C 732A>G), A212D (735C>A), R220K (759G>A), Y222F (765A>T), I224V (770A>G 772A>C), K227D (779A>G 781G>T), N229E (785A>G 787T>A), D230E (790T>G), F231W (792T>G 793T>G), A233L (797G>C 798C>T 799A>T), F237C (810T>G), D241E (823C>G), H242T (824C>A 825A>C 826C>T), S244A (830A>G 831G>C), L245I (833C>A), T248S (843C>G 844A>T), L249V (845C>G 847A>C), C252V (854T>G 855G>T), Q257K (869C>A), V260W (878G>T 879T>G 880A>G), M262T (885T>C 886G>T), E263D (889A>T), C264K (890T>A 891G>A 892T>G), L265M (893T>A 895A>G), T266R (896A>C 897C>G 898A>C), N267S (900A>G 901T>C), N269D (905A>G), F270H (908T>C 909T>A), A272V (915C>T), S274A (920A>G 921G>C 922C>A), I275T (924T>C 925C>T), S280D (938T>G 939C>A), Q282N (944C>A 946A>C), E283T (947G>A 948A>C 949A>T), F286I (956T>A 958T>C), K289R (965A>C 966A>G 967A>G), D293S (977G>T 978A>C), K295S (983A>T 984A>C), T296S (986A>T), I298V (992A>G 994A>G), L300I (998C>A 1000T>C), S301T (1001T>A), N303D (1007A>G), I314V (1040A>G 1042A>C), H324T (1070C>A 1071A>C), N325Q (1073A>C 1075T>A), R326P (1076A>C 1077G>C), I330L (1088A>C 1090A>G), I344V (1130A>G), I349V (1145A>G 1147A>C), E351K (1151G>A 1153A>G), E353D (1159A>T), L354E (1160T>G 1161T>A), P355R (1163C>A 1164C>G 1165A>G), K356M (1167A>T), A358F (1172G>T 1173C>T), E359D (1177A>C), L360I (1178T>A), E361Q (1181G>C 1183A>G), T362K (1185C>A 1186G>A), T366V (1196A>G 1197C>T), K367V (1199A>G 1200A>T), D369E (1207T>G), M371L (1211A>C 1213G>C), E373A (1218A>C 1219G>C), I375V (1223A>G), V376A (1227T>C), I379L (1235A>C 1237A>G)

	Begin	End	Coverage	Score	Concordance	Matches	Identities	I/D/M/F*	Stop Codons
**NT**	**112**	**1237**	**84.0%**	**442**	**19.8%**	**1123 (99.5%)**	**678 (60.1%)**	**3/3**	
Codon mutations:	GAA7GAT (121A>T), GAC9GCT (126A>C 127C>T), CAT10GAG (128C>G 130T>G), GGC12GGG (136C>G), CTC13ATT (137C>A 139C>T), AAG14AAA (142G>A), AAA15GAT (143A>G 145A>T), AAT16GAC (146A>G 148T>C), ATC17CTT (149A>C 151C>T), CTA18CTT (154A>T), AAG19AGA (156A>G 157G>A), GGT20GGG (160T>G), ATA21ATT (163A>T), TAT22TAC (166T>C), TCT23CAG (167T>C 168C>A 169T>G), TGT24TAC (171G>A 172T>C), GGA25GGG (175A>G), TTT26TTC (178T>C), CCT29CCA (187T>A), TCA30TCT (190A>T), ACT31GCC (191A>G 193T>C), AAA35CGA (203A>C 204A>G), GCC36GCT (208C>T), ATT37GTA (209A>G 211T>A), TTT38TTG (214T>G), CCA39CCT (217A>T), TGT40ATT (218T>A 219G>T), ATT41ATA (223T>A), TCT42TCG (226T>G), AAA44CGT (230A>C 231A>G 232A>T), GTG46GTC (238G>C), ATA47ATT (241A>T), GTC48GCT (243T>C 244C>T), CAA49CAG (247A>G), TCG52TCC (256G>C), GGA53GGT (259A>T), ACA54ACC (262A>C), ACA57ACC (271A>C), GCA58TCC (272G>T 274A>C), ACA59ATG (276C>T 277A>G), TAT60ATT (278T>A 279A>T), GCA61GCT (283A>T), ATC62CTT (284A>C 286C>T), AGT63ACC (288G>C 289T>C), TTA65TGC (294T>G 295A>C), CAG66CAA (298G>A), CAA67GTT (299C>G 300A>T 301A>T), ATA68GTG (302A>G 304A>G), ACT70ACC (310T>C), TCT71AAA (311T>A 312C>A 313T>A), AAT74GAG (320A>G 322T>G), ATC75GTA (323A>G 325C>A), GCT77GCA (331T>A), ATC79ATA (337C>A), ACC81TCT (341A>T 343C>T), CCA82CCT (346A>T), CGT84CGG (352T>G), GAA85GAG (355A>G), TTG86CTT (356T>C 358G>T), GCT87GCA (361T>A), CTC88GCT (362C>G 363T>C 364C>T), GCT90ACG (368G>A 370T>G), CAA91GAA (371C>G), CGT92AAG (374C>A 375G>A 376T>G), GTA93GTG (379A>G), TTG94ATT (380T>A 382G>T), CAA95TTG (383C>T 384A>T 385A>G), ACA96GCA (386A>G), GGG98GGA (394G>A), AAT99GAT (395A>G), TAT100TAC (400T>C), CTA101ATT (401C>A 403A>T), TAT102del (404_406delTAT), AAT103AAC (409T>C), TTT104ATA (410T>A 412T>A), AAA105CAG (413A>C 415A>G), TGT106GCA (416T>G 417G>C 418T>A), CAA107CAT (421A>T), GTT108GCG (423T>C 424T>G), ACA113AAA (438C>A), AGT114AGC (442T>C), ATC115GTG (443A>G 445C>G), AAG116GGT (446A>G 447A>G 448G>T), AGC118GAT (452A>G 453G>A 454C>T), CAG119ATC (455C>A 456A>T 457G>C), GAA120AGA (458G>A 459A>G), ACT121AAG (462C>A 463T>G), AAG123GAG (467A>G), AAG123_AAA124insCAT (469_470insCAT), AAA124GGA (470A>G 471A>G), GCC125GTA (474C>T 475C>A), CAG126CAA (478G>A), TTG128GTC (482T>G 484G>C), ATT129TCT (485A>T 486T>C), CGA134AGA (500C>A), ATG135GTC (503A>G 505G>C), ATT136TGT (506A>T 507T>G), GAT137GAC (511T>C), CTA138ATG (512C>A 514A>G), CTC139ATT (515C>A 517C>T), ACC140AAA (519C>A 520C>A), CGA141AGG (521C>A 523A>G), AAA142AGA (525A>G), AGT143ACT (528G>C), ATC144TTA (530A>T 532C>A), GAT145CGA (533G>C 534A>G 535T>A), AAA147AGG (540A>G 541A>G), GCA148GCC (544A>C), ATT149ATC (547T>C), AAA150AAG (550A>G), ATA151ATG (553A>G), GTT152TTT (554G>T), GTA153GTT (559A>T), ATA154TTG (560A>T 562A>G), GAT158GAC (574T>C), GAG159GAA (577G>A), TTG161CTC (581T>C 583G>C), ATA162TCT (584A>T 585T>C 586A>T), GAT163AGA (587G>A 588A>G 589T>A), AAT164GGT (590A>G 591A>G), TTT165TTC (595T>C), TTG166AAG (596T>A 597T>A), AAA168CAG (602A>C 604A>G), ATA169ATT (607A>T), CAA170TAT (608C>T 610A>T), ATT172ATA (616T>A), GAA174CAG (620G>C 622A>G), TTT175CTT (623T>C), TTT176TTG (628T>G), GAA177CCA (629G>C 630A>C), AGT178CCA (632A>C 633G>C 634T>A), CAT179AAA (635C>A 637T>A), GTC180ATC (638G>A), GTT182GTG (646T>G), ATT183GGT (647A>G 648T>G), TTA184GTT (650T>G 652A>T), TTA185TTC (655A>C), TCA186TCT (658A>T), GCA187GCC (661A>C), ACC188ACT (664C>T), GTT189ATG (665G>A 667T>G), TCA191CCA (671T>C), AGG192GAG (674A>G 675G>A), GTT193GCT (678T>C), ATC194CTT (680A>C 682C>T), AAT195GAG (683A>G 685T>G), ACA196ATT (687C>T 688A>T), TCT197ACT (689T>A), CAG198AGA (692C>A 693A>G 694G>A), GTC199AAG (695G>A 696T>A 697C>G), TTT200TTC (700T>C), AGA202AAC (705G>A 706A>C), GAT203AAG (707G>A 709T>G), GTA205GTG (715A>G), AAA206AGG (717A>G 718A>G), CTG208CTT (724G>T), GTA209GTG (727A>G), AAA210AAG (730A>G), AAT211CGT (731A>C 732A>G), GCT212GAT (735C>A), GAA213GAG (739A>G), TTG214CTC (740T>C 742G>C), ACA215ACT (745A>T), CTC216CTT (748C>T), GGC218GGT (754C>T), AGG220AAG (759G>A), TAT222TTT (765A>T), ATA224GTC (770A>G 772A>C), GTA226GTC (778A>C), AAG227GAT (779A>G 781G>T), AAA228AAG (784A>G), AAT229GAA (785A>G 787T>A), GAT230GAG (790T>G), TTT231TGG (792T>G 793T>G), AAA232AAG (796A>G), GCA233CTT (797G>C 798C>T 799A>T), TTT237TGT (810T>G), TAT240TAC (820T>C), GAC241GAG (823C>G), CAC242ACT (824C>A 825A>C 826C>T), TTA243TTG (829A>G), AGC244GCC (830A>G 831G>C), CTC245ATC (833C>A), ACT246ACC (838T>C), CAA247CAG (841A>G), ACA248AGT (843C>G 844A>T), CTA249GTC (845C>G 847A>C), ATA250ATC (850A>C), TGT252GTT (854T>G 855G>T), AAT253AAC (859T>C), ACA254ACT (862A>T), CGT255AGG (863C>A 865T>G), AGA256CGA (866A>C), CAA257AAA (869C>A), GTA260TGG (878G>T 879T>G 880A>G), ATG262ACT (885T>C 886G>T), GAA263GAT (889A>T), TGT264AAG (890T>A 891G>A 892T>G), TTA265ATG (893T>A 895A>G), ACA266CGC (896A>C 897C>G 898A>C), AAT267AGC (900A>G 901T>C), AGA268CGT (902A>C 904A>T), AAT269GAT (905A>G), TTC270CAC (908T>C 909T>A), GCA272GTA (915C>T), TCA273TCT (919A>T), AGC274GCA (920A>G 921G>C 922C>A), ATC275ACT (924T>C 925C>T), GAT278GAC (934T>C), TCC280GAC (938T>G 939C>A), CAA281CAG (943A>G), CAA282AAC (944C>A 946A>C), GAA283ACT (947G>A 948A>C 949A>T), CGT284AGA (950C>A 952T>A), TTT286ATC (956T>A 958T>C), AAA289CGG (965A>C 966A>G 967A>G), GAA290GAG (970A>G), TTT291TTC (973T>C), AGA292CGA (974A>C), GAT293TCT (977G>T 978A>C), GGT294GGC (982T>C), AAA295TCA (983A>T 984A>C), ACT296TCT (986A>T), AGA297CGT (989A>C 991A>T), ATA298GTG (992A>G 994A>G), CTT300ATC (998C>A 1000T>C), TCA301ACA (1001T>A), AAT303GAT (1007A>G), CTA304CTC (1012A>C), TTA305CTT (1013T>C 1015A>T), GCA306GCT (1018A>T), AGA307CGT (1019A>C 1021A>T), GGC308GGT (1024C>T), GTT311GTA (1033T>A), CAA312CAG (1036A>G), ATA314GTC (1040A>G 1042A>C), TCT315TCC (1045T>C), TTA316CTT (1046T>C 1048A>T), GTT317GTC (1051T>C), ATC318ATT (1054C>T), AAT319AAC (1057T>C), GAT321GAC (1063T>C), TTG322CTG (1064T>C), CAT324ACT (1070C>A 1071A>C), AAT325CAA (1073A>C 1075T>A), AGA326CCA (1076A>C 1077G>C), TAT329TAC (1087T>C), ATA330CTG (1088A>C 1090A>G), AGA335CGT (1103A>C 1105A>T), GGT337GGA (1111T>A), AGA338CGA (1112A>C), GGC340GGA (1120C>A), CGA341AGG (1121C>A 1123A>G), AAA342AAG (1126A>G), GGG343GGT (1129G>T), ATT344GTT (1130A>G), AAT347AAC (1141T>C), ATA349GTC (1145A>G 1147A>C), ACT350ACC (1150T>C), GAA351AAG (1151G>A 1153A>G), GAC352GAT (1156C>T), GAA353GAT (1159A>T), TTA354GAA (1160T>G 1161T>A), CCA355AGG (1163C>A 1164C>G 1165A>G), AAG356ATG (1167A>T), GCT358TTT (1172G>T 1173C>T), GAA359GAC (1177A>C), TTA360ATA (1178T>A), GAA361CAG (1181G>C 1183A>G), ACG362AAA (1185C>A 1186G>A), TAT364TAC (1192T>C), AAT365AAC (1195T>C), ACC366GTC (1196A>G 1197C>T), AAA367GTA (1199A>G 1200A>T), ATA368ATT (1204A>T), GAT369GAG (1207T>G), GAA370GAG (1210A>G), ATG371CTC (1211A>C 1213G>C), GAG373GCC (1218A>C 1219G>C), ATT375GTT (1223A>G), GTT376GCT (1227T>C), TTG378CTC (1232T>C 1234G>C), ATA379CTG (1235A>C 1237A>G)								

*: Inserts / Deletes / Misaligned / Frameshifts

Analysis details

This analysis was performed with panviral2.64

NGS Details (UN24): Bracoviriform glomeratae (segment NC_043292.1)

Assembly

**Coverage Length**	310 (1 contig(s))
**Depth Of Coverage**	2222.6
**Number Of Reads**	5765
**Reads Per Million**	115.40 rpm (after QC)
**Ambiguities**	0
**Assembly Method**	de novo + reference guided assembly
**Consensus Caller**	Bcf Tools

Coverage Map

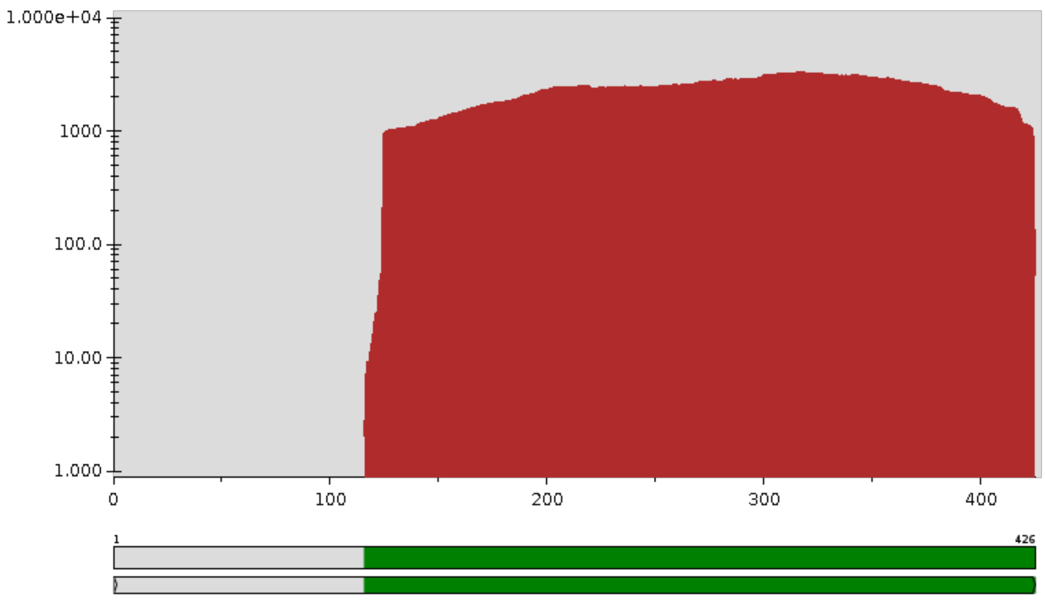

Assignment

**Type**	Bracoviriform glomeratae (Taxonomy ID: 257816)
**Reference Genome**	NC_043292.1
**NT Identity (%)**	72.9032
**AA Identity (%)**	84.466
**Number Of Stop Codons**	1
**Number Of CDS**	1

Alignment

**Alignment Score**	284.0 (NT) + 567.0 (AA) = 851.0
**Concordance (%)**	66.6406

**Alignment Method**	Global, seeded, nucleotide + amino acids (AGA)

Genome Region

Sequence starts at position 117 and ends at position 426 relative to NC_043292.1 reference sequence.

Alignment Detailed Statistics

	Begin	End	Coverage	Score	Concordance	Matches	Identities	I/D/M/F*	Stop Codons
**NT**	**117**	**426**	**72.8%**	**284**	**45.8%**	**310 (100%)**	**226 (72.9%)**	**0/0**	
Mutations:	126G>T, 129C>A, 132A>G, 136A>G, 137C>G, 138G>C, 150A>C, 153A>G, 155C>G, 156G>A, 159C>A, 166C>A, 168T>G, 169T>C, 171T>C, 172C>A, 174C>G, 177A>G, 183T>A, 184C>A, 189T>C, 198A>G, 201A>C, 207C>T, 214A>G, 219C>T, 222T>C, 223C>A, 225T>G, 228G>T, 235C>A, 237T>G, 243A>T, 246C>G, 255C>T, 261A>G, 262T>C, 264A>G, 265G>A, 273A>G, 278T>C, 279T>C, 284A>G, 285T>A, 288T>G, 289T>C, 300C>T, 301C>T, 303C>G, 309C>T, 312T>G, 315C>T, 322G>T, 329T>C, 333T>C, 336C>T, 339A>G, 345A>C, 347A>G, 348A>G, 349C>A, 351T>A, 360C>T, 363C>T, 366C>T, 375C>T, 378C>T, 384A>C, 385T>C, 387G>C, 390A>G, 391C>A, 392A>G, 393T>A, 394A>C, 399C>A, 400C>A, 402T>G, 404T>C, 405G>T, 406A>C, 414T>A, 416A>T, 420A>T								

CDS

	Begin	End	Coverage	Score	Concordance	Matches	Identities	I/D/M/F*	Stop Codons
FK954_p501	40	142	72.5%	567	85.3%	103 (100%)	87 (84.5%)	0/0/0/0	1
Protein mutations:	L42F (126G>T), T46G (136A>G 137C>G 138G>C), A52G (155C>G 156G>A), Y57H (169T>C 171T>C), T72A (214A>G), V89I (265G>A), I93T (278T>C 279T>C), D95G (284A>G 285T>A), A108S (322G>T), I110T (329T>C), K116R (347A>G 348A>G), H131R (391C>A 392A>G 393T>A), K132Q (394A>C), M135T (404T>C 405G>T), I136L (406A>C), Y139F (416A>T)								
Codon mutations:	TTG42TTT (126G>T), GGC43GGA (129C>A), AAA44AAG (132A>G), ACG46GGC (136A>G 137C>G 138G>C), GGA50GGC (150A>C), AAA51AAG (153A>G), GCG52GGA (155C>G 156G>A), GGC53GGA (159C>A), CGT56AGG (166C>A 168T>G), TAT57CAC (169T>C 171T>C), CGC58AGG (172C>A 174C>G), AAA59AAG (177A>G), CTT61CTA (183T>A), CGA62AGA (184C>A), GAT63GAC (189T>C), CAA66CAG (198A>G), GGA67GGC (201A>C), ACC69ACT (207C>T), ACT72GCT (214A>G), ATC73ATT (219C>T), CGT74CGC (222T>C), CGT75AGG (223C>A 225T>G), CTG76CTT (228G>T), CGT79AGG (235C>A 237T>G), GGA81GGT (243A>T), GTC82GTG (246C>G), ATC85ATT (255C>T), GGA87GGG (261A>G), TTA88CTG (262T>C 264A>G), GTC89ATC (265G>A), GAA91GAG (273A>G), ATT93ACC (278T>C 279T>C), GAT95GGA (284A>G 285T>A), GTT96GTG (288T>G), TTG97CTG (289T>C), TTC100TTT (300C>T), CTC101TTG (301C>T 303C>G), AAC103AAT (309C>T), GTT104GTG (312T>G), ATC105ATT (315C>T), GCT108TCT (322G>T), ATC110ACC (329T>C), TAT111TAC (333T>C), ACC112ACT (336C>T), GAA113GAG (339A>G), GCA115GCC (345A>C), AAA116AGG (347A>G 348A>G), CGT117AGA (349C>A 351T>A), GTC120GTT (360C>T), ACC121ACT (363C>T), GCC122GCT (366C>T), GTC125GTT (375C>T), GTC126GTT (378C>T), GCA128GCC (384A>C), TTG129CTC (385T>C 387G>C), AAA130AAG (390A>G), CAT131AGA (391C>A 392A>G 393T>A), AAA132CAA (394A>C), GGC133GGA (399C>A), CGT134AGG (400C>A 402T>G), ATG135ACT (404T>C 405G>T), ATC136CTC (406A>C), GGT138GGA (414T>A), TAT139TTT (416A>T), GGA140GGT (420A>T)								

Proteins

	Begin	End	Coverage	Score	Concordance	Matches	Identities	I/D/M/F*	Stop Codons
putative histone 4 (YP_009665791.1)	40	142	72.5%	567	85.3%	103 (100%)	87 (84.5%)	0/0/0/0	1
Protein mutations:	L42F (126G>T), T46G (136A>G 137C>G 138G>C), A52G (155C>G 156G>A), Y57H (169T>C 171T>C), T72A (214A>G), V89I (265G>A), I93T (278T>C 279T>C), D95G (284A>G 285T>A), A108S (322G>T), I110T (329T>C), K116R (347A>G 348A>G), H131R (391C>A 392A>G 393T>A), K132Q (394A>C), M135T (404T>C 405G>T), I136L (406A>C), Y139F (416A>T)								
Codon mutations:	TTG42TTT (126G>T), GGC43GGA (129C>A), AAA44AAG (132A>G), ACG46GGC (136A>G 137C>G 138G>C), GGA50GGC (150A>C), AAA51AAG (153A>G), GCG52GGA (155C>G 156G>A), GGC53GGA (159C>A), CGT56AGG (166C>A 168T>G), TAT57CAC (169T>C 171T>C), CGC58AGG (172C>A 174C>G), AAA59AAG (177A>G), CTT61CTA (183T>A), CGA62AGA (184C>A), GAT63GAC (189T>C), CAA66CAG (198A>G), GGA67GGC (201A>C), ACC69ACT (207C>T), ACT72GCT (214A>G), ATC73ATT (219C>T), CGT74CGC (222T>C), CGT75AGG (223C>A 225T>G), CTG76CTT (228G>T), CGT79AGG (235C>A 237T>G), GGA81GGT (243A>T), GTC82GTG (246C>G), ATC85ATT (255C>T), GGA87GGG (261A>G), TTA88CTG (262T>C 264A>G), GTC89ATC (265G>A), GAA91GAG (273A>G), ATT93ACC (278T>C 279T>C), GAT95GGA (284A>G 285T>A), GTT96GTG (288T>G), TTG97CTG (289T>C), TTC100TTT (300C>T), CTC101TTG (301C>T 303C>G), AAC103AAT (309C>T), GTT104GTG (312T>G), ATC105ATT (315C>T), GCT108TCT (322G>T), ATC110ACC (329T>C), TAT111TAC (333T>C), ACC112ACT (336C>T), GAA113GAG (339A>G), GCA115GCC (345A>C), AAA116AGG (347A>G 348A>G), CGT117AGA (349C>A 351T>A), GTC120GTT (360C>T), ACC121ACT (363C>T), GCC122GCT (366C>T), GTC125GTT (375C>T), GTC126GTT (378C>T), GCA128GCC (384A>C), TTG129CTC (385T>C 387G>C), AAA130AAG (390A>G), CAT131AGA (391C>A 392A>G 393T>A), AAA132CAA (394A>C), GGC133GGA (399C>A), CGT134AGG (400C>A 402T>G), ATG135ACT (404T>C 405G>T), ATC136CTC (406A>C), GGT138GGA (414T>A), TAT139TTT (416A>T), GGA140GGT (420A>T)								

*: Inserts / Deletes / Misaligned / Frameshifts

Analysis details

This analysis was performed with panviral2.64

NGS Details (UN24): Duamitovirus soch1

Assembly

**Coverage Length**	1876 (2 contig(s))
**Depth Of Coverage**	27.9
**Number Of Reads**	434
**Reads Per Million**	8.69 rpm (after QC)
**Ambiguities**	0
**Assembly Method**	de novo + reference guided assembly
**Consensus Caller**	Bcf Tools

Coverage Map

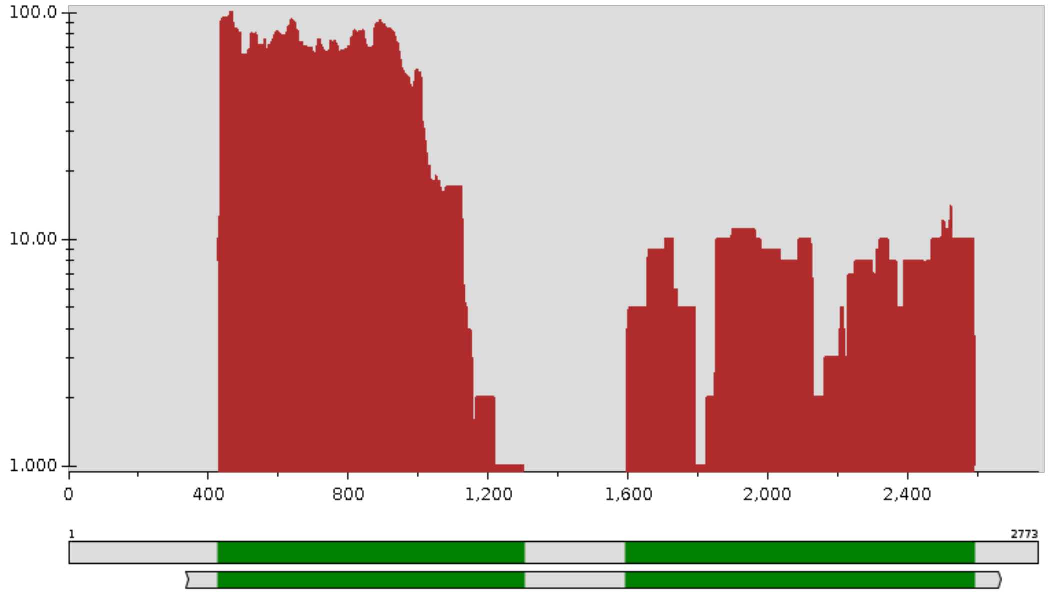

Assignment

**Type**	Duamitovirus soch1 (Taxonomy ID: 2955838)
**Reference Genome**	NC_076524.1
**NT Identity (%)**	68.6065
**AA Identity (%)**	70.1278
**Number Of Stop Codons**	3
**Number Of CDS**	1

Alignment

**Alignment Score**	1364.0 (NT) + 2912.0 (AA) = 4276.0
**Concordance (%)**	53.93

**Alignment Method**	Global, seeded, nucleotide + amino acids (AGA)

Genome Region

Sequence starts at position 428 and ends at position 2593 relative to NC_076524.1 reference sequence.

Alignment Detailed Statistics

	Begin	End	Coverage	Score	Concordance	Matches	Identities	I/D/M/F*	Stop Codons
**NT**	**428**	**2593**	**67.7%**	**1364**	**36.6%**	**1872 (99.7%)**	**1285 (68.5%)**	**1/4**	
Mutations:	437A>G, 441A>C, 443C>T, 447C>T, 459C>T, 466C>T, 470C>T, 471G>A, 472C>T, 473T>G, 477T>A, 480A>C, 481C>A, 482A>T, 485A>G, 489A>G, 494T>C, 497T>A, 500T>G, 503A>C, 506T>C, 509C>T, 512T>C, 513G>A, 516T>A, 517C>A, 518T>A, 520A>C, 524G>T, 525A>G, 526C>T, 527T>G, 529C>A, 530C>A, 531T>C, 533A>G, 534C>A, 535G>A, 536C>A, 539G>A, 540A>C, 541A>G, 542G>T, 545G>A, 548C>T, 550A>T, 556T>A, 557C>T, 560A>T, 569C>T, 575G>A, 581C>T, 582G>A, 587A>T, 589G>C, 591T>C, 593A>T, 599A>T, 602C>T, 612T>A, 613C>G, 614C>T, 616T>A, 618C>T, 619G>C, 620A>T, 624G>A, 626A>T, 629A>C, 630C>A, 632A>C, 633C>A, 635T>G, 638G>T, 641G>T, 644T>A, 647G>T, 651C>T, 653C>G, 656A>C, 657A>C, 661C>G, 662G>T, 665T>A, 668C>T, 672C>A, 673G>A, 674T>G, 680C>A, 683C>T, 684A>G, 685A>C, 686A>C, 692G>A, 695T>A, 699G>C, 700C>A, 701A>C, 702A>G, 705C>A, 707T>G, 708A>G, 709G>C, 710G>A, 713A>G, 714G>T, 715A>C, 719A>T, 722C>T, 737T>C, 746C>T, 752A>T, 761G>A, 765G>T, 767T>A, 770G>A, 771G>T, 772T>A, 773T>G, 774T>A, 776G>T, 777A>G, 778A>C, 779A>G, 782T>G, 796G>A, 797T>G, 803A>C, 809T>C, 810G>C, 815C>A, 818A>T, 821T>G, 824C>T, 827A>T, 831C>T, 833T>A, 841G>A, 842G>T, 850T>G, 851A>T, 857G>T, 860G>T, 862A>G, 869T>G, 875G>T, 880T>G, 881C>A, 885C>A, 887T>G, 892A>G, 905A>T, 908C>A, 909T>C, 910T>A, 916G>A, 918G>T, 919G>C, 923T>A, 926A>T, 927A>C, 929G>T, 934T>A, 935T>G, 938G>T, 942T>A, 943C>A, 948C>A, 971C>A, 972C>T, 975A>C, 976A>T, 977T>C, 983T>C, 990A>C, 992G>C, 993T>C, 994C>G, 995A>G, 997A>T, 998C>T, 999G>C, 1000G>A, 1001T>A, 1003T>A, 1004A>T, 1005G>T, 1007C>G, 1010A>G, 1016C>T, 1017A>G, 1018A>T, 1019A>T, 1022T>A, 1025T>C, 1026C>G, 1027A>G, 1030A>C, 1031G>A, 1034T>G, 1035A>G, 1036G>C, 1044T>G, 1046C>T, 1049G>A, 1050T>G, 1051T>A, 1052T>A, 1054C>A, 1055A>C, 1061T>C, 1067T>G, 1073G>A, 1076A>G, 1083C>A, 1088G>T, 1091C>G, 1099G>T, 1104A>G, 1105C>T, 1106A>G, 1115T>G, 1121A>C, 1122T>A, 1124C>T, 1127G>A, 1130C>T, 1133C>G, 1142A>C, 1145C>T, 1148G>T, 1151G>T, 1152T>C, 1154G>T, 1160C>T, 1161A>C, 1164T>A, 1165C>T, 1166T>G, 1169C>T, 1172C>A, 1174T>A, 1175T>C, 1178T>A, 1181A>T, 1184C>T, 1185A>T, 1186G>C, 1187G>T, 1188G>A, 1189C>A, 1191G>A, 1192C>A, 1193A>T, 1196T>C, 1197_1199delGAA, 1205A>T, 1211C>T, 1212T>G, 1213T>A, 1217T>C, 1220C>T, 1223A>T, 1224C>T, 1225A>T, 1226C>A, 1232G>A, 1236A>C, 1237G>A, 1238T>A, 1241C>A, 1247A>C, 1248T>C, 1249G>T, 1250G>T, 1253T>C, 1256A>T, 1258C>G, 1265G>A, 1266A>G, 1268T>G, 1269C>G, 1271T>C, 1274T>C, 1276G>A, 1280G>A, 1283G>T, 1292T>G, 1596T>A, 1598T>A, 1607T>C, 1613G>T, 1614A>T, 1617C>T, 1619T>G, 1620G>A, 1622T>C, 1623A>G, 1624A>T, 1626A>C, 1627A>C, 1628A>T, 1632T>A, 1634A>G, 1635A>C, 1636C>T, 1637A>C, 1643A>G, 1647T>A, 1648A>G, 1655A>T, 1661T>C, 1662C>T, 1664G>A, 1667T>A, 1676T>G, 1679G>A, 1682C>T, 1684T>A, 1686C>T, 1688C>T, 1691T>A, 1694T>A, 1699C>T, 1700A>T, 1700_1701insC, 1703T>A, 1706C>T, 1707T>G, 1712G>T, 1715T>C, 1721C>T, 1727A>G, 1734T>C, 1739A>T, 1744G>A, 1745C>A, 1746A>T, 1748A>T, 1754T>C, 1758G>A, 1759G>C, 1760A>G, 1761T>A, 1765A>C, 1766C>T, 1767T>A, 1769T>C, 1772A>T, 1777G>C, 1778G>T, 1787T>C, 1788T>C, 1790A>G, 1791C>T, 1819G>C, 1820T>A, 1821A>G, 1823A>G, 1829C>G, 1830A>C, 1831A>G, 1832T>G, 1833G>C, 1840A>G, 1847A>G, 1848T>C, 1856A>G, 1859C>T, 1860A>G, 1861A>G, 1865C>T, 1867A>C, 1871T>C, 1874G>T, 1875delA, 1878C>G, 1880A>T, 1886T>G, 1887T>A, 1888T>G, 1889G>A, 1895T>C, 1898T>G, 1901T>C, 1910T>C, 1919G>T, 1925G>A, 1934T>C, 1937A>G, 1938T>G, 1939C>A, 1940A>C, 1950G>T, 1952T>C, 1953T>C, 1955A>T, 1964T>C, 1967C>T, 1976T>A, 1980T>A, 1981T>C, 1982A>T, 1985T>C, 1989C>A, 1991T>G, 1994T>A, 2000T>G, 2003A>T, 2006T>G, 2009C>T, 2010G>C, 2011C>A, 2012C>A, 2020T>C, 2028G>T, 2029A>C, 2030T>G, 2034A>T, 2035A>C, 2036C>A, 2038T>C, 2042A>G, 2043G>A, 2050A>C, 2051A>C, 2053G>T, 2054A>T, 2057G>T, 2058G>T, 2063T>G, 2069G>A, 2071A>T, 2074A>G, 2075G>A, 2078A>T, 2082G>T, 2084G>T, 2087C>T, 2090A>G, 2091T>A, 2093C>G, 2094A>T, 2099C>T, 2103A>T, 2104G>C, 2105C>T, 2108G>A, 2117G>A, 2118C>A, 2120T>A, 2123A>G, 2129T>G, 2133C>A, 2134A>G, 2135T>G, 2138T>C, 2141G>A, 2146T>G, 2148C>A, 2152C>T, 2158T>C, 2167A>T, 2168C>A, 2173G>T, 2174G>T, 2186G>A, 2191T>A, 2192G>A, 2195C>T, 2204C>T, 2208T>C, 2210G>A, 2213A>G, 2225A>G, 2230A>T, 2231C>T, 2234C>T, 2237T>G, 2239A>G, 2240A>G, 2243A>G, 2250G>C, 2251T>C, 2252T>C, 2264G>T, 2273A>T, 2274A>G, 2276G>A, 2280C>A, 2285G>A, 2288C>T, 2298G>C, 2299T>G, 2300T>G, 2303A>G, 2306A>C, 2309A>T, 2315T>C, 2322C>T, 2326G>T, 2328C>A, 2330A>T, 2336A>G, 2337G>A, 2346T>C, 2348A>T, 2349A>C, 2350A>G, 2355C>G, 2357C>T, 2358A>T, 2359A>C, 2360C>T, 2369T>C, 2372G>T, 2377G>A, 2378T>G, 2382A>T, 2383A>C, 2384C>T, 2385C>T, 2386C>T, 2387A>T, 2390T>A, 2396C>T, 2399T>C, 2400A>G, 2402G>A, 2403A>C, 2404G>A, 2405T>A, 2406C>T, 2408C>G, 2411C>G, 2414C>T, 2415T>G, 2417C>T, 2420T>C, 2423A>T, 2431C>A, 2432A>G, 2435C>A, 2441A>G, 2442A>C, 2444G>A, 2445C>T, 2446T>C, 2447C>G, 2448G>A, 2450A>T, 2451A>G, 2452C>A, 2453T>A, 2456T>C, 2457T>C, 2459T>G, 2460G>A, 2462T>A, 2465A>G, 2466A>G, 2477C>T, 2478C>T, 2489A>G, 2492T>C, 2495C>T, 2504T>A, 2510T>C, 2514A>G, 2518C>T, 2519C>G, 2525C>T, 2528T>A, 2532C>A, 2533A>G, 2536G>A, 2537G>T, 2541T>C, 2543T>C, 2551A>G, 2552C>T, 2555T>C, 2556G>T, 2557T>C, 2561T>C, 2568A>G, 2571A>C, 2572A>G, 2582A>G, 2585G>A								
**CDS**									
RdRp	32	753	80.6%	2912	66.2%	625 (99.7%)	439 (70.0%)	1/1/2/2	3
Protein mutations:	I36L (441A>C 443C>T), L38F (447C>T), A44V (466C>T), A46M (471G>A 472C>T 473T>G), L48M (477T>A), T49H (480A>C 481C>A 482A>T), I52V (489A>G), V60I (513G>A), S61K (516T>A 517C>A 518T>A), K62T (520A>C), T64V (525A>G 526C>T 527T>G), T65K (529C>A 530C>A), R67K (534C>A 535G>A 536C>A), K69R (540A>C 541A>G 542G>T), Y72F (550A>T), F74Y (556T>A 557C>T), A83T (582G>A), C85S (589G>C), F94Y (616T>A), R95S (618C>T 619G>C 620A>T), E97N (624G>A 626A>T), E98D (629A>C), P99T (630C>A 632A>C), L100M (633C>A 635T>G), S109C (661C>G 662G>T), R113K (672C>A 673G>A 674T>G), K117A (684A>G 685A>C 686A>C), A122H (699G>C 700C>A 701A>C), I123V (702A>G), R125A (708A>G 709G>C 710G>A), D127S (714G>T 715A>C), E128D (719A>T), A144S (765G>T 767T>A), V146* (771G>T 772T>A 773T>G), L147I (774T>A 776G>T), K148A (777A>G 778A>C 779A>G), S154K (796G>A 797T>G), E159Q (810G>C), P166S (831C>T 833T>A), G169D (841G>A 842G>T), L172R (850T>G 851A>T), E176G (862A>G), I178M (869T>G), F182* (880T>G 881C>A), H184K (885C>A 887T>G), Q186R (892A>G), F192H (909T>C 910T>A), R194Q (916G>A), G195S (918G>T 919G>C), M198L (927A>C 929G>T), I200K (934T>A 935T>G), S203N (942T>A 943C>A), Q205K (948C>A), N214L (975A>C 976A>T 977T>C), S220R (993T>C 994C>G 995A>G), Y221F (997A>T 998C>T), G222Q (999G>C 1000G>A 1001T>A), L223Y (1003T>A 1004A>T), A224S (1005G>T 1007C>G), K228V (1017A>G 1018A>T 1019A>T), Q231G (1026C>G 1027A>G), E232A (1030A>C 1031G>A), S234A (1035A>G 1036G>C), F237V (1044T>G 1046C>T), F239E (1050T>G 1051T>A 1052T>A), P240H (1054C>A 1055A>C), L250I (1083C>A), W255L (1099G>T), T257V (1104A>G 1105C>T 1106A>G), I260M (1115T>G), F263I (1122T>A 1124C>T), M272I (1151G>T), K276Q (1161A>C), S277M (1164T>A 1165C>T 1166T>G), F279L (1172C>A), F280Y (1174T>A 1175T>C), L282F (1181A>T), R284S (1185A>T 1186G>C 1187G>T), A285N (1188G>A 1189C>A), A286N (1191G>A 1192C>A 1193A>T), E288del (1197_1199delGAA), L290F (1205A>T), L293E (1212T>G 1213T>A), E296D (1223A>T), H297L (1224C>T 1225A>T 1226C>A), S301Q (1236A>C 1237G>A 1238T>A), W305L (1248T>C 1249G>T 1250G>T), T308S (1258C>G), S311G (1266A>G 1268T>G), P312A (1269C>G 1271T>C), R314Q (1276G>A), S421T (1596T>A 1598T>A), I427F (1614A>T), V429I (1620G>A 1622T>C), N430V (1623A>G 1624A>T), K431P (1626A>C 1627A>C 1628A>T), L433M (1632T>A 1634A>G), T434L (1635A>C 1636C>T 1637A>C), Y438S (1647T>A 1648A>G), F450Y (1684T>A), H451Y (1686C>T 1688C>T), A455V (1699C>T 1700A>T), A455_L456insX (1700_1701insC), S458A (1707T>G), L459F (1712G>T), I464M (1727A>G), S470K (1744G>A 1745C>A), K471Y (1746A>T 1748A>T), G475T (1758G>A 1759G>C 1760A>G), C476S (1761T>A), N477T (1765A>C 1766C>T), S478T (1767T>A 1769T>C), G481A (1777G>C 1778G>T), S495T (1819G>C 1820T>A), K496E (1821A>G 1823A>G), N499R (1830A>C 1831A>G 1832T>G), E500Q (1833G>C), K502R (1840A>G), N509G (1860A>G 1861A>G), K511T (1867A>C), P515A (1878C>G 1880A>T), L518R (1887T>A 1888T>G 1889G>A), D521E (1898T>G), S535D (1938T>G 1939C>A 1940A>C), D539Y (1950G>T 1952T>C), L549T (1980T>A 1981T>C 1982A>T), A559Q (2010G>C 2011C>A 2012C>A), I562T (2020T>C), D565S (2028G>T 2029A>C 2030T>G), N567S (2034A>T 2035A>C 2036C>A), I568T (2038T>C), V570I (2043G>A), Q572P (2050A>C 2051A>C), R573I (2053G>T 2054A>T), L574F (2057G>T), G575W (2058G>T), Y579F (2071A>T), K580R (2074A>G 2075G>A), A583S (2082G>T 2084G>T), F586M (2091T>A 2093C>G), T587S (2094A>T), H600R (2133C>A 2134A>G 2135T>G), V604G (2146T>G), S606L (2152C>T), L608S (2158T>C), Y611L (2167A>T 2168C>A), W613F (2173G>T 2174G>T), M619K (2191T>A 2192G>A), Y632F (2230A>T 2231C>T), K635R (2239A>G 2240A>G), V639P (2250G>C 2251T>C 2252T>C), Q643H (2264G>T), K647E (2274A>G 2276G>A), L649M (2280C>A), V655R (2298G>C 2299T>G 2300T>G), R664L (2326G>T), Q665N (2328C>A 2330A>T), E668K (2337G>A), K672R (2349A>C 2350A>G), L674V (2355C>G 2357C>T), N675S (2358A>T 2359A>C 2360C>T), C681* (2377G>A 2378T>G), N683S (2382A>T 2383A>C 2384C>T), P684F (2385C>T 2386C>T 2387A>T), D685E (2390T>A), K689E (2400A>G 2402G>A), S690Q (2403A>C 2404G>A 2405T>A), I692M (2411C>G), S694A (2415T>G 2417C>T), T699K (2431C>A 2432A>G), L704S (2445C>T 2446T>C 2447C>G), E705N (2448G>A 2450A>T), T706E (2451A>G 2452C>A 2453T>A), F708L (2457T>C 2459T>G), D709K (2460G>A 2462T>A), I711V (2466A>G), N727D (2514A>G), P728L (2518C>T 2519C>G), Q733R (2532C>A 2533A>G), W734Y (2536G>A 2537G>T), F736L (2541T>C 2543T>C), N739S (2551A>G 2552C>T), V741S (2556G>T 2557T>C), N745D (2568A>G), K746R (2571A>C 2572A>G)								

	Begin	End	Coverage	Score	Concordance	Matches	Identities	I/D/M/F*	Stop Codons
**NT**	**428**	**2593**	**67.7%**	**1364**	**36.6%**	**1872 (99.7%)**	**1285 (68.5%)**	**1/4**	
Codon mutations:	AAA34AAG (437A>G), ATC36CTT (441A>C 443C>T), CTT38TTT (447C>T), CTA42TTA (459C>T), GCC44GTC (466C>T), TCC45TCT (470C>T), GCT46ATG (471G>A 472C>T 473T>G), TTG48ATG (477T>A), ACA49CAT (480A>C 481C>A 482A>T), AAA50AAG (485A>G), ATC52GTC (489A>G), GCT53GCC (494T>C), CTT54CTA (497T>A), GCT55GCG (500T>G), GGA56GGC (503A>C), TGT57TGC (506T>C), GAC58GAT (509C>T), TTT59TTC (512T>C), GTA60ATA (513G>A), TCT61AAA (516T>A 517C>A 518T>A), AAA62ACA (520A>C), GTG63GTT (524G>T), ACT64GTG (525A>G 526C>T 527T>G), ACC65AAA (529C>A 530C>A), TTA66CTG (531T>C 533A>G), CGC67AAA (534C>A 535G>A 536C>A), CGG68CGA (539G>A), AAG69CGT (540A>C 541A>G 542G>T), TCG70TCA (545G>A), GGC71GGT (548C>T), TAT72TTT (550A>T), TTC74TAT (556T>A 557C>T), ACA75ACT (560A>T), TAC78TAT (569C>T), AAG80AAA (575G>A), TGC82TGT (581C>T), GCA83ACA (582G>A), GTA84GTT (587A>T), TGC85TCC (589G>C), TTA86CTT (591T>C 593A>T), CGA88CGT (599A>T), TAC89TAT (602C>T), TCC93AGT (612T>A 613C>G 614C>T), TTT94TAT (616T>A), CGA95TCT (618C>T 619G>C 620A>T), GAA97AAT (624G>A 626A>T), GAA98GAC (629A>C), CCA99ACC (630C>A 632A>C), CTT100ATG (633C>A 635T>G), TCG101TCT (638G>T), GTG102GTT (641G>T), CCT103CCA (644T>A), GTG104GTT (647G>T), CTC106TTG (651C>T 653C>G), ACA107ACC (656A>C), AGA108CGA (657A>C), TCG109TGT (661C>G 662G>T), GGT110GGA (665T>A), ATC111ATT (668C>T), CGT113AAG (672C>A 673G>A 674T>G), ATC115ATA (680C>A), CCC116CCT (683C>T), AAA117GCC (684A>G 685A>C 686A>C), TTG119TTA (692G>A), CGT120CGA (695T>A), GCA122CAC (699G>C 700C>A 701A>C), ATA123GTA (702A>G), CGT124AGG (705C>A 707T>G), AGG125GCA (708A>G 709G>C 710G>A), AAA126AAG (713A>G), GAT127TCT (714G>T 715A>C), GAA128GAT (719A>T), CAC129CAT (722C>T), GTT134GTC (737T>C), TAC137TAT (746C>T), TCA139TCT (752A>T), GGG142GGA (761G>A), GCT144TCA (765G>T 767T>A), AAG145AAA (770G>A), GTT146TAG (771G>T 772T>A 773T>G), TTG147ATT (774T>A 776G>T), AAA148GCG (777A>G 778A>C 779A>G), GTT149GTG (782T>G), AGT154AAG (796G>A 797T>G), GCA156GCC (803A>C), TTT158TTC (809T>C), GAA159CAA (810G>C), TCC160TCA (815C>A), ATA161ATT (818A>T), ACT162ACG (821T>G), ACC163ACT (824C>T), CCA164CCT (827A>T), CCT166TCA (831C>T 833T>A), GGG169GAT (841G>A 842G>T), CTA172CGT (850T>G 851A>T), GTG174GTT (857G>T), CTG175CTT (860G>T), GAA176GGA (862A>G), ATT178ATG (869T>G), ACG180ACT (875G>T), TTC182TGA (880T>G 881C>A), CAT184AAG (885C>A 887T>G), CAA186CGA (892A>G), CTA190CTT (905A>T), CCC191CCA (908C>A), TTT192CAT (909T>C 910T>A), CGA194CAA (916G>A), GGC195TCC (918G>T 919G>C), ATT196ATA (923T>A), CCA197CCT (926A>T), ATG198CTT (927A>C 929G>T), ATT200AAG (934T>A 935T>G), GGG201GGT (938G>T), TCT203AAT (942T>A 943C>A), CAA205AAA (948C>A), CCC212CCA (971C>A), CTA213TTA (972C>T), AAT214CTC (975A>C 976A>T 977T>C), AAT216AAC (983T>C), AGG219CGC (990A>C 992G>C), TCA220CGG (993T>C 994C>G 995A>G), TAC221TTT (997A>T 998C>T), GGT222CAA (999G>C 1000G>A 1001T>A), TTA223TAT (1003T>A 1004A>T), GCC224TCG (1005G>T 1007C>G), GTA225GTG (1010A>G), GAC227GAT (1016C>T), AAA228GTT (1017A>G 1018A>T 1019A>T), GTT229GTA (1022T>A), GAT230GAC (1025T>C), CAA231GGA (1026C>G 1027A>G), GAG232GCA (1030A>C 1031G>A), GCT233GCG (1034T>G), AGT234GCT (1035A>G 1036G>C), TTC237GTT (1044T>G 1046C>T), AAG238AAA (1049G>A), TTT239GAA (1050T>G 1051T>A 1052T>A), CCA240CAC (1054C>A 1055A>C), ATT242ATC (1061T>C), GTT244GTG (1067T>G), TTG246TTA (1073G>A), AAA247AAG (1076A>G), CTA250ATA (1083C>A), GCG251GCT (1088G>T), GCC252GCG (1091C>G), TGG255TTG (1099G>T), ACA257GTG (1104A>G 1105C>T 1106A>G), ATT260ATG (1115T>G), TCA262TCC (1121A>C), TTC263ATT (1122T>A 1124C>T), CAG264CAA (1127G>A), GAC265GAT (1130C>T), GGC266GGG (1133C>G), TCA269TCC (1142A>C), CCC270CCT (1145C>T), GGG271GGT (1148G>T), ATG272ATT (1151G>T), TTG273CTT (1152T>C 1154G>T), TCC275TCT (1160C>T), AAA276CAA (1161A>C), TCT277ATG (1164T>A 1165C>T 1166T>G), GTC278GTT (1169C>T), TTC279TTA (1172C>A), TTT280TAC (1174T>A 1175T>C), CCT281CCA (1178T>A), TTA282TTT (1181A>T), GAC283GAT (1184C>T), AGG284TCT (1185A>T 1186G>C 1187G>T), GCT285AAT (1188G>A 1189C>A), GCA286AAT (1191G>A 1192C>A 1193A>T), ACT287ACC (1196T>C), GAA288del (1197_1199delGAA), TTA290TTT (1205A>T), AAC292AAT (1211C>T), TTA293GAA (1212T>G 1213T>A), TCT294TCC (1217T>C), CTC295CTT (1220C>T), GAA296GAT (1223A>T), CAC297TTA (1224C>T 1225A>T 1226C>A), GAG299GAA (1232G>A), AGT301CAA (1236A>C 1237G>A 1238T>A), GTC302GTA (1241C>A), CCA304CCC (1247A>C), TGG305CTT (1248T>C 1249G>T 1250G>T), TTT306TTC (1253T>C), TCA307TCT (1256A>T), ACC308AGC (1258C>G), TTG310TTA (1265G>A), AGT311GGG (1266A>G 1268T>G), CCT312GCC (1269C>G 1271T>C), TAT313TAC (1274T>C), CGA314CAA (1276G>A), GGG315GGA (1280G>A), GTG316GTT (1283G>T), GTT319GTG (1292T>G), TCT421ACA (1596T>A 1598T>A), CTT424CTC (1607T>C), ACG426ACT (1613G>T), ATC427TTC (1614A>T), CTT428TTG (1617C>T 1619T>G), GTT429ATC (1620G>A 1622T>C), AAC430GTC (1623A>G 1624A>T), AAA431CCT (1626A>C 1627A>C 1628A>T), TTA433ATG (1632T>A 1634A>G), ACA434CTC (1635A>C 1636C>T 1637A>C), AGA436AGG (1643A>G), TAT438AGT (1647T>A 1648A>G), ATA440ATT (1655A>T), TTT442TTC (1661T>C), CTG443TTA (1662C>T 1664G>A), ACT444ACA (1667T>A), CCT447CCG (1676T>G), TTG448TTA (1679G>A), GGC449GGT (1682C>T), TTC450TAC (1684T>A), CAC451TAT (1686C>T 1688C>T), GGT452GGA (1691T>A), TCT453TCA (1694T>A), GCA455GTT (1699C>T 1700A>T), GCA455_CTT456insC-- (1700_1701insC), CTT456CTA (1703T>A), TTC457TTT (1706C>T), TCG458GCG (1707T>G), TTG459TTT (1712G>T), TCT460TCC (1715T>C), CAC462CAT (1721C>T), ATA464ATG (1727A>G), TTA467CTA (1734T>C), GCA468GCT (1739A>T), AGC470AAA (1744G>A 1745C>A), AAA471TAT (1746A>T 1748A>T), TAT473TAC (1754T>C), GGA475ACG (1758G>A 1759G>C 1760A>G), TGT476AGT (1761T>A), AAC477ACT (1765A>C 1766C>T), TCT478ACC (1767T>A 1769T>C), CCA479CCT (1772A>T), GGG481GCT (1777G>C 1778G>T), GCT484GCC (1787T>C), TTA485CTG (1788T>C 1790A>G), CTA486TTA (1791C>T), AGT495ACA (1819G>C 1820T>A), AAA496GAG (1821A>G 1823A>G), GCC498GCG (1829C>G), AAT499CGG (1830A>C 1831A>G 1832T>G), GAG500CAG (1833G>C), AAG502AGG (1840A>G), TTA504TTG (1847A>G), TTG505CTG (1848T>C), AGA507AGG (1856A>G), CTC508CTT (1859C>T), AAT509GGT (1860A>G 1861A>G), GTC510GTT (1865C>T), AAA511ACA (1867A>C), ATT512ATC (1871T>C), TCG513TCT (1874G>T), ATA514-TA (1875delA), CCA515GCT (1878C>G 1880A>T), TCT517TCG (1886T>G), TTG518AGA (1887T>A 1888T>G 1889G>A), TCT520TCC (1895T>C), GAT521GAG (1898T>G), AAT522AAC (1901T>C), ATT525ATC (1910T>C), GCG528GCT (1919G>T), AGG530AGA (1925G>A), ACT533ACC (1934T>C), AAA534AAG (1937A>G), TCA535GAC (1938T>G 1939C>A 1940A>C), GAT539TAC (1950G>T 1952T>C), TTA540CTT (1953T>C 1955A>T), ATT543ATC (1964T>C), TCC544TCT (1967C>T), GCT547GCA (1976T>A), TTA549ACT (1980T>A 1981T>C 1982A>T), TCT550TCC (1985T>C), CGT552AGG (1989C>A 1991T>G), ACT553ACA (1994T>A), GTT555GTG (2000T>G), GGA556GGT (2003A>T), CTT557CTG (2006T>G), TGC558TGT (2009C>T), GCC559CAA (2010G>C 2011C>A 2012C>A), ATC562ACC (2020T>C), GAT565TCG (2028G>T 2029A>C 2030T>G), AAC567TCA (2034A>T 2035A>C 2036C>A), ATA568ACA (2038T>C), TCA569TCG (2042A>G), GTT570ATT (2043G>A), CAA572CCC (2050A>C 2051A>C), AGA573ATT (2053G>T 2054A>T), TTG574TTT (2057G>T), GGG575TGG (2058G>T), GGT576GGG (2063T>G), GGG578GGA (2069G>A), TAC579TTC (2071A>T), AAG580AGA (2074A>G 2075G>A), GTA581GTT (2078A>T), GCG583TCT (2082G>T 2084G>T), CGC584CGT (2087C>T), TTA585TTG (2090A>G), TTC586ATG (2091T>A 2093C>G), ACT587TCT (2094A>T), ACC588ACT (2099C>T), AGC590TCT (2103A>T 2104G>C 2105C>T), AAG591AAA (2108G>A), GAG594GAA (2117G>A), CGT595AGA (2118C>A 2120T>A), CTA596CTG (2123A>G), GCT598GCG (2129T>G), CAT600AGG (2133C>A 2134A>G 2135T>G), CTT601CTC (2138T>C), AAG602AAA (2141G>A), GTT604GGT (2146T>G), CGG605AGG (2148C>A), TCA606TTA (2152C>T), TTA608TCA (2158T>C), TAC611TTA (2167A>T 2168C>A), TGG613TTT (2173G>T 2174G>T), AGG617AGA (2186G>A), ATG619AAA (2191T>A 2192G>A), CCC620CCT (2195C>T), CCC623CCT (2204C>T), TTG625CTA (2208T>C 2210G>A), AAA626AAG (2213A>G), GTA630GTG (2225A>G), TAC632TTT (2230A>T 2231C>T), CTC633CTT (2234C>T), CTT634CTG (2237T>G), AAA635AGG (2239A>G 2240A>G), GAA636GAG (2243A>G), GTT639CCC (2250G>C 2251T>C 2252T>C), CAG643CAT (2264G>T), CCA646CCT (2273A>T), AAG647GAA (2274A>G 2276G>A), CTG649ATG (2280C>A), GTG650GTA (2285G>A), TTC651TTT (2288C>T), GTT655CGG (2298G>C 2299T>G 2300T>G), GAA656GAG (2303A>G), ATA657ATC (2306A>C), CTA658CTT (2309A>T), CGT660CGC (2315T>C), CTA663TTA (2322C>T), CGC664CTC (2326G>T), CAA665AAT (2328C>A 2330A>T), GTA667GTG (2336A>G), GAA668AAA (2337G>A), TTA671CTT (2346T>C 2348A>T), AAG672CGG (2349A>C 2350A>G), CTC674GTT (2355C>G 2357C>T), AAC675TCT (2358A>T 2359A>C 2360C>T), CAT678CAC (2369T>C), ACG679ACT (2372G>T), TGT681TAG (2377G>A 2378T>G), AAC683TCT (2382A>T 2383A>C 2384C>T), CCA684TTT (2385C>T 2386C>T 2387A>T), GAT685GAA (2390T>A), TCC687TCT (2396C>T), ATT688ATC (2399T>C), AAG689GAA (2400A>G 2402G>A), AGT690CAA (2403A>C 2404G>A 2405T>A), CTC691TTG (2406C>T 2408C>G), ATC692ATG (2411C>G), GAC693GAT (2414C>T), TCC694GCT (2415T>G 2417C>T), CCT695CCC (2420T>C), ATA696ATT (2423A>T), ACA699AAG (2431C>A 2432A>G), TCC700TCA (2435C>A), AAA702AAG (2441A>G), AGG703CGA (2442A>C 2444G>A), CTC704TCG (2445C>T 2446T>C 2447C>G), GAA705AAT (2448G>A 2450A>T), ACT706GAA (2451A>G 2452C>A 2453T>A), GAT707GAC (2456T>C), TTT708CTG (2457T>C 2459T>G), GAT709AAA (2460G>A 2462T>A), TTA710TTG (2465A>G), ATC711GTC (2466A>G), GGC714GGT (2477C>T), CTA715TTA (2478C>T), AAA718AAG (2489A>G), TGT719TGC (2492T>C), TAC720TAT (2495C>T), GGT723GGA (2504T>A), GGT725GGC (2510T>C), AAT727GAT (2514A>G), CCC728CTG (2518C>T 2519C>G), ACC730ACT (2525C>T), ACT731ACA (2528T>A), CAG733AGG (2532C>A 2533A>G), TGG734TAT (2536G>A 2537G>T), TTT736CTC (2541T>C 2543T>C), AAC739AGT (2551A>G 2552C>T), ACT740ACC (2555T>C), GTT741TCT (2556G>T 2557T>C), ATT742ATC (2561T>C), AAT745GAT (2568A>G), AAA746CGA (2571A>C 2572A>G), TTA749TTG (2582A>G), GGG750GGA (2585G>A)								

Proteins

	Begin	End	Coverage	Score	Concordance	Matches	Identities	I/D/M/F*	Stop Codons
RNA-dependent RNA polymerase (YP_010798874.1)	32	753	80.6%	2912	66.2%	625 (99.7%)	439 (70.0%)	1/1/2/2	3
Protein mutations:	I36L (441A>C 443C>T), L38F (447C>T), A44V (466C>T), A46M (471G>A 472C>T 473T>G), L48M (477T>A), T49H (480A>C 481C>A 482A>T), I52V (489A>G), V60I (513G>A), S61K (516T>A 517C>A 518T>A), K62T (520A>C), T64V (525A>G 526C>T 527T>G), T65K (529C>A 530C>A), R67K (534C>A 535G>A 536C>A), K69R (540A>C 541A>G 542G>T), Y72F (550A>T), F74Y (556T>A 557C>T), A83T (582G>A), C85S (589G>C), F94Y (616T>A), R95S (618C>T 619G>C 620A>T), E97N (624G>A 626A>T), E98D (629A>C), P99T (630C>A 632A>C), L100M (633C>A 635T>G), S109C (661C>G 662G>T), R113K (672C>A 673G>A 674T>G), K117A (684A>G 685A>C 686A>C), A122H (699G>C 700C>A 701A>C), I123V (702A>G), R125A (708A>G 709G>C 710G>A), D127S (714G>T 715A>C), E128D (719A>T), A144S (765G>T 767T>A), V146* (771G>T 772T>A 773T>G), L147I (774T>A 776G>T), K148A (777A>G 778A>C 779A>G), S154K (796G>A 797T>G), E159Q (810G>C), P166S (831C>T 833T>A), G169D (841G>A 842G>T), L172R (850T>G 851A>T), E176G (862A>G), I178M (869T>G), F182* (880T>G 881C>A), H184K (885C>A 887T>G), Q186R (892A>G), F192H (909T>C 910T>A), R194Q (916G>A), G195S (918G>T 919G>C), M198L (927A>C 929G>T), I200K (934T>A 935T>G), S203N (942T>A 943C>A), Q205K (948C>A), N214L (975A>C 976A>T 977T>C), S220R (993T>C 994C>G 995A>G), Y221F (997A>T 998C>T), G222Q (999G>C 1000G>A 1001T>A), L223Y (1003T>A 1004A>T), A224S (1005G>T 1007C>G), K228V (1017A>G 1018A>T 1019A>T), Q231G (1026C>G 1027A>G), E232A (1030A>C 1031G>A), S234A (1035A>G 1036G>C), F237V (1044T>G 1046C>T), F239E (1050T>G 1051T>A 1052T>A), P240H (1054C>A 1055A>C), L250I (1083C>A), W255L (1099G>T), T257V (1104A>G 1105C>T 1106A>G), I260M (1115T>G), F263I (1122T>A 1124C>T), M272I (1151G>T), K276Q (1161A>C), S277M (1164T>A 1165C>T 1166T>G), F279L (1172C>A), F280Y (1174T>A 1175T>C), L282F (1181A>T), R284S (1185A>T 1186G>C 1187G>T), A285N (1188G>A 1189C>A), A286N (1191G>A 1192C>A 1193A>T), E288del (1197_1199delGAA), L290F (1205A>T), L293E (1212T>G 1213T>A), E296D (1223A>T), H297L (1224C>T 1225A>T 1226C>A), S301Q (1236A>C 1237G>A 1238T>A), W305L (1248T>C 1249G>T 1250G>T), T308S (1258C>G), S311G (1266A>G 1268T>G), P312A (1269C>G 1271T>C), R314Q (1276G>A), S421T (1596T>A 1598T>A), I427F (1614A>T), V429I (1620G>A 1622T>C), N430V (1623A>G 1624A>T), K431P (1626A>C 1627A>C 1628A>T), L433M (1632T>A 1634A>G), T434L (1635A>C 1636C>T 1637A>C), Y438S (1647T>A 1648A>G), F450Y (1684T>A), H451Y (1686C>T 1688C>T), A455V (1699C>T 1700A>T), A455_L456insX (1700_1701insC), S458A (1707T>G), L459F (1712G>T), I464M (1727A>G), S470K (1744G>A 1745C>A), K471Y (1746A>T 1748A>T), G475T (1758G>A 1759G>C 1760A>G), C476S (1761T>A), N477T (1765A>C 1766C>T), S478T (1767T>A 1769T>C), G481A (1777G>C 1778G>T), S495T (1819G>C 1820T>A), K496E (1821A>G 1823A>G), N499R (1830A>C 1831A>G 1832T>G), E500Q (1833G>C), K502R (1840A>G), N509G (1860A>G 1861A>G), K511T (1867A>C), P515A (1878C>G 1880A>T), L518R (1887T>A 1888T>G 1889G>A), D521E (1898T>G), S535D (1938T>G 1939C>A 1940A>C), D539Y (1950G>T 1952T>C), L549T (1980T>A 1981T>C 1982A>T), A559Q (2010G>C 2011C>A 2012C>A), I562T (2020T>C), D565S (2028G>T 2029A>C 2030T>G), N567S (2034A>T 2035A>C 2036C>A), I568T (2038T>C), V570I (2043G>A), Q572P (2050A>C 2051A>C), R573I (2053G>T 2054A>T), L574F (2057G>T), G575W (2058G>T), Y579F (2071A>T), K580R (2074A>G 2075G>A), A583S (2082G>T 2084G>T), F586M (2091T>A 2093C>G), T587S (2094A>T), H600R (2133C>A 2134A>G 2135T>G), V604G (2146T>G), S606L (2152C>T), L608S (2158T>C), Y611L (2167A>T 2168C>A), W613F (2173G>T 2174G>T), M619K (2191T>A 2192G>A), Y632F (2230A>T 2231C>T), K635R (2239A>G 2240A>G), V639P (2250G>C 2251T>C 2252T>C), Q643H (2264G>T), K647E (2274A>G 2276G>A), L649M (2280C>A), V655R (2298G>C 2299T>G 2300T>G), R664L (2326G>T), Q665N (2328C>A 2330A>T), E668K (2337G>A), K672R (2349A>C 2350A>G), L674V (2355C>G 2357C>T), N675S (2358A>T 2359A>C 2360C>T), C681* (2377G>A 2378T>G), N683S (2382A>T 2383A>C 2384C>T), P684F (2385C>T 2386C>T 2387A>T), D685E (2390T>A), K689E (2400A>G 2402G>A), S690Q (2403A>C 2404G>A 2405T>A), I692M (2411C>G), S694A (2415T>G 2417C>T), T699K (2431C>A 2432A>G), L704S (2445C>T 2446T>C 2447C>G), E705N (2448G>A 2450A>T), T706E (2451A>G 2452C>A 2453T>A), F708L (2457T>C 2459T>G), D709K (2460G>A 2462T>A), I711V (2466A>G), N727D (2514A>G), P728L (2518C>T 2519C>G), Q733R (2532C>A 2533A>G), W734Y (2536G>A 2537G>T), F736L (2541T>C 2543T>C), N739S (2551A>G 2552C>T), V741S (2556G>T 2557T>C), N745D (2568A>G), K746R (2571A>C 2572A>G)								

	Begin	End	Coverage	Score	Concordance	Matches	Identities	I/D/M/F*	Stop Codons
**NT**	**428**	**2593**	**67.7%**	**1364**	**36.6%**	**1872 (99.7%)**	**1285 (68.5%)**	**1/4**	
Codon mutations:	AAA34AAG (437A>G), ATC36CTT (441A>C 443C>T), CTT38TTT (447C>T), CTA42TTA (459C>T), GCC44GTC (466C>T), TCC45TCT (470C>T), GCT46ATG (471G>A 472C>T 473T>G), TTG48ATG (477T>A), ACA49CAT (480A>C 481C>A 482A>T), AAA50AAG (485A>G), ATC52GTC (489A>G), GCT53GCC (494T>C), CTT54CTA (497T>A), GCT55GCG (500T>G), GGA56GGC (503A>C), TGT57TGC (506T>C), GAC58GAT (509C>T), TTT59TTC (512T>C), GTA60ATA (513G>A), TCT61AAA (516T>A 517C>A 518T>A), AAA62ACA (520A>C), GTG63GTT (524G>T), ACT64GTG (525A>G 526C>T 527T>G), ACC65AAA (529C>A 530C>A), TTA66CTG (531T>C 533A>G), CGC67AAA (534C>A 535G>A 536C>A), CGG68CGA (539G>A), AAG69CGT (540A>C 541A>G 542G>T), TCG70TCA (545G>A), GGC71GGT (548C>T), TAT72TTT (550A>T), TTC74TAT (556T>A 557C>T), ACA75ACT (560A>T), TAC78TAT (569C>T), AAG80AAA (575G>A), TGC82TGT (581C>T), GCA83ACA (582G>A), GTA84GTT (587A>T), TGC85TCC (589G>C), TTA86CTT (591T>C 593A>T), CGA88CGT (599A>T), TAC89TAT (602C>T), TCC93AGT (612T>A 613C>G 614C>T), TTT94TAT (616T>A), CGA95TCT (618C>T 619G>C 620A>T), GAA97AAT (624G>A 626A>T), GAA98GAC (629A>C), CCA99ACC (630C>A 632A>C), CTT100ATG (633C>A 635T>G), TCG101TCT (638G>T), GTG102GTT (641G>T), CCT103CCA (644T>A), GTG104GTT (647G>T), CTC106TTG (651C>T 653C>G), ACA107ACC (656A>C), AGA108CGA (657A>C), TCG109TGT (661C>G 662G>T), GGT110GGA (665T>A), ATC111ATT (668C>T), CGT113AAG (672C>A 673G>A 674T>G), ATC115ATA (680C>A), CCC116CCT (683C>T), AAA117GCC (684A>G 685A>C 686A>C), TTG119TTA (692G>A), CGT120CGA (695T>A), GCA122CAC (699G>C 700C>A 701A>C), ATA123GTA (702A>G), CGT124AGG (705C>A 707T>G), AGG125GCA (708A>G 709G>C 710G>A), AAA126AAG (713A>G), GAT127TCT (714G>T 715A>C), GAA128GAT (719A>T), CAC129CAT (722C>T), GTT134GTC (737T>C), TAC137TAT (746C>T), TCA139TCT (752A>T), GGG142GGA (761G>A), GCT144TCA (765G>T 767T>A), AAG145AAA (770G>A), GTT146TAG (771G>T 772T>A 773T>G), TTG147ATT (774T>A 776G>T), AAA148GCG (777A>G 778A>C 779A>G), GTT149GTG (782T>G), AGT154AAG (796G>A 797T>G), GCA156GCC (803A>C), TTT158TTC (809T>C), GAA159CAA (810G>C), TCC160TCA (815C>A), ATA161ATT (818A>T), ACT162ACG (821T>G), ACC163ACT (824C>T), CCA164CCT (827A>T), CCT166TCA (831C>T 833T>A), GGG169GAT (841G>A 842G>T), CTA172CGT (850T>G 851A>T), GTG174GTT (857G>T), CTG175CTT (860G>T), GAA176GGA (862A>G), ATT178ATG (869T>G), ACG180ACT (875G>T), TTC182TGA (880T>G 881C>A), CAT184AAG (885C>A 887T>G), CAA186CGA (892A>G), CTA190CTT (905A>T), CCC191CCA (908C>A), TTT192CAT (909T>C 910T>A), CGA194CAA (916G>A), GGC195TCC (918G>T 919G>C), ATT196ATA (923T>A), CCA197CCT (926A>T), ATG198CTT (927A>C 929G>T), ATT200AAG (934T>A 935T>G), GGG201GGT (938G>T), TCT203AAT (942T>A 943C>A), CAA205AAA (948C>A), CCC212CCA (971C>A), CTA213TTA (972C>T), AAT214CTC (975A>C 976A>T 977T>C), AAT216AAC (983T>C), AGG219CGC (990A>C 992G>C), TCA220CGG (993T>C 994C>G 995A>G), TAC221TTT (997A>T 998C>T), GGT222CAA (999G>C 1000G>A 1001T>A), TTA223TAT (1003T>A 1004A>T), GCC224TCG (1005G>T 1007C>G), GTA225GTG (1010A>G), GAC227GAT (1016C>T), AAA228GTT (1017A>G 1018A>T 1019A>T), GTT229GTA (1022T>A), GAT230GAC (1025T>C), CAA231GGA (1026C>G 1027A>G), GAG232GCA (1030A>C 1031G>A), GCT233GCG (1034T>G), AGT234GCT (1035A>G 1036G>C), TTC237GTT (1044T>G 1046C>T), AAG238AAA (1049G>A), TTT239GAA (1050T>G 1051T>A 1052T>A), CCA240CAC (1054C>A 1055A>C), ATT242ATC (1061T>C), GTT244GTG (1067T>G), TTG246TTA (1073G>A), AAA247AAG (1076A>G), CTA250ATA (1083C>A), GCG251GCT (1088G>T), GCC252GCG (1091C>G), TGG255TTG (1099G>T), ACA257GTG (1104A>G 1105C>T 1106A>G), ATT260ATG (1115T>G), TCA262TCC (1121A>C), TTC263ATT (1122T>A 1124C>T), CAG264CAA (1127G>A), GAC265GAT (1130C>T), GGC266GGG (1133C>G), TCA269TCC (1142A>C), CCC270CCT (1145C>T), GGG271GGT (1148G>T), ATG272ATT (1151G>T), TTG273CTT (1152T>C 1154G>T), TCC275TCT (1160C>T), AAA276CAA (1161A>C), TCT277ATG (1164T>A 1165C>T 1166T>G), GTC278GTT (1169C>T), TTC279TTA (1172C>A), TTT280TAC (1174T>A 1175T>C), CCT281CCA (1178T>A), TTA282TTT (1181A>T), GAC283GAT (1184C>T), AGG284TCT (1185A>T 1186G>C 1187G>T), GCT285AAT (1188G>A 1189C>A), GCA286AAT (1191G>A 1192C>A 1193A>T), ACT287ACC (1196T>C), GAA288del (1197_1199delGAA), TTA290TTT (1205A>T), AAC292AAT (1211C>T), TTA293GAA (1212T>G 1213T>A), TCT294TCC (1217T>C), CTC295CTT (1220C>T), GAA296GAT (1223A>T), CAC297TTA (1224C>T 1225A>T 1226C>A), GAG299GAA (1232G>A), AGT301CAA (1236A>C 1237G>A 1238T>A), GTC302GTA (1241C>A), CCA304CCC (1247A>C), TGG305CTT (1248T>C 1249G>T 1250G>T), TTT306TTC (1253T>C), TCA307TCT (1256A>T), ACC308AGC (1258C>G), TTG310TTA (1265G>A), AGT311GGG (1266A>G 1268T>G), CCT312GCC (1269C>G 1271T>C), TAT313TAC (1274T>C), CGA314CAA (1276G>A), GGG315GGA (1280G>A), GTG316GTT (1283G>T), GTT319GTG (1292T>G), TCT421ACA (1596T>A 1598T>A), CTT424CTC (1607T>C), ACG426ACT (1613G>T), ATC427TTC (1614A>T), CTT428TTG (1617C>T 1619T>G), GTT429ATC (1620G>A 1622T>C), AAC430GTC (1623A>G 1624A>T), AAA431CCT (1626A>C 1627A>C 1628A>T), TTA433ATG (1632T>A 1634A>G), ACA434CTC (1635A>C 1636C>T 1637A>C), AGA436AGG (1643A>G), TAT438AGT (1647T>A 1648A>G), ATA440ATT (1655A>T), TTT442TTC (1661T>C), CTG443TTA (1662C>T 1664G>A), ACT444ACA (1667T>A), CCT447CCG (1676T>G), TTG448TTA (1679G>A), GGC449GGT (1682C>T), TTC450TAC (1684T>A), CAC451TAT (1686C>T 1688C>T), GGT452GGA (1691T>A), TCT453TCA (1694T>A), GCA455GTT (1699C>T 1700A>T), GCA455_CTT456insC-- (1700_1701insC), CTT456CTA (1703T>A), TTC457TTT (1706C>T), TCG458GCG (1707T>G), TTG459TTT (1712G>T), TCT460TCC (1715T>C), CAC462CAT (1721C>T), ATA464ATG (1727A>G), TTA467CTA (1734T>C), GCA468GCT (1739A>T), AGC470AAA (1744G>A 1745C>A), AAA471TAT (1746A>T 1748A>T), TAT473TAC (1754T>C), GGA475ACG (1758G>A 1759G>C 1760A>G), TGT476AGT (1761T>A), AAC477ACT (1765A>C 1766C>T), TCT478ACC (1767T>A 1769T>C), CCA479CCT (1772A>T), GGG481GCT (1777G>C 1778G>T), GCT484GCC (1787T>C), TTA485CTG (1788T>C 1790A>G), CTA486TTA (1791C>T), AGT495ACA (1819G>C 1820T>A), AAA496GAG (1821A>G 1823A>G), GCC498GCG (1829C>G), AAT499CGG (1830A>C 1831A>G 1832T>G), GAG500CAG (1833G>C), AAG502AGG (1840A>G), TTA504TTG (1847A>G), TTG505CTG (1848T>C), AGA507AGG (1856A>G), CTC508CTT (1859C>T), AAT509GGT (1860A>G 1861A>G), GTC510GTT (1865C>T), AAA511ACA (1867A>C), ATT512ATC (1871T>C), TCG513TCT (1874G>T), ATA514-TA (1875delA), CCA515GCT (1878C>G 1880A>T), TCT517TCG (1886T>G), TTG518AGA (1887T>A 1888T>G 1889G>A), TCT520TCC (1895T>C), GAT521GAG (1898T>G), AAT522AAC (1901T>C), ATT525ATC (1910T>C), GCG528GCT (1919G>T), AGG530AGA (1925G>A), ACT533ACC (1934T>C), AAA534AAG (1937A>G), TCA535GAC (1938T>G 1939C>A 1940A>C), GAT539TAC (1950G>T 1952T>C), TTA540CTT (1953T>C 1955A>T), ATT543ATC (1964T>C), TCC544TCT (1967C>T), GCT547GCA (1976T>A), TTA549ACT (1980T>A 1981T>C 1982A>T), TCT550TCC (1985T>C), CGT552AGG (1989C>A 1991T>G), ACT553ACA (1994T>A), GTT555GTG (2000T>G), GGA556GGT (2003A>T), CTT557CTG (2006T>G), TGC558TGT (2009C>T), GCC559CAA (2010G>C 2011C>A 2012C>A), ATC562ACC (2020T>C), GAT565TCG (2028G>T 2029A>C 2030T>G), AAC567TCA (2034A>T 2035A>C 2036C>A), ATA568ACA (2038T>C), TCA569TCG (2042A>G), GTT570ATT (2043G>A), CAA572CCC (2050A>C 2051A>C), AGA573ATT (2053G>T 2054A>T), TTG574TTT (2057G>T), GGG575TGG (2058G>T), GGT576GGG (2063T>G), GGG578GGA (2069G>A), TAC579TTC (2071A>T), AAG580AGA (2074A>G 2075G>A), GTA581GTT (2078A>T), GCG583TCT (2082G>T 2084G>T), CGC584CGT (2087C>T), TTA585TTG (2090A>G), TTC586ATG (2091T>A 2093C>G), ACT587TCT (2094A>T), ACC588ACT (2099C>T), AGC590TCT (2103A>T 2104G>C 2105C>T), AAG591AAA (2108G>A), GAG594GAA (2117G>A), CGT595AGA (2118C>A 2120T>A), CTA596CTG (2123A>G), GCT598GCG (2129T>G), CAT600AGG (2133C>A 2134A>G 2135T>G), CTT601CTC (2138T>C), AAG602AAA (2141G>A), GTT604GGT (2146T>G), CGG605AGG (2148C>A), TCA606TTA (2152C>T), TTA608TCA (2158T>C), TAC611TTA (2167A>T 2168C>A), TGG613TTT (2173G>T 2174G>T), AGG617AGA (2186G>A), ATG619AAA (2191T>A 2192G>A), CCC620CCT (2195C>T), CCC623CCT (2204C>T), TTG625CTA (2208T>C 2210G>A), AAA626AAG (2213A>G), GTA630GTG (2225A>G), TAC632TTT (2230A>T 2231C>T), CTC633CTT (2234C>T), CTT634CTG (2237T>G), AAA635AGG (2239A>G 2240A>G), GAA636GAG (2243A>G), GTT639CCC (2250G>C 2251T>C 2252T>C), CAG643CAT (2264G>T), CCA646CCT (2273A>T), AAG647GAA (2274A>G 2276G>A), CTG649ATG (2280C>A), GTG650GTA (2285G>A), TTC651TTT (2288C>T), GTT655CGG (2298G>C 2299T>G 2300T>G), GAA656GAG (2303A>G), ATA657ATC (2306A>C), CTA658CTT (2309A>T), CGT660CGC (2315T>C), CTA663TTA (2322C>T), CGC664CTC (2326G>T), CAA665AAT (2328C>A 2330A>T), GTA667GTG (2336A>G), GAA668AAA (2337G>A), TTA671CTT (2346T>C 2348A>T), AAG672CGG (2349A>C 2350A>G), CTC674GTT (2355C>G 2357C>T), AAC675TCT (2358A>T 2359A>C 2360C>T), CAT678CAC (2369T>C), ACG679ACT (2372G>T), TGT681TAG (2377G>A 2378T>G), AAC683TCT (2382A>T 2383A>C 2384C>T), CCA684TTT (2385C>T 2386C>T 2387A>T), GAT685GAA (2390T>A), TCC687TCT (2396C>T), ATT688ATC (2399T>C), AAG689GAA (2400A>G 2402G>A), AGT690CAA (2403A>C 2404G>A 2405T>A), CTC691TTG (2406C>T 2408C>G), ATC692ATG (2411C>G), GAC693GAT (2414C>T), TCC694GCT (2415T>G 2417C>T), CCT695CCC (2420T>C), ATA696ATT (2423A>T), ACA699AAG (2431C>A 2432A>G), TCC700TCA (2435C>A), AAA702AAG (2441A>G), AGG703CGA (2442A>C 2444G>A), CTC704TCG (2445C>T 2446T>C 2447C>G), GAA705AAT (2448G>A 2450A>T), ACT706GAA (2451A>G 2452C>A 2453T>A), GAT707GAC (2456T>C), TTT708CTG (2457T>C 2459T>G), GAT709AAA (2460G>A 2462T>A), TTA710TTG (2465A>G), ATC711GTC (2466A>G), GGC714GGT (2477C>T), CTA715TTA (2478C>T), AAA718AAG (2489A>G), TGT719TGC (2492T>C), TAC720TAT (2495C>T), GGT723GGA (2504T>A), GGT725GGC (2510T>C), AAT727GAT (2514A>G), CCC728CTG (2518C>T 2519C>G), ACC730ACT (2525C>T), ACT731ACA (2528T>A), CAG733AGG (2532C>A 2533A>G), TGG734TAT (2536G>A 2537G>T), TTT736CTC (2541T>C 2543T>C), AAC739AGT (2551A>G 2552C>T), ACT740ACC (2555T>C), GTT741TCT (2556G>T 2557T>C), ATT742ATC (2561T>C), AAT745GAT (2568A>G), AAA746CGA (2571A>C 2572A>G), TTA749TTG (2582A>G), GGG750GGA (2585G>A)								

*: Inserts / Deletes / Misaligned / Frameshifts

Analysis details

This analysis was performed with panviral2.64

NGS Details (UN24): Colombian datura virus

Assembly

**Coverage Length**	5854 (11 contig(s))
**Depth Of Coverage**	5.7
**Number Of Reads**	252
**Reads Per Million**	5.04 rpm (after QC)
**Ambiguities**	0
**Assembly Method**	de novo + reference guided assembly
**Consensus Caller**	Bcf Tools

Coverage Map

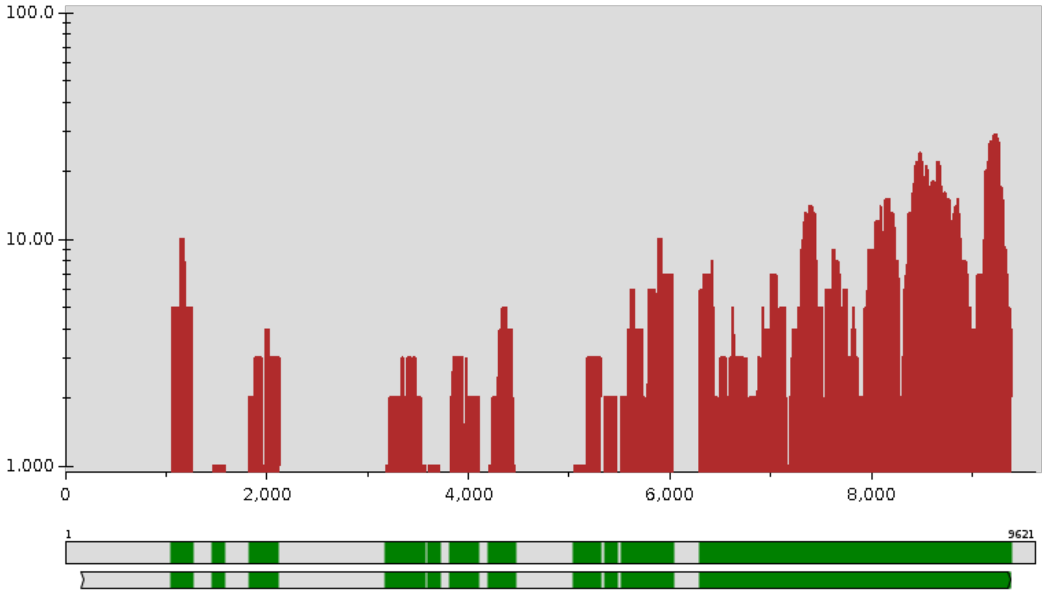

Assignment

**Type**	Colombian datura virus (Taxonomy ID: 91613)
**Reference Genome**	NC_020072.1
**NT Identity (%)**	76.3496
**AA Identity (%)**	85.0
**Number Of Stop Codons**	1
**Number Of CDS**	1

Alignment

**Alignment Score**	6095.0 (NT) + 11399.0 (AA) = 17494.0
**Concordance (%)**	71.3983

Alignment Method	Global, seeded, nucleotide + amino acids (AGA)

Genome Region

Sequence starts at position 1045 and ends at position 9396 relative to NC_020072.1 reference sequence.

Alignment Detailed Statistics

	Begin	End	Coverage	Score	Concordance	Matches	Identities	I/D/M/F*	Stop Codons
**NT**	**1045**	**9396**	**60.8%**	**6095**	**52.5%**	**5835 (99.7%)**	**4455 (76.1%)**	**0/19**	
Mutations:	1045T>A, 1048T>C, 1052T>A, 1057T>C, 1063T>C, 1064C>T, 1066T>A, 1072C>T, 1079A>G, 1080A>C, 1081G>A, 1084T>C, 1087T>C, 1090A>T, 1099A>G, 1102T>A, 1105A>G, 1118C>T, 1120G>A, 1129T>C, 1132A>G, 1138C>T, 1142G>A, 1144G>A, 1147A>G, 1149G>A, 1156A>T, 1157A>T, 1159T>A, 1160G>A, 1186G>C, 1189T>A, 1192T>C, 1193T>C, 1194T>A, 1198T>C, 1201T>A, 1207C>T, 1210G>T, 1216A>G, 1219G>A, 1225G>A, 1226T>C, 1237A>G, 1243C>G, 1255C>T, 1257C>T, 1258A>T, 1463C>A, 1465G>A, 1468C>T, 1471G>A, 1477C>A, 1489T>C, 1490C>T, 1492T>G, 1504T>A, 1505C>T, 1506T>G, 1507A>T, 1508T>C, 1510A>T, 1511T>C, 1516G>A, 1520T>A, 1522T>A, 1523T>A, 1524T>C, 1526A>T, 1529A>G, 1530G>A, 1540G>A, 1546T>C, 1547G>A, 1556A>G, 1562T>C, 1564G>A, 1567T>G, 1571G>A, 1576A>T, 1577C>A, 1583T>C, 1585G>C, 1588C>T, 1823A>C, 1825A>C, 1828A>G, 1829T>C, 1831A>T, 1834A>G, 1840C>A, 1844T>C, 1846G>T, 1847A>G, 1849T>G, 1868T>A, 1870T>A, 1871C>T, 1873T>A, 1876G>A, 1888C>T, 1891G>T, 1894G>A, 1896A>G, 1900A>T, 1901A>G, 1902C>A, 1903A>G, 1904G>T, 1910C>G, 1912C>A, 1933C>T, 1937C>A, 1939T>A, 1945T>C, 1951C>T, 1952T>C, 1954A>T, 1957C>T, 1966C>T, 1975T>A, 1987C>G, 1994T>C, 1996G>T, 1999A>T, 2006T>C, 2008G>T, 2020C>A, 2026G>A, 2029T>C, 2030C>A, 2032T>A, 2041T>C, 2045T>A, 2047T>C, 2050T>A, 2053T>C, 2054G>T, 2056A>T, 2059A>G, 2062T>C, 2065A>T, 2068T>C, 2069C>T, 2071T>A, 2077G>C, 2078G>T, 2079A>C, 2081A>T, 2082T>C, 2084A>G, 2085G>A, 2086T>A, 2087G>C, 2089A>G, 2092G>A, 2097A>T, 2098C>T, 2110A>G, 2113A>T, 3172T>A, 3174G>A, 3177T>A, 3181C>A, 3184C>T, 3186A>G, 3189A>C, 3190A>G, 3199A>T, 3200G>A, 3201C>T, 3203G>C, 3208T>A, 3211T>C, 3214T>C, 3215A>G, 3232A>G, 3241G>A, 3244T>G, 3245T>C, 3249G>C, 3257C>T, 3259G>A, 3263C>T, 3265A>G, 3268C>A, 3274C>A, 3280A>T, 3286T>G, 3289T>C, 3295C>T, 3299G>A, 3301G>C, 3309G>A, 3310G>A, 3313T>C, 3320G>A, 3328G>A, 3336A>C, 3337C>A, 3338A>C, 3340G>T, 3343A>T, 3347G>A, 3348C>T, 3352C>T, 3361A>G, 3362A>G, 3367T>G, 3373C>T, 3376C>T, 3385C>T, 3391C>T, 3395A>G, 3397T>A, 3400T>G, 3401C>A, 3403T>G, 3404T>C, 3405T>C, 3406A>C, 3409G>C, 3412T>A, 3416T>G, 3418T>A, 3421A>G, 3426G>A, 3428A>G, 3429G>A, 3430C>G, 3431A>C, 3432A>G, 3433G>A, 3434G>A, 3436G>C, 3439C>T, 3440T>C, 3445T>A, 3446G>A, 3448T>C, 3450C>A, 3452C>G, 3453A>C, 3454G>T, 3457A>C, 3460C>G, 3461A>G, 3463G>T, 3467A>G, 3469T>G, 3470G>A, 3475T>C, 3481T>C, 3482C>T, 3483A>G, 3484G>C, 3492A>G, 3493A>T, 3496T>G, 3497A>C, 3498C>A, 3499T>A, 3505G>A, 3508G>A, 3511T>A, 3515C>T, 3516A>C, 3517G>T, 3523C>T, 3526C>A, 3528A>T, 3538C>A, 3544A>T, 3548G>A, 3550C>T, 3551A>G, 3556T>A, 3559G>T, 3562T>C, 3563G>A, 3565A>C, 3571T>C, 3598T>C, 3600A>T, 3602A>C, 3603A>G, 3608C>T, 3610C>G, 3612G>A, 3616A>G, 3619T>C, 3622A>G, 3625A>C, 3626T>C, 3628A>G, 3631G>T, 3634T>C, 3637T>A, 3646G>A, 3649A>G, 3650A>G, 3652T>G, 3655T>A, 3656C>A, 3658T>A, 3659T>C, 3661G>T, 3664G>A, 3665A>T, 3666G>C, 3667T>A, 3670T>A, 3671C>T, 3673T>G, 3679C>T, 3691C>A, 3694A>T, 3700T>C, 3706A>G, 3712A>C, 3715G>T, 3812C>A, 3814T>G, 3817A>T, 3820C>T, 3823C>T, 3824C>A, 3826C>G, 3832A>G, 3835A>C, 3836A>C, 3838G>A, 3844T>A, 3850T>C, 3853T>C, 3854C>A, 3862T>A, 3868C>T, 3871A>T, 3874G>A, 3883G>A, 3886T>C, 3895A>T, 3896G>A, 3897A>G, 3898G>T, 3899A>G, 3900C>T, 3901C>G, 3904G>A, 3911T>C, 3922T>A, 3931T>A, 3934T>A, 3937T>G, 3946T>A, 3961T>C, 3962T>C, 3965T>A, 3970G>A, 3973A>G, 3982C>T, 3991G>A, 4001A>C, 4006T>C, 4009G>A, 4015T>C, 4021G>T, 4023T>A, 4024T>C, 4027G>A, 4030A>G, 4031T>C, 4033G>A, 4035A>G, 4036G>A, 4039A>T, 4042G>A, 4053C>T, 4054T>A, 4057T>C, 4060A>C, 4064T>C, 4066G>A, 4072A>C, 4073A>C, 4081A>G, 4084G>C, 4093T>A, 4108C>A, 4111T>C, 4195C>T, 4201G>A, 4213T>A, 4216A>G, 4222T>A, 4231T>C, 4232T>C, 4234G>C, 4235C>T, 4237T>G, 4240T>C, 4252T>C, 4260G>A, 4265T>C, 4267G>T, 4273G>T, 4285G>A, 4288A>T, 4291C>G, 4303A>G, 4307T>A, 4309A>C, 4312A>T, 4318T>C, 4328A>G, 4330C>G, 4336T>G, 4339A>G, 4344A>G, 4348A>C, 4351T>A, 4354C>T, 4357G>A, 4366G>T, 4367T>G, 4378T>G, 4384G>A, 4387A>T, 4392C>T, 4393T>G, 4400G>A, 4405A>T, 4411A>T, 4414C>T, 4420A>T, 4421T>C, 4423A>C, 4438T>C, 4450T>G, 4453T>C, 4454T>A, 4455C>A, 4456A>T, 4457C>T, 4459T>A, 4465A>G, 4466T>C, 4468A>G, 4469C>T, 4471T>G, 5038G>C, 5045C>T, 5050A>T, 5056A>C, 5057C>A, 5059C>T, 5062G>A, 5063A>G, 5065C>G, 5068T>A, 5069G>A, 5070G>A, 5071A>T, 5072G>T, 5073T>C, 5074T>A, 5080T>C, 5083A>G, 5092T>A, 5095T>C, 5096A>C, 5098A>T, 5099A>C, 5101G>C, 5102A>C, 5103C>T, 5104A>T, 5107C>A, 5113T>A, 5116T>A, 5117G>C, 5125A>G, 5128T>C, 5137T>A, 5138G>C, 5140T>A, 5146C>T, 5147A>T, 5155A>T, 5158C>A, 5161G>A, 5166A>G, 5170T>G, 5171_5178delCACAGGGA, 5179G>C, 5182A>C, 5191A>T, 5194T>G, 5195T>A, 5196G>A, 5224T>A, 5225A>C, 5227A>C, 5228C>T, 5233T>A, 5236T>C, 5239C>T, 5245T>C, 5248A>C, 5254G>C, 5260T>C, 5266G>A, 5269G>A, 5272T>A, 5284A>T, 5287T>C, 5288G>T, 5291A>C, 5293G>A, 5302T>A, 5308A>C, 5311C>T, 5314G>A, 5356T>A, 5365T>C, 5371A>T, 5372A>T, 5373C>G, 5374A>T, 5386C>T, 5392G>T, 5393C>T, 5395A>G, 5404C>T, 5416T>C, 5419A>T, 5425A>G, 5431T>A, 5432A>C, 5434G>T, 5446G>A, 5453T>C, 5455G>T, 5515G>A, 5530G>T, 5531C>A, 5533C>A, 5534G>T, 5536C>T, 5539T>C, 5545C>A, 5555G>T, 5557A>C, 5566C>T, 5569A>G, 5573T>C, 5574C>A, 5580G>A, 5585T>G, 5588A>C, 5589A>G, 5590G>C, 5593T>C, 5597G>A, 5598G>A, 5599T>A, 5600T>C, 5602A>T, 5608A>T, 5611A>G, 5619A>G, 5620G>T, 5623T>C, 5624T>C, 5626G>C, 5629C>T, 5632T>A, 5635A>G, 5639G>A, 5640C>T, 5641T>C, 5645A>G, 5647A>T, 5648A>G, 5650A>T, 5668A>T, 5674T>A, 5681C>T, 5683T>A, 5686C>T, 5687A>T, 5688A>C, 5689G>T, 5696C>A, 5698A>G, 5699A>G, 5700G>A, 5701T>G, 5703A>G, 5704T>G, 5705T>G, 5706C>T, 5707T>A, 5709A>G, 5710A>T, 5713A>G, 5714G>A, 5715A>C, 5716A>G, 5724T>C, 5725G>C, 5728A>G, 5734G>A, 5744G>A, 5746A>C, 5750_5754delAAGTT, 5755G>A, 5758A>C, 5764A>T, 5770C>A, 5776G>A, 5779G>A, 5782A>G, 5788T>C, 5794C>T, 5797C>T, 5800A>G, 5803A>C, 5812C>A, 5815C>A, 5818C>A, 5821A>T, 5824C>T, 5827T>C, 5839A>T, 5845C>A, 5846T>A, 5847C>A, 5848A>G, 5851A>G, 5863A>G, 5869A>T, 5878A>C, 5887T>C, 5890G>A, 5896A>G, 5899G>A, 5902C>T, 5917A>T, 5926T>A, 5929C>T, 5932C>T, 5935T>C, 5938A>G, 5944T>A, 5947T>A, 5953G>A, 5959A>T, 5962A>G, 5968T>A, 5969C>A, 5970G>A, 5971C>G, 5976C>T, 5977A>G, 5983A>G, 5986T>A, 5989G>A, 5995T>A, 5998C>T, 6016G>A, 6025A>C, 6028A>G, 6031A>C, 6036C>A, 6037T>A, 6289G>A, 6295A>G, 6296G>T, 6298G>A, 6301A>T, 6302C>T, 6304T>A, 6307A>G, 6313A>G, 6316T>G, 6319A>T, 6334C>T, 6337T>A, 6340T>C, 6341G>T, 6346T>C, 6349T>C, 6352A>G, 6355G>A, 6358A>C, 6364T>C, 6373T>A, 6376G>T, 6377A>C, 6379G>A, 6382C>G, 6391T>A, 6394C>G, 6400T>A, 6406A>G, 6411T>A, 6412C>T, 6415A>T, 6418T>C, 6421G>A, 6427G>A, 6433A>G, 6436C>T, 6437A>C, 6439A>T, 6451T>G, 6455A>C, 6460A>G, 6461A>G, 6463C>T, 6472A>T, 6478T>A, 6479T>C, 6481G>T, 6490T>C, 6491C>A, 6493C>A, 6497C>T, 6499T>G, 6502G>A, 6505G>A, 6506T>C, 6508G>T, 6520G>A, 6526G>T, 6527A>G, 6528A>G, 6529C>T, 6535C>T, 6541A>T, 6542A>T, 6547T>C, 6550A>G, 6562T>C, 6565T>C, 6577T>C, 6586G>A, 6589G>A, 6592C>T, 6593C>A, 6595A>G, 6596C>A, 6598T>G, 6610A>G, 6613G>A, 6616A>G, 6619C>A, 6626G>A, 6628G>T, 6643G>A, 6646A>C, 6652C>T, 6653A>C, 6663A>T, 6667T>G, 6681C>T, 6683A>G, 6685A>T, 6688T>A, 6691A>T, 6700A>G, 6703A>T, 6718T>C, 6724C>T, 6725A>T, 6739C>G, 6740C>A, 6742G>T, 6748T>G, 6752_6756delCCATT, 6757A>C, 6763T>A, 6766G>A, 6769A>G, 6778G>C, 6784T>A, 6787A>G, 6790T>A, 6799T>A, 6802G>A, 6809T>C, 6814A>G, 6820T>C, 6823C>T, 6829C>A, 6832C>T, 6835T>C, 6841A>G, 6850A>T, 6851A>G, 6853T>C, 6854T>A, 6856G>C, 6857A>T, 6859A>G, 6861T>A, 6862G>A, 6866G>T, 6869C>A, 6871C>G, 6874A>T, 6875A>C, 6877G>T, 6880T>G, 6886G>T, 6898A>G, 6901C>T, 6904T>C, 6905T>C, 6907G>A, 6910T>C, 6919C>T, 6925C>T, 6929C>T, 6931C>G, 6934A>G, 6935A>C, 6937T>C, 6940A>G, 6955G>A, 6956C>G, 6958G>A, 6970T>A, 6972A>C, 6973G>A, 6979T>A, 6980G>A, 6982T>A, 6988T>C, 6989C>T, 6991T>G, 6992T>C, 6994T>C, 6997T>A, 7000G>A, 7005T>C, 7006T>A, 7010A>G, 7011T>C, 7016G>T, 7018C>T, 7019A>C, 7021T>A, 7024G>T, 7027A>G, 7030T>G, 7036G>T, 7038A>T, 7039C>T, 7048T>A, 7049T>C, 7051T>G, 7054C>T, 7058T>C, 7060G>T, 7063C>A, 7066A>T, 7067A>G, 7069T>C, 7070G>T, 7072T>C, 7073A>T, 7075A>C, 7078C>T, 7088T>C, 7090A>T, 7093C>T, 7099A>G, 7105T>G, 7108T>G, 7111G>A, 7117G>A, 7121A>C, 7122A>C, 7123T>A, 7124C>T, 7129C>T, 7132C>T, 7133T>G, 7135G>C, 7138T>C, 7139T>C, 7141A>G, 7144C>T, 7151G>A, 7152A>G, 7153A>T, 7156G>T, 7158A>C, 7159G>A, 7162A>G, 7163A>C, 7168T>C, 7172C>A, 7177A>T, 7178C>T, 7180A>G, 7186A>C, 7192T>C, 7194T>A, 7201C>T, 7202A>G, 7203A>C, 7205T>C, 7207G>A, 7212G>A, 7213A>G, 7216G>A, 7219A>T, 7225G>T, 7234A>T, 7240A>G, 7243C>T, 7246A>G, 7248G>A, 7253A>G, 7254C>T, 7258G>A, 7264A>T, 7265T>G, 7270G>C, 7276A>T, 7282C>T, 7285T>C, 7287A>G, 7289A>C, 7290G>A, 7291A>G, 7294A>C, 7297G>C, 7299A>C, 7304T>G, 7306G>C, 7309T>A, 7312T>C, 7318C>T, 7320A>T, 7321A>T, 7330T>C, 7351G>T, 7361A>C, 7366G>A, 7369A>C, 7372C>T, 7378T>A, 7381A>G, 7384T>C, 7396A>T, 7405T>C, 7408A>T, 7414A>G, 7423A>G, 7426A>G, 7429T>C, 7432C>T, 7438C>A, 7439A>T, 7440A>C, 7443G>C, 7444C>A, 7445T>A, 7453T>C, 7456T>C, 7457T>C, 7459G>T, 7462A>G, 7464A>G, 7465T>G, 7467T>A, 7471G>A, 7473G>T, 7474A>G, 7477C>A, 7483T>C, 7484G>A, 7486G>A, 7487A>C, 7489A>C, 7492C>T, 7494T>A, 7498T>C, 7501C>T, 7507T>C, 7510G>A, 7513G>A, 7522C>A, 7525A>C, 7528T>A, 7537A>G, 7538T>C, 7541C>A, 7543G>A, 7547A>T, 7549T>G, 7555G>A, 7558T>G, 7559G>C, 7561G>A, 7567C>T, 7570G>A, 7571A>T, 7576A>C, 7582T>C, 7591A>T, 7595G>A, 7597T>A, 7600T>C, 7604T>C, 7606A>T, 7609G>A, 7612T>A, 7615A>G, 7627G>A, 7636T>C, 7645A>G, 7648C>T, 7651T>C, 7654T>A, 7655C>T, 7657C>A, 7661T>C, 7663G>C, 7664C>A, 7672C>A, 7675A>T, 7681C>T, 7684A>T, 7693A>G, 7696T>C, 7700A>G, 7705G>T, 7711T>C, 7714A>G, 7715T>C, 7717G>T, 7723A>G, 7724G>A, 7726G>C, 7727C>T, 7729T>A, 7732T>G, 7742A>G, 7743C>T, 7750T>C, 7756T>A, 7759C>T, 7768G>A, 7774T>C, 7781C>T, 7783A>G, 7789A>T, 7795T>C, 7796T>A, 7798G>C, 7805A>C, 7807T>C, 7810T>C, 7814G>A, 7820C>T, 7822C>A, 7824A>T, 7825C>T, 7834A>G, 7843T>C, 7846T>A, 7853G>A, 7854T>C, 7855G>C, 7863A>G, 7864G>A, 7873C>T, 7876C>T, 7896T>A, 7897A>C, 7900C>A, 7915T>G, 7918A>T, 7921A>T, 7930T>C, 7936C>T, 7939T>C, 7942T>C, 7944G>C, 7948A>G, 7951A>G, 7957T>A, 7960A>T, 7963C>T, 7966T>G, 7969C>T, 7978G>T, 7984T>A, 7987C>T, 7988A>C, 7999T>C, 8002C>T, 8008G>T, 8011C>T, 8014A>T, 8015C>A, 8017T>A, 8018G>A, 8023T>C, 8026G>T, 8028A>T, 8029A>T, 8032G>T, 8033T>A, 8034C>T, 8035C>G, 8041A>G, 8044T>C, 8046A>T, 8050T>A, 8056A>G, 8063C>T, 8074T>A, 8077A>G, 8080G>A, 8084C>T, 8085A>C, 8086G>T, 8095C>T, 8099T>C, 8101G>T, 8104G>A, 8110T>C, 8113A>G, 8114G>T, 8116T>A, 8119A>G, 8124T>G, 8125A>T, 8134T>A, 8135C>A, 8137T>G, 8138A>T, 8139T>C, 8140T>A, 8146T>A, 8161A>T, 8163A>G, 8164A>G, 8167C>T, 8168C>A, 8170C>A, 8171A>C, 8172G>T, 8173T>A, 8179G>T, 8191A>C, 8194C>T, 8200T>A, 8201G>A, 8203T>G, 8206C>G, 8215T>A, 8216A>G, 8218T>C, 8221C>T, 8224A>C, 8227T>A, 8242G>A, 8243C>A, 8245G>A, 8251T>A, 8254C>A, 8260T>C, 8269C>T, 8270A>C, 8275A>T, 8278A>T, 8281A>G, 8284A>C, 8285T>A, 8291C>A, 8294C>T, 8299A>G, 8323G>A, 8341A>G, 8342C>T, 8347T>A, 8349A>T, 8353G>A, 8357A>C, 8369C>T, 8371T>G, 8383G>A, 8395C>T, 8411G>C, 8416G>A, 8418G>A, 8422T>A, 8428C>T, 8431A>G, 8434T>C, 8437A>G, 8440T>G, 8443T>A, 8444C>T, 8455A>G, 8461T>A, 8464T>C, 8465G>A, 8467T>C, 8473C>A, 8476T>G, 8479A>T, 8480T>A, 8481T>A, 8482G>A, 8485A>G, 8488G>A, 8489C>T, 8491T>G, 8499C>T, 8500A>T, 8503G>T, 8506C>T, 8507A>G, 8515T>A, 8518T>G, 8520A>C, 8524T>C, 8525G>T, 8526A>T, 8527T>G, 8528A>G, 8529G>A, 8530C>T, 8531T>G, 8533G>A, 8535C>A, 8537T>C, 8538C>A, 8539T>A, 8542A>G, 8543G>C, 8545T>C, 8548A>G, 8549T>A, 8550A>T, 8551T>A, 8552C>T, 8560T>A, 8563T>C, 8565C>A, 8578T>A, 8581T>C, 8587A>C, 8591G>A, 8596A>T, 8599G>A, 8601A>G, 8602C>T, 8606T>G, 8607C>G, 8624A>C, 8625G>T, 8626T>C, 8628T>C, 8634C>A, 8635G>A, 8636A>C, 8640G>A, 8641A>C, 8642G>C, 8644G>A, 8647A>G, 8650G>C, 8654C>T, 8655T>C, 8658T>C, 8668A>G, 8680G>T, 8686T>A, 8701C>G, 8710A>T, 8722A>G, 8728T>A, 8731T>C, 8734T>A, 8746A>C, 8761T>G, 8763A>G, 8770T>C, 8773T>C, 8775G>A, 8779G>T, 8782C>T, 8786C>T, 8791G>C, 8792A>C, 8793A>T, 8794C>G, 8803A>T, 8815T>C, 8821A>T, 8839C>A, 8851A>G, 8861A>G, 8864C>A, 8865G>A, 8866T>A, 8869A>G, 8870C>A, 8887T>C, 8890T>A, 8896A>G, 8899G>A, 8905G>A, 8911C>T, 8921T>C, 8923A>T, 8938C>T, 8959C>T, 8962A>T, 8968T>C, 8977T>C, 8992G>C, 9013A>G, 9025T>A, 9028A>G, 9040C>A, 9046T>C, 9052A>G, 9061G>A, 9064T>C, 9073T>C, 9074C>T, 9109T>C, 9124A>T, 9125C>A, 9127T>A, 9142C>T, 9156T>A, 9157G>T, 9160T>C, 9163G>T, 9181C>T, 9191G>A, 9193T>C, 9196T>C, 9197C>T, 9200C>A, 9211C>T, 9244T>A, 9256A>T, 9261A>C, 9262C>A, 9265A>G, 9268G>C, 9271G>A, 9272T>C, 9274A>C, 9283G>C, 9307A>G, 9316C>A, 9325C>T, 9332C>A, 9333A>C, 9334A>T, 9340T>G, 9346A>G, 9347G>A, 9348G>A, 9349T>C, 9364T>A, 9382G>A, 9383A>T, 9384G>T, 9387delT									
**CDS**									
G357_gp1	298	3076	63.2%	11399	85.2%	1940 (99.8%)	1649 (84.8%)	0/4/3/3	1

	Begin	End	Coverage	Score	Concordance	Matches	Identities	I/D/M/F*	Stop Codons
**NT**	**1045**	**9396**	**60.8%**	**6095**	**52.5%**	**5835 (99.7%)**	**4455 (76.1%)**	**0/19**	

Protein mutations:	L300M (1052T>A), K309A (1079A>G 1080A>C 1081G>A), A330T (1142G>A 1144G>A), R332K (1149G>A), E334D (1156A>T), I335L (1157A>T 1159T>A), D336N (1160G>A), L347Q (1193T>C 1194T>A), T368I (1257C>T 1258A>T), D450E (1504T>A), L451C (1505C>T 1506T>G 1507A>T), S456T (1520T>A 1522T>A), L457T (1523T>A 1524T>C), M458L (1526A>T), S459D (1529A>G 1530G>A), V465I (1547G>A), S468G (1556A>G), V473I (1571G>A), I565V (1847A>G 1849T>G), S572T (1868T>A 1870T>A), K581R (1896A>G), T583E (1901A>G 1902C>A 1903A>G), A584S (1904G>T), P586A (1910C>G 1912C>A), L626I (2030C>A 2032T>A), S631T (2045T>A 2047T>C), A634S (2054G>T 2056A>T), K641N (2077G>C), E642S (2078G>T 2079A>C), I643S (2081A>T 2082T>C), S644E (2084A>G 2085G>A 2086T>A), E645Q (2087G>C 2089A>G), Y648F (2097A>T 2098C>T), R1007K (3174G>A), F1008Y (3177T>A), K1011R (3186A>G), K1012T (3189A>C 3190A>G), A1016I (3200G>A 3201C>T), V1017L (3203G>C), S1018R (3208T>A), I1021V (3215A>G), S1032T (3249G>C), V1049I (3299G>A 3301G>C), R1052K (3309G>A 3310G>A), A1056T (3320G>A), N1061T (3336A>C 3337C>A), K1062H (3338A>C 3340G>T), A1065M (3347G>A 3348C>T), I1070V (3362A>G), N1081E (3395A>G 3397T>A), L1084P (3404T>C 3405T>C 3406A>C), S1088A (3416T>G 3418T>A), R1091K (3426G>A), S1092E (3428A>G 3429G>A 3430C>G), K1093R (3431A>C 3432A>G 3433G>A), V1094I (3434G>A 3436G>C), S1096P (3440T>C), D1098N (3446G>A 3448T>C), T1099K (3450C>A), Q1100A (3452C>G 3453A>C 3454G>T), I1102M (3460C>G), K1103D (3461A>G 3463G>T), I1105V (3467A>G 3469T>G), D1106N (3470G>A), Q1110C (3482C>T 3483A>G 3484G>C), E1113G (3492A>G 3493A>T), T1115Q (3497A>C 3498C>A 3499T>A), Q1121S (3515C>T 3516A>C 3517G>T), Y1125F (3528A>T), V1132I (3548G>A 3550C>T), I1133V (3551A>G), V1137I (3563G>A 3565A>C), Y1149F (3600A>T), K1150R (3602A>C 3603A>G), S1153N (3612G>A), T1166A (3650A>G 3652T>G), K1228Q (3836A>C 3838G>A), E1248S (3896G>A 3897A>G 3898G>T), T1249V (3899A>G 3900C>T 3901C>G), C1271S (3965T>A), F1290Y (4023T>A 4024T>C), K1294R (4035A>G 4036G>A), A1300V (4053C>T 4054T>A), R1369K (4260G>A), S1385T (4307T>A 4309A>C), I1392V (4328A>G 4330C>G), I1394M (4336T>G), N1397S (4344A>G), S1405A (4367T>G), A1413V (4392C>T 4393T>G), V1416I (4400G>A), S1434N (4454T>A 4455C>A 4456A>T), H1635N (5057C>A 5059C>T), S1637G (5063A>G 5065C>G), G1639N (5069G>A 5070G>A 5071A>T), V1640S (5072G>T 5073T>C 5074T>A), T1650L (5102A>C 5103C>T 5104A>T), H1653Q (5113T>A), E1655Q (5117G>C), A1662P (5138G>C 5140T>A), T1665S (5147A>T), E1667D (5155A>T), K1671R (5166A>G), H1673_R1674del (5171_5178delCACAGGGA), C1681N (5195T>A 5196G>A), A1712S (5288G>T), T1740C (5372A>T 5373C>G 5374A>T), L1793I (5531C>A 5533C>A), A1794S (5534G>T 5536C>T), A1801S (5555G>T 5557A>C), S1807Q (5573T>C 5574C>A), G1809E (5580G>A), S1811A (5585T>G), K1812R (5588A>C 5589A>G 5590G>C), G1815K (5597G>A 5598G>A 5599T>A), K1822S (5619A>G 5620G>T), A1829I (5639G>A 5640C>T 5641T>C), I1831V (5645A>G 5647A>T), I1832V (5648A>G 5650A>T), K1845S (5687A>T 5688A>C 5689G>T), Q1848K (5696C>A 5698A>G), S1849E (5699A>G 5700G>A 5701T>G), N1850R (5703A>G 5704T>G), S1851V (5705T>G 5706C>T 5707T>A), K1852S (5709A>G 5710A>T), E1854T (5714G>A 5715A>C 5716A>G), L1857P (5724T>C 5725G>C), A1864T (5744G>A 5746A>C), K1866del (5750_5754delAAGTT), S1898K (5846T>A 5847C>A 5848A>G), R1939K (5969C>A 5970G>A 5971C>G), A1941V (5976C>T 5977A>G), A1961E (6036C>A 6037T>A), A2048S (6296G>T 6298G>A), A2063S (6341G>T), I2076M (6382C>G), F2086Y (6411T>A 6412C>T), M2101L (6455A>C), I2103V (6461A>G 6463C>T), Q2106H (6472A>T), E2124D (6526G>T), N2125G (6527A>G 6528A>G 6529C>T), I2130L (6542A>T), L2148M (6596C>A 6598T>G), V2158I (6626G>A 6628G>T), M2167L (6653A>C), Q2170L (6663A>T), T2176I (6681C>T), I2177V (6683A>G 6685A>T), T2191S (6725A>T), Q2196N (6740C>A 6742G>T), P2200del (6752_6756delCCATT), K2208N (6778G>C), Q2232H (6850A>T), N2233D (6851A>G 6853T>C), L2234I (6854T>A 6856G>C), I2235L (6857A>T 6859A>G), M2236K (6861T>A 6862G>A), A2238S (6866G>T), L2239M (6869C>A 6871C>G), E2240D (6874A>T), M2241L (6875A>C 6877G>T), D2242E (6880T>G), I2261L (6935A>C 6937T>C), Q2268E (6956C>G 6958G>A), E2273A (6972A>C 6973G>A), V2276I (6980G>A 6982T>A), F2280L (6992T>C 6994T>C), D2281E (6997T>A), I2284T (7005T>C 7006T>A), I2286A (7010A>G 7011T>C), A2288S (7016G>T 7018C>T), N2289Q (7019A>C 7021T>A), M2290I (7024G>T), N2292K (7030T>G), Y2295F (7038A>T 7039C>T), C2299R (7049T>C 7051T>G), I2305V (7067A>G 7069T>C), A2306S (7070G>T 7072T>C), T2307S (7073A>T 7075A>C), N2323P (7121A>C 7122A>C 7123T>A), L2327V (7133T>G 7135G>C), E2333S (7151G>A 7152A>G 7153A>T), E2334D (7156G>T), E2335A (7158A>C 7159G>A), K2337Q (7163A>C), L2347Q (7194T>A), K2350A (7202A>G 7203A>C), R2353K (7212G>A 7213A>G), S2365N (7248G>A), T2367V (7253A>G 7254C>T), S2371A (7265T>G), D2378G (7287A>G), R2379Q (7289A>C 7290G>A 7291A>G), N2382T (7299A>C), L2384V (7304T>G 7306G>C), E2389V (7320A>T 7321A>T), M2403L (7361A>C), N2429S (7439A>T 7440A>C), C2430S (7443G>C 7444C>A), S2431T (7445T>A), N2437R (7464A>G 7465T>G), F2438Y (7467T>A), R2440M (7473G>T 7474A>G), E2444K (7484G>A 7486G>A), F2447Y (7494T>A), I2465L (7547A>T 7549T>G), E2469Q (7559G>C 7561G>A), T2473S (7571A>T), V2481I (7595G>A 7597T>A), Q2504K (7664C>A), R2516G (7700A>G), G2524S (7724G>A 7726G>C), T2530V (7742A>G 7743C>T), L2548I (7796T>A 7798G>C), I2551L (7805A>C 7807T>C), V2554I (7814G>A), Y2557F (7824A>T 7825C>T), V2567T (7853G>A 7854T>C 7855G>C), K2570R (7863A>G 7864G>A), I2581N (7896T>A 7897A>C), S2597T (7944G>C), I2612L (7988A>C), M2618I (8008G>T), D2622N (8018G>A), K2625I (8028A>T 8029A>T), E2626D (8032G>T), S2627M (8033T>A 8034C>T 8035C>G), Y2631F (8046A>T), I2641M (8077A>G), Q2644S (8084C>T 8085A>C 8086G>T), A2654S (8114G>T 8116T>A), L2657C (8124T>G 8125A>T), L2661M (8135C>A 8137T>G), I2662S (8138A>T 8139T>C 8140T>A), K2670R (8163A>G 8164A>G), S2673L (8171A>C 8172G>T 8173T>A), V2683M (8201G>A 8203T>G), I2688V (8216A>G 8218T>C), S2711T (8285T>A), K2732M (8349A>T), E2753Q (8411G>C), R2755K (8418G>A), A2771T (8465G>A 8467T>C), E2775D (8479A>T), L2776K (8480T>A 8481T>A 8482G>A), A2782V (8499C>T 8500A>T), I2785V (8507A>G), D2788E (8518T>G), Y2789S (8520A>C), D2791L (8525G>T 8526A>T 8527T>G), S2792D (8528A>G 8529G>A 8530C>T), L2793V (8531T>G 8533G>A), A2794E (8535C>A), S2795Q (8537T>C 8538C>A 8539T>A), D2797H (8543G>C 8545T>C), I2798M (8548A>G), Y2799I (8549T>A 8550A>T 8551T>A), L2800F (8552C>T), T2804K (8565C>A), A2813T (8591G>A), N2816S (8601A>G 8602C>T), S2818G (8606T>G 8607C>G), S2824L (8624A>C 8625G>T 8626T>C), V2825A (8628T>C), T2827K (8634C>A 8635G>A), T2828P (8636A>C), G2829D (8640G>A 8641A>C), E2830Q (8642G>C 8644G>A), K2832N (8650G>C), L2834S (8654C>T 8655T>C), V2835A (8658T>C), N2869K (8761T>G), K2870R (8763A>G), S2874N (8775G>A), N2880L (8792A>C 8793A>T 8794C>G), H2895Q (8839C>A), N2903D (8861A>G), R2904K (8864C>A 8865G>A 8866T>A), E2946D (8992G>C), M3001N (9156T>A 9157G>T), V3013I (9191G>A 9193T>C), P3015S (9197C>T), N3036T (9261A>C 9262C>A), Q3060T (9332C>A 9333A>C 9334A>T), G3065N (9347G>A 9348G>A 9349T>C)

	Begin	End	Coverage	Score	Concordance	Matches	Identities	I/D/M/F*	Stop Codons
**NT**	**1045**	**9396**	**60.8%**	**6095**	**52.5%**	**5835 (99.7%)**	**4455 (76.1%)**	**0/19**	

Codon mutations:

CGT297..A (1045T>A), GAT298GAC (1048T>C), TTG300ATG (1052T>A), TGT301TGC (1057T>C), GAT303GAC (1063T>C), CTT304TTA (1064C>T 1066T>A), TAC306TAT (1072C>T), AAG309GCA (1079A>G 1080A>C 1081G>A), CAT310CAC (1084T>C), TTT311TTC (1087T>C), TCA312TCT (1090A>T), CAA315CAG (1099A>G), ATT316ATA (1102T>A), TCA317TCG (1105A>G), CTG322TTA (1118C>T 1120G>A), TGT325TGC (1129T>C), AAA326AAG (1132A>G), TAC328TAT (1138C>T), GCG330ACA (1142G>A 1144G>A), GGA331GGG (1147A>G), AGA332AAA (1149G>A), GAA334GAT (1156A>T), ATT335TTA (1157A>T 1159T>A), GAT336AAT (1160G>A), TCG344TCC (1186G>C), GTT345GTA (1189T>A), TCT346TCC (1192T>C), TTA347CAA (1193T>C 1194T>A), TGT348TGC (1198T>C), GGT349GGA (1201T>A), GTC351GTT (1207C>T), GCG352GCT (1210G>T), TTA354TTG (1216A>G), GTG355GTA (1219G>A), CAG357CAA (1225G>A), TTA358CTA (1226T>C), CCA361CCG (1237A>G), GGC363GGG (1243C>G), TGC367TGT (1255C>T), ACA368ATT (1257C>T 1258A>T), CGG437AGA (1463C>A 1465G>A), AAC438AAT (1468C>T), AAG439AAA (1471G>A), CCC441CCA (1477C>A), GTT445GTC (1489T>C), CTT446TTG (1490C>T 1492T>G), GAT450GAA (1504T>A), CTA451TGT (1505C>T 1506T>G 1507A>T), TTA452CTT (1508T>C 1510A>T), TTG453CTG (1511T>C), AAG454AAA (1516G>A), TCT456ACA (1520T>A 1522T>A), TTA457ACA (1523T>A 1524T>C), ATG458TTG (1526A>T), AGT459GAT (1529A>G 1530G>A), GAG462GAA (1540G>A), GGT464GGC (1546T>C), GTA465ATA (1547G>A), AGT468GGT (1556A>G), TTG470CTA (1562T>C 1564G>A), CTT471CTG (1567T>G), GTT473ATT (1571G>A), GCA474GCT (1576A>T), CGA475AGA (1577C>A), TTG477CTC (1583T>C 1585G>C), AAC478AAT (1588C>T), AGA557CGC (1823A>C 1825A>C), AAA558AAG (1828A>G), TTA559CTT (1829T>C 1831A>T), GCA560GCG (1834A>G), GGC562GGA (1840C>A), TTG564CTT (1844T>C 1846G>T), ATT565GTG (1847A>G 1849T>G), TCT572ACA (1868T>A 1870T>A), CTT573TTA (1871C>T 1873T>A), CGG574CGA (1876G>A), GTC578GTT (1888C>T), GGG579GGT (1891G>T), GAG580GAA (1894G>A), AAG581AGG (1896A>G), ATA582ATT (1900A>T), ACA583GAG (1901A>G 1902C>A 1903A>G), GCA584TCA (1904G>T), CCC586GCA (1910C>G 1912C>A), AGC593AGT (1933C>T), CGT595AGA (1937C>A 1939T>A), GAT597GAC (1945T>C), TTC599TTT (1951C>T), TTA600CTT (1952T>C 1954A>T), TAC601TAT (1957C>T), TGC604TGT (1966C>T), ACT607ACA (1975T>A), GGC611GGG (1987C>G), TTG614CTT (1994T>C 1996G>T), CTA615CTT (1999A>T), TTG618CTT (2006T>C 2008G>T), ACC622ACA (2020C>A), AAG624AAA (2026G>A), CAT625CAC (2029T>C), CTT626ATA (2030C>A 2032T>A), GGT629GGC (2041T>C), TCT631ACC (2045T>A 2047T>C), GGT632GGA (2050T>A), GAT633GAC (2053T>C), GCA634TCT (2054G>T 2056A>T), AAA635AAG (2059A>G), TTT636TTC (2062T>C), ATA637ATT (2065A>T), GAT638GAC (2068T>C), CTT639TTA (2069C>T 2071T>A), AAG641AAC (2077G>C), GAA642TCA (2078G>T 2079A>C), ATT643TCT (2081A>T 2082T>C), AGT644GAA (2084A>G 2085G>A 2086T>A), GAA645CAG (2087G>C 2089A>G), GGG646GGA (2092G>A), TAC648TTT (2097A>T 2098C>T), GAA652GAG (2110A>G), GGA653GGT (2113A>T), GCT1006..A (3172T>A), AGG1007AAG (3174G>A), TTC1008TAC (3177T>A), ACC1009ACA (3181C>A), AAC1010AAT (3184C>T), AAG1011AGG (3186A>G), AAA1012ACG (3189A>C 3190A>G), TCA1015TCT (3199A>T), GCT1016ATT (3200G>A 3201C>T), GTT1017CTT (3203G>C), AGT1018AGA (3208T>A), TAT1019TAC (3211T>C), TGT1020TGC (3214T>C), ATA1021GTA (3215A>G), AAA1026AAG (3232A>G), ACG1029ACA (3241G>A), GTT1030GTG (3244T>G), TTG1031CTG (3245T>C), AGT1032ACT (3249G>C), CTG1035TTA (3257C>T 3259G>A), CTA1037TTG (3263C>T 3265A>G), ACC1038ACA (3268C>A), GTC1040GTA (3274C>A), CGA1042CGT (3280A>T), GTT1044GTG (3286T>G), CAT1045CAC (3289T>C), TAC1047TAT (3295C>T), GTG1049ATC (3299G>A 3301G>C), AGG1052AAA (3309G>A 3310G>A), AAT1053AAC (3313T>C), GCA1056ACA (3320G>A), GAG1058GAA (3328G>A), AAC1061ACA (3336A>C 3337C>A), AAG1062CAT (3338A>C 3340G>T), GCA1063GCT (3343A>T), GCG1065ATG (3347G>A 3348C>T), TGC1066TGT (3352C>T), GAA1069GAG (3361A>G), ATT1070GTT (3362A>G), GGT1071GGG (3367T>G), ATC1073ATT (3373C>T), ATC1074ATT (3376C>T), TTC1077TTT (3385C>T), TTC1079TTT (3391C>T), AAT1081GAA (3395A>G 3397T>A), GGT1082GGG (3400T>G), CGT1083AGG (3401C>A 3403T>G), TTA1084CCC (3404T>C 3405T>C 3406A>C), CCG1085CCC (3409G>C), ACT1086ACA (3412T>A), TCT1088GCA (3416T>G 3418T>A), GAA1089GAG (3421A>G), AGA1091AAA (3426G>A), AGC1092GAG (3428A>G 3429G>A 3430C>G), AAG1093CGA (3431A>C 3432A>G 3433G>A), GTG1094ATC (3434G>A 3436G>C), AAC1095AAT (3439C>T), TCA1096CCA (3440T>C), TCT1097TCA (3445T>A), GAT1098AAC (3446G>A 3448T>C), ACA1099AAA (3450C>A), CAG1100GCT (3452C>G 3453A>C 3454G>T), CTA1101CTC (3457A>C), ATC1102ATG (3460C>G), AAG1103GAT (3461A>G 3463G>T), ATT1105GTG (3467A>G 3469T>G), GAC1106AAC (3470G>A), CAT1107CAC (3475T>C), TAT1109TAC (3481T>C), CAG1110TGC (3482C>T 3483A>G 3484G>C), GAA1113GGT (3492A>G 3493A>T), ACT1114ACG (3496T>G), ACT1115CAA (3497A>C 3498C>A 3499T>A), CAG1117CAA (3505G>A), AAG1118AAA (3508G>A), GGT1119GGA (3511T>A), CAG1121TCT (3515C>T 3516A>C 3517G>T), TTC1123TTT (3523C>T), GCC1124GCA (3526C>A), TAC1125TTC (3528A>T), CGC1128CGA (3538C>A), ATA1130ATT (3544A>T), GTC1132ATT (3548G>A 3550C>T), ATA1133GTA (3551A>G), ACT1134ACA (3556T>A), CTG1135CTT (3559G>T), CTT1136CTC (3562T>C), GTA1137ATC (3563G>A 3565A>C), GCT1139GCC (3571T>C), GTT1148GTC (3598T>C), TAC1149TTC (3600A>T), AAA1150CGA (3602A>C 3603A>G), CTC1152TTG (3608C>T 3610C>G), AGC1153AAC (3612G>A), AAA1154AAG (3616A>G), TTT1155TTC (3619T>C), AAA1156AAG (3622A>G), GGA1157GGC (3625A>C), TTA1158CTG (3626T>C 3628A>G), GTG1159GTT (3631G>T), GTT1160GTC (3634T>C), TCT1161TCA (3637T>A), AAG1164AAA (3646G>A), GAA1165GAG (3649A>G), ACT1166GCG (3650A>G 3652T>G), ACT1167ACA (3655T>A), CGT1168AGA (3656C>A 3658T>A), TTG1169CTT (3659T>C 3661G>T), GAG1170GAA (3664G>A), AGT1171TCA (3665A>T 3666G>C 3667T>A), TCT1172TCA (3670T>A), CTT1173TTG (3671C>T 3673T>G), GAC1175GAT (3679C>T), ATC1179ATA (3691C>A), GCA1180GCT (3694A>T), AAT1182AAC (3700T>C), CAA1184CAG (3706A>G), ACA1186ACC (3712A>C), GTG1187GTT (3715G>T), CGT1220AGG (3812C>A 3814T>G), CCA1221CCT (3817A>T), CAC1222CAT (3820C>T), TAC1223TAT (3823C>T), CGC1224AGG (3824C>A 3826C>G), GAA1226GAG (3832A>G), GGA1227GGC (3835A>C), AAG1228CAA (3836A>C 3838G>A), ATT1230ATA (3844T>A), TTT1232TTC (3850T>C), ACT1233ACC (3853T>C), CGA1234AGA (3854C>A), ACT1236ACA (3862T>A), AGC1238AGT (3868C>T), TCA1239TCT (3871A>T), GTG1240GTA (3874G>A), GAG1243GAA (3883G>A), ATT1244ATC (3886T>C), TCA1247TCT (3895A>T), GAG1248AGT (3896G>A 3897A>G 3898G>T), ACC1249GTG (3899A>G 3900C>T 3901C>G), AAG1250AAA (3904G>A), TTA1253CTA (3911T>C), GGT1256GGA (3922T>A), GGT1259GGA (3931T>A), TCT1260TCA (3934T>A), GGT1261GGG (3937T>G), ACT1264ACA (3946T>A), AAT1269AAC (3961T>C), TTG1270CTG (3962T>C), TGC1271AGC (3965T>A), AAG1272AAA (3970G>A), AGA1273AGG (3973A>G), GTC1276GTT (3982C>T), CTG1279CTA (3991G>A), AGA1283CGA (4001A>C), CCT1284CCC (4006T>C), TTG1285TTA (4009G>A), AAT1287AAC (4015T>C), GTG1289GTT (4021G>T), TTT1290TAC (4023T>A 4024T>C), CAG1291CAA (4027G>A), CAA1292CAG (4030A>G), TTG1293CTA (4031T>C 4033G>A), AAG1294AGA (4035A>G 4036G>A), GCA1295GCT (4039A>T), CAG1296CAA (4042G>A), GCT1300GTA (4053C>T 4054T>A), AGT1301AGC (4057T>C), CCA1302CCC (4060A>C), TTG1304CTA (4064T>C 4066G>A), ATA1306ATC (4072A>C), AGA1307CGA (4073A>C), AAA1309AAG (4081A>G), TCG1310TCC (4084G>C), GGT1313GGA (4093T>A), ACC1318ACA (4108C>A), ATT1319ATC (4111T>C), TGC1347TGT (4195C>T), GTG1349GTA (4201G>A), TCT1353TCA (4213T>A), GGA1354GGG (4216A>G), GCT1356GCA (4222T>A), AAT1359AAC (4231T>C), TTG1360CTC (4232T>C 4234G>C), CTT1361TTG (4235C>T 4237T>G), TTT1362TTC (4240T>C), TTT1366TTC (4252T>C), AGA1369AAA (4260G>A), TTG1371CTT (4265T>C 4267G>T), GTG1373GTT (4273G>T), CCG1377CCA (4285G>A), CCA1378CCT (4288A>T), GGC1379GGG (4291C>G), GAA1383GAG (4303A>G), TCA1385ACC (4307T>A 4309A>C), ACA1386ACT (4312A>T), TAT1388TAC (4318T>C), ATC1392GTG (4328A>G 4330C>G), ATT1394ATG (4336T>G), GAA1395GAG (4339A>G), AAC1397AGC (4344A>G), CTA1398CTC (4348A>C), TCT1399TCA (4351T>A), TTC1400TTT (4354C>T), CAG1401CAA (4357G>A), GTG1404GTT (4366G>T), TCA1405GCA (4367T>G), GGT1408GGG (4378T>G), CAG1410CAA (4384G>A), TCA1411TCT (4387A>T), GCT1413GTG (4392C>T 4393T>G), GTC1416ATC (4400G>A), CGA1417CGT (4405A>T), GGA1419GGT (4411A>T), AAC1420AAT (4414C>T), ATA1422ATT (4420A>T), TTA1423CTC (4421T>C 4423A>C), AGT1428AGC (4438T>C), GTT1432GTG (4450T>G), GAT1433GAC (4453T>C), TCA1434AAT (4454T>A 4455C>A 4456A>T), CTT1435TTA (4457C>T 4459T>A), AAA1437AAG (4465A>G), TTA1438CTG (4466T>C 4468A>G), CTT1439TTG (4469C>T 4471T>G), TTG1628..C (5038G>C), CTG1631TTG (5045C>T), GCA1632GCT (5050A>T), CCA1634CCC (5056A>C), CAC1635AAT (5057C>A 5059C>T), AAG1636AAA (5062G>A), AGC1637GGG (5063A>G 5065C>G), ATT1638ATA (5068T>A), GGA1639AAT (5069G>A 5070G>A 5071A>T), GTT1640TCA (5072G>T 5073T>C 5074T>A), ATT1642ATC (5080T>C), CCA1643CCG (5083A>G), ACT1646ACA (5092T>A), TAT1647TAC (5095T>C), AGA1648CGT (5096A>C 5098A>T), AGG1649CGC (5099A>C 5101G>C), ACA1650CTT (5102A>C 5103C>T 5104A>T), GGC1651GGA (5107C>A), CAT1653CAA (5113T>A), ACT1654ACA (5116T>A), GAA1655CAA (5117G>C), GAA1657GAG (5125A>G), AAT1658AAC (5128T>C), ATT1661ATA (5137T>A), GCT1662CCA (5138G>C 5140T>A), CTC1664CTT (5146C>T), ACA1665TCA (5147A>T), GAA1667GAT (5155A>T), GTC1668GTA (5158C>A), CCG1669CCA (5161G>A), AAA1671AGA (5166A>G), GTT1672GTG (5170T>G), CAC1673_AGG1674del (5171_5178delCACAGGGA), GAG1675--C (5171_5178delCACAGGGA 5179G>C), GTA1676GTC (5182A>C), GCA1679GCT (5191A>T), GTT1680GTG (5194T>G), TGT1681AAT (5195T>A 5196G>A), GGT1690GGA (5224T>A), AGA1691CGC (5225A>C 5227A>C), CTA1692TTA (5228C>T), ACT1693ACA (5233T>A), AGT1694AGC (5236T>C), CAC1695CAT (5239C>T), GTT1697GTC (5245T>C), TCA1698TCC (5248A>C), GTG1700GTC (5254G>C), TAT1702TAC (5260T>C), TTG1704TTA (5266G>A), CAG1705CAA (5269G>A), ACT1706ACA (5272T>A), TCA1710TCT (5284A>T), ATT1711ATC (5287T>C), GCA1712TCA (5288G>T), AGG1713CGA (5291A>C 5293G>A), ACT1716ACA (5302T>A), ATA1718ATC (5308A>C), AAC1719AAT (5311C>T), CAG1720CAA (5314G>A), GCT1734GCA (5356T>A), AGT1737AGC (5365T>C), ACA1739ACT (5371A>T), ACA1740TGT (5372A>T 5373C>G 5374A>T), AAC1744AAT (5386C>T), TCG1746TCT (5392G>T), CTA1747TTG (5393C>T 5395A>G), GTC1750GTT (5404C>T), ATT1754ATC (5416T>C), CGA1755CGT (5419A>T), CGA1757CGG (5425A>G), GCT1759GCA (5431T>A), AGG1760CGT (5432A>C 5434G>T), CAG1764CAA (5446G>A), TTG1767CTT (5453T>C 5455G>T), CGG1787CGA (5515G>A), GTG1792GTT (5530G>T), CTC1793ATA (5531C>A 5533C>A), GCC1794TCT (5534G>T 5536C>T), AAT1795AAC (5539T>C), GGC1797GGA (5545C>A), GCA1801TCC (5555G>T 5557A>C), TTC1804TTT (5566C>T), CAA1805CAG (5569A>G), TCA1807CAA (5573T>C 5574C>A), GGA1809GAA (5580G>A), TCA1811GCA (5585T>G), AAG1812CGC (5588A>C 5589A>G 5590G>C), TTT1813TTC (5593T>C), GGT1815AAA (5597G>A 5598G>A 5599T>A), TTA1816CTT (5600T>C 5602A>T), GGA1818GGT (5608A>T), CAA1819CAG (5611A>G), AAG1822AGT (5619A>G 5620G>T), TCT1823TCC (5623T>C), TTG1824CTC (5624T>C 5626G>C), ATC1825ATT (5629C>T), ACT1826ACA (5632T>A), AGA1827AGG (5635A>G), GCT1829ATC (5639G>A 5640C>T 5641T>C), ATA1831GTT (5645A>G 5647A>T), ATA1832GTT (5648A>G 5650A>T), GGA1838GGT (5668A>T), GCT1840GCA (5674T>A), CTT1843TTA (5681C>T 5683T>A), TAC1844TAT (5686C>T), AAG1845TCT (5687A>T 5688A>C 5689G>T), CAA1848AAG (5696C>A 5698A>G), AGT1849GAG (5699A>G 5700G>A 5701T>G), AAT1850AGG (5703A>G 5704T>G), TCT1851GTA (5705T>G 5706C>T 5707T>A), AAA1852AGT (5709A>G 5710A>T), GAA1853GAG (5713A>G), GAA1854ACG (5714G>A 5715A>C 5716A>G), CTG1857CCC (5724T>C 5725G>C), CAA1858CAG (5728A>G), AGG1860AGA (5734G>A), GCA1864ACC (5744G>A 5746A>C), AAG1866del (5750_5754delAAGTT), TTG1867--A (5750_5754delAAGTT 5755G>A), CGA1868CGC (5758A>C), GCA1870GCT (5764A>T), TCC1872TCA (5770C>A), GCG1874GCA (5776G>A), GAG1875GAA (5779G>A), AAA1876AAG (5782A>G), AAT1878AAC (5788T>C), GTC1880GTT (5794C>T), GTC1881GTT (5797C>T), GAA1882GAG (5800A>G), GGA1883GGC (5803A>C), GCC1886GCA (5812C>A), GCC1887GCA (5815C>A), CTC1888CTA (5818C>A), ACA1889ACT (5821A>T), CAC1890CAT (5824C>T), TAT1891TAC (5827T>C), GCA1895GCT (5839A>T), GCC1897GCA (5845C>A), TCA1898AAG (5846T>A 5847C>A 5848A>G), AAA1899AAG (5851A>G), AAA1903AAG (5863A>G), ACA1905ACT (5869A>T), GGA1908GGC (5878A>C), CAT1911CAC (5887T>C), AAG1912AAA (5890G>A), AGA1914AGG (5896A>G), AAG1915AAA (5899G>A), TTC1916TTT (5902C>T), GGA1921GGT (5917A>T), CCT1924CCA (5926T>A), ACC1925ACT (5929C>T), GAC1926GAT (5932C>T), TTT1927TTC (5935T>C), CAA1928CAG (5938A>G), GTT1930GTA (5944T>A), CGT1931CGA (5947T>A), GTG1933GTA (5953G>A), CCA1935CCT (5959A>T), TTA1936TTG (5962A>G), GGT1938GGA (5968T>A), CGC1939AAG (5969C>A 5970G>A 5971C>G), GCA1941GTG (5976C>T 5977A>G), GAA1943GAG (5983A>G), TCT1944TCA (5986T>A), CCG1945CCA (5989G>A), ACT1947ACA (5995T>A), GAC1948GAT (5998C>T), GAG1954GAA (6016G>A), GGA1957GGC (6025A>C), AAA1958AAG (6028A>G), ATA1959ATC (6031A>C), GCT1961GAA (6036C>A 6037T>A), TTG2045TTA (6289G>A), GGA2047GGG (6295A>G), GCG2048TCA (6296G>T 6298G>A), TCA2049TCT (6301A>T), CTT2050TTA (6302C>T 6304T>A), GCA2051GCG (6307A>G), GGA2053GGG (6313A>G), GTT2054GTG (6316T>G), CGA2055CGT (6319A>T), ATC2060ATT (6334C>T), TCT2061TCA (6337T>A), GCT2062GCC (6340T>C), GCA2063TCA (6341G>T), CTT2064CTC (6346T>C), TGT2065TGC (6349T>C), AAA2066AAG (6352A>G), CTG2067CTA (6355G>A), ACA2068ACC (6358A>C), GAT2070GAC (6364T>C), GGT2073GGA (6373T>A), GTG2074GTT (6376G>T), AGG2075CGA (6377A>C 6379G>A), ATC2076ATG (6382C>G), TCT2079TCA (6391T>A), GGC2080GGG (6394C>G), GGT2082GGA (6400T>A), GGA2084GGG (6406A>G), TTC2086TAT (6411T>A 6412C>T), ATA2087ATT (6415A>T), ATT2088ATC (6418T>C), ACG2089ACA (6421G>A), AAG2091AAA (6427G>A), TTA2093TTG (6433A>G), TTC2094TTT (6436C>T), AGA2095CGT (6437A>C 6439A>T), GGT2099GGG (6451T>G), ATG2101CTG (6455A>C), GAA2102GAG (6460A>G), ATC2103GTT (6461A>G 6463C>T), CAA2106CAT (6472A>T), GGT2108GGA (6478T>A), TTG2109CTT (6479T>C 6481G>T), ATT2112ATC (6490T>C), CGC2113AGA (6491C>A 6493C>A), CTT2115TTG (6497C>T 6499T>G), AAG2116AAA (6502G>A), ACG2117ACA (6505G>A), TTG2118CTT (6506T>C 6508G>T), TTG2122TTA (6520G>A), GAG2124GAT (6526G>T), AAC2125GGT (6527A>G 6528A>G 6529C>T), GAC2127GAT (6535C>T), CTA2129CTT (6541A>T), ATA2130TTA (6542A>T), ATT2131ATC (6547T>C), AAA2132AAG (6550A>G), GAT2136GAC (6562T>C), TTT2137TTC (6565T>C), GCT2141GCC (6577T>C), TTG2144TTA (6586G>A), AAG2145AAA (6589G>A), TTC2146TTT (6592C>T), CGA2147AGG (6593C>A 6595A>G), CTT2148ATG (6596C>A 6598T>G), GAA2152GAG (6610A>G), GAG2153GAA (6613G>A), AAA2154AAG (6616A>G), GCC2155GCA (6619C>A), GTG2158ATT (6626G>A 6628G>T), CAG2163CAA (6643G>A), ACA2164ACC (6646A>C), AGC2166AGT (6652C>T), ATG2167CTG (6653A>C), CAA2170CTA (6663A>T), GTT2171GTG (6667T>G), ACA2176ATA (6681C>T), ATA2177GTT (6683A>G 6685A>T), GTT2178GTA (6688T>A), CCA2179CCT (6691A>T), AAA2182AAG (6700A>G), TCA2183TCT (6703A>T), CAT2188CAC (6718T>C), ATC2190ATT (6724C>T), ACA2191TCA (6725A>T), GGC2195GGG (6739C>G), CAG2196AAT (6740C>A 6742G>T), GGT2198GGG (6748T>G), CCA2200del (6752_6756delCCATT), TTA2201--C (6752_6756delCCATT 6757A>C), GCT2203GCA (6763T>A), TTG2204TTA (6766G>A), AAA2205AAG (6769A>G), AAG2208AAC (6778G>C), CTT2210CTA (6784T>A), GGA2211GGG (6787A>G), ATT2212ATA (6790T>A), GGT2215GGA (6799T>A), TCG2216TCA (6802G>A), TTA2219CTA (6809T>C), ACA2220ACG (6814A>G), AAT2222AAC (6820T>C), CAC2223CAT (6823C>T), ACC2225ACA (6829C>A), TAC2226TAT (6832C>T), ATT2227ATC (6835T>C), GAA2229GAG (6841A>G), CAA2232CAT (6850A>T), AAT2233GAC (6851A>G 6853T>C), TTG2234ATC (6854T>A 6856G>C), ATA2235TTG (6857A>T 6859A>G), ATG2236AAA (6861T>A 6862G>A), GCA2238TCA (6866G>T), CTC2239ATG (6869C>A 6871C>G), GAA2240GAT (6874A>T), ATG2241CTT (6875A>C 6877G>T), GAT2242GAG (6880T>G), GTG2244GTT (6886G>T), AAA2248AAG (6898A>G), TTC2249TTT (6901C>T), AAT2250AAC (6904T>C), TTG2251CTA (6905T>C 6907G>A), AAT2252AAC (6910T>C), AGC2255AGT (6919C>T), GGC2257GGT (6925C>T), CTC2259TTG (6929C>T 6931C>G), AAA2260AAG (6934A>G), ATT2261CTC (6935A>C 6937T>C), CAA2262CAG (6940A>G), GAG2267GAA (6955G>A), CAG2268GAA (6956C>G 6958G>A), ACT2272ACA (6970T>A), GAG2273GCA (6972A>C 6973G>A), ATT2275ATA (6979T>A), GTT2276ATA (6980G>A 6982T>A), GAT2278GAC (6988T>C), CTT2279TTG (6989C>T 6991T>G), TTT2280CTC (6992T>C 6994T>C), GAT2281GAA (6997T>A), GAG2282GAA (7000G>A), ATT2284ACA (7005T>C 7006T>A), ATA2286GCA (7010A>G 7011T>C), GCC2288TCT (7016G>T 7018C>T), AAT2289CAA (7019A>C 7021T>A), ATG2290ATT (7024G>T), CAA2291CAG (7027A>G), AAT2292AAG (7030T>G), GTG2294GTT (7036G>T), TAC2295TTT (7038A>T 7039C>T), ATT2298ATA (7048T>A), TGT2299CGG (7049T>C

	7051T>G), GAC2300GAT (7054C>T), TTG2302CTT (7058T>C 7060G>T), GTC2303GTA (7063C>A), CCA2304CCT (7066A>T), ATT2305GTC (7067A>G 7069T>C), GCT2306TCC (7070G>T 7072T>C), ACA2307TCC (7073A>T 7075A>C), TGC2308TGT (7078C>T), TTA2312CTT (7088T>C 7090A>T), GTC2313GTT (7093C>T), AAA2315AAG (7099A>G), GTT2317GTG (7105T>G), GTT2318GTG (7108T>G), AAG2319AAA (7111G>A), AAG2321AAA (7117G>A), AAT2323CCA (7121A>C 7122A>C 7123T>A), CTG2324TTG (7124C>T), TTC2325TTT (7129C>T), AGC2326AGT (7132C>T), TTG2327GTC (7133T>G 7135G>C), TAT2328TAC (7138T>C), TTA2329CTG (7139T>C 7141A>G), GCC2330GCT (7144C>T), GAA2333AGT (7151G>A 7152A>G 7153A>T), GAG2334GAT (7156G>T), GAG2335GCA (7158A>C 7159G>A), GAA2336GAG (7162A>G), AAA2337CAA (7163A>C), TAT2338TAC (7168T>C), CGA2340AGA (7172C>A), CCA2341CCT (7177A>T), CTA2342TTG (7178C>T 7180A>G), GGA2344GGC (7186A>C), TAT2346TAC (7192T>C), CTA2347CAA (7194T>A), AGC2349AGT (7201C>T), AAA2350GCA (7202A>G 7203A>C), TTG2351CTA (7205T>C 7207G>A), AGA2353AAG (7212G>A 7213A>G), GAG2354GAA (7216G>A), GCA2355GCT (7219A>T), CTG2357CTT (7225G>T), ATA2360ATT (7234A>T), AAA2362AAG (7240A>G), TAC2363TAT (7243C>T), TCA2364TCG (7246A>G), AGT2365AAT (7248G>A), ACA2367GTA (7253A>G 7254C>T), GAG2368GAA (7258G>A), GGA2370GGT (7264A>T), TCT2371GCT (7265T>G), GTG2372GTC (7270G>C), ACA2374ACT (7276A>T), ATC2376ATT (7282C>T), TTT2377TTC (7285T>C), GAT2378GGT (7287A>G), AGA2379CAG (7289A>C 7290G>A 7291A>G), GCA2380GCC (7294A>C), GTG2381GTC (7297G>C), AAT2382ACT (7299A>C), TTG2384GTC (7304T>G 7306G>C), ATT2385ATA (7309T>A), AAT2386AAC (7312T>C), CTC2388CTT (7318C>T), GAA2389GTT (7320A>T 7321A>T), GGT2392GGC (7330T>C), GTG2399GTT (7351G>T), ATG2403CTG (7361A>C), CAG2404CAA (7366G>A), ATA2405ATC (7369A>C), TTC2406TTT (7372C>T), GCT2408GCA (7378T>A), TTA2409TTG (7381A>G), AAT2410AAC (7384T>C), GCA2414GCT (7396A>T), GCT2417GCC (7405T>C), CTA2418CTT (7408A>T), AAA2420AAG (7414A>G), AAA2423AAG (7423A>G), AGA2424AGG (7426A>G), GAT2425GAC (7429T>C), TAC2426TAT (7432C>T), GCC2428GCA (7438C>A), AAT2429TCT (7439A>T 7440A>C), TGC2430TCA (7443G>C 7444C>A), TCA2431ACA (7445T>A), GCT2433GCC (7453T>C), GAT2434GAC (7456T>C), TTG2435CTT (7457T>C 7459G>T), GAA2436GAG (7462A>G), AAT2437AGG (7464A>G 7465T>G), TTC2438TAC (7467T>A), TTG2439TTA (7471G>A), AGA2440ATG (7473G>T 7474A>G), GCC2441GCA (7477C>A), TGT2443TGC (7483T>C), GAG2444AAA (7484G>A 7486G>A), AGA2445CGC (7487A>C 7489A>C), CTC2446CTT (7492C>T), TTT2447TAT (7494T>A), CTT2448CTC (7498T>C), GGC2449GGT (7501C>T), CTT2451CTC (7507T>C), GGG2452GGA (7510G>A), TTG2453TTA (7513G>A), GGC2456GGA (7522C>A), TCA2457TCC (7525A>C), CTT2458CTA (7528T>A), GAA2461GAG (7537A>G), TTG2462CTG (7538T>C), CGG2463AGA (7541C>A 7543G>A), ATT2465TTG (7547A>T 7549T>G), AAG2467AAA (7555G>A), GTT2468GTG (7558T>G), GAG2469CAA (7559G>C 7561G>A), AAC2471AAT (7567C>T), AAG2472AAA (7570G>A), ACA2473TCA (7571A>T), CGA2474CGC (7576A>C), TTT2476TTC (7582T>C), GCA2479GCT (7591A>T), GTT2481ATA (7595G>A 7597T>A), GAT2482GAC (7600T>C), TTA2484CTT (7604T>C 7606A>T), CTG2485CTA (7609G>A), GCT2486GCA (7612T>A), GGA2487GGG (7615A>G), GTG2491GTA (7627G>A), TTT2494TTC (7636T>C), CAA2497CAG (7645A>G), TTC2498TTT (7648C>T), TAT2499TAC (7651T>C), TCT2500TCA (7654T>A), CTC2501TTA (7655C>T 7657C>A), TTG2503CTC (7661T>C 7663G>C), CAG2504AAG (7664C>A), CCC2506CCA (7672C>A), TCA2507TCT (7675A>T), GTC2509GTT (7681C>T), GGA2510GGT (7684A>T), AAA2513AAG (7693A>G), TTT2514TTC (7696T>C), AGA2516GGA (7700A>G), GGG2517GGT (7705G>T), GAT2519GAC (7711T>C), AAA2520AAG (7714A>G), TTG2521CTT (7715T>C 7717G>T), AGA2523AGG (7723A>G), GGG2524AGC (7724G>A 7726G>C), CTT2525TTA (7727C>T 7729T>A), CCT2526CCG (7732T>G), ACT2530GTT (7742A>G 7743C>T), TGT2532TGC (7750T>C), GCT2534GCA (7756T>A), GAC2535GAT (7759C>T), AGG2538AGA (7768G>A), GAT2540GAC (7774T>C), CTA2543TTG (7781C>T 7783A>G), CCA2545CCT (7789A>T), CTT2547CTC (7795T>C), TTG2548ATC (7796T>A 7798G>C), ATT2551CTC (7805A>C 7807T>C), ATT2552ATC (7810T>C), GTC2554ATC (7814G>A), CTC2556TTA (7820C>T 7822C>A), TAC2557TTT (7824A>T 7825C>T), GAA2560GAG (7834A>G), GAT2563GAC (7843T>C), ATT2564ATA (7846T>A), GTG2567ACC (7853G>A 7854T>C 7855G>C), AAG2570AGA (7863A>G 7864G>A), TAC2573TAT (7873C>T), ACC2574ACT (7876C>T), ATA2581AAC (7896T>A 7897A>C), TCC2582TCA (7900C>A), ACT2587ACG (7915T>G), GTA2588GTT (7918A>T), ATA2589ATT (7921A>T), CAT2592CAC (7930T>C), GGC2594GGT (7936C>T), AAT2595AAC (7939T>C), AAT2596AAC (7942T>C), AGT2597ACT (7944G>C), GGA2598GGG (7948A>G), CAA2599CAG (7951A>G), TCT2601TCA (7957T>A), ACA2602ACT (7960A>T), GTC2603GTT (7963C>T), GTT2604GTG (7966T>G), GAC2605GAT (7969C>T), CTG2608CTT (7978G>T), GTT2610GTA (7984T>A), AAC2611AAT (7987C>T), ATA2612CTA (7988A>C), TTT2615TTC (7999T>C), TAC2616TAT (8002C>T), ATG2618ATT (8008G>T), CAC2619CAT (8011C>T), TCA2620TCT (8014A>T), CGT2621AGA (8015C>A 8017T>A), GAC2622AAC (8018G>A), ATT2623ATC (8023T>C), CCG2624CCT (8026G>T), AAA2625ATT (8028A>T 8029A>T), GAG2626GAT (8032G>T), TCC2627ATG (8033T>A 8034C>T 8035C>G), AGA2629AGG (8041A>G), TTT2630TTC (8044T>C), TAT2631TTT (8046A>T), GTT2632GTA (8050T>A), GGA2634GGG (8056A>G), CTG2637TTG (8063C>T), GCT2640GCA (8074T>A), ATA2641ATG (8077A>G), GAG2642GAA (8080G>A), CAG2644TCT (8084C>T 8085A>C 8086G>T), GAC2647GAT (8095C>T), TTG2649CTT (8099T>C 8101G>T), AAG2650AAA (8104G>A), TTT2652TTC (8110T>C), CAA2653CAG (8113A>G), GCT2654TCA (8114G>T 8116T>A), TTA2655TTG (8119A>G), TTA2657TGT (8124T>G 8125A>T), GGT2660GGA (8134T>A), CTT2661ATG (8135C>A 8137T>G), ATT2662TCA (8138A>T 8139T>C 8140T>A), ACT2664ACA (8146T>A), ACA2669ACT (8161A>T), AAA2670AGG (8163A>G 8164A>G), GAC2671GAT (8167C>T), CGC2672AGA (8168C>A 8170C>A), AGT2673CTA (8171A>C 8172G>T 8173T>A), GTG2675GTT (8179G>T), TCA2679TCC (8191A>C), CAC2680CAT (8194C>T), GCT2682GCA (8200T>A), GTT2683ATG (8201G>A 8203T>G), CTC2684CTG (8206C>G), GGT2687GGA (8215T>A), ATT2688GTC (8216A>G 8218T>C), TAC2689TAT (8221C>T), ATA2690ATC (8224A>C), CCT2691CCA (8227T>A), GAG2696GAA (8242G>A), CGG2697AGA (8243C>A 8245G>A), GTT2699GTA (8251T>A), TCC2700TCA (8254C>A), CTT2702CTC (8260T>C), GAC2705GAT (8269C>T), AGA2706CGA (8270A>C), TCA2707TCT (8275A>T), GGA2708GGT (8278A>T), GAA2709GAG (8281A>G), CCA2710CCC (8284A>C), TCT2711ACT (8285T>A), CGG2713AGG (8291C>A), CTG2714TTG (8294C>T), GAA2715GAG (8299A>G), GAG2723GAA (8323G>A), GAA2729GAG (8341A>G), CTG2730TTG (8342C>T), ACT2731ACA (8347T>A), AAG2732ATG (8349A>T), AGG2733AGA (8353G>A), AGA2735CGA (8357A>C), CTT2739TTG (8369C>T 8371T>G), GAG2743GAA (8383G>A), TAC2747TAT (8395C>T), GAA2753CAA (8411G>C), GGG2754GGA (8416G>A), AGA2755AAA (8418G>A), GCT2756GCA (8422T>A), TAC2758TAT (8428C>T), CTA2759CTG (8431A>G), GCT2760GCC (8434T>C), GAA2761GAG (8437A>G), ACT2762ACG (8440T>G), GCT2763GCA (8443T>A), CTG2764TTG (8444C>T), TTA2767TTG (8455A>G), CTT2769CTA (8461T>A), GAT2770GAC (8464T>C), GCT2771ACC (8465G>A 8467T>C), CCC2773CCA (8473C>A), ACT2774ACG (8476T>G), GAA2775GAT (8479A>T), TTG2776AAA (8480T>A 8481T>A 8482G>A), GAA2777GAG (8485A>G), TTG2778TTA (8488G>A), CTT2779TTG (8489C>T 8491T>G), GCA2782GTT (8499C>T 8500A>T), GGG2783GGT (8503G>T), TGC2784TGT (8506C>T), ATC2785GTC (8507A>G), GGT2787GGA (8515T>A), GAT2788GAG (8518T>G), TAT2789TCT (8520A>C), GAT2790GAC (8524T>C), GAT2791TTG (8525G>T 8526A>T 8527T>G), AGC2792GAT (8528A>G 8529G>A 8530C>T), TTG2793GTA (8531T>G 8533G>A), GCA2794GAA (8535C>A), TCT2795CAA (8537T>C 8538C>A 8539T>A), GAA2796GAG (8542A>G), GAT2797CAC (8543G>C 8545T>C), ATA2798ATG (8548A>G), TAT2799ATA (8549T>A 8550A>T 8551T>A), CTC2800TTC (8552C>T), GCT2802GCA (8560T>A), GAT2803GAC (8563T>C), ACA2804AAA (8565C>A), GGT2808GGA (8578T>A), AGT2809AGC (8581T>C), ACA2811ACC (8587A>C), GCC2813ACC (8591G>A), GGA2814GGT (8596A>T), AAG2815AAA (8599G>A), AAC2816AGT (8601A>G 8602C>T), TCA2818GGA (8606T>G 8607C>G), AGT2824CTC (8624A>C 8625G>T 8626T>C), GTT2825GCT (8628T>C), ACG2827AAA (8634C>A 8635G>A), ACT2828CCT (8636A>C), GGA2829GAC (8640G>A 8641A>C), GAG2830CAA (8642G>C 8644G>A), AAA2831AAG (8647A>G), AAG2832AAC (8650G>C), CTT2834TCT (8654C>T 8655T>C), GTC2835GCC (8658T>C), CAA2838CAG (8668A>G), GTG2842GTT (8680G>T), GCT2844GCA (8686T>A), ACC2849ACG (8701C>G), GTA2852GTT (8710A>T), AAA2856AAG (8722A>G), ATT2858ATA (8728T>A), CCT2859CCC (8731T>C), ACT2860ACA (8734T>A), CTA2864CTC (8746A>C), AAT2869AAG (8761T>G), AAA2870AGA (8763A>G), GTT2872GTC (8770T>C), ATT2873ATC (8773T>C), AGT2874AAT (8775G>A), CTG2875CTT (8779G>T), GAC2876GAT (8782C>T), CTG2878TTG (8786C>T), CTG2879CTC (8791G>C), AAC2880CTG (8792A>C 8793A>T 8794C>G), CCA2883CCT (8803A>T), GAT2887GAC (8815T>C), TCA2889TCT (8821A>T), CAC2895CAA (8839C>A), GAA2899GAG (8851A>G), AAT2903GAT (8861A>G), CGT2904AAA (8864C>A 8865G>A 8866T>A), GTA2905GTG (8869A>G), CGA2906AGA (8870C>A), GTT2911GTC (8887T>C), ACT2912ACA (8890T>A), GAA2914GAG (8896A>G), CAG2915CAA (8899G>A), CAG2917CAA (8905G>A), ATC2919ATT (8911C>T), TTA2923CTT (8921T>C 8923A>T), ATC2928ATT (8938C>T), AAC2935AAT (8959C>T), ATA2936ATT (8962A>T), GGT2938GGC (8968T>C), ACT2941ACC (8977T>C), GAG2946GAC (8992G>C), TTA2953TTG (9013A>G), ATT2957ATA (9025T>A), GAA2958GAG (9028A>G), CCC2962CCA (9040C>A), TTT2964TTC (9046T>C), CAA2966CAG (9052A>G), GCG2969GCA (9061G>A), CAT2970CAC (9064T>C), GAT2973GAC (9073T>C), CTA2974TTA (9074C>T), GAT2985GAC (9109T>C), CCA2990CCT (9124A>T), CGT2991AGA (9125C>A 9127T>A), CGC2996CGT (9142C>T), ATG3001AAT (9156T>A 9157G>T), AGT3002AGC (9160T>C), CTG3003CTT (9163G>T), GAC3009GAT (9181C>T), GTT3013ATC (9191G>A 9193T>C), ACT3014ACC (9196T>C), CCG3015TCG (9197C>T), CGG3016AGG (9200C>A), ACC3019ACT (9211C>T), GCT3030GCA (9244T>A), CGA3034CGT (9256A>T), AAC3036ACA (9261A>C 9262C>A), ACA3037ACG (9265A>G), ACG3038ACC (9268G>C), AAG3039AAA (9271G>A), TTA3040CTC (9272T>C 9274A>C), CTG3043CTC (9283G>C), GAA3051GAG (9307A>G), ACC3054ACA (9316C>A), CAC3057CAT (9325C>T), CAA3060ACT (9332C>A 9333A>C 9334A>T), GTT3062GTG (9340T>G), AGA3064AGG (9346A>G), GGT3065AAC (9347G>A 9348G>A 9349T>C), CTT3070CTA (9364T>A), TAG3076TAA (9382G>A)

Proteins

polyprotein (YP_007346986.1)	298	3076	63.2%	11399	85.2%	1940 (99.8%)	1649 (84.8%)	0/4/3/3	1

	Begin	End	Coverage	Score	Concordance	Matches	Identities	I/D/M/F*	Stop Codons
**NT**	**1045**	**9396**	**60.8%**	**6095**	**52.5%**	**5835 (99.7%)**	**4455 (76.1%)**	**0/19**	
Protein mutations:	L300M (1052T>A), K309A (1079A>G 1080A>C 1081G>A), A330T (1142G>A 1144G>A), R332K (1149G>A), E334D (1156A>T), I335L (1157A>T 1159T>A), D336N (1160G>A), L347Q (1193T>C 1194T>A), T368I (1257C>T 1258A>T), D450E (1504T>A), L451C (1505C>T 1506T>G 1507A>T), S456T (1520T>A 1522T>A), L457T (1523T>A 1524T>C), M458L (1526A>T), S459D (1529A>G 1530G>A), V465I (1547G>A), S468G (1556A>G), V473I (1571G>A), I565V (1847A>G 1849T>G), S572T (1868T>A 1870T>A), K581R (1896A>G), T583E (1901A>G 1902C>A 1903A>G), A584S (1904G>T), P586A (1910C>G 1912C>A), L626I (2030C>A 2032T>A), S631T (2045T>A 2047T>C), A634S (2054G>T 2056A>T), K641N (2077G>C), E642S (2078G>T 2079A>C), I643S (2081A>T 2082T>C), S644E (2084A>G 2085G>A 2086T>A), E645Q (2087G>C 2089A>G), Y648F (2097A>T 2098C>T), R1007K (3174G>A), F1008Y (3177T>A), K1011R (3186A>G), K1012T (3189A>C 3190A>G), A1016I (3200G>A 3201C>T), V1017L (3203G>C), S1018R (3208T>A), I1021V (3215A>G), S1032T (3249G>C), V1049I (3299G>A 3301G>C), R1052K (3309G>A 3310G>A), A1056T (3320G>A), N1061T (3336A>C 3337C>A), K1062H (3338A>C 3340G>T), A1065M (3347G>A 3348C>T), I1070V (3362A>G), N1081E (3395A>G 3397T>A), L1084P (3404T>C 3405T>C 3406A>C), S1088A (3416T>G 3418T>A), R1091K (3426G>A), S1092E (3428A>G 3429G>A 3430C>G), K1093R (3431A>C 3432A>G 3433G>A), V1094I (3434G>A 3436G>C), S1096P (3440T>C), D1098N (3446G>A 3448T>C), T1099K (3450C>A), Q1100A (3452C>G 3453A>C 3454G>T), I1102M (3460C>G), K1103D (3461A>G 3463G>T), I1105V (3467A>G 3469T>G), D1106N (3470G>A), Q1110C (3482C>T 3483A>G 3484G>C), E1113G (3492A>G 3493A>T), T1115Q (3497A>C 3498C>A 3499T>A), Q1121S (3515C>T 3516A>C 3517G>T), Y1125F (3528A>T), V1132I (3548G>A 3550C>T), I1133V (3551A>G), V1137I (3563G>A 3565A>C), Y1149F (3600A>T), K1150R (3602A>C 3603A>G), S1153N (3612G>A), T1166A (3650A>G 3652T>G), K1228Q (3836A>C 3838G>A), E1248S (3896G>A 3897A>G 3898G>T), T1249V (3899A>G 3900C>T 3901C>G), C1271S (3965T>A), F1290Y (4023T>A 4024T>C), K1294R (4035A>G 4036G>A), A1300V (4053C>T 4054T>A), R1369K (4260G>A), S1385T (4307T>A 4309A>C), I1392V (4328A>G 4330C>G), I1394M (4336T>G), N1397S (4344A>G), S1405A (4367T>G), A1413V (4392C>T 4393T>G), V1416I (4400G>A), S1434N (4454T>A 4455C>A 4456A>T), H1635N (5057C>A 5059C>T), S1637G (5063A>G 5065C>G), G1639N (5069G>A 5070G>A 5071A>T), V1640S (5072G>T 5073T>C 5074T>A), T1650L (5102A>C 5103C>T 5104A>T), H1653Q (5113T>A), E1655Q (5117G>C), A1662P (5138G>C 5140T>A), T1665S (5147A>T), E1667D (5155A>T), K1671R (5166A>G), H1673_R1674del (5171_5178delCACAGGGA), C1681N (5195T>A 5196G>A), A1712S (5288G>T), T1740C (5372A>T 5373C>G 5374A>T), L1793I (5531C>A 5533C>A), A1794S (5534G>T 5536C>T), A1801S (5555G>T 5557A>C), S1807Q (5573T>C 5574C>A), G1809E (5580G>A), S1811A (5585T>G), K1812R (5588A>C 5589A>G 5590G>C), G1815K (5597G>A 5598G>A 5599T>A), K1822S (5619A>G 5620G>T), A1829I (5639G>A 5640C>T 5641T>C), I1831V (5645A>G 5647A>T), I1832V (5648A>G 5650A>T), K1845S (5687A>T 5688A>C 5689G>T), Q1848K (5696C>A 5698A>G), S1849E (5699A>G 5700G>A 5701T>G), N1850R (5703A>G 5704T>G), S1851V (5705T>G 5706C>T 5707T>A), K1852S (5709A>G 5710A>T), E1854T (5714G>A 5715A>C 5716A>G), L1857P (5724T>C 5725G>C), A1864T (5744G>A 5746A>C), K1866del (5750_5754delAAGTT), S1898K (5846T>A 5847C>A 5848A>G), R1939K (5969C>A 5970G>A 5971C>G), A1941V (5976C>T 5977A>G), A1961E (6036C>A 6037T>A), A2048S (6296G>T 6298G>A), A2063S (6341G>T), I2076M (6382C>G), F2086Y (6411T>A 6412C>T), M2101L (6455A>C), I2103V (6461A>G 6463C>T), Q2106H (6472A>T), E2124D (6526G>T), N2125G (6527A>G 6528A>G 6529C>T), I2130L (6542A>T), L2148M (6596C>A 6598T>G), V2158I (6626G>A 6628G>T), M2167L (6653A>C), Q2170L (6663A>T), T2176I (6681C>T), I2177V (6683A>G 6685A>T), T2191S (6725A>T), Q2196N (6740C>A 6742G>T), P2200del (6752_6756delCCATT), K2208N (6778G>C), Q2232H (6850A>T), N2233D (6851A>G 6853T>C), L2234I (6854T>A 6856G>C), I2235L (6857A>T 6859A>G), M2236K (6861T>A 6862G>A), A2238S (6866G>T), L2239M (6869C>A 6871C>G), E2240D (6874A>T), M2241L (6875A>C 6877G>T), D2242E (6880T>G), I2261L (6935A>C 6937T>C), Q2268E (6956C>G 6958G>A), E2273A (6972A>C 6973G>A), V2276I (6980G>A 6982T>A), F2280L (6992T>C 6994T>C), D2281E (6997T>A), I2284T (7005T>C 7006T>A), I2286A (7010A>G 7011T>C), A2288S (7016G>T 7018C>T), N2289Q (7019A>C 7021T>A), M2290I (7024G>T), N2292K (7030T>G), Y2295F (7038A>T 7039C>T), C2299R (7049T>C 7051T>G), I2305V (7067A>G 7069T>C), A2306S (7070G>T 7072T>C), T2307S (7073A>T 7075A>C), N2323P (7121A>C 7122A>C 7123T>A), L2327V (7133T>G 7135G>C), E2333S (7151G>A 7152A>G 7153A>T), E2334D (7156G>T), E2335A (7158A>C 7159G>A), K2337Q (7163A>C), L2347Q (7194T>A), K2350A (7202A>G 7203A>C), R2353K (7212G>A 7213A>G), S2365N (7248G>A), T2367V (7253A>G 7254C>T), S2371A (7265T>G), D2378G (7287A>G), R2379Q (7289A>C 7290G>A 7291A>G), N2382T (7299A>C), L2384V (7304T>G 7306G>C), E2389V (7320A>T 7321A>T), M2403L (7361A>C), N2429S (7439A>T 7440A>C), C2430S (7443G>C 7444C>A), S2431T (7445T>A), N2437R (7464A>G 7465T>G), F2438Y (7467T>A), R2440M (7473G>T 7474A>G), E2444K (7484G>A 7486G>A), F2447Y (7494T>A), I2465L (7547A>T 7549T>G), E2469Q (7559G>C 7561G>A), T2473S (7571A>T), V2481I (7595G>A 7597T>A), Q2504K (7664C>A), R2516G (7700A>G), G2524S (7724G>A 7726G>C), T2530V (7742A>G 7743C>T), L2548I (7796T>A 7798G>C), I2551L (7805A>C 7807T>C), V2554I (7814G>A), Y2557F (7824A>T 7825C>T), V2567T (7853G>A 7854T>C 7855G>C), K2570R (7863A>G 7864G>A), I2581N (7896T>A 7897A>C), S2597T (7944G>C), I2612L (7988A>C), M2618I (8008G>T), D2622N (8018G>A), K2625I (8028A>T 8029A>T), E2626D (8032G>T), S2627M (8033T>A 8034C>T 8035C>G), Y2631F (8046A>T), I2641M (8077A>G), Q2644S (8084C>T 8085A>C 8086G>T), A2654S (8114G>T 8116T>A), L2657C (8124T>G 8125A>T), L2661M (8135C>A 8137T>G), I2662S (8138A>T 8139T>C 8140T>A), K2670R (8163A>G 8164A>G), S2673L (8171A>C 8172G>T 8173T>A), V2683M (8201G>A 8203T>G), I2688V (8216A>G 8218T>C), S2711T (8285T>A), K2732M (8349A>T), E2753Q (8411G>C), R2755K (8418G>A), A2771T (8465G>A 8467T>C), E2775D (8479A>T), L2776K (8480T>A 8481T>A 8482G>A), A2782V (8499C>T 8500A>T), I2785V (8507A>G), D2788E (8518T>G), Y2789S (8520A>C), D2791L (8525G>T 8526A>T 8527T>G), S2792D (8528A>G 8529G>A 8530C>T), L2793V (8531T>G 8533G>A), A2794E (8535C>A), S2795Q (8537T>C 8538C>A 8539T>A), D2797H (8543G>C 8545T>C), I2798M (8548A>G), Y2799I (8549T>A 8550A>T 8551T>A), L2800F (8552C>T), T2804K (8565C>A), A2813T (8591G>A), N2816S (8601A>G 8602C>T), S2818G (8606T>G 8607C>G), S2824L (8624A>C 8625G>T 8626T>C), V2825A (8628T>C), T2827K (8634C>A 8635G>A), T2828P (8636A>C), G2829D (8640G>A 8641A>C), E2830Q (8642G>C 8644G>A), K2832N (8650G>C), L2834S (8654C>T 8655T>C), V2835A (8658T>C), N2869K (8761T>G), K2870R (8763A>G), S2874N (8775G>A), N2880L (8792A>C 8793A>T 8794C>G), H2895Q (8839C>A), N2903D (8861A>G), R2904K (8864C>A 8865G>A 8866T>A), E2946D (8992G>C), M3001N (9156T>A 9157G>T), V3013I (9191G>A 9193T>C), P3015S (9197C>T), N3036T (9261A>C 9262C>A), Q3060T (9332C>A 9333A>C 9334A>T), G3065N (9347G>A 9348G>A 9349T>C)								

	Begin	End	Coverage	Score	Concordance	Matches	Identities	I/D/M/F*	Stop Codons
**NT**	**1045**	**9396**	**60.8%**	**6095**	**52.5%**	**5835 (99.7%)**	**4455 (76.1%)**	**0/19**	

Codon mutations:

CGT297..A (1045T>A), GAT298GAC (1048T>C), TTG300ATG (1052T>A), TGT301TGC (1057T>C), GAT303GAC (1063T>C), CTT304TTA (1064C>T 1066T>A), TAC306TAT (1072C>T), AAG309GCA (1079A>G 1080A>C 1081G>A), CAT310CAC (1084T>C), TTT311TTC (1087T>C), TCA312TCT (1090A>T), CAA315CAG (1099A>G), ATT316ATA (1102T>A), TCA317TCG (1105A>G), CTG322TTA (1118C>T 1120G>A), TGT325TGC (1129T>C), AAA326AAG (1132A>G), TAC328TAT (1138C>T), GCG330ACA (1142G>A 1144G>A), GGA331GGG (1147A>G), AGA332AAA (1149G>A), GAA334GAT (1156A>T), ATT335TTA (1157A>T 1159T>A), GAT336AAT (1160G>A), TCG344TCC (1186G>C), GTT345GTA (1189T>A), TCT346TCC (1192T>C), TTA347CAA (1193T>C 1194T>A), TGT348TGC (1198T>C), GGT349GGA (1201T>A), GTC351GTT (1207C>T), GCG352GCT (1210G>T), TTA354TTG (1216A>G), GTG355GTA (1219G>A), CAG357CAA (1225G>A), TTA358CTA (1226T>C), CCA361CCG (1237A>G), GGC363GGG (1243C>G), TGC367TGT (1255C>T), ACA368ATT (1257C>T 1258A>T), CGG437AGA (1463C>A 1465G>A), AAC438AAT (1468C>T), AAG439AAA (1471G>A), CCC441CCA (1477C>A), GTT445GTC (1489T>C), CTT446TTG (1490C>T 1492T>G), GAT450GAA (1504T>A), CTA451TGT (1505C>T 1506T>G 1507A>T), TTA452CTT (1508T>C 1510A>T), TTG453CTG (1511T>C), AAG454AAA (1516G>A), TCT456ACA (1520T>A 1522T>A), TTA457ACA (1523T>A 1524T>C), ATG458TTG (1526A>T), AGT459GAT (1529A>G 1530G>A), GAG462GAA (1540G>A), GGT464GGC (1546T>C), GTA465ATA (1547G>A), AGT468GGT (1556A>G), TTG470CTA (1562T>C 1564G>A), CTT471CTG (1567T>G), GTT473ATT (1571G>A), GCA474GCT (1576A>T), CGA475AGA (1577C>A), TTG477CTC (1583T>C 1585G>C), AAC478AAT (1588C>T), AGA557CGC (1823A>C 1825A>C), AAA558AAG (1828A>G), TTA559CTT (1829T>C 1831A>T), GCA560GCG (1834A>G), GGC562GGA (1840C>A), TTG564CTT (1844T>C 1846G>T), ATT565GTG (1847A>G 1849T>G), TCT572ACA (1868T>A 1870T>A), CTT573TTA (1871C>T 1873T>A), CGG574CGA (1876G>A), GTC578GTT (1888C>T), GGG579GGT (1891G>T), GAG580GAA (1894G>A), AAG581AGG (1896A>G), ATA582ATT (1900A>T), ACA583GAG (1901A>G 1902C>A 1903A>G), GCA584TCA (1904G>T), CCC586GCA (1910C>G 1912C>A), AGC593AGT (1933C>T), CGT595AGA (1937C>A 1939T>A), GAT597GAC (1945T>C), TTC599TTT (1951C>T), TTA600CTT (1952T>C 1954A>T), TAC601TAT (1957C>T), TGC604TGT (1966C>T), ACT607ACA (1975T>A), GGC611GGG (1987C>G), TTG614CTT (1994T>C 1996G>T), CTA615CTT (1999A>T), TTG618CTT (2006T>C 2008G>T), ACC622ACA (2020C>A), AAG624AAA (2026G>A), CAT625CAC (2029T>C), CTT626ATA (2030C>A 2032T>A), GGT629GGC (2041T>C), TCT631ACC (2045T>A 2047T>C), GGT632GGA (2050T>A), GAT633GAC (2053T>C), GCA634TCT (2054G>T 2056A>T), AAA635AAG (2059A>G), TTT636TTC (2062T>C), ATA637ATT (2065A>T), GAT638GAC (2068T>C), CTT639TTA (2069C>T 2071T>A), AAG641AAC (2077G>C), GAA642TCA (2078G>T 2079A>C), ATT643TCT (2081A>T 2082T>C), AGT644GAA (2084A>G 2085G>A 2086T>A), GAA645CAG (2087G>C 2089A>G), GGG646GGA (2092G>A), TAC648TTT (2097A>T 2098C>T), GAA652GAG (2110A>G), GGA653GGT (2113A>T), GCT1006..A (3172T>A), AGG1007AAG (3174G>A), TTC1008TAC (3177T>A), ACC1009ACA (3181C>A), AAC1010AAT (3184C>T), AAG1011AGG (3186A>G), AAA1012ACG (3189A>C 3190A>G), TCA1015TCT (3199A>T), GCT1016ATT (3200G>A 3201C>T), GTT1017CTT (3203G>C), AGT1018AGA (3208T>A), TAT1019TAC (3211T>C), TGT1020TGC (3214T>C), ATA1021GTA (3215A>G), AAA1026AAG (3232A>G), ACG1029ACA (3241G>A), GTT1030GTG (3244T>G), TTG1031CTG (3245T>C), AGT1032ACT (3249G>C), CTG1035TTA (3257C>T 3259G>A), CTA1037TTG (3263C>T 3265A>G), ACC1038ACA (3268C>A), GTC1040GTA (3274C>A), CGA1042CGT (3280A>T), GTT1044GTG (3286T>G), CAT1045CAC (3289T>C), TAC1047TAT (3295C>T), GTG1049ATC (3299G>A 3301G>C), AGG1052AAA (3309G>A 3310G>A), AAT1053AAC (3313T>C), GCA1056ACA (3320G>A), GAG1058GAA (3328G>A), AAC1061ACA (3336A>C 3337C>A), AAG1062CAT (3338A>C 3340G>T), GCA1063GCT (3343A>T), GCG1065ATG (3347G>A 3348C>T), TGC1066TGT (3352C>T), GAA1069GAG (3361A>G), ATT1070GTT (3362A>G), GGT1071GGG (3367T>G), ATC1073ATT (3373C>T), ATC1074ATT (3376C>T), TTC1077TTT (3385C>T), TTC1079TTT (3391C>T), AAT1081GAA (3395A>G 3397T>A), GGT1082GGG (3400T>G), CGT1083AGG (3401C>A 3403T>G), TTA1084CCC (3404T>C 3405T>C 3406A>C), CCG1085CCC (3409G>C), ACT1086ACA (3412T>A), TCT1088GCA (3416T>G 3418T>A), GAA1089GAG (3421A>G), AGA1091AAA (3426G>A), AGC1092GAG (3428A>G 3429G>A 3430C>G), AAG1093CGA (3431A>C 3432A>G 3433G>A), GTG1094ATC (3434G>A 3436G>C), AAC1095AAT (3439C>T), TCA1096CCA (3440T>C), TCT1097TCA (3445T>A), GAT1098AAC (3446G>A 3448T>C), ACA1099AAA (3450C>A), CAG1100GCT (3452C>G 3453A>C 3454G>T), CTA1101CTC (3457A>C), ATC1102ATG (3460C>G), AAG1103GAT (3461A>G 3463G>T), ATT1105GTG (3467A>G 3469T>G), GAC1106AAC (3470G>A), CAT1107CAC (3475T>C), TAT1109TAC (3481T>C), CAG1110TGC (3482C>T 3483A>G 3484G>C), GAA1113GGT (3492A>G 3493A>T), ACT1114ACG (3496T>G), ACT1115CAA (3497A>C 3498C>A 3499T>A), CAG1117CAA (3505G>A), AAG1118AAA (3508G>A), GGT1119GGA (3511T>A), CAG1121TCT (3515C>T 3516A>C 3517G>T), TTC1123TTT (3523C>T), GCC1124GCA (3526C>A), TAC1125TTC (3528A>T), CGC1128CGA (3538C>A), ATA1130ATT (3544A>T), GTC1132ATT (3548G>A 3550C>T), ATA1133GTA (3551A>G), ACT1134ACA (3556T>A), CTG1135CTT (3559G>T), CTT1136CTC (3562T>C), GTA1137ATC (3563G>A 3565A>C), GCT1139GCC (3571T>C), GTT1148GTC (3598T>C), TAC1149TTC (3600A>T), AAA1150CGA (3602A>C 3603A>G), CTC1152TTG (3608C>T 3610C>G), AGC1153AAC (3612G>A), AAA1154AAG (3616A>G), TTT1155TTC (3619T>C), AAA1156AAG (3622A>G), GGA1157GGC (3625A>C), TTA1158CTG (3626T>C 3628A>G), GTG1159GTT (3631G>T), GTT1160GTC (3634T>C), TCT1161TCA (3637T>A), AAG1164AAA (3646G>A), GAA1165GAG (3649A>G), ACT1166GCG (3650A>G 3652T>G), ACT1167ACA (3655T>A), CGT1168AGA (3656C>A 3658T>A), TTG1169CTT (3659T>C 3661G>T), GAG1170GAA (3664G>A), AGT1171TCA (3665A>T 3666G>C 3667T>A), TCT1172TCA (3670T>A), CTT1173TTG (3671C>T 3673T>G), GAC1175GAT (3679C>T), ATC1179ATA (3691C>A), GCA1180GCT (3694A>T), AAT1182AAC (3700T>C), CAA1184CAG (3706A>G), ACA1186ACC (3712A>C), GTG1187GTT (3715G>T), CGT1220AGG (3812C>A 3814T>G), CCA1221CCT (3817A>T), CAC1222CAT (3820C>T), TAC1223TAT (3823C>T), CGC1224AGG (3824C>A 3826C>G), GAA1226GAG (3832A>G), GGA1227GGC (3835A>C), AAG1228CAA (3836A>C 3838G>A), ATT1230ATA (3844T>A), TTT1232TTC (3850T>C), ACT1233ACC (3853T>C), CGA1234AGA (3854C>A), ACT1236ACA (3862T>A), AGC1238AGT (3868C>T), TCA1239TCT (3871A>T), GTG1240GTA (3874G>A), GAG1243GAA (3883G>A), ATT1244ATC (3886T>C), TCA1247TCT (3895A>T), GAG1248AGT (3896G>A 3897A>G 3898G>T), ACC1249GTG (3899A>G 3900C>T 3901C>G), AAG1250AAA (3904G>A), TTA1253CTA (3911T>C), GGT1256GGA (3922T>A), GGT1259GGA (3931T>A), TCT1260TCA (3934T>A), GGT1261GGG (3937T>G), ACT1264ACA (3946T>A), AAT1269AAC (3961T>C), TTG1270CTG (3962T>C), TGC1271AGC (3965T>A), AAG1272AAA (3970G>A), AGA1273AGG (3973A>G), GTC1276GTT (3982C>T), CTG1279CTA (3991G>A), AGA1283CGA (4001A>C), CCT1284CCC (4006T>C), TTG1285TTA (4009G>A), AAT1287AAC (4015T>C), GTG1289GTT (4021G>T), TTT1290TAC (4023T>A 4024T>C), CAG1291CAA (4027G>A), CAA1292CAG (4030A>G), TTG1293CTA (4031T>C 4033G>A), AAG1294AGA (4035A>G 4036G>A), GCA1295GCT (4039A>T), CAG1296CAA (4042G>A), GCT1300GTA (4053C>T 4054T>A), AGT1301AGC (4057T>C), CCA1302CCC (4060A>C), TTG1304CTA (4064T>C 4066G>A), ATA1306ATC (4072A>C), AGA1307CGA (4073A>C), AAA1309AAG (4081A>G), TCG1310TCC (4084G>C), GGT1313GGA (4093T>A), ACC1318ACA (4108C>A), ATT1319ATC (4111T>C), TGC1347TGT (4195C>T), GTG1349GTA (4201G>A), TCT1353TCA (4213T>A), GGA1354GGG (4216A>G), GCT1356GCA (4222T>A), AAT1359AAC (4231T>C), TTG1360CTC (4232T>C 4234G>C), CTT1361TTG (4235C>T 4237T>G), TTT1362TTC (4240T>C), TTT1366TTC (4252T>C), AGA1369AAA (4260G>A), TTG1371CTT (4265T>C 4267G>T), GTG1373GTT (4273G>T), CCG1377CCA (4285G>A), CCA1378CCT (4288A>T), GGC1379GGG (4291C>G), GAA1383GAG (4303A>G), TCA1385ACC (4307T>A 4309A>C), ACA1386ACT (4312A>T), TAT1388TAC (4318T>C), ATC1392GTG (4328A>G 4330C>G), ATT1394ATG (4336T>G), GAA1395GAG (4339A>G), AAC1397AGC (4344A>G), CTA1398CTC (4348A>C), TCT1399TCA (4351T>A), TTC1400TTT (4354C>T), CAG1401CAA (4357G>A), GTG1404GTT (4366G>T), TCA1405GCA (4367T>G), GGT1408GGG (4378T>G), CAG1410CAA (4384G>A), TCA1411TCT (4387A>T), GCT1413GTG (4392C>T 4393T>G), GTC1416ATC (4400G>A), CGA1417CGT (4405A>T), GGA1419GGT (4411A>T), AAC1420AAT (4414C>T), ATA1422ATT (4420A>T), TTA1423CTC (4421T>C 4423A>C), AGT1428AGC (4438T>C), GTT1432GTG (4450T>G), GAT1433GAC (4453T>C), TCA1434AAT (4454T>A 4455C>A 4456A>T), CTT1435TTA (4457C>T 4459T>A), AAA1437AAG (4465A>G), TTA1438CTG (4466T>C 4468A>G), CTT1439TTG (4469C>T 4471T>G), TTG1628..C (5038G>C), CTG1631TTG (5045C>T), GCA1632GCT (5050A>T), CCA1634CCC (5056A>C), CAC1635AAT (5057C>A 5059C>T), AAG1636AAA (5062G>A), AGC1637GGG (5063A>G 5065C>G), ATT1638ATA (5068T>A), GGA1639AAT (5069G>A 5070G>A 5071A>T), GTT1640TCA (5072G>T 5073T>C 5074T>A), ATT1642ATC (5080T>C), CCA1643CCG (5083A>G), ACT1646ACA (5092T>A), TAT1647TAC (5095T>C), AGA1648CGT (5096A>C 5098A>T), AGG1649CGC (5099A>C 5101G>C), ACA1650CTT (5102A>C 5103C>T 5104A>T), GGC1651GGA (5107C>A), CAT1653CAA (5113T>A), ACT1654ACA (5116T>A), GAA1655CAA (5117G>C), GAA1657GAG (5125A>G), AAT1658AAC (5128T>C), ATT1661ATA (5137T>A), GCT1662CCA (5138G>C 5140T>A), CTC1664CTT (5146C>T), ACA1665TCA (5147A>T), GAA1667GAT (5155A>T), GTC1668GTA (5158C>A), CCG1669CCA (5161G>A), AAA1671AGA (5166A>G), GTT1672GTG (5170T>G), CAC1673_AGG1674del (5171_5178delCACAGGGA), GAG1675--C (5171_5178delCACAGGGA 5179G>C), GTA1676GTC (5182A>C), GCA1679GCT (5191A>T), GTT1680GTG (5194T>G), TGT1681AAT (5195T>A 5196G>A), GGT1690GGA (5224T>A), AGA1691CGC (5225A>C 5227A>C), CTA1692TTA (5228C>T), ACT1693ACA (5233T>A), AGT1694AGC (5236T>C), CAC1695CAT (5239C>T), GTT1697GTC (5245T>C), TCA1698TCC (5248A>C), GTG1700GTC (5254G>C), TAT1702TAC (5260T>C), TTG1704TTA (5266G>A), CAG1705CAA (5269G>A), ACT1706ACA (5272T>A), TCA1710TCT (5284A>T), ATT1711ATC (5287T>C), GCA1712TCA (5288G>T), AGG1713CGA (5291A>C 5293G>A), ACT1716ACA (5302T>A), ATA1718ATC (5308A>C), AAC1719AAT (5311C>T), CAG1720CAA (5314G>A), GCT1734GCA (5356T>A), AGT1737AGC (5365T>C), ACA1739ACT (5371A>T), ACA1740TGT (5372A>T 5373C>G 5374A>T), AAC1744AAT (5386C>T), TCG1746TCT (5392G>T), CTA1747TTG (5393C>T 5395A>G), GTC1750GTT (5404C>T), ATT1754ATC (5416T>C), CGA1755CGT (5419A>T), CGA1757CGG (5425A>G), GCT1759GCA (5431T>A), AGG1760CGT (5432A>C 5434G>T), CAG1764CAA (5446G>A), TTG1767CTT (5453T>C 5455G>T), CGG1787CGA (5515G>A), GTG1792GTT (5530G>T), CTC1793ATA (5531C>A 5533C>A), GCC1794TCT (5534G>T 5536C>T), AAT1795AAC (5539T>C), GGC1797GGA (5545C>A), GCA1801TCC (5555G>T 5557A>C), TTC1804TTT (5566C>T), CAA1805CAG (5569A>G), TCA1807CAA (5573T>C 5574C>A), GGA1809GAA (5580G>A), TCA1811GCA (5585T>G), AAG1812CGC (5588A>C 5589A>G 5590G>C), TTT1813TTC (5593T>C), GGT1815AAA (5597G>A 5598G>A 5599T>A), TTA1816CTT (5600T>C 5602A>T), GGA1818GGT (5608A>T), CAA1819CAG (5611A>G), AAG1822AGT (5619A>G 5620G>T), TCT1823TCC (5623T>C), TTG1824CTC (5624T>C 5626G>C), ATC1825ATT (5629C>T), ACT1826ACA (5632T>A), AGA1827AGG (5635A>G), GCT1829ATC (5639G>A 5640C>T 5641T>C), ATA1831GTT (5645A>G 5647A>T), ATA1832GTT (5648A>G 5650A>T), GGA1838GGT (5668A>T), GCT1840GCA (5674T>A), CTT1843TTA (5681C>T 5683T>A), TAC1844TAT (5686C>T), AAG1845TCT (5687A>T 5688A>C 5689G>T), CAA1848AAG (5696C>A 5698A>G), AGT1849GAG (5699A>G 5700G>A 5701T>G), AAT1850AGG (5703A>G 5704T>G), TCT1851GTA (5705T>G 5706C>T 5707T>A), AAA1852AGT (5709A>G 5710A>T), GAA1853GAG (5713A>G), GAA1854ACG (5714G>A 5715A>C 5716A>G), CTG1857CCC (5724T>C 5725G>C), CAA1858CAG (5728A>G), AGG1860AGA (5734G>A), GCA1864ACC (5744G>A 5746A>C), AAG1866del (5750_5754delAAGTT), TTG1867--A (5750_5754delAAGTT 5755G>A), CGA1868CGC (5758A>C), GCA1870GCT (5764A>T), TCC1872TCA (5770C>A), GCG1874GCA (5776G>A), GAG1875GAA (5779G>A), AAA1876AAG (5782A>G), AAT1878AAC (5788T>C), GTC1880GTT (5794C>T), GTC1881GTT (5797C>T), GAA1882GAG (5800A>G), GGA1883GGC (5803A>C), GCC1886GCA (5812C>A), GCC1887GCA (5815C>A), CTC1888CTA (5818C>A), ACA1889ACT (5821A>T), CAC1890CAT (5824C>T), TAT1891TAC (5827T>C), GCA1895GCT (5839A>T), GCC1897GCA (5845C>A), TCA1898AAG (5846T>A 5847C>A 5848A>G), AAA1899AAG (5851A>G), AAA1903AAG (5863A>G), ACA1905ACT (5869A>T), GGA1908GGC (5878A>C), CAT1911CAC (5887T>C), AAG1912AAA (5890G>A), AGA1914AGG (5896A>G), AAG1915AAA (5899G>A), TTC1916TTT (5902C>T), GGA1921GGT (5917A>T), CCT1924CCA (5926T>A), ACC1925ACT (5929C>T), GAC1926GAT (5932C>T), TTT1927TTC (5935T>C), CAA1928CAG (5938A>G), GTT1930GTA (5944T>A), CGT1931CGA (5947T>A), GTG1933GTA (5953G>A), CCA1935CCT (5959A>T), TTA1936TTG (5962A>G), GGT1938GGA (5968T>A), CGC1939AAG (5969C>A 5970G>A 5971C>G), GCA1941GTG (5976C>T 5977A>G), GAA1943GAG (5983A>G), TCT1944TCA (5986T>A), CCG1945CCA (5989G>A), ACT1947ACA (5995T>A), GAC1948GAT (5998C>T), GAG1954GAA (6016G>A), GGA1957GGC (6025A>C), AAA1958AAG (6028A>G), ATA1959ATC (6031A>C), GCT1961GAA (6036C>A 6037T>A), TTG2045TTA (6289G>A), GGA2047GGG (6295A>G), GCG2048TCA (6296G>T 6298G>A), TCA2049TCT (6301A>T), CTT2050TTA (6302C>T 6304T>A), GCA2051GCG (6307A>G), GGA2053GGG (6313A>G), GTT2054GTG (6316T>G), CGA2055CGT (6319A>T), ATC2060ATT (6334C>T), TCT2061TCA (6337T>A), GCT2062GCC (6340T>C), GCA2063TCA (6341G>T), CTT2064CTC (6346T>C), TGT2065TGC (6349T>C), AAA2066AAG (6352A>G), CTG2067CTA (6355G>A), ACA2068ACC (6358A>C), GAT2070GAC (6364T>C), GGT2073GGA (6373T>A), GTG2074GTT (6376G>T), AGG2075CGA (6377A>C 6379G>A), ATC2076ATG (6382C>G), TCT2079TCA (6391T>A), GGC2080GGG (6394C>G), GGT2082GGA (6400T>A), GGA2084GGG (6406A>G), TTC2086TAT (6411T>A 6412C>T), ATA2087ATT (6415A>T), ATT2088ATC (6418T>C), ACG2089ACA (6421G>A), AAG2091AAA (6427G>A), TTA2093TTG (6433A>G), TTC2094TTT (6436C>T), AGA2095CGT (6437A>C 6439A>T), GGT2099GGG (6451T>G), ATG2101CTG (6455A>C), GAA2102GAG (6460A>G), ATC2103GTT (6461A>G 6463C>T), CAA2106CAT (6472A>T), GGT2108GGA (6478T>A), TTG2109CTT (6479T>C 6481G>T), ATT2112ATC (6490T>C), CGC2113AGA (6491C>A 6493C>A), CTT2115TTG (6497C>T 6499T>G), AAG2116AAA (6502G>A), ACG2117ACA (6505G>A), TTG2118CTT (6506T>C 6508G>T), TTG2122TTA (6520G>A), GAG2124GAT (6526G>T), AAC2125GGT (6527A>G 6528A>G 6529C>T), GAC2127GAT (6535C>T), CTA2129CTT (6541A>T), ATA2130TTA (6542A>T), ATT2131ATC (6547T>C), AAA2132AAG (6550A>G), GAT2136GAC (6562T>C), TTT2137TTC (6565T>C), GCT2141GCC (6577T>C), TTG2144TTA (6586G>A), AAG2145AAA (6589G>A), TTC2146TTT (6592C>T), CGA2147AGG (6593C>A 6595A>G), CTT2148ATG (6596C>A 6598T>G), GAA2152GAG (6610A>G), GAG2153GAA (6613G>A), AAA2154AAG (6616A>G), GCC2155GCA (6619C>A), GTG2158ATT (6626G>A 6628G>T), CAG2163CAA (6643G>A), ACA2164ACC (6646A>C), AGC2166AGT (6652C>T), ATG2167CTG (6653A>C), CAA2170CTA (6663A>T), GTT2171GTG (6667T>G), ACA2176ATA (6681C>T), ATA2177GTT (6683A>G 6685A>T), GTT2178GTA (6688T>A), CCA2179CCT (6691A>T), AAA2182AAG (6700A>G), TCA2183TCT (6703A>T), CAT2188CAC (6718T>C), ATC2190ATT (6724C>T), ACA2191TCA (6725A>T), GGC2195GGG (6739C>G), CAG2196AAT (6740C>A 6742G>T), GGT2198GGG (6748T>G), CCA2200del (6752_6756delCCATT), TTA2201--C (6752_6756delCCATT 6757A>C), GCT2203GCA (6763T>A), TTG2204TTA (6766G>A), AAA2205AAG (6769A>G), AAG2208AAC (6778G>C), CTT2210CTA (6784T>A), GGA2211GGG (6787A>G), ATT2212ATA (6790T>A), GGT2215GGA (6799T>A), TCG2216TCA (6802G>A), TTA2219CTA (6809T>C), ACA2220ACG (6814A>G), AAT2222AAC (6820T>C), CAC2223CAT (6823C>T), ACC2225ACA (6829C>A), TAC2226TAT (6832C>T), ATT2227ATC (6835T>C), GAA2229GAG (6841A>G), CAA2232CAT (6850A>T), AAT2233GAC (6851A>G 6853T>C), TTG2234ATC (6854T>A 6856G>C), ATA2235TTG (6857A>T 6859A>G), ATG2236AAA (6861T>A 6862G>A), GCA2238TCA (6866G>T), CTC2239ATG (6869C>A 6871C>G), GAA2240GAT (6874A>T), ATG2241CTT (6875A>C 6877G>T), GAT2242GAG (6880T>G), GTG2244GTT (6886G>T), AAA2248AAG (6898A>G), TTC2249TTT (6901C>T), AAT2250AAC (6904T>C), TTG2251CTA (6905T>C 6907G>A), AAT2252AAC (6910T>C), AGC2255AGT (6919C>T), GGC2257GGT (6925C>T), CTC2259TTG (6929C>T 6931C>G), AAA2260AAG (6934A>G), ATT2261CTC (6935A>C 6937T>C), CAA2262CAG (6940A>G), GAG2267GAA (6955G>A), CAG2268GAA (6956C>G 6958G>A), ACT2272ACA (6970T>A), GAG2273GCA (6972A>C 6973G>A), ATT2275ATA (6979T>A), GTT2276ATA (6980G>A 6982T>A), GAT2278GAC (6988T>C), CTT2279TTG (6989C>T 6991T>G), TTT2280CTC (6992T>C 6994T>C), GAT2281GAA (6997T>A), GAG2282GAA (7000G>A), ATT2284ACA (7005T>C 7006T>A), ATA2286GCA (7010A>G 7011T>C), GCC2288TCT (7016G>T 7018C>T), AAT2289CAA (7019A>C 7021T>A), ATG2290ATT (7024G>T), CAA2291CAG (7027A>G), AAT2292AAG (7030T>G), GTG2294GTT (7036G>T), TAC2295TTT (7038A>T 7039C>T), ATT2298ATA (7048T>A), TGT2299CGG (7049T>C

7051T>G), GAC2300GAT (7054C>T), TTG2302CTT (7058T>C 7060G>T), GTC2303GTA (7063C>A), CCA2304CCT (7066A>T), ATT2305GTC (7067A>G 7069T>C), GCT2306TCC (7070G>T 7072T>C), ACA2307TCC (7073A>T 7075A>C), TGC2308TGT (7078C>T), TTA2312CTT (7088T>C 7090A>T), GTC2313GTT (7093C>T), AAA2315AAG (7099A>G), GTT2317GTG (7105T>G), GTT2318GTG (7108T>G), AAG2319AAA (7111G>A), AAG2321AAA (7117G>A), AAT2323CCA (7121A>C 7122A>C 7123T>A), CTG2324TTG (7124C>T), TTC2325TTT (7129C>T), AGC2326AGT (7132C>T), TTG2327GTC (7133T>G 7135G>C), TAT2328TAC (7138T>C), TTA2329CTG (7139T>C 7141A>G), GCC2330GCT (7144C>T), GAA2333AGT (7151G>A 7152A>G 7153A>T), GAG2334GAT (7156G>T), GAG2335GCA (7158A>C 7159G>A), GAA2336GAG (7162A>G), AAA2337CAA (7163A>C), TAT2338TAC (7168T>C), CGA2340AGA (7172C>A), CCA2341CCT (7177A>T), CTA2342TTG (7178C>T 7180A>G), GGA2344GGC (7186A>C), TAT2346TAC (7192T>C), CTA2347CAA (7194T>A), AGC2349AGT (7201C>T), AAA2350GCA (7202A>G 7203A>C), TTG2351CTA (7205T>C 7207G>A), AGA2353AAG (7212G>A 7213A>G), GAG2354GAA (7216G>A), GCA2355GCT (7219A>T), CTG2357CTT (7225G>T), ATA2360ATT (7234A>T), AAA2362AAG (7240A>G), TAC2363TAT (7243C>T), TCA2364TCG (7246A>G), AGT2365AAT (7248G>A), ACA2367GTA (7253A>G 7254C>T), GAG2368GAA (7258G>A), GGA2370GGT (7264A>T), TCT2371GCT (7265T>G), GTG2372GTC (7270G>C), ACA2374ACT (7276A>T), ATC2376ATT (7282C>T), TTT2377TTC (7285T>C), GAT2378GGT (7287A>G), AGA2379CAG (7289A>C 7290G>A 7291A>G), GCA2380GCC (7294A>C), GTG2381GTC (7297G>C), AAT2382ACT (7299A>C), TTG2384GTC (7304T>G 7306G>C), ATT2385ATA (7309T>A), AAT2386AAC (7312T>C), CTC2388CTT (7318C>T), GAA2389GTT (7320A>T 7321A>T), GGT2392GGC (7330T>C), GTG2399GTT (7351G>T), ATG2403CTG (7361A>C), CAG2404CAA (7366G>A), ATA2405ATC (7369A>C), TTC2406TTT (7372C>T), GCT2408GCA (7378T>A), TTA2409TTG (7381A>G), AAT2410AAC (7384T>C), GCA2414GCT (7396A>T), GCT2417GCC (7405T>C), CTA2418CTT (7408A>T), AAA2420AAG (7414A>G), AAA2423AAG (7423A>G), AGA2424AGG (7426A>G), GAT2425GAC (7429T>C), TAC2426TAT (7432C>T), GCC2428GCA (7438C>A), AAT2429TCT (7439A>T 7440A>C), TGC2430TCA (7443G>C 7444C>A), TCA2431ACA (7445T>A), GCT2433GCC (7453T>C), GAT2434GAC (7456T>C), TTG2435CTT (7457T>C 7459G>T), GAA2436GAG (7462A>G), AAT2437AGG (7464A>G 7465T>G), TTC2438TAC (7467T>A), TTG2439TTA (7471G>A), AGA2440ATG (7473G>T 7474A>G), GCC2441GCA (7477C>A), TGT2443TGC (7483T>C), GAG2444AAA (7484G>A 7486G>A), AGA2445CGC (7487A>C 7489A>C), CTC2446CTT (7492C>T), TTT2447TAT (7494T>A), CTT2448CTC (7498T>C), GGC2449GGT (7501C>T), CTT2451CTC (7507T>C), GGG2452GGA (7510G>A), TTG2453TTA (7513G>A), GGC2456GGA (7522C>A), TCA2457TCC (7525A>C), CTT2458CTA (7528T>A), GAA2461GAG (7537A>G), TTG2462CTG (7538T>C), CGG2463AGA (7541C>A 7543G>A), ATT2465TTG (7547A>T 7549T>G), AAG2467AAA (7555G>A), GTT2468GTG (7558T>G), GAG2469CAA (7559G>C 7561G>A), AAC2471AAT (7567C>T), AAG2472AAA (7570G>A), ACA2473TCA (7571A>T), CGA2474CGC (7576A>C), TTT2476TTC (7582T>C), GCA2479GCT (7591A>T), GTT2481ATA (7595G>A 7597T>A), GAT2482GAC (7600T>C), TTA2484CTT (7604T>C 7606A>T), CTG2485CTA (7609G>A), GCT2486GCA (7612T>A), GGA2487GGG (7615A>G), GTG2491GTA (7627G>A), TTT2494TTC (7636T>C), CAA2497CAG (7645A>G), TTC2498TTT (7648C>T), TAT2499TAC (7651T>C), TCT2500TCA (7654T>A), CTC2501TTA (7655C>T 7657C>A), TTG2503CTC (7661T>C 7663G>C), CAG2504AAG (7664C>A), CCC2506CCA (7672C>A), TCA2507TCT (7675A>T), GTC2509GTT (7681C>T), GGA2510GGT (7684A>T), AAA2513AAG (7693A>G), TTT2514TTC (7696T>C), AGA2516GGA (7700A>G), GGG2517GGT (7705G>T), GAT2519GAC (7711T>C), AAA2520AAG (7714A>G), TTG2521CTT (7715T>C 7717G>T), AGA2523AGG (7723A>G), GGG2524AGC (7724G>A 7726G>C), CTT2525TTA (7727C>T 7729T>A), CCT2526CCG (7732T>G), ACT2530GTT (7742A>G 7743C>T), TGT2532TGC (7750T>C), GCT2534GCA (7756T>A), GAC2535GAT (7759C>T), AGG2538AGA (7768G>A), GAT2540GAC (7774T>C), CTA2543TTG (7781C>T 7783A>G), CCA2545CCT (7789A>T), CTT2547CTC (7795T>C), TTG2548ATC (7796T>A 7798G>C), ATT2551CTC (7805A>C 7807T>C), ATT2552ATC (7810T>C), GTC2554ATC (7814G>A), CTC2556TTA (7820C>T 7822C>A), TAC2557TTT (7824A>T 7825C>T), GAA2560GAG (7834A>G), GAT2563GAC (7843T>C), ATT2564ATA (7846T>A), GTG2567ACC (7853G>A 7854T>C 7855G>C), AAG2570AGA (7863A>G 7864G>A), TAC2573TAT (7873C>T), ACC2574ACT (7876C>T), ATA2581AAC (7896T>A 7897A>C), TCC2582TCA (7900C>A), ACT2587ACG (7915T>G), GTA2588GTT (7918A>T), ATA2589ATT (7921A>T), CAT2592CAC (7930T>C), GGC2594GGT (7936C>T), AAT2595AAC (7939T>C), AAT2596AAC (7942T>C), AGT2597ACT (7944G>C), GGA2598GGG (7948A>G), CAA2599CAG (7951A>G), TCT2601TCA (7957T>A), ACA2602ACT (7960A>T), GTC2603GTT (7963C>T), GTT2604GTG (7966T>G), GAC2605GAT (7969C>T), CTG2608CTT (7978G>T), GTT2610GTA (7984T>A), AAC2611AAT (7987C>T), ATA2612CTA (7988A>C), TTT2615TTC (7999T>C), TAC2616TAT (8002C>T), ATG2618ATT (8008G>T), CAC2619CAT (8011C>T), TCA2620TCT (8014A>T), CGT2621AGA (8015C>A 8017T>A), GAC2622AAC (8018G>A), ATT2623ATC (8023T>C), CCG2624CCT (8026G>T), AAA2625ATT (8028A>T 8029A>T), GAG2626GAT (8032G>T), TCC2627ATG (8033T>A 8034C>T 8035C>G), AGA2629AGG (8041A>G), TTT2630TTC (8044T>C), TAT2631TTT (8046A>T), GTT2632GTA (8050T>A), GGA2634GGG (8056A>G), CTG2637TTG (8063C>T), GCT2640GCA (8074T>A), ATA2641ATG (8077A>G), GAG2642GAA (8080G>A), CAG2644TCT (8084C>T 8085A>C 8086G>T), GAC2647GAT (8095C>T), TTG2649CTT (8099T>C 8101G>T), AAG2650AAA (8104G>A), TTT2652TTC (8110T>C), CAA2653CAG (8113A>G), GCT2654TCA (8114G>T 8116T>A), TTA2655TTG (8119A>G), TTA2657TGT (8124T>G 8125A>T), GGT2660GGA (8134T>A), CTT2661ATG (8135C>A 8137T>G), ATT2662TCA (8138A>T 8139T>C 8140T>A), ACT2664ACA (8146T>A), ACA2669ACT (8161A>T), AAA2670AGG (8163A>G 8164A>G), GAC2671GAT (8167C>T), CGC2672AGA (8168C>A 8170C>A), AGT2673CTA (8171A>C 8172G>T 8173T>A), GTG2675GTT (8179G>T), TCA2679TCC (8191A>C), CAC2680CAT (8194C>T), GCT2682GCA (8200T>A), GTT2683ATG (8201G>A 8203T>G), CTC2684CTG (8206C>G), GGT2687GGA (8215T>A), ATT2688GTC (8216A>G 8218T>C), TAC2689TAT (8221C>T), ATA2690ATC (8224A>C), CCT2691CCA (8227T>A), GAG2696GAA (8242G>A), CGG2697AGA (8243C>A 8245G>A), GTT2699GTA (8251T>A), TCC2700TCA (8254C>A), CTT2702CTC (8260T>C), GAC2705GAT (8269C>T), AGA2706CGA (8270A>C), TCA2707TCT (8275A>T), GGA2708GGT (8278A>T), GAA2709GAG (8281A>G), CCA2710CCC (8284A>C), TCT2711ACT (8285T>A), CGG2713AGG (8291C>A), CTG2714TTG (8294C>T), GAA2715GAG (8299A>G), GAG2723GAA (8323G>A), GAA2729GAG (8341A>G), CTG2730TTG (8342C>T), ACT2731ACA (8347T>A), AAG2732ATG (8349A>T), AGG2733AGA (8353G>A), AGA2735CGA (8357A>C), CTT2739TTG (8369C>T 8371T>G), GAG2743GAA (8383G>A), TAC2747TAT (8395C>T), GAA2753CAA (8411G>C), GGG2754GGA (8416G>A), AGA2755AAA (8418G>A), GCT2756GCA (8422T>A), TAC2758TAT (8428C>T), CTA2759CTG (8431A>G), GCT2760GCC (8434T>C), GAA2761GAG (8437A>G), ACT2762ACG (8440T>G), GCT2763GCA (8443T>A), CTG2764TTG (8444C>T), TTA2767TTG (8455A>G), CTT2769CTA (8461T>A), GAT2770GAC (8464T>C), GCT2771ACC (8465G>A 8467T>C), CCC2773CCA (8473C>A), ACT2774ACG (8476T>G), GAA2775GAT (8479A>T), TTG2776AAA (8480T>A 8481T>A 8482G>A), GAA2777GAG (8485A>G), TTG2778TTA (8488G>A), CTT2779TTG (8489C>T 8491T>G), GCA2782GTT (8499C>T 8500A>T), GGG2783GGT (8503G>T), TGC2784TGT (8506C>T), ATC2785GTC (8507A>G), GGT2787GGA (8515T>A), GAT2788GAG (8518T>G), TAT2789TCT (8520A>C), GAT2790GAC (8524T>C), GAT2791TTG (8525G>T 8526A>T 8527T>G), AGC2792GAT (8528A>G 8529G>A 8530C>T), TTG2793GTA (8531T>G 8533G>A), GCA2794GAA (8535C>A), TCT2795CAA (8537T>C 8538C>A 8539T>A), GAA2796GAG (8542A>G), GAT2797CAC (8543G>C 8545T>C), ATA2798ATG (8548A>G), TAT2799ATA (8549T>A 8550A>T 8551T>A), CTC2800TTC (8552C>T), GCT2802GCA (8560T>A), GAT2803GAC (8563T>C), ACA2804AAA (8565C>A), GGT2808GGA (8578T>A), AGT2809AGC (8581T>C), ACA2811ACC (8587A>C), GCC2813ACC (8591G>A), GGA2814GGT (8596A>T), AAG2815AAA (8599G>A), AAC2816AGT (8601A>G 8602C>T), TCA2818GGA (8606T>G 8607C>G), AGT2824CTC (8624A>C 8625G>T 8626T>C), GTT2825GCT (8628T>C), ACG2827AAA (8634C>A 8635G>A), ACT2828CCT (8636A>C), GGA2829GAC (8640G>A 8641A>C), GAG2830CAA (8642G>C 8644G>A), AAA2831AAG (8647A>G), AAG2832AAC (8650G>C), CTT2834TCT (8654C>T 8655T>C), GTC2835GCC (8658T>C), CAA2838CAG (8668A>G), GTG2842GTT (8680G>T), GCT2844GCA (8686T>A), ACC2849ACG (8701C>G), GTA2852GTT (8710A>T), AAA2856AAG (8722A>G), ATT2858ATA (8728T>A), CCT2859CCC (8731T>C), ACT2860ACA (8734T>A), CTA2864CTC (8746A>C), AAT2869AAG (8761T>G), AAA2870AGA (8763A>G), GTT2872GTC (8770T>C), ATT2873ATC (8773T>C), AGT2874AAT (8775G>A), CTG2875CTT (8779G>T), GAC2876GAT (8782C>T), CTG2878TTG (8786C>T), CTG2879CTC (8791G>C), AAC2880CTG (8792A>C 8793A>T 8794C>G), CCA2883CCT (8803A>T), GAT2887GAC (8815T>C), TCA2889TCT (8821A>T), CAC2895CAA (8839C>A), GAA2899GAG (8851A>G), AAT2903GAT (8861A>G), CGT2904AAA (8864C>A 8865G>A 8866T>A), GTA2905GTG (8869A>G), CGA2906AGA (8870C>A), GTT2911GTC (8887T>C), ACT2912ACA (8890T>A), GAA2914GAG (8896A>G), CAG2915CAA (8899G>A), CAG2917CAA (8905G>A), ATC2919ATT (8911C>T), TTA2923CTT (8921T>C 8923A>T), ATC2928ATT (8938C>T), AAC2935AAT (8959C>T), ATA2936ATT (8962A>T), GGT2938GGC (8968T>C), ACT2941ACC (8977T>C), GAG2946GAC (8992G>C), TTA2953TTG (9013A>G), ATT2957ATA (9025T>A), GAA2958GAG (9028A>G), CCC2962CCA (9040C>A), TTT2964TTC (9046T>C), CAA2966CAG (9052A>G), GCG2969GCA (9061G>A), CAT2970CAC (9064T>C), GAT2973GAC (9073T>C), CTA2974TTA (9074C>T), GAT2985GAC (9109T>C), CCA2990CCT (9124A>T), CGT2991AGA (9125C>A 9127T>A), CGC2996CGT (9142C>T), ATG3001AAT (9156T>A 9157G>T), AGT3002AGC (9160T>C), CTG3003CTT (9163G>T), GAC3009GAT (9181C>T), GTT3013ATC (9191G>A 9193T>C), ACT3014ACC (9196T>C), CCG3015TCG (9197C>T), CGG3016AGG (9200C>A), ACC3019ACT (9211C>T), GCT3030GCA (9244T>A), CGA3034CGT (9256A>T), AAC3036ACA (9261A>C 9262C>A), ACA3037ACG (9265A>G), ACG3038ACC (9268G>C), AAG3039AAA (9271G>A), TTA3040CTC (9272T>C 9274A>C), CTG3043CTC (9283G>C), GAA3051GAG (9307A>G), ACC3054ACA (9316C>A), CAC3057CAT (9325C>T), CAA3060ACT (9332C>A 9333A>C 9334A>T), GTT3062GTG (9340T>G), AGA3064AGG (9346A>G), GGT3065AAC (9347G>A 9348G>A 9349T>C), CTT3070CTA (9364T>A), TAG3076TAA (9382G>A)

*: Inserts / Deletes / Misaligned / Frameshifts

Analysis details

This analysis was performed with panviral2.64

NGS Details (UN24): Solendovirus venanicotianae

Assembly

**Coverage Length**	2027 (5 contig(s))
**Depth Of Coverage**	11.2
**Number Of Reads**	201
**Reads Per Million**	4.02 rpm (after QC)
**Ambiguities**	26
**Assembly Method**	de novo + reference guided assembly
**Consensus Caller**	Bcf Tools

Coverage Map

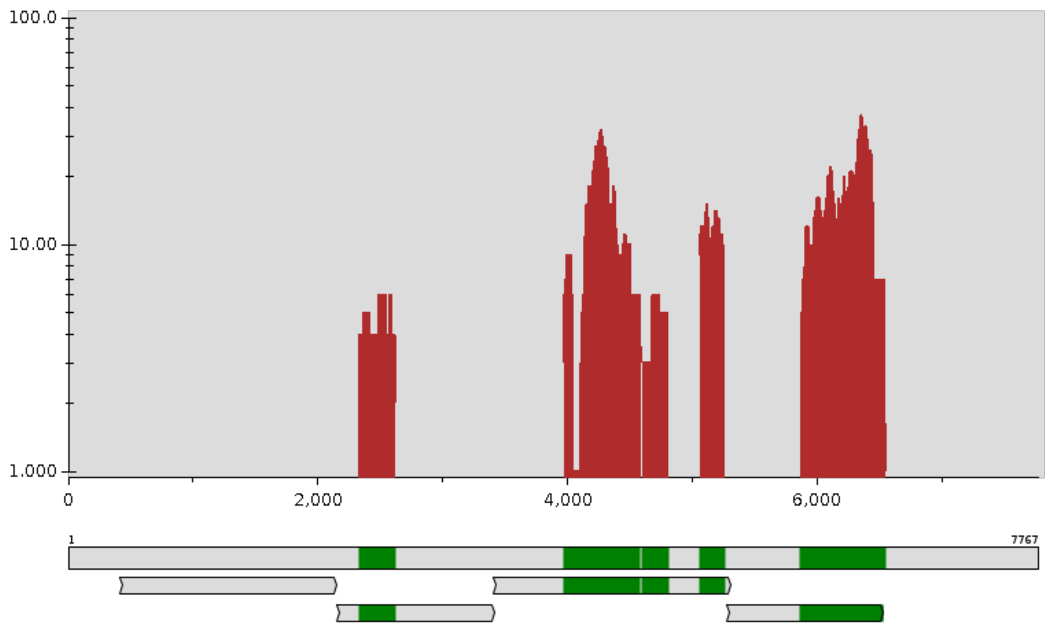

Assignment

**Type**	Solendovirus venanicotianae (Taxonomy ID: 3048371)
**Reference Genome**	NC_003378.1
**NT Identity (%)**	78.1728
**AA Identity (%)**	75.2994
**Number Of Stop Codons**	3
**Number Of CDS**	4

Alignment

**Alignment Score**	2323.0 (NT) + 3342.0 (AA) = 5665.0
**Concordance (%)**	66.0564

Alignment Method	Global, seeded, nucleotide + amino acids (AGA)

Genome Region

Sequence starts at position 2326 and ends at position 6548 relative to NC_003378.1 reference sequence.

Alignment Detailed Statistics

	Begin	End	Coverage	Score	Concordance	Matches	Identities	I/D/M/F*	Stop Codons
**NT**	**2326**	**6548**	**26.1%**	**2323**	**57.5%**	**2025 (99.9%)**	**1583 (78.1%)**	**0/2**	
Mutations:	2326A>T, 2329C>A, 2333A>T, 2347G>A, 2350G>C, 2373T>A, 2379C>T, 2381A>G, 2385T>C, 2388A>T, 2389G>A, 2394T>A, 2397G>A, 2400C>A, 2409A>W, 2410A>C, 2412A>W, 2415A>R, 2417A>S, 2418C>Y, 2420A>R, 2421A>R, 2423G>A, 2424A>R, 2425A>R, 2426T>Y, 2428A>R, 2432A>M, 2433T>M, 2434A>M, 2442A>W, 2443C>R, 2446G>R, 2448T>C, 2454G>A, 2457A>T, 2461A>G, 2463C>A, 2469T>A, 2472G>A, 2475T>C, 2481C>T, 2482T>C, 2493T>C, 2500T>C, 2501T>A, 2505A>T, 2538T>A, 2541A>M, 2545A>M, 2550T>Y, 2562T>Y, 2565A>R, 2569A>R, 2571C>A, 2572C>K, 2574A>W, 2577T>W, 2589C>T, 2602A>G, 2610A>T, 2616T>A, 3974G>T, 3977G>T, 3989A>T, 3991A>C, 4010T>A, 4011G>A, 4013A>T, 4014G>A, 4016G>A, 4017A>G, 4025A>T, 4037A>G, 4040T>A, 4045A>G, 4056A>G, 4059G>A, 4061T>C, 4065A>G, 4066C>A, 4076A>G, 4088A>T, 4091T>A, 4094A>G, 4098G>A, 4100T>A, 4103T>G, 4104A>G, 4105A>G, 4115A>G, 4116G>A, 4121T>C, 4122A>G, 4124T>A, 4127C>T, 4133T>C, 4181A>T, 4187C>T, 4190G>A, 4196C>T, 4199A>T, 4212T>C, 4241T>C, 4247A>T, 4250C>A, 4253T>C, 4265G>A, 4274A>G, 4286T>C, 4292C>T, 4298T>C, 4307C>T, 4313G>A, 4325T>C, 4328T>C, 4331G>A, 4338G>A, 4340G>A, 4362G>A, 4364T>A, 4388T>C, 4400T>A, 4406A>T, 4409T>C, 4415T>C, 4421T>C, 4436C>T, 4451C>T, 4456A>G, 4460T>C, 4481A>T, 4484A>T, 4500C>T, 4503C>T, 4508T>C, 4510C>A, 4513G>A, 4516C>T, 4517A>G, 4518C>A, 4519A>T, 4523T>A, 4524G>C, 4532A>C, 4533A>T, 4534A>T, 4542G>C, 4551G>A, 4552C>T, 4559T>A, 4567A>T, 4568C>A, 4569T>G, 4571T>G, 4574T>A, 4596G>T, 4599G>A, 4604T>A, 4612A>C, 4613T>A, 4616G>A, 4619A>G, 4626T>C, 4637A>G, 4652A>G, 4666C>A, 4667T>A, 4670T>C, 4688C>T, 4692C>T, 4693T>C, 4694T>A, 4695G>A, 4697T>C, 4701A>G, 4703C>T, 4706T>A, 4709T>C, 4722T>C, 4730A>C, 4733T>C, 4734C>T, 4740A>C, 4742A>G, 4745A>C, 4752G>A, 4755A>C, 4757G>T, 4763T>C, 4769T>A, 4784C>T, 4785T>C, 4791C>T, 4793T>A, 4794T>A, 4797C>T, 4799G>T, 4806C>T, 5076T>C, 5077T>A, 5079G>T, 5094C>T, 5117A>T, 5120T>G, 5123C>T, 5125C>A, 5128G>A, 5135T>A, 5136G>A, 5147C>T, 5150T>C, 5156A>G, 5174T>A, 5180A>G, 5189T>C, 5195C>T, 5211C>A, 5217C>T, 5219G>A, 5230T>A, 5234A>G, 5237C>T, 5242C>A, 5256A>T, 5859A>G, 5865C>T, 5868T>C, 5872T>C, 5874G>T, 5877A>T, 5880T>A, 5881T>C, 5882T>A, 5886A>T, 5889T>A, 5892A>C, 5894G>A, 5901A>C, 5902T>C, 5908G>A, 5909C>A, 5910C>A, 5913C>A, 5916C>T, 5921A>G, 5925C>T, 5929C>T, 5931A>G, 5932C>A, 5935G>A, 5937T>C, 5950C>T, 5952G>T, 5953A>G, 5954C>A, 5955A>C, 5956G>A, 5959C>G, 5964G>A, 5968G>T, 5970C>G, 5972A>C, 5973T>C, 5978C>T, 5979T>A, 5986T>C, 5991G>A, 5997C>T, 6001C>A, 6002A>T, 6009G>A, 6012G>A, 6015A>T, 6018T>A, 6022G>A, 6023G>A, 6024G>T, 6026C>A, 6027A>T, 6028T>C, 6033C>T, 6036C>T, 6037G>A, 6040C>A, 6041G>A, 6045C>T, 6049T>A, 6051G>A, 6054T>A, 6055A>C, 6067C>T, 6075A>T, 6078G>A, 6081A>C, 6084G>A, 6087T>A, 6090C>A, 6105A>G, 6108T>A, 6112C>T, 6114C>G, 6120G>A, 6123A>T, 6127A>T, 6129C>A, 6139A>G, 6141G>C, 6145G>A, 6146A>G, 6148G>C, 6150A>G, 6154G>C, 6155A>C, 6157G>A, 6163G>C, 6164A>C, 6169G>A, 6171A>T, 6172A>G, 6174T>G, 6177T>A, 6181G>A, 6182A>G, 6183A>T, 6186C>T, 6189T>C, 6201G>A, 6204A>T, 6211A>C, 6219T>G, 6223C>A, 6225A>C, 6232C>T, 6246T>C, 6247C>A, 6248T>C, 6253C>G, 6254A>G, 6258T>C, 6259G>C, 6264T>G, 6269G>A, 6270C>T, 6271T>C, 6277T>A, 6279A>C, 6281G>A, 6282G>A, 6285A>C, 6290C>T, 6299A>T, 6300T>C, 6301T>G, 6302C>A, 6305A>C, 6306T>G, 6307T>G, 6308G>C, 6309C>A, 6316A>T, 6319A>C, 6321A>G, 6322G>C, 6325G>A, 6340C>G, 6341T>C, 6352G>A, 6354C>A, 6355T>A, 6357A>G, 6359G>C, 6360T>A, 6361C>T, 6363T>G, 6369A>T, 6375A>G, 6376C>A, 6380C>A, 6381T>A, 6382A>C, 6384A>T, 6386C>T, 6387C>T, 6389C>T, 6401G>A, 6402G>A, 6404G>A, 6405G>T, 6406A>G, 6407A>G, 6408T>A, 6411C>T, 6414C>T, 6417C>G, 6418C>G, 6419C>A, 6420A>C, 6423T>A, 6424C>T, 6426G>A, 6427T>C, 6442A>T, 6443A>C, 6444G>A, 6445C>A, 6447A>T, 6450T>C, 6451A>G, 6453T>A, 6454C>A, 6455A>G, 6456C>A, 6459A>T, 6463C>G, 6464C>A, 6465A>T, 6468C>A, 6471C>T, 6472C>A, 6478T>A, 6479C>A, 6480A>C, 6482A>G, 6488A>G, 6489A>T, 6493G>A, 6494A>C, 6495A>C, 6496G>A, 6497A>G, 6501C>T, 6502A>T, 6503T>A, 6505G>A, 6513C>A, 6514G>A, 6516A>C, 6517C>A, 6519A>C, 6520C>T, 6529T>A, 6530_6531delTC								

CDS

	Begin	End	Coverage	Score	Concordance	Matches	Identities	I/D/M/F*	Stop Codons
Tvvgp2	58	156	23.6%	310	46.3%	99 (100%)	74 (74.7%)	0/0/10/0	1
Protein mutations:	R58* (2326A>T), Q59K (2329C>A), K60I (2333A>T), V65I (2347G>A), V66L (2350G>C), K76R (2381A>G), G79R (2389G>A), I86L (2410A>C 2412A>W), R90K (2423G>A 2424A>R), D98B (2446G>R 2448T>C), N103E (2461A>G 2463C>A), L116Q (2500T>C 2501T>A), I131J (2545A>M), I150V (2602A>G)								
Codon mutations:	AGA58TGA (2326A>T), CAA59AAA (2329C>A), AAA60ATA (2333A>T), GTA65ATA (2347G>A), GTT66CTT (2350G>C), ATT73ATA (2373T>A), TGC75TGT (2379C>T), AAA76AGA (2381A>G), TAT77TAC (2385T>C), ACA78ACT (2388A>T), GGA79AGA (2389G>A), GGT80GGA (2394T>A), AAG81AAA (2397G>A), GCC82GCA (2400C>A), CCA85CCW (2409A>W), ATA86CTW (2410A>C 2412A>W), ATA87ATR (2415A>R), AAC88ASY (2417A>S 2418C>Y), AAA89ARR (2420A>R 2421A>R), AGA90AAR (2423G>A 2424A>R), ATA91RYA (2425A>R 2426T>Y), ATA92RTA (2428A>R), AAT93AMM (2432A>M 2433T>M), AAA94MAA (2434A>M), ATA96ATW (2442A>W), CAA97RAA (2443C>R), GAT98RAC (2446G>R 2448T>C), AAG100AAA (2454G>A), GCA101GCT (2457A>T), AAC103GAA (2461A>G 2463C>A), ATT105ATA (2469T>A), AAG106AAA (2472G>A), TAT107TAC (2475T>C), CAC109CAT (2481C>T), TTA110CTA (2482T>C), ACT113ACC (2493T>C), TTA116CAA (2500T>C 2501T>A), ATA117ATT (2505A>T), CCT128CCA (2538T>A), ATA129ATM (2541A>M), ATA131MTA (2545A>M), TAT132TAY (2550T>Y), GAT136GAY (2562T>Y), AGA137AGR (2565A>R), ATC139RTA (2569A>R 2571C>A), CAA140KAW (2572C>K 2574A>W), CCT141CCW (2577T>W), AGC145AGT (2589C>T), ATA150GTA (2602A>G), GGA152GGT (2610A>T), CTT154CTA (2616T>A)								
Tvvgp3	188	617	54.4%	2026	85.1%	346 (100%)	293 (84.7%)	0/0/0/0	1
Protein mutations:	W188C (3974G>T), E189D (3977G>T), K194T (3991A>C), E201N (4011G>A 4013A>T), E202K (4014G>A 4016G>A), N203D (4017A>G), K212R (4045A>G), N216D (4056A>G), D217N (4059G>A 4061T>C), T219E (4065A>G 4066C>A), K226N (4088A>T), D227E (4091T>A), D230K (4098G>A 4100T>A), N231K (4103T>G), K232G (4104A>G 4105A>G), E236K (4116G>A), N238E (4122A>G 4124T>A), E310K (4338G>A 4340G>A), A318T (4362G>A 4364T>A), Y349C (4456A>G), S367* (4510C>A), R368K (4513G>A), T369M (4516C>T 4517A>G), Q370I (4518C>A 4519A>T), D371E (4523T>A), E372Q (4524G>C), K375L (4533A>T 4534A>T), E378Q (4542G>C), A381I (4551G>A 4552C>T), N386I (4567A>T 4568C>A), S387A (4569T>G 4571T>G), A396S (4596G>T), E397K (4599G>A), N401T (4612A>C 4613T>A), I403M (4619A>G), T419K (4666C>A 4667T>A), L428S (4692C>T 4693T>C 4694T>A), D429N (4695G>A 4697T>C), N431D (4701A>G 4703C>T), I444L (4740A>C 4742A>G), V448I (4752G>A), P461S (4791C>T 4793T>A), L462I (4794T>A), Q463Y (4797C>T 4799G>T), F556H (5076T>C 5077T>A), A557S (5079G>T), T572N (5125C>A), R573K (5128G>A), V576I (5136G>A), I607K (5230T>A), T611K (5242C>A), I616L (5256A>T)								
Codon mutations:	TGG188TGT (3974G>T), GAG189GAT (3977G>T), ACA193ACT (3989A>T), AAA194ACA (3991A>C), ATT200ATA (4010T>A), GAA201AAT (4011G>A 4013A>T), GAG202AAA (4014G>A 4016G>A), AAT203GAT (4017A>G), ATA205ATT (4025A>T), AAA209AAG (4037A>G), CCT210CCA (4040T>A), AAA212AGA (4045A>G), AAT216GAT (4056A>G), GAT217AAC (4059G>A 4061T>C), ACA219GAA (4065A>G 4066C>A), AAA222AAG (4076A>G), AAA226AAT (4088A>T), GAT227GAA (4091T>A), TTA228TTG (4094A>G), GAT230AAA (4098G>A 4100T>A), AAT231AAG (4103T>G), AAA232GGA (4104A>G 4105A>G), CAA235CAG (4115A>G), GAA236AAA (4116G>A), AGT237AGC (4121T>C), AAT238GAA (4122A>G 4124T>A), AGC239AGT (4127C>T), CAT241CAC (4133T>C), GGA257GGT (4181A>T), AGC259AGT (4187C>T), AGG260AGA (4190G>A), GTC262GTT (4196C>T), ATA263ATT (4199A>T), TTA268CTA (4212T>C), TAT277TAC (4241T>C), ATA279ATT (4247A>T), CCC280CCA (4250C>A), AAT281AAC (4253T>C), AAG285AAA (4265G>A), CAA288CAG (4274A>G), TAT292TAC (4286T>C), TAC294TAT (4292C>T), AGT296AGC (4298T>C), GAC299GAT (4307C>T), AAG301AAA (4313G>A), TAT305TAC (4325T>C), CAT306CAC (4328T>C), TTG307TTA (4331G>A), GAG310AAA (4338G>A 4340G>A), GCT318ACA (4362G>A 4364T>A), TAT326TAC (4388T>C), GTT330GTA (4400T>A), CCA332CCT (4406A>T), TTT333TTC (4409T>C), TAT335TAC (4415T>C), AAT337AAC (4421T>C), TAC342TAT (4436C>T), GAC347GAT (4451C>T), TAT349TGT (4456A>G), TTT350TTC (4460T>C), ATA357ATT (4481A>T), GTA358GTT (4484A>T), CTA364TTA (4500C>T), CTA365TTA (4503C>T), TAT366TAC (4508T>C), TCA367TAA (4510C>A), AGA368AAA (4513G>A), ACA369ATG (4516C>T 4517A>G), CAA370ATA (4518C>A 4519A>T), GAT371GAA (4523T>A), GAA372CAA (4524G>C), ATA374ATC (4532A>C), AAA375TTA (4533A>T 4534A>T), GAA378CAA (4542G>C), GCA381ATA (4551G>A 4552C>T), ATT383ATA (4559T>A), AAC386ATA (4567A>T 4568C>A), TCT387GCG (4569T>G 4571T>G), GGT388GGA (4574T>A), GCA396TCA (4596G>T), GAA397AAA (4599G>A), ATT398ATA (4604T>A), AAT401ACA (4612A>C 4613T>A), CAG402CAA (4616G>A), ATA403ATG (4619A>G), TTA406CTA (4626T>C), CAA409CAG (4637A>G), GGA414GGG (4652A>G), ACT419AAA (4666C>A 4667T>A), CAT420CAC (4670T>C), ATC426ATT (4688C>T), CTT428TCA (4692C>T 4693T>C 4694T>A), GAT429AAC (4695G>A 4697T>C), AAC431GAT (4701A>G 4703C>T), ATT432ATA (4706T>A), GAT433GAC (4709T>C), TTA438CTA (4722T>C), TCA440TCC (4730A>C), TTT441TTC (4733T>C), CTA442TTA (4734C>T), ATA444CTG (4740A>C 4742A>G), GTA445GTC (4745A>C), GTA448ATA (4752G>A), AGG449CGT (4755A>C 4757G>T), TAT451TAC (4763T>C), CCT453CCA (4769T>A), AAC458AAT (4784C>T), TTA459CTA (4785T>C), CCT461TCA (4791C>T 4793T>A), TTA462ATA (4794T>A), CAG463TAT (4797C>T 4799G>T), CTA466TTA (4806C>T), TTT556CAT (5076T>C 5077T>A), GCA557TCA (5079G>T), CTA562TTA (5094C>T), ATA569ATT (5117A>T), GTT570GTG (5120T>G), TAC571TAT (5123C>T), ACC572AAC (5125C>A), AGA573AAA (5128G>A), ATT575ATA (5135T>A), GTA576ATA (5136G>A), GAC579GAT (5147C>T), AAT580AAC (5150T>C), CAA582CAG (5156A>G), ACT588ACA (5174T>A), AAA590AAG (5180A>G), GAT593GAC (5189T>C), GTC595GTT (5195C>T), CGG601AGG (5211C>A), CTG603TTA (5217C>T 5219G>A), ATA607AAA (5230T>A), TTA608TTG (5234A>G), AAC609AAT (5237C>T), ACA611AAA (5242C>A), ATA616TTA (5256A>T)								
Tvvgp4	196	418	53.3%	1006	66.1%	223 (100%)	136 (61.0%)	0/0/0/0	1
Protein mutations:	L203Q (5881T>C 5882T>A), N205K (5889T>A), S207N (5894G>A), K209N (5901A>C), A212K (5908G>A 5909C>A 5910C>A), N216S (5921A>G), L220I (5932C>A), D221N (5935G>A 5937T>C), Q226Y (5950C>T 5952G>T), T227D (5953A>G 5954C>A 5955A>C), G228R (5956G>A), Q229E (5959C>G), A232S (5968G>T 5970C>G), N233T (5972A>C 5973T>C), P235L (5978C>T 5979T>A), Y238H (5986T>C), Q243I (6001C>A 6002A>T), G250N (6022G>A 6023G>A 6024G>T), T251N (6026C>A 6027A>T), V255I (6037G>A), R256K (6040C>A 6041G>A), S259T (6049T>A 6051G>A), T261P (6055A>C), E267D (6075A>T), E283D (6123A>T), I285L (6127A>T 6129C>A), K289D (6139A>G 6141G>C), E291R (6145G>A 6146A>G), E292Q (6148G>C 6150A>G), E294P (6154G>C 6155A>C), E295K (6157G>A), E297P (6163G>C 6164A>C), V299I (6169G>A 6171A>T), N300E (6172A>G 6174T>G), E303S (6181G>A 6182A>G 6183A>T), I313L (6211A>C), L317I (6223C>A 6225A>C), L325T (6247C>A 6248T>C), Q327G (6253C>G 6254A>G), A329P (6259G>C), I330M (6264T>G), S332N (6269G>A 6270C>T), Y333H (6271T>C), S335T (6277T>A 6279A>C), R336K (6281G>A 6282G>A), E337D (6285A>C), T339I (6290C>T), Y342F (6299A>T 6300T>C), S343E (6301T>G 6302C>A), N344T (6305A>C 6306T>G), C345A (6307T>G 6308G>C 6309C>A), I348L (6316A>T), E350Q (6322G>C), A351T (6325G>A), L356A (6340C>G 6341T>C), V360I (6352G>A 6354C>A), L361M (6355T>A 6357A>G), S362T (6359G>C 6360T>A), K365N (6369A>T), Q368K (6376C>A), P369Q (6380C>A 6381T>A), T370P (6382A>C 6384A>T), T371I (6386C>T 6387C>T), T372I (6389C>T), R376K (6401G>A 6402G>A), R377N (6404G>A 6405G>T), N378G (6406A>G 6407A>G 6408T>A), P382D (6418C>G 6419C>A 6420A>C), D383E (6423T>A), K390S (6442A>T 6443A>C 6444G>A), L391I (6445C>A 6447A>T), S393G (6451A>G 6453T>A), H394R (6454C>A 6455A>G 6456C>A), K395N (6459A>T), P397D (6463C>G 6464C>A 6465A>T), D398E (6468C>A), L400I (6472C>A), S402N (6478T>A 6479C>A 6480A>C), K403R (6482A>G), K405S (6488A>G 6489A>T), E407T (6493G>A 6494A>C 6495A>C), D408S (6496G>A 6497A>G), I410Y (6502A>T 6503T>A), V411I (6505G>A), D413E (6513C>A), V414I (6514G>A 6516A>C), Q415N (6517C>A 6519A>C)								

	Begin	End	Coverage	Score	Concordance	Matches	Identities	I/D/M/F*	Stop Codons
**NT**	**2326**	**6548**	**26.1%**	**2323**	**57.5%**	**2025 (99.9%)**	**1583 (78.1%)**	**0/2**	
Codon mutations:	ACA195..G (5859A>G), TAC197TAT (5865C>T), AAT198AAC (5868T>C), TTG200CTT (5872T>C 5874G>T), ATA201ATT (5877A>T), GCT202GCA (5880T>A), TTA203CAA (5881T>C 5882T>A), CCA204CCT (5886A>T), AAT205AAA (5889T>A), ACA206ACC (5892A>C), AGT207AAT (5894G>A), AAA209AAC (5901A>C), TTA210CTA (5902T>C), GCC212AAA (5908G>A 5909C>A 5910C>A), ACC213ACA (5913C>A), TGC214TGT (5916C>T), AAC216AGC (5921A>G), TAC217TAT (5925C>T), CTA219TTG (5929C>T 5931A>G), CTA220ATA (5932C>A), GAT221AAC (5935G>A 5937T>C), CAG226TAT (5950C>T 5952G>T), ACA227GAC (5953A>G 5954C>A 5955A>C), GGA228AGA (5956G>A), CAA229GAA (5959C>G), GAG230GAA (5964G>A), GCC232TCG (5968G>T 5970C>G), AAT233ACC (5972A>C 5973T>C), CCT235CTA (5978C>T 5979T>A), TAC238CAC (5986T>C), AAG239AAA (5991G>A), TTC241TTT (5997C>T), CAA243ATA (6001C>A 6002A>T), AAG245AAA (6009G>A), AGG246AGA (6012G>A), ATA247ATT (6015A>T), ACT248ACA (6018T>A), GGG250AAT (6022G>A 6023G>A 6024G>T), ACA251AAT (6026C>A 6027A>T), TTA252CTA (6028T>C), TTC253TTT (6033C>T), TAC254TAT (6036C>T), GTA255ATA (6037G>A), CGA256AAA (6040C>A 6041G>A), TTC257TTT (6045C>T), TCG259ACA (6049T>A 6051G>A), GCT260GCA (6054T>A), ACA261CCA (6055A>C), CTA265TTA (6067C>T), GAA267GAT (6075A>T), GAG268GAA (6078G>A), ATA269ATC (6081A>C), AAG270AAA (6084G>A), CCT271CCA (6087T>A), ATC272ATA (6090C>A), AAA277AAG (6105A>G), ATT278ATA (6108T>A), CTC280TTG (6112C>T 6114C>G), AGG282AGA (6120G>A), GAA283GAT (6123A>T), ATC285TTA (6127A>T 6129C>A), AAG289GAC (6139A>G 6141G>C), GAA291AGA (6145G>A 6146A>G), GAA292CAG (6148G>C 6150A>G), GAA294CCA (6154G>C 6155A>C), GAG295AAG (6157G>A), GAA297CCA (6163G>C 6164A>C), GTA299ATT (6169G>A 6171A>T), AAT300GAG (6172A>G 6174T>G), ATT301ATA (6177T>A), GAA303AGT (6181G>A 6182A>G 6183A>T), TTC304TTT (6186C>T), TAT305TAC (6189T>C), AGG309AGA (6201G>A), ATA310ATT (6204A>T), ATA313CTA (6211A>C), ACT315ACG (6219T>G), CTA317ATC (6223C>A 6225A>C), CTA320TTA (6232C>T), TAT324TAC (6246T>C), CTA325ACA (6247C>A 6248T>C), CAG327GGG (6253C>G 6254A>G), AAT328AAC (6258T>C), GCA329CCA (6259G>C), ATT330ATG (6264T>G), AGC332AAT (6269G>A 6270C>T), TAT333CAT (6271T>C), TCA335ACC (6277T>A 6279A>C), AGG336AAA (6281G>A 6282G>A), GAA337GAC (6285A>C), ACA339ATA (6290C>T), TAT342TTC (6299A>T 6300T>C), TCA343GAA (6301T>G 6302C>A), AAT344ACG (6305A>C 6306T>G), TGC345GCA (6307T>G 6308G>C 6309C>A), ATA348TTA (6316A>T), AGA349CGG (6319A>C 6321A>G), GAA350CAA (6322G>C), GCA351ACA (6325G>A), CTA356GCA (6340C>G 6341T>C), GTC360ATA (6352G>A 6354C>A), TTA361ATG (6355T>A 6357A>G), AGT362ACA (6359G>C 6360T>A), CTT363TTG (6361C>T 6363T>G), AAA365AAT (6369A>T), GAA367GAG (6375A>G), CAA368AAA (6376C>A), CCT369CAA (6380C>A 6381T>A), ACA370CCT (6382A>C 6384A>T), ACC371ATT (6386C>T 6387C>T), ACA372ATA (6389C>T), AGG376AAA (6401G>A 6402G>A), AGG377AAT (6404G>A 6405G>T), AAT378GGA (6406A>G 6407A>G 6408T>A), TTC379TTT (6411C>T), ATC380ATT (6414C>T), TCC381TCG (6417C>G), CCA382GAC (6418C>G 6419C>A 6420A>C), GAT383GAA (6423T>A), CTG384TTA (6424C>T 6426G>A), TTA385CTA (6427T>C), AAG390TCA (6442A>T 6443A>C 6444G>A), CTA391ATT (6445C>A 6447A>T), ATT392ATC (6450T>C), AGT393GGA (6451A>G 6453T>A), CAC394AGA (6454C>A 6455A>G 6456C>A), AAA395AAT (6459A>T), CCA397GAT (6463C>G 6464C>A 6465A>T), GAC398GAA (6468C>A), CAC399CAT (6471C>T), CTA400ATA (6472C>A), TCA402AAC (6478T>A 6479C>A 6480A>C), AAA403AGA (6482A>G), AAA405AGT (6488A>G 6489A>T), GAA407ACC (6493G>A 6494A>C 6495A>C), GAT408AGT (6496G>A 6497A>G), AAC409AAT (6501C>T), ATT410TAT (6502A>T 6503T>A), GTT411ATT (6505G>A), GAC413GAA (6513C>A), GTA414ATC (6514G>A 6516A>C), CAA415AAC (6517C>A 6519A>C), CTG416TTG (6520C>T)								

Proteins

	Begin	End	Coverage	Score	Concordance	Matches	Identities	I/D/M/F*	Stop Codons
putative cell-to-cell movement protein (NP_569140.1)	58	156	23.6%	310	46.3%	99 (100%)	74 (74.7%)	0/0/10/0	1
Protein mutations:	R58* (2326A>T), Q59K (2329C>A), K60I (2333A>T), V65I (2347G>A), V66L (2350G>C), K76R (2381A>G), G79R (2389G>A), I86L (2410A>C 2412A>W), R90K (2423G>A 2424A>R), D98B (2446G>R 2448T>C), N103E (2461A>G 2463C>A), L116Q (2500T>C 2501T>A), I131J (2545A>M), I150V (2602A>G)								
Codon mutations:	AGA58TGA (2326A>T), CAA59AAA (2329C>A), AAA60ATA (2333A>T), GTA65ATA (2347G>A), GTT66CTT (2350G>C), ATT73ATA (2373T>A), TGC75TGT (2379C>T), AAA76AGA (2381A>G), TAT77TAC (2385T>C), ACA78ACT (2388A>T), GGA79AGA (2389G>A), GGT80GGA (2394T>A), AAG81AAA (2397G>A), GCC82GCA (2400C>A), CCA85CCW (2409A>W), ATA86CTW (2410A>C 2412A>W), ATA87ATR (2415A>R), AAC88ASY (2417A>S 2418C>Y), AAA89ARR (2420A>R 2421A>R), AGA90AAR (2423G>A 2424A>R), ATA91RYA (2425A>R 2426T>Y), ATA92RTA (2428A>R), AAT93AMM (2432A>M 2433T>M), AAA94MAA (2434A>M), ATA96ATW (2442A>W), CAA97RAA (2443C>R), GAT98RAC (2446G>R 2448T>C), AAG100AAA (2454G>A), GCA101GCT (2457A>T), AAC103GAA (2461A>G 2463C>A), ATT105ATA (2469T>A), AAG106AAA (2472G>A), TAT107TAC (2475T>C), CAC109CAT (2481C>T), TTA110CTA (2482T>C), ACT113ACC (2493T>C), TTA116CAA (2500T>C 2501T>A), ATA117ATT (2505A>T), CCT128CCA (2538T>A), ATA129ATM (2541A>M), ATA131MTA (2545A>M), TAT132TAY (2550T>Y), GAT136GAY (2562T>Y), AGA137AGR (2565A>R), ATC139RTA (2569A>R 2571C>A), CAA140KAW (2572C>K 2574A>W), CCT141CCW (2577T>W), AGC145AGT (2589C>T), ATA150GTA (2602A>G), GGA152GGT (2610A>T), CTT154CTA (2616T>A)								
polyprotein (NP_569141.1)	188	617	54.4%	2026	85.1%	346 (100%)	293 (84.7%)	0/0/0/0	1
Protein mutations:	W188C (3974G>T), E189D (3977G>T), K194T (3991A>C), E201N (4011G>A 4013A>T), E202K (4014G>A 4016G>A), N203D (4017A>G), K212R (4045A>G), N216D (4056A>G), D217N (4059G>A 4061T>C), T219E (4065A>G 4066C>A), K226N (4088A>T), D227E (4091T>A), D230K (4098G>A 4100T>A), N231K (4103T>G), K232G (4104A>G 4105A>G), E236K (4116G>A), N238E (4122A>G 4124T>A), E310K (4338G>A 4340G>A), A318T (4362G>A 4364T>A), Y349C (4456A>G), S367* (4510C>A), R368K (4513G>A), T369M (4516C>T 4517A>G), Q370I (4518C>A 4519A>T), D371E (4523T>A), E372Q (4524G>C), K375L (4533A>T 4534A>T), E378Q (4542G>C), A381I (4551G>A 4552C>T), N386I (4567A>T 4568C>A), S387A (4569T>G 4571T>G), A396S (4596G>T), E397K (4599G>A), N401T (4612A>C 4613T>A), I403M (4619A>G), T419K (4666C>A 4667T>A), L428S (4692C>T 4693T>C 4694T>A), D429N (4695G>A 4697T>C), N431D (4701A>G 4703C>T), I444L (4740A>C 4742A>G), V448I (4752G>A), P461S (4791C>T 4793T>A), L462I (4794T>A), Q463Y (4797C>T 4799G>T), F556H (5076T>C 5077T>A), A557S (5079G>T), T572N (5125C>A), R573K (5128G>A), V576I (5136G>A), I607K (5230T>A), T611K (5242C>A), I616L (5256A>T)								
Codon mutations:	TGG188TGT (3974G>T), GAG189GAT (3977G>T), ACA193ACT (3989A>T), AAA194ACA (3991A>C), ATT200ATA (4010T>A), GAA201AAT (4011G>A 4013A>T), GAG202AAA (4014G>A 4016G>A), AAT203GAT (4017A>G), ATA205ATT (4025A>T), AAA209AAG (4037A>G), CCT210CCA (4040T>A), AAA212AGA (4045A>G), AAT216GAT (4056A>G), GAT217AAC (4059G>A 4061T>C), ACA219GAA (4065A>G 4066C>A), AAA222AAG (4076A>G), AAA226AAT (4088A>T), GAT227GAA (4091T>A), TTA228TTG (4094A>G), GAT230AAA (4098G>A 4100T>A), AAT231AAG (4103T>G), AAA232GGA (4104A>G 4105A>G), CAA235CAG (4115A>G), GAA236AAA (4116G>A), AGT237AGC (4121T>C), AAT238GAA (4122A>G 4124T>A), AGC239AGT (4127C>T), CAT241CAC (4133T>C), GGA257GGT (4181A>T), AGC259AGT (4187C>T), AGG260AGA (4190G>A), GTC262GTT (4196C>T), ATA263ATT (4199A>T), TTA268CTA (4212T>C), TAT277TAC (4241T>C), ATA279ATT (4247A>T), CCC280CCA (4250C>A), AAT281AAC (4253T>C), AAG285AAA (4265G>A), CAA288CAG (4274A>G), TAT292TAC (4286T>C), TAC294TAT (4292C>T), AGT296AGC (4298T>C), GAC299GAT (4307C>T), AAG301AAA (4313G>A), TAT305TAC (4325T>C), CAT306CAC (4328T>C), TTG307TTA (4331G>A), GAG310AAA (4338G>A 4340G>A), GCT318ACA (4362G>A 4364T>A), TAT326TAC (4388T>C), GTT330GTA (4400T>A), CCA332CCT (4406A>T), TTT333TTC (4409T>C), TAT335TAC (4415T>C), AAT337AAC (4421T>C), TAC342TAT (4436C>T), GAC347GAT (4451C>T), TAT349TGT (4456A>G), TTT350TTC (4460T>C), ATA357ATT (4481A>T), GTA358GTT (4484A>T), CTA364TTA (4500C>T), CTA365TTA (4503C>T), TAT366TAC (4508T>C), TCA367TAA (4510C>A), AGA368AAA (4513G>A), ACA369ATG (4516C>T 4517A>G), CAA370ATA (4518C>A 4519A>T), GAT371GAA (4523T>A), GAA372CAA (4524G>C), ATA374ATC (4532A>C), AAA375TTA (4533A>T 4534A>T), GAA378CAA (4542G>C), GCA381ATA (4551G>A 4552C>T), ATT383ATA (4559T>A), AAC386ATA (4567A>T 4568C>A), TCT387GCG (4569T>G 4571T>G), GGT388GGA (4574T>A), GCA396TCA (4596G>T), GAA397AAA (4599G>A), ATT398ATA (4604T>A), AAT401ACA (4612A>C 4613T>A), CAG402CAA (4616G>A), ATA403ATG (4619A>G), TTA406CTA (4626T>C), CAA409CAG (4637A>G), GGA414GGG (4652A>G), ACT419AAA (4666C>A 4667T>A), CAT420CAC (4670T>C), ATC426ATT (4688C>T), CTT428TCA (4692C>T 4693T>C 4694T>A), GAT429AAC (4695G>A 4697T>C), AAC431GAT (4701A>G 4703C>T), ATT432ATA (4706T>A), GAT433GAC (4709T>C), TTA438CTA (4722T>C), TCA440TCC (4730A>C), TTT441TTC (4733T>C), CTA442TTA (4734C>T), ATA444CTG (4740A>C 4742A>G), GTA445GTC (4745A>C), GTA448ATA (4752G>A), AGG449CGT (4755A>C 4757G>T), TAT451TAC (4763T>C), CCT453CCA (4769T>A), AAC458AAT (4784C>T), TTA459CTA (4785T>C), CCT461TCA (4791C>T 4793T>A), TTA462ATA (4794T>A), CAG463TAT (4797C>T 4799G>T), CTA466TTA (4806C>T), TTT556CAT (5076T>C 5077T>A), GCA557TCA (5079G>T), CTA562TTA (5094C>T), ATA569ATT (5117A>T), GTT570GTG (5120T>G), TAC571TAT (5123C>T), ACC572AAC (5125C>A), AGA573AAA (5128G>A), ATT575ATA (5135T>A), GTA576ATA (5136G>A), GAC579GAT (5147C>T), AAT580AAC (5150T>C), CAA582CAG (5156A>G), ACT588ACA (5174T>A), AAA590AAG (5180A>G), GAT593GAC (5189T>C), GTC595GTT (5195C>T), CGG601AGG (5211C>A), CTG603TTA (5217C>T 5219G>A), ATA607AAA (5230T>A), TTA608TTG (5234A>G), AAC609AAT (5237C>T), ACA611AAA (5242C>A), ATA616TTA (5256A>T)								
putative transactivator factor (NP_569142.1)	196	418	53.3%	1006	66.1%	223 (100%)	136 (61.0%)	0/0/0/0	1
Protein mutations:	L203Q (5881T>C 5882T>A), N205K (5889T>A), S207N (5894G>A), K209N (5901A>C), A212K (5908G>A 5909C>A 5910C>A), N216S (5921A>G), L220I (5932C>A), D221N (5935G>A 5937T>C), Q226Y (5950C>T 5952G>T), T227D (5953A>G 5954C>A 5955A>C), G228R (5956G>A), Q229E (5959C>G), A232S (5968G>T 5970C>G), N233T (5972A>C 5973T>C), P235L (5978C>T 5979T>A), Y238H (5986T>C), Q243I (6001C>A 6002A>T), G250N (6022G>A 6023G>A 6024G>T), T251N (6026C>A 6027A>T), V255I (6037G>A), R256K (6040C>A 6041G>A), S259T (6049T>A 6051G>A), T261P (6055A>C), E267D (6075A>T), E283D (6123A>T), I285L (6127A>T 6129C>A), K289D (6139A>G 6141G>C), E291R (6145G>A 6146A>G), E292Q (6148G>C 6150A>G), E294P (6154G>C 6155A>C), E295K (6157G>A), E297P (6163G>C 6164A>C), V299I (6169G>A 6171A>T), N300E (6172A>G 6174T>G), E303S (6181G>A 6182A>G 6183A>T), I313L (6211A>C), L317I (6223C>A 6225A>C), L325T (6247C>A 6248T>C), Q327G (6253C>G 6254A>G), A329P (6259G>C), I330M (6264T>G), S332N (6269G>A 6270C>T), Y333H (6271T>C), S335T (6277T>A 6279A>C), R336K (6281G>A 6282G>A), E337D (6285A>C), T339I (6290C>T), Y342F (6299A>T 6300T>C), S343E (6301T>G 6302C>A), N344T (6305A>C 6306T>G), C345A (6307T>G 6308G>C 6309C>A), I348L (6316A>T), E350Q (6322G>C), A351T (6325G>A), L356A (6340C>G 6341T>C), V360I (6352G>A 6354C>A), L361M (6355T>A 6357A>G), S362T (6359G>C 6360T>A), K365N (6369A>T), Q368K (6376C>A), P369Q (6380C>A 6381T>A), T370P (6382A>C 6384A>T), T371I (6386C>T 6387C>T), T372I (6389C>T), R376K (6401G>A 6402G>A), R377N (6404G>A 6405G>T), N378G (6406A>G 6407A>G 6408T>A), P382D (6418C>G 6419C>A 6420A>C), D383E (6423T>A), K390S (6442A>T 6443A>C 6444G>A), L391I (6445C>A 6447A>T), S393G (6451A>G 6453T>A), H394R (6454C>A 6455A>G 6456C>A), K395N (6459A>T), P397D (6463C>G 6464C>A 6465A>T), D398E (6468C>A), L400I (6472C>A), S402N (6478T>A 6479C>A 6480A>C), K403R (6482A>G), K405S (6488A>G 6489A>T), E407T (6493G>A 6494A>C 6495A>C), D408S (6496G>A 6497A>G), I410Y (6502A>T 6503T>A), V411I (6505G>A), D413E (6513C>A), V414I (6514G>A 6516A>C), Q415N (6517C>A 6519A>C)								

	Begin	End	Coverage	Score	Concordance	Matches	Identities	I/D/M/F*	Stop Codons
**NT**	**2326**	**6548**	**26.1%**	**2323**	**57.5%**	**2025 (99.9%)**	**1583 (78.1%)**	**0/2**	
Codon mutations:	ACA195..G (5859A>G), TAC197TAT (5865C>T), AAT198AAC (5868T>C), TTG200CTT (5872T>C 5874G>T), ATA201ATT (5877A>T), GCT202GCA (5880T>A), TTA203CAA (5881T>C 5882T>A), CCA204CCT (5886A>T), AAT205AAA (5889T>A), ACA206ACC (5892A>C), AGT207AAT (5894G>A), AAA209AAC (5901A>C), TTA210CTA (5902T>C), GCC212AAA (5908G>A 5909C>A 5910C>A), ACC213ACA (5913C>A), TGC214TGT (5916C>T), AAC216AGC (5921A>G), TAC217TAT (5925C>T), CTA219TTG (5929C>T 5931A>G), CTA220ATA (5932C>A), GAT221AAC (5935G>A 5937T>C), CAG226TAT (5950C>T 5952G>T), ACA227GAC (5953A>G 5954C>A 5955A>C), GGA228AGA (5956G>A), CAA229GAA (5959C>G), GAG230GAA (5964G>A), GCC232TCG (5968G>T 5970C>G), AAT233ACC (5972A>C 5973T>C), CCT235CTA (5978C>T 5979T>A), TAC238CAC (5986T>C), AAG239AAA (5991G>A), TTC241TTT (5997C>T), CAA243ATA (6001C>A 6002A>T), AAG245AAA (6009G>A), AGG246AGA (6012G>A), ATA247ATT (6015A>T), ACT248ACA (6018T>A), GGG250AAT (6022G>A 6023G>A 6024G>T), ACA251AAT (6026C>A 6027A>T), TTA252CTA (6028T>C), TTC253TTT (6033C>T), TAC254TAT (6036C>T), GTA255ATA (6037G>A), CGA256AAA (6040C>A 6041G>A), TTC257TTT (6045C>T), TCG259ACA (6049T>A 6051G>A), GCT260GCA (6054T>A), ACA261CCA (6055A>C), CTA265TTA (6067C>T), GAA267GAT (6075A>T), GAG268GAA (6078G>A), ATA269ATC (6081A>C), AAG270AAA (6084G>A), CCT271CCA (6087T>A), ATC272ATA (6090C>A), AAA277AAG (6105A>G), ATT278ATA (6108T>A), CTC280TTG (6112C>T 6114C>G), AGG282AGA (6120G>A), GAA283GAT (6123A>T), ATC285TTA (6127A>T 6129C>A), AAG289GAC (6139A>G 6141G>C), GAA291AGA (6145G>A 6146A>G), GAA292CAG (6148G>C 6150A>G), GAA294CCA (6154G>C 6155A>C), GAG295AAG (6157G>A), GAA297CCA (6163G>C 6164A>C), GTA299ATT (6169G>A 6171A>T), AAT300GAG (6172A>G 6174T>G), ATT301ATA (6177T>A), GAA303AGT (6181G>A 6182A>G 6183A>T), TTC304TTT (6186C>T), TAT305TAC (6189T>C), AGG309AGA (6201G>A), ATA310ATT (6204A>T), ATA313CTA (6211A>C), ACT315ACG (6219T>G), CTA317ATC (6223C>A 6225A>C), CTA320TTA (6232C>T), TAT324TAC (6246T>C), CTA325ACA (6247C>A 6248T>C), CAG327GGG (6253C>G 6254A>G), AAT328AAC (6258T>C), GCA329CCA (6259G>C), ATT330ATG (6264T>G), AGC332AAT (6269G>A 6270C>T), TAT333CAT (6271T>C), TCA335ACC (6277T>A 6279A>C), AGG336AAA (6281G>A 6282G>A), GAA337GAC (6285A>C), ACA339ATA (6290C>T), TAT342TTC (6299A>T 6300T>C), TCA343GAA (6301T>G 6302C>A), AAT344ACG (6305A>C 6306T>G), TGC345GCA (6307T>G 6308G>C 6309C>A), ATA348TTA (6316A>T), AGA349CGG (6319A>C 6321A>G), GAA350CAA (6322G>C), GCA351ACA (6325G>A), CTA356GCA (6340C>G 6341T>C), GTC360ATA (6352G>A 6354C>A), TTA361ATG (6355T>A 6357A>G), AGT362ACA (6359G>C 6360T>A), CTT363TTG (6361C>T 6363T>G), AAA365AAT (6369A>T), GAA367GAG (6375A>G), CAA368AAA (6376C>A), CCT369CAA (6380C>A 6381T>A), ACA370CCT (6382A>C 6384A>T), ACC371ATT (6386C>T 6387C>T), ACA372ATA (6389C>T), AGG376AAA (6401G>A 6402G>A), AGG377AAT (6404G>A 6405G>T), AAT378GGA (6406A>G 6407A>G 6408T>A), TTC379TTT (6411C>T), ATC380ATT (6414C>T), TCC381TCG (6417C>G), CCA382GAC (6418C>G 6419C>A 6420A>C), GAT383GAA (6423T>A), CTG384TTA (6424C>T 6426G>A), TTA385CTA (6427T>C), AAG390TCA (6442A>T 6443A>C 6444G>A), CTA391ATT (6445C>A 6447A>T), ATT392ATC (6450T>C), AGT393GGA (6451A>G 6453T>A), CAC394AGA (6454C>A 6455A>G 6456C>A), AAA395AAT (6459A>T), CCA397GAT (6463C>G 6464C>A 6465A>T), GAC398GAA (6468C>A), CAC399CAT (6471C>T), CTA400ATA (6472C>A), TCA402AAC (6478T>A 6479C>A 6480A>C), AAA403AGA (6482A>G), AAA405AGT (6488A>G 6489A>T), GAA407ACC (6493G>A 6494A>C 6495A>C), GAT408AGT (6496G>A 6497A>G), AAC409AAT (6501C>T), ATT410TAT (6502A>T 6503T>A), GTT411ATT (6505G>A), GAC413GAA (6513C>A), GTA414ATC (6514G>A 6516A>C), CAA415AAC (6517C>A 6519A>C), CTG416TTG (6520C>T)								

*: Inserts / Deletes / Misaligned / Frameshifts

Analysis details

This analysis was performed with panviral2.64

NGS Details (UN24): Potato virus Y

Assembly

**Coverage Length**	3067 (7 contig(s))
**Depth Of Coverage**	3.3
**Number Of Reads**	77
**Reads Per Million**	1.54 rpm (after QC)
**Ambiguities**	0
**Assembly Method**	de novo + reference guided assembly
**Consensus Caller**	Bcf Tools

Coverage Map

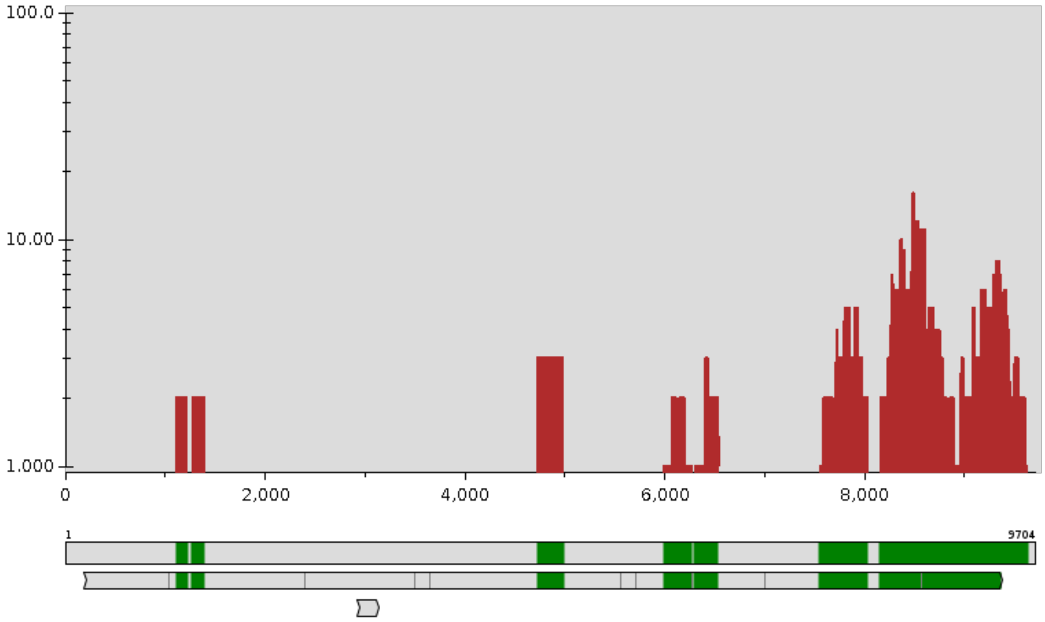

Assignment

**Type**	Potato virus Y (Taxonomy ID: 12216)
**Reference Genome**	NC_001616.1
**NT Identity (%)**	84.5098
**AA Identity (%)**	90.7824
**Number Of Stop Codons**	1
**Number Of CDS**	2

Alignment

**Alignment Score**	4154.0 (NT) + 6023.0 (AA) = 10177.0
**Concordance (%)**	81.0916

Alignment Method	Global, seeded, nucleotide + amino acids (AGA)

Genome Region

Sequence starts at position 1103 and ends at position 9641 relative to NC_001616.1 reference sequence.

Alignment Detailed Statistics

	Begin	End	Coverage	Score	Concordance	Matches	Identities	I/D/M/F*	Stop Codons
**NT**	**1103**	**9641**	**31.6%**	**4154**	**68.5%**	**3057 (99.6%)**	**2586 (84.2%)**	**3/10**	

Mutations: 1105C>T, 1108A>G, 1114A>G, 1117T>G, 1123A>G, 1126T>A, 1129C>T, 1143G>A, 1147A>T, 1150T>A, 1154T>A, 1156G>A, 1165C>T, 1168T>C, 1172C>T, 1174T>A, 1201C>T, 1219C>T, 1221G>A, 1265A>G, 1267A>G, 1273T>C, 1276G>A, 1278A>G, 1283T>C, 1290C>T, 1291G>A, 1298G>A, 1299A>C, 1300C>T, 1306T>C, 1307A>G, 1309A>G, 1312T>C, 1316A>G, 1318T>A, 1327G>A, 1329T>C, 1333G>C, 1336A>G, 1337G>A, 1343C>T, 1345A>G, 1354G>A, 1357G>T, 1359A>T, 1360C>T, 1361C>T, 1363G>A, 1367C>T, 1369C>G, 1373C>A, 1378C>T, 1384G>A, 1385A>G, 1393A>G, 1396C>T, 4738A>G, 4744A>T, 4747A>G, 4748T>C, 4765T>C, 4796G>A, 4798C>T, 4803C>G, 4804T>C, 4807G>A, 4810G>T, 4813T>G, 4816T>C, 4831T>C, 4834C>T, 4840A>T, 4843G>A, 4852A>G, 4853_4858delGGCGTT, 4859T>G, 4864T>C, 4867T>C, 4870G>C, 4873T>A, 4882T>C, 4903A>T, 4909G>A, 4919T>C, 4921G>A, 4924T>C, 4927C>G, 4933T>C, 4936C>A, 4939G>A, 4942T>C, 4945C>T, 4963A>G, 4972T>C, 4990T>C, 5986T>A, 5989C>T, 5990A>G, 6008A>C, 6010A>T, 6019A>T, 6022G>T, 6025A>T, 6030A>C, 6049C>A, 6058G>A, 6061A>G, 6064C>A, 6070T>C, 6072G>A, 6073T>C, 6074A>C, 6079G>A, 6081C>A, 6085A>T, 6091A>T, 6094C>T, 6111G>C, 6112T>C, 6118A>G, 6124G>A, 6127G>A, 6128A>G, 6130T>C, 6133T>C, 6136A>G, 6148C>T, 6157A>G, 6158G>A, 6163T>C, 6169A>G, 6170A>T, 6172A>G, 6178C>T, 6187A>G, 6190T>C, 6196G>A, 6203C>T, 6205C>T, 6208A>G, 6209C>T, 6211A>G, 6217G>A, 6229T>A, 6230G>A, 6238C>T, 6247G>A, 6253A>T, 6257G>A, 6258C>A, 6274T>C, 6298C>T, 6301G>A, 6304A>G, 6307C>T, 6313C>T, 6322C>T, 6328A>T, 6331T>G, 6334T>C, 6337G>A, 6338C>T, 6346A>G, 6358T>C, 6361G>A, 6362G>A, 6367A>C, 6370G>A, 6376C>T, 6379T>G, 6385A>C, 6391A>T, 6394A>G, 6397C>T, 6401G>A, 6405C>T, 6406G>A, 6412C>T, 6418A>G, 6421T>C, 6429A>T, 6436T>A, 6445G>A, 6450A>G, 6454C>A, 6460C>T, 6463T>A, 6466A>G, 6469C>T, 6472G>A, 6475G>A, 6481T>C, 6485C>A, 6487C>T, 6490T>C, 6496C>T, 6502G>A, 6514T>C, 6517G>A, 6527C>A, 6532C>A, 7546T>C, 7570G>A, 7573G>A, 7579G>A, 7588T>C, 7595C>T, 7604T>C, 7606G>A, 7615T>A, 7624T>C, 7633C>T, 7636C>T, 7639T>C, 7642T>C, 7648T>C, 7666A>G, 7672T>C, 7678A>T, 7684G>A, 7690T>C, 7696T>C, 7702T>G, 7709G>A, 7713A>G, 7714A>G, 7715C>T, 7726T>C, 7735G>A, 7744A>G, 7747C>T, 7762C>T, 7768G>A, 7771T>C, 7783A>C, 7786T>C, 7793C>T, 7795A>G, 7798C>T, 7804T>C, 7812C>T, 7834A>G, 7837C>T, 7844G>T, 7846G>A, 7855G>A, 7861G>A, 7870A>G, 7873C>T, 7876T>A, 7879G>A, 7882T>A, 7883G>A, 7894A>T, 7897T>C, 7921C>T, 7927G>A, 7936T>C, 7939T>C, 7942T>C, 7954T>C, 7966G>A, 7969T>C, 7975T>C, 7987C>T, 7990T>C, 8005T>C, 8032A>G, 8038C>T, 8151A>G, 8152C>T, 8164G>A, 8178G>A, 8182G>A, 8191G>A, 8197C>T, 8208G>A, 8213C>T, 8218A>G, 8221C>T, 8224G>A, 8239A>G, 8260T>C, 8263A>G, 8281T>C, 8284A>G, 8294C>G, 8296A>G, 8299G>A, 8302C>T, 8311A>G, 8317T>C, 8320C>T, 8326T>A, 8335G>A, 8338C>T, 8349C>T, 8353A>G, 8362C>T, 8365A>G, 8368C>T, 8380C>T, 8383A>G, 8389A>G, 8404T>C, 8408G>T, 8413A>G, 8419G>T, 8434T>C, 8440T>C, 8452G>A, 8458A>G, 8460G>A, 8464A>G, 8467G>A, 8480G>A, 8482T>A, 8497G>A, 8498C>T, 8500A>G, 8502G>A, 8503A>G, 8506C>T, 8515A>T, 8524C>T, 8527A>C, 8528T>C, 8530A>G, 8539G>C, 8546C>T, 8547T>G, 8552T>A, 8563A>G, 8574C>G, 8585A>G, 8598G>A, 8602C>T, 8603A>T, 8604A>C, 8605C>T, 8620A>G, 8622C>A, 8625A>G, 8626G>A, 8629G>A, 8632C>T, 8638C>T, 8641G>A, 8647C>T, 8648C>T, 8649C>T, 8650G>C, 8653C>T, 8656A>G, 8658G>A, 8664A>C, 8665T>G, 8674T>G, 8679C>T, 8680A>T, 8683C>A, 8692G>A, 8695A>T, 8707G>A, 8708A>C, 8716G>A, 8722C>T, 8725G>A, 8731A>G, 8737A>G, 8743C>T, 8745C>A, 8746A>G, 8749C>T, 8752G>A, 8755A>T, 8770C>T, 8771T>C, 8776A>G, 8779T>C, 8782G>A, 8791T>C, 8797A>G, 8800A>G, 8803A>G, 8809T>C, 8812T>C, 8818T>C, 8827A>G, 8838A>C, 8848G>A, 8854T>C, 8857G>A, 8863G>A, 8865G>A, 8867A>C, 8869G>T, 8872A>G, 8882G>A, 8883G>A, 8888A>T, 8929C>T, 8954G>A, 8959C>T, 8962A>T, 8983G>A, 8984A>G, 8988A>T, 8998G>A, 9004G>A, 9007G>A, 9016C>T, 9037C>A, 9085A>T, 9097C>T, 9131A>G, 9136A>T, 9145G>T, 9149A>G, 9150T>G, 9151G>A, 9152G>A, 9157A>G, 9160G>T, 9163T>C, 9184G>A, 9196A>G, 9214G>A, 9217A>G, 9220G>A, 9244A>T, 9247G>A, 9256C>T, 9260C>T, 9262T>C, 9274G>A, 9280C>T, 9289C>T, 9304G>A, 9334C>T, 9364C>G, 9377_9378insT, 9387C>T, 9404G>A, 9411C>T, 9412A>G, 9414A>T, 9423G>A, 9427T>G, 9454A>G, 9458T>C, 9467G>A, 9470_9471insT, 9482G>A, 9483C>T, 9496T>C, 9516T>G, 9517delA, 9533G>A, 9534G>T, 9548A>T, 9551C>T, 9553_9554delTA, 9556A>T, 9557T>A, 9558A>T, 9574T>A, 9581G>T, 9582T>G, 9583G>C, 9584_9585insG, 9590C>T, 9591T>C, 9607C>T, 9610T>C, 9615delC, 9620A>G

CDS

	Begin	End	Coverage	Score	Concordance	Matches	Identities	I/D/M/F*	Stop Codons
PVYgp1	307	3064	30.5%	6023	91.8%	933 (99.8%)	847 (90.6%)	0/2/0/0	1

Protein mutations: R320K (1143G>A), L324I (1154T>A 1156G>A), S346N (1221G>A), R361G (1265A>G 1267A>G), N365S (1278A>G), A369V (1290C>T 1291G>A), D372T (1298G>A 1299A>C 1300C>T), I375V (1307A>G 1309A>G), N378E (1316A>G 1318T>A), I382T (1329T>C), E385K (1337G>A), D392V (1359A>T 1360C>T), L397I (1373C>A), I401V (1385A>G), V1538I (4796G>A 4798C>T), T1540S (4803C>G 4804T>C), G1557_V1558del (4853_4858delGGCGTT), S1559A (4859T>G), I1936V (5990A>G), E1945D (6019A>T), K1949T (6030A>C), D1955E (6049C>A), M1958I (6058G>A), S1963N (6072G>A 6073T>C), N1964H (6074A>C), T1966N (6081C>A), C1976S (6111G>C 6112T>C), I1982V (6128A>G 6130T>C), V1992I (6158G>A), T1996S (6170A>T 6172A>G), L2007F (6203C>T 6205C>T), V2016I (6230G>A), A2025K (6257G>A 6258C>A), A2060T (6362G>A), V2073I (6401G>A), A2074V (6405C>T 6406G>A), Y2082F (6429A>T), Q2089R (6450A>G), H2101N (6485C>A 6487C>T), L2115I (6527C>A), D2509N (7709G>A), K2510R (7713A>G 7714A>G), T2543I (7812C>T), V2554L (7844G>T 7846G>A), V2567I (7883G>A), N2656S (8151A>G 8152C>T), R2665K (8178G>A), R2675K (8208G>A), P2704A (8294C>G 8296A>G), S2722F (8349C>T), A2742S (8408G>T), M2756I (8452G>A), R2759K (8460G>A), A2766T (8480G>A 8482T>A), R2773K (8502G>A 8503A>G), E2777D (8515A>T), E2785D (8539G>C), L2788C (8546C>T 8547T>G), S2790T (8552T>A), A2797G (8574C>G), I2801V (8585A>G), G2805E (8598G>A), N2807S (8603A>T 8604A>C 8605C>T), P2813Q (8622C>A), E2814G (8625A>G 8626G>A), P2822F (8648C>T 8649C>T 8650G>C), G2825E (8658G>A), D2827A (8664A>C 8665T>G), A2832V (8679C>T 8680A>T), T2854K (8745C>A 8746A>G), Q2885P (8838A>C), R2894Q (8865G>A), M2895L (8867A>C 8869G>T), G2900K (8882G>A 8883G>A), T2902S (8888A>T), V2924I (8954G>A), N2934D (8984A>G), E2935V (8988A>T), I2983V (9131A>G), M2989G (9149A>G 9150T>G 9151G>A), G2990S (9152G>A), P3026S (9260C>T 9262T>C)

	Begin	End	Coverage	Score	Concordance	Matches	Identities	I/D/M/F*	Stop Codons
**NT**	**1103**	**9641**	**31.6%**	**4154**	**68.5%**	**3057 (99.6%)**	**2586 (84.2%)**	**3/10**	

Codon mutations: CAC307CAT (1105C>T), ACA308ACG (1108A>G), GTA310GTG (1114A>G), GCT311GCG (1117T>G), TTA313TTG (1123A>G), CCT314CCA (1126T>A), GTC315GTT (1129C>T), AGG320AAG (1143G>A), GTA321GTT (1147A>T), GCT322GCA (1150T>A), TTG324ATA (1154T>A 1156G>A), CAC327CAT (1165C>T), AGT328AGC (1168T>C), CTT330TTA (1172C>T 1174T>A), ACC339ACT (1201C>T), GCC345GCT (1219C>T), AGC346AAC (1221G>A), AGA361GGG (1265A>G 1267A>G), GGT363GGC (1273T>C), TTG364TTA (1276G>A), AAT365AGT (1278A>G), TTG367CTG (1283T>C), GCG369GTA (1290C>T 1291G>A), GAC372ACT (1298G>A 1299A>C 1300C>T), TTT374TTC (1306T>C), ATA375GTG (1307A>G 1309A>G), CAT376CAC (1312T>C), AAT378GAA (1316A>G 1318T>A), TTG381TTA (1327G>A), ATA382ACA (1329T>C), GCG383GCC (1333G>C), TTA384TTG (1336A>G), GAG385AAG (1337G>A), CTA387TTG (1343C>T 1345A>G), CCG390CCA (1354G>A), GTG391GTT (1357G>T), GAC392GTT (1359A>T 1360C>T), CTG393TTA (1361C>T 1363G>A), CTC395TTG (1367C>T 1369C>G), CTT397ATT (1373C>A), TTC398TTT (1378C>T), GAG400GAA (1384G>A), ATA401GTA (1385A>G), AAA403AAG (1393A>G), TCC404TCT (1396C>T), AAA1518AAG (4738A>G), GTA1520GTT (4744A>T), GCA1521GCG (4747A>G), TTG1522CTG (4748T>C), ACT1527ACC (4765T>C), GTC1538ATT (4796G>A 4798C>T), ACT1540AGC (4803C>G 4804T>C), GAG1541GAA (4807G>A), GCG1542GCT (4810G>T), GCT1543GCG (4813T>G), CTT1544CTC (4816T>C), TAT1549TAC (4831T>C), AAC1550AAT (4834C>T), CCA1552CCT (4840A>T), GTG1553GTA (4843G>A), GGA1556GGG (4852A>G), GGC1557_GTT1558del (4853_4858delGGCGTT), TCA1559GCA (4859T>G), ACT1560ACC (4864T>C), AGT1561AGC (4867T>C), CTG1562CTC (4870G>C), ATT1563ATA (4873T>A), TGT1566TGC (4882T>C), ACA1573ACT (4903A>T), CAG1575CAA (4909G>A), TTG1579CTA (4919T>C 4921G>A), AGT1580AGC (4924T>C), CCC1581CCG (4927C>G), TTT1583TTC (4933T>C), ATC1584ATA (4936C>A), CAG1585CAA (4939G>A), AAT1586AAC (4942T>C), TTC1587TTT (4945C>T), TCA1593TCG (4963A>G), CCT1596CCC (4972T>C), CTT1602CTC (4990T>C), GCT1934GCA (5986T>A), GAC1935GAT (5989C>T), ATT1936GTT (5990A>G), AGA1942CGT (6008A>C 6010A>T), GAA1945GAT (6019A>T), GTG1946GTT (6022G>T), CGA1947CGT (6025A>T), AAA1949ACA (6030A>C), GAC1955GAA (6049C>A), ATG1958ATA (6058G>A), CAA1959CAG (6061A>G), GCC1960GCA (6064C>A), GGT1962GGC (6070T>C), AGT1963AAC (6072G>A 6073T>C), AAC1964CAC (6074A>C), ACG1965ACA (6079G>A), ACC1966AAC (6081C>A), ATA1967ATT (6085A>T), GCA1969GCT (6091A>T), TAC1970TAT (6094C>T), TGT1976TCC (6111G>C 6112T>C), AAA1978AAG (6118A>G), TTG1980TTA (6124G>A), AAG1981AAA (6127G>A), ATT1982GTC (6128A>G 6130T>C), GAT1983GAC (6133T>C), TTA1984TTG (6136A>G), AAC1988AAT (6148C>T), AAA1991AAG (6157A>G), GTT1992ATT (6158G>A), TGT1993TGC (6163T>C), AAA1995AAG (6169A>G), ACA1996TCG (6170A>T 6172A>G), GGC1998GGT (6178C>T), AAA2001AAG (6187A>G), TTT2002TTC (6190T>C), GAG2004GAA (6196G>A), CTC2007TTT (6203C>T 6205C>T), GAA2008GAG (6208A>G), CTA2009TTG (6209C>T 6211A>G), CAG2011CAA (6217G>A), GCT2015GCA (6229T>A), GTA2016ATA (6230G>A), GTC2018GTT (6238C>T), AAG2021AAA (6247G>A), ATA2023ATT (6253A>T), GCA2025AAA (6257G>A 6258C>A), CAT2030CAC (6274T>C), GGC2038GGT (6298C>T), TTG2039TTA (6301G>A), AGA2040AGG (6304A>G), GAC2041GAT (6307C>T), AAC2043AAT (6313C>T), GCC2046GCT (6322C>T), ACA2048ACT (6328A>T), GTT2049GTG (6331T>G), TGT2050TGC (6334T>C), AGG2051AGA (6337G>A), CTG2052TTG (6338C>T), GTA2054GTG (6346A>G), TAT2058TAC (6358T>C), GGG2059GGA (6361G>A), GCA2060ACA (6362G>A), TCA2061TCC (6367A>C), GAG2062GAA (6370G>A), TAC2064TAT (6376C>T), GGT2065GGG (6379T>G), GGA2067GGC (6385A>C), GGA2069GGT (6391A>T), GCA2070GCG (6394A>G), TAC2071TAT (6397C>T), GTA2073ATA (6401G>A), GCG2074GTA (6405C>T 6406G>A), CAC2076CAT (6412C>T), TTA2078TTG (6418A>G), TTT2079TTC (6421T>C), TAC2082TTC (6429A>T), GGT2084GGA (6436T>A), GAG2087GAA (6445G>A), CAA2089CGA (6450A>G), TCC2090TCA (6454C>A), CAC2092CAT (6460C>T), GGT2093GGA (6463T>A), ACA2094ACG (6466A>G), TTC2095TTT (6469C>T), AGG2096AGA (6472G>A), GTG2097GTA (6475G>A), AAT2099AAC (6481T>C), CAC2101AAT (6485C>A 6487C>T), AGT2102AGC (6490T>C), AGC2104AGT (6496C>T), CTG2106CTA (6502G>A), GGT2110GGC (6514T>C), AGG2111AGA (6517G>A), CTC2115ATC (6527C>A), ATC2116ATA (6532C>A), TGT2454..C (7546T>C), AAG2462AAA (7570G>A), ACG2463ACA (7573G>A), ACG2465ACA (7579G>A), GCT2468GCC (7588T>C), CTA2471TTA (7595C>T), TTG2474CTA (7604T>C 7606G>A), GGT2477GGA (7615T>A), TGT2480TGC (7624T>C), GAC2483GAT (7633C>T), TTC2484TTT (7636C>T), AAT2485AAC (7639T>C), AAT2486AAC (7642T>C), TTT2488TTC (7648T>C), GAA2494GAG (7666A>G), TGT2496TGC (7672T>C), ACA2498ACT (7678A>T), GGG2500GGA (7684G>A), ACT2502ACC (7690T>C), TTT2504TTC (7696T>C), GGT2506GGG (7702T>G), GAT2509AAT (7709G>A), AAA2510AGG (7713A>G 7714A>G), CTG2511TTG (7715C>T), CGT2514CGC (7726T>C), GAG2517GAA (7735G>A), GTA2520GTG (7744A>G), TAC2521TAT (7747C>T), GGC2526GGT (7762C>T), CAG2528CAA (7768G>A), TTT2529TTC (7771T>C), CTA2533CTC (7783A>C), ACT2534ACC (7786T>C), CTA2537TTG (7793C>T 7795A>G), ATC2538ATT (7798C>T), GCT2540GCC (7804T>C), ACC2543ATC (7812C>T), GAA2550GAG (7834A>G), GAC2551GAT (7837C>T), GTG2554TTA (7844G>T 7846G>A), CAG2557CAA (7855G>A), CTG2559CTA (7861G>A), TTA2562TTG (7870A>G), TAC2563TAT (7873C>T), ACT2564ACA (7876T>A), GAG2565GAA (7879G>A), ATT2566ATA (7882T>A), GTT2567ATT (7883G>A), CCA2570CCT (7894A>T), ATT2571ATC (7897T>C), GTC2579GTT (7921C>T), AAG2581AAA (7927G>A), GGT2584GGC (7936T>C), AAT2585AAC (7939T>C), AAT2586AAC (7942T>C), CCT2590CCC (7954T>C), GTG2594GTA (7966G>A), GAT2595GAC (7969T>C), TCT2597TCC (7975T>C), GTC2601GTT (7987C>T), CTT2602CTC (7990T>C), GCT2607GCC (8005T>C), GAA2616GAG (8032A>G), ATC2618ATT (8038C>T), AAC2656AGT (8151A>G 8152C>T), TCG2660TCA (8164G>A), AGG2665AAG (8178G>A), AAG2666AAA (8182G>A), TTG2669TTA (8191G>A), TTC2671TTT (8197C>T), AGA2675AAA (8208G>A), CTG2677TTG (8213C>T), CTA2678CTG (8218A>G), ATC2679ATT (8221C>T), GAG2680GAA (8224G>A), CCA2685CCG (8239A>G), ATT2692ATC (8260T>C), GTA2693GTG (8263A>G), GAT2699GAC (8281T>C), AGA2700AGG (8284A>G), CCA2704GCG (8294C>G 8296A>G), GAG2705GAA (8299G>A), CAC2706CAT (8302C>T), GAA2709GAG (8311A>G), ATT2711ATC (8317T>C), TGC2712TGT (8320C>T), GCT2714GCA (8326T>A), GAG2717GAA (8335G>A), TCC2718TCT (8338C>T), TCT2722TTT (8349C>T), GAA2723GAG (8353A>G), CAC2726CAT (8362C>T), CAA2727CAG (8365A>G), ATC2728ATT (8368C>T), TAC2732TAT (8380C>T), TCA2733TCG (8383A>G), TTA2735TTG (8389A>G), CCT2740CCC (8404T>C), GCA2742TCA (8408G>T), ACA2743ACG (8413A>G), GCG2745GCT (8419G>T), GCT2750GCC (8434T>C), TAT2752TAC (8440T>C), ATG2756ATA (8452G>A), CTA2758CTG (8458A>G), AGG2759AAG (8460G>A), AAA2760AAG (8464A>G), CTG2761CTA (8467G>A), GCT2766ACA (8480G>A 8482T>A), GAG2771GAA (8497G>A), CTA2772TTG (8498C>T 8500A>G), AGA2773AAG (8502G>A 8503A>G), GCC2774GCT (8506C>T), GAA2777GAT (8515A>T), GTC2780GTT (8524C>T), GCA2781GCC (8527A>C), TTA2782CTG (8528T>C 8530A>G), GAG2785GAC (8539G>C), CTT2788TGT (8546C>T 8547T>G), TCT2790ACT (8552T>A), GTA2793GTG (8563A>G), GCA2797GGA (8574C>G), ATT2801GTT (8585A>G), GGA2805GAA (8598G>A), AGC2806AGT (8602C>T), AAC2807TCT (8603A>T 8604A>C 8605C>T), AAA2812AAG (8620A>G), CCA2813CAA (8622C>A), GAG2814GGA (8625A>G 8626G>A), CAG2815CAA (8629G>A), GGC2816GGT (8632C>T), ATC2818ATT (8638C>T), CAG2819CAA (8641G>A), AAC2821AAT (8647C>T), CCG2822TTC (8648C>T 8649C>T 8650G>C), AAC2823AAT (8653C>T), AAA2824AAG (8656A>G), GGA2825GAA (8658G>A), GAT2827GCG (8664A>C 8665T>G), GTT2830GTG (8674T>G), GCA2832GTT (8679C>T 8680A>T), GGC2833GGA (8683C>A), GGG2836GGA (8692G>A), ACA2837ACT (8695A>T), CCG2841CCA (8707G>A), AGA2842CGA (8708A>C), AAG2844AAA (8716G>A), ATC2846ATT (8722C>T), ACG2847ACA (8725G>A), AAA2849AAG (8731A>G), AGA2851AGG (8737A>G), CCC2853CCT (8743C>T), ACA2854AAG (8745C>A 8746A>G), AGC2855AGT (8749C>T), AAG2856AAA (8752G>A), GGA2857GGT (8755A>T), AAC2862AAT (8770C>T), TTA2863CTA (8771T>C), GAA2864GAG (8776A>G), CAT2865CAC (8779T>C), TTG2866TTA (8782G>A), TAT2869TAC (8791T>C), CCA2871CCG (8797A>G), CAA2872CAG (8800A>G), CAA2873CAG (8803A>G), GAT2875GAC (8809T>C), ATT2876ATC (8812T>C), AAT2878AAC (8818T>C), GCA2881GCG (8827A>G), CAG2885CCG (8838A>C), ACG2888ACA (8848G>A), TAT2890TAC (8854T>C), GAG2891GAA (8857G>A), GTG2893GTA (8863G>A), CGG2894CAG (8865G>A), ATG2895CTT (8867A>C 8869G>T), GCA2896GCG (8872A>G), GGA2900AAA (8882G>A 8883G>A), ACT2902TCT (8888A>T), TGC2915TGT (8929C>T), GTC2924ATC (8954G>A), AAC2925AAT (8959C>T), GGA2926GGT (8962A>T), GGG2933GGA (8983G>A), AAT2934GAT (8984A>G), GAA2935GTA (8988A>T), GAG2938GAA (8998G>A), CCG2940CCA (9004G>A), TTG2941TTA (9007G>A), ATC2944ATT (9016C>T), ACC2951ACA (9037C>A), ATA2967ATT (9085A>T), AAC2971AAT (9097C>T), ATT2983GTT (9131A>G), CGA2984CGT (9136A>T), CGG2987CGT (9145G>T), ATG2989GGA (9149A>G 9150T>G 9151G>A), GGT2990AGT (9152G>A), TTA2991TTG (9157A>G), GCG2992GCT (9160G>T), CGT2993CGC (9163T>C), GAG3000GAA (9184G>A), CGA3004CGG (9196A>G), AGG3010AGA (9214G>A), GAA3011GAG (9217A>G), GCG3012GCA (9220G>A), GCA3020GCT (9244A>T), TTG3021TTA (9247G>A), GCC3024GCT (9256C>T), CCT3026TCC (9260C>T 9262T>C), GGG3030GGA (9274G>A), GAC3032GAT (9280C>T), ATC3035ATT (9289C>T), GAG3040GAA (9304G>A), GTC3050GTT (9334C>T), GTC3060GTG (9364C>G)

Proteins

	Begin	End	Coverage	Score	Concordance	Matches	Identities	I/D/M/F*	Stop Codons
polyprotein (NP_056759.1)	307	3064	30.5%	6023	91.8%	933 (99.8%)	847 (90.6%)	0/2/0/0	1

Protein mutations: R320K (1143G>A), L324I (1154T>A 1156G>A), S346N (1221G>A), R361G (1265A>G 1267A>G), N365S (1278A>G), A369V (1290C>T 1291G>A), D372T (1298G>A 1299A>C 1300C>T), I375V (1307A>G 1309A>G), N378E (1316A>G 1318T>A), I382T (1329T>C), E385K (1337G>A), D392V (1359A>T 1360C>T), L397I (1373C>A), I401V (1385A>G), V1538I (4796G>A 4798C>T), T1540S (4803C>G 4804T>C), G1557_V1558del (4853_4858delGGCGTT), S1559A (4859T>G), I1936V (5990A>G), E1945D (6019A>T), K1949T (6030A>C), D1955E (6049C>A), M1958I (6058G>A), S1963N (6072G>A 6073T>C), N1964H (6074A>C), T1966N (6081C>A), C1976S (6111G>C 6112T>C), I1982V (6128A>G 6130T>C), V1992I (6158G>A), T1996S (6170A>T 6172A>G), L2007F (6203C>T 6205C>T), V2016I (6230G>A), A2025K (6257G>A 6258C>A), A2060T (6362G>A), V2073I (6401G>A), A2074V (6405C>T 6406G>A), Y2082F (6429A>T), Q2089R (6450A>G), H2101N (6485C>A 6487C>T), L2115I (6527C>A), D2509N (7709G>A), K2510R (7713A>G 7714A>G), T2543I (7812C>T), V2554L (7844G>T 7846G>A), V2567I (7883G>A), N2656S (8151A>G 8152C>T), R2665K (8178G>A), R2675K (8208G>A), P2704A (8294C>G 8296A>G), S2722F (8349C>T), A2742S (8408G>T), M2756I (8452G>A), R2759K (8460G>A), A2766T (8480G>A 8482T>A), R2773K (8502G>A 8503A>G), E2777D (8515A>T), E2785D (8539G>C), L2788C (8546C>T 8547T>G), S2790T (8552T>A), A2797G (8574C>G), I2801V (8585A>G), G2805E (8598G>A), N2807S (8603A>T 8604A>C 8605C>T), P2813Q (8622C>A), E2814G (8625A>G 8626G>A), P2822F (8648C>T 8649C>T 8650G>C), G2825E (8658G>A), D2827A (8664A>C 8665T>G), A2832V (8679C>T 8680A>T), T2854K (8745C>A 8746A>G), Q2885P (8838A>C), R2894Q (8865G>A), M2895L (8867A>C 8869G>T), G2900K (8882G>A 8883G>A), T2902S (8888A>T), V2924I (8954G>A), N2934D (8984A>G), E2935V (8988A>T), I2983V (9131A>G), M2989G (9149A>G 9150T>G 9151G>A), G2990S (9152G>A), P3026S (9260C>T 9262T>C)

	Begin	End	Coverage	Score	Concordance	Matches	Identities	I/D/M/F*	Stop Codons
**NT**	**1103**	**9641**	**31.6%**	**4154**	**68.5%**	**3057 (99.6%)**	**2586 (84.2%)**	**3/10**	
Codon mutations:	CAC307CAT (1105C>T), ACA308ACG (1108A>G), GTA310GTG (1114A>G), GCT311GCG (1117T>G), TTA313TTG (1123A>G), CCT314CCA (1126T>A), GTC315GTT (1129C>T), AGG320AAG (1143G>A), GTA321GTT (1147A>T), GCT322GCA (1150T>A), TTG324ATA (1154T>A 1156G>A), CAC327CAT (1165C>T), AGT328AGC (1168T>C), CTT330TTA (1172C>T 1174T>A), ACC339ACT (1201C>T), GCC345GCT (1219C>T), AGC346AAC (1221G>A), AGA361GGG (1265A>G 1267A>G), GGT363GGC (1273T>C), TTG364TTA (1276G>A), AAT365AGT (1278A>G), TTG367CTG (1283T>C), GCG369GTA (1290C>T 1291G>A), GAC372ACT (1298G>A 1299A>C 1300C>T), TTT374TTC (1306T>C), ATA375GTG (1307A>G 1309A>G), CAT376CAC (1312T>C), AAT378GAA (1316A>G 1318T>A), TTG381TTA (1327G>A), ATA382ACA (1329T>C), GCG383GCC (1333G>C), TTA384TTG (1336A>G), GAG385AAG (1337G>A), CTA387TTG (1343C>T 1345A>G), CCG390CCA (1354G>A), GTG391GTT (1357G>T), GAC392GTT (1359A>T 1360C>T), CTG393TTA (1361C>T 1363G>A), CTC395TTG (1367C>T 1369C>G), CTT397ATT (1373C>A), TTC398TTT (1378C>T), GAG400GAA (1384G>A), ATA401GTA (1385A>G), AAA403AAG (1393A>G), TCC404TCT (1396C>T), AAA1518AAG (4738A>G), GTA1520GTT (4744A>T), GCA1521GCG (4747A>G), TTG1522CTG (4748T>C), ACT1527ACC (4765T>C), GTC1538ATT (4796G>A 4798C>T), ACT1540AGC (4803C>G 4804T>C), GAG1541GAA (4807G>A), GCG1542GCT (4810G>T), GCT1543GCG (4813T>G), CTT1544CTC (4816T>C), TAT1549TAC (4831T>C), AAC1550AAT (4834C>T), CCA1552CCT (4840A>T), GTG1553GTA (4843G>A), GGA1556GGG (4852A>G), GGC1557_GTT1558del (4853_4858delGGCGTT), TCA1559GCA (4859T>G), ACT1560ACC (4864T>C), AGT1561AGC (4867T>C), CTG1562CTC (4870G>C), ATT1563ATA (4873T>A), TGT1566TGC (4882T>C), ACA1573ACT (4903A>T), CAG1575CAA (4909G>A), TTG1579CTA (4919T>C 4921G>A), AGT1580AGC (4924T>C), CCC1581CCG (4927C>G), TTT1583TTC (4933T>C), ATC1584ATA (4936C>A), CAG1585CAA (4939G>A), AAT1586AAC (4942T>C), TTC1587TTT (4945C>T), TCA1593TCG (4963A>G), CCT1596CCC (4972T>C), CTT1602CTC (4990T>C), GCT1934GCA (5986T>A), GAC1935GAT (5989C>T), ATT1936GTT (5990A>G), AGA1942CGT (6008A>C 6010A>T), GAA1945GAT (6019A>T), GTG1946GTT (6022G>T), CGA1947CGT (6025A>T), AAA1949ACA (6030A>C), GAC1955GAA (6049C>A), ATG1958ATA (6058G>A), CAA1959CAG (6061A>G), GCC1960GCA (6064C>A), GGT1962GGC (6070T>C), AGT1963AAC (6072G>A 6073T>C), AAC1964CAC (6074A>C), ACG1965ACA (6079G>A), ACC1966AAC (6081C>A), ATA1967ATT (6085A>T), GCA1969GCT (6091A>T), TAC1970TAT (6094C>T), TGT1976TCC (6111G>C 6112T>C), AAA1978AAG (6118A>G), TTG1980TTA (6124G>A), AAG1981AAA (6127G>A), ATT1982GTC (6128A>G 6130T>C), GAT1983GAC (6133T>C), TTA1984TTG (6136A>G), AAC1988AAT (6148C>T), AAA1991AAG (6157A>G), GTT1992ATT (6158G>A), TGT1993TGC (6163T>C), AAA1995AAG (6169A>G), ACA1996TCG (6170A>T 6172A>G), GGC1998GGT (6178C>T), AAA2001AAG (6187A>G), TTT2002TTC (6190T>C), GAG2004GAA (6196G>A), CTC2007TTT (6203C>T 6205C>T), GAA2008GAG (6208A>G), CTA2009TTG (6209C>T 6211A>G), CAG2011CAA (6217G>A), GCT2015GCA (6229T>A), GTA2016ATA (6230G>A), GTC2018GTT (6238C>T), AAG2021AAA (6247G>A), ATA2023ATT (6253A>T), GCA2025AAA (6257G>A 6258C>A), CAT2030CAC (6274T>C), GGC2038GGT (6298C>T), TTG2039TTA (6301G>A), AGA2040AGG (6304A>G), GAC2041GAT (6307C>T), AAC2043AAT (6313C>T), GCC2046GCT (6322C>T), ACA2048ACT (6328A>T), GTT2049GTG (6331T>G), TGT2050TGC (6334T>C), AGG2051AGA (6337G>A), CTG2052TTG (6338C>T), GTA2054GTG (6346A>G), TAT2058TAC (6358T>C), GGG2059GGA (6361G>A), GCA2060ACA (6362G>A), TCA2061TCC (6367A>C), GAG2062GAA (6370G>A), TAC2064TAT (6376C>T), GGT2065GGG (6379T>G), GGA2067GGC (6385A>C), GGA2069GGT (6391A>T), GCA2070GCG (6394A>G), TAC2071TAT (6397C>T), GTA2073ATA (6401G>A), GCG2074GTA (6405C>T 6406G>A), CAC2076CAT (6412C>T), TTA2078TTG (6418A>G), TTT2079TTC (6421T>C), TAC2082TTC (6429A>T), GGT2084GGA (6436T>A), GAG2087GAA (6445G>A), CAA2089CGA (6450A>G), TCC2090TCA (6454C>A), CAC2092CAT (6460C>T), GGT2093GGA (6463T>A), ACA2094ACG (6466A>G), TTC2095TTT (6469C>T), AGG2096AGA (6472G>A), GTG2097GTA (6475G>A), AAT2099AAC (6481T>C), CAC2101AAT (6485C>A 6487C>T), AGT2102AGC (6490T>C), AGC2104AGT (6496C>T), CTG2106CTA (6502G>A), GGT2110GGC (6514T>C), AGG2111AGA (6517G>A), CTC2115ATC (6527C>A), ATC2116ATA (6532C>A), TGT2454..C (7546T>C), AAG2462AAA (7570G>A), ACG2463ACA (7573G>A), ACG2465ACA (7579G>A), GCT2468GCC (7588T>C), CTA2471TTA (7595C>T), TTG2474CTA (7604T>C 7606G>A), GGT2477GGA (7615T>A), TGT2480TGC (7624T>C), GAC2483GAT (7633C>T), TTC2484TTT (7636C>T), AAT2485AAC (7639T>C), AAT2486AAC (7642T>C), TTT2488TTC (7648T>C), GAA2494GAG (7666A>G), TGT2496TGC (7672T>C), ACA2498ACT (7678A>T), GGG2500GGA (7684G>A), ACT2502ACC (7690T>C), TTT2504TTC (7696T>C), GGT2506GGG (7702T>G), GAT2509AAT (7709G>A), AAA2510AGG (7713A>G 7714A>G), CTG2511TTG (7715C>T), CGT2514CGC (7726T>C), GAG2517GAA (7735G>A), GTA2520GTG (7744A>G), TAC2521TAT (7747C>T), GGC2526GGT (7762C>T), CAG2528CAA (7768G>A), TTT2529TTC (7771T>C), CTA2533CTC (7783A>C), ACT2534ACC (7786T>C), CTA2537TTG (7793C>T 7795A>G), ATC2538ATT (7798C>T), GCT2540GCC (7804T>C), ACC2543ATC (7812C>T), GAA2550GAG (7834A>G), GAC2551GAT (7837C>T), GTG2554TTA (7844G>T 7846G>A), CAG2557CAA (7855G>A), CTG2559CTA (7861G>A), TTA2562TTG (7870A>G), TAC2563TAT (7873C>T), ACT2564ACA (7876T>A), GAG2565GAA (7879G>A), ATT2566ATA (7882T>A), GTT2567ATT (7883G>A), CCA2570CCT (7894A>T), ATT2571ATC (7897T>C), GTC2579GTT (7921C>T), AAG2581AAA (7927G>A), GGT2584GGC (7936T>C), AAT2585AAC (7939T>C), AAT2586AAC (7942T>C), CCT2590CCC (7954T>C), GTG2594GTA (7966G>A), GAT2595GAC (7969T>C), TCT2597TCC (7975T>C), GTC2601GTT (7987C>T), CTT2602CTC (7990T>C), GCT2607GCC (8005T>C), GAA2616GAG (8032A>G), ATC2618ATT (8038C>T), AAC2656AGT (8151A>G 8152C>T), TCG2660TCA (8164G>A), AGG2665AAG (8178G>A), AAG2666AAA (8182G>A), TTG2669TTA (8191G>A), TTC2671TTT (8197C>T), AGA2675AAA (8208G>A), CTG2677TTG (8213C>T), CTA2678CTG (8218A>G), ATC2679ATT (8221C>T), GAG2680GAA (8224G>A), CCA2685CCG (8239A>G), ATT2692ATC (8260T>C), GTA2693GTG (8263A>G), GAT2699GAC (8281T>C), AGA2700AGG (8284A>G), CCA2704GCG (8294C>G 8296A>G), GAG2705GAA (8299G>A), CAC2706CAT (8302C>T), GAA2709GAG (8311A>G), ATT2711ATC (8317T>C), TGC2712TGT (8320C>T), GCT2714GCA (8326T>A), GAG2717GAA (8335G>A), TCC2718TCT (8338C>T), TCT2722TTT (8349C>T), GAA2723GAG (8353A>G), CAC2726CAT (8362C>T), CAA2727CAG (8365A>G), ATC2728ATT (8368C>T), TAC2732TAT (8380C>T), TCA2733TCG (8383A>G), TTA2735TTG (8389A>G), CCT2740CCC (8404T>C), GCA2742TCA (8408G>T), ACA2743ACG (8413A>G), GCG2745GCT (8419G>T), GCT2750GCC (8434T>C), TAT2752TAC (8440T>C), ATG2756ATA (8452G>A), CTA2758CTG (8458A>G), AGG2759AAG (8460G>A), AAA2760AAG (8464A>G), CTG2761CTA (8467G>A), GCT2766ACA (8480G>A 8482T>A), GAG2771GAA (8497G>A), CTA2772TTG (8498C>T 8500A>G), AGA2773AAG (8502G>A 8503A>G), GCC2774GCT (8506C>T), GAA2777GAT (8515A>T), GTC2780GTT (8524C>T), GCA2781GCC (8527A>C), TTA2782CTG (8528T>C 8530A>G), GAG2785GAC (8539G>C), CTT2788TGT (8546C>T 8547T>G), TCT2790ACT (8552T>A), GTA2793GTG (8563A>G), GCA2797GGA (8574C>G), ATT2801GTT (8585A>G), GGA2805GAA (8598G>A), AGC2806AGT (8602C>T), AAC2807TCT (8603A>T 8604A>C 8605C>T), AAA2812AAG (8620A>G), CCA2813CAA (8622C>A), GAG2814GGA (8625A>G 8626G>A), CAG2815CAA (8629G>A), GGC2816GGT (8632C>T), ATC2818ATT (8638C>T), CAG2819CAA (8641G>A), AAC2821AAT (8647C>T), CCG2822TTC (8648C>T 8649C>T 8650G>C), AAC2823AAT (8653C>T), AAA2824AAG (8656A>G), GGA2825GAA (8658G>A), GAT2827GCG (8664A>C 8665T>G), GTT2830GTG (8674T>G), GCA2832GTT (8679C>T 8680A>T), GGC2833GGA (8683C>A), GGG2836GGA (8692G>A), ACA2837ACT (8695A>T), CCG2841CCA (8707G>A), AGA2842CGA (8708A>C), AAG2844AAA (8716G>A), ATC2846ATT (8722C>T), ACG2847ACA (8725G>A), AAA2849AAG (8731A>G), AGA2851AGG (8737A>G), CCC2853CCT (8743C>T), ACA2854AAG (8745C>A 8746A>G), AGC2855AGT (8749C>T), AAG2856AAA (8752G>A), GGA2857GGT (8755A>T), AAC2862AAT (8770C>T), TTA2863CTA (8771T>C), GAA2864GAG (8776A>G), CAT2865CAC (8779T>C), TTG2866TTA (8782G>A), TAT2869TAC (8791T>C), CCA2871CCG (8797A>G), CAA2872CAG (8800A>G), CAA2873CAG (8803A>G), GAT2875GAC (8809T>C), ATT2876ATC (8812T>C), AAT2878AAC (8818T>C), GCA2881GCG (8827A>G), CAG2885CCG (8838A>C), ACG2888ACA (8848G>A), TAT2890TAC (8854T>C), GAG2891GAA (8857G>A), GTG2893GTA (8863G>A), CGG2894CAG (8865G>A), ATG2895CTT (8867A>C 8869G>T), GCA2896GCG (8872A>G), GGA2900AAA (8882G>A 8883G>A), ACT2902TCT (8888A>T), TGC2915TGT (8929C>T), GTC2924ATC (8954G>A), AAC2925AAT (8959C>T), GGA2926GGT (8962A>T), GGG2933GGA (8983G>A), AAT2934GAT (8984A>G), GAA2935GTA (8988A>T), GAG2938GAA (8998G>A), CCG2940CCA (9004G>A), TTG2941TTA (9007G>A), ATC2944ATT (9016C>T), ACC2951ACA (9037C>A), ATA2967ATT (9085A>T), AAC2971AAT (9097C>T), ATT2983GTT (9131A>G), CGA2984CGT (9136A>T), CGG2987CGT (9145G>T), ATG2989GGA (9149A>G 9150T>G 9151G>A), GGT2990AGT (9152G>A), TTA2991TTG (9157A>G), GCG2992GCT (9160G>T), CGT2993CGC (9163T>C), GAG3000GAA (9184G>A), CGA3004CGG (9196A>G), AGG3010AGA (9214G>A), GAA3011GAG (9217A>G), GCG3012GCA (9220G>A), GCA3020GCT (9244A>T), TTG3021TTA (9247G>A), GCC3024GCT (9256C>T), CCT3026TCC (9260C>T 9262T>C), GGG3030GGA (9274G>A), GAC3032GAT (9280C>T), ATC3035ATT (9289C>T), GAG3040GAA (9304G>A), GTC3050GTT (9334C>T), GTC3060GTG (9364C>G)								
HC-Pro protein (NP_734244.1)	23	121	19.1%	538	84.1%	87 (100%)	71 (81.6%)	0/0/0/0	0
Protein mutations:	R36K (1143G>A), L40I (1154T>A 1156G>A), S62N (1221G>A), R77G (1265A>G 1267A>G), N81S (1278A>G), A85V (1290C>T 1291G>A), D88T (1298G>A 1299A>C 1300C>T), I91V (1307A>G 1309A>G), N94E (1316A>G 1318T>A), I98T (1329T>C), E101K (1337G>A), D108V (1359A>T 1360C>T), L113I (1373C>A), I117V (1385A>G)								
Codon mutations:	CAC23CAT (1105C>T), ACA24ACG (1108A>G), GTA26GTG (1114A>G), GCT27GCG (1117T>G), TTA29TTG (1123A>G), CCT30CCA (1126T>A), GTC31GTT (1129C>T), AGG36AAG (1143G>A), GTA37GTT (1147A>T), GCT38GCA (1150T>A), TTG40ATA (1154T>A 1156G>A), CAC43CAT (1165C>T), AGT44AGC (1168T>C), CTT46TTA (1172C>T 1174T>A), ACC55ACT (1201C>T), GCC61GCT (1219C>T), AGC62AAC (1221G>A), AGA77GGG (1265A>G 1267A>G), GGT79GGC (1273T>C), TTG80TTA (1276G>A), AAT81AGT (1278A>G), TTG83CTG (1283T>C), GCG85GTA (1290C>T 1291G>A), GAC88ACT (1298G>A 1299A>C 1300C>T), TTT90TTC (1306T>C), ATA91GTG (1307A>G 1309A>G), CAT92CAC (1312T>C), AAT94GAA (1316A>G 1318T>A), TTG97TTA (1327G>A), ATA98ACA (1329T>C), GCG99GCC (1333G>C), TTA100TTG (1336A>G), GAG101AAG (1337G>A), CTA103TTG (1343C>T 1345A>G), CCG106CCA (1354G>A), GTG107GTT (1357G>T), GAC108GTT (1359A>T 1360C>T), CTG109TTA (1361C>T 1363G>A), CTC111TTG (1367C>T 1369C>G), CTT113ATT (1373C>A), TTC114TTT (1378C>T), GAG116GAA (1384G>A), ATA117GTA (1385A>G), AAA119AAG (1393A>G), TCC120TCT (1396C>T)								
CI protein (NP_734246.1)	356	447	14.5%	600	98.2%	90 (97.8%)	86 (93.5%)	0/2/0/0	0
Protein mutations:	V381I (4796G>A 4798C>T), T383S (4803C>G 4804T>C), G400_V401del (4853_4858delGGCGTT), S402A (4859T>G)								
Codon mutations:	AAA361AAG (4738A>G), GTA363GTT (4744A>T), GCA364GCG (4747A>G), TTG365CTG (4748T>C), ACT370ACC (4765T>C), GTC381ATT (4796G>A 4798C>T), ACT383AGC (4803C>G 4804T>C), GAG384GAA (4807G>A), GCG385GCT (4810G>T), GCT386GCG (4813T>G), CTT387CTC (4816T>C), TAT392TAC (4831T>C), AAC393AAT (4834C>T), CCA395CCT (4840A>T), GTG396GTA (4843G>A), GGA399GGG (4852A>G), GGC400_GTT401del (4853_4858delGGCGTT), TCA402GCA (4859T>G), ACT403ACC (4864T>C), AGT404AGC (4867T>C), CTG405CTC (4870G>C), ATT406ATA (4873T>A), TGT409TGC (4882T>C), ACA416ACT (4903A>T), CAG418CAA (4909G>A), TTG422CTA (4919T>C 4921G>A), AGT423AGC (4924T>C), CCC424CCG (4927C>G), TTT426TTC (4933T>C), ATC427ATA (4936C>A), CAG428CAA (4939G>A), AAT429AAC (4942T>C), TTC430TTT (4945C>T), TCA436TCG (4963A>G), CCT439CCC (4972T>C), CTT445CTC (4990T>C)								
NIa-VPg protein (NP_734252.1)	91	188	52.1%	619	88.2%	98 (100%)	83 (84.7%)	0/0/0/0	0
Protein mutations:	I93V (5990A>G), E102D (6019A>T), K106T (6030A>C), D112E (6049C>A), M115I (6058G>A), S120N (6072G>A 6073T>C), N121H (6074A>C), T123N (6081C>A), C133S (6111G>C 6112T>C), I139V (6128A>G 6130T>C), V149I (6158G>A), T153S (6170A>T 6172A>G), L164F (6203C>T 6205C>T), V173I (6230G>A), A182K (6257G>A 6258C>A)								
Codon mutations:	GCT91GCA (5986T>A), GAC92GAT (5989C>T), ATT93GTT (5990A>G), AGA99CGT (6008A>C 6010A>T), GAA102GAT (6019A>T), GTG103GTT (6022G>T), CGA104CGT (6025A>T), AAA106ACA (6030A>C), GAC112GAA (6049C>A), ATG115ATA (6058G>A), CAA116CAG (6061A>G), GCC117GCA (6064C>A), GGT119GGC (6070T>C), AGT120AAC (6072G>A 6073T>C), AAC121CAC (6074A>C), ACG122ACA (6079G>A), ACC123AAC (6081C>A), ATA124ATT (6085A>T), GCA126GCT (6091A>T), TAC127TAT (6094C>T), TGT133TCC (6111G>C 6112T>C), AAA135AAG (6118A>G), TTG137TTA (6124G>A), AAG138AAA (6127G>A), ATT139GTC (6128A>G 6130T>C), GAT140GAC (6133T>C), TTA141TTG (6136A>G), AAC145AAT (6148C>T), AAA148AAG (6157A>G), GTT149ATT (6158G>A), TGT150TGC (6163T>C), AAA152AAG (6169A>G), ACA153TCG (6170A>T 6172A>G), GGC155GGT (6178C>T), AAA158AAG (6187A>G), TTT159TTC (6190T>C), GAG161GAA (6196G>A), CTC164TTT (6203C>T 6205C>T), GAA165GAG (6208A>G), CTA166TTG (6209C>T 6211A>G), CAG168CAA (6217G>A), GCT172GCA (6229T>A), GTA173ATA (6230G>A), GTC175GTT (6238C>T), AAG178AAA (6247G>A), ATA180ATT (6253A>T), GCA182AAA (6257G>A 6258C>A), CAT187CAC (6274T>C)								
NIa-Pro protein (NP_734248.1)	1	88	34.4%	538	92.0%	84 (100%)	76 (90.5%)	0/0/0/0	0
Protein mutations:	A29T (6362G>A), V42I (6401G>A), A43V (6405C>T 6406G>A), Y51F (6429A>T), Q58R (6450A>G), H70N (6485C>A 6487C>T), L84I (6527C>A)								

	Begin	End	Coverage	Score	Concordance	Matches	Identities	I/D/M/F*	Stop Codons
**NT**	**1103**	**9641**	**31.6%**	**4154**	**68.5%**	**3057 (99.6%)**	**2586 (84.2%)**	**3/10**	
Codon mutations:	GGC7GGT (6298C>T), TTG8TTA (6301G>A), AGA9AGG (6304A>G), GAC10GAT (6307C>T), AAC12AAT (6313C>T), GCC15GCT (6322C>T), ACA17ACT (6328A>T), GTT18GTG (6331T>G), TGT19TGC (6334T>C), AGG20AGA (6337G>A), CTG21TTG (6338C>T), GTA23GTG (6346A>G), TAT27TAC (6358T>C), GGG28GGA (6361G>A), GCA29ACA (6362G>A), TCA30TCC (6367A>C), GAG31GAA (6370G>A), TAC33TAT (6376C>T), GGT34GGG (6379T>G), GGA36GGC (6385A>C), GGA38GGT (6391A>T), GCA39GCG (6394A>G), TAC40TAT (6397C>T), GTA42ATA (6401G>A), GCG43GTA (6405C>T 6406G>A), CAC45CAT (6412C>T), TTA47TTG (6418A>G), TTT48TTC (6421T>C), TAC51TTC (6429A>T), GGT53GGA (6436T>A), GAG56GAA (6445G>A), CAA58CGA (6450A>G), TCC59TCA (6454C>A), CAC61CAT (6460C>T), GGT62GGA (6463T>A), ACA63ACG (6466A>G), TTC64TTT (6469C>T), AGG65AGA (6472G>A), GTG66GTA (6475G>A), AAT68AAC (6481T>C), CAC70AAT (6485C>A 6487C>T), AGT71AGC (6490T>C), AGC73AGT (6496C>T), CTG75CTA (6502G>A), GGT79GGC (6514T>C), AGG80AGA (6517G>A), CTC84ATC (6527C>A), ATC85ATA (6532C>A)								
NIb protein (NP_734249.1)	180	521	58.7%	2066	94.0%	306 (100%)	286 (93.5%)	0/0/0/0	0
Protein mutations:	D234N (7709G>A), K235R (7713A>G 7714A>G), T268I (7812C>T), V279L (7844G>T 7846G>A), V292I (7883G>A), N381S (8151A>G 8152C>T), R390K (8178G>A), R400K (8208G>A), P429A (8294C>G 8296A>G), S447F (8349C>T), A467S (8408G>T), M481I (8452G>A), R484K (8460G>A), A491T (8480G>A 8482T>A), R498K (8502G>A 8503A>G), E502D (8515A>T), E510D (8539G>C), L513C (8546C>T 8547T>G), S515T (8552T>A)								
Codon mutations:	TGT179..C (7546T>C), AAG187AAA (7570G>A), ACG188ACA (7573G>A), ACG190ACA (7579G>A), GCT193GCC (7588T>C), CTA196TTA (7595C>T), TTG199CTA (7604T>C 7606G>A), GGT202GGA (7615T>A), TGT205TGC (7624T>C), GAC208GAT (7633C>T), TTC209TTT (7636C>T), AAT210AAC (7639T>C), AAT211AAC (7642T>C), TTT213TTC (7648T>C), GAA219GAG (7666A>G), TGT221TGC (7672T>C), ACA223ACT (7678A>T), GGG225GGA (7684G>A), ACT227ACC (7690T>C), TTT229TTC (7696T>C), GGT231GGG (7702T>G), GAT234AAT (7709G>A), AAA235AGG (7713A>G 7714A>G), CTG236TTG (7715C>T), CGT239CGC (7726T>C), GAG242GAA (7735G>A), GTA245GTG (7744A>G), TAC246TAT (7747C>T), GGC251GGT (7762C>T), CAG253CAA (7768G>A), TTT254TTC (7771T>C), CTA258CTC (7783A>C), ACT259ACC (7786T>C), CTA262TTG (7793C>T 7795A>G), ATC263ATT (7798C>T), GCT265GCC (7804T>C), ACC268ATC (7812C>T), GAA275GAG (7834A>G), GAC276GAT (7837C>T), GTG279TTA (7844G>T 7846G>A), CAG282CAA (7855G>A), CTG284CTA (7861G>A), TTA287TTG (7870A>G), TAC288TAT (7873C>T), ACT289ACA (7876T>A), GAG290GAA (7879G>A), ATT291ATA (7882T>A), GTT292ATT (7883G>A), CCA295CCT (7894A>T), ATT296ATC (7897T>C), GTC304GTT (7921C>T), AAG306AAA (7927G>A), GGT309GGC (7936T>C), AAT310AAC (7939T>C), AAT311AAC (7942T>C), CCT315CCC (7954T>C), GTG319GTA (7966G>A), GAT320GAC (7969T>C), TCT322TCC (7975T>C), GTC326GTT (7987C>T), CTT327CTC (7990T>C), GCT332GCC (8005T>C), GAA341GAG (8032A>G), ATC343ATT (8038C>T), AAC381AGT (8151A>G 8152C>T), TCG385TCA (8164G>A), AGG390AAG (8178G>A), AAG391AAA (8182G>A), TTG394TTA (8191G>A), TTC396TTT (8197C>T), AGA400AAA (8208G>A), CTG402TTG (8213C>T), CTA403CTG (8218A>G), ATC404ATT (8221C>T), GAG405GAA (8224G>A), CCA410CCG (8239A>G), ATT417ATC (8260T>C), GTA418GTG (8263A>G), GAT424GAC (8281T>C), AGA425AGG (8284A>G), CCA429GCG (8294C>G 8296A>G), GAG430GAA (8299G>A), CAC431CAT (8302C>T), GAA434GAG (8311A>G), ATT436ATC (8317T>C), TGC437TGT (8320C>T), GCT439GCA (8326T>A), GAG442GAA (8335G>A), TCC443TCT (8338C>T), TCT447TTT (8349C>T), GAA448GAG (8353A>G), CAC451CAT (8362C>T), CAA452CAG (8365A>G), ATC453ATT (8368C>T), TAC457TAT (8380C>T), TCA458TCG (8383A>G), TTA460TTG (8389A>G), CCT465CCC (8404T>C), GCA467TCA (8408G>T), ACA468ACG (8413A>G), GCG470GCT (8419G>T), GCT475GCC (8434T>C), TAT477TAC (8440T>C), ATG481ATA (8452G>A), CTA483CTG (8458A>G), AGG484AAG (8460G>A), AAA485AAG (8464A>G), CTG486CTA (8467G>A), GCT491ACA (8480G>A 8482T>A), GAG496GAA (8497G>A), CTA497TTG (8498C>T 8500A>G), AGA498AAG (8502G>A 8503A>G), GCC499GCT (8506C>T), GAA502GAT (8515A>T), GTC505GTT (8524C>T), GCA506GCC (8527A>C), TTA507CTG (8528T>C 8530A>G), GAG510GAC (8539G>C), CTT513TGT (8546C>T 8547T>G), TCT515ACT (8552T>A), GTA518GTG (8563A>G)								
coat protein (NP_734250.1)	1	267	100%	1661	91.1%	267 (100%)	244 (91.4%)	0/0/0/0	0
Protein mutations:	A1G (8574C>G), I5V (8585A>G), G9E (8598G>A), N11S (8603A>T 8604A>C 8605C>T), P17Q (8622C>A), E18G (8625A>G 8626G>A), P26F (8648C>T 8649C>T 8650G>C), G29E (8658G>A), D31A (8664A>C 8665T>G), A36V (8679C>T 8680A>T), T58K (8745C>A 8746A>G), Q89P (8838A>C), R98Q (8865G>A), M99L (8867A>C 8869G>T), G104K (8882G>A 8883G>A), T106S (8888A>T), V128I (8954G>A), N138D (8984A>G), E139V (8988A>T), I187V (9131A>G), M193G (9149A>G 9150T>G 9151G>A), G194S (9152G>A), P230S (9260C>T 9262T>C)								
Codon mutations:	GCA1GGA (8574C>G), ATT5GTT (8585A>G), GGA9GAA (8598G>A), AGC10AGT (8602C>T), AAC11TCT (8603A>T 8604A>C 8605C>T), AAA16AAG (8620A>G), CCA17CAA (8622C>A), GAG18GGA (8625A>G 8626G>A), CAG19CAA (8629G>A), GGC20GGT (8632C>T), ATC22ATT (8638C>T), CAG23CAA (8641G>A), AAC25AAT (8647C>T), CCG26TTC (8648C>T 8649C>T 8650G>C), AAC27AAT (8653C>T), AAA28AAG (8656A>G), GGA29GAA (8658G>A), GAT31GCG (8664A>C 8665T>G), GTT34GTG (8674T>G), GCA36GTT (8679C>T 8680A>T), GGC37GGA (8683C>A), GGG40GGA (8692G>A), ACA41ACT (8695A>T), CCG45CCA (8707G>A), AGA46CGA (8708A>C), AAG48AAA (8716G>A), ATC50ATT (8722C>T), ACG51ACA (8725G>A), AAA53AAG (8731A>G), AGA55AGG (8737A>G), CCC57CCT (8743C>T), ACA58AAG (8745C>A 8746A>G), AGC59AGT (8749C>T), AAG60AAA (8752G>A), GGA61GGT (8755A>T), AAC66AAT (8770C>T), TTA67CTA (8771T>C), GAA68GAG (8776A>G), CAT69CAC (8779T>C), TTG70TTA (8782G>A), TAT73TAC (8791T>C), CCA75CCG (8797A>G), CAA76CAG (8800A>G), CAA77CAG (8803A>G), GAT79GAC (8809T>C), ATT80ATC (8812T>C), AAT82AAC (8818T>C), GCA85GCG (8827A>G), CAG89CCG (8838A>C), ACG92ACA (8848G>A), TAT94TAC (8854T>C), GAG95GAA (8857G>A), GTG97GTA (8863G>A), CGG98CAG (8865G>A), ATG99CTT (8867A>C 8869G>T), GCA100GCG (8872A>G), GGA104AAA (8882G>A 8883G>A), ACT106TCT (8888A>T), TGC119TGT (8929C>T), GTC128ATC (8954G>A), AAC129AAT (8959C>T), GGA130GGT (8962A>T), GGG137GGA (8983G>A), AAT138GAT (8984A>G), GAA139GTA (8988A>T), GAG142GAA (8998G>A), CCG144CCA (9004G>A), TTG145TTA (9007G>A), ATC148ATT (9016C>T), ACC155ACA (9037C>A), ATA171ATT (9085A>T), AAC175AAT (9097C>T), ATT187GTT (9131A>G), CGA188CGT (9136A>T), CGG191CGT (9145G>T), ATG193GGA (9149A>G 9150T>G 9151G>A), GGT194AGT (9152G>A), TTA195TTG (9157A>G), GCG196GCT (9160G>T), CGT197CGC (9163T>C), GAG204GAA (9184G>A), CGA208CGG (9196A>G), AGG214AGA (9214G>A), GAA215GAG (9217A>G), GCG216GCA (9220G>A), GCA224GCT (9244A>T), TTG225TTA (9247G>A), GCC228GCT (9256C>T), CCT230TCC (9260C>T 9262T>C), GGG234GGA (9274G>A), GAC236GAT (9280C>T), ATC239ATT (9289C>T), GAG244GAA (9304G>A), GTC254GTT (9334C>T), GTC264GTG (9364C>G)								

*: Inserts / Deletes / Misaligned / Frameshifts

Analysis details

This analysis was performed with panviral2.64

NGS Details (UN24): Lausannevirus

Assembly

**Coverage Length**	174 (1 contig(s))
**Depth Of Coverage**	11097.3
**Number Of Reads**	20011
**Reads Per Million**	400.57 rpm (after QC)
**Ambiguities**	0
**Assembly Method**	de novo + reference guided assembly
**Consensus Caller**	Bcf Tools

Coverage Map

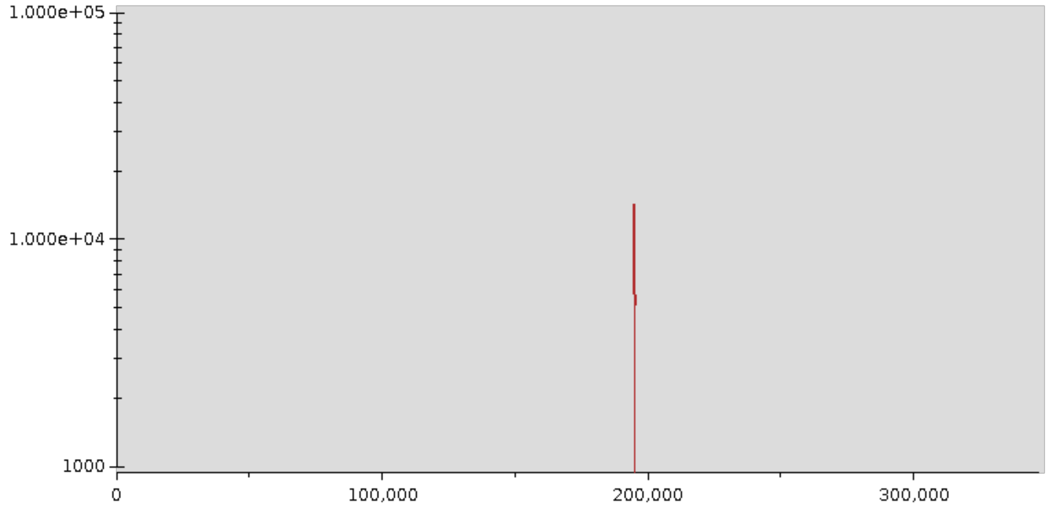

Assignment

**Type**	Lausannevirus (Taxonomy ID: 999883)
**Reference Genome**	NC_015326.1
**NT Identity (%)**	80.4598
**AA Identity (%)**	96.5517
**Number Of Stop Codons**	0
**Number Of CDS**	444

Alignment

**Alignment Score**	212.0 (NT) + 362.0 (AA) = 574.0
**Concordance (%)**	79.5014
**Alignment Method**	Local, heuristic, nucleotide (BLASTN)

Genome Region

Sequence starts at position 194931 and ends at position 195104 relative to NC_015326.1 reference sequence.

Alignment Detailed Statistics

	Begin	End	Coverage	Score	Concordance	Matches	Identities	I/D/M/F*	Stop Codons
NT	194931	195104	0.1%	212	60.9%	174 (100%)	140 (80.5%)	0/0	

Mutations: 194931A>G, 194940A>C, 194946G>T, 194952T>C, 194955T>C, 194958A>G, 194967A>G, 194970G>C, 194979T>G, 194981T>G, 194982C>G, 194988T>C, 195003T>A, 195012A>C, 195015T>C, 195017G>T, 195018T>C, 195021T>C, 195033A>G, 195036G>C, 195042T>C, 195048T>C, 195054T>C, 195057T>A, 195058T>G, 195059G>C, 195060C>T, 195063G>C, 195066G>A, 195069C>G, 195072A>G, 195081C>A, 195087T>C, 195093T>C

*: Inserts / Deletes / Misaligned / Frameshifts

Analysis details

This analysis was performed with panviral2.64

NGS Details (UN24): Noumeavirus

Assembly

**Coverage Length**	217 (1 contig(s))
**Depth Of Coverage**	5688.3
**Number Of Reads**	14282
**Reads Per Million**	285.89 rpm (after QC)
**Ambiguities**	0
**Assembly Method**	de novo + reference guided assembly
**Consensus Caller**	Bcf Tools

Coverage Map

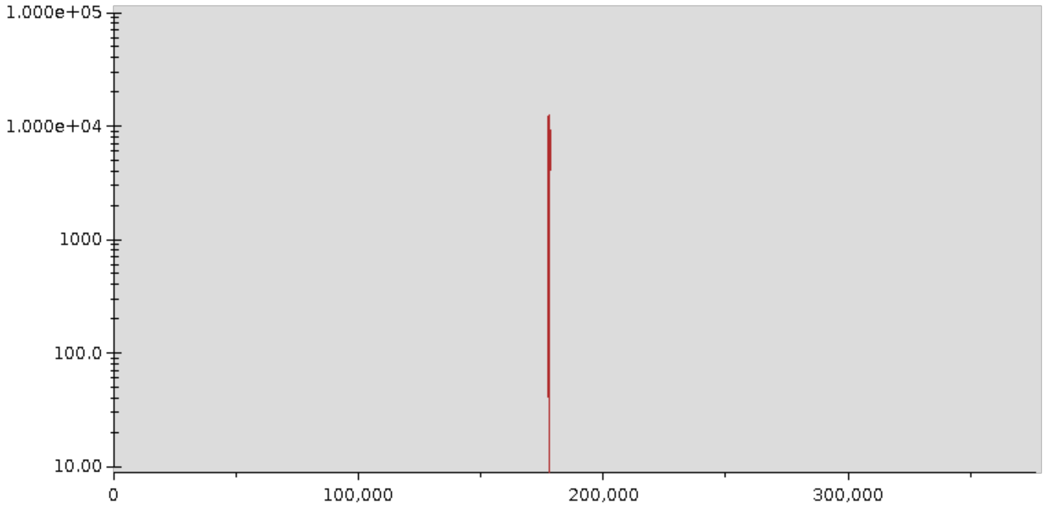

Assignment

**Type**	Noumeavirus (Taxonomy ID: 1955558)
**Reference Genome**	NC_033775.1
**NT Identity (%)**	82.0276
**AA Identity (%)**	95.7746
**Number Of Stop Codons**	0
**Number Of CDS**	452

Alignment

**Alignment Score**	278.0 (NT) + 435.0 (AA) = 713.0
**Concordance (%)**	80.839
**Alignment Method**	Local, heuristic, nucleotide (BLASTN)

Genome Region

Sequence starts at position 177915 and ends at position 178136 relative to NC_033775.1 reference sequence.

Alignment Detailed Statistics

	Begin	End	Coverage	Score	Concordance	Matches	Identities	I/D/M/F*	Stop Codons
**NT**	**177915**	**178136**	**0.1%**	**278**	**64.1%**	**217 (100%)**	**178 (82.0%)**	**0/0**	

Mutations: 177926A>G, 177929T>C, 177941T>A, 177947A>C, 177950A>G, 177953A>G, 177980A>C, 177989C>T, 177992G>C, 177995C>T, 178001A>G, 178002C>G, 178003A>C, 178004A>T, 178019A>G, 178025A>G, 178028C>T, 178034G>A, 178043A>G, 178044C>A, 178047C>T, 178049T>G, 178055C>T, 178064A>G, 178070C>T, 178079A>C, 178080A>C, 178082G>T, 178088T>C, 178091C>T, 178097T>C, 178101G>A, 178106A>G, 178112A>G, 178118A>T, 178121G>C, 178124T>C, 178127T>G, 178136A>G

*: Inserts / Deletes / Misaligned / Frameshifts

Analysis details

This analysis was performed with panviral2.64

NGS Details (UN24): Makelovirus prm1

Assembly

**Coverage Length**	1010 (1 contig(s))
**Depth Of Coverage**	894.1
**Number Of Reads**	7006
**Reads Per Million**	140.24 rpm (after QC)
**Ambiguities**	0
**Assembly Method**	de novo + reference guided assembly
**Consensus Caller**	Bcf Tools

Coverage Map

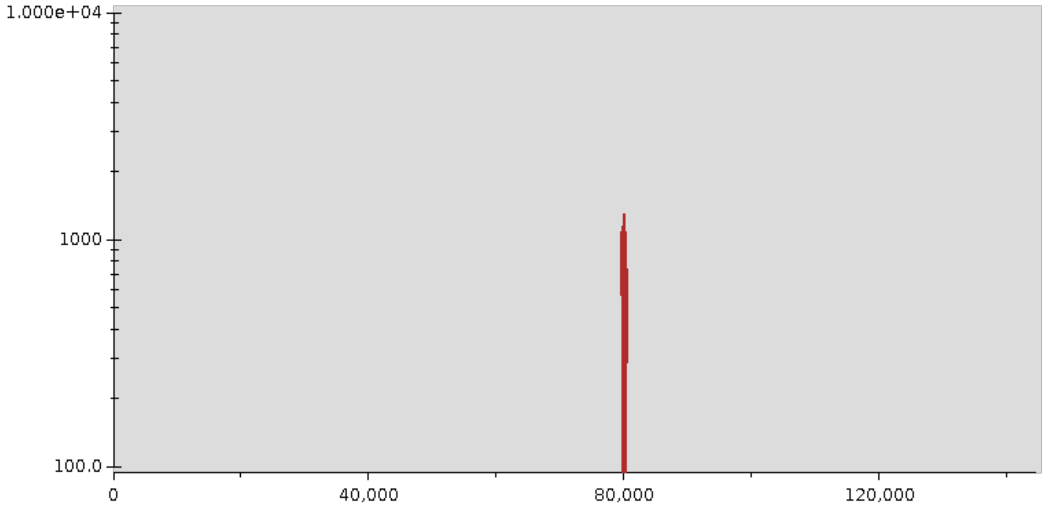

Assignment

**Type**	Makelovirus prm1 (Taxonomy ID: 2956181)
**Reference Genome**	NC_055761.1
**NT Identity (%)**	76.7327
**AA Identity (%)**	87.2404
**Number Of Stop Codons**	0
**Number Of CDS**	190

Alignment

**Alignment Score**	1080.0 (NT) + 2246.0 (AA) = 3326.0
**Concordance (%)**	74.3406
**Alignment Method**	Local, heuristic, nucleotide (BLASTN)

Genome Region

Sequence starts at position 79508 and ends at position 80517 relative to NC_055761.1 reference sequence.

Alignment Detailed Statistics

	Begin	End	Coverage	Score	Concordance	Matches	Identities	I/D/M/F*	Stop Codons
**NT**	**79508**	**80517**	**0.7%**	**1080**	**53.5%**	**1010 (100%)**	**775 (76.7%)**	**0/0**	
Mutations:	79519A>G, 79520A>T, 79522A>G, 79523A>C, 79529C>T, 79530G>A, 79532G>C, 79536G>A, 79541G>T, 79542T>A, 79543C>G, 79544T>C, 79547A>T, 79548A>G, 79550C>A, 79563G>A, 79565C>T, 79568T>A, 79574C>T, 79580G>T, 79581C>T, 79592A>T, 79595T>C, 79596C>T, 79598G>A, 79602G>A, 79604A>G, 79610C>T, 79611G>T, 79612T>C, 79613C>T, 79614T>G, 79615G>T, 79616C>A, 79619C>T, 79624C>T, 79628G>C, 79632G>A, 79643C>T, 79646T>A, 79649G>A, 79655C>T, 79658C>T, 79664C>T, 79673A>T, 79676C>T, 79677G>T, 79679T>A, 79682T>G, 79688G>A, 79689A>C, 79691G>T, 79694T>C, 79703C>T, 79712T>C, 79718T>C, 79719G>A, 79722G>A, 79724A>T, 79728T>A, 79733C>T, 79734A>G, 79735A>C, 79736C>A, 79739A>T, 79742T>A, 79746C>T, 79748T>A, 79751C>T, 79754C>T, 79760C>A, 79763T>C, 79772T>G, 79779C>G, 79791C>T, 79793C>A, 79799T>C, 79811C>T, 79812C>G, 79817G>A, 79818G>A, 79820C>T, 79823A>T, 79824T>C, 79826T>A, 79833C>T, 79835G>A, 79836A>C, 79838C>T, 79842A>G, 79844C>A, 79845T>G, 79846T>C, 79847C>T, 79850C>T, 79871A>G, 79874C>T, 79875T>A, 79876C>G, 79879A>T, 79880T>C, 79895T>A, 79898C>T, 79904C>T, 79905T>G, 79906G>C, 79907C>T, 79916C>T, 79919T>A, 79922A>T, 79931T>A, 79937A>T, 79938T>A, 79940T>C, 79946C>T, 79950C>T, 79953G>A, 79958T>C, 79961T>A, 79962T>A, 79973T>A, 79974T>A, 79975C>G, 79976G>T, 79979C>T, 79985C>T, 79994C>T, 79997C>A, 80000C>A, 80018C>T, 80025C>A, 80030C>A, 80039A>T, 80042A>G, 80054G>T, 80063C>A, 80066C>T, 80069T>C, 80073C>T, 80075T>A, 80078T>C, 80090C>A, 80096T>C, 80099T>C, 80105G>A, 80114A>T, 80120C>T, 80126T>C, 80127C>T, 80132T>A, 80139T>A, 80140C>G, 80141G>T, 80142C>T, 80145G>A, 80147T>C, 80148C>A, 80150T>G, 80159C>A, 80162G>A, 80165C>T, 80168G>A, 80171C>T, 80172C>G, 80173A>C, 80174G>T, 80177C>T, 80178T>G, 80180T>A, 80188A>G, 80189G>A, 80201A>G, 80207G>A, 80210C>T, 80213C>T, 80216C>T, 80222G>A, 80225T>C, 80237C>T, 80240C>T, 80243T>C, 80246C>A, 80247C>T, 80261C>T, 80265T>A, 80266C>G, 80267C>T, 80279C>T, 80286C>T, 80288G>A, 80291T>C, 80300T>A, 80306A>T, 80315T>A, 80318C>A, 80333T>C, 80336A>T, 80337C>T, 80339T>A, 80343G>A, 80346T>A, 80347C>G, 80351C>T, 80357A>T, 80366G>A, 80369C>T, 80378C>T, 80387G>A, 80390C>T, 80391A>G, 80393C>A, 80394A>G, 80396G>T, 80399T>C, 80400G>A, 80409A>C, 80410A>G, 80411A>T, 80414C>A, 80415C>A, 80417C>T, 80433G>A, 80435T>C, 80436C>A, 80438A>C, 80447C>T, 80453G>T, 80456G>T, 80462G>A, 80465A>T, 80471C>T, 80474G>A, 80486C>T, 80498G>A, 80504G>A								

*: Inserts / Deletes / Misaligned / Frameshifts

Analysis details

This analysis was performed with panviral2.64

NGS Details (UN24): Brazilian marseillevirus

Assembly

**Coverage Length**	242 (1 contig(s))
**Depth Of Coverage**	2168.2
**Number Of Reads**	4824
**Reads Per Million**	96.56 rpm (after QC)
**Ambiguities**	0
**Assembly Method**	de novo + reference guided assembly
**Consensus Caller**	Bcf Tools

Coverage Map

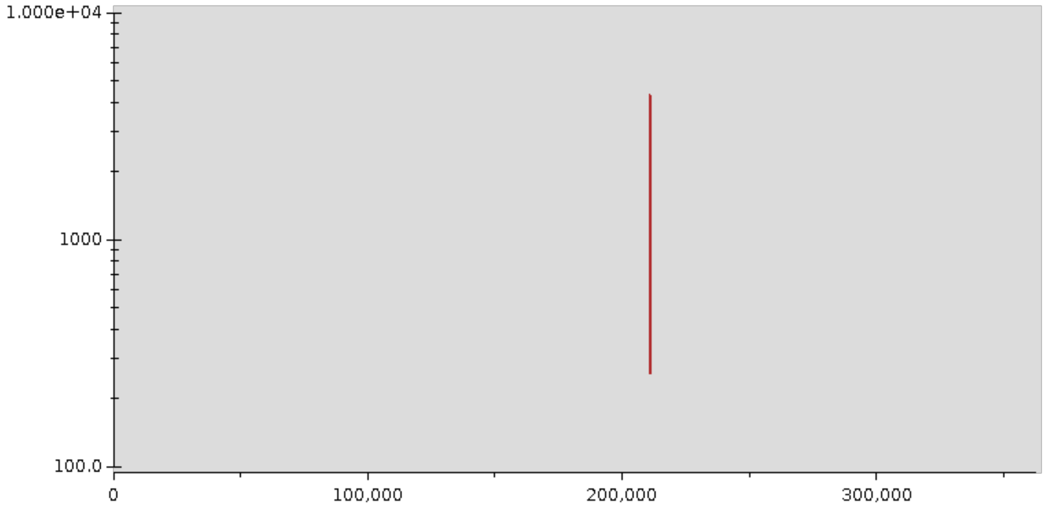

Assignment

**Type**	Brazilian marseillevirus (Taxonomy ID: 1813599)
**Reference Genome**	NC_029692.1
**NT Identity (%)**	82.2314
**AA Identity (%)**	93.3333
**Number Of Stop Codons**	1
**Number Of CDS**	491

Alignment

**Alignment Score**	312.0 (NT) + 452.0 (AA) = 764.0
**Concordance (%)**	80.2521
**Alignment Method**	Local, heuristic, nucleotide (BLASTN)

Genome Region

Sequence starts at position 210884 and ends at position 211125 relative to NC_029692.1 reference sequence.

Alignment Detailed Statistics

	Begin	End	Coverage	Score	Concordance	Matches	Identities	I/D/M/F*	Stop Codons
**NT**	**210884**	**211125**	**0.1%**	**312**	**64.5%**	**242 (100%)**	**199 (82.2%)**	**0/0**	

Mutations: 210899T>G, 210901T>G, 210902T>G, 210917A>G, 210926T>C, 210938A>G, 210941A>G, 210944C>G, 210950G>T, 210953C>G, 210955T>G, 210956T>G, 210965C>G, 210974G>A, 210977T>G, 210986A>C, 210989G>C, 210991G>T, 210992T>C, 210995T>C, 211001A>T, 211004A>G, 211007A>G, 211009C>T, 211010T>C, 211025G>A, 211031T>A, 211032T>G, 211033G>C, 211040G>A, 211043T>G, 211058G>A, 211061T>C, 211064A>C, 211070G>A, 211072G>A, 211079C>G, 211088C>G, 211094A>C, 211100T>A, 211106C>G, 211108C>T, 211115T>C

*: Inserts / Deletes / Misaligned / Frameshifts

Analysis details

This analysis was performed with panviral2.64

NGS Details (UN24): Tokyovirus A1

Assembly

**Coverage Length**	180 (1 contig(s))
**Depth Of Coverage**	1769.2
**Number Of Reads**	3678
**Reads Per Million**	73.62 rpm (after QC)
**Ambiguities**	0
**Assembly Method**	de novo + reference guided assembly
**Consensus Caller**	Bcf Tools

Coverage Map

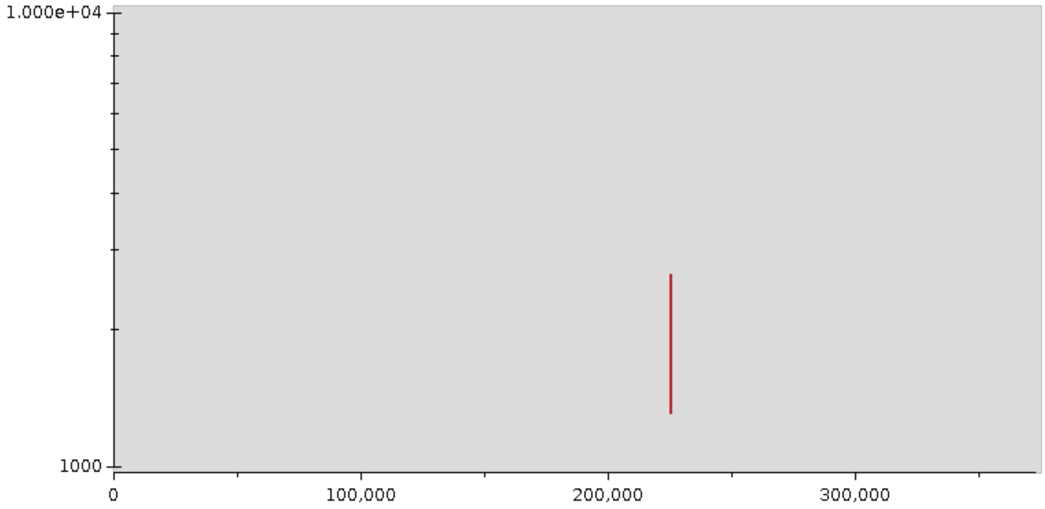

Assignment

**Type**	Tokyovirus A1 (Taxonomy ID: 1826170)
**Reference Genome**	NC_030230.1
**NT Identity (%)**	80.5556
**AA Identity (%)**	93.1034
**Number Of Stop Codons**	1
**Number Of CDS**	470

Alignment

**Alignment Score**	220.0 (NT) + 353.0 (AA) = 573.0
**Concordance (%)**	78.4932
**Alignment Method**	Local, heuristic, nucleotide (BLASTN)

Genome Region

Sequence starts at position 225483 and ends at position 225662 relative to NC_030230.1 reference sequence.

Alignment Detailed Statistics

	Begin	End	Coverage	Score	Concordance	Matches	Identities	I/D/M/F*	Stop Codons
**NT**	**225483**	**225662**	**0.1%**	**220**	**61.1%**	**180 (100%)**	**145 (80.6%)**	**0/0**	
Mutations:	225498T>C, 225504G>C, 225507A>C, 225510G>A, 225513G>T, 225519G>T, 225528T>C, 225533C>T, 225534A>G, 225543G>C, 225549A>G, 225552T>G, 225554T>G, 225555C>G, 225564T>G, 225573C>A, 225579G>A, 225582G>A, 225585A>C, 225590G>T, 225594T>C, 225600G>C, 225603C>T, 225609A>T, 225621T>C, 225624G>T, 225630T>G, 225631T>G, 225632G>C, 225636C>G, 225640G>T, 225641C>T, 225642T>G, 225648G>T, 225651G>A								

*: Inserts / Deletes / Misaligned / Frameshifts

Analysis details

This analysis was performed with panviral2.64

NGS Details (UN24): Marseillevirus marseillevirus

Assembly

**Coverage Length**	110 (1 contig(s))
**Depth Of Coverage**	2178.8
**Number Of Reads**	2867
**Reads Per Million**	57.39 rpm (after QC)
**Ambiguities**	0
**Assembly Method**	de novo + reference guided assembly
**Consensus Caller**	Bcf Tools

Coverage Map

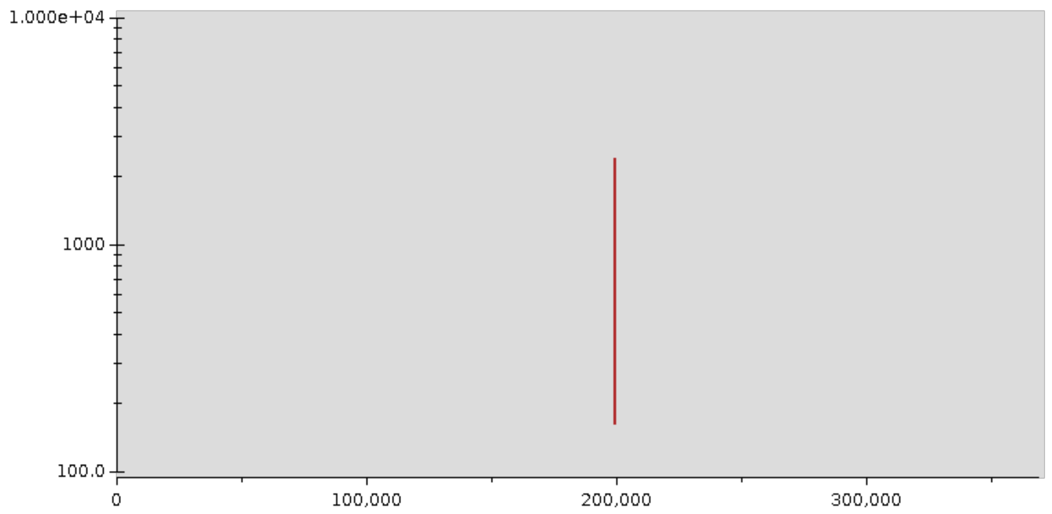

Assignment

**Type**	Marseillevirus marseillevirus (Taxonomy ID: 694581)
**Reference Genome**	NC_013756.1
**NT Identity (%)**	83.6364
**AA Identity (%)**	94.2857
**Number Of Stop Codons**	1
**Number Of CDS**	428

Alignment

**Alignment Score**	148.0 (NT) + 204.0 (AA) = 352.0
**Concordance (%)**	80.3653
**Alignment Method**	Local, heuristic, nucleotide (BLASTN)

Genome Region

Sequence starts at position 199400 and ends at position 199509 relative to NC_013756.1 reference sequence.

Alignment Detailed Statistics

	Begin	End	Coverage	Score	Concordance	Matches	Identities	I/D/M/F*	Stop Codons
NT	199400	199509	0.1%	148	67.3%	110 (100%)	92 (83.6%)	0/0	

Mutations: 199411A>G, 199414T>A, 199416T>G, 199417C>G, 199421A>T, 199429G>C, 199431G>A, 199432A>T, 199435C>G, 199448A>T, 199449C>T, 199459A>C, 199464A>G, 199471T>A, 199489T>C, 199492G>T, 199495G>A, 199498G>A

*: Inserts / Deletes / Misaligned / Frameshifts

Analysis details

This analysis was performed with panviral2.64

NGS Details (UN24): Tunisvirus fontaine2

Assembly

Coverage Length	197 (1 contig(s))
Depth Of Coverage	946.0
Number Of Reads	1907
Reads Per Million	38.17 rpm (after QC)
Ambiguities	0
Assembly Method	de novo + reference guided assembly
Consensus Caller	Bcf Tools

Coverage Map

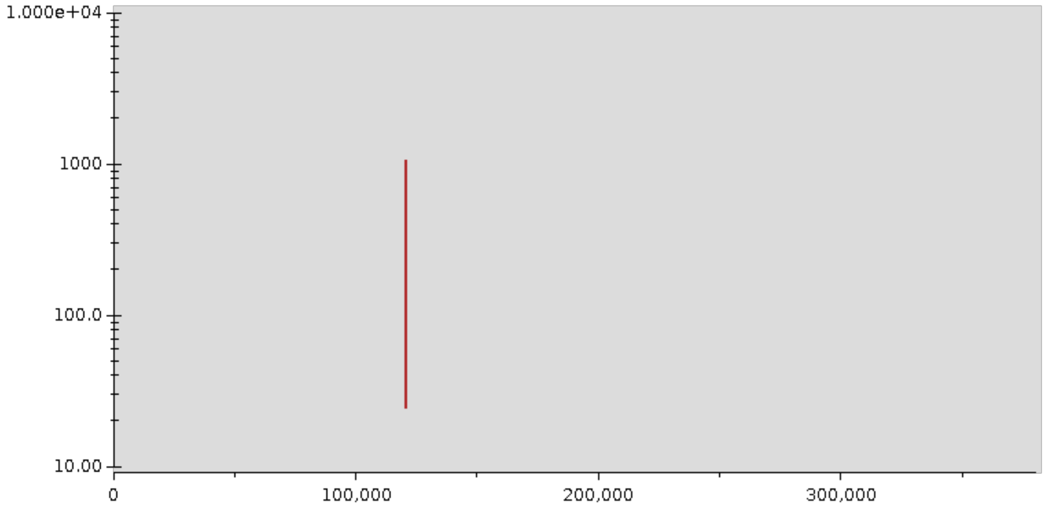

Assignment

Type	Tunisvirus fontaine2 (Taxonomy ID: 1421067)
Reference Genome	NC_038511.1
NT Identity (%)	78.1726
AA Identity (%)	90.4762
Number Of Stop Codons	1
Number Of CDS	484

Alignment

Alignment Score	222.0 (NT) + 378.0 (AA) = 600.0
Concordance (%)	74.813
Alignment Method	Local, heuristic, nucleotide (BLASTN)

Genome Region

Sequence starts at position 120425 and ends at position 120621 relative to NC_038511.1 reference sequence.

Alignment Detailed Statistics

	Begin	End	Coverage	Score	Concordance	Matches	Identities	I/D/M/F*	Stop Codons
**NT**	**120425**	**120621**	**0.1%**	**222**	**56.3%**	**197 (100%)**	**154 (78.2%)**	**0/0**	

Mutations: 120440T>C, 120449A>C, 120451G>A, 120452G>A, 120455A>C, 120457G>A, 120461G>T, 120464T>C, 120470T>C, 120475C>T, 120482A>G, 120485G>A, 120488A>T, 120494C>A, 120496T>G, 120497C>T, 120506C>G, 120509A>C, 120510C>T, 120511T>G, 120515C>T, 120527A>C, 120529G>A, 120536T>C, 120545A>T, 120551G>A, 120557T>C, 120569T>C, 120572T>A, 120573T>G, 120574G>C, 120575C>T, 120582C>T, 120584C>G, 120587G>T, 120590G>A, 120596C>A, 120597G>C, 120598A>T, 120600C>G, 120601T>A, 120605C>A, 120608A>C

*: Inserts / Deletes / Misaligned / Frameshifts

Analysis details

This analysis was performed with panviral2.64

NGS Details (UN24): Yellowstone lake phycodnavirus 1

Assembly

**Coverage Length**	185 (1 contig(s))
**Depth Of Coverage**	856.7
**Number Of Reads**	1645
**Reads Per Million**	32.93 rpm (after QC)
**Ambiguities**	0
**Assembly Method**	de novo + reference guided assembly
**Consensus Caller**	Bcf Tools

Coverage Map

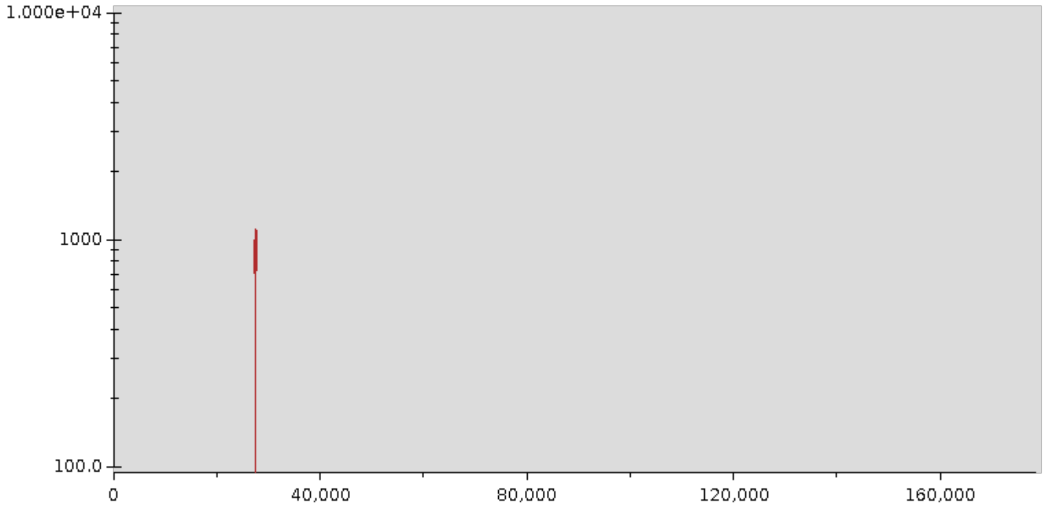

Assignment

**Type**	Yellowstone lake phycodnavirus 1 (Taxonomy ID: 1586713)
**Reference Genome**	NC_028112.1
**NT Identity (%)**	81.6216
**AA Identity (%)**	90.3226
**Number Of Stop Codons**	0
**Number Of CDS**	248

Alignment

**Alignment Score**	234.0 (NT) + 371.0 (AA) = 605.0
**Concordance (%)**	79.5007
**Alignment Method**	Local, heuristic, nucleotide (BLASTN)

Genome Region

Sequence starts at position 27457 and ends at position 27641 relative to NC_028112.1 reference sequence.

Alignment Detailed Statistics

	Begin	End	Coverage	Score	Concordance	Matches	Identities	I/D/M/F*	Stop Codons
**NT**	**27457**	**27641**	**0.1%**	**234**	**63.2%**	**185 (100%)**	**151 (81.6%)**	**0/0**	

Mutations: 27470G>C, 27476C>A, 27477A>T, 27481T>G, 27489T>A, 27492A>G, 27501C>T, 27510A>T, 27513T>C, 27516T>A, 27529C>A, 27531T>G, 27534A>G, 27552A>G, 27555C>G, 27563A>G, 27564T>G, 27567T>C, 27573A>T, 27576A>T, 27579C>T, 27585T>C, 27586G>A, 27588C>T, 27591A>G, 27594G>A, 27600C>A, 27609T>C, 27612A>T, 27615C>T, 27616T>C, 27618G>T, 27624T>C, 27627C>T

*: Inserts / Deletes / Misaligned / Frameshifts

Analysis details

This analysis was performed with panviral2.64

NGS Details (UN24): Amsacta moorei entomopoxvirus

Assembly

**Coverage Length**	131 (1 contig(s))
**Depth Of Coverage**	860.5
**Number Of Reads**	1423
**Reads Per Million**	28.48 rpm (after QC)
**Ambiguities**	0
**Assembly Method**	de novo + reference guided assembly
**Consensus Caller**	Bcf Tools

Coverage Map

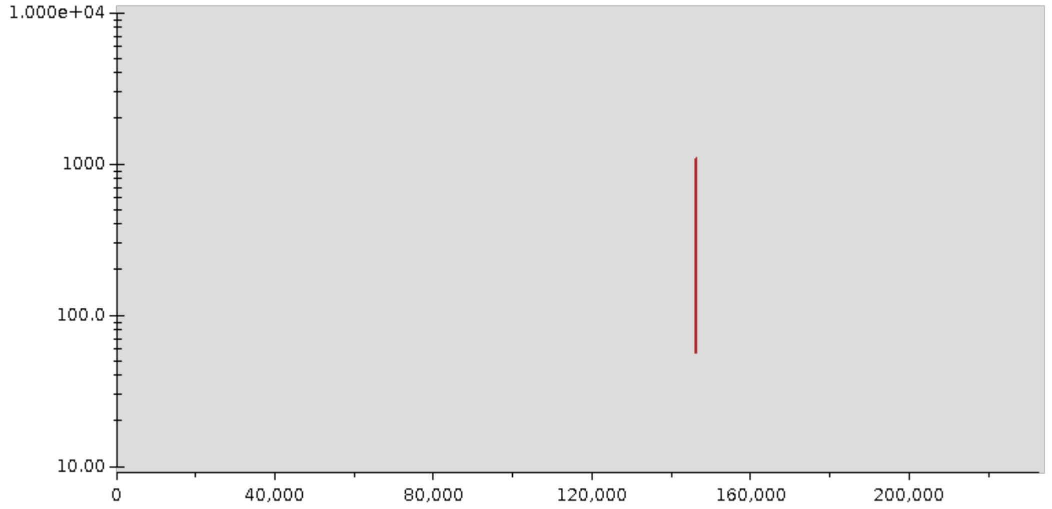

Assignment

**Type**	Amsacta moorei entomopoxvirus (Taxonomy ID: 28321)
**Reference Genome**	NC_002520.1
**NT Identity (%)**	80.1527
**AA Identity (%)**	81.8182
**Number Of Stop Codons**	0
**Number Of CDS**	294

Alignment

**Alignment Score**	158.0 (NT) + 251.0 (AA) = 409.0
**Concordance (%)**	74.4991
**Alignment Method**	Local, heuristic, nucleotide (BLASTN)

Genome Region

Sequence starts at position 146093 and ends at position 146223 relative to NC_002520.1 reference sequence.

Alignment Detailed Statistics

	Begin	End	Coverage	Score	Concordance	Matches	Identities	I/D/M/F*	Stop Codons
**NT**	**146093**	**146223**	**0.1%**	**158**	**60.3%**	**131 (100%)**	**105 (80.2%)**	**0/0**	

Mutations: 146104A>G, 146107T>A, 146109A>C, 146113A>T, 146119G>T, 146121T>C, 146125A>C, 146128T>C, 146131T>C, 146140A>C, 146143A>G, 146144A>T, 146146A>G, 146155T>C, 146179T>C, 146188T>G, 146194A>T, 146195T>G, 146196T>C, 146197T>C, 146200A>G, 146202T>C, 146206T>G, 146207G>T, 146208A>C, 146209T>A

*: Inserts / Deletes / Misaligned / Frameshifts

Analysis details

This analysis was performed with panviral2.64

NGS Details (UN24): Yellowstone lake phycodnavirus 1

Assembly

**Coverage Length**	144 (1 contig(s))
**Depth Of Coverage**	648.7
**Number Of Reads**	1029
**Reads Per Million**	20.60 rpm (after QC)
**Ambiguities**	0
**Assembly Method**	de novo + reference guided assembly
**Consensus Caller**	Bcf Tools

Coverage Map

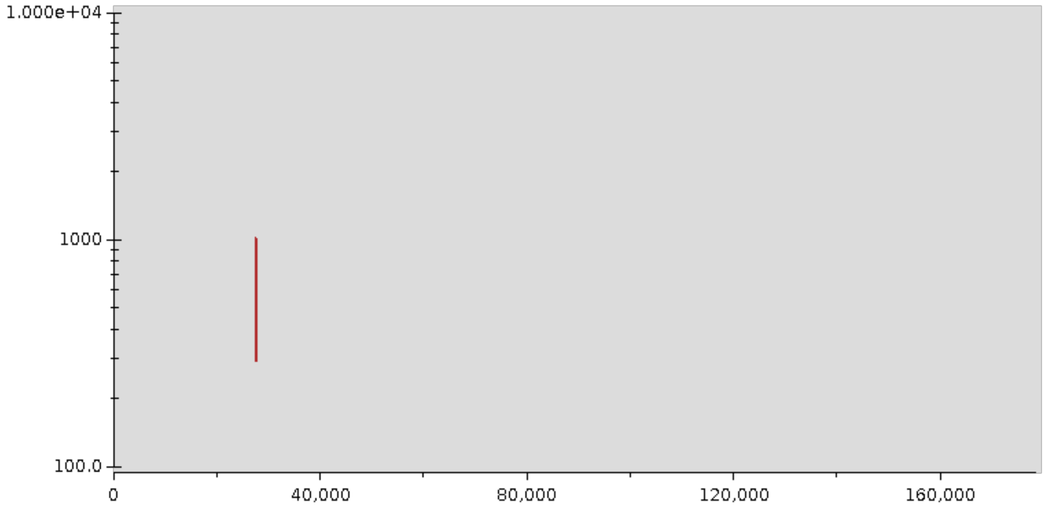

Assignment

**Type**	Yellowstone lake phycodnavirus 1 (Taxonomy ID: 1586713)
**Reference Genome**	NC_028112.1
**NT Identity (%)**	81.9444
**AA Identity (%)**	83.3333
**Number Of Stop Codons**	0
**Number Of CDS**	248

Alignment

**Alignment Score**	184.0 (NT) + 267.0 (AA) = 451.0
**Concordance (%)**	75.2922
**Alignment Method**	Local, heuristic, nucleotide (BLASTN)

Genome Region

Sequence starts at position 27486 and ends at position 27629 relative to NC_028112.1 reference sequence.

Alignment Detailed Statistics

	Begin	End	Coverage	Score	Concordance	Matches	Identities	I/D/M/F*	Stop Codons
**NT**	**27486**	**27629**	**0.1%**	**184**	**63.9%**	**144 (100%)**	**118 (81.9%)**	**0/0**	

Mutations: 27498G>A, 27508G>T, 27510A>T, 27516T>C, 27534A>G, 27549G>A, 27550C>A, 27555C>T, 27558G>T, 27563A>G, 27564T>C, 27565C>A, 27567T>G, 27570C>A, 27573A>T, 27574G>T, 27585T>C, 27586G>A, 27588C>A, 27591A>G, 27597A>T, 27600C>A, 27606T>C, 27610C>T, 27612A>G, 27615C>G

*: Inserts / Deletes / Misaligned / Frameshifts

Analysis details

This analysis was performed with panviral2.64

NGS Details (UN24): Cladosporium fulvum T-1 virus

Assembly

**Coverage Length**	1401 (3 contig(s))
**Depth Of Coverage**	56.9
**Number Of Reads**	749
**Reads Per Million**	14.99 rpm (after QC)
**Ambiguities**	0
**Assembly Method**	de novo + reference guided assembly
**Consensus Caller**	Bcf Tools

Coverage Map

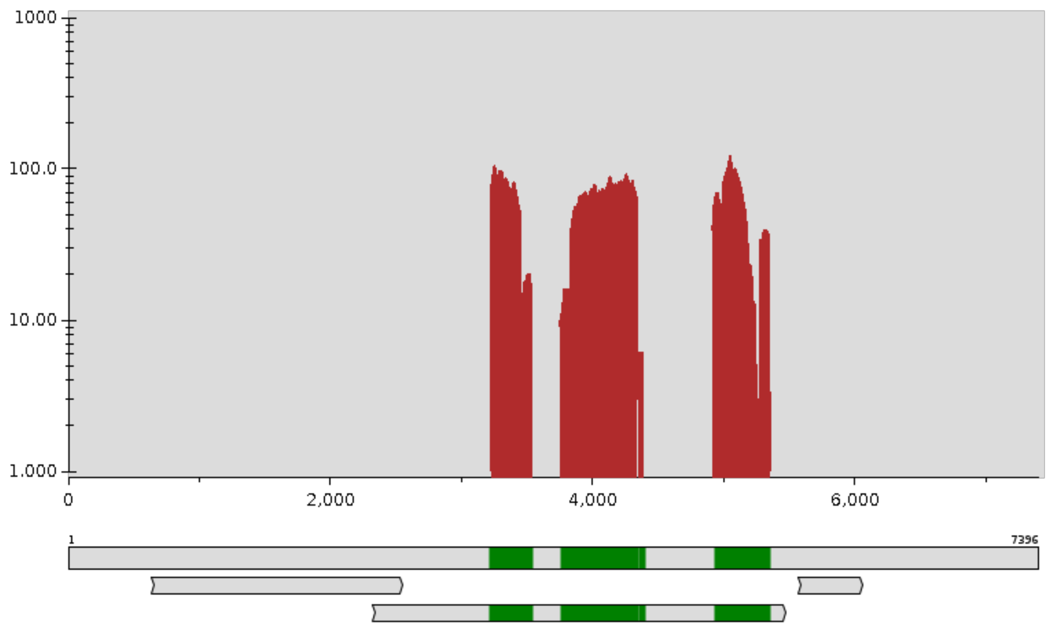

Assignment

**Type**	Cladosporium fulvum T-1 virus (Taxonomy ID: 2052899)
**Reference Genome**	NC_043491.1
**NT Identity (%)**	53.0786
**AA Identity (%)**	47.5584
**Number Of Stop Codons**	1
**Number Of CDS**	3

Alignment

**Alignment Score**	123.0 (NT) + 1606.0 (AA) = 1729.0
**Concordance (%)**	29.2505

**Alignment Method**	Global, seeded, nucleotide + amino acids (AGA)

Genome Region

Sequence starts at position 3223 and ends at position 5354 relative to NC_043491.1 reference sequence.

Alignment Detailed Statistics

	Begin	End	Coverage	Score	Concordance	Matches	Identities	I/D/M/F*	Stop Codons
**NT**	**3223**	**5354**	**18.9%**	**123**	**4.5%**	**1383 (96.6%)**	**750 (52.4%)**	**30/18**	
Mutations:	3223C>G, 3224A>C, 3225A>T, 3229C>T, 3231C>T, 3232C>G, 3233T>A, 3235A>G, 3238T>A, 3238_3239insCCTGAGTTGCCAAAG, 3243A>T, 3244C>G, 3246A>C, 3251T>A, 3253_3254insAGGGATATT, 3261A>G, 3262G>A, 3265A>T, 3266A>G, 3268C>G, 3269A>T, 3271T>G, 3272C>A, 3273A>T, 3280G>T, 3281A>T, 3282A>C, 3283A>T, 3284G>A, 3285A>C, 3289T>G, 3292A>T, 3293T>G, 3294G>C, 3295G>A, 3296G>C, 3297G>A, 3299C>G, 3301C>T, 3303T>C, 3304A>T, 3307T>C, 3308C>A, 3309A>G, 3314T>G, 3317G>C, 3318A>C, 3319G>T, 3326C>T, 3328A>G, 3329C>G, 3330A>C, 3331G>T, 3332A>G, 3333C>A, 3334C>A, 3337A>G, 3338C>A, 3340A>G, 3341G>A, 3343A>G, 3344T>C, 3345G>A, 3352G>C, 3355G>A, 3356A>T, 3357A>T, 3361A>T, 3363C>A, 3364C>T, 3365A>T, 3366A>C, 3371T>C, 3372G>T, 3376A>T, 3378G>A, 3379A>G, 3381G>C, 3382A>T, 3385C>T, 3387C>A, 3388C>G, 3389T>G, 3391A>T, 3392A>C, 3393G>C, 3394T>C, 3395G>T, 3396C>A, 3397A>T, 3400A>T, 3401A>G, 3403T>A, 3406A>T, 3407T>G, 3408G>T, 3409C>T, 3410A>T, 3412G>A, 3415C>T, 3416G>C, 3417T>A, 3418T>G, 3419C>A, 3420C>A, 3425G>C, 3426C>A, 3427A>G, 3428A>G, 3430C>T, 3434A>T, 3435A>C, 3437C>T, 3440C>A, 3442A>G, 3443C>A, 3445C>G, 3446G>T, 3447T>G, 3448A>C, 3449C>A, 3450A>T, 3451A>T, 3460A>G, 3461A>C, 3464T>C, 3470G>A, 3473A>G, 3475C>G, 3478G>T, 3481C>A, 3487C>T, 3488C>A, 3489G>A, 3490A>G, 3493T>C, 3497C>T, 3499A>G, 3502C>T, 3504A>G, 3505C>G, 3508C>T, 3511A>T, 3514A>T, 3515G>T, 3516C>T, 3517A>G, 3518C>T, 3519A>T, 3520A>T, 3523C>T, 3524A>C, 3525G>A, 3529A>T, 3530A>C, 3531C>A, 3532C>G, 3536T>G, 3538A>T, 3751C>T, 3757A>T, 3758C>G, 3760G>C, 3761G>A, 3763C>A, 3766C>T, 3768C>G, 3769A>T, 3774G>A, 3775A>T, 3776T>A, 3778C>G, 3779C>T, 3781C>G, 3782C>G, 3784G>T, 3791A>G, 3792C>T, 3793C>G, 3796G>C, 3799A>T, 3800G>T, 3802T>G, 3803C>A, 3804A>G, 3805A>G, 3806G>T, 3807A>T, 3808T>G, 3811G>A, 3814C>A, 3815G>C, 3819G>T, 3820A>T, 3821C>T, 3823C>A, 3825C>G, 3827A>G, 3829G>A, 3831C>A, 3833G>A, 3834G>A, 3835A>G, 3838C>G, 3839A>T, 3841G>T, 3842A>G, 3843C>T, 3844A>T, 3845G>A, 3846C>A, 3848C>A, 3849C>T, 3850C>G, 3856A>G, 3859C>T, 3862A>G, 3865C>T, 3866C>G, 3867A>C, 3869A>C, 3871G>A, 3880C>A, 3886T>C, 3887T>C, 3893T>C, 3894T>A, 3898C>A, 3899A>G, 3901C>T, 3905A>G, 3906C>A, 3908A>G, 3909C>A, 3910A>C, 3912G>A, 3913G>A, 3914A>G, 3918C>G, 3922C>G, 3925C>T, 3929G>A, 3930C>A, 3931A>G, 3934G>A, 3935A>G, 3936C>T, 3937A>G, 3940G>A, 3941T>G, 3943A>C, 3946C>T, 3947A>G, 3948G>T, 3952A>T, 3957C>A, 3960A>C, 3964G>C, 3967G>A, 3970A>T, 3973C>G, 3975A>C, 3980G>C, 3982A>G, 3983C>A, 3984A>G, 3988A>T, 3992C>T, 3994T>G, 3997A>G, 4000C>A, 4003C>T, 4010A>T, 4012C>T, 4025A>G, 4027G>T, 4029A>G, 4030C>A, 4033T>C, 4038A>C, 4041C>T, 4048A>T, 4052A>C, 4057G>T, 4058A>G, 4059T>A, 4060G>T, 4063T>G, 4064A>C, 4065C>T, 4066A>G, 4068G>A, 4069A>G, 4072A>G, 4073G>A, 4075C>A, 4081C>G, 4085A>G, 4086A>T, 4091G>T, 4092G>C, 4093A>G, 4094A>G, 4095A>T, 4097G>A, 4098A>G, 4100C>T, 4101A>G, 4102G>C, 4103A>G, 4104C>A, 4105C>A, 4111G>T, 4114C>T, 4115A>C, 4117A>G, 4120A>T, 4121C>T, 4123C>G, 4130C>G, 4131A>C, 4132G>T, 4133T>A, 4134G>T, 4135C>T, 4138T>A, 4143C>A, 4144C>A, 4149C>T, 4150G>A, 4153T>G, 4154C>A, 4155G>A, 4156A>G, 4159A>G, 4159_4160insCCAAAT, 4162C>T, 4165T>G, 4166G>C, 4167G>T, 4168T>A, 4169A>C, 4170G>C, 4171C>T, 4172A>T, 4173A>T, 4174G>T, 4177A>G, 4180C>A, 4184A>G, 4186C>G, 4187G>C, 4192C>T, 4195C>T, 4201T>G, 4204T>C, 4206T>A, 4207G>A, 4210A>T, 4211A>G, 4213A>G, 4216C>A, 4218C>G, 4219A>C, 4220T>G, 4221G>T, 4222T>A, 4223C>T, 4225A>G, 4226A>G, 4227C>T, 4228A>G, 4231G>A, 4232_4237delACACAC, 4240T>A, 4243G>T, 4244_4249delAAAAGA, 4252C>T, 4255A>G, 4263A>T, 4265T>G, 4267T>A, 4268T>A, 4269C>G, 4271C>A, 4277A>T, 4279G>A, 4281C>A, 4282C>T, 4283A>G, 4284C>A, 4285A>T, 4288G>T, 4291A>G, 4296A>G, 4297C>G, 4301G>T, 4302A>C, 4303C>T, 4305T>C, 4306C>T, 4312C>A, 4319C>A, 4321T>G, 4322C>G, 4324A>T, 4327C>A, 4328A>G, 4330T>G, 4333T>G, 4334G>C, 4335C>A, 4337G>T, 4338C>G, 4340A>C, 4346C>A, 4347A>C, 4348T>C, 4355G>C, 4356T>A, 4357G>C, 4361G>T, 4363C>G, 4364G>C, 4365A>T, 4366G>T, 4369C>T, 4370C>T, 4372A>T, 4373_4375delCCG, 4381A>C, 4382A>T, 4383C>T, 4384G>A, 4385A>G, 4387T>C, 4388C>A, 4389T>A, 4390T>A, 4391T>A, 4393A>C, 4915A>T, 4918A>T, 4921G>T, 4922A>T, 4924A>G, 4925C>A, 4926C>A, 4927A>G, 4933C>A, 4939T>G, 4941C>A, 4942G>T, 4951C>T, 4952A>G, 4955A>G, 4956C>T, 4957G>A, 4958A>G, 4959A>G, 4961C>T, 4963C>G, 4966G>A, 4968G>A, 4972A>T, 4974A>G, 4976G>A, 4977A>G, 4978T>G, 4980G>A, 4982G>A, 4983T>C, 4984C>T, 4986C>G, 4988G>A, 4990A>G, 4994_4996delGCC, 4997T>C, 5002C>T, 5003A>T, 5004T>C, 5005G>T, 5009C>T, 5010T>G, 5011A>G, 5014C>G, 5017G>A, 5020C>T, 5023C>T, 5024A>C, 5026A>G, 5027C>A, 5029C>G, 5032A>G, 5037A>C, 5038T>A, 5041A>C, 5044C>T, 5047C>T, 5048A>C, 5050T>A, 5053T>A, 5055C>T, 5056A>C, 5057T>A, 5058C>A, 5059A>G, 5060G>A, 5061A>C, 5062A>T, 5064T>C, 5065A>T, 5067A>T, 5070C>G, 5074A>T, 5077G>A, 5078C>G, 5080G>T, 5081C>T, 5082T>A, 5083C>T, 5085G>C, 5086A>C, 5087T>A, 5089C>A, 5092C>G, 5093G>T, 5094T>A, 5095A>C, 5096C>A, 5098G>T, 5099G>C, 5100A>G, 5101C>T, 5102A>G, 5103G>A, 5104A>G, 5108A>G, 5110C>T, 5114T>C, 5115A>T, 5119C>T, 5122A>T, 5123T>A, 5125C>T, 5128G>T, 5129G>T, 5130A>T, 5131A>G, 5132G>T, 5133T>C, 5134A>C, 5135T>A, 5137C>T, 5141A>T, 5143A>T, 5146C>T, 5151A>G, 5152C>T, 5153A>G, 5154A>C, 5155G>T, 5157T>A, 5158C>G, 5161C>T, 5164A>T, 5168A>C, 5172A>T, 5173C>T, 5178A>G, 5179G>A, 5180A>T, 5182G>A, 5183C>T, 5185C>T, 5186A>C, 5187T>A, 5188G>T, 5189G>A, 5190G>A, 5191A>G, 5192A>G, 5193C>G, 5194G>A, 5195A>C, 5200A>T, 5202T>C, 5207C>G, 5208A>T, 5209C>A, 5216T>A, 5217C>G, 5218A>C, 5221A>C, 5224A>T, 5226A>T, 5227C>T, 5233A>T, 5234G>C, 5236G>A, 5239G>T, 5245G>A, 5251G>A, 5257A>G, 5260G>C, 5262A>T, 5263C>T, 5272C>T, 5276C>G, 5278A>C, 5284A>T, 5285C>A, 5292A>T, 5293C>T, 5294A>G, 5296C>G, 5298A>G, 5301A>C, 5302C>T, 5303G>C, 5304C>A, 5305A>T, 5309G>A, 5311C>A, 5312A>G, 5314C>T, 5319T>C, 5321T>A, 5322C>A, 5323A>G, 5326A>G, 5329G>A, 5330C>G, 5331C>A, 5332A>T, 5333A>G, 5338G>C, 5341G>A, 5342A>T, 5345G>T								
**CDS**									
homologue_of_retroviral_POL_genes	300	1010	44.6%	1606	49.8%	461 (96.6%)	224 (47.0%)	10/6/0/0	1

	Begin	End	Coverage	Score	Concordance	Matches	Identities	I/D/M/F*	Stop Codons
**NT**	**3223**	**5354**	**18.9%**	**123**	**4.5%**	**1383 (96.6%)**	**750 (52.4%)**	**30/18**	
Protein mutations:	K300L (3224A>C 3225A>T), A302V (3231C>T 3232C>G), L303M (3233T>A 3235A>G), P304_K305insPELPK (3238_3239insCCTGAGTTGCCAAAG), H306L (3243A>T 3244C>G), Q307P (3246A>C), W309R (3251T>A), W309_D310insRDI (3253_3254insAGGGATATT), K312R (3261A>G 3262G>A), N314E (3266A>G 3268C>G), I315L (3269A>T 3271T>G), Q316M (3272C>A 3273A>T), K319S (3281A>T 3282A>C 3283A>T), E320T (3284G>A 3285A>C), W323A (3293T>G 3294G>C 3295G>A), G324Q (3296G>C 3297G>A), P325A (3299C>G 3301C>T), L326P (3303T>C 3304A>T), Q328R (3308C>A 3309A>G), S330A (3314T>G), E331P (3317G>C 3318A>C 3319G>T), Q335A (3329C>G 3330A>C 3331G>T), T336E (3332A>G 3333C>A 3334C>A), E339K (3341G>A 3343A>G), W340Q (3344T>C 3345G>A), K342N (3352G>C), K344L (3356A>T 3357A>T), A346D (3363C>A 3364C>T), K347S (3365A>T 3366A>C), W349L (3371T>C 3372G>T), R351Q (3378G>A 3379A>G), R352P (3381G>C 3382A>T), T354K (3387C>A 3388C>G), S355A (3389T>G 3391A>T), S356P (3392A>C 3393G>C 3394T>C), A357Y (3395G>T 3396C>A 3397A>T), T359A (3401A>G 3403T>A), C361V (3407T>G 3408G>T 3409C>T), M362L (3410A>T 3412G>A), V364Q (3416G>C 3417T>A 3418T>G), P365K (3419C>A 3420C>A), A367Q (3425G>C 3426C>A 3427A>G), N368D (3428A>G 3430C>T), K370S (3434A>T 3435A>C), L373M (3443C>A 3445C>G), V374C (3446G>T 3447T>G 3448A>C), Q375I (3449C>A 3450A>T 3451A>T), K379Q (3461A>C), E382K (3470G>A), I383V (3473A>G 3475C>G), R388K (3488C>A 3489G>A 3490A>G), N393R (3504A>G 3505C>G), E395D (3511A>T), E396D (3514A>T), A397L (3515G>T 3516C>T 3517A>G), Q398F (3518C>T 3519A>T 3520A>T), R400Q (3524A>C 3525G>A), L401F (3529A>T), T402Q (3530A>C 3531C>A 3532C>G), S404A (3536T>G 3538A>T), L478V (3758C>G 3760G>C), V479I (3761G>A 3763C>A), T481S (3768C>G 3769A>T), G483D (3774G>A 3775A>T), S484T (3776T>A 3778C>G), Q486D (3782C>G 3784G>T), T489V (3791A>G 3792C>T 3793C>G), K490N (3796G>C), Q491H (3799A>T), V492L (3800G>T 3802T>G), Q493R (3803C>A 3804A>G 3805A>G), D494L (3806G>T 3807A>T 3808T>G), F496L (3814C>A), E497Q (3815G>C), R498L (3819G>T 3820A>T), T500R (3825C>G), K501E (3827A>G 3829G>A), S502Y (3831C>A), G503K (3833G>A 3834G>A 3835A>G), F504L (3838C>G), K505Y (3839A>T 3841G>T), T506V (3842A>G 3843C>T 3844A>T), A507K (3845G>A 3846C>A), P508M (3848C>A 3849C>T 3850C>G), H514A (3866C>G 3867A>C), K515Q (3869A>C 3871G>A), F523H (3893T>C 3894T>A), I525V (3899A>G 3901C>T), T527E (3905A>G 3906C>A), T528D (3908A>G 3909C>A 3910A>C), G529E (3912G>A 3913G>A), I530V (3914A>G), T531R (3918C>G), I532M (3922C>G), A535K (3929G>A 3930C>A 3931A>G), T537V (3935A>G 3936C>T 3937A>G), S539A (3941T>G 3943A>C), R541V (3947A>G 3948G>T), E542D (3952A>T), P544Q (3957C>A), E545A (3960A>C), K550T (3975A>C), V552L (3980G>C 3982A>G), Q553R (3983C>A 3984A>G), N562Y (4010A>T 4012C>T), K567D (4025A>G 4027G>T), D568G (4029A>G 4030C>A), K571T (4038A>C), T572I (4041C>T), M576L (4052A>C), M578D (4058A>G 4059T>A 4060G>T), T580L (4064A>C 4065C>T 4066A>G), R581K (4068G>A 4069A>G), D583K (4073G>A 4075C>A), N585K (4081C>G), K587V (4085A>G 4086A>T), G589S (4091G>T 4092G>C 4093A>G), K590V (4094A>G 4095A>T), E591R (4097G>A 4098A>G), Q592C (4100C>T 4101A>G 4102G>C), T593E (4103A>G 4104C>A 4105C>A), K597Q (4115A>C 4117A>G), R598S (4120A>T), Q602A (4130C>G 4131A>C 4132G>T), C603I (4133T>A 4134G>T 4135C>T), A606E (4143C>A 4144C>A), T608I (4149C>T 4150G>A), R610K (4154C>A 4155G>A 4156A>G), L611_F612insPN (4159_4160insCCAAAT), D613E (4165T>G), G614L (4166G>C 4167G>T 4168T>A), S615P (4169A>C 4170G>C 4171C>T), K616F (4172A>T 4173A>T 4174G>T), I620V (4184A>G 4186C>G), E621Q (4187G>C), M627K (4206T>A 4207G>A), I629V (4211A>G 4213A>G), A631G (4218C>G 4219A>C), C632V (4220T>G 4221G>T 4222T>A), T634V (4226A>G 4227C>T 4228A>G), T636_H637del (4232_4237delACACAC), D638E (4240T>A), K640_R641del (4244_4249delAAAAGA), Y646F (4263A>T), Y647E (4265T>G 4267T>A), M651L (4277A>T 4279G>A), T652N (4281C>A 4282C>T), T653D (4283A>G 4284C>A 4285A>T), N657R (4296A>G 4297C>G), D659S (4301G>T 4302A>C 4303C>T), I660T (4305T>C 4306C>T), D662E (4312C>A), L665M (4319C>A 4321T>G), L666V (4322C>G 4324A>T), I668V (4328A>G 4330T>G), A670H (4334G>C 4335C>A), A671C (4337G>T 4338C>G), M672L (4340A>C), H674T (4346C>A 4347A>C 4348T>C), V677H (4355G>C 4356T>A 4357G>C), V679L (4361G>T 4363C>G), E680L (4364G>C 4365A>T 4366G>T), P682S (4370C>T 4372A>T), P683del (4373_4375delCCG), L685F (4381A>C), T686L (4382A>T 4383C>T 4384G>A), I687V (4385A>G 4387T>C), L688K (4388C>A 4389T>A 4390T>A), S689T (4391T>A 4393A>C), K866* (4922A>T 4924A>G), P867K (4925C>A 4926C>A 4927A>G), D869E (4933C>A), T872N (4941C>A 4942G>T), I876V (4952A>G), T877V (4955A>G 4956C>T 4957G>A), K878G (4958A>G 4959A>G), R881K (4968G>A), K883R (4974A>G), D884R (4976G>A 4977A>G 4978T>G), R885Q (4980G>A), V886T (4982G>A 4983T>C 4984C>T), T887R (4986C>G), G888R (4988G>A 4990A>G), A890del (4994_4996delGCC), Y891H (4997T>C), M893S (5003A>T 5004T>C 5005G>T), L895W (5009C>T 5010T>G 5011A>G), M897I (5017G>A), L901M (5027C>A 5029C>G), Y904S (5037A>C 5038T>A), I908L (5048A>C 5050T>A), A910V (5055C>T 5056A>C), S911K (5057T>A 5058C>A 5059A>G), E912T (5060G>A 5061A>C 5062A>T), I913T (5064T>C 5065A>T), Y914F (5067A>T), T915S (5070C>G), Q918D (5078C>G 5080G>T), L919Y (5081C>T 5082T>A 5083C>T), G920A (5085G>C 5086A>C), Y921K (5087T>A 5089C>A), V923Y (5093G>T 5094T>A 5095A>C), L924I (5096C>A 5098G>T), D925R (5099G>C 5100A>G 5101C>T), R926E (5102A>G 5103G>A 5104A>G), I928V (5108A>G 5110C>T), Y930L (5114T>C 5115A>T), F933I (5123T>A 5125C>T), E935L (5129G>T 5130A>T 5131A>G), V936S (5132G>T 5133T>C 5134A>C), F937I (5135T>A 5137C>T), T939S (5141A>T 5143A>T), D942G (5151A>G 5152C>T), K943A (5153A>G 5154A>C 5155G>T), L944Q (5157T>A 5158C>G), N948H (5168A>C), Y949F (5172A>T 5173C>T), K951R (5178A>G 5179G>A), T952S (5180A>T 5182G>A), L953F (5183C>T 5185C>T), M954H (5186A>C 5187T>A 5188G>T), G955K (5189G>A 5190G>A 5191A>G), T956G (5192A>G 5193C>G 5194G>A), I957L (5195A>C), I959T (5202T>C), H961V (5207C>G 5208A>T 5209C>A), Y967F (5226A>T 5227C>T), E970Q (5234G>C 5236G>A), N979I (5262A>T 5263C>T), Q984D (5276C>G 5278A>C), Y989F (5292A>T 5293C>T), I990V (5294A>G 5296C>G), N991S (5298A>G), Y992S (5301A>C 5302C>T), A993H (5303G>C 5304C>A 5305A>T), D995K (5309G>A 5311C>A), N996D (5312A>G 5314C>T), V998A (5319T>C), S999K (5321T>A 5322C>A 5323A>G), P1002D (5330C>G 5331C>A 5332A>T), M1003V (5333A>G), I1006F (5342A>T), A1007S (5345G>T)								

	Begin	End	Coverage	Score	Concordance	Matches	Identities	I/D/M/F*	Stop Codons
**NT**	**3223**	**5354**	**18.9%**	**123**	**4.5%**	**1383 (96.6%)**	**750 (52.4%)**	**30/18**	

Codon mutations:

GGC299..G (3223C>G), AAG300CTG (3224A>C 3225A>T), GAC301GAT (3229C>T), GCC302GTG (3231C>T 3232C>G), TTA303ATG (3233T>A 3235A>G), CCT304CCA (3238T>A), CCT304_AAG305insCCTGAGTTGCCAAAG (3238_3239insCCTGAGTTGCCAAAG), CAC306CTG (3243A>T 3244C>G), CAA307CCA (3246A>C), TGG309AGG (3251T>A), TGG309_GAT310insAGGGATATT (3253_3254insAGGGATATT), AAG312AGA (3261A>G 3262G>A), ATA313ATT (3265A>T), AAC314GAG (3266A>G 3268C>G), ATT315TTG (3269A>T 3271T>G), CAG316ATG (3272C>A 3273A>T), GGG318GGT (3280G>T), AAA319TCT (3281A>T 3282A>C 3283A>T), GAG320ACG (3284G>A 3285A>C), CCT321CCG (3289T>G), CCA322CCT (3292A>T), TGG323GCA (3293T>G 3294G>C 3295G>A), GGA324CAA (3296G>C 3297G>A), CCC325GCT (3299C>G 3301C>T), CTA326CCT (3303T>C 3304A>T), TAT327TAC (3307T>C), CAA328AGA (3308C>A 3309A>G), TCT330GCT (3314T>G), GAG331CCT (3317G>C 3318A>C 3319G>T), CTA334TTG (3326C>T 3328A>G), CAG335GCT (3329C>G 3330A>C 3331G>T), ACC336GAA (3332A>G 3333C>A 3334C>A), CTA337CTG (3337A>G), CGA338AGG (3338C>A 3340A>G), GAA339AAG (3341G>A 3343A>G), TGG340CAG (3344T>C 3345G>A), AAG342AAC (3352G>C), GAG343GAA (3355G>A), AAG344TTG (3356A>T 3357A>T), CTA345CTT (3361A>T), GCC346GAT (3363C>A 3364C>T), AAA347TCA (3365A>T 3366A>C), TGG349CTG (3371T>C 3372G>T), ATA350ATT (3376A>T), CGA351CAG (3378G>A 3379A>G), CGA352CCT (3381G>C 3382A>T), TCC353TCT (3385C>T), ACC354AAG (3387C>A 3388C>G), TCA355GCT (3389T>G 3391A>T), AGT356CCC (3392A>C 3393G>C 3394T>C), GCA357TAT (3395G>T 3396C>A 3397A>T), GGA358GGT (3400A>T), ACT359GCA (3401A>G 3403T>A), CCA360CCT (3406A>T), TGC361GTT (3407T>G 3408G>T 3409C>T), ATG362TTA (3410A>T 3412G>A), TTC363TTT (3415C>T), GTT364CAG (3416G>C 3417T>A 3418T>G), CCA365AAA (3419C>A 3420C>A), GCA367CAG (3425G>C 3426C>A 3427A>G), AAC368GAT (3428A>G 3430C>T), AAA370TCA (3434A>T 3435A>C), CTA371TTA (3437C>T), CGA372AGG (3440C>A 3442A>G), CTC373ATG (3443C>A 3445C>G), GTA374TGC (3446G>T 3447T>G 3448A>C), CAA375ATT (3449C>A 3450A>T 3451A>T), CGA378CGG (3460A>G), AAG379CAG (3461A>C), TTG380CTG (3464T>C), GAG382AAG (3470G>A), ATC383GTG (3473A>G 3475C>G), ACG384ACT (3478G>T), ATC385ATA (3481C>A), AAC387AAT (3487C>T), CGA388AAG (3488C>A 3489G>A 3490A>G), TAT389TAC (3493T>C), CTA391TTG (3497C>T 3499A>G), CCC392CCT (3502C>T), AAC393AGG (3504A>G 3505C>G), ATC394ATT (3508C>T), GAA395GAT (3511A>T), GAA396GAT (3514A>T), GCA397TTG (3515G>T 3516C>T 3517A>G), CAA398TTT (3518C>T 3519A>T 3520A>T), GAC399GAT (3523C>T), AGA400CAA (3524A>C 3525G>A), TTA401TTT (3529A>T), ACC402CAG (3530A>C 3531C>A 3532C>G), TCA404GCT (3536T>G 3538A>T), GAC475GAT (3751C>T), ATA477ATT (3757A>T), CTG478GTC (3758C>G 3760G>C), GTC479ATA (3761G>A 3763C>A), TAC480TAT (3766C>T), ACA481AGT (3768C>G 3769A>T), GGA483GAT (3774G>A 3775A>T), TCC484ACG (3776T>A 3778C>G), CTC485TTG (3779C>T 3781C>G), CAG486GAT (3782C>G 3784G>T), ACC489GTG (3791A>G 3792C>T 3793C>G), AAG490AAC (3796G>C), CAA491CAT (3799A>T), GTT492TTG (3800G>T 3802T>G), CAA493AGG (3803C>A 3804A>G 3805A>G), GAT494TTG (3806G>T 3807A>T 3808T>G), GTG495GTA (3811G>A), TTC496TTA (3814C>A), GAA497CAA (3815G>C), CGA498CTT (3819G>T 3820A>T), CTC499TTA (3821C>T 3823C>A), ACG500AGG (3825C>G), AAG501GAA (3827A>G 3829G>A), TCC502TAC (3831C>A), GGA503AAG (3833G>A 3834G>A 3835A>G), TTC504TTG (3838C>G), AAG505TAT (3839A>T 3841G>T), ACA506GTT (3842A>G 3843C>T 3844A>T), GCA507AAA (3845G>A 3846C>A), CCC508ATG (3848C>A 3849C>T 3850C>G), AAA510AAG (3856A>G), TGC511TGT (3859C>T), GAA512GAG (3862A>G), TTC513TTT (3865C>T), CAC514GCC (3866C>G 3867A>C), AAG515CAA (3869A>C 3871G>A), GTC518GTA (3880C>A), TTT520TTC (3886T>C), TTA521CTA (3887T>C), TTT523CAT (3893T>C 3894T>A), ATC524ATA (3898C>A), ATC525GTT (3899A>G 3901C>T), ACA527GAA (3905A>G 3906C>A), ACA528GAC (3908A>G 3909C>A 3910A>C), GGG529GAA (3912G>A 3913G>A), ATA530GTA (3914A>G), ACG531AGG (3918C>G), ATC532ATG (3922C>G), GAC533GAT (3925C>T), GCA535AAG (3929G>A 3930C>A 3931A>G), AAG536AAA (3934G>A), ACA537GTG (3935A>G 3936C>T 3937A>G), CAG538CAA (3940G>A), TCA539GCC (3941T>G 3943A>C), ATC540ATT (3946C>T), AGA541GTA (3947A>G 3948G>T), GAA542GAT (3952A>T), CCA544CAA (3957C>A), GAA545GCA (3960A>C), CCG546CCC (3964G>C), AAG547AAA (3967G>A), ACA548ACT (3970A>T), GTC549GTG (3973C>G), AAG550ACG (3975A>C), GTA552CTG (3980G>C 3982A>G), CAG553AGG (3983C>A 3984A>G), TCA554TCT (3988A>T), CTT556TTG (3992C>T 3994T>G), GGA557GGG (3997A>G), CTC558CTA (4000C>A), GCC559GCT (4003C>T), AAC562TAT (4010A>T 4012C>T), AAG567GAT (4025A>G 4027G>T), GAC568GGA (4029A>G 4030C>A), TAT569TAC (4033T>C), AAG571ACG (4038A>C), ACA572ATA (4041C>T), GCA574GCT (4048A>T), ATG576CTG (4052A>C), ACG577ACT (4057G>T), ATG578GAT (4058A>G 4059T>A 4060G>T), CTT579CTG (4063T>G), ACA580CTG (4064A>C 4065C>T 4066A>G), AGA581AAG (4068G>A 4069A>G), AAA582AAG (4072A>G), GAC583AAA (4073G>A 4075C>A), AAC585AAG (4081C>G), AAA587GTA (4085A>G 4086A>T), GGA589TCG (4091G>T 4092G>C 4093A>G), AAA590GTA (4094A>G 4095A>T), GAA591AGA (4097G>A 4098A>G), CAG592TGC (4100C>T 4101A>G 4102G>C), ACC593GAA (4103A>G 4104C>A 4105C>A), GCG595GCT (4111G>T), TTC596TTT (4114C>T), AAA597CAG (4115A>C 4117A>G), AGA598AGT (4120A>T), CTC599TTG (4121C>T 4123C>G), CAG602GCT (4130C>G 4131A>C 4132G>T), TGC603ATT (4133T>A 4134G>T 4135C>T), GCT604GCA (4138T>A), GCC606GAA (4143C>A 4144C>A), ACG608ATA (4149C>T 4150G>A), CTT609CTG (4153T>G), CGA610AAG (4154C>A 4155G>A 4156A>G), CTA611CTG (4159A>G), CTA611_TTC612insCCAAAT (4159_4160insCCAAAT), TTC612TTT (4162C>T), GAT613GAG (4165T>G), GGT614CTA (4166G>C 4167G>T 4168T>A), AGC615CCT (4169A>C 4170G>C 4171C>T), AAG616TTT (4172A>T 4173A>T 4174G>T), GAA617GAG (4177A>G), GTC618GTA (4180C>A), ATC620GTG (4184A>G 4186C>G), GAG621CAG (4187G>C), ACC622ACT (4192C>T), GAC623GAT (4195C>T), TCT625TCG (4201T>G), GAT626GAC (4204T>C), ATG627AAA (4206T>A 4207G>A), GCA628GCT (4210A>T), ATA629GTG (4211A>G 4213A>G), GGC630GGA (4216C>A), GCA631GGC (4218C>G 4219A>C), TGT632GTA (4220T>G 4221G>T 4222T>A), CTA633TTG (4223C>T 4225A>G), ACA634GTG (4226A>G 4227C>T 4228A>G), CAG635CAA (4231G>A), ACA636_CAC637del (4232_4237delACACAC), GAT638GAA (4240T>A), GGG639GGT (4243G>T), AAA640_AGA641del (4244_4249delAAAAGA), CAC642CAT (4252C>T), CCA643CCG (4255A>G), TAT646TTT (4263A>T), TAT647GAA (4265T>G 4267T>A), TCC648AGC (4268T>A 4269C>G), CGG649AGG (4271C>A), ATG651TTA (4277A>T 4279G>A), ACC652AAT (4281C>A 4282C>T), ACA653GAT (4283A>G 4284C>A 4285A>T), GCG654GCT (4288G>T), GAA655GAG (4291A>G), AAC657AGG (4296A>G 4297C>G), GAC659TCT (4301G>T 4302A>C 4303C>T), ATC660ACT (4305T>C 4306C>T), GAC662GAA (4312C>A), CTT665ATG (4319C>A 4321T>G), CTA666GTT (4322C>G 4324A>T), GCC667GCA (4327C>A), ATT668GTG (4328A>G 4330T>G), GTT669GTG (4333T>G), GCC670CAC (4334G>C 4335C>A), GCC671TGC (4337G>T 4338C>G), ATG672CTG (4340A>C), CAT674ACC (4346C>A 4347A>C 4348T>C), GTG677CAC (4355G>C 4356T>A 4357G>C), GTC679TTG (4361G>T 4363C>G), GAG680CTT (4364G>C 4365A>T 4366G>T), GGC681GGT (4369C>T), CCA682TCT (4370C>T 4372A>T), CCG683del (4373_4375delCCG), TTA685TTC (4381A>C), ACG686TTA (4382A>T 4383C>T 4384G>A), ATT687GTC (4385A>G 4387T>C), CTT688AAA (4388C>A 4389T>A 4390T>A), TCA689ACC (4391T>A 4393A>C), CCA863..T (4915A>T), CCA864CCT (4918A>T), ACG865ACT (4921G>T), AAA866TAG (4922A>T 4924A>G), CCA867AAG (4925C>A 4926C>A 4927A>G), GAC869GAA (4933C>A), GTT871GTG (4939T>G), ACG872AAT (4941C>A 4942G>T), TTC875TTT (4951C>T), ATT876GTT (4952A>G), ACG877GTA (4955A>G 4956C>T 4957G>A), AAA878GGA (4958A>G 4959A>G), CTC879TTG (4961C>T 4963C>G), CCG880CCA (4966G>A), AGG881AAG (4968G>A), TCA882TCT (4972A>T), AAG883AGG (4974A>G), GAT884AGG (4976G>A 4977A>G 4978T>G), CGA885CAA (4980G>A), GTC886ACT (4982G>A 4983T>C 4984C>T), ACA887AGA (4986C>G), GGA888AGG (4988G>A 4990A>G), GCC890del (4994_4996delGCC), TAT891CAT (4997T>C), GAC892GAT (5002C>T), ATG893TCT (5003A>T 5004T>C 5005G>T), CTA895TGG (5009C>T 5010T>G 5011A>G), GTC896GTG (5014C>G), ATG897ATA (5017G>A), GTC898GTT (5020C>T), GAC899GAT (5023C>T), AGA900CGG (5024A>C 5026A>G), CTC901ATG (5027C>A 5029C>G), ACA902ACG (5032A>G), TAT904TCA (5037A>C 5038T>A), GCA905GCC (5041A>C), CAC906CAT (5044C>T), TTC907TTT (5047C>T), ATT908CTA (5048A>C 5050T>A), CCT909CCA (5053T>A), GCA910GTC (5055C>T 5056A>C), TCA911AAG (5057T>A 5058C>A 5059A>G), GAA912ACT (5060G>A 5061A>C 5062A>T), ATA913ACT (5064T>C 5065A>T), TAC914TTC (5067A>T), ACT915AGT (5070C>G), GCA916GCT (5074A>T), GAG917GAA (5077G>A), CAG918GAT (5078C>G 5080G>T), CTC919TAT (5081C>T 5082T>A 5083C>T), GGA920GCC (5085G>C 5086A>C), TAC921AAA (5087T>A 5089C>A), CTC922CTG (5092C>G), GTA923TAC (5093G>T 5094T>A 5095A>C), CTG924ATT (5096C>A 5098G>T), GAC925CGT (5099G>C 5100A>G 5101C>T), AGA926GAG (5102A>G 5103G>A 5104A>G), ATC928GTT (5108A>G 5110C>T), TAT930CTT (5114T>C 5115A>T), CAC931CAT (5119C>T), GGA932GGT (5122A>T), TTC933ATT (5123T>A 5125C>T), CCG934CCT (5128G>T), GAA935TTG (5129G>T 5130A>T 5131A>G), GTA936TCC (5132G>T 5133T>C 5134A>C), TTC937ATT (5135T>A 5137C>T), ACA939TCT (5141A>T 5143A>T), GAC940GAT (5146C>T), GAC942GGT (5151A>G 5152C>T), AAG943GCT (5153A>G 5154A>C 5155G>T), CTC944CAG (5157T>A 5158C>G), TTC945TTT (5161C>T), ACA946ACT (5164A>T), AAC948CAC (5168A>C), TAC949TTT (5172A>T 5173C>T), AAG951AGA (5178A>G 5179G>A), ACG952TCA (5180A>T 5182G>A), CTC953TTT (5183C>T 5185C>T), ATG954CAT (5186A>C 5187T>A 5188G>T), GGA955AAG (5189G>A 5190G>A 5191A>G), ACG956GGA (5192A>G 5193C>G 5194G>A), ATT957CTT (5195A>C), GGA958GGT (5200A>T), ATC959ACC (5202T>C), CAC961GTA (5207C>G 5208A>T 5209C>A), TCA964AGC (5216T>A 5217C>G 5218A>C), ACA965ACC (5221A>C), GCA966GCT (5224A>T), TAC967TTT (5226A>T 5227C>T), CCA969CCT (5233A>T), GAG970CAA (5234G>C 5236G>A), ACG971ACT (5239G>T), GGG973GGA (5245G>A), ACG975ACA (5251G>A), AGA977AGG (5257A>G), ACG978ACC (5260G>C), AAC979ATT (5262A>T 5263C>T), CTC982CTT (5272C>T), CAA984GAC (5276C>G 5278A>C), CTA986CTT (5284A>T), CGG987AGG (5285C>A), TAC989TTT (5292A>T 5293C>T), ATC990GTG (5294A>G 5296C>G), AAC991AGC (5298A>G), TAC992TCT (5301A>C 5302C>T), GCA993CAT (5303G>C 5304C>A 5305A>T), GAC995AAA (5309G>A 5311C>A), AAC996GAT (5312A>G 5314C>T), GTT998GCT (5319T>C), TCA999AAG (5321T>A 5322C>A 5323A>G), TTA1000TTG (5326A>G), CTG1001CTA (5329G>A), CCA1002GAT (5330C>G 5331C>A 5332A>T), ATG1003GTG (5333A>G), GCG1004GCC (5338G>C), CAG1005CAA (5341G>A), ATC1006TTC (5342A>T), GCA1007TCA (5345G>T)

Proteins

	Begin	End	Coverage	Score	Concordance	Matches	Identities	I/D/M/F*	Stop Codons
Reverse Transcriptase (YP_009666308.1)	300	1010	44.6%	1606	49.8%	461 (96.6%)	224 (47.0%)	10/6/0/0	1

	Begin	End	Coverage	Score	Concordance	Matches	Identities	I/D/M/F*	Stop Codons
**NT**	**3223**	**5354**	**18.9%**	**123**	**4.5%**	**1383 (96.6%)**	**750 (52.4%)**	**30/18**	
Protein mutations:	K300L (3224A>C 3225A>T), A302V (3231C>T 3232C>G), L303M (3233T>A 3235A>G), P304_K305insPELPK (3238_3239insCCTGAGTTGCCAAAG), H306L (3243A>T 3244C>G), Q307P (3246A>C), W309R (3251T>A), W309_D310insRDI (3253_3254insAGGGATATT), K312R (3261A>G 3262G>A), N314E (3266A>G 3268C>G), I315L (3269A>T 3271T>G), Q316M (3272C>A 3273A>T), K319S (3281A>T 3282A>C 3283A>T), E320T (3284G>A 3285A>C), W323A (3293T>G 3294G>C 3295G>A), G324Q (3296G>C 3297G>A), P325A (3299C>G 3301C>T), L326P (3303T>C 3304A>T), Q328R (3308C>A 3309A>G), S330A (3314T>G), E331P (3317G>C 3318A>C 3319G>T), Q335A (3329C>G 3330A>C 3331G>T), T336E (3332A>G 3333C>A 3334C>A), E339K (3341G>A 3343A>G), W340Q (3344T>C 3345G>A), K342N (3352G>C), K344L (3356A>T 3357A>T), A346D (3363C>A 3364C>T), K347S (3365A>T 3366A>C), W349L (3371T>C 3372G>T), R351Q (3378G>A 3379A>G), R352P (3381G>C 3382A>T), T354K (3387C>A 3388C>G), S355A (3389T>G 3391A>T), S356P (3392A>C 3393G>C 3394T>C), A357Y (3395G>T 3396C>A 3397A>T), T359A (3401A>G 3403T>A), C361V (3407T>G 3408G>T 3409C>T), M362L (3410A>T 3412G>A), V364Q (3416G>C 3417T>A 3418T>G), P365K (3419C>A 3420C>A), A367Q (3425G>C 3426C>A 3427A>G), N368D (3428A>G 3430C>T), K370S (3434A>T 3435A>C), L373M (3443C>A 3445C>G), V374C (3446G>T 3447T>G 3448A>C), Q375I (3449C>A 3450A>T 3451A>T), K379Q (3461A>C), E382K (3470G>A), I383V (3473A>G 3475C>G), R388K (3488C>A 3489G>A 3490A>G), N393R (3504A>G 3505C>G), E395D (3511A>T), E396D (3514A>T), A397L (3515G>T 3516C>T 3517A>G), Q398F (3518C>T 3519A>T 3520A>T), R400Q (3524A>C 3525G>A), L401F (3529A>T), T402Q (3530A>C 3531C>A 3532C>G), S404A (3536T>G 3538A>T), L478V (3758C>G 3760G>C), V479I (3761G>A 3763C>A), T481S (3768C>G 3769A>T), G483D (3774G>A 3775A>T), S484T (3776T>A 3778C>G), Q486D (3782C>G 3784G>T), T489V (3791A>G 3792C>T 3793C>G), K490N (3796G>C), Q491H (3799A>T), V492L (3800G>T 3802T>G), Q493R (3803C>A 3804A>G 3805A>G), D494L (3806G>T 3807A>T 3808T>G), F496L (3814C>A), E497Q (3815G>C), R498L (3819G>T 3820A>T), T500R (3825C>G), K501E (3827A>G 3829G>A), S502Y (3831C>A), G503K (3833G>A 3834G>A 3835A>G), F504L (3838C>G), K505Y (3839A>T 3841G>T), T506V (3842A>G 3843C>T 3844A>T), A507K (3845G>A 3846C>A), P508M (3848C>A 3849C>T 3850C>G), H514A (3866C>G 3867A>C), K515Q (3869A>C 3871G>A), F523H (3893T>C 3894T>A), I525V (3899A>G 3901C>T), T527E (3905A>G 3906C>A), T528D (3908A>G 3909C>A 3910A>C), G529E (3912G>A 3913G>A), I530V (3914A>G), T531R (3918C>G), I532M (3922C>G), A535K (3929G>A 3930C>A 3931A>G), T537V (3935A>G 3936C>T 3937A>G), S539A (3941T>G 3943A>C), R541V (3947A>G 3948G>T), E542D (3952A>T), P544Q (3957C>A), E545A (3960A>C), K550T (3975A>C), V552L (3980G>C 3982A>G), Q553R (3983C>A 3984A>G), N562Y (4010A>T 4012C>T), K567D (4025A>G 4027G>T), D568G (4029A>G 4030C>A), K571T (4038A>C), T572I (4041C>T), M576L (4052A>C), M578D (4058A>G 4059T>A 4060G>T), T580L (4064A>C 4065C>T 4066A>G), R581K (4068G>A 4069A>G), D583K (4073G>A 4075C>A), N585K (4081C>G), K587V (4085A>G 4086A>T), G589S (4091G>T 4092G>C 4093A>G), K590V (4094A>G 4095A>T), E591R (4097G>A 4098A>G), Q592C (4100C>T 4101A>G 4102G>C), T593E (4103A>G 4104C>A 4105C>A), K597Q (4115A>C 4117A>G), R598S (4120A>T), Q602A (4130C>G 4131A>C 4132G>T), C603I (4133T>A 4134G>T 4135C>T), A606E (4143C>A 4144C>A), T608I (4149C>T 4150G>A), R610K (4154C>A 4155G>A 4156A>G), L611_F612insPN (4159_4160insCCAAAT), D613E (4165T>G), G614L (4166G>C 4167G>T 4168T>A), S615P (4169A>C 4170G>C 4171C>T), K616F (4172A>T 4173A>T 4174G>T), I620V (4184A>G 4186C>G), E621Q (4187G>C), M627K (4206T>A 4207G>A), I629V (4211A>G 4213A>G), A631G (4218C>G 4219A>C), C632V (4220T>G 4221G>T 4222T>A), T634V (4226A>G 4227C>T 4228A>G), T636_H637del (4232_4237delACACAC), D638E (4240T>A), K640_R641del (4244_4249delAAAAGA), Y646F (4263A>T), Y647E (4265T>G 4267T>A), M651L (4277A>T 4279G>A), T652N (4281C>A 4282C>T), T653D (4283A>G 4284C>A 4285A>T), N657R (4296A>G 4297C>G), D659S (4301G>T 4302A>C 4303C>T), I660T (4305T>C 4306C>T), D662E (4312C>A), L665M (4319C>A 4321T>G), L666V (4322C>G 4324A>T), I668V (4328A>G 4330T>G), A670H (4334G>C 4335C>A), A671C (4337G>T 4338C>G), M672L (4340A>C), H674T (4346C>A 4347A>C 4348T>C), V677H (4355G>C 4356T>A 4357G>C), V679L (4361G>T 4363C>G), E680L (4364G>C 4365A>T 4366G>T), P682S (4370C>T 4372A>T), P683del (4373_4375delCCG), L685F (4381A>C), T686L (4382A>T 4383C>T 4384G>A), I687V (4385A>G 4387T>C), L688K (4388C>A 4389T>A 4390T>A), S689T (4391T>A 4393A>C), K866* (4922A>T 4924A>G), P867K (4925C>A 4926C>A 4927A>G), D869E (4933C>A), T872N (4941C>A 4942G>T), I876V (4952A>G), T877V (4955A>G 4956C>T 4957G>A), K878G (4958A>G 4959A>G), R881K (4968G>A), K883R (4974A>G), D884R (4976G>A 4977A>G 4978T>G), R885Q (4980G>A), V886T (4982G>A 4983T>C 4984C>T), T887R (4986C>G), G888R (4988G>A 4990A>G), A890del (4994_4996delGCC), Y891H (4997T>C), M893S (5003A>T 5004T>C 5005G>T), L895W (5009C>T 5010T>G 5011A>G), M897I (5017G>A), L901M (5027C>A 5029C>G), Y904S (5037A>C 5038T>A), I908L (5048A>C 5050T>A), A910V (5055C>T 5056A>C), S911K (5057T>A 5058C>A 5059A>G), E912T (5060G>A 5061A>C 5062A>T), I913T (5064T>C 5065A>T), Y914F (5067A>T), T915S (5070C>G), Q918D (5078C>G 5080G>T), L919Y (5081C>T 5082T>A 5083C>T), G920A (5085G>C 5086A>C), Y921K (5087T>A 5089C>A), V923Y (5093G>T 5094T>A 5095A>C), L924I (5096C>A 5098G>T), D925R (5099G>C 5100A>G 5101C>T), R926E (5102A>G 5103G>A 5104A>G), I928V (5108A>G 5110C>T), Y930L (5114T>C 5115A>T), F933I (5123T>A 5125C>T), E935L (5129G>T 5130A>T 5131A>G), V936S (5132G>T 5133T>C 5134A>C), F937I (5135T>A 5137C>T), T939S (5141A>T 5143A>T), D942G (5151A>G 5152C>T), K943A (5153A>G 5154A>C 5155G>T), L944Q (5157T>A 5158C>G), N948H (5168A>C), Y949F (5172A>T 5173C>T), K951R (5178A>G 5179G>A), T952S (5180A>T 5182G>A), L953F (5183C>T 5185C>T), M954H (5186A>C 5187T>A 5188G>T), G955K (5189G>A 5190G>A 5191A>G), T956G (5192A>G 5193C>G 5194G>A), I957L (5195A>C), I959T (5202T>C), H961V (5207C>G 5208A>T 5209C>A), Y967F (5226A>T 5227C>T), E970Q (5234G>C 5236G>A), N979I (5262A>T 5263C>T), Q984D (5276C>G 5278A>C), Y989F (5292A>T 5293C>T), I990V (5294A>G 5296C>G), N991S (5298A>G), Y992S (5301A>C 5302C>T), A993H (5303G>C 5304C>A 5305A>T), D995K (5309G>A 5311C>A), N996D (5312A>G 5314C>T), V998A (5319T>C), S999K (5321T>A 5322C>A 5323A>G), P1002D (5330C>G 5331C>A 5332A>T), M1003V (5333A>G), I1006F (5342A>T), A1007S (5345G>T)								

	Begin	End	Coverage	Score	Concordance	Matches	Identities	I/D/M/F*	Stop Codons
NT	3223	5354	18.9%	123	4.5%	1383 (96.6%)	750 (52.4%)	30/18	

Codon mutations:

GGC299..G (3223C>G), AAG300CTG (3224A>C 3225A>T), GAC301GAT (3229C>T), GCC302GTG (3231C>T 3232C>G), TTA303ATG (3233T>A 3235A>G), CCT304CCA (3238T>A), CCT304_AAG305insCCTGAGTTGCCAAAG (3238_3239insCCTGAGTTGCCAAAG), CAC306CTG (3243A>T 3244C>G), CAA307CCA (3246A>C), TGG309AGG (3251T>A), TGG309_GAT310insAGGGATATT (3253_3254insAGGGATATT), AAG312AGA (3261A>G 3262G>A), ATA313ATT (3265A>T), AAC314GAG (3266A>G 3268C>G), ATT315TTG (3269A>T 3271T>G), CAG316ATG (3272C>A 3273A>T), GGG318GGT (3280G>T), AAA319TCT (3281A>T 3282A>C 3283A>T), GAG320ACG (3284G>A 3285A>C), CCT321CCG (3289T>G), CCA322CCT (3292A>T), TGG323GCA (3293T>G 3294G>C 3295G>A), GGA324CAA (3296G>C 3297G>A), CCC325GCT (3299C>G 3301C>T), CTA326CCT (3303T>C 3304A>T), TAT327TAC (3307T>C), CAA328AGA (3308C>A 3309A>G), TCT330GCT (3314T>G), GAG331CCT (3317G>C 3318A>C 3319G>T), CTA334TTG (3326C>T 3328A>G), CAG335GCT (3329C>G 3330A>C 3331G>T), ACC336GAA (3332A>G 3333C>A 3334C>A), CTA337CTG (3337A>G), CGA338AGG (3338C>A 3340A>G), GAA339AAG (3341G>A 3343A>G), TGG340CAG (3344T>C 3345G>A), AAG342AAC (3352G>C), GAG343GAA (3355G>A), AAG344TTG (3356A>T 3357A>T), CTA345CTT (3361A>T), GCC346GAT (3363C>A 3364C>T), AAA347TCA (3365A>T 3366A>C), TGG349CTG (3371T>C 3372G>T), ATA350ATT (3376A>T), CGA351CAG (3378G>A 3379A>G), CGA352CCT (3381G>C 3382A>T), TCC353TCT (3385C>T), ACC354AAG (3387C>A 3388C>G), TCA355GCT (3389T>G 3391A>T), AGT356CCC (3392A>C 3393G>C 3394T>C), GCA357TAT (3395G>T 3396C>A 3397A>T), GGA358GGT (3400A>T), ACT359GCA (3401A>G 3403T>A), CCA360CCT (3406A>T), TGC361GTT (3407T>G 3408G>T 3409C>T), ATG362TTA (3410A>T 3412G>A), TTC363TTT (3415C>T), GTT364CAG (3416G>C 3417T>A 3418T>G), CCA365AAA (3419C>A 3420C>A), GCA367CAG (3425G>C 3426C>A 3427A>G), AAC368GAT (3428A>G 3430C>T), AAA370TCA (3434A>T 3435A>C), CTA371TTA (3437C>T), CGA372AGG (3440C>A 3442A>G), CTC373ATG (3443C>A 3445C>G), GTA374TGC (3446G>T 3447T>G 3448A>C), CAA375ATT (3449C>A 3450A>T 3451A>T), CGA378CGG (3460A>G), AAG379CAG (3461A>C), TTG380CTG (3464T>C), GAG382AAG (3470G>A), ATC383GTG (3473A>G 3475C>G), ACG384ACT (3478G>T), ATC385ATA (3481C>A), AAC387AAT (3487C>T), CGA388AAG (3488C>A 3489G>A 3490A>G), TAT389TAC (3493T>C), CTA391TTG (3497C>T 3499A>G), CCC392CCT (3502C>T), AAC393AGG (3504A>G 3505C>G), ATC394ATT (3508C>T), GAA395GAT (3511A>T), GAA396GAT (3514A>T), GCA397TTG (3515G>T 3516C>T 3517A>G), CAA398TTT (3518C>T 3519A>T 3520A>T), GAC399GAT (3523C>T), AGA400CAA (3524A>C 3525G>A), TTA401TTT (3529A>T), ACC402CAG (3530A>C 3531C>A 3532C>G), TCA404GCT (3536T>G 3538A>T), GAC475GAT (3751C>T), ATA477ATT (3757A>T), CTG478GTC (3758C>G 3760G>C), GTC479ATA (3761G>A 3763C>A), TAC480TAT (3766C>T), ACA481AGT (3768C>G 3769A>T), GGA483GAT (3774G>A 3775A>T), TCC484ACG (3776T>A 3778C>G), CTC485TTG (3779C>T 3781C>G), CAG486GAT (3782C>G 3784G>T), ACC489GTG (3791A>G 3792C>T 3793C>G), AAG490AAC (3796G>C), CAA491CAT (3799A>T), GTT492TTG (3800G>T 3802T>G), CAA493AGG (3803C>A 3804A>G 3805A>G), GAT494TTG (3806G>T 3807A>T 3808T>G), GTG495GTA (3811G>A), TTC496TTA (3814C>A), GAA497CAA (3815G>C), CGA498CTT (3819G>T 3820A>T), CTC499TTA (3821C>T 3823C>A), ACG500AGG (3825C>G), AAG501GAA (3827A>G 3829G>A), TCC502TAC (3831C>A), GGA503AAG (3833G>A 3834G>A 3835A>G), TTC504TTG (3838C>G), AAG505TAT (3839A>T 3841G>T), ACA506GTT (3842A>G 3843C>T 3844A>T), GCA507AAA (3845G>A 3846C>A), CCC508ATG (3848C>A 3849C>T 3850C>G), AAA510AAG (3856A>G), TGC511TGT (3859C>T), GAA512GAG (3862A>G), TTC513TTT (3865C>T), CAC514GCC (3866C>G 3867A>C), AAG515CAA (3869A>C 3871G>A), GTC518GTA (3880C>A), TTT520TTC (3886T>C), TTA521CTA (3887T>C), TTT523CAT (3893T>C 3894T>A), ATC524ATA (3898C>A), ATC525GTT (3899A>G 3901C>T), ACA527GAA (3905A>G 3906C>A), ACA528GAC (3908A>G 3909C>A 3910A>C), GGG529GAA (3912G>A 3913G>A), ATA530GTA (3914A>G), ACG531AGG (3918C>G), ATC532ATG (3922C>G), GAC533GAT (3925C>T), GCA535AAG (3929G>A 3930C>A 3931A>G), AAG536AAA (3934G>A), ACA537GTG (3935A>G 3936C>T 3937A>G), CAG538CAA (3940G>A), TCA539GCC (3941T>G 3943A>C), ATC540ATT (3946C>T), AGA541GTA (3947A>G 3948G>T), GAA542GAT (3952A>T), CCA544CAA (3957C>A), GAA545GCA (3960A>C), CCG546CCC (3964G>C), AAG547AAA (3967G>A), ACA548ACT (3970A>T), GTC549GTG (3973C>G), AAG550ACG (3975A>C), GTA552CTG (3980G>C 3982A>G), CAG553AGG (3983C>A 3984A>G), TCA554TCT (3988A>T), CTT556TTG (3992C>T 3994T>G), GGA557GGG (3997A>G), CTC558CTA (4000C>A), GCC559GCT (4003C>T), AAC562TAT (4010A>T 4012C>T), AAG567GAT (4025A>G 4027G>T), GAC568GGA (4029A>G 4030C>A), TAT569TAC (4033T>C), AAG571ACG (4038A>C), ACA572ATA (4041C>T), GCA574GCT (4048A>T), ATG576CTG (4052A>C), ACG577ACT (4057G>T), ATG578GAT (4058A>G 4059T>A 4060G>T), CTT579CTG (4063T>G), ACA580CTG (4064A>C 4065C>T 4066A>G), AGA581AAG (4068G>A 4069A>G), AAA582AAG (4072A>G), GAC583AAA (4073G>A 4075C>A), AAC585AAG (4081C>G), AAA587GTA (4085A>G 4086A>T), GGA589TCG (4091G>T 4092G>C 4093A>G), AAA590GTA (4094A>G 4095A>T), GAA591AGA (4097G>A 4098A>G), CAG592TGC (4100C>T 4101A>G 4102G>C), ACC593GAA (4103A>G 4104C>A 4105C>A), GCG595GCT (4111G>T), TTC596TTT (4114C>T), AAA597CAG (4115A>C 4117A>G), AGA598AGT (4120A>T), CTC599TTG (4121C>T 4123C>G), CAG602GCT (4130C>G 4131A>C 4132G>T), TGC603ATT (4133T>A 4134G>T 4135C>T), GCT604GCA (4138T>A), GCC606GAA (4143C>A 4144C>A), ACG608ATA (4149C>T 4150G>A), CTT609CTG (4153T>G), CGA610AAG (4154C>A 4155G>A 4156A>G), CTA611CTG (4159A>G), CTA611_TTC612insCCAAAT (4159_4160insCCAAAT), TTC612TTT (4162C>T), GAT613GAG (4165T>G), GGT614CTA (4166G>C 4167G>T 4168T>A), AGC615CCT (4169A>C 4170G>C 4171C>T), AAG616TTT (4172A>T 4173A>T 4174G>T), GAA617GAG (4177A>G), GTC618GTA (4180C>A), ATC620GTG (4184A>G 4186C>G), GAG621CAG (4187G>C), ACC622ACT (4192C>T), GAC623GAT (4195C>T), TCT625TCG (4201T>G), GAT626GAC (4204T>C), ATG627AAA (4206T>A 4207G>A), GCA628GCT (4210A>T), ATA629GTG (4211A>G 4213A>G), GGC630GGA (4216C>A), GCA631GGC (4218C>G 4219A>C), TGT632GTA (4220T>G 4221G>T 4222T>A), CTA633TTG (4223C>T 4225A>G), ACA634GTG (4226A>G 4227C>T 4228A>G), CAG635CAA (4231G>A), ACA636_CAC637del (4232_4237delACACAC), GAT638GAA (4240T>A), GGG639GGT (4243G>T), AAA640_AGA641del (4244_4249delAAAAGA), CAC642CAT (4252C>T), CCA643CCG (4255A>G), TAT646TTT (4263A>T), TAT647GAA (4265T>G 4267T>A), TCC648AGC (4268T>A 4269C>G), CGG649AGG (4271C>A), ATG651TTA (4277A>T 4279G>A), ACC652AAT (4281C>A 4282C>T), ACA653GAT (4283A>G 4284C>A 4285A>T), GCG654GCT (4288G>T), GAA655GAG (4291A>G), AAC657AGG (4296A>G 4297C>G), GAC659TCT (4301G>T 4302A>C 4303C>T), ATC660ACT (4305T>C 4306C>T), GAC662GAA (4312C>A), CTT665ATG (4319C>A 4321T>G), CTA666GTT (4322C>G 4324A>T), GCC667GCA (4327C>A), ATT668GTG (4328A>G 4330T>G), GTT669GTG (4333T>G), GCC670CAC (4334G>C 4335C>A), GCC671TGC (4337G>T 4338C>G), ATG672CTG (4340A>C), CAT674ACC (4346C>A 4347A>C 4348T>C), GTG677CAC (4355G>C 4356T>A 4357G>C), GTC679TTG (4361G>T 4363C>G), GAG680CTT (4364G>C 4365A>T 4366G>T), GGC681GGT (4369C>T), CCA682TCT (4370C>T 4372A>T), CCG683del (4373_4375delCCG), TTA685TTC (4381A>C), ACG686TTA (4382A>T 4383C>T 4384G>A), ATT687GTC (4385A>G 4387T>C), CTT688AAA (4388C>A 4389T>A 4390T>A), TCA689ACC (4391T>A 4393A>C), CCA863..T (4915A>T), CCA864CCT (4918A>T), ACG865ACT (4921G>T), AAA866TAG (4922A>T 4924A>G), CCA867AAG (4925C>A 4926C>A 4927A>G), GAC869GAA (4933C>A), GTT871GTG (4939T>G), ACG872AAT (4941C>A 4942G>T), TTC875TTT (4951C>T), ATT876GTT (4952A>G), ACG877GTA (4955A>G 4956C>T 4957G>A), AAA878GGA (4958A>G 4959A>G), CTC879TTG (4961C>T 4963C>G), CCG880CCA (4966G>A), AGG881AAG (4968G>A), TCA882TCT (4972A>T), AAG883AGG (4974A>G), GAT884AGG (4976G>A 4977A>G 4978T>G), CGA885CAA (4980G>A), GTC886ACT (4982G>A 4983T>C 4984C>T), ACA887AGA (4986C>G), GGA888AGG (4988G>A 4990A>G), GCC890del (4994_4996delGCC), TAT891CAT (4997T>C), GAC892GAT (5002C>T), ATG893TCT (5003A>T 5004T>C 5005G>T), CTA895TGG (5009C>T 5010T>G 5011A>G), GTC896GTG (5014C>G), ATG897ATA (5017G>A), GTC898GTT (5020C>T), GAC899GAT (5023C>T), AGA900CGG (5024A>C 5026A>G), CTC901ATG (5027C>A 5029C>G), ACA902ACG (5032A>G), TAT904TCA (5037A>C 5038T>A), GCA905GCC (5041A>C), CAC906CAT (5044C>T), TTC907TTT (5047C>T), ATT908CTA (5048A>C 5050T>A), CCT909CCA (5053T>A), GCA910GTC (5055C>T 5056A>C), TCA911AAG (5057T>A 5058C>A 5059A>G), GAA912ACT (5060G>A 5061A>C 5062A>T), ATA913ACT (5064T>C 5065A>T), TAC914TTC (5067A>T), ACT915AGT (5070C>G), GCA916GCT (5074A>T), GAG917GAA (5077G>A), CAG918GAT (5078C>G 5080G>T), CTC919TAT (5081C>T 5082T>A 5083C>T), GGA920GCC (5085G>C 5086A>C), TAC921AAA (5087T>A 5089C>A), CTC922CTG (5092C>G), GTA923TAC (5093G>T 5094T>A 5095A>C), CTG924ATT (5096C>A 5098G>T), GAC925CGT (5099G>C 5100A>G 5101C>T), AGA926GAG (5102A>G 5103G>A 5104A>G), ATC928GTT (5108A>G 5110C>T), TAT930CTT (5114T>C 5115A>T), CAC931CAT (5119C>T), GGA932GGT (5122A>T), TTC933ATT (5123T>A 5125C>T), CCG934CCT (5128G>T), GAA935TTG (5129G>T 5130A>T 5131A>G), GTA936TCC (5132G>T 5133T>C 5134A>C), TTC937ATT (5135T>A 5137C>T), ACA939TCT (5141A>T 5143A>T), GAC940GAT (5146C>T), GAC942GGT (5151A>G 5152C>T), AAG943GCT (5153A>G 5154A>C 5155G>T), CTC944CAG (5157T>A 5158C>G), TTC945TTT (5161C>T), ACA946ACT (5164A>T), AAC948CAC (5168A>C), TAC949TTT (5172A>T 5173C>T), AAG951AGA (5178A>G 5179G>A), ACG952TCA (5180A>T 5182G>A), CTC953TTT (5183C>T 5185C>T), ATG954CAT (5186A>C 5187T>A 5188G>T), GGA955AAG (5189G>A 5190G>A 5191A>G), ACG956GGA (5192A>G 5193C>G 5194G>A), ATT957CTT (5195A>C), GGA958GGT (5200A>T), ATC959ACC (5202T>C), CAC961GTA (5207C>G 5208A>T 5209C>A), TCA964AGC (5216T>A 5217C>G 5218A>C), ACA965ACC (5221A>C), GCA966GCT (5224A>T), TAC967TTT (5226A>T 5227C>T), CCA969CCT (5233A>T), GAG970CAA (5234G>C 5236G>A), ACG971ACT (5239G>T), GGG973GGA (5245G>A), ACG975ACA (5251G>A), AGA977AGG (5257A>G), ACG978ACC (5260G>C), AAC979ATT (5262A>T 5263C>T), CTC982CTT (5272C>T), CAA984GAC (5276C>G 5278A>C), CTA986CTT (5284A>T), CGG987AGG (5285C>A), TAC989TTT (5292A>T 5293C>T), ATC990GTG (5294A>G 5296C>G), AAC991AGC (5298A>G), TAC992TCT (5301A>C 5302C>T), GCA993CAT (5303G>C 5304C>A 5305A>T), GAC995AAA (5309G>A 5311C>A), AAC996GAT (5312A>G 5314C>T), GTT998GCT (5319T>C), TCA999AAG (5321T>A 5322C>A 5323A>G), TTA1000TTG (5326A>G), CTG1001CTA (5329G>A), CCA1002GAT (5330C>G 5331C>A 5332A>T), ATG1003GTG (5333A>G), GCG1004GCC (5338G>C), CAG1005CAA (5341G>A), ATC1006TTC (5342A>T), GCA1007TCA (5345G>T)

*: Inserts / Deletes / Misaligned / Frameshifts

Analysis details

This analysis was performed with panviral2.64

NGS Details (UN24): Errantivirus

Assembly

**Coverage Length**	1115 (2 contig(s))
**Depth Of Coverage**	36.2
**Number Of Reads**	503
**Reads Per Million**	10.07 rpm (after QC)
**Ambiguities**	0
**Assembly Method**	de novo + reference guided assembly
**Consensus Caller**	Bcf Tools

Coverage Map

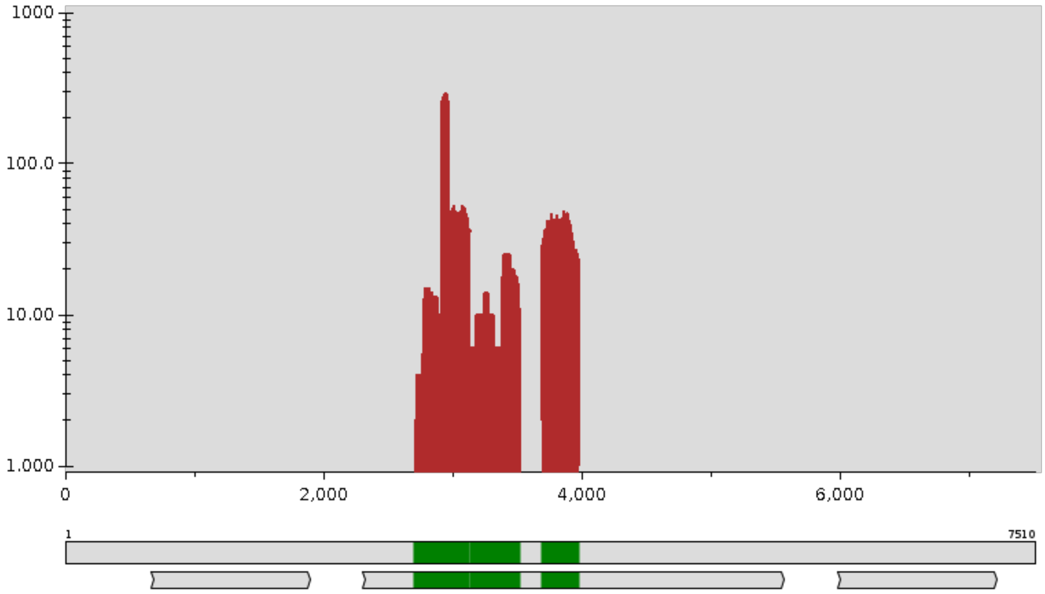

Assignment

**Type**	Errantivirus (Taxonomy ID: 186666)
**Reference Genome**	NC_038512.1
**NT Identity (%)**	52.1429
**AA Identity (%)**	41.2869
**Number Of Stop Codons**	3
**Number Of CDS**	3

Alignment

**Alignment Score**	74.0 (NT) + 958.0 (AA) = 1032.0
**Concordance (%)**	22.3726

Alignment Method	Global, seeded, nucleotide + amino acids (AGA)

Genome Region

Sequence starts at position 2706 and ends at position 3983 relative to NC_038512.1 reference sequence.

Alignment Detailed Statistics

	Begin	End	Coverage	Score	Concordance	Matches	Identities	I/D/M/F*	Stop Codons
**NT**	**2706**	**3983**	**14.8%**	**74**	**3.4%**	**1109 (98.5%)**	**584 (51.9%)**	**11/6**	

Mutations: 2708G>T, 2710C>G, 2712A>T, 2715T>G, 2719C>A, 2722G>T, 2724A>C, 2725C>A, 2726A>G, 2727C>T, 2730A>G, 2731A>G, 2732A>C, 2733G>T, 2737C>T, 2738C>A, 2740G>A, 2744_2749delCCCTTC, 2751T>A, 2756C>T, 2757G>T, 2761G>A, 2762G>A, 2764A>G, 2765G>C, 2767T>A, 2768A>G, 2769G>A, 2771G>C, 2772A>T, 2772_2773insGA, 2773C>G, 2774C>A, 2777A>T, 2779C>G, 2780A>G, 2781C>A, 2782G>A, 2783A>G, 2785A>T, 2786A>T, 2788G>A, 2792G>A, 2794C>G, 2795C>A, 2797A>G, 2799G>A, 2800A>G, 2801A>T, 2806A>T, 2807A>T, 2812A>T, 2813T>A, 2814C>G, 2815A>T, 2817A>G, 2818C>A, 2821T>A, 2822G>C, 2824A>T, 2828A>G, 2830C>T, 2831T>G, 2836C>A, 2837A>G, 2841G>T, 2843G>T, 2846G>A, 2849C>A, 2850C>G, 2851C>A, 2857A>G, 2859T>A, 2860C>G, 2863C>T, 2865C>G, 2866T>G, 2869T>G, 2872G>T, 2874A>T, 2876C>A, 2877A>T, 2880A>G, 2882T>A, 2883G>T, 2885C>T, 2888C>A, 2890C>G, 2891G>T, 2892T>A, 2893A>T, 2894G>A, 2899C>T, 2901T>A, 2902C>T, 2903C>T, 2905T>A, 2907A>G, 2915G>A, 2917G>A, 2919A>T, 2920G>C, 2924A>G, 2926C>G, 2927G>A, 2928A>G, 2929T>G, 2932C>T, 2934A>G, 2935A>G, 2937A>T, 2941G>A, 2944A>C, 2949A>C, 2950C>T, 2953A>T, 2954A>G, 2955G>A, 2959C>G, 2960G>C, 2963C>T, 2968C>T, 2969A>G, 2972T>G, 2975G>A, 2976G>A, 2978A>G, 2979A>G, 2980G>C, 2981T>G, 2982G>C, 2983C>T, 2984C>G, 2985A>T, 2986A>T, 2989C>T, 2993A>T, 2995C>A, 2997C>A, 2998C>G, 2999T>C, 3001A>T, 3008G>T, 3009C>T, 3011A>T, 3012G>C, 3016G>A, 3018T>A, 3019T>C, 3020T>G, 3025G>A, 3026G>A, 3028G>T, 3029G>C, 3030A>G, 3032A>G, 3034G>T, 3035G>A, 3037C>G, 3038C>G, 3039C>T, 3040T>G, 3041C>G, 3042A>C, 3050T>C, 3052G>C, 3058C>T, 3061G>T, 3066A>G, 3067C>G, 3068G>A, 3069T>C, 3071G>C, 3073A>C, 3074C>G, 3076C>T, 3079G>T, 3084T>A, 3085T>C, 3091C>T, 3092C>T, 3094T>A, 3095C>G, 3096G>T, 3097A>C, 3104A>T, 3106G>T, 3109A>G, 3112A>G, 3113A>T, 3114A>C, 3115A>T, 3119T>G, 3121A>G, 3122C>T, 3124A>G, 3127T>C, 3130T>A, 3142T>G, 3146G>A, 3154C>T, 3155C>T, 3157A>T, 3159G>A, 3161G>C, 3162G>C, 3164C>T, 3166C>T, 3168A>T, 3169A>C, 3171A>G, 3172T>G, 3175C>A, 3176A>T, 3178C>T, 3179T>A, 3180G>T, 3181T>A, 3182C>T, 3184C>G, 3187C>A, 3189A>T, 3190C>T, 3191C>T, 3196C>T, 3202T>A, 3203A>C, 3206G>A, 3215A>G, 3216C>T, 3217T>A, 3220C>A, 3221C>T, 3224C>A, 3226G>A, 3229A>G, 3232C>T, 3233C>G, 3235G>T, 3238G>A, 3239A>C, 3242C>T, 3244G>A, 3246A>T, 3247A>T, 3248C>G, 3249G>C, 3250A>T, 3256C>T, 3257C>G, 3259A>T, 3262A>C, 3263C>T, 3265T>G, 3268A>G, 3269G>A, 3271A>C, 3272A>C, 3273G>A, 3274T>C, 3275A>T, 3276A>C, 3277C>T, 3280C>G, 3281A>T, 3283A>T, 3284A>G, 3285T>C, 3287C>A, 3291T>G, 3292G>A, 3293G>T, 3294A>C, 3295C>T, 3298G>A, 3300C>G, 3301C>T, 3302G>T, 3303A>C, 3304A>T, 3307C>T, 3308T>G, 3309T>G, 3310G>A, 3311A>C, 3313G>A, 3315T>C, 3316C>T, 3317G>A, 3319A>G, 3320A>G, 3321C>T, 3324C>A, 3325T>G, 3328T>C, 3329C>T, 3331T>A, 3337C>T, 3338A>G, 3340C>T, 3343A>T, 3345G>C, 3347A>G, 3349G>A, 3352C>A, 3355T>A, 3356A>G, 3358C>G, 3360A>G, 3361G>T, 3362C>A, 3365A>G, 3367C>T, 3372A>C, 3376G>A, 3379T>C, 3380T>G, 3381C>A, 3382C>A, 3388T>G, 3389C>A, 3394A>C, 3396A>G, 3397T>G, 3399T>C, 3400G>A, 3402T>G, 3403T>A, 3406A>T, 3409G>A, 3412C>A, 3417A>T, 3421A>G, 3422A>G, 3424A>T, 3425A>C, 3426A>G, 3427A>T, 3428C>A, 3429A>G, 3430A>T, 3434T>C, 3436A>T, 3439C>A, 3440C>T, 3442T>G, 3443C>G, 3445C>A, 3454C>T, 3455C>A, 3459A>T, 3463C>T, 3464A>G, 3466T>G, 3467C>G, 3468C>A, 3471A>G, 3472T>G, 3476G>T, 3478A>T, 3479C>T, 3480G>C, 3482C>A, 3484C>T, 3485A>T, 3487A>T, 3488A>G, 3489A>C, 3493C>T, 3494C>T, 3496T>A, 3500C>A, 3503T>C, 3504G>T, 3505C>A, 3506T>A, 3507T>C, 3509A>C, 3511A>C, 3514A>G, 3517T>A, 3518A>G, 3519G>C, 3520T>C, 3524G>T, 3526A>T, 3527A>C, 3528C>A, 3694A>G, 3694_3695insATCTTTCAT, 3695C>T, 3698G>A, 3702G>A, 3706C>T, 3710T>A, 3711C>G, 3712T>C, 3713C>A, 3714G>A, 3715A>G, 3718A>C, 3719C>T, 3721C>G, 3722A>G, 3726A>C, 3727G>A, 3728A>G, 3729G>C, 3731G>C, 3733A>G, 3734C>G, 3735T>C, 3742T>C, 3744G>C, 3745C>T, 3746A>G, 3747C>T, 3748A>T, 3750T>C, 3751A>T, 3754G>A, 3757A>G, 3760A>G, 3761T>A, 3763A>G, 3766G>A, 3769T>C, 3770A>G, 3775T>G, 3777G>T, 3778G>T, 3781T>C, 3782A>T, 3783C>T, 3784A>T, 3785A>G, 3787A>C, 3788T>A, 3790T>A, 3793T>C, 3799C>A, 3802C>T, 3803T>C, 3805A>G, 3806T>G, 3808C>G, 3813G>C, 3814A>C, 3818T>G, 3820T>C, 3822A>T, 3823G>T, 3824A>G, 3826A>T, 3828T>A, 3829G>T, 3835C>T, 3838C>T, 3839A>G, 3840A>C, 3841A>T, 3842C>G, 3844A>T, 3847A>T, 3848C>A, 3849A>G, 3852G>A, 3853G>C, 3854A>C, 3856G>A, 3857A>T, 3859G>T, 3861A>G, 3863T>A, 3864T>A, 3865A>G, 3868A>T, 3871C>T, 3872C>G, 3877C>A, 3878T>C, 3881C>A, 3883A>G, 3884A>C, 3888C>T, 3896C>A, 3897G>T, 3898A>T, 3899C>T, 3903G>T, 3907A>C, 3908A>C, 3909G>A, 3910T>A, 3915A>T, 3916T>C, 3920T>A, 3922C>T, 3923T>A, 3924C>A, 3925T>G, 3928A>T, 3929G>A, 3930T>A, 3931G>A, 3932T>G, 3936A>G, 3937G>A, 3940A>G, 3943A>G, 3944A>T, 3945A>G, 3946G>T, 3947T>G, 3948C>A, 3949T>G, 3952T>C, 3953A>C, 3954C>A, 3955C>A, 3957A>T, 3958C>T, 3964T>C, 3965G>C, 3966C>A, 3970T>A, 3973T>C, 3974C>A

CDS

	Begin	End	Coverage	Score	Concordance	Matches	Identities	I/D/M/F*	Stop Codons
D1R33_gp2	135	560	34.2%	958	37.4%	369 (98.4%)	154 (41.1%)	4/2/1/1	3

Protein mutations: D135* (2708G>T 2710C>G), E136V (2712A>T), V137G (2715T>G), Y140S (2724A>C 2725C>A), T141V (2726A>G 2727C>T), K142R (2730A>G 2731A>G), S143L (2732A>C 2733G>T), P147_F148del (2744_2749delCCCTTC), I149N (2751T>A), R151F (2756C>T 2757G>T), E153K (2762G>A 2764A>G), V154L (2765G>C 2767T>A), R155E (2768A>G 2769G>A), D156L (2771G>C 2772A>T 2772_2773insGA), D156_Q157insX (2772_2773insGA 2773C>G), Q157K (2774C>A), I158L (2777A>T 2779C>G), T159E (2780A>G 2781C>A 2782G>A), K160D (2783A>G 2785A>T), M161L (2786A>T 2788G>A), D163K (2792G>A 2794C>G), Q164K (2795C>A 2797A>G), G165E (2799G>A 2800A>G), I166F (2801A>T), R168* (2807A>T), D171G (2817A>G 2818C>A), A173P (2822G>C 2824A>T), S175G (2828A>G 2830C>T), S176A (2831T>G), I178V (2837A>G), W179L (2841G>T), V180F (2843G>T), V181M (2846G>A), P182R (2849C>A 2850C>G 2851C>A), I185K (2859T>A 2860C>G), A187G (2865C>G 2866T>G), K190I (2874A>T), Q191I (2876C>A 2877A>T), K192R (2880A>G), W193M (2882T>A 2883G>T), R194C (2885C>T), L195M (2888C>A 2890C>G), V196Y (2891G>T 2892T>A 2893A>T), V197I (2894G>A), F199Y (2901T>A 2902C>T), R200* (2903C>T 2905T>A), K201R (2907A>G), E204K (2915G>A 2917G>A), K205I (2919A>T 2920G>C), I207V (2924A>G 2926C>G), D208R (2927G>A 2928A>G 2929T>G), K210R (2934A>G 2935A>G), Y211F (2937A>T), N215T (2949A>C 2950C>T), S217D (2954A>G 2955G>A), D218E (2959C>G), V219L (2960G>C), L220F (2963C>T), K222E (2969A>G), L223V (2972T>G), G224N (2975G>A 2976G>A), K225G (2978A>G 2979A>G 2980G>C), C226A (2981T>G 2982G>C 2983C>T), Q227V (2984C>G 2985A>T 2986A>T), T230S (2993A>T 2995C>A), T231K (2997C>A 2998C>G), A235L (3008G>T 3009C>T), F238Y (3018T>A 3019T>C), Y239D (3020T>G), V241I (3026G>A 3028G>T), E242R (3029G>C 3030A>G), M243V (3032A>G 3034G>T), D244K (3035G>A 3037C>G), P245V (3038C>G 3039C>T 3040T>G), Q246A (3041C>G 3042A>C), S249P (3050T>C 3052G>C), N254R (3066A>G 3067C>G), V255T (3068G>A 3069T>C), E256H (3071G>C 3073A>C), H257D (3074C>G 3076C>T), F260Y (3084T>A 3085T>C), R264V (3095C>G 3096G>T 3097A>C), M267F (3104A>T 3106G>T), K270S (3113A>T 3114A>C 3115A>T), S272A (3119T>G 3121A>G), P273S (3122C>T 3124A>G), D281N (3146G>A), L284F (3155C>T 3157A>T), R285K (3159G>A), G286P (3161G>C 3162G>C), L287F (3164C>T 3166C>T), Q288L (3168A>T 3169A>C), N289R (3171A>G 3172T>G), N290K (3175C>A), I291F (3176A>T 3178C>T), C292I (3179T>A 3180G>T 3181T>A), Y295F (3189A>T 3190C>T), L296F (3191C>T), I300L (3203A>C), V301I (3206G>A), T304V (3215A>G 3216C>T 3217T>A), Q307K (3224C>A 3226G>A), L310V (3233C>G 3235G>T), N312H (3239A>C), E314V (3246A>T 3247A>T), R315A (3248C>G 3249G>C 3250A>T), Q318D (3257C>G 3259A>T), R319S (3262A>C), E322N (3269G>A 3271A>C), S323H (3272A>C 3273G>A 3274T>C), N324S (3275A>T 3276A>C 3277C>T), F325L (3280C>G), K326Y (3281A>T 3283A>T), I327A (3284A>G 3285T>C), Q328K (3287C>A), M329R (3291T>G 3292G>A), D330S (3293G>T 3294A>C 3295C>T), S332C (3300C>G 3301C>T), E333S (3302G>T 3303A>C 3304A>T), L335G (3308T>G 3309T>G 3310G>A), K336Q (3311A>C 3313G>A), L337P (3315T>C 3316C>T), E338K (3317G>A 3319A>G), T339V (3320A>G 3321C>T), A340E (3324C>A 3325T>G), I345V (3338A>G 3340C>T), S347T (3345G>C), R348G (3347A>G 3349G>A), D349E (3352C>A), I351V (3356A>G 3358C>G), K352S (3360A>G 3361G>T), P353T (3362C>A), N354D (3365A>G 3367C>T), D356A (3372A>C), S359E (3380T>G 3381C>A 3382C>A), I361M (3388T>G), Q362K (3389C>A), K363N (3394A>C), Y364W (3396A>G 3397T>G), L365P (3399T>C 3400G>A), I366R (3402T>G 3403T>A), K371M (3417A>T), I373V (3422A>G 3424A>T), K374R (3425A>C 3426A>G 3427A>T), Q375S (3428C>A 3429A>G 3430A>T), L380V (3443C>G 3445C>A), K385I (3459A>T), I387V (3464A>G 3466T>G), P388E (3467C>G 3468C>A), D389G (3471A>G 3472T>G), A391S (3476G>T 3478A>T), R392S (3479C>T 3480G>C), L393I (3482C>A 3484C>T), T394S (3485A>T 3487A>T), K395A (3488A>G 3489A>C), Q399K (3500C>A), C400L (3503T>C 3504G>T 3505C>A), L401T (3506T>A 3507T>C), K402H (3509A>C 3511A>C), S405A (3518A>G 3519G>C 3520T>C), V407F (3524G>T 3526A>T), K463_P464insIFH (3694_3695insATCTTTCAT), P464S (3695C>T), V465I (3698G>A), C466Y (3702G>A), R470K (3713C>A 3714G>A 3715A>G), N473D (3722A>G), E474A (3726A>C 3727G>A), S475A (3728A>G 3729G>C), E476Q (3731G>C 3733A>G), L477A (3734C>G 3735T>C), S480T (3744G>C 3745C>T), T481V (3746A>G 3747C>T 3748A>T), I482T (3750T>C 3751A>T), L486M (3761T>A 3763A>G), I489V (3770A>G), W491F (3777G>T 3778G>T), T493F (3782A>T 3783C>T 3784A>T), K494D (3785A>G 3787A>C), Y495K (3788T>A 3790T>A), F501V (3806T>G 3808C>G), R503T (3813G>C 3814A>C), F505V (3818T>G 3820T>C), K506I (3822A>T 3823G>T), I507V (3824A>G 3826A>T), L508Y (3828T>A 3829G>T), K512A (3839A>G 3840A>C 3841A>T), P513A (3842C>G 3844A>T), Q515R (3848C>A 3849A>G), W516Y (3852G>A 3853G>C), M517L (3854A>C 3856G>A), M518F (3857A>T 3859G>T), N519S (3861A>G), L520K (3863T>A 3864T>A 3865A>G), K521N (3868A>T), P523A (3872C>G), N524K (3877C>A), S525P (3878T>C), M527L (3884A>C), T528I (3888C>T), R531I (3896C>A 3897G>T 3898A>T), R533L (3903G>T), S535Q (3908A>C 3909G>A 3910T>A), Y537F (3915A>T 3916T>C), F539I (3920T>A 3922C>T), S540K (3923T>A 3924C>A 3925T>G), V542K (3929G>A 3930T>A 3931G>A), Y543D (3932T>G), K544R (3936A>G 3937G>A), K547C (3944A>T 3945A>G 3946G>T), S548E (3947T>G 3948C>A 3949T>G), T550Q (3953A>C 3954C>A 3955C>A), N551I (3957A>T 3958C>T), A554H (3965G>C 3966C>A), R557S (3974C>A)

	Begin	End	Coverage	Score	Concordance	Matches	Identities	I/D/M/F*	Stop Codons
**NT**	**2706**	**3983**	**14.8%**	**74**	**3.4%**	**1109 (98.5%)**	**584 (51.9%)**	**11/6**	

Codon mutations:

GAC135TAG (2708G>T 2710C>G), GAA136GTA (2712A>T), GTA137GGA (2715T>G), CCC138CCA (2719C>A), GTG139GTT (2722G>T), TAC140TCA (2724A>C 2725C>A), ACC141GTC (2726A>G 2727C>T), AAA142AGG (2730A>G 2731A>G), AGT143CTT (2732A>C 2733G>T), TAC144TAT (2737C>T), CGG145AGA (2738C>A 2740G>A), CCC147_TTC148del (2744_2749delCCCTTC), ATC149AAC (2751T>A), CGC151TTC (2756C>T 2757G>T), CAG152CAA (2761G>A), GAA153AAG (2762G>A 2764A>G), GTT154CTA (2765G>C 2767T>A), AGG155GAG (2768A>G 2769G>A), GAC156CTG (2771G>C 2772A>T 2772_2773insGA), GAC156_CAA157insA-G (2772_2773insGA 2773C>G), CAA157AAA (2774C>A), ATC158TTG (2777A>T 2779C>G), ACG159GAA (2780A>G 2781C>A 2782G>A), AAA160GAT (2783A>G 2785A>T), ATG161TTA (2786A>T 2788G>A), GAC163AAG (2792G>A 2794C>G), CAA164AAG (2795C>A 2797A>G), GGA165GAG (2799G>A 2800A>G), ATT166TTT (2801A>T), ATA167ATT (2806A>T), AGA168TGA (2807A>T), CCA169CCT (2812A>T), TCA170AGT (2813T>A 2814C>G 2815A>T), GAC171GGA (2817A>G 2818C>A), TCT172TCA (2821T>A), GCA173CCT (2822G>C 2824A>T), AGC175GGT (2828A>G 2830C>T), TCA176GCA (2831T>G), CCC177CCA (2836C>A), ATA178GTA (2837A>G), TGG179TTG (2841G>T), GTT180TTT (2843G>T), GTG181ATG (2846G>A), CCC182AGA (2849C>A 2850C>G 2851C>A), AAA184AAG (2857A>G), ATC185AAG (2859T>A 2860C>G), GAC186GAT (2863C>T), GCT187GGG (2865C>G 2866T>G), TCT188TCG (2869T>G), GGG189GGT (2872G>T), AAA190ATA (2874A>T), CAA191ATA (2876C>A 2877A>T), AAG192AGG (2880A>G), TGG193ATG (2882T>A 2883G>T), CGT194TGT (2885C>T), CTC195ATG (2888C>A 2890C>G), GTA196TAT (2891G>T 2892T>A 2893A>T), GTT197ATT (2894G>A), GAC198GAT (2899C>T), TTC199TAT (2901T>A 2902C>T), CGT200TGA (2903C>T 2905T>A), AAG201AGG (2907A>G), GAG204AAA (2915G>A 2917G>A), AAG205ATC (2919A>T 2920G>C), ATC207GTG (2924A>G 2926C>G), GAT208AGG (2927G>A 2928A>G 2929T>G), GAC209GAT (2932C>T), AAA210AGG (2934A>G 2935A>G), TAC211TTC (2937A>T), CCG212CCA (2941G>A), ATA213ATC (2944A>C), AAC215ACT (2949A>C 2950C>T), ATA216ATT (2953A>T), AGT217GAT (2954A>G 2955G>A), GAC218GAG (2959C>G), GTA219CTA (2960G>C), CTT220TTT (2963C>T), GAC221GAT (2968C>T), AAG222GAG (2969A>G), TTA223GTA (2972T>G), GGT224AAT (2975G>A 2976G>A), AAG225GGC (2978A>G 2979A>G 2980G>C), TGC226GCT (2981T>G 2982G>C 2983C>T), CAA227GTT (2984C>G 2985A>T 2986A>T), TAC228TAT (2989C>T), ACC230TCA (2993A>T 2995C>A), ACC231AAG (2997C>A 2998C>G), TTA232CTT (2999T>C 3001A>T), GCA235TTA (3008G>T 3009C>T), AGT236TCT (3011A>T 3012G>C), GGG237GGA (3016G>A), TTT238TAC (3018T>A 3019T>C), TAT239GAT (3020T>G), CAG240CAA (3025G>A), GTG241ATT (3026G>A 3028G>T), GAG242CGG (3029G>C 3030A>G), ATG243GTT (3032A>G 3034G>T), GAC244AAG (3035G>A 3037C>G), CCT245GTG (3038C>G 3039C>T 3040T>G), CAA246GCA (3041C>G 3042A>C), TCG249CCC (3050T>C 3052G>C), ACC251ACT (3058C>T), GCG252GCT (3061G>T), AAC254AGG (3066A>G 3067C>G), GTA255ACA (3068G>A 3069T>C), GAA256CAC (3071G>C 3073A>C), CAC257GAT (3074C>G 3076C>T), GGG258GGT (3079G>T), TTT260TAC (3084T>A 3085T>C), TTC262TTT (3091C>T), CTT263TTA (3092C>T 3094T>A), CGA264GTC (3095C>G 3096G>T 3097A>C), ATG267TTT (3104A>T 3106G>T), GGA268GGG (3109A>G), TTA269TTG (3112A>G), AAA270TCT (3113A>T 3114A>C 3115A>T), TCA272GCG (3119T>G 3121A>G), CCA273TCG (3122C>T 3124A>G), TCT274TCC (3127T>C), ACT275ACA (3130T>A), GTT279.TG (3142T>G), GAC281AAC (3146G>A), GTC283GTT (3154C>T), CTA284TTT (3155C>T 3157A>T), AGA285AAA (3159G>A), GGT286CCT (3161G>C 3162G>C), CTC287TTT (3164C>T 3166C>T), CAA288CTC (3168A>T 3169A>C), AAT289AGG (3171A>G 3172T>G), AAC290AAA (3175C>A), ATC291TTT (3176A>T 3178C>T), TGT292ATA (3179T>A 3180G>T 3181T>A), CTC293TTG (3182C>T 3184C>G), GTC294GTA (3187C>A), TAC295TTT (3189A>T 3190C>T), CTT296TTT (3191C>T), GAC297GAT (3196C>T), ATT299ATA (3202T>A), ATT300CTT (3203A>C), GTC301ATC (3206G>A), ACT304GTA (3215A>G 3216C>T 3217T>A), TCC305TCA (3220C>A), CTA306TTA (3221C>T), CAG307AAA (3224C>A 3226G>A), GAA308GAG (3229A>G), CAC309CAT (3232C>T), CTG310GTT (3233C>G 3235G>T), GAG311GAA (3238G>A), AAC312CAC (3239A>C), CTG313TTA (3242C>T 3244G>A), GAA314GTT (3246A>T 3247A>T), CGA315GCT (3248C>G 3249G>C 3250A>T), TTC317TTT (3256C>T), CAA318GAT (3257C>G 3259A>T), AGA319AGC (3262A>C), CTT320TTG (3263C>T 3265T>G), AGA321AGG (3268A>G), GAA322AAC (3269G>A 3271A>C), AGT323CAC (3272A>C 3273G>A 3274T>C), AAC324TCT (3275A>T 3276A>C 3277C>T), TTC325TTG (3280C>G), AAA326TAT (3281A>T 3283A>T), ATT327GCT (3284A>G 3285T>C), CAA328AAA (3287C>A), ATG329AGA (3291T>G 3292G>A), GAC330TCT (3293G>T 3294A>C 3295C>T), AAG331AAA (3298G>A), TCC332TGT (3300C>G 3301C>T), GAA333TCT (3302G>T 3303A>C 3304A>T), TTC334TTT (3307C>T), TTG335GGA (3308T>G 3309T>G 3310G>A), AAG336CAA (3311A>C 3313G>A), CTC337CCT (3315T>C 3316C>T), GAA338AAG (3317G>A 3319A>G), ACT339GTT (3320A>G 3321C>T), GCT340GAG (3324C>A 3325T>G), TAT341TAC (3328T>C), CTT342TTA (3329C>T 3331T>A), CAC344CAT (3337C>T), ATC345GTT (3338A>G 3340C>T), ATA346ATT (3343A>T), AGC347ACC (3345G>C), AGG348GGA (3347A>G 3349G>A), GAC349GAA (3352C>A), GGT350GGA (3355T>A), ATC351GTG (3356A>G 3358C>G), AAG352AGT (3360A>G 3361G>T), CCT353ACT (3362C>A), AAC354GAT (3365A>G 3367C>T), GAT356GCT (3372A>C), AAG357AAA (3376G>A), ATT358ATC (3379T>C), TCC359GAA (3380T>G 3381C>A 3382C>A), ATT361ATG (3388T>G), CAA362AAA (3389C>A), AAA363AAC (3394A>C), TAT364TGG (3396A>G 3397T>G), CTG365CCA (3399T>C 3400G>A), ATT366AGA (3402T>G 3403T>A), CCA367CCT (3406A>T), AAG368AAA (3409G>A), ACC369ACA (3412C>A), AAG371ATG (3417A>T), GAA372GAG (3421A>G), ATA373GTT (3422A>G 3424A>T), AAA374CGT (3425A>C 3426A>G 3427A>T), CAA375AGT (3428C>A 3429A>G 3430A>T), TTA377CTT (3434T>C 3436A>T), GGC378GGA (3439C>A), CTT379TTG (3440C>T 3442T>G), CTC380GTA (3443C>G 3445C>A), TAC383TAT (3454C>T), CGA384AGA (3455C>A), AAA385ATA (3459A>T), TTC386TTT (3463C>T), ATT387GTG (3464A>G 3466T>G), CCA388GAA (3467C>G 3468C>A), GAT389GGG (3471A>G 3472T>G), GCA391TCT (3476G>T 3478A>T), CGA392TCA (3479C>T 3480G>C), CTC393ATT (3482C>A 3484C>T), ACA394TCT (3485A>T 3487A>T), AAA395GCA (3488A>G 3489A>C), CCC396CCT (3493C>T), CTT397TTA (3494C>T 3496T>A), CAG399AAG (3500C>A), TGC400CTA (3503T>C 3504G>T 3505C>A), TTA401ACA (3506T>A 3507T>C), AAA402CAC (3509A>C 3511A>C), AAA403AAG (3514A>G), GGT404GGA (3517T>A), AGT405GCC (3518A>G 3519G>C 3520T>C), GTA407TTT (3524G>T 3526A>T), ACT408CA. (3527A>C 3528C>A), AAA463AAG (3694A>G), AAA463_CCC464insATCTTTCAT (3694_3695insATCTTTCAT), CCC464TCC (3695C>T), GTC465ATC (3698G>A), TGT466TAT (3702G>A), TAC467TAT (3706C>T), TCT469AGC (3710T>A 3711C>G 3712T>C), CGA470AAG (3713C>A 3714G>A 3715A>G), ACA471ACC (3718A>C), CTC472TTG (3719C>T 3721C>G), AAT473GAT (3722A>G), GAG474GCA (3726A>C 3727G>A), AGC475GCC (3728A>G 3729G>C), GAA476CAG (3731G>C 3733A>G), CTA477GCA (3734C>G 3735T>C), TAT479TAC (3742T>C), AGC480ACT (3744G>C 3745C>T), ACA481GTT (3746A>G 3747C>T 3748A>T), ATA482ACT (3750T>C 3751A>T), GAG483GAA (3754G>A), AAA484AAG (3757A>G), GAA485GAG (3760A>G), TTA486ATG (3761T>A 3763A>G), CTG487CTA (3766G>A), GCT488GCC (3769T>C), ATA489GTA (3770A>G), GTT490GTG (3775T>G), TGG491TTT (3777G>T 3778G>T), GCT492GCC (3781T>C), ACA493TTT (3782A>T 3783C>T 3784A>T), AAA494GAC (3785A>G 3787A>C), TAT495AAA (3788T>A 3790T>A), TTT496TTC (3793T>C), CCC498CCA (3799C>A), TAC499TAT (3802C>T), TTA500CTG (3803T>C 3805A>G), TTC501GTG (3806T>G 3808C>G), AGA503ACC (3813G>C 3814A>C), TTT505GTC (3818T>G 3820T>C), AAG506ATT (3822A>T 3823G>T), ATA507GTT (3824A>G 3826A>T), TTG508TAT (3828T>A 3829G>T), GAC510GAT (3835C>T), CAC511CAT (3838C>T), AAA512GCT (3839A>G 3840A>C 3841A>T), CCA513GCT (3842C>G 3844A>T), CTA514CTT (3847A>T), CAG515AGG (3848C>A 3849A>G), TGG516TAC (3852G>A 3853G>C), ATG517CTA (3854A>C 3856G>A), ATG518TTT (3857A>T 3859G>T), AAC519AGC (3861A>G), TTA520AAG (3863T>A 3864T>A 3865A>G), AAA521AAT (3868A>T), GAC522GAT (3871C>T), CCA523GCA (3872C>G), AAC524AAA (3877C>A), TCA525CCA (3878T>C), CGA526AGG (3881C>A 3883A>G), ATG527CTG (3884A>C), ACT528ATT (3888C>T), CGA531ATT (3896C>A 3897G>T 3898A>T), CTA532TTA (3899C>T), CGA533CTA (3903G>T), CTA534CTC (3907A>C), AGT535CAA (3908A>C 3909G>A 3910T>A), TAT537TTC (3915A>T 3916T>C), TTC539ATT (3920T>A 3922C>T), TCT540AAG (3923T>A 3924C>A 3925T>G), GTA541GTT (3928A>T), GTG542AAA (3929G>A 3930T>A 3931G>A), TAC543GAC (3932T>G), AAG544AGA (3936A>G 3937G>A), AAA545AAG (3940A>G), GGA546GGG (3943A>G), AAG547TGT (3944A>T 3945A>G 3946G>T), TCT548GAG (3947T>G 3948C>A 3949T>G), AAT549AAC (3952T>C), ACC550CAA (3953A>C 3954C>A 3955C>A), AAC551ATT (3957A>T 3958C>T), GAT553GAC (3964T>C), GCC554CAC (3965G>C 3966C>A), CTT555CTA (3970T>A), TCT556TCC (3973T>C), CGT557AGT (3974C>A)

Proteins

	Begin	End	Coverage	Score	Concordance	Matches	Identities	I/D/M/F*	Stop Codons
ORF B (YP_009507248.1)	135	560	34.2%	958	37.4%	369 (98.4%)	154 (41.1%)	4/2/1/1	3

Protein mutations:

D135* (2708G>T 2710C>G), E136V (2712A>T), V137G (2715T>G), Y140S (2724A>C 2725C>A), T141V (2726A>G 2727C>T), K142R (2730A>G 2731A>G), S143L (2732A>C 2733G>T), P147_F148del (2744_2749delCCCTTC), I149N (2751T>A), R151F (2756C>T 2757G>T), E153K (2762G>A 2764A>G), V154L (2765G>C 2767T>A), R155E (2768A>G 2769G>A), D156L (2771G>C 2772A>T 2772_2773insGA), D156_Q157insX (2772_2773insGA 2773C>G), Q157K (2774C>A), I158L (2777A>T 2779C>G), T159E (2780A>G 2781C>A 2782G>A), K160D (2783A>G 2785A>T), M161L (2786A>T 2788G>A), D163K (2792G>A 2794C>G), Q164K (2795C>A 2797A>G), G165E (2799G>A 2800A>G), I166F (2801A>T), R168* (2807A>T), D171G (2817A>G 2818C>A), A173P (2822G>C 2824A>T), S175G (2828A>G 2830C>T), S176A (2831T>G), I178V (2837A>G), W179L (2841G>T), V180F (2843G>T), V181M (2846G>A), P182R (2849C>A 2850C>G 2851C>A), I185K (2859T>A 2860C>G), A187G (2865C>G 2866T>G), K190I (2874A>T), Q191I (2876C>A 2877A>T), K192R (2880A>G), W193M (2882T>A 2883G>T), R194C (2885C>T), L195M (2888C>A 2890C>G), V196Y (2891G>T 2892T>A 2893A>T), V197I (2894G>A), F199Y (2901T>A 2902C>T), R200* (2903C>T 2905T>A), K201R (2907A>G), E204K (2915G>A 2917G>A), K205I (2919A>T 2920G>C), I207V (2924A>G 2926C>G), D208R (2927G>A 2928A>G 2929T>G), K210R (2934A>G 2935A>G), Y211F (2937A>T), N215T (2949A>C 2950C>T), S217D (2954A>G 2955G>A), D218E (2959C>G), V219L (2960G>C), L220F (2963C>T), K222E (2969A>G), L223V (2972T>G), G224N (2975G>A 2976G>A), K225G (2978A>G 2979A>G 2980G>C), C226A (2981T>G 2982G>C 2983C>T), Q227V (2984C>G 2985A>T 2986A>T), T230S (2993A>T 2995C>A), T231K (2997C>A 2998C>G), A235L (3008G>T 3009C>T), F238Y (3018T>A 3019T>C), Y239D (3020T>G), V241I (3026G>A 3028G>T), E242R (3029G>C 3030A>G), M243V (3032A>G 3034G>T), D244K (3035G>A 3037C>G), P245V (3038C>G 3039C>T 3040T>G), Q246A (3041C>G 3042A>C), S249P (3050T>C 3052G>C), N254R (3066A>G 3067C>G), V255T (3068G>A 3069T>C), E256H (3071G>C 3073A>C), H257D (3074C>G 3076C>T), F260Y (3084T>A 3085T>C), R264V (3095C>G 3096G>T 3097A>C), M267F (3104A>T 3106G>T), K270S (3113A>T 3114A>C 3115A>T), S272A (3119T>G 3121A>G), P273S (3122C>T 3124A>G), D281N (3146G>A), L284F (3155C>T 3157A>T), R285K (3159G>A), G286P (3161G>C 3162G>C), L287F (3164C>T 3166C>T), Q288L (3168A>T 3169A>C), N289R (3171A>G 3172T>G), N290K (3175C>A), I291F (3176A>T 3178C>T), C292I (3179T>A 3180G>T 3181T>A), Y295F (3189A>T 3190C>T), L296F (3191C>T), I300L (3203A>C), V301I (3206G>A), T304V (3215A>G 3216C>T 3217T>A), Q307K (3224C>A 3226G>A), L310V (3233C>G 3235G>T), N312H (3239A>C), E314V (3246A>T 3247A>T), R315A (3248C>G 3249G>C 3250A>T), Q318D (3257C>G 3259A>T), R319S (3262A>C), E322N (3269G>A 3271A>C), S323H (3272A>C 3273G>A 3274T>C), N324S (3275A>T 3276A>C 3277C>T), F325L (3280C>G), K326Y (3281A>T 3283A>T), I327A (3284A>G 3285T>C), Q328K (3287C>A), M329R (3291T>G 3292G>A), D330S (3293G>T 3294A>C 3295C>T), S332C (3300C>G 3301C>T), E333S (3302G>T 3303A>C 3304A>T), L335G (3308T>G 3309T>G 3310G>A), K336Q (3311A>C 3313G>A), L337P (3315T>C 3316C>T), E338K (3317G>A 3319A>G), T339V (3320A>G 3321C>T), A340E (3324C>A 3325T>G), I345V (3338A>G 3340C>T), S347T (3345G>C), R348G (3347A>G 3349G>A), D349E (3352C>A), I351V (3356A>G 3358C>G), K352S (3360A>G 3361G>T), P353T (3362C>A), N354D (3365A>G 3367C>T), D356A (3372A>C), S359E (3380T>G 3381C>A 3382C>A), I361M (3388T>G), Q362K (3389C>A), K363N (3394A>C), Y364W (3396A>G 3397T>G), L365P (3399T>C 3400G>A), I366R (3402T>G 3403T>A), K371M (3417A>T), I373V (3422A>G 3424A>T), K374R (3425A>C 3426A>G 3427A>T), Q375S (3428C>A 3429A>G 3430A>T), L380V (3443C>G 3445C>A), K385I (3459A>T), I387V (3464A>G 3466T>G), P388E (3467C>G 3468C>A), D389G (3471A>G 3472T>G), A391S (3476G>T 3478A>T), R392S (3479C>T 3480G>C), L393I (3482C>A 3484C>T), T394S (3485A>T 3487A>T), K395A (3488A>G 3489A>C), Q399K (3500C>A), C400L (3503T>C 3504G>T 3505C>A), L401T (3506T>A 3507T>C), K402H (3509A>C 3511A>C), S405A (3518A>G 3519G>C 3520T>C), V407F (3524G>T 3526A>T), K463_P464insIFH (3694_3695insATCTTTCAT), P464S (3695C>T), V465I (3698G>A), C466Y (3702G>A), R470K (3713C>A 3714G>A 3715A>G), N473D (3722A>G), E474A (3726A>C 3727G>A), S475A (3728A>G 3729G>C), E476Q (3731G>C 3733A>G), L477A (3734C>G 3735T>C), S480T (3744G>C 3745C>T), T481V (3746A>G 3747C>T 3748A>T), I482T (3750T>C 3751A>T), L486M (3761T>A 3763A>G), I489V (3770A>G), W491F (3777G>T 3778G>T), T493F (3782A>T 3783C>T 3784A>T), K494D (3785A>G 3787A>C), Y495K (3788T>A 3790T>A), F501V (3806T>G 3808C>G), R503T (3813G>C 3814A>C), F505V (3818T>G 3820T>C), K506I (3822A>T 3823G>T), I507V (3824A>G 3826A>T), L508Y (3828T>A 3829G>T), K512A (3839A>G 3840A>C 3841A>T), P513A (3842C>G 3844A>T), Q515R (3848C>A 3849A>G), W516Y (3852G>A 3853G>C), M517L (3854A>C 3856G>A), M518F (3857A>T 3859G>T), N519S (3861A>G), L520K (3863T>A 3864T>A 3865A>G), K521N (3868A>T), P523A (3872C>G), N524K (3877C>A), S525P (3878T>C), M527L (3884A>C), T528I (3888C>T), R531I (3896C>A 3897G>T 3898A>T), R533L (3903G>T), S535Q (3908A>C 3909G>A 3910T>A), Y537F (3915A>T 3916T>C), F539I (3920T>A 3922C>T), S540K (3923T>A 3924C>A 3925T>G), V542K (3929G>A 3930T>A 3931G>A), Y543D (3932T>G), K544R (3936A>G 3937G>A), K547C (3944A>T 3945A>G 3946G>T), S548E (3947T>G 3948C>A 3949T>G), T550Q (3953A>C 3954C>A 3955C>A), N551I (3957A>T 3958C>T), A554H (3965G>C 3966C>A), R557S (3974C>A)

	Begin	End	Coverage	Score	Concordance	Matches	Identities	I/D/M/F*	Stop Codons
**NT**	**2706**	**3983**	**14.8%**	**74**	**3.4%**	**1109 (98.5%)**	**584 (51.9%)**	**11/6**	
Codon mutations:	GAC135TAG (2708G>T 2710C>G), GAA136GTA (2712A>T), GTA137GGA (2715T>G), CCC138CCA (2719C>A), GTG139GTT (2722G>T), TAC140TCA (2724A>C 2725C>A), ACC141GTC (2726A>G 2727C>T), AAA142AGG (2730A>G 2731A>G), AGT143CTT (2732A>C 2733G>T), TAC144TAT (2737C>T), CGG145AGA (2738C>A 2740G>A), CCC147_TTC148del (2744_2749delCCCTTC), ATC149AAC (2751T>A), CGC151TTC (2756C>T 2757G>T), CAG152CAA (2761G>A), GAA153AAG (2762G>A 2764A>G), GTT154CTA (2765G>C 2767T>A), AGG155GAG (2768A>G 2769G>A), GAC156CTG (2771G>C 2772A>T 2772_2773insGA), GAC156_CAA157insA-G (2772_2773insGA 2773C>G), CAA157AAA (2774C>A), ATC158TTG (2777A>T 2779C>G), ACG159GAA (2780A>G 2781C>A 2782G>A), AAA160GAT (2783A>G 2785A>T), ATG161TTA (2786A>T 2788G>A), GAC163AAG (2792G>A 2794C>G), CAA164AAG (2795C>A 2797A>G), GGA165GAG (2799G>A 2800A>G), ATT166TTT (2801A>T), ATA167ATT (2806A>T), AGA168TGA (2807A>T), CCA169CCT (2812A>T), TCA170AGT (2813T>A 2814C>G 2815A>T), GAC171GGA (2817A>G 2818C>A), TCT172TCA (2821T>A), GCA173CCT (2822G>C 2824A>T), AGC175GGT (2828A>G 2830C>T), TCA176GCA (2831T>G), CCC177CCA (2836C>A), ATA178GTA (2837A>G), TGG179TTG (2841G>T), GTT180TTT (2843G>T), GTG181ATG (2846G>A), CCC182AGA (2849C>A 2850C>G 2851C>A), AAA184AAG (2857A>G), ATC185AAG (2859T>A 2860C>G), GAC186GAT (2863C>T), GCT187GGG (2865C>G 2866T>G), TCT188TCG (2869T>G), GGG189GGT (2872G>T), AAA190ATA (2874A>T), CAA191ATA (2876C>A 2877A>T), AAG192AGG (2880A>G), TGG193ATG (2882T>A 2883G>T), CGT194TGT (2885C>T), CTC195ATG (2888C>A 2890C>G), GTA196TAT (2891G>T 2892T>A 2893A>T), GTT197ATT (2894G>A), GAC198GAT (2899C>T), TTC199TAT (2901T>A 2902C>T), CGT200TGA (2903C>T 2905T>A), AAG201AGG (2907A>G), GAG204AAA (2915G>A 2917G>A), AAG205ATC (2919A>T 2920G>C), ATC207GTG (2924A>G 2926C>G), GAT208AGG (2927G>A 2928A>G 2929T>G), GAC209GAT (2932C>T), AAA210AGG (2934A>G 2935A>G), TAC211TTC (2937A>T), CCG212CCA (2941G>A), ATA213ATC (2944A>C), AAC215ACT (2949A>C 2950C>T), ATA216ATT (2953A>T), AGT217GAT (2954A>G 2955G>A), GAC218GAG (2959C>G), GTA219CTA (2960G>C), CTT220TTT (2963C>T), GAC221GAT (2968C>T), AAG222GAG (2969A>G), TTA223GTA (2972T>G), GGT224AAT (2975G>A 2976G>A), AAG225GGC (2978A>G 2979A>G 2980G>C), TGC226GCT (2981T>G 2982G>C 2983C>T), CAA227GTT (2984C>G 2985A>T 2986A>T), TAC228TAT (2989C>T), ACC230TCA (2993A>T 2995C>A), ACC231AAG (2997C>A 2998C>G), TTA232CTT (2999T>C 3001A>T), GCA235TTA (3008G>T 3009C>T), AGT236TCT (3011A>T 3012G>C), GGG237GGA (3016G>A), TTT238TAC (3018T>A 3019T>C), TAT239GAT (3020T>G), CAG240CAA (3025G>A), GTG241ATT (3026G>A 3028G>T), GAG242CGG (3029G>C 3030A>G), ATG243GTT (3032A>G 3034G>T), GAC244AAG (3035G>A 3037C>G), CCT245GTG (3038C>G 3039C>T 3040T>G), CAA246GCA (3041C>G 3042A>C), TCG249CCC (3050T>C 3052G>C), ACC251ACT (3058C>T), GCG252GCT (3061G>T), AAC254AGG (3066A>G 3067C>G), GTA255ACA (3068G>A 3069T>C), GAA256CAC (3071G>C 3073A>C), CAC257GAT (3074C>G 3076C>T), GGG258GGT (3079G>T), TTT260TAC (3084T>A 3085T>C), TTC262TTT (3091C>T), CTT263TTA (3092C>T 3094T>A), CGA264GTC (3095C>G 3096G>T 3097A>C), ATG267TTT (3104A>T 3106G>T), GGA268GGG (3109A>G), TTA269TTG (3112A>G), AAA270TCT (3113A>T 3114A>C 3115A>T), TCA272GCG (3119T>G 3121A>G), CCA273TCG (3122C>T 3124A>G), TCT274TCC (3127T>C), ACT275ACA (3130T>A), GTT279.TG (3142T>G), GAC281AAC (3146G>A), GTC283GTT (3154C>T), CTA284TTT (3155C>T 3157A>T), AGA285AAA (3159G>A), GGT286CCT (3161G>C 3162G>C), CTC287TTT (3164C>T 3166C>T), CAA288CTC (3168A>T 3169A>C), AAT289AGG (3171A>G 3172T>G), AAC290AAA (3175C>A), ATC291TTT (3176A>T 3178C>T), TGT292ATA (3179T>A 3180G>T 3181T>A), CTC293TTG (3182C>T 3184C>G), GTC294GTA (3187C>A), TAC295TTT (3189A>T 3190C>T), CTT296TTT (3191C>T), GAC297GAT (3196C>T), ATT299ATA (3202T>A), ATT300CTT (3203A>C), GTC301ATC (3206G>A), ACT304GTA (3215A>G 3216C>T 3217T>A), TCC305TCA (3220C>A), CTA306TTA (3221C>T), CAG307AAA (3224C>A 3226G>A), GAA308GAG (3229A>G), CAC309CAT (3232C>T), CTG310GTT (3233C>G 3235G>T), GAG311GAA (3238G>A), AAC312CAC (3239A>C), CTG313TTA (3242C>T 3244G>A), GAA314GTT (3246A>T 3247A>T), CGA315GCT (3248C>G 3249G>C 3250A>T), TTC317TTT (3256C>T), CAA318GAT (3257C>G 3259A>T), AGA319AGC (3262A>C), CTT320TTG (3263C>T 3265T>G), AGA321AGG (3268A>G), GAA322AAC (3269G>A 3271A>C), AGT323CAC (3272A>C 3273G>A 3274T>C), AAC324TCT (3275A>T 3276A>C 3277C>T), TTC325TTG (3280C>G), AAA326TAT (3281A>T 3283A>T), ATT327GCT (3284A>G 3285T>C), CAA328AAA (3287C>A), ATG329AGA (3291T>G 3292G>A), GAC330TCT (3293G>T 3294A>C 3295C>T), AAG331AAA (3298G>A), TCC332TGT (3300C>G 3301C>T), GAA333TCT (3302G>T 3303A>C 3304A>T), TTC334TTT (3307C>T), TTG335GGA (3308T>G 3309T>G 3310G>A), AAG336CAA (3311A>C 3313G>A), CTC337CCT (3315T>C 3316C>T), GAA338AAG (3317G>A 3319A>G), ACT339GTT (3320A>G 3321C>T), GCT340GAG (3324C>A 3325T>G), TAT341TAC (3328T>C), CTT342TTA (3329C>T 3331T>A), CAC344CAT (3337C>T), ATC345GTT (3338A>G 3340C>T), ATA346ATT (3343A>T), AGC347ACC (3345G>C), AGG348GGA (3347A>G 3349G>A), GAC349GAA (3352C>A), GGT350GGA (3355T>A), ATC351GTG (3356A>G 3358C>G), AAG352AGT (3360A>G 3361G>T), CCT353ACT (3362C>A), AAC354GAT (3365A>G 3367C>T), GAT356GCT (3372A>C), AAG357AAA (3376G>A), ATT358ATC (3379T>C), TCC359GAA (3380T>G 3381C>A 3382C>A), ATT361ATG (3388T>G), CAA362AAA (3389C>A), AAA363AAC (3394A>C), TAT364TGG (3396A>G 3397T>G), CTG365CCA (3399T>C 3400G>A), ATT366AGA (3402T>G 3403T>A), CCA367CCT (3406A>T), AAG368AAA (3409G>A), ACC369ACA (3412C>A), AAG371ATG (3417A>T), GAA372GAG (3421A>G), ATA373GTT (3422A>G 3424A>T), AAA374CGT (3425A>C 3426A>G 3427A>T), CAA375AGT (3428C>A 3429A>G 3430A>T), TTA377CTT (3434T>C 3436A>T), GGC378GGA (3439C>A), CTT379TTG (3440C>T 3442T>G), CTC380GTA (3443C>G 3445C>A), TAC383TAT (3454C>T), CGA384AGA (3455C>A), AAA385ATA (3459A>T), TTC386TTT (3463C>T), ATT387GTG (3464A>G 3466T>G), CCA388GAA (3467C>G 3468C>A), GAT389GGG (3471A>G 3472T>G), GCA391TCT (3476G>T 3478A>T), CGA392TCA (3479C>T 3480G>C), CTC393ATT (3482C>A 3484C>T), ACA394TCT (3485A>T 3487A>T), AAA395GCA (3488A>G 3489A>C), CCC396CCT (3493C>T), CTT397TTA (3494C>T 3496T>A), CAG399AAG (3500C>A), TGC400CTA (3503T>C 3504G>T 3505C>A), TTA401ACA (3506T>A 3507T>C), AAA402CAC (3509A>C 3511A>C), AAA403AAG (3514A>G), GGT404GGA (3517T>A), AGT405GCC (3518A>G 3519G>C 3520T>C), GTA407TTT (3524G>T 3526A>T), ACT408CA. (3527A>C 3528C>A), AAA463AAG (3694A>G), AAA463_CCC464insATCTTTCAT (3694_3695insATCTTTCAT), CCC464TCC (3695C>T), GTC465ATC (3698G>A), TGT466TAT (3702G>A), TAC467TAT (3706C>T), TCT469AGC (3710T>A 3711C>G 3712T>C), CGA470AAG (3713C>A 3714G>A 3715A>G), ACA471ACC (3718A>C), CTC472TTG (3719C>T 3721C>G), AAT473GAT (3722A>G), GAG474GCA (3726A>C 3727G>A), AGC475GCC (3728A>G 3729G>C), GAA476CAG (3731G>C 3733A>G), CTA477GCA (3734C>G 3735T>C), TAT479TAC (3742T>C), AGC480ACT (3744G>C 3745C>T), ACA481GTT (3746A>G 3747C>T 3748A>T), ATA482ACT (3750T>C 3751A>T), GAG483GAA (3754G>A), AAA484AAG (3757A>G), GAA485GAG (3760A>G), TTA486ATG (3761T>A 3763A>G), CTG487CTA (3766G>A), GCT488GCC (3769T>C), ATA489GTA (3770A>G), GTT490GTG (3775T>G), TGG491TTT (3777G>T 3778G>T), GCT492GCC (3781T>C), ACA493TTT (3782A>T 3783C>T 3784A>T), AAA494GAC (3785A>G 3787A>C), TAT495AAA (3788T>A 3790T>A), TTT496TTC (3793T>C), CCC498CCA (3799C>A), TAC499TAT (3802C>T), TTA500CTG (3803T>C 3805A>G), TTC501GTG (3806T>G 3808C>G), AGA503ACC (3813G>C 3814A>C), TTT505GTC (3818T>G 3820T>C), AAG506ATT (3822A>T 3823G>T), ATA507GTT (3824A>G 3826A>T), TTG508TAT (3828T>A 3829G>T), GAC510GAT (3835C>T), CAC511CAT (3838C>T), AAA512GCT (3839A>G 3840A>C 3841A>T), CCA513GCT (3842C>G 3844A>T), CTA514CTT (3847A>T), CAG515AGG (3848C>A 3849A>G), TGG516TAC (3852G>A 3853G>C), ATG517CTA (3854A>C 3856G>A), ATG518TTT (3857A>T 3859G>T), AAC519AGC (3861A>G), TTA520AAG (3863T>A 3864T>A 3865A>G), AAA521AAT (3868A>T), GAC522GAT (3871C>T), CCA523GCA (3872C>G), AAC524AAA (3877C>A), TCA525CCA (3878T>C), CGA526AGG (3881C>A 3883A>G), ATG527CTG (3884A>C), ACT528ATT (3888C>T), CGA531ATT (3896C>A 3897G>T 3898A>T), CTA532TTA (3899C>T), CGA533CTA (3903G>T), CTA534CTC (3907A>C), AGT535CAA (3908A>C 3909G>A 3910T>A), TAT537TTC (3915A>T 3916T>C), TTC539ATT (3920T>A 3922C>T), TCT540AAG (3923T>A 3924C>A 3925T>G), GTA541GTT (3928A>T), GTG542AAA (3929G>A 3930T>A 3931G>A), TAC543GAC (3932T>G), AAG544AGA (3936A>G 3937G>A), AAA545AAG (3940A>G), GGA546GGG (3943A>G), AAG547TGT (3944A>T 3945A>G 3946G>T), TCT548GAG (3947T>G 3948C>A 3949T>G), AAT549AAC (3952T>C), ACC550CAA (3953A>C 3954C>A 3955C>A), AAC551ATT (3957A>T 3958C>T), GAT553GAC (3964T>C), GCC554CAC (3965G>C 3966C>A), CTT555CTA (3970T>A), TCT556TCC (3973T>C), CGT557AGT (3974C>A)								

*: Inserts / Deletes / Misaligned / Frameshifts

Analysis details

This analysis was performed with panviral2.64

NGS Details (UN24): Cassava brown streak virus

Assembly

**Coverage Length**	525 (1 contig(s))
**Depth Of Coverage**	94.4
**Number Of Reads**	421
**Reads Per Million**	8.43 rpm (after QC)
**Ambiguities**	0
**Assembly Method**	de novo + reference guided assembly
**Consensus Caller**	Bcf Tools

Coverage Map

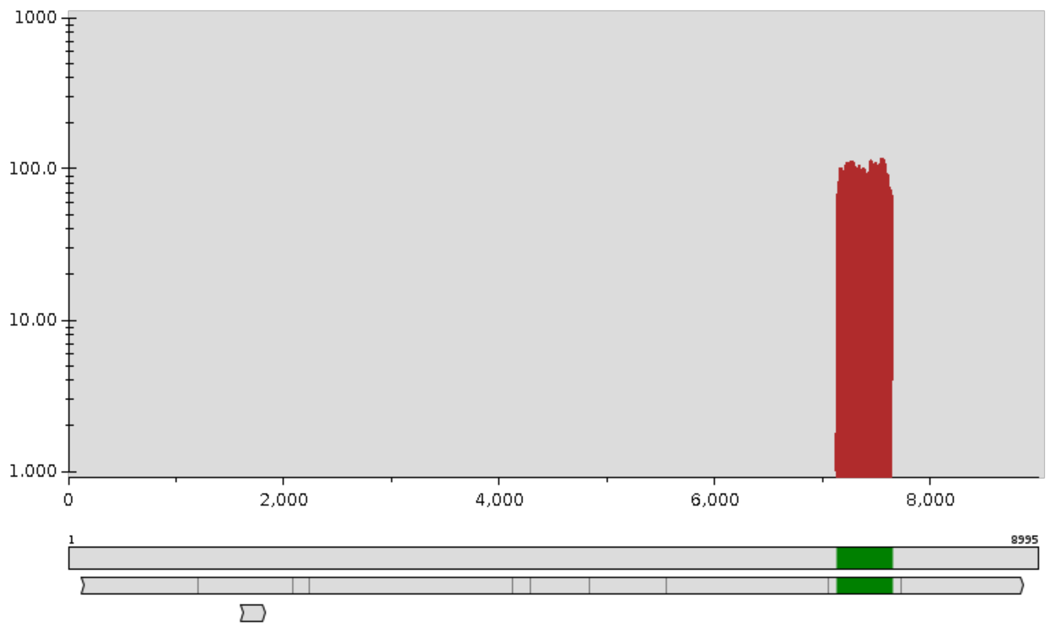

Assignment

**Type**	Cassava brown streak virus (Taxonomy ID: 137758)
**Reference Genome**	NC_012698.2
**NT Identity (%)**	60.7619
**AA Identity (%)**	57.7143
**Number Of Stop Codons**	0
**Number Of CDS**	2

Alignment

**Alignment Score**	226.0 (NT) + 736.0 (AA) = 962.0
**Concordance (%)**	42.4724

**Alignment Method**	Global, seeded, nucleotide + amino acids (AGA)

Genome Region

Sequence starts at position 7126 and ends at position 7650 relative to NC_012698.2 reference sequence.

Alignment Detailed Statistics

	Begin	End	Coverage	Score	Concordance	Matches	Identities	I/D/M/F*	Stop Codons
**NT**	**7126**	**7650**	**5.8%**	**226**	**21.5%**	**525 (100%)**	**319 (60.8%)**	**0/0**	
Mutations:	7130A>G, 7133A>G, 7134A>G, 7136T>G, 7142C>T, 7145A>T, 7148A>C, 7151G>A, 7157A>T, 7158C>A, 7163A>G, 7167A>G, 7175A>T, 7177A>G, 7178A>G, 7179C>G, 7180A>C, 7184A>T, 7185T>C, 7190T>C, 7192C>A, 7193C>G, 7194A>T, 7202A>C, 7203A>T, 7205C>T, 7206A>C, 7207T>A, 7208C>G, 7211T>C, 7213G>T, 7214G>T, 7217A>G, 7218G>C, 7229A>T, 7234C>T, 7235A>T, 7241A>G, 7242A>G, 7243C>A, 7244A>G, 7245G>C, 7246T>C, 7250G>A, 7253A>T, 7256T>A, 7257A>T, 7258T>C, 7270T>C, 7271A>T, 7272C>A, 7273G>A, 7274T>A, 7275G>A, 7277A>T, 7280G>T, 7281T>G, 7283T>C, 7284G>A, 7286G>A, 7287T>G, 7288T>A, 7290A>G, 7292T>G, 7293G>A, 7294G>A, 7295A>T, 7298G>A, 7304T>G, 7307C>A, 7310A>T, 7313A>G, 7319A>T, 7320A>T, 7323T>C, 7325G>C, 7331T>C, 7332G>A, 7334T>C, 7337T>C, 7340C>T, 7343T>G, 7355A>G, 7358A>T, 7361C>T, 7364T>C, 7374A>T, 7377G>C, 7380G>A, 7381G>A, 7385T>C, 7388A>T, 7390T>A, 7391A>T, 7397A>T, 7398T>C, 7400A>T, 7401T>A, 7403T>C, 7406G>C, 7409G>A, 7410G>T, 7413G>A, 7414A>T, 7416C>G, 7418G>T, 7422C>G, 7424A>G, 7425A>G, 7432T>C, 7433G>A, 7436T>A, 7437A>T, 7438G>A, 7442T>C, 7443C>A, 7445C>G, 7448C>T, 7449G>A, 7451G>C, 7455G>T, 7457T>A, 7458T>C, 7462T>C, 7463A>T, 7464A>C, 7465A>T, 7467A>G, 7468A>G, 7469A>G, 7470G>C, 7471T>C, 7472T>A, 7473G>A, 7474G>A, 7476G>A, 7477A>C, 7481T>G, 7484T>G, 7487A>G, 7489T>C, 7490C>T, 7494A>G, 7495A>T, 7496G>T, 7499T>A, 7500G>A, 7501T>A, 7502G>A, 7503C>A, 7504T>C, 7505A>G, 7506A>C, 7507G>T, 7508A>G, 7511T>A, 7512G>A, 7517T>A, 7520A>G, 7521A>C, 7522T>C, 7523G>A, 7524C>G, 7526A>T, 7527C>A, 7529C>G, 7535A>C, 7539T>A, 7540C>A, 7541A>T, 7544T>C, 7547G>A, 7553C>T, 7556A>G, 7559T>A, 7562C>T, 7568A>T, 7570T>A, 7572A>G, 7573A>G, 7576G>A, 7577G>T, 7578A>G, 7579G>A, 7580A>C, 7581A>C, 7586A>T, 7588T>A, 7589T>C, 7599A>C, 7600T>C, 7601G>C, 7602G>A, 7603C>A, 7604C>G, 7607G>A, 7610G>A, 7616T>C, 7618T>A, 7622A>T, 7628T>C, 7631A>C, 7632T>G, 7633T>G, 7635C>A, 7636G>A								

CDS

	Begin	End	Coverage	Score	Concordance	Matches	Identities	I/D/M/F*	Stop Codons
CBSV_gp1	2335	2509	6.0%	736	60.0%	175 (100%)	101 (57.7%)	0/0/0/0	0
Protein mutations:	I2337V (7134A>G 7136T>G), Q2345K (7158C>A), K2348E (7167A>G), K2351R (7177A>G 7178A>G), Q2352A (7179C>G 7180A>C), F2354L (7185T>C), P2356Q (7192C>A 7193C>G), T2357S (7194A>T), I2360F (7203A>T 7205C>T), I2361Q (7206A>C 7207T>A 7208C>G), R2363L (7213G>T 7214G>T), V2365L (7218G>C), P2370L (7234C>T 7235A>T), T2373E (7242A>G 7243C>A 7244A>G), V2374P (7245G>C 7246T>C), E2376D (7253A>T), I2378S (7257A>T 7258T>C), V2382A (7270T>C 7271A>T), R2383K (7272C>A 7273G>A 7274T>A), V2384I (7275G>A 7277A>T), S2386A (7281T>G 7283T>C), E2387K (7284G>A 7286G>A), L2388E (7287T>G 7288T>A), I2389V (7290A>G 7292T>G), G2390N (7293G>A 7294G>A 7295A>T), S2399C (7320A>T), D2403N (7332G>A 7334T>C), N2406K (7343T>G), M2417L (7374A>T), E2418Q (7377G>C), G2419K (7380G>A 7381G>A), L2422H (7390T>A 7391A>T), Y2426N (7401T>A 7403T>C), K2427N (7406G>C), V2429L (7410G>T), E2430M (7413G>A 7414A>T), P2431A (7416C>G 7418G>T), Q2433E (7422C>G 7424A>G), N2434D (7425A>G), M2436T (7432T>C 7433G>A), S2438Y (7437A>T 7438G>A), L2440M (7443C>A 7445C>G), V2442I (7449G>A 7451G>C), A2444S (7455G>T 7457T>A), F2445L (7458T>C), V2446A (7462T>C 7463A>T), N2447L (7464A>C 7465A>T), K2448G (7467A>G 7468A>G 7469A>G), V2449P (7470G>C 7471T>C 7472T>A), G2450N (7473G>A 7474G>A), D2451T (7476G>A 7477A>C), D2452E (7481T>G), I2454M (7487A>G), I2455T (7489T>C 7490C>T), K2457V (7494A>G 7495A>T 7496G>T), V2459K (7500G>A 7501T>A 7502G>A), L2460T (7503C>A 7504T>C 7505A>G), R2461L (7506A>C 7507G>T 7508A>G), E2463K (7512G>A), M2466P (7521A>C 7522T>C 7523G>A), P2467A (7524C>G 7526A>T), S2472N (7539T>A 7540C>A 7541A>T), L2482H (7570T>A), N2483G (7572A>G 7573A>G), W2484Y (7576G>A 7577G>T), R2485D (7578A>G 7579G>A 7580A>C), K2486Q (7581A>C), F2488Y (7588T>A 7589T>C), M2492P (7599A>C 7600T>C 7601G>C), A2493K (7602G>A 7603C>A 7604C>G), M2498K (7618T>A), F2503G (7632T>G 7633T>G), R2504K (7635C>A 7636G>A)								
Codon mutations:	GCA2335GCG (7130A>G), CCA2336CCG (7133A>G), ATT2337GTG (7134A>G 7136T>G), TTC2339TTT (7142C>T), GTA2340GTT (7145A>T), ACA2341ACC (7148A>C), GGG2342GGA (7151G>A), GCA2344GCT (7157A>T), CAA2345AAA (7158C>A), AAA2346AAG (7163A>G), AAG2348GAG (7167A>G), GTA2350GTT (7175A>T), AAA2351AGG (7177A>G 7178A>G), CAA2352GCA (7179C>G 7180A>C), ATA2353ATT (7184A>T), TTT2354CTT (7185T>C), GGT2355GGC (7190T>C), CCC2356CAG (7192C>A 7193C>G), ACT2357TCT (7194A>T), CCA2359CCC (7202A>C), ATC2360TTT (7203A>T 7205C>T), ATC2361CAG (7206A>C 7207T>A 7208C>G), TCT2362TCC (7211T>C), CGG2363CTT (7213G>T 7214G>T), AAA2364AAG (7217A>G), GTT2365CTT (7218G>C), CCA2368CCT (7229A>T), CCA2370CTT (7234C>T 7235A>T), GGA2372GGG (7241A>G), ACA2373GAG (7242A>G 7243C>A 7244A>G), GTT2374CCT (7245G>C 7246T>C), GAG2375GAA (7250G>A), GAA2376GAT (7253A>T), ATT2377ATA (7256T>A), ATC2378TCC (7257A>T 7258T>C), GTA2382GCT (7270T>C 7271A>T), CGT2383AAA (7272C>A 7273G>A 7274T>A), GTA2384ATT (7275G>A 7277A>T), GCG2385GCT (7280G>T), TCT2386GCC (7281T>G 7283T>C), GAG2387AAA (7284G>A 7286G>A), TTG2388GAG (7287T>G 7288T>A), ATT2389GTG (7290A>G 7292T>G), GGA2390AAT (7293G>A 7294G>A 7295A>T), GGG2391GGA (7298G>A), GTT2393GTG (7304T>G), CTC2394CTA (7307C>A), GTA2395GTT (7310A>T), GAA2396GAG (7313A>G), ACA2398ACT (7319A>T), AGT2399TGT (7320A>T), TTG2400CTC (7323T>C 7325G>C), TTT2402TTC (7331T>C), GAT2403AAC (7332G>A 7334T>C), GCT2404GCC (7337T>C), CTC2405CTT (7340C>T), AAT2406AAG (7343T>G), GGA2410GGG (7355A>G), CCA2411CCT (7358A>T), TAC2412TAT (7361C>T), ATT2413ATC (7364T>C), ATG2417TTG (7374A>T), GAA2418CAA (7377G>C), GGG2419AAG (7380G>A 7381G>A), ATT2420ATC (7385T>C), GGA2421GGT (7388A>T), CTA2422CAT (7390T>A 7391A>T), GGA2424GGT (7397A>T), TTA2425CTT (7398T>C 7400A>T), TAT2426AAC (7401T>A 7403T>C), AAG2427AAC (7406G>C), TTG2428TTA (7409G>A), GTG2429TTG (7410G>T), GAG2430ATG (7413G>A 7414A>T), CCG2431GCT (7416C>G 7418G>T), CAA2433GAG (7422C>G 7424A>G), AAT2434GAT (7425A>G), ATG2436ACA (7432T>C 7433G>A), GCT2437GCA (7436T>A), AGT2438TAT (7437A>T 7438G>A), GCT2439GCC (7442T>C), CTC2440ATG (7443C>A 7445C>G), TGC2441TGT (7448C>T), GTG2442ATC (7449G>A 7451G>C), GCT2444TCA (7455G>T 7457T>A), TTT2445CTT (7458T>C), GTA2446GCT (7462T>C 7463A>T), AAT2447CTT (7464A>C 7465A>T), AAA2448GGG (7467A>G 7468A>G 7469A>G), GTT2449CCA (7470G>C 7471T>C 7472T>A), GGT2450AAT (7473G>A 7474G>A), GAT2451ACT (7476G>A 7477A>C), GAT2452GAG (7481T>G), CCT2453CCG (7484T>G), ATA2454ATG (7487A>G), ATC2455ACT (7489T>C 7490C>T), AAG2457GTT (7494A>G 7495A>T 7496G>T), GGT2458GGA (7499T>A), GTG2459AAA (7500G>A 7501T>A 7502G>A), CTA2460ACG (7503C>A 7504T>C 7505A>G), AGA2461CTG (7506A>C 7507G>T 7508A>G), GGT2462GGA (7511T>A), GAG2463AAG (7512G>A), ATT2464ATA (7517T>A), GTA2465GTG (7520A>G), ATG2466CCA (7521A>C 7522T>C 7523G>A), CCA2467GCT (7524C>G 7526A>T), CGC2468AGG (7527C>A 7529C>G), CCA2470CCC (7535A>C), TCA2472AAT (7539T>A 7540C>A 7541A>T), TTT2473TTC (7544T>C), GGG2474GGA (7547G>A), GAC2476GAT (7553C>T), CCA2477CCG (7556A>G), ATT2478ATA (7559T>A), TTC2479TTT (7562C>T), CCA2481CCT (7568A>T), CTT2482CAT (7570T>A), AAC2483GGC (7572A>G 7573A>G), TGG2484TAT (7576G>A 7577G>T), AGA2485GAC (7578A>G 7579G>A 7580A>C), AAG2486CAG (7581A>C), ACA2487ACT (7586A>T), TTT2488TAC (7588T>A 7589T>C), ATG2492CCC (7599A>C 7600T>C 7601G>C), GCC2493AAG (7602G>A 7603C>A 7604C>G), GAG2494GAA (7607G>A), GAG2495GAA (7610G>A), AAT2497AAC (7616T>C), ATG2498AAG (7618T>A), ATA2499ATT (7622A>T), CAT2501CAC (7628T>C), CGA2502CGC (7631A>C), TTT2503GGT (7632T>G 7633T>G), CGA2504AAA (7635C>A 7636G>A)								

Proteins

	Begin	End	Coverage	Score	Concordance	Matches	Identities	I/D/M/F*	Stop Codons
polyprotein (YP_007027011.1)	2335	2509	6.0%	736	60.0%	175 (100%)	101 (57.7%)	0/0/0/0	0
Protein mutations:	I2337V (7134A>G 7136T>G), Q2345K (7158C>A), K2348E (7167A>G), K2351R (7177A>G 7178A>G), Q2352A (7179C>G 7180A>C), F2354L (7185T>C), P2356Q (7192C>A 7193C>G), T2357S (7194A>T), I2360F (7203A>T 7205C>T), I2361Q (7206A>C 7207T>A 7208C>G), R2363L (7213G>T 7214G>T), V2365L (7218G>C), P2370L (7234C>T 7235A>T), T2373E (7242A>G 7243C>A 7244A>G), V2374P (7245G>C 7246T>C), E2376D (7253A>T), I2378S (7257A>T 7258T>C), V2382A (7270T>C 7271A>T), R2383K (7272C>A 7273G>A 7274T>A), V2384I (7275G>A 7277A>T), S2386A (7281T>G 7283T>C), E2387K (7284G>A 7286G>A), L2388E (7287T>G 7288T>A), I2389V (7290A>G 7292T>G), G2390N (7293G>A 7294G>A 7295A>T), S2399C (7320A>T), D2403N (7332G>A 7334T>C), N2406K (7343T>G), M2417L (7374A>T), E2418Q (7377G>C), G2419K (7380G>A 7381G>A), L2422H (7390T>A 7391A>T), Y2426N (7401T>A 7403T>C), K2427N (7406G>C), V2429L (7410G>T), E2430M (7413G>A 7414A>T), P2431A (7416C>G 7418G>T), Q2433E (7422C>G 7424A>G), N2434D (7425A>G), M2436T (7432T>C 7433G>A), S2438Y (7437A>T 7438G>A), L2440M (7443C>A 7445C>G), V2442I (7449G>A 7451G>C), A2444S (7455G>T 7457T>A), F2445L (7458T>C), V2446A (7462T>C 7463A>T), N2447L (7464A>C 7465A>T), K2448G (7467A>G 7468A>G 7469A>G), V2449P (7470G>C 7471T>C 7472T>A), G2450N (7473G>A 7474G>A), D2451T (7476G>A 7477A>C), D2452E (7481T>G), I2454M (7487A>G), I2455T (7489T>C 7490C>T), K2457V (7494A>G 7495A>T 7496G>T), V2459K (7500G>A 7501T>A 7502G>A), L2460T (7503C>A 7504T>C 7505A>G), R2461L (7506A>C 7507G>T 7508A>G), E2463K (7512G>A), M2466P (7521A>C 7522T>C 7523G>A), P2467A (7524C>G 7526A>T), S2472N (7539T>A 7540C>A 7541A>T), L2482H (7570T>A), N2483G (7572A>G 7573A>G), W2484Y (7576G>A 7577G>T), R2485D (7578A>G 7579G>A 7580A>C), K2486Q (7581A>C), F2488Y (7588T>A 7589T>C), M2492P (7599A>C 7600T>C 7601G>C), A2493K (7602G>A 7603C>A 7604C>G), M2498K (7618T>A), F2503G (7632T>G 7633T>G), R2504K (7635C>A 7636G>A)								

	Begin	End	Coverage	Score	Concordance	Matches	Identities	I/D/M/F*	Stop Codons
**NT**	**7126**	**7650**	**5.8%**	**226**	**21.5%**	**525 (100%)**	**319 (60.8%)**	**0/0**	
Codon mutations:	GCA2335GCG (7130A>G), CCA2336CCG (7133A>G), ATT2337GTG (7134A>G 7136T>G), TTC2339TTT (7142C>T), GTA2340GTT (7145A>T), ACA2341ACC (7148A>C), GGG2342GGA (7151G>A), GCA2344GCT (7157A>T), CAA2345AAA (7158C>A), AAA2346AAG (7163A>G), AAG2348GAG (7167A>G), GTA2350GTT (7175A>T), AAA2351AGG (7177A>G 7178A>G), CAA2352GCA (7179C>G 7180A>C), ATA2353ATT (7184A>T), TTT2354CTT (7185T>C), GGT2355GGC (7190T>C), CCC2356CAG (7192C>A 7193C>G), ACT2357TCT (7194A>T), CCA2359CCC (7202A>C), ATC2360TTT (7203A>T 7205C>T), ATC2361CAG (7206A>C 7207T>A 7208C>G), TCT2362TCC (7211T>C), CGG2363CTT (7213G>T 7214G>T), AAA2364AAG (7217A>G), GTT2365CTT (7218G>C), CCA2368CCT (7229A>T), CCA2370CTT (7234C>T 7235A>T), GGA2372GGG (7241A>G), ACA2373GAG (7242A>G 7243C>A 7244A>G), GTT2374CCT (7245G>C 7246T>C), GAG2375GAA (7250G>A), GAA2376GAT (7253A>T), ATT2377ATA (7256T>A), ATC2378TCC (7257A>T 7258T>C), GTA2382GCT (7270T>C 7271A>T), CGT2383AAA (7272C>A 7273G>A 7274T>A), GTA2384ATT (7275G>A 7277A>T), GCG2385GCT (7280G>T), TCT2386GCC (7281T>G 7283T>C), GAG2387AAA (7284G>A 7286G>A), TTG2388GAG (7287T>G 7288T>A), ATT2389GTG (7290A>G 7292T>G), GGA2390AAT (7293G>A 7294G>A 7295A>T), GGG2391GGA (7298G>A), GTT2393GTG (7304T>G), CTC2394CTA (7307C>A), GTA2395GTT (7310A>T), GAA2396GAG (7313A>G), ACA2398ACT (7319A>T), AGT2399TGT (7320A>T), TTG2400CTC (7323T>C 7325G>C), TTT2402TTC (7331T>C), GAT2403AAC (7332G>A 7334T>C), GCT2404GCC (7337T>C), CTC2405CTT (7340C>T), AAT2406AAG (7343T>G), GGA2410GGG (7355A>G), CCA2411CCT (7358A>T), TAC2412TAT (7361C>T), ATT2413ATC (7364T>C), ATG2417TTG (7374A>T), GAA2418CAA (7377G>C), GGG2419AAG (7380G>A 7381G>A), ATT2420ATC (7385T>C), GGA2421GGT (7388A>T), CTA2422CAT (7390T>A 7391A>T), GGA2424GGT (7397A>T), TTA2425CTT (7398T>C 7400A>T), TAT2426AAC (7401T>A 7403T>C), AAG2427AAC (7406G>C), TTG2428TTA (7409G>A), GTG2429TTG (7410G>T), GAG2430ATG (7413G>A 7414A>T), CCG2431GCT (7416C>G 7418G>T), CAA2433GAG (7422C>G 7424A>G), AAT2434GAT (7425A>G), ATG2436ACA (7432T>C 7433G>A), GCT2437GCA (7436T>A), AGT2438TAT (7437A>T 7438G>A), GCT2439GCC (7442T>C), CTC2440ATG (7443C>A 7445C>G), TGC2441TGT (7448C>T), GTG2442ATC (7449G>A 7451G>C), GCT2444TCA (7455G>T 7457T>A), TTT2445CTT (7458T>C), GTA2446GCT (7462T>C 7463A>T), AAT2447CTT (7464A>C 7465A>T), AAA2448GGG (7467A>G 7468A>G 7469A>G), GTT2449CCA (7470G>C 7471T>C 7472T>A), GGT2450AAT (7473G>A 7474G>A), GAT2451ACT (7476G>A 7477A>C), GAT2452GAG (7481T>G), CCT2453CCG (7484T>G), ATA2454ATG (7487A>G), ATC2455ACT (7489T>C 7490C>T), AAG2457GTT (7494A>G 7495A>T 7496G>T), GGT2458GGA (7499T>A), GTG2459AAA (7500G>A 7501T>A 7502G>A), CTA2460ACG (7503C>A 7504T>C 7505A>G), AGA2461CTG (7506A>C 7507G>T 7508A>G), GGT2462GGA (7511T>A), GAG2463AAG (7512G>A), ATT2464ATA (7517T>A), GTA2465GTG (7520A>G), ATG2466CCA (7521A>C 7522T>C 7523G>A), CCA2467GCT (7524C>G 7526A>T), CGC2468AGG (7527C>A 7529C>G), CCA2470CCC (7535A>C), TCA2472AAT (7539T>A 7540C>A 7541A>T), TTT2473TTC (7544T>C), GGG2474GGA (7547G>A), GAC2476GAT (7553C>T), CCA2477CCG (7556A>G), ATT2478ATA (7559T>A), TTC2479TTT (7562C>T), CCA2481CCT (7568A>T), CTT2482CAT (7570T>A), AAC2483GGC (7572A>G 7573A>G), TGG2484TAT (7576G>A 7577G>T), AGA2485GAC (7578A>G 7579G>A 7580A>C), AAG2486CAG (7581A>C), ACA2487ACT (7586A>T), TTT2488TAC (7588T>A 7589T>C), ATG2492CCC (7599A>C 7600T>C 7601G>C), GCC2493AAG (7602G>A 7603C>A 7604C>G), GAG2494GAA (7607G>A), GAG2495GAA (7610G>A), AAT2497AAC (7616T>C), ATG2498AAG (7618T>A), ATA2499ATT (7622A>T), CAT2501CAC (7628T>C), CGA2502CGC (7631A>C), TTT2503GGT (7632T>G 7633T>G), CGA2504AAA (7635C>A 7636G>A)								
HAM1-like protein (YP_007032446.1)	27	201	77.4%	736	60.0%	175 (100%)	101 (57.7%)	0/0/0/0	0
Protein mutations:	I29V (7134A>G 7136T>G), Q37K (7158C>A), K40E (7167A>G), K43R (7177A>G 7178A>G), Q44A (7179C>G 7180A>C), F46L (7185T>C), P48Q (7192C>A 7193C>G), T49S (7194A>T), I52F (7203A>T 7205C>T), I53Q (7206A>C 7207T>A 7208C>G), R55L (7213G>T 7214G>T), V57L (7218G>C), P62L (7234C>T 7235A>T), T65E (7242A>G 7243C>A 7244A>G), V66P (7245G>C 7246T>C), E68D (7253A>T), I70S (7257A>T 7258T>C), V74A (7270T>C 7271A>T), R75K (7272C>A 7273G>A 7274T>A), V76I (7275G>A 7277A>T), S78A (7281T>G 7283T>C), E79K (7284G>A 7286G>A), L80E (7287T>G 7288T>A), I81V (7290A>G 7292T>G), G82N (7293G>A 7294G>A 7295A>T), S91C (7320A>T), D95N (7332G>A 7334T>C), N98K (7343T>G), M109L (7374A>T), E110Q (7377G>C), G111K (7380G>A 7381G>A), L114H (7390T>A 7391A>T), Y118N (7401T>A 7403T>C), K119N (7406G>C), V121L (7410G>T), E122M (7413G>A 7414A>T), P123A (7416C>G 7418G>T), Q125E (7422C>G 7424A>G), N126D (7425A>G), M128T (7432T>C 7433G>A), S130Y (7437A>T 7438G>A), L132M (7443C>A 7445C>G), V134I (7449G>A 7451G>C), A136S (7455G>T 7457T>A), F137L (7458T>C), V138A (7462T>C 7463A>T), N139L (7464A>C 7465A>T), K140G (7467A>G 7468A>G 7469A>G), V141P (7470G>C 7471T>C 7472T>A), G142N (7473G>A 7474G>A), D143T (7476G>A 7477A>C), D144E (7481T>G), I146M (7487A>G), I147T (7489T>C 7490C>T), K149V (7494A>G 7495A>T 7496G>T), V151K (7500G>A 7501T>A 7502G>A), L152T (7503C>A 7504T>C 7505A>G), R153L (7506A>C 7507G>T 7508A>G), E155K (7512G>A), M158P (7521A>C 7522T>C 7523G>A), P159A (7524C>G 7526A>T), S164N (7539T>A 7540C>A 7541A>T), L174H (7570T>A), N175G (7572A>G 7573A>G), W176Y (7576G>A 7577G>T), R177D (7578A>G 7579G>A 7580A>C), K178Q (7581A>C), F180Y (7588T>A 7589T>C), M184P (7599A>C 7600T>C 7601G>C), A185K (7602G>A 7603C>A 7604C>G), M190K (7618T>A), F195G (7632T>G 7633T>G), R196K (7635C>A 7636G>A)								
Codon mutations:	GCA27GCG (7130A>G), CCA28CCG (7133A>G), ATT29GTG (7134A>G 7136T>G), TTC31TTT (7142C>T), GTA32GTT (7145A>T), ACA33ACC (7148A>C), GGG34GGA (7151G>A), GCA36GCT (7157A>T), CAA37AAA (7158C>A), AAA38AAG (7163A>G), AAG40GAG (7167A>G), GTA42GTT (7175A>T), AAA43AGG (7177A>G 7178A>G), CAA44GCA (7179C>G 7180A>C), ATA45ATT (7184A>T), TTT46CTT (7185T>C), GGT47GGC (7190T>C), CCC48CAG (7192C>A 7193C>G), ACT49TCT (7194A>T), CCA51CCC (7202A>C), ATC52TTT (7203A>T 7205C>T), ATC53CAG (7206A>C 7207T>A 7208C>G), TCT54TCC (7211T>C), CGG55CTT (7213G>T 7214G>T), AAA56AAG (7217A>G), GTT57CTT (7218G>C), CCA60CCT (7229A>T), CCA62CTT (7234C>T 7235A>T), GGA64GGG (7241A>G), ACA65GAG (7242A>G 7243C>A 7244A>G), GTT66CCT (7245G>C 7246T>C), GAG67GAA (7250G>A), GAA68GAT (7253A>T), ATT69ATA (7256T>A), ATC70TCC (7257A>T 7258T>C), GTA74GCT (7270T>C 7271A>T), CGT75AAA (7272C>A 7273G>A 7274T>A), GTA76ATT (7275G>A 7277A>T), GCG77GCT (7280G>T), TCT78GCC (7281T>G 7283T>C), GAG79AAA (7284G>A 7286G>A), TTG80GAG (7287T>G 7288T>A), ATT81GTG (7290A>G 7292T>G), GGA82AAT (7293G>A 7294G>A 7295A>T), GGG83GGA (7298G>A), GTT85GTG (7304T>G), CTC86CTA (7307C>A), GTA87GTT (7310A>T), GAA88GAG (7313A>G), ACA90ACT (7319A>T), AGT91TGT (7320A>T), TTG92CTC (7323T>C 7325G>C), TTT94TTC (7331T>C), GAT95AAC (7332G>A 7334T>C), GCT96GCC (7337T>C), CTC97CTT (7340C>T), AAT98AAG (7343T>G), GGA102GGG (7355A>G), CCA103CCT (7358A>T), TAC104TAT (7361C>T), ATT105ATC (7364T>C), ATG109TTG (7374A>T), GAA110CAA (7377G>C), GGG111AAG (7380G>A 7381G>A), ATT112ATC (7385T>C), GGA113GGT (7388A>T), CTA114CAT (7390T>A 7391A>T), GGA116GGT (7397A>T), TTA117CTT (7398T>C 7400A>T), TAT118AAC (7401T>A 7403T>C), AAG119AAC (7406G>C), TTG120TTA (7409G>A), GTG121TTG (7410G>T), GAG122ATG (7413G>A 7414A>T), CCG123GCT (7416C>G 7418G>T), CAA125GAG (7422C>G 7424A>G), AAT126GAT (7425A>G), ATG128ACA (7432T>C 7433G>A), GCT129GCA (7436T>A), AGT130TAT (7437A>T 7438G>A), GCT131GCC (7442T>C), CTC132ATG (7443C>A 7445C>G), TGC133TGT (7448C>T), GTG134ATC (7449G>A 7451G>C), GCT136TCA (7455G>T 7457T>A), TTT137CTT (7458T>C), GTA138GCT (7462T>C 7463A>T), AAT139CTT (7464A>C 7465A>T), AAA140GGG (7467A>G 7468A>G 7469A>G), GTT141CCA (7470G>C 7471T>C 7472T>A), GGT142AAT (7473G>A 7474G>A), GAT143ACT (7476G>A 7477A>C), GAT144GAG (7481T>G), CCT145CCG (7484T>G), ATA146ATG (7487A>G), ATC147ACT (7489T>C 7490C>T), AAG149GTT (7494A>G 7495A>T 7496G>T), GGT150GGA (7499T>A), GTG151AAA (7500G>A 7501T>A 7502G>A), CTA152ACG (7503C>A 7504T>C 7505A>G), AGA153CTG (7506A>C 7507G>T 7508A>G), GGT154GGA (7511T>A), GAG155AAG (7512G>A), ATT156ATA (7517T>A), GTA157GTG (7520A>G), ATG158CCA (7521A>C 7522T>C 7523G>A), CCA159GCT (7524C>G 7526A>T), CGC160AGG (7527C>A 7529C>G), CCA162CCC (7535A>C), TCA164AAT (7539T>A 7540C>A 7541A>T), TTT165TTC (7544T>C), GGG166GGA (7547G>A), GAC168GAT (7553C>T), CCA169CCG (7556A>G), ATT170ATA (7559T>A), TTC171TTT (7562C>T), CCA173CCT (7568A>T), CTT174CAT (7570T>A), AAC175GGC (7572A>G 7573A>G), TGG176TAT (7576G>A 7577G>T), AGA177GAC (7578A>G 7579G>A 7580A>C), AAG178CAG (7581A>C), ACA179ACT (7586A>T), TTT180TAC (7588T>A 7589T>C), ATG184CCC (7599A>C 7600T>C 7601G>C), GCC185AAG (7602G>A 7603C>A 7604C>G), GAG186GAA (7607G>A), GAG187GAA (7610G>A), AAT189AAC (7616T>C), ATG190AAG (7618T>A), ATA191ATT (7622A>T), CAT193CAC (7628T>C), CGA194CGC (7631A>C), TTT195GGT (7632T>G 7633T>G), CGA196AAA (7635C>A 7636G>A)								

*: Inserts / Deletes / Misaligned / Frameshifts

Analysis details

This analysis was performed with panviral2.64

NGS Details (UN24): Nodensvirus spm2

Assembly

**Coverage Length**	169 (1 contig(s))
**Depth Of Coverage**	194.4
**Number Of Reads**	374
**Reads Per Million**	7.49 rpm (after QC)
**Ambiguities**	0
**Assembly Method**	de novo + reference guided assembly
**Consensus Caller**	Bcf Tools

Coverage Map

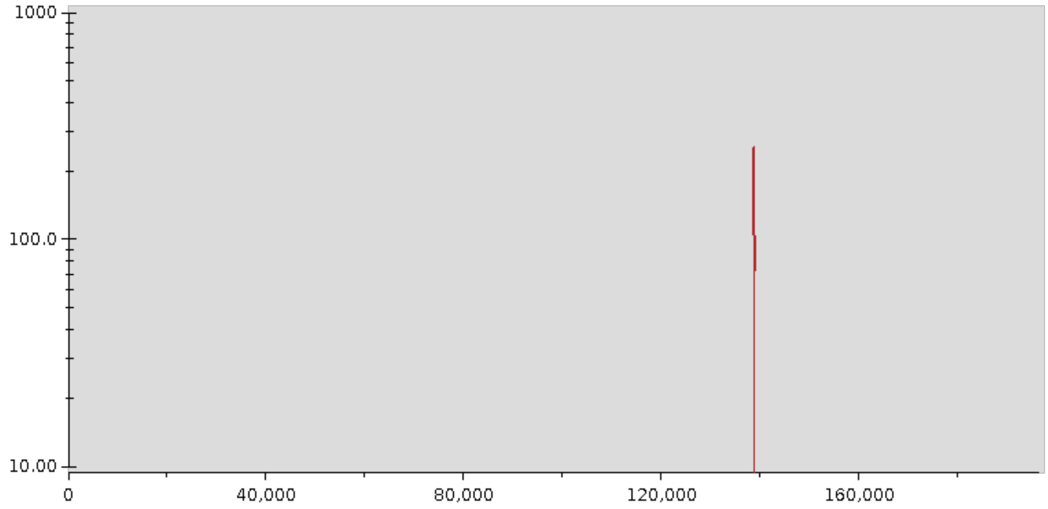

Assignment

**Type**	Nodensvirus spm2 (Taxonomy ID: 2734126)
**Reference Genome**	NC_006820.1
**NT Identity (%)**	81.4371
**AA Identity (%)**	86.7925
**Number Of Stop Codons**	1
**Number Of CDS**	243

Alignment

**Alignment Score**	199.0 (NT) + 223.0 (AA) = 422.0
**Concordance (%)**	73.9635
**Alignment Method**	Local, heuristic, nucleotide (BLASTN)

Genome Region

Sequence starts at position 138722 and ends at position 138890 relative to NC_006820.1 reference sequence.

Alignment Detailed Statistics

	Begin	End	Coverage	Score	Concordance	Matches	Identities	I/D/M/F*	Stop Codons
**NT**	**138722**	**138890**	**0.1%**	**199**	**61.6%**	**167 (98.8%)**	**136 (80.5%)**	**0/2**	

Mutations: 138734T>C, 138740A>G, 138755C>T, 138756G>A, 138761C>T, 138773G>A, 138776C>T, 138778T>C, 138783_138784delCT, 138785G>A, 138788T>C, 138795C>A, 138797G>T, 138803C>T, 138812T>G, 138813C>G, 138816G>C, 138817T>A, 138824A>G, 138830C>T, 138836C>T, 138840G>A, 138842T>A, 138851A>G, 138854A>G, 138857A>T, 138858T>C, 138860G>A, 138864A>C, 138866A>T, 138869C>A, 138878G>T

*: Inserts / Deletes / Misaligned / Frameshifts

Analysis details

This analysis was performed with panviral2.64

NGS Details (UN24): Rahariannevirus raharianne

Assembly

**Coverage Length**	4366 (11 contig(s))
**Depth Of Coverage**	5.0
**Number Of Reads**	176
**Reads Per Million**	3.52 rpm (after QC)
**Ambiguities**	0
**Assembly Method**	de novo + reference guided assembly
**Consensus Caller**	Bcf Tools

Coverage Map

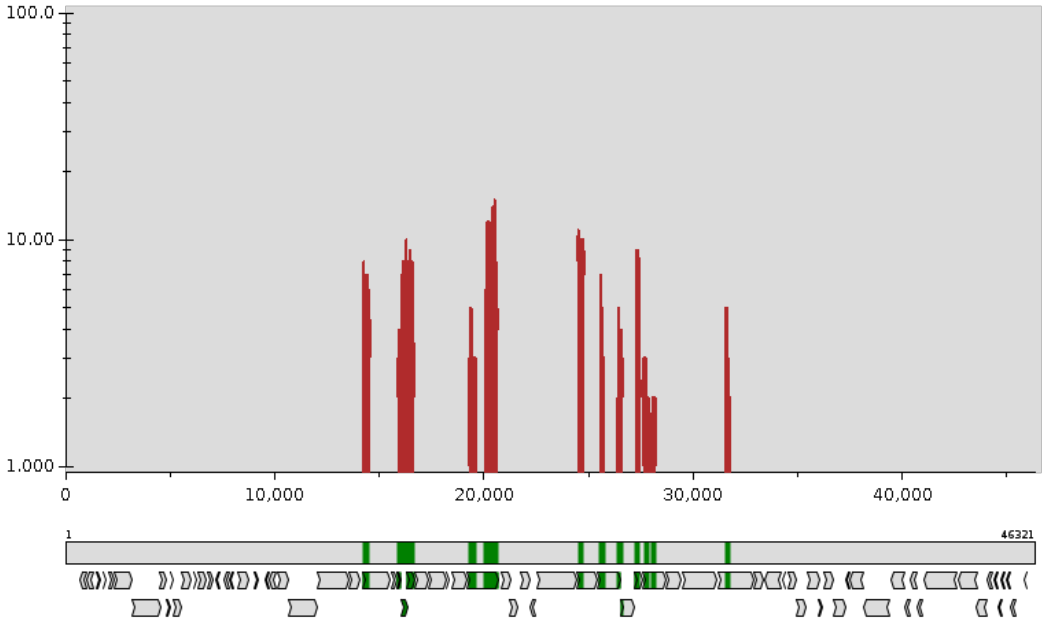

Assignment

**Type**	Rahariannevirus raharianne (Taxonomy ID: 2846050)
**Reference Genome**	NC_054955.1
**NT Identity (%)**	70.758
**AA Identity (%)**	68.0603
**Number Of Stop Codons**	3
**Number Of CDS**	75

Alignment

**Alignment Score**	3582.0 (NT) + 6867.0 (AA) = 10449.0
**Concordance (%)**	57.8415

Alignment Method	Local, heuristic, nucleotide (BLASTN)

Genome Region

Sequence starts at position 14196 and ends at position 31794 relative to NC_054955.1 reference sequence.

Alignment Detailed Statistics

	Begin	End	Coverage	Score	Concordance	Matches	Identities	I/D/M/F*	Stop Codons
**NT**	**14196**	**31794**	**9.4%**	**3582**	**41.3%**	**4358 (99.6%)**	**3090 (70.6%)**	**9/8**	
Mutations:	14204C>T, 14205A>C, 14206G>A, 14207C>A, 14210T>C, 14219C>T, 14222T>C, 14231G>C, 14264T>C, 14270T>A, 14272C>G, 14285C>G, 14288C>G, 14289G>A, 14293T>A, 14294C>T, 14298C>T, 14299A>C, 14306A>C, 14309T>C, 14317G>A, 14318T>G, 14336T>C, 14339A>G, 14342C>A, 14357T>C, 14363T>C, 14366G>C, 14372G>T, 14375G>C, 14378C>G, 14381C>G, 14394C>G, 14397T>G, 14399G>C, 14400G>A, 14401C>G, 14402A>C, 14427T>A, 14432G>C, 14433T>A, 14434C>G, 14435G>C, 14447G>C, 14457T>A, 14462C>G, 14486G>C, 14500C>T, 14504G>C, 14516C>G, 14525G>C, 15865C>G, 15868T>C, 15870C>T, 15871C>G, 15872A>G, 15875C>G, 15878T>C, 15883C>G, 15884G>C, 15885A>G, 15886G>A, 15887T>G, 15888C>A, 15890A>T, 15895G>C, 15897G>T, 15898G>C, 15900T>G, 15901G>C, 15903A>T, 15904A>C, 15911A>C, 15913C>G, 15919A>C, 15920C>G, 15921A>T, 15924T>G, 15925C>G, 15926C>G, 15927A>C, 15930A>C, 15931A>T, 15934T>C, 15936C>A, 15939A>C, 15941G>A, 15947A>G, 15949C>A, 15950C>G, 15951C>T, 15952T>G, 15953G>C, 15954T>G, 15959G>A, 15961G>C, 15962G>C, 15963C>G, 15966C>A, 15974C>G, 15975A>C, 15976G>C, 15977C>A, 15981G>A, 15987G>C, 15988T>G, 15988_15989insATCAAGGCC, 15992T>C, 15993T>G, 15998G>A, 16001delA, 16002G>T, 16003C>G, 16014C>T, 16018G>A, 16025T>C, 16026T>C, 16035C>G, 16036A>C, 16037A>G, 16038A>C, 16040G>A, 16045A>G, 16047C>G, 16053T>C, 16057A>G, 16059G>T, 16061C>G, 16062C>G, 16065G>C, 16066C>A, 16067A>C, 16071G>C, 16074T>C, 16078T>A, 16086C>A, 16087A>G, 16088A>C, 16089G>C, 16091A>G, 16095T>C, 16099A>C, 16101C>G, 16113A>C, 16126A>C, 16128T>G, 16131T>G, 16134C>G, 16137T>G, 16140G>C, 16143G>C, 16146T>C, 16147G>A, 16149G>T, 16150G>C, 16151G>C, 16158C>G, 16161G>C, 16164C>T, 16170G>A, 16176G>C, 16177A>G, 16178C>T, 16179G>C, 16182C>T, 16183A>G, 16184C>G, 16190C>T, 16191T>C, 16198A>G, 16199A>T, 16204A>G, 16208G>C, 16209C>G, 16212G>C, 16218G>C, 16227G>C, 16231T>A, 16233A>C, 16239C>G, 16245T>C, 16251G>C, 16255C>T, 16258A>G, 16259A>C, 16260C>A, 16263C>G, 16270A>G, 16278T>C, 16282G>A, 16284T>C, 16293C>G, 16296T>C, 16297T>G, 16302T>C, 16305T>C, 16317C>T, 16319G>C, 16321G>C, 16323delG, 16335A>C, 16338C>G, 16339G>C, 16340C>A, 16341C>G, 16345A>C, 16346A>G, 16365T>C, 16368C>T, 16369G>T, 16371A>G, 16378A>G, 16380G>C, 16382T>A, 16383C>T, 16389G>C, 16395A>C, 16396T>A, 16397C>G, 16398G>T, 16399T>G, 16413T>C, 16414C>A, 16428G>C, 16431C>T, 16440T>G, 16443A>G, 16450A>C, 16453A>T, 16454A>C, 16455C>G, 16459A>C, 16464G>C, 16465G>C, 16467C>G, 16473A>G, 16478C>G, 16479T>C, 16483G>A, 16485G>C, 16494G>C, 16497T>C, 16500G>C, 16503T>C, 16512C>G, 16515T>G, 16518C>G, 16524G>C, 16527A>C, 16534T>G, 16535C>G, 16536T>C, 16537G>A, 16540A>T, 16541G>C, 16542C>G, 16545G>A, 16551C>T, 16557A>G, 16561C>A, 16566G>C, 16569C>G, 16576G>A, 16577T>C, 16578G>C, 16593A>G, 16594G>C, 16599A>C, 16602C>T, 16605C>T, 16608C>T, 16617T>C, 16618G>A, 16619C>G, 16629G>C, 16631G>T, 16632C>G, 16642G>C, 16648A>C, 16650G>C, 16654A>G, 16656T>G, 16662C>G, 16665G>C, 16668C>G, 16670G>C, 16671G>C, 16672C>T, 19263T>C, 19271A>G, 19274A>G, 19280G>C, 19283T>G, 19286A>G, 19290G>A, 19291T>C, 19292T>C, 19293T>C, 19294C>A, 19295C>G, 19296C>T, 19297T>A, 19298G>C, 19301C>G, 19319T>C, 19320G>A, 19322G>C, 19323C>T, 19325T>G, 19328T>C, 19329G>A, 19331T>C, 19334G>C, 19340C>G, 19343A>G, 19344A>T, 19352C>G, 19358C>G, 19362T>A, 19363C>G, 19364G>C, 19367G>C, 19370G>C, 19371C>A, 19372T>C, 19374A>G, 19381A>G, 19383A>C, 19384T>C, 19388G>T, 19389A>C, 19390T>A, 19392T>G, 19393A>C, 19397C>G, 19398C>G, 19399A>C, 19400G>C, 19401G>A, 19402C>T, 19410G>C, 19411C>A, 19413A>G, 19414T>C, 19424T>C, 19426C>A, 19427C>G, 19430T>C, 19434A>C, 19437G>A, 19438G>T, 19439C>G, 19445T>C, 19446G>A, 19448G>C, 19449G>T, 19452A>G, 19457A>G, 19458G>A, 19459T>C, 19460G>C, 19461C>A, 19462A>T, 19472G>C, 19482C>A, 19483G>A, 19484C>A, 19485T>G, 19486G>C, 19493C>G, 19496C>G, 19512A>G, 19513C>T, 19520G>C, 19521T>A, 19522C>G, 19523G>C, 19525C>T, 19526G>C, 19527A>G, 19528T>C, 19529C>G, 19534A>T, 19542G>T, 19545G>A, 19547G>C, 19550G>C, 19551C>T, 19553G>C, 19557G>T, 19559G>C, 19574G>C, 19575C>T, 19577G>C, 19584T>G, 19585C>G, 19586G>C, 19587G>A, 19589G>C, 19595C>G, 19596G>A, 19601C>G, 19602G>T, 19604C>G, 19607G>C, 19611A>T, 19612G>C, 19613C>G, 19614G>C, 19615C>A, 19616C>G, 19620A>T, 19621A>C, 19622T>G, 19626T>C, 19627G>A, 19633T>C, 19634A>C, 19635A>G, 19636C>T, 19640C>T, 19641G>T, 20005T>C, 20006C>G, 20013A>G, 20016G>A, 20017G>T, 20018C>G, 20019G>A, 20020T>C, 20028G>A, 20029C>G, 20039G>C, 20043A>G, 20044G>C, 20045C>G, 20051C>G, 20053C>T, 20056C>G, 20057G>C, 20058A>G, 20064G>A, 20065C>G, 20076A>G, 20078C>G, 20091T>G, 20092T>C, 20102G>T, 20108T>C, 20115G>T, 20116T>G, 20117G>C, 20124T>C, 20132G>C, 20133G>T, 20134T>A, 20139A>C, 20140G>A, 20141C>G, 20142G>A, 20144T>C, 20145A>C, 20146C>T, 20147A>T, 20151C>T, 20157T>A, 20158C>G, 20159G>C, 20165A>G, 20166G>T, 20168G>C, 20174C>G, 20177T>C, 20180T>C, 20187T>G, 20189G>C, 20211A>T, 20229T>C, 20231T>G, 20235G>A, 20237G>C, 20240G>C, 20253T>A, 20254C>G, 20255G>C, 20268A>C, 20269C>A, 20279C>G, 20282C>T, 20286T>C, 20292T>C, 20293C>A, 20298A>G, 20299T>G, 20301C>A, 20302A>T, 20303G>C, 20306G>C, 20309T>C, 20336C>G, 20343T>A, 20344C>G, 20345G>C, 20355T>G, 20356G>C, 20357C>G, 20360G>C, 20362C>T, 20363G>C, 20369T>C, 20385G>C, 20388C>G, 20394C>A, 20396G>C, 20411C>G, 20433A>T, 20434G>A, 20439G>C, 20444T>C, 20461A>T, 20463G>A, 20468G>T, 20477A>G, 20478T>A, 20479C>G, 20480G>C, 20481G>T, 20483C>G, 20489G>C, 20493G>A, 20495G>T, 20501G>C, 20502G>A, 20503C>T, 20504C>G, 20511C>A, 20513G>C, 20514T>A, 20515C>G, 20516G>C, 20517A>G, 20518A>C, 20526T>G, 20527C>A, 20530G>A, 20532A>C, 20535G>C, 20546C>A, 20547A>C, 20557C>A, 20559G>C, 20560T>C, 20561C>G, 20564T>C, 20571C>G, 20573A>G, 20574T>G, 20576G>C, 20578T>A, 20580T>A, 20581C>G, 20582G>C, 20588A>G, 20589A>G, 20591C>G, 20597T>G, 20598A>G, 20599A>C, 20610A>G, 20611T>C, 20613A>T, 20614A>C, 20615C>G, 20618G>C, 20624G>C, 20627T>G, 20634C>A, 20636T>G, 20642T>C, 20648C>G, 20656T>A, 20657T>G, 20664T>G, 20666G>C, 20669T>C, 20682A>C, 20685G>A, 20687G>C, 20690T>C, 20691C>A, 20693T>C, 24489A>G, 24491C>T, 24492G>C, 24495A>T, 24496G>C, 24500A>G, 24502T>G, 24505C>G, 24507A>C, 24508G>C, 24509T>G, 24510C>A, 24515C>G, 24516C>A, 24526G>C, 24527A>G, 24529C>G, 24532A>G, 24541G>C, 24542C>G, 24543G>T, 24544C>G, 24550C>G, 24553T>G, 24554G>A, 24562A>C, 24568G>C, 24572G>A, 24580G>C, 24587G>C, 24588C>G, 24591T>C, 24592C>G, 24593A>G, 24594C>A, 24595G>T, 24596G>C, 24597A>G, 24601C>G, 24602A>C, 24605A>G, 24606A>G, 24613T>C, 24616C>G, 24617T>A, 24618G>T, 24620C>A, 24625T>C, 24628C>T, 24629C>G, 24630A>T, 24631A>C, 24633A>G, 24634T>C, 24637T>C, 24638T>A, 24642C>A, 24643G>C, 24646A>C, 24651G>T, 24653C>T, 24655G>C, 24658T>G, 24661T>C, 24663C>G, 24664G>C, 24665A>C, 24670G>C, 24671G>A, 24673T>C, 24675C>G, 24676G>C, 24680A>C, 24681C>A, 24682C>G, 24685C>G, 24688G>A, 24690T>A, 24691G>A, 24695C>A, 24696A>G, 24697G>C, 24701T>G, 24702C>G, 24703A>C, 24704G>C, 24707T>C, 24710T>C, 24711C>G, 24714T>A, 24715T>C, 24716T>A, 24717C>A, 24718C>G, 24721A>G, 24722G>A, 24724G>C, 24725A>G, 24730G>C, 24733C>G, 24737C>T, 24738G>C, 24739C>G, 24743G>A, 24744A>C, 24745T>C, 24747T>A, 24748C>T, 24754T>C, 24755T>G, 24756T>C, 24757C>T, 24760T>C, 24775A>C, 24779A>G, 24780C>T, 24781G>C, 24787G>A, 25498C>G, 25506A>T, 25507G>C, 25508T>C, 25510T>G, 25511T>G, 25517C>G, 25520A>C, 25521A>G, 25522A>C, 25523C>G, 25531T>A, 25535T>C, 25536C>G, 25541T>C, 25553T>C, 25565A>C, 25566G>A, 25568G>T, 25571T>G, 25575C>A, 25578C>G, 25580G>C, 25582C>A, 25583G>C, 25584G>C, 25589A>C, 25590_25592delGAC, 25595G>C, 25596A>G, 25604C>T, 25607C>T, 25608A>C, 25610C>G, 25614A>G, 25615A>C, 25616A>C, 25620G>A, 25621C>T, 25622G>C, 25624T>A, 25634T>C, 25635T>C, 25637C>G, 25650T>A, 25651C>G, 25653G>A, 25659A>G, 25662A>G, 25665T>C, 25666A>C, 25667C>G, 25672A>C, 25673G>C, 25675C>A, 25676G>C, 25679C>A, 25682C>G, 25683A>T, 25685C>G, 25691G>C, 25697T>C, 25700G>C, 25706G>C, 25709T>C, 25720A>T, 25724C>T, 25727C>G, 25733G>C, 25737C>A, 25744C>G, 25771C>T, 25772G>C, 26324G>A, 26329C>T, 26330G>C, 26331C>G, 26332A>C, 26336C>T, 26339A>G, 26343A>C, 26344A>G, 26345G>C, 26346C>A, 26354G>C, 26366A>C, 26373A>C, 26375C>G, 26379A>C, 26380C>A, 26382C>G, 26383A>C, 26384G>C, 26386C>A, 26388G>T, 26390C>G, 26391A>G, 26393G>C, 26394C>A, 26396T>C, 26397C>A, 26398G>A, 26400C>G, 26403T>C, 26404C>A, 26405T>G, 26408G>C, 26418A>G, 26419A>C, 26420G>T, 26426T>C, 26430G>A, 26431A>C, 26437T>A, 26439G>C, 26440T>C, 26441A>C, 26442G>A, 26443A>T, 26444C>G, 26450T>C, 26453G>C, 26456G>A, 26457T>A, 26458C>T, 26466C>A, 26468C>G, 26471T>C, 26480T>C, 26481G>A, 26482T>C, 26483G>C, 26487G>T, 26488A>T, 26489A>G, 26493T>G, 26494C>A, 26505A>C, 26506T>G, 26510T>C, 26513T>C, 26516A>C, 26520_26522delGTG, 26525C>G, 26526T>C, 26527C>T, 26528C>G, 26529A>C, 26530T>A, 26537C>G, 26538A>G, 26539G>A, 26541A>C, 26542T>A, 26543T>C, 26547G>A, 26548A>G, 26551T>C, 26552C>G, 26553A>C, 26558G>C, 26559C>G, 26560T>C, 26563C>G, 26564G>C, 26565C>A, 26566G>A, 26569C>G, 26571A>T, 26572A>G, 26574T>G, 26575A>G, 26578G>C, 26579C>A, 26580A>C, 26582G>T, 26584T>C, 26586G>A, 26587C>T, 26589G>A, 26590C>G, 26593T>C, 26595A>C, 26597A>G, 26598G>C, 26599C>A, 26602G>C, 26605T>G, 26608C>G, 26611T>C, 26612G>A, 26616C>G, 26626C>G, 26629A>C, 26631G>C, 26632C>G, 26635G>C, 26636G>T, 26637C>T, 26639T>A, 26640C>G, 26641T>C, 26643C>T, 26644T>A, 26650C>G, 26652G>A, 26654A>C, 26656T>G, 26659G>A, 27222C>G, 27225T>C, 27228G>T, 27229T>C, 27230G>T, 27231G>T, 27232G>T, 27233T>G, 27234T>G, 27237T>C, 27244A>C, 27246C>G, 27249C>A, 27251C>G, 27252G>C, 27253T>A, 27254C>G, 27256T>C, 27258A>C, 27260C>G, 27261G>C, 27267T>C, 27268C>A, 27270G>C, 27272G>C, 27273C>G, 27274A>C, 27275C>T, 27277G>T, 27281A>G, 27284C>G, 27286G>A, 27287A>C, 27288C>G, 27289A>C, 27290C>A, 27295A>G, 27296C>G, 27297A>C, 27307A>G, 27309A>G, 27310G>C, 27318C>G, 27321T>G, 27322G>C, 27324G>C, 27327G>C, 27330T>C, 27333C>G, 27339G>C, 27343A>G, 27345C>A, 27351C>A, 27354T>G, 27355G>A, 27356A>G, 27357G>C, 27360T>C, 27379A>T, 27381G>C, 27382A>G, 27387T>C, 27391A>C, 27392C>T, 27399C>T, 27402G>C, 27406G>A, 27407C>A, 27409A>G, 27411T>G, 27412C>G, 27413G>T, 27416G>C, 27417A>C, 27425T>G, 27426G>C, 27432G>A, 27433G>A, 27436A>C, 27437G>T, 27439A>C, 27447G>C, 27448G>A, 27453T>G, 27456G>C, 27457C>A, 27459A>G, 27466G>C, 27467A>C, 27474A>G, 27475G>A, 27477G>C, 27478G>A, 27641T>G, 27642A>C, 27643C>A, 27646T>G, 27650G>C, 27651C>A, 27652C>G, 27653T>C, 27655A>C, 27658T>C, 27662C>A, 27668G>A, 27676G>C, 27677A>G, 27679C>G, 27685C>G, 27689G>T, 27691G>T, 27701A>G, 27702A>T, 27703T>G, 27706T>G, 27709C>A, 27710G>A, 27712C>T, 27714T>C, 27716A>C, 27731C>A, 27734A>G, 27735T>C, 27736C>G, 27737A>C, 27742A>G, 27743G>A, 27745G>T, 27746C>G, 27747T>C, 27748G>A, 27749G>T, 27752A>C, 27754C>G, 27761G>T, 27762C>T, 27763G>T, 27766T>G, 27769G>C, 27770G>A, 27771C>G, 27772G>C, 27774C>A, 27775C>T, 27779A>G, 27781G>C, 27785C>G, 27786T>C, 27787T>G, 27792C>G, 27793G>T, 27798T>C, 27802G>C, 27812G>T, 27814G>C, 27815A>G, 27820T>C, 27823T>G, 27826T>G, 27829G>C, 27830G>C, 27831T>A, 27833G>C, 27834C>A, 27841T>C, 27844A>C, 27845A>C, 27846C>A, 27850C>G, 27851G>A, 27857T>G, 27868G>T, 27869A>T, 27874C>G, 27875C>A, 27876A>C, 27877G>C, 27883C>T, 27886T>G, 27895T>C, 27896A>G, 27898A>G, 27899G>T, 27901C>T, 27904T>C, 27913A>G, 27922G>C, 27923A>T, 27929C>A, 27931A>G, 27933T>A, 27964T>C, 27967T>C, 27971T>G, 27976A>G, 27985C>G, 27988C>T, 27994T>G, 27997T>G, 28000G>C, 28006G>C, 28007G>A, 28009G>C, 28012C>T, 28013G>T, 28014T>G, 28015G>C, 28016A>G, 28017A>T, 28018T>G, 28020T>C, 28021A>G, 28022C>A, 28024G>C, 28028G>A, 28029T>A, 28031G>T, 28036G>C, 28038C>A, 28039G>C, 28042C>A, 28044G>C, 28045C>G, 28051T>G, 28053G>C, 28054C>G, 28062T>C, 28064G>A, 28065C>T, 28066T>C, 28068G>C, 28072C>G, 28073G>A, 28075A>C, 28076T>A, 28077C>A, 28078G>C, 28081C>G, 28082A>C, 28086T>G, 28088G>C, 28089G>A, 28090C>G, 28093A>C, 28096T>C, 28097T>G, 28099C>G, 28108T>C, 28114C>G, 28117G>C, 28123C>G, 28126A>G, 28137A>C, 28138C>G, 28141C>T, 28147T>C, 28154G>A, 28156A>G, 28159C>T, 28162T>C, 28165G>C, 28166C>T, 28168G>C, 28171T>C, 28172A>T, 28177C>G, 28181A>G, 28185C>G, 28190G>A, 28192A>C, 28193G>A, 28194C>A, 28195G>C, 28207G>A, 28213G>C, 28215A>G, 28216A>G, 28217T>G, 28219C>T, 28222T>C, 28223G>A, 31501T>C, 31504G>C, 31508C>T, 31510G>C, 31528T>C, 31531G>A, 31535A>G, 31537A>C, 31540C>T, 31543A>G, 31546T>C, 31547C>A, 31548A>G, 31549G>C, 31556G>A, 31559C>T, 31562A>G, 31563C>T, 31564T>G, 31565G>A, 31605G>A, 31606G>T, 31607A>C, 31608G>A, 31609C>G, 31616A>G, 31617A>C, 31618C>G, 31619A>G, 31620A>G, 31621C>A, 31623G>A, 31631G>A, 31632C>G, 31633A>T, 31639G>C, 31643A>G, 31660C>G, 31663T>G, 31672C>G, 31678T>A, 31682G>A, 31684A>C, 31687G>C, 31690T>A, 31693C>G, 31699C>A, 31701G>C, 31702C>G, 31708C>G, 31711C>G, 31714A>G, 31717C>A, 31724T>G, 31725C>T, 31729C>T, 31733A>G, 31734C>T, 31735C>G, 31738G>T, 31741G>C, 31743G>A, 31744G>T, 31745G>T, 31746T>C, 31753C>G, 31756C>T, 31760A>T, 31765T>C, 31771G>C, 31774C>G, 31783G>C, 31786G>C, 31789C>T								

CDS

KMC43_gp27	1	117	26.4%	715	90.2%	117 (100%)	103 (88.0%)	0/0/0/0	0
Protein mutations:	S4Q (14205A>C 14206G>A 14207C>A), D25E (14270T>A), A26G (14272C>G), I30M (14285C>G), G32S (14289G>A), F33Y (14293T>A 14294C>T), Q35S (14298C>T 14299A>C), R41Q (14317G>A 14318T>G), P67A (14394C>G), S68A (14397T>G 14399G>C), A69S (14400G>A 14401C>G 14402A>C), S78T (14427T>A), S88T (14457T>A), T102M (14500C>T)								

	Begin	End	Coverage	Score	Concordance	Matches	Identities	I/D/M/F*	Stop Codons
**NT**	**14196**	**31794**	**9.4%**	**3582**	**41.3%**	**4358 (99.6%)**	**3090 (70.6%)**	**9/8**	
Codon mutations:	ATC3ATT (14204C>T), AGC4CAA (14205A>C 14206G>A 14207C>A), AAT5AAC (14210T>C), GCC8GCT (14219C>T), GCT9GCC (14222T>C), CCG12CCC (14231G>C), TTT23TTC (14264T>C), GAT25GAA (14270T>A), GCC26GGC (14272C>G), ATC30ATG (14285C>G), CTC31CTG (14288C>G), GGC32AGC (14289G>A), TTC33TAT (14293T>A 14294C>T), CAG35TCG (14298C>T 14299A>C), GCA37GCC (14306A>C), CGT38CGC (14309T>C), CGT41CAG (14317G>A 14318T>G), GGT47GGC (14336T>C), GAA48GAG (14339A>G), ACC49ACA (14342C>A), CGT54CGC (14357T>C), GGT56GGC (14363T>C), CTG57CTC (14366G>C), GCG59GCT (14372G>T), CCG60CCC (14375G>C), GTC61GTG (14378C>G), ACC62ACG (14381C>G), CCG67GCG (14394C>G), TCG68GCC (14397T>G 14399G>C), GCA69AGC (14400G>A 14401C>G 14402A>C), TCG78ACG (14427T>A), CCG79CCC (14432G>C), TCG80AGC (14433T>A 14434C>G 14435G>C), GTG84GTC (14447G>C), TCG88ACG (14457T>A), CTC89CTG (14462C>G), ACG97ACC (14486G>C), ACG102ATG (14500C>T), GTG103GTC (14504G>C), GTC107GTG (14516C>G), GTG110GTC (14525G>C)								
KMC43_gp29	15	64	78.1%	-12	-3.5%	50 (94.3%)	19 (35.8%)	3/0/1/1	1
Protein mutations:	S18L (15870C>T 15871C>G), I19V (15872A>G), L20V (15875C>G), D22E (15883C>G), E23R (15884G>C 15885A>G 15886G>A), S24D (15887T>G 15888C>A), I25F (15890A>T), W27F (15897G>T 15898G>C), M28S (15900T>G 15901G>C), E29V (15903A>T 15904A>C), I32L (15911A>C 15913C>G), Q35V (15920C>G 15921A>T), F36W (15924T>G 15925C>G), H37A (15926C>G 15927A>C), E38A (15930A>C 15931A>T), T40K (15936C>A), H41P (15939A>C), V42I (15941G>A), N44E (15947A>G 15949C>A), P45V (15950C>G 15951C>T 15952T>G), V46R (15953G>C 15954T>G), V48I (15959G>A 15961G>C), A49R (15962G>C 15963C>G), A50E (15966C>A), Q53A (15974C>G 15975A>C 15976G>C), H54N (15977C>A), G55D (15981G>A), R57P (15987G>C 15988T>G), R57_Y58insIKA (15988_15989insATCAAGGCC), L59R (15992T>C 15993T>G), V61I (15998G>A)								
Codon mutations:	CCC16CCG (15865C>G), GAT17GAC (15868T>C), TCC18TTG (15870C>T 15871C>G), ATC19GTC (15872A>G), CTG20GTG (15875C>G), TTG21CTG (15878T>C), GAC22GAG (15883C>G), GAG23CGA (15884G>C 15885A>G 15886G>A), TCC24GAC (15887T>G 15888C>A), ATC25TTC (15890A>T), GGG26GGC (15895G>C), TGG27TTC (15897G>T 15898G>C), ATG28AGC (15900T>G 15901G>C), GAA29GTC (15903A>T 15904A>C), ATC32CTG (15911A>C 15913C>G), CGA34CGC (15919A>C), CAG35GTG (15920C>G 15921A>T), TTC36TGG (15924T>G 15925C>G), CAC37GCC (15926C>G 15927A>C), GAA38GCT (15930A>C 15931A>T), GGT39GGC (15934T>C), ACG40AAG (15936C>A), CAC41CCC (15939A>C), GTC42ATC (15941G>A), AAC44GAA (15947A>G 15949C>A), CCT45GTG (15950C>G 15951C>T 15952T>G), GTC46CGC (15953G>C 15954T>G), GTG48ATC (15959G>A 15961G>C), GCC49CGC (15962G>C 15963C>G), GCG50GAG (15966C>A), CAG53GCC (15974C>G 15975A>C 15976G>C), CAC54AAC (15977C>A), GGC55GAC (15981G>A), CGT57CCG (15987G>C 15988T>G), CGT57_TAC58insATCAAGGCC (15988_15989insATCAAGGCC), TTG59CGG (15992T>C 15993T>G), GTC61ATC (15998G>A), AGC62-TG (16001delA 16002G>T 16003C>G)								
KMC43_gp30	1	103	100%	538	80.9%	103 (100%)	77 (74.8%)	0/0/0/0	1
Protein mutations:	V5I (16018G>A), I7T (16025T>C 16026T>C), K11R (16036A>C 16037A>G 16038A>C), S12N (16040G>A), T14A (16045A>G 16047C>G), T18A (16057A>G 16059G>T), A19G (16061C>G 16062C>G), Q21T (16066C>A 16067A>C), S25T (16078T>A), K28A (16087A>G 16088A>C 16089G>C), D29G (16091A>G), I32L (16099A>C 16101C>G), N41Q (16126A>C 16128T>G), A48T (16147G>A 16149G>T), G49P (16150G>C 16151G>C), T58V (16177A>G 16178C>T 16179G>C), T60G (16183A>G 16184C>G), A62V (16190C>T 16191T>C), N65V (16198A>G 16199A>T), I67V (16204A>G), G68A (16208G>C 16209C>G), S76T (16231T>A 16233A>C), N85A (16258A>G 16259A>C 16260C>A), I89V (16270A>G), A93T (16282G>A 16284T>C), S98A (16297T>G)								
Codon mutations:	GAC3GAT (16014C>T), GTC5ATC (16018G>A), ATT7ACC (16025T>C 16026T>C), GGC10GGG (16035C>G), AAA11CGC (16036A>C 16037A>G 16038A>C), AGC12AAC (16040G>A), ACC14GCG (16045A>G 16047C>G), AAT16AAC (16053T>C), ACG18GCT (16057A>G 16059G>T), GCC19GGG (16061C>G 16062C>G), GCG20GCC (16065G>C), CAG21ACG (16066C>A 16067A>C), GTG22GTC (16071G>C), GTT23GTC (16074T>C), TCG25ACG (16078T>A), CCC27CCA (16086C>A), AAG28GCC (16087A>G 16088A>C 16089G>C), GAC29GGC (16091A>G), TTT30TTC (16095T>C), ATC32CTG (16099A>C 16101C>G), CGA36CGC (16113A>C), AAT41CAG (16126A>C 16128T>G), GTT42GTG (16131T>G), CTC43CTG (16134C>G), GTT44GTG (16137T>G), GCG45GCC (16140G>C), GGG46GGC (16143G>C), ACT47ACC (16146T>C), GCG48ACT (16147G>A 16149G>T), GGG49CCG (16150G>C 16151G>C), GCC51GCG (16158C>G), GCG52GCC (16161G>C), TAC53TAT (16164C>T), GCG55GCA (16170G>A), ACG57ACC (16176G>C), ACG58GTC (16177A>G 16178C>T 16179G>C), GCC59GCT (16182C>T), ACC60GGC (16183A>G 16184C>G), GCT62GTC (16190C>T 16191T>C), AAC65GTC (16198A>G 16199A>T), ATC67GTC (16204A>G), GGC68GCG (16208G>C 16209C>G), GCG69GCC (16212G>C), CCG71CCC (16218G>C), GTG74GTC (16227G>C), TCA76ACC (16231T>A 16233A>C), CTC78CTG (16239C>G), GAT80GAC (16245T>C), CCG82CCC (16251G>C), CTG84TTG (16255C>T), AAC85GCA (16258A>G 16259A>C 16260C>A), GGC86GGG (16263C>G), ATC89GTC (16270A>G), CCT91CCC (16278T>C), GCT93ACC (16282G>A 16284T>C), ACC96ACG (16293C>G), GTT97GTC (16296T>C), TCT98GCT (16297T>G), GTT99GTC (16302T>C), TCT100TCC (16305T>C)								
KMC43_gp31	1	117	91.4%	710	86.3%	117 (100%)	93 (79.5%)	0/0/0/0	0
Protein mutations:	A6Q (16339G>C 16340C>A 16341C>G), K8R (16345A>C 16346A>G), G16W (16369G>T 16371A>G), M19V (16378A>G 16380G>C), F20Y (16382T>A 16383C>T), S26A (16399T>G), M43L (16450A>C), N44S (16453A>T 16454A>C 16455C>G), M46L (16459A>C), A48P (16465G>C 16467C>G), A52G (16478C>G 16479T>C), V54I (16483G>A 16485G>C), S71G (16534T>G 16535C>G 16536T>C), A72T (16537G>A), Q80K (16561C>A), V85T (16576G>A 16577T>C 16578G>C), V91L (16594G>C), A99S (16618G>A 16619C>G), R103L (16631G>T 16632C>G), E107Q (16642G>C), M109L (16648A>C 16650G>C), I111V (16654A>G 16656T>G), G116A (16670G>C 16671G>C)								
Codon mutations:	ACA4ACC (16335A>C), ACC5ACG (16338C>G), GCC6CAG (16339G>C 16340C>A 16341C>G), AAG8CGG (16345A>C 16346A>G), TAT14TAC (16365T>C), TGC15TGT (16368C>T), GGA16TGG (16369G>T 16371A>G), ATG19GTC (16378A>G 16380G>C), TTC20TAT (16382T>A 16383C>T), GGG22GGC (16389G>C), CCA24CCC (16395A>C), TCG25AGT (16396T>A 16397C>G 16398G>T), TCG26GCG (16399T>G), TAT30TAC (16413T>C), CGG31AGG (16414C>A), GCG35GCC (16428G>C), TAC36TAT (16431C>T), CTT39CTG (16440T>G), GAA40GAG (16443A>G), ATG43CTG (16450A>C), AAC44TCG (16453A>T 16454A>C 16455C>G), ATG46CTG (16459A>C), CTG47CTC (16464G>C), GCC48CCG (16465G>C 16467C>G), GAA50GAG (16473A>G), GCT52GGC (16478C>G 16479T>C), GTG54ATC (16483G>A 16485G>C), ACG57ACC (16494G>C), TAT58TAC (16497T>C), CTG59CTC (16500G>C), GCT60GCC (16503T>C), ACC63ACG (16512C>G), ACT64ACG (16515T>G), CTC65CTG (16518C>G), ACG67ACC (16524G>C), GCA68GCC (16527A>C), TCT71GGC (16534T>G 16535C>G 16536T>C), GCG72ACG (16537G>A), AGC73TCG (16540A>T 16541G>C 16542C>G), GCG74GCA (16545G>A), CTC76CTT (16551C>T), ACA78ACG (16557A>G), CAG80AAG (16561C>A), GCG81GCC (16566G>C), GCC82GCG (16569C>G), GTG85ACC (16576G>A 16577T>C 16578G>C), GAA90GAG (16593A>G), GTG91CTG (16594G>C), GCA92GCC (16599A>C), GAC93GAT (16602C>T), CGC94CGT (16605C>T), GAC95GAT (16608C>T), TTT98TTC (16617T>C), GCC99AGC (16618G>A 16619C>G), CGG102CGC (16629G>C), CGC103CTG (16631G>T 16632C>G), GAG107CAG (16642G>C), ATG109CTC (16648A>C 16650G>C), ATT111GTG (16654A>G 16656T>G), CCC113CCG (16662C>G), GGG114GGC (16665G>C), CCC115CCG (16668C>G), GGG116GCC (16670G>C 16671G>C), CTC117T.. (16672C>T)								
KMC43_gp36	17	497	72.0%	1825	76.6%	368 (100%)	262 (71.2%)	0/0/0/0	0
Protein mutations:	V28T (19290G>A 19291T>C 19292T>C), S29Q (19293T>C 19294C>A 19295C>G), L30Y (19296C>T 19297T>A 19298G>C), V38I (19320G>A 19322G>C), V41I (19329G>A 19331T>C), T46S (19344A>T), L55T (19371C>A 19372T>C), I56V (19374A>G), N58S (19381A>G), M59P (19383A>C 19384T>C), M61Q (19389A>C 19390T>A), Y62A (19392T>G 19393A>C), Q64A (19398C>G 19399A>C 19400G>C), A65I (19401G>A 19402C>T), A68Q (19410G>C 19411C>A), I69A (19413A>G 19414T>C), T73K (19426C>A 19427C>G), M76L (19434A>C), G77M (19437G>A 19438G>T 19439C>G), V80I (19446G>A 19448G>C), A81S (19449G>T), I82V (19452A>G), V84T (19458G>A 19459T>C 19460G>C), Q85M (19461C>A 19462A>T), R92K (19482C>A 19483G>A 19484C>A), C93A (19485T>G 19486G>C), T102V (19512A>G 19513C>T), T106I (19525C>T 19526G>C), I107A (19527A>G 19528T>C 19529C>G), Q109L (19534A>T), G112C (19542G>T), V113I (19545G>A 19547G>C), L115F (19551C>T 19553G>C), A117S (19557G>T 19559G>C), L123F (19575C>T 19577G>C), S126G (19584T>G 19585C>G 19586G>C), V127I (19587G>A 19589G>C), A130T (19596G>A), A132S (19602G>T 19604C>G), A136Q (19614G>C 19615C>A 19616C>G), N138S (19620A>T 19621A>C 19622T>G), W140Q (19626T>C 19627G>A), V142A (19633T>C 19634A>C), T143V (19635A>G 19636C>T), A145S (19641G>T), I266T (20005T>C 20006C>G), I269V (20013A>G), G270M (20016G>A 20017G>T 20018C>G), V271T (20019G>A 20020T>C), A274S (20028G>A 20029C>G), S279A (20043A>G 20044G>C 20045C>G), T282I (20053C>T), T283S (20056C>G 20057G>C), T284A (20058A>G), A286S (20064G>A 20065C>G), I290V (20076A>G 20078C>G), F295A (20091T>G 20092T>C), V303C (20115G>T 20116T>G 20117G>C), V309Y (20133G>T 20134T>A), S311Q (20139A>C 20140G>A 20141C>G), V312I (20142G>A 20144T>C), T313L (20145A>C 20146C>T 20147A>T), A320S (20166G>T 20168G>C), S327A (20187T>G 20189G>C), T335S (20211A>T), Y341Q (20229T>C 20231T>G), V343I (20235G>A 20237G>C), T354Q (20268A>C 20269C>A), S362Q (20292T>C 20293C>A), I364G (20298A>G 20299T>G), Q365I (20301C>A 20302A>T 20303G>C), C383A (20355T>G 20356G>C 20357C>G), S385F (20362C>T 20363G>C), A393P (20385G>C), L394V (20388C>G), Q396N (20394C>A 20396G>C), S409Y (20433A>T 20434G>A), V411L (20439G>C), Y418F (20461A>T), V419I (20463G>A), A425S (20481G>T 20483C>G), A429T (20493G>A 20495G>T), A432M (20502G>A 20503C>T 20504C>G), L435I (20511C>A 20513G>C), N437A (20517A>G 20518A>C), S440D (20526T>G 20527C>A), S441N (20530G>A), M442L (20532A>C), E443Q (20535G>C), M447L (20547A>C), A450D (20557C>A), V451P (20559G>C 20560T>C 20561C>G), P455A (20571C>G 20573A>G), S456A (20574T>G 20576G>C), F457Y (20578T>A), I461V (20589A>G 20591C>G), N464A (20598A>G 20599A>C), M468A (20610A>G 20611T>C), N469S (20613A>T 20614A>C 20615C>G), L476M (20634C>A 20636T>G), I483K (20656T>A 20657T>G), S486A (20664T>G 20666G>C), I492L (20682A>C), V493I (20685G>A 20687G>C), L495I (20691C>A 20693T>C)								

	Begin	End	Coverage	Score	Concordance	Matches	Identities	I/D/M/F*	Stop Codons
**NT**	**14196**	**31794**	**9.4%**	**3582**	**41.3%**	**4358 (99.6%)**	**3090 (70.6%)**	**9/8**	
Codon mutations:	TTG19CTG (19263T>C), GTA21GTG (19271A>G), CAA22CAG (19274A>G), GTG24GTC (19280G>C), CCT25CCG (19283T>G), CCA26CCG (19286A>G), GTT28ACC (19290G>A 19291T>C 19292T>C), TCC29CAG (19293T>C 19294C>A 19295C>G), CTG30TAC (19296C>T 19297T>A 19298G>C), CTC31CTG (19301C>G), AAT37AAC (19319T>C), GTG38ATC (19320G>A 19322G>C), CTT39TTG (19323C>T 19325T>G), GGT40GGC (19328T>C), GTT41ATC (19329G>A 19331T>C), GTG42GTC (19334G>C), ACC44ACG (19340C>G), GCA45GCG (19343A>G), ACC46TCC (19344A>T), GGC48GGG (19352C>G), ACC50ACG (19358C>G), TCG52AGC (19362T>A 19363C>G 19364G>C), CCG53CCC (19367G>C), ACG54ACC (19370G>C), CTG55ACG (19371C>A 19372T>C), ATC56GTC (19374A>G), AAC58AGC (19381A>G), ATG59CCG (19383A>C 19384T>C), GCG60GCT (19388G>T), ATG61CAG (19389A>C 19390T>A), TAC62GCC (19392T>G 19393A>C), GCC63GCG (19397C>G), CAG64GCC (19398C>G 19399A>C 19400G>C), GCC65ATC (19401G>A 19402C>T), GCG68CAG (19410G>C 19411C>A), ATC69GCC (19413A>G 19414T>C), CGT72CGC (19424T>C), ACC73AAG (19426C>A 19427C>G), TAT74TAC (19430T>C), ATG76CTG (19434A>C), GGC77ATG (19437G>A 19438G>T 19439C>G), GCT79GCC (19445T>C), GTG80ATC (19446G>A 19448G>C), GCG81TCG (19449G>T), ATC82GTC (19452A>G), GCA83GCG (19457A>G), GTG84ACC (19458G>A 19459T>C 19460G>C), CAG85ATG (19461C>A 19462A>T), GCG88GCC (19472G>C), CGC92AAA (19482C>A 19483G>A 19484C>A), TGC93GCC (19485T>G 19486G>C), CGC95CGG (19493C>G), GTC96GTG (19496C>G), ACG102GTG (19512A>G 19513C>T), GCG104GCC (19520G>C), TCG105AGC (19521T>A 19522C>G 19523G>C), ACG106ATC (19525C>T 19526G>C), ATC107GCG (19527A>G 19528T>C 19529C>G), CAG109CTG (19534A>T), GGC112TGC (19542G>T), GTG113ATC (19545G>A 19547G>C), ACG114ACC (19550G>C), CTG115TTC (19551C>T 19553G>C), GCG117TCC (19557G>T 19559G>C), ACG122ACC (19574G>C), CTG123TTC (19575C>T 19577G>C), TCG126GGC (19584T>G 19585C>G 19586G>C), GTG127ATC (19587G>A 19589G>C), GTC129GTG (19595C>G), GCG130ACG (19596G>A), CTC131CTG (19601C>G), GCC132TCG (19602G>T 19604C>G), GCG133GCC (19607G>C), AGC135TCG (19611A>T 19612G>C 19613C>G), GCC136CAG (19614G>C 19615C>A 19616C>G), AAT138TCG (19620A>T 19621A>C 19622T>G), TGG140CAG (19626T>C 19627G>A), GTA142GCC (19633T>C 19634A>C), ACG143GTG (19635A>G 19636C>T), GTC144GTT (19640C>T), GCG145TCG (19641G>T), ATC266ACG (20005T>C 20006C>G), ATC269GTC (20013A>G), GGC270ATG (20016G>A 20017G>T 20018C>G), GTC271ACC (20019G>A 20020T>C), GCT274AGT (20028G>A 20029C>G), ACG277ACC (20039G>C), AGC279GCG (20043A>G 20044G>C 20045C>G), GCC281GCG (20051C>G), ACC282ATC (20053C>T), ACG283AGC (20056C>G 20057G>C), ACC284GCC (20058A>G), GCC286AGC (20064G>A 20065C>G), ATC290GTG (20076A>G 20078C>G), TTC295GCC (20091T>G 20092T>C), CTG298CTT (20102G>T), GGT300GGC (20108T>C), GTG303TGC (20115G>T 20116T>G 20117G>C), TTG306CTG (20124T>C), ACG308ACC (20132G>C), GTC309TAC (20133G>T 20134T>A), AGC311CAG (20139A>C 20140G>A 20141C>G), GTT312ATC (20142G>A 20144T>C), ACA313CTT (20145A>C 20146C>T 20147A>T), CTG315TTG (20151C>T), TCG317AGC (20157T>A 20158C>G 20159G>C), CAA319CAG (20165A>G), GCG320TCC (20166G>T 20168G>C), GTC322GTG (20174C>G), GCT323GCC (20177T>C), GGT324GGC (20180T>C), TCG327GCC (20187T>G 20189G>C), ACG335TCG (20211A>T), TAT341CAG (20229T>C 20231T>G), GTG343ATC (20235G>A 20237G>C), GTG344GTC (20240G>C), TCG349AGC (20253T>A 20254C>G 20255G>C), ACG354CAG (20268A>C 20269C>A), TCC357TCG (20279C>G), GCC358GCT (20282C>T), TTG360CTG (20286T>C), TCG362CAG (20292T>C 20293C>A), ATC364GGC (20298A>G 20299T>G), CAG365ATC (20301C>A 20302A>T 20303G>C), GCG366GCC (20306G>C), GGT367GGC (20309T>C), CCC376CCG (20336C>G), TCG379AGC (20343T>A 20344C>G 20345G>C), TGC383GCG (20355T>G 20356G>C 20357C>G), CGG384CGC (20360G>C), TCG385TTC (20362C>T 20363G>C), CAT387CAC (20369T>C), GCG393CCG (20385G>C), CTG394GTG (20388C>G), CAG396AAC (20394C>A 20396G>C), ACC401ACG (20411C>G), AGC409TAC (20433A>T 20434G>A), GTC411CTC (20439G>C), AAT412AAC (20444T>C), TAC418TTC (20461A>T), GTC419ATC (20463G>A), GGG420GGT (20468G>T), CAA423CAG (20477A>G), TCG424AGC (20478T>A 20479C>G 20480G>C), GCC425TCG (20481G>T 20483C>G), GTG427GTC (20489G>C), GCG429ACT (20493G>A 20495G>T), GCG431GCC (20501G>C), GCC432ATG (20502G>A 20503C>T 20504C>G), CTG435ATC (20511C>A 20513G>C), TCG436AGC (20514T>A 20515C>G 20516G>C), AAC437GCC (20517A>G 20518A>C), TCC440GAC (20526T>G 20527C>A), AGC441AAC (20530G>A), ATG442CTG (20532A>C), GAG443CAG (20535G>C), GGC446GGA (20546C>A), ATG447CTG (20547A>C), GCC450GAC (20557C>A), GTC451CCG (20559G>C 20560T>C 20561C>G), AAT452AAC (20564T>C), CCA455GCG (20571C>G 20573A>G), TCG456GCC (20574T>G 20576G>C), TTC457TAC (20578T>A), TCG458AGC (20580T>A 20581C>G 20582G>C), CAA460CAG (20588A>G), ATC461GTG (20589A>G 20591C>G), GCT463GCG (20597T>G), AAC464GCC (20598A>G 20599A>C), ATG468GCG (20610A>G 20611T>C), AAC469TCG (20613A>T 20614A>C 20615C>G), CGG470CGC (20618G>C), GCG472GCC (20624G>C), CTT473CTG (20627T>G), CTT476ATG (20634C>A 20636T>G), GCT478GCC (20642T>C), GTC480GTG (20648C>G), ATT483AAG (20656T>A 20657T>G), TCG486GCC (20664T>G 20666G>C), GTT487GTC (20669T>C), ATC492CTC (20682A>C), GTG493ATC (20685G>A 20687G>C), AAT494AAC (20690T>C), CTT495ATC (20691C>A 20693T>C)									
KMC43_gp42	12	115	32.4%	453	59.9%	104 (100%)	57 (54.8%)	0/0/0/0	0
Protein mutations:	D14G (24489A>G), R15S (24491C>T 24492G>C), Q16L (24495A>T 24496G>C), I18V (24500A>G 24502T>G), E20A (24507A>C 24508G>C), S21E (24509T>G 24510C>A), P23E (24515C>G 24516C>A), T27A (24527A>G 24529C>G), R32V (24542C>G 24543G>T 24544C>G), D34E (24550C>G), V36I (24554G>A), V42I (24572G>A), A47R (24587G>C 24588C>G), F48S (24591T>C 24592C>G), T49D (24593A>G 24594C>A 24595G>T), E50R (24596G>C 24597A>G), I52L (24602A>C), N53G (24605A>G 24606A>G), W57M (24617T>A 24618G>T), L58M (24620C>A), Q61V (24629C>G 24630A>T 24631A>C), D62G (24633A>G 24634T>C), L64M (24638T>A), A65D (24642C>A 24643G>C), R68L (24651G>T), Q69Y (24653C>T 24655G>C), A72G (24663C>G 24664G>C), M73L (24665A>C), V75I (24671G>A 24673T>C), A76G (24675C>G 24676G>C), T78Q (24680A>C 24681C>A 24682C>G), V81E (24690T>A 24691G>A), Q83S (24695C>A 24696A>G 24697G>C), S85G (24701T>G 24702C>G 24703A>C), E86Q (24704G>C), S88R (24710T>C 24711C>G), F89Y (24714T>A 24715T>C), S90K (24716T>A 24717C>A 24718C>G), V92I (24722G>A 24724G>C), I93V (24725A>G), D95E (24733C>G), R97S (24737C>T 24738G>C 24739C>G), D99T (24743G>A 24744A>C 24745T>C), F100Y (24747T>A 24748C>T), F103A (24755T>G 24756T>C 24757C>T), T111V (24779A>G 24780C>T 24781G>C)									
Codon mutations:	GAC14GGC (24489A>G), CGG15TCG (24491C>T 24492G>C), CAG16CTC (24495A>T 24496G>C), ATT18GTG (24500A>G 24502T>G), CCC19CCG (24505C>G), GAG20GCC (24507A>C 24508G>C), TCG21GAG (24509T>G 24510C>A), CCG23GAG (24515C>G 24516C>A), GGG26GGC (24526G>C), ACC27GCG (24527A>G 24529C>G), CAA28CAG (24532A>G), GCG31GCC (24541G>C), CGC32GTG (24542C>G 24543G>T 24544C>G), GAC34GAG (24550C>G), CTT35CTG (24553T>G), GTC36ATC (24554G>A), GGA38GGC (24562A>C), CGG40CGC (24568G>C), GTC42ATC (24572G>A), GCG44GCC (24580G>C), GCC47CGC (24587G>C 24588C>G), TTC48TCG (24591T>C 24592C>G), ACG49GAT (24593A>G 24594C>A 24595G>T), GAG50CGG (24596G>C 24597A>G), CCC51CCG (24601C>G), ATC52CTC (24602A>C), AAC53GGC (24605A>G 24606A>G), TCT55TCC (24613T>C), GGC56GGG (24616C>G), TGG57ATG (24617T>A 24618G>T), CTG58ATG (24620C>A), CTT59CTC (24625T>C), GGC60GGT (24628C>T), CAA61GTC (24629C>G 24630A>T 24631A>C), GAT62GGC (24633A>G 24634T>C), GCT63GCC (24637T>C), TTG64ATG (24638T>A), GCG65GAC (24642C>A 24643G>C), CGA66CGC (24646A>C), CGG68CTG (24651G>T), CAG69TAC (24653C>T 24655G>C), CTT70CTG (24658T>G), GAT71GAC (24661T>C), GCG72GGC (24663C>G 24664G>C), ATG73CTG (24665A>C), CGG74CGC (24670G>C), GTT75ATC (24671G>A 24673T>C), GCG76GGC (24675C>G 24676G>C), ACC78CAG (24680A>C 24681C>A 24682C>G), GTC79GTG (24685C>G), CAG80CAA (24688G>A), GTG81GAA (24690T>A 24691G>A), CAG83AGC (24695C>A 24696A>G 24697G>C), TCA85GGC (24701T>G 24702C>G 24703A>C), GAG86CAG (24704G>C), TTG87CTG (24707T>C), TCC88CGC (24710T>C 24711C>G), TTT89TAC (24714T>A 24715T>C), TCC90AAG (24716T>A 24717C>A 24718C>G), GTA91GTG (24721A>G), GTG92ATC (24722G>A 24724G>C), ATT93GTT (24725A>G), CGG94CGC (24730G>C), GAC95GAG (24733C>G), CGC97TCG (24737C>T 24738G>C 24739C>G), GAT99ACC (24743G>A 24744A>C 24745T>C), TTC100TAT (24747T>A 24748C>T), CGT102CGC (24754T>C), TTC103GCT (24755T>G 24756T>C 24757C>T), TAT104TAC (24760T>C), CGA109CGC (24775A>C), ACG111GTC (24779A>G 24780C>T 24781G>C), GAG113GAA (24787G>A)									
KMC43_gp43	25	373	46.4%	790	70.5%	171 (98.8%)	114 (65.9%)	0/2/0/0	1
Protein mutations:	A28G (25498C>G), F32W (25510T>G 25511T>G), N36A (25521A>G 25522A>C 25523C>G), F39Y (25531T>A), Q41E (25536C>G), A51T (25566G>A 25568G>T), L54M (25575C>A), P55A (25578C>G 25580G>C), A56D (25582C>A 25583G>C), E57Q (25584G>C), D59del (25590_25592delGAC), N61D (25596A>G), N65Q (25608A>C 25610C>G), K67A (25614A>G 25615A>C 25616A>C), A69I (25620G>A 25621C>T 25622G>C), F70Y (25624T>A), F74L (25635T>C 25637C>G), D80N (25653G>A), T82A (25659A>G), N83D (25662A>G), Y84P (25665T>C 25666A>C 25667C>G), E86A (25672A>C 25673G>C), T87N (25675C>A 25676G>C), D88E (25679C>A), T90S (25683A>T 25685C>G), Y102F (25720A>T), L108M (25737C>A), T110S (25744C>G), A119V (25771C>T 25772G>C), T305I (26329C>T 26330G>C), Q306A (26331C>G 26332A>C), K310R (26343A>C 26344A>G 26345G>C), L311M (26346C>A), I320L (26373A>C 26375C>G), T322Q (26379A>C 26380C>A), Q323A (26382C>G 26383A>C 26384G>C), T324K (26386C>A), A325S (26388G>T 26390C>G), M326V (26391A>G 26393G>C), L327I (26394C>A 26396T>C), R328K (26397C>A 26398G>A), L329V (26400C>G), S330Q (26403T>C 26404C>A 26405T>G), K335A (26418A>G 26419A>C 26420G>T), D339T (26430G>A 26431A>C), F341Y (26437T>A), V342P (26439G>C 26440T>C 26441A>C), D343M (26442G>A 26443A>T 26444C>G), S348M (26457T>A 26458C>T), L351M (26466C>A 26468C>G), V356T (26481G>A 26482T>C 26483G>C), E358L (26487G>T 26488A>T 26489A>G), S360D (26493T>G 26494C>A), I364R (26505A>C 26506T>G), E367D (26516A>C), V369del (26520_26522delGTG), S371L (26526T>C 26527C>T 26528C>G), I372Q (26529A>C 26530T>A)									
Codon mutations:	GCC28GGC (25498C>G), AGT31TCC (25506A>T 25507G>C 25508T>C), TTT32TGG (25510T>G 25511T>G), GTC34GTG (25517C>G), ACA35ACC (25520A>C), AAC36GCG (25521A>G 25522A>C 25523C>G), TTC39TAC (25531T>A), TAT40TAC (25535T>C), CAG41GAG (25536C>G), GCT42GCC (25541T>C), CGT46CGC (25553T>C), GCA50GCC (25565A>C), GCG51ACT (25566G>A 25568G>T), TCT52TCG (25571T>G), CTG54ATG (25575C>A), CCG55GCC (25578C>G 25580G>C), GCG56GAC (25582C>A 25583G>C), GAG57CAG (25584G>C), ACA58ACC (25589A>C), GAC59del (25590_25592delGAC), GCG60GCC (25595G>C), AAC61GAC (25596A>G), TTC63TTT (25604C>T), TCC64TCT (25607C>T), AAC65CAG (25608A>C 25610C>G), AAA67GCC (25614A>G 25615A>C 25616A>C), GCG69ATC (25620G>A 25621C>T 25622G>C), TTC70TAC (25624T>A), ATT73ATC (25634T>C), TTC74CTG (25635T>C 25637C>G), TCC79AGC (25650T>A 25651C>G), GAT80AAT (25653G>A), ACG82GCG (25659A>G), AAC83GAC (25662A>G), TAC84CCG (25665T>C 25666A>C 25667C>G), GAG86GCC (25672A>C 25673G>C), ACG87AAC (25675C>A 25676G>C), GAC88GAA (25679C>A), CTC89CTG (25682C>G), ACC90TCG (25683A>T 25685C>G), CTG92CTC (25691G>C), TAT94TAC (25697T>C), GGG95GGC (25700G>C), GTG97GTC (25706G>C), GAT98GAC (25709T>C), TAC102TTC (25720A>T), GAC103GAT (25724C>T), CCC104CCG (25727C>G), TCG106TCC (25733G>C), CTG108ATG (25737C>A), ACC110AGC (25744C>G), GCG119GTC (25771C>T 25772G>C), GAG303GAA (26324G>A), ACG305ATC (26329C>T 26330G>C), CAG306GCG (26331C>G 26332A>C), CAC307CAT (26336C>T), GAA308GAG (26339A>G), AAG310CGC (26343A>C 26344A>G 26345G>C), CTG311ATG (26346C>A), GCG313GCC (26354G>C), GCA317GCC (26366A>C), ATC320CTG (26373A>C 26375C>G), ACG322CAG (26379A>C 26380C>A), CAG323GCC (26382C>G 26383A>C 26384G>C), ACG324AAG (26386C>A), GCC325TCG (26388G>T 26390C>G), ATG326GTC (26391A>G 26393G>C), CTT327ATC (26394C>A 26396T>C), CGG328AAG (26397C>A 26398G>A), CTG329GTG (26400C>G), TCT330CAG (26403T>C 26404C>A 26405T>G), GGG331GGC (26408G>C), AAG335GCT (26418A>G 26419A>C 26420G>T), GAT337GAC (26426T>C), GAC339ACC (26430G>A 26431A>C), TTC341TAC (26437T>A), GTA342CCC (26439G>C 26440T>C 26441A>C), GAC343ATG (26442G>A 26443A>T 26444C>G), ATT345ATC (26450T>C), ACG346ACC (26453G>C), CGG347CGA (26456G>A), TCG348ATG (26457T>A 26458C>T), CTC351ATG (26466C>A 26468C>G), GAT352GAC (26471T>C), TAT355TAC (26480T>C), GTG356ACC (26481G>A 26482T>C 26483G>C), GAA358TTG (26487G>T 26488A>T 26489A>G), TCC360GAC (26493T>G 26494C>A), ATC364CGC (26505A>C 26506T>G), AAT365AAC (26510T>C), CCT366CCC (26513T>C), GAA367GAC (26516A>C), GTG369del (26520_26522delGTG), CCC370CCG (26525C>G), TCC371CTG (26526T>C 26527C>T 26528C>G), ATA372CAA (26529A>C 26530T>A)									
KMC43_gp44	1	44	19.7%	136	47.1%	44 (100%)	20 (45.5%)	0/0/0/0	0
Protein mutations:	Q3G (26537C>G 26538A>G 26539G>A), H4P (26541A>C 26542T>A), F5L (26543T>C), R6Q (26547G>A 26548A>G), Q8A (26552C>G 26553A>C), A10R (26558G>C 26559C>G 26560T>C), A12Q (26564G>C 26565C>A 26566G>A), Q14L (26571A>T 26572A>G), L15R (26574T>G 26575A>G), Q17T (26579C>A 26580A>C), A18S (26582G>T 26584T>C), G19D (26586G>A 26587C>T), R20Q (26589G>A 26590C>G), E22A (26595A>C), S23A (26597A>G 26598G>C 26599C>A), V28I (26612G>A), T29S (26616C>G), D32E (26626C>G), G34A (26631G>C 26632C>G), A36F (26636G>T 26637C>T), A38V (26643C>T 26644T>A), R41K (26652G>A), I42L (26654A>C 26656T>G)									
Codon mutations:	CAG3GGA (26537C>G 26538A>G 26539G>A), CAT4CCA (26541A>C 26542T>A), TTC5CTC (26543T>C), CGA6CAG (26547G>A 26548A>G), AAT7AAC (26551T>C), CAG8GCG (26552C>G 26553A>C), GCT10CGC (26558G>C 26559C>G 26560T>C), CTC11CTG (26563C>G), GCG12CAA (26564G>C 26565C>A 26566G>A), GCC13GCG (26569C>G), CAA14CTG (26571A>T 26572A>G), CTA15CGG (26574T>G 26575A>G), GCG16GCC (26578G>C), CAG17ACG (26579C>A 26580A>C), GCT18TCC (26582G>T 26584T>C), GGC19GAT (26586G>A 26587C>T), CGC20CAG (26589G>A 26590C>G), GCT21GCC (26593T>C), GAG22GCG (26595A>C), AGC23GCA (26597A>G 26598G>C 26599C>A), CGG24CGC (26602G>C), GTT25GTG (26605T>G), GGC26GGG (26608C>G), ATT27ATC (26611T>C), GTC28ATC (26612G>A), ACC29AGC (26616C>G), GAC32GAG (26626C>G), CCA33CCC (26629A>C), GGC34GCG (26631G>C 26632C>G), ACG35ACC (26635G>C), GCC36TTC (26636G>T 26637C>T), TCT37AGC (26639T>A 26640C>G 26641T>C), GCT38GTA (26643C>T 26644T>A), GTC40GTG (26650C>G), AGG41AAG (26652G>A), ATT42CTG (26654A>C 26656T>G), CAG43CAA (26659G>A)									
KMC43_gp45	8	96	70.1%	381	60.8%	89 (100%)	48 (53.9%)	0/0/0/0	0

	Begin	End	Coverage	Score	Concordance	Matches	Identities	I/D/M/F*	Stop Codons
**NT**	**14196**	**31794**	**9.4%**	**3582**	**41.3%**	**4358 (99.6%)**	**3090 (70.6%)**	**9/8**	
Protein mutations:	Q11H (27228G>T), W12L (27229T>C 27230G>T 27231G>T), V13W (27232G>T 27233T>G 27234T>G), I17L (27244A>C 27246C>G), T19S (27251C>G 27252G>C), S21P (27256T>C 27258A>C), T22S (27260C>G 27261G>C), L25I (27268C>A 27270G>C), G26A (27272G>C 27273C>G), T27L (27274A>C 27275C>T), A28S (27277G>T), N29S (27281A>G), A30G (27284C>G), D31T (27286G>A 27287A>C 27288C>G), T32Q (27289A>C 27290C>A), T34G (27295A>G 27296C>G 27297A>C), I38V (27307A>G 27309A>G), V39L (27310G>C), V43L (27322G>C 27324G>C), E48D (27339G>C), I50V (27343A>G 27345C>A), H52Q (27351C>A), E54S (27355G>A 27356A>G 27357G>C), K62Y (27379A>T 27381G>C), I63V (27382A>G), T66L (27391A>C 27392C>T), A71K (27406G>A 27407C>A), I72V (27409A>G 27411T>G), R73V (27412C>G 27413G>T), G74A (27416G>C 27417A>C), L77R (27425T>G 27426G>C), V80I (27433G>A), R81L (27436A>C 27437G>T), M82L (27439A>C), E84D (27447G>C), G85S (27448G>A), Q88K (27457C>A 27459A>G), E91P (27466G>C 27467A>C), V94I (27475G>A 27477G>C), D95N (27478G>A)								
Codon mutations:	CTC9CTG (27222C>G), AAT10AAC (27225T>C), CAG11CAT (27228G>T), TGG12CTT (27229T>C 27230G>T 27231G>T), GTT13TGG (27232G>T 27233T>G 27234T>G), GGT14GGC (27237T>C), ATC17CTG (27244A>C 27246C>G), GGC18GGA (27249C>A), ACG19AGC (27251C>G 27252G>C), TCC20AGC (27253T>A 27254C>G), TCA21CCC (27256T>C 27258A>C), ACG22AGC (27260C>G 27261G>C), GAT24GAC (27267T>C), CTG25ATC (27268C>A 27270G>C), GGC26GCG (27272G>C 27273C>G), ACC27CTC (27274A>C 27275C>T), GCC28TCC (27277G>T), AAC29AGC (27281A>G), GCC30GGC (27284C>G), GAC31ACG (27286G>A 27287A>C 27288C>G), ACG32CAG (27289A>C 27290C>A), ACA34GGC (27295A>G 27296C>G 27297A>C), ATA38GTG (27307A>G 27309A>G), GTG39CTG (27310G>C), CGC41CGG (27318C>G), CTT42CTG (27321T>G), GTG43CTC (27322G>C 27324G>C), ACG44ACC (27327G>C), AAT45AAC (27330T>C), CCC46CCG (27333C>G), GAG48GAC (27339G>C), ATC50GTA (27343A>G 27345C>A), CAC52CAA (27351C>A), CCT53CCG (27354T>G), GAG54AGC (27355G>A 27356A>G 27357G>C), TAT55TAC (27360T>C), AAG62TAC (27379A>T 27381G>C), ATC63GTC (27382A>G), GGT64GGC (27387T>C), ACG66CTG (27391A>C 27392C>T), GAC68GAT (27399C>T), GTG69GTC (27402G>C), GCG71AAG (27406G>A 27407C>A), ATT72GTG (27409A>G 27411T>G), CGC73GTC (27412C>G 27413G>T), GGA74GCC (27416G>C 27417A>C), CTG77CGC (27425T>G 27426G>C), CAG79CAA (27432G>A), GTC80ATC (27433G>A), AGG81CTG (27436A>C 27437G>T), ATG82CTG (27439A>C), GAG84GAC (27447G>C), GGC85AGC (27448G>A), GTT86GTG (27453T>G), GCG87GCC (27456G>C), CAA88AAG (27457C>A 27459A>G), GAG91CCG (27466G>C 27467A>C), CAA93CAG (27474A>G), GTG94ATC (27475G>A 27477G>C), GAC95AAC (27478G>A)								
KMC43_gp46	18	218	51.5%	814	71.2%	193 (100%)	121 (62.7%)	0/0/0/0	0
Protein mutations:	Y22A (27641T>G 27642A>C 27643C>A), A25Q (27650G>C 27651C>A 27652C>G), L29M (27662C>A), V31I (27668G>A), I34V (27677A>G 27679C>G), V38F (27689G>T 27691G>T), N42V (27701A>G 27702A>T 27703T>G), V45I (27710G>A 27712C>T), V46A (27714T>C), I47L (27716A>C), L52I (27731C>A), I53A (27734A>G 27735T>C 27736C>G), M54L (27737A>C), V56I (27743G>A 27745G>T), L57A (27746C>G 27747T>C 27748G>A), A58S (27749G>T), I59L (27752A>C 27754C>G), A62F (27761G>T 27762C>T 27763G>T), A65S (27770G>A 27771C>G 27772G>C), T66N (27774C>A 27775C>T), T68A (27779A>G 27781G>C), L70A (27785C>G 27786T>C 27787T>G), T72S (27792C>G 27793G>T), V74A (27798T>C), V79F (27812G>T 27814G>C), T80A (27815A>G), V85Q (27830G>C 27831T>A), A86Q (27833G>C 27834C>A), T90Q (27845A>C 27846C>A), A92T (27851G>A), S94A (27857T>G), T98S (27869A>T), Q100T (27875C>A 27876A>C 27877G>C), T107A (27896A>G 27898A>G), A108S (27899G>T 27901C>T), T116S (27923A>T), Q118K (27929C>A 27931A>G), F119Y (27933T>A), S132A (27971T>G), I139M (27994T>G), A144T (28007G>A 28009G>C), V146C (28013G>T 28014T>G 28015G>C), N147V (28016A>G 28017A>T 28018T>G), V148A (28020T>C 28021A>G), P149T (28022C>A 28024G>C), V151K (28028G>A 28029T>A), A152S (28031G>T), T154N (28038C>A 28039G>C), G156A (28044G>C 28045C>G), G159A (28053G>C 28054C>G), V162A (28062T>C), A163I (28064G>A 28065C>T 28066T>C), G164A (28068G>C), V166I (28073G>A 28075A>C), S167N (28076T>A 28077C>A 28078G>C), I169L (28082A>C), V170G (28086T>G), G171Q (28088G>C 28089G>A 28090C>G), S174A (28097T>G 28099C>G), N187T (28137A>C 28138C>G), A193T (28154G>A 28156A>G), L197F (28166C>T 28168G>C), T199S (28172A>T), I202V (28181A>G), A203G (28185C>G), V205I (28190G>A 28192A>C), A206N (28193G>A 28194C>A 28195G>C), K213R (28215A>G 28216A>G), S214A (28217T>G 28219C>T), V216I (28223G>A)								
Codon mutations:	TAC22GCA (27641T>G 27642A>C 27643C>A), TCT23TCG (27646T>G), GCC25CAG (27650G>C 27651C>A 27652C>G), TTA26CTC (27653T>C 27655A>C), GTT27GTC (27658T>C), CTG29ATG (27662C>A), GTC31ATC (27668G>A), TCG33TCC (27676G>C), ATC34GTG (27677A>G 27679C>G), CGC36CGG (27685C>G), GTG38TTT (27689G>T 27691G>T), AAT42GTG (27701A>G 27702A>T 27703T>G), GCT43GCG (27706T>G), GCC44GCA (27709C>A), GTC45ATT (27710G>A 27712C>T), GTG46GCG (27714T>C), ATC47CTC (27716A>C), CTC52ATC (27731C>A), ATC53GCG (27734A>G 27735T>C 27736C>G), ATG54CTG (27737A>C), CAA55CAG (27742A>G), GTG56ATT (27743G>A 27745G>T), CTG57GCA (27746C>G 27747T>C 27748G>A), GCG58TCG (27749G>T), ATC59CTG (27752A>C 27754C>G), GCG62TTT (27761G>T 27762C>T 27763G>T), GCT63GCG (27766T>G), ACG64ACC (27769G>C), GCG65AGC (27770G>A 27771C>G 27772G>C), ACC66AAT (27774C>A 27775C>T), ACG68GCC (27779A>G 27781G>C), CTT70GCG (27785C>G 27786T>C 27787T>G), ACG72AGT (27792C>G 27793G>T), GTG74GCG (27798T>C), GCG75GCC (27802G>C), GTG79TTC (27812G>T 27814G>C), ACG80GCG (27815A>G), CGT81CGC (27820T>C), CTT82CTG (27823T>G), GCT83GCG (27826T>G), GCG84GCC (27829G>C), GTG85CAG (27830G>C 27831T>A), GCG86CAG (27833G>C 27834C>A), ACT88ACC (27841T>C), GGA89GGC (27844A>C), ACG90CAG (27845A>C 27846C>A), GTC91GTG (27850C>G), GCC92ACC (27851G>A), TCG94GCG (27857T>G), ACG97ACT (27868G>T), ACG98TCG (27869A>T), ACC99ACG (27874C>G), CAG100ACC (27875C>A 27876A>C 27877G>C), GTC102GTT (27883C>T), GTT103GTG (27886T>G), CTT106CTC (27895T>C), ACA107GCG (27896A>G 27898A>G), GCC108TCT (27899G>T 27901C>T), GTT109GTC (27904T>C), ACA112ACG (27913A>G), GGG115GGC (27922G>C), ACG116TCG (27923A>T), CAA118AAG (27929C>A 27931A>G), TTC119TAC (27933T>A), GCT129GCC (27964T>C), TAT130TAC (27967T>C), TCG132GCG (27971T>G), ACA133ACG (27976A>G), GGC136GGG (27985C>G), TAC137TAT (27988C>T), ATT139ATG (27994T>G), GCT140GCG (27997T>G), GCG141GCC (28000G>C), GTG143GTC (28006G>C), GCG144ACC (28007G>A 28009G>C), AGC145AGT (28012C>T), GTG146TGC (28013G>T 28014T>G 28015G>C), AAT147GTG (28016A>G 28017A>T 28018T>G), GTA148GCG (28020T>C 28021A>G), CCG149ACC (28022C>A 28024G>C), GTG151AAG (28028G>A 28029T>A), GCA152TCA (28031G>T), GTG153GTC (28036G>C), ACG154AAC (28038C>A 28039G>C), GCC155GCA (28042C>A), GGC156GCG (28044G>C 28045C>G), GCT158GCG (28051T>G), GGC159GCG (28053G>C 28054C>G), GTG162GCG (28062T>C), GCT163ATC (28064G>A 28065C>T 28066T>C), GGC164GCC (28068G>C), GCC165GCG (28072C>G), GTA166ATC (28073G>A 28075A>C), TCG167AAC (28076T>A 28077C>A 28078G>C), ACC168ACG (28081C>G), ATC169CTC (28082A>C), GTC170GGC (28086T>G), GGC171CAG (28088G>C 28089G>A 28090C>G), GCA172GCC (28093A>C), ATT173ATC (28096T>C), TCC174GCG (28097T>G 28099C>G), GAT177GAC (28108T>C), GTC179GTG (28114C>G), ACG180ACC (28117G>C), GCC182GCG (28123C>G), GCA183GCG (28126A>G), AAC187ACG (28137A>C 28138C>G), GGC188GGT (28141C>T), GAT190GAC (28147T>C), GCA193ACG (28154G>A 28156A>G), GAC194GAT (28159C>T), GCT195GCC (28162T>C), GCG196GCC (28165G>C), CTG197TTC (28166C>T 28168G>C), CGT198CGC (28171T>C), ACG199TCG (28172A>T), CGC200CGG (28177C>G), ATC202GTC (28181A>G), GCC203GGC (28185C>G), GTA205ATC (28190G>A 28192A>C), GCG206AAC (28193G>A 28194C>A 28195G>C), AAG210AAA (28207G>A), ACG212ACC (28213G>C), AAA213AGG (28215A>G 28216A>G), TCC214GCT (28217T>G 28219C>T), GCT215GCC (28222T>C), GTC216ATC (28223G>A)								
KMC43_gp49	105	204	17.3%	517	79.4%	100 (100%)	79 (79.0%)	0/0/0/0	0
Protein mutations:	Q109Y (31508C>T 31510G>C), T118A (31535A>G 31537A>C), Q122S (31547C>A 31548A>G 31549G>C), D125N (31556G>A), T127V (31562A>G 31563C>T 31564T>G), A128T (31565G>A), W141Y (31605G>A 31606G>T), S142Q (31607A>C 31608G>A 31609C>G), N145A (31616A>G 31617A>C 31618C>G), N146G (31619A>G 31620A>G 31621C>A), S147N (31623G>A), A150S (31631G>A 31632C>G 31633A>T), T154A (31643A>G), V167I (31682G>A 31684A>C), S173T (31701G>C 31702C>G), S181V (31724T>G 31725C>T), T184V (31733A>G 31734C>T 31735C>G), W187Y (31743G>A 31744G>T), V188S (31745G>T 31746T>C), N193Y (31760A>T)								
Codon mutations:	ACT106ACC (31501T>C), GTG107GTC (31504G>C), CAG109TAC (31508C>T 31510G>C), ATT115ATC (31528T>C), CAG116CAA (31531G>A), ACA118GCC (31535A>G 31537A>C), TAC119TAT (31540C>T), CAA120CAG (31543A>G), GAT121GAC (31546T>C), CAG122AGC (31547C>A 31548A>G 31549G>C), GAC125AAC (31556G>A), CTG126TTG (31559C>T), ACT127GTG (31562A>G 31563C>T 31564T>G), GCG128ACG (31565G>A), TGG141TAT (31605G>A 31606G>T), AGC142CAG (31607A>C 31608G>A 31609C>G), AAC145GCG (31616A>G 31617A>C 31618C>G), AAC146GGA (31619A>G 31620A>G 31621C>A), AGC147AAC (31623G>A), GCA150AGT (31631G>A 31632C>G 31633A>T), GCG152GCC (31639G>C), ACT154GCT (31643A>G), GTC159GTG (31660C>G), GTT160GTG (31663T>G), GCC163GCG (31672C>G), GCT165GCA (31678T>A), GTA167ATC (31682G>A 31684A>C), GCG168GCC (31687G>C), GCT169GCA (31690T>A), ACC170ACG (31693C>G), GGC172GGA (31699C>A), AGC173ACG (31701G>C 31702C>G), ACC175ACG (31708C>G), ACC176ACG (31711C>G), CCA177CCG (31714A>G), GCC178GCA (31717C>A), TCC181GTC (31724T>G 31725C>T), GGC182GGT (31729C>T), ACC184GTG (31733A>G 31734C>T 31735C>G), GGG185GGT (31738G>T), CTG186CTC (31741G>C), TGG187TAT (31743G>A 31744G>T), GTG188TCG (31745G>T 31746T>C), ACC190ACG (31753C>G), GTC191GTT (31756C>T), AAT193TAT (31760A>T), GGT194GGC (31765T>C), ACG196ACC (31771G>C), ACC197ACG (31774C>G), GCG200GCC (31783G>C), CCG201CCC (31786G>C), AAC202AAT (31789C>T)								

Proteins

	Begin	End	Coverage	Score	Concordance	Matches	Identities	I/D/M/F*	Stop Codons
DUF4043 domain-containing protein (YP_010078151.1)	1	117	26.4%	715	90.2%	117 (100%)	103 (88.0%)	0/0/0/0	0
Protein mutations:	S4Q (14205A>C 14206G>A 14207C>A), D25E (14270T>A), A26G (14272C>G), I30M (14285C>G), G32S (14289G>A), F33Y (14293T>A 14294C>T), Q35S (14298C>T 14299A>C), R41Q (14317G>A 14318T>G), P67A (14394C>G), S68A (14397T>G 14399G>C), A69S (14400G>A 14401C>G 14402A>C), S78T (14427T>A), S88T (14457T>A), T102M (14500C>T)								
Codon mutations:	ATC3ATT (14204C>T), AGC4CAA (14205A>C 14206G>A 14207C>A), AAT5AAC (14210T>C), GCC8GCT (14219C>T), GCT9GCC (14222T>C), CCG12CCC (14231G>C), TTT23TTC (14264T>C), GAT25GAA (14270T>A), GCC26GGC (14272C>G), ATC30ATG (14285C>G), CTC31CTG (14288C>G), GGC32AGC (14289G>A), TTC33TAT (14293T>A 14294C>T), CAG35TCG (14298C>T 14299A>C), GCA37GCC (14306A>C), CGT38CGC (14309T>C), CGT41CAG (14317G>A 14318T>G), GGT47GGC (14336T>C), GAA48GAG (14339A>G), ACC49ACA (14342C>A), CGT54CGC (14357T>C), GGT56GGC (14363T>C), CTG57CTC (14366G>C), GCG59GCT (14372G>T), CCG60CCC (14375G>C), GTC61GTG (14378C>G), ACC62ACG (14381C>G), CCG67GCG (14394C>G), TCG68GCC (14397T>G 14399G>C), GCA69AGC (14400G>A 14401C>G 14402A>C), TCG78ACG (14427T>A), CCG79CCC (14432G>C), TCG80AGC (14433T>A 14434C>G 14435G>C), GTG84GTC (14447G>C), TCG88ACG (14457T>A), CTC89CTG (14462C>G), ACG97ACC (14486G>C), ACG102ATG (14500C>T), GTG103GTC (14504G>C), GTC107GTG (14516C>G), GTG110GTC (14525G>C)								
hypothetical protein (YP_010078153.1)	15	64	78.1%	-12	-3.5%	50 (94.3%)	19 (35.8%)	3/0/1/1	1
Protein mutations:	S18L (15870C>T 15871C>G), I19V (15872A>G), L20V (15875C>G), D22E (15883C>G), E23R (15884G>C 15885A>G 15886G>A), S24D (15887T>G 15888C>A), I25F (15890A>T), W27F (15897G>T 15898G>C), M28S (15900T>G 15901G>C), E29V (15903A>T 15904A>C), I32L (15911A>C 15913C>G), Q35V (15920C>G 15921A>T), F36W (15924T>G 15925C>G), H37A (15926C>G 15927A>C), E38A (15930A>C 15931A>T), T40K (15936C>A), H41P (15939A>C), V42I (15941G>A), N44E (15947A>G 15949C>A), P45V (15950C>G 15951C>T 15952T>G), V46R (15953G>C 15954T>G), V48I (15959G>A 15961G>C), A49R (15962G>C 15963C>G), A50E (15966C>A), Q53A (15974C>G 15975A>C 15976G>C), H54N (15977C>A), G55D (15981G>A), R57P (15987G>C 15988T>G), R57_Y58insIKA (15988_15989insATCAAGGCC), L59R (15992T>C 15993T>G), V61I (15998G>A)								

	Begin	End	Coverage	Score	Concordance	Matches	Identities	I/D/M/F*	Stop Codons
**NT**	**14196**	**31794**	**9.4%**	**3582**	**41.3%**	**4358 (99.6%)**	**3090 (70.6%)**	**9/8**	
Codon mutations:	CCC16CCG (15865C>G), GAT17GAC (15868T>C), TCC18TTG (15870C>T 15871C>G), ATC19GTC (15872A>G), CTG20GTG (15875C>G), TTG21CTG (15878T>C), GAC22GAG (15883C>G), GAG23CGA (15884G>C 15885A>G 15886G>A), TCC24GAC (15887T>G 15888C>A), ATC25TTC (15890A>T), GGG26GGC (15895G>C), TGG27TTC (15897G>T 15898G>C), ATG28AGC (15900T>G 15901G>C), GAA29GTC (15903A>T 15904A>C), ATC32CTG (15911A>C 15913C>G), CGA34CGC (15919A>C), CAG35GTG (15920C>G 15921A>T), TTC36TGG (15924T>G 15925C>G), CAC37GCC (15926C>G 15927A>C), GAA38GCT (15930A>C 15931A>T), GGT39GGC (15934T>C), ACG40AAG (15936C>A), CAC41CCC (15939A>C), GTC42ATC (15941G>A), AAC44GAA (15947A>G 15949C>A), CCT45GTG (15950C>G 15951C>T 15952T>G), GTC46CGC (15953G>C 15954T>G), GTG48ATC (15959G>A 15961G>C), GCC49CGC (15962G>C 15963C>G), GCG50GAG (15966C>A), CAG53GCC (15974C>G 15975A>C 15976G>C), CAC54AAC (15977C>A), GGC55GAC (15981G>A), CGT57CCG (15987G>C 15988T>G), CGT57_TAC58insATCAAGGCC (15988_15989insATCAAGGCC), TTG59CGG (15992T>C 15993T>G), GTC61ATC (15998G>A), AGC62-TG (16001delA 16002G>T 16003C>G)								
hypothetical protein (YP_010078154.1)	1	103	100%	538	80.9%	103 (100%)	77 (74.8%)	0/0/0/0	1
Protein mutations:	V5I (16018G>A), I7T (16025T>C 16026T>C), K11R (16036A>C 16037A>G 16038A>C), S12N (16040G>A), T14A (16045A>G 16047C>G), T18A (16057A>G 16059G>T), A19G (16061C>G 16062C>G), Q21T (16066C>A 16067A>C), S25T (16078T>A), K28A (16087A>G 16088A>C 16089G>C), D29G (16091A>G), I32L (16099A>C 16101C>G), N41Q (16126A>C 16128T>G), A48T (16147G>A 16149G>T), G49P (16150G>C 16151G>C), T58V (16177A>G 16178C>T 16179G>C), T60G (16183A>G 16184C>G), A62V (16190C>T 16191T>C), N65V (16198A>G 16199A>T), I67V (16204A>G), G68A (16208G>C 16209C>G), S76T (16231T>A 16233A>C), N85A (16258A>G 16259A>C 16260C>A), I89V (16270A>G), A93T (16282G>A 16284T>C), S98A (16297T>G)								
Codon mutations:	GAC3GAT (16014C>T), GTC5ATC (16018G>A), ATT7ACC (16025T>C 16026T>C), GGC10GGG (16035C>G), AAA11CGC (16036A>C 16037A>G 16038A>C), AGC12AAC (16040G>A), ACC14GCG (16045A>G 16047C>G), AAT16AAC (16053T>C), ACG18GCT (16057A>G 16059G>T), GCC19GGG (16061C>G 16062C>G), GCG20GCC (16065G>C), CAG21ACG (16066C>A 16067A>C), GTG22GTC (16071G>C), GTT23GTC (16074T>C), TCG25ACG (16078T>A), CCC27CCA (16086C>A), AAG28GCC (16087A>G 16088A>C 16089G>C), GAC29GGC (16091A>G), TTT30TTC (16095T>C), ATC32CTG (16099A>C 16101C>G), CGA36CGC (16113A>C), AAT41CAG (16126A>C 16128T>G), GTT42GTG (16131T>G), CTC43CTG (16134C>G), GTT44GTG (16137T>G), GCG45GCC (16140G>C), GGG46GGC (16143G>C), ACT47ACC (16146T>C), GCG48ACT (16147G>A 16149G>T), GGG49CCG (16150G>C 16151G>C), GCC51GCG (16158C>G), GCG52GCC (16161G>C), TAC53TAT (16164C>T), GCG55GCA (16170G>A), ACG57ACC (16176G>C), ACG58GTC (16177A>G 16178C>T 16179G>C), GCC59GCT (16182C>T), ACC60GGC (16183A>G 16184C>G), GCT62GTC (16190C>T 16191T>C), AAC65GTC (16198A>G 16199A>T), ATC67GTC (16204A>G), GGC68GCG (16208G>C 16209C>G), GCG69GCC (16212G>C), CCG71CCC (16218G>C), GTG74GTC (16227G>C), TCA76ACC (16231T>A 16233A>C), CTC78CTG (16239C>G), GAT80GAC (16245T>C), CCG82CCC (16251G>C), CTG84TTG (16255C>T), AAC85GCA (16258A>G 16259A>C 16260C>A), GGC86GGG (16263C>G), ATC89GTC (16270A>G), CCT91CCC (16278T>C), GCT93ACC (16282G>A 16284T>C), ACC96ACG (16293C>G), GTT97GTC (16296T>C), TCT98GCT (16297T>G), GTT99GTC (16302T>C), TCT100TCC (16305T>C)								
hypothetical protein (YP_010078155.1)	1	117	91.4%	710	86.3%	117 (100%)	93 (79.5%)	0/0/0/0	0
Protein mutations:	A6Q (16339G>C 16340C>A 16341C>G), K8R (16345A>C 16346A>G), G16W (16369G>T 16371A>G), M19V (16378A>G 16380G>C), F20Y (16382T>A 16383C>T), S26A (16399T>G), M43L (16450A>C), N44S (16453A>T 16454A>C 16455C>G), M46L (16459A>C), A48P (16465G>C 16467C>G), A52G (16478C>G 16479T>C), V54I (16483G>A 16485G>C), S71G (16534T>G 16535C>G 16536T>C), A72T (16537G>A), Q80K (16561C>A), V85T (16576G>A 16577T>C 16578G>C), V91L (16594G>C), A99S (16618G>A 16619C>G), R103L (16631G>T 16632C>G), E107Q (16642G>C), M109L (16648A>C 16650G>C), I111V (16654A>G 16656T>G), G116A (16670G>C 16671G>C)								
Codon mutations:	ACA4ACC (16335A>C), ACC5ACG (16338C>G), GCC6CAG (16339G>C 16340C>A 16341C>G), AAG8CGG (16345A>C 16346A>G), TAT14TAC (16365T>C), TGC15TGT (16368C>T), GGA16TGG (16369G>T 16371A>G), ATG19GTC (16378A>G 16380G>C), TTC20TAT (16382T>A 16383C>T), GGG22GGC (16389G>C), CCA24CCC (16395A>C), TCG25AGT (16396T>A 16397C>G 16398G>T), TCG26GCG (16399T>G), TAT30TAC (16413T>C), CGG31AGG (16414C>A), GCG35GCC (16428G>C), TAC36TAT (16431C>T), CTT39CTG (16440T>G), GAA40GAG (16443A>G), ATG43CTG (16450A>C), AAC44TCG (16453A>T 16454A>C 16455C>G), ATG46CTG (16459A>C), CTG47CTC (16464G>C), GCC48CCG (16465G>C 16467C>G), GAA50GAG (16473A>G), GCT52GGC (16478C>G 16479T>C), GTG54ATC (16483G>A 16485G>C), ACG57ACC (16494G>C), TAT58TAC (16497T>C), CTG59CTC (16500G>C), GCT60GCC (16503T>C), ACC63ACG (16512C>G), ACT64ACG (16515T>G), CTC65CTG (16518C>G), ACG67ACC (16524G>C), GCA68GCC (16527A>C), TCT71GGC (16534T>G 16535C>G 16536T>C), GCG72ACG (16537G>A), AGC73TCG (16540A>T 16541G>C 16542C>G), GCG74GCA (16545G>A), CTC76CTT (16551C>T), ACA78ACG (16557A>G), CAG80AAG (16561C>A), GCG81GCC (16566G>C), GCC82GCG (16569C>G), GTG85ACC (16576G>A 16577T>C 16578G>C), GAA90GAG (16593A>G), GTG91CTG (16594G>C), GCA92GCC (16599A>C), GAC93GAT (16602C>T), CGC94CGT (16605C>T), GAC95GAT (16608C>T), TTT98TTC (16617T>C), GCC99AGC (16618G>A 16619C>G), CGG102CGC (16629G>C), CGC103CTG (16631G>T 16632C>G), GAG107CAG (16642G>C), ATG109CTC (16648A>C 16650G>C), ATT111GTG (16654A>G 16656T>G), CCC113CCG (16662C>G), GGG114GGC (16665G>C), CCC115CCG (16668C>G), GGG116GCC (16670G>C 16671G>C), CTC117T.. (16672C>T)								
tail protein (YP_010078160.1)	17	497	72.0%	1825	76.6%	368 (100%)	262 (71.2%)	0/0/0/0	0
Protein mutations:	V28T (19290G>A 19291T>C 19292T>C), S29Q (19293T>C 19294C>A 19295C>G), L30Y (19296C>T 19297T>A 19298G>C), V38I (19320G>A 19322G>C), V41I (19329G>A 19331T>C), T46S (19344A>T), L55T (19371C>A 19372T>C), I56V (19374A>G), N58S (19381A>G), M59P (19383A>C 19384T>C), M61Q (19389A>C 19390T>A), Y62A (19392T>G 19393A>C), Q64A (19398C>G 19399A>C 19400G>C), A65I (19401G>A 19402C>T), A68Q (19410G>C 19411C>A), I69A (19413A>G 19414T>C), T73K (19426C>A 19427C>G), M76L (19434A>C), G77M (19437G>A 19438G>T 19439C>G), V80I (19446G>A 19448G>C), A81S (19449G>T), I82V (19452A>G), V84T (19458G>A 19459T>C 19460G>C), Q85M (19461C>A 19462A>T), R92K (19482C>A 19483G>A 19484C>A), C93A (19485T>G 19486G>C), T102V (19512A>G 19513C>T), T106I (19525C>T 19526G>C), I107A (19527A>G 19528T>C 19529C>G), Q109L (19534A>T), G112C (19542G>T), V113I (19545G>A 19547G>C), L115F (19551C>T 19553G>C), A117S (19557G>T 19559G>C), L123F (19575C>T 19577G>C), S126G (19584T>G 19585C>G 19586G>C), V127I (19587G>A 19589G>C), A130T (19596G>A), A132S (19602G>T 19604C>G), A136Q (19614G>C 19615C>A 19616C>G), N138S (19620A>T 19621A>C 19622T>G), W140Q (19626T>C 19627G>A), V142A (19633T>C 19634A>C), T143V (19635A>G 19636C>T), A145S (19641G>T), I266T (20005T>C 20006C>G), I269V (20013A>G), G270M (20016G>A 20017G>T 20018C>G), V271T (20019G>A 20020T>C), A274S (20028G>A 20029C>G), S279A (20043A>G 20044G>C 20045C>G), T282I (20053C>T), T283S (20056C>G 20057G>C), T284A (20058A>G), A286S (20064G>A 20065C>G), I290V (20076A>G 20078C>G), F295A (20091T>G 20092T>C), V303C (20115G>T 20116T>G 20117G>C), V309Y (20133G>T 20134T>A), S311Q (20139A>C 20140G>A 20141C>G), V312I (20142G>A 20144T>C), T313L (20145A>C 20146C>T 20147A>T), A320S (20166G>T 20168G>C), S327A (20187T>G 20189G>C), T335S (20211A>T), Y341Q (20229T>C 20231T>G), V343I (20235G>A 20237G>C), T354Q (20268A>C 20269C>A), S362Q (20292T>C 20293C>A), I364G (20298A>G 20299T>G), Q365I (20301C>A 20302A>T 20303G>C), C383A (20355T>G 20356G>C 20357C>G), S385F (20362C>T 20363G>C), A393P (20385G>C), L394V (20388C>G), Q396N (20394C>A 20396G>C), S409Y (20433A>T 20434G>A), V411L (20439G>C), Y418F (20461A>T), V419I (20463G>A), A425S (20481G>T 20483C>G), A429T (20493G>A 20495G>T), A432M (20502G>A 20503C>T 20504C>G), L435I (20511C>A 20513G>C), N437A (20517A>G 20518A>C), S440D (20526T>G 20527C>A), S441N (20530G>A), M442L (20532A>C), E443Q (20535G>C), M447L (20547A>C), A450D (20557C>A), V451P (20559G>C 20560T>C 20561C>G), P455A (20571C>G 20573A>G), S456A (20574T>G 20576G>C), F457Y (20578T>A), I461V (20589A>G 20591C>G), N464A (20598A>G 20599A>C), M468A (20610A>G 20611T>C), N469S (20613A>T 20614A>C 20615C>G), L476M (20634C>A 20636T>G), I483K (20656T>A 20657T>G), S486A (20664T>G 20666G>C), I492L (20682A>C), V493I (20685G>A 20687G>C), L495I (20691C>A 20693T>C)								

	Begin	End	Coverage	Score	Concordance	Matches	Identities	I/D/M/F*	Stop Codons
**NT**	**14196**	**31794**	**9.4%**	**3582**	**41.3%**	**4358 (99.6%)**	**3090 (70.6%)**	**9/8**	
Codon mutations:	TTG19CTG (19263T>C), GTA21GTG (19271A>G), CAA22CAG (19274A>G), GTG24GTC (19280G>C), CCT25CCG (19283T>G), CCA26CCG (19286A>G), GTT28ACC (19290G>A 19291T>C 19292T>C), TCC29CAG (19293T>C 19294C>A 19295C>G), CTG30TAC (19296C>T 19297T>A 19298G>C), CTC31CTG (19301C>G), AAT37AAC (19319T>C), GTG38ATC (19320G>A 19322G>C), CTT39TTG (19323C>T 19325T>G), GGT40GGC (19328T>C), GTT41ATC (19329G>A 19331T>C), GTG42GTC (19334G>C), ACC44ACG (19340C>G), GCA45GCG (19343A>G), ACC46TCC (19344A>T), GGC48GGG (19352C>G), ACC50ACG (19358C>G), TCG52AGC (19362T>A 19363C>G 19364G>C), CCG53CCC (19367G>C), ACG54ACC (19370G>C), CTG55ACG (19371C>A 19372T>C), ATC56GTC (19374A>G), AAC58AGC (19381A>G), ATG59CCG (19383A>C 19384T>C), GCG60GCT (19388G>T), ATG61CAG (19389A>C 19390T>A), TAC62GCC (19392T>G 19393A>C), GCC63GCG (19397C>G), CAG64GCC (19398C>G 19399A>C 19400G>C), GCC65ATC (19401G>A 19402C>T), GCG68CAG (19410G>C 19411C>A), ATC69GCC (19413A>G 19414T>C), CGT72CGC (19424T>C), ACC73AAG (19426C>A 19427C>G), TAT74TAC (19430T>C), ATG76CTG (19434A>C), GGC77ATG (19437G>A 19438G>T 19439C>G), GCT79GCC (19445T>C), GTG80ATC (19446G>A 19448G>C), GCG81TCG (19449G>T), ATC82GTC (19452A>G), GCA83GCG (19457A>G), GTG84ACC (19458G>A 19459T>C 19460G>C), CAG85ATG (19461C>A 19462A>T), GCG88GCC (19472G>C), CGC92AAA (19482C>A 19483G>A 19484C>A), TGC93GCC (19485T>G 19486G>C), CGC95CGG (19493C>G), GTC96GTG (19496C>G), ACG102GTG (19512A>G 19513C>T), GCG104GCC (19520G>C), TCG105AGC (19521T>A 19522C>G 19523G>C), ACG106ATC (19525C>T 19526G>C), ATC107GCG (19527A>G 19528T>C 19529C>G), CAG109CTG (19534A>T), GGC112TGC (19542G>T), GTG113ATC (19545G>A 19547G>C), ACG114ACC (19550G>C), CTG115TTC (19551C>T 19553G>C), GCG117TCC (19557G>T 19559G>C), ACG122ACC (19574G>C), CTG123TTC (19575C>T 19577G>C), TCG126GGC (19584T>G 19585C>G 19586G>C), GTG127ATC (19587G>A 19589G>C), GTC129GTG (19595C>G), GCG130ACG (19596G>A), CTC131CTG (19601C>G), GCC132TCG (19602G>T 19604C>G), GCG133GCC (19607G>C), AGC135TCG (19611A>T 19612G>C 19613C>G), GCC136CAG (19614G>C 19615C>A 19616C>G), AAT138TCG (19620A>T 19621A>C 19622T>G), TGG140CAG (19626T>C 19627G>A), GTA142GCC (19633T>C 19634A>C), ACG143GTG (19635A>G 19636C>T), GTC144GTT (19640C>T), GCG145TCG (19641G>T), ATC266ACG (20005T>C 20006C>G), ATC269GTC (20013A>G), GGC270ATG (20016G>A 20017G>T 20018C>G), GTC271ACC (20019G>A 20020T>C), GCT274AGT (20028G>A 20029C>G), ACG277ACC (20039G>C), AGC279GCG (20043A>G 20044G>C 20045C>G), GCC281GCG (20051C>G), ACC282ATC (20053C>T), ACG283AGC (20056C>G 20057G>C), ACC284GCC (20058A>G), GCC286AGC (20064G>A 20065C>G), ATC290GTG (20076A>G 20078C>G), TTC295GCC (20091T>G 20092T>C), CTG298CTT (20102G>T), GGT300GGC (20108T>C), GTG303TGC (20115G>T 20116T>G 20117G>C), TTG306CTG (20124T>C), ACG308ACC (20132G>C), GTC309TAC (20133G>T 20134T>A), AGC311CAG (20139A>C 20140G>A 20141C>G), GTT312ATC (20142G>A 20144T>C), ACA313CTT (20145A>C 20146C>T 20147A>T), CTG315TTG (20151C>T), TCG317AGC (20157T>A 20158C>G 20159G>C), CAA319CAG (20165A>G), GCG320TCC (20166G>T 20168G>C), GTC322GTG (20174C>G), GCT323GCC (20177T>C), GGT324GGC (20180T>C), TCG327GCC (20187T>G 20189G>C), ACG335TCG (20211A>T), TAT341CAG (20229T>C 20231T>G), GTG343ATC (20235G>A 20237G>C), GTG344GTC (20240G>C), TCG349AGC (20253T>A 20254C>G 20255G>C), ACG354CAG (20268A>C 20269C>A), TCC357TCG (20279C>G), GCC358GCT (20282C>T), TTG360CTG (20286T>C), TCG362CAG (20292T>C 20293C>A), ATC364GGC (20298A>G 20299T>G), CAG365ATC (20301C>A 20302A>T 20303G>C), GCG366GCC (20306G>C), GGT367GGC (20309T>C), CCC376CCG (20336C>G), TCG379AGC (20343T>A 20344C>G 20345G>C), TGC383GCG (20355T>G 20356G>C 20357C>G), CGG384CGC (20360G>C), TCG385TTC (20362C>T 20363G>C), CAT387CAC (20369T>C), GCG393CCG (20385G>C), CTG394GTG (20388C>G), CAG396AAC (20394C>A 20396G>C), ACC401ACG (20411C>G), AGC409TAC (20433A>T 20434G>A), GTC411CTC (20439G>C), AAT412AAC (20444T>C), TAC418TTC (20461A>T), GTC419ATC (20463G>A), GGG420GGT (20468G>T), CAA423CAG (20477A>G), TCG424AGC (20478T>A 20479C>G 20480G>C), GCC425TCG (20481G>T 20483C>G), GTG427GTC (20489G>C), GCG429ACT (20493G>A 20495G>T), GCG431GCC (20501G>C), GCC432ATG (20502G>A 20503C>T 20504C>G), CTG435ATC (20511C>A 20513G>C), TCG436AGC (20514T>A 20515C>G 20516G>C), AAC437GCC (20517A>G 20518A>C), TCC440GAC (20526T>G 20527C>A), AGC441AAC (20530G>A), ATG442CTG (20532A>C), GAG443CAG (20535G>C), GGC446GGA (20546C>A), ATG447CTG (20547A>C), GCC450GAC (20557C>A), GTC451CCG (20559G>C 20560T>C 20561C>G), AAT452AAC (20564T>C), CCA455GCG (20571C>G 20573A>G), TCG456GCC (20574T>G 20576G>C), TTC457TAC (20578T>A), TCG458AGC (20580T>A 20581C>G 20582G>C), CAA460CAG (20588A>G), ATC461GTG (20589A>G 20591C>G), GCT463GCG (20597T>G), AAC464GCC (20598A>G 20599A>C), ATG468GCG (20610A>G 20611T>C), AAC469TCG (20613A>T 20614A>C 20615C>G), CGG470CGC (20618G>C), GCG472GCC (20624G>C), CTT473CTG (20627T>G), CTT476ATG (20634C>A 20636T>G), GCT478GCC (20642T>C), GTC480GTG (20648C>G), ATT483AAG (20656T>A 20657T>G), TCG486GCC (20664T>G 20666G>C), GTT487GTC (20669T>C), ATC492CTC (20682A>C), GTG493ATC (20685G>A 20687G>C), AAT494AAC (20690T>C), CTT495ATC (20691C>A 20693T>C)								
hypothetical protein (YP_010078166.1)	12	115	32.4%	453	59.9%	104 (100%)	57 (54.8%)	0/0/0/0	0
Protein mutations:	D14G (24489A>G), R15S (24491C>T 24492G>C), Q16L (24495A>T 24496G>C), I18V (24500A>G 24502T>G), E20A (24507A>C 24508G>C), S21E (24509T>G 24510C>A), P23E (24515C>G 24516C>A), T27A (24527A>G 24529C>G), R32V (24542C>G 24543G>T 24544C>G), D34E (24550C>G), V36I (24554G>A), V42I (24572G>A), A47R (24587G>C 24588C>G), F48S (24591T>C 24592C>G), T49D (24593A>G 24594C>A 24595G>T), E50R (24596G>C 24597A>G), I52L (24602A>C), N53G (24605A>G 24606A>G), W57M (24617T>A 24618G>T), L58M (24620C>A), Q61V (24629C>G 24630A>T 24631A>C), D62G (24633A>G 24634T>C), L64M (24638T>A), A65D (24642C>A 24643G>C), R68L (24651G>T), Q69Y (24653C>T 24655G>C), A72G (24663C>G 24664G>C), M73L (24665A>C), V75I (24671G>A 24673T>C), A76G (24675C>G 24676G>C), T78Q (24680A>C 24681C>A 24682C>G), V81E (24690T>A 24691G>A), Q83S (24695C>A 24696A>G 24697G>C), S85G (24701T>G 24702C>G 24703A>C), E86Q (24704G>C), S88R (24710T>C 24711C>G), F89Y (24714T>A 24715T>C), S90K (24716T>A 24717C>A 24718C>G), V92I (24722G>A 24724G>C), I93V (24725A>G), D95E (24733C>G), R97S (24737C>T 24738G>C 24739C>G), D99T (24743G>A 24744A>C 24745T>C), F100Y (24747T>A 24748C>T), F103A (24755T>G 24756T>C 24757C>T), T111V (24779A>G 24780C>T 24781G>C)								
Codon mutations:	GAC14GGC (24489A>G), CGG15TCG (24491C>T 24492G>C), CAG16CTC (24495A>T 24496G>C), ATT18GTG (24500A>G 24502T>G), CCC19CCG (24505C>G), GAG20GCC (24507A>C 24508G>C), TCG21GAG (24509T>G 24510C>A), CCG23GAG (24515C>G 24516C>A), GGG26GGC (24526G>C), ACC27GCG (24527A>G 24529C>G), CAA28CAG (24532A>G), GCG31GCC (24541G>C), CGC32GTG (24542C>G 24543G>T 24544C>G), GAC34GAG (24550C>G), CTT35CTG (24553T>G), GTC36ATC (24554G>A), GGA38GGC (24562A>C), CGG40CGC (24568G>C), GTC42ATC (24572G>A), GCG44GCC (24580G>C), GCC47CGC (24587G>C 24588C>G), TTC48TCG (24591T>C 24592C>G), ACG49GAT (24593A>G 24594C>A 24595G>T), GAG50CGG (24596G>C 24597A>G), CCC51CCG (24601C>G), ATC52CTC (24602A>C), AAC53GGC (24605A>G 24606A>G), TCT55TCC (24613T>C), GGC56GGG (24616C>G), TGG57ATG (24617T>A 24618G>T), CTG58ATG (24620C>A), CTT59CTC (24625T>C), GGC60GGT (24628C>T), CAA61GTC (24629C>G 24630A>T 24631A>C), GAT62GGC (24633A>G 24634T>C), GCT63GCC (24637T>C), TTG64ATG (24638T>A), GCG65GAC (24642C>A 24643G>C), CGA66CGC (24646A>C), CGG68CTG (24651G>T), CAG69TAC (24653C>T 24655G>C), CTT70CTG (24658T>G), GAT71GAC (24661T>C), GCG72GGC (24663C>G 24664G>C), ATG73CTG (24665A>C), CGG74CGC (24670G>C), GTT75ATC (24671G>A 24673T>C), GCG76GGC (24675C>G 24676G>C), ACC78CAG (24680A>C 24681C>A 24682C>G), GTC79GTG (24685C>G), CAG80CAA (24688G>A), GTG81GAA (24690T>A 24691G>A), CAG83AGC (24695C>A 24696A>G 24697G>C), TCA85GGC (24701T>G 24702C>G 24703A>C), GAG86CAG (24704G>C), TTG87CTG (24707T>C), TCC88CGC (24710T>C 24711C>G), TTT89TAC (24714T>A 24715T>C), TCC90AAG (24716T>A 24717C>A 24718C>G), GTA91GTG (24721A>G), GTG92ATC (24722G>A 24724G>C), ATT93GTT (24725A>G), CGG94CGC (24730G>C), GAC95GAG (24733C>G), CGC97TCG (24737C>T 24738G>C 24739C>G), GAT99ACC (24743G>A 24744A>C 24745T>C), TTC100TAT (24747T>A 24748C>T), CGT102CGC (24754T>C), TTC103GCT (24755T>G 24756T>C 24757C>T), TAT104TAC (24760T>C), CGA109CGC (24775A>C), ACG111GTC (24779A>G 24780C>T 24781G>C), GAG113GAA (24787G>A)								
hypothetical protein (YP_010078167.1)	25	373	46.4%	790	70.5%	171 (98.8%)	114 (65.9%)	0/2/0/0	1
Protein mutations:	A28G (25498C>G), F32W (25510T>G 25511T>G), N36A (25521A>G 25522A>C 25523C>G), F39Y (25531T>A), Q41E (25536C>G), A51T (25566G>A 25568G>T), L54M (25575C>A), P55A (25578C>G 25580G>C), A56D (25582C>A 25583G>C), E57Q (25584G>C), D59del (25590_25592delGAC), N61D (25596A>G), N65Q (25608A>C 25610C>G), K67A (25614A>G 25615A>C 25616A>C), A69I (25620G>A 25621C>T 25622G>C), F70Y (25624T>A), F74L (25635T>C 25637C>G), D80N (25653G>A), T82A (25659A>G), N83D (25662A>G), Y84P (25665T>C 25666A>C 25667C>G), E86A (25672A>C 25673G>C), T87N (25675C>A 25676G>C), D88E (25679C>A), T90S (25683A>T 25685C>G), Y102F (25720A>T), L108M (25737C>A), T110S (25744C>G), A119V (25771C>T 25772G>C), T305I (26329C>T 26330G>C), Q306A (26331C>G 26332A>C), K310R (26343A>C 26344A>G 26345G>C), L311M (26346C>A), I320L (26373A>C 26375C>G), T322Q (26379A>C 26380C>A), Q323A (26382C>G 26383A>C 26384G>C), T324K (26386C>A), A325S (26388G>T 26390C>G), M326V (26391A>G 26393G>C), L327I (26394C>A 26396T>C), R328K (26397C>A 26398G>A), L329V (26400C>G), S330Q (26403T>C 26404C>A 26405T>G), K335A (26418A>G 26419A>C 26420G>T), D339T (26430G>A 26431A>C), F341Y (26437T>A), V342P (26439G>C 26440T>C 26441A>C), D343M (26442G>A 26443A>T 26444C>G), S348M (26457T>A 26458C>T), L351M (26466C>A 26468C>G), V356T (26481G>A 26482T>C 26483G>C), E358L (26487G>T 26488A>T 26489A>G), S360D (26493T>G 26494C>A), I364R (26505A>C 26506T>G), E367D (26516A>C), V369del (26520_26522delGTG), S371L (26526T>C 26527C>T 26528C>G), I372Q (26529A>C 26530T>A)								
Codon mutations:	GCC28GGC (25498C>G), AGT31TCC (25506A>T 25507G>C 25508T>C), TTT32TGG (25510T>G 25511T>G), GTC34GTG (25517C>G), ACA35ACC (25520A>C), AAC36GCG (25521A>G 25522A>C 25523C>G), TTC39TAC (25531T>A), TAT40TAC (25535T>C), CAG41GAG (25536C>G), GCT42GCC (25541T>C), CGT46CGC (25553T>C), GCA50GCC (25565A>C), GCG51ACT (25566G>A 25568G>T), TCT52TCG (25571T>G), CTG54ATG (25575C>A), CCG55GCC (25578C>G 25580G>C), GCG56GAC (25582C>A 25583G>C), GAG57CAG (25584G>C), ACA58ACC (25589A>C), GAC59del (25590_25592delGAC), GCG60GCC (25595G>C), AAC61GAC (25596A>G), TTC63TTT (25604C>T), TCC64TCT (25607C>T), AAC65CAG (25608A>C 25610C>G), AAA67GCC (25614A>G 25615A>C 25616A>C), GCG69ATC (25620G>A 25621C>T 25622G>C), TTC70TAC (25624T>A), ATT73ATC (25634T>C), TTC74CTG (25635T>C 25637C>G), TCC79AGC (25650T>A 25651C>G), GAT80AAT (25653G>A), ACG82GCG (25659A>G), AAC83GAC (25662A>G), TAC84CCG (25665T>C 25666A>C 25667C>G), GAG86GCC (25672A>C 25673G>C), ACG87AAC (25675C>A 25676G>C), GAC88GAA (25679C>A), CTC89CTG (25682C>G), ACC90TCG (25683A>T 25685C>G), CTG92CTC (25691G>C), TAT94TAC (25697T>C), GGG95GGC (25700G>C), GTG97GTC (25706G>C), GAT98GAC (25709T>C), TAC102TTC (25720A>T), GAC103GAT (25724C>T), CCC104CCG (25727C>G), TCG106TCC (25733G>C), CTG108ATG (25737C>A), ACC110AGC (25744C>G), GCG119GTC (25771C>T 25772G>C), GAG303GAA (26324G>A), ACG305ATC (26329C>T 26330G>C), CAG306GCG (26331C>G 26332A>C), CAC307CAT (26336C>T), GAA308GAG (26339A>G), AAG310CGC (26343A>C 26344A>G 26345G>C), CTG311ATG (26346C>A), GCG313GCC (26354G>C), GCA317GCC (26366A>C), ATC320CTG (26373A>C 26375C>G), ACG322CAG (26379A>C 26380C>A), CAG323GCC (26382C>G 26383A>C 26384G>C), ACG324AAG (26386C>A), GCC325TCG (26388G>T 26390C>G), ATG326GTC (26391A>G 26393G>C), CTT327ATC (26394C>A 26396T>C), CGG328AAG (26397C>A 26398G>A), CTG329GTG (26400C>G), TCT330CAG (26403T>C 26404C>A 26405T>G), GGG331GGC (26408G>C), AAG335GCT (26418A>G 26419A>C 26420G>T), GAT337GAC (26426T>C), GAC339ACC (26430G>A 26431A>C), TTC341TAC (26437T>A), GTA342CCC (26439G>C 26440T>C 26441A>C), GAC343ATG (26442G>A 26443A>T 26444C>G), ATT345ATC (26450T>C), ACG346ACC (26453G>C), CGG347CGA (26456G>A), TCG348ATG (26457T>A 26458C>T), CTC351ATG (26466C>A 26468C>G), GAT352GAC (26471T>C), TAT355TAC (26480T>C), GTG356ACC (26481G>A 26482T>C 26483G>C), GAA358TTG (26487G>T 26488A>T 26489A>G), TCC360GAC (26493T>G 26494C>A), ATC364CGC (26505A>C 26506T>G), AAT365AAC (26510T>C), CCT366CCC (26513T>C), GAA367GAC (26516A>C), GTG369del (26520_26522delGTG), CCC370CCG (26525C>G), TCC371CTG (26526T>C 26527C>T 26528C>G), ATA372CAA (26529A>C 26530T>A)								
baseplate assembly protein V (YP_010078168.1)	1	44	19.7%	136	47.1%	44 (100%)	20 (45.5%)	0/0/0/0	0
Protein mutations:	Q3G (26537C>G 26538A>G 26539G>A), H4P (26541A>C 26542T>A), F5L (26543T>C), R6Q (26547G>A 26548A>G), Q8A (26552C>G 26553A>C), A10R (26558G>C 26559C>G 26560T>C), A12Q (26564G>C 26565C>A 26566G>A), Q14L (26571A>T 26572A>G), L15R (26574T>G 26575A>G), Q17T (26579C>A 26580A>C), A18S (26582G>T 26584T>C), G19D (26586G>A 26587C>T), R20Q (26589G>A 26590C>G), E22A (26595A>C), S23A (26597A>G 26598G>C 26599C>A), V28I (26612G>A), T29S (26616C>G), D32E (26626C>G), G34A (26631G>C 26632C>G), A36F (26636G>T 26637C>T), A38V (26643C>T 26644T>A), R41K (26652G>A), I42L (26654A>C 26656T>G)								

	Begin	End	Coverage	Score	Concordance	Matches	Identities	I/D/M/F*	Stop Codons
**NT**	**14196**	**31794**	**9.4%**	**3582**	**41.3%**	**4358 (99.6%)**	**3090 (70.6%)**	**9/8**	
Codon mutations:	CAG3GGA (26537C>G 26538A>G 26539G>A), CAT4CCA (26541A>C 26542T>A), TTC5CTC (26543T>C), CGA6CAG (26547G>A 26548A>G), AAT7AAC (26551T>C), CAG8GCG (26552C>G 26553A>C), GCT10CGC (26558G>C 26559C>G 26560T>C), CTC11CTG (26563C>G), GCG12CAA (26564G>C 26565C>A 26566G>A), GCC13GCG (26569C>G), CAA14CTG (26571A>T 26572A>G), CTA15CGG (26574T>G 26575A>G), GCG16GCC (26578G>C), CAG17ACG (26579C>A 26580A>C), GCT18TCC (26582G>T 26584T>C), GGC19GAT (26586G>A 26587C>T), CGC20CAG (26589G>A 26590C>G), GCT21GCC (26593T>C), GAG22GCG (26595A>C), AGC23GCA (26597A>G 26598G>C 26599C>A), CGG24CGC (26602G>C), GTT25GTG (26605T>G), GGC26GGG (26608C>G), ATT27ATC (26611T>C), GTC28ATC (26612G>A), ACC29AGC (26616C>G), GAC32GAG (26626C>G), CCA33CCC (26629A>C), GGC34GCG (26631G>C 26632C>G), ACG35ACC (26635G>C), GCC36TTC (26636G>T 26637C>T), TCT37AGC (26639T>A 26640C>G 26641T>C), GCT38GTA (26643C>T 26644T>A), GTC40GTG (26650C>G), AGG41AAG (26652G>A), ATT42CTG (26654A>C 26656T>G), CAG43CAA (26659G>A)								
tail protein (YP_010078169.1)	8	96	70.1%	381	60.8%	89 (100%)	48 (53.9%)	0/0/0/0	0
Protein mutations:	Q11H (27228G>T), W12L (27229T>C 27230G>T 27231G>T), V13W (27232G>T 27233T>G 27234T>G), I17L (27244A>C 27246C>G), T19S (27251C>G 27252G>C), S21P (27256T>C 27258A>C), T22S (27260C>G 27261G>C), L25I (27268C>A 27270G>C), G26A (27272G>C 27273C>G), T27L (27274A>C 27275C>T), A28S (27277G>T), N29S (27281A>G), A30G (27284C>G), D31T (27286G>A 27287A>C 27288C>G), T32Q (27289A>C 27290C>A), T34G (27295A>G 27296C>G 27297A>C), I38V (27307A>G 27309A>G), V39L (27310G>C), V43L (27322G>C 27324G>C), E48D (27339G>C), I50V (27343A>G 27345C>A), H52Q (27351C>A), E54S (27355G>A 27356A>G 27357G>C), K62Y (27379A>T 27381G>C), I63V (27382A>G), T66L (27391A>C 27392C>T), A71K (27406G>A 27407C>A), I72V (27409A>G 27411T>G), R73V (27412C>G 27413G>T), G74A (27416G>C 27417A>C), L77R (27425T>G 27426G>C), V80I (27433G>A), R81L (27436A>C 27437G>T), M82L (27439A>C), E84D (27447G>C), G85S (27448G>A), Q88K (27457C>A 27459A>G), E91P (27466G>C 27467A>C), V94I (27475G>A 27477G>C), D95N (27478G>A)								
Codon mutations:	CTC9CTG (27222C>G), AAT10AAC (27225T>C), CAG11CAT (27228G>T), TGG12CTT (27229T>C 27230G>T 27231G>T), GTT13TGG (27232G>T 27233T>G 27234T>G), GGT14GGC (27237T>C), ATC17CTG (27244A>C 27246C>G), GGC18GGA (27249C>A), ACG19AGC (27251C>G 27252G>C), TCC20AGC (27253T>A 27254C>G), TCA21CCC (27256T>C 27258A>C), ACG22AGC (27260C>G 27261G>C), GAT24GAC (27267T>C), CTG25ATC (27268C>A 27270G>C), GGC26GCG (27272G>C 27273C>G), ACC27CTC (27274A>C 27275C>T), GCC28TCC (27277G>T), AAC29AGC (27281A>G), GCC30GGC (27284C>G), GAC31ACG (27286G>A 27287A>C 27288C>G), ACG32CAG (27289A>C 27290C>A), ACA34GGC (27295A>G 27296C>G 27297A>C), ATA38GTG (27307A>G 27309A>G), GTG39CTG (27310G>C), CGC41CGG (27318C>G), CTT42CTG (27321T>G), GTG43CTC (27322G>C 27324G>C), ACG44ACC (27327G>C), AAT45AAC (27330T>C), CCC46CCG (27333C>G), GAG48GAC (27339G>C), ATC50GTA (27343A>G 27345C>A), CAC52CAA (27351C>A), CCT53CCG (27354T>G), GAG54AGC (27355G>A 27356A>G 27357G>C), TAT55TAC (27360T>C), AAG62TAC (27379A>T 27381G>C), ATC63GTC (27382A>G), GGT64GGC (27387T>C), ACG66CTG (27391A>C 27392C>T), GAC68GAT (27399C>T), GTG69GTC (27402G>C), GCG71AAG (27406G>A 27407C>A), ATT72GTG (27409A>G 27411T>G), CGC73GTC (27412C>G 27413G>T), GGA74GCC (27416G>C 27417A>C), CTG77CGC (27425T>G 27426G>C), CAG79CAA (27432G>A), GTC80ATC (27433G>A), AGG81CTG (27436A>C 27437G>T), ATG82CTG (27439A>C), GAG84GAC (27447G>C), GGC85AGC (27448G>A), GTT86GTG (27453T>G), GCG87GCC (27456G>C), CAA88AAG (27457C>A 27459A>G), GAG91CCG (27466G>C 27467A>C), CAA93CAG (27474A>G), GTG94ATC (27475G>A 27477G>C), GAC95AAC (27478G>A)								
hypothetical protein (YP_010078170.1)	18	218	51.5%	814	71.2%	193 (100%)	121 (62.7%)	0/0/0/0	0
Protein mutations:	Y22A (27641T>G 27642A>C 27643C>A), A25Q (27650G>C 27651C>A 27652C>G), L29M (27662C>A), V31I (27668G>A), I34V (27677A>G 27679C>G), V38F (27689G>T 27691G>T), N42V (27701A>G 27702A>T 27703T>G), V45I (27710G>A 27712C>T), V46A (27714T>C), I47L (27716A>C), L52I (27731C>A), I53A (27734A>G 27735T>C 27736C>G), M54L (27737A>C), V56I (27743G>A 27745G>T), L57A (27746C>G 27747T>C 27748G>A), A58S (27749G>T), I59L (27752A>C 27754C>G), A62F (27761G>T 27762C>T 27763G>T), A65S (27770G>A 27771C>G 27772G>C), T66N (27774C>A 27775C>T), T68A (27779A>G 27781G>C), L70A (27785C>G 27786T>C 27787T>G), T72S (27792C>G 27793G>T), V74A (27798T>C), V79F (27812G>T 27814G>C), T80A (27815A>G), V85Q (27830G>C 27831T>A), A86Q (27833G>C 27834C>A), T90Q (27845A>C 27846C>A), A92T (27851G>A), S94A (27857T>G), T98S (27869A>T), Q100T (27875C>A 27876A>C 27877G>C), T107A (27896A>G 27898A>G), A108S (27899G>T 27901C>T), T116S (27923A>T), Q118K (27929C>A 27931A>G), F119Y (27933T>A), S132A (27971T>G), I139M (27994T>G), A144T (28007G>A 28009G>C), V146C (28013G>T 28014T>G 28015G>C), N147V (28016A>G 28017A>T 28018T>G), V148A (28020T>C 28021A>G), P149T (28022C>A 28024G>C), V151K (28028G>A 28029T>A), A152S (28031G>T), T154N (28038C>A 28039G>C), G156A (28044G>C 28045C>G), G159A (28053G>C 28054C>G), V162A (28062T>C), A163I (28064G>A 28065C>T 28066T>C), G164A (28068G>C), V166I (28073G>A 28075A>C), S167N (28076T>A 28077C>A 28078G>C), I169L (28082A>C), V170G (28086T>G), G171Q (28088G>C 28089G>A 28090C>G), S174A (28097T>G 28099C>G), N187T (28137A>C 28138C>G), A193T (28154G>A 28156A>G), L197F (28166C>T 28168G>C), T199S (28172A>T), I202V (28181A>G), A203G (28185C>G), V205I (28190G>A 28192A>C), A206N (28193G>A 28194C>A 28195G>C), K213R (28215A>G 28216A>G), S214A (28217T>G 28219C>T), V216I (28223G>A)								
Codon mutations:	TAC22GCA (27641T>G 27642A>C 27643C>A), TCT23TCG (27646T>G), GCC25CAG (27650G>C 27651C>A 27652C>G), TTA26CTC (27653T>C 27655A>C), GTT27GTC (27658T>C), CTG29ATG (27662C>A), GTC31ATC (27668G>A), TCG33TCC (27676G>C), ATC34GTG (27677A>G 27679C>G), CGC36CGG (27685C>G), GTG38TTT (27689G>T 27691G>T), AAT42GTG (27701A>G 27702A>T 27703T>G), GCT43GCG (27706T>G), GCC44GCA (27709C>A), GTC45ATT (27710G>A 27712C>T), GTG46GCG (27714T>C), ATC47CTC (27716A>C), CTC52ATC (27731C>A), ATC53GCG (27734A>G 27735T>C 27736C>G), ATG54CTG (27737A>C), CAA55CAG (27742A>G), GTG56ATT (27743G>A 27745G>T), CTG57GCA (27746C>G 27747T>C 27748G>A), GCG58TCG (27749G>T), ATC59CTG (27752A>C 27754C>G), GCG62TTT (27761G>T 27762C>T 27763G>T), GCT63GCG (27766T>G), ACG64ACC (27769G>C), GCG65AGC (27770G>A 27771C>G 27772G>C), ACC66AAT (27774C>A 27775C>T), ACG68GCC (27779A>G 27781G>C), CTT70GCG (27785C>G 27786T>C 27787T>G), ACG72AGT (27792C>G 27793G>T), GTG74GCG (27798T>C), GCG75GCC (27802G>C), GTG79TTC (27812G>T 27814G>C), ACG80GCG (27815A>G), CGT81CGC (27820T>C), CTT82CTG (27823T>G), GCT83GCG (27826T>G), GCG84GCC (27829G>C), GTG85CAG (27830G>C 27831T>A), GCG86CAG (27833G>C 27834C>A), ACT88ACC (27841T>C), GGA89GGC (27844A>C), ACG90CAG (27845A>C 27846C>A), GTC91GTG (27850C>G), GCC92ACC (27851G>A), TCG94GCG (27857T>G), ACG97ACT (27868G>T), ACG98TCG (27869A>T), ACC99ACG (27874C>G), CAG100ACC (27875C>A 27876A>C 27877G>C), GTC102GTT (27883C>T), GTT103GTG (27886T>G), CTT106CTC (27895T>C), ACA107GCG (27896A>G 27898A>G), GCC108TCT (27899G>T 27901C>T), GTT109GTC (27904T>C), ACA112ACG (27913A>G), GGG115GGC (27922G>C), ACG116TCG (27923A>T), CAA118AAG (27929C>A 27931A>G), TTC119TAC (27933T>A), GCT129GCC (27964T>C), TAT130TAC (27967T>C), TCG132GCG (27971T>G), ACA133ACG (27976A>G), GGC136GGG (27985C>G), TAC137TAT (27988C>T), ATT139ATG (27994T>G), GCT140GCG (27997T>G), GCG141GCC (28000G>C), GTG143GTC (28006G>C), GCG144ACC (28007G>A 28009G>C), AGC145AGT (28012C>T), GTG146TGC (28013G>T 28014T>G 28015G>C), AAT147GTG (28016A>G 28017A>T 28018T>G), GTA148GCG (28020T>C 28021A>G), CCG149ACC (28022C>A 28024G>C), GTG151AAG (28028G>A 28029T>A), GCA152TCA (28031G>T), GTG153GTC (28036G>C), ACG154AAC (28038C>A 28039G>C), GCC155GCA (28042C>A), GGC156GCG (28044G>C 28045C>G), GCT158GCG (28051T>G), GGC159GCG (28053G>C 28054C>G), GTG162GCG (28062T>C), GCT163ATC (28064G>A 28065C>T 28066T>C), GGC164GCC (28068G>C), GCC165GCG (28072C>G), GTA166ATC (28073G>A 28075A>C), TCG167AAC (28076T>A 28077C>A 28078G>C), ACC168ACG (28081C>G), ATC169CTC (28082A>C), GTC170GGC (28086T>G), GGC171CAG (28088G>C 28089G>A 28090C>G), GCA172GCC (28093A>C), ATT173ATC (28096T>C), TCC174GCG (28097T>G 28099C>G), GAT177GAC (28108T>C), GTC179GTG (28114C>G), ACG180ACC (28117G>C), GCC182GCG (28123C>G), GCA183GCG (28126A>G), AAC187ACG (28137A>C 28138C>G), GGC188GGT (28141C>T), GAT190GAC (28147T>C), GCA193ACG (28154G>A 28156A>G), GAC194GAT (28159C>T), GCT195GCC (28162T>C), GCG196GCC (28165G>C), CTG197TTC (28166C>T 28168G>C), CGT198CGC (28171T>C), ACG199TCG (28172A>T), CGC200CGG (28177C>G), ATC202GTC (28181A>G), GCC203GGC (28185C>G), GTA205ATC (28190G>A 28192A>C), GCG206AAC (28193G>A 28194C>A 28195G>C), AAG210AAA (28207G>A), ACG212ACC (28213G>C), AAA213AGG (28215A>G 28216A>G), TCC214GCT (28217T>G 28219C>T), GCT215GCC (28222T>C), GTC216ATC (28223G>A)								
hypothetical protein (YP_010078173.1)	105	204	17.3%	517	79.4%	100 (100%)	79 (79.0%)	0/0/0/0	0
Protein mutations:	Q109Y (31508C>T 31510G>C), T118A (31535A>G 31537A>C), Q122S (31547C>A 31548A>G 31549G>C), D125N (31556G>A), T127V (31562A>G 31563C>T 31564T>G), A128T (31565G>A), W141Y (31605G>A 31606G>T), S142Q (31607A>C 31608G>A 31609C>G), N145A (31616A>G 31617A>C 31618C>G), N146G (31619A>G 31620A>G 31621C>A), S147N (31623G>A), A150S (31631G>A 31632C>G 31633A>T), T154A (31643A>G), V167I (31682G>A 31684A>C), S173T (31701G>C 31702C>G), S181V (31724T>G 31725C>T), T184V (31733A>G 31734C>T 31735C>G), W187Y (31743G>A 31744G>T), V188S (31745G>T 31746T>C), N193Y (31760A>T)								
Codon mutations:	ACT106ACC (31501T>C), GTG107GTC (31504G>C), CAG109TAC (31508C>T 31510G>C), ATT115ATC (31528T>C), CAG116CAA (31531G>A), ACA118GCC (31535A>G 31537A>C), TAC119TAT (31540C>T), CAA120CAG (31543A>G), GAT121GAC (31546T>C), CAG122AGC (31547C>A 31548A>G 31549G>C), GAC125AAC (31556G>A), CTG126TTG (31559C>T), ACT127GTG (31562A>G 31563C>T 31564T>G), GCG128ACG (31565G>A), TGG141TAT (31605G>A 31606G>T), AGC142CAG (31607A>C 31608G>A 31609C>G), AAC145GCG (31616A>G 31617A>C 31618C>G), AAC146GGA (31619A>G 31620A>G 31621C>A), AGC147AAC (31623G>A), GCA150AGT (31631G>A 31632C>G 31633A>T), GCG152GCC (31639G>C), ACT154GCT (31643A>G), GTC159GTG (31660C>G), GTT160GTG (31663T>G), GCC163GCG (31672C>G), GCT165GCA (31678T>A), GTA167ATC (31682G>A 31684A>C), GCG168GCC (31687G>C), GCT169GCA (31690T>A), ACC170ACG (31693C>G), GGC172GGA (31699C>A), AGC173ACG (31701G>C 31702C>G), ACC175ACG (31708C>G), ACC176ACG (31711C>G), CCA177CCG (31714A>G), GCC178GCA (31717C>A), TCC181GTC (31724T>G 31725C>T), GGC182GGT (31729C>T), ACC184GTG (31733A>G 31734C>T 31735C>G), GGG185GGT (31738G>T), CTG186CTC (31741G>C), TGG187TAT (31743G>A 31744G>T), GTG188TCG (31745G>T 31746T>C), ACC190ACG (31753C>G), GTC191GTT (31756C>T), AAT193TAT (31760A>T), GGT194GGC (31765T>C), ACG196ACC (31771G>C), ACC197ACG (31774C>G), GCG200GCC (31783G>C), CCG201CCC (31786G>C), AAC202AAT (31789C>T)								

*: Inserts / Deletes / Misaligned / Frameshifts

Analysis details

This analysis was performed with panviral2.64

NGS Details (UN24): Caulimovirus venafragariae

Assembly

**Coverage Length**	857 (2 contig(s))
**Depth Of Coverage**	21.5
**Number Of Reads**	168
**Reads Per Million**	3.36 rpm (after QC)
**Ambiguities**	0
**Assembly Method**	de novo + reference guided assembly
**Consensus Caller**	Bcf Tools

Coverage Map

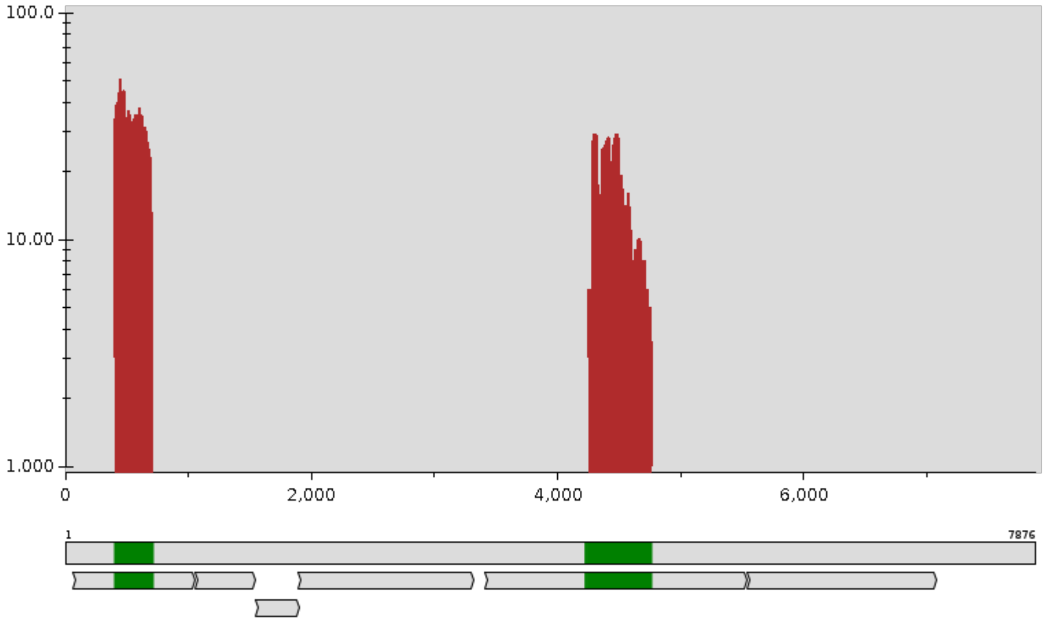

Assignment

**Type**	Caulimovirus venafragariae (Taxonomy ID: 3048344)
**Reference Genome**	NC_001725.1
**NT Identity (%)**	58.4597
**AA Identity (%)**	54.4484
**Number Of Stop Codons**	1
**Number Of CDS**	6

Alignment

**Alignment Score**	272.0 (NT) + 1125.0 (AA) = 1397.0
**Concordance (%)**	38.159

Alignment Method	Global, seeded, nucleotide + amino acids (AGA)

Genome Region

Sequence starts at position 396 and ends at position 4770 relative to NC_001725.1 reference sequence.

Alignment Detailed Statistics

	Begin	End	Coverage	Score	Concordance	Matches	Identities	I/D/M/F*	Stop Codons
**NT**	**396**	**4770**	**10.9%**	**272**	**16.0%**	**854 (99.3%)**	**501 (58.3%)**	**3/3**	
Mutations:	403A>C, 405A>T, 411A>G, 412G>A, 416T>A, 418A>G, 424A>T, 426A>G, 430T>G, 431C>G, 432T>A, 433G>A, 435T>A, 436A>C, 441T>C, 445A>G, 447C>G, 451A>T, 452G>T, 456C>T, 459T>C, 461G>A, 462G>A, 463A>G, 464C>A, 468A>T, 472G>A, 474C>T, 476C>G, 483T>A, 485G>T, 486T>A, 487G>A, 490G>A, 491C>A, 492A>C, 495C>T, 496C>T, 497T>C, 501C>T, 502C>G, 503G>A, 504G>A, 508A>T, 510G>T, 512A>T, 513A>T, 516C>T, 517G>A, 521A>G, 525C>A, 527C>G, 529G>A, 530T>A, 531C>T, 532T>C, 534C>T, 537C>A, 538G>A, 539G>T, 544A>G, 552C>T, 553C>T, 555T>A, 556T>G, 558C>A, 562G>A, 563G>C, 564G>T, 567A>G, 568C>T, 570T>A, 571A>C, 573C>T, 579C>A, 582C>T, 583A>T, 585C>T, 588A>C, 590A>G, 592A>T, 593T>A, 594C>T, 596C>G, 598G>A, 600T>A, 601A>T, 602G>C, 603T>A, 604C>T, 606T>A, 608G>A, 612C>T, 613C>G, 617C>A, 618G>C, 619A>T, 624C>T, 625A>G, 627G>A, 628A>G, 630T>A, 633A>G, 635C>G, 636A>T, 637C>T, 639A>G, 640_642delGCT, 648C>T, 648_649insCAG, 649T>G, 651T>C, 652G>A, 656A>G, 658G>A, 663A>T, 666G>A, 667A>T, 669G>C, 670C>A, 672T>G, 673G>C, 674A>C, 675G>A, 678A>T, 685C>A, 686C>T, 687C>T, 688T>A, 689A>T, 690T>G, 692C>G, 693T>C, 696A>T, 698C>A, 699C>T, 702C>T, 703A>C, 705A>G, 706A>G, 707T>C, 4219T>C, 4222T>A, 4223T>A, 4227C>T, 4228A>C, 4230T>C, 4232A>G, 4235A>T, 4236A>C, 4239T>A, 4242T>A, 4244A>G, 4245G>A, 4246T>G, 4247G>T, 4249A>C, 4254A>C, 4258C>T, 4259A>T, 4260G>T, 4264T>A, 4265C>A, 4266A>C, 4267C>G, 4268C>A, 4270C>G, 4275C>T, 4276C>A, 4277G>A, 4279G>A, 4281A>T, 4282G>A, 4284G>A, 4287T>C, 4289A>G, 4292C>T, 4299C>T, 4300G>C, 4308A>T, 4314G>A, 4315C>T, 4317T>A, 4318G>A, 4319G>A, 4320T>A, 4321A>T, 4322T>A, 4323C>A, 4326C>A, 4330C>G, 4331C>A, 4332T>A, 4335C>T, 4348T>A, 4349C>G, 4351T>A, 4352C>G, 4353A>T, 4356A>T, 4359C>A, 4362C>T, 4366G>A, 4371G>A, 4380C>G, 4383G>A, 4384A>C, 4385T>A, 4386C>A, 4390C>A, 4395C>A, 4398A>G, 4401A>T, 4402A>C, 4410A>T, 4413T>A, 4423A>G, 4426T>C, 4428A>T, 4432G>A, 4434C>A, 4435C>A, 4437T>C, 4438A>T, 4439C>G, 4440A>T, 4442A>T, 4443G>T, 4444G>T, 4445G>T, 4446A>T, 4449T>C, 4452C>A, 4456C>T, 4458A>T, 4459C>A, 4461T>C, 4467C>T, 4470G>A, 4471G>A, 4472A>C, 4473G>T, 4474C>A, 4476A>T, 4479G>T, 4480C>A, 4482T>A, 4483C>A, 4485A>T, 4486A>C, 4487G>T, 4489A>G, 4492G>A, 4493G>A, 4496G>T, 4497T>A, 4500G>A, 4505T>A, 4506T>C, 4508A>T, 4509C>T, 4512T>C, 4513T>A, 4514C>A, 4515C>A, 4521C>T, 4528T>A, 4529C>G, 4543G>A, 4546C>A, 4548C>A, 4551T>C, 4552G>T, 4554T>A, 4555C>G, 4556C>A, 4557A>G, 4561A>T, 4568A>C, 4569G>T, 4575C>A, 4578T>A, 4585T>A, 4586G>C, 4590C>T, 4591C>A, 4593A>T, 4599C>T, 4602C>T, 4614C>A, 4618C>T, 4620T>A, 4633C>A, 4635A>T, 4638C>A, 4641T>A, 4642G>C, 4643C>A, 4644T>A, 4650C>T, 4656G>A, 4657C>A, 4659C>A, 4663G>T, 4665C>T, 4666G>A, 4670G>T, 4671T>A, 4672C>T, 4674C>T, 4675A>T, 4676G>C, 4677C>T, 4678A>G, 4680C>T, 4681A>C, 4684T>A, 4686T>A, 4687C>A, 4688C>A, 4689A>G, 4690C>A, 4692G>A, 4697G>T, 4698T>G, 4699G>A, 4700C>T, 4701T>A, 4707T>C, 4708G>A, 4710T>A, 4719C>T, 4720A>T, 4722C>G, 4725C>A, 4727T>G, 4729A>T, 4730G>C, 4731C>A, 4733A>C, 4737T>A, 4738G>T, 4740A>T, 4741G>C, 4743A>G, 4750T>C, 4752A>T, 4753G>A, 4754G>C, 4759G>C, 4761C>T, 4765A>G, 4766T>A, 4767T>A, 4768G>T								
**CDS**									
ORF_I	111	216	32.2%	349	49.8%	105 (98.1%)	46 (43.0%)	1/1/0/0	0
Protein mutations:	K113H (403A>C 405A>T), I115M (411A>G), D116N (412G>A), F117Y (416T>A), I118V (418A>G), I120L (424A>T 426A>G), S122G (430T>G 431C>G 432T>A), V123I (433G>A 435T>A), K124Q (436A>C), I127V (445A>G 447C>G), S129F (451A>T 452G>T), R132K (461G>A 462G>A), T133E (463A>G 464C>A), D136N (472G>A 474C>T), S137C (476C>G), S140I (485G>T 486T>A), V141I (487G>A), A142N (490G>A 491C>A 492A>C), L144S (496C>T 497T>C), R146E (502C>G 503G>A 504G>A), M148F (508A>T 510G>T), K149I (512A>T 513A>T), A151T (517G>A), K152R (521A>G), D153E (525C>A), A154G (527C>G), V155N (529G>A 530T>A 531C>T), F156L (532T>C 534C>T), G158I (538G>A 539G>T), K160E (544A>G), S164A (556T>G 558C>A), G166T (562G>A 563G>C 564G>T), I169L (571A>C 573C>T), N173Y (583A>T 585C>T), K175R (590A>G), I176Y (592A>T 593T>A 594C>T), S177C (596C>G), V178I (598G>A 600T>A), R181K (608G>A), P183A (613C>G), T184N (617C>A 618G>C), I185F (619A>T), K187E (625A>G 627G>A), T188A (628A>G 630T>A), T190S (635C>G 636A>T), A192del (640_642delGCT), F194_F195insQ (648_649insCAG), F195V (649T>G 651T>C), E196K (652G>A), K197R (656A>G), E198K (658G>A), E199D (663A>T), M201F (667A>T 669G>C), H202K (670C>A 672T>G), E203P (673G>C 674A>C 675G>A), P207I (685C>A 686C>T 687C>T), Y208M (688T>A 689A>T 690T>G), T209S (692C>G 693T>C), S211Y (698C>A 699C>T), K213Q (703A>C 705A>G), I214A (706A>G 707T>C)								
Codon mutations:	AAA113CAT (403A>C 405A>T), ATA115ATG (411A>G), GAT116AAT (412G>A), TTT117TAT (416T>A), ATT118GTT (418A>G), ATA120TTG (424A>T 426A>G), TCT122GGA (430T>G 431C>G 432T>A), GTT123ATA (433G>A 435T>A), AAA124CAA (436A>C), ATT125ATC (441T>C), ATC127GTG (445A>G 447C>G), AGC129TTC (451A>T 452G>T), ACC130ACT (456C>T), TTT131TTC (459T>C), AGG132AAA (461G>A 462G>A), ACA133GAA (463A>G 464C>A), GGA134GGT (468A>T), GAC136AAT (472G>A 474C>T), TCT137TGT (476C>G), ATT139ATA (483T>A), AGT140ATA (485G>T 486T>A), GTA141ATA (487G>A), GCA142AAC (490G>A 491C>A 492A>C), CTC143CTT (495C>T), CTT144TCT (496C>T 497T>C), GAC145GAT (501C>T), CGG146GAA (502C>G 503G>A 504G>A), ATG148TTT (508A>T 510G>T), AAA149ATT (512A>T 513A>T), AAC150AAT (516C>T), GCT151ACT (517G>A), AAA152AGA (521A>G), GAC153GAA (525C>A), GCA154GGA (527C>G), GTC155AAT (529G>A 530T>A 531C>T), TTC156CTT (532T>C 534C>T), GGC157GGA (537C>A), GGA158ATA (538G>A 539G>T), AAA160GAA (544A>G), AAC162AAT (552C>T), CTT163TTA (553C>T 555T>A), TCC164GCA (556T>G 558C>A), GGG166ACT (562G>A 563G>C 564G>T), AAA167AAG (567A>G), CTT168TTA (568C>T 570T>A), ATC169CTT (571A>C 573C>T), ACC171ACA (579C>A), TAC172TAT (582C>T), AAC173TAT (583A>T 585C>T), CCA174CCC (588A>C), AAA175AGA (590A>G), ATC176TAT (592A>T 593T>A 594C>T), TCT177TGT (596C>G), GTT178ATA (598G>A 600T>A), AGT179TCA (601A>T 602G>C 603T>A), CTT180TTA (604C>T 606T>A), AGA181AAA (608G>A), GAC182GAT (612C>T), CCT183GCT (613C>G), ACG184AAC (617C>A 618G>C), ATT185TTT (619A>T), AAC186AAT (624C>T), AAG187GAA (625A>G 627G>A), ACT188GCA (628A>G 630T>A), TTA189TTG (633A>G), ACA190AGT (635C>G 636A>T), CTA191TTG (637C>T 639A>G), GCT192del (640_642delGCT), TTC194TTT (648C>T), TTC194_TTT195insCAG (648_649insCAG), TTT195GTC (649T>G 651T>C), GAA196AAA (652G>A), AAA197AGA (656A>G), GAA198AAA (658G>A), GAA199GAT (663A>T), TTG200TTA (666G>A), ATG201TTC (667A>T 669G>C), CAT202AAG (670C>A 672T>G), GAG203CCA (673G>C 674A>C 675G>A), GGA204GGT (678A>T), CCC207ATT (685C>A 686C>T 687C>T), TAT208ATG (688T>A 689A>T 690T>G), ACT209AGC (692C>G 693T>C), ATA210ATT (696A>T), TCC211TAT (698C>A 699C>T), TAC212TAT (702C>T), AAA213CAG (703A>C 705A>G), ATA214GCA (706A>G 707T>C)								
ORF_V	281	455	24.7%	776	58.8%	175 (100%)	107 (61.1%)	0/0/0/0	1
Protein mutations:	C281V (4246T>G 4247G>T), K282Q (4249A>C), Q285F (4258C>T 4259A>T 4260G>T), S287N (4264T>A 4265C>A 4266A>C), P288D (4267C>G 4268C>A), Q289E (4270C>G), R291K (4276C>A 4277G>A), E292N (4279G>A 4281A>T), E293K (4282G>A 4284G>A), K295R (4289A>G), T296I (4292C>T), E299Q (4300G>C), G305K (4318G>A 4319G>A 4320T>A), I306* (4321A>T 4322T>A 4323C>A), P309E (4330C>G 4331C>A 4332T>A), V321I (4366G>A), I327Q (4384A>C 4385T>A 4386C>A), K340E (4423A>G), D343K (4432G>A 4434C>A), H344N (4435C>A 4437T>C), T345C (4438A>T 4439C>G 4440A>T), K346I (4442A>T 4443G>T), G347F (4444G>T 4445G>T 4446A>T), L351F (4456C>T 4458A>T), L352I (4459C>A 4461T>C), E356T (4471G>A 4472A>C 4473G>T), Q357N (4474C>A 4476A>T), L359I (4480C>A 4482T>A), Q360N (4483C>A 4485A>T), R361L (4486A>C 4487G>T), I362V (4489A>G), G363K (4492G>A 4493G>A), G364V (4496G>T 4497T>A), F367Y (4505T>A 4506T>C), Y368F (4508A>T 4509C>T), S370K (4513T>A 4514C>A 4515C>A), V380I (4543G>A), A383S (4552G>T 4554T>A), P384E (4555C>G 4556C>A 4557A>G), T386S (4561A>T), Q388P (4568A>C 4569G>T), C394T (4585T>A 4586G>C), Q396N (4591C>A 4593A>T), P405S (4618C>T 4620T>A), Q410N (4633C>A 4635A>T), A413Q (4642G>C 4643C>A 4644T>A), H418K (4657C>A 4659C>A), D420Y (4663G>T 4665C>T), E421K (4666G>A), S422I (4670G>T 4671T>A), L423F (4672C>T 4674C>T), N425D (4678A>G 4680C>T), M426L (4681A>C), Y427K (4684T>A 4686T>A), P428K (4687C>A 4688C>A 4689A>G), Q429K (4690C>A 4692G>A), C431L (4697G>T 4698T>G), A432I (4699G>A 4700C>T 4701T>A), V435I (4708G>A 4710T>A), I439L (4720A>T 4722C>G), F441C (4727T>G), K443T (4733A>C), E445Y (4738G>T 4740A>T), E446Q (4741G>C 4743A>G), G450T (4753G>A 4754G>C), V452L (4759G>C 4761C>T), I454E (4765A>G 4766T>A 4767T>A), V455F (4768G>T)								
Codon mutations:	TTA272C.. (4219T>C), TTA273AA. (4222T>A 4223T>A), GAC274..T (4227C>T), ACT275C.C (4228A>C 4230T>C), AAC276.G. (4232A>G), AAA277.TC (4235A>T 4236A>C), ATT278..A (4239T>A), ATT279..A (4242T>A), AAG280.GA (4244A>G 4245G>A), TGT281GTT (4246T>G 4247G>T), AAA282CAA (4249A>C), CCA283CCC (4254A>C), CAG285TTT (4258C>T 4259A>T 4260G>T), TCA287AAC (4264T>A 4265C>A 4266A>C), CCT288GAT (4267C>G 4268C>A), CAA289GAA (4270C>G), GAC290GAT (4275C>T), CGA291AAA (4276C>A 4277G>A), GAA292AAT (4279G>A 4281A>T), GAG293AAA (4282G>A 4284G>A), TTT294TTC (4287T>C), AAA295AGA (4289A>G), ACT296ATT (4292C>T), ATC298ATT (4299C>T), GAA299CAA (4300G>C), CTA301CTT (4308A>T), AAG303AAA (4314G>A), CTT304TTA (4315C>T 4317T>A), GGT305AAA (4318G>A 4319G>A 4320T>A), ATC306TAA (4321A>T 4322T>A 4323C>A), ATC307ATA (4326C>A), CCT309GAA (4330C>G 4331C>A 4332T>A), AGC310AGT (4335C>T), TCC315AGC (4348T>A 4349C>G), TCA316AGT (4351T>A 4352C>G 4353A>T), CCA317CCT (4356A>T), GCC318GCA (4359C>A), TTC319TTT (4362C>T), GTA321ATA (4366G>A), AGG322AGA (4371G>A), GCC325GCG (4380C>G), GAG326GAA (4383G>A), ATC327CAA (4384A>C 4385T>A 4386C>A), CGA329AGA (4390C>A), GGC330GGA (4395C>A), AAA331AAG (4398A>G), GCA332GCT (4401A>T), AGA333CGA (4402A>C), GTA335GTT (4410A>T), ATT336ATA (4413T>A), AAG340GAG (4423A>G), TTA341CTT (4426T>C 4428A>T), GAC343AAA (4432G>A 4434C>A), CAT344AAC (4435C>A 4437T>C), ACA345TGT (4438A>T 4439C>G 4440A>T), AAG346ATT (4442A>T 4443G>T), GGA347TTT (4444G>T 4445G>T 4446A>T), GAT348GAC (4449T>C), GGC349GGA (4452C>A), CTA351TTT (4456C>T 4458A>T), CTT352ATC (4459C>A 4461T>C), AAC354AAT (4467C>T), AAG355AAA (4470G>A), GAG356ACT (4471G>A 4472A>C 4473G>T), CAA357AAT (4474C>A 4476A>T), CTG358CTT (4479G>T), CTT359ATA (4480C>A 4482T>A), CAA360AAT (4483C>A 4485A>T), AGA361CTA (4486A>C 4487G>T), ATC362GTC (4489A>G), GGA363AAA (4492G>A 4493G>A), GGT364GTA (4496G>T 4497T>A), AAG365AAA (4500G>A), TTT367TAC (4505T>A 4506T>C), TAC368TTT (4508A>T 4509C>T), TCT369TCC (4512T>C), TCC370AAA (4513T>A 4514C>A 4515C>A), GAC372GAT (4521C>T), TCT375AGT (4528T>A 4529C>G), GTA380ATA (4543G>A), CGC381AGA (4546C>A 4548C>A), CTT382CTC (4551T>C), GCT383TCA (4552G>T 4554T>A), CCA384GAG (4555C>G 4556C>A 4557A>G), ACA386TCA (4561A>T), CAG388CCT (4568A>C 4569G>T), ACC390ACA (4575C>A), GCT391GCA (4578T>A), TGT394ACT (4585T>A 4586G>C), CCC395CCT (4590C>T), CAA396AAT (4591C>A 4593A>T), CAC398CAT (4599C>T), TAC399TAT (4602C>T), GTC403GTA (4614C>A), CCT405TCA (4618C>T 4620T>A), CAA410AAT (4633C>A 4635A>T), GCC411GCA (4638C>A), CCT412CCA (4641T>A), GCT413CAA (4642G>C 4643C>A 4644T>A), TTC415TTT (4650C>T), AGG417AGA (4656G>A), CAC418AAA (4657C>A 4659C>A), GAC420TAT (4663G>T 4665C>T), GAA421AAA (4666G>A), AGT422ATA (4670G>T 4671T>A), CTC423TTT (4672C>T 4674C>T), AGC424TCT (4675A>T 4676G>C 4677C>T), AAC425GAT (4678A>G 4680C>T), ATG426CTG (4681A>C), TAT427AAA (4684T>A 4686T>A), CCA428AAG (4687C>A 4688C>A 4689A>G), CAG429AAA (4690C>A 4692G>A), TGT431TTG (4697G>T 4698T>G), GCT432ATA (4699G>A 4700C>T 4701T>A), TAT434TAC (4707T>C), GTT435ATA (4708G>A 4710T>A), ATC438ATT (4719C>T), ATC439TTG (4720A>T 4722C>G), GTC440GTA (4725C>A), TTC441TGC (4727T>G), AGC442TCA (4729A>T 4730G>C 4731C>A), AAA443ACA (4733A>C), ACT444ACA (4737T>A), GAA445TAT (4738G>T 4740A>T), GAA446CAG (4741G>C 4743A>G), TTA449CTT (4750T>C 4752A>T), GGA450ACA (4753G>A 4754G>C), GTC452CTT (4759G>C 4761C>T), ATT454GAA (4765A>G 4766T>A 4767T>A), GTT455TTT (4768G>T)								
**Proteins**									
hypothetical protein (NP_043929.1)	111	216	32.2%	349	49.8%	105 (98.1%)	46 (43.0%)	1/1/0/0	0

	Begin	End	Coverage	Score	Concordance	Matches	Identities	I/D/M/F*	Stop Codons
**NT**	**396**	**4770**	**10.9%**	**272**	**16.0%**	**854 (99.3%)**	**501 (58.3%)**	**3/3**	
Protein mutations:	K113H (403A>C 405A>T), I115M (411A>G), D116N (412G>A), F117Y (416T>A), I118V (418A>G), I120L (424A>T 426A>G), S122G (430T>G 431C>G 432T>A), V123I (433G>A 435T>A), K124Q (436A>C), I127V (445A>G 447C>G), S129F (451A>T 452G>T), R132K (461G>A 462G>A), T133E (463A>G 464C>A), D136N (472G>A 474C>T), S137C (476C>G), S140I (485G>T 486T>A), V141I (487G>A), A142N (490G>A 491C>A 492A>C), L144S (496C>T 497T>C), R146E (502C>G 503G>A 504G>A), M148F (508A>T 510G>T), K149I (512A>T 513A>T), A151T (517G>A), K152R (521A>G), D153E (525C>A), A154G (527C>G), V155N (529G>A 530T>A 531C>T), F156L (532T>C 534C>T), G158I (538G>A 539G>T), K160E (544A>G), S164A (556T>G 558C>A), G166T (562G>A 563G>C 564G>T), I169L (571A>C 573C>T), N173Y (583A>T 585C>T), K175R (590A>G), I176Y (592A>T 593T>A 594C>T), S177C (596C>G), V178I (598G>A 600T>A), R181K (608G>A), P183A (613C>G), T184N (617C>A 618G>C), I185F (619A>T), K187E (625A>G 627G>A), T188A (628A>G 630T>A), T190S (635C>G 636A>T), A192del (640_642delGCT), F194_F195insQ (648_649insCAG), F195V (649T>G 651T>C), E196K (652G>A), K197R (656A>G), E198K (658G>A), E199D (663A>T), M201F (667A>T 669G>C), H202K (670C>A 672T>G), E203P (673G>C 674A>C 675G>A), P207I (685C>A 686C>T 687C>T), Y208M (688T>A 689A>T 690T>G), T209S (692C>G 693T>C), S211Y (698C>A 699C>T), K213Q (703A>C 705A>G), I214A (706A>G 707T>C)								
Codon mutations:	AAA113CAT (403A>C 405A>T), ATA115ATG (411A>G), GAT116AAT (412G>A), TTT117TAT (416T>A), ATT118GTT (418A>G), ATA120TTG (424A>T 426A>G), TCT122GGA (430T>G 431C>G 432T>A), GTT123ATA (433G>A 435T>A), AAA124CAA (436A>C), ATT125ATC (441T>C), ATC127GTG (445A>G 447C>G), AGC129TTC (451A>T 452G>T), ACC130ACT (456C>T), TTT131TTC (459T>C), AGG132AAA (461G>A 462G>A), ACA133GAA (463A>G 464C>A), GGA134GGT (468A>T), GAC136AAT (472G>A 474C>T), TCT137TGT (476C>G), ATT139ATA (483T>A), AGT140ATA (485G>T 486T>A), GTA141ATA (487G>A), GCA142AAC (490G>A 491C>A 492A>C), CTC143CTT (495C>T), CTT144TCT (496C>T 497T>C), GAC145GAT (501C>T), CGG146GAA (502C>G 503G>A 504G>A), ATG148TTT (508A>T 510G>T), AAA149ATT (512A>T 513A>T), AAC150AAT (516C>T), GCT151ACT (517G>A), AAA152AGA (521A>G), GAC153GAA (525C>A), GCA154GGA (527C>G), GTC155AAT (529G>A 530T>A 531C>T), TTC156CTT (532T>C 534C>T), GGC157GGA (537C>A), GGA158ATA (538G>A 539G>T), AAA160GAA (544A>G), AAC162AAT (552C>T), CTT163TTA (553C>T 555T>A), TCC164GCA (556T>G 558C>A), GGG166ACT (562G>A 563G>C 564G>T), AAA167AAG (567A>G), CTT168TTA (568C>T 570T>A), ATC169CTT (571A>C 573C>T), ACC171ACA (579C>A), TAC172TAT (582C>T), AAC173TAT (583A>T 585C>T), CCA174CCC (588A>C), AAA175AGA (590A>G), ATC176TAT (592A>T 593T>A 594C>T), TCT177TGT (596C>G), GTT178ATA (598G>A 600T>A), AGT179TCA (601A>T 602G>C 603T>A), CTT180TTA (604C>T 606T>A), AGA181AAA (608G>A), GAC182GAT (612C>T), CCT183GCT (613C>G), ACG184AAC (617C>A 618G>C), ATT185TTT (619A>T), AAC186AAT (624C>T), AAG187GAA (625A>G 627G>A), ACT188GCA (628A>G 630T>A), TTA189TTG (633A>G), ACA190AGT (635C>G 636A>T), CTA191TTG (637C>T 639A>G), GCT192del (640_642delGCT), TTC194TTT (648C>T), TTC194_TTT195insCAG (648_649insCAG), TTT195GTC (649T>G 651T>C), GAA196AAA (652G>A), AAA197AGA (656A>G), GAA198AAA (658G>A), GAA199GAT (663A>T), TTG200TTA (666G>A), ATG201TTC (667A>T 669G>C), CAT202AAG (670C>A 672T>G), GAG203CCA (673G>C 674A>C 675G>A), GGA204GGT (678A>T), CCC207ATT (685C>A 686C>T 687C>T), TAT208ATG (688T>A 689A>T 690T>G), ACT209AGC (692C>G 693T>C), ATA210ATT (696A>T), TCC211TAT (698C>A 699C>T), TAC212TAT (702C>T), AAA213CAG (703A>C 705A>G), ATA214GCA (706A>G 707T>C)								
hypothetical protein (NP_043933.1)	281	455	24.7%	776	58.8%	175 (100%)	107 (61.1%)	0/0/0/0	1
Protein mutations:	C281V (4246T>G 4247G>T), K282Q (4249A>C), Q285F (4258C>T 4259A>T 4260G>T), S287N (4264T>A 4265C>A 4266A>C), P288D (4267C>G 4268C>A), Q289E (4270C>G), R291K (4276C>A 4277G>A), E292N (4279G>A 4281A>T), E293K (4282G>A 4284G>A), K295R (4289A>G), T296I (4292C>T), E299Q (4300G>C), G305K (4318G>A 4319G>A 4320T>A), I306* (4321A>T 4322T>A 4323C>A), P309E (4330C>G 4331C>A 4332T>A), V321I (4366G>A), I327Q (4384A>C 4385T>A 4386C>A), K340E (4423A>G), D343K (4432G>A 4434C>A), H344N (4435C>A 4437T>C), T345C (4438A>T 4439C>G 4440A>T), K346I (4442A>T 4443G>T), G347F (4444G>T 4445G>T 4446A>T), L351F (4456C>T 4458A>T), L352I (4459C>A 4461T>C), E356T (4471G>A 4472A>C 4473G>T), Q357N (4474C>A 4476A>T), L359I (4480C>A 4482T>A), Q360N (4483C>A 4485A>T), R361L (4486A>C 4487G>T), I362V (4489A>G), G363K (4492G>A 4493G>A), G364V (4496G>T 4497T>A), F367Y (4505T>A 4506T>C), Y368F (4508A>T 4509C>T), S370K (4513T>A 4514C>A 4515C>A), V380I (4543G>A), A383S (4552G>T 4554T>A), P384E (4555C>G 4556C>A 4557A>G), T386S (4561A>T), Q388P (4568A>C 4569G>T), C394T (4585T>A 4586G>C), Q396N (4591C>A 4593A>T), P405S (4618C>T 4620T>A), Q410N (4633C>A 4635A>T), A413Q (4642G>C 4643C>A 4644T>A), H418K (4657C>A 4659C>A), D420Y (4663G>T 4665C>T), E421K (4666G>A), S422I (4670G>T 4671T>A), L423F (4672C>T 4674C>T), N425D (4678A>G 4680C>T), M426L (4681A>C), Y427K (4684T>A 4686T>A), P428K (4687C>A 4688C>A 4689A>G), Q429K (4690C>A 4692G>A), C431L (4697G>T 4698T>G), A432I (4699G>A 4700C>T 4701T>A), V435I (4708G>A 4710T>A), I439L (4720A>T 4722C>G), F441C (4727T>G), K443T (4733A>C), E445Y (4738G>T 4740A>T), E446Q (4741G>C 4743A>G), G450T (4753G>A 4754G>C), V452L (4759G>C 4761C>T), I454E (4765A>G 4766T>A 4767T>A), V455F (4768G>T)								
Codon mutations:	TTA272C.. (4219T>C), TTA273AA. (4222T>A 4223T>A), GAC274..T (4227C>T), ACT275C.C (4228A>C 4230T>C), AAC276.G. (4232A>G), AAA277.TC (4235A>T 4236A>C), ATT278..A (4239T>A), ATT279..A (4242T>A), AAG280.GA (4244A>G 4245G>A), TGT281GTT (4246T>G 4247G>T), AAA282CAA (4249A>C), CCA283CCC (4254A>C), CAG285TTT (4258C>T 4259A>T 4260G>T), TCA287AAC (4264T>A 4265C>A 4266A>C), CCT288GAT (4267C>G 4268C>A), CAA289GAA (4270C>G), GAC290GAT (4275C>T), CGA291AAA (4276C>A 4277G>A), GAA292AAT (4279G>A 4281A>T), GAG293AAA (4282G>A 4284G>A), TTT294TTC (4287T>C), AAA295AGA (4289A>G), ACT296ATT (4292C>T), ATC298ATT (4299C>T), GAA299CAA (4300G>C), CTA301CTT (4308A>T), AAG303AAA (4314G>A), CTT304TTA (4315C>T 4317T>A), GGT305AAA (4318G>A 4319G>A 4320T>A), ATC306TAA (4321A>T 4322T>A 4323C>A), ATC307ATA (4326C>A), CCT309GAA (4330C>G 4331C>A 4332T>A), AGC310AGT (4335C>T), TCC315AGC (4348T>A 4349C>G), TCA316AGT (4351T>A 4352C>G 4353A>T), CCA317CCT (4356A>T), GCC318GCA (4359C>A), TTC319TTT (4362C>T), GTA321ATA (4366G>A), AGG322AGA (4371G>A), GCC325GCG (4380C>G), GAG326GAA (4383G>A), ATC327CAA (4384A>C 4385T>A 4386C>A), CGA329AGA (4390C>A), GGC330GGA (4395C>A), AAA331AAG (4398A>G), GCA332GCT (4401A>T), AGA333CGA (4402A>C), GTA335GTT (4410A>T), ATT336ATA (4413T>A), AAG340GAG (4423A>G), TTA341CTT (4426T>C 4428A>T), GAC343AAA (4432G>A 4434C>A), CAT344AAC (4435C>A 4437T>C), ACA345TGT (4438A>T 4439C>G 4440A>T), AAG346ATT (4442A>T 4443G>T), GGA347TTT (4444G>T 4445G>T 4446A>T), GAT348GAC (4449T>C), GGC349GGA (4452C>A), CTA351TTT (4456C>T 4458A>T), CTT352ATC (4459C>A 4461T>C), AAC354AAT (4467C>T), AAG355AAA (4470G>A), GAG356ACT (4471G>A 4472A>C 4473G>T), CAA357AAT (4474C>A 4476A>T), CTG358CTT (4479G>T), CTT359ATA (4480C>A 4482T>A), CAA360AAT (4483C>A 4485A>T), AGA361CTA (4486A>C 4487G>T), ATC362GTC (4489A>G), GGA363AAA (4492G>A 4493G>A), GGT364GTA (4496G>T 4497T>A), AAG365AAA (4500G>A), TTT367TAC (4505T>A 4506T>C), TAC368TTT (4508A>T 4509C>T), TCT369TCC (4512T>C), TCC370AAA (4513T>A 4514C>A 4515C>A), GAC372GAT (4521C>T), TCT375AGT (4528T>A 4529C>G), GTA380ATA (4543G>A), CGC381AGA (4546C>A 4548C>A), CTT382CTC (4551T>C), GCT383TCA (4552G>T 4554T>A), CCA384GAG (4555C>G 4556C>A 4557A>G), ACA386TCA (4561A>T), CAG388CCT (4568A>C 4569G>T), ACC390ACA (4575C>A), GCT391GCA (4578T>A), TGT394ACT (4585T>A 4586G>C), CCC395CCT (4590C>T), CAA396AAT (4591C>A 4593A>T), CAC398CAT (4599C>T), TAC399TAT (4602C>T), GTC403GTA (4614C>A), CCT405TCA (4618C>T 4620T>A), CAA410AAT (4633C>A 4635A>T), GCC411GCA (4638C>A), CCT412CCA (4641T>A), GCT413CAA (4642G>C 4643C>A 4644T>A), TTC415TTT (4650C>T), AGG417AGA (4656G>A), CAC418AAA (4657C>A 4659C>A), GAC420TAT (4663G>T 4665C>T), GAA421AAA (4666G>A), AGT422ATA (4670G>T 4671T>A), CTC423TTT (4672C>T 4674C>T), AGC424TCT (4675A>T 4676G>C 4677C>T), AAC425GAT (4678A>G 4680C>T), ATG426CTG (4681A>C), TAT427AAA (4684T>A 4686T>A), CCA428AAG (4687C>A 4688C>A 4689A>G), CAG429AAA (4690C>A 4692G>A), TGT431TTG (4697G>T 4698T>G), GCT432ATA (4699G>A 4700C>T 4701T>A), TAT434TAC (4707T>C), GTT435ATA (4708G>A 4710T>A), ATC438ATT (4719C>T), ATC439TTG (4720A>T 4722C>G), GTC440GTA (4725C>A), TTC441TGC (4727T>G), AGC442TCA (4729A>T 4730G>C 4731C>A), AAA443ACA (4733A>C), ACT444ACA (4737T>A), GAA445TAT (4738G>T 4740A>T), GAA446CAG (4741G>C 4743A>G), TTA449CTT (4750T>C 4752A>T), GGA450ACA (4753G>A 4754G>C), GTC452CTT (4759G>C 4761C>T), ATT454GAA (4765A>G 4766T>A 4767T>A), GTT455TTT (4768G>T)								

*: Inserts / Deletes / Misaligned / Frameshifts

Analysis details

This analysis was performed with panviral2.64

NGS Details (UN24): Dioscovirus dioscoreae

Assembly

**Coverage Length**	1376 (1 contig(s))
**Depth Of Coverage**	12.2
**Number Of Reads**	161
**Reads Per Million**	3.22 rpm (after QC)
**Ambiguities**	0
**Assembly Method**	de novo + reference guided assembly
**Consensus Caller**	Bcf Tools

Coverage Map

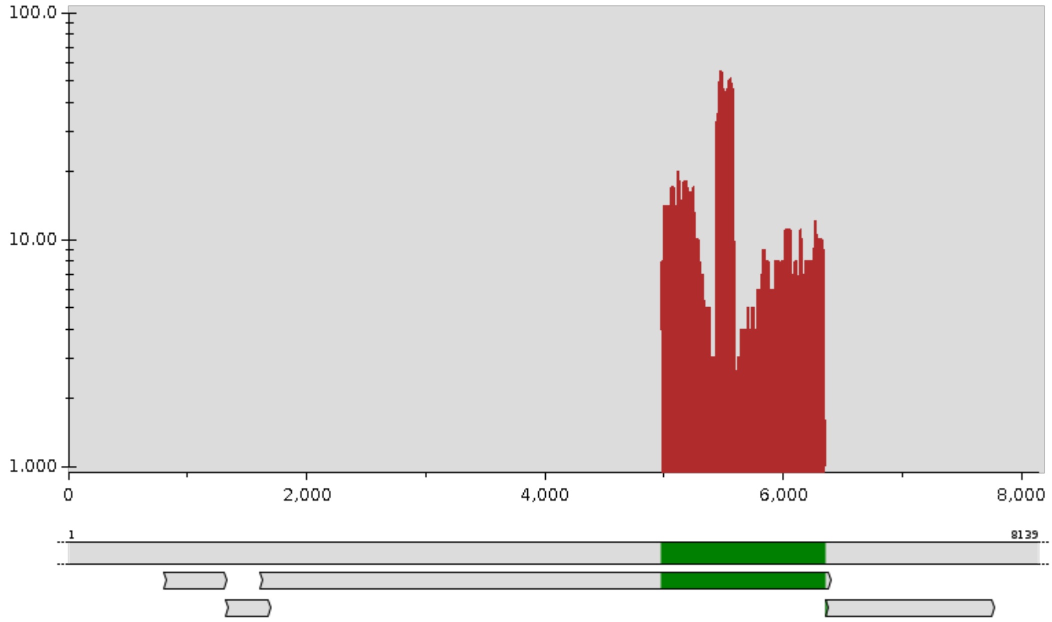

Assignment

**Type**	Dioscovirus dioscoreae (Taxonomy ID: 3052184)
**Reference Genome**	NC_040712.1
**NT Identity (%)**	59.7242
**AA Identity (%)**	49.5652
**Number Of Stop Codons**	0
**Number Of CDS**	4

Alignment

**Alignment Score**	493.0 (NT) + 1519.0 (AA) = 2012.0
**Concordance (%)**	35.1314

Alignment Method	Global, seeded, nucleotide + amino acids (AGA)

Genome Region

Sequence starts at position 4980 and ends at position 6355 relative to NC_040712.1 reference sequence.

Alignment Detailed Statistics

	Begin	End	Coverage	Score	Concordance	Matches	Identities	I/D/M/F*	Stop Codons
**NT**	**4980**	**6355**	**16.9%**	**493**	**18.2%**	**1369 (98.8%)**	**823 (59.4%)**	**9/7**	
Mutations:	4983C>A, 4986C>A, 4988T>A, 4989A>T, 4990A>T, 4992T>C, 4994G>A, 4997A>T, 5001A>T, 5003C>A, 5004C>G, 5009T>A, 5012T>A, 5015G>A, 5016A>G, 5018T>A, 5019G>A, 5021C>A, 5024A>C, 5025T>C, 5029A>T, 5030G>A, 5031T>C, 5038A>G, 5040A>G, 5041A>C, 5042G>C, 5046C>A, 5047A>G, 5048G>A, 5051T>A, 5053G>T, 5054A>G, 5057C>T, 5063G>A, 5066C>T, 5069C>A, 5078T>A, 5081T>A, 5082C>T, 5083T>A, 5091C>A, 5093G>A, 5094G>A, 5095A>T, 5096C>A, 5097A>G, 5098A>C, 5099G>A, 5100C>G, 5102T>A, 5105T>A, 5106A>G, 5107T>A, 5109C>G, 5112T>A, 5115C>A, 5116C>A, 5117A>T, 5118G>C, 5121A>G, 5122T>A, 5123G>C, 5124G>A, 5125C>T, 5126G>A, 5127A>G, 5132A>T, 5134A>T, 5136G>C, 5138A>G, 5140A>T, 5141G>A, 5144T>A, 5147T>A, 5149C>A, 5157C>T, 5159G>A, 5161C>A, 5162A>T, 5163C>T, 5165C>A, 5166A>G, 5167A>G, 5168G>A, 5171T>A, 5174C>A, 5178C>A, 5179C>G, 5180T>A, 5183C>A, 5184C>A, 5185A>C, 5187A>T, 5188G>C, 5190C>A, 5192C>A, 5195T>C, 5198G>A, 5199A>T, 5207T>A, 5213T>G, 5216C>A, 5219C>A, 5220delA, 5222A>C, 5226T>A, 5227C>A, 5228T>C, 5232C>A, 5233A>T, 5237A>C, 5243T>A, 5247A>G, 5248G>C, 5249T>C, 5250C>A, 5252T>A, 5259T>A, 5260A>T, 5261C>A, 5271C>A, 5276A>C, 5288A>T, 5289T>A, 5290A>G, 5291C>A, 5293A>T, 5294A>G, 5297C>T, 5298C>G, 5299A>C, 5300A>T, 5303C>T, 5305C>A, 5307C>T, 5309C>A, 5313A>G, 5314G>A, 5315T>C, 5317T>A, 5318T>A, 5319G>A, 5321C>T, 5322T>G, 5324T>A, 5327C>A, 5331C>A, 5332T>A, 5335A>G, 5339T>A, 5340A>C, 5344A>G, 5345C>A, 5348G>A, 5350T>G, 5351T>A, 5352G>A, 5354C>T, 5356A>T, 5360C>T, 5369T>C, 5370C>T, 5371T>G, 5372A>T, 5375G>A, 5381A>G, 5383T>A, 5384C>T, 5385C>T, 5386A>G, 5387C>G, 5390G>A, 5391A>G, 5393T>A, 5395T>A, 5396G>A, 5400G>C, 5402T>C, 5405A>T, 5406C>G, 5408G>T, 5424G>A, 5430G>A, 5431T>C, 5432C>T, 5435C>T, 5439C>G, 5441G>A, 5444T>A, 5447T>C, 5449T>A, 5455G>T, 5456G>T, 5457A>C, 5459A>G, 5462A>T, 5471C>T, 5474A>C, 5477C>T, 5479A>C, 5492A>C, 5493G>T, 5494T>C, 5498C>T, 5501A>G, 5502C>A, 5503G>A, 5504A>T, 5505A>C, 5506A>T, 5507A>T, 5511G>A, 5514A>G, 5515A>C, 5517A>G, 5519A>C, 5522C>T, 5526A>C, 5527A>C, 5528_5529insCTC, 5529T>C, 5530A>T, 5532T>A, 5533C>G, 5535G>A, 5536A>G, 5539T>C, 5540T>A, 5543C>A, 5545G>T, 5546T>G, 5549T>C, 5551A>T, 5553A>T, 5555A>T, 5558C>T, 5564T>A, 5568A>G, 5570A>T, 5572T>A, 5573T>C, 5574T>A, 5575C>G, 5576T>C, 5577G>A, 5579A>G, 5580A>G, 5581G>A, 5585A>G, 5589C>G, 5595G>C, 5598C>G, 5607T>C, 5608T>A, 5610C>A, 5611A>C, 5612G>A, 5613T>A, 5615C>T, 5618C>T, 5619C>A, 5620A>G, 5621A>G, 5622G>T, 5626G>T, 5627T>C, 5628A>G, 5629A>T, 5630G>A, 5631G>A, 5632A>G, 5633A>T, 5634G>A, 5639G>A, 5640C>A, 5642T>A, 5645T>A, 5646T>A, 5648G>A, 5649T>A, 5651A>C, 5656C>A, 5657A>G, 5661T>A, 5664A>G, 5667A>T, 5670G>T, 5672A>T, 5673G>A, 5674T>A, 5676G>A, 5679A>C, 5685G>A, 5687A>C, 5691C>T, 5693T>A, 5696C>A, 5697C>G, 5700G>A, 5701A>C, 5702A>T, 5703A>T, 5711A>T, 5714A>G, 5717A>G, 5718G>A, 5720T>A, 5721C>A, 5726C>A, 5739T>G, 5740T>C, 5741A>T, 5748A>G, 5756A>T, 5757T>A, 5759T>G, 5762T>A, 5763_5765delGAA, 5768C>T, 5769C>A, 5777A>T, 5779C>A, 5781T>A, 5782T>C, 5786G>A, 5788G>A, 5790C>T, 5795G>A, 5798G>A, 5807G>A, 5808A>T, 5811T>G, 5813A>G, 5816C>T, 5822C>T, 5828T>C, 5830A>T, 5834C>A, 5836C>A, 5843G>A, 5844A>G, 5846T>A, 5850T>A, 5851A>T, 5853A>G, 5855T>A, 5856A>G, 5859A>C, 5860T>C, 5864T>A, 5868A>G, 5869A>C, 5870T>A, 5873G>A, 5874T>A, 5875G>T, 5876T>A, 5877T>G, 5878C>G, 5879C>A, 5882C>T, 5889G>C, 5893G>A, 5895A>C, 5897A>C, 5903T>C, 5904T>A, 5906T>A, 5907C>G, 5910G>A, 5912C>T, 5913T>A, 5914G>T, 5915G>T, 5918G>A, 5919A>C, 5924T>A, 5925A>C, 5926G>A, 5928A>C, 5933C>T, 5940G>A, 5941T>A, 5945T>A, 5947C>A, 5948C>A, 5951A>C, 5952T>A, 5957C>A, 5958C>G, 5959C>A, 5960T>C, 5963G>A, 5965T>G, 5966T>C, 5967T>A, 5973A>C, 5974A>T, 5976C>G, 5977C>A, 5979C>T, 5980A>C, 5982A>G, 5983G>A, 5988A>C, 5990T>A, 5999G>A, 6005T>C, 6009T>A, 6010C>G, 6011T>C, 6014A>C, 6015G>C, 6026G>A, 6028G>C, 6029A>T, 6030A>G, 6032T>A, 6039T>G, 6040G>C, 6041G>A, 6047C>A, 6048T>A, 6049C>A, 6050T>C, 6051C>A, 6054C>T, 6056A>C, 6057G>A, 6058A>G, 6060T>C, 6062A>T, 6063C>A, 6064C>A, 6065T>A, 6066T>A, 6067C>A, 6069A>G, 6070C>A, 6082C>G, 6083A>T, 6084C>G, 6085G>C, 6086G>T, 6089T>C, 6090T>C, 6091G>A, 6092T>A, 6100G>A, 6101C>A, 6104T>C, 6107_6108insGAAAAG, 6108T>G, 6109C>G, 6111G>A, 6112C>A, 6116C>G, 6117T>A, 6118C>G, 6120G>A, 6121C>G, 6122A>T, 6130C>T, 6131T>C, 6135A>G, 6138A>T, 6140G>A, 6144T>A, 6145G>T, 6146T>A, 6154T>G, 6156T>C, 6159G>A, 6163A>G, 6164G>T, 6167T>C, 6171A>T, 6175T>A, 6177T>A, 6179A>T, 6181A>T, 6182T>A, 6183G>A, 6185G>C, 6190A>G, 6191A>T, 6195T>A, 6197T>A, 6198A>G, 6199C>T, 6200C>A, 6201C>G, 6203T>A, 6206G>A, 6212C>T, 6215T>C, 6216A>G, 6217T>A, 6218T>A, 6221A>T, 6224T>A, 6230T>A, 6234T>C, 6237A>C, 6239C>A, 6243C>A, 6245T>A, 6246A>G, 6248T>C, 6249A>C, 6251T>G, 6257A>T, 6258T>A, 6259T>G, 6260G>T, 6262A>G, 6265T>A, 6268A>G, 6269T>A, 6270C>A, 6272T>A, 6276G>C, 6284C>T, 6285T>A, 6286G>T, 6287T>A, 6290T>C, 6291T>A, 6292T>C, 6293A>C, 6297A>T, 6299A>T, 6300G>A, 6301G>T, 6304T>A, 6305A>C, 6306G>C, 6307G>C, 6309_6311delTTA, 6313C>A, 6318A>G, 6319A>T, 6320A>T, 6321A>T, 6323A>C, 6327C>T, 6332C>A, 6333C>A, 6335G>A, 6338T>A									

CDS

	Begin	End	Coverage	Score	Concordance	Matches	Identities	I/D/M/F*	Stop Codons
EXK67_gp3	1124	1582	28.8%	1519	47.7%	457 (98.9%)	228 (49.4%)	3/2/1/1	0
Protein mutations:	L1125I (4983C>A), H1126K (4986C>A 4988T>A), K1127L (4989A>T 4990A>T), E1129D (4997A>T), I1131L (5001A>T 5003C>A), Q1132E (5004C>G), N1136E (5016A>G 5018T>A), D1137K (5019G>A 5021C>A), K1140I (5029A>T 5030G>A), Y1141H (5031T>C), E1143G (5038A>G), K1144A (5040A>G 5041A>C 5042G>C), Q1146R (5046C>A 5047A>G 5048G>A), R1148M (5053G>T 5054A>G), D1157E (5081T>A), L1158Y (5082C>T 5083T>A), Q1161K (5091C>A 5093G>A), D1162I (5094G>A 5095A>T 5096C>A), K1163A (5097A>G 5098A>C 5099G>A), P1164A (5100C>G 5102T>A), I1166E (5106A>G 5107T>A), P1167A (5109C>G), S1168T (5112T>A), P1169N (5115C>A 5116C>A 5117A>T), E1170Q (5118G>C), M1171D (5121A>G 5122T>A 5123G>C), A1172I (5124G>A 5125C>T 5126G>A), K1173E (5127A>G), E1174D (5132A>T), Y1175F (5134A>T), E1176Q (5136G>C 5138A>G), K1177I (5140A>T 5141G>A), H1178Q (5144T>A), T1180K (5149C>A), A1184D (5161C>A 5162A>T), K1186G (5166A>G 5167A>G 5168G>A), P1190R (5178C>A 5179C>G 5180T>A), Q1192T (5184C>A 5185A>C), T1197S (5199A>T), I1201M (5213T>G), N1203K (5219C>A), S1206N (5226T>A 5227C>A 5228T>C), Q1208I (5232C>A 5233A>T), S1213A (5247A>G 5248G>C 5249T>C), Y1217I (5259T>A 5260A>T 5261C>A), Y1227R (5289T>A 5290A>G 5291C>A), K1228M (5293A>T 5294A>G), Q1230A (5298C>G 5299A>C 5300A>T), T1232K (5305C>A), S1235D (5313A>G 5314G>A 5315T>C), I1236K (5317T>A 5318T>A), D1237N (5319G>A 5321C>T), Y1238E (5322T>G 5324T>A), L1241N (5331C>A 5332T>A), K1242R (5335A>G), K1244Q (5340A>C), D1245G (5344A>G 5345C>A), I1247R (5350T>G 5351T>A), V1248I (5352G>A 5354C>T), Y1249F (5356A>T), L1254C (5370C>T 5371T>G 5372A>T), F1258Y (5383T>A 5384C>T), H1259W (5385C>T 5386A>G 5387C>G), I1261V (5391A>G 5393T>A), M1262K (5395T>A 5396G>A), D1264H (5400G>C 5402T>C), Q1266D (5406C>G 5408G>T), A1272T (5424G>A), V1274T (5430G>A 5431T>C 5432C>T), Q1277E (5439C>G 5441G>A), F1280Y (5449T>A), W1282F (5455G>T 5456G>T), I1283L (5457A>C 5459A>G), K1290T (5479A>C), V1295S (5493G>T 5494T>C), R1298N (5502C>A 5503G>A 5504A>T), K1299L (5505A>C 5506A>T 5507A>T), D1301N (5511G>A), N1302A (5514A>G 5515A>C), I1303V (5517A>G 5519A>C), K1306P (5526A>C 5527A>C), K1306_Y1307insL (5528_5529insCTC), Y1307L (5529T>C 5530A>T), S1308R (5532T>A 5533C>G), E1309R (5535G>A 5536A>G), F1310S (5539T>C 5540T>A), C1312L (5545G>T 5546T>G), Y1314F (5551A>T), I1315F (5553A>T 5555A>T), I1320V (5568A>G 5570A>T), F1321Y (5572T>A 5573T>C), E1323K (5577G>A 5579A>G), S1324D (5580A>G 5581G>A), I1325M (5585A>G), Q1327E (5589C>G), V1329L (5595G>C), Q1330E (5598C>G), L1333Q (5607T>C 5608T>A), Q1334T (5610C>A 5611A>C 5612G>A), F1335I (5613T>A 5615C>T), Q1337R (5619C>A 5620A>G 5621A>G), V1338L (5622G>T), C1339F (5626G>T 5627T>C), K1340V (5628A>G 5629A>T 5630G>A), E1341S (5631G>A 5632A>G 5633A>T), E1342K (5634G>A), L1344I (5640C>A 5642T>A), L1346I (5646T>A 5648G>A), S1347T (5649T>A 5651A>C), T1349K (5656C>A 5657A>G), L1351M (5661T>A), K1352E (5664A>G), I1353L (5667A>T), G1354C (5670G>T 5672A>T), V1355K (5673G>A 5674T>A), A1356T (5676G>A), N1357H (5679A>C), E1359N (5685G>A 5687A>C), L1363V (5697C>G), E1364T (5700G>A 5701A>C 5702A>T), I1365L (5703A>T), E1367D (5711A>T), V1370I (5718G>A 5720T>A), Q1371K (5721C>A), L1377A (5739T>G 5740T>C 5741A>T), I1380V (5748A>G), E1382D (5756A>T), F1383M (5757T>A 5759T>G), E1385del (5763_5765delGAA), Q1387K (5769C>A), E1389D (5777A>T), T1390K (5779C>A), L1391T (5781T>A 5782T>C), G1393D (5788G>A), I1400L (5808A>T), L1401V (5811T>G 5813A>G), Y1407F (5830A>T), P1409Q (5836C>A), S1412G (5844A>G 5846T>A), Y1414I (5850T>A 5851A>T), T1415A (5853A>G 5855T>A), R1416G (5856A>G), I1417P (5859A>C 5860T>C), F1418L (5864T>A), N1420A (5868A>G 5869A>C 5870T>A), C1422I (5874T>A 5875G>T 5876T>A), S1423G (5877T>G 5878C>G 5879C>A), E1427Q (5889G>C), R1428K (5893G>A), K1429H (5895A>C 5897A>C), S1432T (5904T>A 5906T>A), Q1433E (5907C>G), D1434N (5910G>A 5912C>T), W1435I (5913T>A 5914G>T 5915G>T), M1437L (5919A>C), R1439Q (5925A>C 5926G>A), K1440Q (5928A>C), V1444K (5940G>A 5941T>A), T1446K (5947C>A 5948C>A), K1447N (5951A>C), L1448I (5952T>A), P1450D (5958C>G 5959C>A 5960T>C), I1452S (5965T>G 5966T>C), L1453I (5967T>A), K1455L (5973A>C 5974A>T), P1456E (5976C>G 5977C>A), Q1457S (5979C>T 5980A>C), S1458D (5982A>G 5983G>A), I1460L (5988A>C 5990T>A), E1469Q (6015G>C), G1473A (6028G>C 6029A>T), I1474V (6030A>G 6032T>A), W1477A (6039T>G 6040G>C 6041G>A), S1480N (6048T>A 6049C>A 6050T>C), Q1481K (6051C>A), Q1482Y (6054C>T 6056A>C), D1483S (6057G>A 6058A>G), S1484P (6060T>C 6062A>T), P1485K (6063C>A 6064C>A 6065T>A), S1486K (6066T>A 6067C>A), T1487E (6069A>G 6070C>A), S1491C (6082C>G 6083A>T), R1492A (6084C>G 6085G>C 6086G>T), C1494Q (6090T>C 6091G>A 6092T>A), S1497K (6100G>A 6101C>A), K1499_S1500insEK (6107_6108insGAAAAG), S1500G (6108T>G 6109C>G), A1501N (6111G>A 6112C>A), I1502M (6116C>G), A1504S (6120G>A 6121C>G 6122A>T), A1507V (6130C>T 6131T>C), I1509V (6135A>G), M1510L (6138A>T 6140G>A), C1512I (6144T>A 6145G>T 6146T>A), V1515G (6154T>G), D1517N (6159G>A), K1518S (6163A>G 6164G>T), I1521L (6171A>T), F1522Y (6175T>A), L1523I (6177T>A 6179A>T), Y1524L (6181A>T 6182T>A), E1525N (6183G>A 6185G>C), K1527S (6190A>G 6191A>T), F1529I (6195T>A 6197T>A), T1530V (6198A>G 6199C>T 6200C>A), L1531V (6201C>G 6203T>A), I1536E (6216A>G 6217T>A 6218T>A), N1540K (6230T>A), Y1542H (6234T>C), N1543Q (6237A>C 6239C>A), L1545I (6243C>A 6245T>A), N1546D (6246A>G 6248T>C), N1547Q (6249A>C 6251T>G), K1549N (6257A>T), L1550S (6258T>A 6259T>G 6260G>T), N1551S (6262A>G), I1552K (6265T>A), N1553R (6268A>G 6269T>A), V1556L (6276G>C), C1559I (6285T>A 6286G>T 6287T>A), L1561T (6291T>A 6292T>C 6293A>C), T1563S (6297A>T 6299A>T), G1564I (6300G>A 6301G>T), L1565Y (6304T>A 6305A>C), G1566P (6306G>C 6307G>C), L1567del (6309_6311delTTA), T1568N (6313C>A), K1570V (6318A>G 6319A>T 6320A>T), I1571F (6321A>T 6323A>C), H1573Y (6327C>T), Q1575K (6333C>A 6335G>A)									

	Begin	End	Coverage	Score	Concordance	Matches	Identities	I/D/M/F*	Stop Codons
**NT**	**4980**	**6355**	**16.9%**	**493**	**18.2%**	**1369 (98.8%)**	**823 (59.4%)**	**9/7**	
Codon mutations:	CTA1125ATA (4983C>A), CAT1126AAA (4986C>A 4988T>A), AAA1127TTA (4989A>T 4990A>T), TTG1128CTA (4992T>C 4994G>A), GAA1129GAT (4997A>T), ATC1131TTA (5001A>T 5003C>A), CAA1132GAA (5004C>G), ATT1133ATA (5009T>A), ATT1134ATA (5012T>A), GGG1135GGA (5015G>A), AAT1136GAA (5016A>G 5018T>A), GAC1137AAA (5019G>A 5021C>A), CCA1138CCC (5024A>C), TTA1139CTA (5025T>C), AAG1140ATA (5029A>T 5030G>A), TAT1141CAT (5031T>C), GAA1143GGA (5038A>G), AAG1144GCC (5040A>G 5041A>C 5042G>C), CAG1146AGA (5046C>A 5047A>G 5048G>A), ATT1147ATA (5051T>A), AGA1148ATG (5053G>T 5054A>G), TGC1149TGT (5057C>T), CTG1151CTA (5063G>A), GAC1152GAT (5066C>T), ATC1153ATA (5069C>A), CCT1156CCA (5078T>A), GAT1157GAA (5081T>A), CTT1158TAT (5082C>T 5083T>A), CAG1161AAA (5091C>A 5093G>A), GAC1162ATA (5094G>A 5095A>T 5096C>A), AAG1163GCA (5097A>G 5098A>C 5099G>A), CCT1164GCA (5100C>G 5102T>A), ATT1165ATA (5105T>A), ATA1166GAA (5106A>G 5107T>A), CCT1167GCT (5109C>G), TCA1168ACA (5112T>A), CCA1169AAT (5115C>A 5116C>A 5117A>T), GAA1170CAA (5118G>C), ATG1171GAC (5121A>G 5122T>A 5123G>C), GCG1172ATA (5124G>A 5125C>T 5126G>A), AAA1173GAA (5127A>G), GAA1174GAT (5132A>T), TAT1175TTT (5134A>T), GAA1176CAG (5136G>C 5138A>G), AAG1177ATA (5140A>T 5141G>A), CAT1178CAA (5144T>A), ATT1179ATA (5147T>A), ACA1180AAA (5149C>A), CTG1183TTA (5157C>T 5159G>A), GCA1184GAT (5161C>A 5162A>T), CTC1185TTA (5163C>T 5165C>A), AAG1186GGA (5166A>G 5167A>G 5168G>A), GTT1187GTA (5171T>A), ATC1188ATA (5174C>A), CCT1190AGA (5178C>A 5179C>G 5180T>A), TCC1191TCA (5183C>A), CAA1192ACA (5184C>A 5185A>C), AGT1193TCT (5187A>T 5188G>C), CGC1194AGA (5190C>A 5192C>A), CAT1195CAC (5195T>C), AGG1196AGA (5198G>A), ACA1197TCA (5199A>T), GCT1199GCA (5207T>A), ATT1201ATG (5213T>G), GTC1202GTA (5216C>A), AAC1203AAA (5219C>A), AAA1204-AC (5220delA 5222A>C), TCT1206AAC (5226T>A 5227C>A 5228T>C), CAA1208ATA (5232C>A 5233A>T), GTA1209GTC (5237A>C), GGT1211GGA (5243T>A), AGT1213GCC (5247A>G 5248G>C 5249T>C), CGT1214AGA (5250C>A 5252T>A), TAC1217ATA (5259T>A 5260A>T 5261C>A), CGA1221AGA (5271C>A), CTA1222CTC (5276A>C), ACA1226ACT (5288A>T), TAC1227AGA (5289T>A 5290A>G 5291C>A), AAA1228ATG (5293A>T 5294A>G), GAC1229GAT (5297C>T), CAA1230GCT (5298C>G 5299A>C 5300A>T), TAC1231TAT (5303C>T), ACA1232AAA (5305C>A), CTC1233TTA (5307C>T 5309C>A), AGT1235GAC (5313A>G 5314G>A 5315T>C), ATT1236AAA (5317T>A 5318T>A), GAC1237AAT (5319G>A 5321C>T), TAT1238GAA (5322T>G 5324T>A), CTC1239CTA (5327C>A), CTT1241AAT (5331C>A 5332T>A), AAA1242AGA (5335A>G), ATT1243ATA (5339T>A), AAA1244CAA (5340A>C), GAC1245GGA (5344A>G 5345C>A), AAG1246AAA (5348G>A), ATT1247AGA (5350T>G 5351T>A), GTC1248ATT (5352G>A 5354C>T), TAC1249TTC (5356A>T), AGC1250AGT (5360C>T), GAT1253GAC (5369T>C), CTA1254TGT (5370C>T 5371T>G 5372A>T), AAG1255AAA (5375G>A), GGA1257GGG (5381A>G), TTC1258TAT (5383T>A 5384C>T), CAC1259TGG (5385C>T 5386A>G 5387C>G), CAG1260CAA (5390G>A), ATT1261GTA (5391A>G 5393T>A), ATG1262AAA (5395T>A 5396G>A), GAT1264CAC (5400G>C 5402T>C), CCA1265CCT (5405A>T), CAG1266GAT (5406C>G 5408G>T), GCA1272ACA (5424G>A), GTC1274ACT (5430G>A 5431T>C 5432C>T), TGC1275TGT (5435C>T), CAG1277GAA (5439C>G 5441G>A), GGT1278GGA (5444T>A), CAT1279CAC (5447T>C), TTT1280TAT (5449T>A), TGG1282TTT (5455G>T 5456G>T), ATA1283CTG (5457A>C 5459A>G), GTA1284GTT (5462A>T), TTC1287TTT (5471C>T), GGA1288GGC (5474A>C), CTC1289CTT (5477C>T), AAA1290ACA (5479A>C), TCA1294TCC (5492A>C), GTA1295TCA (5493G>T 5494T>C), TTC1296TTT (5498C>T), CAA1297CAG (5501A>G), CGA1298AAT (5502C>A 5503G>A 5504A>T), AAA1299CTT (5505A>C 5506A>T 5507A>T), GAT1301AAT (5511G>A), AAT1302GCT (5514A>G 5515A>C), ATA1303GTC (5517A>G 5519A>C), TTC1304TTT (5522C>T), AAA1306CCA (5526A>C 5527A>C), AAA1306_TAT1307insCTC (5528_5529insCTC), TAT1307CTT (5529T>C 5530A>T), TCA1308AGA (5532T>A 5533C>G), GAA1309AGA (5535G>A 5536A>G), TTT1310TCA (5539T>C 5540T>A), GTC1311GTA (5543C>A), TGT1312TTG (5545G>T 5546T>G), GTT1313GTC (5549T>C), TAC1314TTC (5551A>T), ATA1315TTT (5553A>T 5555A>T), GAC1316GAT (5558C>T), ATT1318ATA (5564T>A), ATA1320GTT (5568A>G 5570A>T), TTT1321TAC (5572T>A 5573T>C), TCT1322AGC (5574T>A 5575C>G 5576T>C), GAA1323AAG (5577G>A 5579A>G), AGT1324GAT (5580A>G 5581G>A), ATA1325ATG (5585A>G), CAA1327GAA (5589C>G), GTA1329CTA (5595G>C), CAA1330GAA (5598C>G), TTA1333CAA (5607T>C 5608T>A), CAG1334ACA (5610C>A 5611A>C 5612G>A), TTC1335ATT (5613T>A 5615C>T), TTC1336TTT (5618C>T), CAA1337AGG (5619C>A 5620A>G 5621A>G), GTA1338TTA (5622G>T), TGT1339TTC (5626G>T 5627T>C), AAG1340GTA (5628A>G 5629A>T 5630G>A), GAA1341AGT (5631G>A 5632A>G 5633A>T), GAA1342AAA (5634G>A), GGG1343GGA (5639G>A), CTT1344ATA (5640C>A 5642T>A), ATT1345ATA (5645T>A), TTG1346ATA (5646T>A 5648G>A), TCA1347ACC (5649T>A 5651A>C), ACA1349AAG (5656C>A 5657A>G), TTG1351ATG (5661T>A), AAA1352GAA (5664A>G), ATA1353TTA (5667A>T), GGA1354TGT (5670G>T 5672A>T), GTA1355AAA (5673G>A 5674T>A), GCA1356ACA (5676G>A), AAT1357CAT (5679A>C), GAA1359AAC (5685G>A 5687A>C), CTT1361TTA (5691C>T 5693T>A), GGC1362GGA (5696C>A), CTA1363GTA (5697C>G), GAA1364ACT (5700G>A 5701A>C 5702A>T), ATA1365TTA (5703A>T), GAA1367GAT (5711A>T), GGA1368GGG (5714A>G), AAA1369AAG (5717A>G), GTT1370ATA (5718G>A 5720T>A), CAA1371AAA (5721C>A), CTC1372CTA (5726C>A), TTA1377GCT (5739T>G 5740T>C 5741A>T), ATA1380GTA (5748A>G), GAA1382GAT (5756A>T), TTT1383ATG (5757T>A 5759T>G), CCT1384CCA (5762T>A), GAA1385del (5763_5765delGAA), GAC1386GAT (5768C>T), CAA1387AAA (5769C>A), GAA1389GAT (5777A>T), ACA1390AAA (5779C>A), TTA1391ACA (5781T>A 5782T>C), AAG1392AAA (5786G>A), GGT1393GAT (5788G>A), CTA1394TTA (5790C>T), CAG1395CAA (5795G>A), AAG1396AAA (5798G>A), GGG1399GGA (5807G>A), ATA1400TTA (5808A>T), TTA1401GTG (5811T>G 5813A>G), AAC1402AAT (5816C>T), GCC1404GCT (5822C>T), AAT1406AAC (5828T>C), TAT1407TTT (5830A>T), ATC1408ATA (5834C>A), CCA1409CAA (5836C>A), TTG1411TTA (5843G>A), AGT1412GGA (5844A>G 5846T>A), TAT1414ATT (5850T>A 5851A>T), ACT1415GCA (5853A>G 5855T>A), AGA1416GGA (5856A>G), ATA1417CCA (5859A>C 5860T>C), TTT1418TTA (5864T>A), AAT1420GCA (5868A>G 5869A>C 5870T>A), AAG1421AAA (5873G>A), TGT1422ATA (5874T>A 5875G>T 5876T>A), TCC1423GGA (5877T>G 5878C>G 5879C>A), AGC1424AGT (5882C>T), GAA1427CAA (5889G>C), AGA1428AAA (5893G>A), AAA1429CAC (5895A>C 5897A>C), AAT1431AAC (5903T>C), TCT1432ACA (5904T>A 5906T>A), CAA1433GAA (5907C>G), GAC1434AAT (5910G>A 5912C>T), TGG1435ATT (5913T>A 5914G>T 5915G>T), AAG1436AAA (5918G>A), ATG1437CTG (5919A>C), GTT1438GTA (5924T>A), AGA1439CAA (5925A>C 5926G>A), AAA1440CAA (5928A>C), ATC1441ATT (5933C>T), GTA1444AAA (5940G>A 5941T>A), ATT1445ATA (5945T>A), ACC1446AAA (5947C>A 5948C>A), AAA1447AAC (5951A>C), TTA1448ATA (5952T>A), CCC1449CCA (5957C>A), CCT1450GAC (5958C>G 5959C>A 5960T>C), TTG1451TTA (5963G>A), ATT1452AGC (5965T>G 5966T>C), TTA1453ATA (5967T>A), AAA1455CTA (5973A>C 5974A>T), CCA1456GAA (5976C>G 5977C>A), CAA1457TCA (5979C>T 5980A>C), AGT1458GAT (5982A>G 5983G>A), ATT1460CTA (5988A>C 5990T>A), GAG1463GAA (5999G>A), GAT1465GAC (6005T>C), TCT1467AGC (6009T>A 6010C>G 6011T>C), CTA1468CTC (6014A>C), GAA1469CAA (6015G>C), GGG1472GGA (6026G>A), GGA1473GCT (6028G>C 6029A>T), ATT1474GTA (6030A>G 6032T>A), TGG1477GCA (6039T>G 6040G>C 6041G>A), CCC1479CCA (6047C>A), TCT1480AAC (6048T>A 6049C>A 6050T>C), CAA1481AAA (6051C>A), CAA1482TAC (6054C>T 6056A>C), GAT1483AGT (6057G>A 6058A>G), TCA1484CCT (6060T>C 6062A>T), CCT1485AAA (6063C>A 6064C>A 6065T>A), TCA1486AAA (6066T>A 6067C>A), ACA1487GAA (6069A>G 6070C>A), TCA1491TGT (6082C>G 6083A>T), CGG1492GCT (6084C>G 6085G>C 6086G>T), TAT1493TAC (6089T>C), TGT1494CAA (6090T>C 6091G>A 6092T>A), AGC1497AAA (6100G>A 6101C>A), TAT1498TAC (6104T>C), AAA1499_TCT1500insGAAAAG (6107_6108insGAAAAG), TCT1500GGT (6108T>G 6109C>G), GCC1501AAC (6111G>A 6112C>A), ATC1502ATG (6116C>G), TCT1503AGT (6117T>A 6118C>G), GCA1504AGT (6120G>A 6121C>G 6122A>T), GCT1507GTC (6130C>T 6131T>C), ATA1509GTA (6135A>G), ATG1510TTA (6138A>T 6140G>A), TGT1512ATA (6144T>A 6145G>T 6146T>A), GTA1515GGA (6154T>G), TTA1516CTA (6156T>C), GAT1517AAT (6159G>A), AAG1518AGT (6163A>G 6164G>T), TTT1519TTC (6167T>C), ATA1521TTA (6171A>T), TTT1522TAT (6175T>A), TTA1523ATT (6177T>A 6179A>T), TAT1524TTA (6181A>T 6182T>A), GAG1525AAC (6183G>A 6185G>C), AAA1527AGT (6190A>G 6191A>T), TTT1529ATA (6195T>A 6197T>A), ACC1530GTA (6198A>G 6199C>T 6200C>A), CTT1531GTA (6201C>G 6203T>A), AGG1532AGA (6206G>A), GAC1534GAT (6212C>T), TGT1535TGC (6215T>C), ATT1536GAA (6216A>G 6217T>A 6218T>A), GCA1537GCT (6221A>T), ATT1538ATA (6224T>A), AAT1540AAA (6230T>A), TAT1542CAT (6234T>C), AAC1543CAA (6237A>C 6239C>A), CTT1545ATA (6243C>A 6245T>A), AAT1546GAC (6246A>G 6248T>C), AAT1547CAG (6249A>C 6251T>G), AAA1549AAT (6257A>T), TTG1550AGT (6258T>A 6259T>G 6260G>T), AAT1551AGT (6262A>G), ATA1552AAA (6265T>A), AAT1553AGA (6268A>G 6269T>A), CGT1554AGA (6270C>A 6272T>A), GTA1556CTA (6276G>C), TTC1558TTT (6284C>T), TGT1559ATA (6285T>A 6286G>T 6287T>A), GAT1560GAC (6290T>C), TTA1561ACC (6291T>A 6292T>C 6293A>C), ACA1563TCT (6297A>T 6299A>T), GGA1564ATA (6300G>A 6301G>T), TTA1565TAC (6304T>A 6305A>C), GGT1566CCT (6306G>C 6307G>C), TTA1567del (6309_6311delTTA), ACT1568AAT (6313C>A), AAA1570GTT (6318A>G 6319A>T 6320A>T), ATA1571TTC (6321A>T 6323A>C), CAT1573TAT (6327C>T), ATC1574ATA (6332C>A), CAG1575AAA (6333C>A 6335G>A), GGT1576GGA (6338T>A)								

Proteins

ORF3 (YP_009553219.1)	1124	1582	28.8%	1519	47.7%	457 (98.9%)	228 (49.4%)	3/2/1/1	0
Protein mutations:	L1125I (4983C>A), H1126K (4986C>A 4988T>A), K1127L (4989A>T 4990A>T), E1129D (4997A>T), I1131L (5001A>T 5003C>A), Q1132E (5004C>G), N1136E (5016A>G 5018T>A), D1137K (5019G>A 5021C>A), K1140I (5029A>T 5030G>A), Y1141H (5031T>C), E1143G (5038A>G), K1144A (5040A>G 5041A>C 5042G>C), Q1146R (5046C>A 5047A>G 5048G>A), R1148M (5053G>T 5054A>G), D1157E (5081T>A), L1158Y (5082C>T 5083T>A), Q1161K (5091C>A 5093G>A), D1162I (5094G>A 5095A>T 5096C>A), K1163A (5097A>G 5098A>C 5099G>A), P1164A (5100C>G 5102T>A), I1166E (5106A>G 5107T>A), P1167A (5109C>G), S1168T (5112T>A), P1169N (5115C>A 5116C>A 5117A>T), E1170Q (5118G>C), M1171D (5121A>G 5122T>A 5123G>C), A1172I (5124G>A 5125C>T 5126G>A), K1173E (5127A>G), E1174D (5132A>T), Y1175F (5134A>T), E1176Q (5136G>C 5138A>G), K1177I (5140A>T 5141G>A), H1178Q (5144T>A), T1180K (5149C>A), A1184D (5161C>A 5162A>T), K1186G (5166A>G 5167A>G 5168G>A), P1190R (5178C>A 5179C>G 5180T>A), Q1192T (5184C>A 5185A>C), T1197S (5199A>T), I1201M (5213T>G), N1203K (5219C>A), S1206N (5226T>A 5227C>A 5228T>C), Q1208I (5232C>A 5233A>T), S1213A (5247A>G 5248G>C 5249T>C), Y1217I (5259T>A 5260A>T 5261C>A), Y1227R (5289T>A 5290A>G 5291C>A), K1228M (5293A>T 5294A>G), Q1230A (5298C>G 5299A>C 5300A>T), T1232K (5305C>A), S1235D (5313A>G 5314G>A 5315T>C), I1236K (5317T>A 5318T>A), D1237N (5319G>A 5321C>T), Y1238E (5322T>G 5324T>A), L1241N (5331C>A 5332T>A), K1242R (5335A>G), K1244Q (5340A>C), D1245G (5344A>G 5345C>A), I1247R (5350T>G 5351T>A), V1248I (5352G>A 5354C>T), Y1249F (5356A>T), L1254C (5370C>T 5371T>G 5372A>T), F1258Y (5383T>A 5384C>T), H1259W (5385C>T 5386A>G 5387C>G), I1261V (5391A>G 5393T>A), M1262K (5395T>A 5396G>A), D1264H (5400G>C 5402T>C), Q1266D (5406C>G 5408G>T), A1272T (5424G>A), V1274T (5430G>A 5431T>C 5432C>T), Q1277E (5439C>G 5441G>A), F1280Y (5449T>A), W1282F (5455G>T 5456G>T), I1283L (5457A>C 5459A>G), K1290T (5479A>C), V1295S (5493G>T 5494T>C), R1298N (5502C>A 5503G>A 5504A>T), K1299L (5505A>C 5506A>T 5507A>T), D1301N (5511G>A), N1302A (5514A>G 5515A>C), I1303V (5517A>G 5519A>C), K1306P (5526A>C 5527A>C), K1306_Y1307insL (5528_5529insCTC), Y1307L (5529T>C 5530A>T), S1308R (5532T>A 5533C>G), E1309R (5535G>A 5536A>G), F1310S (5539T>C 5540T>A), C1312L (5545G>T 5546T>G), Y1314F (5551A>T), I1315F (5553A>T 5555A>T), I1320V (5568A>G 5570A>T), F1321Y (5572T>A 5573T>C), E1323K (5577G>A 5579A>G), S1324D (5580A>G 5581G>A), I1325M (5585A>G), Q1327E (5589C>G), V1329L (5595G>C), Q1330E (5598C>G), L1333Q (5607T>C 5608T>A), Q1334T (5610C>A 5611A>C 5612G>A), F1335I (5613T>A 5615C>T), Q1337R (5619C>A 5620A>G 5621A>G), V1338L (5622G>T), C1339F (5626G>T 5627T>C), K1340V (5628A>G 5629A>T 5630G>A), E1341S (5631G>A 5632A>G 5633A>T), E1342K (5634G>A), L1344I (5640C>A 5642T>A), L1346I (5646T>A 5648G>A), S1347T (5649T>A 5651A>C), T1349K (5656C>A 5657A>G), L1351M (5661T>A), K1352E (5664A>G), I1353L (5667A>T), G1354C (5670G>T 5672A>T), V1355K (5673G>A 5674T>A), A1356T (5676G>A), N1357H (5679A>C), E1359N (5685G>A 5687A>C), L1363V (5697C>G), E1364T (5700G>A 5701A>C 5702A>T), I1365L (5703A>T), E1367D (5711A>T), V1370I (5718G>A 5720T>A), Q1371K (5721C>A), L1377A (5739T>G 5740T>C 5741A>T), I1380V (5748A>G), E1382D (5756A>T), F1383M (5757T>A 5759T>G), E1385del (5763_5765delGAA), Q1387K (5769C>A), E1389D (5777A>T), T1390K (5779C>A), L1391T (5781T>A 5782T>C), G1393D (5788G>A), I1400L (5808A>T), L1401V (5811T>G 5813A>G), Y1407F (5830A>T), P1409Q (5836C>A), S1412G (5844A>G 5846T>A), Y1414I (5850T>A 5851A>T), T1415A (5853A>G 5855T>A), R1416G (5856A>G), I1417P (5859A>C 5860T>C), F1418L (5864T>A), N1420A (5868A>G 5869A>C 5870T>A), C1422I (5874T>A 5875G>T 5876T>A), S1423G (5877T>G 5878C>G 5879C>A), E1427Q (5889G>C), R1428K (5893G>A), K1429H (5895A>C 5897A>C), S1432T (5904T>A 5906T>A), Q1433E (5907C>G), D1434N (5910G>A 5912C>T), W1435I (5913T>A 5914G>T 5915G>T), M1437L (5919A>C), R1439Q (5925A>C 5926G>A), K1440Q (5928A>C), V1444K (5940G>A 5941T>A), T1446K (5947C>A 5948C>A), K1447N (5951A>C), L1448I (5952T>A), P1450D (5958C>G 5959C>A 5960T>C), I1452S (5965T>G 5966T>C), L1453I (5967T>A), K1455L (5973A>C 5974A>T), P1456E (5976C>G 5977C>A), Q1457S (5979C>T 5980A>C), S1458D (5982A>G 5983G>A), I1460L (5988A>C 5990T>A), E1469Q (6015G>C), G1473A (6028G>C 6029A>T), I1474V (6030A>G 6032T>A), W1477A (6039T>G 6040G>C 6041G>A), S1480N (6048T>A 6049C>A 6050T>C), Q1481K (6051C>A), Q1482Y (6054C>T 6056A>C), D1483S (6057G>A 6058A>G), S1484P (6060T>C 6062A>T), P1485K (6063C>A 6064C>A 6065T>A), S1486K (6066T>A 6067C>A), T1487E (6069A>G 6070C>A), S1491C (6082C>G 6083A>T), R1492A (6084C>G 6085G>C 6086G>T), C1494Q (6090T>C 6091G>A 6092T>A), S1497K (6100G>A 6101C>A), K1499_S1500insEK (6107_6108insGAAAAG), S1500G (6108T>G 6109C>G), A1501N (6111G>A 6112C>A), I1502M (6116C>G), A1504S (6120G>A 6121C>G 6122A>T), A1507V (6130C>T 6131T>C), I1509V (6135A>G), M1510L (6138A>T 6140G>A), C1512I (6144T>A 6145G>T 6146T>A), V1515G (6154T>G), D1517N (6159G>A), K1518S (6163A>G 6164G>T), I1521L (6171A>T), F1522Y (6175T>A), L1523I (6177T>A 6179A>T), Y1524L (6181A>T 6182T>A), E1525N (6183G>A 6185G>C), K1527S (6190A>G 6191A>T), F1529I (6195T>A 6197T>A), T1530V (6198A>G 6199C>T 6200C>A), L1531V (6201C>G 6203T>A), I1536E (6216A>G 6217T>A 6218T>A), N1540K (6230T>A), Y1542H (6234T>C), N1543Q (6237A>C 6239C>A), L1545I (6243C>A 6245T>A), N1546D (6246A>G 6248T>C), N1547Q (6249A>C 6251T>G), K1549N (6257A>T), L1550S (6258T>A 6259T>G 6260G>T), N1551S (6262A>G), I1552K (6265T>A), N1553R (6268A>G 6269T>A), V1556L (6276G>C), C1559I (6285T>A 6286G>T 6287T>A), L1561T (6291T>A 6292T>C 6293A>C), T1563S (6297A>T 6299A>T), G1564I (6300G>A 6301G>T), L1565Y (6304T>A 6305A>C), G1566P (6306G>C 6307G>C), L1567del (6309_6311delTTA), T1568N (6313C>A), K1570V (6318A>G 6319A>T 6320A>T), I1571F (6321A>T 6323A>C), H1573Y (6327C>T), Q1575K (6333C>A 6335G>A)								

	Begin	End	Coverage	Score	Concordance	Matches	Identities	I/D/M/F*	Stop Codons
**NT**	**4980**	**6355**	**16.9%**	**493**	**18.2%**	**1369 (98.8%)**	**823 (59.4%)**	**9/7**	
Codon mutations:	CTA1125ATA (4983C>A), CAT1126AAA (4986C>A 4988T>A), AAA1127TTA (4989A>T 4990A>T), TTG1128CTA (4992T>C 4994G>A), GAA1129GAT (4997A>T), ATC1131TTA (5001A>T 5003C>A), CAA1132GAA (5004C>G), ATT1133ATA (5009T>A), ATT1134ATA (5012T>A), GGG1135GGA (5015G>A), AAT1136GAA (5016A>G 5018T>A), GAC1137AAA (5019G>A 5021C>A), CCA1138CCC (5024A>C), TTA1139CTA (5025T>C), AAG1140ATA (5029A>T 5030G>A), TAT1141CAT (5031T>C), GAA1143GGA (5038A>G), AAG1144GCC (5040A>G 5041A>C 5042G>C), CAG1146AGA (5046C>A 5047A>G 5048G>A), ATT1147ATA (5051T>A), AGA1148ATG (5053G>T 5054A>G), TGC1149TGT (5057C>T), CTG1151CTA (5063G>A), GAC1152GAT (5066C>T), ATC1153ATA (5069C>A), CCT1156CCA (5078T>A), GAT1157GAA (5081T>A), CTT1158TAT (5082C>T 5083T>A), CAG1161AAA (5091C>A 5093G>A), GAC1162ATA (5094G>A 5095A>T 5096C>A), AAG1163GCA (5097A>G 5098A>C 5099G>A), CCT1164GCA (5100C>G 5102T>A), ATT1165ATA (5105T>A), ATA1166GAA (5106A>G 5107T>A), CCT1167GCT (5109C>G), TCA1168ACA (5112T>A), CCA1169AAT (5115C>A 5116C>A 5117A>T), GAA1170CAA (5118G>C), ATG1171GAC (5121A>G 5122T>A 5123G>C), GCG1172ATA (5124G>A 5125C>T 5126G>A), AAA1173GAA (5127A>G), GAA1174GAT (5132A>T), TAT1175TTT (5134A>T), GAA1176CAG (5136G>C 5138A>G), AAG1177ATA (5140A>T 5141G>A), CAT1178CAA (5144T>A), ATT1179ATA (5147T>A), ACA1180AAA (5149C>A), CTG1183TTA (5157C>T 5159G>A), GCA1184GAT (5161C>A 5162A>T), CTC1185TTA (5163C>T 5165C>A), AAG1186GGA (5166A>G 5167A>G 5168G>A), GTT1187GTA (5171T>A), ATC1188ATA (5174C>A), CCT1190AGA (5178C>A 5179C>G 5180T>A), TCC1191TCA (5183C>A), CAA1192ACA (5184C>A 5185A>C), AGT1193TCT (5187A>T 5188G>C), CGC1194AGA (5190C>A 5192C>A), CAT1195CAC (5195T>C), AGG1196AGA (5198G>A), ACA1197TCA (5199A>T), GCT1199GCA (5207T>A), ATT1201ATG (5213T>G), GTC1202GTA (5216C>A), AAC1203AAA (5219C>A), AAA1204-AC (5220delA 5222A>C), TCT1206AAC (5226T>A 5227C>A 5228T>C), CAA1208ATA (5232C>A 5233A>T), GTA1209GTC (5237A>C), GGT1211GGA (5243T>A), AGT1213GCC (5247A>G 5248G>C 5249T>C), CGT1214AGA (5250C>A 5252T>A), TAC1217ATA (5259T>A 5260A>T 5261C>A), CGA1221AGA (5271C>A), CTA1222CTC (5276A>C), ACA1226ACT (5288A>T), TAC1227AGA (5289T>A 5290A>G 5291C>A), AAA1228ATG (5293A>T 5294A>G), GAC1229GAT (5297C>T), CAA1230GCT (5298C>G 5299A>C 5300A>T), TAC1231TAT (5303C>T), ACA1232AAA (5305C>A), CTC1233TTA (5307C>T 5309C>A), AGT1235GAC (5313A>G 5314G>A 5315T>C), ATT1236AAA (5317T>A 5318T>A), GAC1237AAT (5319G>A 5321C>T), TAT1238GAA (5322T>G 5324T>A), CTC1239CTA (5327C>A), CTT1241AAT (5331C>A 5332T>A), AAA1242AGA (5335A>G), ATT1243ATA (5339T>A), AAA1244CAA (5340A>C), GAC1245GGA (5344A>G 5345C>A), AAG1246AAA (5348G>A), ATT1247AGA (5350T>G 5351T>A), GTC1248ATT (5352G>A 5354C>T), TAC1249TTC (5356A>T), AGC1250AGT (5360C>T), GAT1253GAC (5369T>C), CTA1254TGT (5370C>T 5371T>G 5372A>T), AAG1255AAA (5375G>A), GGA1257GGG (5381A>G), TTC1258TAT (5383T>A 5384C>T), CAC1259TGG (5385C>T 5386A>G 5387C>G), CAG1260CAA (5390G>A), ATT1261GTA (5391A>G 5393T>A), ATG1262AAA (5395T>A 5396G>A), GAT1264CAC (5400G>C 5402T>C), CCA1265CCT (5405A>T), CAG1266GAT (5406C>G 5408G>T), GCA1272ACA (5424G>A), GTC1274ACT (5430G>A 5431T>C 5432C>T), TGC1275TGT (5435C>T), CAG1277GAA (5439C>G 5441G>A), GGT1278GGA (5444T>A), CAT1279CAC (5447T>C), TTT1280TAT (5449T>A), TGG1282TTT (5455G>T 5456G>T), ATA1283CTG (5457A>C 5459A>G), GTA1284GTT (5462A>T), TTC1287TTT (5471C>T), GGA1288GGC (5474A>C), CTC1289CTT (5477C>T), AAA1290ACA (5479A>C), TCA1294TCC (5492A>C), GTA1295TCA (5493G>T 5494T>C), TTC1296TTT (5498C>T), CAA1297CAG (5501A>G), CGA1298AAT (5502C>A 5503G>A 5504A>T), AAA1299CTT (5505A>C 5506A>T 5507A>T), GAT1301AAT (5511G>A), AAT1302GCT (5514A>G 5515A>C), ATA1303GTC (5517A>G 5519A>C), TTC1304TTT (5522C>T), AAA1306CCA (5526A>C 5527A>C), AAA1306_TAT1307insCTC (5528_5529insCTC), TAT1307CTT (5529T>C 5530A>T), TCA1308AGA (5532T>A 5533C>G), GAA1309AGA (5535G>A 5536A>G), TTT1310TCA (5539T>C 5540T>A), GTC1311GTA (5543C>A), TGT1312TTG (5545G>T 5546T>G), GTT1313GTC (5549T>C), TAC1314TTC (5551A>T), ATA1315TTT (5553A>T 5555A>T), GAC1316GAT (5558C>T), ATT1318ATA (5564T>A), ATA1320GTT (5568A>G 5570A>T), TTT1321TAC (5572T>A 5573T>C), TCT1322AGC (5574T>A 5575C>G 5576T>C), GAA1323AAG (5577G>A 5579A>G), AGT1324GAT (5580A>G 5581G>A), ATA1325ATG (5585A>G), CAA1327GAA (5589C>G), GTA1329CTA (5595G>C), CAA1330GAA (5598C>G), TTA1333CAA (5607T>C 5608T>A), CAG1334ACA (5610C>A 5611A>C 5612G>A), TTC1335ATT (5613T>A 5615C>T), TTC1336TTT (5618C>T), CAA1337AGG (5619C>A 5620A>G 5621A>G), GTA1338TTA (5622G>T), TGT1339TTC (5626G>T 5627T>C), AAG1340GTA (5628A>G 5629A>T 5630G>A), GAA1341AGT (5631G>A 5632A>G 5633A>T), GAA1342AAA (5634G>A), GGG1343GGA (5639G>A), CTT1344ATA (5640C>A 5642T>A), ATT1345ATA (5645T>A), TTG1346ATA (5646T>A 5648G>A), TCA1347ACC (5649T>A 5651A>C), ACA1349AAG (5656C>A 5657A>G), TTG1351ATG (5661T>A), AAA1352GAA (5664A>G), ATA1353TTA (5667A>T), GGA1354TGT (5670G>T 5672A>T), GTA1355AAA (5673G>A 5674T>A), GCA1356ACA (5676G>A), AAT1357CAT (5679A>C), GAA1359AAC (5685G>A 5687A>C), CTT1361TTA (5691C>T 5693T>A), GGC1362GGA (5696C>A), CTA1363GTA (5697C>G), GAA1364ACT (5700G>A 5701A>C 5702A>T), ATA1365TTA (5703A>T), GAA1367GAT (5711A>T), GGA1368GGG (5714A>G), AAA1369AAG (5717A>G), GTT1370ATA (5718G>A 5720T>A), CAA1371AAA (5721C>A), CTC1372CTA (5726C>A), TTA1377GCT (5739T>G 5740T>C 5741A>T), ATA1380GTA (5748A>G), GAA1382GAT (5756A>T), TTT1383ATG (5757T>A 5759T>G), CCT1384CCA (5762T>A), GAA1385del (5763_5765delGAA), GAC1386GAT (5768C>T), CAA1387AAA (5769C>A), GAA1389GAT (5777A>T), ACA1390AAA (5779C>A), TTA1391ACA (5781T>A 5782T>C), AAG1392AAA (5786G>A), GGT1393GAT (5788G>A), CTA1394TTA (5790C>T), CAG1395CAA (5795G>A), AAG1396AAA (5798G>A), GGG1399GGA (5807G>A), ATA1400TTA (5808A>T), TTA1401GTG (5811T>G 5813A>G), AAC1402AAT (5816C>T), GCC1404GCT (5822C>T), AAT1406AAC (5828T>C), TAT1407TTT (5830A>T), ATC1408ATA (5834C>A), CCA1409CAA (5836C>A), TTG1411TTA (5843G>A), AGT1412GGA (5844A>G 5846T>A), TAT1414ATT (5850T>A 5851A>T), ACT1415GCA (5853A>G 5855T>A), AGA1416GGA (5856A>G), ATA1417CCA (5859A>C 5860T>C), TTT1418TTA (5864T>A), AAT1420GCA (5868A>G 5869A>C 5870T>A), AAG1421AAA (5873G>A), TGT1422ATA (5874T>A 5875G>T 5876T>A), TCC1423GGA (5877T>G 5878C>G 5879C>A), AGC1424AGT (5882C>T), GAA1427CAA (5889G>C), AGA1428AAA (5893G>A), AAA1429CAC (5895A>C 5897A>C), AAT1431AAC (5903T>C), TCT1432ACA (5904T>A 5906T>A), CAA1433GAA (5907C>G), GAC1434AAT (5910G>A 5912C>T), TGG1435ATT (5913T>A 5914G>T 5915G>T), AAG1436AAA (5918G>A), ATG1437CTG (5919A>C), GTT1438GTA (5924T>A), AGA1439CAA (5925A>C 5926G>A), AAA1440CAA (5928A>C), ATC1441ATT (5933C>T), GTA1444AAA (5940G>A 5941T>A), ATT1445ATA (5945T>A), ACC1446AAA (5947C>A 5948C>A), AAA1447AAC (5951A>C), TTA1448ATA (5952T>A), CCC1449CCA (5957C>A), CCT1450GAC (5958C>G 5959C>A 5960T>C), TTG1451TTA (5963G>A), ATT1452AGC (5965T>G 5966T>C), TTA1453ATA (5967T>A), AAA1455CTA (5973A>C 5974A>T), CCA1456GAA (5976C>G 5977C>A), CAA1457TCA (5979C>T 5980A>C), AGT1458GAT (5982A>G 5983G>A), ATT1460CTA (5988A>C 5990T>A), GAG1463GAA (5999G>A), GAT1465GAC (6005T>C), TCT1467AGC (6009T>A 6010C>G 6011T>C), CTA1468CTC (6014A>C), GAA1469CAA (6015G>C), GGG1472GGA (6026G>A), GGA1473GCT (6028G>C 6029A>T), ATT1474GTA (6030A>G 6032T>A), TGG1477GCA (6039T>G 6040G>C 6041G>A), CCC1479CCA (6047C>A), TCT1480AAC (6048T>A 6049C>A 6050T>C), CAA1481AAA (6051C>A), CAA1482TAC (6054C>T 6056A>C), GAT1483AGT (6057G>A 6058A>G), TCA1484CCT (6060T>C 6062A>T), CCT1485AAA (6063C>A 6064C>A 6065T>A), TCA1486AAA (6066T>A 6067C>A), ACA1487GAA (6069A>G 6070C>A), TCA1491TGT (6082C>G 6083A>T), CGG1492GCT (6084C>G 6085G>C 6086G>T), TAT1493TAC (6089T>C), TGT1494CAA (6090T>C 6091G>A 6092T>A), AGC1497AAA (6100G>A 6101C>A), TAT1498TAC (6104T>C), AAA1499_TCT1500insGAAAAG (6107_6108insGAAAAG), TCT1500GGT (6108T>G 6109C>G), GCC1501AAC (6111G>A 6112C>A), ATC1502ATG (6116C>G), TCT1503AGT (6117T>A 6118C>G), GCA1504AGT (6120G>A 6121C>G 6122A>T), GCT1507GTC (6130C>T 6131T>C), ATA1509GTA (6135A>G), ATG1510TTA (6138A>T 6140G>A), TGT1512ATA (6144T>A 6145G>T 6146T>A), GTA1515GGA (6154T>G), TTA1516CTA (6156T>C), GAT1517AAT (6159G>A), AAG1518AGT (6163A>G 6164G>T), TTT1519TTC (6167T>C), ATA1521TTA (6171A>T), TTT1522TAT (6175T>A), TTA1523ATT (6177T>A 6179A>T), TAT1524TTA (6181A>T 6182T>A), GAG1525AAC (6183G>A 6185G>C), AAA1527AGT (6190A>G 6191A>T), TTT1529ATA (6195T>A 6197T>A), ACC1530GTA (6198A>G 6199C>T 6200C>A), CTT1531GTA (6201C>G 6203T>A), AGG1532AGA (6206G>A), GAC1534GAT (6212C>T), TGT1535TGC (6215T>C), ATT1536GAA (6216A>G 6217T>A 6218T>A), GCA1537GCT (6221A>T), ATT1538ATA (6224T>A), AAT1540AAA (6230T>A), TAT1542CAT (6234T>C), AAC1543CAA (6237A>C 6239C>A), CTT1545ATA (6243C>A 6245T>A), AAT1546GAC (6246A>G 6248T>C), AAT1547CAG (6249A>C 6251T>G), AAA1549AAT (6257A>T), TTG1550AGT (6258T>A 6259T>G 6260G>T), AAT1551AGT (6262A>G), ATA1552AAA (6265T>A), AAT1553AGA (6268A>G 6269T>A), CGT1554AGA (6270C>A 6272T>A), GTA1556CTA (6276G>C), TTC1558TTT (6284C>T), TGT1559ATA (6285T>A 6286G>T 6287T>A), GAT1560GAC (6290T>C), TTA1561ACC (6291T>A 6292T>C 6293A>C), ACA1563TCT (6297A>T 6299A>T), GGA1564ATA (6300G>A 6301G>T), TTA1565TAC (6304T>A 6305A>C), GGT1566CCT (6306G>C 6307G>C), TTA1567del (6309_6311delTTA), ACT1568AAT (6313C>A), AAA1570GTT (6318A>G 6319A>T 6320A>T), ATA1571TTC (6321A>T 6323A>C), CAT1573TAT (6327C>T), ATC1574ATA (6332C>A), CAG1575AAA (6333C>A 6335G>A), GGT1576GGA (6338T>A)								

*: Inserts / Deletes / Misaligned / Frameshifts

Analysis details

This analysis was performed with panviral2.64

NGS Details (UN24): Badnavirus occultipomeae

Assembly

**Coverage Length**	319 (1 contig(s))
**Depth Of Coverage**	53.6
**Number Of Reads**	157
**Reads Per Million**	3.14 rpm (after QC)
**Ambiguities**	0
**Assembly Method**	de novo + reference guided assembly
**Consensus Caller**	Bcf Tools

Coverage Map

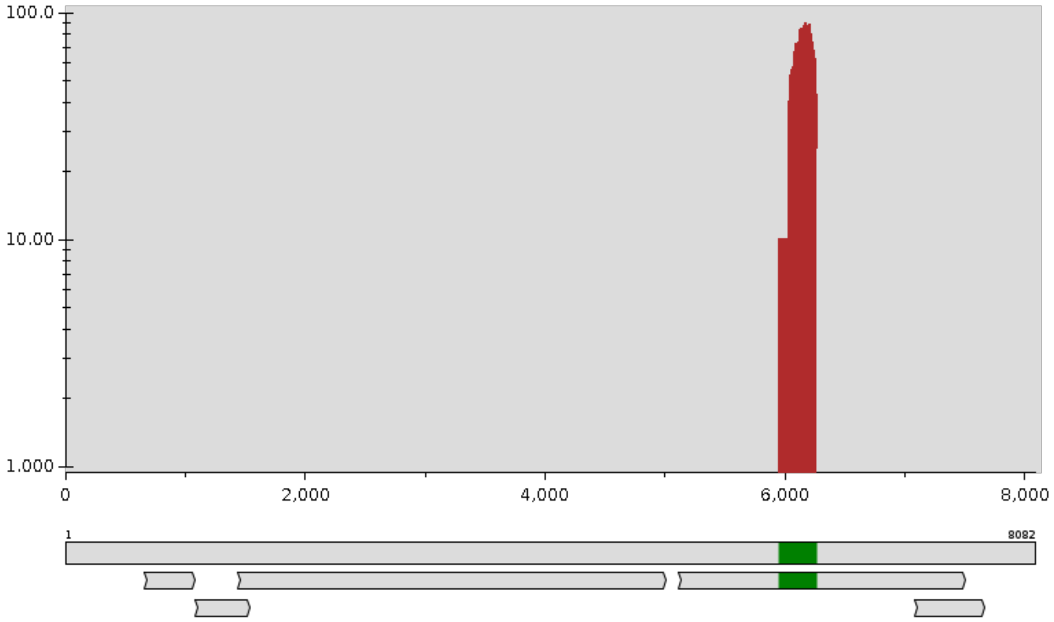

Assignment

**Type**	Badnavirus occultipomeae (Taxonomy ID: 3048353)
**Reference Genome**	NC_015655.1
**NT Identity (%)**	58.0745
**AA Identity (%)**	52.3364
**Number Of Stop Codons**	0
**Number Of CDS**	5

Alignment

**Alignment Score**	98.0 (NT) + 408.0 (AA) = 506.0
**Concordance (%)**	36.5871

**Alignment Method**	Global, seeded, nucleotide + amino acids (AGA)

Genome Region

Sequence starts at position 5946 and ends at position 6264 relative to NC_015655.1 reference sequence.

Alignment Detailed Statistics

	Begin	End	Coverage	Score	Concordance	Matches	Identities	I/D/M/F*	Stop Codons
**NT**	**5946**	**6264**	**3.9%**	**98**	**15.4%**	**319 (99.1%)**	**187 (58.1%)**	**3/0**	
Mutations:	5949G>A, 5950C>A, 5951G>A, 5952C>A, 5955C>T, 5961C>T, 5962A>G, 5963A>C, 5967A>C, 5968G>C, 5970A>G, 5973A>G, 5976T>C, 5979G>C, 5982T>C, 5983T>C, 5985C>A, 5986C>G, 5988A>G, 5991G>C, 5992G>T, 5993G>T, 5994A>C, 5997C>T, 6002C>A, 6003T>A, 6006T>G, 6007G>C, 6009C>T, 6010A>G, 6011G>A, 6012T>C, 6016A>T, 6018A>G, 6019T>G, 6024A>G, 6025A>C, 6028A>G, 6029A>C, 6031A>G, 6033A>G, 6039A>T, 6042A>G, 6043T>A, 6048T>C, 6049C>T, 6051C>G, 6053A>G, 6054A>G, 6055G>T, 6057A>G, 6060C>T, 6062T>A, 6070A>T, 6072A>G, 6076A>G, 6078G>T, 6079G>C, 6080A>G, 6084A>G, 6085A>T, 6086A>C, 6087G>T, 6088T>G, 6089C>A, 6090C>T, 6092A>T, 6093A>C, 6096A>G, 6097T>A, 6098G>A, 6109T>C, 6114T>A, 6116C>G, 6117A>C, 6118G>T, 6120A>T, 6123G>T, 6125T>A, 6126C>T, 6137A>T, 6138A>G, 6141C>T, 6145C>T, 6153A>G, 6156G>T, 6158T>C, 6159G>C, 6165A>C, 6168A>T, 6171A>G, 6172G>A, 6173A>C, 6174T>A, 6177T>C, 6178C>A, 6179A>T, 6181A>G, 6182G>A, 6183A>G, 6184A>T, 6185A>T, 6190G>A, 6193A>C, 6194A>G, 6195T>G, 6197C>T, 6198A>G, 6203G>A, 6205G>T, 6206G>C, 6207A>C, 6207_6208insTAT, 6208A>T, 6209C>T, 6210A>G, 6214G>C, 6215C>A, 6216A>G, 6220A>G, 6222C>G, 6223G>A, 6224C>T, 6225A>T, 6228A>G, 6230A>T, 6234T>A, 6243A>C, 6249A>G, 6251T>A, 6255T>C, 6256G>A, 6264A>G								

CDS

	Begin	End	Coverage	Score	Concordance	Matches	Identities	I/D/M/F*	Stop Codons
SPBVa_gp4	278	383	13.3%	408	54.1%	106 (99.1%)	56 (52.3%)	1/0/0/0	0
Protein mutations:	R279K (5950C>A 5951G>A 5952C>A), N283A (5962A>G 5963A>C), E285Q (5968G>C 5970A>G), Q288H (5979G>C), S290P (5983T>C 5985C>A), L291V (5986C>G 5988A>G), G293F (5992G>T 5993G>T 5994A>C), T296K (6002C>A 6003T>A), I297M (6006T>G), V298L (6007G>C 6009C>T), S299D (6010A>G 6011G>A 6012T>C), I301L (6016A>T 6018A>G), S302A (6019T>G), K304Q (6025A>C), K305A (6028A>G 6029A>C), I306V (6031A>G 6033A>G), F310I (6043T>A), K313R (6053A>G 6054A>G), A314S (6055G>T 6057A>G), F316Y (6062T>A), I319L (6070A>T 6072A>G), M321V (6076A>G 6078G>T), E322R (6079G>C 6080A>G), K324S (6085A>T 6086A>C 6087G>T), S325D (6088T>G 6089C>A 6090C>T), K326I (6092A>T 6093A>C), W328K (6097T>A 6098G>A), W332R (6109T>C), P334R (6116C>G 6117A>C), E335Y (6118G>T 6120A>T), L337H (6125T>A 6126C>T), E341V (6137A>T 6138A>G), P344S (6145C>T), M348T (6158T>C 6159G>C), D353T (6172G>A 6173A>C 6174T>A), Q355M (6178C>A 6179A>T), R356E (6181A>G 6182G>A 6183A>G), K357L (6184A>T 6185A>T), D359N (6190G>A), N360R (6193A>C 6194A>G 6195T>G), A361V (6197C>T 6198A>G), R363K (6203G>A), G364S (6205G>T 6206G>C 6207A>C), G364_T365insY (6207_6208insTAT), T365L (6208A>T 6209C>T 6210A>G), A367Q (6214G>C 6215C>A 6216A>G), I369V (6220A>G 6222C>G), A370I (6223G>A 6224C>T 6225A>T), Y372F (6230A>T), F379Y (6251T>A), E381K (6256G>A)								
Codon mutations:	AAG278AAA (5949G>A), CGC279AAA (5950C>A 5951G>A 5952C>A), CTC280CTT (5955C>T), GAC282GAT (5961C>T), AAT283GCT (5962A>G 5963A>C), ACA284ACC (5967A>C), GAA285CAG (5968G>C 5970A>G), AAA286AAG (5973A>G), GAT287GAC (5976T>C), CAG288CAC (5979G>C), TAT289TAC (5982T>C), TCC290CCA (5983T>C 5985C>A), CTA291GTG (5986C>G 5988A>G), CCG292CCC (5991G>C), GGA293TTC (5992G>T 5993G>T 5994A>C), ATC294ATT (5997C>T), ACT296AAA (6002C>A 6003T>A), ATT297ATG (6006T>G), GTC298CTT (6007G>C 6009C>T), AGT299GAC (6010A>G 6011G>A 6012T>C), ATA301TTG (6016A>T 6018A>G), TCT302GCT (6019T>G), GGA303GGG (6024A>G), AAA304CAA (6025A>C), AAG305GCG (6028A>G 6029A>C), ATA306GTG (6031A>G 6033A>G), TCA308TCT (6039A>T), AAA309AAG (6042A>G), TTT310ATT (6043T>A), GAT311GAC (6048T>C), CTC312TTG (6049C>T 6051C>G), AAA313AGG (6053A>G 6054A>G), GCA314TCG (6055G>T 6057A>G), GGC315GGT (6060C>T), TTT316TAT (6062T>A), ATA319TTG (6070A>T 6072A>G), ATG321GTT (6076A>G 6078G>T), GAG322CGG (6079G>C 6080A>G), GAA323GAG (6084A>G), AAG324TCT (6085A>T 6086A>C 6087G>T), TCC325GAT (6088T>G 6089C>A 6090C>T), AAA326ATC (6092A>T 6093A>C), CCA327CCG (6096A>G), TGG328AAG (6097T>A 6098G>A), TGG332CGG (6109T>C), ACT333ACA (6114T>A), CCA334CGC (6116C>G 6117A>C), GAA335TAT (6118G>T 6120A>T), GGG336GGT (6123G>T), CTC337CAT (6125T>A 6126C>T), GAA341GTG (6137A>T 6138A>G), GTC342GTT (6141C>T), CCC344TCC (6145C>T), GGA346GGG (6153A>G), CTG347CTT (6156G>T), ATG348ACC (6158T>C 6159G>C), GCA350GCC (6165A>C), CCA351CCT (6168A>T), GCA352GCG (6171A>G), GAT353ACA (6172G>A 6173A>C 6174T>A), TTT354TTC (6177T>C), CAG355ATG (6178C>A 6179A>T), AGA356GAG (6181A>G 6182G>A 6183A>G), AAG357TTG (6184A>T 6185A>T), GAT359AAT (6190G>A), AAT360CGG (6193A>C 6194A>G 6195T>G), GCA361GTG (6197C>T 6198A>G), AGG363AAG (6203G>A), GGA364TCC (6205G>T 6206G>C 6207A>C), GGA364_ACA365insTAT (6207_6208insTAT), ACA365TTG (6208A>T 6209C>T 6210A>G), GCA367CAG (6214G>C 6215C>A 6216A>G), ATC369GTG (6220A>G 6222C>G), GCA370ATT (6223G>A 6224C>T 6225A>T), GTA371GTG (6228A>G), TAT372TTT (6230A>T), ATT373ATA (6234T>A), ATA376ATC (6243A>C), GTA378GTG (6249A>G), TTC379TAC (6251T>A), TCT380TCC (6255T>C), GAA381AAA (6256G>A), GAA383GAG (6264A>G)								

Proteins

	Begin	End	Coverage	Score	Concordance	Matches	Identities	I/D/M/F*	Stop Codons
RNaseH/reverse transcriptase (YP_004581513.1)	278	383	13.3%	408	54.1%	106 (99.1%)	56 (52.3%)	1/0/0/0	0
Protein mutations:	R279K (5950C>A 5951G>A 5952C>A), N283A (5962A>G 5963A>C), E285Q (5968G>C 5970A>G), Q288H (5979G>C), S290P (5983T>C 5985C>A), L291V (5986C>G 5988A>G), G293F (5992G>T 5993G>T 5994A>C), T296K (6002C>A 6003T>A), I297M (6006T>G), V298L (6007G>C 6009C>T), S299D (6010A>G 6011G>A 6012T>C), I301L (6016A>T 6018A>G), S302A (6019T>G), K304Q (6025A>C), K305A (6028A>G 6029A>C), I306V (6031A>G 6033A>G), F310I (6043T>A), K313R (6053A>G 6054A>G), A314S (6055G>T 6057A>G), F316Y (6062T>A), I319L (6070A>T 6072A>G), M321V (6076A>G 6078G>T), E322R (6079G>C 6080A>G), K324S (6085A>T 6086A>C 6087G>T), S325D (6088T>G 6089C>A 6090C>T), K326I (6092A>T 6093A>C), W328K (6097T>A 6098G>A), W332R (6109T>C), P334R (6116C>G 6117A>C), E335Y (6118G>T 6120A>T), L337H (6125T>A 6126C>T), E341V (6137A>T 6138A>G), P344S (6145C>T), M348T (6158T>C 6159G>C), D353T (6172G>A 6173A>C 6174T>A), Q355M (6178C>A 6179A>T), R356E (6181A>G 6182G>A 6183A>G), K357L (6184A>T 6185A>T), D359N (6190G>A), N360R (6193A>C 6194A>G 6195T>G), A361V (6197C>T 6198A>G), R363K (6203G>A), G364S (6205G>T 6206G>C 6207A>C), G364_T365insY (6207_6208insTAT), T365L (6208A>T 6209C>T 6210A>G), A367Q (6214G>C 6215C>A 6216A>G), I369V (6220A>G 6222C>G), A370I (6223G>A 6224C>T 6225A>T), Y372F (6230A>T), F379Y (6251T>A), E381K (6256G>A)								
Codon mutations:	AAG278AAA (5949G>A), CGC279AAA (5950C>A 5951G>A 5952C>A), CTC280CTT (5955C>T), GAC282GAT (5961C>T), AAT283GCT (5962A>G 5963A>C), ACA284ACC (5967A>C), GAA285CAG (5968G>C 5970A>G), AAA286AAG (5973A>G), GAT287GAC (5976T>C), CAG288CAC (5979G>C), TAT289TAC (5982T>C), TCC290CCA (5983T>C 5985C>A), CTA291GTG (5986C>G 5988A>G), CCG292CCC (5991G>C), GGA293TTC (5992G>T 5993G>T 5994A>C), ATC294ATT (5997C>T), ACT296AAA (6002C>A 6003T>A), ATT297ATG (6006T>G), GTC298CTT (6007G>C 6009C>T), AGT299GAC (6010A>G 6011G>A 6012T>C), ATA301TTG (6016A>T 6018A>G), TCT302GCT (6019T>G), GGA303GGG (6024A>G), AAA304CAA (6025A>C), AAG305GCG (6028A>G 6029A>C), ATA306GTG (6031A>G 6033A>G), TCA308TCT (6039A>T), AAA309AAG (6042A>G), TTT310ATT (6043T>A), GAT311GAC (6048T>C), CTC312TTG (6049C>T 6051C>G), AAA313AGG (6053A>G 6054A>G), GCA314TCG (6055G>T 6057A>G), GGC315GGT (6060C>T), TTT316TAT (6062T>A), ATA319TTG (6070A>T 6072A>G), ATG321GTT (6076A>G 6078G>T), GAG322CGG (6079G>C 6080A>G), GAA323GAG (6084A>G), AAG324TCT (6085A>T 6086A>C 6087G>T), TCC325GAT (6088T>G 6089C>A 6090C>T), AAA326ATC (6092A>T 6093A>C), CCA327CCG (6096A>G), TGG328AAG (6097T>A 6098G>A), TGG332CGG (6109T>C), ACT333ACA (6114T>A), CCA334CGC (6116C>G 6117A>C), GAA335TAT (6118G>T 6120A>T), GGG336GGT (6123G>T), CTC337CAT (6125T>A 6126C>T), GAA341GTG (6137A>T 6138A>G), GTC342GTT (6141C>T), CCC344TCC (6145C>T), GGA346GGG (6153A>G), CTG347CTT (6156G>T), ATG348ACC (6158T>C 6159G>C), GCA350GCC (6165A>C), CCA351CCT (6168A>T), GCA352GCG (6171A>G), GAT353ACA (6172G>A 6173A>C 6174T>A), TTT354TTC (6177T>C), CAG355ATG (6178C>A 6179A>T), AGA356GAG (6181A>G 6182G>A 6183A>G), AAG357TTG (6184A>T 6185A>T), GAT359AAT (6190G>A), AAT360CGG (6193A>C 6194A>G 6195T>G), GCA361GTG (6197C>T 6198A>G), AGG363AAG (6203G>A), GGA364TCC (6205G>T 6206G>C 6207A>C), GGA364_ACA365insTAT (6207_6208insTAT), ACA365TTG (6208A>T 6209C>T 6210A>G), GCA367CAG (6214G>C 6215C>A 6216A>G), ATC369GTG (6220A>G 6222C>G), GCA370ATT (6223G>A 6224C>T 6225A>T), GTA371GTG (6228A>G), TAT372TTT (6230A>T), ATT373ATA (6234T>A), ATA376ATC (6243A>C), GTA378GTG (6249A>G), TTC379TAC (6251T>A), TCT380TCC (6255T>C), GAA381AAA (6256G>A), GAA383GAG (6264A>G)								

*: Inserts / Deletes / Misaligned / Frameshifts

Analysis details

This analysis was performed with panviral2.64

NGS Details (UN24): Pseudomonas phage phi297

Assembly

**Coverage Length**	2114 (3 contig(s))
**Depth Of Coverage**	7.3
**Number Of Reads**	122
**Reads Per Million**	2.44 rpm (after QC)
**Ambiguities**	0
**Assembly Method**	de novo + reference guided assembly
**Consensus Caller**	Bcf Tools

Coverage Map

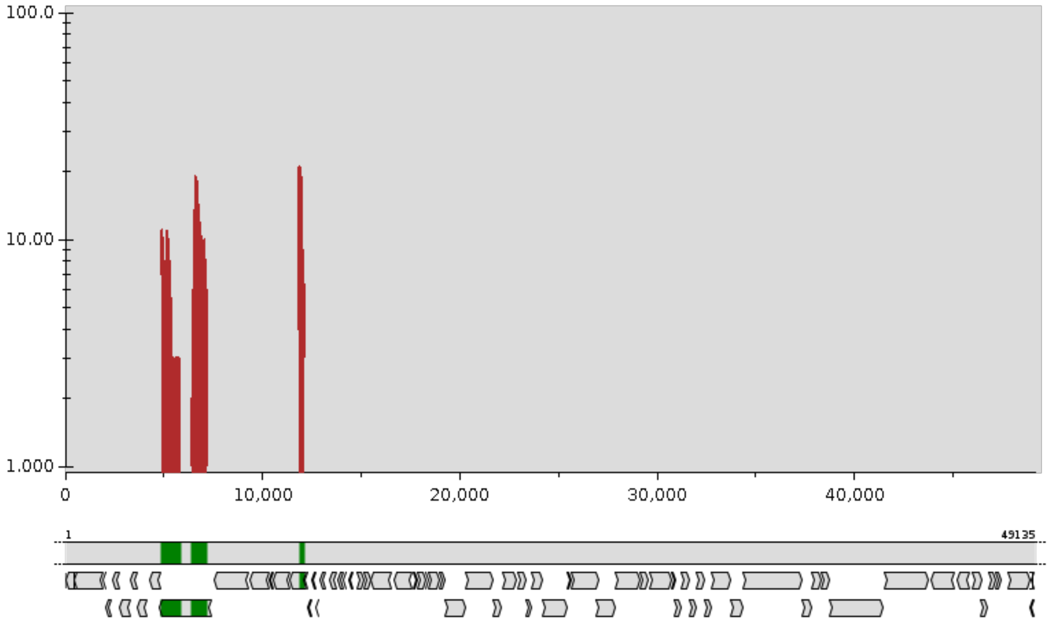

Assignment

**Type**	Pseudomonas phage phi297 (Taxonomy ID: 1129145)
**Reference Genome**	NC_016762.1
**NT Identity (%)**	72.6802
**AA Identity (%)**	67.1853
**Number Of Stop Codons**	0
**Number Of CDS**	69

Alignment

**Alignment Score**	1899.0 (NT) + 3530.0 (AA) = 5429.0
**Concordance (%)**	58.7872

**Alignment Method**	Local, heuristic, nucleotide (BLASTN)

Genome Region

Sequence starts at position 4836 and ends at position 12136 relative to NC_016762.1 reference sequence.

Alignment Detailed Statistics

	Begin	End	Coverage	Score	Concordance	Matches	Identities	I/D/M/F*	Stop Codons
**NT**	**4836**	**12136**	**4.3%**	**1899**	**45.2%**	**2108 (99.0%)**	**1543 (72.5%)**	**15/6**	
Mutations:	4841A>T, 4842G>C, 4843C>A, 4847G>T, 4849G>A, 4855G>C, 4856T>G, 4857C>A, 4858G>C, 4862A>G, 4864G>C, 4866C>A, 4871T>G, 4872C>A, 4873T>G, 4874T>C, 4875C>G, 4877T>A, 4879C>G, 4882G>C, 4883G>T, 4884A>G, 4887T>C, 4891A>G, 4898G>A, 4900A>G, 4903G>A, 4916C>G, 4918G>C, 4919G>T, 4920A>T, 4922T>G, 4923T>C, 4927T>C, 4931T>C, 4932G>A, 4934G>C, 4940G>C, 4941T>C, 4942T>G, 4944T>G, 4945G>C, 4948C>G, 4949T>C, 4950T>C, 4951G>C, 4955T>A, 4956T>G, 4959G>C, 4960C>A, 4963G>C, 4965A>G, 4969C>G, 4972G>C, 4975G>C, 4981G>C, 4983T>G, 4984C>G, 4996A>G, 5000A>T, 5006A>T, 5007G>C, 5008G>C, 5011T>G, 5014T>C, 5016C>T, 5029C>G, 5030A>G, 5031C>T, 5035G>C, 5044G>C, 5045A>G, 5047G>A, 5061G>T, 5077T>C, 5080C>G, 5081T>C, 5083G>C, 5086C>G, 5087T>G, 5088T>C, 5089G>T, 5090C>G, 5091T>C, 5092G>C, 5093T>G, 5099T>G, 5100G>C, 5102G>C, 5103C>T, 5113C>G, 5122G>C, 5124C>T, 5125G>C, 5137T>G, 5146G>A, 5153G>C, 5154A>T, 5161T>C, 5162G>T, 5177C>G, 5178C>T, 5189T>A, 5191C>G, 5195G>C, 5196T>G, 5197G>C, 5198A>T, 5200C>G, 5201T>G, 5208A>C, 5209T>C, 5221G>C, 5226G>C, 5246G>C, 5247T>G, 5248G>C, 5249G>T, 5250A>C, 5254G>C, 5255C>G, 5256T>A, 5259A>G, 5260G>C, 5262C>T, 5267T>A, 5278C>G, 5281C>G, 5282A>T, 5285T>G, 5287G>A, 5290C>G, 5292G>T, 5293A>G, 5295T>G, 5302G>C, 5304A>G, 5305G>C, 5309G>T, 5312T>G, 5313C>G, 5314A>C, 5321G>A, 5323A>C, 5328T>C, 5332G>C, 5341G>C, 5343T>C, 5345A>T, 5359C>G, 5371T>C, 5373C>G, 5465G>T, 5470G>C, 5476C>G, 5479A>C, 5481T>C, 5486C>G, 5488G>A, 5492G>C, 5495T>C, 5496T>G, 5498G>C, 5499A>T, 5501T>G, 5502T>C, 5509G>C, 5512G>T, 5514G>C, 5540G>C, 5541G>A, 5550C>A, 5551A>G, 5557C>G, 5558T>C, 5559T>G, 5560G>C, 5562T>A, 5566G>A, 5575C>T, 5578T>C, 5581C>G, 5582G>C, 5584C>G, 5585C>T, 5587C>G, 5588G>C, 5589A>C, 5590G>C, 5606G>T, 5607C>T, 5617A>C, 5641C>G, 5642A>C, 5643T>G, 5649T>C, 5650A>G, 5651T>A, 5654G>T, 5655C>G, 5656C>G, 5657C>A, 5662A>G, 5663T>G, 5664G>C, 5666T>C, 5667T>G, 5669A>C, 5670C>G, 5675G>C, 5676C>A, 5677T>G, 5678C>G, 5679G>C, 5680A>G, 5681G>A, 5683C>G, 5684A>G, 5685C>T, 5686T>G, 5691T>C, 5694G>C, 5704G>C, 5705A>T, 5719A>G, 5721G>C, 5724_5725insCTC, 5725G>C, 5726G>A, 5727T>C, 5731T>G, 5736C>T, 5740A>C, 5742T>C, 5749A>G, 5751C>T, 5755C>G, 5765C>G, 5766A>C, 5772G>C, 5773A>C, 5775G>T, 5776C>G, 5779G>C, 5782G>C, 5785G>C, 5786G>A, 5787T>G, 5800T>C, 5806G>C, 5808T>C, 5812A>G, 5813T>A, 5826C>G, 5843T>C, 5844C>T, 6398A>G, 6399T>C, 6403G>C, 6406G>C, 6412A>G, 6416G>C, 6417C>G, 6424C>G, 6428T>C, 6433T>C, 6436C>A, 6437G>T, 6442G>C, 6448A>G, 6457G>C, 6459T>C, 6460C>A, 6466A>G, 6469A>G, 6470T>G, 6472T>C, 6475A>G, 6476G>T, 6484C>G, 6487G>C, 6489T>G, 6498G>T, 6499T>C, 6505G>C, 6507T>C, 6508C>G, 6511G>C, 6515G>T, 6516G>C, 6517A>C, 6523C>G, 6535T>G, 6536G>C, 6537C>G, 6538G>C, 6541G>C, 6548T>A, 6549G>C, 6556A>G, 6562G>C, 6563G>T, 6564T>G, 6566T>C, 6567T>G, 6568G>C, 6569C>T, 6570G>T, 6571A>G, 6573G>A, 6576G>T, 6577G>C, 6578C>T, 6580G>C, 6581C>G, 6591T>C, 6592A>C, 6593C>A, 6594G>T, 6595C>A, 6597C>T, 6598A>G, 6599T>G, 6600G>T, 6601G>A, 6609G>C, 6610C>G, 6612A>C, 6619G>C, 6621A>C, 6625T>G, 6627C>T, 6628T>C, 6646C>G, 6648G>T, 6649T>G, 6650T>G, 6651C>G, 6655T>C, 6659G>T, 6667T>G, 6677A>T, 6679C>G, 6688G>C, 6689C>G, 6690T>A, 6694G>C, 6695C>T, 6696G>T, 6700A>C, 6706G>C, 6708T>G, 6709C>G, 6716G>C, 6717A>T, 6718C>T, 6729T>G, 6736C>G, 6741C>G, 6745T>G, 6746T>C, 6747T>G, 6748G>C, 6749T>G, 6750G>C, 6751G>T, 6753C>G, 6754A>G, 6755T>C, 6756G>T, 6757G>C, 6759T>G, 6760G>C, 6763G>C, 6766T>G, 6768G>T, 6781A>G, 6782G>C, 6783A>T, 6790T>C, 6799C>A, 6801T>G, 6802G>C, 6817G>A, 6820A>G, 6832G>C, 6841C>G, 6842G>C, 6843A>T, 6847A>G, 6853T>C, 6861T>C, 6862G>A, 6863T>A, 6865T>C, 6871C>T, 6874G>A, 6877T>G, 6886A>C, 6896T>A, 6898C>G, 6901G>A, 6905C>G, 6907G>C, 6914T>G, 6918C>T, 6919G>C, 6920C>T, 6921G>T, 6922G>C, 6927C>G, 6929G>T, 6930C>T, 6933_6934insCATGTCGCG, 6934G>C, 6937A>G, 6943A>G, 6946A>G, 6955A>C, 6973C>G, 6997A>C, 7003A>T, 7004C>G, 7005T>C, 7006G>C, 7009T>C, 7021T>G, 7034C>A, 7036G>C, 7037C>A, 7039A>C, 7045G>C, 7053C>G, 7062C>T, 7063G>C, 7066C>T, 7072A>G, 7078C>G, 7088G>C, 7089T>C, 7091G>T, 7093G>C, 7102T>G, 7103A>G, 7105C>T, 7108G>C, 7113A>T, 7114A>G, 7116T>G, 7127C>T, 7128G>T, 7132G>C, 7141G>A, 7144C>G, 7145A>C, 7146T>G, 7147C>T, 7150G>C, 7160T>A, 7161A>T, 11839C>G, 11840T>C, 11841T>G, 11842C>G, 11853T>C, 11857T>C, 11858C>G, 11861G>A, 11864G>C, 11865A>G, 11872T>G, 11881G>C, 11882C>G, 11883T>C, 11885A>G, 11887G>T, 11890G>T, 11891G>T, 11892A>C, 11893_11898delGCCGCG, 11902G>A, 11911G>T, 11912A>T, 11913A>C, 11914C>T, 11917C>G, 11922G>T, 11923C>G, 11926C>G, 11928G>T, 11931T>C, 11934G>C, 11938C>G, 11940G>C, 11941C>A, 11942A>G, 11943G>C, 11944T>G, 11945T>C, 11947C>G, 11949A>C, 11956G>A, 11958A>C, 11965G>C, 11967T>A, 11968C>G, 11970_11971insAGT, 11971C>T, 11972T>C, 11977G>C, 11978T>G, 11979T>G, 11980G>C, 11982C>G, 11983C>G, 11984C>T, 11986T>G, 11988C>A, 11990G>T, 11991C>G, 11998C>G, 11999G>A, 12000C>G, 12009T>C, 12016G>C, 12018T>C, 12022G>C, 12025A>G, 12028G>C, 12032A>T, 12037C>G, 12038C>G, 12039G>C, 12042G>C, 12044G>C, 12045A>T, 12046T>G, 12047G>C, 12048A>G, 12049A>C, 12054T>C, 12061C>G, 12062T>C, 12063T>C, 12069G>T, 12073T>C, 12074A>T, 12075C>T, 12076G>C, 12077C>G, 12079C>T, 12086T>C, 12088G>T, 12089A>G, 12090A>C, 12091C>A, 12093C>G, 12107G>T, 12110C>G, 12113C>A, 12114A>G, 12115G>C, 12117A>T, 12118C>G, 12119G>C, 12120A>T, 12127C>G, 12129C>T, 12132T>G								

CDS

phi297_00010	89	865	68.5%	3140	72.2%	601 (99.3%)	423 (69.9%)	4/0/0/0	0
Protein mutations:	Y90I (7160T>A 7161A>T), M95R (7144C>G 7145A>C 7146T>G), R101K (7127C>T 7128G>T), S105R (7114A>G 7116T>G), F106I (7113A>T), D107E (7108G>C), V109A (7102T>G 7103A>G), T113N (7091G>T), T114G (7088G>C 7089T>C), V123I (7062C>T), G126R (7053C>G), R131L (7036G>C 7037C>A), R132L (7034C>A), S142A (7003A>T 7004C>G 7005T>C), R165_A166insRDM (6933_6934insCATGTCGCG), A167N (6929G>T 6930C>T), G168R (6927C>G), R170K (6919G>C 6920C>T 6921G>T), G171S (6918C>T), D172A (6914T>G), R175P (6905C>G), Y178F (6896T>A), Y189F (6862G>A 6863T>A), I190V (6861T>C), Q221N (6766T>G 6768G>T), I224L (6757G>C 6759T>G), H225S (6754A>G 6755T>C 6756G>T), A226P (6751G>T 6753C>G), H227A (6748G>C 6749T>G 6750G>C), K228R (6745T>G 6746T>C 6747T>G), G230R (6741C>G), E231D (6736C>G), M234L (6729T>G), I241L (6706G>C 6708T>G), R245K (6694G>C 6695C>T 6696G>T), F251Y (6677A>T), E254D (6667T>G), A257E (6659G>T), E260P (6649T>G 6650T>G 6651C>G), L261I (6646C>G 6648G>T), V268I (6625T>G 6627C>T), S270A (6619G>C 6621A>C), S273A (6610C>G 6612A>C), H274D (6609G>C), H277T (6598A>G 6599T>G 6600G>T), E278N (6595C>A 6597C>T), R279M (6592A>C 6593C>A 6594G>T), N280D (6591T>C), G283A (6580G>C 6581C>G), R284Q (6577G>C 6578C>T), L285M (6576G>T), L286F (6571A>G 6573G>A), R287K (6568G>C 6569C>T 6570G>T), N288R (6566T>C 6567T>G), T289Q (6562G>C 6563G>T 6564T>G), Q294V (6548T>A 6549G>C), A298R (6535T>G 6536G>C 6537C>G), E302D (6523C>G), P305D (6515G>T 6516G>C), I308V (6505G>C 6507T>C), L311M (6498G>T), I314L (6487G>C 6489T>G), A318D (6475A>G 6476G>T), D320A (6469A>G 6470T>G), I324V (6457G>C 6459T>C), A331D (6436C>A 6437G>T), H334R (6428T>C), A338R (6416G>C 6417C>G), I344A (6398A>G 6399T>C), D529S (5843T>C 5844C>T), E535Q (5826C>G), Y539F (5812A>G 5813T>A), I541V (5806G>C 5808T>C), T548L (5785G>C 5786G>A 5787T>G), I549M (5782G>C), L553V (5772G>C), C555A (5765C>G 5766A>C), D560N (5749A>G 5751C>T), I563V (5740A>C 5742T>C), G565S (5736C>T), T568V (5725G>C 5726G>A 5727T>C), T568_G569insE (5724_5725insCTC), L570V (5719A>G 5721G>C), V575E (5704G>C 5705A>T), H579D (5694G>C), M580V (5691T>C), E581D (5686T>G), V582T (5683C>G 5684A>G 5685C>T), T583I (5680A>G 5681G>A), R584A (5677T>G 5678C>G 5679G>C), A585C (5675G>C 5676C>A), V587R (5669A>C 5670C>G), K588R (5666T>C 5667T>G), H589A (5662A>G 5663T>G 5664G>C), W591F (5656C>G 5657C>A), A592H (5654G>T 5655C>G), Y593F (5650A>G 5651T>A), M594V (5649T>C), M596R (5641C>G 5642A>C 5643T>G), A608N (5606G>T 5607C>T), S614G (5587C>G 5588G>C 5589A>C), W615Y (5584C>G 5585C>T), T616S (5581C>G 5582G>C), T623S (5560G>C 5562T>A), K624R (5557C>G 5558T>C 5559T>G), V627F (5550C>A), P630C (5540G>C 5541G>A), P639A (5512G>T 5514G>C), N643A (5501T>G 5502T>C), N645R (5495T>C 5496T>G), A646G (5492G>C), G648A (5486C>G), I650V (5479A>C 5481T>C), A655D (5465G>T), A686P (5371T>C 5373C>G), L690F (5359C>G), F695Y (5345A>T), T696A (5341G>C 5343T>C), N699K (5332G>C), N701D (5328T>C), P703L (5321G>A), E706P (5312T>G 5313C>G), A707E (5309G>T), D708E (5305G>C), F709L (5302G>C 5304A>G), I712L (5293A>G 5295T>G), P713T (5290C>G 5292G>T), D715A (5285T>G), V716D (5281C>G 5282A>T), M717I (5278C>G), Y721F (5267T>A), D723K (5260G>C 5262C>T), Y724H (5259A>G), S727E (5248G>C 5249G>T 5250A>C), T728R (5246G>C 5247T>G), H735D (5226G>C), S741A (5208A>C), E743A (5200C>G 5201T>G), V744E (5197G>C 5198A>T), T745R (5195G>C 5196T>G), K747M (5189T>A), G751T (5177C>G 5178C>T), P756Q (5161T>C 5162G>T), A769T (5122G>C 5124C>T), A776S (5102G>C 5103C>T), Q777A (5099T>G 5100G>C), Y779S (5092G>C 5093T>G), S780A (5089G>T 5090C>G 5091T>C), K781A (5086C>G 5087T>G 5088T>C), Q783R (5080C>G 5081T>C), L790M (5061G>T), V795A (5044G>C 5045A>G), V800T (5029C>G 5030A>G 5031C>T), E805K (5014T>C 5016C>T), D807E (5008G>C), L808E (5006A>T 5007G>C), F810Y (5000A>T), I816L (4981G>C 4983T>G), C822R (4963G>C 4965A>G), L824V (4959G>C), K825L (4955T>A 4956T>G), K827G (4948C>G 4949T>C 4950T>C), T830G (4940G>C 4941T>C), A832G (4934G>C), H833C (4931T>C 4932G>A), N836A (4922T>G 4923T>C), S837K (4918G>C 4919G>T 4920A>T), R838P (4916C>G), A844V (4898G>A), K848E (4887T>C), S849Q (4882G>C 4883G>T 4884A>G), E851V (4877T>A), E852R (4873T>G 4874T>C 4875C>G), E853S (4871T>G 4872C>A), A855S (4864G>C 4866C>A), M856T (4862A>G), D858S (4855G>C 4856T>G 4857C>A), A861D (4847G>T), L863D (4841A>T 4842G>C)								

	Begin	End	Coverage	Score	Concordance	Matches	Identities	I/D/M/F*	Stop Codons
**NT**	**4836**	**12136**	**4.3%**	**1899**	**45.2%**	**2108 (99.0%)**	**1543 (72.5%)**	**15/6**	
Codon mutations:	TAC90ATC (7160T>A 7161A>T), GCC93GCG (7150G>C), GCG94GCA (7147C>T), ATG95CGC (7144C>G 7145A>C 7146T>G), CTC96CTT (7141G>A), ACC99ACG (7132G>C), CGG101AAG (7127C>T 7128G>T), AGT105CGC (7114A>G 7116T>G), TTC106ATC (7113A>T), GAC107GAG (7108G>C), GAG108GAA (7105C>T), GTA109GCC (7102T>G 7103A>G), CCC112CCG (7093G>C), ACC113AAC (7091G>T), ACC114GGC (7088G>C 7089T>C), CTG117CTC (7078C>G), AAT119AAC (7072A>G), GAG121GAA (7066C>T), ACC122ACG (7063G>C), GTC123ATC (7062C>T), GGC126CGC (7053C>G), CTC128CTG (7045G>C), CCT130CCG (7039A>C), CGC131CTG (7036G>C 7037C>A), CGG132CTG (7034C>A), GCA136GCC (7021T>G), GAA140GAG (7009T>C), GCC141GCG (7006G>C), AGT142GCA (7003A>T 7004C>G 7005T>C), CTT144CTG (6997A>C), ACG152ACC (6973C>G), GCT158GCG (6955A>C), GCT161GCC (6946A>G), GGT162GGC (6943A>G), GAT164GAC (6937A>G), CGC165CGG (6934G>C), CGC165_GCC166insCGCGACATG (6933_6934insCATGTCGCG), GCC167AAC (6929G>T 6930C>T), GGC168CGC (6927C>G), GTC169GTG (6922G>C), CGC170AAG (6919G>C 6920C>T 6921G>T), GGC171AGC (6918C>T), GAT172GCT (6914T>G), GTC174GTG (6907G>C), CGG175CCG (6905C>G), TAC176TAT (6901G>A), CGG177CGC (6898C>G), TAC178TTC (6896T>A), ACT181ACG (6886A>C), CCA184CCC (6877T>G), AGC185AGT (6874G>A), CCG186CCA (6871C>T), GAA188GAG (6865T>C), TAC189TTT (6862G>A 6863T>A), ATC190GTC (6861T>C), CTA192CTG (6853T>C), GCT194GCC (6847A>G), TCG196AGC (6841C>G 6842G>C 6843A>T), CTC199CTG (6832G>C), GAT203GAC (6820A>G), GTC204GTT (6817G>A), ACC209ACG (6802G>C), AGG210CGT (6799C>A 6801T>G), AAA213AAG (6790T>C), TCT216AGC (6781A>G 6782G>C 6783A>T), CAA221AAC (6766T>G 6768G>T), CTC222CTG (6763G>C), ACC223ACG (6760G>C), ATC224CTG (6757G>C 6759T>G), CAT225AGC (6754A>G 6755T>C 6756G>T), GCC226CCA (6751G>T 6753C>G), CAC227GCG (6748G>C 6749T>G 6750G>C), AAA228CGC (6745T>G 6746T>C 6747T>G), GGC230CGC (6741C>G), GAG231GAC (6736C>G), ATG234CTG (6729T>G), GCG237GCA (6718C>T), TCC238AGC (6716G>C 6717A>T), GCG240GCC (6709C>G), ATC241CTG (6706G>C 6708T>G), GTT243GTG (6700A>C), CGC245AAG (6694G>C 6695C>T 6696G>T), AGC247TCG (6688G>C 6689C>G 6690T>A), GGG250GGC (6679C>G), TTC251TAC (6677A>T), GAA254GAC (6667T>G), GCG257GAG (6659G>T), CTA258CTG (6655T>C), GAA260CCC (6649T>G 6650T>G 6651C>G), CTG261ATC (6646C>G 6648G>T), GAA267GAG (6628T>C), GTA268ATC (6625T>G 6627C>T), TCC270GCG (6619G>C 6621A>C), TCG273GCC (6610C>G 6612A>C), CAC274GAC (6609G>C), GGC276GGT (6601G>A), CAT277ACC (6598A>G 6599T>G 6600G>T), GAG278AAT (6595C>A 6597C>T), CGT279ATG (6592A>C 6593C>A 6594G>T), AAC280GAC (6591T>C), GGC283GCG (6580G>C 6581C>G), CGC284CAG (6577G>C 6578C>T), CTG285ATG (6576G>T), CTT286TTC (6571A>G 6573G>A), CGC287AAG (6568G>C 6569C>T 6570G>T), AAC288CGC (6566T>C 6567T>G), ACC289CAG (6562G>C 6563G>T 6564T>G), ATT291ATC (6556A>G), CAG294GTG (6548T>A 6549G>C), GCC296GCG (6541G>C), GCC297GCG (6538G>C), GCA298CGC (6535T>G 6536G>C 6537C>G), GAG302GAC (6523C>G), CTT304CTG (6517A>C), CCT305GAT (6515G>T 6516G>C), GCC306GCG (6511G>C), CGG307CGC (6508C>G), ATC308GTG (6505G>C 6507T>C), AAA310AAG (6499T>C), CTG311ATG (6498G>T), ATC314CTG (6487G>C 6489T>G), CGG315CGC (6484C>G), GCT318GAC (6475A>G 6476G>T), CCA319CCG (6472T>C), GAT320GCC (6469A>G 6470T>G), GCT321GCC (6466A>G), CGG323CGT (6460C>A), ATC324GTG (6457G>C 6459T>C), CAT327CAC (6448A>G), CTC329CTG (6442G>C), GCG331GAT (6436C>A 6437G>T), GAA332GAG (6433T>C), CAC334CGC (6428T>C), GCG335GCC (6424C>G), GCC338CGC (6416G>C 6417C>G), GCT339GCC (6412A>G), CCC341CCG (6406G>C), ACC342ACG (6403G>C), ATC344GCC (6398A>G 6399T>C), GAC529AGC (5843T>C 5844C>T), GAG535CAG (5826C>G), TAT539TTC (5812A>G 5813T>A), ATC541GTG (5806G>C 5808T>C), GAA543GAG (5800T>C), ACC548CTG (5785G>C 5786G>A 5787T>G), ATC549ATG (5782G>C), CCC550CCG (5779G>C), GGG551GGC (5776C>G), CGT552AGG (5773A>C 5775G>T), CTG553GTG (5772G>C), TGC555GCC (5765C>G 5766A>C), GTG558GTC (5755C>G), GAT560AAC (5749A>G 5751C>T), ATT563GTG (5740A>C 5742T>C), GGC565AGC (5736C>T), GGA566GGC (5731T>G), ACC568GTG (5725G>C 5726G>A 5727T>C), ACC568_GGC569insGAG (5724_5725insCTC), CTT570GTC (5719A>G 5721G>C), GTC575GAG (5704G>C 5705A>T), CAC579GAC (5694G>C), ATG580GTG (5691T>C), GAA581GAC (5686T>G), GTG582ACC (5683C>G 5684A>G 5685C>T), ACT583ATC (5680A>G 5681G>A), CGA584GCC (5677T>G 5678C>G 5679G>C), GCC585TGC (5675G>C 5676C>A), GTC587CGC (5669A>C 5670C>G), AAG588CGG (5666T>C 5667T>G), CAT589GCC (5662A>G 5663T>G 5664G>C), TGG591TTC (5656C>G 5657C>A), GCC592CAC (5654G>T 5655C>G), TAT593TTC (5650A>G 5651T>A), ATG594GTG (5649T>C), ATG596CGC (5641C>G 5642A>C 5643T>G), GTT604GTG (5617A>C), GCC608AAC (5606G>T 5607C>T), CTC613CTG (5590G>C), TCG614GGC (5587C>G 5588G>C 5589A>C), TGG615TAC (5584C>G 5585C>T), ACG616AGC (5581C>G 5582G>C), GAA617GAG (5578T>C), CAG618CAA (5575C>T), GAC621GAT (5566G>A), ACC623TCG (5560G>C 5562T>A), AAG624CGC (5557C>G 5558T>C 5559T>G), GGT626GGC (5551A>G), GTC627TTC (5550C>A), CCC630TGC (5540G>C 5541G>A), CCC639GCA (5512G>T 5514G>C), CCC640CCG (5509G>C), AAC643GCC (5501T>G 5502T>C), TCC644AGC (5498G>C 5499A>T), AAC645CGC (5495T>C 5496T>G), GCC646GGC (5492G>C), TAC647TAT (5488G>A), GGC648GCC (5486C>G), ATT650GTG (5479A>C 5481T>C), CCG651CCC (5476C>G), GTC653GTG (5470G>C), GCC655GAC (5465G>T), GCA686CCG (5371T>C 5373C>G), TTG690TTC (5359C>G), TTC695TAC (5345A>T), ACC696GCG (5341G>C 5343T>C), AAC699AAG (5332G>C), AAC701GAC (5328T>C), CGT702CGG (5323A>C), CCG703CTG (5321G>A), ACT705ACG (5314A>C), GAA706CCA (5312T>G 5313C>G), GCG707GAG (5309G>T), GAC708GAG (5305G>C), TTC709CTG (5302G>C 5304A>G), ATT712CTC (5293A>G 5295T>G), CCG713ACC (5290C>G 5292G>T), CAC714CAT (5287G>A), GAC715GCC (5285T>G), GTG716GAC (5281C>G 5282A>T), ATG717ATC (5278C>G), TAC721TTC (5267T>A), GAC723AAG (5260G>C 5262C>T), TAC724CAC (5259A>G), AGC725TCG (5254G>C 5255C>G 5256T>A), TCC727GAG (5248G>C 5249G>T 5250A>C), ACC728CGC (5246G>C 5247T>G), CAC735GAC (5226G>C), GTC736GTG (5221G>C), GAA740GAG (5209T>C), TCG741GCG (5208A>C), GAG743GCC (5200C>G 5201T>G), GTC744GAG (5197G>C 5198A>T), ACC745CGC (5195G>C 5196T>G), GGG746GGC (5191C>G), AAG747ATG (5189T>A), GGC751ACC (5177C>G 5178C>T), CCA756CAG (5161T>C 5162G>T), TCC759AGC (5153G>C 5154A>T), CAC761CAT (5146G>A), GTA764GTC (5137T>G), GTC768GTG (5125G>C), GCC769ACG (5122G>C 5124C>T), CGG772CGC (5113C>G), GCC776AGC (5102G>C 5103C>T), CAG777GCG (5099T>G 5100G>C), TAC779TCG (5092G>C 5093T>G), AGC780GCA (5089G>T 5090C>G 5091T>C), AAG781GCC (5086C>G 5087T>G 5088T>C), GGC782GGG (5083G>C), CAG783CGC (5080C>G 5081T>C), GAA784GAG (5077T>C), CTG790ATG (5061G>T), ATC794ATT (5047G>A), GTC795GCG (5044G>C 5045A>G), GCC798GCG (5035G>C), GTG800ACC (5029C>G 5030A>G 5031C>T), GAA805AAG (5014T>C 5016C>T), GGA806GGC (5011T>G), GAC807GAG (5008G>C), CTG808GAG (5006A>T 5007G>C), TTC810TAC (5000A>T), GAT811GAC (4996A>G), GGG815GGC (4984C>G), ATC816CTG (4981G>C 4983T>G), ACC818ACG (4975G>C), GTC819GTG (4972G>C), CCG820CCC (4969C>G), TGC822CGG (4963G>C 4965A>G), GCG823GCT (4960C>A), CTG824GTG (4959G>C), AAG825CTG (4955T>A 4956T>G), CTC826CTG (4951G>C), AAG827GGC (4948C>G 4949T>C 4950T>C), CGC828CGG (4945G>C), AGA829CGC (4942T>G 4944T>G), ACC830GGC (4940G>C 4941T>C), GCC832GGC (4934G>C), CAC833TGC (4931T>C 4932G>A), GAA834GAG (4927T>C), AAC836GCC (4922T>G 4923T>C), TCC837AAG (4918G>C 4919G>T 4920A>T), CGC838CCC (4916C>G), GAC842GAT (4903G>A), GGT843GGC (4900A>G), GCC844GTC (4898G>A), TAT846TAC (4891A>G), AAG848GAG (4887T>C), TCC849CAG (4882G>C 4883G>T 4884A>G), GCG850GCC (4879C>G), GAG851GTG (4877T>A), GAA852CGC (4873T>G 4874T>C 4875C>G), GAG853TCG (4871T>G 4872C>A), GCC855TCG (4864G>C 4866C>A), ATG856ACG (4862A>G), CCC857CCG (4858G>C), GAC858TCG (4855G>C 4856T>G 4857C>A), TTC860TTT (4849G>A), GCC861GAC (4847G>T), CTG862CTT (4843C>A), CTC863GAC (4841A>T 4842G>C)								
phi297_00015	4	106	38.4%	390	55.2%	101 (97.1%)	52 (50.0%)	1/2/0/0	0
Protein mutations:	T5P (12132T>G), V6I (12127C>G 12129C>T), S10T (12115G>C 12117A>T), W11L (12113C>A 12114A>G), G12A (12110C>G), A13E (12107G>T), A18P (12091C>A 12093C>G), F19A (12088G>T 12089A>G 12090A>C), K20R (12086T>C), G23A (12076G>C 12077C>G), V24K (12073T>C 12074A>T 12075C>T), L26I (12069G>T), K28G (12061C>G 12062T>C 12063T>C), S31G (12054T>C), S33R (12046T>G 12047G>C 12048A>G), P35A (12042G>C), R36A (12037C>G 12038C>G 12039G>C), L38Q (12032A>T), I43V (12016G>C 12018T>C), S46G (12009T>C), A49L (11998C>G 11999G>A 12000C>G), A52Q (11990G>T 11991C>G), A53S (11986T>G 11988C>A), R54H (11983C>G 11984C>T), V55L (11980G>C 11982C>G), N56P (11977G>C 11978T>G 11979T>G), E58G (11971C>T 11972T>C), E58_P59insT (11970_11971insAGT), I60L (11965G>C 11967T>A), Y63D (11956G>A 11958A>C), S66A (11947C>G 11949A>C), E67G (11944T>G 11945T>C), L68A (11941C>A 11942A>G 11943G>C), L69V (11938C>G 11940G>C), H71D (11934G>C), T72A (11931T>C), L73I (11926C>G 11928G>T), Q74H (11923C>G), Q75K (11922G>T), F78E (11911G>T 11912A>T 11913A>C), R83_G84del (11893_11898delGCCGCG), S85E (11890G>T 11891G>T 11892A>C), D86E (11887G>T), I87T (11885A>G), S88A (11881G>C 11882C>G 11883T>C), E91D (11872T>G), S94R (11864G>C 11865A>G), T95I (11861G>A), G96A (11857T>C 11858C>G), T98A (11853T>C), K102R (11839C>G 11840T>C 11841T>G)								
Codon mutations:	ACC5CCC (12132T>G), GTG6ATC (12127C>G 12129C>T), TCG9AGC (12118C>G 12119G>C 12120A>T), TCC10ACG (12115G>C 12117A>T), TGG11CTG (12113C>A 12114A>G), GGC12GCC (12110C>G), GCG13GAG (12107G>T), GCG18CCT (12091C>A 12093C>G), TTC19GCA (12088G>T 12089A>G 12090A>C), AAG20AGG (12086T>C), GAG22GAA (12079C>T), GGC23GCG (12076G>C 12077C>G), GTA24AAG (12073T>C 12074A>T 12075C>T), CTC26ATC (12069G>T), AAG28GGC (12061C>G 12062T>C 12063T>C), AGC31GGC (12054T>C), CCT32CCG (12049A>C), TCA33CGC (12046T>G 12047G>C 12048A>G), TCC34AGC (12044G>C 12045A>T), CCC35GCC (12042G>C), CGG36GCC (12037C>G 12038C>G 12039G>C), CTG38CAG (12032A>T), CTC39CTG (12028G>C), GGT40GGC (12025A>G), ACC41ACG (12022G>C), ATC43GTG (12016G>C 12018T>C), AGC46GGC (12009T>C), GCG49CTC (11998C>G 11999G>A 12000C>G), GCG52CAG (11990G>T 11991C>G), GCA53TCC (11986T>G 11988C>A), CGG54CAC (11983C>G 11984C>T), GTC55CTG (11980G>C 11982C>G), AAC56CCG (11977G>C 11978T>G 11979T>G), GAG58GGA (11971C>T 11972T>C), GAG58_CCG59insACT (11970_11971insAGT), CCG59CCC (11968C>G), ATC60TTG (11965G>C 11967T>A), TAC63GAT (11956G>A 11958A>C), TCG66GCC (11947C>G 11949A>C), GAA67GGC (11944T>G 11945T>C), CTG68GCT (11941C>A 11942A>G 11943G>C), CTG69GTC (11938C>G 11940G>C), CAC71GAC (11934G>C), ACG72GCG (11931T>C), CTG73ATC (11926C>G 11928G>T), CAG74CAC (11923C>G), CAG75AAG (11922G>T), CCG76CCC (11917C>G), GAG77GAA (11914C>T), TTC78GAA (11911G>T 11912A>T 11913A>C), GAC81GAT (11902G>A), CGC83_GGC84del (11893_11898delGCCGCG), TCC85GAA (11890G>T 11891G>T 11892A>C), GAC86GAA (11887G>T), ATC87ACC (11885A>G), AGC88GCG (11881G>C 11882C>G 11883T>C), GAA91GAC (11872T>G), TCC94CGC (11864G>C 11865A>G), ACC95ATC (11861G>A), GGA96GCG (11857T>C 11858C>G), ACG98GCG (11853T>C), ACG101ACC (11842C>G), AAG102CGC (11839C>G 11840T>C 11841T>G)								

Proteins

putative DNA methylase N-4/N-6 domain protein (YP_005098037.1)	89	865	68.5%	3140	72.2%	601 (99.3%)	423 (69.9%)	4/0/0/0	0

	Begin	End	Coverage	Score	Concordance	Matches	Identities	I/D/M/F*	Stop Codons
**NT**	**4836**	**12136**	**4.3%**	**1899**	**45.2%**	**2108 (99.0%)**	**1543 (72.5%)**	**15/6**	
Protein mutations:	Y90I (7160T>A 7161A>T), M95R (7144C>G 7145A>C 7146T>G), R101K (7127C>T 7128G>T), S105R (7114A>G 7116T>G), F106I (7113A>T), D107E (7108G>C), V109A (7102T>G 7103A>G), T113N (7091G>T), T114G (7088G>C 7089T>C), V123I (7062C>T), G126R (7053C>G), R131L (7036G>C 7037C>A), R132L (7034C>A), S142A (7003A>T 7004C>G 7005T>C), R165_A166insRDM (6933_6934insCATGTCGCG), A167N (6929G>T 6930C>T), G168R (6927C>G), R170K (6919G>C 6920C>T 6921G>T), G171S (6918C>T), D172A (6914T>G), R175P (6905C>G), Y178F (6896T>A), Y189F (6862G>A 6863T>A), I190V (6861T>C), Q221N (6766T>G 6768G>T), I224L (6757G>C 6759T>G), H225S (6754A>G 6755T>C 6756G>T), A226P (6751G>T 6753C>G), H227A (6748G>C 6749T>G 6750G>C), K228R (6745T>G 6746T>C 6747T>G), G230R (6741C>G), E231D (6736C>G), M234L (6729T>G), I241L (6706G>C 6708T>G), R245K (6694G>C 6695C>T 6696G>T), F251Y (6677A>T), E254D (6667T>G), A257E (6659G>T), E260P (6649T>G 6650T>G 6651C>G), L261I (6646C>G 6648G>T), V268I (6625T>G 6627C>T), S270A (6619G>C 6621A>C), S273A (6610C>G 6612A>C), H274D (6609G>C), H277T (6598A>G 6599T>G 6600G>T), E278N (6595C>A 6597C>T), R279M (6592A>C 6593C>A 6594G>T), N280D (6591T>C), G283A (6580G>C 6581C>G), R284Q (6577G>C 6578C>T), L285M (6576G>T), L286F (6571A>G 6573G>A), R287K (6568G>C 6569C>T 6570G>T), N288R (6566T>C 6567T>G), T289Q (6562G>C 6563G>T 6564T>G), Q294V (6548T>A 6549G>C), A298R (6535T>G 6536G>C 6537C>G), E302D (6523C>G), P305D (6515G>T 6516G>C), I308V (6505G>C 6507T>C), L311M (6498G>T), I314L (6487G>C 6489T>G), A318D (6475A>G 6476G>T), D320A (6469A>G 6470T>G), I324V (6457G>C 6459T>C), A331D (6436C>A 6437G>T), H334R (6428T>C), A338R (6416G>C 6417C>G), I344A (6398A>G 6399T>C), D529S (5843T>C 5844C>T), E535Q (5826C>G), Y539F (5812A>G 5813T>A), I541V (5806G>C 5808T>C), T548L (5785G>C 5786G>A 5787T>G), I549M (5782G>C), L553V (5772G>C), C555A (5765C>G 5766A>C), D560N (5749A>G 5751C>T), I563V (5740A>C 5742T>C), G565S (5736C>T), T568V (5725G>C 5726G>A 5727T>C), T568_G569insE (5724_5725insCTC), L570V (5719A>G 5721G>C), V575E (5704G>C 5705A>T), H579D (5694G>C), M580V (5691T>C), E581D (5686T>G), V582T (5683C>G 5684A>G 5685C>T), T583I (5680A>G 5681G>A), R584A (5677T>G 5678C>G 5679G>C), A585C (5675G>C 5676C>A), V587R (5669A>C 5670C>G), K588R (5666T>C 5667T>G), H589A (5662A>G 5663T>G 5664G>C), W591F (5656C>G 5657C>A), A592H (5654G>T 5655C>G), Y593F (5650A>G 5651T>A), M594V (5649T>C), M596R (5641C>G 5642A>C 5643T>G), A608N (5606G>T 5607C>T), S614G (5587C>G 5588G>C 5589A>C), W615Y (5584C>G 5585C>T), T616S (5581C>G 5582G>C), T623S (5560G>C 5562T>A), K624R (5557C>G 5558T>C 5559T>G), V627F (5550C>A), P630C (5540G>C 5541G>A), P639A (5512G>T 5514G>C), N643A (5501T>G 5502T>C), N645R (5495T>C 5496T>G), A646G (5492G>C), G648A (5486C>G), I650V (5479A>C 5481T>C), A655D (5465G>T), A686P (5371T>C 5373C>G), L690F (5359C>G), F695Y (5345A>T), T696A (5341G>C 5343T>C), N699K (5332G>C), N701D (5328T>C), P703L (5321G>A), E706P (5312T>G 5313C>G), A707E (5309G>T), D708E (5305G>C), F709L (5302G>C 5304A>G), I712L (5293A>G 5295T>G), P713T (5290C>G 5292G>T), D715A (5285T>G), V716D (5281C>G 5282A>T), M717I (5278C>G), Y721F (5267T>A), D723K (5260G>C 5262C>T), Y724H (5259A>G), S727E (5248G>C 5249G>T 5250A>C), T728R (5246G>C 5247T>G), H735D (5226G>C), S741A (5208A>C), E743A (5200C>G 5201T>G), V744E (5197G>C 5198A>T), T745R (5195G>C 5196T>G), K747M (5189T>A), G751T (5177C>G 5178C>T), P756Q (5161T>C 5162G>T), A769T (5122G>C 5124C>T), A776S (5102G>C 5103C>T), Q777A (5099T>G 5100G>C), Y779S (5092G>C 5093T>G), S780A (5089G>T 5090C>G 5091T>C), K781A (5086C>G 5087T>G 5088T>C), Q783R (5080C>G 5081T>C), L790M (5061G>T), V795A (5044G>C 5045A>G), V800T (5029C>G 5030A>G 5031C>T), E805K (5014T>C 5016C>T), D807E (5008G>C), L808E (5006A>T 5007G>C), F810Y (5000A>T), I816L (4981G>C 4983T>G), C822R (4963G>C 4965A>G), L824V (4959G>C), K825L (4955T>A 4956T>G), K827G (4948C>G 4949T>C 4950T>C), T830G (4940G>C 4941T>C), A832G (4934G>C), H833C (4931T>C 4932G>A), N836A (4922T>G 4923T>C), S837K (4918G>C 4919G>T 4920A>T), R838P (4916C>G), A844V (4898G>A), K848E (4887T>C), S849Q (4882G>C 4883G>T 4884A>G), E851V (4877T>A), E852R (4873T>G 4874T>C 4875C>G), E853S (4871T>G 4872C>A), A855S (4864G>C 4866C>A), M856T (4862A>G), D858S (4855G>C 4856T>G 4857C>A), A861D (4847G>T), L863D (4841A>T 4842G>C)								
Codon mutations:	TAC90ATC (7160T>A 7161A>T), GCC93GCG (7150G>C), GCG94GCA (7147C>T), ATG95CGC (7144C>G 7145A>C 7146T>G), CTC96CTT (7141G>A), ACC99ACG (7132G>C), CGG101AAG (7127C>T 7128G>T), AGT105CGC (7114A>G 7116T>G), TTC106ATC (7113A>T), GAC107GAG (7108G>C), GAG108GAA (7105C>T), GTA109GCC (7102T>G 7103A>G), CCC112CCG (7093G>C), ACC113AAC (7091G>T), ACC114GGC (7088G>C 7089T>C), CTG117CTC (7078C>G), AAT119AAC (7072A>G), GAG121GAA (7066C>T), ACC122ACG (7063G>C), GTC123ATC (7062C>T), GGC126CGC (7053C>G), CTC128CTG (7045G>C), CCT130CCG (7039A>C), CGC131CTG (7036G>C 7037C>A), CGG132CTG (7034C>A), GCA136GCC (7021T>G), GAA140GAG (7009T>C), GCC141GCG (7006G>C), AGT142GCA (7003A>T 7004C>G 7005T>C), CTT144CTG (6997A>C), ACG152ACC (6973C>G), GCT158GCG (6955A>C), GCT161GCC (6946A>G), GGT162GGC (6943A>G), GAT164GAC (6937A>G), CGC165CGG (6934G>C), CGC165_GCC166insCGCGACATG (6933_6934insCATGTCGCG), GCC167AAC (6929G>T 6930C>T), GGC168CGC (6927C>G), GTC169GTG (6922G>C), CGC170AAG (6919G>C 6920C>T 6921G>T), GGC171AGC (6918C>T), GAT172GCT (6914T>G), GTC174GTG (6907G>C), CGG175CCG (6905C>G), TAC176TAT (6901G>A), CGG177CGC (6898C>G), TAC178TTC (6896T>A), ACT181ACG (6886A>C), CCA184CCC (6877T>G), AGC185AGT (6874G>A), CCG186CCA (6871C>T), GAA188GAG (6865T>C), TAC189TTT (6862G>A 6863T>A), ATC190GTC (6861T>C), CTA192CTG (6853T>C), GCT194GCC (6847A>G), TCG196AGC (6841C>G 6842G>C 6843A>T), CTC199CTG (6832G>C), GAT203GAC (6820A>G), GTC204GTT (6817G>A), ACC209ACG (6802G>C), AGG210CGT (6799C>A 6801T>G), AAA213AAG (6790T>C), TCT216AGC (6781A>G 6782G>C 6783A>T), CAA221AAC (6766T>G 6768G>T), CTC222CTG (6763G>C), ACC223ACG (6760G>C), ATC224CTG (6757G>C 6759T>G), CAT225AGC (6754A>G 6755T>C 6756G>T), GCC226CCA (6751G>T 6753C>G), CAC227GCG (6748G>C 6749T>G 6750G>C), AAA228CGC (6745T>G 6746T>C 6747T>G), GGC230CGC (6741C>G), GAG231GAC (6736C>G), ATG234CTG (6729T>G), GCG237GCA (6718C>T), TCC238AGC (6716G>C 6717A>T), GCG240GCC (6709C>G), ATC241CTG (6706G>C 6708T>G), GTT243GTG (6700A>C), CGC245AAG (6694G>C 6695C>T 6696G>T), AGC247TCG (6688G>C 6689C>G 6690T>A), GGG250GGC (6679C>G), TTC251TAC (6677A>T), GAA254GAC (6667T>G), GCG257GAG (6659G>T), CTA258CTG (6655T>C), GAA260CCC (6649T>G 6650T>G 6651C>G), CTG261ATC (6646C>G 6648G>T), GAA267GAG (6628T>C), GTA268ATC (6625T>G 6627C>T), TCC270GCG (6619G>C 6621A>C), TCG273GCC (6610C>G 6612A>C), CAC274GAC (6609G>C), GGC276GGT (6601G>A), CAT277ACC (6598A>G 6599T>G 6600G>T), GAG278AAT (6595C>A 6597C>T), CGT279ATG (6592A>C 6593C>A 6594G>T), AAC280GAC (6591T>C), GGC283GCG (6580G>C 6581C>G), CGC284CAG (6577G>C 6578C>T), CTG285ATG (6576G>T), CTT286TTC (6571A>G 6573G>A), CGC287AAG (6568G>C 6569C>T 6570G>T), AAC288CGC (6566T>C 6567T>G), ACC289CAG (6562G>C 6563G>T 6564T>G), ATT291ATC (6556A>G), CAG294GTG (6548T>A 6549G>C), GCC296GCG (6541G>C), GCC297GCG (6538G>C), GCA298CGC (6535T>G 6536G>C 6537C>G), GAG302GAC (6523C>G), CTT304CTG (6517A>C), CCT305GAT (6515G>T 6516G>C), GCC306GCG (6511G>C), CGG307CGC (6508C>G), ATC308GTG (6505G>C 6507T>C), AAA310AAG (6499T>C), CTG311ATG (6498G>T), ATC314CTG (6487G>C 6489T>G), CGG315CGC (6484C>G), GCT318GAC (6475A>G 6476G>T), CCA319CCG (6472T>C), GAT320GCC (6469A>G 6470T>G), GCT321GCC (6466A>G), CGG323CGT (6460C>A), ATC324GTG (6457G>C 6459T>C), CAT327CAC (6448A>G), CTC329CTG (6442G>C), GCG331GAT (6436C>A 6437G>T), GAA332GAG (6433T>C), CAC334CGC (6428T>C), GCG335GCC (6424C>G), GCC338CGC (6416G>C 6417C>G), GCT339GCC (6412A>G), CCC341CCG (6406G>C), ACC342ACG (6403G>C), ATC344GCC (6398A>G 6399T>C), GAC529AGC (5843T>C 5844C>T), GAG535CAG (5826C>G), TAT539TTC (5812A>G 5813T>A), ATC541GTG (5806G>C 5808T>C), GAA543GAG (5800T>C), ACC548CTG (5785G>C 5786G>A 5787T>G), ATC549ATG (5782G>C), CCC550CCG (5779G>C), GGG551GGC (5776C>G), CGT552AGG (5773A>C 5775G>T), CTG553GTG (5772G>C), TGC555GCC (5765C>G 5766A>C), GTG558GTC (5755C>G), GAT560AAC (5749A>G 5751C>T), ATT563GTG (5740A>C 5742T>C), GGC565AGC (5736C>T), GGA566GGC (5731T>G), ACC568GTG (5725G>C 5726G>A 5727T>C), ACC568_GGC569insGAG (5724_5725insCTC), CTT570GTC (5719A>G 5721G>C), GTC575GAG (5704G>C 5705A>T), CAC579GAC (5694G>C), ATG580GTG (5691T>C), GAA581GAC (5686T>G), GTG582ACC (5683C>G 5684A>G 5685C>T), ACT583ATC (5680A>G 5681G>A), CGA584GCC (5677T>G 5678C>G 5679G>C), GCC585TGC (5675G>C 5676C>A), GTC587CGC (5669A>C 5670C>G), AAG588CGG (5666T>C 5667T>G), CAT589GCC (5662A>G 5663T>G 5664G>C), TGG591TTC (5656C>G 5657C>A), GCC592CAC (5654G>T 5655C>G), TAT593TTC (5650A>G 5651T>A), ATG594GTG (5649T>C), ATG596CGC (5641C>G 5642A>C 5643T>G), GTT604GTG (5617A>C), GCC608AAC (5606G>T 5607C>T), CTC613CTG (5590G>C), TCG614GGC (5587C>G 5588G>C 5589A>C), TGG615TAC (5584C>G 5585C>T), ACG616AGC (5581C>G 5582G>C), GAA617GAG (5578T>C), CAG618CAA (5575C>T), GAC621GAT (5566G>A), ACC623TCG (5560G>C 5562T>A), AAG624CGC (5557C>G 5558T>C 5559T>G), GGT626GGC (5551A>G), GTC627TTC (5550C>A), CCC630TGC (5540G>C 5541G>A), CCC639GCA (5512G>T 5514G>C), CCC640CCG (5509G>C), AAC643GCC (5501T>G 5502T>C), TCC644AGC (5498G>C 5499A>T), AAC645CGC (5495T>C 5496T>G), GCC646GGC (5492G>C), TAC647TAT (5488G>A), GGC648GCC (5486C>G), ATT650GTG (5479A>C 5481T>C), CCG651CCC (5476C>G), GTC653GTG (5470G>C), GCC655GAC (5465G>T), GCA686CCG (5371T>C 5373C>G), TTG690TTC (5359C>G), TTC695TAC (5345A>T), ACC696GCG (5341G>C 5343T>C), AAC699AAG (5332G>C), AAC701GAC (5328T>C), CGT702CGG (5323A>C), CCG703CTG (5321G>A), ACT705ACG (5314A>C), GAA706CCA (5312T>G 5313C>G), GCG707GAG (5309G>T), GAC708GAG (5305G>C), TTC709CTG (5302G>C 5304A>G), ATT712CTC (5293A>G 5295T>G), CCG713ACC (5290C>G 5292G>T), CAC714CAT (5287G>A), GAC715GCC (5285T>G), GTG716GAC (5281C>G 5282A>T), ATG717ATC (5278C>G), TAC721TTC (5267T>A), GAC723AAG (5260G>C 5262C>T), TAC724CAC (5259A>G), AGC725TCG (5254G>C 5255C>G 5256T>A), TCC727GAG (5248G>C 5249G>T 5250A>C), ACC728CGC (5246G>C 5247T>G), CAC735GAC (5226G>C), GTC736GTG (5221G>C), GAA740GAG (5209T>C), TCG741GCG (5208A>C), GAG743GCC (5200C>G 5201T>G), GTC744GAG (5197G>C 5198A>T), ACC745CGC (5195G>C 5196T>G), GGG746GGC (5191C>G), AAG747ATG (5189T>A), GGC751ACC (5177C>G 5178C>T), CCA756CAG (5161T>C 5162G>T), TCC759AGC (5153G>C 5154A>T), CAC761CAT (5146G>A), GTA764GTC (5137T>G), GTC768GTG (5125G>C), GCC769ACG (5122G>C 5124C>T), CGG772CGC (5113C>G), GCC776AGC (5102G>C 5103C>T), CAG777GCG (5099T>G 5100G>C), TAC779TCG (5092G>C 5093T>G), AGC780GCA (5089G>T 5090C>G 5091T>C), AAG781GCC (5086C>G 5087T>G 5088T>C), GGC782GGG (5083G>C), CAG783CGC (5080C>G 5081T>C), GAA784GAG (5077T>C), CTG790ATG (5061G>T), ATC794ATT (5047G>A), GTC795GCG (5044G>C 5045A>G), GCC798GCG (5035G>C), GTG800ACC (5029C>G 5030A>G 5031C>T), GAA805AAG (5014T>C 5016C>T), GGA806GGC (5011T>G), GAC807GAG (5008G>C), CTG808GAG (5006A>T 5007G>C), TTC810TAC (5000A>T), GAT811GAC (4996A>G), GGG815GGC (4984C>G), ATC816CTG (4981G>C 4983T>G), ACC818ACG (4975G>C), GTC819GTG (4972G>C), CCG820CCC (4969C>G), TGC822CGG (4963G>C 4965A>G), GCG823GCT (4960C>A), CTG824GTG (4959G>C), AAG825CTG (4955T>A 4956T>G), CTC826CTG (4951G>C), AAG827GGC (4948C>G 4949T>C 4950T>C), CGC828CGG (4945G>C), AGA829CGC (4942T>G 4944T>G), ACC830GGC (4940G>C 4941T>C), GCC832GGC (4934G>C), CAC833TGC (4931T>C 4932G>A), GAA834GAG (4927T>C), AAC836GCC (4922T>G 4923T>C), TCC837AAG (4918G>C 4919G>T 4920A>T), CGC838CCC (4916C>G), GAC842GAT (4903G>A), GGT843GGC (4900A>G), GCC844GTC (4898G>A), TAT846TAC (4891A>G), AAG848GAG (4887T>C), TCC849CAG (4882G>C 4883G>T 4884A>G), GCG850GCC (4879C>G), GAG851GTG (4877T>A), GAA852CGC (4873T>G 4874T>C 4875C>G), GAG853TCG (4871T>G 4872C>A), GCC855TCG (4864G>C 4866C>A), ATG856ACG (4862A>G), CCC857CCG (4858G>C), GAC858TCG (4855G>C 4856T>G 4857C>A), TTC860TTT (4849G>A), GCC861GAC (4847G>T), CTG862CTT (4843C>A), CTC863GAC (4841A>T 4842G>C)								
PD-(D/E)XK nuclease family protein (YP_005098042.1)	4	106	38.4%	390	55.2%	101 (97.1%)	52 (50.0%)	1/2/0/0	0
Protein mutations:	T5P (12132T>G), V6I (12127C>G 12129C>T), S10T (12115G>C 12117A>T), W11L (12113C>A 12114A>G), G12A (12110C>G), A13E (12107G>T), A18P (12091C>A 12093C>G), F19A (12088G>T 12089A>G 12090A>C), K20R (12086T>C), G23A (12076G>C 12077C>G), V24K (12073T>C 12074A>T 12075C>T), L26I (12069G>T), K28G (12061C>G 12062T>C 12063T>C), S31G (12054T>C), S33R (12046T>G 12047G>C 12048A>G), P35A (12042G>C), R36A (12037C>G 12038C>G 12039G>C), L38Q (12032A>T), I43V (12016G>C 12018T>C), S46G (12009T>C), A49L (11998C>G 11999G>A 12000C>G), A52Q (11990G>T 11991C>G), A53S (11986T>G 11988C>A), R54H (11983C>G 11984C>T), V55L (11980G>C 11982C>G), N56P (11977G>C 11978T>G 11979T>G), E58G (11971C>T 11972T>C), E58_P59insT (11970_11971insAGT), I60L (11965G>C 11967T>A), Y63D (11956G>A 11958A>C), S66A (11947C>G 11949A>C), E67G (11944T>G 11945T>C), L68A (11941C>A 11942A>G 11943G>C), L69V (11938C>G 11940G>C), H71D (11934G>C), T72A (11931T>C), L73I (11926C>G 11928G>T), Q74H (11923C>G), Q75K (11922G>T), F78E (11911G>T 11912A>T 11913A>C), R83_G84del (11893_11898delGCCGCG), S85E (11890G>T 11891G>T 11892A>C), D86E (11887G>T), I87T (11885A>G), S88A (11881G>C 11882C>G 11883T>C), E91D (11872T>G), S94R (11864G>C 11865A>G), T95I (11861G>A), G96A (11857T>C 11858C>G), T98A (11853T>C), K102R (11839C>G 11840T>C 11841T>G)								

	Begin	End	Coverage	Score	Concordance	Matches	Identities	I/D/M/F*	Stop Codons
**NT**	**4836**	**12136**	**4.3%**	**1899**	**45.2%**	**2108 (99.0%)**	**1543 (72.5%)**	**15/6**	
Codon mutations:	ACC5CCC (12132T>G), GTG6ATC (12127C>G 12129C>T), TCG9AGC (12118C>G 12119G>C 12120A>T), TCC10ACG (12115G>C 12117A>T), TGG11CTG (12113C>A 12114A>G), GGC12GCC (12110C>G), GCG13GAG (12107G>T), GCG18CCT (12091C>A 12093C>G), TTC19GCA (12088G>T 12089A>G 12090A>C), AAG20AGG (12086T>C), GAG22GAA (12079C>T), GGC23GCG (12076G>C 12077C>G), GTA24AAG (12073T>C 12074A>T 12075C>T), CTC26ATC (12069G>T), AAG28GGC (12061C>G 12062T>C 12063T>C), AGC31GGC (12054T>C), CCT32CCG (12049A>C), TCA33CGC (12046T>G 12047G>C 12048A>G), TCC34AGC (12044G>C 12045A>T), CCC35GCC (12042G>C), CGG36GCC (12037C>G 12038C>G 12039G>C), CTG38CAG (12032A>T), CTC39CTG (12028G>C), GGT40GGC (12025A>G), ACC41ACG (12022G>C), ATC43GTG (12016G>C 12018T>C), AGC46GGC (12009T>C), GCG49CTC (11998C>G 11999G>A 12000C>G), GCG52CAG (11990G>T 11991C>G), GCA53TCC (11986T>G 11988C>A), CGG54CAC (11983C>G 11984C>T), GTC55CTG (11980G>C 11982C>G), AAC56CCG (11977G>C 11978T>G 11979T>G), GAG58GGA (11971C>T 11972T>C), GAG58_CCG59insACT (11970_11971insAGT), CCG59CCC (11968C>G), ATC60TTG (11965G>C 11967T>A), TAC63GAT (11956G>A 11958A>C), TCG66GCC (11947C>G 11949A>C), GAA67GGC (11944T>G 11945T>C), CTG68GCT (11941C>A 11942A>G 11943G>C), CTG69GTC (11938C>G 11940G>C), CAC71GAC (11934G>C), ACG72GCG (11931T>C), CTG73ATC (11926C>G 11928G>T), CAG74CAC (11923C>G), CAG75AAG (11922G>T), CCG76CCC (11917C>G), GAG77GAA (11914C>T), TTC78GAA (11911G>T 11912A>T 11913A>C), GAC81GAT (11902G>A), CGC83_GGC84del (11893_11898delGCCGCG), TCC85GAA (11890G>T 11891G>T 11892A>C), GAC86GAA (11887G>T), ATC87ACC (11885A>G), AGC88GCG (11881G>C 11882C>G 11883T>C), GAA91GAC (11872T>G), TCC94CGC (11864G>C 11865A>G), ACC95ATC (11861G>A), GGA96GCG (11857T>C 11858C>G), ACG98GCG (11853T>C), ACG101ACC (11842C>G), AAG102CGC (11839C>G 11840T>C 11841T>G)								

*: Inserts / Deletes / Misaligned / Frameshifts

Analysis details

This analysis was performed with panviral2.64

NGS Details (UN24): Epiphyllum badnavirus 1

Assembly

**Coverage Length**	279 (1 contig(s))
**Depth Of Coverage**	35.8
**Number Of Reads**	90
**Reads Per Million**	1.80 rpm (after QC)
**Ambiguities**	0
**Assembly Method**	de novo + reference guided assembly
**Consensus Caller**	Bcf Tools

Coverage Map

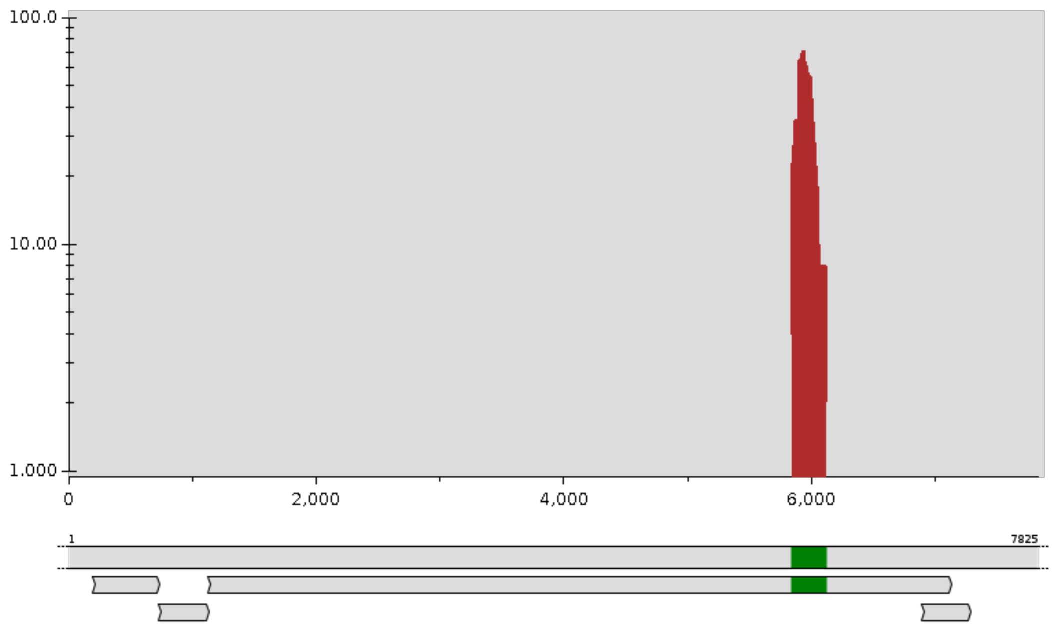

Assignment

**Type**	Epiphyllum badnavirus 1 (Taxonomy ID: 2518008)
**Reference Genome**	NC_076247.1
**NT Identity (%)**	53.9007
**AA Identity (%)**	51.0638
**Number Of Stop Codons**	0
**Number Of CDS**	4

Alignment

**Alignment Score**	38.0 (NT) + 339.0 (AA) = 377.0
**Concordance (%)**	30.4278

Alignment Method	Global, seeded, nucleotide + amino acids (AGA)

Genome Region

Sequence starts at position 5840 and ends at position 6118 relative to NC_076247.1 reference sequence.

Alignment Detailed Statistics

	Begin	End	Coverage	Score	Concordance	Matches	Identities	I/D/M/F*	Stop Codons
**NT**	**5840**	**6118**	**3.6%**	**38**	**6.8%**	**279 (98.9%)**	**152 (53.9%)**	**3/0**	
Mutations:	5841C>T, 5847C>A, 5851T>A, 5853C>A, 5859G>T, 5860A>C, 5861A>G, 5862G>T, 5863A>T, 5864G>C, 5865C>G, 5868A>T, 5870T>A, 5871C>T, 5878A>T, 5880T>G, 5882T>A, 5884A>G, 5886G>T, 5887G>A, 5889C>G, 5892A>G, 5893G>T, 5894A>G, 5895A>T, 5896T>G, 5897C>A, 5898C>T, 5901C>T, 5904T>C, 5905T>A, 5906G>A, 5910C>A, 5913C>A, 5918T>G, 5922C>G, 5923C>A, 5924C>G, 5925A>G, 5926G>T, 5933T>A, 5936A>T, 5937C>T, 5938G>A, 5942G>T, 5943G>C, 5944T>C, 5946A>T, 5953C>T, 5955A>C, 5958C>T, 5961A>T, 5964G>A, 5966A>C, 5967G>T, 5970T>C, 5973C>T, 5976T>G, 5977G>A, 5982G>A, 5985C>T, 5986C>A, 5987A>T, 5988A>G, 5989C>G, 5990G>A, 5991A>C, 5992A>C, 5993A>T, 5994G>T, 5998G>A, 6000C>T, 6002A>G, 6003C>G, 6004T>A, 6005G>T, 6006T>A, 6011G>A, 6012A>G, 6013G>C, 6014A>C, 6015_6016insTAT, 6018G>C, 6019T>G, 6020C>A, 6021A>C, 6022G>A, 6023G>T, 6024C>G, 6026T>A, 6030A>G, 6032C>T, 6033A>T, 6036T>G, 6038A>T, 6059T>A, 6060C>T, 6061A>T, 6062G>C, 6064A>C, 6065A>G, 6066C>A, 6068C>G, 6069T>C, 6072A>G, 6073G>C, 6075T>A, 6078C>T, 6081T>C, 6082G>A, 6083C>T, 6084A>G, 6085C>G, 6087G>A, 6090C>T, 6091C>T, 6095A>G, 6097T>A, 6099C>G, 6100A>T, 6102G>A, 6103C>T, 6108G>A, 6113G>T, 6114T>G, 6115G>A, 6116A>G, 6118A>G								

CDS

	Begin	End	Coverage	Score	Concordance	Matches	Identities	I/D/M/F*	Stop Codons
QKM20_gp3	1572	1664	4.6%	339	48.8%	93 (98.9%)	48 (51.1%)	1/0/0/0	0
Protein mutations:	F1575I (5851T>A 5853C>A), K1578R (5860A>C 5861A>G 5862G>T), F1581Y (5870T>A 5871C>T), I1584L (5878A>T 5880T>G), M1585K (5882T>A), M1586V (5884A>G 5886G>T), D1587K (5887G>A 5889C>G), E1589C (5893G>T 5894A>G 5895A>T), S1590D (5896T>G 5897C>A 5898C>T), W1593K (5905T>A 5906G>A), L1597R (5918T>G), P1599R (5923C>A 5924C>G 5925A>G), D1600Y (5926G>T), L1602H (5933T>A), Y1603F (5936A>T 5937C>T), E1604K (5938G>A), W1605F (5942G>T 5943G>C), P1609S (5953C>T 5955A>C), K1613T (5966A>C 5967G>T), A1617T (5977G>A), Q1620M (5986C>A 5987A>T 5988A>G), R1621D (5989C>G 5990G>A 5991A>C), K1622L (5992A>C 5993A>T 5994G>T), D1624N (5998G>A 6000C>T), N1625R (6002A>G 6003C>G), C1626I (6004T>A 6005G>T 6006T>A), R1628K (6011G>A 6012A>G), D1629P (6013G>C 6014A>C), D1629_L1630insY (6015_6016insTAT), S1631D (6019T>G 6020C>A 6021A>C), G1632M (6022G>A 6023G>T 6024C>G), F1633Y (6026T>A), A1635V (6032C>T 6033A>T), Y1637F (6038A>T), F1644Y (6059T>A 6060C>T), N1646R (6064A>C 6065A>G 6066C>A), T1647S (6068C>G 6069T>C), D1649Q (6073G>C 6075T>A), A1652M (6082G>A 6083C>T 6084A>G), Q1653E (6085C>G 6087G>A), K1656R (6095A>G), S1657T (6097T>A 6099C>G), M1658L (6100A>T 6102G>A), C1662L (6113G>T 6114T>G), E1663R (6115G>A 6116A>G)								
Codon mutations:	GTC1571.TT (5841C>T), TCC1573TCA (5847C>A), TTC1575ATA (5851T>A 5853C>A), CTG1577CTT (5859G>T), AAG1578CGT (5860A>C 5861A>G 5862G>T), AGC1579TCG (5863A>T 5864G>C 5865C>G), GGA1580GGT (5868A>T), TTC1581TAT (5870T>A 5871C>T), ATT1584TTG (5878A>T 5880T>G), ATG1585AAG (5882T>A), ATG1586GTT (5884A>G 5886G>T), GAC1587AAG (5887G>A 5889C>G), GAA1588GAG (5892A>G), GAA1589TGT (5893G>T 5894A>G 5895A>T), TCC1590GAT (5896T>G 5897C>A 5898C>T), ATC1591ATT (5901C>T), CCT1592CCC (5904T>C), TGG1593AAG (5905T>A 5906G>A), ACC1594ACA (5910C>A), GCC1595GCA (5913C>A), CTG1597CGG (5918T>G), ACC1598ACG (5922C>G), CCA1599AGG (5923C>A 5924C>G 5925A>G), GAT1600TAT (5926G>T), CTT1602CAT (5933T>A), TAC1603TTT (5936A>T 5937C>T), GAA1604AAA (5938G>A), TGG1605TTC (5942G>T 5943G>C), TTA1606CTT (5944T>C 5946A>T), CCA1609TCC (5953C>T 5955A>C), TTC1610TTT (5958C>T), GGA1611GGT (5961A>T), TTG1612TTA (5964G>A), AAG1613ACT (5966A>C 5967G>T), AAT1614AAC (5970T>C), GCC1615GCT (5973C>T), CCT1616CCG (5976T>G), GCT1617ACT (5977G>A), GTG1618GTA (5982G>A), TTC1619TTT (5985C>T), CAA1620ATG (5986C>A 5987A>T 5988A>G), CGA1621GAC (5989C>G 5990G>A 5991A>C), AAG1622CTT (5992A>C 5993A>T 5994G>T), GAC1624AAT (5998G>A 6000C>T), AAC1625AGG (6002A>G 6003C>G), TGT1626ATA (6004T>A 6005G>T 6006T>A), AGA1628AAG (6011G>A 6012A>G), GAC1629CCC (6013G>C 6014A>C), GAC1629_CTG1630insTAT (6015_6016insTAT), CTG1630CTC (6018G>C), TCA1631GAC (6019T>G 6020C>A 6021A>C), GGC1632ATG (6022G>A 6023G>T 6024C>G), TTT1633TAT (6026T>A), GTA1634GTG (6030A>G), GCA1635GTT (6032C>T 6033A>T), GTT1636GTG (6036T>G), TAC1637TTC (6038A>T), TTC1644TAT (6059T>A 6060C>T), AGT1645TCT (6061A>T 6062G>C), AAC1646CGA (6064A>C 6065A>G 6066C>A), ACT1647AGC (6068C>G 6069T>C), GAA1648GAG (6072A>G), GAT1649CAA (6073G>C 6075T>A), GAC1650GAT (6078C>T), CAT1651CAC (6081T>C), GCA1652ATG (6082G>A 6083C>T 6084A>G), CAG1653GAA (6085C>G 6087G>A), CAC1654CAT (6090C>T), CTG1655TTG (6091C>T), AAG1656AGG (6095A>G), TCC1657ACG (6097T>A 6099C>G), ATG1658TTA (6100A>T 6102G>A), CTG1659TTG (6103C>T), CAG1660CAA (6108G>A), TGT1662TTG (6113G>T 6114T>G), GAG1663AGG (6115G>A 6116A>G), AGA1664G.. (6118A>G)								

Proteins

	Begin	End	Coverage	Score	Concordance	Matches	Identities	I/D/M/F*	Stop Codons
polyprotein (YP_010797894.1)	1572	1664	4.6%	339	48.8%	93 (98.9%)	48 (51.1%)	1/0/0/0	0
Protein mutations:	F1575I (5851T>A 5853C>A), K1578R (5860A>C 5861A>G 5862G>T), F1581Y (5870T>A 5871C>T), I1584L (5878A>T 5880T>G), M1585K (5882T>A), M1586V (5884A>G 5886G>T), D1587K (5887G>A 5889C>G), E1589C (5893G>T 5894A>G 5895A>T), S1590D (5896T>G 5897C>A 5898C>T), W1593K (5905T>A 5906G>A), L1597R (5918T>G), P1599R (5923C>A 5924C>G 5925A>G), D1600Y (5926G>T), L1602H (5933T>A), Y1603F (5936A>T 5937C>T), E1604K (5938G>A), W1605F (5942G>T 5943G>C), P1609S (5953C>T 5955A>C), K1613T (5966A>C 5967G>T), A1617T (5977G>A), Q1620M (5986C>A 5987A>T 5988A>G), R1621D (5989C>G 5990G>A 5991A>C), K1622L (5992A>C 5993A>T 5994G>T), D1624N (5998G>A 6000C>T), N1625R (6002A>G 6003C>G), C1626I (6004T>A 6005G>T 6006T>A), R1628K (6011G>A 6012A>G), D1629P (6013G>C 6014A>C), D1629_L1630insY (6015_6016insTAT), S1631D (6019T>G 6020C>A 6021A>C), G1632M (6022G>A 6023G>T 6024C>G), F1633Y (6026T>A), A1635V (6032C>T 6033A>T), Y1637F (6038A>T), F1644Y (6059T>A 6060C>T), N1646R (6064A>C 6065A>G 6066C>A), T1647S (6068C>G 6069T>C), D1649Q (6073G>C 6075T>A), A1652M (6082G>A 6083C>T 6084A>G), Q1653E (6085C>G 6087G>A), K1656R (6095A>G), S1657T (6097T>A 6099C>G), M1658L (6100A>T 6102G>A), C1662L (6113G>T 6114T>G), E1663R (6115G>A 6116A>G)								
Codon mutations:	GTC1571.TT (5841C>T), TCC1573TCA (5847C>A), TTC1575ATA (5851T>A 5853C>A), CTG1577CTT (5859G>T), AAG1578CGT (5860A>C 5861A>G 5862G>T), AGC1579TCG (5863A>T 5864G>C 5865C>G), GGA1580GGT (5868A>T), TTC1581TAT (5870T>A 5871C>T), ATT1584TTG (5878A>T 5880T>G), ATG1585AAG (5882T>A), ATG1586GTT (5884A>G 5886G>T), GAC1587AAG (5887G>A 5889C>G), GAA1588GAG (5892A>G), GAA1589TGT (5893G>T 5894A>G 5895A>T), TCC1590GAT (5896T>G 5897C>A 5898C>T), ATC1591ATT (5901C>T), CCT1592CCC (5904T>C), TGG1593AAG (5905T>A 5906G>A), ACC1594ACA (5910C>A), GCC1595GCA (5913C>A), CTG1597CGG (5918T>G), ACC1598ACG (5922C>G), CCA1599AGG (5923C>A 5924C>G 5925A>G), GAT1600TAT (5926G>T), CTT1602CAT (5933T>A), TAC1603TTT (5936A>T 5937C>T), GAA1604AAA (5938G>A), TGG1605TTC (5942G>T 5943G>C), TTA1606CTT (5944T>C 5946A>T), CCA1609TCC (5953C>T 5955A>C), TTC1610TTT (5958C>T), GGA1611GGT (5961A>T), TTG1612TTA (5964G>A), AAG1613ACT (5966A>C 5967G>T), AAT1614AAC (5970T>C), GCC1615GCT (5973C>T), CCT1616CCG (5976T>G), GCT1617ACT (5977G>A), GTG1618GTA (5982G>A), TTC1619TTT (5985C>T), CAA1620ATG (5986C>A 5987A>T 5988A>G), CGA1621GAC (5989C>G 5990G>A 5991A>C), AAG1622CTT (5992A>C 5993A>T 5994G>T), GAC1624AAT (5998G>A 6000C>T), AAC1625AGG (6002A>G 6003C>G), TGT1626ATA (6004T>A 6005G>T 6006T>A), AGA1628AAG (6011G>A 6012A>G), GAC1629CCC (6013G>C 6014A>C), GAC1629_CTG1630insTAT (6015_6016insTAT), CTG1630CTC (6018G>C), TCA1631GAC (6019T>G 6020C>A 6021A>C), GGC1632ATG (6022G>A 6023G>T 6024C>G), TTT1633TAT (6026T>A), GTA1634GTG (6030A>G), GCA1635GTT (6032C>T 6033A>T), GTT1636GTG (6036T>G), TAC1637TTC (6038A>T), TTC1644TAT (6059T>A 6060C>T), AGT1645TCT (6061A>T 6062G>C), AAC1646CGA (6064A>C 6065A>G 6066C>A), ACT1647AGC (6068C>G 6069T>C), GAA1648GAG (6072A>G), GAT1649CAA (6073G>C 6075T>A), GAC1650GAT (6078C>T), CAT1651CAC (6081T>C), GCA1652ATG (6082G>A 6083C>T 6084A>G), CAG1653GAA (6085C>G 6087G>A), CAC1654CAT (6090C>T), CTG1655TTG (6091C>T), AAG1656AGG (6095A>G), TCC1657ACG (6097T>A 6099C>G), ATG1658TTA (6100A>T 6102G>A), CTG1659TTG (6103C>T), CAG1660CAA (6108G>A), TGT1662TTG (6113G>T 6114T>G), GAG1663AGG (6115G>A 6116A>G), AGA1664G.. (6118A>G)								

*: Inserts / Deletes / Misaligned / Frameshifts

Analysis details

This analysis was performed with panviral2.64

NGS Details (UN24): Caulimovirus venafragariae

Assembly

**Coverage Length**	262 (1 contig(s))
**Depth Of Coverage**	31.5
**Number Of Reads**	74
**Reads Per Million**	1.48 rpm (after QC)
**Ambiguities**	0
**Assembly Method**	de novo + reference guided assembly
**Consensus Caller**	Bcf Tools

Coverage Map

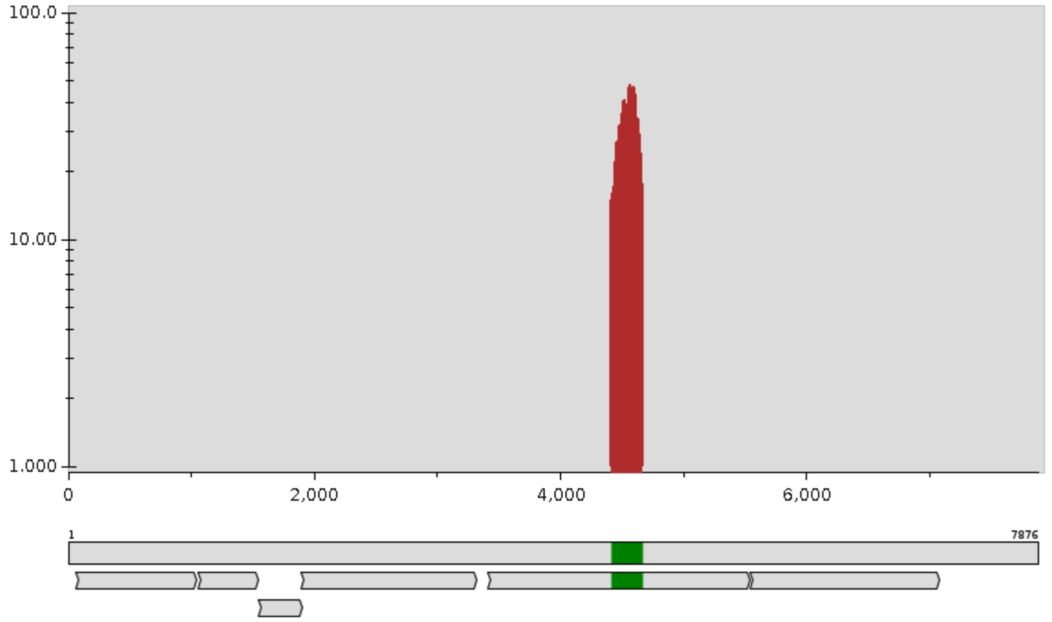

Assignment

**Type**	Caulimovirus venafragariae (Taxonomy ID: 3048344)
**Reference Genome**	NC_001725.1
**NT Identity (%)**	57.6336
**AA Identity (%)**	47.1264
**Number Of Stop Codons**	0
**Number Of CDS**	6

Alignment

**Alignment Score**	80.0 (NT) + 295.0 (AA) = 375.0
**Concordance (%)**	31.9693

Alignment Method	Global, seeded, nucleotide + amino acids (AGA)

Genome Region

Sequence starts at position 4409 and ends at position 4670 relative to NC_001725.1 reference sequence.

Alignment Detailed Statistics

	Begin	End	Coverage	Score	Concordance	Matches	Identities	I/D/M/F*	Stop Codons
**NT**	**4409**	**4670**	**3.3%**	**80**	**15.3%**	**262 (100%)**	**151 (57.6%)**	**0/0**	
Mutations:	4410A>C, 4413T>G, 4414A>G, 4419T>C, 4421A>G, 4426T>C, 4428A>T, 4434C>T, 4435C>G, 4436A>C, 4442A>G, 4443G>A, 4444G>A, 4445G>A, 4450G>C, 4451G>A, 4457T>C, 4459C>G, 4461T>A, 4464T>C, 4465A>T, 4466A>T, 4469A>T, 4470G>T, 4472A>G, 4473G>T, 4477C>A, 4482T>A, 4483C>G, 4485A>C, 4489A>T, 4491C>G, 4493G>C, 4494A>T, 4497T>G, 4498A>C, 4500G>A, 4501A>G, 4502C>A, 4503C>T, 4505T>A, 4509C>T, 4511C>G, 4512T>C, 4514C>T, 4516T>C, 4518T>A, 4521C>T, 4522T>G, 4524T>G, 4525A>T, 4527A>C, 4530T>A, 4535T>A, 4537T>A, 4538G>A, 4539G>T, 4543G>A, 4545A>C, 4546C>A, 4547G>C, 4548C>A, 4549C>A, 4554T>G, 4560A>G, 4561A>G, 4562C>A, 4563A>C, 4564A>C, 4565T>A, 4566T>G, 4567C>G, 4569G>A, 4570C>A, 4571T>A, 4572A>G, 4576G>A, 4583G>C, 4584C>T, 4587T>C, 4590C>T, 4591C>T, 4593A>T, 4597C>A, 4598A>C, 4599C>A, 4602C>T, 4604A>C, 4607G>T, 4608G>C, 4609T>A, 4610T>A, 4611A>G, 4612G>C, 4613T>G, 4614C>G, 4626A>G, 4630A>T, 4631A>G, 4632A>C, 4633C>A, 4635A>T, 4638C>A, 4641T>G, 4644T>G, 4646T>C, 4647T>A, 4650C>T, 4656G>A, 4657C>T, 4658A>G								
**CDS**									
ORF_V	336	422	12.3%	295	44.8%	87 (100%)	41 (47.1%)	0/0/0/0	0
Protein mutations:	I336M (4413T>G), N337D (4414A>G), K339R (4421A>G), H344A (4435C>G 4436A>C), K346R (4442A>G 4443G>A), G347K (4444G>A 4445G>A), G349H (4450G>C 4451G>A), L351P (4457T>C), L352V (4459C>G 4461T>A), N354F (4465A>T 4466A>T), K355I (4469A>T 4470G>T), E356G (4472A>G 4473G>T), L358M (4477C>A), Q360D (4483C>G 4485A>C), I362L (4489A>T 4491C>G), G363A (4493G>C 4494A>T), K365Q (4498A>C 4500G>A), T366D (4501A>G 4502C>A 4503C>T), F367Y (4505T>A), S369C (4511C>G 4512T>C), S370F (4514C>T), F371L (4516T>C 4518T>A), C373G (4522T>G 4524T>G), K374Y (4525A>T 4527A>C), F377Y (4535T>A), W378N (4537T>A 4538G>A 4539G>T), V380I (4543G>A 4545A>C), R381T (4546C>A 4547G>C 4548C>A), L382I (4549C>A), T386D (4561A>G 4562C>A 4563A>C), I387Q (4564A>C 4565T>A 4566T>G), Q388E (4567C>G 4569G>A), L389K (4570C>A 4571T>A 4572A>G), A391T (4576G>A), S393T (4583G>C 4584C>T), Q396Y (4591C>T 4593A>T), H398T (4597C>A 4598A>C 4599C>A), E400A (4604A>C), W401F (4607G>T 4608G>C), L402K (4609T>A 4610T>A 4611A>G), V403R (4612G>C 4613T>G 4614C>G), K409C (4630A>T 4631A>G 4632A>C), Q410N (4633C>A 4635A>T), I414T (4646T>C 4647T>A), H418C (4657C>T 4658A>G)								
Codon mutations:	GTA335.TC (4410A>C), ATT336ATG (4413T>G), AAC337GAC (4414A>G), TAT338TAC (4419T>C), AAA339AGA (4421A>G), TTA341CTT (4426T>C 4428A>T), GAC343GAT (4434C>T), CAT344GCT (4435C>G 4436A>C), AAG346AGA (4442A>G 4443G>A), GGA347AAA (4444G>A 4445G>A), GGC349CAC (4450G>C 4451G>A), CTA351CCA (4457T>C), CTT352GTA (4459C>G 4461T>A), CCT353CCC (4464T>C), AAC354TTC (4465A>T 4466A>T), AAG355ATT (4469A>T 4470G>T), GAG356GGT (4472A>G 4473G>T), CTG358ATG (4477C>A), CTT359CTA (4482T>A), CAA360GAC (4483C>G 4485A>C), ATC362TTG (4489A>T 4491C>G), GGA363GCT (4493G>C 4494A>T), GGT364GGG (4497T>G), AAG365CAA (4498A>C 4500G>A), ACC366GAT (4501A>G 4502C>A 4503C>T), TTT367TAT (4505T>A), TAC368TAT (4509C>T), TCT369TGC (4511C>G 4512T>C), TCC370TTC (4514C>T), TTT371CTA (4516T>C 4518T>A), GAC372GAT (4521C>T), TGT373GGG (4522T>G 4524T>G), AAA374TAC (4525A>T 4527A>C), TCT375TCA (4530T>A), TTT377TAT (4535T>A), TGG378AAT (4537T>A 4538G>A 4539G>T), GTA380ATC (4543G>A 4545A>C), CGC381ACA (4546C>A 4547G>C 4548C>A), CTT382ATT (4549C>A), GCT383GCG (4554T>G), GAA385GAG (4560A>G), ACA386GAC (4561A>G 4562C>A 4563A>C), ATT387CAG (4564A>C 4565T>A 4566T>G), CAG388GAA (4567C>G 4569G>A), CTA389AAG (4570C>A 4571T>A 4572A>G), GCT391ACT (4576G>A), AGC393ACT (4583G>C 4584C>T), TGT394TGC (4587T>C), CCC395CCT (4590C>T), CAA396TAT (4591C>T 4593A>T), CAC398ACA (4597C>A 4598A>C 4599C>A), TAC399TAT (4602C>T), GAA400GCA (4604A>C), TGG401TTC (4607G>T 4608G>C), TTA402AAG (4609T>A 4610T>A 4611A>G), GTC403CGG (4612G>C 4613T>G 4614C>G), GGA407GGG (4626A>G), AAA409TGC (4630A>T 4631A>G 4632A>C), CAA410AAT (4633C>A 4635A>T), GCC411GCA (4638C>A), CCT412CCG (4641T>G), GCT413GCG (4644T>G), ATT414ACA (4646T>C 4647T>A), TTC415TTT (4650C>T), AGG417AGA (4656G>A), CAC418TGC (4657C>T 4658A>G)								
**Proteins**									
hypothetical protein (NP_043933.1)	336	422	12.3%	295	44.8%	87 (100%)	41 (47.1%)	0/0/0/0	0
Protein mutations:	I336M (4413T>G), N337D (4414A>G), K339R (4421A>G), H344A (4435C>G 4436A>C), K346R (4442A>G 4443G>A), G347K (4444G>A 4445G>A), G349H (4450G>C 4451G>A), L351P (4457T>C), L352V (4459C>G 4461T>A), N354F (4465A>T 4466A>T), K355I (4469A>T 4470G>T), E356G (4472A>G 4473G>T), L358M (4477C>A), Q360D (4483C>G 4485A>C), I362L (4489A>T 4491C>G), G363A (4493G>C 4494A>T), K365Q (4498A>C 4500G>A), T366D (4501A>G 4502C>A 4503C>T), F367Y (4505T>A), S369C (4511C>G 4512T>C), S370F (4514C>T), F371L (4516T>C 4518T>A), C373G (4522T>G 4524T>G), K374Y (4525A>T 4527A>C), F377Y (4535T>A), W378N (4537T>A 4538G>A 4539G>T), V380I (4543G>A 4545A>C), R381T (4546C>A 4547G>C 4548C>A), L382I (4549C>A), T386D (4561A>G 4562C>A 4563A>C), I387Q (4564A>C 4565T>A 4566T>G), Q388E (4567C>G 4569G>A), L389K (4570C>A 4571T>A 4572A>G), A391T (4576G>A), S393T (4583G>C 4584C>T), Q396Y (4591C>T 4593A>T), H398T (4597C>A 4598A>C 4599C>A), E400A (4604A>C), W401F (4607G>T 4608G>C), L402K (4609T>A 4610T>A 4611A>G), V403R (4612G>C 4613T>G 4614C>G), K409C (4630A>T 4631A>G 4632A>C), Q410N (4633C>A 4635A>T), I414T (4646T>C 4647T>A), H418C (4657C>T 4658A>G)								
Codon mutations:	GTA335.TC (4410A>C), ATT336ATG (4413T>G), AAC337GAC (4414A>G), TAT338TAC (4419T>C), AAA339AGA (4421A>G), TTA341CTT (4426T>C 4428A>T), GAC343GAT (4434C>T), CAT344GCT (4435C>G 4436A>C), AAG346AGA (4442A>G 4443G>A), GGA347AAA (4444G>A 4445G>A), GGC349CAC (4450G>C 4451G>A), CTA351CCA (4457T>C), CTT352GTA (4459C>G 4461T>A), CCT353CCC (4464T>C), AAC354TTC (4465A>T 4466A>T), AAG355ATT (4469A>T 4470G>T), GAG356GGT (4472A>G 4473G>T), CTG358ATG (4477C>A), CTT359CTA (4482T>A), CAA360GAC (4483C>G 4485A>C), ATC362TTG (4489A>T 4491C>G), GGA363GCT (4493G>C 4494A>T), GGT364GGG (4497T>G), AAG365CAA (4498A>C 4500G>A), ACC366GAT (4501A>G 4502C>A 4503C>T), TTT367TAT (4505T>A), TAC368TAT (4509C>T), TCT369TGC (4511C>G 4512T>C), TCC370TTC (4514C>T), TTT371CTA (4516T>C 4518T>A), GAC372GAT (4521C>T), TGT373GGG (4522T>G 4524T>G), AAA374TAC (4525A>T 4527A>C), TCT375TCA (4530T>A), TTT377TAT (4535T>A), TGG378AAT (4537T>A 4538G>A 4539G>T), GTA380ATC (4543G>A 4545A>C), CGC381ACA (4546C>A 4547G>C 4548C>A), CTT382ATT (4549C>A), GCT383GCG (4554T>G), GAA385GAG (4560A>G), ACA386GAC (4561A>G 4562C>A 4563A>C), ATT387CAG (4564A>C 4565T>A 4566T>G), CAG388GAA (4567C>G 4569G>A), CTA389AAG (4570C>A 4571T>A 4572A>G), GCT391ACT (4576G>A), AGC393ACT (4583G>C 4584C>T), TGT394TGC (4587T>C), CCC395CCT (4590C>T), CAA396TAT (4591C>T 4593A>T), CAC398ACA (4597C>A 4598A>C 4599C>A), TAC399TAT (4602C>T), GAA400GCA (4604A>C), TGG401TTC (4607G>T 4608G>C), TTA402AAG (4609T>A 4610T>A 4611A>G), GTC403CGG (4612G>C 4613T>G 4614C>G), GGA407GGG (4626A>G), AAA409TGC (4630A>T 4631A>G 4632A>C), CAA410AAT (4633C>A 4635A>T), GCC411GCA (4638C>A), CCT412CCG (4641T>G), GCT413GCG (4644T>G), ATT414ACA (4646T>C 4647T>A), TTC415TTT (4650C>T), AGG417AGA (4656G>A), CAC418TGC (4657C>T 4658A>G)								

*: Inserts / Deletes / Misaligned / Frameshifts

Analysis details

This analysis was performed with panviral2.64

NGS Details (UN24): Badnavirus volubetulae

Assembly

**Coverage Length**	244 (1 contig(s))
**Depth Of Coverage**	27.0
**Number Of Reads**	63
**Reads Per Million**	1.26 rpm (after QC)
**Ambiguities**	0
**Assembly Method**	de novo + reference guided assembly
**Consensus Caller**	Bcf Tools

Coverage Map

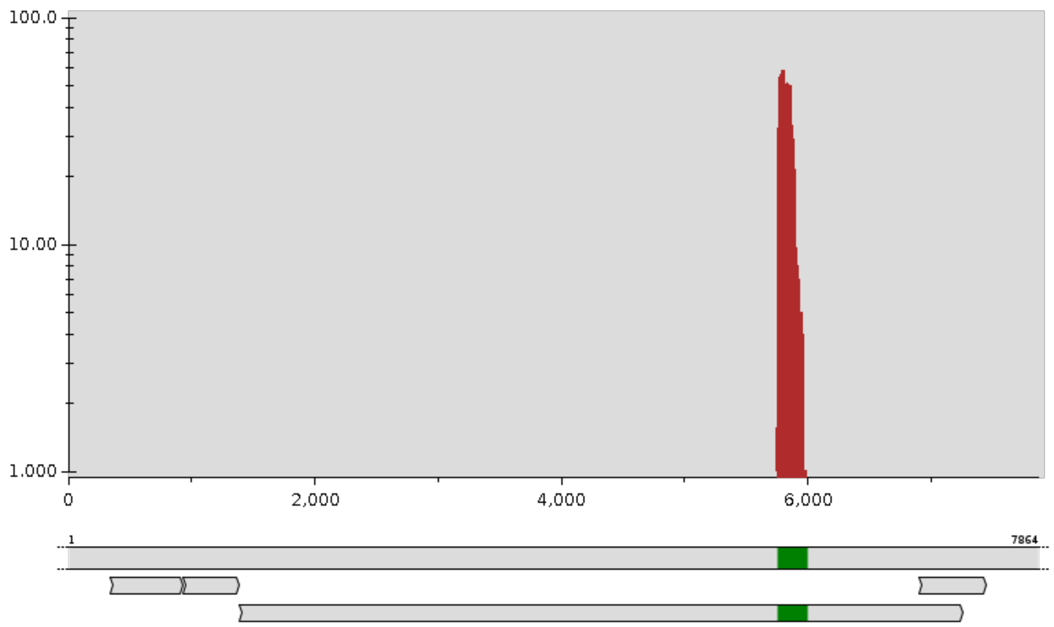

Assignment

**Type**	Badnavirus volubetulae (Taxonomy ID: 3047683)
**Reference Genome**	NC_040635.1
**NT Identity (%)**	54.0984
**AA Identity (%)**	49.3827
**Number Of Stop Codons**	1
**Number Of CDS**	4

Alignment

**Alignment Score**	40.0 (NT) + 267.0 (AA) = 307.0
**Concordance (%)**	29.0445

Alignment Method	Global, seeded, nucleotide + amino acids (AGA)

Genome Region

Sequence starts at position 5753 and ends at position 5996 relative to NC_040635.1 reference sequence.

Alignment Detailed Statistics

	Begin	End	Coverage	Score	Concordance	Matches	Identities	I/D/M/F*	Stop Codons
**NT**	**5753**	**5996**	**3.1%**	**40**	**8.2%**	**244 (100%)**	**132 (54.1%)**	**0/0**	
Mutations:	5755G>T, 5756T>A, 5757C>T, 5758C>A, 5760C>T, 5770A>C, 5772T>G, 5773C>T, 5779C>A, 5781G>A, 5782A>G, 5783A>T, 5784C>G, 5788C>A, 5789A>T, 5790C>A, 5794G>A, 5797C>A, 5799A>G, 5802C>T, 5803A>C, 5804G>C, 5806C>T, 5808A>G, 5811A>G, 5812G>C, 5814A>T, 5817C>T, 5820C>T, 5821A>G, 5822C>A, 5823T>C, 5824A>T, 5826T>G, 5827G>T, 5829C>T, 5830C>G, 5832G>T, 5834G>A, 5835C>G, 5836A>C, 5839G>C, 5840G>A, 5841T>G, 5842A>G, 5843A>G, 5844T>A, 5847A>T, 5848A>T, 5849A>C, 5850G>C, 5853A>G, 5855T>C, 5859A>T, 5862A>G, 5863T>A, 5868T>C, 5869C>T, 5871T>G, 5873A>G, 5875A>T, 5876G>C, 5877T>G, 5880C>T, 5882T>A, 5889A>G, 5890G>T, 5891T>A, 5892C>G, 5894C>G, 5895C>T, 5896A>G, 5898G>T, 5899G>C, 5900A>G, 5901T>G, 5904A>G, 5905G>T, 5906A>C, 5908T>G, 5909C>A, 5910A>T, 5913A>C, 5916A>G, 5917T>A, 5918G>A, 5922A>T, 5923G>A, 5925A>T, 5928C>T, 5930T>G, 5931C>G, 5935C>T, 5936C>G, 5937A>C, 5938G>T, 5943G>T, 5944T>C, 5945T>A, 5946G>T, 5949C>T, 5954G>T, 5955G>T, 5956C>A, 5958A>G, 5965C>T, 5967A>C, 5973T>G, 5974C>T, 5978A>C, 5979A>C, 5988A>T								
**CDS**									
BLRaV_gp3	1456	1536	4.1%	267	45.6%	81 (100%)	40 (49.4%)	0/0/0/0	1
Protein mutations:	V1456Y (5755G>T 5756T>A 5757C>T), L1457I (5758C>A 5760C>T), S1461R (5770A>C 5772T>G), Q1464K (5779C>A 5781G>A), N1465V (5782A>G 5783A>T 5784C>G), H1467I (5788C>A 5789A>T 5790C>A), D1469N (5794G>A), Q1470K (5797C>A 5799A>G), S1472P (5803A>C 5804G>C), G1475R (5812G>C 5814A>T), T1478D (5821A>G 5822C>A 5823T>C), I1479L (5824A>T 5826T>G), V1480F (5827G>T 5829C>T), Q1481D (5830C>G 5832G>T), R1482Q (5834G>A 5835C>G), I1483L (5836A>C), G1484Q (5839G>C 5840G>A 5841T>G), N1485G (5842A>G 5843A>G 5844T>A), K1487S (5848A>T 5849A>C 5850G>C), F1489S (5855T>C), F1492I (5863T>A), K1495R (5873A>G), F1498Y (5882T>A), V1501* (5890G>T 5891T>A 5892C>G), T1502S (5894C>G 5895C>T), M1503V (5896A>G 5898G>T), D1504R (5899G>C 5900A>G 5901T>G), E1506S (5905G>T 5906A>C), S1507D (5908T>G 5909C>A 5910A>T), W1510K (5917T>A 5918G>A), A1512T (5923G>A 5925A>T), L1514R (5930T>G 5931C>G), P1516C (5935C>T 5936C>G 5937A>C), D1517Y (5938G>T), L1519H (5944T>C 5945T>A 5946G>T), W1522F (5954G>T 5955G>T), L1523M (5956C>A 5958A>G), P1526S (5965C>T 5967A>C), L1529F (5974C>T), K1530T (5978A>C 5979A>C)								
Codon mutations:	GTC1456TAT (5755G>T 5756T>A 5757C>T), CTC1457ATT (5758C>A 5760C>T), AGT1461CGG (5770A>C 5772T>G), CTG1462TTG (5773C>T), CAG1464AAA (5779C>A 5781G>A), AAC1465GTG (5782A>G 5783A>T 5784C>G), CAC1467ATA (5788C>A 5789A>T 5790C>A), GAT1469AAT (5794G>A), CAA1470AAG (5797C>A 5799A>G), TAC1471TAT (5802C>T), AGC1472CCC (5803A>C 5804G>C), CTA1473TTG (5806C>T 5808A>G), CCA1474CCG (5811A>G), GGA1475CGT (5812G>C 5814A>T), ATC1476ATT (5817C>T), AAC1477AAT (5820C>T), ACT1478GAC (5821A>G 5822C>A 5823T>C), ATT1479TTG (5824A>T 5826T>G), GTC1480TTT (5827G>T 5829C>T), CAG1481GAT (5830C>G 5832G>T), CGC1482CAG (5834G>A 5835C>G), ATA1483CTA (5836A>C), GGT1484CAG (5839G>C 5840G>A 5841T>G), AAT1485GGA (5842A>G 5843A>G 5844T>A), GCA1486GCT (5847A>T), AAG1487TCC (5848A>T 5849A>C 5850G>C), GTA1488GTG (5853A>G), TTC1489TCC (5855T>C), TCA1490TCT (5859A>T), AAA1491AAG (5862A>G), TTC1492ATC (5863T>A), GAT1493GAC (5868T>C), CTT1494TTG (5869C>T 5871T>G), AAG1495AGG (5873A>G), AGT1496TCG (5875A>T 5876G>C 5877T>G), GGC1497GGT (5880C>T), TTT1498TAT (5882T>A), CAA1500CAG (5889A>G), GTC1501TAG (5890G>T 5891T>A 5892C>G), ACC1502AGT (5894C>G 5895C>T), ATG1503GTT (5896A>G 5898G>T), GAT1504CGG (5899G>C 5900A>G 5901T>G), GAA1505GAG (5904A>G), GAA1506TCA (5905G>T 5906A>C), TCA1507GAT (5908T>G 5909C>A 5910A>T), ATA1508ATC (5913A>C), CCA1509CCG (5916A>G), TGG1510AAG (5917T>A 5918G>A), ACA1511ACT (5922A>T), GCA1512ACT (5923G>A 5925A>T), TTC1513TTT (5928C>T), CTC1514CGG (5930T>G 5931C>G), CCA1516TGC (5935C>T 5936C>G 5937A>C), GAT1517TAT (5938G>T), GGG1518GGT (5943G>T), TTG1519CAT (5944T>C 5945T>A 5946G>T), TAC1520TAT (5949C>T), TGG1522TTT (5954G>T 5955G>T), CTA1523ATG (5956C>A 5958A>G), CCA1526TCC (5965C>T 5967A>C), GGT1528GGG (5973T>G), CTT1529TTT (5974C>T), AAA1530ACC (5978A>C 5979A>C), CCA1533CCT (5988A>T)								
**Proteins**									
polyprotein (YP_009552737.1)	1456	1536	4.1%	267	45.6%	81 (100%)	40 (49.4%)	0/0/0/0	1
Protein mutations:	V1456Y (5755G>T 5756T>A 5757C>T), L1457I (5758C>A 5760C>T), S1461R (5770A>C 5772T>G), Q1464K (5779C>A 5781G>A), N1465V (5782A>G 5783A>T 5784C>G), H1467I (5788C>A 5789A>T 5790C>A), D1469N (5794G>A), Q1470K (5797C>A 5799A>G), S1472P (5803A>C 5804G>C), G1475R (5812G>C 5814A>T), T1478D (5821A>G 5822C>A 5823T>C), I1479L (5824A>T 5826T>G), V1480F (5827G>T 5829C>T), Q1481D (5830C>G 5832G>T), R1482Q (5834G>A 5835C>G), I1483L (5836A>C), G1484Q (5839G>C 5840G>A 5841T>G), N1485G (5842A>G 5843A>G 5844T>A), K1487S (5848A>T 5849A>C 5850G>C), F1489S (5855T>C), F1492I (5863T>A), K1495R (5873A>G), F1498Y (5882T>A), V1501* (5890G>T 5891T>A 5892C>G), T1502S (5894C>G 5895C>T), M1503V (5896A>G 5898G>T), D1504R (5899G>C 5900A>G 5901T>G), E1506S (5905G>T 5906A>C), S1507D (5908T>G 5909C>A 5910A>T), W1510K (5917T>A 5918G>A), A1512T (5923G>A 5925A>T), L1514R (5930T>G 5931C>G), P1516C (5935C>T 5936C>G 5937A>C), D1517Y (5938G>T), L1519H (5944T>C 5945T>A 5946G>T), W1522F (5954G>T 5955G>T), L1523M (5956C>A 5958A>G), P1526S (5965C>T 5967A>C), L1529F (5974C>T), K1530T (5978A>C 5979A>C)								
Codon mutations:	GTC1456TAT (5755G>T 5756T>A 5757C>T), CTC1457ATT (5758C>A 5760C>T), AGT1461CGG (5770A>C 5772T>G), CTG1462TTG (5773C>T), CAG1464AAA (5779C>A 5781G>A), AAC1465GTG (5782A>G 5783A>T 5784C>G), CAC1467ATA (5788C>A 5789A>T 5790C>A), GAT1469AAT (5794G>A), CAA1470AAG (5797C>A 5799A>G), TAC1471TAT (5802C>T), AGC1472CCC (5803A>C 5804G>C), CTA1473TTG (5806C>T 5808A>G), CCA1474CCG (5811A>G), GGA1475CGT (5812G>C 5814A>T), ATC1476ATT (5817C>T), AAC1477AAT (5820C>T), ACT1478GAC (5821A>G 5822C>A 5823T>C), ATT1479TTG (5824A>T 5826T>G), GTC1480TTT (5827G>T 5829C>T), CAG1481GAT (5830C>G 5832G>T), CGC1482CAG (5834G>A 5835C>G), ATA1483CTA (5836A>C), GGT1484CAG (5839G>C 5840G>A 5841T>G), AAT1485GGA (5842A>G 5843A>G 5844T>A), GCA1486GCT (5847A>T), AAG1487TCC (5848A>T 5849A>C 5850G>C), GTA1488GTG (5853A>G), TTC1489TCC (5855T>C), TCA1490TCT (5859A>T), AAA1491AAG (5862A>G), TTC1492ATC (5863T>A), GAT1493GAC (5868T>C), CTT1494TTG (5869C>T 5871T>G), AAG1495AGG (5873A>G), AGT1496TCG (5875A>T 5876G>C 5877T>G), GGC1497GGT (5880C>T), TTT1498TAT (5882T>A), CAA1500CAG (5889A>G), GTC1501TAG (5890G>T 5891T>A 5892C>G), ACC1502AGT (5894C>G 5895C>T), ATG1503GTT (5896A>G 5898G>T), GAT1504CGG (5899G>C 5900A>G 5901T>G), GAA1505GAG (5904A>G), GAA1506TCA (5905G>T 5906A>C), TCA1507GAT (5908T>G 5909C>A 5910A>T), ATA1508ATC (5913A>C), CCA1509CCG (5916A>G), TGG1510AAG (5917T>A 5918G>A), ACA1511ACT (5922A>T), GCA1512ACT (5923G>A 5925A>T), TTC1513TTT (5928C>T), CTC1514CGG (5930T>G 5931C>G), CCA1516TGC (5935C>T 5936C>G 5937A>C), GAT1517TAT (5938G>T), GGG1518GGT (5943G>T), TTG1519CAT (5944T>C 5945T>A 5946G>T), TAC1520TAT (5949C>T), TGG1522TTT (5954G>T 5955G>T), CTA1523ATG (5956C>A 5958A>G), CCA1526TCC (5965C>T 5967A>C), GGT1528GGG (5973T>G), CTT1529TTT (5974C>T), AAA1530ACC (5978A>C 5979A>C), CCA1533CCT (5988A>T)								

*: Inserts / Deletes / Misaligned / Frameshifts

Analysis details

This analysis was performed with panviral2.64

NGS Details (UN24): Yellowstone lake phycodnavirus 1

Assembly

**Coverage Length**	135 (1 contig(s))
**Depth Of Coverage**	35.6
**Number Of Reads**	55
**Reads Per Million**	1.10 rpm (after QC)
**Ambiguities**	0
**Assembly Method**	de novo + reference guided assembly
**Consensus Caller**	Bcf Tools

Coverage Map

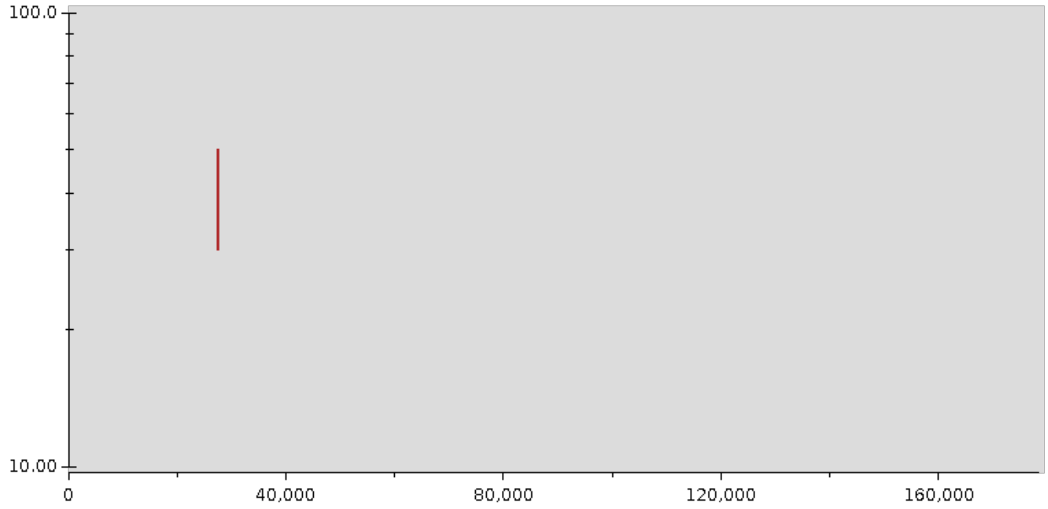

Assignment

**Type**	Yellowstone lake phycodnavirus 1 (Taxonomy ID: 1586713)
**Reference Genome**	NC_028112.1
**NT Identity (%)**	83.7037
**AA Identity (%)**	93.3333
**Number Of Stop Codons**	0
**Number Of CDS**	248

Alignment

**Alignment Score**	182.0 (NT) + 273.0 (AA) = 455.0
**Concordance (%)**	82.13
**Alignment Method**	Local, heuristic, nucleotide (BLASTN)

Genome Region

Sequence starts at position 27473 and ends at position 27607 relative to NC_028112.1 reference sequence.

Alignment Detailed Statistics

	Begin	End	Coverage	Score	Concordance	Matches	Identities	I/D/M/F*	Stop Codons
NT	27473	27607	0.1%	182	67.4%	135 (100%)	113 (83.7%)	0/0	

Mutations: 27486G>A, 27492A>G, 27507G>A, 27510A>G, 27513T>C, 27529C>A, 27531T>G, 27534A>G, 27537T>C, 27540T>C, 27552A>G, 27563A>G, 27564T>C, 27567T>C, 27570C>T, 27573A>T, 27576A>G, 27582T>C, 27586G>A, 27591A>G, 27597A>G, 27600C>A

*: Inserts / Deletes / Misaligned / Frameshifts

Analysis details

This analysis was performed with panviral2.64

NGS Details (UN24): Caulimovirus tessellobrassicae

Assembly

**Coverage Length**	812 (1 contig(s))
**Depth Of Coverage**	4.8
**Number Of Reads**	32
**Reads Per Million**	0.64 rpm (after QC)
**Ambiguities**	0
**Assembly Method**	de novo + reference guided assembly
**Consensus Caller**	Bcf Tools

Coverage Map

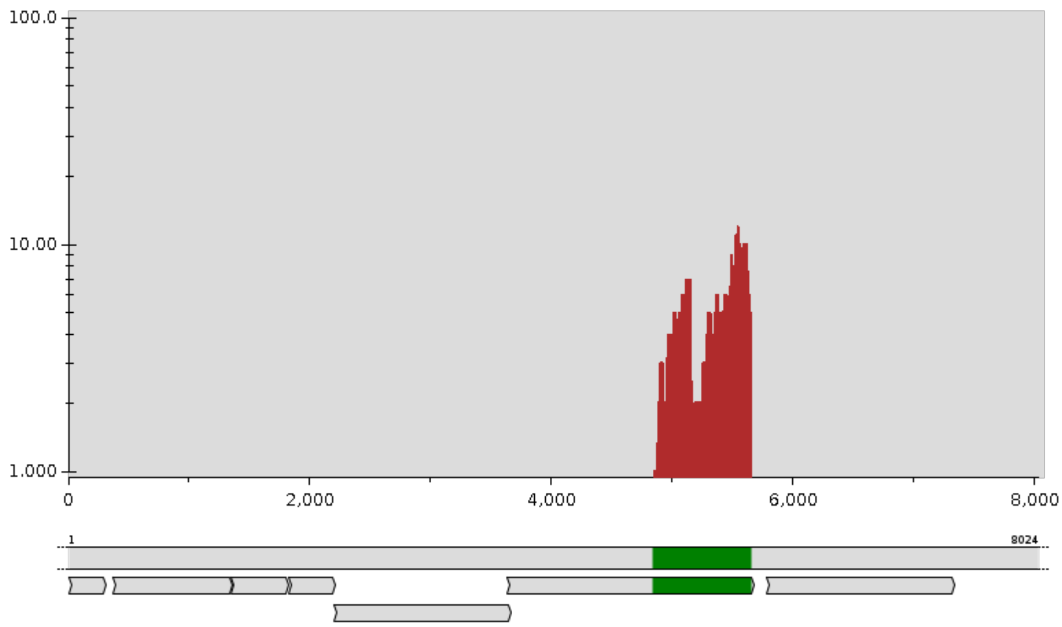

Assignment

**Type**	Caulimovirus tessellobrassicae (Taxonomy ID: 3047738)
**Reference Genome**	NC_001497.2
**NT Identity (%)**	55.4293
**AA Identity (%)**	44.9057
**Number Of Stop Codons**	1
**Number Of CDS**	7

Alignment

**Alignment Score**	116.0 (NT) + 668.0 (AA) = 784.0
**Concordance (%)**	23.3403

**Alignment Method**	Global, seeded, nucleotide + amino acids (AGA)

Genome Region

Sequence starts at position 4845 and ends at position 5656 relative to NC_001497.2 reference sequence.

Alignment Detailed Statistics

	Begin	End	Coverage	Score	Concordance	Matches	Identities	I/D/M/F*	Stop Codons
**NT**	**4845**	**5656**	**10.1%**	**116**	**7.6%**	**788 (96.6%)**	**439 (53.8%)**	**4/24**	
Mutations:	4849C>T, 4851C>G, 4856T>G, 4858A>T, 4859G>C, 4860T>A, 4862A>C, 4863C>A, 4865A>C, 4866C>A, 4867G>T, 4869A>T, 4870G>C, 4872A>G, 4875T>A, 4881A>T, 4882C>A, 4883T>C, 4884T>A, 4887C>T, 4888G>C, 4890A>T, 4891G>A, 4892C>A, 4894A>G, 4895T>A, 4896G>A, 4897A>T, 4899C>T, 4901T>C, 4903C>A, 4906A>C, 4907A>T, 4908G>C, 4912A>G, 4913A>T, 4914T>C, 4915C>A, 4917A>T, 4918C>A, 4922G>A, 4923A>C, 4926T>A, 4927A>G, 4929C>A, 4930C>T, 4932T>A, 4933T>A, 4934C>G, 4935C>T, 4936A>C, 4941G>A, 4948C>G, 4951C>A, 4953C>A, 4954T>A, 4955T>A, 4956C>T, 4959G>A, 4962G>T, 4965G>A, 4969A>G, 4971C>A, 4980T>A, 4981C>A, 4983A>G, 4984G>A, 4985A>T, 4986A>T, 4989A>T, 4990G>T, 4991A>C, 4992T>A, 4993G>A, 4996G>A, 4997G>A, 4998A>T, 4999A>G, 5000C>G, 5002C>A, 5003A>T, 5004T>A, 5005A>G, 5007G>A, 5008C>T, 5009C>T, 5010T>A, 5014G>A, 5015G>C, 5016A>T, 5019T>C, 5022C>A, 5024T>C, 5025G>C, 5026G>A, 5029C>A, 5031C>G, 5034C>A, 5035A>T, 5036A>T, 5037C>A, 5038A>G, 5043C>T, 5046C>T, 5049T>C, 5051C>A, 5052C>G, 5053C>T, 5055T>A, 5056G>A, 5057A>T, 5059G>A, 5060A>C, 5064G>A, 5065A>C, 5067G>A, 5068C>G, 5071C>A, 5073T>A, 5076G>A, 5082C>T, 5083T>C, 5085A>T, 5088C>A, 5089A>T, 5090T>G, 5091A>C, 5092C>T, 5096C>A, 5097A>C, 5103C>A, 5104T>G, 5105C>G, 5106G>A, 5109T>A, 5111A>T, 5115C>A, 5118G>A, 5119A>G, 5121G>A, 5122C>T, 5127T>A, 5128C>A, 5130A>G, 5132T>A, 5133C>G, 5139G>T, 5141C>T, 5145G>T, 5149_5151delGCC, 5154G>A, 5155C>A, 5156T>C, 5158A>C, 5161G>C, 5166C>A, 5167G>A, 5169T>C, 5170C>A, 5171C>A, 5174G>T, 5175_5176insATTA, 5176A>G, 5187A>T, 5190G>A, 5191G>C, 5196C>T, 5199C>T, 5201A>G, 5202C>T, 5203A>G, 5205G>A, 5206C>A, 5211G>A, 5212G>C, 5214G>C, 5217G>A, 5218A>G, 5221A>G, 5223T>A, 5224C>T, 5225T>G, 5226G>T, 5227C>A, 5228A>T, 5229A>G, 5230G>A, 5231G>A, 5232A>G, 5233T>C, 5238T>A, 5239C>A, 5240C>A, 5241A>G, 5242C>A, 5244A>T, 5245C>A, 5246A>G, 5247T>A, 5248C>T, 5249A>C, 5250T>A, 5253C>A, 5254T>G, 5255T>A, 5262G>A, 5265G>C, 5268G>T, 5272A>G, 5274C>A, 5275A>T, 5277C>A, 5278G>C, 5280G>A, 5283C>A, 5289A>C, 5292A>C, 5295C>T, 5296G>T, 5298C>T, 5299T>C, 5301C>T, 5309G>C, 5310T>A, 5311A>T, 5314T>C, 5317A>C, 5320G>A, 5322T>A, 5323_5325delATC, 5326A>G, 5328A>C, 5329A>C, 5331T>A, 5338_5355delGGTACTAATACTGAGTTA, 5361C>T, 5363G>A, 5364A>G, 5367C>T, 5368G>A, 5371T>A, 5372C>G, 5378G>C, 5379C>T, 5385A>C, 5387C>A, 5388T>A, 5392G>A, 5395A>G, 5396A>T, 5397G>T, 5403C>T, 5404C>T, 5405A>C, 5408G>C, 5409C>A, 5415C>A, 5421G>A, 5422A>T, 5423C>T, 5427G>A, 5430G>A, 5434A>G, 5438A>G, 5439T>A, 5440A>G, 5441C>G, 5442T>A, 5446A>C, 5448G>A, 5455A>T, 5456G>C, 5457T>G, 5458A>G, 5459T>C, 5462A>G, 5463T>C, 5464C>T, 5467A>T, 5468C>T, 5469T>A, 5472T>A, 5473G>A, 5474T>A, 5475T>A, 5476C>T, 5477A>C, 5478T>A, 5482C>G, 5484G>T, 5489G>A, 5490G>A, 5505T>A, 5506T>A, 5508C>A, 5509A>T, 5510A>C, 5511G>A, 5512A>G, 5514T>G, 5517C>G, 5518G>A, 5520T>A, 5521A>T, 5522A>T, 5524C>A, 5525T>A, 5529T>A, 5530T>C, 5531A>T, 5533A>C, 5534A>C, 5535A>T, 5536G>T, 5537G>C, 5538A>C, 5541T>A, 5542T>C, 5544G>C, 5545A>C, 5548C>T, 5549T>A, 5551G>A, 5557A>C, 5558A>T, 5559C>G, 5560A>C, 5561T>A, 5562C>T, 5568G>A, 5573C>T, 5574A>T, 5576G>T, 5581A>T, 5582G>C, 5583C>G, 5584C>T, 5585A>T, 5592A>T, 5596G>A, 5598T>A, 5599G>A, 5605C>A, 5607C>T, 5610T>A, 5619C>T, 5622C>T, 5625C>T, 5626C>T, 5627A>T, 5628C>T, 5634G>A, 5637C>T								
**CDS**									
CaMVgp6	405	675	39.9%	668	36.1%	263 (96.3%)	119 (43.6%)	2/8/1/1	1
Protein mutations:	F408C (4856T>G), N410T (4862A>C 4863C>A), N411T (4865A>C 4866C>A), E412Y (4867G>T 4869A>T), E413Q (4870G>C 4872A>G), D414E (4875T>A), L417T (4882C>A 4883T>C 4884T>A), V419L (4888G>C 4890A>T), A420K (4891G>A 4892C>A), M421E (4894A>G 4895T>A 4896G>A), I422F (4897A>T 4899C>T), L423S (4901T>C), Q424K (4903C>A), K425L (4906A>C 4907A>T 4908G>C), N427V (4912A>G 4913A>T 4914T>C), Q428N (4915C>A 4917A>T), H429N (4918C>A), G430D (4922G>A 4923A>C), I432V (4927A>G 4929C>A), K435Q (4936A>C), Q439E (4948C>G), L440I (4951C>A 4953C>A), F441N (4954T>A 4955T>A 4956C>T), K443N (4962G>T), N446E (4969A>G 4971C>A), L450M (4981C>A 4983A>G), E451I (4984G>A 4985A>T 4986A>T), D453S (4990G>T 4991A>C 4992T>A), E454K (4993G>A), G455N (4996G>A 4997G>A 4998A>T), T456G (4999A>G 5000C>G), H457I (5002C>A 5003A>T 5004T>A), K458E (5005A>G 5007G>A), P459L (5008C>T 5009C>T 5010T>A), G461T (5014G>A 5015G>C 5016A>T), L464S (5024T>C 5025G>C), E465K (5026G>A), H466K (5029C>A 5031C>G), N468L (5035A>T 5036A>T 5037C>A), K469E (5038A>G), T473K (5051C>A 5052C>G), E475I (5056G>A 5057A>T), D476T (5059G>A 5060A>C), K478Q (5065A>C 5067G>A), Q479E (5068C>G), L480I (5071C>A 5073T>A), I486C (5089A>T 5090T>G 5091A>C), T488N (5096C>A 5097A>C), S491G (5104T>G 5105C>G 5106G>A), D492E (5109T>A), Y493F (5111A>T), K496E (5119A>G 5121G>A), Q499K (5128C>A 5130A>G), I500K (5132T>A 5133C>G), K502N (5139G>T), P503L (5141C>T), A506del (5149_5151delGCC), L508T (5155C>A 5156T>C), K509Q (5158A>C), E510Q (5161G>C), N511K (5166C>A), V512I (5167G>A 5169T>C), P513K (5170C>A 5171C>A), W514L (5174G>T), W514_R515insIX (5175_5176insATTA), R515G (5176A>G), K518N (5187A>T), D520H (5191G>C), Y523C (5201A>G 5202C>T), M524V (5203A>G 5205G>A), Q525K (5206C>A), V527L (5212G>C 5214G>C), K529E (5218A>G), N530E (5221A>G 5223T>A), L531C (5224C>T 5225T>G 5226G>T), Q532M (5227C>A 5228A>T 5229A>G), G533K (5230G>A 5231G>A 5232A>G), F534L (5233T>C), P536K (5239C>A 5240C>A 5241A>G), L537I (5242C>A 5244A>T), H538R (5245C>A 5246A>G 5247T>A), H539S (5248C>T 5249A>C 5250T>A), L541E (5254T>G 5255T>A), E544D (5265G>C), K545N (5268G>T), I547V (5272A>G 5274C>A), I548L (5275A>T 5277C>A), E549Q (5278G>C 5280G>A), D555Y (5296G>T 5298C>T), Y556H (5299T>C 5301C>T), G559A (5309G>C 5310T>A), M560L (5311A>T), K562Q (5317A>C), A563T (5320G>A 5322T>A), I564del (5323_5325delATC), K565D (5326A>G 5328A>C), I566L (5329A>C 5331T>A), G569_L574del (5338_5355delGGTACTAATACTGAGTTA), R577K (5363G>A 5364A>G), A579T (5368G>A), S582T (5378G>C 5379C>T), K584N (5385A>C), A585E (5387C>A 5388T>A), E587K (5392G>A), K588V (5395A>G 5396A>T 5397G>T), H591S (5404C>T 5405A>C), S592T (5408G>C 5409C>A), D594E (5415C>A), T597L (5422A>T 5423C>T), I601V (5434A>G), N602R (5438A>G 5439T>A), T603G (5440A>G 5441C>G 5442T>A), K605Q (5446A>C 5448G>A), I609A (5458A>G 5459T>C), Y610C (5462A>G 5463T>C), T612L (5467A>T 5468C>T 5469T>A), V614K (5473G>A 5474T>A 5475T>A), H615S (5476C>T 5477A>C 5478T>A), L617V (5482C>G 5484G>T), R619K (5489G>A 5490G>A), H624Q (5505T>A), F625I (5506T>A 5508C>A), K626S (5509A>T 5510A>C 5511G>A), S627G (5512A>G 5514T>G), F628L (5517C>G), V629I (5518G>A 5520T>A), N630F (5521A>T 5522A>T), L631N (5524C>A 5525T>A), N632K (5529T>A), Y633L (5530T>C 5531A>T), K634P (5533A>C 5534A>C 5535A>T), G635S (5536G>T 5537G>C 5538A>C), D636E (5541T>A), S637P (5542T>C 5544G>C), K638Q (5545A>C), L639Y (5548C>T 5549T>A), G640R (5551G>A), N642L (5557A>C 5558A>T 5559C>G), I643H (5560A>C 5561T>A 5562C>T), W645* (5568G>A), A647V (5573C>T 5574A>T), W648L (5576G>T), H651F (5584C>T 5585A>T), D655K (5596G>A 5598T>A), V656I (5599G>A), H658N (5605C>A 5607C>T), H665F (5626C>T 5627A>T 5628C>T)								
Codon mutations:	CTC406TTG (4849C>T 4851C>G), TTC408TGC (4856T>G), AGT409TCA (4858A>T 4859G>C 4860T>A), AAC410ACA (4862A>C 4863C>A), AAC411ACA (4865A>C 4866C>A), GAA412TAT (4867G>T 4869A>T), GAA413CAG (4870G>C 4872A>G), GAT414GAA (4875T>A), CTA416CTT (4881A>T), CTT417ACA (4882C>A 4883T>C 4884T>A), CAC418CAT (4887C>T), GTA419CTT (4888G>C 4890A>T), GCA420AAA (4891G>A 4892C>A), ATG421GAA (4894A>G 4895T>A 4896G>A), ATC422TTT (4897A>T 4899C>T), TTA423TCA (4901T>C), CAA424AAA (4903C>A), AAG425CTC (4906A>C 4907A>T 4908G>C), AAT427GTC (4912A>G 4913A>T 4914T>C), CAA428AAT (4915C>A 4917A>T), CAT429AAT (4918C>A), GGA430GAC (4922G>A 4923A>C), ATT431ATA (4926T>A), ATC432GTA (4927A>G 4929C>A), CTT433TTA (4930C>T 4932T>A), TCC434AGT (4933T>A 4934C>G 4935C>T), AAG435CAG (4936A>C), AAG436AAA (4941G>A), CAA439GAA (4948C>G), CTC440ATA (4951C>A 4953C>A), TTC441AAT (4954T>A 4955T>A 4956C>T), AAG442AAA (4959G>A), AAG443AAT (4962G>T), AAG444AAA (4965G>A), AAC446GAA (4969A>G 4971C>A), GGT449GGA (4980T>A), CTA450ATG (4981C>A 4983A>G), GAA451ATT (4984G>A 4985A>T 4986A>T), ATA452ATT (4989A>T), GAT453TCA (4990G>T 4991A>C 4992T>A), GAA454AAA (4993G>A), GGA455AAT (4996G>A 4997G>A 4998A>T), ACA456GGA (4999A>G 5000C>G), CAT457ATA (5002C>A 5003A>T 5004T>A), AAG458GAA (5005A>G 5007G>A), CCT459TTA (5008C>T 5009C>T 5010T>A), GGA461ACT (5014G>A 5015G>C 5016A>T), CAT462CAC (5019T>C), ATC463ATA (5022C>A), TTG464TCC (5024T>C 5025G>C), GAA465AAA (5026G>A), CAC466AAG (5029C>A 5031C>G), ATC467ATA (5034C>A), AAC468TTA (5035A>T 5036A>T 5037C>A), AAG469GAG (5038A>G), TTC470TTT (5043C>T), CCC471CCT (5046C>T), GAT472GAC (5049T>C), ACC473AAG (5051C>A 5052C>G), CTT474TTA (5053C>T 5055T>A), GAA475ATA (5056G>A 5057A>T), GAC476ACC (5059G>A 5060A>C), AAG477AAA (5064G>A), AAG478CAA (5065A>C 5067G>A), CAA479GAA (5068C>G), CTT480ATA (5071C>A 5073T>A), CAG481CAA (5076G>A), TTC483TTT (5082C>T), TTA484CTT (5083T>C 5085A>T), GGC485GGA (5088C>A), ATA486TGC (5089A>T 5090T>G 5091A>C), CTA487TTA (5092C>T), ACA488AAC (5096C>A 5097A>C), GCC490GCA (5103C>A), TCG491GGA (5104T>G 5105C>G 5106G>A), GAT492GAA (5109T>A), TAC493TTC (5111A>T), ATC494ATA (5115C>A), CCG495CCA (5118G>A), AAG496GAA (5119A>G 5121G>A), CTA497TTA (5122C>T), GCT498GCA (5127T>A), CAA499AAG (5128C>A 5130A>G), ATC500AAG (5132T>A 5133C>G), AAG502AAT (5139G>T), CCT503CTT (5141C>T), CTG504CTT (5145G>T), GCC506del (5149_5151delGCC), AAG507AAA (5154G>A), CTT508ACT (5155C>A 5156T>C), AAA509CAA (5158A>C), GAA510CAA (5161G>C), AAC511AAA (5166C>A), GTT512ATC (5167G>A 5169T>C), CCA513AAA (5170C>A 5171C>A), TGG514TTG (5174G>T), TGG514_AGA515insATTA-- (5175_5176insATTA), AGA515GGA (5176A>G), AAA518AAT (5187A>T), GAG519GAA (5190G>A), GAT520CAT (5191G>C), ACC521ACT (5196C>T), CTC522CTT (5199C>T), TAC523TGT (5201A>G 5202C>T), ATG524GTA (5203A>G 5205G>A), CAA525AAA (5206C>A), AAG526AAA (5211G>A), GTG527CTC (5212G>C 5214G>C), AAG528AAA (5217G>A), AAA529GAA (5218A>G), AAT530GAA (5221A>G 5223T>A), CTG531TGT (5224C>T 5225T>G 5226G>T), CAA532ATG (5227C>A 5228A>T 5229A>G), GGA533AAG (5230G>A 5231G>A 5232A>G), TTT534CTT (5233T>C), CCT535CCA (5238T>A), CCA536AAG (5239C>A 5240C>A 5241A>G), CTA537ATT (5242C>A 5244A>T), CAT538AGA (5245C>A 5246A>G 5247T>A), CAT539TCA (5248C>T 5249A>C 5250T>A), CCC540CCA (5253C>A), TTA541GAA (5254T>G 5255T>A), GAG543GAA (5262G>A), GAG544GAC (5265G>C), AAG545AAT (5268G>T), ATC547GTA (5272A>G 5274C>A), ATC548TTA (5275A>T 5277C>A), GAG549CAA (5278G>C 5280G>A), ACC550ACA (5283C>A), GCA552GCC (5289A>C), TCA553TCC (5292A>C), GAC554GAT (5295C>T), GAC555TAT (5296G>T 5298C>T), TAC556CAT (5299T>C 5301C>T), GGT559GCA (5309G>C 5310T>A), ATG560TTG (5311A>T), TTA561CTA (5314T>C), AAA562CAA (5317A>C), GCT563ACA (5320G>A 5322T>A), ATC564del (5323_5325delATC), AAA565GAC (5326A>G 5328A>C), ATT566CTA (5329A>C 5331T>A), GGT569_TTA574del (5338_5355delGGTACTAATACTGAGTTA), TGC576TGT (5361C>T), AGA577AAG (5363G>A 5364A>G), TAC578TAT (5367C>T), GCA579ACA (5368G>A), TCT580AGT (5371T>A 5372C>G), AGC582ACT (5378G>C 5379C>T), AAA584AAC (5385A>C), GCT585GAA (5387C>A 5388T>A), GAA587AAA (5392G>A), AAG588GTT (5395A>G 5396A>T 5397G>T), TAC590TAT (5403C>T), CAC591TCC (5404C>T 5405A>C), AGC592ACA (5408G>C 5409C>A), GAC594GAA (5415C>A), GAG596GAA (5421G>A), ACA597TTA (5422A>T 5423C>T), TTG598TTA (5427G>A), GCG599GCA (5430G>A), ATA601GTA (5434A>G), AAT602AGA (5438A>G 5439T>A), ACT603GGA (5440A>G 5441C>G 5442T>A), AAG605CAA (5446A>C 5448G>A), AGT608TCG (5455A>T 5456G>C 5457T>G), ATT609GCT (5458A>G 5459T>C), TAT610TGC (5462A>G 5463T>C), CTA611TTA (5464C>T), ACT612TTA (5467A>T 5468C>T 5469T>A), CCT613CCA (5472T>A), GTT614AAA (5473G>A 5474T>A 5475T>A), CAT615TCA (5476C>T 5477A>C 5478T>A), CTG617GTT (5482C>G 5484G>T), AGG619AAA (5489G>A 5490G>A), CAT624CAA (5505T>A), TTC625ATA (5506T>A 5508C>A), AAG626TCA (5509A>T 5510A>C 5511G>A), AGT627GGG (5512A>G 5514T>G), TTC628TTG (5517C>G), GTT629ATA (5518G>A 5520T>A), AAT630TTT (5521A>T 5522A>T), CTC631AAC (5524C>A 5525T>A), AAT632AAA (5529T>A), TAC633CTC (5530T>C 5531A>T), AAA634CCT (5533A>C 5534A>C 5535A>T), GGA635TCC (5536G>T 5537G>C 5538A>C), GAT636GAA (5541T>A), TCG637CCC (5542T>C 5544G>C), AAA638CAA (5545A>C), CTT639TAT (5548C>T 5549T>A), GGA640AGA (5551G>A), AAC642CTG (5557A>C 5558A>T 5559C>G), ATC643CAT (5560A>C 5561T>A 5562C>T), TGG645TGA (5568G>A), GCA647GTT (5573C>T 5574A>T), TGG648TTG (5576G>T), AGC650TCG (5581A>T 5582G>C 5583C>G), CAC651TTC (5584C>T 5585A>T), TCA653TCT (5592A>T), GAT655AAA (5596G>A 5598T>A), GTT656ATT (5599G>A), CAC658AAT (5605C>A 5607C>T), ATT659ATA (5610T>A), ACC662ACT (5619C>T), GAC663GAT (5622C>T), AAC664AAT (5625C>T), CAC665TTT (5626C>T 5627A>T 5628C>T), GCG667GCA (5634G>A), GAC668GAT (5637C>T)								
**Proteins**									
reverse transcriptase (NP_056728.1)	405	675	39.9%	668	36.1%	263 (96.3%)	119 (43.6%)	2/8/1/1	1

	Begin	End	Coverage	Score	Concordance	Matches	Identities	I/D/M/F*	Stop Codons
**NT**	**4845**	**5656**	**10.1%**	**116**	**7.6%**	**788 (96.6%)**	**439 (53.8%)**	**4/24**	
Protein mutations:	F408C (4856T>G), N410T (4862A>C 4863C>A), N411T (4865A>C 4866C>A), E412Y (4867G>T 4869A>T), E413Q (4870G>C 4872A>G), D414E (4875T>A), L417T (4882C>A 4883T>C 4884T>A), V419L (4888G>C 4890A>T), A420K (4891G>A 4892C>A), M421E (4894A>G 4895T>A 4896G>A), I422F (4897A>T 4899C>T), L423S (4901T>C), Q424K (4903C>A), K425L (4906A>C 4907A>T 4908G>C), N427V (4912A>G 4913A>T 4914T>C), Q428N (4915C>A 4917A>T), H429N (4918C>A), G430D (4922G>A 4923A>C), I432V (4927A>G 4929C>A), K435Q (4936A>C), Q439E (4948C>G), L440I (4951C>A 4953C>A), F441N (4954T>A 4955T>A 4956C>T), K443N (4962G>T), N446E (4969A>G 4971C>A), L450M (4981C>A 4983A>G), E451I (4984G>A 4985A>T 4986A>T), D453S (4990G>T 4991A>C 4992T>A), E454K (4993G>A), G455N (4996G>A 4997G>A 4998A>T), T456G (4999A>G 5000C>G), H457I (5002C>A 5003A>T 5004T>A), K458E (5005A>G 5007G>A), P459L (5008C>T 5009C>T 5010T>A), G461T (5014G>A 5015G>C 5016A>T), L464S (5024T>C 5025G>C), E465K (5026G>A), H466K (5029C>A 5031C>G), N468L (5035A>T 5036A>T 5037C>A), K469E (5038A>G), T473K (5051C>A 5052C>G), E475I (5056G>A 5057A>T), D476T (5059G>A 5060A>C), K478Q (5065A>C 5067G>A), Q479E (5068C>G), L480I (5071C>A 5073T>A), I486C (5089A>T 5090T>G 5091A>C), T488N (5096C>A 5097A>C), S491G (5104T>G 5105C>G 5106G>A), D492E (5109T>A), Y493F (5111A>T), K496E (5119A>G 5121G>A), Q499K (5128C>A 5130A>G), I500K (5132T>A 5133C>G), K502N (5139G>T), P503L (5141C>T), A506del (5149_5151delGCC), L508T (5155C>A 5156T>C), K509Q (5158A>C), E510Q (5161G>C), N511K (5166C>A), V512I (5167G>A 5169T>C), P513K (5170C>A 5171C>A), W514L (5174G>T), W514_R515insIX (5175_5176insATTA), R515G (5176A>G), K518N (5187A>T), D520H (5191G>C), Y523C (5201A>G 5202C>T), M524V (5203A>G 5205G>A), Q525K (5206C>A), V527L (5212G>C 5214G>C), K529E (5218A>G), N530E (5221A>G 5223T>A), L531C (5224C>T 5225T>G 5226G>T), Q532M (5227C>A 5228A>T 5229A>G), G533K (5230G>A 5231G>A 5232A>G), F534L (5233T>C), P536K (5239C>A 5240C>A 5241A>G), L537I (5242C>A 5244A>T), H538R (5245C>A 5246A>G 5247T>A), H539S (5248C>T 5249A>C 5250T>A), L541E (5254T>G 5255T>A), E544D (5265G>C), K545N (5268G>T), I547V (5272A>G 5274C>A), I548L (5275A>T 5277C>A), E549Q (5278G>C 5280G>A), D555Y (5296G>T 5298C>T), Y556H (5299T>C 5301C>T), G559A (5309G>C 5310T>A), M560L (5311A>T), K562Q (5317A>C), A563T (5320G>A 5322T>A), I564del (5323_5325delATC), K565D (5326A>G 5328A>C), I566L (5329A>C 5331T>A), G569_L574del (5338_5355delGGTACTAATACTGAGTTA), R577K (5363G>A 5364A>G), A579T (5368G>A), S582T (5378G>C 5379C>T), K584N (5385A>C), A585E (5387C>A 5388T>A), E587K (5392G>A), K588V (5395A>G 5396A>T 5397G>T), H591S (5404C>T 5405A>C), S592T (5408G>C 5409C>A), D594E (5415C>A), T597L (5422A>T 5423C>T), I601V (5434A>G), N602R (5438A>G 5439T>A), T603G (5440A>G 5441C>G 5442T>A), K605Q (5446A>C 5448G>A), I609A (5458A>G 5459T>C), Y610C (5462A>G 5463T>C), T612L (5467A>T 5468C>T 5469T>A), V614K (5473G>A 5474T>A 5475T>A), H615S (5476C>T 5477A>C 5478T>A), L617V (5482C>G 5484G>T), R619K (5489G>A 5490G>A), H624Q (5505T>A), F625I (5506T>A 5508C>A), K626S (5509A>T 5510A>C 5511G>A), S627G (5512A>G 5514T>G), F628L (5517C>G), V629I (5518G>A 5520T>A), N630F (5521A>T 5522A>T), L631N (5524C>A 5525T>A), N632K (5529T>A), Y633L (5530T>C 5531A>T), K634P (5533A>C 5534A>C 5535A>T), G635S (5536G>T 5537G>C 5538A>C), D636E (5541T>A), S637P (5542T>C 5544G>C), K638Q (5545A>C), L639Y (5548C>T 5549T>A), G640R (5551G>A), N642L (5557A>C 5558A>T 5559C>G), I643H (5560A>C 5561T>A 5562C>T), W645* (5568G>A), A647V (5573C>T 5574A>T), W648L (5576G>T), H651F (5584C>T 5585A>T), D655K (5596G>A 5598T>A), V656I (5599G>A), H658N (5605C>A 5607C>T), H665F (5626C>T 5627A>T 5628C>T)								
Codon mutations:	CTC406TTG (4849C>T 4851C>G), TTC408TGC (4856T>G), AGT409TCA (4858A>T 4859G>C 4860T>A), AAC410ACA (4862A>C 4863C>A), AAC411ACA (4865A>C 4866C>A), GAA412TAT (4867G>T 4869A>T), GAA413CAG (4870G>C 4872A>G), GAT414GAA (4875T>A), CTA416CTT (4881A>T), CTT417ACA (4882C>A 4883T>C 4884T>A), CAC418CAT (4887C>T), GTA419CTT (4888G>C 4890A>T), GCA420AAA (4891G>A 4892C>A), ATG421GAA (4894A>G 4895T>A 4896G>A), ATC422TTT (4897A>T 4899C>T), TTA423TCA (4901T>C), CAA424AAA (4903C>A), AAG425CTC (4906A>C 4907A>T 4908G>C), AAT427GTC (4912A>G 4913A>T 4914T>C), CAA428AAT (4915C>A 4917A>T), CAT429AAT (4918C>A), GGA430GAC (4922G>A 4923A>C), ATT431ATA (4926T>A), ATC432GTA (4927A>G 4929C>A), CTT433TTA (4930C>T 4932T>A), TCC434AGT (4933T>A 4934C>G 4935C>T), AAG435CAG (4936A>C), AAG436AAA (4941G>A), CAA439GAA (4948C>G), CTC440ATA (4951C>A 4953C>A), TTC441AAT (4954T>A 4955T>A 4956C>T), AAG442AAA (4959G>A), AAG443AAT (4962G>T), AAG444AAA (4965G>A), AAC446GAA (4969A>G 4971C>A), GGT449GGA (4980T>A), CTA450ATG (4981C>A 4983A>G), GAA451ATT (4984G>A 4985A>T 4986A>T), ATA452ATT (4989A>T), GAT453TCA (4990G>T 4991A>C 4992T>A), GAA454AAA (4993G>A), GGA455AAT (4996G>A 4997G>A 4998A>T), ACA456GGA (4999A>G 5000C>G), CAT457ATA (5002C>A 5003A>T 5004T>A), AAG458GAA (5005A>G 5007G>A), CCT459TTA (5008C>T 5009C>T 5010T>A), GGA461ACT (5014G>A 5015G>C 5016A>T), CAT462CAC (5019T>C), ATC463ATA (5022C>A), TTG464TCC (5024T>C 5025G>C), GAA465AAA (5026G>A), CAC466AAG (5029C>A 5031C>G), ATC467ATA (5034C>A), AAC468TTA (5035A>T 5036A>T 5037C>A), AAG469GAG (5038A>G), TTC470TTT (5043C>T), CCC471CCT (5046C>T), GAT472GAC (5049T>C), ACC473AAG (5051C>A 5052C>G), CTT474TTA (5053C>T 5055T>A), GAA475ATA (5056G>A 5057A>T), GAC476ACC (5059G>A 5060A>C), AAG477AAA (5064G>A), AAG478CAA (5065A>C 5067G>A), CAA479GAA (5068C>G), CTT480ATA (5071C>A 5073T>A), CAG481CAA (5076G>A), TTC483TTT (5082C>T), TTA484CTT (5083T>C 5085A>T), GGC485GGA (5088C>A), ATA486TGC (5089A>T 5090T>G 5091A>C), CTA487TTA (5092C>T), ACA488AAC (5096C>A 5097A>C), GCC490GCA (5103C>A), TCG491GGA (5104T>G 5105C>G 5106G>A), GAT492GAA (5109T>A), TAC493TTC (5111A>T), ATC494ATA (5115C>A), CCG495CCA (5118G>A), AAG496GAA (5119A>G 5121G>A), CTA497TTA (5122C>T), GCT498GCA (5127T>A), CAA499AAG (5128C>A 5130A>G), ATC500AAG (5132T>A 5133C>G), AAG502AAT (5139G>T), CCT503CTT (5141C>T), CTG504CTT (5145G>T), GCC506del (5149_5151delGCC), AAG507AAA (5154G>A), CTT508ACT (5155C>A 5156T>C), AAA509CAA (5158A>C), GAA510CAA (5161G>C), AAC511AAA (5166C>A), GTT512ATC (5167G>A 5169T>C), CCA513AAA (5170C>A 5171C>A), TGG514TTG (5174G>T), TGG514_AGA515insATTA-- (5175_5176insATTA), AGA515GGA (5176A>G), AAA518AAT (5187A>T), GAG519GAA (5190G>A), GAT520CAT (5191G>C), ACC521ACT (5196C>T), CTC522CTT (5199C>T), TAC523TGT (5201A>G 5202C>T), ATG524GTA (5203A>G 5205G>A), CAA525AAA (5206C>A), AAG526AAA (5211G>A), GTG527CTC (5212G>C 5214G>C), AAG528AAA (5217G>A), AAA529GAA (5218A>G), AAT530GAA (5221A>G 5223T>A), CTG531TGT (5224C>T 5225T>G 5226G>T), CAA532ATG (5227C>A 5228A>T 5229A>G), GGA533AAG (5230G>A 5231G>A 5232A>G), TTT534CTT (5233T>C), CCT535CCA (5238T>A), CCA536AAG (5239C>A 5240C>A 5241A>G), CTA537ATT (5242C>A 5244A>T), CAT538AGA (5245C>A 5246A>G 5247T>A), CAT539TCA (5248C>T 5249A>C 5250T>A), CCC540CCA (5253C>A), TTA541GAA (5254T>G 5255T>A), GAG543GAA (5262G>A), GAG544GAC (5265G>C), AAG545AAT (5268G>T), ATC547GTA (5272A>G 5274C>A), ATC548TTA (5275A>T 5277C>A), GAG549CAA (5278G>C 5280G>A), ACC550ACA (5283C>A), GCA552GCC (5289A>C), TCA553TCC (5292A>C), GAC554GAT (5295C>T), GAC555TAT (5296G>T 5298C>T), TAC556CAT (5299T>C 5301C>T), GGT559GCA (5309G>C 5310T>A), ATG560TTG (5311A>T), TTA561CTA (5314T>C), AAA562CAA (5317A>C), GCT563ACA (5320G>A 5322T>A), ATC564del (5323_5325delATC), AAA565GAC (5326A>G 5328A>C), ATT566CTA (5329A>C 5331T>A), GGT569_TTA574del (5338_5355delGGTACTAATACTGAGTTA), TGC576TGT (5361C>T), AGA577AAG (5363G>A 5364A>G), TAC578TAT (5367C>T), GCA579ACA (5368G>A), TCT580AGT (5371T>A 5372C>G), AGC582ACT (5378G>C 5379C>T), AAA584AAC (5385A>C), GCT585GAA (5387C>A 5388T>A), GAA587AAA (5392G>A), AAG588GTT (5395A>G 5396A>T 5397G>T), TAC590TAT (5403C>T), CAC591TCC (5404C>T 5405A>C), AGC592ACA (5408G>C 5409C>A), GAC594GAA (5415C>A), GAG596GAA (5421G>A), ACA597TTA (5422A>T 5423C>T), TTG598TTA (5427G>A), GCG599GCA (5430G>A), ATA601GTA (5434A>G), AAT602AGA (5438A>G 5439T>A), ACT603GGA (5440A>G 5441C>G 5442T>A), AAG605CAA (5446A>C 5448G>A), AGT608TCG (5455A>T 5456G>C 5457T>G), ATT609GCT (5458A>G 5459T>C), TAT610TGC (5462A>G 5463T>C), CTA611TTA (5464C>T), ACT612TTA (5467A>T 5468C>T 5469T>A), CCT613CCA (5472T>A), GTT614AAA (5473G>A 5474T>A 5475T>A), CAT615TCA (5476C>T 5477A>C 5478T>A), CTG617GTT (5482C>G 5484G>T), AGG619AAA (5489G>A 5490G>A), CAT624CAA (5505T>A), TTC625ATA (5506T>A 5508C>A), AAG626TCA (5509A>T 5510A>C 5511G>A), AGT627GGG (5512A>G 5514T>G), TTC628TTG (5517C>G), GTT629ATA (5518G>A 5520T>A), AAT630TTT (5521A>T 5522A>T), CTC631AAC (5524C>A 5525T>A), AAT632AAA (5529T>A), TAC633CTC (5530T>C 5531A>T), AAA634CCT (5533A>C 5534A>C 5535A>T), GGA635TCC (5536G>T 5537G>C 5538A>C), GAT636GAA (5541T>A), TCG637CCC (5542T>C 5544G>C), AAA638CAA (5545A>C), CTT639TAT (5548C>T 5549T>A), GGA640AGA (5551G>A), AAC642CTG (5557A>C 5558A>T 5559C>G), ATC643CAT (5560A>C 5561T>A 5562C>T), TGG645TGA (5568G>A), GCA647GTT (5573C>T 5574A>T), TGG648TTG (5576G>T), AGC650TCG (5581A>T 5582G>C 5583C>G), CAC651TTC (5584C>T 5585A>T), TCA653TCT (5592A>T), GAT655AAA (5596G>A 5598T>A), GTT656ATT (5599G>A), CAC658AAT (5605C>A 5607C>T), ATT659ATA (5610T>A), ACC662ACT (5619C>T), GAC663GAT (5622C>T), AAC664AAT (5625C>T), CAC665TTT (5626C>T 5627A>T 5628C>T), GCG667GCA (5634G>A), GAC668GAT (5637C>T)								

*: Inserts / Deletes / Misaligned / Frameshifts

Analysis details

This analysis was performed with panviral2.64

NGS Details (UN24): Pinus nigra virus 1

Assembly

**Coverage Length**	246 (1 contig(s))
**Depth Of Coverage**	10.8
**Number Of Reads**	24
**Reads Per Million**	0.48 rpm (after QC)
**Ambiguities**	0
**Assembly Method**	de novo + reference guided assembly
**Consensus Caller**	Bcf Tools

Coverage Map

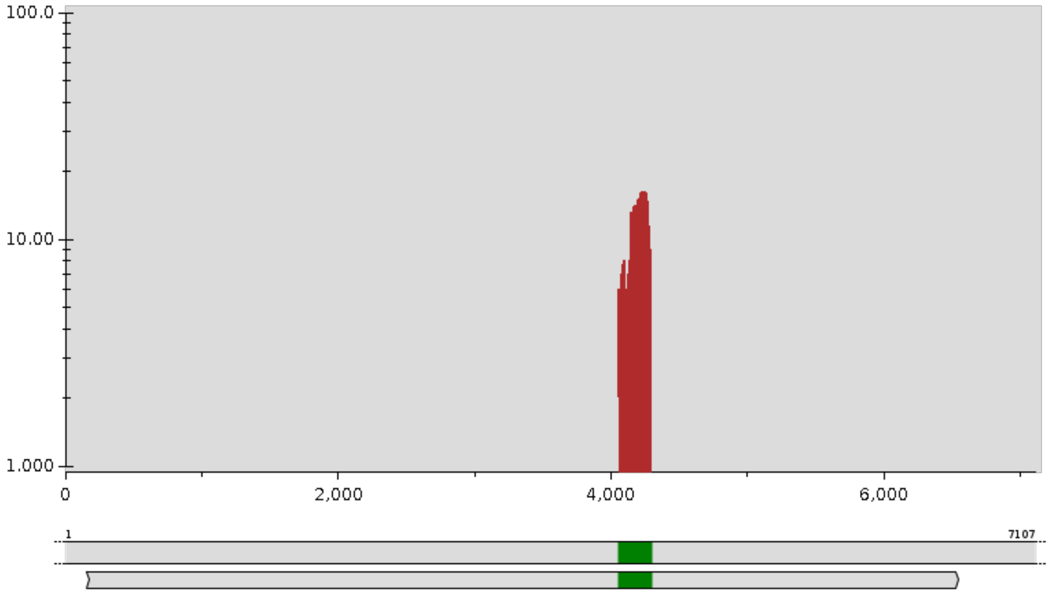

Assignment

**Type**	Pinus nigra virus 1 (Taxonomy ID: 2267679)
**Reference Genome**	NC_040841.1
**NT Identity (%)**	54.3651
**AA Identity (%)**	40.4762
**Number Of Stop Codons**	0
**Number Of CDS**	1

Alignment

**Alignment Score**	41.0 (NT) + 281.0 (AA) = 322.0
**Concordance (%)**	28.9308

Alignment Method	Global, seeded, nucleotide + amino acids (AGA)

Genome Region

Sequence starts at position 4054 and ends at position 4299 relative to NC_040841.1 reference sequence.

Alignment Detailed Statistics

	Begin	End	Coverage	Score	Concordance	Matches	Identities	I/D/M/F*	Stop Codons
**NT**	**4054**	**4299**	**3.5%**	**41**	**8.3%**	**246 (97.6%)**	**137 (54.4%)**	**6/0**	
Mutations:	4056A>G, 4060T>G, 4061C>A, 4064C>T, 4065C>T, 4066A>G, 4073A>G, 4074G>T, 4075A>G, 4084A>T, 4090C>T, 4092T>G, 4094T>A, 4095G>C, 4098C>A, 4099A>T, 4100G>A, 4101C>T, 4102A>G, 4106T>C, 4107T>A, 4111T>C, 4112C>G, 4116G>T, 4117G>C, 4120A>G, 4123T>C, 4124G>C, 4126T>G, 4132T>C, 4135T>A, 4138G>A, 4140A>C, 4141G>A, 4147G>C, 4153C>A, 4154T>A, 4155T>C, 4156C>A, 4159T>C, 4163A>G, 4164G>C, 4165A>C, 4166A>T, 4167G>T, 4172G>A, 4175T>C, 4177T>G, 4178A>G, 4180C>A, 4184G>C, 4185C>A, 4186T>A, 4187A>C, 4188A>C, 4189A>C, 4192_4193insCTCAGA, 4193G>A, 4195T>G, 4199A>G, 4201A>G, 4202G>C, 4204T>G, 4205A>G, 4206C>T, 4207A>T, 4209A>T, 4211A>T, 4213A>T, 4219T>C, 4226A>G, 4228T>G, 4229C>T, 4231T>C, 4234T>C, 4235C>T, 4236C>T, 4237A>G, 4238G>T, 4239A>C, 4240T>C, 4241G>C, 4244C>G, 4246G>A, 4247A>G, 4253T>A, 4255G>T, 4256A>C, 4262T>C, 4264A>C, 4265G>A, 4266A>T, 4267A>T, 4268A>G, 4271T>G, 4273T>G, 4274C>T, 4277G>T, 4278A>C, 4279A>T, 4280G>A, 4281A>C, 4282A>C, 4283G>T, 4285C>G, 4287A>G, 4289A>G, 4295G>T, 4296G>C, 4298A>T								
**CDS**									
EXL67_gp1	1301	1382	3.8%	281	44.4%	82 (97.6%)	34 (40.5%)	2/0/0/0	0
Protein mutations:	Q1301R (4056A>G), I1302M (4060T>G), Q1303K (4061C>A), P1304L (4064C>T 4065C>T 4066A>G), R1307V (4073A>G 4074G>T 4075A>G), I1313R (4092T>G), C1314T (4094T>A 4095G>C), P1315H (4098C>A 4099A>T), A1316M (4100G>A 4101C>T 4102A>G), F1318H (4106T>C 4107T>A), Q1320E (4112C>G), W1321F (4116G>T 4117G>C), V1324L (4124G>C 4126T>G), K1329T (4140A>C 4141G>A), F1334T (4154T>A 4155T>C 4156C>A), R1337A (4163A>G 4164G>C 4165A>C), R1338L (4166A>T 4167G>T), D1340N (4172G>A), Y1341Q (4175T>C 4177T>G), I1342V (4178A>G 4180C>A), A1344Q (4184G>C 4185C>A 4186T>A), K1345P (4187A>C 4188A>C 4189A>C), Y1346_D1347insLR (4192_4193insCTCAGA), D1347K (4193G>A 4195T>G), I1349V (4199A>G 4201A>G), V1350L (4202G>C 4204T>G), T1351V (4205A>G 4206C>T 4207A>T), Y1352F (4209A>T), I1353F (4211A>T 4213A>T), I1358V (4226A>G 4228T>G), H1359Y (4229C>T 4231T>C), P1361L (4235C>T 4236C>T 4237A>G), D1362S (4238G>T 4239A>C 4240T>C), V1363L (4241G>C), Q1364E (4244C>G 4246G>A), N1365D (4247A>G), L1367I (4253T>A 4255G>T), K1368Q (4256A>C), E1371I (4265G>A 4266A>T 4267A>T), I1372V (4268A>G), F1373V (4271T>G 4273T>G), L1374F (4274C>T), E1375S (4277G>T 4278A>C 4279A>T), E1376T (4280G>A 4281A>C 4282A>C), V1377L (4283G>T 4285C>G), K1378R (4287A>G), K1379E (4289A>G), G1381S (4295G>T 4296G>C)								
Codon mutations:	CAA1301CGA (4056A>G), ATT1302ATG (4060T>G), CAG1303AAG (4061C>A), CCA1304TTG (4064C>T 4065C>T 4066A>G), AGA1307GTG (4073A>G 4074G>T 4075A>G), ACA1310ACT (4084A>T), TTC1312TTT (4090C>T), ATA1313AGA (4092T>G), TGT1314ACT (4094T>A 4095G>C), CCA1315CAT (4098C>A 4099A>T), GCA1316ATG (4100G>A 4101C>T 4102A>G), TTC1318CAC (4106T>C 4107T>A), TAT1319TAC (4111T>C), CAG1320GAG (4112C>G), TGG1321TTC (4116G>T 4117G>C), AAA1322AAG (4120A>G), GTT1323GTC (4123T>C), GTT1324CTG (4124G>C 4126T>G), TTT1326TTC (4132T>C), GGT1327GGA (4135T>A), TTG1328TTA (4138G>A), AAG1329ACA (4140A>C 4141G>A), GCG1331GCC (4147G>C), GCC1333GCA (4153C>A), TTC1334ACA (4154T>A 4155T>C 4156C>A), TTT1335TTC (4159T>C), AGA1337GCC (4163A>G 4164G>C 4165A>C), AGG1338TTG (4166A>T 4167G>T), GAT1340AAT (4172G>A), TAT1341CAG (4175T>C 4177T>G), ATC1342GTA (4178A>G 4180C>A), GCT1344CAA (4184G>C 4185C>A 4186T>A), AAA1345CCC (4187A>C 4188A>C 4189A>C), TAT1346_GAT1347insCTCAGA (4192_4193insCTCAGA), GAT1347AAG (4193G>A 4195T>G), ATA1349GTG (4199A>G 4201A>G), GTT1350CTG (4202G>C 4204T>G), ACA1351GTT (4205A>G 4206C>T 4207A>T), TAC1352TTC (4209A>T), ATA1353TTT (4211A>T 4213A>T), GAT1355GAC (4219T>C), ATT1358GTG (4226A>G 4228T>G), CAT1359TAC (4229C>T 4231T>C), AGT1360AGC (4234T>C), CCA1361TTG (4235C>T 4236C>T 4237A>G), GAT1362TCC (4238G>T 4239A>C 4240T>C), GTA1363CTA (4241G>C), CAG1364GAA (4244C>G 4246G>A), AAT1365GAT (4247A>G), TTG1367ATT (4253T>A 4255G>T), AAG1368CAG (4256A>C), TTA1370CTC (4262T>C 4264A>C), GAA1371ATT (4265G>A 4266A>T 4267A>T), ATT1372GTT (4268A>G), TTT1373GTG (4271T>G 4273T>G), CTT1374TTT (4274C>T), GAA1375TCT (4277G>T 4278A>C 4279A>T), GAA1376ACC (4280G>A 4281A>C 4282A>C), GTC1377TTG (4283G>T 4285C>G), AAG1378AGG (4287A>G), AAA1379GAA (4289A>G), GGA1381TCA (4295G>T 4296G>C), ATT1382TT. (4298A>T)								
**Proteins**									
polyprotein (YP_009553669.1)	1301	1382	3.8%	281	44.4%	82 (97.6%)	34 (40.5%)	2/0/0/0	0
Protein mutations:	Q1301R (4056A>G), I1302M (4060T>G), Q1303K (4061C>A), P1304L (4064C>T 4065C>T 4066A>G), R1307V (4073A>G 4074G>T 4075A>G), I1313R (4092T>G), C1314T (4094T>A 4095G>C), P1315H (4098C>A 4099A>T), A1316M (4100G>A 4101C>T 4102A>G), F1318H (4106T>C 4107T>A), Q1320E (4112C>G), W1321F (4116G>T 4117G>C), V1324L (4124G>C 4126T>G), K1329T (4140A>C 4141G>A), F1334T (4154T>A 4155T>C 4156C>A), R1337A (4163A>G 4164G>C 4165A>C), R1338L (4166A>T 4167G>T), D1340N (4172G>A), Y1341Q (4175T>C 4177T>G), I1342V (4178A>G 4180C>A), A1344Q (4184G>C 4185C>A 4186T>A), K1345P (4187A>C 4188A>C 4189A>C), Y1346_D1347insLR (4192_4193insCTCAGA), D1347K (4193G>A 4195T>G), I1349V (4199A>G 4201A>G), V1350L (4202G>C 4204T>G), T1351V (4205A>G 4206C>T 4207A>T), Y1352F (4209A>T), I1353F (4211A>T 4213A>T), I1358V (4226A>G 4228T>G), H1359Y (4229C>T 4231T>C), P1361L (4235C>T 4236C>T 4237A>G), D1362S (4238G>T 4239A>C 4240T>C), V1363L (4241G>C), Q1364E (4244C>G 4246G>A), N1365D (4247A>G), L1367I (4253T>A 4255G>T), K1368Q (4256A>C), E1371I (4265G>A 4266A>T 4267A>T), I1372V (4268A>G), F1373V (4271T>G 4273T>G), L1374F (4274C>T), E1375S (4277G>T 4278A>C 4279A>T), E1376T (4280G>A 4281A>C 4282A>C), V1377L (4283G>T 4285C>G), K1378R (4287A>G), K1379E (4289A>G), G1381S (4295G>T 4296G>C)								
Codon mutations:	CAA1301CGA (4056A>G), ATT1302ATG (4060T>G), CAG1303AAG (4061C>A), CCA1304TTG (4064C>T 4065C>T 4066A>G), AGA1307GTG (4073A>G 4074G>T 4075A>G), ACA1310ACT (4084A>T), TTC1312TTT (4090C>T), ATA1313AGA (4092T>G), TGT1314ACT (4094T>A 4095G>C), CCA1315CAT (4098C>A 4099A>T), GCA1316ATG (4100G>A 4101C>T 4102A>G), TTC1318CAC (4106T>C 4107T>A), TAT1319TAC (4111T>C), CAG1320GAG (4112C>G), TGG1321TTC (4116G>T 4117G>C), AAA1322AAG (4120A>G), GTT1323GTC (4123T>C), GTT1324CTG (4124G>C 4126T>G), TTT1326TTC (4132T>C), GGT1327GGA (4135T>A), TTG1328TTA (4138G>A), AAG1329ACA (4140A>C 4141G>A), GCG1331GCC (4147G>C), GCC1333GCA (4153C>A), TTC1334ACA (4154T>A 4155T>C 4156C>A), TTT1335TTC (4159T>C), AGA1337GCC (4163A>G 4164G>C 4165A>C), AGG1338TTG (4166A>T 4167G>T), GAT1340AAT (4172G>A), TAT1341CAG (4175T>C 4177T>G), ATC1342GTA (4178A>G 4180C>A), GCT1344CAA (4184G>C 4185C>A 4186T>A), AAA1345CCC (4187A>C 4188A>C 4189A>C), TAT1346_GAT1347insCTCAGA (4192_4193insCTCAGA), GAT1347AAG (4193G>A 4195T>G), ATA1349GTG (4199A>G 4201A>G), GTT1350CTG (4202G>C 4204T>G), ACA1351GTT (4205A>G 4206C>T 4207A>T), TAC1352TTC (4209A>T), ATA1353TTT (4211A>T 4213A>T), GAT1355GAC (4219T>C), ATT1358GTG (4226A>G 4228T>G), CAT1359TAC (4229C>T 4231T>C), AGT1360AGC (4234T>C), CCA1361TTG (4235C>T 4236C>T 4237A>G), GAT1362TCC (4238G>T 4239A>C 4240T>C), GTA1363CTA (4241G>C), CAG1364GAA (4244C>G 4246G>A), AAT1365GAT (4247A>G), TTG1367ATT (4253T>A 4255G>T), AAG1368CAG (4256A>C), TTA1370CTC (4262T>C 4264A>C), GAA1371ATT (4265G>A 4266A>T 4267A>T), ATT1372GTT (4268A>G), TTT1373GTG (4271T>G 4273T>G), CTT1374TTT (4274C>T), GAA1375TCT (4277G>T 4278A>C 4279A>T), GAA1376ACC (4280G>A 4281A>C 4282A>C), GTC1377TTG (4283G>T 4285C>G), AAG1378AGG (4287A>G), AAA1379GAA (4289A>G), GGA1381TCA (4295G>T 4296G>C), ATT1382TT. (4298A>T)								

*: Inserts / Deletes / Misaligned / Frameshifts

Analysis details

This analysis was performed with panviral2.64

NGS Details (UN24): Badnavirus venatheobromae

Assembly

**Coverage Length**	273 (1 contig(s))
**Depth Of Coverage**	8.9
**Number Of Reads**	21
**Reads Per Million**	0.42 rpm (after QC)
**Ambiguities**	0
**Assembly Method**	de novo + reference guided assembly
**Consensus Caller**	Bcf Tools

Coverage Map

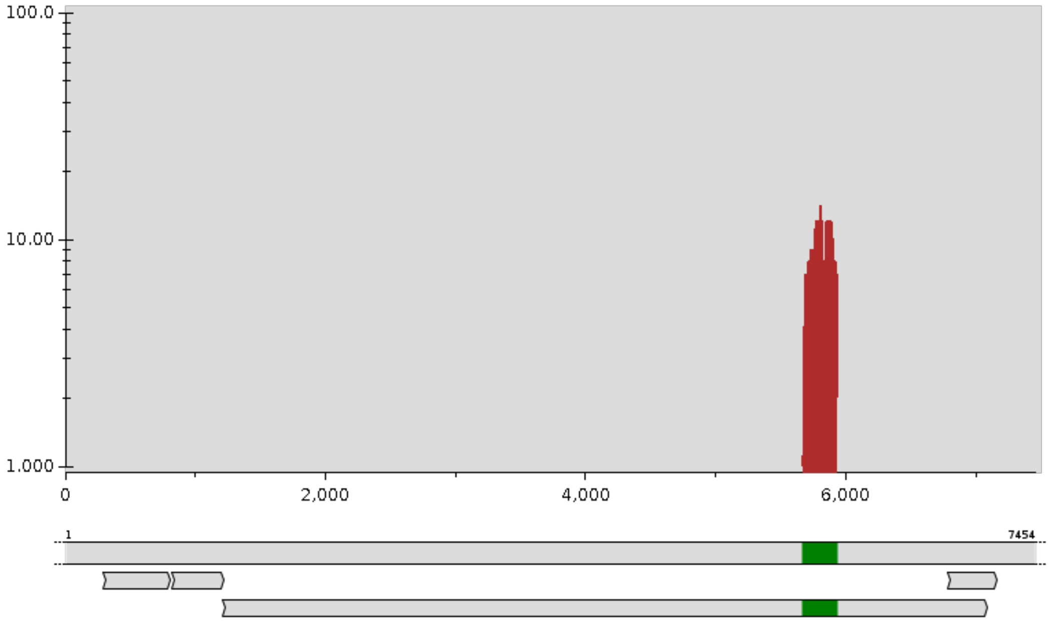

Assignment

**Type**	Badnavirus venatheobromae (Taxonomy ID: 3047715)
**Reference Genome**	NC_033739.1
**NT Identity (%)**	57.971
**AA Identity (%)**	48.913
**Number Of Stop Codons**	1
**Number Of CDS**	4

Alignment

**Alignment Score**	82.0 (NT) + 324.0 (AA) = 406.0
**Concordance (%)**	33.1699

**Alignment Method**	Global, seeded, nucleotide + amino acids (AGA)

Genome Region

Sequence starts at position 5666 and ends at position 5938 relative to NC_033739.1 reference sequence.

Alignment Detailed Statistics

	Begin	End	Coverage	Score	Concordance	Matches	Identities	I/D/M/F*	Stop Codons
**NT**	**5666**	**5938**	**3.7%**	**82**	**15.0%**	**273 (98.9%)**	**160 (58.0%)**	**3/0**	
Mutations:	5666G>C, 5669T>C, 5670T>A, 5674C>G, 5675C>A, 5677A>G, 5679T>A, 5685T>C, 5687A>T, 5688A>C, 5689A>G, 5692C>T, 5698T>A, 5706G>T, 5708A>G, 5709G>A, 5710C>A, 5711A>G, 5712A>G, 5715G>A, 5720A>G, 5722C>G, 5723A>T, 5724T>G, 5725C>A, 5726A>T, 5732A>C, 5733T>A, 5734G>A, 5738A>G, 5741G>C, 5745T>C, 5747G>A, 5748G>A, 5749T>C, 5750C>T, 5751C>A, 5752C>G, 5753T>A, 5754G>T, 5759A>T, 5761T>A, 5762A>T, 5764A>T, 5770G>T, 5771G>C, 5772C>T, 5774A>G, 5777C>T, 5781C>T, 5783G>A, 5786C>T, 5789A>T, 5792A>G, 5794A>C, 5795G>C, 5801T>A, 5804A>G, 5807C>G, 5810T>A, 5814C>A, 5815A>T, 5816A>G, 5817A>G, 5818G>A, 5819A>C, 5820A>C, 5821A>T, 5822G>C, 5826G>A, 5828C>T, 5830A>G, 5831C>G, 5832T>G, 5833G>T, 5837T>C, 5841G>C, 5842G>C, 5843A>C, 5843_5844insTTC, 5844A>C, 5845C>T, 5846A>T, 5849A>T, 5850G>A, 5851A>T, 5852A>G, 5856A>G, 5858A>T, 5860C>T, 5861A>G, 5864T>A, 5866A>T, 5880C>T, 5882A>G, 5883G>A, 5887T>A, 5888C>T, 5891A>C, 5893A>G, 5896C>A, 5906A>G, 5909T>C, 5910G>A, 5912C>G, 5913C>A, 5915T>G, 5919C>T, 5921A>G, 5922C>A, 5926G>T, 5928T>G, 5930C>G, 5933C>G								

CDS

	Begin	End	Coverage	Score	Concordance	Matches	Identities	I/D/M/F*	Stop Codons
B1U04_gp3	1486	1576	4.6%	324	47.1%	91 (98.9%)	45 (48.9%)	1/0/0/0	1
Protein mutations:	Y1487N (5670T>A), S1488* (5674C>G 5675C>A), K1489R (5677A>G), F1490I (5679T>A), K1493R (5688A>C 5689A>G), S1494F (5692C>T), F1496Y (5698T>A), V1499L (5706G>T 5708A>G), A1500K (5709G>A 5710C>A 5711A>G), M1501V (5712A>G), E1502K (5715G>A), S1504C (5722C>G 5723A>T), S1505D (5724T>G 5725C>A 5726A>T), W1508K (5733T>A 5734G>A), W1512R (5745T>C 5747G>A), V1513T (5748G>A 5749T>C 5750C>T), P1514R (5751C>A 5752C>G 5753T>A), D1515Y (5754G>T), L1517H (5761T>A 5762A>T), Y1518F (5764A>T), W1520F (5770G>T 5771G>C), P1524S (5781C>T 5783G>A), K1528T (5794A>C 5795G>C), Q1535M (5814C>A 5815A>T 5816A>G), R1536D (5817A>G 5818G>A 5819A>C), K1537L (5820A>C 5821A>T 5822G>C), D1539N (5826G>A 5828C>T), N1540R (5830A>G 5831C>G), C1541V (5832T>G 5833G>T), G1544P (5841G>C 5842G>C 5843A>C), G1544_T1545insF (5843_5844insTTC), T1545L (5844A>C 5845C>T 5846A>T), E1546D (5849A>T), E1547M (5850G>A 5851A>T 5852A>G), I1549V (5856A>G 5858A>T), A1550V (5860C>T 5861A>G), Y1552F (5866A>T), V1558I (5883G>A), F1559Y (5887T>A 5888C>T), Q1561R (5893A>G), T1562N (5896C>A), V1567M (5910G>A 5912C>G), Q1571K (5922C>A), R1572M (5926G>T), F1573V (5928T>G 5930C>G), F1574L (5933C>G)								
Codon mutations:	AAG1485..C (5666G>C), ATT1486ATC (5669T>C), TAT1487AAT (5670T>A), TCC1488TGA (5674C>G 5675C>A), AAG1489AGG (5677A>G), TTT1490ATT (5679T>A), TTA1492CTT (5685T>C 5687A>T), AAG1493CGG (5688A>C 5689A>G), TCC1494TTC (5692C>T), TTT1496TAT (5698T>A), GTA1499TTG (5706G>T 5708A>G), GCA1500AAG (5709G>A 5710C>A 5711A>G), ATG1501GTG (5712A>G), GAG1502AAG (5715G>A), GAA1503GAG (5720A>G), TCA1504TGT (5722C>G 5723A>T), TCA1505GAT (5724T>G 5725C>A 5726A>T), CCA1507CCC (5732A>C), TGG1508AAG (5733T>A 5734G>A), ACA1509ACG (5738A>G), GCG1510GCC (5741G>C), TGG1512CGA (5745T>C 5747G>A), GTC1513ACT (5748G>A 5749T>C 5750C>T), CCT1514AGA (5751C>A 5752C>G 5753T>A), GAT1515TAT (5754G>T), GGA1516GGT (5759A>T), CTA1517CAT (5761T>A 5762A>T), TAT1518TTT (5764A>T), TGG1520TTC (5770G>T 5771G>C), CTA1521TTG (5772C>T 5774A>G), GTC1522GTT (5777C>T), CCG1524TCA (5781C>T 5783G>A), TTC1525TTT (5786C>T), GGA1526GGT (5789A>T), TTA1527TTG (5792A>G), AAG1528ACC (5794A>C 5795G>C), GCT1530GCA (5801T>A), CCA1531CCG (5804A>G), GCC1532GCG (5807C>G), ATT1533ATA (5810T>A), CAA1535ATG (5814C>A 5815A>T 5816A>G), AGA1536GAC (5817A>G 5818G>A 5819A>C), AAG1537CTC (5820A>C 5821A>T 5822G>C), GAC1539AAT (5826G>A 5828C>T), AAC1540AGG (5830A>G 5831C>G), TGC1541GTC (5832T>G 5833G>T), TTT1542TTC (5837T>C), GGA1544CCC (5841G>C 5842G>C 5843A>C), GGA1544_ACA1545insTTC (5843_5844insTTC), ACA1545CTT (5844A>C 5845C>T 5846A>T), GAA1546GAT (5849A>T), GAA1547ATG (5850G>A 5851A>T 5852A>G), ATA1549GTT (5856A>G 5858A>T), GCA1550GTG (5860C>T 5861A>G), GTT1551GTA (5864T>A), TAT1552TTT (5866A>T), CTA1557TTG (5880C>T 5882A>G), GTC1558ATC (5883G>A), TTC1559TAT (5887T>A 5888C>T), TCA1560TCC (5891A>C), CAG1561CGG (5893A>G), ACT1562AAT (5896C>A), GAA1565GAG (5906A>G), CAT1566CAC (5909T>C), GTC1567ATG (5910G>A 5912C>G), CGT1568AGG (5913C>A 5915T>G), CTA1570TTG (5919C>T 5921A>G), CAA1571AAA (5922C>A), AGG1572ATG (5926G>T), TTC1573GTG (5928T>G 5930C>G), TTC1574TTG (5933C>G)								

Proteins

	Begin	End	Coverage	Score	Concordance	Matches	Identities	I/D/M/F*	Stop Codons
polyprotein (YP_009345075.1)	1486	1576	4.6%	324	47.1%	91 (98.9%)	45 (48.9%)	1/0/0/0	1
Protein mutations:	Y1487N (5670T>A), S1488* (5674C>G 5675C>A), K1489R (5677A>G), F1490I (5679T>A), K1493R (5688A>C 5689A>G), S1494F (5692C>T), F1496Y (5698T>A), V1499L (5706G>T 5708A>G), A1500K (5709G>A 5710C>A 5711A>G), M1501V (5712A>G), E1502K (5715G>A), S1504C (5722C>G 5723A>T), S1505D (5724T>G 5725C>A 5726A>T), W1508K (5733T>A 5734G>A), W1512R (5745T>C 5747G>A), V1513T (5748G>A 5749T>C 5750C>T), P1514R (5751C>A 5752C>G 5753T>A), D1515Y (5754G>T), L1517H (5761T>A 5762A>T), Y1518F (5764A>T), W1520F (5770G>T 5771G>C), P1524S (5781C>T 5783G>A), K1528T (5794A>C 5795G>C), Q1535M (5814C>A 5815A>T 5816A>G), R1536D (5817A>G 5818G>A 5819A>C), K1537L (5820A>C 5821A>T 5822G>C), D1539N (5826G>A 5828C>T), N1540R (5830A>G 5831C>G), C1541V (5832T>G 5833G>T), G1544P (5841G>C 5842G>C 5843A>C), G1544_T1545insF (5843_5844insTTC), T1545L (5844A>C 5845C>T 5846A>T), E1546D (5849A>T), E1547M (5850G>A 5851A>T 5852A>G), I1549V (5856A>G 5858A>T), A1550V (5860C>T 5861A>G), Y1552F (5866A>T), V1558I (5883G>A), F1559Y (5887T>A 5888C>T), Q1561R (5893A>G), T1562N (5896C>A), V1567M (5910G>A 5912C>G), Q1571K (5922C>A), R1572M (5926G>T), F1573V (5928T>G 5930C>G), F1574L (5933C>G)								
Codon mutations:	AAG1485..C (5666G>C), ATT1486ATC (5669T>C), TAT1487AAT (5670T>A), TCC1488TGA (5674C>G 5675C>A), AAG1489AGG (5677A>G), TTT1490ATT (5679T>A), TTA1492CTT (5685T>C 5687A>T), AAG1493CGG (5688A>C 5689A>G), TCC1494TTC (5692C>T), TTT1496TAT (5698T>A), GTA1499TTG (5706G>T 5708A>G), GCA1500AAG (5709G>A 5710C>A 5711A>G), ATG1501GTG (5712A>G), GAG1502AAG (5715G>A), GAA1503GAG (5720A>G), TCA1504TGT (5722C>G 5723A>T), TCA1505GAT (5724T>G 5725C>A 5726A>T), CCA1507CCC (5732A>C), TGG1508AAG (5733T>A 5734G>A), ACA1509ACG (5738A>G), GCG1510GCC (5741G>C), TGG1512CGA (5745T>C 5747G>A), GTC1513ACT (5748G>A 5749T>C 5750C>T), CCT1514AGA (5751C>A 5752C>G 5753T>A), GAT1515TAT (5754G>T), GGA1516GGT (5759A>T), CTA1517CAT (5761T>A 5762A>T), TAT1518TTT (5764A>T), TGG1520TTC (5770G>T 5771G>C), CTA1521TTG (5772C>T 5774A>G), GTC1522GTT (5777C>T), CCG1524TCA (5781C>T 5783G>A), TTC1525TTT (5786C>T), GGA1526GGT (5789A>T), TTA1527TTG (5792A>G), AAG1528ACC (5794A>C 5795G>C), GCT1530GCA (5801T>A), CCA1531CCG (5804A>G), GCC1532GCG (5807C>G), ATT1533ATA (5810T>A), CAA1535ATG (5814C>A 5815A>T 5816A>G), AGA1536GAC (5817A>G 5818G>A 5819A>C), AAG1537CTC (5820A>C 5821A>T 5822G>C), GAC1539AAT (5826G>A 5828C>T), AAC1540AGG (5830A>G 5831C>G), TGC1541GTC (5832T>G 5833G>T), TTT1542TTC (5837T>C), GGA1544CCC (5841G>C 5842G>C 5843A>C), GGA1544_ACA1545insTTC (5843_5844insTTC), ACA1545CTT (5844A>C 5845C>T 5846A>T), GAA1546GAT (5849A>T), GAA1547ATG (5850G>A 5851A>T 5852A>G), ATA1549GTT (5856A>G 5858A>T), GCA1550GTG (5860C>T 5861A>G), GTT1551GTA (5864T>A), TAT1552TTT (5866A>T), CTA1557TTG (5880C>T 5882A>G), GTC1558ATC (5883G>A), TTC1559TAT (5887T>A 5888C>T), TCA1560TCC (5891A>C), CAG1561CGG (5893A>G), ACT1562AAT (5896C>A), GAA1565GAG (5906A>G), CAT1566CAC (5909T>C), GTC1567ATG (5910G>A 5912C>G), CGT1568AGG (5913C>A 5915T>G), CTA1570TTG (5919C>T 5921A>G), CAA1571AAA (5922C>A), AGG1572ATG (5926G>T), TTC1573GTG (5928T>G 5930C>G), TTC1574TTG (5933C>G)								

*: Inserts / Deletes / Misaligned / Frameshifts

Analysis details

This analysis was performed with panviral2.64

NGS Details (UN24): Unknown

Assembly

**Coverage Length**	314 (1 contig(s))
**Depth Of Coverage**	7.4
**Number Of Reads**	21
**Reads Per Million**	0.42 rpm (after QC)
**Ambiguities**	0
**Assembly Method**	de novo + reference guided assembly
**Consensus Caller**	Bcf Tools

Coverage Map

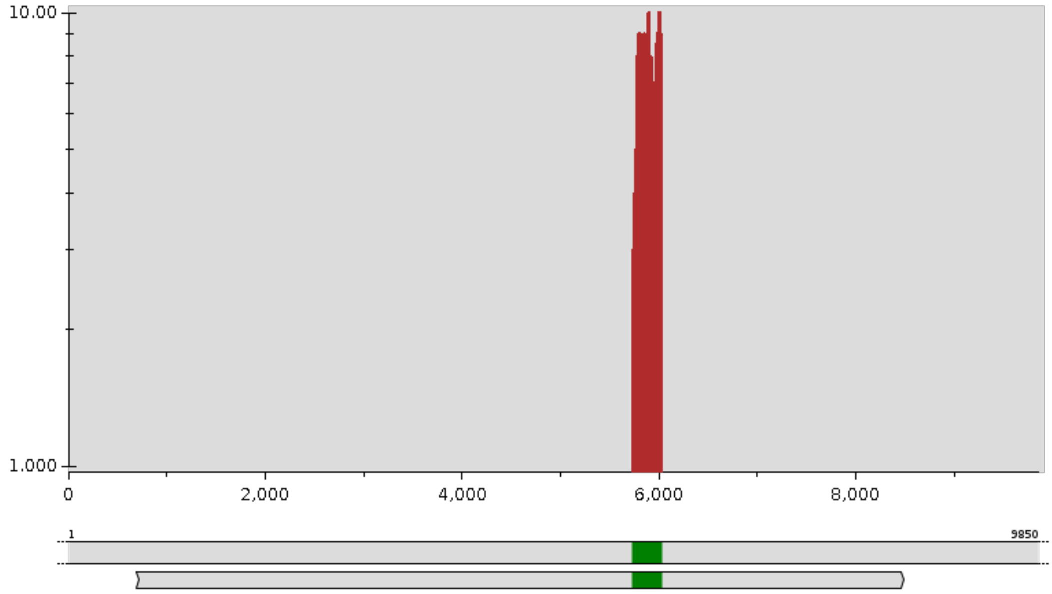

Assignment

**Type**	Unknown (Taxonomy ID: -1)
**Reference Genome**	NC_028462.1
**NT Identity (%)**	57.9618
**AA Identity (%)**	55.7692
**Number Of Stop Codons**	0
**Number Of CDS**	1

Alignment

**Alignment Score**	100.0 (NT) + 411.0 (AA) = 511.0
**Concordance (%)**	37.8238

**Alignment Method**	Global, seeded, nucleotide + amino acids (AGA)

Genome Region

Sequence starts at position 5722 and ends at position 6035 relative to NC_028462.1 reference sequence.

Alignment Detailed Statistics

	Begin	End	Coverage	Score	Concordance	Matches	Identities	I/D/M/F*	Stop Codons
**NT**	**5722**	**6035**	**3.2%**	**100**	**15.9%**	**314 (100%)**	**182 (58.0%)**	**0/0**	
Mutations:	5727A>T, 5729C>A, 5730C>G, 5731A>C, 5735G>A, 5741T>A, 5743T>A, 5744C>A, 5745A>T, 5747C>T, 5750A>T, 5751A>G, 5752C>A, 5753C>A, 5754T>A, 5755A>G, 5756T>A, 5759T>A, 5760G>A, 5761A>G, 5762C>G, 5763A>T, 5766C>A, 5768A>T, 5771C>A, 5774G>A, 5780G>C, 5781T>A, 5782A>G, 5783T>C, 5784G>A, 5785C>T, 5786A>C, 5787C>A, 5789C>A, 5793C>A, 5795T>A, 5798A>T, 5801G>A, 5804C>T, 5809C>T, 5815G>A, 5818C>A, 5819T>A, 5820G>C, 5826G>A, 5829G>A, 5834G>A, 5838G>A, 5842G>T, 5843A>T, 5846A>G, 5847C>A, 5849C>T, 5850G>C, 5855G>A, 5856C>T, 5857A>T, 5859C>T, 5861C>A, 5864A>T, 5865C>A, 5868G>A, 5871C>T, 5873C>A, 5876T>A, 5877C>A, 5878A>G, 5879G>A, 5881C>A, 5882C>T, 5888G>A, 5898T>A, 5899C>G, 5900C>T, 5906A>C, 5912C>T, 5913T>A, 5914G>T, 5915T>G, 5918C>A, 5924T>C, 5929C>G, 5930T>C, 5933G>A, 5934A>C, 5935T>A, 5936C>A, 5939G>A, 5940A>C, 5942A>T, 5943C>G, 5944A>G, 5945G>A, 5951C>T, 5954C>A, 5957T>G, 5960C>A, 5963C>A, 5969C>T, 5975A>G, 5978C>T, 5982C>A, 5983G>A, 5985G>A, 5986T>A, 5987C>T, 5988A>T, 5989C>G, 5990C>T, 5991A>G, 5992A>T, 5993A>C, 5994G>T, 5995A>T, 5999T>C, 6002C>A, 6005C>T, 6008T>C, 6009C>A, 6011A>C, 6014C>A, 6018T>A, 6019T>A, 6020G>A, 6021G>A, 6023G>T, 6025C>A, 6027T>C, 6029A>T, 6030G>A, 6033T>A								

CDS

	Begin	End	Coverage	Score	Concordance	Matches	Identities	I/D/M/F*	Stop Codons
ATL80_gp1	1678	1781	4.0%	411	56.2%	104 (100%)	58 (55.8%)	0/0/0/0	0
Protein mutations:	I1679L (5727A>T 5729C>A), H1680A (5730C>G 5731A>C), D1683E (5741T>A), V1684E (5743T>A 5744C>A), T1685S (5745A>T 5747C>T), Q1686H (5750A>T), T1687E (5751A>G 5752C>A 5753C>A), Y1688R (5754T>A 5755A>G 5756T>A), D1690R (5760G>A 5761A>G 5762C>G), I1691L (5763A>T), Q1692N (5766C>A 5768A>T), Y1697S (5781T>A 5782A>G 5783T>C), A1698I (5784G>A 5785C>T 5786A>C), L1699I (5787C>A 5789C>A), P1706L (5809C>T), S1708N (5815G>A), A1709E (5818C>A 5819T>A), E1710Q (5820G>C), E1712K (5826G>A), A1713T (5829G>A), E1716K (5838G>A), R1717I (5842G>T 5843A>T), L1719I (5847C>A 5849C>T), E1720Q (5850G>C), Q1722L (5856C>T 5857A>T), K1724N (5864A>T), L1725M (5865C>A), E1726K (5868G>A), Q1729R (5877C>A 5878A>G 5879G>A), P1730H (5881C>A 5882C>T), C1741M (5913T>A 5914G>T 5915T>G), A1746G (5929C>G 5930T>C), I1748Q (5934A>C 5935T>A 5936C>A), Q1751G (5943C>G 5944A>G 5945G>A), I1755M (5957T>G), R1764K (5982C>A 5983G>A), V1765N (5985G>A 5986T>A 5987C>T), T1766C (5988A>T 5989C>G 5990C>T), K1767V (5991A>G 5992A>T 5993A>C), D1768F (5994G>T 5995A>T), L1773I (6009C>A 6011A>C), L1776K (6018T>A 6019T>A 6020G>A), E1777N (6021G>A 6023G>T), T1778N (6025C>A), V1780I (6030G>A), Y1781N (6033T>A)								
Codon mutations:	ATC1679TTA (5727A>T 5729C>A), CAC1680GCC (5730C>G 5731A>C), AAG1681AAA (5735G>A), GAT1683GAA (5741T>A), GTC1684GAA (5743T>A 5744C>A), ACC1685TCT (5745A>T 5747C>T), CAA1686CAT (5750A>T), ACC1687GAA (5751A>G 5752C>A 5753C>A), TAT1688AGA (5754T>A 5755A>G 5756T>A), GCT1689GCA (5759T>A), GAC1690AGG (5760G>A 5761A>G 5762C>G), ATA1691TTA (5763A>T), CAA1692AAT (5766C>A 5768A>T), CTC1693CTA (5771C>A), AAG1694AAA (5774G>A), CCG1696CCC (5780G>C), TAT1697AGC (5781T>A 5782A>G 5783T>C), GCA1698ATC (5784G>A 5785C>T 5786A>C), CTC1699ATA (5787C>A 5789C>A), CGT1701AGA (5793C>A 5795T>A), GTA1702GTT (5798A>T), AAG1703AAA (5801G>A), CCC1704CCT (5804C>T), CCA1706CTA (5809C>T), AGT1708AAT (5815G>A), GCT1709GAA (5818C>A 5819T>A), GAA1710CAA (5820G>C), GAA1712AAA (5826G>A), GCT1713ACT (5829G>A), GAG1714GAA (5834G>A), GAA1716AAA (5838G>A), AGA1717ATT (5842G>T 5843A>T), CAA1718CAG (5846A>G), CTC1719ATT (5847C>A 5849C>T), GAA1720CAA (5850G>C), GAG1721GAA (5855G>A), CAA1722TTA (5856C>T 5857A>T), CTC1723TTA (5859C>T 5861C>A), AAA1724AAT (5864A>T), CTG1725ATG (5865C>A), GAA1726AAA (5868G>A), CTC1727TTA (5871C>T 5873C>A), ATT1728ATA (5876T>A), CAG1729AGA (5877C>A 5878A>G 5879G>A), CCC1730CAT (5881C>A 5882C>T), AAG1732AAA (5888G>A), TCC1736AGT (5898T>A 5899C>G 5900C>T), CCA1738CCC (5906A>C), TTC1740TTT (5912C>T), TGT1741ATG (5913T>A 5914G>T 5915T>G), GTC1742GTA (5918C>A), AAT1744AAC (5924T>C), GCT1746GGC (5929C>G 5930T>C), GAG1747GAA (5933G>A), ATC1748CAA (5934A>C 5935T>A 5936C>A), AAG1749AAA (5939G>A), AGA1750CGT (5940A>C 5942A>T), CAG1751GGA (5943C>G 5944A>G 5945G>A), GCC1753GCT (5951C>T), CGC1754CGA (5954C>A), ATT1755ATG (5957T>G), GTC1756GTA (5960C>A), ATC1757ATA (5963C>A), TAC1759TAT (5969C>T), GAA1761GAG (5975A>G), CTC1762CTT (5978C>T), CGA1764AAA (5982C>A 5983G>A), GTC1765AAT (5985G>A 5986T>A 5987C>T), ACC1766TGT (5988A>T 5989C>G 5990C>T), AAA1767GTC (5991A>G 5992A>T 5993A>C), GAC1768TTC (5994G>T 5995A>T), GAT1769GAC (5999T>C), GGC1770GGA (6002C>A), TAC1771TAT (6005C>T), TTT1772TTC (6008T>C), CTA1773ATC (6009C>A 6011A>C), CCC1774CCA (6014C>A), TTG1776AAA (6018T>A 6019T>A 6020G>A), GAG1777AAT (6021G>A 6023G>T), ACC1778AAC (6025C>A), TTA1779CTT (6027T>C 6029A>T), GTA1780ATA (6030G>A), TAT1781AAT (6033T>A)								

Proteins

	Begin	End	Coverage	Score	Concordance	Matches	Identities	I/D/M/F*	Stop Codons
polyprotein (YP_009182100.1)	1678	1781	4.0%	411	56.2%	104 (100%)	58 (55.8%)	0/0/0/0	0
Protein mutations:	I1679L (5727A>T 5729C>A), H1680A (5730C>G 5731A>C), D1683E (5741T>A), V1684E (5743T>A 5744C>A), T1685S (5745A>T 5747C>T), Q1686H (5750A>T), T1687E (5751A>G 5752C>A 5753C>A), Y1688R (5754T>A 5755A>G 5756T>A), D1690R (5760G>A 5761A>G 5762C>G), I1691L (5763A>T), Q1692N (5766C>A 5768A>T), Y1697S (5781T>A 5782A>G 5783T>C), A1698I (5784G>A 5785C>T 5786A>C), L1699I (5787C>A 5789C>A), P1706L (5809C>T), S1708N (5815G>A), A1709E (5818C>A 5819T>A), E1710Q (5820G>C), E1712K (5826G>A), A1713T (5829G>A), E1716K (5838G>A), R1717I (5842G>T 5843A>T), L1719I (5847C>A 5849C>T), E1720Q (5850G>C), Q1722L (5856C>T 5857A>T), K1724N (5864A>T), L1725M (5865C>A), E1726K (5868G>A), Q1729R (5877C>A 5878A>G 5879G>A), P1730H (5881C>A 5882C>T), C1741M (5913T>A 5914G>T 5915T>G), A1746G (5929C>G 5930T>C), I1748Q (5934A>C 5935T>A 5936C>A), Q1751G (5943C>G 5944A>G 5945G>A), I1755M (5957T>G), R1764K (5982C>A 5983G>A), V1765N (5985G>A 5986T>A 5987C>T), T1766C (5988A>T 5989C>G 5990C>T), K1767V (5991A>G 5992A>T 5993A>C), D1768F (5994G>T 5995A>T), L1773I (6009C>A 6011A>C), L1776K (6018T>A 6019T>A 6020G>A), E1777N (6021G>A 6023G>T), T1778N (6025C>A), V1780I (6030G>A), Y1781N (6033T>A)								
Codon mutations:	ATC1679TTA (5727A>T 5729C>A), CAC1680GCC (5730C>G 5731A>C), AAG1681AAA (5735G>A), GAT1683GAA (5741T>A), GTC1684GAA (5743T>A 5744C>A), ACC1685TCT (5745A>T 5747C>T), CAA1686CAT (5750A>T), ACC1687GAA (5751A>G 5752C>A 5753C>A), TAT1688AGA (5754T>A 5755A>G 5756T>A), GCT1689GCA (5759T>A), GAC1690AGG (5760G>A 5761A>G 5762C>G), ATA1691TTA (5763A>T), CAA1692AAT (5766C>A 5768A>T), CTC1693CTA (5771C>A), AAG1694AAA (5774G>A), CCG1696CCC (5780G>C), TAT1697AGC (5781T>A 5782A>G 5783T>C), GCA1698ATC (5784G>A 5785C>T 5786A>C), CTC1699ATA (5787C>A 5789C>A), CGT1701AGA (5793C>A 5795T>A), GTA1702GTT (5798A>T), AAG1703AAA (5801G>A), CCC1704CCT (5804C>T), CCA1706CTA (5809C>T), AGT1708AAT (5815G>A), GCT1709GAA (5818C>A 5819T>A), GAA1710CAA (5820G>C), GAA1712AAA (5826G>A), GCT1713ACT (5829G>A), GAG1714GAA (5834G>A), GAA1716AAA (5838G>A), AGA1717ATT (5842G>T 5843A>T), CAA1718CAG (5846A>G), CTC1719ATT (5847C>A 5849C>T), GAA1720CAA (5850G>C), GAG1721GAA (5855G>A), CAA1722TTA (5856C>T 5857A>T), CTC1723TTA (5859C>T 5861C>A), AAA1724AAT (5864A>T), CTG1725ATG (5865C>A), GAA1726AAA (5868G>A), CTC1727TTA (5871C>T 5873C>A), ATT1728ATA (5876T>A), CAG1729AGA (5877C>A 5878A>G 5879G>A), CCC1730CAT (5881C>A 5882C>T), AAG1732AAA (5888G>A), TCC1736AGT (5898T>A 5899C>G 5900C>T), CCA1738CCC (5906A>C), TTC1740TTT (5912C>T), TGT1741ATG (5913T>A 5914G>T 5915T>G), GTC1742GTA (5918C>A), AAT1744AAC (5924T>C), GCT1746GGC (5929C>G 5930T>C), GAG1747GAA (5933G>A), ATC1748CAA (5934A>C 5935T>A 5936C>A), AAG1749AAA (5939G>A), AGA1750CGT (5940A>C 5942A>T), CAG1751GGA (5943C>G 5944A>G 5945G>A), GCC1753GCT (5951C>T), CGC1754CGA (5954C>A), ATT1755ATG (5957T>G), GTC1756GTA (5960C>A), ATC1757ATA (5963C>A), TAC1759TAT (5969C>T), GAA1761GAG (5975A>G), CTC1762CTT (5978C>T), CGA1764AAA (5982C>A 5983G>A), GTC1765AAT (5985G>A 5986T>A 5987C>T), ACC1766TGT (5988A>T 5989C>G 5990C>T), AAA1767GTC (5991A>G 5992A>T 5993A>C), GAC1768TTC (5994G>T 5995A>T), GAT1769GAC (5999T>C), GGC1770GGA (6002C>A), TAC1771TAT (6005C>T), TTT1772TTC (6008T>C), CTA1773ATC (6009C>A 6011A>C), CCC1774CCA (6014C>A), TTG1776AAA (6018T>A 6019T>A 6020G>A), GAG1777AAT (6021G>A 6023G>T), ACC1778AAC (6025C>A), TTA1779CTT (6027T>C 6029A>T), GTA1780ATA (6030G>A), TAT1781AAT (6033T>A)								

*: Inserts / Deletes / Misaligned / Frameshifts

Analysis details

This analysis was performed with panviral2.64

NGS Details (UN24): Bracoviriform congregatae (segment Circle 7)

Assembly

**Coverage Length**	145 (1 contig(s))
**Depth Of Coverage**	4.4
**Number Of Reads**	5
**Reads Per Million**	0.10 rpm (after QC)
**Ambiguities**	0
**Assembly Method**	de novo + reference guided assembly
**Consensus Caller**	Bcf Tools

Coverage Map

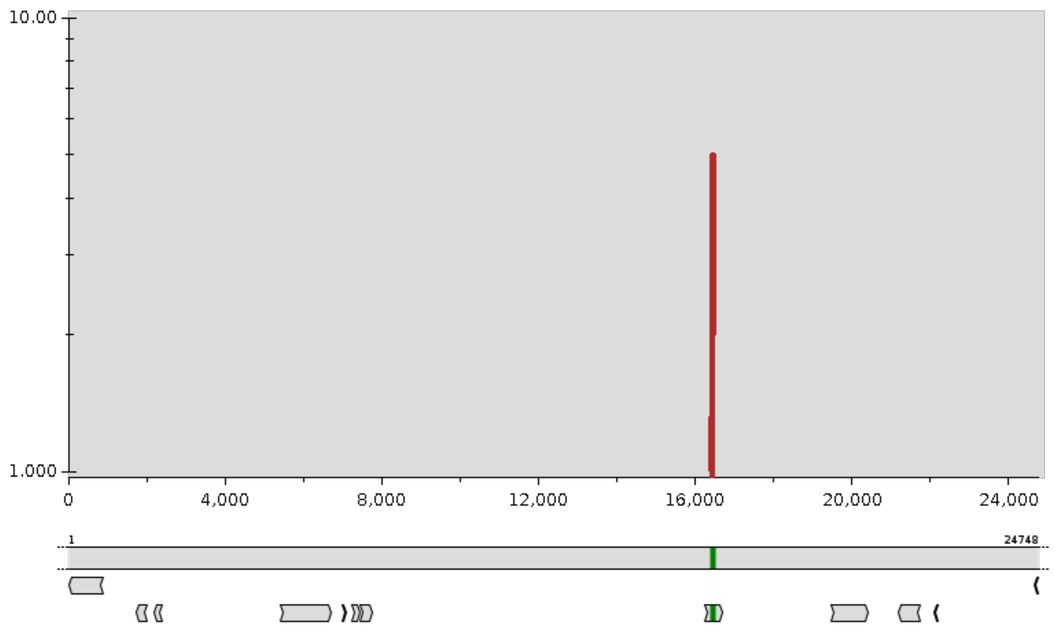

Assignment

**Type**	Bracoviriform congregatae (Taxonomy ID: 39640)
**Reference Genome**	NC_006639.1
**NT Identity (%)**	80.0
**AA Identity (%)**	87.7551
**Number Of Stop Codons**	0
**Number Of CDS**	5

Alignment

**Alignment Score**	174.0 (NT) + 285.0 (AA) = 459.0
**Concordance (%)**	75.9934

**Alignment Method**	Global, seeded, nucleotide + amino acids (AGA)

Genome Region

Sequence starts at position 16385 and ends at position 16529 relative to NC_006639.1 reference sequence.

Alignment Detailed Statistics

	Begin	End	Coverage	Score	Concordance	Matches	Identities	I/D/M/F*	Stop Codons
**NT**	**16385**	**16529**	**0.6%**	**174**	**60.0%**	**145 (100%)**	**116 (80.0%)**	**0/0**	
Mutations:	16393A>T, 16397T>C, 16398C>G, 16399G>T, 16401T>G, 16408A>T, 16411G>A, 16414A>G, 16418T>C, 16420G>C, 16426A>G, 16429G>A, 16432C>A, 16435T>C, 16444T>C, 16449G>A, 16450A>G, 16451T>A, 16452C>T, 16453T>C, 16459A>T, 16462T>C, 16471A>G, 16474A>T, 16477C>T, 16486A>C, 16492C>T, 16501G>C, 16516A>T								
**CDS**									
Histone	52	100	31.4%	285	89.6%	49 (100%)	43 (87.8%)	0/0/0/0	0
Protein mutations:	K54N (16393A>T), S56R (16397T>C 16398C>G 16399G>T), V57G (16401T>G), R73K (16449G>A 16450A>G), S74I (16451T>A 16452C>T 16453T>C)								
Codon mutations:	AAA54AAT (16393A>T), TCG56CGT (16397T>C 16398C>G 16399G>T), GTA57GGA (16401T>G), GGA59GGT (16408A>T), GGG60GGA (16411G>A), AAA61AAG (16414A>G), TTG63CTC (16418T>C 16420G>C), AAA65AAG (16426A>G), GGG66GGA (16429G>A), GGC67GGA (16432C>A), GCT68GCC (16435T>C), CAT71CAC (16444T>C), AGA73AAG (16449G>A 16450A>G), TCT74ATC (16451T>A 16452C>T 16453T>C), CGA76CGT (16459A>T), GAT77GAC (16462T>C), CAA80CAG (16471A>G), GGA81GGT (16474A>T), ATC82ATT (16477C>T), CCA85CCC (16486A>C), ATC87ATT (16492C>T), CTG90CTC (16501G>C), GGA95GGT (16516A>T)								
**Proteins**									
hypothetical protein (YP_184795.1)	52	100	31.4%	285	89.6%	49 (100%)	43 (87.8%)	0/0/0/0	0
Protein mutations:	K54N (16393A>T), S56R (16397T>C 16398C>G 16399G>T), V57G (16401T>G), R73K (16449G>A 16450A>G), S74I (16451T>A 16452C>T 16453T>C)								
Codon mutations:	AAA54AAT (16393A>T), TCG56CGT (16397T>C 16398C>G 16399G>T), GTA57GGA (16401T>G), GGA59GGT (16408A>T), GGG60GGA (16411G>A), AAA61AAG (16414A>G), TTG63CTC (16418T>C 16420G>C), AAA65AAG (16426A>G), GGG66GGA (16429G>A), GGC67GGA (16432C>A), GCT68GCC (16435T>C), CAT71CAC (16444T>C), AGA73AAG (16449G>A 16450A>G), TCT74ATC (16451T>A 16452C>T 16453T>C), CGA76CGT (16459A>T), GAT77GAC (16462T>C), CAA80CAG (16471A>G), GGA81GGT (16474A>T), ATC82ATT (16477C>T), CCA85CCC (16486A>C), ATC87ATT (16492C>T), CTG90CTC (16501G>C), GGA95GGT (16516A>T)								

*: Inserts / Deletes / Misaligned / Frameshifts

Analysis details

This analysis was performed with panviral2.64

NGS Details (UN24): Bracoviriform congregatae (segment Circle 7)

Assembly

**Coverage Length**	135 (1 contig(s))
**Depth Of Coverage**	12.2
**Number Of Reads**	20
**Reads Per Million**	0.40 rpm (after QC)
**Ambiguities**	0
**Assembly Method**	de novo + reference guided assembly
**Consensus Caller**	Bcf Tools

Coverage Map

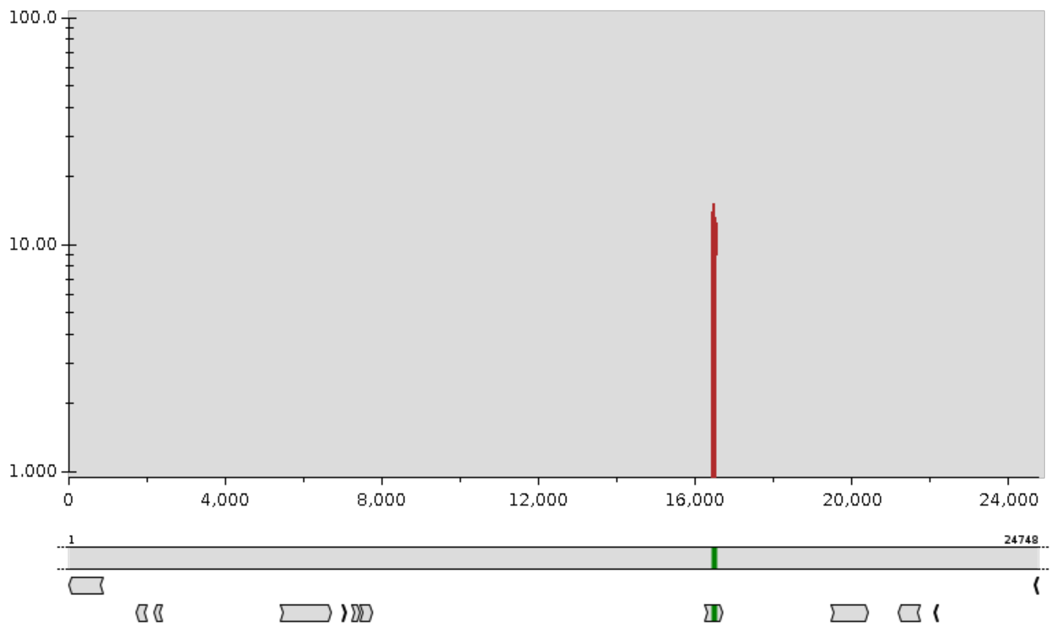

Assignment

**Type**	Bracoviriform congregatae (Taxonomy ID: 39640)
**Reference Genome**	NC_006639.1
**NT Identity (%)**	77.7778
**AA Identity (%)**	84.4444
**Number Of Stop Codons**	0
**Number Of CDS**	5

Alignment

**Alignment Score**	150.0 (NT) + 266.0 (AA) = 416.0
**Concordance (%)**	74.2857

Alignment Method	Global, seeded, nucleotide + amino acids (AGA)

Genome Region

Sequence starts at position 16414 and ends at position 16548 relative to NC_006639.1 reference sequence.

Alignment Detailed Statistics

	Begin	End	Coverage	Score	Concordance	Matches	Identities	I/D/M/F*	Stop Codons
**NT**	**16414**	**16548**	**0.5%**	**150**	**55.6%**	**135 (100%)**	**105 (77.8%)**	**0/0**	
Mutations:	16414A>G, 16417A>T, 16418T>C, 16420G>C, 16423A>C, 16426A>G, 16429G>C, 16435T>C, 16444T>C, 16449G>A, 16450A>G, 16451T>A, 16452C>T, 16459A>T, 16462T>C, 16471A>G, 16474A>T, 16486A>C, 16495T>C, 16501G>C, 16516A>T, 16526G>A, 16528C>T, 16531T>A, 16533G>C, 16534A>C, 16535T>A, 16538G>A, 16543T>C, 16546A>G								
**CDS**									
Histone	62	106	28.8%	266	88.4%	45 (100%)	38 (84.4%)	0/0/0/0	0
Protein mutations:	R73K (16449G>A 16450A>G), S74I (16451T>A 16452C>T), V99I (16526G>A 16528C>T), G101A (16533G>C 16534A>C), L102M (16535T>A), V103I (16538G>A)								
Codon mutations:	AAA61..G (16414A>G), GGA62GGT (16417A>T), TTG63CTC (16418T>C 16420G>C), GGA64GGC (16423A>C), AAA65AAG (16426A>G), GGG66GGC (16429G>C), GCT68GCC (16435T>C), CAT71CAC (16444T>C), AGA73AAG (16449G>A 16450A>G), TCT74ATT (16451T>A 16452C>T), CGA76CGT (16459A>T), GAT77GAC (16462T>C), CAA80CAG (16471A>G), GGA81GGT (16474A>T), CCA85CCC (16486A>C), CGT88CGC (16495T>C), CTG90CTC (16501G>C), GGA95GGT (16516A>T), GTC99ATT (16526G>A 16528C>T), TCT100TCA (16531T>A), GGA101GCC (16533G>C 16534A>C), TTG102ATG (16535T>A), GTC103ATC (16538G>A), TAT104TAC (16543T>C), GAA105GAG (16546A>G)								
**Proteins**									
hypothetical protein (YP_184795.1)	62	106	28.8%	266	88.4%	45 (100%)	38 (84.4%)	0/0/0/0	0
Protein mutations:	R73K (16449G>A 16450A>G), S74I (16451T>A 16452C>T), V99I (16526G>A 16528C>T), G101A (16533G>C 16534A>C), L102M (16535T>A), V103I (16538G>A)								
Codon mutations:	AAA61..G (16414A>G), GGA62GGT (16417A>T), TTG63CTC (16418T>C 16420G>C), GGA64GGC (16423A>C), AAA65AAG (16426A>G), GGG66GGC (16429G>C), GCT68GCC (16435T>C), CAT71CAC (16444T>C), AGA73AAG (16449G>A 16450A>G), TCT74ATT (16451T>A 16452C>T), CGA76CGT (16459A>T), GAT77GAC (16462T>C), CAA80CAG (16471A>G), GGA81GGT (16474A>T), CCA85CCC (16486A>C), CGT88CGC (16495T>C), CTG90CTC (16501G>C), GGA95GGT (16516A>T), GTC99ATT (16526G>A 16528C>T), TCT100TCA (16531T>A), GGA101GCC (16533G>C 16534A>C), TTG102ATG (16535T>A), GTC103ATC (16538G>A), TAT104TAC (16543T>C), GAA105GAG (16546A>G)								

*: Inserts / Deletes / Misaligned / Frameshifts

Analysis details

This analysis was performed with panviral2.64

NGS Details (UN24): Petuvirus venapetuniae

Assembly

**Coverage Length**	316 (1 contig(s))
**Depth Of Coverage**	6.3
**Number Of Reads**	20
**Reads Per Million**	0.40 rpm (after QC)
**Ambiguities**	0
**Assembly Method**	de novo + reference guided assembly
**Consensus Caller**	Bcf Tools

Coverage Map

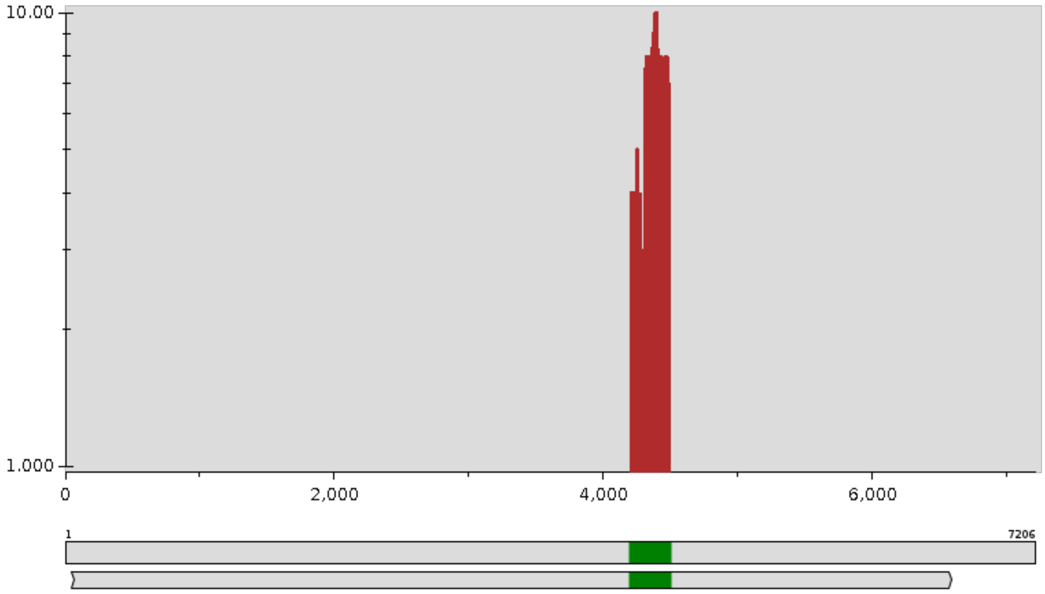

Assignment

**Type**	Petuvirus venapetuniae (Taxonomy ID: 3048265)
**Reference Genome**	NC_001839.2
**NT Identity (%)**	61.0759
**AA Identity (%)**	54.2857
**Number Of Stop Codons**	0
**Number Of CDS**	1

Alignment

**Alignment Score**	140.0 (NT) + 387.0 (AA) = 527.0
**Concordance (%)**	37.8864

**Alignment Method**	Global, seeded, nucleotide + amino acids (AGA)

Genome Region

Sequence starts at position 4190 and ends at position 4505 relative to NC_001839.2 reference sequence.

Alignment Detailed Statistics

	Begin	End	Coverage	Score	Concordance	Matches	Identities	I/D/M/F*	Stop Codons
**NT**	**4190**	**4505**	**4.4%**	**140**	**22.2%**	**316 (100%)**	**193 (61.1%)**	**0/0**	
Mutations:	4196C>T, 4197A>G, 4198T>A, 4199C>T, 4201A>T, 4208A>T, 4211G>A, 4212G>T, 4215A>C, 4217C>T, 4219A>C, 4220T>C, 4222C>G, 4224G>C, 4225G>A, 4233C>G, 4234C>T, 4235A>T, 4239C>T, 4240A>T, 4241T>A, 4242C>G, 4244C>G, 4245C>G, 4247G>A, 4250A>C, 4251G>T, 4252C>G, 4253A>C, 4255T>A, 4259G>A, 4260G>A, 4262A>G, 4263T>A, 4264G>T, 4269G>A, 4271G>T, 4275C>T, 4276A>T, 4281T>A, 4282T>A, 4283T>G, 4285A>G, 4287C>T, 4292C>A, 4293G>A, 4298C>A, 4301T>A, 4302G>A, 4304T>G, 4307A>T, 4309A>C, 4310A>T, 4314G>T, 4319T>C, 4320G>A, 4321A>C, 4324C>T, 4334C>T, 4337C>T, 4340G>T, 4341A>G, 4342G>C, 4343G>T, 4344T>A, 4345C>G, 4346A>T, 4349G>A, 4350C>A, 4352A>G, 4354T>A, 4355G>A, 4356A>C, 4361A>T, 4363A>T, 4364A>T, 4365T>C, 4367A>C, 4368A>C, 4370G>A, 4376C>T, 4386C>A, 4397C>T, 4398C>A, 4400T>A, 4403C>T, 4406C>T, 4407C>A, 4410G>T, 4411A>G, 4412T>G, 4413G>A, 4414A>T, 4415C>T, 4417A>G, 4420T>A, 4430A>G, 4436G>A, 4437C>A, 4438T>G, 4439C>G, 4440A>G, 4441C>A, 4442A>T, 4443C>T, 4445C>A, 4448C>A, 4452C>A, 4453A>G, 4454C>A, 4459C>A, 4460C>T, 4461A>G, 4463A>T, 4466A>C, 4469G>T, 4470C>A, 4472C>A, 4481G>T, 4488C>T, 4490T>A, 4493G>A, 4501T>A								

CDS

	Begin	End	Coverage	Score	Concordance	Matches	Identities	I/D/M/F*	Stop Codons
PVCVgp1	1381	1485	4.8%	387	50.5%	105 (100%)	57 (54.3%)	0/0/0/0	0
Protein mutations:	I1383D (4197A>G 4198T>A 4199C>T), N1384I (4201A>T), A1388S (4212G>T), S1389R (4215A>C 4217C>T), H1390P (4219A>C 4220T>C), S1391C (4222C>G), G1392Q (4224G>C 4225G>A), P1395V (4233C>G 4234C>T 4235A>T), H1397L (4239C>T 4240A>T 4241T>A), L1398V (4242C>G 4244C>G), Q1399E (4245C>G 4247G>A), L1400F (4250A>C), A1401C (4251G>T 4252C>G 4253A>C), I1402K (4255T>A), E1404K (4260G>A 4262A>G), C1405I (4263T>A 4264G>T), E1407N (4269G>A 4271G>T), Q1409L (4275C>T 4276A>T), F1411K (4281T>A 4282T>A 4283T>G), D1412G (4285A>G), E1415K (4293G>A), D1418K (4302G>A 4304T>G), Q1420P (4309A>C 4310A>T), A1422S (4314G>T), E1424T (4320G>A 4321A>C), A1425V (4324C>T), K1430N (4340G>T), R1431A (4341A>G 4342G>C 4343G>T), Q1434K (4350C>A 4352A>G), V1435E (4354T>A 4355G>A), K1438I (4363A>T 4364A>T), Q1446K (4386C>A), H1450K (4398C>A 4400T>A), Q1453K (4407C>A), D1454W (4410G>T 4411A>G 4412T>G), D1455I (4413G>A 4414A>T 4415C>T), K1456R (4417A>G), F1457Y (4420T>A), L1463R (4437C>A 4438T>G 4439C>G), T1464D (4440A>G 4441C>A 4442A>T), F1466L (4448C>A), H1468R (4452C>A 4453A>G 4454C>A), S1470Y (4459C>A 4460C>T), K1471D (4461A>G 4463A>T), K1473N (4469G>T), L1474I (4470C>A 4472C>A), K1477N (4481G>T), F1484Y (4501T>A)								
Codon mutations:	AAC1382AAT (4196C>T), ATC1383GAT (4197A>G 4198T>A 4199C>T), AAT1384ATT (4201A>T), ACA1386ACT (4208A>T), AAG1387AAA (4211G>A), GCA1388TCA (4212G>T), AGC1389CGT (4215A>C 4217C>T), CAT1390CCC (4219A>C 4220T>C), TCT1391TGT (4222C>G), GGA1392CAA (4224G>C 4225G>A), CCA1395GTT (4233C>G 4234C>T 4235A>T), CAT1397TTA (4239C>T 4240A>T 4241T>A), CTC1398GTG (4242C>G 4244C>G), CAG1399GAA (4245C>G 4247G>A), TTA1400TTC (4250A>C), GCA1401TGC (4251G>T 4252C>G 4253A>C), ATA1402AAA (4255T>A), AAG1403AAA (4259G>A), GAA1404AAG (4260G>A 4262A>G), TGT1405ATT (4263T>A 4264G>T), GAG1407AAT (4269G>A 4271G>T), CAA1409TTA (4275C>T 4276A>T), TTT1411AAG (4281T>A 4282T>A 4283T>G), GAT1412GGT (4285A>G), CTA1413TTA (4287C>T), ATC1414ATA (4292C>A), GAA1415AAA (4293G>A), CCC1416CCA (4298C>A), TCT1417TCA (4301T>A), GAT1418AAG (4302G>A 4304T>G), TCA1419TCT (4307A>T), CAA1420CCT (4309A>C 4310A>T), GCA1422TCA (4314G>T), TGT1423TGC (4319T>C), GAA1424ACA (4320G>A 4321A>C), GCT1425GTT (4324C>T), GTC1428GTT (4334C>T), AAC1429AAT (4337C>T), AAG1430AAT (4340G>T), AGG1431GCT (4341A>G 4342G>C 4343G>T), TCA1432AGT (4344T>A 4345C>G 4346A>T), GAG1433GAA (4349G>A), CAA1434AAG (4350C>A 4352A>G), GTG1435GAA (4354T>A 4355G>A), AGA1436CGA (4356A>C), GGA1437GGT (4361A>T), AAA1438ATT (4363A>T 4364A>T), TTA1439CTC (4365T>C 4367A>C), AGG1440CGA (4368A>C 4370G>A), GTC1442GTT (4376C>T), CAA1446AAA (4386C>A), AAC1449AAT (4397C>T), CAT1450AAA (4398C>A 4400T>A), TTC1451TTT (4403C>T), CTC1452CTT (4406C>T), CAA1453AAA (4407C>A), GAT1454TGG (4410G>T 4411A>G 4412T>G), GAC1455ATT (4413G>A 4414A>T 4415C>T), AAG1456AGG (4417A>G), TTT1457TAT (4420T>A), CCA1460CCG (4430A>G), AAG1462AAA (4436G>A), CTC1463AGG (4437C>A 4438T>G 4439C>G), ACA1464GAT (4440A>G 4441C>A 4442A>T), CTC1465TTA (4443C>T 4445C>A), TTC1466TTA (4448C>A), CAC1468AGA (4452C>A 4453A>G 4454C>A), TCC1470TAT (4459C>A 4460C>T), AAA1471GAT (4461A>G 4463A>T), GCA1472GCC (4466A>C), AAG1473AAT (4469G>T), CTC1474ATA (4470C>A 4472C>A), AAG1477AAT (4481G>T), CTT1480TTA (4488C>T 4490T>A), AAG1481AAA (4493G>A), TTT1484TAT (4501T>A)								

Proteins

	Begin	End	Coverage	Score	Concordance	Matches	Identities	I/D/M/F*	Stop Codons
ORF I polyprotein (NP_127504.1)	1381	1485	4.8%	387	50.5%	105 (100%)	57 (54.3%)	0/0/0/0	0
Protein mutations:	I1383D (4197A>G 4198T>A 4199C>T), N1384I (4201A>T), A1388S (4212G>T), S1389R (4215A>C 4217C>T), H1390P (4219A>C 4220T>C), S1391C (4222C>G), G1392Q (4224G>C 4225G>A), P1395V (4233C>G 4234C>T 4235A>T), H1397L (4239C>T 4240A>T 4241T>A), L1398V (4242C>G 4244C>G), Q1399E (4245C>G 4247G>A), L1400F (4250A>C), A1401C (4251G>T 4252C>G 4253A>C), I1402K (4255T>A), E1404K (4260G>A 4262A>G), C1405I (4263T>A 4264G>T), E1407N (4269G>A 4271G>T), Q1409L (4275C>T 4276A>T), F1411K (4281T>A 4282T>A 4283T>G), D1412G (4285A>G), E1415K (4293G>A), D1418K (4302G>A 4304T>G), Q1420P (4309A>C 4310A>T), A1422S (4314G>T), E1424T (4320G>A 4321A>C), A1425V (4324C>T), K1430N (4340G>T), R1431A (4341A>G 4342G>C 4343G>T), Q1434K (4350C>A 4352A>G), V1435E (4354T>A 4355G>A), K1438I (4363A>T 4364A>T), Q1446K (4386C>A), H1450K (4398C>A 4400T>A), Q1453K (4407C>A), D1454W (4410G>T 4411A>G 4412T>G), D1455I (4413G>A 4414A>T 4415C>T), K1456R (4417A>G), F1457Y (4420T>A), L1463R (4437C>A 4438T>G 4439C>G), T1464D (4440A>G 4441C>A 4442A>T), F1466L (4448C>A), H1468R (4452C>A 4453A>G 4454C>A), S1470Y (4459C>A 4460C>T), K1471D (4461A>G 4463A>T), K1473N (4469G>T), L1474I (4470C>A 4472C>A), K1477N (4481G>T), F1484Y (4501T>A)								
Codon mutations:	AAC1382AAT (4196C>T), ATC1383GAT (4197A>G 4198T>A 4199C>T), AAT1384ATT (4201A>T), ACA1386ACT (4208A>T), AAG1387AAA (4211G>A), GCA1388TCA (4212G>T), AGC1389CGT (4215A>C 4217C>T), CAT1390CCC (4219A>C 4220T>C), TCT1391TGT (4222C>G), GGA1392CAA (4224G>C 4225G>A), CCA1395GTT (4233C>G 4234C>T 4235A>T), CAT1397TTA (4239C>T 4240A>T 4241T>A), CTC1398GTG (4242C>G 4244C>G), CAG1399GAA (4245C>G 4247G>A), TTA1400TTC (4250A>C), GCA1401TGC (4251G>T 4252C>G 4253A>C), ATA1402AAA (4255T>A), AAG1403AAA (4259G>A), GAA1404AAG (4260G>A 4262A>G), TGT1405ATT (4263T>A 4264G>T), GAG1407AAT (4269G>A 4271G>T), CAA1409TTA (4275C>T 4276A>T), TTT1411AAG (4281T>A 4282T>A 4283T>G), GAT1412GGT (4285A>G), CTA1413TTA (4287C>T), ATC1414ATA (4292C>A), GAA1415AAA (4293G>A), CCC1416CCA (4298C>A), TCT1417TCA (4301T>A), GAT1418AAG (4302G>A 4304T>G), TCA1419TCT (4307A>T), CAA1420CCT (4309A>C 4310A>T), GCA1422TCA (4314G>T), TGT1423TGC (4319T>C), GAA1424ACA (4320G>A 4321A>C), GCT1425GTT (4324C>T), GTC1428GTT (4334C>T), AAC1429AAT (4337C>T), AAG1430AAT (4340G>T), AGG1431GCT (4341A>G 4342G>C 4343G>T), TCA1432AGT (4344T>A 4345C>G 4346A>T), GAG1433GAA (4349G>A), CAA1434AAG (4350C>A 4352A>G), GTG1435GAA (4354T>A 4355G>A), AGA1436CGA (4356A>C), GGA1437GGT (4361A>T), AAA1438ATT (4363A>T 4364A>T), TTA1439CTC (4365T>C 4367A>C), AGG1440CGA (4368A>C 4370G>A), GTC1442GTT (4376C>T), CAA1446AAA (4386C>A), AAC1449AAT (4397C>T), CAT1450AAA (4398C>A 4400T>A), TTC1451TTT (4403C>T), CTC1452CTT (4406C>T), CAA1453AAA (4407C>A), GAT1454TGG (4410G>T 4411A>G 4412T>G), GAC1455ATT (4413G>A 4414A>T 4415C>T), AAG1456AGG (4417A>G), TTT1457TAT (4420T>A), CCA1460CCG (4430A>G), AAG1462AAA (4436G>A), CTC1463AGG (4437C>A 4438T>G 4439C>G), ACA1464GAT (4440A>G 4441C>A 4442A>T), CTC1465TTA (4443C>T 4445C>A), TTC1466TTA (4448C>A), CAC1468AGA (4452C>A 4453A>G 4454C>A), TCC1470TAT (4459C>A 4460C>T), AAA1471GAT (4461A>G 4463A>T), GCA1472GCC (4466A>C), AAG1473AAT (4469G>T), CTC1474ATA (4470C>A 4472C>A), AAG1477AAT (4481G>T), CTT1480TTA (4488C>T 4490T>A), AAG1481AAA (4493G>A), TTT1484TAT (4501T>A)								

*: Inserts / Deletes / Misaligned / Frameshifts

Analysis details

This analysis was performed with panviral2.64

NGS Details (UN24): Marseillevirus marseillevirus

Assembly

**Coverage Length**	117 (1 contig(s))
**Depth Of Coverage**	11.4
**Number Of Reads**	18
**Reads Per Million**	0.36 rpm (after QC)
**Ambiguities**	0
**Assembly Method**	de novo + reference guided assembly
**Consensus Caller**	Bcf Tools

Coverage Map

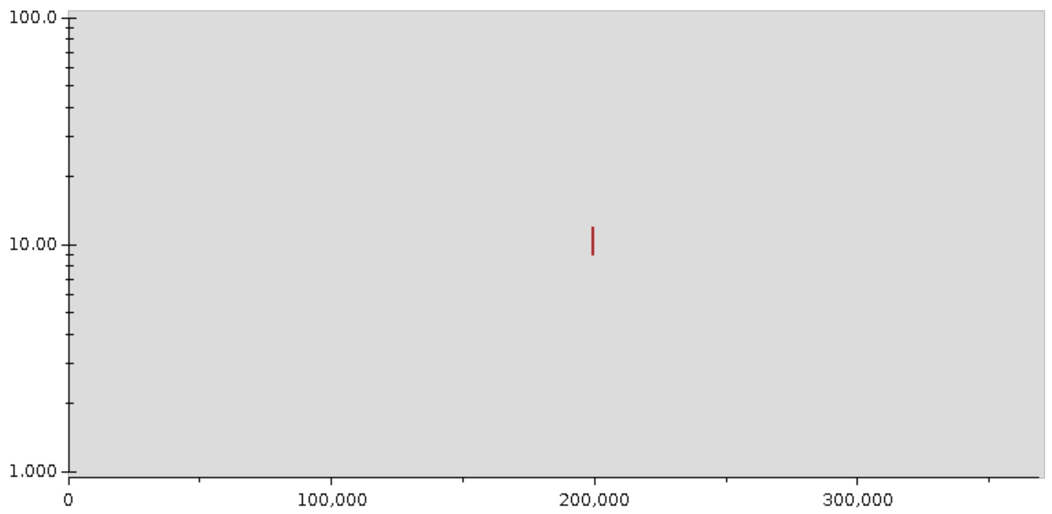

Assignment

**Type**	Marseillevirus marseillevirus (Taxonomy ID: 694581)
**Reference Genome**	NC_013756.1
**NT Identity (%)**	82.0513
**AA Identity (%)**	92.3077
**Number Of Stop Codons**	0
**Number Of CDS**	428

Alignment

**Alignment Score**	150.0 (NT) + 248.0 (AA) = 398.0
**Concordance (%)**	81.893
**Alignment Method**	Local, heuristic, nucleotide (BLASTN)

Genome Region

Sequence starts at position 199409 and ends at position 199525 relative to NC_013756.1 reference sequence.

Alignment Detailed Statistics

	Begin	End	Coverage	Score	Concordance	Matches	Identities	I/D/M/F*	Stop Codons
NT	199409	199525	0.1%	150	64.1%	117 (100%)	96 (82.1%)	0/0	

Mutations: 199414T>A, 199416T>G, 199417C>G, 199420G>A, 199423A>C, 199425G>A, 199429G>A, 199432A>C, 199444T>C, 199449C>T, 199461C>A, 199462C>G, 199464A>G, 199468C>G, 199470T>G, 199471T>G, 199477T>C, 199480C>G, 199489T>G, 199513G>A, 199516C>G

*: Inserts / Deletes / Misaligned / Frameshifts

Analysis details

This analysis was performed with panviral2.64

NGS Details (UN24): Metaplexis yellow mottle-associated virus

Assembly

**Coverage Length**	293 (1 contig(s))
**Depth Of Coverage**	6.8
**Number Of Reads**	17
**Reads Per Million**	0.34 rpm (after QC)
**Ambiguities**	0
**Assembly Method**	de novo + reference guided assembly
**Consensus Caller**	Bcf Tools

Coverage Map

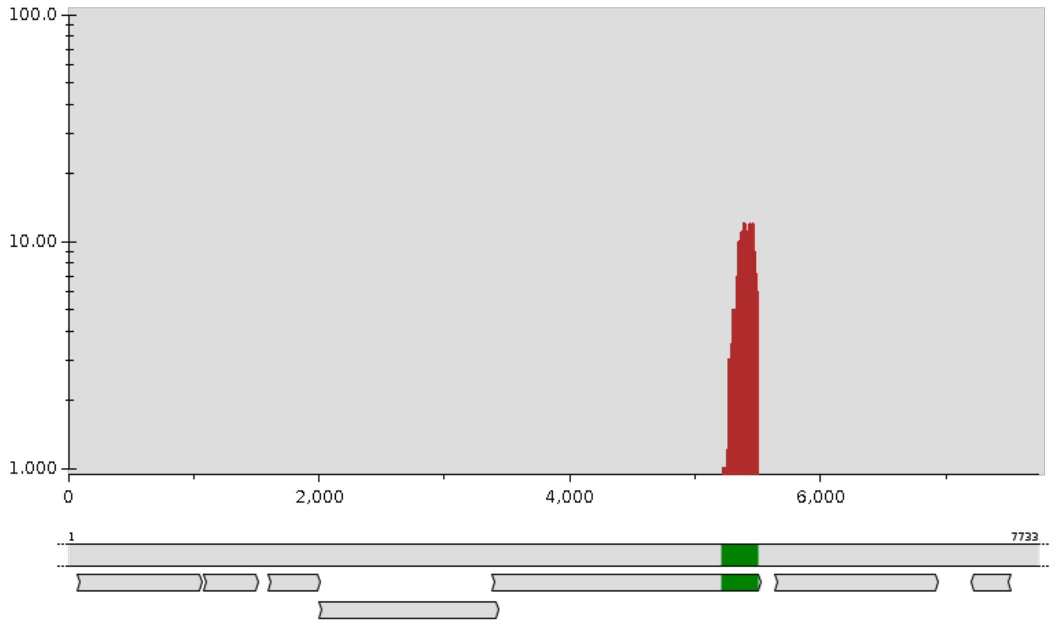

Assignment

**Type**	Metaplexis yellow mottle-associated virus (Taxonomy ID: 2878269)
**Reference Genome**	NC_077108.1
**NT Identity (%)**	58.7031
**AA Identity (%)**	45.9184
**Number Of Stop Codons**	0
**Number Of CDS**	7

Alignment

**Alignment Score**	102.0 (NT) + 323.0 (AA) = 425.0
**Concordance (%)**	34.219

Alignment Method	Global, seeded, nucleotide + amino acids (AGA)

Genome Region

Sequence starts at position 5214 and ends at position 5506 relative to NC_077108.1 reference sequence.

Alignment Detailed Statistics

	Begin	End	Coverage	Score	Concordance	Matches	Identities	I/D/M/F*	Stop Codons
**NT**	**5214**	**5506**	**3.8%**	**102**	**17.4%**	**293 (100%)**	**172 (58.7%)**	**0/0**	
Mutations:	5217A>T, 5219C>T, 5226T>A, 5227G>C, 5229T>A, 5230C>G, 5231C>T, 5233G>A, 5234T>C, 5235T>A, 5243A>T, 5244C>G, 5246G>A, 5247G>A, 5252G>A, 5254A>C, 5255A>T, 5258T>A, 5262C>T, 5263A>C, 5264T>A, 5266G>C, 5267T>A, 5273G>A, 5276A>G, 5280A>T, 5285A>G, 5288T>C, 5289T>A, 5291G>A, 5292A>G, 5294T>A, 5298A>G, 5299C>G, 5300A>C, 5303T>A, 5304A>C, 5305A>G, 5306G>A, 5307G>A, 5308C>A, 5309T>A, 5313A>T, 5314A>C, 5315A>T, 5318A>T, 5320A>T, 5324G>A, 5331G>A, 5332T>A, 5333A>G, 5334A>C, 5335G>A, 5339T>C, 5342T>C, 5343G>A, 5344C>T, 5345T>C, 5349A>T, 5351A>G, 5354T>C, 5360T>A, 5361A>C, 5363T>G, 5366T>A, 5367A>T, 5368A>C, 5370T>G, 5371A>G, 5375C>G, 5378T>A, 5379A>T, 5380G>T, 5383C>A, 5387C>A, 5388A>C, 5390C>T, 5391T>C, 5394G>T, 5395G>C, 5399T>A, 5400T>C, 5401A>C, 5402C>T, 5403A>C, 5406C>T, 5408A>T, 5409G>A, 5410C>G, 5414A>G, 5415C>T, 5417T>A, 5418G>C, 5419T>A, 5420G>T, 5430C>G, 5431A>T, 5434A>T, 5435C>G, 5436C>T, 5438T>A, 5439A>T, 5440A>C, 5443A>T, 5444C>T, 5447C>T, 5449A>C, 5454G>A, 5461T>A, 5463C>A, 5465C>T, 5475A>T, 5476A>C, 5478A>G, 5479C>A, 5487C>T, 5495C>T, 5496A>T, 5499A>C, 5501G>A, 5504C>A								

CDS

	Begin	End	Coverage	Score	Concordance	Matches	Identities	I/D/M/F*	Stop Codons
QKU89_gp5	611	708	13.7%	323	48.6%	98 (100%)	45 (45.9%)	0/0/0/0	0
Protein mutations:	S612C (5217A>T 5219C>T), C615T (5226T>A 5227G>C), G617D (5233G>A 5234T>C), S618T (5235T>A), K620N (5243A>T), Q621E (5244C>G 5246G>A), A622T (5247G>A), K624T (5254A>C 5255A>T), N625K (5258T>A), H627S (5262C>T 5263A>C 5264T>A), S628T (5266G>C 5267T>A), I633L (5280A>T), L636I (5289T>A 5291G>A), I637V (5292A>G 5294T>A), T639G (5298A>G 5299C>G 5300A>C), K641R (5304A>C 5305A>G 5306G>A), A642K (5307G>A 5308C>A 5309T>A), K644S (5313A>T 5314A>C 5315A>T), Y646F (5320A>T), V650K (5331G>A 5332T>A 5333A>G), R651Q (5334A>C 5335G>A), A654I (5343G>A 5344C>T 5345T>C), T656S (5349A>T 5351A>G), N660Q (5361A>C 5363T>G), K662S (5367A>T 5368A>C), Y663G (5370T>G 5371A>G), F664L (5375C>G), S666F (5379A>T 5380G>T), T667N (5383C>A), N668K (5387C>A), I669L (5388A>C 5390C>T), S670P (5391T>C), G671S (5394G>T 5395G>C), D672E (5399T>A), Y673P (5400T>C 5401A>C 5402C>T), K674Q (5403A>C), Q675Y (5406C>T 5408A>T), A676R (5409G>A 5410C>G), V679H (5418G>C 5419T>A 5420G>T), Q683V (5430C>G 5431A>T), Y684L (5434A>T 5435C>G), N686S (5439A>T 5440A>C), Y687F (5443A>T 5444C>T), N689T (5449A>C), E691K (5454G>A), V693E (5461T>A), H694N (5463C>A 5465C>T), N698S (5475A>T 5476A>C), T699D (5478A>G 5479C>A), L702F (5487C>T), I705F (5496A>T), M706L (5499A>C 5501G>A)								
Codon mutations:	AGC612TGT (5217A>T 5219C>T), TGT615ACT (5226T>A 5227G>C), TCC616AGT (5229T>A 5230C>G 5231C>T), GGT617GAC (5233G>A 5234T>C), TCA618ACA (5235T>A), AAA620AAT (5243A>T), CAG621GAA (5244C>G 5246G>A), GCC622ACC (5247G>A), GAG623GAA (5252G>A), AAA624ACT (5254A>C 5255A>T), AAT625AAA (5258T>A), CAT627TCA (5262C>T 5263A>C 5264T>A), AGT628ACA (5266G>C 5267T>A), GAG630GAA (5273G>A), AAA631AAG (5276A>G), ATA633TTA (5280A>T), TTA634TTG (5285A>G), GCT635GCC (5288T>C), TTG636ATA (5289T>A 5291G>A), ATT637GTA (5292A>G 5294T>A), ACA639GGC (5298A>G 5299C>G 5300A>C), ATT640ATA (5303T>A), AAG641CGA (5304A>C 5305A>G 5306G>A), GCT642AAA (5307G>A 5308C>A 5309T>A), AAA644TCT (5313A>T 5314A>C 5315A>T), GCA645GCT (5318A>T), TAT646TTT (5320A>T), CTG647CTA (5324G>A), GTA650AAG (5331G>A 5332T>A 5333A>G), AGA651CAA (5334A>C 5335G>A), TTT652TTC (5339T>C), ATT653ATC (5342T>C), GCT654ATC (5343G>A 5344C>T 5345T>C), ACA656TCG (5349A>T 5351A>G), GAT657GAC (5354T>C), ACT659ACA (5360T>A), AAT660CAG (5361A>C 5363T>G), GTT661GTA (5366T>A), AAA662TCA (5367A>T 5368A>C), TAT663GGT (5370T>G 5371A>G), TTC664TTG (5375C>G), ATT665ATA (5378T>A), AGT666TTT (5379A>T 5380G>T), ACT667AAT (5383C>A), AAC668AAA (5387C>A), ATC669CTT (5388A>C 5390C>T), TCA670CCA (5391T>C), GGT671TCT (5394G>T 5395G>C), GAT672GAA (5399T>A), TAC673CCT (5400T>C 5401A>C 5402C>T), AAA674CAA (5403A>C), CAA675TAT (5406C>T 5408A>T), GCA676AGA (5409G>A 5410C>G), AGA677AGG (5414A>G), CTT678TTA (5415C>T 5417T>A), GTG679CAT (5418G>C 5419T>A 5420G>T), CAA683GTA (5430C>G 5431A>T), TAC684TTG (5434A>T 5435C>G), CTT685TTA (5436C>T 5438T>A), AAT686TCT (5439A>T 5440A>C), TAC687TTT (5443A>T 5444C>T), TAC688TAT (5447C>T), AAT689ACT (5449A>C), GAA691AAA (5454G>A), GTA693GAA (5461T>A), CAC694AAT (5463C>A 5465C>T), AAT698TCT (5475A>T 5476A>C), ACT699GAT (5478A>G 5479C>A), CTT702TTT (5487C>T), GAC704GAT (5495C>T), ATC705TTC (5496A>T), ATG706CTA (5499A>C 5501G>A), TCC707TCA (5504C>A)								

Proteins

	Begin	End	Coverage	Score	Concordance	Matches	Identities	I/D/M/F*	Stop Codons
polymerase polyprotein (YP_010805169.1)	611	708	13.7%	323	48.6%	98 (100%)	45 (45.9%)	0/0/0/0	0
Protein mutations:	S612C (5217A>T 5219C>T), C615T (5226T>A 5227G>C), G617D (5233G>A 5234T>C), S618T (5235T>A), K620N (5243A>T), Q621E (5244C>G 5246G>A), A622T (5247G>A), K624T (5254A>C 5255A>T), N625K (5258T>A), H627S (5262C>T 5263A>C 5264T>A), S628T (5266G>C 5267T>A), I633L (5280A>T), L636I (5289T>A 5291G>A), I637V (5292A>G 5294T>A), T639G (5298A>G 5299C>G 5300A>C), K641R (5304A>C 5305A>G 5306G>A), A642K (5307G>A 5308C>A 5309T>A), K644S (5313A>T 5314A>C 5315A>T), Y646F (5320A>T), V650K (5331G>A 5332T>A 5333A>G), R651Q (5334A>C 5335G>A), A654I (5343G>A 5344C>T 5345T>C), T656S (5349A>T 5351A>G), N660Q (5361A>C 5363T>G), K662S (5367A>T 5368A>C), Y663G (5370T>G 5371A>G), F664L (5375C>G), S666F (5379A>T 5380G>T), T667N (5383C>A), N668K (5387C>A), I669L (5388A>C 5390C>T), S670P (5391T>C), G671S (5394G>T 5395G>C), D672E (5399T>A), Y673P (5400T>C 5401A>C 5402C>T), K674Q (5403A>C), Q675Y (5406C>T 5408A>T), A676R (5409G>A 5410C>G), V679H (5418G>C 5419T>A 5420G>T), Q683V (5430C>G 5431A>T), Y684L (5434A>T 5435C>G), N686S (5439A>T 5440A>C), Y687F (5443A>T 5444C>T), N689T (5449A>C), E691K (5454G>A), V693E (5461T>A), H694N (5463C>A 5465C>T), N698S (5475A>T 5476A>C), T699D (5478A>G 5479C>A), L702F (5487C>T), I705F (5496A>T), M706L (5499A>C 5501G>A)								
Codon mutations:	AGC612TGT (5217A>T 5219C>T), TGT615ACT (5226T>A 5227G>C), TCC616AGT (5229T>A 5230C>G 5231C>T), GGT617GAC (5233G>A 5234T>C), TCA618ACA (5235T>A), AAA620AAT (5243A>T), CAG621GAA (5244C>G 5246G>A), GCC622ACC (5247G>A), GAG623GAA (5252G>A), AAA624ACT (5254A>C 5255A>T), AAT625AAA (5258T>A), CAT627TCA (5262C>T 5263A>C 5264T>A), AGT628ACA (5266G>C 5267T>A), GAG630GAA (5273G>A), AAA631AAG (5276A>G), ATA633TTA (5280A>T), TTA634TTG (5285A>G), GCT635GCC (5288T>C), TTG636ATA (5289T>A 5291G>A), ATT637GTA (5292A>G 5294T>A), ACA639GGC (5298A>G 5299C>G 5300A>C), ATT640ATA (5303T>A), AAG641CGA (5304A>C 5305A>G 5306G>A), GCT642AAA (5307G>A 5308C>A 5309T>A), AAA644TCT (5313A>T 5314A>C 5315A>T), GCA645GCT (5318A>T), TAT646TTT (5320A>T), CTG647CTA (5324G>A), GTA650AAG (5331G>A 5332T>A 5333A>G), AGA651CAA (5334A>C 5335G>A), TTT652TTC (5339T>C), ATT653ATC (5342T>C), GCT654ATC (5343G>A 5344C>T 5345T>C), ACA656TCG (5349A>T 5351A>G), GAT657GAC (5354T>C), ACT659ACA (5360T>A), AAT660CAG (5361A>C 5363T>G), GTT661GTA (5366T>A), AAA662TCA (5367A>T 5368A>C), TAT663GGT (5370T>G 5371A>G), TTC664TTG (5375C>G), ATT665ATA (5378T>A), AGT666TTT (5379A>T 5380G>T), ACT667AAT (5383C>A), AAC668AAA (5387C>A), ATC669CTT (5388A>C 5390C>T), TCA670CCA (5391T>C), GGT671TCT (5394G>T 5395G>C), GAT672GAA (5399T>A), TAC673CCT (5400T>C 5401A>C 5402C>T), AAA674CAA (5403A>C), CAA675TAT (5406C>T 5408A>T), GCA676AGA (5409G>A 5410C>G), AGA677AGG (5414A>G), CTT678TTA (5415C>T 5417T>A), GTG679CAT (5418G>C 5419T>A 5420G>T), CAA683GTA (5430C>G 5431A>T), TAC684TTG (5434A>T 5435C>G), CTT685TTA (5436C>T 5438T>A), AAT686TCT (5439A>T 5440A>C), TAC687TTT (5443A>T 5444C>T), TAC688TAT (5447C>T), AAT689ACT (5449A>C), GAA691AAA (5454G>A), GTA693GAA (5461T>A), CAC694AAT (5463C>A 5465C>T), AAT698TCT (5475A>T 5476A>C), ACT699GAT (5478A>G 5479C>A), CTT702TTT (5487C>T), GAC704GAT (5495C>T), ATC705TTC (5496A>T), ATG706CTA (5499A>C 5501G>A), TCC707TCA (5504C>A)								

*: Inserts / Deletes / Misaligned / Frameshifts

Analysis details

This analysis was performed with panviral2.64

NGS Details (UN24): Cavemovirus venamanihotis

Assembly

**Coverage Length**	577 (1 contig(s))
**Depth Of Coverage**	3.3
**Number Of Reads**	15
**Reads Per Million**	0.30 rpm (after QC)
**Ambiguities**	0
**Assembly Method**	de novo + reference guided assembly
**Consensus Caller**	Bcf Tools

Coverage Map

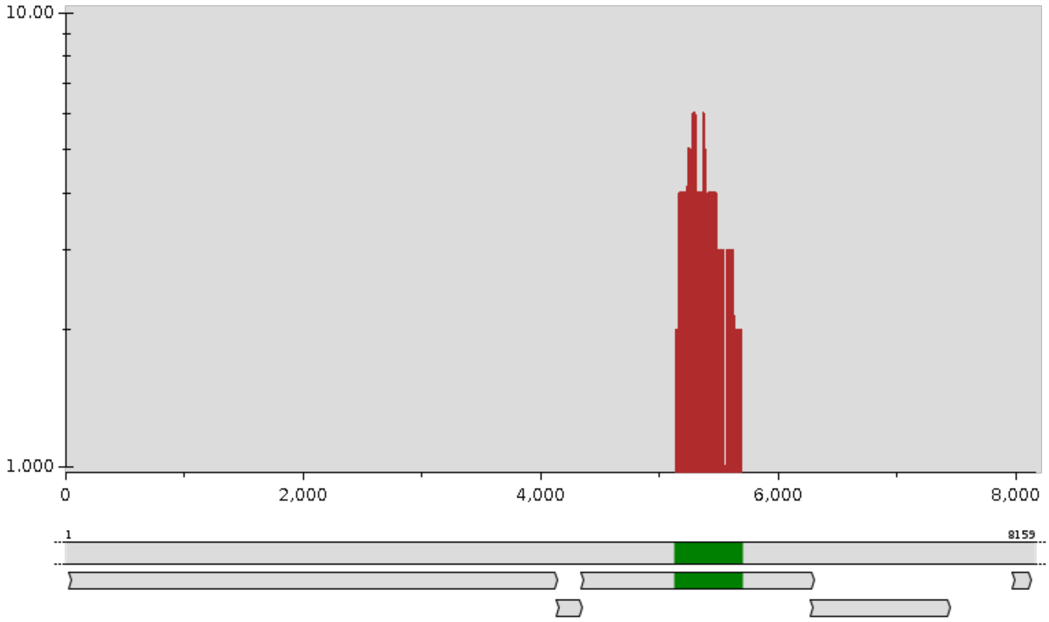

Assignment

**Type**	Cavemovirus venamanihotis (Taxonomy ID: 3047736)
**Reference Genome**	NC_001648.1
**NT Identity (%)**	57.1924
**AA Identity (%)**	47.3958
**Number Of Stop Codons**	0
**Number Of CDS**	5

Alignment

**Alignment Score**	148.0 (NT) + 625.0 (AA) = 773.0
**Concordance (%)**	31.929

**Alignment Method**	Global, seeded, nucleotide + amino acids (AGA)

Genome Region

Sequence starts at position 5128 and ends at position 5704 relative to NC_001648.1 reference sequence.

Alignment Detailed Statistics

	Begin	End	Coverage	Score	Concordance	Matches	Identities	I/D/M/F*	Stop Codons
**NT**	**5128**	**5704**	**7.1%**	**148**	**13.2%**	**568 (96.9%)**	**330 (56.3%)**	**9/9**	
Mutations:	5130A>C, 5132G>T, 5135A>T, 5139G>A, 5148G>C, 5149A>C, 5151T>C, 5153A>G, 5156T>C, 5158A>C, 5159A>T, 5160A>G, 5161A>C, 5162A>T, 5163G>C, 5164C>T, 5165T>A, 5169G>T, 5170T>G, 5171A>G, 5172G>A, 5176A>G, 5180T>C, 5183T>G, 5186C>T, 5189A>T, 5196G>A, 5197A>G, 5198T>A, 5199A>G, 5200C>A, 5201A>T, 5202T>C, 5204A>T, 5205A>T, 5208C>A, 5210T>A, 5211A>C, 5215G>C, 5216T>C, 5217A>T, 5219A>T, 5220C>A, 5222G>A, 5224C>A, 5225A>C, 5227G>A, 5228A>T, 5229T>A, 5230A>T, 5231C>T, 5246T>C, 5247T>A, 5248G>T, 5249T>G, 5258A>C, 5263A>G, 5264T>G, 5267T>A, 5270A>C, 5271A>C, 5273G>A, 5274T>A, 5276A>T, 5277G>T, 5278A>T, 5279A>C, 5283G>A, 5285T>G, 5286A>G, 5287G>A, 5292A>T, 5294A>T, 5295T>A, 5297T>A, 5300A>T, 5306T>C, 5308C>A, 5309A>T, 5312T>C, 5316C>T, 5317A>T, 5318G>T, 5322T>C, 5324T>A, 5328C>G, 5330G>A, 5336A>T, 5338T>C, 5339A>T, 5340T>A, 5342A>G, 5352T>C, 5353A>T, 5354T>A, 5355C>A, 5357T>A, 5361T>G, 5363A>C, 5366A>T, 5367A>T, 5368G>T, 5369T>C, 5370A>G, 5371T>A, 5372T>A, 5375C>T, 5379C>A, 5381A>T, 5382T>A, 5384T>C, 5388G>A, 5392G>A, 5393A>T, 5396A>T, 5401G>A, 5402A>T, 5410A>T, 5411T>A, 5420T>C, 5426G>T, 5438T>C, 5441A>T, 5444A>G, 5445G>A, 5447C>T, 5449T>A, 5456G>A, 5459T>G, 5460A>T, 5466G>A, 5467A>T, 5473A>T, 5474A>C, 5476T>A, 5481A>C, 5485C>A, 5486A>T, 5488A>T, 5489A>C, 5492T>C, 5494G>A, 5496G>A, 5498T>A, 5501A>T, 5502A>G, 5503C>T, 5504A>T, 5505C>A, 5507T>A, 5508G>C, 5509C>A, 5515G>A, 5516A>T, 5519A>G, 5520A>G, 5522T>A, 5526A>T, 5527G>C, 5531G>A, 5532A>C, 5533A>C, 5539C>T, 5540A>G, 5541G>A, 5542A>G, 5543A>T, 5548G>T, 5550A>C, 5553G>A, 5554A>C, 5556A>G, 5558A>T, 5559A>G, 5561A>C, 5562G>C, 5563A>G, 5564T>A, 5567C>T, 5570T>A, 5574G>C, 5575T>A, 5576C>T, 5577C>A, 5579A>T, 5582A>T, 5583G>T, 5584A>G, 5585A>C, 5590G>A, 5592G>A, 5593G>A, 5594T>A, 5598_5606delGAATTACAG, 5607C>A, 5609T>C, 5615_5616insCAGAGATCT, 5619A>G, 5622A>T, 5623A>T, 5624A>T, 5625A>G, 5626T>C, 5629T>C, 5631G>A, 5634A>T, 5635A>T, 5636A>T, 5638A>T, 5640A>G, 5641C>A, 5642G>T, 5644A>T, 5645A>T, 5650A>C, 5651A>T, 5652A>G, 5654T>C, 5656A>G, 5657A>G, 5661G>A, 5662A>T, 5663A>T, 5664T>C, 5666A>C, 5671G>C, 5672T>A, 5673A>T, 5675A>T, 5678A>G, 5682T>A, 5683T>A, 5684A>T, 5690C>T, 5691C>T, 5693G>T, 5697A>T, 5698G>T, 5699A>T, 5701A>T								

CDS

	Begin	End	Coverage	Score	Concordance	Matches	Identities	I/D/M/F*	Stop Codons
CsVMVgp3	263	454	29.4%	625	47.3%	189 (96.9%)	91 (46.7%)	3/3/0/0	0
Protein mutations:	M263L (5130A>C 5132G>T), D266N (5139G>A), D269P (5148G>C 5149A>C), K272T (5158A>C 5159A>T), K273A (5160A>G 5161A>C 5162A>T), A274L (5163G>C 5164C>T 5165T>A), V276W (5169G>T 5170T>G 5171A>G), V277I (5172G>A), K278R (5176A>G), D285R (5196G>A 5197A>G 5198T>A), T286D (5199A>G 5200C>A 5201A>T), I288L (5205A>T), H289K (5208C>A 5210T>A), S291T (5215G>C 5216T>C), I292F (5217A>T 5219A>T), Q293K (5220C>A 5222G>A), A294D (5224C>A 5225A>C), R295N (5227G>A 5228A>T), Y296I (5229T>A 5230A>T 5231C>T), C302M (5247T>A 5248G>T 5249T>G), Y307W (5263A>G 5264T>G), H308Q (5267T>A), K310Q (5271A>C 5273G>A), L311I (5274T>A 5276A>T), E312F (5277G>T 5278A>T 5279A>C), D314K (5283G>A 5285T>G), S315D (5286A>G 5287G>A), K317Y (5292A>T 5294A>T), Y318K (5295T>A 5297T>A), T322N (5308C>A 5309A>T), Q325F (5316C>T 5317A>T 5318G>T), Y327Q (5322T>C 5324T>A), Q329E (5328C>G 5330G>A), K331N (5336A>T), V332A (5338T>C 5339A>T), L333M (5340T>A 5342A>G), Y337L (5352T>C 5353A>T 5354T>A), H338K (5355C>A 5357T>A), S340A (5361T>G 5363A>C), S342F (5367A>T 5368G>T 5369T>C), I343E (5370A>G 5371T>A 5372T>A), Q346N (5379C>A 5381A>T), F347I (5382T>A 5384T>C), D349N (5388G>A), R350N (5392G>A 5393A>T), R353N (5401G>A 5402A>T), Y356L (5410A>T 5411T>A), V368I (5445G>A 5447C>T), F369Y (5449T>A), I373F (5460A>T), E375M (5466G>A 5467A>T), K377I (5473A>T 5474A>C), I378K (5476T>A), I380L (5481A>C), A381D (5485C>A 5486A>T), K382I (5488A>T 5489A>C), R384K (5494G>A), D385K (5496G>A 5498T>A), T387V (5502A>G 5503C>T 5504A>T), L388I (5505C>A 5507T>A), A389Q (5508G>C 5509C>A), G391D (5515G>A 5516A>T), I393V (5520A>G 5522T>A), K397P (5532A>C 5533A>C), T399M (5539C>T 5540A>G), E400S (5541G>A 5542A>G 5543A>T), C402F (5548G>T), K403Q (5550A>C), E404T (5553G>A 5554A>C), K405D (5556A>G 5558A>T), I406V (5559A>G 5561A>C), D407R (5562G>C 5563A>G 5564T>A), V411H (5574G>C 5575T>A 5576C>T), Q412N (5577C>A 5579A>T), E414C (5583G>T 5584A>G 5585A>C), G416E (5590G>A), G417K (5592G>A 5593G>A 5594T>A), E419_Q421del (5598_5606delGAATTACAG), P422T (5607C>A 5609T>C), I424_I425insQRS (5615_5616insCAGAGATCT), N426D (5619A>G), K427F (5622A>T 5623A>T 5624A>T), I428A (5625A>G 5626T>C), L429S (5629T>C), E430K (5631G>A), K431F (5634A>T 5635A>T 5636A>T), H432L (5638A>T), T433D (5640A>G 5641C>A 5642G>T), K434I (5644A>T 5645A>T), K436T (5650A>C 5651A>T), N437D (5652A>G 5654T>C), K438R (5656A>G 5657A>G), E440I (5661G>A 5662A>T 5663A>T), S443T (5671G>C 5672T>A), I444F (5673A>T 5675A>T), L447N (5682T>A 5683T>A 5684A>T), Q450Y (5691C>T 5693G>T), R452F (5697A>T 5698G>T 5699A>T), H453L (5701A>T)								
Codon mutations:	ATG263CTT (5130A>C 5132G>T), GTA264GTT (5135A>T), GAT266AAT (5139G>A), GAT269CCT (5148G>C 5149A>C), TTA270CTG (5151T>C 5153A>G), AAT271AAC (5156T>C), AAA272ACT (5158A>C 5159A>T), AAA273GCT (5160A>G 5161A>C 5162A>T), GCT274CTA (5163G>C 5164C>T 5165T>A), GTA276TGG (5169G>T 5170T>G 5171A>G), GTA277ATA (5172G>A), AAA278AGA (5176A>G), TAT279TAC (5180T>C), CCT280CCG (5183T>G), ATC281ATT (5186C>T), CCA282CCT (5189A>T), GAT285AGA (5196G>A 5197A>G 5198T>A), ACA286GAT (5199A>G 5200C>A 5201A>T), TTA287CTT (5202T>C 5204A>T), ATA288TTA (5205A>T), CAT289AAA (5208C>A 5210T>A), AGA290CGA (5211A>C), AGT291ACC (5215G>C 5216T>C), ATA292TTT (5217A>T 5219A>T), CAG293AAA (5220C>A 5222G>A), GCA294GAC (5224C>A 5225A>C), AGA295AAT (5227G>A 5228A>T), TAC296ATT (5229T>A 5230A>T 5231C>T), GAT301GAC (5246T>C), TGT302ATG (5247T>A 5248G>T 5249T>G), GGA305GGC (5258A>C), TAT307TGG (5263A>G 5264T>G), CAT308CAA (5267T>A), ATA309ATC (5270A>C), AAG310CAA (5271A>C 5273G>A), TTA311ATT (5274T>A 5276A>T), GAA312TTC (5277G>T 5278A>T 5279A>C), GAT314AAG (5283G>A 5285T>G), AGT315GAT (5286A>G 5287G>A), AAA317TAT (5292A>T 5294A>T), TAT318AAA (5295T>A 5297T>A), ACA319ACT (5300A>T), TTT321TTC (5306T>C), ACA322AAT (5308C>A 5309A>T), GTT323GTC (5312T>C), CAG325TTT (5316C>T 5317A>T 5318G>T), TAT327CAA (5322T>C 5324T>A), CAG329GAA (5328C>G 5330G>A), AAA331AAT (5336A>T), GTA332GCT (5338T>C 5339A>T), TTA333ATG (5340T>A 5342A>G), TAT337CTA (5352T>C 5353A>T 5354T>A), CAT338AAA (5355C>A 5357T>A), TCA340GCC (5361T>G 5363A>C), CCA341CCT (5366A>T), AGT342TTC (5367A>T 5368G>T 5369T>C), ATT343GAA (5370A>G 5371T>A 5372T>A), TTC344TTT (5375C>T), CAA346AAT (5379C>A 5381A>T), TTT347ATC (5382T>A 5384T>C), GAT349AAT (5388G>A), AGA350AAT (5392G>A 5393A>T), ATA351ATT (5396A>T), AGA353AAT (5401G>A 5402A>T), TAT356TTA (5410A>T 5411T>A), ATT359ATC (5420T>C), GTG361GTT (5426G>T), GAT365GAC (5438T>C), ATA366ATT (5441A>T), TTA367TTG (5444A>G), GTC368ATT (5445G>A 5447C>T), TTT369TAT (5449T>A), AAG371AAA (5456G>A), ACT372ACG (5459T>G), ATT373TTT (5460A>T), GAG375ATG (5466G>A 5467A>T), AAA377ATC (5473A>T 5474A>C), ATA378AAA (5476T>A), ATT380CTT (5481A>C), GCA381GAT (5485C>A 5486A>T), AAA382ATC (5488A>T 5489A>C), TTT383TTC (5492T>C), AGA384AAA (5494G>A), GAT385AAA (5496G>A 5498T>A), ATA386ATT (5501A>T), ACA387GTT (5502A>G 5503C>T 5504A>T), CTT388ATA (5505C>A 5507T>A), GCA389CAA (5508G>C 5509C>A), GGA391GAT (5515G>A 5516A>T), TTA392TTG (5519A>G), ATT393GTA (5520A>G 5522T>A), AGT395TCT (5526A>T 5527G>C), AAG396AAA (5531G>A), AAA397CCA (5532A>C 5533A>C), ACA399ATG (5539C>T 5540A>G), GAA400AGT (5541G>A 5542A>G 5543A>T), TGT402TTT (5548G>T), AAA403CAA (5550A>C), GAA404ACA (5553G>A 5554A>C), AAA405GAT (5556A>G 5558A>T), ATA406GTC (5559A>G 5561A>C), GAT407CGA (5562G>C 5563A>G 5564T>A), TTC408TTT (5567C>T), CTT409CTA (5570T>A), GTC411CAT (5574G>C 5575T>A 5576C>T), CAA412AAT (5577C>A 5579A>T), ATA413ATT (5582A>T), GAA414TGC (5583G>T 5584A>G 5585A>C), GGA416GAA (5590G>A), GGT417AAA (5592G>A 5593G>A 5594T>A), GAA419_CAG421del (5598_5606delGAATTACAG), CCT422ACC (5607C>A 5609T>C), ATT424_ATT425insCAGAGATCT (5615_5616insCAGAGATCT), AAT426GAT (5619A>G), AAA427TTT (5622A>T 5623A>T 5624A>T), ATA428GCA (5625A>G 5626T>C), TTA429TCA (5629T>C), GAA430AAA (5631G>A), AAA431TTT (5634A>T 5635A>T 5636A>T), CAT432CTT (5638A>T), ACG433GAT (5640A>G 5641C>A 5642G>T), AAA434ATT (5644A>T 5645A>T), AAA436ACT (5650A>C 5651A>T), AAT437GAC (5652A>G 5654T>C), AAA438AGG (5656A>G 5657A>G), GAA440ATT (5661G>A 5662A>T 5663A>T), TTA441CTC (5664T>C 5666A>C), AGT443ACA (5671G>C 5672T>A), ATA444TTT (5673A>T 5675A>T), TTA445TTG (5678A>G), TTA447AAT (5682T>A 5683T>A 5684A>T), AAC449AAT (5690C>T), CAG450TAT (5691C>T 5693G>T), AGA452TTT (5697A>T 5698G>T 5699A>T), CAT453CTT (5701A>T)								

Proteins

	Begin	End	Coverage	Score	Concordance	Matches	Identities	I/D/M/F*	Stop Codons
aspartic protease/reverse transcriptase (NP_056848.1)	263	454	29.4%	625	47.3%	189 (96.9%)	91 (46.7%)	3/3/0/0	0
Protein mutations:	M263L (5130A>C 5132G>T), D266N (5139G>A), D269P (5148G>C 5149A>C), K272T (5158A>C 5159A>T), K273A (5160A>G 5161A>C 5162A>T), A274L (5163G>C 5164C>T 5165T>A), V276W (5169G>T 5170T>G 5171A>G), V277I (5172G>A), K278R (5176A>G), D285R (5196G>A 5197A>G 5198T>A), T286D (5199A>G 5200C>A 5201A>T), I288L (5205A>T), H289K (5208C>A 5210T>A), S291T (5215G>C 5216T>C), I292F (5217A>T 5219A>T), Q293K (5220C>A 5222G>A), A294D (5224C>A 5225A>C), R295N (5227G>A 5228A>T), Y296I (5229T>A 5230A>T 5231C>T), C302M (5247T>A 5248G>T 5249T>G), Y307W (5263A>G 5264T>G), H308Q (5267T>A), K310Q (5271A>C 5273G>A), L311I (5274T>A 5276A>T), E312F (5277G>T 5278A>T 5279A>C), D314K (5283G>A 5285T>G), S315D (5286A>G 5287G>A), K317Y (5292A>T 5294A>T), Y318K (5295T>A 5297T>A), T322N (5308C>A 5309A>T), Q325F (5316C>T 5317A>T 5318G>T), Y327Q (5322T>C 5324T>A), Q329E (5328C>G 5330G>A), K331N (5336A>T), V332A (5338T>C 5339A>T), L333M (5340T>A 5342A>G), Y337L (5352T>C 5353A>T 5354T>A), H338K (5355C>A 5357T>A), S340A (5361T>G 5363A>C), S342F (5367A>T 5368G>T 5369T>C), I343E (5370A>G 5371T>A 5372T>A), Q346N (5379C>A 5381A>T), F347I (5382T>A 5384T>C), D349N (5388G>A), R350N (5392G>A 5393A>T), R353N (5401G>A 5402A>T), Y356L (5410A>T 5411T>A), V368I (5445G>A 5447C>T), F369Y (5449T>A), I373F (5460A>T), E375M (5466G>A 5467A>T), K377I (5473A>T 5474A>C), I378K (5476T>A), I380L (5481A>C), A381D (5485C>A 5486A>T), K382I (5488A>T 5489A>C), R384K (5494G>A), D385K (5496G>A 5498T>A), T387V (5502A>G 5503C>T 5504A>T), L388I (5505C>A 5507T>A), A389Q (5508G>C 5509C>A), G391D (5515G>A 5516A>T), I393V (5520A>G 5522T>A), K397P (5532A>C 5533A>C), T399M (5539C>T 5540A>G), E400S (5541G>A 5542A>G 5543A>T), C402F (5548G>T), K403Q (5550A>C), E404T (5553G>A 5554A>C), K405D (5556A>G 5558A>T), I406V (5559A>G 5561A>C), D407R (5562G>C 5563A>G 5564T>A), V411H (5574G>C 5575T>A 5576C>T), Q412N (5577C>A 5579A>T), E414C (5583G>T 5584A>G 5585A>C), G416E (5590G>A), G417K (5592G>A 5593G>A 5594T>A), E419_Q421del (5598_5606delGAATTACAG), P422T (5607C>A 5609T>C), I424_I425insQRS (5615_5616insCAGAGATCT), N426D (5619A>G), K427F (5622A>T 5623A>T 5624A>T), I428A (5625A>G 5626T>C), L429S (5629T>C), E430K (5631G>A), K431F (5634A>T 5635A>T 5636A>T), H432L (5638A>T), T433D (5640A>G 5641C>A 5642G>T), K434I (5644A>T 5645A>T), K436T (5650A>C 5651A>T), N437D (5652A>G 5654T>C), K438R (5656A>G 5657A>G), E440I (5661G>A 5662A>T 5663A>T), S443T (5671G>C 5672T>A), I444F (5673A>T 5675A>T), L447N (5682T>A 5683T>A 5684A>T), Q450Y (5691C>T 5693G>T), R452F (5697A>T 5698G>T 5699A>T), H453L (5701A>T)								

	Begin	End	Coverage	Score	Concordance	Matches	Identities	I/D/M/F*	Stop Codons
**NT**	**5128**	**5704**	**7.1%**	**148**	**13.2%**	**568 (96.9%)**	**330 (56.3%)**	**9/9**	

Codon mutations: ATG263CTT (5130A>C 5132G>T), GTA264GTT (5135A>T), GAT266AAT (5139G>A), GAT269CCT (5148G>C 5149A>C), TTA270CTG (5151T>C 5153A>G), AAT271AAC (5156T>C), AAA272ACT (5158A>C 5159A>T), AAA273GCT (5160A>G 5161A>C 5162A>T), GCT274CTA (5163G>C 5164C>T 5165T>A), GTA276TGG (5169G>T 5170T>G 5171A>G), GTA277ATA (5172G>A), AAA278AGA (5176A>G), TAT279TAC (5180T>C), CCT280CCG (5183T>G), ATC281ATT (5186C>T), CCA282CCT (5189A>T), GAT285AGA (5196G>A 5197A>G 5198T>A), ACA286GAT (5199A>G 5200C>A 5201A>T), TTA287CTT (5202T>C 5204A>T), ATA288TTA (5205A>T), CAT289AAA (5208C>A 5210T>A), AGA290CGA (5211A>C), AGT291ACC (5215G>C 5216T>C), ATA292TTT (5217A>T 5219A>T), CAG293AAA (5220C>A 5222G>A), GCA294GAC (5224C>A 5225A>C), AGA295AAT (5227G>A 5228A>T), TAC296ATT (5229T>A 5230A>T 5231C>T), GAT301GAC (5246T>C), TGT302ATG (5247T>A 5248G>T 5249T>G), GGA305GGC (5258A>C), TAT307TGG (5263A>G 5264T>G), CAT308CAA (5267T>A), ATA309ATC (5270A>C), AAG310CAA (5271A>C 5273G>A), TTA311ATT (5274T>A 5276A>T), GAA312TTC (5277G>T 5278A>T 5279A>C), GAT314AAG (5283G>A 5285T>G), AGT315GAT (5286A>G 5287G>A), AAA317TAT (5292A>T 5294A>T), TAT318AAA (5295T>A 5297T>A), ACA319ACT (5300A>T), TTT321TTC (5306T>C), ACA322AAT (5308C>A 5309A>T), GTT323GTC (5312T>C), CAG325TTT (5316C>T 5317A>T 5318G>T), TAT327CAA (5322T>C 5324T>A), CAG329GAA (5328C>G 5330G>A), AAA331AAT (5336A>T), GTA332GCT (5338T>C 5339A>T), TTA333ATG (5340T>A 5342A>G), TAT337CTA (5352T>C 5353A>T 5354T>A), CAT338AAA (5355C>A 5357T>A), TCA340GCC (5361T>G 5363A>C), CCA341CCT (5366A>T), AGT342TTC (5367A>T 5368G>T 5369T>C), ATT343GAA (5370A>G 5371T>A 5372T>A), TTC344TTT (5375C>T), CAA346AAT (5379C>A 5381A>T), TTT347ATC (5382T>A 5384T>C), GAT349AAT (5388G>A), AGA350AAT (5392G>A 5393A>T), ATA351ATT (5396A>T), AGA353AAT (5401G>A 5402A>T), TAT356TTA (5410A>T 5411T>A), ATT359ATC (5420T>C), GTG361GTT (5426G>T), GAT365GAC (5438T>C), ATA366ATT (5441A>T), TTA367TTG (5444A>G), GTC368ATT (5445G>A 5447C>T), TTT369TAT (5449T>A), AAG371AAA (5456G>A), ACT372ACG (5459T>G), ATT373TTT (5460A>T), GAG375ATG (5466G>A 5467A>T), AAA377ATC (5473A>T 5474A>C), ATA378AAA (5476T>A), ATT380CTT (5481A>C), GCA381GAT (5485C>A 5486A>T), AAA382ATC (5488A>T 5489A>C), TTT383TTC (5492T>C), AGA384AAA (5494G>A), GAT385AAA (5496G>A 5498T>A), ATA386ATT (5501A>T), ACA387GTT (5502A>G 5503C>T 5504A>T), CTT388ATA (5505C>A 5507T>A), GCA389CAA (5508G>C 5509C>A), GGA391GAT (5515G>A 5516A>T), TTA392TTG (5519A>G), ATT393GTA (5520A>G 5522T>A), AGT395TCT (5526A>T 5527G>C), AAG396AAA (5531G>A), AAA397CCA (5532A>C 5533A>C), ACA399ATG (5539C>T 5540A>G), GAA400AGT (5541G>A 5542A>G 5543A>T), TGT402TTT (5548G>T), AAA403CAA (5550A>C), GAA404ACA (5553G>A 5554A>C), AAA405GAT (5556A>G 5558A>T), ATA406GTC (5559A>G 5561A>C), GAT407CGA (5562G>C 5563A>G 5564T>A), TTC408TTT (5567C>T), CTT409CTA (5570T>A), GTC411CAT (5574G>C 5575T>A 5576C>T), CAA412AAT (5577C>A 5579A>T), ATA413ATT (5582A>T), GAA414TGC (5583G>T 5584A>G 5585A>C), GGA416GAA (5590G>A), GGT417AAA (5592G>A 5593G>A 5594T>A), GAA419_CAG421del (5598_5606delGAATTACAG), CCT422ACC (5607C>A 5609T>C), ATT424_ATT425insCAGAGATCT (5615_5616insCAGAGATCT), AAT426GAT (5619A>G), AAA427TTT (5622A>T 5623A>T 5624A>T), ATA428GCA (5625A>G 5626T>C), TTA429TCA (5629T>C), GAA430AAA (5631G>A), AAA431TTT (5634A>T 5635A>T 5636A>T), CAT432CTT (5638A>T), ACG433GAT (5640A>G 5641C>A 5642G>T), AAA434ATT (5644A>T 5645A>T), AAA436ACT (5650A>C 5651A>T), AAT437GAC (5652A>G 5654T>C), AAA438AGG (5656A>G 5657A>G), GAA440ATT (5661G>A 5662A>T 5663A>T), TTA441CTC (5664T>C 5666A>C), AGT443ACA (5671G>C 5672T>A), ATA444TTT (5673A>T 5675A>T), TTA445TTG (5678A>G), TTA447AAT (5682T>A 5683T>A 5684A>T), AAC449AAT (5690C>T), CAG450TAT (5691C>T 5693G>T), AGA452TTT (5697A>T 5698G>T 5699A>T), CAT453CTT (5701A>T)

*: Inserts / Deletes / Misaligned / Frameshifts

Analysis details

This analysis was performed with panviral2.64

NGS Details (UN24): Caulimovirus tessellobrassicae

Assembly

**Coverage Length**	385 (1 contig(s))
**Depth Of Coverage**	4.5
**Number Of Reads**	13
**Reads Per Million**	0.26 rpm (after QC)
**Ambiguities**	0
**Assembly Method**	de novo + reference guided assembly
**Consensus Caller**	Bcf Tools

Coverage Map

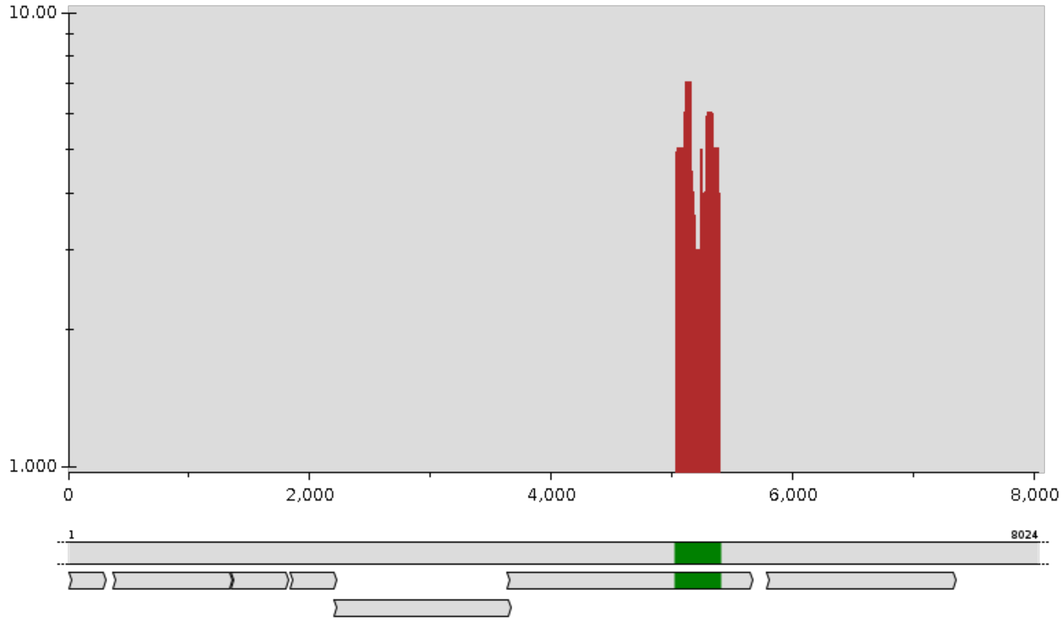

Assignment

**Type**	Caulimovirus tessellobrassicae (Taxonomy ID: 3047738)
**Reference Genome**	NC_001497.2
**NT Identity (%)**	52.3936
**AA Identity (%)**	48.4127
**Number Of Stop Codons**	1
**Number Of CDS**	7

Alignment

**Alignment Score**	0.0 (NT) + 371.0 (AA) = 371.0
**Concordance (%)**	23.7973

**Alignment Method**	Global, seeded, nucleotide + amino acids (AGA)

Genome Region

Sequence starts at position 5020 and ends at position 5404 relative to NC_001497.2 reference sequence.

Alignment Detailed Statistics

	Begin	End	Coverage	Score	Concordance	Matches	Identities	I/D/M/F*	Stop Codons
**NT**	**5020**	**5404**	**4.8%**	**0**	**0%**	**376 (97.7%)**	**197 (51.2%)**	**0/9**	
Mutations:	5020A>G, 5021T>C, 5022C>T, 5023T>A, 5025G>T, 5029C>T, 5030A>T, 5032A>T, 5033T>C, 5034C>T, 5035A>G, 5036A>G, 5037C>T, 5043C>T, 5046C>T, 5047G>A, 5050A>G, 5051C>A, 5052C>A, 5053C>A, 5055T>A, 5056G>A, 5057A>C, 5058A>T, 5061C>T, 5064G>A, 5066A>C, 5067G>T, 5070A>G, 5071C>T, 5073T>G, 5079A>G, 5088C>A, 5090T>G, 5091A>C, 5094A>T, 5096C>A, 5097A>C, 5100T>C, 5102C>T, 5103C>T, 5104T>G, 5106G>A, 5109T>C, 5111A>T, 5112C>T, 5115C>T, 5118G>A, 5122C>G, 5125G>A, 5126C>G, 5127T>G, 5134A>T, 5136A>C, 5139G>A, 5142T>C, 5143C>T, 5145G>A, 5146C>T, 5148A>T, 5150C>A, 5153A>G, 5154G>A, 5155C>T, 5157T>G, 5160A>G, 5161G>A, 5163A>G, 5166C>T, 5167_5169delGTT, 5172A>G, 5173T>G, 5174G>T, 5175G>T, 5176A>C, 5177G>C, 5178A>C, 5179T>C, 5183C>A, 5187A>G, 5190G>A, 5193T>G, 5196C>T, 5199C>G, 5200T>G, 5201A>C, 5202C>T, 5203A>G, 5205G>C, 5214G>T, 5217G>A, 5218A>G, 5220A>T, 5222A>T, 5223T>G, 5224C>G, 5226G>C, 5227C>A, 5230G>A, 5231G>A, 5232A>C, 5233T>C, 5239C>T, 5240C>G, 5241A>T, 5242C>T, 5244A>G, 5245C>G, 5246A>G, 5247T>A, 5248C>A, 5249A>T, 5253C>T, 5254T>A, 5255T>A, 5256A>C, 5259A>C, 5262G>T, 5263G>T, 5265G>T, 5266A>T, 5267A>T, 5268G>T, 5269C>A, 5274C>T, 5275A>G, 5277C>T, 5280G>A, 5283C>T, 5289A>T, 5292A>C, 5296G>A, 5297A>T, 5298C>T, 5299T>G, 5301C>T, 5303G>A, 5304G>C, 5310T>A, 5313G>T, 5314T>C, 5320G>C, 5321C>A, 5322T>A, 5324T>A, 5325C>G, 5330T>C, 5332A>C, 5333A>C, 5334C>A, 5335G>A, 5338G>T, 5339G>C, 5340T>A, 5341A>G, 5343T>C, 5344A>G, 5346T>A, 5347_5349delACT, 5350G>C, 5352G>A, 5356A>G, 5358T>A, 5359_5361delTGC, 5362A>C, 5364A>G, 5367C>T, 5368G>A, 5370A>T, 5373T>G, 5377A>G, 5378G>T, 5379C>T, 5381T>G, 5382T>G, 5385A>T, 5386G>T, 5387C>T, 5392G>T, 5400T>C, 5403C>T, 5404C>A								

CDS

	Begin	End	Coverage	Score	Concordance	Matches	Identities	I/D/M/F*	Stop Codons
CaMVgp6	463	591	19.0%	371	43.2%	126 (97.7%)	61 (47.3%)	0/3/0/0	1
Protein mutations:	I463A (5020A>G 5021T>C 5022C>T), L464I (5023T>A 5025G>T), H466F (5029C>T 5030A>T), I467S (5032A>T 5033T>C 5034C>T), N468G (5035A>G 5036A>G 5037C>T), D472N (5047G>A), T473E (5050A>G 5051C>A 5052C>A), L474I (5053C>A 5055T>A), E475T (5056G>A 5057A>C 5058A>T), K478T (5066A>C 5067G>T), I486S (5090T>G 5091A>C), T488N (5096C>A 5097A>C), A490V (5102C>T 5103C>T), S491A (5104T>G 5106G>A), Y493F (5111A>T 5112C>T), L497V (5122C>G), A498R (5125G>A 5126C>G 5127T>G), R501C (5134A>T 5136A>C), Q505Y (5146C>T 5148A>T), A506D (5150C>A), K507R (5153A>G 5154G>A), E510K (5161G>A 5163A>G), V512del (5167_5169delGTT), W514V (5173T>G 5174G>T 5175G>T), R515P (5176A>C 5177G>C 5178A>C), W516R (5179T>C), T517K (5183C>A), D520E (5193T>G), Y523A (5200T>G 5201A>C 5202C>T), M524V (5203A>G 5205G>C), K529D (5218A>G 5220A>T), N530M (5222A>T 5223T>G), L531V (5224C>G 5226G>C), Q532K (5227C>A), G533N (5230G>A 5231G>A 5232A>C), F534L (5233T>C), P536C (5239C>T 5240C>G 5241A>T), H538G (5245C>G 5246A>G 5247T>A), H539I (5248C>A 5249A>T), L541N (5254T>A 5255T>A 5256A>C), E543D (5262G>T), E544Y (5263G>T 5265G>T), K545F (5266A>T 5267A>T 5268G>T), L546M (5269C>A), I548V (5275A>G 5277C>T), D555I (5296G>A 5297A>T 5298C>T), Y556D (5299T>G 5301C>T), W557Y (5303G>A 5304G>C), M560I (5313G>T), A563Q (5320G>C 5321C>A 5322T>A), I564K (5324T>A 5325C>G), I566T (5330T>C), N567P (5332A>C 5333A>C 5334C>A), E568K (5335G>A), G569S (5338G>T 5339G>C 5340T>A), T570A (5341A>G 5343T>C), N571E (5344A>G 5346T>A), T572del (5347_5349delACT), E573Q (5350G>C 5352G>A), I575V (5356A>G 5358T>A), C576del (5359_5361delTGC), A579T (5368G>A 5370A>T), S582V (5377A>G 5378G>T 5379C>T), F583W (5381T>G 5382T>G), K584N (5385A>T), A585F (5386G>T 5387C>T), E587* (5392G>T)								
Codon mutations:	ATC463GCT (5020A>G 5021T>C 5022C>T), TTG464ATT (5023T>A 5025G>T), CAC466TTC (5029C>T 5030A>T), ATC467TCT (5032A>T 5033T>C 5034C>T), AAC468GGT (5035A>G 5036A>G 5037C>T), TTC470TTT (5043C>T), CCC471CCT (5046C>T), GAT472AAT (5047G>A), ACC473GAA (5050A>G 5051C>A 5052C>A), CTT474ATA (5053C>A 5055T>A), GAA475ACT (5056G>A 5057A>C 5058A>T), GAC476GAT (5061C>T), AAG477AAA (5064G>A), AAG478ACT (5066A>C 5067G>T), CAA479CAG (5070A>G), CTT480TTG (5071C>T 5073T>G), AGA482AGG (5079A>G), GGC485GGA (5088C>A), ATA486AGC (5090T>G 5091A>C), CTA487CTT (5094A>T), ACA488AAC (5096C>A 5097A>C), TAT489TAC (5100T>C), GCC490GTT (5102C>T 5103C>T), TCG491GCA (5104T>G 5106G>A), GAT492GAC (5109T>C), TAC493TTT (5111A>T 5112C>T), ATC494ATT (5115C>T), CCG495CCA (5118G>A), CTA497GTA (5122C>G), GCT498AGG (5125G>A 5126C>G 5127T>G), AGA501TGC (5134A>T 5136A>C), AAG502AAA (5139G>A), CCT503CCC (5142T>C), CTG504TTA (5143C>T 5145G>A), CAA505TAT (5146C>T 5148A>T), GCC506GAC (5150C>A), AAG507AGA (5153A>G 5154G>A), CTT508TTG (5155C>T 5157T>G), AAA509AAG (5160A>G), GAA510AAG (5161G>A 5163A>G), AAC511AAT (5166C>T), GTT512del (5167_5169delGTT), CCA513CCG (5172A>G), TGG514GTT (5173T>G 5174G>T 5175G>T), AGA515CCC (5176A>C 5177G>C 5178A>C), TGG516CGG (5179T>C), ACA517AAA (5183C>A), AAA518AAG (5187A>G), GAG519GAA (5190G>A), GAT520GAG (5193T>G), ACC521ACT (5196C>T), CTC522CTG (5199C>G), TAC523GCT (5200T>G 5201A>C 5202C>T), ATG524GTC (5203A>G 5205G>C), GTG527GTT (5214G>T), AAG528AAA (5217G>A), AAA529GAT (5218A>G 5220A>T), AAT530ATG (5222A>T 5223T>G), CTG531GTC (5224C>G 5226G>C), CAA532AAA (5227C>A), GGA533AAC (5230G>A 5231G>A 5232A>C), TTT534CTT (5233T>C), CCA536TGT (5239C>T 5240C>G 5241A>T), CTA537TTG (5242C>T 5244A>G), CAT538GGA (5245C>G 5246A>G 5247T>A), CAT539ATT (5248C>A 5249A>T), CCC540CCT (5253C>T), TTA541AAC (5254T>A 5255T>A 5256A>C), CCA542CCC (5259A>C), GAG543GAT (5262G>T), GAG544TAT (5263G>T 5265G>T), AAG545TTT (5266A>T 5267A>T 5268G>T), CTG546ATG (5269C>A), ATC547ATT (5274C>T), ATC548GTT (5275A>G 5277C>T), GAG549GAA (5280G>A), ACC550ACT (5283C>T), GCA552GCT (5289A>T), TCA553TCC (5292A>C), GAC555ATT (5296G>A 5297A>T 5298C>T), TAC556GAT (5299T>G 5301C>T), TGG557TAC (5303G>A 5304G>C), GGT559GGA (5310T>A), ATG560ATT (5313G>T), TTA561CTA (5314T>C), GCT563CAA (5320G>C 5321C>A 5322T>A), ATC564AAG (5324T>A 5325C>G), ATT566ACT (5330T>C), AAC567CCA (5332A>C 5333A>C 5334C>A), GAA568AAA (5335G>A), GGT569TCA (5338G>T 5339G>C 5340T>A), ACT570GCC (5341A>G 5343T>C), AAT571GAA (5344A>G 5346T>A), ACT572del (5347_5349delACT), GAG573CAA (5350G>C 5352G>A), ATT575GTA (5356A>G 5358T>A), TGC576del (5359_5361delTGC), AGA577CGG (5362A>C 5364A>G), TAC578TAT (5367C>T), GCA579ACT (5368G>A 5370A>T), TCT580TCG (5373T>G), AGC582GTT (5377A>G 5378G>T 5379C>T), TTT583TGG (5381T>G 5382T>G), AAA584AAT (5385A>T), GCT585TTT (5386G>T 5387C>T), GAA587TAA (5392G>T), AAT589AAC (5400T>C), TAC590TAT (5403C>T), CAC591A.. (5404C>A)								

Proteins

	Begin	End	Coverage	Score	Concordance	Matches	Identities	I/D/M/F*	Stop Codons
reverse transcriptase (NP_056728.1)	463	591	19.0%	371	43.2%	126 (97.7%)	61 (47.3%)	0/3/0/0	1
Protein mutations:	I463A (5020A>G 5021T>C 5022C>T), L464I (5023T>A 5025G>T), H466F (5029C>T 5030A>T), I467S (5032A>T 5033T>C 5034C>T), N468G (5035A>G 5036A>G 5037C>T), D472N (5047G>A), T473E (5050A>G 5051C>A 5052C>A), L474I (5053C>A 5055T>A), E475T (5056G>A 5057A>C 5058A>T), K478T (5066A>C 5067G>T), I486S (5090T>G 5091A>C), T488N (5096C>A 5097A>C), A490V (5102C>T 5103C>T), S491A (5104T>G 5106G>A), Y493F (5111A>T 5112C>T), L497V (5122C>G), A498R (5125G>A 5126C>G 5127T>G), R501C (5134A>T 5136A>C), Q505Y (5146C>T 5148A>T), A506D (5150C>A), K507R (5153A>G 5154G>A), E510K (5161G>A 5163A>G), V512del (5167_5169delGTT), W514V (5173T>G 5174G>T 5175G>T), R515P (5176A>C 5177G>C 5178A>C), W516R (5179T>C), T517K (5183C>A), D520E (5193T>G), Y523A (5200T>G 5201A>C 5202C>T), M524V (5203A>G 5205G>C), K529D (5218A>G 5220A>T), N530M (5222A>T 5223T>G), L531V (5224C>G 5226G>C), Q532K (5227C>A), G533N (5230G>A 5231G>A 5232A>C), F534L (5233T>C), P536C (5239C>T 5240C>G 5241A>T), H538G (5245C>G 5246A>G 5247T>A), H539I (5248C>A 5249A>T), L541N (5254T>A 5255T>A 5256A>C), E543D (5262G>T), E544Y (5263G>T 5265G>T), K545F (5266A>T 5267A>T 5268G>T), L546M (5269C>A), I548V (5275A>G 5277C>T), D555I (5296G>A 5297A>T 5298C>T), Y556D (5299T>G 5301C>T), W557Y (5303G>A 5304G>C), M560I (5313G>T), A563Q (5320G>C 5321C>A 5322T>A), I564K (5324T>A 5325C>G), I566T (5330T>C), N567P (5332A>C 5333A>C 5334C>A), E568K (5335G>A), G569S (5338G>T 5339G>C 5340T>A), T570A (5341A>G 5343T>C), N571E (5344A>G 5346T>A), T572del (5347_5349delACT), E573Q (5350G>C 5352G>A), I575V (5356A>G 5358T>A), C576del (5359_5361delTGC), A579T (5368G>A 5370A>T), S582V (5377A>G 5378G>T 5379C>T), F583W (5381T>G 5382T>G), K584N (5385A>T), A585F (5386G>T 5387C>T), E587* (5392G>T)								
Codon mutations:	ATC463GCT (5020A>G 5021T>C 5022C>T), TTG464ATT (5023T>A 5025G>T), CAC466TTC (5029C>T 5030A>T), ATC467TCT (5032A>T 5033T>C 5034C>T), AAC468GGT (5035A>G 5036A>G 5037C>T), TTC470TTT (5043C>T), CCC471CCT (5046C>T), GAT472AAT (5047G>A), ACC473GAA (5050A>G 5051C>A 5052C>A), CTT474ATA (5053C>A 5055T>A), GAA475ACT (5056G>A 5057A>C 5058A>T), GAC476GAT (5061C>T), AAG477AAA (5064G>A), AAG478ACT (5066A>C 5067G>T), CAA479CAG (5070A>G), CTT480TTG (5071C>T 5073T>G), AGA482AGG (5079A>G), GGC485GGA (5088C>A), ATA486AGC (5090T>G 5091A>C), CTA487CTT (5094A>T), ACA488AAC (5096C>A 5097A>C), TAT489TAC (5100T>C), GCC490GTT (5102C>T 5103C>T), TCG491GCA (5104T>G 5106G>A), GAT492GAC (5109T>C), TAC493TTT (5111A>T 5112C>T), ATC494ATT (5115C>T), CCG495CCA (5118G>A), CTA497GTA (5122C>G), GCT498AGG (5125G>A 5126C>G 5127T>G), AGA501TGC (5134A>T 5136A>C), AAG502AAA (5139G>A), CCT503CCC (5142T>C), CTG504TTA (5143C>T 5145G>A), CAA505TAT (5146C>T 5148A>T), GCC506GAC (5150C>A), AAG507AGA (5153A>G 5154G>A), CTT508TTG (5155C>T 5157T>G), AAA509AAG (5160A>G), GAA510AAG (5161G>A 5163A>G), AAC511AAT (5166C>T), GTT512del (5167_5169delGTT), CCA513CCG (5172A>G), TGG514GTT (5173T>G 5174G>T 5175G>T), AGA515CCC (5176A>C 5177G>C 5178A>C), TGG516CGG (5179T>C), ACA517AAA (5183C>A), AAA518AAG (5187A>G), GAG519GAA (5190G>A), GAT520GAG (5193T>G), ACC521ACT (5196C>T), CTC522CTG (5199C>G), TAC523GCT (5200T>G 5201A>C 5202C>T), ATG524GTC (5203A>G 5205G>C), GTG527GTT (5214G>T), AAG528AAA (5217G>A), AAA529GAT (5218A>G 5220A>T), AAT530ATG (5222A>T 5223T>G), CTG531GTC (5224C>G 5226G>C), CAA532AAA (5227C>A), GGA533AAC (5230G>A 5231G>A 5232A>C), TTT534CTT (5233T>C), CCA536TGT (5239C>T 5240C>G 5241A>T), CTA537TTG (5242C>T 5244A>G), CAT538GGA (5245C>G 5246A>G 5247T>A), CAT539ATT (5248C>A 5249A>T), CCC540CCT (5253C>T), TTA541AAC (5254T>A 5255T>A 5256A>C), CCA542CCC (5259A>C), GAG543GAT (5262G>T), GAG544TAT (5263G>T 5265G>T), AAG545TTT (5266A>T 5267A>T 5268G>T), CTG546ATG (5269C>A), ATC547ATT (5274C>T), ATC548GTT (5275A>G 5277C>T), GAG549GAA (5280G>A), ACC550ACT (5283C>T), GCA552GCT (5289A>T), TCA553TCC (5292A>C), GAC555ATT (5296G>A 5297A>T 5298C>T), TAC556GAT (5299T>G 5301C>T), TGG557TAC (5303G>A 5304G>C), GGT559GGA (5310T>A), ATG560ATT (5313G>T), TTA561CTA (5314T>C), GCT563CAA (5320G>C 5321C>A 5322T>A), ATC564AAG (5324T>A 5325C>G), ATT566ACT (5330T>C), AAC567CCA (5332A>C 5333A>C 5334C>A), GAA568AAA (5335G>A), GGT569TCA (5338G>T 5339G>C 5340T>A), ACT570GCC (5341A>G 5343T>C), AAT571GAA (5344A>G 5346T>A), ACT572del (5347_5349delACT), GAG573CAA (5350G>C 5352G>A), ATT575GTA (5356A>G 5358T>A), TGC576del (5359_5361delTGC), AGA577CGG (5362A>C 5364A>G), TAC578TAT (5367C>T), GCA579ACT (5368G>A 5370A>T), TCT580TCG (5373T>G), AGC582GTT (5377A>G 5378G>T 5379C>T), TTT583TGG (5381T>G 5382T>G), AAA584AAT (5385A>T), GCT585TTT (5386G>T 5387C>T), GAA587TAA (5392G>T), AAT589AAC (5400T>C), TAC590TAT (5403C>T), CAC591A.. (5404C>A)								

*: Inserts / Deletes / Misaligned / Frameshifts

Analysis details

This analysis was performed with panviral2.64

NGS Details (UN24): Duamitovirus peex1

Assembly

**Coverage Length**	135 (1 contig(s))
**Depth Of Coverage**	8.0
**Number Of Reads**	12
**Reads Per Million**	0.24 rpm (after QC)
**Ambiguities**	0
**Assembly Method**	de novo + reference guided assembly
**Consensus Caller**	Bcf Tools

Coverage Map

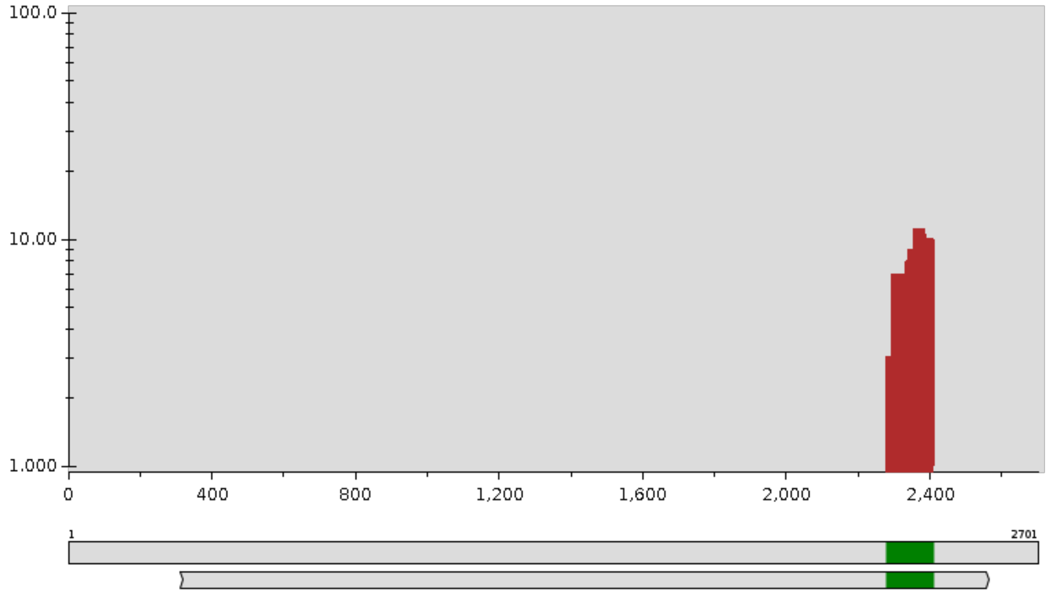

Assignment

**Type**	Duamitovirus peex1 (Taxonomy ID: 2955799)
**Reference Genome**	NC_076525.1
**NT Identity (%)**	70.3704
**AA Identity (%)**	66.6667
**Number Of Stop Codons**	0
**Number Of CDS**	1

Alignment

**Alignment Score**	110.0 (NT) + 251.0 (AA) = 361.0
**Concordance (%)**	58.4142

Alignment Method	Global, seeded, nucleotide + amino acids (AGA)

Genome Region

Sequence starts at position 2278 and ends at position 2412 relative to NC_076525.1 reference sequence.

Alignment Detailed Statistics

	Begin	End	Coverage	Score	Concordance	Matches	Identities	I/D/M/F*	Stop Codons
**NT**	**2278**	**2412**	**5.0%**	**110**	**40.7%**	**135 (100%)**	**95 (70.4%)**	**0/0**	
Mutations:	2278G>A, 2281T>A, 2283G>A, 2294T>G, 2295C>A, 2296G>A, 2301C>G, 2303T>C, 2306A>T, 2311A>G, 2312T>C, 2315C>T, 2321G>T, 2325A>G, 2326A>C, 2327T>A, 2330C>A, 2331C>G, 2333G>T, 2339G>A, 2345A>T, 2348C>T, 2351A>G, 2352C>T, 2354T>C, 2363G>T, 2373G>A, 2374C>G, 2375G>T, 2377C>A, 2378T>A, 2379T>A, 2388C>A, 2390T>A, 2393T>A, 2394C>A, 2396G>C, 2398C>A, 2399T>A, 2406C>G								
**CDS**									
RdRp	657	701	6.0%	251	67.3%	45 (100%)	30 (66.7%)	0/0/0/0	0
Protein mutations:	M657K (2281T>A), E658K (2283G>A), R662K (2295C>A 2296G>A), L664V (2301C>G 2303T>C), Y667C (2311A>G 2312T>C), N672A (2325A>G 2326A>C 2327T>A), P674A (2331C>G 2333G>T), L681F (2352C>T 2354T>C), A688S (2373G>A 2374C>G 2375G>T), T689K (2377C>A 2378T>A), S690T (2379T>A), Q695N (2394C>A 2396G>C), T696K (2398C>A 2399T>A), H699D (2406C>G)								
Codon mutations:	TGG656.AG (2278G>A), ATG657AAG (2281T>A), GAA658AAA (2283G>A), CTT661CTG (2294T>G), CGA662AAA (2295C>A 2296G>A), CTT664GTC (2301C>G 2303T>C), TCA665TCT (2306A>T), TAT667TGC (2311A>G 2312T>C), CAC668CAT (2315C>T), GTG670GTT (2321G>T), AAT672GCA (2325A>G 2326A>C 2327T>A), TCC673TCA (2330C>A), CCG674GCT (2331C>G 2333G>T), GTG676GTA (2339G>A), ATA678ATT (2345A>T), GAC679GAT (2348C>T), CAA680CAG (2351A>G), CTT681TTC (2352C>T 2354T>C), GTG684GTT (2363G>T), GCG688AGT (2373G>A 2374C>G 2375G>T), ACT689AAA (2377C>A 2378T>A), TCT690ACT (2379T>A), CGT693AGA (2388C>A 2390T>A), ACT694ACA (2393T>A), CAG695AAC (2394C>A 2396G>C), ACT696AAA (2398C>A 2399T>A), CAT699GAT (2406C>G)								
**Proteins**									
RNA-dependent RNA polymerase (YP_010798875.1)	657	701	6.0%	251	67.3%	45 (100%)	30 (66.7%)	0/0/0/0	0
Protein mutations:	M657K (2281T>A), E658K (2283G>A), R662K (2295C>A 2296G>A), L664V (2301C>G 2303T>C), Y667C (2311A>G 2312T>C), N672A (2325A>G 2326A>C 2327T>A), P674A (2331C>G 2333G>T), L681F (2352C>T 2354T>C), A688S (2373G>A 2374C>G 2375G>T), T689K (2377C>A 2378T>A), S690T (2379T>A), Q695N (2394C>A 2396G>C), T696K (2398C>A 2399T>A), H699D (2406C>G)								
Codon mutations:	TGG656.AG (2278G>A), ATG657AAG (2281T>A), GAA658AAA (2283G>A), CTT661CTG (2294T>G), CGA662AAA (2295C>A 2296G>A), CTT664GTC (2301C>G 2303T>C), TCA665TCT (2306A>T), TAT667TGC (2311A>G 2312T>C), CAC668CAT (2315C>T), GTG670GTT (2321G>T), AAT672GCA (2325A>G 2326A>C 2327T>A), TCC673TCA (2330C>A), CCG674GCT (2331C>G 2333G>T), GTG676GTA (2339G>A), ATA678ATT (2345A>T), GAC679GAT (2348C>T), CAA680CAG (2351A>G), CTT681TTC (2352C>T 2354T>C), GTG684GTT (2363G>T), GCG688AGT (2373G>A 2374C>G 2375G>T), ACT689AAA (2377C>A 2378T>A), TCT690ACT (2379T>A), CGT693AGA (2388C>A 2390T>A), ACT694ACA (2393T>A), CAG695AAC (2394C>A 2396G>C), ACT696AAA (2398C>A 2399T>A), CAT699GAT (2406C>G)								

*: Inserts / Deletes / Misaligned / Frameshifts

Analysis details

This analysis was performed with panviral2.64

NGS Details (UN24): Caulimovirus tessellobrassicae

Assembly

**Coverage Length**	330 (1 contig(s))
**Depth Of Coverage**	4.9
**Number Of Reads**	12
**Reads Per Million**	0.24 rpm (after QC)
**Ambiguities**	0
**Assembly Method**	de novo + reference guided assembly
**Consensus Caller**	Bcf Tools

Coverage Map

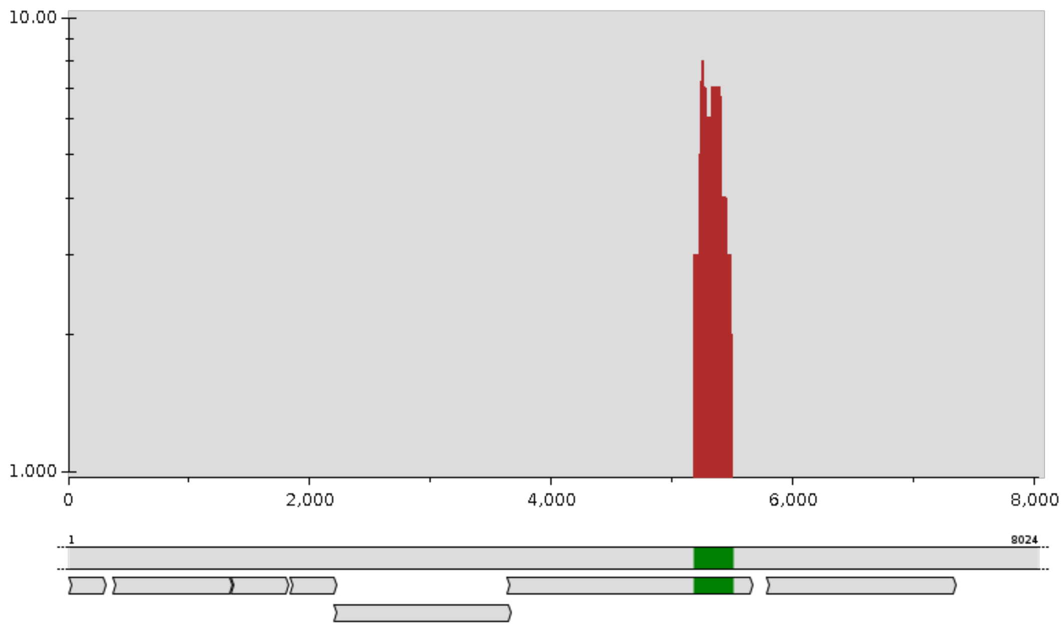

Assignment

**Type**	Caulimovirus tessellobrassicae (Taxonomy ID: 3047738)
**Reference Genome**	NC_001497.2
**NT Identity (%)**	58.2524
**AA Identity (%)**	45.6311
**Number Of Stop Codons**	0
**Number Of CDS**	7

Alignment

**Alignment Score**	63.0 (NT) + 343.0 (AA) = 406.0
**Concordance (%)**	32.0189

**Alignment Method**	Global, seeded, nucleotide + amino acids (AGA)

Genome Region

Sequence starts at position 5174 and ends at position 5503 relative to NC_001497.2 reference sequence.

Alignment Detailed Statistics

	Begin	End	Coverage	Score	Concordance	Matches	Identities	I/D/M/F*	Stop Codons
**NT**	**5174**	**5503**	**4.1%**	**63**	**10.9%**	**309 (93.6%)**	**180 (54.5%)**	**0/21**	
Mutations:	5174G>T, 5176A>G, 5185A>G, 5187A>T, 5190G>A, 5191G>C, 5197C>A, 5198T>A, 5199C>A, 5201A>G, 5202C>T, 5203A>G, 5205G>T, 5210A>G, 5211G>C, 5212G>C, 5214G>A, 5217G>A, 5218A>C, 5221A>G, 5223T>A, 5224C>T, 5225T>G, 5226G>T, 5227C>T, 5228A>C, 5229A>C, 5230G>A, 5231G>A, 5235T>A, 5238T>A, 5239C>A, 5240C>A, 5241A>G, 5245C>A, 5246A>G, 5247T>A, 5249A>T, 5253C>A, 5254T>G, 5255T>A, 5259A>G, 5262G>A, 5265G>C, 5268G>C, 5269C>T, 5271G>A, 5272A>G, 5274C>T, 5275A>C, 5277C>A, 5278G>C, 5280G>A, 5283C>A, 5289A>T, 5293G>A, 5295C>T, 5296G>T, 5299T>C, 5301C>T, 5309G>C, 5310T>A, 5311A>T, 5313G>A, 5317A>C, 5320G>A, 5323_5325delATC, 5326A>G, 5328A>C, 5329A>C, 5334C>T, 5338_5355delGGTACTAATACTGAGTTA, 5361C>T, 5364A>G, 5367C>T, 5368G>A, 5371T>A, 5372C>G, 5376A>T, 5378G>C, 5379C>A, 5385A>T, 5387C>A, 5388T>A, 5389G>A, 5391A>C, 5396A>C, 5397G>T, 5400T>A, 5404C>T, 5405A>C, 5406C>A, 5408G>C, 5409C>A, 5415C>A, 5421G>A, 5422A>T, 5423C>T, 5430G>C, 5431G>A, 5434A>G, 5438A>G, 5439T>A, 5440A>G, 5441C>G, 5442T>C, 5446A>C, 5447A>G, 5448G>A, 5455A>T, 5456G>C, 5458A>G, 5459T>C, 5462A>T, 5463T>C, 5467A>C, 5468C>T, 5469T>A, 5472T>A, 5473G>A, 5474T>A, 5475T>G, 5478T>A, 5481T>C, 5482C>A, 5484G>C, 5487T>C, 5490G>A, 5491A>T, 5496T>C, 5502T>A								
**CDS**									
CaMVgp6	515	624	16.2%	343	47.4%	103 (93.6%)	47 (42.7%)	0/7/0/0	0
Protein mutations:	R515G (5176A>G), K518D (5185A>G 5187A>T), D520H (5191G>C), L522K (5197C>A 5198T>A 5199C>A), Y523C (5201A>G 5202C>T), M524V (5203A>G 5205G>T), K526S (5210A>G 5211G>C), V527L (5212G>C 5214G>A), K529Q (5218A>C), N530E (5221A>G 5223T>A), L531C (5224C>T 5225T>G 5226G>T), Q532S (5227C>T 5228A>C 5229A>C), G533K (5230G>A 5231G>A), F534L (5235T>A), P536K (5239C>A 5240C>A 5241A>G), H538R (5245C>A 5246A>G 5247T>A), H539L (5249A>T), L541E (5254T>G 5255T>A), E544D (5265G>C), K545N (5268G>C), I547V (5272A>G 5274C>T), I548L (5275A>C 5277C>A), E549Q (5278G>C 5280G>A), D554N (5293G>A 5295C>T), D555Y (5296G>T), Y556H (5299T>C 5301C>T), G559A (5309G>C 5310T>A), M560L (5311A>T 5313G>A), K562Q (5317A>C), A563T (5320G>A), I564del (5323_5325delATC), K565D (5326A>G 5328A>C), I566L (5329A>C), G569_L574del (5338_5355delGGTACTAATACTGAGTTA), A579T (5368G>A), S582T (5378G>C 5379C>A), K584N (5385A>T), A585E (5387C>A 5388T>A), A586T (5389G>A 5391A>C), K588T (5396A>C 5397G>T), N589K (5400T>A), H591S (5404C>T 5405A>C 5406C>A), S592T (5408G>C 5409C>A), D594E (5415C>A), T597L (5422A>T 5423C>T), V600I (5431G>A), I601V (5434A>G), N602R (5438A>G 5439T>A), T603G (5440A>G 5441C>G 5442T>C), K605R (5446A>C 5447A>G 5448G>A), I609A (5458A>G 5459T>C), Y610F (5462A>T 5463T>C), T612L (5467A>C 5468C>T 5469T>A), V614K (5473G>A 5474T>A 5475T>G), H615Q (5478T>A), L617I (5482C>A 5484G>C), T620S (5491A>T)								
Codon mutations:	TGG514.TG (5174G>T), AGA515GGA (5176A>G), AAA518GAT (5185A>G 5187A>T), GAG519GAA (5190G>A), GAT520CAT (5191G>C), CTC522AAA (5197C>A 5198T>A 5199C>A), TAC523TGT (5201A>G 5202C>T), ATG524GTT (5203A>G 5205G>T), AAG526AGC (5210A>G 5211G>C), GTG527CTA (5212G>C 5214G>A), AAG528AAA (5217G>A), AAA529CAA (5218A>C), AAT530GAA (5221A>G 5223T>A), CTG531TGT (5224C>T 5225T>G 5226G>T), CAA532TCC (5227C>T 5228A>C 5229A>C), GGA533AAA (5230G>A 5231G>A), TTT534TTA (5235T>A), CCT535CCA (5238T>A), CCA536AAG (5239C>A 5240C>A 5241A>G), CAT538AGA (5245C>A 5246A>G 5247T>A), CAT539CTT (5249A>T), CCC540CCA (5253C>A), TTA541GAA (5254T>G 5255T>A), CCA542CCG (5259A>G), GAG543GAA (5262G>A), GAG544GAC (5265G>C), AAG545AAC (5268G>C), CTG546TTA (5269C>T 5271G>A), ATC547GTT (5272A>G 5274C>T), ATC548CTA (5275A>C 5277C>A), GAG549CAA (5278G>C 5280G>A), ACC550ACA (5283C>A), GCA552GCT (5289A>T), GAC554AAT (5293G>A 5295C>T), GAC555TAC (5296G>T), TAC556CAT (5299T>C 5301C>T), GGT559GCA (5309G>C 5310T>A), ATG560TTA (5311A>T 5313G>A), AAA562CAA (5317A>C), GCT563ACT (5320G>A), ATC564del (5323_5325delATC), AAA565GAC (5326A>G 5328A>C), ATT566CTT (5329A>C), AAC567AAT (5334C>T), GGT569_TTA574del (5338_5355delGGTACTAATACTGAGTTA), TGC576TGT (5361C>T), AGA577AGG (5364A>G), TAC578TAT (5367C>T), GCA579ACA (5368G>A), TCT580AGT (5371T>A 5372C>G), GGA581GGT (5376A>T), AGC582ACA (5378G>C 5379C>A), AAA584AAT (5385A>T), GCT585GAA (5387C>A 5388T>A), GCA586ACC (5389G>A 5391A>C), AAG588ACT (5396A>C 5397G>T), AAT589AAA (5400T>A), CAC591TCA (5404C>T 5405A>C 5406C>A), AGC592ACA (5408G>C 5409C>A), GAC594GAA (5415C>A), GAG596GAA (5421G>A), ACA597TTA (5422A>T 5423C>T), GCG599GCC (5430G>C), GTA600ATA (5431G>A), ATA601GTA (5434A>G), AAT602AGA (5438A>G 5439T>A), ACT603GGC (5440A>G 5441C>G 5442T>C), AAG605CGA (5446A>C 5447A>G 5448G>A), AGT608TCT (5455A>T 5456G>C), ATT609GCT (5458A>G 5459T>C), TAT610TTC (5462A>T 5463T>C), ACT612CTA (5467A>C 5468C>T 5469T>A), CCT613CCA (5472T>A), GTT614AAG (5473G>A 5474T>A 5475T>G), CAT615CAA (5478T>A), TTT616TTC (5481T>C), CTG617ATC (5482C>A 5484G>C), ATT618ATC (5487T>C), AGG619AGA (5490G>A), ACA620TCA (5491A>T), GAT621GAC (5496T>C), ACT623ACA (5502T>A)								
**Proteins**									
reverse transcriptase (NP_056728.1)	515	624	16.2%	343	47.4%	103 (93.6%)	47 (42.7%)	0/7/0/0	0
Protein mutations:	R515G (5176A>G), K518D (5185A>G 5187A>T), D520H (5191G>C), L522K (5197C>A 5198T>A 5199C>A), Y523C (5201A>G 5202C>T), M524V (5203A>G 5205G>T), K526S (5210A>G 5211G>C), V527L (5212G>C 5214G>A), K529Q (5218A>C), N530E (5221A>G 5223T>A), L531C (5224C>T 5225T>G 5226G>T), Q532S (5227C>T 5228A>C 5229A>C), G533K (5230G>A 5231G>A), F534L (5235T>A), P536K (5239C>A 5240C>A 5241A>G), H538R (5245C>A 5246A>G 5247T>A), H539L (5249A>T), L541E (5254T>G 5255T>A), E544D (5265G>C), K545N (5268G>C), I547V (5272A>G 5274C>T), I548L (5275A>C 5277C>A), E549Q (5278G>C 5280G>A), D554N (5293G>A 5295C>T), D555Y (5296G>T), Y556H (5299T>C 5301C>T), G559A (5309G>C 5310T>A), M560L (5311A>T 5313G>A), K562Q (5317A>C), A563T (5320G>A), I564del (5323_5325delATC), K565D (5326A>G 5328A>C), I566L (5329A>C), G569_L574del (5338_5355delGGTACTAATACTGAGTTA), A579T (5368G>A), S582T (5378G>C 5379C>A), K584N (5385A>T), A585E (5387C>A 5388T>A), A586T (5389G>A 5391A>C), K588T (5396A>C 5397G>T), N589K (5400T>A), H591S (5404C>T 5405A>C 5406C>A), S592T (5408G>C 5409C>A), D594E (5415C>A), T597L (5422A>T 5423C>T), V600I (5431G>A), I601V (5434A>G), N602R (5438A>G 5439T>A), T603G (5440A>G 5441C>G 5442T>C), K605R (5446A>C 5447A>G 5448G>A), I609A (5458A>G 5459T>C), Y610F (5462A>T 5463T>C), T612L (5467A>C 5468C>T 5469T>A), V614K (5473G>A 5474T>A 5475T>G), H615Q (5478T>A), L617I (5482C>A 5484G>C), T620S (5491A>T)								
Codon mutations:	TGG514.TG (5174G>T), AGA515GGA (5176A>G), AAA518GAT (5185A>G 5187A>T), GAG519GAA (5190G>A), GAT520CAT (5191G>C), CTC522AAA (5197C>A 5198T>A 5199C>A), TAC523TGT (5201A>G 5202C>T), ATG524GTT (5203A>G 5205G>T), AAG526AGC (5210A>G 5211G>C), GTG527CTA (5212G>C 5214G>A), AAG528AAA (5217G>A), AAA529CAA (5218A>C), AAT530GAA (5221A>G 5223T>A), CTG531TGT (5224C>T 5225T>G 5226G>T), CAA532TCC (5227C>T 5228A>C 5229A>C), GGA533AAA (5230G>A 5231G>A), TTT534TTA (5235T>A), CCT535CCA (5238T>A), CCA536AAG (5239C>A 5240C>A 5241A>G), CAT538AGA (5245C>A 5246A>G 5247T>A), CAT539CTT (5249A>T), CCC540CCA (5253C>A), TTA541GAA (5254T>G 5255T>A), CCA542CCG (5259A>G), GAG543GAA (5262G>A), GAG544GAC (5265G>C), AAG545AAC (5268G>C), CTG546TTA (5269C>T 5271G>A), ATC547GTT (5272A>G 5274C>T), ATC548CTA (5275A>C 5277C>A), GAG549CAA (5278G>C 5280G>A), ACC550ACA (5283C>A), GCA552GCT (5289A>T), GAC554AAT (5293G>A 5295C>T), GAC555TAC (5296G>T), TAC556CAT (5299T>C 5301C>T), GGT559GCA (5309G>C 5310T>A), ATG560TTA (5311A>T 5313G>A), AAA562CAA (5317A>C), GCT563ACT (5320G>A), ATC564del (5323_5325delATC), AAA565GAC (5326A>G 5328A>C), ATT566CTT (5329A>C), AAC567AAT (5334C>T), GGT569_TTA574del (5338_5355delGGTACTAATACTGAGTTA), TGC576TGT (5361C>T), AGA577AGG (5364A>G), TAC578TAT (5367C>T), GCA579ACA (5368G>A), TCT580AGT (5371T>A 5372C>G), GGA581GGT (5376A>T), AGC582ACA (5378G>C 5379C>A), AAA584AAT (5385A>T), GCT585GAA (5387C>A 5388T>A), GCA586ACC (5389G>A 5391A>C), AAG588ACT (5396A>C 5397G>T), AAT589AAA (5400T>A), CAC591TCA (5404C>T 5405A>C 5406C>A), AGC592ACA (5408G>C 5409C>A), GAC594GAA (5415C>A), GAG596GAA (5421G>A), ACA597TTA (5422A>T 5423C>T), GCG599GCC (5430G>C), GTA600ATA (5431G>A), ATA601GTA (5434A>G), AAT602AGA (5438A>G 5439T>A), ACT603GGC (5440A>G 5441C>G 5442T>C), AAG605CGA (5446A>C 5447A>G 5448G>A), AGT608TCT (5455A>T 5456G>C), ATT609GCT (5458A>G 5459T>C), TAT610TTC (5462A>T 5463T>C), ACT612CTA (5467A>C 5468C>T 5469T>A), CCT613CCA (5472T>A), GTT614AAG (5473G>A 5474T>A 5475T>G), CAT615CAA (5478T>A), TTT616TTC (5481T>C), CTG617ATC (5482C>A 5484G>C), ATT618ATC (5487T>C), AGG619AGA (5490G>A), ACA620TCA (5491A>T), GAT621GAC (5496T>C), ACT623ACA (5502T>A)								

*: Inserts / Deletes / Misaligned / Frameshifts

Analysis details

This analysis was performed with panviral2.64

NGS Details (UN24): Badnavirus venaribis

Assembly

**Coverage Length**	238 (1 contig(s))
**Depth Of Coverage**	6.4
**Number Of Reads**	12
**Reads Per Million**	0.24 rpm (after QC)
**Ambiguities**	0
**Assembly Method**	de novo + reference guided assembly
**Consensus Caller**	Bcf Tools

Coverage Map

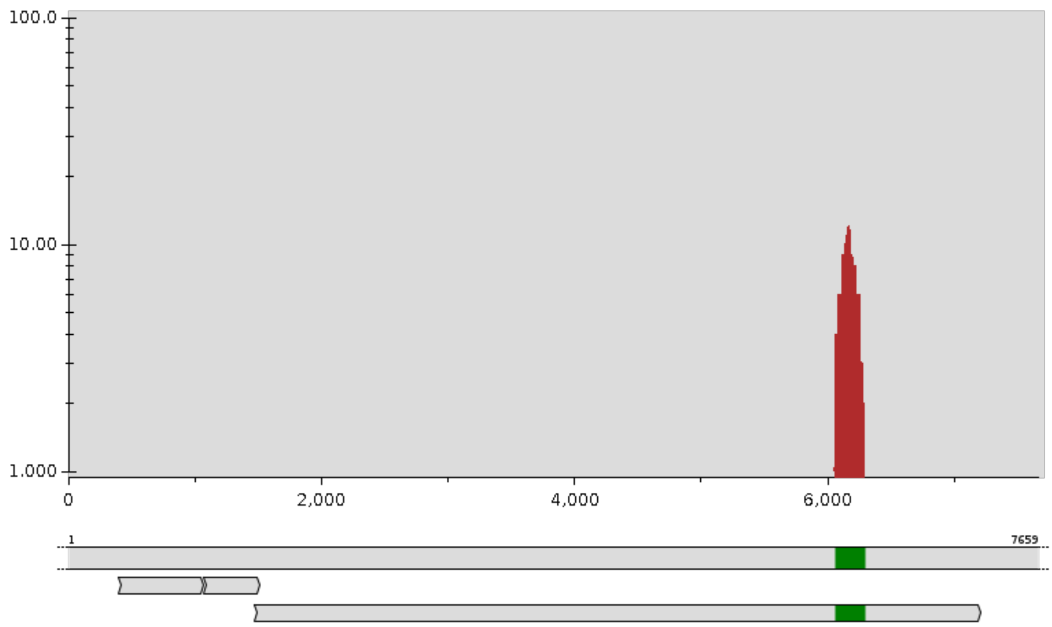

Assignment

**Type**	Badnavirus venaribis (Taxonomy ID: 3047843)
**Reference Genome**	NC_018105.1
**NT Identity (%)**	53.9419
**AA Identity (%)**	48.1481
**Number Of Stop Codons**	0
**Number Of CDS**	3

Alignment

**Alignment Score**	32.0 (NT) + 266.0 (AA) = 298.0
**Concordance (%)**	27.9288

Alignment Method	Global, seeded, nucleotide + amino acids (AGA)

Genome Region

Sequence starts at position 6058 and ends at position 6295 relative to NC_018105.1 reference sequence.

Alignment Detailed Statistics

	Begin	End	Coverage	Score	Concordance	Matches	Identities	I/D/M/F*	Stop Codons
**NT**	**6058**	**6295**	**3.1%**	**32**	**6.7%**	**238 (98.8%)**	**130 (53.9%)**	**3/0**	
Mutations:	6061C>G, 6064T>A, 6065G>A, 6072G>C, 6076A>C, 6077T>G, 6078C>T, 6081G>C, 6083C>A, 6086T>A, 6087A>T, 6090A>C, 6092T>A, 6093C>T, 6101G>T, 6102G>T, 6113C>T, 6114T>G, 6117C>T, 6120T>A, 6121A>T, 6123T>G, 6124G>A, 6126T>A, 6129C>T, 6132T>C, 6135A>T, 6136G>T, 6139G>A, 6141T>A, 6144C>T, 6147G>A, 6148A>G, 6149G>C, 6150G>T, 6152A>C, 6153A>T, 6157G>A, 6158A>C, 6160A>G, 6162C>A, 6163T>A, 6164G>T, 6165C>T, 6168C>T, 6171C>T, 6171_6172insCCA, 6172A>C, 6174G>T, 6175C>A, 6178A>C, 6180A>T, 6181G>C, 6182A>G, 6183G>C, 6186C>T, 6189C>T, 6190G>T, 6191C>T, 6195T>A, 6197A>T, 6199A>T, 6201C>T, 6204C>T, 6207C>T, 6211C>T, 6213C>G, 6218T>A, 6219C>T, 6222C>T, 6223A>G, 6224A>C, 6225T>A, 6228C>T, 6229C>T, 6230T>G, 6234A>T, 6237A>T, 6241G>T, 6242A>T, 6244A>G, 6245G>A, 6246C>A, 6253C>G, 6254G>C, 6255G>A, 6256C>G, 6257A>T, 6258A>G, 6259A>G, 6261G>A, 6262C>T, 6265G>C, 6267A>G, 6268G>T, 6270T>G, 6272G>T, 6273C>G, 6277A>C, 6280A>C, 6283G>C, 6284G>A, 6285G>A, 6286T>C, 6288G>T, 6292C>G, 6293T>C, 6294G>C, 6295T>A								

CDS

	Begin	End	Coverage	Score	Concordance	Matches	Identities	I/D/M/F*	Stop Codons
B227_gp3	1531	1610	4.2%	266	44.7%	80 (98.8%)	39 (48.1%)	1/0/0/0	0
Protein mutations:	P1532A (6061C>G), W1533K (6064T>A 6065G>A), I1537R (6076A>C 6077T>G 6078C>T), P1539H (6083C>A), V1540D (6086T>A 6087A>T), F1542Y (6092T>A 6093C>T), W1545F (6101G>T 6102G>T), P1549L (6113C>T 6114T>G), I1552L (6121A>T 6123T>G), A1553T (6124G>A 6126T>A), A1557S (6136G>T), V1558I (6139G>A 6141T>A), R1561A (6148A>G 6149G>C 6150G>T), K1562T (6152A>C 6153A>T), D1564T (6157G>A 6158A>C), N1565E (6160A>G 6162C>A), C1566I (6163T>A 6164G>T 6165C>T), H1568_K1569insP (6171_6172insCCA), K1569H (6172A>C 6174G>T), L1570M (6175C>A), E1572R (6181G>C 6182A>G 6183G>C), A1575L (6190G>T 6191C>T), Y1577F (6197A>T), I1578F (6199A>T 6201C>T), F1584Y (6218T>A 6219C>T), N1586A (6223A>G 6224A>C 6225T>A), L1588W (6229C>T 6230T>G), Q1589H (6234A>T), E1590D (6237A>T), E1592L (6241G>T 6242A>T), S1593E (6244A>G 6245G>A 6246C>A), R1596A (6253C>G 6254G>C 6255G>A), Q1597V (6256C>G 6257A>T 6258A>G), M1598V (6259A>G 6261G>A), E1600Q (6265G>C 6267A>G), V1601L (6268G>T 6270T>G), C1602L (6272G>T 6273C>G), K1604Q (6277A>C), N1605H (6280A>C), G1606Q (6283G>C 6284G>A 6285G>A), L1609A (6292C>G 6293T>C 6294G>C)								
Codon mutations:	CCT1532GCT (6061C>G), TGG1533AAG (6064T>A 6065G>A), GCG1535GCC (6072G>C), ATC1537CGT (6076A>C 6077T>G 6078C>T), ACG1538ACC (6081G>C), CCC1539CAC (6083C>A), GTA1540GAT (6086T>A 6087A>T), GGA1541GGC (6090A>C), TTC1542TAT (6092T>A 6093C>T), TGG1545TTT (6101G>T 6102G>T), CCT1549CTG (6113C>T 6114T>G), TTC1550TTT (6117C>T), GGT1551GGA (6120T>A), ATT1552TTG (6121A>T 6123T>G), GCT1553ACA (6124G>A 6126T>A), AAC1554AAT (6129C>T), GCT1555GCC (6132T>C), CCA1556CCT (6135A>T), GCG1557TCG (6136G>T), GTT1558ATA (6139G>A 6141T>A), TTC1559TTT (6144C>T), CAG1560CAA (6147G>A), AGG1561GCT (6148A>G 6149G>C 6150G>T), AAA1562ACT (6152A>C 6153A>T), GAT1564ACT (6157G>A 6158A>C), AAC1565GAA (6160A>G 6162C>A), TGC1566ATT (6163T>A 6164G>T 6165C>T), TTC1567TTT (6168C>T), CAC1568CAT (6171C>T), CAC1568_AAG1569insCCA (6171_6172insCCA), AAG1569CAT (6172A>C 6174G>T), CTG1570ATG (6175C>A), AGA1571CGT (6178A>C 6180A>T), GAG1572CGC (6181G>C 6182A>G 6183G>C), TTC1573TTT (6186C>T), GTC1574GTT (6189C>T), GCG1575TTG (6190G>T 6191C>T), GTT1576GTA (6195T>A), TAT1577TTT (6197A>T), ATC1578TTT (6199A>T 6201C>T), GAC1579GAT (6204C>T), GAC1580GAT (6207C>T), CTC1582TTG (6211C>T 6213C>G), TTC1584TAT (6218T>A 6219C>T), AGC1585AGT (6222C>T), AAT1586GCA (6223A>G 6224A>C 6225T>A), TCC1587TCT (6228C>T), CTG1588TGG (6229C>T 6230T>G), CAA1589CAT (6234A>T), GAA1590GAT (6237A>T), GAG1592TTG (6241G>T 6242A>T), AGC1593GAA (6244A>G 6245G>A 6246C>A), CGG1596GCA (6253C>G 6254G>C 6255G>A), CAA1597GTG (6256C>G 6257A>T 6258A>G), ATG1598GTA (6259A>G 6261G>A), CTG1599TTG (6262C>T), GAA1600CAG (6265G>C 6267A>G), GTT1601TTG (6268G>T 6270T>G), TGC1602TTG (6272G>T 6273C>G), AAG1604CAG (6277A>C), AAT1605CAT (6280A>C), GGG1606CAA (6283G>C 6284G>A 6285G>A), TTG1607CTT (6286T>C 6288G>T), CTG1609GCC (6292C>G 6293T>C 6294G>C), TCA1610A.. (6295T>A)								

Proteins

	Begin	End	Coverage	Score	Concordance	Matches	Identities	I/D/M/F*	Stop Codons
polyprotein (YP_006495799.1)	1531	1610	4.2%	266	44.7%	80 (98.8%)	39 (48.1%)	1/0/0/0	0
Protein mutations:	P1532A (6061C>G), W1533K (6064T>A 6065G>A), I1537R (6076A>C 6077T>G 6078C>T), P1539H (6083C>A), V1540D (6086T>A 6087A>T), F1542Y (6092T>A 6093C>T), W1545F (6101G>T 6102G>T), P1549L (6113C>T 6114T>G), I1552L (6121A>T 6123T>G), A1553T (6124G>A 6126T>A), A1557S (6136G>T), V1558I (6139G>A 6141T>A), R1561A (6148A>G 6149G>C 6150G>T), K1562T (6152A>C 6153A>T), D1564T (6157G>A 6158A>C), N1565E (6160A>G 6162C>A), C1566I (6163T>A 6164G>T 6165C>T), H1568_K1569insP (6171_6172insCCA), K1569H (6172A>C 6174G>T), L1570M (6175C>A), E1572R (6181G>C 6182A>G 6183G>C), A1575L (6190G>T 6191C>T), Y1577F (6197A>T), I1578F (6199A>T 6201C>T), F1584Y (6218T>A 6219C>T), N1586A (6223A>G 6224A>C 6225T>A), L1588W (6229C>T 6230T>G), Q1589H (6234A>T), E1590D (6237A>T), E1592L (6241G>T 6242A>T), S1593E (6244A>G 6245G>A 6246C>A), R1596A (6253C>G 6254G>C 6255G>A), Q1597V (6256C>G 6257A>T 6258A>G), M1598V (6259A>G 6261G>A), E1600Q (6265G>C 6267A>G), V1601L (6268G>T 6270T>G), C1602L (6272G>T 6273C>G), K1604Q (6277A>C), N1605H (6280A>C), G1606Q (6283G>C 6284G>A 6285G>A), L1609A (6292C>G 6293T>C 6294G>C)								
Codon mutations:	CCT1532GCT (6061C>G), TGG1533AAG (6064T>A 6065G>A), GCG1535GCC (6072G>C), ATC1537CGT (6076A>C 6077T>G 6078C>T), ACG1538ACC (6081G>C), CCC1539CAC (6083C>A), GTA1540GAT (6086T>A 6087A>T), GGA1541GGC (6090A>C), TTC1542TAT (6092T>A 6093C>T), TGG1545TTT (6101G>T 6102G>T), CCT1549CTG (6113C>T 6114T>G), TTC1550TTT (6117C>T), GGT1551GGA (6120T>A), ATT1552TTG (6121A>T 6123T>G), GCT1553ACA (6124G>A 6126T>A), AAC1554AAT (6129C>T), GCT1555GCC (6132T>C), CCA1556CCT (6135A>T), GCG1557TCG (6136G>T), GTT1558ATA (6139G>A 6141T>A), TTC1559TTT (6144C>T), CAG1560CAA (6147G>A), AGG1561GCT (6148A>G 6149G>C 6150G>T), AAA1562ACT (6152A>C 6153A>T), GAT1564ACT (6157G>A 6158A>C), AAC1565GAA (6160A>G 6162C>A), TGC1566ATT (6163T>A 6164G>T 6165C>T), TTC1567TTT (6168C>T), CAC1568CAT (6171C>T), CAC1568_AAG1569insCCA (6171_6172insCCA), AAG1569CAT (6172A>C 6174G>T), CTG1570ATG (6175C>A), AGA1571CGT (6178A>C 6180A>T), GAG1572CGC (6181G>C 6182A>G 6183G>C), TTC1573TTT (6186C>T), GTC1574GTT (6189C>T), GCG1575TTG (6190G>T 6191C>T), GTT1576GTA (6195T>A), TAT1577TTT (6197A>T), ATC1578TTT (6199A>T 6201C>T), GAC1579GAT (6204C>T), GAC1580GAT (6207C>T), CTC1582TTG (6211C>T 6213C>G), TTC1584TAT (6218T>A 6219C>T), AGC1585AGT (6222C>T), AAT1586GCA (6223A>G 6224A>C 6225T>A), TCC1587TCT (6228C>T), CTG1588TGG (6229C>T 6230T>G), CAA1589CAT (6234A>T), GAA1590GAT (6237A>T), GAG1592TTG (6241G>T 6242A>T), AGC1593GAA (6244A>G 6245G>A 6246C>A), CGG1596GCA (6253C>G 6254G>C 6255G>A), CAA1597GTG (6256C>G 6257A>T 6258A>G), ATG1598GTA (6259A>G 6261G>A), CTG1599TTG (6262C>T), GAA1600CAG (6265G>C 6267A>G), GTT1601TTG (6268G>T 6270T>G), TGC1602TTG (6272G>T 6273C>G), AAG1604CAG (6277A>C), AAT1605CAT (6280A>C), GGG1606CAA (6283G>C 6284G>A 6285G>A), TTG1607CTT (6286T>C 6288G>T), CTG1609GCC (6292C>G 6293T>C 6294G>C), TCA1610A.. (6295T>A)								

*: Inserts / Deletes / Misaligned / Frameshifts

Analysis details

This analysis was performed with panviral2.64

NGS Details (UN24): Badnavirus rutilanscamelliae

Assembly

**Coverage Length**	364 (1 contig(s))
**Depth Of Coverage**	3.5
**Number Of Reads**	11
**Reads Per Million**	0.22 rpm (after QC)
**Ambiguities**	0
**Assembly Method**	de novo + reference guided assembly
**Consensus Caller**	Bcf Tools

Coverage Map

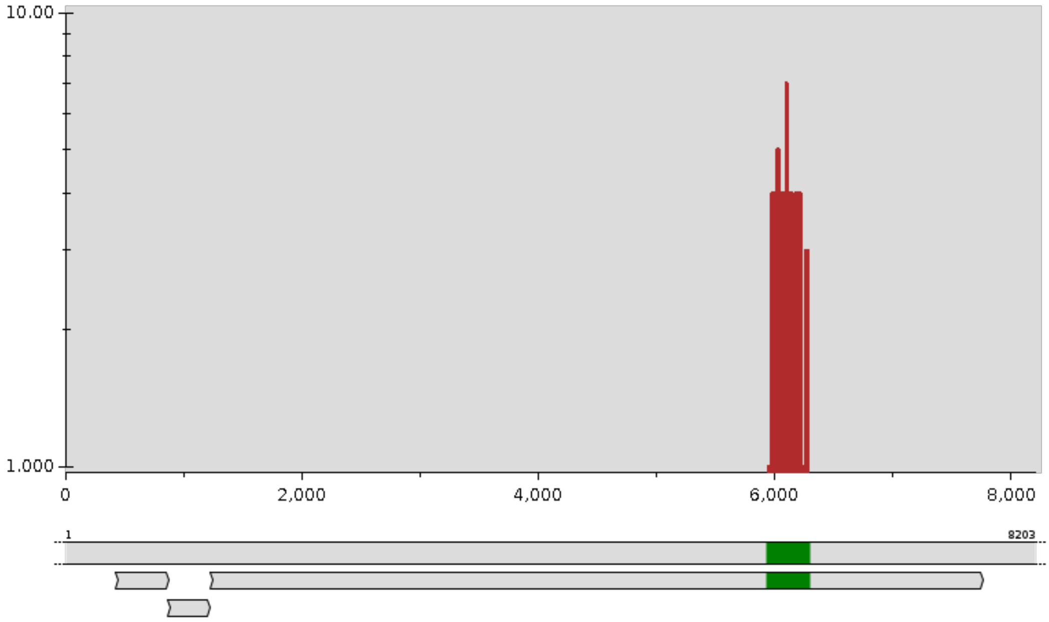

Assignment

**Type**	Badnavirus rutilanscamelliae (Taxonomy ID: 3047719)
**Reference Genome**	NC_055598.1
**NT Identity (%)**	56.044
**AA Identity (%)**	53.719
**Number Of Stop Codons**	1
**Number Of CDS**	3

Alignment

**Alignment Score**	88.0 (NT) + 446.0 (AA) = 534.0
**Concordance (%)**	33.1883

Alignment Method	Global, seeded, nucleotide + amino acids (AGA)

Genome Region

Sequence starts at position 5938 and ends at position 6301 relative to NC_055598.1 reference sequence.

Alignment Detailed Statistics

	Begin	End	Coverage	Score	Concordance	Matches	Identities	I/D/M/F*	Stop Codons
**NT**	**5938**	**6301**	**4.4%**	**88**	**12.1%**	**364 (100%)**	**204 (56.0%)**	**0/0**	
Mutations:	5938C>A, 5941A>C, 5944C>T, 5946A>T, 5950G>A, 5953G>A, 5956C>T, 5959C>T, 5960C>T, 5962G>A, 5965G>A, 5971G>A, 5973T>A, 5975C>T, 5976A>G, 5977T>G, 5981G>A, 5984G>C, 5985C>A, 5986T>G, 5989G>C, 5990G>T, 5991A>T, 5993C>A, 5994C>A, 5995T>A, 5996C>G, 5998A>G, 6001C>T, 6003T>C, 6005G>T, 6007A>T, 6008T>A, 6010G>A, 6013C>T, 6020T>A, 6021G>A, 6022G>T, 6023A>G, 6024C>T, 6025T>C, 6029G>T, 6031G>T, 6034C>A, 6035T>C, 6036T>A, 6046G>A, 6047C>A, 6048T>A, 6049G>T, 6064G>T, 6067T>A, 6070G>A, 6072A>T, 6079A>T, 6080G>T, 6082A>T, 6084T>A, 6091A>G, 6093G>A, 6094G>A, 6096A>T, 6097G>C, 6101G>A, 6103C>T, 6104C>G, 6105G>A, 6106A>T, 6109C>T, 6121C>T, 6122G>A, 6123C>T, 6124T>A, 6125G>A, 6127A>T, 6130T>C, 6133T>A, 6134G>A, 6135C>T, 6139G>C, 6151T>C, 6154C>A, 6155C>T, 6157C>G, 6160A>T, 6162T>A, 6163C>T, 6164A>T, 6165G>C, 6166C>T, 6167C>A, 6168C>A, 6172A>T, 6173C>T, 6174C>T, 6179G>A, 6180C>T, 6181C>G, 6186A>T, 6187A>C, 6193C>T, 6196G>A, 6198C>A, 6199C>A, 6200C>A, 6201A>T, 6202A>C, 6205C>T, 6206G>A, 6207T>A, 6209G>A, 6211G>A, 6214A>T, 6215T>G, 6216G>T, 6217C>T, 6219A>T, 6220G>A, 6221G>C, 6224C>A, 6229G>T, 6230C>T, 6232T>G, 6235C>A, 6236T>A, 6238G>A, 6239A>T, 6240G>C, 6242C>A, 6243C>A, 6245A>C, 6250G>A, 6255A>G, 6256G>T, 6257G>T, 6259G>A, 6260G>T, 6261C>T, 6265G>A, 6266G>A, 6268C>A, 6269C>G, 6272A>G, 6275G>C, 6276A>G, 6277C>A, 6280T>C, 6283G>A, 6286C>A, 6287T>C, 6288C>A, 6289A>T, 6291C>A, 6292A>T, 6295T>A, 6296A>T, 6297C>G, 6298A>T, 6299A>C, 6301T>A								
**CDS**									
KM754_gp3	1573	1693	5.6%	446	50.4%	121 (100%)	65 (53.7%)	0/0/0/0	1
Protein mutations:	K1573N (5941A>C), Y1575F (5946A>T), F1584Y (5973T>A), H1585W (5975C>T 5976A>G 5977T>G), V1587I (5981G>A), A1588Q (5984G>C 5985C>A 5986T>G), M1589I (5989G>C), D1590F (5990G>T 5991A>T), P1591K (5993C>A 5994C>A 5995T>A), Q1592E (5996C>G 5998A>G), I1594T (6003T>C), E1595Y (6005G>T 6007A>T), W1596R (6008T>A 6010G>A), W1600N (6020T>A 6021G>A 6022G>T), T1601V (6023A>G 6024C>T 6025T>C), V1603F (6029G>T 6031G>T), L1605Q (6035T>C 6036T>A), W1608* (6046G>A), L1609N (6047C>A 6048T>A 6049G>T), N1617I (6072A>T), A1620S (6080G>T 6082A>T), V1621E (6084T>A), R1624K (6093G>A 6094G>A), K1625I (6096A>T 6097G>C), D1627N (6101G>A 6103C>T), R1628D (6104C>G 6105G>A 6106A>T), A1634I (6122G>A 6123C>T 6124T>A), G1635S (6125G>A 6127A>T), A1638I (6134G>A 6135C>T), F1647Y (6162T>A 6163C>T), P1649K (6167C>A 6168C>A), P1651F (6173C>T 6174C>T), A1653M (6179G>A 6180C>T 6181C>G), K1655I (6186A>T 6187A>C), A1659E (6198C>A 6199C>A), Q1660I (6200C>A 6201A>T 6202A>C), V1662K (6206G>A 6207T>A), E1663K (6209G>A 6211G>A), C1665V (6215T>G 6216G>T 6217C>T), K1666I (6219A>T 6220G>A), E1667Q (6221G>C), H1668N (6224C>A), L1672I (6236T>A 6238G>A), P1674K (6242C>A 6243C>A), T1675P (6245A>C), K1678S (6255A>G 6256G>T), V1679L (6257G>T 6259G>A), A1680F (6260G>T 6261C>T), A1682T (6266G>A 6268C>A), Q1683E (6269C>G), I1684V (6272A>G), D1685R (6275G>C 6276A>G 6277C>A), S1689H (6287T>C 6288C>A 6289A>T), T1690N (6291C>A 6292A>T), T1692C (6296A>T 6297C>G 6298A>T), N1693Q (6299A>C 6301T>A)								
Codon mutations:	AGC1572..A (5938C>A), AAA1573AAC (5941A>C), ATC1574ATT (5944C>T), TAT1575TTT (5946A>T), TCG1576TCA (5950G>A), AAG1577AAA (5953G>A), TTC1578TTT (5956C>T), GAC1579GAT (5959C>T), CTG1580TTA (5960C>T 5962G>A), AAG1581AAA (5965G>A), GGG1583GGA (5971G>A), TTT1584TAT (5973T>A), CAT1585TGG (5975C>T 5976A>G 5977T>G), GTT1587ATT (5981G>A), GCT1588CAG (5984G>C 5985C>A 5986T>G), ATG1589ATC (5989G>C), GAT1590TTT (5990G>T 5991A>T), CCT1591AAA (5993C>A 5994C>A 5995T>A), CAA1592GAG (5996C>G 5998A>G), CAC1593CAT (6001C>T), ATT1594ACT (6003T>C), GAA1595TAT (6005G>T 6007A>T), TGG1596AGA (6008T>A 6010G>A), ACC1597ACT (6013C>T), TGG1600AAT (6020T>A 6021G>A 6022G>T), ACT1601GTC (6023A>G 6024C>T 6025T>C), GTG1603TTT (6029G>T 6031G>T), GGC1604GGA (6034C>A), TTA1605CAA (6035T>C 6036T>A), TGG1608TGA (6046G>A), CTG1609AAT (6047C>A 6048T>A 6049G>T), GGG1614GGT (6064G>T), CTT1615CTA (6067T>A), AAG1616AAA (6070G>A), AAT1617ATT (6072A>T), CCA1619CCT (6079A>T), GCA1620TCT (6080G>T 6082A>T), GTA1621GAA (6084T>A), CAA1623CAG (6091A>G), AGG1624AAA (6093G>A 6094G>A), AAG1625ATC (6096A>T 6097G>C), GAC1627AAT (6101G>A 6103C>T), CGA1628GAT (6104C>G 6105G>A 6106A>T), ATC1629ATT (6109C>T), TAC1633TAT (6121C>T), GCT1634ATA (6122G>A 6123C>T 6124T>A), GGA1635AGT (6125G>A 6127A>T), TTT1636TTC (6130T>C), ATT1637ATA (6133T>A), GCT1638ATT (6134G>A 6135C>T), GTG1639GTC (6139G>C), GAT1643GAC (6151T>C), ATC1644ATA (6154C>A), CTC1645TTG (6155C>T 6157C>G), ATA1646ATT (6160A>T), TTC1647TAT (6162T>A 6163C>T), AGC1648TCT (6164A>T 6165G>C 6166C>T), CCA1649AAA (6167C>A 6168C>A), ACA1650ACT (6172A>T), CCT1651TTT (6173C>T 6174C>T), GCC1653ATG (6179G>A 6180C>T 6181C>G), AAA1655ATC (6186A>T 6187A>C), CAC1657CAT (6193C>T), TTG1658TTA (6196G>A), GCC1659GAA (6198C>A 6199C>A), CAA1660ATC (6200C>A 6201A>T 6202A>C), TTC1661TTT (6205C>T), GTG1662AAG (6206G>A 6207T>A), GAG1663AAA (6209G>A 6211G>A), ATA1664ATT (6214A>T), TGC1665GTT (6215T>G 6216G>T 6217C>T), AAG1666ATA (6219A>T 6220G>A), GAA1667CAA (6221G>C), CAT1668AAT (6224C>A), GGG1669GGT (6229G>T), CTT1670TTG (6230C>T 6232T>G), GTC1671GTA (6235C>A), TTG1672ATA (6236T>A 6238G>A), AGT1673TCT (6239A>T 6240G>C), CCA1674AAA (6242C>A 6243C>A), ACA1675CCA (6245A>C), AAG1676AAA (6250G>A), AAG1678AGT (6255A>G 6256G>T), GTG1679TTA (6257G>T 6259G>A), GCC1680TTC (6260G>T 6261C>T), CAG1681CAA (6265G>A), GCC1682ACA (6266G>A 6268C>A), CAA1683GAA (6269C>G), ATT1684GTT (6272A>G), GAC1685CGA (6275G>C 6276A>G 6277C>A), TTT1686TTC (6280T>C), TTG1687TTA (6283G>A), GGC1688GGA (6286C>A), TCA1689CAT (6287T>C 6288C>A 6289A>T), ACA1690AAT (6291C>A 6292A>T), ATT1691ATA (6295T>A), ACA1692TGT (6296A>T 6297C>G 6298A>T), AAT1693CAA (6299A>C 6301T>A)								
**Proteins**									
polyprotein (YP_010087856.1)	1573	1693	5.6%	446	50.4%	121 (100%)	65 (53.7%)	0/0/0/0	1
Protein mutations:	K1573N (5941A>C), Y1575F (5946A>T), F1584Y (5973T>A), H1585W (5975C>T 5976A>G 5977T>G), V1587I (5981G>A), A1588Q (5984G>C 5985C>A 5986T>G), M1589I (5989G>C), D1590F (5990G>T 5991A>T), P1591K (5993C>A 5994C>A 5995T>A), Q1592E (5996C>G 5998A>G), I1594T (6003T>C), E1595Y (6005G>T 6007A>T), W1596R (6008T>A 6010G>A), W1600N (6020T>A 6021G>A 6022G>T), T1601V (6023A>G 6024C>T 6025T>C), V1603F (6029G>T 6031G>T), L1605Q (6035T>C 6036T>A), W1608* (6046G>A), L1609N (6047C>A 6048T>A 6049G>T), N1617I (6072A>T), A1620S (6080G>T 6082A>T), V1621E (6084T>A), R1624K (6093G>A 6094G>A), K1625I (6096A>T 6097G>C), D1627N (6101G>A 6103C>T), R1628D (6104C>G 6105G>A 6106A>T), A1634I (6122G>A 6123C>T 6124T>A), G1635S (6125G>A 6127A>T), A1638I (6134G>A 6135C>T), F1647Y (6162T>A 6163C>T), P1649K (6167C>A 6168C>A), P1651F (6173C>T 6174C>T), A1653M (6179G>A 6180C>T 6181C>G), K1655I (6186A>T 6187A>C), A1659E (6198C>A 6199C>A), Q1660I (6200C>A 6201A>T 6202A>C), V1662K (6206G>A 6207T>A), E1663K (6209G>A 6211G>A), C1665V (6215T>G 6216G>T 6217C>T), K1666I (6219A>T 6220G>A), E1667Q (6221G>C), H1668N (6224C>A), L1672I (6236T>A 6238G>A), P1674K (6242C>A 6243C>A), T1675P (6245A>C), K1678S (6255A>G 6256G>T), V1679L (6257G>T 6259G>A), A1680F (6260G>T 6261C>T), A1682T (6266G>A 6268C>A), Q1683E (6269C>G), I1684V (6272A>G), D1685R (6275G>C 6276A>G 6277C>A), S1689H (6287T>C 6288C>A 6289A>T), T1690N (6291C>A 6292A>T), T1692C (6296A>T 6297C>G 6298A>T), N1693Q (6299A>C 6301T>A)								
Codon mutations:	AGC1572..A (5938C>A), AAA1573AAC (5941A>C), ATC1574ATT (5944C>T), TAT1575TTT (5946A>T), TCG1576TCA (5950G>A), AAG1577AAA (5953G>A), TTC1578TTT (5956C>T), GAC1579GAT (5959C>T), CTG1580TTA (5960C>T 5962G>A), AAG1581AAA (5965G>A), GGG1583GGA (5971G>A), TTT1584TAT (5973T>A), CAT1585TGG (5975C>T 5976A>G 5977T>G), GTT1587ATT (5981G>A), GCT1588CAG (5984G>C 5985C>A 5986T>G), ATG1589ATC (5989G>C), GAT1590TTT (5990G>T 5991A>T), CCT1591AAA (5993C>A 5994C>A 5995T>A), CAA1592GAG (5996C>G 5998A>G), CAC1593CAT (6001C>T), ATT1594ACT (6003T>C), GAA1595TAT (6005G>T 6007A>T), TGG1596AGA (6008T>A 6010G>A), ACC1597ACT (6013C>T), TGG1600AAT (6020T>A 6021G>A 6022G>T), ACT1601GTC (6023A>G 6024C>T 6025T>C), GTG1603TTT (6029G>T 6031G>T), GGC1604GGA (6034C>A), TTA1605CAA (6035T>C 6036T>A), TGG1608TGA (6046G>A), CTG1609AAT (6047C>A 6048T>A 6049G>T), GGG1614GGT (6064G>T), CTT1615CTA (6067T>A), AAG1616AAA (6070G>A), AAT1617ATT (6072A>T), CCA1619CCT (6079A>T), GCA1620TCT (6080G>T 6082A>T), GTA1621GAA (6084T>A), CAA1623CAG (6091A>G), AGG1624AAA (6093G>A 6094G>A), AAG1625ATC (6096A>T 6097G>C), GAC1627AAT (6101G>A 6103C>T), CGA1628GAT (6104C>G 6105G>A 6106A>T), ATC1629ATT (6109C>T), TAC1633TAT (6121C>T), GCT1634ATA (6122G>A 6123C>T 6124T>A), GGA1635AGT (6125G>A 6127A>T), TTT1636TTC (6130T>C), ATT1637ATA (6133T>A), GCT1638ATT (6134G>A 6135C>T), GTG1639GTC (6139G>C), GAT1643GAC (6151T>C), ATC1644ATA (6154C>A), CTC1645TTG (6155C>T 6157C>G), ATA1646ATT (6160A>T), TTC1647TAT (6162T>A 6163C>T), AGC1648TCT (6164A>T 6165G>C 6166C>T), CCA1649AAA (6167C>A 6168C>A), ACA1650ACT (6172A>T), CCT1651TTT (6173C>T 6174C>T), GCC1653ATG (6179G>A 6180C>T 6181C>G), AAA1655ATC (6186A>T 6187A>C), CAC1657CAT (6193C>T), TTG1658TTA (6196G>A), GCC1659GAA (6198C>A 6199C>A), CAA1660ATC (6200C>A 6201A>T 6202A>C), TTC1661TTT (6205C>T), GTG1662AAG (6206G>A 6207T>A), GAG1663AAA (6209G>A 6211G>A), ATA1664ATT (6214A>T), TGC1665GTT (6215T>G 6216G>T 6217C>T), AAG1666ATA (6219A>T 6220G>A), GAA1667CAA (6221G>C), CAT1668AAT (6224C>A), GGG1669GGT (6229G>T), CTT1670TTG (6230C>T 6232T>G), GTC1671GTA (6235C>A), TTG1672ATA (6236T>A 6238G>A), AGT1673TCT (6239A>T 6240G>C), CCA1674AAA (6242C>A 6243C>A), ACA1675CCA (6245A>C), AAG1676AAA (6250G>A), AAG1678AGT (6255A>G 6256G>T), GTG1679TTA (6257G>T 6259G>A), GCC1680TTC (6260G>T 6261C>T), CAG1681CAA (6265G>A), GCC1682ACA (6266G>A 6268C>A), CAA1683GAA (6269C>G), ATT1684GTT (6272A>G), GAC1685CGA (6275G>C 6276A>G 6277C>A), TTT1686TTC (6280T>C), TTG1687TTA (6283G>A), GGC1688GGA (6286C>A), TCA1689CAT (6287T>C 6288C>A 6289A>T), ACA1690AAT (6291C>A 6292A>T), ATT1691ATA (6295T>A), ACA1692TGT (6296A>T 6297C>G 6298A>T), AAT1693CAA (6299A>C 6301T>A)								

*: Inserts / Deletes / Misaligned / Frameshifts

Analysis details

This analysis was performed with panviral2.64

NGS Details (UN24): Soymovirus virgarachidis

Assembly

**Coverage Length**	334 (1 contig(s))
**Depth Of Coverage**	3.9
**Number Of Reads**	10
**Reads Per Million**	0.20 rpm (after QC)
**Ambiguities**	0
**Assembly Method**	de novo + reference guided assembly
**Consensus Caller**	Bcf Tools

Coverage Map

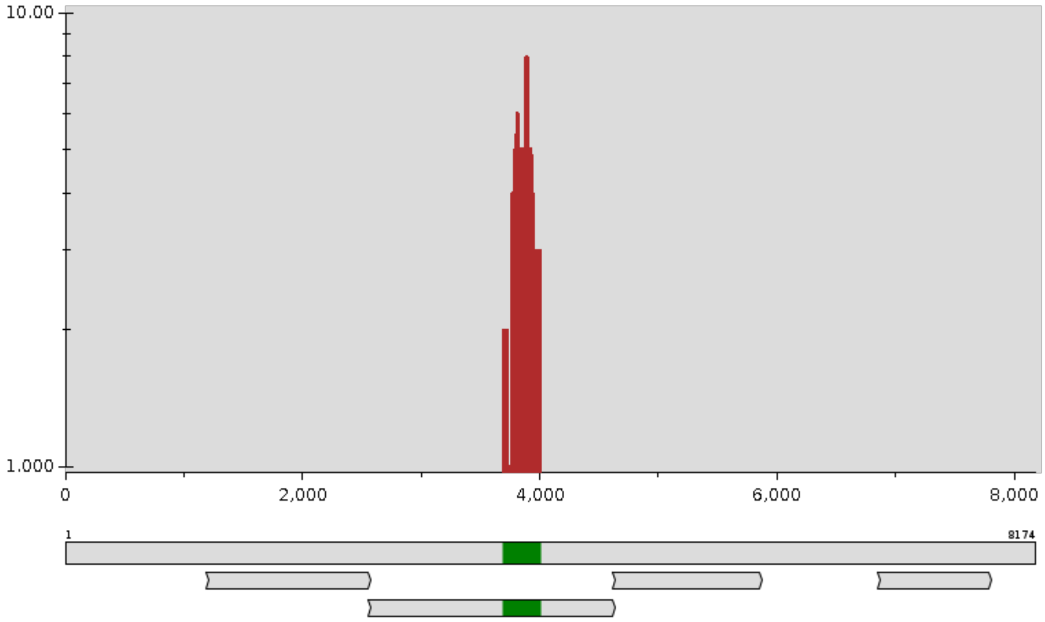

Assignment

**Type**	Soymovirus virgarachidis (Taxonomy ID: 3048252)
**Reference Genome**	NC_001634.1
**NT Identity (%)**	59.4427
**AA Identity (%)**	48.1481
**Number Of Stop Codons**	2
**Number Of CDS**	4

Alignment

**Alignment Score**	84.0 (NT) + 215.0 (AA) = 299.0
**Concordance (%)**	25.4902

Alignment Method	Global, seeded, nucleotide + amino acids (AGA)

Genome Region

Sequence starts at position 3684 and ends at position 4017 relative to NC_001634.1 reference sequence.

Alignment Detailed Statistics

	Begin	End	Coverage	Score	Concordance	Matches	Identities	I/D/M/F*	Stop Codons
**NT**	**3684**	**4017**	**4.1%**	**84**	**13.8%**	**323 (96.7%)**	**192 (57.5%)**	**0/11**	
Mutations:	3684G>C, 3689G>A, 3693A>G, 3695G>C, 3696C>T, 3697A>G, 3701A>T, 3702T>A, 3703C>A, 3704G>T, 3706T>A, 3707T>A, 3708T>A, 3709T>A, 3710G>A, 3712A>T, 3718T>A, 3721G>A, 3724T>C, 3727C>T, 3730T>A, 3731T>A, 3733A>G, 3735A>T, 3736G>T, 3741T>C, 3742G>A, 3751A>G, 3752_3754delTTT, 3757T>A, 3760A>G, 3761C>T, 3762C>T, 3766G>A, 3768A>C, 3769A>T, 3775T>C, 3776_3777delTT, 3778A>C, 3779G>A, 3784G>A, 3788G>T, 3789C>T, 3790T>A, 3799A>T, 3803C>A, 3805G>A, 3806C>T, 3809C>A, 3810A>C, 3812G>A, 3813A>C, 3816G>A, 3818A>C, 3820G>A, 3821C>G, 3826T>A, 3829G>A, 3831A>G, 3838C>T, 3852A>T, 3853C>T, 3854A>G, 3855T>C, 3856T>A, 3863_3868delAAAGGA, 3871T>C, 3872T>A, 3873T>C, 3874T>A, 3875A>C, 3876A>C, 3877G>A, 3890G>A, 3892A>G, 3898A>T, 3899G>C, 3902T>C, 3907G>A, 3911A>C, 3913G>C, 3914C>A, 3916T>C, 3917T>A, 3918C>G, 3920G>A, 3923A>T, 3924A>C, 3926C>A, 3927T>A, 3930C>G, 3933G>T, 3935A>T, 3936A>G, 3942A>C, 3943T>C, 3944G>A, 3947T>G, 3948T>A, 3950G>C, 3951C>A, 3952C>T, 3957T>G, 3958G>T, 3959G>T, 3960C>G, 3961A>T, 3964T>A, 3965A>C, 3969G>A, 3970G>A, 3971T>C, 3973A>C, 3977C>G, 3979G>A, 3981T>A, 3982T>A, 3985C>T, 3986A>G, 3987A>T, 3988A>T, 3991C>G, 3992T>C, 3994A>T, 3999G>A, 4003G>T, 4004T>A, 4005A>G, 4006T>G, 4007G>C, 4009T>A, 4010G>C, 4013A>G, 4015G>T								

CDS

	Begin	End	Coverage	Score	Concordance	Matches	Identities	I/D/M/F*	Stop Codons
PCSVgp2	378	488	16.0%	215	30.5%	108 (97.3%)	52 (46.8%)	0/3/1/1	2
Protein mutations:	E379K (3689G>A), K380R (3693A>G), A381L (3695G>C 3696C>T 3697A>G), I383* (3701A>T 3702T>A 3703C>A), A384S (3704G>T 3706T>A), F385K (3707T>A 3708T>A 3709T>A), E386N (3710G>A 3712A>T), L393M (3731T>A 3733A>G), K394I (3735A>T 3736G>T), L396S (3741T>C 3742G>A), F400del (3752_3754delTTT), P403L (3761C>T 3762C>T), K405T (3768A>C 3769A>T), E409K (3779G>A), A412L (3788G>T 3789C>T 3790T>A), Q417K (3803C>A 3805G>A), Q419T (3809C>A 3810A>C), D420T (3812G>A 3813A>C), R421K (3816G>A), K422Q (3818A>C 3820G>A), Q423E (3821C>G), K426R (3831A>G), Y433F (3852A>T 3853C>T), I434A (3854A>G 3855T>C 3856T>A), K437_G438del (3863_3868delAAAGGA), F440T (3872T>A 3873T>C 3874T>A), K441P (3875A>C 3876A>C 3877G>A), E446K (3890G>A 3892A>G), K448N (3898A>T), V449L (3899G>C), M453L (3911A>C 3913G>C), L454I (3914C>A 3916T>C), S455R (3917T>A 3918C>G), E456K (3920G>A), K457S (3923A>T 3924A>C), L458N (3926C>A 3927T>A), P459R (3930C>G), W460L (3933G>T), K461* (3935A>T 3936A>G), N463T (3942A>C 3943T>C), D464N (3944G>A), L465E (3947T>G 3948T>A), A466H (3950G>C 3951C>A 3952C>T), L468R (3957T>G 3958G>T), A469C (3959G>T 3960C>G 3961A>T), K471Q (3965A>C), R472K (3969G>A 3970G>A), Q475E (3977C>G 3979G>A), V476E (3981T>A 3982T>A), K478V (3986A>G 3987A>T 3988A>T), N479K (3991C>G), R482K (3999G>A), Y484R (4004T>A 4005A>G 4006T>G), V485L (4007G>C 4009T>A), A486P (4010G>C), K487D (4013A>G 4015G>T)								
Codon mutations:	AGC377.CC (3684G>C), GAA379AAA (3689G>A), AAG380AGG (3693A>G), GCA381CTG (3695G>C 3696C>T 3697A>G), ATC383TAA (3701A>T 3702T>A 3703C>A), GCT384TCA (3704G>T 3706T>A), TTT385AAA (3707T>A 3708T>A 3709T>A), GAA386AAT (3710G>A 3712A>T), ATT388ATA (3718T>A), GAG389GAA (3721G>A), TTT390TTC (3724T>C), CTC391CTT (3727C>T), GGT392GGA (3730T>A), TTA393ATG (3731T>A 3733A>G), AAG394ATT (3735A>T 3736G>T), TTG396TCA (3741T>C 3742G>A), GGA399GGG (3751A>G), TTT400del (3752_3754delTTT), ATT401ATA (3757T>A), GAA402GAG (3760A>G), CCA403TTA (3761C>T 3762C>T), CAG404CAA (3766G>A), AAA405ACT (3768A>C 3769A>T), CTT407CTC (3775T>C), TTA408--C (3776_3777delTT 3778A>C), GAA409AAA (3779G>A), AAG410AAA (3784G>A), GCT412TTA (3788G>T 3789C>T 3790T>A), CCA415CCT (3799A>T), CAG417AAA (3803C>A 3805G>A), CTA418TTA (3806C>T), CAA419ACA (3809C>A 3810A>C), GAC420ACC (3812G>A 3813A>C), AGA421AAA (3816G>A), AAG422CAA (3818A>C 3820G>A), CAA423GAA (3821C>G), ATT424ATA (3826T>A), CAG425CAA (3829G>A), AAA426AGA (3831A>G), CTC428CTT (3838C>T), TAC433TTT (3852A>T 3853C>T), ATT434GCA (3854A>G 3855T>C 3856T>A), AAA437_GGA438del (3863_3868delAAAGGA), TTT439TTC (3871T>C), TTT440ACA (3872T>A 3873T>C 3874T>A), AAG441CCA (3875A>C 3876A>C 3877G>A), GAA446AAG (3890G>A 3892A>G), AAA448AAT (3898A>T), GTT449CTT (3899G>C), TTA450CTA (3902T>C), CAG451CAA (3907G>A), ATG453CTC (3911A>C 3913G>C), CTT454ATC (3914C>A 3916T>C), TCA455AGA (3917T>A 3918C>G), GAA456AAA (3920G>A), AAA457TCA (3923A>T 3924A>C), CTT458AAT (3926C>A 3927T>A), CCA459CGA (3930C>G), TGG460TTG (3933G>T), AAA461TGA (3935A>T 3936A>G), AAT463ACC (3942A>C 3943T>C), GAT464AAT (3944G>A), TTA465GAA (3947T>G 3948T>A), GCC466CAT (3950G>C 3951C>A 3952C>T), CTG468CGT (3957T>G 3958G>T), GCA469TGT (3959G>T 3960C>G 3961A>T), GTT470GTA (3964T>A), AAA471CAA (3965A>C), AGG472AAA (3969G>A 3970G>A), TTA473CTC (3971T>C 3973A>C), CAG475GAA (3977C>G 3979G>A), GTT476GAA (3981T>A 3982T>A), TGC477TGT (3985C>T), AAA478GTT (3986A>G 3987A>T 3988A>T), AAC479AAG (3991C>G), TTA480CTT (3992T>C 3994A>T), AGG482AAG (3999G>A), CTG483CTT (4003G>T), TAT484AGG (4004T>A 4005A>G 4006T>G), GTT485CTA (4007G>C 4009T>A), GCA486CCA (4010G>C), AAG487GAT (4013A>G 4015G>T)								

Proteins

	Begin	End	Coverage	Score	Concordance	Matches	Identities	I/D/M/F*	Stop Codons
reverse transcriptase (NP_042513.1)	378	488	16.0%	215	30.5%	108 (97.3%)	52 (46.8%)	0/3/1/1	2
Protein mutations:	E379K (3689G>A), K380R (3693A>G), A381L (3695G>C 3696C>T 3697A>G), I383* (3701A>T 3702T>A 3703C>A), A384S (3704G>T 3706T>A), F385K (3707T>A 3708T>A 3709T>A), E386N (3710G>A 3712A>T), L393M (3731T>A 3733A>G), K394I (3735A>T 3736G>T), L396S (3741T>C 3742G>A), F400del (3752_3754delTTT), P403L (3761C>T 3762C>T), K405T (3768A>C 3769A>T), E409K (3779G>A), A412L (3788G>T 3789C>T 3790T>A), Q417K (3803C>A 3805G>A), Q419T (3809C>A 3810A>C), D420T (3812G>A 3813A>C), R421K (3816G>A), K422Q (3818A>C 3820G>A), Q423E (3821C>G), K426R (3831A>G), Y433F (3852A>T 3853C>T), I434A (3854A>G 3855T>C 3856T>A), K437_G438del (3863_3868delAAAGGA), F440T (3872T>A 3873T>C 3874T>A), K441P (3875A>C 3876A>C 3877G>A), E446K (3890G>A 3892A>G), K448N (3898A>T), V449L (3899G>C), M453L (3911A>C 3913G>C), L454I (3914C>A 3916T>C), S455R (3917T>A 3918C>G), E456K (3920G>A), K457S (3923A>T 3924A>C), L458N (3926C>A 3927T>A), P459R (3930C>G), W460L (3933G>T), K461* (3935A>T 3936A>G), N463T (3942A>C 3943T>C), D464N (3944G>A), L465E (3947T>G 3948T>A), A466H (3950G>C 3951C>A 3952C>T), L468R (3957T>G 3958G>T), A469C (3959G>T 3960C>G 3961A>T), K471Q (3965A>C), R472K (3969G>A 3970G>A), Q475E (3977C>G 3979G>A), V476E (3981T>A 3982T>A), K478V (3986A>G 3987A>T 3988A>T), N479K (3991C>G), R482K (3999G>A), Y484R (4004T>A 4005A>G 4006T>G), V485L (4007G>C 4009T>A), A486P (4010G>C), K487D (4013A>G 4015G>T)								
Codon mutations:	AGC377.CC (3684G>C), GAA379AAA (3689G>A), AAG380AGG (3693A>G), GCA381CTG (3695G>C 3696C>T 3697A>G), ATC383TAA (3701A>T 3702T>A 3703C>A), GCT384TCA (3704G>T 3706T>A), TTT385AAA (3707T>A 3708T>A 3709T>A), GAA386AAT (3710G>A 3712A>T), ATT388ATA (3718T>A), GAG389GAA (3721G>A), TTT390TTC (3724T>C), CTC391CTT (3727C>T), GGT392GGA (3730T>A), TTA393ATG (3731T>A 3733A>G), AAG394ATT (3735A>T 3736G>T), TTG396TCA (3741T>C 3742G>A), GGA399GGG (3751A>G), TTT400del (3752_3754delTTT), ATT401ATA (3757T>A), GAA402GAG (3760A>G), CCA403TTA (3761C>T 3762C>T), CAG404CAA (3766G>A), AAA405ACT (3768A>C 3769A>T), CTT407CTC (3775T>C), TTA408--C (3776_3777delTT 3778A>C), GAA409AAA (3779G>A), AAG410AAA (3784G>A), GCT412TTA (3788G>T 3789C>T 3790T>A), CCA415CCT (3799A>T), CAG417AAA (3803C>A 3805G>A), CTA418TTA (3806C>T), CAA419ACA (3809C>A 3810A>C), GAC420ACC (3812G>A 3813A>C), AGA421AAA (3816G>A), AAG422CAA (3818A>C 3820G>A), CAA423GAA (3821C>G), ATT424ATA (3826T>A), CAG425CAA (3829G>A), AAA426AGA (3831A>G), CTC428CTT (3838C>T), TAC433TTT (3852A>T 3853C>T), ATT434GCA (3854A>G 3855T>C 3856T>A), AAA437_GGA438del (3863_3868delAAAGGA), TTT439TTC (3871T>C), TTT440ACA (3872T>A 3873T>C 3874T>A), AAG441CCA (3875A>C 3876A>C 3877G>A), GAA446AAG (3890G>A 3892A>G), AAA448AAT (3898A>T), GTT449CTT (3899G>C), TTA450CTA (3902T>C), CAG451CAA (3907G>A), ATG453CTC (3911A>C 3913G>C), CTT454ATC (3914C>A 3916T>C), TCA455AGA (3917T>A 3918C>G), GAA456AAA (3920G>A), AAA457TCA (3923A>T 3924A>C), CTT458AAT (3926C>A 3927T>A), CCA459CGA (3930C>G), TGG460TTG (3933G>T), AAA461TGA (3935A>T 3936A>G), AAT463ACC (3942A>C 3943T>C), GAT464AAT (3944G>A), TTA465GAA (3947T>G 3948T>A), GCC466CAT (3950G>C 3951C>A 3952C>T), CTG468CGT (3957T>G 3958G>T), GCA469TGT (3959G>T 3960C>G 3961A>T), GTT470GTA (3964T>A), AAA471CAA (3965A>C), AGG472AAA (3969G>A 3970G>A), TTA473CTC (3971T>C 3973A>C), CAG475GAA (3977C>G 3979G>A), GTT476GAA (3981T>A 3982T>A), TGC477TGT (3985C>T), AAA478GTT (3986A>G 3987A>T 3988A>T), AAC479AAG (3991C>G), TTA480CTT (3992T>C 3994A>T), AGG482AAG (3999G>A), CTG483CTT (4003G>T), TAT484AGG (4004T>A 4005A>G 4006T>G), GTT485CTA (4007G>C 4009T>A), GCA486CCA (4010G>C), AAG487GAT (4013A>G 4015G>T)								

*: Inserts / Deletes / Misaligned / Frameshifts

Analysis details

This analysis was performed with panviral2.64

NGS Details (UN24): Dioscovirus dioscoreae

Assembly

**Coverage Length**	513 (1 contig(s))
**Depth Of Coverage**	2.6
**Number Of Reads**	10
**Reads Per Million**	0.20 rpm (after QC)
**Ambiguities**	0
**Assembly Method**	de novo + reference guided assembly
**Consensus Caller**	Bcf Tools

Coverage Map

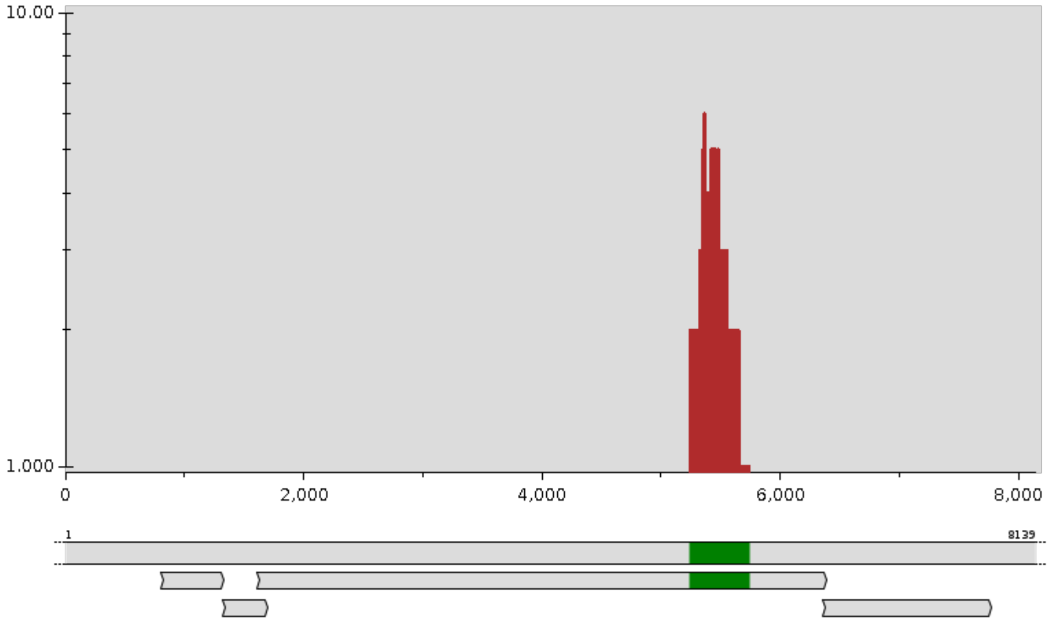

Assignment

**Type**	Dioscovirus dioscoreae (Taxonomy ID: 3052184)
**Reference Genome**	NC_040712.1
**NT Identity (%)**	63.9376
**AA Identity (%)**	57.3099
**Number Of Stop Codons**	0
**Number Of CDS**	4

Alignment

**Alignment Score**	286.0 (NT) + 755.0 (AA) = 1041.0
**Concordance (%)**	46.7026

Alignment Method	Global, seeded, nucleotide + amino acids (AGA)

Genome Region

Sequence starts at position 5237 and ends at position 5749 relative to NC_040712.1 reference sequence.

Alignment Detailed Statistics

	Begin	End	Coverage	Score	Concordance	Matches	Identities	I/D/M/F*	Stop Codons
**NT**	**5237**	**5749**	**6.3%**	**286**	**27.9%**	**513 (100%)**	**328 (63.9%)**	**0/0**	
Mutations:	5237A>T, 5243T>A, 5247A>G, 5248G>C, 5249T>C, 5250C>A, 5252T>A, 5258A>C, 5259T>A, 5260A>T, 5261C>T, 5264T>A, 5270G>A, 5271C>A, 5274C>T, 5289T>G, 5291C>A, 5293A>T, 5294A>G, 5298C>G, 5299A>G, 5303C>T, 5305C>A, 5309C>A, 5313A>G, 5314G>A, 5315T>C, 5317T>A, 5318T>A, 5319G>A, 5320A>C, 5321C>A, 5322T>A, 5324T>A, 5325C>T, 5327C>A, 5328C>A, 5331C>A, 5332T>A, 5335A>G, 5339T>A, 5342A>T, 5344A>G, 5345C>A, 5348G>A, 5350T>G, 5351T>A, 5352G>A, 5354C>T, 5356A>T, 5357C>T, 5363A>G, 5370C>T, 5371T>G, 5372A>T, 5381A>C, 5383T>A, 5384C>T, 5385C>T, 5386A>G, 5387C>G, 5390G>A, 5391A>G, 5393T>A, 5395T>A, 5396G>A, 5400G>C, 5406C>G, 5408G>C, 5414A>T, 5417A>G, 5426A>C, 5430G>T, 5431T>A, 5432C>T, 5439C>G, 5441G>A, 5444T>A, 5450T>C, 5457A>T, 5461T>C, 5471C>T, 5475C>T, 5477C>A, 5482A>C, 5486A>T, 5489T>A, 5490T>C, 5493G>A, 5495A>T, 5498C>T, 5501A>G, 5502C>A, 5519A>T, 5522C>T, 5523A>G, 5524A>G, 5527A>G, 5532T>A, 5533C>A, 5535G>C, 5537A>T, 5541G>A, 5543C>T, 5545G>T, 5546T>A, 5552C>T, 5558C>T, 5561T>C, 5565T>C, 5567G>A, 5568A>G, 5570A>T, 5574T>A, 5575C>G, 5577G>A, 5579A>T, 5581G>C, 5582T>C, 5585A>G, 5589C>G, 5591A>G, 5595G>T, 5598C>G, 5599A>G, 5604T>C, 5606A>C, 5607T>C, 5608T>A, 5610C>A, 5613T>G, 5615C>T, 5618C>T, 5619C>A, 5621A>T, 5622G>C, 5626G>T, 5629A>T, 5630G>A, 5633A>C, 5634G>A, 5636A>T, 5639G>T, 5640C>T, 5642T>A, 5646T>A, 5648G>A, 5649T>A, 5650C>G, 5651A>T, 5656C>A, 5657A>G, 5660A>G, 5661T>A, 5663G>A, 5664A>G, 5666A>G, 5667A>T, 5670G>T, 5672A>C, 5673G>A, 5674T>A, 5676G>A, 5679A>T, 5684A>T, 5685G>A, 5687A>T, 5691C>T, 5693T>A, 5696C>A, 5697C>A, 5700G>A, 5701A>C, 5702A>C, 5718G>A, 5720T>A, 5721C>A, 5724C>T, 5726C>A, 5732C>T, 5739T>G, 5740T>C, 5741A>T, 5743A>G, 5747T>G								

CDS

	Begin	End	Coverage	Score	Concordance	Matches	Identities	I/D/M/F*	Stop Codons
EXK67_gp3	1210	1380	10.7%	755	62.2%	171 (100%)	98 (57.3%)	0/0/0/0	0
Protein mutations:	S1213A (5247A>G 5248G>C 5249T>C), Y1217I (5259T>A 5260A>T 5261C>T), N1218K (5264T>A), Y1227E (5289T>G 5291C>A), K1228M (5293A>T 5294A>G), Q1230G (5298C>G 5299A>G), T1232K (5305C>A), S1235D (5313A>G 5314G>A 5315T>C), I1236K (5317T>A 5318T>A), D1237T (5319G>A 5320A>C 5321C>A), Y1238K (5322T>A 5324T>A), L1240I (5328C>A), L1241N (5331C>A 5332T>A), K1242R (5335A>G), K1244N (5342A>T), D1245G (5344A>G 5345C>A), I1247R (5350T>G 5351T>A), V1248I (5352G>A 5354C>T), Y1249F (5356A>T 5357C>T), L1254C (5370C>T 5371T>G 5372A>T), F1258Y (5383T>A 5384C>T), H1259W (5385C>T 5386A>G 5387C>G), I1261V (5391A>G 5393T>A), M1262K (5395T>A 5396G>A), D1264H (5400G>C), Q1266D (5406C>G 5408G>C), V1274Y (5430G>T 5431T>A 5432C>T), Q1277E (5439C>G 5441G>A), I1283L (5457A>T), V1284A (5461T>C), N1291T (5482A>C), S1294P (5490T>C), V1295I (5493G>A 5495A>T), K1305G (5523A>G 5524A>G), K1306R (5527A>G), S1308K (5532T>A 5533C>A), E1309H (5535G>C 5537A>T), V1311I (5541G>A 5543C>T), C1312L (5545G>T 5546T>A), I1320V (5568A>G 5570A>T), E1323N (5577G>A 5579A>T), S1324T (5581G>C 5582T>C), I1325M (5585A>G), Q1327E (5589C>G 5591A>G), V1329L (5595G>T), Q1330G (5598C>G 5599A>G), L1333Q (5607T>C 5608T>A), Q1334K (5610C>A), F1335V (5613T>G 5615C>T), Q1337N (5619C>A 5621A>T), V1338L (5622G>C), C1339F (5626G>T), K1340I (5629A>T 5630G>A), E1341D (5633A>C), E1342N (5634G>A 5636A>T), L1346I (5646T>A 5648G>A), T1349K (5656C>A 5657A>G), L1351I (5661T>A 5663G>A), K1352E (5664A>G 5666A>G), I1353L (5667A>T), G1354C (5670G>T 5672A>C), V1355K (5673G>A 5674T>A), A1356T (5676G>A), N1357Y (5679A>T), E1359N (5685G>A 5687A>T), L1363I (5697C>A), E1364T (5700G>A 5701A>C 5702A>C), V1370I (5718G>A 5720T>A), Q1371K (5721C>A), L1377A (5739T>G 5740T>C 5741A>T), K1378R (5743A>G), N1379K (5747T>G)								
Codon mutations:	GTA1209..T (5237A>T), GGT1211GGA (5243T>A), AGT1213GCC (5247A>G 5248G>C 5249T>C), CGT1214AGA (5250C>A 5252T>A), GTA1216GTC (5258A>C), TAC1217ATT (5259T>A 5260A>T 5261C>T), AAT1218AAA (5264T>A), AAG1220AAA (5270G>A), CGA1221AGA (5271C>A), CTA1222TTA (5274C>T), TAC1227GAA (5289T>G 5291C>A), AAA1228ATG (5293A>T 5294A>G), CAA1230GGA (5298C>G 5299A>G), TAC1231TAT (5303C>T), ACA1232AAA (5305C>A), CTC1233CTA (5309C>A), AGT1235GAC (5313A>G 5314G>A 5315T>C), ATT1236AAA (5317T>A 5318T>A), GAC1237ACA (5319G>A 5320A>C 5321C>A), TAT1238AAA (5322T>A 5324T>A), CTC1239TTA (5325C>T 5327C>A), CTA1240ATA (5328C>A), CTT1241AAT (5331C>A 5332T>A), AAA1242AGA (5335A>G), ATT1243ATA (5339T>A), AAA1244AAT (5342A>T), GAC1245GGA (5344A>G 5345C>A), AAG1246AAA (5348G>A), ATT1247AGA (5350T>G 5351T>A), GTC1248ATT (5352G>A 5354C>T), TAC1249TTT (5356A>T 5357C>T), AAA1251AAG (5363A>G), CTA1254TGT (5370C>T 5371T>G 5372A>T), GGA1257GGC (5381A>C), TTC1258TAT (5383T>A 5384C>T), CAC1259TGG (5385C>T 5386A>G 5387C>G), CAG1260CAA (5390G>A), ATT1261GTA (5391A>G 5393T>A), ATG1262AAA (5395T>A 5396G>A), GAT1264CAT (5400G>C), CAG1266GAC (5406C>G 5408G>C), ATA1268ATT (5414A>T), GAA1269GAG (5417A>G), GCA1272GCC (5426A>C), GTC1274TAT (5430G>T 5431T>A 5432C>T), CAG1277GAA (5439C>G 5441G>A), GGT1278GGA (5444T>A), TTT1280TTC (5450T>C), ATA1283TTA (5457A>T), GTA1284GCA (5461T>C), TTC1287TTT (5471C>T), CTC1289TTA (5475C>T 5477C>A), AAT1291ACT (5482A>C), GCA1292GCT (5486A>T), CCT1293CCA (5489T>A), TCA1294CCA (5490T>C), GTA1295ATT (5493G>A 5495A>T), TTC1296TTT (5498C>T), CAA1297CAG (5501A>G), CGA1298AGA (5502C>A), ATA1303ATT (5519A>T), TTC1304TTT (5522C>T), AAA1305GGA (5523A>G 5524A>G), AAA1306AGA (5527A>G), TCA1308AAA (5532T>A 5533C>A), GAA1309CAT (5535G>C 5537A>T), GTC1311ATT (5541G>A 5543C>T), TGT1312TTA (5545G>T 5546T>A), TAC1314TAT (5552C>T), GAC1316GAT (5558C>T), GAT1317GAC (5561T>C), TTG1319CTA (5565T>C 5567G>A), ATA1320GTT (5568A>G 5570A>T), TCT1322AGT (5574T>A 5575C>G), GAA1323AAT (5577G>A 5579A>T), AGT1324ACC (5581G>C 5582T>C), ATA1325ATG (5585A>G), CAA1327GAG (5589C>G 5591A>G), GTA1329TTA (5595G>T), CAA1330GGA (5598C>G 5599A>G), TTA1332CTC (5604T>C 5606A>C), TTA1333CAA (5607T>C 5608T>A), CAG1334AAG (5610C>A), TTC1335GTT (5613T>G 5615C>T), TTC1336TTT (5618C>T), CAA1337AAT (5619C>A 5621A>T), GTA1338CTA (5622G>C), TGT1339TTT (5626G>T), AAG1340ATA (5629A>T 5630G>A), GAA1341GAC (5633A>C), GAA1342AAT (5634G>A 5636A>T), GGG1343GGT (5639G>T), CTT1344TTA (5640C>T 5642T>A), TTG1346ATA (5646T>A 5648G>A), TCA1347AGT (5649T>A 5650C>G 5651A>T), ACA1349AAG (5656C>A 5657A>G), AAA1350AAG (5660A>G), TTG1351ATA (5661T>A 5663G>A), AAA1352GAG (5664A>G 5666A>G), ATA1353TTA (5667A>T), GGA1354TGC (5670G>T 5672A>C), GTA1355AAA (5673G>A 5674T>A), GCA1356ACA (5676G>A), AAT1357TAT (5679A>T), ATA1358ATT (5684A>T), GAA1359AAT (5685G>A 5687A>T), CTT1361TTA (5691C>T 5693T>A), GGC1362GGA (5696C>A), CTA1363ATA (5697C>A), GAA1364ACC (5700G>A 5701A>C 5702A>C), GTT1370ATA (5718G>A 5720T>A), CAA1371AAA (5721C>A), CTC1372TTA (5724C>T 5726C>A), CCC1374CCT (5732C>T), TTA1377GCT (5739T>G 5740T>C 5741A>T), AAA1378AGA (5743A>G), AAT1379AAG (5747T>G)								

Proteins

	Begin	End	Coverage	Score	Concordance	Matches	Identities	I/D/M/F*	Stop Codons
ORF3 (YP_009553219.1)	1210	1380	10.7%	755	62.2%	171 (100%)	98 (57.3%)	0/0/0/0	0
Protein mutations:	S1213A (5247A>G 5248G>C 5249T>C), Y1217I (5259T>A 5260A>T 5261C>T), N1218K (5264T>A), Y1227E (5289T>G 5291C>A), K1228M (5293A>T 5294A>G), Q1230G (5298C>G 5299A>G), T1232K (5305C>A), S1235D (5313A>G 5314G>A 5315T>C), I1236K (5317T>A 5318T>A), D1237T (5319G>A 5320A>C 5321C>A), Y1238K (5322T>A 5324T>A), L1240I (5328C>A), L1241N (5331C>A 5332T>A), K1242R (5335A>G), K1244N (5342A>T), D1245G (5344A>G 5345C>A), I1247R (5350T>G 5351T>A), V1248I (5352G>A 5354C>T), Y1249F (5356A>T 5357C>T), L1254C (5370C>T 5371T>G 5372A>T), F1258Y (5383T>A 5384C>T), H1259W (5385C>T 5386A>G 5387C>G), I1261V (5391A>G 5393T>A), M1262K (5395T>A 5396G>A), D1264H (5400G>C), Q1266D (5406C>G 5408G>C), V1274Y (5430G>T 5431T>A 5432C>T), Q1277E (5439C>G 5441G>A), I1283L (5457A>T), V1284A (5461T>C), N1291T (5482A>C), S1294P (5490T>C), V1295I (5493G>A 5495A>T), K1305G (5523A>G 5524A>G), K1306R (5527A>G), S1308K (5532T>A 5533C>A), E1309H (5535G>C 5537A>T), V1311I (5541G>A 5543C>T), C1312L (5545G>T 5546T>A), I1320V (5568A>G 5570A>T), E1323N (5577G>A 5579A>T), S1324T (5581G>C 5582T>C), I1325M (5585A>G), Q1327E (5589C>G 5591A>G), V1329L (5595G>T), Q1330G (5598C>G 5599A>G), L1333Q (5607T>C 5608T>A), Q1334K (5610C>A), F1335V (5613T>G 5615C>T), Q1337N (5619C>A 5621A>T), V1338L (5622G>C), C1339F (5626G>T), K1340I (5629A>T 5630G>A), E1341D (5633A>C), E1342N (5634G>A 5636A>T), L1346I (5646T>A 5648G>A), T1349K (5656C>A 5657A>G), L1351I (5661T>A 5663G>A), K1352E (5664A>G 5666A>G), I1353L (5667A>T), G1354C (5670G>T 5672A>C), V1355K (5673G>A 5674T>A), A1356T (5676G>A), N1357Y (5679A>T), E1359N (5685G>A 5687A>T), L1363I (5697C>A), E1364T (5700G>A 5701A>C 5702A>C), V1370I (5718G>A 5720T>A), Q1371K (5721C>A), L1377A (5739T>G 5740T>C 5741A>T), K1378R (5743A>G), N1379K (5747T>G)								
Codon mutations:	GTA1209..T (5237A>T), GGT1211GGA (5243T>A), AGT1213GCC (5247A>G 5248G>C 5249T>C), CGT1214AGA (5250C>A 5252T>A), GTA1216GTC (5258A>C), TAC1217ATT (5259T>A 5260A>T 5261C>T), AAT1218AAA (5264T>A), AAG1220AAA (5270G>A), CGA1221AGA (5271C>A), CTA1222TTA (5274C>T), TAC1227GAA (5289T>G 5291C>A), AAA1228ATG (5293A>T 5294A>G), CAA1230GGA (5298C>G 5299A>G), TAC1231TAT (5303C>T), ACA1232AAA (5305C>A), CTC1233CTA (5309C>A), AGT1235GAC (5313A>G 5314G>A 5315T>C), ATT1236AAA (5317T>A 5318T>A), GAC1237ACA (5319G>A 5320A>C 5321C>A), TAT1238AAA (5322T>A 5324T>A), CTC1239TTA (5325C>T 5327C>A), CTA1240ATA (5328C>A), CTT1241AAT (5331C>A 5332T>A), AAA1242AGA (5335A>G), ATT1243ATA (5339T>A), AAA1244AAT (5342A>T), GAC1245GGA (5344A>G 5345C>A), AAG1246AAA (5348G>A), ATT1247AGA (5350T>G 5351T>A), GTC1248ATT (5352G>A 5354C>T), TAC1249TTT (5356A>T 5357C>T), AAA1251AAG (5363A>G), CTA1254TGT (5370C>T 5371T>G 5372A>T), GGA1257GGC (5381A>C), TTC1258TAT (5383T>A 5384C>T), CAC1259TGG (5385C>T 5386A>G 5387C>G), CAG1260CAA (5390G>A), ATT1261GTA (5391A>G 5393T>A), ATG1262AAA (5395T>A 5396G>A), GAT1264CAT (5400G>C), CAG1266GAC (5406C>G 5408G>C), ATA1268ATT (5414A>T), GAA1269GAG (5417A>G), GCA1272GCC (5426A>C), GTC1274TAT (5430G>T 5431T>A 5432C>T), CAG1277GAA (5439C>G 5441G>A), GGT1278GGA (5444T>A), TTT1280TTC (5450T>C), ATA1283TTA (5457A>T), GTA1284GCA (5461T>C), TTC1287TTT (5471C>T), CTC1289TTA (5475C>T 5477C>A), AAT1291ACT (5482A>C), GCA1292GCT (5486A>T), CCT1293CCA (5489T>A), TCA1294CCA (5490T>C), GTA1295ATT (5493G>A 5495A>T), TTC1296TTT (5498C>T), CAA1297CAG (5501A>G), CGA1298AGA (5502C>A), ATA1303ATT (5519A>T), TTC1304TTT (5522C>T), AAA1305GGA (5523A>G 5524A>G), AAA1306AGA (5527A>G), TCA1308AAA (5532T>A 5533C>A), GAA1309CAT (5535G>C 5537A>T), GTC1311ATT (5541G>A 5543C>T), TGT1312TTA (5545G>T 5546T>A), TAC1314TAT (5552C>T), GAC1316GAT (5558C>T), GAT1317GAC (5561T>C), TTG1319CTA (5565T>C 5567G>A), ATA1320GTT (5568A>G 5570A>T), TCT1322AGT (5574T>A 5575C>G), GAA1323AAT (5577G>A 5579A>T), AGT1324ACC (5581G>C 5582T>C), ATA1325ATG (5585A>G), CAA1327GAG (5589C>G 5591A>G), GTA1329TTA (5595G>T), CAA1330GGA (5598C>G 5599A>G), TTA1332CTC (5604T>C 5606A>C), TTA1333CAA (5607T>C 5608T>A), CAG1334AAG (5610C>A), TTC1335GTT (5613T>G 5615C>T), TTC1336TTT (5618C>T), CAA1337AAT (5619C>A 5621A>T), GTA1338CTA (5622G>C), TGT1339TTT (5626G>T), AAG1340ATA (5629A>T 5630G>A), GAA1341GAC (5633A>C), GAA1342AAT (5634G>A 5636A>T), GGG1343GGT (5639G>T), CTT1344TTA (5640C>T 5642T>A), TTG1346ATA (5646T>A 5648G>A), TCA1347AGT (5649T>A 5650C>G 5651A>T), ACA1349AAG (5656C>A 5657A>G), AAA1350AAG (5660A>G), TTG1351ATA (5661T>A 5663G>A), AAA1352GAG (5664A>G 5666A>G), ATA1353TTA (5667A>T), GGA1354TGC (5670G>T 5672A>C), GTA1355AAA (5673G>A 5674T>A), GCA1356ACA (5676G>A), AAT1357TAT (5679A>T), ATA1358ATT (5684A>T), GAA1359AAT (5685G>A 5687A>T), CTT1361TTA (5691C>T 5693T>A), GGC1362GGA (5696C>A), CTA1363ATA (5697C>A), GAA1364ACC (5700G>A 5701A>C 5702A>C), GTT1370ATA (5718G>A 5720T>A), CAA1371AAA (5721C>A), CTC1372TTA (5724C>T 5726C>A), CCC1374CCT (5732C>T), TTA1377GCT (5739T>G 5740T>C 5741A>T), AAA1378AGA (5743A>G), AAT1379AAG (5747T>G)								

*: Inserts / Deletes / Misaligned / Frameshifts

Analysis details

This analysis was performed with panviral2.64

NGS Details (UN24): Caulimovirus minutangelicae

Assembly

**Coverage Length**	270 (1 contig(s))
**Depth Of Coverage**	3.2
**Number Of Reads**	9
**Reads Per Million**	0.18 rpm (after QC)
**Ambiguities**	0
**Assembly Method**	de novo + reference guided assembly
**Consensus Caller**	Bcf Tools

Coverage Map

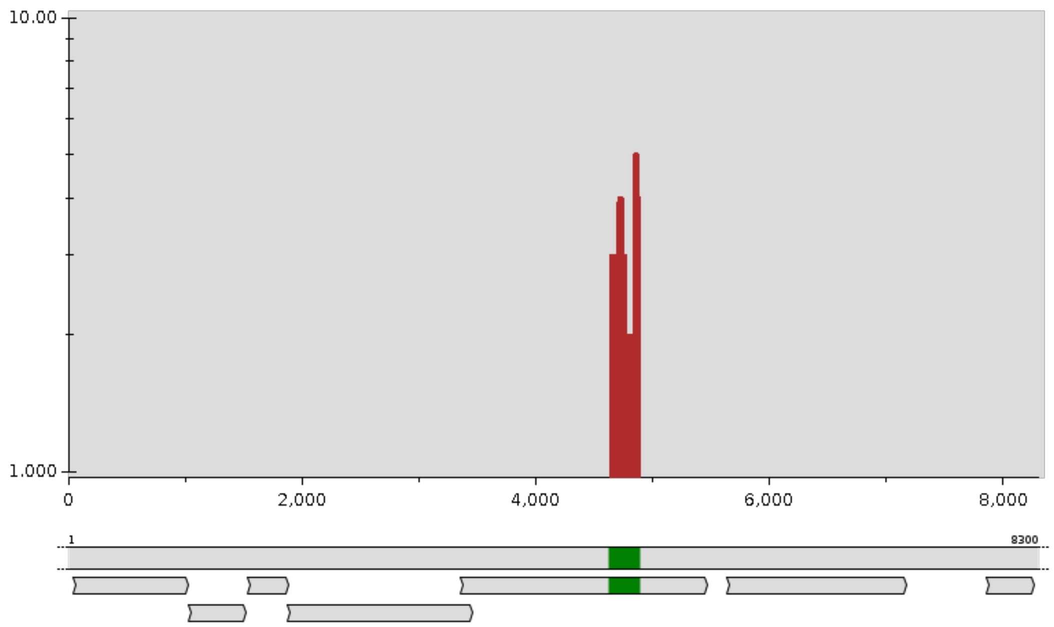

Assignment

**Type**	Caulimovirus minutangelicae (Taxonomy ID: 3047363)
**Reference Genome**	NC_043523.1
**NT Identity (%)**	56.6667
**AA Identity (%)**	50.0
**Number Of Stop Codons**	0
**Number Of CDS**	7

Alignment

**Alignment Score**	72.0 (NT) + 314.0 (AA) = 386.0
**Concordance (%)**	32.7674

Alignment Method	Global, seeded, nucleotide + amino acids (AGA)

Genome Region

Sequence starts at position 4631 and ends at position 4900 relative to NC_043523.1 reference sequence.

Alignment Detailed Statistics

	Begin	End	Coverage	Score	Concordance	Matches	Identities	I/D/M/F*	Stop Codons
**NT**	**4631**	**4900**	**3.3%**	**72**	**13.3%**	**270 (100%)**	**153 (56.7%)**	**0/0**	
Mutations:	4631A>C, 4633A>T, 4638G>C, 4640T>A, 4641G>T, 4645T>C, 4648T>C, 4649G>A, 4651A>T, 4657T>C, 4658A>G, 4661C>T, 4666C>A, 4675T>A, 4676A>G, 4679G>A, 4680A>T, 4681A>T, 4684A>T, 4685G>T, 4686A>C, 4690C>T, 4691C>T, 4692A>T, 4693G>C, 4696A>G, 4702A>C, 4703G>A, 4704C>A, 4705A>T, 4708A>T, 4709G>T, 4711T>C, 4714A>C, 4717G>A, 4719A>C, 4720A>T, 4721T>A, 4722G>T, 4723C>T, 4724G>A, 4727C>A, 4728A>T, 4729A>C, 4730C>A, 4731T>A, 4732G>C, 4735A>C, 4736A>C, 4739A>G, 4741T>C, 4742C>A, 4745T>A, 4746C>G, 4747A>T, 4748A>G, 4749A>C, 4757G>A, 4758C>T, 4759T>A, 4760C>A, 4763T>C, 4765A>T, 4768C>T, 4769A>C, 4774A>T, 4775A>C, 4777A>G, 4780T>A, 4782A>G, 4783T>A, 4787T>C, 4789A>T, 4792T>A, 4794T>A, 4795A>T, 4798A>T, 4799A>T, 4801T>A, 4802G>T, 4810A>G, 4814C>T, 4817C>A, 4818G>A, 4819T>G, 4823C>A, 4824A>T, 4825A>T, 4826A>T, 4827A>G, 4829C>A, 4830A>G, 4831T>A, 4833T>C, 4834C>T, 4835C>A, 4837A>T, 4841C>T, 4842A>T, 4844A>G, 4845T>C, 4846T>A, 4848C>A, 4849A>T, 4852G>A, 4855C>T, 4858G>T, 4863A>C, 4867T>C, 4868G>A, 4869A>C, 4870A>T, 4873C>T, 4878A>C, 4894C>T, 4895T>C, 4897A>T								

CDS

	Begin	End	Coverage	Score	Concordance	Matches	Identities	I/D/M/F*	Stop Codons
FLA58_gp5	424	513	12.8%	314	49.2%	90 (100%)	45 (50.0%)	0/0/0/0	0
Protein mutations:	K424H (4631A>C 4633A>T), C426S (4638G>C), C427I (4640T>A 4641G>T), V430I (4649G>A 4651A>T), I433V (4658A>G), D438E (4675T>A), N439D (4676A>G), E440I (4679G>A 4680A>T 4681A>T), E441D (4684A>T), D442S (4685G>T 4686A>C), Q444F (4691C>T 4692A>T 4693G>C), A448N (4703G>A 4704C>A 4705A>T), V450F (4709G>T 4711T>C), L451F (4714A>C), K453T (4719A>C 4720A>T), C454I (4721T>A 4722G>T 4723C>T), E455K (4724G>A), Q456I (4727C>A 4728A>T 4729A>C), L457N (4730C>A 4731T>A 4732G>C), I459L (4736A>C), I460V (4739A>G 4741T>C), L461I (4742C>A), K463A (4748A>G 4749A>C), A466I (4757G>A 4758C>T 4759T>A), Q467K (4760C>A), K470Q (4769A>C), K472Q (4775A>C 4777A>G), N474R (4782A>G 4783T>A), L478H (4794T>A 4795A>T), E479D (4798A>T), I480L (4799A>T 4801T>A), D481Y (4802G>T), H485Y (4814C>T), R486K (4817C>A 4818G>A 4819T>G), Q488I (4823C>A 4824A>T 4825A>T), N489C (4826A>T 4827A>G), H490R (4829C>A 4830A>G 4831T>A), I491T (4833T>C 4834C>T), L492I (4835C>A 4837A>T), H494F (4841C>T 4842A>T), I495A (4844A>G 4845T>C 4846T>A), A496D (4848C>A 4849A>T), K501T (4863A>C), E503T (4868G>A 4869A>C 4870A>T), K506T (4878A>C)								
Codon mutations:	AAA424CAT (4631A>C 4633A>T), TGT426TCT (4638G>C), TGT427ATT (4640T>A 4641G>T), GTT428GTC (4645T>C), TAT429TAC (4648T>C), GTA430ATT (4649G>A 4651A>T), GAT432GAC (4657T>C), ATT433GTT (4658A>G), CTA434TTA (4661C>T), ATC435ATA (4666C>A), GAT438GAA (4675T>A), AAT439GAT (4676A>G), GAA440ATT (4679G>A 4680A>T 4681A>T), GAA441GAT (4684A>T), GAT442TCT (4685G>T 4686A>C), CAC443CAT (4690C>T), CAG444TTC (4691C>T 4692A>T 4693G>C), AAA445AAG (4696A>G), CTA447CTC (4702A>C), GCA448AAT (4703G>A 4704C>A 4705A>T), GCA449GCT (4708A>T), GTT450TTC (4709G>T 4711T>C), TTA451TTC (4714A>C), AAG452AAA (4717G>A), AAA453ACT (4719A>C 4720A>T), TGC454ATT (4721T>A 4722G>T 4723C>T), GAA455AAA (4724G>A), CAA456ATC (4727C>A 4728A>T 4729A>C), CTG457AAC (4730C>A 4731T>A 4732G>C), GGA458GGC (4735A>C), ATT459CTT (4736A>C), ATT460GTC (4739A>G 4741T>C), CTT461ATT (4742C>A), TCA462AGT (4745T>A 4746C>G 4747A>T), AAA463GCA (4748A>G 4749A>C), GCT466ATA (4757G>A 4758C>T 4759T>A), CAG467AAG (4760C>A), TTA468CTT (4763T>C 4765A>T), TTC469TTT (4768C>T), AAA470CAA (4769A>C), ACA471ACT (4774A>T), AAA472CAG (4775A>C 4777A>G), ATT473ATA (4780T>A), AAT474AGA (4782A>G 4783T>A), TTA476CTT (4787T>C 4789A>T), GGT477GGA (4792T>A), CTA478CAT (4794T>A 4795A>T), GAA479GAT (4798A>T), ATT480TTA (4799A>T 4801T>A), GAT481TAT (4802G>T), GGA483GGG (4810A>G), CAT485TAT (4814C>T), CGT486AAG (4817C>A 4818G>A 4819T>G), CAA488ATT (4823C>A 4824A>T 4825A>T), AAC489TGC (4826A>T 4827A>G), CAT490AGA (4829C>A 4830A>G 4831T>A), ATC491ACT (4833T>C 4834C>T), CTA492ATT (4835C>A 4837A>T), CAT494TTT (4841C>T 4842A>T), ATT495GCA (4844A>G 4845T>C 4846T>A), GCA496GAT (4848C>A 4849A>T), AAG497AAA (4852G>A), TTC498TTT (4855C>T), CCG499CCT (4858G>T), AAA501ACA (4863A>C), ATT502ATC (4867T>C), GAA503ACT (4868G>A 4869A>C 4870A>T), GAC504GAT (4873C>T), AAA506ACA (4878A>C), TTC511TTT (4894C>T), TTA512CTT (4895T>C 4897A>T)								

Proteins

	Begin	End	Coverage	Score	Concordance	Matches	Identities	I/D/M/F*	Stop Codons
reverse transcriptase (YP_009666503.1)	424	513	12.8%	314	49.2%	90 (100%)	45 (50.0%)	0/0/0/0	0
Protein mutations:	K424H (4631A>C 4633A>T), C426S (4638G>C), C427I (4640T>A 4641G>T), V430I (4649G>A 4651A>T), I433V (4658A>G), D438E (4675T>A), N439D (4676A>G), E440I (4679G>A 4680A>T 4681A>T), E441D (4684A>T), D442S (4685G>T 4686A>C), Q444F (4691C>T 4692A>T 4693G>C), A448N (4703G>A 4704C>A 4705A>T), V450F (4709G>T 4711T>C), L451F (4714A>C), K453T (4719A>C 4720A>T), C454I (4721T>A 4722G>T 4723C>T), E455K (4724G>A), Q456I (4727C>A 4728A>T 4729A>C), L457N (4730C>A 4731T>A 4732G>C), I459L (4736A>C), I460V (4739A>G 4741T>C), L461I (4742C>A), K463A (4748A>G 4749A>C), A466I (4757G>A 4758C>T 4759T>A), Q467K (4760C>A), K470Q (4769A>C), K472Q (4775A>C 4777A>G), N474R (4782A>G 4783T>A), L478H (4794T>A 4795A>T), E479D (4798A>T), I480L (4799A>T 4801T>A), D481Y (4802G>T), H485Y (4814C>T), R486K (4817C>A 4818G>A 4819T>G), Q488I (4823C>A 4824A>T 4825A>T), N489C (4826A>T 4827A>G), H490R (4829C>A 4830A>G 4831T>A), I491T (4833T>C 4834C>T), L492I (4835C>A 4837A>T), H494F (4841C>T 4842A>T), I495A (4844A>G 4845T>C 4846T>A), A496D (4848C>A 4849A>T), K501T (4863A>C), E503T (4868G>A 4869A>C 4870A>T), K506T (4878A>C)								
Codon mutations:	AAA424CAT (4631A>C 4633A>T), TGT426TCT (4638G>C), TGT427ATT (4640T>A 4641G>T), GTT428GTC (4645T>C), TAT429TAC (4648T>C), GTA430ATT (4649G>A 4651A>T), GAT432GAC (4657T>C), ATT433GTT (4658A>G), CTA434TTA (4661C>T), ATC435ATA (4666C>A), GAT438GAA (4675T>A), AAT439GAT (4676A>G), GAA440ATT (4679G>A 4680A>T 4681A>T), GAA441GAT (4684A>T), GAT442TCT (4685G>T 4686A>C), CAC443CAT (4690C>T), CAG444TTC (4691C>T 4692A>T 4693G>C), AAA445AAG (4696A>G), CTA447CTC (4702A>C), GCA448AAT (4703G>A 4704C>A 4705A>T), GCA449GCT (4708A>T), GTT450TTC (4709G>T 4711T>C), TTA451TTC (4714A>C), AAG452AAA (4717G>A), AAA453ACT (4719A>C 4720A>T), TGC454ATT (4721T>A 4722G>T 4723C>T), GAA455AAA (4724G>A), CAA456ATC (4727C>A 4728A>T 4729A>C), CTG457AAC (4730C>A 4731T>A 4732G>C), GGA458GGC (4735A>C), ATT459CTT (4736A>C), ATT460GTC (4739A>G 4741T>C), CTT461ATT (4742C>A), TCA462AGT (4745T>A 4746C>G 4747A>T), AAA463GCA (4748A>G 4749A>C), GCT466ATA (4757G>A 4758C>T 4759T>A), CAG467AAG (4760C>A), TTA468CTT (4763T>C 4765A>T), TTC469TTT (4768C>T), AAA470CAA (4769A>C), ACA471ACT (4774A>T), AAA472CAG (4775A>C 4777A>G), ATT473ATA (4780T>A), AAT474AGA (4782A>G 4783T>A), TTA476CTT (4787T>C 4789A>T), GGT477GGA (4792T>A), CTA478CAT (4794T>A 4795A>T), GAA479GAT (4798A>T), ATT480TTA (4799A>T 4801T>A), GAT481TAT (4802G>T), GGA483GGG (4810A>G), CAT485TAT (4814C>T), CGT486AAG (4817C>A 4818G>A 4819T>G), CAA488ATT (4823C>A 4824A>T 4825A>T), AAC489TGC (4826A>T 4827A>G), CAT490AGA (4829C>A 4830A>G 4831T>A), ATC491ACT (4833T>C 4834C>T), CTA492ATT (4835C>A 4837A>T), CAT494TTT (4841C>T 4842A>T), ATT495GCA (4844A>G 4845T>C 4846T>A), GCA496GAT (4848C>A 4849A>T), AAG497AAA (4852G>A), TTC498TTT (4855C>T), CCG499CCT (4858G>T), AAA501ACA (4863A>C), ATT502ATC (4867T>C), GAA503ACT (4868G>A 4869A>C 4870A>T), GAC504GAT (4873C>T), AAA506ACA (4878A>C), TTC511TTT (4894C>T), TTA512CTT (4895T>C 4897A>T)								

*: Inserts / Deletes / Misaligned / Frameshifts

Analysis details

This analysis was performed with panviral2.64

NGS Details (UN24): Badnavirus maculaucubae

Assembly

**Coverage Length**	279 (1 contig(s))
**Depth Of Coverage**	3.6
**Number Of Reads**	9
**Reads Per Million**	0.18 rpm (after QC)
**Ambiguities**	0
**Assembly Method**	de novo + reference guided assembly
**Consensus Caller**	Bcf Tools

Coverage Map

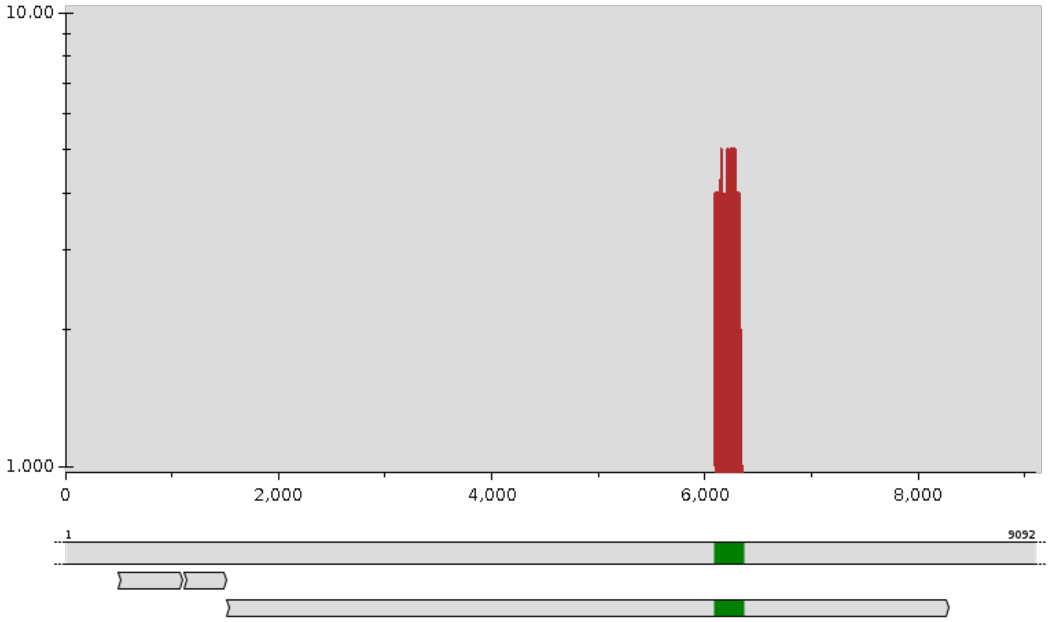

Assignment

**Type**	Badnavirus maculaucubae (Taxonomy ID: 3051986)
**Reference Genome**	NC_076606.1
**NT Identity (%)**	56.7376
**AA Identity (%)**	45.7447
**Number Of Stop Codons**	1
**Number Of CDS**	3

Alignment

**Alignment Score**	70.0 (NT) + 300.0 (AA) = 370.0
**Concordance (%)**	30.3777

Alignment Method	Global, seeded, nucleotide + amino acids (AGA)

Genome Region

Sequence starts at position 6090 and ends at position 6368 relative to NC_076606.1 reference sequence.

Alignment Detailed Statistics

	Begin	End	Coverage	Score	Concordance	Matches	Identities	I/D/M/F*	Stop Codons
**NT**	**6090**	**6368**	**3.1%**	**70**	**12.5%**	**279 (98.9%)**	**160 (56.7%)**	**3/0**	
Mutations:	6093A>T, 6100A>T, 6101G>C, 6106T>A, 6116A>G, 6119C>T, 6120T>C, 6125T>A, 6131A>G, 6133G>C, 6135G>A, 6136G>A, 6137C>A, 6141G>A, 6142G>C, 6143A>G, 6144A>G, 6146A>C, 6148T>G, 6151A>G, 6152G>A, 6153C>T, 6156T>G, 6159A>T, 6160T>A, 6161G>A, 6162G>A, 6164C>T, 6165G>T, 6171C>T, 6172A>T, 6173T>G, 6177T>A, 6178C>A, 6179C>G, 6180A>G, 6181G>A, 6185G>A, 6186A>G, 6188T>A, 6189A>C, 6192C>T, 6195A>G, 6197G>T, 6198G>C, 6199C>T, 6201A>G, 6208C>T, 6210A>T, 6216T>A, 6219A>T, 6221A>C, 6222G>A, 6228A>C, 6234A>T, 6235G>A, 6236T>C, 6237G>T, 6241C>A, 6242A>T, 6243A>G, 6246G>C, 6247A>C, 6248A>T, 6249A>T, 6253G>A, 6255C>A, 6256A>G, 6257A>G, 6258C>A, 6259T>A, 6260G>T, 6261T>C, 6264C>T, 6266G>A, 6267_6268insCCA, 6269G>A, 6271A>T, 6272C>T, 6273A>G, 6274G>A, 6276A>T, 6277G>C, 6278A>T, 6279T>C, 6282C>T, 6285C>T, 6286G>A, 6287C>T, 6291T>G, 6293A>T, 6294C>T, 6306T>A, 6307C>G, 6310G>A, 6312A>C, 6314T>A, 6318T>A, 6319G>A, 6321A>G, 6323A>G, 6324T>C, 6325C>A, 6326T>G, 6327T>A, 6328C>A, 6333G>A, 6339G>A, 6345T>C, 6346C>T, 6348C>G, 6350A>G, 6354A>T, 6355A>G, 6357G>T, 6360G>A, 6362A>T, 6363A>T, 6364A>C, 6367T>G								

CDS

	Begin	End	Coverage	Score	Concordance	Matches	Identities	I/D/M/F*	Stop Codons
QKP79_gp3	1528	1620	4.1%	300	43.9%	93 (98.9%)	43 (45.7%)	1/0/0/0	1
Protein mutations:	K1528N (6093A>T), F1533I (6106T>A), K1536R (6116A>G), S1537F (6119C>T 6120T>C), F1539Y (6125T>A), Q1541R (6131A>G), V1542L (6133G>C 6135G>A), A1543K (6136G>A 6137C>A), M1544I (6141G>A), E1545R (6142G>C 6143A>G 6144A>G), E1546A (6146A>C), S1547A (6148T>G), S1548D (6151A>G 6152G>A 6153C>T), I1549M (6156T>G), W1551K (6160T>A 6161G>A 6162G>A), T1552I (6164C>T 6165G>T), I1555* (6172A>T 6173T>G), P1557R (6178C>A 6179C>G 6180A>G), D1558N (6181G>A), G1559E (6185G>A 6186A>G), L1560H (6188T>A 6189A>C), W1563F (6197G>T 6198G>C), P1567S (6208C>T 6210A>T), K1571T (6221A>C 6222G>A), V1576T (6235G>A 6236T>C 6237G>T), Q1578M (6241C>A 6242A>T 6243A>G), R1579S (6246G>C), K1580L (6247A>C 6248A>T 6249A>T), D1582K (6253G>A 6255C>A), N1583G (6256A>G 6257A>G 6258C>A), C1584I (6259T>A 6260G>T 6261T>C), R1586K (6266G>A), R1586_G1587insP (6267_6268insCCA), G1587D (6269G>A), T1588L (6271A>T 6272C>T 6273A>G), E1589N (6274G>A 6276A>T), D1590L (6277G>C 6278A>T 6279T>C), A1593I (6286G>A 6287C>T), Y1595F (6293A>T 6294C>T), L1600V (6307C>G), V1601I (6310G>A 6312A>C), F1602Y (6314T>A), E1604K (6319G>A 6321A>G), N1605S (6323A>G 6324T>C), L1606R (6325C>A 6326T>G 6327T>A), Q1607K (6328C>A), K1614R (6350A>G), M1616V (6355A>G 6357G>T), E1618V (6362A>T 6363A>T), I1619L (6364A>C)								
Codon mutations:	AAA1528AAT (6093A>T), AGC1531TCC (6100A>T 6101G>C), TTT1533ATT (6106T>A), AAA1536AGA (6116A>G), TCT1537TTC (6119C>T 6120T>C), TTT1539TAT (6125T>A), CAG1541CGG (6131A>G), GTG1542CTA (6133G>C 6135G>A), GCA1543AAA (6136G>A 6137C>A), ATG1544ATA (6141G>A), GAA1545CGG (6142G>C 6143A>G 6144A>G), GAA1546GCA (6146A>C), TCA1547GCA (6148T>G), AGC1548GAT (6151A>G 6152G>A 6153C>T), ATT1549ATG (6156T>G), CCA1550CCT (6159A>T), TGG1551AAA (6160T>A 6161G>A 6162G>A), ACG1552ATT (6164C>T 6165G>T), TTC1554TTT (6171C>T), ATA1555TGA (6172A>T 6173T>G), ACT1556ACA (6177T>A), CCA1557AGG (6178C>A 6179C>G 6180A>G), GAT1558AAT (6181G>A), GGA1559GAG (6185G>A 6186A>G), CTA1560CAC (6188T>A 6189A>C), TAC1561TAT (6192C>T), GAA1562GAG (6195A>G), TGG1563TTC (6197G>T 6198G>C), CTA1564TTG (6199C>T 6201A>G), CCA1567TCT (6208C>T 6210A>T), GGT1569GGA (6216T>A), CTA1570CTT (6219A>T), AAG1571ACA (6221A>C 6222G>A), GCA1573GCC (6228A>C), GCA1575GCT (6234A>T), GTG1576ACT (6235G>A 6236T>C 6237G>T), CAA1578ATG (6241C>A 6242A>T 6243A>G), AGG1579AGC (6246G>C), AAA1580CTT (6247A>C 6248A>T 6249A>T), GAC1582AAA (6253G>A 6255C>A), AAC1583GGA (6256A>G 6257A>G 6258C>A), TGT1584ATC (6259T>A 6260G>T 6261T>C), TTC1585TTT (6264C>T), AGG1586AAG (6266G>A), AGG1586_GGT1587insCCA (6267_6268insCCA), GGT1587GAT (6269G>A), ACA1588TTG (6271A>T 6272C>T 6273A>G), GAA1589AAT (6274G>A 6276A>T), GAT1590CTC (6277G>C 6278A>T 6279T>C), TTC1591TTT (6282C>T), ATC1592ATT (6285C>T), GCT1593ATT (6286G>A 6287C>T), GTT1594GTG (6291T>G), TAC1595TTT (6293A>T 6294C>T), ATT1599ATA (6306T>A), CTG1600GTG (6307C>G), GTA1601ATC (6310G>A 6312A>C), TTC1602TAC (6314T>A), TCT1603TCA (6318T>A), GAA1604AAG (6319G>A 6321A>G), AAT1605AGC (6323A>G 6324T>C), CTT1606AGA (6325C>A 6326T>G 6327T>A), CAA1607AAA (6328C>A), GAG1608GAA (6333G>A), GAG1610GAA (6339G>A), CAT1612CAC (6345T>C), CTC1613TTG (6346C>T 6348C>G), AAA1614AGA (6350A>G), ATA1615ATT (6354A>T), ATG1616GTT (6355A>G 6357G>T), CTG1617CTA (6360G>A), GAA1618GTT (6362A>T 6363A>T), ATA1619CTA (6364A>C), TGC1620GG. (6367T>G)								

Proteins

	Begin	End	Coverage	Score	Concordance	Matches	Identities	I/D/M/F*	Stop Codons
hypothetical protein (YP_010799265.1)	1528	1620	4.1%	300	43.9%	93 (98.9%)	43 (45.7%)	1/0/0/0	1
Protein mutations:	K1528N (6093A>T), F1533I (6106T>A), K1536R (6116A>G), S1537F (6119C>T 6120T>C), F1539Y (6125T>A), Q1541R (6131A>G), V1542L (6133G>C 6135G>A), A1543K (6136G>A 6137C>A), M1544I (6141G>A), E1545R (6142G>C 6143A>G 6144A>G), E1546A (6146A>C), S1547A (6148T>G), S1548D (6151A>G 6152G>A 6153C>T), I1549M (6156T>G), W1551K (6160T>A 6161G>A 6162G>A), T1552I (6164C>T 6165G>T), I1555* (6172A>T 6173T>G), P1557R (6178C>A 6179C>G 6180A>G), D1558N (6181G>A), G1559E (6185G>A 6186A>G), L1560H (6188T>A 6189A>C), W1563F (6197G>T 6198G>C), P1567S (6208C>T 6210A>T), K1571T (6221A>C 6222G>A), V1576T (6235G>A 6236T>C 6237G>T), Q1578M (6241C>A 6242A>T 6243A>G), R1579S (6246G>C), K1580L (6247A>C 6248A>T 6249A>T), D1582K (6253G>A 6255C>A), N1583G (6256A>G 6257A>G 6258C>A), C1584I (6259T>A 6260G>T 6261T>C), R1586K (6266G>A), R1586_G1587insP (6267_6268insCCA), G1587D (6269G>A), T1588L (6271A>T 6272C>T 6273A>G), E1589N (6274G>A 6276A>T), D1590L (6277G>C 6278A>T 6279T>C), A1593I (6286G>A 6287C>T), Y1595F (6293A>T 6294C>T), L1600V (6307C>G), V1601I (6310G>A 6312A>C), F1602Y (6314T>A), E1604K (6319G>A 6321A>G), N1605S (6323A>G 6324T>C), L1606R (6325C>A 6326T>G 6327T>A), Q1607K (6328C>A), K1614R (6350A>G), M1616V (6355A>G 6357G>T), E1618V (6362A>T 6363A>T), I1619L (6364A>C)								
Codon mutations:	AAA1528AAT (6093A>T), AGC1531TCC (6100A>T 6101G>C), TTT1533ATT (6106T>A), AAA1536AGA (6116A>G), TCT1537TTC (6119C>T 6120T>C), TTT1539TAT (6125T>A), CAG1541CGG (6131A>G), GTG1542CTA (6133G>C 6135G>A), GCA1543AAA (6136G>A 6137C>A), ATG1544ATA (6141G>A), GAA1545CGG (6142G>C 6143A>G 6144A>G), GAA1546GCA (6146A>C), TCA1547GCA (6148T>G), AGC1548GAT (6151A>G 6152G>A 6153C>T), ATT1549ATG (6156T>G), CCA1550CCT (6159A>T), TGG1551AAA (6160T>A 6161G>A 6162G>A), ACG1552ATT (6164C>T 6165G>T), TTC1554TTT (6171C>T), ATA1555TGA (6172A>T 6173T>G), ACT1556ACA (6177T>A), CCA1557AGG (6178C>A 6179C>G 6180A>G), GAT1558AAT (6181G>A), GGA1559GAG (6185G>A 6186A>G), CTA1560CAC (6188T>A 6189A>C), TAC1561TAT (6192C>T), GAA1562GAG (6195A>G), TGG1563TTC (6197G>T 6198G>C), CTA1564TTG (6199C>T 6201A>G), CCA1567TCT (6208C>T 6210A>T), GGT1569GGA (6216T>A), CTA1570CTT (6219A>T), AAG1571ACA (6221A>C 6222G>A), GCA1573GCC (6228A>C), GCA1575GCT (6234A>T), GTG1576ACT (6235G>A 6236T>C 6237G>T), CAA1578ATG (6241C>A 6242A>T 6243A>G), AGG1579AGC (6246G>C), AAA1580CTT (6247A>C 6248A>T 6249A>T), GAC1582AAA (6253G>A 6255C>A), AAC1583GGA (6256A>G 6257A>G 6258C>A), TGT1584ATC (6259T>A 6260G>T 6261T>C), TTC1585TTT (6264C>T), AGG1586AAG (6266G>A), AGG1586_GGT1587insCCA (6267_6268insCCA), GGT1587GAT (6269G>A), ACA1588TTG (6271A>T 6272C>T 6273A>G), GAA1589AAT (6274G>A 6276A>T), GAT1590CTC (6277G>C 6278A>T 6279T>C), TTC1591TTT (6282C>T), ATC1592ATT (6285C>T), GCT1593ATT (6286G>A 6287C>T), GTT1594GTG (6291T>G), TAC1595TTT (6293A>T 6294C>T), ATT1599ATA (6306T>A), CTG1600GTG (6307C>G), GTA1601ATC (6310G>A 6312A>C), TTC1602TAC (6314T>A), TCT1603TCA (6318T>A), GAA1604AAG (6319G>A 6321A>G), AAT1605AGC (6323A>G 6324T>C), CTT1606AGA (6325C>A 6326T>G 6327T>A), CAA1607AAA (6328C>A), GAG1608GAA (6333G>A), GAG1610GAA (6339G>A), CAT1612CAC (6345T>C), CTC1613TTG (6346C>T 6348C>G), AAA1614AGA (6350A>G), ATA1615ATT (6354A>T), ATG1616GTT (6355A>G 6357G>T), CTG1617CTA (6360G>A), GAA1618GTT (6362A>T 6363A>T), ATA1619CTA (6364A>C), TGC1620GG. (6367T>G)								

*: Inserts / Deletes / Misaligned / Frameshifts

Analysis details

This analysis was performed with panviral2.64

NGS Details (UN24): Caulimovirus venafragariae

Assembly

**Coverage Length**	265 (1 contig(s))
**Depth Of Coverage**	3.8
**Number Of Reads**	8
**Reads Per Million**	0.16 rpm (after QC)
**Ambiguities**	0
**Assembly Method**	de novo + reference guided assembly
**Consensus Caller**	Bcf Tools

Coverage Map

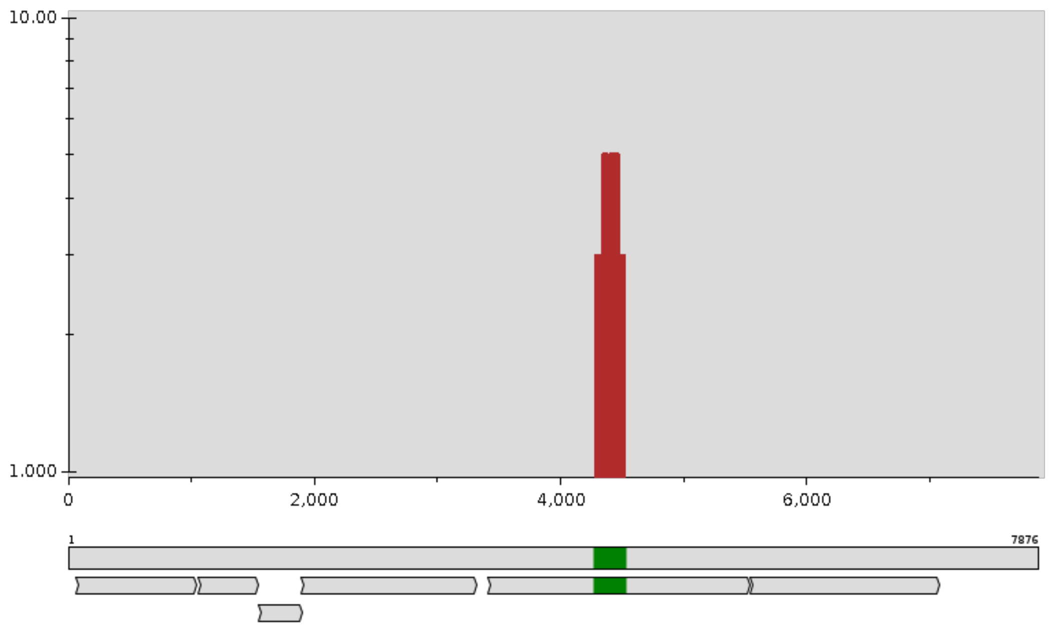

Assignment

**Type**	Caulimovirus venafragariae (Taxonomy ID: 3048344)
**Reference Genome**	NC_001725.1
**NT Identity (%)**	61.5094
**AA Identity (%)**	57.9545
**Number Of Stop Codons**	1
**Number Of CDS**	6

Alignment

**Alignment Score**	122.0 (NT) + 343.0 (AA) = 465.0
**Concordance (%)**	41.5179

Alignment Method	Global, seeded, nucleotide + amino acids (AGA)

Genome Region

Sequence starts at position 4268 and ends at position 4532 relative to NC_001725.1 reference sequence.

Alignment Detailed Statistics

	Begin	End	Coverage	Score	Concordance	Matches	Identities	I/D/M/F*	Stop Codons
**NT**	**4268**	**4532**	**3.4%**	**122**	**23.0%**	**265 (100%)**	**163 (61.5%)**	**0/0**	
Mutations:	4268C>A, 4275C>T, 4276C>A, 4277G>T, 4284G>C, 4287T>C, 4290A>C, 4293T>A, 4296G>A, 4299C>T, 4300G>A, 4306C>T, 4309C>T, 4311C>A, 4314G>A, 4315C>T, 4317T>A, 4320T>A, 4321A>G, 4323C>T, 4326C>A, 4330C>A, 4331C>G, 4332T>A, 4333A>T, 4334G>C, 4337A>T, 4338G>T, 4339A>T, 4340G>C, 4342C>A, 4343C>A, 4348T>A, 4349C>G, 4350C>A, 4353A>T, 4354C>G, 4357G>A, 4359C>A, 4362C>T, 4370G>A, 4371G>A, 4378G>A, 4379C>G, 4388A>T, 4390C>A, 4394G>A, 4395C>A, 4397A>G, 4401A>T, 4407G>A, 4413T>A, 4416C>T, 4424A>G, 4425G>A, 4426T>C, 4428A>T, 4432G>A, 4434C>T, 4435C>A, 4440A>T, 4442A>G, 4443G>A, 4444G>A, 4445G>C, 4446A>G, 4451G>C, 4452C>A, 4455C>T, 4456C>A, 4457T>A, 4459C>T, 4461T>A, 4464T>A, 4465A>G, 4467C>T, 4470G>A, 4471G>A, 4472A>G, 4473G>T, 4474C>G, 4476A>T, 4477C>T, 4479G>A, 4480C>A, 4483C>A, 4485A>T, 4491C>G, 4492G>T, 4493G>A, 4496G>A, 4500G>A, 4502C>A, 4503C>A, 4506T>A, 4508A>T, 4510T>A, 4511C>G, 4513T>A, 4514C>A, 4515C>A, 4521C>T								
**CDS**									
ORF_V	289	376	12.4%	343	56.2%	88 (100%)	51 (58.0%)	0/0/0/0	1
Protein mutations:	R291I (4276C>A 4277G>T), E293D (4284G>C), K295N (4290A>C), E299K (4300G>A), I306V (4321A>G 4323C>T), P309R (4330C>A 4331C>G 4332T>A), K311I (4337A>T 4338G>T), P313K (4342C>A 4343C>A), S315R (4348T>A 4349C>G 4350C>A), P317A (4354C>G), A318T (4357G>A 4359C>A), R322K (4370G>A 4371G>A), A325S (4378G>A 4379C>G), K328I (4388A>T), G330E (4394G>A 4395C>A), K331R (4397A>G), M334I (4407G>A), K340R (4424A>G 4425G>A), D343N (4432G>A 4434C>T), H344N (4435C>A), K346R (4442A>G 4443G>A), G347T (4444G>A 4445G>C 4446A>G), G349A (4451G>C 4452C>A), L351K (4456C>A 4457T>A), N354D (4465A>G 4467C>T), E356S (4471G>A 4472A>G 4473G>T), Q357D (4474C>G 4476A>T), L359I (4480C>A), Q360N (4483C>A 4485A>T), I362M (4491C>G), G363* (4492G>T 4493G>A), G364D (4496G>A), T366K (4502C>A 4503C>A), F367L (4506T>A), Y368F (4508A>T), S370K (4513T>A 4514C>A 4515C>A)								
Codon mutations:	CCT288.AT (4268C>A), GAC290GAT (4275C>T), CGA291ATA (4276C>A 4277G>T), GAG293GAC (4284G>C), TTT294TTC (4287T>C), AAA295AAC (4290A>C), ACT296ACA (4293T>A), CAG297CAA (4296G>A), ATC298ATT (4299C>T), GAA299AAA (4300G>A), CTA301TTA (4306C>T), CTC302TTA (4309C>T 4311C>A), AAG303AAA (4314G>A), CTT304TTA (4315C>T 4317T>A), GGT305GGA (4320T>A), ATC306GTT (4321A>G 4323C>T), ATC307ATA (4326C>A), CCT309AGA (4330C>A 4331C>G 4332T>A), AGC310TCC (4333A>T 4334G>C), AAG311ATT (4337A>T 4338G>T), AGT312TCT (4339A>T 4340G>C), CCA313AAA (4342C>A 4343C>A), TCC315AGA (4348T>A 4349C>G 4350C>A), TCA316TCT (4353A>T), CCA317GCA (4354C>G), GCC318ACA (4357G>A 4359C>A), TTC319TTT (4362C>T), AGG322AAA (4370G>A 4371G>A), GCC325AGC (4378G>A 4379C>G), AAA328ATA (4388A>T), CGA329AGA (4390C>A), GGC330GAA (4394G>A 4395C>A), AAA331AGA (4397A>G), GCA332GCT (4401A>T), ATG334ATA (4407G>A), ATT336ATA (4413T>A), AAC337AAT (4416C>T), AAG340AGA (4424A>G 4425G>A), TTA341CTT (4426T>C 4428A>T), GAC343AAT (4432G>A 4434C>T), CAT344AAT (4435C>A), ACA345ACT (4440A>T), AAG346AGA (4442A>G 4443G>A), GGA347ACG (4444G>A 4445G>C 4446A>G), GGC349GCA (4451G>C 4452C>A), TAC350TAT (4455C>T), CTA351AAA (4456C>A 4457T>A), CTT352TTA (4459C>T 4461T>A), CCT353CCA (4464T>A), AAC354GAT (4465A>G 4467C>T), AAG355AAA (4470G>A), GAG356AGT (4471G>A 4472A>G 4473G>T), CAA357GAT (4474C>G 4476A>T), CTG358TTA (4477C>T 4479G>A), CTT359ATT (4480C>A), CAA360AAT (4483C>A 4485A>T), ATC362ATG (4491C>G), GGA363TAA (4492G>T 4493G>A), GGT364GAT (4496G>A), AAG365AAA (4500G>A), ACC366AAA (4502C>A 4503C>A), TTT367TTA (4506T>A), TAC368TTC (4508A>T), TCT369AGT (4510T>A 4511C>G), TCC370AAA (4513T>A 4514C>A 4515C>A), GAC372GAT (4521C>T)								
**Proteins**									
hypothetical protein (NP_043933.1)	289	376	12.4%	343	56.2%	88 (100%)	51 (58.0%)	0/0/0/0	1
Protein mutations:	R291I (4276C>A 4277G>T), E293D (4284G>C), K295N (4290A>C), E299K (4300G>A), I306V (4321A>G 4323C>T), P309R (4330C>A 4331C>G 4332T>A), K311I (4337A>T 4338G>T), P313K (4342C>A 4343C>A), S315R (4348T>A 4349C>G 4350C>A), P317A (4354C>G), A318T (4357G>A 4359C>A), R322K (4370G>A 4371G>A), A325S (4378G>A 4379C>G), K328I (4388A>T), G330E (4394G>A 4395C>A), K331R (4397A>G), M334I (4407G>A), K340R (4424A>G 4425G>A), D343N (4432G>A 4434C>T), H344N (4435C>A), K346R (4442A>G 4443G>A), G347T (4444G>A 4445G>C 4446A>G), G349A (4451G>C 4452C>A), L351K (4456C>A 4457T>A), N354D (4465A>G 4467C>T), E356S (4471G>A 4472A>G 4473G>T), Q357D (4474C>G 4476A>T), L359I (4480C>A), Q360N (4483C>A 4485A>T), I362M (4491C>G), G363* (4492G>T 4493G>A), G364D (4496G>A), T366K (4502C>A 4503C>A), F367L (4506T>A), Y368F (4508A>T), S370K (4513T>A 4514C>A 4515C>A)								
Codon mutations:	CCT288.AT (4268C>A), GAC290GAT (4275C>T), CGA291ATA (4276C>A 4277G>T), GAG293GAC (4284G>C), TTT294TTC (4287T>C), AAA295AAC (4290A>C), ACT296ACA (4293T>A), CAG297CAA (4296G>A), ATC298ATT (4299C>T), GAA299AAA (4300G>A), CTA301TTA (4306C>T), CTC302TTA (4309C>T 4311C>A), AAG303AAA (4314G>A), CTT304TTA (4315C>T 4317T>A), GGT305GGA (4320T>A), ATC306GTT (4321A>G 4323C>T), ATC307ATA (4326C>A), CCT309AGA (4330C>A 4331C>G 4332T>A), AGC310TCC (4333A>T 4334G>C), AAG311ATT (4337A>T 4338G>T), AGT312TCT (4339A>T 4340G>C), CCA313AAA (4342C>A 4343C>A), TCC315AGA (4348T>A 4349C>G 4350C>A), TCA316TCT (4353A>T), CCA317GCA (4354C>G), GCC318ACA (4357G>A 4359C>A), TTC319TTT (4362C>T), AGG322AAA (4370G>A 4371G>A), GCC325AGC (4378G>A 4379C>G), AAA328ATA (4388A>T), CGA329AGA (4390C>A), GGC330GAA (4394G>A 4395C>A), AAA331AGA (4397A>G), GCA332GCT (4401A>T), ATG334ATA (4407G>A), ATT336ATA (4413T>A), AAC337AAT (4416C>T), AAG340AGA (4424A>G 4425G>A), TTA341CTT (4426T>C 4428A>T), GAC343AAT (4432G>A 4434C>T), CAT344AAT (4435C>A), ACA345ACT (4440A>T), AAG346AGA (4442A>G 4443G>A), GGA347ACG (4444G>A 4445G>C 4446A>G), GGC349GCA (4451G>C 4452C>A), TAC350TAT (4455C>T), CTA351AAA (4456C>A 4457T>A), CTT352TTA (4459C>T 4461T>A), CCT353CCA (4464T>A), AAC354GAT (4465A>G 4467C>T), AAG355AAA (4470G>A), GAG356AGT (4471G>A 4472A>G 4473G>T), CAA357GAT (4474C>G 4476A>T), CTG358TTA (4477C>T 4479G>A), CTT359ATT (4480C>A), CAA360AAT (4483C>A 4485A>T), ATC362ATG (4491C>G), GGA363TAA (4492G>T 4493G>A), GGT364GAT (4496G>A), AAG365AAA (4500G>A), ACC366AAA (4502C>A 4503C>A), TTT367TTA (4506T>A), TAC368TTC (4508A>T), TCT369AGT (4510T>A 4511C>G), TCC370AAA (4513T>A 4514C>A 4515C>A), GAC372GAT (4521C>T)								

*: Inserts / Deletes / Misaligned / Frameshifts

Analysis details

This analysis was performed with panviral2.64

NGS Details (UN24): Caulimovirus venafragariae

Assembly

**Coverage Length**	257 (1 contig(s))
**Depth Of Coverage**	3.5
**Number Of Reads**	8
**Reads Per Million**	0.16 rpm (after QC)
**Ambiguities**	0
**Assembly Method**	de novo + reference guided assembly
**Consensus Caller**	Bcf Tools

Coverage Map

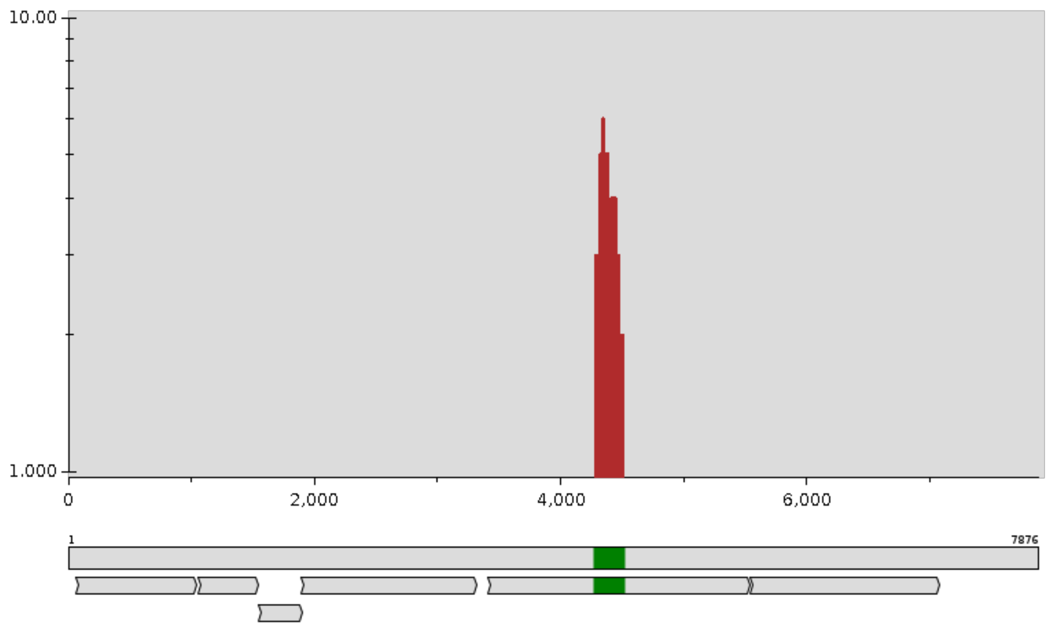

Assignment

**Type**	Caulimovirus venafragariae (Taxonomy ID: 3048344)
**Reference Genome**	NC_001725.1
**NT Identity (%)**	62.2568
**AA Identity (%)**	64.7059
**Number Of Stop Codons**	0
**Number Of CDS**	6

Alignment

**Alignment Score**	126.0 (NT) + 389.0 (AA) = 515.0
**Concordance (%)**	46.9462

Alignment Method	Global, seeded, nucleotide + amino acids (AGA)

Genome Region

Sequence starts at position 4268 and ends at position 4524 relative to NC_001725.1 reference sequence.

Alignment Detailed Statistics

	Begin	End	Coverage	Score	Concordance	Matches	Identities	I/D/M/F*	Stop Codons
**NT**	**4268**	**4524**	**3.3%**	**126**	**24.5%**	**257 (100%)**	**160 (62.3%)**	**0/0**	
Mutations:	4268C>A, 4276C>G, 4277G>T, 4284G>T, 4289A>G, 4290A>T, 4292C>A, 4296G>A, 4299C>T, 4300G>A, 4311C>A, 4313A>C, 4314G>T, 4315C>T, 4317T>A, 4320T>A, 4321A>G, 4323C>T, 4326C>A, 4330C>A, 4331C>G, 4332T>A, 4333A>T, 4334G>C, 4335C>T, 4337A>C, 4338G>A, 4339A>T, 4340G>C, 4341T>A, 4342C>A, 4343C>A, 4348T>A, 4349C>G, 4350C>A, 4354C>G, 4362C>T, 4368A>T, 4371G>A, 4378G>A, 4379C>G, 4383G>A, 4386C>A, 4387A>G, 4388A>T, 4390C>A, 4395C>T, 4398A>G, 4402A>C, 4413T>A, 4419T>C, 4424A>G, 4425G>A, 4426T>C, 4428A>T, 4434C>T, 4435C>A, 4437T>C, 4440A>T, 4442A>G, 4443G>A, 4444G>A, 4445G>C, 4446A>G, 4451G>C, 4455C>T, 4456C>A, 4457T>A, 4458A>G, 4461T>A, 4464T>A, 4465A>G, 4467C>T, 4470G>A, 4471G>A, 4472A>G, 4473G>T, 4474C>G, 4477C>T, 4479G>A, 4480C>A, 4482T>A, 4483C>A, 4485A>T, 4491C>A, 4492G>C, 4493G>A, 4503C>A, 4504T>G, 4506T>A, 4508A>T, 4510T>A, 4511C>A, 4513T>A, 4514C>A, 4515C>A, 4521C>T								
**CDS**									
ORF_V	289	373	12.0%	389	65.5%	85 (100%)	55 (64.7%)	0/0/0/0	0
Protein mutations:	R291V (4276C>G 4277G>T), E293D (4284G>T), K295S (4289A>G 4290A>T), T296N (4292C>A), E299K (4300G>A), K303T (4313A>C 4314G>T), I306V (4321A>G 4323C>T), P309R (4330C>A 4331C>G 4332T>A), K311T (4337A>C 4338G>A), P313K (4342C>A 4343C>A), S315R (4348T>A 4349C>G 4350C>A), P317A (4354C>G), A325S (4378G>A 4379C>G), K328V (4387A>G 4388A>T), K340R (4424A>G 4425G>A), H344N (4435C>A 4437T>C), K346R (4442A>G 4443G>A), G347T (4444G>A 4445G>C 4446A>G), G349A (4451G>C), L351K (4456C>A 4457T>A 4458A>G), N354D (4465A>G 4467C>T), E356S (4471G>A 4472A>G 4473G>T), Q357E (4474C>G), L359I (4480C>A 4482T>A), Q360N (4483C>A 4485A>T), G363Q (4492G>C 4493G>A), F367V (4504T>G 4506T>A), Y368F (4508A>T), S369N (4510T>A 4511C>A), S370K (4513T>A 4514C>A 4515C>A)								
Codon mutations:	CCT288.AT (4268C>A), CGA291GTA (4276C>G 4277G>T), GAG293GAT (4284G>T), AAA295AGT (4289A>G 4290A>T), ACT296AAT (4292C>A), CAG297CAA (4296G>A), ATC298ATT (4299C>T), GAA299AAA (4300G>A), CTC302CTA (4311C>A), AAG303ACT (4313A>C 4314G>T), CTT304TTA (4315C>T 4317T>A), GGT305GGA (4320T>A), ATC306GTT (4321A>G 4323C>T), ATC307ATA (4326C>A), CCT309AGA (4330C>A 4331C>G 4332T>A), AGC310TCT (4333A>T 4334G>C 4335C>T), AAG311ACA (4337A>C 4338G>A), AGT312TCA (4339A>T 4340G>C 4341T>A), CCA313AAA (4342C>A 4343C>A), TCC315AGA (4348T>A 4349C>G 4350C>A), CCA317GCA (4354C>G), TTC319TTT (4362C>T), GTA321GTT (4368A>T), AGG322AGA (4371G>A), GCC325AGC (4378G>A 4379C>G), GAG326GAA (4383G>A), ATC327ATA (4386C>A), AAA328GTA (4387A>G 4388A>T), CGA329AGA (4390C>A), GGC330GGT (4395C>T), AAA331AAG (4398A>G), AGA333CGA (4402A>C), ATT336ATA (4413T>A), TAT338TAC (4419T>C), AAG340AGA (4424A>G 4425G>A), TTA341CTT (4426T>C 4428A>T), GAC343GAT (4434C>T), CAT344AAC (4435C>A 4437T>C), ACA345ACT (4440A>T), AAG346AGA (4442A>G 4443G>A), GGA347ACG (4444G>A 4445G>C 4446A>G), GGC349GCC (4451G>C), TAC350TAT (4455C>T), CTA351AAG (4456C>A 4457T>A 4458A>G), CTT352CTA (4461T>A), CCT353CCA (4464T>A), AAC354GAT (4465A>G 4467C>T), AAG355AAA (4470G>A), GAG356AGT (4471G>A 4472A>G 4473G>T), CAA357GAA (4474C>G), CTG358TTA (4477C>T 4479G>A), CTT359ATA (4480C>A 4482T>A), CAA360AAT (4483C>A 4485A>T), ATC362ATA (4491C>A), GGA363CAA (4492G>C 4493G>A), ACC366ACA (4503C>A), TTT367GTA (4504T>G 4506T>A), TAC368TTC (4508A>T), TCT369AAT (4510T>A 4511C>A), TCC370AAA (4513T>A 4514C>A 4515C>A), GAC372GAT (4521C>T)								
**Proteins**									
hypothetical protein (NP_043933.1)	289	373	12.0%	389	65.5%	85 (100%)	55 (64.7%)	0/0/0/0	0
Protein mutations:	R291V (4276C>G 4277G>T), E293D (4284G>T), K295S (4289A>G 4290A>T), T296N (4292C>A), E299K (4300G>A), K303T (4313A>C 4314G>T), I306V (4321A>G 4323C>T), P309R (4330C>A 4331C>G 4332T>A), K311T (4337A>C 4338G>A), P313K (4342C>A 4343C>A), S315R (4348T>A 4349C>G 4350C>A), P317A (4354C>G), A325S (4378G>A 4379C>G), K328V (4387A>G 4388A>T), K340R (4424A>G 4425G>A), H344N (4435C>A 4437T>C), K346R (4442A>G 4443G>A), G347T (4444G>A 4445G>C 4446A>G), G349A (4451G>C), L351K (4456C>A 4457T>A 4458A>G), N354D (4465A>G 4467C>T), E356S (4471G>A 4472A>G 4473G>T), Q357E (4474C>G), L359I (4480C>A 4482T>A), Q360N (4483C>A 4485A>T), G363Q (4492G>C 4493G>A), F367V (4504T>G 4506T>A), Y368F (4508A>T), S369N (4510T>A 4511C>A), S370K (4513T>A 4514C>A 4515C>A)								
Codon mutations:	CCT288.AT (4268C>A), CGA291GTA (4276C>G 4277G>T), GAG293GAT (4284G>T), AAA295AGT (4289A>G 4290A>T), ACT296AAT (4292C>A), CAG297CAA (4296G>A), ATC298ATT (4299C>T), GAA299AAA (4300G>A), CTC302CTA (4311C>A), AAG303ACT (4313A>C 4314G>T), CTT304TTA (4315C>T 4317T>A), GGT305GGA (4320T>A), ATC306GTT (4321A>G 4323C>T), ATC307ATA (4326C>A), CCT309AGA (4330C>A 4331C>G 4332T>A), AGC310TCT (4333A>T 4334G>C 4335C>T), AAG311ACA (4337A>C 4338G>A), AGT312TCA (4339A>T 4340G>C 4341T>A), CCA313AAA (4342C>A 4343C>A), TCC315AGA (4348T>A 4349C>G 4350C>A), CCA317GCA (4354C>G), TTC319TTT (4362C>T), GTA321GTT (4368A>T), AGG322AGA (4371G>A), GCC325AGC (4378G>A 4379C>G), GAG326GAA (4383G>A), ATC327ATA (4386C>A), AAA328GTA (4387A>G 4388A>T), CGA329AGA (4390C>A), GGC330GGT (4395C>T), AAA331AAG (4398A>G), AGA333CGA (4402A>C), ATT336ATA (4413T>A), TAT338TAC (4419T>C), AAG340AGA (4424A>G 4425G>A), TTA341CTT (4426T>C 4428A>T), GAC343GAT (4434C>T), CAT344AAC (4435C>A 4437T>C), ACA345ACT (4440A>T), AAG346AGA (4442A>G 4443G>A), GGA347ACG (4444G>A 4445G>C 4446A>G), GGC349GCC (4451G>C), TAC350TAT (4455C>T), CTA351AAG (4456C>A 4457T>A 4458A>G), CTT352CTA (4461T>A), CCT353CCA (4464T>A), AAC354GAT (4465A>G 4467C>T), AAG355AAA (4470G>A), GAG356AGT (4471G>A 4472A>G 4473G>T), CAA357GAA (4474C>G), CTG358TTA (4477C>T 4479G>A), CTT359ATA (4480C>A 4482T>A), CAA360AAT (4483C>A 4485A>T), ATC362ATA (4491C>A), GGA363CAA (4492G>C 4493G>A), ACC366ACA (4503C>A), TTT367GTA (4504T>G 4506T>A), TAC368TTC (4508A>T), TCT369AAT (4510T>A 4511C>A), TCC370AAA (4513T>A 4514C>A 4515C>A), GAC372GAT (4521C>T)								

*: Inserts / Deletes / Misaligned / Frameshifts

Analysis details

This analysis was performed with panviral2.64

NGS Details (UN24): Petuvirus venapetuniae

Assembly

**Coverage Length**	403 (2 contig(s))
**Depth Of Coverage**	1.9
**Number Of Reads**	8
**Reads Per Million**	0.16 rpm (after QC)
**Ambiguities**	0
**Assembly Method**	de novo + reference guided assembly
**Consensus Caller**	Bcf Tools

Coverage Map

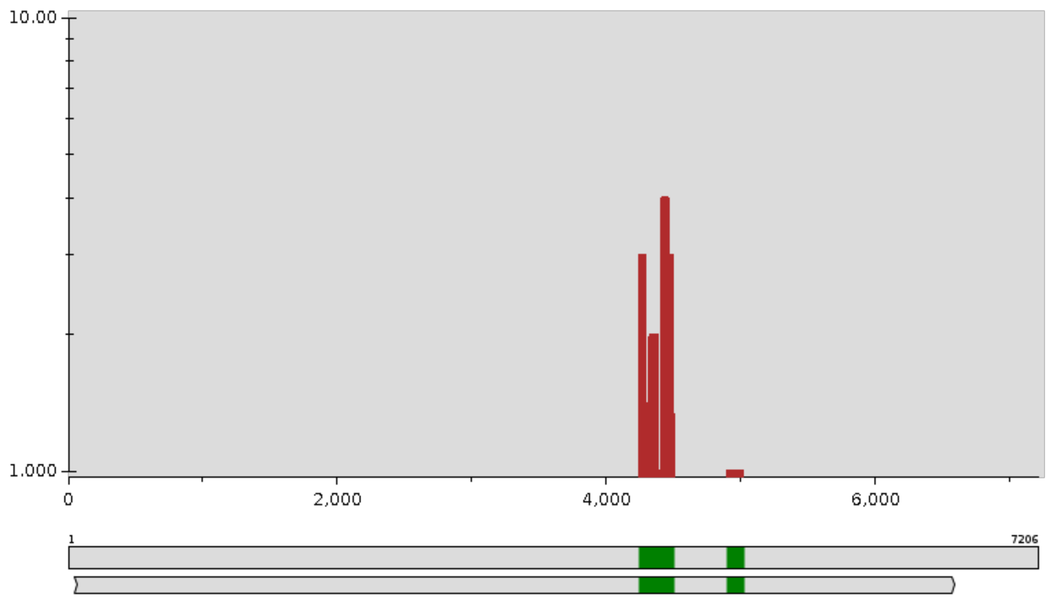

Assignment

**Type**	Petuvirus venapetuniae (Taxonomy ID: 3048265)
**Reference Genome**	NC_001839.2
**NT Identity (%)**	59.5533
**AA Identity (%)**	51.4925
**Number Of Stop Codons**	1
**Number Of CDS**	1

Alignment

**Alignment Score**	154.0 (NT) + 522.0 (AA) = 676.0
**Concordance (%)**	38.106

	Alignment Method	Global, seeded, nucleotide + amino acids (AGA)

Genome Region

Sequence starts at position 4238 and ends at position 5024 relative to NC_001839.2 reference sequence.

Alignment Detailed Statistics

	Begin	End	Coverage	Score	Concordance	Matches	Identities	I/D/M/F*	Stop Codons
**NT**	**4238**	**5024**	**5.6%**	**154**	**19.1%**	**403 (100%)**	**240 (59.6%)**	**0/0**	
Mutations:	4240A>T, 4242C>A, 4244C>G, 4245C>G, 4247G>A, 4250A>T, 4251G>T, 4252C>G, 4253A>T, 4255T>A, 4263T>A, 4264G>T, 4265T>C, 4266G>A, 4269G>T, 4270A>C, 4271G>T, 4275C>T, 4276A>T, 4278C>G, 4281T>A, 4282T>A, 4283T>C, 4284G>A, 4286T>A, 4287C>A, 4289A>T, 4293G>A, 4294A>G, 4298C>T, 4302G>A, 4304T>A, 4307A>T, 4309A>C, 4310A>T, 4314G>A, 4315C>G, 4316A>C, 4320G>T, 4321A>C, 4325T>C, 4328T>C, 4334C>T, 4337C>T, 4340G>A, 4342G>C, 4343G>T, 4344T>G, 4346A>T, 4349G>A, 4350C>A, 4354T>A, 4355G>A, 4361A>C, 4362A>T, 4363A>C, 4364A>C, 4365T>C, 4366T>C, 4367A>T, 4376C>T, 4379A>T, 4386C>A, 4388A>G, 4391A>T, 4397C>T, 4398C>T, 4399A>C, 4401T>G, 4403C>T, 4404C>T, 4406C>A, 4407C>A, 4410G>T, 4411A>G, 4412T>G, 4413G>A, 4414A>T, 4415C>T, 4417A>G, 4418G>A, 4420T>A, 4421T>C, 4424C>G, 4430A>T, 4436G>A, 4437C>A, 4439C>G, 4440A>G, 4441C>A, 4442A>T, 4445C>T, 4448C>G, 4449T>A, 4450C>A, 4451T>A, 4452C>A, 4453A>G, 4454C>A, 4455T>A, 4456T>C, 4457A>T, 4459C>T, 4460C>T, 4466A>C, 4469G>T, 4470C>A, 4472C>T, 4474T>A, 4475T>C, 4476T>A, 4477C>G, 4478A>T, 4481G>A, 4488C>A, 4490T>G, 4493G>A, 4499A>T, 4502T>C, 4894C>T, 4896G>A, 4898A>C, 4904G>A, 4905A>C, 4907A>C, 4908C>T, 4910A>G, 4916T>C, 4922G>T, 4923A>G, 4925T>G, 4926G>A, 4935A>G, 4938A>C, 4939G>A, 4952A>T, 4953G>T, 4955G>T, 4956G>A, 4958T>C, 4959A>T, 4961T>G, 4962G>A, 4965C>T, 4973C>T, 4976T>A, 4981G>T, 4982T>G, 4983G>A, 4985T>G, 4988G>T, 4992A>C, 4994G>A, 5001C>T, 5002C>A, 5003A>T, 5006G>T, 5008C>T, 5009A>T, 5015A>G, 5018A>G, 5019T>G, 5020G>A								

CDS

	Begin	End	Coverage	Score	Concordance	Matches	Identities	I/D/M/F*	Stop Codons
PVCVgp1	1397	1658	6.1%	522	52.9%	134 (100%)	69 (51.5%)	0/0/0/0	1
Protein mutations:	H1397L (4240A>T), L1398M (4242C>A 4244C>G), Q1399E (4245C>G 4247G>A), L1400F (4250A>T), A1401C (4251G>T 4252C>G 4253A>T), I1402K (4255T>A), C1405I (4263T>A 4264G>T 4265T>C), D1406N (4266G>A), E1407S (4269G>T 4270A>C 4271G>T), Q1409L (4275C>T 4276A>T), Q1410E (4278C>G), F1411N (4281T>A 4282T>A 4283T>C), D1412K (4284G>A 4286T>A), L1413I (4287C>A 4289A>T), E1415R (4293G>A 4294A>G), D1418K (4302G>A 4304T>A), Q1420P (4309A>C 4310A>T), A1422S (4314G>A 4315C>G 4316A>C), E1424S (4320G>T 4321A>C), R1431T (4342G>C 4343G>T), S1432A (4344T>G 4346A>T), Q1434K (4350C>A), V1435E (4354T>A 4355G>A), K1438S (4362A>T 4363A>C 4364A>C), L1439P (4365T>C 4366T>C 4367A>T), Q1446K (4386C>A 4388A>G), H1450S (4398C>T 4399A>C), F1451V (4401T>G 4403C>T), Q1453K (4407C>A), D1454W (4410G>T 4411A>G 4412T>G), D1455I (4413G>A 4414A>T 4415C>T), K1456R (4417A>G 4418G>A), F1457Y (4420T>A 4421T>C), L1463M (4437C>A 4439C>G), T1464D (4440A>G 4441C>A 4442A>T), F1466L (4448C>G), S1467K (4449T>A 4450C>A 4451T>A), H1468R (4452C>A 4453A>G 4454C>A), L1469T (4455T>A 4456T>C 4457A>T), S1470F (4459C>T 4460C>T), K1473N (4469G>T), L1474I (4470C>A 4472C>T), F1475Y (4474T>A 4475T>C), L1480M (4488C>A 4490T>G), S1615L (4894C>T), V1616I (4896G>A 4898A>C), I1619L (4905A>C 4907A>C), Q1620* (4908C>T 4910A>G), I1625V (4923A>G 4925T>G), V1626I (4926G>A), I1629V (4935A>G), R1630Q (4938A>C 4939G>A), E1635Y (4953G>T 4955G>T), V1636I (4956G>A 4958T>C), T1637S (4959A>T 4961T>G), E1638K (4962G>A), H1639Y (4965C>T), S1644M (4981G>T 4982T>G), D1645K (4983G>A 4985T>G), M1646I (4988G>T), K1648Q (4992A>C 4994G>A), P1651Y (5001C>T 5002C>A 5003A>T), A1653V (5008C>T 5009A>T), C1657D (5019T>G 5020G>A)								
Codon mutations:	CAT1397CTT (4240A>T), CTC1398ATG (4242C>A 4244C>G), CAG1399GAA (4245C>G 4247G>A), TTA1400TTT (4250A>T), GCA1401TGT (4251G>T 4252C>G 4253A>T), ATA1402AAA (4255T>A), TGT1405ATC (4263T>A 4264G>T 4265T>C), GAT1406AAT (4266G>A), GAG1407TCT (4269G>T 4270A>C 4271G>T), CAA1409TTA (4275C>T 4276A>T), CAA1410GAA (4278C>G), TTT1411AAC (4281T>A 4282T>A 4283T>C), GAT1412AAA (4284G>A 4286T>A), CTA1413ATT (4287C>A 4289A>T), GAA1415AGA (4293G>A 4294A>G), CCC1416CCT (4298C>T), GAT1418AAA (4302G>A 4304T>A), TCA1419TCT (4307A>T), CAA1420CCT (4309A>C 4310A>T), GCA1422AGC (4314G>A 4315C>G 4316A>C), GAA1424TCA (4320G>T 4321A>C), GCT1425GCC (4325T>C), TTT1426TTC (4328T>C), GTC1428GTT (4334C>T), AAC1429AAT (4337C>T), AAG1430AAA (4340G>A), AGG1431ACT (4342G>C 4343G>T), TCA1432GCT (4344T>G 4346A>T), GAG1433GAA (4349G>A), CAA1434AAA (4350C>A), GTG1435GAA (4354T>A 4355G>A), GGA1437GGC (4361A>C), AAA1438TCC (4362A>T 4363A>C 4364A>C), TTA1439CCT (4365T>C 4366T>C 4367A>T), GTC1442GTT (4376C>T), ATA1443ATT (4379A>T), CAA1446AAG (4386C>A 4388A>G), CCA1447CCT (4391A>T), AAC1449AAT (4397C>T), CAT1450TCT (4398C>T 4399A>C), TTC1451GTT (4401T>G 4403C>T), CTC1452TTA (4404C>T 4406C>A), CAA1453AAA (4407C>A), GAT1454TGG (4410G>T 4411A>G 4412T>G), GAC1455ATT (4413G>A 4414A>T 4415C>T), AAG1456AGA (4417A>G 4418G>A), TTT1457TAC (4420T>A 4421T>C), CCC1458CCG (4424C>G), CCA1460CCT (4430A>T), AAG1462AAA (4436G>A), CTC1463ATG (4437C>A 4439C>G), ACA1464GAT (4440A>G 4441C>A 4442A>T), CTC1465CTT (4445C>T), TTC1466TTG (4448C>G), TCT1467AAA (4449T>A 4450C>A 4451T>A), CAC1468AGA (4452C>A 4453A>G 4454C>A), TTA1469ACT (4455T>A 4456T>C 4457A>T), TCC1470TTT (4459C>T 4460C>T), GCA1472GCC (4466A>C), AAG1473AAT (4469G>T), CTC1474ATT (4470C>A 4472C>T), TTT1475TAC (4474T>A 4475T>C), TCA1476AGT (4476T>A 4477C>G 4478A>T), AAG1477AAA (4481G>A), CTT1480ATG (4488C>A 4490T>G), AAG1481AAA (4493G>A), GGA1483GGT (4499A>T), TTT1484TTC (4502T>C), TCG1615TTG (4894C>T), GTA1616ATC (4896G>A 4898A>C), CAG1618CAA (4904G>A), ATA1619CTC (4905A>C 4907A>C), CAA1620TAG (4908C>T 4910A>G), TTT1622TTC (4916T>C), GGG1624GGT (4922G>T), ATT1625GTG (4923A>G 4925T>G), GTT1626ATT (4926G>A), ATC1629GTC (4935A>G), AGA1630CAA (4938A>C 4939G>A), CCA1634CCT (4952A>T), GAG1635TAT (4953G>T 4955G>T), GTT1636ATC (4956G>A 4958T>C), ACT1637TCG (4959A>T 4961T>G), GAG1638AAG (4962G>A), CAT1639TAT (4965C>T), TCC1641TCT (4973C>T), CCT1642CCA (4976T>A), AGT1644ATG (4981G>T 4982T>G), GAT1645AAG (4983G>A 4985T>G), ATG1646ATT (4988G>T), AAG1648CAA (4992A>C 4994G>A), CCA1651TAT (5001C>T 5002C>A 5003A>T), CCG1652CCT (5006G>T), GCA1653GTT (5008C>T 5009A>T), GGA1655GGG (5015A>G), AAA1656AAG (5018A>G), TGT1657GAT (5019T>G 5020G>A)								

Proteins

	Begin	End	Coverage	Score	Concordance	Matches	Identities	I/D/M/F*	Stop Codons
ORF I polyprotein (NP_127504.1)	1397	1658	6.1%	522	52.9%	134 (100%)	69 (51.5%)	0/0/0/0	1
Protein mutations:	H1397L (4240A>T), L1398M (4242C>A 4244C>G), Q1399E (4245C>G 4247G>A), L1400F (4250A>T), A1401C (4251G>T 4252C>G 4253A>T), I1402K (4255T>A), C1405I (4263T>A 4264G>T 4265T>C), D1406N (4266G>A), E1407S (4269G>T 4270A>C 4271G>T), Q1409L (4275C>T 4276A>T), Q1410E (4278C>G), F1411N (4281T>A 4282T>A 4283T>C), D1412K (4284G>A 4286T>A), L1413I (4287C>A 4289A>T), E1415R (4293G>A 4294A>G), D1418K (4302G>A 4304T>A), Q1420P (4309A>C 4310A>T), A1422S (4314G>A 4315C>G 4316A>C), E1424S (4320G>T 4321A>C), R1431T (4342G>C 4343G>T), S1432A (4344T>G 4346A>T), Q1434K (4350C>A), V1435E (4354T>A 4355G>A), K1438S (4362A>T 4363A>C 4364A>C), L1439P (4365T>C 4366T>C 4367A>T), Q1446K (4386C>A 4388A>G), H1450S (4398C>T 4399A>C), F1451V (4401T>G 4403C>T), Q1453K (4407C>A), D1454W (4410G>T 4411A>G 4412T>G), D1455I (4413G>A 4414A>T 4415C>T), K1456R (4417A>G 4418G>A), F1457Y (4420T>A 4421T>C), L1463M (4437C>A 4439C>G), T1464D (4440A>G 4441C>A 4442A>T), F1466L (4448C>G), S1467K (4449T>A 4450C>A 4451T>A), H1468R (4452C>A 4453A>G 4454C>A), L1469T (4455T>A 4456T>C 4457A>T), S1470F (4459C>T 4460C>T), K1473N (4469G>T), L1474I (4470C>A 4472C>T), F1475Y (4474T>A 4475T>C), L1480M (4488C>A 4490T>G), S1615L (4894C>T), V1616I (4896G>A 4898A>C), I1619L (4905A>C 4907A>C), Q1620* (4908C>T 4910A>G), I1625V (4923A>G 4925T>G), V1626I (4926G>A), I1629V (4935A>G), R1630Q (4938A>C 4939G>A), E1635Y (4953G>T 4955G>T), V1636I (4956G>A 4958T>C), T1637S (4959A>T 4961T>G), E1638K (4962G>A), H1639Y (4965C>T), S1644M (4981G>T 4982T>G), D1645K (4983G>A 4985T>G), M1646I (4988G>T), K1648Q (4992A>C 4994G>A), P1651Y (5001C>T 5002C>A 5003A>T), A1653V (5008C>T 5009A>T), C1657D (5019T>G 5020G>A)								
Codon mutations:	CAT1397CTT (4240A>T), CTC1398ATG (4242C>A 4244C>G), CAG1399GAA (4245C>G 4247G>A), TTA1400TTT (4250A>T), GCA1401TGT (4251G>T 4252C>G 4253A>T), ATA1402AAA (4255T>A), TGT1405ATC (4263T>A 4264G>T 4265T>C), GAT1406AAT (4266G>A), GAG1407TCT (4269G>T 4270A>C 4271G>T), CAA1409TTA (4275C>T 4276A>T), CAA1410GAA (4278C>G), TTT1411AAC (4281T>A 4282T>A 4283T>C), GAT1412AAA (4284G>A 4286T>A), CTA1413ATT (4287C>A 4289A>T), GAA1415AGA (4293G>A 4294A>G), CCC1416CCT (4298C>T), GAT1418AAA (4302G>A 4304T>A), TCA1419TCT (4307A>T), CAA1420CCT (4309A>C 4310A>T), GCA1422AGC (4314G>A 4315C>G 4316A>C), GAA1424TCA (4320G>T 4321A>C), GCT1425GCC (4325T>C), TTT1426TTC (4328T>C), GTC1428GTT (4334C>T), AAC1429AAT (4337C>T), AAG1430AAA (4340G>A), AGG1431ACT (4342G>C 4343G>T), TCA1432GCT (4344T>G 4346A>T), GAG1433GAA (4349G>A), CAA1434AAA (4350C>A), GTG1435GAA (4354T>A 4355G>A), GGA1437GGC (4361A>C), AAA1438TCC (4362A>T 4363A>C 4364A>C), TTA1439CCT (4365T>C 4366T>C 4367A>T), GTC1442GTT (4376C>T), ATA1443ATT (4379A>T), CAA1446AAG (4386C>A 4388A>G), CCA1447CCT (4391A>T), AAC1449AAT (4397C>T), CAT1450TCT (4398C>T 4399A>C), TTC1451GTT (4401T>G 4403C>T), CTC1452TTA (4404C>T 4406C>A), CAA1453AAA (4407C>A), GAT1454TGG (4410G>T 4411A>G 4412T>G), GAC1455ATT (4413G>A 4414A>T 4415C>T), AAG1456AGA (4417A>G 4418G>A), TTT1457TAC (4420T>A 4421T>C), CCC1458CCG (4424C>G), CCA1460CCT (4430A>T), AAG1462AAA (4436G>A), CTC1463ATG (4437C>A 4439C>G), ACA1464GAT (4440A>G 4441C>A 4442A>T), CTC1465CTT (4445C>T), TTC1466TTG (4448C>G), TCT1467AAA (4449T>A 4450C>A 4451T>A), CAC1468AGA (4452C>A 4453A>G 4454C>A), TTA1469ACT (4455T>A 4456T>C 4457A>T), TCC1470TTT (4459C>T 4460C>T), GCA1472GCC (4466A>C), AAG1473AAT (4469G>T), CTC1474ATT (4470C>A 4472C>T), TTT1475TAC (4474T>A 4475T>C), TCA1476AGT (4476T>A 4477C>G 4478A>T), AAG1477AAA (4481G>A), CTT1480ATG (4488C>A 4490T>G), AAG1481AAA (4493G>A), GGA1483GGT (4499A>T), TTT1484TTC (4502T>C), TCG1615TTG (4894C>T), GTA1616ATC (4896G>A 4898A>C), CAG1618CAA (4904G>A), ATA1619CTC (4905A>C 4907A>C), CAA1620TAG (4908C>T 4910A>G), TTT1622TTC (4916T>C), GGG1624GGT (4922G>T), ATT1625GTG (4923A>G 4925T>G), GTT1626ATT (4926G>A), ATC1629GTC (4935A>G), AGA1630CAA (4938A>C 4939G>A), CCA1634CCT (4952A>T), GAG1635TAT (4953G>T 4955G>T), GTT1636ATC (4956G>A 4958T>C), ACT1637TCG (4959A>T 4961T>G), GAG1638AAG (4962G>A), CAT1639TAT (4965C>T), TCC1641TCT (4973C>T), CCT1642CCA (4976T>A), AGT1644ATG (4981G>T 4982T>G), GAT1645AAG (4983G>A 4985T>G), ATG1646ATT (4988G>T), AAG1648CAA (4992A>C 4994G>A), CCA1651TAT (5001C>T 5002C>A 5003A>T), CCG1652CCT (5006G>T), GCA1653GTT (5008C>T 5009A>T), GGA1655GGG (5015A>G), AAA1656AAG (5018A>G), TGT1657GAT (5019T>G 5020G>A)								

*: Inserts / Deletes / Misaligned / Frameshifts

Analysis details

This analysis was performed with panviral2.64

NGS Details (UN24): Epiphyllum badnavirus 1

Assembly

**Coverage Length**	261 (1 contig(s))
**Depth Of Coverage**	3.9
**Number Of Reads**	8
**Reads Per Million**	0.16 rpm (after QC)
**Ambiguities**	0
**Assembly Method**	de novo + reference guided assembly
**Consensus Caller**	Bcf Tools

Coverage Map

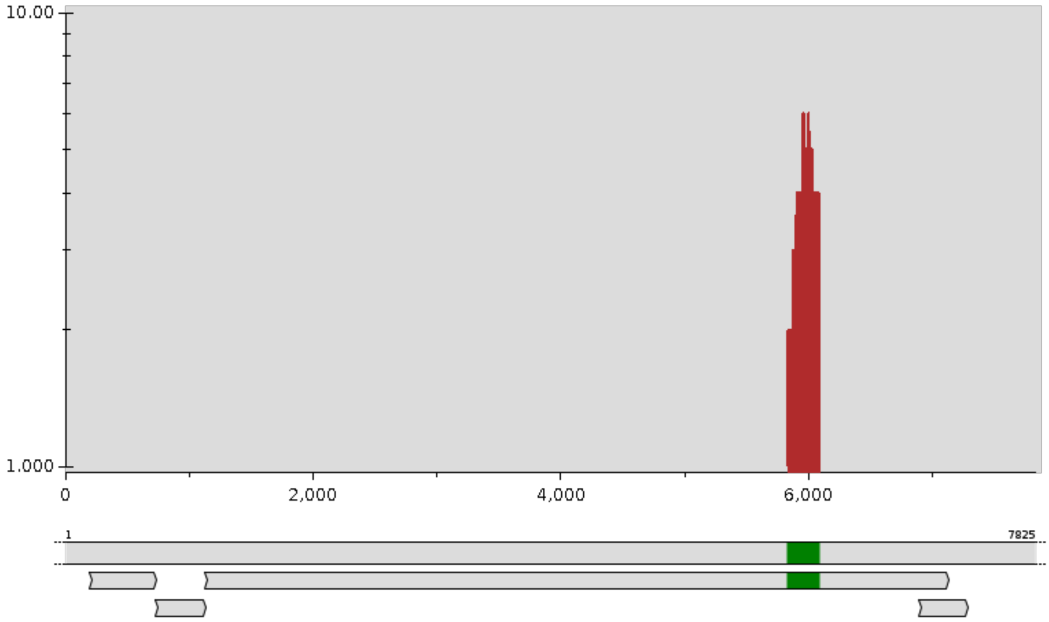

Assignment

**Type**	Epiphyllum badnavirus 1 (Taxonomy ID: 2518008)
**Reference Genome**	NC_076247.1
**NT Identity (%)**	53.7879
**AA Identity (%)**	46.5909
**Number Of Stop Codons**	1
**Number Of CDS**	4

Alignment

**Alignment Score**	34.0 (NT) + 271.0 (AA) = 305.0
**Concordance (%)**	26.3158

**Alignment Method**	Global, seeded, nucleotide + amino acids (AGA)

Genome Region

Sequence starts at position 5831 and ends at position 6091 relative to NC_076247.1 reference sequence.

Alignment Detailed Statistics

	Begin	End	Coverage	Score	Concordance	Matches	Identities	I/D/M/F*	Stop Codons
**NT**	**5831**	**6091**	**3.3%**	**34**	**6.5%**	**261 (98.9%)**	**142 (53.8%)**	**3/0**	
Mutations:	5832T>G, 5835C>T, 5839G>C, 5840T>A, 5850G>A, 5851T>A, 5853C>T, 5859G>T, 5860A>T, 5863A>T, 5864G>C, 5865C>T, 5868A>C, 5870T>A, 5871C>T, 5874C>T, 5876A>T, 5878A>T, 5880T>G, 5882T>G, 5884A>G, 5887G>A, 5888A>G, 5889C>G, 5892A>G, 5893G>A, 5894A>G, 5895A>T, 5896T>G, 5897C>A, 5898C>T, 5901C>T, 5904T>A, 5905T>A, 5906G>A, 5910C>A, 5912C>T, 5913C>A, 5916C>T, 5918T>A, 5922C>G, 5923C>A, 5924C>G, 5926G>T, 5931A>C, 5933T>A, 5936A>T, 5937C>T, 5940A>G, 5942G>T, 5943G>C, 5944T>C, 5947G>A, 5949T>C, 5953C>T, 5955A>C, 5958C>T, 5960G>A, 5961A>G, 5964G>A, 5966A>C, 5967G>C, 5973C>T, 5976T>G, 5979T>A, 5982G>T, 5986C>A, 5987A>T, 5989C>G, 5990G>A, 5991A>C, 5992A>C, 5993A>T, 5994G>T, 5998G>A, 6000C>T, 6002A>G, 6003C>G, 6004T>G, 6005G>T, 6006T>G, 6011G>A, 6012A>T, 6012_6013insTTC, 6013G>T, 6015C>T, 6018G>C, 6019T>A, 6020C>A, 6021A>T, 6023G>C, 6024C>A, 6028G>A, 6030A>G, 6032C>T, 6033A>G, 6038A>T, 6042T>A, 6046G>T, 6051C>T, 6052C>T, 6055G>A, 6059T>A, 6061A>T, 6062G>C, 6063T>C, 6064A>C, 6065A>G, 6066C>T, 6068C>G, 6072A>G, 6075T>A, 6078C>A, 6082G>A, 6083C>T, 6084A>G, 6085C>A, 6086A>G, 6087G>T, 6091C>T								
**CDS**									
QKM20_gp3	1569	1655	4.3%	271	41.8%	87 (98.9%)	41 (46.6%)	1/0/0/0	1
Protein mutations:	V1571H (5839G>C 5840T>A), F1575I (5851T>A 5853C>T), K1578* (5860A>T), F1581Y (5870T>A 5871C>T), Q1583L (5876A>T), I1584L (5878A>T 5880T>G), M1585R (5882T>G), M1586V (5884A>G), D1587R (5887G>A 5888A>G 5889C>G), E1589S (5893G>A 5894A>G 5895A>T), S1590D (5896T>G 5897C>A 5898C>T), W1593K (5905T>A 5906G>A), A1595V (5912C>T 5913C>A), L1597Q (5918T>A), P1599R (5923C>A 5924C>G), D1600Y (5926G>T), L1602H (5933T>A), Y1603F (5936A>T 5937C>T), W1605F (5942G>T 5943G>C), V1607I (5947G>A 5949T>C), P1609S (5953C>T 5955A>C), G1611E (5960G>A 5961A>G), K1613T (5966A>C 5967G>C), Q1620I (5986C>A 5987A>T), R1621D (5989C>G 5990G>A 5991A>C), K1622L (5992A>C 5993A>T 5994G>T), D1624N (5998G>A 6000C>T), N1625R (6002A>G 6003C>G), C1626V (6004T>G 6005G>T 6006T>G), R1628N (6011G>A 6012A>T), R1628_D1629insF (6012_6013insTTC), D1629Y (6013G>T 6015C>T), S1631N (6019T>A 6020C>A 6021A>T), G1632A (6023G>C 6024C>A), V1634M (6028G>A 6030A>G), A1635V (6032C>T 6033A>G), Y1637F (6038A>T), D1640Y (6046G>T), V1643I (6055G>A), F1644Y (6059T>A), N1646R (6064A>C 6065A>G 6066C>T), T1647S (6068C>G), D1649E (6075T>A), D1650E (6078C>A), A1652M (6082G>A 6083C>T 6084A>G), Q1653S (6085C>A 6086A>G 6087G>T)								
Codon mutations:	AAT1568.AG (5832T>G), GCC1569GCT (5835C>T), GTC1571CAC (5839G>C 5840T>A), AAG1574AAA (5850G>A), TTC1575ATT (5851T>A 5853C>T), CTG1577CTT (5859G>T), AAG1578TAG (5860A>T), AGC1579TCT (5863A>T 5864G>C 5865C>T), GGA1580GGC (5868A>C), TTC1581TAT (5870T>A 5871C>T), CAC1582CAT (5874C>T), CAG1583CTG (5876A>T), ATT1584TTG (5878A>T 5880T>G), ATG1585AGG (5882T>G), ATG1586GTG (5884A>G), GAC1587AGG (5887G>A 5888A>G 5889C>G), GAA1588GAG (5892A>G), GAA1589AGT (5893G>A 5894A>G 5895A>T), TCC1590GAT (5896T>G 5897C>A 5898C>T), ATC1591ATT (5901C>T), CCT1592CCA (5904T>A), TGG1593AAG (5905T>A 5906G>A), ACC1594ACA (5910C>A), GCC1595GTA (5912C>T 5913C>A), TTC1596TTT (5916C>T), CTG1597CAG (5918T>A), ACC1598ACG (5922C>G), CCA1599AGA (5923C>A 5924C>G), GAT1600TAT (5926G>T), GGA1601GGC (5931A>C), CTT1602CAT (5933T>A), TAC1603TTT (5936A>T 5937C>T), GAA1604GAG (5940A>G), TGG1605TTC (5942G>T 5943G>C), TTA1606CTA (5944T>C), GTT1607ATC (5947G>A 5949T>C), CCA1609TCC (5953C>T 5955A>C), TTC1610TTT (5958C>T), GGA1611GAG (5960G>A 5961A>G), TTG1612TTA (5964G>A), AAG1613ACC (5966A>C 5967G>C), GCC1615GCT (5973C>T), CCT1616CCG (5976T>G), GCT1617GCA (5979T>A), GTG1618GTT (5982G>T), CAA1620ATA (5986C>A 5987A>T), CGA1621GAC (5989C>G 5990G>A 5991A>C), AAG1622CTT (5992A>C 5993A>T 5994G>T), GAC1624AAT (5998G>A 6000C>T), AAC1625AGG (6002A>G 6003C>G), TGT1626GTG (6004T>G 6005G>T 6006T>G), AGA1628AAT (6011G>A 6012A>T), AGA1628_GAC1629insTTC (6012_6013insTTC), GAC1629TAT (6013G>T 6015C>T), CTG1630CTC (6018G>C), TCA1631AAT (6019T>A 6020C>A 6021A>T), GGC1632GCA (6023G>C 6024C>A), GTA1634ATG (6028G>A 6030A>G), GCA1635GTG (6032C>T 6033A>G), TAC1637TTC (6038A>T), ATT1638ATA (6042T>A), GAC1640TAC (6046G>T), ATC1641ATT (6051C>T), CTG1642TTG (6052C>T), GTC1643ATC (6055G>A), TTC1644TAC (6059T>A), AGT1645TCC (6061A>T 6062G>C 6063T>C), AAC1646CGT (6064A>C 6065A>G 6066C>T), ACT1647AGT (6068C>G), GAA1648GAG (6072A>G), GAT1649GAA (6075T>A), GAC1650GAA (6078C>A), GCA1652ATG (6082G>A 6083C>T 6084A>G), CAG1653AGT (6085C>A 6086A>G 6087G>T), CTG1655T.. (6091C>T)								
**Proteins**									
polyprotein (YP_010797894.1)	1569	1655	4.3%	271	41.8%	87 (98.9%)	41 (46.6%)	1/0/0/0	1
Protein mutations:	V1571H (5839G>C 5840T>A), F1575I (5851T>A 5853C>T), K1578* (5860A>T), F1581Y (5870T>A 5871C>T), Q1583L (5876A>T), I1584L (5878A>T 5880T>G), M1585R (5882T>G), M1586V (5884A>G), D1587R (5887G>A 5888A>G 5889C>G), E1589S (5893G>A 5894A>G 5895A>T), S1590D (5896T>G 5897C>A 5898C>T), W1593K (5905T>A 5906G>A), A1595V (5912C>T 5913C>A), L1597Q (5918T>A), P1599R (5923C>A 5924C>G), D1600Y (5926G>T), L1602H (5933T>A), Y1603F (5936A>T 5937C>T), W1605F (5942G>T 5943G>C), V1607I (5947G>A 5949T>C), P1609S (5953C>T 5955A>C), G1611E (5960G>A 5961A>G), K1613T (5966A>C 5967G>C), Q1620I (5986C>A 5987A>T), R1621D (5989C>G 5990G>A 5991A>C), K1622L (5992A>C 5993A>T 5994G>T), D1624N (5998G>A 6000C>T), N1625R (6002A>G 6003C>G), C1626V (6004T>G 6005G>T 6006T>G), R1628N (6011G>A 6012A>T), R1628_D1629insF (6012_6013insTTC), D1629Y (6013G>T 6015C>T), S1631N (6019T>A 6020C>A 6021A>T), G1632A (6023G>C 6024C>A), V1634M (6028G>A 6030A>G), A1635V (6032C>T 6033A>G), Y1637F (6038A>T), D1640Y (6046G>T), V1643I (6055G>A), F1644Y (6059T>A), N1646R (6064A>C 6065A>G 6066C>T), T1647S (6068C>G), D1649E (6075T>A), D1650E (6078C>A), A1652M (6082G>A 6083C>T 6084A>G), Q1653S (6085C>A 6086A>G 6087G>T)								
Codon mutations:	AAT1568.AG (5832T>G), GCC1569GCT (5835C>T), GTC1571CAC (5839G>C 5840T>A), AAG1574AAA (5850G>A), TTC1575ATT (5851T>A 5853C>T), CTG1577CTT (5859G>T), AAG1578TAG (5860A>T), AGC1579TCT (5863A>T 5864G>C 5865C>T), GGA1580GGC (5868A>C), TTC1581TAT (5870T>A 5871C>T), CAC1582CAT (5874C>T), CAG1583CTG (5876A>T), ATT1584TTG (5878A>T 5880T>G), ATG1585AGG (5882T>G), ATG1586GTG (5884A>G), GAC1587AGG (5887G>A 5888A>G 5889C>G), GAA1588GAG (5892A>G), GAA1589AGT (5893G>A 5894A>G 5895A>T), TCC1590GAT (5896T>G 5897C>A 5898C>T), ATC1591ATT (5901C>T), CCT1592CCA (5904T>A), TGG1593AAG (5905T>A 5906G>A), ACC1594ACA (5910C>A), GCC1595GTA (5912C>T 5913C>A), TTC1596TTT (5916C>T), CTG1597CAG (5918T>A), ACC1598ACG (5922C>G), CCA1599AGA (5923C>A 5924C>G), GAT1600TAT (5926G>T), GGA1601GGC (5931A>C), CTT1602CAT (5933T>A), TAC1603TTT (5936A>T 5937C>T), GAA1604GAG (5940A>G), TGG1605TTC (5942G>T 5943G>C), TTA1606CTA (5944T>C), GTT1607ATC (5947G>A 5949T>C), CCA1609TCC (5953C>T 5955A>C), TTC1610TTT (5958C>T), GGA1611GAG (5960G>A 5961A>G), TTG1612TTA (5964G>A), AAG1613ACC (5966A>C 5967G>C), GCC1615GCT (5973C>T), CCT1616CCG (5976T>G), GCT1617GCA (5979T>A), GTG1618GTT (5982G>T), CAA1620ATA (5986C>A 5987A>T), CGA1621GAC (5989C>G 5990G>A 5991A>C), AAG1622CTT (5992A>C 5993A>T 5994G>T), GAC1624AAT (5998G>A 6000C>T), AAC1625AGG (6002A>G 6003C>G), TGT1626GTG (6004T>G 6005G>T 6006T>G), AGA1628AAT (6011G>A 6012A>T), AGA1628_GAC1629insTTC (6012_6013insTTC), GAC1629TAT (6013G>T 6015C>T), CTG1630CTC (6018G>C), TCA1631AAT (6019T>A 6020C>A 6021A>T), GGC1632GCA (6023G>C 6024C>A), GTA1634ATG (6028G>A 6030A>G), GCA1635GTG (6032C>T 6033A>G), TAC1637TTC (6038A>T), ATT1638ATA (6042T>A), GAC1640TAC (6046G>T), ATC1641ATT (6051C>T), CTG1642TTG (6052C>T), GTC1643ATC (6055G>A), TTC1644TAC (6059T>A), AGT1645TCC (6061A>T 6062G>C 6063T>C), AAC1646CGT (6064A>C 6065A>G 6066C>T), ACT1647AGT (6068C>G), GAA1648GAG (6072A>G), GAT1649GAA (6075T>A), GAC1650GAA (6078C>A), GCA1652ATG (6082G>A 6083C>T 6084A>G), CAG1653AGT (6085C>A 6086A>G 6087G>T), CTG1655T.. (6091C>T)								

*: Inserts / Deletes / Misaligned / Frameshifts

Analysis details

This analysis was performed with panviral2.64

NGS Details (UN24): Chinaberry tree badnavirus 1

Assembly

**Coverage Length**	261 (1 contig(s))
**Depth Of Coverage**	2.7
**Number Of Reads**	7
**Reads Per Million**	0.14 rpm (after QC)
**Ambiguities**	0
**Assembly Method**	de novo + reference guided assembly
**Consensus Caller**	Bcf Tools

Coverage Map

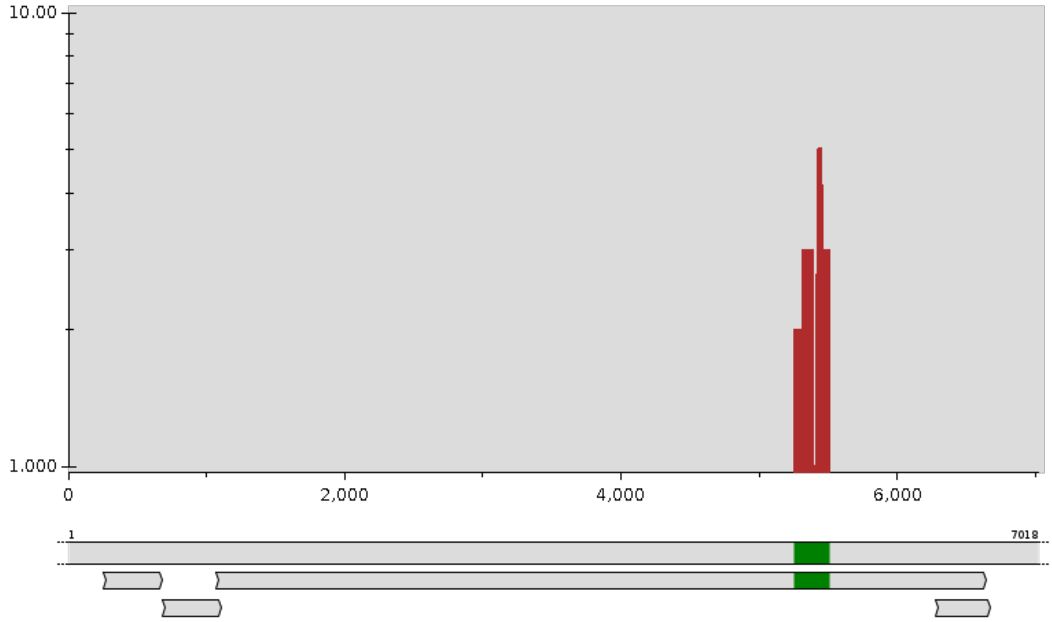

Assignment

**Type**	Chinaberry tree badnavirus 1 (Taxonomy ID: 2908099)
**Reference Genome**	NC_077165.1
**NT Identity (%)**	59.8485
**AA Identity (%)**	47.7273
**Number Of Stop Codons**	1
**Number Of CDS**	4

Alignment

**Alignment Score**	98.0 (NT) + 310.0 (AA) = 408.0
**Concordance (%)**	34.9914

**Alignment Method**	Global, seeded, nucleotide + amino acids (AGA)

Genome Region

Sequence starts at position 5253 and ends at position 5513 relative to NC_077165.1 reference sequence.

Alignment Detailed Statistics

	Begin	End	Coverage	Score	Concordance	Matches	Identities	I/D/M/F*	Stop Codons
**NT**	**5253**	**5513**	**3.7%**	**98**	**18.8%**	**261 (98.9%)**	**158 (59.8%)**	**3/0**	
Mutations:	5255G>A, 5256T>A, 5264G>T, 5265A>T, 5266A>G, 5270G>C, 5273C>T, 5275T>A, 5282A>G, 5283G>T, 5285A>C, 5286G>A, 5287C>G, 5288C>G, 5289A>G, 5292G>A, 5293A>G, 5294C>G, 5295C>G, 5296C>A, 5298G>T, 5299A>G, 5300G>C, 5301T>A, 5302C>A, 5304G>A, 5306A>T, 5307G>C, 5308A>T, 5309A>C, 5310T>A, 5311G>A, 5316G>A, 5325G>A, 5326T>C, 5327T>A, 5328C>A, 5329C>G, 5331G>T, 5336G>C, 5338T>A, 5341A>T, 5347G>T, 5348G>T, 5349C>T, 5357T>G, 5358C>T, 5360A>G, 5363C>T, 5369A>G, 5371A>C, 5374A>C, 5380C>T, 5381A>C, 5387A>C, 5390C>T, 5391C>A, 5392A>T, 5393A>G, 5395G>A, 5396A>C, 5397A>T, 5398A>T, 5403G>A, 5405C>T, 5407A>G, 5409T>A, 5410G>T, 5411C>T, 5417G>A, 5418G>T, 5419G>C, 5420_5421insTAT, 5421A>C, 5422C>T, 5424G>T, 5426G>T, 5428A>T, 5429G>A, 5432C>T, 5433A>G, 5435T>G, 5437C>T, 5443A>T, 5444C>T, 5447A>T, 5453C>T, 5456C>A, 5460G>A, 5462C>T, 5464T>A, 5469A>C, 5471G>A, 5474C>T, 5480G>A, 5487G>A, 5488C>T, 5489A>G, 5492C>T, 5496G>T, 5498A>G, 5503A>T, 5504A>T, 5511G>C								

CDS

	Begin	End	Coverage	Score	Concordance	Matches	Identities	I/D/M/F*	Stop Codons
QKV46_gp3	1395	1481	4.7%	310	48.1%	87 (98.9%)	42 (47.7%)	1/0/0/0	1
Protein mutations:	F1396I (5256T>A), K1399* (5265A>T 5266A>G), F1402Y (5275T>A), V1405F (5283G>T 5285A>C), A1406R (5286G>A 5287C>G 5288C>G), M1407V (5289A>G), D1408R (5292G>A 5293A>G 5294C>G), P1409E (5295C>G 5296C>A), E1410C (5298G>T 5299A>G 5300G>C), S1411N (5301T>A 5302C>A), V1412I (5304G>A 5306A>T), E1413L (5307G>C 5308A>T 5309A>C), W1414K (5310T>A 5311G>A), A1416T (5316G>A), V1419T (5325G>A 5326T>C 5327T>A), P1420R (5328C>A 5329C>G), D1421Y (5331G>T), L1423H (5338T>A), Y1424F (5341A>T), W1426F (5347G>T 5348G>T), I1429M (5357T>G), P1430S (5358C>T 5360A>G), K1434T (5371A>C), N1435T (5374A>C), P1437L (5380C>T 5381A>C), Q1441M (5391C>A 5392A>T 5393A>G), R1442N (5395G>A 5396A>C), K1443L (5397A>T 5398A>T), D1445N (5403G>A 5405C>T), Q1446R (5407A>G), C1447I (5409T>A 5410G>T 5411C>T), G1450S (5418G>T 5419G>C), G1450_T1451insY (5420_5421insTAT), T1451L (5421A>C 5422C>T), E1452Y (5424G>T 5426G>T), K1453I (5428A>T 5429G>A), I1455V (5433A>G 5435T>G), A1456V (5437C>T), Y1458F (5443A>T 5444C>T), V1464I (5460G>A 5462C>T), F1465Y (5464T>A), K1467Q (5469A>C 5471G>A), A1473M (5487G>A 5488C>T 5489A>G), V1476L (5496G>T 5498A>G), K1478I (5503A>T 5504A>T), E1481Q (5511G>C)								
Codon mutations:	AAG1395AAA (5255G>A), TTT1396ATT (5256T>A), CTG1398CTT (5264G>T), AAA1399TGA (5265A>T 5266A>G), TCG1400TCC (5270G>C), GGC1401GGT (5273C>T), TTT1402TAT (5275T>A), CAA1404CAG (5282A>G), GTA1405TTC (5283G>T 5285A>C), GCC1406AGG (5286G>A 5287C>G 5288C>G), ATG1407GTG (5289A>G), GAC1408AGG (5292G>A 5293A>G 5294C>G), CCG1409GAG (5295C>G 5296C>A), GAG1410TGC (5298G>T 5299A>G 5300G>C), TCT1411AAT (5301T>A 5302C>A), GTA1412ATT (5304G>A 5306A>T), GAA1413CTC (5307G>C 5308A>T 5309A>C), TGG1414AAG (5310T>A 5311G>A), GCA1416ACA (5316G>A), GTT1419ACA (5325G>A 5326T>C 5327T>A), CCA1420AGA (5328C>A 5329C>G), GAT1421TAT (5331G>T), GGG1422GGC (5336G>C), CTT1423CAT (5338T>A), TAT1424TTT (5341A>T), TGG1426TTT (5347G>T 5348G>T), CTG1427TTG (5349C>T), ATT1429ATG (5357T>G), CCA1430TCG (5358C>T 5360A>G), TTC1431TTT (5363C>T), TTA1433TTG (5369A>G), AAA1434ACA (5371A>C), AAT1435ACT (5374A>C), CCA1437CTC (5380C>T 5381A>C), ATA1439ATC (5387A>C), TTC1440TTT (5390C>T), CAA1441ATG (5391C>A 5392A>T 5393A>G), AGA1442AAC (5395G>A 5396A>C), AAG1443TTG (5397A>T 5398A>T), GAC1445AAT (5403G>A 5405C>T), CAA1446CGA (5407A>G), TGC1447ATT (5409T>A 5410G>T 5411C>T), AAG1449AAA (5417G>A), GGA1450TCA (5418G>T 5419G>C), GGA1450_ACA1451insTAT (5420_5421insTAT), ACA1451CTA (5421A>C 5422C>T), GAG1452TAT (5424G>T 5426G>T), AAG1453ATA (5428A>T 5429G>A), TTC1454TTT (5432C>T), ATT1455GTG (5433A>G 5435T>G), GCG1456GTG (5437C>T), TAC1458TTT (5443A>T 5444C>T), ATA1459ATT (5447A>T), GAC1461GAT (5453C>T), ATC1462ATA (5456C>A), GTC1464ATT (5460G>A 5462C>T), TTT1465TAT (5464T>A), AAG1467CAA (5469A>C 5471G>A), AAC1468AAT (5474C>T), GAG1470GAA (5480G>A), GCA1473ATG (5487G>A 5488C>T 5489A>G), GGC1474GGT (5492C>T), GTA1476TTG (5496G>T 5498A>G), AAA1478ATT (5503A>T 5504A>T), GAA1481CAA (5511G>C)								

Proteins

	Begin	End	Coverage	Score	Concordance	Matches	Identities	I/D/M/F*	Stop Codons
ORF3 (YP_010805662.1)	1395	1481	4.7%	310	48.1%	87 (98.9%)	42 (47.7%)	1/0/0/0	1
Protein mutations:	F1396I (5256T>A), K1399* (5265A>T 5266A>G), F1402Y (5275T>A), V1405F (5283G>T 5285A>C), A1406R (5286G>A 5287C>G 5288C>G), M1407V (5289A>G), D1408R (5292G>A 5293A>G 5294C>G), P1409E (5295C>G 5296C>A), E1410C (5298G>T 5299A>G 5300G>C), S1411N (5301T>A 5302C>A), V1412I (5304G>A 5306A>T), E1413L (5307G>C 5308A>T 5309A>C), W1414K (5310T>A 5311G>A), A1416T (5316G>A), V1419T (5325G>A 5326T>C 5327T>A), P1420R (5328C>A 5329C>G), D1421Y (5331G>T), L1423H (5338T>A), Y1424F (5341A>T), W1426F (5347G>T 5348G>T), I1429M (5357T>G), P1430S (5358C>T 5360A>G), K1434T (5371A>C), N1435T (5374A>C), P1437L (5380C>T 5381A>C), Q1441M (5391C>A 5392A>T 5393A>G), R1442N (5395G>A 5396A>C), K1443L (5397A>T 5398A>T), D1445N (5403G>A 5405C>T), Q1446R (5407A>G), C1447I (5409T>A 5410G>T 5411C>T), G1450S (5418G>T 5419G>C), G1450_T1451insY (5420_5421insTAT), T1451L (5421A>C 5422C>T), E1452Y (5424G>T 5426G>T), K1453I (5428A>T 5429G>A), I1455V (5433A>G 5435T>G), A1456V (5437C>T), Y1458F (5443A>T 5444C>T), V1464I (5460G>A 5462C>T), F1465Y (5464T>A), K1467Q (5469A>C 5471G>A), A1473M (5487G>A 5488C>T 5489A>G), V1476L (5496G>T 5498A>G), K1478I (5503A>T 5504A>T), E1481Q (5511G>C)								
Codon mutations:	AAG1395AAA (5255G>A), TTT1396ATT (5256T>A), CTG1398CTT (5264G>T), AAA1399TGA (5265A>T 5266A>G), TCG1400TCC (5270G>C), GGC1401GGT (5273C>T), TTT1402TAT (5275T>A), CAA1404CAG (5282A>G), GTA1405TTC (5283G>T 5285A>C), GCC1406AGG (5286G>A 5287C>G 5288C>G), ATG1407GTG (5289A>G), GAC1408AGG (5292G>A 5293A>G 5294C>G), CCG1409GAG (5295C>G 5296C>A), GAG1410TGC (5298G>T 5299A>G 5300G>C), TCT1411AAT (5301T>A 5302C>A), GTA1412ATT (5304G>A 5306A>T), GAA1413CTC (5307G>C 5308A>T 5309A>C), TGG1414AAG (5310T>A 5311G>A), GCA1416ACA (5316G>A), GTT1419ACA (5325G>A 5326T>C 5327T>A), CCA1420AGA (5328C>A 5329C>G), GAT1421TAT (5331G>T), GGG1422GGC (5336G>C), CTT1423CAT (5338T>A), TAT1424TTT (5341A>T), TGG1426TTT (5347G>T 5348G>T), CTG1427TTG (5349C>T), ATT1429ATG (5357T>G), CCA1430TCG (5358C>T 5360A>G), TTC1431TTT (5363C>T), TTA1433TTG (5369A>G), AAA1434ACA (5371A>C), AAT1435ACT (5374A>C), CCA1437CTC (5380C>T 5381A>C), ATA1439ATC (5387A>C), TTC1440TTT (5390C>T), CAA1441ATG (5391C>A 5392A>T 5393A>G), AGA1442AAC (5395G>A 5396A>C), AAG1443TTG (5397A>T 5398A>T), GAC1445AAT (5403G>A 5405C>T), CAA1446CGA (5407A>G), TGC1447ATT (5409T>A 5410G>T 5411C>T), AAG1449AAA (5417G>A), GGA1450TCA (5418G>T 5419G>C), GGA1450_ACA1451insTAT (5420_5421insTAT), ACA1451CTA (5421A>C 5422C>T), GAG1452TAT (5424G>T 5426G>T), AAG1453ATA (5428A>T 5429G>A), TTC1454TTT (5432C>T), ATT1455GTG (5433A>G 5435T>G), GCG1456GTG (5437C>T), TAC1458TTT (5443A>T 5444C>T), ATA1459ATT (5447A>T), GAC1461GAT (5453C>T), ATC1462ATA (5456C>A), GTC1464ATT (5460G>A 5462C>T), TTT1465TAT (5464T>A), AAG1467CAA (5469A>C 5471G>A), AAC1468AAT (5474C>T), GAG1470GAA (5480G>A), GCA1473ATG (5487G>A 5488C>T 5489A>G), GGC1474GGT (5492C>T), GTA1476TTG (5496G>T 5498A>G), AAA1478ATT (5503A>T 5504A>T), GAA1481CAA (5511G>C)								

*: Inserts / Deletes / Misaligned / Frameshifts

Analysis details

This analysis was performed with panviral2.64

NGS Details (UN24): Gihfavirus pelohabitans

Assembly

**Coverage Length**	278 (1 contig(s))
**Depth Of Coverage**	2.4
**Number Of Reads**	6
**Reads Per Million**	0.12 rpm (after QC)
**Ambiguities**	0
**Assembly Method**	de novo + reference guided assembly
**Consensus Caller**	Bcf Tools

Coverage Map

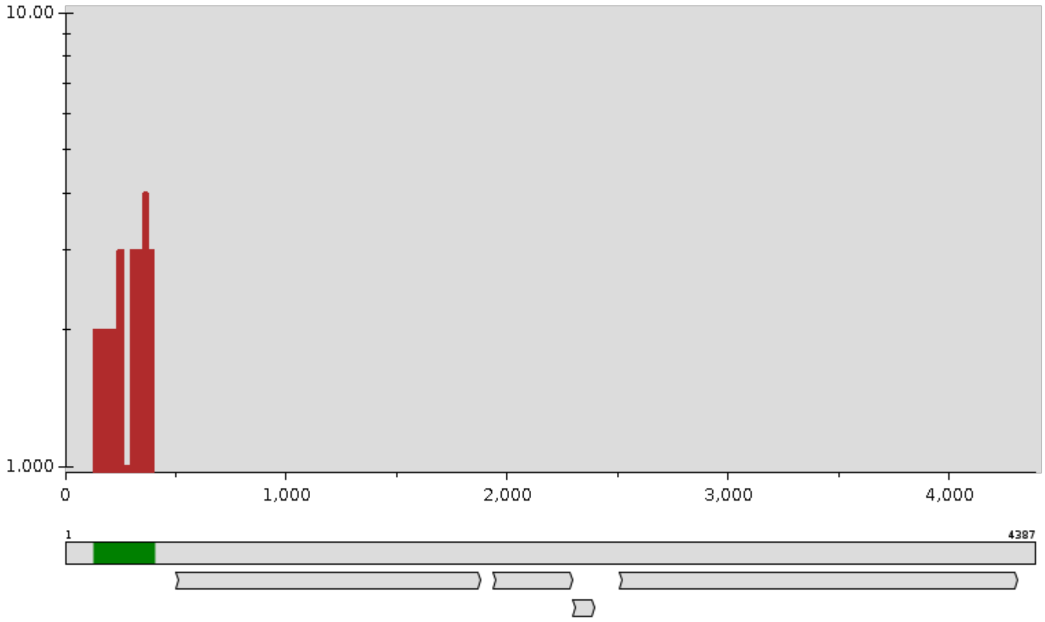

Assignment

**Type**	Gihfavirus pelohabitans (Taxonomy ID: 2844652)
**Reference Genome**	NC_074583.1
**NT Identity (%)**	98.2014
**AA Identity (%)**	0.0
**Number Of Stop Codons**	0
**Number Of CDS**	4

Alignment

**Alignment Score**	536.0 (NT) + 0.0 (AA) = 536.0
**Concordance (%)**	96.4029

Alignment Method	Global, seeded, nucleotide + amino acids (AGA)

Genome Region

Sequence starts at position 127 and ends at position 404 relative to NC_074583.1 reference sequence.

Alignment Detailed Statistics

	Begin	End	Coverage	Score	Concordance	Matches	Identities	I/D/M/F*	Stop Codons
**NT**	**127**	**404**	**6.3%**	**536**	**96.4%**	**278 (100%)**	**273 (98.2%)**	**0/0**	
Mutations:	168G>A, 169A>C, 178T>G, 179C>T, 182T>C								

CDS

Proteins

*: Inserts / Deletes / Misaligned / Frameshifts

Analysis details

This analysis was performed with panviral2.64

NGS Details (UN24): Duamitovirus peex1

Assembly

**Coverage Length**	264 (1 contig(s))
**Depth Of Coverage**	3.1
**Number Of Reads**	6
**Reads Per Million**	0.12 rpm (after QC)
**Ambiguities**	0
**Assembly Method**	de novo + reference guided assembly
**Consensus Caller**	Bcf Tools

Coverage Map

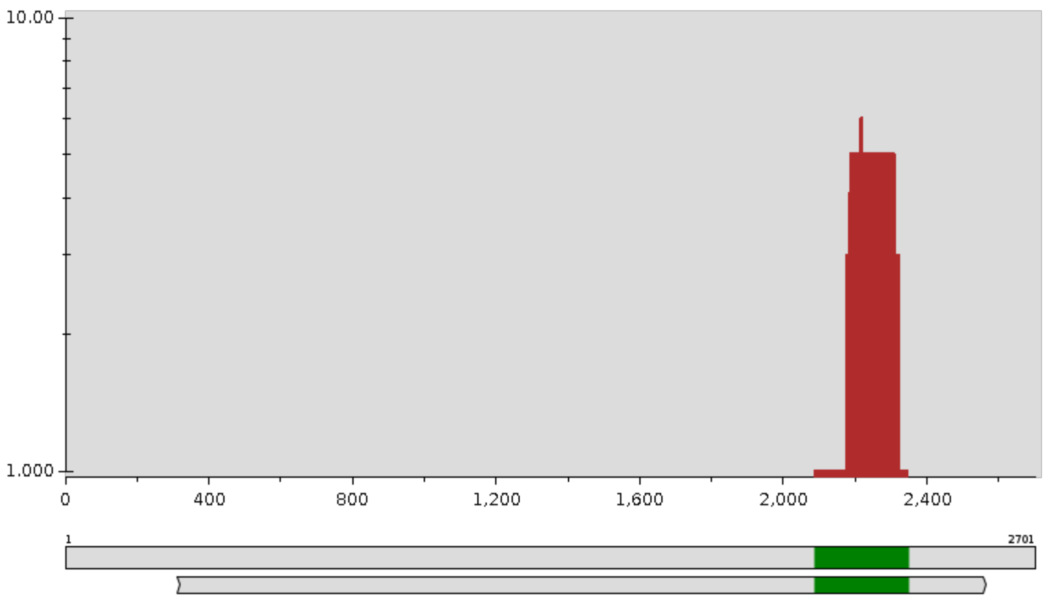

Assignment

**Type**	Duamitovirus peex1 (Taxonomy ID: 2955799)
**Reference Genome**	NC_076525.1
**NT Identity (%)**	72.3485
**AA Identity (%)**	64.7727
**Number Of Stop Codons**	0
**Number Of CDS**	1

Alignment

**Alignment Score**	236.0 (NT) + 421.0 (AA) = 657.0
**Concordance (%)**	55.7725

Alignment Method	Global, seeded, nucleotide + amino acids (AGA)

Genome Region

Sequence starts at position 2087 and ends at position 2350 relative to NC_076525.1 reference sequence.

Alignment Detailed Statistics

	Begin	End	Coverage	Score	Concordance	Matches	Identities	I/D/M/F*	Stop Codons
**NT**	**2087**	**2350**	**9.8%**	**236**	**44.7%**	**264 (100%)**	**191 (72.3%)**	**0/0**	
Mutations:	2091T>A, 2095T>G, 2096A>G, 2098C>T, 2102G>T, 2104G>T, 2105G>A, 2109T>C, 2111T>A, 2119G>T, 2120G>T, 2133G>T, 2134G>T, 2135A>T, 2136A>G, 2137A>G, 2138G>A, 2139C>A, 2145G>A, 2156A>G, 2158G>A, 2159A>G, 2161G>C, 2162T>G, 2165G>A, 2171C>A, 2173C>A, 2180T>G, 2184A>G, 2188A>C, 2189G>T, 2190T>G, 2195A>G, 2202G>C, 2210G>A, 2211T>A, 2213G>C, 2214A>T, 2216A>T, 2223C>G, 2225A>T, 2226C>T, 2231G>A, 2232T>A, 2234A>T, 2240T>A, 2245A>G, 2246T>G, 2249G>A, 2255A>T, 2258A>G, 2261C>G, 2268G>A, 2270A>C, 2272G>T, 2274C>A, 2283G>A, 2292C>T, 2294T>G, 2295C>A, 2296G>C, 2297A>T, 2306A>T, 2315C>T, 2321G>A, 2325A>T, 2326A>T, 2327T>A, 2330C>A, 2333G>A, 2336A>T, 2345A>T, 2348C>T								
**CDS**									
RdRp	593	680	11.7%	421	63.6%	88 (100%)	57 (64.8%)	0/0/0/0	0
Protein mutations:	Y594N (2091T>A), I595R (2095T>G 2096A>G), S596L (2098C>T), Q597H (2102G>T), R598L (2104G>T 2105G>A), S600P (2109T>C 2111T>A), W603F (2119G>T 2120G>T), G608F (2133G>T 2134G>T 2135A>T), K609G (2136A>G 2137A>G 2138G>A), P610T (2139C>A), D612N (2145G>A), R616K (2158G>A 2159A>G), G617A (2161G>C 2162T>G), A621D (2173C>A), K625E (2184A>G), E626A (2188A>C 2189G>T), L627V (2190T>G), E631Q (2202G>C), L634I (2211T>A 2213G>C), I635F (2214A>T 2216A>T), Q638D (2223C>G 2225A>T), L641I (2232T>A 2234A>T), H645R (2245A>G 2246T>G), V653I (2268G>A 2270A>C), R654L (2272G>T), H655N (2274C>A), E658K (2283G>A), R662T (2295C>A 2296G>C 2297A>T), N672L (2325A>T 2326A>T 2327T>A), E675D (2336A>T)								
Codon mutations:	TAC594AAC (2091T>A), ATA595AGG (2095T>G 2096A>G), TCA596TTA (2098C>T), CAG597CAT (2102G>T), CGG598CTA (2104G>T 2105G>A), TCT600CCA (2109T>C 2111T>A), TGG603TTT (2119G>T 2120G>T), GGA608TTT (2133G>T 2134G>T 2135A>T), AAG609GGA (2136A>G 2137A>G 2138G>A), CCC610ACC (2139C>A), GAT612AAT (2145G>A), TTA615TTG (2156A>G), AGA616AAG (2158G>A 2159A>G), GGT617GCG (2161G>C 2162T>G), AAG618AAA (2165G>A), ATC620ATA (2171C>A), GCT621GAT (2173C>A), CTT623CTG (2180T>G), AAG625GAG (2184A>G), GAG626GCT (2188A>C 2189G>T), TTA627GTA (2190T>G), AAA628AAG (2195A>G), GAG631CAG (2202G>C), CAG633CAA (2210G>A), TTG634ATC (2211T>A 2213G>C), ATA635TTT (2214A>T 2216A>T), CAA638GAT (2223C>G 2225A>T), CTG639TTG (2226C>T), GTG640GTA (2231G>A), TTA641ATT (2232T>A 2234A>T), GGT643GGA (2240T>A), CAT645CGG (2245A>G 2246T>G), GAG646GAA (2249G>A), CTA648CTT (2255A>T), GAA649GAG (2258A>G), CGC650CGG (2261C>G), GTA653ATC (2268G>A 2270A>C), CGT654CTT (2272G>T), CAT655AAT (2274C>A), GAA658AAA (2283G>A), CTT661TTG (2292C>T 2294T>G), CGA662ACT (2295C>A 2296G>C 2297A>T), TCA665TCT (2306A>T), CAC668CAT (2315C>T), GTG670GTA (2321G>A), AAT672TTA (2325A>T 2326A>T 2327T>A), TCC673TCA (2330C>A), CCG674CCA (2333G>A), GAA675GAT (2336A>T), ATA678ATT (2345A>T), GAC679GAT (2348C>T)								
**Proteins**									
RNA-dependent RNA polymerase (YP_010798875.1)	593	680	11.7%	421	63.6%	88 (100%)	57 (64.8%)	0/0/0/0	0
Protein mutations:	Y594N (2091T>A), I595R (2095T>G 2096A>G), S596L (2098C>T), Q597H (2102G>T), R598L (2104G>T 2105G>A), S600P (2109T>C 2111T>A), W603F (2119G>T 2120G>T), G608F (2133G>T 2134G>T 2135A>T), K609G (2136A>G 2137A>G 2138G>A), P610T (2139C>A), D612N (2145G>A), R616K (2158G>A 2159A>G), G617A (2161G>C 2162T>G), A621D (2173C>A), K625E (2184A>G), E626A (2188A>C 2189G>T), L627V (2190T>G), E631Q (2202G>C), L634I (2211T>A 2213G>C), I635F (2214A>T 2216A>T), Q638D (2223C>G 2225A>T), L641I (2232T>A 2234A>T), H645R (2245A>G 2246T>G), V653I (2268G>A 2270A>C), R654L (2272G>T), H655N (2274C>A), E658K (2283G>A), R662T (2295C>A 2296G>C 2297A>T), N672L (2325A>T 2326A>T 2327T>A), E675D (2336A>T)								
Codon mutations:	TAC594AAC (2091T>A), ATA595AGG (2095T>G 2096A>G), TCA596TTA (2098C>T), CAG597CAT (2102G>T), CGG598CTA (2104G>T 2105G>A), TCT600CCA (2109T>C 2111T>A), TGG603TTT (2119G>T 2120G>T), GGA608TTT (2133G>T 2134G>T 2135A>T), AAG609GGA (2136A>G 2137A>G 2138G>A), CCC610ACC (2139C>A), GAT612AAT (2145G>A), TTA615TTG (2156A>G), AGA616AAG (2158G>A 2159A>G), GGT617GCG (2161G>C 2162T>G), AAG618AAA (2165G>A), ATC620ATA (2171C>A), GCT621GAT (2173C>A), CTT623CTG (2180T>G), AAG625GAG (2184A>G), GAG626GCT (2188A>C 2189G>T), TTA627GTA (2190T>G), AAA628AAG (2195A>G), GAG631CAG (2202G>C), CAG633CAA (2210G>A), TTG634ATC (2211T>A 2213G>C), ATA635TTT (2214A>T 2216A>T), CAA638GAT (2223C>G 2225A>T), CTG639TTG (2226C>T), GTG640GTA (2231G>A), TTA641ATT (2232T>A 2234A>T), GGT643GGA (2240T>A), CAT645CGG (2245A>G 2246T>G), GAG646GAA (2249G>A), CTA648CTT (2255A>T), GAA649GAG (2258A>G), CGC650CGG (2261C>G), GTA653ATC (2268G>A 2270A>C), CGT654CTT (2272G>T), CAT655AAT (2274C>A), GAA658AAA (2283G>A), CTT661TTG (2292C>T 2294T>G), CGA662ACT (2295C>A 2296G>C 2297A>T), TCA665TCT (2306A>T), CAC668CAT (2315C>T), GTG670GTA (2321G>A), AAT672TTA (2325A>T 2326A>T 2327T>A), TCC673TCA (2330C>A), CCG674CCA (2333G>A), GAA675GAT (2336A>T), ATA678ATT (2345A>T), GAC679GAT (2348C>T)								

*: Inserts / Deletes / Misaligned / Frameshifts

Analysis details

This analysis was performed with panviral2.64

NGS Details (UN24): Badnavirus venatheobromae

Assembly

**Coverage Length**	354 (1 contig(s))
**Depth Of Coverage**	2.3
**Number Of Reads**	6
**Reads Per Million**	0.12 rpm (after QC)
**Ambiguities**	0
**Assembly Method**	de novo + reference guided assembly
**Consensus Caller**	Bcf Tools

Coverage Map

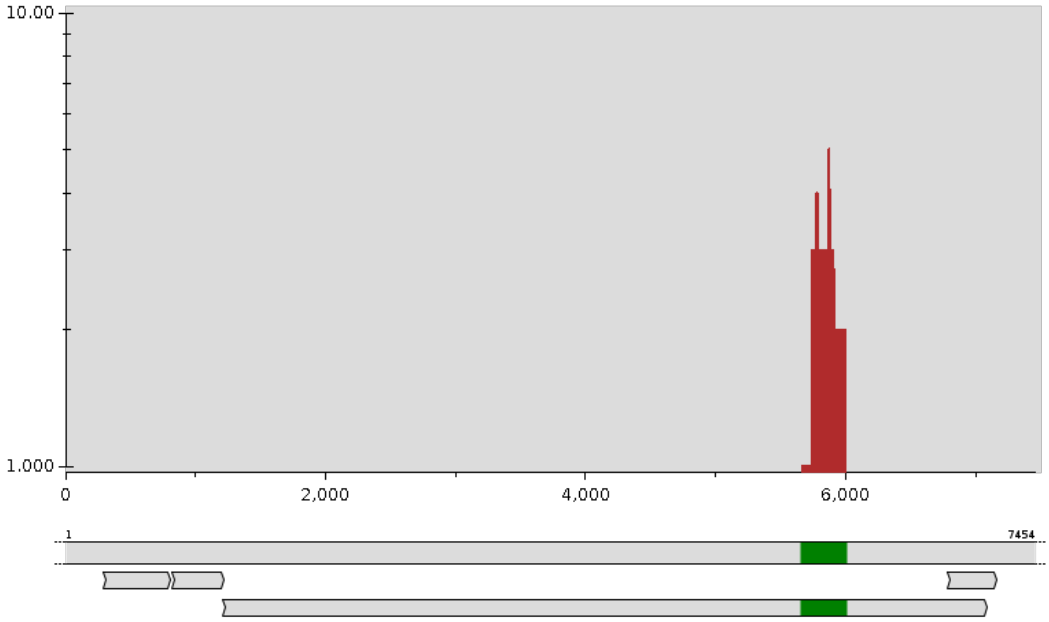

Assignment

**Type**	Badnavirus venatheobromae (Taxonomy ID: 3047715)
**Reference Genome**	NC_033739.1
**NT Identity (%)**	54.0616
**AA Identity (%)**	42.8571
**Number Of Stop Codons**	0
**Number Of CDS**	4

Alignment

**Alignment Score**	52.0 (NT) + 369.0 (AA) = 421.0
**Concordance (%)**	26.9526

Alignment Method	Global, seeded, nucleotide + amino acids (AGA)

Genome Region

Sequence starts at position 5656 and ends at position 6009 relative to NC_033739.1 reference sequence.

Alignment Detailed Statistics

	Begin	End	Coverage	Score	Concordance	Matches	Identities	I/D/M/F*	Stop Codons
**NT**	**5656**	**6009**	**4.7%**	**52**	**7.3%**	**354 (99.2%)**	**193 (54.1%)**	**3/0**	
Mutations:	5659A>G, 5663A>C, 5666G>A, 5667A>G, 5673T>A, 5679T>C, 5681T>G, 5689A>G, 5690G>A, 5693C>A, 5696T>C, 5698T>A, 5702T>C, 5705A>G, 5706G>A, 5709G>A, 5710C>G, 5716A>C, 5717G>T, 5718G>C, 5721T>G, 5724T>G, 5725C>A, 5726A>T, 5727A>G, 5729T>G, 5731C>A, 5733T>A, 5734G>A, 5738A>T, 5741G>C, 5742T>A, 5744C>G, 5745T>A, 5748G>A, 5749T>C, 5750C>A, 5752C>A, 5753T>C, 5754G>T, 5755A>C, 5756T>A, 5759A>T, 5761T>A, 5762A>C, 5763T>A, 5765T>C, 5766G>A, 5768A>G, 5770G>A, 5771G>C, 5777C>G, 5783G>T, 5786C>T, 5789A>T, 5790T>C, 5792A>C, 5794A>C, 5795G>A, 5801T>C, 5805G>T, 5807C>T, 5809T>G, 5810T>C, 5813T>C, 5816A>G, 5817A>G, 5820A>C, 5821A>T, 5822G>C, 5826G>A, 5829A>C, 5831C>T, 5832T>G, 5833G>T, 5837T>C, 5840A>G, 5842G>A, 5843A>C, 5843_5844insCAT, 5844A>C, 5845C>T, 5846A>T, 5847G>A, 5848A>G, 5849A>G, 5850G>A, 5855T>C, 5858A>C, 5859G>C, 5860C>T, 5864T>A, 5866A>T, 5867T>C, 5868A>G, 5869T>C, 5876T>C, 5882A>C, 5885C>T, 5887T>A, 5888C>T, 5889T>A, 5890C>G, 5891A>T, 5896C>A, 5898G>A, 5899A>T, 5903A>G, 5907C>T, 5910G>C, 5912C>A, 5914G>A, 5915T>A, 5918T>C, 5919C>T, 5921A>G, 5922C>A, 5923A>G, 5926G>T, 5927G>T, 5928T>G, 5934C>A, 5936G>C, 5937A>C, 5939A>G, 5941G>T, 5942C>G, 5943C>A, 5944A>T, 5945G>C, 5948G>A, 5951T>C, 5952G>C, 5953G>A, 5955T>C, 5957A>C, 5958G>T, 5960C>A, 5961C>G, 5963G>C, 5966T>G, 5969G>A, 5970G>A, 5972T>C, 5976A>T, 5977T>G, 5978G>T, 5979G>T, 5981A>T, 5982A>T, 5984A>T, 5986C>G, 5989T>C, 5990C>T, 5991C>T, 5993A>T, 5994C>A, 5996A>G, 5997A>G, 5999T>G, 6002G>A, 6004T>A								

CDS

	Begin	End	Coverage	Score	Concordance	Matches	Identities	I/D/M/F*	Stop Codons
B1U04_gp3	1483	1600	6.0%	369	42.4%	118 (99.2%)	51 (42.9%)	1/0/0/0	0
Protein mutations:	N1483S (5659A>G), I1486V (5667A>G), S1488T (5673T>A), F1490L (5679T>C 5681T>G), K1493R (5689A>G 5690G>A), F1496Y (5698T>A), V1499I (5706G>A), A1500R (5709G>A 5710C>G), E1502A (5716A>C 5717G>T), E1503Q (5718G>C), S1504A (5721T>G), S1505D (5724T>G 5725C>A 5726A>T), I1506V (5727A>G 5729T>G), P1507Q (5731C>A), W1508K (5733T>A 5734G>A), F1511M (5742T>A 5744C>G), W1512R (5745T>A), V1513T (5748G>A 5749T>C 5750C>A), P1514H (5752C>A 5753T>C), D1515S (5754G>T 5755A>C 5756T>A), L1517H (5761T>A 5762A>C), Y1518N (5763T>A 5765T>C), E1519K (5766G>A 5768A>G), W1520Y (5770G>A 5771G>C), K1528T (5794A>C 5795G>A), A1532S (5805G>T 5807C>T), I1533S (5809T>G 5810T>C), R1536G (5817A>G), K1537L (5820A>C 5821A>T 5822G>C), D1539N (5826G>A), N1540H (5829A>C 5831C>T), C1541V (5832T>G 5833G>T), G1544D (5842G>A 5843A>C), G1544_T1545insH (5843_5844insCAT), T1545L (5844A>C 5845C>T 5846A>T), E1546R (5847G>A 5848A>G 5849A>G), E1547K (5850G>A), A1550L (5859G>C 5860C>T), Y1552F (5866A>T 5867T>C), I1553A (5868A>G 5869T>C), F1559Y (5887T>A 5888C>T), T1562N (5896C>A), E1563M (5898G>A 5899A>T), H1566Y (5907C>T), V1567L (5910G>C 5912C>A), R1568Q (5914G>A 5915T>A), Q1571R (5922C>A 5923A>G), R1572I (5926G>T 5927G>T), F1573V (5928T>G), Q1575N (5934C>A 5936G>C), I1576L (5937A>C 5939A>G), C1577L (5941G>T 5942C>G), Q1578I (5943C>A 5944A>T 5945G>C), G1581Q (5952G>C 5953G>A), V1583L (5958G>T 5960C>A), L1584V (5961C>G 5963G>C), S1585R (5966T>G), D1587N (5970G>A 5972T>C), M1589C (5976A>T 5977T>G 5978G>T), A1590S (5979G>T 5981A>T), I1591F (5982A>T 5984A>T), A1592G (5986C>G), V1593A (5989T>C 5990C>T), P1594S (5991C>T 5993A>T), Q1595K (5994C>A 5996A>G), I1596V (5997A>G 5999T>G), F1598Y (6004T>A)								
Codon mutations:	AAT1483AGT (5659A>G), TCA1484TCC (5663A>C), AAG1485AAA (5666G>A), ATT1486GTT (5667A>G), TCC1488ACC (5673T>A), TTT1490CTG (5679T>C 5681T>G), AAG1493AGA (5689A>G 5690G>A), TCC1494TCA (5693C>A), GGT1495GGC (5696T>C), TTT1496TAT (5698T>A), CAT1497CAC (5702T>C), CAA1498CAG (5705A>G), GTA1499ATA (5706G>A), GCA1500AGA (5709G>A 5710C>G), GAG1502GCT (5716A>C 5717G>T), GAA1503CAA (5718G>C), TCA1504GCA (5721T>G), TCA1505GAT (5724T>G 5725C>A 5726A>T), ATT1506GTG (5727A>G 5729T>G), CCA1507CAA (5731C>A), TGG1508AAG (5733T>A 5734G>A), ACA1509ACT (5738A>T), GCG1510GCC (5741G>C), TTC1511ATG (5742T>A 5744C>G), TGG1512AGG (5745T>A), GTC1513ACA (5748G>A 5749T>C 5750C>A), CCT1514CAC (5752C>A 5753T>C), GAT1515TCA (5754G>T 5755A>C 5756T>A), GGA1516GGT (5759A>T), CTA1517CAC (5761T>A 5762A>C), TAT1518AAC (5763T>A 5765T>C), GAA1519AAG (5766G>A 5768A>G), TGG1520TAC (5770G>A 5771G>C), GTC1522GTG (5777C>G), CCG1524CCT (5783G>T), TTC1525TTT (5786C>T), GGA1526GGT (5789A>T), TTA1527CTC (5790T>C 5792A>C), AAG1528ACA (5794A>C 5795G>A), GCT1530GCC (5801T>C), GCC1532TCT (5805G>T 5807C>T), ATT1533AGC (5809T>G 5810T>C), TTT1534TTC (5813T>C), CAA1535CAG (5816A>G), AGA1536GGA (5817A>G), AAG1537CTC (5820A>C 5821A>T 5822G>C), GAC1539AAC (5826G>A), AAC1540CAT (5829A>C 5831C>T), TGC1541GTC (5832T>G 5833G>T), TTT1542TTC (5837T>C), AAA1543AAG (5840A>G), GGA1544GAC (5842G>A 5843A>C), GGA1544_ACA1545insCAT (5843_5844insCAT), ACA1545CTT (5844A>C 5845C>T 5846A>T), GAA1546AGG (5847G>A 5848A>G 5849A>G), GAA1547AAA (5850G>A), TTT1548TTC (5855T>C), ATA1549ATC (5858A>C), GCA1550CTA (5859G>C 5860C>T), GTT1551GTA (5864T>A), TAT1552TTC (5866A>T 5867T>C), ATT1553GCT (5868A>G 5869T>C), GAT1555GAC (5876T>C), CTA1557CTC (5882A>C), GTC1558GTT (5885C>T), TTC1559TAT (5887T>A 5888C>T), TCA1560AGT (5889T>A 5890C>G 5891A>T), ACT1562AAT (5896C>A), GAG1563ATG (5898G>A 5899A>T), GAA1564GAG (5903A>G), CAT1566TAT (5907C>T), GTC1567CTA (5910G>C 5912C>A), CGT1568CAA (5914G>A 5915T>A), CAT1569CAC (5918T>C), CTA1570TTG (5919C>T 5921A>G), CAA1571AGA (5922C>A 5923A>G), AGG1572ATT (5926G>T 5927G>T), TTC1573GTC (5928T>G), CAG1575AAC (5934C>A 5936G>C), ATA1576CTG (5937A>C 5939A>G), TGC1577TTG (5941G>T 5942C>G), CAG1578ATC (5943C>A 5944A>T 5945G>C), AAG1579AAA (5948G>A), CAT1580CAC (5951T>C), GGG1581CAG (5952G>C 5953G>A), TTA1582CTC (5955T>C 5957A>C), GTC1583TTA (5958G>T 5960C>A), CTG1584GTC (5961C>G 5963G>C), AGT1585AGG (5966T>G), AAG1586AAA (5969G>A), GAT1587AAC (5970G>A 5972T>C), ATG1589TGT (5976A>T 5977T>G 5978G>T), GCA1590TCT (5979G>T 5981A>T), ATA1591TTT (5982A>T 5984A>T), GCA1592GGA (5986C>G), GTC1593GCT (5989T>C 5990C>T), CCA1594TCT (5991C>T 5993A>T), CAA1595AAG (5994C>A 5996A>G), ATT1596GTG (5997A>G 5999T>G), GAG1597GAA (6002G>A), TTC1598TAC (6004T>A)								

Proteins

	Begin	End	Coverage	Score	Concordance	Matches	Identities	I/D/M/F*	Stop Codons
polyprotein (YP_009345075.1)	1483	1600	6.0%	369	42.4%	118 (99.2%)	51 (42.9%)	1/0/0/0	0
Protein mutations:	N1483S (5659A>G), I1486V (5667A>G), S1488T (5673T>A), F1490L (5679T>C 5681T>G), K1493R (5689A>G 5690G>A), F1496Y (5698T>A), V1499I (5706G>A), A1500R (5709G>A 5710C>G), E1502A (5716A>C 5717G>T), E1503Q (5718G>C), S1504A (5721T>G), S1505D (5724T>G 5725C>A 5726A>T), I1506V (5727A>G 5729T>G), P1507Q (5731C>A), W1508K (5733T>A 5734G>A), F1511M (5742T>A 5744C>G), W1512R (5745T>A), V1513T (5748G>A 5749T>C 5750C>A), P1514H (5752C>A 5753T>C), D1515S (5754G>T 5755A>C 5756T>A), L1517H (5761T>A 5762A>C), Y1518N (5763T>A 5765T>C), E1519K (5766G>A 5768A>G), W1520Y (5770G>A 5771G>C), K1528T (5794A>C 5795G>A), A1532S (5805G>T 5807C>T), I1533S (5809T>G 5810T>C), R1536G (5817A>G), K1537L (5820A>C 5821A>T 5822G>C), D1539N (5826G>A), N1540H (5829A>C 5831C>T), C1541V (5832T>G 5833G>T), G1544D (5842G>A 5843A>C), G1544_T1545insH (5843_5844insCAT), T1545L (5844A>C 5845C>T 5846A>T), E1546R (5847G>A 5848A>G 5849A>G), E1547K (5850G>A), A1550L (5859G>C 5860C>T), Y1552F (5866A>T 5867T>C), I1553A (5868A>G 5869T>C), F1559Y (5887T>A 5888C>T), T1562N (5896C>A), E1563M (5898G>A 5899A>T), H1566Y (5907C>T), V1567L (5910G>C 5912C>A), R1568Q (5914G>A 5915T>A), Q1571R (5922C>A 5923A>G), R1572I (5926G>T 5927G>T), F1573V (5928T>G), Q1575N (5934C>A 5936G>C), I1576L (5937A>C 5939A>G), C1577L (5941G>T 5942C>G), Q1578I (5943C>A 5944A>T 5945G>C), G1581Q (5952G>C 5953G>A), V1583L (5958G>T 5960C>A), L1584V (5961C>G 5963G>C), S1585R (5966T>G), D1587N (5970G>A 5972T>C), M1589C (5976A>T 5977T>G 5978G>T), A1590S (5979G>T 5981A>T), I1591F (5982A>T 5984A>T), A1592G (5986C>G), V1593A (5989T>C 5990C>T), P1594S (5991C>T 5993A>T), Q1595K (5994C>A 5996A>G), I1596V (5997A>G 5999T>G), F1598Y (6004T>A)								
Codon mutations:	AAT1483AGT (5659A>G), TCA1484TCC (5663A>C), AAG1485AAA (5666G>A), ATT1486GTT (5667A>G), TCC1488ACC (5673T>A), TTT1490CTG (5679T>C 5681T>G), AAG1493AGA (5689A>G 5690G>A), TCC1494TCA (5693C>A), GGT1495GGC (5696T>C), TTT1496TAT (5698T>A), CAT1497CAC (5702T>C), CAA1498CAG (5705A>G), GTA1499ATA (5706G>A), GCA1500AGA (5709G>A 5710C>G), GAG1502GCT (5716A>C 5717G>T), GAA1503CAA (5718G>C), TCA1504GCA (5721T>G), TCA1505GAT (5724T>G 5725C>A 5726A>T), ATT1506GTG (5727A>G 5729T>G), CCA1507CAA (5731C>A), TGG1508AAG (5733T>A 5734G>A), ACA1509ACT (5738A>T), GCG1510GCC (5741G>C), TTC1511ATG (5742T>A 5744C>G), TGG1512AGG (5745T>A), GTC1513ACA (5748G>A 5749T>C 5750C>A), CCT1514CAC (5752C>A 5753T>C), GAT1515TCA (5754G>T 5755A>C 5756T>A), GGA1516GGT (5759A>T), CTA1517CAC (5761T>A 5762A>C), TAT1518AAC (5763T>A 5765T>C), GAA1519AAG (5766G>A 5768A>G), TGG1520TAC (5770G>A 5771G>C), GTC1522GTG (5777C>G), CCG1524CCT (5783G>T), TTC1525TTT (5786C>T), GGA1526GGT (5789A>T), TTA1527CTC (5790T>C 5792A>C), AAG1528ACA (5794A>C 5795G>A), GCT1530GCC (5801T>C), GCC1532TCT (5805G>T 5807C>T), ATT1533AGC (5809T>G 5810T>C), TTT1534TTC (5813T>C), CAA1535CAG (5816A>G), AGA1536GGA (5817A>G), AAG1537CTC (5820A>C 5821A>T 5822G>C), GAC1539AAC (5826G>A), AAC1540CAT (5829A>C 5831C>T), TGC1541GTC (5832T>G 5833G>T), TTT1542TTC (5837T>C), AAA1543AAG (5840A>G), GGA1544GAC (5842G>A 5843A>C), GGA1544_ACA1545insCAT (5843_5844insCAT), ACA1545CTT (5844A>C 5845C>T 5846A>T), GAA1546AGG (5847G>A 5848A>G 5849A>G), GAA1547AAA (5850G>A), TTT1548TTC (5855T>C), ATA1549ATC (5858A>C), GCA1550CTA (5859G>C 5860C>T), GTT1551GTA (5864T>A), TAT1552TTC (5866A>T 5867T>C), ATT1553GCT (5868A>G 5869T>C), GAT1555GAC (5876T>C), CTA1557CTC (5882A>C), GTC1558GTT (5885C>T), TTC1559TAT (5887T>A 5888C>T), TCA1560AGT (5889T>A 5890C>G 5891A>T), ACT1562AAT (5896C>A), GAG1563ATG (5898G>A 5899A>T), GAA1564GAG (5903A>G), CAT1566TAT (5907C>T), GTC1567CTA (5910G>C 5912C>A), CGT1568CAA (5914G>A 5915T>A), CAT1569CAC (5918T>C), CTA1570TTG (5919C>T 5921A>G), CAA1571AGA (5922C>A 5923A>G), AGG1572ATT (5926G>T 5927G>T), TTC1573GTC (5928T>G), CAG1575AAC (5934C>A 5936G>C), ATA1576CTG (5937A>C 5939A>G), TGC1577TTG (5941G>T 5942C>G), CAG1578ATC (5943C>A 5944A>T 5945G>C), AAG1579AAA (5948G>A), CAT1580CAC (5951T>C), GGG1581CAG (5952G>C 5953G>A), TTA1582CTC (5955T>C 5957A>C), GTC1583TTA (5958G>T 5960C>A), CTG1584GTC (5961C>G 5963G>C), AGT1585AGG (5966T>G), AAG1586AAA (5969G>A), GAT1587AAC (5970G>A 5972T>C), ATG1589TGT (5976A>T 5977T>G 5978G>T), GCA1590TCT (5979G>T 5981A>T), ATA1591TTT (5982A>T 5984A>T), GCA1592GGA (5986C>G), GTC1593GCT (5989T>C 5990C>T), CCA1594TCT (5991C>T 5993A>T), CAA1595AAG (5994C>A 5996A>G), ATT1596GTG (5997A>G 5999T>G), GAG1597GAA (6002G>A), TTC1598TAC (6004T>A)								

*: Inserts / Deletes / Misaligned / Frameshifts

Analysis details

This analysis was performed with panviral2.64

NGS Details (UN24): Petuvirus venapetuniae

Assembly

**Coverage Length**	281 (1 contig(s))
**Depth Of Coverage**	2.5
**Number Of Reads**	6
**Reads Per Million**	0.12 rpm (after QC)
**Ambiguities**	0
**Assembly Method**	de novo + reference guided assembly
**Consensus Caller**	Bcf Tools

Coverage Map

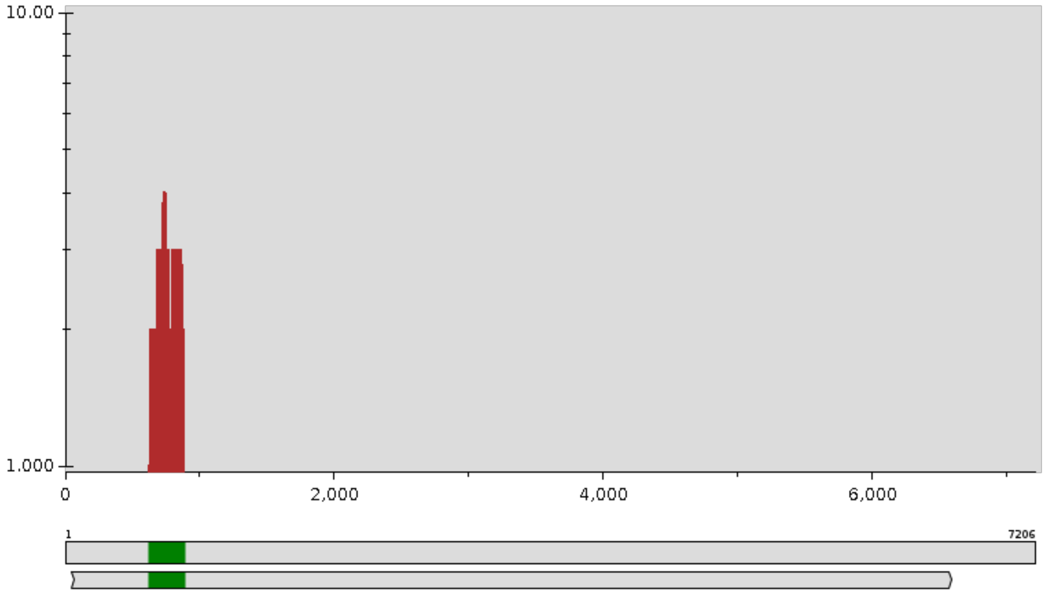

Assignment

**Type**	Petuvirus venapetuniae (Taxonomy ID: 3048265)
**Reference Genome**	NC_001839.2
**NT Identity (%)**	62.5
**AA Identity (%)**	53.1915
**Number Of Stop Codons**	5
**Number Of CDS**	1

Alignment

**Alignment Score**	130.0 (NT) + 219.0 (AA) = 349.0
**Concordance (%)**	34.4186

**Alignment Method**	Global, seeded, nucleotide + amino acids (AGA)

Genome Region

Sequence starts at position 614 and ends at position 894 relative to NC_001839.2 reference sequence.

Alignment Detailed Statistics

	Begin	End	Coverage	Score	Concordance	Matches	Identities	I/D/M/F*	Stop Codons
**NT**	**614**	**894**	**3.9%**	**130**	**23.6%**	**280 (99.6%)**	**175 (62.3%)**	**0/1**	
Mutations:	615G>A, 618G>C, 620C>A, 621A>C, 627T>A, 629C>A, 630A>G, 631C>A, 632A>G, 635C>A, 636T>A, 638A>G, 641A>G, 644C>A, 645C>T, 647A>G, 656C>A, 658A>T, 659A>T, 668C>G, 670T>A, 672A>G, 674A>T, 676A>G, 680C>T, 681T>G, 682C>T, 686C>A, 693delA, 694C>A, 696T>C, 698A>T, 701T>C, 704C>T, 705C>T, 707A>G, 708A>C, 710T>A, 712C>T, 717C>A, 719T>A, 722C>T, 723C>T, 725A>G, 726A>G, 728T>C, 729C>A, 733C>T, 734T>C, 740T>C, 741T>C, 743G>A, 744A>T, 749C>T, 752T>A, 755A>T, 758T>C, 759G>A, 761G>T, 764A>T, 765G>T, 767C>T, 774C>T, 776C>A, 777A>C, 779A>C, 782T>A, 786G>T, 789G>A, 790G>T, 793G>A, 798G>A, 800C>T, 804C>T, 806A>G, 809T>C, 815A>G, 816G>A, 818C>G, 819C>T, 821T>A, 823G>A, 827G>C, 828T>A, 830G>A, 835A>G, 836A>G, 839A>C, 840G>A, 842T>G, 845A>T, 846A>T, 849A>T, 851A>G, 854A>T, 855T>A, 857G>C, 861G>A, 863T>C, 866T>A, 872A>C, 875A>T, 878T>A, 884A>G, 890A>G, 893G>A								
**CDS**									
PVCVgp1	189	282	4.3%	219	34.4%	94 (100%)	50 (53.2%)	0/0/1/1	5
Protein mutations:	D189N (615G>A), A190P (618G>C 620C>A), N191H (621A>C), S193T (627T>A 629C>A), T194E (630A>G 631C>A 632A>G), L196M (636T>A 638A>G), Q199* (645C>T 647A>G), Q203L (658A>T 659A>T), L207H (670T>A), T208A (672A>G 674A>T), K209R (676A>G), S211V (681T>G 682C>T), Q219* (705C>T 707A>G), I220L (708A>C 710T>A), A221V (712C>T), Q225* (723C>T 725A>G), N226D (726A>G 728T>C), H227N (729C>A), A228V (733C>T 734T>C), T232S (744A>T), E237N (759G>A 761G>T), E238D (764A>T), A239S (765G>T 767C>T), K243H (777A>C 779A>C), A246S (786G>T), G247I (789G>A 790G>T), S248N (793G>A), A250T (798G>A 800C>T), Q252* (804C>T 806A>G), V256M (816G>A 818C>G), P257S (819C>T 821T>A), R258K (823G>A), Q259H (827G>C), L260I (828T>A 830G>A), K262R (835A>G 836A>G), E263D (839A>C), D264K (840G>A 842T>G), I266F (846A>T), K267* (849A>T 851A>G), L269I (855T>A 857G>C), D271N (861G>A 863T>C), N276K (878T>A)								
Codon mutations:	GAC189AAC (615G>A), GCC190CCA (618G>C 620C>A), AAT191CAT (621A>C), TCC193ACA (627T>A 629C>A), ACA194GAG (630A>G 631C>A 632A>G), GCC195GCA (635C>A), TTA196ATG (636T>A 638A>G), AAA197AAG (641A>G), ATC198ATA (644C>A), CAA199TAG (645C>T 647A>G), ATC202ATA (656C>A), CAA203CTT (658A>T 659A>T), CCC206CCG (668C>G), CTT207CAT (670T>A), ACA208GCT (672A>G 674A>T), AAA209AGA (676A>G), GAC210GAT (680C>T), TCC211GTC (681T>G 682C>T), ATC212ATA (686C>A), ACT215-AT (693delA 694C>A), TTA216CTT (696T>C 698A>T), CAT217CAC (701T>C), TAC218TAT (704C>T), CAA219TAG (705C>T 707A>G), ATT220CTA (708A>C 710T>A), GCT221GTT (712C>T), CGT223AGA (717C>A 719T>A), GTC224GTT (722C>T), CAA225TAG (723C>T 725A>G), AAT226GAC (726A>G 728T>C), CAT227AAT (729C>A), GCT228GTC (733C>T 734T>C), GAT230GAC (740T>C), TTG231CTA (741T>C 743G>A), ACT232TCT (744A>T), CTC233CTT (749C>T), CCT234CCA (752T>A), GGA235GGT (755A>T), GGT236GGC (758T>C), GAG237AAT (759G>A 761G>T), GAA238GAT (764A>T), GCC239TCT (765G>T 767C>T), CTC242TTA (774C>T 776C>A), AAA243CAC (777A>C 779A>C), ATT244ATA (782T>A), GCC246TCC (786G>T), GGA247ATA (789G>A 790G>T), AGT248AAT (793G>A), GCC250ACT (798G>A 800C>T), CAA252TAG (804C>T 806A>G), TGT253TGC (809T>C), CAA255CAG (815A>G), GTC256ATG (816G>A 818C>G), CCT257TCA (819C>T 821T>A), AGA258AAA (823G>A), CAG259CAC (827G>C), TTG260ATA (828T>A 830G>A), AAA262AGG (835A>G 836A>G), GAA263GAC (839A>C), GAT264AAG (840G>A 842T>G), CTA265CTT (845A>T), ATC266TTC (846A>T), AAA267TAG (849A>T 851A>G), ATA268ATT (854A>T), TTG269ATC (855T>A 857G>C), GAT271AAC (861G>A 863T>C), TCT272TCA (866T>A), GTA274GTC (872A>C), ACA275ACT (875A>T), AAT276AAA (878T>A), GAA278GAG (884A>G), TTA280TTG (890A>G), AGG281AGA (893G>A)								
**Proteins**									
ORF I polyprotein (NP_127504.1)	189	282	4.3%	219	34.4%	94 (100%)	50 (53.2%)	0/0/1/1	5
Protein mutations:	D189N (615G>A), A190P (618G>C 620C>A), N191H (621A>C), S193T (627T>A 629C>A), T194E (630A>G 631C>A 632A>G), L196M (636T>A 638A>G), Q199* (645C>T 647A>G), Q203L (658A>T 659A>T), L207H (670T>A), T208A (672A>G 674A>T), K209R (676A>G), S211V (681T>G 682C>T), Q219* (705C>T 707A>G), I220L (708A>C 710T>A), A221V (712C>T), Q225* (723C>T 725A>G), N226D (726A>G 728T>C), H227N (729C>A), A228V (733C>T 734T>C), T232S (744A>T), E237N (759G>A 761G>T), E238D (764A>T), A239S (765G>T 767C>T), K243H (777A>C 779A>C), A246S (786G>T), G247I (789G>A 790G>T), S248N (793G>A), A250T (798G>A 800C>T), Q252* (804C>T 806A>G), V256M (816G>A 818C>G), P257S (819C>T 821T>A), R258K (823G>A), Q259H (827G>C), L260I (828T>A 830G>A), K262R (835A>G 836A>G), E263D (839A>C), D264K (840G>A 842T>G), I266F (846A>T), K267* (849A>T 851A>G), L269I (855T>A 857G>C), D271N (861G>A 863T>C), N276K (878T>A)								
Codon mutations:	GAC189AAC (615G>A), GCC190CCA (618G>C 620C>A), AAT191CAT (621A>C), TCC193ACA (627T>A 629C>A), ACA194GAG (630A>G 631C>A 632A>G), GCC195GCA (635C>A), TTA196ATG (636T>A 638A>G), AAA197AAG (641A>G), ATC198ATA (644C>A), CAA199TAG (645C>T 647A>G), ATC202ATA (656C>A), CAA203CTT (658A>T 659A>T), CCC206CCG (668C>G), CTT207CAT (670T>A), ACA208GCT (672A>G 674A>T), AAA209AGA (676A>G), GAC210GAT (680C>T), TCC211GTC (681T>G 682C>T), ATC212ATA (686C>A), ACT215-AT (693delA 694C>A), TTA216CTT (696T>C 698A>T), CAT217CAC (701T>C), TAC218TAT (704C>T), CAA219TAG (705C>T 707A>G), ATT220CTA (708A>C 710T>A), GCT221GTT (712C>T), CGT223AGA (717C>A 719T>A), GTC224GTT (722C>T), CAA225TAG (723C>T 725A>G), AAT226GAC (726A>G 728T>C), CAT227AAT (729C>A), GCT228GTC (733C>T 734T>C), GAT230GAC (740T>C), TTG231CTA (741T>C 743G>A), ACT232TCT (744A>T), CTC233CTT (749C>T), CCT234CCA (752T>A), GGA235GGT (755A>T), GGT236GGC (758T>C), GAG237AAT (759G>A 761G>T), GAA238GAT (764A>T), GCC239TCT (765G>T 767C>T), CTC242TTA (774C>T 776C>A), AAA243CAC (777A>C 779A>C), ATT244ATA (782T>A), GCC246TCC (786G>T), GGA247ATA (789G>A 790G>T), AGT248AAT (793G>A), GCC250ACT (798G>A 800C>T), CAA252TAG (804C>T 806A>G), TGT253TGC (809T>C), CAA255CAG (815A>G), GTC256ATG (816G>A 818C>G), CCT257TCA (819C>T 821T>A), AGA258AAA (823G>A), CAG259CAC (827G>C), TTG260ATA (828T>A 830G>A), AAA262AGG (835A>G 836A>G), GAA263GAC (839A>C), GAT264AAG (840G>A 842T>G), CTA265CTT (845A>T), ATC266TTC (846A>T), AAA267TAG (849A>T 851A>G), ATA268ATT (854A>T), TTG269ATC (855T>A 857G>C), GAT271AAC (861G>A 863T>C), TCT272TCA (866T>A), GTA274GTC (872A>C), ACA275ACT (875A>T), AAT276AAA (878T>A), GAA278GAG (884A>G), TTA280TTG (890A>G), AGG281AGA (893G>A)								

*: Inserts / Deletes / Misaligned / Frameshifts

Analysis details

This analysis was performed with panviral2.64

NGS Details (UN24): Marseillevirus marseillevirus

Assembly

**Coverage Length**	110 (1 contig(s))
**Depth Of Coverage**	4.7
**Number Of Reads**	6
**Reads Per Million**	0.12 rpm (after QC)
**Ambiguities**	0
**Assembly Method**	de novo + reference guided assembly
**Consensus Caller**	Bcf Tools

Coverage Map

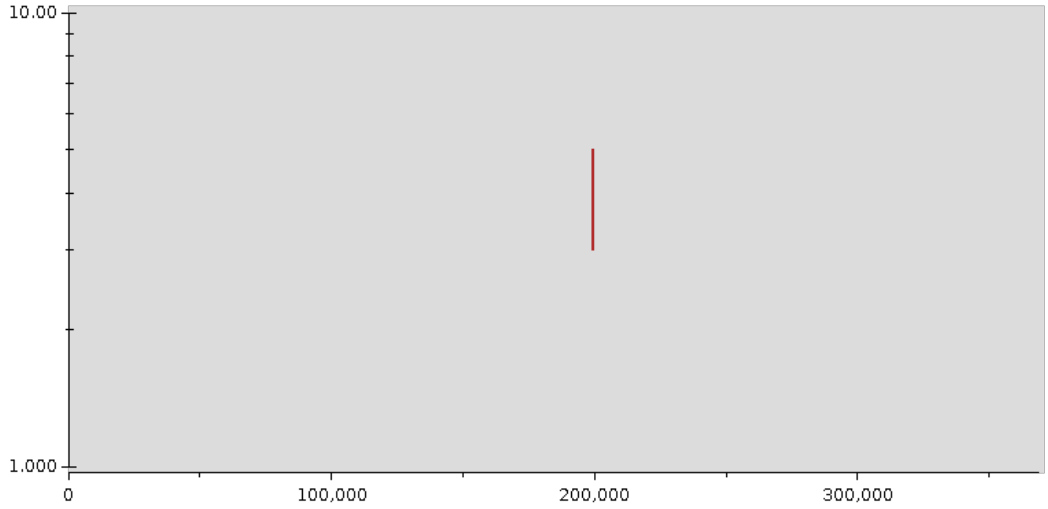

Assignment

**Type**	Marseillevirus marseillevirus (Taxonomy ID: 694581)
**Reference Genome**	NC_013756.1
**NT Identity (%)**	80.9091
**AA Identity (%)**	94.5946
**Number Of Stop Codons**	0
**Number Of CDS**	428

Alignment

**Alignment Score**	136.0 (NT) + 235.0 (AA) = 371.0
**Concordance (%)**	81.3596
**Alignment Method**	Local, heuristic, nucleotide (BLASTN)

Genome Region

Sequence starts at position 199409 and ends at position 199518 relative to NC_013756.1 reference sequence.

Alignment Detailed Statistics

	Begin	End	Coverage	Score	Concordance	Matches	Identities	I/D/M/F*	Stop Codons
NT	199409	199518	0.1%	136	61.8%	110 (100%)	89 (80.9%)	0/0	

Mutations: 199414T>C, 199419G>A, 199420G>A, 199423A>C, 199425G>A, 199426G>A, 199429G>C, 199431G>A, 199435C>T, 199441C>T, 199444T>C, 199449C>T, 199453G>A, 199462C>T, 199464A>G, 199468C>A, 199470T>G, 199480C>G, 199501G>C, 199503G>A, 199504A>T

*: Inserts / Deletes / Misaligned / Frameshifts

Analysis details

This analysis was performed with panviral2.64

NGS Details (UN24): Punavirus P1

Assembly

**Coverage Length**	211 (1 contig(s))
**Depth Of Coverage**	2.2
**Number Of Reads**	4
**Reads Per Million**	0.08 rpm (after QC)
**Ambiguities**	0
**Assembly Method**	de novo + reference guided assembly
**Consensus Caller**	Bcf Tools

Coverage Map

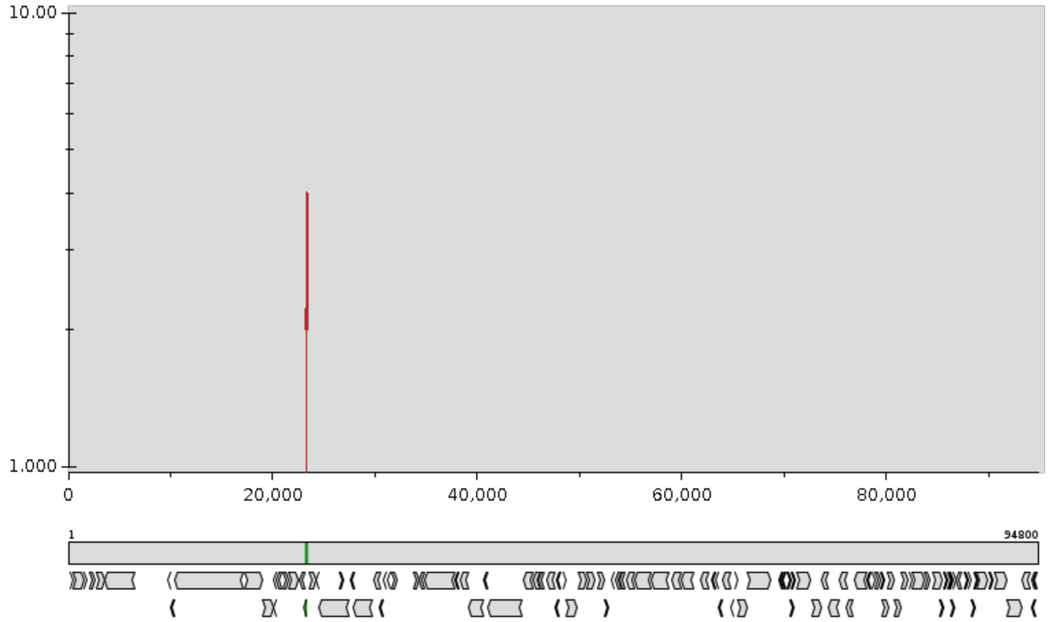

Assignment

**Type**	Punavirus P1 (Taxonomy ID: 10678)
**Reference Genome**	NC_005856.1
**NT Identity (%)**	100.0
**AA Identity (%)**	98.0392
**Number Of Stop Codons**	0
**Number Of CDS**	110

Alignment

**Alignment Score**	422.0 (NT) + 377.0 (AA) = 799.0
**Concordance (%)**	100.0

Alignment Method	Local, heuristic, nucleotide (BLASTN)

Genome Region

Sequence starts at position 23187 and ends at position 23397 relative to NC_005856.1 reference sequence.

Alignment Detailed Statistics

	Begin	End	Coverage	Score	Concordance	Matches	Identities	I/D/M/F*	Stop Codons
**NT**	**23187**	**23397**	**0.2%**	**422**	**100%**	**211 (100%)**	**211 (100%)**	**0/0**	
Mutations:	*none*								
**CDS**									
insA	1	51	55.4%	377	97.7%	51 (100%)	50 (98.0%)	0/0/0/0	0
**Proteins**									
IS1 family transposase (YP_006488.1)	1	51	55.4%	377	97.7%	51 (100%)	50 (98.0%)	0/0/0/0	0
Protein mutations:	*none*								
Codon mutations:	*none*								

*: Inserts / Deletes / Misaligned / Frameshifts

Analysis details

This analysis was performed with panviral2.64

NGS Details (UN24): Sugarcane chlorotic streak virus

Assembly

**Coverage Length**	134 (1 contig(s))
**Depth Of Coverage**	2.0
**Number Of Reads**	2
**Reads Per Million**	0.04 rpm (after QC)
**Ambiguities**	0
**Assembly Method**	de novo + reference guided assembly
**Consensus Caller**	Bcf Tools

Coverage Map

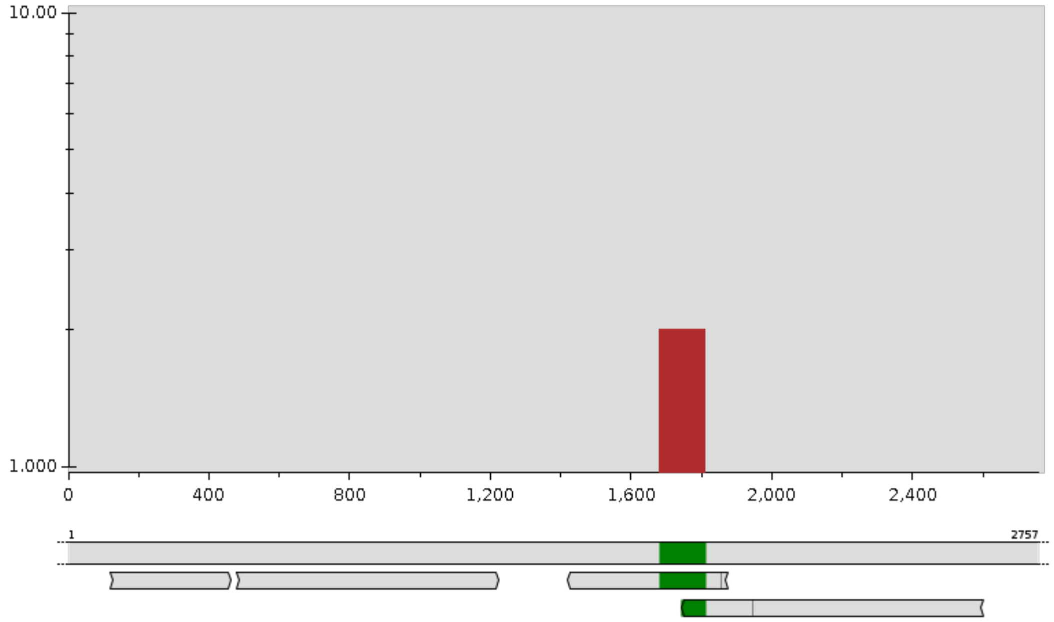

Assignment

**Type**	Sugarcane chlorotic streak virus (Taxonomy ID: 1919062)
**Reference Genome**	NC_032004.1
**NT Identity (%)**	64.9254
**AA Identity (%)**	58.4071
**Number Of Stop Codons**	1
**Number Of CDS**	5

Alignment

**Alignment Score**	80.0 (NT) + 509.0 (AA) = 589.0
**Concordance (%)**	53.5455

Alignment Method	Global, seeded, nucleotide + amino acids (AGA)

Genome Region

Sequence starts at position 1681 and ends at position 1814 relative to NC_032004.1 reference sequence.

Alignment Detailed Statistics

	Begin	End	Coverage	Score	Concordance	Matches	Identities	I/D/M/F*	Stop Codons
**NT**	**1681**	**1814**	**4.9%**	**80**	**29.9%**	**134 (100%)**	**87 (64.9%)**	**0/0**	
Mutations:	1684T>A, 1689G>A, 1692A>C, 1695T>G, 1701A>G, 1704A>T, 1710G>A, 1713G>A, 1714A>T, 1715C>A, 1716G>C, 1718C>A, 1721C>T, 1722C>G, 1723T>A, 1724C>T, 1725T>A, 1734A>G, 1735G>T, 1736A>T, 1737G>A, 1738G>C, 1740C>G, 1741C>A, 1744G>T, 1755T>G, 1756T>C, 1757G>A, 1758C>A, 1759C>A, 1761G>A, 1767A>G, 1776C>A, 1779G>A, 1784T>G, 1785A>T, 1791A>T, 1793A>T, 1794T>C, 1796A>T, 1797C>T, 1800T>A, 1803T>A, 1806C>T, 1809T>A, 1811T>A, 1812T>A								
**CDS**									
BS327_gp3	234	278	12.4%	240	67.4%	45 (100%)	29 (64.4%)	0/0/0/0	0
Protein mutations:	T235S (1809T>A 1811T>A), S240T (1794T>C 1796A>T), S241T (1791A>T 1793A>T), W252F (1758C>A 1759C>A), Q253C (1755T>G 1756T>C 1757G>A), A257D (1744G>T), W258F (1740C>G 1741C>A), T259S (1737G>A 1738G>C), S260N (1734A>G 1735G>T 1736A>T), E263D (1725T>A), E264I (1722C>G 1723T>A 1724C>T), A265T (1721C>T), V266L (1716G>C 1718C>A), V267Y (1713G>A 1714A>T 1715C>A), Y277F (1684T>A)								
Codon mutations:	CCA234CCT (1812T>A), ACA235TCT (1809T>A 1811T>A), AGG236AGA (1806C>T), ACA237ACT (1803T>A), GGA238GGT (1800T>A), AAG239AAA (1797C>T), TCA240ACG (1794T>C 1796A>T), TCT241ACA (1791A>T 1793A>T), GCT243GCA (1785A>T), AGA244CGA (1784T>G), AGC245AGT (1779G>A), CTG246CTT (1776C>A), CAT249CAC (1767A>G), TAC251TAT (1761G>A), TGG252TTT (1758C>A 1759C>A), CAA253TGC (1755T>G 1756T>C 1757G>A), GCC257GAC (1744G>T), TGG258TTC (1740C>G 1741C>A), ACC259AGT (1737G>A 1738G>C), TCT260AAC (1734A>G 1735G>T 1736A>T), GAA263GAT (1725T>A), GAG264ATC (1722C>G 1723T>A 1724C>T), GCA265ACA (1721C>T), GTC266TTG (1716G>C 1718C>A), GTC267TAT (1713G>A 1714A>T 1715C>A), AAC268AAT (1710G>A), ATT270ATA (1704A>T), GAT271GAC (1701A>G), ATA273ATC (1695T>G), CCT274CCG (1692A>C), TTC275TTT (1689G>A), TAC277TTC (1684T>A)								
BS327_gp4	22	66	29.6%	240	67.4%	45 (100%)	29 (64.4%)	0/0/0/0	0
Protein mutations:	T23S (1809T>A 1811T>A), S28T (1794T>C 1796A>T), S29T (1791A>T 1793A>T), W40F (1758C>A 1759C>A), Q41C (1755T>G 1756T>C 1757G>A), A45D (1744G>T), W46F (1740C>G 1741C>A), T47S (1737G>A 1738G>C), S48N (1734A>G 1735G>T 1736A>T), E51D (1725T>A), E52I (1722C>G 1723T>A 1724C>T), A53T (1721C>T), V54L (1716G>C 1718C>A), V55Y (1713G>A 1714A>T 1715C>A), Y65F (1684T>A)								
Codon mutations:	CCA22CCT (1812T>A), ACA23TCT (1809T>A 1811T>A), AGG24AGA (1806C>T), ACA25ACT (1803T>A), GGA26GGT (1800T>A), AAG27AAA (1797C>T), TCA28ACG (1794T>C 1796A>T), TCT29ACA (1791A>T 1793A>T), GCT31GCA (1785A>T), AGA32CGA (1784T>G), AGC33AGT (1779G>A), CTG34CTT (1776C>A), CAT37CAC (1767A>G), TAC39TAT (1761G>A), TGG40TTT (1758C>A 1759C>A), CAA41TGC (1755T>G 1756T>C 1757G>A), GCC45GAC (1744G>T), TGG46TTC (1740C>G 1741C>A), ACC47AGT (1737G>A 1738G>C), TCT48AAC (1734A>G 1735G>T 1736A>T), GAA51GAT (1725T>A), GAG52ATC (1722C>G 1723T>A 1724C>T), GCA53ACA (1721C>T), GTC54TTG (1716G>C 1718C>A), GTC55TAT (1713G>A 1714A>T 1715C>A), AAC56AAT (1710G>A), ATT58ATA (1704A>T), GAT59GAC (1701A>G), ATA61ATC (1695T>G), CCT62CCG (1692A>C), TTC63TTT (1689G>A), TAC65TTC (1684T>A)								
BS327_gp5	265	287	8.0%	29	17.9%	23 (100%)	8 (34.8%)	0/0/0/0	1
Protein mutations:	Q265L (1811T>A 1812T>A), Q266L (1809T>A), G267E (1806C>T), Q268L (1803T>A), E269V (1800T>A), S270K (1796A>T 1797C>T), H271R (1793A>T 1794T>C), L272H (1791A>T), L274H (1784T>G 1785A>T), A276V (1779G>A), W277L (1776C>A), I280T (1767A>G), T282I (1761G>A), G283F (1757G>A 1758C>A 1759C>A), K284A (1755T>G 1756T>C)								
Codon mutations:	CAA265CTT (1811T>A 1812T>A), CAA266CTA (1809T>A), GGA267GAA (1806C>T), CAG268CTG (1803T>A), GAA269GTA (1800T>A), AGT270AAA (1796A>T 1797C>T), CAT271CGA (1793A>T 1794T>C), CTT272CAT (1791A>T), CTA274CAC (1784T>G 1785A>T), GCC276GTC (1779G>A), TGG277TTG (1776C>A), ATA280ACA (1767A>G), ACT282ATT (1761G>A), GGC283TTT (1757G>A 1758C>A 1759C>A), AAA284GCA (1755T>G 1756T>C)								
**Proteins**									
Rep-associated protein (YP_009325925.1)	234	278	12.4%	240	67.4%	45 (100%)	29 (64.4%)	0/0/0/0	0
Protein mutations:	T235S (1809T>A 1811T>A), S240T (1794T>C 1796A>T), S241T (1791A>T 1793A>T), W252F (1758C>A 1759C>A), Q253C (1755T>G 1756T>C 1757G>A), A257D (1744G>T), W258F (1740C>G 1741C>A), T259S (1737G>A 1738G>C), S260N (1734A>G 1735G>T 1736A>T), E263D (1725T>A), E264I (1722C>G 1723T>A 1724C>T), A265T (1721C>T), V266L (1716G>C 1718C>A), V267Y (1713G>A 1714A>T 1715C>A), Y277F (1684T>A)								
Codon mutations:	CCA234CCT (1812T>A), ACA235TCT (1809T>A 1811T>A), AGG236AGA (1806C>T), ACA237ACT (1803T>A), GGA238GGT (1800T>A), AAG239AAA (1797C>T), TCA240ACG (1794T>C 1796A>T), TCT241ACA (1791A>T 1793A>T), GCT243GCA (1785A>T), AGA244CGA (1784T>G), AGC245AGT (1779G>A), CTG246CTT (1776C>A), CAT249CAC (1767A>G), TAC251TAT (1761G>A), TGG252TTT (1758C>A 1759C>A), CAA253TGC (1755T>G 1756T>C 1757G>A), GCC257GAC (1744G>T), TGG258TTC (1740C>G 1741C>A), ACC259AGT (1737G>A 1738G>C), TCT260AAC (1734A>G 1735G>T 1736A>T), GAA263GAT (1725T>A), GAG264ATC (1722C>G 1723T>A 1724C>T), GCA265ACA (1721C>T), GTC266TTG (1716G>C 1718C>A), GTC267TAT (1713G>A 1714A>T 1715C>A), AAC268AAT (1710G>A), ATT270ATA (1704A>T), GAT271GAC (1701A>G), ATA273ATC (1695T>G), CCT274CCG (1692A>C), TTC275TTT (1689G>A), TAC277TTC (1684T>A)								
RepB (YP_009325926.1)	22	66	29.6%	240	67.4%	45 (100%)	29 (64.4%)	0/0/0/0	0
Protein mutations:	T23S (1809T>A 1811T>A), S28T (1794T>C 1796A>T), S29T (1791A>T 1793A>T), W40F (1758C>A 1759C>A), Q41C (1755T>G 1756T>C 1757G>A), A45D (1744G>T), W46F (1740C>G 1741C>A), T47S (1737G>A 1738G>C), S48N (1734A>G 1735G>T 1736A>T), E51D (1725T>A), E52I (1722C>G 1723T>A 1724C>T), A53T (1721C>T), V54L (1716G>C 1718C>A), V55Y (1713G>A 1714A>T 1715C>A), Y65F (1684T>A)								
Codon mutations:	CCA22CCT (1812T>A), ACA23TCT (1809T>A 1811T>A), AGG24AGA (1806C>T), ACA25ACT (1803T>A), GGA26GGT (1800T>A), AAG27AAA (1797C>T), TCA28ACG (1794T>C 1796A>T), TCT29ACA (1791A>T 1793A>T), GCT31GCA (1785A>T), AGA32CGA (1784T>G), AGC33AGT (1779G>A), CTG34CTT (1776C>A), CAT37CAC (1767A>G), TAC39TAT (1761G>A), TGG40TTT (1758C>A 1759C>A), CAA41TGC (1755T>G 1756T>C 1757G>A), GCC45GAC (1744G>T), TGG46TTC (1740C>G 1741C>A), ACC47AGT (1737G>A 1738G>C), TCT48AAC (1734A>G 1735G>T 1736A>T), GAA51GAT (1725T>A), GAG52ATC (1722C>G 1723T>A 1724C>T), GCA53ACA (1721C>T), GTC54TTG (1716G>C 1718C>A), GTC55TAT (1713G>A 1714A>T 1715C>A), AAC56AAT (1710G>A), ATT58ATA (1704A>T), GAT59GAC (1701A>G), ATA61ATC (1695T>G), CCT62CCG (1692A>C), TTC63TTT (1689G>A), TAC65TTC (1684T>A)								
RepA (YP_009325927.1)	265	287	8.0%	29	17.9%	23 (100%)	8 (34.8%)	0/0/0/0	1
Protein mutations:	Q265L (1811T>A 1812T>A), Q266L (1809T>A), G267E (1806C>T), Q268L (1803T>A), E269V (1800T>A), S270K (1796A>T 1797C>T), H271R (1793A>T 1794T>C), L272H (1791A>T), L274H (1784T>G 1785A>T), A276V (1779G>A), W277L (1776C>A), I280T (1767A>G), T282I (1761G>A), G283F (1757G>A 1758C>A 1759C>A), K284A (1755T>G 1756T>C)								
Codon mutations:	CAA265CTT (1811T>A 1812T>A), CAA266CTA (1809T>A), GGA267GAA (1806C>T), CAG268CTG (1803T>A), GAA269GTA (1800T>A), AGT270AAA (1796A>T 1797C>T), CAT271CGA (1793A>T 1794T>C), CTT272CAT (1791A>T), CTA274CAC (1784T>G 1785A>T), GCC276GTC (1779G>A), TGG277TTG (1776C>A), ATA280ACA (1767A>G), ACT282ATT (1761G>A), GGC283TTT (1757G>A 1758C>A 1759C>A), AAA284GCA (1755T>G 1756T>C)								

*: Inserts / Deletes / Misaligned / Frameshifts

Analysis details

This analysis was performed with panviral2.64

NGS Details (UN24): Caulimovirus venafragariae

Assembly

**Coverage Length**	244 (1 contig(s))
**Depth Of Coverage**	1.1
**Number Of Reads**	2
**Reads Per Million**	0.04 rpm (after QC)
**Ambiguities**	0
**Assembly Method**	de novo + reference guided assembly
**Consensus Caller**	Bcf Tools

Coverage Map

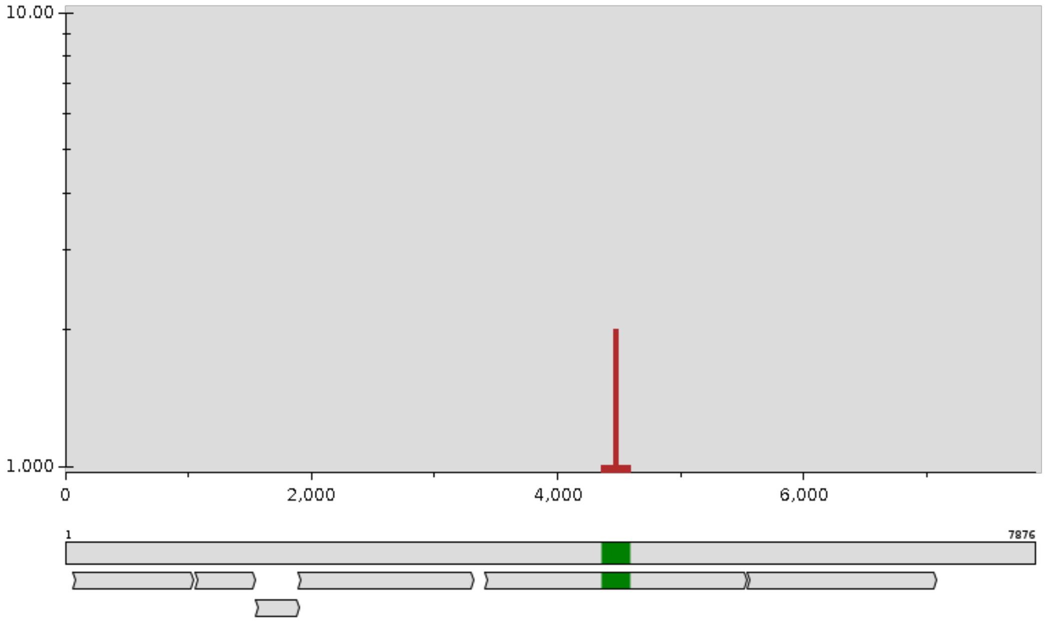

Assignment

**Type**	Caulimovirus venafragariae (Taxonomy ID: 3048344)
**Reference Genome**	NC_001725.1
**NT Identity (%)**	59.4262
**AA Identity (%)**	51.8519
**Number Of Stop Codons**	3
**Number Of CDS**	6

Alignment

**Alignment Score**	92.0 (NT) + 295.0 (AA) = 387.0
**Concordance (%)**	36.4407

Alignment Method	Global, seeded, nucleotide + amino acids (AGA)

Genome Region

Sequence starts at position 4352 and ends at position 4595 relative to NC_001725.1 reference sequence.

Alignment Detailed Statistics

	Begin	End	Coverage	Score	Concordance	Matches	Identities	I/D/M/F*	Stop Codons
**NT**	**4352**	**4595**	**3.1%**	**92**	**18.9%**	**244 (100%)**	**145 (59.4%)**	**0/0**	
Mutations:	4354C>A, 4357G>A, 4359C>A, 4362C>T, 4371G>A, 4378G>A, 4379C>G, 4380C>T, 4383G>A, 4386C>A, 4387A>G, 4388A>T, 4390C>A, 4395C>A, 4398A>G, 4399G>A, 4407G>A, 4413T>A, 4416C>T, 4424A>G, 4425G>A, 4426T>C, 4428A>T, 4432G>A, 4434C>T, 4435C>A, 4440A>T, 4442A>G, 4443G>A, 4444G>A, 4445G>C, 4447G>T, 4451G>C, 4452C>A, 4455C>T, 4456C>A, 4457T>A, 4458A>T, 4459C>T, 4461T>A, 4462C>T, 4464T>A, 4465A>G, 4467C>T, 4470G>A, 4471G>A, 4472A>G, 4473G>T, 4474C>G, 4477C>T, 4479G>A, 4480C>A, 4483C>A, 4485A>T, 4491C>A, 4492G>C, 4493G>A, 4497T>A, 4502C>A, 4503C>G, 4504T>C, 4506T>G, 4508A>T, 4510T>A, 4511C>G, 4513T>A, 4514C>A, 4515C>A, 4530T>A, 4533A>G, 4535T>A, 4538G>A, 4539G>A, 4540C>T, 4542G>A, 4545A>C, 4548C>A, 4549C>A, 4551T>G, 4552G>C, 4553C>A, 4555C>A, 4556C>A, 4560A>T, 4562C>G, 4563A>C, 4566T>C, 4567C>G, 4569G>A, 4570C>T, 4571T>G, 4575C>G, 4576G>A, 4578T>A, 4583G>T, 4584C>G, 4587T>C, 4590C>T, 4591C>A								
**CDS**									
ORF_V	317	397	11.4%	295	50.3%	81 (100%)	42 (51.9%)	0/0/0/0	3
Protein mutations:	P317T (4354C>A), A318T (4357G>A 4359C>A), A325S (4378G>A 4379C>G 4380C>T), K328V (4387A>G 4388A>T), A332T (4399G>A), M334I (4407G>A), K340R (4424A>G 4425G>A), D343N (4432G>A 4434C>T), H344N (4435C>A), K346R (4442A>G 4443G>A), G347T (4444G>A 4445G>C), D348Y (4447G>T), G349A (4451G>C 4452C>A), L351N (4456C>A 4457T>A 4458A>T), P353S (4462C>T 4464T>A), N354D (4465A>G 4467C>T), E356S (4471G>A 4472A>G 4473G>T), Q357E (4474C>G), L359I (4480C>A), Q360N (4483C>A 4485A>T), G363Q (4492G>C 4493G>A), T366K (4502C>A 4503C>G), F367L (4504T>C 4506T>G), Y368F (4508A>T), S370K (4513T>A 4514C>A 4515C>A), F377Y (4535T>A), W378* (4538G>A 4539G>A), Q379* (4540C>T 4542G>A), L382M (4549C>A 4551T>G), A383H (4552G>C 4553C>A), P384K (4555C>A 4556C>A), E385D (4560A>T), T386S (4562C>G 4563A>C), Q388E (4567C>G 4569G>A), L389* (4570C>T 4571T>G), A391T (4576G>A 4578T>A), S393M (4583G>T 4584C>G), Q396K (4591C>A)								
Codon mutations:	CCA317ACA (4354C>A), GCC318ACA (4357G>A 4359C>A), TTC319TTT (4362C>T), AGG322AGA (4371G>A), GCC325AGT (4378G>A 4379C>G 4380C>T), GAG326GAA (4383G>A), ATC327ATA (4386C>A), AAA328GTA (4387A>G 4388A>T), CGA329AGA (4390C>A), GGC330GGA (4395C>A), AAA331AAG (4398A>G), GCA332ACA (4399G>A), ATG334ATA (4407G>A), ATT336ATA (4413T>A), AAC337AAT (4416C>T), AAG340AGA (4424A>G 4425G>A), TTA341CTT (4426T>C 4428A>T), GAC343AAT (4432G>A 4434C>T), CAT344AAT (4435C>A), ACA345ACT (4440A>T), AAG346AGA (4442A>G 4443G>A), GGA347ACA (4444G>A 4445G>C), GAT348TAT (4447G>T), GGC349GCA (4451G>C 4452C>A), TAC350TAT (4455C>T), CTA351AAT (4456C>A 4457T>A 4458A>T), CTT352TTA (4459C>T 4461T>A), CCT353TCA (4462C>T 4464T>A), AAC354GAT (4465A>G 4467C>T), AAG355AAA (4470G>A), GAG356AGT (4471G>A 4472A>G 4473G>T), CAA357GAA (4474C>G), CTG358TTA (4477C>T 4479G>A), CTT359ATT (4480C>A), CAA360AAT (4483C>A 4485A>T), ATC362ATA (4491C>A), GGA363CAA (4492G>C 4493G>A), GGT364GGA (4497T>A), ACC366AAG (4502C>A 4503C>G), TTT367CTG (4504T>C 4506T>G), TAC368TTC (4508A>T), TCT369AGT (4510T>A 4511C>G), TCC370AAA (4513T>A 4514C>A 4515C>A), TCT375TCA (4530T>A), GGA376GGG (4533A>G), TTT377TAT (4535T>A), TGG378TAA (4538G>A 4539G>A), CAG379TAA (4540C>T 4542G>A), GTA380GTC (4545A>C), CGC381CGA (4548C>A), CTT382ATG (4549C>A 4551T>G), GCT383CAT (4552G>C 4553C>A), CCA384AAA (4555C>A 4556C>A), GAA385GAT (4560A>T), ACA386AGC (4562C>G 4563A>C), ATT387ATC (4566T>C), CAG388GAA (4567C>G 4569G>A), CTA389TGA (4570C>T 4571T>G), ACC390ACG (4575C>G), GCT391ACA (4576G>A 4578T>A), AGC393ATG (4583G>T 4584C>G), TGT394TGC (4587T>C), CCC395CCT (4590C>T), CAA396AAA (4591C>A)								
**Proteins**									
hypothetical protein (NP_043933.1)	317	397	11.4%	295	50.3%	81 (100%)	42 (51.9%)	0/0/0/0	3
Protein mutations:	P317T (4354C>A), A318T (4357G>A 4359C>A), A325S (4378G>A 4379C>G 4380C>T), K328V (4387A>G 4388A>T), A332T (4399G>A), M334I (4407G>A), K340R (4424A>G 4425G>A), D343N (4432G>A 4434C>T), H344N (4435C>A), K346R (4442A>G 4443G>A), G347T (4444G>A 4445G>C), D348Y (4447G>T), G349A (4451G>C 4452C>A), L351N (4456C>A 4457T>A 4458A>T), P353S (4462C>T 4464T>A), N354D (4465A>G 4467C>T), E356S (4471G>A 4472A>G 4473G>T), Q357E (4474C>G), L359I (4480C>A), Q360N (4483C>A 4485A>T), G363Q (4492G>C 4493G>A), T366K (4502C>A 4503C>G), F367L (4504T>C 4506T>G), Y368F (4508A>T), S370K (4513T>A 4514C>A 4515C>A), F377Y (4535T>A), W378* (4538G>A 4539G>A), Q379* (4540C>T 4542G>A), L382M (4549C>A 4551T>G), A383H (4552G>C 4553C>A), P384K (4555C>A 4556C>A), E385D (4560A>T), T386S (4562C>G 4563A>C), Q388E (4567C>G 4569G>A), L389* (4570C>T 4571T>G), A391T (4576G>A 4578T>A), S393M (4583G>T 4584C>G), Q396K (4591C>A)								
Codon mutations:	CCA317ACA (4354C>A), GCC318ACA (4357G>A 4359C>A), TTC319TTT (4362C>T), AGG322AGA (4371G>A), GCC325AGT (4378G>A 4379C>G 4380C>T), GAG326GAA (4383G>A), ATC327ATA (4386C>A), AAA328GTA (4387A>G 4388A>T), CGA329AGA (4390C>A), GGC330GGA (4395C>A), AAA331AAG (4398A>G), GCA332ACA (4399G>A), ATG334ATA (4407G>A), ATT336ATA (4413T>A), AAC337AAT (4416C>T), AAG340AGA (4424A>G 4425G>A), TTA341CTT (4426T>C 4428A>T), GAC343AAT (4432G>A 4434C>T), CAT344AAT (4435C>A), ACA345ACT (4440A>T), AAG346AGA (4442A>G 4443G>A), GGA347ACA (4444G>A 4445G>C), GAT348TAT (4447G>T), GGC349GCA (4451G>C 4452C>A), TAC350TAT (4455C>T), CTA351AAT (4456C>A 4457T>A 4458A>T), CTT352TTA (4459C>T 4461T>A), CCT353TCA (4462C>T 4464T>A), AAC354GAT (4465A>G 4467C>T), AAG355AAA (4470G>A), GAG356AGT (4471G>A 4472A>G 4473G>T), CAA357GAA (4474C>G), CTG358TTA (4477C>T 4479G>A), CTT359ATT (4480C>A), CAA360AAT (4483C>A 4485A>T), ATC362ATA (4491C>A), GGA363CAA (4492G>C 4493G>A), GGT364GGA (4497T>A), ACC366AAG (4502C>A 4503C>G), TTT367CTG (4504T>C 4506T>G), TAC368TTC (4508A>T), TCT369AGT (4510T>A 4511C>G), TCC370AAA (4513T>A 4514C>A 4515C>A), TCT375TCA (4530T>A), GGA376GGG (4533A>G), TTT377TAT (4535T>A), TGG378TAA (4538G>A 4539G>A), CAG379TAA (4540C>T 4542G>A), GTA380GTC (4545A>C), CGC381CGA (4548C>A), CTT382ATG (4549C>A 4551T>G), GCT383CAT (4552G>C 4553C>A), CCA384AAA (4555C>A 4556C>A), GAA385GAT (4560A>T), ACA386AGC (4562C>G 4563A>C), ATT387ATC (4566T>C), CAG388GAA (4567C>G 4569G>A), CTA389TGA (4570C>T 4571T>G), ACC390ACG (4575C>G), GCT391ACA (4576G>A 4578T>A), AGC393ATG (4583G>T 4584C>G), TGT394TGC (4587T>C), CCC395CCT (4590C>T), CAA396AAA (4591C>A)								

*: Inserts / Deletes / Misaligned / Frameshifts

Analysis details

This analysis was performed with panviral2.64

NGS Details (UN24): Tomato associated geminivirus 1

Assembly

**Coverage Length**	258 (2 contig(s))
**Depth Of Coverage**	1.0
**Number Of Reads**	2
**Reads Per Million**	0.04 rpm (after QC)
**Ambiguities**	0
**Assembly Method**	de novo + reference guided assembly
**Consensus Caller**	Bcf Tools

Coverage Map

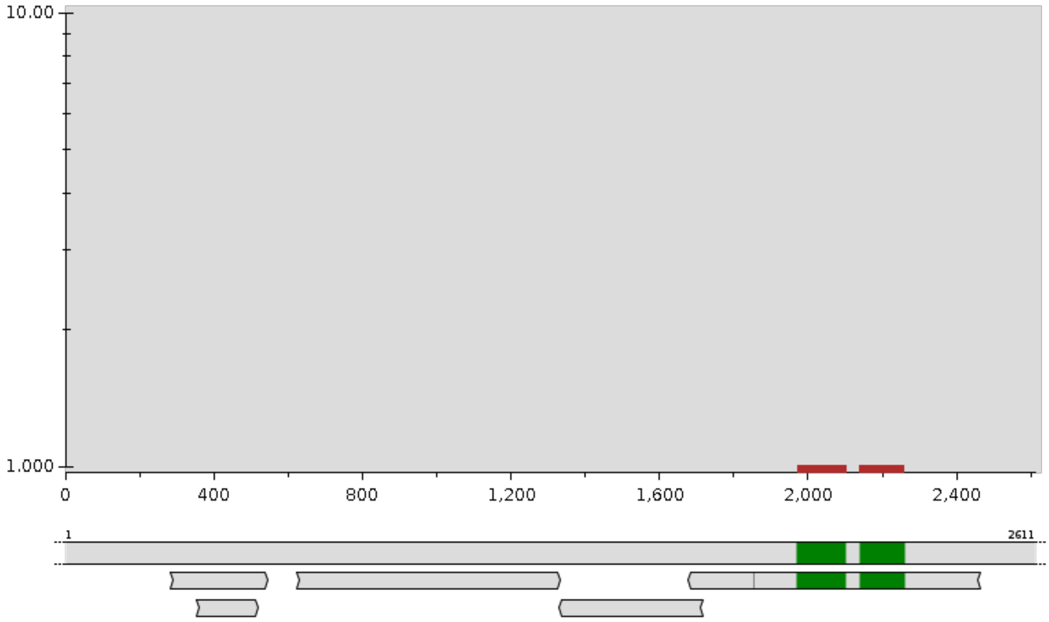

Assignment

**Type**	Tomato associated geminivirus 1 (Taxonomy ID: 2027645)
**Reference Genome**	NC_035676.1
**NT Identity (%)**	75.969
**AA Identity (%)**	79.0698
**Number Of Stop Codons**	4
**Number Of CDS**	5

Alignment

**Alignment Score**	268.0 (NT) + 976.0 (AA) = 1244.0
**Concordance (%)**	71.8245

Alignment Method	Global, seeded, nucleotide + amino acids (AGA)

Genome Region

Sequence starts at position 1970 and ends at position 2261 relative to NC_035676.1 reference sequence.

Alignment Detailed Statistics

	Begin	End	Coverage	Score	Concordance	Matches	Identities	I/D/M/F*	Stop Codons
**NT**	**1970**	**2261**	**9.9%**	**268**	**51.9%**	**258 (100%)**	**196 (76.0%)**	**0/0**	
Mutations:	1981C>T, 1989G>A, 1992G>A, 1998C>G, 1999G>C, 2001G>A, 2007C>T, 2018G>T, 2019T>G, 2024T>G, 2027T>A, 2034G>A, 2037A>G, 2043G>T, 2049G>T, 2055T>A, 2056G>T, 2057A>T, 2061A>T, 2065C>G, 2067T>A, 2073G>T, 2076A>T, 2078C>A, 2079G>A, 2082T>C, 2084T>G, 2086T>G, 2087T>A, 2088C>T, 2094C>A, 2102T>A, 2139A>C, 2150A>C, 2151A>G, 2157G>A, 2160C>G, 2163A>G, 2175C>T, 2177T>A, 2190G>A, 2191A>C, 2192T>A, 2196T>G, 2205A>G, 2209C>G, 2210C>T, 2216C>T, 2220A>T, 2223G>A, 2227G>A, 2228T>C, 2229G>A, 2241C>G, 2243T>G, 2244C>T, 2247G>A, 2251T>C, 2252G>T, 2253C>G, 2256C>G, 2258G>T								
**CDS**									
CK833_gp4	69	166	25.8%	488	78.0%	86 (100%)	68 (79.1%)	0/0/0/0	2
Protein mutations:	Q70N (2256C>G 2258G>T), Q72R (2251T>C 2252G>T), T80V (2227G>A 2228T>C), G84R (2216C>T), G86T (2209C>G 2210C>T), I92C (2190G>A 2191A>C 2192T>A), R97* (2175C>T 2177T>A), S106A (2150A>C), R122* (2102T>A), N127S (2086T>G 2087T>A), A130S (2076A>T 2078C>A), R134T (2065C>G), S137N (2055T>A 2056G>T 2057A>T), T147S (2027T>A), L150M (2018G>T), T156S (1998C>G 1999G>C), R162K (1981C>T)								
Codon mutations:	CAG70AAC (2256C>G 2258G>T), ACG71ACC (2253C>G), CAG72AGG (2251T>C 2252G>T), AAC73AAT (2247G>A), CAG74CAA (2244C>T), AGG75CGC (2241C>G 2243T>G), ATC79ATT (2229G>A), ACC80GTC (2227G>A 2228T>C), GAC81GAT (2223G>A), CCT82CCA (2220A>T), GGA84AGA (2216C>T), GGA86ACA (2209C>G 2210C>T), ATT87ATC (2205A>G), CCA90CCC (2196T>G), ATC92TGT (2190G>A 2191A>C 2192T>A), AGG97TGA (2175C>T 2177T>A), GTT101GTC (2163A>G), GCG102GCC (2160C>G), GAC103GAT (2157G>A), ATT105ATC (2151A>G), TCC106GCC (2150A>C), GGT109GGG (2139A>C), AGA122TGA (2102T>A), CCG124CCT (2094C>A), AAG126AAA (2088C>T), AAT127TCT (2086T>G 2087T>A), AGA128CGG (2082T>C 2084T>G), GAC129GAT (2079G>A), GCT130TCA (2076A>T 2078C>A), ATC131ATA (2073G>T), ACA133ACT (2067T>A), AGA134ACA (2065C>G), ATT135ATA (2061A>T), TCA137AAT (2055T>A 2056G>T 2057A>T), TCC139TCA (2049G>T), TCC141TCA (2043G>T), TCT143TCC (2037A>G), GAC144GAT (2034G>A), ACC147TCC (2027T>A), AGA148CGA (2024T>G), GTA149GTC (2019T>G), CTG150ATG (2018G>T), CAG153CAA (2007C>T), TAC155TAT (2001G>A), ACG156AGC (1998C>G 1999G>C), GCC158GCT (1992G>A), AAC159AAT (1989G>A), AGA162AAA (1981C>T)								
CK833_gp5	69	166	32.8%	488	78.0%	86 (100%)	68 (79.1%)	0/0/0/0	2
Protein mutations:	Q70N (2256C>G 2258G>T), Q72R (2251T>C 2252G>T), T80V (2227G>A 2228T>C), G84R (2216C>T), G86T (2209C>G 2210C>T), I92C (2190G>A 2191A>C 2192T>A), R97* (2175C>T 2177T>A), S106A (2150A>C), R122* (2102T>A), N127S (2086T>G 2087T>A), A130S (2076A>T 2078C>A), R134T (2065C>G), S137N (2055T>A 2056G>T 2057A>T), T147S (2027T>A), L150M (2018G>T), T156S (1998C>G 1999G>C), R162K (1981C>T)								
Codon mutations:	CAG70AAC (2256C>G 2258G>T), ACG71ACC (2253C>G), CAG72AGG (2251T>C 2252G>T), AAC73AAT (2247G>A), CAG74CAA (2244C>T), AGG75CGC (2241C>G 2243T>G), ATC79ATT (2229G>A), ACC80GTC (2227G>A 2228T>C), GAC81GAT (2223G>A), CCT82CCA (2220A>T), GGA84AGA (2216C>T), GGA86ACA (2209C>G 2210C>T), ATT87ATC (2205A>G), CCA90CCC (2196T>G), ATC92TGT (2190G>A 2191A>C 2192T>A), AGG97TGA (2175C>T 2177T>A), GTT101GTC (2163A>G), GCG102GCC (2160C>G), GAC103GAT (2157G>A), ATT105ATC (2151A>G), TCC106GCC (2150A>C), GGT109GGG (2139A>C), AGA122TGA (2102T>A), CCG124CCT (2094C>A), AAG126AAA (2088C>T), AAT127TCT (2086T>G 2087T>A), AGA128CGG (2082T>C 2084T>G), GAC129GAT (2079G>A), GCT130TCA (2076A>T 2078C>A), ATC131ATA (2073G>T), ACA133ACT (2067T>A), AGA134ACA (2065C>G), ATT135ATA (2061A>T), TCA137AAT (2055T>A 2056G>T 2057A>T), TCC139TCA (2049G>T), TCC141TCA (2043G>T), TCT143TCC (2037A>G), GAC144GAT (2034G>A), ACC147TCC (2027T>A), AGA148CGA (2024T>G), GTA149GTC (2019T>G), CTG150ATG (2018G>T), CAG153CAA (2007C>T), TAC155TAT (2001G>A), ACG156AGC (1998C>G 1999G>C), GCC158GCT (1992G>A), AAC159AAT (1989G>A), AGA162AAA (1981C>T)								
**Proteins**									
replication-associated protein (YP_009417307.1)	69	166	25.8%	488	78.0%	86 (100%)	68 (79.1%)	0/0/0/0	2
Protein mutations:	Q70N (2256C>G 2258G>T), Q72R (2251T>C 2252G>T), T80V (2227G>A 2228T>C), G84R (2216C>T), G86T (2209C>G 2210C>T), I92C (2190G>A 2191A>C 2192T>A), R97* (2175C>T 2177T>A), S106A (2150A>C), R122* (2102T>A), N127S (2086T>G 2087T>A), A130S (2076A>T 2078C>A), R134T (2065C>G), S137N (2055T>A 2056G>T 2057A>T), T147S (2027T>A), L150M (2018G>T), T156S (1998C>G 1999G>C), R162K (1981C>T)								
Codon mutations:	CAG70AAC (2256C>G 2258G>T), ACG71ACC (2253C>G), CAG72AGG (2251T>C 2252G>T), AAC73AAT (2247G>A), CAG74CAA (2244C>T), AGG75CGC (2241C>G 2243T>G), ATC79ATT (2229G>A), ACC80GTC (2227G>A 2228T>C), GAC81GAT (2223G>A), CCT82CCA (2220A>T), GGA84AGA (2216C>T), GGA86ACA (2209C>G 2210C>T), ATT87ATC (2205A>G), CCA90CCC (2196T>G), ATC92TGT (2190G>A 2191A>C 2192T>A), AGG97TGA (2175C>T 2177T>A), GTT101GTC (2163A>G), GCG102GCC (2160C>G), GAC103GAT (2157G>A), ATT105ATC (2151A>G), TCC106GCC (2150A>C), GGT109GGG (2139A>C), AGA122TGA (2102T>A), CCG124CCT (2094C>A), AAG126AAA (2088C>T), AAT127TCT (2086T>G 2087T>A), AGA128CGG (2082T>C 2084T>G), GAC129GAT (2079G>A), GCT130TCA (2076A>T 2078C>A), ATC131ATA (2073G>T), ACA133ACT (2067T>A), AGA134ACA (2065C>G), ATT135ATA (2061A>T), TCA137AAT (2055T>A 2056G>T 2057A>T), TCC139TCA (2049G>T), TCC141TCA (2043G>T), TCT143TCC (2037A>G), GAC144GAT (2034G>A), ACC147TCC (2027T>A), AGA148CGA (2024T>G), GTA149GTC (2019T>G), CTG150ATG (2018G>T), CAG153CAA (2007C>T), TAC155TAT (2001G>A), ACG156AGC (1998C>G 1999G>C), GCC158GCT (1992G>A), AAC159AAT (1989G>A), AGA162AAA (1981C>T)								
RepA (YP_009417308.1)	69	166	32.8%	488	78.0%	86 (100%)	68 (79.1%)	0/0/0/0	2
Protein mutations:	Q70N (2256C>G 2258G>T), Q72R (2251T>C 2252G>T), T80V (2227G>A 2228T>C), G84R (2216C>T), G86T (2209C>G 2210C>T), I92C (2190G>A 2191A>C 2192T>A), R97* (2175C>T 2177T>A), S106A (2150A>C), R122* (2102T>A), N127S (2086T>G 2087T>A), A130S (2076A>T 2078C>A), R134T (2065C>G), S137N (2055T>A 2056G>T 2057A>T), T147S (2027T>A), L150M (2018G>T), T156S (1998C>G 1999G>C), R162K (1981C>T)								
Codon mutations:	CAG70AAC (2256C>G 2258G>T), ACG71ACC (2253C>G), CAG72AGG (2251T>C 2252G>T), AAC73AAT (2247G>A), CAG74CAA (2244C>T), AGG75CGC (2241C>G 2243T>G), ATC79ATT (2229G>A), ACC80GTC (2227G>A 2228T>C), GAC81GAT (2223G>A), CCT82CCA (2220A>T), GGA84AGA (2216C>T), GGA86ACA (2209C>G 2210C>T), ATT87ATC (2205A>G), CCA90CCC (2196T>G), ATC92TGT (2190G>A 2191A>C 2192T>A), AGG97TGA (2175C>T 2177T>A), GTT101GTC (2163A>G), GCG102GCC (2160C>G), GAC103GAT (2157G>A), ATT105ATC (2151A>G), TCC106GCC (2150A>C), GGT109GGG (2139A>C), AGA122TGA (2102T>A), CCG124CCT (2094C>A), AAG126AAA (2088C>T), AAT127TCT (2086T>G 2087T>A), AGA128CGG (2082T>C 2084T>G), GAC129GAT (2079G>A), GCT130TCA (2076A>T 2078C>A), ATC131ATA (2073G>T), ACA133ACT (2067T>A), AGA134ACA (2065C>G), ATT135ATA (2061A>T), TCA137AAT (2055T>A 2056G>T 2057A>T), TCC139TCA (2049G>T), TCC141TCA (2043G>T), TCT143TCC (2037A>G), GAC144GAT (2034G>A), ACC147TCC (2027T>A), AGA148CGA (2024T>G), GTA149GTC (2019T>G), CTG150ATG (2018G>T), CAG153CAA (2007C>T), TAC155TAT (2001G>A), ACG156AGC (1998C>G 1999G>C), GCC158GCT (1992G>A), AAC159AAT (1989G>A), AGA162AAA (1981C>T)								

*: Inserts / Deletes / Misaligned / Frameshifts

Analysis details

This analysis was performed with panviral2.64
