# Supplementary material for: Nucleotide sequence analysis reveals the presence of PVY-Tam isolates affecting tamarillo in Colombia
Source: Virol J. 2026 Apr 20;23:145. doi: 10.1186/s12985-026-03166-6 (PMC13234967; doi:10.1186/s12985-026-03166-6)
Supplement: Supplementary file 5 — Additional file 5. [file 12985_2026_3166_MOESM5_ESM.pdf]

Analysis of UN59

**Technology**	Paired-end short reads
**Input Files**	UN59_R1.fq.gz (1.47 GB), UN59_R2.fq.gz (1.52 GB)
**Submitted On**	2023-09-26 13:15:02 UTC
**Duration**	2h 52m 57s
**Tool Version**	panviral2.64
**Location**	https://www.genomedetective.com/db/ui/analysis/2e79870b-faad-478b-bd65-8d012eb0827f

Statistics

**Original Read Length**	20 - 150
**Trimmed Read Length**	50 - 135

	# Reads	% of Reads
**Input file**	46314422	100.0%
**After QC**	46000056	99.3%
**After filtering**	2552086	5.5%
**Mapped back to contigs**	725798	1.6%

Assignments

Assignment	No. of Reads	Depth of Coverage	Identity		Genome Coverage	
			NT	AA		
Torradovirus lycopersici (2 segments out of 2)	415162	4267.6	89.2%	95.0%	99.8%	
Torradovirus lycopersici (segment RNA 2)	251860	6024.7	85.2%	89.6%	99.6%	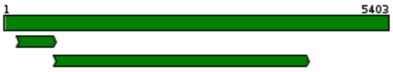
Torradovirus lycopersici (segment RNA 1)	163302	3055.8	92.0%	98.4%	99.9%	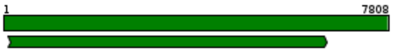
Torradovirus marchitezum (2 segments out of 2)	188453	2391.1	65.6%	69.1%	84.2%	
Torradovirus marchitezum (segment RNA 2)	128651	3539.8	64.5%	68.0%	94.9%	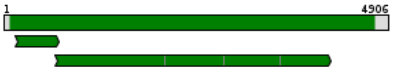
Torradovirus marchitezum (segment RNA 1)	59802	1430.4	66.5%	69.9%	77.0%	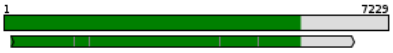
Bracoviriform glomeratae (segment NC_043292.1)	12058	4640.4	72.7%	84.3%	72.3%	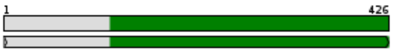
Diachasmimorpha longicaudata entomopoxvirus (segment NC_043455.1)	7928	876.9	59.1%	55.3%	83.7%	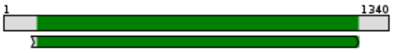

Assignment	No. of Reads	Depth of Coverage	Identity		Genome Coverage	
			NT	AA		
Potato virus Y	757	11.7	83.3%	90.2%	89.3%	
Duamitovirus soch1	734	39.0	67.5%	68.0%	82.0%	
Solendovirus venanicotianae	285	14.6	79.8%	76.8%	29.4%	

Discoveries

Similar to	No. of Reads	Depth of Coverage	Identity		Genome Coverage	
			NT	AA		
Tomato chocolate spot virus (3 segments out of 2)	71567	4220.5	70.5%	73.2%	19.7%	
Tomato chocolate spot virus (segment RNA 1)	56205	7250.8	67.5%	66.2%	16.2%	
Tomato chocolate spot virus (segment RNA2)	9765	1360.0	69.7%	75.4%	15.9%	
Tomato chocolate spot virus (segment RNA 1)	5597	1311.6	79.5%	80.4%	6.2%	
Lausannevirus	18309	9728.0	81.8%	94.9%	0.1%	
Makelovirus prm1	4406	557.0	76.8%	87.6%	0.7%	
Lausannevirus	1051	709.4	79.8%	95.3%	0.0%	
Betabaculovirus disaccharalis	905	510.6	83.9%	91.3%	0.1%	
Betabaculovirus disaccharalis	820	486.0	82.8%	88.9%	0.1%	
Cladosporium fulvum T-1 virus	529	42.1	54.1%	47.2%	19.8%	
Epiphyllum badnavirus 1	512	143.5	52.5%	46.8%	5.3%	
Human gammaherpesvirus 8 (subtype: Could not assign)	421	265.0	83.7%	84.4%	0.1%	
Tomato necrotic dwarf virus (segment RNA1)	407	176.1	75.2%	86.1%	4.2%	

Similar to	No. of Reads	Depth of Coverage	Identity		Genome Coverage	
			NT	AA		
Badnavirus maculasmallanthi	339	121.8	56.5%	53.5%	3.9%	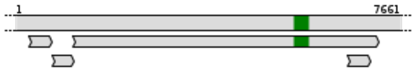
Cassava brown streak virus	273	61.7	61.1%	58.2%	5.9%	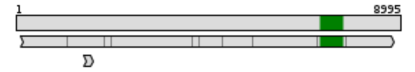
Caulimovirus venafragariae	134	12.6	56.6%	48.5%	15.5%	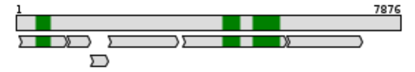
Dioscovirus dioscoreae	81	8.0	62.0%	52.2%	14.7%	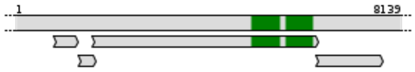
Badnavirus occultipomeae	80	30.9	58.1%	50.0%	3.2%	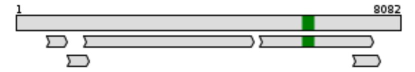
Badnavirus venabougainvilleae	73	25.3	55.2%	46.9%	3.3%	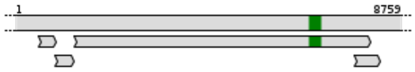
Badnavirus occultipomeae	50	18.2	57.8%	49.0%	3.7%	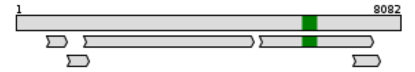
Dioscorea bacilliform virus	35	14.2	55.2%	45.2%	3.8%	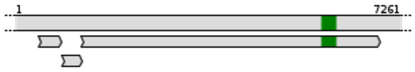
Dioscorea bacilliform virus	11	3.5	53.1%	39.7%	5.8%	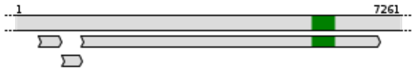
Badnavirus venaribis	36	9.7	52.9%	45.9%	5.8%	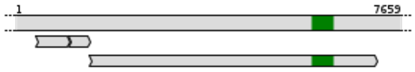
Errantivirus	32	8.5	31.4%	30.3%	6.6%	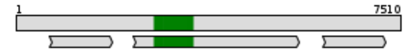
Pinus nigra virus 1	27	6.5	56.6%	43.5%	7.5%	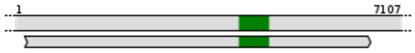
Duamitovirus peex1	26	6.5	71.9%	65.9%	14.4%	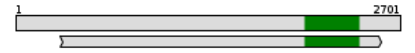
Caulimovirus latensarmoraciae	26	9.8	59.9%	56.1%	4.3%	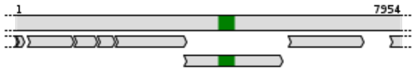
Badnavirus volubetulae	25	7.7	50.9%	47.1%	5.0%	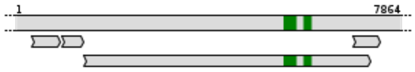
Petuvirus venapetuniae	25	5.3	51.9%	42.9%	8.5%	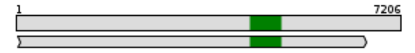
Badnavirus maculasmallanthi	23	5.8	58.7%	60.4%	6.3%	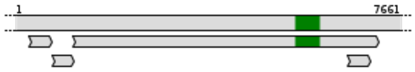

Similar to	No. of Reads	Depth of Coverage	Identity		Genome Coverage	
			NT	AA		
Epiphyllum badnavirus 1	22	7.4	51.0%	42.0%	4.5%	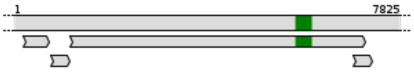
Badnavirus rutilanscamelliae	21	9.1	52.4%	38.5%	3.4%	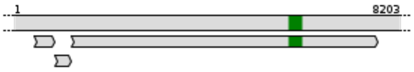
Unknown	20	5.6	56.7%	53.6%	4.3%	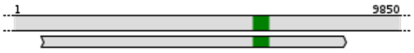
Badnavirus venabougainvilleae	18	6.9	53.3%	46.1%	3.5%	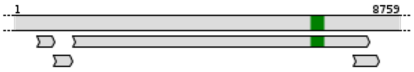
Epiphyllum badnavirus 1	16	6.7	58.0%	50.0%	3.5%	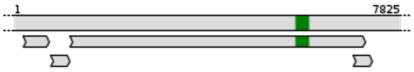
Cavemovirus deltaepiphylli	15	4.4	60.0%	52.9%	5.7%	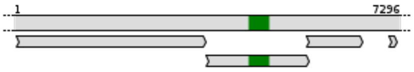
Alphabaculovirus lydisparis	8	4.3	78.6%	89.1%	0.1%	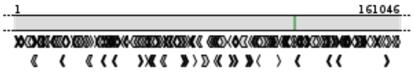
Trialeurodes vaporariorum mononega-like virus 2	8	4.0	99.6%	97.8%	2.4%	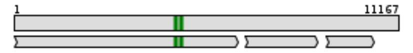
Caulimovirus venafragariae	7	2.2	65.1%	60.9%	5.0%	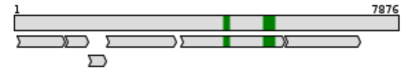
Burkholderia phage Magia	4	2.6	86.8%	84.8%	0.4%	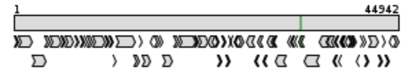
Sugarcane chlorotic streak virus	3	1.6	59.9%	56.2%	7.3%	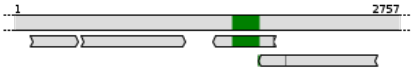
Alphafusarivirus pleosporae	2	1.1	75.2%	85.7%	3.8%	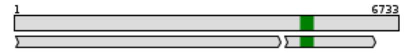
Potato virus X	2	1.2	79.1%	97.3%	3.5%	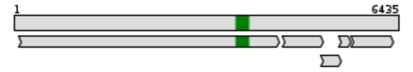
Colombian datura virus	2	1.0	98.1%	97.8%	2.8%	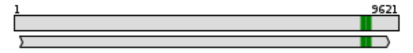
Rahariannevirus raharianne	2	1.0	82.1%	82.1%	0.5%	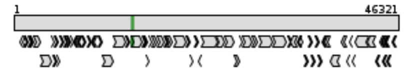
Euphorbia caput-medusae latent virus	1	1.0	64.2%	66.3%	4.6%	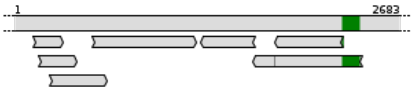
Escherichia virus DE3	1	1.0	100.0%	96.3%	0.3%	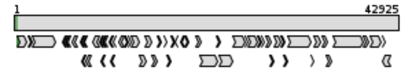

NGS Details (UN59): Torradovirus lycopersici (segment RNA 1)

Assembly

**Coverage Length**	7799 (1 contig(s))
**Depth Of Coverage**	3055.8
**Number Of Reads**	163302
**Reads Per Million**	3550.04 rpm (after QC)
**Ambiguities**	0
**Assembly Method**	de novo + reference guided assembly
**Consensus Caller**	Bcf Tools

Coverage Map

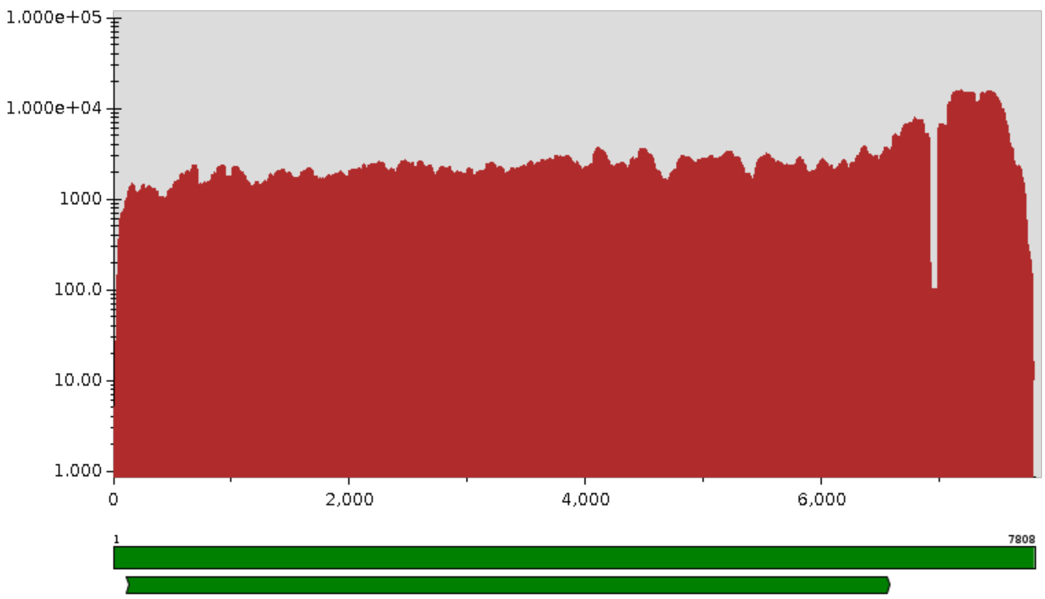

Assignment

**Type**	Torradovirus lycopersici (Taxonomy ID: 3048378)
**Reference Genome**	NC_009013.1
**NT Identity (%)**	92.0064
**AA Identity (%)**	98.4252
**Number Of Stop Codons**	1
**Number Of CDS**	1

Alignment

**Alignment Score**	12080.0 (NT) + 14905.0 (AA) = 26985.0
**Concordance (%)**	91.0702

Alignment Method	Global, seeded, nucleotide + amino acids (AGA)

Genome Region

Sequence starts at position 1 and ends at position 7799 relative to NC_009013.1 reference sequence.

Alignment Detailed Statistics

	Begin	End	Coverage	Score	Concordance	Matches	Identities	I/D/M/F*	Stop Codons
**NT**	**1**	**7799**	**99.9%**	**12080**	**83.0%**	**7519 (96.3%)**	**6929 (88.7%)**	**12/280**	
Mutations:	2T>C, 12A>C, 15T>A, 76_77insT, 83C>T, 83_84insT, 87C>T, 115T>C, 127C>T, 128C>T, 130C>T, 135T>C, 148T>C, 158G>A, 160T>C, 161A>G, 187A>T, 199T>A, 208T>C, 220C>T, 224C>T, 253A>G, 259T>C, 262T>A, 265C>T, 340G>A, 349G>A, 373C>T, 376C>T, 445C>T, 487C>T, 502G>A, 541T>C, 559C>T, 578C>T, 628C>T, 655C>T, 676A>G, 694A>G, 736A>G, 842C>T, 880A>G, 898G>A, 925C>T, 952A>G, 964T>G, 970G>A, 997C>T, 1006T>C, 1033G>A, 1060T>C, 1078A>T, 1081C>T, 1093T>C, 1105A>G, 1108T>C, 1109C>T, 1165C>T, 1180G>A, 1196A>G, 1231G>A, 1240C>T, 1255G>A, 1267T>C, 1276A>G, 1300G>A, 1351A>G, 1372C>T, 1411T>C, 1417T>C, 1429C>T, 1450T>C, 1451C>T, 1480C>T, 1481A>G, 1489T>C, 1522C>T, 1552C>T, 1555C>T, 1570A>G, 1573T>C, 1582C>T, 1585A>T, 1591C>T, 1594T>C, 1607G>T, 1612G>A, 1618T>G, 1624C>A, 1645A>G, 1669C>T, 1681T>A, 1684G>A, 1699T>C, 1705T>C, 1737A>G, 1738A>G, 1771C>T, 1781T>C, 1789A>G, 1897A>G, 1910T>C, 1954T>C, 1960A>G, 1984C>T, 2014A>G, 2023T>C, 2056T>C, 2077C>A, 2082T>A, 2092A>G, 2107A>G, 2128T>C, 2138C>T, 2143T>C, 2149T>C, 2155T>C, 2174C>T, 2179A>C, 2182G>A, 2194T>A, 2200T>C, 2215G>A, 2218C>T, 2224C>T, 2236A>G, 2241A>G, 2250G>A, 2251C>T, 2257C>T, 2263G>A, 2275T>C, 2287G>A, 2290C>T, 2314C>T, 2323A>T, 2347C>T, 2350A>G, 2356C>T, 2359G>A, 2362C>T, 2377G>A, 2380T>C, 2392A>G, 2407T>C, 2409G>A, 2443T>C, 2446G>A, 2479G>A, 2485C>T, 2497G>A, 2500T>C, 2503G>A, 2509C>T, 2512T>C, 2542G>A, 2548C>T, 2557C>T, 2620G>A, 2626T>C, 2650G>A, 2665T>C, 2675T>C, 2683A>G, 2692G>A, 2702C>T, 2710A>G, 2719T>C, 2725T>C, 2731C>T, 2734C>T, 2743A>G, 2744T>C, 2765T>C, 2773A>G, 2809C>T, 2812G>A, 2827A>G, 2869C>T, 2881T>C, 2929C>T, 2930T>C, 2935G>A, 2941A>G, 2950C>T, 2951C>T, 3037C>T, 3040T>C, 3049A>G, 3064A>G, 3109T>C, 3163T>C, 3169C>T, 3202T>C, 3241T>C, 3253C>T, 3256G>A, 3265A>G, 3313T>C, 3406A>C, 3418C>A, 3421T>C, 3463A>G, 3466G>A, 3481C>T, 3484A>T, 3534G>A, 3541C>T, 3580T>C, 3583C>T, 3610A>G, 3613T>C, 3625C>T, 3628G>A, 3643A>G, 3646C>T, 3661A>G, 3664T>C, 3673G>A, 3676C>T, 3686C>T, 3706T>C, 3716T>C, 3721T>A, 3739A>T, 3751A>G, 3754G>A, 3758G>A, 3766G>A, 3808T>C, 3814C>T, 3817G>C, 3824T>C, 3835T>C, 3844T>C, 3857T>C, 3859G>A, 3868C>T, 3871T>C, 3883G>A, 3898T>C, 3907A>T, 3916C>T, 3925A>G, 3928G>A, 3949C>T, 3955G>A, 3961G>A, 3970A>G, 3973T>C, 4034T>C, 4066T>C, 4075A>G, 4084C>T, 4100T>C, 4118C>T, 4129C>T, 4147C>A, 4159G>A, 4162A>T, 4168A>G, 4186A>T, 4195A>G, 4207G>A, 4210T>C, 4228C>T, 4294A>G, 4339T>C, 4343C>T, 4348C>T, 4354T>C, 4396T>C, 4408C>T, 4414T>G, 4417T>C, 4420G>A, 4459T>C, 4462C>T, 4465C>T, 4468C>T, 4471T>C, 4474T>C, 4591T>C, 4636T>C, 4640C>T, 4660A>G, 4684A>C, 4696C>T, 4702G>A, 4705C>T, 4714T>C, 4735C>T, 4747A>G, 4759C>T, 4768T>C, 4774G>A, 4777C>T, 4783A>G, 4786A>G, 4789C>T, 4813A>G, 4816T>C, 4843G>A, 4898G>A, 4906G>A, 4969G>A, 4988A>G, 4993A>T, 5005T>C, 5012T>C, 5029G>A, 5065C>T, 5074T>C, 5077T>C, 5079T>C, 5102T>C, 5118C>T, 5119T>C, 5120G>A, 5125G>C, 5146T>C, 5148C>A, 5200G>A, 5218T>G, 5244G>A, 5266C>T, 5281C>T, 5329C>T, 5341T>A, 5344A>G, 5347C>T, 5369G>A, 5371G>A, 5386C>T, 5392G>A, 5407A>G, 5413C>T, 5419A>T, 5437T>C, 5440C>T, 5461T>C, 5471T>C, 5482A>G, 5533T>A, 5572T>C, 5578T>C, 5584T>C, 5617A>T, 5641T>C, 5647T>C, 5686G>A, 5690T>C, 5710T>A, 5716G>T, 5734T>C, 5737T>C, 5739G>A, 5746T>C, 5750T>C, 5758C>T, 5773G>A, 5784T>C, 5786T>C, 5797A>G, 5806C>T, 5836A>T, 5849T>C, 5850T>A, 5851G>A, 5878G>T, 5884T>C, 5887C>T, 5890C>T, 5914T>C, 5923T>C, 5927C>T, 5932C>T, 5955A>G, 5965G>A, 5998A>G, 6004C>T, 6016A>G, 6028C>T, 6031A>T, 6033C>T, 6035G>T, 6055T>A, 6067C>A, 6070T>C, 6094T>G, 6103G>A, 6106G>A, 6139T>C, 6145T>C, 6148T>C, 6154C>T, 6184A>G, 6187T>C, 6193C>T, 6196A>G, 6208A>G, 6226G>A, 6230A>G, 6232A>G, 6235G>A, 6256C>A, 6262A>G, 6265G>A, 6292T>A, 6298A>G, 6304A>G, 6308T>C, 6313T>C, 6319A>C, 6322G>A, 6323A>G, 6358T>C, 6372C>T, 6487T>C, 6493T>C, 6517C>T, 6545C>T, 6571C>T, 6587G>A, 6660C>T, 6661T>C, 6670C>T, 6685A>G, 6742A>T, 6766delC, 6786T>C, 6798_6799insA, 6806_6852delTAACGGAGAGTACTGACTTTAACTAGTTGGGAGTCCGGCTCCATTTG, 6854A>T, 6857A>G, 6858C>A, 6859C>T, 6860A>G, 6861A>G, 6864A>T, 6867T>A, 6871_6894delATTGGTTAAAGAGATTTGCACGCC, 6897T>A, 6905T>G, 6907T>A, 6908G>T, 6911T>C, 6912C>G, 6915G>C, 6918C>T, 6921T>G, 6923C>G, 6924T>A, 6927G>T, 6928T>G, 6931G>T, 6933_6935delAAG, 6938C>A, 6947A>C, 6948_6951delGGTT, 6957A>T, 6959_6968delTACGGGGAGC, 6984T>C, 6984_6985insG, 6988G>A, 6994G>A, 6997A>T, 6998T>C, 7002A>T, 7003T>A, 7005_7012delATAGTCCT, 7014T>A, 7020_7026delAAGGGTT, 7031T>A, 7033G>A, 7035T>G, 7037T>A, 7038G>C, 7043T>C, 7045T>G, 7047_7060delGCCGTGATGAAAGA, 7063A>T, 7069C>A, 7071T>A, 7074C>A, 7077T>C, 7083G>T, 7085A>G, 7086G>T, 7087_7099delGCTGAAACTACAC, 7101T>G, 7108T>A, 7109G>T, 7113T>G, 7117C>T, 7118T>G, 7120A>T, 7123_7125delCCT, 7129_7133delATCAG, 7136C>A, 7137G>C, 7141G>A, 7144_7169delATTCGATAATTTTCCGTAGCTTGCGT, 7173_7177delGCTGC, 7179C>G, 7182G>A, 7183T>C, 7184T>C, 7185A>C, 7189G>A, 7194_7216delAGTGAAGATGCGCCGTACCACGT, 7221C>T, 7224_7270delGGCAATGCCAGTGGTTCAGAGCGGGCCCTCAGAATAGAGGTTAAAAC, 7275_7285delGTGATGGTGTA, 7288T>A, 7294T>G, 7295A>C, 7297A>C, 7300T>G, 7303C>A, 7304_7315delACCCGGGTTGTG, 7320delG, 7325_7326delGT, 7332C>G, 7333G>T, 7336C>T, 7337A>G, 7339_7348delAGGTCCCACC, 7350T>A, 7351A>T, 7353A>T, 7354G>A, 7358A>C, 7360G>A, 7361A>C, 7362A>C, 7364C>T, 7368G>A, 7372A>T, 7375T>G, 7376T>G, 7377T>A, 7379A>T, 7388C>T, 7397G>A, 7398C>A, 7399G>A, 7400A>T, 7404C>T, 7407A>T, 7408G>T, 7423A>T, 7425G>A, 7435T>C, 7436G>A, 7444C>T, 7445A>G, 7457A>G, 7458T>C, 7460G>A, 7498C>T, 7503C>T, 7505A>G, 7511_7512insC, 7513A>C, 7514A>T, 7515A>C, 7522T>C, 7525_7526insG, 7526T>A, 7526_7527insG, 7545T>A, 7554A>G, 7569G>A, 7585T>C, 7589T>A, 7600T>G, 7601T>A, 7602T>G, 7603_7604insC, 7610C>T, 7612G>A, 7615A>G, 7616A>C, 7617A>T, 7618C>T, 7625A>T, 7628_7631delGCTT, 7637G>T, 7641T>A, 7644G>T, 7647T>G, 7648G>T, 7649T>C, 7657G>C, 7660T>A, 7665G>T, 7666T>C, 7667G>T, 7678C>T, 7686T>G, 7687T>A, 7689G>T, 7698G>A, 7717A>C, 7719T>A, 7723T>C, 7739T>A, 7742_7743insC, 7749T>C, 7751T>C, 7752G>A, 7754_7755insG, 7756_7757insT, 7762C>T, 7763A>C, 7764A>T, 7767C>T, 7773G>T, 7775G>A, 7778T>A, 7782A>T, 7783A>C, 7784_7785insT									
**CDS**									
ToTV_sRNA1gp1	1	2159	100%	14905	98.9%	2159 (100%)	2125 (98.4%)	0/0/0/0	1
Protein mutations:	P8S (128C>T 130C>T), F10S (135T>C), A18T (158G>A 160T>C), T19A (161A>G), T364A (1196A>G), I459V (1481A>G), A501S (1607G>T), K544R (1737A>G 1738A>G), F659Y (2082T>A), N712S (2241A>G), S715N (2250G>A 2251C>T), R768K (2409G>A), R1143K (3534G>A), V1218I (3758G>A), P1413S (4343C>T), V1598I (4898G>A), T1628A (4988A>G), I1658T (5079T>C), T1671I (5118C>T 5119T>C), V1672I (5120G>A), T1681N (5148C>A), R1713K (5244G>A), V1755I (5369G>A 5371G>A), I1792M (5482A>G), R1878K (5739G>A), I1893T (5784T>C), L1915Q (5849T>C 5850T>A 5851G>A), N1950S (5955A>G), T1976I (6033C>T), A1977S (6035G>T), I2042V (6230A>G 6232A>G), I2073V (6323A>G), A2089V (6372C>T), L2147F (6545C>T)									

	Begin	End	Coverage	Score	Concordance	Matches	Identities	I/D/M/F*	Stop Codons
**NT**	**1**	**7799**	**99.9%**	**12080**	**83.0%**	**7519 (96.3%)**	**6929 (88.7%)**	**12/280**	
Codon mutations:	TTT3TTC (115T>C), TTC7TTT (127C>T), CCC8TCT (128C>T 130C>T), TTC10TCC (135T>C), ACT14ACC (148T>C), GCT18ACC (158G>A 160T>C), ACC19GCC (161A>G), TCA27TCT (187A>T), GCT31GCA (199T>A), AGT34AGC (208T>C), CGC38CGT (220C>T), CTG40TTG (224C>T), CAA49CAG (253A>G), TCT51TCC (259T>C), TCT52TCA (262T>A), CAC53CAT (265C>T), TTG78TTA (340G>A), AAG81AAA (349G>A), TTC89TTT (373C>T), ATC90ATT (376C>T), AGC113AGT (445C>T), GAC127GAT (487C>T), GTG132GTA (502G>A), TCT145TCC (541T>C), TTC151TTT (559C>T), CTA158TTA (578C>T), AAC174AAT (628C>T), GAC183GAT (655C>T), CAA190CAG (676A>G), GCA196GCG (694A>G), CCA210CCG (736A>G), CTG246TTG (842C>T), CAA258CAG (880A>G), AGG264AGA (898G>A), GCC273GCT (925C>T), GTA282GTG (952A>G), GGT286GGG (964T>G), AAG288AAA (970G>A), GCC297GCT (997C>T), TCT300TCC (1006T>C), GAG309GAA (1033G>A), CAT318CAC (1060T>C), GCA324GCT (1078A>T), AAC325AAT (1081C>T), ACT329ACC (1093T>C), CAA333CAG (1105A>G), CAT334CAC (1108T>C), CTA335TTA (1109C>T), GCC353GCT (1165C>T), AGG358AGA (1180G>A), ACC364GCC (1196A>G), CAG375CAA (1231G>A), GGC378GGT (1240C>T), AGG383AGA (1255G>A), TTT387TTC (1267T>C), CAA390CAG (1276A>G), GGG398GGA (1300G>A), GAA415GAG (1351A>G), CTC422CTT (1372C>T), TAT435TAC (1411T>C), CAT437CAC (1417T>C), ATC441ATT (1429C>T), GCT448GCC (1450T>C), CTG449TTG (1451C>T), GAC458GAT (1480C>T), ATT459GTT (1481A>G), GAT461GAC (1489T>C), CCC472CCT (1522C>T), GGC482GGT (1552C>T), CGC483CGT (1555C>T), CGA488CGG (1570A>G), TAT489TAC (1573T>C), GCC492GCT (1582C>T), ACA493ACT (1585A>T), AAC495AAT (1591C>T), CTT496CTC (1594T>C), GCT501TCT (1607G>T), CAG502CAA (1612G>A), GGT504GGG (1618T>G), ACC506ACA (1624C>A), AGA513AGG (1645A>G), TGC521TGT (1669C>T), GGT525GGA (1681T>A), GAG526GAA (1684G>A), GCT531GCC (1699T>C), ACT533ACC (1705T>C), AAA544AGG (1737A>G 1738A>G), AGC555AGT (1771C>T), TTG559CTG (1781T>C), GAA561GAG (1789A>G), GCA597GCG (1897A>G), TTG602CTG (1910T>C), GTT616GTC (1954T>C), GTA618GTG (1960A>G), TAC626TAT (1984C>T), AAA636AAG (2014A>G), TAT639TAC (2023T>C), CCT650CCC (2056T>C), GCC657GCA (2077C>A), TTT659TAT (2082T>A), CTA662CTG (2092A>G), AAA667AAG (2107A>G), GTT674GTC (2128T>C), CTG678TTG (2138C>T), TAT679TAC (2143T>C), GGT681GGC (2149T>C), TGT683TGC (2155T>C), CTG690TTG (2174C>T), ATA691ATC (2179A>C), AAG692AAA (2182G>A), GCT696GCA (2194T>A), TAT698TAC (2200T>C), AAG703AAA (2215G>A), AGC704AGT (2218C>T), ATC706ATT (2224C>T), ACA710ACG (2236A>G), AAT712AGT (2241A>G), AGC715AAT (2250G>A 2251C>T), GAC717GAT (2257C>T), CAG719CAA (2263G>A), CCT723CCC (2275T>C), GCG727GCA (2287G>A), CAC728CAT (2290C>T), GAC736GAT (2314C>T), ATA739ATT (2323A>T), CAC747CAT (2347C>T), GTA748GTG (2350A>G), TTC750TTT (2356C>T), TTG751TTA (2359G>A), TAC752TAT (2362C>T), AGG757AGA (2377G>A), TAT758TAC (2380T>C), AGA762AGG (2392A>G), CCT767CCC (2407T>C), AGG768AAG (2409G>A), ATT779ATC (2443T>C), GTG780GTA (2446G>A), CCG791CCA (2479G>A), ATC793ATT (2485C>T), CTG797CTA (2497G>A), ATT798ATC (2500T>C), AAG799AAA (2503G>A), GGC801GGT (2509C>T), CTT802CTC (2512T>C), GGG812GGA (2542G>A), GTC814GTT (2548C>T), TTC817TTT (2557C>T), GAG838GAA (2620G>A), AAT840AAC (2626T>C), AAG848AAA (2650G>A), TTT853TTC (2665T>C), TTG857CTG (2675T>C), GCA859GCG (2683A>G), AAG862AAA (2692G>A), CTG866TTG (2702C>T), AAA868AAG (2710A>G), AGT871AGC (2719T>C), GAT873GAC (2725T>C), TTC875TTT (2731C>T), ATC876ATT (2734C>T), GGA879GGG (2743A>G), TTG880CTG (2744T>C), TTG887CTG (2765T>C), CTA889CTG (2773A>G), TGC901TGT (2809C>T), TCG902TCA (2812G>A), ACA907ACG (2827A>G), TTC921TTT (2869C>T), CAT925CAC (2881T>C), TTC941TTT (2929C>T), TTG942CTG (2930T>C), AAG943AAA (2935G>A), CAA945CAG (2941A>G), GTC948GTT (2950C>T), CTG949TTG (2951C>T), GGC977GGT (3037C>T), ACT978ACC (3040T>C), GTA981GTG (3049A>G), GTA986GTG (3064A>G), TTT1001TTC (3109T>C), TCT1019TCC (3163T>C), TCC1021TCT (3169C>T), TAT1032TAC (3202T>C), CTT1045CTC (3241T>C), AAC1049AAT (3253C>T), GTG1050GTA (3256G>A), AAA1053AAG (3265A>G), GCT1069GCC (3313T>C), GTA1100GTC (3406A>C), CTC1104CTA (3418C>A), CTT1105CTC (3421T>C), AAA1119AAG (3463A>G), CAG1120CAA (3466G>A), GGC1125GGT (3481C>T), ACA1126ACT (3484A>T), AGG1143AAG (3534G>A), GGC1145GGT (3541C>T), TCT1158TCC (3580T>C), ATC1159ATT (3583C>T), GAA1168GAG (3610A>G), GGT1169GGC (3613T>C), TAC1173TAT (3625C>T), GTG1174GTA (3628G>A), AAA1179AAG (3643A>G), TAC1180TAT (3646C>T), CAA1185CAG (3661A>G), AAT1186AAC (3664T>C), GAG1189GAA (3673G>A), GAC1190GAT (3676C>T), CTA1194TTA (3686C>T), CGT1200CGC (3706T>C), TTG1204CTG (3716T>C), CCT1205CCA (3721T>A), CGA1211CGT (3739A>T), GGA1215GGG (3751A>G), AAG1216AAA (3754G>A), GTC1218ATC (3758G>A), CAG1220CAA (3766G>A), TAT1234TAC (3808T>C), GAC1236GAT (3814C>T), GGG1237GGC (3817G>C), TTG1240CTG (3824T>C), TCT1243TCC (3835T>C), TAT1246TAC (3844T>C), TTG1251CTA (3857T>C 3859G>A), GGC1254GGT (3868C>T), ACT1255ACC (3871T>C), GAG1259GAA (3883G>A), AGT1264AGC (3898T>C), GGA1267GGT (3907A>T), GTC1270GTT (3916C>T), AAA1273AAG (3925A>G), AGG1274AGA (3928G>A), GAC1281GAT (3949C>T), GAG1283GAA (3955G>A), TTG1285TTA (3961G>A), GAA1288GAG (3970A>G), ATT1289ATC (3973T>C), TTG1310CTG (4034T>C), CGT1320CGC (4066T>C), GAA1323GAG (4075A>G), CCC1326CCT (4084C>T), TTG1332CTG (4100T>C), CTG1338TTG (4118C>T), TCC1341TCT (4129C>T), TCC1347TCA (4147C>A), CTG1351CTA (4159G>A), CCA1352CCT (4162A>T), AAA1354AAG (4168A>G), GGA1360GGT (4186A>T), GAA1363GAG (4195A>G), TTG1367TTA (4207G>A), GCT1368GCC (4210T>C), GTC1374GTT (4228C>T), CAA1396CAG (4294A>G), GGT1411GGC (4339T>C), CCA1413TCA (4343C>T), GAC1414GAT (4348C>T), AAT1416AAC (4354T>C), ACT1430ACC (4396T>C), AAC1434AAT (4408C>T), GTT1436GTG (4414T>G), TTT1437TTC (4417T>C), AGG1438AGA (4420G>A), GTT1451GTC (4459T>C), ATC1452ATT (4462C>T), TTC1453TTT (4465C>T), AAC1454AAT (4468C>T), TCT1455TCC (4471T>C), CTT1456CTC (4474T>C), TAT1495TAC (4591T>C), TAT1510TAC (4636T>C), CTG1512TTG (4640C>T), GAA1518GAG (4660A>G), ATA1526ATC (4684A>C), ATC1530ATT (4696C>T), AAG1532AAA (4702G>A), AAC1533AAT (4705C>T), GTT1536GTC (4714T>C), GAC1543GAT (4735C>T), CTA1547CTG (4747A>G), AGC1551AGT (4759C>T), TTT1554TTC (4768T>C), AGG1556AGA (4774G>A), CAC1557CAT (4777C>T), CTA1559CTG (4783A>G), GGA1560GGG (4786A>G), TAC1561TAT (4789C>T), GTA1569GTG (4813A>G), TCT1570TCC (4816T>C), AGG1579AGA (4843G>A), GTT1598ATT (4898G>A), GAG1600GAA (4906G>A), AAG1621AAA (4969G>A), ACC1628GCC (4988A>G), ACA1629ACT (4993A>T), TAT1633TAC (5005T>C), TTG1636CTG (5012T>C), CTG1641CTA (5029G>A), ATC1653ATT (5065C>T), CTT1656CTC (5074T>C), CAT1657CAC (5077T>C), ATT1658ACT (5079T>C), TTG1666CTG (5102T>C), ACT1671ATC (5118C>T 5119T>C), GTT1672ATT (5120G>A), CTG1673CTC (5125G>C), CAT1680CAC (5146T>C), ACT1681AAT (5148C>A), CAG1698CAA (5200G>A), GTT1704GTG (5218T>G), AGA1713AAA (5244G>A), CCC1720CCT (5266C>T), GAC1725GAT (5281C>T), CAC1741CAT (5329C>T), ATT1745ATA (5341T>A), GTA1746GTG (5344A>G), ACC1747ACT (5347C>T), GTG1755ATA (5369G>A 5371G>A), GGC1760GGT (5386C>T), GGG1762GGA (5392G>A), CCA1767CCG (5407A>G), TGC1769TGT (5413C>T), GCA1771GCT (5419A>T), ACT1777ACC (5437T>C), GGC1778GGT (5440C>T), GTT1785GTC (5461T>C), TTG1789CTG (5471T>C), ATA1792ATG (5482A>G), CTT1809CTA (5533T>A), GAT1822GAC (5572T>C), TGT1824TGC (5578T>C), ATT1826ATC (5584T>C), ATA1837ATT (5617A>T), GGT1845GGC (5641T>C), TTT1847TTC (5647T>C), CAG1860CAA (5686G>A), TTG1862CTG (5690T>C), CCT1868CCA (5710T>A), GGG1870GGT (5716G>T), ACT1876ACC (5734T>C), ACT1877ACC (5737T>C), AGA1878AAA (5739G>A), CTT1880CTC (5746T>C), TTG1882CTG (5750T>C), CTC1884CTT (5758C>T), AAG1889AAA (5773G>A), ATT1893ACT (5784T>C), TTA1894CTA (5786T>C), AGA1897AGG (5797A>G), TTC1900TTT (5806C>T), TCA1910TCT (5836A>T), TTG1915CAA (5849T>C 5850T>A 5851G>A), GGG1924GGT (5878G>T), TTT1926TTC (5884T>C), CCC1927CCT (5887C>T), CGC1928CGT (5890C>T), CTT1936CTC (5914T>C), TTT1939TTC (5923T>C), CTG1941TTG (5927C>T), AGC1942AGT (5932C>T), AAC1950AGC (5955A>G), ACG1953ACA (5965G>A), AAA1964AAG (5998A>G), ACC1966ACT (6004C>T), AAA1970AAG (6016A>G), TCC1974TCT (6028C>T), GCA1975GCT (6031A>T), ACA1976ATA (6033C>T), GCT1977TCT (6035G>T), GGT1983GGA (6055T>A), TCC1987TCA (6067C>A), GCT1988GCC (6070T>C), CTT1996CTG (6094T>G), CAG1999CAA (6103G>A), AAG2000AAA (6106G>A), TTT2011TTC (6139T>C), ATT2013ATC (6145T>C), TAT2014TAC (6148T>C), GTC2016GTT (6154C>T), GTA2026GTG (6184A>G), ACT2027ACC (6187T>C), TTC2029TTT (6193C>T), CAA2030CAG (6196A>G), CAA2034CAG (6208A>G), GGG2040GGA (6226G>A), ATA2042GTG (6230A>G 6232A>G), GAG2043GAA (6235G>A), ACC2050ACA (6256C>A), ACA2052ACG (6262A>G), CTG2053CTA (6265G>A), GTT2062GTA (6292T>A), GTA2064GTG (6298A>G), TTA2066TTG (6304A>G), TTG2068CTG (6308T>C), GAT2069GAC (6313T>C), ATA2071ATC (6319A>C), GAG2072GAA (6322G>A), ATC2073GTC (6323A>G), AAT2084AAC (6358T>C), GCA2089GTA (6372C>T), CAT2127CAC (6487T>C), CAT2129CAC (6493T>C), TCC2137TCT (6517C>T), CTT2147TTT (6545C>T), TAC2155TAT (6571C>T)									

Proteins

	Begin	End	Coverage	Score	Concordance	Matches	Identities	I/D/M/F*	Stop Codons
polyprotein (YP_001039627.1)	1	2159	100%	14905	98.9%	2159 (100%)	2125 (98.4%)	0/0/0/0	1
Protein mutations:	P8S (128C>T 130C>T), F10S (135T>C), A18T (158G>A 160T>C), T19A (161A>G), T364A (1196A>G), I459V (1481A>G), A501S (1607G>T), K544R (1737A>G 1738A>G), F659Y (2082T>A), N712S (2241A>G), S715N (2250G>A 2251C>T), R768K (2409G>A), R1143K (3534G>A), V1218I (3758G>A), P1413S (4343C>T), V1598I (4898G>A), T1628A (4988A>G), I1658T (5079T>C), T1671I (5118C>T 5119T>C), V1672I (5120G>A), T1681N (5148C>A), R1713K (5244G>A), V1755I (5369G>A 5371G>A), I1792M (5482A>G), R1878K (5739G>A), I1893T (5784T>C), L1915Q (5849T>C 5850T>A 5851G>A), N1950S (5955A>G), T1976I (6033C>T), A1977S (6035G>T), I2042V (6230A>G 6232A>G), I2073V (6323A>G), A2089V (6372C>T), L2147F (6545C>T)									

	Begin	End	Coverage	Score	Concordance	Matches	Identities	I/D/M/F*	Stop Codons
**NT**	**1**	**7799**	**99.9%**	**12080**	**83.0%**	**7519 (96.3%)**	**6929 (88.7%)**	**12/280**	

Codon mutations: TTT3TTC (115T>C), TTC7TTT (127C>T), CCC8TCT (128C>T 130C>T), TTC10TCC (135T>C), ACT14ACC (148T>C), GCT18ACC (158G>A 160T>C), ACC19GCC (161A>G), TCA27TCT (187A>T), GCT31GCA (199T>A), AGT34AGC (208T>C), CGC38CGT (220C>T), CTG40TTG (224C>T), CAA49CAG (253A>G), TCT51TCC (259T>C), TCT52TCA (262T>A), CAC53CAT (265C>T), TTG78TTA (340G>A), AAG81AAA (349G>A), TTC89TTT (373C>T), ATC90ATT (376C>T), AGC113AGT (445C>T), GAC127GAT (487C>T), GTG132GTA (502G>A), TCT145TCC (541T>C), TTC151TTT (559C>T), CTA158TTA (578C>T), AAC174AAT (628C>T), GAC183GAT (655C>T), CAA190CAG (676A>G), GCA196GCG (694A>G), CCA210CCG (736A>G), CTG246TTG (842C>T), CAA258CAG (880A>G), AGG264AGA (898G>A), GCC273GCT (925C>T), GTA282GTG (952A>G), GGT286GGG (964T>G), AAG288AAA (970G>A), GCC297GCT (997C>T), TCT300TCC (1006T>C), GAG309GAA (1033G>A), CAT318CAC (1060T>C), GCA324GCT (1078A>T), AAC325AAT (1081C>T), ACT329ACC (1093T>C), CAA333CAG (1105A>G), CAT334CAC (1108T>C), CTA335TTA (1109C>T), GCC353GCT (1165C>T), AGG358AGA (1180G>A), ACC364GCC (1196A>G), CAG375CAA (1231G>A), GGC378GGT (1240C>T), AGG383AGA (1255G>A), TTT387TTC (1267T>C), CAA390CAG (1276A>G), GGG398GGA (1300G>A), GAA415GAG (1351A>G), CTC422CTT (1372C>T), TAT435TAC (1411T>C), CAT437CAC (1417T>C), ATC441ATT (1429C>T), GCT448GCC (1450T>C), CTG449TTG (1451C>T), GAC458GAT (1480C>T), ATT459GTT (1481A>G), GAT461GAC (1489T>C), CCC472CCT (1522C>T), GGC482GGT (1552C>T), CGC483CGT (1555C>T), CGA488CGG (1570A>G), TAT489TAC (1573T>C), GCC492GCT (1582C>T), ACA493ACT (1585A>T), AAC495AAT (1591C>T), CTT496CTC (1594T>C), GCT501TCT (1607G>T), CAG502CAA (1612G>A), GGT504GGG (1618T>G), ACC506ACA (1624C>A), AGA513AGG (1645A>G), TGC521TGT (1669C>T), GGT525GGA (1681T>A), GAG526GAA (1684G>A), GCT531GCC (1699T>C), ACT533ACC (1705T>C), AAA544AGG (1737A>G 1738A>G), AGC555AGT (1771C>T), TTG559CTG (1781T>C), GAA561GAG (1789A>G), GCA597GCG (1897A>G), TTG602CTG (1910T>C), GTT616GTC (1954T>C), GTA618GTG (1960A>G), TAC626TAT (1984C>T), AAA636AAG (2014A>G), TAT639TAC (2023T>C), CCT650CCC (2056T>C), GCC657GCA (2077C>A), TTT659TAT (2082T>A), CTA662CTG (2092A>G), AAA667AAG (2107A>G), GTT674GTC (2128T>C), CTG678TTG (2138C>T), TAT679TAC (2143T>C), GGT681GGC (2149T>C), TGT683TGC (2155T>C), CTG690TTG (2174C>T), ATA691ATC (2179A>C), AAG692AAA (2182G>A), GCT696GCA (2194T>A), TAT698TAC (2200T>C), AAG703AAA (2215G>A), AGC704AGT (2218C>T), ATC706ATT (2224C>T), ACA710ACG (2236A>G), AAT712AGT (2241A>G), AGC715AAT (2250G>A 2251C>T), GAC717GAT (2257C>T), CAG719CAA (2263G>A), CCT723CCC (2275T>C), GCG727GCA (2287G>A), CAC728CAT (2290C>T), GAC736GAT (2314C>T), ATA739ATT (2323A>T), CAC747CAT (2347C>T), GTA748GTG (2350A>G), TTC750TTT (2356C>T), TTG751TTA (2359G>A), TAC752TAT (2362C>T), AGG757AGA (2377G>A), TAT758TAC (2380T>C), AGA762AGG (2392A>G), CCT767CCC (2407T>C), AGG768AAG (2409G>A), ATT779ATC (2443T>C), GTG780GTA (2446G>A), CCG791CCA (2479G>A), ATC793ATT (2485C>T), CTG797CTA (2497G>A), ATT798ATC (2500T>C), AAG799AAA (2503G>A), GGC801GGT (2509C>T), CTT802CTC (2512T>C), GGG812GGA (2542G>A), GTC814GTT (2548C>T), TTC817TTT (2557C>T), GAG838GAA (2620G>A), AAT840AAC (2626T>C), AAG848AAA (2650G>A), TTT853TTC (2665T>C), TTG857CTG (2675T>C), GCA859GCG (2683A>G), AAG862AAA (2692G>A), CTG866TTG (2702C>T), AAA868AAG (2710A>G), AGT871AGC (2719T>C), GAT873GAC (2725T>C), TTC875TTT (2731C>T), ATC876ATT (2734C>T), GGA879GGG (2743A>G), TTG880CTG (2744T>C), TTG887CTG (2765T>C), CTA889CTG (2773A>G), TGC901TGT (2809C>T), TCG902TCA (2812G>A), ACA907ACG (2827A>G), TTC921TTT (2869C>T), CAT925CAC (2881T>C), TTC941TTT (2929C>T), TTG942CTG (2930T>C), AAG943AAA (2935G>A), CAA945CAG (2941A>G), GTC948GTT (2950C>T), CTG949TTG (2951C>T), GGC977GGT (3037C>T), ACT978ACC (3040T>C), GTA981GTG (3049A>G), GTA986GTG (3064A>G), TTT1001TTC (3109T>C), TCT1019TCC (3163T>C), TCC1021TCT (3169C>T), TAT1032TAC (3202T>C), CTT1045CTC (3241T>C), AAC1049AAT (3253C>T), GTG1050GTA (3256G>A), AAA1053AAG (3265A>G), GCT1069GCC (3313T>C), GTA1100GTC (3406A>C), CTC1104CTA (3418C>A), CTT1105CTC (3421T>C), AAA1119AAG (3463A>G), CAG1120CAA (3466G>A), GGC1125GGT (3481C>T), ACA1126ACT (3484A>T), AGG1143AAG (3534G>A), GGC1145GGT (3541C>T), TCT1158TCC (3580T>C), ATC1159ATT (3583C>T), GAA1168GAG (3610A>G), GGT1169GGC (3613T>C), TAC1173TAT (3625C>T), GTG1174GTA (3628G>A), AAA1179AAG (3643A>G), TAC1180TAT (3646C>T), CAA1185CAG (3661A>G), AAT1186AAC (3664T>C), GAG1189GAA (3673G>A), GAC1190GAT (3676C>T), CTA1194TTA (3686C>T), CGT1200CGC (3706T>C), TTG1204CTG (3716T>C), CCT1205CCA (3721T>A), CGA1211CGT (3739A>T), GGA1215GGG (3751A>G), AAG1216AAA (3754G>A), GTC1218ATC (3758G>A), CAG1220CAA (3766G>A), TAT1234TAC (3808T>C), GAC1236GAT (3814C>T), GGG1237GGC (3817G>C), TTG1240CTG (3824T>C), TCT1243TCC (3835T>C), TAT1246TAC (3844T>C), TTG1251CTA (3857T>C 3859G>A), GGC1254GGT (3868C>T), ACT1255ACC (3871T>C), GAG1259GAA (3883G>A), AGT1264AGC (3898T>C), GGA1267GGT (3907A>T), GTC1270GTT (3916C>T), AAA1273AAG (3925A>G), AGG1274AGA (3928G>A), GAC1281GAT (3949C>T), GAG1283GAA (3955G>A), TTG1285TTA (3961G>A), GAA1288GAG (3970A>G), ATT1289ATC (3973T>C), TTG1310CTG (4034T>C), CGT1320CGC (4066T>C), GAA1323GAG (4075A>G), CCC1326CCT (4084C>T), TTG1332CTG (4100T>C), CTG1338TTG (4118C>T), TCC1341TCT (4129C>T), TCC1347TCA (4147C>A), CTG1351CTA (4159G>A), CCA1352CCT (4162A>T), AAA1354AAG (4168A>G), GGA1360GGT (4186A>T), GAA1363GAG (4195A>G), TTG1367TTA (4207G>A), GCT1368GCC (4210T>C), GTC1374GTT (4228C>T), CAA1396CAG (4294A>G), GGT1411GGC (4339T>C), CCA1413TCA (4343C>T), GAC1414GAT (4348C>T), AAT1416AAC (4354T>C), ACT1430ACC (4396T>C), AAC1434AAT (4408C>T), GTT1436GTG (4414T>G), TTT1437TTC (4417T>C), AGG1438AGA (4420G>A), GTT1451GTC (4459T>C), ATC1452ATT (4462C>T), TTC1453TTT (4465C>T), AAC1454AAT (4468C>T), TCT1455TCC (4471T>C), CTT1456CTC (4474T>C), TAT1495TAC (4591T>C), TAT1510TAC (4636T>C), CTG1512TTG (4640C>T), GAA1518GAG (4660A>G), ATA1526ATC (4684A>C), ATC1530ATT (4696C>T), AAG1532AAA (4702G>A), AAC1533AAT (4705C>T), GTT1536GTC (4714T>C), GAC1543GAT (4735C>T), CTA1547CTG (4747A>G), AGC1551AGT (4759C>T), TTT1554TTC (4768T>C), AGG1556AGA (4774G>A), CAC1557CAT (4777C>T), CTA1559CTG (4783A>G), GGA1560GGG (4786A>G), TAC1561TAT (4789C>T), GTA1569GTG (4813A>G), TCT1570TCC (4816T>C), AGG1579AGA (4843G>A), GTT1598ATT (4898G>A), GAG1600GAA (4906G>A), AAG1621AAA (4969G>A), ACC1628GCC (4988A>G), ACA1629ACT (4993A>T), TAT1633TAC (5005T>C), TTG1636CTG (5012T>C), CTG1641CTA (5029G>A), ATC1653ATT (5065C>T), CTT1656CTC (5074T>C), CAT1657CAC (5077T>C), ATT1658ACT (5079T>C), TTG1666CTG (5102T>C), ACT1671ATC (5118C>T 5119T>C), GTT1672ATT (5120G>A), CTG1673CTC (5125G>C), CAT1680CAC (5146T>C), ACT1681AAT (5148C>A), CAG1698CAA (5200G>A), GTT1704GTG (5218T>G), AGA1713AAA (5244G>A), CCC1720CCT (5266C>T), GAC1725GAT (5281C>T), CAC1741CAT (5329C>T), ATT1745ATA (5341T>A), GTA1746GTG (5344A>G), ACC1747ACT (5347C>T), GTG1755ATA (5369G>A 5371G>A), GGC1760GGT (5386C>T), GGG1762GGA (5392G>A), CCA1767CCG (5407A>G), TGC1769TGT (5413C>T), GCA1771GCT (5419A>T), ACT1777ACC (5437T>C), GGC1778GGT (5440C>T), GTT1785GTC (5461T>C), TTG1789CTG (5471T>C), ATA1792ATG (5482A>G), CTT1809CTA (5533T>A), GAT1822GAC (5572T>C), TGT1824TGC (5578T>C), ATT1826ATC (5584T>C), ATA1837ATT (5617A>T), GGT1845GGC (5641T>C), TTT1847TTC (5647T>C), CAG1860CAA (5686G>A), TTG1862CTG (5690T>C), CCT1868CCA (5710T>A), GGG1870GGT (5716G>T), ACT1876ACC (5734T>C), ACT1877ACC (5737T>C), AGA1878AAA (5739G>A), CTT1880CTC (5746T>C), TTG1882CTG (5750T>C), CTC1884CTT (5758C>T), AAG1889AAA (5773G>A), ATT1893ACT (5784T>C), TTA1894CTA (5786T>C), AGA1897AGG (5797A>G), TTC1900TTT (5806C>T), TCA1910TCT (5836A>T), TTG1915CAA (5849T>C 5850T>A 5851G>A), GGG1924GGT (5878G>T), TTT1926TTC (5884T>C), CCC1927CCT (5887C>T), CGC1928CGT (5890C>T), CTT1936CTC (5914T>C), TTT1939TTC (5923T>C), CTG1941TTG (5927C>T), AGC1942AGT (5932C>T), AAC1950AGC (5955A>G), ACG1953ACA (5965G>A), AAA1964AAG (5998A>G), ACC1966ACT (6004C>T), AAA1970AAG (6016A>G), TCC1974TCT (6028C>T), GCA1975GCT (6031A>T), ACA1976ATA (6033C>T), GCT1977TCT (6035G>T), GGT1983GGA (6055T>A), TCC1987TCA (6067C>A), GCT1988GCC (6070T>C), CTT1996CTG (6094T>G), CAG1999CAA (6103G>A), AAG2000AAA (6106G>A), TTT2011TTC (6139T>C), ATT2013ATC (6145T>C), TAT2014TAC (6148T>C), GTC2016GTT (6154C>T), GTA2026GTG (6184A>G), ACT2027ACC (6187T>C), TTC2029TTT (6193C>T), CAA2030CAG (6196A>G), CAA2034CAG (6208A>G), GGG2040GGA (6226G>A), ATA2042GTG (6230A>G 6232A>G), GAG2043GAA (6235G>A), ACC2050ACA (6256C>A), ACA2052ACG (6262A>G), CTG2053CTA (6265G>A), GTT2062GTA (6292T>A), GTA2064GTG (6298A>G), TTA2066TTG (6304A>G), TTG2068CTG (6308T>C), GAT2069GAC (6313T>C), ATA2071ATC (6319A>C), GAG2072GAA (6322G>A), ATC2073GTC (6323A>G), AAT2084AAC (6358T>C), GCA2089GTA (6372C>T), CAT2127CAC (6487T>C), CAT2129CAC (6493T>C), TCC2137TCT (6517C>T), CTT2147TTT (6545C>T), TAC2155TAT (6571C>T)

*: Inserts / Deletes / Misaligned / Frameshifts

Analysis details

This analysis was performed with panviral2.64

NGS Details (UN59): Torradovirus lycopersici (segment RNA 2)

Assembly

**Coverage Length**	5379 (1 contig(s))
**Depth Of Coverage**	6024.7
**Number Of Reads**	251860
**Reads Per Million**	5475.21 rpm (after QC)
**Ambiguities**	0
**Assembly Method**	de novo + reference guided assembly
**Consensus Caller**	Bcf Tools

Coverage Map

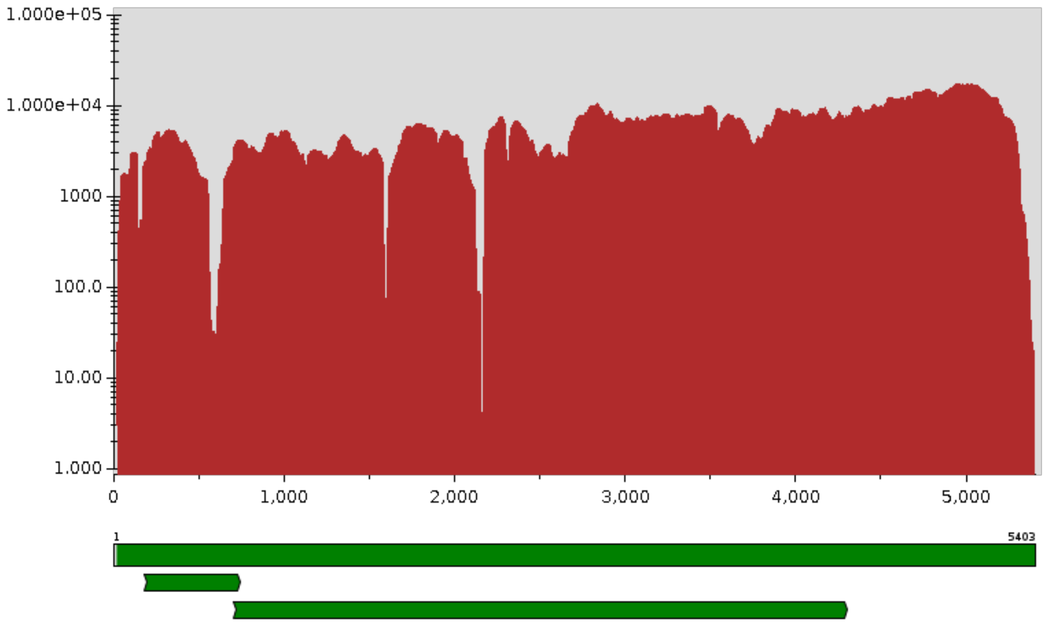

Assignment

**Type**	Torradovirus lycopersici (Taxonomy ID: 3048378)
**Reference Genome**	NC_009032.1
**NT Identity (%)**	85.17
**AA Identity (%)**	89.6179
**Number Of Stop Codons**	5
**Number Of CDS**	2

Alignment

**Alignment Score**	7554.0 (NT) + 8679.0 (AA) = 16233.0
**Concordance (%)**	80.1828

**Alignment Method**	Global, seeded, nucleotide + amino acids (AGA)

Genome Region

Sequence starts at position 25 and ends at position 5403 relative to NC_009032.1 reference sequence.

Alignment Detailed Statistics

	Begin	End	Coverage	Score	Concordance	Matches	Identities	I/D/M/F*	Stop Codons
**NT**	**25**	**5403**	**99.6%**	**7554**	**70.2%**	**5379 (99.9%)**	**4583 (85.2%)**	**2/0**	
Mutations:	27T>G, 52C>T, 58T>C, 60G>A, 69A>G, 79A>G, 91T>A, 96C>T, 99T>G, 103G>C, 104A>G, 105G>T, 107G>A, 108C>G, 111C>A, 112G>A, 117C>T, 120A>G, 121A>C, 132C>T, 137T>C, 142G>A, 143G>T, 149G>T, 161A>T, 164T>A, 170G>T, 181T>C, 187A>C, 194T>G, 202G>T, 212C>G, 223G>A, 232C>T, 235G>A, 238A>G, 244A>T, 247C>T, 265G>A, 268T>A, 277C>T, 280T>C, 286A>C, 289A>C, 292C>T, 301A>T, 304T>C, 305C>T, 307A>G, 313C>T, 316G>A, 325C>T, 329A>G, 334T>A, 338G>A, 340A>T, 352A>T, 356G>A, 370C>T, 372A>C, 376C>T, 388T>A, 400T>C, 401T>C, 403A>C, 418T>G, 433C>T, 439G>A, 445T>A, 448C>A, 451A>T, 461A>C, 481C>T, 484C>A, 487A>G, 496A>G, 502T>A, 505G>C, 507G>A, 508G>A, 509A>G, 511T>A, 515G>A, 520C>T, 541C>T, 547T>G, 552C>A, 553T>G, 554G>A, 555C>A, 564A>G, 565A>T, 568A>T, 570A>G, 576C>A, 578C>G, 586A>G, 587T>C, 589A>T, 595G>T, 599C>T, 601T>A, 603A>C, 604C>T, 607G>A, 610C>T, 613A>G, 618G>A, 619C>A, 622A>G, 631A>G, 632G>T, 637A>G, 644C>G, 645T>C, 654T>A, 658C>A, 665T>G, 670A>G, 673A>G, 679G>A, 683T>A, 685G>A, 689A>T, 690A>C, 696T>C, 706T>C, 731G>A, 733A>G, 750A>G, 755A>T, 758T>A, 761A>G, 771T>C, 782G>A, 788A>T, 791T>A, 818G>A, 821T>C, 827A>G, 833C>T, 844A>T, 854C>A, 860C>G, 863C>T, 866A>G, 878C>T, 884A>G, 887G>T, 893C>T, 911T>C, 929T>A, 932A>T, 968A>G, 1010T>G, 1061T>C, 1073C>A, 1076T>C, 1083C>T, 1085G>A, 1095G>A, 1100G>A, 1109C>T, 1115G>A, 1118A>C, 1130A>G, 1139G>A, 1142C>T, 1151A>G, 1163A>G, 1169C>G, 1178C>T, 1187A>T, 1205T>A, 1220T>C, 1235C>A, 1247A>G, 1262T>C, 1277A>G, 1313T>C, 1316C>T, 1317C>A, 1319A>G, 1325A>G, 1328T>C, 1331C>T, 1343G>A, 1355G>A, 1361A>T, 1367A>T, 1370A>T, 1376T>A, 1383A>G, 1385C>T, 1388C>T, 1390C>G, 1394A>T, 1395A>T, 1397T>A, 1400T>C, 1403A>T, 1406G>A, 1407C>T, 1409T>A, 1412G>T, 1418G>A, 1426A>C, 1427G>T, 1434G>A, 1439A>C, 1451G>T, 1454A>T, 1456G>C, 1457C>T, 1460A>G, 1461G>T, 1465G>A, 1472C>G, 1475G>C, 1478C>G, 1481C>T, 1487C>G, 1490G>A, 1493G>A, 1496C>T, 1502C>T, 1514T>C, 1524T>C, 1526C>T, 1529A>T, 1538A>T, 1543C>T, 1545A>G, 1547C>T, 1553T>C, 1559C>A, 1560C>T, 1562C>A, 1568C>G, 1574G>A, 1575C>A, 1577T>G, 1589A>G, 1593C>G, 1595G>T, 1603G>A, 1605A>G, 1608T>C, 1610C>G, 1613A>G, 1616G>A, 1621G>C, 1622T>A, 1626G>T, 1627A>C, 1628T>C, 1631G>A, 1634T>A, 1646T>C, 1651A>G, 1655T>A, 1661T>C, 1662C>T, 1664G>T, 1667C>T, 1676T>C, 1677G>A, 1679G>T, 1685T>C, 1694T>C, 1700G>A, 1721G>C, 1724C>T, 1727A>G, 1730C>T, 1740C>T, 1745G>A, 1748G>A, 1749A>C, 1754G>A, 1757A>G, 1760T>G, 1763T>A, 1766A>G, 1769A>T, 1778A>C, 1784A>T, 1790G>A, 1793T>A, 1794G>A, 1797T>G, 1799C>G, 1805C>T, 1811A>C, 1814T>C, 1817A>T, 1819T>A, 1820C>G, 1823C>T, 1832G>C, 1841C>T, 1843A>G, 1845T>C, 1847G>T, 1850G>A, 1856C>T, 1863C>T, 1865C>T, 1875G>A, 1878A>G, 1892C>T, 1898T>G, 1899G>A, 1900T>C, 1901G>A, 1906C>T, 1907C>G, 1913T>A, 1928A>C, 1931C>T, 1938A>G, 1941C>T, 1943T>G, 1946C>A, 1947T>C, 1952A>C, 1955G>A, 1958A>G, 1967G>A, 1970A>G, 1979A>G, 1986G>A, 1994T>C, 2002T>A, 2012C>T, 2024T>C, 2027T>A, 2049T>A, 2051A>T, 2054G>T, 2059A>G, 2060A>C, 2066C>G, 2074T>A, 2083T>C, 2084G>A, 2087A>C, 2094C>G, 2096G>C, 2098C>A, 2099G>A, 2100C>A, 2101G>T, 2102C>G, 2106A>G, 2107G>A, 2111T>G, 2114C>T, 2115A>C, 2116A>G, 2119A>C, 2120G>A, 2123G>T, 2125C>T, 2126C>T, 2127A>G, 2128G>T, 2130G>A, 2132T>C, 2135G>A, 2136G>C, 2138G>T, 2144C>G, 2159T>A, 2162T>C, 2164T>G, 2170A>T, 2171A>T, 2174A>T, 2177A>T, 2178T>G, 2183C>T, 2186A>C, 2189C>T, 2195A>C, 2198G>A, 2199G>A, 2200T>C, 2204C>T, 2205A>G, 2210G>A, 2216C>G, 2219T>C, 2225T>A, 2228C>T, 2229A>G, 2230T>A, 2231G>T, 2240T>A, 2243T>C, 2252A>C, 2256G>A, 2259A>C, 2261T>A, 2262G>C, 2270A>G, 2285T>C, 2294A>C, 2303A>G, 2312T>C, 2315T>A, 2320G>C, 2321T>C, 2324G>C, 2326C>T, 2339A>G, 2345A>G, 2375C>T, 2393G>A, 2414T>C, 2420T>G, 2429G>A, 2432G>A, 2436T>G, 2444G>C, 2447C>A, 2453G>A, 2459C>G, 2469T>A, 2471C>G, 2474G>A, 2489A>C, 2492G>A, 2495A>G, 2501A>G, 2503G>A, 2507T>C, 2516C>T, 2523A>C, 2546T>C, 2552C>T, 2561T>G, 2567T>A, 2570G>A, 2573T>A, 2588T>A, 2609C>A, 2627C>T, 2630C>G, 2645G>C, 2660T>C, 2672A>T, 2693G>T, 2702T>A, 2708A>G, 2717T>C, 2720C>A, 2729C>T, 2747C>T, 2753G>T, 2774A>T, 2792C>T, 2795C>G, 2798G>A, 2799G>A, 2804T>C, 2835A>G, 2837C>T, 2840T>C, 2841C>T, 2846G>A, 2852A>G, 2861G>A, 2864C>A, 2867C>T, 2870C>A, 2879T>A, 2882T>A, 2886T>C, 2888G>A, 2891A>G, 2894A>G, 2900A>T, 2909T>C, 2915A>G, 2921C>T, 2927A>G, 2936A>C, 2939A>G, 2948A>G, 2954G>A, 2957A>T, 2960G>A, 2966C>T, 2972T>C, 2981G>T, 2982G>A, 2996T>G, 2999T>A, 3003T>C, 3011C>T, 3012A>G, 3014C>T, 3020T>C, 3023G>C, 3029C>T, 3032A>T, 3044C>T, 3047T>A, 3050G>C, 3051C>T, 3059A>T, 3062C>T, 3063T>C, 3065G>A, 3071A>G, 3077A>T, 3083C>A, 3101T>G, 3104C>T, 3107T>G, 3113G>C, 3122T>G, 3128T>C, 3131G>A, 3143C>T, 3146T>C, 3152T>A, 3158G>A, 3161C>A, 3167A>T, 3176A>C, 3179G>T, 3182C>T, 3187G>A, 3194C>T, 3195A>G, 3197C>T, 3200T>C, 3206C>T, 3212A>G, 3227T>G, 3230T>A, 3236C>T, 3239T>G, 3242G>A, 3248A>C, 3251A>T, 3253A>G, 3265G>A, 3269T>C, 3270A>T, 3272G>T, 3278C>T, 3293A>G, 3299T>A, 3303A>T, 3305C>T, 3311T>A, 3324C>T, 3329G>T, 3350C>T, 3354A>C, 3359G>A, 3368A>G, 3371C>G, 3372T>C, 3386A>T, 3404A>G, 3410G>A, 3413C>T, 3422A>G, 3428G>A, 3431T>G, 3437C>T, 3446T>C, 3449G>A, 3452C>A, 3455T>A, 3458G>A, 3461C>T, 3470T>C, 3473T>C, 3479C>T, 3482T>C, 3485C>T, 3488A>G, 3491C>T, 3503A>G, 3508A>G, 3512T>C, 3515G>T, 3522C>T, 3524A>G, 3527C>T, 3533G>A, 3543T>A, 3551C>T, 3560T>C, 3564A>C, 3567T>A, 3569A>G, 3575G>A, 3578A>G, 3581C>T, 3590A>C, 3605A>G, 3611G>A, 3617A>T, 3620G>A, 3623A>T, 3629G>C, 3631C>A, 3634T>C, 3635C>T, 3638A>G, 3641C>T, 3650A>T, 3656G>T, 3659T>C, 3662A>G, 3674G>C, 3689C>T, 3698A>C, 3702T>C, 3708T>C, 3716C>T, 3719A>G, 3722C>A, 3728C>G, 3742A>C, 3743G>C, 3746G>A, 3749A>T, 3752G>A, 3755A>G, 3770A>C, 3773C>T, 3776A>T, 3779C>T, 3800C>T, 3815A>T, 3827C>T, 3833T>A, 3839A>G, 3845A>G, 3848G>A, 3851G>A, 3852A>G, 3854T>G, 3857C>A, 3866A>C, 3869A>T, 3872A>T, 3878G>C, 3881C>T, 3882A>G, 3884C>A, 3885A>G, 3890A>G, 3893T>C, 3896A>T, 3920A>T, 3923C>T, 3927A>C, 3932C>A, 3933A>G, 3935C>T, 3941T>G, 3944C>A, 3947T>C, 3950T>A, 3953T>A, 3960G>A, 3971T>A, 3983T>A, 3984A>G, 3986G>T, 3988G>A, 3989C>T, 3990G>A, 3991A>G, 3995C>T, 3998C>T, 4001T>C, 4016T>G, 4025G>A, 4031T>C, 4034T>G, 4035A>C, 4037A>T, 4040C>T, 4046C>T, 4052C>T, 4059A>G, 4061A>T, 4067A>T, 4069A>G, 4070T>C, 4072C>T, 4077A>C, 4100A>C, 4103A>T, 4106A>C, 4112C>T, 4142A>C, 4149C>A, 4151G>A, 4181C>T, 4187G>A, 4188C>G, 4196T>A, 4208A>T, 4211A>T, 4223G>A, 4224C>T, 4227T>C, 4229G>T, 4250C>T, 4253C>T, 4256T>G, 4262T>C, 4265T>A, 4274A>T, 4281A>C, 4284A>G, 4286T>A, 4289T>C, 4300T>C, 4302T>G, 4316T>C, 4354G>A, 4355T>G, 4382A>T, 4383C>T, 4388A>T, 4391A>C, 4393A>G, 4424C>T, 4425T>C, 4429T>C, 4447T>A, 4457A>G, 4468T>C, 4496T>A, 4506A>T, 4525T>C, 4567_4568insT, 4605T>C, 4633T>A, 4634G>A, 4643G>A, 4648G>A, 4650T>C, 4651G>A, 4659A>G, 4672C>T, 4675A>T, 4812C>T, 4813C>T, 4860T>C, 4915G>A, 5020C>T, 5068T>C, 5118T>C, 5139T>C, 5141T>C, 5206C>T, 5213A>G, 5214C>T, 5218C>T, 5222C>T, 5224G>A, 5225C>A, 5226T>G, 5231A>T, 5233G>T, 5235T>A, 5236G>A, 5239_5240insA, 5242T>G, 5244G>A, 5249A>G, 5258A>G, 5259T>A, 5262A>T, 5282T>C, 5311C>T, 5313A>T, 5315T>C, 5325G>A, 5343C>T, 5350A>G, 5358C>T, 5370T>A, 5371G>A, 5377G>C, 5379A>G								

CDS

	Begin	End	Coverage	Score	Concordance	Matches	Identities	I/D/M/F*	Stop Codons
ToTV_sRNA2gp1	1	188	100%	1072	88.7%	188 (100%)	159 (84.6%)	0/0/0/0	2
Protein mutations:	S5A (194T>G), L7F (202G>T), L11V (212C>G), T50A (329A>G), A53T (338G>A 340A>T), V59I (356G>A), N64T (372A>C), L90F (451A>T), Q108H (505G>C), R109K (507G>A 508G>A), I110V (509A>G 511T>A), V112I (515G>A), A124E (552C>A 553T>G), A125K (554G>A 555C>A), Q128R (564A>G 565A>T), Q130R (570A>G), T132K (576C>A), L133V (578C>G), E138D (595G>T), D141A (603A>C 604C>T), R146Q (618G>A 619C>A), E151* (632G>T), L155A (644C>G 645T>C), L158Q (654T>A), S162A (665T>G), L168I (683T>A 685G>A), N170S (689A>T 690A>C), L172P (696T>C), E184K (731G>A 733A>G)								
Codon mutations:	TCA2TCC (187A>C), TCT5GCT (194T>G), TTG7TTT (202G>T), CTT11GTT (212C>G), GAG14GAA (223G>A), CAC17CAT (232C>T), AAG18AAA (235G>A), CAA19CAG (238A>G), GCA21GCT (244A>T), GAC22GAT (247C>T), TCG28TCA (265G>A), GTT29GTA (268T>A), GGC32GGT (277C>T), TCT33TCC (280T>C), ATA35ATC (286A>C), ATA36ATC (289A>C), AAC37AAT (292C>T), CCA40CCT (301A>T), ACT41ACC (304T>C), CTA42TTG (305C>T 307A>G), TTC44TTT (313C>T), AAG45AAA (316G>A), CCC48CCT (325C>T), ACA50GCA (329A>G), GGT51GGA (334T>A), GCA53ACT (338G>A 340A>T), CTA57CTT (352A>T), GTT59ATT (356G>A), AAC63AAT (370C>T), AAT64ACT (372A>C), AGC65AGT (376C>T), GTT69GTA (388T>A), TAT73TAC (400T>C), TTA74CTC (401T>C 403A>C), ACT79ACG (418T>G), GGC84GGT (433C>T), AAG86AAA (439G>A), TCT88TCA (445T>A), GGC89GGA (448C>A), TTA90TTT (451A>T), AGA94CGA (461A>C), ACC100ACT (481C>T), ACC101ACA (484C>A), AGA102AGG (487A>G), CTA105CTG (496A>G), GCT107GCA (502T>A), CAG108CAC (505G>C), AGG109AAA (507G>A 508G>A), ATT110GTA (509A>G 511T>A), GTA112ATA (515G>A), TCC113TCT (520C>T), TCC120TCT (541C>T), GCT122GCG (547T>G), GCT124GAG (552C>A 553T>G), GCA125AAA (554G>A 555C>A), CAA128CGT (564A>G 565A>T), CCA129CCT (568A>T), CAA130CGA (570A>G), ACG132AAG (576C>A), CTT133GTT (578C>G), CAA135CAG (586A>G), TTA136CTT (587T>C 589A>T), GAG138GAT (595G>T), CTT140TTA (599C>T 601T>A), GAC141GCT (603A>C 604C>T), GAG142GAA (607G>A), GCC143GCT (610C>T), AAA144AAG (613A>G), CGC146CAA (618G>A 619C>A), TTA147TTG (622A>G), AAA150AAG (631A>G), GAG151TAG (632G>T), AAA152AAG (637A>G), CTT155GCT (644C>G 645T>C), CTA158CAA (654T>A), TCC159TCA (658C>A), TCC162GCC (665T>G), AAA163AAG (670A>G), TTA164TTG (673A>G), TTG166TTA (679G>A), TTG168ATA (683T>A 685G>A), AAC170TCC (689A>T 690A>C), CTA172CCA (696T>C), GAT175GAC (706T>C), GAA184AAG (731G>A 733A>G)								
ToTV_sRNA2gp2	1	1199	100%	7607	91.9%	1199 (100%)	1084 (90.4%)	0/0/0/0	3
Protein mutations:	M2T (706T>C), K11R (733A>G), S17G (750A>G), Y48F (844A>T), V132I (1095G>A), T228A (1383A>G 1385C>T), S230* (1390C>G), T232S (1395A>T 1397T>A), Q242P (1426A>C 1427G>T), D245N (1434G>A), S252T (1456G>C 1457C>T), A254S (1461G>T), R255K (1465G>A), I257M (1472C>G), S275P (1524T>C 1526C>T), S281F (1543C>T), T282A (1545A>G 1547C>T), H286Q (1559C>A), Q298D (1593C>G 1595G>T), S301N (1603G>A), R302G (1605A>G), Y303Q (1608T>C 1610C>G), G307A (1621G>C 1622T>A), D309S (1626G>T 1627A>C 1628T>C), K317R (1651A>G), L321F (1662C>T 1664G>T), V326I (1677G>A 1679G>T), V365I (1794G>A), C366G (1797T>G 1799C>G), K370N (1811A>C), I373K (1819T>A 1820C>G), K377N (1832G>C), H381R (1843A>G), H388Y (1863C>T 1865C>T), V392M (1875G>A), I393V (1878A>G), I399M (1898T>G), V400T (1899G>A 1900T>C 1901G>A), A402V (1906C>T 1907C>G), E409D (1928A>C), N413D (1938A>G), D429N (1986G>A), F434Y (2002T>A), L450I (2049T>A 2051A>T), K453S (2059A>G 2060A>C), L458Q (2074T>A), V461A (2083T>C 2084G>A), Q465D (2094C>G 2096G>C), A466E (2098C>A 2099G>A), R467M (2100C>A 2101G>T 2102C>G), R469E (2106A>G 2107G>A), F470L (2111T>G), K472R (2115A>C 2116A>G), E473A (2119A>C 2120G>A), A475V (2125C>T 2126C>T), R476V (2127A>G 2128G>T), A477T (2130G>A 2132T>C), V479L (2136G>C 2138G>T), N481K (2144C>G), V488G (2164T>G), E490V (2170A>T 2171A>T), S493A (2178T>G), V500T (2199G>A 2200T>C), M502V (2205A>G), D508E (2225T>A), M510D (2229A>G 2230T>A 2231G>T), E517D (2252A>C), A519T (2256G>A), N520Q (2259A>C 2261T>A), E521Q (2262G>C), E531D (2294A>C), N538K (2315T>A), G540A (2320G>C 2321T>C), T542I (2326C>T), S579A (2436T>G), S590T (2469T>A 2471C>G), R601K (2503G>A), I608L (2523A>C), I643M (2630C>G), C667* (2702T>A), A700T (2799G>A), I712V (2835A>G 2837C>T), A761T (2982G>A), I771V (3012A>G 3014C>T), I807M (3122T>G), S829N (3187G>A), T832A (3195A>G 3197C>T), K851R (3253A>G), R855K (3265G>A), T857S (3270A>T 3272G>T), T868S (3303A>T 3305C>T), N936S (3508A>G), S948T (3543T>A), K955Q (3564A>C), L956M (3567T>A 3569A>G), P977Q (3631C>A), L978P (3634T>C 3635C>T), Q1014P (3742A>C 3743G>C), T1051A (3852A>G 3854T>G), I1061V (3882A>G 3884C>A), T1062A (3885A>G), K1076Q (3927A>C), T1078A (3933A>G 3935C>T), A1087T (3960G>A), M1095V (3984A>G 3986G>T), S1096N (3988G>A 3989C>T), D1097S (3990G>A 3991A>G), H1105Q (4016T>G), I1120V (4059A>G 4061A>T), N1123S (4069A>G 4070T>C), A1124V (4072C>T), L1150I (4149C>A 4151G>A), Q1163E (4188C>G), H1175Y (4224C>T), S1195G (4284A>G 4286T>A)								

	Begin	End	Coverage	Score	Concordance	Matches	Identities	I/D/M/F*	Stop Codons
**NT**	**25**	**5403**	**99.6%**	**7554**	**70.2%**	**5379 (99.9%)**	**4583 (85.2%)**	**2/0**	
Codon mutations:	ATG2ACG (706T>C), AGG10AGA (731G>A), AAG11AGG (733A>G), AGT17GGT (750A>G), GCA18GCT (755A>T), GCT19GCA (758T>A), GTA20GTG (761A>G), TTA24CTA (771T>C), CAG27CAA (782G>A), ACA29ACT (788A>T), GCT30GCA (791T>A), CTG39CTA (818G>A), CTT40CTC (821T>C), AAA42AAG (827A>G), GGC44GGT (833C>T), TAT48TTT (844A>T), CTC51CTA (854C>A), GGC53GGG (860C>G), GCC54GCT (863C>T), CAA55CAG (866A>G), GCC59GCT (878C>T), GAA61GAG (884A>G), CTG62CTT (887G>T), GAC64GAT (893C>T), AAT70AAC (911T>C), TCT76TCA (929T>A), TCA77TCT (932A>T), GGA89GGG (968A>G), GTT103GTG (1010T>G), AGT120AGC (1061T>C), ACC124ACA (1073C>A), AAT125AAC (1076T>C), CTG128TTA (1083C>T 1085G>A), GTT132ATT (1095G>A), TTG133TTA (1100G>A), TCC136TCT (1109C>T), GGG138GGA (1115G>A), GCA139GCC (1118A>C), TTA143TTG (1130A>G), CCG146CCA (1139G>A), ACC147ACT (1142C>T), CTA150CTG (1151A>G), AAA154AAG (1163A>G), GGC156GGG (1169C>G), TTC159TTT (1178C>T), ACA162ACT (1187A>T), ACT168ACA (1205T>A), CCT173CCC (1220T>C), GCC178GCA (1235C>A), CAA182CAG (1247A>G), CGT187CGC (1262T>C), TCA192TCG (1277A>G), CTT204CTC (1313T>C), CAC205CAT (1316C>T), CGA206AGG (1317C>A 1319A>G), CAA208CAG (1325A>G), TTT209TTC (1328T>C), TTC210TTT (1331C>T), CAG214CAA (1343G>A), CAG218CAA (1355G>A), ATA220ATT (1361A>T), TCA222TCT (1367A>T), GTA223GTT (1370A>T), TCT225TCA (1376T>A), ACC228GCT (1383A>G 1385C>T), TCC229TCT (1388C>T), TCA230TGA (1390C>G), GCA231GCT (1394A>T), ACT232TCA (1395A>T 1397T>A), GAT233GAC (1400T>C), TCA234TCT (1403A>T), GTG235GTA (1406G>A), CTT236TTA (1407C>T 1409T>A), CCG237CCT (1412G>T), GTG239GTA (1418G>A), CAG242CCT (1426A>C 1427G>T), GAC245AAC (1434G>A), ACA246ACC (1439A>C), GTG250GTT (1451G>T), CCA251CCT (1454A>T), AGC252ACT (1456G>C 1457C>T), GAA253GAG (1460A>G), GCA254TCA (1461G>T), AGA255AAA (1465G>A), ATC257ATG (1472C>G), CGG258CGC (1475G>C), TCC259TCG (1478C>G), TCC260TCT (1481C>T), CTC262CTG (1487C>G), CGG263CGA (1490G>A), GTG264GTA (1493G>A), CCC265CCT (1496C>T), CTC267CTT (1502C>T), AGT271AGC (1514T>C), TCC275CCT (1524T>C 1526C>T), TCA276TCT (1529A>T), ATA279ATT (1538A>T), TCT281TTT (1543C>T), ACC282GCT (1545A>G 1547C>T), ACT284ACC (1553T>C), CAC286CAA (1559C>A), CTC287TTA (1560C>T 1562C>A), GGC289GGG (1568C>G), ACG291ACA (1574G>A), CGT292AGG (1575C>A 1577T>G), GTA296GTG (1589A>G), CAG298GAT (1593C>G 1595G>T), AGT301AAT (1603G>A), AGG302GGG (1605A>G), TAC303CAG (1608T>C 1610C>G), GAA304GAG (1613A>G), GAG305GAA (1616G>A), GGT307GCA (1621G>C 1622T>A), GAT309TCC (1626G>T 1627A>C 1628T>C), GGG310GGA (1631G>A), GTT311GTA (1634T>A), GTT315GTC (1646T>C), AAG317AGG (1651A>G), GCT318GCA (1655T>A), GGT320GGC (1661T>C), CTG321TTT (1662C>T 1664G>T), AAC322AAT (1667C>T), CAT325CAC (1676T>C), GTG326ATT (1677G>A 1679G>T), GAT328GAC (1685T>C), GGT331GGC (1694T>C), GAG333GAA (1700G>A), CTG340CTC (1721G>C), CCC341CCT (1724C>T), GAA342GAG (1727A>G), AAC343AAT (1730C>T), CTG347TTG (1740C>T), TCG348TCA (1745G>A), GTG349GTA (1748G>A), AGA350CGA (1749A>C), CAG351CAA (1754G>A), CAA352CAG (1757A>G), GTT353GTG (1760T>G), GCT354GCA (1763T>A), GCA355GCG (1766A>G), GCA356GCT (1769A>T), GCA359GCC (1778A>C), GCA361GCT (1784A>T), AAG363AAA (1790G>A), GGT364GGA (1793T>A), GTT365ATT (1794G>A), TGC366GGG (1797T>G 1799C>G), GTC368GTT (1805C>T), AAA370AAC (1811A>C), CAT371CAC (1814T>C), ATA372ATT (1817A>T), ATC373AAG (1819T>A 1820C>G), AAC374AAT (1823C>T), AAG377AAC (1832G>C), ATC380ATT (1841C>T), CAC381CGC (1843A>G), TTG382CTT (1845T>C 1847G>T), GAG383GAA (1850G>A), ATC385ATT (1856C>T), CAC388TAT (1863C>T 1865C>T), GTG392ATG (1875G>A), ATA393GTA (1878A>G), TAC397TAT (1892C>T), ATT399ATG (1898T>G), GTG400ACA (1899G>A 1900T>C 1901G>A), GCC402GTG (1906C>T 1907C>G), TCT404TCA (1913T>A), GAA409GAC (1928A>C), AAC410AAT (1931C>T), AAT413GAT (1938A>G), CTT414TTG (1941C>T 1943T>G), ACC415ACA (1946C>A), TTG416CTG (1947T>C), GCA417GCC (1952A>C), CAG418CAA (1955G>A), AGA419AGG (1958A>G), GAG422GAA (1967G>A), CTA423CTG (1970A>G), CAA426CAG (1979A>G), GAT429AAT (1986G>A), TAT431TAC (1994T>C), TTT434TAT (2002T>A), AAC437AAT (2012C>T), TAT441TAC (2024T>C), CCT442CCA (2027T>A), TTA450ATT (2049T>A 2051A>T), ACG451ACT (2054G>T), AAA453AGC (2059A>G 2060A>C), CGC455CGG (2066C>G), CTA458CAA (2074T>A), GTG461GCA (2083T>C 2084G>A), GCA462GCC (2087A>C), CAG465GAC (2094C>G 2096G>C), GCG466GAA (2098C>A 2099G>A), CGC467ATG (2100C>A 2101G>T 2102C>G), AGA469GAA (2106A>G 2107G>A), TTT470TTG (2111T>G), TAC471TAT (2114C>T), AAG472CGG (2115A>C 2116A>G), GAG473GCA (2119A>C 2120G>A), GCG474GCT (2123G>T), GCC475GTT (2125C>T 2126C>T), AGG476GTG (2127A>G 2128G>T), GCT477ACC (2130G>A 2132T>C), CAG478CAA (2135G>A), GTG479CTT (2136G>C 2138G>T), AAC481AAG (2144C>G), ACT486ACA (2159T>A), AGT487AGC (2162T>C), GTG488GGG (2164T>G), GAA490GTT (2170A>T 2171A>T), ATA491ATT (2174A>T), CCA492CCT (2177A>T), TCC493GCC (2178T>G), ACC494ACT (2183C>T), TCA495TCC (2186A>C), TTC496TTT (2189C>T), ACA498ACC (2195A>C), AAG499AAA (2198G>A), GTT500ACT (2199G>A 2200T>C), GCC501GCT (2204C>T), ATG502GTG (2205A>G), GAG503GAA (2210G>A), GGC505GGG (2216C>G), AGT506AGC (2219T>C), GAT508GAA (2225T>A), AGC509AGT (2228C>T), ATG510GAT (2229A>G 2230T>A 2231G>T), GCT513GCA (2240T>A), ATT514ATC (2243T>C), GAA517GAC (2252A>C), GCC519ACC (2256G>A), AAT520CAA (2259A>C 2261T>A), GAA521CAA (2262G>C), GTA523GTG (2270A>G), GTT528GTC (2285T>C), GAA531GAC (2294A>C), CAA534CAG (2303A>G), AAT537AAC (2312T>C), AAT538AAA (2315T>A), GGT540GCC (2320G>C 2321T>C), GCG541GCC (2324G>C), ACA542ATA (2326C>T), TTA546TTG (2339A>G), CAA548CAG (2345A>G), TTC558TTT (2375C>T), GAG564GAA (2393G>A), AGT571AGC (2414T>C), GTT573GTG (2420T>G), GAG576GAA (2429G>A), GTG577GTA (2432G>A), TCA579GCA (2436T>G), GCG581GCC (2444G>C), CTC582CTA (2447C>A), GCG584GCA (2453G>A), CTC586CTG (2459C>G), TCC590ACG (2469T>A 2471C>G), AAG591AAA (2474G>A), GGA596GGC (2489A>C), TCG597TCA (2492G>A), CAA598CAG (2495A>G), CTA600CTG (2501A>G), AGG601AAG (2503G>A), TAT602TAC (2507T>C), GCC605GCT (2516C>T), ATT608CTT (2523A>C), TAT615TAC (2546T>C), TCC617TCT (2552C>T), GGT620GGG (2561T>G), ATT622ATA (2567T>A), TCG623TCA (2570G>A), GGT624GGA (2573T>A), GTT629GTA (2588T>A), CTC636CTA (2609C>A), TTC642TTT (2627C>T), ATC643ATG (2630C>G), CTG648CTC (2645G>C), CAT653CAC (2660T>C), TCA657TCT (2672A>T), GGG664GGT (2693G>T), TGT667TGA (2702T>A), ACA669ACG (2708A>G), GGT672GGC (2717T>C), ATC673ATA (2720C>A), TTC676TTT (2729C>T), GCC682GCT (2747C>T), GGG684GGT (2753G>T), ATA691ATT (2774A>T), CAC697CAT (2792C>T), CCC698CCG (2795C>G), TTG699TTA (2798G>A), GCT700ACT (2799G>A), AGT701AGC (2804T>C), ATC712GTT (2835A>G 2837C>T), CAT713CAC (2840T>C), CTG714TTG (2841C>T), CAG715CAA (2846G>A), AAA717AAG (2852A>G), TCG720TCA (2861G>A), ACC721ACA (2864C>A), AAC722AAT (2867C>T), ATC723ATA (2870C>A), CCT726CCA (2879T>A), CCT727CCA (2882T>A), TTG729CTA (2886T>C 2888G>A), CGA730CGG (2891A>G), GCA731GCG (2894A>G), GCA733GCT (2900A>T), GGT736GGC (2909T>C), AAA738AAG (2915A>G), GAC740GAT (2921C>T), ACA742ACG (2927A>G), CCA745CCC (2936A>C), CGA746CGG (2939A>G), ACA749ACG (2948A>G), CAG751CAA (2954G>A), GTA752GTT (2957A>T), CTG753CTA (2960G>A), CAC755CAT (2966C>T), AAT757AAC (2972T>C), GTG760GTT (2981G>T), GCT761ACT (2982G>A), GGT765GGG (2996T>G), ACT766ACA (2999T>A), TTA768CTA (3003T>C), AGC770AGT (3011C>T), ATC771GTT (3012A>G 3014C>T), TCT773TCC (3020T>C), CCG774CCC (3023G>C), GGC776GGT (3029C>T), ATA777ATT (3032A>T), GAC781GAT (3044C>T), GGT782GGA (3047T>A), ACG783ACC (3050G>C), CTG784TTG (3051C>T), CCA786CCT (3059A>T), TCC787TCT (3062C>T), TTG788CTA (3063T>C 3065G>A), GGA790GGG (3071A>G), ATA792ATT (3077A>T), CGC794CGA (3083C>A), ACT800ACG (3101T>G), GGC801GGT (3104C>T), ACT802ACG (3107T>G), GTG804GTC (3113G>C), ATT807ATG (3122T>G), ATT809ATC (3128T>C), GAG810GAA (3131G>A), TTC814TTT (3143C>T), CAT815CAC (3146T>C), GGT817GGA (3152T>A), TTG819TTA (3158G>A), GCC820GCA (3161C>A), GGA822GGT (3167A>T), ACA825ACC (3176A>C), CTG826CTT (3179G>T), AAC827AAT (3182C>T), AGC829AAC (3187G>A), TCC831TCT (3194C>T), ACC832GCT (3195A>G 3197C>T), CCT833CCC (3200T>C), GAC835GAT (3206C>T), TTA837TTG (3212A>G), GTT842GTG (3227T>G), ATT843ATA (3230T>A), AAC845AAT (3236C>T), CTT846CTG (3239T>G), GAG847GAA (3242G>A), GGA849GGC (3248A>C), CGA850CGT (3251A>T), AAG851AGG (3253A>G), AGG855AAG (3265G>A), TGT856TGC (3269T>C), ACG857TCT (3270A>T 3272G>T), ACC859ACT (3278C>T), AAA864AAG (3293A>G), CTT866CTA (3299T>A), ACC868TCT (3303A>T 3305C>T), GGT870GGA (3311T>A), CTA875TTA (3324C>T), CCG876CCT (3329G>T), CAC883CAT (3350C>T), AGG885CGG (3354A>C), TTG886TTA (3359G>A), ACA889ACG (3368A>G), GTC890GTG (3371C>G), TTG891CTG (3372T>C), GTA895GTT (3386A>T), CTA901CTG (3404A>G), ACG903ACA (3410G>A), GTC904GTT (3413C>T), ACA907ACG (3422A>G), CAG909CAA (3428G>A), CTT910CTG (3431T>G), TGC912TGT (3437C>T), GAT915GAC (3446T>C), TTG916TTA (3449G>A), GTC917GTA (3452C>A), CTT918CTA (3455T>A), GGG919GGA (3458G>A), GGC920GGT (3461C>T), TCT923TCC (3470T>C), GTT924GTC (3473T>C), CCC926CCT (3479C>T), ATT927ATC (3482T>C), TAC928TAT (3485C>T), GGA929GGG (3488A>G), CAC930CAT (3491C>T), GGA934GGG (3503A>G), AAT936AGT (3508A>G), GCT937GCC (3512T>C), GTG938GTT (3515G>T), CTA941TTG (3522C>T 3524A>G), TTC942TTT (3527C>T), GAG944GAA (3533G>A), TCT948ACT (3543T>A), CGC950CGT (3551C>T), ATT953ATC (3560T>C), AAA955CAA (3564A>C), TTA956ATG (3567T>A 3569A>G), AAG958AAA (3575G>A), GAA959GAG (3578A>G), AAC960AAT (3581C>T), ACA963ACC (3590A>C), GGA968GGG (3605A>G), AAG970AAA (3611G>A), CCA972CCT (3617A>T), CAG973CAA (3620G>A), GTA974GTT (3623A>T), GTG976GTC (3629G>C), CCG977CAG (3631C>A), CTC978CCT (3634T>C 3635C>T), AGA979AGG (3638A>G), GAC980GAT (3641C>T), TCA983TCT (3650A>T), GGG985GGT (3656G>T), GCT986GCC (3659T>C), GTA987GTG (3662A>G), GTG991GTC (3674G>C), GAC996GAT (3689C>T), CGA999CGC (3698A>C), TTG1001CTG (3702T>C), TTA1003CTA (3708T>C), TGC1005TGT (3716C>T), GCA1006GCG (3719A>G), CCC1007CCA (3722C>A), TCC1009TCG (3728C>G), CAG1014CCC (3742A>C 3743G>C), GGG1015GGA (3746G>A), GCA1016GCT (3749A>T), CTG1017CTA (3752G>A), GTA1018GTG (3755A>G), ACA1023ACC (3770A>C), TGC1024TGT (3773C>T), CCA1025CCT (3776A>T), TTC1026TTT (3779C>T), TTC1033TTT (3800C>T), GGA1038GGT (3815A>T), TAC1042TAT (3827C>T), ATT1044ATA (3833T>A), GTA1046GTG (3839A>G), AGA1048AGG (3845A>G), GTG1049GTA (3848G>A), CAG1050CAA (3851G>A), ACT1051GCG (3852A>G 3854T>G), TCC1052TCA (3857C>A), ATA1055ATC (3866A>C), GGA1056GGT (3869A>T), GGA1057GGT (3872A>T), CTG1059CTC (3878G>C), AAC1060AAT (3881C>T), ATC1061GTA (3882A>G 3884C>A), ACC1062GCC (3885A>G), TTA1063TTG (3890A>G), GAT1064GAC (3893T>C), TCA1065TCT (3896A>T), GGA1073GGT (3920A>T), ATC1074ATT (3923C>T), AAG1076CAG (3927A>C), GGC1077GGA (3932C>A), ACC1078GCT (3933A>G 3935C>T), GTT1080GTG (3941T>G), GTC1081GTA (3944C>A), TCT1082TCC (3947T>C), GCT1083GCA (3950T>A), GGT1084GGA (3953T>A), GCA1087ACA (3960G>A), GCT1090GCA (3971T>A), GGT1094GGA (3983T>A), ATG1095GTT (3984A>G 3986G>T), AGC1096AAT (3988G>A 3989C>T), GAC1097AGC (3990G>A 3991A>G), AAC1098AAT (3995C>T), ATC1099ATT (3998C>T), TTT1100TTC (4001T>C), CAT1105CAG (4016T>G), GAG1108GAA (4025G>A), TTT1110TTC (4031T>C), CCT1111CCG (4034T>G), AGA1112CGT (4035A>C 4037A>T), CGC1113CGT (4040C>T), ACC1115ACT (4046C>T), GCC1117GCT (4052C>T), ATA1120GTT (4059A>G 4061A>T), CCA1122CCT (4067A>T), AAT1123AGC (4069A>G 4070T>C), GCT1124GTT (4072C>T), AGA1126CGA (4077A>C), CGA1133CGC (4100A>C), CTA1134CTT (4103A>T), GGA1135GGC (4106A>C), CTC1137CTT (4112C>T), ATA1147ATC (4142A>C), CTG1150ATA (4149C>A 4151G>A), TTC1160TTT (4181C>T), TTG1162TTA (4187G>A), CAA1163GAA (4188C>G), GCT1165GCA (4196T>A), TCA1169TCT (4208A>T), GCA1170GCT (4211A>T), AAG1174AAA (4223G>A), CAT1175TAT (4224C>T), TTG1176CTT (4227T>C 4229G>T), ACC1183ACT (4250C>T), TAC1184TAT (4253C>T), CTT1185CTG (4256T>G), ACT1187ACC (4262T>C), CCT1188CCA (4265T>A), TCA1191TCT (4274A>T), AGG1194CGG (4281A>C), AGT1195GGA (4284A>G 4286T>A), TTT1196TTC (4289T>C)								

Proteins

	Begin	End	Coverage	Score	Concordance	Matches	Identities	I/D/M/F*	Stop Codons
Orf1 (YP_001040017.1)	1	188	100%	1072	88.7%	188 (100%)	159 (84.6%)	0/0/0/0	2
Protein mutations:	S5A (194T>G), L7F (202G>T), L11V (212C>G), T50A (329A>G), A53T (338G>A 340A>T), V59I (356G>A), N64T (372A>C), L90F (451A>T), Q108H (505G>C), R109K (507G>A 508G>A), I110V (509A>G 511T>A), V112I (515G>A), A124E (552C>A 553T>G), A125K (554G>A 555C>A), Q128R (564A>G 565A>T), Q130R (570A>G), T132K (576C>A), L133V (578C>G), E138D (595G>T), D141A (603A>C 604C>T), R146Q (618G>A 619C>A), E151* (632G>T), L155A (644C>G 645T>C), L158Q (654T>A), S162A (665T>G), L168I (683T>A 685G>A), N170S (689A>T 690A>C), L172P (696T>C), E184K (731G>A 733A>G)								
Codon mutations:	TCA2TCC (187A>C), TCT5GCT (194T>G), TTG7TTT (202G>T), CTT11GTT (212C>G), GAG14GAA (223G>A), CAC17CAT (232C>T), AAG18AAA (235G>A), CAA19CAG (238A>G), GCA21GCT (244A>T), GAC22GAT (247C>T), TCG28TCA (265G>A), GTT29GTA (268T>A), GGC32GGT (277C>T), TCT33TCC (280T>C), ATA35ATC (286A>C), ATA36ATC (289A>C), AAC37AAT (292C>T), CCA40CCT (301A>T), ACT41ACC (304T>C), CTA42TTG (305C>T 307A>G), TTC44TTT (313C>T), AAG45AAA (316G>A), CCC48CCT (325C>T), ACA50GCA (329A>G), GGT51GGA (334T>A), GCA53ACT (338G>A 340A>T), CTA57CTT (352A>T), GTT59ATT (356G>A), AAC63AAT (370C>T), AAT64ACT (372A>C), AGC65AGT (376C>T), GTT69GTA (388T>A), TAT73TAC (400T>C), TTA74CTC (401T>C 403A>C), ACT79ACG (418T>G), GGC84GGT (433C>T), AAG86AAA (439G>A), TCT88TCA (445T>A), GGC89GGA (448C>A), TTA90TTT (451A>T), AGA94CGA (461A>C), ACC100ACT (481C>T), ACC101ACA (484C>A), AGA102AGG (487A>G), CTA105CTG (496A>G), GCT107GCA (502T>A), CAG108CAC (505G>C), AGG109AAA (507G>A 508G>A), ATT110GTA (509A>G 511T>A), GTA112ATA (515G>A), TCC113TCT (520C>T), TCC120TCT (541C>T), GCT122GCG (547T>G), GCT124GAG (552C>A 553T>G), GCA125AAA (554G>A 555C>A), CAA128CGT (564A>G 565A>T), CCA129CCT (568A>T), CAA130CGA (570A>G), ACG132AAG (576C>A), CTT133GTT (578C>G), CAA135CAG (586A>G), TTA136CTT (587T>C 589A>T), GAG138GAT (595G>T), CTT140TTA (599C>T 601T>A), GAC141GCT (603A>C 604C>T), GAG142GAA (607G>A), GCC143GCT (610C>T), AAA144AAG (613A>G), CGC146CAA (618G>A 619C>A), TTA147TTG (622A>G), AAA150AAG (631A>G), GAG151TAG (632G>T), AAA152AAG (637A>G), CTT155GCT (644C>G 645T>C), CTA158CAA (654T>A), TCC159TCA (658C>A), TCC162GCC (665T>G), AAA163AAG (670A>G), TTA164TTG (673A>G), TTG166TTA (679G>A), TTG168ATA (683T>A 685G>A), AAC170TCC (689A>T 690A>C), CTA172CCA (696T>C), GAT175GAC (706T>C), GAA184AAG (731G>A 733A>G)								
polyprotein (YP_001040018.1)	1	1199	100%	7607	91.9%	1199 (100%)	1084 (90.4%)	0/0/0/0	3

	Begin	End	Coverage	Score	Concordance	Matches	Identities	I/D/M/F*	Stop Codons
**NT**	**25**	**5403**	**99.6%**	**7554**	**70.2%**	**5379 (99.9%)**	**4583 (85.2%)**	**2/0**	
Protein mutations:	M2T (706T>C), K11R (733A>G), S17G (750A>G), Y48F (844A>T), V132I (1095G>A), T228A (1383A>G 1385C>T), S230* (1390C>G), T232S (1395A>T 1397T>A), Q242P (1426A>C 1427G>T), D245N (1434G>A), S252T (1456G>C 1457C>T), A254S (1461G>T), R255K (1465G>A), I257M (1472C>G), S275P (1524T>C 1526C>T), S281F (1543C>T), T282A (1545A>G 1547C>T), H286Q (1559C>A), Q298D (1593C>G 1595G>T), S301N (1603G>A), R302G (1605A>G), Y303Q (1608T>C 1610C>G), G307A (1621G>C 1622T>A), D309S (1626G>T 1627A>C 1628T>C), K317R (1651A>G), L321F (1662C>T 1664G>T), V326I (1677G>A 1679G>T), V365I (1794G>A), C366G (1797T>G 1799C>G), K370N (1811A>C), I373K (1819T>A 1820C>G), K377N (1832G>C), H381R (1843A>G), H388Y (1863C>T 1865C>T), V392M (1875G>A), I393V (1878A>G), I399M (1898T>G), V400T (1899G>A 1900T>C 1901G>A), A402V (1906C>T 1907C>G), E409D (1928A>C), N413D (1938A>G), D429N (1986G>A), F434Y (2002T>A), L450I (2049T>A 2051A>T), K453S (2059A>G 2060A>C), L458Q (2074T>A), V461A (2083T>C 2084G>A), Q465D (2094C>G 2096G>C), A466E (2098C>A 2099G>A), R467M (2100C>A 2101G>T 2102C>G), R469E (2106A>G 2107G>A), F470L (2111T>G), K472R (2115A>C 2116A>G), E473A (2119A>C 2120G>A), A475V (2125C>T 2126C>T), R476V (2127A>G 2128G>T), A477T (2130G>A 2132T>C), V479L (2136G>C 2138G>T), N481K (2144C>G), V488G (2164T>G), E490V (2170A>T 2171A>T), S493A (2178T>G), V500T (2199G>A 2200T>C), M502V (2205A>G), D508E (2225T>A), M510D (2229A>G 2230T>A 2231G>T), E517D (2252A>C), A519T (2256G>A), N520Q (2259A>C 2261T>A), E521Q (2262G>C), E531D (2294A>C), N538K (2315T>A), G540A (2320G>C 2321T>C), T542I (2326C>T), S579A (2436T>G), S590T (2469T>A 2471C>G), R601K (2503G>A), I608L (2523A>C), I643M (2630C>G), C667* (2702T>A), A700T (2799G>A), I712V (2835A>G 2837C>T), A761T (2982G>A), I771V (3012A>G 3014C>T), I807M (3122T>G), S829N (3187G>A), T832A (3195A>G 3197C>T), K851R (3253A>G), R855K (3265G>A), T857S (3270A>T 3272G>T), T868S (3303A>T 3305C>T), N936S (3508A>G), S948T (3543T>A), K955Q (3564A>C), L956M (3567T>A 3569A>G), P977Q (3631C>A), L978P (3634T>C 3635C>T), Q1014P (3742A>C 3743G>C), T1051A (3852A>G 3854T>G), I1061V (3882A>G 3884C>A), T1062A (3885A>G), K1076Q (3927A>C), T1078A (3933A>G 3935C>T), A1087T (3960G>A), M1095V (3984A>G 3986G>T), S1096N (3988G>A 3989C>T), D1097S (3990G>A 3991A>G), H1105Q (4016T>G), I1120V (4059A>G 4061A>T), N1123S (4069A>G 4070T>C), A1124V (4072C>T), L1150I (4149C>A 4151G>A), Q1163E (4188C>G), H1175Y (4224C>T), S1195G (4284A>G 4286T>A)								
Codon mutations:	ATG2ACG (706T>C), AGG10AGA (731G>A), AAG11AGG (733A>G), AGT17GGT (750A>G), GCA18GCT (755A>T), GCT19GCA (758T>A), GTA20GTG (761A>G), TTA24CTA (771T>C), CAG27CAA (782G>A), ACA29ACT (788A>T), GCT30GCA (791T>A), CTG39CTA (818G>A), CTT40CTC (821T>C), AAA42AAG (827A>G), GGC44GGT (833C>T), TAT48TTT (844A>T), CTC51CTA (854C>A), GGC53GGG (860C>G), GCC54GCT (863C>T), CAA55CAG (866A>G), GCC59GCT (878C>T), GAA61GAG (884A>G), CTG62CTT (887G>T), GAC64GAT (893C>T), AAT70AAC (911T>C), TCT76TCA (929T>A), TCA77TCT (932A>T), GGA89GGG (968A>G), GTT103GTG (1010T>G), AGT120AGC (1061T>C), ACC124ACA (1073C>A), AAT125AAC (1076T>C), CTG128TTA (1083C>T 1085G>A), GTT132ATT (1095G>A), TTG133TTA (1100G>A), TCC136TCT (1109C>T), GGG138GGA (1115G>A), GCA139GCC (1118A>C), TTA143TTG (1130A>G), CCG146CCA (1139G>A), ACC147ACT (1142C>T), CTA150CTG (1151A>G), AAA154AAG (1163A>G), GGC156GGG (1169C>G), TTC159TTT (1178C>T), ACA162ACT (1187A>T), ACT168ACA (1205T>A), CCT173CCC (1220T>C), GCC178GCA (1235C>A), CAA182CAG (1247A>G), CGT187CGC (1262T>C), TCA192TCG (1277A>G), CTT204CTC (1313T>C), CAC205CAT (1316C>T), CGA206AGG (1317C>A 1319A>G), CAA208CAG (1325A>G), TTT209TTC (1328T>C), TTC210TTT (1331C>T), CAG214CAA (1343G>A), CAG218CAA (1355G>A), ATA220ATT (1361A>T), TCA222TCT (1367A>T), GTA223GTT (1370A>T), TCT225TCA (1376T>A), ACC228GCT (1383A>G 1385C>T), TCC229TCT (1388C>T), TCA230TGA (1390C>G), GCA231GCT (1394A>T), ACT232TCA (1395A>T 1397T>A), GAT233GAC (1400T>C), TCA234TCT (1403A>T), GTG235GTA (1406G>A), CTT236TTA (1407C>T 1409T>A), CCG237CCT (1412G>T), GTG239GTA (1418G>A), CAG242CCT (1426A>C 1427G>T), GAC245AAC (1434G>A), ACA246ACC (1439A>C), GTG250GTT (1451G>T), CCA251CCT (1454A>T), AGC252ACT (1456G>C 1457C>T), GAA253GAG (1460A>G), GCA254TCA (1461G>T), AGA255AAA (1465G>A), ATC257ATG (1472C>G), CGG258CGC (1475G>C), TCC259TCG (1478C>G), TCC260TCT (1481C>T), CTC262CTG (1487C>G), CGG263CGA (1490G>A), GTG264GTA (1493G>A), CCC265CCT (1496C>T), CTC267CTT (1502C>T), AGT271AGC (1514T>C), TCC275CCT (1524T>C 1526C>T), TCA276TCT (1529A>T), ATA279ATT (1538A>T), TCT281TTT (1543C>T), ACC282GCT (1545A>G 1547C>T), ACT284ACC (1553T>C), CAC286CAA (1559C>A), CTC287TTA (1560C>T 1562C>A), GGC289GGG (1568C>G), ACG291ACA (1574G>A), CGT292AGG (1575C>A 1577T>G), GTA296GTG (1589A>G), CAG298GAT (1593C>G 1595G>T), AGT301AAT (1603G>A), AGG302GGG (1605A>G), TAC303CAG (1608T>C 1610C>G), GAA304GAG (1613A>G), GAG305GAA (1616G>A), GGT307GCA (1621G>C 1622T>A), GAT309TCC (1626G>T 1627A>C 1628T>C), GGG310GGA (1631G>A), GTT311GTA (1634T>A), GTT315GTC (1646T>C), AAG317AGG (1651A>G), GCT318GCA (1655T>A), GGT320GGC (1661T>C), CTG321TTT (1662C>T 1664G>T), AAC322AAT (1667C>T), CAT325CAC (1676T>C), GTG326ATT (1677G>A 1679G>T), GAT328GAC (1685T>C), GGT331GGC (1694T>C), GAG333GAA (1700G>A), CTG340CTC (1721G>C), CCC341CCT (1724C>T), GAA342GAG (1727A>G), AAC343AAT (1730C>T), CTG347TTG (1740C>T), TCG348TCA (1745G>A), GTG349GTA (1748G>A), AGA350CGA (1749A>C), CAG351CAA (1754G>A), CAA352CAG (1757A>G), GTT353GTG (1760T>G), GCT354GCA (1763T>A), GCA355GCG (1766A>G), GCA356GCT (1769A>T), GCA359GCC (1778A>C), GCA361GCT (1784A>T), AAG363AAA (1790G>A), GGT364GGA (1793T>A), GTT365ATT (1794G>A), TGC366GGG (1797T>G 1799C>G), GTC368GTT (1805C>T), AAA370AAC (1811A>C), CAT371CAC (1814T>C), ATA372ATT (1817A>T), ATC373AAG (1819T>A 1820C>G), AAC374AAT (1823C>T), AAG377AAC (1832G>C), ATC380ATT (1841C>T), CAC381CGC (1843A>G), TTG382CTT (1845T>C 1847G>T), GAG383GAA (1850G>A), ATC385ATT (1856C>T), CAC388TAT (1863C>T 1865C>T), GTG392ATG (1875G>A), ATA393GTA (1878A>G), TAC397TAT (1892C>T), ATT399ATG (1898T>G), GTG400ACA (1899G>A 1900T>C 1901G>A), GCC402GTG (1906C>T 1907C>G), TCT404TCA (1913T>A), GAA409GAC (1928A>C), AAC410AAT (1931C>T), AAT413GAT (1938A>G), CTT414TTG (1941C>T 1943T>G), ACC415ACA (1946C>A), TTG416CTG (1947T>C), GCA417GCC (1952A>C), CAG418CAA (1955G>A), AGA419AGG (1958A>G), GAG422GAA (1967G>A), CTA423CTG (1970A>G), CAA426CAG (1979A>G), GAT429AAT (1986G>A), TAT431TAC (1994T>C), TTT434TAT (2002T>A), AAC437AAT (2012C>T), TAT441TAC (2024T>C), CCT442CCA (2027T>A), TTA450ATT (2049T>A 2051A>T), ACG451ACT (2054G>T), AAA453AGC (2059A>G 2060A>C), CGC455CGG (2066C>G), CTA458CAA (2074T>A), GTG461GCA (2083T>C 2084G>A), GCA462GCC (2087A>C), CAG465GAC (2094C>G 2096G>C), GCG466GAA (2098C>A 2099G>A), CGC467ATG (2100C>A 2101G>T 2102C>G), AGA469GAA (2106A>G 2107G>A), TTT470TTG (2111T>G), TAC471TAT (2114C>T), AAG472CGG (2115A>C 2116A>G), GAG473GCA (2119A>C 2120G>A), GCG474GCT (2123G>T), GCC475GTT (2125C>T 2126C>T), AGG476GTG (2127A>G 2128G>T), GCT477ACC (2130G>A 2132T>C), CAG478CAA (2135G>A), GTG479CTT (2136G>C 2138G>T), AAC481AAG (2144C>G), ACT486ACA (2159T>A), AGT487AGC (2162T>C), GTG488GGG (2164T>G), GAA490GTT (2170A>T 2171A>T), ATA491ATT (2174A>T), CCA492CCT (2177A>T), TCC493GCC (2178T>G), ACC494ACT (2183C>T), TCA495TCC (2186A>C), TTC496TTT (2189C>T), ACA498ACC (2195A>C), AAG499AAA (2198G>A), GTT500ACT (2199G>A 2200T>C), GCC501GCT (2204C>T), ATG502GTG (2205A>G), GAG503GAA (2210G>A), GGC505GGG (2216C>G), AGT506AGC (2219T>C), GAT508GAA (2225T>A), AGC509AGT (2228C>T), ATG510GAT (2229A>G 2230T>A 2231G>T), GCT513GCA (2240T>A), ATT514ATC (2243T>C), GAA517GAC (2252A>C), GCC519ACC (2256G>A), AAT520CAA (2259A>C 2261T>A), GAA521CAA (2262G>C), GTA523GTG (2270A>G), GTT528GTC (2285T>C), GAA531GAC (2294A>C), CAA534CAG (2303A>G), AAT537AAC (2312T>C), AAT538AAA (2315T>A), GGT540GCC (2320G>C 2321T>C), GCG541GCC (2324G>C), ACA542ATA (2326C>T), TTA546TTG (2339A>G), CAA548CAG (2345A>G), TTC558TTT (2375C>T), GAG564GAA (2393G>A), AGT571AGC (2414T>C), GTT573GTG (2420T>G), GAG576GAA (2429G>A), GTG577GTA (2432G>A), TCA579GCA (2436T>G), GCG581GCC (2444G>C), CTC582CTA (2447C>A), GCG584GCA (2453G>A), CTC586CTG (2459C>G), TCC590ACG (2469T>A 2471C>G), AAG591AAA (2474G>A), GGA596GGC (2489A>C), TCG597TCA (2492G>A), CAA598CAG (2495A>G), CTA600CTG (2501A>G), AGG601AAG (2503G>A), TAT602TAC (2507T>C), GCC605GCT (2516C>T), ATT608CTT (2523A>C), TAT615TAC (2546T>C), TCC617TCT (2552C>T), GGT620GGG (2561T>G), ATT622ATA (2567T>A), TCG623TCA (2570G>A), GGT624GGA (2573T>A), GTT629GTA (2588T>A), CTC636CTA (2609C>A), TTC642TTT (2627C>T), ATC643ATG (2630C>G), CTG648CTC (2645G>C), CAT653CAC (2660T>C), TCA657TCT (2672A>T), GGG664GGT (2693G>T), TGT667TGA (2702T>A), ACA669ACG (2708A>G), GGT672GGC (2717T>C), ATC673ATA (2720C>A), TTC676TTT (2729C>T), GCC682GCT (2747C>T), GGG684GGT (2753G>T), ATA691ATT (2774A>T), CAC697CAT (2792C>T), CCC698CCG (2795C>G), TTG699TTA (2798G>A), GCT700ACT (2799G>A), AGT701AGC (2804T>C), ATC712GTT (2835A>G 2837C>T), CAT713CAC (2840T>C), CTG714TTG (2841C>T), CAG715CAA (2846G>A), AAA717AAG (2852A>G), TCG720TCA (2861G>A), ACC721ACA (2864C>A), AAC722AAT (2867C>T), ATC723ATA (2870C>A), CCT726CCA (2879T>A), CCT727CCA (2882T>A), TTG729CTA (2886T>C 2888G>A), CGA730CGG (2891A>G), GCA731GCG (2894A>G), GCA733GCT (2900A>T), GGT736GGC (2909T>C), AAA738AAG (2915A>G), GAC740GAT (2921C>T), ACA742ACG (2927A>G), CCA745CCC (2936A>C), CGA746CGG (2939A>G), ACA749ACG (2948A>G), CAG751CAA (2954G>A), GTA752GTT (2957A>T), CTG753CTA (2960G>A), CAC755CAT (2966C>T), AAT757AAC (2972T>C), GTG760GTT (2981G>T), GCT761ACT (2982G>A), GGT765GGG (2996T>G), ACT766ACA (2999T>A), TTA768CTA (3003T>C), AGC770AGT (3011C>T), ATC771GTT (3012A>G 3014C>T), TCT773TCC (3020T>C), CCG774CCC (3023G>C), GGC776GGT (3029C>T), ATA777ATT (3032A>T), GAC781GAT (3044C>T), GGT782GGA (3047T>A), ACG783ACC (3050G>C), CTG784TTG (3051C>T), CCA786CCT (3059A>T), TCC787TCT (3062C>T), TTG788CTA (3063T>C 3065G>A), GGA790GGG (3071A>G), ATA792ATT (3077A>T), CGC794CGA (3083C>A), ACT800ACG (3101T>G), GGC801GGT (3104C>T), ACT802ACG (3107T>G), GTG804GTC (3113G>C), ATT807ATG (3122T>G), ATT809ATC (3128T>C), GAG810GAA (3131G>A), TTC814TTT (3143C>T), CAT815CAC (3146T>C), GGT817GGA (3152T>A), TTG819TTA (3158G>A), GCC820GCA (3161C>A), GGA822GGT (3167A>T), ACA825ACC (3176A>C), CTG826CTT (3179G>T), AAC827AAT (3182C>T), AGC829AAC (3187G>A), TCC831TCT (3194C>T), ACC832GCT (3195A>G 3197C>T), CCT833CCC (3200T>C), GAC835GAT (3206C>T), TTA837TTG (3212A>G), GTT842GTG (3227T>G), ATT843ATA (3230T>A), AAC845AAT (3236C>T), CTT846CTG (3239T>G), GAG847GAA (3242G>A), GGA849GGC (3248A>C), CGA850CGT (3251A>T), AAG851AGG (3253A>G), AGG855AAG (3265G>A), TGT856TGC (3269T>C), ACG857TCT (3270A>T 3272G>T), ACC859ACT (3278C>T), AAA864AAG (3293A>G), CTT866CTA (3299T>A), ACC868TCT (3303A>T 3305C>T), GGT870GGA (3311T>A), CTA875TTA (3324C>T), CCG876CCT (3329G>T), CAC883CAT (3350C>T), AGG885CGG (3354A>C), TTG886TTA (3359G>A), ACA889ACG (3368A>G), GTC890GTG (3371C>G), TTG891CTG (3372T>C), GTA895GTT (3386A>T), CTA901CTG (3404A>G), ACG903ACA (3410G>A), GTC904GTT (3413C>T), ACA907ACG (3422A>G), CAG909CAA (3428G>A), CTT910CTG (3431T>G), TGC912TGT (3437C>T), GAT915GAC (3446T>C), TTG916TTA (3449G>A), GTC917GTA (3452C>A), CTT918CTA (3455T>A), GGG919GGA (3458G>A), GGC920GGT (3461C>T), TCT923TCC (3470T>C), GTT924GTC (3473T>C), CCC926CCT (3479C>T), ATT927ATC (3482T>C), TAC928TAT (3485C>T), GGA929GGG (3488A>G), CAC930CAT (3491C>T), GGA934GGG (3503A>G), AAT936AGT (3508A>G), GCT937GCC (3512T>C), GTG938GTT (3515G>T), CTA941TTG (3522C>T 3524A>G), TTC942TTT (3527C>T), GAG944GAA (3533G>A), TCT948ACT (3543T>A), CGC950CGT (3551C>T), ATT953ATC (3560T>C), AAA955CAA (3564A>C), TTA956ATG (3567T>A 3569A>G), AAG958AAA (3575G>A), GAA959GAG (3578A>G), AAC960AAT (3581C>T), ACA963ACC (3590A>C), GGA968GGG (3605A>G), AAG970AAA (3611G>A), CCA972CCT (3617A>T), CAG973CAA (3620G>A), GTA974GTT (3623A>T), GTG976GTC (3629G>C), CCG977CAG (3631C>A), CTC978CCT (3634T>C 3635C>T), AGA979AGG (3638A>G), GAC980GAT (3641C>T), TCA983TCT (3650A>T), GGG985GGT (3656G>T), GCT986GCC (3659T>C), GTA987GTG (3662A>G), GTG991GTC (3674G>C), GAC996GAT (3689C>T), CGA999CGC (3698A>C), TTG1001CTG (3702T>C), TTA1003CTA (3708T>C), TGC1005TGT (3716C>T), GCA1006GCG (3719A>G), CCC1007CCA (3722C>A), TCC1009TCG (3728C>G), CAG1014CCC (3742A>C 3743G>C), GGG1015GGA (3746G>A), GCA1016GCT (3749A>T), CTG1017CTA (3752G>A), GTA1018GTG (3755A>G), ACA1023ACC (3770A>C), TGC1024TGT (3773C>T), CCA1025CCT (3776A>T), TTC1026TTT (3779C>T), TTC1033TTT (3800C>T), GGA1038GGT (3815A>T), TAC1042TAT (3827C>T), ATT1044ATA (3833T>A), GTA1046GTG (3839A>G), AGA1048AGG (3845A>G), GTG1049GTA (3848G>A), CAG1050CAA (3851G>A), ACT1051GCG (3852A>G 3854T>G), TCC1052TCA (3857C>A), ATA1055ATC (3866A>C), GGA1056GGT (3869A>T), GGA1057GGT (3872A>T), CTG1059CTC (3878G>C), AAC1060AAT (3881C>T), ATC1061GTA (3882A>G 3884C>A), ACC1062GCC (3885A>G), TTA1063TTG (3890A>G), GAT1064GAC (3893T>C), TCA1065TCT (3896A>T), GGA1073GGT (3920A>T), ATC1074ATT (3923C>T), AAG1076CAG (3927A>C), GGC1077GGA (3932C>A), ACC1078GCT (3933A>G 3935C>T), GTT1080GTG (3941T>G), GTC1081GTA (3944C>A), TCT1082TCC (3947T>C), GCT1083GCA (3950T>A), GGT1084GGA (3953T>A), GCA1087ACA (3960G>A), GCT1090GCA (3971T>A), GGT1094GGA (3983T>A), ATG1095GTT (3984A>G 3986G>T), AGC1096AAT (3988G>A 3989C>T), GAC1097AGC (3990G>A 3991A>G), AAC1098AAT (3995C>T), ATC1099ATT (3998C>T), TTT1100TTC (4001T>C), CAT1105CAG (4016T>G), GAG1108GAA (4025G>A), TTT1110TTC (4031T>C), CCT1111CCG (4034T>G), AGA1112CGT (4035A>C 4037A>T), CGC1113CGT (4040C>T), ACC1115ACT (4046C>T), GCC1117GCT (4052C>T), ATA1120GTT (4059A>G 4061A>T), CCA1122CCT (4067A>T), AAT1123AGC (4069A>G 4070T>C), GCT1124GTT (4072C>T), AGA1126CGA (4077A>C), CGA1133CGC (4100A>C), CTA1134CTT (4103A>T), GGA1135GGC (4106A>C), CTC1137CTT (4112C>T), ATA1147ATC (4142A>C), CTG1150ATA (4149C>A 4151G>A), TTC1160TTT (4181C>T), TTG1162TTA (4187G>A), CAA1163GAA (4188C>G), GCT1165GCA (4196T>A), TCA1169TCT (4208A>T), GCA1170GCT (4211A>T), AAG1174AAA (4223G>A), CAT1175TAT (4224C>T), TTG1176CTT (4227T>C 4229G>T), ACC1183ACT (4250C>T), TAC1184TAT (4253C>T), CTT1185CTG (4256T>G), ACT1187ACC (4262T>C), CCT1188CCA (4265T>A), TCA1191TCT (4274A>T), AGG1194CGG (4281A>C), AGT1195GGA (4284A>G 4286T>A), TTT1196TTC (4289T>C)								

*: Inserts / Deletes / Misaligned / Frameshifts

Analysis details

This analysis was performed with panviral2.64

NGS Details (UN59): Torradovirus marchitezum (segment RNA 1)

Assembly

**Coverage Length**	5567 (1 contig(s))
**Depth Of Coverage**	1430.4
**Number Of Reads**	59802
**Reads Per Million**	1300.04 rpm (after QC)
**Ambiguities**	0
**Assembly Method**	de novo + reference guided assembly
**Consensus Caller**	Bcf Tools

Coverage Map

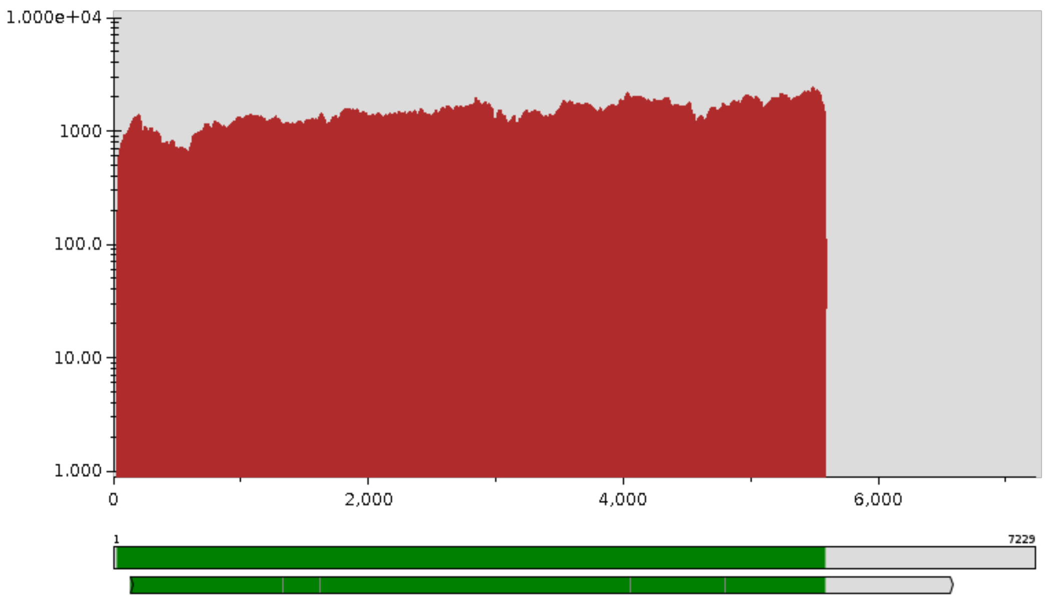

Assignment

**Type**	Torradovirus marchitezum (Taxonomy ID: 3048376)
**Reference Genome**	NC_010987.1
**NT Identity (%)**	66.5281
**AA Identity (%)**	69.9448
**Number Of Stop Codons**	0
**Number Of CDS**	1

Alignment

**Alignment Score**	3552.0 (NT) + 9297.0 (AA) = 12849.0
**Concordance (%)**	54.4311

Alignment Method	Global, seeded, nucleotide + amino acids (AGA)

Genome Region

Sequence starts at position 23 and ends at position 5589 relative to NC_010987.1 reference sequence.

Alignment Detailed Statistics

	Begin	End	Coverage	Score	Concordance	Matches	Identities	I/D/M/F*	Stop Codons
**NT**	**23**	**5589**	**77.0%**	**3552**	**32.4%**	**5524 (99.1%)**	**3681 (66.0%)**	**9/43**	

	Begin	End	Coverage	Score	Concordance	Matches	Identities	I/D/M/F*	Stop Codons
NT	23	5589	77.0%	3552	32.4%	5524 (99.1%)	3681 (66.0%)	9/43	

Mutations:

32T>C, 37G>A, 40C>T, 59A>C, 61T>C, 62T>A, 63G>C, 66A>C, 67T>A, 68T>A, 70G>A, 71A>C, 74C>T, 75T>G, 76T>C, 77G>T, 79A>T, 81A>C, 82C>T, 85T>C, 87C>A, 89_99delAACATCTTCTT, 108A>T, 112G>C, 114C>T, 115C>T, 116A>C, 118T>C, 119A>T, 122T>G, 125A>T, 128C>A, 129C>A, 130T>A, 133_134delAT, 136T>A, 146T>A, 160T>G, 161C>G, 162T>A, 163C>G, 164C>T, 165_188delAAGGTTGCTGGTGTTGATGACGCC, 189A>C, 190G>C, 192T>C, 195T>G, 200C>T, 201A>T, 203C>A, 204A>C, 217G>C, 218T>A, 222C>G, 227A>T, 228A>T, 229A>C, 234A>T, 239C>T, 240A>T, 241A>C, 242A>T, 243T>C, 251A>G, 252G>A, 254G>T, 255G>C, 257T>C, 258A>T, 259G>C, 260T>C, 264A>G, 268C>A, 269C>T, 271C>A, 272T>A, 273A>G, 275G>T, 283A>C, 284G>C, 288T>G, 289C>T, 296T>C, 297C>A, 299T>A, 306C>G, 308G>C, 314A>G, 315T>C, 317T>A, 320G>T, 322C>T, 323T>G, 325A>C, 326T>A, 328T>C, 329G>A, 330T>G, 332C>A, 338T>A, 343T>C, 344G>C, 345G>T, 347C>T, 350T>A, 353T>C, 355G>A, 357T>G, 359A>T, 360C>G, 362T>A, 363A>G, 365T>A, 367A>G, 374G>A, 377C>T, 380A>G, 385G>C, 386T>A, 387G>A, 388C>A, 390C>A, 392T>A, 395A>G, 398T>A, 401A>C, 404T>C, 405C>A, 407T>C, 410A>G, 412C>G, 413G>T, 416C>T, 417T>G, 418C>A, 422C>T, 423T>G, 425G>T, 426T>A, 427C>A, 428C>A, 431A>T, 437C>T, 441T>A, 446C>A, 447A>G, 448A>T, 449G>A, 453A>C, 455G>T, 458T>A, 459T>C, 461A>T, 465T>A, 468T>G, 471G>T, 472A>T, 473G>A, 477T>G, 479G>C, 480T>A, 481C>A, 482C>G, 483A>G, 485T>C, 489G>T, 491T>C, 493A>T, 494A>C, 495T>C, 502T>A, 503T>C, 505C>A, 506T>A, 510G>T, 512G>A, 516A>G, 518T>A, 522A>G, 523G>T, 527A>G, 528G>T, 529A>C, 530T>A, 531G>T, 542T>G, 543G>T, 545A>T, 546T>A, 547A>T, 548T>A, 550G>C, 555C>T, 557T>A, 560A>T, 567A>T, 569A>G, 570A>G, 572A>C, 573T>A, 575G>T, 578A>G, 579G>C, 581G>A, 582C>T, 584T>A, 587A>G, 589T>A, 593T>A, 596T>C, 600A>G, 601G>T, 602T>A, 611T>A, 612G>A, 613A>C, 615G>C, 616G>C, 618G>T, 621T>A, 622C>A, 626C>T, 627A>G, 628C>T, 629T>A, 632C>T, 633A>G, 634A>T, 635A>T, 637A>C, 639A>T, 641C>G, 646T>C, 647G>A, 648A>G, 650A>T, 653C>T, 659C>T, 660A>T, 662G>T, 665T>C, 668A>T, 669A>T, 670G>C, 671T>A, 672C>T, 674A>G, 677C>T, 697A>C, 702G>T, 704C>T, 706A>G, 707A>G, 708C>A, 709G>A, 710A>G, 713C>T, 714A>T, 717A>C, 718T>A, 720G>C, 725C>G, 731T>A, 732A>G, 733G>C, 734T>A, 735A>G, 737G>A, 746G>T, 750T>A, 751C>G, 752A>T, 753G>T, 755T>A, 756T>A, 758A>G, 770G>T, 771T>A, 772C>G, 773T>C, 776G>A, 777T>C, 779G>T, 780A>T, 782G>A, 786A>T, 789A>T, 791A>G, 803G>T, 806T>C, 809G>A, 812G>T, 815G>A, 818G>A, 821C>T, 822A>G, 827A>T, 830G>T, 836T>G, 848C>T, 849T>C, 851G>T, 852A>T, 853G>C, 854C>A, 861T>C, 866C>A, 872T>C, 873C>T, 875C>G, 878T>C, 881G>T, 882A>G, 884A>T, 896T>G, 899G>A, 911A>G, 914A>G, 918A>C, 919C>A, 920T>G, 921C>T, 923T>G, 927C>A, 929T>G, 932T>C, 938A>G, 944C>T, 947C>A, 950C>T, 953G>A, 956T>C, 974A>G, 978A>G, 980A>G, 983C>T, 984G>A, 988C>T, 989C>T, 995A>C, 998C>T, 1010G>A, 1019T>G, 1024G>A, 1028A>T, 1031G>C, 1032C>T, 1034G>A, 1040T>C, 1046G>T, 1050G>C, 1059C>A, 1060A>C, 1064G>A, 1068G>T, 1069A>C, 1070T>A, 1072C>A, 1073A>G, 1076G>T, 1077T>G, 1078G>C, 1082T>A, 1085T>A, 1086G>T, 1088A>T, 1090C>T, 1100G>A, 1103C>T, 1109C>A, 1113A>C, 1114A>C, 1115G>T, 1118T>A, 1121C>T, 1124T>A, 1126C>T, 1127C>T, 1130A>G, 1134A>C, 1136C>A, 1140C>T, 1142G>A, 1144G>A, 1145G>A, 1148A>G, 1149G>C, 1151A>G, 1154T>C, 1155C>G, 1160G>A, 1163C>A, 1178A>C, 1193G>A, 1199C>T, 1200A>T, 1215A>T, 1216G>C, 1226T>A, 1227G>A, 1232G>A, 1233T>C, 1238A>T, 1242G>T, 1244T>G, 1245T>G, 1246A>T, 1253A>G, 1254A>G, 1255G>C, 1256T>C, 1257T>A, 1259T>C, 1260A>C, 1261G>A, 1262G>A, 1265C>T, 1266A>T, 1267T>C, 1268G>T, 1271C>T, 1280G>A, 1284C>A, 1286T>G, 1292A>G, 1298T>C, 1304A>G, 1313C>T, 1316G>A, 1317T>G, 1319C>T, 1325G>A, 1337T>A, 1340A>G, 1342C>G, 1350T>C, 1352A>T, 1355T>C, 1367G>T, 1368T>C, 1370G>C, 1374A>G, 1375G>C, 1385G>A, 1391A>G, 1394C>G, 1397T>C, 1400G>C, 1401C>T, 1403A>G, 1409A>G, 1418G>A, 1421G>A, 1445T>A, 1448C>T, 1457T>A, 1458A>G, 1460C>G, 1463G>T, 1472C>T, 1478A>T, 1481A>T, 1482T>C, 1484G>T, 1487A>G, 1496T>A, 1505C>T, 1508A>G, 1511T>C, 1514T>A, 1517A>G, 1529T>C, 1538A>T, 1541A>C, 1544A>G, 1547A>T, 1553C>T, 1554T>C, 1556A>T, 1565C>T, 1571T>C, 1577C>T, 1583C>A, 1589T>C, 1592C>T, 1595C>A, 1598C>A, 1604T>C, 1607A>C, 1616A>C, 1623C>T, 1628C>A, 1631A>T, 1637T>A, 1638G>A, 1640C>T, 1643A>G, 1649T>G, 1652T>A, 1655A>G, 1661A>T, 1667T>A, 1673G>A, 1674C>A, 1679A>G, 1682G>A, 1685C>T, 1688C>T, 1689C>T, 1691G>A, 1694G>T, 1700T>C, 1702T>G, 1703G>A, 1708A>T, 1715G>T, 1719T>G, 1720C>A, 1721C>T, 1722A>C, 1724T>A, 1725G>A, 1727A>T, 1729A>C, 1733T>A, 1736G>A, 1738G>C, 1739A>C, 1745C>A, 1746A>G, 1747G>T, 1748A>T, 1752A>G, 1754C>T, 1755A>G, 1757A>G, 1763G>A, 1781T>C, 1787T>A, 1790C>T, 1793G>A, 1794A>G, 1795A>C, 1799G>A, 1800A>T, 1806A>G, 1807G>A, 1811T>C, 1814T>C, 1818G>C, 1820G>A, 1829A>G, 1832C>A, 1835A>G, 1838T>C, 1840A>G, 1844A>G, 1847T>C, 1848G>T, 1849C>A, 1850A>T, 1853A>G, 1856A>G, 1861A>G, 1862G>C, 1866T>C, 1868G>T, 1870T>A, 1872G>A, 1874A>T, 1875G>A, 1878G>A, 1879A>C, 1883T>A, 1886C>A, 1887A>T, 1889A>G, 1890A>G, 1891G>C, 1893C>G, 1894C>A, 1895T>C, 1899A>G, 1901A>T, 1910T>C, 1911C>A, 1913A>T, 1917T>G, 1921C>T, 1922A>T, 1928T>A, 1932A>C, 1934A>G, 1936C>A, 1937G>T, 1941G>T, 1943G>C, 1944G>C, 1946T>A, 1948G>A, 1949G>T, 1952T>C, 1953_1955delAGA, 1958G>A, 1960G>A, 1961C>A, 1962A>G, 1963C>T, 1964C>T, 1965A>T, 1966A>T, 1969A>C, 1970A>T, 1971G>C, 1972A>C, 1976A>G, 1980A>G, 1981T>C, 1983T>A, 1984T>C, 1985G>T, 1986G>T, 1987T>A, 1988G>T, 1989C>G, 1990C>A, 1991C>G, 1992A>C, 1993C>A, 1994T>G, 1995G>A, 1996T>C, 1997T>A, 1998G>A, 2000G>T, 2002G>C, 2003T>A, 2007A>G, 2008A>C, 2012T>A, 2014G>A, 2016G>A, 2018G>T, 2019A>G, 2021C>T, 2024T>C, 2025G>T, 2027A>C, 2028A>C, 2029G>A, 2030A>G, 2033G>A, 2039G>A, 2042A>T, 2048A>C, 2049T>C, 2051G>C, 2060A>T, 2066G>A, 2069C>G, 2073T>A, 2076G>A, 2079C>A, 2080C>A, 2081T>A, 2082G>T, 2083A>T, 2084G>T, 2087A>G, 2088C>T, 2093T>C, 2096T>A, 2104A>T, 2105T>C, 2106C>T, 2107A>C, 2108T>C, 2117C>T, 2120G>C, 2127T>G, 2128C>T, 2129T>G, 2132T>A, 2133G>T, 2134A>C, 2136G>T, 2137A>C, 2141A>T, 2142C>G, 2143A>C, 2144T>C, 2148G>C, 2152T>A, 2156C>T, 2157A>G, 2159C>A, 2161A>T, 2162A>G, 2166A>G, 2167G>T, 2169G>A, 2170T>A, 2171C>T, 2172A>G, 2174C>T, 2175A>T, 2181T>G, 2182A>T, 2183T>C, 2185G>A, 2193G>A, 2194A>G, 2196G>C, 2199G>C, 2201A>G, 2208C>G, 2209G>C, 2210A>T, 2219A>G, 2237C>T, 2241A>G, 2243A>C, 2249C>T, 2252T>C, 2268G>A, 2269C>G, 2270A>C, 2271T>A, 2279C>T, 2285G>A, 2286A>G, 2289A>T, 2290A>T, 2291A>G, 2294A>G, 2296C>A, 2297A>T, 2300G>C, 2303G>A, 2304A>C, 2305C>G, 2306A>C, 2307G>A, 2309G>T, 2310A>C, 2312G>T, 2313A>G, 2314G>A, 2316A>C, 2317C>A, 2318T>A, 2321A>G, 2322T>A, 2323T>A, 2324T>A, 2327A>G, 2328C>T, 2331T>A, 2332C>A, 2333T>G, 2334T>G, 2335G>C, 2336C>G, 2337A>C, 2340G>C, 2342T>G, 2345A>T, 2350T>C, 2351T>A, 2354C>T, 2357C>A, 2360C>T, 2362T>C, 2363A>G, 2369C>T, 2372G>A, 2381A>G, 2385T>A, 2386T>G, 2387T>A, 2390T>A, 2396T>C, 2397T>C, 2399A>G, 2405A>T, 2408A>T, 2415A>C, 2417A>C, 2426C>T, 2428A>G, 2429C>G, 2435A>T, 2436T>G, 2438_2439insGGT, 2440A>C, 2441A>T, 2444G>C, 2445C>A, 2446C>A, 2447A>G, 2451G>A, 2452A>G, 2454T>A, 2455C>A, 2456A>T, 2462C>T, 2464A>T, 2465A>C, 2466A>G, 2467T>A, 2468T>A, 2472T>A, 2473A>T, 2474C>G, 2475C>A, 2476A>T, 2478A>G, 2479A>G, 2480A>T, 2484G>A, 2485T>A, 2486T>G, 2489A>C, 2490T>A, 2492T>A, 2495C>G, 2497A>T, 2498G>C, 2499G>C, 2501T>G, 2503C>T, 2504A>G, 2508T>A, 2509G>A, 2510G>T, 2511A>G, 2512G>A, 2514C>T, 2516T>G, 2521A>T, 2522T>C, 2523G>A, 2524C>T, 2525A>C, 2526C>T, 2530G>A, 2531A>G, 2537C>T, 2546A>C, 2549T>A, 2556A>T, 2558A>G, 2561G>A, 2564T>A, 2573A>C, 2574G>A, 2575T>C, 2576T>C, 2577T>A, 2579T>G, 2580G>A, 2582A>T, 2584G>C, 2586A>G, 2591A>T, 2594A>T, 2597T>C, 2598T>A, 2600G>T, 2601G>A, 2603G>T, 2607T>C, 2608G>T, 2609G>C, 2610C>A, 2613A>G, 2614G>C, 2615T>A, 2616A>G, 2617A>C, 2618C>A, 2619T>A, 2623T>C, 2627C>T, 2628A>T, 2629A>T, 2630T>A, 2633A>T, 2634T>G, 2635C>A, 2636G>T, 2645A>G, 2648T>C, 2651G>A, 2660A>G, 2672A>C, 2678T>C, 2684G>A, 2687C>T, 2690G>A, 2693T>G, 2696T>G, 2698A>C, 2699G>T, 2699_2700insCAT, 2700C>T, 2703A>G, 2704G>C, 2708T>A, 2709C>T, 2710T>C, 2711T>C, 2712C>T, 2714C>G, 2715G>C, 2717T>A, 2720T>G, 2724A>G, 2725G>C, 2726T>G, 2727T>G, 2729T>A, 2730C>G, 2732A>C, 2733T>A, 2734C>A, 2735A>C, 2736T>A, 2737T>A, 2738G>C, 2739G>C, 2740C>A, 2741A>T, 2742C>G, 2743A>T, 2744A>G, 2747T>C, 2747_2748insCGA, 2753T>A, 2755C>A, 2756T>A, 2757G>C, 2759A>G, 2771A>T, 2775C>G, 2777G>A, 2780C>T, 2783C>G, 2784A>C, 2786G>C, 2792T>C, 2798G>T, 2801G>T, 2804A>G, 2808C>A, 2812G>C, 2822G>C, 2825C>T, 2828T>C, 2834C>T, 2837C>G, 2840C>T, 2843G>T, 2858A>T, 2859A>C, 2861G>C, 2864C>T, 2867A>G, 2870T>A, 2876A>C, 2879C>T, 2880C>A, 2881A>G, 2882C>A, 2885T>C, 2886G>A, 2887C>T, 2888T>G, 2889C>A, 2891A>G, 2897T>C, 2903A>T, 2907A>T, 2909A>C, 2915C>T, 2920C>A, 2921C>G, 2922A>C, 2927T>C, 2928C>A, 2929G>T, 2930A>T, 2932C>G, 2936A>T, 2939T>G, 2942A>G, 2944A>T, 2945C>T, 2948C>T, 2953G>C, 2954A>C, 2955G>C, 2964A>T, 2966T>G, 2969T>A, 2975C>T, 2981C>T, 2982A>G, 2984C>G, 2988C>T, 2994C>A, 2996C>G, 2999T>A, 3002T>G, 3017A>C, 3020G>A, 3021C>T, 3023T>A, 3026C>T, 3029C>A, 3030C>A, 3032T>A, 3038C>T, 3044A>T, 3045A>C, 3047A>C, 3050G>A, 3056G>T, 3062C>T, 3064T>A, 3066A>T, 3068A>C, 3078C>A, 3079A>T, 3080A>G, 3083A>C, 3090T>G, 3091G>T, 3092T>G, 3095A>G, 3101T>A, 3104G>T, 3107T>A, 3109C>A, 3110A>G, 3116A>C, 3117C>A, 3118G>A, 3119T>G, 3120C>A, 3121T>A, 3122T>A, 3123G>T, 3124G>C, 3131A>G, 3135T>G, 3137T>G, 3140C>T, 3143G>A, 3144A>T, 3145T>A, 3146G>T, 3167A>C, 3173A>C, 3175T>A, 3176T>A, 3182G>C, 3183G>A, 3184A>C, 3190A>G, 3192C>T, 3194G>A, 3195A>G, 3197C>T, 3200C>G, 3201T>A, 3202C>A, 3203C>T, 3204A>G, 3205G>C, 3206T>A, 3210A>G, 3211A>C, 3212G>T, 3213A>C, 3216T>A, 3221T>A, 3225C>G, 3226A>T, 3227A>T, 3228T>G, 3230T>A, 3236A>G, 3245A>T, 3249G>A, 3251G>T, 3254A>G, 3260T>A, 3263C>T, 3269C>T, 3272G>T, 3273T>C, 3275G>T, 3278A>C, 3279C>T, 3281G>A, 3284A>G, 3289G>C, 3293T>G, 3299T>C, 3305A>G, 3308T>G, 3309G>T, 3310T>G, 3311G>C, 3320C>A, 3323C>A, 3326C>T, 3335A>T, 3343G>A, 3344C>T, 3345G>A, 3346T>C, 3347A>C, 3350A>T, 3353T>A, 3356A>G, 3357C>A, 3358G>A, 3359T>G, 3362A>T, 3364T>A, 3365T>C, 3366A>G, 3368C>G, 3369T>G, 3377T>C, 3380T>A, 3386T>A, 3387A>G, 3389C>G, 3391A>T, 3392C>T, 3395G>A, 3398A>T, 3402C>T, 3404A>G, 3407G>A, 3416A>C, 3419T>G, 3422G>A, 3425G>C, 3426G>A, 3428C>T, 3433C>T, 3434A>G, 3437A>T, 3452T>A, 3453A>C, 3455T>G, 3456C>T, 3462T>C, 3463C>A, 3464A>C, 3465A>C, 3467T>G, 3470A>G, 3471A>T, 3472G>C, 3475G>A, 3476A>G, 3479A>G, 3480A>G, 3481C>A, 3483C>A, 3484A>G, 3485G>A, 3486C>T, 3488T>C, 3497A>G, 3500C>A, 3503A>G, 3507T>G, 3508A>C, 3509C>T, 3511A>T, 3513C>T, 3515T>G, 3518A>C, 3521A>T, 3524T>A, 3527A>G, 3528C>T, 3529A>T, 3533G>A, 3534T>A, 3539C>T, 3542C>T, 3548T>G, 3551C>A, 3557G>A, 3560C>A, 3563T>C, 3566G>T, 3567C>A, 3568G>A, 3569G>A, 3570A>C, 3571A>G, 3572A>C, 3575C>T, 3577A>C, 3578A>C, 3579T>C, 3587A>G, 3590A>T, 3593C>T, 3596A>G, 3597C>G, 3598C>A, 3602A>T, 3603G>C, 3608A>T, 3611A>C, 3620T>C, 3624A>T, 3626A>T, 3628T>C, 3629T>C, 3630C>A, 3632T>A, 3641A>G, 3644T>A, 3647T>A, 3650T>G, 3656C>T, 3671G>T, 3674A>C, 3675A>G, 3676T>A, 3680A>G, 3683C>T, 3697G>C, 3698T>C, 3699G>A, 3700G>A, 3701A>C, 3707T>G, 3710T>G, 3711G>C, 3712A>C, 3713C>A, 3714A>G, 3719C>A, 3722A>G, 3725A>G, 3728A>C, 3731C>T, 3734A>G, 3735A>G, 3736A>G, 3740C>T, 3743G>A, 3744T>G, 3745G>C, 3746C>A, 3749G>C, 3750C>A, 3752G>T, 3755A>T, 3759A>G, 3761C>G, 3762A>C, 3764G>T, 3765C>A, 3766G>A, 3767T>G, 3769G>T, 3770C>G, 3774A>C, 3775A>G, 3776G>A, 3777A>G, 3779C>G, 3782T>C, 3788A>G, 3789C>A, 3790G>A, 3791T>A, 3794A>T, 3795T>G, 3796G>C, 3797T>A, 3798G>A, 3799A>T, 3800A>T, 3803G>A, 3804G>A, 3827A>G, 3838G>T, 3839T>C, 3845C>T, 3847A>T, 3853G>C, 3854C>A, 3860G>A, 3861C>T, 3869A>G, 3872T>A, 3875C>T, 3878A>T, 3884C>T, 3887C>T, 3894A>T, 3899G>A, 3900A>C, 3902G>T, 3905G>T, 3906A>T, 3907T>C, 3908G>T, 3914G>A, 3917A>G, 3927C>G, 3930T>G, 3933A>C, 3934C>A, 3935T>A, 3936G>C, 3937G>C, 3938C>A, 3941A>C, 3942G>T, 3943G>C, 3944A>G, 3945G>C, 3950A>G, 3953G>T, 3954G>C, 3955A>T, 3956A>T, 3965A>G, 3966G>A, 3971T>A, 3977A>C, 3983A>T, 3996C>T, 3998A>G, 4001T>C, 4002G>T, 4003T>C, 4008A>G, 4010T>A, 4014A>C, 4019C>A, 4021T>C, 4022C>A, 4023C>T, 4024A>T, 4028G>T, 4031T>C, 4034G>T, 4040C>A, 4046A>G, 4049C>T, 4052C>G, 4064C>G, 4065C>T, 4067C>G, 4079A>G, 4088C>G, 4094A>G, 4101A>C, 4103G>C, 4106C>G, 4109T>C, 4112G>A, 4115A>C, 4116C>T, 4118T>G, 4130C>T, 4137T>C, 4142C>G, 4143A>C, 4148G>A, 4151T>C, 4154T>C, 4160T>C, 4163C>T, 4166A>T, 4169C>T, 4170A>T, 4171C>G, 4172A>T, 4182T>G, 4183T>C, 4184G>A, 4190T>C, 4192C>T, 4193T>A, 4194A>C, 4196A>C, 4199A>T, 4205A>G, 4208T>A, 4214T>C, 4215G>A, 4220T>A, 4223A>G, 4226T>C, 4227T>C, 4228C>A, 4239T>G, 4242T>C, 4247A>T, 4249A>G, 4251G>A, 4253G>C, 4259A>G, 4260G>T, 4262T>A, 4265G>A, 4268A>T, 4271C>T, 4273T>A, 4280T>C, 4286T>C, 4292T>C, 4295C>G, 4316T>C, 4317G>A, 4319T>C, 4325T>A, 4331G>A, 4344T>C, 4346G>A, 4354A>C, 4355A>T, 4357A>T, 4358C>T, 4361C>T, 4364A>T, 4365T>G, 4366C>A, 4367A>T, 4368A>G, 4376C>G, 4385T>G, 4388C>A, 4395C>A, 4397A>G, 4400T>C, 4401G>A, 4404C>T, 4406T>A, 4418A>C, 4424T>C, 4430C>T, 4433A>C, 4436G>C, 4439C>T, 4443G>A, 4445T>C, 4448A>G, 4449G>T, 4455A>C, 4457G>C, 4458T>C, 4460G>A, 4463T>G, 4466G>T, 4469C>A, 4475A>C, 4478A>T, 4481A>T, 4487A>T, 4488C>T, 4490A>G, 4493G>T, 4499C>A, 4502C>T, 4508T>A, 4509C>T, 4511C>G, 4514C>A, 4530C>A, 4532A>G, 4535T>C, 4538C>G, 4548T>C, 4553A>G, 4556A>G, 4562C>A, 4566A>G, 4568T>C, 4569G>A, 4570C>A, 4571T>A, 4576G>A, 4577G>A, 4578G>T, 4581A>G, 4583A>G, 4586A>C, 4587G>A, 4592A>G, 4597G>A, 4601T>C, 4611C>T, 4613A>G, 4622T>A, 4625T>A, 4631A>T, 4637C>T, 4640T>C, 4641C>T, 4643T>G, 4649T>G, 4652A>T, 4656T>A, 4658A>G, 4662C>T, 4664C>G, 4665C>A, 4666A>G, 4673T>C, 4674T>G, 4675C>A, 4679G>A, 4683A>T, 4684A>C, 4685T>C, 4688A>T, 4692C>A, 4693A>G, 4694T>A, 4697G>A, 4698T>C, 4700A>G, 4704C>G, 4705T>C, 4706G>T, 4709C>T, 4712T>C, 4715A>C, 4716A>G, 4731C>T, 4733T>G, 4739A>G, 4742G>A, 4751T>A, 4752A>G, 4754T>G, 4755G>A, 4758G>A, 4759T>C, 4761C>A, 4768A>T, 4769A>G, 4784A>C, 4787T>C, 4793G>C, 4799A>G, 4805T>C, 4808A>G, 4817T>A, 4818C>T, 4820T>G, 4826C>T, 4829C>A, 4835T>A, 4838T>A, 4844G>A, 4854A>G, 4868G>T, 4871T>C, 4877A>T, 4880A>G, 4883T>A, 4886C>A, 4887C>G, 4888C>T, 4889A>G, 4892T>A, 4901C>T, 4902A>G, 4903C>T, 4904T>A, 4907G>A, 4910C>T, 4914A>T, 4915T>A, 4916G>T, 4923G>A, 4925G>T, 4934A>C, 4937A>C, 4938C>A, 4940T>A, 4946T>C, 4955C>T, 4958T>G, 4960G>A, 4961T>G, 4963T>C, 4964G>C, 4965G>A, 4973C>A, 4975A>G, 4976G>T, 4977C>T, 4982C>A, 4983T>A, 4984T>A, 4985A>T, 4993A>T, 4994G>T, 4995A>G, 4996C>G, 4997G>A, 5000G>T, 5001T>C, 5003G>C, 5004G>A, 5006T>G, 5007G>C, 5009A>G, 5012G>A, 5013G>A, 5014G>A, 5015T>C, 5016T>C, 5019A>C, 5020G>C, 5021A>C, 5022G>A, 5023C>T, 5024T>C, 5025G>A, 5031T>A, 5033T>G, 5036T>C, 5037T>A, 5039C>T, 5042T>C, 5044T>A, 5045G>T, 5049G>C, 5051G>T, 5055C>A, 5056A>G, 5057G>T, 5058G>C, 5064A>T, 5066T>G, 5067G>A, 5070A>G, 5075A>C, 5081G>A, 5085T>G, 5086C>A, 5087A>G, 5091G>A, 5092C>T, 5093C>T, 5094C>G, 5095T>G, 5097A>G, 5098C>T, 5099A>T, 5100A>T, 5102T>G, 5103T>A, 5104G>T, 5105T>G, 5108A>G, 5109A>C, 5111G>A, 5112G>T, 5115A>G, 5117A>T, 5120A>T, 5121G>A, 5122C>A, 5123T>A, 5125T>A, 5127T>A, 5129C>A, 5132T>C, 5135G>C, 5138A>T, 5141G>A, 5142G>C, 5143A>C, 5144C>T, 5147A>T, 5150T>G, 5153G>A, 5159C>T, 5160_5162delACA, 5172C>G, 5173G>A, 5174T>G, 5176A>G, 5178C>G, 5180G>C, 5181A>C, 5184T>G, 5185G>A, 5186G>A, 5192A>G, 5195A>T, 5196A>G, 5198C>T, 5199A>G, 5201T>A, 5202G>T, 5203G>T, 5207T>C, 5211T>A, 5212C>G, 5213A>T, 5216A>T, 5219T>G, 5226G>T, 5228C>A, 5229C>A, 5232T>C, 5234C>A, 5235A>C, 5236C>A, 5237G>A, 5241G>T, 5242A>C, 5243T>A, 5244G>T, 5245A>C, 5249G>T, 5250T>A, 5255T>C, 5256A>C, 5258T>G, 5264C>T, 5267T>C, 5270A>T, 5273T>A, 5276A>C, 5277A>C, 5278A>G, 5279G>C, 5281A>G, 5282G>A, 5283T>A, 5285C>A, 5287A>C, 5288T>A, 5294T>G, 5300T>G, 5303A>T, 5309C>T, 5318C>T, 5333G>A, 5339G>C, 5346A>T, 5349T>G, 5354T>A, 5357A>C, 5358A>C, 5359T>A, 5364G>C, 5365C>A, 5366C>T, 5370A>C, 5371A>G, 5372G>C, 5375T>G, 5376G>A, 5377T>G, 5378G>T, 5379T>C, 5383C>A, 5386G>T, 5387A>T, 5390A>T, 5391C>G, 5395T>G, 5396A>G, 5399A>T, 5403A>T, 5404A>C, 5409G>A, 5410T>C, 5411T>C, 5417G>T, 5423A>C, 5429A>T, 5435T>A, 5436G>A, 5438C>A, 5440G>C, 5441C>T, 5447T>C, 5456G>A, 5457C>T, 5460A>T, 5462G>T, 5465C>A, 5468A>G, 5471A>C, 5474A>T, 5475A>C, 5476G>A, 5477G>T, 5483T>C, 5484T>A, 5486T>G, 5488T>C, 5489G>C, 5490G>A, 5491A>C, 5492G>A, 5496A>C, 5498A>T, 5501A>T, 5504T>G, 5505A>C, 5507A>C, 5508T>C, 5510G>A, 5513C>A, 5516A>C, 5519T>G, 5521G>C, 5522A>T, 5523A>G, 5524A>G, 5525T>G, 5531T>G, 5534C>T, 5537C>G, 5540T>C, 5546T>C, 5549T>C, 5555C>T, 5560T>C, 5564G>A, 5567T>C, 5570T>G, 5573T>C, 5574C>A, 5576A>G

CDS

	Begin	End	Coverage	Score	Concordance	Matches	Identities	I/D/M/F*	Stop Codons
**NT**	**23**	**5589**	**77.0%**	**3552**	**32.4%**	**5524 (99.1%)**	**3681 (66.0%)**	**9/43**	
ToMarV_RNA1gp1	1	1817	84.4%	9297	73.5%	1807 (99.3%)	1266 (69.6%)	3/10/0/0	0
Protein mutations:	F7W (160T>G 161C>G), K9_A16del (165_188delAAGGTTGCTGGTGTTGATGACGCC), S17P (189A>C 190G>C), S18P (192T>C), S19A (195T>G), T21S (201A>T 203C>A), T22P (204A>C), G26A (217G>C 218T>A), L28V (222C>G), N30S (228A>T 229A>C), I32F (234A>T), K34S (240A>T 241A>C 242A>T), F35L (243T>C), A38T (252G>A 254G>T), V39L (255G>C 257T>C), I42V (264A>G), T43N (268C>A 269C>T), T44K (271C>A 272T>A), M45V (273A>G 275G>T), K48T (283A>C 284G>C), S50V (288T>G 289C>T), L53I (297C>A 299T>A), L56V (306C>G 308G>C), S59P (315T>C 317T>A), T61M (322C>T 323T>G), D62A (325A>C 326T>A), V63A (328T>C 329G>A), S64A (330T>G 332C>A), V68A (343T>C 344G>C), A69S (345G>T 347C>T), S72N (355G>A), S73A (357T>G 359A>T), L74V (360C>G 362T>A), N75E (363A>G 365T>A), K76R (367A>G), G82A (385G>C 386T>A), A83K (387G>A 388C>A), L84I (390C>A 392T>A), I85M (395A>G), D86E (398T>A), L89I (405C>A 407T>C), A91G (412C>G 413G>T), S93D (417T>G 418C>A), L95V (423T>G 425G>T), S96K (426T>A 427C>A 428C>A), S101T (441T>A), K103V (447A>G 448A>T 449G>A), M105L (453A>C 455G>T), L109M (465T>A), F110V (468T>G), E111L (471G>T 472A>T 473G>A), L113V (477T>G 479G>C), S114K (480T>A 481C>A 482C>G), N115D (483A>G 485T>C), V117F (489G>T 491T>C), K118I (493A>T 494A>C), F121Y (502T>A 503T>C), A122E (505C>A 506T>A), V124L (510G>T 512G>A), N126E (516A>G 518T>A), S128V (522A>G 523G>T), D130S (528G>T 529A>C 530T>A), V131L (531G>T), V135F (543G>T 545A>T), Y136I (546T>A 547A>T 548T>A), G137A (550G>C), I143L (567A>T 569A>G), T144A (570A>G 572A>C), L145I (573T>A 575G>T), V147L (579G>C 581G>A), F150Y (589T>A), S154V (600A>G 601G>T 602T>A), E158T (612G>A 613A>C), G159P (615G>C 616G>C), V160F (618G>T), S161N (621T>A 622C>A), T163V (627A>G 628C>T 629T>A), K165V (633A>G 634A>T 635A>T), N166T (637A>C), I167L (639A>T 641C>G), L169S (646T>C 647G>A), I170V (648A>G 650A>T), M174F (660A>T 662G>T), K186T (697A>C), A188S (702G>T 704C>T), E189G (706A>G 707A>G), R190K (708C>A 709G>A 710A>G), T192S (714A>T), M193Q (717A>C 718T>A), V194L (720G>C), S198A (732A>G 733G>C 734T>A), M199V (735A>G 737G>A), V205L (753G>T 755T>A), S206T (756T>A 758A>G), M214L (780A>T 782G>A), T216S (786A>T), I217L (789A>T 791A>G), I228V (822A>G), T248A (882A>G 884A>T), T260Q (918A>C 919C>A 920T>G), I280V (978A>G 980A>G), G282S (984G>A), T283I (988C>T 989C>T), S295N (1024G>A), E304Q (1050G>C), Q307T (1059C>A 1060A>C), D310S (1068G>T 1069A>C 1070T>A), A311E (1072C>A 1073A>G), C313A (1077T>G 1078G>C), D314E (1082T>A), A316S (1086G>T 1088A>T), T317M (1090C>T), K325P (1113A>C 1114A>C 1115G>T), T329I (1126C>T 1127C>T), N332Q (1134A>C 1136C>A), R335K (1144G>A 1145G>A), E337Q (1149G>C 1151A>G), L339V (1155C>G), M354L (1200A>T), A363T (1227G>A), E366D (1238A>T), V368L (1242G>T 1244T>G), Y369V (1245T>G 1246A>T), S372A (1254A>G 1255G>C 1256T>C), C373S (1257T>A 1259T>C), R374Q (1260A>C 1261G>A 1262G>A), M376S (1266A>T 1267T>C 1268G>T), S393A (1317T>G 1319C>T), S401C (1342C>G), R412A (1374A>G 1375G>C), I440V (1458A>G 1460C>G), A500T (1638G>A 1640C>T), M521R (1702T>G 1703G>A), E523V (1708A>T), E525D (1715G>T), S527D (1719T>G 1720C>A 1721C>T), T528P (1722A>C 1724T>A), E529N (1725G>A 1727A>T), D530A (1729A>C), G533A (1738G>C 1739A>C), R536V (1746A>G 1747G>T 1748A>T), N538D (1752A>G 1754C>T), I539V (1755A>G 1757A>G), N552A (1794A>G 1795A>C), T554S (1800A>T), S556D (1806A>G 1807G>A), E560Q (1818G>C 1820G>A), Q567R (1840A>G), A570Y (1848G>T 1849C>A 1850A>T), K574S (1861A>G 1862G>C), F577Y (1870T>A), E578N (1872G>A 1874A>T), A579T (1875G>A), E580T (1878G>A 1879A>C), I583L (1887A>T 1889A>G), S584A (1890A>G 1891G>C), P585D (1893C>G 1894C>A 1895T>C), I587V (1899A>G 1901A>T), Q591N (1911C>A 1913A>T), S593A (1917T>G), A594V (1921C>T 1922A>T), D596E (1928T>A), I598L (1932A>C 1934A>G), A599D (1936C>A 1937G>T), V601F (1941G>T 1943G>C), D602Q (1944G>C 1946T>A), G603D (1948G>A 1949G>T), R605del (1953_1955delAGA), R607Q (1960G>A 1961C>A), T608V (1962A>G 1963C>T 1964C>T), K609L (1965A>T 1966A>T), Q610P (1969A>C 1970A>T), D611P (1971G>C 1972A>C), I614A (1980A>G 1981T>C), L615T (1983T>A 1984T>C 1985G>T), V616Y (1986G>T 1987T>A 1988G>T), P617E (1989C>G 1990C>A 1991C>G), T618Q (1992A>C 1993C>A 1994T>G), V619T (1995G>A 1996T>C 1997T>A), V620I (1998G>A 2000G>T), S621T (2002G>C 2003T>A), N623A (2007A>G 2008A>C), D624E (2012T>A), R625K (2014G>A), V626I (2016G>A 2018G>T), I627V (2019A>G 2021C>T), A629S (2025G>T 2027A>C), R630Q (2028A>C 2029G>A 2030A>G), L645M (2073T>A), D646N (2076G>A), P647K (2079C>A 2080C>A 2081T>A), E648F (2082G>T 2083A>T 2084G>T), L650F (2088C>T), H655L (2104A>T 2105T>C), H656S (2106C>T 2107A>C 2108T>C), M660I (2120G>C), S663V (2127T>G 2128C>T 2129T>G), D665S (2133G>T 2134A>C), D666S (2136G>T 2137A>C), E667D (2141A>T), H668A (2142C>G 2143A>C 2144T>C), E670Q (2148G>C), I671N (2152T>A), N673E (2157A>G 2159C>A), K674M (2161A>T 2162A>G), R676V (2166A>G 2167G>T), V677N (2169G>A 2170T>A 2171C>T), N678D (2172A>G 2174C>T), M679L (2175A>T), Y681V (2181T>G 2182A>T 2183T>C), R682K (2185G>A), D685S (2193G>A 2194A>G), A686P (2196G>C), E687Q (2199G>C 2201A>G), R690A (2208C>G 2209G>C 2210A>T), I701V (2241A>G 2243A>C), A710S (2268G>A 2269C>G 2270A>C), S711T (2271T>A), K716E (2286A>G), K717L (2289A>T 2290A>T 2291A>G), A719D (2296C>A 2297A>T), T722R (2304A>C 2305C>G 2306A>C), E723N (2307G>A 2309G>T), M724L (2310A>C 2312G>T), R725E (2313A>G 2314G>A), T726Q (2316A>C 2317C>A 2318T>A), F728K (2322T>A 2323T>A 2324T>A), H730Y (2328C>T), S731K (2331T>A 2332C>A 2333T>G), C732A (2334T>G 2335G>C 2336C>G), I733L (2337A>C), D734Q (2340G>C 2342T>G), V737A (2350T>C 2351T>A), I741T (2362T>C 2363A>G), F749R (2385T>A 2386T>G 2387T>A), N763R (2428A>G 2429C>G), S766A (2436T>G), S766_K767insG (2438_2439insGGT), K767T (2440A>C 2441A>T), E768D (2444G>C), P769K (2445C>A 2446C>A 2447A>G), E771R (2451G>A 2452A>G), S772N (2454T>A 2455C>A 2456A>T), K775I (2464A>T 2465A>C), I776E (2466A>G 2467T>A 2468T>A), Y778M (2472T>A 2473A>T 2474C>G), H779I (2475C>A 2476A>T), K780G (2478A>G 2479A>G 2480A>T), V782K (2484G>A 2485T>A 2486T>G), E783D (2489A>C), Y784K (2490T>A 2492T>A), I785M (2495C>G), K786I (2497A>T 2498G>C), D787Q (2499G>C 2501T>G), T788M (2503C>T 2504A>G), W790N (2508T>A 2509G>A 2510G>T), S791D (2511A>G 2512G>A), Y794F (2521A>T 2522T>C), A795I (2523G>A 2524C>T 2525A>C), R797K (2530G>A 2531A>G), I806L (2556A>T 2558A>G), M807I (2561G>A), V812T (2574G>A 2575T>C 2576T>C), F813M (2577T>A 2579T>G), G814S (2580G>A 2582A>T), G815A (2584G>C), I816V (2586A>G), L820I (2598T>A 2600G>T), A821T (2601G>A 2603G>T), W823L (2607T>C 2608G>T 2609G>C), Q824K (2610C>A), S825A (2613A>G 2614G>C 2615T>A), N826A (2616A>G 2617A>C 2618C>A), S827T (2619T>A), F828S (2623T>C), N830L (2628A>T 2629A>T 2630T>A), S832D (2634T>G 2635C>A 2636G>T), N852K (2696T>G), K853T (2698A>C 2699G>T), K853_L854insH (2699_2700insCAT), L854F (2700C>T), R855A (2703A>G 2704G>C), N856K (2708T>A), L857S (2709C>T 2710T>C 2711T>C), V859L (2715G>C 2717T>A), S862A (2724A>G 2725G>C 2726T>G), S863A (2727T>G 2729T>A), Q864D (2730C>G 2732A>C), S865N (2733T>A 2734C>A 2735A>C), L866N (2736T>A 2737T>A 2738G>C), A867H (2739G>C 2740C>A 2741A>T), Q868V (2742C>G 2743A>T 2744A>G), D869_W870insR (2747_2748insCGA), A872E (2755C>A 2756T>A), E873Q (2757G>C 2759A>G), Q879E (2775C>G 2777G>A), K882H (2784A>C 2786G>C), L890I (2808C>A), G891A (2812G>C), M907L (2859A>C 2861G>C), H914R (2880C>A 2881A>G 2882C>A), A916M (2886G>A 2887C>T 2888T>G), T923S (2907A>T 2909A>C), A927E (2920C>A 2921C>G), N928H (2922A>C), R930I (2928C>A 2929G>T 2930A>T), S931C (2932C>G), K932N (2936A>T), Y935F (2944A>T 2945C>T), R938T (2953G>C 2954A>C), E939Q (2955G>C), I942L (2964A>T 2966T>G), I948V (2982A>G 2984C>G), P961S (3021C>T 3023T>A), E972D (3056G>T), F975Y (3064T>A), T976S (3066A>T 3068A>C), Q980M (3078C>A 3079A>T 3080A>G), C984V (3090T>G 3091G>T 3092T>G), T990K (3109C>A 3110A>G), R993K (3117C>A 3118G>A 3119T>G), L994K (3120C>A 3121T>A 3122T>A), G995S (3123G>T 3124G>C), F999V (3135T>G 3137T>G), M1002Y (3144A>T 3145T>A 3146G>T), V1012E (3175T>A 3176T>A), E1015T (3183G>A 3184A>C), E1017G (3190A>G), N1019D (3195A>G 3197C>T), S1021N (3201T>A 3202C>A 3203C>T), S1022A (3204A>G 3205G>C 3206T>A), K1024A (3210A>G 3211A>C 3212G>T), I1025L (3213A>C), C1026S (3216T>A), Q1029V (3225C>G 3226A>T 3227A>T), S1030A (3228T>G 3230T>A), G1037S (3249G>A 3251G>T), S1050T (3289G>C), V1057C (3309G>T 3310T>G 3311G>C), G1068D (3343G>A 3344C>T), V1069T (3345G>A 3346T>C 3347A>C), R1073K (3357C>A 3358G>A 3359T>G), F1075Y (3364T>A 3365T>C), I1076V (3366A>G 3368C>G), S1077A (3369T>G), I1083V (3387A>G 3389C>G), Y1084F (3391A>T 3392C>T), E1086D (3398A>T), V1096I (3426G>A 3428C>T), A1098V (3433C>T 3434A>G), D1104E (3452T>A), I1105L (3453A>C 3455T>G), S1108H (3462T>C 3463C>A 3464A>C), I1109L (3465A>C 3467T>G), R1112K (3475G>A 3476A>G), T1114E (3480A>G 3481C>A), Q1115R (3483C>A 3484A>G 3485G>A), L1116F (3486C>T 3488T>C), N1120K (3500C>A), Y1123A (3507T>G 3508A>C 3509C>T), Y1124F (3511A>T), Q1130L (3528C>T 3529A>T), L1132M (3534T>A), R1143K (3567C>A 3568G>A 3569G>A), K1144R (3570A>C 3571A>G 3572A>C), Q1146P (3577A>C 3578A>C), P1153D (3597C>G 3598C>A), E1155Q (3603G>C), T1162S (3624A>T 3626A>T), V1163A (3628T>C 3629T>C), H1164K (3630C>A 3632T>A), D1169E (3647T>A), M1179E (3675A>G 3676T>A), C1186S (3697G>C 3698T>C), G1187N (3699G>A 3700G>A 3701A>C), D1189E (3707T>G), D1190E (3710T>G), D1191P (3711G>C 3712A>C 3713C>A), I1192V (3714A>G), N1199G (3735A>G 3736A>G), C1202A (3744T>G 3745G>C 3746C>A), L1204I (3750C>A 3752G>T), I1207V (3759A>G 3761C>G), M1208L (3762A>C 3764G>T), R1209K (3765C>A 3766G>A 3767T>G), S1210M (3769G>T 3770C>G), K1212R (3774A>C 3775A>G 3776G>A), I1213V (3777A>G 3779C>G), R1217K (3789C>A 3790G>A 3791T>A), C1219A (3795T>G 3796G>C 3797T>A), E1220I (3798G>A 3799A>T 3800A>T), D1222N (3804G>A), C1233F (3838G>T 3839T>C), Y1236F (3847A>T), G1238A (3853G>C 3854C>A), M1252L (3894A>T), M1254L (3900A>C 3902G>T), M1256S (3906A>T 3907T>C 3908G>T), Q1263E (3927C>G), S1264A (3930T>G), T1265Q (3933A>C 3934C>A 3935T>A), G1266P (3936G>C 3937G>C 3938C>A), E1267D (3941A>C), G1268S (3942G>T 3943G>C 3944A>G), E1269Q (3945G>C), E1272L (3954G>C 3955A>T 3956A>T), D1276N (3966G>A), E1281D (3983A>T), V1288S (4002G>T 4003T>C), I1290V (4008A>G 4010T>A), K1292Q (4014A>C), I1294T (4021T>C 4022C>A), H1295F (4023C>T 4024A>T), T1344C (4170A>T 4171C>G 4172A>T), L1348A (4182T>G 4183T>C 4184G>A), A1351V (4192C>T 4193T>A), I1352L (4194A>C 4196A>C), D1359N (4215G>A), S1363H (4227T>C 4228C>A), L1367V (4239T>G), N1370S (4249A>G), G1371S (4251G>A 4253G>C), A1374S (4260G>T 4262T>A), V1378E (4273T>A), V1393I (4317G>A 4319T>C), E1405A (4354A>C 4355A>T), Y1406F (4357A>T 4358C>T), S1409D (4365T>G 4366C>A 4367A>T), K1410E (4368A>G), D1415E (4385T>G), A1421T (4401G>A), D1435N (4443G>A 4445T>C), V1437L (4449G>T), I1476V (4566A>G 4568T>C), A1477K (4569G>A 4570C>A 4571T>A), R1479K (4576G>A 4577G>A), A1480S (4578G>T), I1481V (4581A>G 4583A>G), V1483I (4587G>A), S1486N (4597G>A), L1506M (4656T>A 4658A>G), Q1509R (4665C>A 4666A>G), S1512D (4674T>G 4675C>A), N1515S (4683A>T 4684A>C 4685T>C), H1518R (4692C>A 4693A>G 4694T>A), L1522A (4704C>G 4705T>C 4706G>T), I1526V (4716A>G), N1538E (4752A>G 4754T>G), E1539K (4755G>A), V1540T (4758G>A 4759T>C), Q1541K (4761C>A), Q1543L (4768A>T 4769A>G), H1563Q (4829C>A), I1572V (4854A>G), M1576I (4868G>T), P1583V (4887C>G 4888C>T 4889A>G), T1588V (4902A>G 4903C>T 4904T>A), M1592Y (4914A>T 4915T>A 4916G>T), V1595I (4923G>A 4925G>T), R1607Q (4960G>A 4961T>G), V1608A (4963T>C 4964G>C), A1609T (4965G>A), D1611E (4973C>A), K1612S (4975A>G 4976G>T), L1613F (4977C>T), L1615N (4983T>A 4984T>A 4985A>T), Q1618L (4993A>T 4994G>T), T1619G (4995A>G 4996C>G 4997G>A), D1622K (5004G>A 5006T>G), E1623Q (5007G>C 5009A>G), G1625N (5013G>A 5014G>A 5015T>C), F1626L (5016T>C), R1627P (5019A>C 5020G>C 5021A>C), A1628I (5022G>A 5023C>T 5024T>C), V1629M (5025G>A), F1631M (5031T>A 5033T>G), S1633T (5037T>A 5039C>T), L1635Y (5044T>A 5045G>T), V1637L (5049G>C 5051G>T), Q1639S (5055C>A 5056A>G 5057G>T), E1640Q (5058G>C), I1642L (5064A>T 5066T>G), A1643T (5067G>A), K1644E (5070A>G), S1649E (5085T>G 5086C>A 5087A>G), A1651I (5091G>A 5092C>T 5093C>T), L1652G (5094C>G 5095T>G), T1653V (5097A>G 5098C>T 5099A>T), I1654L (5100A>T 5102T>G), C1655M (5103T>A 5104G>T 5105T>G), M1657L (5109A>C 5111G>A), A1658S (5112G>T), K1659D (5115A>G 5117A>T), A1661K (5121G>A 5122C>A 5123T>A), I1662N (5125T>A), S1663T (5127T>A 5129C>A), D1668P (5142G>C 5143A>C 5144C>T), T1674del (5160_5162delACA), R1678E (5172C>G 5173G>A 5174T>G), D1679G (5176A>G), L1680V (5178C>G 5180G>C), N1681H (5181A>C), W1682E (5184T>G 5185G>A 5186G>A), N1686D (5196A>G 5198C>T), I1687V (5199A>G 5201T>A), G1688F (5202G>T 5203G>T), A1696S (5226G>T 5228C>A), H1697N (5229C>A), Y1698Q (5232T>C 5234C>A), T1699Q (5235A>C 5236C>A 5237G>A), D1701S (5241G>T 5242A>C 5243T>A), E1702S (5244G>T 5245A>C), Y1704N (5250T>A), I1706L (5256A>C 5258T>G), K1713R (5277A>C 5278A>G 5279G>C), K1714R (5281A>G 5282G>A), S1715T (5283T>A 5285C>A), N1716T (5287A>C 5288T>A), M1731I (5333G>A), M1736L (5346A>T), S1737A (5349T>G), M1740Q (5358A>C 5359T>A), A1742H (5364G>C 5365C>A 5366C>T), K1744R (5370A>C 5371A>G 5372G>C), N1745K (5375T>G), V1746S (5376G>A 5377T>G 5378G>T), A1748E (5383C>A), G1749V (5386G>T 5387A>T), H1751D (5391C>G), V1752G (5395T>G 5396A>G), N1755S (5403A>T 5404A>C), V1757T (5409G>A 5410T>C 5411T>C), V1766I (5436G>A 5438C>A), G1767A (5440G>C 5441C>T), M1774F (5460A>T 5462G>T), R1779H (5475A>C 5476G>A 5477G>T), C1782R (5484T>A 5486T>G), V1783A (5488T>C 5489G>C), E1784T (5490G>A 5491A>C 5492G>A), I1786L (5496A>C 5498A>T), I1793M (5519T>G), G1794A (5521G>C 5522A>T), N1795G (5523A>G 5524A>G 5525T>G), V1807A (5560T>C), F1810L (5570T>G), Q1812K (5574C>A 5576A>G)								

	Begin	End	Coverage	Score	Concordance	Matches	Identities	I/D/M/F*	Stop Codons
**NT**	**23**	**5589**	**77.0%**	**3552**	**32.4%**	**5524 (99.1%)**	**3681 (66.0%)**	**9/43**	

Codon mutations:

GCT2GCA (146T>A), TTC7TGG (160T>G 161C>G), TCC8AGT (162T>A 163C>G 164C>T), AAG9_GCC16del (165_188delAAGGTTGCTGGTGTTGATGACGCC), AGT17CCT (189A>C 190G>C), TCT18CCT (192T>C), TCT19GCT (195T>G), TCC20TCT (200C>T), ACC21TCA (201A>T 203C>A), ACT22CCT (204A>C), GGT26GCA (217G>C 218T>A), CTT28GTT (222C>G), TCA29TCT (227A>T), AAT30TCT (228A>T 229A>C), ATT32TTT (234A>T), TCC33TCT (239C>T), AAA34TCT (240A>T 241A>C 242A>T), TTT35CTT (243T>C), AAA37AAG (251A>G), GCG38ACT (252G>A 254G>T), GTT39CTC (255G>C 257T>C), AGT40TCC (258A>T 259G>C 260T>C), ATT42GTT (264A>G), ACC43AAT (268C>A 269C>T), ACT44AAA (271C>A 272T>A), ATG45GTT (273A>G 275G>T), AAG48ACC (283A>C 284G>C), TCA50GTA (288T>G 289C>T), CAT52CAC (296T>C), CTT53ATA (297C>A 299T>A), CTG56GTC (306C>G 308G>C), CCA58CCG (314A>G), TCT59CCA (315T>C 317T>A), GTG60GTT (320G>T), ACT61ATG (322C>T 323T>G), GAT62GCA (325A>C 326T>A), GTG63GCA (328T>C 329G>A), TCC64GCA (330T>G 332C>A), TCT66TCA (338T>A), GTG68GCC (343T>C 344G>C), GCC69TCT (345G>T 347C>T), ACT70ACA (350T>A), TGT71TGC (353T>C), AGT72AAT (355G>A), TCA73GCT (357T>G 359A>T), CTT74GTA (360C>G 362T>A), AAT75GAA (363A>G 365T>A), AAA76AGA (367A>G), TTG78TTA (374G>A), GAC79GAT (377C>T), AAA80AAG (380A>G), GGT82GCA (385G>C 386T>A), GCA83AAA (387G>A 388C>A), CTT84ATA (390C>A 392T>A), ATA85ATG (395A>G), GAT86GAA (398T>A), CCA87CCC (401A>C), TTT88TTC (404T>C), CTT89ATC (405C>A 407T>C), AAA90AAG (410A>G), GCG91GGT (412C>G 413G>T), TAC92TAT (416C>T), TCT93GAT (417T>G 418C>A), TTC94TTT (422C>T), TTG95GTT (423T>G 425G>T), TCC96AAA (426T>A 427C>A 428C>A), ACA97ACT (431A>T), TAC99TAT (437C>T), TCT101ACT (441T>A), ATC102ATA (446C>A), AAG103GTA (447A>G 448A>T 449G>A), ATG105CTT (453A>C 455G>T), GTT106GTA (458T>A), TTA107CTT (459T>C 461A>T), TTG109ATG (465T>A), TTT110GTT (468T>G), GAG111TTA (471G>T 472A>T 473G>A), TTG113GTC (477T>G 479G>C), TCC114AAG (480T>A 481C>A 482C>G), AAT115GAC (483A>G 485T>C), GTT117TTC (489G>T 491T>C), AAA118ATC (493A>T 494A>C), TTA119CTA (495T>C), TTT121TAC (502T>A 503T>C), GCT122GAA (505C>A 506T>A), GTG124TTA (510G>T 512G>A), AAT126GAA (516A>G 518T>A), AGT128GTT (522A>G 523G>T), GAA129GAG (527A>G), GAT130TCA (528G>T 529A>C 530T>A), GTA131TTA (531G>T), GTT134GTG (542T>G), GTA135TTT (543G>T 545A>T), TAT136ATA (546T>A 547A>T 548T>A), GGT137GCT (550G>C), CTT139TTA (555C>T 557T>A), GTA140GTT (560A>T), ATA143TTG (567A>T 569A>G), ACA144GCC (570A>G 572A>C), TTG145ATT (573T>A 575G>T), TTA146TTG (578A>G), GTG147CTA (579G>C 581G>A), CTT148TTA (582C>T 584T>A), CTA149CTG (587A>G), TTT150TAT (589T>A), GTT151GTA (593T>A), TGT152TGC (596T>C), AGT154GTA (600A>G 601G>T 602T>A), GTT157GTA (611T>A), GAG158ACG (612G>A 613A>C), GGT159CCT (615G>C 616G>C), GTT160TTT (618G>T), TCT161AAT (621T>A 622C>A), AAC162AAT (626C>T), ACT163GTA (627A>G 628C>T 629T>A), GTC164GTT (632C>T), AAA165GTT (633A>G 634A>T 635A>T), AAT166ACT (637A>C), ATC167TTG (639A>T 641C>G), TTG169TCA (646T>C 647G>A), ATA170GTT (648A>G 650A>T), GTC171GTT (653C>T), AAC173AAT (659C>T), ATG174TTT (660A>T 662G>T), TTT175TTC (665T>C), TCA176TCT (668A>T), AGT177TCA (669A>T 670G>C 671T>A), CTA178TTG (672C>T 674A>G), TAC179TAT (677C>T), AAA186ACA (697A>C), GCC188TCT (702G>T 704C>T), GAA189GGG (706A>G 707A>G), CGA190AAG (708C>A 709G>A 710A>G), TTC191TTT (713C>T), ACC192TCC (714A>T), ATG193CAG (717A>C 718T>A), GTG194CTG (720G>C), GCC195GCG (725C>G), GCT197GCA (731T>A), AGT198GCA (732A>G 733G>C 734T>A), ATG199GTA (735A>G 737G>A), GGG202GGT (746G>T), TCA204AGT (750T>A 751C>G 752A>T), GTT205TTA (753G>T 755T>A), TCA206ACG (756T>A 758A>G), ACG210ACT (770G>T), TCT211AGC (771T>A 772C>G 773T>C), CAG212CAA (776G>A), TTG213CTT (777T>C 779G>T), ATG214TTA (780A>T 782G>A), ACA216TCA (786A>T), ATA217TTG (789A>T 791A>G), GGG221GGT (803G>T), ATT222ATC (806T>C), TCG223TCA (809G>A), ACG224ACT (812G>T), TTG225TTA (815G>A), GTG226GTA (818G>A), TTC227TTT (821C>T), ATT228GTT (822A>G), GCA229GCT (827A>T), GTG230GTT (830G>T), GGT232GGG (836T>G), GGC236GGT (848C>T), TTG237CTT (849T>C 851G>T), AGC238TCA (852A>T 853G>C 854C>A), TTG241CTG (861T>C), TCC242TCA (866C>A), ATT244ATC (872T>C), CTC245TTG (873C>T 875C>G), TAT246TAC (878T>C), TCG247TCT (881G>T), ACA248GCT (882A>G 884A>T), GCT252GCG (896T>G), CAG253CAA (899G>A), CAA257CAG (911A>G), CTA258CTG (914A>G), ACT260CAG (918A>C 919C>A 920T>G), CTT261TTG (921C>T 923T>G), CGT263AGG (927C>A 929T>G), AAT264AAC (932T>C), AAA266AAG (938A>G), TGC268TGT (944C>T), ACC269ACA (947C>A), TCC270TCT (950C>T), CAG271CAA (953G>A), GCT272GCC (956T>C), GAA278GAG (974A>G), ATA280GTG (978A>G 980A>G), GTC281GTT (983C>T), GGC282AGC (984G>A), ACC283ATT (988C>T 989C>T), GGA285GGC (995A>C), TTC286TTT (998C>T), GTG290GTA (1010G>A), GCT293GCG (1019T>G), AGT295AAT (1024G>A), GCA296GCT (1028A>T), ACG297ACC (1031G>C), CTG298TTA (1032C>T 1034G>A), ACT300ACC (1040T>C), CTG302CTT (1046G>T), GAG304CAG (1050G>C), CAG307ACG (1059C>A 1060A>C), GAG308GAA (1064G>A), GAT310TCA (1068G>T 1069A>C 1070T>A), GCA311GAG (1072C>A 1073A>G), GTG312GTT (1076G>T), TGT313GCT (1077T>G 1078G>C), GAT314GAA (1082T>A), CCT315CCA (1085T>A), GCA316TCT (1086G>T 1088A>T), ACG317ATG (1090C>T), GAG320GAA (1100G>A), AAC321AAT (1103C>T), GCC323GCA (1109C>A), AAG325CCT (1113A>C 1114A>C 1115G>T), GCT326GCA (1118T>A), TTC327TTT (1121C>T), CCT328CCA (1124T>A), ACC329ATT (1126C>T 1127C>T), AAA330AAG (1130A>G), AAC332CAA (1134A>C 1136C>A), CTG334TTA (1140C>T 1142G>A), AGG335AAA (1144G>A 1145G>A), GAA336GAG (1148A>G), GAA337CAG (1149G>C 1151A>G), GCT338GCC (1154T>C), CTC339GTC (1155C>G), AAG340AAA (1160G>A), ATC341ATA (1163C>A), GCA346GCC (1178A>C), GCG351GCA (1193G>A), TTC353TTT (1199C>T), ATG354TTG (1200A>T), AGT359TCT (1215A>T 1216G>C), ATT362ATA (1226T>A), GCG363ACG (1227G>A), CAG364CAA (1232G>A), TTA365CTA (1233T>C), GAA366GAT (1238A>T), GTT368TTG (1242G>T 1244T>G), TAT369GTT (1245T>G 1246A>T), GAA371GAG (1253A>G), AGT372GCC (1254A>G 1255G>C 1256T>C), TGT373AGC (1257T>A 1259T>C), AGG374CAA (1260A>C 1261G>A 1262G>A), CAC375CAT (1265C>T), ATG376TCT (1266A>T 1267T>C 1268G>T), GGC377GGT (1271C>T), CAG380CAA (1280G>A), CGT382AGG (1284C>A 1286T>G), GAA384GAG (1292A>G), TTT386TTC (1298T>C), GTA388GTG (1304A>G), TTC391TTT (1313C>T), GGG392GGA (1316G>A), TCC393GCT (1317T>G 1319C>T), GGG395GGA (1325G>A), TCT399TCA (1337T>A), ACA400ACG (1340A>G), TCC401TGC (1342C>G), TTA404CTT (1350T>C 1352A>T), TTT405TTC (1355T>C), GTG409GTT (1367G>T), TTG410CTC (1368T>C 1370G>C), AGA412GCA (1374A>G 1375G>C), GAG415GAA (1385G>A), AAA417AAG (1391A>G), CTC418CTG (1394C>G), AAT419AAC (1397T>C), CGG420CGC (1400G>C), CTA421TTG (1401C>T 1403A>G), GCA423GCG (1409A>G), AAG426AAA (1418G>A), AGG427AGA (1421G>A), GCT435GCA (1445T>A), CAC436CAT (1448C>T), GCT439GCA (1457T>A), ATC440GTG (1458A>G 1460C>G), CTG441CTT (1463G>T), GAC444GAT (1472C>T), GGA446GGT (1478A>T), GCA447GCT (1481A>T), TTG448CTT (1482T>C 1484G>T), CGA449CGG (1487A>G), GCT452GCA (1496T>A), TGC455TGT (1505C>T), CAA456CAG (1508A>G), GAT457GAC (1511T>C), ATT458ATA (1514T>A), AAA459AAG (1517A>G), GAT463GAC (1529T>C), TCA466TCT (1538A>T), ACA467ACC (1541A>C), CAA468CAG (1544A>G), CCA469CCT (1547A>T), CCC471CCT (1553C>T), TTA472CTT (1554T>C 1556A>T), GCC475GCT (1565C>T), GTT477GTC (1571T>C), GAC479GAT (1577C>T), GGC481GGA (1583C>A), CAT483CAC (1589T>C), TTC484TTT (1592C>T), ACC485ACA (1595C>A), TCC486TCA (1598C>A), TAT488TAC (1604T>C), ATA489ATC (1607A>C), ACA492ACC (1616A>C), CTG495TTG (1623C>T), ATC496ATA (1628C>A), TCA497TCT (1631A>T), CCT499CCA (1637T>A), GCC500ACT (1638G>A 1640C>T), CAA501CAG (1643A>G), GGT503GGG (1649T>G), CTT504CTA (1652T>A), ACA505ACG (1655A>G), CCA507CCT (1661A>T), GCT509GCA (1667T>A), GAG511GAA (1673G>A), CGG512AGG (1674C>A), AGA513AGG (1679A>G), AGG514AGA (1682G>A), GAC515GAT (1685C>T), ATC516ATT (1688C>T), CTG517TTA (1689C>T 1691G>A), GTG518GTT (1694G>T), TGT520TGC (1700T>C), ATG521AGA (1702T>G 1703G>A), GAG523GTG (1708A>T), GAG525GAT (1715G>T), TCC527GAT (1719T>G 1720C>A 1721C>T), ACT528CCA (1722A>C 1724T>A), GAA529AAT (1725G>A 1727A>T), GAT530GCT (1729A>C), CCT531CCA (1733T>A), ACG532ACA (1736G>A), GGA533GCC (1738G>C 1739A>C), CTC535CTA (1745C>A), AGA536GTT (1746A>G 1747G>T 1748A>T), AAC538GAT (1752A>G 1754C>T), ATA539GTG (1755A>G 1757A>G), GAG541GAA (1763G>A), GCT547GCC (1781T>C), ACT549ACA (1787T>A), CAC550CAT (1790C>T), AGG551AGA (1793G>A), AAT552GCT (1794A>G 1795A>C), TTG553TTA (1799G>A), ACC554TCC (1800A>T), AGT556GAT (1806A>G 1807G>A), GAT557GAC (1811T>C), CTT558CTC (1814T>C), GAG560CAA (1818G>C 1820G>A), GTA563GTG (1829A>G), GCC564GCA (1832C>A), AAA565AAG (1835A>G), TGT566TGC (1838T>C), CAG567CGG (1840A>G), GTA568GTG (1844A>G), CAT569CAC (1847T>C), GCA570TAT (1848G>T 1849C>A 1850A>T), GAA571GAG (1853A>G), GTA572GTG (1856A>G), AAG574AGC (1861A>G 1862G>C), TTG576CTT (1866T>C 1868G>T), TTT577TAT (1870T>A), GAA578AAT (1872G>A 1874A>T), GCT579ACT (1875G>A), GAA580ACA (1878G>A 1879A>C), TCT581TCA (1883T>A), GGC582GGA (1886C>A), ATA583TTG (1887A>T 1889A>G), AGT584GCT (1890A>G 1891G>C), CCT585GAC (1893C>G 1894C>A 1895T>C), ATA587GTT (1899A>G 1901A>T), GTT590GTC (1910T>C), CAA591AAT (1911C>A 1913A>T), TCT593GCT (1917T>G), GCA594GTT (1921C>T 1922A>T), GAT596GAA (1928T>A), ATA598CTG (1932A>C 1934A>G), GCG599GAT (1936C>A 1937G>T), GTG601TTC (1941G>T 1943G>C), GAT602CAA (1944G>C 1946T>A), GGG603GAT (1948G>A 1949G>T), GCT604GCC (1952T>C), AGA605del (1953_1955delAGA), TTG606TTA (1958G>A), CGC607CAA (1960G>A 1961C>A), ACC608GTT (1962A>G 1963C>T 1964C>T), AAG609TTG (1965A>T 1966A>T), CAA610CCT (1969A>C 1970A>T), GAT611CCT (1971G>C 1972A>C), GAA612GAG (1976A>G), ATA614GCA (1980A>G 1981T>C), TTG615ACT (1983T>A 1984T>C 1985G>T), GTG616TAT (1986G>T 1987T>A 1988G>T), CCC617GAG (1989C>G 1990C>A 1991C>G), ACT618CAG (1992A>C 1993C>A 1994T>G), GTT619ACA (1995G>A 1996T>C 1997T>A), GTG620ATT (1998G>A 2000G>T), AGT621ACA (2002G>C 2003T>A), AAT623GCT (2007A>G 2008A>C), GAT624GAA (2012T>A), AGA625AAA (2014G>A), GTG626ATT (2016G>A 2018G>T), ATC627GTT (2019A>G 2021C>T), TAT628TAC (2024T>C), GCA629TCC (2025G>T 2027A>C), AGA630CAG (2028A>C 2029G>A 2030A>G), GAG631GAA (2033G>A), ACG633ACA (2039G>A), GTA634GTT (2042A>T), GCA636GCC (2048A>C), TTG637CTC (2049T>C 2051G>C), GCA640GCT (2060A>T), CAG642CAA (2066G>A), GGC643GGG (2069C>G), TTG645ATG (2073T>A), GAC646AAC (2076G>A), CCT647AAA (2079C>A 2080C>A 2081T>A), GAG648TTT (2082G>T 2083A>T 2084G>T), GAA649GAG (2087A>G), CTT650TTT (2088C>T), TTT651TTC (2093T>C), CCT652CCA (2096T>A), CAT655CTC (2104A>T 2105T>C), CAT656TCC (2106C>T 2107A>C 2108T>C), GCC659GCT (2117C>T), ATG660ATC (2120G>C), TCT663GTG (2127T>G 2128C>T 2129T>G), CTT664CTA (2132T>A), GAT665TCT (2133G>T 2134A>C), GAT666TCT (2136G>T 2137A>C), GAA667GAT (2141A>T), CAT668GCC (2142C>G 2143A>C 2144T>C), GAG670CAG (2148G>C), ATT671AAT (2152T>A), TTC672TTT (2156C>T), AAC673GAA (2157A>G 2159C>A), AAA674ATG (2161A>T 2162A>G), AGA676GTA (2166A>G 2167G>T), GTC677AAT (2169G>A 2170T>A 2171C>T), AAC678GAT (2172A>G 2174C>T), ATG679TTG (2175A>T), TAT681GTC (2181T>G 2182A>T 2183T>C), AGA682AAA (2185G>A), GAT685AGT (2193G>A 2194A>G), GCT686CCT (2196G>C), GAA687CAG (2199G>C 2201A>G), CGA690GCT (2208C>G 2209G>C 2210A>T), GTA693GTG (2219A>G), GAC699GAT (2237C>T), ATA701GTC (2241A>G 2243A>C), CAC703CAT (2249C>T), TTT704TTC (2252T>C), GCA710AGC (2268G>A 2269C>G 2270A>C), TCA711ACA (2271T>A), ATC713ATT (2279C>T), GAG715GAA (2285G>A), AAG716GAG (2286A>G), AAA717TTG (2289A>T 2290A>T 2291A>G), CTA718CTG (2294A>G), GCA719GAT (2296C>A 2297A>T), GTG720GTC (2300G>C), CAG721CAA (2303G>A), ACA722CGC (2304A>C 2305C>G 2306A>C), GAG723AAT (2307G>A 2309G>T), ATG724CTT (2310A>C 2312G>T), AGG725GAG (2313A>G 2314G>A), ACT726CAA (2316A>C 2317C>A 2318T>A), GGA727GGG (2321A>G), TTT728AAA (2322T>A 2323T>A 2324T>A), GCA729GCG (2327A>G), CAT730TAT (2328C>T), TCT731AAG (2331T>A 2332C>A 2333T>G), TGC732GCG (2334T>G 2335G>C 2336C>G), ATT733CTT (2337A>C), GAT734CAG (2340G>C 2342T>G), GCA735GCT (2345A>T), GTT737GCA (2350T>C 2351T>A), GAC738GAT (2354C>T), ACC739ACA (2357C>A), CTC740CTT (2360C>T), ATA741ACG (2362T>C 2363A>G), ATC743ATT (2369C>T), GAG744GAA (2372G>A), CCA747CCG (2381A>G), TTT749AGA (2385T>A 2386T>G 2387T>A), GTT750GTA (2390T>A), TTT752TTC (2396T>C), TTA753CTG (2397T>C 2399A>G), ACA755ACT (2405A>T), GCA756GCT (2408A>T), AGA759CGC (2415A>C 2417A>C), TGC762TGT (2426C>T), AAC763AGG (2428A>G 2429C>G), GTA765GTT (2435A>T), TCA766GCA (2436T>G), TCA766_AAA767insGGT (2438_2439insGGT), AAA767ACT (2440A>C 2441A>T), GAG768GAC (2444G>C), CCA769AAG (2445C>A 2446C>A 2447A>G), GAA771AGA (2451G>A 2452A>G), TCA772AAT (2454T>A 2455C>A 2456A>T), ATC774ATT (2462C>T), AAA775ATC (2464A>T 2465A>C), ATT776GAA (2466A>G 2467T>A 2468T>A), TAC778ATG (2472T>A 2473A>T 2474C>G), CAT779ATT (2475C>A 2476A>T), AAA780GGT (2478A>G 2479A>G 2480A>T), GTT782AAG (2484G>A 2485T>A 2486T>G), GAA783GAC (2489A>C), TAT784AAA (2490T>A 2492T>A), ATC785ATG (2495C>G), AAG786ATC (2497A>T 2498G>C), GAT787CAG (2499G>C 2501T>G), ACA788ATG (2503C>T 2504A>G), TGG790AAT (2508T>A 2509G>A 2510G>T), AGC791GAC (2511A>G 2512G>A), CTT792TTG (2514C>T 2516T>G), TAT794TTC (2521A>T 2522T>C), GCA795ATC (2523G>A 2524C>T 2525A>C), CTG796TTG (2526C>T), AGA797AAG (2530G>A 2531A>G), CTC799CTT (2537C>T), GCA802GCC (2546A>C), GGT803GGA (2549T>A), ATA806TTG (2556A>T 2558A>G), ATG807ATA (2561G>A), GCT808GCA (2564T>A), GGA811GGC (2573A>C), GTT812ACC (2574G>A 2575T>C 2576T>C), TTT813ATG (2577T>A 2579T>G), GGA814AGT (2580G>A 2582A>T), GGC815GCC (2584G>C), ATT816GTT (2586A>G), ACA817ACT (2591A>T), GCA818GCT (2594A>T), TTT819TTC (2597T>C), TTG820ATT (2598T>A 2600G>T), GCG821ACT (2601G>A 2603G>T), TGG823CTC (2607T>C 2608G>T 2609G>C), CAG824AAG (2610C>A), AGT825GCA (2613A>G 2614G>C 2615T>A), AAC826GCA (2616A>G 2617A>C 2618C>A), TCT827ACT (2619T>A), TTC828TCC (2623T>C), CCC829CCT (2627C>T), AAT830TTA (2628A>T 2629A>T 2630T>A), GCA831GCT (2633A>T), TCG832GAT (2634T>G 2635C>A 2636G>T), GGA835GGG (2645A>G), GGT836GGC (2648T>C), GTG837GTA (2651G>A), GAA840GAG (2660A>G), ATA844ATC (2672A>C), AGT846AGC (2678T>C), AAG848AAA (2684G>A), AAC849AAT (2687C>T), AAG850AAA (2690G>A), GGT851GGG (2693T>G), AAT852AAG (2696T>G), AAG853ACT (2698A>C 2699G>T), AAG853_CTC854insCAT (2699_2700insCAT), CTC854TTC (2700C>T), AGA855GCA (2703A>G 2704G>C), AAT856AAA (2708T>A), CTT857TCC (2709C>T 2710T>C 2711T>C), CTC858TTG (2712C>T 2714C>G), GTT859CTA (2715G>C 2717T>A), GGT860GGG (2720T>G), AGT862GCG (2724A>G 2725G>C 2726T>G), TCT863GCA (2727T>G 2729T>A), CAA864GAC (2730C>G 2732A>C), TCA865AAC (2733T>A 2734C>A 2735A>C), TTG866AAC (2736T>A 2737T>A 2738G>C), GCA867CAT (2739G>C 2740C>A 2741A>T), CAA868GTG (2742C>G 2743A>T 2744A>G), GAT869GAC (2747T>C), GAT869_TGG870insCGA (2747_2748insCGA), GCT871GCA (2753T>A), GCT872GAA (2755C>A 2756T>A), GAA873CAG (2757G>C 2759A>G), GTA877GTT (2771A>T), CAG879GAA (2775C>G 2777G>A), AGC880AGT (2780C>T), CTC881CTG (2783C>G), AAG882CAC (2784A>C 2786G>C), AAT884AAC (2792T>C), GTG886GTT (2798G>T), GTG887GTT (2801G>T), TTA888TTG (2804A>G), CTT890ATT (2808C>A), GGT891GCT (2812G>C), GTG894GTC (2822G>C), TAC895TAT (2825C>T), TTT896TTC (2828T>C), GGC898GGT (2834C>T), ACC899ACG (2837C>G), TAC900TAT (2840C>T), GTG901GTT (2843G>T), ATA906ATT (2858A>T), ATG907CTC (2859A>C 2861G>C), ACC908ACT (2864C>T), GTA909GTG (2867A>G), GCT910GCA (2870T>A), GCA912GCC (2876A>C), TTC913TTT (2879C>T), CAC914AGA (2880C>A 2881A>G 2882C>A), AAT915AAC (2885T>C), GCT916ATG (2886G>A 2887C>T 2888T>G), CGA917AGG (2889C>A 2891A>G), GGT919GGC (2897T>C), CCA921CCT (2903A>T), ACA923TCC (2907A>T 2909A>C), ATC925ATT (2915C>T), GCC927GAG (2920C>A 2921C>G), AAT928CAT (2922A>C), TCT929TCC (2927T>C), CGA930ATT (2928C>A 2929G>T 2930A>T), TCT931TGT (2932C>G), AAA932AAT (2936A>T), GTT933GTG (2939T>G), CAA934CAG (2942A>G), TAC935TTT (2944A>T 2945C>T), AAC936AAT (2948C>T), AGA938ACC (2953G>C 2954A>C), GAA939CAA (2955G>C), ATT942TTG (2964A>T 2966T>G), ATT943ATA (2969T>A), GGC945GGT (2975C>T), GAC947GAT (2981C>T), ATC948GTG (2982A>G 2984C>G), CTG950TTG (2988C>T), CGC952AGG (2994C>A 2996C>G), GTT953GTA (2999T>A), GGT954GGG (3002T>G), CCA959CCC (3017A>C), AAG960AAA (3020G>A), CCT961TCA (3021C>T 3023T>A), GAC962GAT (3026C>T), ATC963ATA (3029C>A), CGT964AGA (3030C>A 3032T>A), CAC966CAT (3038C>T), CCA968CCT (3044A>T), AGA969CGC (3045A>C 3047A>C), AGG970AGA (3050G>A), GAG972GAT (3056G>T), TGC974TGT (3062C>T), TTC975TAC (3064T>A), ACA976TCC (3066A>T 3068A>C), CAA980ATG

	(3078C>A 3079A>T 3080A>G), GGA981GGC (3083A>C), TGT984GTG (3090T>G 3091G>T 3092T>G), AGA985AGG (3095A>G), GTT987GTA (3101T>A), GCG988GCT (3104G>T), TCT989TCA (3107T>A), ACA990AAG (3109C>A 3110A>G), CCA992CCC (3116A>C), CGT993AAG (3117C>A 3118G>A 3119T>G), CTT994AAA (3120C>A 3121T>A 3122T>A), GGC995TCC (3123G>T 3124G>C), TTA997TTG (3131A>G), TTT999GTG (3135T>G 3137T>G), CTC1000CTT (3140C>T), AAG1001AAA (3143G>A), ATG1002TAT (3144A>T 3145T>A 3146G>T), GGA1009GGC (3167A>C), ACA1011ACC (3173A>C), GTT1012GAA (3175T>A 3176T>A), GTG1014GTC (3182G>C), GAA1015ACA (3183G>A 3184A>C), GAA1017GGA (3190A>G), CTG1018TTA (3192C>T 3194G>A), AAC1019GAT (3195A>G 3197C>T), TCC1020TCG (3200C>G), TCC1021AAT (3201T>A 3202C>A 3203C>T), AGT1022GCA (3204A>G 3205G>C 3206T>A), AAG1024GCT (3210A>G 3211A>C 3212G>T), ATT1025CTT (3213A>C), TGC1026AGC (3216T>A), TCT1027TCA (3221T>A), CAA1029GTT (3225C>G 3226A>T 3227A>T), TCT1030GCA (3228T>G 3230T>A), GAA1032GAG (3236A>G), ATA1035ATT (3245A>T), GGG1037AGT (3249G>A 3251G>T), GAA1038GAG (3254A>G), GGT1040GGA (3260T>A), GAC1041GAT (3263C>T), GGC1043GGT (3269C>T), ACG1044ACT (3272G>T), TTG1045CTT (3273T>C 3275G>T), CTA1046CTC (3278A>C), CTG1047TTA (3279C>T 3281G>A), TTA1048TTG (3284A>G), AGT1050ACT (3289G>C), GTT1051GTG (3293T>G), AAT1053AAC (3299T>C), CAA1055CAG (3305A>G), CCT1056CCG (3308T>G), GTG1057TGC (3309G>T 3310T>G 3311G>C), GGC1060GGA (3320C>A), ATC1061ATA (3323C>A), CAC1062CAT (3326C>T), TCA1065TCT (3335A>T), GGC1068GAT (3343G>A 3344C>T), GTA1069ACC (3345G>A 3346T>C 3347A>C), GCA1070GCT (3350A>T), GCT1071GCA (3353T>A), GAA1072GAG (3356A>G), CGT1073AAG (3357C>A 3358G>A 3359T>G), GGA1074GGT (3362A>T), TTT1075TAC (3364T>A 3365T>C), ATC1076GTG (3366A>G 3368C>G), TCT1077GCT (3369T>G), AAT1079AAC (3377T>C), GCT1080GCA (3380T>A), GCT1082GCA (3386T>A), ATC1083GTG (3387A>G 3389C>G), TAC1084TTT (3391A>T 3392C>T), AGG1085AGA (3395G>A), GAA1086GAT (3398A>T), CTA1088TTG (3402C>T 3404A>G), GAG1089GAA (3407G>A), CCA1092CCC (3416A>C), ACT1093ACG (3419T>G), GGG1094GGA (3422G>A), CCG1095CCC (3425G>C), GTC1096ATT (3426G>A 3428C>T), GCA1098GTG (3433C>T 3434A>G), GCA1099GCT (3437A>T), GAT1104GAA (3452T>A), ATT1105CTG (3453A>C 3455T>G), CTG1106TTG (3456C>T), TCA1108CAC (3462T>C 3463C>A 3464A>C), ATT1109CTG (3465A>C 3467T>G), AGA1110AGG (3470A>G), AGC1111TCC (3471A>T 3472G>C), AGA1112AAG (3475G>A 3476A>G), GAA1113GAG (3479A>G), ACA1114GAA (3480A>G 3481C>A), CAG1115AGA (3483C>A 3484A>G 3485G>A), CTT1116TTC (3486C>T 3488T>C), GAA1119GAG (3497A>G), AAC1120AAA (3500C>A), CAA1121CAG (3503A>G), TAC1123GCT (3507T>G 3508A>C 3509C>T), TAC1124TTC (3511A>T), CTT1125TTG (3513C>T 3515T>G), GGA1126GGC (3518A>C), ACA1127ACT (3521A>T), GTT1128GTA (3524T>A), CCA1129CCG (3527A>G), CAG1130TTG (3528C>T 3529A>T), GAG1131GAA (3533G>A), TTG1132ATG (3534T>A), GCC1133GCT (3539C>T), GCC1134GCT (3542C>T), GTT1136GTG (3548T>G), CCC1137CCA (3551C>A), AAG1139AAA (3557G>A), ACC1140ACA (3560C>A), ACT1141ACC (3563T>C), CTG1142CTT (3566G>T), CGG1143AAA (3567C>A 3568G>A 3569G>A), AAA1144CGC (3570A>C 3571A>G 3572A>C), AGC1145AGT (3575C>T), CAA1146CCC (3577A>C 3578A>C), TTG1147CTG (3579T>C), GAA1149GAG (3587A>G), GCA1150GCT (3590A>T), TTC1151TTT (3593C>T), GGA1152GGG (3596A>G), CCT1153GAT (3597C>G 3598C>A), GCA1154GCT (3602A>T), GAG1155CAG (3603G>C), ACA1156ACT (3608A>T), GCA1157GCC (3611A>C), ATT1160ATC (3620T>C), ACA1162TCT (3624A>T 3626A>T), GTT1163GCC (3628T>C 3629T>C), CAT1164AAA (3630C>A 3632T>A), AGA1167AGG (3641A>G), GGT1168GGA (3644T>A), GAT1169GAA (3647T>A), GGT1170GGG (3650T>G), GAC1172GAT (3656C>T), GGG1177GGT (3671G>T), GTA1178GTC (3674A>C), ATG1179GAG (3675A>G 3676T>A), AAA1180AAG (3680A>G), TAC1181TAT (3683C>T), TGT1186TCC (3697G>C 3698T>C), GGA1187AAC (3699G>A 3700G>A 3701A>C), GAT1189GAG (3707T>G), GAT1190GAG (3710T>G), GAC1191CCA (3711G>C 3712A>C 3713C>A), ATT1192GTT (3714A>G), GCC1193GCA (3719C>A), AAA1194AAG (3722A>G), CTA1195CTG (3725A>G), GCA1196GCC (3728A>C), TTC1197TTT (3731C>T), GAA1198GAG (3734A>G), AAT1199GGT (3735A>G 3736A>G), CTC1200CTT (3740C>T), AAG1201AAA (3743G>A), TGC1202GCA (3744T>G 3745G>C 3746C>A), TCG1203TCC (3749G>C), CTG1204ATT (3750C>A 3752G>T), CTA1205CTT (3755A>T), ATC1207GTG (3759A>G 3761C>G), ATG1208CTT (3762A>C 3764G>T), CGT1209AAG (3765C>A 3766G>A 3767T>G), AGC1210ATG (3769G>T 3770C>G), AAG1212CGA (3774A>C 3775A>G 3776G>A), ATC1213GTG (3777A>G 3779C>G), CCT1214CCC (3782T>C), GGA1216GGG (3788A>G), CGT1217AAA (3789C>A 3790G>A 3791T>A), CCA1218CCT (3794A>T), TGT1219GCA (3795T>G 3796G>C 3797T>A), GAA1220ATT (3798G>A 3799A>T 3800A>T), AGG1221AGA (3803G>A), GAT1222AAT (3804G>A), GGA1229GGG (3827A>G), TGT1233TTC (3838G>T 3839T>C), TAC1235TAT (3845C>T), TAT1236TTT (3847A>T), GGC1238GCA (3853G>C 3854C>A), GAG1240GAA (3860G>A), CTG1241TTG (3861C>T), ACA1243ACG (3869A>G), TCT1244TCA (3872T>A), TGC1245TGT (3875C>T), GGA1246GGT (3878A>T), CCC1248CCT (3884C>T), TTC1249TTT (3887C>T), ATG1252TTG (3894A>T), GGG1253GGA (3899G>A), ATG1254CTT (3900A>C 3902G>T), GGG1255GGT (3905G>T), ATG1256TCT (3906A>T 3907T>C 3908G>T), AAG1258AAA (3914G>A), AGA1259AGG (3917A>G), CAA1263GAA (3927C>G), TCC1264GCC (3930T>G), ACT1265CAA (3933A>C 3934C>A 3935T>A), GGC1266CCA (3936G>C 3937G>C 3938C>A), GAA1267GAC (3941A>C), GGA1268TCG (3942G>T 3943G>C 3944A>G), GAG1269CAG (3945G>C), AGA1270AGG (3950A>G), GTG1271GTT (3953G>T), GAA1272CTT (3954G>C 3955A>T 3956A>T), AGA1275AGG (3965A>G), GAC1276AAC (3966G>A), ACT1277ACA (3971T>A), GTA1279GTC (3977A>C), GAA1281GAT (3983A>T), CTA1286TTG (3996C>T 3998A>G), GAT1287GAC (4001T>C), GTG1288TCG (4002G>T 4003T>C), ATT1290GTA (4008A>G 4010T>A), AAA1292CAA (4014A>C), GGC1293GGA (4019C>A), ATC1294ACA (4021T>C 4022C>A), CAT1295TTT (4023C>T 4024A>T), GTG1296GTT (4028G>T), GAT1297GAC (4031T>C), CTG1298CTT (4034G>T), ACC1300ACA (4040C>A), CAA1302CAG (4046A>G), TGC1303TGT (4049C>T), GCC1304GCG (4052C>G), CGC1308CGG (4064C>G), CTC1309TTG (4065C>T 4067C>G), AAA1313AAG (4079A>G), GGC1316GGG (4088C>G), AGA1318AGG (4094A>G), AGG1321CGC (4101A>C 4103G>C), CTC1322CTG (4106C>G), TTT1323TTC (4109T>C), GAG1324GAA (4112G>A), ATA1325ATC (4115A>C), CTT1326TTG (4116C>T 4118T>G), TAC1330TAT (4130C>T), TTG1333CTG (4137T>C), GTC1334GTG (4142C>G), AGG1335CGG (4143A>C), AAG1336AAA (4148G>A), TAT1337TAC (4151T>C), TTT1338TTC (4154T>C), GAT1340GAC (4160T>C), TTC1341TTT (4163C>T), TCA1342TCT (4166A>T), GCC1343GCT (4169C>T), ACA1344TGT (4170A>T 4171C>G 4172A>T), TTG1348GCA (4182T>G 4183T>C 4184G>A), AAT1350AAC (4190T>C), GCT1351GTA (4192C>T 4193T>A), ATA1352CTC (4194A>C 4196A>C), CCA1353CCT (4199A>T), AAA1355AAG (4205A>G), GTT1356GTA (4208T>A), ATT1358ATC (4214T>C), GAT1359AAT (4215G>A), CCT1360CCA (4220T>A), ACA1361ACG (4223A>G), AGT1362AGC (4226T>C), TCT1363CAT (4227T>C 4228C>A), TTG1367GTG (4239T>G), TTG1368CTG (4242T>C), GCA1369GCT (4247A>T), AAT1370AGT (4249A>G), GGG1371AGC (4251G>A 4253G>C), AGA1373AGG (4259A>G), GCT1374TCA (4260G>T 4262T>A), GTG1375GTA (4265G>A), TCA1376TCT (4268A>T), GAC1377GAT (4271C>T), GTG1378GAG (4273T>A), TTT1380TTC (4280T>C), GCT1382GCC (4286T>C), TAT1384TAC (4292T>C), TCC1385TCG (4295C>G), CCT1392CCC (4316T>C), GTT1393ATC (4317G>A 4319T>C), GCT1395GCA (4325T>A), CAG1397CAA (4331G>A), TTG1402CTA (4344T>C 4346G>A), GAA1405GCT (4354A>C 4355A>T), TAC1406TTT (4357A>T 4358C>T), TAC1407TAT (4361C>T), GGA1408GGT (4364A>T), TCA1409GAT (4365T>G 4366C>A 4367A>T), AAG1410GAG (4368A>G), GGC1412GGG (4376C>G), GAT1415GAG (4385T>G), TCC1416TCA (4388C>A), CGA1419AGG (4395C>A 4397A>G), CAT1420CAC (4400T>C), GCA1421ACA (4401G>A), CTT1422TTA (4404C>T 4406T>A), GCA1426GCC (4418A>C), TGT1428TGC (4424T>C), TAC1430TAT (4430C>T), ACA1431ACC (4433A>C), CTG1432CTC (4436G>C), TGC1433TGT (4439C>T), GAT1435AAC (4443G>A 4445T>C), AAA1436AAG (4448A>G), GTG1437TTG (4449G>T), AGG1439CGC (4455A>C 4457G>C), TTG1440CTA (4458T>C 4460G>A), GTT1441GTG (4463T>G), GGG1442GGT (4466G>T), GGC1443GGA (4469C>A), CCA1445CCC (4475A>C), TCA1446TCT (4478A>T), GGA1447GGT (4481A>T), GCA1449GCT (4487A>T), CTA1450TTG (4488C>T 4490A>G), ACG1451ACT (4493G>T), ATC1453ATA (4499C>A), TTC1454TTT (4502C>T), TCT1456TCA (4508T>A), CTC1457TTG (4509C>T 4511C>G), CTC1458CTA (4514C>A), CGA1464AGG (4530C>A 4532A>G), TAT1465TAC (4535T>C), GCC1466GCG (4538C>G), TTG1470CTG (4548T>C), TTA1471TTG (4553A>G), AGA1472AGG (4556A>G), CCC1474CCA (4562C>A), ATT1476GTC (4566A>G 4568T>C), GCT1477AAA (4569G>A 4570C>A 4571T>A), AGG1479AAA (4576G>A 4577G>A), GCT1480TCT (4578G>T), ATA1481GTG (4581A>G 4583A>G), GGA1482GGC (4586A>C), GTT1483ATT (4587G>A), AAA1484AAG (4592A>G), AGT1486AAT (4597G>A), GAT1487GAC (4601T>C), CTA1491TTG (4611C>T 4613A>G), GCT1494GCA (4622T>A), GTT1495GTA (4625T>A), GGA1497GGT (4631A>T), GAC1499GAT (4637C>T), AAT1500AAC (4640T>C), CTT1501TTG (4641C>T 4643T>G), GCT1503GCG (4649T>G), GTA1504GTT (4652A>T), TTA1506ATG (4656T>A 4658A>G), CTC1508TTG (4662C>T 4664C>G), CAG1509AGG (4665C>A 4666A>G), TAT1511TAC (4673T>C), TCT1512GAT (4674T>G 4675C>A), CTG1513CTA (4679G>A), AAT1515TCC (4683A>T 4684A>C 4685T>C), ATA1516ATT (4688A>T), CAT1518AGA (4692C>A 4693A>G 4694T>A), GAG1519GAA (4697G>A), TTA1520CTG (4698T>C 4700A>G), CTG1522GCT (4704C>G 4705T>C 4706G>T), GTC1523GTT (4709C>T), AAT1524AAC (4712T>C), GTA1525GTC (4715A>C), ATC1526GTC (4716A>G), CTT1531TTG (4731C>T 4733T>G), AAA1533AAG (4739A>G), TCG1534TCA (4742G>A), GTT1537GTA (4751T>A), AAT1538GAG (4752A>G 4754T>G), GAG1539AAG (4755G>A), GTA1540ACA (4758G>A 4759T>C), CAA1541AAA (4761C>A), CAA1543CTG (4768A>T 4769A>G), CTA1548CTC (4784A>C), ACT1549ACC (4787T>C), CTG1551CTC (4793G>C), AGA1553AGG (4799A>G), TTT1555TTC (4805T>C), AAA1556AAG (4808A>G), GCT1559GCA (4817T>A), CTT1560TTG (4818C>T 4820T>G), TAC1562TAT (4826C>T), CAC1563CAA (4829C>A), GCT1565GCA (4835T>A), CCT1566CCA (4838T>A), AAG1568AAA (4844G>A), ATC1572GTC (4854A>G), ATG1576ATT (4868G>T), TAT1577TAC (4871T>C), ATA1579ATT (4877A>T), AGA1580AGG (4880A>G), CCT1581CCA (4883T>A), GCC1582GCA (4886C>A), CCA1583GTG (4887C>G 4888C>T 4889A>G), GGT1584GGA (4892T>A), GAC1587GAT (4901C>T), ACT1588GTA (4902A>G 4903C>T 4904T>A), CAG1589CAA (4907G>A), GCC1590GCT (4910C>T), ATG1592TAT (4914A>T 4915T>A 4916G>T), GTG1595ATT (4923G>A 4925G>T), GGA1598GGC (4934A>C), ATA1599ATC (4937A>C), CGT1600AGA (4938C>A 4940T>A), GCT1602GCC (4946T>C), CAC1605CAT (4955C>T), GGT1606GGG (4958T>G), CGT1607CAG (4960G>A 4961T>G), GTG1608GCC (4963T>C 4964G>C), GCT1609ACT (4965G>A), GAC1611GAA (4973C>A), AAG1612AGT (4975A>G 4976G>T), CTT1613TTT (4977C>T), GTC1614GTA (4982C>A), TTA1615AAT (4983T>A 4984T>A 4985A>T), CAG1618CTT (4993A>T 4994G>T), ACG1619GGA (4995A>G 4996C>G 4997G>A), GCG1620GCT (5000G>T), TTG1621CTC (5001T>C 5003G>C), GAT1622AAG (5004G>A 5006T>G), GAA1623CAG (5007G>C 5009A>G), AGG1624AGA (5012G>A), GGT1625AAC (5013G>A 5014G>A 5015T>C), TTC1626CTC (5016T>C), AGA1627CCC (5019A>C 5020G>C 5021A>C), GCT1628ATC (5022G>A 5023C>T 5024T>C), GTG1629ATG (5025G>A), TTT1631ATG (5031T>A 5033T>G), CCT1632CCC (5036T>C), TCC1633ACT (5037T>A 5039C>T), TAT1634TAC (5042T>C), TTG1635TAT (5044T>A 5045G>T), GTG1637CTT (5049G>C 5051G>T), CAG1639AGT (5055C>A 5056A>G 5057G>T), GAA1640CAA (5058G>C), ATT1642TTG (5064A>T 5066T>G), GCA1643ACA (5067G>A), AAG1644GAG (5070A>G), GTA1645GTC (5075A>C), GGG1647GGA (5081G>A), TCA1649GAG (5085T>G 5086C>A 5087A>G), GCC1651ATT (5091G>A 5092C>T 5093C>T), CTG1652GGG (5094C>G 5095T>G), ACA1653GTT (5097A>G 5098C>T 5099A>T), ATT1654TTG (5100A>T 5102T>G), TGT1655ATG (5103T>A 5104G>T 5105T>G), GAA1656GAG (5108A>G), ATG1657CTA (5109A>C 5111G>A), GCA1658TCA (5112G>T), AAA1659GAT (5115A>G 5117A>T), GCA1660GCT (5120A>T), GCT1661AAA (5121G>A 5122C>A 5123T>A), ATT1662AAT (5125T>A), TCC1663ACA (5127T>A 5129C>A), TAT1664TAC (5132T>C), ACG1665ACC (5135G>C), CCA1666CCT (5138A>T), TTG1667TTA (5141G>A), GAC1668CCT (5142G>C 5143A>C 5144C>T), GCA1669GCT (5147A>T), GGT1670GGG (5150T>G), GAG1671GAA (5153G>A), ATC1673ATT (5159C>T), ACA1674del (5160_5162delACA), CGT1678GAG (5172C>G 5173G>A 5174T>G), GAT1679GGT (5176A>G), CTG1680GTC (5178C>G 5180G>C), AAT1681CAT (5181A>C), TGG1682GAA (5184T>G 5185G>A 5186G>A), GCA1684GCG (5192A>G), CCA1685CCT (5195A>T), AAC1686GAT (5196A>G 5198C>T), ATT1687GTA (5199A>G 5201T>A), GGT1688TTT (5202G>T 5203G>T), TTT1689TTC (5207T>C), TCA1691AGT (5211T>A 5212C>G 5213A>T), GCA1692GCT (5216A>T), CGT1693CGG (5219T>G), GCC1696TCA (5226G>T 5228C>A), CAC1697AAC (5229C>A), TAC1698CAA (5232T>C 5234C>A), ACG1699CAA (5235A>C 5236C>A 5237G>A), GAT1701TCA (5241G>T 5242A>C 5243T>A), GAA1702TCA (5244G>T 5245A>C), GGG1703GGT (5249G>T), TAC1704AAC (5250T>A), ATT1705ATC (5255T>C), ATT1706CTG (5256A>C 5258T>G), AAC1708AAT (5264C>T), TGT1709TGC (5267T>C), ACA1710ACT (5270A>T), GGT1711GGA (5273T>A), GCA1712GCC (5276A>C), AAG1713CGC (5277A>C 5278A>G 5279G>C), AAG1714AGA (5281A>G 5282G>A), TCC1715ACA (5283T>A 5285C>A), AAT1716ACA (5287A>C 5288T>A), GTT1718GTG (5294T>G), GGT1720GGG (5300T>G), CCA1721CCT (5303A>T), AAC1723AAT (5309C>T), GAC1726GAT (5318C>T), ATG1731ATA (5333G>A), CCG1733CCC (5339G>C), ATG1736TTG (5346A>T), TCA1737GCA (5349T>G), GCT1738GCA (5354T>A), ATA1739ATC (5357A>C), ATG1740CAG (5358A>C 5359T>A), GCC1742CAT (5364G>C 5365C>A 5366C>T), AAG1744CGC (5370A>C 5371A>G 5372G>C), AAT1745AAG (5375T>G), GTG1746AGT (5376G>A 5377T>G 5378G>T), TTG1747CTG (5379T>C), GCA1748GAA (5383C>A), GGA1749GTT (5386G>T 5387A>T), GGA1750GGT (5390A>T), CAT1751GAT (5391C>G), GTA1752GGG (5395T>G 5396A>G), GCA1753GCT (5399A>T), AAT1755TCT (5403A>T 5404A>C), GTT1757ACC (5409G>A 5410T>C 5411T>C), GTG1759GTT (5417G>T), GGA1761GGC (5423A>C), GGA1763GGT (5429A>T), GCT1765GCA (5435T>A), GTC1766ATA (5436G>A 5438C>A), GGC1767GCT (5440G>C 5441C>T), ATT1769ATC (5447T>C), GCG1772GCA (5456G>A), CTG1773TTG (5457C>T), ATG1774TTT (5460A>T 5462G>T), GCC1775GCA (5465C>A), TTA1776TTG (5468A>G), GCA1777GCC (5471A>C), ACA1778ACT (5474A>T), AGG1779CAT (5475A>C 5476G>A 5477G>T), TAT1781TAC (5483T>C), TGT1782AGG (5484T>A 5486T>G), GTG1783GCC (5488T>C 5489G>C), GAG1784ACA (5490G>A 5491A>C 5492G>A), ATA1786CTT (5496A>C 5498A>T), ATA1787ATT (5501A>T), GTT1788GTG (5504T>G), AGA1789CGC (5505A>C 5507A>C), TTG1790CTA (5508T>C 5510G>A), CGC1791CGA (5513C>A), ACA1792ACC (5516A>C), ATT1793ATG (5519T>G), GGA1794GCT (5521G>C 5522A>T), AAT1795GGG (5523A>G 5524A>G 5525T>G), CTT1797CTG (5531T>G), GAC1798GAT (5534C>T), CTC1799CTG (5537C>G), AAT1800AAC (5540T>C), TAT1802TAC (5546T>C), CCT1803CCC (5549T>C), GGC1805GGT (5555C>T), GTG1807GCG (5560T>C), CAG1808CAA (5564G>A), TAT1809TAC (5567T>C), TTT1810TTG (5570T>G), CTT1811CTC (5573T>C), CAA1812AAG (5574C>A 5576A>G)

Proteins

polyprotein (YP_001976147.1)	1	1817	84.4%	9297	73.5%	1807 (99.3%)	1266 (69.6%)	3/10/0/0	0

	Begin	End	Coverage	Score	Concordance	Matches	Identities	I/D/M/F*	Stop Codons
**NT**	**23**	**5589**	**77.0%**	**3552**	**32.4%**	**5524 (99.1%)**	**3681 (66.0%)**	**9/43**	

Protein mutations:

F7W (160T>G 161C>G), K9_A16del (165_188delAAGGTTGCTGGTGTTGATGACGCC), S17P (189A>C 190G>C), S18P (192T>C), S19A (195T>G), T21S (201A>T 203C>A), T22P (204A>C), G26A (217G>C 218T>A), L28V (222C>G), N30S (228A>T 229A>C), I32F (234A>T), K34S (240A>T 241A>C 242A>T), F35L (243T>C), A38T (252G>A 254G>T), V39L (255G>C 257T>C), I42V (264A>G), T43N (268C>A 269C>T), T44K (271C>A 272T>A), M45V (273A>G 275G>T), K48T (283A>C 284G>C), S50V (288T>G 289C>T), L53I (297C>A 299T>A), L56V (306C>G 308G>C), S59P (315T>C 317T>A), T61M (322C>T 323T>G), D62A (325A>C 326T>A), V63A (328T>C 329G>A), S64A (330T>G 332C>A), V68A (343T>C 344G>C), A69S (345G>T 347C>T), S72N (355G>A), S73A (357T>G 359A>T), L74V (360C>G 362T>A), N75E (363A>G 365T>A), K76R (367A>G), G82A (385G>C 386T>A), A83K (387G>A 388C>A), L84I (390C>A 392T>A), I85M (395A>G), D86E (398T>A), L89I (405C>A 407T>C), A91G (412C>G 413G>T), S93D (417T>G 418C>A), L95V (423T>G 425G>T), S96K (426T>A 427C>A 428C>A), S101T (441T>A), K103V (447A>G 448A>T 449G>A), M105L (453A>C 455G>T), L109M (465T>A), F110V (468T>G), E111L (471G>T 472A>T 473G>A), L113V (477T>G 479G>C), S114K (480T>A 481C>A 482C>G), N115D (483A>G 485T>C), V117F (489G>T 491T>C), K118I (493A>T 494A>C), F121Y (502T>A 503T>C), A122E (505C>A 506T>A), V124L (510G>T 512G>A), N126E (516A>G 518T>A), S128V (522A>G 523G>T), D130S (528G>T 529A>C 530T>A), V131L (531G>T), V135F (543G>T 545A>T), Y136I (546T>A 547A>T 548T>A), G137A (550G>C), I143L (567A>T 569A>G), T144A (570A>G 572A>C), L145I (573T>A 575G>T), V147L (579G>C 581G>A), F150Y (589T>A), S154V (600A>G 601G>T 602T>A), E158T (612G>A 613A>C), G159P (615G>C 616G>C), V160F (618G>T), S161N (621T>A 622C>A), T163V (627A>G 628C>T 629T>A), K165V (633A>G 634A>T 635A>T), N166T (637A>C), I167L (639A>T 641C>G), L169S (646T>C 647G>A), I170V (648A>G 650A>T), M174F (660A>T 662G>T), K186T (697A>C), A188S (702G>T 704C>T), E189G (706A>G 707A>G), R190K (708C>A 709G>A 710A>G), T192S (714A>T), M193Q (717A>C 718T>A), V194L (720G>C), S198A (732A>G 733G>C 734T>A), M199V (735A>G 737G>A), V205L (753G>T 755T>A), S206T (756T>A 758A>G), M214L (780A>T 782G>A), T216S (786A>T), I217L (789A>T 791A>G), I228V (822A>G), T248A (882A>G 884A>T), T260Q (918A>C 919C>A 920T>G), I280V (978A>G 980A>G), G282S (984G>A), T283I (988C>T 989C>T), S295N (1024G>A), E304Q (1050G>C), Q307T (1059C>A 1060A>C), D310S (1068G>T 1069A>C 1070T>A), A311E (1072C>A 1073A>G), C313A (1077T>G 1078G>C), D314E (1082T>A), A316S (1086G>T 1088A>T), T317M (1090C>T), K325P (1113A>C 1114A>C 1115G>T), T329I (1126C>T 1127C>T), N332Q (1134A>C 1136C>A), R335K (1144G>A 1145G>A), E337Q (1149G>C 1151A>G), L339V (1155C>G), M354L (1200A>T), A363T (1227G>A), E366D (1238A>T), V368L (1242G>T 1244T>G), Y369V (1245T>G 1246A>T), S372A (1254A>G 1255G>C 1256T>C), C373S (1257T>A 1259T>C), R374Q (1260A>C 1261G>A 1262G>A), M376S (1266A>T 1267T>C 1268G>T), S393A (1317T>G 1319C>T), S401C (1342C>G), R412A (1374A>G 1375G>C), I440V (1458A>G 1460C>G), A500T (1638G>A 1640C>T), M521R (1702T>G 1703G>A), E523V (1708A>T), E525D (1715G>T), S527D (1719T>G 1720C>A 1721C>T), T528P (1722A>C 1724T>A), E529N (1725G>A 1727A>T), D530A (1729A>C), G533A (1738G>C 1739A>C), R536V (1746A>G 1747G>T 1748A>T), N538D (1752A>G 1754C>T), I539V (1755A>G 1757A>G), N552A (1794A>G 1795A>C), T554S (1800A>T), S556D (1806A>G 1807G>A), E560Q (1818G>C 1820G>A), Q567R (1840A>G), A570Y (1848G>T 1849C>A 1850A>T), K574S (1861A>G 1862G>C), F577Y (1870T>A), E578N (1872G>A 1874A>T), A579T (1875G>A), E580T (1878G>A 1879A>C), I583L (1887A>T 1889A>G), S584A (1890A>G 1891G>C), P585D (1893C>G 1894C>A 1895T>C), I587V (1899A>G 1901A>T), Q591N (1911C>A 1913A>T), S593A (1917T>G), A594V (1921C>T 1922A>T), D596E (1928T>A), I598L (1932A>C 1934A>G), A599D (1936C>A 1937G>T), V601F (1941G>T 1943G>C), D602Q (1944G>C 1946T>A), G603D (1948G>A 1949G>T), R605del (1953_1955delAGA), R607Q (1960G>A 1961C>A), T608V (1962A>G 1963C>T 1964C>T), K609L (1965A>T 1966A>T), Q610P (1969A>C 1970A>T), D611P (1971G>C 1972A>C), I614A (1980A>G 1981T>C), L615T (1983T>A 1984T>C 1985G>T), V616Y (1986G>T 1987T>A 1988G>T), P617E (1989C>G 1990C>A 1991C>G), T618Q (1992A>C 1993C>A 1994T>G), V619T (1995G>A 1996T>C 1997T>A), V620I (1998G>A 2000G>T), S621T (2002G>C 2003T>A), N623A (2007A>G 2008A>C), D624E (2012T>A), R625K (2014G>A), V626I (2016G>A 2018G>T), I627V (2019A>G 2021C>T), A629S (2025G>T 2027A>C), R630Q (2028A>C 2029G>A 2030A>G), L645M (2073T>A), D646N (2076G>A), P647K (2079C>A 2080C>A 2081T>A), E648F (2082G>T 2083A>T 2084G>T), L650F (2088C>T), H655L (2104A>T 2105T>C), H656S (2106C>T 2107A>C 2108T>C), M660I (2120G>C), S663V (2127T>G 2128C>T 2129T>G), D665S (2133G>T 2134A>C), D666S (2136G>T 2137A>C), E667D (2141A>T), H668A (2142C>G 2143A>C 2144T>C), E670Q (2148G>C), I671N (2152T>A), N673E (2157A>G 2159C>A), K674M (2161A>T 2162A>G), R676V (2166A>G 2167G>T), V677N (2169G>A 2170T>A 2171C>T), N678D (2172A>G 2174C>T), M679L (2175A>T), Y681V (2181T>G 2182A>T 2183T>C), R682K (2185G>A), D685S (2193G>A 2194A>G), A686P (2196G>C), E687Q (2199G>C 2201A>G), R690A (2208C>G 2209G>C 2210A>T), I701V (2241A>G 2243A>C), A710S (2268G>A 2269C>G 2270A>C), S711T (2271T>A), K716E (2286A>G), K717L (2289A>T 2290A>T 2291A>G), A719D (2296C>A 2297A>T), T722R (2304A>C 2305C>G 2306A>C), E723N (2307G>A 2309G>T), M724L (2310A>C 2312G>T), R725E (2313A>G 2314G>A), T726Q (2316A>C 2317C>A 2318T>A), F728K (2322T>A 2323T>A 2324T>A), H730Y (2328C>T), S731K (2331T>A 2332C>A 2333T>G), C732A (2334T>G 2335G>C 2336C>G), I733L (2337A>C), D734Q (2340G>C 2342T>G), V737A (2350T>C 2351T>A), I741T (2362T>C 2363A>G), F749R (2385T>A 2386T>G 2387T>A), N763R (2428A>G 2429C>G), S766A (2436T>G), S766_K767insG (2438_2439insGGT), K767T (2440A>C 2441A>T), E768D (2444G>C), P769K (2445C>A 2446C>A 2447A>G), E771R (2451G>A 2452A>G), S772N (2454T>A 2455C>A 2456A>T), K775I (2464A>T 2465A>C), I776E (2466A>G 2467T>A 2468T>A), Y778M (2472T>A 2473A>T 2474C>G), H779I (2475C>A 2476A>T), K780G (2478A>G 2479A>G 2480A>T), V782K (2484G>A 2485T>A 2486T>G), E783D (2489A>C), Y784K (2490T>A 2492T>A), I785M (2495C>G), K786I (2497A>T 2498G>C), D787Q (2499G>C 2501T>G), T788M (2503C>T 2504A>G), W790N (2508T>A 2509G>A 2510G>T), S791D (2511A>G 2512G>A), Y794F (2521A>T 2522T>C), A795I (2523G>A 2524C>T 2525A>C), R797K (2530G>A 2531A>G), I806L (2556A>T 2558A>G), M807I (2561G>A), V812T (2574G>A 2575T>C 2576T>C), F813M (2577T>A 2579T>G), G814S (2580G>A 2582A>T), G815A (2584G>C), I816V (2586A>G), L820I (2598T>A 2600G>T), A821T (2601G>A 2603G>T), W823L (2607T>C 2608G>T 2609G>C), Q824K (2610C>A), S825A (2613A>G 2614G>C 2615T>A), N826A (2616A>G 2617A>C 2618C>A), S827T (2619T>A), F828S (2623T>C), N830L (2628A>T 2629A>T 2630T>A), S832D (2634T>G 2635C>A 2636G>T), N852K (2696T>G), K853T (2698A>C 2699G>T), K853_L854insH (2699_2700insCAT), L854F (2700C>T), R855A (2703A>G 2704G>C), N856K (2708T>A), L857S (2709C>T 2710T>C 2711T>C), V859L (2715G>C 2717T>A), S862A (2724A>G 2725G>C 2726T>G), S863A (2727T>G 2729T>A), Q864D (2730C>G 2732A>C), S865N (2733T>A 2734C>A 2735A>C), L866N (2736T>A 2737T>A 2738G>C), A867H (2739G>C 2740C>A 2741A>T), Q868V (2742C>G 2743A>T 2744A>G), D869_W870insR (2747_2748insCGA), A872E (2755C>A 2756T>A), E873Q (2757G>C 2759A>G), Q879E (2775C>G 2777G>A), K882H (2784A>C 2786G>C), L890I (2808C>A), G891A (2812G>C), M907L (2859A>C 2861G>C), H914R (2880C>A 2881A>G 2882C>A), A916M (2886G>A 2887C>T 2888T>G), T923S (2907A>T 2909A>C), A927E (2920C>A 2921C>G), N928H (2922A>C), R930I (2928C>A 2929G>T 2930A>T), S931C (2932C>G), K932N (2936A>T), Y935F (2944A>T 2945C>T), R938T (2953G>C 2954A>C), E939Q (2955G>C), I942L (2964A>T 2966T>G), I948V (2982A>G 2984C>G), P961S (3021C>T 3023T>A), E972D (3056G>T), F975Y (3064T>A), T976S (3066A>T 3068A>C), Q980M (3078C>A 3079A>T 3080A>G), C984V (3090T>G 3091G>T 3092T>G), T990K (3109C>A 3110A>G), R993K (3117C>A 3118G>A 3119T>G), L994K (3120C>A 3121T>A 3122T>A), G995S (3123G>T 3124G>C), F999V (3135T>G 3137T>G), M1002Y (3144A>T 3145T>A 3146G>T), V1012E (3175T>A 3176T>A), E1015T (3183G>A 3184A>C), E1017G (3190A>G), N1019D (3195A>G 3197C>T), S1021N (3201T>A 3202C>A 3203C>T), S1022A (3204A>G 3205G>C 3206T>A), K1024A (3210A>G 3211A>C 3212G>T), I1025L (3213A>C), C1026S (3216T>A), Q1029V (3225C>G 3226A>T 3227A>T), S1030A (3228T>G 3230T>A), G1037S (3249G>A 3251G>T), S1050T (3289G>C), V1057C (3309G>T 3310T>G 3311G>C), G1068D (3343G>A 3344C>T), V1069T (3345G>A 3346T>C 3347A>C), R1073K (3357C>A 3358G>A 3359T>G), F1075Y (3364T>A 3365T>C), I1076V (3366A>G 3368C>G), S1077A (3369T>G), I1083V (3387A>G 3389C>G), Y1084F (3391A>T 3392C>T), E1086D (3398A>T), V1096I (3426G>A 3428C>T), A1098V (3433C>T 3434A>G), D1104E (3452T>A), I1105L (3453A>C 3455T>G), S1108H (3462T>C 3463C>A 3464A>C), I1109L (3465A>C 3467T>G), R1112K (3475G>A 3476A>G), T1114E (3480A>G 3481C>A), Q1115R (3483C>A 3484A>G 3485G>A), L1116F (3486C>T 3488T>C), N1120K (3500C>A), Y1123A (3507T>G 3508A>C 3509C>T), Y1124F (3511A>T), Q1130L (3528C>T 3529A>T), L1132M (3534T>A), R1143K (3567C>A 3568G>A 3569G>A), K1144R (3570A>C 3571A>G 3572A>C), Q1146P (3577A>C 3578A>C), P1153D (3597C>G 3598C>A), E1155Q (3603G>C), T1162S (3624A>T 3626A>T), V1163A (3628T>C 3629T>C), H1164K (3630C>A 3632T>A), D1169E (3647T>A), M1179E (3675A>G 3676T>A), C1186S (3697G>C 3698T>C), G1187N (3699G>A 3700G>A 3701A>C), D1189E (3707T>G), D1190E (3710T>G), D1191P (3711G>C 3712A>C 3713C>A), I1192V (3714A>G), N1199G (3735A>G 3736A>G), C1202A (3744T>G 3745G>C 3746C>A), L1204I (3750C>A 3752G>T), I1207V (3759A>G 3761C>G), M1208L (3762A>C 3764G>T), R1209K (3765C>A 3766G>A 3767T>G), S1210M (3769G>T 3770C>G), K1212R (3774A>C 3775A>G 3776G>A), I1213V (3777A>G 3779C>G), R1217K (3789C>A 3790G>A 3791T>A), C1219A (3795T>G 3796G>C 3797T>A), E1220I (3798G>A 3799A>T 3800A>T), D1222N (3804G>A), C1233F (3838G>T 3839T>C), Y1236F (3847A>T), G1238A (3853G>C 3854C>A), M1252L (3894A>T), M1254L (3900A>C 3902G>T), M1256S (3906A>T 3907T>C 3908G>T), Q1263E (3927C>G), S1264A (3930T>G), T1265Q (3933A>C 3934C>A 3935T>A), G1266P (3936G>C 3937G>C 3938C>A), E1267D (3941A>C), G1268S (3942G>T 3943G>C 3944A>G), E1269Q (3945G>C), E1272L (3954G>C 3955A>T 3956A>T), D1276N (3966G>A), E1281D (3983A>T), V1288S (4002G>T 4003T>C), I1290V (4008A>G 4010T>A), K1292Q (4014A>C), I1294T (4021T>C 4022C>A), H1295F (4023C>T 4024A>T), T1344C (4170A>T 4171C>G 4172A>T), L1348A (4182T>G 4183T>C 4184G>A), A1351V (4192C>T 4193T>A), I1352L (4194A>C 4196A>C), D1359N (4215G>A), S1363H (4227T>C 4228C>A), L1367V (4239T>G), N1370S (4249A>G), G1371S (4251G>A 4253G>C), A1374S (4260G>T 4262T>A), V1378E (4273T>A), V1393I (4317G>A 4319T>C), E1405A (4354A>C 4355A>T), Y1406F (4357A>T 4358C>T), S1409D (4365T>G 4366C>A 4367A>T), K1410E (4368A>G), D1415E (4385T>G), A1421T (4401G>A), D1435N (4443G>A 4445T>C), V1437L (4449G>T), I1476V (4566A>G 4568T>C), A1477K (4569G>A 4570C>A 4571T>A), R1479K (4576G>A 4577G>A), A1480S (4578G>T), I1481V (4581A>G 4583A>G), V1483I (4587G>A), S1486N (4597G>A), L1506M (4656T>A 4658A>G), Q1509R (4665C>A 4666A>G), S1512D (4674T>G 4675C>A), N1515S (4683A>T 4684A>C 4685T>C), H1518R (4692C>A 4693A>G 4694T>A), L1522A (4704C>G 4705T>C 4706G>T), I1526V (4716A>G), N1538E (4752A>G 4754T>G), E1539K (4755G>A), V1540T (4758G>A 4759T>C), Q1541K (4761C>A), Q1543L (4768A>T 4769A>G), H1563Q (4829C>A), I1572V (4854A>G), M1576I (4868G>T), P1583V (4887C>G 4888C>T 4889A>G), T1588V (4902A>G 4903C>T 4904T>A), M1592Y (4914A>T 4915T>A 4916G>T), V1595I (4923G>A 4925G>T), R1607Q (4960G>A 4961T>G), V1608A (4963T>C 4964G>C), A1609T (4965G>A), D1611E (4973C>A), K1612S (4975A>G 4976G>T), L1613F (4977C>T), L1615N (4983T>A 4984T>A 4985A>T), Q1618L (4993A>T 4994G>T), T1619G (4995A>G 4996C>G 4997G>A), D1622K (5004G>A 5006T>G), E1623Q (5007G>C 5009A>G), G1625N (5013G>A 5014G>A 5015T>C), F1626L (5016T>C), R1627P (5019A>C 5020G>C 5021A>C), A1628I (5022G>A 5023C>T 5024T>C), V1629M (5025G>A), F1631M (5031T>A 5033T>G), S1633T (5037T>A 5039C>T), L1635Y (5044T>A 5045G>T), V1637L (5049G>C 5051G>T), Q1639S (5055C>A 5056A>G 5057G>T), E1640Q (5058G>C), I1642L (5064A>T 5066T>G), A1643T (5067G>A), K1644E (5070A>G), S1649E (5085T>G 5086C>A 5087A>G), A1651I (5091G>A 5092C>T 5093C>T), L1652G (5094C>G 5095T>G), T1653V (5097A>G 5098C>T 5099A>T), I1654L (5100A>T 5102T>G), C1655M (5103T>A 5104G>T 5105T>G), M1657L (5109A>C 5111G>A), A1658S (5112G>T), K1659D (5115A>G 5117A>T), A1661K (5121G>A 5122C>A 5123T>A), I1662N (5125T>A), S1663T (5127T>A 5129C>A), D1668P (5142G>C 5143A>C 5144C>T), T1674del (5160_5162delACA), R1678E (5172C>G 5173G>A 5174T>G), D1679G (5176A>G), L1680V (5178C>G 5180G>C), N1681H (5181A>C), W1682E (5184T>G 5185G>A 5186G>A), N1686D (5196A>G 5198C>T), I1687V (5199A>G 5201T>A), G1688F (5202G>T 5203G>T), A1696S (5226G>T 5228C>A), H1697N (5229C>A), Y1698Q (5232T>C 5234C>A), T1699Q (5235A>C 5236C>A 5237G>A), D1701S (5241G>T 5242A>C 5243T>A), E1702S (5244G>T 5245A>C), Y1704N (5250T>A), I1706L (5256A>C 5258T>G), K1713R (5277A>C 5278A>G 5279G>C), K1714R (5281A>G 5282G>A), S1715T (5283T>A 5285C>A), N1716T (5287A>C 5288T>A), M1731I (5333G>A), M1736L (5346A>T), S1737A (5349T>G), M1740Q (5358A>C 5359T>A), A1742H (5364G>C 5365C>A 5366C>T), K1744R (5370A>C 5371A>G 5372G>C), N1745K (5375T>G), V1746S (5376G>A 5377T>G 5378G>T), A1748E (5383C>A), G1749V (5386G>T 5387A>T), H1751D (5391C>G), V1752G (5395T>G 5396A>G), N1755S (5403A>T 5404A>C), V1757T (5409G>A 5410T>C 5411T>C), V1766I (5436G>A 5438C>A), G1767A (5440G>C 5441C>T), M1774F (5460A>T 5462G>T), R1779H (5475A>C 5476G>A 5477G>T), C1782R (5484T>A 5486T>G), V1783A (5488T>C 5489G>C), E1784T (5490G>A 5491A>C 5492G>A), I1786L (5496A>C 5498A>T), I1793M (5519T>G), G1794A (5521G>C 5522A>T), N1795G (5523A>G 5524A>G 5525T>G), V1807A (5560T>C), F1810L (5570T>G), Q1812K (5574C>A 5576A>G)

	Begin	End	Coverage	Score	Concordance	Matches	Identities	I/D/M/F*	Stop Codons
**NT**	**23**	**5589**	**77.0%**	**3552**	**32.4%**	**5524 (99.1%)**	**3681 (66.0%)**	**9/43**	

Codon mutations:

GCT2GCA (146T>A), TTC7TGG (160T>G 161C>G), TCC8AGT (162T>A 163C>G 164C>T), AAG9_GCC16del (165_188delAAGGTTGCTGGTGTTGATGACGCC), AGT17CCT (189A>C 190G>C), TCT18CCT (192T>C), TCT19GCT (195T>G), TCC20TCT (200C>T), ACC21TCA (201A>T 203C>A), ACT22CCT (204A>C), GGT26GCA (217G>C 218T>A), CTT28GTT (222C>G), TCA29TCT (227A>T), AAT30TCT (228A>T 229A>C), ATT32TTT (234A>T), TCC33TCT (239C>T), AAA34TCT (240A>T 241A>C 242A>T), TTT35CTT (243T>C), AAA37AAG (251A>G), GCG38ACT (252G>A 254G>T), GTT39CTC (255G>C 257T>C), AGT40TCC (258A>T 259G>C 260T>C), ATT42GTT (264A>G), ACC43AAT (268C>A 269C>T), ACT44AAA (271C>A 272T>A), ATG45GTT (273A>G 275G>T), AAG48ACC (283A>C 284G>C), TCA50GTA (288T>G 289C>T), CAT52CAC (296T>C), CTT53ATA (297C>A 299T>A), CTG56GTC (306C>G 308G>C), CCA58CCG (314A>G), TCT59CCA (315T>C 317T>A), GTG60GTT (320G>T), ACT61ATG (322C>T 323T>G), GAT62GCA (325A>C 326T>A), GTG63GCA (328T>C 329G>A), TCC64GCA (330T>G 332C>A), TCT66TCA (338T>A), GTG68GCC (343T>C 344G>C), GCC69TCT (345G>T 347C>T), ACT70ACA (350T>A), TGT71TGC (353T>C), AGT72AAT (355G>A), TCA73GCT (357T>G 359A>T), CTT74GTA (360C>G 362T>A), AAT75GAA (363A>G 365T>A), AAA76AGA (367A>G), TTG78TTA (374G>A), GAC79GAT (377C>T), AAA80AAG (380A>G), GGT82GCA (385G>C 386T>A), GCA83AAA (387G>A 388C>A), CTT84ATA (390C>A 392T>A), ATA85ATG (395A>G), GAT86GAA (398T>A), CCA87CCC (401A>C), TTT88TTC (404T>C), CTT89ATC (405C>A 407T>C), AAA90AAG (410A>G), GCG91GGT (412C>G 413G>T), TAC92TAT (416C>T), TCT93GAT (417T>G 418C>A), TTC94TTT (422C>T), TTG95GTT (423T>G 425G>T), TCC96AAA (426T>A 427C>A 428C>A), ACA97ACT (431A>T), TAC99TAT (437C>T), TCT101ACT (441T>A), ATC102ATA (446C>A), AAG103GTA (447A>G 448A>T 449G>A), ATG105CTT (453A>C 455G>T), GTT106GTA (458T>A), TTA107CTT (459T>C 461A>T), TTG109ATG (465T>A), TTT110GTT (468T>G), GAG111TTA (471G>T 472A>T 473G>A), TTG113GTC (477T>G 479G>C), TCC114AAG (480T>A 481C>A 482C>G), AAT115GAC (483A>G 485T>C), GTT117TTC (489G>T 491T>C), AAA118ATC (493A>T 494A>C), TTA119CTA (495T>C), TTT121TAC (502T>A 503T>C), GCT122GAA (505C>A 506T>A), GTG124TTA (510G>T 512G>A), AAT126GAA (516A>G 518T>A), AGT128GTT (522A>G 523G>T), GAA129GAG (527A>G), GAT130TCA (528G>T 529A>C 530T>A), GTA131TTA (531G>T), GTT134GTG (542T>G), GTA135TTT (543G>T 545A>T), TAT136ATA (546T>A 547A>T 548T>A), GGT137GCT (550G>C), CTT139TTA (555C>T 557T>A), GTA140GTT (560A>T), ATA143TTG (567A>T 569A>G), ACA144GCC (570A>G 572A>C), TTG145ATT (573T>A 575G>T), TTA146TTG (578A>G), GTG147CTA (579G>C 581G>A), CTT148TTA (582C>T 584T>A), CTA149CTG (587A>G), TTT150TAT (589T>A), GTT151GTA (593T>A), TGT152TGC (596T>C), AGT154GTA (600A>G 601G>T 602T>A), GTT157GTA (611T>A), GAG158ACG (612G>A 613A>C), GGT159CCT (615G>C 616G>C), GTT160TTT (618G>T), TCT161AAT (621T>A 622C>A), AAC162AAT (626C>T), ACT163GTA (627A>G 628C>T 629T>A), GTC164GTT (632C>T), AAA165GTT (633A>G 634A>T 635A>T), AAT166ACT (637A>C), ATC167TTG (639A>T 641C>G), TTG169TCA (646T>C 647G>A), ATA170GTT (648A>G 650A>T), GTC171GTT (653C>T), AAC173AAT (659C>T), ATG174TTT (660A>T 662G>T), TTT175TTC (665T>C), TCA176TCT (668A>T), AGT177TCA (669A>T 670G>C 671T>A), CTA178TTG (672C>T 674A>G), TAC179TAT (677C>T), AAA186ACA (697A>C), GCC188TCT (702G>T 704C>T), GAA189GGG (706A>G 707A>G), CGA190AAG (708C>A 709G>A 710A>G), TTC191TTT (713C>T), ACC192TCC (714A>T), ATG193CAG (717A>C 718T>A), GTG194CTG (720G>C), GCC195GCG (725C>G), GCT197GCA (731T>A), AGT198GCA (732A>G 733G>C 734T>A), ATG199GTA (735A>G 737G>A), GGG202GGT (746G>T), TCA204AGT (750T>A 751C>G 752A>T), GTT205TTA (753G>T 755T>A), TCA206ACG (756T>A 758A>G), ACG210ACT (770G>T), TCT211AGC (771T>A 772C>G 773T>C), CAG212CAA (776G>A), TTG213CTT (777T>C 779G>T), ATG214TTA (780A>T 782G>A), ACA216TCA (786A>T), ATA217TTG (789A>T 791A>G), GGG221GGT (803G>T), ATT222ATC (806T>C), TCG223TCA (809G>A), ACG224ACT (812G>T), TTG225TTA (815G>A), GTG226GTA (818G>A), TTC227TTT (821C>T), ATT228GTT (822A>G), GCA229GCT (827A>T), GTG230GTT (830G>T), GGT232GGG (836T>G), GGC236GGT (848C>T), TTG237CTT (849T>C 851G>T), AGC238TCA (852A>T 853G>C 854C>A), TTG241CTG (861T>C), TCC242TCA (866C>A), ATT244ATC (872T>C), CTC245TTG (873C>T 875C>G), TAT246TAC (878T>C), TCG247TCT (881G>T), ACA248GCT (882A>G 884A>T), GCT252GCG (896T>G), CAG253CAA (899G>A), CAA257CAG (911A>G), CTA258CTG (914A>G), ACT260CAG (918A>C 919C>A 920T>G), CTT261TTG (921C>T 923T>G), CGT263AGG (927C>A 929T>G), AAT264AAC (932T>C), AAA266AAG (938A>G), TGC268TGT (944C>T), ACC269ACA (947C>A), TCC270TCT (950C>T), CAG271CAA (953G>A), GCT272GCC (956T>C), GAA278GAG (974A>G), ATA280GTG (978A>G 980A>G), GTC281GTT (983C>T), GGC282AGC (984G>A), ACC283ATT (988C>T 989C>T), GGA285GGC (995A>C), TTC286TTT (998C>T), GTG290GTA (1010G>A), GCT293GCG (1019T>G), AGT295AAT (1024G>A), GCA296GCT (1028A>T), ACG297ACC (1031G>C), CTG298TTA (1032C>T 1034G>A), ACT300ACC (1040T>C), CTG302CTT (1046G>T), GAG304CAG (1050G>C), CAG307ACG (1059C>A 1060A>C), GAG308GAA (1064G>A), GAT310TCA (1068G>T 1069A>C 1070T>A), GCA311GAG (1072C>A 1073A>G), GTG312GTT (1076G>T), TGT313GCT (1077T>G 1078G>C), GAT314GAA (1082T>A), CCT315CCA (1085T>A), GCA316TCT (1086G>T 1088A>T), ACG317ATG (1090C>T), GAG320GAA (1100G>A), AAC321AAT (1103C>T), GCC323GCA (1109C>A), AAG325CCT (1113A>C 1114A>C 1115G>T), GCT326GCA (1118T>A), TTC327TTT (1121C>T), CCT328CCA (1124T>A), ACC329ATT (1126C>T 1127C>T), AAA330AAG (1130A>G), AAC332CAA (1134A>C 1136C>A), CTG334TTA (1140C>T 1142G>A), AGG335AAA (1144G>A 1145G>A), GAA336GAG (1148A>G), GAA337CAG (1149G>C 1151A>G), GCT338GCC (1154T>C), CTC339GTC (1155C>G), AAG340AAA (1160G>A), ATC341ATA (1163C>A), GCA346GCC (1178A>C), GCG351GCA (1193G>A), TTC353TTT (1199C>T), ATG354TTG (1200A>T), AGT359TCT (1215A>T 1216G>C), ATT362ATA (1226T>A), GCG363ACG (1227G>A), CAG364CAA (1232G>A), TTA365CTA (1233T>C), GAA366GAT (1238A>T), GTT368TTG (1242G>T 1244T>G), TAT369GTT (1245T>G 1246A>T), GAA371GAG (1253A>G), AGT372GCC (1254A>G 1255G>C 1256T>C), TGT373AGC (1257T>A 1259T>C), AGG374CAA (1260A>C 1261G>A 1262G>A), CAC375CAT (1265C>T), ATG376TCT (1266A>T 1267T>C 1268G>T), GGC377GGT (1271C>T), CAG380CAA (1280G>A), CGT382AGG (1284C>A 1286T>G), GAA384GAG (1292A>G), TTT386TTC (1298T>C), GTA388GTG (1304A>G), TTC391TTT (1313C>T), GGG392GGA (1316G>A), TCC393GCT (1317T>G 1319C>T), GGG395GGA (1325G>A), TCT399TCA (1337T>A), ACA400ACG (1340A>G), TCC401TGC (1342C>G), TTA404CTT (1350T>C 1352A>T), TTT405TTC (1355T>C), GTG409GTT (1367G>T), TTG410CTC (1368T>C 1370G>C), AGA412GCA (1374A>G 1375G>C), GAG415GAA (1385G>A), AAA417AAG (1391A>G), CTC418CTG (1394C>G), AAT419AAC (1397T>C), CGG420CGC (1400G>C), CTA421TTG (1401C>T 1403A>G), GCA423GCG (1409A>G), AAG426AAA (1418G>A), AGG427AGA (1421G>A), GCT435GCA (1445T>A), CAC436CAT (1448C>T), GCT439GCA (1457T>A), ATC440GTG (1458A>G 1460C>G), CTG441CTT (1463G>T), GAC444GAT (1472C>T), GGA446GGT (1478A>T), GCA447GCT (1481A>T), TTG448CTT (1482T>C 1484G>T), CGA449CGG (1487A>G), GCT452GCA (1496T>A), TGC455TGT (1505C>T), CAA456CAG (1508A>G), GAT457GAC (1511T>C), ATT458ATA (1514T>A), AAA459AAG (1517A>G), GAT463GAC (1529T>C), TCA466TCT (1538A>T), ACA467ACC (1541A>C), CAA468CAG (1544A>G), CCA469CCT (1547A>T), CCC471CCT (1553C>T), TTA472CTT (1554T>C 1556A>T), GCC475GCT (1565C>T), GTT477GTC (1571T>C), GAC479GAT (1577C>T), GGC481GGA (1583C>A), CAT483CAC (1589T>C), TTC484TTT (1592C>T), ACC485ACA (1595C>A), TCC486TCA (1598C>A), TAT488TAC (1604T>C), ATA489ATC (1607A>C), ACA492ACC (1616A>C), CTG495TTG (1623C>T), ATC496ATA (1628C>A), TCA497TCT (1631A>T), CCT499CCA (1637T>A), GCC500ACT (1638G>A 1640C>T), CAA501CAG (1643A>G), GGT503GGG (1649T>G), CTT504CTA (1652T>A), ACA505ACG (1655A>G), CCA507CCT (1661A>T), GCT509GCA (1667T>A), GAG511GAA (1673G>A), CGG512AGG (1674C>A), AGA513AGG (1679A>G), AGG514AGA (1682G>A), GAC515GAT (1685C>T), ATC516ATT (1688C>T), CTG517TTA (1689C>T 1691G>A), GTG518GTT (1694G>T), TGT520TGC (1700T>C), ATG521AGA (1702T>G 1703G>A), GAG523GTG (1708A>T), GAG525GAT (1715G>T), TCC527GAT (1719T>G 1720C>A 1721C>T), ACT528CCA (1722A>C 1724T>A), GAA529AAT (1725G>A 1727A>T), GAT530GCT (1729A>C), CCT531CCA (1733T>A), ACG532ACA (1736G>A), GGA533GCC (1738G>C 1739A>C), CTC535CTA (1745C>A), AGA536GTT (1746A>G 1747G>T 1748A>T), AAC538GAT (1752A>G 1754C>T), ATA539GTG (1755A>G 1757A>G), GAG541GAA (1763G>A), GCT547GCC (1781T>C), ACT549ACA (1787T>A), CAC550CAT (1790C>T), AGG551AGA (1793G>A), AAT552GCT (1794A>G 1795A>C), TTG553TTA (1799G>A), ACC554TCC (1800A>T), AGT556GAT (1806A>G 1807G>A), GAT557GAC (1811T>C), CTT558CTC (1814T>C), GAG560CAA (1818G>C 1820G>A), GTA563GTG (1829A>G), GCC564GCA (1832C>A), AAA565AAG (1835A>G), TGT566TGC (1838T>C), CAG567CGG (1840A>G), GTA568GTG (1844A>G), CAT569CAC (1847T>C), GCA570TAT (1848G>T 1849C>A 1850A>T), GAA571GAG (1853A>G), GTA572GTG (1856A>G), AAG574AGC (1861A>G 1862G>C), TTG576CTT (1866T>C 1868G>T), TTT577TAT (1870T>A), GAA578AAT (1872G>A 1874A>T), GCT579ACT (1875G>A), GAA580ACA (1878G>A 1879A>C), TCT581TCA (1883T>A), GGC582GGA (1886C>A), ATA583TTG (1887A>T 1889A>G), AGT584GCT (1890A>G 1891G>C), CCT585GAC (1893C>G 1894C>A 1895T>C), ATA587GTT (1899A>G 1901A>T), GTT590GTC (1910T>C), CAA591AAT (1911C>A 1913A>T), TCT593GCT (1917T>G), GCA594GTT (1921C>T 1922A>T), GAT596GAA (1928T>A), ATA598CTG (1932A>C 1934A>G), GCG599GAT (1936C>A 1937G>T), GTG601TTC (1941G>T 1943G>C), GAT602CAA (1944G>C 1946T>A), GGG603GAT (1948G>A 1949G>T), GCT604GCC (1952T>C), AGA605del (1953_1955delAGA), TTG606TTA (1958G>A), CGC607CAA (1960G>A 1961C>A), ACC608GTT (1962A>G 1963C>T 1964C>T), AAG609TTG (1965A>T 1966A>T), CAA610CCT (1969A>C 1970A>T), GAT611CCT (1971G>C 1972A>C), GAA612GAG (1976A>G), ATA614GCA (1980A>G 1981T>C), TTG615ACT (1983T>A 1984T>C 1985G>T), GTG616TAT (1986G>T 1987T>A 1988G>T), CCC617GAG (1989C>G 1990C>A 1991C>G), ACT618CAG (1992A>C 1993C>A 1994T>G), GTT619ACA (1995G>A 1996T>C 1997T>A), GTG620ATT (1998G>A 2000G>T), AGT621ACA (2002G>C 2003T>A), AAT623GCT (2007A>G 2008A>C), GAT624GAA (2012T>A), AGA625AAA (2014G>A), GTG626ATT (2016G>A 2018G>T), ATC627GTT (2019A>G 2021C>T), TAT628TAC (2024T>C), GCA629TCC (2025G>T 2027A>C), AGA630CAG (2028A>C 2029G>A 2030A>G), GAG631GAA (2033G>A), ACG633ACA (2039G>A), GTA634GTT (2042A>T), GCA636GCC (2048A>C), TTG637CTC (2049T>C 2051G>C), GCA640GCT (2060A>T), CAG642CAA (2066G>A), GGC643GGG (2069C>G), TTG645ATG (2073T>A), GAC646AAC (2076G>A), CCT647AAA (2079C>A 2080C>A 2081T>A), GAG648TTT (2082G>T 2083A>T 2084G>T), GAA649GAG (2087A>G), CTT650TTT (2088C>T), TTT651TTC (2093T>C), CCT652CCA (2096T>A), CAT655CTC (2104A>T 2105T>C), CAT656TCC (2106C>T 2107A>C 2108T>C), GCC659GCT (2117C>T), ATG660ATC (2120G>C), TCT663GTG (2127T>G 2128C>T 2129T>G), CTT664CTA (2132T>A), GAT665TCT (2133G>T 2134A>C), GAT666TCT (2136G>T 2137A>C), GAA667GAT (2141A>T), CAT668GCC (2142C>G 2143A>C 2144T>C), GAG670CAG (2148G>C), ATT671AAT (2152T>A), TTC672TTT (2156C>T), AAC673GAA (2157A>G 2159C>A), AAA674ATG (2161A>T 2162A>G), AGA676GTA (2166A>G 2167G>T), GTC677AAT (2169G>A 2170T>A 2171C>T), AAC678GAT (2172A>G 2174C>T), ATG679TTG (2175A>T), TAT681GTC (2181T>G 2182A>T 2183T>C), AGA682AAA (2185G>A), GAT685AGT (2193G>A 2194A>G), GCT686CCT (2196G>C), GAA687CAG (2199G>C 2201A>G), CGA690GCT (2208C>G 2209G>C 2210A>T), GTA693GTG (2219A>G), GAC699GAT (2237C>T), ATA701GTC (2241A>G 2243A>C), CAC703CAT (2249C>T), TTT704TTC (2252T>C), GCA710AGC (2268G>A 2269C>G 2270A>C), TCA711ACA (2271T>A), ATC713ATT (2279C>T), GAG715GAA (2285G>A), AAG716GAG (2286A>G), AAA717TTG (2289A>T 2290A>T 2291A>G), CTA718CTG (2294A>G), GCA719GAT (2296C>A 2297A>T), GTG720GTC (2300G>C), CAG721CAA (2303G>A), ACA722CGC (2304A>C 2305C>G 2306A>C), GAG723AAT (2307G>A 2309G>T), ATG724CTT (2310A>C 2312G>T), AGG725GAG (2313A>G 2314G>A), ACT726CAA (2316A>C 2317C>A 2318T>A), GGA727GGG (2321A>G), TTT728AAA (2322T>A 2323T>A 2324T>A), GCA729GCG (2327A>G), CAT730TAT (2328C>T), TCT731AAG (2331T>A 2332C>A 2333T>G), TGC732GCG (2334T>G 2335G>C 2336C>G), ATT733CTT (2337A>C), GAT734CAG (2340G>C 2342T>G), GCA735GCT (2345A>T), GTT737GCA (2350T>C 2351T>A), GAC738GAT (2354C>T), ACC739ACA (2357C>A), CTC740CTT (2360C>T), ATA741ACG (2362T>C 2363A>G), ATC743ATT (2369C>T), GAG744GAA (2372G>A), CCA747CCG (2381A>G), TTT749AGA (2385T>A 2386T>G 2387T>A), GTT750GTA (2390T>A), TTT752TTC (2396T>C), TTA753CTG (2397T>C 2399A>G), ACA755ACT (2405A>T), GCA756GCT (2408A>T), AGA759CGC (2415A>C 2417A>C), TGC762TGT (2426C>T), AAC763AGG (2428A>G 2429C>G), GTA765GTT (2435A>T), TCA766GCA (2436T>G), TCA766_AAA767insGGT (2438_2439insGGT), AAA767ACT (2440A>C 2441A>T), GAG768GAC (2444G>C), CCA769AAG (2445C>A 2446C>A 2447A>G), GAA771AGA (2451G>A 2452A>G), TCA772AAT (2454T>A 2455C>A 2456A>T), ATC774ATT (2462C>T), AAA775ATC (2464A>T 2465A>C), ATT776GAA (2466A>G 2467T>A 2468T>A), TAC778ATG (2472T>A 2473A>T 2474C>G), CAT779ATT (2475C>A 2476A>T), AAA780GGT (2478A>G 2479A>G 2480A>T), GTT782AAG (2484G>A 2485T>A 2486T>G), GAA783GAC (2489A>C), TAT784AAA (2490T>A 2492T>A), ATC785ATG (2495C>G), AAG786ATC (2497A>T 2498G>C), GAT787CAG (2499G>C 2501T>G), ACA788ATG (2503C>T 2504A>G), TGG790AAT (2508T>A 2509G>A 2510G>T), AGC791GAC (2511A>G 2512G>A), CTT792TTG (2514C>T 2516T>G), TAT794TTC (2521A>T 2522T>C), GCA795ATC (2523G>A 2524C>T 2525A>C), CTG796TTG (2526C>T), AGA797AAG (2530G>A 2531A>G), CTC799CTT (2537C>T), GCA802GCC (2546A>C), GGT803GGA (2549T>A), ATA806TTG (2556A>T 2558A>G), ATG807ATA (2561G>A), GCT808GCA (2564T>A), GGA811GGC (2573A>C), GTT812ACC (2574G>A 2575T>C 2576T>C), TTT813ATG (2577T>A 2579T>G), GGA814AGT (2580G>A 2582A>T), GGC815GCC (2584G>C), ATT816GTT (2586A>G), ACA817ACT (2591A>T), GCA818GCT (2594A>T), TTT819TTC (2597T>C), TTG820ATT (2598T>A 2600G>T), GCG821ACT (2601G>A 2603G>T), TGG823CTC (2607T>C 2608G>T 2609G>C), CAG824AAG (2610C>A), AGT825GCA (2613A>G 2614G>C 2615T>A), AAC826GCA (2616A>G 2617A>C 2618C>A), TCT827ACT (2619T>A), TTC828TCC (2623T>C), CCC829CCT (2627C>T), AAT830TTA (2628A>T 2629A>T 2630T>A), GCA831GCT (2633A>T), TCG832GAT (2634T>G 2635C>A 2636G>T), GGA835GGG (2645A>G), GGT836GGC (2648T>C), GTG837GTA (2651G>A), GAA840GAG (2660A>G), ATA844ATC (2672A>C), AGT846AGC (2678T>C), AAG848AAA (2684G>A), AAC849AAT (2687C>T), AAG850AAA (2690G>A), GGT851GGG (2693T>G), AAT852AAG (2696T>G), AAG853ACT (2698A>C 2699G>T), AAG853_CTC854insCAT (2699_2700insCAT), CTC854TTC (2700C>T), AGA855GCA (2703A>G 2704G>C), AAT856AAA (2708T>A), CTT857TCC (2709C>T 2710T>C 2711T>C), CTC858TTG (2712C>T 2714C>G), GTT859CTA (2715G>C 2717T>A), GGT860GGG (2720T>G), AGT862GCG (2724A>G 2725G>C 2726T>G), TCT863GCA (2727T>G 2729T>A), CAA864GAC (2730C>G 2732A>C), TCA865AAC (2733T>A 2734C>A 2735A>C), TTG866AAC (2736T>A 2737T>A 2738G>C), GCA867CAT (2739G>C 2740C>A 2741A>T), CAA868GTG (2742C>G 2743A>T 2744A>G), GAT869GAC (2747T>C), GAT869_TGG870insCGA (2747_2748insCGA), GCT871GCA (2753T>A), GCT872GAA (2755C>A 2756T>A), GAA873CAG (2757G>C 2759A>G), GTA877GTT (2771A>T), CAG879GAA (2775C>G 2777G>A), AGC880AGT (2780C>T), CTC881CTG (2783C>G), AAG882CAC (2784A>C 2786G>C), AAT884AAC (2792T>C), GTG886GTT (2798G>T), GTG887GTT (2801G>T), TTA888TTG (2804A>G), CTT890ATT (2808C>A), GGT891GCT (2812G>C), GTG894GTC (2822G>C), TAC895TAT (2825C>T), TTT896TTC (2828T>C), GGC898GGT (2834C>T), ACC899ACG (2837C>G), TAC900TAT (2840C>T), GTG901GTT (2843G>T), ATA906ATT (2858A>T), ATG907CTC (2859A>C 2861G>C), ACC908ACT (2864C>T), GTA909GTG (2867A>G), GCT910GCA (2870T>A), GCA912GCC (2876A>C), TTC913TTT (2879C>T), CAC914AGA (2880C>A 2881A>G 2882C>A), AAT915AAC (2885T>C), GCT916ATG (2886G>A 2887C>T 2888T>G), CGA917AGG (2889C>A 2891A>G), GGT919GGC (2897T>C), CCA921CCT (2903A>T), ACA923TCC (2907A>T 2909A>C), ATC925ATT (2915C>T), GCC927GAG (2920C>A 2921C>G), AAT928CAT (2922A>C), TCT929TCC (2927T>C), CGA930ATT (2928C>A 2929G>T 2930A>T), TCT931TGT (2932C>G), AAA932AAT (2936A>T), GTT933GTG (2939T>G), CAA934CAG (2942A>G), TAC935TTT (2944A>T 2945C>T), AAC936AAT (2948C>T), AGA938ACC (2953G>C 2954A>C), GAA939CAA (2955G>C), ATT942TTG (2964A>T 2966T>G), ATT943ATA (2969T>A), GGC945GGT (2975C>T), GAC947GAT (2981C>T), ATC948GTG (2982A>G 2984C>G), CTG950TTG (2988C>T), CGC952AGG (2994C>A 2996C>G), GTT953GTA (2999T>A), GGT954GGG (3002T>G), CCA959CCC (3017A>C), AAG960AAA (3020G>A), CCT961TCA (3021C>T 3023T>A), GAC962GAT (3026C>T), ATC963ATA (3029C>A), CGT964AGA (3030C>A 3032T>A), CAC966CAT (3038C>T), CCA968CCT (3044A>T), AGA969CGC (3045A>C 3047A>C), AGG970AGA (3050G>A), GAG972GAT (3056G>T), TGC974TGT (3062C>T), TTC975TAC (3064T>A), ACA976TCC (3066A>T 3068A>C), CAA980ATG

	(3078C>A 3079A>T 3080A>G), GGA981GGC (3083A>C), TGT984GTG (3090T>G 3091G>T 3092T>G), AGA985AGG (3095A>G), GTT987GTA (3101T>A), GCG988GCT (3104G>T), TCT989TCA (3107T>A), ACA990AAG (3109C>A 3110A>G), CCA992CCC (3116A>C), CGT993AAG (3117C>A 3118G>A 3119T>G), CTT994AAA (3120C>A 3121T>A 3122T>A), GGC995TCC (3123G>T 3124G>C), TTA997TTG (3131A>G), TTT999GTG (3135T>G 3137T>G), CTC1000CTT (3140C>T), AAG1001AAA (3143G>A), ATG1002TAT (3144A>T 3145T>A 3146G>T), GGA1009GGC (3167A>C), ACA1011ACC (3173A>C), GTT1012GAA (3175T>A 3176T>A), GTG1014GTC (3182G>C), GAA1015ACA (3183G>A 3184A>C), GAA1017GGA (3190A>G), CTG1018TTA (3192C>T 3194G>A), AAC1019GAT (3195A>G 3197C>T), TCC1020TCG (3200C>G), TCC1021AAT (3201T>A 3202C>A 3203C>T), AGT1022GCA (3204A>G 3205G>C 3206T>A), AAG1024GCT (3210A>G 3211A>C 3212G>T), ATT1025CTT (3213A>C), TGC1026AGC (3216T>A), TCT1027TCA (3221T>A), CAA1029GTT (3225C>G 3226A>T 3227A>T), TCT1030GCA (3228T>G 3230T>A), GAA1032GAG (3236A>G), ATA1035ATT (3245A>T), GGG1037AGT (3249G>A 3251G>T), GAA1038GAG (3254A>G), GGT1040GGA (3260T>A), GAC1041GAT (3263C>T), GGC1043GGT (3269C>T), ACG1044ACT (3272G>T), TTG1045CTT (3273T>C 3275G>T), CTA1046CTC (3278A>C), CTG1047TTA (3279C>T 3281G>A), TTA1048TTG (3284A>G), AGT1050ACT (3289G>C), GTT1051GTG (3293T>G), AAT1053AAC (3299T>C), CAA1055CAG (3305A>G), CCT1056CCG (3308T>G), GTG1057TGC (3309G>T 3310T>G 3311G>C), GGC1060GGA (3320C>A), ATC1061ATA (3323C>A), CAC1062CAT (3326C>T), TCA1065TCT (3335A>T), GGC1068GAT (3343G>A 3344C>T), GTA1069ACC (3345G>A 3346T>C 3347A>C), GCA1070GCT (3350A>T), GCT1071GCA (3353T>A), GAA1072GAG (3356A>G), CGT1073AAG (3357C>A 3358G>A 3359T>G), GGA1074GGT (3362A>T), TTT1075TAC (3364T>A 3365T>C), ATC1076GTG (3366A>G 3368C>G), TCT1077GCT (3369T>G), AAT1079AAC (3377T>C), GCT1080GCA (3380T>A), GCT1082GCA (3386T>A), ATC1083GTG (3387A>G 3389C>G), TAC1084TTT (3391A>T 3392C>T), AGG1085AGA (3395G>A), GAA1086GAT (3398A>T), CTA1088TTG (3402C>T 3404A>G), GAG1089GAA (3407G>A), CCA1092CCC (3416A>C), ACT1093ACG (3419T>G), GGG1094GGA (3422G>A), CCG1095CCC (3425G>C), GTC1096ATT (3426G>A 3428C>T), GCA1098GTG (3433C>T 3434A>G), GCA1099GCT (3437A>T), GAT1104GAA (3452T>A), ATT1105CTG (3453A>C 3455T>G), CTG1106TTG (3456C>T), TCA1108CAC (3462T>C 3463C>A 3464A>C), ATT1109CTG (3465A>C 3467T>G), AGA1110AGG (3470A>G), AGC1111TCC (3471A>T 3472G>C), AGA1112AAG (3475G>A 3476A>G), GAA1113GAG (3479A>G), ACA1114GAA (3480A>G 3481C>A), CAG1115AGA (3483C>A 3484A>G 3485G>A), CTT1116TTC (3486C>T 3488T>C), GAA1119GAG (3497A>G), AAC1120AAA (3500C>A), CAA1121CAG (3503A>G), TAC1123GCT (3507T>G 3508A>C 3509C>T), TAC1124TTC (3511A>T), CTT1125TTG (3513C>T 3515T>G), GGA1126GGC (3518A>C), ACA1127ACT (3521A>T), GTT1128GTA (3524T>A), CCA1129CCG (3527A>G), CAG1130TTG (3528C>T 3529A>T), GAG1131GAA (3533G>A), TTG1132ATG (3534T>A), GCC1133GCT (3539C>T), GCC1134GCT (3542C>T), GTT1136GTG (3548T>G), CCC1137CCA (3551C>A), AAG1139AAA (3557G>A), ACC1140ACA (3560C>A), ACT1141ACC (3563T>C), CTG1142CTT (3566G>T), CGG1143AAA (3567C>A 3568G>A 3569G>A), AAA1144CGC (3570A>C 3571A>G 3572A>C), AGC1145AGT (3575C>T), CAA1146CCC (3577A>C 3578A>C), TTG1147CTG (3579T>C), GAA1149GAG (3587A>G), GCA1150GCT (3590A>T), TTC1151TTT (3593C>T), GGA1152GGG (3596A>G), CCT1153GAT (3597C>G 3598C>A), GCA1154GCT (3602A>T), GAG1155CAG (3603G>C), ACA1156ACT (3608A>T), GCA1157GCC (3611A>C), ATT1160ATC (3620T>C), ACA1162TCT (3624A>T 3626A>T), GTT1163GCC (3628T>C 3629T>C), CAT1164AAA (3630C>A 3632T>A), AGA1167AGG (3641A>G), GGT1168GGA (3644T>A), GAT1169GAA (3647T>A), GGT1170GGG (3650T>G), GAC1172GAT (3656C>T), GGG1177GGT (3671G>T), GTA1178GTC (3674A>C), ATG1179GAG (3675A>G 3676T>A), AAA1180AAG (3680A>G), TAC1181TAT (3683C>T), TGT1186TCC (3697G>C 3698T>C), GGA1187AAC (3699G>A 3700G>A 3701A>C), GAT1189GAG (3707T>G), GAT1190GAG (3710T>G), GAC1191CCA (3711G>C 3712A>C 3713C>A), ATT1192GTT (3714A>G), GCC1193GCA (3719C>A), AAA1194AAG (3722A>G), CTA1195CTG (3725A>G), GCA1196GCC (3728A>C), TTC1197TTT (3731C>T), GAA1198GAG (3734A>G), AAT1199GGT (3735A>G 3736A>G), CTC1200CTT (3740C>T), AAG1201AAA (3743G>A), TGC1202GCA (3744T>G 3745G>C 3746C>A), TCG1203TCC (3749G>C), CTG1204ATT (3750C>A 3752G>T), CTA1205CTT (3755A>T), ATC1207GTG (3759A>G 3761C>G), ATG1208CTT (3762A>C 3764G>T), CGT1209AAG (3765C>A 3766G>A 3767T>G), AGC1210ATG (3769G>T 3770C>G), AAG1212CGA (3774A>C 3775A>G 3776G>A), ATC1213GTG (3777A>G 3779C>G), CCT1214CCC (3782T>C), GGA1216GGG (3788A>G), CGT1217AAA (3789C>A 3790G>A 3791T>A), CCA1218CCT (3794A>T), TGT1219GCA (3795T>G 3796G>C 3797T>A), GAA1220ATT (3798G>A 3799A>T 3800A>T), AGG1221AGA (3803G>A), GAT1222AAT (3804G>A), GGA1229GGG (3827A>G), TGT1233TTC (3838G>T 3839T>C), TAC1235TAT (3845C>T), TAT1236TTT (3847A>T), GGC1238GCA (3853G>C 3854C>A), GAG1240GAA (3860G>A), CTG1241TTG (3861C>T), ACA1243ACG (3869A>G), TCT1244TCA (3872T>A), TGC1245TGT (3875C>T), GGA1246GGT (3878A>T), CCC1248CCT (3884C>T), TTC1249TTT (3887C>T), ATG1252TTG (3894A>T), GGG1253GGA (3899G>A), ATG1254CTT (3900A>C 3902G>T), GGG1255GGT (3905G>T), ATG1256TCT (3906A>T 3907T>C 3908G>T), AAG1258AAA (3914G>A), AGA1259AGG (3917A>G), CAA1263GAA (3927C>G), TCC1264GCC (3930T>G), ACT1265CAA (3933A>C 3934C>A 3935T>A), GGC1266CCA (3936G>C 3937G>C 3938C>A), GAA1267GAC (3941A>C), GGA1268TCG (3942G>T 3943G>C 3944A>G), GAG1269CAG (3945G>C), AGA1270AGG (3950A>G), GTG1271GTT (3953G>T), GAA1272CTT (3954G>C 3955A>T 3956A>T), AGA1275AGG (3965A>G), GAC1276AAC (3966G>A), ACT1277ACA (3971T>A), GTA1279GTC (3977A>C), GAA1281GAT (3983A>T), CTA1286TTG (3996C>T 3998A>G), GAT1287GAC (4001T>C), GTG1288TCG (4002G>T 4003T>C), ATT1290GTA (4008A>G 4010T>A), AAA1292CAA (4014A>C), GGC1293GGA (4019C>A), ATC1294ACA (4021T>C 4022C>A), CAT1295TTT (4023C>T 4024A>T), GTG1296GTT (4028G>T), GAT1297GAC (4031T>C), CTG1298CTT (4034G>T), ACC1300ACA (4040C>A), CAA1302CAG (4046A>G), TGC1303TGT (4049C>T), GCC1304GCG (4052C>G), CGC1308CGG (4064C>G), CTC1309TTG (4065C>T 4067C>G), AAA1313AAG (4079A>G), GGC1316GGG (4088C>G), AGA1318AGG (4094A>G), AGG1321CGC (4101A>C 4103G>C), CTC1322CTG (4106C>G), TTT1323TTC (4109T>C), GAG1324GAA (4112G>A), ATA1325ATC (4115A>C), CTT1326TTG (4116C>T 4118T>G), TAC1330TAT (4130C>T), TTG1333CTG (4137T>C), GTC1334GTG (4142C>G), AGG1335CGG (4143A>C), AAG1336AAA (4148G>A), TAT1337TAC (4151T>C), TTT1338TTC (4154T>C), GAT1340GAC (4160T>C), TTC1341TTT (4163C>T), TCA1342TCT (4166A>T), GCC1343GCT (4169C>T), ACA1344TGT (4170A>T 4171C>G 4172A>T), TTG1348GCA (4182T>G 4183T>C 4184G>A), AAT1350AAC (4190T>C), GCT1351GTA (4192C>T 4193T>A), ATA1352CTC (4194A>C 4196A>C), CCA1353CCT (4199A>T), AAA1355AAG (4205A>G), GTT1356GTA (4208T>A), ATT1358ATC (4214T>C), GAT1359AAT (4215G>A), CCT1360CCA (4220T>A), ACA1361ACG (4223A>G), AGT1362AGC (4226T>C), TCT1363CAT (4227T>C 4228C>A), TTG1367GTG (4239T>G), TTG1368CTG (4242T>C), GCA1369GCT (4247A>T), AAT1370AGT (4249A>G), GGG1371AGC (4251G>A 4253G>C), AGA1373AGG (4259A>G), GCT1374TCA (4260G>T 4262T>A), GTG1375GTA (4265G>A), TCA1376TCT (4268A>T), GAC1377GAT (4271C>T), GTG1378GAG (4273T>A), TTT1380TTC (4280T>C), GCT1382GCC (4286T>C), TAT1384TAC (4292T>C), TCC1385TCG (4295C>G), CCT1392CCC (4316T>C), GTT1393ATC (4317G>A 4319T>C), GCT1395GCA (4325T>A), CAG1397CAA (4331G>A), TTG1402CTA (4344T>C 4346G>A), GAA1405GCT (4354A>C 4355A>T), TAC1406TTT (4357A>T 4358C>T), TAC1407TAT (4361C>T), GGA1408GGT (4364A>T), TCA1409GAT (4365T>G 4366C>A 4367A>T), AAG1410GAG (4368A>G), GGC1412GGG (4376C>G), GAT1415GAG (4385T>G), TCC1416TCA (4388C>A), CGA1419AGG (4395C>A 4397A>G), CAT1420CAC (4400T>C), GCA1421ACA (4401G>A), CTT1422TTA (4404C>T 4406T>A), GCA1426GCC (4418A>C), TGT1428TGC (4424T>C), TAC1430TAT (4430C>T), ACA1431ACC (4433A>C), CTG1432CTC (4436G>C), TGC1433TGT (4439C>T), GAT1435AAC (4443G>A 4445T>C), AAA1436AAG (4448A>G), GTG1437TTG (4449G>T), AGG1439CGC (4455A>C 4457G>C), TTG1440CTA (4458T>C 4460G>A), GTT1441GTG (4463T>G), GGG1442GGT (4466G>T), GGC1443GGA (4469C>A), CCA1445CCC (4475A>C), TCA1446TCT (4478A>T), GGA1447GGT (4481A>T), GCA1449GCT (4487A>T), CTA1450TTG (4488C>T 4490A>G), ACG1451ACT (4493G>T), ATC1453ATA (4499C>A), TTC1454TTT (4502C>T), TCT1456TCA (4508T>A), CTC1457TTG (4509C>T 4511C>G), CTC1458CTA (4514C>A), CGA1464AGG (4530C>A 4532A>G), TAT1465TAC (4535T>C), GCC1466GCG (4538C>G), TTG1470CTG (4548T>C), TTA1471TTG (4553A>G), AGA1472AGG (4556A>G), CCC1474CCA (4562C>A), ATT1476GTC (4566A>G 4568T>C), GCT1477AAA (4569G>A 4570C>A 4571T>A), AGG1479AAA (4576G>A 4577G>A), GCT1480TCT (4578G>T), ATA1481GTG (4581A>G 4583A>G), GGA1482GGC (4586A>C), GTT1483ATT (4587G>A), AAA1484AAG (4592A>G), AGT1486AAT (4597G>A), GAT1487GAC (4601T>C), CTA1491TTG (4611C>T 4613A>G), GCT1494GCA (4622T>A), GTT1495GTA (4625T>A), GGA1497GGT (4631A>T), GAC1499GAT (4637C>T), AAT1500AAC (4640T>C), CTT1501TTG (4641C>T 4643T>G), GCT1503GCG (4649T>G), GTA1504GTT (4652A>T), TTA1506ATG (4656T>A 4658A>G), CTC1508TTG (4662C>T 4664C>G), CAG1509AGG (4665C>A 4666A>G), TAT1511TAC (4673T>C), TCT1512GAT (4674T>G 4675C>A), CTG1513CTA (4679G>A), AAT1515TCC (4683A>T 4684A>C 4685T>C), ATA1516ATT (4688A>T), CAT1518AGA (4692C>A 4693A>G 4694T>A), GAG1519GAA (4697G>A), TTA1520CTG (4698T>C 4700A>G), CTG1522GCT (4704C>G 4705T>C 4706G>T), GTC1523GTT (4709C>T), AAT1524AAC (4712T>C), GTA1525GTC (4715A>C), ATC1526GTC (4716A>G), CTT1531TTG (4731C>T 4733T>G), AAA1533AAG (4739A>G), TCG1534TCA (4742G>A), GTT1537GTA (4751T>A), AAT1538GAG (4752A>G 4754T>G), GAG1539AAG (4755G>A), GTA1540ACA (4758G>A 4759T>C), CAA1541AAA (4761C>A), CAA1543CTG (4768A>T 4769A>G), CTA1548CTC (4784A>C), ACT1549ACC (4787T>C), CTG1551CTC (4793G>C), AGA1553AGG (4799A>G), TTT1555TTC (4805T>C), AAA1556AAG (4808A>G), GCT1559GCA (4817T>A), CTT1560TTG (4818C>T 4820T>G), TAC1562TAT (4826C>T), CAC1563CAA (4829C>A), GCT1565GCA (4835T>A), CCT1566CCA (4838T>A), AAG1568AAA (4844G>A), ATC1572GTC (4854A>G), ATG1576ATT (4868G>T), TAT1577TAC (4871T>C), ATA1579ATT (4877A>T), AGA1580AGG (4880A>G), CCT1581CCA (4883T>A), GCC1582GCA (4886C>A), CCA1583GTG (4887C>G 4888C>T 4889A>G), GGT1584GGA (4892T>A), GAC1587GAT (4901C>T), ACT1588GTA (4902A>G 4903C>T 4904T>A), CAG1589CAA (4907G>A), GCC1590GCT (4910C>T), ATG1592TAT (4914A>T 4915T>A 4916G>T), GTG1595ATT (4923G>A 4925G>T), GGA1598GGC (4934A>C), ATA1599ATC (4937A>C), CGT1600AGA (4938C>A 4940T>A), GCT1602GCC (4946T>C), CAC1605CAT (4955C>T), GGT1606GGG (4958T>G), CGT1607CAG (4960G>A 4961T>G), GTG1608GCC (4963T>C 4964G>C), GCT1609ACT (4965G>A), GAC1611GAA (4973C>A), AAG1612AGT (4975A>G 4976G>T), CTT1613TTT (4977C>T), GTC1614GTA (4982C>A), TTA1615AAT (4983T>A 4984T>A 4985A>T), CAG1618CTT (4993A>T 4994G>T), ACG1619GGA (4995A>G 4996C>G 4997G>A), GCG1620GCT (5000G>T), TTG1621CTC (5001T>C 5003G>C), GAT1622AAG (5004G>A 5006T>G), GAA1623CAG (5007G>C 5009A>G), AGG1624AGA (5012G>A), GGT1625AAC (5013G>A 5014G>A 5015T>C), TTC1626CTC (5016T>C), AGA1627CCC (5019A>C 5020G>C 5021A>C), GCT1628ATC (5022G>A 5023C>T 5024T>C), GTG1629ATG (5025G>A), TTT1631ATG (5031T>A 5033T>G), CCT1632CCC (5036T>C), TCC1633ACT (5037T>A 5039C>T), TAT1634TAC (5042T>C), TTG1635TAT (5044T>A 5045G>T), GTG1637CTT (5049G>C 5051G>T), CAG1639AGT (5055C>A 5056A>G 5057G>T), GAA1640CAA (5058G>C), ATT1642TTG (5064A>T 5066T>G), GCA1643ACA (5067G>A), AAG1644GAG (5070A>G), GTA1645GTC (5075A>C), GGG1647GGA (5081G>A), TCA1649GAG (5085T>G 5086C>A 5087A>G), GCC1651ATT (5091G>A 5092C>T 5093C>T), CTG1652GGG (5094C>G 5095T>G), ACA1653GTT (5097A>G 5098C>T 5099A>T), ATT1654TTG (5100A>T 5102T>G), TGT1655ATG (5103T>A 5104G>T 5105T>G), GAA1656GAG (5108A>G), ATG1657CTA (5109A>C 5111G>A), GCA1658TCA (5112G>T), AAA1659GAT (5115A>G 5117A>T), GCA1660GCT (5120A>T), GCT1661AAA (5121G>A 5122C>A 5123T>A), ATT1662AAT (5125T>A), TCC1663ACA (5127T>A 5129C>A), TAT1664TAC (5132T>C), ACG1665ACC (5135G>C), CCA1666CCT (5138A>T), TTG1667TTA (5141G>A), GAC1668CCT (5142G>C 5143A>C 5144C>T), GCA1669GCT (5147A>T), GGT1670GGG (5150T>G), GAG1671GAA (5153G>A), ATC1673ATT (5159C>T), ACA1674del (5160_5162delACA), CGT1678GAG (5172C>G 5173G>A 5174T>G), GAT1679GGT (5176A>G), CTG1680GTC (5178C>G 5180G>C), AAT1681CAT (5181A>C), TGG1682GAA (5184T>G 5185G>A 5186G>A), GCA1684GCG (5192A>G), CCA1685CCT (5195A>T), AAC1686GAT (5196A>G 5198C>T), ATT1687GTA (5199A>G 5201T>A), GGT1688TTT (5202G>T 5203G>T), TTT1689TTC (5207T>C), TCA1691AGT (5211T>A 5212C>G 5213A>T), GCA1692GCT (5216A>T), CGT1693CGG (5219T>G), GCC1696TCA (5226G>T 5228C>A), CAC1697AAC (5229C>A), TAC1698CAA (5232T>C 5234C>A), ACG1699CAA (5235A>C 5236C>A 5237G>A), GAT1701TCA (5241G>T 5242A>C 5243T>A), GAA1702TCA (5244G>T 5245A>C), GGG1703GGT (5249G>T), TAC1704AAC (5250T>A), ATT1705ATC (5255T>C), ATT1706CTG (5256A>C 5258T>G), AAC1708AAT (5264C>T), TGT1709TGC (5267T>C), ACA1710ACT (5270A>T), GGT1711GGA (5273T>A), GCA1712GCC (5276A>C), AAG1713CGC (5277A>C 5278A>G 5279G>C), AAG1714AGA (5281A>G 5282G>A), TCC1715ACA (5283T>A 5285C>A), AAT1716ACA (5287A>C 5288T>A), GTT1718GTG (5294T>G), GGT1720GGG (5300T>G), CCA1721CCT (5303A>T), AAC1723AAT (5309C>T), GAC1726GAT (5318C>T), ATG1731ATA (5333G>A), CCG1733CCC (5339G>C), ATG1736TTG (5346A>T), TCA1737GCA (5349T>G), GCT1738GCA (5354T>A), ATA1739ATC (5357A>C), ATG1740CAG (5358A>C 5359T>A), GCC1742CAT (5364G>C 5365C>A 5366C>T), AAG1744CGC (5370A>C 5371A>G 5372G>C), AAT1745AAG (5375T>G), GTG1746AGT (5376G>A 5377T>G 5378G>T), TTG1747CTG (5379T>C), GCA1748GAA (5383C>A), GGA1749GTT (5386G>T 5387A>T), GGA1750GGT (5390A>T), CAT1751GAT (5391C>G), GTA1752GGG (5395T>G 5396A>G), GCA1753GCT (5399A>T), AAT1755TCT (5403A>T 5404A>C), GTT1757ACC (5409G>A 5410T>C 5411T>C), GTG1759GTT (5417G>T), GGA1761GGC (5423A>C), GGA1763GGT (5429A>T), GCT1765GCA (5435T>A), GTC1766ATA (5436G>A 5438C>A), GGC1767GCT (5440G>C 5441C>T), ATT1769ATC (5447T>C), GCG1772GCA (5456G>A), CTG1773TTG (5457C>T), ATG1774TTT (5460A>T 5462G>T), GCC1775GCA (5465C>A), TTA1776TTG (5468A>G), GCA1777GCC (5471A>C), ACA1778ACT (5474A>T), AGG1779CAT (5475A>C 5476G>A 5477G>T), TAT1781TAC (5483T>C), TGT1782AGG (5484T>A 5486T>G), GTG1783GCC (5488T>C 5489G>C), GAG1784ACA (5490G>A 5491A>C 5492G>A), ATA1786CTT (5496A>C 5498A>T), ATA1787ATT (5501A>T), GTT1788GTG (5504T>G), AGA1789CGC (5505A>C 5507A>C), TTG1790CTA (5508T>C 5510G>A), CGC1791CGA (5513C>A), ACA1792ACC (5516A>C), ATT1793ATG (5519T>G), GGA1794GCT (5521G>C 5522A>T), AAT1795GGG (5523A>G 5524A>G 5525T>G), CTT1797CTG (5531T>G), GAC1798GAT (5534C>T), CTC1799CTG (5537C>G), AAT1800AAC (5540T>C), TAT1802TAC (5546T>C), CCT1803CCC (5549T>C), GGC1805GGT (5555C>T), GTG1807GCG (5560T>C), CAG1808CAA (5564G>A), TAT1809TAC (5567T>C), TTT1810TTG (5570T>G), CTT1811CTC (5573T>C), CAA1812AAG (5574C>A 5576A>G)								
helicase (YP_001976154.1)	1	98	100%	654	97.5%	98 (100%)	95 (96.9%)	0/0/0/0	0
Protein mutations:	S5C (1342C>G), R16A (1374A>G 1375G>C), I44V (1458A>G 1460C>G)								
Codon mutations:	TCT3TCA (1337T>A), ACA4ACG (1340A>G), TCC5TGC (1342C>G), TTA8CTT (1350T>C 1352A>T), TTT9TTC (1355T>C), GTG13GTT (1367G>T), TTG14CTC (1368T>C 1370G>C), AGA16GCA (1374A>G 1375G>C), GAG19GAA (1385G>A), AAA21AAG (1391A>G), CTC22CTG (1394C>G), AAT23AAC (1397T>C), CGG24CGC (1400G>C), CTA25TTG (1401C>T 1403A>G), GCA27GCG (1409A>G), AAG30AAA (1418G>A), AGG31AGA (1421G>A), GCT39GCA (1445T>A), CAC40CAT (1448C>T), GCT43GCA (1457T>A), ATC44GTG (1458A>G 1460C>G), CTG45CTT (1463G>T), GAC48GAT (1472C>T), GGA50GGT (1478A>T), GCA51GCT (1481A>T), TTG52CTT (1482T>C 1484G>T), CGA53CGG (1487A>G), GCT56GCA (1496T>A), TGC59TGT (1505C>T), CAA60CAG (1508A>G), GAT61GAC (1511T>C), ATT62ATA (1514T>A), AAA63AAG (1517A>G), GAT67GAC (1529T>C), TCA70TCT (1538A>T), ACA71ACC (1541A>C), CAA72CAG (1544A>G), CCA73CCT (1547A>T), CCC75CCT (1553C>T), TTA76CTT (1554T>C 1556A>T), GCC79GCT (1565C>T), GTT81GTC (1571T>C), GAC83GAT (1577C>T), GGC85GGA (1583C>A), CAT87CAC (1589T>C), TTC88TTT (1592C>T), ACC89ACA (1595C>A), TCC90TCA (1598C>A), TAT92TAC (1604T>C), ATA93ATC (1607A>C), ACA96ACC (1616A>C)								

	Begin	End	Coverage	Score	Concordance	Matches	Identities	I/D/M/F*	Stop Codons
**NT**	**23**	**5589**	**77.0%**	**3552**	**32.4%**	**5524 (99.1%)**	**3681 (66.0%)**	**9/43**	
RNA-dependent RNA polymerase (YP_001976153.1)	1	249	100%	1552	88.4%	249 (100%)	210 (84.3%)	0/0/0/0	0
Protein mutations:	T40C (4170A>T 4171C>G 4172A>T), L44A (4182T>G 4183T>C 4184G>A), A47V (4192C>T 4193T>A), I48L (4194A>C 4196A>C), D55N (4215G>A), S59H (4227T>C 4228C>A), L63V (4239T>G), N66S (4249A>G), G67S (4251G>A 4253G>C), A70S (4260G>T 4262T>A), V74E (4273T>A), V89I (4317G>A 4319T>C), E101A (4354A>C 4355A>T), Y102F (4357A>T 4358C>T), S105D (4365T>G 4366C>A 4367A>T), K106E (4368A>G), D111E (4385T>G), A117T (4401G>A), D131N (4443G>A 4445T>C), V133L (4449G>T), I172V (4566A>G 4568T>C), A173K (4569G>A 4570C>A 4571T>A), R175K (4576G>A 4577G>A), A176S (4578G>T), I177V (4581A>G 4583A>G), V179I (4587G>A), S182N (4597G>A), L202M (4656T>A 4658A>G), Q205R (4665C>A 4666A>G), S208D (4674T>G 4675C>A), N211S (4683A>T 4684A>C 4685T>C), H214R (4692C>A 4693A>G 4694T>A), L218A (4704C>G 4705T>C 4706G>T), I222V (4716A>G), N234E (4752A>G 4754T>G), E235K (4755G>A), V236T (4758G>A 4759T>C), Q237K (4761C>A), Q239L (4768A>T 4769A>G)								
Codon mutations:	CGC4CGG (4064C>G), CTC5TTG (4065C>T 4067C>G), AAA9AAG (4079A>G), GGC12GGG (4088C>G), AGA14AGG (4094A>G), AGG17CGC (4101A>C 4103G>C), CTC18CTG (4106C>G), TTT19TTC (4109T>C), GAG20GAA (4112G>A), ATA21ATC (4115A>C), CTT22TTG (4116C>T 4118T>G), TAC26TAT (4130C>T), TTG29CTG (4137T>C), GTC30GTG (4142C>G), AGG31CGG (4143A>C), AAG32AAA (4148G>A), TAT33TAC (4151T>C), TTT34TTC (4154T>C), GAT36GAC (4160T>C), TTC37TTT (4163C>T), TCA38TCT (4166A>T), GCC39GCT (4169C>T), ACA40TGT (4170A>T 4171C>G 4172A>T), TTG44GCA (4182T>G 4183T>C 4184G>A), AAT46AAC (4190T>C), GCT47GTA (4192C>T 4193T>A), ATA48CTC (4194A>C 4196A>C), CCA49CCT (4199A>T), AAA51AAG (4205A>G), GTT52GTA (4208T>A), ATT54ATC (4214T>C), GAT55AAT (4215G>A), CCT56CCA (4220T>A), ACA57ACG (4223A>G), AGT58AGC (4226T>C), TCT59CAT (4227T>C 4228C>A), TTG63GTG (4239T>G), TTG64CTG (4242T>C), GCA65GCT (4247A>T), AAT66AGT (4249A>G), GGG67AGC (4251G>A 4253G>C), AGA69AGG (4259A>G), GCT70TCA (4260G>T 4262T>A), GTG71GTA (4265G>A), TCA72TCT (4268A>T), GAC73GAT (4271C>T), GTG74GAG (4273T>A), TTT76TTC (4280T>C), GCT78GCC (4286T>C), TAT80TAC (4292T>C), TCC81TCG (4295C>G), CCT88CCC (4316T>C), GTT89ATC (4317G>A 4319T>C), GCT91GCA (4325T>A), CAG93CAA (4331G>A), TTG98CTA (4344T>C 4346G>A), GAA101GCT (4354A>C 4355A>T), TAC102TTT (4357A>T 4358C>T), TAC103TAT (4361C>T), GGA104GGT (4364A>T), TCA105GAT (4365T>G 4366C>A 4367A>T), AAG106GAG (4368A>G), GGC108GGG (4376C>G), GAT111GAG (4385T>G), TCC112TCA (4388C>A), CGA115AGG (4395C>A 4397A>G), CAT116CAC (4400T>C), GCA117ACA (4401G>A), CTT118TTA (4404C>T 4406T>A), GCA122GCC (4418A>C), TGT124TGC (4424T>C), TAC126TAT (4430C>T), ACA127ACC (4433A>C), CTG128CTC (4436G>C), TGC129TGT (4439C>T), GAT131AAC (4443G>A 4445T>C), AAA132AAG (4448A>G), GTG133TTG (4449G>T), AGG135CGC (4455A>C 4457G>C), TTG136CTA (4458T>C 4460G>A), GTT137GTG (4463T>G), GGG138GGT (4466G>T), GGC139GGA (4469C>A), CCA141CCC (4475A>C), TCA142TCT (4478A>T), GGA143GGT (4481A>T), GCA145GCT (4487A>T), CTA146TTG (4488C>T 4490A>G), ACG147ACT (4493G>T), ATC149ATA (4499C>A), TTC150TTT (4502C>T), TCT152TCA (4508T>A), CTC153TTG (4509C>T 4511C>G), CTC154CTA (4514C>A), CGA160AGG (4530C>A 4532A>G), TAT161TAC (4535T>C), GCC162GCG (4538C>G), TTG166CTG (4548T>C), TTA167TTG (4553A>G), AGA168AGG (4556A>G), CCC170CCA (4562C>A), ATT172GTC (4566A>G 4568T>C), GCT173AAA (4569G>A 4570C>A 4571T>A), AGG175AAA (4576G>A 4577G>A), GCT176TCT (4578G>T), ATA177GTG (4581A>G 4583A>G), GGA178GGC (4586A>C), GTT179ATT (4587G>A), AAA180AAG (4592A>G), AGT182AAT (4597G>A), GAT183GAC (4601T>C), CTA187TTG (4611C>T 4613A>G), GCT190GCA (4622T>A), GTT191GTA (4625T>A), GGA193GGT (4631A>T), GAC195GAT (4637C>T), AAT196AAC (4640T>C), CTT197TTG (4641C>T 4643T>G), GCT199GCG (4649T>G), GTA200GTT (4652A>T), TTA202ATG (4656T>A 4658A>G), CTC204TTG (4662C>T 4664C>G), CAG205AGG (4665C>A 4666A>G), TAT207TAC (4673T>C), TCT208GAT (4674T>G 4675C>A), CTG209CTA (4679G>A), AAT211TCC (4683A>T 4684A>C 4685T>C), ATA212ATT (4688A>T), CAT214AGA (4692C>A 4693A>G 4694T>A), GAG215GAA (4697G>A), TTA216CTG (4698T>C 4700A>G), CTG218GCT (4704C>G 4705T>C 4706G>T), GTC219GTT (4709C>T), AAT220AAC (4712T>C), GTA221GTC (4715A>C), ATC222GTC (4716A>G), CTT227TTG (4731C>T 4733T>G), AAA229AAG (4739A>G), TCG230TCA (4742G>A), GTT233GTA (4751T>A), AAT234GAG (4752A>G 4754T>G), GAG235AAG (4755G>A), GTA236ACA (4758G>A 4759T>C), CAA237AAA (4761C>A), CAA239CTG (4768A>T 4769A>G), CTA244CTC (4784A>C), ACT245ACC (4787T>C), CTG247CTC (4793G>C), AGA249AGG (4799A>G)								

*: Inserts / Deletes / Misaligned / Frameshifts

Analysis details

This analysis was performed with panviral2.64

NGS Details (UN59): Torradovirus marchitezum (segment RNA 2)

Assembly

**Coverage Length**	4656 (1 contig(s))
**Depth Of Coverage**	3539.8
**Number Of Reads**	128651
**Reads Per Million**	2796.76 rpm (after QC)
**Ambiguities**	0
**Assembly Method**	de novo + reference guided assembly
**Consensus Caller**	Bcf Tools

Coverage Map

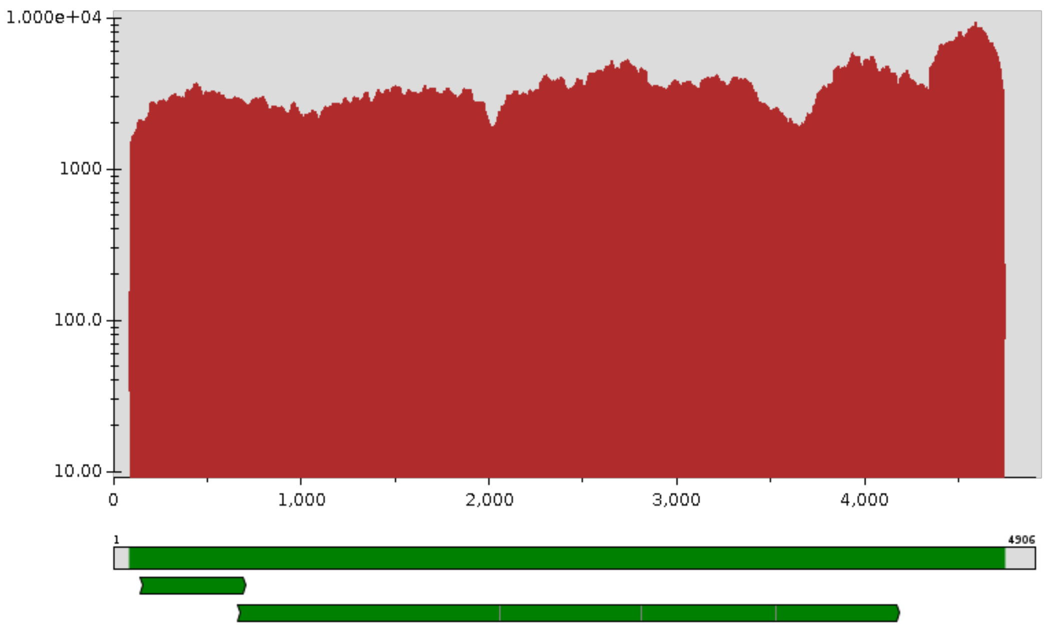

Assignment

**Type**	Torradovirus marchitezum (Taxonomy ID: 3048376)
**Reference Genome**	NC_010988.1
**NT Identity (%)**	64.4751
**AA Identity (%)**	67.9739
**Number Of Stop Codons**	2
**Number Of CDS**	2

Alignment

**Alignment Score**	2600.0 (NT) + 6793.0 (AA) = 9393.0
**Concordance (%)**	50.8032

**Alignment Method**	Global, seeded, nucleotide + amino acids (AGA)

Genome Region

Sequence starts at position 89 and ends at position 4744 relative to NC_010988.1 reference sequence.

Alignment Detailed Statistics

	Begin	End	Coverage	Score	Concordance	Matches	Identities	I/D/M/F*	Stop Codons
**NT**	**89**	**4744**	**94.9%**	**2600**	**28.6%**	**4608 (96.7%)**	**3040 (63.8%)**	**107/48**	

Mutations:

101G>T, 106C>A, 109A>C, 110T>C, 111T>C, 118T>C, 120C>A, 121T>A, 122T>A, 124G>C, 128T>A, 129G>A, 130T>A, 132G>T, 133_134insCACTCT, 135C>T, 137T>G, 144A>T, 147C>T, 150T>C, 151G>A, 153G>T, 154C>A, 155G>A, 156T>G, 157T>C, 159G>C, 162T>C, 165A>C, 166T>C, 167C>A, 168T>A, 169G>A, 174C>G, 177A>T, 182C>A, 183T>A, 186T>C, 190C>A, 198T>A, 201C>A, 202A>G, 204C>A, 207G>T, 209A>C, 210T>C, 214A>G, 220T>A, 221C>G, 229G>A, 230T>C, 234C>T, 235A>G, 236C>T, 237C>T, 241T>A, 243G>T, 246A>T, 261C>T, 262C>T, 264T>A, 267C>T, 273A>G, 274G>A, 277G>T, 278T>G, 282T>C, 291T>A, 298G>A, 300C>T, 303A>G, 306T>G, 307T>C, 309A>T, 310A>T, 312C>T, 315T>G, 316T>A, 317C>G, 324G>A, 333G>T, 336A>C, 340T>G, 342A>T, 348T>A, 351A>C, 354C>T, 357T>C, 358T>C, 360G>A, 361T>C, 363G>A, 374A>C, 375T>G, 379C>G, 380C>A, 381C>T, 384T>C, 387G>A, 388A>G, 389A>C, 390C>A, 402T>A, 405T>C, 408A>G, 409C>A, 414C>G, 416A>G, 417T>A, 423A>C, 424G>A, 426G>T, 427C>A, 428G>A, 429T>A, 430C>T, 432T>G, 441C>A, 442A>C, 444A>G, 448G>T, 450C>G, 451C>T, 453A>G, 456G>A, 458A>C, 459A>C, 462C>T, 465G>A, 468T>G, 469T>C, 470C>A, 477A>T, 478G>T, 480A>T, 481T>A, 483A>C, 489A>T, 492T>A, 495T>C, 496C>A, 497C>A, 498T>C, 499T>C, 501T>C, 508_525delGACAGTAAAGGCAAGAGT, 527A>C, 529G>T, 532G>T, 533A>C, 534G>A, 535C>G, 538C>A, 540C>T, 541A>G, 544_549delCGAGAG, 550G>A, 551A>G, 553C>T, 555C>A, 557T>G, 558T>C, 559A>G, 560A>G, 564A>G, 565G>A, 566T>A, 567T>C, 568G>A, 572T>C, 573T>A, 576C>T, 579A>G, 580C>G, 581G>A, 582T>A, 584A>C, 585G>C, 586T>C, 588A>T, 591G>A, 592A>G, 593G>T, 594A>T, 595T>G, 596T>C, 597T>C, 598C>A, 599A>G, 600G>A, 606G>A, 607T>C, 610G>C, 611C>A, 613A>G, 614G>A, 615T>A, 616C>G, 617A>C, 619A>C, 620A>G, 621A>G, 629A>T, 631C>G, 632A>T, 633G>C, 639A>C, 640C>A, 641A>G, 642A>G, 643C>T, 645T>G, 649C>A, 651C>T, 652T>C, 653C>A, 654C>A, 655A>G, 656A>G, 661G>C, 662T>C, 663T>A, 666T>C, 669T>C, 670A>G, 672T>G, 675C>T, 676A>G, 681C>T, 682T>G, 687T>C, 693T>G, 694G>A, 695A>G, 696A>T, 697A>G, 698G>A, 702G>C, 706C>A, 710A>G, 712T>C, 714A>G, 716C>T, 717A>C, 718G>A, 719_721delCAG, 722G>A, 724G>A, 729C>T, 730C>A, 733C>T, 736G>C, 738G>A, 739G>A, 745A>T, 748A>T, 751A>G, 754A>C, 760T>G, 766G>T, 769G>A, 771C>A, 772C>A, 775A>T, 784T>C, 785T>C, 787G>T, 790C>T, 792G>A, 796G>A, 797C>T, 799T>G, 802C>A, 811C>A, 814C>T, 817G>C, 818G>A, 820T>A, 821C>T, 823T>G, 828G>C, 829T>A, 835A>G, 841G>A, 844A>G, 853G>T, 854T>C, 856G>T, 858T>A, 862C>T, 874A>T, 875T>A, 876C>G, 877A>T, 880G>A, 881A>G, 883C>G, 884A>G, 892T>A, 899A>G, 901A>T, 904C>T, 908C>A, 909A>G, 910T>G, 912A>C, 913G>C, 914T>A, 916T>A, 917G>A, 918T>C, 919T>A, 922G>A, 925T>A, 926A>C, 927A>G, 928G>T, 931C>T, 936C>G, 937A>G, 940C>T, 943G>A, 949T>C, 953C>T, 955G>A, 956T>C, 958T>C, 961T>G, 965C>T, 967T>G, 970C>T, 973C>T, 979C>T, 982A>C, 987T>C, 988T>A, 993G>A, 997G>A, 1003A>T, 1004A>G, 1005G>C, 1006G>C, 1009T>C, 1012G>A, 1015A>C, 1018T>C, 1019C>T, 1021T>G, 1024T>C, 1027A>T, 1030T>C, 1033T>C, 1040C>A, 1041T>C, 1042C>T, 1043T>G, 1044C>A, 1045A>T, 1048G>T, 1057A>G, 1063G>A, 1065C>T, 1066A>T, 1067C>T, 1071C>A, 1075G>T, 1076G>T, 1078C>T, 1080A>G, 1081T>A, 1087G>A, 1090G>A, 1093A>C, 1099C>A, 1105C>T, 1108C>T, 1111C>T, 1127C>T, 1129C>G, 1132A>G, 1135A>G, 1138G>C, 1156C>A, 1162G>A, 1165C>T, 1167A>T, 1168A>C, 1174C>G, 1177G>A, 1180A>T, 1183G>A, 1189G>T, 1195C>T, 1198G>A, 1204C>A, 1207A>T, 1223G>A, 1224A>G, 1225C>T, 1226A>C, 1228A>T, 1229A>C, 1231G>T, 1237A>T, 1240T>A, 1244T>A, 1247C>G, 1249G>C, 1252A>T, 1255A>C, 1258A>T, 1262T>A, 1263C>G, 1270C>T, 1273A>T, 1276A>G, 1279A>T, 1282C>T, 1285T>C, 1286C>A, 1288T>A, 1291G>T, 1294A>G, 1306T>C, 1309A>G, 1315G>A, 1318T>A, 1321T>A, 1327T>C, 1330A>T, 1333T>A, 1339T>G, 1342T>C, 1345G>C, 1347G>A, 1351_1352insGTA, 1354A>G, 1357T>A, 1359C>G, 1363A>G, 1364A>C, 1367_1369delGCT, 1370C>T, 1372T>G, 1375A>G, 1380A>G, 1382G>C, 1384C>T, 1390T>G, 1393A>T, 1395A>C, 1396A>C, 1397C>G, 1398A>C, 1399G>A, 1405G>T, 1407G>A, 1408C>T, 1411A>C, 1418G>A, 1420C>T, 1423C>G, 1424A>C, 1425T>C, 1427A>C, 1429T>G, 1432A>G, 1433A>G, 1434G>C, 1435C>A, 1437C>A, 1442A>G, 1444C>G, 1447G>C, 1448A>T, 1449G>C, 1456T>A, 1462A>G, 1471T>C, 1474C>A, 1477A>G, 1480G>A, 1488A>G, 1492C>A, 1496C>G, 1498G>A, 1501C>A, 1508C>T, 1513G>T, 1513_1514insGACTTT, 1517G>T, 1518T>C, 1521G>C, 1523A>T, 1525A>T, 1526T>A, 1527C>G, 1529T>G, 1531C>G, 1533T>A, 1534A>T, 1536T>A, 1537C>G, 1538A>G, 1540C>A, 1541A>G, 1542C>T, 1543A>G, 1544C>A, 1546T>A, 1549A>G, 1555A>T, 1557T>G, 1559C>A, 1560A>T, 1565_1567delGAG, 1574T>A, 1575G>C, 1576T>G, 1577G>T, 1578G>C, 1579G>T, 1580G>C, 1581G>T, 1583T>C, 1591A>T, 1594A>C, 1595_1600delCCCAAG, 1603A>G, 1605A>T, 1606G>A, 1607G>T, 1608G>C, 1609C>A, 1615C>A, 1616T>G, 1617T>G, 1618C>A, 1620A>C, 1621C>A, 1622A>C, 1624G>C, 1625G>C, 1626G>A, 1627T>A, 1629C>A, 1630C>A, 1631A>G, 1633T>C, 1639T>C, 1644T>A, 1645G>T, 1651G>T, 1652G>C, 1654G>A, 1655T>A, 1656C>G, 1657G>T, 1660C>T, 1662C>A, 1664C>G, 1666C>T, 1667A>T, 1671A>C, 1672T>C, 1679G>A, 1680A>G, 1682G>A, 1684T>C, 1692A>C, 1693A>C, 1695G>T, 1696A>T, 1700A>G, 1702A>G, 1703G>C, 1705T>C, 1706C>G, 1711G>A, 1712G>A, 1714A>T, 1715C>G, 1717A>T, 1719T>A, 1721A>G, 1723C>T, 1726C>T, 1727T>G, 1729T>G, 1730A>C, 1731G>A, 1732T>G, 1733G>A, 1740A>G, 1742A>G, 1743G>C, 1744A>T, 1747A>T, 1750C>T, 1751G>C, 1753T>C, 1754A>T, 1755T>G, 1759G>C, 1764G>A, 1769G>A, 1773C>A, 1774A>G, 1776A>T, 1778C>A, 1781C>G, 1783T>C, 1784C>T, 1789T>C, 1790A>T, 1791A>G, 1795C>A, 1799A>T, 1800G>T, 1801G>T, 1804G>A, 1807C>T, 1810A>C, 1813C>T, 1818A>C, 1828A>T, 1831A>T, 1834A>T, 1835C>T, 1837T>G, 1839A>C, 1840A>G, 1843T>A, 1849T>A, 1858T>G, 1859A>G, 1861C>T, 1862C>G, 1863A>G, 1864A>G, 1865G>A, 1866G>T, 1867C>G, 1871A>G, 1872C>A, 1873T>A, 1874C>A, 1876C>A, 1879T>A, 1880T>C, 1881T>C, 1882C>A, 1891T>C, 1892G>T, 1895A>T, 1898T>A, 1899C>G, 1900T>C, 1903C>G, 1907G>C, 1915T>G, 1918G>T, 1924C>A, 1930T>C, 1931C>A, 1932A>T, 1933A>G, 1936A>G, 1939T>C, 1943T>C, 1944C>A, 1945C>A, 1948C>T, 1953T>G, 1954T>G, 1955C>T, 1957A>T, 1958G>T, 1961C>G, 1963G>T, 1964A>T, 1965C>T, 1966T>A, 1968A>C, 1969C>A, 1972T>C, 1975A>G, 1981G>T, 1982A>G, 1984T>C, 1991T>G, 1992G>T, 1993C>G, 2002A>T, 2003T>C, 2005A>T, 2008C>A, 2009T>C, 2012A>G, 2013A>C, 2017G>T, 2018C>A, 2020T>G, 2020_2021insGTGAAA, 2023G>T, 2024T>C, 2025T>C, 2026G>C, 2027A>T, 2029T>G, 2030G>A, 2033G>A, 2034A>G, 2035G>T, 2038G>T, 2040A>T, 2043T>C, 2044G>A, 2045G>T, 2046C>G, 2050_2051insTTGAAT, 2051A>C, 2053A>C, 2054A>T, 2055G>T, 2057T>G, 2058C>G, 2059T>A, 2060G>A, 2063A>C, 2064G>C, 2066C>A, 2067A>G, 2068A>C, 2069G>A, 2070C>T, 2071A>G, 2072C>A, 2073A>G, 2074G>T, 2078A>G, 2081G>C, 2082C>A, 2085G>C, 2086G>T, 2089C>A, 2091T>A, 2092A>T, 2095T>C, 2095_2096insAGC, 2096G>A, 2098A>G, 2099A>T, 2101T>G, 2104G>A, 2105C>A, 2112A>G, 2113C>A, 2115G>A, 2116A>G, 2120A>C, 2121C>T, 2122T>G, 2128C>A, 2134G>T, 2135T>A, 2140T>C, 2143C>A, 2144A>G, 2146G>A, 2147A>C, 2149G>T, 2152A>G, 2152_2153insAGT, 2155T>G, 2156C>G, 2157C>G, 2158C>T, 2160C>T, 2162A>C, 2164G>A, 2165C>A, 2166C>G, 2168G>A, 2170T>G, 2171A>G, 2174A>G, 2176A>G, 2177G>T, 2178A>C, 2184T>A, 2185T>G, 2188A>T, 2191G>A, 2193G>A, 2194A>T, 2195G>C, 2197A>G, 2202A>G, 2203A>G, 2204G>A, 2209T>A, 2210C>G, 2212A>G, 2216G>A, 2217G>A, 2221T>C, 2222G>A, 2225A>T, 2227T>C, 2233C>T, 2239A>G, 2240C>G, 2241C>A, 2242A>G, 2249G>A, 2250A>T, 2251C>G, 2252A>G, 2253C>G, 2254A>T, 2255T>G, 2256C>A, 2257C>T, 2258A>G, 2259T>C, 2260G>C, 2261G>C, 2263A>G, 2264G>T, 2266T>A, 2267G>A, 2269A>T, 2275T>G, 2276A>T, 2281G>A, 2284A>G, 2287G>A, 2289T>C, 2290G>A, 2296A>G, 2302C>T, 2308T>G, 2311C>T, 2323A>G, 2324G>A, 2335C>T, 2336G>C, 2338T>G, 2342G>A, 2344G>A, 2348T>A, 2349C>G, 2351G>A, 2352C>A, 2353T>C, 2365G>A, 2368A>G, 2371A>T, 2372A>C, 2374G>T, 2375A>G, 2376G>C, 2377C>A, 2378T>C, 2380G>A, 2381G>T, 2383T>C, 2384T>C, 2386G>C, 2389C>A, 2390G>A, 2391C>T, 2392T>G, 2395C>A, 2396C>T, 2398A>G, 2401C>T, 2402T>A, 2403C>G, 2404A>C, 2407A>G, 2409G>A, 2410C>G, 2413A>G, 2417A>G, 2422A>C, 2428T>G, 2429G>T, 2431G>C, 2440T>C, 2446T>C, 2449C>T, 2452T>C, 2455A>T, 2458T>A, 2459C>A, 2460T>A, 2461A>C, 2462A>C, 2464C>T, 2469A>T, 2470C>T, 2474A>G, 2476A>C, 2482T>G, 2485T>C, 2486G>A, 2495G>A, 2497T>G, 2503T>G, 2504A>G, 2506C>G, 2509T>G, 2512T>A, 2515A>G, 2518G>A, 2521T>C, 2536A>G, 2539T>C, 2544T>C, 2545G>A, 2552C>A, 2556A>C, 2557G>C, 2572C>T, 2575C>A, 2581A>C, 2582T>A, 2584G>T, 2596G>A, 2600A>C, 2601C>A, 2602A>T, 2603T>C, 2608T>G, 2618C>G, 2624A>T, 2625A>C, 2631A>G, 2633G>A, 2639T>G, 2640G>T, 2641C>G, 2644T>C, 2645A>T, 2647C>A, 2650A>T, 2651A>C, 2652C>A, 2656G>T, 2662C>T, 2664A>C, 2665G>C, 2667A>T, 2668C>T, 2671G>A, 2674T>A, 2675C>T, 2681G>A, 2682A>C, 2683A>T, 2684G>T, 2685G>C, 2686T>A, 2687A>C, 2688C>T, 2689A>G, 2692A>T, 2694C>T, 2695C>T, 2696A>C, 2701T>A, 2702T>C, 2704A>T, 2707T>C, 2713G>T, 2728A>C, 2731C>T, 2737A>G, 2738T>G, 2740T>A, 2746A>C, 2752G>A, 2756A>G, 2758T>G, 2759A>T, 2760G>C, 2761T>A, 2762G>A, 2767A>T, 2770C>T, 2773T>C, 2774T>A, 2776G>A, 2780T>C, 2783C>T, 2784A>C, 2785G>C, 2791A>G, 2795T>C, 2797A>C, 2803C>A, 2810C>A, 2812T>G, 2822C>A, 2824A>G, 2825A>T, 2829T>G, 2832C>G, 2833A>G, 2836G>A, 2842A>G, 2844A>T, 2848C>A, 2852A>C, 2853A>G, 2855G>C, 2857G>A, 2860G>C, 2861C>A, 2862A>C, 2863G>A, 2866A>C, 2868A>T, 2869T>C, 2873C>A, 2874C>G, 2875A>T, 2876A>C, 2878G>C, 2881T>C, 2884A>T, 2887T>A, 2896T>G, 2897T>C, 2899G>T, 2900T>C, 2902A>G, 2905T>C, 2908T>C, 2915G>A, 2917G>T, 2918A>G, 2919C>T, 2921T>G, 2923A>T, 2924T>G, 2926C>A, 2927C>G, 2929A>C, 2941A>T, 2950C>T, 2951A>G, 2953A>C, 2956T>C, 2959A>C, 2971A>T, 2977G>A, 2986C>T, 2988C>T, 2992G>A, 2998G>A, 3001T>A, 3002T>C, 3010T>A, 3016A>T, 3019C>A, 3022G>A, 3031G>A, 3039C>G, 3040T>A, 3043A>C, 3046T>C, 3059A>G, 3061T>G, 3064C>T, 3065A>G, 3076C>T, 3077T>C, 3088A>T, 3091T>G, 3097G>T, 3100A>T, 3103T>A, 3106A>G, 3107C>T, 3109T>G, 3112A>C, 3115T>G, 3119A>G, 3120A>C, 3121C>A, 3124C>A, 3125A>G, 3127A>G, 3131A>T, 3135A>C, 3136T>A, 3139T>G, 3151T>G, 3160A>C, 3169A>G, 3172C>T, 3176C>T, 3178T>G, 3187T>G, 3192A>G, 3193A>G, 3198G>A, 3199G>A, 3203C>A, 3204G>A, 3205G>A, 3208T>C, 3211T>A, 3212A>C, 3214T>G, 3217A>C, 3229A>G, 3230A>T, 3231A>G, 3232A>C, 3235T>C, 3236T>C, 3238G>C, 3241T>G, 3244C>T, 3247A>C, 3250G>A, 3253A>C, 3256G>A, 3262C>A, 3265G>A, 3275C>A, 3280C>T, 3281T>C, 3282T>A, 3283T>A, 3286C>T, 3290T>A, 3295C>T, 3296T>C, 3301T>C, 3307G>T, 3310A>T, 3322C>G, 3331G>A, 3335A>T, 3337A>G, 3340T>C, 3347T>A, 3349C>T, 3355T>A, 3358C>A, 3367G>A, 3373G>A, 3374T>C, 3377C>A, 3383A>G, 3388T>C, 3391T>G, 3392T>C, 3394G>T, 3397T>G, 3400C>T, 3401A>T, 3403A>T, 3406A>G, 3409T>A, 3412G>T, 3415A>G, 3421A>T, 3427G>T, 3436A>C, 3439A>G, 3442C>G, 3444A>G, 3448C>T, 3449T>G, 3451A>T, 3454T>G, 3460C>T, 3463T>G, 3466C>T, 3476G>A, 3478T>C, 3479T>C, 3481A>G, 3482T>A, 3483C>A, 3484A>T, 3490T>C, 3497A>C, 3502A>G, 3505G>A, 3506T>C, 3514G>A, 3517C>A, 3520C>T, 3521A>G, 3526G>T, 3527G>A, 3528A>T, 3529G>C, 3531A>T, 3532C>T, 3533C>G, 3535G>C, 3536G>C, 3544C>G, 3548_3553delGATCCG, 3554C>A, 3555C>G, 3561A>C, 3562G>A, 3563G>T, 3569A>G, 3570G>T, 3572A>T, 3573T>C, 3574T>C, 3575C>T, 3577G>C, 3578T>C, 3579C>A, 3580C>G, 3583A>C, 3586A>G, 3589G>A, 3592A>G, 3595T>C, 3596T>A, 3598C>A, 3604T>A, 3607T>A, 3610A>G, 3619C>T, 3622C>T, 3623A>G, 3624T>C, 3625G>T, 3627G>C, 3628T>A, 3638G>A, 3639A>C, 3640C>A, 3643A>C, 3650T>G, 3652G>A, 3655A>T, 3656A>T, 3658T>G, 3661T>A, 3667A>C, 3670A>T, 3676C>A, 3677A>G, 3679A>G, 3680A>C, 3682G>C, 3688A>T, 3690G>C, 3691G>A, 3692C>T, 3693A>C, 3700T>A, 3701G>A, 3706G>A, 3715C>A, 3721T>C, 3727T>C, 3733T>C, 3740A>T, 3742A>C, 3745A>T, 3760A>T, 3763T>A, 3770A>G, 3772T>A, 3776T>A, 3779A>T, 3784G>A, 3787A>G, 3791A>C, 3794G>A, 3795T>C, 3796A>T, 3799A>G, 3800T>A, 3801C>G, 3803A>T, 3804G>C, 3805T>C, 3806C>A, 3807C>G, 3808T>C, 3810A>G, 3812G>A, 3814T>A, 3817A>T, 3821G>A, 3823A>C, 3824C>T, 3826A>G, 3829T>C, 3830G>A, 3835T>A, 3836T>C, 3841T>C, 3842G>A, 3844C>A, 3850C>G, 3859T>C, 3861C>A, 3863G>T, 3864C>T, 3868G>T, 3869C>G, 3871T>A, 3872A>T, 3873A>C, 3875A>G, 3876A>C, 3877A>G, 3881A>G, 3882A>C, 3883T>A, 3884T>C, 3890G>T, 3901T>C, 3904A>T, 3907C>A, 3910A>T, 3925A>C, 3928C>T, 3931T>G, 3934G>T, 3935G>A, 3937A>C, 3940A>C, 3943T>C, 3945C>T, 3946G>C, 3952A>T, 3955C>T, 3956A>G, 3957C>T, 3958T>A, 3971G>C, 3976T>C, 3979C>T, 3982A>T, 3983A>C, 3985G>C, 3988G>T, 3989C>T, 3990A>T, 3991T>C, 3996G>A, 3998A>G, 3999T>C, 4000G>C, 4002G>A, 4003G>A, 4004G>T, 4005A>C, 4006A>C, 4008T>A, 4009C>T, 4010T>G, 4011C>A, 4012A>T, 4014G>A, 4015C>G, 4016A>G, 4018G>A, 4019C>A, 4020A>G, 4021A>T, 4024C>A, 4025C>A, 4027C>A, 4030C>T, 4031A>G, 4033G>T, 4036A>T, 4037C>T, 4039A>G, 4045T>C, 4047G>A, 4051T>A, 4054A>C, 4055A>T, 4056A>T, 4057T>G, 4058C>T, 4061A>C, 4063C>A, 4066A>C, 4070T>C, 4072G>T, 4075T>A, 4078T>A, 4079T>G, 4080C>T, 4081C>T, 4083C>A, 4084C>A, 4085A>C, 4087A>T, 4088G>A, 4090G>T, 4096C>A, 4102G>A, 4105A>C, 4106C>A, 4108T>A, 4109A>G, 4111A>C, 4112T>A, 4113C>A, 4114T>G, 4117T>A, 4120A>T, 4122T>C, 4129C>T, 4132G>A, 4144C>T, 4150T>C, 4153T>A, 4154T>C, 4155C>T, 4159C>A, 4163C>A, 4168A>G, 4171A>G, 4177T>C, 4180C>A, 4181A>G, 4189A>G, 4192T>G, 4195C>T, 4198C>T, 4210C>A, 4213A>T, 4226C>T, 4228A>G, 4231T>C, 4242T>A, 4247A>G, 4248A>C, 4259C>T, 4261A>G, 4267T>A, 4274T>C, 4276G>A, 4279A>T, 4280A>T, 4281A>T, 4283A>C, 4284C>T, 4285A>T, 4286C>T, 4287T>G, 4289A>T, 4291C>A, 4291_4292insCGTA, 4294C>T, 4304T>G, 4306G>A, 4309G>A, 4312G>T, 4312_4313insAGTAAATAAATAAAGT, 4317G>T, 4320G>T, 4324T>G, 4325T>G, 4330G>C, 4331C>A, 4333A>G, 4336A>T, 4339A>G, 4340G>T, 4342G>T, 4343A>T, 4345A>T, 4349A>G, 4351G>A, 4363T>C, 4364C>T, 4365T>C, 4370A>T, 4372A>G, 4373G>A, 4374A>G, 4384C>T, 4385C>G, 4386T>A, 4391T>G, 4393C>T, 4394A>G, 4398A>T, 4399G>T, 4400A>G, 4404A>G, 4415T>C, 4420delA, 4422A>G, 4426G>A, 4437C>T, 4441T>C, 4449A>T, 4451T>G, 4477_4478insTCCTCCACA, 4479A>C, 4480T>A, 4482T>A, 4486G>T, 4488T>A, 4489T>A, 4490A>C, 4491A>G, 4492C>G, 4493A>T, 4497C>G, 4499C>G, 4500G>T, 4501T>G, 4502T>A, 4506A>T, 4510T>A, 4510_4511insTCACTTA, 4518A>G, 4519G>A, 4520A>C, 4521C>A, 4523T>C, 4525A>G, 4526T>G, 4532C>G, 4534A>T, 4535A>T, 4539A>C, 4540_4541insAGTTAACGCGAACACAGGTTGTTTCCCTATAGAAACTA, 4542C>A, 4544T>C, 4548T>G, 4552C>G, 4553A>C, 4554T>A, 4556T>C, 4557A>T, 4559G>A, 4562A>G, 4563_4564delTA, 4566A>G, 4568G>A, 4569A>G, 4570G>C, 4581C>T, 4594T>C, 4595T>A, 4602C>G, 4606A>T, 4613T>C, 4614C>T, 4616C>T, 4618A>G, 4626T>C, 4628G>A, 4630G>A, 4632C>T, 4637T>A, 4639G>A, 4642A>T, 4643G>T, 4646C>A, 4654C>T, 4675G>C, 4680A>G, 4684T>A, 4685T>A, 4688C>T, 4689C>G, 4704G>C, 4706G>A, 4708T>A, 4722T>G

CDS

	Begin	End	Coverage	Score	Concordance	Matches	Identities	I/D/M/F*	Stop Codons
**NT**	**89**	**4744**	**94.9%**	**2600**	**28.6%**	**4608 (96.7%)**	**3040 (63.8%)**	**107/48**	
ToMarV_RNA2gp1	1	191	100%	784	66.7%	183 (95.8%)	116 (60.7%)	0/8/0/0	1
Protein mutations:	G5S (151G>A 153G>T), R6K (154C>A 155G>A 156T>G), S10Q (166T>C 167C>A 168T>A), V11I (169G>A), D12E (174C>G), E13D (177A>T), A15E (182C>A 183T>A), H18N (190C>A), T22A (202A>G 204C>A), N24T (209A>C 210T>C), I26V (214A>G), V31T (229G>A 230T>C), T33V (235A>G 236C>T 237C>T), L35I (241T>A 243G>T), V46I (274G>A), V47C (277G>T 278T>G), V54I (298G>A 300C>T), T58S (310A>T 312C>T), L68V (340T>G 342A>T), N79T (374A>C 375T>G), P81D (379C>G 380C>A 381C>T), N84A (388A>G 389A>C 390C>A), L91I (409C>A), H93R (416A>G 417T>A), V96I (424G>A 426G>T), R97K (427C>A 428G>A 429T>A), V104L (448G>T 450C>G), E107A (458A>C 459A>C), S111H (469T>C 470C>A), A114S (478G>T 480A>T), S115T (481T>A 483A>C), P120N (496C>A 497C>A 498T>C), S121P (499T>C 501T>C), D124_S129del (508_525delGACAGTAAAGGCAAGAGT), K130T (527A>C), V131L (529G>T), E132S (532G>T 533A>C 534G>A), Q133E (535C>G), P134T (538C>A 540C>T), T135A (541A>G), R136_E137del (544_549delCGAGAG), D138S (550G>A 551A>G), I140S (557T>G 558T>C), K141G (559A>G 560A>G), V143N (565G>A 566T>A 567T>C), E144K (568G>A), V145A (572T>C 573T>A), R148E (580C>G 581G>A 582T>A), E149A (584A>C 585G>C), R152V (592A>G 593G>T 594A>T), F153A (595T>G 596T>C 597T>C), Q154R (598C>A 599A>G 600G>A), A158Q (610G>C 611C>A), S159E (613A>G 614G>A 615T>A), Q160A (616C>G 617A>C), K161R (619A>C 620A>G 621A>G), N164I (629A>T), Q165V (631C>G 632A>T 633G>C), Q168R (640C>A 641A>G 642A>G), L171I (649C>A 651C>T), S172Q (652T>C 653C>A 654C>A), N173G (655A>G 656A>G), V175P (661G>C 662T>C 663T>A), N178E (670A>G 672T>G), I180V (676A>G), S182A (682T>G), E186S (694G>A 695A>G 696A>T), S187D (697A>G 698G>A), Q190K (706C>A)								
Codon mutations:	TCA2TCT (144A>T), TTC3TTT (147C>T), ATT4ATC (150T>C), GGG5AGT (151G>A 153G>T), CGT6AAG (154C>A 155G>A 156T>G), TTG7CTC (157T>C 159G>C), AAT8AAC (162T>C), ACA9ACC (165A>C), TCT10CAA (166T>C 167C>A 168T>A), GTA11ATA (169G>A), GAC12GAG (174C>G), GAA13GAT (177A>T), GCT15GAA (182C>A 183T>A), TTT16TTC (186T>C), CAT18AAT (190C>A), GTT20GTA (198T>A), GCC21GCA (201C>A), ACC22GCA (202A>G 204C>A), TCG23TCT (207G>T), AAT24ACC (209A>C 210T>C), ATT26GTT (214A>G), TCT28AGT (220T>A 221C>G), GTT31ACT (229G>A 230T>C), GGC32GGT (234C>T), ACC33GTT (235A>G 236C>T 237C>T), TTG35ATT (241T>A 243G>T), ATA36ATT (246A>T), ACC41ACT (261C>T), CTT42TTA (262C>T 264T>A), GAC43GAT (267C>T), AAA45AAG (273A>G), GTT46ATT (274G>A), GTT47TGT (277G>T 278T>G), CCT48CCC (282T>C), GGT51GGA (291T>A), GTC54ATT (298G>A 300C>T), TCA55TCG (303A>G), GTT56GTG (306T>G), TTA57CTT (307T>C 309A>T), ACC58TCT (310A>T 312C>T), GTT59GTG (315T>G), TCT60AGT (316T>A 317C>G), GAG62GAA (324G>A), ACG65ACT (333G>T), CCA66CCC (336A>C), TTA68GTT (340T>G 342A>T), CCT70CCA (348T>A), GGA71GGC (351A>C), CAC72CAT (354C>T), TAT73TAC (357T>C), TTG74CTA (358T>C 360G>A), TTG75CTA (361T>C 363G>A), AAT79ACG (374A>C 375T>G), CCC81GAT (379C>G 380C>A 381C>T), ATT82ATC (384T>C), AAG83AAA (387G>A), AAC84GCA (388A>G 389A>C 390C>A), TCT88TCA (402T>A), GGT89GGC (405T>C), TTA90TTG (408A>G), CTT91ATT (409C>A), GTC92GTG (414C>G), CAT93CGA (416A>G 417T>A), TCA95TCC (423A>C), GTG96ATT (424G>A 426G>T), CGT97AAA (427C>A 428G>A 429T>A), CTT98TTG (430C>T 432T>G), ACC101ACA (441C>A), AGA102CGG (442A>C 444A>G), GTC104TTG (448G>T 450C>G), CTA105TTG (451C>T 453A>G), GAG106GAA (456G>A), GAA107GCC (458A>C 459A>C), AAC108AAT (462C>T), AAG109AAA (465G>A), GTT110GTG (468T>G), TCT111CAT (469T>C 470C>A), TCA113TCT (477A>T), GCA114TCT (478G>T 480A>T), TCA115ACC (481T>A 483A>C), TCA117TCT (489A>T), TCT118TCA (492T>A), TCT119TCC (495T>C), CCT120AAC (496C>A 497C>A 498T>C), TCT121CCC (499T>C 501T>C), GAC124_AGT129del (508_525delGACAGTAAAGGCAAGAGT), AAA130ACA (527A>C), GTA131TTA (529G>T), GAG132TCA (532G>T 533A>C 534G>A), CAA133GAA (535C>G), CCC134ACT (538C>A 540C>T), ACA135GCA (541A>G), CGA136_GAG137del (544_549delCGAGAG), GAT138AGT (550G>A 551A>G), CTC139TTA (553C>T 555C>A), ATT140AGC (557T>G 558T>C), AAA141GGA (559A>G 560A>G), GAA142GAG (564A>G), GTT143AAC (565G>A 566T>A 567T>C), GAG144AAG (568G>A), GTT145GCA (572T>C 573T>A), CTC146CTT (576C>T), AAA147AAG (579A>G), CGT148GAA (580C>G 581G>A 582T>A), GAG149GCC (584A>C 585G>C), TTA150CTT (586T>C 588A>T), GAG151GAA (591G>A), AGA152GTT (592A>G 593G>T 594A>T), TTT153GCC (595T>G 596T>C 597T>C), CAG154AGA (598C>A 599A>G 600G>A), GAG156GAA (606G>A), TTG157CTG (607T>C), GCA158CAA (610G>C 611C>A), AGT159GAA (613A>G 614G>A 615T>A), CAA160GCA (616C>G 617A>C), AAA161CGG (619A>C 620A>G 621A>G), AAT164ATT (629A>T), CAG165GTC (631C>G 632A>T 633G>C), CTA167CTC (639A>C), CAA168AGG (640C>A 641A>G 642A>G), CTT169TTG (643C>T 645T>G), CTC171ATT (649C>A 651C>T), TCC172CAA (652T>C 653C>A 654C>A), AAT173GGT (655A>G 656A>G), GTT175CCA (661G>C 662T>C 663T>A), AGT176AGC (666T>C), AAT177AAC (669T>C), AAT178GAG (670A>G 672T>G), GAC179GAT (675C>T), ATC180GTC (676A>G), TTC181TTT (681C>T), TCA182GCA (682T>G), GGT183GGC (687T>C), ACT185ACG (693T>G), GAA186AGT (694G>A 695A>G 696A>T), AGT187GAT (697A>G 698G>A), GGG188GGC (702G>C), CAG190AAG (706C>A), TAA191TGA (710A>G)								
ToMarV_RNA2gp2	1	1192	100%	6009	73.0%	1185 (98.7%)	820 (68.3%)	9/7/0/0	1
Protein mutations:	M1R (672T>G), T2M (675C>T 676A>G), S4L (681C>T 682T>G), V6A (687T>C), L8R (693T>G 694G>A), K9V (695A>G 696A>T 697A>G), V10M (698G>A), G11A (702G>C), N14D (710A>G 712T>C), H15R (714A>G), Q16S (716C>T 717A>C 718G>A), Q17del (719_721delCAG), E18K (722G>A 724G>A), A20V (729C>T 730C>A), E22D (736G>C), R23K (738G>A 739G>A), A34E (771C>A 772C>A), R41K (792G>A), V50I (818G>A 820T>A), G53A (828G>C 829T>A), E61D (853G>T), F63Y (858T>A), I71V (881A>G 883C>G), T72A (884A>G), T77A (899A>G 901A>T), H80R (908C>A 909A>G 910T>G), K81T (912A>C 913G>C), F82I (914T>A 916T>A), V83T (917G>A 918T>C 919T>A), K86R (926A>C 927A>G 928G>T), A89G (936C>G 937A>G), Y96H (956T>C 958T>C), V106A (987T>C 988T>A), S108N (993G>A), R112A (1004A>G 1005G>C 1006G>C), L124T (1040C>A 1041T>C 1042C>T), S125D (1043T>G 1044C>A 1045A>T), T132I (1065C>T 1066A>T), S134Y (1071C>A), L135F (1075G>T), A136S (1076G>T 1078C>T), D137G (1080A>G 1081T>A), Q166L (1167A>T 1168A>C), D185S (1223G>A 1224A>G 1225C>T), T186P (1226A>C 1228A>T), S192T (1244T>A), Q193D (1247C>G 1249G>C), Q194H (1252A>T), E195D (1255A>C), S226N (1347G>A), G227_Q228insV (1351_1352insGTA), S229R (1357T>A), T230S (1359C>G), M232L (1364A>C), A233del (1367_1369delGCT), K237R (1380A>G), A238P (1382G>C 1384C>T), E242A (1395A>C 1396A>C), Q243A (1397C>G 1398A>C 1399G>A), G246D (1407G>A 1408C>T), D250N (1418G>A 1420C>T), M252P (1424A>C 1425T>C), N253Q (1427A>C 1429T>G), S255A (1433A>G 1434G>C 1435C>A), T256N (1437C>A), I258V (1442A>G 1444C>G), K273R (1488A>G), P276A (1496C>G 1498G>A), P281_A282insDF (1513_1514insGACTTT), V283S (1517G>T 1518T>C), S284T (1521G>C), T285S (1523A>T 1525A>T), S286R (1526T>A 1527C>G), S287A (1529T>G 1531C>G), L288Y (1533T>A 1534A>T), L289Q (1536T>A 1537C>G), S290G (1538A>G 1540C>A), T291V (1541A>G 1542C>T 1543A>G), V296G (1557T>G), H297I (1559C>A 1560A>T), E299del (1565_1567delGAG), C302T (1574T>A 1575G>C 1576T>G), G303S (1577G>T 1578G>C 1579G>T), G304L (1580G>C 1581G>T), P309_K310del (1595_1600delCCCAAG), K312I (1605A>T 1606G>A), G313S (1607G>T 1608G>C 1609C>A), F316G (1616T>G 1617T>G 1618C>A), N317T (1620A>C 1621C>A), M318L (1622A>C 1624G>C), G319Q (1625G>C 1626G>A 1627T>A), A320E (1629C>A 1630C>A), I321V (1631A>G 1633T>C), L325Y (1644T>A 1645G>T), M327I (1651G>T), E328Q (1652G>C 1654G>A), A331E (1662C>A), H332D (1664C>G 1666C>T), I333F (1667A>T), D334A (1671A>C 1672T>C), D337S (1679G>A 1680A>G), D338N (1682G>A 1684T>C), E341A (1692A>C 1693A>C), R342L (1695G>T 1696A>T), I344V (1700A>G 1702A>G), A345P (1703G>C 1705T>C), Q346E (1706C>G), V348I (1712G>A 1714A>T), Q349D (1715C>G 1717A>T), F350Y (1719T>A), I351V (1721A>G 1723C>T), F353V (1727T>G 1729T>G), S354Q (1730A>C 1731G>A 1732T>G), E355K (1733G>A), K357R (1740A>G), R358A (1742A>G 1743G>C 1744A>T), V361L (1751G>C 1753T>C), I362C (1754A>T 1755T>G), R365K (1764G>A), V367I (1769G>A), A368E (1773C>A 1774A>G), K369M (1776A>T), H371D (1781C>G 1783T>C), N374C (1790A>T 1791A>G), R377F (1799A>T 1800G>T 1801G>T), D383A (1818A>C), E387D (1831A>T), E390A (1839A>C 1840A>G), N396K (1858T>G), I397V (1859A>G 1861C>T), Q398G (1862C>G 1863A>G 1864A>G), G399M (1865G>A 1866G>T 1867C>G), T401E (1871A>G 1872C>A 1873T>A), F404P (1880T>C 1881T>C 1882C>A), A408S (1892G>T), M409L (1895A>T), E413Q (1907G>C), Q421M (1931C>A 1932A>T 1933A>G), S425Q (1943T>C 1944C>A 1945C>A), I428R (1953T>G 1954T>G), Q429Y (1955C>T 1957A>T), G430W (1958G>T), Q431D (1961C>G 1963G>T), T432L (1964A>T 1965C>T 1966T>A), N433T (1968A>C 1969C>A), N438D (1982A>G 1984T>C), C441V (1991T>G 1992G>T 1993C>G), K448A (2012A>G 2013A>C), E449D (2017G>T), R450_Q451insVK (2020_2021insGTGAAA), Q451H (2023G>T), L452P (2024T>C 2025T>C 2026G>C), I453L (2027A>T 2029T>G), V454M (2030G>A), E455S (2033G>A 2034A>G 2035G>T), E456D (2038G>T), Q457L (2040A>T), V458A (2043T>C 2044G>A), A459C (2045G>T 2046C>G), Q460_R461insLN (2050_2051insTTGAAT), R462L (2054A>T 2055G>T), S463G (2057T>G 2058C>G 2059T>A), E464K (2060G>A), R465P (2063A>C 2064G>C), Q466S (2066C>A 2067A>G 2068A>C), A467M (2069G>A 2070C>T 2071A>G), Q468S (2072C>A 2073A>G 2074G>T), T470A (2078A>G), A471Q (2081G>C 2082C>A), R472T (2085G>C 2086G>T), I474N (2091T>A 2092A>T), A475_E476insS (2095_2096insAGC), E476K (2096G>A 2098A>G), S477W (2099A>T 2101T>G), P479T (2105C>A), D481G (2112A>G 2113C>A), R482K (2115G>A 2116A>G), T484L (2120A>C 2121C>T 2122T>G), S489T (2135T>A), T492A (2144A>G 2146G>A), M493L (2147A>C 2149G>T), E494_D495insS (2152_2153insAGT), D495E (2155T>G), P496G (2156C>G 2157C>G 2158C>T), T497I (2160C>T), K498Q (2162A>C 2164G>A), P499R (2165C>A 2166C>G), D500K (2168G>A 2170T>G), K501E (2171A>G), I502V (2174A>G 2176A>G), E503S (2177G>T 2178A>C), V505E (2184T>A 2185T>G), G508D (2193G>A 2194A>T), A509P (2195G>C 2197A>G), E511G (2202A>G 2203A>G), E512K (2204G>A), Q514E (2210C>G 2212A>G), G516N (2216G>A 2217G>A), V518I (2222G>A), I519F (2225A>T 2227T>C), P524E (2240C>G 2241C>A 2242A>G), D527M (2249G>A 2250A>T 2251C>G), T528G (2252A>G 2253C>G 2254A>T), S529D (2255T>G 2256C>A 2257C>T), M530A (2258A>G 2259T>C 2260G>C), A531P (2261G>C 2263A>G), V532L (2264G>T 2266T>A), E533N (2267G>A 2269A>T), D535E (2275T>G), M536L (2276A>T), V540A (2289T>C 2290G>A), D546E (2308T>G), V552I (2324G>A), V556L (2336G>C 2338T>G), E558K (2342G>A 2344G>A), A561N (2351G>A 2352C>A 2353T>C), M568L (2372A>C 2374G>T), S569A (2375A>G 2376G>C 2377C>A), A571S (2381G>T 2383T>C), A574M (2390G>A 2391C>T 2392T>G), S580K (2409G>A 2410C>G), T583A (2417A>G), A587S (2429G>T 2431G>C), L597N (2459C>A 2460T>A 2461A>C), I598L (2462A>C 2464C>T), Y600F (2469A>T 2470C>T), I602V (2474A>G 2476A>C), V606I (2486G>A), V609M (2495G>A 2497T>G), I612V (2504A>G 2506C>G), V625A (2544T>C 2545G>A), K629T (2556A>C 2557G>C), L638I (2582T>A 2584G>T), T644H (2600A>C 2601C>A 2602A>T), Q650E (2618C>G), N652S (2624A>T 2625A>C), E654G (2631A>G), E655K (2633G>A), C657V (2639T>G 2640G>T 2641C>G), T659S (2645A>T 2647C>A), T661Q (2651A>C 2652C>A), K665T (2664A>C 2665G>C), Y666F (2667A>T 2668C>T), E671T (2681G>A 2682A>C 2683A>T), G672S (2684G>T 2685G>C 2686T>A), T673L (2687A>C 2688C>T 2689A>G), A675V (2694C>T 2695C>T), T676P (2696A>C), D677E (2701T>A), S690A (2738T>G 2740T>A), N696E (2756A>G 2758T>G), V698I (2762G>A), L702I (2774T>A 2776G>A), Q705S (2783C>T 2784A>C 2785G>C), L714M (2810C>A 2812T>G), M719L (2825A>T), I720R (2829T>G), A721G (2832C>G 2833A>G), Y725F (2844A>T), K728R (2852A>C 2853A>G), A729P (2855G>C 2857G>A), Q731T (2861C>A 2862A>C 2863G>A), Y733F (2868A>T 2869T>C), P735S (2873C>A 2874C>G 2875A>T), G749S (2915G>A 2917G>T), T750V (2918A>G 2919C>T), S751A (2921T>G 2923A>T), S752A (2924T>G 2926C>A), P753A (2927C>G 2929A>C), I761V (2951A>G 2953A>C), T773I (2988C>T), T790R (3039C>G 3040T>A), I797V (3059A>G 3061T>G), I799V (3065A>G), N817A (3119A>G 3120A>C 3121C>A), K819E (3125A>G 3127A>G), T821S (3131A>T), N822T (3135A>C 3136T>A), F827L (3151T>G), K841R (3192A>G 3193A>G), R843Q (3198G>A 3199G>A), R845K (3203C>A 3204G>A 3205G>A), I848L (3212A>C 3214T>G), K854C (3230A>T 3231A>G 3232A>C), Q869K (3275C>A), F871Q (3281T>C 3282T>A 3283T>A), L874M (3290T>A), I889L (3335A>T 3337A>G), S893T (3347T>A 3349C>T), C902R (3374T>C), L903I (3377C>A), N905D (3383A>G), T911S (3401A>T 3403A>T), K925R (3444A>G), S927A (3449T>G 3451A>T), D936N (3476G>A 3478T>C), S938N (3482T>A 3483C>A 3484A>T), I943L (3497A>C), D949E (3517C>A), I951V (3521A>G), E952D (3526G>T), E953I (3527G>A 3528A>T 3529G>C), Y954F (3531A>T 3532C>T), Q955D (3533C>G 3535G>C), E956Q (3536G>C), D960_P961del (3548_3553delGATCCG), P962S (3554C>A 3555C>G), K964T (3561A>C 3562G>A), A965S (3563G>T), S967V (3569A>G 3570G>T), I968S (3572A>T 3573T>C 3574T>C), L969F (3575C>T 3577G>C), S970Q (3578T>C 3579C>A 3580C>G), S976T (3596T>A 3598C>A), M985A (3623A>G 3624T>C 3625G>T), G986A (3627G>C 3628T>A), D990T (3638G>A 3639A>C 3640C>A), E991D (3643A>C), L994V (3650T>G 3652G>A), I996L (3656A>T 3658T>G), I1003V (3677A>G 3679A>G), E1006D (3688A>T), G1007A (3690G>C 3691G>A), H1008S (3692C>T 3693A>C), V1011I (3701G>A), T1024S (3740A>T 3742A>C), N1034E (3770A>G 3772T>A), S1036T (3776T>A), I1037F (3779A>T), I1039M (3787A>G), V1042T (3794G>A 3795T>C 3796A>T), P1046S (3806C>A 3807C>G 3808T>C), N1047S (3810A>G), V1048I (3812G>A 3814T>A), V1051I (3821G>A 3823A>C), V1054I (3830G>A), F1056L (3836T>C), A1058T (3842G>A 3844C>A), P1064Q (3861C>A), A1065F (3863G>T 3864C>T), L1067V (3869C>G 3871T>A), N1068S (3872A>T 3873A>C), K1069A (3875A>G 3876A>C 3877A>G), N1071A (3881A>G 3882A>C 3883T>A), Y1072H (3884T>C), V1074L (3890G>T), A1089T (3935G>A 3937A>C), T1092I (3945C>T 3946G>C), T1096V (3956A>G 3957C>T 3958T>A), E1101Q (3971G>C), H1107F (3989C>T 3990A>T 3991T>C), R1109K (3996G>A), M1110A (3998A>G 3999T>C 4000G>C), R1111K (4002G>A 4003G>A), E1112S (4004G>T 4005A>C 4006A>C), F1113Y (4008T>A 4009C>T), S1114D (4010T>G 4011C>A 4012A>T), S1115K (4014G>A 4015C>G), K1116E (4016A>G 4018G>A), Q1117S (4019C>A 4020A>G 4021A>T), M1121V (4031A>G 4033G>T), R1126K (4047G>A), N1129L (4055A>T 4056A>T 4057T>G), I1131L (4061A>C 4063C>A), S1137V (4079T>G 4080C>T 4081C>T), A1138E (4083C>A 4084C>A), I1139L (4085A>C 4087A>T), V1140I (4088G>A 4090G>T), L1146I (4106C>A 4108T>A), I1147V (4109A>G 4111A>C), S1148K (4112T>A 4113C>A 4114T>G), L1151P (4122T>C), S1162L (4154T>C 4155C>T), H1165N (4163C>A), N1171D (4181A>G), I1191N (4242T>A)								

	Begin	End	Coverage	Score	Concordance	Matches	Identities	I/D/M/F*	Stop Codons
**NT**	**89**	**4744**	**94.9%**	**2600**	**28.6%**	**4608 (96.7%)**	**3040 (63.8%)**	**107/48**	

Codon mutations:

ATG1AGG (672T>G), ACA2ATG (675C>T 676A>G), TCT4TTG (681C>T 682T>G), GTT6GCT (687T>C), CTG8CGA (693T>G 694G>A), AAA9GTG (695A>G 696A>T 697A>G), GTG10ATG (698G>A), GGC11GCC (702G>C), CCC12CCA (706C>A), AAT14GAC (710A>G 712T>C), CAC15CGC (714A>G), CAG16TCA (716C>T 717A>C 718G>A), CAG17del (719_721delCAG), GAG18AAA (722G>A 724G>A), GCC20GTA (729C>T 730C>A), TCC21TCT (733C>T), GAG22GAC (736G>C), AGG23AAA (738G>A 739G>A), ATA25ATT (745A>T), TCA26TCT (748A>T), CAA27CAG (751A>G), ATA28ATC (754A>C), GCT30GCG (760T>G), GTG32GTT (766G>T), GAG33GAA (769G>A), GCC34GAA (771C>A 772C>A), GGA35GGT (775A>T), AAT38AAC (784T>C), TTG39CTT (785T>C 787G>T), CTC40CTT (790C>T), AGA41AAA (792G>A), AAG42AAA (796G>A), CTT43TTG (797C>T 799T>G), GGC44GGA (802C>A), TCC47TCA (811C>A), TAC48TAT (814C>T), GGG49GGC (817G>C), GTT50ATA (818G>A 820T>A), CTT51TTG (821C>T 823T>G), GGT53GCA (828G>C 829T>A), CAA55CAG (835A>G), AAG57AAA (841G>A), AGA58AGG (844A>G), GAG61GAT (853G>T), TTG62CTT (854T>C 856G>T), TTT63TAT (858T>A), GAC64GAT (862C>T), GTA68GTT (874A>T), TCA69AGT (875T>A 876C>G 877A>T), AAG70AAA (880G>A), ATC71GTG (881A>G 883C>G), ACA72GCA (884A>G), CTT74CTA (892T>A), ACA77GCT (899A>G 901A>T), TTC78TTT (904C>T), CAT80AGG (908C>A 909A>G 910T>G), AAG81ACC (912A>C 913G>C), TTT82ATA (914T>A 916T>A), GTT83ACA (917G>A 918T>C 919T>A), GAG84GAA (922G>A), TCT85TCA (925T>A), AAG86CGT (926A>C 927A>G 928G>T), GAC87GAT (931C>T), GCA89GGG (936C>G 937A>G), AAC90AAT (940C>T), TTG91TTA (943G>A), TTT93TTC (949T>C), CTG95TTA (953C>T 955G>A), TAT96CAC (956T>C 958T>C), GGT97GGG (961T>G), CTT99TTG (965C>T 967T>G), TTC100TTT (970C>T), TTC101TTT (973C>T), GTC103GTT (979C>T), CCA104CCC (982A>C), GTT106GCA (987T>C 988T>A), AGT108AAT (993G>A), GGG109GGA (997G>A), GGA111GGT (1003A>T), AGG112GCC (1004A>G 1005G>C 1006G>C), GTT113GTC (1009T>C), AAG114AAA (1012G>A), ATA115ATC (1015A>C), AGT116AGC (1018T>C), CTT117TTG (1019C>T 1021T>G), TGT118TGC (1024T>C), TCA119TCT (1027A>T), AGT120AGC (1030T>C), AAT121AAC (1033T>C), CTC124ACT (1040C>A 1041T>C 1042C>T), TCA125GAT (1043T>G 1044C>A 1045A>T), CCG126CCT (1048G>T), CAA129CAG (1057A>G), AAG131AAA (1063G>A), ACA132ATT (1065C>T 1066A>T), CTG133TTG (1067C>T), TCC134TAC (1071C>A), TTG135TTT (1075G>T), GCC136TCT (1076G>T 1078C>T), GAT137GGA (1080A>G 1081T>A), GCG139GCA (1087G>A), CAG140CAA (1090G>A), GCA141GCC (1093A>C), CTC143CTA (1099C>A), AGC145AGT (1105C>T), CCC146CCT (1108C>T), AGC147AGT (1111C>T), CTC153TTG (1127C>T 1129C>G), AAA154AAG (1132A>G), AGA155AGG (1135A>G), GGG156GGC (1138G>C), ACC162ACA (1156C>A), GAG164GAA (1162G>A), TGC165TGT (1165C>T), CAA166CTC (1167A>T 1168A>C), ACC168ACG (1174C>G), AGG169AGA (1177G>A), GCA170GCT (1180A>T), CAG171CAA (1183G>A), CCG173CCT (1189G>T), TCC175TCT (1195C>T), GTG176GTA (1198G>A), GCC178GCA (1204C>A), ATA179ATT (1207A>T), GAC185AGT (1223G>A 1224A>G 1225C>T), ACA186CCT (1226A>C 1228A>T), AGG187CGT (1229A>C 1231G>T), GCA189GCT (1237A>T), GTT190GTA (1240T>A), TCA192ACA (1244T>A), CAG193GAC (1247C>G 1249G>C), CAA194CAT (1252A>T), GAA195GAC (1255A>C), ACA196ACT (1258A>T), TCT198AGT (1262T>A 1263C>G), GCC200GCT (1270C>T), ATA201ATT (1273A>T), GAA202GAG (1276A>G), GCA203GCT (1279A>T), CTC204CTT (1282C>T), AAT205AAC (1285T>C), CGT206AGA (1286C>A 1288T>A), CCG207CCT (1291G>T), CAA208CAG (1294A>G), GAT212GAC (1306T>C), AGA213AGG (1309A>G), GAG215GAA (1315G>A), GCT216GCA (1318T>A), GCT217GCA (1321T>A), TAT219TAC (1327T>C), ATA220ATT (1330A>T), GCT221GCA (1333T>A), GTT223GTG (1339T>G), TAT224TAC (1342T>C), TCG225TCC (1345G>C), AGT226AAT (1347G>A), GGG227_CAA228insGTA (1351_1352insGTA), CAA228CAG (1354A>G), AGT229AGA (1357T>A), ACT230AGT (1359C>G), CAA231CAG (1363A>G), ATG232CTG (1364A>C), GCT233del (1367_1369delGCT), CTT234TTG (1370C>T 1372T>G), GAA235GAG (1375A>G), AAA237AGA (1380A>G), GCC238CCT (1382G>C 1384C>T), GTT240GTG (1390T>G), GGA241GGT (1393A>T), GAA242GCC (1395A>C 1396A>C), CAG243GCA (1397C>G 1398A>C 1399G>A), GGG245GGT (1405G>T), GGC246GAT (1407G>A 1408C>T), ACA247ACC (1411A>C), GAC250AAT (1418G>A 1420C>T), GTC251GTG (1423C>G), ATG252CCG (1424A>C 1425T>C), AAT253CAG (1427A>C 1429T>G), GAA254GAG (1432A>G), AGC255GCA (1433A>G 1434G>C 1435C>A), ACT256AAT (1437C>A), ATC258GTG (1442A>G 1444C>G), CGG259CGC (1447G>C), AGT260TCT (1448A>T 1449G>C), TCT262TCA (1456T>A), AGA264AGG (1462A>G), ACT267ACC (1471T>C), CTC268CTA (1474C>A), AAA269AAG (1477A>G), GTG270GTA (1480G>A), AAG273AGG (1488A>G), CGC274CGA (1492C>A), CCG276GCA (1496C>G 1498G>A), TCC277TCA (1501C>A), CTG280TTG (1508C>T), CCG281CCT (1513G>T), CCG281_GCT282insGACTTT (1513_1514insGACTTT), GTA283TCA (1517G>T 1518T>C), AGT284ACT (1521G>C), ACA285TCT (1523A>T 1525A>T), TCA286AGA (1526T>A 1527C>G), TCC287GCG (1529T>G 1531C>G), TTA288TAT (1533T>A 1534A>T), CTC289CAG (1536T>A 1537C>G), AGC290GGA (1538A>G 1540C>A), ACA291GTG (1541A>G 1542C>T 1543A>G), CGT292AGA (1544C>A 1546T>A), GAA293GAG (1549A>G), ACA295ACT (1555A>T), GTT296GGT (1557T>G), CAT297ATT (1559C>A 1560A>T), GAG299del (1565_1567delGAG), TGT302ACG (1574T>A 1575G>C 1576T>G), GGG303TCT (1577G>T 1578G>C 1579G>T), GGA304CTA (1580G>C 1581G>T), TTG305CTG (1583T>C), CCA307CCT (1591A>T), GCA308GCC (1594A>C), CCC309_AAG310del (1595_1600delCCCAAG), AAA311AAG (1603A>G), AAG312ATA (1605A>T 1606G>A), GGC313TCA (1607G>T 1608G>C 1609C>A), GCC315GCA (1615C>A), TTC316GGA (1616T>G 1617T>G 1618C>A), AAC317ACA (1620A>C 1621C>A), ATG318CTC (1622A>C 1624G>C), GGT319CAA (1625G>C 1626G>A 1627T>A), GCC320GAA (1629C>A 1630C>A), ATT321GTC (1631A>G 1633T>C), GAT323GAC (1639T>C), TTG325TAT (1644T>A 1645G>T), ATG327ATT (1651G>T), GAG328CAA (1652G>C 1654G>A), TCG329AGT (1655T>A 1656C>G 1657G>T), TTC330TTT (1660C>T), GCA331GAA (1662C>A), CAC332GAT (1664C>G 1666C>T), ATT333TTT (1667A>T), GAT334GCC (1671A>C 1672T>C), GAT337AGT (1679G>A 1680A>G), GAT338AAC (1682G>A 1684T>C), GAA341GCC (1692A>C 1693A>C), CGA342CTT (1695G>T 1696A>T), ATA344GTG (1700A>G 1702A>G), GCT345CCC (1703G>C 1705T>C), CAG346GAG (1706C>G), CAG347CAA (1711G>A), GTA348ATT (1712G>A 1714A>T), CAA349GAT (1715C>G 1717A>T), TTT350TAT (1719T>A), ATC351GTT (1721A>G 1723C>T), CTC352CTT (1726C>T), TTT353GTG (1727T>G 1729T>G), AGT354CAG (1730A>C 1731G>A 1732T>G), GAG355AAG (1733G>A), AAA357AGA (1740A>G), AGA358GCT (1742A>G 1743G>C 1744A>T), GGA359GGT (1747A>T), AAC360AAT (1750C>T), GTT361CTC (1751G>C 1753T>C), ATT362TGT (1754A>T 1755T>G), GTG363GTC (1759G>C), AGA365AAA (1764G>A), GTT367ATT (1769G>A), GCA368GAG (1773C>A 1774A>G), AAG369ATG (1776A>T), CGG370AGG (1778C>A), CAT371GAC (1781C>G 1783T>C), CTG372TTG (1784C>T), CAT373CAC (1789T>C), AAT374TGT (1790A>T 1791A>G), ATC375ATA (1795C>A), AGG377TTT (1799A>T 1800G>T 1801G>T), GAG378GAA (1804G>A), CAC379CAT (1807C>T), ATA380ATC (1810A>C), ACC381ACT (1813C>T), GAC383GCC (1818A>C), GTA386GTT (1828A>T), GAA387GAT (1831A>T), ATA388ATT (1834A>T), CTT389TTG (1835C>T 1837T>G), GAA390GCG (1839A>C 1840A>G), GGT391GGA (1843T>A), GGT393GGA (1849T>A), AAT396AAG (1858T>G), ATC397GTT (1859A>G 1861C>T), CAA398GGG (1862C>G 1863A>G 1864A>G), GGC399ATG (1865G>A 1866G>T 1867C>G), ACT401GAA (1871A>G 1872C>A 1873T>A), CGC402AGA (1874C>A 1876C>A), ACT403ACA (1879T>A), TTC404CCA (1880T>C 1881T>C 1882C>A), TAT407TAC (1891T>C), GCT408TCT (1892G>T), ATG409TTG (1895A>T), TCT410AGC (1898T>A 1899C>G 1900T>C), CTC411CTG (1903C>G), GAG413CAG (1907G>C), GTT415GTG (1915T>G), GTG416GTT (1918G>T), CTC418CTA (1924C>A), CAT420CAC (1930T>C), CAA421ATG (1931C>A 1932A>T 1933A>G), AGA422AGG (1936A>G), GAT423GAC (1939T>C), TCC425CAA (1943T>C 1944C>A 1945C>A), TTC426TTT (1948C>T), ATT428AGG (1953T>G 1954T>G), CAA429TAT (1955C>T 1957A>T), GGG430TGG (1958G>T), CAG431GAT (1961C>G 1963G>T), ACT432TTA (1964A>T 1965C>T 1966T>A), AAC433ACA (1968A>C 1969C>A), AAT434AAC (1972T>C), CCA435CCG (1975A>G), CCG437CCT (1981G>T), AAT438GAC (1982A>G 1984T>C), TGC441GTG (1991T>G 1992G>T 1993C>G), GGA444GGT (2002A>T), TTA445CTT (2003T>C 2005A>T), ACC446ACA (2008C>A), TTG447CTG (2009T>C), AAA448GCA (2012A>G 2013A>C), GAG449GAT (2017G>T), CGT450AGG (2018C>A 2020T>G), CGT450_CAG451insGTGAAA (2020_2021insGTGAAA), CAG451CAT (2023G>T), TTG452CCC (2024T>C 2025T>C 2026G>C), ATT453TTG (2027A>T 2029T>G), GTG454ATG (2030G>A), GAG455AGT (2033G>A 2034A>G 2035G>T), GAG456GAT (2038G>T), CAA457CTA (2040A>T), GTG458GCA (2043T>C 2044G>A), GCC459TGC (2045G>T 2046C>G), CAA460_AGA461insTTGAAT (2050_2051insTTGAAT), AGA461CGC (2051A>C 2053A>C), AGG462TTG (2054A>T 2055G>T), TCT463GGA (2057T>G 2058C>G 2059T>A), GAA464AAA (2060G>A), AGA465CCA (2063A>C 2064G>C), CAA466AGC (2066C>A 2067A>G 2068A>C), GCA467ATG (2069G>A 2070C>T 2071A>G), CAG468AGT (2072C>A 2073A>G 2074G>T), ACA470GCA (2078A>G), GCA471CAA (2081G>C 2082C>A), AGG472ACT (2085G>C 2086G>T), GGC473GGA (2089C>A), ATA474AAT (2091T>A 2092A>T), GCT475GCC (2095T>C), GCT475_GAA476insAGC (2095_2096insAGC), GAA476AAG (2096G>A 2098A>G), AGT477TGG (2099A>T 2101T>G), CAG478CAA (2104G>A), CCT479ACT (2105C>A), GAC481GGA (2112A>G 2113C>A), AGA482AAG (2115G>A 2116A>G), ACT484CTG (2120A>C 2121C>T 2122T>G), TCC486TCA (2128C>A), GTG488GTT (2134G>T), TCA489ACA (2135T>A), ACT490ACC (2140T>C), ACC491ACA (2143C>A), ACG492GCA (2144A>G 2146G>A), ATG493CTT (2147A>C 2149G>T), GAA494GAG (2152A>G), GAA494_GAT495insAGT (2152_2153insAGT), GAT495GAG (2155T>G), CCC496GGT (2156C>G 2157C>G 2158C>T), ACC497ATC (2160C>T), AAG498CAA (2162A>C 2164G>A), CCA499AGA (2165C>A 2166C>G), GAT500AAG (2168G>A 2170T>G), AAG501GAG (2171A>G), ATA502GTG (2174A>G 2176A>G), GAA503TCA (2177G>T 2178A>C), GTT505GAG (2184T>A 2185T>G), GCA506GCT (2188A>T), GAG507GAA (2191G>A), GGA508GAT (2193G>A 2194A>T), GCA509CCG (2195G>C 2197A>G), GAA511GGG (2202A>G 2203A>G), GAA512AAA (2204G>A), ACT513ACA (2209T>A), CAA514GAG (2210C>G 2212A>G), GGT516AAT (2216G>A 2217G>A), GAT517GAC (2221T>C), GTT518ATT (2222G>A), ATT519TTC (2225A>T 2227T>C), GAC521GAT (2233C>T), GGA523GGG (2239A>G), CCA524GAG (2240C>G 2241C>A 2242A>G), GAC527ATG (2249G>A 2250A>T 2251C>G), ACA528GGT (2252A>G 2253C>G 2254A>T), TCC529GAT (2255T>G 2256C>A 2257C>T), ATG530GCC (2258A>G 2259T>C 2260G>C), GCA531CCG (2261G>C 2263A>G), GTT532TTA (2264G>T 2266T>A), GAA533AAT (2267G>A 2269A>T), GAT535GAG (2275T>G), ATG536TTG (2276A>T), CAG537CAA (2281G>A), CAA538CAG (2284A>G), CCG539CCA (2287G>A), GTG540GCA (2289T>C 2290G>A), GTA542GTG (2296A>G), AGC544AGT (2302C>T), GAT546GAG (2308T>G), TTC547TTT (2311C>T), GGA551GGG (2323A>G), GTT552ATT (2324G>A), TTC555TTT (2335C>T), GTT556CTG (2336G>C 2338T>G), GAG558AAA (2342G>A 2344G>A), TCC560AGC (2348T>A 2349C>G), GCT561AAC (2351G>A 2352C>A 2353T>C), GAG565GAA (2365G>A), CAA566CAG (2368A>G), GTA567GTT (2371A>T), ATG568CTT (2372A>C 2374G>T), AGC569GCA (2375A>G 2376G>C 2377C>A), TTG570CTA (2378T>C 2380G>A), GCT571TCC (2381G>T 2383T>C), TTG572CTC (2384T>C 2386G>C), CCC573CCA (2389C>A), GCT574ATG (2390G>A 2391C>T 2392T>G), GCC575GCA (2395C>A), CTA576TTG (2396C>T 2398A>G), TTC577TTT (2401C>T), TCA578AGC (2402T>A 2403C>G 2404A>C), AAA579AAG (2407A>G), ACG580AAG (2409G>A 2410C>G), AAA581AAG (2413A>G), ACT583GCT (2417A>G), TCA584TCC (2422A>C), GGT586GGG (2428T>G), GCG587TCC (2429G>T 2431G>C), CTT590CTC (2440T>C), TAT592TAC (2446T>C), TAC593TAT (2449C>T), GAT594GAC (2452T>C), GCA595GCT (2455A>T), GCT596GCA (2458T>A), CTA597AAC (2459C>A 2460T>A 2461A>C), ATC598CTT (2462A>C 2464C>T), TAC600TTT (2469A>T 2470C>T), ATA602GTC (2474A>G 2476A>C), CTT604CTG (2482T>G), TAT605TAC (2485T>C), GTT606ATT (2486G>A), GTT609ATG (2495G>A 2497T>G), GCT611GCG (2503T>G), ATC612GTG (2504A>G 2506C>G), TCT613TCG (2509T>G), GGT614GGA (2512T>A), CAA615CAG (2515A>G), CTG616CTA (2518G>A), GCT617GCC (2521T>C), GAA622GAG (2536A>G), TGT623TGC (2539T>C), GTG625GCA (2544T>C 2545G>A), CGA628AGA (2552C>A), AAG629ACC (2556A>C 2557G>C), AAC634AAT (2572C>T), ATC635ATA (2575C>A), ACA637ACC (2581A>C), TTG638ATT (2582T>A 2584G>T), AAG642AAA (2596G>A), ACA644CAT (2600A>C 2601C>A 2602A>T), TTG645CTG (2603T>C), GTT646GTG (2608T>G), CAA650GAA (2618C>G), AAC652TCC (2624A>T 2625A>C), GAG654GGG (2631A>G), GAG655AAG (2633G>A), TGC657GTG (2639T>G 2640G>T 2641C>G), TTT658TTC (2644T>C), ACC659TCA (2645A>T 2647C>A), CCA660CCT (2650A>T), ACA661CAA (2651A>C 2652C>A), GGG662GGT (2656G>T), GGC664GGT (2662C>T), AAG665ACC (2664A>C 2665G>C), TAC666TTT (2667A>T 2668C>T), GTG667GTA (2671G>A), CCT668CCA (2674T>A), CTG669TTG (2675C>T), GAA671ACT (2681G>A 2682A>C 2683A>T), GGT672TCA (2684G>T 2685G>C 2686T>A), ACA673CTG (2687A>C 2688C>T 2689A>G), GGA674GGT (2692A>T), GCC675GTT (2694C>T 2695C>T), ACT676CCT (2696A>C), GAT677GAA (2701T>A), TTA678CTT (2702T>C 2704A>T), GGT679GGC (2707T>C), GTG681GTT (2713G>T), ACA686ACC (2728A>C), CAC687CAT (2731C>T), TTA689TTG (2737A>G), TCT690GCA (2738T>G 2740T>A), GCA692GCC (2746A>C), GAG694GAA (2752G>A), AAT696GAG (2756A>G 2758T>G), AGT697TCA (2759A>T 2760G>C 2761T>A), GTA698ATA (2762G>A), CCA699CCT (2767A>T), TGC700TGT (2770C>T), CAT701CAC (2773T>C), TTG702ATA (2774T>A 2776G>A), TTA704CTA (2780T>C), CAG705TCC (2783C>T 2784A>C 2785G>C), AAA707AAG (2791A>G), TTA709CTC (2795T>C 2797A>C), ACC711ACA (2803C>A), CTT714ATG (2810C>A 2812T>G), CGA718AGG (2822C>A 2824A>G), ATG719TTG (2825A>T), ATA720AGA (2829T>G), GCA721GGG (2832C>G 2833A>G), CAG722CAA (2836G>A), CAA724CAG (2842A>G), TAT725TTT (2844A>T), GGC726GGA (2848C>A), AAG728CGG (2852A>C 2853A>G), GCG729CCA (2855G>C 2857G>A), GGG730GGC (2860G>C), CAG731ACA (2861C>A 2862A>C 2863G>A), ACA732ACC (2866A>C), TAT733TTC (2868A>T 2869T>C), CCA735AGT (2873C>A 2874C>G 2875A>T), AGG736CGC (2876A>C 2878G>C), TTT737TTC (2881T>C), CCA738CCT (2884A>T), ACT739ACA (2887T>A), GTT742GTG (2896T>G), TTG743CTT (2897T>C 2899G>T), TTA744CTG (2900T>C 2902A>G), CAT745CAC (2905T>C), TAT746TAC (2908T>C), GGG749AGT (2915G>A 2917G>T), ACA750GTA (2918A>G 2919C>T), TCA751GCT (2921T>G 2923A>T), TCC752GCA (2924T>G 2926C>A), CCA753GCC (2927C>G 2929A>C), ACA757ACT (2941A>T), AGC760AGT (2950C>T), ATA761GTC (2951A>G 2953A>C), TTT762TTC (2956T>C), TCA763TCC (2959A>C), ATA767ATT (2971A>T), GAG769GAA (2977G>A), GGC772GGT (2986C>T), ACC773ATC (2988C>T), TTG774TTA (2992G>A), CCG776CCA (2998G>A), TCT777TCA (3001T>A), TTG778CTG (3002T>C), GGT780GGA (3010T>A), ATA782ATT (3016A>T), GCC783GCA (3019C>A), AGG784AGA (3022G>A), AAG787AAA (3031G>A), ACT790AGA (3039C>G 3040T>A), GGA791GGC (3043A>C), ACT792ACC (3046T>C), ATT797GTG (3059A>G 3061T>G), TGC798TGT (3064C>T), ATT799GTT (3065A>G), ACC802ACT (3076C>T), TTG803CTG (3077T>C), TCA806TCT (3088A>T), GGT807GGG (3091T>G), CTG809CTT (3097G>T), GCA810GCT (3100A>T), ATT811ATA (3103T>A), GGA812GGG (3106A>G), CTT813TTG (3107C>T 3109T>G), GGA814GGC (3112A>C), ACT815ACG (3115T>G), AAC817GCA (3119A>G 3120A>C 3121C>A), ACC818ACA (3124C>A), AAA819GAG (3125A>G 3127A>G), ACC821TCC (3131A>T), AAT822ACA (3135A>C 3136T>A), GCT823GCG (3139T>G), TTT827TTG (3151T>G), CCA830CCC (3160A>C), GTA833GTG (3169A>G), TGC834TGT (3172C>T), CTT836TTG (3176C>T 3178T>G), GGT839GGG (3187T>G), AAA841AGG (3192A>G 3193A>G), CGG843CAA (3198G>A 3199G>A), CGG845AAA (3203C>A 3204G>A 3205G>A), TGT846TGC (3208T>C), TCT847TCA (3211T>A), ATT848CTG (3212A>C 3214T>G), ACA849ACC (3217A>C), GGA853GGG (3229A>G), AAA854TGC (3230A>T 3231A>G 3232A>C), AAT855AAC (3235T>C), TTG856CTC (3236T>C 3238G>C), CTT857CTG (3241T>G), TCC858TCT (3244C>T), ACA859ACC (3247A>C), GGG860GGA (3250G>A), CGA861CGC (3253A>C), AAG862AAA (3256G>A), TCC864TCA (3262C>A), TTG865TTA (3265G>A), CAG869AAG (3275C>A), CAC870CAT (3280C>T), TTT871CAA (3281T>C 3282T>A 3283T>A), TCC872TCT (3286C>T), TTG874ATG (3290T>A), CGC875CGT (3295C>T), TTG876CTG (3296T>C), TTT877TTC (3301T>C), ACG879ACT (3307G>T), GTA880GTT (3310A>T), CTC884CTG (3322C>G), ACG887ACA (3331G>A), ATA889TTG (3335A>T 3337A>G), CAT890CAC (3340T>C), TCC893ACT (3347T>A 3349C>T), GGT895GGA (3355T>A), GTC896GTA (3358C>A), CAG899CAA (3367G>A), AAG901AAA (3373G>A), TGC902CGC (3374T>C), CTT903ATT (3377C>A), AAT905GAT (3383A>G), CTT906CTC (3388T>C), ACT907ACG (3391T>G), TTG908CTT (3392T>C 3394G>T), GGT909GGG (3397T>G), GGC910GGT (3400C>T), ACA911TCT (3401A>T 3403A>T), GTA912GTG (3406A>G), TCT913TCA (3409T>A), GTG914GTT (3412G>T), AAA915AAG (3415A>G), ATA917ATT (3421A>T), GGG919GGT (3427G>T), ACA922ACC (3436A>C), AAA923AAG (3439A>G), GGC924GGG (3442C>G), AAA925AGA (3444A>G), AGC926AGT (3448C>T), TCA927GCT (3449T>G 3451A>T), GTT928GTG (3454T>G), TTC930TTT (3460C>T),

	CTT931CTG (3463T>G), TTC932TTT (3466C>T), GAT936AAC (3476G>A 3478T>C), TTA937CTG (3479T>C 3481A>G), TCA938AAT (3482T>A 3483C>A 3484A>T), CGT940CGC (3490T>C), ATT943CTT (3497A>C), GAA944GAG (3502A>G), AAG945AAA (3505G>A), TTG946CTG (3506T>C), AAG948AAA (3514G>A), GAC949GAA (3517C>A), AAC950AAT (3520C>T), ATT951GTT (3521A>G), GAG952GAT (3526G>T), GAG953ATC (3527G>A 3528A>T 3529G>C), TAC954TTT (3531A>T 3532C>T), CAG955GAC (3533C>G 3535G>C), GAG956CAG (3536G>C), GGC958GGG (3544C>G), GAT960_CCG961del (3548_3553delGATCCG), CCC962AGC (3554C>A 3555C>G), AAG964ACA (3561A>C 3562G>A), GCT965TCT (3563G>T), AGT967GTT (3569A>G 3570G>T), ATT968TCC (3572A>T 3573T>C 3574T>C), CTG969TTC (3575C>T 3577G>C), TCC970CAG (3578T>C 3579C>A 3580C>G), ATA971ATC (3583A>C), AGA972AGG (3586A>G), GAG973GAA (3589G>A), AAA974AAG (3592A>G), TTT975TTC (3595T>C), TCC976ACA (3596T>A 3598C>A), GGT978GGA (3604T>A), GCT979GCA (3607T>A), GTA980GTG (3610A>G), TTC983TTT (3619C>T), TGC984TGT (3622C>T), ATG985GCT (3623A>G 3624T>C 3625G>T), GGT986GCA (3627G>C 3628T>A), GAC990ACA (3638G>A 3639A>C 3640C>A), GAA991GAC (3643A>C), TTG994GTA (3650T>G 3652G>A), GTA995GTT (3655A>T), ATT996TTG (3656A>T 3658T>G), CCT997CCA (3661T>A), GCA999GCC (3667A>C), CCA1000CCT (3670A>T), TCC1002TCA (3676C>A), ATA1003GTG (3677A>G 3679A>G), AGG1004CGC (3680A>C 3682G>C), GAA1006GAT (3688A>T), GGG1007GCA (3690G>C 3691G>A), CAC1008TCC (3692C>T 3693A>C), CCT1010CCA (3700T>A), GTT1011ATT (3701G>A), AAG1012AAA (3706G>A), ATC1015ATA (3715C>A), TGT1017TGC (3721T>C), TTT1019TTC (3727T>C), GAT1021GAC (3733T>C), ACA1024TCC (3740A>T 3742A>C), TCA1025TCT (3745A>T), TCA1030TCT (3760A>T), GGT1031GGA (3763T>A), AAT1034GAA (3770A>G 3772T>A), TCA1036ACA (3776T>A), ATT1037TTT (3779A>T), GTG1038GTA (3784G>A), ATA1039ATG (3787A>G), AGA1041CGA (3791A>C), GTA1042ACT (3794G>A 3795T>C 3796A>T), CAA1043CAG (3799A>G), TCC1044AGC (3800T>A 3801C>G), AGT1045TCC (3803A>T 3804G>C 3805T>C), CCT1046AGC (3806C>A 3807C>G 3808T>C), AAT1047AGT (3810A>G), GTT1048ATA (3812G>A 3814T>A), GGA1049GGT (3817A>T), GTA1051ATC (3821G>A 3823A>C), CTA1052TTG (3824C>T 3826A>G), AAT1053AAC (3829T>C), GTT1054ATT (3830G>A), GCT1055GCA (3835T>A), TTT1056CTT (3836T>C), GAT1057GAC (3841T>C), GCC1058ACA (3842G>A 3844C>A), GGC1060GGG (3850C>G), TTT1063TTC (3859T>C), CCA1064CAA (3861C>A), GCT1065TTT (3863G>T 3864C>T), GGG1066GGT (3868G>T), CTT1067GTA (3869C>G 3871T>A), AAT1068TCT (3872A>T 3873A>C), AAA1069GCG (3875A>G 3876A>C 3877A>G), AAT1071GCA (3881A>G 3882A>C 3883T>A), TAT1072CAT (3884T>C), GTA1074TTA (3890G>T), GGT1077GGC (3901T>C), GGA1078GGT (3904A>T), GGC1079GGA (3907C>A), ACA1080ACT (3910A>T), TCA1085TCC (3925A>C), TAC1086TAT (3928C>T), GGT1087GGG (3931T>G), GTG1088GTT (3934G>T), GCA1089ACC (3935G>A 3937A>C), ACA1090ACC (3940A>C), AAT1091AAC (3943T>C), ACG1092ATC (3945C>T 3946G>C), TCA1094TCT (3952A>T), TTC1095TTT (3955C>T), ACT1096GTA (3956A>G 3957C>T 3958T>A), GAG1101CAG (3971G>C), TTT1102TTC (3976T>C), TTC1103TTT (3979C>T), CCA1104CCT (3982A>T), AGG1105CGC (3983A>C 3985G>C), CGG1106CGT (3988G>T), CAT1107TTC (3989C>T 3990A>T 3991T>C), AGG1109AAG (3996G>A), ATG1110GCC (3998A>G 3999T>C 4000G>C), AGG1111AAA (4002G>A 4003G>A), GAA1112TCC (4004G>T 4005A>C 4006A>C), TTC1113TAT (4008T>A 4009C>T), TCA1114GAT (4010T>G 4011C>A 4012A>T), AGC1115AAG (4014G>A 4015C>G), AAG1116GAA (4016A>G 4018G>A), CAA1117AGT (4019C>A 4020A>G 4021A>T), TCC1118TCA (4024C>A), CGC1119AGA (4025C>A 4027C>A), ATC1120ATT (4030C>T), ATG1121GTT (4031A>G 4033G>T), TCA1122TCT (4036A>T), CTA1123TTG (4037C>T 4039A>G), GAT1125GAC (4045T>C), AGG1126AAG (4047G>A), CTT1127CTA (4051T>A), GGA1128GGC (4054A>C), AAT1129TTG (4055A>T 4056A>T 4057T>G), CTG1130TTG (4058C>T), ATC1131CTA (4061A>C 4063C>A), ATA1132ATC (4066A>C), TTG1134CTT (4070T>C 4072G>T), CCT1135CCA (4075T>A), CCT1136CCA (4078T>A), TCC1137GTT (4079T>G 4080C>T 4081C>T), GCC1138GAA (4083C>A 4084C>A), ATA1139CTT (4085A>C 4087A>T), GTG1140ATT (4088G>A 4090G>T), TCC1142TCA (4096C>A), GAG1144GAA (4102G>A), ATA1145ATC (4105A>C), CTT1146ATA (4106C>A 4108T>A), ATA1147GTC (4109A>G 4111A>C), TCT1148AAG (4112T>A 4113C>A 4114T>G), CCT1149CCA (4117T>A), GGA1150GGT (4120A>T), CTT1151CCT (4122T>C), TTC1153TTT (4129C>T), AAG1154AAA (4132G>A), GCC1158GCT (4144C>T), CCT1160CCC (4150T>C), CCT1161CCA (4153T>A), TCT1162CTT (4154T>C 4155C>T), GCC1163GCA (4159C>A), CAT1165AAT (4163C>A), GAA1166GAG (4168A>G), AAA1167AAG (4171A>G), CTT1169CTC (4177T>C), GGC1170GGA (4180C>A), AAT1171GAT (4181A>G), CAA1173CAG (4189A>G), ACT1174ACG (4192T>G), CAC1175CAT (4195C>T), ACC1176ACT (4198C>T), ACC1180ACA (4210C>A), TCA1181TCT (4213A>T), CTA1186TTG (4226C>T 4228A>G), CGT1187CGC (4231T>C), ATT1191AAT (4242T>A)

Proteins

hypothetical protein (YP_001976148.1)	1	191	100%	784	66.7%	183 (95.8%)	116 (60.7%)	0/8/0/0	1
Protein mutations:	G5S (151G>A 153G>T), R6K (154C>A 155G>A 156T>G), S10Q (166T>C 167C>A 168T>A), V11I (169G>A), D12E (174C>G), E13D (177A>T), A15E (182C>A 183T>A), H18N (190C>A), T22A (202A>G 204C>A), N24T (209A>C 210T>C), I26V (214A>G), V31T (229G>A 230T>C), T33V (235A>G 236C>T 237C>T), L35I (241T>A 243G>T), V46I (274G>A), V47C (277G>T 278T>G), V54I (298G>A 300C>T), T58S (310A>T 312C>T), L68V (340T>G 342A>T), N79T (374A>C 375T>G), P81D (379C>G 380C>A 381C>T), N84A (388A>G 389A>C 390C>A), L91I (409C>A), H93R (416A>G 417T>A), V96I (424G>A 426G>T), R97K (427C>A 428G>A 429T>A), V104L (448G>T 450C>G), E107A (458A>C 459A>C), S111H (469T>C 470C>A), A114S (478G>T 480A>T), S115T (481T>A 483A>C), P120N (496C>A 497C>A 498T>C), S121P (499T>C 501T>C), D124_S129del (508_525delGACAGTAAAGGCAAGAGT), K130T (527A>C), V131L (529G>T), E132S (532G>T 533A>C 534G>A), Q133E (535C>G), P134T (538C>A 540C>T), T135A (541A>G), R136_E137del (544_549delCGAGAG), D138S (550G>A 551A>G), I140S (557T>G 558T>C), K141G (559A>G 560A>G), V143N (565G>A 566T>A 567T>C), E144K (568G>A), V145A (572T>C 573T>A), R148E (580C>G 581G>A 582T>A), E149A (584A>C 585G>C), R152V (592A>G 593G>T 594A>T), F153A (595T>G 596T>C 597T>C), Q154R (598C>A 599A>G 600G>A), A158Q (610G>C 611C>A), S159E (613A>G 614G>A 615T>A), Q160A (616C>G 617A>C), K161R (619A>C 620A>G 621A>G), N164I (629A>T), Q165V (631C>G 632A>T 633G>C), Q168R (640C>A 641A>G 642A>G), L171I (649C>A 651C>T), S172Q (652T>C 653C>A 654C>A), N173G (655A>G 656A>G), V175P (661G>C 662T>C 663T>A), N178E (670A>G 672T>G), I180V (676A>G), S182A (682T>G), E186S (694G>A 695A>G 696A>T), S187D (697A>G 698G>A), Q190K (706C>A)								
Codon mutations:	TCA2TCT (144A>T), TTC3TTT (147C>T), ATT4ATC (150T>C), GGG5AGT (151G>A 153G>T), CGT6AAG (154C>A 155G>A 156T>G), TTG7CTC (157T>C 159G>C), AAT8AAC (162T>C), ACA9ACC (165A>C), TCT10CAA (166T>C 167C>A 168T>A), GTA11ATA (169G>A), GAC12GAG (174C>G), GAA13GAT (177A>T), GCT15GAA (182C>A 183T>A), TTT16TTC (186T>C), CAT18AAT (190C>A), GTT20GTA (198T>A), GCC21GCA (201C>A), ACC22GCA (202A>G 204C>A), TCG23TCT (207G>T), AAT24ACC (209A>C 210T>C), ATT26GTT (214A>G), TCT28AGT (220T>A 221C>G), GTT31ACT (229G>A 230T>C), GGC32GGT (234C>T), ACC33GTT (235A>G 236C>T 237C>T), TTG35ATT (241T>A 243G>T), ATA36ATT (246A>T), ACC41ACT (261C>T), CTT42TTA (262C>T 264T>A), GAC43GAT (267C>T), AAA45AAG (273A>G), GTT46ATT (274G>A), GTT47TGT (277G>T 278T>G), CCT48CCC (282T>C), GGT51GGA (291T>A), GTC54ATT (298G>A 300C>T), TCA55TCG (303A>G), GTT56GTG (306T>G), TTA57CTT (307T>C 309A>T), ACC58TCT (310A>T 312C>T), GTT59GTG (315T>G), TCT60AGT (316T>A 317C>G), GAG62GAA (324G>A), ACG65ACT (333G>T), CCA66CCC (336A>C), TTA68GTT (340T>G 342A>T), CCT70CCA (348T>A), GGA71GGC (351A>C), CAC72CAT (354C>T), TAT73TAC (357T>C), TTG74CTA (358T>C 360G>A), TTG75CTA (361T>C 363G>A), AAT79ACG (374A>C 375T>G), CCC81GAT (379C>G 380C>A 381C>T), ATT82ATC (384T>C), AAG83AAA (387G>A), AAC84GCA (388A>G 389A>C 390C>A), TCT88TCA (402T>A), GGT89GGC (405T>C), TTA90TTG (408A>G), CTT91ATT (409C>A), GTC92GTG (414C>G), CAT93CGA (416A>G 417T>A), TCA95TCC (423A>C), GTG96ATT (424G>A 426G>T), CGT97AAA (427C>A 428G>A 429T>A), CTT98TTG (430C>T 432T>G), ACC101ACA (441C>A), AGA102CGG (442A>C 444A>G), GTC104TTG (448G>T 450C>G), CTA105TTG (451C>T 453A>G), GAG106GAA (456G>A), GAA107GCC (458A>C 459A>C), AAC108AAT (462C>T), AAG109AAA (465G>A), GTT110GTG (468T>G), TCT111CAT (469T>C 470C>A), TCA113TCT (477A>T), GCA114TCT (478G>T 480A>T), TCA115ACC (481T>A 483A>C), TCA117TCT (489A>T), TCT118TCA (492T>A), TCT119TCC (495T>C), CCT120AAC (496C>A 497C>A 498T>C), TCT121CCC (499T>C 501T>C), GAC124_AGT129del (508_525delGACAGTAAAGGCAAGAGT), AAA130ACA (527A>C), GTA131TTA (529G>T), GAG132TCA (532G>T 533A>C 534G>A), CAA133GAA (535C>G), CCC134ACT (538C>A 540C>T), ACA135GCA (541A>G), CGA136_GAG137del (544_549delCGAGAG), GAT138AGT (550G>A 551A>G), CTC139TTA (553C>T 555C>A), ATT140AGC (557T>G 558T>C), AAA141GGA (559A>G 560A>G), GAA142GAG (564A>G), GTT143AAC (565G>A 566T>A 567T>C), GAG144AAG (568G>A), GTT145GCA (572T>C 573T>A), CTC146CTT (576C>T), AAA147AAG (579A>G), CGT148GAA (580C>G 581G>A 582T>A), GAG149GCC (584A>C 585G>C), TTA150CTT (586T>C 588A>T), GAG151GAA (591G>A), AGA152GTT (592A>G 593G>T 594A>T), TTT153GCC (595T>G 596T>C 597T>C), CAG154AGA (598C>A 599A>G 600G>A), GAG156GAA (606G>A), TTG157CTG (607T>C), GCA158CAA (610G>C 611C>A), AGT159GAA (613A>G 614G>A 615T>A), CAA160GCA (616C>G 617A>C), AAA161CGG (619A>C 620A>G 621A>G), AAT164ATT (629A>T), CAG165GTC (631C>G 632A>T 633G>C), CTA167CTC (639A>C), CAA168AGG (640C>A 641A>G 642A>G), CTT169TTG (643C>T 645T>G), CTC171ATT (649C>A 651C>T), TCC172CAA (652T>C 653C>A 654C>A), AAT173GGT (655A>G 656A>G), GTT175CCA (661G>C 662T>C 663T>A), AGT176AGC (666T>C), AAT177AAC (669T>C), AAT178GAG (670A>G 672T>G), GAC179GAT (675C>T), ATC180GTC (676A>G), TTC181TTT (681C>T), TCA182GCA (682T>G), GGT183GGC (687T>C), ACT185ACG (693T>G), GAA186AGT (694G>A 695A>G 696A>T), AGT187GAT (697A>G 698G>A), GGG188GGC (702G>C), CAG190AAG (706C>A), TAA191TGA (710A>G)								
polyprotein (YP_001976149.1)	1	1192	100%	6009	73.0%	1185 (98.7%)	820 (68.3%)	9/7/0/0	1

	Begin	End	Coverage	Score	Concordance	Matches	Identities	I/D/M/F*	Stop Codons
**NT**	**89**	**4744**	**94.9%**	**2600**	**28.6%**	**4608 (96.7%)**	**3040 (63.8%)**	**107/48**	
Protein mutations:	M1R (672T>G), T2M (675C>T 676A>G), S4L (681C>T 682T>G), V6A (687T>C), L8R (693T>G 694G>A), K9V (695A>G 696A>T 697A>G), V10M (698G>A), G11A (702G>C), N14D (710A>G 712T>C), H15R (714A>G), Q16S (716C>T 717A>C 718G>A), Q17del (719_721delCAG), E18K (722G>A 724G>A), A20V (729C>T 730C>A), E22D (736G>C), R23K (738G>A 739G>A), A34E (771C>A 772C>A), R41K (792G>A), V50I (818G>A 820T>A), G53A (828G>C 829T>A), E61D (853G>T), F63Y (858T>A), I71V (881A>G 883C>G), T72A (884A>G), T77A (899A>G 901A>T), H80R (908C>A 909A>G 910T>G), K81T (912A>C 913G>C), F82I (914T>A 916T>A), V83T (917G>A 918T>C 919T>A), K86R (926A>C 927A>G 928G>T), A89G (936C>G 937A>G), Y96H (956T>C 958T>C), V106A (987T>C 988T>A), S108N (993G>A), R112A (1004A>G 1005G>C 1006G>C), L124T (1040C>A 1041T>C 1042C>T), S125D (1043T>G 1044C>A 1045A>T), T132I (1065C>T 1066A>T), S134Y (1071C>A), L135F (1075G>T), A136S (1076G>T 1078C>T), D137G (1080A>G 1081T>A), Q166L (1167A>T 1168A>C), D185S (1223G>A 1224A>G 1225C>T), T186P (1226A>C 1228A>T), S192T (1244T>A), Q193D (1247C>G 1249G>C), Q194H (1252A>T), E195D (1255A>C), S226N (1347G>A), G227_Q228insV (1351_1352insGTA), S229R (1357T>A), T230S (1359C>G), M232L (1364A>C), A233del (1367_1369delGCT), K237R (1380A>G), A238P (1382G>C 1384C>T), E242A (1395A>C 1396A>C), Q243A (1397C>G 1398A>C 1399G>A), G246D (1407G>A 1408C>T), D250N (1418G>A 1420C>T), M252P (1424A>C 1425T>C), N253Q (1427A>C 1429T>G), S255A (1433A>G 1434G>C 1435C>A), T256N (1437C>A), I258V (1442A>G 1444C>G), K273R (1488A>G), P276A (1496C>G 1498G>A), P281_A282insDF (1513_1514insGACTTT), V283S (1517G>T 1518T>C), S284T (1521G>C), T285S (1523A>T 1525A>T), S286R (1526T>A 1527C>G), S287A (1529T>G 1531C>G), L288Y (1533T>A 1534A>T), L289Q (1536T>A 1537C>G), S290G (1538A>G 1540C>A), T291V (1541A>G 1542C>T 1543A>G), V296G (1557T>G), H297I (1559C>A 1560A>T), E299del (1565_1567delGAG), C302T (1574T>A 1575G>C 1576T>G), G303S (1577G>T 1578G>C 1579G>T), G304L (1580G>C 1581G>T), P309_K310del (1595_1600delCCCAAG), K312I (1605A>T 1606G>A), G313S (1607G>T 1608G>C 1609C>A), F316G (1616T>G 1617T>G 1618C>A), N317T (1620A>C 1621C>A), M318L (1622A>C 1624G>C), G319Q (1625G>C 1626G>A 1627T>A), A320E (1629C>A 1630C>A), I321V (1631A>G 1633T>C), L325Y (1644T>A 1645G>T), M327I (1651G>T), E328Q (1652G>C 1654G>A), A331E (1662C>A), H332D (1664C>G 1666C>T), I333F (1667A>T), D334A (1671A>C 1672T>C), D337S (1679G>A 1680A>G), D338N (1682G>A 1684T>C), E341A (1692A>C 1693A>C), R342L (1695G>T 1696A>T), I344V (1700A>G 1702A>G), A345P (1703G>C 1705T>C), Q346E (1706C>G), V348I (1712G>A 1714A>T), Q349D (1715C>G 1717A>T), F350Y (1719T>A), I351V (1721A>G 1723C>T), F353V (1727T>G 1729T>G), S354Q (1730A>C 1731G>A 1732T>G), E355K (1733G>A), K357R (1740A>G), R358A (1742A>G 1743G>C 1744A>T), V361L (1751G>C 1753T>C), I362C (1754A>T 1755T>G), R365K (1764G>A), V367I (1769G>A), A368E (1773C>A 1774A>G), K369M (1776A>T), H371D (1781C>G 1783T>C), N374C (1790A>T 1791A>G), R377F (1799A>T 1800G>T 1801G>T), D383A (1818A>C), E387D (1831A>T), E390A (1839A>C 1840A>G), N396K (1858T>G), I397V (1859A>G 1861C>T), Q398G (1862C>G 1863A>G 1864A>G), G399M (1865G>A 1866G>T 1867C>G), T401E (1871A>G 1872C>A 1873T>A), F404P (1880T>C 1881T>C 1882C>A), A408S (1892G>T), M409L (1895A>T), E413Q (1907G>C), Q421M (1931C>A 1932A>T 1933A>G), S425Q (1943T>C 1944C>A 1945C>A), I428R (1953T>G 1954T>G), Q429Y (1955C>T 1957A>T), G430W (1958G>T), Q431D (1961C>G 1963G>T), T432L (1964A>T 1965C>T 1966T>A), N433T (1968A>C 1969C>A), N438D (1982A>G 1984T>C), C441V (1991T>G 1992G>T 1993C>G), K448A (2012A>G 2013A>C), E449D (2017G>T), R450_Q451insVK (2020_2021insGTGAAA), Q451H (2023G>T), L452P (2024T>C 2025T>C 2026G>C), I453L (2027A>T 2029T>G), V454M (2030G>A), E455S (2033G>A 2034A>G 2035G>T), E456D (2038G>T), Q457L (2040A>T), V458A (2043T>C 2044G>A), A459C (2045G>T 2046C>G), Q460_R461insLN (2050_2051insTTGAAT), R462L (2054A>T 2055G>T), S463G (2057T>G 2058C>G 2059T>A), E464K (2060G>A), R465P (2063A>C 2064G>C), Q466S (2066C>A 2067A>G 2068A>C), A467M (2069G>A 2070C>T 2071A>G), Q468S (2072C>A 2073A>G 2074G>T), T470A (2078A>G), A471Q (2081G>C 2082C>A), R472T (2085G>C 2086G>T), I474N (2091T>A 2092A>T), A475_E476insS (2095_2096insAGC), E476K (2096G>A 2098A>G), S477W (2099A>T 2101T>G), P479T (2105C>A), D481G (2112A>G 2113C>A), R482K (2115G>A 2116A>G), T484L (2120A>C 2121C>T 2122T>G), S489T (2135T>A), T492A (2144A>G 2146G>A), M493L (2147A>C 2149G>T), E494_D495insS (2152_2153insAGT), D495E (2155T>G), P496G (2156C>G 2157C>G 2158C>T), T497I (2160C>T), K498Q (2162A>C 2164G>A), P499R (2165C>A 2166C>G), D500K (2168G>A 2170T>G), K501E (2171A>G), I502V (2174A>G 2176A>G), E503S (2177G>T 2178A>C), V505E (2184T>A 2185T>G), G508D (2193G>A 2194A>T), A509P (2195G>C 2197A>G), E511G (2202A>G 2203A>G), E512K (2204G>A), Q514E (2210C>G 2212A>G), G516N (2216G>A 2217G>A), V518I (2222G>A), I519F (2225A>T 2227T>C), P524E (2240C>G 2241C>A 2242A>G), D527M (2249G>A 2250A>T 2251C>G), T528G (2252A>G 2253C>G 2254A>T), S529D (2255T>G 2256C>A 2257C>T), M530A (2258A>G 2259T>C 2260G>C), A531P (2261G>C 2263A>G), V532L (2264G>T 2266T>A), E533N (2267G>A 2269A>T), D535E (2275T>G), M536L (2276A>T), V540A (2289T>C 2290G>A), D546E (2308T>G), V552I (2324G>A), V556L (2336G>C 2338T>G), E558K (2342G>A 2344G>A), A561N (2351G>A 2352C>A 2353T>C), M568L (2372A>C 2374G>T), S569A (2375A>G 2376G>C 2377C>A), A571S (2381G>T 2383T>C), A574M (2390G>A 2391C>T 2392T>G), S580K (2409G>A 2410C>G), T583A (2417A>G), A587S (2429G>T 2431G>C), L597N (2459C>A 2460T>A 2461A>C), I598L (2462A>C 2464C>T), Y600F (2469A>T 2470C>T), I602V (2474A>G 2476A>C), V606I (2486G>A), V609M (2495G>A 2497T>G), I612V (2504A>G 2506C>G), V625A (2544T>C 2545G>A), K629T (2556A>C 2557G>C), L638I (2582T>A 2584G>T), T644H (2600A>C 2601C>A 2602A>T), Q650E (2618C>G), N652S (2624A>T 2625A>C), E654G (2631A>G), E655K (2633G>A), C657V (2639T>G 2640G>T 2641C>G), T659S (2645A>T 2647C>A), T661Q (2651A>C 2652C>A), K665T (2664A>C 2665G>C), Y666F (2667A>T 2668C>T), E671T (2681G>A 2682A>C 2683A>T), G672S (2684G>T 2685G>C 2686T>A), T673L (2687A>C 2688C>T 2689A>G), A675V (2694C>T 2695C>T), T676P (2696A>C), D677E (2701T>A), S690A (2738T>G 2740T>A), N696E (2756A>G 2758T>G), V698I (2762G>A), L702I (2774T>A 2776G>A), Q705S (2783C>T 2784A>C 2785G>C), L714M (2810C>A 2812T>G), M719L (2825A>T), I720R (2829T>G), A721G (2832C>G 2833A>G), Y725F (2844A>T), K728R (2852A>C 2853A>G), A729P (2855G>C 2857G>A), Q731T (2861C>A 2862A>C 2863G>A), Y733F (2868A>T 2869T>C), P735S (2873C>A 2874C>G 2875A>T), G749S (2915G>A 2917G>T), T750V (2918A>G 2919C>T), S751A (2921T>G 2923A>T), S752A (2924T>G 2926C>A), P753A (2927C>G 2929A>C), I761V (2951A>G 2953A>C), T773I (2988C>T), T790R (3039C>G 3040T>A), I797V (3059A>G 3061T>G), I799V (3065A>G), N817A (3119A>G 3120A>C 3121C>A), K819E (3125A>G 3127A>G), T821S (3131A>T), N822T (3135A>C 3136T>A), F827L (3151T>G), K841R (3192A>G 3193A>G), R843Q (3198G>A 3199G>A), R845K (3203C>A 3204G>A 3205G>A), I848L (3212A>C 3214T>G), K854C (3230A>T 3231A>G 3232A>C), Q869K (3275C>A), F871Q (3281T>C 3282T>A 3283T>A), L874M (3290T>A), I889L (3335A>T 3337A>G), S893T (3347T>A 3349C>T), C902R (3374T>C), L903I (3377C>A), N905D (3383A>G), T911S (3401A>T 3403A>T), K925R (3444A>G), S927A (3449T>G 3451A>T), D936N (3476G>A 3478T>C), S938N (3482T>A 3483C>A 3484A>T), I943L (3497A>C), D949E (3517C>A), I951V (3521A>G), E952D (3526G>T), E953I (3527G>A 3528A>T 3529G>C), Y954F (3531A>T 3532C>T), Q955D (3533C>G 3535G>C), E956Q (3536G>C), D960_P961del (3548_3553delGATCCG), P962S (3554C>A 3555C>G), K964T (3561A>C 3562G>A), A965S (3563G>T), S967V (3569A>G 3570G>T), I968S (3572A>T 3573T>C 3574T>C), L969F (3575C>T 3577G>C), S970Q (3578T>C 3579C>A 3580C>G), S976T (3596T>A 3598C>A), M985A (3623A>G 3624T>C 3625G>T), G986A (3627G>C 3628T>A), D990T (3638G>A 3639A>C 3640C>A), E991D (3643A>C), L994V (3650T>G 3652G>A), I996L (3656A>T 3658T>G), I1003V (3677A>G 3679A>G), E1006D (3688A>T), G1007A (3690G>C 3691G>A), H1008S (3692C>T 3693A>C), V1011I (3701G>A), T1024S (3740A>T 3742A>C), N1034E (3770A>G 3772T>A), S1036T (3776T>A), I1037F (3779A>T), I1039M (3787A>G), V1042T (3794G>A 3795T>C 3796A>T), P1046S (3806C>A 3807C>G 3808T>C), N1047S (3810A>G), V1048I (3812G>A 3814T>A), V1051I (3821G>A 3823A>C), V1054I (3830G>A), F1056L (3836T>C), A1058T (3842G>A 3844C>A), P1064Q (3861C>A), A1065F (3863G>T 3864C>T), L1067V (3869C>G 3871T>A), N1068S (3872A>T 3873A>C), K1069A (3875A>G 3876A>C 3877A>G), N1071A (3881A>G 3882A>C 3883T>A), Y1072H (3884T>C), V1074L (3890G>T), A1089T (3935G>A 3937A>C), T1092I (3945C>T 3946G>C), T1096V (3956A>G 3957C>T 3958T>A), E1101Q (3971G>C), H1107F (3989C>T 3990A>T 3991T>C), R1109K (3996G>A), M1110A (3998A>G 3999T>C 4000G>C), R1111K (4002G>A 4003G>A), E1112S (4004G>T 4005A>C 4006A>C), F1113Y (4008T>A 4009C>T), S1114D (4010T>G 4011C>A 4012A>T), S1115K (4014G>A 4015C>G), K1116E (4016A>G 4018G>A), Q1117S (4019C>A 4020A>G 4021A>T), M1121V (4031A>G 4033G>T), R1126K (4047G>A), N1129L (4055A>T 4056A>T 4057T>G), I1131L (4061A>C 4063C>A), S1137V (4079T>G 4080C>T 4081C>T), A1138E (4083C>A 4084C>A), I1139L (4085A>C 4087A>T), V1140I (4088G>A 4090G>T), L1146I (4106C>A 4108T>A), I1147V (4109A>G 4111A>C), S1148K (4112T>A 4113C>A 4114T>G), L1151P (4122T>C), S1162L (4154T>C 4155C>T), H1165N (4163C>A), N1171D (4181A>G), I1191N (4242T>A)								

	Begin	End	Coverage	Score	Concordance	Matches	Identities	I/D/M/F*	Stop Codons
**NT**	**89**	**4744**	**94.9%**	**2600**	**28.6%**	**4608 (96.7%)**	**3040 (63.8%)**	**107/48**	

Codon mutations:

ATG1AGG (672T>G), ACA2ATG (675C>T 676A>G), TCT4TTG (681C>T 682T>G), GTT6GCT (687T>C), CTG8CGA (693T>G 694G>A), AAA9GTG (695A>G 696A>T 697A>G), GTG10ATG (698G>A), GGC11GCC (702G>C), CCC12CCA (706C>A), AAT14GAC (710A>G 712T>C), CAC15CGC (714A>G), CAG16TCA (716C>T 717A>C 718G>A), CAG17del (719_721delCAG), GAG18AAA (722G>A 724G>A), GCC20GTA (729C>T 730C>A), TCC21TCT (733C>T), GAG22GAC (736G>C), AGG23AAA (738G>A 739G>A), ATA25ATT (745A>T), TCA26TCT (748A>T), CAA27CAG (751A>G), ATA28ATC (754A>C), GCT30GCG (760T>G), GTG32GTT (766G>T), GAG33GAA (769G>A), GCC34GAA (771C>A 772C>A), GGA35GGT (775A>T), AAT38AAC (784T>C), TTG39CTT (785T>C 787G>T), CTC40CTT (790C>T), AGA41AAA (792G>A), AAG42AAA (796G>A), CTT43TTG (797C>T 799T>G), GGC44GGA (802C>A), TCC47TCA (811C>A), TAC48TAT (814C>T), GGG49GGC (817G>C), GTT50ATA (818G>A 820T>A), CTT51TTG (821C>T 823T>G), GGT53GCA (828G>C 829T>A), CAA55CAG (835A>G), AAG57AAA (841G>A), AGA58AGG (844A>G), GAG61GAT (853G>T), TTG62CTT (854T>C 856G>T), TTT63TAT (858T>A), GAC64GAT (862C>T), GTA68GTT (874A>T), TCA69AGT (875T>A 876C>G 877A>T), AAG70AAA (880G>A), ATC71GTG (881A>G 883C>G), ACA72GCA (884A>G), CTT74CTA (892T>A), ACA77GCT (899A>G 901A>T), TTC78TTT (904C>T), CAT80AGG (908C>A 909A>G 910T>G), AAG81ACC (912A>C 913G>C), TTT82ATA (914T>A 916T>A), GTT83ACA (917G>A 918T>C 919T>A), GAG84GAA (922G>A), TCT85TCA (925T>A), AAG86CGT (926A>C 927A>G 928G>T), GAC87GAT (931C>T), GCA89GGG (936C>G 937A>G), AAC90AAT (940C>T), TTG91TTA (943G>A), TTT93TTC (949T>C), CTG95TTA (953C>T 955G>A), TAT96CAC (956T>C 958T>C), GGT97GGG (961T>G), CTT99TTG (965C>T 967T>G), TTC100TTT (970C>T), TTC101TTT (973C>T), GTC103GTT (979C>T), CCA104CCC (982A>C), GTT106GCA (987T>C 988T>A), AGT108AAT (993G>A), GGG109GGA (997G>A), GGA111GGT (1003A>T), AGG112GCC (1004A>G 1005G>C 1006G>C), GTT113GTC (1009T>C), AAG114AAA (1012G>A), ATA115ATC (1015A>C), AGT116AGC (1018T>C), CTT117TTG (1019C>T 1021T>G), TGT118TGC (1024T>C), TCA119TCT (1027A>T), AGT120AGC (1030T>C), AAT121AAC (1033T>C), CTC124ACT (1040C>A 1041T>C 1042C>T), TCA125GAT (1043T>G 1044C>A 1045A>T), CCG126CCT (1048G>T), CAA129CAG (1057A>G), AAG131AAA (1063G>A), ACA132ATT (1065C>T 1066A>T), CTG133TTG (1067C>T), TCC134TAC (1071C>A), TTG135TTT (1075G>T), GCC136TCT (1076G>T 1078C>T), GAT137GGA (1080A>G 1081T>A), GCG139GCA (1087G>A), CAG140CAA (1090G>A), GCA141GCC (1093A>C), CTC143CTA (1099C>A), AGC145AGT (1105C>T), CCC146CCT (1108C>T), AGC147AGT (1111C>T), CTC153TTG (1127C>T 1129C>G), AAA154AAG (1132A>G), AGA155AGG (1135A>G), GGG156GGC (1138G>C), ACC162ACA (1156C>A), GAG164GAA (1162G>A), TGC165TGT (1165C>T), CAA166CTC (1167A>T 1168A>C), ACC168ACG (1174C>G), AGG169AGA (1177G>A), GCA170GCT (1180A>T), CAG171CAA (1183G>A), CCG173CCT (1189G>T), TCC175TCT (1195C>T), GTG176GTA (1198G>A), GCC178GCA (1204C>A), ATA179ATT (1207A>T), GAC185AGT (1223G>A 1224A>G 1225C>T), ACA186CCT (1226A>C 1228A>T), AGG187CGT (1229A>C 1231G>T), GCA189GCT (1237A>T), GTT190GTA (1240T>A), TCA192ACA (1244T>A), CAG193GAC (1247C>G 1249G>C), CAA194CAT (1252A>T), GAA195GAC (1255A>C), ACA196ACT (1258A>T), TCT198AGT (1262T>A 1263C>G), GCC200GCT (1270C>T), ATA201ATT (1273A>T), GAA202GAG (1276A>G), GCA203GCT (1279A>T), CTC204CTT (1282C>T), AAT205AAC (1285T>C), CGT206AGA (1286C>A 1288T>A), CCG207CCT (1291G>T), CAA208CAG (1294A>G), GAT212GAC (1306T>C), AGA213AGG (1309A>G), GAG215GAA (1315G>A), GCT216GCA (1318T>A), GCT217GCA (1321T>A), TAT219TAC (1327T>C), ATA220ATT (1330A>T), GCT221GCA (1333T>A), GTT223GTG (1339T>G), TAT224TAC (1342T>C), TCG225TCC (1345G>C), AGT226AAT (1347G>A), GGG227_CAA228insGTA (1351_1352insGTA), CAA228CAG (1354A>G), AGT229AGA (1357T>A), ACT230AGT (1359C>G), CAA231CAG (1363A>G), ATG232CTG (1364A>C), GCT233del (1367_1369delGCT), CTT234TTG (1370C>T 1372T>G), GAA235GAG (1375A>G), AAA237AGA (1380A>G), GCC238CCT (1382G>C 1384C>T), GTT240GTG (1390T>G), GGA241GGT (1393A>T), GAA242GCC (1395A>C 1396A>C), CAG243GCA (1397C>G 1398A>C 1399G>A), GGG245GGT (1405G>T), GGC246GAT (1407G>A 1408C>T), ACA247ACC (1411A>C), GAC250AAT (1418G>A 1420C>T), GTC251GTG (1423C>G), ATG252CCG (1424A>C 1425T>C), AAT253CAG (1427A>C 1429T>G), GAA254GAG (1432A>G), AGC255GCA (1433A>G 1434G>C 1435C>A), ACT256AAT (1437C>A), ATC258GTG (1442A>G 1444C>G), CGG259CGC (1447G>C), AGT260TCT (1448A>T 1449G>C), TCT262TCA (1456T>A), AGA264AGG (1462A>G), ACT267ACC (1471T>C), CTC268CTA (1474C>A), AAA269AAG (1477A>G), GTG270GTA (1480G>A), AAG273AGG (1488A>G), CGC274CGA (1492C>A), CCG276GCA (1496C>G 1498G>A), TCC277TCA (1501C>A), CTG280TTG (1508C>T), CCG281CCT (1513G>T), CCG281_GCT282insGACTTT (1513_1514insGACTTT), GTA283TCA (1517G>T 1518T>C), AGT284ACT (1521G>C), ACA285TCT (1523A>T 1525A>T), TCA286AGA (1526T>A 1527C>G), TCC287GCG (1529T>G 1531C>G), TTA288TAT (1533T>A 1534A>T), CTC289CAG (1536T>A 1537C>G), AGC290GGA (1538A>G 1540C>A), ACA291GTG (1541A>G 1542C>T 1543A>G), CGT292AGA (1544C>A 1546T>A), GAA293GAG (1549A>G), ACA295ACT (1555A>T), GTT296GGT (1557T>G), CAT297ATT (1559C>A 1560A>T), GAG299del (1565_1567delGAG), TGT302ACG (1574T>A 1575G>C 1576T>G), GGG303TCT (1577G>T 1578G>C 1579G>T), GGA304CTA (1580G>C 1581G>T), TTG305CTG (1583T>C), CCA307CCT (1591A>T), GCA308GCC (1594A>C), CCC309_AAG310del (1595_1600delCCCAAG), AAA311AAG (1603A>G), AAG312ATA (1605A>T 1606G>A), GGC313TCA (1607G>T 1608G>C 1609C>A), GCC315GCA (1615C>A), TTC316GGA (1616T>G 1617T>G 1618C>A), AAC317ACA (1620A>C 1621C>A), ATG318CTC (1622A>C 1624G>C), GGT319CAA (1625G>C 1626G>A 1627T>A), GCC320GAA (1629C>A 1630C>A), ATT321GTC (1631A>G 1633T>C), GAT323GAC (1639T>C), TTG325TAT (1644T>A 1645G>T), ATG327ATT (1651G>T), GAG328CAA (1652G>C 1654G>A), TCG329AGT (1655T>A 1656C>G 1657G>T), TTC330TTT (1660C>T), GCA331GAA (1662C>A), CAC332GAT (1664C>G 1666C>T), ATT333TTT (1667A>T), GAT334GCC (1671A>C 1672T>C), GAT337AGT (1679G>A 1680A>G), GAT338AAC (1682G>A 1684T>C), GAA341GCC (1692A>C 1693A>C), CGA342CTT (1695G>T 1696A>T), ATA344GTG (1700A>G 1702A>G), GCT345CCC (1703G>C 1705T>C), CAG346GAG (1706C>G), CAG347CAA (1711G>A), GTA348ATT (1712G>A 1714A>T), CAA349GAT (1715C>G 1717A>T), TTT350TAT (1719T>A), ATC351GTT (1721A>G 1723C>T), CTC352CTT (1726C>T), TTT353GTG (1727T>G 1729T>G), AGT354CAG (1730A>C 1731G>A 1732T>G), GAG355AAG (1733G>A), AAA357AGA (1740A>G), AGA358GCT (1742A>G 1743G>C 1744A>T), GGA359GGT (1747A>T), AAC360AAT (1750C>T), GTT361CTC (1751G>C 1753T>C), ATT362TGT (1754A>T 1755T>G), GTG363GTC (1759G>C), AGA365AAA (1764G>A), GTT367ATT (1769G>A), GCA368GAG (1773C>A 1774A>G), AAG369ATG (1776A>T), CGG370AGG (1778C>A), CAT371GAC (1781C>G 1783T>C), CTG372TTG (1784C>T), CAT373CAC (1789T>C), AAT374TGT (1790A>T 1791A>G), ATC375ATA (1795C>A), AGG377TTT (1799A>T 1800G>T 1801G>T), GAG378GAA (1804G>A), CAC379CAT (1807C>T), ATA380ATC (1810A>C), ACC381ACT (1813C>T), GAC383GCC (1818A>C), GTA386GTT (1828A>T), GAA387GAT (1831A>T), ATA388ATT (1834A>T), CTT389TTG (1835C>T 1837T>G), GAA390GCG (1839A>C 1840A>G), GGT391GGA (1843T>A), GGT393GGA (1849T>A), AAT396AAG (1858T>G), ATC397GTT (1859A>G 1861C>T), CAA398GGG (1862C>G 1863A>G 1864A>G), GGC399ATG (1865G>A 1866G>T 1867C>G), ACT401GAA (1871A>G 1872C>A 1873T>A), CGC402AGA (1874C>A 1876C>A), ACT403ACA (1879T>A), TTC404CCA (1880T>C 1881T>C 1882C>A), TAT407TAC (1891T>C), GCT408TCT (1892G>T), ATG409TTG (1895A>T), TCT410AGC (1898T>A 1899C>G 1900T>C), CTC411CTG (1903C>G), GAG413CAG (1907G>C), GTT415GTG (1915T>G), GTG416GTT (1918G>T), CTC418CTA (1924C>A), CAT420CAC (1930T>C), CAA421ATG (1931C>A 1932A>T 1933A>G), AGA422AGG (1936A>G), GAT423GAC (1939T>C), TCC425CAA (1943T>C 1944C>A 1945C>A), TTC426TTT (1948C>T), ATT428AGG (1953T>G 1954T>G), CAA429TAT (1955C>T 1957A>T), GGG430TGG (1958G>T), CAG431GAT (1961C>G 1963G>T), ACT432TTA (1964A>T 1965C>T 1966T>A), AAC433ACA (1968A>C 1969C>A), AAT434AAC (1972T>C), CCA435CCG (1975A>G), CCG437CCT (1981G>T), AAT438GAC (1982A>G 1984T>C), TGC441GTG (1991T>G 1992G>T 1993C>G), GGA444GGT (2002A>T), TTA445CTT (2003T>C 2005A>T), ACC446ACA (2008C>A), TTG447CTG (2009T>C), AAA448GCA (2012A>G 2013A>C), GAG449GAT (2017G>T), CGT450AGG (2018C>A 2020T>G), CGT450_CAG451insGTGAAA (2020_2021insGTGAAA), CAG451CAT (2023G>T), TTG452CCC (2024T>C 2025T>C 2026G>C), ATT453TTG (2027A>T 2029T>G), GTG454ATG (2030G>A), GAG455AGT (2033G>A 2034A>G 2035G>T), GAG456GAT (2038G>T), CAA457CTA (2040A>T), GTG458GCA (2043T>C 2044G>A), GCC459TGC (2045G>T 2046C>G), CAA460_AGA461insTTGAAT (2050_2051insTTGAAT), AGA461CGC (2051A>C 2053A>C), AGG462TTG (2054A>T 2055G>T), TCT463GGA (2057T>G 2058C>G 2059T>A), GAA464AAA (2060G>A), AGA465CCA (2063A>C 2064G>C), CAA466AGC (2066C>A 2067A>G 2068A>C), GCA467ATG (2069G>A 2070C>T 2071A>G), CAG468AGT (2072C>A 2073A>G 2074G>T), ACA470GCA (2078A>G), GCA471CAA (2081G>C 2082C>A), AGG472ACT (2085G>C 2086G>T), GGC473GGA (2089C>A), ATA474AAT (2091T>A 2092A>T), GCT475GCC (2095T>C), GCT475_GAA476insAGC (2095_2096insAGC), GAA476AAG (2096G>A 2098A>G), AGT477TGG (2099A>T 2101T>G), CAG478CAA (2104G>A), CCT479ACT (2105C>A), GAC481GGA (2112A>G 2113C>A), AGA482AAG (2115G>A 2116A>G), ACT484CTG (2120A>C 2121C>T 2122T>G), TCC486TCA (2128C>A), GTG488GTT (2134G>T), TCA489ACA (2135T>A), ACT490ACC (2140T>C), ACC491ACA (2143C>A), ACG492GCA (2144A>G 2146G>A), ATG493CTT (2147A>C 2149G>T), GAA494GAG (2152A>G), GAA494_GAT495insAGT (2152_2153insAGT), GAT495GAG (2155T>G), CCC496GGT (2156C>G 2157C>G 2158C>T), ACC497ATC (2160C>T), AAG498CAA (2162A>C 2164G>A), CCA499AGA (2165C>A 2166C>G), GAT500AAG (2168G>A 2170T>G), AAG501GAG (2171A>G), ATA502GTG (2174A>G 2176A>G), GAA503TCA (2177G>T 2178A>C), GTT505GAG (2184T>A 2185T>G), GCA506GCT (2188A>T), GAG507GAA (2191G>A), GGA508GAT (2193G>A 2194A>T), GCA509CCG (2195G>C 2197A>G), GAA511GGG (2202A>G 2203A>G), GAA512AAA (2204G>A), ACT513ACA (2209T>A), CAA514GAG (2210C>G 2212A>G), GGT516AAT (2216G>A 2217G>A), GAT517GAC (2221T>C), GTT518ATT (2222G>A), ATT519TTC (2225A>T 2227T>C), GAC521GAT (2233C>T), GGA523GGG (2239A>G), CCA524GAG (2240C>G 2241C>A 2242A>G), GAC527ATG (2249G>A 2250A>T 2251C>G), ACA528GGT (2252A>G 2253C>G 2254A>T), TCC529GAT (2255T>G 2256C>A 2257C>T), ATG530GCC (2258A>G 2259T>C 2260G>C), GCA531CCG (2261G>C 2263A>G), GTT532TTA (2264G>T 2266T>A), GAA533AAT (2267G>A 2269A>T), GAT535GAG (2275T>G), ATG536TTG (2276A>T), CAG537CAA (2281G>A), CAA538CAG (2284A>G), CCG539CCA (2287G>A), GTG540GCA (2289T>C 2290G>A), GTA542GTG (2296A>G), AGC544AGT (2302C>T), GAT546GAG (2308T>G), TTC547TTT (2311C>T), GGA551GGG (2323A>G), GTT552ATT (2324G>A), TTC555TTT (2335C>T), GTT556CTG (2336G>C 2338T>G), GAG558AAA (2342G>A 2344G>A), TCC560AGC (2348T>A 2349C>G), GCT561AAC (2351G>A 2352C>A 2353T>C), GAG565GAA (2365G>A), CAA566CAG (2368A>G), GTA567GTT (2371A>T), ATG568CTT (2372A>C 2374G>T), AGC569GCA (2375A>G 2376G>C 2377C>A), TTG570CTA (2378T>C 2380G>A), GCT571TCC (2381G>T 2383T>C), TTG572CTC (2384T>C 2386G>C), CCC573CCA (2389C>A), GCT574ATG (2390G>A 2391C>T 2392T>G), GCC575GCA (2395C>A), CTA576TTG (2396C>T 2398A>G), TTC577TTT (2401C>T), TCA578AGC (2402T>A 2403C>G 2404A>C), AAA579AAG (2407A>G), AGC580AAG (2409G>A 2410C>G), AAA581AAG (2413A>G), ACT583GCT (2417A>G), TCA584TCC (2422A>C), GGT586GGG (2428T>G), GCG587TCC (2429G>T 2431G>C), CTT590CTC (2440T>C), TAT592TAC (2446T>C), TAC593TAT (2449C>T), GAT594GAC (2452T>C), GCA595GCT (2455A>T), GCT596GCA (2458T>A), CTA597AAC (2459C>A 2460T>A 2461A>C), ATC598CTT (2462A>C 2464C>T), TAC600TTT (2469A>T 2470C>T), ATA602GTC (2474A>G 2476A>C), CTT604CTG (2482T>G), TAT605TAC (2485T>C), GTT606ATT (2486G>A), GTT609ATG (2495G>A 2497T>G), GCT611GCG (2503T>G), ATC612GTG (2504A>G 2506C>G), TCT613TCG (2509T>G), GGT614GGA (2512T>A), CAA615CAG (2515A>G), CTG616CTA (2518G>A), GCT617GCC (2521T>C), GAA622GAG (2536A>G), TGT623TGC (2539T>C), GTG625GCA (2544T>C 2545G>A), CGA628AGA (2552C>A), AAG629ACC (2556A>C 2557G>C), AAC634AAT (2572C>T), ATC635ATA (2575C>A), ACA637ACC (2581A>C), TTG638ATT (2582T>A 2584G>T), AAG642AAA (2596G>A), ACA644CAT (2600A>C 2601C>A 2602A>T), TTG645CTG (2603T>C), GTT646GTG (2608T>G), CAA650GAA (2618C>G), AAC652TCC (2624A>T 2625A>C), GAG654GGG (2631A>G), GAG655AAG (2633G>A), TGC657GTG (2639T>G 2640G>T 2641C>G), TTT658TTC (2644T>C), ACC659TCA (2645A>T 2647C>A), CCA660CCT (2650A>T), ACA661CAA (2651A>C 2652C>A), GGG662GGT (2656G>T), GGC664GGT (2662C>T), AAG665ACC (2664A>C 2665G>C), TAC666TTT (2667A>T 2668C>T), GTG667GTA (2671G>A), CCT668CCA (2674T>A), CTG669TTG (2675C>T), GAA671ACT (2681G>A 2682A>C 2683A>T), GGT672TCA (2684G>T 2685G>C 2686T>A), ACA673CTG (2687A>C 2688C>T 2689A>G), GGA674GGT (2692A>T), GCC675GTT (2694C>T 2695C>T), ACT676CCT (2696A>C), GAT677GAA (2701T>A), TTA678CTT (2702T>C 2704A>T), GGT679GGC (2707T>C), GTG681GTT (2713G>T), ACA686ACC (2728A>C), CAC687CAT (2731C>T), TTA689TTG (2737A>G), TCT690GCA (2738T>G 2740T>A), GCA692GCC (2746A>C), GAG694GAA (2752G>A), AAT696GAG (2756A>G 2758T>G), AGT697TCA (2759A>T 2760G>C 2761T>A), GTA698ATA (2762G>A), CCA699CCT (2767A>T), TGC700TGT (2770C>T), CAT701CAC (2773T>C), TTG702ATA (2774T>A 2776G>A), TTA704CTA (2780T>C), CAG705TCC (2783C>T 2784A>C 2785G>C), AAA707AAG (2791A>G), TTA709CTC (2795T>C 2797A>C), ACC711ACA (2803C>A), CTT714ATG (2810C>A 2812T>G), CGA718AGG (2822C>A 2824A>G), ATG719TTG (2825A>T), ATA720AGA (2829T>G), GCA721GGG (2832C>G 2833A>G), CAG722CAA (2836G>A), CAA724CAG (2842A>G), TAT725TTT (2844A>T), GGC726GGA (2848C>A), AAG728CGG (2852A>C 2853A>G), GCG729CCA (2855G>C 2857G>A), GGG730GGC (2860G>C), CAG731ACA (2861C>A 2862A>C 2863G>A), ACA732ACC (2866A>C), TAT733TTC (2868A>T 2869T>C), CCA735AGT (2873C>A 2874C>G 2875A>T), AGG736CGC (2876A>C 2878G>C), TTT737TTC (2881T>C), CCA738CCT (2884A>T), ACT739ACA (2887T>A), GTT742GTG (2896T>G), TTG743CTT (2897T>C 2899G>T), TTA744CTG (2900T>C 2902A>G), CAT745CAC (2905T>C), TAT746TAC (2908T>C), GGG749AGT (2915G>A 2917G>T), ACA750GTA (2918A>G 2919C>T), TCA751GCT (2921T>G 2923A>T), TCC752GCA (2924T>G 2926C>A), CCA753GCC (2927C>G 2929A>C), ACA757ACT (2941A>T), AGC760AGT (2950C>T), ATA761GTC (2951A>G 2953A>C), TTT762TTC (2956T>C), TCA763TCC (2959A>C), ATA767ATT (2971A>T), GAG769GAA (2977G>A), GGC772GGT (2986C>T), ACC773ATC (2988C>T), TTG774TTA (2992G>A), CCG776CCA (2998G>A), TCT777TCA (3001T>A), TTG778CTG (3002T>C), GGT780GGA (3010T>A), ATA782ATT (3016A>T), GCC783GCA (3019C>A), AGG784AGA (3022G>A), AAG787AAA (3031G>A), ACT790AGA (3039C>G 3040T>A), GGA791GGC (3043A>C), ACT792ACC (3046T>C), ATT797GTG (3059A>G 3061T>G), TGC798TGT (3064C>T), ATT799GTT (3065A>G), ACC802ACT (3076C>T), TTG803CTG (3077T>C), TCA806TCT (3088A>T), GGT807GGG (3091T>G), CTG809CTT (3097G>T), GCA810GCT (3100A>T), ATT811ATA (3103T>A), GGA812GGG (3106A>G), CTT813TTG (3107C>T 3109T>G), GGA814GGC (3112A>C), ACT815ACG (3115T>G), AAC817GCA (3119A>G 3120A>C 3121C>A), ACC818ACA (3124C>A), AAA819GAG (3125A>G 3127A>G), ACC821TCC (3131A>T), AAT822ACA (3135A>C 3136T>A), GCT823GCG (3139T>G), TTT827TTG (3151T>G), CCA830CCC (3160A>C), GTA833GTG (3169A>G), TGC834TGT (3172C>T), CTT836TTG (3176C>T 3178T>G), GGT839GGG (3187T>G), AAA841AGG (3192A>G 3193A>G), CGG843CAA (3198G>A 3199G>A), CGG845AAA (3203C>A 3204G>A 3205G>A), TGT846TGC (3208T>C), TCT847TCA (3211T>A), ATT848CTG (3212A>C 3214T>G), ACA849ACC (3217A>C), GGA853GGG (3229A>G), AAA854TGC (3230A>T 3231A>G 3232A>C), AAT855AAC (3235T>C), TTG856CTC (3236T>C 3238G>C), CTT857CTG (3241T>G), TCC858TCT (3244C>T), ACA859ACC (3247A>C), GGG860GGA (3250G>A), CGA861CGC (3253A>C), AAG862AAA (3256G>A), TCC864TCA (3262C>A), TTG865TTA (3265G>A), CAG869AAG (3275C>A), CAC870CAT (3280C>T), TTT871CAA (3281T>C 3282T>A 3283T>A), TCC872TCT (3286C>T), TTG874ATG (3290T>A), CGC875CGT (3295C>T), TTG876CTG (3296T>C), TTT877TTC (3301T>C), ACG879ACT (3307G>T), GTA880GTT (3310A>T), CTC884CTG (3322C>G), ACG887ACA (3331G>A), ATA889TTG (3335A>T 3337A>G), CAT890CAC (3340T>C), TCC893ACT (3347T>A 3349C>T), GGT895GGA (3355T>A), GTC896GTA (3358C>A), CAG899CAA (3367G>A), AAG901AAA (3373G>A), TGC902CGC (3374T>C), CTT903ATT (3377C>A), AAT905GAT (3383A>G), CTT906CTC (3388T>C), ACT907ACG (3391T>G), TTG908CTT (3392T>C 3394G>T), GGT909GGG (3397T>G), GGC910GGT (3400C>T), ACA911TCT (3401A>T 3403A>T), GTA912GTG (3406A>G), TCT913TCA (3409T>A), GTG914GTT (3412G>T), AAA915AAG (3415A>G), ATA917ATT (3421A>T), GGG919GGT (3427G>T), ACA922ACC (3436A>C), AAA923AAG (3439A>G), GGC924GGG (3442C>G), AAA925AGA (3444A>G), AGC926AGT (3448C>T), TCA927GCT (3449T>G 3451A>T), GTT928GTG (3454T>G), TTC930TTT (3460C>T),

	CTT931CTG (3463T>G), TTC932TTT (3466C>T), GAT936AAC (3476G>A 3478T>C), TTA937CTG (3479T>C 3481A>G), TCA938AAT (3482T>A 3483C>A 3484A>T), CGT940CGC (3490T>C), ATT943CTT (3497A>C), GAA944GAG (3502A>G), AAG945AAA (3505G>A), TTG946CTG (3506T>C), AAG948AAA (3514G>A), GAC949GAA (3517C>A), AAC950AAT (3520C>T), ATT951GTT (3521A>G), GAG952GAT (3526G>T), GAG953ATC (3527G>A 3528A>T 3529G>C), TAC954TTT (3531A>T 3532C>T), CAG955GAC (3533C>G 3535G>C), GAG956CAG (3536G>C), GGC958GGG (3544C>G), GAT960_CCG961del (3548_3553delGATCCG), CCC962AGC (3554C>A 3555C>G), AAG964ACA (3561A>C 3562G>A), GCT965TCT (3563G>T), AGT967GTT (3569A>G 3570G>T), ATT968TCC (3572A>T 3573T>C 3574T>C), CTG969TTC (3575C>T 3577G>C), TCC970CAG (3578T>C 3579C>A 3580C>G), ATA971ATC (3583A>C), AGA972AGG (3586A>G), GAG973GAA (3589G>A), AAA974AAG (3592A>G), TTT975TTC (3595T>C), TCC976ACA (3596T>A 3598C>A), GGT978GGA (3604T>A), GCT979GCA (3607T>A), GTA980GTG (3610A>G), TTC983TTT (3619C>T), TGC984TGT (3622C>T), ATG985GCT (3623A>G 3624T>C 3625G>T), GGT986GCA (3627G>C 3628T>A), GAC990ACA (3638G>A 3639A>C 3640C>A), GAA991GAC (3643A>C), TTG994GTA (3650T>G 3652G>A), GTA995GTT (3655A>T), ATT996TTG (3656A>T 3658T>G), CCT997CCA (3661T>A), GCA999GCC (3667A>C), CCA1000CCT (3670A>T), TCC1002TCA (3676C>A), ATA1003GTG (3677A>G 3679A>G), AGG1004CGC (3680A>C 3682G>C), GAA1006GAT (3688A>T), GGG1007GCA (3690G>C 3691G>A), CAC1008TCC (3692C>T 3693A>C), CCT1010CCA (3700T>A), GTT1011ATT (3701G>A), AAG1012AAA (3706G>A), ATC1015ATA (3715C>A), TGT1017TGC (3721T>C), TTT1019TTC (3727T>C), GAT1021GAC (3733T>C), ACA1024TCC (3740A>T 3742A>C), TCA1025TCT (3745A>T), TCA1030TCT (3760A>T), GGT1031GGA (3763T>A), AAT1034GAA (3770A>G 3772T>A), TCA1036ACA (3776T>A), ATT1037TTT (3779A>T), GTG1038GTA (3784G>A), ATA1039ATG (3787A>G), AGA1041CGA (3791A>C), GTA1042ACT (3794G>A 3795T>C 3796A>T), CAA1043CAG (3799A>G), TCC1044AGC (3800T>A 3801C>G), AGT1045TCC (3803A>T 3804G>C 3805T>C), CCT1046AGC (3806C>A 3807C>G 3808T>C), AAT1047AGT (3810A>G), GTT1048ATA (3812G>A 3814T>A), GGA1049GGT (3817A>T), GTA1051ATC (3821G>A 3823A>C), CTA1052TTG (3824C>T 3826A>G), AAT1053AAC (3829T>C), GTT1054ATT (3830G>A), GCT1055GCA (3835T>A), TTT1056CTT (3836T>C), GAT1057GAC (3841T>C), GCC1058ACA (3842G>A 3844C>A), GGC1060GGG (3850C>G), TTT1063TTC (3859T>C), CCA1064CAA (3861C>A), GCT1065TTT (3863G>T 3864C>T), GGG1066GGT (3868G>T), CTT1067GTA (3869C>G 3871T>A), AAT1068TCT (3872A>T 3873A>C), AAA1069GCG (3875A>G 3876A>C 3877A>G), AAT1071GCA (3881A>G 3882A>C 3883T>A), TAT1072CAT (3884T>C), GTA1074TTA (3890G>T), GGT1077GGC (3901T>C), GGA1078GGT (3904A>T), GGC1079GGA (3907C>A), ACA1080ACT (3910A>T), TCA1085TCC (3925A>C), TAC1086TAT (3928C>T), GGT1087GGG (3931T>G), GTG1088GTT (3934G>T), GCA1089ACC (3935G>A 3937A>C), ACA1090ACC (3940A>C), AAT1091AAC (3943T>C), ACG1092ATC (3945C>T 3946G>C), TCA1094TCT (3952A>T), TTC1095TTT (3955C>T), ACT1096GTA (3956A>G 3957C>T 3958T>A), GAG1101CAG (3971G>C), TTT1102TTC (3976T>C), TTC1103TTT (3979C>T), CCA1104CCT (3982A>T), AGG1105CGC (3983A>C 3985G>C), CGG1106CGT (3988G>T), CAT1107TTC (3989C>T 3990A>T 3991T>C), AGG1109AAG (3996G>A), ATG1110GCC (3998A>G 3999T>C 4000G>C), AGG1111AAA (4002G>A 4003G>A), GAA1112TCC (4004G>T 4005A>C 4006A>C), TTC1113TAT (4008T>A 4009C>T), TCA1114GAT (4010T>G 4011C>A 4012A>T), AGC1115AAG (4014G>A 4015C>G), AAG1116GAA (4016A>G 4018G>A), CAA1117AGT (4019C>A 4020A>G 4021A>T), TCC1118TCA (4024C>A), CGC1119AGA (4025C>A 4027C>A), ATC1120ATT (4030C>T), ATG1121GTT (4031A>G 4033G>T), TCA1122TCT (4036A>T), CTA1123TTG (4037C>T 4039A>G), GAT1125GAC (4045T>C), AGG1126AAG (4047G>A), CTT1127CTA (4051T>A), GGA1128GGC (4054A>C), AAT1129TTG (4055A>T 4056A>T 4057T>G), CTG1130TTG (4058C>T), ATC1131CTA (4061A>C 4063C>A), ATA1132ATC (4066A>C), TTG1134CTT (4070T>C 4072G>T), CCT1135CCA (4075T>A), CCT1136CCA (4078T>A), TCC1137GTT (4079T>G 4080C>T 4081C>T), GCC1138GAA (4083C>A 4084C>A), ATA1139CTT (4085A>C 4087A>T), GTG1140ATT (4088G>A 4090G>T), TCC1142TCA (4096C>A), GAG1144GAA (4102G>A), ATA1145ATC (4105A>C), CTT1146ATA (4106C>A 4108T>A), ATA1147GTC (4109A>G 4111A>C), TCT1148AAG (4112T>A 4113C>A 4114T>G), CCT1149CCA (4117T>A), GGA1150GGT (4120A>T), CTT1151CCT (4122T>C), TTC1153TTT (4129C>T), AAG1154AAA (4132G>A), GCC1158GCT (4144C>T), CCT1160CCC (4150T>C), CCT1161CCA (4153T>A), TCT1162CTT (4154T>C 4155C>T), GCC1163GCA (4159C>A), CAT1165AAT (4163C>A), GAA1166GAG (4168A>G), AAA1167AAG (4171A>G), CTT1169CTC (4177T>C), GGC1170GGA (4180C>A), AAT1171GAT (4181A>G), CAA1173CAG (4189A>G), ACT1174ACG (4192T>G), CAC1175CAT (4195C>T), ACC1176ACT (4198C>T), ACC1180ACA (4210C>A), TCA1181TCT (4213A>T), CTA1186TTG (4226C>T 4228A>G), CGT1187CGC (4231T>C), ATT1191AAT (4242T>A)									
Coat protein A (YP_001976155.1)	1	254	100%	1189	70.1%	254 (99.2%)	165 (64.5%)	2/0/0/0	0	
Protein mutations:	T2A (2078A>G), A3Q (2081G>C 2082C>A), R4T (2085G>C 2086G>T), I6N (2091T>A 2092A>T), A7_E8insS (2095_2096insAGC), E8K (2096G>A 2098A>G), S9W (2099A>T 2101T>G), P11T (2105C>A), D13G (2112A>G 2113C>A), R14K (2115G>A 2116A>G), T16L (2120A>C 2121C>T 2122T>G), S21T (2135T>A), T24A (2144A>G 2146G>A), M25L (2147A>C 2149G>T), E26_D27insS (2152_2153insAGT), D27E (2155T>G), P28G (2156C>G 2157C>G 2158C>T), T29I (2160C>T), K30Q (2162A>C 2164G>A), P31R (2165C>A 2166C>G), D32K (2168G>A 2170T>G), K33E (2171A>G), I34V (2174A>G 2176A>G), E35S (2177G>T 2178A>C), V37E (2184T>A 2185T>G), G40D (2193G>A 2194A>T), A41P (2195G>C 2197A>G), E43G (2202A>G 2203A>G), E44K (2204G>A), Q46E (2210C>G 2212A>G), G48N (2216G>A 2217G>A), V50I (2222G>A), I51F (2225A>T 2227T>C), P56E (2240C>G 2241C>A 2242A>G), D59M (2249G>A 2250A>T 2251C>G), T60G (2252A>G 2253C>G 2254A>T), S61D (2255T>G 2256C>A 2257C>T), M62A (2258A>G 2259T>C 2260G>C), A63P (2261G>C 2263A>G), V64L (2264G>T 2266T>A), E65N (2267G>A 2269A>T), D67E (2275T>G), M68L (2276A>T), V72A (2289T>C 2290G>A), D78E (2308T>G), V84I (2324G>A), V88L (2336G>C 2338T>G), E90K (2342G>A 2344G>A), A93N (2351G>A 2352C>A 2353T>C), M100L (2372A>C 2374G>T), S101A (2375A>G 2376G>C 2377C>A), A103S (2381G>T 2383T>C), A106M (2390G>A 2391C>T 2392T>G), S112K (2409G>A 2410C>G), T115A (2417A>G), A119S (2429G>T 2431G>C), L129N (2459C>A 2460T>A 2461A>C), I130L (2462A>C 2464C>T), Y132F (2469A>T 2470C>T), I134V (2474A>G 2476A>C), V138I (2486G>A), V141M (2495G>A 2497T>G), I144V (2504A>G 2506C>G), V157A (2544T>C 2545G>A), K161T (2556A>C 2557G>C), L170I (2582T>A 2584G>T), T176H (2600A>C 2601C>A 2602A>T), Q182E (2618C>G), N184S (2624A>T 2625A>C), E186G (2631A>G), E187K (2633G>A), C189V (2639T>G 2640G>T 2641C>G), T191S (2645A>T 2647C>A), T193Q (2651A>C 2652C>A), K197T (2664A>C 2665G>C), Y198F (2667A>T 2668C>T), E203T (2681G>A 2682A>C 2683A>T), G204S (2684G>T 2685G>C 2686T>A), T205L (2687A>C 2688C>T 2689A>G), A207V (2694C>T 2695C>T), T208P (2696A>C), D209E (2701T>A), S222A (2738T>G 2740T>A), N228E (2756A>G 2758T>G), V230I (2762G>A), L234I (2774T>A 2776G>A), Q237S (2783C>T 2784A>C 2785G>C), L246M (2810C>A 2812T>G), M251L (2825A>T), I252R (2829T>G), A253G (2832C>G 2833A>G)									
Codon mutations:	ACA2GCA (2078A>G), GCA3CAA (2081G>C 2082C>A), AGG4ACT (2085G>C 2086G>T), GGC5GGA (2089C>A), ATA6AAT (2091T>A 2092A>T), GCT7GCC (2095T>C), GCT7_GAA8insAGC (2095_2096insAGC), GAA8AAG (2096G>A 2098A>G), AGT9TGG (2099A>T 2101T>G), CAG10CAA (2104G>A), CCT11ACT (2105C>A), GAC13GGA (2112A>G 2113C>A), AGA14AAG (2115G>A 2116A>G), ACT16CTG (2120A>C 2121C>T 2122T>G), TCC18TCA (2128C>A), GTG20GTT (2134G>T), TCA21ACA (2135T>A), ACT22ACC (2140T>C), ACC23ACA (2143C>A), ACG24GCA (2144A>G 2146G>A), ATG25CTT (2147A>C 2149G>T), GAA26GAG (2152A>G), GAA26_GAT27insAGT (2152_2153insAGT), GAT27GAG (2155T>G), CCC28GGT (2156C>G 2157C>G 2158C>T), ACC29ATC (2160C>T), AAG30CAA (2162A>C 2164G>A), CCA31AGA (2165C>A 2166C>G), GAT32AAG (2168G>A 2170T>G), AAG33GAG (2171A>G), ATA34GTG (2174A>G 2176A>G), GAA35TCA (2177G>T 2178A>C), GTT37GAG (2184T>A 2185T>G), GCA38GCT (2188A>T), GAG39GAA (2191G>A), GGA40GAT (2193G>A 2194A>T), GCA41CCG (2195G>C 2197A>G), GAA43GGG (2202A>G 2203A>G), GAA44AAA (2204G>A), ACT45ACA (2209T>A), CAA46GAG (2210C>G 2212A>G), GGT48AAT (2216G>A 2217G>A), GAT49GAC (2221T>C), GTT50ATT (2222G>A), ATT51TTC (2225A>T 2227T>C), GAC53GAT (2233C>T), GGA55GGG (2239A>G), CCA56GAG (2240C>G 2241C>A 2242A>G), GAC59ATG (2249G>A 2250A>T 2251C>G), ACA60GGT (2252A>G 2253C>G 2254A>T), TCC61GAT (2255T>G 2256C>A 2257C>T), ATG62GCC (2258A>G 2259T>C 2260G>C), GCA63CCG (2261G>C 2263A>G), GTT64TTA (2264G>T 2266T>A), GAA65AAT (2267G>A 2269A>T), GAT67GAG (2275T>G), ATG68TTG (2276A>T), CAG69CAA (2281G>A), CAA70CAG (2284A>G), CCG71CCA (2287G>A), GTG72GCA (2289T>C 2290G>A), GTA74GTG (2296A>G), AGC76AGT (2302C>T), GAT78GAG (2308T>G), TTC79TTT (2311C>T), GGA83GGG (2323A>G), GTT84ATT (2324G>A), TTC87TTT (2335C>T), GTT88CTG (2336G>C 2338T>G), GAG90AAA (2342G>A 2344G>A), TCC92AGC (2348T>A 2349C>G), GCT93AAC (2351G>A 2352C>A 2353T>C), GAG97GAA (2365G>A), CAA98CAG (2368A>G), GTA99GTT (2371A>T), ATG100CTT (2372A>C 2374G>T), AGC101GCA (2375A>G 2376G>C 2377C>A), TTG102CTA (2378T>C 2380G>A), GCT103TCC (2381G>T 2383T>C), TTG104CTC (2384T>C 2386G>C), CCC105CCA (2389C>A), GCT106ATG (2390G>A 2391C>T 2392T>G), GCC107GCA (2395C>A), CTA108TTG (2396C>T 2398A>G), TTC109TTT (2401C>T), TCA110AGC (2402T>A 2403C>G 2404A>C), AAA111AAG (2407A>G), AGC112AAG (2409G>A 2410C>G), AAA113AAG (2413A>G), ACT115GCT (2417A>G), TCA116TCC (2422A>C), GGT118GGG (2428T>G), GCG119TCC (2429G>T 2431G>C), CTT122CTC (2440T>C), TAT124TAC (2446T>C), TAC125TAT (2449C>T), GAT126GAC (2452T>C), GCA127GCT (2455A>T), GCT128GCA (2458T>A), CTA129AAC (2459C>A 2460T>A 2461A>C), ATC130CTT (2462A>C 2464C>T), TAC132TTT (2469A>T 2470C>T), ATA134GTC (2474A>G 2476A>C), CTT136CTG (2482T>G), TAT137TAC (2485T>C), GTT138ATT (2486G>A), GTT141ATG (2495G>A 2497T>G), GCT143GCG (2503T>G), ATC144GTG (2504A>G 2506C>G), TCT145TCG (2509T>G), GGT146GGA (2512T>A), CAA147CAG (2515A>G), CTG148CTA (2518G>A), GCT149GCC (2521T>C), GAA154GAG (2536A>G), TGT155TGC (2539T>C), GTG157GCA (2544T>C 2545G>A), CGA160AGA (2552C>A), AAG161ACC (2556A>C 2557G>C), AAC166AAT (2572C>T), ATC167ATA (2575C>A), ACA169ACC (2581A>C), TTG170ATT (2582T>A 2584G>T), AAG174AAA (2596G>A), ACA176CAT (2600A>C 2601C>A 2602A>T), TTG177CTG (2603T>C), GTT178GTG (2608T>G), CAA182GAA (2618C>G), AAC184TCC (2624A>T 2625A>C), GAG186GGG (2631A>G), GAG187AAG (2633G>A), TGC189GTG (2639T>G 2640G>T 2641C>G), TTT190TTC (2644T>C), ACC191TCA (2645A>T 2647C>A), CCA192CCT (2650A>T), ACA193CAA (2651A>C 2652C>A), GGG194GGT (2656G>T), GGC196GGT (2662C>T), AAG197ACC (2664A>C 2665G>C), TAC198TTT (2667A>T 2668C>T), GTG199GTA (2671G>A), CCT200CCA (2674T>A), CTG201TTG (2675C>T), GAA203ACT (2681G>A 2682A>C 2683A>T), GGT204TCA (2684G>T 2685G>C 2686T>A), ACA205CTG (2687A>C 2688C>T 2689A>G), GGA206GGT (2692A>T), GCC207GTT (2694C>T 2695C>T), ACT208CCT (2696A>C), GAT209GAA (2701T>A), TTA210CTT (2702T>C 2704A>T), GGT211GGC (2707T>C), GTG213GTT (2713G>T), ACA218ACC (2728A>C), CAC219CAT (2731C>T), TTA221TTG (2737A>G), TCT222GCA (2738T>G 2740T>A), GCA224GCC (2746A>C), GAG226GAA (2752G>A), AAT228GAG (2756A>G 2758T>G), AGT229TCA (2759A>T 2760G>C 2761T>A), GTA230ATA (2762G>A), CCA231CCT (2767A>T), TGC232TGT (2770C>T), CAT233CAC (2773T>C), TTG234ATA (2774T>A 2776G>A), TTA236CTA (2780T>C), CAG237TCC (2783C>T 2784A>C 2785G>C), AAA239AAG (2791A>G), TTA241CTC (2795T>C 2797A>C), ACC243ACA (2803C>A), CTT246ATG (2810C>A 2812T>G), CGA250AGG (2822C>A 2824A>G), ATG251TTG (2825A>T), ATA252AGA (2829T>G), GCA253GGG (2832C>G 2833A>G), CAG254CAA (2836G>A)									
Coat protein B (YP_001976156.1)	1	244	100%	1420	83.1%	242 (99.2%)	192 (78.7%)	0/2/0/0	0	
Protein mutations:	Y3F (2844A>T), K6R (2852A>C 2853A>G), A7P (2855G>C 2857G>A), Q9T (2861C>A 2862A>C 2863G>A), Y11F (2868A>T 2869T>C), P13S (2873C>A 2874C>G 2875A>T), G27S (2915G>A 2917G>T), T28V (2918A>G 2919C>T), S29A (2921T>G 2923A>T), S30A (2924T>G 2926C>A), P31A (2927C>G 2929A>C), I39V (2951A>G 2953A>C), T51I (2988C>T), T68R (3039C>G 3040T>A), I75V (3059A>G 3061T>G), I77V (3065A>G), N95A (3119A>G 3120A>C 3121C>A), K97E (3125A>G 3127A>G), T99S (3131A>T), N100T (3135A>C 3136T>A), F105L (3151T>G), K119R (3192A>G 3193A>G), R121Q (3198G>A 3199G>A), R123K (3203C>A 3204G>A 3205G>A), I126L (3212A>C 3214T>G), K132C (3230A>T 3231A>G 3232A>C), Q147K (3275C>A), F149Q (3281T>C 3282T>A 3283T>A), L152M (3290T>A), I167L (3335A>T 3337A>G), S171T (3347T>A 3349C>T), C180R (3374T>C), L181I (3377C>A), N183D (3383A>G), T189S (3401A>T 3403A>T), K203R (3444A>G), S205A (3449T>G 3451A>T), D214N (3476G>A 3478T>C), S216N (3482T>A 3483C>A 3484A>T), I221L (3497A>C), D227E (3517C>A), I229V (3521A>G), E230D (3526G>T), E231I (3527G>A 3528A>T 3529G>C), Y232F (3531A>T 3532C>T), Q233D (3533C>G 3535G>C), E234Q (3536G>C), D238_P239del (3548_3553delGATCCG), P240S (3554C>A 3555C>G), K242T (3561A>C 3562G>A), A243S (3563G>T)									

	Begin	End	Coverage	Score	Concordance	Matches	Identities	I/D/M/F*	Stop Codons
**NT**	**89**	**4744**	**94.9%**	**2600**	**28.6%**	**4608 (96.7%)**	**3040 (63.8%)**	**107/48**	
Codon mutations:	CAA2CAG (2842A>G), TAT3TTT (2844A>T), GGC4GGA (2848C>A), AAG6CGG (2852A>C 2853A>G), GCG7CCA (2855G>C 2857G>A), GGG8GGC (2860G>C), CAG9ACA (2861C>A 2862A>C 2863G>A), ACA10ACC (2866A>C), TAT11TTC (2868A>T 2869T>C), CCA13AGT (2873C>A 2874C>G 2875A>T), AGG14CGC (2876A>C 2878G>C), TTT15TTC (2881T>C), CCA16CCT (2884A>T), ACT17ACA (2887T>A), GTT20GTG (2896T>G), TTG21CTT (2897T>C 2899G>T), TTA22CTG (2900T>C 2902A>G), CAT23CAC (2905T>C), TAT24TAC (2908T>C), GGG27AGT (2915G>A 2917G>T), ACA28GTA (2918A>G 2919C>T), TCA29GCT (2921T>G 2923A>T), TCC30GCA (2924T>G 2926C>A), CCA31GCC (2927C>G 2929A>C), ACA35ACT (2941A>T), AGC38AGT (2950C>T), ATA39GTC (2951A>G 2953A>C), TTT40TTC (2956T>C), TCA41TCC (2959A>C), ATA45ATT (2971A>T), GAG47GAA (2977G>A), GGC50GGT (2986C>T), ACC51ATC (2988C>T), TTG52TTA (2992G>A), CCG54CCA (2998G>A), TCT55TCA (3001T>A), TTG56CTG (3002T>C), GGT58GGA (3010T>A), ATA60ATT (3016A>T), GCC61GCA (3019C>A), AGG62AGA (3022G>A), AAG65AAA (3031G>A), ACT68AGA (3039C>G 3040T>A), GGA69GGC (3043A>C), ACT70ACC (3046T>C), ATT75GTG (3059A>G 3061T>G), TGC76TGT (3064C>T), ATT77GTT (3065A>G), ACC80ACT (3076C>T), TTG81CTG (3077T>C), TCA84TCT (3088A>T), GGT85GGG (3091T>G), CTG87CTT (3097G>T), GCA88GCT (3100A>T), ATT89ATA (3103T>A), GGA90GGG (3106A>G), CTT91TTG (3107C>T 3109T>G), GGA92GGC (3112A>C), ACT93ACG (3115T>G), AAC95GCA (3119A>G 3120A>C 3121C>A), ACC96ACA (3124C>A), AAA97GAG (3125A>G 3127A>G), ACC99TCC (3131A>T), AAT100ACA (3135A>C 3136T>A), GCT101GCG (3139T>G), TTT105TTG (3151T>G), CCA108CCC (3160A>C), GTA111GTG (3169A>G), TGC112TGT (3172C>T), CTT114TTG (3176C>T 3178T>G), GGT117GGG (3187T>G), AAA119AGG (3192A>G 3193A>G), CGG121CAA (3198G>A 3199G>A), CGG123AAA (3203C>A 3204G>A 3205G>A), TGT124TGC (3208T>C), TCT125TCA (3211T>A), ATT126CTG (3212A>C 3214T>G), ACA127ACC (3217A>C), GGA131GGG (3229A>G), AAA132TGC (3230A>T 3231A>G 3232A>C), AAT133AAC (3235T>C), TTG134CTC (3236T>C 3238G>C), CTT135CTG (3241T>G), TCC136TCT (3244C>T), ACA137ACC (3247A>C), GGG138GGA (3250G>A), CGA139CGC (3253A>C), AAG140AAA (3256G>A), TCC142TCA (3262C>A), TTG143TTA (3265G>A), CAG147AAG (3275C>A), CAC148CAT (3280C>T), TTT149CAA (3281T>C 3282T>A 3283T>A), TCC150TCT (3286C>T), TTG152ATG (3290T>A), CGC153CGT (3295C>T), TTG154CTG (3296T>C), TTT155TTC (3301T>C), ACG157ACT (3307G>T), GTA158GTT (3310A>T), CTC162CTG (3322C>G), ACG165ACA (3331G>A), ATA167TTG (3335A>T 3337A>G), CAT168CAC (3340T>C), TCC171ACT (3347T>A 3349C>T), GGT173GGA (3355T>A), GTC174GTA (3358C>A), CAG177CAA (3367G>A), AAG179AAA (3373G>A), TGC180CGC (3374T>C), CTT181ATT (3377C>A), AAT183GAT (3383A>G), CTT184CTC (3388T>C), ACT185ACG (3391T>G), TTG186CTT (3392T>C 3394G>T), GGT187GGG (3397T>G), GGC188GGT (3400C>T), ACA189TCT (3401A>T 3403A>T), GTA190GTG (3406A>G), TCT191TCA (3409T>A), GTG192GTT (3412G>T), AAA193AAG (3415A>G), ATA195ATT (3421A>T), GGG197GGT (3427G>T), ACA200ACC (3436A>C), AAA201AAG (3439A>G), GGC202GGG (3442C>G), AAA203AGA (3444A>G), AGC204AGT (3448C>T), TCA205GCT (3449T>G 3451A>T), GTT206GTG (3454T>G), TTC208TTT (3460C>T), CTT209CTG (3463T>G), TTC210TTT (3466C>T), GAT214AAC (3476G>A 3478T>C), TTA215CTG (3479T>C 3481A>G), TCA216AAT (3482T>A 3483C>A 3484A>T), CGT218CGC (3490T>C), ATT221CTT (3497A>C), GAA222GAG (3502A>G), AAG223AAA (3505G>A), TTG224CTG (3506T>C), AAG226AAA (3514G>A), GAC227GAA (3517C>A), AAC228AAT (3520C>T), ATT229GTT (3521A>G), GAG230GAT (3526G>T), GAG231ATC (3527G>A 3528A>T 3529G>C), TAC232TTT (3531A>T 3532C>T), CAG233GAC (3533C>G 3535G>C), GAG234CAG (3536G>C), GGC236GGG (3544C>G), GAT238_CCG239del (3548_3553delGATCCG), CCC240AGC (3554C>A 3555C>G), AAG242ACA (3561A>C 3562G>A), GCT243TCT (3563G>T)								
Coat protein C (YP_001976157.1)	1	225	100%	1273	77.2%	225 (100%)	158 (70.2%)	0/0/0/0	0
Protein mutations:	S1V (3569A>G 3570G>T), I2S (3572A>T 3573T>C 3574T>C), L3F (3575C>T 3577G>C), S4Q (3578T>C 3579C>A 3580C>G), S10T (3596T>A 3598C>A), M19A (3623A>G 3624T>C 3625G>T), G20A (3627G>C 3628T>A), D24T (3638G>A 3639A>C 3640C>A), E25D (3643A>C), L28V (3650T>G 3652G>A), I30L (3656A>T 3658T>G), I37V (3677A>G 3679A>G), E40D (3688A>T), G41A (3690G>C 3691G>A), H42S (3692C>T 3693A>C), V45I (3701G>A), T58S (3740A>T 3742A>C), N68E (3770A>G 3772T>A), S70T (3776T>A), I71F (3779A>T), I73M (3787A>G), V76T (3794G>A 3795T>C 3796A>T), P80S (3806C>A 3807C>G 3808T>C), N81S (3810A>G), V82I (3812G>A 3814T>A), V85I (3821G>A 3823A>C), V88I (3830G>A), F90L (3836T>C), A92T (3842G>A 3844C>A), P98Q (3861C>A), A99F (3863G>T 3864C>T), L101V (3869C>G 3871T>A), N102S (3872A>T 3873A>C), K103A (3875A>G 3876A>C 3877A>G), N105A (3881A>G 3882A>C 3883T>A), Y106H (3884T>C), V108L (3890G>T), A123T (3935G>A 3937A>C), T126I (3945C>T 3946G>C), T130V (3956A>G 3957C>T 3958T>A), E135Q (3971G>C), H141F (3989C>T 3990A>T 3991T>C), R143K (3996G>A), M144A (3998A>G 3999T>C 4000G>C), R145K (4002G>A 4003G>A), E146S (4004G>T 4005A>C 4006A>C), F147Y (4008T>A 4009C>T), S148D (4010T>G 4011C>A 4012A>T), S149K (4014G>A 4015C>G), K150E (4016A>G 4018G>A), Q151S (4019C>A 4020A>G 4021A>T), M155V (4031A>G 4033G>T), R160K (4047G>A), N163L (4055A>T 4056A>T 4057T>G), I165L (4061A>C 4063C>A), S171V (4079T>G 4080C>T 4081C>T), A172E (4083C>A 4084C>A), I173L (4085A>C 4087A>T), V174I (4088G>A 4090G>T), L180I (4106C>A 4108T>A), I181V (4109A>G 4111A>C), S182K (4112T>A 4113C>A 4114T>G), L185P (4122T>C), S196L (4154T>C 4155C>T), H199N (4163C>A), N205D (4181A>G), I225N (4242T>A)								
Codon mutations:	AGT1GTT (3569A>G 3570G>T), ATT2TCC (3572A>T 3573T>C 3574T>C), CTG3TTC (3575C>T 3577G>C), TCC4CAG (3578T>C 3579C>A 3580C>G), ATA5ATC (3583A>C), AGA6AGG (3586A>G), GAG7GAA (3589G>A), AAA8AAG (3592A>G), TTT9TTC (3595T>C), TCC10ACA (3596T>A 3598C>A), GGT12GGA (3604T>A), GCT13GCA (3607T>A), GTA14GTG (3610A>G), TTC17TTT (3619C>T), TGC18TGT (3622C>T), ATG19GCT (3623A>G 3624T>C 3625G>T), GGT20GCA (3627G>C 3628T>A), GAC24ACA (3638G>A 3639A>C 3640C>A), GAA25GAC (3643A>C), TTG28GTA (3650T>G 3652G>A), GTA29GTT (3655A>T), ATT30TTG (3656A>T 3658T>G), CCT31CCA (3661T>A), GCA33GCC (3667A>C), CCA34CCT (3670A>T), TCC36TCA (3676C>A), ATA37GTG (3677A>G 3679A>G), AGG38CGC (3680A>C 3682G>C), GAA40GAT (3688A>T), GGG41GCA (3690G>C 3691G>A), CAC42TCC (3692C>T 3693A>C), CCT44CCA (3700T>A), GTT45ATT (3701G>A), AAG46AAA (3706G>A), ATC49ATA (3715C>A), TGT51TGC (3721T>C), TTT53TTC (3727T>C), GAT55GAC (3733T>C), ACA58TCC (3740A>T 3742A>C), TCA59TCT (3745A>T), TCA64TCT (3760A>T), GGT65GGA (3763T>A), AAT68GAA (3770A>G 3772T>A), TCA70ACA (3776T>A), ATT71TTT (3779A>T), GTG72GTA (3784G>A), ATA73ATG (3787A>G), AGA75CGA (3791A>C), GTA76ACT (3794G>A 3795T>C 3796A>T), CAA77CAG (3799A>G), TCC78AGC (3800T>A 3801C>G), AGT79TCC (3803A>T 3804G>C 3805T>C), CCT80AGC (3806C>A 3807C>G 3808T>C), AAT81AGT (3810A>G), GTT82ATA (3812G>A 3814T>A), GGA83GGT (3817A>T), GTA85ATC (3821G>A 3823A>C), CTA86TTG (3824C>T 3826A>G), AAT87AAC (3829T>C), GTT88ATT (3830G>A), GCT89GCA (3835T>A), TTT90CTT (3836T>C), GAT91GAC (3841T>C), GCC92ACA (3842G>A 3844C>A), GGC94GGG (3850C>G), TTT97TTC (3859T>C), CCA98CAA (3861C>A), GCT99TTT (3863G>T 3864C>T), GGG100GGT (3868G>T), CTT101GTA (3869C>G 3871T>A), AAT102TCT (3872A>T 3873A>C), AAA103GCG (3875A>G 3876A>C 3877A>G), AAT105GCA (3881A>G 3882A>C 3883T>A), TAT106CAT (3884T>C), GTA108TTA (3890G>T), GGT111GGC (3901T>C), GGA112GGT (3904A>T), GGC113GGA (3907C>A), ACA114ACT (3910A>T), TCA119TCC (3925A>C), TAC120TAT (3928C>T), GGT121GGG (3931T>G), GTG122GTT (3934G>T), GCA123ACC (3935G>A 3937A>C), ACA124ACC (3940A>C), AAT125AAC (3943T>C), ACG126ATC (3945C>T 3946G>C), TCA128TCT (3952A>T), TTC129TTT (3955C>T), ACT130GTA (3956A>G 3957C>T 3958T>A), GAG135CAG (3971G>C), TTT136TTC (3976T>C), TTC137TTT (3979C>T), CCA138CCT (3982A>T), AGG139CGC (3983A>C 3985G>C), CGG140CGT (3988G>T), CAT141TTC (3989C>T 3990A>T 3991T>C), AGG143AAG (3996G>A), ATG144GCC (3998A>G 3999T>C 4000G>C), AGG145AAA (4002G>A 4003G>A), GAA146TCC (4004G>T 4005A>C 4006A>C), TTC147TAT (4008T>A 4009C>T), TCA148GAT (4010T>G 4011C>A 4012A>T), AGC149AAG (4014G>A 4015C>G), AAG150GAA (4016A>G 4018G>A), CAA151AGT (4019C>A 4020A>G 4021A>T), TCC152TCA (4024C>A), CGC153AGA (4025C>A 4027C>A), ATC154ATT (4030C>T), ATG155GTT (4031A>G 4033G>T), TCA156TCT (4036A>T), CTA157TTG (4037C>T 4039A>G), GAT159GAC (4045T>C), AGG160AAG (4047G>A), CTT161CTA (4051T>A), GGA162GGC (4054A>C), AAT163TTG (4055A>T 4056A>T 4057T>G), CTG164TTG (4058C>T), ATC165CTA (4061A>C 4063C>A), ATA166ATC (4066A>C), TTG168CTT (4070T>C 4072G>T), CCT169CCA (4075T>A), CCT170CCA (4078T>A), TCC171GTT (4079T>G 4080C>T 4081C>T), GCC172GAA (4083C>A 4084C>A), ATA173CTT (4085A>C 4087A>T), GTG174ATT (4088G>A 4090G>T), TCC176TCA (4096C>A), GAG178GAA (4102G>A), ATA179ATC (4105A>C), CTT180ATA (4106C>A 4108T>A), ATA181GTC (4109A>G 4111A>C), TCT182AAG (4112T>A 4113C>A 4114T>G), CCT183CCA (4117T>A), GGA184GGT (4120A>T), CTT185CCT (4122T>C), TTC187TTT (4129C>T), AAG188AAA (4132G>A), GCC192GCT (4144C>T), CCT194CCC (4150T>C), CCT195CCA (4153T>A), TCT196CTT (4154T>C 4155C>T), GCC197GCA (4159C>A), CAT199AAT (4163C>A), GAA200GAG (4168A>G), AAA201AAG (4171A>G), CTT203CTC (4177T>C), GGC204GGA (4180C>A), AAT205GAT (4181A>G), CAA207CAG (4189A>G), ACT208ACG (4192T>G), CAC209CAT (4195C>T), ACC210ACT (4198C>T), ACC214ACA (4210C>A), TCA215TCT (4213A>T), CTA220TTG (4226C>T 4228A>G), CGT221CGC (4231T>C), ATT225AAT (4242T>A)								

*: Inserts / Deletes / Misaligned / Frameshifts

Analysis details

This analysis was performed with panviral2.64

NGS Details (UN59): Bracoviriform glomeratae (segment NC_043292.1)

Assembly

**Coverage Length**	308 (1 contig(s))
**Depth Of Coverage**	4640.4
**Number Of Reads**	12058
**Reads Per Million**	262.13 rpm (after QC)
**Ambiguities**	0
**Assembly Method**	de novo + reference guided assembly
**Consensus Caller**	Bcf Tools

Coverage Map

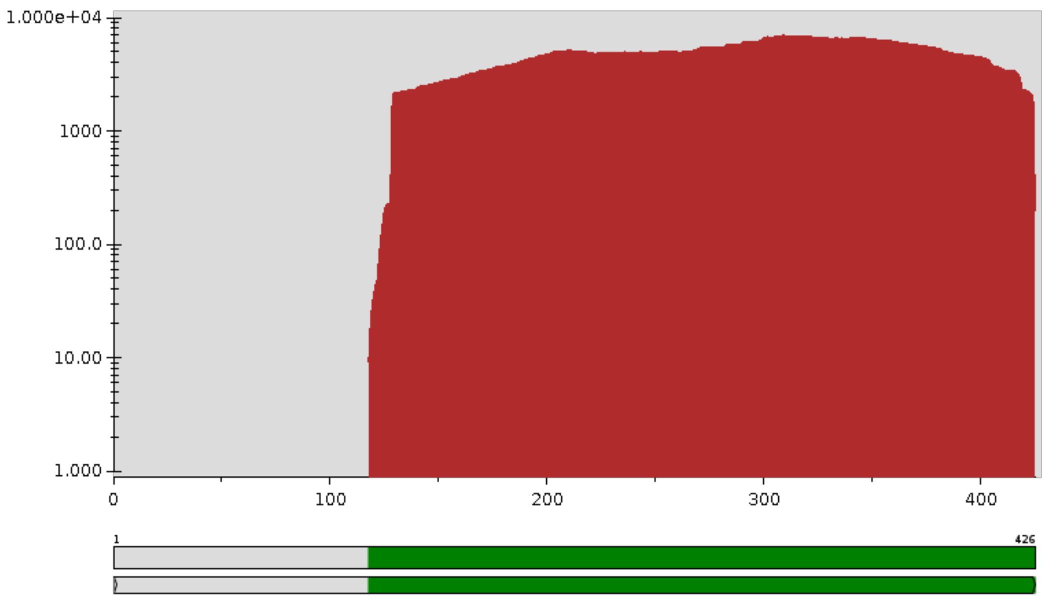

Assignment

**Type**	Bracoviriform glomeratae (Taxonomy ID: 257816)
**Reference Genome**	NC_043292.1
**NT Identity (%)**	72.7273
**AA Identity (%)**	84.3137
**Number Of Stop Codons**	1
**Number Of CDS**	1

Alignment

**Alignment Score**	280.0 (NT) + 559.0 (AA) = 839.0
**Concordance (%)**	66.3241

**Alignment Method**	Global, seeded, nucleotide + amino acids (AGA)

Genome Region

Sequence starts at position 119 and ends at position 426 relative to NC_043292.1 reference sequence.

Alignment Detailed Statistics

	Begin	End	Coverage	Score	Concordance	Matches	Identities	I/D/M/F*	Stop Codons
**NT**	**119**	**426**	**72.3%**	**280**	**45.5%**	**308 (100%)**	**224 (72.7%)**	**0/0**	
Mutations:	126G>T, 129C>A, 132A>G, 136A>G, 137C>G, 138G>C, 150A>C, 153A>G, 155C>G, 156G>A, 159C>A, 166C>A, 168T>G, 169T>C, 171T>C, 172C>A, 174C>G, 177A>G, 183T>A, 184C>A, 189T>C, 198A>G, 201A>C, 207C>T, 214A>G, 219C>T, 222T>C, 223C>A, 225T>G, 228G>T, 235C>A, 237T>G, 243A>T, 246C>G, 255C>T, 261A>G, 262T>C, 264A>G, 265G>A, 273A>G, 278T>C, 279T>C, 284A>G, 285T>A, 288T>G, 289T>C, 300C>T, 301C>T, 303C>G, 309C>T, 312T>G, 315C>T, 322G>T, 324T>C, 329T>C, 333T>C, 336C>T, 339A>G, 345A>C, 347A>G, 348A>G, 349C>A, 351T>A, 360C>T, 363C>T, 366C>T, 375C>T, 384A>C, 385T>C, 387G>T, 390A>G, 391C>A, 392A>G, 393T>A, 394A>C, 399C>A, 400C>A, 402T>G, 404T>C, 405G>T, 406A>C, 414T>A, 416A>T, 420A>G								
**CDS**									
FK954_p501	41	142	71.8%	559	85.1%	102 (100%)	86 (84.3%)	0/0/0/0	1
Protein mutations:	L42F (126G>T), T46G (136A>G 137C>G 138G>C), A52G (155C>G 156G>A), Y57H (169T>C 171T>C), T72A (214A>G), V89I (265G>A), I93T (278T>C 279T>C), D95G (284A>G 285T>A), A108S (322G>T 324T>C), I110T (329T>C), K116R (347A>G 348A>G), H131R (391C>A 392A>G 393T>A), K132Q (394A>C), M135T (404T>C 405G>T), I136L (406A>C), Y139F (416A>T)								
Codon mutations:	TTG42TTT (126G>T), GGC43GGA (129C>A), AAA44AAG (132A>G), ACG46GGC (136A>G 137C>G 138G>C), GGA50GGC (150A>C), AAA51AAG (153A>G), GCG52GGA (155C>G 156G>A), GGC53GGA (159C>A), CGT56AGG (166C>A 168T>G), TAT57CAC (169T>C 171T>C), CGC58AGG (172C>A 174C>G), AAA59AAG (177A>G), CTT61CTA (183T>A), CGA62AGA (184C>A), GAT63GAC (189T>C), CAA66CAG (198A>G), GGA67GGC (201A>C), ACC69ACT (207C>T), ACT72GCT (214A>G), ATC73ATT (219C>T), CGT74CGC (222T>C), CGT75AGG (223C>A 225T>G), CTG76CTT (228G>T), CGT79AGG (235C>A 237T>G), GGA81GGT (243A>T), GTC82GTG (246C>G), ATC85ATT (255C>T), GGA87GGG (261A>G), TTA88CTG (262T>C 264A>G), GTC89ATC (265G>A), GAA91GAG (273A>G), ATT93ACC (278T>C 279T>C), GAT95GGA (284A>G 285T>A), GTT96GTG (288T>G), TTG97CTG (289T>C), TTC100TTT (300C>T), CTC101TTG (301C>T 303C>G), AAC103AAT (309C>T), GTT104GTG (312T>G), ATC105ATT (315C>T), GCT108TCC (322G>T 324T>C), ATC110ACC (329T>C), TAT111TAC (333T>C), ACC112ACT (336C>T), GAA113GAG (339A>G), GCA115GCC (345A>C), AAA116AGG (347A>G 348A>G), CGT117AGA (349C>A 351T>A), GTC120GTT (360C>T), ACC121ACT (363C>T), GCC122GCT (366C>T), GTC125GTT (375C>T), GCA128GCC (384A>C), TTG129CTT (385T>C 387G>T), AAA130AAG (390A>G), CAT131AGA (391C>A 392A>G 393T>A), AAA132CAA (394A>C), GGC133GGA (399C>A), CGT134AGG (400C>A 402T>G), ATG135ACT (404T>C 405G>T), ATC136CTC (406A>C), GGT138GGA (414T>A), TAT139TTT (416A>T), GGA140GGG (420A>G)								
**Proteins**									
putative histone 4 (YP_009665791.1)	41	142	71.8%	559	85.1%	102 (100%)	86 (84.3%)	0/0/0/0	1
Protein mutations:	L42F (126G>T), T46G (136A>G 137C>G 138G>C), A52G (155C>G 156G>A), Y57H (169T>C 171T>C), T72A (214A>G), V89I (265G>A), I93T (278T>C 279T>C), D95G (284A>G 285T>A), A108S (322G>T 324T>C), I110T (329T>C), K116R (347A>G 348A>G), H131R (391C>A 392A>G 393T>A), K132Q (394A>C), M135T (404T>C 405G>T), I136L (406A>C), Y139F (416A>T)								
Codon mutations:	TTG42TTT (126G>T), GGC43GGA (129C>A), AAA44AAG (132A>G), ACG46GGC (136A>G 137C>G 138G>C), GGA50GGC (150A>C), AAA51AAG (153A>G), GCG52GGA (155C>G 156G>A), GGC53GGA (159C>A), CGT56AGG (166C>A 168T>G), TAT57CAC (169T>C 171T>C), CGC58AGG (172C>A 174C>G), AAA59AAG (177A>G), CTT61CTA (183T>A), CGA62AGA (184C>A), GAT63GAC (189T>C), CAA66CAG (198A>G), GGA67GGC (201A>C), ACC69ACT (207C>T), ACT72GCT (214A>G), ATC73ATT (219C>T), CGT74CGC (222T>C), CGT75AGG (223C>A 225T>G), CTG76CTT (228G>T), CGT79AGG (235C>A 237T>G), GGA81GGT (243A>T), GTC82GTG (246C>G), ATC85ATT (255C>T), GGA87GGG (261A>G), TTA88CTG (262T>C 264A>G), GTC89ATC (265G>A), GAA91GAG (273A>G), ATT93ACC (278T>C 279T>C), GAT95GGA (284A>G 285T>A), GTT96GTG (288T>G), TTG97CTG (289T>C), TTC100TTT (300C>T), CTC101TTG (301C>T 303C>G), AAC103AAT (309C>T), GTT104GTG (312T>G), ATC105ATT (315C>T), GCT108TCC (322G>T 324T>C), ATC110ACC (329T>C), TAT111TAC (333T>C), ACC112ACT (336C>T), GAA113GAG (339A>G), GCA115GCC (345A>C), AAA116AGG (347A>G 348A>G), CGT117AGA (349C>A 351T>A), GTC120GTT (360C>T), ACC121ACT (363C>T), GCC122GCT (366C>T), GTC125GTT (375C>T), GCA128GCC (384A>C), TTG129CTT (385T>C 387G>T), AAA130AAG (390A>G), CAT131AGA (391C>A 392A>G 393T>A), AAA132CAA (394A>C), GGC133GGA (399C>A), CGT134AGG (400C>A 402T>G), ATG135ACT (404T>C 405G>T), ATC136CTC (406A>C), GGT138GGA (414T>A), TAT139TTT (416A>T), GGA140GGG (420A>G)								

*: Inserts / Deletes / Misaligned / Frameshifts

Analysis details

This analysis was performed with panviral2.64

NGS Details (UN59): Diachasmimorpha longicaudata entomopoxvirus (segment NC_043455.1)

Assembly

**Coverage Length**	1121 (1 contig(s))
**Depth Of Coverage**	876.9
**Number Of Reads**	7928
**Reads Per Million**	172.35 rpm (after QC)
**Ambiguities**	0
**Assembly Method**	de novo + reference guided assembly
**Consensus Caller**	Bcf Tools

Coverage Map

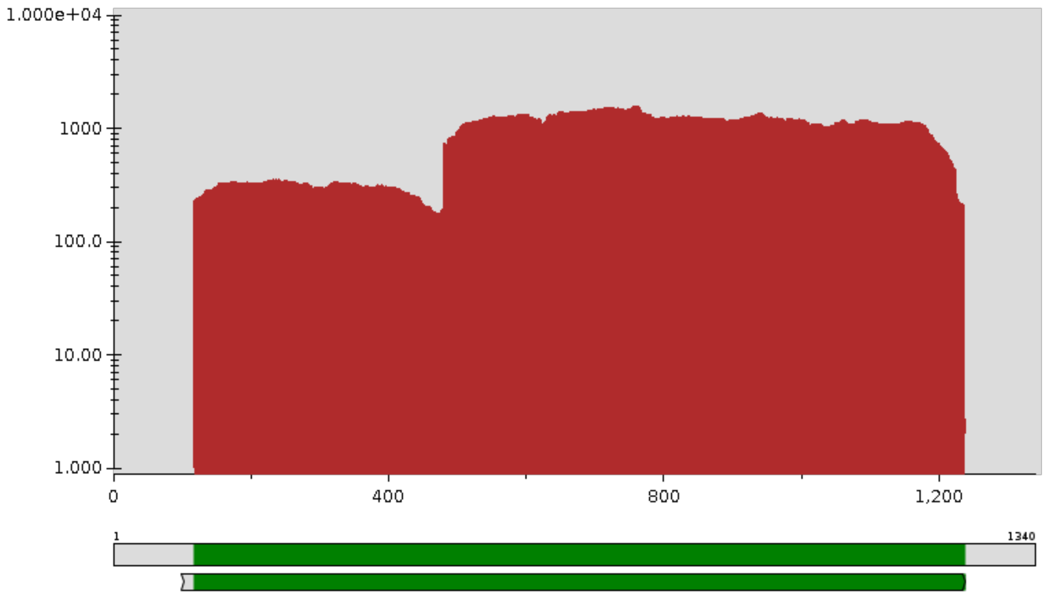

Assignment

**Type**	Diachasmimorpha longicaudata entomopoxvirus (Taxonomy ID: 109981)
**Reference Genome**	NC_043455.1
**NT Identity (%)**	59.1436
**AA Identity (%)**	55.3476
**Number Of Stop Codons**	0
**Number Of CDS**	1

Alignment

**Alignment Score**	392.0 (NT) + 1456.0 (AA) = 1848.0
**Concordance (%)**	39.4535

**Alignment Method**	Global, seeded, nucleotide + amino acids (AGA)

Genome Region

Sequence starts at position 118 and ends at position 1238 relative to NC_043455.1 reference sequence.

Alignment Detailed Statistics

	Begin	End	Coverage	Score	Concordance	Matches	Identities	I/D/M/F*	Stop Codons
**NT**	**118**	**1238**	**83.7%**	**392**	**17.6%**	**1118 (99.5%)**	**663 (59.0%)**	**3/3**	
Mutations:	119G>A, 120A>G, 121A>T, 126A>C, 127C>T, 128C>G, 130T>G, 136C>G, 137C>A, 139C>T, 142G>A, 143A>G, 145A>T, 146A>G, 148T>C, 149A>C, 151C>T, 154A>T, 156A>G, 157G>A, 160T>G, 163A>T, 166T>C, 167T>C, 168C>A, 169T>G, 171G>A, 172T>C, 175A>G, 178T>C, 187T>A, 190A>T, 191A>G, 193T>C, 203A>C, 204A>G, 208C>T, 209A>G, 211T>A, 214T>G, 217A>T, 218T>A, 219G>T, 223T>A, 226T>G, 230A>C, 231A>G, 232A>T, 238G>C, 241A>T, 243T>C, 244C>T, 247A>G, 256G>C, 259A>T, 262A>C, 271A>C, 272G>T, 274A>C, 276C>T, 277A>G, 278T>A, 279A>T, 283A>T, 284A>C, 286C>T, 288G>C, 289T>C, 294T>G, 295A>C, 298G>A, 299C>G, 300A>T, 301A>T, 302A>G, 304A>G, 310T>C, 311T>A, 312C>A, 313T>A, 320A>G, 322T>G, 323A>G, 325C>A, 331T>A, 337C>A, 341A>T, 343C>T, 346A>T, 352T>G, 355A>G, 356T>C, 358G>T, 361T>A, 362C>G, 363T>C, 364C>T, 368G>A, 370T>G, 371C>G, 374C>A, 375G>A, 376T>G, 379A>G, 380T>A, 382G>T, 383C>T, 384A>T, 385A>G, 386A>G, 394G>A, 395A>G, 400T>C, 401C>A, 403A>T, 404_406delTAT, 409T>C, 410T>A, 412T>A, 413A>C, 415A>G, 416T>G, 417G>C, 418T>A, 421A>T, 423T>C, 424T>G, 438C>A, 442T>C, 443A>G, 445C>G, 446A>G, 447A>G, 448G>T, 452A>G, 453G>A, 454C>T, 455C>A, 456A>T, 457G>C, 458G>A, 459A>G, 462C>A, 463T>G, 467A>G, 469_470insCAT, 470A>G, 471A>G, 474C>T, 475C>A, 478G>A, 481T>A, 482T>G, 485A>G, 490A>T, 496T>A, 499A>C, 502A>T, 503A>G, 505G>C, 506A>T, 511T>C, 512C>A, 514A>G, 517C>G, 518A>C, 519C>G, 521C>A, 523A>G, 524A>C, 526A>G, 527A>T, 528G>C, 530A>C, 532C>T, 533G>C, 534A>G, 535T>C, 536A>C, 538C>T, 539A>G, 541A>C, 542G>C, 543C>A, 544A>C, 547T>C, 550A>G, 553A>G, 554G>T, 559A>T, 560A>T, 562A>G, 574T>C, 577G>A, 581T>C, 583G>C, 584A>T, 585T>C, 586A>T, 587G>A, 588A>G, 589T>A, 590A>G, 591A>G, 595T>C, 596T>A, 597T>A, 602A>C, 604A>G, 607A>T, 608C>T, 610A>T, 616T>A, 620G>C, 622A>G, 623T>C, 628T>G, 629G>C, 630A>C, 632A>C, 633G>C, 634T>A, 635C>A, 637T>A, 638G>A, 646T>G, 647A>G, 648T>G, 650T>G, 652A>T, 655A>C, 658A>T, 661A>T, 664C>T, 665G>A, 667T>G, 671T>C, 674A>G, 675G>A, 678T>C, 680A>C, 682C>T, 683A>G, 685T>A, 687C>T, 688A>T, 689T>A, 692C>A, 693A>G, 695G>A, 696T>A, 697C>G, 700T>C, 705G>A, 706A>C, 707G>A, 709T>A, 712C>T, 715A>G, 717A>G, 718A>G, 724G>T, 727A>G, 730A>G, 731A>C, 732A>G, 735C>A, 739A>G, 740T>C, 742G>C, 745A>T, 748C>T, 754C>T, 757T>C, 759G>A, 765A>T, 770A>G, 772A>C, 778A>C, 779A>G, 781G>T, 784A>G, 785A>G, 787T>A, 790T>A, 792T>G, 793T>G, 796A>G, 797G>C, 798C>T, 799A>T, 810T>G, 823C>G, 824C>A, 825A>C, 826C>T, 829A>G, 830A>G, 831G>C, 833C>A, 838T>C, 841A>G, 843C>G, 844A>T, 845C>G, 847A>C, 850A>C, 854T>G, 855G>T, 856T>G, 859T>C, 862A>C, 863C>A, 865T>G, 866A>C, 868A>T, 869C>A, 878G>T, 879T>G, 880A>G, 885T>C, 886G>T, 889A>T, 890T>A, 891G>A, 892T>G, 893T>A, 895A>G, 896A>C, 897C>G, 898A>C, 900A>G, 901T>C, 902A>C, 904A>T, 905A>G, 908T>C, 909T>A, 915C>T, 919A>T, 920A>G, 921G>C, 922C>A, 924T>C, 925C>T, 934T>C, 938T>G, 939C>A, 943A>G, 944C>A, 946A>C, 947G>A, 948A>C, 949A>T, 950C>A, 952T>A, 956T>A, 958T>C, 965A>C, 966A>G, 970A>G, 973T>C, 974A>C, 977G>T, 978A>C, 982T>G, 983A>T, 984A>C, 986A>T, 989A>C, 991A>T, 992A>G, 994A>G, 997T>C, 998C>A, 1000T>C, 1001T>A, 1007A>G, 1012A>C, 1013T>C, 1015A>T, 1018A>T, 1019A>C, 1021A>T, 1024C>T, 1033T>A, 1036A>G, 1040A>G, 1042A>C, 1045T>C, 1046T>C, 1048A>T, 1051T>C, 1054C>T, 1057T>C, 1063T>C, 1064T>C, 1070C>A, 1071A>C, 1073A>C, 1075T>A, 1076A>C, 1077G>C, 1087T>C, 1088A>C, 1090A>G, 1103A>C, 1105A>T, 1111T>A, 1112A>C, 1120C>A, 1121C>A, 1123A>G, 1126A>G, 1129G>T, 1130A>G, 1141T>C, 1145A>G, 1147A>C, 1150T>C, 1151G>A, 1153A>G, 1156C>T, 1159A>T, 1160T>G, 1161T>A, 1163C>A, 1164C>G, 1165A>G, 1167A>T, 1172G>T, 1173C>T, 1177A>C, 1178T>A, 1181G>C, 1183A>G, 1185C>A, 1186G>A, 1192T>C, 1195T>C, 1196A>G, 1197C>T, 1199A>G, 1200A>T, 1204A>C, 1207T>G, 1210A>G, 1211A>C, 1213G>C, 1218A>C, 1219G>C, 1223A>G, 1227T>C, 1232T>C, 1234G>C, 1235A>C, 1237A>G								

CDS

	Begin	End	Coverage	Score	Concordance	Matches	Identities	I/D/M/F*	Stop Codons
FLA14_p101	7	380	98.4%	1456	58.9%	373 (99.5%)	207 (55.2%)	1/1/0/0	0
Protein mutations:	E7S (119G>A 120A>G 121A>T), D9A (126A>C 127C>T), H10E (128C>G 130T>G), L13I (137C>A 139C>T), K15D (143A>G 145A>T), N16D (146A>G 148T>C), I17L (149A>C 151C>T), K19R (156A>G 157G>A), S23Q (167T>C 168C>A 169T>G), C24Y (171G>A 172T>C), T31A (191A>G 193T>C), K35R (203A>C 204A>G), I37V (209A>G 211T>A), F38L (214T>G), C40I (218T>A 219G>T), K44R (230A>C 231A>G 232A>T), V48A (243T>C 244C>T), A58S (272G>T 274A>C), T59M (276C>T 277A>G), Y60I (278T>A 279A>T), I62L (284A>C 286C>T), S63T (288G>C 289T>C), L65C (294T>G 295A>C), Q67V (299C>G 300A>T 301A>T), I68V (302A>G 304A>G), S71K (311T>A 312C>A 313T>A), N74E (320A>G 322T>G), I75V (323A>G 325C>A), T81S (341A>T 343C>T), L88A (362C>G 363T>C 364C>T), A90T (368G>A 370T>G), Q91E (371C>G), R92K (374C>A 375G>A 376T>G), L94I (380T>A 382G>T), Q95L (383C>T 384A>T 385A>G), T96A (386A>G), N99D (395A>G), L101I (401C>A 403A>T), Y102del (404_406delTAT), F104I (410T>A 412T>A), K105Q (413A>C 415A>G), C106A (416T>G 417G>C 418T>A), Q107H (421A>T), V108A (423T>C 424T>G), T113K (438C>A), I115V (443A>G 445C>G), K116G (446A>G 447A>G 448G>T), S118D (452A>G 453G>A 454C>T), Q119I (455C>A 456A>T 457G>C), E120R (458G>A 459A>G), T121K (462C>A 463T>G), K123E (467A>G), K123_K124insH (469_470insCAT), K124G (470A>G 471A>G), A125V (474C>T 475C>A), L128V (482T>G), I129V (485A>G), M135V (503A>G 505G>C), I136F (506A>T), L138M (512C>A 514A>G), T140R (518A>C 519C>G), K142Q (524A>C 526A>G), I144L (530A>C 532C>T), D145R (533G>C 534A>G 535T>C), T146P (536A>C 538C>T), K147D (539A>G 541A>C), A148H (542G>C 543C>A 544A>C), I151M (553A>G), V152F (554G>T), I154L (560A>T 562A>G), I162S (584A>T 585T>C 586A>T), D163R (587G>A 588A>G 589T>A), N164G (590A>G 591A>G), L166K (596T>A 597T>A), K168Q (602A>C 604A>G), Q170Y (608C>T 610A>T), E174Q (620G>C 622A>G), F175L (623T>C), F176L (628T>G), E177P (629G>C 630A>C), S178P (632A>C 633G>C 634T>A), H179K (635C>A 637T>A), V180I (638G>A), I183G (647A>G 648T>G), L184V (650T>G 652A>T), L185F (655A>C), V189M (665G>A 667T>G), S191P (671T>C), R192E (674A>G 675G>A), V193A (678T>C), I194L (680A>C 682C>T), N195E (683A>G 685T>A), T196I (687C>T 688A>T), S197T (689T>A), Q198R (692C>A 693A>G), V199K (695G>A 696T>A 697C>G), R202N (705G>A 706A>C), D203K (707G>A 709T>A), K206R (717A>G 718A>G), N211R (731A>C 732A>G), A212D (735C>A), R220K (759G>A), Y222F (765A>T), I224V (770A>G 772A>C), K227D (779A>G 781G>T), N229E (785A>G 787T>A), D230E (790T>A), F231W (792T>G 793T>G), A233L (797G>C 798C>T 799A>T), F237C (810T>G), D241E (823C>G), H242T (824C>A 825A>C 826C>T), S244A (830A>G 831G>C), L245I (833C>A), T248S (843C>G 844A>T), L249V (845C>G 847A>C), C252V (854T>G 855G>T 856T>G), Q257K (869C>A), V260W (878G>T 879T>G 880A>G), M262T (885T>C 886G>T), E263D (889A>T), C264K (890T>A 891G>A 892T>G), L265M (893T>A 895A>G), T266R (896A>C 897C>G 898A>C), N267S (900A>G 901T>C), N269D (905A>G), F270H (908T>C 909T>A), A272V (915C>T), S274A (920A>G 921G>C 922C>A), I275T (924T>C 925C>T), S280D (938T>G 939C>A), Q282N (944C>A 946A>C), E283T (947G>A 948A>C 949A>T), F286I (956T>A 958T>C), K289R (965A>C 966A>G), D293S (977G>T 978A>C), K295S (983A>T 984A>C), T296S (986A>T), I298V (992A>G 994A>G), L300I (998C>A 1000T>C), S301T (1001T>A), N303D (1007A>G), I314V (1040A>G 1042A>C), H324T (1070C>A 1071A>C), N325Q (1073A>C 1075T>A), R326P (1076A>C 1077G>C), I330L (1088A>C 1090A>G), I344V (1130A>G), I349V (1145A>G 1147A>C), E351K (1151G>A 1153A>G), E353D (1159A>T), L354E (1160T>G 1161T>A), P355R (1163C>A 1164C>G 1165A>G), K356M (1167A>T), A358F (1172G>T 1173C>T), E359D (1177A>C), L360I (1178T>A), E361Q (1181G>C 1183A>G), T362K (1185C>A 1186G>A), T366V (1196A>G 1197C>T), K367V (1199A>G 1200A>T), D369E (1207T>G), M371L (1211A>C 1213G>C), E373A (1218A>C 1219G>C), I375V (1223A>G), V376A (1227T>C), I379L (1235A>C 1237A>G)								

	Begin	End	Coverage	Score	Concordance	Matches	Identities	I/D/M/F*	Stop Codons
**NT**	**118**	**1238**	**83.7%**	**392**	**17.6%**	**1118 (99.5%)**	**663 (59.0%)**	**3/3**	
Codon mutations:	GAA7AGT (119G>A 120A>G 121A>T), GAC9GCT (126A>C 127C>T), CAT10GAG (128C>G 130T>G), GGC12GGG (136C>G), CTC13ATT (137C>A 139C>T), AAG14AAA (142G>A), AAA15GAT (143A>G 145A>T), AAT16GAC (146A>G 148T>C), ATC17CTT (149A>C 151C>T), CTA18CTT (154A>T), AAG19AGA (156A>G 157G>A), GGT20GGG (160T>G), ATA21ATT (163A>T), TAT22TAC (166T>C), TCT23CAG (167T>C 168C>A 169T>G), TGT24TAC (171G>A 172T>C), GGA25GGG (175A>G), TTT26TTC (178T>C), CCT29CCA (187T>A), TCA30TCT (190A>T), ACT31GCC (191A>G 193T>C), AAA35CGA (203A>C 204A>G), GCC36GCT (208C>T), ATT37GTA (209A>G 211T>A), TTT38TTG (214T>G), CCA39CCT (217A>T), TGT40ATT (218T>A 219G>T), ATT41ATA (223T>A), TCT42TCG (226T>G), AAA44CGT (230A>C 231A>G 232A>T), GTG46GTC (238G>C), ATA47ATT (241A>T), GTC48GCT (243T>C 244C>T), CAA49CAG (247A>G), TCG52TCC (256G>C), GGA53GGT (259A>T), ACA54ACC (262A>C), ACA57ACC (271A>C), GCA58TCC (272G>T 274A>C), ACA59ATG (276C>T 277A>G), TAT60ATT (278T>A 279A>T), GCA61GCT (283A>T), ATC62CTT (284A>C 286C>T), AGT63ACC (288G>C 289T>C), TTA65TGC (294T>G 295A>C), CAG66CAA (298G>A), CAA67GTT (299C>G 300A>T 301A>T), ATA68GTG (302A>G 304A>G), ACT70ACC (310T>C), TCT71AAA (311T>A 312C>A 313T>A), AAT74GAG (320A>G 322T>G), ATC75GTA (323A>G 325C>A), GCT77GCA (331T>A), ATC79ATA (337C>A), ACC81TCT (341A>T 343C>T), CCA82CCT (346A>T), CGT84CGG (352T>G), GAA85GAG (355A>G), TTG86CTT (356T>C 358G>T), GCT87GCA (361T>A), CTC88GCT (362C>G 363T>C 364C>T), GCT90ACG (368G>A 370T>G), CAA91GAA (371C>G), CGT92AAG (374C>A 375G>A 376T>G), GTA93GTG (379A>G), TTG94ATT (380T>A 382G>T), CAA95TTG (383C>T 384A>T 385A>G), ACA96GCA (386A>G), GGG98GGA (394G>A), AAT99GAT (395A>G), TAT100TAC (400T>C), CTA101ATT (401C>A 403A>T), TAT102del (404_406delTAT), AAT103AAC (409T>C), TTT104ATA (410T>A 412T>A), AAA105CAG (413A>C 415A>G), TGT106GCA (416T>G 417G>C 418T>A), CAA107CAT (421A>T), GTT108GCG (423T>C 424T>G), ACA113AAA (438C>A), AGT114AGC (442T>C), ATC115GTG (443A>G 445C>G), AAG116GGT (446A>G 447A>G 448G>T), AGC118GAT (452A>G 453G>A 454C>T), CAG119ATC (455C>A 456A>T 457G>C), GAA120AGA (458G>A 459A>G), ACT121AAG (462C>A 463T>G), AAG123GAG (467A>G), AAG123_AAA124insCAT (469_470insCAT), AAA124GGA (470A>G 471A>G), GCC125GTA (474C>T 475C>A), CAG126CAA (478G>A), GTT127GTA (481T>A), TTG128GTG (482T>G), ATT129GTT (485A>G), GGA130GGT (490A>T), CCT132CCA (496T>A), GGA133GGC (499A>C), CGA134CGT (502A>T), ATG135GTC (503A>G 505G>C), ATT136TTT (506A>T), GAT137GAC (511T>C), CTA138ATG (512C>A 514A>G), CTC139CTG (517C>G), ACC140CGC (518A>C 519C>G), CGA141AGG (521C>A 523A>G), AAA142CAG (524A>C 526A>G), AGT143TCT (527A>T 528G>C), ATC144CTT (530A>C 532C>T), GAT145CGC (533G>C 534A>G 535T>C), ACC146CCT (536A>C 538C>T), AAA147GAC (539A>G 541A>C), GCA148CAC (542G>C 543C>A 544A>C), ATT149ATC (547T>C), AAA150AAG (550A>G), ATA151ATG (553A>G), GTT152TTT (554G>T), GTA153GTT (559A>T), ATA154TTG (560A>T 562A>G), GAT158GAC (574T>C), GAG159GAA (577G>A), TTG161CTC (581T>C 583G>C), ATA162TCT (584A>T 585T>C 586A>T), GAT163AGA (587G>A 588A>G 589T>A), AAT164GGT (590A>G 591A>G), TTT165TTC (595T>C), TTG166AAG (596T>A 597T>A), AAA168CAG (602A>C 604A>G), ATA169ATT (607A>T), CAA170TAT (608C>T 610A>T), ATT172ATA (616T>A), GAA174CAG (620G>C 622A>G), TTT175CTT (623T>C), TTT176TTG (628T>G), GAA177CCA (629G>C 630A>C), AGT178CCA (632A>C 633G>C 634T>A), CAT179AAA (635C>A 637T>A), GTC180ATC (638G>A), GTT182GTG (646T>G), ATT183GGT (647A>G 648T>G), TTA184GTT (650T>G 652A>T), TTA185TTC (655A>C), TCA186TCT (658A>T), GCA187GCT (661A>T), ACC188ACT (664C>T), GTT189ATG (665G>A 667T>G), TCA191CCA (671T>C), AGG192GAG (674A>G 675G>A), GTT193GCT (678T>C), ATC194CTT (680A>C 682C>T), AAT195GAA (683A>G 685T>A), ACA196ATT (687C>T 688A>T), TCT197ACT (689T>A), CAG198AGG (692C>A 693A>G), GTC199AAG (695G>A 696T>A 697C>G), TTT200TTC (700T>C), AGA202AAC (705G>A 706A>C), GAT203AAA (707G>A 709T>A), CCC204CCT (712C>T), GTA205GTG (715A>G), AAA206AGG (717A>G 718A>G), CTG208CTT (724G>T), GTA209GTG (727A>G), AAA210AAG (730A>G), AAT211CGT (731A>C 732A>G), GCT212GAT (735C>A), GAA213GAG (739A>G), TTG214CTC (740T>C 742G>C), ACA215ACT (745A>T), CTC216CTT (748C>T), GGC218GGT (754C>T), ATT219ATC (757T>C), AGG220AAG (759G>A), TAT222TTT (765A>T), ATA224GTC (770A>G 772A>C), GTA226GTC (778A>C), AAG227GAT (779A>G 781G>T), AAA228AAG (784A>G), AAT229GAA (785A>G 787T>A), GAT230GAA (790T>A), TTT231TGG (792T>G 793T>G), AAA232AAG (796A>G), GCA233CTT (797G>C 798C>T 799A>T), TTT237TGT (810T>G), GAC241GAG (823C>G), CAC242ACT (824C>A 825A>C 826C>T), TTA243TTG (829A>G), AGC244GCC (830A>G 831G>C), CTC245ATC (833C>A), ACT246ACC (838T>C), CAA247CAG (841A>G), ACA248AGT (843C>G 844A>T), CTA249GTC (845C>G 847A>C), ATA250ATC (850A>C), TGT252GTG (854T>G 855G>T 856T>G), AAT253AAC (859T>C), ACA254ACC (862A>C), CGT255AGG (863C>A 865T>G), AGA256CGT (866A>C 868A>T), CAA257AAA (869C>A), GTA260TGG (878G>T 879T>G 880A>G), ATG262ACT (885T>C 886G>T), GAA263GAT (889A>T), TGT264AAG (890T>A 891G>A 892T>G), TTA265ATG (893T>A 895A>G), ACA266CGC (896A>C 897C>G 898A>C), AAT267AGC (900A>G 901T>C), AGA268CGT (902A>C 904A>T), AAT269GAT (905A>G), TTC270CAC (908T>C 909T>A), GCA272GTA (915C>T), TCA273TCT (919A>T), AGC274GCA (920A>G 921G>C 922C>A), ATC275ACT (924T>C 925C>T), GAT278GAC (934T>C), TCC280GAC (938T>G 939C>A), CAA281CAG (943A>G), CAA282AAC (944C>A 946A>C), GAA283ACT (947G>A 948A>C 949A>T), CGT284AGA (950C>A 952T>A), TTT286ATC (956T>A 958T>C), AAA289CGA (965A>C 966A>G), GAA290GAG (970A>G), TTT291TTC (973T>C), AGA292CGA (974A>C), GAT293TCT (977G>T 978A>C), GGT294GGG (982T>G), AAA295TCA (983A>T 984A>C), ACT296TCT (986A>T), AGA297CGT (989A>C 991A>T), ATA298GTG (992A>G 994A>G), CTT299CTC (997T>C), CTT300ATC (998C>A 1000T>C), TCA301ACA (1001T>A), AAT303GAT (1007A>G), CTA304CTC (1012A>C), TTA305CTT (1013T>C 1015A>T), GCA306GCT (1018A>T), AGA307CGT (1019A>C 1021A>T), GGC308GGT (1024C>T), GTT311GTA (1033T>A), CAA312CAG (1036A>G), ATA314GTC (1040A>G 1042A>C), TCT315TCC (1045T>C), TTA316CTT (1046T>C 1048A>T), GTT317GTC (1051T>C), ATC318ATT (1054C>T), AAT319AAC (1057T>C), GAT321GAC (1063T>C), TTG322CTG (1064T>C), CAT324ACT (1070C>A 1071A>C), AAT325CAA (1073A>C 1075T>A), AGA326CCA (1076A>C 1077G>C), TAT329TAC (1087T>C), ATA330CTG (1088A>C 1090A>G), AGA335CGT (1103A>C 1105A>T), GGT337GGA (1111T>A), AGA338CGA (1112A>C), GGC340GGA (1120C>A), CGA341AGG (1121C>A 1123A>G), AAA342AAG (1126A>G), GGG343GGT (1129G>T), ATT344GTT (1130A>G), AAT347AAC (1141T>C), ATA349GTC (1145A>G 1147A>C), ACT350ACC (1150T>C), GAA351AAG (1151G>A 1153A>G), GAC352GAT (1156C>T), GAA353GAT (1159A>T), TTA354GAA (1160T>G 1161T>A), CCA355AGG (1163C>A 1164C>G 1165A>G), AAG356ATG (1167A>T), GCT358TTT (1172G>T 1173C>T), GAA359GAC (1177A>C), TTA360ATA (1178T>A), GAA361CAG (1181G>C 1183A>G), ACG362AAA (1185C>A 1186G>A), TAT364TAC (1192T>C), AAT365AAC (1195T>C), ACC366GTC (1196A>G 1197C>T), AAA367GTA (1199A>G 1200A>T), ATA368ATC (1204A>C), GAT369GAG (1207T>G), GAA370GAG (1210A>G), ATG371CTC (1211A>C 1213G>C), GAG373GCC (1218A>C 1219G>C), ATT375GTT (1223A>G), GTT376GCT (1227T>C), TTG378CTC (1232T>C 1234G>C), ATA379CTG (1235A>C 1237A>G)								

Proteins

hypothetical protein (YP_009666148.1)	7	380	98.4%	1456	58.9%	373 (99.5%)	207 (55.2%)	1/1/0/0	0
Protein mutations:	E7S (119G>A 120A>G 121A>T), D9A (126A>C 127C>T), H10E (128C>G 130T>G), L13I (137C>A 139C>T), K15D (143A>G 145A>T), N16D (146A>G 148T>C), I17L (149A>C 151C>T), K19R (156A>G 157G>A), S23Q (167T>C 168C>A 169T>G), C24Y (171G>A 172T>C), T31A (191A>G 193T>C), K35R (203A>C 204A>G), I37V (209A>G 211T>A), F38L (214T>G), C40I (218T>A 219G>T), K44R (230A>C 231A>G 232A>T), V48A (243T>C 244C>T), A58S (272G>T 274A>C), T59M (276C>T 277A>G), Y60I (278T>A 279A>T), I62L (284A>C 286C>T), S63T (288G>C 289T>C), L65C (294T>G 295A>C), Q67V (299C>G 300A>T 301A>T), I68V (302A>G 304A>G), S71K (311T>A 312C>A 313T>A), N74E (320A>G 322T>G), I75V (323A>G 325C>A), T81S (341A>T 343C>T), L88A (362C>G 363T>C 364C>T), A90T (368G>A 370T>G), Q91E (371C>G), R92K (374C>A 375G>A 376T>G), L94I (380T>A 382G>T), Q95L (383C>T 384A>T 385A>G), T96A (386A>G), N99D (395A>G), L101I (401C>A 403A>T), Y102del (404_406delTAT), F104I (410T>A 412T>A), K105Q (413A>C 415A>G), C106A (416T>G 417G>C 418T>A), Q107H (421A>T), V108A (423T>C 424T>G), T113K (438C>A), I115V (443A>G 445C>G), K116G (446A>G 447A>G 448G>T), S118D (452A>G 453G>A 454C>T), Q119I (455C>A 456A>T 457G>C), E120R (458G>A 459A>G), T121K (462C>A 463T>G), K123E (467A>G), K123_K124insH (469_470insCAT), K124G (470A>G 471A>G), A125V (474C>T 475C>A), L128V (482T>G), I129V (485A>G), M135V (503A>G 505G>C), I136F (506A>T), L138M (512C>A 514A>G), T140R (518A>C 519C>G), K142Q (524A>C 526A>G), I144L (530A>C 532C>T), D145R (533G>C 534A>G 535T>C), T146P (536A>C 538C>T), K147D (539A>G 541A>C), A148H (542G>C 543C>A 544A>C), I151M (553A>G), V152F (554G>T), I154L (560A>T 562A>G), I162S (584A>T 585T>C 586A>T), D163R (587G>A 588A>G 589T>A), N164G (590A>G 591A>G), L166K (596T>A 597T>A), K168Q (602A>C 604A>G), Q170Y (608C>T 610A>T), E174Q (620G>C 622A>G), F175L (623T>C), F176L (628T>G), E177P (629G>C 630A>C), S178P (632A>C 633G>C 634T>A), H179K (635C>A 637T>A), V180I (638G>A), I183G (647A>G 648T>G), L184V (650T>G 652A>T), L185F (655A>C), V189M (665G>A 667T>G), S191P (671T>C), R192E (674A>G 675G>A), V193A (678T>C), I194L (680A>C 682C>T), N195E (683A>G 685T>A), T196I (687C>T 688A>T), S197T (689T>A), Q198R (692C>A 693A>G), V199K (695G>A 696T>A 697C>G), R202N (705G>A 706A>C), D203K (707G>A 709T>A), K206R (717A>G 718A>G), N211R (731A>C 732A>G), A212D (735C>A), R220K (759G>A), Y222F (765A>T), I224V (770A>G 772A>C), K227D (779A>G 781G>T), N229E (785A>G 787T>A), D230E (790T>A), F231W (792T>G 793T>G), A233L (797G>C 798C>T 799A>T), F237C (810T>G), D241E (823C>G), H242T (824C>A 825A>C 826C>T), S244A (830A>G 831G>C), L245I (833C>A), T248S (843C>G 844A>T), L249V (845C>G 847A>C), C252V (854T>G 855G>T 856T>G), Q257K (869C>A), V260W (878G>T 879T>G 880A>G), M262T (885T>C 886G>T), E263D (889A>T), C264K (890T>A 891G>A 892T>G), L265M (893T>A 895A>G), T266R (896A>C 897C>G 898A>C), N267S (900A>G 901T>C), N269D (905A>G), F270H (908T>C 909T>A), A272V (915C>T), S274A (920A>G 921G>C 922C>A), I275T (924T>C 925C>T), S280D (938T>G 939C>A), Q282N (944C>A 946A>C), E283T (947G>A 948A>C 949A>T), F286I (956T>A 958T>C), K289R (965A>C 966A>G), D293S (977G>T 978A>C), K295S (983A>T 984A>C), T296S (986A>T), I298V (992A>G 994A>G), L300I (998C>A 1000T>C), S301T (1001T>A), N303D (1007A>G), I314V (1040A>G 1042A>C), H324T (1070C>A 1071A>C), N325Q (1073A>C 1075T>A), R326P (1076A>C 1077G>C), I330L (1088A>C 1090A>G), I344V (1130A>G), I349V (1145A>G 1147A>C), E351K (1151G>A 1153A>G), E353D (1159A>T), L354E (1160T>G 1161T>A), P355R (1163C>A 1164C>G 1165A>G), K356M (1167A>T), A358F (1172G>T 1173C>T), E359D (1177A>C), L360I (1178T>A), E361Q (1181G>C 1183A>G), T362K (1185C>A 1186G>A), T366V (1196A>G 1197C>T), K367V (1199A>G 1200A>T), D369E (1207T>G), M371L (1211A>C 1213G>C), E373A (1218A>C 1219G>C), I375V (1223A>G), V376A (1227T>C), I379L (1235A>C 1237A>G)								

	Begin	End	Coverage	Score	Concordance	Matches	Identities	I/D/M/F*	Stop Codons
**NT**	**118**	**1238**	**83.7%**	**392**	**17.6%**	**1118 (99.5%)**	**663 (59.0%)**	**3/3**	
Codon mutations:	GAA7AGT (119G>A 120A>G 121A>T), GAC9GCT (126A>C 127C>T), CAT10GAG (128C>G 130T>G), GGC12GGG (136C>G), CTC13ATT (137C>A 139C>T), AAG14AAA (142G>A), AAA15GAT (143A>G 145A>T), AAT16GAC (146A>G 148T>C), ATC17CTT (149A>C 151C>T), CTA18CTT (154A>T), AAG19AGA (156A>G 157G>A), GGT20GGG (160T>G), ATA21ATT (163A>T), TAT22TAC (166T>C), TCT23CAG (167T>C 168C>A 169T>G), TGT24TAC (171G>A 172T>C), GGA25GGG (175A>G), TTT26TTC (178T>C), CCT29CCA (187T>A), TCA30TCT (190A>T), ACT31GCC (191A>G 193T>C), AAA35CGA (203A>C 204A>G), GCC36GCT (208C>T), ATT37GTA (209A>G 211T>A), TTT38TTG (214T>G), CCA39CCT (217A>T), TGT40ATT (218T>A 219G>T), ATT41ATA (223T>A), TCT42TCG (226T>G), AAA44CGT (230A>C 231A>G 232A>T), GTG46GTC (238G>C), ATA47ATT (241A>T), GTC48GCT (243T>C 244C>T), CAA49CAG (247A>G), TCG52TCC (256G>C), GGA53GGT (259A>T), ACA54ACC (262A>C), ACA57ACC (271A>C), GCA58TCC (272G>T 274A>C), ACA59ATG (276C>T 277A>G), TAT60ATT (278T>A 279A>T), GCA61GCT (283A>T), ATC62CTT (284A>C 286C>T), AGT63ACC (288G>C 289T>C), TTA65TGC (294T>G 295A>C), CAG66CAA (298G>A), CAA67GTT (299C>G 300A>T 301A>T), ATA68GTG (302A>G 304A>G), ACT70ACC (310T>C), TCT71AAA (311T>A 312C>A 313T>A), AAT74GAG (320A>G 322T>G), ATC75GTA (323A>G 325C>A), GCT77GCA (331T>A), ATC79ATA (337C>A), ACC81TCT (341A>T 343C>T), CCA82CCT (346A>T), CGT84CGG (352T>G), GAA85GAG (355A>G), TTG86CTT (356T>C 358G>T), GCT87GCA (361T>A), CTC88GCT (362C>G 363T>C 364C>T), GCT90ACG (368G>A 370T>G), CAA91GAA (371C>G), CGT92AAG (374C>A 375G>A 376T>G), GTA93GTG (379A>G), TTG94ATT (380T>A 382G>T), CAA95TTG (383C>T 384A>T 385A>G), ACA96GCA (386A>G), GGG98GGA (394G>A), AAT99GAT (395A>G), TAT100TAC (400T>C), CTA101ATT (401C>A 403A>T), TAT102del (404_406delTAT), AAT103AAC (409T>C), TTT104ATA (410T>A 412T>A), AAA105CAG (413A>C 415A>G), TGT106GCA (416T>G 417G>C 418T>A), CAA107CAT (421A>T), GTT108GCG (423T>C 424T>G), ACA113AAA (438C>A), AGT114AGC (442T>C), ATC115GTG (443A>G 445C>G), AAG116GGT (446A>G 447A>G 448G>T), AGC118GAT (452A>G 453G>A 454C>T), CAG119ATC (455C>A 456A>T 457G>C), GAA120AGA (458G>A 459A>G), ACT121AAG (462C>A 463T>G), AAG123GAG (467A>G), AAG123_AAA124insCAT (469_470insCAT), AAA124GGA (470A>G 471A>G), GCC125GTA (474C>T 475C>A), CAG126CAA (478G>A), GTT127GTA (481T>A), TTG128GTG (482T>G), ATT129GTT (485A>G), GGA130GGT (490A>T), CCT132CCA (496T>A), GGA133GGC (499A>C), CGA134CGT (502A>T), ATG135GTC (503A>G 505G>C), ATT136TTT (506A>T), GAT137GAC (511T>C), CTA138ATG (512C>A 514A>G), CTC139CTG (517C>G), ACC140CGC (518A>C 519C>G), CGA141AGG (521C>A 523A>G), AAA142CAG (524A>C 526A>G), AGT143TCT (527A>T 528G>C), ATC144CTT (530A>C 532C>T), GAT145CGC (533G>C 534A>G 535T>C), ACC146CCT (536A>C 538C>T), AAA147GAC (539A>G 541A>C), GCA148CAC (542G>C 543C>A 544A>C), ATT149ATC (547T>C), AAA150AAG (550A>G), ATA151ATG (553A>G), GTT152TTT (554G>T), GTA153GTT (559A>T), ATA154TTG (560A>T 562A>G), GAT158GAC (574T>C), GAG159GAA (577G>A), TTG161CTC (581T>C 583G>C), ATA162TCT (584A>T 585T>C 586A>T), GAT163AGA (587G>A 588A>G 589T>A), AAT164GGT (590A>G 591A>G), TTT165TTC (595T>C), TTG166AAG (596T>A 597T>A), AAA168CAG (602A>C 604A>G), ATA169ATT (607A>T), CAA170TAT (608C>T 610A>T), ATT172ATA (616T>A), GAA174CAG (620G>C 622A>G), TTT175CTT (623T>C), TTT176TTG (628T>G), GAA177CCA (629G>C 630A>C), AGT178CCA (632A>C 633G>C 634T>A), CAT179AAA (635C>A 637T>A), GTC180ATC (638G>A), GTT182GTG (646T>G), ATT183GGT (647A>G 648T>G), TTA184GTT (650T>G 652A>T), TTA185TTC (655A>C), TCA186TCT (658A>T), GCA187GCT (661A>T), ACC188ACT (664C>T), GTT189ATG (665G>A 667T>G), TCA191CCA (671T>C), AGG192GAG (674A>G 675G>A), GTT193GCT (678T>C), ATC194CTT (680A>C 682C>T), AAT195GAA (683A>G 685T>A), ACA196ATT (687C>T 688A>T), TCT197ACT (689T>A), CAG198AGG (692C>A 693A>G), GTC199AAG (695G>A 696T>A 697C>G), TTT200TTC (700T>C), AGA202AAC (705G>A 706A>C), GAT203AAA (707G>A 709T>A), CCC204CCT (712C>T), GTA205GTG (715A>G), AAA206AGG (717A>G 718A>G), CTG208CTT (724G>T), GTA209GTG (727A>G), AAA210AAG (730A>G), AAT211CGT (731A>C 732A>G), GCT212GAT (735C>A), GAA213GAG (739A>G), TTG214CTC (740T>C 742G>C), ACA215ACT (745A>T), CTC216CTT (748C>T), GGC218GGT (754C>T), ATT219ATC (757T>C), AGG220AAG (759G>A), TAT222TTT (765A>T), ATA224GTC (770A>G 772A>C), GTA226GTC (778A>C), AAG227GAT (779A>G 781G>T), AAA228AAG (784A>G), AAT229GAA (785A>G 787T>A), GAT230GAA (790T>A), TTT231TGG (792T>G 793T>G), AAA232AAG (796A>G), GCA233CTT (797G>C 798C>T 799A>T), TTT237TGT (810T>G), GAC241GAG (823C>G), CAC242ACT (824C>A 825A>C 826C>T), TTA243TTG (829A>G), AGC244GCC (830A>G 831G>C), CTC245ATC (833C>A), ACT246ACC (838T>C), CAA247CAG (841A>G), ACA248AGT (843C>G 844A>T), CTA249GTC (845C>G 847A>C), ATA250ATC (850A>C), TGT252GTG (854T>G 855G>T 856T>G), AAT253AAC (859T>C), ACA254ACC (862A>C), CGT255AGG (863C>A 865T>G), AGA256CGT (866A>C 868A>T), CAA257AAA (869C>A), GTA260TGG (878G>T 879T>G 880A>G), ATG262ACT (885T>C 886G>T), GAA263GAT (889A>T), TGT264AAG (890T>A 891G>A 892T>G), TTA265ATG (893T>A 895A>G), ACA266CGC (896A>C 897C>G 898A>C), AAT267AGC (900A>G 901T>C), AGA268CGT (902A>C 904A>T), AAT269GAT (905A>G), TTC270CAC (908T>C 909T>A), GCA272GTA (915C>T), TCA273TCT (919A>T), AGC274GCA (920A>G 921G>C 922C>A), ATC275ACT (924T>C 925C>T), GAT278GAC (934T>C), TCC280GAC (938T>G 939C>A), CAA281CAG (943A>G), CAA282AAC (944C>A 946A>C), GAA283ACT (947G>A 948A>C 949A>T), CGT284AGA (950C>A 952T>A), TTT286ATC (956T>A 958T>C), AAA289CGA (965A>C 966A>G), GAA290GAG (970A>G), TTT291TTC (973T>C), AGA292CGA (974A>C), GAT293TCT (977G>T 978A>C), GGT294GGG (982T>G), AAA295TCA (983A>T 984A>C), ACT296TCT (986A>T), AGA297CGT (989A>C 991A>T), ATA298GTG (992A>G 994A>G), CTT299CTC (997T>C), CTT300ATC (998C>A 1000T>C), TCA301ACA (1001T>A), AAT303GAT (1007A>G), CTA304CTC (1012A>C), TTA305CTT (1013T>C 1015A>T), GCA306GCT (1018A>T), AGA307CGT (1019A>C 1021A>T), GGC308GGT (1024C>T), GTT311GTA (1033T>A), CAA312CAG (1036A>G), ATA314GTC (1040A>G 1042A>C), TCT315TCC (1045T>C), TTA316CTT (1046T>C 1048A>T), GTT317GTC (1051T>C), ATC318ATT (1054C>T), AAT319AAC (1057T>C), GAT321GAC (1063T>C), TTG322CTG (1064T>C), CAT324ACT (1070C>A 1071A>C), AAT325CAA (1073A>C 1075T>A), AGA326CCA (1076A>C 1077G>C), TAT329TAC (1087T>C), ATA330CTG (1088A>C 1090A>G), AGA335CGT (1103A>C 1105A>T), GGT337GGA (1111T>A), AGA338CGA (1112A>C), GGC340GGA (1120C>A), CGA341AGG (1121C>A 1123A>G), AAA342AAG (1126A>G), GGG343GGT (1129G>T), ATT344GTT (1130A>G), AAT347AAC (1141T>C), ATA349GTC (1145A>G 1147A>C), ACT350ACC (1150T>C), GAA351AAG (1151G>A 1153A>G), GAC352GAT (1156C>T), GAA353GAT (1159A>T), TTA354GAA (1160T>G 1161T>A), CCA355AGG (1163C>A 1164C>G 1165A>G), AAG356ATG (1167A>T), GCT358TTT (1172G>T 1173C>T), GAA359GAC (1177A>C), TTA360ATA (1178T>A), GAA361CAG (1181G>C 1183A>G), ACG362AAA (1185C>A 1186G>A), TAT364TAC (1192T>C), AAT365AAC (1195T>C), ACC366GTC (1196A>G 1197C>T), AAA367GTA (1199A>G 1200A>T), ATA368ATC (1204A>C), GAT369GAG (1207T>G), GAA370GAG (1210A>G), ATG371CTC (1211A>C 1213G>C), GAG373GCC (1218A>C 1219G>C), ATT375GTT (1223A>G), GTT376GCT (1227T>C), TTG378CTC (1232T>C 1234G>C), ATA379CTG (1235A>C 1237A>G)								

*: Inserts / Deletes / Misaligned / Frameshifts

Analysis details

This analysis was performed with panviral2.64

NGS Details (UN59): Potato virus Y

Assembly

**Coverage Length**	8670 (6 contig(s))
**Depth Of Coverage**	11.7
**Number Of Reads**	757
**Reads Per Million**	16.46 rpm (after QC)
**Ambiguities**	0
**Assembly Method**	de novo + reference guided assembly
**Consensus Caller**	Bcf Tools

Coverage Map

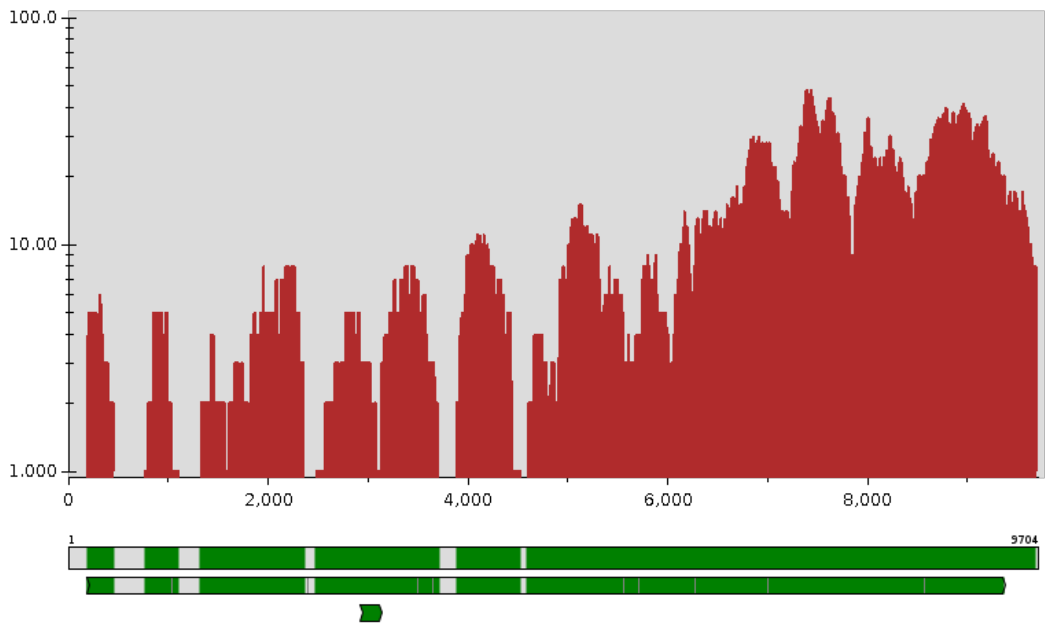

Assignment

**Type**	Potato virus Y (Taxonomy ID: 12216)
**Reference Genome**	NC_001616.1
**NT Identity (%)**	83.3353
**AA Identity (%)**	90.1748
**Number Of Stop Codons**	4
**Number Of CDS**	2

Alignment

**Alignment Score**	11427.0 (NT) + 17126.0 (AA) = 28553.0
**Concordance (%)**	78.8456

Alignment Method	Global, seeded, nucleotide + amino acids (AGA)

Genome Region

Sequence starts at position 183 and ends at position 9687 relative to NC_001616.1 reference sequence.

Alignment Detailed Statistics

	Begin	End	Coverage	Score	Concordance	Matches	Identities	I/D/M/F*	Stop Codons
**NT**	**183**	**9687**	**89.3%**	**11427**	**66.3%**	**8654 (99.8%)**	**7216 (83.2%)**	**5/16**	
Mutations:	183C>T, 193T>C, 194T>C, 196C>A, 202A>G, 208C>T, 209T>C, 210G>T, 211T>G, 220G>A, 221T>A, 223T>G, 226A>G, 235A>T, 241C>T, 247A>T, 251T>C, 255G>T, 256C>T, 258A>G, 262T>C, 263A>G, 265T>G, 267T>C, 268G>A, 273A>T, 277A>T, 280A>G, 283G>T, 286G>A, 287G>A, 289T>C, 293G>A, 294T>C, 301T>C, 304C>T, 305G>A, 306C>T, 309A>G, 311C>T, 316A>T, 317A>G, 318C>T, 319A>G, 322A>G, 331A>T, 339T>A, 340C>A, 342A>G, 347A>G, 348A>C, 349A>G, 350T>C, 355T>A, 356A>G, 357C>T, 364T>C, 365G>A, 366T>C, 367G>A, 368C>T, 369T>C, 373A>G, 376C>T, 384T>G, 388G>A, 391C>T, 394A>G, 397C>T, 400G>A, 406T>C, 408C>T, 409C>T, 412G>A, 415A>T, 416A>G, 417T>A, 424T>C, 427G>A, 433A>G, 436G>C, 437G>A, 440A>G, 441G>A, 442G>A, 443A>C, 444A>G, 445G>A, 448T>A, 454A>G, 765T>A, 770T>C, 773C>A, 774G>A, 775A>G, 776C>A, 790C>T, 801T>A, 813G>C, 814A>G, 816T>A, 818T>C, 823A>G, 825G>A, 826T>A, 833C>A, 835G>A, 841C>T, 853T>C, 859C>T, 860C>T, 862C>T, 863A>G, 865T>C, 867T>C, 869A>G, 871C>T, 872A>T, 874A>G, 879G>A, 886T>C, 896A>G, 898C>T, 903A>G, 906C>A, 907A>C, 909A>C, 910A>C, 912G>A, 913C>T, 922C>A, 931T>A, 938T>C, 939C>T, 940A>T, 943A>G, 952C>T, 967A>G, 973A>G, 976G>C, 980T>A, 982G>C, 991A>G, 994T>C, 999G>A, 1003T>C, 1007C>A, 1008A>G, 1009G>A, 1010A>G, 1012T>G, 1013A>G, 1018A>G, 1019A>C, 1021C>G, 1030C>T, 1039G>A, 1042T>C, 1045C>A, 1048C>A, 1051T>C, 1060G>A, 1063T>A, 1064C>T, 1069C>T, 1083G>A, 1092A>G, 1093T>C, 1097T>G, 1099G>A, 1105C>T, 1318T>A, 1321G>A, 1327G>A, 1329T>C, 1333G>T, 1336A>G, 1343C>T, 1345A>G, 1354G>A, 1357G>T, 1360C>T, 1367C>T, 1369C>G, 1373C>A, 1378C>T, 1384G>A, 1385A>G, 1393A>G, 1396C>T, 1405G>T, 1408A>G, 1411G>A, 1415G>T, 1417A>T, 1420G>C, 1429T>C, 1432A>G, 1436G>A, 1438C>T, 1439T>C, 1441A>G, 1444T>C, 1453C>T, 1456G>A, 1468A>G, 1492A>G, 1499T>C, 1501G>A, 1504T>C, 1507G>A, 1510C>T, 1513A>G, 1516A>G, 1519A>T, 1522G>A, 1525C>T, 1528G>A, 1534C>T, 1540T>A, 1546C>T, 1555A>G, 1561T>C, 1564A>C, 1565_1568delTCTT, 1573C>T, 1576A>G, 1583T>C, 1588T>A, 1594G>A, 1600C>T, 1618G>A, 1621C>T, 1624C>T, 1631C>T, 1639A>G, 1651C>T, 1654C>G, 1675T>C, 1693A>G, 1702T>C, 1707A>G, 1718G>A, 1726A>G, 1729C>T, 1732A>C, 1738T>C, 1742A>G, 1744C>T, 1747C>T, 1753T>C, 1758G>A, 1768G>A, 1786C>T, 1789A>G, 1790G>A, 1795C>T, 1805T>C, 1807A>G, 1813G>A, 1819G>A, 1825G>A, 1837C>T, 1840T>C, 1849A>G, 1858C>G, 1864A>G, 1870C>T, 1876T>C, 1879G>T, 1882A>G, 1888T>A, 1891T>C, 1906T>C, 1915A>T, 1930T>C, 1936C>T, 1937A>G, 1939T>C, 1945A>G, 1951C>T, 1960A>T, 1978A>G, 1981T>A, 1984C>T, 1987T>C, 1996C>T, 2004T>A, 2020A>G, 2023G>A, 2026T>C, 2029G>T, 2032G>A, 2059T>C, 2068T>G, 2071C>T, 2072G>A, 2074G>A, 2087C>T, 2089G>A, 2101C>T, 2104G>A, 2107G>A, 2116G>A, 2122C>T, 2137C>T, 2158T>C, 2164C>T, 2170A>G, 2182T>C, 2188G>A, 2191C>A, 2208G>A, 2218T>C, 2224C>T, 2227A>T, 2242G>A, 2245C>T, 2254G>A, 2257T>C, 2260C>T, 2266C>G, 2272A>G, 2275G>A, 2293A>G, 2299C>T, 2302G>A, 2305G>A, 2314A>T, 2317T>C, 2323T>C, 2326A>G, 2338C>T, 2341G>A, 2347A>G, 2350T>C, 2356G>A, 2365T>C, 2476T>C, 2482G>A, 2491G>A, 2497G>T, 2503G>A, 2518A>G, 2521T>C, 2523A>G, 2533A>G, 2539G>A, 2545A>G, 2551G>A, 2554T>A, 2558C>T, 2561T>C, 2563G>C, 2569A>G, 2572A>G, 2584T>C, 2587C>T, 2593G>C, 2599T>C, 2605T>C, 2608T>C, 2620T>C, 2629G>A, 2632G>A, 2634G>A, 2638G>A, 2644T>A, 2653A>G, 2664C>T, 2665T>C, 2678C>T, 2683A>G, 2689T>C, 2698A>C, 2707A>G, 2710G>A, 2716A>G, 2719A>G, 2722C>T, 2723G>A, 2728A>G, 2734G>A, 2737T>A, 2747G>A, 2749T>A, 2752A>T, 2755T>A, 2761C>T, 2764C>T, 2779T>C, 2785G>T, 2791C>T, 2795C>A, 2806C>T, 2816T>C, 2818G>C, 2822G>A, 2824G>A, 2827G>A, 2836T>C, 2839G>A, 2842T>C, 2863C>A, 2864C>T, 2882C>A, 2885A>C, 2886G>A, 2893C>T, 2895C>T, 2902C>T, 2905A>G, 2911T>C, 2923T>C, 2931A>G, 2940A>G, 2947T>C, 2962T>C, 2971_2972insA, 2989T>C, 2991C>T, 2994A>G, 2999G>A, 3000A>G, 3016delC, 3021G>A, 3024A>T, 3025C>T, 3036C>T, 3039A>G, 3040A>C, 3042A>C, 3043G>A, 3052G>A, 3061A>G, 3082G>A, 3091C>T, 3099C>T, 3100C>A, 3104G>A, 3105T>G, 3106G>A, 3109C>T, 3112A>G, 3118T>C, 3121C>T, 3127A>G, 3132G>A, 3137C>A, 3138G>A, 3139A>G, 3151T>C, 3152T>A, 3154C>T, 3165G>A, 3169A>G, 3199C>T, 3203A>C, 3208T>C, 3214A>G, 3217C>T, 3218T>C, 3223C>A, 3230G>A, 3235C>T, 3257T>C, 3262C>T, 3265C>T, 3266G>A, 3268A>G, 3271G>A, 3275G>A, 3284G>A, 3301G>A, 3315G>A, 3316A>G, 3318A>C, 3322C>T, 3325G>A, 3328G>A, 3337A>T, 3349G>A, 3355C>T, 3358C>T, 3359A>G, 3367A>G, 3370T>C, 3377T>C, 3379A>G, 3394A>G, 3397C>T, 3400T>G, 3406A>G, 3418C>T, 3421T>A, 3427T>C, 3433G>A, 3436A>T, 3439A>G, 3442C>T, 3445T>C, 3457G>A, 3460T>C, 3463T>A, 3469G>T, 3472G>A, 3496C>T, 3499G>A, 3505C>T, 3513T>G, 3520A>G, 3529A>G, 3532A>G, 3545A>G, 3548G>A, 3551T>C, 3554G>A, 3556C>T, 3583T>C, 3586T>C, 3589C>T, 3595C>T, 3607T>C, 3613T>C, 3616G>A, 3619T>C, 3623C>G, 3628C>T, 3632C>A, 3637T>C, 3638T>C, 3643A>G, 3661A>G, 3670G>A, 3676G>C, 3679T>A, 3688G>A, 3694T>A, 3697G>A, 3698A>G, 3704G>T, 3706T>A, 3709T>A, 3883C>T, 3886A>G, 3892A>G, 3895C>T, 3898T>C, 3904A>G, 3905C>A, 3907G>A, 3913T>C, 3928G>A, 3931A>T, 3937G>A, 3955T>C, 3958G>A, 3961C>T, 3964A>G, 3967T>C, 3976A>G, 3979T>C, 3985A>T, 3997A>T, 4000G>A, 4006C>T, 4009T>G, 4012C>T, 4015A>G, 4024T>C, 4030A>G, 4033A>T, 4042G>A, 4052C>T, 4054G>A, 4057T>C, 4063T>A, 4066A>G, 4073A>G, 4078T>C, 4081C>T, 4084T>C, 4096C>T, 4108C>T, 4111A>T, 4117G>A, 4118C>T, 4120A>G, 4126C>T, 4128T>A, 4129C>T, 4132C>T, 4135T>C, 4147A>G, 4148T>C, 4151G>T, 4162C>T, 4166G>A, 4171A>C, 4180G>A, 4190C>T, 4198T>C, 4201C>T, 4210G>T, 4213C>T, 4219T>C, 4220C>T, 4223C>A, 4225G>A, 4231T>G, 4237T>C, 4252A>G, 4253T>C, 4255A>G, 4258A>G, 4270T>C, 4282A>G, 4285G>A, 4291A>G, 4303G>A, 4312C>T, 4319A>G, 4321A>G, 4324G>T, 4327G>A, 4336G>T, 4339T>C, 4348A>T, 4360A>G, 4366T>C, 4369T>A, 4375T>C, 4387T>C, 4395T>A, 4406G>A, 4444C>A, 4450C>T, 4456C>T, 4465C>T, 4475A>G, 4477G>T, 4480C>T, 4481A>T, 4495C>T, 4507G>A, 4510C>T, 4513T>G, 4514T>C, 4517C>T, 4528C>T, 4534A>G, 4591G>A, 4597G>A, 4600C>T, 4606C>T, 4609G>A, 4615G>A, 4618C>T, 4625T>C, 4627G>T, 4648C>T, 4651T>C, 4660G>A, 4663C>T, 4669T>A, 4679G>A, 4681G>C, 4684T>C, 4687T>G, 4699G>A, 4702A>G, 4708A>G, 4711G>A, 4712C>T, 4738A>G, 4747A>G, 4748T>C, 4765T>C, 4796G>A, 4798C>T, 4803C>G, 4804T>C, 4807G>A, 4810G>T, 4813T>G, 4816T>C, 4831T>C, 4834C>T, 4840A>T, 4852A>G, 4855C>T, 4867T>C, 4870G>C, 4873T>A, 4903A>T, 4909G>A, 4915T>C, 4918A>G, 4919T>C, 4921G>A, 4924T>C, 4927C>G, 4933T>C, 4936C>A, 4939G>A, 4942T>C, 4945C>T, 4963A>G, 4972T>C, 4990T>C, 5002A>G, 5023T>C, 5024T>C, 5026G>A, 5029C>T, 5032T>C, 5038T>C, 5044A>T, 5053A>T, 5056G>A, 5057A>G, 5060A>G, 5071G>T, 5090C>A, 5095A>G, 5098G>T, 5100C>T, 5104A>G, 5107A>T, 5110T>C, 5113A>G, 5116G>A, 5119A>G, 5120G>T, 5122C>T, 5126A>G, 5137T>C, 5140C>T, 5143A>G, 5146G>A, 5161C>A, 5167A>G, 5173T>G, 5182G>A, 5183G>A, 5188C>T, 5191G>A, 5197A>G, 5200C>T, 5206C>T, 5209A>G, 5230G>A, 5236C>T, 5239C>T, 5245C>T, 5263A>T, 5269C>T, 5272C>T, 5275C>A, 5278C>T, 5281A>C, 5284A>G, 5287T>C, 5288C>T, 5293A>T, 5294T>C, 5296G>A, 5302G>A, 5311T>A, 5314A>G, 5323A>T, 5344A>G, 5347T>C, 5353C>T, 5365C>T, 5386T>C, 5395C>A, 5422A>G, 5425T>C, 5431C>T, 5437G>A, 5452T>C, 5458G>A, 5467T>C, 5470A>G, 5471C>T, 5473A>G, 5482C>T, 5485A>T, 5488T>C, 5489T>C, 5491G>A, 5497T>C, 5506T>C, 5509G>A, 5512G>A, 5516T>C, 5530T>C, 5533G>A, 5536G>T, 5539G>A, 5548T>G, 5554C>T, 5560T>A, 5561G>A, 5563G>A, 5566G>C, 5569A>G, 5584C>G, 5593G>A, 5596G>A, 5597A>G, 5605C>A, 5617G>T, 5619C>T, 5620T>G, 5629G>A, 5630A>C, 5641C>T, 5650A>T, 5662A>T, 5665A>G, 5689A>G, 5693G>A, 5698G>A, 5713A>G, 5716G>C, 5719A>G, 5743C>A, 5755C>A, 5758T>C, 5764T>C, 5770A>G, 5779C>T, 5782T>C, 5791C>T, 5803C>T, 5836G>A, 5839A>G, 5845A>G, 5848A>G, 5857T>C, 5860C>T, 5863A>T, 5869T>A, 5875T>C, 5884C>T, 5893C>T, 5894A>G, 5896C>G, 5899C>T, 5908G>A, 5911T>C, 5923G>A, 5938A>G, 5941C>T, 5944T>C, 5947T>C, 5959G>A, 5960C>G, 5961G>C, 5962G>C, 5974A>G, 5986T>A, 5989C>T, 5990A>G, 5998T>C, 6008A>C, 6010A>T, 6019A>T, 6022G>T, 6025A>T, 6030A>C, 6049C>A, 6058G>A, 6061A>G, 6064C>A, 6070T>C, 6072G>A, 6073T>C, 6074A>C, 6079G>A, 6081C>A, 6085A>T, 6091A>T, 6094C>T, 6111G>C, 6112T>C, 6118A>G, 6124G>A, 6127G>A, 6128A>G, 6130T>C, 6133T>C, 6136A>G, 6148C>T, 6157A>G, 6158G>A, 6163T>C, 6169A>G, 6170A>T, 6172A>G, 6178C>T, 6187A>G, 6190T>C, 6196G>A, 6203C>T, 6205C>T, 6208A>G, 6209C>T, 6211A>G, 6217G>A, 6229T>A, 6230G>A, 6238C>T, 6247G>A, 6253A>T, 6257G>A, 6258C>A, 6274T>C, 6282A>G, 6286G>A, 6298C>T, 6301G>A, 6304A>G, 6307C>T, 6313C>T, 6322C>T, 6328A>T, 6331T>G, 6334T>C, 6337G>A, 6338C>T, 6358T>C, 6361G>A, 6362G>A, 6367A>T, 6370G>A, 6376C>T, 6379T>G, 6385A>C, 6391A>T, 6394A>G, 6397C>T, 6401G>A, 6405C>T, 6412C>T, 6418A>G, 6421T>C, 6429A>T, 6436T>A, 6445G>A, 6450A>G, 6454C>A, 6460C>T, 6463T>A, 6469C>T, 6472G>A, 6475G>A, 6481T>C, 6485C>A, 6487C>T, 6490T>C, 6496C>T, 6502G>A, 6514T>C, 6517G>A, 6527C>A, 6532C>A, 6541G>A, 6544A>G, 6556C>T, 6559T>C, 6565G>A, 6569T>C, 6571G>A, 6586T>A, 6588C>T, 6598A>G, 6601A>G, 6604T>C, 6607T>C, 6616A>G, 6622C>T, 6631G>A, 6634G>A, 6635T>C, 6640T>A, 6646G>C, 6647A>G, 6652C>T, 6664C>T, 6670T>G, 6677A>G, 6679A>G, 6685C>T, 6691A>T, 6694C>T, 6715A>G, 6716G>A, 6719A>G, 6724A>G, 6734C>T, 6742G>A, 6745G>A, 6754C>T, 6760A>G, 6764A>T, 6766A>G, 6769C>T, 6775T>A, 6782C>T, 6787A>T, 6790C>T, 6795C>T, 6796A>G, 6799C>A, 6802C>A, 6808C>T, 6811C>T, 6814C>T, 6823C>T, 6829A>G, 6843A>G, 6847C>T, 6853A>C, 6857A>G, 6862G>A, 6871A>G, 6875G>A, 6876T>C, 6877C>A, 6880G>A, 6883T>A, 6889T>G, 6901C>T, 6916C>T, 6919G>A, 6925A>G, 6928T>C, 6934C>A, 6949G>C, 6955C>T, 6958A>G, 6976A>G, 6980C>T, 6985C>T, 6994T>C, 6997A>T, 7000G>T, 7001G>A, 7003G>A, 7012T>C, 7015G>A, 7018T>C, 7032T>A, 7039C>T, 7040T>C, 7042G>A, 7048A>G, 7060T>A, 7063C>A, 7066A>G, 7078C>T, 7081A>G, 7082T>C, 7084A>G, 7087A>G, 7090C>A, 7096T>C, 7099A>G, 7102T>C, 7111G>A, 7117A>G, 7125C>A, 7129A>G, 7133C>T, 7135G>A, 7141G>A, 7144T>C, 7145G>T, 7147A>G, 7150G>A, 7156G>A, 7159A>T, 7160_7165delGAGGCA, 7171C>T, 7189G>T, 7198A>G, 7201C>T, 7205C>T, 7210T>C, 7216T>A, 7219G>A, 7225C>A, 7228G>A, 7240G>A, 7243T>C, 7249A>G, 7252T>G, 7258T>A, 7261T>C, 7264T>A, 7267C>G, 7270G>A, 7273_7274insT, 7274C>G, 7275G>T, 7281A>T, 7284T>C, 7296C>T, 7311C>T, 7314C>T, 7317G>A, 7321T>A, 7322delG, 7324G>A, 7327C>T, 7330C>T, 7333C>T, 7334A>C, 7342T>C, 7343G>A, 7344C>A, 7345A>C, 7354T>C, 7363A>G, 7369T>C, 7372C>T, 7378G>T, 7381C>T, 7396A>T, 7399C>T, 7405C>T, 7407G>T, 7408T>G, 7414G>T, 7415T>G, 7420A>G, 7429C>T, 7432T>C, 7441T>C, 7459G>A, 7462A>G, 7468A>T, 7471C>A, 7477A>G, 7480C>T, 7486G>T, 7495T>C, 7498A>G, 7501T>C, 7503T>C, 7504G>A, 7513T>A, 7519C>T, 7522A>G, 7531G>A, 7537G>A, 7540C>T, 7546T>C, 7570G>A, 7573G>A, 7579G>A, 7588T>C, 7595C>T, 7604T>C, 7606G>A, 7615T>A, 7624T>C, 7633C>T, 7636C>T, 7639T>C, 7648T>C, 7666A>G, 7672T>C, 7678A>T, 7684G>A, 7690T>C, 7696T>C, 7702T>G, 7709G>A, 7713A>G, 7714A>G, 7715C>T, 7726T>C, 7735G>A, 7744A>G, 7747C>T, 7762C>T, 7768G>A, 7771T>C, 7780A>G, 7783A>C, 7786T>C, 7793C>T, 7795A>G, 7798C>T, 7804T>C, 7812C>T, 7834A>G, 7837C>T, 7844G>T, 7846G>A, 7855G>A, 7861G>A, 7870A>G, 7873C>T, 7876T>A, 7879G>A, 7882T>A, 7883G>A, 7894A>T, 7897T>C, 7921C>T, 7927G>A, 7936T>C, 7939T>C, 7942T>C, 7966G>A, 7969T>C, 7975T>C, 7987C>T, 7990T>C, 8005T>C, 8032A>G, 8038C>T, 8041C>T, 8043G>A, 8080G>A, 8089G>A, 8095G>A, 8098G>A, 8107C>T, 8110T>C, 8113C>T, 8116T>C, 8128A>G, 8146T>C, 8151A>G, 8152C>T, 8164G>A, 8178G>A, 8182G>A, 8191G>A, 8197C>T, 8203C>T, 8208G>A, 8213C>T, 8218A>G, 8221C>T, 8224G>A, 8239A>G, 8260T>C, 8263A>G, 8281T>C, 8284A>G, 8294C>G, 8296A>G, 8299G>A, 8302C>T, 8314G>A, 8317T>C, 8320C>T, 8326T>A, 8335G>A, 8338C>T, 8349C>T, 8353A>G, 8362C>T, 8365A>G, 8368C>T, 8380C>T, 8383A>G, 8389A>G, 8404T>C, 8408G>T, 8413A>G, 8419G>T, 8434T>C, 8440T>C, 8452G>A, 8458A>G, 8460G>A, 8464A>G, 8467G>A, 8480G>A, 8482T>A, 8497G>A, 8498C>T, 8500A>G, 8502G>A, 8503A>G, 8506C>T, 8515A>T, 8524C>T, 8527A>C, 8528T>C, 8530A>G, 8539G>C, 8546C>T, 8547T>G, 8552T>A, 8563A>G, 8574C>G, 8585A>G, 8598G>A, 8602C>T, 8603A>T, 8604A>C, 8605C>T, 8620A>G, 8622C>A, 8625A>G, 8626G>A, 8629G>A, 8632C>T, 8638C>T, 8641G>A, 8647C>T, 8648C>T, 8649C>T, 8650G>C, 8653C>T, 8656A>G, 8658G>A, 8664A>C, 8665T>G, 8674T>G, 8679C>T, 8680A>T, 8683C>A, 8692G>A, 8695A>T, 8707G>A, 8708A>C, 8716G>A, 8722C>T, 8725G>A, 8731A>G, 8737A>G, 8743C>T, 8745C>A, 8746A>G, 8749C>T, 8752G>A, 8755A>T, 8770C>T, 8771T>C, 8776A>G, 8779T>C, 8782G>A, 8791T>C, 8797A>G, 8800A>G, 8803A>G, 8809T>C, 8812T>C, 8818T>C, 8824G>A, 8827A>G, 8848G>A, 8854T>C, 8856A>G, 8857G>A, 8863G>A, 8865G>A, 8867A>C, 8869G>T, 8872A>G, 8883G>A, 8888A>T, 8929C>T, 8954G>A, 8959C>T, 8962A>T, 8969G>A, 8983G>A, 8984A>G, 8986T>C, 8988A>T, 8998G>A, 9004G>A, 9007G>A, 9016C>T, 9037C>A, 9085A>T, 9097C>T, 9131A>G, 9136A>T, 9145G>T, 9149A>G, 9150T>G, 9151G>A, 9152G>A, 9157A>G, 9160G>T, 9163T>C, 9184G>A, 9196A>G, 9214G>A, 9217A>G, 9220G>A, 9244A>T, 9247G>A, 9256C>T, 9260C>T, 9262T>C, 9274G>A, 9280C>T, 9289C>T, 9304G>A, 9334C>T, 9364C>G, 9377_9378insT, 9387C>T, 9404G>A, 9411C>T, 9412A>G, 9414A>T, 9432C>T, 9437C>T, 9454A>G, 9458T>C, 9467G>A, 9470_9471insT, 9482G>A, 9483C>T, 9498T>C, 9516T>G, 9517delA, 9533G>A, 9534G>T, 9548A>T, 9551C>T, 9553_9554delTA, 9556A>T, 9557T>A, 9558A>T, 9574T>A, 9580_9581insT, 9582T>C, 9583G>A, 9590C>T, 9591T>C, 9607C>T, 9610T>C, 9615delC, 9620A>G, 9648A>G								
**CDS**									
PVYgp1	1	3064	90.9%	17031	88.8%	2782 (99.8%)	2525 (90.6%)	2/3/5/5	2

	Begin	End	Coverage	Score	Concordance	Matches	Identities	I/D/M/F*	Stop Codons
**NT**	**183**	**9687**	**89.3%**	**11427**	**66.3%**	**8654 (99.8%)**	**7216 (83.2%)**	**5/16**	
Protein mutations:	Y4Q (194T>C 196C>A), C9L (209T>C 210G>T 211T>G), F13M (221T>A 223T>G), S23P (251T>C), C24F (255G>T 256C>T), E25G (258A>G), I27V (263A>G 265T>G), V28A (267T>C 268G>A), E30V (273A>T), A35T (287G>A 289T>C), V37T (293G>A 294T>C), A41I (305G>A 306C>T), D42G (309A>G), E44D (316A>T), T45V (317A>G 318C>T 319A>G), L52Q (339T>A 340C>A), K53R (342A>G), K55A (347A>G 348A>C 349A>G), Y56H (350T>C), T58V (356A>G 357C>T), V61T (365G>A 366T>C 367G>A), L62S (368C>T 369T>C), F67C (384T>G), A75V (408C>T 409C>T), M78E (416A>G 417T>A), E85K (437G>A), R86E (440A>G 441G>A 442G>A), K87R (443A>C 444A>G 445G>A), D88E (448T>A), M194K (765T>A), R197K (773C>A 774G>A 775A>G), M206K (801T>A), G210A (813G>C 814A>G), L211H (816T>A), R214Q (825G>A 826T>A), R226C (860C>T 862C>T), T227A (863A>G 865T>C), I228T (867T>C), N229D (869A>G 871C>T), I230L (872A>T 874A>G), R232K (879G>A), I238V (896A>G 898C>T), N240S (903A>G), T241N (906C>A 907A>C), K242T (909A>C 910A>C), S243N (912G>A 913C>T), S252L (938T>C 939C>T 940A>T), L266I (980T>A 982G>C), R272K (999G>A), Q275R (1007C>A 1008A>G 1009G>A), S276G (1010A>G 1012T>G), I277V (1013A>G), N279Q (1019A>C 1021C>G), D288E (1048C>A), R300Q (1083G>A), Y303C (1092A>G 1093T>C), S305A (1097T>G 1099G>A), I382T (1329T>C), L397I (1373C>A), I401V (1385A>G), E407D (1405G>T), A411S (1415G>T 1417A>T), V418I (1436G>A 1438C>T), S461del (1565_1568delTCTT), E508G (1707A>G), A512T (1718G>A), I520V (1742A>G 1744C>T), S525N (1758G>A), V536I (1790G>A), I585V (1937A>G 1939T>C), F607Y (2004T>A), I628M (2068T>G), V630I (2072G>A 2074G>A), R675K (2208G>A), D694E (2266C>G), K780R (2523A>G), R817K (2634G>A), A827V (2664C>T 2665T>C), V847I (2723G>A), A855T (2747G>A 2749T>A), H871N (2795C>A), V880I (2822G>A 2824G>A), P900T (2882C>A), S901H (2885A>C 2886G>A), T904M (2895C>T), N916S (2931A>G), N919S (2940A>G), E929_N930insX (2971_2972insA), T936I (2991C>T), H937R (2994A>G), E939R (2999G>A 3000A>G), R946Q (3021G>A), Y947F (3024A>T 3025C>T), T951I (3036C>T), E952G (3039A>G 3040A>C), K953T (3042A>C 3043G>A), A972V (3099C>T 3100C>A), V974R (3104G>A 3105T>G 3106G>A), S983N (3132G>A), R985K (3137C>A 3138G>A 3139A>G), F990I (3152T>A 3154C>T), C994Y (3165G>A), F1012L (3218T>C), V1016I (3230G>A), V1028M (3266G>A 3268A>G), V1031M (3275G>A), A1034T (3284G>A), R1044K (3315G>A 3316A>G), E1045A (3318A>C), M1059V (3359A>G), D1072E (3400T>G), V1110G (3513T>G), M1121V (3545A>G), A1122T (3548G>A), V1124I (3554G>A 3556C>T), L1147V (3623C>G), L1150M (3632C>A), Y1152H (3638T>C), K1164N (3676G>C), N1165K (3679T>A), N1170K (3694T>A), I1172V (3698A>G), D1174* (3704G>T 3706T>A), F1175L (3709T>A), I1297V (4073A>G), F1315Y (4128T>A 4129C>T), A1323S (4151G>T), V1328I (4166G>A), L1347I (4223C>A 4225G>A), I1379V (4319A>G 4321A>G), F1404Y (4395T>A), V1408I (4406G>A), M1431V (4475A>G 4477G>T), T1433S (4481A>T), C1444R (4514T>C), V1499I (4679G>A 4681G>C), V1538I (4796G>A 4798C>T), T1540S (4803C>G 4804T>C), S1625G (5057A>G), T1626A (5060A>G), L1636I (5090C>A), A1639V (5100C>T), E1641D (5107A>T), V1646F (5120G>T 5122C>T), I1648V (5126A>G), V1667I (5183G>A), A1793T (5561G>A 5563G>A), I1805V (5597A>G), N1807K (5605C>A), A1812V (5619C>T 5620T>G), I1816L (5630A>C), V1837I (5693G>A), I1904V (5894A>G 5896C>G), R1926A (5960C>G 5961G>C 5962G>C), I1936V (5990A>G), E1945D (6019A>T), K1949T (6030A>C), D1955E (6049C>A), M1958I (6058G>A), S1963N (6072G>A 6073T>C), N1964H (6074A>C), T1966N (6081C>A), C1976S (6111G>C 6112T>C), I1982V (6128A>G 6130T>C), V1992I (6158G>A), T1996S (6170A>T 6172A>G), L2007F (6203C>T 6205C>T), V2016I (6230G>A), A2025K (6257G>A 6258C>A), K2033R (6282A>G), A2060T (6362G>A), V2073I (6401G>A), A2074V (6405C>T), Y2082F (6429A>T), Q2089R (6450A>G), H2101N (6485C>A 6487C>T), L2115I (6527C>A), T2135I (6588C>T), Y2151H (6635T>C), I2155V (6647A>G), I2165V (6677A>G 6679A>G), D2178N (6716G>A), N2179D (6719A>G), I2194L (6764A>T 6766A>G), A2204V (6795C>T 6796A>G), H2205Q (6799C>A), K2220R (6843A>G), N2225D (6857A>G), V2231T (6875G>A 6876T>C 6877C>A), D2250E (6934C>A), V2273I (7001G>A 7003G>A), F2283Y (7032T>A), T2314K (7125C>A), A2321S (7145G>T 7147A>G), E2326_A2327del (7160_7165delGAGGCA), D2344E (7216T>A), D2358E (7258T>A), D2363_R2364insX (7273_7274insT), R2364V (7274C>G 7275G>T), H2366L (7281A>T), L2367S (7284T>C), S2371L (7296C>T), S2376F (7311C>T), T2377I (7314C>T), C2378Y (7317G>A), N2379K (7321T>A), K2384Q (7334A>C), A2387N (7343G>A 7344C>A 7345A>C), S2408M (7407G>T 7408T>G), C2411G (7415T>G), L2440S (7503T>C 7504G>A), D2509N (7709G>A), K2510R (7713A>G 7714A>G), T2543I (7812C>T), V2554L (7844G>T 7846G>A), V2567I (7883G>A), S2620N (8043G>A), N2656S (8151A>G 8152C>T), R2665K (8178G>A), R2675K (8208G>A), P2704A (8294C>G 8296A>G), S2722F (8349C>T), A2742S (8408G>T), M2756I (8452G>A), R2759K (8460G>A), A2766T (8480G>A 8482T>A), R2773K (8502G>A 8503A>G), E2777D (8515A>T), E2785D (8539G>C), L2788C (8546C>T 8547T>G), S2790T (8552T>A), A2797G (8574C>G), I2801V (8585A>G), G2805E (8598G>A), N2807S (8603A>T 8604A>C 8605C>T), P2813Q (8622C>A), E2814G (8625A>G 8626G>A), P2822F (8648C>T 8649C>T 8650G>C), G2825E (8658G>A), D2827A (8664A>C 8665T>G), A2832V (8679C>T 8680A>T), T2854K (8745C>A 8746A>G), E2891G (8856A>G 8857G>A), R2894Q (8865G>A), M2895L (8867A>C 8869G>T), G2900E (8883G>A), T2902S (8888A>T), V2924I (8954G>A), V2929I (8969G>A), N2934D (8984A>G 8986T>C), E2935V (8988A>T), I2983V (9131A>G), M2989G (9149A>G 9150T>G 9151G>A), G2990S (9152G>A), P3026S (9260C>T 9262T>C)								

	Begin	End	Coverage	Score	Concordance	Matches	Identities	I/D/M/F*	Stop Codons
**NT**	**183**	**9687**	**89.3%**	**11427**	**66.3%**	**8654 (99.8%)**	**7216 (83.2%)**	**5/16**	

Codon mutations:

ACT3ACC (193T>C), TAC4CAA (194T>C 196C>A), TCA6TCG (202A>G), ATC8ATT (208C>T), TGT9CTG (209T>C 210G>T 211T>G), TCG12TCA (220G>A), TTT13ATG (221T>A 223T>G), GAA14GAG (226A>G), CTA17CTT (235A>T), TAC19TAT (241C>T), CCA21CCT (247A>T), TCT23CCT (251T>C), TGC24TTT (255G>T 256C>T), GAG25GGG (258A>G), CAT26CAC (262T>C), ATT27GTG (263A>G 265T>G), GTG28GCA (267T>C 268G>A), GAA30GTA (273A>T), CGA31CGT (277A>T), GAA32GAG (280A>G), GTG33GTT (283G>T), CCG34CCA (286G>A), GCT35ACC (287G>A 289T>C), GTT37ACT (293G>A 294T>C), CCT39CCC (301T>C), TTC40TTT (304C>T), GCA41ATA (305G>A 306C>T), GAT42GGT (309A>G), CTG43TTG (311C>T), GAA44GAT (316A>T), ACA45GTG (317A>G 318C>T 319A>G), CAA46CAG (322A>G), GCA49GCT (331A>T), CTC52CAA (339T>A 340C>A), AAG53AGG (342A>G), AAA55GCG (347A>G 348A>C 349A>G), TAT56CAT (350T>C), GCT57GCA (355T>A), ACT58GTT (356A>G 357C>T), CGT60CGC (364T>C), GTG61ACA (365G>A 366T>C 367G>A), CTC62TCC (368C>T 369T>C), AAA63AAG (373A>G), AAC64AAT (376C>T), TTT67TGT (384T>G), ACG68ACA (388G>A), TAC69TAT (391C>T), CGA70CGG (394A>G), TAC71TAT (397C>T), AAG72AAA (400G>A), GAT74GAC (406T>C), GCC75GTT (408C>T 409C>T), CAG76CAA (412G>A), ATA77ATT (415A>T), ATG78GAG (416A>G 417T>A), ATT80ATC (424T>C), CAG81CAA (427G>A), AAA83AAG (433A>G), CTG84CTC (436G>C), GAG85AAG (437G>A), AGG86GAA (440A>G 441G>A 442G>A), AAG87CGA (443A>C 444A>G 445G>A), GAT88GAA (448T>A), GAA90GAG (454A>G), ATG194AAG (765T>A), TTG196CTG (770T>C), CGA197AAG (773C>A 774G>A 775A>G), CGG198AGG (776C>A), TTC202TTT (790C>T), ATG206AAG (801T>A), GGA210GCG (813G>C 814A>G), CTT211CAT (816T>A), TTG212CTG (818T>C), CAA213CAG (823A>G), CGT214CAA (825G>A 826T>A), CGG217AGA (833C>A 835G>A), GAC219GAT (841C>T), AAT223AAC (853T>C), GTC225GTT (859C>T), CGC226TGT (860C>T 862C>T), ACT227GCC (863A>G 865T>C), ATC228ACC (867T>C), AAC229GAT (869A>G 871C>T), ATA230TTG (872A>T 874A>G), AGG232AAG (879G>A), GAT234GAC (886T>C), ATC238GTT (896A>G 898C>T), AAC240AGC (903A>G), ACA241AAC (906C>A 907A>C), AAA242ACC (909A>C 910A>C), AGC243AAT (912G>A 913C>T), GGC246GGA (922C>A), GGT249GGA (931T>A), TCA252CTT (938T>C 939C>T 940A>T), GGA253GGG (943A>G), TTC256TTT (952C>T), TCA261TCG (967A>G), GAA263GAG (973A>G), GGG264GGC (976G>C), TTG266ATC (980T>A 982G>C), GCA269GCG (991A>G), CGT270CGC (994T>C), AGA272AAA (999G>A), GTT273GTC (1003T>C), CAG275AGA (1007C>A 1008A>G 1009G>A), AGT276GGG (1010A>G 1012T>G), ATT277GTT (1013A>G), TTA278TTG (1018A>G), AAC279CAG (1019A>C 1021C>G), ATC282ATT (1030C>T), TCG285TCA (1039G>A), AAT286AAC (1042T>C), GCC287GCA (1045C>A), GAC288GAA (1048C>A), AAT289AAC (1051T>C), AAG292AAA (1060G>A), GGT293GGA (1063T>A), CTG294TTG (1064C>T), GAC295GAT (1069C>T), CGA300CAA (1083G>A), TAT303TGC (1092A>G 1093T>C), TCG305GCA (1097T>G 1099G>A), CAC307CAT (1105C>T), AAT378..A (1318T>A), AAG379AAA (1321G>A), TTG381TTA (1327G>A), ATA382ACA (1329T>C), GCG383GCT (1333G>T), TTA384TTG (1336A>G), CTA387TTG (1343C>T 1345A>G), CCG390CCA (1354G>A), GTG391GTT (1357G>T), GAC392GAT (1360C>T), CTC395TTG (1367C>T 1369C>G), CTT397ATT (1373C>A), TTC398TTT (1378C>T), GAG400GAA (1384G>A), ATA401GTA (1385A>G), AAA403AAG (1393A>G), TCC404TCT (1396C>T), GAG407GAT (1405G>T), AAA408AAG (1408A>G), CAG409CAA (1411G>A), GCA411TCT (1415G>T 1417A>T), CCG412CCC (1420G>C), AAT415AAC (1429T>C), TTA416TTG (1432A>G), GTC418ATT (1436G>A 1438C>T), TTA419CTG (1439T>C 1441A>G), AAT420AAC (1444T>C), TTC423TTT (1453C>T), CTG424CTA (1456G>A), GAA428GAG (1468A>G), GTA436GTG (1492A>G), TTG439CTA (1499T>C 1501G>A), AGT440AGC (1504T>C), TTG441TTA (1507G>A), CTC442CTT (1510C>T), GAA443GAG (1513A>G), TTA444TTG (1516A>G), GCA445GCT (1519A>T), AGG446AGA (1522G>A), TTC447TTT (1525C>T), CAG448CAA (1528G>A), AAC450AAT (1534C>T), ACT452ACA (1540T>A), AAC454AAT (1546C>T), AAA457AAG (1555A>G), GAT459GAC (1561T>C), ATA460ATC (1564A>C), TCT461del (1565_1568delTCTT), TTC462-TC (1565_1568delTCTT), TTC463TTT (1573C>T), AGA464AGG (1576A>G), TTA467CTA (1583T>C), TCT468TCA (1588T>A), AAG470AAA (1594G>A), AAC472AAT (1600C>T), TCG478TCA (1618G>A), TGC479TGT (1621C>T), GAC480GAT (1624C>T), CTG483TTG (1631C>T), AAA485AAG (1639A>G), TTC489TTT (1651C>T), CTC490CTG (1654C>G), CAT497CAC (1675T>C), TCA503TCG (1693A>G), TTT506TTC (1702T>C), GAA508GGA (1707A>G), GCA512ACA (1718G>A), GGA514GGG (1726A>G), TAC515TAT (1729C>T), TCA516TCC (1732A>C), TAT518TAC (1738T>C), ATC520GTT (1742A>G 1744C>T), CGC521CGT (1747C>T), CAT523CAC (1753T>C), AGT525AAT (1758G>A), AGG528AGA (1768G>A), AAC534AAT (1786C>T), TTA535TTG (1789A>G), GTT536ATT (1790G>A), GTC537GTT (1795C>T), TTA541CTG (1805T>C 1807A>G), GAG543GAA (1813G>A), AGG545AGA (1819G>A), AAG547AAA (1825G>A), GAC551GAT (1837C>T), TAT552TAC (1840T>C), CAA555CAG (1849A>G), GTC558GTG (1858C>G), AAA560AAG (1864A>G), TGC562TGT (1870C>T), AGT564AGC (1876T>C), TCG565TCT (1879G>T), AAA566AAG (1882A>G), GGT568GGA (1888T>A), AAT569AAC (1891T>C), TGT574TGC (1906T>C), ACA577ACT (1915A>T), GGT582GGC (1930T>C), GCC584GCT (1936C>T), ATT585GTC (1937A>G 1939T>C), TCA587TCG (1945A>G), TTC589TTT (1951C>T), CCA592CCT (1960A>T), GTA598GTG (1978A>G), ATT599ATA (1981T>A), GGC600GGT (1984C>T), AAT601AAC (1987T>C), GAC604GAT (1996C>T), TTT607TAT (2004T>A), AAA612AAG (2020A>G), GGG613GGA (2023G>A), GAT614GAC (2026T>C), TCG615TCT (2029G>T), GAG616GAA (2032G>A), TAT625TAC (2059T>C), ATT628ATG (2068T>G), AAC629AAT (2071C>T), GTG630ATA (2072G>A 2074G>A), CTG635TTA (2087C>T 2089G>A), AGC639AGT (2101C>T), GAG640GAA (2104G>A), GAG641GAA (2107G>A), AAG644AAA (2116G>A), TTC646TTT (2122C>T), CGC651CGT (2137C>T), CTT658CTC (2158T>C), ACC660ACT (2164C>T), CCA662CCG (2170A>G), GAT666GAC (2182T>C), GCG668GCA (2188G>A), ACC669ACA (2191C>A), AGA675AAA (2208G>A), TAT678TAC (2218T>C), GAC680GAT (2224C>T), GTA681GTT (2227A>T), TTG686TTA (2242G>A), CCC687CCT (2245C>T), TTG690TTA (2254G>A), GTT691GTC (2257T>C), GAC692GAT (2260C>T), GAC694GAG (2266C>G), CAA696CAG (2272A>G), ACG697ACA (2275G>A), TCA703TCG (2293A>G), GGC705GGT (2299C>T), TCG706TCA (2302G>A), CAG707CAA (2305G>A), GGA710GGT (2314A>T), TAT711TAC (2317T>C), ATT713ATC (2323T>C), CTA714CTG (2326A>G), AGC718AGT (2338C>T), GTG719GTA (2341G>A), CAA721CAG (2347A>G), CTT722CTC (2350T>C), TTG724TTA (2356G>A), AAT727AAC (2365T>C), TCT764.CC (2476T>C), TCG766TCA (2482G>A), CTG769CTA (2491G>A), CTG771CTT (2497G>T), TTG773TTA (2503G>A), AGA778AGG (2518A>G), CCT779CCC (2521T>C), AAG780AGG (2523A>G), AGA783AGG (2533A>G), TTG785TTA (2539G>A), TTA787TTG (2545A>G), GAG789GAA (2551G>A), CCT790CCA (2554T>A), CTG792TTG (2558C>T), TTG793CTC (2561T>C 2563G>C), CTA795CTG (2569A>G), TCA796TCG (2572A>G), CCT800CCC (2584T>C), GGC801GGT (2587C>T), CTG803CTC (2593G>C), GCT805GCC (2599T>C), TAT807TAC (2605T>C), AAT808AAC (2608T>C), TTT812TTC (2620T>C), GCG815GCA (2629G>A), GTG816GTA (2632G>A), AGG817AAG (2634G>A), TTG818TTA (2638G>A), ATT820ATA (2644T>A), AAA823AAG (2653A>G), GCT827GTC (2664C>T 2665T>C), CTA832TTA (2678C>T), CTA833CTG (2683A>G), GCT835GCC (2689T>C), CTA838CTC (2698A>C), TCA841TCG (2707A>G), GCG842GCA (2710G>A), GAA844GAG (2716A>G), ACA845ACG (2719A>G), CTC846CTT (2722C>T), GTC847ATC (2723G>A), GCA848GCG (2728A>G), AGG850AGA (2734G>A), ATT851ATA (2737T>A), GCT855ACA (2747G>A 2749T>A), GCA856GCT (2752A>T), GCT857GCA (2755T>A), GAC859GAT (2761C>T), CTC860CTT (2764C>T), TGT865TGC (2779T>C), GGG867GGT (2785G>T), AAC869AAT (2791C>T), CAT871AAT (2795C>A), TAC874TAT (2806C>T), TTG878CTC (2816T>C 2818G>C), GTG880ATA (2822G>A 2824G>A), TTG881TTA (2827G>A), GTT884GTC (2836T>C), AAG885AAA (2839G>A), AAT886AAC (2842T>C), ACC893ACA (2863C>A), CTA894TTA (2864C>T), CCA900ACA (2882C>A), AGT901CAT (2885A>C 2886G>A), AAC903AAT (2893C>T), ACG904ATG (2895C>T), GTC906GTT (2902C>T), GTA907GTG (2905A>G), ATT909ATC (2911T>C), AAT913AAC (2923T>C), AAT916AGT (2931A>G), AAC919AGC (2940A>G), GCT921GCC (2947T>C), ACT926ACC (2962T>C), GAA929_AAT930insA-- (2971_2972insA), GGT935GGC (2989T>C), ACT936ATT (2991C>T), CAT937CGT (2994A>G), GAG939AGG (2999G>A 3000A>G), TCC944TC- (3016delC), CGG946CAG (3021G>A), TAC947TTT (3024A>T 3025C>T), ACA951ATA (3036C>T), GAA952GGC (3039A>G 3040A>C), AAG953ACA (3042A>C 3043G>A), TTG956TTA (3052G>A), TTA959TTG (3061A>G), GCG966GCA (3082G>A), GGC969GGT (3091C>T), GCC972GTA (3099C>T 3100C>A), GTG974AGA (3104G>A 3105T>G 3106G>A), GTC975GTT (3109C>T), AAA976AAG (3112A>G), ACT978ACC (3118T>C), GCC979GCT (3121C>T), GGA981GGG (3127A>G), AGC983AAC (3132G>A), CGA985AAG (3137C>A 3138G>A 3139A>G), TAT989TAC (3151T>C), TTC990ATT (3152T>A 3154C>T), TGT994TAT (3165G>A), GTA995GTG (3169A>G), ATC1005ATT (3199C>T), AGG1007CGG (3203A>C), CGT1008CGC (3208T>C), CCA1010CCG (3214A>G), ACC1011ACT (3217C>T), TTT1012CTT (3218T>C), GTC1013GTA (3223C>A), GTT1016ATT (3230G>A), AAC1017AAT (3235C>T), TTA1025CTA (3257T>C), ACC1026ACT (3262C>T), AGC1027AGT (3265C>T), GTA1028ATG (3266G>A 3268A>G), GTG1029GTA (3271G>A), GTG1031ATG (3275G>A), GCA1034ACA (3284G>A), CAG1039CAA (3301G>A), AGA1044AAG (3315G>A 3316A>G), GAA1045GCA (3318A>C), ATC1046ATT (3322C>T), GAG1047GAA (3325G>A), TTG1048TTA (3328G>A), ATA1051ATT (3337A>T), GAG1055GAA (3349G>A), GTC1057GTT (3355C>T), TGC1058TGT (3358C>T), ATG1059GTG (3359A>G), CTA1061CTG (3367A>G), TAT1062TAC (3370T>C), TTA1065CTG (3377T>C 3379A>G), GAA1070GAG (3394A>G), CGC1071CGT (3397C>T), GAT1072GAG (3400T>G), ACA1074ACG (3406A>G), TAC1078TAT (3418C>T), ATT1079ATA (3421T>A), TAT1081TAC (3427T>C), AAG1083AAA (3433G>A), TCA1084TCT (3436A>T), GTA1085GTG (3439A>G), AAC1086AAT (3442C>T), CCT1087CCC (3445T>C), CAG1091CAA (3457G>A), TTT1092TTC (3460T>C), GCT1093GCA (3463T>A), GCG1095GCT (3469G>T), CAG1096CAA (3472G>A), CAC1104CAT (3496C>T), CAG1105CAA (3499G>A), TCC1107TCT (3505C>T), GTT1110GGT (3513T>G), AAA1112AAG (3520A>G), GAA1115GAG (3529A>G), CAA1116CAG (3532A>G), ATG1121GTG (3545A>G), GCT1122ACT (3548G>A), TTA1123CTA (3551T>C), GTC1124ATT (3554G>A 3556C>T), AGT1133AGC (3583T>C), GAT1134GAC (3586T>C), TGC1135TGT (3589C>T), TTC1137TTT (3595C>T), AAT1141AAC (3607T>C), TTT1143TTC (3613T>C), AAG1144AAA (3616G>A), GGT1145GGC (3619T>C), CTT1147GTT (3623C>G), TCC1148TCT (3628C>T), CTG1150ATG (3632C>A), GAT1151GAC (3637T>C), TAT1152CAT (3638T>C), GAA1153GAG (3643A>G), TTA1159TTG (3661A>G), GTG1162GTA (3670G>A), AAG1164AAC (3676G>C), AAT1165AAA (3679T>A), GAG1168GAA (3688G>A), AAT1170AAA (3694T>A), GAG1171GAA (3697G>A), ATT1172GTT (3698A>G), GAT1174TAA (3704G>T 3706T>A), TTT1175TTA (3709T>A), AGC1233..T (3883C>T), GAA1234GAG (3886A>G), CTA1236CTG (3892A>G), GAC1237GAT (3895C>T), TTT1238TTC (3898T>C), GTA1240GTG (3904A>G), CGG1241AGA (3905C>A 3907G>A), GCT1243GCC (3913T>C), AAG1248AAA (3928G>A), TCA1249TCT (3931A>T), GGG1251GGA (3937G>A), AGT1257AGC (3955T>C), GTG1258GTA (3958G>A), GCC1259GCT (3961C>T), GGA1260GGG (3964A>G), TCT1261TCC (3967T>C), TTA1264TTG (3976A>G), ATT1265ATC (3979T>C), CCA1267CCT (3985A>T), CTA1271CTT (3997A>T), GCG1272GCA (4000G>A), AAC1274AAT (4006C>T), GTT1275GTG (4009T>G), TTC1276TTT (4012C>T), AAA1277AAG (4015A>G), TCT1280TCC (4024T>C), GAA1282GAG (4030A>G), CCA1283CCT (4033A>T), AAG1286AAA (4042G>A), CTG1290TTA (4052C>T 4054G>A), CGT1291CGC (4057T>C), CGT1293CGA (4063T>A), GGA1294GGG (4066A>G), ATA1297GTA (4073A>G), TTT1298TTC (4078T>C), GGC1299GGT (4081C>T), TCT1300TCC (4084T>C), TCC1304TCT (4096C>T), AGC1308AGT (4108C>T), GGA1309GGT (4111A>T), GCG1311GCA (4117G>A), CTA1312TTG (4118C>T 4120A>G), TAC1314TAT (4126C>T), TTC1315TAT (4128T>A 4129C>T), GCC1316GCT (4132C>T), AAT1317AAC (4135T>C), CAA1321CAG (4147A>G), TTA1322CTA (4148T>C), GCT1323TCT (4151G>T), AAC1326AAT (4162C>T), GTA1328ATA (4166G>A), ATA1329ATC (4171A>C), GAG1332GAA (4180G>A), CTG1336TTG (4190C>T), CCT1338CCC (4198T>C), TCC1339TCT (4201C>T), GCG1342GCT (4210G>T), TTC1343TTT (4213C>T), AGT1345AGC (4219T>C), CTG1346TTG (4220C>T), CTG1347ATA (4223C>A 4225G>A), GTT1349GTG (4231T>G), CAT1351CAC (4237T>C), GTA1356GTG (4252A>G), TTA1357CTG (4253T>C 4255A>G), AAA1358AAG (4258A>G), ACT1362ACC (4270T>C), AGA1366AGG (4282A>G), GAG1367GAA (4285G>A), GAA1369GAG (4291A>G), CAG1373CAA (4303G>A), GTC1376GTT (4312C>T), ATA1379GTG (4319A>G 4321A>G), GTG1380GTT (4324G>T), GAG1381GAA (4327G>A), CTG1384CTT (4336G>T), TCT1385TCC (4339T>C), TCA1388TCT (4348A>T), GCA1392GCG (4360A>G), GGT1394GGC (4366T>C), TCT1395TCA (4369T>A), ACT1397ACC (4375T>C), GTT1401GTC (4387T>C), TTT1404TAT (4395T>A), GTA1408ATA (4406G>A), ACC1420ACA (4444C>A), GCC1422GCT (4450C>T), CTC1424CTT (4456C>T), GAC1427GAT (4465C>T), ATG1431GTT (4475A>G 4477G>T), GTC1432GTT (4480C>T), ACA1433TCA (4481A>T), GGC1437GGT (4495C>T), AAG1441AAA (4507G>A), CAC1442CAT (4510C>T), GGT1443GGG (4513T>G), TGC1444CGC (4514T>C), CTA1445TTA (4517C>T), GTC1448GTT (4528C>T), AAA1450AAG (4534A>G), GTG1469GTA (4591G>A), TTG1471TTA (4597G>A), GAC1472GAT (4600C>T), GAC1474GAT (4606C>T), GTG1475GTA (4609G>A), GTG1477GTA (4615G>A), GAC1478GAT (4618C>T), TTG1481CTT (4625T>C 4627G>T), GAC1488GAT (4648C>T), ATT1489ATC (4651T>C), AGG1492AGA (4660G>A), AGC1493AGT (4663C>T), GCT1495GCA (4669T>A), GTG1499ATC (4679G>A 4681G>C), AGT1500AGC (4684T>C), GTT1501GTG (4687T>G), GAG1505GAA (4699G>A), AGA1506AGG (4702A>G), CAA1508CAG (4708A>G), AGG1509AGA (4711G>A), CTG1510TTG (4712C>T), AAA1518AAG (4738A>G), GCA1521GCG (4747A>G), TTG1522CTG (4748T>C), ACT1527ACC (4765T>C), GTC1538ATT (4796G>A 4798C>T), ACT1540AGC (4803C>G 4804T>C), GAG1541GAA (4807G>A), GCG1542GCT (4810G>T), GCT1543GCG (4813T>G), CTT1544CTC (4816T>C), TAT1549TAC (4831T>C), AAC1550AAT (4834C>T), CCA1552CCT (4840A>T), GGA1556GGG (4852A>G), GGC1557GGT (4855C>T), AGT1561AGC (4867T>C), CTG1562CTC (4870G>C), ATT1563ATA (4873T>A), ACA1573ACT (4903A>T), CAG1575CAA (4909G>A), TTT1577TTC (4915T>C), GAA1578GAG (4918A>G), TTG1579CTA (4919T>C 4921G>A), AGT1580AGC (4924T>C), CCC1581CCG (4927C>G), TTT1583TTC (4933T>C), ATC1584ATA (4936C>A), CAG1585CAA (4939G>A), AAT1586AAC (4942T>C), TTC1587TTT (4945C>T), TCA1593TCG (4963A>G), CCT1596CCC (4972T>C), CTT1602CTC (4990T>C), AAA1606AAG (5002A>G), CCT1613CCC (5023T>C), TTG1614CTA (5024T>C 5026G>A), TGC1615TGT (5029C>T), GAT1616GAC (5032T>C), TCT1618TCC (5038T>C), CCA1620CCT (5044A>T), GCA1623GCT (5053A>T), TCG1624TCA (5056G>A), AGT1625GGT (5057A>G), ACT1626GCT (5060A>G), TCG1629TCT (5071G>T), CTT1636ATT (5090C>A), GGA1637GGG (5095A>G), GTG1638GTT (5098G>T), GCC1639GTC (5100C>T), TTA1640TTG (5104A>G), GAA1641GAT (5107A>T), ATT1642ATC (5110T>C), CCA1643CCG (5113A>G), AAG1644AAA (5116G>A), CAA1645CAG (5119A>G), GTC1646TTT (5120G>T 5122C>T), ATT1648GTT (5126A>G), CAT1651CAC (5137T>C), ATC1652ATT (5140C>T), AAA1653AAG (5143A>G), GAG1654GAA (5146G>A), CTC1659CTA (5161C>A), GAA1661GAG (5167A>G), CTT1663CTG (5173T>G), ACG1666ACA (5182G>A), GTT1667ATT (5183G>A), GTC1668GTT (5188C>T), AAG1669AAA (5191G>A), AAA1671AAG (5197A>G), GAC1672GAT (5200C>T), TGC1674TGT (5206C>T), TTA1675TTG (5209A>G), TCG1682TCA (5230G>A), ATC1684ATT (5236C>T), AGC1685AGT (5239C>T), ATC1687ATT (5245C>T), ACA1693ACT (5263A>T), CTC1695CTT (5269C>T), TTC1696TTT (5272C>T), GCC1697GCA (5275C>A), ATC1698ATT (5278C>T), CCA1699CCC (5281A>C), AGA1700AGG (5284A>G), ACT1701ACC (5287T>C), CTA1702TTA (5288C>T), ATA1703ATT (5293A>T), TTG1704CTA (5294T>C 5296G>A), GAG1706GAA (5302G>A), CTT1709CTA (5311T>A), GAA1710GAG (5314A>G), CGA1713CGT (5323A>T), AGA1720AGG (5344A>G), AGT1721AGC (5347T>C), ATC1723ATT (5353C>T), TGC1727TGT (5365C>T), GTT1734GTC (5386T>C), ACC1737ACA (5395C>A), AAA1746AAG (5422A>G), GAT1747GAC (5425T>C), ACC1749ACT (5431C>T), GAG1751GAA (5437G>A), CTT1756CTC (5452T>C), AAG1758AAA (5458G>A), AGT1761AGC (5467T>C), CAA1762CAG (5470A>G), CTA1763TTG (5471C>T 5473A>G), TTC1766TTT (5482C>T), TCA1767TCT (5485A>T), AAT1768AAC (5488T>C), TTG1769CTA (5489T>C 5491G>A), GGT1771GGC (5497T>C), TGT1774TGC (5506T>C), GAG1775GAA (5509G>A), GAG1776GAA (5512G>A), TTA1778CTA (5516T>C), TAT1782TAC (5530T>C), GAG1783GAA (5533G>A), TCG1784TCT (5536G>T), TTG1785TTA (5539G>A), GTT1788GTG (5548T>G), CAC1790CAT (5554C>T), GCT1792GCA (5560T>A), GCG1793ACA (5561G>A 5563G>A), ACG1794ACC (5566G>C), TCA1795TCG (5569A>G), CTC1800CTG (5584C>G), AAG1803AAA (5593G>A), GGG1804GGA (5596G>A), ATT1805GTT (5597A>G), AAC1807AAA (5605C>A), GTG1811GTT (5617G>T), GCT1812GTG (5619C>T 5620T>G), TTG1815TTA (5629G>A), ATC1816CTC (5630A>C), GGC1819GGT (5641C>T), GCA1822GCT (5650A>T), ATA1826ATT (5662A>T), GGA1827GGG (5665A>G), CAA1835CAG (5689A>G), GTT1837ATT (5693G>A), GAG1838GAA

	(5698G>A), CAA1843CAG (5713A>G), GGG1844GGC (5716G>C), AAA1845AAG (5719A>G), GCC1853GCA (5743C>A), CGC1857CGA (5755C>A), CAT1858CAC (5758T>C), CGT1860CGC (5764T>C), AAA1862AAG (5770A>G), GGC1865GGT (5779C>T), TTT1866TTC (5782T>C), GAC1869GAT (5791C>T), GAC1873GAT (5803C>T), AGG1884AGA (5836G>A), AAA1885AAG (5839A>G), GGA1887GGG (5845A>G), AAA1888AAG (5848A>G), GGT1891GGC (5857T>C), ACC1892ACT (5860C>T), ACA1893ACT (5863A>T), GGT1895GGA (5869T>A), GGT1897GGC (5875T>C), AGC1900AGT (5884C>T), TTC1903TTT (5893C>T), ATC1904GTG (5894A>G 5896C>G), AAC1905AAT (5899C>T), GGG1908GGA (5908G>A), TTT1909TTC (5911T>C), GAG1913GAA (5923G>A), CAA1918CAG (5938A>G), TTC1919TTT (5941C>T), GTT1920GTC (5944T>C), GAT1921GAC (5947T>C), GGG1925GGA (5959G>A), CGG1926GCC (5960C>G 5961G>C 5962G>C), GAA1930GAG (5974A>G), GCT1934GCA (5986T>A), GAC1935GAT (5989C>T), ATT1936GTT (5990A>G), GAT1938GAC (5998T>C), AGA1942CGT (6008A>C 6010A>T), GAA1945GAT (6019A>T), GTG1946GTT (6022G>T), CGA1947CGT (6025A>T), AAA1949ACA (6030A>C), GAC1955GAA (6049C>A), ATG1958ATA (6058G>A), CAA1959CAG (6061A>G), GCC1960GCA (6064C>A), GGT1962GGC (6070T>C), AGT1963AAC (6072G>A 6073T>C), AAC1964CAC (6074A>C), ACG1965ACA (6079G>A), ACC1966AAC (6081C>A), ATA1967ATT (6085A>T), GCA1969GCT (6091A>T), TAC1970TAT (6094C>T), TGT1976TCC (6111G>C 6112T>C), AAA1978AAG (6118A>G), TTG1980TTA (6124G>A), AAG1981AAA (6127G>A), ATT1982GTC (6128A>G 6130T>C), GAT1983GAC (6133T>C), TTA1984TTG (6136A>G), AAC1988AAT (6148C>T), AAA1991AAG (6157A>G), GTT1992ATT (6158G>A), TGT1993TGC (6163T>C), AAA1995AAG (6169A>G), ACA1996TCG (6170A>T 6172A>G), GGC1998GGT (6178C>T), AAA2001AAG (6187A>G), TTT2002TTC (6190T>C), GAG2004GAA (6196G>A), CTC2007TTT (6203C>T 6205C>T), GAA2008GAG (6208A>G), CTA2009TTG (6209C>T 6211A>G), CAG2011CAA (6217G>A), GCT2015GCA (6229T>A), GTA2016ATA (6230G>A), GTC2018GTT (6238C>T), AAG2021AAA (6247G>A), ATA2023ATT (6253A>T), GCA2025AAA (6257G>A 6258C>A), CAT2030CAC (6274T>C), AAA2033AGA (6282A>G), TCG2034TCA (6286G>A), GGC2038GGT (6298C>T), TTG2039TTA (6301G>A), AGA2040AGG (6304A>G), GAC2041GAT (6307C>T), AAC2043AAT (6313C>T), GCC2046GCT (6322C>T), ACA2048ACT (6328A>T), GTT2049GTG (6331T>G), TGT2050TGC (6334T>C), AGG2051AGA (6337G>A), CTG2052TTG (6338C>T), TAT2058TAC (6358T>C), GGG2059GGA (6361G>A), GCA2060ACA (6362G>A), TCA2061TCT (6367A>T), GAG2062GAA (6370G>A), TAC2064TAT (6376C>T), GGT2065GGG (6379T>G), GGA2067GGC (6385A>C), GGA2069GGT (6391A>T), GCA2070GCG (6394A>G), TAC2071TAT (6397C>T), GTA2073ATA (6401G>A), GCG2074GTG (6405C>T), CAC2076CAT (6412C>T), TTA2078TTG (6418A>G), TTT2079TTC (6421T>C), TAC2082TTC (6429A>T), GGT2084GGA (6436T>A), GAG2087GAA (6445G>A), CAA2089CGA (6450A>G), TCC2090TCA (6454C>A), CAC2092CAT (6460C>T), GGT2093GGA (6463T>A), TTC2095TTT (6469C>T), AGG2096AGA (6472G>A), GTG2097GTA (6475G>A), AAT2099AAC (6481T>C), CAC2101AAT (6485C>A 6487C>T), AGT2102AGC (6490T>C), AGC2104AGT (6496C>T), CTG2106CTA (6502G>A), GGT2110GGC (6514T>C), AGG2111AGA (6517G>A), CTC2115ATC (6527C>A), ATC2116ATA (6532C>A), CCG2119CCA (6541G>A), AAA2120AAG (6544A>G), GTC2124GTT (6556C>T), TTT2125TTC (6559T>C), CAG2127CAA (6565G>A), TTG2129CTA (6569T>C 6571G>A), CCT2134CCA (6586T>A), ACA2135ATA (6588C>T), GAA2138GAG (6598A>G), AGA2139AGG (6601A>G), ATT2140ATC (6604T>C), TGT2141TGC (6607T>C), GGA2144GGG (6616A>G), AAC2146AAT (6622C>T), GAG2149GAA (6631G>A), AAG2150AAA (6634G>A), TAT2151CAT (6635T>C), GCT2152GCA (6640T>A), TCG2154TCC (6646G>C), ATC2155GTC (6647A>G), ATC2156ATT (6652C>T), AGC2160AGT (6664C>T), ACT2162ACG (6670T>G), ATA2165GTG (6677A>G 6679A>G), GGC2167GGT (6685C>T), ACA2169ACT (6691A>T), TTC2170TTT (6694C>T), ACA2177ACG (6715A>G), GAT2178AAT (6716G>A), AAT2179GAT (6719A>G), GGA2180GGG (6724A>G), CTA2184TTA (6734C>T), GTG2186GTA (6742G>A), GTG2187GTA (6745G>A), GCC2190GCT (6754C>T), GGA2192GGG (6760A>G), ATA2194TTG (6764A>T 6766A>G), GTC2195GTT (6769C>T), ATT2197ATA (6775T>A), CTG2200TTG (6782C>T), GCA2201GCT (6787A>T), AAC2202AAT (6790C>T), GCA2204GTG (6795C>T 6796A>G), CAC2205CAA (6799C>A), ACC2206ACA (6802C>A), AAC2208AAT (6808C>T), TAC2209TAT (6811C>T), TAC2210TAT (6814C>T), TTC2213TTT (6823C>T), GAA2215GAG (6829A>G), AAG2220AGG (6843A>G), TAC2221TAT (6847C>T), CGA2223CGC (6853A>C), AAT2225GAT (6857A>G), GAG2226GAA (6862G>A), GAA2229GAG (6871A>G), GTC2231ACA (6875G>A 6876T>C 6877C>A), AAG2232AAA (6880G>A), TCT2233TCA (6883T>A), GTT2235GTG (6889T>G), GAC2239GAT (6901C>T), GGC2244GGT (6916C>T), CCG2245CCA (6919G>A), AAA2247AAG (6925A>G), CTT2248CTC (6928T>C), GAC2250GAA (6934C>A), GGG2255GGC (6949G>C), TTC2257TTT (6955C>T), AAA2258AAG (6958A>G), CAA2264CAG (6976A>G), CTA2266TTA (6980C>T), ATC2267ATT (6985C>T), GAT2270GAC (6994T>C), GTA2271GTT (6997A>T), GTG2272GTT (7000G>T), GTG2273ATA (7001G>A 7003G>A), GCT2276GCC (7012T>C), AAG2277AAA (7015G>A), CAT2278CAC (7018T>C), TTT2283TAT (7032T>A), GCC2285GCT (7039C>T), TTG2286CTA (7040T>C 7042G>A), GGA2288GGG (7048A>G), GCT2292GCA (7060T>A), GTC2293GTA (7063C>A), GCA2294GCG (7066A>G), AGC2298AGT (7078C>T), CAA2299CAG (7081A>G), TTA2300CTG (7082T>C 7084A>G), GTA2301GTG (7087A>G), ACC2302ACA (7090C>A), CAT2304CAC (7096T>C), GTA2305GTG (7099A>G), GTT2306GTC (7102T>C), GAG2309GAA (7111G>A), CGA2311CGG (7117A>G), ACA2314AAA (7125C>A), GAA2315GAG (7129A>G), CTG2317TTA (7133C>T 7135G>A), GTG2319GTA (7141G>A), GAT2320GAC (7144T>C), GCA2321TCG (7145G>T 7147A>G), GAG2322GAA (7150G>A), GAG2324GAA (7156G>A), GCA2325GCT (7159A>T), GAG2326_GCA2327del (7160_7165delGAGGCA), TTC2329TTT (7171C>T), GCG2335GCT (7189G>T), AAA2338AAG (7198A>G), AGC2339AGT (7201C>T), CTA2341TTA (7205C>T), AAT2342AAC (7210T>C), GAT2344GAA (7216T>A), GCG2345GCA (7219G>A), ATC2347ATA (7225C>A), AAG2348AAA (7228G>A), AAG2352AAA (7240G>A), TAT2353TAC (7243T>C), AAA2355AAG (7249A>G), CCT2356CCG (7252T>G), GAT2358GAA (7258T>A), GTT2359GTC (7261T>C), GGT2360GGA (7264T>A), GTC2361GTG (7267C>G), GTG2362GTA (7270G>A), GAT2363_CGG2364insT-- (7273_7274insT), CGG2364GTG (7274C>G 7275G>T), CAT2366CTT (7281A>T), TTG2367TCG (7284T>C), TCA2371TTA (7296C>T), TCT2376TTT (7311C>T), ACC2377ATC (7314C>T), TGC2378TAC (7317G>A), AAT2379AAA (7321T>A), GTG2380-TA (7322delG 7324G>A), CAC2381CAT (7327C>T), GGC2382GGT (7330C>T), TTC2383TTT (7333C>T), AAG2384CAG (7334A>C), TGT2386TGC (7342T>C), GCA2387AAC (7343G>A 7344C>A 7345A>C), ACT2390ACC (7354T>C), CAA2393CAG (7363A>G), ATT2395ATC (7369T>C), TTC2396TTT (7372C>T), GCG2398GCT (7378G>T), CTC2399CTT (7381C>T), GCA2404GCT (7396A>T), GTC2405GTT (7399C>T), GCC2407GCT (7405C>T), AGT2408ATG (7407G>T 7408T>G), GGG2410GGT (7414G>T), TGC2411GGC (7415T>G), AAA2412AAG (7420A>G), GAC2415GAT (7429C>T), TAT2416TAC (7432T>C), CAT2419CAC (7441T>C), AAG2425AAA (7459G>A), GAA2426GAG (7462A>G), ATA2428ATT (7468A>T), GTC2429GTA (7471C>A), CAA2431CAG (7477A>G), AGC2432AGT (7480C>T), CTG2434CTT (7486G>T), TAT2437TAC (7495T>C), AAA2438AAG (7498A>G), GGT2439GGC (7501T>C), TTG2440TCA (7503T>C 7504G>A), ATT2443ATA (7513T>A), AAC2445AAT (7519C>T), GGA2446GGG (7522A>G), AAG2449AAA (7531G>A), GAG2451GAA (7537G>A), CTC2452CTT (7540C>T), TGT2454TGC (7546T>C), AAG2462AAA (7570G>A), ACG2463ACA (7573G>A), ACG2465ACA (7579G>A), GCT2468GCC (7588T>C), CTA2471TTA (7595C>T), TTG2474CTA (7604T>C 7606G>A), GGT2477GGA (7615T>A), TGT2480TGC (7624T>C), GAC2483GAT (7633C>T), TTC2484TTT (7636C>T), AAT2485AAC (7639T>C), TTT2488TTC (7648T>C), GAA2494GAG (7666A>G), TGT2496TGC (7672T>C), ACA2498ACT (7678A>T), GGG2500GGA (7684G>A), ACT2502ACC (7690T>C), TTT2504TTC (7696T>C), GGT2506GGG (7702T>G), GAT2509AAT (7709G>A), AAA2510AGG (7713A>G 7714A>G), CTG2511TTG (7715C>T), CGT2514CGC (7726T>C), GAG2517GAA (7735G>A), GTA2520GTG (7744A>G), TAC2521TAT (7747C>T), GGC2526GGT (7762C>T), CAG2528CAA (7768G>A), TTT2529TTC (7771T>C), TCA2532TCG (7780A>G), CTA2533CTC (7783A>C), ACT2534ACC (7786T>C), CTA2537TTG (7793C>T 7795A>G), ATC2538ATT (7798C>T), GCT2540GCC (7804T>C), ACC2543ATC (7812C>T), GAA2550GAG (7834A>G), GAC2551GAT (7837C>T), GTG2554TTA (7844G>T 7846G>A), CAG2557CAA (7855G>A), CTG2559CTA (7861G>A), TTA2562TTG (7870A>G), TAC2563TAT (7873C>T), ACT2564ACA (7876T>A), GAG2565GAA (7879G>A), ATT2566ATA (7882T>A), GTT2567ATT (7883G>A), CCA2570CCT (7894A>T), ATT2571ATC (7897T>C), GTC2579GTT (7921C>T), AAG2581AAA (7927G>A), GGT2584GGC (7936T>C), AAT2585AAC (7939T>C), AAT2586AAC (7942T>C), GTG2594GTA (7966G>A), GAT2595GAC (7969T>C), TCT2597TCC (7975T>C), GTC2601GTT (7987C>T), CTT2602CTC (7990T>C), GCT2607GCC (8005T>C), GAA2616GAG (8032A>G), ATC2618ATT (8038C>T), GAC2619GAT (8041C>T), AGC2620AAC (8043G>A), TTG2632TTA (8080G>A), GTG2635GTA (8089G>A), CCG2637CCA (8095G>A), GAG2638GAA (8098G>A), AGC2641AGT (8107C>T), ATT2642ATC (8110T>C), CTC2643CTT (8113C>T), GAT2644GAC (8116T>C), CAA2648CAG (8128A>G), GGT2654GGC (8146T>C), AAC2656AGT (8151A>G 8152C>T), TCG2660TCA (8164G>A), AGG2665AAG (8178G>A), AAG2666AAA (8182G>A), TTG2669TTA (8191G>A), TTC2671TTT (8197C>T), TCC2673TCT (8203C>T), AGA2675AAA (8208G>A), CTG2677TTG (8213C>T), CTA2678CTG (8218A>G), ATC2679ATT (8221C>T), GAG2680GAA (8224G>A), CCA2685CCG (8239A>G), ATT2692ATC (8260T>C), GTA2693GTG (8263A>G), GAT2699GAC (8281T>C), AGA2700AGG (8284A>G), CCA2704GCG (8294C>G 8296A>G), GAG2705GAA (8299G>A), CAC2706CAT (8302C>T), GCG2710GCA (8314G>A), ATT2711ATC (8317T>C), TGC2712TGT (8320C>T), GCT2714GCA (8326T>A), GAG2717GAA (8335G>A), TCC2718TCT (8338C>T), TCT2722TTT (8349C>T), GAA2723GAG (8353A>G), CAC2726CAT (8362C>T), CAA2727CAG (8365A>G), ATC2728ATT (8368C>T), TAC2732TAT (8380C>T), TCA2733TCG (8383A>G), TTA2735TTG (8389A>G), CCT2740CCC (8404T>C), GCA2742TCA (8408G>T), ACA2743ACG (8413A>G), GCG2745GCT (8419G>T), GCT2750GCC (8434T>C), TAT2752TAC (8440T>C), ATG2756ATA (8452G>A), CTA2758CTG (8458A>G), AGG2759AAG (8460G>A), AAA2760AAG (8464A>G), CTG2761CTA (8467G>A), GCT2766ACA (8480G>A 8482T>A), GAG2771GAA (8497G>A), CTA2772TTG (8498C>T 8500A>G), AGA2773AAG (8502G>A 8503A>G), GCC2774GCT (8506C>T), GAA2777GAT (8515A>T), GTC2780GTT (8524C>T), GCA2781GCC (8527A>C), TTA2782CTG (8528T>C 8530A>G), GAG2785GAC (8539G>C), CTT2788TGT (8546C>T 8547T>G), TCT2790ACT (8552T>A), GTA2793GTG (8563A>G), GCA2797GGA (8574C>G), ATT2801GTT (8585A>G), GGA2805GAA (8598G>A), AGC2806AGT (8602C>T), AAC2807TCT (8603A>T 8604A>C 8605C>T), AAA2812AAG (8620A>G), CCA2813CAA (8622C>A), GAG2814GGA (8625A>G 8626G>A), CAG2815CAA (8629G>A), GGC2816GGT (8632C>T), ATC2818ATT (8638C>T), CAG2819CAA (8641G>A), AAC2821AAT (8647C>T), CCG2822TTC (8648C>T 8649C>T 8650G>C), AAC2823AAT (8653C>T), AAA2824AAG (8656A>G), GGA2825GAA (8658G>A), GAT2827GCG (8664A>C 8665T>G), GTT2830GTG (8674T>G), GCA2832GTT (8679C>T 8680A>T), GGC2833GGA (8683C>A), GGG2836GGA (8692G>A), ACA2837ACT (8695A>T), CCG2841CCA (8707G>A), AGA2842CGA (8708A>C), AAG2844AAA (8716G>A), ATC2846ATT (8722C>T), ACG2847ACA (8725G>A), AAA2849AAG (8731A>G), AGA2851AGG (8737A>G), CCC2853CCT (8743C>T), ACA2854AAG (8745C>A 8746A>G), AGC2855AGT (8749C>T), AAG2856AAA (8752G>A), GGA2857GGT (8755A>T), AAC2862AAT (8770C>T), TTA2863CTA (8771T>C), GAA2864GAG (8776A>G), CAT2865CAC (8779T>C), TTG2866TTA (8782G>A), TAT2869TAC (8791T>C), CCA2871CCG (8797A>G), CAA2872CAG (8800A>G), CAA2873CAG (8803A>G), GAT2875GAC (8809T>C), ATT2876ATC (8812T>C), AAT2878AAC (8818T>C), CGG2880CGA (8824G>A), GCA2881GCG (8827A>G), ACG2888ACA (8848G>A), TAT2890TAC (8854T>C), GAG2891GGA (8856A>G 8857G>A), GTG2893GTA (8863G>A), CGG2894CAG (8865G>A), ATG2895CTT (8867A>C 8869G>T), GCA2896GCG (8872A>G), GGA2900GAA (8883G>A), ACT2902TCT (8888A>T), TGC2915TGT (8929C>T), GTC2924ATC (8954G>A), AAC2925AAT (8959C>T), GGA2926GGT (8962A>T), GTT2929ATT (8969G>A), GGG2933GGA (8983G>A), AAT2934GAC (8984A>G 8986T>C), GAA2935GTA (8988A>T), GAG2938GAA (8998G>A), CCG2940CCA (9004G>A), TTG2941TTA (9007G>A), ATC2944ATT (9016C>T), ACC2951ACA (9037C>A), ATA2967ATT (9085A>T), AAC2971AAT (9097C>T), ATT2983GTT (9131A>G), CGA2984CGT (9136A>T), CGG2987CGT (9145G>T), ATG2989GGA (9149A>G 9150T>G 9151G>A), GGT2990AGT (9152G>A), TTA2991TTG (9157A>G), GCG2992GCT (9160G>T), CGT2993CGC (9163T>C), GAG3000GAA (9184G>A), CGA3004CGG (9196A>G), AGG3010AGA (9214G>A), GAA3011GAG (9217A>G), GCG3012GCA (9220G>A), GCA3020GCT (9244A>T), TTG3021TTA (9247G>A), GCC3024GCT (9256C>T), CCT3026TCC (9260C>T 9262T>C), GGG3030GGA (9274G>A), GAC3032GAT (9280C>T), ATC3035ATT (9289C>T), GAG3040GAA (9304G>A), GTC3050GTT (9334C>T), GTC3060GTG (9364C>G)								
PVYgp2	1	75	100%	95	18.1%	75 (98.7%)	54 (71.1%)	1/0/3/2	2
Protein mutations:	R17_K17insX (2971_2972insA), Y23H (2989T>C 2991C>T), R26K (2999G>A 3000A>G), H35Y (3025C>T), K40H (3040A>C 3042A>C), G41S (3043G>A), E44K (3052G>A), I47V (3061A>G), V54I (3082G>A), P57S (3091C>T), P60T (3100C>A), G61E (3104G>A 3105T>G), G62S (3106G>A), Q63* (3109C>T), R64G (3112A>G), C66R (3118T>C), L67F (3121C>T), I69V (3127A>G), A72E (3137C>A 3138G>A), I73V (3139A>G)								
Codon mutations:	TTA2CTA (2923T>C), AAA4AAG (2931A>G), GAA7GAG (2940A>G), TTG10CTG (2947T>C), TTG15CTG (2962T>C), AGA17_AAA17insAA- (2971_2972insA), TAC23CAT (2989T>C 2991C>T), TCA24TCG (2994A>G), AGA26AAG (2999G>A 3000A>G), CAC32-AC (3016delC), TCG33TCA (3021G>A), GTA34GTT (3024A>T), CAT35TAT (3025C>T), CAC38CAT (3036C>T), AGA39AGG (3039A>G), AAA40CAC (3040A>C 3042A>C), GGC41AGC (3043G>A), GAA44AAA (3052G>A), ATA47GTA (3061A>G), GTT54ATT (3082G>A), CCG57TCG (3091C>T), CGC59CGT (3099C>T), CCA60ACA (3100C>A), GGT61GAG (3104G>A 3105T>G), GGT62AGT (3106G>A), CAA63TAA (3109C>T), AGG64GGG (3112A>G), TGC66CGC (3118T>C), CTC67TTC (3121C>T), ATT69GTT (3127A>G), GAG70GAA (3132G>A), GCG72GAA (3137C>A 3138G>A), ATT73GTT (3139A>G)								

Proteins

polyprotein (NP_056759.1)	1	3064	90.9%	17031	88.8%	2782 (99.8%)	2525 (90.6%)	2/3/5/5	2

	Begin	End	Coverage	Score	Concordance	Matches	Identities	I/D/M/F*	Stop Codons
**NT**	**183**	**9687**	**89.3%**	**11427**	**66.3%**	**8654 (99.8%)**	**7216 (83.2%)**	**5/16**	
Protein mutations:	Y4Q (194T>C 196C>A), C9L (209T>C 210G>T 211T>G), F13M (221T>A 223T>G), S23P (251T>C), C24F (255G>T 256C>T), E25G (258A>G), I27V (263A>G 265T>G), V28A (267T>C 268G>A), E30V (273A>T), A35T (287G>A 289T>C), V37T (293G>A 294T>C), A41I (305G>A 306C>T), D42G (309A>G), E44D (316A>T), T45V (317A>G 318C>T 319A>G), L52Q (339T>A 340C>A), K53R (342A>G), K55A (347A>G 348A>C 349A>G), Y56H (350T>C), T58V (356A>G 357C>T), V61T (365G>A 366T>C 367G>A), L62S (368C>T 369T>C), F67C (384T>G), A75V (408C>T 409C>T), M78E (416A>G 417T>A), E85K (437G>A), R86E (440A>G 441G>A 442G>A), K87R (443A>C 444A>G 445G>A), D88E (448T>A), M194K (765T>A), R197K (773C>A 774G>A 775A>G), M206K (801T>A), G210A (813G>C 814A>G), L211H (816T>A), R214Q (825G>A 826T>A), R226C (860C>T 862C>T), T227A (863A>G 865T>C), I228T (867T>C), N229D (869A>G 871C>T), I230L (872A>T 874A>G), R232K (879G>A), I238V (896A>G 898C>T), N240S (903A>G), T241N (906C>A 907A>C), K242T (909A>C 910A>C), S243N (912G>A 913C>T), S252L (938T>C 939C>T 940A>T), L266I (980T>A 982G>C), R272K (999G>A), Q275R (1007C>A 1008A>G 1009G>A), S276G (1010A>G 1012T>G), I277V (1013A>G), N279Q (1019A>C 1021C>G), D288E (1048C>A), R300Q (1083G>A), Y303C (1092A>G 1093T>C), S305A (1097T>G 1099G>A), I382T (1329T>C), L397I (1373C>A), I401V (1385A>G), E407D (1405G>T), A411S (1415G>T 1417A>T), V418I (1436G>A 1438C>T), S461del (1565_1568delTCTT), E508G (1707A>G), A512T (1718G>A), I520V (1742A>G 1744C>T), S525N (1758G>A), V536I (1790G>A), I585V (1937A>G 1939T>C), F607Y (2004T>A), I628M (2068T>G), V630I (2072G>A 2074G>A), R675K (2208G>A), D694E (2266C>G), K780R (2523A>G), R817K (2634G>A), A827V (2664C>T 2665T>C), V847I (2723G>A), A855T (2747G>A 2749T>A), H871N (2795C>A), V880I (2822G>A 2824G>A), P900T (2882C>A), S901H (2885A>C 2886G>A), T904M (2895C>T), N916S (2931A>G), N919S (2940A>G), E929_N930insX (2971_2972insA), T936I (2991C>T), H937R (2994A>G), E939R (2999G>A 3000A>G), R946Q (3021G>A), Y947F (3024A>T 3025C>T), T951I (3036C>T), E952G (3039A>G 3040A>C), K953T (3042A>C 3043G>A), A972V (3099C>T 3100C>A), V974R (3104G>A 3105T>G 3106G>A), S983N (3132G>A), R985K (3137C>A 3138G>A 3139A>G), F990I (3152T>A 3154C>T), C994Y (3165G>A), F1012L (3218T>C), V1016I (3230G>A), V1028M (3266G>A 3268A>G), V1031M (3275G>A), A1034T (3284G>A), R1044K (3315G>A 3316A>G), E1045A (3318A>C), M1059V (3359A>G), D1072E (3400T>G), V1110G (3513T>G), M1121V (3545A>G), A1122T (3548G>A), V1124I (3554G>A 3556C>T), L1147V (3623C>G), L1150M (3632C>A), Y1152H (3638T>C), K1164N (3676G>C), N1165K (3679T>A), N1170K (3694T>A), I1172V (3698A>G), D1174* (3704G>T 3706T>A), F1175L (3709T>A), I1297V (4073A>G), F1315Y (4128T>A 4129C>T), A1323S (4151G>T), V1328I (4166G>A), L1347I (4223C>A 4225G>A), I1379V (4319A>G 4321A>G), F1404Y (4395T>A), V1408I (4406G>A), M1431V (4475A>G 4477G>T), T1433S (4481A>T), C1444R (4514T>C), V1499I (4679G>A 4681G>C), V1538I (4796G>A 4798C>T), T1540S (4803C>G 4804T>C), S1625G (5057A>G), T1626A (5060A>G), L1636I (5090C>A), A1639V (5100C>T), E1641D (5107A>T), V1646F (5120G>T 5122C>T), I1648V (5126A>G), V1667I (5183G>A), A1793T (5561G>A 5563G>A), I1805V (5597A>G), N1807K (5605C>A), A1812V (5619C>T 5620T>G), I1816L (5630A>C), V1837I (5693G>A), I1904V (5894A>G 5896C>G), R1926A (5960C>G 5961G>C 5962G>C), I1936V (5990A>G), E1945D (6019A>T), K1949T (6030A>C), D1955E (6049C>A), M1958I (6058G>A), S1963N (6072G>A 6073T>C), N1964H (6074A>C), T1966N (6081C>A), C1976S (6111G>C 6112T>C), I1982V (6128A>G 6130T>C), V1992I (6158G>A), T1996S (6170A>T 6172A>G), L2007F (6203C>T 6205C>T), V2016I (6230G>A), A2025K (6257G>A 6258C>A), K2033R (6282A>G), A2060T (6362G>A), V2073I (6401G>A), A2074V (6405C>T), Y2082F (6429A>T), Q2089R (6450A>G), H2101N (6485C>A 6487C>T), L2115I (6527C>A), T2135I (6588C>T), Y2151H (6635T>C), I2155V (6647A>G), I2165V (6677A>G 6679A>G), D2178N (6716G>A), N2179D (6719A>G), I2194L (6764A>T 6766A>G), A2204V (6795C>T 6796A>G), H2205Q (6799C>A), K2220R (6843A>G), N2225D (6857A>G), V2231T (6875G>A 6876T>C 6877C>A), D2250E (6934C>A), V2273I (7001G>A 7003G>A), F2283Y (7032T>A), T2314K (7125C>A), A2321S (7145G>T 7147A>G), E2326_A2327del (7160_7165delGAGGCA), D2344E (7216T>A), D2358E (7258T>A), D2363_R2364insX (7273_7274insT), R2364V (7274C>G 7275G>T), H2366L (7281A>T), L2367S (7284T>C), S2371L (7296C>T), S2376F (7311C>T), T2377I (7314C>T), C2378Y (7317G>A), N2379K (7321T>A), K2384Q (7334A>C), A2387N (7343G>A 7344C>A 7345A>C), S2408M (7407G>T 7408T>G), C2411G (7415T>G), L2440S (7503T>C 7504G>A), D2509N (7709G>A), K2510R (7713A>G 7714A>G), T2543I (7812C>T), V2554L (7844G>T 7846G>A), V2567I (7883G>A), S2620N (8043G>A), N2656S (8151A>G 8152C>T), R2665K (8178G>A), R2675K (8208G>A), P2704A (8294C>G 8296A>G), S2722F (8349C>T), A2742S (8408G>T), M2756I (8452G>A), R2759K (8460G>A), A2766T (8480G>A 8482T>A), R2773K (8502G>A 8503A>G), E2777D (8515A>T), E2785D (8539G>C), L2788C (8546C>T 8547T>G), S2790T (8552T>A), A2797G (8574C>G), I2801V (8585A>G), G2805E (8598G>A), N2807S (8603A>T 8604A>C 8605C>T), P2813Q (8622C>A), E2814G (8625A>G 8626G>A), P2822F (8648C>T 8649C>T 8650G>C), G2825E (8658G>A), D2827A (8664A>C 8665T>G), A2832V (8679C>T 8680A>T), T2854K (8745C>A 8746A>G), E2891G (8856A>G 8857G>A), R2894Q (8865G>A), M2895L (8867A>C 8869G>T), G2900E (8883G>A), T2902S (8888A>T), V2924I (8954G>A), V2929I (8969G>A), N2934D (8984A>G 8986T>C), E2935V (8988A>T), I2983V (9131A>G), M2989G (9149A>G 9150T>G 9151G>A), G2990S (9152G>A), P3026S (9260C>T 9262T>C)								

	Begin	End	Coverage	Score	Concordance	Matches	Identities	I/D/M/F*	Stop Codons
NT	183	9687	89.3%	11427	66.3%	8654 (99.8%)	7216 (83.2%)	5/16	

Codon mutations:

ACT3ACC (193T>C), TAC4CAA (194T>C 196C>A), TCA6TCG (202A>G), ATC8ATT (208C>T), TGT9CTG (209T>C 210G>T 211T>G), TCG12TCA (220G>A), TTT13ATG (221T>A 223T>G), GAA14GAG (226A>G), CTA17CTT (235A>T), TAC19TAT (241C>T), CCA21CCT (247A>T), TCT23CCT (251T>C), TGC24TTT (255G>T 256C>T), GAG25GGG (258A>G), CAT26CAC (262T>C), ATT27GTG (263A>G 265T>G), GTG28GCA (267T>C 268G>A), GAA30GTA (273A>T), CGA31CGT (277A>T), GAA32GAG (280A>G), GTG33GTT (283G>T), CCG34CCA (286G>A), GCT35ACC (287G>A 289T>C), GTT37ACT (293G>A 294T>C), CCT39CCC (301T>C), TTC40TTT (304C>T), GCA41ATA (305G>A 306C>T), GAT42GGT (309A>G), CTG43TTG (311C>T), GAA44GAT (316A>T), ACA45GTG (317A>G 318C>T 319A>G), CAA46CAG (322A>G), GCA49GCT (331A>T), CTC52CAA (339T>A 340C>A), AAG53AGG (342A>G), AAA55GCG (347A>G 348A>C 349A>G), TAT56CAT (350T>C), GCT57GCA (355T>A), ACT58GTT (356A>G 357C>T), CGT60CGC (364T>C), GTG61ACA (365G>A 366T>C 367G>A), CTC62TCC (368C>T 369T>C), AAA63AAG (373A>G), AAC64AAT (376C>T), TTT67TGT (384T>G), ACG68ACA (388G>A), TAC69TAT (391C>T), CGA70CGG (394A>G), TAC71TAT (397C>T), AAG72AAA (400G>A), GAT74GAC (406T>C), GCC75GTT (408C>T 409C>T), CAG76CAA (412G>A), ATA77ATT (415A>T), ATG78GAG (416A>G 417T>A), ATT80ATC (424T>C), CAG81CAA (427G>A), AAA83AAG (433A>G), CTG84CTC (436G>C), GAG85AAG (437G>A), AGG86GAA (440A>G 441G>A 442G>A), AAG87CGA (443A>C 444A>G 445G>A), GAT88GAA (448T>A), GAA90GAG (454A>G), ATG194AAG (765T>A), TTG196CTG (770T>C), CGA197AAG (773C>A 774G>A 775A>G), CGG198AGG (776C>A), TTC202TTT (790C>T), ATG206AAG (801T>A), GGA210GCG (813G>C 814A>G), CTT211CAT (816T>A), TTG212CTG (818T>C), CAA213CAG (823A>G), CGT214CAA (825G>A 826T>A), CGG217AGA (833C>A 835G>A), GAC219GAT (841C>T), AAT223AAC (853T>C), GTC225GTT (859C>T), CGC226TGT (860C>T 862C>T), ACT227GCC (863A>G 865T>C), ATC228ACC (867T>C), AAC229GAT (869A>G 871C>T), ATA230TTG (872A>T 874A>G), AGG232AAG (879G>A), GAT234GAC (886T>C), ATC238GTT (896A>G 898C>T), AAC240AGC (903A>G), ACA241AAC (906C>A 907A>C), AAA242ACC (909A>C 910A>C), AGC243AAT (912G>A 913C>T), GGC246GGA (922C>A), GGT249GGA (931T>A), TCA252CTT (938T>C 939C>T 940A>T), GGA253GGG (943A>G), TTC256TTT (952C>T), TCA261TCG (967A>G), GAA263GAG (973A>G), GGG264GGC (976G>C), TTG266ATC (980T>A 982G>C), GCA269GCG (991A>G), CGT270CGC (994T>C), AGA272AAA (999G>A), GTT273GTC (1003T>C), CAG275AGA (1007C>A 1008A>G 1009G>A), AGT276GGG (1010A>G 1012T>G), ATT277GTT (1013A>G), TTA278TTG (1018A>G), AAC279CAG (1019A>C 1021C>G), ATC282ATT (1030C>T), TCG285TCA (1039G>A), AAT286AAC (1042T>C), GCC287GCA (1045C>A), GAC288GAA (1048C>A), AAT289AAC (1051T>C), AAG292AAA (1060G>A), GGT293GGA (1063T>A), CTG294TTG (1064C>T), GAC295GAT (1069C>T), CGA300CAA (1083G>A), TAT303TGC (1092A>G 1093T>C), TCG305GCA (1097T>G 1099G>A), CAC307CAT (1105C>T), AAT378..A (1318T>A), AAG379AAA (1321G>A), TTG381TTA (1327G>A), ATA382ACA (1329T>C), GCG383GCT (1333G>T), TTA384TTG (1336A>G), CTA387TTG (1343C>T 1345A>G), CCG390CCA (1354G>A), GTG391GTT (1357G>T), GAC392GAT (1360C>T), CTC395TTG (1367C>T 1369C>G), CTT397ATT (1373C>A), TTC398TTT (1378C>T), GAG400GAA (1384G>A), ATA401GTA (1385A>G), AAA403AAG (1393A>G), TCC404TCT (1396C>T), GAG407GAT (1405G>T), AAA408AAG (1408A>G), CAG409CAA (1411G>A), GCA411TCT (1415G>T 1417A>T), CCG412CCC (1420G>C), AAT415AAC (1429T>C), TTA416TTG (1432A>G), GTC418ATT (1436G>A 1438C>T), TTA419CTG (1439T>C 1441A>G), AAT420AAC (1444T>C), TTC423TTT (1453C>T), CTG424CTA (1456G>A), GAA428GAG (1468A>G), GTA436GTG (1492A>G), TTG439CTA (1499T>C 1501G>A), AGT440AGC (1504T>C), TTG441TTA (1507G>A), CTC442CTT (1510C>T), GAA443GAG (1513A>G), TTA444TTG (1516A>G), GCA445GCT (1519A>T), AGG446AGA (1522G>A), TTC447TTT (1525C>T), CAG448CAA (1528G>A), AAC450AAT (1534C>T), ACT452ACA (1540T>A), AAC454AAT (1546C>T), AAA457AAG (1555A>G), GAT459GAC (1561T>C), ATA460ATC (1564A>C), TCT461del (1565_1568delTCTT), TTC462-TC (1565_1568delTCTT), TTC463TTT (1573C>T), AGA464AGG (1576A>G), TTA467CTA (1583T>C), TCT468TCA (1588T>A), AAG470AAA (1594G>A), AAC472AAT (1600C>T), TCG478TCA (1618G>A), TGC479TGT (1621C>T), GAC480GAT (1624C>T), CTG483TTG (1631C>T), AAA485AAG (1639A>G), TTC489TTT (1651C>T), CTC490CTG (1654C>G), CAT497CAC (1675T>C), TCA503TCG (1693A>G), TTT506TTC (1702T>C), GAA508GGA (1707A>G), GCA512ACA (1718G>A), GGA514GGG (1726A>G), TAC515TAT (1729C>T), TCA516TCC (1732A>C), TAT518TAC (1738T>C), ATC520GTT (1742A>G 1744C>T), CGC521CGT (1747C>T), CAT523CAC (1753T>C), AGT525AAT (1758G>A), AGG528AGA (1768G>A), AAC534AAT (1786C>T), TTA535TTG (1789A>G), GTT536ATT (1790G>A), GTC537GTT (1795C>T), TTA541CTG (1805T>C 1807A>G), GAG543GAA (1813G>A), AGG545AGA (1819G>A), AAG547AAA (1825G>A), GAC551GAT (1837C>T), TAT552TAC (1840T>C), CAA555CAG (1849A>G), GTC558GTG (1858C>G), AAA560AAG (1864A>G), TGC562TGT (1870C>T), AGT564AGC (1876T>C), TCG565TCT (1879G>T), AAA566AAG (1882A>G), GGT568GGA (1888T>A), AAT569AAC (1891T>C), TGT574TGC (1906T>C), ACA577ACT (1915A>T), GGT582GGC (1930T>C), GCC584GCT (1936C>T), ATT585GTC (1937A>G 1939T>C), TCA587TCG (1945A>G), TTC589TTT (1951C>T), CCA592CCT (1960A>T), GTA598GTG (1978A>G), ATT599ATA (1981T>A), GGC600GGT (1984C>T), AAT601AAC (1987T>C), GAC604GAT (1996C>T), TTT607TAT (2004T>A), AAA612AAG (2020A>G), GGG613GGA (2023G>A), GAT614GAC (2026T>C), TCG615TCT (2029G>T), GAG616GAA (2032G>A), TAT625TAC (2059T>C), ATT628ATG (2068T>G), AAC629AAT (2071C>T), GTG630ATA (2072G>A 2074G>A), CTG635TTA (2087C>T 2089G>A), AGC639AGT (2101C>T), GAG640GAA (2104G>A), GAG641GAA (2107G>A), AAG644AAA (2116G>A), TTC646TTT (2122C>T), CGC651CGT (2137C>T), CTT658CTC (2158T>C), ACC660ACT (2164C>T), CCA662CCG (2170A>G), GAT666GAC (2182T>C), GCG668GCA (2188G>A), ACC669ACA (2191C>A), AGA675AAA (2208G>A), TAT678TAC (2218T>C), GAC680GAT (2224C>T), GTA681GTT (2227A>T), TTG686TTA (2242G>A), CCC687CCT (2245C>T), TTG690TTA (2254G>A), GTT691GTC (2257T>C), GAC692GAT (2260C>T), GAC694GAG (2266C>G), CAA696CAG (2272A>G), ACG697ACA (2275G>A), TCA703TCG (2293A>G), GGC705GGT (2299C>T), TCG706TCA (2302G>A), CAG707CAA (2305G>A), GGA710GGT (2314A>T), TAT711TAC (2317T>C), ATT713ATC (2323T>C), CTA714CTG (2326A>G), AGC718AGT (2338C>T), GTG719GTA (2341G>A), CAA721CAG (2347A>G), CTT722CTC (2350T>C), TTG724TTA (2356G>A), AAT727AAC (2365T>C), TCT764.CC (2476T>C), TCG766TCA (2482G>A), CTG769CTA (2491G>A), CTG771CTT (2497G>T), TTG773TTA (2503G>A), AGA778AGG (2518A>G), CCT779CCC (2521T>C), AAG780AGG (2523A>G), AGA783AGG (2533A>G), TTG785TTA (2539G>A), TTA787TTG (2545A>G), GAG789GAA (2551G>A), CCT790CCA (2554T>A), CTG792TTG (2558C>T), TTG793CTC (2561T>C 2563G>C), CTA795CTG (2569A>G), TCA796TCG (2572A>G), CCT800CCC (2584T>C), GGC801GGT (2587C>T), CTG803CTC (2593G>C), GCT805GCC (2599T>C), TAT807TAC (2605T>C), AAT808AAC (2608T>C), TTT812TTC (2620T>C), GCG815GCA (2629G>A), GTG816GTA (2632G>A), AGG817AAG (2634G>A), TTG818TTA (2638G>A), ATT820ATA (2644T>A), AAA823AAG (2653A>G), GCT827GTC (2664C>T 2665T>C), CTA832TTA (2678C>T), CTA833CTG (2683A>G), GCT835GCC (2689T>C), CTA838CTC (2698A>C), TCA841TCG (2707A>G), GCG842GCA (2710G>A), GAA844GAG (2716A>G), ACA845ACG (2719A>G), CTC846CTT (2722C>T), GTC847ATC (2723G>A), GCA848GCG (2728A>G), AGG850AGA (2734G>A), ATT851ATA (2737T>A), GCT855ACA (2747G>A 2749T>A), GCA856GCT (2752A>T), GCT857GCA (2755T>A), GAC859GAT (2761C>T), CTC860CTT (2764C>T), TGT865TGC (2779T>C), GGG867GGT (2785G>T), AAC869AAT (2791C>T), CAT871AAT (2795C>A), TAC874TAT (2806C>T), TTG878CTC (2816T>C 2818G>C), GTG880ATA (2822G>A 2824G>A), TTG881TTA (2827G>A), GTT884GTC (2836T>C), AAG885AAA (2839G>A), AAT886AAC (2842T>C), ACC893ACA (2863C>A), CTA894TTA (2864C>T), CCA900ACA (2882C>A), AGT901CAT (2885A>C 2886G>A), AAC903AAT (2893C>T), ACG904ATG (2895C>T), GTC906GTT (2902C>T), GTA907GTG (2905A>G), ATT909ATC (2911T>C), AAT913AAC (2923T>C), AAT916AGT (2931A>G), AAC919AGC (2940A>G), GCT921GCC (2947T>C), ACT926ACC (2962T>C), GAA929_AAT930insA-- (2971_2972insA), GGT935GGC (2989T>C), ACT936ATT (2991C>T), CAT937CGT (2994A>G), GAG939AGG (2999G>A 3000A>G), TCC944TC- (3016delC), CGG946CAG (3021G>A), TAC947TTT (3024A>T 3025C>T), ACA951ATA (3036C>T), GAA952GGC (3039A>G 3040A>C), AAG953ACA (3042A>C 3043G>A), TTG956TTA (3052G>A), TTA959TTG (3061A>G), GCG966GCA (3082G>A), GGC969GGT (3091C>T), GCC972GTA (3099C>T 3100C>A), GTG974AGA (3104G>A 3105T>G 3106G>A), GTC975GTT (3109C>T), AAA976AAG (3112A>G), ACT978ACC (3118T>C), GCC979GCT (3121C>T), GGA981GGG (3127A>G), AGC983AAC (3132G>A), CGA985AAG (3137C>A 3138G>A 3139A>G), TAT989TAC (3151T>C), TTC990ATT (3152T>A 3154C>T), TGT994TAT (3165G>A), GTA995GTG (3169A>G), ATC1005ATT (3199C>T), AGG1007CGG (3203A>C), CGT1008CGC (3208T>C), CCA1010CCG (3214A>G), ACC1011ACT (3217C>T), TTT1012CTT (3218T>C), GTC1013GTA (3223C>A), GTT1016ATT (3230G>A), AAC1017AAT (3235C>T), TTA1025CTA (3257T>C), ACC1026ACT (3262C>T), AGC1027AGT (3265C>T), GTA1028ATG (3266G>A 3268A>G), GTG1029GTA (3271G>A), GTG1031ATG (3275G>A), GCA1034ACA (3284G>A), CAG1039CAA (3301G>A), AGA1044AAG (3315G>A 3316A>G), GAA1045GCA (3318A>C), ATC1046ATT (3322C>T), GAG1047GAA (3325G>A), TTG1048TTA (3328G>A), ATA1051ATT (3337A>T), GAG1055GAA (3349G>A), GTC1057GTT (3355C>T), TGC1058TGT (3358C>T), ATG1059GTG (3359A>G), CTA1061CTG (3367A>G), TAT1062TAC (3370T>C), TTA1065CTG (3377T>C 3379A>G), GAA1070GAG (3394A>G), CGC1071CGT (3397C>T), GAT1072GAG (3400T>G), ACA1074ACG (3406A>G), TAC1078TAT (3418C>T), ATT1079ATA (3421T>A), TAT1081TAC (3427T>C), AAG1083AAA (3433G>A), TCA1084TCT (3436A>T), GTA1085GTG (3439A>G), AAC1086AAT (3442C>T), CCT1087CCC (3445T>C), CAG1091CAA (3457G>A), TTT1092TTC (3460T>C), GCT1093GCA (3463T>A), GCG1095GCT (3469G>T), CAG1096CAA (3472G>A), CAC1104CAT (3496C>T), CAG1105CAA (3499G>A), TCC1107TCT (3505C>T), GTT1110GGT (3513T>G), AAA1112AAG (3520A>G), GAA1115GAG (3529A>G), CAA1116CAG (3532A>G), ATG1121GTG (3545A>G), GCT1122ACT (3548G>A), TTA1123CTA (3551T>C), GTC1124ATT (3554G>A 3556C>T), AGT1133AGC (3583T>C), GAT1134GAC (3586T>C), TGC1135TGT (3589C>T), TTC1137TTT (3595C>T), AAT1141AAC (3607T>C), TTT1143TTC (3613T>C), AAG1144AAA (3616G>A), GGT1145GGC (3619T>C), CTT1147GTT (3623C>G), TCC1148TCT (3628C>T), CTG1150ATG (3632C>A), GAT1151GAC (3637T>C), TAT1152CAT (3638T>C), GAA1153GAG (3643A>G), TTA1159TTG (3661A>G), GTG1162GTA (3670G>A), AAG1164AAC (3676G>C), AAT1165AAA (3679T>A), GAG1168GAA (3688G>A), AAT1170AAA (3694T>A), GAG1171GAA (3697G>A), ATT1172GTT (3698A>G), GAT1174TAA (3704G>T 3706T>A), TTT1175TTA (3709T>A), AGC1233..T (3883C>T), GAA1234GAG (3886A>G), CTA1236CTG (3892A>G), GAC1237GAT (3895C>T), TTT1238TTC (3898T>C), GTA1240GTG (3904A>G), CGG1241AGA (3905C>A 3907G>A), GCT1243GCC (3913T>C), AAG1248AAA (3928G>A), TCA1249TCT (3931A>T), GGG1251GGA (3937G>A), AGT1257AGC (3955T>C), GTG1258GTA (3958G>A), GCC1259GCT (3961C>T), GGA1260GGG (3964A>G), TCT1261TCC (3967T>C), TTA1264TTG (3976A>G), ATT1265ATC (3979T>C), CCA1267CCT (3985A>T), CTA1271CTT (3997A>T), GCG1272GCA (4000G>A), AAC1274AAT (4006C>T), GTT1275GTG (4009T>G), TTC1276TTT (4012C>T), AAA1277AAG (4015A>G), TCT1280TCC (4024T>C), GAA1282GAG (4030A>G), CCA1283CCT (4033A>T), AAG1286AAA (4042G>A), CTG1290TTA (4052C>T 4054G>A), CGT1291CGC (4057T>C), CGT1293CGA (4063T>A), GGA1294GGG (4066A>G), ATA1297GTA (4073A>G), TTT1298TTC (4078T>C), GGC1299GGT (4081C>T), TCT1300TCC (4084T>C), TCC1304TCT (4096C>T), AGC1308AGT (4108C>T), GGA1309GGT (4111A>T), GCG1311GCA (4117G>A), CTA1312TTG (4118C>T 4120A>G), TAC1314TAT (4126C>T), TTC1315TAT (4128T>A 4129C>T), GCC1316GCT (4132C>T), AAT1317AAC (4135T>C), CAA1321CAG (4147A>G), TTA1322CTA (4148T>C), GCT1323TCT (4151G>T), AAC1326AAT (4162C>T), GTA1328ATA (4166G>A), ATA1329ATC (4171A>C), GAG1332GAA (4180G>A), CTG1336TTG (4190C>T), CCT1338CCC (4198T>C), TCC1339TCT (4201C>T), GCG1342GCT (4210G>T), TTC1343TTT (4213C>T), AGT1345AGC (4219T>C), CTG1346TTG (4220C>T), CTG1347ATA (4223C>A 4225G>A), GTT1349GTG (4231T>G), CAT1351CAC (4237T>C), GTA1356GTG (4252A>G), TTA1357CTG (4253T>C 4255A>G), AAA1358AAG (4258A>G), ACT1362ACC (4270T>C), AGA1366AGG (4282A>G), GAG1367GAA (4285G>A), GAA1369GAG (4291A>G), CAG1373CAA (4303G>A), GTC1376GTT (4312C>T), ATA1379GTG (4319A>G 4321A>G), GTG1380GTT (4324G>T), GAG1381GAA (4327G>A), CTG1384CTT (4336G>T), TCT1385TCC (4339T>C), TCA1388TCT (4348A>T), GCA1392GCG (4360A>G), GGT1394GGC (4366T>C), TCT1395TCA (4369T>A), ACT1397ACC (4375T>C), GTT1401GTC (4387T>C), TTT1404TAT (4395T>A), GTA1408ATA (4406G>A), ACC1420ACA (4444C>A), GCC1422GCT (4450C>T), CTC1424CTT (4456C>T), GAC1427GAT (4465C>T), ATG1431GTT (4475A>G 4477G>T), GTC1432GTT (4480C>T), ACA1433TCA (4481A>T), GGC1437GGT (4495C>T), AAG1441AAA (4507G>A), CAC1442CAT (4510C>T), GGT1443GGG (4513T>G), TGC1444CGC (4514T>C), CTA1445TTA (4517C>T), GTC1448GTT (4528C>T), AAA1450AAG (4534A>G), GTG1469GTA (4591G>A), TTG1471TTA (4597G>A), GAC1472GAT (4600C>T), GAC1474GAT (4606C>T), GTG1475GTA (4609G>A), GTG1477GTA (4615G>A), GAC1478GAT (4618C>T), TTG1481CTT (4625T>C 4627G>T), GAC1488GAT (4648C>T), ATT1489ATC (4651T>C), AGG1492AGA (4660G>A), AGC1493AGT (4663C>T), GCT1495GCA (4669T>A), GTG1499ATC (4679G>A 4681G>C), AGT1500AGC (4684T>C), GTT1501GTG (4687T>G), GAG1505GAA (4699G>A), AGA1506AGG (4702A>G), CAA1508CAG (4708A>G), AGG1509AGA (4711G>A), CTG1510TTG (4712C>T), AAA1518AAG (4738A>G), GCA1521GCG (4747A>G), TTG1522CTG (4748T>C), ACT1527ACC (4765T>C), GTC1538ATT (4796G>A 4798C>T), ACT1540AGC (4803C>G 4804T>C), GAG1541GAA (4807G>A), GCG1542GCT (4810G>T), GCT1543GCG (4813T>G), CTT1544CTC (4816T>C), TAT1549TAC (4831T>C), AAC1550AAT (4834C>T), CCA1552CCT (4840A>T), GGA1556GGG (4852A>G), GGC1557GGT (4855C>T), AGT1561AGC (4867T>C), CTG1562CTC (4870G>C), ATT1563ATA (4873T>A), ACA1573ACT (4903A>T), CAG1575CAA (4909G>A), TTT1577TTC (4915T>C), GAA1578GAG (4918A>G), TTG1579CTA (4919T>C 4921G>A), AGT1580AGC (4924T>C), CCC1581CCG (4927C>G), TTT1583TTC (4933T>C), ATC1584ATA (4936C>A), CAG1585CAA (4939G>A), AAT1586AAC (4942T>C), TTC1587TTT (4945C>T), TCA1593TCG (4963A>G), CCT1596CCC (4972T>C), CTT1602CTC (4990T>C), AAA1606AAG (5002A>G), CCT1613CCC (5023T>C), TTG1614CTA (5024T>C 5026G>A), TGC1615TGT (5029C>T), GAT1616GAC (5032T>C), TCT1618TCC (5038T>C), CCA1620CCT (5044A>T), GCA1623GCT (5053A>T), TCG1624TCA (5056G>A), AGT1625GGT (5057A>G), ACT1626GCT (5060A>G), TCG1629TCT (5071G>T), CTT1636ATT (5090C>A), GGA1637GGG (5095A>G), GTG1638GTT (5098G>T), GCC1639GTC (5100C>T), TTA1640TTG (5104A>G), GAA1641GAT (5107A>T), ATT1642ATC (5110T>C), CCA1643CCG (5113A>G), AAG1644AAA (5116G>A), CAA1645CAG (5119A>G), GTC1646TTT (5120G>T 5122C>T), ATT1648GTT (5126A>G), CAT1651CAC (5137T>C), ATC1652ATT (5140C>T), AAA1653AAG (5143A>G), GAG1654GAA (5146G>A), CTC1659CTA (5161C>A), GAA1661GAG (5167A>G), CTT1663CTG (5173T>G), ACG1666ACA (5182G>A), GTT1667ATT (5183G>A), GTC1668GTT (5188C>T), AAG1669AAA (5191G>A), AAA1671AAG (5197A>G), GAC1672GAT (5200C>T), TGC1674TGT (5206C>T), TTA1675TTG (5209A>G), TCG1682TCA (5230G>A), ATC1684ATT (5236C>T), AGC1685AGT (5239C>T), ATC1687ATT (5245C>T), ACA1693ACT (5263A>T), CTC1695CTT (5269C>T), TTC1696TTT (5272C>T), GCC1697GCA (5275C>A), ATC1698ATT (5278C>T), CCA1699CCC (5281A>C), AGA1700AGG (5284A>G), ACT1701ACC (5287T>C), CTA1702TTA (5288C>T), ATA1703ATT (5293A>T), TTG1704CTA (5294T>C 5296G>A), GAG1706GAA (5302G>A), CTT1709CTA (5311T>A), GAA1710GAG (5314A>G), CGA1713CGT (5323A>T), AGA1720AGG (5344A>G), AGT1721AGC (5347T>C), ATC1723ATT (5353C>T), TGC1727TGT (5365C>T), GTT1734GTC (5386T>C), ACC1737ACA (5395C>A), AAA1746AAG (5422A>G), GAT1747GAC (5425T>C), ACC1749ACT (5431C>T), GAG1751GAA (5437G>A), CTT1756CTC (5452T>C), AAG1758AAA (5458G>A), AGT1761AGC (5467T>C), CAA1762CAG (5470A>G), CTA1763TTG (5471C>T 5473A>G), TTC1766TTT (5482C>T), TCA1767TCT (5485A>T), AAT1768AAC (5488T>C), TTG1769CTA (5489T>C 5491G>A), GGT1771GGC (5497T>C), TGT1774TGC (5506T>C), GAG1775GAA (5509G>A), GAG1776GAA (5512G>A), TTA1778CTA (5516T>C), TAT1782TAC (5530T>C), GAG1783GAA (5533G>A), TCG1784TCT (5536G>T), TTG1785TTA (5539G>A), GTT1788GTG (5548T>G), CAC1790CAT (5554C>T), GCT1792GCA (5560T>A), GCG1793ACA (5561G>A 5563G>A), ACG1794ACC (5566G>C), TCA1795TCG (5569A>G), CTC1800CTG (5584C>G), AAG1803AAA (5593G>A), GGG1804GGA (5596G>A), ATT1805GTT (5597A>G), AAC1807AAA (5605C>A), GTG1811GTT (5617G>T), GCT1812GTG (5619C>T 5620T>G), TTG1815TTA (5629G>A), ATC1816CTC (5630A>C), GGC1819GGT (5641C>T), GCA1822GCT (5650A>T), ATA1826ATT (5662A>T), GGA1827GGG (5665A>G), CAA1835CAG (5689A>G), GTT1837ATT (5693G>A), GAG1838GAA

	(5698G>A), CAA1843CAG (5713A>G), GGG1844GGC (5716G>C), AAA1845AAG (5719A>G), GCC1853GCA (5743C>A), CGC1857CGA (5755C>A), CAT1858CAC (5758T>C), CGT1860CGC (5764T>C), AAA1862AAG (5770A>G), GGC1865GGT (5779C>T), TTT1866TTC (5782T>C), GAC1869GAT (5791C>T), GAC1873GAT (5803C>T), AGG1884AGA (5836G>A), AAA1885AAG (5839A>G), GGA1887GGG (5845A>G), AAA1888AAG (5848A>G), GGT1891GGC (5857T>C), ACC1892ACT (5860C>T), ACA1893ACT (5863A>T), GGT1895GGA (5869T>A), GGT1897GGC (5875T>C), AGC1900AGT (5884C>T), TTC1903TTT (5893C>T), ATC1904GTG (5894A>G 5896C>G), AAC1905AAT (5899C>T), GGG1908GGA (5908G>A), TTT1909TTC (5911T>C), GAG1913GAA (5923G>A), CAA1918CAG (5938A>G), TTC1919TTT (5941C>T), GTT1920GTC (5944T>C), GAT1921GAC (5947T>C), GGG1925GGA (5959G>A), CGG1926GCC (5960C>G 5961G>C 5962G>C), GAA1930GAG (5974A>G), GCT1934GCA (5986T>A), GAC1935GAT (5989C>T), ATT1936GTT (5990A>G), GAT1938GAC (5998T>C), AGA1942CGT (6008A>C 6010A>T), GAA1945GAT (6019A>T), GTG1946GTT (6022G>T), CGA1947CGT (6025A>T), AAA1949ACA (6030A>C), GAC1955GAA (6049C>A), ATG1958ATA (6058G>A), CAA1959CAG (6061A>G), GCC1960GCA (6064C>A), GGT1962GGC (6070T>C), AGT1963AAC (6072G>A 6073T>C), AAC1964CAC (6074A>C), ACG1965ACA (6079G>A), ACC1966AAC (6081C>A), ATA1967ATT (6085A>T), GCA1969GCT (6091A>T), TAC1970TAT (6094C>T), TGT1976TCC (6111G>C 6112T>C), AAA1978AAG (6118A>G), TTG1980TTA (6124G>A), AAG1981AAA (6127G>A), ATT1982GTC (6128A>G 6130T>C), GAT1983GAC (6133T>C), TTA1984TTG (6136A>G), AAC1988AAT (6148C>T), AAA1991AAG (6157A>G), GTT1992ATT (6158G>A), TGT1993TGC (6163T>C), AAA1995AAG (6169A>G), ACA1996TCG (6170A>T 6172A>G), GGC1998GGT (6178C>T), AAA2001AAG (6187A>G), TTT2002TTC (6190T>C), GAG2004GAA (6196G>A), CTC2007TTT (6203C>T 6205C>T), GAA2008GAG (6208A>G), CTA2009TTG (6209C>T 6211A>G), CAG2011CAA (6217G>A), GCT2015GCA (6229T>A), GTA2016ATA (6230G>A), GTC2018GTT (6238C>T), AAG2021AAA (6247G>A), ATA2023ATT (6253A>T), GCA2025AAA (6257G>A 6258C>A), CAT2030CAC (6274T>C), AAA2033AGA (6282A>G), TCG2034TCA (6286G>A), GGC2038GGT (6298C>T), TTG2039TTA (6301G>A), AGA2040AGG (6304A>G), GAC2041GAT (6307C>T), AAC2043AAT (6313C>T), GCC2046GCT (6322C>T), ACA2048ACT (6328A>T), GTT2049GTG (6331T>G), TGT2050TGC (6334T>C), AGG2051AGA (6337G>A), CTG2052TTG (6338C>T), TAT2058TAC (6358T>C), GGG2059GGA (6361G>A), GCA2060ACA (6362G>A), TCA2061TCT (6367A>T), GAG2062GAA (6370G>A), TAC2064TAT (6376C>T), GGT2065GGG (6379T>G), GGA2067GGC (6385A>C), GGA2069GGT (6391A>T), GCA2070GCG (6394A>G), TAC2071TAT (6397C>T), GTA2073ATA (6401G>A), GCG2074GTG (6405C>T), CAC2076CAT (6412C>T), TTA2078TTG (6418A>G), TTT2079TTC (6421T>C), TAC2082TTC (6429A>T), GGT2084GGA (6436T>A), GAG2087GAA (6445G>A), CAA2089CGA (6450A>G), TCC2090TCA (6454C>A), CAC2092CAT (6460C>T), GGT2093GGA (6463T>A), TTC2095TTT (6469C>T), AGG2096AGA (6472G>A), GTG2097GTA (6475G>A), AAT2099AAC (6481T>C), CAC2101AAT (6485C>A 6487C>T), AGT2102AGC (6490T>C), AGC2104AGT (6496C>T), CTG2106CTA (6502G>A), GGT2110GGC (6514T>C), AGG2111AGA (6517G>A), CTC2115ATC (6527C>A), ATC2116ATA (6532C>A), CCG2119CCA (6541G>A), AAA2120AAG (6544A>G), GTC2124GTT (6556C>T), TTT2125TTC (6559T>C), CAG2127CAA (6565G>A), TTG2129CTA (6569T>C 6571G>A), CCT2134CCA (6586T>A), ACA2135ATA (6588C>T), GAA2138GAG (6598A>G), AGA2139AGG (6601A>G), ATT2140ATC (6604T>C), TGT2141TGC (6607T>C), GGA2144GGG (6616A>G), AAC2146AAT (6622C>T), GAG2149GAA (6631G>A), AAG2150AAA (6634G>A), TAT2151CAT (6635T>C), GCT2152GCA (6640T>A), TCG2154TCC (6646G>C), ATC2155GTC (6647A>G), ATC2156ATT (6652C>T), AGC2160AGT (6664C>T), ACT2162ACG (6670T>G), ATA2165GTG (6677A>G 6679A>G), GGC2167GGT (6685C>T), ACA2169ACT (6691A>T), TTC2170TTT (6694C>T), ACA2177ACG (6715A>G), GAT2178AAT (6716G>A), AAT2179GAT (6719A>G), GGA2180GGG (6724A>G), CTA2184TTA (6734C>T), GTG2186GTA (6742G>A), GTG2187GTA (6745G>A), GCC2190GCT (6754C>T), GGA2192GGG (6760A>G), ATA2194TTG (6764A>T 6766A>G), GTC2195GTT (6769C>T), ATT2197ATA (6775T>A), CTG2200TTG (6782C>T), GCA2201GCT (6787A>T), AAC2202AAT (6790C>T), GCA2204GTG (6795C>T 6796A>G), CAC2205CAA (6799C>A), ACC2206ACA (6802C>A), AAC2208AAT (6808C>T), TAC2209TAT (6811C>T), TAC2210TAT (6814C>T), TTC2213TTT (6823C>T), GAA2215GAG (6829A>G), AAG2220AGG (6843A>G), TAC2221TAT (6847C>T), CGA2223CGC (6853A>C), AAT2225GAT (6857A>G), GAG2226GAA (6862G>A), GAA2229GAG (6871A>G), GTC2231ACA (6875G>A 6876T>C 6877C>A), AAG2232AAA (6880G>A), TCT2233TCA (6883T>A), GTT2235GTG (6889T>G), GAC2239GAT (6901C>T), GGC2244GGT (6916C>T), CCG2245CCA (6919G>A), AAA2247AAG (6925A>G), CTT2248CTC (6928T>C), GAC2250GAA (6934C>A), GGG2255GGC (6949G>C), TTC2257TTT (6955C>T), AAA2258AAG (6958A>G), CAA2264CAG (6976A>G), CTA2266TTA (6980C>T), ATC2267ATT (6985C>T), GAT2270GAC (6994T>C), GTA2271GTT (6997A>T), GTG2272GTT (7000G>T), GTG2273ATA (7001G>A 7003G>A), GCT2276GCC (7012T>C), AAG2277AAA (7015G>A), CAT2278CAC (7018T>C), TTT2283TAT (7032T>A), GCC2285GCT (7039C>T), TTG2286CTA (7040T>C 7042G>A), GGA2288GGG (7048A>G), GCT2292GCA (7060T>A), GTC2293GTA (7063C>A), GCA2294GCG (7066A>G), AGC2298AGT (7078C>T), CAA2299CAG (7081A>G), TTA2300CTG (7082T>C 7084A>G), GTA2301GTG (7087A>G), ACC2302ACA (7090C>A), CAT2304CAC (7096T>C), GTA2305GTG (7099A>G), GTT2306GTC (7102T>C), GAG2309GAA (7111G>A), CGA2311CGG (7117A>G), ACA2314AAA (7125C>A), GAA2315GAG (7129A>G), CTG2317TTA (7133C>T 7135G>A), GTG2319GTA (7141G>A), GAT2320GAC (7144T>C), GCA2321TCG (7145G>T 7147A>G), GAG2322GAA (7150G>A), GAG2324GAA (7156G>A), GCA2325GCT (7159A>T), GAG2326_GCA2327del (7160_7165delGAGGCA), TTC2329TTT (7171C>T), GCG2335GCT (7189G>T), AAA2338AAG (7198A>G), AGC2339AGT (7201C>T), CTA2341TTA (7205C>T), AAT2342AAC (7210T>C), GAT2344GAA (7216T>A), GCG2345GCA (7219G>A), ATC2347ATA (7225C>A), AAG2348AAA (7228G>A), AAG2352AAA (7240G>A), TAT2353TAC (7243T>C), AAA2355AAG (7249A>G), CCT2356CCG (7252T>G), GAT2358GAA (7258T>A), GTT2359GTC (7261T>C), GGT2360GGA (7264T>A), GTC2361GTG (7267C>G), GTG2362GTA (7270G>A), GAT2363_CGG2364insT-- (7273_7274insT), CGG2364GTG (7274C>G 7275G>T), CAT2366CTT (7281A>T), TTG2367TCG (7284T>C), TCA2371TTA (7296C>T), TCT2376TTT (7311C>T), ACC2377ATC (7314C>T), TGC2378TAC (7317G>A), AAT2379AAA (7321T>A), GTG2380-TA (7322delG 7324G>A), CAC2381CAT (7327C>T), GGC2382GGT (7330C>T), TTC2383TTT (7333C>T), AAG2384CAG (7334A>C), TGT2386TGC (7342T>C), GCA2387AAC (7343G>A 7344C>A 7345A>C), ACT2390ACC (7354T>C), CAA2393CAG (7363A>G), ATT2395ATC (7369T>C), TTC2396TTT (7372C>T), GCG2398GCT (7378G>T), CTC2399CTT (7381C>T), GCA2404GCT (7396A>T), GTC2405GTT (7399C>T), GCC2407GCT (7405C>T), AGT2408ATG (7407G>T 7408T>G), GGG2410GGT (7414G>T), TGC2411GGC (7415T>G), AAA2412AAG (7420A>G), GAC2415GAT (7429C>T), TAT2416TAC (7432T>C), CAT2419CAC (7441T>C), AAG2425AAA (7459G>A), GAA2426GAG (7462A>G), ATA2428ATT (7468A>T), GTC2429GTA (7471C>A), CAA2431CAG (7477A>G), AGC2432AGT (7480C>T), CTG2434CTT (7486G>T), TAT2437TAC (7495T>C), AAA2438AAG (7498A>G), GGT2439GGC (7501T>C), TTG2440TCA (7503T>C 7504G>A), ATT2443ATA (7513T>A), AAC2445AAT (7519C>T), GGA2446GGG (7522A>G), AAG2449AAA (7531G>A), GAG2451GAA (7537G>A), CTC2452CTT (7540C>T), TGT2454TGC (7546T>C), AAG2462AAA (7570G>A), ACG2463ACA (7573G>A), ACG2465ACA (7579G>A), GCT2468GCC (7588T>C), CTA2471TTA (7595C>T), TTG2474CTA (7604T>C 7606G>A), GGT2477GGA (7615T>A), TGT2480TGC (7624T>C), GAC2483GAT (7633C>T), TTC2484TTT (7636C>T), AAT2485AAC (7639T>C), TTT2488TTC (7648T>C), GAA2494GAG (7666A>G), TGT2496TGC (7672T>C), ACA2498ACT (7678A>T), GGG2500GGA (7684G>A), ACT2502ACC (7690T>C), TTT2504TTC (7696T>C), GGT2506GGG (7702T>G), GAT2509AAT (7709G>A), AAA2510AGG (7713A>G 7714A>G), CTG2511TTG (7715C>T), CGT2514CGC (7726T>C), GAG2517GAA (7735G>A), GTA2520GTG (7744A>G), TAC2521TAT (7747C>T), GGC2526GGT (7762C>T), CAG2528CAA (7768G>A), TTT2529TTC (7771T>C), TCA2532TCG (7780A>G), CTA2533CTC (7783A>C), ACT2534ACC (7786T>C), CTA2537TTG (7793C>T 7795A>G), ATC2538ATT (7798C>T), GCT2540GCC (7804T>C), ACC2543ATC (7812C>T), GAA2550GAG (7834A>G), GAC2551GAT (7837C>T), GTG2554TTA (7844G>T 7846G>A), CAG2557CAA (7855G>A), CTG2559CTA (7861G>A), TTA2562TTG (7870A>G), TAC2563TAT (7873C>T), ACT2564ACA (7876T>A), GAG2565GAA (7879G>A), ATT2566ATA (7882T>A), GTT2567ATT (7883G>A), CCA2570CCT (7894A>T), ATT2571ATC (7897T>C), GTC2579GTT (7921C>T), AAG2581AAA (7927G>A), GGT2584GGC (7936T>C), AAT2585AAC (7939T>C), AAT2586AAC (7942T>C), GTG2594GTA (7966G>A), GAT2595GAC (7969T>C), TCT2597TCC (7975T>C), GTC2601GTT (7987C>T), CTT2602CTC (7990T>C), GCT2607GCC (8005T>C), GAA2616GAG (8032A>G), ATC2618ATT (8038C>T), GAC2619GAT (8041C>T), AGC2620AAC (8043G>A), TTG2632TTA (8080G>A), GTG2635GTA (8089G>A), CCG2637CCA (8095G>A), GAG2638GAA (8098G>A), AGC2641AGT (8107C>T), ATT2642ATC (8110T>C), CTC2643CTT (8113C>T), GAT2644GAC (8116T>C), CAA2648CAG (8128A>G), GGT2654GGC (8146T>C), AAC2656AGT (8151A>G 8152C>T), TCG2660TCA (8164G>A), AGG2665AAG (8178G>A), AAG2666AAA (8182G>A), TTG2669TTA (8191G>A), TTC2671TTT (8197C>T), TCC2673TCT (8203C>T), AGA2675AAA (8208G>A), CTG2677TTG (8213C>T), CTA2678CTG (8218A>G), ATC2679ATT (8221C>T), GAG2680GAA (8224G>A), CCA2685CCG (8239A>G), ATT2692ATC (8260T>C), GTA2693GTG (8263A>G), GAT2699GAC (8281T>C), AGA2700AGG (8284A>G), CCA2704GCG (8294C>G 8296A>G), GAG2705GAA (8299G>A), CAC2706CAT (8302C>T), GCG2710GCA (8314G>A), ATT2711ATC (8317T>C), TGC2712TGT (8320C>T), GCT2714GCA (8326T>A), GAG2717GAA (8335G>A), TCC2718TCT (8338C>T), TCT2722TTT (8349C>T), GAA2723GAG (8353A>G), CAC2726CAT (8362C>T), CAA2727CAG (8365A>G), ATC2728ATT (8368C>T), TAC2732TAT (8380C>T), TCA2733TCG (8383A>G), TTA2735TTG (8389A>G), CCT2740CCC (8404T>C), GCA2742TCA (8408G>T), ACA2743ACG (8413A>G), GCG2745GCT (8419G>T), GCT2750GCC (8434T>C), TAT2752TAC (8440T>C), ATG2756ATA (8452G>A), CTA2758CTG (8458A>G), AGG2759AAG (8460G>A), AAA2760AAG (8464A>G), CTG2761CTA (8467G>A), GCT2766ACA (8480G>A 8482T>A), GAG2771GAA (8497G>A), CTA2772TTG (8498C>T 8500A>G), AGA2773AAG (8502G>A 8503A>G), GCC2774GCT (8506C>T), GAA2777GAT (8515A>T), GTC2780GTT (8524C>T), GCA2781GCC (8527A>C), TTA2782CTG (8528T>C 8530A>G), GAG2785GAC (8539G>C), CTT2788TGT (8546C>T 8547T>G), TCT2790ACT (8552T>A), GTA2793GTG (8563A>G), GCA2797GGA (8574C>G), ATT2801GTT (8585A>G), GGA2805GAA (8598G>A), AGC2806AGT (8602C>T), AAC2807TCT (8603A>T 8604A>C 8605C>T), AAA2812AAG (8620A>G), CCA2813CAA (8622C>A), GAG2814GGA (8625A>G 8626G>A), CAG2815CAA (8629G>A), GGC2816GGT (8632C>T), ATC2818ATT (8638C>T), CAG2819CAA (8641G>A), AAC2821AAT (8647C>T), CCG2822TTC (8648C>T 8649C>T 8650G>C), AAC2823AAT (8653C>T), AAA2824AAG (8656A>G), GGA2825GAA (8658G>A), GAT2827GCG (8664A>C 8665T>G), GTT2830GTG (8674T>G), GCA2832GTT (8679C>T 8680A>T), GGC2833GGA (8683C>A), GGG2836GGA (8692G>A), ACA2837ACT (8695A>T), CCG2841CCA (8707G>A), AGA2842CGA (8708A>C), AAG2844AAA (8716G>A), ATC2846ATT (8722C>T), ACG2847ACA (8725G>A), AAA2849AAG (8731A>G), AGA2851AGG (8737A>G), CCC2853CCT (8743C>T), ACA2854AAG (8745C>A 8746A>G), AGC2855AGT (8749C>T), AAG2856AAA (8752G>A), GGA2857GGT (8755A>T), AAC2862AAT (8770C>T), TTA2863CTA (8771T>C), GAA2864GAG (8776A>G), CAT2865CAC (8779T>C), TTG2866TTA (8782G>A), TAT2869TAC (8791T>C), CCA2871CCG (8797A>G), CAA2872CAG (8800A>G), CAA2873CAG (8803A>G), GAT2875GAC (8809T>C), ATT2876ATC (8812T>C), AAT2878AAC (8818T>C), CGG2880CGA (8824G>A), GCA2881GCG (8827A>G), ACG2888ACA (8848G>A), TAT2890TAC (8854T>C), GAG2891GGA (8856A>G 8857G>A), GTG2893GTA (8863G>A), CGG2894CAG (8865G>A), ATG2895CTT (8867A>C 8869G>T), GCA2896GCG (8872A>G), GGA2900GAA (8883G>A), ACT2902TCT (8888A>T), TGC2915TGT (8929C>T), GTC2924ATC (8954G>A), AAC2925AAT (8959C>T), GGA2926GGT (8962A>T), GTT2929ATT (8969G>A), GGG2933GGA (8983G>A), AAT2934GAC (8984A>G 8986T>C), GAA2935GTA (8988A>T), GAG2938GAA (8998G>A), CCG2940CCA (9004G>A), TTG2941TTA (9007G>A), ATC2944ATT (9016C>T), ACC2951ACA (9037C>A), ATA2967ATT (9085A>T), AAC2971AAT (9097C>T), ATT2983GTT (9131A>G), CGA2984CGT (9136A>T), CGG2987CGT (9145G>T), ATG2989GGA (9149A>G 9150T>G 9151G>A), GGT2990AGT (9152G>A), TTA2991TTG (9157A>G), GCG2992GCT (9160G>T), CGT2993CGC (9163T>C), GAG3000GAA (9184G>A), CGA3004CGG (9196A>G), AGG3010AGA (9214G>A), GAA3011GAG (9217A>G), GCG3012GCA (9220G>A), GCA3020GCT (9244A>T), TTG3021TTA (9247G>A), GCC3024GCT (9256C>T), CCT3026TCC (9260C>T 9262T>C), GGG3030GGA (9274G>A), GAC3032GAT (9280C>T), ATC3035ATT (9289C>T), GAG3040GAA (9304G>A), GTC3050GTT (9334C>T), GTC3060GTG (9364C>G)								
P1 protein (NP_734243.1)	1	284	64.1%	917	72.8%	182 (100%)	129 (70.9%)	0/0/0/0	0
Protein mutations:	Y4Q (194T>C 196C>A), C9L (209T>C 210G>T 211T>G), F13M (221T>A 223T>G), S23P (251T>C), C24F (255G>T 256C>T), E25G (258A>G), I27V (263A>G 265T>G), V28A (267T>C 268G>A), E30V (273A>T), A35T (287G>A 289T>C), V37T (293G>A 294T>C), A41I (305G>A 306C>T), D42G (309A>G), E44D (316A>T), T45V (317A>G 318C>T 319A>G), L52Q (339T>A 340C>A), K53R (342A>G), K55A (347A>G 348A>C 349A>G), Y56H (350T>C), T58V (356A>G 357C>T), V61T (365G>A 366T>C 367G>A), L62S (368C>T 369T>C), F67C (384T>G), A75V (408C>T 409C>T), M78E (416A>G 417T>A), E85K (437G>A), R86E (440A>G 441G>A 442G>A), K87R (443A>C 444A>G 445G>A), D88E (448T>A), M194K (765T>A), R197K (773C>A 774G>A 775A>G), M206K (801T>A), G210A (813G>C 814A>G), L211H (816T>A), R214Q (825G>A 826T>A), R226C (860C>T 862C>T), T227A (863A>G 865T>C), I228T (867T>C), N229D (869A>G 871C>T), I230L (872A>T 874A>G), R232K (879G>A), I238V (896A>G 898C>T), N240S (903A>G), T241N (906C>A 907A>C), K242T (909A>C 910A>C), S243N (912G>A 913C>T), S252L (938T>C 939C>T 940A>T), L266I (980T>A 982G>C), R272K (999G>A), Q275R (1007C>A 1008A>G 1009G>A), S276G (1010A>G 1012T>G), I277V (1013A>G), N279Q (1019A>C 1021C>G)								
Codon mutations:	ACT3ACC (193T>C), TAC4CAA (194T>C 196C>A), TCA6TCG (202A>G), ATC8ATT (208C>T), TGT9CTG (209T>C 210G>T 211T>G), TCG12TCA (220G>A), TTT13ATG (221T>A 223T>G), GAA14GAG (226A>G), CTA17CTT (235A>T), TAC19TAT (241C>T), CCA21CCT (247A>T), TCT23CCT (251T>C), TGC24TTT (255G>T 256C>T), GAG25GGG (258A>G), CAT26CAC (262T>C), ATT27GTG (263A>G 265T>G), GTG28GCA (267T>C 268G>A), GAA30GTA (273A>T), CGA31CGT (277A>T), GAA32GAG (280A>G), GTG33GTT (283G>T), CCG34CCA (286G>A), GCT35ACC (287G>A 289T>C), GTT37ACT (293G>A 294T>C), CCT39CCC (301T>C), TTC40TTT (304C>T), GCA41ATA (305G>A 306C>T), GAT42GGT (309A>G), CTG43TTG (311C>T), GAA44GAT (316A>T), ACA45GTG (317A>G 318C>T 319A>G), CAA46CAG (322A>G), GCA49GCT (331A>T), CTC52CAA (339T>A 340C>A), AAG53AGG (342A>G), AAA55GCG (347A>G 348A>C 349A>G), TAT56CAT (350T>C), GCT57GCA (355T>A), ACT58GTT (356A>G 357C>T), CGT60CGC (364T>C), GTG61ACA (365G>A 366T>C 367G>A), CTC62TCC (368C>T 369T>C), AAA63AAG (373A>G), AAC64AAT (376C>T), TTT67TGT (384T>G), ACG68ACA (388G>A), TAC69TAT (391C>T), CGA70CGG (394A>G), TAC71TAT (397C>T), AAG72AAA (400G>A), GAT74GAC (406T>C), GCC75GTT (408C>T 409C>T), CAG76CAA (412G>A), ATA77ATT (415A>T), ATG78GAG (416A>G 417T>A), ATT80ATC (424T>C), CAG81CAA (427G>A), AAA83AAG (433A>G), CTG84CTC (436G>C), GAG85AAG (437G>A), AGG86GAA (440A>G 441G>A 442G>A), AAG87CGA (443A>C 444A>G 445G>A), GAT88GAA (448T>A), GAA90GAG (454A>G), ATG194AAG (765T>A), TTG196CTG (770T>C), CGA197AAG (773C>A 774G>A 775A>G), CGG198AGG (776C>A), TTC202TTT (790C>T), ATG206AAG (801T>A), GGA210GCG (813G>C 814A>G), CTT211CAT (816T>A), TTG212CTG (818T>C), CAA213CAG (823A>G), CGT214CAA (825G>A 826T>A), CGG217AGA (833C>A 835G>A), GAC219GAT (841C>T), AAT223AAC (853T>C), GTC225GTT (859C>T), CGC226TGT (860C>T 862C>T), ACT227GCC (863A>G 865T>C), ATC228ACC (867T>C), AAC229GAT (869A>G 871C>T), ATA230TTG (872A>T 874A>G), AGG232AAG (879G>A), GAT234GAC (886T>C), ATC238GTT (896A>G 898C>T), AAC240AGC (903A>G), ACA241AAC (906C>A 907A>C), AAA242ACC (909A>C 910A>C), AGC243AAT (912G>A 913C>T), GGC246GGA (922C>A), GGT249GGA (931T>A), TCA252CTT (938T>C 939C>T 940A>T), GGA253GGG (943A>G), TTC256TTT (952C>T), TCA261TCG (967A>G), GAA263GAG (973A>G), GGG264GGC (976G>C), TTG266ATC (980T>A 982G>C), GCA269GCG (991A>G), CGT270CGC (994T>C), AGA272AAA (999G>A), GTT273GTC (1003T>C), CAG275AGA (1007C>A 1008A>G 1009G>A), AGT276GGG (1010A>G 1012T>G), ATT277GTT (1013A>G), TTA278TTG (1018A>G), AAC279CAG (1019A>C 1021C>G), ATC282ATT (1030C>T)								
HC-Pro protein (NP_734244.1)	1	444	82.2%	2446	90.7%	374 (99.7%)	351 (93.6%)	0/1/1/1	0

	Begin	End	Coverage	Score	Concordance	Matches	Identities	I/D/M/F*	Stop Codons
**NT**	**183**	**9687**	**89.3%**	**11427**	**66.3%**	**8654 (99.8%)**	**7216 (83.2%)**	**5/16**	
Protein mutations:	D4E (1048C>A), R16Q (1083G>A), Y19C (1092A>G 1093T>C), S21A (1097T>G 1099G>A), I98T (1329T>C), L113I (1373C>A), I117V (1385A>G), E123D (1405G>T), A127S (1415G>T 1417A>T), V134I (1436G>A 1438C>T), S177del (1565_1568delTCTT), E224G (1707A>G), A228T (1718G>A), I236V (1742A>G 1744C>T), S241N (1758G>A), V252I (1790G>A), I301V (1937A>G 1939T>C), F323Y (2004T>A), I344M (2068T>G), V346I (2072G>A 2074G>A), R391K (2208G>A), D410E (2266C>G)								
Codon mutations:	TCG1TCA (1039G>A), AAT2AAC (1042T>C), GCC3GCA (1045C>A), GAC4GAA (1048C>A), AAT5AAC (1051T>C), AAG8AAA (1060G>A), GGT9GGA (1063T>A), CTG10TTG (1064C>T), GAC11GAT (1069C>T), CGA16CAA (1083G>A), TAT19TGC (1092A>G 1093T>C), TCG21GCA (1097T>G 1099G>A), CAC23CAT (1105C>T), AAT94..A (1318T>A), AAG95AAA (1321G>A), TTG97TTA (1327G>A), ATA98ACA (1329T>C), GCG99GCT (1333G>T), TTA100TTG (1336A>G), CTA103TTG (1343C>T 1345A>G), CCG106CCA (1354G>A), GTG107GTT (1357G>T), GAC108GAT (1360C>T), CTC111TTG (1367C>T 1369C>G), CTT113ATT (1373C>A), TTC114TTT (1378C>T), GAG116GAA (1384G>A), ATA117GTA (1385A>G), AAA119AAG (1393A>G), TCC120TCT (1396C>T), GAG123GAT (1405G>T), AAA124AAG (1408A>G), CAG125CAA (1411G>A), GCA127TCT (1415G>T 1417A>T), CCG128CCC (1420G>C), AAT131AAC (1429T>C), TTA132TTG (1432A>G), GTC134ATT (1436G>A 1438C>T), TTA135CTG (1439T>C 1441A>G), AAT136AAC (1444T>C), TTC139TTT (1453C>T), CTG140CTA (1456G>A), GAA144GAG (1468A>G), GTA152GTG (1492A>G), TTG155CTA (1499T>C 1501G>A), AGT156AGC (1504T>C), TTG157TTA (1507G>A), CTC158CTT (1510C>T), GAA159GAG (1513A>G), TTA160TTG (1516A>G), GCA161GCT (1519A>T), AGG162AGA (1522G>A), TTC163TTT (1525C>T), CAG164CAA (1528G>A), AAC166AAT (1534C>T), ACT168ACA (1540T>A), AAC170AAT (1546C>T), AAA173AAG (1555A>G), GAT175GAC (1561T>C), ATA176ATC (1564A>C), TCT177del (1565_1568delTCTT), TTC178-TC (1565_1568delTCTT), TTC179TTT (1573C>T), AGA180AGG (1576A>G), TTA183CTA (1583T>C), TCT184TCA (1588T>A), AAG186AAA (1594G>A), AAC188AAT (1600C>T), TCG194TCA (1618G>A), TGC195TGT (1621C>T), GAC196GAT (1624C>T), CTG199TTG (1631C>T), AAA201AAG (1639A>G), TTC205TTT (1651C>T), CTC206CTG (1654C>G), CAT213CAC (1675T>C), TCA219TCG (1693A>G), TTT222TTC (1702T>C), GAA224GGA (1707A>G), GCA228ACA (1718G>A), GGA230GGG (1726A>G), TAC231TAT (1729C>T), TCA232TCC (1732A>C), TAT234TAC (1738T>C), ATC236GTT (1742A>G 1744C>T), CGC237CGT (1747C>T), CAT239CAC (1753T>C), AGT241AAT (1758G>A), AGG244AGA (1768G>A), AAC250AAT (1786C>T), TTA251TTG (1789A>G), GTT252ATT (1790G>A), GTC253GTT (1795C>T), TTA257CTG (1805T>C 1807A>G), GAG259GAA (1813G>A), AGG261AGA (1819G>A), AAG263AAA (1825G>A), GAC267GAT (1837C>T), TAT268TAC (1840T>C), CAA271CAG (1849A>G), GTC274GTG (1858C>G), AAA276AAG (1864A>G), TGC278TGT (1870C>T), AGT280AGC (1876T>C), TCG281TCT (1879G>T), AAA282AAG (1882A>G), GGT284GGA (1888T>A), AAT285AAC (1891T>C), TGT290TGC (1906T>C), ACA293ACT (1915A>T), GGT298GGC (1930T>C), GCC300GCT (1936C>T), ATT301GTC (1937A>G 1939T>C), TCA303TCG (1945A>G), TTC305TTT (1951C>T), CCA308CCT (1960A>T), GTA314GTG (1978A>G), ATT315ATA (1981T>A), GGC316GGT (1984C>T), AAT317AAC (1987T>C), GAC320GAT (1996C>T), TTT323TAT (2004T>A), AAA328AAG (2020A>G), GGG329GGA (2023G>A), GAT330GAC (2026T>C), TCG331TCT (2029G>T), GAG332GAA (2032G>A), TAT341TAC (2059T>C), ATT344ATG (2068T>G), AAC345AAT (2071C>T), GTG346ATA (2072G>A 2074G>A), CTG351TTA (2087C>T 2089G>A), AGC355AGT (2101C>T), GAG356GAA (2104G>A), GAG357GAA (2107G>A), AAG360AAA (2116G>A), TTC362TTT (2122C>T), CGC367CGT (2137C>T), CTT374CTC (2158T>C), ACC376ACT (2164C>T), CCA378CCG (2170A>G), GAT382GAC (2182T>C), GCG384GCA (2188G>A), ACC385ACA (2191C>A), AGA391AAA (2208G>A), TAT394TAC (2218T>C), GAC396GAT (2224C>T), GTA397GTT (2227A>T), TTG402TTA (2242G>A), CCC403CCT (2245C>T), TTG406TTA (2254G>A), GTT407GTC (2257T>C), GAC408GAT (2260C>T), GAC410GAG (2266C>G), CAA412CAG (2272A>G), ACG413ACA (2275G>A), TCA419TCG (2293A>G), GGC421GGT (2299C>T), TCG422TCA (2302G>A), CAG423CAA (2305G>A), GGA426GGT (2314A>T), TAT427TAC (2317T>C), ATT429ATC (2323T>C), CTA430CTG (2326A>G), AGC434AGT (2338C>T), GTG435GTA (2341G>A), CAA437CAG (2347A>G), CTT438CTC (2350T>C), TTG440TTA (2356G>A), AAT443AAC (2365T>C)								
P3 protein (NP_734251.1)	25	365	93.4%	1779	78.7%	341 (99.7%)	305 (89.2%)	1/0/2/2	0
Protein mutations:	K40R (2523A>G), R77K (2634G>A), A87V (2664C>T 2665T>C), V107I (2723G>A), A115T (2747G>A 2749T>A), H131N (2795C>A), V140I (2822G>A 2824G>A), P160T (2882C>A), S161H (2885A>C 2886G>A), T164M (2895C>T), N176S (2931A>G), N179S (2940A>G), E189_N190insX (2971_2972insA), T196I (2991C>T), H197R (2994A>G), E199R (2999G>A 3000A>G), R206Q (3021G>A), Y207F (3024A>T 3025C>T), T211I (3036C>T), E212G (3039A>G 3040A>C), K213T (3042A>C 3043G>A), A232V (3099C>T 3100C>A), V234R (3104G>A 3105T>G 3106G>A), S243N (3132G>A), R245K (3137C>A 3138G>A 3139A>G), F250I (3152T>A 3154C>T), C254Y (3165G>A), F272L (3218T>C), V276I (3230G>A), V288M (3266G>A 3268A>G), V291M (3275G>A), A294T (3284G>A), R304K (3315G>A 3316A>G), E305A (3318A>C), M319V (3359A>G), D332E (3400T>G)								
Codon mutations:	TCT24.CC (2476T>C), TCG26TCA (2482G>A), CTG29CTA (2491G>A), CTG31CTT (2497G>T), TTG33TTA (2503G>A), AGA38AGG (2518A>G), CCT39CCC (2521T>C), AAG40AGG (2523A>G), AGA43AGG (2533A>G), TTG45TTA (2539G>A), TTA47TTG (2545A>G), GAG49GAA (2551G>A), CCT50CCA (2554T>A), CTG52TTG (2558C>T), TTG53CTC (2561T>C 2563G>C), CTA55CTG (2569A>G), TCA56TCG (2572A>G), CCT60CCC (2584T>C), GGC61GGT (2587C>T), CTG63CTC (2593G>C), GCT65GCC (2599T>C), TAT67TAC (2605T>C), AAT68AAC (2608T>C), TTT72TTC (2620T>C), GCG75GCA (2629G>A), GTG76GTA (2632G>A), AGG77AAG (2634G>A), TTG78TTA (2638G>A), ATT80ATA (2644T>A), AAA83AAG (2653A>G), GCT87GTC (2664C>T 2665T>C), CTA92TTA (2678C>T), CTA93CTG (2683A>G), GCT95GCC (2689T>C), CTA98CTC (2698A>C), TCA101TCG (2707A>G), GCG102GCA (2710G>A), GAA104GAG (2716A>G), ACA105ACG (2719A>G), CTC106CTT (2722C>T), GTC107ATC (2723G>A), GCA108GCG (2728A>G), AGG110AGA (2734G>A), ATT111ATA (2737T>A), GCT115ACA (2747G>A 2749T>A), GCA116GCT (2752A>T), GCT117GCA (2755T>A), GAC119GAT (2761C>T), CTC120CTT (2764C>T), TGT125TGC (2779T>C), GGG127GGT (2785G>T), AAC129AAT (2791C>T), CAT131AAT (2795C>A), TAC134TAT (2806C>T), TTG138CTC (2816T>C 2818G>C), GTG140ATA (2822G>A 2824G>A), TTG141TTA (2827G>A), GTT144GTC (2836T>C), AAG145AAA (2839G>A), AAT146AAC (2842T>C), ACC153ACA (2863C>A), CTA154TTA (2864C>T), CCA160ACA (2882C>A), AGT161CAT (2885A>C 2886G>A), AAC163AAT (2893C>T), ACG164ATG (2895C>T), GTC166GTT (2902C>T), GTA167GTG (2905A>G), ATT169ATC (2911T>C), AAT173AAC (2923T>C), AAT176AGT (2931A>G), AAC179AGC (2940A>G), GCT181GCC (2947T>C), ACT186ACC (2962T>C), GAA189_AAT190insA-- (2971_2972insA), GGT195GGC (2989T>C), ACT196ATT (2991C>T), CAT197CGT (2994A>G), GAG199AGG (2999G>A 3000A>G), TCC204TC- (3016delC), CGG206CAG (3021G>A), TAC207TTT (3024A>T 3025C>T), ACA211ATA (3036C>T), GAA212GGC (3039A>G 3040A>C), AAG213ACA (3042A>C 3043G>A), TTG216TTA (3052G>A), TTA219TTG (3061A>G), GCG226GCA (3082G>A), GGC229GGT (3091C>T), GCC232GTA (3099C>T 3100C>A), GTG234AGA (3104G>A 3105T>G 3106G>A), GTC235GTT (3109C>T), AAA236AAG (3112A>G), ACT238ACC (3118T>C), GCC239GCT (3121C>T), GGA241GGG (3127A>G), AGC243AAC (3132G>A), CGA245AAG (3137C>A 3138G>A 3139A>G), TAT249TAC (3151T>C), TTC250ATT (3152T>A 3154C>T), TGT254TAT (3165G>A), GTA255GTG (3169A>G), ATC265ATT (3199C>T), AGG267CGG (3203A>C), CGT268CGC (3208T>C), CCA270CCG (3214A>G), ACC271ACT (3217C>T), TTT272CTT (3218T>C), GTC273GTA (3223C>A), GTT276ATT (3230G>A), AAC277AAT (3235C>T), TTA285CTA (3257T>C), ACC286ACT (3262C>T), AGC287AGT (3265C>T), GTA288ATG (3266G>A 3268A>G), GTG289GTA (3271G>A), GTG291ATG (3275G>A), GCA294ACA (3284G>A), CAG299CAA (3301G>A), AGA304AAG (3315G>A 3316A>G), GAA305GCA (3318A>C), ATC306ATT (3322C>T), GAG307GAA (3325G>A), TTG308TTA (3328G>A), ATA311ATT (3337A>T), GAG315GAA (3349G>A), GTC317GTT (3355C>T), TGC318TGT (3358C>T), ATG319GTG (3359A>G), CTA321CTG (3367A>G), TAT322TAC (3370T>C), TTA325CTG (3377T>C 3379A>G), GAA330GAG (3394A>G), CGC331CGT (3397C>T), GAT332GAG (3400T>G), ACA334ACG (3406A>G), TAC338TAT (3418C>T), ATT339ATA (3421T>A), TAT341TAC (3427T>C), AAG343AAA (3433G>A), TCA344TCT (3436A>T), GTA345GTG (3439A>G), AAC346AAT (3442C>T), CCT347CCC (3445T>C), CAG351CAA (3457G>A), TTT352TTC (3460T>C), GCT353GCA (3463T>A), GCG355GCT (3469G>T), CAG356CAA (3472G>A), CAC364CAT (3496C>T), CAG365CAA (3499G>A)								
6K1 protein (NP_734245.1)	1	52	100%	305	90.5%	52 (100%)	45 (86.5%)	0/0/0/0	0
Protein mutations:	V5G (3513T>G), M16V (3545A>G), A17T (3548G>A), V19I (3554G>A 3556C>T), L42V (3623C>G), L45M (3632C>A), Y47H (3638T>C)								
Codon mutations:	TCC2TCT (3505C>T), GTT5GGT (3513T>G), AAA7AAG (3520A>G), GAA10GAG (3529A>G), CAA11CAG (3532A>G), ATG16GTG (3545A>G), GCT17ACT (3548G>A), TTA18CTA (3551T>C), GTC19ATT (3554G>A 3556C>T), AGT28AGC (3583T>C), GAT29GAC (3586T>C), TGC30TGT (3589C>T), TTC32TTT (3595C>T), AAT36AAC (3607T>C), TTT38TTC (3613T>C), AAG39AAA (3616G>A), GGT40GGC (3619T>C), CTT42GTT (3623C>G), TCC43TCT (3628C>T), CTG45ATG (3632C>A), GAT46GAC (3637T>C), TAT47CAT (3638T>C), GAA48GAG (3643A>G)								
CI protein (NP_734246.1)	1	634	88.6%	3611	96.1%	562 (100%)	532 (94.7%)	0/0/0/0	1
Protein mutations:	K7N (3676G>C), N8K (3679T>A), N13K (3694T>A), I15V (3698A>G), D17* (3704G>T 3706T>A), F18L (3709T>A), I140V (4073A>G), F158Y (4128T>A 4129C>T), A166S (4151G>T), V171I (4166G>A), L190I (4223C>A 4225G>A), I222V (4319A>G 4321A>G), F247Y (4395T>A), V251I (4406G>A), M274V (4475A>G 4477G>T), T276S (4481A>T), C287R (4514T>C), V342I (4679G>A 4681G>C), V381I (4796G>A 4798C>T), T383S (4803C>G 4804T>C), S468G (5057A>G), T469A (5060A>G), L479I (5090C>A), A482V (5100C>T), E484D (5107A>T), V489F (5120G>T 5122C>T), I491V (5126A>G), V510I (5183G>A)								

	Begin	End	Coverage	Score	Concordance	Matches	Identities	I/D/M/F*	Stop Codons
**NT**	**183**	**9687**	**89.3%**	**11427**	**66.3%**	**8654 (99.8%)**	**7216 (83.2%)**	**5/16**	
Codon mutations:	TTA2TTG (3661A>G), GTG5GTA (3670G>A), AAG7AAC (3676G>C), AAT8AAA (3679T>A), GAG11GAA (3688G>A), AAT13AAA (3694T>A), GAG14GAA (3697G>A), ATT15GTT (3698A>G), GAT17TAA (3704G>T 3706T>A), TTT18TTA (3709T>A), AGC76..T (3883C>T), GAA77GAG (3886A>G), CTA79CTG (3892A>G), GAC80GAT (3895C>T), TTT81TTC (3898T>C), GTA83GTG (3904A>G), CGG84AGA (3905C>A 3907G>A), GCT86GCC (3913T>C), AAG91AAA (3928G>A), TCA92TCT (3931A>T), GGG94GGA (3937G>A), AGT100AGC (3955T>C), GTG101GTA (3958G>A), GCC102GCT (3961C>T), GGA103GGG (3964A>G), TCT104TCC (3967T>C), TTA107TTG (3976A>G), ATT108ATC (3979T>C), CCA110CCT (3985A>T), CTA114CTT (3997A>T), GCG115GCA (4000G>A), AAC117AAT (4006C>T), GTT118GTG (4009T>G), TTC119TTT (4012C>T), AAA120AAG (4015A>G), TCT123TCC (4024T>C), GAA125GAG (4030A>G), CCA126CCT (4033A>T), AAG129AAA (4042G>A), CTG133TTA (4052C>T 4054G>A), CGT134CGC (4057T>C), CGT136CGA (4063T>A), GGA137GGG (4066A>G), ATA140GTA (4073A>G), TTT141TTC (4078T>C), GGC142GGT (4081C>T), TCT143TCC (4084T>C), TCC147TCT (4096C>T), AGC151AGT (4108C>T), GGA152GGT (4111A>T), GCG154GCA (4117G>A), CTA155TTG (4118C>T 4120A>G), TAC157TAT (4126C>T), TTC158TAT (4128T>A 4129C>T), GCC159GCT (4132C>T), AAT160AAC (4135T>C), CAA164CAG (4147A>G), TTA165CTA (4148T>C), GCT166TCT (4151G>T), AAC169AAT (4162C>T), GTA171ATA (4166G>A), ATA172ATC (4171A>C), GAG175GAA (4180G>A), CTG179TTG (4190C>T), CCT181CCC (4198T>C), TCC182TCT (4201C>T), GCG185GCT (4210G>T), TTC186TTT (4213C>T), AGT188AGC (4219T>C), CTG189TTG (4220C>T), CTG190ATA (4223C>A 4225G>A), GTT192GTG (4231T>G), CAT194CAC (4237T>C), GTA199GTG (4252A>G), TTA200CTG (4253T>C 4255A>G), AAA201AAG (4258A>G), ACT205ACC (4270T>C), AGA209AGG (4282A>G), GAG210GAA (4285G>A), GAA212GAG (4291A>G), CAG216CAA (4303G>A), GTC219GTT (4312C>T), ATA222GTG (4319A>G 4321A>G), GTG223GTT (4324G>T), GAG224GAA (4327G>A), CTG227CTT (4336G>T), TCT228TCC (4339T>C), TCA231TCT (4348A>T), GCA235GCG (4360A>G), GGT237GGC (4366T>C), TCT238TCA (4369T>A), ACT240ACC (4375T>C), GTT244GTC (4387T>C), TTT247TAT (4395T>A), GTA251ATA (4406G>A), ACC263ACA (4444C>A), GCC265GCT (4450C>T), CTC267CTT (4456C>T), GAC270GAT (4465C>T), ATG274GTT (4475A>G 4477G>T), GTC275GTT (4480C>T), ACA276TCA (4481A>T), GGC280GGT (4495C>T), AAG284AAA (4507G>A), CAC285CAT (4510C>T), GGT286GGG (4513T>G), TGC287CGC (4514T>C), CTA288TTA (4517C>T), GTC291GTT (4528C>T), AAA293AAG (4534A>G), GTG312GTA (4591G>A), TTG314TTA (4597G>A), GAC315GAT (4600C>T), GAC317GAT (4606C>T), GTG318GTA (4609G>A), GTG320GTA (4615G>A), GAC321GAT (4618C>T), TTG324CTT (4625T>C 4627G>T), GAC331GAT (4648C>T), ATT332ATC (4651T>C), AGG335AGA (4660G>A), AGC336AGT (4663C>T), GCT338GCA (4669T>A), GTG342ATC (4679G>A 4681G>C), AGT343AGC (4684T>C), GTT344GTG (4687T>G), GAG348GAA (4699G>A), AGA349AGG (4702A>G), CAA351CAG (4708A>G), AGG352AGA (4711G>A), CTG353TTG (4712C>T), AAA361AAG (4738A>G), GCA364GCG (4747A>G), TTG365CTG (4748T>C), ACT370ACC (4765T>C), GTC381ATT (4796G>A 4798C>T), ACT383AGC (4803C>G 4804T>C), GAG384GAA (4807G>A), GCG385GCT (4810G>T), GCT386GCG (4813T>G), CTT387CTC (4816T>C), TAT392TAC (4831T>C), AAC393AAT (4834C>T), CCA395CCT (4840A>T), GGA399GGG (4852A>G), GGC400GGT (4855C>T), AGT404AGC (4867T>C), CTG405CTC (4870G>C), ATT406ATA (4873T>A), ACA416ACT (4903A>T), CAG418CAA (4909G>A), TTT420TTC (4915T>C), GAA421GAG (4918A>G), TTG422CTA (4919T>C 4921G>A), AGT423AGC (4924T>C), CCC424CCG (4927C>G), TTT426TTC (4933T>C), ATC427ATA (4936C>A), CAG428CAA (4939G>A), AAT429AAC (4942T>C), TTC430TTT (4945C>T), TCA436TCG (4963A>G), CCT439CCC (4972T>C), CTT445CTC (4990T>C), AAA449AAG (5002A>G), CCT456CCC (5023T>C), TTG457CTA (5024T>C 5026G>A), TGC458TGT (5029C>T), GAT459GAC (5032T>C), TCT461TCC (5038T>C), CCA463CCT (5044A>T), GCA466GCT (5053A>T), TCG467TCA (5056G>A), AGT468GGT (5057A>G), ACT469GCT (5060A>G), TCG472TCT (5071G>T), CTT479ATT (5090C>A), GGA480GGG (5095A>G), GTG481GTT (5098G>T), GCC482GTC (5100C>T), TTA483TTG (5104A>G), GAA484GAT (5107A>T), ATT485ATC (5110T>C), CCA486CCG (5113A>G), AAG487AAA (5116G>A), CAA488CAG (5119A>G), GTC489TTT (5120G>T 5122C>T), ATT491GTT (5126A>G), CAT494CAC (5137T>C), ATC495ATT (5140C>T), AAA496AAG (5143A>G), GAG497GAA (5146G>A), CTC502CTA (5161C>A), GAA504GAG (5167A>G), CTT506CTG (5173T>G), ACG509ACA (5182G>A), GTT510ATT (5183G>A), GTC511GTT (5188C>T), AAG512AAA (5191G>A), AAA514AAG (5197A>G), GAC515GAT (5200C>T), TGC517TGT (5206C>T), TTA518TTG (5209A>G), TCG525TCA (5230G>A), ATC527ATT (5236C>T), AGC528AGT (5239C>T), ATC530ATT (5245C>T), ACA536ACT (5263A>T), CTC538CTT (5269C>T), TTC539TTT (5272C>T), GCC540GCA (5275C>A), ATC541ATT (5278C>T), CCA542CCC (5281A>C), AGA543AGG (5284A>G), ACT544ACC (5287T>C), CTA545TTA (5288C>T), ATA546ATT (5293A>T), TTG547CTA (5294T>C 5296G>A), GAG549GAA (5302G>A), CTT552CTA (5311T>A), GAA553GAG (5314A>G), CGA556CGT (5323A>T), AGA563AGG (5344A>G), AGT564AGC (5347T>C), ATC566ATT (5353C>T), TGC570TGT (5365C>T), GTT577GTC (5386T>C), ACC580ACA (5395C>A), AAA589AAG (5422A>G), GAT590GAC (5425T>C), ACC592ACT (5431C>T), GAG594GAA (5437G>A), CTT599CTC (5452T>C), AAG601AAA (5458G>A), AGT604AGC (5467T>C), CAA605CAG (5470A>G), CTA606TTG (5471C>T 5473A>G), TTC609TTT (5482C>T), TCA610TCT (5485A>T), AAT611AAC (5488T>C), TTG612CTA (5489T>C 5491G>A), GGT614GGC (5497T>C), TGT617TGC (5506T>C), GAG618GAA (5509G>A), GAG619GAA (5512G>A), TTA621CTA (5516T>C), TAT625TAC (5530T>C), GAG626GAA (5533G>A), TCG627TCT (5536G>T), TTG628TTA (5539G>A), GTT631GTG (5548T>G), CAC633CAT (5554C>T)								
6K2 protein (NP_734247.1)	1	52	100%	303	93.5%	52 (100%)	46 (88.5%)	0/0/0/0	0
Protein mutations:	A2T (5561G>A 5563G>A), I14V (5597A>G), N16K (5605C>A), A21V (5619C>T 5620T>G), I25L (5630A>C), V46I (5693G>A)								
Codon mutations:	GCT1GCA (5560T>A), GCG2ACA (5561G>A 5563G>A), ACG3ACC (5566G>C), TCA4TCG (5569A>G), CTC9CTG (5584C>G), AAG12AAA (5593G>A), GGG13GGA (5596G>A), ATT14GTT (5597A>G), AAC16AAA (5605C>A), GTG20GTT (5617G>T), GCT21GTG (5619C>T 5620T>G), TTG24TTA (5629G>A), ATC25CTC (5630A>C), GGC28GGT (5641C>T), GCA31GCT (5650A>T), ATA35ATT (5662A>T), GGA36GGG (5665A>G), CAA44CAG (5689A>G), GTT46ATT (5693G>A), GAG47GAA (5698G>A), CAA52CAG (5713A>G)								
NIa-VPg protein (NP_734252.1)	1	188	100%	1230	93.5%	188 (100%)	171 (91.0%)	0/0/0/0	0
Protein mutations:	I61V (5894A>G 5896C>G), R83A (5960C>G 5961G>C 5962G>C), I93V (5990A>G), E102D (6019A>T), K106T (6030A>C), D112E (6049C>A), M115I (6058G>A), S120N (6072G>A 6073T>C), N121H (6074A>C), T123N (6081C>A), C133S (6111G>C 6112T>C), I139V (6128A>G 6130T>C), V149I (6158G>A), T153S (6170A>T 6172A>G), L164F (6203C>T 6205C>T), V173I (6230G>A), A182K (6257G>A 6258C>A)								
Codon mutations:	GGG1GGC (5716G>C), AAA2AAG (5719A>G), GCC10GCA (5743C>A), CGC14CGA (5755C>A), CAT15CAC (5758T>C), CGT17CGC (5764T>C), AAA19AAG (5770A>G), GGC22GGT (5779C>T), TTT23TTC (5782T>C), GAC26GAT (5791C>T), GAC30GAT (5803C>T), AGG41AGA (5836G>A), AAA42AAG (5839A>G), GGA44GGG (5845A>G), AAA45AAG (5848A>G), GGT48GGC (5857T>C), ACC49ACT (5860C>T), ACA50ACT (5863A>T), GGT52GGA (5869T>A), GGT54GGC (5875T>C), AGC57AGT (5884C>T), TTC60TTT (5893C>T), ATC61GTG (5894A>G 5896C>G), AAC62AAT (5899C>T), GGG65GGA (5908G>A), TTT66TTC (5911T>C), GAG70GAA (5923G>A), CAA75CAG (5938A>G), TTC76TTT (5941C>T), GTT77GTC (5944T>C), GAT78GAC (5947T>C), GGG82GGA (5959G>A), CGG83GCC (5960C>G 5961G>C 5962G>C), GAA87GAG (5974A>G), GCT91GCA (5986T>A), GAC92GAT (5989C>T), ATT93GTT (5990A>G), GAT95GAC (5998T>C), AGA99CGT (6008A>C 6010A>T), GAA102GAT (6019A>T), GTG103GTT (6022G>T), CGA104CGT (6025A>T), AAA106ACA (6030A>C), GAC112GAA (6049C>A), ATG115ATA (6058G>A), CAA116CAG (6061A>G), GCC117GCA (6064C>A), GGT119GGC (6070T>C), AGT120AAC (6072G>A 6073T>C), AAC121CAC (6074A>C), ACG122ACA (6079G>A), ACC123AAC (6081C>A), ATA124ATT (6085A>T), GCA126GCT (6091A>T), TAC127TAT (6094C>T), TGT133TCC (6111G>C 6112T>C), AAA135AAG (6118A>G), TTG137TTA (6124G>A), AAG138AAA (6127G>A), ATT139GTC (6128A>G 6130T>C), GAT140GAC (6133T>C), TTA141TTG (6136A>G), AAC145AAT (6148C>T), AAA148AAG (6157A>G), GTT149ATT (6158G>A), TGT150TGC (6163T>C), AAA152AAG (6169A>G), ACA153TCG (6170A>T 6172A>G), GGC155GGT (6178C>T), AAA158AAG (6187A>G), TTT159TTC (6190T>C), GAG161GAA (6196G>A), CTC164TTT (6203C>T 6205C>T), GAA165GAG (6208A>G), CTA166TTG (6209C>T 6211A>G), CAG168CAA (6217G>A), GCT172GCA (6229T>A), GTA173ATA (6230G>A), GTC175GTT (6238C>T), AAG178AAA (6247G>A), ATA180ATT (6253A>T), GCA182AAA (6257G>A 6258C>A), CAT187CAC (6274T>C)								
NIa-Pro protein (NP_734248.1)	1	244	100%	1651	93.5%	244 (100%)	222 (91.0%)	0/0/0/0	0
Protein mutations:	K2R (6282A>G), A29T (6362G>A), V42I (6401G>A), A43V (6405C>T), Y51F (6429A>T), Q58R (6450A>G), H70N (6485C>A 6487C>T), L84I (6527C>A), T104I (6588C>T), Y120H (6635T>C), I124V (6647A>G), I134V (6677A>G 6679A>G), D147N (6716G>A), N148D (6719A>G), I163L (6764A>T 6766A>G), A173V (6795C>T 6796A>G), H174Q (6799C>A), K189R (6843A>G), N194D (6857A>G), V200T (6875G>A 6876T>C 6877C>A), D219E (6934C>A), V242I (7001G>A 7003G>A)								
Codon mutations:	AAA2AGA (6282A>G), TCG3TCA (6286G>A), GGC7GGT (6298C>T), TTG8TTA (6301G>A), AGA9AGG (6304A>G), GAC10GAT (6307C>T), AAC12AAT (6313C>T), GCC15GCT (6322C>T), ACA17ACT (6328A>T), GTT18GTG (6331T>G), TGT19TGC (6334T>C), AGG20AGA (6337G>A), CTG21TTG (6338C>T), TAT27TAC (6358T>C), GGG28GGA (6361G>A), GCA29ACA (6362G>A), TCA30TCT (6367A>T), GAG31GAA (6370G>A), TAC33TAT (6376C>T), GGT34GGG (6379T>G), GGA36GGC (6385A>C), GGA38GGT (6391A>T), GCA39GCG (6394A>G), TAC40TAT (6397C>T), GTA42ATA (6401G>A), GCG43GTG (6405C>T), CAC45CAT (6412C>T), TTA47TTG (6418A>G), TTT48TTC (6421T>C), TAC51TTC (6429A>T), GGT53GGA (6436T>A), GAG56GAA (6445G>A), CAA58CGA (6450A>G), TCC59TCA (6454C>A), CAC61CAT (6460C>T), GGT62GGA (6463T>A), TTC64TTT (6469C>T), AGG65AGA (6472G>A), GTG66GTA (6475G>A), AAT68AAC (6481T>C), CAC70AAT (6485C>A 6487C>T), AGT71AGC (6490T>C), AGC73AGT (6496C>T), CTG75CTA (6502G>A), GGT79GGC (6514T>C), AGG80AGA (6517G>A), CTC84ATC (6527C>A), ATC85ATA (6532C>A), CCG88CCA (6541G>A), AAA89AAG (6544A>G), GTC93GTT (6556C>T), TTT94TTC (6559T>C), CAG96CAA (6565G>A), TTG98CTA (6569T>C 6571G>A), CCT103CCA (6586T>A), ACA104ATA (6588C>T), GAA107GAG (6598A>G), AGA108AGG (6601A>G), ATT109ATC (6604T>C), TGT110TGC (6607T>C), GGA113GGG (6616A>G), AAC115AAT (6622C>T), GAG118GAA (6631G>A), AAG119AAA (6634G>A), TAT120CAT (6635T>C), GCT121GCA (6640T>A), TCG123TCC (6646G>C), ATC124GTC (6647A>G), ATC125ATT (6652C>T), AGC129AGT (6664C>T), ACT131ACG (6670T>G), ATA134GTG (6677A>G 6679A>G), GGC136GGT (6685C>T), ACA138ACT (6691A>T), TTC139TTT (6694C>T), ACA146ACG (6715A>G), GAT147AAT (6716G>A), AAT148GAT (6719A>G), GGA149GGG (6724A>G), CTA153TTA (6734C>T), GTG155GTA (6742G>A), GTG156GTA (6745G>A), GCC159GCT (6754C>T), GGA161GGG (6760A>G), ATA163TTG (6764A>T 6766A>G), GTC164GTT (6769C>T), ATT166ATA (6775T>A), CTG169TTG (6782C>T), GCA170GCT (6787A>T), AAC171AAT (6790C>T), GCA173GTG (6795C>T 6796A>G), CAC174CAA (6799C>A), ACC175ACA (6802C>A), AAC177AAT (6808C>T), TAC178TAT (6811C>T), TAC179TAT (6814C>T), TTC182TTT (6823C>T), GAA184GAG (6829A>G), AAG189AGG (6843A>G), TAC190TAT (6847C>T), CGA192CGC (6853A>C), AAT194GAT (6857A>G), GAG195GAA (6862G>A), GAA198GAG (6871A>G), GTC200ACA (6875G>A 6876T>C 6877C>A), AAG201AAA (6880G>A), TCT202TCA (6883T>A), GTT204GTG (6889T>G), GAC208GAT (6901C>T), GGC213GGT (6916C>T), CCG214CCA (6919G>A), AAA216AAG (6925A>G), CTT217CTC (6928T>C), GAC219GAA (6934C>A), GGG224GGC (6949G>C), TTC226TTT (6955C>T), AAA227AAG (6958A>G), CAA233CAG (6976A>G), CTA235TTA (6980C>T), ATC236ATT (6985C>T), GAT239GAC (6994T>C), GTA240GTT (6997A>T), GTG241GTT (7000G>T), GTG242ATA (7001G>A 7003G>A)								
NIb protein (NP_734249.1)	1	521	100%	3129	86.1%	519 (99.4%)	480 (92.0%)	1/2/2/2	0
Protein mutations:	F8Y (7032T>A), T39K (7125C>A), A46S (7145G>T 7147A>G), E51_A52del (7160_7165delGAGGCA), D69E (7216T>A), D83E (7258T>A), D88_R89insX (7273_7274insT), R89V (7274C>G 7275G>T), H91L (7281A>T), L92S (7284T>C), S96L (7296C>T), S101F (7311C>T), T102I (7314C>T), C103Y (7317G>A), N104K (7321T>A), K109Q (7334A>C), A112N (7343G>A 7344C>A 7345A>C), S133M (7407G>T 7408T>G), C136G (7415T>G), L165S (7503T>C 7504G>A), D234N (7709G>A), K235R (7713A>G 7714A>G), T268I (7812C>T), V279L (7844G>T 7846G>A), V292I (7883G>A), S345N (8043G>A), N381S (8151A>G 8152C>T), R390K (8178G>A), R400K (8208G>A), P429A (8294C>G 8296A>G), S447F (8349C>T), A467S (8408G>T), M481I (8452G>A), R484K (8460G>A), A491T (8480G>A 8482T>A), R498K (8502G>A 8503A>G), E502D (8515A>T), E510D (8539G>C), L513C (8546C>T 8547T>G), S515T (8552T>A)								

	Begin	End	Coverage	Score	Concordance	Matches	Identities	I/D/M/F*	Stop Codons
**NT**	**183**	**9687**	**89.3%**	**11427**	**66.3%**	**8654 (99.8%)**	**7216 (83.2%)**	**5/16**	
Codon mutations:	GCT1GCC (7012T>C), AAG2AAA (7015G>A), CAT3CAC (7018T>C), TTT8TAT (7032T>A), GCC10GCT (7039C>T), TTG11CTA (7040T>C 7042G>A), GGA13GGG (7048A>G), GCT17GCA (7060T>A), GTC18GTA (7063C>A), GCA19GCG (7066A>G), AGC23AGT (7078C>T), CAA24CAG (7081A>G), TTA25CTG (7082T>C 7084A>G), GTA26GTG (7087A>G), ACC27ACA (7090C>A), CAT29CAC (7096T>C), GTA30GTG (7099A>G), GTT31GTC (7102T>C), GAG34GAA (7111G>A), CGA36CGG (7117A>G), ACA39AAA (7125C>A), GAA40GAG (7129A>G), CTG42TTA (7133C>T 7135G>A), GTG44GTA (7141G>A), GAT45GAC (7144T>C), GCA46TCG (7145G>T 7147A>G), GAG47GAA (7150G>A), GAG49GAA (7156G>A), GCA50GCT (7159A>T), GAG51_GCA52del (7160_7165delGAGGCA), TTC54TTT (7171C>T), GCG60GCT (7189G>T), AAA63AAG (7198A>G), AGC64AGT (7201C>T), CTA66TTA (7205C>T), AAT67AAC (7210T>C), GAT69GAA (7216T>A), GCG70GCA (7219G>A), ATC72ATA (7225C>A), AAG73AAA (7228G>A), AAG77AAA (7240G>A), TAT78TAC (7243T>C), AAA80AAG (7249A>G), CCT81CCG (7252T>G), GAT83GAA (7258T>A), GTT84GTC (7261T>C), GGT85GGA (7264T>A), GTC86GTG (7267C>G), GTG87GTA (7270G>A), GAT88_CGG89insT-- (7273_7274insT), CGG89GTG (7274C>G 7275G>T), CAT91CTT (7281A>T), TTG92TCG (7284T>C), TCA96TTA (7296C>T), TCT101TTT (7311C>T), ACC102ATC (7314C>T), TGC103TAC (7317G>A), AAT104AAA (7321T>A), GTG105-TA (7322delG 7324G>A), CAC106CAT (7327C>T), GGC107GGT (7330C>T), TTC108TTT (7333C>T), AAG109CAG (7334A>C), TGT111TGC (7342T>C), GCA112AAC (7343G>A 7344C>A 7345A>C), ACT115ACC (7354T>C), CAA118CAG (7363A>G), ATT120ATC (7369T>C), TTC121TTT (7372C>T), GCG123GCT (7378G>T), CTC124CTT (7381C>T), GCA129GCT (7396A>T), GTC130GTT (7399C>T), GCC132GCT (7405C>T), AGT133ATG (7407G>T 7408T>G), GGG135GGT (7414G>T), TGC136GGC (7415T>G), AAA137AAG (7420A>G), GAC140GAT (7429C>T), TAT141TAC (7432T>C), CAT144CAC (7441T>C), AAG150AAA (7459G>A), GAA151GAG (7462A>G), ATA153ATT (7468A>T), GTC154GTA (7471C>A), CAA156CAG (7477A>G), AGC157AGT (7480C>T), CTG159CTT (7486G>T), TAT162TAC (7495T>C), AAA163AAG (7498A>G), GGT164GGC (7501T>C), TTG165TCA (7503T>C 7504G>A), ATT168ATA (7513T>A), AAC170AAT (7519C>T), GGA171GGG (7522A>G), AAG174AAA (7531G>A), GAG176GAA (7537G>A), CTC177CTT (7540C>T), TGT179TGC (7546T>C), AAG187AAA (7570G>A), ACG188ACA (7573G>A), ACG190ACA (7579G>A), GCT193GCC (7588T>C), CTA196TTA (7595C>T), TTG199CTA (7604T>C 7606G>A), GGT202GGA (7615T>A), TGT205TGC (7624T>C), GAC208GAT (7633C>T), TTC209TTT (7636C>T), AAT210AAC (7639T>C), TTT213TTC (7648T>C), GAA219GAG (7666A>G), TGT221TGC (7672T>C), ACA223ACT (7678A>T), GGG225GGA (7684G>A), ACT227ACC (7690T>C), TTT229TTC (7696T>C), GGT231GGG (7702T>G), GAT234AAT (7709G>A), AAA235AGG (7713A>G 7714A>G), CTG236TTG (7715C>T), CGT239CGC (7726T>C), GAG242GAA (7735G>A), GTA245GTG (7744A>G), TAC246TAT (7747C>T), GGC251GGT (7762C>T), CAG253CAA (7768G>A), TTT254TTC (7771T>C), TCA257TCG (7780A>G), CTA258CTC (7783A>C), ACT259ACC (7786T>C), CTA262TTG (7793C>T 7795A>G), ATC263ATT (7798C>T), GCT265GCC (7804T>C), ACC268ATC (7812C>T), GAA275GAG (7834A>G), GAC276GAT (7837C>T), GTG279TTA (7844G>T 7846G>A), CAG282CAA (7855G>A), CTG284CTA (7861G>A), TTA287TTG (7870A>G), TAC288TAT (7873C>T), ACT289ACA (7876T>A), GAG290GAA (7879G>A), ATT291ATA (7882T>A), GTT292ATT (7883G>A), CCA295CCT (7894A>T), ATT296ATC (7897T>C), GTC304GTT (7921C>T), AAG306AAA (7927G>A), GGT309GGC (7936T>C), AAT310AAC (7939T>C), AAT311AAC (7942T>C), GTG319GTA (7966G>A), GAT320GAC (7969T>C), TCT322TCC (7975T>C), GTC326GTT (7987C>T), CTT327CTC (7990T>C), GCT332GCC (8005T>C), GAA341GAG (8032A>G), ATC343ATT (8038C>T), GAC344GAT (8041C>T), AGC345AAC (8043G>A), TTG357TTA (8080G>A), GTG360GTA (8089G>A), CCG362CCA (8095G>A), GAG363GAA (8098G>A), AGC366AGT (8107C>T), ATT367ATC (8110T>C), CTC368CTT (8113C>T), GAT369GAC (8116T>C), CAA373CAG (8128A>G), GGT379GGC (8146T>C), AAC381AGT (8151A>G 8152C>T), TCG385TCA (8164G>A), AGG390AAG (8178G>A), AAG391AAA (8182G>A), TTG394TTA (8191G>A), TTC396TTT (8197C>T), TCC398TCT (8203C>T), AGA400AAA (8208G>A), CTG402TTG (8213C>T), CTA403CTG (8218A>G), ATC404ATT (8221C>T), GAG405GAA (8224G>A), CCA410CCG (8239A>G), ATT417ATC (8260T>C), GTA418GTG (8263A>G), GAT424GAC (8281T>C), AGA425AGG (8284A>G), CCA429GCG (8294C>G 8296A>G), GAG430GAA (8299G>A), CAC431CAT (8302C>T), GCG435GCA (8314G>A), ATT436ATC (8317T>C), TGC437TGT (8320C>T), GCT439GCA (8326T>A), GAG442GAA (8335G>A), TCC443TCT (8338C>T), TCT447TTT (8349C>T), GAA448GAG (8353A>G), CAC451CAT (8362C>T), CAA452CAG (8365A>G), ATC453ATT (8368C>T), TAC457TAT (8380C>T), TCA458TCG (8383A>G), TTA460TTG (8389A>G), CCT465CCC (8404T>C), GCA467TCA (8408G>T), ACA468ACG (8413A>G), GCG470GCT (8419G>T), GCT475GCC (8434T>C), TAT477TAC (8440T>C), ATG481ATA (8452G>A), CTA483CTG (8458A>G), AGG484AAG (8460G>A), AAA485AAG (8464A>G), CTG486CTA (8467G>A), GCT491ACA (8480G>A 8482T>A), GAG496GAA (8497G>A), CTA497TTG (8498C>T 8500A>G), AGA498AAG (8502G>A 8503A>G), GCC499GCT (8506C>T), GAA502GAT (8515A>T), GTC505GTT (8524C>T), GCA506GCC (8527A>C), TTA507CTG (8528T>C 8530A>G), GAG510GAC (8539G>C), CTT513TGT (8546C>T 8547T>G), TCT515ACT (8552T>A), GTA518GTG (8563A>G)								
coat protein (NP_734250.1)	1	267	100%	1659	91.0%	267 (100%)	243 (91.0%)	0/0/0/0	0
Protein mutations:	A1G (8574C>G), I5V (8585A>G), G9E (8598G>A), N11S (8603A>T 8604A>C 8605C>T), P17Q (8622C>A), E18G (8625A>G 8626G>A), P26F (8648C>T 8649C>T 8650G>C), G29E (8658G>A), D31A (8664A>C 8665T>G), A36V (8679C>T 8680A>T), T58K (8745C>A 8746A>G), E95G (8856A>G 8857G>A), R98Q (8865G>A), M99L (8867A>C 8869G>T), G104E (8883G>A), T106S (8888A>T), V128I (8954G>A), V133I (8969G>A), N138D (8984A>G 8986T>C), E139V (8988A>T), I187V (9131A>G), M193G (9149A>G 9150T>G 9151G>A), G194S (9152G>A), P230S (9260C>T 9262T>C)								
Codon mutations:	GCA1GGA (8574C>G), ATT5GTT (8585A>G), GGA9GAA (8598G>A), AGC10AGT (8602C>T), AAC11TCT (8603A>T 8604A>C 8605C>T), AAA16AAG (8620A>G), CCA17CAA (8622C>A), GAG18GGA (8625A>G 8626G>A), CAG19CAA (8629G>A), GGC20GGT (8632C>T), ATC22ATT (8638C>T), CAG23CAA (8641G>A), AAC25AAT (8647C>T), CCG26TTC (8648C>T 8649C>T 8650G>C), AAC27AAT (8653C>T), AAA28AAG (8656A>G), GGA29GAA (8658G>A), GAT31GCG (8664A>C 8665T>G), GTT34GTG (8674T>G), GCA36GTT (8679C>T 8680A>T), GGC37GGA (8683C>A), GGG40GGA (8692G>A), ACA41ACT (8695A>T), CCG45CCA (8707G>A), AGA46CGA (8708A>C), AAG48AAA (8716G>A), ATC50ATT (8722C>T), ACG51ACA (8725G>A), AAA53AAG (8731A>G), AGA55AGG (8737A>G), CCC57CCT (8743C>T), ACA58AAG (8745C>A 8746A>G), AGC59AGT (8749C>T), AAG60AAA (8752G>A), GGA61GGT (8755A>T), AAC66AAT (8770C>T), TTA67CTA (8771T>C), GAA68GAG (8776A>G), CAT69CAC (8779T>C), TTG70TTA (8782G>A), TAT73TAC (8791T>C), CCA75CCG (8797A>G), CAA76CAG (8800A>G), CAA77CAG (8803A>G), GAT79GAC (8809T>C), ATT80ATC (8812T>C), AAT82AAC (8818T>C), CGG84CGA (8824G>A), GCA85GCG (8827A>G), ACG92ACA (8848G>A), TAT94TAC (8854T>C), GAG95GGA (8856A>G 8857G>A), GTG97GTA (8863G>A), CGG98CAG (8865G>A), ATG99CTT (8867A>C 8869G>T), GCA100GCG (8872A>G), GGA104GAA (8883G>A), ACT106TCT (8888A>T), TGC119TGT (8929C>T), GTC128ATC (8954G>A), AAC129AAT (8959C>T), GGA130GGT (8962A>T), GTT133ATT (8969G>A), GGG137GGA (8983G>A), AAT138GAC (8984A>G 8986T>C), GAA139GTA (8988A>T), GAG142GAA (8998G>A), CCG144CCA (9004G>A), TTG145TTA (9007G>A), ATC148ATT (9016C>T), ACC155ACA (9037C>A), ATA171ATT (9085A>T), AAC175AAT (9097C>T), ATT187GTT (9131A>G), CGA188CGT (9136A>T), CGG191CGT (9145G>T), ATG193GGA (9149A>G 9150T>G 9151G>A), GGT194AGT (9152G>A), TTA195TTG (9157A>G), GCG196GCT (9160G>T), CGT197CGC (9163T>C), GAG204GAA (9184G>A), CGA208CGG (9196A>G), AGG214AGA (9214G>A), GAA215GAG (9217A>G), GCG216GCA (9220G>A), GCA224GCT (9244A>T), TTG225TTA (9247G>A), GCC228GCT (9256C>T), CCT230TCC (9260C>T 9262T>C), GGG234GGA (9274G>A), GAC236GAT (9280C>T), ATC239ATT (9289C>T), GAG244GAA (9304G>A), GTC254GTT (9334C>T), GTC264GTG (9364C>G)								
PIPO (YP_006393460.1)	1	75	100%	95	18.1%	75 (98.7%)	54 (71.1%)	1/0/3/2	2
Protein mutations:	R17_K17insX (2971_2972insA), Y23H (2989T>C 2991C>T), R26K (2999G>A 3000A>G), H35Y (3025C>T), K40H (3040A>C 3042A>C), G41S (3043G>A), E44K (3052G>A), I47V (3061A>G), V54I (3082G>A), P57S (3091C>T), P60T (3100C>A), G61E (3104G>A 3105T>G), G62S (3106G>A), Q63* (3109C>T), R64G (3112A>G), C66R (3118T>C), L67F (3121C>T), I69V (3127A>G), A72E (3137C>A 3138G>A), I73V (3139A>G)								
Codon mutations:	TTA2CTA (2923T>C), AAA4AAG (2931A>G), GAA7GAG (2940A>G), TTG10CTG (2947T>C), TTG15CTG (2962T>C), AGA17_AAA17insAA- (2971_2972insA), TAC23CAT (2989T>C 2991C>T), TCA24TCG (2994A>G), AGA26AAG (2999G>A 3000A>G), CAC32-AC (3016delC), TCG33TCA (3021G>A), GTA34GTT (3024A>T), CAT35TAT (3025C>T), CAC38CAT (3036C>T), AGA39AGG (3039A>G), AAA40CAC (3040A>C 3042A>C), GGC41AGC (3043G>A), GAA44AAA (3052G>A), ATA47GTA (3061A>G), GTT54ATT (3082G>A), CCG57TCG (3091C>T), CGC59CGT (3099C>T), CCA60ACA (3100C>A), GGT61GAG (3104G>A 3105T>G), GGT62AGT (3106G>A), CAA63TAA (3109C>T), AGG64GGG (3112A>G), TGC66CGC (3118T>C), CTC67TTC (3121C>T), ATT69GTT (3127A>G), GAG70GAA (3132G>A), GCG72GAA (3137C>A 3138G>A), ATT73GTT (3139A>G)								

*: Inserts / Deletes / Misaligned / Frameshifts

Analysis details

This analysis was performed with panviral2.64

NGS Details (UN59): Duamitovirus soch1

Assembly

**Coverage Length**	2273 (1 contig(s))
**Depth Of Coverage**	39.0
**Number Of Reads**	734
**Reads Per Million**	15.96 rpm (after QC)
**Ambiguities**	0
**Assembly Method**	de novo + reference guided assembly
**Consensus Caller**	Bcf Tools

Coverage Map

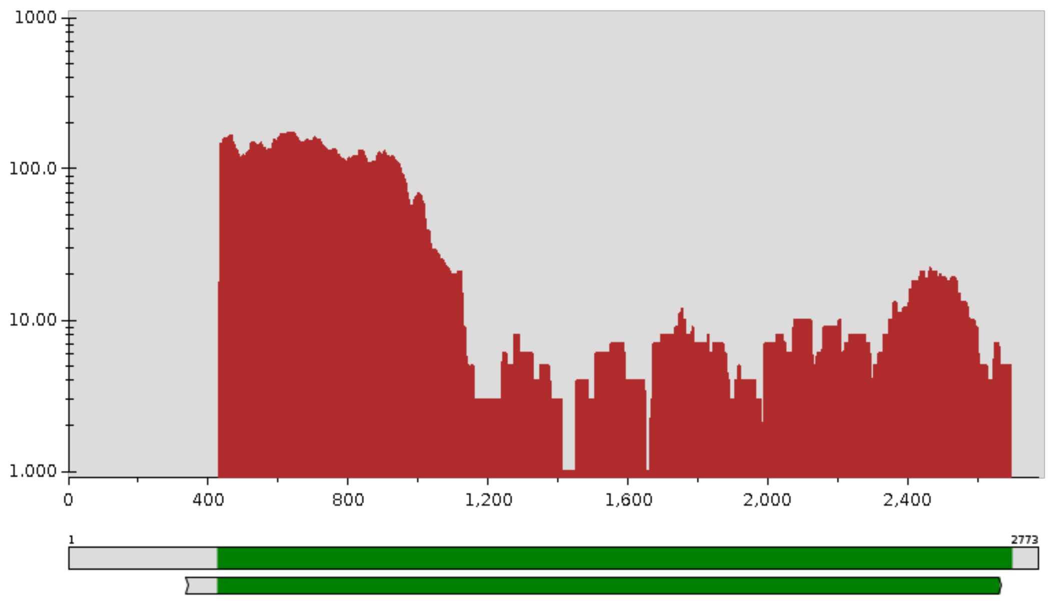

Assignment

**Type**	Duamitovirus soch1 (Taxonomy ID: 2955838)
**Reference Genome**	NC_076524.1
**NT Identity (%)**	67.5497
**AA Identity (%)**	68.0108
**Number Of Stop Codons**	4
**Number Of CDS**	1

Alignment

**Alignment Score**	1546.0 (NT) + 3373.0 (AA) = 4919.0
**Concordance (%)**	51.6287

Alignment Method	Global, seeded, nucleotide + amino acids (AGA)

Genome Region

Sequence starts at position 428 and ends at position 2700 relative to NC_076524.1 reference sequence.

Alignment Detailed Statistics

	Begin	End	Coverage	Score	Concordance	Matches	Identities	I/D/M/F*	Stop Codons
**NT**	**428**	**2700**	**82.0%**	**1546**	**34.4%**	**2264 (99.6%)**	**1530 (67.3%)**	**1/9**	
Mutations:	437A>G, 441A>C, 443C>T, 447C>T, 459C>T, 466C>T, 470C>T, 471G>A, 472C>T, 473T>G, 477T>A, 480A>C, 481C>A, 482A>T, 485A>G, 489A>G, 494T>C, 497T>A, 500T>G, 503A>C, 506T>C, 509C>T, 512T>C, 513G>A, 516T>A, 517C>A, 518T>A, 520A>C, 524G>T, 525A>G, 526C>T, 527T>G, 529C>A, 530C>A, 531T>C, 533A>G, 534C>A, 535G>A, 536C>A, 539G>A, 540A>C, 541A>G, 542G>T, 545G>A, 548C>T, 550A>T, 556T>A, 557C>T, 560A>T, 569C>T, 575G>A, 581C>T, 582G>A, 587A>T, 589G>C, 591T>C, 593A>T, 599A>T, 602C>T, 612T>A, 613C>G, 614C>T, 616T>A, 618C>T, 619G>C, 620A>T, 624G>A, 626A>T, 629A>C, 630C>A, 632A>C, 633C>A, 635T>G, 638G>T, 641G>T, 644T>A, 647G>T, 651C>T, 653C>G, 656A>C, 657A>C, 661C>G, 662G>T, 665T>A, 668C>T, 672C>A, 673G>A, 674T>G, 680C>A, 683C>T, 684A>G, 685A>C, 686A>C, 692G>A, 695T>A, 699G>C, 700C>A, 701A>C, 702A>G, 705C>A, 707T>G, 708A>G, 709G>C, 710G>A, 713A>G, 714G>T, 715A>C, 719A>T, 722C>T, 737T>C, 746C>T, 752A>T, 761G>A, 765G>T, 767T>A, 770G>A, 771G>T, 772T>A, 773T>G, 774T>A, 776G>T, 777A>G, 778A>C, 779A>G, 782T>G, 796G>A, 797T>G, 803A>C, 809T>C, 810G>C, 815C>A, 818A>T, 821T>G, 824C>T, 827A>T, 831C>T, 833T>A, 841G>A, 842G>T, 850T>G, 851A>T, 857G>T, 860G>T, 862A>G, 869T>G, 875G>T, 880T>G, 881C>A, 885C>A, 887T>G, 892A>G, 905A>T, 908C>A, 909T>C, 910T>A, 916G>A, 918G>T, 919G>C, 923T>A, 926A>T, 927A>C, 929G>T, 934T>A, 935T>G, 938G>T, 942T>A, 943C>A, 948C>A, 971C>A, 972C>T, 975A>C, 976A>T, 977T>C, 983T>C, 990A>C, 992G>C, 993T>C, 994C>G, 995A>G, 997A>T, 998C>T, 999G>C, 1000G>A, 1001T>A, 1003T>A, 1004A>T, 1005G>T, 1007C>G, 1010A>G, 1016C>T, 1017A>G, 1018A>T, 1019A>T, 1022T>A, 1025T>C, 1026C>G, 1027A>G, 1030A>C, 1031G>A, 1034T>G, 1035A>G, 1036G>C, 1044T>G, 1046C>T, 1049G>A, 1050T>G, 1051T>A, 1052T>A, 1054C>A, 1055A>C, 1061T>C, 1067T>G, 1073G>A, 1076A>G, 1083C>A, 1088G>T, 1091C>G, 1099G>T, 1104A>G, 1105C>T, 1106A>G, 1115T>G, 1121A>C, 1122T>A, 1124C>T, 1127G>A, 1130C>T, 1133C>G, 1142A>C, 1145C>T, 1148G>T, 1151G>T, 1152T>C, 1154G>T, 1160C>T, 1161A>C, 1164T>A, 1165C>T, 1166T>G, 1169C>T, 1172C>A, 1174T>A, 1175T>C, 1178T>A, 1181A>T, 1184C>T, 1185A>T, 1186G>C, 1187G>T, 1188G>A, 1189C>A, 1191G>A, 1192C>A, 1193A>T, 1196T>C, 1197_1199delGAA, 1205A>T, 1211C>T, 1212T>G, 1213T>A, 1217T>C, 1220C>T, 1223A>T, 1224C>T, 1225A>T, 1226C>A, 1232G>A, 1236A>C, 1237G>A, 1238T>A, 1241C>A, 1247A>C, 1248T>C, 1249G>T, 1250G>T, 1253T>C, 1256A>T, 1258C>G, 1265G>A, 1266A>G, 1268T>G, 1269C>G, 1271T>C, 1274T>C, 1276G>A, 1280G>A, 1283G>T, 1292T>G, 1301G>A, 1302C>T, 1304T>G, 1306G>C, 1308G>C, 1309C>A, 1310A>G, 1312T>C, 1313G>C, 1316A>T, 1319G>A, 1322C>T, 1328A>G, 1332A>C, 1334A>G, 1338G>A, 1340A>C, 1345T>C, 1346A>T, 1349G>A, 1355C>T, 1357A>C, 1358C>T, 1376C>G, 1379C>T, 1382T>C, 1385T>C, 1388C>T, 1389G>A, 1390A>T, 1403A>T, 1406C>A, 1409G>A, 1412T>A, 1414A>G, 1415A>T, 1416A>C, 1418A>T, 1424A>T, 1427C>T, 1430G>T, 1433T>A, 1437G>A, 1445G>A, 1446A>C, 1447A>G, 1448A>T, 1451T>G, 1455T>C, 1456T>A, 1458G>T, 1459C>A, 1461T>C, 1465A>G, 1466G>A, 1468A>T, 1469A>T, 1472G>A, 1474A>G, 1475C>A, 1476T>C, 1477T>C, 1478T>G, 1481A>G, 1482A>T, 1483A>C, 1490T>C, 1502A>C, 1505G>A, 1514G>A, 1515G>A, 1526G>A, 1535C>T, 1538G>A, 1541T>C, 1545C>T, 1547T>A, 1550G>C, 1551T>G, 1552C>A, 1553T>A, 1559A>T, 1565G>T, 1566C>A, 1567C>G, 1571C>T, 1574G>A, 1580C>T, 1589T>C, 1593_1597delAGCTC, 1598T>A, 1613G>T, 1614A>T, 1616C>T, 1619T>C, 1622T>A, 1623A>T, 1624A>C, 1625C>A, 1626A>C, 1627A>C, 1628A>T, 1631G>T, 1632T>A, 1634A>G, 1635A>G, 1636C>T, 1637A>G, 1642G>A, 1643A>G, 1644G>A, 1646G>T, 1647T>A, 1648A>G, 1652G>A, 1653A>G, 1664G>T, 1670G>A, 1673G>A, 1676T>G, 1679G>A, 1682C>T, 1684T>A, 1686C>T, 1694T>A, 1698G>T, 1700A>C, 1703T>A, 1706C>T, 1707T>G, 1710T>C, 1712G>T, 1715T>C, 1721C>T, 1727A>G, 1734T>C, 1739A>T, 1744G>A, 1745C>A, 1746A>T, 1748A>T, 1754T>C, 1758G>A, 1759G>C, 1760A>G, 1761T>A, 1765A>C, 1766C>T, 1767T>A, 1769T>C, 1772A>T, 1777G>C, 1778G>T, 1787T>C, 1788T>C, 1790A>G, 1791C>T, 1819G>C, 1820T>A, 1821A>G, 1823A>G, 1829C>G, 1830A>C, 1831A>G, 1832T>G, 1833G>C, 1840A>G, 1847A>G, 1848T>C, 1856A>G, 1859C>T, 1860A>G, 1861A>G, 1865C>T, 1867A>C, 1871T>C, 1874G>T, 1875A>T, 1878C>G, 1880A>T, 1886T>G, 1887T>A, 1888T>G, 1889G>A, 1895T>C, 1898T>G, 1901T>C, 1910T>C, 1919G>T, 1925G>A, 1934T>C, 1937A>G, 1938T>G, 1939C>A, 1940A>C, 1950G>T, 1952T>C, 1953T>C, 1955A>T, 1964T>C, 1967C>T, 1976T>A, 1980T>A, 1981T>C, 1982A>T, 1985T>C, 1989C>A, 1991T>G, 1994T>A, 2000T>G, 2003A>T, 2006T>G, 2009C>T, 2010G>C, 2011C>A, 2012C>A, 2020T>C, 2028G>T, 2029A>C, 2030T>G, 2034A>T, 2035A>C, 2036C>A, 2038T>C, 2042A>G, 2043G>A, 2050A>C, 2051A>C, 2053G>T, 2054A>T, 2057G>T, 2058G>T, 2063T>G, 2069G>A, 2071A>T, 2074A>G, 2075G>A, 2078A>T, 2082G>T, 2084G>T, 2087C>T, 2090A>G, 2091T>A, 2093C>G, 2094A>T, 2099C>T, 2103A>T, 2104G>C, 2105C>T, 2108G>A, 2117G>A, 2118C>A, 2120T>A, 2123A>G, 2129T>G, 2133C>G, 2134A>C, 2135T>A, 2136C>A, 2137T>G, 2138T>C, 2141G>A, 2145G>C, 2146T>A, 2147T>C, 2148C>A, 2156C>T, 2157T>C, 2160C>T, 2166T>C, 2167A>T, 2173G>A, 2174G>T, 2183A>G, 2186G>A, 2187G>T, 2188G>T, 2191T>A, 2192G>A, 2195C>T, 2204C>T, 2206A>T, 2207_2208insT, 2213A>G, 2216G>A, 2223G>A, 2226A>G, 2227G>A, 2231C>T, 2236T>G, 2237T>G, 2240A>G, 2248A>C, 2250G>C, 2251T>C, 2259T>A, 2263A>G, 2264G>A, 2265A>C, 2276G>T, 2279T>G, 2280C>T, 2288C>T, 2297A>G, 2298G>A, 2299T>G, 2300T>A, 2306A>C, 2309A>T, 2313C>T, 2315T>C, 2317C>T, 2322C>T, 2325C>G, 2326G>T, 2328C>A, 2330A>T, 2336A>T, 2337G>A, 2346T>C, 2348A>T, 2349A>C, 2350A>G, 2355C>G, 2357C>T, 2358A>T, 2359A>C, 2360C>T, 2362G>A, 2369T>C, 2372G>T, 2377G>T, 2378T>G, 2382A>T, 2383A>C, 2384C>T, 2385C>T, 2386C>T, 2387A>T, 2390T>A, 2396C>T, 2399T>C, 2400A>G, 2402G>A, 2404G>A, 2405T>A, 2406C>T, 2408C>G, 2411C>G, 2414C>T, 2415T>G, 2417C>T, 2420T>C, 2423A>T, 2425G>A, 2430A>T, 2431C>A, 2432A>G, 2435C>A, 2441A>G, 2442A>C, 2444G>A, 2445C>T, 2446T>C, 2447C>G, 2448G>A, 2450A>T, 2451A>G, 2452C>A, 2453T>A, 2456T>C, 2457T>C, 2459T>G, 2462T>A, 2465A>G, 2466A>G, 2477C>T, 2478C>T, 2489A>G, 2492T>C, 2495C>T, 2504T>A, 2510T>C, 2514A>G, 2518C>T, 2519C>G, 2524C>T, 2525C>T, 2528T>A, 2532C>A, 2533A>G, 2536G>A, 2537G>T, 2543T>G, 2551A>G, 2552C>T, 2555T>C, 2556G>T, 2557T>C, 2561T>C, 2568A>G, 2571A>C, 2572A>G, 2582A>T, 2585G>A, 2597G>A, 2598A>G, 2599A>C, 2600C>T, 2601G>A, 2609T>A, 2613T>G, 2615T>A, 2618T>A, 2622G>T, 2624A>T, 2626A>T, 2627C>A, 2629A>C, 2631A>T, 2634A>C, 2635C>T, 2638G>T, 2639A>G, 2644A>G, 2646G>T, 2647A>G, 2648A>T, 2649G>A, 2650T>G, 2652G>T, 2653G>C, 2654C>A, 2656T>A, 2657A>T, 2658A>G, 2659G>A, 2660A>C, 2661C>T, 2664T>A, 2665A>G, 2667delG, 2674T>A, 2678C>T, 2681G>A, 2686T>A, 2689T>C, 2690G>C, 2694A>G, 2695T>A								

CDS

RdRp	32	777	95.9%	3373	64.7%	743 (99.6%)	506 (67.8%)	1/2/2/2	4
Protein mutations:	I36L (441A>C 443C>T), L38F (447C>T), A44V (466C>T), A46M (471G>A 472C>T 473T>G), L48M (477T>A), T49H (480A>C 481C>A 482A>T), I52V (489A>G), V60I (513G>A), S61K (516T>A 517C>A 518T>A), K62T (520A>C), T64V (525A>G 526C>T 527T>G), T65K (529C>A 530C>A), R67K (534C>A 535G>A 536C>A), K69R (540A>C 541A>G 542G>T), Y72F (550A>T), F74Y (556T>A 557C>T), A83T (582G>A), C85S (589G>C), F94Y (616T>A), R95S (618C>T 619G>C 620A>T), E97N (624G>A 626A>T), E98D (629A>C), P99T (630C>A 632A>C), L100M (633C>A 635T>G), S109C (661C>G 662G>T), R113K (672C>A 673G>A 674T>G), K117A (684A>G 685A>C 686A>C), A122H (699G>C 700C>A 701A>C), I123V (702A>G), R125A (708A>G 709G>C 710G>A), D127S (714G>T 715A>C), E128D (719A>T), A144S (765G>T 767T>A), V146* (771G>T 772T>A 773T>G), L147I (774T>A 776G>T), K148A (777A>G 778A>C 779A>G), S154K (796G>A 797T>G), E159Q (810G>C), P166S (831C>T 833T>A), G169D (841G>A 842G>T), L172R (850T>G 851A>T), E176G (862A>G), I178M (869T>G), F182* (880T>G 881C>A), H184K (885C>A 887T>G), Q186R (892A>G), F192H (909T>C 910T>A), R194Q (916G>A), G195S (918G>T 919G>C), M198L (927A>C 929G>T), I200K (934T>A 935T>G), S203N (942T>A 943C>A), Q205K (948C>A), N214L (975A>C 976A>T 977T>C), S220R (993T>C 994C>G 995A>G), Y221F (997A>T 998C>T), G222Q (999G>C 1000G>A 1001T>A), L223Y (1003T>A 1004A>T), A224S (1005G>T 1007C>G), K228V (1017A>G 1018A>T 1019A>T), Q231G (1026C>G 1027A>G), E232A (1030A>C 1031G>A), S234A (1035A>G 1036G>C), F237V (1044T>G 1046C>T), F239E (1050T>G 1051T>A 1052T>A), P240H (1054C>A 1055A>C), L250I (1083C>A), W255L (1099G>T), T257V (1104A>G 1105C>T 1106A>G), I260M (1115T>G), F263I (1122T>A 1124C>T), M272I (1151G>T), K276Q (1161A>C), S277M (1164T>A 1165C>T 1166T>G), F279L (1172C>A), F280Y (1174T>A 1175T>C), L282F (1181A>T), R284S (1185A>T 1186G>C 1187G>T), A285N (1188G>A 1189C>A), A286N (1191G>A 1192C>A 1193A>T), E288del (1197_1199delGAA), L290F (1205A>T), L293E (1212T>G 1213T>A), E296D (1223A>T), H297L (1224C>T 1225A>T 1226C>A), S301Q (1236A>C 1237G>A 1238T>A), W305L (1248T>C 1249G>T 1250G>T), T308S (1258C>G), S311G (1266A>G 1268T>G), P312A (1269C>G 1271T>C), R314Q (1276G>A), G324A (1306G>C), A325Q (1308G>C 1309C>A 1310A>G), V326A (1312T>C 1313G>C), V335I (1338G>A 1340A>C), V337A (1345T>C 1346A>T), M338I (1349G>A), Y341S (1357A>C 1358C>T), D352I (1389G>A 1390A>T), K360S (1414A>G 1415A>T), I361L (1416A>C 1418A>T), D368N (1437G>A), K371R (1446A>C 1447A>G 1448A>T), L374Q (1455T>C 1456T>A), A375Y (1458G>T 1459C>A), K377R (1465A>G 1466G>A), Q378L (1468A>T 1469A>T), N380R (1474A>G 1475C>A), F381P (1476T>C 1477T>C 1478T>G), N383S (1482A>T 1483A>C), L389F (1502A>C), D394N (1515G>A), M401I (1538G>A), M405I (1550G>C), S406E (1551T>G 1552C>A 1553T>A), P411S (1566C>A 1567C>G), S420del (1593_1597delAGCTC), I427F (1614A>T 1616C>T), N430S (1623A>T 1624A>C 1625C>A), K431P (1626A>C 1627A>C 1628A>T), L433M (1632T>A 1634A>G), T434V (1635A>G 1636C>T 1637A>G), R436K (1642G>A 1643A>G), V437I (1644G>A 1646G>T), Y438S (1647T>A 1648A>G), I440V (1653A>G), F450Y (1684T>A), H451Y (1686C>T), A455S (1698G>T 1700A>C), S458A (1707T>G), I464M (1727A>G), S470K (1744G>A 1745C>A), K471Y (1746A>T 1748A>T), G475T (1758G>A 1759G>C 1760A>G), C476S (1761T>A), N477T (1765A>C 1766C>T), S478T (1767T>A 1769T>C), G481A (1777G>C 1778G>T), S495T (1819G>C 1820T>A), K496E (1821A>G 1823A>G), N499R (1830A>C 1831A>G 1832T>G), E500Q (1833G>C), K502R (1840A>G), N509G (1860A>G 1861A>G), K511T (1867A>C), I514L (1875A>T), P515A (1878C>G 1880A>T), L518R (1887T>A 1888T>G 1889G>A), D521E (1898T>G), S535D (1938T>G 1939C>A 1940A>C), D539Y (1950G>T 1952T>C), L549T (1980T>A 1981T>C 1982A>T), A559Q (2010G>C 2011C>A 2012C>A), I562T (2020T>C), D565S (2028G>T 2029A>C 2030T>G), N567S (2034A>T 2035A>C 2036C>A), I568T (2038T>C), V570I (2043G>A), Q572P (2050A>C 2051A>C), R573I (2053G>T 2054A>T), L574F (2057G>T), G575W (2058G>T), Y579F (2071A>T), K580R (2074A>G 2075G>A), A583S (2082G>T 2084G>T), F586M (2091T>A 2093C>G), T587S (2094A>T), H600A (2133C>G 2134A>C 2135T>A), L601S (2136C>A 2137T>G 2138T>C), V604H (2145G>C 2146T>A 2147T>C), Y611L (2166T>C 2167A>T), W613Y (2173G>A 2174G>T), G618F (2187G>T 2188G>T), M619K (2191T>A 2192G>A), Y624F (2206A>T), Y624_L625insX (2207_2208insT), V630I (2223G>A), S631D (2226A>G 2227G>A), L634R (2236T>G 2237T>G), K638T (2248A>C), V639P (2250G>C 2251T>C), L642I (2259T>A), Q643R (2263A>G 2264G>A), I644L (2265A>C), K647N (2276G>T), D648E (2279T>G), V655R (2298G>A 2299T>G 2300T>A), R660C (2313C>T 2315T>C), T661I (2317C>T), R664V (2325C>G 2326G>T), Q665N (2328C>A 2330A>T), E668K (2337G>A), K672R (2349A>C 2350A>G), L674V (2355C>G 2357C>T), N675S (2358A>T 2359A>C 2360C>T), W676* (2362G>A), C681L (2377G>T 2378T>G), N683S (2382A>T 2383A>C 2384C>T), P684F (2385C>T 2386C>T 2387A>T), D685E (2390T>A), K689E (2400A>G 2402G>A), S690K (2404G>A 2405T>A), I692M (2411C>G), S694A (2415T>G 2417C>T), C697Y (2425G>A), T699* (2430A>T 2431C>A 2432A>G), L704S (2445C>T 2446T>C 2447C>G), E705N (2448G>A 2450A>T), T706E (2451A>G 2452C>A 2453T>A), F708L (2457T>C 2459T>G), D709E (2462T>A), I711V (2466A>G), N727D (2514A>G), P728L (2518C>T 2519C>G), T730I (2524C>T 2525C>T), Q733R (2532C>A 2533A>G), W734Y (2536G>A 2537G>T), F736L (2543T>G), N739S (2551A>G 2552C>T), V741S (2556G>T 2557T>C), N745D (2568A>G), K746R (2571A>C 2572A>G), L749F (2582A>T), N755A (2598A>G 2599A>C 2600C>T), D756N (2601G>A), S760A (2613T>G 2615T>A), E763Y (2622G>T 2624A>T), Y764L (2626A>T 2627C>A), Q765P (2629A>C), T766S (2631A>T), T767L (2634A>C 2635C>T), G768V (2638G>T 2639A>G), E770G (2644A>G), E771C (2646G>T 2647A>G 2648A>T), V772R (2649G>A 2650T>G), G773S (2652G>T 2653G>C 2654C>A), L774Y (2656T>A 2657A>T), R775D (2658A>G 2659G>A 2660A>C), L776F (2661C>T), *777R (2664T>A 2665A>G)								

	Begin	End	Coverage	Score	Concordance	Matches	Identities	I/D/M/F*	Stop Codons
**NT**	**428**	**2700**	**82.0%**	**1546**	**34.4%**	**2264 (99.6%)**	**1530 (67.3%)**	**1/9**	
Codon mutations:	AAA34AAG (437A>G), ATC36CTT (441A>C 443C>T), CTT38TTT (447C>T), CTA42TTA (459C>T), GCC44GTC (466C>T), TCC45TCT (470C>T), GCT46ATG (471G>A 472C>T 473T>G), TTG48ATG (477T>A), ACA49CAT (480A>C 481C>A 482A>T), AAA50AAG (485A>G), ATC52GTC (489A>G), GCT53GCC (494T>C), CTT54CTA (497T>A), GCT55GCG (500T>G), GGA56GGC (503A>C), TGT57TGC (506T>C), GAC58GAT (509C>T), TTT59TTC (512T>C), GTA60ATA (513G>A), TCT61AAA (516T>A 517C>A 518T>A), AAA62ACA (520A>C), GTG63GTT (524G>T), ACT64GTG (525A>G 526C>T 527T>G), ACC65AAA (529C>A 530C>A), TTA66CTG (531T>C 533A>G), CGC67AAA (534C>A 535G>A 536C>A), CGG68CGA (539G>A), AAG69CGT (540A>C 541A>G 542G>T), TCG70TCA (545G>A), GGC71GGT (548C>T), TAT72TTT (550A>T), TTC74TAT (556T>A 557C>T), ACA75ACT (560A>T), TAC78TAT (569C>T), AAG80AAA (575G>A), TGC82TGT (581C>T), GCA83ACA (582G>A), GTA84GTT (587A>T), TGC85TCC (589G>C), TTA86CTT (591T>C 593A>T), CGA88CGT (599A>T), TAC89TAT (602C>T), TCC93AGT (612T>A 613C>G 614C>T), TTT94TAT (616T>A), CGA95TCT (618C>T 619G>C 620A>T), GAA97AAT (624G>A 626A>T), GAA98GAC (629A>C), CCA99ACC (630C>A 632A>C), CTT100ATG (633C>A 635T>G), TCG101TCT (638G>T), GTG102GTT (641G>T), CCT103CCA (644T>A), GTG104GTT (647G>T), CTC106TTG (651C>T 653C>G), ACA107ACC (656A>C), AGA108CGA (657A>C), TCG109TGT (661C>G 662G>T), GGT110GGA (665T>A), ATC111ATT (668C>T), CGT113AAG (672C>A 673G>A 674T>G), ATC115ATA (680C>A), CCC116CCT (683C>T), AAA117GCC (684A>G 685A>C 686A>C), TTG119TTA (692G>A), CGT120CGA (695T>A), GCA122CAC (699G>C 700C>A 701A>C), ATA123GTA (702A>G), CGT124AGG (705C>A 707T>G), AGG125GCA (708A>G 709G>C 710G>A), AAA126AAG (713A>G), GAT127TCT (714G>T 715A>C), GAA128GAT (719A>T), CAC129CAT (722C>T), GTT134GTC (737T>C), TAC137TAT (746C>T), TCA139TCT (752A>T), GGG142GGA (761G>A), GCT144TCA (765G>T 767T>A), AAG145AAA (770G>A), GTT146TAG (771G>T 772T>A 773T>G), TTG147ATT (774T>A 776G>T), AAA148GCG (777A>G 778A>C 779A>G), GTT149GTG (782T>G), AGT154AAG (796G>A 797T>G), GCA156GCC (803A>C), TTT158TTC (809T>C), GAA159CAA (810G>C), TCC160TCA (815C>A), ATA161ATT (818A>T), ACT162ACG (821T>G), ACC163ACT (824C>T), CCA164CCT (827A>T), CCT166TCA (831C>T 833T>A), GGG169GAT (841G>A 842G>T), CTA172CGT (850T>G 851A>T), GTG174GTT (857G>T), CTG175CTT (860G>T), GAA176GGA (862A>G), ATT178ATG (869T>G), ACG180ACT (875G>T), TTC182TGA (880T>G 881C>A), CAT184AAG (885C>A 887T>G), CAA186CGA (892A>G), CTA190CTT (905A>T), CCC191CCA (908C>A), TTT192CAT (909T>C 910T>A), CGA194CAA (916G>A), GGC195TCC (918G>T 919G>C), ATT196ATA (923T>A), CCA197CCT (926A>T), ATG198CTT (927A>C 929G>T), ATT200AAG (934T>A 935T>G), GGG201GGT (938G>T), TCT203AAT (942T>A 943C>A), CAA205AAA (948C>A), CCC212CCA (971C>A), CTA213TTA (972C>T), AAT214CTC (975A>C 976A>T 977T>C), AAT216AAC (983T>C), AGG219CGC (990A>C 992G>C), TCA220CGG (993T>C 994C>G 995A>G), TAC221TTT (997A>T 998C>T), GGT222CAA (999G>C 1000G>A 1001T>A), TTA223TAT (1003T>A 1004A>T), GCC224TCG (1005G>T 1007C>G), GTA225GTG (1010A>G), GAC227GAT (1016C>T), AAA228GTT (1017A>G 1018A>T 1019A>T), GTT229GTA (1022T>A), GAT230GAC (1025T>C), CAA231GGA (1026C>G 1027A>G), GAG232GCA (1030A>C 1031G>A), GCT233GCG (1034T>G), AGT234GCT (1035A>G 1036G>C), TTC237GTT (1044T>G 1046C>T), AAG238AAA (1049G>A), TTT239GAA (1050T>G 1051T>A 1052T>A), CCA240CAC (1054C>A 1055A>C), ATT242ATC (1061T>C), GTT244GTG (1067T>G), TTG246TTA (1073G>A), AAA247AAG (1076A>G), CTA250ATA (1083C>A), GCG251GCT (1088G>T), GCC252GCG (1091C>G), TGG255TTG (1099G>T), ACA257GTG (1104A>G 1105C>T 1106A>G), ATT260ATG (1115T>G), TCA262TCC (1121A>C), TTC263ATT (1122T>A 1124C>T), CAG264CAA (1127G>A), GAC265GAT (1130C>T), GGC266GGG (1133C>G), TCA269TCC (1142A>C), CCC270CCT (1145C>T), GGG271GGT (1148G>T), ATG272ATT (1151G>T), TTG273CTT (1152T>C 1154G>T), TCC275TCT (1160C>T), AAA276CAA (1161A>C), TCT277ATG (1164T>A 1165C>T 1166T>G), GTC278GTT (1169C>T), TTC279TTA (1172C>A), TTT280TAC (1174T>A 1175T>C), CCT281CCA (1178T>A), TTA282TTT (1181A>T), GAC283GAT (1184C>T), AGG284TCT (1185A>T 1186G>C 1187G>T), GCT285AAT (1188G>A 1189C>A), GCA286AAT (1191G>A 1192C>A 1193A>T), ACT287ACC (1196T>C), GAA288del (1197_1199delGAA), TTA290TTT (1205A>T), AAC292AAT (1211C>T), TTA293GAA (1212T>G 1213T>A), TCT294TCC (1217T>C), CTC295CTT (1220C>T), GAA296GAT (1223A>T), CAC297TTA (1224C>T 1225A>T 1226C>A), GAG299GAA (1232G>A), AGT301CAA (1236A>C 1237G>A 1238T>A), GTC302GTA (1241C>A), CCA304CCC (1247A>C), TGG305CTT (1248T>C 1249G>T 1250G>T), TTT306TTC (1253T>C), TCA307TCT (1256A>T), ACC308AGC (1258C>G), TTG310TTA (1265G>A), AGT311GGG (1266A>G 1268T>G), CCT312GCC (1269C>G 1271T>C), TAT313TAC (1274T>C), CGA314CAA (1276G>A), GGG315GGA (1280G>A), GTG316GTT (1283G>T), GTT319GTG (1292T>G), AGG322AGA (1301G>A), CTT323TTG (1302C>T 1304T>G), GGT324GCT (1306G>C), GCA325CAG (1308G>C 1309C>A 1310A>G), GTG326GCC (1312T>C 1313G>C), ATA327ATT (1316A>T), GAG328GAA (1319G>A), GGC329GGT (1322C>T), GGA331GGG (1328A>G), AGA333CGG (1332A>C 1334A>G), GTA335ATC (1338G>A 1340A>C), GTA337GCT (1345T>C 1346A>T), ATG338ATA (1349G>A), AAC340AAT (1355C>T), TAC341TCT (1357A>C 1358C>T), CTC347CTG (1376C>G), CAC348CAT (1379C>T), CCT349CCC (1382T>C), GTT350GTC (1385T>C), CAC351CAT (1388C>T), GAT352ATT (1389G>A 1390A>T), ACA356ACT (1403A>T), GTC357GTA (1406C>A), CTG358CTA (1409G>A), TCT359TCA (1412T>A), AAA360AGT (1414A>G 1415A>T), ATA361CTT (1416A>C 1418A>T), ACA363ACT (1424A>T), GAC364GAT (1427C>T), GGG365GGT (1430G>T), ACT366ACA (1433T>A), GAC368AAC (1437G>A), GAG370GAA (1445G>A), AAA371CGT (1446A>C 1447A>G 1448A>T), CCT372CCG (1451T>G), TTG374CAG (1455T>C 1456T>A), GCT375TAT (1458G>T 1459C>A), TTA376CTA (1461T>C), AAG377AGA (1465A>G 1466G>A), CAA378CTT (1468A>T 1469A>T), AAG379AAA (1472G>A), AAC380AGA (1474A>G 1475C>A), TTT381CCG (1476T>C 1477T>C 1478T>G), AAA382AAG (1481A>G), AAT383TCT (1482A>T 1483A>C), TAT385TAC (1490T>C), TTA389TTC (1502A>C), AAG390AAA (1505G>A), ACG393ACA (1514G>A), GAC394AAC (1515G>A), CCG397CCA (1526G>A), GTC400GTT (1535C>T), ATG401ATA (1538G>A), TAT402TAC (1541T>C), CTT404TTA (1545C>T 1547T>A), ATG405ATC (1550G>C), TCT406GAA (1551T>G 1552C>A 1553T>A), ATA408ATT (1559A>T), GGG410GGT (1565G>T), CCT411AGT (1566C>A 1567C>G), ACC412ACT (1571C>T), TTG413TTA (1574G>A), TCC415TCT (1580C>T), GTT418GTC (1589T>C), AGC420del (1593_1597delAGCTC), TCT421--A (1593_1597delAGCTC 1598T>A), ACG426ACT (1613G>T), ATC427TTT (1614A>T 1616C>T), CTT428CTC (1619T>C), GTT429GTA (1622T>A), AAC430TCA (1623A>T 1624A>C 1625C>A), AAA431CCT (1626A>C 1627A>C 1628A>T), CCG432CCT (1631G>T), TTA433ATG (1632T>A 1634A>G), ACA434GTG (1635A>G 1636C>T 1637A>G), AGA436AAG (1642G>A 1643A>G), GTG437ATT (1644G>A 1646G>T), TAT438AGT (1647T>A 1648A>G), GAG439GAA (1652G>A), ATA440GTA (1653A>G), CTG443CTT (1664G>T), GGG445GGA (1670G>A), CAG446CAA (1673G>A), CCT447CCG (1676T>G), TTG448TTA (1679G>A), GGC449GGT (1682C>T), TTC450TAC (1684T>A), CAC451TAC (1686C>T), TCT453TCA (1694T>A), GCA455TCC (1698G>T 1700A>C), CTT456CTA (1703T>A), TTC457TTT (1706C>T), TCG458GCG (1707T>G), TTG459CTT (1710T>C 1712G>T), TCT460TCC (1715T>C), CAC462CAT (1721C>T), ATA464ATG (1727A>G), TTA467CTA (1734T>C), GCA468GCT (1739A>T), AGC470AAA (1744G>A 1745C>A), AAA471TAT (1746A>T 1748A>T), TAT473TAC (1754T>C), GGA475ACG (1758G>A 1759G>C 1760A>G), TGT476AGT (1761T>A), AAC477ACT (1765A>C 1766C>T), TCT478ACC (1767T>A 1769T>C), CCA479CCT (1772A>T), GGG481GCT (1777G>C 1778G>T), GCT484GCC (1787T>C), TTA485CTG (1788T>C 1790A>G), CTA486TTA (1791C>T), AGT495ACA (1819G>C 1820T>A), AAA496GAG (1821A>G 1823A>G), GCC498GCG (1829C>G), AAT499CGG (1830A>C 1831A>G 1832T>G), GAG500CAG (1833G>C), AAG502AGG (1840A>G), TTA504TTG (1847A>G), TTG505CTG (1848T>C), AGA507AGG (1856A>G), CTC508CTT (1859C>T), AAT509GGT (1860A>G 1861A>G), GTC510GTT (1865C>T), AAA511ACA (1867A>C), ATT512ATC (1871T>C), TCG513TCT (1874G>T), ATA514TTA (1875A>T), CCA515GCT (1878C>G 1880A>T), TCT517TCG (1886T>G), TTG518AGA (1887T>A 1888T>G 1889G>A), TCT520TCC (1895T>C), GAT521GAG (1898T>G), AAT522AAC (1901T>C), ATT525ATC (1910T>C), GCG528GCT (1919G>T), AGG530AGA (1925G>A), ACT533ACC (1934T>C), AAA534AAG (1937A>G), TCA535GAC (1938T>G 1939C>A 1940A>C), GAT539TAC (1950G>T 1952T>C), TTA540CTT (1953T>C 1955A>T), ATT543ATC (1964T>C), TCC544TCT (1967C>T), GCT547GCA (1976T>A), TTA549ACT (1980T>A 1981T>C 1982A>T), TCT550TCC (1985T>C), CGT552AGG (1989C>A 1991T>G), ACT553ACA (1994T>A), GTT555GTG (2000T>G), GGA556GGT (2003A>T), CTT557CTG (2006T>G), TGC558TGT (2009C>T), GCC559CAA (2010G>C 2011C>A 2012C>A), ATC562ACC (2020T>C), GAT565TCG (2028G>T 2029A>C 2030T>G), AAC567TCA (2034A>T 2035A>C 2036C>A), ATA568ACA (2038T>C), TCA569TCG (2042A>G), GTT570ATT (2043G>A), CAA572CCC (2050A>C 2051A>C), AGA573ATT (2053G>T 2054A>T), TTG574TTT (2057G>T), GGG575TGG (2058G>T), GGT576GGG (2063T>G), GGG578GGA (2069G>A), TAC579TTC (2071A>T), AAG580AGA (2074A>G 2075G>A), GTA581GTT (2078A>T), GCG583TCT (2082G>T 2084G>T), CGC584CGT (2087C>T), TTA585TTG (2090A>G), TTC586ATG (2091T>A 2093C>G), ACT587TCT (2094A>T), ACC588ACT (2099C>T), AGC590TCT (2103A>T 2104G>C 2105C>T), AAG591AAA (2108G>A), GAG594GAA (2117G>A), CGT595AGA (2118C>A 2120T>A), CTA596CTG (2123A>G), GCT598GCG (2129T>G), CAT600GCA (2133C>G 2134A>C 2135T>A), CTT601AGC (2136C>A 2137T>G 2138T>C), AAG602AAA (2141G>A), GTT604CAC (2145G>C 2146T>A 2147T>C), CGG605AGG (2148C>A), CAC607CAT (2156C>T), TTA608CTA (2157T>C), CTA609TTA (2160C>T), TAC611CTC (2166T>C 2167A>T), TGG613TAT (2173G>A 2174G>T), GGA616GGG (2183A>G), AGG617AGA (2186G>A), GGT618TTT (2187G>T 2188G>T), ATG619AAA (2191T>A 2192G>A), CCC620CCT (2195C>T), CCC623CCT (2204C>T), TAT624TTT (2206A>T), TAT624_TTG625insT-- (2207_2208insT), AAA626AAG (2213A>G), GGG627GGA (2216G>A), GTA630ATA (2223G>A), AGT631GAT (2226A>G 2227G>A), TAC632TAT (2231C>T), CTT634CGG (2236T>G 2237T>G), AAA635AAG (2240A>G), AAA638ACA (2248A>C), GTT639CCT (2250G>C 2251T>C), TTA642ATA (2259T>A), CAG643CGA (2263A>G 2264G>A), ATC644CTC (2265A>C), AAG647AAT (2276G>T), GAT648GAG (2279T>G), CTG649TTG (2280C>T), TTC651TTT (2288C>T), GAA654GAG (2297A>G), GTT655AGA (2298G>A 2299T>G 2300T>A), ATA657ATC (2306A>C), CTA658CTT (2309A>T), CGT660TGC (2313C>T 2315T>C), ACT661ATT (2317C>T), CTA663TTA (2322C>T), CGC664GTC (2325C>G 2326G>T), CAA665AAT (2328C>A 2330A>T), GTA667GTT (2336A>T), GAA668AAA (2337G>A), TTA671CTT (2346T>C 2348A>T), AAG672CGG (2349A>C 2350A>G), CTC674GTT (2355C>G 2357C>T), AAC675TCT (2358A>T 2359A>C 2360C>T), TGG676TAG (2362G>A), CAT678CAC (2369T>C), ACG679ACT (2372G>T), TGT681TTG (2377G>T 2378T>G), AAC683TCT (2382A>T 2383A>C 2384C>T), CCA684TTT (2385C>T 2386C>T 2387A>T), GAT685GAA (2390T>A), TCC687TCT (2396C>T), ATT688ATC (2399T>C), AAG689GAA (2400A>G 2402G>A), AGT690AAA (2404G>A 2405T>A), CTC691TTG (2406C>T 2408C>G), ATC692ATG (2411C>G), GAC693GAT (2414C>T), TCC694GCT (2415T>G 2417C>T), CCT695CCC (2420T>C), ATA696ATT (2423A>T), TGC697TAC (2425G>A), ACA699TAG (2430A>T 2431C>A 2432A>G), TCC700TCA (2435C>A), AAA702AAG (2441A>G), AGG703CGA (2442A>C 2444G>A), CTC704TCG (2445C>T 2446T>C 2447C>G), GAA705AAT (2448G>A 2450A>T), ACT706GAA (2451A>G 2452C>A 2453T>A), GAT707GAC (2456T>C), TTT708CTG (2457T>C 2459T>G), GAT709GAA (2462T>A), TTA710TTG (2465A>G), ATC711GTC (2466A>G), GGC714GGT (2477C>T), CTA715TTA (2478C>T), AAA718AAG (2489A>G), TGT719TGC (2492T>C), TAC720TAT (2495C>T), GGT723GGA (2504T>A), GGT725GGC (2510T>C), AAT727GAT (2514A>G), CCC728CTG (2518C>T 2519C>G), ACC730ATT (2524C>T 2525C>T), ACT731ACA (2528T>A), CAG733AGG (2532C>A 2533A>G), TGG734TAT (2536G>A 2537G>T), TTT736TTG (2543T>G), AAC739AGT (2551A>G 2552C>T), ACT740ACC (2555T>C), GTT741TCT (2556G>T 2557T>C), ATT742ATC (2561T>C), AAT745GAT (2568A>G), AAA746CGA (2571A>C 2572A>G), TTA749TTT (2582A>T), GGG750GGA (2585G>A), GGG754GGA (2597G>A), AAC755GCT (2598A>G 2599A>C 2600C>T), GAT756AAT (2601G>A), ATT758ATA (2609T>A), TCT760GCA (2613T>G 2615T>A), CCT761CCA (2618T>A), GAA763TAT (2622G>T 2624A>T), TAC764TTA (2626A>T 2627C>A), CAA765CCA (2629A>C), ACT766TCT (2631A>T), ACA767CTA (2634A>C 2635C>T), GGA768GTG (2638G>T 2639A>G), GAG770GGG (2644A>G), GAA771TGT (2646G>T 2647A>G 2648A>T), GTG772AGG (2649G>A 2650T>G), GGC773TCA (2652G>T 2653G>C 2654C>A), TTA774TAT (2656T>A 2657A>T), AGA775GAC (2658A>G 2659G>A 2660A>C), CTT776TTT (2661C>T), TAG777AGG (2664T>A 2665A>G)								

Proteins

RNA-dependent RNA polymerase (YP_010798874.1)	32	777	95.9%	3373	64.7%	743 (99.6%)	506 (67.8%)	1/2/2/2	4

	Begin	End	Coverage	Score	Concordance	Matches	Identities	I/D/M/F*	Stop Codons
**NT**	**428**	**2700**	**82.0%**	**1546**	**34.4%**	**2264 (99.6%)**	**1530 (67.3%)**	**1/9**	

Protein mutations:

I36L (441A>C 443C>T), L38F (447C>T), A44V (466C>T), A46M (471G>A 472C>T 473T>G), L48M (477T>A), T49H (480A>C 481C>A 482A>T), I52V (489A>G), V60I (513G>A), S61K (516T>A 517C>A 518T>A), K62T (520A>C), T64V (525A>G 526C>T 527T>G), T65K (529C>A 530C>A), R67K (534C>A 535G>A 536C>A), K69R (540A>C 541A>G 542G>T), Y72F (550A>T), F74Y (556T>A 557C>T), A83T (582G>A), C85S (589G>C), F94Y (616T>A), R95S (618C>T 619G>C 620A>T), E97N (624G>A 626A>T), E98D (629A>C), P99T (630C>A 632A>C), L100M (633C>A 635T>G), S109C (661C>G 662G>T), R113K (672C>A 673G>A 674T>G), K117A (684A>G 685A>C 686A>C), A122H (699G>C 700C>A 701A>C), I123V (702A>G), R125A (708A>G 709G>C 710G>A), D127S (714G>T 715A>C), E128D (719A>T), A144S (765G>T 767T>A), V146* (771G>T 772T>A 773T>G), L147I (774T>A 776G>T), K148A (777A>G 778A>C 779A>G), S154K (796G>A 797T>G), E159Q (810G>C), P166S (831C>T 833T>A), G169D (841G>A 842G>T), L172R (850T>G 851A>T), E176G (862A>G), I178M (869T>G), F182* (880T>G 881C>A), H184K (885C>A 887T>G), Q186R (892A>G), F192H (909T>C 910T>A), R194Q (916G>A), G195S (918G>T 919G>C), M198L (927A>C 929G>T), I200K (934T>A 935T>G), S203N (942T>A 943C>A), Q205K (948C>A), N214L (975A>C 976A>T 977T>C), S220R (993T>C 994C>G 995A>G), Y221F (997A>T 998C>T), G222Q (999G>C 1000G>A 1001T>A), L223Y (1003T>A 1004A>T), A224S (1005G>T 1007C>G), K228V (1017A>G 1018A>T 1019A>T), Q231G (1026C>G 1027A>G), E232A (1030A>C 1031G>A), S234A (1035A>G 1036G>C), F237V (1044T>G 1046C>T), F239E (1050T>G 1051T>A 1052T>A), P240H (1054C>A 1055A>C), L250I (1083C>A), W255L (1099G>T), T257V (1104A>G 1105C>T 1106A>G), I260M (1115T>G), F263I (1122T>A 1124C>T), M272I (1151G>T), K276Q (1161A>C), S277M (1164T>A 1165C>T 1166T>G), F279L (1172C>A), F280Y (1174T>A 1175T>C), L282F (1181A>T), R284S (1185A>T 1186G>C 1187G>T), A285N (1188G>A 1189C>A), A286N (1191G>A 1192C>A 1193A>T), E288del (1197_1199delGAA), L290F (1205A>T), L293E (1212T>G 1213T>A), E296D (1223A>T), H297L (1224C>T 1225A>T 1226C>A), S301Q (1236A>C 1237G>A 1238T>A), W305L (1248T>C 1249G>T 1250G>T), T308S (1258C>G), S311G (1266A>G 1268T>G), P312A (1269C>G 1271T>C), R314Q (1276G>A), G324A (1306G>C), A325Q (1308G>C 1309C>A 1310A>G), V326A (1312T>C 1313G>C), V335I (1338G>A 1340A>C), V337A (1345T>C 1346A>T), M338I (1349G>A), Y341S (1357A>C 1358C>T), D352I (1389G>A 1390A>T), K360S (1414A>G 1415A>T), I361L (1416A>C 1418A>T), D368N (1437G>A), K371R (1446A>C 1447A>G 1448A>T), L374Q (1455T>C 1456T>A), A375Y (1458G>T 1459C>A), K377R (1465A>G 1466G>A), Q378L (1468A>T 1469A>T), N380R (1474A>G 1475C>A), F381P (1476T>C 1477T>C 1478T>G), N383S (1482A>T 1483A>C), L389F (1502A>C), D394N (1515G>A), M401I (1538G>A), M405I (1550G>C), S406E (1551T>G 1552C>A 1553T>A), P411S (1566C>A 1567C>G), S420del (1593_1597delAGCTC), I427F (1614A>T 1616C>T), N430S (1623A>T 1624A>C 1625C>A), K431P (1626A>C 1627A>C 1628A>T), L433M (1632T>A 1634A>G), T434V (1635A>G 1636C>T 1637A>G), R436K (1642G>A 1643A>G), V437I (1644G>A 1646G>T), Y438S (1647T>A 1648A>G), I440V (1653A>G), F450Y (1684T>A), H451Y (1686C>T), A455S (1698G>T 1700A>C), S458A (1707T>G), I464M (1727A>G), S470K (1744G>A 1745C>A), K471Y (1746A>T 1748A>T), G475T (1758G>A 1759G>C 1760A>G), C476S (1761T>A), N477T (1765A>C 1766C>T), S478T (1767T>A 1769T>C), G481A (1777G>C 1778G>T), S495T (1819G>C 1820T>A), K496E (1821A>G 1823A>G), N499R (1830A>C 1831A>G 1832T>G), E500Q (1833G>C), K502R (1840A>G), N509G (1860A>G 1861A>G), K511T (1867A>C), I514L (1875A>T), P515A (1878C>G 1880A>T), L518R (1887T>A 1888T>G 1889G>A), D521E (1898T>G), S535D (1938T>G 1939C>A 1940A>C), D539Y (1950G>T 1952T>C), L549T (1980T>A 1981T>C 1982A>T), A559Q (2010G>C 2011C>A 2012C>A), I562T (2020T>C), D565S (2028G>T 2029A>C 2030T>G), N567S (2034A>T 2035A>C 2036C>A), I568T (2038T>C), V570I (2043G>A), Q572P (2050A>C 2051A>C), R573I (2053G>T 2054A>T), L574F (2057G>T), G575W (2058G>T), Y579F (2071A>T), K580R (2074A>G 2075G>A), A583S (2082G>T 2084G>T), F586M (2091T>A 2093C>G), T587S (2094A>T), H600A (2133C>G 2134A>C 2135T>A), L601S (2136C>A 2137T>G 2138T>C), V604H (2145G>C 2146T>A 2147T>C), Y611L (2166T>C 2167A>T), W613Y (2173G>A 2174G>T), G618F (2187G>T 2188G>T), M619K (2191T>A 2192G>A), Y624F (2206A>T), Y624_L625insX (2207_2208insT), V630I (2223G>A), S631D (2226A>G 2227G>A), L634R (2236T>G 2237T>G), K638T (2248A>C), V639P (2250G>C 2251T>C), L642I (2259T>A), Q643R (2263A>G 2264G>A), I644L (2265A>C), K647N (2276G>T), D648E (2279T>G), V655R (2298G>A 2299T>G 2300T>A), R660C (2313C>T 2315T>C), T661I (2317C>T), R664V (2325C>G 2326G>T), Q665N (2328C>A 2330A>T), E668K (2337G>A), K672R (2349A>C 2350A>G), L674V (2355C>G 2357C>T), N675S (2358A>T 2359A>C 2360C>T), W676* (2362G>A), C681L (2377G>T 2378T>G), N683S (2382A>T 2383A>C 2384C>T), P684F (2385C>T 2386C>T 2387A>T), D685E (2390T>A), K689E (2400A>G 2402G>A), S690K (2404G>A 2405T>A), I692M (2411C>G), S694A (2415T>G 2417C>T), C697Y (2425G>A), T699* (2430A>T 2431C>A 2432A>G), L704S (2445C>T 2446T>C 2447C>G), E705N (2448G>A 2450A>T), T706E (2451A>G 2452C>A 2453T>A), F708L (2457T>C 2459T>G), D709E (2462T>A), I711V (2466A>G), N727D (2514A>G), P728L (2518C>T 2519C>G), T730I (2524C>T 2525C>T), Q733R (2532C>A 2533A>G), W734Y (2536G>A 2537G>T), F736L (2543T>G), N739S (2551A>G 2552C>T), V741S (2556G>T 2557T>C), N745D (2568A>G), K746R (2571A>C 2572A>G), L749F (2582A>T), N755A (2598A>G 2599A>C 2600C>T), D756N (2601G>A), S760A (2613T>G 2615T>A), E763Y (2622G>T 2624A>T), Y764L (2626A>T 2627C>A), Q765P (2629A>C), T766S (2631A>T), T767L (2634A>C 2635C>T), G768V (2638G>T 2639A>G), E770G (2644A>G), E771C (2646G>T 2647A>G 2648A>T), V772R (2649G>A 2650T>G), G773S (2652G>T 2653G>C 2654C>A), L774Y (2656T>A 2657A>T), R775D (2658A>G 2659G>A 2660A>C), L776F (2661C>T), *777R (2664T>A 2665A>G)

	Begin	End	Coverage	Score	Concordance	Matches	Identities	I/D/M/F*	Stop Codons
**NT**	**428**	**2700**	**82.0%**	**1546**	**34.4%**	**2264 (99.6%)**	**1530 (67.3%)**	**1/9**	

Codon mutations: AAA34AAG (437A>G), ATC36CTT (441A>C 443C>T), CTT38TTT (447C>T), CTA42TTA (459C>T), GCC44GTC (466C>T), TCC45TCT (470C>T), GCT46ATG (471G>A 472C>T 473T>G), TTG48ATG (477T>A), ACA49CAT (480A>C 481C>A 482A>T), AAA50AAG (485A>G), ATC52GTC (489A>G), GCT53GCC (494T>C), CTT54CTA (497T>A), GCT55GCG (500T>G), GGA56GGC (503A>C), TGT57TGC (506T>C), GAC58GAT (509C>T), TTT59TTC (512T>C), GTA60ATA (513G>A), TCT61AAA (516T>A 517C>A 518T>A), AAA62ACA (520A>C), GTG63GTT (524G>T), ACT64GTG (525A>G 526C>T 527T>G), ACC65AAA (529C>A 530C>A), TTA66CTG (531T>C 533A>G), CGC67AAA (534C>A 535G>A 536C>A), CGG68CGA (539G>A), AAG69CGT (540A>C 541A>G 542G>T), TCG70TCA (545G>A), GGC71GGT (548C>T), TAT72TTT (550A>T), TTC74TAT (556T>A 557C>T), ACA75ACT (560A>T), TAC78TAT (569C>T), AAG80AAA (575G>A), TGC82TGT (581C>T), GCA83ACA (582G>A), GTA84GTT (587A>T), TGC85TCC (589G>C), TTA86CTT (591T>C 593A>T), CGA88CGT (599A>T), TAC89TAT (602C>T), TCC93AGT (612T>A 613C>G 614C>T), TTT94TAT (616T>A), CGA95TCT (618C>T 619G>C 620A>T), GAA97AAT (624G>A 626A>T), GAA98GAC (629A>C), CCA99ACC (630C>A 632A>C), CTT100ATG (633C>A 635T>G), TCG101TCT (638G>T), GTG102GTT (641G>T), CCT103CCA (644T>A), GTG104GTT (647G>T), CTC106TTG (651C>T 653C>G), ACA107ACC (656A>C), AGA108CGA (657A>C), TCG109TGT (661C>G 662G>T), GGT110GGA (665T>A), ATC111ATT (668C>T), CGT113AAG (672C>A 673G>A 674T>G), ATC115ATA (680C>A), CCC116CCT (683C>T), AAA117GCC (684A>G 685A>C 686A>C), TTG119TTA (692G>A), CGT120CGA (695T>A), GCA122CAC (699G>C 700C>A 701A>C), ATA123GTA (702A>G), CGT124AGG (705C>A 707T>G), AGG125GCA (708A>G 709G>C 710G>A), AAA126AAG (713A>G), GAT127TCT (714G>T 715A>C), GAA128GAT (719A>T), CAC129CAT (722C>T), GTT134GTC (737T>C), TAC137TAT (746C>T), TCA139TCT (752A>T), GGG142GGA (761G>A), GCT144TCA (765G>T 767T>A), AAG145AAA (770G>A), GTT146TAG (771G>T 772T>A 773T>G), TTG147ATT (774T>A 776G>T), AAA148GCG (777A>G 778A>C 779A>G), GTT149GTG (782T>G), AGT154AAG (796G>A 797T>G), GCA156GCC (803A>C), TTT158TTC (809T>C), GAA159CAA (810G>C), TCC160TCA (815C>A), ATA161ATT (818A>T), ACT162ACG (821T>G), ACC163ACT (824C>T), CCA164CCT (827A>T), CCT166TCA (831C>T 833T>A), GGG169GAT (841G>A 842G>T), CTA172CGT (850T>G 851A>T), GTG174GTT (857G>T), CTG175CTT (860G>T), GAA176GGA (862A>G), ATT178ATG (869T>G), ACG180ACT (875G>T), TTC182TGA (880T>G 881C>A), CAT184AAG (885C>A 887T>G), CAA186CGA (892A>G), CTA190CTT (905A>T), CCC191CCA (908C>A), TTT192CAT (909T>C 910T>A), CGA194CAA (916G>A), GGC195TCC (918G>T 919G>C), ATT196ATA (923T>A), CCA197CCT (926A>T), ATG198CTT (927A>C 929G>T), ATT200AAG (934T>A 935T>G), GGG201GGT (938G>T), TCT203AAT (942T>A 943C>A), CAA205AAA (948C>A), CCC212CCA (971C>A), CTA213TTA (972C>T), AAT214CTC (975A>C 976A>T 977T>C), AAT216AAC (983T>C), AGG219CGC (990A>C 992G>C), TCA220CGG (993T>C 994C>G 995A>G), TAC221TTT (997A>T 998C>T), GGT222CAA (999G>C 1000G>A 1001T>A), TTA223TAT (1003T>A 1004A>T), GCC224TCG (1005G>T 1007C>G), GTA225GTG (1010A>G), GAC227GAT (1016C>T), AAA228GTT (1017A>G 1018A>T 1019A>T), GTT229GTA (1022T>A), GAT230GAC (1025T>C), CAA231GGA (1026C>G 1027A>G), GAG232GCA (1030A>C 1031G>A), GCT233GCG (1034T>G), AGT234GCT (1035A>G 1036G>C), TTC237GTT (1044T>G 1046C>T), AAG238AAA (1049G>A), TTT239GAA (1050T>G 1051T>A 1052T>A), CCA240CAC (1054C>A 1055A>C), ATT242ATC (1061T>C), GTT244GTG (1067T>G), TTG246TTA (1073G>A), AAA247AAG (1076A>G), CTA250ATA (1083C>A), GCG251GCT (1088G>T), GCC252GCG (1091C>G), TGG255TTG (1099G>T), ACA257GTG (1104A>G 1105C>T 1106A>G), ATT260ATG (1115T>G), TCA262TCC (1121A>C), TTC263ATT (1122T>A 1124C>T), CAG264CAA (1127G>A), GAC265GAT (1130C>T), GGC266GGG (1133C>G), TCA269TCC (1142A>C), CCC270CCT (1145C>T), GGG271GGT (1148G>T), ATG272ATT (1151G>T), TTG273CTT (1152T>C 1154G>T), TCC275TCT (1160C>T), AAA276CAA (1161A>C), TCT277ATG (1164T>A 1165C>T 1166T>G), GTC278GTT (1169C>T), TTC279TTA (1172C>A), TTT280TAC (1174T>A 1175T>C), CCT281CCA (1178T>A), TTA282TTT (1181A>T), GAC283GAT (1184C>T), AGG284TCT (1185A>T 1186G>C 1187G>T), GCT285AAT (1188G>A 1189C>A), GCA286AAT (1191G>A 1192C>A 1193A>T), ACT287ACC (1196T>C), GAA288del (1197_1199delGAA), TTA290TTT (1205A>T), AAC292AAT (1211C>T), TTA293GAA (1212T>G 1213T>A), TCT294TCC (1217T>C), CTC295CTT (1220C>T), GAA296GAT (1223A>T), CAC297TTA (1224C>T 1225A>T 1226C>A), GAG299GAA (1232G>A), AGT301CAA (1236A>C 1237G>A 1238T>A), GTC302GTA (1241C>A), CCA304CCC (1247A>C), TGG305CTT (1248T>C 1249G>T 1250G>T), TTT306TTC (1253T>C), TCA307TCT (1256A>T), ACC308AGC (1258C>G), TTG310TTA (1265G>A), AGT311GGG (1266A>G 1268T>G), CCT312GCC (1269C>G 1271T>C), TAT313TAC (1274T>C), CGA314CAA (1276G>A), GGG315GGA (1280G>A), GTG316GTT (1283G>T), GTT319GTG (1292T>G), AGG322AGA (1301G>A), CTT323TTG (1302C>T 1304T>G), GGT324GCT (1306G>C), GCA325CAG (1308G>C 1309C>A 1310A>G), GTG326GCC (1312T>C 1313G>C), ATA327ATT (1316A>T), GAG328GAA (1319G>A), GGC329GGT (1322C>T), GGA331GGG (1328A>G), AGA333CGG (1332A>C 1334A>G), GTA335ATC (1338G>A 1340A>C), GTA337GCT (1345T>C 1346A>T), ATG338ATA (1349G>A), AAC340AAT (1355C>T), TAC341TCT (1357A>C 1358C>T), CTC347CTG (1376C>G), CAC348CAT (1379C>T), CCT349CCC (1382T>C), GTT350GTC (1385T>C), CAC351CAT (1388C>T), GAT352ATT (1389G>A 1390A>T), ACA356ACT (1403A>T), GTC357GTA (1406C>A), CTG358CTA (1409G>A), TCT359TCA (1412T>A), AAA360AGT (1414A>G 1415A>T), ATA361CTT (1416A>C 1418A>T), ACA363ACT (1424A>T), GAC364GAT (1427C>T), GGG365GGT (1430G>T), ACT366ACA (1433T>A), GAC368AAC (1437G>A), GAG370GAA (1445G>A), AAA371CGT (1446A>C 1447A>G 1448A>T), CCT372CCG (1451T>G), TTG374CAG (1455T>C 1456T>A), GCT375TAT (1458G>T 1459C>A), TTA376CTA (1461T>C), AAG377AGA (1465A>G 1466G>A), CAA378CTT (1468A>T 1469A>T), AAG379AAA (1472G>A), AAC380AGA (1474A>G 1475C>A), TTT381CCG (1476T>C 1477T>C 1478T>G), AAA382AAG (1481A>G), AAT383TCT (1482A>T 1483A>C), TAT385TAC (1490T>C), TTA389TTC (1502A>C), AAG390AAA (1505G>A), ACG393ACA (1514G>A), GAC394AAC (1515G>A), CCG397CCA (1526G>A), GTC400GTT (1535C>T), ATG401ATA (1538G>A), TAT402TAC (1541T>C), CTT404TTA (1545C>T 1547T>A), ATG405ATC (1550G>C), TCT406GAA (1551T>G 1552C>A 1553T>A), ATA408ATT (1559A>T), GGG410GGT (1565G>T), CCT411AGT (1566C>A 1567C>G), ACC412ACT (1571C>T), TTG413TTA (1574G>A), TCC415TCT (1580C>T), GTT418GTC (1589T>C), AGC420del (1593_1597delAGCTC), TCT421--A (1593_1597delAGCTC 1598T>A), ACG426ACT (1613G>T), ATC427TTT (1614A>T 1616C>T), CTT428CTC (1619T>C), GTT429GTA (1622T>A), AAC430TCA (1623A>T 1624A>C 1625C>A), AAA431CCT (1626A>C 1627A>C 1628A>T), CCG432CCT (1631G>T), TTA433ATG (1632T>A 1634A>G), ACA434GTG (1635A>G 1636C>T 1637A>G), AGA436AAG (1642G>A 1643A>G), GTG437ATT (1644G>A 1646G>T), TAT438AGT (1647T>A 1648A>G), GAG439GAA (1652G>A), ATA440GTA (1653A>G), CTG443CTT (1664G>T), GGG445GGA (1670G>A), CAG446CAA (1673G>A), CCT447CCG (1676T>G), TTG448TTA (1679G>A), GGC449GGT (1682C>T), TTC450TAC (1684T>A), CAC451TAC (1686C>T), TCT453TCA (1694T>A), GCA455TCC (1698G>T 1700A>C), CTT456CTA (1703T>A), TTC457TTT (1706C>T), TCG458GCG (1707T>G), TTG459CTT (1710T>C 1712G>T), TCT460TCC (1715T>C), CAC462CAT (1721C>T), ATA464ATG (1727A>G), TTA467CTA (1734T>C), GCA468GCT (1739A>T), AGC470AAA (1744G>A 1745C>A), AAA471TAT (1746A>T 1748A>T), TAT473TAC (1754T>C), GGA475ACG (1758G>A 1759G>C 1760A>G), TGT476AGT (1761T>A), AAC477ACT (1765A>C 1766C>T), TCT478ACC (1767T>A 1769T>C), CCA479CCT (1772A>T), GGG481GCT (1777G>C 1778G>T), GCT484GCC (1787T>C), TTA485CTG (1788T>C 1790A>G), CTA486TTA (1791C>T), AGT495ACA (1819G>C 1820T>A), AAA496GAG (1821A>G 1823A>G), GCC498GCG (1829C>G), AAT499CGG (1830A>C 1831A>G 1832T>G), GAG500CAG (1833G>C), AAG502AGG (1840A>G), TTA504TTG (1847A>G), TTG505CTG (1848T>C), AGA507AGG (1856A>G), CTC508CTT (1859C>T), AAT509GGT (1860A>G 1861A>G), GTC510GTT (1865C>T), AAA511ACA (1867A>C), ATT512ATC (1871T>C), TCG513TCT (1874G>T), ATA514TTA (1875A>T), CCA515GCT (1878C>G 1880A>T), TCT517TCG (1886T>G), TTG518AGA (1887T>A 1888T>G 1889G>A), TCT520TCC (1895T>C), GAT521GAG (1898T>G), AAT522AAC (1901T>C), ATT525ATC (1910T>C), GCG528GCT (1919G>T), AGG530AGA (1925G>A), ACT533ACC (1934T>C), AAA534AAG (1937A>G), TCA535GAC (1938T>G 1939C>A 1940A>C), GAT539TAC (1950G>T 1952T>C), TTA540CTT (1953T>C 1955A>T), ATT543ATC (1964T>C), TCC544TCT (1967C>T), GCT547GCA (1976T>A), TTA549ACT (1980T>A 1981T>C 1982A>T), TCT550TCC (1985T>C), CGT552AGG (1989C>A 1991T>G), ACT553ACA (1994T>A), GTT555GTG (2000T>G), GGA556GGT (2003A>T), CTT557CTG (2006T>G), TGC558TGT (2009C>T), GCC559CAA (2010G>C 2011C>A 2012C>A), ATC562ACC (2020T>C), GAT565TCG (2028G>T 2029A>C 2030T>G), AAC567TCA (2034A>T 2035A>C 2036C>A), ATA568ACA (2038T>C), TCA569TCG (2042A>G), GTT570ATT (2043G>A), CAA572CCC (2050A>C 2051A>C), AGA573ATT (2053G>T 2054A>T), TTG574TTT (2057G>T), GGG575TGG (2058G>T), GGT576GGG (2063T>G), GGG578GGA (2069G>A), TAC579TTC (2071A>T), AAG580AGA (2074A>G 2075G>A), GTA581GTT (2078A>T), GCG583TCT (2082G>T 2084G>T), CGC584CGT (2087C>T), TTA585TTG (2090A>G), TTC586ATG (2091T>A 2093C>G), ACT587TCT (2094A>T), ACC588ACT (2099C>T), AGC590TCT (2103A>T 2104G>C 2105C>T), AAG591AAA (2108G>A), GAG594GAA (2117G>A), CGT595AGA (2118C>A 2120T>A), CTA596CTG (2123A>G), GCT598GCG (2129T>G), CAT600GCA (2133C>G 2134A>C 2135T>A), CTT601AGC (2136C>A 2137T>G 2138T>C), AAG602AAA (2141G>A), GTT604CAC (2145G>C 2146T>A 2147T>C), CGG605AGG (2148C>A), CAC607CAT (2156C>T), TTA608CTA (2157T>C), CTA609TTA (2160C>T), TAC611CTC (2166T>C 2167A>T), TGG613TAT (2173G>A 2174G>T), GGA616GGG (2183A>G), AGG617AGA (2186G>A), GGT618TTT (2187G>T 2188G>T), ATG619AAA (2191T>A 2192G>A), CCC620CCT (2195C>T), CCC623CCT (2204C>T), TAT624TTT (2206A>T), TAT624_TTG625insT-- (2207_2208insT), AAA626AAG (2213A>G), GGG627GGA (2216G>A), GTA630ATA (2223G>A), AGT631GAT (2226A>G 2227G>A), TAC632TAT (2231C>T), CTT634CGG (2236T>G 2237T>G), AAA635AAG (2240A>G), AAA638ACA (2248A>C), GTT639CCT (2250G>C 2251T>C), TTA642ATA (2259T>A), CAG643CGA (2263A>G 2264G>A), ATC644CTC (2265A>C), AAG647AAT (2276G>T), GAT648GAG (2279T>G), CTG649TTG (2280C>T), TTC651TTT (2288C>T), GAA654GAG (2297A>G), GTT655AGA (2298G>A 2299T>G 2300T>A), ATA657ATC (2306A>C), CTA658CTT (2309A>T), CGT660TGC (2313C>T 2315T>C), ACT661ATT (2317C>T), CTA663TTA (2322C>T), CGC664GTC (2325C>G 2326G>T), CAA665AAT (2328C>A 2330A>T), GTA667GTT (2336A>T), GAA668AAA (2337G>A), TTA671CTT (2346T>C 2348A>T), AAG672CGG (2349A>C 2350A>G), CTC674GTT (2355C>G 2357C>T), AAC675TCT (2358A>T 2359A>C 2360C>T), TGG676TAG (2362G>A), CAT678CAC (2369T>C), ACG679ACT (2372G>T), TGT681TTG (2377G>T 2378T>G), AAC683TCT (2382A>T 2383A>C 2384C>T), CCA684TTT (2385C>T 2386C>T 2387A>T), GAT685GAA (2390T>A), TCC687TCT (2396C>T), ATT688ATC (2399T>C), AAG689GAA (2400A>G 2402G>A), AGT690AAA (2404G>A 2405T>A), CTC691TTG (2406C>T 2408C>G), ATC692ATG (2411C>G), GAC693GAT (2414C>T), TCC694GCT (2415T>G 2417C>T), CCT695CCC (2420T>C), ATA696ATT (2423A>T), TGC697TAC (2425G>A), ACA699TAG (2430A>T 2431C>A 2432A>G), TCC700TCA (2435C>A), AAA702AAG (2441A>G), AGG703CGA (2442A>C 2444G>A), CTC704TCG (2445C>T 2446T>C 2447C>G), GAA705AAT (2448G>A 2450A>T), ACT706GAA (2451A>G 2452C>A 2453T>A), GAT707GAC (2456T>C), TTT708CTG (2457T>C 2459T>G), GAT709GAA (2462T>A), TTA710TTG (2465A>G), ATC711GTC (2466A>G), GGC714GGT (2477C>T), CTA715TTA (2478C>T), AAA718AAG (2489A>G), TGT719TGC (2492T>C), TAC720TAT (2495C>T), GGT723GGA (2504T>A), GGT725GGC (2510T>C), AAT727GAT (2514A>G), CCC728CTG (2518C>T 2519C>G), ACC730ATT (2524C>T 2525C>T), ACT731ACA (2528T>A), CAG733AGG (2532C>A 2533A>G), TGG734TAT (2536G>A 2537G>T), TTT736TTG (2543T>G), AAC739AGT (2551A>G 2552C>T), ACT740ACC (2555T>C), GTT741TCT (2556G>T 2557T>C), ATT742ATC (2561T>C), AAT745GAT (2568A>G), AAA746CGA (2571A>C 2572A>G), TTA749TTT (2582A>T), GGG750GGA (2585G>A), GGG754GGA (2597G>A), AAC755GCT (2598A>G 2599A>C 2600C>T), GAT756AAT (2601G>A), ATT758ATA (2609T>A), TCT760GCA (2613T>G 2615T>A), CCT761CCA (2618T>A), GAA763TAT (2622G>T 2624A>T), TAC764TTA (2626A>T 2627C>A), CAA765CCA (2629A>C), ACT766TCT (2631A>T), ACA767CTA (2634A>C 2635C>T), GGA768GTG (2638G>T 2639A>G), GAG770GGG (2644A>G), GAA771TGT (2646G>T 2647A>G 2648A>T), GTG772AGG (2649G>A 2650T>G), GGC773TCA (2652G>T 2653G>C 2654C>A), TTA774TAT (2656T>A 2657A>T), AGA775GAC (2658A>G 2659G>A 2660A>C), CTT776TTT (2661C>T), TAG777AGG (2664T>A 2665A>G)

*: Inserts / Deletes / Misaligned / Frameshifts

Analysis details

This analysis was performed with panviral2.64

NGS Details (UN59): Solendovirus venanicotianae

Assembly

**Coverage Length**	2286 (5 contig(s))
**Depth Of Coverage**	14.6
**Number Of Reads**	285
**Reads Per Million**	6.20 rpm (after QC)
**Ambiguities**	0
**Assembly Method**	de novo + reference guided assembly
**Consensus Caller**	Bcf Tools

Coverage Map

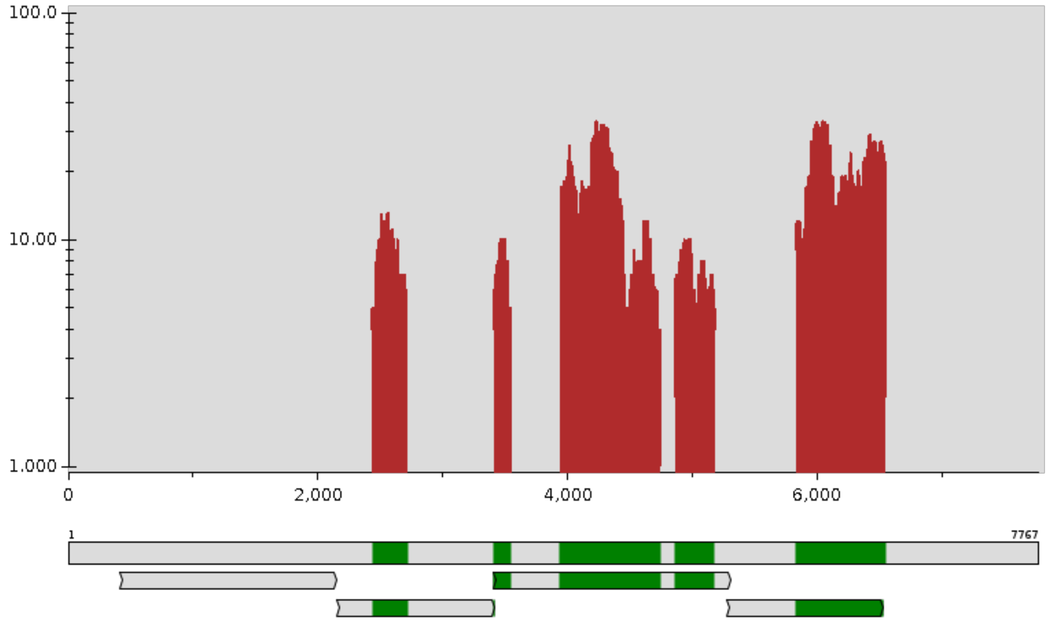

Assignment

**Type**	Solendovirus venanicotianae (Taxonomy ID: 3048371)
**Reference Genome**	NC_003378.1
**NT Identity (%)**	79.8494
**AA Identity (%)**	76.8097
**Number Of Stop Codons**	4
**Number Of CDS**	4

Alignment

**Alignment Score**	2623.0 (NT) + 3754.0 (AA) = 6377.0
**Concordance (%)**	68.6585

Alignment Method	Global, seeded, nucleotide + amino acids (AGA)

Genome Region

Sequence starts at position 2433 and ends at position 6550 relative to NC_003378.1 reference sequence.

Alignment Detailed Statistics

	Begin	End	Coverage	Score	Concordance	Matches	Identities	I/D/M/F*	Stop Codons
**NT**	**2433**	**6550**	**29.4%**	**2623**	**59.0%**	**2257 (98.7%)**	**1803 (78.8%)**	**1/29**	
Mutations:	2442A>C, 2443C>A, 2446G>A, 2448T>G, 2454G>A, 2455G>A, 2456C>T, 2457A>G, 2463C>A, 2464C>G, 2465C>A, 2472G>A, 2482T>C, 2493T>A, 2511A>T, 2514T>C, 2535C>A, 2551C>T, 2571C>A, 2590A>G, 2598T>C, 2602A>G, 2604A>T, 2622T>C, 2628G>A, 2637T>C, 2643A>G, 2646C>T, 2648C>T, 2652C>T, 2665C>A, 2668A>G, 2670T>A, 2676A>T, 2687A>G, 2692T>C, 2709G>A, 2717G>A, 3406G>A, 3409G>T, 3410A>T, 3419A>T, 3422C>T, 3431A>C, 3434A>G, 3437C>A, 3443A>T, 3452T>C, 3456A>G, 3464C>T, 3467T>C, 3470A>T, 3500T>C, 3506T>C, 3518T>C, 3525A>G, 3526C>A, 3530T>A, 3536G>A, 3537G>A, 3547A>C, 3550C>T, 3936T>C, 3942A>G, 3952A>G, 3956T>A, 3957A>G, 3962T>A, 3963C>T, 3966C>A, 3977G>T, 3989A>T, 3991A>C, 4010T>A, 4011G>A, 4013A>T, 4014G>A, 4016G>A, 4017A>G, 4025A>T, 4028A>C, 4040T>C, 4041C>T, 4045A>G, 4046A>T, 4049T>C, 4051A>G, 4052C>T, 4056A>G, 4061T>C, 4062T>C, 4063T>A, 4066C>A, 4070A>G, 4073T>C, 4079A>G, 4086A>G, 4088A>T, 4091T>A, 4097G>A, 4100T>A, 4103T>A, 4104A>G, 4105A>G, 4115A>G, 4116G>A, 4121T>C, 4122A>G, 4124T>A, 4125A>G, 4127C>T, 4133T>C, 4161C>T, 4181A>T, 4190G>A, 4196C>T, 4199A>T, 4202C>T, 4212T>C, 4241T>C, 4250C>A, 4253T>C, 4265G>A, 4275A>G, 4286T>C, 4292C>T, 4298T>C, 4307C>T, 4313G>A, 4325T>C, 4328T>C, 4329T>C, 4331G>A, 4334A>G, 4340G>A, 4343T>A, 4355A>G, 4362G>A, 4364T>A, 4388T>C, 4400T>A, 4406A>T, 4409T>C, 4415T>C, 4421T>C, 4423C>T, 4436C>T, 4442T>C, 4448G>T, 4449G>A, 4451C>T, 4454C>T, 4457T>C, 4463T>C, 4475T>C, 4484A>T, 4487T>C, 4493T>C, 4500C>T, 4503C>T, 4508T>C, 4513G>A, 4540T>C, 4545A>C, 4550C>T, 4551G>A, 4552C>T, 4554C>A, 4559T>A, 4560A>G, 4568C>A, 4571T>A, 4574T>A, 4581T>C, 4591C>A, 4592C>G, 4598A>T, 4601A>G, 4604T>A, 4605A>T, 4607G>T, 4626T>C, 4643T>C, 4653A>G, 4660T>C, 4665A>C, 4666C>A, 4667T>A, 4679A>G, 4687T>A, 4691T>C, 4692C>T, 4693T>C, 4694T>A, 4697T>A, 4701A>G, 4703C>A, 4704A>T, 4706T>A, 4721A>G, 4722T>C, 4725C>T, 4734C>T, 4740A>T, 4868G>A, 4869A>T, 4872T>C, 4874G>T, 4897A>G, 4898C>T, 4907T>C, 4913C>T, 4919A>T, 4924C>T, 4925C>A, 4931T>A, 4938A>G, 4944T>G, 4946A>G, 4950A>G, 4953T>C, 4955T>A, 4970T>A, 4971C>T, 4973T>A, 4976G>A, 4979T>C, 4982A>G, 4986A>G, 4988T>A, 5011G>A, 5012C>T, 5018C>T, 5021T>C, 5027A>T, 5028T>A, 5030T>A, 5032A>T, 5033T>C, 5034A>T, 5037G>A, 5039A>C, 5040C>T, 5043C>G, 5046G>A, 5047A>T, 5053G>A, 5055G>A, 5076T>C, 5077T>A, 5079G>T, 5094C>T, 5117A>T, 5120T>G, 5123C>T, 5125C>A, 5128G>A, 5135T>A, 5136G>A, 5147C>T, 5150T>C, 5156A>G, 5174T>A, 5180A>G, 5833G>C, 5841T>C, 5844T>A, 5850C>A, 5851C>A, 5853T>A, 5857A>C, 5858C>A, 5859A>G, 5865C>T, 5868T>C, 5872T>C, 5874G>T, 5877A>T, 5880T>A, 5881T>C, 5882T>A, 5885C>T, 5886A>C, 5889T>A, 5892A>T, 5894G>A, 5897C>T, 5898A>C, 5901A>T, 5902T>C, 5907A>T, 5908G>A, 5909C>G, 5910C>A, 5913C>A, 5916C>T, 5921A>G, 5922C>T, 5925C>T, 5935G>A, 5936A>G, 5940T>C, 5941G>A, 5950C>T, 5952G>C, 5953A>G, 5954C>A, 5955A>T, 5959C>G, 5964G>A, 5968G>T, 5970C>A, 5972A>C, 5979T>A, 5991G>A, 5997C>T, 5999T>C, 6000G>A, 6001C>A, 6002A>C, 6009G>A, 6012G>A, 6015A>T, 6018T>A, 6024G>C, 6026C>A, 6027A>C, 6033C>T, 6036C>T, 6037G>A, 6040C>A, 6041G>A, 6042A>G, 6045C>T, 6049T>A, 6051G>A, 6054T>A, 6055A>C, 6063G>T, 6067C>T, 6071A>T, 6075A>T, 6078G>A, 6081A>T, 6084G>A, 6087T>A, 6088A>G, 6090C>G, 6105A>G, 6108T>A, 6114C>A, 6120G>A, 6121G>A, 6123A>T, 6127A>T, 6129C>A, 6139A>G, 6141G>C, 6148G>C, 6154G>C, 6155A>C, 6163G>C, 6164A>C, 6169G>A, 6171A>T, 6172A>G, 6174T>G, 6177T>A, 6181G>A, 6182A>G, 6183A>T, 6186C>T, 6188A>G, 6189T>C, 6201G>A, 6204A>T, 6211A>C, 6219T>G, 6223C>A, 6225A>C, 6232C>T, 6246T>C, 6247C>A, 6248T>C, 6253C>G, 6254A>G, 6258T>C, 6259G>C, 6264T>G, 6269G>A, 6270C>T, 6271T>C, 6277_6283delTCAAGGG, 6285A>C, 6290C>T, 6298_6315delTATTCAAATTGCAGAGAT, 6318A>C, 6319_6321delAGA, 6325G>A, 6326C>G, 6340C>G, 6341T>C, 6344G>T, 6345A>G, 6352G>A, 6354C>A, 6355T>A, 6357A>G, 6359G>C, 6360T>A, 6361C>T, 6363T>G, 6369A>T, 6375A>G, 6376C>A, 6380C>A, 6381T>A, 6382A>C, 6384A>T, 6386C>T, 6387C>T, 6401G>A, 6402G>A, 6404G>A, 6406delA, 6407A>G, 6408T>A, 6411C>T, 6414C>T, 6417C>G, 6418C>G, 6419C>A, 6420A>T, 6423T>A, 6424C>T, 6426G>A, 6427T>C, 6446T>A, 6451A>G, 6453T>G, 6456C>A, 6462T>C, 6471C>T, 6477C>T, 6480A>G, 6489A>C, 6502A>G, 6504T>A, 6505G>A, 6507T>C, 6513C>T, 6522G>A, 6525A>C, 6526T>G, 6530T>A, 6531C>A, 6532_6533insA, 6537G>T, 6542A>T								
**CDS**									
Tvvgp2	94	420	23.6%	549	83.6%	99 (100%)	82 (82.8%)	0/0/0/0	1
Protein mutations:	Q97K (2443C>A), D98K (2446G>A 2448T>G), A101M (2455G>A 2456C>T 2457A>G), N103K (2463C>A), P104E (2464C>G 2465C>A), I146V (2590A>G), I150V (2602A>G 2604A>T), I163M (2643A>G), A165V (2648C>T), L171I (2665C>A), N172E (2668A>G 2670T>A), R174S (2676A>T), K178R (2687A>G), G188E (2717G>A), E418K (3406G>A), E419L (3409G>T 3410A>T)								
Codon mutations:	ATA96ATC (2442A>C), CAA97AAA (2443C>A), GAT98AAG (2446G>A 2448T>G), AAG100AAA (2454G>A), GCA101ATG (2455G>A 2456C>T 2457A>G), AAC103AAA (2463C>A), CCA104GAA (2464C>G 2465C>A), AAG106AAA (2472G>A), TTA110CTA (2482T>C), ACT113ACA (2493T>A), GCA119GCT (2511A>T), TGT120TGC (2514T>C), ACC127ACA (2535C>A), CTA133TTA (2551C>T), ATC139ATA (2571C>A), ATA146GTA (2590A>G), AGT148AGC (2598T>C), ATA150GTT (2602A>G 2604A>T), TAT156TAC (2622T>C), AAG158AAA (2628G>A), TTT161TTC (2637T>C), ATA163ATG (2643A>G), AGC164AGT (2646C>T), GCC165GTC (2648C>T), AAC166AAT (2652C>T), CTA171ATA (2665C>A), AAT172GAA (2668A>G 2670T>A), AGA174AGT (2676A>T), AAA178AGA (2687A>G), TTA180CTA (2692T>C), AAG185AAA (2709G>A), GGA188GAA (2717G>A), GAA418AAA (3406G>A), GAA419TTA (3409G>T 3410A>T)								
Tvvgp3	1	591	66.8%	2399	80.4%	425 (100%)	342 (80.5%)	0/0/0/0	3
Protein mutations:	N16D (3456A>G), T39E (3525A>G 3526C>A), D40E (3530T>A), W42* (3536G>A), E43K (3537G>A), K46T (3547A>C), T47I (3550C>T), K178E (3942A>G), N181S (3952A>G), D182E (3956T>A), N183D (3957A>G), Q186K (3966C>A), E189D (3977G>T), K194T (3991A>C), E201N (4011G>A 4013A>T), E202K (4014G>A 4016G>A), N203D (4017A>G), K212S (4045A>G 4046A>T), N214S (4051A>G 4052C>T), N216D (4056A>G), L218Q (4062T>C 4063T>A), T219K (4066C>A), I223M (4079A>G), K226D (4086A>G 4088A>T), D227E (4091T>A), D230E (4100T>A), N231K (4103T>A), K232G (4104A>G 4105A>G), E236K (4116G>A), N238E (4122A>G 4124T>A), S239G (4125A>G 4127C>T), H251Y (4161C>T), I289V (4275A>G), D311E (4343T>A), A318T (4362G>A 4364T>A), A338V (4423C>T), M346I (4448G>T), D347N (4449G>A 4451C>T), R368K (4513G>A), L377S (4540T>C), K379Q (4545A>C), A381I (4551G>A 4552C>T), H382N (4554C>A), I384V (4560A>G), N386K (4568C>A), T394K (4591C>A 4592C>G), M399F (4605A>T 4607G>T), I415V (4653A>G), M417T (4660T>C), T419Q (4665A>C 4666C>A 4667T>A), I426N (4687T>A), L428S (4692C>T 4693T>C 4694T>A), D429E (4697T>A), N431E (4701A>G 4703C>A), I432L (4704A>T 4706T>A), Q439* (4725C>T), I444L (4740A>T), I487L (4869A>T), Y496C (4897A>G 4898C>T), K503N (4919A>T), T505I (4924C>T 4925C>A), T510A (4938A>G), S512A (4944T>G 4946A>G), N514D (4950A>G), Y515Q (4953T>C 4955T>A), N526E (4986A>G 4988T>A), C534Y (5011G>A 5012C>T), S540T (5028T>A 5030T>A), Y541F (5032A>T 5033T>C), T542S (5034A>T), E543N (5037G>A 5039A>C), P544S (5040C>T), Q545E (5043C>G), E546I (5046G>A 5047A>T), W548* (5053G>A), E549K (5055G>A), F556H (5076T>C 5077T>A), A557S (5079G>T), T572N (5125C>A), R573K (5128G>A), V576I (5136G>A)								
Codon mutations:	ATA3ATT (3419A>T), TAC4TAT (3422C>T), GCA7GCC (3431A>C), AAA8AAG (3434A>G), ATC9ATA (3437C>A), GTA11GTT (3443A>T), TAT14TAC (3452T>C), AAT16GAT (3456A>G), TAC18TAT (3464C>T), TAT19TAC (3467T>C), ACA20ACT (3470A>T), AAT30AAC (3500T>C), TGT32TGC (3506T>C), TGT36TGC (3518T>C), ACA39GAA (3525A>G 3526C>A), GAT40GAA (3530T>A), TGG42TGA (3536G>A), GAA43AAA (3537G>A), AAA46ACA (3547A>C), ACA47ATA (3550C>T), TTA176CTA (3936T>C), AAA178GAA (3942A>G), AAT181AGT (3952A>G), GAT182GAA (3956T>A), AAC183GAC (3957A>G), CCT184CCA (3962T>A), CTA185TTA (3963C>T), CAA186AAA (3966C>A), GAG189GAT (3977G>T), ACA193ACT (3989A>T), AAA194ACA (3991A>C), ATT200ATA (4010T>A), GAA201AAT (4011G>A 4013A>T), GAG202AAA (4014G>A 4016G>A), AAT203GAT (4017A>G), ATA205ATT (4025A>T), ATA206ATC (4028A>C), CCT210CCC (4040T>C), CTA211TTA (4041C>T), AAA212AGT (4045A>G 4046A>T), TAT213TAC (4049T>C), AAC214AGT (4051A>G 4052C>T), AAT216GAT (4056A>G), GAT217GAC (4061T>C), TTA218CAA (4062T>C 4063T>A), ACA219AAA (4066C>A), GAA220GAG (4070A>G), TTT221TTC (4073T>C), ATA223ATG (4079A>G), AAA226GAT (4086A>G 4088A>T), GAT227GAA (4091T>A), TTG229TTA (4097G>A), GAT230GAA (4100T>A), AAT231AAA (4103T>A), AAA232GGA (4104A>G 4105A>G), CAA235CAG (4115A>G), GAA236AAA (4116G>A), AGT237AGC (4121T>C), AAT238GAA (4122A>G 4124T>A), AGC239GGT (4125A>G 4127C>T), CAT241CAC (4133T>C), CAT251TAT (4161C>T), GGA257GGT (4181A>T), AGG260AGA (4190G>A), GTC262GTT (4196C>T), ATA263ATT (4199A>T), GAC264GAT (4202C>T), TTA268CTA (4212T>C), TAT277TAC (4241T>C), CCC280CCA (4250C>A), AAT281AAC (4253T>C), AAG285AAA (4265G>A), ATA289GTA (4275A>G), TAT292TAC (4286T>C), TAC294TAT (4292C>T), AGT296AGC (4298T>C), GAC299GAT (4307C>T), AAG301AAA (4313G>A), TAT305TAC (4325T>C), CAT306CAC (4328T>C), TTG307CTA (4329T>C 4331G>A), AAA308AAG (4334A>G), GAG310GAA (4340G>A), GAT311GAA (4343T>A), AAA315AAG (4355A>G), GCT318ACA (4362G>A 4364T>A), TAT326TAC (4388T>C), GTT330GTA (4400T>A), CCA332CCT (4406A>T), TTT333TTC (4409T>C), TAT335TAC (4415T>C), AAT337AAC (4421T>C), GCA338GTA (4423C>T), TAC342TAT (4436C>T), CAT344CAC (4442T>C), ATG346ATT (4448G>T), GAC347AAT (4449G>A 4451C>T), AAC348AAT (4454C>T), TAT349TAC (4457T>C), AAT351AAC (4463T>C), AAT355AAC (4475T>C), GTA358GTT (4484A>T), TAT359TAC (4487T>C), GAT361GAC (4493T>C), CTA364TTA (4500C>T), CTA365TTA (4503C>T), TAT366TAC (4508T>C), AGA368AAA (4513G>A), TTA377TCA (4540T>C), AAA379CAA (4545A>C), TTC380TTT (4550C>T), GCA381ATA (4551G>A 4552C>T), CAT382AAT (4554C>A), ATT383ATA (4559T>A), ATA384GTA (4560A>G), AAC386AAA (4568C>A), TCT387TCA (4571T>A), GGT388GGA (4574T>A), TTA391CTA (4581T>C), ACC394AAG (4591C>A 4592C>G), GCA396GCT (4598A>T), GAA397GAG (4601A>G), ATT398ATA (4604T>A), ATG399TTT (4605A>T 4607G>T), TTA406CTA (4626T>C), GAT411GAC (4643T>C), ATA415GTA (4653A>G), ATG417ACG (4660T>C), ACT419CAA (4665A>C 4666C>A 4667T>A), CAA423CAG (4679A>G), ATC426AAC (4687T>A), AAT427AAC (4691T>C), CTT428TCA (4692C>T 4693T>C 4694T>A), GAT429GAA (4697T>A), AAC431GAA (4701A>G 4703C>A), ATT432TTA (4704A>T 4706T>A), AAA437AAG (4721A>G), TTA438CTA (4722T>C), CAA439TAA (4725C>T), CTA442TTA (4734C>T), ATA444TTA (4740A>T), AAG486AAA (4868G>A), ATA487TTA (4869A>T), TTG488CTT (4872T>C 4874G>T), TAC496TGT (4897A>G 4898C>T), GAT499GAC (4907T>C), AAC501AAT (4913C>T), AAA503AAT (4919A>T), ACC505ATA (4924C>T 4925C>A), ATT507ATA (4931T>A), ACA510GCA (4938A>G), TCA512GCG (4944T>G 4946A>G), AAT514GAT (4950A>G), TAT515CAA (4953T>C 4955T>A), GTT520GTA (4970T>A), CTT521TTA (4971C>T 4973T>A), AAG522AAA (4976G>A), TAT523TAC (4979T>C), AGA524AGG (4982A>G), AAT526GAA (4986A>G 4988T>A), TGC534TAT (5011G>A 5012C>T), TAC536TAT (5018C>T), TAT537TAC (5021T>C), GGA539GGT (5027A>T), TCT540ACA (5028T>A 5030T>A), TAT541TTC (5032A>T 5033T>C), ACA542TCA (5034A>T), GAA543AAC (5037G>A 5039A>C), CCA544TCA (5040C>T), CAA545GAA (5043C>G), GAA546ATA (5046G>A 5047A>T), TGG548TAG (5053G>A), GAA549AAA (5055G>A), TTT556CAT (5076T>C 5077T>A), GCA557TCA (5079G>T), CTA562TTA (5094C>T), ATA569ATT (5117A>T), GTT570GTG (5120T>G), TAC571TAT (5123C>T), ACC572AAC (5125C>A), AGA573AAA (5128G>A), ATT575ATA (5135T>A), GTA576ATA (5136G>A), GAC579GAT (5147C>T), AAT580AAC (5150T>C), CAA582CAG (5156A>G), ACT588ACA (5174T>A), AAA590AAG (5180A>G)								
Tvvgp4	187	418	55.3%	806	54.0%	222 (96.1%)	149 (64.5%)	0/9/2/2	0

	Begin	End	Coverage	Score	Concordance	Matches	Identities	I/D/M/F*	Stop Codons
**NT**	**2433**	**6550**	**29.4%**	**2623**	**59.0%**	**2257 (98.7%)**	**1803 (78.8%)**	**1/29**	
Protein mutations:	E187Q (5833G>C), H192Q (5850C>A), H193K (5851C>A 5853T>A), T195Q (5857A>C 5858C>A 5859A>G), L203Q (5881T>C 5882T>A), P204L (5885C>T 5886A>C), N205K (5889T>A), S207N (5894G>A), A208V (5897C>T 5898A>C), K209N (5901A>T), A212R (5908G>A 5909C>G 5910C>A), N216S (5921A>G 5922C>T), D221S (5935G>A 5936A>G), V223I (5941G>A), Q226Y (5950C>T 5952G>C), T227D (5953A>G 5954C>A 5955A>T), Q229E (5959C>G), A232S (5968G>T 5970C>A), N233T (5972A>C), M242T (5999T>C 6000G>A), Q243T (6001C>A 6002A>C), T251N (6026C>A 6027A>C), V255I (6037G>A), R256K (6040C>A 6041G>A 6042A>G), S259T (6049T>A 6051G>A), T261P (6055A>C), E263D (6063G>T), Y266F (6071A>T), E267D (6075A>T), I272V (6088A>G 6090C>G), E283N (6121G>A 6123A>T), I285L (6127A>T 6129C>A), K289D (6139A>G 6141G>C), E292Q (6148G>C), E294P (6154G>C 6155A>C), E297P (6163G>C 6164A>C), V299I (6169G>A 6171A>T), N300E (6172A>G 6174T>G), E303S (6181G>A 6182A>G 6183A>T), Y305C (6188A>G 6189T>C), I313L (6211A>C), L317I (6223C>A 6225A>C), L325T (6247C>A 6248T>C), Q327G (6253C>G 6254A>G), A329P (6259G>C), I330M (6264T>G), S332N (6269G>A 6270C>T), Y333H (6271T>C), S335_R336del (6277_6283delTCAAGGG), T339I (6290C>T), Y342_D347del (6298_6315delTATTCAAATTGCAGAGAT), R349del (6319_6321delAGA), A351R (6325G>A 6326C>G), L356A (6340C>G 6341T>C), R357M (6344G>T 6345A>G), V360I (6352G>A 6354C>A), L361M (6355T>A 6357A>G), S362T (6359G>C 6360T>A), K365N (6369A>T), Q368K (6376C>A), P369Q (6380C>A 6381T>A), T370P (6382A>C 6384A>T), T371I (6386C>T 6387C>T), R376K (6401G>A 6402G>A), R377K (6404G>A), P382D (6418C>G 6419C>A 6420A>T), D383E (6423T>A), L391Q (6446T>A), S393G (6451A>G 6453T>G), H394Q (6456C>A), K405N (6489A>C), I410V (6502A>G 6504T>A), V411I (6505G>A 6507T>C), E417D (6525A>C), *418E (6526T>G)								
Codon mutations:	GAA187CAA (5833G>C), TAT189TAC (5841T>C), ATT190ATA (5844T>A), CAC192CAA (5850C>A), CAT193AAA (5851C>A 5853T>A), ACA195CAG (5857A>C 5858C>A 5859A>G), TAC197TAT (5865C>T), AAT198AAC (5868T>C), TTG200CTT (5872T>C 5874G>T), ATA201ATT (5877A>T), GCT202GCA (5880T>A), TTA203CAA (5881T>C 5882T>A), CCA204CTC (5885C>T 5886A>C), AAT205AAA (5889T>A), ACA206ACT (5892A>T), AGT207AAT (5894G>A), GCA208GTC (5897C>T 5898A>C), AAA209AAT (5901A>T), TTA210CTA (5902T>C), GTA211GTT (5907A>T), GCC212AGA (5908G>A 5909C>G 5910C>A), ACC213ACA (5913C>A), TGC214TGT (5916C>T), AAC216AGT (5921A>G 5922C>T), TAC217TAT (5925C>T), GAT221AGT (5935G>A 5936A>G), ACT222ACC (5940T>C), GTA223ATA (5941G>A), CAG226TAC (5950C>T 5952G>C), ACA227GAT (5953A>G 5954C>A 5955A>T), CAA229GAA (5959C>G), GAG230GAA (5964G>A), GCC232TCA (5968G>T 5970C>A), AAT233ACT (5972A>C), CCT235CCA (5979T>A), AAG239AAA (5991G>A), TTC241TTT (5997C>T), ATG242ACA (5999T>C 6000G>A), CAA243ACA (6001C>A 6002A>C), AAG245AAA (6009G>A), AGG246AGA (6012G>A), ATA247ATT (6015A>T), ACT248ACA (6018T>A), GGG250GGC (6024G>C), ACA251AAC (6026C>A 6027A>C), TTC253TTT (6033C>T), TAC254TAT (6036C>T), GTA255ATA (6037G>A), CGA256AAG (6040C>A 6041G>A 6042A>G), TTC257TTT (6045C>T), TCG259ACA (6049T>A 6051G>A), GCT260GCA (6054T>A), ACA261CCA (6055A>C), GAG263GAT (6063G>T), CTA265TTA (6067C>T), TAT266TTT (6071A>T), GAA267GAT (6075A>T), GAG268GAA (6078G>A), ATA269ATT (6081A>T), AAG270AAA (6084G>A), CCT271CCA (6087T>A), ATC272GTG (6088A>G 6090C>G), AAA277AAG (6105A>G), ATT278ATA (6108T>A), CTC280CTA (6114C>A), AGG282AGA (6120G>A), GAA283AAT (6121G>A 6123A>T), ATC285TTA (6127A>T 6129C>A), AAG289GAC (6139A>G 6141G>C), GAA292CAA (6148G>C), GAA294CCA (6154G>C 6155A>C), GAA297CCA (6163G>C 6164A>C), GTA299ATT (6169G>A 6171A>T), AAT300GAG (6172A>G 6174T>G), ATT301ATA (6177T>A), GAA303AGT (6181G>A 6182A>G 6183A>T), TTC304TTT (6186C>T), TAT305TGC (6188A>G 6189T>C), AGG309AGA (6201G>A), ATA310ATT (6204A>T), ATA313CTA (6211A>C), ACT315ACG (6219T>G), CTA317ATC (6223C>A 6225A>C), CTA320TTA (6232C>T), TAT324TAC (6246T>C), CTA325ACA (6247C>A 6248T>C), CAG327GGG (6253C>G 6254A>G), AAT328AAC (6258T>C), GCA329CCA (6259G>C), ATT330ATG (6264T>G), AGC332AAT (6269G>A 6270C>T), TAT333CAT (6271T>C), TCA335_AGG336del (6277_6283delTCAAGGG), GAA337-AC (6277_6283delTCAAGGG 6285A>C), ACA339ATA (6290C>T), TAT342_GAT347del (6298_6315delTATTCAAATTGCAGAGAT), ATA348ATC (6318A>C), AGA349del (6319_6321delAGA), GCA351AGA (6325G>A 6326C>G), CTA356GCA (6340C>G 6341T>C), AGA357ATG (6344G>T 6345A>G), GTC360ATA (6352G>A 6354C>A), TTA361ATG (6355T>A 6357A>G), AGT362ACA (6359G>C 6360T>A), CTT363TTG (6361C>T 6363T>G), AAA365AAT (6369A>T), GAA367GAG (6375A>G), CAA368AAA (6376C>A), CCT369CAA (6380C>A 6381T>A), ACA370CCT (6382A>C 6384A>T), ACC371ATT (6386C>T 6387C>T), AGG376AAA (6401G>A 6402G>A), AGG377AAG (6404G>A), AAT378-GA (6406delA 6407A>G 6408T>A), TTC379TTT (6411C>T), ATC380ATT (6414C>T), TCC381TCG (6417C>G), CCA382GAT (6418C>G 6419C>A 6420A>T), GAT383GAA (6423T>A), CTG384TTA (6424C>T 6426G>A), TTA385CTA (6427T>C), CTA391CAA (6446T>A), AGT393GGG (6451A>G 6453T>G), CAC394CAA (6456C>A), TAT396TAC (6462T>C), CAC399CAT (6471C>T), TGC401TGT (6477C>T), TCA402TCG (6480A>G), AAA405AAC (6489A>C), ATT410GTA (6502A>G 6504T>A), GTT411ATC (6505G>A 6507T>C), GAC413GAT (6513C>T), CTG416CTA (6522G>A), GAA417GAC (6525A>C), TAA418GAA (6526T>G)								

Proteins

	Begin	End	Coverage	Score	Concordance	Matches	Identities	I/D/M/F*	Stop Codons
putative cell-to-cell movement protein (NP_569140.1)	94	420	23.6%	549	83.6%	99 (100%)	82 (82.8%)	0/0/0/0	1
Protein mutations:	Q97K (2443C>A), D98K (2446G>A 2448T>G), A101M (2455G>A 2456C>T 2457A>G), N103K (2463C>A), P104E (2464C>G 2465C>A), I146V (2590A>G), I150V (2602A>G 2604A>T), I163M (2643A>G), A165V (2648C>T), L171I (2665C>A), N172E (2668A>G 2670T>A), R174S (2676A>T), K178R (2687A>G), G188E (2717G>A), E418K (3406G>A), E419L (3409G>T 3410A>T)								
Codon mutations:	ATA96ATC (2442A>C), CAA97AAA (2443C>A), GAT98AAG (2446G>A 2448T>G), AAG100AAA (2454G>A), GCA101ATG (2455G>A 2456C>T 2457A>G), AAC103AAA (2463C>A), CCA104GAA (2464C>G 2465C>A), AAG106AAA (2472G>A), TTA110CTA (2482T>C), ACT113ACA (2493T>A), GCA119GCT (2511A>T), TGT120TGC (2514T>C), ACC127ACA (2535C>A), CTA133TTA (2551C>T), ATC139ATA (2571C>A), ATA146GTA (2590A>G), AGT148AGC (2598T>C), ATA150GTT (2602A>G 2604A>T), TAT156TAC (2622T>C), AAG158AAA (2628G>A), TTT161TTC (2637T>C), ATA163ATG (2643A>G), AGC164AGT (2646C>T), GCC165GTC (2648C>T), AAC166AAT (2652C>T), CTA171ATA (2665C>A), AAT172GAA (2668A>G 2670T>A), AGA174AGT (2676A>T), AAA178AGA (2687A>G), TTA180CTA (2692T>C), AAG185AAA (2709G>A), GGA188GAA (2717G>A), GAA418AAA (3406G>A), GAA419TTA (3409G>T 3410A>T)								
polyprotein (NP_569141.1)	1	591	66.8%	2399	80.4%	425 (100%)	342 (80.5%)	0/0/0/0	3
Protein mutations:	N16D (3456A>G), T39E (3525A>G 3526C>A), D40E (3530T>A), W42* (3536G>A), E43K (3537G>A), K46T (3547A>C), T47I (3550C>T), K178E (3942A>G), N181S (3952A>G), D182E (3956T>A), N183D (3957A>G), Q186K (3966C>A), E189D (3977G>T), K194T (3991A>C), E201N (4011G>A 4013A>T), E202K (4014G>A 4016G>A), N203D (4017A>G), K212S (4045A>G 4046A>T), N214S (4051A>G 4052C>T), N216D (4056A>G), L218Q (4062T>C 4063T>A), T219K (4066C>A), I223M (4079A>G), K226D (4086A>G 4088A>T), D227E (4091T>A), D230E (4100T>A), N231K (4103T>A), K232G (4104A>G 4105A>G), E236K (4116G>A), N238E (4122A>G 4124T>A), S239G (4125A>G 4127C>T), H251Y (4161C>T), I289V (4275A>G), D311E (4343T>A), A318T (4362G>A 4364T>A), A338V (4423C>T), M346I (4448G>T), D347N (4449G>A 4451C>T), R368K (4513G>A), L377S (4540T>C), K379Q (4545A>C), A381I (4551G>A 4552C>T), H382N (4554C>A), I384V (4560A>G), N386K (4568C>A), T394K (4591C>A 4592C>G), M399F (4605A>T 4607G>T), I415V (4653A>G), M417T (4660T>C), T419Q (4665A>C 4666C>A 4667T>A), I426N (4687T>A), L428S (4692C>T 4693T>C 4694T>A), D429E (4697T>A), N431E (4701A>G 4703C>A), I432L (4704A>T 4706T>A), Q439* (4725C>T), I444L (4740A>T), I487L (4869A>T), Y496C (4897A>G 4898C>T), K503N (4919A>T), T505I (4924C>T 4925C>A), T510A (4938A>G), S512A (4944T>G 4946A>G), N514D (4950A>G), Y515Q (4953T>C 4955T>A), N526E (4986A>G 4988T>A), C534Y (5011G>A 5012C>T), S540T (5028T>A 5030T>A), Y541F (5032A>T 5033T>C), T542S (5034A>T), E543N (5037G>A 5039A>C), P544S (5040C>T), Q545E (5043C>G), E546I (5046G>A 5047A>T), W548* (5053G>A), E549K (5055G>A), F556H (5076T>C 5077T>A), A557S (5079G>T), T572N (5125C>A), R573K (5128G>A), V576I (5136G>A)								
Codon mutations:	ATA3ATT (3419A>T), TAC4TAT (3422C>T), GCA7GCC (3431A>C), AAA8AAG (3434A>G), ATC9ATA (3437C>A), GTA11GTT (3443A>T), TAT14TAC (3452T>C), AAT16GAT (3456A>G), TAC18TAT (3464C>T), TAT19TAC (3467T>C), ACA20ACT (3470A>T), AAT30AAC (3500T>C), TGT32TGC (3506T>C), TGT36TGC (3518T>C), ACA39GAA (3525A>G 3526C>A), GAT40GAA (3530T>A), TGG42TGA (3536G>A), GAA43AAA (3537G>A), AAA46ACA (3547A>C), ACA47ATA (3550C>T), TTA176CTA (3936T>C), AAA178GAA (3942A>G), AAT181AGT (3952A>G), GAT182GAA (3956T>A), AAC183GAC (3957A>G), CCT184CCA (3962T>A), CTA185TTA (3963C>T), CAA186AAA (3966C>A), GAG189GAT (3977G>T), ACA193ACT (3989A>T), AAA194ACA (3991A>C), ATT200ATA (4010T>A), GAA201AAT (4011G>A 4013A>T), GAG202AAA (4014G>A 4016G>A), AAT203GAT (4017A>G), ATA205ATT (4025A>T), ATA206ATC (4028A>C), CCT210CCC (4040T>C), CTA211TTA (4041C>T), AAA212AGT (4045A>G 4046A>T), TAT213TAC (4049T>C), AAC214AGT (4051A>G 4052C>T), AAT216GAT (4056A>G), GAT217GAC (4061T>C), TTA218CAA (4062T>C 4063T>A), ACA219AAA (4066C>A), GAA220GAG (4070A>G), TTT221TTC (4073T>C), ATA223ATG (4079A>G), AAA226GAT (4086A>G 4088A>T), GAT227GAA (4091T>A), TTG229TTA (4097G>A), GAT230GAA (4100T>A), AAT231AAA (4103T>A), AAA232GGA (4104A>G 4105A>G), CAA235CAG (4115A>G), GAA236AAA (4116G>A), AGT237AGC (4121T>C), AAT238GAA (4122A>G 4124T>A), AGC239GGT (4125A>G 4127C>T), CAT241CAC (4133T>C), CAT251TAT (4161C>T), GGA257GGT (4181A>T), AGG260AGA (4190G>A), GTC262GTT (4196C>T), ATA263ATT (4199A>T), GAC264GAT (4202C>T), TTA268CTA (4212T>C), TAT277TAC (4241T>C), CCC280CCA (4250C>A), AAT281AAC (4253T>C), AAG285AAA (4265G>A), ATA289GTA (4275A>G), TAT292TAC (4286T>C), TAC294TAT (4292C>T), AGT296AGC (4298T>C), GAC299GAT (4307C>T), AAG301AAA (4313G>A), TAT305TAC (4325T>C), CAT306CAC (4328T>C), TTG307CTA (4329T>C 4331G>A), AAA308AAG (4334A>G), GAG310GAA (4340G>A), GAT311GAA (4343T>A), AAA315AAG (4355A>G), GCT318ACA (4362G>A 4364T>A), TAT326TAC (4388T>C), GTT330GTA (4400T>A), CCA332CCT (4406A>T), TTT333TTC (4409T>C), TAT335TAC (4415T>C), AAT337AAC (4421T>C), GCA338GTA (4423C>T), TAC342TAT (4436C>T), CAT344CAC (4442T>C), ATG346ATT (4448G>T), GAC347AAT (4449G>A 4451C>T), AAC348AAT (4454C>T), TAT349TAC (4457T>C), AAT351AAC (4463T>C), AAT355AAC (4475T>C), GTA358GTT (4484A>T), TAT359TAC (4487T>C), GAT361GAC (4493T>C), CTA364TTA (4500C>T), CTA365TTA (4503C>T), TAT366TAC (4508T>C), AGA368AAA (4513G>A), TTA377TCA (4540T>C), AAA379CAA (4545A>C), TTC380TTT (4550C>T), GCA381ATA (4551G>A 4552C>T), CAT382AAT (4554C>A), ATT383ATA (4559T>A), ATA384GTA (4560A>G), AAC386AAA (4568C>A), TCT387TCA (4571T>A), GGT388GGA (4574T>A), TTA391CTA (4581T>C), ACC394AAG (4591C>A 4592C>G), GCA396GCT (4598A>T), GAA397GAG (4601A>G), ATT398ATA (4604T>A), ATG399TTT (4605A>T 4607G>T), TTA406CTA (4626T>C), GAT411GAC (4643T>C), ATA415GTA (4653A>G), ATG417ACG (4660T>C), ACT419CAA (4665A>C 4666C>A 4667T>A), CAA423CAG (4679A>G), ATC426AAC (4687T>A), AAT427AAC (4691T>C), CTT428TCA (4692C>T 4693T>C 4694T>A), GAT429GAA (4697T>A), AAC431GAA (4701A>G 4703C>A), ATT432TTA (4704A>T 4706T>A), AAA437AAG (4721A>G), TTA438CTA (4722T>C), CAA439TAA (4725C>T), CTA442TTA (4734C>T), ATA444TTA (4740A>T), AAG486AAA (4868G>A), ATA487TTA (4869A>T), TTG488CTT (4872T>C 4874G>T), TAC496TGT (4897A>G 4898C>T), GAT499GAC (4907T>C), AAC501AAT (4913C>T), AAA503AAT (4919A>T), ACC505ATA (4924C>T 4925C>A), ATT507ATA (4931T>A), ACA510GCA (4938A>G), TCA512GCG (4944T>G 4946A>G), AAT514GAT (4950A>G), TAT515CAA (4953T>C 4955T>A), GTT520GTA (4970T>A), CTT521TTA (4971C>T 4973T>A), AAG522AAA (4976G>A), TAT523TAC (4979T>C), AGA524AGG (4982A>G), AAT526GAA (4986A>G 4988T>A), TGC534TAT (5011G>A 5012C>T), TAC536TAT (5018C>T), TAT537TAC (5021T>C), GGA539GGT (5027A>T), TCT540ACA (5028T>A 5030T>A), TAT541TTC (5032A>T 5033T>C), ACA542TCA (5034A>T), GAA543AAC (5037G>A 5039A>C), CCA544TCA (5040C>T), CAA545GAA (5043C>G), GAA546ATA (5046G>A 5047A>T), TGG548TAG (5053G>A), GAA549AAA (5055G>A), TTT556CAT (5076T>C 5077T>A), GCA557TCA (5079G>T), CTA562TTA (5094C>T), ATA569ATT (5117A>T), GTT570GTG (5120T>G), TAC571TAT (5123C>T), ACC572AAC (5125C>A), AGA573AAA (5128G>A), ATT575ATA (5135T>A), GTA576ATA (5136G>A), GAC579GAT (5147C>T), AAT580AAC (5150T>C), CAA582CAG (5156A>G), ACT588ACA (5174T>A), AAA590AAG (5180A>G)								
putative transactivator factor (NP_569142.1)	187	418	55.3%	806	54.0%	222 (96.1%)	149 (64.5%)	0/9/2/2	0

	Begin	End	Coverage	Score	Concordance	Matches	Identities	I/D/M/F*	Stop Codons
**NT**	**2433**	**6550**	**29.4%**	**2623**	**59.0%**	**2257 (98.7%)**	**1803 (78.8%)**	**1/29**	
Protein mutations:	E187Q (5833G>C), H192Q (5850C>A), H193K (5851C>A 5853T>A), T195Q (5857A>C 5858C>A 5859A>G), L203Q (5881T>C 5882T>A), P204L (5885C>T 5886A>C), N205K (5889T>A), S207N (5894G>A), A208V (5897C>T 5898A>C), K209N (5901A>T), A212R (5908G>A 5909C>G 5910C>A), N216S (5921A>G 5922C>T), D221S (5935G>A 5936A>G), V223I (5941G>A), Q226Y (5950C>T 5952G>C), T227D (5953A>G 5954C>A 5955A>T), Q229E (5959C>G), A232S (5968G>T 5970C>A), N233T (5972A>C), M242T (5999T>C 6000G>A), Q243T (6001C>A 6002A>C), T251N (6026C>A 6027A>C), V255I (6037G>A), R256K (6040C>A 6041G>A 6042A>G), S259T (6049T>A 6051G>A), T261P (6055A>C), E263D (6063G>T), Y266F (6071A>T), E267D (6075A>T), I272V (6088A>G 6090C>G), E283N (6121G>A 6123A>T), I285L (6127A>T 6129C>A), K289D (6139A>G 6141G>C), E292Q (6148G>C), E294P (6154G>C 6155A>C), E297P (6163G>C 6164A>C), V299I (6169G>A 6171A>T), N300E (6172A>G 6174T>G), E303S (6181G>A 6182A>G 6183A>T), Y305C (6188A>G 6189T>C), I313L (6211A>C), L317I (6223C>A 6225A>C), L325T (6247C>A 6248T>C), Q327G (6253C>G 6254A>G), A329P (6259G>C), I330M (6264T>G), S332N (6269G>A 6270C>T), Y333H (6271T>C), S335_R336del (6277_6283delTCAAGGG), T339I (6290C>T), Y342_D347del (6298_6315delTATTCAAATTGCAGAGAT), R349del (6319_6321delAGA), A351R (6325G>A 6326C>G), L356A (6340C>G 6341T>C), R357M (6344G>T 6345A>G), V360I (6352G>A 6354C>A), L361M (6355T>A 6357A>G), S362T (6359G>C 6360T>A), K365N (6369A>T), Q368K (6376C>A), P369Q (6380C>A 6381T>A), T370P (6382A>C 6384A>T), T371I (6386C>T 6387C>T), R376K (6401G>A 6402G>A), R377K (6404G>A), P382D (6418C>G 6419C>A 6420A>T), D383E (6423T>A), L391Q (6446T>A), S393G (6451A>G 6453T>G), H394Q (6456C>A), K405N (6489A>C), I410V (6502A>G 6504T>A), V411I (6505G>A 6507T>C), E417D (6525A>C), *418E (6526T>G)								
Codon mutations:	GAA187CAA (5833G>C), TAT189TAC (5841T>C), ATT190ATA (5844T>A), CAC192CAA (5850C>A), CAT193AAA (5851C>A 5853T>A), ACA195CAG (5857A>C 5858C>A 5859A>G), TAC197TAT (5865C>T), AAT198AAC (5868T>C), TTG200CTT (5872T>C 5874G>T), ATA201ATT (5877A>T), GCT202GCA (5880T>A), TTA203CAA (5881T>C 5882T>A), CCA204CTC (5885C>T 5886A>C), AAT205AAA (5889T>A), ACA206ACT (5892A>T), AGT207AAT (5894G>A), GCA208GTC (5897C>T 5898A>C), AAA209AAT (5901A>T), TTA210CTA (5902T>C), GTA211GTT (5907A>T), GCC212AGA (5908G>A 5909C>G 5910C>A), ACC213ACA (5913C>A), TGC214TGT (5916C>T), AAC216AGT (5921A>G 5922C>T), TAC217TAT (5925C>T), GAT221AGT (5935G>A 5936A>G), ACT222ACC (5940T>C), GTA223ATA (5941G>A), CAG226TAC (5950C>T 5952G>C), ACA227GAT (5953A>G 5954C>A 5955A>T), CAA229GAA (5959C>G), GAG230GAA (5964G>A), GCC232TCA (5968G>T 5970C>A), AAT233ACT (5972A>C), CCT235CCA (5979T>A), AAG239AAA (5991G>A), TTC241TTT (5997C>T), ATG242ACA (5999T>C 6000G>A), CAA243ACA (6001C>A 6002A>C), AAG245AAA (6009G>A), AGG246AGA (6012G>A), ATA247ATT (6015A>T), ACT248ACA (6018T>A), GGG250GGC (6024G>C), ACA251AAC (6026C>A 6027A>C), TTC253TTT (6033C>T), TAC254TAT (6036C>T), GTA255ATA (6037G>A), CGA256AAG (6040C>A 6041G>A 6042A>G), TTC257TTT (6045C>T), TCG259ACA (6049T>A 6051G>A), GCT260GCA (6054T>A), ACA261CCA (6055A>C), GAG263GAT (6063G>T), CTA265TTA (6067C>T), TAT266TTT (6071A>T), GAA267GAT (6075A>T), GAG268GAA (6078G>A), ATA269ATT (6081A>T), AAG270AAA (6084G>A), CCT271CCA (6087T>A), ATC272GTG (6088A>G 6090C>G), AAA277AAG (6105A>G), ATT278ATA (6108T>A), CTC280CTA (6114C>A), AGG282AGA (6120G>A), GAA283AAT (6121G>A 6123A>T), ATC285TTA (6127A>T 6129C>A), AAG289GAC (6139A>G 6141G>C), GAA292CAA (6148G>C), GAA294CCA (6154G>C 6155A>C), GAA297CCA (6163G>C 6164A>C), GTA299ATT (6169G>A 6171A>T), AAT300GAG (6172A>G 6174T>G), ATT301ATA (6177T>A), GAA303AGT (6181G>A 6182A>G 6183A>T), TTC304TTT (6186C>T), TAT305TGC (6188A>G 6189T>C), AGG309AGA (6201G>A), ATA310ATT (6204A>T), ATA313CTA (6211A>C), ACT315ACG (6219T>G), CTA317ATC (6223C>A 6225A>C), CTA320TTA (6232C>T), TAT324TAC (6246T>C), CTA325ACA (6247C>A 6248T>C), CAG327GGG (6253C>G 6254A>G), AAT328AAC (6258T>C), GCA329CCA (6259G>C), ATT330ATG (6264T>G), AGC332AAT (6269G>A 6270C>T), TAT333CAT (6271T>C), TCA335_AGG336del (6277_6283delTCAAGGG), GAA337-AC (6277_6283delTCAAGGG 6285A>C), ACA339ATA (6290C>T), TAT342_GAT347del (6298_6315delTATTCAAATTGCAGAGAT), ATA348ATC (6318A>C), AGA349del (6319_6321delAGA), GCA351AGA (6325G>A 6326C>G), CTA356GCA (6340C>G 6341T>C), AGA357ATG (6344G>T 6345A>G), GTC360ATA (6352G>A 6354C>A), TTA361ATG (6355T>A 6357A>G), AGT362ACA (6359G>C 6360T>A), CTT363TTG (6361C>T 6363T>G), AAA365AAT (6369A>T), GAA367GAG (6375A>G), CAA368AAA (6376C>A), CCT369CAA (6380C>A 6381T>A), ACA370CCT (6382A>C 6384A>T), ACC371ATT (6386C>T 6387C>T), AGG376AAA (6401G>A 6402G>A), AGG377AAG (6404G>A), AAT378-GA (6406delA 6407A>G 6408T>A), TTC379TTT (6411C>T), ATC380ATT (6414C>T), TCC381TCG (6417C>G), CCA382GAT (6418C>G 6419C>A 6420A>T), GAT383GAA (6423T>A), CTG384TTA (6424C>T 6426G>A), TTA385CTA (6427T>C), CTA391CAA (6446T>A), AGT393GGG (6451A>G 6453T>G), CAC394CAA (6456C>A), TAT396TAC (6462T>C), CAC399CAT (6471C>T), TGC401TGT (6477C>T), TCA402TCG (6480A>G), AAA405AAC (6489A>C), ATT410GTA (6502A>G 6504T>A), GTT411ATC (6505G>A 6507T>C), GAC413GAT (6513C>T), CTG416CTA (6522G>A), GAA417GAC (6525A>C), TAA418GAA (6526T>G)								

*: Inserts / Deletes / Misaligned / Frameshifts

Analysis details

This analysis was performed with panviral2.64

NGS Details (UN59): Tomato chocolate spot virus (segment RNA 1)

Assembly

**Coverage Length**	464 (2 contig(s))
**Depth Of Coverage**	1311.6
**Number Of Reads**	5597
**Reads Per Million**	121.67 rpm (after QC)
**Ambiguities**	0
**Assembly Method**	de novo + reference guided assembly
**Consensus Caller**	Bcf Tools

Coverage Map

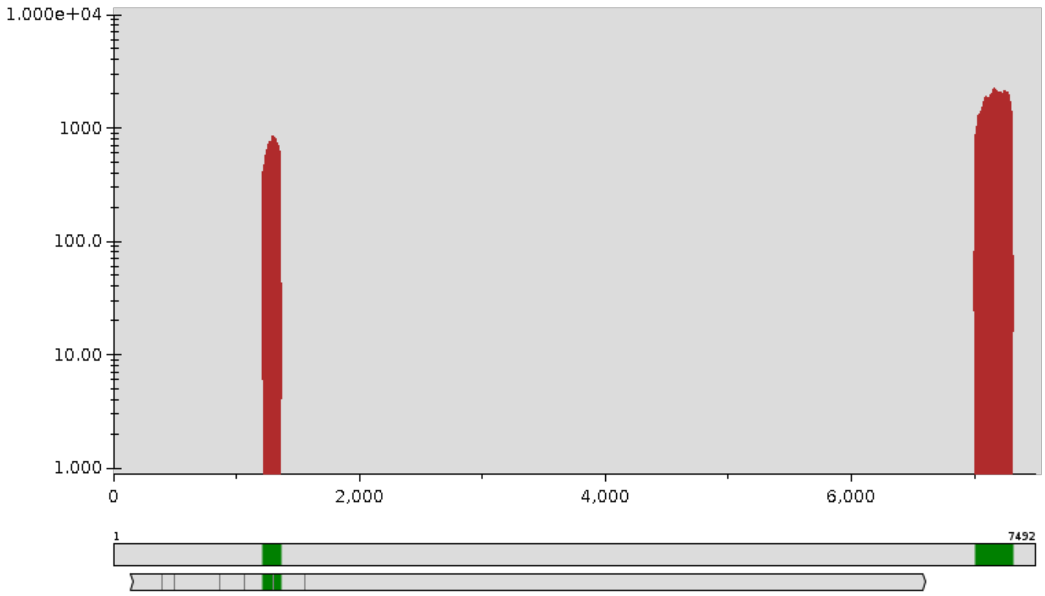

Assignment

**Type**	Tomato chocolate spot virus (Taxonomy ID: 661101)
**Reference Genome**	NC_013075.1
**NT Identity (%)**	79.5259
**AA Identity (%)**	80.3922
**Number Of Stop Codons**	0
**Number Of CDS**	1

Alignment

**Alignment Score**	548.0 (NT) + 310.0 (AA) = 858.0
**Concordance (%)**	66.5632

Alignment Method	Global, seeded, nucleotide + amino acids (AGA)

Genome Region

Sequence starts at position 1213 and ends at position 7314 relative to NC_013075.1 reference sequence.

Alignment Detailed Statistics

	Begin	End	Coverage	Score	Concordance	Matches	Identities	I/D/M/F*	Stop Codons
**NT**	**1213**	**7314**	**6.2%**	**548**	**59.1%**	**464 (100%)**	**369 (79.5%)**	**0/0**	
Mutations:	1222G>A, 1228A>G, 1229A>T, 1230T>G, 1233G>A, 1234C>T, 1236T>G, 1239A>T, 1243G>C, 1245G>C, 1246T>A, 1247A>T, 1248T>A, 1251T>A, 1254G>A, 1256G>A, 1260C>T, 1261A>C, 1262G>A, 1266C>T, 1268T>C, 1269G>A, 1275C>G, 1278A>T, 1281A>G, 1287G>A, 1293G>A, 1305G>T, 1317A>T, 1319C>A, 1320T>G, 1326C>A, 1329T>C, 1335G>A, 1338A>C, 1341A>G, 1342T>A, 1343C>G, 1348A>C, 1351T>C, 7013A>T, 7014T>A, 7018C>G, 7019A>G, 7020A>T, 7023C>A, 7042G>T, 7044A>T, 7062G>A, 7070T>C, 7071C>T, 7084A>G, 7087T>C, 7098C>T, 7099T>C, 7107G>A, 7116C>T, 7118T>A, 7121G>A, 7122T>C, 7123C>T, 7124T>G, 7137A>T, 7138G>A, 7139A>G, 7140C>T, 7148A>G, 7151A>T, 7159A>T, 7162T>C, 7165G>A, 7171T>G, 7173C>T, 7177A>C, 7185G>A, 7199C>T, 7208A>T, 7209T>A, 7210C>T, 7211A>G, 7213C>T, 7214A>T, 7229T>C, 7245T>C, 7250G>A, 7253T>A, 7254G>A, 7256T>C, 7257G>A, 7258G>T, 7259C>G, 7274G>C, 7275T>C, 7292T>G, 7303T>A								
**CDS**									
ToChSV_s1_gp1	359	409	2.4%	310	83.6%	51 (100%)	41 (80.4%)	0/0/0/0	0
Protein mutations:	A362T (1222G>A), N364V (1228A>G 1229A>T 1230T>G), E367D (1239A>T), V369L (1243G>C 1245G>C), Y370I (1246T>A 1247A>T 1248T>A), S373N (1256G>A), R375Q (1261A>C 1262G>A), M377T (1268T>C 1269G>A), A394E (1319C>A 1320T>G)								
Codon mutations:	GCA362ACA (1222G>A), AAT364GTG (1228A>G 1229A>T 1230T>G), CAG365CAA (1233G>A), CTT366TTG (1234C>T 1236T>G), GAA367GAT (1239A>T), GTG369CTC (1243G>C 1245G>C), TAT370ATA (1246T>A 1247A>T 1248T>A), GCT371GCA (1251T>A), GAG372GAA (1254G>A), AGC373AAC (1256G>A), TGC374TGT (1260C>T), AGA375CAA (1261A>C 1262G>A), CAC376CAT (1266C>T), ATG377ACA (1268T>C 1269G>A), GTC379GTG (1275C>G), GGA380GGT (1278A>T), CAA381CAG (1281A>G), AGG383AGA (1287G>A), GAG385GAA (1293G>A), GTG389GTT (1305G>T), GGA393GGT (1317A>T), GCT394GAG (1319C>A 1320T>G), GGC396GGA (1326C>A), TGT397TGC (1329T>C), AAG399AAA (1335G>A), TCA400TCC (1338A>C), ACA401ACG (1341A>G), TCC402AGC (1342T>A 1343C>G), AGA404CGA (1348A>C), TTG405CTG (1351T>C)								
**Proteins**									
polyprotein (YP_003097229.1)	359	409	2.4%	310	83.6%	51 (100%)	41 (80.4%)	0/0/0/0	0
Protein mutations:	A362T (1222G>A), N364V (1228A>G 1229A>T 1230T>G), E367D (1239A>T), V369L (1243G>C 1245G>C), Y370I (1246T>A 1247A>T 1248T>A), S373N (1256G>A), R375Q (1261A>C 1262G>A), M377T (1268T>C 1269G>A), A394E (1319C>A 1320T>G)								
Codon mutations:	GCA362ACA (1222G>A), AAT364GTG (1228A>G 1229A>T 1230T>G), CAG365CAA (1233G>A), CTT366TTG (1234C>T 1236T>G), GAA367GAT (1239A>T), GTG369CTC (1243G>C 1245G>C), TAT370ATA (1246T>A 1247A>T 1248T>A), GCT371GCA (1251T>A), GAG372GAA (1254G>A), AGC373AAC (1256G>A), TGC374TGT (1260C>T), AGA375CAA (1261A>C 1262G>A), CAC376CAT (1266C>T), ATG377ACA (1268T>C 1269G>A), GTC379GTG (1275C>G), GGA380GGT (1278A>T), CAA381CAG (1281A>G), AGG383AGA (1287G>A), GAG385GAA (1293G>A), GTG389GTT (1305G>T), GGA393GGT (1317A>T), GCT394GAG (1319C>A 1320T>G), GGC396GGA (1326C>A), TGT397TGC (1329T>C), AAG399AAA (1335G>A), TCA400TCC (1338A>C), ACA401ACG (1341A>G), TCC402AGC (1342T>A 1343C>G), AGA404CGA (1348A>C), TTG405CTG (1351T>C)								
RNA-dependent RNA polymerase (YP_003097234.1)	1	20	23.8%	141	91.0%	20 (100%)	18 (90.0%)	0/0/0/0	0
Protein mutations:	A5E (1319C>A 1320T>G)								
Codon mutations:	GGA4GGT (1317A>T), GCT5GAG (1319C>A 1320T>G), GGC7GGA (1326C>A), TGT8TGC (1329T>C), AAG10AAA (1335G>A), TCA11TCC (1338A>C), ACA12ACG (1341A>G), TCC13AGC (1342T>A 1343C>G), AGA15CGA (1348A>C), TTG16CTG (1351T>C)								

*: Inserts / Deletes / Misaligned / Frameshifts

Analysis details

This analysis was performed with panviral2.64

NGS Details (UN59): Tomato chocolate spot virus (segment RNA2)

Assembly

**Coverage Length**	810 (2 contig(s))
**Depth Of Coverage**	1360.0
**Number Of Reads**	9765
**Reads Per Million**	212.28 rpm (after QC)
**Ambiguities**	0
**Assembly Method**	de novo + reference guided assembly
**Consensus Caller**	Bcf Tools

Coverage Map

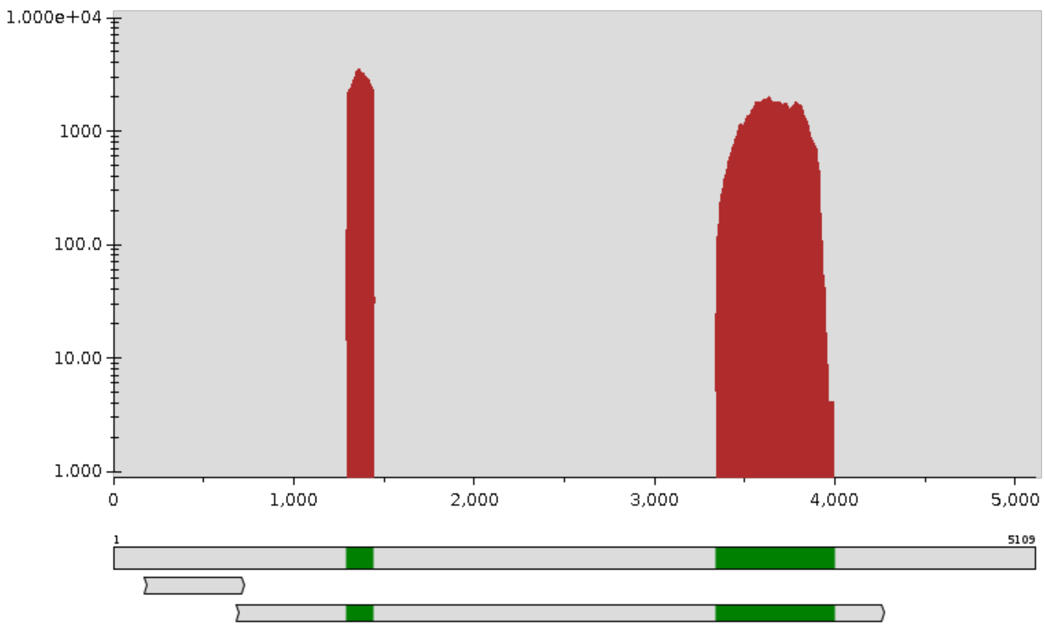

Assignment

**Type**	Tomato chocolate spot virus (Taxonomy ID: 661101)
**Reference Genome**	NC_013076.1
**NT Identity (%)**	69.7385
**AA Identity (%)**	75.3731
**Number Of Stop Codons**	0
**Number Of CDS**	2

Alignment

**Alignment Score**	609.0 (NT) + 1363.0 (AA) = 1972.0
**Concordance (%)**	59.8301

Alignment Method	Global, seeded, nucleotide + amino acids (AGA)

Genome Region

Sequence starts at position 1293 and ends at position 3998 relative to NC_013076.1 reference sequence.

Alignment Detailed Statistics

	Begin	End	Coverage	Score	Concordance	Matches	Identities	I/D/M/F*	Stop Codons
**NT**	**1293**	**3998**	**15.9%**	**609**	**38.5%**	**803 (99.1%)**	**560 (69.1%)**	**0/7**	
Mutations:	1306T>A, 1309A>G, 1312T>C, 1315C>T, 1316C>T, 1317A>C, 1318A>T, 1327G>A, 1333T>A, 1336C>A, 1351A>T, 1354A>C, 1360T>G, 1366G>A, 1367C>G, 1369A>G, 1372T>G, 1373T>G, 1375C>G, 1376C>T, 1377A>C, 1378G>T, 1379A>C, 1380T>A, 1381G>A, 1385T>C, 1387G>A, 1394C>A, 1396T>A, 1397G>C, 1405T>A, 1408A>T, 1410A>C, 1411G>A, 1412A>C, 1413G>T, 1414G>T, 1423T>C, 1426A>T, 1429A>C, 1433G>A, 3355T>G, 3358G>T, 3361C>A, 3367A>C, 3368G>T, 3370T>G, 3376T>G, 3379C>T, 3380T>A, 3382A>T, 3388T>A, 3391C>A, 3394G>A, 3400G>A, 3401C>T, 3403T>G, 3407T>C, 3410C>A, 3416A>G, 3419T>C, 3421G>C, 3422G>A, 3423T>C, 3424C>G, 3427A>T, 3430T>G, 3434A>T, 3436G>T, 3439T>G, 3442T>A, 3445G>T, 3454C>T, 3460A>T, 3463C>T, 3469A>C, 3472A>G, 3475A>G, 3477A>G, 3478G>A, 3484G>T, 3490C>T, 3496T>G, 3499C>T, 3505G>A, 3509G>A, 3511T>C, 3512A>C, 3516C>A, 3517C>T, 3530A>C, 3535A>G, 3538G>A, 3541C>G, 3544G>A, 3547G>A, 3550T>A, 3553C>T, 3556G>T, 3559A>T, 3560C>A, 3561A>T, 3562A>C, 3564A>T, 3568T>C, 3569G>C, 3570C>A, 3571C>G, 3574G>A, 3577T>G, 3581_3586delCAGCCA, 3587G>A, 3588T>G, 3593C>A, 3594A>C, 3596A>T, 3597G>C, 3598C>T, 3601G>A, 3604G>T, 3605A>T, 3606A>C, 3607T>C, 3610G>C, 3611T>C, 3612C>A, 3613C>G, 3616T>C, 3622G>A, 3625A>G, 3628T>C, 3629T>A, 3637T>A, 3643C>G, 3646G>A, 3652C>T, 3655C>T, 3656A>G, 3657T>C, 3658G>T, 3660G>C, 3661C>A, 3671G>A, 3672A>C, 3676A>C, 3679G>A, 3683C>G, 3685T>A, 3688A>T, 3689A>T, 3691A>G, 3700T>C, 3703C>T, 3721A>T, 3724T>A, 3725C>T, 3726A>C, 3727G>C, 3731G>C, 3733T>A, 3734G>A, 3739G>A, 3751C>T, 3754T>C, 3757T>A, 3763C>A, 3766T>C, 3773A>T, 3775A>C, 3778C>T, 3787C>T, 3793A>T, 3796C>A, 3799C>T, 3800C>T, 3802C>G, 3803A>G, 3805T>A, 3809T>A, 3811C>A, 3812A>T, 3814A>T, 3817T>A, 3818G>A, 3827G>A, 3828T>C, 3833T>A, 3834C>G, 3836A>T, 3837G>C, 3839C>A, 3840C>G, 3841T>C, 3843A>G, 3845G>A, 3847C>A, 3850A>T, 3853C>T, 3856T>C, 3857C>T, 3862T>C, 3863G>A, 3865A>T, 3868T>A, 3875T>A, 3877C>A, 3880T>A, 3883T>G, 3889A>T, 3892T>C, 3894C>A, 3896G>T, 3897C>T, 3898A>T, 3901C>T, 3902C>G, 3904G>A, 3905A>T, 3906G>C, 3908A>G, 3909A>C, 3914A>G, 3915A>C, 3916T>A, 3917T>C, 3922A>G, 3923G>T, 3925T>A, 3934A>C, 3937A>T, 3941T>A, 3943C>T, 3946G>A, 3950delA, 3951A>G, 3952C>A, 3964T>G, 3970A>C, 3973A>C, 3979A>C, 3982T>C, 3989A>G, 3990C>T, 3991G>A, 3994T>G								

CDS

	Begin	End	Coverage	Score	Concordance	Matches	Identities	I/D/M/F*	Stop Codons
ToChV_s2_gp2	204	1105	22.5%	1363	72.9%	268 (99.3%)	202 (74.8%)	0/2/1/1	0
Protein mutations:	Q211S (1316C>T 1317A>C 1318A>T), Q228E (1367C>G 1369A>G), S229R (1372T>G), S230A (1373T>G 1375C>G), Q231S (1376C>T 1377A>C 1378G>T), M232Q (1379A>C 1380T>A 1381G>A), A238P (1397G>C), E242A (1410A>C 1411G>A), R243L (1412A>C 1413G>T 1414G>T), D250N (1433G>A), V895L (3368G>T 3370T>G), S899T (3380T>A 3382A>T), C908R (3407T>C), L909I (3410C>A), N911D (3416A>G), V913T (3422G>A 3423T>C 3424C>G), T917S (3434A>T 3436G>T), K931R (3477A>G 3478G>A), D942N (3509G>A 3511T>C), M943L (3512A>C), T944N (3516C>A 3517C>T), I949L (3530A>C), D955E (3550T>A), E958D (3559A>T), Q959I (3560C>A 3561A>T 3562A>C), Y960F (3564A>T), A962Q (3569G>C 3570C>A 3571C>G), Q966_P967del (3581_3586delCAGCCA), V968S (3587G>A 3588T>G), Q970T (3593C>A 3594A>C), N974S (3605A>T 3606A>C 3607T>C), L975F (3610G>C), S976Q (3611T>C 3612C>A 3613C>G), S982T (3629T>A), M991A (3656A>G 3657T>C 3658G>T), G992A (3660G>C 3661C>A), E996T (3671G>A 3672A>C), E997D (3676A>C), L1000V (3683C>G 3685T>A), I1002L (3689A>T 3691A>G), E1012D (3721A>T), Q1014S (3725C>T 3726A>C 3727G>C), A1016P (3731G>C 3733T>A), V1017I (3734G>A), T1030S (3773A>T 3775A>C), N1040E (3803A>G 3805T>A), S1042T (3809T>A 3811C>A), I1043F (3812A>T 3814A>T), V1045M (3818G>A), V1048T (3827G>A 3828T>C), P1052S (3839C>A 3840C>G 3841T>C), N1053S (3843A>G), V1054I (3845G>A 3847C>A), V1060I (3863G>A 3865A>T), S1064T (3875T>A 3877C>A), P1070Q (3894C>A), A1071F (3896G>T 3897C>T 3898A>T), L1073V (3902C>G 3904G>A), K1075A (3908A>G 3909A>C), N1077A (3914A>G 3915A>C 3916T>A), Y1078H (3917T>C), V1080L (3923G>T 3925T>A), S1086T (3941T>A 3943C>T), T1102V (3989A>G 3990C>T 3991G>A)								
Codon mutations:	CCT207CCA (1306T>A), CAA208CAG (1309A>G), TTT209TTC (1312T>C), TTC210TTT (1315C>T), CAA211TCT (1316C>T 1317A>C 1318A>T), CAG214CAA (1327G>A), GCT216GCA (1333T>A), GCC217GCA (1336C>A), TCA222TCT (1351A>T), GTA223GTC (1354A>C), TCT225TCG (1360T>G), GGG227GGA (1366G>A), CAA228GAG (1367C>G 1369A>G), AGT229AGG (1372T>G), TCC230GCG (1373T>G 1375C>G), CAG231TCT (1376C>T 1377A>C 1378G>T), ATG232CAA (1379A>C 1380T>A 1381G>A), TTG234CTA (1385T>C 1387G>A), CGT237AGA (1394C>A 1396T>A), GCT238CCT (1397G>C), GTT240GTA (1405T>A), GGA241GGT (1408A>T), GAG242GCA (1410A>C 1411G>A), AGG243CTT (1412A>C 1413G>T 1414G>T), AAT246AAC (1423T>C), ACA247ACT (1426A>T), CGA248CGC (1429A>C), GAT250AAT (1433G>A), CTT890CTG (3355T>G), GTG891GTT (3358G>T), TCC892TCA (3361C>A), TCA894TCC (3367A>C), GTT895TTG (3368G>T 3370T>G), CTT897CTG (3376T>G), GAC898GAT (3379C>T), TCA899ACT (3380T>A 3382A>T), GGT901GGA (3388T>A), GTC902GTA (3391C>A), ACG903ACA (3394G>A), CAG905CAA (3400G>A), CTT906TTG (3401C>T 3403T>G), TGC908CGC (3407T>C), CTT909ATT (3410C>A), AAT911GAT (3416A>G), TTG912CTC (3419T>C 3421G>C), GTC913ACG (3422G>A 3423T>C 3424C>G), CTA914CTT (3427A>T), GGT915GGG (3430T>G), ACG917TCT (3434A>T 3436G>T), GTT918GTG (3439T>G), TCT919TCA (3442T>A), GTG920GTT (3445G>T), ATC923ATT (3454C>T), GGA925GGT (3460A>T), CAC926CAT (3463C>T), ACA928ACC (3469A>C), AAA929AAG (3472A>G), GGA930GGG (3475A>G), AAG931AGA (3477A>G 3478G>A), GCG933GCT (3484G>T), GAC935GAT (3490C>T), CTT937CTG (3496T>G), TTC938TTT (3499C>T), GAG940GAA (3505G>A), GAT942AAC (3509G>A 3511T>C), ATG943CTG (3512A>C), ACC944AAT (3516C>A 3517C>T), ATT949CTT (3530A>C), GAA950GAG (3535A>G), AAG951AAA (3538G>A), CTC952CTG (3541C>G), AGG953AGA (3544G>A), AAG954AAA (3547G>A), GAT955GAA (3550T>A), AAC956AAT (3553C>T), GTG957GTT (3556G>T), GAA958GAT (3559A>T), CAA959ATC (3560C>A 3561A>T 3562A>C), TAT960TTT (3564A>T), GAT961GAC (3568T>C), GCC962CAG (3569G>C 3570C>A 3571C>G), AAG963AAA (3574G>A), GGT964GGG (3577T>G), CAG966_CCA967del (3581_3586delCAGCCA), GTC968AGC (3587G>A 3588T>G), CAA970ACA (3593C>A 3594A>C), AGC971TCT (3596A>T 3597G>C 3598C>T), CAG972CAA (3601G>A), GTG973GTT (3604G>T), AAT974TCC (3605A>T 3606A>C 3607T>C), TTG975TTC (3610G>C), TCC976CAG (3611T>C 3612C>A 3613C>G), ATT977ATC (3616T>C), GAG979GAA (3622G>A), AAA980AAG (3625A>G), TTT981TTC (3628T>C), TCA982ACA (3629T>A), GGT984GGA (3637T>A), GTC986GTG (3643C>G), CAG987CAA (3646G>A), TTC989TTT (3652C>T), TGC990TGT (3655C>T), ATG991GCT (3656A>G 3657T>C 3658G>T), GGC992GCA (3660G>C 3661C>A), GAA996ACA (3671G>A 3672A>C), GAA997GAC (3676A>C), AGG998AGA (3679G>A), CTT1000GTA (3683C>G 3685T>A), GTA1001GTT (3688A>T), ATA1002TTG (3689A>T 3691A>G), GCT1005GCC (3700T>C), CCC1006CCT (3703C>T), GAA1012GAT (3721A>T), GCT1013GCA (3724T>A), CAG1014TCC (3725C>T 3726A>C 3727G>C), GCT1016CCA (3731G>C 3733T>A), GTT1017ATT (3734G>A), AAG1018AAA (3739G>A), ACC1022ACT (3751C>T), TGT1023TGC (3754T>C), CCT1024CCA (3757T>A), ATC1026ATA (3763C>A), GAT1027GAC (3766T>C), ACA1030TCC (3773A>T 3775A>C), TCC1031TCT (3778C>T), TAC1034TAT (3787C>T), TCA1036TCT (3793A>T), GGC1037GGA (3796C>A), AGC1038AGT (3799C>T), CTC1039TTG (3800C>T 3802C>G), AAT1040GAA (3803A>G 3805T>A), TCC1042ACA (3809T>A 3811C>A), ATA1043TTT (3812A>T 3814A>T), GTT1044GTA (3817T>A), GTG1045ATG (3818G>A), GTT1048ACT (3827G>A 3828T>C), TCC1050AGC (3833T>A 3834C>G), AGC1051TCC (3836A>T 3837G>C), CCT1052AGC (3839C>A 3840C>G 3841T>C), AAT1053AGT (3843A>G), GTC1054ATA (3845G>A 3847C>A), GGA1055GGT (3850A>T), GGC1056GGT (3853C>T), ATT1057ATC (3856T>C), CTG1058TTG (3857C>T), AAT1059AAC (3862T>C), GTA1060ATT (3863G>A 3865A>T), GCT1061GCA (3868T>A), TCC1064ACA (3875T>A 3877C>A), TCT1065TCA (3880T>A), GGT1066GGG (3883T>G), CCA1068CCT (3889A>T), TTT1069TTC (3892T>C), CCA1070CAA (3894C>A), GCA1071TTT (3896G>T 3897C>T 3898A>T), GGC1072GGT (3901C>T), CTG1073GTA (3902C>G 3904G>A), AGT1074TCT (3905A>T 3906G>C), AAG1075GCG (3908A>G 3909A>C), AAT1077GCA (3914A>G 3915A>C 3916T>A), TAT1078CAT (3917T>C), GTA1079GTG (3922A>G), GTT1080TTA (3923G>T 3925T>A), GGA1083GGC (3934A>C), GGA1084GGT (3937A>T), TCC1086ACT (3941T>A 3943C>T), AAG1087AAA (3946G>A), AAC1089-GA (3950delA 3951A>G 3952C>A), GGT1093GGG (3964T>G), ACA1095ACC (3970A>C), ACA1096ACC (3973A>C), ATA1098ATC (3979A>C), TTT1099TTC (3982T>C), ACG1102GTA (3989A>G 3990C>T 3991G>A), GTT1103GTG (3994T>G)								

Proteins

	Begin	End	Coverage	Score	Concordance	Matches	Identities	I/D/M/F*	Stop Codons
polyprotein (YP_003097231.1)	204	1105	22.5%	1363	72.9%	268 (99.3%)	202 (74.8%)	0/2/1/1	0
Protein mutations:	Q211S (1316C>T 1317A>C 1318A>T), Q228E (1367C>G 1369A>G), S229R (1372T>G), S230A (1373T>G 1375C>G), Q231S (1376C>T 1377A>C 1378G>T), M232Q (1379A>C 1380T>A 1381G>A), A238P (1397G>C), E242A (1410A>C 1411G>A), R243L (1412A>C 1413G>T 1414G>T), D250N (1433G>A), V895L (3368G>T 3370T>G), S899T (3380T>A 3382A>T), C908R (3407T>C), L909I (3410C>A), N911D (3416A>G), V913T (3422G>A 3423T>C 3424C>G), T917S (3434A>T 3436G>T), K931R (3477A>G 3478G>A), D942N (3509G>A 3511T>C), M943L (3512A>C), T944N (3516C>A 3517C>T), I949L (3530A>C), D955E (3550T>A), E958D (3559A>T), Q959I (3560C>A 3561A>T 3562A>C), Y960F (3564A>T), A962Q (3569G>C 3570C>A 3571C>G), Q966_P967del (3581_3586delCAGCCA), V968S (3587G>A 3588T>G), Q970T (3593C>A 3594A>C), N974S (3605A>T 3606A>C 3607T>C), L975F (3610G>C), S976Q (3611T>C 3612C>A 3613C>G), S982T (3629T>A), M991A (3656A>G 3657T>C 3658G>T), G992A (3660G>C 3661C>A), E996T (3671G>A 3672A>C), E997D (3676A>C), L1000V (3683C>G 3685T>A), I1002L (3689A>T 3691A>G), E1012D (3721A>T), Q1014S (3725C>T 3726A>C 3727G>C), A1016P (3731G>C 3733T>A), V1017I (3734G>A), T1030S (3773A>T 3775A>C), N1040E (3803A>G 3805T>A), S1042T (3809T>A 3811C>A), I1043F (3812A>T 3814A>T), V1045M (3818G>A), V1048T (3827G>A 3828T>C), P1052S (3839C>A 3840C>G 3841T>C), N1053S (3843A>G), V1054I (3845G>A 3847C>A), V1060I (3863G>A 3865A>T), S1064T (3875T>A 3877C>A), P1070Q (3894C>A), A1071F (3896G>T 3897C>T 3898A>T), L1073V (3902C>G 3904G>A), K1075A (3908A>G 3909A>C), N1077A (3914A>G 3915A>C 3916T>A), Y1078H (3917T>C), V1080L (3923G>T 3925T>A), S1086T (3941T>A 3943C>T), T1102V (3989A>G 3990C>T 3991G>A)								

	Begin	End	Coverage	Score	Concordance	Matches	Identities	I/D/M/F*	Stop Codons
**NT**	**1293**	**3998**	**15.9%**	**609**	**38.5%**	**803 (99.1%)**	**560 (69.1%)**	**0/7**	
Codon mutations:	CCT207CCA (1306T>A), CAA208CAG (1309A>G), TTT209TTC (1312T>C), TTC210TTT (1315C>T), CAA211TCT (1316C>T 1317A>C 1318A>T), CAG214CAA (1327G>A), GCT216GCA (1333T>A), GCC217GCA (1336C>A), TCA222TCT (1351A>T), GTA223GTC (1354A>C), TCT225TCG (1360T>G), GGG227GGA (1366G>A), CAA228GAG (1367C>G 1369A>G), AGT229AGG (1372T>G), TCC230GCG (1373T>G 1375C>G), CAG231TCT (1376C>T 1377A>C 1378G>T), ATG232CAA (1379A>C 1380T>A 1381G>A), TTG234CTA (1385T>C 1387G>A), CGT237AGA (1394C>A 1396T>A), GCT238CCT (1397G>C), GTT240GTA (1405T>A), GGA241GGT (1408A>T), GAG242GCA (1410A>C 1411G>A), AGG243CTT (1412A>C 1413G>T 1414G>T), AAT246AAC (1423T>C), ACA247ACT (1426A>T), CGA248CGC (1429A>C), GAT250AAT (1433G>A), CTT890CTG (3355T>G), GTG891GTT (3358G>T), TCC892TCA (3361C>A), TCA894TCC (3367A>C), GTT895TTG (3368G>T 3370T>G), CTT897CTG (3376T>G), GAC898GAT (3379C>T), TCA899ACT (3380T>A 3382A>T), GGT901GGA (3388T>A), GTC902GTA (3391C>A), ACG903ACA (3394G>A), CAG905CAA (3400G>A), CTT906TTG (3401C>T 3403T>G), TGC908CGC (3407T>C), CTT909ATT (3410C>A), AAT911GAT (3416A>G), TTG912CTC (3419T>C 3421G>C), GTC913ACG (3422G>A 3423T>C 3424C>G), CTA914CTT (3427A>T), GGT915GGG (3430T>G), ACG917TCT (3434A>T 3436G>T), GTT918GTG (3439T>G), TCT919TCA (3442T>A), GTG920GTT (3445G>T), ATC923ATT (3454C>T), GGA925GGT (3460A>T), CAC926CAT (3463C>T), ACA928ACC (3469A>C), AAA929AAG (3472A>G), GGA930GGG (3475A>G), AAG931AGA (3477A>G 3478G>A), GCG933GCT (3484G>T), GAC935GAT (3490C>T), CTT937CTG (3496T>G), TTC938TTT (3499C>T), GAG940GAA (3505G>A), GAT942AAC (3509G>A 3511T>C), ATG943CTG (3512A>C), ACC944AAT (3516C>A 3517C>T), ATT949CTT (3530A>C), GAA950GAG (3535A>G), AAG951AAA (3538G>A), CTC952CTG (3541C>G), AGG953AGA (3544G>A), AAG954AAA (3547G>A), GAT955GAA (3550T>A), AAC956AAT (3553C>T), GTG957GTT (3556G>T), GAA958GAT (3559A>T), CAA959ATC (3560C>A 3561A>T 3562A>C), TAT960TTT (3564A>T), GAT961GAC (3568T>C), GCC962CAG (3569G>C 3570C>A 3571C>G), AAG963AAA (3574G>A), GGT964GGG (3577T>G), CAG966_CCA967del (3581_3586delCAGCCA), GTC968AGC (3587G>A 3588T>G), CAA970ACA (3593C>A 3594A>C), AGC971TCT (3596A>T 3597G>C 3598C>T), CAG972CAA (3601G>A), GTG973GTT (3604G>T), AAT974TCC (3605A>T 3606A>C 3607T>C), TTG975TTC (3610G>C), TCC976CAG (3611T>C 3612C>A 3613C>G), ATT977ATC (3616T>C), GAG979GAA (3622G>A), AAA980AAG (3625A>G), TTT981TTC (3628T>C), TCA982ACA (3629T>A), GGT984GGA (3637T>A), GTC986GTG (3643C>G), CAG987CAA (3646G>A), TTC989TTT (3652C>T), TGC990TGT (3655C>T), ATG991GCT (3656A>G 3657T>C 3658G>T), GGC992GCA (3660G>C 3661C>A), GAA996ACA (3671G>A 3672A>C), GAA997GAC (3676A>C), AGG998AGA (3679G>A), CTT1000GTA (3683C>G 3685T>A), GTA1001GTT (3688A>T), ATA1002TTG (3689A>T 3691A>G), GCT1005GCC (3700T>C), CCC1006CCT (3703C>T), GAA1012GAT (3721A>T), GCT1013GCA (3724T>A), CAG1014TCC (3725C>T 3726A>C 3727G>C), GCT1016CCA (3731G>C 3733T>A), GTT1017ATT (3734G>A), AAG1018AAA (3739G>A), ACC1022ACT (3751C>T), TGT1023TGC (3754T>C), CCT1024CCA (3757T>A), ATC1026ATA (3763C>A), GAT1027GAC (3766T>C), ACA1030TCC (3773A>T 3775A>C), TCC1031TCT (3778C>T), TAC1034TAT (3787C>T), TCA1036TCT (3793A>T), GGC1037GGA (3796C>A), AGC1038AGT (3799C>T), CTC1039TTG (3800C>T 3802C>G), AAT1040GAA (3803A>G 3805T>A), TCC1042ACA (3809T>A 3811C>A), ATA1043TTT (3812A>T 3814A>T), GTT1044GTA (3817T>A), GTG1045ATG (3818G>A), GTT1048ACT (3827G>A 3828T>C), TCC1050AGC (3833T>A 3834C>G), AGC1051TCC (3836A>T 3837G>C), CCT1052AGC (3839C>A 3840C>G 3841T>C), AAT1053AGT (3843A>G), GTC1054ATA (3845G>A 3847C>A), GGA1055GGT (3850A>T), GGC1056GGT (3853C>T), ATT1057ATC (3856T>C), CTG1058TTG (3857C>T), AAT1059AAC (3862T>C), GTA1060ATT (3863G>A 3865A>T), GCT1061GCA (3868T>A), TCC1064ACA (3875T>A 3877C>A), TCT1065TCA (3880T>A), GGT1066GGG (3883T>G), CCA1068CCT (3889A>T), TTT1069TTC (3892T>C), CCA1070CAA (3894C>A), GCA1071TTT (3896G>T 3897C>T 3898A>T), GGC1072GGT (3901C>T), CTG1073GTA (3902C>G 3904G>A), AGT1074TCT (3905A>T 3906G>C), AAG1075GCG (3908A>G 3909A>C), AAT1077GCA (3914A>G 3915A>C 3916T>A), TAT1078CAT (3917T>C), GTA1079GTG (3922A>G), GTT1080TTA (3923G>T 3925T>A), GGA1083GGC (3934A>C), GGA1084GGT (3937A>T), TCC1086ACT (3941T>A 3943C>T), AAG1087AAA (3946G>A), AAC1089-GA (3950delA 3951A>G 3952C>A), GGT1093GGG (3964T>G), ACA1095ACC (3970A>C), ACA1096ACC (3973A>C), ATA1098ATC (3979A>C), TTT1099TTC (3982T>C), ACG1102GTA (3989A>G 3990C>T 3991G>A), GTT1103GTG (3994T>G)								

*: Inserts / Deletes / Misaligned / Frameshifts

Analysis details

This analysis was performed with panviral2.64

NGS Details (UN59): Tomato chocolate spot virus (segment RNA 1)

Assembly

**Coverage Length**	1210 (2 contig(s))
**Depth Of Coverage**	7250.8
**Number Of Reads**	56205
**Reads Per Million**	1221.85 rpm (after QC)
**Ambiguities**	0
**Assembly Method**	de novo + reference guided assembly
**Consensus Caller**	Bcf Tools

Coverage Map

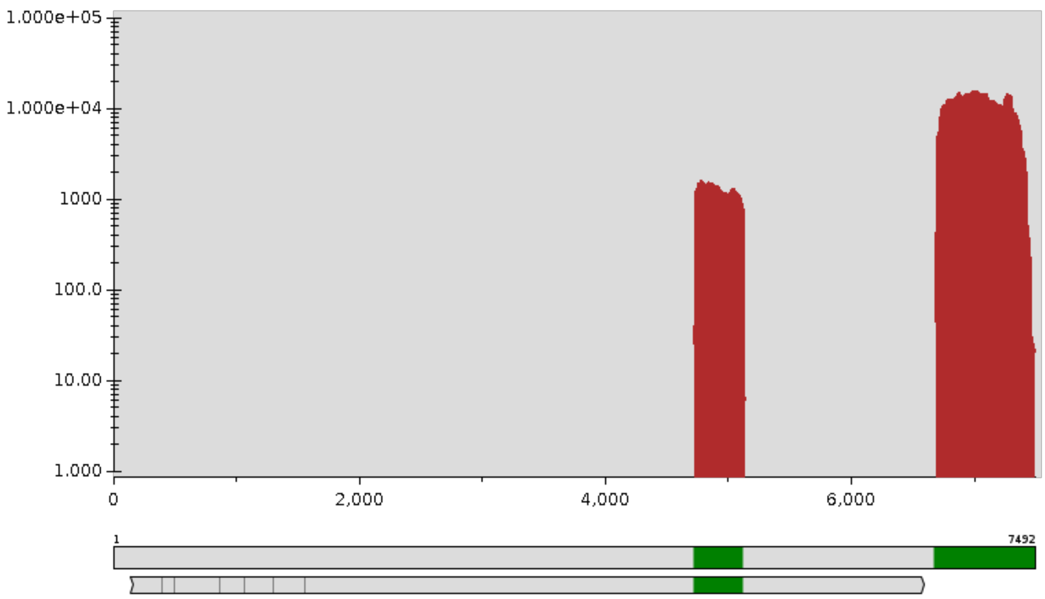

Assignment

**Type**	Tomato chocolate spot virus (Taxonomy ID: 661101)
**Reference Genome**	NC_013075.1
**NT Identity (%)**	67.5094
**AA Identity (%)**	66.1765
**Number Of Stop Codons**	0
**Number Of CDS**	1

Alignment

**Alignment Score**	327.0 (NT) + 683.0 (AA) = 1010.0
**Concordance (%)**	37.9699

Alignment Method	Global, seeded, nucleotide + amino acids (AGA)

Genome Region

Sequence starts at position 4726 and ends at position 7492 relative to NC_013075.1 reference sequence.

Alignment Detailed Statistics

	Begin	End	Coverage	Score	Concordance	Matches	Identities	I/D/M/F*	Stop Codons
**NT**	**4726**	**7492**	**16.2%**	**327**	**19.2%**	**1049 (85.4%)**	**721 (58.7%)**	**19/161**	
Mutations:	4735T>C, 4737G>T, 4740C>T, 4744T>A, 4745C>A, 4746A>C, 4750G>A, 4755G>C, 4761G>T, 4764G>C, 4765C>A, 4767G>A, 4770T>C, 4785A>G, 4786C>T, 4791T>G, 4795C>T, 4806G>C, 4813A>C, 4815A>C, 4818T>C, 4822C>T, 4827C>T, 4833T>A, 4834A>C, 4836G>A, 4839A>C, 4842G>C, 4845T>C, 4854A>T, 4860A>T, 4861A>G, 4863A>G, 4881A>C, 4882C>A, 4891C>G, 4892C>T, 4893C>T, 4896T>G, 4902T>A, 4906G>A, 4907C>T, 4908A>C, 4909C>A, 4910A>C, 4911G>T, 4914A>C, 4918A>T, 4919T>A, 4920G>T, 4929T>G, 4935T>G, 4938C>T, 4939A>G, 4950C>T, 4953T>C, 4956T>G, 4962T>A, 4965A>G, 4968T>C, 4970C>T, 4971A>T, 4977G>C, 4979A>C, 4984G>A, 4985T>C, 4986G>A, 4988C>A, 4989A>C, 4990G>A, 4995G>C, 4999G>A, 5005C>A, 5007G>A, 5009A>T, 5010T>G, 5011G>C, 5013A>G, 5016T>A, 5019A>C, 5022T>G, 5024G>C, 5025T>A, 5026G>A, 5027C>T, 5029A>G, 5030C>T, 5031A>G, 5032A>C, 5033T>A, 5037C>T, 5040T>A, 5043C>T, 5046C>T, 5047C>G, 5048T>C, 5049G>T, 5053G>C, 5054T>A, 5055C>A, 5059C>A, 5060A>G, 5061A>T, 5064A>G, 5068A>C, 5070T>A, 5071G>C, 5072C>A, 5073C>A, 5074A>G, 5075A>C, 5076G>T, 5086A>G, 5088T>C, 5089G>A, 5090C>G, 5092G>A, 5094T>C, 5095G>A, 5097G>A, 5098A>G, 5099T>G, 5101A>G, 5103A>T, 5104A>C, 5106C>G, 5107T>C, 5108G>T, 5109C>G, 5113A>C, 5116G>A, 5117C>G, 5118A>C, 5120G>A, 5121A>G, 6706A>G, 6707T>A, 6721G>C, 6722G>C, 6729T>C, 6731G>A, 6735G>A, 6736T>G, 6740C>A, 6741G>A, 6753G>T, 6757T>G, 6758_6759insT, 6759_6760insG, 6772C>T, 6777T>C, 6795A>G, 6806C>T, 6814C>T, 6815_6834delAATTGTTTGGTGTTGTCAAC, 6837G>T, 6840G>A, 6842C>A, 6843T>C, 6845T>G, 6847_6850delGCCA, 6853A>G, 6855A>T, 6857C>A, 6859C>T, 6860C>G, 6861C>G, 6862C>T, 6863T>C, 6867_6868delGC, 6870T>G, 6872A>G, 6873T>A, 6878A>G, 6879A>T, 6881G>A, 6885A>C, 6886G>T, 6887A>T, 6889_6894delGTGGAG, 6897G>A, 6903C>A, 6906G>C, 6908A>C, 6909A>G, 6911C>G, 6912A>G, 6914A>G, 6915A>T, 6918A>T, 6919_6921delGAG, 6923C>T, 6924T>C, 6926T>C, 6930_6937delGGTCGGGC, 6942G>C, 6944_6946delTGG, 6949G>A, 6950C>G, 6951C>T, 6953G>C, 6955A>G, 6956T>G, 6958A>T, 6960A>T, 6961G>T, 6962C>T, 6964T>C, 6965T>C, 6967G>A, 6969G>A, 6970T>G, 6972T>A, 6973G>A, 6976T>A, 6978T>A, 6982_6985delCATC, 6987G>T, 6989T>A, 6991T>C, 6996A>C, 7001_7012delCTCGTTTGGAGT, 7014T>G, 7016A>G, 7018C>G, 7020A>T, 7022delT, 7026G>T, 7030_7035delTGGTGT, 7040A>C, 7041C>G, 7042G>A, 7047_7067delGCAATGACACCCGGGGTGTGC, 7073C>G, 7077T>C, 7079A>T, 7080A>G, 7084_7088delAAGTA, 7091G>A, 7093T>C, 7097T>C, 7098C>T, 7099T>G, 7101C>A, 7103A>T, 7104T>A, 7105A>G, 7108A>G, 7109A>T, 7110C>G, 7111T>C, 7115_7123delGCGTTGGTC, 7125C>G, 7128C>G, 7130C>T, 7131A>G, 7132A>T, 7134delG, 7136G>C, 7138G>C, 7142C>A, 7143G>C, 7146T>C, 7147G>C, 7148_7161delAAAATCGATAAATT, 7165G>C, 7169C>G, 7172G>T, 7173delC, 7177A>C, 7180_7183delCTGC, 7186T>G, 7188G>T, 7190T>C, 7193G>A, 7195_7218delGCAACAGTGAAGAATCATCACACC, 7220C>G, 7223C>A, 7224T>C, 7225T>C, 7227C>T, 7228C>G, 7230A>C, 7231G>T, 7234T>G, 7235C>G, 7238C>T, 7241G>C, 7244G>A, 7245T>A, 7247_7248delTC, 7251A>T, 7259_7260delCT, 7262C>G, 7264T>A, 7266G>A, 7270A>C, 7273_7274delAG, 7288G>C, 7289T>C, 7291T>G, 7292T>G, 7293G>A, 7294C>T, 7296_7297insGCTTCTCCTAGTTAA, 7298G>A, 7299A>C, 7300C>G, 7302A>G, 7303T>C, 7304G>A, 7308C>T, 7310T>A, 7311A>T, 7314G>C, 7315T>C, 7316T>A, 7317T>G, 7318G>T, 7319G>A, 7321T>A, 7325C>A, 7327T>A, 7328A>T, 7330A>T, 7335_7336insT, 7342T>G, 7346A>T, 7348A>T, 7350G>C, 7351G>T, 7352_7353insC, 7358C>G, 7367C>T, 7369G>A, 7372G>T, 7373delA, 7375T>C, 7376G>C, 7378A>C, 7381C>G, 7385C>T, 7387T>G, 7388T>C, 7389C>A, 7390A>T, 7392T>C, 7393_7396delTATT, 7407G>T, 7410delG, 7425A>C, 7428delC, 7430T>C, 7431C>A, 7447A>C, 7453A>G, 7454G>A, 7455A>T, 7460C>G								

CDS

	Begin	End	Coverage	Score	Concordance	Matches	Identities	I/D/M/F*	Stop Codons
ToChSV_s1_gp1	1530	1665	6.3%	683	71.1%	136 (100%)	90 (66.2%)	0/0/0/0	0
Protein mutations:	S1536N (4744T>A 4745C>A 4746A>C), D1538N (4750G>A), E1541D (4761G>T), Q1543K (4765C>A 4767G>A), H1565Q (4833T>A), M1566L (4834A>C 4836G>A), I1575V (4861A>G 4863A>G), P1585V (4891C>G 4892C>T 4893C>T), A1590I (4906G>A 4907C>T 4908A>C), Q1591T (4909C>A 4910A>C 4911G>T), M1594Y (4918A>T 4919T>A 4920G>T), I1601V (4939A>G), H1606Q (4956T>G), A1611V (4970C>T 4971A>T), E1613D (4977G>C), K1614T (4979A>C), V1616T (4984G>A 4985T>C 4986G>A), A1617D (4988C>A 4989A>C), D1618N (4990G>A), E1621K (4999G>A), L1623I (5005C>A 5007G>A), N1624M (5009A>T 5010T>G), E1625Q (5011G>C 5013A>G), F1628L (5022T>G), R1629P (5024G>C 5025T>A), A1630I (5026G>A 5027C>T), T1631V (5029A>G 5030C>T 5031A>G), I1632H (5032A>C 5033T>A), L1637A (5047C>G 5048T>C 5049G>T), V1639Q (5053G>C 5054T>A 5055C>A), Q1641S (5059C>A 5060A>G 5061A>T), I1644L (5068A>C 5070T>A), A1645Q (5071G>C 5072C>A 5073C>A), K1646A (5074A>G 5075A>C 5076G>T), N1650D (5086A>G 5088T>C), A1651S (5089G>A 5090C>G), D1652N (5092G>A 5094T>C), V1653I (5095G>A 5097G>A), M1654G (5098A>G 5099T>G), T1655A (5101A>G 5103A>T), I1656L (5104A>C 5106C>G), C1657L (5107T>C 5108G>T 5109C>G), M1659L (5113A>C), A1660S (5116G>A 5117C>G 5118A>C), R1661K (5120G>A 5121A>G)								
Codon mutations:	TTG1533CTT (4735T>C 4737G>T), GAC1534GAT (4740C>T), TCA1536AAC (4744T>A 4745C>A 4746A>C), GAT1538AAT (4750G>A), GTG1539GTC (4755G>C), GAG1541GAT (4761G>T), GTG1542GTC (4764G>C), CAG1543AAA (4765C>A 4767G>A), TTT1544TTC (4770T>C), GAA1549GAG (4785A>G), CTG1550TTG (4786C>T), ACT1551ACG (4791T>G), CTG1553TTG (4795C>T), GGG1556GGC (4806G>C), AGA1559CGC (4813A>C 4815A>C), CAT1560CAC (4818T>C), CTG1562TTG (4822C>T), GGC1563GGT (4827C>T), CAT1565CAA (4833T>A), ATG1566CTA (4834A>C 4836G>A), GCA1567GCC (4839A>C), CCG1568CCC (4842G>C), CTT1569CTC (4845T>C), GTA1572GTT (4854A>T), ATA1574ATT (4860A>T), ATA1575GTG (4861A>G 4863A>G), ATA1581ATC (4881A>C), CGA1582AGA (4882C>A), CCC1585GTT (4891C>G 4892C>T 4893C>T), GGT1586GGG (4896T>G), CCT1588CCA (4902T>A), GCA1590ATC (4906G>A 4907C>T 4908A>C), CAG1591ACT (4909C>A 4910A>C 4911G>T), GCA1592GCC (4914A>C), ATG1594TAT (4918A>T 4919T>A 4920G>T), GTT1597GTG (4929T>G), ACT1599ACG (4935T>G), GGC1600GGT (4938C>T), ATA1601GTA (4939A>G), GCC1604GCT (4950C>T), TTT1605TTC (4953T>C), CAT1606CAG (4956T>G), GGT1608GGA (4962T>A), CAA1609CAG (4965A>G), GTT1610GTC (4968T>C), GCA1611GTT (4970C>T 4971A>T), GAG1613GAC (4977G>C), AAA1614ACA (4979A>C), GTG1616ACA (4984G>A 4985T>C 4986G>A), GCA1617GAC (4988C>A 4989A>C), GAT1618AAT (4990G>A), GTG1619GTC (4995G>C), GAA1621AAA (4999G>A), CTG1623ATA (5005C>A 5007G>A), AAT1624ATG (5009A>T 5010T>G), GAA1625CAG (5011G>C 5013A>G), CGT1626CGA (5016T>A), GGA1627GGC (5019A>C), TTT1628TTG (5022T>G), CGT1629CCA (5024G>C 5025T>A), GCC1630ATC (5026G>A 5027C>T), ACA1631GTG (5029A>G 5030C>T 5031A>G), ATT1632CAT (5032A>C 5033T>A), TTC1633TTT (5037C>T), CCT1634CCA (5040T>A), TCC1635TCT (5043C>T), TAC1636TAT (5046C>T), CTG1637GCT (5047C>G 5048T>C 5049G>T), GTC1639CAA (5053G>C 5054T>A 5055C>A), CAA1641AGT (5059C>A 5060A>G 5061A>T), GAA1642GAG (5064A>G), ATT1644CTA (5068A>C 5070T>A), GCC1645CAA (5071G>C 5072C>A 5073C>A), AAG1646GCT (5074A>G 5075A>C 5076G>T), AAT1650GAC (5086A>G 5088T>C), GCT1651AGT (5089G>A 5090C>G), GAT1652AAC (5092G>A 5094T>C), GTG1653ATA (5095G>A 5097G>A), ATG1654GGG (5098A>G 5099T>G), ACA1655GCT (5101A>G 5103A>T), ATC1656CTG (5104A>C 5106C>G), TGC1657CTG (5107T>C 5108G>T 5109C>G), ATG1659CTG (5113A>C), GCA1660AGC (5116G>A 5117C>G 5118A>C), AGA1661AAG (5120G>A 5121A>G)								

Proteins

	Begin	End	Coverage	Score	Concordance	Matches	Identities	I/D/M/F*	Stop Codons
polyprotein (YP_003097229.1)	1530	1665	6.3%	683	71.1%	136 (100%)	90 (66.2%)	0/0/0/0	0
Protein mutations:	S1536N (4744T>A 4745C>A 4746A>C), D1538N (4750G>A), E1541D (4761G>T), Q1543K (4765C>A 4767G>A), H1565Q (4833T>A), M1566L (4834A>C 4836G>A), I1575V (4861A>G 4863A>G), P1585V (4891C>G 4892C>T 4893C>T), A1590I (4906G>A 4907C>T 4908A>C), Q1591T (4909C>A 4910A>C 4911G>T), M1594Y (4918A>T 4919T>A 4920G>T), I1601V (4939A>G), H1606Q (4956T>G), A1611V (4970C>T 4971A>T), E1613D (4977G>C), K1614T (4979A>C), V1616T (4984G>A 4985T>C 4986G>A), A1617D (4988C>A 4989A>C), D1618N (4990G>A), E1621K (4999G>A), L1623I (5005C>A 5007G>A), N1624M (5009A>T 5010T>G), E1625Q (5011G>C 5013A>G), F1628L (5022T>G), R1629P (5024G>C 5025T>A), A1630I (5026G>A 5027C>T), T1631V (5029A>G 5030C>T 5031A>G), I1632H (5032A>C 5033T>A), L1637A (5047C>G 5048T>C 5049G>T), V1639Q (5053G>C 5054T>A 5055C>A), Q1641S (5059C>A 5060A>G 5061A>T), I1644L (5068A>C 5070T>A), A1645Q (5071G>C 5072C>A 5073C>A), K1646A (5074A>G 5075A>C 5076G>T), N1650D (5086A>G 5088T>C), A1651S (5089G>A 5090C>G), D1652N (5092G>A 5094T>C), V1653I (5095G>A 5097G>A), M1654G (5098A>G 5099T>G), T1655A (5101A>G 5103A>T), I1656L (5104A>C 5106C>G), C1657L (5107T>C 5108G>T 5109C>G), M1659L (5113A>C), A1660S (5116G>A 5117C>G 5118A>C), R1661K (5120G>A 5121A>G)								
Codon mutations:	TTG1533CTT (4735T>C 4737G>T), GAC1534GAT (4740C>T), TCA1536AAC (4744T>A 4745C>A 4746A>C), GAT1538AAT (4750G>A), GTG1539GTC (4755G>C), GAG1541GAT (4761G>T), GTG1542GTC (4764G>C), CAG1543AAA (4765C>A 4767G>A), TTT1544TTC (4770T>C), GAA1549GAG (4785A>G), CTG1550TTG (4786C>T), ACT1551ACG (4791T>G), CTG1553TTG (4795C>T), GGG1556GGC (4806G>C), AGA1559CGC (4813A>C 4815A>C), CAT1560CAC (4818T>C), CTG1562TTG (4822C>T), GGC1563GGT (4827C>T), CAT1565CAA (4833T>A), ATG1566CTA (4834A>C 4836G>A), GCA1567GCC (4839A>C), CCG1568CCC (4842G>C), CTT1569CTC (4845T>C), GTA1572GTT (4854A>T), ATA1574ATT (4860A>T), ATA1575GTG (4861A>G 4863A>G), ATA1581ATC (4881A>C), CGA1582AGA (4882C>A), CCC1585GTT (4891C>G 4892C>T 4893C>T), GGT1586GGG (4896T>G), CCT1588CCA (4902T>A), GCA1590ATC (4906G>A 4907C>T 4908A>C), CAG1591ACT (4909C>A 4910A>C 4911G>T), GCA1592GCC (4914A>C), ATG1594TAT (4918A>T 4919T>A 4920G>T), GTT1597GTG (4929T>G), ACT1599ACG (4935T>G), GGC1600GGT (4938C>T), ATA1601GTA (4939A>G), GCC1604GCT (4950C>T), TTT1605TTC (4953T>C), CAT1606CAG (4956T>G), GGT1608GGA (4962T>A), CAA1609CAG (4965A>G), GTT1610GTC (4968T>C), GCA1611GTT (4970C>T 4971A>T), GAG1613GAC (4977G>C), AAA1614ACA (4979A>C), GTG1616ACA (4984G>A 4985T>C 4986G>A), GCA1617GAC (4988C>A 4989A>C), GAT1618AAT (4990G>A), GTG1619GTC (4995G>C), GAA1621AAA (4999G>A), CTG1623ATA (5005C>A 5007G>A), AAT1624ATG (5009A>T 5010T>G), GAA1625CAG (5011G>C 5013A>G), CGT1626CGA (5016T>A), GGA1627GGC (5019A>C), TTT1628TTG (5022T>G), CGT1629CCA (5024G>C 5025T>A), GCC1630ATC (5026G>A 5027C>T), ACA1631GTG (5029A>G 5030C>T 5031A>G), ATT1632CAT (5032A>C 5033T>A), TTC1633TTT (5037C>T), CCT1634CCA (5040T>A), TCC1635TCT (5043C>T), TAC1636TAT (5046C>T), CTG1637GCT (5047C>G 5048T>C 5049G>T), GTC1639CAA (5053G>C 5054T>A 5055C>A), CAA1641AGT (5059C>A 5060A>G 5061A>T), GAA1642GAG (5064A>G), ATT1644CTA (5068A>C 5070T>A), GCC1645CAA (5071G>C 5072C>A 5073C>A), AAG1646GCT (5074A>G 5075A>C 5076G>T), AAT1650GAC (5086A>G 5088T>C), GCT1651AGT (5089G>A 5090C>G), GAT1652AAC (5092G>A 5094T>C), GTG1653ATA (5095G>A 5097G>A), ATG1654GGG (5098A>G 5099T>G), ACA1655GCT (5101A>G 5103A>T), ATC1656CTG (5104A>C 5106C>G), TGC1657CTG (5107T>C 5108G>T 5109C>G), ATG1659CTG (5113A>C), GCA1660AGC (5116G>A 5117C>G 5118A>C), AGA1661AAG (5120G>A 5121A>G)								

*: Inserts / Deletes / Misaligned / Frameshifts

Analysis details

This analysis was performed with panviral2.64

NGS Details (UN59): Lausannevirus

Assembly

**Coverage Length**	176 (1 contig(s))
**Depth Of Coverage**	9728.0
**Number Of Reads**	18309
**Reads Per Million**	398.02 rpm (after QC)
**Ambiguities**	0
**Assembly Method**	de novo + reference guided assembly
**Consensus Caller**	Bcf Tools

Coverage Map

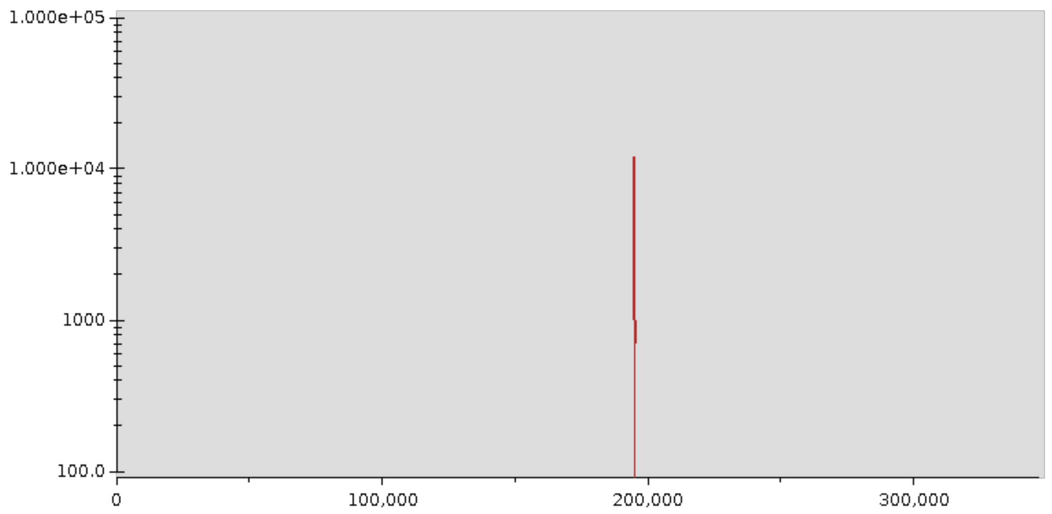

Assignment

**Type**	Lausannevirus (Taxonomy ID: 999883)
**Reference Genome**	NC_015326.1
**NT Identity (%)**	81.8182
**AA Identity (%)**	94.9153
**Number Of Stop Codons**	0
**Number Of CDS**	444

Alignment

**Alignment Score**	224.0 (NT) + 364.0 (AA) = 588.0
**Concordance (%)**	80.7692
**Alignment Method**	Local, heuristic, nucleotide (BLASTN)

Genome Region

Sequence starts at position 194923 and ends at position 195098 relative to NC_015326.1 reference sequence.

Alignment Detailed Statistics

	Begin	End	Coverage	Score	Concordance	Matches	Identities	I/D/M/F*	Stop Codons
**NT**	**194923**	**195098**	**0.1%**	**224**	**63.6%**	**176 (100%)**	**144 (81.8%)**	**0/0**	

Mutations: 194940A>C, 194946G>T, 194952T>C, 194955T>C, 194958A>G, 194967A>G, 194970G>C, 194979T>G, 194981T>G, 194982C>G, 194988T>C, 195003T>A, 195012A>C, 195015T>C, 195017G>T, 195018T>C, 195021T>C, 195033A>G, 195036G>C, 195042T>C, 195048T>C, 195054T>C, 195057T>A, 195058T>G, 195059G>C, 195060C>T, 195063G>C, 195066G>A, 195069C>G, 195072A>G, 195081C>A, 195087T>C

*: Inserts / Deletes / Misaligned / Frameshifts

Analysis details

This analysis was performed with panviral2.64

NGS Details (UN59): Makelovirus prm1

Assembly

**Coverage Length**	1014 (1 contig(s))
**Depth Of Coverage**	557.0
**Number Of Reads**	4406
**Reads Per Million**	95.78 rpm (after QC)
**Ambiguities**	0
**Assembly Method**	de novo + reference guided assembly
**Consensus Caller**	Bcf Tools

Coverage Map

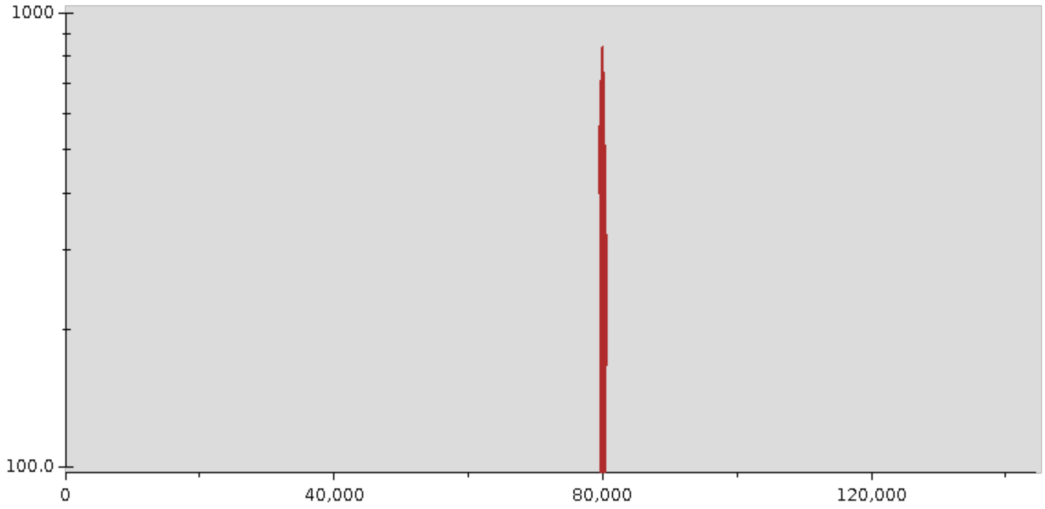

Assignment

**Type**	Makelovirus prm1 (Taxonomy ID: 2956181)
**Reference Genome**	NC_055761.1
**NT Identity (%)**	76.8245
**AA Identity (%)**	87.574
**Number Of Stop Codons**	0
**Number Of CDS**	190

Alignment

**Alignment Score**	1088.0 (NT) + 2258.0 (AA) = 3346.0
**Concordance (%)**	74.4548
**Alignment Method**	Local, heuristic, nucleotide (BLASTN)

Genome Region

Sequence starts at position 79506 and ends at position 80519 relative to NC_055761.1 reference sequence.

Alignment Detailed Statistics

	Begin	End	Coverage	Score	Concordance	Matches	Identities	I/D/M/F*	Stop Codons
**NT**	**79506**	**80519**	**0.7%**	**1088**	**53.6%**	**1014 (100%)**	**779 (76.8%)**	**0/0**	

Mutations: 79519A>G, 79520A>T, 79522A>G, 79523A>C, 79529C>T, 79530G>A, 79532G>C, 79536G>A, 79541G>T, 79542T>A, 79543C>G, 79544T>C, 79547A>T, 79548A>G, 79550C>A, 79563G>A, 79565C>T, 79568T>A, 79574C>T, 79580G>T, 79581C>T, 79592A>T, 79595T>C, 79596C>T, 79598G>A, 79602G>A, 79604A>G, 79610C>T, 79611G>T, 79612T>C, 79613C>T, 79614T>G, 79615G>T, 79616C>A, 79619C>T, 79624C>T, 79628G>C, 79632G>A, 79643C>T, 79646T>A, 79649G>A, 79655C>T, 79658C>T, 79664C>T, 79673A>T, 79676C>T, 79677G>T, 79679T>A, 79682T>G, 79688G>A, 79689A>C, 79691G>T, 79694T>C, 79703C>T, 79712T>C, 79718T>C, 79719G>A, 79722G>A, 79724A>T, 79728T>A, 79733C>T, 79734A>G, 79735A>C, 79736C>A, 79739A>T, 79742T>A, 79746C>T, 79748T>A, 79751C>T, 79754C>T, 79760C>A, 79763T>C, 79772T>G, 79779C>G, 79791C>T, 79793C>A, 79799T>C, 79811C>T, 79812C>G, 79817G>A, 79818G>A, 79820C>T, 79823A>T, 79824T>C, 79826T>A, 79833C>T, 79835G>A, 79836A>C, 79838C>T, 79842A>G, 79844C>A, 79845T>G, 79846T>C, 79847C>T, 79850C>T, 79871A>G, 79874C>T, 79875T>A, 79876C>G, 79879A>T, 79880T>C, 79895T>A, 79898C>T, 79904C>T, 79905T>G, 79906G>C, 79907C>T, 79916C>T, 79919T>A, 79922A>T, 79931T>A, 79937A>T, 79938T>A, 79940T>C, 79946C>T, 79950C>T, 79953G>A, 79958T>C, 79961T>A, 79962T>A, 79973T>A, 79974T>A, 79975C>G, 79976G>T, 79979C>T, 79985C>T, 79994C>T, 79997C>A, 80000C>A, 80018C>T, 80025C>A, 80030C>A, 80039A>T, 80042A>G, 80054G>T, 80063C>A, 80066C>T, 80069T>C, 80073C>T, 80075T>A, 80078T>C, 80090C>A, 80096T>C, 80099T>C, 80105G>A, 80114A>T, 80120C>T, 80126T>C, 80127C>T, 80132T>A, 80139T>A, 80140C>G, 80141G>T, 80142C>T, 80145G>A, 80147T>C, 80148C>A, 80150T>G, 80159C>A, 80162G>A, 80165C>T, 80168G>A, 80171C>T, 80172C>G, 80173A>C, 80174G>T, 80177C>T, 80178T>G, 80180T>A, 80188A>G, 80189G>A, 80201A>G, 80207G>A, 80210C>T, 80213C>T, 80216C>T, 80222G>A, 80225T>C, 80237C>T, 80240C>T, 80243T>C, 80246C>A, 80247C>T, 80261C>T, 80265T>A, 80266C>G, 80267C>T, 80279C>T, 80286C>T, 80288G>A, 80291T>C, 80300T>A, 80306A>T, 80315T>A, 80318C>A, 80333T>C, 80336A>T, 80337C>T, 80339T>A, 80343G>A, 80346T>A, 80347C>G, 80351C>T, 80357A>T, 80366G>A, 80369C>T, 80378C>T, 80387G>A, 80390C>T, 80391A>G, 80393C>A, 80394A>G, 80396G>T, 80399T>C, 80400G>A, 80409A>C, 80410A>G, 80411A>T, 80414C>A, 80415C>A, 80417C>T, 80433G>A, 80435T>C, 80436C>A, 80438A>C, 80447C>T, 80453G>T, 80456G>T, 80462G>A, 80465A>T, 80471C>T, 80474G>A, 80486C>T, 80498G>A, 80504G>A

*: Inserts / Deletes / Misaligned / Frameshifts

Analysis details

This analysis was performed with panviral2.64

NGS Details (UN59): Lausannevirus

Assembly

**Coverage Length**	129 (1 contig(s))
**Depth Of Coverage**	709.4
**Number Of Reads**	1051
**Reads Per Million**	22.85 rpm (after QC)
**Ambiguities**	0
**Assembly Method**	de novo + reference guided assembly
**Consensus Caller**	Bcf Tools

Coverage Map

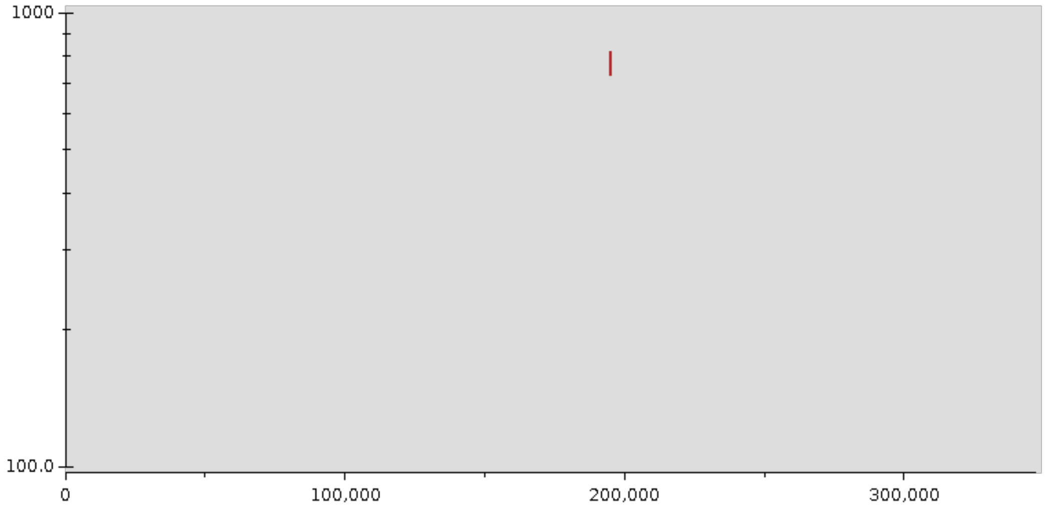

Assignment

**Type**	Lausannevirus (Taxonomy ID: 999883)
**Reference Genome**	NC_015326.1
**NT Identity (%)**	79.845
**AA Identity (%)**	95.3488
**Number Of Stop Codons**	1
**Number Of CDS**	444

Alignment

**Alignment Score**	154.0 (NT) + 273.0 (AA) = 427.0
**Concordance (%)**	77.9197
**Alignment Method**	Local, heuristic, nucleotide (BLASTN)

Genome Region

Sequence starts at position 194922 and ends at position 195050 relative to NC_015326.1 reference sequence.

Alignment Detailed Statistics

	Begin	End	Coverage	Score	Concordance	Matches	Identities	I/D/M/F*	Stop Codons
**NT**	**194922**	**195050**	**0.1%**	**154**	**59.7%**	**129 (100%)**	**103 (79.8%)**	**0/0**	

Mutations: 194937G>A, 194940A>T, 194941A>C, 194942G>A, 194943A>G, 194946G>T, 194953T>A, 194955T>C, 194967A>G, 194973A>C, 194976A>G, 194979T>C, 194982C>T, 194988T>C, 194991G>A, 194994C>T, 195000A>C, 195003T>G, 195006G>A, 195012A>C, 195014G>A, 195015T>C, 195017G>T, 195018T>C, 195036G>A, 195039T>C

*: Inserts / Deletes / Misaligned / Frameshifts

Analysis details

This analysis was performed with panviral2.64

NGS Details (UN59): Betabaculovirus disaccharalis

Assembly

**Coverage Length**	137 (1 contig(s))
**Depth Of Coverage**	510.6
**Number Of Reads**	905
**Reads Per Million**	19.67 rpm (after QC)
**Ambiguities**	0
**Assembly Method**	de novo + reference guided assembly
**Consensus Caller**	Bcf Tools

Coverage Map

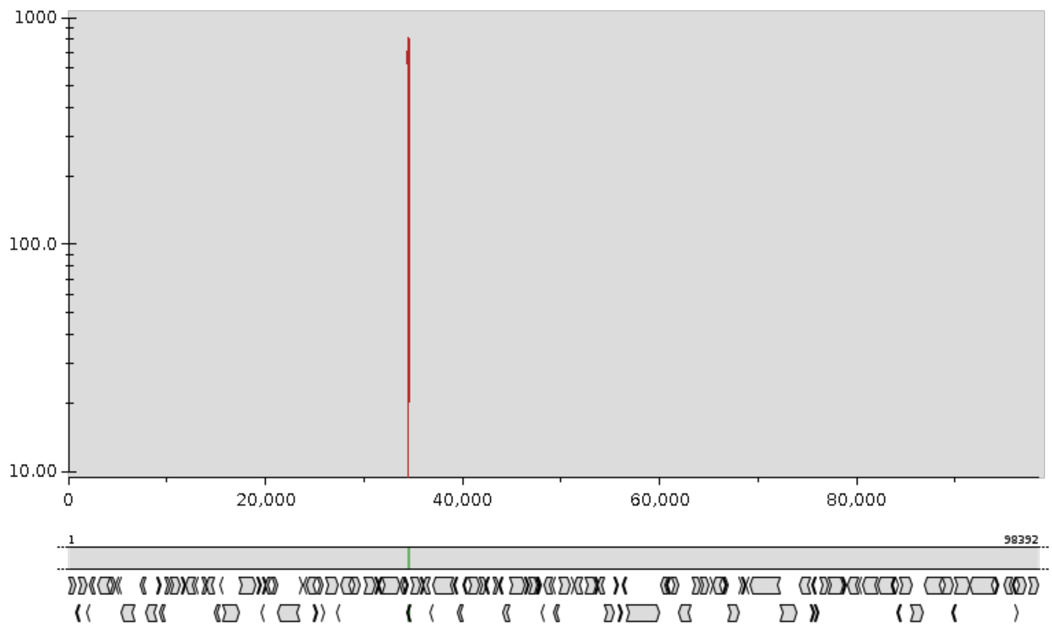

Assignment

**Type**	Betabaculovirus disaccharalis (Taxonomy ID: 3047795)
**Reference Genome**	NC_028491.1
**NT Identity (%)**	83.9416
**AA Identity (%)**	91.3043
**Number Of Stop Codons**	0
**Number Of CDS**	125

Alignment

**Alignment Score**	186.0 (NT) + 285.0 (AA) = 471.0
**Concordance (%)**	81.2069

Alignment Method	Local, heuristic, nucleotide (BLASTN)

Genome Region

Sequence starts at position 34471 and ends at position 34607 relative to NC_028491.1 reference sequence.

Alignment Detailed Statistics

	Begin	End	Coverage	Score	Concordance	Matches	Identities	I/D/M/F*	Stop Codons
**NT**	**34471**	**34607**	**0.1%**	**186**	**67.9%**	**137 (100%)**	**115 (83.9%)**	**0/0**	
Mutations:	34482G>T, 34485T>C, 34488A>C, 34490G>A, 34513C>G, 34514T>A, 34521T>C, 34524A>G, 34533A>G, 34538A>G, 34542T>A, 34545T>C, 34547G>T, 34549T>C, 34554T>C, 34561T>G, 34569G>A, 34575T>G, 34578T>C, 34587T>C, 34593C>T, 34596C>G								
**CDS**									
v-ubq	57	102	38.7%	285	86.9%	46 (100%)	42 (91.3%)	0/0/0/0	0
Protein mutations:	Q72P (34561T>G), E76G (34549T>C), S80P (34538A>G)								
Codon mutations:	CCG60CCC (34596C>G), CCG61CCA (34593C>T), CAA63CAG (34587T>C), TTA66TTG (34578T>C), ATA67ATC (34575T>G), GCC69GCT (34569G>A), CAA72CCA (34561T>G), GAA74GAG (34554T>C), GAG76GGG (34549T>C), CGA77AGG (34545T>C 34547G>T), ACA78ACT (34542T>A), TCA80CCA (34538A>G), GAT81GAC (34533A>G), ATT84ATC (34524A>G), CAA85CAG (34521T>C), AGC88TCC (34513C>G 34514T>A), CTT96TTG (34488A>C 34490G>A), AGA97AGG (34485T>C), GGC98GGA (34482G>T)								
**Proteins**									
ubiquitin-like protein (YP_009182246.1)	57	102	38.7%	285	86.9%	46 (100%)	42 (91.3%)	0/0/0/0	0
Protein mutations:	Q72P (34561T>G), E76G (34549T>C), S80P (34538A>G)								
Codon mutations:	CCG60CCC (34596C>G), CCG61CCA (34593C>T), CAA63CAG (34587T>C), TTA66TTG (34578T>C), ATA67ATC (34575T>G), GCC69GCT (34569G>A), CAA72CCA (34561T>G), GAA74GAG (34554T>C), GAG76GGG (34549T>C), CGA77AGG (34545T>C 34547G>T), ACA78ACT (34542T>A), TCA80CCA (34538A>G), GAT81GAC (34533A>G), ATT84ATC (34524A>G), CAA85CAG (34521T>C), AGC88TCC (34513C>G 34514T>A), CTT96TTG (34488A>C 34490G>A), AGA97AGG (34485T>C), GGC98GGA (34482G>T)								

*: Inserts / Deletes / Misaligned / Frameshifts

Analysis details

This analysis was performed with panviral2.64

NGS Details (UN59): Betabaculovirus disaccharalis

Assembly

**Coverage Length**	134 (1 contig(s))
**Depth Of Coverage**	486.0
**Number Of Reads**	820
**Reads Per Million**	17.83 rpm (after QC)
**Ambiguities**	0
**Assembly Method**	de novo + reference guided assembly
**Consensus Caller**	Bcf Tools

Coverage Map

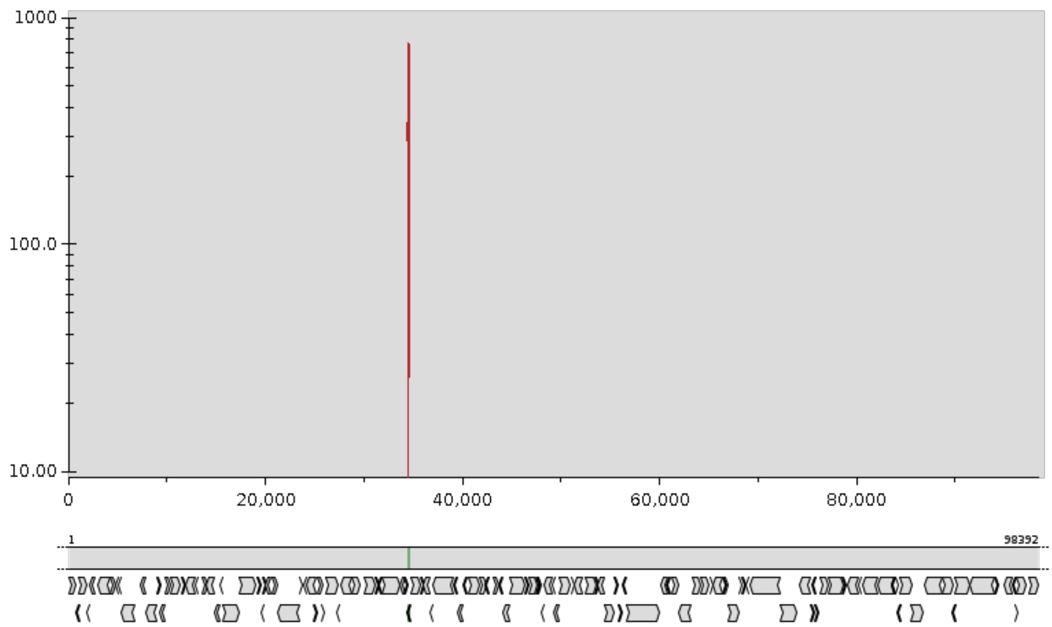

Assignment

**Type**	Betabaculovirus disaccharalis (Taxonomy ID: 3047795)
**Reference Genome**	NC_028491.1
**NT Identity (%)**	82.8358
**AA Identity (%)**	88.8889
**Number Of Stop Codons**	0
**Number Of CDS**	125

Alignment

**Alignment Score**	176.0 (NT) + 279.0 (AA) = 455.0
**Concordance (%)**	80.3887

Alignment Method	Local, heuristic, nucleotide (BLASTN)

Genome Region

Sequence starts at position 34474 and ends at position 34607 relative to NC_028491.1 reference sequence.

Alignment Detailed Statistics

	Begin	End	Coverage	Score	Concordance	Matches	Identities	I/D/M/F*	Stop Codons
**NT**	**34474**	**34607**	**0.1%**	**176**	**65.7%**	**134 (100%)**	**111 (82.8%)**	**0/0**	
Mutations:	34482G>T, 34485T>C, 34488A>C, 34490G>A, 34510G>T, 34513C>G, 34514T>A, 34518C>A, 34521T>C, 34524A>G, 34533A>G, 34538A>C, 34542T>A, 34545T>C, 34547G>T, 34549T>C, 34554T>C, 34569G>A, 34575T>G, 34578T>C, 34587T>C, 34593C>T, 34596C>G								
**CDS**									
v-ubq	57	101	37.8%	279	90.6%	45 (100%)	40 (88.9%)	0/0/0/0	0
Protein mutations:	E76G (34549T>C), S80A (34538A>C), K86N (34518C>A), T89N (34510G>T)								
Codon mutations:	CCG60CCC (34596C>G), CCG61CCA (34593C>T), CAA63CAG (34587T>C), TTA66TTG (34578T>C), ATA67ATC (34575T>G), GCC69GCT (34569G>A), GAA74GAG (34554T>C), GAG76GGG (34549T>C), CGA77AGG (34545T>C 34547G>T), ACA78ACT (34542T>A), TCA80GCA (34538A>C), GAT81GAC (34533A>G), ATT84ATC (34524A>G), CAA85CAG (34521T>C), AAG86AAT (34518C>A), AGC88TCC (34513C>G 34514T>A), ACT89AAT (34510G>T), CTT96TTG (34488A>C 34490G>A), AGA97AGG (34485T>C), GGC98GGA (34482G>T)								
**Proteins**									
ubiquitin-like protein (YP_009182246.1)	57	101	37.8%	279	90.6%	45 (100%)	40 (88.9%)	0/0/0/0	0
Protein mutations:	E76G (34549T>C), S80A (34538A>C), K86N (34518C>A), T89N (34510G>T)								
Codon mutations:	CCG60CCC (34596C>G), CCG61CCA (34593C>T), CAA63CAG (34587T>C), TTA66TTG (34578T>C), ATA67ATC (34575T>G), GCC69GCT (34569G>A), GAA74GAG (34554T>C), GAG76GGG (34549T>C), CGA77AGG (34545T>C 34547G>T), ACA78ACT (34542T>A), TCA80GCA (34538A>C), GAT81GAC (34533A>G), ATT84ATC (34524A>G), CAA85CAG (34521T>C), AAG86AAT (34518C>A), AGC88TCC (34513C>G 34514T>A), ACT89AAT (34510G>T), CTT96TTG (34488A>C 34490G>A), AGA97AGG (34485T>C), GGC98GGA (34482G>T)								

*: Inserts / Deletes / Misaligned / Frameshifts

Analysis details

This analysis was performed with panviral2.64

NGS Details (UN59): Cladosporium fulvum T-1 virus

Assembly

**Coverage Length**	1462 (4 contig(s))
**Depth Of Coverage**	42.1
**Number Of Reads**	529
**Reads Per Million**	11.50 rpm (after QC)
**Ambiguities**	0
**Assembly Method**	de novo + reference guided assembly
**Consensus Caller**	Bcf Tools

Coverage Map

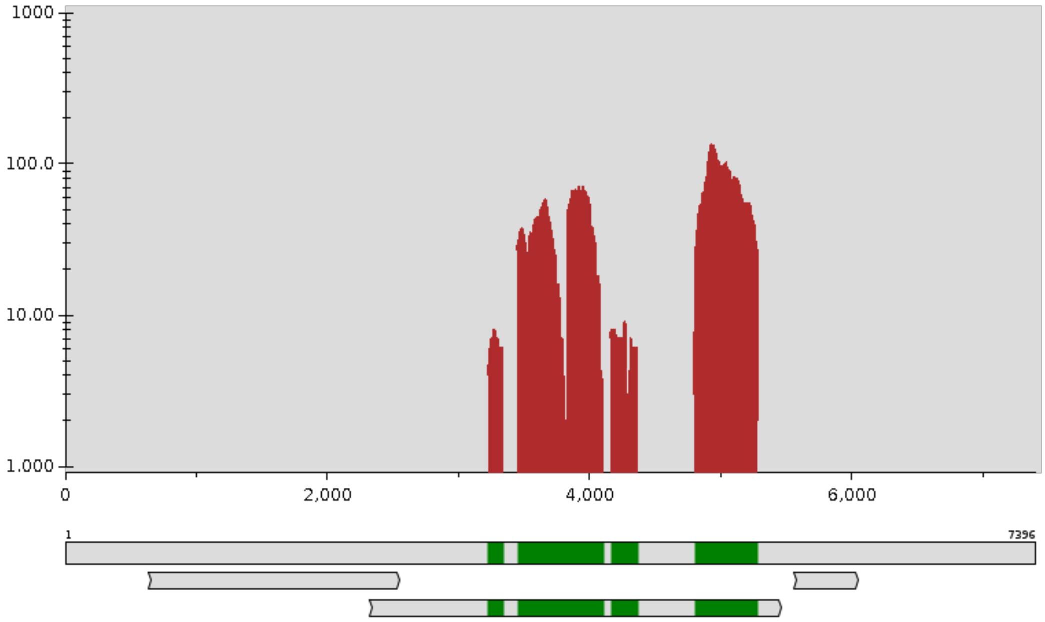

Assignment

**Type**	Cladosporium fulvum T-1 virus (Taxonomy ID: 2052899)
**Reference Genome**	NC_043491.1
**NT Identity (%)**	54.095
**AA Identity (%)**	47.205
**Number Of Stop Codons**	3
**Number Of CDS**	3

Alignment

**Alignment Score**	193.0 (NT) + 1658.0 (AA) = 1851.0
**Concordance (%)**	29.7924

Alignment Method	Global, seeded, nucleotide + amino acids (AGA)

Genome Region

Sequence starts at position 3231 and ends at position 5281 relative to NC_043491.1 reference sequence.

Alignment Detailed Statistics

	Begin	End	Coverage	Score	Concordance	Matches	Identities	I/D/M/F*	Stop Codons
**NT**	**3231**	**5281**	**19.8%**	**193**	**6.8%**	**1441 (97.8%)**	**786 (53.3%)**	**12/21**	
Mutations:	3231C>A, 3232C>G, 3233T>C, 3235A>G, 3238T>A, 3242C>A, 3244C>G, 3245C>T, 3246A>T, 3251T>C, 3252G>C, 3253_3254insAAGAGGGAGGTG, 3256T>C, 3266A>G, 3268C>G, 3269A>C, 3271T>C, 3272C>G, 3274G>A, 3280G>T, 3281A>G, 3282A>C, 3283A>C, 3284G>A, 3285A>C, 3286G>A, 3289T>G, 3293T>G, 3294G>C, 3297G>C, 3298A>T, 3299C>G, 3300C>T, 3301C>G, 3303T>C, 3307T>C, 3309A>G, 3310A>C, 3314T>G, 3316T>A, 3317G>C, 3318A>C, 3319G>T, 3320A>C, 3321A>C, 3322A>G, 3328A>T, 3329C>G, 3331G>A, 3332A>G, 3333C>A, 3334C>A, 3335C>T, 3338C>A, 3340A>G, 3443C>A, 3445C>G, 3446G>T, 3447T>G, 3448A>C, 3449C>A, 3450A>T, 3451A>G, 3454C>T, 3457C>T, 3460A>G, 3461A>G, 3462A>C, 3464T>C, 3470G>A, 3473A>G, 3475C>A, 3478G>T, 3479A>G, 3481C>G, 3488C>A, 3489G>A, 3490A>G, 3493T>C, 3496G>A, 3497C>G, 3499A>T, 3502C>T, 3503A>T, 3504A>T, 3505C>G, 3508C>G, 3511A>T, 3514A>T, 3515G>C, 3516C>T, 3517A>G, 3518C>T, 3519A>T, 3520A>G, 3525G>A, 3526A>G, 3529A>G, 3531C>G, 3534G>A, 3536T>G, 3538A>G, 3539G>T, 3540A>G, 3546A>T, 3548A>T, 3550G>T, 3556T>A, 3559C>T, 3562A>T, 3563C>A, 3565A>G, 3566G>T, 3567A>C, 3568C>A, 3570C>G, 3573T>A, 3574C>T, 3578G>C, 3579C>A, 3580T>G, 3581A>G, 3583C>T, 3584C>A, 3586A>G, 3589G>A, 3598A>G, 3601A>T, 3603A>T, 3604A>G, 3605T>C, 3606G>C, 3607G>T, 3610A>G, 3613C>T, 3614G>A, 3619C>T, 3620A>G, 3621G>T, 3622G>A, 3625A>T, 3628A>G, 3631C>T, 3634A>G, 3635C>T, 3636T>A, 3637C>A, 3640C>T, 3647T>C, 3649G>T, 3652C>A, 3658A>G, 3659A>T, 3661G>T, 3664A>G, 3670C>T, 3673C>T, 3676A>C, 3679C>G, 3682A>T, 3683T>A, 3687G>T, 3688C>T, 3689C>T, 3690A>G, 3692G>A, 3694C>T, 3695C>T, 3697T>A, 3698G>A, 3700C>G, 3703C>T, 3706A>C, 3707A>G, 3708C>T, 3712T>G, 3713A>T, 3714G>A, 3715A>T, 3719C>T, 3720T>A, 3721A>T, 3724C>T, 3727C>T, 3728G>A, 3729T>A, 3732G>T, 3733C>T, 3736C>A, 3739T>A, 3741C>T, 3742T>G, 3744A>T, 3748G>A, 3751C>T, 3757A>T, 3758C>G, 3760G>C, 3761G>A, 3763C>A, 3766C>T, 3768C>G, 3769A>T, 3771A>G, 3774G>A, 3775A>T, 3776T>A, 3778C>G, 3779C>T, 3781C>G, 3782C>G, 3784G>T, 3791A>G, 3792C>T, 3793C>G, 3796G>C, 3799A>T, 3800G>T, 3802T>G, 3803C>A, 3804A>G, 3805A>T, 3806G>A, 3807A>T, 3808T>G, 3814C>G, 3815G>C, 3818C>A, 3820A>T, 3821C>T, 3823C>G, 3825C>G, 3827A>G, 3829G>A, 3831C>A, 3833G>C, 3834G>A, 3836T>C, 3839A>T, 3841G>T, 3842A>G, 3843C>T, 3844A>T, 3845G>A, 3846C>A, 3848C>A, 3849C>T, 3850C>G, 3856A>G, 3859C>T, 3862A>G, 3865C>T, 3866C>G, 3867A>C, 3869A>C, 3871G>A, 3877A>G, 3878G>A, 3880C>A, 3886T>C, 3887T>C, 3892C>G, 3893T>C, 3894T>A, 3896A>C, 3898C>A, 3899A>G, 3901C>T, 3905A>G, 3906C>A, 3909C>A, 3910A>C, 3911G>C, 3912G>A, 3913G>A, 3914A>G, 3918C>G, 3922C>G, 3925C>T, 3929G>A, 3930C>A, 3931A>G, 3934G>A, 3935A>G, 3936C>T, 3937A>G, 3940G>A, 3941T>G, 3943A>C, 3946C>T, 3947A>G, 3948G>T, 3949A>T, 3952A>T, 3957C>A, 3960A>C, 3964G>C, 3967G>A, 3970A>T, 3973C>G, 3980G>C, 3982A>G, 3983C>A, 3984A>G, 3988A>T, 3992C>T, 3994T>G, 3997A>G, 4000C>G, 4003C>A, 4006C>T, 4009C>T, 4010A>T, 4012C>T, 4025A>G, 4026A>C, 4027G>T, 4029A>G, 4030C>A, 4033T>C, 4036A>G, 4041C>A, 4052A>T, 4057G>T, 4058A>G, 4059T>A, 4060G>T, 4061C>T, 4063T>G, 4064A>C, 4065C>T, 4066A>G, 4068G>A, 4069A>G, 4072A>G, 4073G>A, 4075C>G, 4081C>G, 4085A>G, 4086A>T, 4091G>T, 4092G>C, 4093A>G, 4094A>G, 4097G>A, 4100C>T, 4101A>G, 4102G>C, 4103A>G, 4104C>A, 4165T>C, 4166G>C, 4167G>A, 4168T>C, 4170G>C, 4175G>C, 4176A>C, 4178G>T, 4179T>A, 4181C>G, 4183C>A, 4184A>G, 4186C>G, 4187G>C, 4192C>G, 4195C>T, 4198G>C, 4201T>A, 4204T>C, 4205A>T, 4207G>C, 4210A>C, 4211A>T, 4216C>A, 4218C>G, 4219A>G, 4220T>G, 4221G>T, 4222T>C, 4225A>T, 4227C>T, 4228A>G, 4231G>A, 4232_4237delACACAC, 4240T>A, 4244A>G, 4248G>A, 4252C>T, 4255A>G, 4261C>T, 4264T>C, 4265T>G, 4267T>G, 4268T>A, 4269C>G, 4273G>C, 4276A>G, 4277A>C, 4279G>A, 4281C>A, 4283A>G, 4284C>A, 4285A>G, 4286G>A, 4288G>A, 4293A>G, 4294G>A, 4295A>C, 4296A>G, 4297C>G, 4301G>A, 4302A>C, 4303C>A, 4304A>G, 4309T>A, 4312C>G, 4319C>A, 4321T>G, 4322C>A, 4323T>C, 4330T>C, 4333T>C, 4334G>C, 4335C>A, 4337G>T, 4338C>G, 4340A>C, 4344A>G, 4345A>C, 4346C>A, 4347A>C, 4348T>C, 4355G>C, 4356T>A, 4357G>C, 4361G>T, 4363C>G, 4364G>C, 4365A>T, 4366G>T, 4804A>G, 4806T>G, 4807C>G, 4808C>A, 4809C>A, 4810A>T, 4811C>G, 4812A>G, 4813G>T, 4818G>A, 4820C>A, 4821T>G, 4822T>G, 4823A>G, 4825G>C, 4826G>A, 4828G>T, 4829C>G, 4830T>C, 4832C>G, 4833G>A, 4834C>A, 4836A>T, 4837C>T, 4838A>G, 4840C>A, 4841A>G, 4842A>C, 4843G>C, 4844A>C, 4849C>T, 4850G>C, 4851T>C, 4853C>A, 4855C>T, 4865A>G, 4866A>T, 4870A>G, 4872C>T, 4873T>A, 4875C>A, 4878G>A, 4879G>C, 4882C>A, 4883G>A, 4884C>G, 4886A>C, 4887A>C, 4889T>G, 4890A>G, 4894T>A, 4895C>T, 4896A>T, 4897C>A, 4903G>A, 4904T>G, 4905T>A, 4908G>T, 4909G>T, 4910A>G, 4911C>A, 4913C>A, 4914C>T, 4915A>T, 4918A>T, 4921G>T, 4922A>T, 4923A>G, 4924A>G, 4925C>A, 4926C>A, 4927A>G, 4933C>A, 4939T>A, 4941C>A, 4942G>T, 4951C>T, 4952A>G, 4955A>G, 4956C>T, 4958A>G, 4959A>G, 4961C>T, 4963C>G, 4966G>A, 4968G>A, 4970T>A, 4972A>T, 4974A>G, 4976_4987delGATCGAGTCACA, 4988G>A, 4993A>G, 4994_4996delGCC, 4997T>C, 5002C>T, 5003A>T, 5004T>C, 5005G>T, 5009C>T, 5010T>G, 5011A>G, 5014C>G, 5017G>A, 5020C>T, 5024A>C, 5027C>A, 5029C>G, 5032A>C, 5037A>C, 5038T>A, 5041A>C, 5048A>C, 5050T>A, 5053T>A, 5055C>T, 5056A>C, 5057T>A, 5058C>A, 5059A>G, 5060G>A, 5061A>C, 5062A>T, 5064T>C, 5065A>T, 5067A>T, 5070C>G, 5074A>T, 5077G>A, 5078C>A, 5080G>T, 5081C>T, 5082T>A, 5083C>T, 5085G>C, 5086A>C, 5087T>A, 5089C>A, 5092C>G, 5093G>T, 5094T>A, 5095A>T, 5096C>A, 5098G>T, 5099G>C, 5100A>G, 5101C>T, 5102A>G, 5103G>A, 5108A>G, 5111C>A, 5114T>C, 5115A>T, 5119C>T, 5122A>T, 5123T>A, 5125C>T, 5128G>C, 5129G>T, 5130A>T, 5131A>G, 5132G>T, 5133T>C, 5134A>C, 5135T>A, 5141A>T, 5143A>T, 5146C>T, 5151A>G, 5154A>C, 5155G>T, 5156C>T, 5157T>A, 5158C>A, 5167A>G, 5168A>C, 5172A>T, 5173C>T, 5179G>A, 5180A>T, 5182G>A, 5183C>T, 5185C>T, 5186A>T, 5187T>A, 5189G>A, 5190G>A, 5191A>G, 5192A>G, 5193C>G, 5194G>A, 5195A>C, 5200A>C, 5202T>C, 5206G>A, 5207C>G, 5208A>T, 5209C>T, 5213T>C, 5215G>A, 5216T>A, 5217C>G, 5218A>C, 5221A>T, 5226A>T, 5227C>T, 5230T>C, 5233A>T, 5234G>C, 5238C>T, 5239G>A, 5245G>A, 5248A>G, 5249A>G, 5251G>A, 5255A>C, 5262A>T, 5263C>T, 5269A>C, 5276C>G								
**CDS**									
homologue_of_retroviral_POL_genes	303	985	46.5%	1658	48.7%	479 (97.8%)	228 (46.5%)	4/7/0/0	3

	Begin	End	Coverage	Score	Concordance	Matches	Identities	I/D/M/F*	Stop Codons
**NT**	**3231**	**5281**	**19.8%**	**193**	**6.8%**	**1441 (97.8%)**	**786 (53.3%)**	**12/21**	
Protein mutations:	H306K (3242C>A 3244C>G), Q307L (3245C>T 3246A>T), W309P (3251T>C 3252G>C), W309_D310insKREV (3253_3254insAAGAGGGAGGTG), N314E (3266A>G 3268C>G), I315L (3269A>C 3271T>C), Q316E (3272C>G 3274G>A), K319A (3281A>G 3282A>C 3283A>C), E320T (3284G>A 3285A>C 3286G>A), W323A (3293T>G 3294G>C), G324A (3297G>C 3298A>T), P325V (3299C>G 3300C>T 3301C>G), L326P (3303T>C), Q328R (3309A>G 3310A>C), S330A (3314T>G 3316T>A), E331P (3317G>C 3318A>C 3319G>T), K332P (3320A>C 3321A>C 3322A>G), Q335E (3329C>G 3331G>A), T336E (3332A>G 3333C>A 3334C>A), L373M (3443C>A 3445C>G), V374C (3446G>T 3447T>G 3448A>C), Q375M (3449C>A 3450A>T 3451A>G), K379A (3461A>G 3462A>C), E382K (3470G>A), I383V (3473A>G 3475C>A), I385V (3479A>G 3481C>G), R388K (3488C>A 3489G>A 3490A>G), L391V (3497C>G 3499A>T), N393L (3503A>T 3504A>T 3505C>G), I394M (3508C>G), E395D (3511A>T), E396D (3514A>T), A397L (3515G>C 3516C>T 3517A>G), Q398L (3518C>T 3519A>T 3520A>G), R400K (3525G>A 3526A>G), T402S (3531C>G), G403E (3534G>A), S404A (3536T>G 3538A>G), D405C (3539G>T 3540A>G), Y407F (3546A>T), T408S (3548A>T 3550G>T), D414S (3566G>T 3567A>C 3568C>A), A415G (3570C>G), F416Y (3573T>A 3574C>T), A418Q (3578G>C 3579C>A 3580T>G), I419V (3581A>G 3583C>T), M421I (3589G>A), E425D (3601A>T), E426V (3603A>T 3604A>G), W427P (3605T>C 3606G>C 3607G>T), A430T (3614G>A), R432V (3620A>G 3621G>T 3622G>A), L437* (3635C>T 3636T>A 3637C>A), M445F (3659A>T 3661G>T), S453T (3683T>A), C454F (3687G>T 3688C>T), Q455W (3689C>T 3690A>G), D456N (3692G>A 3694C>T), V458M (3698G>A 3700C>G), E460D (3706A>C), T461V (3707A>G 3708C>T), R463Y (3713A>T 3714G>A 3715A>T), L465Y (3719C>T 3720T>A 3721A>T), V468K (3728G>A 3729T>A), C469F (3732G>T 3733C>T), A472V (3741C>T 3742T>G), Y473F (3744A>T), M474I (3748G>A), L478V (3758C>G 3760G>C), V479I (3761G>A 3763C>A), T481S (3768C>G 3769A>T), K482R (3771A>G), G483D (3774G>A 3775A>T), S484T (3776T>A 3778C>G), Q486D (3782C>G 3784G>T), T489V (3791A>G 3792C>T 3793C>G), K490N (3796G>C), Q491H (3799A>T), V492L (3800G>T 3802T>G), Q493S (3803C>A 3804A>G 3805A>T), D494M (3806G>A 3807A>T 3808T>G), F496L (3814C>G), E497Q (3815G>C), R498S (3818C>A 3820A>T), T500R (3825C>G), K501E (3827A>G 3829G>A), S502Y (3831C>A), G503Q (3833G>C 3834G>A), F504L (3836T>C), K505Y (3839A>T 3841G>T), T506V (3842A>G 3843C>T 3844A>T), A507K (3845G>A 3846C>A), P508M (3848C>A 3849C>T 3850C>G), H514A (3866C>G 3867A>C), K515Q (3869A>C 3871G>A), V518I (3878G>A 3880C>A), F523H (3893T>C 3894T>A), I524L (3896A>C 3898C>A), I525V (3899A>G 3901C>T), T527E (3905A>G 3906C>A), T528N (3909C>A 3910A>C), G529Q (3911G>C 3912G>A 3913G>A), I530V (3914A>G), T531R (3918C>G), I532M (3922C>G), A535K (3929G>A 3930C>A 3931A>G), T537V (3935A>G 3936C>T 3937A>G), S539A (3941T>G 3943A>C), R541V (3947A>G 3948G>T 3949A>T), E542D (3952A>T), P544Q (3957C>A), E545A (3960A>C), V552L (3980G>C 3982A>G), Q553R (3983C>A 3984A>G), N562Y (4010A>T 4012C>T), K567A (4025A>G 4026A>C 4027G>T), D568G (4029A>G 4030C>A), T572K (4041C>A), M576L (4052A>T), M578D (4058A>G 4059T>A 4060G>T), T580L (4064A>C 4065C>T 4066A>G), R581K (4068G>A 4069A>G), D583K (4073G>A 4075C>G), N585K (4081C>G), K587V (4085A>G 4086A>T), G589S (4091G>T 4092G>C 4093A>G), K590E (4094A>G), E591K (4097G>A), Q592C (4100C>T 4101A>G 4102G>C), G614H (4166G>C 4167G>A 4168T>C), S615T (4170G>C), E617P (4175G>C 4176A>C), V618Y (4178G>T 4179T>A), H619E (4181C>G 4183C>A), I620V (4184A>G 4186C>G), E621Q (4187G>C), M627F (4205A>T 4207G>C), I629L (4211A>T), A631G (4218C>G 4219A>G), C632V (4220T>G 4221G>T 4222T>C), T634M (4227C>T 4228A>G), T636_H637del (4232_4237delACACAC), D638E (4240T>A), K640E (4244A>G), R641K (4248G>A), Y647E (4265T>G 4267T>G), M651L (4277A>C 4279G>A), T652N (4281C>A), T653E (4283A>G 4284C>A 4285A>G), A654T (4286G>A 4288G>A), Q656R (4293A>G 4294G>A), N657R (4295A>C 4296A>G 4297C>G), D659T (4301G>A 4302A>C 4303C>A), I660V (4304A>G), H661Q (4309T>A), D662E (4312C>G), L665M (4319C>A 4321T>G), L666T (4322C>A 4323T>C), A670H (4334G>C 4335C>A), A671C (4337G>T 4338C>G), M672L (4340A>C), Q673R (4344A>G 4345A>C), H674T (4346C>A 4347A>C 4348T>C), V677H (4355G>C 4356T>A 4357G>C), V679L (4361G>T 4363C>G), E680L (4364G>C 4365A>T 4366G>T), F827W (4806T>G 4807C>G), P828N (4808C>A 4809C>A 4810A>T), Q829G (4811C>G 4812A>G 4813G>T), R831K (4818G>A), L832R (4820C>A 4821T>G 4822T>G), K833D (4823A>G 4825G>C), V834I (4826G>A 4828G>T), L835A (4829C>G 4830T>C), R836E (4832C>G 4833G>A 4834C>A), Y837F (4836A>T 4837C>T), I838V (4838A>G 4840C>A), K839A (4841A>G 4842A>C 4843G>C), K840Q (4844A>C), V842P (4850G>C 4851T>C), H843N (4853C>A 4855C>T), N847V (4865A>G 4866A>T), A849V (4872C>T 4873T>A), A850E (4875C>A), R851H (4878G>A 4879G>C), H852Q (4882C>A), A853R (4883G>A 4884C>G), K854P (4886A>C 4887A>C), Y855G (4889T>G 4890A>G), H857L (4895C>T 4896A>T 4897C>A), F860D (4904T>G 4905T>A), R861I (4908G>T 4909G>T), T862E (4910A>G 4911C>A), P863I (4913C>A 4914C>T 4915A>T), K866W (4922A>T 4923A>G 4924A>G), P867K (4925C>A 4926C>A 4927A>G), D869E (4933C>A), T872N (4941C>A 4942G>T), I876V (4952A>G), T877V (4955A>G 4956C>T), K878G (4958A>G 4959A>G), R881K (4968G>A), S882T (4970T>A 4972A>T), K883R (4974A>G), D884_T887del (4976_4987delGATCGAGTCACA), G888R (4988G>A), A890del (4994_4996delGCC), Y891H (4997T>C), M893S (5003A>T 5004T>C 5005G>T), L895W (5009C>T 5010T>G 5011A>G), M897I (5017G>A), L901M (5027C>A 5029C>G), Y904S (5037A>C 5038T>A), I908L (5048A>C 5050T>A), A910V (5055C>T 5056A>C), S911K (5057T>A 5058C>A 5059A>G), E912T (5060G>A 5061A>C 5062A>T), I913T (5064T>C 5065A>T), Y914F (5067A>T), T915S (5070C>G), Q918N (5078C>A 5080G>T), L919Y (5081C>T 5082T>A 5083C>T), G920A (5085G>C 5086A>C), Y921K (5087T>A 5089C>A), V923Y (5093G>T 5094T>A 5095A>T), L924I (5096C>A 5098G>T), D925R (5099G>C 5100A>G 5101C>T), R926E (5102A>G 5103G>A), I928V (5108A>G), Y930L (5114T>C 5115A>T), F933I (5123T>A 5125C>T), E935L (5129G>T 5130A>T 5131A>G), V936S (5132G>T 5133T>C 5134A>C), F937I (5135T>A), T939S (5141A>T 5143A>T), D942G (5151A>G), K943T (5154A>C 5155G>T), L944* (5156C>T 5157T>A 5158C>A), N948H (5168A>C), Y949F (5172A>T 5173C>T), T952S (5180A>T 5182G>A), L953F (5183C>T 5185C>T), M954* (5186A>T 5187T>A), G955K (5189G>A 5190G>A 5191A>G), T956G (5192A>G 5193C>G 5194G>A), I957L (5195A>C), I959T (5202T>C), H961V (5207C>G 5208A>T 5209C>T), Y967F (5226A>T 5227C>T), E970Q (5234G>C), T971I (5238C>T 5239G>A), T975A (5249A>G 5251G>A), N979I (5262A>T 5263C>T), Q984E (5276C>G)								

	Begin	End	Coverage	Score	Concordance	Matches	Identities	I/D/M/F*	Stop Codons
**NT**	**3231**	**5281**	**19.8%**	**193**	**6.8%**	**1441 (97.8%)**	**786 (53.3%)**	**12/21**	
Codon mutations:	GCC302.AG (3231C>A 3232C>G), TTA303CTG (3233T>C 3235A>G), CCT304CCA (3238T>A), CAC306AAG (3242C>A 3244C>G), CAA307TTA (3245C>T 3246A>T), TGG309CCG (3251T>C 3252G>C), TGG309_GAT310insAAGAGGGAGGTG (3253_3254insAAGAGGGAGGTG), GAT310GAC (3256T>C), AAC314GAG (3266A>G 3268C>G), ATT315CTC (3269A>C 3271T>C), CAG316GAA (3272C>G 3274G>A), GGG318GGT (3280G>T), AAA319GCC (3281A>G 3282A>C 3283A>C), GAG320ACA (3284G>A 3285A>C 3286G>A), CCT321CCG (3289T>G), TGG323GCG (3293T>G 3294G>C), GGA324GCT (3297G>C 3298A>T), CCC325GTG (3299C>G 3300C>T 3301C>G), CTA326CCA (3303T>C), TAT327TAC (3307T>C), CAA328CGC (3309A>G 3310A>C), TCT330GCA (3314T>G 3316T>A), GAG331CCT (3317G>C 3318A>C 3319G>T), AAA332CCG (3320A>C 3321A>C 3322A>G), CTA334CTT (3328A>T), CAG335GAA (3329C>G 3331G>A), ACC336GAA (3332A>G 3333C>A 3334C>A), CTA337TTA (3335C>T), CGA338AGG (3338C>A 3340A>G), CTC373ATG (3443C>A 3445C>G), GTA374TGC (3446G>T 3447T>G 3448A>C), CAA375ATG (3449C>A 3450A>T 3451A>G), GAC376GAT (3454C>T), TAC377TAT (3457C>T), CGA378CGG (3460A>G), AAG379GCG (3461A>G 3462A>C), TTG380CTG (3464T>C), GAG382AAG (3470G>A), ATC383GTA (3473A>G 3475C>A), ACG384ACT (3478G>T), ATC385GTG (3479A>G 3481C>G), CGA388AAG (3488C>A 3489G>A 3490A>G), TAT389TAC (3493T>C), CCG390CCA (3496G>A), CTA391GTT (3497C>G 3499A>T), CCC392CCT (3502C>T), AAC393TTG (3503A>T 3504A>T 3505C>G), ATC394ATG (3508C>G), GAA395GAT (3511A>T), GAA396GAT (3514A>T), GCA397CTG (3515G>C 3516C>T 3517A>G), CAA398TTG (3518C>T 3519A>T 3520A>G), AGA400AAG (3525G>A 3526A>G), TTA401TTG (3529A>G), ACC402AGC (3531C>G), GGA403GAA (3534G>A), TCA404GCG (3536T>G 3538A>G), GAC405TGC (3539G>T 3540A>G), TAC407TTC (3546A>T), ACG408TCT (3548A>T 3550G>T), ATT410ATA (3556T>A), GAC411GAT (3559C>T), CTA412CTT (3562A>T), CGA413AGG (3563C>A 3565A>G), GAC414TCA (3566G>T 3567A>C 3568C>A), GCC415GGC (3570C>G), TTC416TAT (3573T>A 3574C>T), GCT418CAG (3578G>C 3579C>A 3580T>G), ATC419GTT (3581A>G 3583C>T), CGA420AGG (3584C>A 3586A>G), ATG421ATA (3589G>A), GGA424GGG (3598A>G), GAA425GAT (3601A>T), GAA426GTG (3603A>T 3604A>G), TGG427CCT (3605T>C 3606G>C 3607G>T), AAA428AAG (3610A>G), ACC429ACT (3613C>T), GCT430ACT (3614G>A), TTC431TTT (3619C>T), AGG432GTA (3620A>G 3621G>T 3622G>A), ACA433ACT (3625A>T), AGA434AGG (3628A>G), TAC435TAT (3631C>T), GGA436GGG (3634A>G), CTC437TAA (3635C>T 3636T>A 3637C>A), TAC438TAT (3640C>T), TTG441CTT (3647T>C 3649G>T), GTC442GTA (3652C>A), CCA444CCG (3658A>G), ATG445TTT (3659A>T 3661G>T), GGA446GGG (3664A>G), ACC448ACT (3670C>T), AAC449AAT (3673C>T), GCA450GCC (3676A>C), CCC451CCG (3679C>G), GCA452GCT (3682A>T), TCC453ACC (3683T>A), TGC454TTT (3687G>T 3688C>T), CAG455TGG (3689C>T 3690A>G), GAC456AAT (3692G>A 3694C>T), CTT457TTA (3695C>T 3697T>A), GTC458ATG (3698G>A 3700C>G), AAC459AAT (3703C>T), GAA460GAC (3706A>C), ACA461GTA (3707A>G 3708C>T), CTT462CTG (3712T>G), AGA463TAT (3713A>T 3714G>A 3715A>T), CTA465TAT (3719C>T 3720T>A 3721A>T), CTC466CTT (3724C>T), GAC467GAT (3727C>T), GTG468AAG (3728G>A 3729T>A), TGC469TTT (3732G>T 3733C>T), GTC470GTA (3736C>A), GTT471GTA (3739T>A), GCT472GTG (3741C>T 3742T>G), TAC473TTC (3744A>T), ATG474ATA (3748G>A), GAC475GAT (3751C>T), ATA477ATT (3757A>T), CTG478GTC (3758C>G 3760G>C), GTC479ATA (3761G>A 3763C>A), TAC480TAT (3766C>T), ACA481AGT (3768C>G 3769A>T), AAA482AGA (3771A>G), GGA483GAT (3774G>A 3775A>T), TCC484ACG (3776T>A 3778C>G), CTC485TTG (3779C>T 3781C>G), CAG486GAT (3782C>G 3784G>T), ACC489GTG (3791A>G 3792C>T 3793C>G), AAG490AAC (3796G>C), CAA491CAT (3799A>T), GTT492TTG (3800G>T 3802T>G), CAA493AGT (3803C>A 3804A>G 3805A>T), GAT494ATG (3806G>A 3807A>T 3808T>G), TTC496TTG (3814C>G), GAA497CAA (3815G>C), CGA498AGT (3818C>A 3820A>T), CTC499TTG (3821C>T 3823C>G), ACG500AGG (3825C>G), AAG501GAA (3827A>G 3829G>A), TCC502TAC (3831C>A), GGA503CAA (3833G>C 3834G>A), TTC504CTC (3836T>C), AAG505TAT (3839A>T 3841G>T), ACA506GTT (3842A>G 3843C>T 3844A>T), GCA507AAA (3845G>A 3846C>A), CCC508ATG (3848C>A 3849C>T 3850C>G), AAA510AAG (3856A>G), TGC511TGT (3859C>T), GAA512GAG (3862A>G), TTC513TTT (3865C>T), CAC514GCC (3866C>G 3867A>C), AAG515CAA (3869A>C 3871G>A), GAA517GAG (3877A>G), GTC518ATA (3878G>A 3880C>A), TTT520TTC (3886T>C), TTA521CTA (3887T>C), GGC522GGG (3892C>G), TTT523CAT (3893T>C 3894T>A), ATC524CTA (3896A>C 3898C>A), ATC525GTT (3899A>G 3901C>T), ACA527GAA (3905A>G 3906C>A), ACA528AAC (3909C>A 3910A>C), GGG529CAA (3911G>C 3912G>A 3913G>A), ATA530GTA (3914A>G), ACG531AGG (3918C>G), ATC532ATG (3922C>G), GAC533GAT (3925C>T), GCA535AAG (3929G>A 3930C>A 3931A>G), AAG536AAA (3934G>A), ACA537GTG (3935A>G 3936C>T 3937A>G), CAG538CAA (3940G>A), TCA539GCC (3941T>G 3943A>C), ATC540ATT (3946C>T), AGA541GTT (3947A>G 3948G>T 3949A>T), GAA542GAT (3952A>T), CCA544CAA (3957C>A), GAA545GCA (3960A>C), CCG546CCC (3964G>C), AAG547AAA (3967G>A), ACA548ACT (3970A>T), GTC549GTG (3973C>G), GTA552CTG (3980G>C 3982A>G), CAG553AGG (3983C>A 3984A>G), TCA554TCT (3988A>T), CTT556TTG (3992C>T 3994T>G), GGA557GGG (3997A>G), CTC558CTG (4000C>G), GCC559GCA (4003C>A), AAC560AAT (4006C>T), TAC561TAT (4009C>T), AAC562TAT (4010A>T 4012C>T), AAG567GCT (4025A>G 4026A>C 4027G>T), GAC568GGA (4029A>G 4030C>A), TAT569TAC (4033T>C), TCA570TCG (4036A>G), ACA572AAA (4041C>A), ATG576TTG (4052A>T), ACG577ACT (4057G>T), ATG578GAT (4058A>G 4059T>A 4060G>T), CTT579TTG (4061C>T 4063T>G), ACA580CTG (4064A>C 4065C>T 4066A>G), AGA581AAG (4068G>A 4069A>G), AAA582AAG (4072A>G), GAC583AAG (4073G>A 4075C>G), AAC585AAG (4081C>G), AAA587GTA (4085A>G 4086A>T), GGA589TCG (4091G>T 4092G>C 4093A>G), AAA590GAA (4094A>G), GAA591AAA (4097G>A), CAG592TGC (4100C>T 4101A>G 4102G>C), ACC593GA. (4103A>G 4104C>A), GAT613GAC (4165T>C), GGT614CAC (4166G>C 4167G>A 4168T>C), AGC615ACC (4170G>C), GAA617CCA (4175G>C 4176A>C), GTC618TAC (4178G>T 4179T>A), CAC619GAA (4181C>G 4183C>A), ATC620GTG (4184A>G 4186C>G), GAG621CAG (4187G>C), ACC622ACG (4192C>G), GAC623GAT (4195C>T), GCG624GCC (4198G>C), TCT625TCA (4201T>A), GAT626GAC (4204T>C), ATG627TTC (4205A>T 4207G>C), GCA628GCC (4210A>C), ATA629TTA (4211A>T), GGC630GGA (4216C>A), GCA631GGG (4218C>G 4219A>G), TGT632GTC (4220T>G 4221G>T 4222T>C), CTA633CTT (4225A>T), ACA634ATG (4227C>T 4228A>G), CAG635CAA (4231G>A), ACA636_CAC637del (4232_4237delACACAC), GAT638GAA (4240T>A), AAA640GAA (4244A>G), AGA641AAA (4248G>A), CAC642CAT (4252C>T), CCA643CCG (4255A>G), GCC645GCT (4261C>T), TAT646TAC (4264T>C), TAT647GAG (4265T>G 4267T>G), TCC648AGC (4268T>A 4269C>G), CGG649CGC (4273G>C), AAA650AAG (4276A>G), ATG651CTA (4277A>C 4279G>A), ACC652AAC (4281C>A), ACA653GAG (4283A>G 4284C>A 4285A>G), GCG654ACA (4286G>A 4288G>A), CAG656CGA (4293A>G 4294G>A), AAC657CGG (4295A>C 4296A>G 4297C>G), GAC659ACA (4301G>A 4302A>C 4303C>A), ATC660GTC (4304A>G), CAT661CAA (4309T>A), GAC662GAG (4312C>G), CTT665ATG (4319C>A 4321T>G), CTA666ACA (4322C>A 4323T>C), ATT668ATC (4330T>C), GTT669GTC (4333T>C), GCC670CAC (4334G>C 4335C>A), GCC671TGC (4337G>T 4338C>G), ATG672CTG (4340A>C), CAA673CGC (4344A>G 4345A>C), CAT674ACC (4346C>A 4347A>C 4348T>C), GTG677CAC (4355G>C 4356T>A 4357G>C), GTC679TTG (4361G>T 4363C>G), GAG680CTT (4364G>C 4365A>T 4366G>T), TCA826TCG (4804A>G), TTC827TGG (4806T>G 4807C>G), CCA828AAT (4808C>A 4809C>A 4810A>T), CAG829GGT (4811C>G 4812A>G 4813G>T), AGA831AAA (4818G>A), CTT832AGG (4820C>A 4821T>G 4822T>G), AAG833GAC (4823A>G 4825G>C), GTG834ATT (4826G>A 4828G>T), CTA835GCA (4829C>G 4830T>C), CGC836GAA (4832C>G 4833G>A 4834C>A), TAC837TTT (4836A>T 4837C>T), ATC838GTA (4838A>G 4840C>A), AAG839GCC (4841A>G 4842A>C 4843G>C), AAA840CAA (4844A>C), TGC841TGT (4849C>T), GTA842CCA (4850G>C 4851T>C), CAC843AAT (4853C>A 4855C>T), AAC847GTC (4865A>G 4866A>T), AAA848AAG (4870A>G), GCT849GTA (4872C>T 4873T>A), GCA850GAA (4875C>A), CGG851CAC (4878G>A 4879G>C), CAC852CAA (4882C>A), GCG853AGG (4883G>A 4884C>G), AAA854CCA (4886A>C 4887A>C), TAC855GGC (4889T>G 4890A>G), GGT856GGA (4894T>A), CAC857TTA (4895C>T 4896A>T 4897C>A), CAG859CAA (4903G>A), TTC860GAC (4904T>G 4905T>A), AGG861ATT (4908G>T 4909G>T), ACA862GAA (4910A>G 4911C>A), CCA863ATT (4913C>A 4914C>T 4915A>T), CCA864CCT (4918A>T), ACG865ACT (4921G>T), AAA866TGG (4922A>T 4923A>G 4924A>G), CCA867AAG (4925C>A 4926C>A 4927A>G), GAC869GAA (4933C>A), GTT871GTA (4939T>A), ACG872AAT (4941C>A 4942G>T), TTC875TTT (4951C>T), ATT876GTT (4952A>G), ACG877GTG (4955A>G 4956C>T), AAA878GGA (4958A>G 4959A>G), CTC879TTG (4961C>T 4963C>G), CCG880CCA (4966G>A), AGG881AAG (4968G>A), TCA882ACT (4970T>A 4972A>T), AAG883AGG (4974A>G), GAT884_ACA887del (4976_4987delGATCGAGTCACA), GGA888AGA (4988G>A), CAA889CAG (4993A>G), GCC890del (4994_4996delGCC), TAT891CAT (4997T>C), GAC892GAT (5002C>T), ATG893TCT (5003A>T 5004T>C 5005G>T), CTA895TGG (5009C>T 5010T>G 5011A>G), GTC896GTG (5014C>G), ATG897ATA (5017G>A), GTC898GTT (5020C>T), AGA900CGA (5024A>C), CTC901ATG (5027C>A 5029C>G), ACA902ACC (5032A>C), TAT904TCA (5037A>C 5038T>A), GCA905GCC (5041A>C), ATT908CTA (5048A>C 5050T>A), CCT909CCA (5053T>A), GCA910GTC (5055C>T 5056A>C), TCA911AAG (5057T>A 5058C>A 5059A>G), GAA912ACT (5060G>A 5061A>C 5062A>T), ATA913ACT (5064T>C 5065A>T), TAC914TTC (5067A>T), ACT915AGT (5070C>G), GCA916GCT (5074A>T), GAG917GAA (5077G>A), CAG918AAT (5078C>A 5080G>T), CTC919TAT (5081C>T 5082T>A 5083C>T), GGA920GCC (5085G>C 5086A>C), TAC921AAA (5087T>A 5089C>A), CTC922CTG (5092C>G), GTA923TAT (5093G>T 5094T>A 5095A>T), CTG924ATT (5096C>A 5098G>T), GAC925CGT (5099G>C 5100A>G 5101C>T), AGA926GAA (5102A>G 5103G>A), ATC928GTC (5108A>G), CGA929AGA (5111C>A), TAT930CTT (5114T>C 5115A>T), CAC931CAT (5119C>T), GGA932GGT (5122A>T), TTC933ATT (5123T>A 5125C>T), CCG934CCC (5128G>C), GAA935TTG (5129G>T 5130A>T 5131A>G), GTA936TCC (5132G>T 5133T>C 5134A>C), TTC937ATC (5135T>A), ACA939TCT (5141A>T 5143A>T), GAC940GAT (5146C>T), GAC942GGC (5151A>G), AAG943ACT (5154A>C 5155G>T), CTC944TAA (5156C>T 5157T>A 5158C>A), TCA947TCG (5167A>G), AAC948CAC (5168A>C), TAC949TTT (5172A>T 5173C>T), AAG951AAA (5179G>A), ACG952TCA (5180A>T 5182G>A), CTC953TTT (5183C>T 5185C>T), ATG954TAG (5186A>T 5187T>A), GGA955AAG (5189G>A 5190G>A 5191A>G), ACG956GGA (5192A>G 5193C>G 5194G>A), ATT957CTT (5195A>C), GGA958GGC (5200A>C), ATC959ACC (5202T>C), AAG960AAA (5206G>A), CAC961GTT (5207C>G 5208A>T 5209C>T), TTG963CTA (5213T>C 5215G>A), TCA964AGC (5216T>A 5217C>G 5218A>C), ACA965ACT (5221A>T), TAC967TTT (5226A>T 5227C>T), CAT968CAC (5230T>C), CCA969CCT (5233A>T), GAG970CAG (5234G>C), ACG971ATA (5238C>T 5239G>A), GGG973GGA (5245G>A), CAA974CAG (5248A>G), ACG975GCA (5249A>G 5251G>A), AGA977CGA (5255A>C), AAC979ATT (5262A>T 5263C>T), ACA981ACC (5269A>C), CAA984GAA (5276C>G)								

Proteins

	Begin	End	Coverage	Score	Concordance	Matches	Identities	I/D/M/F*	Stop Codons
Reverse Transcriptase (YP_009666308.1)	303	985	46.5%	1658	48.7%	479 (97.8%)	228 (46.5%)	4/7/0/0	3

	Begin	End	Coverage	Score	Concordance	Matches	Identities	I/D/M/F*	Stop Codons
**NT**	**3231**	**5281**	**19.8%**	**193**	**6.8%**	**1441 (97.8%)**	**786 (53.3%)**	**12/21**	
Protein mutations:	H306K (3242C>A 3244C>G), Q307L (3245C>T 3246A>T), W309P (3251T>C 3252G>C), W309_D310insKREV (3253_3254insAAGAGGGAGGTG), N314E (3266A>G 3268C>G), I315L (3269A>C 3271T>C), Q316E (3272C>G 3274G>A), K319A (3281A>G 3282A>C 3283A>C), E320T (3284G>A 3285A>C 3286G>A), W323A (3293T>G 3294G>C), G324A (3297G>C 3298A>T), P325V (3299C>G 3300C>T 3301C>G), L326P (3303T>C), Q328R (3309A>G 3310A>C), S330A (3314T>G 3316T>A), E331P (3317G>C 3318A>C 3319G>T), K332P (3320A>C 3321A>C 3322A>G), Q335E (3329C>G 3331G>A), T336E (3332A>G 3333C>A 3334C>A), L373M (3443C>A 3445C>G), V374C (3446G>T 3447T>G 3448A>C), Q375M (3449C>A 3450A>T 3451A>G), K379A (3461A>G 3462A>C), E382K (3470G>A), I383V (3473A>G 3475C>A), I385V (3479A>G 3481C>G), R388K (3488C>A 3489G>A 3490A>G), L391V (3497C>G 3499A>T), N393L (3503A>T 3504A>T 3505C>G), I394M (3508C>G), E395D (3511A>T), E396D (3514A>T), A397L (3515G>C 3516C>T 3517A>G), Q398L (3518C>T 3519A>T 3520A>G), R400K (3525G>A 3526A>G), T402S (3531C>G), G403E (3534G>A), S404A (3536T>G 3538A>G), D405C (3539G>T 3540A>G), Y407F (3546A>T), T408S (3548A>T 3550G>T), D414S (3566G>T 3567A>C 3568C>A), A415G (3570C>G), F416Y (3573T>A 3574C>T), A418Q (3578G>C 3579C>A 3580T>G), I419V (3581A>G 3583C>T), M421I (3589G>A), E425D (3601A>T), E426V (3603A>T 3604A>G), W427P (3605T>C 3606G>C 3607G>T), A430T (3614G>A), R432V (3620A>G 3621G>T 3622G>A), L437* (3635C>T 3636T>A 3637C>A), M445F (3659A>T 3661G>T), S453T (3683T>A), C454F (3687G>T 3688C>T), Q455W (3689C>T 3690A>G), D456N (3692G>A 3694C>T), V458M (3698G>A 3700C>G), E460D (3706A>C), T461V (3707A>G 3708C>T), R463Y (3713A>T 3714G>A 3715A>T), L465Y (3719C>T 3720T>A 3721A>T), V468K (3728G>A 3729T>A), C469F (3732G>T 3733C>T), A472V (3741C>T 3742T>G), Y473F (3744A>T), M474I (3748G>A), L478V (3758C>G 3760G>C), V479I (3761G>A 3763C>A), T481S (3768C>G 3769A>T), K482R (3771A>G), G483D (3774G>A 3775A>T), S484T (3776T>A 3778C>G), Q486D (3782C>G 3784G>T), T489V (3791A>G 3792C>T 3793C>G), K490N (3796G>C), Q491H (3799A>T), V492L (3800G>T 3802T>G), Q493S (3803C>A 3804A>G 3805A>T), D494M (3806G>A 3807A>T 3808T>G), F496L (3814C>G), E497Q (3815G>C), R498S (3818C>A 3820A>T), T500R (3825C>G), K501E (3827A>G 3829G>A), S502Y (3831C>A), G503Q (3833G>C 3834G>A), F504L (3836T>C), K505Y (3839A>T 3841G>T), T506V (3842A>G 3843C>T 3844A>T), A507K (3845G>A 3846C>A), P508M (3848C>A 3849C>T 3850C>G), H514A (3866C>G 3867A>C), K515Q (3869A>C 3871G>A), V518I (3878G>A 3880C>A), F523H (3893T>C 3894T>A), I524L (3896A>C 3898C>A), I525V (3899A>G 3901C>T), T527E (3905A>G 3906C>A), T528N (3909C>A 3910A>C), G529Q (3911G>C 3912G>A 3913G>A), I530V (3914A>G), T531R (3918C>G), I532M (3922C>G), A535K (3929G>A 3930C>A 3931A>G), T537V (3935A>G 3936C>T 3937A>G), S539A (3941T>G 3943A>C), R541V (3947A>G 3948G>T 3949A>T), E542D (3952A>T), P544Q (3957C>A), E545A (3960A>C), V552L (3980G>C 3982A>G), Q553R (3983C>A 3984A>G), N562Y (4010A>T 4012C>T), K567A (4025A>G 4026A>C 4027G>T), D568G (4029A>G 4030C>A), T572K (4041C>A), M576L (4052A>T), M578D (4058A>G 4059T>A 4060G>T), T580L (4064A>C 4065C>T 4066A>G), R581K (4068G>A 4069A>G), D583K (4073G>A 4075C>G), N585K (4081C>G), K587V (4085A>G 4086A>T), G589S (4091G>T 4092G>C 4093A>G), K590E (4094A>G), E591K (4097G>A), Q592C (4100C>T 4101A>G 4102G>C), G614H (4166G>C 4167G>A 4168T>C), S615T (4170G>C), E617P (4175G>C 4176A>C), V618Y (4178G>T 4179T>A), H619E (4181C>G 4183C>A), I620V (4184A>G 4186C>G), E621Q (4187G>C), M627F (4205A>T 4207G>C), I629L (4211A>T), A631G (4218C>G 4219A>G), C632V (4220T>G 4221G>T 4222T>C), T634M (4227C>T 4228A>G), T636_H637del (4232_4237delACACAC), D638E (4240T>A), K640E (4244A>G), R641K (4248G>A), Y647E (4265T>G 4267T>G), M651L (4277A>C 4279G>A), T652N (4281C>A), T653E (4283A>G 4284C>A 4285A>G), A654T (4286G>A 4288G>A), Q656R (4293A>G 4294G>A), N657R (4295A>C 4296A>G 4297C>G), D659T (4301G>A 4302A>C 4303C>A), I660V (4304A>G), H661Q (4309T>A), D662E (4312C>G), L665M (4319C>A 4321T>G), L666T (4322C>A 4323T>C), A670H (4334G>C 4335C>A), A671C (4337G>T 4338C>G), M672L (4340A>C), Q673R (4344A>G 4345A>C), H674T (4346C>A 4347A>C 4348T>C), V677H (4355G>C 4356T>A 4357G>C), V679L (4361G>T 4363C>G), E680L (4364G>C 4365A>T 4366G>T), F827W (4806T>G 4807C>G), P828N (4808C>A 4809C>A 4810A>T), Q829G (4811C>G 4812A>G 4813G>T), R831K (4818G>A), L832R (4820C>A 4821T>G 4822T>G), K833D (4823A>G 4825G>C), V834I (4826G>A 4828G>T), L835A (4829C>G 4830T>C), R836E (4832C>G 4833G>A 4834C>A), Y837F (4836A>T 4837C>T), I838V (4838A>G 4840C>A), K839A (4841A>G 4842A>C 4843G>C), K840Q (4844A>C), V842P (4850G>C 4851T>C), H843N (4853C>A 4855C>T), N847V (4865A>G 4866A>T), A849V (4872C>T 4873T>A), A850E (4875C>A), R851H (4878G>A 4879G>C), H852Q (4882C>A), A853R (4883G>A 4884C>G), K854P (4886A>C 4887A>C), Y855G (4889T>G 4890A>G), H857L (4895C>T 4896A>T 4897C>A), F860D (4904T>G 4905T>A), R861I (4908G>T 4909G>T), T862E (4910A>G 4911C>A), P863I (4913C>A 4914C>T 4915A>T), K866W (4922A>T 4923A>G 4924A>G), P867K (4925C>A 4926C>A 4927A>G), D869E (4933C>A), T872N (4941C>A 4942G>T), I876V (4952A>G), T877V (4955A>G 4956C>T), K878G (4958A>G 4959A>G), R881K (4968G>A), S882T (4970T>A 4972A>T), K883R (4974A>G), D884_T887del (4976_4987delGATCGAGTCACA), G888R (4988G>A), A890del (4994_4996delGCC), Y891H (4997T>C), M893S (5003A>T 5004T>C 5005G>T), L895W (5009C>T 5010T>G 5011A>G), M897I (5017G>A), L901M (5027C>A 5029C>G), Y904S (5037A>C 5038T>A), I908L (5048A>C 5050T>A), A910V (5055C>T 5056A>C), S911K (5057T>A 5058C>A 5059A>G), E912T (5060G>A 5061A>C 5062A>T), I913T (5064T>C 5065A>T), Y914F (5067A>T), T915S (5070C>G), Q918N (5078C>A 5080G>T), L919Y (5081C>T 5082T>A 5083C>T), G920A (5085G>C 5086A>C), Y921K (5087T>A 5089C>A), V923Y (5093G>T 5094T>A 5095A>T), L924I (5096C>A 5098G>T), D925R (5099G>C 5100A>G 5101C>T), R926E (5102A>G 5103G>A), I928V (5108A>G), Y930L (5114T>C 5115A>T), F933I (5123T>A 5125C>T), E935L (5129G>T 5130A>T 5131A>G), V936S (5132G>T 5133T>C 5134A>C), F937I (5135T>A), T939S (5141A>T 5143A>T), D942G (5151A>G), K943T (5154A>C 5155G>T), L944* (5156C>T 5157T>A 5158C>A), N948H (5168A>C), Y949F (5172A>T 5173C>T), T952S (5180A>T 5182G>A), L953F (5183C>T 5185C>T), M954* (5186A>T 5187T>A), G955K (5189G>A 5190G>A 5191A>G), T956G (5192A>G 5193C>G 5194G>A), I957L (5195A>C), I959T (5202T>C), H961V (5207C>G 5208A>T 5209C>T), Y967F (5226A>T 5227C>T), E970Q (5234G>C), T971I (5238C>T 5239G>A), T975A (5249A>G 5251G>A), N979I (5262A>T 5263C>T), Q984E (5276C>G)								

	Begin	End	Coverage	Score	Concordance	Matches	Identities	I/D/M/F*	Stop Codons
**NT**	**3231**	**5281**	**19.8%**	**193**	**6.8%**	**1441 (97.8%)**	**786 (53.3%)**	**12/21**	

Codon mutations:

GCC302.AG (3231C>A 3232C>G), TTA303CTG (3233T>C 3235A>G), CCT304CCA (3238T>A), CAC306AAG (3242C>A 3244C>G), CAA307TTA (3245C>T 3246A>T), TGG309CCG (3251T>C 3252G>C), TGG309_GAT310insAAGAGGGAGGTG (3253_3254insAAGAGGGAGGTG), GAT310GAC (3256T>C), AAC314GAG (3266A>G 3268C>G), ATT315CTC (3269A>C 3271T>C), CAG316GAA (3272C>G 3274G>A), GGG318GGT (3280G>T), AAA319GCC (3281A>G 3282A>C 3283A>C), GAG320ACA (3284G>A 3285A>C 3286G>A), CCT321CCG (3289T>G), TGG323GCG (3293T>G 3294G>C), GGA324GCT (3297G>C 3298A>T), CCC325GTG (3299C>G 3300C>T 3301C>G), CTA326CCA (3303T>C), TAT327TAC (3307T>C), CAA328CGC (3309A>G 3310A>C), TCT330GCA (3314T>G 3316T>A), GAG331CCT (3317G>C 3318A>C 3319G>T), AAA332CCG (3320A>C 3321A>C 3322A>G), CTA334CTT (3328A>T), CAG335GAA (3329C>G 3331G>A), ACC336GAA (3332A>G 3333C>A 3334C>A), CTA337TTA (3335C>T), CGA338AGG (3338C>A 3340A>G), CTC373ATG (3443C>A 3445C>G), GTA374TGC (3446G>T 3447T>G 3448A>C), CAA375ATG (3449C>A 3450A>T 3451A>G), GAC376GAT (3454C>T), TAC377TAT (3457C>T), CGA378CGG (3460A>G), AAG379GCG (3461A>G 3462A>C), TTG380CTG (3464T>C), GAG382AAG (3470G>A), ATC383GTA (3473A>G 3475C>A), ACG384ACT (3478G>T), ATC385GTG (3479A>G 3481C>G), CGA388AAG (3488C>A 3489G>A 3490A>G), TAT389TAC (3493T>C), CCG390CCA (3496G>A), CTA391GTT (3497C>G 3499A>T), CCC392CCT (3502C>T), AAC393TTG (3503A>T 3504A>T 3505C>G), ATC394ATG (3508C>G), GAA395GAT (3511A>T), GAA396GAT (3514A>T), GCA397CTG (3515G>C 3516C>T 3517A>G), CAA398TTG (3518C>T 3519A>T 3520A>G), AGA400AAG (3525G>A 3526A>G), TTA401TTG (3529A>G), ACC402AGC (3531C>G), GGA403GAA (3534G>A), TCA404GCG (3536T>G 3538A>G), GAC405TGC (3539G>T 3540A>G), TAC407TTC (3546A>T), ACG408TCT (3548A>T 3550G>T), ATT410ATA (3556T>A), GAC411GAT (3559C>T), CTA412CTT (3562A>T), CGA413AGG (3563C>A 3565A>G), GAC414TCA (3566G>T 3567A>C 3568C>A), GCC415GGC (3570C>G), TTC416TAT (3573T>A 3574C>T), GCT418CAG (3578G>C 3579C>A 3580T>G), ATC419GTT (3581A>G 3583C>T), CGA420AGG (3584C>A 3586A>G), ATG421ATA (3589G>A), GGA424GGG (3598A>G), GAA425GAT (3601A>T), GAA426GTG (3603A>T 3604A>G), TGG427CCT (3605T>C 3606G>C 3607G>T), AAA428AAG (3610A>G), ACC429ACT (3613C>T), GCT430ACT (3614G>A), TTC431TTT (3619C>T), AGG432GTA (3620A>G 3621G>T 3622G>A), ACA433ACT (3625A>T), AGA434AGG (3628A>G), TAC435TAT (3631C>T), GGA436GGG (3634A>G), CTC437TAA (3635C>T 3636T>A 3637C>A), TAC438TAT (3640C>T), TTG441CTT (3647T>C 3649G>T), GTC442GTA (3652C>A), CCA444CCG (3658A>G), ATG445TTT (3659A>T 3661G>T), GGA446GGG (3664A>G), ACC448ACT (3670C>T), AAC449AAT (3673C>T), GCA450GCC (3676A>C), CCC451CCG (3679C>G), GCA452GCT (3682A>T), TCC453ACC (3683T>A), TGC454TTT (3687G>T 3688C>T), CAG455TGG (3689C>T 3690A>G), GAC456AAT (3692G>A 3694C>T), CTT457TTA (3695C>T 3697T>A), GTC458ATG (3698G>A 3700C>G), AAC459AAT (3703C>T), GAA460GAC (3706A>C), ACA461GTA (3707A>G 3708C>T), CTT462CTG (3712T>G), AGA463TAT (3713A>T 3714G>A 3715A>T), CTA465TAT (3719C>T 3720T>A 3721A>T), CTC466CTT (3724C>T), GAC467GAT (3727C>T), GTG468AAG (3728G>A 3729T>A), TGC469TTT (3732G>T 3733C>T), GTC470GTA (3736C>A), GTT471GTA (3739T>A), GCT472GTG (3741C>T 3742T>G), TAC473TTC (3744A>T), ATG474ATA (3748G>A), GAC475GAT (3751C>T), ATA477ATT (3757A>T), CTG478GTC (3758C>G 3760G>C), GTC479ATA (3761G>A 3763C>A), TAC480TAT (3766C>T), ACA481AGT (3768C>G 3769A>T), AAA482AGA (3771A>G), GGA483GAT (3774G>A 3775A>T), TCC484ACG (3776T>A 3778C>G), CTC485TTG (3779C>T 3781C>G), CAG486GAT (3782C>G 3784G>T), ACC489GTG (3791A>G 3792C>T 3793C>G), AAG490AAC (3796G>C), CAA491CAT (3799A>T), GTT492TTG (3800G>T 3802T>G), CAA493AGT (3803C>A 3804A>G 3805A>T), GAT494ATG (3806G>A 3807A>T 3808T>G), TTC496TTG (3814C>G), GAA497CAA (3815G>C), CGA498AGT (3818C>A 3820A>T), CTC499TTG (3821C>T 3823C>G), ACG500AGG (3825C>G), AAG501GAA (3827A>G 3829G>A), TCC502TAC (3831C>A), GGA503CAA (3833G>C 3834G>A), TTC504CTC (3836T>C), AAG505TAT (3839A>T 3841G>T), ACA506GTT (3842A>G 3843C>T 3844A>T), GCA507AAA (3845G>A 3846C>A), CCC508ATG (3848C>A 3849C>T 3850C>G), AAA510AAG (3856A>G), TGC511TGT (3859C>T), GAA512GAG (3862A>G), TTC513TTT (3865C>T), CAC514GCC (3866C>G 3867A>C), AAG515CAA (3869A>C 3871G>A), GAA517GAG (3877A>G), GTC518ATA (3878G>A 3880C>A), TTT520TTC (3886T>C), TTA521CTA (3887T>C), GGC522GGG (3892C>G), TTT523CAT (3893T>C 3894T>A), ATC524CTA (3896A>C 3898C>A), ATC525GTT (3899A>G 3901C>T), ACA527GAA (3905A>G 3906C>A), ACA528AAC (3909C>A 3910A>C), GGG529CAA (3911G>C 3912G>A 3913G>A), ATA530GTA (3914A>G), ACG531AGG (3918C>G), ATC532ATG (3922C>G), GAC533GAT (3925C>T), GCA535AAG (3929G>A 3930C>A 3931A>G), AAG536AAA (3934G>A), ACA537GTG (3935A>G 3936C>T 3937A>G), CAG538CAA (3940G>A), TCA539GCC (3941T>G 3943A>C), ATC540ATT (3946C>T), AGA541GTT (3947A>G 3948G>T 3949A>T), GAA542GAT (3952A>T), CCA544CAA (3957C>A), GAA545GCA (3960A>C), CCG546CCC (3964G>C), AAG547AAA (3967G>A), ACA548ACT (3970A>T), GTC549GTG (3973C>G), GTA552CTG (3980G>C 3982A>G), CAG553AGG (3983C>A 3984A>G), TCA554TCT (3988A>T), CTT556TTG (3992C>T 3994T>G), GGA557GGG (3997A>G), CTC558CTG (4000C>G), GCC559GCA (4003C>A), AAC560AAT (4006C>T), TAC561TAT (4009C>T), AAC562TAT (4010A>T 4012C>T), AAG567GCT (4025A>G 4026A>C 4027G>T), GAC568GGA (4029A>G 4030C>A), TAT569TAC (4033T>C), TCA570TCG (4036A>G), ACA572AAA (4041C>A), ATG576TTG (4052A>T), ACG577ACT (4057G>T), ATG578GAT (4058A>G 4059T>A 4060G>T), CTT579TTG (4061C>T 4063T>G), ACA580CTG (4064A>C 4065C>T 4066A>G), AGA581AAG (4068G>A 4069A>G), AAA582AAG (4072A>G), GAC583AAG (4073G>A 4075C>G), AAC585AAG (4081C>G), AAA587GTA (4085A>G 4086A>T), GGA589TCG (4091G>T 4092G>C 4093A>G), AAA590GAA (4094A>G), GAA591AAA (4097G>A), CAG592TGC (4100C>T 4101A>G 4102G>C), ACC593GA. (4103A>G 4104C>A), GAT613GAC (4165T>C), GGT614CAC (4166G>C 4167G>A 4168T>C), AGC615ACC (4170G>C), GAA617CCA (4175G>C 4176A>C), GTC618TAC (4178G>T 4179T>A), CAC619GAA (4181C>G 4183C>A), ATC620GTG (4184A>G 4186C>G), GAG621CAG (4187G>C), ACC622ACG (4192C>G), GAC623GAT (4195C>T), GCG624GCC (4198G>C), TCT625TCA (4201T>A), GAT626GAC (4204T>C), ATG627TTC (4205A>T 4207G>C), GCA628GCC (4210A>C), ATA629TTA (4211A>T), GGC630GGA (4216C>A), GCA631GGG (4218C>G 4219A>G), TGT632GTC (4220T>G 4221G>T 4222T>C), CTA633CTT (4225A>T), ACA634ATG (4227C>T 4228A>G), CAG635CAA (4231G>A), ACA636_CAC637del (4232_4237delACACAC), GAT638GAA (4240T>A), AAA640GAA (4244A>G), AGA641AAA (4248G>A), CAC642CAT (4252C>T), CCA643CCG (4255A>G), GCC645GCT (4261C>T), TAT646TAC (4264T>C), TAT647GAG (4265T>G 4267T>G), TCC648AGC (4268T>A 4269C>G), CGG649CGC (4273G>C), AAA650AAG (4276A>G), ATG651CTA (4277A>C 4279G>A), ACC652AAC (4281C>A), ACA653GAG (4283A>G 4284C>A 4285A>G), GCG654ACA (4286G>A 4288G>A), CAG656CGA (4293A>G 4294G>A), AAC657CGG (4295A>C 4296A>G 4297C>G), GAC659ACA (4301G>A 4302A>C 4303C>A), ATC660GTC (4304A>G), CAT661CAA (4309T>A), GAC662GAG (4312C>G), CTT665ATG (4319C>A 4321T>G), CTA666ACA (4322C>A 4323T>C), ATT668ATC (4330T>C), GTT669GTC (4333T>C), GCC670CAC (4334G>C 4335C>A), GCC671TGC (4337G>T 4338C>G), ATG672CTG (4340A>C), CAA673CGC (4344A>G 4345A>C), CAT674ACC (4346C>A 4347A>C 4348T>C), GTG677CAC (4355G>C 4356T>A 4357G>C), GTC679TTG (4361G>T 4363C>G), GAG680CTT (4364G>C 4365A>T 4366G>T), TCA826TCG (4804A>G), TTC827TGG (4806T>G 4807C>G), CCA828AAT (4808C>A 4809C>A 4810A>T), CAG829GGT (4811C>G 4812A>G 4813G>T), AGA831AAA (4818G>A), CTT832AGG (4820C>A 4821T>G 4822T>G), AAG833GAC (4823A>G 4825G>C), GTG834ATT (4826G>A 4828G>T), CTA835GCA (4829C>G 4830T>C), CGC836GAA (4832C>G 4833G>A 4834C>A), TAC837TTT (4836A>T 4837C>T), ATC838GTA (4838A>G 4840C>A), AAG839GCC (4841A>G 4842A>C 4843G>C), AAA840CAA (4844A>C), TGC841TGT (4849C>T), GTA842CCA (4850G>C 4851T>C), CAC843AAT (4853C>A 4855C>T), AAC847GTC (4865A>G 4866A>T), AAA848AAG (4870A>G), GCT849GTA (4872C>T 4873T>A), GCA850GAA (4875C>A), CGG851CAC (4878G>A 4879G>C), CAC852CAA (4882C>A), GCG853AGG (4883G>A 4884C>G), AAA854CCA (4886A>C 4887A>C), TAC855GGC (4889T>G 4890A>G), GGT856GGA (4894T>A), CAC857TTA (4895C>T 4896A>T 4897C>A), CAG859CAA (4903G>A), TTC860GAC (4904T>G 4905T>A), AGG861ATT (4908G>T 4909G>T), ACA862GAA (4910A>G 4911C>A), CCA863ATT (4913C>A 4914C>T 4915A>T), CCA864CCT (4918A>T), ACG865ACT (4921G>T), AAA866TGG (4922A>T 4923A>G 4924A>G), CCA867AAG (4925C>A 4926C>A 4927A>G), GAC869GAA (4933C>A), GTT871GTA (4939T>A), ACG872AAT (4941C>A 4942G>T), TTC875TTT (4951C>T), ATT876GTT (4952A>G), ACG877GTG (4955A>G 4956C>T), AAA878GGA (4958A>G 4959A>G), CTC879TTG (4961C>T 4963C>G), CCG880CCA (4966G>A), AGG881AAG (4968G>A), TCA882ACT (4970T>A 4972A>T), AAG883AGG (4974A>G), GAT884_ACA887del (4976_4987delGATCGAGTCACA), GGA888AGA (4988G>A), CAA889CAG (4993A>G), GCC890del (4994_4996delGCC), TAT891CAT (4997T>C), GAC892GAT (5002C>T), ATG893TCT (5003A>T 5004T>C 5005G>T), CTA895TGG (5009C>T 5010T>G 5011A>G), GTC896GTG (5014C>G), ATG897ATA (5017G>A), GTC898GTT (5020C>T), AGA900CGA (5024A>C), CTC901ATG (5027C>A 5029C>G), ACA902ACC (5032A>C), TAT904TCA (5037A>C 5038T>A), GCA905GCC (5041A>C), ATT908CTA (5048A>C 5050T>A), CCT909CCA (5053T>A), GCA910GTC (5055C>T 5056A>C), TCA911AAG (5057T>A 5058C>A 5059A>G), GAA912ACT (5060G>A 5061A>C 5062A>T), ATA913ACT (5064T>C 5065A>T), TAC914TTC (5067A>T), ACT915AGT (5070C>G), GCA916GCT (5074A>T), GAG917GAA (5077G>A), CAG918AAT (5078C>A 5080G>T), CTC919TAT (5081C>T 5082T>A 5083C>T), GGA920GCC (5085G>C 5086A>C), TAC921AAA (5087T>A 5089C>A), CTC922CTG (5092C>G), GTA923TAT (5093G>T 5094T>A 5095A>T), CTG924ATT (5096C>A 5098G>T), GAC925CGT (5099G>C 5100A>G 5101C>T), AGA926GAA (5102A>G 5103G>A), ATC928GTC (5108A>G), CGA929AGA (5111C>A), TAT930CTT (5114T>C 5115A>T), CAC931CAT (5119C>T), GGA932GGT (5122A>T), TTC933ATT (5123T>A 5125C>T), CCG934CCC (5128G>C), GAA935TTG (5129G>T 5130A>T 5131A>G), GTA936TCC (5132G>T 5133T>C 5134A>C), TTC937ATC (5135T>A), ACA939TCT (5141A>T 5143A>T), GAC940GAT (5146C>T), GAC942GGC (5151A>G), AAG943ACT (5154A>C 5155G>T), CTC944TAA (5156C>T 5157T>A 5158C>A), TCA947TCG (5167A>G), AAC948CAC (5168A>C), TAC949TTT (5172A>T 5173C>T), AAG951AAA (5179G>A), ACG952TCA (5180A>T 5182G>A), CTC953TTT (5183C>T 5185C>T), ATG954TAG (5186A>T 5187T>A), GGA955AAG (5189G>A 5190G>A 5191A>G), ACG956GGA (5192A>G 5193C>G 5194G>A), ATT957CTT (5195A>C), GGA958GGC (5200A>C), ATC959ACC (5202T>C), AAG960AAA (5206G>A), CAC961GTT (5207C>G 5208A>T 5209C>T), TTG963CTA (5213T>C 5215G>A), TCA964AGC (5216T>A 5217C>G 5218A>C), ACA965ACT (5221A>T), TAC967TTT (5226A>T 5227C>T), CAT968CAC (5230T>C), CCA969CCT (5233A>T), GAG970CAG (5234G>C), ACG971ATA (5238C>T 5239G>A), GGG973GGA (5245G>A), CAA974CAG (5248A>G), ACG975GCA (5249A>G 5251G>A), AGA977CGA (5255A>C), AAC979ATT (5262A>T 5263C>T), ACA981ACC (5269A>C), CAA984GAA (5276C>G)

*: Inserts / Deletes / Misaligned / Frameshifts

Analysis details

This analysis was performed with panviral2.64

NGS Details (UN59): Epiphyllum badnavirus 1

Assembly

**Coverage Length**	414 (1 contig(s))
**Depth Of Coverage**	143.5
**Number Of Reads**	512
**Reads Per Million**	11.13 rpm (after QC)
**Ambiguities**	0
**Assembly Method**	de novo + reference guided assembly
**Consensus Caller**	Bcf Tools

Coverage Map

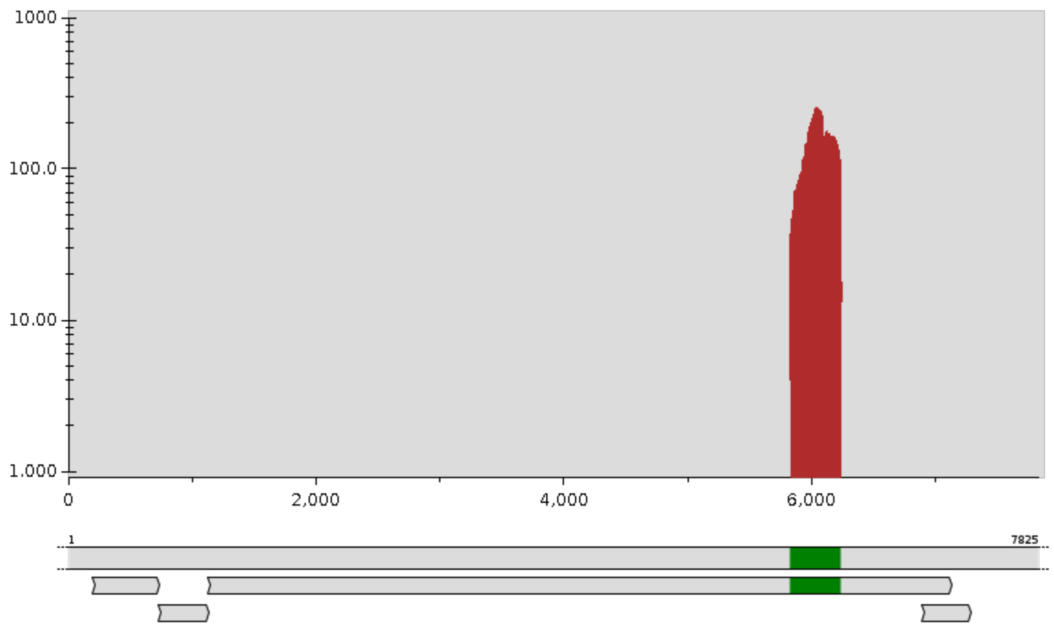

Assignment

**Type**	Epiphyllum badnavirus 1 (Taxonomy ID: 2518008)
**Reference Genome**	NC_076247.1
**NT Identity (%)**	52.518
**AA Identity (%)**	46.7626
**Number Of Stop Codons**	0
**Number Of CDS**	4

Alignment

**Alignment Score**	36.0 (NT) + 441.0 (AA) = 477.0
**Concordance (%)**	26.5295

**Alignment Method**	Global, seeded, nucleotide + amino acids (AGA)

Genome Region

Sequence starts at position 5825 and ends at position 6238 relative to NC_076247.1 reference sequence.

Alignment Detailed Statistics

	Begin	End	Coverage	Score	Concordance	Matches	Identities	I/D/M/F*	Stop Codons
**NT**	**5825**	**6238**	**5.3%**	**36**	**4.3%**	**414 (99.3%)**	**219 (52.5%)**	**3/0**	
Mutations:	5835C>T, 5836A>T, 5837G>C, 5841C>G, 5847C>T, 5851T>A, 5853C>T, 5856T>C, 5857C>T, 5861A>G, 5862G>A, 5863A>T, 5864G>C, 5865C>G, 5868A>T, 5870T>A, 5871C>T, 5874C>T, 5878A>T, 5880T>G, 5882T>G, 5883G>A, 5884A>G, 5886G>T, 5887G>C, 5888A>G, 5889C>A, 5892A>G, 5893G>T, 5894A>C, 5896T>G, 5897C>A, 5898C>T, 5904T>G, 5905T>A, 5906G>A, 5910C>T, 5913C>G, 5917C>A, 5918T>G, 5924C>G, 5925A>C, 5926G>T, 5931A>C, 5933T>A, 5937C>T, 5940A>G, 5942G>T, 5943G>T, 5944T>G, 5946A>G, 5953C>T, 5955A>T, 5958C>T, 5961A>G, 5966A>C, 5967G>T, 5981T>C, 5982G>T, 5986C>A, 5987A>T, 5988A>G, 5989C>G, 5990G>A, 5991A>G, 5992A>T, 5993A>T, 5998G>A, 6001A>C, 6002A>G, 6003C>A, 6004T>G, 6005G>T, 6006T>G, 6011G>A, 6012_6013insTCT, 6013G>T, 6015C>T, 6018G>T, 6019T>G, 6020C>A, 6021A>T, 6022G>C, 6023G>A, 6024C>G, 6030A>G, 6031G>A, 6032C>T, 6033A>T, 6036T>G, 6038A>T, 6042T>A, 6048C>T, 6052C>T, 6057C>T, 6059T>A, 6060C>T, 6061A>T, 6062G>C, 6063T>C, 6066C>G, 6068C>G, 6072A>G, 6073G>A, 6074A>G, 6078C>T, 6083C>T, 6084A>G, 6085C>A, 6090C>T, 6091C>T, 6095A>G, 6096G>A, 6098C>T, 6099C>T, 6100A>G, 6103C>T, 6109G>A, 6110T>G, 6111T>G, 6113G>T, 6114T>G, 6115G>C, 6116A>G, 6118A>G, 6119G>A, 6120A>T, 6124G>A, 6125G>A, 6127C>T, 6130A>T, 6131T>A, 6132C>T, 6133C>G, 6134T>C, 6136T>A, 6137C>A, 6138T>G, 6139C>T, 6140C>T, 6141T>C, 6142A>T, 6144G>A, 6148A>T, 6149T>G, 6150G>T, 6151A>G, 6154A>G, 6156A>T, 6157G>T, 6158C>G, 6159T>G, 6160G>C, 6161C>T, 6165A>G, 6166G>T, 6167A>C, 6168G>A, 6169A>G, 6171T>A, 6172G>T, 6173A>C, 6177C>T, 6180G>A, 6183A>T, 6184A>C, 6185T>A, 6186C>T, 6187A>G, 6188C>T, 6189C>G, 6190C>G, 6192A>G, 6193G>A, 6194G>C, 6195A>T, 6199C>G, 6201G>T, 6202C>G, 6203A>G, 6204G>C, 6205C>A, 6207G>T, 6209A>T, 6211T>G, 6213G>T, 6214C>G, 6216A>T, 6219C>G, 6220C>G, 6221A>C, 6224T>A, 6225C>A, 6226A>G, 6228C>G, 6229A>G								

CDS

	Begin	End	Coverage	Score	Concordance	Matches	Identities	I/D/M/F*	Stop Codons
QKM20_gp3	1567	1704	6.9%	441	45.0%	138 (99.3%)	65 (46.8%)	1/0/0/0	0
Protein mutations:	F1575I (5851T>A 5853C>T), K1578R (5861A>G 5862G>A), F1581Y (5870T>A 5871C>T), I1584L (5878A>T 5880T>G), M1585R (5882T>G 5883G>A), M1586V (5884A>G 5886G>T), D1587R (5887G>C 5888A>G 5889C>A), E1589S (5893G>T 5894A>C), S1590D (5896T>G 5897C>A 5898C>T), W1593K (5905T>A 5906G>A), L1597R (5917C>A 5918T>G), P1599R (5924C>G 5925A>C), D1600Y (5926G>T), L1602H (5933T>A), W1605F (5942G>T 5943G>T), L1606V (5944T>G 5946A>G), P1609S (5953C>T 5955A>T), K1613T (5966A>C 5967G>T), V1618A (5981T>C 5982G>T), Q1620M (5986C>A 5987A>T 5988A>G), R1621E (5989C>G 5990G>A 5991A>G), K1622L (5992A>T 5993A>T), D1624N (5998G>A), N1625R (6001A>C 6002A>G 6003C>A), C1626V (6004T>G 6005G>T 6006T>G), R1628K (6011G>A), R1628_D1629insS (6012_6013insTCT), D1629Y (6013G>T 6015C>T), S1631D (6019T>G 6020C>A 6021A>T), G1632Q (6022G>C 6023G>A 6024C>G), A1635I (6031G>A 6032C>T 6033A>T), Y1637F (6038A>T), F1644Y (6059T>A 6060C>T), N1646K (6066C>G), T1647S (6068C>G), D1649S (6073G>A 6074A>G), A1652V (6083C>T 6084A>G), Q1653K (6085C>A), K1656R (6095A>G 6096G>A), S1657F (6098C>T 6099C>T), M1658V (6100A>G), V1661R (6109G>A 6110T>G 6111T>G), C1662L (6113G>T 6114T>G), E1663R (6115G>C 6116A>G), R1664D (6118A>G 6119G>A 6120A>T), G1666K (6124G>A 6125G>A), I1668Y (6130A>T 6131T>A 6132C>T), L1669A (6133C>G 6134T>C), S1670K (6136T>A 6137C>A 6138T>G), P1671F (6139C>T 6140C>T 6141T>C), T1672S (6142A>T 6144G>A), M1674C (6148A>T 6149T>G 6150G>T), K1675E (6151A>G), I1676V (6154A>G 6156A>T), A1677W (6157G>T 6158C>G 6159T>G), A1678L (6160G>C 6161C>T), E1680S (6166G>T 6167A>C 6168G>A), I1681V (6169A>G 6171T>A), D1682S (6172G>T 6173A>C), I1686H (6184A>C 6185T>A 6186C>T), T1687V (6187A>G 6188C>T 6189C>G), L1688V (6190C>G 6192A>G), G1689T (6193G>A 6194G>C 6195A>T), Q1691D (6199C>G 6201G>T), Q1692G (6202C>G 6203A>G 6204G>C), L1693I (6205C>A 6207G>T), K1694M (6209A>T), L1695V (6211T>G 6213G>T), Q1696D (6214C>G 6216A>T), H1698A (6220C>G 6221A>C), I1699K (6224T>A 6225C>A), I1700V (6226A>G 6228C>G), T1701A (6229A>G)								
Codon mutations:	GCC1569GCT (5835C>T), AGT1570TCT (5836A>T 5837G>C), GTC1571GTG (5841C>G), TCC1573TCT (5847C>T), TTC1575ATT (5851T>A 5853C>T), GAT1576GAC (5856T>C), CTG1577TTG (5857C>T), AAG1578AGA (5861A>G 5862G>A), AGC1579TCG (5863A>T 5864G>C 5865C>G), GGA1580GGT (5868A>T), TTC1581TAT (5870T>A 5871C>T), CAC1582CAT (5874C>T), ATT1584TTG (5878A>T 5880T>G), ATG1585AGA (5882T>G 5883G>A), ATG1586GTT (5884A>G 5886G>T), GAC1587CGA (5887G>C 5888A>G 5889C>A), GAA1588GAG (5892A>G), GAA1589TCA (5893G>T 5894A>C), TCC1590GAT (5896T>G 5897C>A 5898C>T), CCT1592CCG (5904T>G), TGG1593AAG (5905T>A 5906G>A), ACC1594ACT (5910C>T), GCC1595GCG (5913C>G), CTG1597AGG (5917C>A 5918T>G), CCA1599CGC (5924C>G 5925A>C), GAT1600TAT (5926G>T), GGA1601GGC (5931A>C), CTT1602CAT (5933T>A), TAC1603TAT (5937C>T), GAA1604GAG (5940A>G), TGG1605TTT (5942G>T 5943G>T), TTA1606GTG (5944T>G 5946A>G), CCA1609TCT (5953C>T 5955A>T), TTC1610TTT (5958C>T), GGA1611GGG (5961A>G), AAG1613ACT (5966A>C 5967G>T), GTG1618GCT (5981T>C 5982G>T), CAA1620ATG (5986C>A 5987A>T 5988A>G), CGA1621GAG (5989C>G 5990G>A 5991A>G), AAG1622TTG (5992A>T 5993A>T), GAC1624AAC (5998G>A), AAC1625CGA (6001A>C 6002A>G 6003C>A), TGT1626GTG (6004T>G 6005G>T 6006T>G), AGA1628AAA (6011G>A), AGA1628_GAC1629insTCT (6012_6013insTCT), GAC1629TAT (6013G>T 6015C>T), CTG1630CTT (6018G>T), TCA1631GAT (6019T>G 6020C>A 6021A>T), GGC1632CAG (6022G>C 6023G>A 6024C>G), GTA1634GTG (6030A>G), GCA1635ATT (6031G>A 6032C>T 6033A>T), GTT1636GTG (6036T>G), TAC1637TTC (6038A>T), ATT1638ATA (6042T>A), GAC1640GAT (6048C>T), CTG1642TTG (6052C>T), GTC1643GTT (6057C>T), TTC1644TAT (6059T>A 6060C>T), AGT1645TCC (6061A>T 6062G>C 6063T>C), AAC1646AAG (6066C>G), ACT1647AGT (6068C>G), GAA1648GAG (6072A>G), GAT1649AGT (6073G>A 6074A>G), GAC1650GAT (6078C>T), GCA1652GTG (6083C>T 6084A>G), CAG1653AAG (6085C>A), CAC1654CAT (6090C>T), CTG1655TTG (6091C>T), AAG1656AGA (6095A>G 6096G>A), TCC1657TTT (6098C>T 6099C>T), ATG1658GTG (6100A>G), CTG1659TTG (6103C>T), GTT1661AGG (6109G>A 6110T>G 6111T>G), TGT1662TTG (6113G>T 6114T>G), GAG1663CGG (6115G>C 6116A>G), AGA1664GAT (6118A>G 6119G>A 6120A>T), GGG1666AAG (6124G>A 6125G>A), CTG1667TTG (6127C>T), ATC1668TAT (6130A>T 6131T>A 6132C>T), CTC1669GCC (6133C>G 6134T>C), TCT1670AAG (6136T>A 6137C>A 6138T>G), CCT1671TTC (6139C>T 6140C>T 6141T>C), ACG1672TCA (6142A>T 6144G>A), ATG1674TGT (6148A>T 6149T>G 6150G>T), AAG1675GAG (6151A>G), ATA1676GTT (6154A>G 6156A>T), GCT1677TGG (6157G>T 6158C>G 6159T>G), GCA1678CTA (6160G>C 6161C>T), CAA1679CAG (6165A>G), GAG1680TCA (6166G>T 6167A>C 6168G>A), ATT1681GTA (6169A>G 6171T>A), GAC1682TCC (6172G>T 6173A>C), TTC1683TTT (6177C>T), CTG1684CTA (6180G>A), GGA1685GGT (6183A>T), ATC1686CAT (6184A>C 6185T>A 6186C>T), ACC1687GTG (6187A>G 6188C>T 6189C>G), CTA1688GTG (6190C>G 6192A>G), GGA1689ACT (6193G>A 6194G>C 6195A>T), CAG1691GAT (6199C>G 6201G>T), CAG1692GGC (6202C>G 6203A>G 6204G>C), CTG1693ATT (6205C>A 6207G>T), AAG1694ATG (6209A>T), TTG1695GTT (6211T>G 6213G>T), CAA1696GAT (6214C>G 6216A>T), CCC1697CCG (6219C>G), CAC1698GCC (6220C>G 6221A>C), ATC1699AAA (6224T>A 6225C>A), ATC1700GTG (6226A>G 6228C>G), ACC1701GCC (6229A>G)								

Proteins

	Begin	End	Coverage	Score	Concordance	Matches	Identities	I/D/M/F*	Stop Codons
polyprotein (YP_010797894.1)	1567	1704	6.9%	441	45.0%	138 (99.3%)	65 (46.8%)	1/0/0/0	0
Protein mutations:	F1575I (5851T>A 5853C>T), K1578R (5861A>G 5862G>A), F1581Y (5870T>A 5871C>T), I1584L (5878A>T 5880T>G), M1585R (5882T>G 5883G>A), M1586V (5884A>G 5886G>T), D1587R (5887G>C 5888A>G 5889C>A), E1589S (5893G>T 5894A>C), S1590D (5896T>G 5897C>A 5898C>T), W1593K (5905T>A 5906G>A), L1597R (5917C>A 5918T>G), P1599R (5924C>G 5925A>C), D1600Y (5926G>T), L1602H (5933T>A), W1605F (5942G>T 5943G>T), L1606V (5944T>G 5946A>G), P1609S (5953C>T 5955A>T), K1613T (5966A>C 5967G>T), V1618A (5981T>C 5982G>T), Q1620M (5986C>A 5987A>T 5988A>G), R1621E (5989C>G 5990G>A 5991A>G), K1622L (5992A>T 5993A>T), D1624N (5998G>A), N1625R (6001A>C 6002A>G 6003C>A), C1626V (6004T>G 6005G>T 6006T>G), R1628K (6011G>A), R1628_D1629insS (6012_6013insTCT), D1629Y (6013G>T 6015C>T), S1631D (6019T>G 6020C>A 6021A>T), G1632Q (6022G>C 6023G>A 6024C>G), A1635I (6031G>A 6032C>T 6033A>T), Y1637F (6038A>T), F1644Y (6059T>A 6060C>T), N1646K (6066C>G), T1647S (6068C>G), D1649S (6073G>A 6074A>G), A1652V (6083C>T 6084A>G), Q1653K (6085C>A), K1656R (6095A>G 6096G>A), S1657F (6098C>T 6099C>T), M1658V (6100A>G), V1661R (6109G>A 6110T>G 6111T>G), C1662L (6113G>T 6114T>G), E1663R (6115G>C 6116A>G), R1664D (6118A>G 6119G>A 6120A>T), G1666K (6124G>A 6125G>A), I1668Y (6130A>T 6131T>A 6132C>T), L1669A (6133C>G 6134T>C), S1670K (6136T>A 6137C>A 6138T>G), P1671F (6139C>T 6140C>T 6141T>C), T1672S (6142A>T 6144G>A), M1674C (6148A>T 6149T>G 6150G>T), K1675E (6151A>G), I1676V (6154A>G 6156A>T), A1677W (6157G>T 6158C>G 6159T>G), A1678L (6160G>C 6161C>T), E1680S (6166G>T 6167A>C 6168G>A), I1681V (6169A>G 6171T>A), D1682S (6172G>T 6173A>C), I1686H (6184A>C 6185T>A 6186C>T), T1687V (6187A>G 6188C>T 6189C>G), L1688V (6190C>G 6192A>G), G1689T (6193G>A 6194G>C 6195A>T), Q1691D (6199C>G 6201G>T), Q1692G (6202C>G 6203A>G 6204G>C), L1693I (6205C>A 6207G>T), K1694M (6209A>T), L1695V (6211T>G 6213G>T), Q1696D (6214C>G 6216A>T), H1698A (6220C>G 6221A>C), I1699K (6224T>A 6225C>A), I1700V (6226A>G 6228C>G), T1701A (6229A>G)								

	Begin	End	Coverage	Score	Concordance	Matches	Identities	I/D/M/F*	Stop Codons
**NT**	**5825**	**6238**	**5.3%**	**36**	**4.3%**	**414 (99.3%)**	**219 (52.5%)**	**3/0**	
Codon mutations:	GCC1569GCT (5835C>T), AGT1570TCT (5836A>T 5837G>C), GTC1571GTG (5841C>G), TCC1573TCT (5847C>T), TTC1575ATT (5851T>A 5853C>T), GAT1576GAC (5856T>C), CTG1577TTG (5857C>T), AAG1578AGA (5861A>G 5862G>A), AGC1579TCG (5863A>T 5864G>C 5865C>G), GGA1580GGT (5868A>T), TTC1581TAT (5870T>A 5871C>T), CAC1582CAT (5874C>T), ATT1584TTG (5878A>T 5880T>G), ATG1585AGA (5882T>G 5883G>A), ATG1586GTT (5884A>G 5886G>T), GAC1587CGA (5887G>C 5888A>G 5889C>A), GAA1588GAG (5892A>G), GAA1589TCA (5893G>T 5894A>C), TCC1590GAT (5896T>G 5897C>A 5898C>T), CCT1592CCG (5904T>G), TGG1593AAG (5905T>A 5906G>A), ACC1594ACT (5910C>T), GCC1595GCG (5913C>G), CTG1597AGG (5917C>A 5918T>G), CCA1599CGC (5924C>G 5925A>C), GAT1600TAT (5926G>T), GGA1601GGC (5931A>C), CTT1602CAT (5933T>A), TAC1603TAT (5937C>T), GAA1604GAG (5940A>G), TGG1605TTT (5942G>T 5943G>T), TTA1606GTG (5944T>G 5946A>G), CCA1609TCT (5953C>T 5955A>T), TTC1610TTT (5958C>T), GGA1611GGG (5961A>G), AAG1613ACT (5966A>C 5967G>T), GTG1618GCT (5981T>C 5982G>T), CAA1620ATG (5986C>A 5987A>T 5988A>G), CGA1621GAG (5989C>G 5990G>A 5991A>G), AAG1622TTG (5992A>T 5993A>T), GAC1624AAC (5998G>A), AAC1625CGA (6001A>C 6002A>G 6003C>A), TGT1626GTG (6004T>G 6005G>T 6006T>G), AGA1628AAA (6011G>A), AGA1628_GAC1629insTCT (6012_6013insTCT), GAC1629TAT (6013G>T 6015C>T), CTG1630CTT (6018G>T), TCA1631GAT (6019T>G 6020C>A 6021A>T), GGC1632CAG (6022G>C 6023G>A 6024C>G), GTA1634GTG (6030A>G), GCA1635ATT (6031G>A 6032C>T 6033A>T), GTT1636GTG (6036T>G), TAC1637TTC (6038A>T), ATT1638ATA (6042T>A), GAC1640GAT (6048C>T), CTG1642TTG (6052C>T), GTC1643GTT (6057C>T), TTC1644TAT (6059T>A 6060C>T), AGT1645TCC (6061A>T 6062G>C 6063T>C), AAC1646AAG (6066C>G), ACT1647AGT (6068C>G), GAA1648GAG (6072A>G), GAT1649AGT (6073G>A 6074A>G), GAC1650GAT (6078C>T), GCA1652GTG (6083C>T 6084A>G), CAG1653AAG (6085C>A), CAC1654CAT (6090C>T), CTG1655TTG (6091C>T), AAG1656AGA (6095A>G 6096G>A), TCC1657TTT (6098C>T 6099C>T), ATG1658GTG (6100A>G), CTG1659TTG (6103C>T), GTT1661AGG (6109G>A 6110T>G 6111T>G), TGT1662TTG (6113G>T 6114T>G), GAG1663CGG (6115G>C 6116A>G), AGA1664GAT (6118A>G 6119G>A 6120A>T), GGG1666AAG (6124G>A 6125G>A), CTG1667TTG (6127C>T), ATC1668TAT (6130A>T 6131T>A 6132C>T), CTC1669GCC (6133C>G 6134T>C), TCT1670AAG (6136T>A 6137C>A 6138T>G), CCT1671TTC (6139C>T 6140C>T 6141T>C), ACG1672TCA (6142A>T 6144G>A), ATG1674TGT (6148A>T 6149T>G 6150G>T), AAG1675GAG (6151A>G), ATA1676GTT (6154A>G 6156A>T), GCT1677TGG (6157G>T 6158C>G 6159T>G), GCA1678CTA (6160G>C 6161C>T), CAA1679CAG (6165A>G), GAG1680TCA (6166G>T 6167A>C 6168G>A), ATT1681GTA (6169A>G 6171T>A), GAC1682TCC (6172G>T 6173A>C), TTC1683TTT (6177C>T), CTG1684CTA (6180G>A), GGA1685GGT (6183A>T), ATC1686CAT (6184A>C 6185T>A 6186C>T), ACC1687GTG (6187A>G 6188C>T 6189C>G), CTA1688GTG (6190C>G 6192A>G), GGA1689ACT (6193G>A 6194G>C 6195A>T), CAG1691GAT (6199C>G 6201G>T), CAG1692GGC (6202C>G 6203A>G 6204G>C), CTG1693ATT (6205C>A 6207G>T), AAG1694ATG (6209A>T), TTG1695GTT (6211T>G 6213G>T), CAA1696GAT (6214C>G 6216A>T), CCC1697CCG (6219C>G), CAC1698GCC (6220C>G 6221A>C), ATC1699AAA (6224T>A 6225C>A), ATC1700GTG (6226A>G 6228C>G), ACC1701GCC (6229A>G)								

*: Inserts / Deletes / Misaligned / Frameshifts

Analysis details

This analysis was performed with panviral2.64

NGS Details (UN59): Human gammaherpesvirus 8

Assembly

**Coverage Length**	135 (1 contig(s))
**Depth Of Coverage**	265.0
**Number Of Reads**	421
**Reads Per Million**	9.15 rpm (after QC)
**Ambiguities**	0
**Assembly Method**	de novo + reference guided assembly
**Consensus Caller**	Bcf Tools

Coverage Map

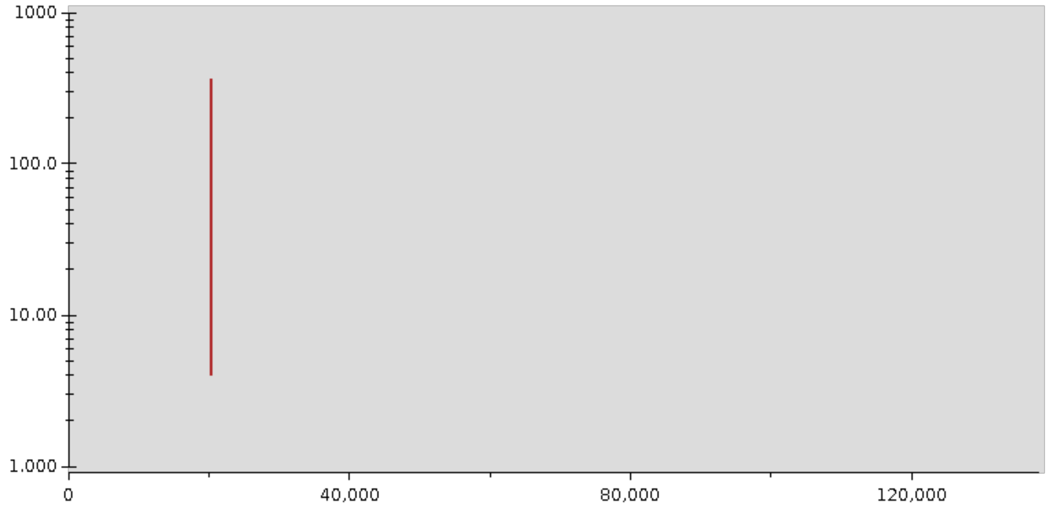

Assignment

**Type**	Human gammaherpesvirus 8 (Taxonomy ID: 37296)
**Subtype**	Could not assign
**Reference Genome**	NC_009333.1
**NT Identity (%)**	83.7037
**AA Identity (%)**	84.4444
**Number Of Stop Codons**	0
**Number Of CDS**	86

Alignment

**Alignment Score**	182.0 (NT) + 287.0 (AA) = 469.0
**Concordance (%)**	78.2972
**Alignment Method**	Local, heuristic, nucleotide (BLASTN)

Genome Region

Sequence starts at position 20273 and ends at position 20407 relative to NC_009333.1 reference sequence.

Alignment Detailed Statistics

	Begin	End	Coverage	Score	Concordance	Matches	Identities	I/D/M/F*	Stop Codons
**NT**	**20273**	**20407**	**0.1%**	**182**	**67.4%**	**135 (100%)**	**113 (83.7%)**	**0/0**	

Mutations: 20293A>T, 20296A>C, 20311G>A, 20314T>G, 20315C>G, 20317C>A, 20320C>T, 20322T>G, 20331G>T, 20341A>C, 20347A>C, 20350G>A, 20352C>T, 20353A>G, 20369C>G, 20370A>C, 20371T>A, 20376A>T, 20383G>A, 20386C>T, 20389A>G, 20392C>T

*: Inserts / Deletes / Misaligned / Frameshifts

Analysis details

This analysis was performed with panviral2.64

NGS Details (UN59): Tomato necrotic dwarf virus (segment RNA1)

Assembly

**Coverage Length**	303 (1 contig(s))
**Depth Of Coverage**	176.1
**Number Of Reads**	407
**Reads Per Million**	8.85 rpm (after QC)
**Ambiguities**	0
**Assembly Method**	de novo + reference guided assembly
**Consensus Caller**	Bcf Tools

Coverage Map

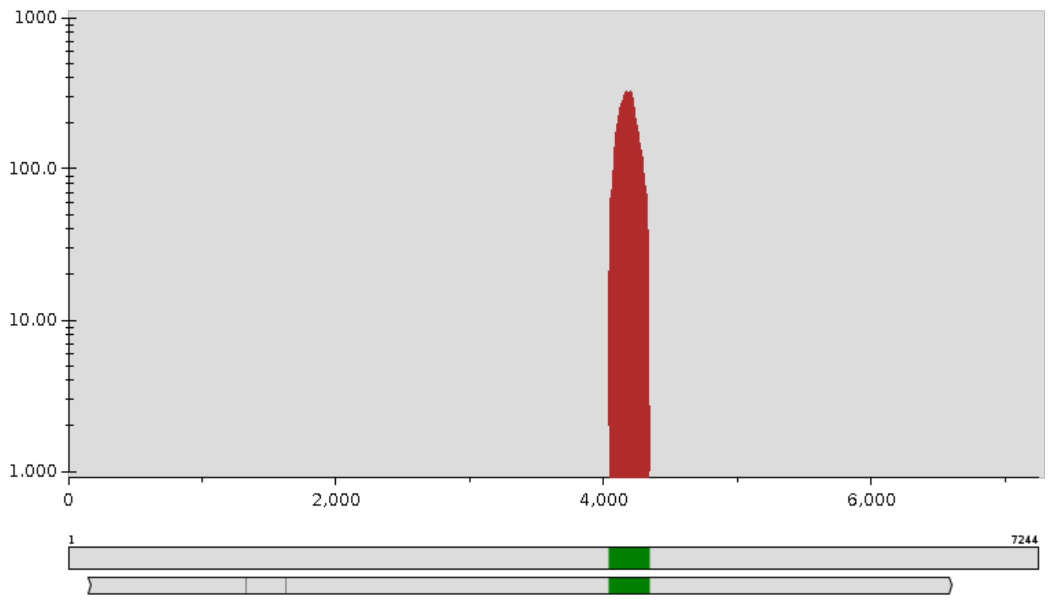

Assignment

**Type**	Tomato necrotic dwarf virus (Taxonomy ID: 1481465)
**Reference Genome**	NC_027926.1
**NT Identity (%)**	75.2475
**AA Identity (%)**	86.1386
**Number Of Stop Codons**	0
**Number Of CDS**	1

Alignment

**Alignment Score**	306.0 (NT) + 643.0 (AA) = 949.0
**Concordance (%)**	71.9484

Alignment Method	Global, seeded, nucleotide + amino acids (AGA)

Genome Region

Sequence starts at position 4040 and ends at position 4342 relative to NC_027926.1 reference sequence.

Alignment Detailed Statistics

	Begin	End	Coverage	Score	Concordance	Matches	Identities	I/D/M/F*	Stop Codons
**NT**	**4040**	**4342**	**4.2%**	**306**	**50.5%**	**303 (100%)**	**228 (75.2%)**	**0/0**	
Mutations:	4050A>C, 4053A>G, 4059C>G, 4062G>A, 4068G>A, 4071C>G, 4072C>T, 4074T>G, 4077T>A, 4083A>G, 4089T>C, 4090T>C, 4104A>G, 4107A>C, 4108A>C, 4110A>C, 4111T>C, 4113A>G, 4116T>C, 4119G>A, 4122A>C, 4137C>T, 4149C>G, 4150A>C, 4161T>C, 4173C>T, 4176C>T, 4177A>T, 4178C>G, 4179C>T, 4188A>T, 4189T>G, 4190T>C, 4197T>C, 4200C>A, 4201A>C, 4206A>T, 4209T>C, 4215T>A, 4218A>T, 4221A>C, 4222G>A, 4230C>G, 4233T>C, 4234T>C, 4235C>A, 4243T>A, 4245T>A, 4246C>G, 4248T>G, 4251T>G, 4254C>T, 4256A>G, 4257C>T, 4259A>G, 4263C>T, 4267G>T, 4269T>A, 4279A>G, 4280C>A, 4281T>G, 4284G>A, 4287T>C, 4293T>C, 4302A>G, 4305T>C, 4311C>T, 4314G>A, 4315C>A, 4317C>A, 4323A>C, 4324G>A, 4326G>C, 4329C>T, 4332T>A								
**CDS**									
APL33_sRA11gp1	1298	1398	4.7%	643	88.3%	101 (100%)	87 (86.1%)	0/0/0/0	0
Protein mutations:	Y1314H (4090T>C), T1343C (4177A>T 4178C>G 4179C>T), L1347A (4189T>G 4190T>C), I1351L (4201A>C), D1358N (4222G>A), S1362H (4234T>C 4235C>A), S1365T (4243T>A 4245T>A), L1366V (4246C>G 4248T>G), N1369S (4256A>G 4257C>T), N1370S (4259A>G), A1373S (4267G>T 4269T>A), T1377E (4279A>G 4280C>A 4281T>G), V1392I (4324G>A 4326G>C)								
Codon mutations:	ACA1300ACC (4050A>C), CAA1301CAG (4053A>G), GCC1303GCG (4059C>G), AAG1304AAA (4062G>A), GAG1306GAA (4068G>A), CGC1307CGG (4071C>G), CTT1308TTG (4072C>T 4074T>G), CCT1309CCA (4077T>A), GAA1311GAG (4083A>G), ATT1313ATC (4089T>C), TAT1314CAT (4090T>C), AAA1318AAG (4104A>G), ACA1319ACC (4107A>C), AGA1320CGC (4108A>C 4110A>C), TTA1321CTG (4111T>C 4113A>G), TTT1322TTC (4116T>C), GAG1323GAA (4119G>A), ATA1324ATC (4122A>C), TAC1329TAT (4137C>T), GTC1333GTG (4149C>G), AGG1334CGG (4150A>C), TTT1337TTC (4161T>C), TCC1341TCT (4173C>T), GCC1342GCT (4176C>T), ACC1343TGT (4177A>T 4178C>G 4179C>T), GCA1346GCT (4188A>T), TTA1347GCA (4189T>G 4190T>C), AAT1349AAC (4197T>C), GTC1350GTA (4200C>A), ATC1351CTC (4201A>C), CCA1352CCT (4206A>T), TGT1353TGC (4209T>C), GTT1355GTA (4215T>A), GGA1356GGT (4218A>T), ATA1357ATC (4221A>C), GAT1358AAT (4222G>A), ACC1360ACG (4230C>G), AGT1361AGC (4233T>C), TCT1362CAT (4234T>C 4235C>A), TCT1365ACA (4243T>A 4245T>A), CTT1366GTG (4246C>G 4248T>G), CTT1367CTG (4251T>G), GCC1368GCT (4254C>T), AAC1369AGT (4256A>G 4257C>T), AAC1370AGC (4259A>G), TTC1371TTT (4263C>T), GCT1373TCA (4267G>T 4269T>A), ACT1377GAG (4279A>G 4280C>A 4281T>G), GGG1378GGA (4284G>A), TTT1379TTC (4287T>C), GCT1381GCC (4293T>C), TCA1384TCG (4302A>G), AGT1385AGC (4305T>C), GAC1387GAT (4311C>T), GGG1388GGA (4314G>A), CGC1389AGA (4315C>A 4317C>A), CCA1391CCC (4323A>C), GTG1392ATC (4324G>A 4326G>C), TTC1393TTT (4329C>T), GCT1394GCA (4332T>A)								
**Proteins**									
polyprotein (YP_009165993.1)	1298	1398	4.7%	643	88.3%	101 (100%)	87 (86.1%)	0/0/0/0	0
Protein mutations:	Y1314H (4090T>C), T1343C (4177A>T 4178C>G 4179C>T), L1347A (4189T>G 4190T>C), I1351L (4201A>C), D1358N (4222G>A), S1362H (4234T>C 4235C>A), S1365T (4243T>A 4245T>A), L1366V (4246C>G 4248T>G), N1369S (4256A>G 4257C>T), N1370S (4259A>G), A1373S (4267G>T 4269T>A), T1377E (4279A>G 4280C>A 4281T>G), V1392I (4324G>A 4326G>C)								
Codon mutations:	ACA1300ACC (4050A>C), CAA1301CAG (4053A>G), GCC1303GCG (4059C>G), AAG1304AAA (4062G>A), GAG1306GAA (4068G>A), CGC1307CGG (4071C>G), CTT1308TTG (4072C>T 4074T>G), CCT1309CCA (4077T>A), GAA1311GAG (4083A>G), ATT1313ATC (4089T>C), TAT1314CAT (4090T>C), AAA1318AAG (4104A>G), ACA1319ACC (4107A>C), AGA1320CGC (4108A>C 4110A>C), TTA1321CTG (4111T>C 4113A>G), TTT1322TTC (4116T>C), GAG1323GAA (4119G>A), ATA1324ATC (4122A>C), TAC1329TAT (4137C>T), GTC1333GTG (4149C>G), AGG1334CGG (4150A>C), TTT1337TTC (4161T>C), TCC1341TCT (4173C>T), GCC1342GCT (4176C>T), ACC1343TGT (4177A>T 4178C>G 4179C>T), GCA1346GCT (4188A>T), TTA1347GCA (4189T>G 4190T>C), AAT1349AAC (4197T>C), GTC1350GTA (4200C>A), ATC1351CTC (4201A>C), CCA1352CCT (4206A>T), TGT1353TGC (4209T>C), GTT1355GTA (4215T>A), GGA1356GGT (4218A>T), ATA1357ATC (4221A>C), GAT1358AAT (4222G>A), ACC1360ACG (4230C>G), AGT1361AGC (4233T>C), TCT1362CAT (4234T>C 4235C>A), TCT1365ACA (4243T>A 4245T>A), CTT1366GTG (4246C>G 4248T>G), CTT1367CTG (4251T>G), GCC1368GCT (4254C>T), AAC1369AGT (4256A>G 4257C>T), AAC1370AGC (4259A>G), TTC1371TTT (4263C>T), GCT1373TCA (4267G>T 4269T>A), ACT1377GAG (4279A>G 4280C>A 4281T>G), GGG1378GGA (4284G>A), TTT1379TTC (4287T>C), GCT1381GCC (4293T>C), TCA1384TCG (4302A>G), AGT1385AGC (4305T>C), GAC1387GAT (4311C>T), GGG1388GGA (4314G>A), CGC1389AGA (4315C>A 4317C>A), CCA1391CCC (4323A>C), GTG1392ATC (4324G>A 4326G>C), TTC1393TTT (4329C>T), GCT1394GCA (4332T>A)								

*: Inserts / Deletes / Misaligned / Frameshifts

Analysis details

This analysis was performed with panviral2.64

NGS Details (UN59): Badnavirus maculasmallanthi

Assembly

**Coverage Length**	296 (1 contig(s))
**Depth Of Coverage**	121.8
**Number Of Reads**	339
**Reads Per Million**	7.37 rpm (after QC)
**Ambiguities**	0
**Assembly Method**	de novo + reference guided assembly
**Consensus Caller**	Bcf Tools

Coverage Map

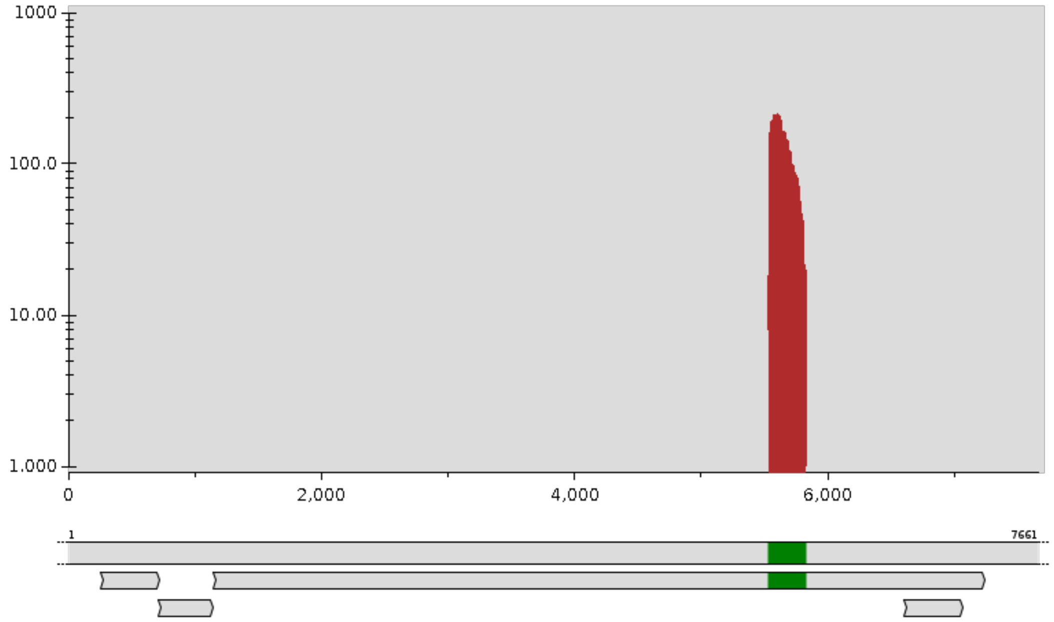

Assignment

**Type**	Badnavirus maculasmallanthi (Taxonomy ID: 3048453)
**Reference Genome**	NC_026472.1
**NT Identity (%)**	56.5217
**AA Identity (%)**	53.5354
**Number Of Stop Codons**	0
**Number Of CDS**	4

Alignment

**Alignment Score**	72.0 (NT) + 371.0 (AA) = 443.0
**Concordance (%)**	34.5824

Alignment Method	Global, seeded, nucleotide + amino acids (AGA)

Genome Region

Sequence starts at position 5534 and ends at position 5829 relative to NC_026472.1 reference sequence.

Alignment Detailed Statistics

	Begin	End	Coverage	Score	Concordance	Matches	Identities	I/D/M/F*	Stop Codons
**NT**	**5534**	**5829**	**3.9%**	**72**	**12.2%**	**296 (99.0%)**	**169 (56.5%)**	**3/0**	
Mutations:	5544A>T, 5545A>G, 5547A>C, 5548A>C, 5549G>A, 5550A>G, 5551A>T, 5554G>C, 5555G>A, 5557A>G, 5558A>G, 5559T>A, 5562A>T, 5563A>T, 5564A>C, 5565G>T, 5566A>G, 5568A>G, 5574A>T, 5578T>A, 5584C>T, 5588A>G, 5589G>A, 5592T>G, 5595A>T, 5597T>A, 5604A>G, 5605G>T, 5607T>G, 5608G>A, 5609C>G, 5610C>A, 5611A>G, 5613G>T, 5614G>C, 5615A>G, 5616A>G, 5619A>G, 5620G>T, 5621A>C, 5623T>G, 5624C>A, 5625C>T, 5628A>C, 5631C>G, 5632T>A, 5633G>A, 5637G>T, 5640C>G, 5643T>C, 5644T>A, 5645T>G, 5646A>G, 5648T>C, 5649C>T, 5651C>G, 5652A>C, 5653G>T, 5654G>A, 5655A>T, 5658A>T, 5659T>C, 5660T>A, 5661A>T, 5664C>T, 5667A>G, 5669G>T, 5670G>T, 5671C>G, 5673T>G, 5680C>T, 5682A>T, 5685C>T, 5688A>G, 5691C>T, 5693A>C, 5694A>C, 5700A>C, 5703C>T, 5707A>G, 5708T>C, 5712T>C, 5713C>A, 5714A>T, 5716A>G, 5717G>A, 5718A>G, 5719A>T, 5720A>T, 5721A>G, 5725G>A, 5728A>C, 5729A>G, 5730A>G, 5731T>G, 5732G>T, 5733C>G, 5736C>T, 5739A>G, 5740G>T, 5741A>C, 5742_5743insTAT, 5743A>T, 5744C>T, 5745A>G, 5748A>T, 5749G>C, 5755A>G, 5757C>G, 5758G>A, 5759C>T, 5765A>T, 5766C>T, 5769C>A, 5772C>T, 5779C>T, 5784A>G, 5787C>T, 5793C>A, 5799A>G, 5800G>A, 5801C>G, 5802G>T, 5813G>A, 5820A>G, 5822A>G, 5823G>A, 5827A>G								

CDS

	Begin	End	Coverage	Score	Concordance	Matches	Identities	I/D/M/F*	Stop Codons
UF61_gp3	1465	1562	4.8%	371	53.2%	98 (99.0%)	53 (53.5%)	1/0/0/0	0
Protein mutations:	K1468D (5545A>G 5547A>C), R1469Q (5548A>C 5549G>A 5550A>G), I1470L (5551A>T), G1471Q (5554G>C 5555G>A), N1472G (5557A>G 5558A>G 5559T>A), K1474S (5563A>T 5564A>C 5565G>T), I1475V (5566A>G 5568A>G), F1479I (5578T>A), K1482R (5588A>G 5589G>A), F1485Y (5597T>A), V1488L (5605G>T 5607T>G), A1489R (5608G>A 5609C>G 5610C>A), M1490V (5611A>G 5613G>T), E1491R (5614G>C 5615A>G 5616A>G), E1493S (5620G>T 5621A>C), S1494D (5623T>G 5624C>A 5625C>T), W1497K (5632T>A 5633G>A), L1501R (5644T>A 5645T>G 5646A>G), I1502T (5648T>C 5649C>T), P1503R (5651C>G 5652A>C), G1504Y (5653G>T 5654G>A 5655A>T), L1506H (5659T>C 5660T>A 5661A>T), W1509F (5669G>T 5670G>T), L1510V (5671C>G 5673T>G), P1513S (5680C>T 5682A>T), K1517T (5693A>C 5694A>C), I1522A (5707A>G 5708T>C), Q1524M (5713C>A 5714A>T), R1525E (5716A>G 5717G>A 5718A>G), K1526L (5719A>T 5720A>T 5721A>G), D1528N (5725G>A), K1529R (5728A>C 5729A>G 5730A>G), C1530V (5731T>G 5732G>T 5733C>G), D1533S (5740G>T 5741A>C), D1533_T1534insY (5742_5743insTAT), T1534L (5743A>T 5744C>T 5745A>G), E1535D (5748A>T), E1536Q (5749G>C), I1538V (5755A>G 5757C>G), A1539I (5758G>A 5759C>T), Y1541F (5765A>T 5766C>T), N1550K (5793C>A), A1553S (5800G>A 5801C>G 5802G>T), R1557K (5813G>A), K1560R (5822A>G 5823G>A), M1562V (5827A>G)								
Codon mutations:	CTA1467CTT (5544A>T), AAA1468GAC (5545A>G 5547A>C), AGA1469CAG (5548A>C 5549G>A 5550A>G), ATA1470TTA (5551A>T), GGG1471CAG (5554G>C 5555G>A), AAT1472GGA (5557A>G 5558A>G 5559T>A), GCA1473GCT (5562A>T), AAG1474TCT (5563A>T 5564A>C 5565G>T), ATA1475GTG (5566A>G 5568A>G), TCA1477TCT (5574A>T), TTT1479ATT (5578T>A), CTG1481TTG (5584C>T), AAG1482AGA (5588A>G 5589G>A), TCT1483TCG (5592T>G), GGA1484GGT (5595A>T), TTT1485TAT (5597T>A), CAA1487CAG (5604A>G), GTT1488TTG (5605G>T 5607T>G), GCC1489AGA (5608G>A 5609C>G 5610C>A), ATG1490GTT (5611A>G 5613G>T), GAA1491CGG (5614G>C 5615A>G 5616A>G), GAA1492GAG (5619A>G), GAA1493TCA (5620G>T 5621A>C), TCC1494GAT (5623T>G 5624C>A 5625C>T), ATA1495ATC (5628A>C), CCC1496CCG (5631C>G), TGG1497AAG (5632T>A 5633G>A), ACG1498ACT (5637G>T), GCC1499GCG (5640C>G), TTT1500TTC (5643T>C), TTA1501AGG (5644T>A 5645T>G 5646A>G), ATC1502ACT (5648T>C 5649C>T), CCA1503CGC (5651C>G 5652A>C), GGA1504TAT (5653G>T 5654G>A 5655A>T), GGA1505GGT (5658A>T), TTA1506CAT (5659T>C 5660T>A 5661A>T), TAC1507TAT (5664C>T), GAA1508GAG (5667A>G), TGG1509TTT (5669G>T 5670G>T), CTT1510GTG (5671C>G 5673T>G), CCA1513TCT (5680C>T 5682A>T), TTC1514TTT (5685C>T), GGA1515GGG (5688A>G), CTC1516CTT (5691C>T), AAA1517ACC (5693A>C 5694A>C), GCA1519GCC (5700A>C), CCC1520CCT (5703C>T), ATT1522GCT (5707A>G 5708T>C), TTT1523TTC (5712T>C), CAG1524ATG (5713C>A 5714A>T), AGA1525GAG (5716A>G 5717G>A 5718A>G), AAA1526TTG (5719A>T 5720A>T 5721A>G), GAT1528AAT (5725G>A), AAA1529CGG (5728A>C 5729A>G 5730A>G), TGC1530GTG (5731T>G 5732G>T 5733C>G), TTC1531TTT (5736C>T), AAA1532AAG (5739A>G), GAC1533TCC (5740G>T 5741A>C), GAC1533_ACA1534insTAT (5742_5743insTAT), ACA1534TTG (5743A>T 5744C>T 5745A>G), GAA1535GAT (5748A>T), GAG1536CAG (5749G>C), ATC1538GTG (5755A>G 5757C>G), GCC1539ATC (5758G>A 5759C>T), TAC1541TTT (5765A>T 5766C>T), ATC1542ATA (5769C>A), GAC1543GAT (5772C>T), CTG1546TTG (5779C>T), GTA1547GTG (5784A>G), TAC1548TAT (5787C>T), AAC1550AAA (5793C>A), GAA1552GAG (5799A>G), GCG1553AGT (5800G>A 5801C>G 5802G>T), AGG1557AAG (5813G>A), TTA1559TTG (5820A>G), AAG1560AGA (5822A>G 5823G>A), ATG1562GTG (5827A>G)								

Proteins

	Begin	End	Coverage	Score	Concordance	Matches	Identities	I/D/M/F*	Stop Codons
ORF3 (YP_009121747.1)	1465	1562	4.8%	371	53.2%	98 (99.0%)	53 (53.5%)	1/0/0/0	0
Protein mutations:	K1468D (5545A>G 5547A>C), R1469Q (5548A>C 5549G>A 5550A>G), I1470L (5551A>T), G1471Q (5554G>C 5555G>A), N1472G (5557A>G 5558A>G 5559T>A), K1474S (5563A>T 5564A>C 5565G>T), I1475V (5566A>G 5568A>G), F1479I (5578T>A), K1482R (5588A>G 5589G>A), F1485Y (5597T>A), V1488L (5605G>T 5607T>G), A1489R (5608G>A 5609C>G 5610C>A), M1490V (5611A>G 5613G>T), E1491R (5614G>C 5615A>G 5616A>G), E1493S (5620G>T 5621A>C), S1494D (5623T>G 5624C>A 5625C>T), W1497K (5632T>A 5633G>A), L1501R (5644T>A 5645T>G 5646A>G), I1502T (5648T>C 5649C>T), P1503R (5651C>G 5652A>C), G1504Y (5653G>T 5654G>A 5655A>T), L1506H (5659T>C 5660T>A 5661A>T), W1509F (5669G>T 5670G>T), L1510V (5671C>G 5673T>G), P1513S (5680C>T 5682A>T), K1517T (5693A>C 5694A>C), I1522A (5707A>G 5708T>C), Q1524M (5713C>A 5714A>T), R1525E (5716A>G 5717G>A 5718A>G), K1526L (5719A>T 5720A>T 5721A>G), D1528N (5725G>A), K1529R (5728A>C 5729A>G 5730A>G), C1530V (5731T>G 5732G>T 5733C>G), D1533S (5740G>T 5741A>C), D1533_T1534insY (5742_5743insTAT), T1534L (5743A>T 5744C>T 5745A>G), E1535D (5748A>T), E1536Q (5749G>C), I1538V (5755A>G 5757C>G), A1539I (5758G>A 5759C>T), Y1541F (5765A>T 5766C>T), N1550K (5793C>A), A1553S (5800G>A 5801C>G 5802G>T), R1557K (5813G>A), K1560R (5822A>G 5823G>A), M1562V (5827A>G)								
Codon mutations:	CTA1467CTT (5544A>T), AAA1468GAC (5545A>G 5547A>C), AGA1469CAG (5548A>C 5549G>A 5550A>G), ATA1470TTA (5551A>T), GGG1471CAG (5554G>C 5555G>A), AAT1472GGA (5557A>G 5558A>G 5559T>A), GCA1473GCT (5562A>T), AAG1474TCT (5563A>T 5564A>C 5565G>T), ATA1475GTG (5566A>G 5568A>G), TCA1477TCT (5574A>T), TTT1479ATT (5578T>A), CTG1481TTG (5584C>T), AAG1482AGA (5588A>G 5589G>A), TCT1483TCG (5592T>G), GGA1484GGT (5595A>T), TTT1485TAT (5597T>A), CAA1487CAG (5604A>G), GTT1488TTG (5605G>T 5607T>G), GCC1489AGA (5608G>A 5609C>G 5610C>A), ATG1490GTT (5611A>G 5613G>T), GAA1491CGG (5614G>C 5615A>G 5616A>G), GAA1492GAG (5619A>G), GAA1493TCA (5620G>T 5621A>C), TCC1494GAT (5623T>G 5624C>A 5625C>T), ATA1495ATC (5628A>C), CCC1496CCG (5631C>G), TGG1497AAG (5632T>A 5633G>A), ACG1498ACT (5637G>T), GCC1499GCG (5640C>G), TTT1500TTC (5643T>C), TTA1501AGG (5644T>A 5645T>G 5646A>G), ATC1502ACT (5648T>C 5649C>T), CCA1503CGC (5651C>G 5652A>C), GGA1504TAT (5653G>T 5654G>A 5655A>T), GGA1505GGT (5658A>T), TTA1506CAT (5659T>C 5660T>A 5661A>T), TAC1507TAT (5664C>T), GAA1508GAG (5667A>G), TGG1509TTT (5669G>T 5670G>T), CTT1510GTG (5671C>G 5673T>G), CCA1513TCT (5680C>T 5682A>T), TTC1514TTT (5685C>T), GGA1515GGG (5688A>G), CTC1516CTT (5691C>T), AAA1517ACC (5693A>C 5694A>C), GCA1519GCC (5700A>C), CCC1520CCT (5703C>T), ATT1522GCT (5707A>G 5708T>C), TTT1523TTC (5712T>C), CAG1524ATG (5713C>A 5714A>T), AGA1525GAG (5716A>G 5717G>A 5718A>G), AAA1526TTG (5719A>T 5720A>T 5721A>G), GAT1528AAT (5725G>A), AAA1529CGG (5728A>C 5729A>G 5730A>G), TGC1530GTG (5731T>G 5732G>T 5733C>G), TTC1531TTT (5736C>T), AAA1532AAG (5739A>G), GAC1533TCC (5740G>T 5741A>C), GAC1533_ACA1534insTAT (5742_5743insTAT), ACA1534TTG (5743A>T 5744C>T 5745A>G), GAA1535GAT (5748A>T), GAG1536CAG (5749G>C), ATC1538GTG (5755A>G 5757C>G), GCC1539ATC (5758G>A 5759C>T), TAC1541TTT (5765A>T 5766C>T), ATC1542ATA (5769C>A), GAC1543GAT (5772C>T), CTG1546TTG (5779C>T), GTA1547GTG (5784A>G), TAC1548TAT (5787C>T), AAC1550AAA (5793C>A), GAA1552GAG (5799A>G), GCG1553AGT (5800G>A 5801C>G 5802G>T), AGG1557AAG (5813G>A), TTA1559TTG (5820A>G), AAG1560AGA (5822A>G 5823G>A), ATG1562GTG (5827A>G)								

*: Inserts / Deletes / Misaligned / Frameshifts

Analysis details

This analysis was performed with panviral2.64

NGS Details (UN59): Cassava brown streak virus

Assembly

**Coverage Length**	530 (1 contig(s))
**Depth Of Coverage**	61.7
**Number Of Reads**	273
**Reads Per Million**	5.93 rpm (after QC)
**Ambiguities**	0
**Assembly Method**	de novo + reference guided assembly
**Consensus Caller**	Bcf Tools

Coverage Map

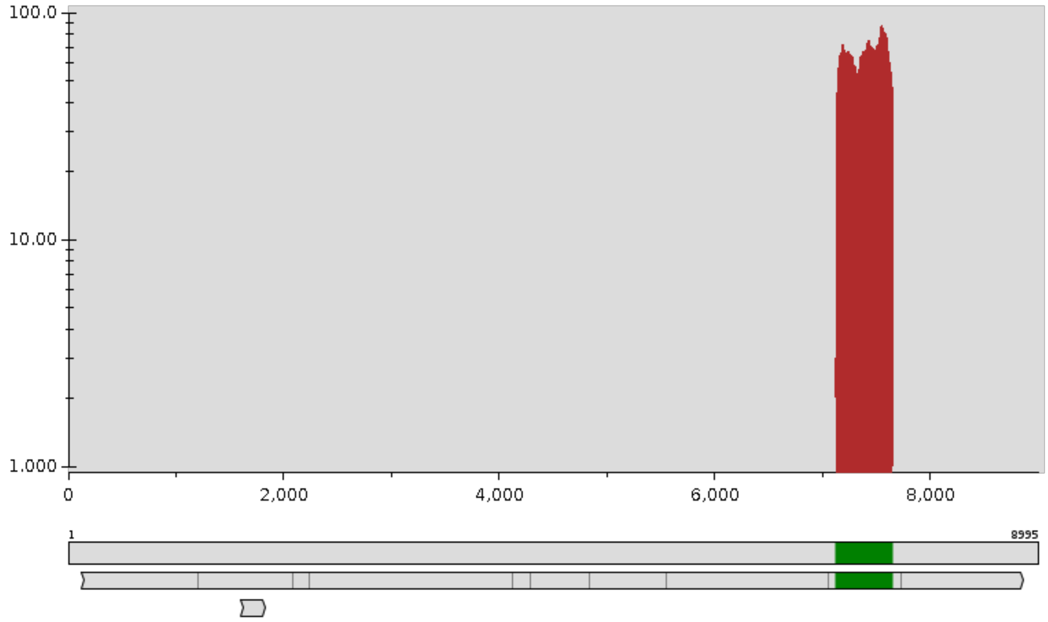

Assignment

**Type**	Cassava brown streak virus (Taxonomy ID: 137758)
**Reference Genome**	NC_012698.2
**NT Identity (%)**	61.1321
**AA Identity (%)**	58.1921
**Number Of Stop Codons**	0
**Number Of CDS**	2

Alignment

**Alignment Score**	236.0 (NT) + 748.0 (AA) = 984.0
**Concordance (%)**	43.0258

Alignment Method	Global, seeded, nucleotide + amino acids (AGA)

Genome Region

Sequence starts at position 7121 and ends at position 7650 relative to NC_012698.2 reference sequence.

Alignment Detailed Statistics

	Begin	End	Coverage	Score	Concordance	Matches	Identities	I/D/M/F*	Stop Codons
**NT**	**7121**	**7650**	**5.9%**	**236**	**22.3%**	**530 (100%)**	**324 (61.1%)**	**0/0**	
Mutations:	7130A>G, 7133A>G, 7134A>G, 7136T>G, 7142C>T, 7145A>T, 7148A>C, 7151G>A, 7157A>T, 7158C>A, 7163A>G, 7167A>G, 7175A>T, 7177A>G, 7178A>G, 7179C>G, 7180A>C, 7184A>T, 7185T>C, 7190T>C, 7192C>A, 7193C>G, 7194A>T, 7202A>C, 7203A>T, 7205C>T, 7206A>C, 7207T>A, 7208C>G, 7211T>C, 7213G>T, 7214G>T, 7217A>G, 7218G>C, 7229A>T, 7234C>T, 7235A>T, 7241A>G, 7242A>G, 7243C>A, 7244A>G, 7245G>C, 7246T>C, 7250G>A, 7253A>T, 7256T>A, 7257A>T, 7258T>C, 7270T>C, 7271A>T, 7272C>A, 7273G>A, 7274T>A, 7275G>A, 7277A>T, 7280G>T, 7281T>G, 7283T>C, 7284G>A, 7286G>A, 7287T>G, 7288T>A, 7290A>G, 7292T>G, 7293G>A, 7294G>A, 7295A>T, 7298G>A, 7304T>G, 7307C>A, 7310A>T, 7313A>G, 7319A>T, 7320A>T, 7323T>C, 7325G>C, 7331T>C, 7332G>A, 7334T>C, 7337T>C, 7340C>T, 7343T>G, 7355A>G, 7358A>T, 7361C>T, 7364T>C, 7374A>T, 7377G>C, 7380G>A, 7381G>A, 7385T>C, 7388A>T, 7390T>A, 7391A>T, 7397A>T, 7398T>C, 7400A>T, 7401T>A, 7403T>C, 7406G>C, 7409G>A, 7410G>T, 7413G>A, 7414A>T, 7416C>G, 7418G>T, 7422C>G, 7424A>G, 7425A>G, 7432T>C, 7433G>A, 7436T>A, 7437A>T, 7438G>A, 7442T>C, 7443C>A, 7445C>G, 7448C>T, 7449G>A, 7451G>C, 7455G>T, 7457T>A, 7458T>C, 7462T>C, 7463A>T, 7464A>C, 7465A>T, 7467A>G, 7468A>G, 7469A>G, 7470G>C, 7471T>C, 7472T>A, 7473G>A, 7474G>A, 7476G>A, 7477A>C, 7481T>G, 7484T>G, 7487A>G, 7489T>C, 7490C>T, 7494A>G, 7495A>T, 7496G>T, 7499T>A, 7500G>A, 7501T>A, 7502G>A, 7503C>A, 7504T>C, 7505A>G, 7506A>C, 7507G>T, 7508A>G, 7511T>A, 7512G>A, 7517T>A, 7520A>G, 7521A>C, 7522T>C, 7523G>A, 7524C>G, 7526A>T, 7527C>A, 7529C>G, 7535A>C, 7539T>A, 7540C>A, 7541A>T, 7544T>C, 7547G>A, 7553C>T, 7556A>G, 7559T>A, 7562C>T, 7568A>T, 7570T>A, 7572A>G, 7573A>G, 7576G>A, 7577G>T, 7578A>G, 7579G>A, 7580A>C, 7581A>C, 7586A>T, 7588T>A, 7589T>C, 7599A>C, 7600T>C, 7601G>C, 7602G>A, 7603C>A, 7604C>G, 7607G>A, 7610G>A, 7616T>C, 7618T>A, 7622A>T, 7628T>C, 7631A>C, 7632T>G, 7633T>G, 7635C>A, 7636G>A								

CDS

	Begin	End	Coverage	Score	Concordance	Matches	Identities	I/D/M/F*	Stop Codons
CBSV_gp1	2333	2509	6.1%	748	60.3%	177 (100%)	103 (58.2%)	0/0/0/0	0
Protein mutations:	I2337V (7134A>G 7136T>G), Q2345K (7158C>A), K2348E (7167A>G), K2351R (7177A>G 7178A>G), Q2352A (7179C>G 7180A>C), F2354L (7185T>C), P2356Q (7192C>A 7193C>G), T2357S (7194A>T), I2360F (7203A>T 7205C>T), I2361Q (7206A>C 7207T>A 7208C>G), R2363L (7213G>T 7214G>T), V2365L (7218G>C), P2370L (7234C>T 7235A>T), T2373E (7242A>G 7243C>A 7244A>G), V2374P (7245G>C 7246T>C), E2376D (7253A>T), I2378S (7257A>T 7258T>C), V2382A (7270T>C 7271A>T), R2383K (7272C>A 7273G>A 7274T>A), V2384I (7275G>A 7277A>T), S2386A (7281T>G 7283T>C), E2387K (7284G>A 7286G>A), L2388E (7287T>G 7288T>A), I2389V (7290A>G 7292T>G), G2390N (7293G>A 7294G>A 7295A>T), S2399C (7320A>T), D2403N (7332G>A 7334T>C), N2406K (7343T>G), M2417L (7374A>T), E2418Q (7377G>C), G2419K (7380G>A 7381G>A), L2422H (7390T>A 7391A>T), Y2426N (7401T>A 7403T>C), K2427N (7406G>C), V2429L (7410G>T), E2430M (7413G>A 7414A>T), P2431A (7416C>G 7418G>T), Q2433E (7422C>G 7424A>G), N2434D (7425A>G), M2436T (7432T>C 7433G>A), S2438Y (7437A>T 7438G>A), L2440M (7443C>A 7445C>G), V2442I (7449G>A 7451G>C), A2444S (7455G>T 7457T>A), F2445L (7458T>C), V2446A (7462T>C 7463A>T), N2447L (7464A>C 7465A>T), K2448G (7467A>G 7468A>G 7469A>G), V2449P (7470G>C 7471T>C 7472T>A), G2450N (7473G>A 7474G>A), D2451T (7476G>A 7477A>C), D2452E (7481T>G), I2454M (7487A>G), I2455T (7489T>C 7490C>T), K2457V (7494A>G 7495A>T 7496G>T), V2459K (7500G>A 7501T>A 7502G>A), L2460T (7503C>A 7504T>C 7505A>G), R2461L (7506A>C 7507G>T 7508A>G), E2463K (7512G>A), M2466P (7521A>C 7522T>C 7523G>A), P2467A (7524C>G 7526A>T), S2472N (7539T>A 7540C>A 7541A>T), L2482H (7570T>A), N2483G (7572A>G 7573A>G), W2484Y (7576G>A 7577G>T), R2485D (7578A>G 7579G>A 7580A>C), K2486Q (7581A>C), F2488Y (7588T>A 7589T>C), M2492P (7599A>C 7600T>C 7601G>C), A2493K (7602G>A 7603C>A 7604C>G), M2498K (7618T>A), F2503G (7632T>G 7633T>G), R2504K (7635C>A 7636G>A)								
Codon mutations:	GCA2335GCG (7130A>G), CCA2336CCG (7133A>G), ATT2337GTG (7134A>G 7136T>G), TTC2339TTT (7142C>T), GTA2340GTT (7145A>T), ACA2341ACC (7148A>C), GGG2342GGA (7151G>A), GCA2344GCT (7157A>T), CAA2345AAA (7158C>A), AAA2346AAG (7163A>G), AAG2348GAG (7167A>G), GTA2350GTT (7175A>T), AAA2351AGG (7177A>G 7178A>G), CAA2352GCA (7179C>G 7180A>C), ATA2353ATT (7184A>T), TTT2354CTT (7185T>C), GGT2355GGC (7190T>C), CCC2356CAG (7192C>A 7193C>G), ACT2357TCT (7194A>T), CCA2359CCC (7202A>C), ATC2360TTT (7203A>T 7205C>T), ATC2361CAG (7206A>C 7207T>A 7208C>G), TCT2362TCC (7211T>C), CGG2363CTT (7213G>T 7214G>T), AAA2364AAG (7217A>G), GTT2365CTT (7218G>C), CCA2368CCT (7229A>T), CCA2370CTT (7234C>T 7235A>T), GGA2372GGG (7241A>G), ACA2373GAG (7242A>G 7243C>A 7244A>G), GTT2374CCT (7245G>C 7246T>C), GAG2375GAA (7250G>A), GAA2376GAT (7253A>T), ATT2377ATA (7256T>A), ATC2378TCC (7257A>T 7258T>C), GTA2382GCT (7270T>C 7271A>T), CGT2383AAA (7272C>A 7273G>A 7274T>A), GTA2384ATT (7275G>A 7277A>T), GCG2385GCT (7280G>T), TCT2386GCC (7281T>G 7283T>C), GAG2387AAA (7284G>A 7286G>A), TTG2388GAG (7287T>G 7288T>A), ATT2389GTG (7290A>G 7292T>G), GGA2390AAT (7293G>A 7294G>A 7295A>T), GGG2391GGA (7298G>A), GTT2393GTG (7304T>G), CTC2394CTA (7307C>A), GTA2395GTT (7310A>T), GAA2396GAG (7313A>G), ACA2398ACT (7319A>T), AGT2399TGT (7320A>T), TTG2400CTC (7323T>C 7325G>C), TTT2402TTC (7331T>C), GAT2403AAC (7332G>A 7334T>C), GCT2404GCC (7337T>C), CTC2405CTT (7340C>T), AAT2406AAG (7343T>G), GGA2410GGG (7355A>G), CCA2411CCT (7358A>T), TAC2412TAT (7361C>T), ATT2413ATC (7364T>C), ATG2417TTG (7374A>T), GAA2418CAA (7377G>C), GGG2419AAG (7380G>A 7381G>A), ATT2420ATC (7385T>C), GGA2421GGT (7388A>T), CTA2422CAT (7390T>A 7391A>T), GGA2424GGT (7397A>T), TTA2425CTT (7398T>C 7400A>T), TAT2426AAC (7401T>A 7403T>C), AAG2427AAC (7406G>C), TTG2428TTA (7409G>A), GTG2429TTG (7410G>T), GAG2430ATG (7413G>A 7414A>T), CCG2431GCT (7416C>G 7418G>T), CAA2433GAG (7422C>G 7424A>G), AAT2434GAT (7425A>G), ATG2436ACA (7432T>C 7433G>A), GCT2437GCA (7436T>A), AGT2438TAT (7437A>T 7438G>A), GCT2439GCC (7442T>C), CTC2440ATG (7443C>A 7445C>G), TGC2441TGT (7448C>T), GTG2442ATC (7449G>A 7451G>C), GCT2444TCA (7455G>T 7457T>A), TTT2445CTT (7458T>C), GTA2446GCT (7462T>C 7463A>T), AAT2447CTT (7464A>C 7465A>T), AAA2448GGG (7467A>G 7468A>G 7469A>G), GTT2449CCA (7470G>C 7471T>C 7472T>A), GGT2450AAT (7473G>A 7474G>A), GAT2451ACT (7476G>A 7477A>C), GAT2452GAG (7481T>G), CCT2453CCG (7484T>G), ATA2454ATG (7487A>G), ATC2455ACT (7489T>C 7490C>T), AAG2457GTT (7494A>G 7495A>T 7496G>T), GGT2458GGA (7499T>A), GTG2459AAA (7500G>A 7501T>A 7502G>A), CTA2460ACG (7503C>A 7504T>C 7505A>G), AGA2461CTG (7506A>C 7507G>T 7508A>G), GGT2462GGA (7511T>A), GAG2463AAG (7512G>A), ATT2464ATA (7517T>A), GTA2465GTG (7520A>G), ATG2466CCA (7521A>C 7522T>C 7523G>A), CCA2467GCT (7524C>G 7526A>T), CGC2468AGG (7527C>A 7529C>G), CCA2470CCC (7535A>C), TCA2472AAT (7539T>A 7540C>A 7541A>T), TTT2473TTC (7544T>C), GGG2474GGA (7547G>A), GAC2476GAT (7553C>T), CCA2477CCG (7556A>G), ATT2478ATA (7559T>A), TTC2479TTT (7562C>T), CCA2481CCT (7568A>T), CTT2482CAT (7570T>A), AAC2483GGC (7572A>G 7573A>G), TGG2484TAT (7576G>A 7577G>T), AGA2485GAC (7578A>G 7579G>A 7580A>C), AAG2486CAG (7581A>C), ACA2487ACT (7586A>T), TTT2488TAC (7588T>A 7589T>C), ATG2492CCC (7599A>C 7600T>C 7601G>C), GCC2493AAG (7602G>A 7603C>A 7604C>G), GAG2494GAA (7607G>A), GAG2495GAA (7610G>A), AAT2497AAC (7616T>C), ATG2498AAG (7618T>A), ATA2499ATT (7622A>T), CAT2501CAC (7628T>C), CGA2502CGC (7631A>C), TTT2503GGT (7632T>G 7633T>G), CGA2504AAA (7635C>A 7636G>A)								

Proteins

	Begin	End	Coverage	Score	Concordance	Matches	Identities	I/D/M/F*	Stop Codons
polyprotein (YP_007027011.1)	2333	2509	6.1%	748	60.3%	177 (100%)	103 (58.2%)	0/0/0/0	0
Protein mutations:	I2337V (7134A>G 7136T>G), Q2345K (7158C>A), K2348E (7167A>G), K2351R (7177A>G 7178A>G), Q2352A (7179C>G 7180A>C), F2354L (7185T>C), P2356Q (7192C>A 7193C>G), T2357S (7194A>T), I2360F (7203A>T 7205C>T), I2361Q (7206A>C 7207T>A 7208C>G), R2363L (7213G>T 7214G>T), V2365L (7218G>C), P2370L (7234C>T 7235A>T), T2373E (7242A>G 7243C>A 7244A>G), V2374P (7245G>C 7246T>C), E2376D (7253A>T), I2378S (7257A>T 7258T>C), V2382A (7270T>C 7271A>T), R2383K (7272C>A 7273G>A 7274T>A), V2384I (7275G>A 7277A>T), S2386A (7281T>G 7283T>C), E2387K (7284G>A 7286G>A), L2388E (7287T>G 7288T>A), I2389V (7290A>G 7292T>G), G2390N (7293G>A 7294G>A 7295A>T), S2399C (7320A>T), D2403N (7332G>A 7334T>C), N2406K (7343T>G), M2417L (7374A>T), E2418Q (7377G>C), G2419K (7380G>A 7381G>A), L2422H (7390T>A 7391A>T), Y2426N (7401T>A 7403T>C), K2427N (7406G>C), V2429L (7410G>T), E2430M (7413G>A 7414A>T), P2431A (7416C>G 7418G>T), Q2433E (7422C>G 7424A>G), N2434D (7425A>G), M2436T (7432T>C 7433G>A), S2438Y (7437A>T 7438G>A), L2440M (7443C>A 7445C>G), V2442I (7449G>A 7451G>C), A2444S (7455G>T 7457T>A), F2445L (7458T>C), V2446A (7462T>C 7463A>T), N2447L (7464A>C 7465A>T), K2448G (7467A>G 7468A>G 7469A>G), V2449P (7470G>C 7471T>C 7472T>A), G2450N (7473G>A 7474G>A), D2451T (7476G>A 7477A>C), D2452E (7481T>G), I2454M (7487A>G), I2455T (7489T>C 7490C>T), K2457V (7494A>G 7495A>T 7496G>T), V2459K (7500G>A 7501T>A 7502G>A), L2460T (7503C>A 7504T>C 7505A>G), R2461L (7506A>C 7507G>T 7508A>G), E2463K (7512G>A), M2466P (7521A>C 7522T>C 7523G>A), P2467A (7524C>G 7526A>T), S2472N (7539T>A 7540C>A 7541A>T), L2482H (7570T>A), N2483G (7572A>G 7573A>G), W2484Y (7576G>A 7577G>T), R2485D (7578A>G 7579G>A 7580A>C), K2486Q (7581A>C), F2488Y (7588T>A 7589T>C), M2492P (7599A>C 7600T>C 7601G>C), A2493K (7602G>A 7603C>A 7604C>G), M2498K (7618T>A), F2503G (7632T>G 7633T>G), R2504K (7635C>A 7636G>A)								

	Begin	End	Coverage	Score	Concordance	Matches	Identities	I/D/M/F*	Stop Codons
**NT**	**7121**	**7650**	**5.9%**	**236**	**22.3%**	**530 (100%)**	**324 (61.1%)**	**0/0**	
Codon mutations:	GCA2335GCG (7130A>G), CCA2336CCG (7133A>G), ATT2337GTG (7134A>G 7136T>G), TTC2339TTT (7142C>T), GTA2340GTT (7145A>T), ACA2341ACC (7148A>C), GGG2342GGA (7151G>A), GCA2344GCT (7157A>T), CAA2345AAA (7158C>A), AAA2346AAG (7163A>G), AAG2348GAG (7167A>G), GTA2350GTT (7175A>T), AAA2351AGG (7177A>G 7178A>G), CAA2352GCA (7179C>G 7180A>C), ATA2353ATT (7184A>T), TTT2354CTT (7185T>C), GGT2355GGC (7190T>C), CCC2356CAG (7192C>A 7193C>G), ACT2357TCT (7194A>T), CCA2359CCC (7202A>C), ATC2360TTT (7203A>T 7205C>T), ATC2361CAG (7206A>C 7207T>A 7208C>G), TCT2362TCC (7211T>C), CGG2363CTT (7213G>T 7214G>T), AAA2364AAG (7217A>G), GTT2365CTT (7218G>C), CCA2368CCT (7229A>T), CCA2370CTT (7234C>T 7235A>T), GGA2372GGG (7241A>G), ACA2373GAG (7242A>G 7243C>A 7244A>G), GTT2374CCT (7245G>C 7246T>C), GAG2375GAA (7250G>A), GAA2376GAT (7253A>T), ATT2377ATA (7256T>A), ATC2378TCC (7257A>T 7258T>C), GTA2382GCT (7270T>C 7271A>T), CGT2383AAA (7272C>A 7273G>A 7274T>A), GTA2384ATT (7275G>A 7277A>T), GCG2385GCT (7280G>T), TCT2386GCC (7281T>G 7283T>C), GAG2387AAA (7284G>A 7286G>A), TTG2388GAG (7287T>G 7288T>A), ATT2389GTG (7290A>G 7292T>G), GGA2390AAT (7293G>A 7294G>A 7295A>T), GGG2391GGA (7298G>A), GTT2393GTG (7304T>G), CTC2394CTA (7307C>A), GTA2395GTT (7310A>T), GAA2396GAG (7313A>G), ACA2398ACT (7319A>T), AGT2399TGT (7320A>T), TTG2400CTC (7323T>C 7325G>C), TTT2402TTC (7331T>C), GAT2403AAC (7332G>A 7334T>C), GCT2404GCC (7337T>C), CTC2405CTT (7340C>T), AAT2406AAG (7343T>G), GGA2410GGG (7355A>G), CCA2411CCT (7358A>T), TAC2412TAT (7361C>T), ATT2413ATC (7364T>C), ATG2417TTG (7374A>T), GAA2418CAA (7377G>C), GGG2419AAG (7380G>A 7381G>A), ATT2420ATC (7385T>C), GGA2421GGT (7388A>T), CTA2422CAT (7390T>A 7391A>T), GGA2424GGT (7397A>T), TTA2425CTT (7398T>C 7400A>T), TAT2426AAC (7401T>A 7403T>C), AAG2427AAC (7406G>C), TTG2428TTA (7409G>A), GTG2429TTG (7410G>T), GAG2430ATG (7413G>A 7414A>T), CCG2431GCT (7416C>G 7418G>T), CAA2433GAG (7422C>G 7424A>G), AAT2434GAT (7425A>G), ATG2436ACA (7432T>C 7433G>A), GCT2437GCA (7436T>A), AGT2438TAT (7437A>T 7438G>A), GCT2439GCC (7442T>C), CTC2440ATG (7443C>A 7445C>G), TGC2441TGT (7448C>T), GTG2442ATC (7449G>A 7451G>C), GCT2444TCA (7455G>T 7457T>A), TTT2445CTT (7458T>C), GTA2446GCT (7462T>C 7463A>T), AAT2447CTT (7464A>C 7465A>T), AAA2448GGG (7467A>G 7468A>G 7469A>G), GTT2449CCA (7470G>C 7471T>C 7472T>A), GGT2450AAT (7473G>A 7474G>A), GAT2451ACT (7476G>A 7477A>C), GAT2452GAG (7481T>G), CCT2453CCG (7484T>G), ATA2454ATG (7487A>G), ATC2455ACT (7489T>C 7490C>T), AAG2457GTT (7494A>G 7495A>T 7496G>T), GGT2458GGA (7499T>A), GTG2459AAA (7500G>A 7501T>A 7502G>A), CTA2460ACG (7503C>A 7504T>C 7505A>G), AGA2461CTG (7506A>C 7507G>T 7508A>G), GGT2462GGA (7511T>A), GAG2463AAG (7512G>A), ATT2464ATA (7517T>A), GTA2465GTG (7520A>G), ATG2466CCA (7521A>C 7522T>C 7523G>A), CCA2467GCT (7524C>G 7526A>T), CGC2468AGG (7527C>A 7529C>G), CCA2470CCC (7535A>C), TCA2472AAT (7539T>A 7540C>A 7541A>T), TTT2473TTC (7544T>C), GGG2474GGA (7547G>A), GAC2476GAT (7553C>T), CCA2477CCG (7556A>G), ATT2478ATA (7559T>A), TTC2479TTT (7562C>T), CCA2481CCT (7568A>T), CTT2482CAT (7570T>A), AAC2483GGC (7572A>G 7573A>G), TGG2484TAT (7576G>A 7577G>T), AGA2485GAC (7578A>G 7579G>A 7580A>C), AAG2486CAG (7581A>C), ACA2487ACT (7586A>T), TTT2488TAC (7588T>A 7589T>C), ATG2492CCC (7599A>C 7600T>C 7601G>C), GCC2493AAG (7602G>A 7603C>A 7604C>G), GAG2494GAA (7607G>A), GAG2495GAA (7610G>A), AAT2497AAC (7616T>C), ATG2498AAG (7618T>A), ATA2499ATT (7622A>T), CAT2501CAC (7628T>C), CGA2502CGC (7631A>C), TTT2503GGT (7632T>G 7633T>G), CGA2504AAA (7635C>A 7636G>A)								
HAM1-like protein (YP_007032446.1)	25	201	78.3%	748	60.3%	177 (100%)	103 (58.2%)	0/0/0/0	0
Protein mutations:	I29V (7134A>G 7136T>G), Q37K (7158C>A), K40E (7167A>G), K43R (7177A>G 7178A>G), Q44A (7179C>G 7180A>C), F46L (7185T>C), P48Q (7192C>A 7193C>G), T49S (7194A>T), I52F (7203A>T 7205C>T), I53Q (7206A>C 7207T>A 7208C>G), R55L (7213G>T 7214G>T), V57L (7218G>C), P62L (7234C>T 7235A>T), T65E (7242A>G 7243C>A 7244A>G), V66P (7245G>C 7246T>C), E68D (7253A>T), I70S (7257A>T 7258T>C), V74A (7270T>C 7271A>T), R75K (7272C>A 7273G>A 7274T>A), V76I (7275G>A 7277A>T), S78A (7281T>G 7283T>C), E79K (7284G>A 7286G>A), L80E (7287T>G 7288T>A), I81V (7290A>G 7292T>G), G82N (7293G>A 7294G>A 7295A>T), S91C (7320A>T), D95N (7332G>A 7334T>C), N98K (7343T>G), M109L (7374A>T), E110Q (7377G>C), G111K (7380G>A 7381G>A), L114H (7390T>A 7391A>T), Y118N (7401T>A 7403T>C), K119N (7406G>C), V121L (7410G>T), E122M (7413G>A 7414A>T), P123A (7416C>G 7418G>T), Q125E (7422C>G 7424A>G), N126D (7425A>G), M128T (7432T>C 7433G>A), S130Y (7437A>T 7438G>A), L132M (7443C>A 7445C>G), V134I (7449G>A 7451G>C), A136S (7455G>T 7457T>A), F137L (7458T>C), V138A (7462T>C 7463A>T), N139L (7464A>C 7465A>T), K140G (7467A>G 7468A>G 7469A>G), V141P (7470G>C 7471T>C 7472T>A), G142N (7473G>A 7474G>A), D143T (7476G>A 7477A>C), D144E (7481T>G), I146M (7487A>G), I147T (7489T>C 7490C>T), K149V (7494A>G 7495A>T 7496G>T), V151K (7500G>A 7501T>A 7502G>A), L152T (7503C>A 7504T>C 7505A>G), R153L (7506A>C 7507G>T 7508A>G), E155K (7512G>A), M158P (7521A>C 7522T>C 7523G>A), P159A (7524C>G 7526A>T), S164N (7539T>A 7540C>A 7541A>T), L174H (7570T>A), N175G (7572A>G 7573A>G), W176Y (7576G>A 7577G>T), R177D (7578A>G 7579G>A 7580A>C), K178Q (7581A>C), F180Y (7588T>A 7589T>C), M184P (7599A>C 7600T>C 7601G>C), A185K (7602G>A 7603C>A 7604C>G), M190K (7618T>A), F195G (7632T>G 7633T>G), R196K (7635C>A 7636G>A)								
Codon mutations:	GCA27GCG (7130A>G), CCA28CCG (7133A>G), ATT29GTG (7134A>G 7136T>G), TTC31TTT (7142C>T), GTA32GTT (7145A>T), ACA33ACC (7148A>C), GGG34GGA (7151G>A), GCA36GCT (7157A>T), CAA37AAA (7158C>A), AAA38AAG (7163A>G), AAG40GAG (7167A>G), GTA42GTT (7175A>T), AAA43AGG (7177A>G 7178A>G), CAA44GCA (7179C>G 7180A>C), ATA45ATT (7184A>T), TTT46CTT (7185T>C), GGT47GGC (7190T>C), CCC48CAG (7192C>A 7193C>G), ACT49TCT (7194A>T), CCA51CCC (7202A>C), ATC52TTT (7203A>T 7205C>T), ATC53CAG (7206A>C 7207T>A 7208C>G), TCT54TCC (7211T>C), CGG55CTT (7213G>T 7214G>T), AAA56AAG (7217A>G), GTT57CTT (7218G>C), CCA60CCT (7229A>T), CCA62CTT (7234C>T 7235A>T), GGA64GGG (7241A>G), ACA65GAG (7242A>G 7243C>A 7244A>G), GTT66CCT (7245G>C 7246T>C), GAG67GAA (7250G>A), GAA68GAT (7253A>T), ATT69ATA (7256T>A), ATC70TCC (7257A>T 7258T>C), GTA74GCT (7270T>C 7271A>T), CGT75AAA (7272C>A 7273G>A 7274T>A), GTA76ATT (7275G>A 7277A>T), GCG77GCT (7280G>T), TCT78GCC (7281T>G 7283T>C), GAG79AAA (7284G>A 7286G>A), TTG80GAG (7287T>G 7288T>A), ATT81GTG (7290A>G 7292T>G), GGA82AAT (7293G>A 7294G>A 7295A>T), GGG83GGA (7298G>A), GTT85GTG (7304T>G), CTC86CTA (7307C>A), GTA87GTT (7310A>T), GAA88GAG (7313A>G), ACA90ACT (7319A>T), AGT91TGT (7320A>T), TTG92CTC (7323T>C 7325G>C), TTT94TTC (7331T>C), GAT95AAC (7332G>A 7334T>C), GCT96GCC (7337T>C), CTC97CTT (7340C>T), AAT98AAG (7343T>G), GGA102GGG (7355A>G), CCA103CCT (7358A>T), TAC104TAT (7361C>T), ATT105ATC (7364T>C), ATG109TTG (7374A>T), GAA110CAA (7377G>C), GGG111AAG (7380G>A 7381G>A), ATT112ATC (7385T>C), GGA113GGT (7388A>T), CTA114CAT (7390T>A 7391A>T), GGA116GGT (7397A>T), TTA117CTT (7398T>C 7400A>T), TAT118AAC (7401T>A 7403T>C), AAG119AAC (7406G>C), TTG120TTA (7409G>A), GTG121TTG (7410G>T), GAG122ATG (7413G>A 7414A>T), CCG123GCT (7416C>G 7418G>T), CAA125GAG (7422C>G 7424A>G), AAT126GAT (7425A>G), ATG128ACA (7432T>C 7433G>A), GCT129GCA (7436T>A), AGT130TAT (7437A>T 7438G>A), GCT131GCC (7442T>C), CTC132ATG (7443C>A 7445C>G), TGC133TGT (7448C>T), GTG134ATC (7449G>A 7451G>C), GCT136TCA (7455G>T 7457T>A), TTT137CTT (7458T>C), GTA138GCT (7462T>C 7463A>T), AAT139CTT (7464A>C 7465A>T), AAA140GGG (7467A>G 7468A>G 7469A>G), GTT141CCA (7470G>C 7471T>C 7472T>A), GGT142AAT (7473G>A 7474G>A), GAT143ACT (7476G>A 7477A>C), GAT144GAG (7481T>G), CCT145CCG (7484T>G), ATA146ATG (7487A>G), ATC147ACT (7489T>C 7490C>T), AAG149GTT (7494A>G 7495A>T 7496G>T), GGT150GGA (7499T>A), GTG151AAA (7500G>A 7501T>A 7502G>A), CTA152ACG (7503C>A 7504T>C 7505A>G), AGA153CTG (7506A>C 7507G>T 7508A>G), GGT154GGA (7511T>A), GAG155AAG (7512G>A), ATT156ATA (7517T>A), GTA157GTG (7520A>G), ATG158CCA (7521A>C 7522T>C 7523G>A), CCA159GCT (7524C>G 7526A>T), CGC160AGG (7527C>A 7529C>G), CCA162CCC (7535A>C), TCA164AAT (7539T>A 7540C>A 7541A>T), TTT165TTC (7544T>C), GGG166GGA (7547G>A), GAC168GAT (7553C>T), CCA169CCG (7556A>G), ATT170ATA (7559T>A), TTC171TTT (7562C>T), CCA173CCT (7568A>T), CTT174CAT (7570T>A), AAC175GGC (7572A>G 7573A>G), TGG176TAT (7576G>A 7577G>T), AGA177GAC (7578A>G 7579G>A 7580A>C), AAG178CAG (7581A>C), ACA179ACT (7586A>T), TTT180TAC (7588T>A 7589T>C), ATG184CCC (7599A>C 7600T>C 7601G>C), GCC185AAG (7602G>A 7603C>A 7604C>G), GAG186GAA (7607G>A), GAG187GAA (7610G>A), AAT189AAC (7616T>C), ATG190AAG (7618T>A), ATA191ATT (7622A>T), CAT193CAC (7628T>C), CGA194CGC (7631A>C), TTT195GGT (7632T>G 7633T>G), CGA196AAA (7635C>A 7636G>A)								

*: Inserts / Deletes / Misaligned / Frameshifts

Analysis details

This analysis was performed with panviral2.64

NGS Details (UN59): Caulimovirus venafragariae

Assembly

**Coverage Length**	1219 (3 contig(s))
**Depth Of Coverage**	12.6
**Number Of Reads**	134
**Reads Per Million**	2.91 rpm (after QC)
**Ambiguities**	0
**Assembly Method**	de novo + reference guided assembly
**Consensus Caller**	Bcf Tools

Coverage Map

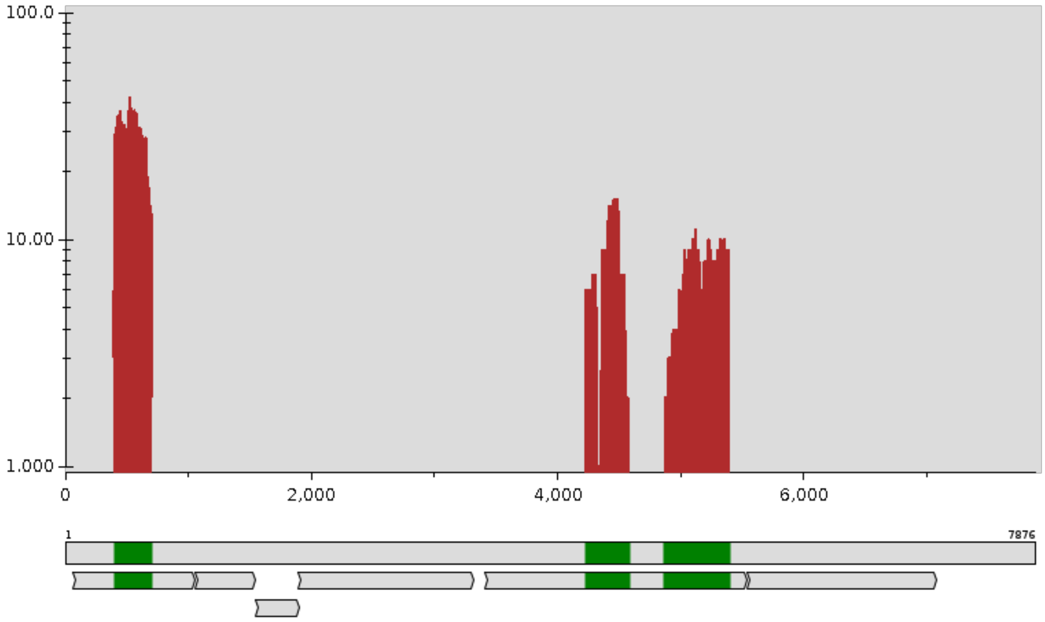

Assignment

**Type**	Caulimovirus venafragariae (Taxonomy ID: 3048344)
**Reference Genome**	NC_001725.1
**NT Identity (%)**	56.6445
**AA Identity (%)**	48.5
**Number Of Stop Codons**	0
**Number Of CDS**	6

Alignment

**Alignment Score**	269.0 (NT) + 1436.0 (AA) = 1705.0
**Concordance (%)**	33.7423

Alignment Method	Global, seeded, nucleotide + amino acids (AGA)

Genome Region

Sequence starts at position 395 and ends at position 5402 relative to NC_001725.1 reference sequence.

Alignment Detailed Statistics

	Begin	End	Coverage	Score	Concordance	Matches	Identities	I/D/M/F*	Stop Codons
**NT**	**395**	**5402**	**15.5%**	**269**	**11.4%**	**1201 (98.3%)**	**682 (55.8%)**	**3/18**	
Mutations:	403A>C, 405A>T, 411A>G, 412G>A, 416T>A, 418A>G, 424A>T, 426A>G, 430T>G, 431C>G, 432T>A, 433G>A, 435T>A, 436A>C, 441T>C, 445A>G, 447C>G, 451A>T, 452G>T, 456C>T, 459T>C, 461G>A, 462G>A, 463A>G, 464C>A, 468A>T, 472G>A, 474C>T, 476C>G, 483T>A, 485G>T, 486T>A, 487G>A, 490G>A, 491C>A, 492A>C, 495C>T, 496C>T, 497T>C, 501C>T, 502C>G, 503G>A, 504G>A, 508A>T, 510G>T, 512A>T, 513A>T, 516C>T, 517G>A, 521A>G, 525C>A, 527C>G, 529G>A, 530T>A, 531C>T, 532T>C, 534C>T, 537C>A, 538G>A, 539G>T, 544A>G, 552C>T, 553C>T, 555T>A, 556T>G, 558C>A, 562G>A, 563G>C, 564G>T, 567A>G, 568C>T, 570T>A, 571A>C, 573C>T, 579C>A, 582C>T, 583A>T, 585C>T, 588A>C, 590A>G, 592A>T, 593T>A, 594C>T, 596C>G, 598G>A, 600T>A, 601A>T, 602G>C, 603T>A, 604C>T, 606T>A, 608G>A, 612C>T, 613C>G, 617C>A, 618G>C, 619A>T, 624C>T, 625A>G, 627G>A, 628A>G, 630T>A, 633A>G, 635C>G, 636A>T, 637C>T, 639A>G, 640_642delGCT, 648C>T, 648_649insCAG, 649T>G, 651T>C, 652G>A, 656A>G, 658G>A, 663A>T, 666G>A, 667A>T, 669G>C, 670C>A, 672T>G, 673G>C, 674A>C, 675G>A, 678A>T, 685C>A, 686C>T, 687C>T, 688T>A, 689A>T, 690T>G, 692C>G, 693T>C, 696A>T, 698C>A, 699C>T, 4222T>A, 4223T>A, 4227C>T, 4228A>C, 4230T>C, 4232A>G, 4235A>T, 4236A>C, 4242T>A, 4244A>G, 4245G>A, 4246T>G, 4247G>T, 4259A>T, 4260G>A, 4264T>A, 4265C>A, 4266A>T, 4267C>G, 4268C>A, 4269T>G, 4275C>T, 4276C>A, 4277G>A, 4279G>A, 4280A>C, 4281A>T, 4284G>A, 4292C>T, 4299C>T, 4300G>C, 4306C>T, 4309C>T, 4311C>A, 4314G>T, 4315C>A, 4317T>G, 4318G>A, 4319G>A, 4320T>A, 4321A>T, 4323C>A, 4326C>A, 4330C>G, 4331C>A, 4332T>A, 4335C>T, 4338G>A, 4348T>A, 4349C>G, 4350C>T, 4351T>A, 4352C>G, 4353A>T, 4356A>T, 4359C>A, 4362C>T, 4371G>A, 4374T>C, 4380C>G, 4383G>A, 4384A>C, 4385T>A, 4386C>A, 4395C>A, 4401A>T, 4402A>C, 4410A>T, 4413T>A, 4423A>G, 4426T>C, 4428A>T, 4432G>A, 4434C>A, 4435C>A, 4437T>C, 4438A>T, 4439C>G, 4440A>T, 4442A>T, 4443G>T, 4444G>T, 4445G>T, 4446A>T, 4449T>C, 4452C>A, 4456C>T, 4458A>T, 4459C>A, 4461T>C, 4467C>T, 4470G>A, 4471G>A, 4472A>C, 4473G>T, 4474C>A, 4476A>T, 4479G>T, 4480C>A, 4482T>A, 4483C>A, 4485A>T, 4486A>C, 4487G>T, 4489A>G, 4492G>A, 4493G>A, 4496G>T, 4497T>A, 4500G>A, 4505T>A, 4506T>C, 4508A>T, 4509C>T, 4512T>C, 4513T>A, 4514C>A, 4515C>A, 4521C>T, 4528T>A, 4529C>G, 4543G>A, 4546C>A, 4548C>A, 4551T>C, 4552G>T, 4554T>A, 4555C>G, 4556C>A, 4557A>G, 4561A>T, 4568A>C, 4569G>T, 4575C>A, 4578T>A, 4859T>C, 4861G>A, 4865G>A, 4866A>T, 4869A>C, 4871A>T, 4873_4875delCTC, 4876A>G, 4879G>T, 4881C>A, 4884G>A, 4886G>C, 4890T>C, 4893C>A, 4894G>T, 4895G>C, 4897C>A, 4898T>A, 4900C>A, 4902C>G, 4903T>A, 4905A>C, 4906G>T, 4907T>G, 4910C>A, 4911T>A, 4915C>T, 4921C>A, 4926T>A, 4928G>A, 4929T>C, 4930G>A, 4931A>C, 4932T>A, 4934G>A, 4935G>A, 4936A>C, 4938T>A, 4940C>A, 4941C>A, 4942T>A, 4944A>C, 4950A>G, 4954T>C, 4956A>T, 4959C>A, 4960C>T, 4961T>G, 4962C>T, 4963C>T, 4971T>C, 4972A>G, 4973T>C, 4974C>A, 4975T>A, 4976C>G, 4977C>T, 4979C>A, 4980T>A, 4982A>T, 4984T>A, 4986T>A, 4990A>G, 4992A>C, 4993A>C, 5001C>A, 5002C>A, 5003T>A, 5004T>G, 5007G>A, 5008T>A, 5009C>A, 5010A>T, 5012C>T, 5019G>A, 5020G>A, 5021T>G, 5022T>G, 5023A>C, 5024A>T, 5025A>T, 5026C>A, 5029A>C, 5030A>G, 5031G>A, 5035G>T, 5036A>C, 5037G>C, 5039T>A, 5040A>C, 5041A>C, 5042C>G, 5043T>A, 5044T>A, 5045G>T, 5047T>G, 5048C>G, 5049T>A, 5055T>A, 5058G>T, 5059A>G, 5062G>C, 5064C>T, 5067G>C, 5068G>A, 5071A>T, 5072C>G, 5076C>T, 5077A>C, 5078G>A, 5081A>G, 5082A>C, 5083A>C, 5088G>A, 5089A>C, 5090G>A, 5091T>A, 5092C>G, 5093T>A, 5095G>T, 5096T>G, 5097A>T, 5098A>T, 5099A>C, 5100A>C, 5102C>A, 5103T>A, 5110G>A, 5112C>G, 5115T>A, 5116T>A, 5117A>G, 5118C>A, 5119A>C, 5120A>T, 5124T>A, 5125T>G, 5126C>A, 5130A>G, 5133G>A, 5136T>C, 5139G>C, 5140C>T, 5141C>T, 5142T>A, 5143A>G, 5145C>T, 5146A>C, 5148C>A, 5149G>C, 5152T>A, 5153G>C, 5154C>A, 5160A>T, 5167G>T, 5182A>T, 5184C>A, 5185C>T, 5187C>A, 5188A>C, 5191G>A, 5193A>T, 5194A>G, 5196A>C, 5199T>A, 5200C>A, 5201C>A, 5202A>T, 5206_5217delGGTAAAGAGGTA, 5220A>T, 5223C>T, 5230G>A, 5233T>A, 5234C>G, 5235A>T, 5238A>C, 5241C>A, 5247A>T, 5248C>G, 5249C>A, 5251G>A, 5253T>C, 5256G>A, 5258A>C, 5259G>T, 5262T>A, 5266C>T, 5267A>C, 5268T>A, 5270G>C, 5271T>A, 5284A>T, 5286C>A, 5287C>T, 5289T>G, 5290T>G, 5295T>A, 5296A>G, 5298T>A, 5300A>G, 5301G>A, 5303C>G, 5304A>C, 5307C>A, 5308A>C, 5309A>G, 5311G>A, 5312C>A, 5313T>A, 5317A>T, 5318G>C, 5319A>T, 5324A>T, 5325T>C, 5326A>C, 5328C>A, 5331T>A, 5334T>A, 5335T>A, 5337T>G, 5338A>C, 5343T>C, 5344C>A, 5346T>C, 5347G>A, 5349A>C, 5352G>A, 5353A>T, 5355T>A, 5358T>C, 5364T>A, 5365A>C, 5367T>G, 5369C>T, 5370T>A, 5371G>T, 5373T>A, 5374T>G, 5375A>G, 5379T>G, 5380G>A, 5382G>A, 5383A>T, 5384G>T, 5385A>T, 5387C>A, 5388A>T, 5391T>A, 5392A>C, 5395G>C, 5398G>T, 5399G>C								
**CDS**									
ORF_I	111	212	31.0%	332	49.3%	101 (98.1%)	44 (42.7%)	1/1/0/0	0
Protein mutations:	K113H (403A>C 405A>T), I115M (411A>G), D116N (412G>A), F117Y (416T>A), I118V (418A>G), I120L (424A>T 426A>G), S122G (430T>G 431C>G 432T>A), V123I (433G>A 435T>A), K124Q (436A>C), I127V (445A>G 447C>G), S129F (451A>T 452G>T), R132K (461G>A 462G>A), T133E (463A>G 464C>A), D136N (472G>A 474C>T), S137C (476C>G), S140I (485G>T 486T>A), V141I (487G>A), A142N (490G>A 491C>A 492A>C), L144S (496C>T 497T>C), R146E (502C>G 503G>A 504G>A), M148F (508A>T 510G>T), K149I (512A>T 513A>T), A151T (517G>A), K152R (521A>G), D153E (525C>A), A154G (527C>G), V155N (529G>A 530T>A 531C>T), F156L (532T>C 534C>T), G158I (538G>A 539G>T), K160E (544A>G), S164A (556T>G 558C>A), G166T (562G>A 563G>C 564G>T), I169L (571A>C 573C>T), N173Y (583A>T 585C>T), K175R (590A>G), I176Y (592A>T 593T>A 594C>T), S177C (596C>G), V178I (598G>A 600T>A), R181K (608G>A), P183A (613C>G), T184N (617C>A 618G>C), I185F (619A>T), K187E (625A>G 627G>A), T188A (628A>G 630T>A), T190S (635C>G 636A>T), A192del (640_642delGCT), F194_F195insQ (648_649insCAG), F195V (649T>G 651T>C), E196K (652G>A), K197R (656A>G), E198K (658G>A), E199D (663A>T), M201F (667A>T 669G>C), H202K (670C>A 672T>G), E203P (673G>C 674A>C 675G>A), P207I (685C>A 686C>T 687C>T), Y208M (688T>A 689A>T 690T>G), T209S (692C>G 693T>C), S211Y (698C>A 699C>T)								
Codon mutations:	AAA113CAT (403A>C 405A>T), ATA115ATG (411A>G), GAT116AAT (412G>A), TTT117TAT (416T>A), ATT118GTT (418A>G), ATA120TTG (424A>T 426A>G), TCT122GGA (430T>G 431C>G 432T>A), GTT123ATA (433G>A 435T>A), AAA124CAA (436A>C), ATT125ATC (441T>C), ATC127GTG (445A>G 447C>G), AGC129TTC (451A>T 452G>T), ACC130ACT (456C>T), TTT131TTC (459T>C), AGG132AAA (461G>A 462G>A), ACA133GAA (463A>G 464C>A), GGA134GGT (468A>T), GAC136AAT (472G>A 474C>T), TCT137TGT (476C>G), ATT139ATA (483T>A), AGT140ATA (485G>T 486T>A), GTA141ATA (487G>A), GCA142AAC (490G>A 491C>A 492A>C), CTC143CTT (495C>T), CTT144TCT (496C>T 497T>C), GAC145GAT (501C>T), CGG146GAA (502C>G 503G>A 504G>A), ATG148TTT (508A>T 510G>T), AAA149ATT (512A>T 513A>T), AAC150AAT (516C>T), GCT151ACT (517G>A), AAA152AGA (521A>G), GAC153GAA (525C>A), GCA154GGA (527C>G), GTC155AAT (529G>A 530T>A 531C>T), TTC156CTT (532T>C 534C>T), GGC157GGA (537C>A), GGA158ATA (538G>A 539G>T), AAA160GAA (544A>G), AAC162AAT (552C>T), CTT163TTA (553C>T 555T>A), TCC164GCA (556T>G 558C>A), GGG166ACT (562G>A 563G>C 564G>T), AAA167AAG (567A>G), CTT168TTA (568C>T 570T>A), ATC169CTT (571A>C 573C>T), ACC171ACA (579C>A), TAC172TAT (582C>T), AAC173TAT (583A>T 585C>T), CCA174CCC (588A>C), AAA175AGA (590A>G), ATC176TAT (592A>T 593T>A 594C>T), TCT177TGT (596C>G), GTT178ATA (598G>A 600T>A), AGT179TCA (601A>T 602G>C 603T>A), CTT180TTA (604C>T 606T>A), AGA181AAA (608G>A), GAC182GAT (612C>T), CCT183GCT (613C>G), ACG184AAC (617C>A 618G>C), ATT185TTT (619A>T), AAC186AAT (624C>T), AAG187GAA (625A>G 627G>A), ACT188GCA (628A>G 630T>A), TTA189TTG (633A>G), ACA190AGT (635C>G 636A>T), CTA191TTG (637C>T 639A>G), GCT192del (640_642delGCT), TTC194TTT (648C>T), TTC194_TTT195insCAG (648_649insCAG), TTT195GTC (649T>G 651T>C), GAA196AAA (652G>A), AAA197AGA (656A>G), GAA198AAA (658G>A), GAA199GAT (663A>T), TTG200TTA (666G>A), ATG201TTC (667A>T 669G>C), CAT202AAG (670C>A 672T>G), GAG203CCA (673G>C 674A>C 675G>A), GGA204GGT (678A>T), CCC207ATT (685C>A 686C>T 687C>T), TAT208ATG (688T>A 689A>T 690T>G), ACT209AGC (692C>G 693T>C), ATA210ATT (696A>T), TCC211TAT (698C>A 699C>T)								
ORF_V	273	666	42.7%	1104	53.9%	298 (98.3%)	150 (49.5%)	0/5/0/0	0
Protein mutations:	L273K (4222T>A 4223T>A), T275P (4228A>C 4230T>C), N276S (4232A>G), K277I (4235A>T 4236A>C), K280R (4244A>G 4245G>A), C281V (4246T>G 4247G>T), Q285L (4259A>T 4260G>A), S287N (4264T>A 4265C>A 4266A>T), P288E (4267C>G 4268C>A 4269T>G), R291K (4276C>A 4277G>A), E292T (4279G>A 4280A>C 4281A>T), T296I (4292C>T), E299Q (4300G>C), K303N (4314G>T), L304M (4315C>A 4317T>G), G305K (4318G>A 4319G>A 4320T>A), I306L (4321A>T 4323C>A), P309E (4330C>G 4331C>A 4332T>A), I327Q (4384A>C 4385T>A 4386C>A), K340E (4423A>G), D343K (4432G>A 4434C>A), H344N (4435C>A 4437T>C), T345C (4438A>T 4439C>G 4440A>T), K346I (4442A>T 4443G>T), G347F (4444G>T 4445G>T 4446A>T), L351F (4456C>T 4458A>T), L352I (4459C>A 4461T>C), E356T (4471G>A 4472A>C 4473G>T), Q357N (4474C>A 4476A>T), L359I (4480C>A 4482T>A), Q360N (4483C>A 4485A>T), R361L (4486A>C 4487G>T), I362V (4489A>G), G363K (4492G>A 4493G>A), G364V (4496G>T 4497T>A), F367Y (4505T>A 4506T>C), Y368F (4508A>T 4509C>T), S370K (4513T>A 4514C>A 4515C>A), V380I (4543G>A), A383S (4552G>T 4554T>A), P384E (4555C>G 4556C>A 4557A>G), T386S (4561A>T), Q388P (4568A>C 4569G>T), E486K (4861G>A), R487N (4865G>A 4866A>T), N489I (4871A>T), L490del (4873_4875delCTC), K491E (4876A>G), V492L (4879G>T 4881C>A), S494T (4886G>C), G497S (4894G>T 4895G>C), L498K (4897C>A 4898T>A), H499K (4900C>A 4902C>G), L500I (4903T>A 4905A>C), V501C (4906G>T 4907T>G), A502E (4910C>A 4911T>A), P504S (4915C>T), Q506K (4921C>A), S508N (4928G>A 4929T>C), D509T (4930G>A 4931A>C 4932T>A), R510K (4934G>A 4935G>A), N511Q (4936A>C 4938T>A), A512E (4940C>A 4941C>A), L513I (4942T>A 4944A>C), L519C (4960C>T 4961T>G 4962C>T), I523A (4972A>G 4973T>C 4974C>A), A525E (4979C>A 4980T>A), Y526F (4982A>T), F527I (4984T>A 4986T>A), K529D (4990A>G 4992A>C), I530L (4993A>C), N532K (5001C>A), L533K (5002C>A 5003T>A 5004T>G), S535N (5008T>A 5009C>A 5010A>T), P536L (5012C>T), V539R (5020G>A 5021T>G 5022T>G), K540L (5023A>C 5024A>T 5025A>T), L541I (5026C>A), K542R (5029A>C 5030A>G 5031G>A), E544S (5035G>T 5036A>C 5037G>C), I545N (5039T>A 5040A>C), T546R (5041A>C 5042C>G 5043T>A), W547M (5044T>A 5045G>T), S548G (5047T>G 5048C>G 5049T>A), E551D (5058G>T), K552E (5059A>G), D553H (5062G>C 5064C>T), E555K (5068G>A), T556C (5071A>T 5072C>G), R558Q (5077A>C 5078G>A), K559S (5081A>G 5082A>C), I560L (5083A>C), S562Q (5089A>C 5090G>A 5091T>A), L563E (5092C>G 5093T>A), V564C (5095G>T 5096T>G 5097A>T), K565S (5098A>T 5099A>C 5100A>C), T566K (5102C>A 5103T>A), D569K (5110G>A 5112C>G), Y571R (5116T>A 5117A>G 5118C>A), N572L (5119A>C 5120A>T), S574E (5125T>G 5126C>A), K578N (5139G>C), P579L (5140C>T 5141C>T 5142T>A), I580V (5143A>G 5145C>T), I581L (5146A>C 5148C>A), E582Q (5149G>C), C583T (5152T>A 5153G>C 5154C>A), D588Y (5167G>T), I593L (5182A>T 5184C>A), K595Q (5188A>C), A596T (5191G>A 5193A>T), K597D (5194A>G 5196A>C), P599N (5200C>A 5201C>A 5202A>T), G601_V604del (5206_5217delGGTAAAGAGGTA), A609T (5230G>A), K614N (5247A>T), P615E (5248C>G 5249C>A), A616T (5251G>A 5253T>C), K618T (5258A>C 5259G>T), N619K (5262T>A), H621S (5266C>T 5267A>C 5268T>A), S622T (5270G>C 5271T>A), I627L (5284A>T 5286C>A), S629A (5290T>G), I631V (5296A>G 5298T>A), K632R (5300A>G 5301G>A), A633G (5303C>G 5304A>C), K635R (5308A>C 5309A>G), A636K (5311G>A 5312C>A 5313T>A), R638S (5317A>T 5318G>C 5319A>T), Y640F (5324A>T 5325T>C), I641L (5326A>C 5328C>A), Y644K (5335T>A 5337T>G), K645Q (5338A>C), L647I (5344C>A 5346T>C), V648I (5347G>A 5349A>C), T650S (5353A>T 5355T>A), N654Q (5365A>C 5367T>G), A655V (5369C>T 5370T>A), A656S (5371G>T 5373T>A), Y657G (5374T>G 5375A>G), F658L (5379T>G), V659I (5380G>A 5382G>A), R660F (5383A>T 5384G>T 5385A>T), T661N (5387C>A 5388A>T), N662K (5391T>A), I663L (5392A>C), A664P (5395G>C), G665S (5398G>T 5399G>C)								

	Begin	End	Coverage	Score	Concordance	Matches	Identities	I/D/M/F*	Stop Codons
**NT**	**395**	**5402**	**15.5%**	**269**	**11.4%**	**1201 (98.3%)**	**682 (55.8%)**	**3/18**	
Codon mutations:	TTA273AAA (4222T>A 4223T>A), GAC274GAT (4227C>T), ACT275CCC (4228A>C 4230T>C), AAC276AGC (4232A>G), AAA277ATC (4235A>T 4236A>C), ATT279ATA (4242T>A), AAG280AGA (4244A>G 4245G>A), TGT281GTT (4246T>G 4247G>T), CAG285CTA (4259A>T 4260G>A), TCA287AAT (4264T>A 4265C>A 4266A>T), CCT288GAG (4267C>G 4268C>A 4269T>G), GAC290GAT (4275C>T), CGA291AAA (4276C>A 4277G>A), GAA292ACT (4279G>A 4280A>C 4281A>T), GAG293GAA (4284G>A), ACT296ATT (4292C>T), ATC298ATT (4299C>T), GAA299CAA (4300G>C), CTA301TTA (4306C>T), CTC302TTA (4309C>T 4311C>A), AAG303AAT (4314G>T), CTT304ATG (4315C>A 4317T>G), GGT305AAA (4318G>A 4319G>A 4320T>A), ATC306TTA (4321A>T 4323C>A), ATC307ATA (4326C>A), CCT309GAA (4330C>G 4331C>A 4332T>A), AGC310AGT (4335C>T), AAG311AAA (4338G>A), TCC315AGT (4348T>A 4349C>G 4350C>T), TCA316AGT (4351T>A 4352C>G 4353A>T), CCA317CCT (4356A>T), GCC318GCA (4359C>A), TTC319TTT (4362C>T), AGG322AGA (4371G>A), AAT323AAC (4374T>C), GCC325GCG (4380C>G), GAG326GAA (4383G>A), ATC327CAA (4384A>C 4385T>A 4386C>A), GGC330GGA (4395C>A), GCA332GCT (4401A>T), AGA333CGA (4402A>C), GTA335GTT (4410A>T), ATT336ATA (4413T>A), AAG340GAG (4423A>G), TTA341CTT (4426T>C 4428A>T), GAC343AAA (4432G>A 4434C>A), CAT344AAC (4435C>A 4437T>C), ACA345TGT (4438A>T 4439C>G 4440A>T), AAG346ATT (4442A>T 4443G>T), GGA347TTT (4444G>T 4445G>T 4446A>T), GAT348GAC (4449T>C), GGC349GGA (4452C>A), CTA351TTT (4456C>T 4458A>T), CTT352ATC (4459C>A 4461T>C), AAC354AAT (4467C>T), AAG355AAA (4470G>A), GAG356ACT (4471G>A 4472A>C 4473G>T), CAA357AAT (4474C>A 4476A>T), CTG358CTT (4479G>T), CTT359ATA (4480C>A 4482T>A), CAA360AAT (4483C>A 4485A>T), AGA361CTA (4486A>C 4487G>T), ATC362GTC (4489A>G), GGA363AAA (4492G>A 4493G>A), GGT364GTA (4496G>T 4497T>A), AAG365AAA (4500G>A), TTT367TAC (4505T>A 4506T>C), TAC368TTT (4508A>T 4509C>T), TCT369TCC (4512T>C), TCC370AAA (4513T>A 4514C>A 4515C>A), GAC372GAT (4521C>T), TCT375AGT (4528T>A 4529C>G), GTA380ATA (4543G>A), CGC381AGA (4546C>A 4548C>A), CTT382CTC (4551T>C), GCT383TCA (4552G>T 4554T>A), CCA384GAG (4555C>G 4556C>A 4557A>G), ACA386TCA (4561A>T), CAG388CCT (4568A>C 4569G>T), ACC390ACA (4575C>A), GCT391GCA (4578T>A), ATA485.CA (4859T>C), GAA486AAA (4861G>A), AGA487AAT (4865G>A 4866A>T), GGA488GGC (4869A>C), AAT489ATT (4871A>T), CTC490del (4873_4875delCTC), AAA491GAA (4876A>G), GTC492TTA (4879G>T 4881C>A), CAG493CAA (4884G>A), AGT494ACT (4886G>C), CAT495CAC (4890T>C), ATC496ATA (4893C>A), GGA497TCA (4894G>T 4895G>C), CTA498AAA (4897C>A 4898T>A), CAC499AAG (4900C>A 4902C>G), TTA500ATC (4903T>A 4905A>C), GTT501TGT (4906G>T 4907T>G), GCT502GAA (4910C>A 4911T>A), CCA504TCA (4915C>T), CAA506AAA (4921C>A), CTT507CTA (4926T>A), AGT508AAC (4928G>A 4929T>C), GAT509ACA (4930G>A 4931A>C 4932T>A), AGG510AAA (4934G>A 4935G>A), AAT511CAA (4936A>C 4938T>A), GCC512GAA (4940C>A 4941C>A), TTA513ATC (4942T>A 4944A>C), AGA515AGG (4950A>G), TTA517CTT (4954T>C 4956A>T), GGC518GGA (4959C>A), CTC519TGT (4960C>T 4961T>G 4962C>T), CTA520TTA (4963C>T), TAT522TAC (4971T>C), ATC523GCA (4972A>G 4973T>C 4974C>A), TCC524AGT (4975T>A 4976C>G 4977C>T), GCT525GAA (4979C>A 4980T>A), TAT526TTT (4982A>T), TTT527ATA (4984T>A 4986T>A), AAA529GAC (4990A>G 4992A>C), ATA530CTA (4993A>C), AAC532AAA (5001C>A), CTT533AAG (5002C>A 5003T>A 5004T>G), AGG534AGA (5007G>A), TCA535AAT (5008T>A 5009C>A 5010A>T), CCT536CTT (5012C>T), CAG538CAA (5019G>A), GTT539AGG (5020G>A 5021T>G 5022T>G), AAA540CTT (5023A>C 5024A>T 5025A>T), CTT541ATT (5026C>A), AAG542CGA (5029A>C 5030A>G 5031G>A), GAG544TCC (5035G>T 5036A>C 5037G>C), ATA545AAC (5039T>A 5040A>C), ACT546CGA (5041A>C 5042C>G 5043T>A), TGG547ATG (5044T>A 5045G>T), TCT548GGA (5047T>G 5048C>G 5049T>A), ACT550ACA (5055T>A), GAG551GAT (5058G>T), AAA552GAA (5059A>G), GAC553CAT (5062G>C 5064C>T), ACG554ACC (5067G>C), GAA555AAA (5068G>A), ACT556TGT (5071A>T 5072C>G), GTC557GTT (5076C>T), AGA558CAA (5077A>C 5078G>A), AAA559AGC (5081A>G 5082A>C), ATA560CTA (5083A>C), AAG561AAA (5088G>A), AGT562CAA (5089A>C 5090G>A 5091T>A), CTA563GAA (5092C>G 5093T>A), GTA564TGT (5095G>T 5096T>G 5097A>T), AAA565TCC (5098A>T 5099A>C 5100A>C), ACT566AAA (5102C>A 5103T>A), GAC569AAG (5110G>A 5112C>G), CTT570CTA (5115T>A), TAC571AGA (5116T>A 5117A>G 5118C>A), AAT572CTT (5119A>C 5120A>T), CCT573CCA (5124T>A), TCA574GAA (5125T>G 5126C>A), CCA575CCG (5130A>G), GAG576GAA (5133G>A), GAT577GAC (5136T>C), AAG578AAC (5139G>C), CCT579TTA (5140C>T 5141C>T 5142T>A), ATC580GTT (5143A>G 5145C>T), ATC581CTA (5146A>C 5148C>A), GAA582CAA (5149G>C), TGC583ACA (5152T>A 5153G>C 5154C>A), GCA585GCT (5160A>T), GAC588TAC (5167G>T), ATC593TTA (5182A>T 5184C>A), CTC594TTA (5185C>T 5187C>A), AAA595CAA (5188A>C), GCA596ACT (5191G>A 5193A>T), AAA597GAC (5194A>G 5196A>C), CTT598CTA (5199T>A), CCA599AAT (5200C>A 5201C>A 5202A>T), GGT601_GTA604del (5206_5217delGGTAAAGAGGTA), ATA605ATT (5220A>T), TGC606TGT (5223C>T), GCT609ACT (5230G>A), TCA610AGT (5233T>A 5234C>G 5235A>T), GGA611GGC (5238A>C), ACC612ACA (5241C>A), AAA614AAT (5247A>T), CCA615GAA (5248C>G 5249C>A), GCT616ACC (5251G>A 5253T>C), GAG617GAA (5256G>A), AAG618ACT (5258A>C 5259G>T), AAT619AAA (5262T>A), CAT621TCA (5266C>T 5267A>C 5268T>A), AGT622ACA (5270G>C 5271T>A), ATC627TTA (5284A>T 5286C>A), CTT628TTG (5287C>T 5289T>G), TCC629GCC (5290T>G), ATT630ATA (5295T>A), ATT631GTA (5296A>G 5298T>A), AAG632AGA (5300A>G 5301G>A), GCA633GGC (5303C>G 5304A>C), ATC634ATA (5307C>A), AAA635CGA (5308A>C 5309A>G), GCT636AAA (5311G>A 5312C>A 5313T>A), AGA638TCT (5317A>T 5318G>C 5319A>T), TAT640TTC (5324A>T 5325T>C), ATC641CTA (5326A>C 5328C>A), CTT642CTA (5331T>A), CCT643CCA (5334T>A), TAT644AAG (5335T>A 5337T>G), AAA645CAA (5338A>C), TTT646TTC (5343T>C), CTT647ATC (5344C>A 5346T>C), GTA648ATC (5347G>A 5349A>C), AGG649AGA (5352G>A), ACT650TCA (5353A>T 5355T>A), GAT651GAC (5358T>C), ACT653ACA (5364T>A), AAT654CAG (5365A>C 5367T>G), GCT655GTA (5369C>T 5370T>A), GCT656TCA (5371G>T 5373T>A), TAT657GGT (5374T>G 5375A>G), TTT658TTG (5379T>G), GTG659ATA (5380G>A 5382G>A), AGA660TTT (5383A>T 5384G>T 5385A>T), ACA661AAT (5387C>A 5388A>T), AAT662AAA (5391T>A), ATT663CTT (5392A>C), GCA664CCA (5395G>C), GGT665TCT (5398G>T 5399G>C)								

Proteins

	Begin	End	Coverage	Score	Concordance	Matches	Identities	I/D/M/F*	Stop Codons
hypothetical protein (NP_043929.1)	111	212	31.0%	332	49.3%	101 (98.1%)	44 (42.7%)	1/1/0/0	0
Protein mutations:	K113H (403A>C 405A>T), I115M (411A>G), D116N (412G>A), F117Y (416T>A), I118V (418A>G), I120L (424A>T 426A>G), S122G (430T>G 431C>G 432T>A), V123I (433G>A 435T>A), K124Q (436A>C), I127V (445A>G 447C>G), S129F (451A>T 452G>T), R132K (461G>A 462G>A), T133E (463A>G 464C>A), D136N (472G>A 474C>T), S137C (476C>G), S140I (485G>T 486T>A), V141I (487G>A), A142N (490G>A 491C>A 492A>C), L144S (496C>T 497T>C), R146E (502C>G 503G>A 504G>A), M148F (508A>T 510G>T), K149I (512A>T 513A>T), A151T (517G>A), K152R (521A>G), D153E (525C>A), A154G (527C>G), V155N (529G>A 530T>A 531C>T), F156L (532T>C 534C>T), G158I (538G>A 539G>T), K160E (544A>G), S164A (556T>G 558C>A), G166T (562G>A 563G>C 564G>T), I169L (571A>C 573C>T), N173Y (583A>T 585C>T), K175R (590A>G), I176Y (592A>T 593T>A 594C>T), S177C (596C>G), V178I (598G>A 600T>A), R181K (608G>A), P183A (613C>G), T184N (617C>A 618G>C), I185F (619A>T), K187E (625A>G 627G>A), T188A (628A>G 630T>A), T190S (635C>G 636A>T), A192del (640_642delGCT), F194_F195insQ (648_649insCAG), F195V (649T>G 651T>C), E196K (652G>A), K197R (656A>G), E198K (658G>A), E199D (663A>T), M201F (667A>T 669G>C), H202K (670C>A 672T>G), E203P (673G>C 674A>C 675G>A), P207I (685C>A 686C>T 687C>T), Y208M (688T>A 689A>T 690T>G), T209S (692C>G 693T>C), S211Y (698C>A 699C>T)								
Codon mutations:	AAA113CAT (403A>C 405A>T), ATA115ATG (411A>G), GAT116AAT (412G>A), TTT117TAT (416T>A), ATT118GTT (418A>G), ATA120TTG (424A>T 426A>G), TCT122GGA (430T>G 431C>G 432T>A), GTT123ATA (433G>A 435T>A), AAA124CAA (436A>C), ATT125ATC (441T>C), ATC127GTG (445A>G 447C>G), AGC129TTC (451A>T 452G>T), ACC130ACT (456C>T), TTT131TTC (459T>C), AGG132AAA (461G>A 462G>A), ACA133GAA (463A>G 464C>A), GGA134GGT (468A>T), GAC136AAT (472G>A 474C>T), TCT137TGT (476C>G), ATT139ATA (483T>A), AGT140ATA (485G>T 486T>A), GTA141ATA (487G>A), GCA142AAC (490G>A 491C>A 492A>C), CTC143CTT (495C>T), CTT144TCT (496C>T 497T>C), GAC145GAT (501C>T), CGG146GAA (502C>G 503G>A 504G>A), ATG148TTT (508A>T 510G>T), AAA149ATT (512A>T 513A>T), AAC150AAT (516C>T), GCT151ACT (517G>A), AAA152AGA (521A>G), GAC153GAA (525C>A), GCA154GGA (527C>G), GTC155AAT (529G>A 530T>A 531C>T), TTC156CTT (532T>C 534C>T), GGC157GGA (537C>A), GGA158ATA (538G>A 539G>T), AAA160GAA (544A>G), AAC162AAT (552C>T), CTT163TTA (553C>T 555T>A), TCC164GCA (556T>G 558C>A), GGG166ACT (562G>A 563G>C 564G>T), AAA167AAG (567A>G), CTT168TTA (568C>T 570T>A), ATC169CTT (571A>C 573C>T), ACC171ACA (579C>A), TAC172TAT (582C>T), AAC173TAT (583A>T 585C>T), CCA174CCC (588A>C), AAA175AGA (590A>G), ATC176TAT (592A>T 593T>A 594C>T), TCT177TGT (596C>G), GTT178ATA (598G>A 600T>A), AGT179TCA (601A>T 602G>C 603T>A), CTT180TTA (604C>T 606T>A), AGA181AAA (608G>A), GAC182GAT (612C>T), CCT183GCT (613C>G), ACG184AAC (617C>A 618G>C), ATT185TTT (619A>T), AAC186AAT (624C>T), AAG187GAA (625A>G 627G>A), ACT188GCA (628A>G 630T>A), TTA189TTG (633A>G), ACA190AGT (635C>G 636A>T), CTA191TTG (637C>T 639A>G), GCT192del (640_642delGCT), TTC194TTT (648C>T), TTC194_TTT195insCAG (648_649insCAG), TTT195GTC (649T>G 651T>C), GAA196AAA (652G>A), AAA197AGA (656A>G), GAA198AAA (658G>A), GAA199GAT (663A>T), TTG200TTA (666G>A), ATG201TTC (667A>T 669G>C), CAT202AAG (670C>A 672T>G), GAG203CCA (673G>C 674A>C 675G>A), GGA204GGT (678A>T), CCC207ATT (685C>A 686C>T 687C>T), TAT208ATG (688T>A 689A>T 690T>G), ACT209AGC (692C>G 693T>C), ATA210ATT (696A>T), TCC211TAT (698C>A 699C>T)								
hypothetical protein (NP_043933.1)	273	666	42.7%	1104	53.9%	298 (98.3%)	150 (49.5%)	0/5/0/0	0
Protein mutations:	L273K (4222T>A 4223T>A), T275P (4228A>C 4230T>C), N276S (4232A>G), K277I (4235A>T 4236A>C), K280R (4244A>G 4245G>A), C281V (4246T>G 4247G>T), Q285L (4259A>T 4260G>A), S287N (4264T>A 4265C>A 4266A>T), P288E (4267C>G 4268C>A 4269T>G), R291K (4276C>A 4277G>A), E292T (4279G>A 4280A>C 4281A>T), T296I (4292C>T), E299Q (4300G>C), K303N (4314G>T), L304M (4315C>A 4317T>G), G305K (4318G>A 4319G>A 4320T>A), I306L (4321A>T 4323C>A), P309E (4330C>G 4331C>A 4332T>A), I327Q (4384A>C 4385T>A 4386C>A), K340E (4423A>G), D343K (4432G>A 4434C>A), H344N (4435C>A 4437T>C), T345C (4438A>T 4439C>G 4440A>T), K346I (4442A>T 4443G>T), G347F (4444G>T 4445G>T 4446A>T), L351F (4456C>T 4458A>T), L352I (4459C>A 4461T>C), E356T (4471G>A 4472A>C 4473G>T), Q357N (4474C>A 4476A>T), L359I (4480C>A 4482T>A), Q360N (4483C>A 4485A>T), R361L (4486A>C 4487G>T), I362V (4489A>G), G363K (4492G>A 4493G>A), G364V (4496G>T 4497T>A), F367Y (4505T>A 4506T>C), Y368F (4508A>T 4509C>T), S370K (4513T>A 4514C>A 4515C>A), V380I (4543G>A), A383S (4552G>T 4554T>A), P384E (4555C>G 4556C>A 4557A>G), T386S (4561A>T), Q388P (4568A>C 4569G>T), E486K (4861G>A), R487N (4865G>A 4866A>T), N489I (4871A>T), L490del (4873_4875delCTC), K491E (4876A>G), V492L (4879G>T 4881C>A), S494T (4886G>C), G497S (4894G>T 4895G>C), L498K (4897C>A 4898T>A), H499K (4900C>A 4902C>G), L500I (4903T>A 4905A>C), V501C (4906G>T 4907T>G), A502E (4910C>A 4911T>A), P504S (4915C>T), Q506K (4921C>A), S508N (4928G>A 4929T>C), D509T (4930G>A 4931A>C 4932T>A), R510K (4934G>A 4935G>A), N511Q (4936A>C 4938T>A), A512E (4940C>A 4941C>A), L513I (4942T>A 4944A>C), L519C (4960C>T 4961T>G 4962C>T), I523A (4972A>G 4973T>C 4974C>A), A525E (4979C>A 4980T>A), Y526F (4982A>T), F527I (4984T>A 4986T>A), K529D (4990A>G 4992A>C), I530L (4993A>C), N532K (5001C>A), L533K (5002C>A 5003T>A 5004T>G), S535N (5008T>A 5009C>A 5010A>T), P536L (5012C>T), V539R (5020G>A 5021T>G 5022T>G), K540L (5023A>C 5024A>T 5025A>T), L541I (5026C>A), K542R (5029A>C 5030A>G 5031G>A), E544S (5035G>T 5036A>C 5037G>C), I545N (5039T>A 5040A>C), T546R (5041A>C 5042C>G 5043T>A), W547M (5044T>A 5045G>T), S548G (5047T>G 5048C>G 5049T>A), E551D (5058G>T), K552E (5059A>G), D553H (5062G>C 5064C>T), E555K (5068G>A), T556C (5071A>T 5072C>G), R558Q (5077A>C 5078G>A), K559S (5081A>G 5082A>C), I560L (5083A>C), S562Q (5089A>C 5090G>A 5091T>A), L563E (5092C>G 5093T>A), V564C (5095G>T 5096T>G 5097A>T), K565S (5098A>T 5099A>C 5100A>C), T566K (5102C>A 5103T>A), D569K (5110G>A 5112C>G), Y571R (5116T>A 5117A>G 5118C>A), N572L (5119A>C 5120A>T), S574E (5125T>G 5126C>A), K578N (5139G>C), P579L (5140C>T 5141C>T 5142T>A), I580V (5143A>G 5145C>T), I581L (5146A>C 5148C>A), E582Q (5149G>C), C583T (5152T>A 5153G>C 5154C>A), D588Y (5167G>T), I593L (5182A>T 5184C>A), K595Q (5188A>C), A596T (5191G>A 5193A>T), K597D (5194A>G 5196A>C), P599N (5200C>A 5201C>A 5202A>T), G601_V604del (5206_5217delGGTAAAGAGGTA), A609T (5230G>A), K614N (5247A>T), P615E (5248C>G 5249C>A), A616T (5251G>A 5253T>C), K618T (5258A>C 5259G>T), N619K (5262T>A), H621S (5266C>T 5267A>C 5268T>A), S622T (5270G>C 5271T>A), I627L (5284A>T 5286C>A), S629A (5290T>G), I631V (5296A>G 5298T>A), K632R (5300A>G 5301G>A), A633G (5303C>G 5304A>C), K635R (5308A>C 5309A>G), A636K (5311G>A 5312C>A 5313T>A), R638S (5317A>T 5318G>C 5319A>T), Y640F (5324A>T 5325T>C), I641L (5326A>C 5328C>A), Y644K (5335T>A 5337T>G), K645Q (5338A>C), L647I (5344C>A 5346T>C), V648I (5347G>A 5349A>C), T650S (5353A>T 5355T>A), N654Q (5365A>C 5367T>G), A655V (5369C>T 5370T>A), A656S (5371G>T 5373T>A), Y657G (5374T>G 5375A>G), F658L (5379T>G), V659I (5380G>A 5382G>A), R660F (5383A>T 5384G>T 5385A>T), T661N (5387C>A 5388A>T), N662K (5391T>A), I663L (5392A>C), A664P (5395G>C), G665S (5398G>T 5399G>C)								

	Begin	End	Coverage	Score	Concordance	Matches	Identities	I/D/M/F*	Stop Codons
**NT**	**395**	**5402**	**15.5%**	**269**	**11.4%**	**1201 (98.3%)**	**682 (55.8%)**	**3/18**	
Codon mutations:	TTA273AAA (4222T>A 4223T>A), GAC274GAT (4227C>T), ACT275CCC (4228A>C 4230T>C), AAC276AGC (4232A>G), AAA277ATC (4235A>T 4236A>C), ATT279ATA (4242T>A), AAG280AGA (4244A>G 4245G>A), TGT281GTT (4246T>G 4247G>T), CAG285CTA (4259A>T 4260G>A), TCA287AAT (4264T>A 4265C>A 4266A>T), CCT288GAG (4267C>G 4268C>A 4269T>G), GAC290GAT (4275C>T), CGA291AAA (4276C>A 4277G>A), GAA292ACT (4279G>A 4280A>C 4281A>T), GAG293GAA (4284G>A), ACT296ATT (4292C>T), ATC298ATT (4299C>T), GAA299CAA (4300G>C), CTA301TTA (4306C>T), CTC302TTA (4309C>T 4311C>A), AAG303AAT (4314G>T), CTT304ATG (4315C>A 4317T>G), GGT305AAA (4318G>A 4319G>A 4320T>A), ATC306TTA (4321A>T 4323C>A), ATC307ATA (4326C>A), CCT309GAA (4330C>G 4331C>A 4332T>A), AGC310AGT (4335C>T), AAG311AAA (4338G>A), TCC315AGT (4348T>A 4349C>G 4350C>T), TCA316AGT (4351T>A 4352C>G 4353A>T), CCA317CCT (4356A>T), GCC318GCA (4359C>A), TTC319TTT (4362C>T), AGG322AGA (4371G>A), AAT323AAC (4374T>C), GCC325GCG (4380C>G), GAG326GAA (4383G>A), ATC327CAA (4384A>C 4385T>A 4386C>A), GGC330GGA (4395C>A), GCA332GCT (4401A>T), AGA333CGA (4402A>C), GTA335GTT (4410A>T), ATT336ATA (4413T>A), AAG340GAG (4423A>G), TTA341CTT (4426T>C 4428A>T), GAC343AAA (4432G>A 4434C>A), CAT344AAC (4435C>A 4437T>C), ACA345TGT (4438A>T 4439C>G 4440A>T), AAG346ATT (4442A>T 4443G>T), GGA347TTT (4444G>T 4445G>T 4446A>T), GAT348GAC (4449T>C), GGC349GGA (4452C>A), CTA351TTT (4456C>T 4458A>T), CTT352ATC (4459C>A 4461T>C), AAC354AAT (4467C>T), AAG355AAA (4470G>A), GAG356ACT (4471G>A 4472A>C 4473G>T), CAA357AAT (4474C>A 4476A>T), CTG358CTT (4479G>T), CTT359ATA (4480C>A 4482T>A), CAA360AAT (4483C>A 4485A>T), AGA361CTA (4486A>C 4487G>T), ATC362GTC (4489A>G), GGA363AAA (4492G>A 4493G>A), GGT364GTA (4496G>T 4497T>A), AAG365AAA (4500G>A), TTT367TAC (4505T>A 4506T>C), TAC368TTT (4508A>T 4509C>T), TCT369TCC (4512T>C), TCC370AAA (4513T>A 4514C>A 4515C>A), GAC372GAT (4521C>T), TCT375AGT (4528T>A 4529C>G), GTA380ATA (4543G>A), CGC381AGA (4546C>A 4548C>A), CTT382CTC (4551T>C), GCT383TCA (4552G>T 4554T>A), CCA384GAG (4555C>G 4556C>A 4557A>G), ACA386TCA (4561A>T), CAG388CCT (4568A>C 4569G>T), ACC390ACA (4575C>A), GCT391GCA (4578T>A), ATA485.CA (4859T>C), GAA486AAA (4861G>A), AGA487AAT (4865G>A 4866A>T), GGA488GGC (4869A>C), AAT489ATT (4871A>T), CTC490del (4873_4875delCTC), AAA491GAA (4876A>G), GTC492TTA (4879G>T 4881C>A), CAG493CAA (4884G>A), AGT494ACT (4886G>C), CAT495CAC (4890T>C), ATC496ATA (4893C>A), GGA497TCA (4894G>T 4895G>C), CTA498AAA (4897C>A 4898T>A), CAC499AAG (4900C>A 4902C>G), TTA500ATC (4903T>A 4905A>C), GTT501TGT (4906G>T 4907T>G), GCT502GAA (4910C>A 4911T>A), CCA504TCA (4915C>T), CAA506AAA (4921C>A), CTT507CTA (4926T>A), AGT508AAC (4928G>A 4929T>C), GAT509ACA (4930G>A 4931A>C 4932T>A), AGG510AAA (4934G>A 4935G>A), AAT511CAA (4936A>C 4938T>A), GCC512GAA (4940C>A 4941C>A), TTA513ATC (4942T>A 4944A>C), AGA515AGG (4950A>G), TTA517CTT (4954T>C 4956A>T), GGC518GGA (4959C>A), CTC519TGT (4960C>T 4961T>G 4962C>T), CTA520TTA (4963C>T), TAT522TAC (4971T>C), ATC523GCA (4972A>G 4973T>C 4974C>A), TCC524AGT (4975T>A 4976C>G 4977C>T), GCT525GAA (4979C>A 4980T>A), TAT526TTT (4982A>T), TTT527ATA (4984T>A 4986T>A), AAA529GAC (4990A>G 4992A>C), ATA530CTA (4993A>C), AAC532AAA (5001C>A), CTT533AAG (5002C>A 5003T>A 5004T>G), AGG534AGA (5007G>A), TCA535AAT (5008T>A 5009C>A 5010A>T), CCT536CTT (5012C>T), CAG538CAA (5019G>A), GTT539AGG (5020G>A 5021T>G 5022T>G), AAA540CTT (5023A>C 5024A>T 5025A>T), CTT541ATT (5026C>A), AAG542CGA (5029A>C 5030A>G 5031G>A), GAG544TCC (5035G>T 5036A>C 5037G>C), ATA545AAC (5039T>A 5040A>C), ACT546CGA (5041A>C 5042C>G 5043T>A), TGG547ATG (5044T>A 5045G>T), TCT548GGA (5047T>G 5048C>G 5049T>A), ACT550ACA (5055T>A), GAG551GAT (5058G>T), AAA552GAA (5059A>G), GAC553CAT (5062G>C 5064C>T), ACG554ACC (5067G>C), GAA555AAA (5068G>A), ACT556TGT (5071A>T 5072C>G), GTC557GTT (5076C>T), AGA558CAA (5077A>C 5078G>A), AAA559AGC (5081A>G 5082A>C), ATA560CTA (5083A>C), AAG561AAA (5088G>A), AGT562CAA (5089A>C 5090G>A 5091T>A), CTA563GAA (5092C>G 5093T>A), GTA564TGT (5095G>T 5096T>G 5097A>T), AAA565TCC (5098A>T 5099A>C 5100A>C), ACT566AAA (5102C>A 5103T>A), GAC569AAG (5110G>A 5112C>G), CTT570CTA (5115T>A), TAC571AGA (5116T>A 5117A>G 5118C>A), AAT572CTT (5119A>C 5120A>T), CCT573CCA (5124T>A), TCA574GAA (5125T>G 5126C>A), CCA575CCG (5130A>G), GAG576GAA (5133G>A), GAT577GAC (5136T>C), AAG578AAC (5139G>C), CCT579TTA (5140C>T 5141C>T 5142T>A), ATC580GTT (5143A>G 5145C>T), ATC581CTA (5146A>C 5148C>A), GAA582CAA (5149G>C), TGC583ACA (5152T>A 5153G>C 5154C>A), GCA585GCT (5160A>T), GAC588TAC (5167G>T), ATC593TTA (5182A>T 5184C>A), CTC594TTA (5185C>T 5187C>A), AAA595CAA (5188A>C), GCA596ACT (5191G>A 5193A>T), AAA597GAC (5194A>G 5196A>C), CTT598CTA (5199T>A), CCA599AAT (5200C>A 5201C>A 5202A>T), GGT601_GTA604del (5206_5217delGGTAAAGAGGTA), ATA605ATT (5220A>T), TGC606TGT (5223C>T), GCT609ACT (5230G>A), TCA610AGT (5233T>A 5234C>G 5235A>T), GGA611GGC (5238A>C), ACC612ACA (5241C>A), AAA614AAT (5247A>T), CCA615GAA (5248C>G 5249C>A), GCT616ACC (5251G>A 5253T>C), GAG617GAA (5256G>A), AAG618ACT (5258A>C 5259G>T), AAT619AAA (5262T>A), CAT621TCA (5266C>T 5267A>C 5268T>A), AGT622ACA (5270G>C 5271T>A), ATC627TTA (5284A>T 5286C>A), CTT628TTG (5287C>T 5289T>G), TCC629GCC (5290T>G), ATT630ATA (5295T>A), ATT631GTA (5296A>G 5298T>A), AAG632AGA (5300A>G 5301G>A), GCA633GGC (5303C>G 5304A>C), ATC634ATA (5307C>A), AAA635CGA (5308A>C 5309A>G), GCT636AAA (5311G>A 5312C>A 5313T>A), AGA638TCT (5317A>T 5318G>C 5319A>T), TAT640TTC (5324A>T 5325T>C), ATC641CTA (5326A>C 5328C>A), CTT642CTA (5331T>A), CCT643CCA (5334T>A), TAT644AAG (5335T>A 5337T>G), AAA645CAA (5338A>C), TTT646TTC (5343T>C), CTT647ATC (5344C>A 5346T>C), GTA648ATC (5347G>A 5349A>C), AGG649AGA (5352G>A), ACT650TCA (5353A>T 5355T>A), GAT651GAC (5358T>C), ACT653ACA (5364T>A), AAT654CAG (5365A>C 5367T>G), GCT655GTA (5369C>T 5370T>A), GCT656TCA (5371G>T 5373T>A), TAT657GGT (5374T>G 5375A>G), TTT658TTG (5379T>G), GTG659ATA (5380G>A 5382G>A), AGA660TTT (5383A>T 5384G>T 5385A>T), ACA661AAT (5387C>A 5388A>T), AAT662AAA (5391T>A), ATT663CTT (5392A>C), GCA664CCA (5395G>C), GGT665TCT (5398G>T 5399G>C)								

*: Inserts / Deletes / Misaligned / Frameshifts

Analysis details

This analysis was performed with panviral2.64

NGS Details (UN59): Dioscovirus dioscoreae

Assembly

**Coverage Length**	1200 (2 contig(s))
**Depth Of Coverage**	8.0
**Number Of Reads**	81
**Reads Per Million**	1.76 rpm (after QC)
**Ambiguities**	0
**Assembly Method**	de novo + reference guided assembly
**Consensus Caller**	Bcf Tools

Coverage Map

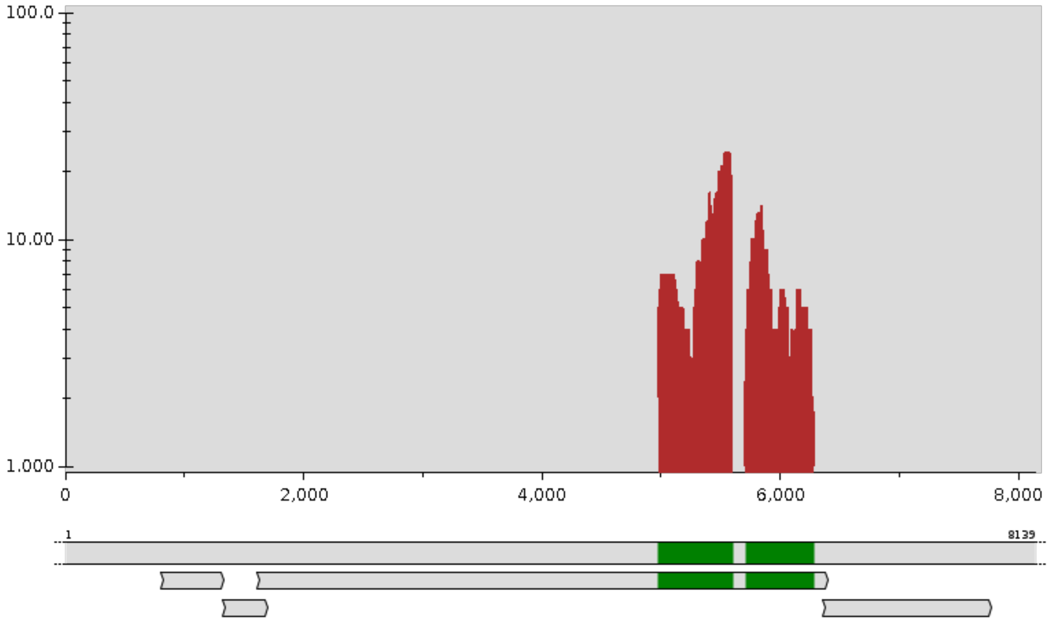

Assignment

**Type**	Dioscovirus dioscoreae (Taxonomy ID: 3052184)
**Reference Genome**	NC_040712.1
**NT Identity (%)**	61.9917
**AA Identity (%)**	52.2388
**Number Of Stop Codons**	1
**Number Of CDS**	4

Alignment

**Alignment Score**	547.0 (NT) + 1411.0 (AA) = 1958.0
**Concordance (%)**	39.0292

Alignment Method	Global, seeded, nucleotide + amino acids (AGA)

Genome Region

Sequence starts at position 4979 and ends at position 6282 relative to NC_040712.1 reference sequence.

Alignment Detailed Statistics

	Begin	End	Coverage	Score	Concordance	Matches	Identities	I/D/M/F*	Stop Codons
**NT**	**4979**	**6282**	**14.7%**	**547**	**23.1%**	**1196 (98.9%)**	**747 (61.8%)**	**9/4**	
Mutations:	4983C>A, 4986C>A, 4988T>A, 4989A>T, 4990A>T, 4992T>C, 4994G>A, 4997A>T, 5001A>T, 5003C>A, 5004C>G, 5009T>A, 5012T>A, 5015G>A, 5016A>G, 5018T>A, 5019G>A, 5021C>A, 5024A>C, 5025T>C, 5029A>T, 5030G>A, 5031T>C, 5038A>G, 5040A>G, 5041A>C, 5042G>C, 5046C>A, 5047A>G, 5048G>A, 5051T>A, 5053G>T, 5054A>G, 5057C>T, 5063G>A, 5066C>T, 5069C>A, 5078T>A, 5081T>A, 5082C>T, 5083T>A, 5091C>A, 5093G>A, 5094G>A, 5095A>T, 5096C>A, 5097A>G, 5098A>C, 5099G>A, 5100C>G, 5102T>A, 5105T>A, 5106A>G, 5107T>A, 5109C>G, 5112T>A, 5115C>A, 5116C>A, 5117A>T, 5118G>C, 5121A>G, 5122T>A, 5123G>C, 5124G>A, 5125C>T, 5126G>A, 5127A>G, 5132A>T, 5134A>T, 5136G>C, 5138A>G, 5140A>T, 5141G>A, 5144T>A, 5147T>A, 5149C>A, 5157C>T, 5159G>A, 5161C>A, 5162A>T, 5163C>T, 5165C>A, 5166A>G, 5167A>G, 5168G>A, 5171T>A, 5174C>A, 5178C>A, 5179C>G, 5180T>A, 5183C>A, 5184C>A, 5185A>C, 5187A>T, 5188G>C, 5190C>A, 5192C>A, 5195T>C, 5198G>A, 5199A>T, 5207T>A, 5213T>G, 5216C>A, 5219C>A, 5220delA, 5222A>C, 5226T>A, 5227C>A, 5228T>C, 5232C>A, 5233A>T, 5237A>C, 5243T>A, 5247A>G, 5248G>C, 5249T>C, 5250C>A, 5252T>A, 5258A>T, 5265T>A, 5266A>G, 5268A>C, 5270G>A, 5273_5274insGAA, 5276A>T, 5288A>T, 5289T>A, 5290A>G, 5291C>A, 5293A>C, 5297C>T, 5298C>G, 5299A>C, 5303C>T, 5305C>A, 5307C>T, 5309C>A, 5310C>T, 5313A>G, 5314G>A, 5317T>A, 5318T>A, 5322T>A, 5324T>A, 5325C>T, 5327C>G, 5328C>A, 5330A>T, 5331C>T, 5332T>G, 5335A>G, 5340A>C, 5344A>G, 5345C>T, 5348G>A, 5350T>A, 5351T>A, 5352G>A, 5356A>T, 5357C>T, 5360C>T, 5361A>G, 5370C>T, 5371T>G, 5372A>T, 5375G>A, 5378A>G, 5383T>A, 5385C>T, 5387C>G, 5390G>A, 5391A>G, 5393T>A, 5395T>A, 5396G>A, 5400G>C, 5402T>C, 5406C>G, 5408G>C, 5429T>C, 5430G>A, 5431T>C, 5432C>T, 5435C>T, 5437C>T, 5438A>T, 5439C>G, 5444T>A, 5457A>T, 5461T>C, 5462A>C, 5471C>T, 5474A>T, 5475C>T, 5477C>A, 5482A>C, 5483T>A, 5486A>G, 5492A>T, 5493G>A, 5498C>T, 5502C>A, 5511G>A, 5514A>G, 5519A>G, 5523A>G, 5524A>G, 5526A>G, 5528A>C, 5532T>A, 5533C>A, 5535G>A, 5537A>C, 5543C>T, 5545G>T, 5546T>A, 5549T>C, 5553A>G, 5558C>T, 5567G>A, 5568A>G, 5574T>A, 5575C>G, 5576T>C, 5577G>A, 5580A>G, 5581G>A, 5589C>G, 5595G>T, 5598C>G, 5599A>G, 5604T>C, 5606A>T, 5711A>T, 5714A>G, 5717A>G, 5718G>A, 5720T>A, 5721C>A, 5726C>A, 5739T>G, 5740T>C, 5741A>T, 5748A>G, 5756A>T, 5757T>A, 5759T>G, 5762T>A, 5763_5765delGAA, 5768C>T, 5769C>A, 5777A>T, 5779C>A, 5781T>A, 5782T>C, 5786G>A, 5788G>A, 5790C>T, 5795G>A, 5798G>A, 5807G>A, 5808A>T, 5811T>G, 5813A>G, 5816C>T, 5822C>T, 5828T>C, 5830A>T, 5834C>A, 5836C>A, 5843G>A, 5844A>G, 5846T>A, 5850T>A, 5851A>T, 5853A>G, 5855T>A, 5856A>G, 5859A>C, 5860T>C, 5864T>A, 5868A>G, 5869A>C, 5870T>A, 5873G>A, 5874T>A, 5875G>T, 5876T>A, 5877T>G, 5878C>G, 5879C>A, 5882C>T, 5889G>C, 5893G>A, 5895A>C, 5897A>C, 5903T>C, 5904T>A, 5906T>A, 5907C>G, 5910G>A, 5912C>T, 5913T>A, 5914G>T, 5915G>T, 5918G>A, 5919A>C, 5924T>A, 5925A>C, 5926G>A, 5928A>C, 5933C>T, 5940G>A, 5941T>A, 5945T>A, 5947C>A, 5948C>A, 5951A>C, 5952T>A, 5957C>A, 5958C>G, 5959C>A, 5960T>C, 5963G>A, 5965T>G, 5966T>C, 5967T>A, 5973A>C, 5974A>T, 5976C>G, 5977C>A, 5979C>T, 5980A>C, 5982A>G, 5983G>A, 5988A>C, 5990T>A, 5999G>A, 6005T>C, 6009T>A, 6010C>G, 6011T>C, 6014A>C, 6015G>C, 6026G>A, 6028G>C, 6029A>T, 6030A>G, 6032T>A, 6039T>G, 6040G>C, 6041G>A, 6047C>A, 6048T>A, 6049C>A, 6050T>C, 6051C>A, 6054C>T, 6056A>C, 6057G>A, 6058A>G, 6060T>C, 6062A>T, 6063C>A, 6064C>A, 6065T>A, 6066T>A, 6067C>A, 6069A>G, 6070C>A, 6082C>G, 6083A>T, 6084C>G, 6085G>C, 6086G>T, 6089T>C, 6090T>C, 6091G>A, 6092T>A, 6100G>A, 6101C>A, 6104T>C, 6107_6108insGAAAAG, 6108T>G, 6109C>G, 6111G>A, 6112C>A, 6116C>G, 6117T>A, 6118C>G, 6120G>A, 6121C>G, 6122A>T, 6130C>T, 6131T>C, 6135A>G, 6138A>T, 6140G>A, 6144T>A, 6145G>T, 6146T>A, 6154T>G, 6156T>C, 6159G>A, 6163A>G, 6164G>T, 6167T>C, 6171A>T, 6175T>A, 6177T>A, 6179A>T, 6181A>T, 6182T>A, 6183G>A, 6185G>C, 6190A>G, 6191A>T, 6195T>A, 6197T>A, 6198A>G, 6199C>T, 6200C>A, 6201C>G, 6203T>A, 6206G>A, 6212C>T, 6215T>C, 6216A>G, 6217T>A, 6218T>A, 6221A>T, 6224T>A, 6230T>A, 6234T>C, 6237A>C, 6239C>A, 6243C>A, 6245T>A, 6246A>G, 6248T>C, 6249A>C, 6251T>G, 6257A>T, 6258T>A, 6259T>G, 6260G>T, 6262A>G, 6265T>A, 6268A>G, 6269T>A, 6270C>A, 6272T>A, 6276G>C								

CDS

	Begin	End	Coverage	Score	Concordance	Matches	Identities	I/D/M/F*	Stop Codons
EXK67_gp3	1124	1558	25.1%	1411	49.9%	399 (99.0%)	210 (52.1%)	3/1/1/1	1
Protein mutations:	L1125I (4983C>A), H1126K (4986C>A 4988T>A), K1127L (4989A>T 4990A>T), E1129D (4997A>T), I1131L (5001A>T 5003C>A), Q1132E (5004C>G), N1136E (5016A>G 5018T>A), D1137K (5019G>A 5021C>A), K1140I (5029A>T 5030G>A), Y1141H (5031T>C), E1143G (5038A>G), K1144A (5040A>G 5041A>C 5042G>C), Q1146R (5046C>A 5047A>G 5048G>A), R1148M (5053G>T 5054A>G), D1157E (5081T>A), L1158Y (5082C>T 5083T>A), Q1161K (5091C>A 5093G>A), D1162I (5094G>A 5095A>T 5096C>A), K1163A (5097A>G 5098A>C 5099G>A), P1164A (5100C>G 5102T>A), I1166E (5106A>G 5107T>A), P1167A (5109C>G), S1168T (5112T>A), P1169N (5115C>A 5116C>A 5117A>T), E1170Q (5118G>C), M1171D (5121A>G 5122T>A 5123G>C), A1172I (5124G>A 5125C>T 5126G>A), K1173E (5127A>G), E1174D (5132A>T), Y1175F (5134A>T), E1176Q (5136G>C 5138A>G), K1177I (5140A>T 5141G>A), H1178Q (5144T>A), T1180K (5149C>A), A1184D (5161C>A 5162A>T), K1186G (5166A>G 5167A>G 5168G>A), P1190R (5178C>A 5179C>G 5180T>A), Q1192T (5184C>A 5185A>C), T1197S (5199A>T), I1201M (5213T>G), N1203K (5219C>A), S1206N (5226T>A 5227C>A 5228T>C), Q1208I (5232C>A 5233A>T), S1213A (5247A>G 5248G>C 5249T>C), Y1219S (5265T>A 5266A>G), K1220Q (5268A>C 5270G>A), R1221_L1222insE (5273_5274insGAA), Y1227R (5289T>A 5290A>G 5291C>A), K1228T (5293A>C), Q1230A (5298C>G 5299A>C), T1232K (5305C>A), P1234S (5310C>T), S1235D (5313A>G 5314G>A), I1236K (5317T>A 5318T>A), Y1238K (5322T>A 5324T>A), L1240I (5328C>A 5330A>T), L1241C (5331C>T 5332T>G), K1242R (5335A>G), K1244Q (5340A>C), D1245G (5344A>G 5345C>T), I1247K (5350T>A 5351T>A), V1248I (5352G>A), Y1249F (5356A>T 5357C>T), K1251E (5361A>G), L1254C (5370C>T 5371T>G 5372A>T), F1258Y (5383T>A), H1259* (5385C>T 5387C>G), I1261V (5391A>G 5393T>A), M1262K (5395T>A 5396G>A), D1264H (5400G>C 5402T>C), Q1266D (5406C>G 5408G>C), V1274T (5430G>A 5431T>C 5432C>T), P1276L (5437C>T 5438A>T), Q1277E (5439C>G), I1283L (5457A>T), V1284A (5461T>C 5462A>C), N1291T (5482A>C 5483T>A), V1295I (5493G>A), D1301N (5511G>A), N1302D (5514A>G), I1303M (5519A>G), K1305G (5523A>G 5524A>G), K1306D (5526A>G 5528A>C), S1308K (5532T>A 5533C>A), E1309N (5535G>A 5537A>C), C1312L (5545G>T 5546T>A), I1315V (5553A>G), I1320V (5568A>G), E1323K (5577G>A), S1324D (5580A>G 5581G>A), Q1327E (5589C>G), V1329L (5595G>T), Q1330G (5598C>G 5599A>G), V1370I (5718G>A 5720T>A), Q1371K (5721C>A), L1377A (5739T>G 5740T>C 5741A>T), I1380V (5748A>G), E1382D (5756A>T), F1383M (5757T>A 5759T>G), E1385del (5763_5765delGAA), Q1387K (5769C>A), E1389D (5777A>T), T1390K (5779C>A), L1391T (5781T>A 5782T>C), G1393D (5788G>A), I1400L (5808A>T), L1401V (5811T>G 5813A>G), Y1407F (5830A>T), P1409Q (5836C>A), S1412G (5844A>G 5846T>A), Y1414I (5850T>A 5851A>T), T1415A (5853A>G 5855T>A), R1416G (5856A>G), I1417P (5859A>C 5860T>C), F1418L (5864T>A), N1420A (5868A>G 5869A>C 5870T>A), C1422I (5874T>A 5875G>T 5876T>A), S1423G (5877T>G 5878C>G 5879C>A), E1427Q (5889G>C), R1428K (5893G>A), K1429H (5895A>C 5897A>C), S1432T (5904T>A 5906T>A), Q1433E (5907C>G), D1434N (5910G>A 5912C>T), W1435I (5913T>A 5914G>T 5915G>T), M1437L (5919A>C), R1439Q (5925A>C 5926G>A), K1440Q (5928A>C), V1444K (5940G>A 5941T>A), T1446K (5947C>A 5948C>A), K1447N (5951A>C), L1448I (5952T>A), P1450D (5958C>G 5959C>A 5960T>C), I1452S (5965T>G 5966T>C), L1453I (5967T>A), K1455L (5973A>C 5974A>T), P1456E (5976C>G 5977C>A), Q1457S (5979C>T 5980A>C), S1458D (5982A>G 5983G>A), I1460L (5988A>C 5990T>A), E1469Q (6015G>C), G1473A (6028G>C 6029A>T), I1474V (6030A>G 6032T>A), W1477A (6039T>G 6040G>C 6041G>A), S1480N (6048T>A 6049C>A 6050T>C), Q1481K (6051C>A), Q1482Y (6054C>T 6056A>C), D1483S (6057G>A 6058A>G), S1484P (6060T>C 6062A>T), P1485K (6063C>A 6064C>A 6065T>A), S1486K (6066T>A 6067C>A), T1487E (6069A>G 6070C>A), S1491C (6082C>G 6083A>T), R1492A (6084C>G 6085G>C 6086G>T), C1494Q (6090T>C 6091G>A 6092T>A), S1497K (6100G>A 6101C>A), K1499_S1500insEK (6107_6108insGAAAAG), S1500G (6108T>G 6109C>G), A1501N (6111G>A 6112C>A), I1502M (6116C>G), A1504S (6120G>A 6121C>G 6122A>T), A1507V (6130C>T 6131T>C), I1509V (6135A>G), M1510L (6138A>T 6140G>A), C1512I (6144T>A 6145G>T 6146T>A), V1515G (6154T>G), D1517N (6159G>A), K1518S (6163A>G 6164G>T), I1521L (6171A>T), F1522Y (6175T>A), L1523I (6177T>A 6179A>T), Y1524L (6181A>T 6182T>A), E1525N (6183G>A 6185G>C), K1527S (6190A>G 6191A>T), F1529I (6195T>A 6197T>A), T1530V (6198A>G 6199C>T 6200C>A), L1531V (6201C>G 6203T>A), I1536E (6216A>G 6217T>A 6218T>A), N1540K (6230T>A), Y1542H (6234T>C), N1543Q (6237A>C 6239C>A), L1545I (6243C>A 6245T>A), N1546D (6246A>G 6248T>C), N1547Q (6249A>C 6251T>G), K1549N (6257A>T), L1550S (6258T>A 6259T>G 6260G>T), N1551S (6262A>G), I1552K (6265T>A), N1553R (6268A>G 6269T>A), V1556L (6276G>C)								

	Begin	End	Coverage	Score	Concordance	Matches	Identities	I/D/M/F*	Stop Codons
**NT**	**4979**	**6282**	**14.7%**	**547**	**23.1%**	**1196 (98.9%)**	**747 (61.8%)**	**9/4**	

Codon mutations:

CTA1125ATA (4983C>A), CAT1126AAA (4986C>A 4988T>A), AAA1127TTA (4989A>T 4990A>T), TTG1128CTA (4992T>C 4994G>A), GAA1129GAT (4997A>T), ATC1131TTA (5001A>T 5003C>A), CAA1132GAA (5004C>G), ATT1133ATA (5009T>A), ATT1134ATA (5012T>A), GGG1135GGA (5015G>A), AAT1136GAA (5016A>G 5018T>A), GAC1137AAA (5019G>A 5021C>A), CCA1138CCC (5024A>C), TTA1139CTA (5025T>C), AAG1140ATA (5029A>T 5030G>A), TAT1141CAT (5031T>C), GAA1143GGA (5038A>G), AAG1144GCC (5040A>G 5041A>C 5042G>C), CAG1146AGA (5046C>A 5047A>G 5048G>A), ATT1147ATA (5051T>A), AGA1148ATG (5053G>T 5054A>G), TGC1149TGT (5057C>T), CTG1151CTA (5063G>A), GAC1152GAT (5066C>T), ATC1153ATA (5069C>A), CCT1156CCA (5078T>A), GAT1157GAA (5081T>A), CTT1158TAT (5082C>T 5083T>A), CAG1161AAA (5091C>A 5093G>A), GAC1162ATA (5094G>A 5095A>T 5096C>A), AAG1163GCA (5097A>G 5098A>C 5099G>A), CCT1164GCA (5100C>G 5102T>A), ATT1165ATA (5105T>A), ATA1166GAA (5106A>G 5107T>A), CCT1167GCT (5109C>G), TCA1168ACA (5112T>A), CCA1169AAT (5115C>A 5116C>A 5117A>T), GAA1170CAA (5118G>C), ATG1171GAC (5121A>G 5122T>A 5123G>C), GCG1172ATA (5124G>A 5125C>T 5126G>A), AAA1173GAA (5127A>G), GAA1174GAT (5132A>T), TAT1175TTT (5134A>T), GAA1176CAG (5136G>C 5138A>G), AAG1177ATA (5140A>T 5141G>A), CAT1178CAA (5144T>A), ATT1179ATA (5147T>A), ACA1180AAA (5149C>A), CTG1183TTA (5157C>T 5159G>A), GCA1184GAT (5161C>A 5162A>T), CTC1185TTA (5163C>T 5165C>A), AAG1186GGA (5166A>G 5167A>G 5168G>A), GTT1187GTA (5171T>A), ATC1188ATA (5174C>A), CCT1190AGA (5178C>A 5179C>G 5180T>A), TCC1191TCA (5183C>A), CAA1192ACA (5184C>A 5185A>C), AGT1193TCT (5187A>T 5188G>C), CGC1194AGA (5190C>A 5192C>A), CAT1195CAC (5195T>C), AGG1196AGA (5198G>A), ACA1197TCA (5199A>T), GCT1199GCA (5207T>A), ATT1201ATG (5213T>G), GTC1202GTA (5216C>A), AAC1203AAA (5219C>A), AAA1204-AC (5220delA 5222A>C), TCT1206AAC (5226T>A 5227C>A 5228T>C), CAA1208ATA (5232C>A 5233A>T), GTA1209GTC (5237A>C), GGT1211GGA (5243T>A), AGT1213GCC (5247A>G 5248G>C 5249T>C), CGT1214AGA (5250C>A 5252T>A), GTA1216GTT (5258A>T), TAT1219AGT (5265T>A 5266A>G), AAG1220CAA (5268A>C 5270G>A), CGA1221_CTA1222insGAA (5273_5274insGAA), CTA1222CTT (5276A>T), ACA1226ACT (5288A>T), TAC1227AGA (5289T>A 5290A>G 5291C>A), AAA1228ACA (5293A>C), GAC1229GAT (5297C>T), CAA1230GCA (5298C>G 5299A>C), TAC1231TAT (5303C>T), ACA1232AAA (5305C>A), CTC1233TTA (5307C>T 5309C>A), CCA1234TCA (5310C>T), AGT1235GAT (5313A>G 5314G>A), ATT1236AAA (5317T>A 5318T>A), TAT1238AAA (5322T>A 5324T>A), CTC1239TTG (5325C>T 5327C>G), CTA1240ATT (5328C>A 5330A>T), CTT1241TGT (5331C>T 5332T>G), AAA1242AGA (5335A>G), AAA1244CAA (5340A>C), GAC1245GGT (5344A>G 5345C>T), AAG1246AAA (5348G>A), ATT1247AAA (5350T>A 5351T>A), GTC1248ATC (5352G>A), TAC1249TTT (5356A>T 5357C>T), AGC1250AGT (5360C>T), AAA1251GAA (5361A>G), CTA1254TGT (5370C>T 5371T>G 5372A>T), AAG1255AAA (5375G>A), TCA1256TCG (5378A>G), TTC1258TAC (5383T>A), CAC1259TAG (5385C>T 5387C>G), CAG1260CAA (5390G>A), ATT1261GTA (5391A>G 5393T>A), ATG1262AAA (5395T>A 5396G>A), GAT1264CAC (5400G>C 5402T>C), CAG1266GAC (5406C>G 5408G>C), TTT1273TTC (5429T>C), GTC1274ACT (5430G>A 5431T>C 5432C>T), TGC1275TGT (5435C>T), CCA1276CTT (5437C>T 5438A>T), CAG1277GAG (5439C>G), GGT1278GGA (5444T>A), ATA1283TTA (5457A>T), GTA1284GCC (5461T>C 5462A>C), TTC1287TTT (5471C>T), GGA1288GGT (5474A>T), CTC1289TTA (5475C>T 5477C>A), AAT1291ACA (5482A>C 5483T>A), GCA1292GCG (5486A>G), TCA1294TCT (5492A>T), GTA1295ATA (5493G>A), TTC1296TTT (5498C>T), CGA1298AGA (5502C>A), GAT1301AAT (5511G>A), AAT1302GAT (5514A>G), ATA1303ATG (5519A>G), AAA1305GGA (5523A>G 5524A>G), AAA1306GAC (5526A>G 5528A>C), TCA1308AAA (5532T>A 5533C>A), GAA1309AAC (5535G>A 5537A>C), GTC1311GTT (5543C>T), TGT1312TTA (5545G>T 5546T>A), GTT1313GTC (5549T>C), ATA1315GTA (5553A>G), GAC1316GAT (5558C>T), TTG1319TTA (5567G>A), ATA1320GTA (5568A>G), TCT1322AGC (5574T>A 5575C>G 5576T>C), GAA1323AAA (5577G>A), AGT1324GAT (5580A>G 5581G>A), CAA1327GAA (5589C>G), GTA1329TTA (5595G>T), CAA1330GGA (5598C>G 5599A>G), TTA1332CTT (5604T>C 5606A>T), GAA1367..T (5711A>T), GGA1368GGG (5714A>G), AAA1369AAG (5717A>G), GTT1370ATA (5718G>A 5720T>A), CAA1371AAA (5721C>A), CTC1372CTA (5726C>A), TTA1377GCT (5739T>G 5740T>C 5741A>T), ATA1380GTA (5748A>G), GAA1382GAT (5756A>T), TTT1383ATG (5757T>A 5759T>G), CCT1384CCA (5762T>A), GAA1385del (5763_5765delGAA), GAC1386GAT (5768C>T), CAA1387AAA (5769C>A), GAA1389GAT (5777A>T), ACA1390AAA (5779C>A), TTA1391ACA (5781T>A 5782T>C), AAG1392AAA (5786G>A), GGT1393GAT (5788G>A), CTA1394TTA (5790C>T), CAG1395CAA (5795G>A), AAG1396AAA (5798G>A), GGG1399GGA (5807G>A), ATA1400TTA (5808A>T), TTA1401GTG (5811T>G 5813A>G), AAC1402AAT (5816C>T), GCC1404GCT (5822C>T), AAT1406AAC (5828T>C), TAT1407TTT (5830A>T), ATC1408ATA (5834C>A), CCA1409CAA (5836C>A), TTG1411TTA (5843G>A), AGT1412GGA (5844A>G 5846T>A), TAT1414ATT (5850T>A 5851A>T), ACT1415GCA (5853A>G 5855T>A), AGA1416GGA (5856A>G), ATA1417CCA (5859A>C 5860T>C), TTT1418TTA (5864T>A), AAT1420GCA (5868A>G 5869A>C 5870T>A), AAG1421AAA (5873G>A), TGT1422ATA (5874T>A 5875G>T 5876T>A), TCC1423GGA (5877T>G 5878C>G 5879C>A), AGC1424AGT (5882C>T), GAA1427CAA (5889G>C), AGA1428AAA (5893G>A), AAA1429CAC (5895A>C 5897A>C), AAT1431AAC (5903T>C), TCT1432ACA (5904T>A 5906T>A), CAA1433GAA (5907C>G), GAC1434AAT (5910G>A 5912C>T), TGG1435ATT (5913T>A 5914G>T 5915G>T), AAG1436AAA (5918G>A), ATG1437CTG (5919A>C), GTT1438GTA (5924T>A), AGA1439CAA (5925A>C 5926G>A), AAA1440CAA (5928A>C), ATC1441ATT (5933C>T), GTA1444AAA (5940G>A 5941T>A), ATT1445ATA (5945T>A), ACC1446AAA (5947C>A 5948C>A), AAA1447AAC (5951A>C), TTA1448ATA (5952T>A), CCC1449CCA (5957C>A), CCT1450GAC (5958C>G 5959C>A 5960T>C), TTG1451TTA (5963G>A), ATT1452AGC (5965T>G 5966T>C), TTA1453ATA (5967T>A), AAA1455CTA (5973A>C 5974A>T), CCA1456GAA (5976C>G 5977C>A), CAA1457TCA (5979C>T 5980A>C), AGT1458GAT (5982A>G 5983G>A), ATT1460CTA (5988A>C 5990T>A), GAG1463GAA (5999G>A), GAT1465GAC (6005T>C), TCT1467AGC (6009T>A 6010C>G 6011T>C), CTA1468CTC (6014A>C), GAA1469CAA (6015G>C), GGG1472GGA (6026G>A), GGA1473GCT (6028G>C 6029A>T), ATT1474GTA (6030A>G 6032T>A), TGG1477GCA (6039T>G 6040G>C 6041G>A), CCC1479CCA (6047C>A), TCT1480AAC (6048T>A 6049C>A 6050T>C), CAA1481AAA (6051C>A), CAA1482TAC (6054C>T 6056A>C), GAT1483AGT (6057G>A 6058A>G), TCA1484CCT (6060T>C 6062A>T), CCT1485AAA (6063C>A 6064C>A 6065T>A), TCA1486AAA (6066T>A 6067C>A), ACA1487GAA (6069A>G 6070C>A), TCA1491TGT (6082C>G 6083A>T), CGG1492GCT (6084C>G 6085G>C 6086G>T), TAT1493TAC (6089T>C), TGT1494CAA (6090T>C 6091G>A 6092T>A), AGC1497AAA (6100G>A 6101C>A), TAT1498TAC (6104T>C), AAA1499_TCT1500insGAAAAG (6107_6108insGAAAAG), TCT1500GGT (6108T>G 6109C>G), GCC1501AAC (6111G>A 6112C>A), ATC1502ATG (6116C>G), TCT1503AGT (6117T>A 6118C>G), GCA1504AGT (6120G>A 6121C>G 6122A>T), GCT1507GTC (6130C>T 6131T>C), ATA1509GTA (6135A>G), ATG1510TTA (6138A>T 6140G>A), TGT1512ATA (6144T>A 6145G>T 6146T>A), GTA1515GGA (6154T>G), TTA1516CTA (6156T>C), GAT1517AAT (6159G>A), AAG1518AGT (6163A>G 6164G>T), TTT1519TTC (6167T>C), ATA1521TTA (6171A>T), TTT1522TAT (6175T>A), TTA1523ATT (6177T>A 6179A>T), TAT1524TTA (6181A>T 6182T>A), GAG1525AAC (6183G>A 6185G>C), AAA1527AGT (6190A>G 6191A>T), TTT1529ATA (6195T>A 6197T>A), ACC1530GTA (6198A>G 6199C>T 6200C>A), CTT1531GTA (6201C>G 6203T>A), AGG1532AGA (6206G>A), GAC1534GAT (6212C>T), TGT1535TGC (6215T>C), ATT1536GAA (6216A>G 6217T>A 6218T>A), GCA1537GCT (6221A>T), ATT1538ATA (6224T>A), AAT1540AAA (6230T>A), TAT1542CAT (6234T>C), AAC1543CAA (6237A>C 6239C>A), CTT1545ATA (6243C>A 6245T>A), AAT1546GAC (6246A>G 6248T>C), AAT1547CAG (6249A>C 6251T>G), AAA1549AAT (6257A>T), TTG1550AGT (6258T>A 6259T>G 6260G>T), AAT1551AGT (6262A>G), ATA1552AAA (6265T>A), AAT1553AGA (6268A>G 6269T>A), CGT1554AGA (6270C>A 6272T>A), GTA1556CTA (6276G>C)

Proteins

	Begin	End	Coverage	Score	Concordance	Matches	Identities	I/D/M/F*	Stop Codons
ORF3 (YP_009553219.1)	1124	1558	25.1%	1411	49.9%	399 (99.0%)	210 (52.1%)	3/1/1/1	1

Protein mutations:

L1125I (4983C>A), H1126K (4986C>A 4988T>A), K1127L (4989A>T 4990A>T), E1129D (4997A>T), I1131L (5001A>T 5003C>A), Q1132E (5004C>G), N1136E (5016A>G 5018T>A), D1137K (5019G>A 5021C>A), K1140I (5029A>T 5030G>A), Y1141H (5031T>C), E1143G (5038A>G), K1144A (5040A>G 5041A>C 5042G>C), Q1146R (5046C>A 5047A>G 5048G>A), R1148M (5053G>T 5054A>G), D1157E (5081T>A), L1158Y (5082C>T 5083T>A), Q1161K (5091C>A 5093G>A), D1162I (5094G>A 5095A>T 5096C>A), K1163A (5097A>G 5098A>C 5099G>A), P1164A (5100C>G 5102T>A), I1166E (5106A>G 5107T>A), P1167A (5109C>G), S1168T (5112T>A), P1169N (5115C>A 5116C>A 5117A>T), E1170Q (5118G>C), M1171D (5121A>G 5122T>A 5123G>C), A1172I (5124G>A 5125C>T 5126G>A), K1173E (5127A>G), E1174D (5132A>T), Y1175F (5134A>T), E1176Q (5136G>C 5138A>G), K1177I (5140A>T 5141G>A), H1178Q (5144T>A), T1180K (5149C>A), A1184D (5161C>A 5162A>T), K1186G (5166A>G 5167A>G 5168G>A), P1190R (5178C>A 5179C>G 5180T>A), Q1192T (5184C>A 5185A>C), T1197S (5199A>T), I1201M (5213T>G), N1203K (5219C>A), S1206N (5226T>A 5227C>A 5228T>C), Q1208I (5232C>A 5233A>T), S1213A (5247A>G 5248G>C 5249T>C), Y1219S (5265T>A 5266A>G), K1220Q (5268A>C 5270G>A), R1221_L1222insE (5273_5274insGAA), Y1227R (5289T>A 5290A>G 5291C>A), K1228T (5293A>C), Q1230A (5298C>G 5299A>C), T1232K (5305C>A), P1234S (5310C>T), S1235D (5313A>G 5314G>A), I1236K (5317T>A 5318T>A), Y1238K (5322T>A 5324T>A), L1240I (5328C>A 5330A>T), L1241C (5331C>T 5332T>G), K1242R (5335A>G), K1244Q (5340A>C), D1245G (5344A>G 5345C>T), I1247K (5350T>A 5351T>A), V1248I (5352G>A), Y1249F (5356A>T 5357C>T), K1251E (5361A>G), L1254C (5370C>T 5371T>G 5372A>T), F1258Y (5383T>A), H1259* (5385C>T 5387C>G), I1261V (5391A>G 5393T>A), M1262K (5395T>A 5396G>A), D1264H (5400G>C 5402T>C), Q1266D (5406C>G 5408G>C), V1274T (5430G>A 5431T>C 5432C>T), P1276L (5437C>T 5438A>T), Q1277E (5439C>G), I1283L (5457A>T), V1284A (5461T>C 5462A>C), N1291T (5482A>C 5483T>A), V1295I (5493G>A), D1301N (5511G>A), N1302D (5514A>G), I1303M (5519A>G), K1305G (5523A>G 5524A>G), K1306D (5526A>G 5528A>C), S1308K (5532T>A 5533C>A), E1309N (5535G>A 5537A>C), C1312L (5545G>T 5546T>A), I1315V (5553A>G), I1320V (5568A>G), E1323K (5577G>A), S1324D (5580A>G 5581G>A), Q1327E (5589C>G), V1329L (5595G>T), Q1330G (5598C>G 5599A>G), V1370I (5718G>A 5720T>A), Q1371K (5721C>A), L1377A (5739T>G 5740T>C 5741A>T), I1380V (5748A>G), E1382D (5756A>T), F1383M (5757T>A 5759T>G), E1385del (5763_5765delGAA), Q1387K (5769C>A), E1389D (5777A>T), T1390K (5779C>A), L1391T (5781T>A 5782T>C), G1393D (5788G>A), I1400L (5808A>T), L1401V (5811T>G 5813A>G), Y1407F (5830A>T), P1409Q (5836C>A), S1412G (5844A>G 5846T>A), Y1414I (5850T>A 5851A>T), T1415A (5853A>G 5855T>A), R1416G (5856A>G), I1417P (5859A>C 5860T>C), F1418L (5864T>A), N1420A (5868A>G 5869A>C 5870T>A), C1422I (5874T>A 5875G>T 5876T>A), S1423G (5877T>G 5878C>G 5879C>A), E1427Q (5889G>C), R1428K (5893G>A), K1429H (5895A>C 5897A>C), S1432T (5904T>A 5906T>A), Q1433E (5907C>G), D1434N (5910G>A 5912C>T), W1435I (5913T>A 5914G>T 5915G>T), M1437L (5919A>C), R1439Q (5925A>C 5926G>A), K1440Q (5928A>C), V1444K (5940G>A 5941T>A), T1446K (5947C>A 5948C>A), K1447N (5951A>C), L1448I (5952T>A), P1450D (5958C>G 5959C>A 5960T>C), I1452S (5965T>G 5966T>C), L1453I (5967T>A), K1455L (5973A>C 5974A>T), P1456E (5976C>G 5977C>A), Q1457S (5979C>T 5980A>C), S1458D (5982A>G 5983G>A), I1460L (5988A>C 5990T>A), E1469Q (6015G>C), G1473A (6028G>C 6029A>T), I1474V (6030A>G 6032T>A), W1477A (6039T>G 6040G>C 6041G>A), S1480N (6048T>A 6049C>A 6050T>C), Q1481K (6051C>A), Q1482Y (6054C>T 6056A>C), D1483S (6057G>A 6058A>G), S1484P (6060T>C 6062A>T), P1485K (6063C>A 6064C>A 6065T>A), S1486K (6066T>A 6067C>A), T1487E (6069A>G 6070C>A), S1491C (6082C>G 6083A>T), R1492A (6084C>G 6085G>C 6086G>T), C1494Q (6090T>C 6091G>A 6092T>A), S1497K (6100G>A 6101C>A), K1499_S1500insEK (6107_6108insGAAAAG), S1500G (6108T>G 6109C>G), A1501N (6111G>A 6112C>A), I1502M (6116C>G), A1504S (6120G>A 6121C>G 6122A>T), A1507V (6130C>T 6131T>C), I1509V (6135A>G), M1510L (6138A>T 6140G>A), C1512I (6144T>A 6145G>T 6146T>A), V1515G (6154T>G), D1517N (6159G>A), K1518S (6163A>G 6164G>T), I1521L (6171A>T), F1522Y (6175T>A), L1523I (6177T>A 6179A>T), Y1524L (6181A>T 6182T>A), E1525N (6183G>A 6185G>C), K1527S (6190A>G 6191A>T), F1529I (6195T>A 6197T>A), T1530V (6198A>G 6199C>T 6200C>A), L1531V (6201C>G 6203T>A), I1536E (6216A>G 6217T>A 6218T>A), N1540K (6230T>A), Y1542H (6234T>C), N1543Q (6237A>C 6239C>A), L1545I (6243C>A 6245T>A), N1546D (6246A>G 6248T>C), N1547Q (6249A>C 6251T>G), K1549N (6257A>T), L1550S (6258T>A 6259T>G 6260G>T), N1551S (6262A>G), I1552K (6265T>A), N1553R (6268A>G 6269T>A), V1556L (6276G>C)

	Begin	End	Coverage	Score	Concordance	Matches	Identities	I/D/M/F*	Stop Codons
**NT**	**4979**	**6282**	**14.7%**	**547**	**23.1%**	**1196 (98.9%)**	**747 (61.8%)**	**9/4**	
Codon mutations:	CTA1125ATA (4983C>A), CAT1126AAA (4986C>A 4988T>A), AAA1127TTA (4989A>T 4990A>T), TTG1128CTA (4992T>C 4994G>A), GAA1129GAT (4997A>T), ATC1131TTA (5001A>T 5003C>A), CAA1132GAA (5004C>G), ATT1133ATA (5009T>A), ATT1134ATA (5012T>A), GGG1135GGA (5015G>A), AAT1136GAA (5016A>G 5018T>A), GAC1137AAA (5019G>A 5021C>A), CCA1138CCC (5024A>C), TTA1139CTA (5025T>C), AAG1140ATA (5029A>T 5030G>A), TAT1141CAT (5031T>C), GAA1143GGA (5038A>G), AAG1144GCC (5040A>G 5041A>C 5042G>C), CAG1146AGA (5046C>A 5047A>G 5048G>A), ATT1147ATA (5051T>A), AGA1148ATG (5053G>T 5054A>G), TGC1149TGT (5057C>T), CTG1151CTA (5063G>A), GAC1152GAT (5066C>T), ATC1153ATA (5069C>A), CCT1156CCA (5078T>A), GAT1157GAA (5081T>A), CTT1158TAT (5082C>T 5083T>A), CAG1161AAA (5091C>A 5093G>A), GAC1162ATA (5094G>A 5095A>T 5096C>A), AAG1163GCA (5097A>G 5098A>C 5099G>A), CCT1164GCA (5100C>G 5102T>A), ATT1165ATA (5105T>A), ATA1166GAA (5106A>G 5107T>A), CCT1167GCT (5109C>G), TCA1168ACA (5112T>A), CCA1169AAT (5115C>A 5116C>A 5117A>T), GAA1170CAA (5118G>C), ATG1171GAC (5121A>G 5122T>A 5123G>C), GCG1172ATA (5124G>A 5125C>T 5126G>A), AAA1173GAA (5127A>G), GAA1174GAT (5132A>T), TAT1175TTT (5134A>T), GAA1176CAG (5136G>C 5138A>G), AAG1177ATA (5140A>T 5141G>A), CAT1178CAA (5144T>A), ATT1179ATA (5147T>A), ACA1180AAA (5149C>A), CTG1183TTA (5157C>T 5159G>A), GCA1184GAT (5161C>A 5162A>T), CTC1185TTA (5163C>T 5165C>A), AAG1186GGA (5166A>G 5167A>G 5168G>A), GTT1187GTA (5171T>A), ATC1188ATA (5174C>A), CCT1190AGA (5178C>A 5179C>G 5180T>A), TCC1191TCA (5183C>A), CAA1192ACA (5184C>A 5185A>C), AGT1193TCT (5187A>T 5188G>C), CGC1194AGA (5190C>A 5192C>A), CAT1195CAC (5195T>C), AGG1196AGA (5198G>A), ACA1197TCA (5199A>T), GCT1199GCA (5207T>A), ATT1201ATG (5213T>G), GTC1202GTA (5216C>A), AAC1203AAA (5219C>A), AAA1204-AC (5220delA 5222A>C), TCT1206AAC (5226T>A 5227C>A 5228T>C), CAA1208ATA (5232C>A 5233A>T), GTA1209GTC (5237A>C), GGT1211GGA (5243T>A), AGT1213GCC (5247A>G 5248G>C 5249T>C), CGT1214AGA (5250C>A 5252T>A), GTA1216GTT (5258A>T), TAT1219AGT (5265T>A 5266A>G), AAG1220CAA (5268A>C 5270G>A), CGA1221_CTA1222insGAA (5273_5274insGAA), CTA1222CTT (5276A>T), ACA1226ACT (5288A>T), TAC1227AGA (5289T>A 5290A>G 5291C>A), AAA1228ACA (5293A>C), GAC1229GAT (5297C>T), CAA1230GCA (5298C>G 5299A>C), TAC1231TAT (5303C>T), ACA1232AAA (5305C>A), CTC1233TTA (5307C>T 5309C>A), CCA1234TCA (5310C>T), AGT1235GAT (5313A>G 5314G>A), ATT1236AAA (5317T>A 5318T>A), TAT1238AAA (5322T>A 5324T>A), CTC1239TTG (5325C>T 5327C>G), CTA1240ATT (5328C>A 5330A>T), CTT1241TGT (5331C>T 5332T>G), AAA1242AGA (5335A>G), AAA1244CAA (5340A>C), GAC1245GGT (5344A>G 5345C>T), AAG1246AAA (5348G>A), ATT1247AAA (5350T>A 5351T>A), GTC1248ATC (5352G>A), TAC1249TTT (5356A>T 5357C>T), AGC1250AGT (5360C>T), AAA1251GAA (5361A>G), CTA1254TGT (5370C>T 5371T>G 5372A>T), AAG1255AAA (5375G>A), TCA1256TCG (5378A>G), TTC1258TAC (5383T>A), CAC1259TAG (5385C>T 5387C>G), CAG1260CAA (5390G>A), ATT1261GTA (5391A>G 5393T>A), ATG1262AAA (5395T>A 5396G>A), GAT1264CAC (5400G>C 5402T>C), CAG1266GAC (5406C>G 5408G>C), TTT1273TTC (5429T>C), GTC1274ACT (5430G>A 5431T>C 5432C>T), TGC1275TGT (5435C>T), CCA1276CTT (5437C>T 5438A>T), CAG1277GAG (5439C>G), GGT1278GGA (5444T>A), ATA1283TTA (5457A>T), GTA1284GCC (5461T>C 5462A>C), TTC1287TTT (5471C>T), GGA1288GGT (5474A>T), CTC1289TTA (5475C>T 5477C>A), AAT1291ACA (5482A>C 5483T>A), GCA1292GCG (5486A>G), TCA1294TCT (5492A>T), GTA1295ATA (5493G>A), TTC1296TTT (5498C>T), CGA1298AGA (5502C>A), GAT1301AAT (5511G>A), AAT1302GAT (5514A>G), ATA1303ATG (5519A>G), AAA1305GGA (5523A>G 5524A>G), AAA1306GAC (5526A>G 5528A>C), TCA1308AAA (5532T>A 5533C>A), GAA1309AAC (5535G>A 5537A>C), GTC1311GTT (5543C>T), TGT1312TTA (5545G>T 5546T>A), GTT1313GTC (5549T>C), ATA1315GTA (5553A>G), GAC1316GAT (5558C>T), TTG1319TTA (5567G>A), ATA1320GTA (5568A>G), TCT1322AGC (5574T>A 5575C>G 5576T>C), GAA1323AAA (5577G>A), AGT1324GAT (5580A>G 5581G>A), CAA1327GAA (5589C>G), GTA1329TTA (5595G>T), CAA1330GGA (5598C>G 5599A>G), TTA1332CTT (5604T>C 5606A>T), GAA1367..T (5711A>T), GGA1368GGG (5714A>G), AAA1369AAG (5717A>G), GTT1370ATA (5718G>A 5720T>A), CAA1371AAA (5721C>A), CTC1372CTA (5726C>A), TTA1377GCT (5739T>G 5740T>C 5741A>T), ATA1380GTA (5748A>G), GAA1382GAT (5756A>T), TTT1383ATG (5757T>A 5759T>G), CCT1384CCA (5762T>A), GAA1385del (5763_5765delGAA), GAC1386GAT (5768C>T), CAA1387AAA (5769C>A), GAA1389GAT (5777A>T), ACA1390AAA (5779C>A), TTA1391ACA (5781T>A 5782T>C), AAG1392AAA (5786G>A), GGT1393GAT (5788G>A), CTA1394TTA (5790C>T), CAG1395CAA (5795G>A), AAG1396AAA (5798G>A), GGG1399GGA (5807G>A), ATA1400TTA (5808A>T), TTA1401GTG (5811T>G 5813A>G), AAC1402AAT (5816C>T), GCC1404GCT (5822C>T), AAT1406AAC (5828T>C), TAT1407TTT (5830A>T), ATC1408ATA (5834C>A), CCA1409CAA (5836C>A), TTG1411TTA (5843G>A), AGT1412GGA (5844A>G 5846T>A), TAT1414ATT (5850T>A 5851A>T), ACT1415GCA (5853A>G 5855T>A), AGA1416GGA (5856A>G), ATA1417CCA (5859A>C 5860T>C), TTT1418TTA (5864T>A), AAT1420GCA (5868A>G 5869A>C 5870T>A), AAG1421AAA (5873G>A), TGT1422ATA (5874T>A 5875G>T 5876T>A), TCC1423GGA (5877T>G 5878C>G 5879C>A), AGC1424AGT (5882C>T), GAA1427CAA (5889G>C), AGA1428AAA (5893G>A), AAA1429CAC (5895A>C 5897A>C), AAT1431AAC (5903T>C), TCT1432ACA (5904T>A 5906T>A), CAA1433GAA (5907C>G), GAC1434AAT (5910G>A 5912C>T), TGG1435ATT (5913T>A 5914G>T 5915G>T), AAG1436AAA (5918G>A), ATG1437CTG (5919A>C), GTT1438GTA (5924T>A), AGA1439CAA (5925A>C 5926G>A), AAA1440CAA (5928A>C), ATC1441ATT (5933C>T), GTA1444AAA (5940G>A 5941T>A), ATT1445ATA (5945T>A), ACC1446AAA (5947C>A 5948C>A), AAA1447AAC (5951A>C), TTA1448ATA (5952T>A), CCC1449CCA (5957C>A), CCT1450GAC (5958C>G 5959C>A 5960T>C), TTG1451TTA (5963G>A), ATT1452AGC (5965T>G 5966T>C), TTA1453ATA (5967T>A), AAA1455CTA (5973A>C 5974A>T), CCA1456GAA (5976C>G 5977C>A), CAA1457TCA (5979C>T 5980A>C), AGT1458GAT (5982A>G 5983G>A), ATT1460CTA (5988A>C 5990T>A), GAG1463GAA (5999G>A), GAT1465GAC (6005T>C), TCT1467AGC (6009T>A 6010C>G 6011T>C), CTA1468CTC (6014A>C), GAA1469CAA (6015G>C), GGG1472GGA (6026G>A), GGA1473GCT (6028G>C 6029A>T), ATT1474GTA (6030A>G 6032T>A), TGG1477GCA (6039T>G 6040G>C 6041G>A), CCC1479CCA (6047C>A), TCT1480AAC (6048T>A 6049C>A 6050T>C), CAA1481AAA (6051C>A), CAA1482TAC (6054C>T 6056A>C), GAT1483AGT (6057G>A 6058A>G), TCA1484CCT (6060T>C 6062A>T), CCT1485AAA (6063C>A 6064C>A 6065T>A), TCA1486AAA (6066T>A 6067C>A), ACA1487GAA (6069A>G 6070C>A), TCA1491TGT (6082C>G 6083A>T), CGG1492GCT (6084C>G 6085G>C 6086G>T), TAT1493TAC (6089T>C), TGT1494CAA (6090T>C 6091G>A 6092T>A), AGC1497AAA (6100G>A 6101C>A), TAT1498TAC (6104T>C), AAA1499_TCT1500insGAAAAG (6107_6108insGAAAAG), TCT1500GGT (6108T>G 6109C>G), GCC1501AAC (6111G>A 6112C>A), ATC1502ATG (6116C>G), TCT1503AGT (6117T>A 6118C>G), GCA1504AGT (6120G>A 6121C>G 6122A>T), GCT1507GTC (6130C>T 6131T>C), ATA1509GTA (6135A>G), ATG1510TTA (6138A>T 6140G>A), TGT1512ATA (6144T>A 6145G>T 6146T>A), GTA1515GGA (6154T>G), TTA1516CTA (6156T>C), GAT1517AAT (6159G>A), AAG1518AGT (6163A>G 6164G>T), TTT1519TTC (6167T>C), ATA1521TTA (6171A>T), TTT1522TAT (6175T>A), TTA1523ATT (6177T>A 6179A>T), TAT1524TTA (6181A>T 6182T>A), GAG1525AAC (6183G>A 6185G>C), AAA1527AGT (6190A>G 6191A>T), TTT1529ATA (6195T>A 6197T>A), ACC1530GTA (6198A>G 6199C>T 6200C>A), CTT1531GTA (6201C>G 6203T>A), AGG1532AGA (6206G>A), GAC1534GAT (6212C>T), TGT1535TGC (6215T>C), ATT1536GAA (6216A>G 6217T>A 6218T>A), GCA1537GCT (6221A>T), ATT1538ATA (6224T>A), AAT1540AAA (6230T>A), TAT1542CAT (6234T>C), AAC1543CAA (6237A>C 6239C>A), CTT1545ATA (6243C>A 6245T>A), AAT1546GAC (6246A>G 6248T>C), AAT1547CAG (6249A>C 6251T>G), AAA1549AAT (6257A>T), TTG1550AGT (6258T>A 6259T>G 6260G>T), AAT1551AGT (6262A>G), ATA1552AAA (6265T>A), AAT1553AGA (6268A>G 6269T>A), CGT1554AGA (6270C>A 6272T>A), GTA1556CTA (6276G>C)								

*: Inserts / Deletes / Misaligned / Frameshifts

Analysis details

This analysis was performed with panviral2.64

NGS Details (UN59): Badnavirus occultipomeae

Assembly

**Coverage Length**	255 (1 contig(s))
**Depth Of Coverage**	30.9
**Number Of Reads**	80
**Reads Per Million**	1.74 rpm (after QC)
**Ambiguities**	0
**Assembly Method**	de novo + reference guided assembly
**Consensus Caller**	Bcf Tools

Coverage Map

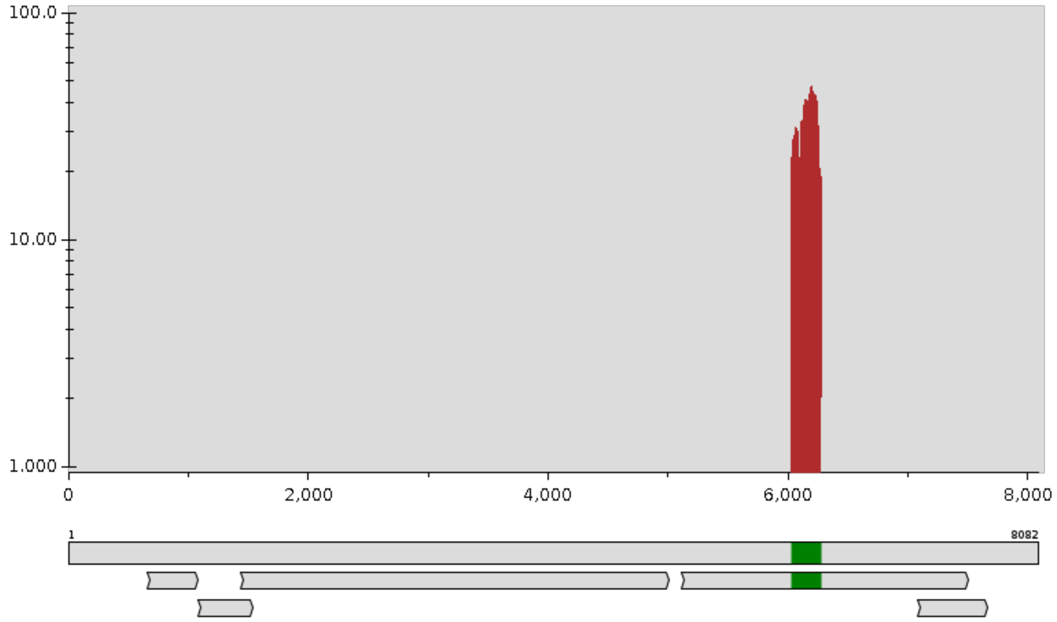

Assignment

**Type**	Badnavirus occultipomeae (Taxonomy ID: 3048353)
**Reference Genome**	NC_015655.1
**NT Identity (%)**	58.1395
**AA Identity (%)**	50.0
**Number Of Stop Codons**	0
**Number Of CDS**	5

Alignment

**Alignment Score**	78.0 (NT) + 347.0 (AA) = 425.0
**Concordance (%)**	37.7107

**Alignment Method**	Global, seeded, nucleotide + amino acids (AGA)

Genome Region

Sequence starts at position 6025 and ends at position 6279 relative to NC_015655.1 reference sequence.

Alignment Detailed Statistics

	Begin	End	Coverage	Score	Concordance	Matches	Identities	I/D/M/F*	Stop Codons
**NT**	**6025**	**6279**	**3.2%**	**78**	**15.3%**	**255 (98.8%)**	**150 (58.1%)**	**3/0**	
Mutations:	6033A>T, 6036C>T, 6042A>G, 6043T>A, 6051C>T, 6052A>C, 6053A>G, 6054A>T, 6055G>T, 6057A>T, 6060C>G, 6062T>A, 6066T>C, 6070A>C, 6072A>T, 6075A>G, 6076A>G, 6079G>A, 6081G>A, 6084A>G, 6087G>T, 6088T>G, 6089C>A, 6090C>T, 6091A>G, 6092A>T, 6093A>T, 6097T>A, 6098G>A, 6099G>A, 6101C>T, 6102T>G, 6114T>G, 6115C>A, 6116C>G, 6117A>G, 6118G>T, 6120A>T, 6125T>A, 6126C>T, 6128A>T, 6129T>C, 6130G>A, 6135T>C, 6136G>C, 6137A>T, 6138A>T, 6145C>T, 6147C>T, 6153A>T, 6154C>T, 6156G>A, 6158T>C, 6159G>T, 6163G>T, 6165A>C, 6168A>T, 6169G>T, 6173A>T, 6174T>G, 6178C>A, 6179A>T, 6181A>G, 6182G>A, 6183A>T, 6184A>T, 6185A>T, 6190G>A, 6192T>C, 6194A>G, 6195T>G, 6197C>T, 6198A>T, 6201T>C, 6203G>A, 6205G>C, 6206G>C, 6207A>T, 6207_6208insTTC, 6208A>C, 6209C>T, 6210A>C, 6213T>C, 6214G>A, 6215C>T, 6216A>G, 6220A>G, 6222C>G, 6224C>T, 6225A>G, 6228A>C, 6230A>T, 6240C>T, 6241A>G, 6243A>G, 6244T>C, 6247G>A, 6251T>A, 6255T>C, 6256G>C, 6259A>G, 6260A>G, 6261T>A, 6262G>A, 6263A>G, 6264A>G								
**CDS**									
SPBVa_gp4	304	388	10.7%	347	56.2%	85 (98.8%)	43 (50.0%)	1/0/0/0	0
Protein mutations:	F310I (6043T>A), K313R (6052A>C 6053A>G 6054A>T), A314S (6055G>T 6057A>T), F316Y (6062T>A), I319L (6070A>C 6072A>T), M321V (6076A>G), E322K (6079G>A 6081G>A), K324N (6087G>T), S325D (6088T>G 6089C>A 6090C>T), K326V (6091A>G 6092A>T 6093A>T), W328K (6097T>A 6098G>A 6099G>A), T329M (6101C>T 6102T>G), P334R (6115C>A 6116C>G 6117A>G), E335Y (6118G>T 6120A>T), L337H (6125T>A 6126C>T), Y338F (6128A>T 6129T>C), E339K (6130G>A), E341L (6136G>C 6137A>T 6138A>T), P344S (6145C>T 6147C>T), M348T (6158T>C 6159G>T), A350S (6163G>T 6165A>C), A352S (6169G>T), D353V (6173A>T 6174T>G), Q355M (6178C>A 6179A>T), R356D (6181A>G 6182G>A 6183A>T), K357L (6184A>T 6185A>T), D359N (6190G>A 6192T>C), N360R (6194A>G 6195T>G), A361V (6197C>T 6198A>T), R363K (6203G>A), G364P (6205G>C 6206G>C 6207A>T), G364_T365insF (6207_6208insTTC), T365L (6208A>C 6209C>T 6210A>C), A367M (6214G>A 6215C>T 6216A>G), I369V (6220A>G 6222C>G), A370V (6224C>T 6225A>G), Y372F (6230A>T), I376V (6241A>G 6243A>G), V378I (6247G>A), F379Y (6251T>A), E381Q (6256G>C), N382G (6259A>G 6260A>G 6261T>A), E383R (6262G>A 6263A>G 6264A>G)								
Codon mutations:	ATA306ATT (6033A>T), TTC307TTT (6036C>T), AAA309AAG (6042A>G), TTT310ATT (6043T>A), CTC312CTT (6051C>T), AAA313CGT (6052A>C 6053A>G 6054A>T), GCA314TCT (6055G>T 6057A>T), GGC315GGG (6060C>G), TTT316TAT (6062T>A), CAT317CAC (6066T>C), ATA319CTT (6070A>C 6072A>T), AGA320AGG (6075A>G), ATG321GTG (6076A>G), GAG322AAA (6079G>A 6081G>A), GAA323GAG (6084A>G), AAG324AAT (6087G>T), TCC325GAT (6088T>G 6089C>A 6090C>T), AAA326GTT (6091A>G 6092A>T 6093A>T), TGG328AAA (6097T>A 6098G>A 6099G>A), ACT329ATG (6101C>T 6102T>G), ACT333ACG (6114T>G), CCA334AGG (6115C>A 6116C>G 6117A>G), GAA335TAT (6118G>T 6120A>T), CTC337CAT (6125T>A 6126C>T), TAT338TTC (6128A>T 6129T>C), GAA339AAA (6130G>A), TTT340TTC (6135T>C), GAA341CTT (6136G>C 6137A>T 6138A>T), CCC344TCT (6145C>T 6147C>T), GGA346GGT (6153A>T), CTG347TTA (6154C>T 6156G>A), ATG348ACT (6158T>C 6159G>T), GCA350TCC (6163G>T 6165A>C), CCA351CCT (6168A>T), GCA352TCA (6169G>T), GAT353GTG (6173A>T 6174T>G), CAG355ATG (6178C>A 6179A>T), AGA356GAT (6181A>G 6182G>A 6183A>T), AAG357TTG (6184A>T 6185A>T), GAT359AAC (6190G>A 6192T>C), AAT360AGG (6194A>G 6195T>G), GCA361GTT (6197C>T 6198A>T), TTT362TTC (6201T>C), AGG363AAG (6203G>A), GGA364CCT (6205G>C 6206G>C 6207A>T), GGA364_ACA365insTTC (6207_6208insTTC), ACA365CTC (6208A>C 6209C>T 6210A>C), GAT366GAC (6213T>C), GCA367ATG (6214G>A 6215C>T 6216A>G), ATC369GTG (6220A>G 6222C>G), GCA370GTG (6224C>T 6225A>G), GTA371GTC (6228A>C), TAT372TTT (6230A>T), GAC375GAT (6240C>T), ATA376GTG (6241A>G 6243A>G), TTG377CTG (6244T>C), GTA378ATA (6247G>A), TTC379TAC (6251T>A), TCT380TCC (6255T>C), GAA381CAA (6256G>C), AAT382GGA (6259A>G 6260A>G 6261T>A), GAA383AGG (6262G>A 6263A>G 6264A>G)								
**Proteins**									
RNaseH/reverse transcriptase (YP_004581513.1)	304	388	10.7%	347	56.2%	85 (98.8%)	43 (50.0%)	1/0/0/0	0
Protein mutations:	F310I (6043T>A), K313R (6052A>C 6053A>G 6054A>T), A314S (6055G>T 6057A>T), F316Y (6062T>A), I319L (6070A>C 6072A>T), M321V (6076A>G), E322K (6079G>A 6081G>A), K324N (6087G>T), S325D (6088T>G 6089C>A 6090C>T), K326V (6091A>G 6092A>T 6093A>T), W328K (6097T>A 6098G>A 6099G>A), T329M (6101C>T 6102T>G), P334R (6115C>A 6116C>G 6117A>G), E335Y (6118G>T 6120A>T), L337H (6125T>A 6126C>T), Y338F (6128A>T 6129T>C), E339K (6130G>A), E341L (6136G>C 6137A>T 6138A>T), P344S (6145C>T 6147C>T), M348T (6158T>C 6159G>T), A350S (6163G>T 6165A>C), A352S (6169G>T), D353V (6173A>T 6174T>G), Q355M (6178C>A 6179A>T), R356D (6181A>G 6182G>A 6183A>T), K357L (6184A>T 6185A>T), D359N (6190G>A 6192T>C), N360R (6194A>G 6195T>G), A361V (6197C>T 6198A>T), R363K (6203G>A), G364P (6205G>C 6206G>C 6207A>T), G364_T365insF (6207_6208insTTC), T365L (6208A>C 6209C>T 6210A>C), A367M (6214G>A 6215C>T 6216A>G), I369V (6220A>G 6222C>G), A370V (6224C>T 6225A>G), Y372F (6230A>T), I376V (6241A>G 6243A>G), V378I (6247G>A), F379Y (6251T>A), E381Q (6256G>C), N382G (6259A>G 6260A>G 6261T>A), E383R (6262G>A 6263A>G 6264A>G)								
Codon mutations:	ATA306ATT (6033A>T), TTC307TTT (6036C>T), AAA309AAG (6042A>G), TTT310ATT (6043T>A), CTC312CTT (6051C>T), AAA313CGT (6052A>C 6053A>G 6054A>T), GCA314TCT (6055G>T 6057A>T), GGC315GGG (6060C>G), TTT316TAT (6062T>A), CAT317CAC (6066T>C), ATA319CTT (6070A>C 6072A>T), AGA320AGG (6075A>G), ATG321GTG (6076A>G), GAG322AAA (6079G>A 6081G>A), GAA323GAG (6084A>G), AAG324AAT (6087G>T), TCC325GAT (6088T>G 6089C>A 6090C>T), AAA326GTT (6091A>G 6092A>T 6093A>T), TGG328AAA (6097T>A 6098G>A 6099G>A), ACT329ATG (6101C>T 6102T>G), ACT333ACG (6114T>G), CCA334AGG (6115C>A 6116C>G 6117A>G), GAA335TAT (6118G>T 6120A>T), CTC337CAT (6125T>A 6126C>T), TAT338TTC (6128A>T 6129T>C), GAA339AAA (6130G>A), TTT340TTC (6135T>C), GAA341CTT (6136G>C 6137A>T 6138A>T), CCC344TCT (6145C>T 6147C>T), GGA346GGT (6153A>T), CTG347TTA (6154C>T 6156G>A), ATG348ACT (6158T>C 6159G>T), GCA350TCC (6163G>T 6165A>C), CCA351CCT (6168A>T), GCA352TCA (6169G>T), GAT353GTG (6173A>T 6174T>G), CAG355ATG (6178C>A 6179A>T), AGA356GAT (6181A>G 6182G>A 6183A>T), AAG357TTG (6184A>T 6185A>T), GAT359AAC (6190G>A 6192T>C), AAT360AGG (6194A>G 6195T>G), GCA361GTT (6197C>T 6198A>T), TTT362TTC (6201T>C), AGG363AAG (6203G>A), GGA364CCT (6205G>C 6206G>C 6207A>T), GGA364_ACA365insTTC (6207_6208insTTC), ACA365CTC (6208A>C 6209C>T 6210A>C), GAT366GAC (6213T>C), GCA367ATG (6214G>A 6215C>T 6216A>G), ATC369GTG (6220A>G 6222C>G), GCA370GTG (6224C>T 6225A>G), GTA371GTC (6228A>C), TAT372TTT (6230A>T), GAC375GAT (6240C>T), ATA376GTG (6241A>G 6243A>G), TTG377CTG (6244T>C), GTA378ATA (6247G>A), TTC379TAC (6251T>A), TCT380TCC (6255T>C), GAA381CAA (6256G>C), AAT382GGA (6259A>G 6260A>G 6261T>A), GAA383AGG (6262G>A 6263A>G 6264A>G)								

*: Inserts / Deletes / Misaligned / Frameshifts

Analysis details

This analysis was performed with panviral2.64

NGS Details (UN59): Badnavirus venabougainvilleae

Assembly

**Coverage Length**	285 (1 contig(s))
**Depth Of Coverage**	25.3
**Number Of Reads**	73
**Reads Per Million**	1.59 rpm (after QC)
**Ambiguities**	0
**Assembly Method**	de novo + reference guided assembly
**Consensus Caller**	Bcf Tools

Coverage Map

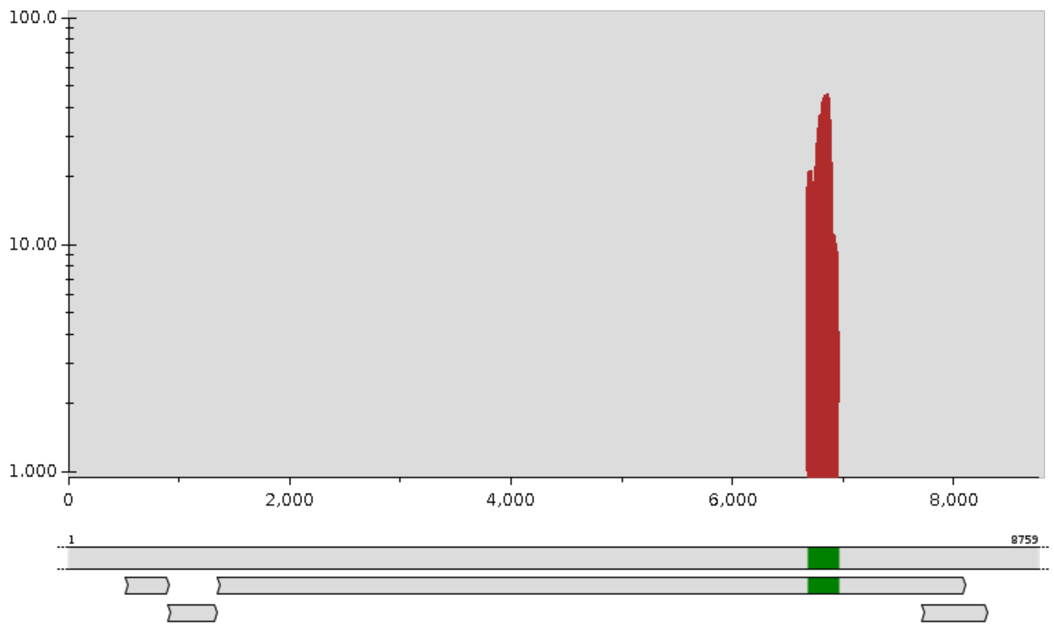

Assignment

**Type**	Badnavirus venabougainvilleae (Taxonomy ID: 3047697)
**Reference Genome**	NC_011592.1
**NT Identity (%)**	55.2083
**AA Identity (%)**	46.875
**Number Of Stop Codons**	1
**Number Of CDS**	4

Alignment

**Alignment Score**	54.0 (NT) + 333.0 (AA) = 387.0
**Concordance (%)**	30.5687

Alignment Method	Global, seeded, nucleotide + amino acids (AGA)

Genome Region

Sequence starts at position 6678 and ends at position 6962 relative to NC_011592.1 reference sequence.

Alignment Detailed Statistics

	Begin	End	Coverage	Score	Concordance	Matches	Identities	I/D/M/F*	Stop Codons
**NT**	**6678**	**6962**	**3.3%**	**54**	**9.5%**	**285 (99.0%)**	**159 (55.2%)**	**3/0**	
Mutations:	6681A>T, 6682A>T, 6683T>G, 6686A>T, 6693A>G, 6694T>A, 6696T>A, 6702G>T, 6703A>C, 6704A>G, 6705G>T, 6706A>T, 6707G>C, 6708T>G, 6711A>T, 6713T>A, 6717T>C, 6721G>T, 6723T>A, 6724G>A, 6725C>A, 6726T>G, 6729G>T, 6730G>A, 6731A>G, 6732T>G, 6733C>G, 6734C>A, 6735A>G, 6736G>A, 6737A>G, 6738A>T, 6739T>G, 6740C>A, 6741C>T, 6744A>T, 6747A>T, 6748T>A, 6749G>A, 6753G>A, 6756C>G, 6761T>G, 6762T>G, 6764C>T, 6765T>G, 6766C>A, 6767C>G, 6768A>G, 6769C>T, 6771A>T, 6776T>A, 6777G>T, 6785G>T, 6786G>T, 6789A>T, 6790G>T, 6796C>T, 6798A>C, 6805C>T, 6809A>C, 6810G>T, 6813T>C, 6816A>T, 6818C>T, 6819A>G, 6821C>T, 6823A>G, 6828C>T, 6829C>A, 6830A>T, 6831A>G, 6832A>G, 6833G>A, 6834G>T, 6835A>C, 6836A>T, 6837G>T, 6841G>A, 6843C>T, 6845A>G, 6846C>G, 6847T>A, 6848G>T, 6849C>A, 6851T>A, 6852T>C, 6853T>A, 6854C>A, 6857G>C, 6858A>T, 6862A>C, 6863G>T, 6864C>T, 6867T>C, 6867_6868insATG, 6869T>A, 6870C>T, 6873T>G, 6875C>T, 6876T>G, 6879A>G, 6880T>C, 6881A>T, 6882C>T, 6894A>T, 6900G>C, 6902T>A, 6907G>C, 6909G>A, 6910A>G, 6913G>T, 6915A>G, 6916A>C, 6917G>A, 6921C>A, 6924T>C, 6925G>A, 6926A>T, 6928A>G, 6930G>A, 6934C>T, 6936C>G, 6940G>A, 6941T>C, 6946C>T, 6948T>G, 6951A>G								

CDS

	Begin	End	Coverage	Score	Concordance	Matches	Identities	I/D/M/F*	Stop Codons
BSCVV_gp3	1779	1873	4.2%	333	47.1%	95 (99.0%)	45 (46.9%)	1/0/0/0	1
Protein mutations:	K1779N (6681A>T), I1780C (6682A>T 6683T>G), Y1781F (6686A>T), F1784I (6694T>A 6696T>A), K1787R (6703A>C 6704A>G 6705G>T), F1790Y (6713T>A), V1793L (6721G>T 6723T>A), A1794K (6724G>A 6725C>A 6726T>G), M1795I (6729G>T), D1796R (6730G>A 6731A>G 6732T>G), P1797E (6733C>G 6734C>A 6735A>G), E1798S (6736G>A 6737A>G 6738A>T), S1799D (6739T>G 6740C>A 6741C>T), W1802K (6748T>A 6749G>A), L1806R (6761T>G 6762T>G), T1807M (6764C>T 6765T>G), P1808R (6766C>A 6767C>G 6768A>G), Q1809Y (6769C>T 6771A>T), L1811Y (6776T>A 6777G>T), W1814F (6785G>T 6786G>T), V1816F (6790G>T), P1818S (6796C>T 6798A>C), K1822T (6809A>C 6810G>T), P1825L (6818C>T 6819A>G), A1826V (6821C>T), I1827V (6823A>G), Q1829M (6829C>A 6830A>T 6831A>G), R1830D (6832A>G 6833G>A 6834G>T), K1831L (6835A>C 6836A>T 6837G>T), D1833N (6841G>A 6843C>T), N1834R (6845A>G 6846C>G), C1835I (6847T>A 6848G>T 6849C>A), F1836Y (6851T>A 6852T>C), S1837K (6853T>A 6854C>A), R1838P (6857G>C 6858A>T), S1840L (6862A>C 6863G>T 6864C>T), D1841_F1842insM (6867_6868insATG), F1842Y (6869T>A 6870C>T), I1843M (6873T>G), A1844V (6875C>T 6876T>G), Y1846L (6880T>C 6881A>T 6882C>T), F1853Y (6902T>A), E1855Q (6907G>C 6909G>A), S1856G (6910A>G), E1857* (6913G>T 6915A>G), R1858Q (6916A>C 6917G>A), D1859E (6921C>A), E1861M (6925G>A 6926A>T), K1862E (6928A>G 6930G>A), V1866T (6940G>A 6941T>C)								
Codon mutations:	AAA1779AAT (6681A>T), ATC1780TGC (6682A>T 6683T>G), TAC1781TTC (6686A>T), AAA1783AAG (6693A>G), TTT1784ATA (6694T>A 6696T>A), CTG1786CTT (6702G>T), AAG1787CGT (6703A>C 6704A>G 6705G>T), AGT1788TCG (6706A>T 6707G>C 6708T>G), GGA1789GGT (6711A>T), TTT1790TAT (6713T>A), CAT1791CAC (6717T>C), GTT1793TTA (6721G>T 6723T>A), GCT1794AAG (6724G>A 6725C>A 6726T>G), ATG1795ATT (6729G>T), GAT1796AGG (6730G>A 6731A>G 6732T>G), CCA1797GAG (6733C>G 6734C>A 6735A>G), GAA1798AGT (6736G>A 6737A>G 6738A>T), TCC1799GAT (6739T>G 6740C>A 6741C>T), ATA1800ATT (6744A>T), CCA1801CCT (6747A>T), TGG1802AAG (6748T>A 6749G>A), ACG1803ACA (6753G>A), GCC1804GCG (6756C>G), CTT1806CGG (6761T>G 6762T>G), ACT1807ATG (6764C>T 6765T>G), CCA1808AGG (6766C>A 6767C>G 6768A>G), CAA1809TAT (6769C>T 6771A>T), TTG1811TAT (6776T>A 6777G>T), TGG1814TTT (6785G>T 6786G>T), CTA1815CTT (6789A>T), GTC1816TTC (6790G>T), CCA1818TCC (6796C>T 6798A>C), CTA1821TTA (6805C>T), AAG1822ACT (6809A>C 6810G>T), AAT1823AAC (6813T>C), GCA1824GCT (6816A>T), CCA1825CTG (6818C>T 6819A>G), GCT1826GTT (6821C>T), ATC1827GTC (6823A>G), TTC1828TTT (6828C>T), CAA1829ATG (6829C>A 6830A>T 6831A>G), AGG1830GAT (6832A>G 6833G>A 6834G>T), AAG1831CTT (6835A>C 6836A>T 6837G>T), GAC1833AAT (6841G>A 6843C>T), AAC1834AGG (6845A>G 6846C>G), TGC1835ATA (6847T>A 6848G>T 6849C>A), TTT1836TAC (6851T>A 6852T>C), TCA1837AAA (6853T>A 6854C>A), CGA1838CCT (6857G>C 6858A>T), AGC1840CTT (6862A>C 6863G>T 6864C>T), GAT1841GAC (6867T>C), GAT1841_TTC1842insATG (6867_6868insATG), TTC1842TAT (6869T>A 6870C>T), ATT1843ATG (6873T>G), GCT1844GTG (6875C>T 6876T>G), GTA1845GTG (6879A>G), TAC1846CTT (6880T>C 6881A>T 6882C>T), ATA1850ATT (6894A>T), GTG1852GTC (6900G>C), TTT1853TAT (6902T>A), GAG1855CAA (6907G>C 6909G>A), AGT1856GGT (6910A>G), GAA1857TAG (6913G>T 6915A>G), AGA1858CAA (6916A>C 6917G>A), GAC1859GAA (6921C>A), CAT1860CAC (6924T>C), GAG1861ATG (6925G>A 6926A>T), AAG1862GAA (6928A>G 6930G>A), CTC1864TTG (6934C>T 6936C>G), GTT1866ACT (6940G>A 6941T>C), CTT1868TTG (6946C>T 6948T>G), CAA1869CAG (6951A>G)								

Proteins

	Begin	End	Coverage	Score	Concordance	Matches	Identities	I/D/M/F*	Stop Codons
polyprotein (YP_002321513.1)	1779	1873	4.2%	333	47.1%	95 (99.0%)	45 (46.9%)	1/0/0/0	1
Protein mutations:	K1779N (6681A>T), I1780C (6682A>T 6683T>G), Y1781F (6686A>T), F1784I (6694T>A 6696T>A), K1787R (6703A>C 6704A>G 6705G>T), F1790Y (6713T>A), V1793L (6721G>T 6723T>A), A1794K (6724G>A 6725C>A 6726T>G), M1795I (6729G>T), D1796R (6730G>A 6731A>G 6732T>G), P1797E (6733C>G 6734C>A 6735A>G), E1798S (6736G>A 6737A>G 6738A>T), S1799D (6739T>G 6740C>A 6741C>T), W1802K (6748T>A 6749G>A), L1806R (6761T>G 6762T>G), T1807M (6764C>T 6765T>G), P1808R (6766C>A 6767C>G 6768A>G), Q1809Y (6769C>T 6771A>T), L1811Y (6776T>A 6777G>T), W1814F (6785G>T 6786G>T), V1816F (6790G>T), P1818S (6796C>T 6798A>C), K1822T (6809A>C 6810G>T), P1825L (6818C>T 6819A>G), A1826V (6821C>T), I1827V (6823A>G), Q1829M (6829C>A 6830A>T 6831A>G), R1830D (6832A>G 6833G>A 6834G>T), K1831L (6835A>C 6836A>T 6837G>T), D1833N (6841G>A 6843C>T), N1834R (6845A>G 6846C>G), C1835I (6847T>A 6848G>T 6849C>A), F1836Y (6851T>A 6852T>C), S1837K (6853T>A 6854C>A), R1838P (6857G>C 6858A>T), S1840L (6862A>C 6863G>T 6864C>T), D1841_F1842insM (6867_6868insATG), F1842Y (6869T>A 6870C>T), I1843M (6873T>G), A1844V (6875C>T 6876T>G), Y1846L (6880T>C 6881A>T 6882C>T), F1853Y (6902T>A), E1855Q (6907G>C 6909G>A), S1856G (6910A>G), E1857* (6913G>T 6915A>G), R1858Q (6916A>C 6917G>A), D1859E (6921C>A), E1861M (6925G>A 6926A>T), K1862E (6928A>G 6930G>A), V1866T (6940G>A 6941T>C)								
Codon mutations:	AAA1779AAT (6681A>T), ATC1780TGC (6682A>T 6683T>G), TAC1781TTC (6686A>T), AAA1783AAG (6693A>G), TTT1784ATA (6694T>A 6696T>A), CTG1786CTT (6702G>T), AAG1787CGT (6703A>C 6704A>G 6705G>T), AGT1788TCG (6706A>T 6707G>C 6708T>G), GGA1789GGT (6711A>T), TTT1790TAT (6713T>A), CAT1791CAC (6717T>C), GTT1793TTA (6721G>T 6723T>A), GCT1794AAG (6724G>A 6725C>A 6726T>G), ATG1795ATT (6729G>T), GAT1796AGG (6730G>A 6731A>G 6732T>G), CCA1797GAG (6733C>G 6734C>A 6735A>G), GAA1798AGT (6736G>A 6737A>G 6738A>T), TCC1799GAT (6739T>G 6740C>A 6741C>T), ATA1800ATT (6744A>T), CCA1801CCT (6747A>T), TGG1802AAG (6748T>A 6749G>A), ACG1803ACA (6753G>A), GCC1804GCG (6756C>G), CTT1806CGG (6761T>G 6762T>G), ACT1807ATG (6764C>T 6765T>G), CCA1808AGG (6766C>A 6767C>G 6768A>G), CAA1809TAT (6769C>T 6771A>T), TTG1811TAT (6776T>A 6777G>T), TGG1814TTT (6785G>T 6786G>T), CTA1815CTT (6789A>T), GTC1816TTC (6790G>T), CCA1818TCC (6796C>T 6798A>C), CTA1821TTA (6805C>T), AAG1822ACT (6809A>C 6810G>T), AAT1823AAC (6813T>C), GCA1824GCT (6816A>T), CCA1825CTG (6818C>T 6819A>G), GCT1826GTT (6821C>T), ATC1827GTC (6823A>G), TTC1828TTT (6828C>T), CAA1829ATG (6829C>A 6830A>T 6831A>G), AGG1830GAT (6832A>G 6833G>A 6834G>T), AAG1831CTT (6835A>C 6836A>T 6837G>T), GAC1833AAT (6841G>A 6843C>T), AAC1834AGG (6845A>G 6846C>G), TGC1835ATA (6847T>A 6848G>T 6849C>A), TTT1836TAC (6851T>A 6852T>C), TCA1837AAA (6853T>A 6854C>A), CGA1838CCT (6857G>C 6858A>T), AGC1840CTT (6862A>C 6863G>T 6864C>T), GAT1841GAC (6867T>C), GAT1841_TTC1842insATG (6867_6868insATG), TTC1842TAT (6869T>A 6870C>T), ATT1843ATG (6873T>G), GCT1844GTG (6875C>T 6876T>G), GTA1845GTG (6879A>G), TAC1846CTT (6880T>C 6881A>T 6882C>T), ATA1850ATT (6894A>T), GTG1852GTC (6900G>C), TTT1853TAT (6902T>A), GAG1855CAA (6907G>C 6909G>A), AGT1856GGT (6910A>G), GAA1857TAG (6913G>T 6915A>G), AGA1858CAA (6916A>C 6917G>A), GAC1859GAA (6921C>A), CAT1860CAC (6924T>C), GAG1861ATG (6925G>A 6926A>T), AAG1862GAA (6928A>G 6930G>A), CTC1864TTG (6934C>T 6936C>G), GTT1866ACT (6940G>A 6941T>C), CTT1868TTG (6946C>T 6948T>G), CAA1869CAG (6951A>G)								

*: Inserts / Deletes / Misaligned / Frameshifts

Analysis details

This analysis was performed with panviral2.64

NGS Details (UN59): Badnavirus occultipomeae

Assembly

**Coverage Length**	301 (1 contig(s))
**Depth Of Coverage**	18.2
**Number Of Reads**	50
**Reads Per Million**	1.09 rpm (after QC)
**Ambiguities**	0
**Assembly Method**	de novo + reference guided assembly
**Consensus Caller**	Bcf Tools

Coverage Map

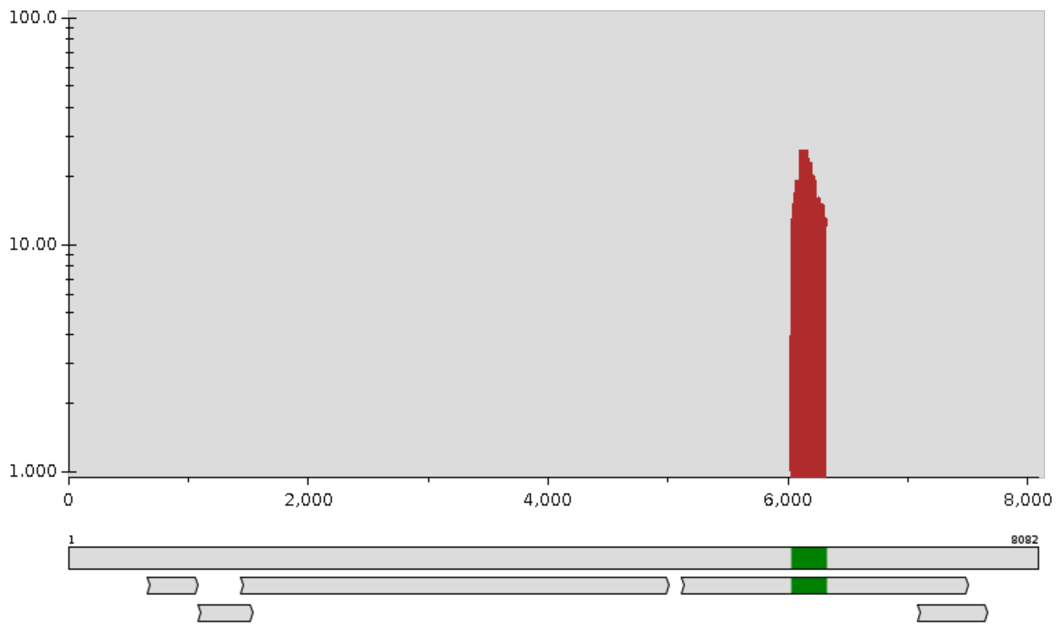

Assignment

**Type**	Badnavirus occultipomeae (Taxonomy ID: 3048353)
**Reference Genome**	NC_015655.1
**NT Identity (%)**	57.7558
**AA Identity (%)**	49.0196
**Number Of Stop Codons**	2
**Number Of CDS**	5

Alignment

**Alignment Score**	78.0 (NT) + 196.0 (AA) = 274.0
**Concordance (%)**	24.2525

Alignment Method	Global, seeded, nucleotide + amino acids (AGA)

Genome Region

Sequence starts at position 6025 and ends at position 6325 relative to NC_015655.1 reference sequence.

Alignment Detailed Statistics

	Begin	End	Coverage	Score	Concordance	Matches	Identities	I/D/M/F*	Stop Codons
**NT**	**6025**	**6325**	**3.7%**	**78**	**13.2%**	**300 (98.7%)**	**175 (57.6%)**	**3/1**	
Mutations:	6031A>G, 6036C>T, 6039A>T, 6042A>G, 6043T>A, 6045T>A, 6048T>C, 6051C>G, 6053A>G, 6060C>T, 6062T>A, 6069G>A, 6072A>T, 6079G>C, 6081G>T, 6084A>G, 6086A>T, 6087G>A, 6088T>G, 6089C>A, 6092A>T, 6094C>T, 6095C>A, 6096A>C, 6097T>A, 6098G>A, 6109T>A, 6114T>A, 6116C>A, 6117A>T, 6118G>C, 6119A>T, 6120A>G, 6125T>A, 6126C>T, 6135T>C, 6136G>A, 6138A>G, 6147C>T, 6153A>T, 6154C>T, 6156G>A, 6158T>C, 6159G>C, 6162T>C, 6165A>C, 6168A>T, 6171A>C, 6172G>A, 6173A>C, 6174T>C, 6178C>T, 6183A>T, 6184A>T, 6185A>T, 6186G>A, 6190G>A, 6191A>G, 6192T>C, 6193A>C, 6197C>T, 6198A>G, 6202delA, 6203G>T, 6204G>A, 6208A>T, 6209C>T, 6210_6211insCTT, 6217T>G, 6220A>T, 6221T>G, 6222C>T, 6224C>T, 6225A>T, 6226G>A, 6227T>G, 6228A>T, 6230A>T, 6232A>T, 6235G>A, 6240C>T, 6243A>T, 6244T>C, 6249A>G, 6251T>A, 6252C>T, 6253T>A, 6254C>G, 6256G>C, 6257A>C, 6258A>T, 6262G>A, 6263A>T, 6264A>C, 6265G>T, 6267A>G, 6268G>A, 6269A>C, 6270G>T, 6275A>T, 6276G>A, 6277G>T, 6278A>G, 6279T>G, 6282T>G, 6283C>T, 6285C>G, 6286T>G, 6287T>A, 6288A>G, 6291T>G, 6294G>C, 6295G>T, 6296C>T, 6297C>G, 6300A>T, 6304G>C, 6306G>T, 6309A>C, 6315A>G, 6316G>A, 6317G>A, 6319C>T, 6320T>G, 6321T>G, 6322A>T, 6325C>T								

CDS

	Begin	End	Coverage	Score	Concordance	Matches	Identities	I/D/M/F*	Stop Codons
SPBVa_gp4	304	404	12.7%	196	27.3%	101 (99.0%)	50 (49.0%)	1/0/1/1	2
Protein mutations:	I306V (6031A>G), F310I (6043T>A 6045T>A), K313R (6053A>G), F316Y (6062T>A), E322H (6079G>C 6081G>T), K324I (6086A>T 6087G>A), S325D (6088T>G 6089C>A), K326I (6092A>T), P327Y (6094C>T 6095C>A 6096A>C), W328K (6097T>A 6098G>A), W332R (6109T>A), P334H (6116C>A 6117A>T), E335L (6118G>C 6119A>T 6120A>G), L337H (6125T>A 6126C>T), E341K (6136G>A 6138A>G), M348T (6158T>C 6159G>C), D353T (6172G>A 6173A>C 6174T>C), Q355* (6178C>T), R356S (6183A>T), K357L (6184A>T 6185A>T 6186G>A), D359S (6190G>A 6191A>G 6192T>C), N360H (6193A>C), A361V (6197C>T 6198A>G), T365L (6208A>T 6209C>T), T365_D366insL (6210_6211insCTT), F368V (6217T>G), I369C (6220A>T 6221T>G 6222C>T), A370V (6224C>T 6225A>T), V371S (6226G>A 6227T>G 6228A>T), Y372F (6230A>T), I373F (6232A>T), D374N (6235G>A), F379Y (6251T>A 6252C>T), E381P (6256G>C 6257A>C 6258A>T), E383I (6262G>A 6263A>T 6264A>C), E384* (6265G>T 6267A>G), E385T (6268G>A 6269A>C 6270G>T), E387V (6275A>T 6276G>A), D388W (6277G>T 6278A>G 6279T>G), H389Q (6282T>G), L391E (6286T>G 6287T>A 6288A>G), N392K (6291T>G), L393F (6294G>C), A394L (6295G>T 6296C>T 6297C>G), Q395H (6300A>T), V397L (6304G>C 6306G>T), R398S (6309A>C), G401K (6316G>A 6317G>A), L402W (6319C>T 6320T>G 6321T>G), I403F (6322A>T)								
Codon mutations:	ATA306GTA (6031A>G), TTC307TTT (6036C>T), TCA308TCT (6039A>T), AAA309AAG (6042A>G), TTT310ATA (6043T>A 6045T>A), GAT311GAC (6048T>C), CTC312CTG (6051C>G), AAA313AGA (6053A>G), GGC315GGT (6060C>T), TTT316TAT (6062T>A), CAG318CAA (6069G>A), ATA319ATT (6072A>T), GAG322CAT (6079G>C 6081G>T), GAA323GAG (6084A>G), AAG324ATA (6086A>T 6087G>A), TCC325GAC (6088T>G 6089C>A), AAA326ATA (6092A>T), CCA327TAC (6094C>T 6095C>A 6096A>C), TGG328AAG (6097T>A 6098G>A), TGG332AGG (6109T>A), ACT333ACA (6114T>A), CCA334CAT (6116C>A 6117A>T), GAA335CTG (6118G>C 6119A>T 6120A>G), CTC337CAT (6125T>A 6126C>T), TTT340TTC (6135T>C), GAA341AAG (6136G>A 6138A>G), CCC344CCT (6147C>T), GGA346GGT (6153A>T), CTG347TTA (6154C>T 6156G>A), ATG348ACC (6158T>C 6159G>C), AAT349AAC (6162T>C), GCA350GCC (6165A>C), CCA351CCT (6168A>T), GCA352GCC (6171A>C), GAT353ACC (6172G>A 6173A>C 6174T>C), CAG355TAG (6178C>T), AGA356AGT (6183A>T), AAG357TTA (6184A>T 6185A>T 6186G>A), GAT359AGC (6190G>A 6191A>G 6192T>C), AAT360CAT (6193A>C), GCA361GTG (6197C>T 6198A>G), AGG363-TA (6202delA 6203G>T 6204G>A), ACA365TTA (6208A>T 6209C>T), ACA365_GAT366insCTT (6210_6211insCTT), TTT368GTT (6217T>G), ATC369TGT (6220A>T 6221T>G 6222C>T), GCA370GTT (6224C>T 6225A>T), GTA371AGT (6226G>A 6227T>G 6228A>T), TAT372TTT (6230A>T), ATT373TTT (6232A>T), GAT374AAT (6235G>A), GAC375GAT (6240C>T), ATA376ATT (6243A>T), TTG377CTG (6244T>C), GTA378GTG (6249A>G), TTC379TAT (6251T>A 6252C>T), TCT380AGT (6253T>A 6254C>G), GAA381CCT (6256G>C 6257A>C 6258A>T), GAA383ATC (6262G>A 6263A>T 6264A>C), GAA384TAG (6265G>T 6267A>G), GAG385ACT (6268G>A 6269A>C 6270G>T), GAG387GTA (6275A>T 6276G>A), GAT388TGG (6277G>T 6278A>G 6279T>G), CAT389CAG (6282T>G), CTC390TTG (6283C>T 6285C>G), TTA391GAG (6286T>G 6287T>A 6288A>G), AAT392AAG (6291T>G), TTG393TTC (6294G>C), GCC394TTG (6295G>T 6296C>T 6297C>G), CAA395CAT (6300A>T), GTG397CTT (6304G>C 6306G>T), AGA398AGC (6309A>C), GAA400GAG (6315A>G), GGG401AAG (6316G>A 6317G>A), CTT402TGG (6319C>T 6320T>G 6321T>G), ATT403TTT (6322A>T), CTA404T.. (6325C>T)								

Proteins

	Begin	End	Coverage	Score	Concordance	Matches	Identities	I/D/M/F*	Stop Codons
RNaseH/reverse transcriptase (YP_004581513.1)	304	404	12.7%	196	27.3%	101 (99.0%)	50 (49.0%)	1/0/1/1	2
Protein mutations:	I306V (6031A>G), F310I (6043T>A 6045T>A), K313R (6053A>G), F316Y (6062T>A), E322H (6079G>C 6081G>T), K324I (6086A>T 6087G>A), S325D (6088T>G 6089C>A), K326I (6092A>T), P327Y (6094C>T 6095C>A 6096A>C), W328K (6097T>A 6098G>A), W332R (6109T>A), P334H (6116C>A 6117A>T), E335L (6118G>C 6119A>T 6120A>G), L337H (6125T>A 6126C>T), E341K (6136G>A 6138A>G), M348T (6158T>C 6159G>C), D353T (6172G>A 6173A>C 6174T>C), Q355* (6178C>T), R356S (6183A>T), K357L (6184A>T 6185A>T 6186G>A), D359S (6190G>A 6191A>G 6192T>C), N360H (6193A>C), A361V (6197C>T 6198A>G), T365L (6208A>T 6209C>T), T365_D366insL (6210_6211insCTT), F368V (6217T>G), I369C (6220A>T 6221T>G 6222C>T), A370V (6224C>T 6225A>T), V371S (6226G>A 6227T>G 6228A>T), Y372F (6230A>T), I373F (6232A>T), D374N (6235G>A), F379Y (6251T>A 6252C>T), E381P (6256G>C 6257A>C 6258A>T), E383I (6262G>A 6263A>T 6264A>C), E384* (6265G>T 6267A>G), E385T (6268G>A 6269A>C 6270G>T), E387V (6275A>T 6276G>A), D388W (6277G>T 6278A>G 6279T>G), H389Q (6282T>G), L391E (6286T>G 6287T>A 6288A>G), N392K (6291T>G), L393F (6294G>C), A394L (6295G>T 6296C>T 6297C>G), Q395H (6300A>T), V397L (6304G>C 6306G>T), R398S (6309A>C), G401K (6316G>A 6317G>A), L402W (6319C>T 6320T>G 6321T>G), I403F (6322A>T)								
Codon mutations:	ATA306GTA (6031A>G), TTC307TTT (6036C>T), TCA308TCT (6039A>T), AAA309AAG (6042A>G), TTT310ATA (6043T>A 6045T>A), GAT311GAC (6048T>C), CTC312CTG (6051C>G), AAA313AGA (6053A>G), GGC315GGT (6060C>T), TTT316TAT (6062T>A), CAG318CAA (6069G>A), ATA319ATT (6072A>T), GAG322CAT (6079G>C 6081G>T), GAA323GAG (6084A>G), AAG324ATA (6086A>T 6087G>A), TCC325GAC (6088T>G 6089C>A), AAA326ATA (6092A>T), CCA327TAC (6094C>T 6095C>A 6096A>C), TGG328AAG (6097T>A 6098G>A), TGG332AGG (6109T>A), ACT333ACA (6114T>A), CCA334CAT (6116C>A 6117A>T), GAA335CTG (6118G>C 6119A>T 6120A>G), CTC337CAT (6125T>A 6126C>T), TTT340TTC (6135T>C), GAA341AAG (6136G>A 6138A>G), CCC344CCT (6147C>T), GGA346GGT (6153A>T), CTG347TTA (6154C>T 6156G>A), ATG348ACC (6158T>C 6159G>C), AAT349AAC (6162T>C), GCA350GCC (6165A>C), CCA351CCT (6168A>T), GCA352GCC (6171A>C), GAT353ACC (6172G>A 6173A>C 6174T>C), CAG355TAG (6178C>T), AGA356AGT (6183A>T), AAG357TTA (6184A>T 6185A>T 6186G>A), GAT359AGC (6190G>A 6191A>G 6192T>C), AAT360CAT (6193A>C), GCA361GTG (6197C>T 6198A>G), AGG363-TA (6202delA 6203G>T 6204G>A), ACA365TTA (6208A>T 6209C>T), ACA365_GAT366insCTT (6210_6211insCTT), TTT368GTT (6217T>G), ATC369TGT (6220A>T 6221T>G 6222C>T), GCA370GTT (6224C>T 6225A>T), GTA371AGT (6226G>A 6227T>G 6228A>T), TAT372TTT (6230A>T), ATT373TTT (6232A>T), GAT374AAT (6235G>A), GAC375GAT (6240C>T), ATA376ATT (6243A>T), TTG377CTG (6244T>C), GTA378GTG (6249A>G), TTC379TAT (6251T>A 6252C>T), TCT380AGT (6253T>A 6254C>G), GAA381CCT (6256G>C 6257A>C 6258A>T), GAA383ATC (6262G>A 6263A>T 6264A>C), GAA384TAG (6265G>T 6267A>G), GAG385ACT (6268G>A 6269A>C 6270G>T), GAG387GTA (6275A>T 6276G>A), GAT388TGG (6277G>T 6278A>G 6279T>G), CAT389CAG (6282T>G), CTC390TTG (6283C>T 6285C>G), TTA391GAG (6286T>G 6287T>A 6288A>G), AAT392AAG (6291T>G), TTG393TTC (6294G>C), GCC394TTG (6295G>T 6296C>T 6297C>G), CAA395CAT (6300A>T), GTG397CTT (6304G>C 6306G>T), AGA398AGC (6309A>C), GAA400GAG (6315A>G), GGG401AAG (6316G>A 6317G>A), CTT402TGG (6319C>T 6320T>G 6321T>G), ATT403TTT (6322A>T), CTA404T.. (6325C>T)								

*: Inserts / Deletes / Misaligned / Frameshifts

Analysis details

This analysis was performed with panviral2.64

NGS Details (UN59): Badnavirus venaribis

Assembly

**Coverage Length**	441 (1 contig(s))
**Depth Of Coverage**	9.7
**Number Of Reads**	36
**Reads Per Million**	0.78 rpm (after QC)
**Ambiguities**	0
**Assembly Method**	de novo + reference guided assembly
**Consensus Caller**	Bcf Tools

Coverage Map

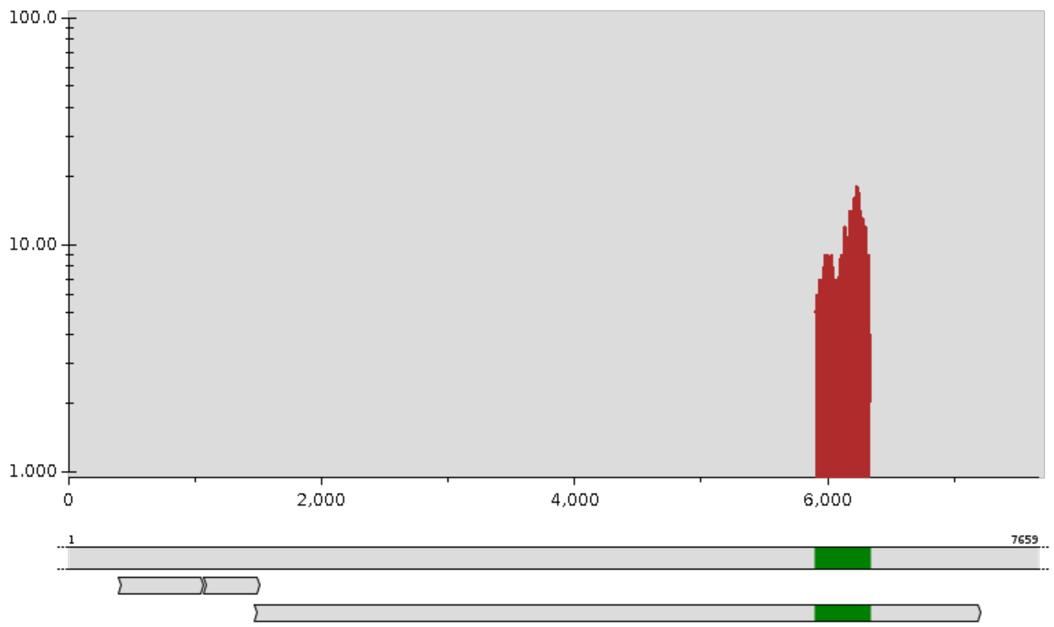

Assignment

**Type**	Badnavirus venaribis (Taxonomy ID: 3047843)
**Reference Genome**	NC_018105.1
**NT Identity (%)**	52.9279
**AA Identity (%)**	45.9459
**Number Of Stop Codons**	0
**Number Of CDS**	3

Alignment

**Alignment Score**	37.0 (NT) + 494.0 (AA) = 531.0
**Concordance (%)**	28.2297

Alignment Method	Global, seeded, nucleotide + amino acids (AGA)

Genome Region

Sequence starts at position 5902 and ends at position 6342 relative to NC_018105.1 reference sequence.

Alignment Detailed Statistics

	Begin	End	Coverage	Score	Concordance	Matches	Identities	I/D/M/F*	Stop Codons
**NT**	**5902**	**6342**	**5.8%**	**37**	**4.3%**	**438 (98.0%)**	**235 (52.6%)**	**6/3**	
Mutations:	5902G>T, 5903T>G, 5904C>T, 5905T>G, 5907C>A, 5913T>C, 5914C>A, 5916C>G, 5918C>A, 5919T>A, 5920C>T, 5922G>A, 5927A>C, 5934G>T, 5935C>G, 5936A>T, 5943C>T, 5944C>A, 5948A>T, 5949C>T, 5950A>C, 5953T>A, 5955G>T, 5958T>A, 5960G>T, 5961C>T, 5964C>T, 5967C>G, 5968T>G, 5969C>A, 5970G>A, 5971A>T, 5973C>A, 5974G>T, 5978C>A, 5979A>T, 5981C>A, 5982T>G, 5983G>T, 5985T>G, 5986G>C, 5987G>A, 5988C>T, 5989A>G, 5990A>G, 5991C>A, 5997A>G, 5998A>T, 5999T>A, 6000C>T, 6002A>T, 6003C>T, 6004A>T, 6005G>C, 6006C>T, 6009A>G, 6010T>C, 6012C>A, 6020A>G, 6021G>A, 6022G>T, 6029T>A, 6037G>A, 6039C>A, 6040C>A, 6041T>G, 6046G>T, 6047A>T, 6048A>T, 6054C>T, 6055T>A, 6056C>A, 6060C>A, 6064T>A, 6065G>A, 6066G>A, 6069A>G, 6070G>A, 6072G>A, 6077T>G, 6078C>A, 6081G>C, 6083C>A, 6084C>T, 6085G>C, 6086T>A, 6087A>G, 6090A>G, 6091T>C, 6092T>A, 6099A>G, 6101G>T, 6102G>C, 6105G>A, 6108A>G, 6114T>C, 6117C>T, 6120T>C, 6121A>C, 6124G>A, 6126T>C, 6129C>T, 6132T>C, 6135A>C, 6136G>T, 6138G>C, 6139G>A, 6140T>C, 6141T>A, 6144C>T, 6150G>C, 6151A>C, 6152A>T, 6153A>C, 6157G>A, 6160A>C, 6162C>A, 6163T>A, 6164G>T, 6165C>A, 6168_6169insAATGCT, 6171C>T, 6172_6174delAAG, 6177G>T, 6181G>A, 6183G>A, 6186C>T, 6187G>A, 6190G>C, 6191C>T, 6197A>T, 6198T>C, 6199A>T, 6201C>T, 6204C>T, 6207C>T, 6210C>A, 6213C>T, 6214G>A, 6216C>A, 6218T>A, 6221G>A, 6224A>G, 6225T>C, 6226T>A, 6227C>A, 6228C>T, 6229C>T, 6230T>G, 6232C>T, 6233A>C, 6234A>T, 6237A>T, 6241G>C, 6242A>T, 6243G>C, 6244A>T, 6245G>C, 6246C>A, 6250T>C, 6252A>G, 6254G>A, 6255G>A, 6260T>C, 6261G>T, 6262C>T, 6264G>C, 6265G>C, 6268G>A, 6271T>C, 6272G>A, 6273C>T, 6279G>C, 6284G>T, 6285G>T, 6286T>C, 6288G>T, 6289G>T, 6291G>T, 6294G>C, 6295T>A, 6296C>A, 6297A>G, 6298C>A, 6299C>A, 6303A>C, 6306A>G, 6307A>T, 6308T>G, 6309G>T, 6310A>G, 6311A>C, 6312G>T, 6313G>T, 6317C>G, 6318T>G, 6324A>T, 6325A>C, 6326C>A, 6327A>G, 6328G>A, 6330A>T, 6333A>C, 6335T>A, 6342G>T								

CDS

	Begin	End	Coverage	Score	Concordance	Matches	Identities	I/D/M/F*	Stop Codons
B227_gp3	1479	1625	7.7%	494	48.6%	146 (98.0%)	68 (45.6%)	2/1/0/0	0
Protein mutations:	V1479C (5902G>T 5903T>G 5904C>T), F1480V (5905T>G 5907C>A), A1484E (5918C>A 5919T>A), N1487T (5927A>C), H1490V (5935C>G 5936A>T), Q1493K (5944C>A), Y1494F (5948A>T 5949C>T), T1495P (5950A>C), L1496I (5953T>A 5955G>T), G1498V (5960G>T 5961C>T), N1500K (5967C>G), S1501E (5968T>G 5969C>A 5970G>A), I1502L (5971A>T 5973C>A), V1503L (5974G>T), A1504D (5978C>A 5979A>T), A1505E (5981C>A 5982T>G), V1506L (5983G>T 5985T>G), G1507H (5986G>C 5987G>A 5988C>T), N1508G (5989A>G 5990A>G 5991C>A), I1511Y (5998A>T 5999T>A 6000C>T), Y1512F (6002A>T 6003C>T), F1515L (6010T>C 6012C>A), K1518R (6020A>G 6021G>A), A1519S (6022G>T), F1521Y (6029T>A), V1524I (6037G>A 6039C>A), L1525R (6040C>A 6041T>G), E1527F (6046G>T 6047A>T 6048A>T), S1530N (6055T>A 6056C>A), W1533K (6064T>A 6065G>A 6066G>A), A1535T (6070G>A 6072G>A), I1537R (6077T>G 6078C>A), P1539H (6083C>A 6084C>T), V1540Q (6085G>C 6086T>A 6087A>G), F1542H (6091T>C 6092T>A), W1545F (6101G>T 6102G>C), I1552L (6121A>C), A1553T (6124G>A 6126T>C), A1557S (6136G>T 6138G>C), V1558T (6139G>A 6140T>C 6141T>A), R1561S (6150G>C), K1562L (6151A>C 6152A>T 6153A>C), D1564N (6157G>A), N1565Q (6160A>C 6162C>A), C1566I (6163T>A 6164G>T 6165C>A), F1567_H1568insNA (6168_6169insAATGCT), K1569del (6172_6174delAAG), E1572K (6181G>A 6183G>A), V1574I (6187G>A), A1575L (6190G>C 6191C>T), Y1577F (6197A>T 6198T>C), I1578F (6199A>T 6201C>T), V1583I (6214G>A 6216C>A), F1584Y (6218T>A), S1585N (6221G>A), N1586S (6224A>G 6225T>C), S1587N (6226T>A 6227C>A 6228C>T), L1588W (6229C>T 6230T>G), Q1589S (6232C>T 6233A>C 6234A>T), E1590D (6237A>T), E1592L (6241G>C 6242A>T 6243G>C), R1596Q (6254G>A 6255G>A), M1598T (6260T>C 6261G>T), L1599F (6262C>T 6264G>C), E1600Q (6265G>C), V1601I (6268G>A), C1602H (6271T>C 6272G>A 6273C>T), K1604N (6279G>C), G1606V (6284G>T 6285G>T), V1608F (6289G>T 6291G>T), S1610K (6295T>A 6296C>A 6297A>G), P1611K (6298C>A 6299C>A), M1614C (6307A>T 6308T>G 6309G>T), K1615A (6310A>G 6311A>C 6312G>T), V1616F (6313G>T), A1617G (6317C>G 6318T>G), T1620Q (6325A>C 6326C>A 6327A>G), V1621I (6328G>A 6330A>T), E1622D (6333A>C), F1623Y (6335T>A)								
Codon mutations:	GTC1479TGT (5902G>T 5903T>G 5904C>T), TTC1480GTA (5905T>G 5907C>A), TAT1482TAC (5913T>C), CGC1483AGG (5914C>A 5916C>G), GCT1484GAA (5918C>A 5919T>A), CTG1485TTA (5920C>T 5922G>A), AAC1487ACC (5927A>C), ACG1489ACT (5934G>T), CAC1490GTC (5935C>G 5936A>T), GAC1492GAT (5943C>T), CAG1493AAG (5944C>A), TAC1494TTT (5948A>T 5949C>T), ACA1495CCA (5950A>C), TTG1496ATT (5953T>A 5955G>T), CCT1497CCA (5958T>A), GGC1498GTT (5960G>T 5961C>T), ATC1499ATT (5964C>T), AAC1500AAG (5967C>G), TCG1501GAA (5968T>G 5969C>A 5970G>A), ATC1502TTA (5971A>T 5973C>A), GTA1503TTA (5974G>T), GCA1504GAT (5978C>A 5979A>T), GCT1505GAG (5981C>A 5982T>G), GTT1506TTG (5983G>T 5985T>G), GGC1507CAT (5986G>C 5987G>A 5988C>T), AAC1508GGA (5989A>G 5990A>G 5991C>A), AAA1510AAG (5997A>G), ATC1511TAT (5998A>T 5999T>A 6000C>T), TAC1512TTT (6002A>T 6003C>T), AGC1513TCT (6004A>T 6005G>C 6006C>T), AAA1514AAG (6009A>G), TTC1515CTA (6010T>C 6012C>A), AAG1518AGA (6020A>G 6021G>A), GCT1519TCT (6022G>T), TTC1521TAC (6029T>A), GTC1524ATA (6037G>A 6039C>A), CTA1525AGA (6040C>A 6041T>G), GAA1527TTT (6046G>T 6047A>T 6048A>T), TCC1529TCT (6054C>T), TCT1530AAT (6055T>A 6056C>A), ATC1531ATA (6060C>A), TGG1533AAA (6064T>A 6065G>A 6066G>A), ACA1534ACG (6069A>G), GCG1535ACA (6070G>A 6072G>A), ATC1537AGA (6077T>G 6078C>A), ACG1538ACC (6081G>C), CCC1539CAT (6083C>A 6084C>T), GTA1540CAG (6085G>C 6086T>A 6087A>G), GGA1541GGG (6090A>G), TTC1542CAC (6091T>C 6092T>A), GAA1544GAG (6099A>G), TGG1545TTC (6101G>T 6102G>C), TTG1546TTA (6105G>A), GTA1547GTG (6108A>G), CCT1549CCC (6114T>C), TTC1550TTT (6117C>T), GGT1551GGC (6120T>C), ATT1552CTT (6121A>C), GCT1553ACC (6124G>A 6126T>C), AAC1554AAT (6129C>T), GCT1555GCC (6132T>C), CCA1556CCC (6135A>C), GCG1557TCC (6136G>T 6138G>C), GTT1558ACA (6139G>A 6140T>C 6141T>A), TTC1559TTT (6144C>T), AGG1561AGC (6150G>C), AAA1562CTC (6151A>C 6152A>T 6153A>C), GAT1564AAT (6157G>A), AAC1565CAA (6160A>C 6162C>A), TGC1566ATA (6163T>A 6164G>T 6165C>A), TTC1567_CAC1568insAATGCT (6168_6169insAATGCT), CAC1568CAT (6171C>T), AAG1569del (6172_6174delAAG), CTG1570CTT (6177G>T), GAG1572AAA (6181G>A 6183G>A), TTC1573TTT (6186C>T), GTC1574ATC (6187G>A), GCG1575CTG (6190G>C 6191C>T), TAT1577TTC (6197A>T 6198T>C), ATC1578TTT (6199A>T 6201C>T), GAC1579GAT (6204C>T), GAC1580GAT (6207C>T), ATC1581ATA (6210C>A), CTC1582CTT (6213C>T), GTC1583ATA (6214G>A 6216C>A), TTC1584TAC (6218T>A), AGC1585AAC (6221G>A), AAT1586AGC (6224A>G 6225T>C), TCC1587AAT (6226T>A 6227C>A 6228C>T), CTG1588TGG (6229C>T 6230T>G), CAA1589TCT (6232C>T 6233A>C 6234A>T), GAA1590GAT (6237A>T), GAG1592CTC (6241G>C 6242A>T 6243G>C), AGC1593TCA (6244A>T 6245G>C 6246C>A), TTA1595CTG (6250T>C 6252A>G), CGG1596CAA (6254G>A 6255G>A), ATG1598ACT (6260T>C 6261G>T), CTG1599TTC (6262C>T 6264G>C), GAA1600CAA (6265G>C), GTT1601ATT (6268G>A), TGC1602CAT (6271T>C 6272G>A 6273C>T), AAG1604AAC (6279G>C), GGG1606GTT (6284G>T 6285G>T), TTG1607CTT (6286T>C 6288G>T), GTG1608TTT (6289G>T 6291G>T), CTG1609CTC (6294G>C), TCA1610AAG (6295T>A 6296C>A 6297A>G), CCA1611AAA (6298C>A 6299C>A), ACA1612ACC (6303A>C), AAA1613AAG (6306A>G), ATG1614TGT (6307A>T 6308T>G 6309G>T), AAG1615GCT (6310A>G 6311A>C 6312G>T), GTT1616TTT (6313G>T), GCT1617GGG (6317C>G 6318T>G), ACA1619ACT (6324A>T), ACA1620CAG (6325A>C 6326C>A 6327A>G), GTA1621ATT (6328G>A 6330A>T), GAA1622GAC (6333A>C), TTC1623TAC (6335T>A), GGG1625GGT (6342G>T)								

Proteins

	Begin	End	Coverage	Score	Concordance	Matches	Identities	I/D/M/F*	Stop Codons
polyprotein (YP_006495799.1)	1479	1625	7.7%	494	48.6%	146 (98.0%)	68 (45.6%)	2/1/0/0	0
Protein mutations:	V1479C (5902G>T 5903T>G 5904C>T), F1480V (5905T>G 5907C>A), A1484E (5918C>A 5919T>A), N1487T (5927A>C), H1490V (5935C>G 5936A>T), Q1493K (5944C>A), Y1494F (5948A>T 5949C>T), T1495P (5950A>C), L1496I (5953T>A 5955G>T), G1498V (5960G>T 5961C>T), N1500K (5967C>G), S1501E (5968T>G 5969C>A 5970G>A), I1502L (5971A>T 5973C>A), V1503L (5974G>T), A1504D (5978C>A 5979A>T), A1505E (5981C>A 5982T>G), V1506L (5983G>T 5985T>G), G1507H (5986G>C 5987G>A 5988C>T), N1508G (5989A>G 5990A>G 5991C>A), I1511Y (5998A>T 5999T>A 6000C>T), Y1512F (6002A>T 6003C>T), F1515L (6010T>C 6012C>A), K1518R (6020A>G 6021G>A), A1519S (6022G>T), F1521Y (6029T>A), V1524I (6037G>A 6039C>A), L1525R (6040C>A 6041T>G), E1527F (6046G>T 6047A>T 6048A>T), S1530N (6055T>A 6056C>A), W1533K (6064T>A 6065G>A 6066G>A), A1535T (6070G>A 6072G>A), I1537R (6077T>G 6078C>A), P1539H (6083C>A 6084C>T), V1540Q (6085G>C 6086T>A 6087A>G), F1542H (6091T>C 6092T>A), W1545F (6101G>T 6102G>C), I1552L (6121A>C), A1553T (6124G>A 6126T>C), A1557S (6136G>T 6138G>C), V1558T (6139G>A 6140T>C 6141T>A), R1561S (6150G>C), K1562L (6151A>C 6152A>T 6153A>C), D1564N (6157G>A), N1565Q (6160A>C 6162C>A), C1566I (6163T>A 6164G>T 6165C>A), F1567_H1568insNA (6168_6169insAATGCT), K1569del (6172_6174delAAG), E1572K (6181G>A 6183G>A), V1574I (6187G>A), A1575L (6190G>C 6191C>T), Y1577F (6197A>T 6198T>C), I1578F (6199A>T 6201C>T), V1583I (6214G>A 6216C>A), F1584Y (6218T>A), S1585N (6221G>A), N1586S (6224A>G 6225T>C), S1587N (6226T>A 6227C>A 6228C>T), L1588W (6229C>T 6230T>G), Q1589S (6232C>T 6233A>C 6234A>T), E1590D (6237A>T), E1592L (6241G>C 6242A>T 6243G>C), R1596Q (6254G>A 6255G>A), M1598T (6260T>C 6261G>T), L1599F (6262C>T 6264G>C), E1600Q (6265G>C), V1601I (6268G>A), C1602H (6271T>C 6272G>A 6273C>T), K1604N (6279G>C), G1606V (6284G>T 6285G>T), V1608F (6289G>T 6291G>T), S1610K (6295T>A 6296C>A 6297A>G), P1611K (6298C>A 6299C>A), M1614C (6307A>T 6308T>G 6309G>T), K1615A (6310A>G 6311A>C 6312G>T), V1616F (6313G>T), A1617G (6317C>G 6318T>G), T1620Q (6325A>C 6326C>A 6327A>G), V1621I (6328G>A 6330A>T), E1622D (6333A>C), F1623Y (6335T>A)								

	Begin	End	Coverage	Score	Concordance	Matches	Identities	I/D/M/F*	Stop Codons
**NT**	**5902**	**6342**	**5.8%**	**37**	**4.3%**	**438 (98.0%)**	**235 (52.6%)**	**6/3**	
Codon mutations:	GTC1479TGT (5902G>T 5903T>G 5904C>T), TTC1480GTA (5905T>G 5907C>A), TAT1482TAC (5913T>C), CGC1483AGG (5914C>A 5916C>G), GCT1484GAA (5918C>A 5919T>A), CTG1485TTA (5920C>T 5922G>A), AAC1487ACC (5927A>C), ACG1489ACT (5934G>T), CAC1490GTC (5935C>G 5936A>T), GAC1492GAT (5943C>T), CAG1493AAG (5944C>A), TAC1494TTT (5948A>T 5949C>T), ACA1495CCA (5950A>C), TTG1496ATT (5953T>A 5955G>T), CCT1497CCA (5958T>A), GGC1498GTT (5960G>T 5961C>T), ATC1499ATT (5964C>T), AAC1500AAG (5967C>G), TCG1501GAA (5968T>G 5969C>A 5970G>A), ATC1502TTA (5971A>T 5973C>A), GTA1503TTA (5974G>T), GCA1504GAT (5978C>A 5979A>T), GCT1505GAG (5981C>A 5982T>G), GTT1506TTG (5983G>T 5985T>G), GGC1507CAT (5986G>C 5987G>A 5988C>T), AAC1508GGA (5989A>G 5990A>G 5991C>A), AAA1510AAG (5997A>G), ATC1511TAT (5998A>T 5999T>A 6000C>T), TAC1512TTT (6002A>T 6003C>T), AGC1513TCT (6004A>T 6005G>C 6006C>T), AAA1514AAG (6009A>G), TTC1515CTA (6010T>C 6012C>A), AAG1518AGA (6020A>G 6021G>A), GCT1519TCT (6022G>T), TTC1521TAC (6029T>A), GTC1524ATA (6037G>A 6039C>A), CTA1525AGA (6040C>A 6041T>G), GAA1527TTT (6046G>T 6047A>T 6048A>T), TCC1529TCT (6054C>T), TCT1530AAT (6055T>A 6056C>A), ATC1531ATA (6060C>A), TGG1533AAA (6064T>A 6065G>A 6066G>A), ACA1534ACG (6069A>G), GCG1535ACA (6070G>A 6072G>A), ATC1537AGA (6077T>G 6078C>A), ACG1538ACC (6081G>C), CCC1539CAT (6083C>A 6084C>T), GTA1540CAG (6085G>C 6086T>A 6087A>G), GGA1541GGG (6090A>G), TTC1542CAC (6091T>C 6092T>A), GAA1544GAG (6099A>G), TGG1545TTC (6101G>T 6102G>C), TTG1546TTA (6105G>A), GTA1547GTG (6108A>G), CCT1549CCC (6114T>C), TTC1550TTT (6117C>T), GGT1551GGC (6120T>C), ATT1552CTT (6121A>C), GCT1553ACC (6124G>A 6126T>C), AAC1554AAT (6129C>T), GCT1555GCC (6132T>C), CCA1556CCC (6135A>C), GCG1557TCC (6136G>T 6138G>C), GTT1558ACA (6139G>A 6140T>C 6141T>A), TTC1559TTT (6144C>T), AGG1561AGC (6150G>C), AAA1562CTC (6151A>C 6152A>T 6153A>C), GAT1564AAT (6157G>A), AAC1565CAA (6160A>C 6162C>A), TGC1566ATA (6163T>A 6164G>T 6165C>A), TTC1567_CAC1568insAATGCT (6168_6169insAATGCT), CAC1568CAT (6171C>T), AAG1569del (6172_6174delAAG), CTG1570CTT (6177G>T), GAG1572AAA (6181G>A 6183G>A), TTC1573TTT (6186C>T), GTC1574ATC (6187G>A), GCG1575CTG (6190G>C 6191C>T), TAT1577TTC (6197A>T 6198T>C), ATC1578TTT (6199A>T 6201C>T), GAC1579GAT (6204C>T), GAC1580GAT (6207C>T), ATC1581ATA (6210C>A), CTC1582CTT (6213C>T), GTC1583ATA (6214G>A 6216C>A), TTC1584TAC (6218T>A), AGC1585AAC (6221G>A), AAT1586AGC (6224A>G 6225T>C), TCC1587AAT (6226T>A 6227C>A 6228C>T), CTG1588TGG (6229C>T 6230T>G), CAA1589TCT (6232C>T 6233A>C 6234A>T), GAA1590GAT (6237A>T), GAG1592CTC (6241G>C 6242A>T 6243G>C), AGC1593TCA (6244A>T 6245G>C 6246C>A), TTA1595CTG (6250T>C 6252A>G), CGG1596CAA (6254G>A 6255G>A), ATG1598ACT (6260T>C 6261G>T), CTG1599TTC (6262C>T 6264G>C), GAA1600CAA (6265G>C), GTT1601ATT (6268G>A), TGC1602CAT (6271T>C 6272G>A 6273C>T), AAG1604AAC (6279G>C), GGG1606GTT (6284G>T 6285G>T), TTG1607CTT (6286T>C 6288G>T), GTG1608TTT (6289G>T 6291G>T), CTG1609CTC (6294G>C), TCA1610AAG (6295T>A 6296C>A 6297A>G), CCA1611AAA (6298C>A 6299C>A), ACA1612ACC (6303A>C), AAA1613AAG (6306A>G), ATG1614TGT (6307A>T 6308T>G 6309G>T), AAG1615GCT (6310A>G 6311A>C 6312G>T), GTT1616TTT (6313G>T), GCT1617GGG (6317C>G 6318T>G), ACA1619ACT (6324A>T), ACA1620CAG (6325A>C 6326C>A 6327A>G), GTA1621ATT (6328G>A 6330A>T), GAA1622GAC (6333A>C), TTC1623TAC (6335T>A), GGG1625GGT (6342G>T)								

*: Inserts / Deletes / Misaligned / Frameshifts

Analysis details

This analysis was performed with panviral2.64

NGS Details (UN59): Dioscorea bacilliform virus

Assembly

**Coverage Length**	278 (1 contig(s))
**Depth Of Coverage**	14.2
**Number Of Reads**	35
**Reads Per Million**	0.76 rpm (after QC)
**Ambiguities**	0
**Assembly Method**	de novo + reference guided assembly
**Consensus Caller**	Bcf Tools

Coverage Map

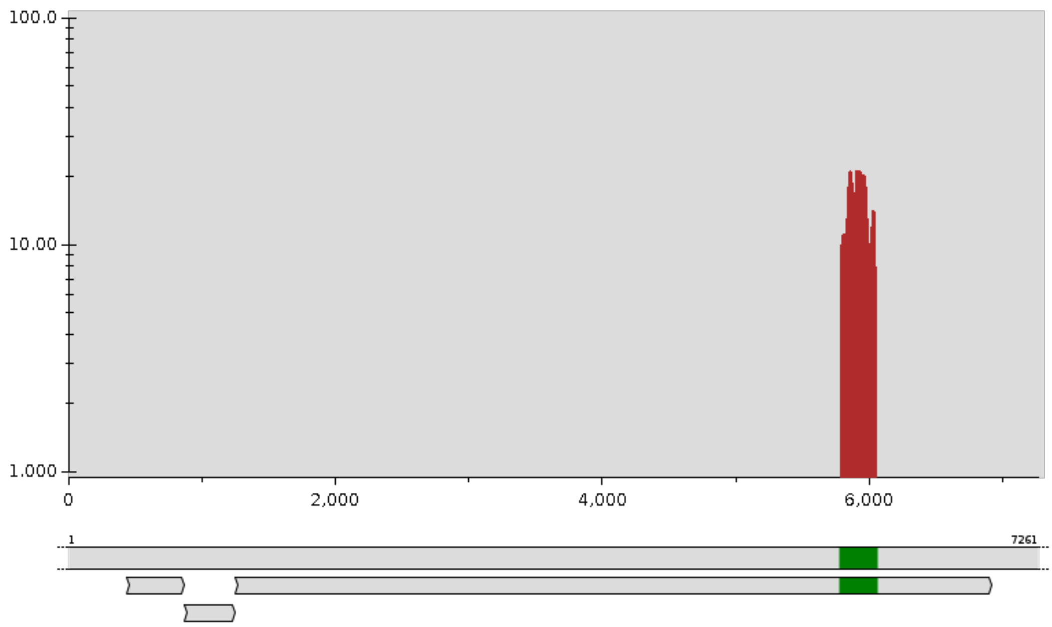

Assignment

**Type**	Dioscorea bacilliform virus (Taxonomy ID: 52996)
**Reference Genome**	NC_009010.1
**NT Identity (%)**	55.1601
**AA Identity (%)**	45.1613
**Number Of Stop Codons**	1
**Number Of CDS**	3

Alignment

**Alignment Score**	52.0 (NT) + 293.0 (AA) = 345.0
**Concordance (%)**	28.6545

Alignment Method	Global, seeded, nucleotide + amino acids (AGA)

Genome Region

Sequence starts at position 5783 and ends at position 6060 relative to NC_009010.1 reference sequence.

Alignment Detailed Statistics

	Begin	End	Coverage	Score	Concordance	Matches	Identities	I/D/M/F*	Stop Codons
**NT**	**5783**	**6060**	**3.8%**	**52**	**9.4%**	**278 (98.9%)**	**155 (55.2%)**	**3/0**	
Mutations:	5790G>A, 5793G>A, 5796G>C, 5800C>A, 5801T>G, 5803G>A, 5805G>T, 5807C>A, 5808A>T, 5809G>T, 5810G>T, 5811A>T, 5816T>A, 5817T>C, 5819A>T, 5820C>T, 5825G>A, 5826G>T, 5827T>G, 5832G>T, 5838A>C, 5841C>T, 5844A>C, 5848A>T, 5849A>C, 5850G>A, 5857C>A, 5859A>T, 5862A>C, 5863G>A, 5864T>C, 5865G>A, 5868C>T, 5871G>A, 5872A>G, 5874G>T, 5875A>C, 5876A>T, 5881G>A, 5884A>T, 5886C>T, 5892C>T, 5894G>A, 5895A>G, 5897G>C, 5898T>C, 5898_5899insTTT, 5899A>C, 5900C>T, 5902G>A, 5903A>G, 5904A>G, 5905G>A, 5908T>C, 5909T>A, 5911A>G, 5913T>A, 5914G>C, 5915C>T, 5919A>T, 5921A>T, 5923A>T, 5931T>C, 5937G>T, 5938G>A, 5940G>C, 5942T>A, 5943T>C, 5944T>A, 5945C>G, 5946T>C, 5947G>A, 5948A>G, 5952A>T, 5953G>C, 5954A>T, 5965T>C, 5970G>A, 5973T>C, 5974C>T, 5976G>A, 5980A>T, 5981T>C, 5982A>T, 5983C>G, 5985G>T, 5986C>T, 5989C>G, 5992A>G, 5994C>T, 5995T>A, 5996G>T, 5997T>G, 6003A>C, 6007G>C, 6008G>A, 6012T>A, 6013G>T, 6016C>G, 6017T>C, 6020G>A, 6021C>G, 6023C>A, 6024A>G, 6025A>T, 6027C>T, 6031A>T, 6032T>G, 6033G>C, 6034A>T, 6035A>T, 6036G>C, 6037A>T, 6039A>T, 6043A>G, 6044C>T, 6045T>G, 6047A>G, 6050C>G, 6051A>G, 6054C>T, 6057G>A, 6059T>A, 6060C>T								

CDS

	Begin	End	Coverage	Score	Concordance	Matches	Identities	I/D/M/F*	Stop Codons
DBV_gp3	1514	1605	4.9%	293	44.9%	92 (98.9%)	42 (45.2%)	1/0/0/0	1
Protein mutations:	W1515* (5790G>A), L1519R (5800C>A 5801T>G), A1520T (5803G>A 5805G>T), P1521H (5807C>A 5808A>T), G1522F (5809G>T 5810G>T 5811A>T), L1524H (5816T>A 5817T>C), Y1525F (5819A>T 5820C>T), W1527Y (5825G>A 5826G>T), L1528V (5827T>G), K1535S (5848A>T 5849A>C 5850G>A), P1538T (5857C>A 5859A>T), V1540T (5863G>A 5864T>C 5865G>A), R1543G (5872A>G 5874G>T), K1544L (5875A>C 5876A>T), D1546N (5881G>A), N1547Y (5884A>T 5886C>T), R1550Q (5894G>A 5895A>G), G1551A (5897G>C 5898T>C), G1551_T1552insF (5898_5899insTTT), T1552L (5899A>C 5900C>T), E1553R (5902G>A 5903A>G 5904A>G), E1554K (5905G>A), F1555H (5908T>C 5909T>A), I1556V (5911A>G 5913T>A), A1557L (5914G>C 5915C>T), Y1559F (5921A>T), I1560F (5923A>T), V1565I (5938G>A 5940G>C), F1566Y (5942T>A 5943T>C), E1568R (5947G>A 5948A>G), E1570L (5953G>C 5954A>T), I1579S (5980A>T 5981T>C 5982A>T), L1580V (5983C>G 5985G>T), L1581F (5986C>T), Q1582E (5989C>G), I1583V (5992A>G 5994C>T), C1584M (5995T>A 5996G>T 5997T>G), Q1586H (6003A>C), G1588Q (6007G>C 6008G>A), V1590F (6013G>T), L1591A (6016C>G 6017T>C), S1592K (6020G>A 6021C>G), P1593Q (6023C>A 6024A>G), T1594S (6025A>T 6027C>T), M1596C (6031A>T 6032T>G 6033G>C), K1597F (6034A>T 6035A>T 6036G>C), I1598F (6037A>T 6039A>T), T1600V (6043A>G 6044C>T 6045T>G), K1601R (6047A>G), T1602R (6050C>G 6051A>G), F1605Y (6059T>A 6060C>T)								
Codon mutations:	TGG1515TGA (5790G>A), ACG1516ACA (5793G>A), GCG1517GCC (5796G>C), CTA1519AGA (5800C>A 5801T>G), GCG1520ACT (5803G>A 5805G>T), CCA1521CAT (5807C>A 5808A>T), GGA1522TTT (5809G>T 5810G>T 5811A>T), CTT1524CAC (5816T>A 5817T>C), TAC1525TTT (5819A>T 5820C>T), TGG1527TAT (5825G>A 5826G>T), TTG1528GTG (5827T>G), GTG1529GTT (5832G>T), CCA1531CCC (5838A>C), TTC1532TTT (5841C>T), GGA1533GGC (5844A>C), AAG1535TCA (5848A>T 5849A>C 5850G>A), CCA1538ACT (5857C>A 5859A>T), GCA1539GCC (5862A>C), GTG1540ACA (5863G>A 5864T>C 5865G>A), TTC1541TTT (5868C>T), CAG1542CAA (5871G>A), AGG1543GGT (5872A>G 5874G>T), AAG1544CTG (5875A>C 5876A>T), GAC1546AAC (5881G>A), AAC1547TAT (5884A>T 5886C>T), TTC1549TTT (5892C>T), CGA1550CAG (5894G>A 5895A>G), GGT1551GCC (5897G>C 5898T>C), GGT1551_ACT1552insTTT (5898_5899insTTT), ACT1552CTT (5899A>C 5900C>T), GAA1553AGG (5902G>A 5903A>G 5904A>G), GAA1554AAA (5905G>A), TTT1555CAT (5908T>C 5909T>A), ATT1556GTA (5911A>G 5913T>A), GCT1557CTT (5914G>C 5915C>T), GTA1558GTT (5919A>T), TAC1559TTC (5921A>T), ATT1560TTT (5923A>T), GAT1562GAC (5931T>C), CTG1564CTT (5937G>T), GTG1565ATC (5938G>A 5940G>C), TTT1566TAC (5942T>A 5943T>C), TCT1567AGC (5944T>A 5945C>G 5946T>C), GAA1568AGA (5947G>A 5948A>G), ACA1569ACT (5952A>T), GAA1570CTA (5953G>C 5954A>T), TTG1574CTG (5965T>C), AAG1575AAA (5970G>A), CAT1576CAC (5973T>C), CTG1577TTA (5974C>T 5976G>A), ATA1579TCT (5980A>T 5981T>C 5982A>T), CTG1580GTT (5983C>G 5985G>T), CTT1581TTT (5986C>T), CAA1582GAA (5989C>G), ATC1583GTT (5992A>G 5994C>T), TGT1584ATG (5995T>A 5996G>T 5997T>G), CAA1586CAC (6003A>C), GGG1588CAG (6007G>C 6008G>A), CTT1589CTA (6012T>A), GTT1590TTT (6013G>T), CTT1591GCT (6016C>G 6017T>C), AGC1592AAG (6020G>A 6021C>G), CCA1593CAG (6023C>A 6024A>G), ACC1594TCT (6025A>T 6027C>T), ATG1596TGC (6031A>T 6032T>G 6033G>C), AAG1597TTC (6034A>T 6035A>T 6036G>C), ATA1598TTT (6037A>T 6039A>T), ACT1600GTG (6043A>G 6044C>T 6045T>G), AAA1601AGA (6047A>G), ACA1602AGG (6050C>G 6051A>G), ATC1603ATT (6054C>T), GAG1604GAA (6057G>A), TTC1605TAT (6059T>A 6060C>T)								

Proteins

	Begin	End	Coverage	Score	Concordance	Matches	Identities	I/D/M/F*	Stop Codons
ORF3 protein (YP_001036293.1)	1514	1605	4.9%	293	44.9%	92 (98.9%)	42 (45.2%)	1/0/0/0	1
Protein mutations:	W1515* (5790G>A), L1519R (5800C>A 5801T>G), A1520T (5803G>A 5805G>T), P1521H (5807C>A 5808A>T), G1522F (5809G>T 5810G>T 5811A>T), L1524H (5816T>A 5817T>C), Y1525F (5819A>T 5820C>T), W1527Y (5825G>A 5826G>T), L1528V (5827T>G), K1535S (5848A>T 5849A>C 5850G>A), P1538T (5857C>A 5859A>T), V1540T (5863G>A 5864T>C 5865G>A), R1543G (5872A>G 5874G>T), K1544L (5875A>C 5876A>T), D1546N (5881G>A), N1547Y (5884A>T 5886C>T), R1550Q (5894G>A 5895A>G), G1551A (5897G>C 5898T>C), G1551_T1552insF (5898_5899insTTT), T1552L (5899A>C 5900C>T), E1553R (5902G>A 5903A>G 5904A>G), E1554K (5905G>A), F1555H (5908T>C 5909T>A), I1556V (5911A>G 5913T>A), A1557L (5914G>C 5915C>T), Y1559F (5921A>T), I1560F (5923A>T), V1565I (5938G>A 5940G>C), F1566Y (5942T>A 5943T>C), E1568R (5947G>A 5948A>G), E1570L (5953G>C 5954A>T), I1579S (5980A>T 5981T>C 5982A>T), L1580V (5983C>G 5985G>T), L1581F (5986C>T), Q1582E (5989C>G), I1583V (5992A>G 5994C>T), C1584M (5995T>A 5996G>T 5997T>G), Q1586H (6003A>C), G1588Q (6007G>C 6008G>A), V1590F (6013G>T), L1591A (6016C>G 6017T>C), S1592K (6020G>A 6021C>G), P1593Q (6023C>A 6024A>G), T1594S (6025A>T 6027C>T), M1596C (6031A>T 6032T>G 6033G>C), K1597F (6034A>T 6035A>T 6036G>C), I1598F (6037A>T 6039A>T), T1600V (6043A>G 6044C>T 6045T>G), K1601R (6047A>G), T1602R (6050C>G 6051A>G), F1605Y (6059T>A 6060C>T)								
Codon mutations:	TGG1515TGA (5790G>A), ACG1516ACA (5793G>A), GCG1517GCC (5796G>C), CTA1519AGA (5800C>A 5801T>G), GCG1520ACT (5803G>A 5805G>T), CCA1521CAT (5807C>A 5808A>T), GGA1522TTT (5809G>T 5810G>T 5811A>T), CTT1524CAC (5816T>A 5817T>C), TAC1525TTT (5819A>T 5820C>T), TGG1527TAT (5825G>A 5826G>T), TTG1528GTG (5827T>G), GTG1529GTT (5832G>T), CCA1531CCC (5838A>C), TTC1532TTT (5841C>T), GGA1533GGC (5844A>C), AAG1535TCA (5848A>T 5849A>C 5850G>A), CCA1538ACT (5857C>A 5859A>T), GCA1539GCC (5862A>C), GTG1540ACA (5863G>A 5864T>C 5865G>A), TTC1541TTT (5868C>T), CAG1542CAA (5871G>A), AGG1543GGT (5872A>G 5874G>T), AAG1544CTG (5875A>C 5876A>T), GAC1546AAC (5881G>A), AAC1547TAT (5884A>T 5886C>T), TTC1549TTT (5892C>T), CGA1550CAG (5894G>A 5895A>G), GGT1551GCC (5897G>C 5898T>C), GGT1551_ACT1552insTTT (5898_5899insTTT), ACT1552CTT (5899A>C 5900C>T), GAA1553AGG (5902G>A 5903A>G 5904A>G), GAA1554AAA (5905G>A), TTT1555CAT (5908T>C 5909T>A), ATT1556GTA (5911A>G 5913T>A), GCT1557CTT (5914G>C 5915C>T), GTA1558GTT (5919A>T), TAC1559TTC (5921A>T), ATT1560TTT (5923A>T), GAT1562GAC (5931T>C), CTG1564CTT (5937G>T), GTG1565ATC (5938G>A 5940G>C), TTT1566TAC (5942T>A 5943T>C), TCT1567AGC (5944T>A 5945C>G 5946T>C), GAA1568AGA (5947G>A 5948A>G), ACA1569ACT (5952A>T), GAA1570CTA (5953G>C 5954A>T), TTG1574CTG (5965T>C), AAG1575AAA (5970G>A), CAT1576CAC (5973T>C), CTG1577TTA (5974C>T 5976G>A), ATA1579TCT (5980A>T 5981T>C 5982A>T), CTG1580GTT (5983C>G 5985G>T), CTT1581TTT (5986C>T), CAA1582GAA (5989C>G), ATC1583GTT (5992A>G 5994C>T), TGT1584ATG (5995T>A 5996G>T 5997T>G), CAA1586CAC (6003A>C), GGG1588CAG (6007G>C 6008G>A), CTT1589CTA (6012T>A), GTT1590TTT (6013G>T), CTT1591GCT (6016C>G 6017T>C), AGC1592AAG (6020G>A 6021C>G), CCA1593CAG (6023C>A 6024A>G), ACC1594TCT (6025A>T 6027C>T), ATG1596TGC (6031A>T 6032T>G 6033G>C), AAG1597TTC (6034A>T 6035A>T 6036G>C), ATA1598TTT (6037A>T 6039A>T), ACT1600GTG (6043A>G 6044C>T 6045T>G), AAA1601AGA (6047A>G), ACA1602AGG (6050C>G 6051A>G), ATC1603ATT (6054C>T), GAG1604GAA (6057G>A), TTC1605TAT (6059T>A 6060C>T)								

*: Inserts / Deletes / Misaligned / Frameshifts

Analysis details

This analysis was performed with panviral2.64

NGS Details (UN59): Errantivirus

Assembly

**Coverage Length**	498 (10 contig(s))
**Depth Of Coverage**	8.5
**Number Of Reads**	32
**Reads Per Million**	0.70 rpm (after QC)
**Ambiguities**	0
**Assembly Method**	de novo + reference guided assembly
**Consensus Caller**	Bcf Tools

Coverage Map

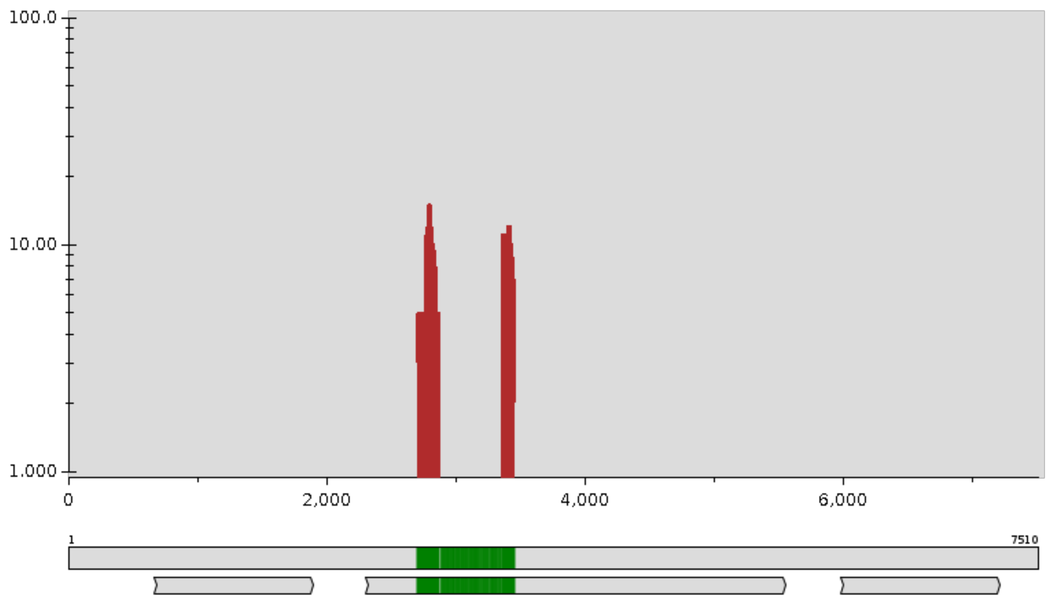

Assignment

**Type**	Errantivirus (Taxonomy ID: 186666)
**Reference Genome**	NC_038512.1
**NT Identity (%)**	31.4168
**AA Identity (%)**	30.3279
**Number Of Stop Codons**	1
**Number Of CDS**	3

Alignment

**Alignment Score**	-391.0 (NT) + -161.0 (AA) = -552.0
**Concordance (%)**	-36.7318

Alignment Method	Global, seeded, nucleotide + amino acids (AGA)

Genome Region

Sequence starts at position 2698 and ends at position 3462 relative to NC_038512.1 reference sequence.

Alignment Detailed Statistics

	Begin	End	Coverage	Score	Concordance	Matches	Identities	I/D/M/F*	Stop Codons
**NT**	**2698**	**3462**	**6.6%**	**-391**	**-41.1%**	**486 (97.4%)**	**153 (30.7%)**	**1/12**	

Mutations: 2699A>T, 2700G>T, 2701A>G, 2702A>C, 2703C>A, 2705A>T, 2709A>G, 2710C>T, 2712A>T, 2713A>G, 2715T>A, 2716A>G, 2719C>T, 2723T>A, 2725C>T, 2727C>A, 2728C>G, 2730A>G, 2732A>C, 2733G>C, 2737C>T, 2738C>A, 2740G>A, 2743C>T, 2745C>T, 2746C>T, 2748T>C, 2750A>G, 2753C>A, 2755T>A, 2756C>A, 2757G>A, 2758C>A, 2759C>G, 2761G>T, 2763A>T, 2764A>T, 2765G>A, 2767T>G, 2769G>A, 2773C>G, 2777A>C, 2779C>G, 2781C>A, 2782G>A, 2783A>G, 2785A>T, 2786A>T, 2788G>A, 2791G>A, 2794C>G, 2795C>A, 2806A>T, 2807A>C, 2809A>G, 2812A>T, 2813T>A, 2814C>G, 2816G>A, 2818C>A, 2821T>C, 2822G>C, 2824A>G, 2828A>G, 2830C>G, 2830_2831insT, 2831T>G, 2833A>T, 2836C>G, 2837A>G, 2839A>G, 2841G>T, 2842G>A, 2843G>T, 2849C>A, 2850C>G, 2851C>A, 2857A>G, 2859T>A, 2860C>A, 2863C>T, 2865C>G, 2869T>C, 2870_2881delGGGAAACAAAAG, 2885C>A, 2887T>G, 2888C>A, 2890C>G, 2891G>T, 2892T>G, 2893A>T, 2896T>A, 2901T>A, 2903C>A, 2905T>G, 2906A>G, 2908G>T, 2915G>A, 2919A>T, 2920G>C, 2924A>G, 2926C>G, 2927G>A, 2929T>G, 2932C>T, 2935A>G, 2937A>T, 2938C>T, 2941G>A, 2944A>T, 2949A>T, 2950C>T, 2953A>T, 2954A>G, 2955G>A, 2959C>G, 2960G>T, 2963C>T, 2968C>T, 2969A>G, 2974A>G, 2975G>A, 2976G>A, 2978A>G, 2979A>G, 2980G>T, 2981T>G, 2982G>C, 2983C>T, 2984C>G, 2985A>T, 2986A>T, 2987T>A, 2988A>T, 2989C>T, 2993A>T, 2995C>A, 2997C>A, 2998C>G, 2999T>C, 3001A>T, 3008G>A, 3009C>G, 3011A>T, 3012G>C, 3016G>C, 3018T>A, 3020T>C, 3025G>A, 3026G>A, 3028G>T, 3029G>A, 3030A>G, 3035G>A, 3037C>G, 3038C>G, 3039C>A, 3040T>G, 3041C>G, 3043A>G, 3047A>G, 3050T>C, 3052G>C, 3058C>T, 3061G>T, 3066A>G, 3067C>G, 3068G>A, 3069T>C, 3071G>C, 3073A>C, 3074C>T, 3076C>T, 3079G>C, 3084T>A, 3092C>T, 3094T>A, 3095C>G, 3096G>T, 3097A>C, 3104A>T, 3106G>T, 3109A>G, 3110T>C, 3112A>C, 3114A>C, 3115A>T, 3118C>T, 3119T>G, 3124A>G, 3127T>C, 3130T>A, 3137A>G, 3139A>G, 3140G>C, 3142T>G, 3146G>A, 3148C>T, 3149A>C, 3154C>G, 3155C>T, 3157A>C, 3159G>A, 3161G>C, 3162G>C, 3164C>T, 3165T>A, 3166C>T, 3168A>T, 3169A>C, 3170A>T, 3171A>G, 3172T>A, 3176A>T, 3178C>T, 3179T>G, 3180G>T, 3181T>A, 3182C>T, 3184C>G, 3189A>T, 3190C>T, 3191C>T, 3196C>T, 3202T>C, 3203A>C, 3205T>G, 3206G>A, 3208C>G, 3215A>T, 3218T>A, 3219C>A, 3220C>T, 3221C>T, 3223A>G, 3224C>G, 3226G>A, 3227G>A, 3232C>T, 3233C>T, 3236G>T, 3237A>T, 3239A>C, 3241C>T, 3242C>T, 3245G>A, 3246A>G, 3248C>A, 3261G>C, 3268A>T, 3271A>C, 3273G>A, 3274T>C, 3277C>A, 3280C>G, 3281A>T, 3283A>T, 3284A>G, 3286T>C, 3287C>A, 3289A>G, 3292G>A, 3293G>A, 3294A>G, 3300C>G, 3301C>T, 3303A>C, 3304A>T, 3307C>T, 3308T>G, 3309T>G, 3311A>T, 3313G>A, 3317G>A, 3318A>G, 3319A>G, 3320A>G, 3321C>T, 3322T>G, 3327A>T, 3331T>A, 3338A>T, 3340C>G, 3343A>T, 3345G>C, 3346C>T, 3347A>T, 3353G>A, 3354G>T, 3359A>C, 3360A>G, 3361G>A, 3362C>G, 3363C>T, 3364T>G, 3365A>G, 3367C>T, 3368C>A, 3371G>C, 3373T>A, 3380T>G, 3381C>A, 3382C>G, 3385T>A, 3386A>G, 3388T>C, 3389C>A, 3394A>C, 3396A>G, 3397T>G, 3399T>C, 3400G>C, 3402T>A, 3403T>A, 3406A>C, 3408A>C, 3409G>C, 3410A>T, 3412C>T, 3415T>A, 3417A>C, 3418G>T, 3426A>G, 3428C>A, 3429A>G, 3430A>T, 3433T>C, 3436A>G, 3439C>T, 3440C>T, 3442T>G, 3443C>G, 3444T>C, 3445C>T, 3448T>G

CDS

	Begin	End	Coverage	Score	Concordance	Matches	Identities	I/D/M/F*	Stop Codons
D1R33_gp2	132	386	11.5%	-161	-10.8%	121 (96.0%)	37 (29.4%)	1/4/14/1	1

Protein mutations: R132L (2699A>T 2700G>T 2701A>G), T133Q (2702A>C 2703C>A), T134S (2705A>T), D135G (2709A>G 2710C>T), E136V (2712A>T 2713A>G), V137E (2715T>A 2716A>G), Y140N (2723T>A 2725C>T), T141K (2727C>A 2728C>G), K142R (2730A>G), S143P (2732A>C 2733G>C), P147L (2745C>T 2746C>T), F148S (2748T>C), I149V (2750A>G), H150K (2753C>A 2755T>A), R151K (2756C>A 2757G>A 2758C>A), Q152D (2759C>G 2761G>T), E153V (2763A>T 2764A>T), V154M (2765G>A 2767T>G), R155K (2769G>A), D156E (2773C>G), I158L (2777A>C 2779C>G), T159K (2781C>A 2782G>A), K160D (2783A>G 2785A>T), M161L (2786A>T 2788G>A), D163E (2794C>G), Q164K (2795C>A), S170R (2813T>A 2814C>G), D171K (2816G>A 2818C>A), A173P (2822G>C 2824A>G), S175G (2828A>G 2830C>G), S175_S176insX (2830_2831insT), S176A (2831T>G 2833A>T), I178V (2837A>G 2839A>G), W179L (2841G>T 2842G>A), V180F (2843G>T), P182R (2849C>A 2850C>G 2851C>A), I185K (2859T>A 2860C>A), A187G (2865C>G), G189_K192del (2870_2881delGGGAAACAAAAG), V196C (2891G>T 2892T>G 2893A>T), K225G (2978A>G 2979A>G 2980G>T), C226A (2981T>G 2982G>C 2983C>T), Q227V (2984C>G 2985A>T 2986A>T), Y228I (2987T>A 2988A>T 2989C>T), P245E (3038C>G 3039C>A 3040T>G), R264V (3095C>G 3096G>T 3097A>C), L287Y (3164C>T 3165T>A 3166C>T), N289* (3170A>T 3171A>G 3172T>A), C292V (3179T>G 3180G>T 3181T>A), S305N (3218T>A 3219C>A 3220C>T), E338R (3317G>A 3318A>G 3319A>G), T339V (3320A>G 3321C>T 3322T>G), G350I (3353G>A 3354G>T), K352R (3359A>C 3360A>G 3361G>A), P353V (3362C>G 3363C>T 3364T>G), N354D (3365A>G 3367C>T), P355T (3368C>A), D356Q (3371G>C 3373T>A), S359E (3380T>G 3381C>A 3382C>G), I361V (3386A>G 3388T>C), Q362K (3389C>A), K363N (3394A>C), Y364W (3396A>G 3397T>G), L365P (3399T>C 3400G>C), I366K (3402T>A 3403T>A), K368T (3408A>C 3409G>C), T369S (3410A>T 3412C>T), K371T (3417A>C 3418G>T), K374R (3426A>G), Q375S (3428C>A 3429A>G 3430A>T), L380A (3443C>G 3444T>C 3445C>T)

Codon mutations: AGA132TTG (2699A>T 2700G>T 2701A>G), ACA133CAA (2702A>C 2703C>A), ACG134TCG (2705A>T), GAC135GGT (2709A>G 2710C>T), GAA136GTG (2712A>T 2713A>G), GTA137GAG (2715T>A 2716A>G), CCC138CCT (2719C>T), TAC140AAT (2723T>A 2725C>T), ACC141AAG (2727C>A 2728C>G), AAA142AGA (2730A>G), AGT143CCT (2732A>C 2733G>C), TAC144TAT (2737C>T), CGG145AGA (2738C>A 2740G>A), TAC146TAT (2743C>T), CCC147CTT (2745C>T 2746C>T), TTC148TCC (2748T>C), ATC149GTC (2750A>G), CAT150AAA (2753C>A 2755T>A), CGC151AAA (2756C>A 2757G>A 2758C>A), CAG152GAT (2759C>G 2761G>T), GAA153GTT (2763A>T 2764A>T), GTT154ATG (2765G>A 2767T>G), AGG155AAG (2769G>A), GAC156GAG (2773C>G), ATC158CTG (2777A>C 2779C>G), ACG159AAA (2781C>A 2782G>A), AAA160GAT (2783A>G 2785A>T), ATG161TTA (2786A>T 2788G>A), TTG162TTA (2791G>A), GAC163GAG (2794C>G), CAA164AAA (2795C>A), ATA167ATT (2806A>T), AGA168CGG (2807A>C 2809A>G), CCA169CCT (2812A>T), TCA170AGA (2813T>A 2814C>G), GAC171AAA (2816G>A 2818C>A), TCT172TCC (2821T>C), GCA173CCG (2822G>C 2824A>G), AGC175GGG (2828A>G 2830C>G), AGC175_TCA176insT-- (2830_2831insT), TCA176GCT (2831T>G 2833A>T), CCC177CCG (2836C>G), ATA178GTG (2837A>G 2839A>G), TGG179TTA (2841G>T 2842G>A), GTT180TTT (2843G>T), CCC182AGA (2849C>A 2850C>G 2851C>A), AAA184AAG (2857A>G), ATC185AAA (2859T>A 2860C>A), GAC186GAT (2863C>T), GCT187GGT (2865C>G), TCT188TCC (2869T>C), GGG189_AAG192del (2870_2881delGGGAAACAAAAG), CGT194A.G (2885C>A 2887T>G), CTC195A.G (2888C>A 2890C>G), GTA196TGT (2891G>T 2892T>G 2893A>T), GTT197..A (2896T>A), TTC199.A. (2901T>A), CGT200A.G (2903C>A 2905T>G), AAG201G.T (2906A>G 2908G>T), GAG204A.. (2915G>A), AAG205.TC (2919A>T 2920G>C), ATC207G.G (2924A>G 2926C>G), GAT208A.G (2927G>A 2929T>G), GAC209..T (2932C>T), AAA210..G (2935A>G), TAC211.TT (2937A>T 2938C>T), CCG212..A (2941G>A), ATA213..T (2944A>T), AAC215.TT (2949A>T 2950C>T), ATA216..T (2953A>T), AGT217GA. (2954A>G 2955G>A), GAC218..G (2959C>G), GTA219T.. (2960G>T), CTT220T.. (2963C>T), GAC221..T (2968C>T), AAG222G.. (2969A>G), TTA223..G (2974A>G), GGT224AA. (2975G>A 2976G>A), AAG225GGT (2978A>G 2979A>G 2980G>T), TGC226GCT (2981T>G 2982G>C 2983C>T), CAA227GTT (2984C>G 2985A>T 2986A>T), TAC228ATT (2987T>A 2988A>T 2989C>T), ACC230T.A (2993A>T 2995C>A), ACC231.AG (2997C>A 2998C>G), TTA232C.T (2999T>C 3001A>T), GCA235AG. (3008G>A 3009C>G), AGT236TC. (3011A>T 3012G>C), GGG237..C (3016G>C), TTT238.A. (3018T>A), TAT239C.. (3020T>C), CAG240..A (3025G>A), GTG241A.T (3026G>A 3028G>T), GAG242AG. (3029G>A 3030A>G), GAC244A.G (3035G>A 3037C>G), CCT245GAG (3038C>G 3039C>A 3040T>G), CAA246G.G (3041C>G 3043A>G), ATA248G.. (3047A>G), TCG249C.C (3050T>C 3052G>C), ACC251..T (3058C>T), GCG252..T (3061G>T), AAC254.GG (3066A>G 3067C>G), GTA255AC. (3068G>A 3069T>C), GAA256C.C (3071G>C 3073A>C), CAC257T.T (3074C>T 3076C>T), GGG258..C (3079G>C), TTT260.A. (3084T>A), CTT263T.A (3092C>T 3094T>A), CGA264GTC (3095C>G 3096G>T 3097A>C), ATG267T.T (3104A>T 3106G>T), GGA268..G (3109A>G), TTA269C.C (3110T>C 3112A>C), AAA270.CT (3114A>C 3115A>T), AAC271..T (3118C>T), TCA272G.. (3119T>G), CCA273..G (3124A>G), TCT274..C (3127T>C), ACT275..A (3130T>A), AGA278G.G (3137A>G 3139A>G), GTT279C.G (3140G>C 3142T>G), GAC281A.T (3146G>A 3148C>T), AAT282C.. (3149A>C), GTC283..G (3154C>G), CTA284T.C (3155C>T 3157A>C), AGA285.A. (3159G>A), GGT286CC. (3161G>C 3162G>C), CTC287TAT (3164C>T 3165T>A 3166C>T), CAA288.TC (3168A>T 3169A>C), AAT289TGA (3170A>T 3171A>G 3172T>A), ATC291T.T (3176A>T 3178C>T), TGT292GTA (3179T>G 3180G>T 3181T>A), CTC293T.G (3182C>T 3184C>G), TAC295.TT (3189A>T 3190C>T), CTT296T.. (3191C>T), GAC297..T (3196C>T), ATT299..C (3202T>C), ATT300C.G (3203A>C 3205T>G), GTC301A.G (3206G>A 3208C>G), ACT304T.. (3215A>T), TCC305AAT (3218T>A 3219C>A 3220C>T), CTA306T.G (3221C>T 3223A>G), CAG307G.A (3224C>G 3226G>A), GAA308A.. (3227G>A), CAC309..T (3232C>T), CTG310T.. (3233C>T), GAG311TT. (3236G>T 3237A>T), AAC312C.T (3239A>C 3241C>T), CTG313T.. (3242C>T), GAA314AG. (3245G>A 3246A>G), CGA315A.. (3248C>A), AGA319.C. (3261G>C), AGA321..T (3268A>T), GAA322..C (3271A>C), AGT323.AC (3273G>A 3274T>C), AAC324..A (3277C>A), TTC325..G (3280C>G), AAA326T.T (3281A>T 3283A>T), ATT327G.C (3284A>G 3286T>C), CAA328A.G (3287C>A 3289A>G), ATG329..A (3292G>A), GAC330AG. (3293G>A 3294A>G), TCC332.GT (3300C>G 3301C>T), GAA333.CT (3303A>C 3304A>T), TTC334..T (3307C>T), TTG335GG. (3308T>G 3309T>G), AAG336T.A (3311A>T 3313G>A), GAA338AGG (3317G>A 3318A>G 3319A>G), ACT339GTG (3320A>G 3321C>T 3322T>G), TAT341.T. (3327A>T), CTT342..A (3331T>A), ATC345T.G (3338A>T 3340C>G), ATA346..T (3343A>T), AGC347.CT (3345G>C 3346C>T), AGG348T.. (3347A>T), GGT350ATT (3353G>A 3354G>T), AAG352CGA (3359A>C 3360A>G 3361G>A), CCT353GTG (3362C>G 3363C>T 3364T>G), AAC354GAT (3365A>G 3367C>T), CCT355ACT (3368C>A), GAT356CAA (3371G>C 3373T>A), TCC359GAG (3380T>G 3381C>A 3382C>G), GCT360GCA (3385T>A), ATT361GTC (3386A>G 3388T>C), CAA362AAA (3389C>A), AAA363AAC (3394A>C), TAT364TGG (3396A>G 3397T>G), CTG365CCC (3399T>C 3400G>C), ATT366AAA (3402T>A 3403T>A), CCA367CCC (3406A>C), AAG368ACC (3408A>C 3409G>C), ACC369TCT (3410A>T 3412C>T), CCT370CCA (3415T>A), AAG371ACT (3417A>C 3418G>T), AAA374AGA (3426A>G), CAA375AGT (3428C>A 3429A>G 3430A>T), TTT376TTC (3433T>C), TTA377TTG (3436A>G), GGC378GGT (3439C>T), CTT379TTG (3440C>T 3442T>G), CTC380GCT (3443C>G 3444T>C 3445C>T), GGT381GGG (3448T>G)

Proteins

	Begin	End	Coverage	Score	Concordance	Matches	Identities	I/D/M/F*	Stop Codons
ORF B (YP_009507248.1)	132	386	11.5%	-161	-10.8%	121 (96.0%)	37 (29.4%)	1/4/14/1	1

Protein mutations: R132L (2699A>T 2700G>T 2701A>G), T133Q (2702A>C 2703C>A), T134S (2705A>T), D135G (2709A>G 2710C>T), E136V (2712A>T 2713A>G), V137E (2715T>A 2716A>G), Y140N (2723T>A 2725C>T), T141K (2727C>A 2728C>G), K142R (2730A>G), S143P (2732A>C 2733G>C), P147L (2745C>T 2746C>T), F148S (2748T>C), I149V (2750A>G), H150K (2753C>A 2755T>A), R151K (2756C>A 2757G>A 2758C>A), Q152D (2759C>G 2761G>T), E153V (2763A>T 2764A>T), V154M (2765G>A 2767T>G), R155K (2769G>A), D156E (2773C>G), I158L (2777A>C 2779C>G), T159K (2781C>A 2782G>A), K160D (2783A>G 2785A>T), M161L (2786A>T 2788G>A), D163E (2794C>G), Q164K (2795C>A), S170R (2813T>A 2814C>G), D171K (2816G>A 2818C>A), A173P (2822G>C 2824A>G), S175G (2828A>G 2830C>G), S175_S176insX (2830_2831insT), S176A (2831T>G 2833A>T), I178V (2837A>G 2839A>G), W179L (2841G>T 2842G>A), V180F (2843G>T), P182R (2849C>A 2850C>G 2851C>A), I185K (2859T>A 2860C>A), A187G (2865C>G), G189_K192del (2870_2881delGGGAAACAAAAG), V196C (2891G>T 2892T>G 2893A>T), K225G (2978A>G 2979A>G 2980G>T), C226A (2981T>G 2982G>C 2983C>T), Q227V (2984C>G 2985A>T 2986A>T), Y228I (2987T>A 2988A>T 2989C>T), P245E (3038C>G 3039C>A 3040T>G), R264V (3095C>G 3096G>T 3097A>C), L287Y (3164C>T 3165T>A 3166C>T), N289* (3170A>T 3171A>G 3172T>A), C292V (3179T>G 3180G>T 3181T>A), S305N (3218T>A 3219C>A 3220C>T), E338R (3317G>A 3318A>G 3319A>G), T339V (3320A>G 3321C>T 3322T>G), G350I (3353G>A 3354G>T), K352R (3359A>C 3360A>G 3361G>A), P353V (3362C>G 3363C>T 3364T>G), N354D (3365A>G 3367C>T), P355T (3368C>A), D356Q (3371G>C 3373T>A), S359E (3380T>G 3381C>A 3382C>G), I361V (3386A>G 3388T>C), Q362K (3389C>A), K363N (3394A>C), Y364W (3396A>G 3397T>G), L365P (3399T>C 3400G>C), I366K (3402T>A 3403T>A), K368T (3408A>C 3409G>C), T369S (3410A>T 3412C>T), K371T (3417A>C 3418G>T), K374R (3426A>G), Q375S (3428C>A 3429A>G 3430A>T), L380A (3443C>G 3444T>C 3445C>T)

	Begin	End	Coverage	Score	Concordance	Matches	Identities	I/D/M/F*	Stop Codons
**NT**	**2698**	**3462**	**6.6%**	**-391**	**-41.1%**	**486 (97.4%)**	**153 (30.7%)**	**1/12**	

Codon mutations: AGA132TTG (2699A>T 2700G>T 2701A>G), ACA133CAA (2702A>C 2703C>A), ACG134TCG (2705A>T), GAC135GGT (2709A>G 2710C>T), GAA136GTG (2712A>T 2713A>G), GTA137GAG (2715T>A 2716A>G), CCC138CCT (2719C>T), TAC140AAT (2723T>A 2725C>T), ACC141AAG (2727C>A 2728C>G), AAA142AGA (2730A>G), AGT143CCT (2732A>C 2733G>C), TAC144TAT (2737C>T), CGG145AGA (2738C>A 2740G>A), TAC146TAT (2743C>T), CCC147CTT (2745C>T 2746C>T), TTC148TCC (2748T>C), ATC149GTC (2750A>G), CAT150AAA (2753C>A 2755T>A), CGC151AAA (2756C>A 2757G>A 2758C>A), CAG152GAT (2759C>G 2761G>T), GAA153GTT (2763A>T 2764A>T), GTT154ATG (2765G>A 2767T>G), AGG155AAG (2769G>A), GAC156GAG (2773C>G), ATC158CTG (2777A>C 2779C>G), ACG159AAA (2781C>A 2782G>A), AAA160GAT (2783A>G 2785A>T), ATG161TTA (2786A>T 2788G>A), TTG162TTA (2791G>A), GAC163GAG (2794C>G), CAA164AAA (2795C>A), ATA167ATT (2806A>T), AGA168CGG (2807A>C 2809A>G), CCA169CCT (2812A>T), TCA170AGA (2813T>A 2814C>G), GAC171AAA (2816G>A 2818C>A), TCT172TCC (2821T>C), GCA173CCG (2822G>C 2824A>G), AGC175GGG (2828A>G 2830C>G), AGC175_TCA176insT-- (2830_2831insT), TCA176GCT (2831T>G 2833A>T), CCC177CCG (2836C>G), ATA178GTG (2837A>G 2839A>G), TGG179TTA (2841G>T 2842G>A), GTT180TTT (2843G>T), CCC182AGA (2849C>A 2850C>G 2851C>A), AAA184AAG (2857A>G), ATC185AAA (2859T>A 2860C>A), GAC186GAT (2863C>T), GCT187GGT (2865C>G), TCT188TCC (2869T>C), GGG189_AAG192del (2870_2881delGGGAAACAAAAG), CGT194A.G (2885C>A 2887T>G), CTC195A.G (2888C>A 2890C>G), GTA196TGT (2891G>T 2892T>G 2893A>T), GTT197..A (2896T>A), TTC199.A. (2901T>A), CGT200A.G (2903C>A 2905T>G), AAG201G.T (2906A>G 2908G>T), GAG204A.. (2915G>A), AAG205.TC (2919A>T 2920G>C), ATC207G.G (2924A>G 2926C>G), GAT208A.G (2927G>A 2929T>G), GAC209..T (2932C>T), AAA210..G (2935A>G), TAC211.TT (2937A>T 2938C>T), CCG212..A (2941G>A), ATA213..T (2944A>T), AAC215.TT (2949A>T 2950C>T), ATA216..T (2953A>T), AGT217GA. (2954A>G 2955G>A), GAC218..G (2959C>G), GTA219T.. (2960G>T), CTT220T.. (2963C>T), GAC221..T (2968C>T), AAG222G.. (2969A>G), TTA223..G (2974A>G), GGT224AA. (2975G>A 2976G>A), AAG225GGT (2978A>G 2979A>G 2980G>T), TGC226GCT (2981T>G 2982G>C 2983C>T), CAA227GTT (2984C>G 2985A>T 2986A>T), TAC228ATT (2987T>A 2988A>T 2989C>T), ACC230T.A (2993A>T 2995C>A), ACC231.AG (2997C>A 2998C>G), TTA232C.T (2999T>C 3001A>T), GCA235AG. (3008G>A 3009C>G), AGT236TC. (3011A>T 3012G>C), GGG237..C (3016G>C), TTT238.A. (3018T>A), TAT239C.. (3020T>C), CAG240..A (3025G>A), GTG241A.T (3026G>A 3028G>T), GAG242AG. (3029G>A 3030A>G), GAC244A.G (3035G>A 3037C>G), CCT245GAG (3038C>G 3039C>A 3040T>G), CAA246G.G (3041C>G 3043A>G), ATA248G.. (3047A>G), TCG249C.C (3050T>C 3052G>C), ACC251..T (3058C>T), GCG252..T (3061G>T), AAC254.GG (3066A>G 3067C>G), GTA255AC. (3068G>A 3069T>C), GAA256C.C (3071G>C 3073A>C), CAC257T.T (3074C>T 3076C>T), GGG258..C (3079G>C), TTT260.A. (3084T>A), CTT263T.A (3092C>T 3094T>A), CGA264GTC (3095C>G 3096G>T 3097A>C), ATG267T.T (3104A>T 3106G>T), GGA268..G (3109A>G), TTA269C.C (3110T>C 3112A>C), AAA270.CT (3114A>C 3115A>T), AAC271..T (3118C>T), TCA272G.. (3119T>G), CCA273..G (3124A>G), TCT274..C (3127T>C), ACT275..A (3130T>A), AGA278G.G (3137A>G 3139A>G), GTT279C.G (3140G>C 3142T>G), GAC281A.T (3146G>A 3148C>T), AAT282C.. (3149A>C), GTC283..G (3154C>G), CTA284T.C (3155C>T 3157A>C), AGA285.A. (3159G>A), GGT286CC. (3161G>C 3162G>C), CTC287TAT (3164C>T 3165T>A 3166C>T), CAA288.TC (3168A>T 3169A>C), AAT289TGA (3170A>T 3171A>G 3172T>A), ATC291T.T (3176A>T 3178C>T), TGT292GTA (3179T>G 3180G>T 3181T>A), CTC293T.G (3182C>T 3184C>G), TAC295.TT (3189A>T 3190C>T), CTT296T.. (3191C>T), GAC297..T (3196C>T), ATT299..C (3202T>C), ATT300C.G (3203A>C 3205T>G), GTC301A.G (3206G>A 3208C>G), ACT304T.. (3215A>T), TCC305AAT (3218T>A 3219C>A 3220C>T), CTA306T.G (3221C>T 3223A>G), CAG307G.A (3224C>G 3226G>A), GAA308A.. (3227G>A), CAC309..T (3232C>T), CTG310T.. (3233C>T), GAG311TT. (3236G>T 3237A>T), AAC312C.T (3239A>C 3241C>T), CTG313T.. (3242C>T), GAA314AG. (3245G>A 3246A>G), CGA315A.. (3248C>A), AGA319.C. (3261G>C), AGA321..T (3268A>T), GAA322..C (3271A>C), AGT323.AC (3273G>A 3274T>C), AAC324..A (3277C>A), TTC325..G (3280C>G), AAA326T.T (3281A>T 3283A>T), ATT327G.C (3284A>G 3286T>C), CAA328A.G (3287C>A 3289A>G), ATG329..A (3292G>A), GAC330AG. (3293G>A 3294A>G), TCC332.GT (3300C>G 3301C>T), GAA333.CT (3303A>C 3304A>T), TTC334..T (3307C>T), TTG335GG. (3308T>G 3309T>G), AAG336T.A (3311A>T 3313G>A), GAA338AGG (3317G>A 3318A>G 3319A>G), ACT339GTG (3320A>G 3321C>T 3322T>G), TAT341.T. (3327A>T), CTT342..A (3331T>A), ATC345T.G (3338A>T 3340C>G), ATA346..T (3343A>T), AGC347.CT (3345G>C 3346C>T), AGG348T.. (3347A>T), GGT350ATT (3353G>A 3354G>T), AAG352CGA (3359A>C 3360A>G 3361G>A), CCT353GTG (3362C>G 3363C>T 3364T>G), AAC354GAT (3365A>G 3367C>T), CCT355ACT (3368C>A), GAT356CAA (3371G>C 3373T>A), TCC359GAG (3380T>G 3381C>A 3382C>G), GCT360GCA (3385T>A), ATT361GTC (3386A>G 3388T>C), CAA362AAA (3389C>A), AAA363AAC (3394A>C), TAT364TGG (3396A>G 3397T>G), CTG365CCC (3399T>C 3400G>C), ATT366AAA (3402T>A 3403T>A), CCA367CCC (3406A>C), AAG368ACC (3408A>C 3409G>C), ACC369TCT (3410A>T 3412C>T), CCT370CCA (3415T>A), AAG371ACT (3417A>C 3418G>T), AAA374AGA (3426A>G), CAA375AGT (3428C>A 3429A>G 3430A>T), TTT376TTC (3433T>C), TTA377TTG (3436A>G), GGC378GGT (3439C>T), CTT379TTG (3440C>T 3442T>G), CTC380GCT (3443C>G 3444T>C 3445C>T), GGT381GGG (3448T>G)

*: Inserts / Deletes / Misaligned / Frameshifts

Analysis details

This analysis was performed with panviral2.64

NGS Details (UN59): Pinus nigra virus 1

Assembly

**Coverage Length**	535 (1 contig(s))
**Depth Of Coverage**	6.5
**Number Of Reads**	27
**Reads Per Million**	0.59 rpm (after QC)
**Ambiguities**	0
**Assembly Method**	de novo + reference guided assembly
**Consensus Caller**	Bcf Tools

Coverage Map

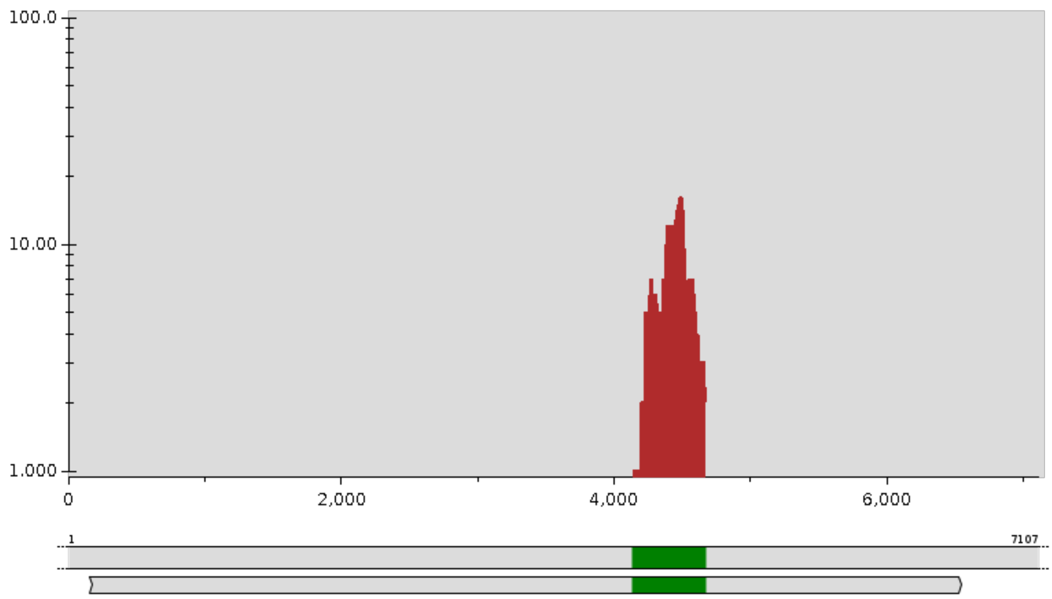

Assignment

**Type**	Pinus nigra virus 1 (Taxonomy ID: 2267679)
**Reference Genome**	NC_040841.1
**NT Identity (%)**	56.6485
**AA Identity (%)**	43.4783
**Number Of Stop Codons**	1
**Number Of CDS**	1

Alignment

**Alignment Score**	124.0 (NT) + 313.0 (AA) = 437.0
**Concordance (%)**	22.1215

**Alignment Method**	Global, seeded, nucleotide + amino acids (AGA)

Genome Region

Sequence starts at position 4141 and ends at position 4675 relative to NC_040841.1 reference sequence.

Alignment Detailed Statistics

	Begin	End	Coverage	Score	Concordance	Matches	Identities	I/D/M/F*	Stop Codons
**NT**	**4141**	**4675**	**7.5%**	**124**	**11.8%**	**533 (96.7%)**	**311 (56.4%)**	**16/2**	
Mutations:	4141G>A, 4147G>A, 4151G>C, 4152C>A, 4153C>A, 4154T>A, 4167G>A, 4168G>A, 4175T>A, 4177T>A, 4180C>A, 4184G>T, 4186T>A, 4187A>G, 4190T>A, 4192T>G, 4193G>A, 4195T>A, 4199A>T, 4202G>A, 4204T>A, 4205A>G, 4206C>T, 4210C>T, 4223T>C, 4226A>G, 4228T>A, 4229C>T, 4230A>G, 4232A>T, 4233G>C, 4234T>A, 4235C>G, 4236C>A, 4238G>A, 4239A>C, 4240T>A, 4241G>T, 4242T>A, 4243A>C, 4246G>A, 4247A>G, 4249T>A, 4253T>C, 4255G>T, 4257A>C, 4258G>A, 4261C>T, 4262T>C, 4264A>T, 4265G>A, 4268A>G, 4269T>A, 4270T>A, 4274C>G, 4275T>C, 4276T>A, 4279A>T, 4280G>C, 4281A>T, 4282A>C, 4283G>T, 4284T>G, 4285C>T, 4286A>G, 4287A>T, 4288G>C, 4291A>C, 4292C>A, 4294T>C, 4296G>A, 4297A>C, 4300T>A, 4306G>A, 4307T>A, 4308C>G, 4309A>C, 4310G>C, 4314G>A, 4319A>G, 4320T>C, 4321G>T, 4322T>G, 4323C>A, 4324C>A, 4325T>A, 4328T>A, 4330T>C, 4331C>A, 4333G>A, 4333_4334insA, 4334G>A, 4337A>G, 4339T>A, 4342T>A, 4345T>A, 4348T>C, 4349T>C, 4351G>T, 4357T>G, 4359A>T, 4361G>A, 4364G>T, 4369T>A, 4370G>A, 4371G>A, 4373T>G, 4374C>G, 4375T>G, 4378T>A, 4379C>G, 4381A>G, 4382A>T, 4384G>A, 4388C>A, 4390C>T, 4394G>C, 4396G>C, 4398T>A, 4400_4401delAC, 4406T>A, 4409A>T, 4410C>T, 4412C>G, 4414G>A, 4429G>A, 4431A>C, 4432G>A, 4433G>A, 4434A>C, 4438G>A, 4439A>C, 4447C>A, 4456C>T, 4457A>C, 4463G>T, 4464T>G, 4466C>T, 4468G>A, 4473A>T, 4475T>G, 4476T>C, 4477G>A, 4478C>G, 4479A>G, 4480C>A, 4481A>G, 4485A>T, 4486T>C, 4488T>C, 4489T>A, 4493A>G, 4495C>A, 4496C>T, 4498C>A, 4499T>G, 4502G>A, 4509C>G, 4511G>A, 4512C>A, 4515C>T, 4516_4517insCTACAAAAACTC, 4519A>C, 4524G>A, 4526C>T, 4527A>C, 4528C>A, 4531C>T, 4532A>C, 4533A>G, 4534T>A, 4535G>T, 4536G>T, 4537A>G, 4538G>T, 4544T>A, 4546A>C, 4547G>A, 4553G>C, 4554C>A, 4558A>T, 4559G>C, 4560C>G, 4561A>T, 4562G>T, 4563C>G, 4564G>T, 4568A>C, 4570G>A, 4573G>A, 4576T>C, 4589C>G, 4590A>T, 4591G>T, 4592C>A, 4594T>G, 4607C>A, 4608A>G, 4609A>G, 4611C>T, 4617G>A, 4618_4619insCCA, 4621T>A, 4623G>A, 4625C>A, 4626T>A, 4627A>T, 4628T>C, 4631A>G, 4633T>A, 4642T>A, 4643G>A, 4647C>T, 4648T>C, 4651A>C, 4652C>G, 4653T>A, 4655G>T, 4658T>C, 4659T>A, 4664T>G, 4665C>G, 4669T>A, 4670G>T, 4672A>G, 4673T>C								

CDS

	Begin	End	Coverage	Score	Concordance	Matches	Identities	I/D/M/F*	Stop Codons
EXL67_gp1	1330	1507	8.4%	313	24.9%	178 (96.7%)	80 (43.5%)	6/0/2/2	1
Protein mutations:	A1333Q (4151G>C 4152C>A 4153C>A), F1334I (4154T>A), R1338K (4167G>A 4168G>A), Y1341K (4175T>A 4177T>A), A1344S (4184G>T 4186T>A), K1345E (4187A>G), Y1346K (4190T>A 4192T>G), D1347K (4193G>A 4195T>A), I1349L (4199A>T), V1350I (4202G>A 4204T>A), T1351V (4205A>G 4206C>T), I1358V (4226A>G 4228T>A), H1359C (4229C>T 4230A>G), P1361E (4235C>G 4236C>A), D1362T (4238G>A 4239A>C 4240T>A), V1363Y (4241G>T 4242T>A 4243A>C), N1365E (4247A>G 4249T>A), K1368T (4257A>C 4258G>A), E1371K (4265G>A), I1372E (4268A>G 4269T>A 4270T>A), L1374A (4274C>G 4275T>C 4276T>A), E1375D (4279A>T), E1376L (4280G>C 4281A>T 4282A>C), V1377C (4283G>T 4284T>G 4285C>T), K1378V (4286A>G 4287A>T 4288G>C), K1379N (4291A>C), H1380N (4292C>A 4294T>C), G1381D (4296G>A 4297A>C), E1386Q (4310G>C), R1387K (4314G>A), M1389A (4319A>G 4320T>C 4321G>T), S1390E (4322T>G 4323C>A 4324C>A), L1391I (4325T>A), F1392I (4328T>A 4330T>C), Q1393K (4331C>A 4333G>A), Q1393_D1394insX (4333_4334insA), D1394N (4334G>A), N1395E (4337A>G 4339T>A), D1397E (4345T>A), I1401M (4357T>G), N1402I (4359A>T), V1403I (4361G>A), A1404S (4364G>T), N1405K (4369T>A), G1406N (4370G>A 4371G>A), S1407G (4373T>G 4374C>G 4375T>G), Q1409E (4379C>G 4381A>G), M1410L (4382A>T 4384G>A), P1412T (4388C>A 4390C>T), V1414L (4394G>C 4396G>C), L1415Q (4398T>A), L1418I (4406T>A), T1419L (4409A>T 4410C>T), Q1420E (4412C>G 4414G>A), K1426T (4431A>C 4432G>A), D1427T (4433G>A 4434A>C), K1429Q (4439A>C), I1435L (4457A>C), V1437C (4463G>T 4464T>G), Y1440F (4473A>T), L1441A (4475T>G 4476T>C 4477G>A), H1442G (4478C>G 4479A>G 4480C>A), K1443E (4481A>G), Y1444F (4485A>T 4486T>C), I1445T (4488T>C 4489T>A), N1447E (4493A>G 4495C>A), S1449A (4499T>G), E1450K (4502G>A), T1452R (4509C>G), A1453N (4511G>A 4512C>A), P1454L (4515C>T), P1454_I1455insLQKL (4516_4517insCTACAAAAACTC), R1457K (4524G>A), H1458S (4526C>T 4527A>C 4528C>A), N1460R (4532A>C 4533A>G 4534T>A), G1461L (4535G>T 4536G>T 4537A>G), G1462* (4538G>T), S1464T (4544T>A 4546A>C), D1465N (4547G>A), A1467H (4553G>C 4554C>A), A1469R (4559G>C 4560C>G 4561A>T), A1470C (4562G>T 4563C>G 4564G>T), K1472Q (4568A>C 4570G>A), Q1479V (4589C>G 4590A>T 4591G>T), H1480K (4592C>A 4594T>G), Q1485R (4607C>A 4608A>G 4609A>G), P1486L (4611C>T), G1488D (4617G>A), G1488_D1489insP (4618_4619insCCA), D1489E (4621T>A), G1490D (4623G>A), L1491N (4625C>A 4626T>A 4627A>T), I1493V (4631A>G 4633T>A), D1497N (4643G>A), A1498V (4647C>T 4648T>C), L1500D (4652C>G 4653T>A), D1501Y (4655G>T), F1502H (4658T>C 4659T>A), S1504G (4664T>G 4665C>G), V1506L (4670G>T 4672A>G)								
Codon mutations:	AAG1329..A (4141G>A), GCG1331GCA (4147G>A), GCC1333CAA (4151G>C 4152C>A 4153C>A), TTC1334ATC (4154T>A), AGG1338AAA (4167G>A 4168G>A), TAT1341AAA (4175T>A 4177T>A), ATC1342ATA (4180C>A), GCT1344TCA (4184G>T 4186T>A), AAA1345GAA (4187A>G), TAT1346AAG (4190T>A 4192T>G), GAT1347AAA (4193G>A 4195T>A), ATA1349TTA (4199A>T), GTT1350ATA (4202G>A 4204T>A), ACA1351GTA (4205A>G 4206C>T), TAC1352TAT (4210C>T), TTA1357CTA (4223T>C), ATT1358GTA (4226A>G 4228T>A), CAT1359TGT (4229C>T 4230A>G), AGT1360TCA (4232A>T 4233G>C 4234T>A), CCA1361GAA (4235C>G 4236C>A), GAT1362ACA (4238G>A 4239A>C 4240T>A), GTA1363TAC (4241G>T 4242T>A 4243A>C), CAG1364CAA (4246G>A), AAT1365GAA (4247A>G 4249T>A), TTG1367CTT (4253T>C 4255G>T), AAG1368ACA (4257A>C 4258G>A), CAC1369CAT (4261C>T), TTA1370CTT (4262T>C 4264A>T), GAA1371AAA (4265G>A), ATT1372GAA (4268A>G 4269T>A 4270T>A), CTT1374GCA (4274C>G 4275T>C 4276T>A), GAA1375GAT (4279A>T), GAA1376CTC (4280G>C 4281A>T 4282A>C), GTC1377TGT (4283G>T 4284T>G 4285C>T), AAG1378GTC (4286A>G 4287A>T 4288G>C), AAA1379AAC (4291A>C), CAT1380AAC (4292C>A 4294T>C), GGA1381GAC (4296G>A 4297A>C), ATT1382ATA (4300T>A), TTG1384TTA (4306G>A), TCA1385AGC (4307T>A 4308C>G 4309A>C), GAA1386CAA (4310G>C), AGA1387AAA (4314G>A), ATG1389GCT (4319A>G 4320T>C 4321G>T), TCC1390GAA (4322T>G 4323C>A 4324C>A), TTA1391ATA (4325T>A), TTT1392ATC (4328T>A 4330T>C), CAG1393AAA (4331C>A 4333G>A), CAG1393_GAT1394insA-- (4333_4334insA), GAT1394AAT (4334G>A), AAT1395GAA (4337A>G 4339T>A), ATT1396ATA (4342T>A), GAT1397GAA (4345T>A), TTT1398TTC (4348T>C), TTG1399CTT (4349T>C 4351G>T), ATT1401ATG (4357T>G), AAT1402ATT (4359A>T), GTT1403ATT (4361G>A), GCA1404TCA (4364G>T), AAT1405AAA (4369T>A), GGT1406AAT (4370G>A 4371G>A), TCT1407GGG (4373T>G 4374C>G 4375T>G), ATT1408ATA (4378T>A), CAA1409GAG (4379C>G 4381A>G), ATG1410TTA (4382A>T 4384G>A), CCC1412ACT (4388C>A 4390C>T), GTG1414CTC (4394G>C 4396G>C), CTA1415CAA (4398T>A), ACA1416--A (4400_4401delAC), TTA1418ATA (4406T>A), ACA1419TTA (4409A>T 4410C>T), CAG1420GAA (4412C>G 4414G>A), TTG1425TTA (4429G>A), AAG1426ACA (4431A>C 4432G>A), GAC1427ACC (4433G>A 4434A>C), AAG1428AAA (4438G>A), AAA1429CAA (4439A>C), ATC1431ATA (4447C>A), TTC1434TTT (4456C>T), ATT1435CTT (4457A>C), GTT1437TGT (4463G>T 4464T>G), CTG1438TTA (4466C>T 4468G>A), TAT1440TTT (4473A>T), TTG1441GCA (4475T>G 4476T>C 4477G>A), CAC1442GGA (4478C>G 4479A>G 4480C>A), AAA1443GAA (4481A>G), TAT1444TTC (4485A>T 4486T>C), ATT1445ACA (4488T>C 4489T>A), AAC1447GAA (4493A>G 4495C>A), CTC1448TTA (4496C>T 4498C>A), TCA1449GCA (4499T>G), GAA1450AAA (4502G>A), ACA1452AGA (4509C>G), GCT1453AAT (4511G>A 4512C>A), CCT1454CTT (4515C>T), CCT1454_ATA1455insCTACAAAAACTC (4516_4517insCTACAAAAACTC), ATA1455ATC (4519A>C), AGA1457AAA (4524G>A), CAC1458TCA (4526C>T 4527A>C 4528C>A), AAC1459AAT (4531C>T), AAT1460CGA (4532A>C 4533A>G 4534T>A), GGA1461TTG (4535G>T 4536G>T 4537A>G), GGA1462TGA (4538G>T), TCA1464ACC (4544T>A 4546A>C), GAT1465AAT (4547G>A), GCT1467CAT (4553G>C 4554C>A), ACA1468ACT (4558A>T), GCA1469CGT (4559G>C 4560C>G 4561A>T), GCG1470TGT (4562G>T 4563C>G 4564G>T), AAG1472CAA (4568A>C 4570G>A), AAG1473AAA (4573G>A), CTT1474CTC (4576T>C), CAG1479GTT (4589C>G 4590A>T 4591G>T), CAT1480AAG (4592C>A 4594T>G), CAA1485AGG (4607C>A 4608A>G 4609A>G), CCA1486CTA (4611C>T), GGT1488GAT (4617G>A), GGT1488_GAT1489insCCA (4618_4619insCCA), GAT1489GAA (4621T>A), GGC1490GAC (4623G>A), CTA1491AAT (4625C>A 4626T>A 4627A>T), TTG1492CTG (4628T>C), ATT1493GTA (4631A>G 4633T>A), ACT1496ACA (4642T>A), GAT1497AAT (4643G>A), GCT1498GTC (4647C>T 4648T>C), TCA1499TCC (4651A>C), CTT1500GAT (4652C>G 4653T>A), GAT1501TAT (4655G>T), TTT1502CAT (4658T>C 4659T>A), TCA1504GGA (4664T>G 4665C>G), GCT1505GCA (4669T>A), GTA1506TTG (4670G>T 4672A>G), TTA1507CTA (4673T>C)								

Proteins

	Begin	End	Coverage	Score	Concordance	Matches	Identities	I/D/M/F*	Stop Codons
polyprotein (YP_009553669.1)	1330	1507	8.4%	313	24.9%	178 (96.7%)	80 (43.5%)	6/0/2/2	1
Protein mutations:	A1333Q (4151G>C 4152C>A 4153C>A), F1334I (4154T>A), R1338K (4167G>A 4168G>A), Y1341K (4175T>A 4177T>A), A1344S (4184G>T 4186T>A), K1345E (4187A>G), Y1346K (4190T>A 4192T>G), D1347K (4193G>A 4195T>A), I1349L (4199A>T), V1350I (4202G>A 4204T>A), T1351V (4205A>G 4206C>T), I1358V (4226A>G 4228T>A), H1359C (4229C>T 4230A>G), P1361E (4235C>G 4236C>A), D1362T (4238G>A 4239A>C 4240T>A), V1363Y (4241G>T 4242T>A 4243A>C), N1365E (4247A>G 4249T>A), K1368T (4257A>C 4258G>A), E1371K (4265G>A), I1372E (4268A>G 4269T>A 4270T>A), L1374A (4274C>G 4275T>C 4276T>A), E1375D (4279A>T), E1376L (4280G>C 4281A>T 4282A>C), V1377C (4283G>T 4284T>G 4285C>T), K1378V (4286A>G 4287A>T 4288G>C), K1379N (4291A>C), H1380N (4292C>A 4294T>C), G1381D (4296G>A 4297A>C), E1386Q (4310G>C), R1387K (4314G>A), M1389A (4319A>G 4320T>C 4321G>T), S1390E (4322T>G 4323C>A 4324C>A), L1391I (4325T>A), F1392I (4328T>A 4330T>C), Q1393K (4331C>A 4333G>A), Q1393_D1394insX (4333_4334insA), D1394N (4334G>A), N1395E (4337A>G 4339T>A), D1397E (4345T>A), I1401M (4357T>G), N1402I (4359A>T), V1403I (4361G>A), A1404S (4364G>T), N1405K (4369T>A), G1406N (4370G>A 4371G>A), S1407G (4373T>G 4374C>G 4375T>G), Q1409E (4379C>G 4381A>G), M1410L (4382A>T 4384G>A), P1412T (4388C>A 4390C>T), V1414L (4394G>C 4396G>C), L1415Q (4398T>A), L1418I (4406T>A), T1419L (4409A>T 4410C>T), Q1420E (4412C>G 4414G>A), K1426T (4431A>C 4432G>A), D1427T (4433G>A 4434A>C), K1429Q (4439A>C), I1435L (4457A>C), V1437C (4463G>T 4464T>G), Y1440F (4473A>T), L1441A (4475T>G 4476T>C 4477G>A), H1442G (4478C>G 4479A>G 4480C>A), K1443E (4481A>G), Y1444F (4485A>T 4486T>C), I1445T (4488T>C 4489T>A), N1447E (4493A>G 4495C>A), S1449A (4499T>G), E1450K (4502G>A), T1452R (4509C>G), A1453N (4511G>A 4512C>A), P1454L (4515C>T), P1454_I1455insLQKL (4516_4517insCTACAAAAACTC), R1457K (4524G>A), H1458S (4526C>T 4527A>C 4528C>A), N1460R (4532A>C 4533A>G 4534T>A), G1461L (4535G>T 4536G>T 4537A>G), G1462* (4538G>T), S1464T (4544T>A 4546A>C), D1465N (4547G>A), A1467H (4553G>C 4554C>A), A1469R (4559G>C 4560C>G 4561A>T), A1470C (4562G>T 4563C>G 4564G>T), K1472Q (4568A>C 4570G>A), Q1479V (4589C>G 4590A>T 4591G>T), H1480K (4592C>A 4594T>G), Q1485R (4607C>A 4608A>G 4609A>G), P1486L (4611C>T), G1488D (4617G>A), G1488_D1489insP (4618_4619insCCA), D1489E (4621T>A), G1490D (4623G>A), L1491N (4625C>A 4626T>A 4627A>T), I1493V (4631A>G 4633T>A), D1497N (4643G>A), A1498V (4647C>T 4648T>C), L1500D (4652C>G 4653T>A), D1501Y (4655G>T), F1502H (4658T>C 4659T>A), S1504G (4664T>G 4665C>G), V1506L (4670G>T 4672A>G)								

	Begin	End	Coverage	Score	Concordance	Matches	Identities	I/D/M/F*	Stop Codons
**NT**	**4141**	**4675**	**7.5%**	**124**	**11.8%**	**533 (96.7%)**	**311 (56.4%)**	**16/2**	
Codon mutations:	AAG1329..A (4141G>A), GCG1331GCA (4147G>A), GCC1333CAA (4151G>C 4152C>A 4153C>A), TTC1334ATC (4154T>A), AGG1338AAA (4167G>A 4168G>A), TAT1341AAA (4175T>A 4177T>A), ATC1342ATA (4180C>A), GCT1344TCA (4184G>T 4186T>A), AAA1345GAA (4187A>G), TAT1346AAG (4190T>A 4192T>G), GAT1347AAA (4193G>A 4195T>A), ATA1349TTA (4199A>T), GTT1350ATA (4202G>A 4204T>A), ACA1351GTA (4205A>G 4206C>T), TAC1352TAT (4210C>T), TTA1357CTA (4223T>C), ATT1358GTA (4226A>G 4228T>A), CAT1359TGT (4229C>T 4230A>G), AGT1360TCA (4232A>T 4233G>C 4234T>A), CCA1361GAA (4235C>G 4236C>A), GAT1362ACA (4238G>A 4239A>C 4240T>A), GTA1363TAC (4241G>T 4242T>A 4243A>C), CAG1364CAA (4246G>A), AAT1365GAA (4247A>G 4249T>A), TTG1367CTT (4253T>C 4255G>T), AAG1368ACA (4257A>C 4258G>A), CAC1369CAT (4261C>T), TTA1370CTT (4262T>C 4264A>T), GAA1371AAA (4265G>A), ATT1372GAA (4268A>G 4269T>A 4270T>A), CTT1374GCA (4274C>G 4275T>C 4276T>A), GAA1375GAT (4279A>T), GAA1376CTC (4280G>C 4281A>T 4282A>C), GTC1377TGT (4283G>T 4284T>G 4285C>T), AAG1378GTC (4286A>G 4287A>T 4288G>C), AAA1379AAC (4291A>C), CAT1380AAC (4292C>A 4294T>C), GGA1381GAC (4296G>A 4297A>C), ATT1382ATA (4300T>A), TTG1384TTA (4306G>A), TCA1385AGC (4307T>A 4308C>G 4309A>C), GAA1386CAA (4310G>C), AGA1387AAA (4314G>A), ATG1389GCT (4319A>G 4320T>C 4321G>T), TCC1390GAA (4322T>G 4323C>A 4324C>A), TTA1391ATA (4325T>A), TTT1392ATC (4328T>A 4330T>C), CAG1393AAA (4331C>A 4333G>A), CAG1393_GAT1394insA-- (4333_4334insA), GAT1394AAT (4334G>A), AAT1395GAA (4337A>G 4339T>A), ATT1396ATA (4342T>A), GAT1397GAA (4345T>A), TTT1398TTC (4348T>C), TTG1399CTT (4349T>C 4351G>T), ATT1401ATG (4357T>G), AAT1402ATT (4359A>T), GTT1403ATT (4361G>A), GCA1404TCA (4364G>T), AAT1405AAA (4369T>A), GGT1406AAT (4370G>A 4371G>A), TCT1407GGG (4373T>G 4374C>G 4375T>G), ATT1408ATA (4378T>A), CAA1409GAG (4379C>G 4381A>G), ATG1410TTA (4382A>T 4384G>A), CCC1412ACT (4388C>A 4390C>T), GTG1414CTC (4394G>C 4396G>C), CTA1415CAA (4398T>A), ACA1416--A (4400_4401delAC), TTA1418ATA (4406T>A), ACA1419TTA (4409A>T 4410C>T), CAG1420GAA (4412C>G 4414G>A), TTG1425TTA (4429G>A), AAG1426ACA (4431A>C 4432G>A), GAC1427ACC (4433G>A 4434A>C), AAG1428AAA (4438G>A), AAA1429CAA (4439A>C), ATC1431ATA (4447C>A), TTC1434TTT (4456C>T), ATT1435CTT (4457A>C), GTT1437TGT (4463G>T 4464T>G), CTG1438TTA (4466C>T 4468G>A), TAT1440TTT (4473A>T), TTG1441GCA (4475T>G 4476T>C 4477G>A), CAC1442GGA (4478C>G 4479A>G 4480C>A), AAA1443GAA (4481A>G), TAT1444TTC (4485A>T 4486T>C), ATT1445ACA (4488T>C 4489T>A), AAC1447GAA (4493A>G 4495C>A), CTC1448TTA (4496C>T 4498C>A), TCA1449GCA (4499T>G), GAA1450AAA (4502G>A), ACA1452AGA (4509C>G), GCT1453AAT (4511G>A 4512C>A), CCT1454CTT (4515C>T), CCT1454_ATA1455insCTACAAAAACTC (4516_4517insCTACAAAAACTC), ATA1455ATC (4519A>C), AGA1457AAA (4524G>A), CAC1458TCA (4526C>T 4527A>C 4528C>A), AAC1459AAT (4531C>T), AAT1460CGA (4532A>C 4533A>G 4534T>A), GGA1461TTG (4535G>T 4536G>T 4537A>G), GGA1462TGA (4538G>T), TCA1464ACC (4544T>A 4546A>C), GAT1465AAT (4547G>A), GCT1467CAT (4553G>C 4554C>A), ACA1468ACT (4558A>T), GCA1469CGT (4559G>C 4560C>G 4561A>T), GCG1470TGT (4562G>T 4563C>G 4564G>T), AAG1472CAA (4568A>C 4570G>A), AAG1473AAA (4573G>A), CTT1474CTC (4576T>C), CAG1479GTT (4589C>G 4590A>T 4591G>T), CAT1480AAG (4592C>A 4594T>G), CAA1485AGG (4607C>A 4608A>G 4609A>G), CCA1486CTA (4611C>T), GGT1488GAT (4617G>A), GGT1488_GAT1489insCCA (4618_4619insCCA), GAT1489GAA (4621T>A), GGC1490GAC (4623G>A), CTA1491AAT (4625C>A 4626T>A 4627A>T), TTG1492CTG (4628T>C), ATT1493GTA (4631A>G 4633T>A), ACT1496ACA (4642T>A), GAT1497AAT (4643G>A), GCT1498GTC (4647C>T 4648T>C), TCA1499TCC (4651A>C), CTT1500GAT (4652C>G 4653T>A), GAT1501TAT (4655G>T), TTT1502CAT (4658T>C 4659T>A), TCA1504GGA (4664T>G 4665C>G), GCT1505GCA (4669T>A), GTA1506TTG (4670G>T 4672A>G), TTA1507CTA (4673T>C)								

*: Inserts / Deletes / Misaligned / Frameshifts

Analysis details

This analysis was performed with panviral2.64

NGS Details (UN59): Duamitovirus peex1

Assembly

**Coverage Length**	388 (1 contig(s))
**Depth Of Coverage**	6.5
**Number Of Reads**	26
**Reads Per Million**	0.57 rpm (after QC)
**Ambiguities**	0
**Assembly Method**	de novo + reference guided assembly
**Consensus Caller**	Bcf Tools

Coverage Map

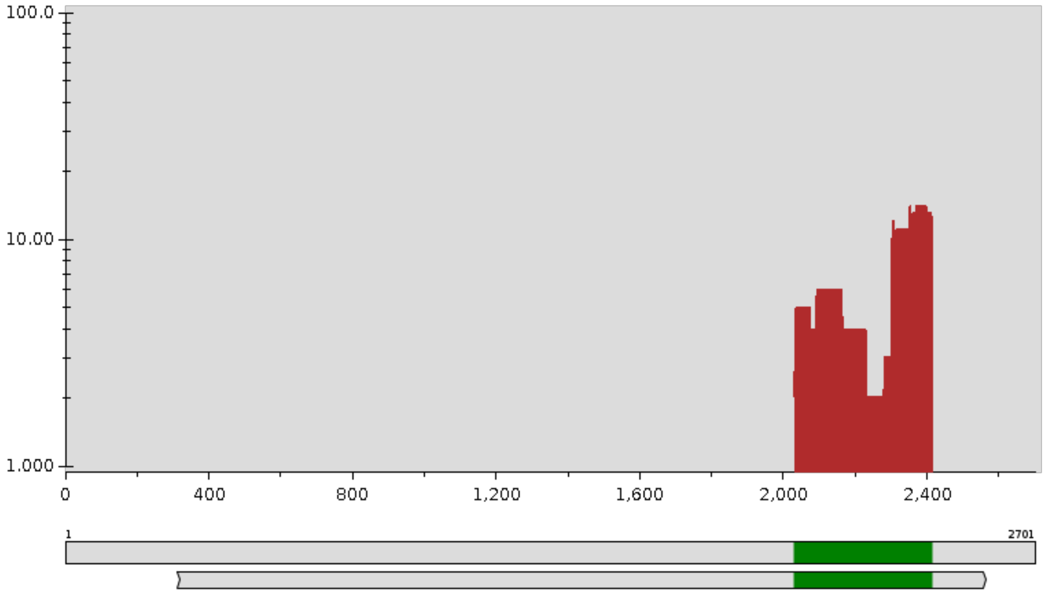

Assignment

**Type**	Duamitovirus peex1 (Taxonomy ID: 2955799)
**Reference Genome**	NC_076525.1
**NT Identity (%)**	71.9072
**AA Identity (%)**	65.8915
**Number Of Stop Codons**	0
**Number Of CDS**	1

Alignment

**Alignment Score**	340.0 (NT) + 633.0 (AA) = 973.0
**Concordance (%)**	56.5041

**Alignment Method**	Global, seeded, nucleotide + amino acids (AGA)

Genome Region

Sequence starts at position 2030 and ends at position 2417 relative to NC_076525.1 reference sequence.

Alignment Detailed Statistics

	Begin	End	Coverage	Score	Concordance	Matches	Identities	I/D/M/F*	Stop Codons
**NT**	**2030**	**2417**	**14.4%**	**340**	**43.8%**	**388 (100%)**	**279 (71.9%)**	**0/0**	
Mutations:	2036T>G, 2040A>T, 2042G>T, 2051T>A, 2052A>C, 2053A>C, 2057C>A, 2066G>A, 2067A>C, 2069T>A, 2075T>G, 2077T>C, 2088T>C, 2090T>A, 2091T>A, 2095T>G, 2096A>G, 2098C>T, 2102G>T, 2104G>T, 2105G>A, 2109T>C, 2119G>T, 2120G>T, 2133G>T, 2134G>T, 2135A>T, 2136A>G, 2137A>G, 2138G>A, 2139C>A, 2145G>A, 2156A>G, 2158G>A, 2159A>G, 2161G>C, 2162T>G, 2165G>A, 2171C>A, 2173C>A, 2180T>G, 2184A>G, 2188A>C, 2189G>T, 2190T>G, 2195A>G, 2202G>C, 2210G>A, 2211T>A, 2213G>C, 2214A>T, 2216A>T, 2223C>G, 2225A>T, 2226C>T, 2231G>A, 2234A>T, 2240T>A, 2245A>G, 2246T>G, 2249G>A, 2255A>T, 2258A>G, 2261C>G, 2268G>A, 2270A>C, 2272G>T, 2274C>A, 2283G>A, 2292C>T, 2294T>G, 2295C>A, 2296G>C, 2297A>T, 2306A>T, 2311A>G, 2312T>C, 2315C>T, 2321G>T, 2325A>G, 2326A>C, 2327T>A, 2330C>A, 2331C>G, 2333G>T, 2339G>A, 2345A>T, 2348C>T, 2351A>G, 2352C>T, 2354T>C, 2363G>T, 2373G>A, 2374C>G, 2375G>T, 2377C>A, 2378T>A, 2379T>A, 2388C>A, 2390T>A, 2393T>A, 2394C>A, 2396G>C, 2398C>A, 2399T>A, 2406C>G, 2411A>G, 2412G>A, 2414G>C								
**CDS**									
RdRp	574	702	17.2%	633	66.6%	129 (100%)	85 (65.9%)	0/0/0/0	0
Protein mutations:	T577S (2040A>T 2042G>T), K581P (2052A>C 2053A>C), I586L (2067A>C 2069T>A), V589A (2077T>C), S593P (2088T>C 2090T>A), Y594N (2091T>A), I595R (2095T>G 2096A>G), S596L (2098C>T), Q597H (2102G>T), R598L (2104G>T 2105G>A), S600P (2109T>C), W603F (2119G>T 2120G>T), G608F (2133G>T 2134G>T 2135A>T), K609G (2136A>G 2137A>G 2138G>A), P610T (2139C>A), D612N (2145G>A), R616K (2158G>A 2159A>G), G617A (2161G>C 2162T>G), A621D (2173C>A), K625E (2184A>G), E626A (2188A>C 2189G>T), L627V (2190T>G), E631Q (2202G>C), L634I (2211T>A 2213G>C), I635F (2214A>T 2216A>T), Q638D (2223C>G 2225A>T), L641F (2234A>T), H645R (2245A>G 2246T>G), V653I (2268G>A 2270A>C), R654L (2272G>T), H655N (2274C>A), E658K (2283G>A), R662T (2295C>A 2296G>C 2297A>T), Y667C (2311A>G 2312T>C), N672A (2325A>G 2326A>C 2327T>A), P674A (2331C>G 2333G>T), L681F (2352C>T 2354T>C), A688S (2373G>A 2374C>G 2375G>T), T689K (2377C>A 2378T>A), S690T (2379T>A), Q695N (2394C>A 2396G>C), T696K (2398C>A 2399T>A), H699D (2406C>G), V701I (2412G>A 2414G>C)								
Codon mutations:	CTT575CTG (2036T>G), ACG577TCT (2040A>T 2042G>T), TCT580TCA (2051T>A), AAA581CCA (2052A>C 2053A>C), CGC582CGA (2057C>A), AGG585AGA (2066G>A), ATT586CTA (2067A>C 2069T>A), GCT588GCG (2075T>G), GTT589GCT (2077T>C), TCT593CCA (2088T>C 2090T>A), TAC594AAC (2091T>A), ATA595AGG (2095T>G 2096A>G), TCA596TTA (2098C>T), CAG597CAT (2102G>T), CGG598CTA (2104G>T 2105G>A), TCT600CCT (2109T>C), TGG603TTT (2119G>T 2120G>T), GGA608TTT (2133G>T 2134G>T 2135A>T), AAG609GGA (2136A>G 2137A>G 2138G>A), CCC610ACC (2139C>A), GAT612AAT (2145G>A), TTA615TTG (2156A>G), AGA616AAG (2158G>A 2159A>G), GGT617GCG (2161G>C 2162T>G), AAG618AAA (2165G>A), ATC620ATA (2171C>A), GCT621GAT (2173C>A), CTT623CTG (2180T>G), AAG625GAG (2184A>G), GAG626GCT (2188A>C 2189G>T), TTA627GTA (2190T>G), AAA628AAG (2195A>G), GAG631CAG (2202G>C), CAG633CAA (2210G>A), TTG634ATC (2211T>A 2213G>C), ATA635TTT (2214A>T 2216A>T), CAA638GAT (2223C>G 2225A>T), CTG639TTG (2226C>T), GTG640GTA (2231G>A), TTA641TTT (2234A>T), GGT643GGA (2240T>A), CAT645CGG (2245A>G 2246T>G), GAG646GAA (2249G>A), CTA648CTT (2255A>T), GAA649GAG (2258A>G), CGC650CGG (2261C>G), GTA653ATC (2268G>A 2270A>C), CGT654CTT (2272G>T), CAT655AAT (2274C>A), GAA658AAA (2283G>A), CTT661TTG (2292C>T 2294T>G), CGA662ACT (2295C>A 2296G>C 2297A>T), TCA665TCT (2306A>T), TAT667TGC (2311A>G 2312T>C), CAC668CAT (2315C>T), GTG670GTT (2321G>T), AAT672GCA (2325A>G 2326A>C 2327T>A), TCC673TCA (2330C>A), CCG674GCT (2331C>G 2333G>T), GTG676GTA (2339G>A), ATA678ATT (2345A>T), GAC679GAT (2348C>T), CAA680CAG (2351A>G), CTT681TTC (2352C>T 2354T>C), GTG684GTT (2363G>T), GCG688AGT (2373G>A 2374C>G 2375G>T), ACT689AAA (2377C>A 2378T>A), TCT690ACT (2379T>A), CGT693AGA (2388C>A 2390T>A), ACT694ACA (2393T>A), CAG695AAC (2394C>A 2396G>C), ACT696AAA (2398C>A 2399T>A), CAT699GAT (2406C>G), TTA700TTG (2411A>G), GTG701ATC (2412G>A 2414G>C)								
**Proteins**									
RNA-dependent RNA polymerase (YP_010798875.1)	574	702	17.2%	633	66.6%	129 (100%)	85 (65.9%)	0/0/0/0	0
Protein mutations:	T577S (2040A>T 2042G>T), K581P (2052A>C 2053A>C), I586L (2067A>C 2069T>A), V589A (2077T>C), S593P (2088T>C 2090T>A), Y594N (2091T>A), I595R (2095T>G 2096A>G), S596L (2098C>T), Q597H (2102G>T), R598L (2104G>T 2105G>A), S600P (2109T>C), W603F (2119G>T 2120G>T), G608F (2133G>T 2134G>T 2135A>T), K609G (2136A>G 2137A>G 2138G>A), P610T (2139C>A), D612N (2145G>A), R616K (2158G>A 2159A>G), G617A (2161G>C 2162T>G), A621D (2173C>A), K625E (2184A>G), E626A (2188A>C 2189G>T), L627V (2190T>G), E631Q (2202G>C), L634I (2211T>A 2213G>C), I635F (2214A>T 2216A>T), Q638D (2223C>G 2225A>T), L641F (2234A>T), H645R (2245A>G 2246T>G), V653I (2268G>A 2270A>C), R654L (2272G>T), H655N (2274C>A), E658K (2283G>A), R662T (2295C>A 2296G>C 2297A>T), Y667C (2311A>G 2312T>C), N672A (2325A>G 2326A>C 2327T>A), P674A (2331C>G 2333G>T), L681F (2352C>T 2354T>C), A688S (2373G>A 2374C>G 2375G>T), T689K (2377C>A 2378T>A), S690T (2379T>A), Q695N (2394C>A 2396G>C), T696K (2398C>A 2399T>A), H699D (2406C>G), V701I (2412G>A 2414G>C)								
Codon mutations:	CTT575CTG (2036T>G), ACG577TCT (2040A>T 2042G>T), TCT580TCA (2051T>A), AAA581CCA (2052A>C 2053A>C), CGC582CGA (2057C>A), AGG585AGA (2066G>A), ATT586CTA (2067A>C 2069T>A), GCT588GCG (2075T>G), GTT589GCT (2077T>C), TCT593CCA (2088T>C 2090T>A), TAC594AAC (2091T>A), ATA595AGG (2095T>G 2096A>G), TCA596TTA (2098C>T), CAG597CAT (2102G>T), CGG598CTA (2104G>T 2105G>A), TCT600CCT (2109T>C), TGG603TTT (2119G>T 2120G>T), GGA608TTT (2133G>T 2134G>T 2135A>T), AAG609GGA (2136A>G 2137A>G 2138G>A), CCC610ACC (2139C>A), GAT612AAT (2145G>A), TTA615TTG (2156A>G), AGA616AAG (2158G>A 2159A>G), GGT617GCG (2161G>C 2162T>G), AAG618AAA (2165G>A), ATC620ATA (2171C>A), GCT621GAT (2173C>A), CTT623CTG (2180T>G), AAG625GAG (2184A>G), GAG626GCT (2188A>C 2189G>T), TTA627GTA (2190T>G), AAA628AAG (2195A>G), GAG631CAG (2202G>C), CAG633CAA (2210G>A), TTG634ATC (2211T>A 2213G>C), ATA635TTT (2214A>T 2216A>T), CAA638GAT (2223C>G 2225A>T), CTG639TTG (2226C>T), GTG640GTA (2231G>A), TTA641TTT (2234A>T), GGT643GGA (2240T>A), CAT645CGG (2245A>G 2246T>G), GAG646GAA (2249G>A), CTA648CTT (2255A>T), GAA649GAG (2258A>G), CGC650CGG (2261C>G), GTA653ATC (2268G>A 2270A>C), CGT654CTT (2272G>T), CAT655AAT (2274C>A), GAA658AAA (2283G>A), CTT661TTG (2292C>T 2294T>G), CGA662ACT (2295C>A 2296G>C 2297A>T), TCA665TCT (2306A>T), TAT667TGC (2311A>G 2312T>C), CAC668CAT (2315C>T), GTG670GTT (2321G>T), AAT672GCA (2325A>G 2326A>C 2327T>A), TCC673TCA (2330C>A), CCG674GCT (2331C>G 2333G>T), GTG676GTA (2339G>A), ATA678ATT (2345A>T), GAC679GAT (2348C>T), CAA680CAG (2351A>G), CTT681TTC (2352C>T 2354T>C), GTG684GTT (2363G>T), GCG688AGT (2373G>A 2374C>G 2375G>T), ACT689AAA (2377C>A 2378T>A), TCT690ACT (2379T>A), CGT693AGA (2388C>A 2390T>A), ACT694ACA (2393T>A), CAG695AAC (2394C>A 2396G>C), ACT696AAA (2398C>A 2399T>A), CAT699GAT (2406C>G), TTA700TTG (2411A>G), GTG701ATC (2412G>A 2414G>C)								

*: Inserts / Deletes / Misaligned / Frameshifts

Analysis details

This analysis was performed with panviral2.64

NGS Details (UN59): Caulimovirus latensarmoraciae

Assembly

**Coverage Length**	342 (1 contig(s))
**Depth Of Coverage**	9.8
**Number Of Reads**	26
**Reads Per Million**	0.57 rpm (after QC)
**Ambiguities**	0
**Assembly Method**	de novo + reference guided assembly
**Consensus Caller**	Bcf Tools

Coverage Map

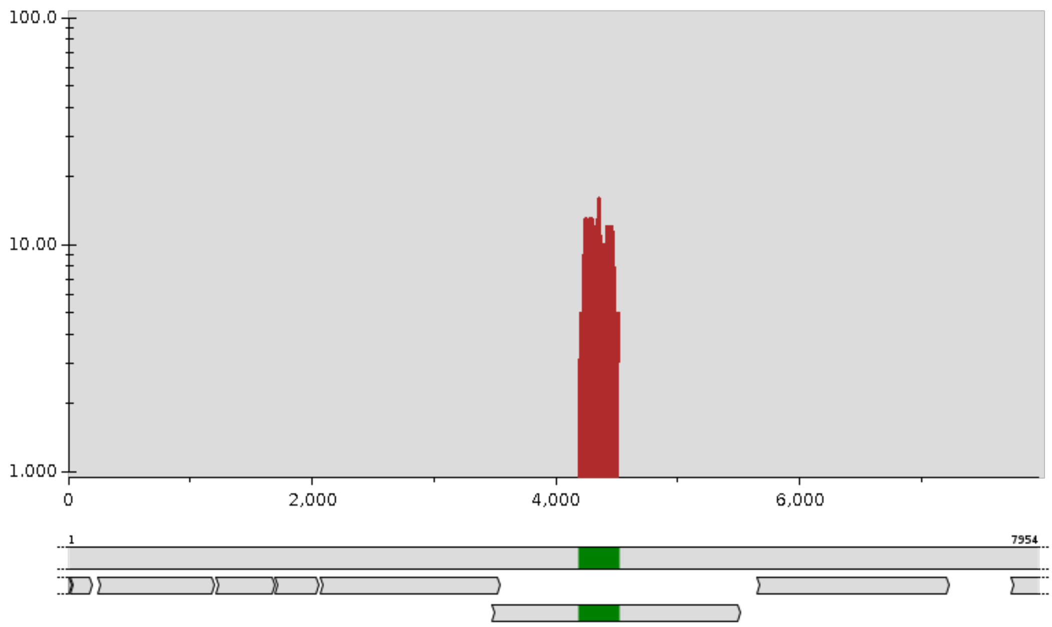

Assignment

**Type**	Caulimovirus latensarmoraciae (Taxonomy ID: 3047955)
**Reference Genome**	NC_018858.1
**NT Identity (%)**	59.9415
**AA Identity (%)**	56.1404
**Number Of Stop Codons**	0
**Number Of CDS**	8

Alignment

**Alignment Score**	136.0 (NT) + 482.0 (AA) = 618.0
**Concordance (%)**	43.338

Alignment Method	Global, seeded, nucleotide + amino acids (AGA)

Genome Region

Sequence starts at position 4182 and ends at position 4523 relative to NC_018858.1 reference sequence.

Alignment Detailed Statistics

	Begin	End	Coverage	Score	Concordance	Matches	Identities	I/D/M/F*	Stop Codons
**NT**	**4182**	**4523**	**4.3%**	**136**	**19.9%**	**342 (100%)**	**205 (59.9%)**	**0/0**	
Mutations:	4182A>G, 4186C>A, 4189C>G, 4190A>T, 4192C>A, 4195A>T, 4199T>A, 4200C>A, 4209C>G, 4210G>C, 4212A>T, 4213G>C, 4214G>A, 4219C>A, 4221A>G, 4222G>A, 4231C>G, 4235A>C, 4236A>T, 4240T>C, 4242G>A, 4243C>T, 4244C>G, 4245C>A, 4247A>C, 4248T>A, 4249G>A, 4252C>T, 4253C>A, 4254G>A, 4256G>A, 4257A>C, 4258A>T, 4265G>A, 4267G>A, 4269A>T, 4270G>T, 4273A>G, 4286C>T, 4288G>A, 4289G>A, 4291C>T, 4292C>A, 4294C>G, 4297G>A, 4298G>T, 4303C>A, 4306G>A, 4308C>A, 4309C>T, 4318C>T, 4326T>G, 4327G>T, 4328G>A, 4329C>G, 4330A>C, 4336G>A, 4339C>T, 4340T>A, 4342A>G, 4345C>A, 4347A>G, 4348C>A, 4352G>C, 4354A>T, 4356C>G, 4357T>C, 4360G>A, 4361A>C, 4364C>A, 4365G>A, 4366C>A, 4367A>C, 4369A>T, 4375G>A, 4376A>G, 4377A>C, 4378G>T, 4381T>A, 4387G>A, 4388G>A, 4390C>A, 4396C>T, 4401C>A, 4402A>G, 4403A>C, 4405G>T, 4409G>A, 4411T>A, 4412G>A, 4413C>A, 4414G>T, 4415A>T, 4416C>G, 4420A>C, 4421G>T, 4422G>T, 4428C>G, 4432C>T, 4433A>T, 4434A>T, 4435T>C, 4436C>A, 4438T>C, 4441C>A, 4444C>T, 4447G>A, 4448G>A, 4451G>A, 4453A>C, 4456C>T, 4457C>A, 4459T>A, 4461C>A, 4462G>T, 4465C>T, 4466A>G, 4467T>C, 4468C>A, 4470G>A, 4477G>A, 4479A>C, 4480A>C, 4481A>T, 4482T>A, 4486C>T, 4489T>A, 4490T>A, 4491C>A, 4492C>A, 4498C>T, 4505T>A, 4506C>G, 4507A>T, 4513C>T, 4520G>A, 4522T>A								

CDS

	Begin	End	Coverage	Score	Concordance	Matches	Identities	I/D/M/F*	Stop Codons
D306_gp7	236	349	16.8%	482	63.5%	114 (100%)	64 (56.1%)	0/0/0/0	0
Protein mutations:	S237R (4189C>G), I238L (4190A>T 4192C>A), K239N (4195A>T), S241K (4199T>A 4200C>A), T244S (4209C>G 4210G>C), K245I (4212A>T 4213G>C), V246I (4214G>A), K248R (4221A>G 4222G>A), K253L (4235A>C 4236A>T), S255N (4242G>A 4243C>T), P256E (4244C>G 4245C>A), M257Q (4247A>C 4248T>A 4249G>A), R259K (4253C>A 4254G>A), E260T (4256G>A 4257A>C 4258A>T), E263K (4265G>A 4267G>A), K264I (4269A>T 4270G>T), D271N (4289G>A 4291C>T), L272M (4292C>A 4294C>G), V274L (4298G>T), P277H (4308C>A 4309C>T), M283S (4326T>G 4327G>T), A284S (4328G>A 4329C>G 4330A>C), L288M (4340T>A 4342A>G), N290R (4347A>G 4348C>A), E292H (4352G>C 4354A>T), A293G (4356C>G 4357T>C), K295Q (4361A>C), R296K (4364C>A 4365G>A 4366C>A), K300A (4376A>G 4377A>C 4378G>T), V304I (4388G>A 4390C>A), A308E (4401C>A 4402A>G), M309L (4403A>C 4405G>T), D311K (4409G>A 4411T>A), A312N (4412G>A 4413C>A 4414G>T), T313C (4415A>T 4416C>G), G315F (4421G>T 4422G>T), A317G (4428C>G), N319F (4433A>T 4434A>T 4435T>C), L320I (4436C>A 4438T>C), D324N (4448G>A), E325N (4451G>A 4453A>C), L327I (4457C>A 4459T>A), T328N (4461C>A 4462G>T), I330A (4466A>G 4467T>C 4468C>A), R331K (4470G>A), K334T (4479A>C 4480A>C), I335Y (4481A>T 4482T>A), S338K (4490T>A 4491C>A 4492C>A), V348I (4520G>A 4522T>A)								
Codon mutations:	CAA235.GA (4182A>G), GCC236GCA (4186C>A), AGC237AGG (4189C>G), ATC238TTA (4190A>T 4192C>A), AAA239AAT (4195A>T), TCG241AAG (4199T>A 4200C>A), ACG244AGC (4209C>G 4210G>C), AAG245ATC (4212A>T 4213G>C), GTT246ATT (4214G>A), ATC247ATA (4219C>A), AAG248AGA (4221A>G 4222G>A), CCC251CCG (4231C>G), AAA253CTA (4235A>C 4236A>T), TAT254TAC (4240T>C), AGC255AAT (4242G>A 4243C>T), CCA256GAA (4244C>G 4245C>A), ATG257CAA (4247A>C 4248T>A 4249G>A), GAC258GAT (4252C>T), CGA259AAA (4253C>A 4254G>A), GAA260ACT (4256G>A 4257A>C 4258A>T), GAG263AAA (4265G>A 4267G>A), AAG264ATT (4269A>T 4270G>T), CAA265CAG (4273A>G), CTG270TTA (4286C>T 4288G>A), GAC271AAT (4289G>A 4291C>T), CTC272ATG (4292C>A 4294C>G), AAG273AAA (4297G>A), GTA274TTA (4298G>T), ATC275ATA (4303C>A), AGG276AGA (4306G>A), CCC277CAT (4308C>A 4309C>T), AGC280AGT (4318C>T), ATG283AGT (4326T>G 4327G>T), GCA284AGC (4328G>A 4329C>G 4330A>C), GCG286GCA (4336G>A), TTC287TTT (4339C>T), TTA288ATG (4340T>A 4342A>G), GTC289GTA (4345C>A), AAC290AGA (4347A>G 4348C>A), GAA292CAT (4352G>C 4354A>T), GCT293GGC (4356C>G 4357T>C), GAG294GAA (4360G>A), AAA295CAA (4361A>C), CGC296AAA (4364C>A 4365G>A 4366C>A), AGA297CGT (4367A>C 4369A>T), AAG299AAA (4375G>A), AAG300GCT (4376A>G 4377A>C 4378G>T), CGT301CGA (4381T>A), GTG303GTA (4387G>A), GTC304ATA (4388G>A 4390C>A), TAC306TAT (4396C>T), GCA308GAG (4401C>A 4402A>G), ATG309CTT (4403A>C 4405G>T), GAT311AAA (4409G>A 4411T>A), GCG312AAT (4412G>A 4413C>A 4414G>T), ACT313TGT (4415A>T 4416C>G), GTA314GTC (4420A>C), GGC315TTC (4421G>T 4422G>T), GCA317GGA (4428C>G), TAC318TAT (4432C>T), AAT319TTC (4433A>T 4434A>T 4435T>C), CTT320ATC (4436C>A 4438T>C), CCC321CCA (4441C>A), AAC322AAT (4444C>T), AAG323AAA (4447G>A), GAT324AAT (4448G>A), GAA325AAC (4451G>A 4453A>C), CTC326CTT (4456C>T), CTT327ATA (4457C>A 4459T>A), ACG328AAT (4461C>A 4462G>T), CTC329CTT (4465C>T), ATC330GCA (4466A>G 4467T>C 4468C>A), AGA331AAA (4470G>A), AAG333AAA (4477G>A), AAA334ACC (4479A>C 4480A>C), ATC335TAC (4481A>T 4482T>A), TTC336TTT (4486C>T), TCT337TCA (4489T>A), TCC338AAA (4490T>A 4491C>A 4492C>A), GAC340GAT (4498C>T), TCA343AGT (4505T>A 4506C>G 4507A>T), TTC345TTT (4513C>T), GTT348ATA (4520G>A 4522T>A)								

Proteins

	Begin	End	Coverage	Score	Concordance	Matches	Identities	I/D/M/F*	Stop Codons
polyprotein (YP_006907834.1)	236	349	16.8%	482	63.5%	114 (100%)	64 (56.1%)	0/0/0/0	0
Protein mutations:	S237R (4189C>G), I238L (4190A>T 4192C>A), K239N (4195A>T), S241K (4199T>A 4200C>A), T244S (4209C>G 4210G>C), K245I (4212A>T 4213G>C), V246I (4214G>A), K248R (4221A>G 4222G>A), K253L (4235A>C 4236A>T), S255N (4242G>A 4243C>T), P256E (4244C>G 4245C>A), M257Q (4247A>C 4248T>A 4249G>A), R259K (4253C>A 4254G>A), E260T (4256G>A 4257A>C 4258A>T), E263K (4265G>A 4267G>A), K264I (4269A>T 4270G>T), D271N (4289G>A 4291C>T), L272M (4292C>A 4294C>G), V274L (4298G>T), P277H (4308C>A 4309C>T), M283S (4326T>G 4327G>T), A284S (4328G>A 4329C>G 4330A>C), L288M (4340T>A 4342A>G), N290R (4347A>G 4348C>A), E292H (4352G>C 4354A>T), A293G (4356C>G 4357T>C), K295Q (4361A>C), R296K (4364C>A 4365G>A 4366C>A), K300A (4376A>G 4377A>C 4378G>T), V304I (4388G>A 4390C>A), A308E (4401C>A 4402A>G), M309L (4403A>C 4405G>T), D311K (4409G>A 4411T>A), A312N (4412G>A 4413C>A 4414G>T), T313C (4415A>T 4416C>G), G315F (4421G>T 4422G>T), A317G (4428C>G), N319F (4433A>T 4434A>T 4435T>C), L320I (4436C>A 4438T>C), D324N (4448G>A), E325N (4451G>A 4453A>C), L327I (4457C>A 4459T>A), T328N (4461C>A 4462G>T), I330A (4466A>G 4467T>C 4468C>A), R331K (4470G>A), K334T (4479A>C 4480A>C), I335Y (4481A>T 4482T>A), S338K (4490T>A 4491C>A 4492C>A), V348I (4520G>A 4522T>A)								
Codon mutations:	CAA235.GA (4182A>G), GCC236GCA (4186C>A), AGC237AGG (4189C>G), ATC238TTA (4190A>T 4192C>A), AAA239AAT (4195A>T), TCG241AAG (4199T>A 4200C>A), ACG244AGC (4209C>G 4210G>C), AAG245ATC (4212A>T 4213G>C), GTT246ATT (4214G>A), ATC247ATA (4219C>A), AAG248AGA (4221A>G 4222G>A), CCC251CCG (4231C>G), AAA253CTA (4235A>C 4236A>T), TAT254TAC (4240T>C), AGC255AAT (4242G>A 4243C>T), CCA256GAA (4244C>G 4245C>A), ATG257CAA (4247A>C 4248T>A 4249G>A), GAC258GAT (4252C>T), CGA259AAA (4253C>A 4254G>A), GAA260ACT (4256G>A 4257A>C 4258A>T), GAG263AAA (4265G>A 4267G>A), AAG264ATT (4269A>T 4270G>T), CAA265CAG (4273A>G), CTG270TTA (4286C>T 4288G>A), GAC271AAT (4289G>A 4291C>T), CTC272ATG (4292C>A 4294C>G), AAG273AAA (4297G>A), GTA274TTA (4298G>T), ATC275ATA (4303C>A), AGG276AGA (4306G>A), CCC277CAT (4308C>A 4309C>T), AGC280AGT (4318C>T), ATG283AGT (4326T>G 4327G>T), GCA284AGC (4328G>A 4329C>G 4330A>C), GCG286GCA (4336G>A), TTC287TTT (4339C>T), TTA288ATG (4340T>A 4342A>G), GTC289GTA (4345C>A), AAC290AGA (4347A>G 4348C>A), GAA292CAT (4352G>C 4354A>T), GCT293GGC (4356C>G 4357T>C), GAG294GAA (4360G>A), AAA295CAA (4361A>C), CGC296AAA (4364C>A 4365G>A 4366C>A), AGA297CGT (4367A>C 4369A>T), AAG299AAA (4375G>A), AAG300GCT (4376A>G 4377A>C 4378G>T), CGT301CGA (4381T>A), GTG303GTA (4387G>A), GTC304ATA (4388G>A 4390C>A), TAC306TAT (4396C>T), GCA308GAG (4401C>A 4402A>G), ATG309CTT (4403A>C 4405G>T), GAT311AAA (4409G>A 4411T>A), GCG312AAT (4412G>A 4413C>A 4414G>T), ACT313TGT (4415A>T 4416C>G), GTA314GTC (4420A>C), GGC315TTC (4421G>T 4422G>T), GCA317GGA (4428C>G), TAC318TAT (4432C>T), AAT319TTC (4433A>T 4434A>T 4435T>C), CTT320ATC (4436C>A 4438T>C), CCC321CCA (4441C>A), AAC322AAT (4444C>T), AAG323AAA (4447G>A), GAT324AAT (4448G>A), GAA325AAC (4451G>A 4453A>C), CTC326CTT (4456C>T), CTT327ATA (4457C>A 4459T>A), ACG328AAT (4461C>A 4462G>T), CTC329CTT (4465C>T), ATC330GCA (4466A>G 4467T>C 4468C>A), AGA331AAA (4470G>A), AAG333AAA (4477G>A), AAA334ACC (4479A>C 4480A>C), ATC335TAC (4481A>T 4482T>A), TTC336TTT (4486C>T), TCT337TCA (4489T>A), TCC338AAA (4490T>A 4491C>A 4492C>A), GAC340GAT (4498C>T), TCA343AGT (4505T>A 4506C>G 4507A>T), TTC345TTT (4513C>T), GTT348ATA (4520G>A 4522T>A)								

*: Inserts / Deletes / Misaligned / Frameshifts

Analysis details

This analysis was performed with panviral2.64

NGS Details (UN59): Badnavirus volubetulae

Assembly

**Coverage Length**	391 (7 contig(s))
**Depth Of Coverage**	7.7
**Number Of Reads**	25
**Reads Per Million**	0.54 rpm (after QC)
**Ambiguities**	0
**Assembly Method**	de novo + reference guided assembly
**Consensus Caller**	Bcf Tools

Coverage Map

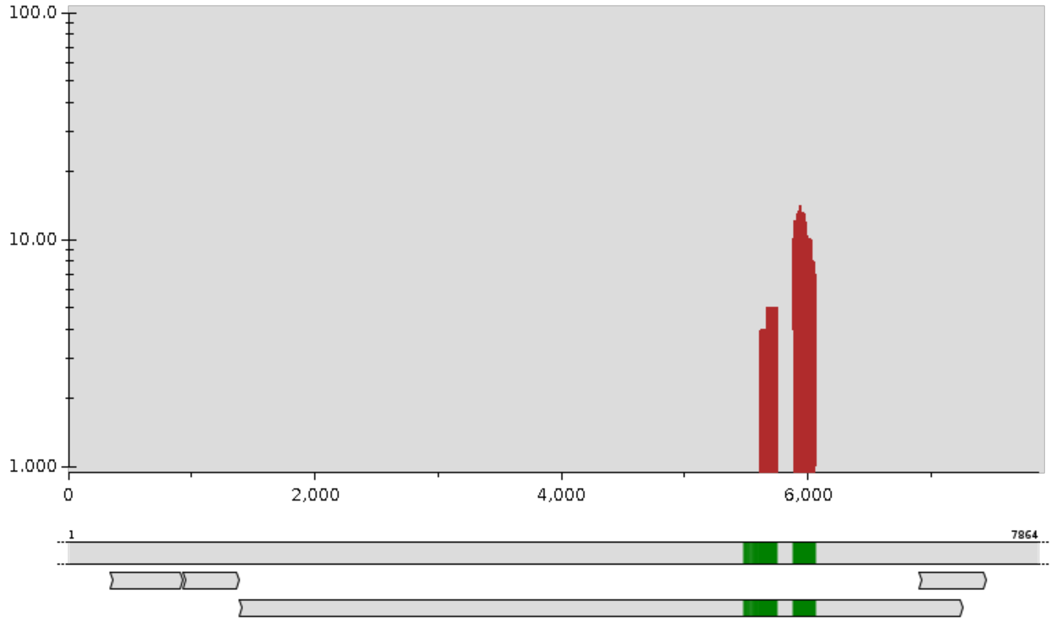

Assignment

**Type**	Badnavirus volubetulae (Taxonomy ID: 3047683)
**Reference Genome**	NC_040635.1
**NT Identity (%)**	50.9485
**AA Identity (%)**	47.0588
**Number Of Stop Codons**	0
**Number Of CDS**	4

Alignment

**Alignment Score**	-35.0 (NT) + 45.0 (AA) = 10.0
**Concordance (%)**	0.896057

**Alignment Method**	Global, seeded, nucleotide + amino acids (AGA)

Genome Region

Sequence starts at position 5482 and ends at position 6063 relative to NC_040635.1 reference sequence.

Alignment Detailed Statistics

	Begin	End	Coverage	Score	Concordance	Matches	Identities	I/D/M/F*	Stop Codons
**NT**	**5482**	**6063**	**5.0%**	**-35**	**-5.1%**	**366 (92.9%)**	**188 (47.7%)**	**3/25**	
Mutations:	5482T>A, 5484C>A, 5487T>A, 5490T>A, 5499A>C, 5500T>C, 5503C>A, 5504A>T, 5512T>G, 5513C>G, 5515C>G, 5516T>C, 5523G>A, 5526T>A, 5527C>A, 5528A>T, 5529A>G, 5538G>A, 5544C>A, 5547C>A, 5554C>G, 5556C>A, 5557C>T, 5558T>A, 5562T>A, 5566G>A, 5569T>A, 5570G>T, 5571T>A, 5572A>G, 5573G>C, 5575C>G, 5578C>A, 5580G>A, 5581A>G, 5583G>A, 5584C>G, 5585A>C, 5587G>A, 5588T>C, 5591C>A, 5592A>T, 5594C>A, 5597C>A, 5598A>C, 5601G>A, 5602A>G, 5604G>A, 5608_5610delCAG, 5613C>T, 5614A>C, 5618G>T, 5622C>A, 5624C>T, 5625T>A, 5626G>A, 5628C>A, 5629C>G, 5631G>A, 5632C>T, 5634G>A, 5635C>T, 5637C>A, 5638A>G, 5640G>T, 5641C>T, 5643C>A, 5646G>A, 5649T>A, 5652C>A, 5656C>A, 5657C>G, 5659A>T, 5660G>C, 5661C>A, 5662T>A, 5665A>T, 5666G>C, 5667C>T, 5670G>A, 5673T>C, 5677A>T, 5680A>G, 5681T>C, 5682G>A, 5685G>A, 5686A>T, 5691A>G, 5694T>A, 5695C>A, 5697G>A, 5698T>A, 5700A>C, 5701G>A, 5702G>T, 5706C>G, 5707_5728delAGTATAGATCCTAAAACAGGCA, 5731G>A, 5732A>T, 5736T>C, 5737C>A, 5747A>C, 5748A>C, 5757C>A, 5758C>A, 5877T>G, 5880C>T, 5882T>A, 5886T>C, 5890G>A, 5892C>T, 5893A>C, 5894C>T, 5895C>G, 5908T>G, 5909C>A, 5910A>T, 5911A>G, 5912T>C, 5914C>A, 5915C>A, 5917T>A, 5918G>A, 5923G>T, 5925A>T, 5929C>A, 5934A>C, 5938G>T, 5939A>G, 5940T>G, 5943G>A, 5944T>G, 5949C>T, 5950G>C, 5952A>C, 5954G>A, 5955G>T, 5957T>G, 5958A>G, 5961T>A, 5967A>G, 5979A>G, 5985C>T, 5986C>G, 5987C>G, 5988A>T, 5991A>T, 5992G>A, 5994A>G, 5996T>A, 5998C>A, 5999A>T, 6004A>G, 6005A>C, 6006A>T, 6010G>A, 6011A>C, 6012T>C, 6014A>C, 6015T>C, 6016T>A, 6017G>T, 6022A>C, 6024A>T, 6026G>A, 6029C>T, 6030_6031insATC, 6031G>C, 6033A>T, 6034G>A, 6036A>G, 6037T>G, 6038T>A, 6039C>G, 6042A>T, 6044C>A, 6045T>A, 6051C>T, 6052A>G, 6054C>G, 6063T>C								
**CDS**									
BLRaV_gp3	1380	1558	6.4%	45	4.7%	118 (92.9%)	56 (44.1%)	1/8/11/1	0
Protein mutations:	Q1380M (5527C>A 5528A>T 5529A>G), C1394I (5569T>A 5570G>T 5571T>A), K1405E (5602A>G 5604G>A), Q1407del (5608_5610delCAG), K1409Q (5614A>C), R1410I (5618G>T), H1411Q (5622C>A), T1412I (5624C>T 5625T>A), D1413K (5626G>A 5628C>A), Q1414E (5629C>G 5631G>A), K1417D (5638A>G 5640G>T), P1423R (5656C>A 5657C>G), S1425T (5662T>A), T1430S (5677A>T), M1431A (5680A>G 5681T>C 5682G>A), I1433F (5686A>T), I1434M (5691A>G), Q1436K (5695C>A 5697G>A), S1437T (5698T>A 5700A>C), G1438I (5701G>A 5702G>T), S1440_G1446del (5707_5728delAGTATAGATCCTAAAACAGGCA), E1448I (5731G>A 5732A>T), E1453A (5747A>C 5748A>C), F1498Y (5882T>A), V1501I (5890G>A 5892C>T), T1502L (5893A>C 5894C>T 5895C>G), S1507D (5908T>G 5909C>A 5910A>T), I1508A (5911A>G 5912T>C), P1509K (5914C>A 5915C>A), W1510K (5917T>A 5918G>A), A1512S (5923G>T 5925A>T), L1514I (5929C>A), D1517W (5938G>T 5939A>G 5940T>G), L1519V (5944T>G), E1521H (5950G>C 5952A>C), W1522Y (5954G>A 5955G>T), L1523R (5957T>G 5958A>G), P1533G (5986C>G 5987C>G 5988A>T), V1535M (5992G>A 5994A>G), F1536Y (5996T>A), Q1537M (5998C>A 5999A>T), K1539A (6004A>G 6005A>C 6006A>T), D1541T (6010G>A 6011A>C 6012T>C), N1542T (6014A>C 6015T>C), C1543I (6016T>A 6017G>T), K1545H (6022A>C 6024A>T), G1546D (6026G>A), T1547M (6029C>T), T1547_E1548insI (6030_6031insATC), E1548H (6031G>C 6033A>T), E1549K (6034G>A 6036A>G), F1550E (6037T>G 6038T>A 6039C>G), A1552E (6044C>A 6045T>A), I1555V (6052A>G 6054C>G)								
Codon mutations:	TTC1365A.A (5482T>A 5484C>A), ATT1366..A (5487T>A), GGT1367..A (5490T>A), CCA1370..C (5499A>C), TTA1371C.. (5500T>C), CAA1372AT. (5503C>A 5504A>T), TCA1375GG. (5512T>G 5513C>G), CTC1376GC. (5515C>G 5516T>C), AGG1378..A (5523G>A), ATT1379..A (5526T>A), CAA1380ATG (5527C>A 5528A>T 5529A>G), CTG1383..A (5538G>A), ATC1385..A (5544C>A), ATC1386..A (5547C>A), CAC1389G.A (5554C>G 5556C>A), CTT1390TA. (5557C>T 5558T>A), ACT1391..A (5562T>A), GAA1393A.. (5566G>A), TGT1394ATA (5569T>A 5570G>T 5571T>A), AGA1395GC. (5572A>G 5573G>C), CCA1396G.. (5575C>G), CTG1397A.A (5578C>A 5580G>A), AAG1398G.A (5581A>G 5583G>A), CAT1399GC. (5584C>G 5585A>C), GTA1400AC. (5587G>A 5588T>C), ACA1401.AT (5591C>A 5592A>T), CCA1402.A. (5594C>A), GCA1403.AC (5597C>A 5598A>C), ATG1404..A (5601G>A), AAG1405GAA (5602A>G 5604G>A), CAG1407del (5608_5610delCAG), TTC1408TTT (5613C>T), AAG1409CAG (5614A>C), AGA1410ATA (5618G>T), CAC1411CAA (5622C>A), ACT1412ATA (5624C>T 5625T>A), GAC1413AAA (5626G>A 5628C>A), CAG1414GAA (5629C>G 5631G>A), CTG1415TTA (5632C>T 5634G>A), CTC1416TTA (5635C>T 5637C>A), AAG1417GAT (5638A>G 5640G>T), CTC1418TTA (5641C>T 5643C>A), GGG1419GGA (5646G>A), GTT1420GTA (5649T>A), ATC1421ATA (5652C>A), CCA1423AGA (5656C>A 5657C>G), AGC1424TCA (5659A>T 5660G>C 5661C>A), TCA1425ACA (5662T>A), AGC1426TCT (5665A>T 5666G>C 5667C>T), AGG1427AGA (5670G>A), CAT1428CAC (5673T>C), ACA1430TCA (5677A>T), ATG1431GCA (5680A>G 5681T>C 5682G>A), GCG1432GCA (5685G>A), ATC1433TTC (5686A>T), ATA1434ATG (5691A>G), GTT1435GTA (5694T>A), CAG1436AAA (5695C>A 5697G>A), TCA1437ACC (5698T>A 5700A>C), GGA1438ATA (5701G>A 5702G>T), ACC1439ACG (5706C>G), AGT1440_GGC1446del (5707_5728delAGTATAGATCCTAAAACAGGCA), AAA1447-AA (5707_5728delAGTATAGATCCTAAAACAGGCA), GAA1448ATA (5731G>A 5732A>T), GTT1449GTC (5736T>C), CGA1450AGA (5737C>A), GAA1453GCC (5747A>C 5748A>C), GTC1456GTA (5757C>A), CTC1457AT. (5758C>A), AGT1496..G (5877T>G), GGC1497GGT (5880C>T), TTT1498TAT (5882T>A), CAT1499CAC (5886T>C), GTC1501ATT (5890G>A 5892C>T), ACC1502CTG (5893A>C 5894C>T 5895C>G), TCA1507GAT (5908T>G 5909C>A 5910A>T), ATA1508GCA (5911A>G 5912T>C), CCA1509AAA (5914C>A 5915C>A), TGG1510AAG (5917T>A 5918G>A), GCA1512TCT (5923G>T 5925A>T), CTC1514ATC (5929C>A), ACA1515ACC (5934A>C), GAT1517TGG (5938G>T 5939A>G 5940T>G), GGG1518GGA (5943G>A), TTG1519GTG (5944T>G), TAC1520TAT (5949C>T), GAA1521CAC (5950G>C 5952A>C), TGG1522TAT (5954G>A 5955G>T), CTA1523CGG (5957T>G 5958A>G), GTT1524GTA (5961T>A), CCA1526CCG (5967A>G), AAA1530AAG (5979A>G), GCC1532GCT (5985C>T), CCA1533GGT (5986C>G 5987C>G 5988A>T), GCA1534GCT (5991A>T), GTA1535ATG (5992G>A 5994A>G), TTC1536TAC (5996T>A), CAG1537ATG (5998C>A 5999A>T), AAA1539GCT (6004A>G 6005A>C 6006A>T), GAT1541ACC (6010G>A 6011A>C 6012T>C), AAT1542ACC (6014A>C 6015T>C), TGC1543ATC (6016T>A 6017G>T), AAA1545CAT (6022A>C 6024A>T), GGT1546GAT (6026G>A), ACG1547ATG (6029C>T), ACG1547_GAA1548insATC (6030_6031insATC), GAA1548CAT (6031G>C 6033A>T), GAA1549AAG (6034G>A 6036A>G), TTC1550GAG (6037T>G 6038T>A 6039C>G), ATA1551ATT (6042A>T), GCT1552GAA (6044C>A 6045T>A), TAC1554TAT (6051C>T), ATC1555GTG (6052A>G 6054C>G), ATT1558ATC (6063T>C)								
**Proteins**									
polyprotein (YP_009552737.1)	1380	1558	6.4%	45	4.7%	118 (92.9%)	56 (44.1%)	1/8/11/1	0
Protein mutations:	Q1380M (5527C>A 5528A>T 5529A>G), C1394I (5569T>A 5570G>T 5571T>A), K1405E (5602A>G 5604G>A), Q1407del (5608_5610delCAG), K1409Q (5614A>C), R1410I (5618G>T), H1411Q (5622C>A), T1412I (5624C>T 5625T>A), D1413K (5626G>A 5628C>A), Q1414E (5629C>G 5631G>A), K1417D (5638A>G 5640G>T), P1423R (5656C>A 5657C>G), S1425T (5662T>A), T1430S (5677A>T), M1431A (5680A>G 5681T>C 5682G>A), I1433F (5686A>T), I1434M (5691A>G), Q1436K (5695C>A 5697G>A), S1437T (5698T>A 5700A>C), G1438I (5701G>A 5702G>T), S1440_G1446del (5707_5728delAGTATAGATCCTAAAACAGGCA), E1448I (5731G>A 5732A>T), E1453A (5747A>C 5748A>C), F1498Y (5882T>A), V1501I (5890G>A 5892C>T), T1502L (5893A>C 5894C>T 5895C>G), S1507D (5908T>G 5909C>A 5910A>T), I1508A (5911A>G 5912T>C), P1509K (5914C>A 5915C>A), W1510K (5917T>A 5918G>A), A1512S (5923G>T 5925A>T), L1514I (5929C>A), D1517W (5938G>T 5939A>G 5940T>G), L1519V (5944T>G), E1521H (5950G>C 5952A>C), W1522Y (5954G>A 5955G>T), L1523R (5957T>G 5958A>G), P1533G (5986C>G 5987C>G 5988A>T), V1535M (5992G>A 5994A>G), F1536Y (5996T>A), Q1537M (5998C>A 5999A>T), K1539A (6004A>G 6005A>C 6006A>T), D1541T (6010G>A 6011A>C 6012T>C), N1542T (6014A>C 6015T>C), C1543I (6016T>A 6017G>T), K1545H (6022A>C 6024A>T), G1546D (6026G>A), T1547M (6029C>T), T1547_E1548insI (6030_6031insATC), E1548H (6031G>C 6033A>T), E1549K (6034G>A 6036A>G), F1550E (6037T>G 6038T>A 6039C>G), A1552E (6044C>A 6045T>A), I1555V (6052A>G 6054C>G)								
Codon mutations:	TTC1365A.A (5482T>A 5484C>A), ATT1366..A (5487T>A), GGT1367..A (5490T>A), CCA1370..C (5499A>C), TTA1371C.. (5500T>C), CAA1372AT. (5503C>A 5504A>T), TCA1375GG. (5512T>G 5513C>G), CTC1376GC. (5515C>G 5516T>C), AGG1378..A (5523G>A), ATT1379..A (5526T>A), CAA1380ATG (5527C>A 5528A>T 5529A>G), CTG1383..A (5538G>A), ATC1385..A (5544C>A), ATC1386..A (5547C>A), CAC1389G.A (5554C>G 5556C>A), CTT1390TA. (5557C>T 5558T>A), ACT1391..A (5562T>A), GAA1393A.. (5566G>A), TGT1394ATA (5569T>A 5570G>T 5571T>A), AGA1395GC. (5572A>G 5573G>C), CCA1396G.. (5575C>G), CTG1397A.A (5578C>A 5580G>A), AAG1398G.A (5581A>G 5583G>A), CAT1399GC. (5584C>G 5585A>C), GTA1400AC. (5587G>A 5588T>C), ACA1401.AT (5591C>A 5592A>T), CCA1402.A. (5594C>A), GCA1403.AC (5597C>A 5598A>C), ATG1404..A (5601G>A), AAG1405GAA (5602A>G 5604G>A), CAG1407del (5608_5610delCAG), TTC1408TTT (5613C>T), AAG1409CAG (5614A>C), AGA1410ATA (5618G>T), CAC1411CAA (5622C>A), ACT1412ATA (5624C>T 5625T>A), GAC1413AAA (5626G>A 5628C>A), CAG1414GAA (5629C>G 5631G>A), CTG1415TTA (5632C>T 5634G>A), CTC1416TTA (5635C>T 5637C>A), AAG1417GAT (5638A>G 5640G>T), CTC1418TTA (5641C>T 5643C>A), GGG1419GGA (5646G>A), GTT1420GTA (5649T>A), ATC1421ATA (5652C>A), CCA1423AGA (5656C>A 5657C>G), AGC1424TCA (5659A>T 5660G>C 5661C>A), TCA1425ACA (5662T>A), AGC1426TCT (5665A>T 5666G>C 5667C>T), AGG1427AGA (5670G>A), CAT1428CAC (5673T>C), ACA1430TCA (5677A>T), ATG1431GCA (5680A>G 5681T>C 5682G>A), GCG1432GCA (5685G>A), ATC1433TTC (5686A>T), ATA1434ATG (5691A>G), GTT1435GTA (5694T>A), CAG1436AAA (5695C>A 5697G>A), TCA1437ACC (5698T>A 5700A>C), GGA1438ATA (5701G>A 5702G>T), ACC1439ACG (5706C>G), AGT1440_GGC1446del (5707_5728delAGTATAGATCCTAAAACAGGCA), AAA1447-AA (5707_5728delAGTATAGATCCTAAAACAGGCA), GAA1448ATA (5731G>A 5732A>T), GTT1449GTC (5736T>C), CGA1450AGA (5737C>A), GAA1453GCC (5747A>C 5748A>C), GTC1456GTA (5757C>A), CTC1457AT. (5758C>A), AGT1496..G (5877T>G), GGC1497GGT (5880C>T), TTT1498TAT (5882T>A), CAT1499CAC (5886T>C), GTC1501ATT (5890G>A 5892C>T), ACC1502CTG (5893A>C 5894C>T 5895C>G), TCA1507GAT (5908T>G 5909C>A 5910A>T), ATA1508GCA (5911A>G 5912T>C), CCA1509AAA (5914C>A 5915C>A), TGG1510AAG (5917T>A 5918G>A), GCA1512TCT (5923G>T 5925A>T), CTC1514ATC (5929C>A), ACA1515ACC (5934A>C), GAT1517TGG (5938G>T 5939A>G 5940T>G), GGG1518GGA (5943G>A), TTG1519GTG (5944T>G), TAC1520TAT (5949C>T), GAA1521CAC (5950G>C 5952A>C), TGG1522TAT (5954G>A 5955G>T), CTA1523CGG (5957T>G 5958A>G), GTT1524GTA (5961T>A), CCA1526CCG (5967A>G), AAA1530AAG (5979A>G), GCC1532GCT (5985C>T), CCA1533GGT (5986C>G 5987C>G 5988A>T), GCA1534GCT (5991A>T), GTA1535ATG (5992G>A 5994A>G), TTC1536TAC (5996T>A), CAG1537ATG (5998C>A 5999A>T), AAA1539GCT (6004A>G 6005A>C 6006A>T), GAT1541ACC (6010G>A 6011A>C 6012T>C), AAT1542ACC (6014A>C 6015T>C), TGC1543ATC (6016T>A 6017G>T), AAA1545CAT (6022A>C 6024A>T), GGT1546GAT (6026G>A), ACG1547ATG (6029C>T), ACG1547_GAA1548insATC (6030_6031insATC), GAA1548CAT (6031G>C 6033A>T), GAA1549AAG (6034G>A 6036A>G), TTC1550GAG (6037T>G 6038T>A 6039C>G), ATA1551ATT (6042A>T), GCT1552GAA (6044C>A 6045T>A), TAC1554TAT (6051C>T), ATC1555GTG (6052A>G 6054C>G), ATT1558ATC (6063T>C)								

*: Inserts / Deletes / Misaligned / Frameshifts

Analysis details

This analysis was performed with panviral2.64

NGS Details (UN59): Petuvirus venapetuniae

Assembly

**Coverage Length**	610 (1 contig(s))
**Depth Of Coverage**	5.3
**Number Of Reads**	25
**Reads Per Million**	0.54 rpm (after QC)
**Ambiguities**	0
**Assembly Method**	de novo + reference guided assembly
**Consensus Caller**	Bcf Tools

Coverage Map

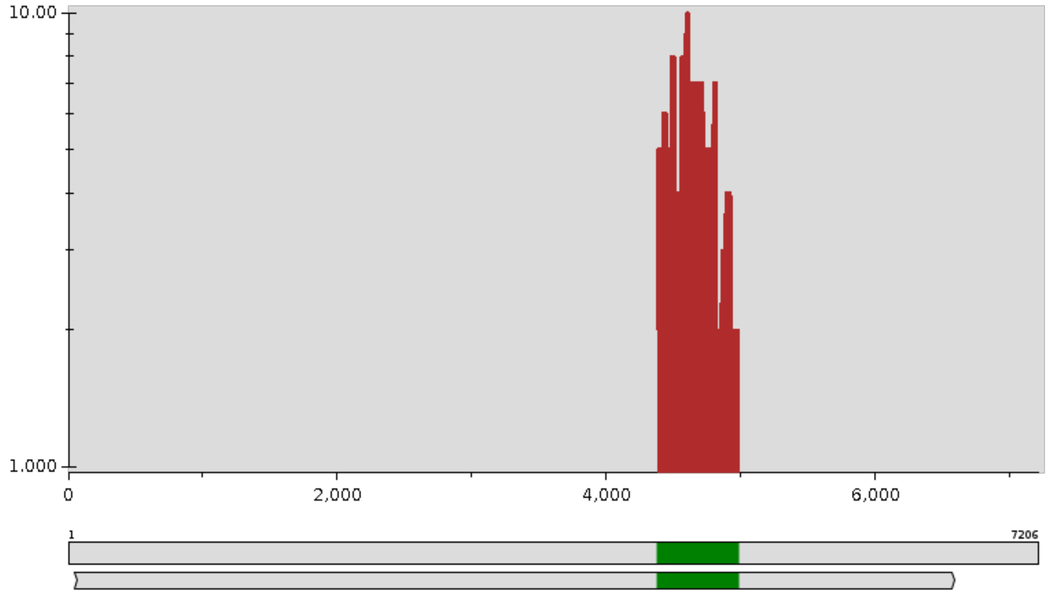

Assignment

**Type**	Petuvirus venapetuniae (Taxonomy ID: 3048265)
**Reference Genome**	NC_001839.2
**NT Identity (%)**	51.8822
**AA Identity (%)**	42.9268
**Number Of Stop Codons**	1
**Number Of CDS**	1

Alignment

**Alignment Score**	38.0 (NT) + 491.0 (AA) = 529.0
**Concordance (%)**	20.983

Alignment Method	Global, seeded, nucleotide + amino acids (AGA)

Genome Region

Sequence starts at position 4380 and ends at position 4989 relative to NC_001839.2 reference sequence.

Alignment Detailed Statistics

	Begin	End	Coverage	Score	Concordance	Matches	Identities	I/D/M/F*	Stop Codons
**NT**	**4380**	**4989**	**8.5%**	**38**	**3.1%**	**610 (99.8%)**	**317 (51.9%)**	**1/0**	
Mutations:	4386C>A, 4391A>T, 4394A>G, 4397C>T, 4398C>A, 4399A>C, 4401T>G, 4402T>C, 4403C>T, 4404C>T, 4406C>A, 4407C>A, 4410G>T, 4411A>G, 4412T>G, 4413G>A, 4414A>T, 4415C>T, 4417A>G, 4418G>A, 4420T>A, 4421T>C, 4424C>T, 4430A>T, 4433T>C, 4436G>A, 4437C>A, 4438T>A, 4439C>G, 4440A>G, 4441C>A, 4442A>T, 4445C>T, 4446T>C, 4448C>A, 4449T>A, 4450C>A, 4451T>A, 4452C>A, 4453A>G, 4454C>A, 4455T>A, 4456T>C, 4457A>C, 4459C>T, 4460C>T, 4469G>C, 4470C>A, 4472C>T, 4474T>A, 4475T>C, 4476T>A, 4477C>G, 4478A>T, 4481G>A, 4488C>A, 4490T>G, 4493G>A, 4499A>G, 4509T>A, 4511A>T, 4512G>C, 4513G>A, 4514A>G, 4517C>T, 4518C>T, 4519A>C, 4521C>G, 4522C>A, 4523T>A, 4526T>A, 4529G>C, 4531G>A, 4533C>T, 4534C>A, 4535A>G, 4538A>G, 4539A>T, 4541A>C, 4543G>C, 4544G>T, 4548T>A, 4549G>A, 4550C>T, 4551A>G, 4556T>G, 4557G>T, 4558A>T, 4560A>G, 4562A>T, 4565T>A, 4566T>C, 4567T>A, 4569C>G, 4577G>C, 4580C>T, 4585C>T, 4586A>T, 4589C>T, 4592T>G, 4600C>A, 4601A>T, 4607T>C, 4610T>A, 4611T>G, 4612T>A, 4613G>A, 4616C>T, 4623G>A, 4624C>A, 4625C>T, 4625_4626insT, 4630T>A, 4631C>T, 4632A>G, 4634A>T, 4638T>A, 4640C>T, 4641C>A, 4643G>T, 4645C>A, 4646C>T, 4647A>T, 4649C>G, 4650C>A, 4651T>G, 4652T>C, 4653T>C, 4654T>A, 4656T>A, 4657C>T, 4658A>G, 4659G>T, 4661T>A, 4662T>A, 4664G>A, 4667C>T, 4670C>T, 4677G>A, 4680A>G, 4682C>T, 4683C>T, 4685T>A, 4686C>A, 4688T>C, 4691T>C, 4697A>G, 4698A>G, 4699C>A, 4700A>C, 4701C>A, 4703G>C, 4706A>T, 4707G>T, 4708A>C, 4712C>T, 4713A>T, 4715A>G, 4719T>C, 4720T>A, 4721G>C, 4724T>A, 4727C>T, 4728C>A, 4729A>T, 4730G>T, 4733C>A, 4734A>T, 4736A>T, 4738G>A, 4739T>C, 4740T>A, 4741T>C, 4742G>C, 4745T>C, 4750A>G, 4752T>A, 4753T>A, 4757A>T, 4758G>T, 4760C>G, 4761A>G, 4763G>A, 4764T>G, 4767T>A, 4768C>G, 4769G>C, 4770G>A, 4772A>T, 4781G>T, 4783T>A, 4784C>A, 4788G>T, 4789C>T, 4795A>C, 4796T>A, 4803C>A, 4804A>G, 4808C>T, 4811A>G, 4814A>T, 4815A>C, 4816T>A, 4817G>T, 4823T>A, 4824G>T, 4825C>A, 4826T>C, 4827G>C, 4829T>A, 4832A>T, 4833A>T, 4837T>G, 4838T>C, 4839T>A, 4840C>G, 4844T>A, 4845G>A, 4846C>T, 4848G>T, 4850T>C, 4851C>A, 4852A>G, 4853C>A, 4854A>G, 4855T>C, 4857T>A, 4858C>T, 4860T>G, 4861T>A, 4863G>T, 4864A>T, 4865A>T, 4866C>T, 4867T>C, 4868C>T, 4869C>A, 4871A>T, 4874G>A, 4880T>C, 4881G>A, 4884A>G, 4885C>T, 4886A>T, 4888A>T, 4890C>A, 4891T>C, 4892C>T, 4893T>G, 4894C>A, 4895G>C, 4896G>A, 4897T>A, 4900A>C, 4901G>C, 4904G>A, 4905A>C, 4907A>G, 4911C>A, 4912A>G, 4913A>G, 4917C>T, 4919T>G, 4922G>A, 4924T>G, 4926G>C, 4931T>C, 4935A>G, 4938A>G, 4939G>C, 4940A>T, 4943T>C, 4946T>C, 4952A>T, 4953G>A, 4960C>G, 4961T>A, 4962G>C, 4964G>A, 4965C>G, 4966A>T, 4968A>T, 4969T>G, 4971T>A, 4972C>A, 4973C>A, 4974C>T, 4977C>T, 4979T>G, 4980A>T, 4981G>T, 4985T>C, 4987T>G, 4989C>T								

CDS

	Begin	End	Coverage	Score	Concordance	Matches	Identities	I/D/M/F*	Stop Codons
PVCVgp1	1444	1647	9.4%	491	34.4%	204 (99.5%)	88 (42.9%)	1/0/1/1	1
Protein mutations:	Q1446K (4386C>A), H1450T (4398C>A 4399A>C), F1451A (4401T>G 4402T>C 4403C>T), Q1453K (4407C>A), D1454W (4410G>T 4411A>G 4412T>G), D1455I (4413G>A 4414A>T 4415C>T), K1456R (4417A>G 4418G>A), F1457Y (4420T>A 4421T>C), L1463K (4437C>A 4438T>A 4439C>G), T1464D (4440A>G 4441C>A 4442A>T), F1466L (4446T>C 4448C>A), S1467K (4449T>A 4450C>A 4451T>A), H1468R (4452C>A 4453A>G 4454C>A), L1469T (4455T>A 4456T>C 4457A>C), S1470F (4459C>T 4460C>T), K1473N (4469G>C), L1474I (4470C>A 4472C>T), F1475Y (4474T>A 4475T>C), L1480M (4488C>A 4490T>G), L1487I (4509T>A 4511A>T), G1488Q (4512G>C 4513G>A 4514A>G), H1490S (4518C>T 4519A>C), P1491E (4521C>G 4522C>A 4523T>A), N1492K (4526T>A), E1493D (4529G>C), R1494K (4531G>A), P1495* (4533C>T 4534C>A 4535A>G), T1497S (4539A>T 4541A>C), G1498A (4543G>C 4544G>T), C1500N (4548T>A 4549G>A 4550C>T), I1501V (4551A>G), D1503F (4557G>T 4558A>T), R1504G (4560A>G 4562A>T), H1505Q (4565T>A), F1506H (4566T>C 4567T>A), Q1507E (4569C>G), K1509N (4577G>C), P1512L (4585C>T 4586A>T), T1517N (4600C>A 4601A>T), L1521E (4611T>G 4612T>A 4613G>A), A1525N (4623G>A 4624C>A 4625C>T), A1525_M1526insX (4625_4626insT), I1527N (4630T>A 4631C>T), K1528D (4632A>G 4634A>T), F1530I (4638T>A 4640C>T), Q1531N (4641C>A 4643G>T), P1532H (4645C>A 4646C>T), I1533L (4647A>T 4649C>G), L1534S (4650C>A 4651T>G 4652T>C), F1535H (4653T>C 4654T>A), S1536M (4656T>A 4657C>T 4658A>G), A1537S (4659G>T 4661T>A), L1538I (4662T>A 4664G>A), D1543N (4677G>A), I1544V (4680A>G 4682C>T), L1546I (4686C>A 4688T>C), T1550D (4698A>G 4699C>A 4700A>C), L1551I (4701C>A 4703G>C), E1552D (4706A>T), D1553S (4707G>T 4708A>C), I1555L (4713A>T 4715A>G), L1557H (4719T>C 4720T>A 4721G>C), Q1560I (4728C>A 4729A>T 4730G>T), F1561L (4733C>A), I1562F (4734A>T 4736A>T), S1563N (4738G>A 4739T>C), L1564T (4740T>A 4741T>C 4742G>C), K1567R (4750A>G), F1568N (4752T>A 4753T>A), V1570L (4758G>T 4760C>G), M1571V (4761A>G 4763G>A), L1572V (4764T>G), A1574T (4770G>A 4772A>T), M1577I (4781G>T), I1578K (4783T>A 4784C>A), A1580F (4788G>T 4789C>T), N1582T (4795A>C 4796T>A), Q1585R (4803C>A 4804A>G), M1589H (4815A>C 4816T>A 4817G>T), F1591L (4823T>A), A1592Y (4824G>T 4825C>A 4826T>C), D1593Q (4827G>C 4829T>A), T1595S (4833A>T), F1596C (4837T>G 4838T>C), S1597R (4839T>A 4840C>G), A1599I (4845G>A 4846C>T), G1600C (4848G>T 4850T>C), H1601R (4851C>A 4852A>G 4853C>A), I1602A (4854A>G 4855T>C), S1603I (4857T>A 4858C>T), L1604E (4860T>G 4861T>A), E1605F (4863G>T 4864A>T 4865A>T), L1606S (4866C>T 4867T>C 4868C>T), Q1607N (4869C>A 4871A>T), D1611N (4881G>A), T1612V (4884A>G 4885C>T 4886A>T), N1613I (4888A>T), L1614T (4890C>A 4891T>C 4892C>T), S1615D (4893T>G 4894C>A 4895G>C), V1616K (4896G>A 4897T>A), K1617T (4900A>C 4901G>C), I1619L (4905A>C 4907A>G), Q1621R (4911C>A 4912A>G 4913A>G), I1625S (4924T>G), V1626L (4926G>C), I1629V (4935A>G), R1630A (4938A>G 4939G>C 4940A>T), E1635K (4953G>A), T1637R (4960C>G 4961T>A), E1638Q (4962G>C 4964G>A), H1639V (4965C>G 4966A>T), I1640C (4968A>T 4969T>G), S1641K (4971T>A 4972C>A 4973C>A), P1642S (4974C>T), S1644F (4980A>T 4981G>T), M1646R (4987T>G)								
Codon mutations:	CAA1446AAA (4386C>A), CCA1447CCT (4391A>T), TTA1448TTG (4394A>G), AAC1449AAT (4397C>T), CAT1450ACT (4398C>A 4399A>C), TTC1451GCT (4401T>G 4402T>C 4403C>T), CTC1452TTA (4404C>T 4406C>A), CAA1453AAA (4407C>A), GAT1454TGG (4410G>T 4411A>G 4412T>G), GAC1455ATT (4413G>A 4414A>T 4415C>T), AAG1456AGA (4417A>G 4418G>A), TTT1457TAC (4420T>A 4421T>C), CCC1458CCT (4424C>T), CCA1460CCT (4430A>T), AAT1461AAC (4433T>C), AAG1462AAA (4436G>A), CTC1463AAG (4437C>A 4438T>A 4439C>G), ACA1464GAT (4440A>G 4441C>A 4442A>T), CTC1465CTT (4445C>T), TTC1466CTA (4446T>C 4448C>A), TCT1467AAA (4449T>A 4450C>A 4451T>A), CAC1468AGA (4452C>A 4453A>G 4454C>A), TTA1469ACC (4455T>A 4456T>C 4457A>C), TCC1470TTT (4459C>T 4460C>T), AAG1473AAC (4469G>C), CTC1474ATT (4470C>A 4472C>T), TTT1475TAC (4474T>A 4475T>C), TCA1476AGT (4476T>A 4477C>G 4478A>T), AAG1477AAA (4481G>A), CTT1480ATG (4488C>A 4490T>G), AAG1481AAA (4493G>A), GGA1483GGG (4499A>G), TTA1487ATT (4509T>A 4511A>T), GGA1488CAG (4512G>C 4513G>A 4514A>G), ATC1489ATT (4517C>T), CAT1490TCT (4518C>T 4519A>C), CCT1491GAA (4521C>G 4522C>A 4523T>A), AAT1492AAA (4526T>A), GAG1493GAC (4529G>C), AGA1494AAA (4531G>A), CCA1495TAG (4533C>T 4534C>A 4535A>G), AAA1496AAG (4538A>G), ACA1497TCC (4539A>T 4541A>C), GGG1498GCT (4543G>C 4544G>T), TGC1500AAT (4548T>A 4549G>A 4550C>T), ATT1501GTT (4551A>G), CCT1502CCG (4556T>G), GAT1503TTT (4557G>T 4558A>T), AGA1504GGT (4560A>G 4562A>T), CAT1505CAA (4565T>A), TTT1506CAT (4566T>C 4567T>A), CAA1507GAA (4569C>G), AAG1509AAC (4577G>C), GTC1510GTT (4580C>T), CCA1512CTT (4585C>T 4586A>T), TTC1513TTT (4589C>T), GGT1514GGG (4592T>G), ACA1517AAT (4600C>A 4601A>T), CCT1519CCC (4607T>C), TCT1520TCA (4610T>A), TTG1521GAA (4611T>G 4612T>A 4613G>A), TTC1522TTT (4616C>T), GCC1525AAT (4623G>A 4624C>A 4625C>T), GCC1525_ATG1526insT-- (4625_4626insT), ATC1527AAT (4630T>A 4631C>T), AAA1528GAT (4632A>G 4634A>T), TTC1530ATT (4638T>A 4640C>T), CAG1531AAT (4641C>A 4643G>T), CCC1532CAT (4645C>A 4646C>T), ATC1533TTG (4647A>T 4649C>G), CTT1534AGC (4650C>A 4651T>G 4652T>C), TTT1535CAT (4653T>C 4654T>A), TCA1536ATG (4656T>A 4657C>T 4658A>G), GCT1537TCA (4659G>T 4661T>A), TTG1538ATA (4662T>A 4664G>A), GTC1539GTT (4667C>T), TAC1540TAT (4670C>T), GAT1543AAT (4677G>A), ATC1544GTT (4680A>G 4682C>T), CTT1545TTA (4683C>T 4685T>A), CTT1546ATC (4686C>A 4688T>C), TTT1547TTC (4691T>C), GAA1549GAG (4697A>G), ACA1550GAC (4698A>G 4699C>A 4700A>C), CTG1551ATC (4701C>A 4703G>C), GAA1552GAT (4706A>T), GAT1553TCT (4707G>T 4708A>C), CAC1554CAT (4712C>T), ATA1555TTG (4713A>T 4715A>G), TTG1557CAC (4719T>C 4720T>A 4721G>C), CTT1558CTA (4724T>A), AAC1559AAT (4727C>T), CAG1560ATT (4728C>A 4729A>T 4730G>T), TTC1561TTA (4733C>A), ATA1562TTT (4734A>T 4736A>T), AGT1563AAC (4738G>A 4739T>C), TTG1564ACC (4740T>A 4741T>C 4742G>C), GTT1565GTC (4745T>C), AAA1567AGA (4750A>G), TTT1568AAT (4752T>A 4753T>A), GGA1569GGT (4757A>T), GTC1570TTG (4758G>T 4760C>G), ATG1571GTA (4761A>G 4763G>A), TTA1572GTA (4764T>G), TCG1573AGC (4767T>A 4768C>G 4769G>C), GCA1574ACT (4770G>A 4772A>T), ATG1577ATT (4781G>T), ATC1578AAA (4783T>A 4784C>A), GCT1580TTT (4788G>T 4789C>T), AAT1582ACA (4795A>C 4796T>A), CAA1585AGA (4803C>A 4804A>G), TTC1586TTT (4808C>T), TTA1587TTG (4811A>G), GGA1588GGT (4814A>T), ATG1589CAT (4815A>C 4816T>A 4817G>T), TTT1591TTA (4823T>A), GCT1592TAC (4824G>T 4825C>A 4826T>C), GAT1593CAA (4827G>C 4829T>A), GGA1594GGT (4832A>T), ACT1595TCT (4833A>T), TTT1596TGC (4837T>G 4838T>C), TCA1597AGA (4839T>A 4840C>G), CCT1598CCA (4844T>A), GCT1599ATT (4845G>A 4846C>T), GGT1600TGC (4848G>T 4850T>C), CAC1601AGA (4851C>A 4852A>G 4853C>A), ATC1602GCC (4854A>G 4855T>C), TCT1603ATT (4857T>A 4858C>T), TTG1604GAG (4860T>G 4861T>A), GAA1605TTT (4863G>T 4864A>T 4865A>T), CTC1606TCT (4866C>T 4867T>C 4868C>T), CAA1607AAT (4869C>A 4871A>T), AAG1608AAA (4874G>A), CCT1610CCC (4880T>C), GAT1611AAT (4881G>A), ACA1612GTT (4884A>G 4885C>T 4886A>T), AAT1613ATT (4888A>T), CTC1614ACT (4890C>A 4891T>C 4892C>T), TCG1615GAC (4893T>G 4894C>A 4895G>C), GTA1616AAA (4896G>A 4897T>A), AAG1617ACC (4900A>C 4901G>C), CAG1618CAA (4904G>A), ATA1619CTG (4905A>C 4907A>G), CAA1621AGG (4911C>A 4912A>G 4913A>G), CTT1623TTG (4917C>T 4919T>G), GGG1624GGA (4922G>A), ATT1625AGT (4924T>G), GTT1626CTT (4926G>C), AAT1627AAC (4931T>C), ATC1629GTC (4935A>G), AGA1630GCT (4938A>G 4939G>C 4940A>T), GAT1631GAC (4943T>C), TTT1632TTC (4946T>C), CCA1634CCT (4952A>T), GAG1635AAG (4953G>A), ACT1637AGA (4960C>G 4961T>A), GAG1638CAA (4962G>C 4964G>A), CAT1639GTT (4965C>G 4966A>T), ATT1640TGT (4968A>T 4969T>G), TCC1641AAA (4971T>A 4972C>A 4973C>A), CCT1642TCT (4974C>T), CTT1643TTG (4977C>T 4979T>G), AGT1644TTT (4980A>T 4981G>T), GAT1645GAC (4985T>C), ATG1646AGG (4987T>G), CTA1647T.. (4989C>T)								

Proteins

	Begin	End	Coverage	Score	Concordance	Matches	Identities	I/D/M/F*	Stop Codons
ORF I polyprotein (NP_127504.1)	1444	1647	9.4%	491	34.4%	204 (99.5%)	88 (42.9%)	1/0/1/1	1

	Begin	End	Coverage	Score	Concordance	Matches	Identities	I/D/M/F*	Stop Codons
**NT**	**4380**	**4989**	**8.5%**	**38**	**3.1%**	**610 (99.8%)**	**317 (51.9%)**	**1/0**	
Protein mutations:	Q1446K (4386C>A), H1450T (4398C>A 4399A>C), F1451A (4401T>G 4402T>C 4403C>T), Q1453K (4407C>A), D1454W (4410G>T 4411A>G 4412T>G), D1455I (4413G>A 4414A>T 4415C>T), K1456R (4417A>G 4418G>A), F1457Y (4420T>A 4421T>C), L1463K (4437C>A 4438T>A 4439C>G), T1464D (4440A>G 4441C>A 4442A>T), F1466L (4446T>C 4448C>A), S1467K (4449T>A 4450C>A 4451T>A), H1468R (4452C>A 4453A>G 4454C>A), L1469T (4455T>A 4456T>C 4457A>C), S1470F (4459C>T 4460C>T), K1473N (4469G>C), L1474I (4470C>A 4472C>T), F1475Y (4474T>A 4475T>C), L1480M (4488C>A 4490T>G), L1487I (4509T>A 4511A>T), G1488Q (4512G>C 4513G>A 4514A>G), H1490S (4518C>T 4519A>C), P1491E (4521C>G 4522C>A 4523T>A), N1492K (4526T>A), E1493D (4529G>C), R1494K (4531G>A), P1495* (4533C>T 4534C>A 4535A>G), T1497S (4539A>T 4541A>C), G1498A (4543G>C 4544G>T), C1500N (4548T>A 4549G>A 4550C>T), I1501V (4551A>G), D1503F (4557G>T 4558A>T), R1504G (4560A>G 4562A>T), H1505Q (4565T>A), F1506H (4566T>C 4567T>A), Q1507E (4569C>G), K1509N (4577G>C), P1512L (4585C>T 4586A>T), T1517N (4600C>A 4601A>T), L1521E (4611T>G 4612T>A 4613G>A), A1525N (4623G>A 4624C>A 4625C>T), A1525_M1526insX (4625_4626insT), I1527N (4630T>A 4631C>T), K1528D (4632A>G 4634A>T), F1530I (4638T>A 4640C>T), Q1531N (4641C>A 4643G>T), P1532H (4645C>A 4646C>T), I1533L (4647A>T 4649C>G), L1534S (4650C>A 4651T>G 4652T>C), F1535H (4653T>C 4654T>A), S1536M (4656T>A 4657C>T 4658A>G), A1537S (4659G>T 4661T>A), L1538I (4662T>A 4664G>A), D1543N (4677G>A), I1544V (4680A>G 4682C>T), L1546I (4686C>A 4688T>C), T1550D (4698A>G 4699C>A 4700A>C), L1551I (4701C>A 4703G>C), E1552D (4706A>T), D1553S (4707G>T 4708A>C), I1555L (4713A>T 4715A>G), L1557H (4719T>C 4720T>A 4721G>C), Q1560I (4728C>A 4729A>T 4730G>T), F1561L (4733C>A), I1562F (4734A>T 4736A>T), S1563N (4738G>A 4739T>C), L1564T (4740T>A 4741T>C 4742G>C), K1567R (4750A>G), F1568N (4752T>A 4753T>A), V1570L (4758G>T 4760C>G), M1571V (4761A>G 4763G>A), L1572V (4764T>G), A1574T (4770G>A 4772A>T), M1577I (4781G>T), I1578K (4783T>A 4784C>A), A1580F (4788G>T 4789C>T), N1582T (4795A>C 4796T>A), Q1585R (4803C>A 4804A>G), M1589H (4815A>C 4816T>A 4817G>T), F1591L (4823T>A), A1592Y (4824G>T 4825C>A 4826T>C), D1593Q (4827G>C 4829T>A), T1595S (4833A>T), F1596C (4837T>G 4838T>C), S1597R (4839T>A 4840C>G), A1599I (4845G>A 4846C>T), G1600C (4848G>T 4850T>C), H1601R (4851C>A 4852A>G 4853C>A), I1602A (4854A>G 4855T>C), S1603I (4857T>A 4858C>T), L1604E (4860T>G 4861T>A), E1605F (4863G>T 4864A>T 4865A>T), L1606S (4866C>T 4867T>C 4868C>T), Q1607N (4869C>A 4871A>T), D1611N (4881G>A), T1612V (4884A>G 4885C>T 4886A>T), N1613I (4888A>T), L1614T (4890C>A 4891T>C 4892C>T), S1615D (4893T>G 4894C>A 4895G>C), V1616K (4896G>A 4897T>A), K1617T (4900A>C 4901G>C), I1619L (4905A>C 4907A>G), Q1621R (4911C>A 4912A>G 4913A>G), I1625S (4924T>G), V1626L (4926G>C), I1629V (4935A>G), R1630A (4938A>G 4939G>C 4940A>T), E1635K (4953G>A), T1637R (4960C>G 4961T>A), E1638Q (4962G>C 4964G>A), H1639V (4965C>G 4966A>T), I1640C (4968A>T 4969T>G), S1641K (4971T>A 4972C>A 4973C>A), P1642S (4974C>T), S1644F (4980A>T 4981G>T), M1646R (4987T>G)								
Codon mutations:	CAA1446AAA (4386C>A), CCA1447CCT (4391A>T), TTA1448TTG (4394A>G), AAC1449AAT (4397C>T), CAT1450ACT (4398C>A 4399A>C), TTC1451GCT (4401T>G 4402T>C 4403C>T), CTC1452TTA (4404C>T 4406C>A), CAA1453AAA (4407C>A), GAT1454TGG (4410G>T 4411A>G 4412T>G), GAC1455ATT (4413G>A 4414A>T 4415C>T), AAG1456AGA (4417A>G 4418G>A), TTT1457TAC (4420T>A 4421T>C), CCC1458CCT (4424C>T), CCA1460CCT (4430A>T), AAT1461AAC (4433T>C), AAG1462AAA (4436G>A), CTC1463AAG (4437C>A 4438T>A 4439C>G), ACA1464GAT (4440A>G 4441C>A 4442A>T), CTC1465CTT (4445C>T), TTC1466CTA (4446T>C 4448C>A), TCT1467AAA (4449T>A 4450C>A 4451T>A), CAC1468AGA (4452C>A 4453A>G 4454C>A), TTA1469ACC (4455T>A 4456T>C 4457A>C), TCC1470TTT (4459C>T 4460C>T), AAG1473AAC (4469G>C), CTC1474ATT (4470C>A 4472C>T), TTT1475TAC (4474T>A 4475T>C), TCA1476AGT (4476T>A 4477C>G 4478A>T), AAG1477AAA (4481G>A), CTT1480ATG (4488C>A 4490T>G), AAG1481AAA (4493G>A), GGA1483GGG (4499A>G), TTA1487ATT (4509T>A 4511A>T), GGA1488CAG (4512G>C 4513G>A 4514A>G), ATC1489ATT (4517C>T), CAT1490TCT (4518C>T 4519A>C), CCT1491GAA (4521C>G 4522C>A 4523T>A), AAT1492AAA (4526T>A), GAG1493GAC (4529G>C), AGA1494AAA (4531G>A), CCA1495TAG (4533C>T 4534C>A 4535A>G), AAA1496AAG (4538A>G), ACA1497TCC (4539A>T 4541A>C), GGG1498GCT (4543G>C 4544G>T), TGC1500AAT (4548T>A 4549G>A 4550C>T), ATT1501GTT (4551A>G), CCT1502CCG (4556T>G), GAT1503TTT (4557G>T 4558A>T), AGA1504GGT (4560A>G 4562A>T), CAT1505CAA (4565T>A), TTT1506CAT (4566T>C 4567T>A), CAA1507GAA (4569C>G), AAG1509AAC (4577G>C), GTC1510GTT (4580C>T), CCA1512CTT (4585C>T 4586A>T), TTC1513TTT (4589C>T), GGT1514GGG (4592T>G), ACA1517AAT (4600C>A 4601A>T), CCT1519CCC (4607T>C), TCT1520TCA (4610T>A), TTG1521GAA (4611T>G 4612T>A 4613G>A), TTC1522TTT (4616C>T), GCC1525AAT (4623G>A 4624C>A 4625C>T), GCC1525_ATG1526insT-- (4625_4626insT), ATC1527AAT (4630T>A 4631C>T), AAA1528GAT (4632A>G 4634A>T), TTC1530ATT (4638T>A 4640C>T), CAG1531AAT (4641C>A 4643G>T), CCC1532CAT (4645C>A 4646C>T), ATC1533TTG (4647A>T 4649C>G), CTT1534AGC (4650C>A 4651T>G 4652T>C), TTT1535CAT (4653T>C 4654T>A), TCA1536ATG (4656T>A 4657C>T 4658A>G), GCT1537TCA (4659G>T 4661T>A), TTG1538ATA (4662T>A 4664G>A), GTC1539GTT (4667C>T), TAC1540TAT (4670C>T), GAT1543AAT (4677G>A), ATC1544GTT (4680A>G 4682C>T), CTT1545TTA (4683C>T 4685T>A), CTT1546ATC (4686C>A 4688T>C), TTT1547TTC (4691T>C), GAA1549GAG (4697A>G), ACA1550GAC (4698A>G 4699C>A 4700A>C), CTG1551ATC (4701C>A 4703G>C), GAA1552GAT (4706A>T), GAT1553TCT (4707G>T 4708A>C), CAC1554CAT (4712C>T), ATA1555TTG (4713A>T 4715A>G), TTG1557CAC (4719T>C 4720T>A 4721G>C), CTT1558CTA (4724T>A), AAC1559AAT (4727C>T), CAG1560ATT (4728C>A 4729A>T 4730G>T), TTC1561TTA (4733C>A), ATA1562TTT (4734A>T 4736A>T), AGT1563AAC (4738G>A 4739T>C), TTG1564ACC (4740T>A 4741T>C 4742G>C), GTT1565GTC (4745T>C), AAA1567AGA (4750A>G), TTT1568AAT (4752T>A 4753T>A), GGA1569GGT (4757A>T), GTC1570TTG (4758G>T 4760C>G), ATG1571GTA (4761A>G 4763G>A), TTA1572GTA (4764T>G), TCG1573AGC (4767T>A 4768C>G 4769G>C), GCA1574ACT (4770G>A 4772A>T), ATG1577ATT (4781G>T), ATC1578AAA (4783T>A 4784C>A), GCT1580TTT (4788G>T 4789C>T), AAT1582ACA (4795A>C 4796T>A), CAA1585AGA (4803C>A 4804A>G), TTC1586TTT (4808C>T), TTA1587TTG (4811A>G), GGA1588GGT (4814A>T), ATG1589CAT (4815A>C 4816T>A 4817G>T), TTT1591TTA (4823T>A), GCT1592TAC (4824G>T 4825C>A 4826T>C), GAT1593CAA (4827G>C 4829T>A), GGA1594GGT (4832A>T), ACT1595TCT (4833A>T), TTT1596TGC (4837T>G 4838T>C), TCA1597AGA (4839T>A 4840C>G), CCT1598CCA (4844T>A), GCT1599ATT (4845G>A 4846C>T), GGT1600TGC (4848G>T 4850T>C), CAC1601AGA (4851C>A 4852A>G 4853C>A), ATC1602GCC (4854A>G 4855T>C), TCT1603ATT (4857T>A 4858C>T), TTG1604GAG (4860T>G 4861T>A), GAA1605TTT (4863G>T 4864A>T 4865A>T), CTC1606TCT (4866C>T 4867T>C 4868C>T), CAA1607AAT (4869C>A 4871A>T), AAG1608AAA (4874G>A), CCT1610CCC (4880T>C), GAT1611AAT (4881G>A), ACA1612GTT (4884A>G 4885C>T 4886A>T), AAT1613ATT (4888A>T), CTC1614ACT (4890C>A 4891T>C 4892C>T), TCG1615GAC (4893T>G 4894C>A 4895G>C), GTA1616AAA (4896G>A 4897T>A), AAG1617ACC (4900A>C 4901G>C), CAG1618CAA (4904G>A), ATA1619CTG (4905A>C 4907A>G), CAA1621AGG (4911C>A 4912A>G 4913A>G), CTT1623TTG (4917C>T 4919T>G), GGG1624GGA (4922G>A), ATT1625AGT (4924T>G), GTT1626CTT (4926G>C), AAT1627AAC (4931T>C), ATC1629GTC (4935A>G), AGA1630GCT (4938A>G 4939G>C 4940A>T), GAT1631GAC (4943T>C), TTT1632TTC (4946T>C), CCA1634CCT (4952A>T), GAG1635AAG (4953G>A), ACT1637AGA (4960C>G 4961T>A), GAG1638CAA (4962G>C 4964G>A), CAT1639GTT (4965C>G 4966A>T), ATT1640TGT (4968A>T 4969T>G), TCC1641AAA (4971T>A 4972C>A 4973C>A), CCT1642TCT (4974C>T), CTT1643TTG (4977C>T 4979T>G), AGT1644TTT (4980A>T 4981G>T), GAT1645GAC (4985T>C), ATG1646AGG (4987T>G), CTA1647T.. (4989C>T)								

*: Inserts / Deletes / Misaligned / Frameshifts

Analysis details

This analysis was performed with panviral2.64

NGS Details (UN59): Badnavirus maculasmallanthi

Assembly

**Coverage Length**	481 (1 contig(s))
**Depth Of Coverage**	5.8
**Number Of Reads**	23
**Reads Per Million**	0.50 rpm (after QC)
**Ambiguities**	0
**Assembly Method**	de novo + reference guided assembly
**Consensus Caller**	Bcf Tools

Coverage Map

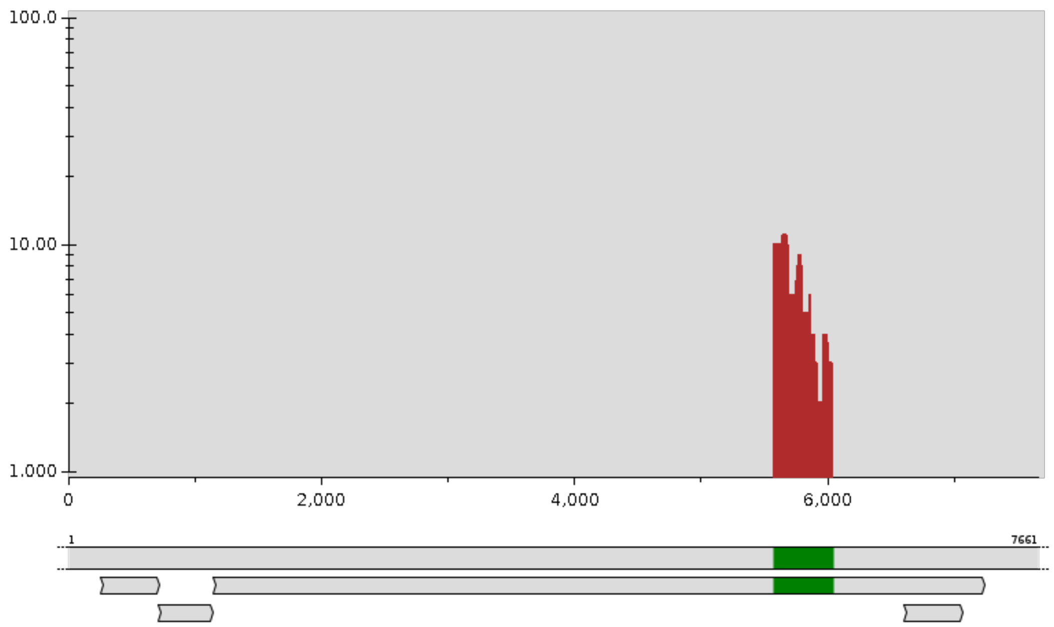

Assignment

**Type**	Badnavirus maculasmallanthi (Taxonomy ID: 3048453)
**Reference Genome**	NC_026472.1
**NT Identity (%)**	58.7368
**AA Identity (%)**	60.3774
**Number Of Stop Codons**	0
**Number Of CDS**	4

Alignment

**Alignment Score**	142.0 (NT) + 654.0 (AA) = 796.0
**Concordance (%)**	39.602

Alignment Method	Global, seeded, nucleotide + amino acids (AGA)

Genome Region

Sequence starts at position 5569 and ends at position 6049 relative to NC_026472.1 reference sequence.

Alignment Detailed Statistics

	Begin	End	Coverage	Score	Concordance	Matches	Identities	I/D/M/F*	Stop Codons
**NT**	**5569**	**6049**	**6.3%**	**142**	**15.3%**	**475 (98.8%)**	**279 (58.0%)**	**0/6**	
Mutations:	5571C>T, 5577G>A, 5584C>T, 5585T>G, 5586G>C, 5589G>A, 5590T>A, 5591C>G, 5599C>T, 5600A>G, 5601T>G, 5605G>A, 5607T>A, 5608G>A, 5609C>A, 5610C>A, 5611A>C, 5613G>A, 5620G>A, 5622A>T, 5625C>T, 5629C>G, 5631C>T, 5633G>T, 5634G>A, 5637G>A, 5640C>A, 5643T>C, 5644T>A, 5645T>G, 5646A>T, 5648T>C, 5649C>A, 5653G>A, 5654G>A, 5655A>T, 5659T>C, 5660T>A, 5661A>C, 5671C>T, 5673T>A, 5676C>G, 5682A>T, 5685C>T, 5689C>T, 5691C>A, 5700A>C, 5703C>T, 5704G>C, 5705C>A, 5706T>A, 5709T>C, 5718A>G, 5731T>A, 5732G>T, 5733C>A, 5736C>T, 5737_5739delAAA, 5740G>T, 5741A>C, 5743A>C, 5744C>A, 5746G>A, 5748A>G, 5749G>A, 5751G>T, 5755A>C, 5757C>A, 5758G>A, 5759C>T, 5763A>C, 5769C>A, 5772C>T, 5775C>T, 5778C>A, 5779C>T, 5781G>A, 5782G>A, 5786A>G, 5787C>T, 5794A>T, 5795G>C, 5796T>A, 5797G>T, 5799A>T, 5800G>C, 5801C>A, 5802G>A, 5805T>A, 5809G>T, 5810A>T, 5813G>C, 5814G>T, 5818T>C, 5820A>T, 5823G>A, 5824A>C, 5825T>A, 5826T>A, 5827A>T, 5829G>T, 5830C>T, 5831T>C, 5832G>T, 5833G>C, 5834G>A, 5835T>A, 5837A>T, 5841C>T, 5842C>T, 5843A>G, 5844A>C, 5854T>A, 5856A>T, 5860C>T, 5863T>A, 5864C>G, 5865T>C, 5867C>A, 5868A>G, 5870C>A, 5874A>G, 5875A>G, 5876T>C, 5877G>A, 5878A>G, 5880G>C, 5884G>A, 5885C>A, 5886C>T, 5887G>A, 5888T>A, 5889C>A, 5890C>A, 5891C>A, 5892C>T, 5896G>A, 5898G>T, 5904C>T, 5907G>C, 5911G>A, 5912C>T, 5913A>G, 5919A>T, 5920G>T, 5921G>C, 5924G>A, 5925A>G, 5928C>T, 5929A>G, 5930A>G, 5931G>C, 5934A>T, 5935A>G, 5937G>A, 5938C>T, 5940G>A, 5943G>A, 5944C>A, 5946A>T, 5952T>A, 5953A>T, 5954T>C, 5955T>A, 5961A>G, 5964A>C, 5970C>A, 5974_5976delGAT, 5977G>C, 5978A>C, 5979G>A, 5982A>T, 5985G>A, 5991G>T, 5994T>A, 5998G>C, 5999C>A, 6000C>A, 6002G>A, 6003C>A, 6004C>A, 6006T>C, 6007A>C, 6008G>A, 6010T>A, 6011C>G, 6012C>G, 6015C>T, 6022A>T, 6023T>G, 6024C>T, 6030C>T, 6036C>A, 6039A>T, 6040A>G, 6042C>A, 6044A>T, 6048T>A								

CDS

	Begin	End	Coverage	Score	Concordance	Matches	Identities	I/D/M/F*	Stop Codons
UF61_gp3	1476	1636	7.9%	654	59.7%	159 (98.8%)	96 (59.6%)	0/2/0/0	0
Protein mutations:	L1481C (5584C>T 5585T>G 5586G>C), H1486W (5599C>T 5600A>G 5601T>G), V1488I (5605G>A 5607T>A), A1489K (5608G>A 5609C>A 5610C>A), M1490L (5611A>C 5613G>A), E1493N (5620G>A 5622A>T), P1496A (5629C>G 5631C>T), W1497L (5633G>T 5634G>A), L1501S (5644T>A 5645T>G 5646A>T), I1502T (5648T>C 5649C>A), G1504N (5653G>A 5654G>A 5655A>T), L1506H (5659T>C 5660T>A 5661A>C), A1521Q (5704G>C 5705C>A 5706T>A), C1530I (5731T>A 5732G>T 5733C>A), K1532del (5737_5739delAAA), D1533S (5740G>T 5741A>C), T1534Q (5743A>C 5744C>A), E1535K (5746G>A 5748A>G), E1536N (5749G>A 5751G>T), I1538L (5755A>C 5757C>A), A1539I (5758G>A 5759C>T), V1547I (5782G>A), Y1548C (5786A>G 5787C>T), E1552Y (5797G>T 5799A>T), A1553Q (5800G>C 5801C>A 5802G>A), D1554E (5805T>A), E1556L (5809G>T 5810A>T), R1557T (5813G>C 5814G>T), I1561Q (5824A>C 5825T>A 5826T>A), M1562F (5827A>T 5829G>T), L1563S (5830C>T 5831T>C 5832G>T), G1564Q (5833G>C 5834G>A 5835T>A), K1565I (5837A>T), Q1567C (5842C>T 5843A>G 5844A>C), L1571I (5854T>A 5856A>T), P1575Q (5867C>A 5868A>G), T1576K (5870C>A), M1578A (5875A>G 5876T>C 5877G>A), K1579D (5878A>G 5880G>C), A1581N (5884G>A 5885C>A 5886C>T), V1582K (5887G>A 5888T>A 5889C>A), P1583N (5890C>A 5891C>A 5892C>T), V1585I (5896G>A 5898G>T), A1590M (5911G>A 5912C>T 5913A>G), G1593S (5920G>T 5921G>C), R1594K (5924G>A 5925A>G), K1596G (5929A>G 5930A>G 5931G>C), K1598E (5935A>G 5937G>A), P1601T (5944C>A 5946A>T), I1604S (5953A>T 5954T>C 5955T>A), D1609E (5970C>A), D1611del (5974_5976delGAT), E1612P (5977G>C 5978A>C 5979G>A), E1613D (5982A>T), K1616N (5991G>T), A1619Q (5998G>C 5999C>A 6000C>A), G1620E (6002G>A 6003C>A), L1621I (6004C>A 6006T>C), R1622Q (6007A>C 6008G>A), S1623R (6010T>A 6011C>G 6012C>G), I1627C (6022A>T 6023T>G 6024C>T), R1632S (6039A>T), N1633E (6040A>G 6042C>A), Y1634F (6044A>T)								
Codon mutations:	TTC1476TTT (5571C>T), AAG1478AAA (5577G>A), CTG1481TGC (5584C>T 5585T>G 5586G>C), AAG1482AAA (5589G>A), TCT1483AGT (5590T>A 5591C>G), CAT1486TGG (5599C>T 5600A>G 5601T>G), GTT1488ATA (5605G>A 5607T>A), GCC1489AAA (5608G>A 5609C>A 5610C>A), ATG1490CTA (5611A>C 5613G>A), GAA1493AAT (5620G>A 5622A>T), TCC1494TCT (5625C>T), CCC1496GCT (5629C>G 5631C>T), TGG1497TTA (5633G>T 5634G>A), ACG1498ACA (5637G>A), GCC1499GCA (5640C>A), TTT1500TTC (5643T>C), TTA1501AGT (5644T>A 5645T>G 5646A>T), ATC1502ACA (5648T>C 5649C>A), GGA1504AAT (5653G>A 5654G>A 5655A>T), TTA1506CAC (5659T>C 5660T>A 5661A>C), CTT1510TTA (5671C>T 5673T>A), GTC1511GTG (5676C>G), CCA1513CCT (5682A>T), TTC1514TTT (5685C>T), CTC1516TTA (5689C>T 5691C>A), GCA1519GCC (5700A>C), CCC1520CCT (5703C>T), GCT1521CAA (5704G>C 5705C>A 5706T>A), ATT1522ATC (5709T>C), AGA1525AGG (5718A>G), TGC1530ATA (5731T>A 5732G>T 5733C>A), TTC1531TTT (5736C>T), AAA1532del (5737_5739delAAA), GAC1533TCC (5740G>T 5741A>C), ACA1534CAA (5743A>C 5744C>A), GAA1535AAG (5746G>A 5748A>G), GAG1536AAT (5749G>A 5751G>T), ATC1538CTA (5755A>C 5757C>A), GCC1539ATC (5758G>A 5759C>T), GTA1540GTC (5763A>C), ATC1542ATA (5769C>A), GAC1543GAT (5772C>T), GAC1544GAT (5775C>T), ATC1545ATA (5778C>A), CTG1546TTA (5779C>T 5781G>A), GTA1547ATA (5782G>A), TAC1548TGT (5786A>G 5787C>T), AGT1551TCA (5794A>T 5795G>C 5796T>A), GAA1552TAT (5797G>T 5799A>T), GCG1553CAA (5800G>C 5801C>A 5802G>A), GAT1554GAA (5805T>A), GAA1556TTA (5809G>T 5810A>T), AGG1557ACT (5813G>C 5814G>T), TTA1559CTT (5818T>C 5820A>T), AAG1560AAA (5823G>A), ATT1561CAA (5824A>C 5825T>A 5826T>A), ATG1562TTT (5827A>T 5829G>T), CTG1563TCT (5830C>T 5831T>C 5832G>T), GGT1564CAA (5833G>C 5834G>A 5835T>A), AAA1565ATA (5837A>T), TGC1566TGT (5841C>T), CAA1567TGC (5842C>T 5843A>G 5844A>C), TTA1571ATT (5854T>A 5856A>T), CTA1573TTA (5860C>T), TCT1574AGC (5863T>A 5864C>G 5865T>C), CCA1575CAG (5867C>A 5868A>G), ACA1576AAA (5870C>A), AAA1577AAG (5874A>G), ATG1578GCA (5875A>G 5876T>C 5877G>A), AAG1579GAC (5878A>G 5880G>C), GCC1581AAT (5884G>A 5885C>A 5886C>T), GTC1582AAA (5887G>A 5888T>A 5889C>A), CCC1583AAT (5890C>A 5891C>A 5892C>T), GTG1585ATT (5896G>A 5898G>T), TTC1587TTT (5904C>T), CTG1588CTC (5907G>C), GCA1590ATG (5911G>A 5912C>T 5913A>G), ATA1592ATT (5919A>T), GGA1593TCA (5920G>T 5921G>C), AGA1594AAG (5924G>A 5925A>G), AAC1595AAT (5928C>T), AAG1596GGC (5929A>G 5930A>G 5931G>C), ATA1597ATT (5934A>T), AAG1598GAA (5935A>G 5937G>A), CTG1599TTA (5938C>T 5940G>A), CAG1600CAA (5943G>A), CCA1601ACT (5944C>A 5946A>T), ATT1603ATA (5952T>A), ATT1604TCA (5953A>T 5954T>C 5955T>A), AAA1606AAG (5961A>G), ATA1607ATC (5964A>C), GAC1609GAA (5970C>A), GAT1611del (5974_5976delGAT), GAG1612CCA (5977G>C 5978A>C 5979G>A), GAA1613GAT (5982A>T), AAG1614AAA (5985G>A), AAG1616AAT (5991G>T), ACT1617ACA (5994T>A), GCC1619CAA (5998G>C 5999C>A 6000C>A), GGC1620GAA (6002G>A 6003C>A), CTT1621ATC (6004C>A 6006T>C), AGA1622CAA (6007A>C 6008G>A), TCC1623AGG (6010T>A 6011C>G 6012C>G), TTC1624TTT (6015C>T), ATC1627TGT (6022A>T 6023T>G 6024C>T), AAC1629AAT (6030C>T), GCC1631GCA (6036C>A), AGA1632AGT (6039A>T), AAC1633GAA (6040A>G 6042C>A), TAC1634TTC (6044A>T), ATT1635ATA (6048T>A)								

Proteins

	Begin	End	Coverage	Score	Concordance	Matches	Identities	I/D/M/F*	Stop Codons
ORF3 (YP_009121747.1)	1476	1636	7.9%	654	59.7%	159 (98.8%)	96 (59.6%)	0/2/0/0	0
Protein mutations:	L1481C (5584C>T 5585T>G 5586G>C), H1486W (5599C>T 5600A>G 5601T>G), V1488I (5605G>A 5607T>A), A1489K (5608G>A 5609C>A 5610C>A), M1490L (5611A>C 5613G>A), E1493N (5620G>A 5622A>T), P1496A (5629C>G 5631C>T), W1497L (5633G>T 5634G>A), L1501S (5644T>A 5645T>G 5646A>T), I1502T (5648T>C 5649C>A), G1504N (5653G>A 5654G>A 5655A>T), L1506H (5659T>C 5660T>A 5661A>C), A1521Q (5704G>C 5705C>A 5706T>A), C1530I (5731T>A 5732G>T 5733C>A), K1532del (5737_5739delAAA), D1533S (5740G>T 5741A>C), T1534Q (5743A>C 5744C>A), E1535K (5746G>A 5748A>G), E1536N (5749G>A 5751G>T), I1538L (5755A>C 5757C>A), A1539I (5758G>A 5759C>T), V1547I (5782G>A), Y1548C (5786A>G 5787C>T), E1552Y (5797G>T 5799A>T), A1553Q (5800G>C 5801C>A 5802G>A), D1554E (5805T>A), E1556L (5809G>T 5810A>T), R1557T (5813G>C 5814G>T), I1561Q (5824A>C 5825T>A 5826T>A), M1562F (5827A>T 5829G>T), L1563S (5830C>T 5831T>C 5832G>T), G1564Q (5833G>C 5834G>A 5835T>A), K1565I (5837A>T), Q1567C (5842C>T 5843A>G 5844A>C), L1571I (5854T>A 5856A>T), P1575Q (5867C>A 5868A>G), T1576K (5870C>A), M1578A (5875A>G 5876T>C 5877G>A), K1579D (5878A>G 5880G>C), A1581N (5884G>A 5885C>A 5886C>T), V1582K (5887G>A 5888T>A 5889C>A), P1583N (5890C>A 5891C>A 5892C>T), V1585I (5896G>A 5898G>T), A1590M (5911G>A 5912C>T 5913A>G), G1593S (5920G>T 5921G>C), R1594K (5924G>A 5925A>G), K1596G (5929A>G 5930A>G 5931G>C), K1598E (5935A>G 5937G>A), P1601T (5944C>A 5946A>T), I1604S (5953A>T 5954T>C 5955T>A), D1609E (5970C>A), D1611del (5974_5976delGAT), E1612P (5977G>C 5978A>C 5979G>A), E1613D (5982A>T), K1616N (5991G>T), A1619Q (5998G>C 5999C>A 6000C>A), G1620E (6002G>A 6003C>A), L1621I (6004C>A 6006T>C), R1622Q (6007A>C 6008G>A), S1623R (6010T>A 6011C>G 6012C>G), I1627C (6022A>T 6023T>G 6024C>T), R1632S (6039A>T), N1633E (6040A>G 6042C>A), Y1634F (6044A>T)								
Codon mutations:	TTC1476TTT (5571C>T), AAG1478AAA (5577G>A), CTG1481TGC (5584C>T 5585T>G 5586G>C), AAG1482AAA (5589G>A), TCT1483AGT (5590T>A 5591C>G), CAT1486TGG (5599C>T 5600A>G 5601T>G), GTT1488ATA (5605G>A 5607T>A), GCC1489AAA (5608G>A 5609C>A 5610C>A), ATG1490CTA (5611A>C 5613G>A), GAA1493AAT (5620G>A 5622A>T), TCC1494TCT (5625C>T), CCC1496GCT (5629C>G 5631C>T), TGG1497TTA (5633G>T 5634G>A), ACG1498ACA (5637G>A), GCC1499GCA (5640C>A), TTT1500TTC (5643T>C), TTA1501AGT (5644T>A 5645T>G 5646A>T), ATC1502ACA (5648T>C 5649C>A), GGA1504AAT (5653G>A 5654G>A 5655A>T), TTA1506CAC (5659T>C 5660T>A 5661A>C), CTT1510TTA (5671C>T 5673T>A), GTC1511GTG (5676C>G), CCA1513CCT (5682A>T), TTC1514TTT (5685C>T), CTC1516TTA (5689C>T 5691C>A), GCA1519GCC (5700A>C), CCC1520CCT (5703C>T), GCT1521CAA (5704G>C 5705C>A 5706T>A), ATT1522ATC (5709T>C), AGA1525AGG (5718A>G), TGC1530ATA (5731T>A 5732G>T 5733C>A), TTC1531TTT (5736C>T), AAA1532del (5737_5739delAAA), GAC1533TCC (5740G>T 5741A>C), ACA1534CAA (5743A>C 5744C>A), GAA1535AAG (5746G>A 5748A>G), GAG1536AAT (5749G>A 5751G>T), ATC1538CTA (5755A>C 5757C>A), GCC1539ATC (5758G>A 5759C>T), GTA1540GTC (5763A>C), ATC1542ATA (5769C>A), GAC1543GAT (5772C>T), GAC1544GAT (5775C>T), ATC1545ATA (5778C>A), CTG1546TTA (5779C>T 5781G>A), GTA1547ATA (5782G>A), TAC1548TGT (5786A>G 5787C>T), AGT1551TCA (5794A>T 5795G>C 5796T>A), GAA1552TAT (5797G>T 5799A>T), GCG1553CAA (5800G>C 5801C>A 5802G>A), GAT1554GAA (5805T>A), GAA1556TTA (5809G>T 5810A>T), AGG1557ACT (5813G>C 5814G>T), TTA1559CTT (5818T>C 5820A>T), AAG1560AAA (5823G>A), ATT1561CAA (5824A>C 5825T>A 5826T>A), ATG1562TTT (5827A>T 5829G>T), CTG1563TCT (5830C>T 5831T>C 5832G>T), GGT1564CAA (5833G>C 5834G>A 5835T>A), AAA1565ATA (5837A>T), TGC1566TGT (5841C>T), CAA1567TGC (5842C>T 5843A>G 5844A>C), TTA1571ATT (5854T>A 5856A>T), CTA1573TTA (5860C>T), TCT1574AGC (5863T>A 5864C>G 5865T>C), CCA1575CAG (5867C>A 5868A>G), ACA1576AAA (5870C>A), AAA1577AAG (5874A>G), ATG1578GCA (5875A>G 5876T>C 5877G>A), AAG1579GAC (5878A>G 5880G>C), GCC1581AAT (5884G>A 5885C>A 5886C>T), GTC1582AAA (5887G>A 5888T>A 5889C>A), CCC1583AAT (5890C>A 5891C>A 5892C>T), GTG1585ATT (5896G>A 5898G>T), TTC1587TTT (5904C>T), CTG1588CTC (5907G>C), GCA1590ATG (5911G>A 5912C>T 5913A>G), ATA1592ATT (5919A>T), GGA1593TCA (5920G>T 5921G>C), AGA1594AAG (5924G>A 5925A>G), AAC1595AAT (5928C>T), AAG1596GGC (5929A>G 5930A>G 5931G>C), ATA1597ATT (5934A>T), AAG1598GAA (5935A>G 5937G>A), CTG1599TTA (5938C>T 5940G>A), CAG1600CAA (5943G>A), CCA1601ACT (5944C>A 5946A>T), ATT1603ATA (5952T>A), ATT1604TCA (5953A>T 5954T>C 5955T>A), AAA1606AAG (5961A>G), ATA1607ATC (5964A>C), GAC1609GAA (5970C>A), GAT1611del (5974_5976delGAT), GAG1612CCA (5977G>C 5978A>C 5979G>A), GAA1613GAT (5982A>T), AAG1614AAA (5985G>A), AAG1616AAT (5991G>T), ACT1617ACA (5994T>A), GCC1619CAA (5998G>C 5999C>A 6000C>A), GGC1620GAA (6002G>A 6003C>A), CTT1621ATC (6004C>A 6006T>C), AGA1622CAA (6007A>C 6008G>A), TCC1623AGG (6010T>A 6011C>G 6012C>G), TTC1624TTT (6015C>T), ATC1627TGT (6022A>T 6023T>G 6024C>T), AAC1629AAT (6030C>T), GCC1631GCA (6036C>A), AGA1632AGT (6039A>T), AAC1633GAA (6040A>G 6042C>A), TAC1634TTC (6044A>T), ATT1635ATA (6048T>A)								

*: Inserts / Deletes / Misaligned / Frameshifts

Analysis details

This analysis was performed with panviral2.64

NGS Details (UN59): Epiphyllum badnavirus 1

Assembly

**Coverage Length**	354 (1 contig(s))
**Depth Of Coverage**	7.4
**Number Of Reads**	22
**Reads Per Million**	0.48 rpm (after QC)
**Ambiguities**	0
**Assembly Method**	de novo + reference guided assembly
**Consensus Caller**	Bcf Tools

Coverage Map

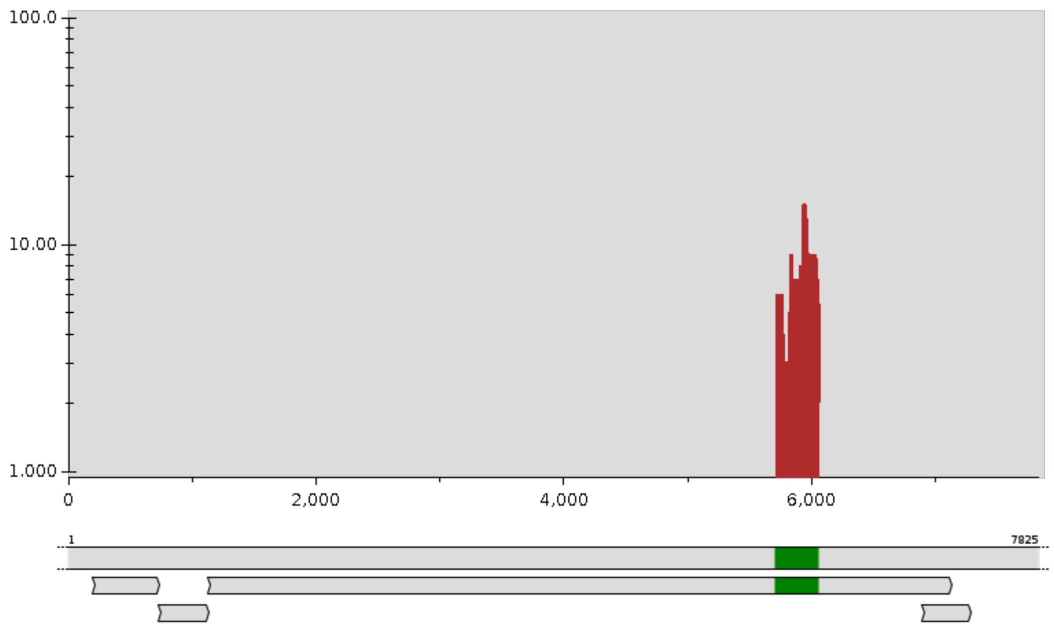

Assignment

**Type**	Epiphyllum badnavirus 1 (Taxonomy ID: 2518008)
**Reference Genome**	NC_076247.1
**NT Identity (%)**	50.9804
**AA Identity (%)**	42.0168
**Number Of Stop Codons**	0
**Number Of CDS**	4

Alignment

**Alignment Score**	8.0 (NT) + 335.0 (AA) = 343.0
**Concordance (%)**	22.215

Alignment Method	Global, seeded, nucleotide + amino acids (AGA)

Genome Region

Sequence starts at position 5709 and ends at position 6062 relative to NC_076247.1 reference sequence.

Alignment Detailed Statistics

	Begin	End	Coverage	Score	Concordance	Matches	Identities	I/D/M/F*	Stop Codons
**NT**	**5709**	**6062**	**4.5%**	**8**	**1.1%**	**354 (99.2%)**	**182 (51.0%)**	**3/0**	
Mutations:	5709A>G, 5710A>T, 5711C>T, 5714G>T, 5715A>T, 5716G>A, 5718A>G, 5719G>A, 5721G>A, 5722A>C, 5724G>A, 5725A>G, 5727A>T, 5730A>T, 5732A>G, 5733G>T, 5734G>A, 5735A>T, 5738G>A, 5742G>A, 5743G>T, 5744T>G, 5745C>T, 5746T>A, 5748C>A, 5752T>A, 5755A>C, 5756A>G, 5758A>C, 5759C>A, 5760C>A, 5763G>A, 5766C>T, 5767G>A, 5768A>C, 5769C>G, 5770A>G, 5771A>T, 5775G>C, 5776T>A, 5778C>T, 5779A>C, 5780A>G, 5782G>A, 5784T>C, 5785C>A, 5793T>A, 5794C>T, 5796A>G, 5800G>C, 5802C>T, 5805T>A, 5806A>G, 5808C>T, 5809A>G, 5810C>A, 5811A>T, 5812A>C, 5815A>T, 5817A>T, 5818C>G, 5820G>C, 5821A>C, 5824G>T, 5826C>A, 5827T>C, 5828G>A, 5829T>G, 5835C>A, 5836A>T, 5837G>C, 5838T>A, 5841C>G, 5847C>A, 5850G>A, 5851T>A, 5853C>T, 5854G>A, 5859G>A, 5861A>G, 5862G>A, 5863A>T, 5864G>C, 5865C>T, 5868A>T, 5870T>A, 5874C>T, 5878A>T, 5882T>A, 5886G>T, 5887G>A, 5889C>G, 5890G>C, 5891A>C, 5892A>G, 5893G>A, 5895A>G, 5896T>G, 5897C>A, 5898C>T, 5899A>G, 5901C>G, 5905T>A, 5910C>T, 5913C>T, 5916C>T, 5917C>A, 5918T>G, 5919G>A, 5924C>G, 5925A>G, 5926G>T, 5931A>G, 5933T>A, 5934T>C, 5942G>T, 5943G>C, 5946A>G, 5947G>A, 5949T>G, 5953C>T, 5955A>G, 5956T>A, 5958C>T, 5961A>G, 5966A>C, 5967G>C, 5973C>G, 5976T>A, 5979T>A, 5981T>C, 5982G>C, 5986C>A, 5987A>T, 5988A>G, 5989C>A, 5991A>T, 5992A>T, 5993A>T, 5998G>A, 6000C>T, 6001A>G, 6002A>G, 6003C>G, 6004T>G, 6005G>T, 6006T>G, 6009C>T, 6011G>A, 6012A>G, 6013G>C, 6014A>C, 6015C>A, 6015_6016insTTT, 6016C>T, 6019T>G, 6020C>A, 6021A>T, 6022G>T, 6023G>C, 6024C>G, 6030A>G, 6031G>A, 6032C>T, 6033A>T, 6035T>G, 6036T>G, 6038A>T, 6039C>T, 6051C>T, 6054G>T, 6055G>A, 6057C>T								

CDS

QKM20_gp3	1528	1645	5.9%	335	39.7%	118 (99.2%)	50 (42.0%)	1/0/0/0	0
Protein mutations:	T1528F (5710A>T 5711C>T), G1529V (5714G>T 5715A>T), E1530K (5716G>A 5718A>G), E1531K (5719G>A 5721G>A), K1532Q (5722A>C 5724G>A), K1533D (5725A>G 5727A>T), K1535S (5732A>G 5733G>T), E1536M (5734G>A 5735A>T), R1537K (5738G>A), M1538I (5742G>A), V1539C (5743G>T 5744T>G 5745C>T), F1540I (5746T>A 5748C>A), Y1542N (5752T>A), K1543R (5755A>C 5756A>G), T1544Q (5758A>C 5759C>A 5760C>A), D1547T (5767G>A 5768A>C 5769C>G), N1548V (5770A>G 5771A>T), F1550I (5776T>A 5778C>T), K1551R (5779A>C 5780A>G), D1552N (5782G>A 5784T>C), Q1553K (5785C>A), G1558R (5800G>C 5802C>T), N1560D (5806A>G 5808C>T), T1561D (5809A>G 5810C>A 5811A>T), I1562L (5812A>C), I1563F (5815A>T 5817A>T), Q1564D (5818C>G 5820G>C), K1565Q (5821A>C), V1566L (5824G>T 5826C>A), C1567Q (5827T>C 5828G>A 5829T>G), F1575I (5851T>A 5853C>T), D1576N (5854G>A), K1578R (5861A>G 5862G>A), F1581Y (5870T>A), I1584F (5878A>T), M1585K (5882T>A), M1586I (5886G>T), D1587K (5887G>A 5889C>G), E1588P (5890G>C 5891A>C 5892A>G), E1589K (5893G>A 5895A>G), S1590D (5896T>G 5897C>A 5898C>T), I1591V (5899A>G 5901C>G), W1593R (5905T>A), L1597R (5917C>A 5918T>G 5919G>A), P1599R (5924C>G 5925A>G), D1600Y (5926G>T), L1602H (5933T>A 5934T>C), W1605F (5942G>T 5943G>C), V1607M (5947G>A 5949T>G), P1609S (5953C>T 5955A>G), F1610I (5956T>A 5958C>T), K1613T (5966A>C 5967G>C), V1618A (5981T>C 5982G>C), Q1620M (5986C>A 5987A>T 5988A>G), R1621S (5989C>A 5991A>T), K1622L (5992A>T 5993A>T), D1624N (5998G>A 6000C>T), N1625G (6001A>G 6002A>G 6003C>G), C1626V (6004T>G 6005G>T 6006T>G), R1628K (6011G>A 6012A>G), D1629P (6013G>C 6014A>C 6015C>A), D1629_L1630insF (6015_6016insTTT), S1631D (6019T>G 6020C>A 6021A>T), G1632S (6022G>T 6023G>C 6024C>G), A1635I (6031G>A 6032C>T 6033A>T), V1636G (6035T>G 6036T>G), Y1637F (6038A>T 6039C>T), V1643I (6055G>A 6057C>T)								
Codon mutations:	GCA1527..G (5709A>G), ACT1528TTT (5710A>T 5711C>T), GGA1529GTT (5714G>T 5715A>T), GAA1530AAG (5716G>A 5718A>G), GAG1531AAA (5719G>A 5721G>A), AAG1532CAA (5722A>C 5724G>A), AAA1533GAT (5725A>G 5727A>T), GGA1534GGT (5730A>T), AAG1535AGT (5732A>G 5733G>T), GAG1536ATG (5734G>A 5735A>T), AGA1537AAA (5738G>A), ATG1538ATA (5742G>A), GTC1539TGT (5743G>T 5744T>G 5745C>T), TTC1540ATA (5746T>A 5748C>A), TAC1542AAC (5752T>A), AAA1543CGA (5755A>C 5756A>G), ACC1544CAA (5758A>C 5759C>A 5760C>A), TTG1545TTA (5763G>A), AAC1546AAT (5766C>T), GAC1547ACG (5767G>A 5768A>C 5769C>G), AAC1548GTC (5770A>G 5771A>T), ACG1549ACC (5775G>C), TTC1550ATT (5776T>A 5778C>T), AAA1551CGA (5779A>C 5780A>G), GAT1552AAC (5782G>A 5784T>C), CAG1553AAG (5785C>A), TCT1555TCA (5793T>A), CTA1556TTG (5794C>T 5796A>G), GGC1558CGT (5800G>C 5802C>T), ATT1559ATA (5805T>A), AAC1560GAT (5806A>G 5808C>T), ACA1561GAT (5809A>G 5810C>A 5811A>T), ATC1562CTC (5812A>C), ATA1563TTT (5815A>T 5817A>T), CAG1564GAC (5818C>G 5820G>C), AAG1565CAG (5821A>C), GTC1566TTA (5824G>T 5826C>A), TGT1567CAG (5827T>C 5828G>A 5829T>G), GCC1569GCA (5835C>A), AGT1570TCA (5836A>T 5837G>C 5838T>A), GTC1571GTG (5841C>G), TCC1573TCA (5847C>A), AAG1574AAA (5850G>A), TTC1575ATT (5851T>A 5853C>T), GAT1576AAT (5854G>A), CTG1577CTA (5859G>A), AAG1578AGA (5861A>G 5862G>A), AGC1579TCT (5863A>T 5864G>C 5865C>T), GGA1580GGT (5868A>T), TTC1581TAC (5870T>A), CAC1582CAT (5874C>T), ATT1584TTT (5878A>T), ATG1585AAG (5882T>A), ATG1586ATT (5886G>T), GAC1587AAG (5887G>A 5889C>G), GAA1588CCG (5890G>C 5891A>C 5892A>G), GAA1589AAG (5893G>A 5895A>G), TCC1590GAT (5896T>G 5897C>A 5898C>T), ATC1591GTG (5899A>G 5901C>G), TGG1593AGG (5905T>A), ACC1594ACT (5910C>T), GCC1595GCT (5913C>T), TTC1596TTT (5916C>T), CTG1597AGA (5917C>A 5918T>G 5919G>A), CCA1599CGG (5924C>G 5925A>G), GAT1600TAT (5926G>T), GGA1601GGG (5931A>G), CTT1602CAC (5933T>A 5934T>C), TGG1605TTC (5942G>T 5943G>C), TTA1606TTG (5946A>G), GTT1607ATG (5947G>A 5949T>G), CCA1609TCG (5953C>T 5955A>G), TTC1610ATT (5956T>A 5958C>T), GGA1611GGG (5961A>G), AAG1613ACC (5966A>C 5967G>C), GCC1615GCG (5973C>G), CCT1616CCA (5976T>A), GCT1617GCA (5979T>A), GTG1618GCC (5981T>C 5982G>C), CAA1620ATG (5986C>A 5987A>T 5988A>G), CGA1621AGT (5989C>A 5991A>T), AAG1622TTG (5992A>T 5993A>T), GAC1624AAT (5998G>A 6000C>T), AAC1625GGG (6001A>G 6002A>G 6003C>G), TGT1626GTG (6004T>G 6005G>T 6006T>G), TTC1627TTT (6009C>T), AGA1628AAG (6011G>A 6012A>G), GAC1629CCA (6013G>C 6014A>C 6015C>A), GAC1629_CTG1630insTTT (6015_6016insTTT), CTG1630TTG (6016C>T), TCA1631GAT (6019T>G 6020C>A 6021A>T), GGC1632TCG (6022G>T 6023G>C 6024C>G), GTA1634GTG (6030A>G), GCA1635ATT (6031G>A 6032C>T 6033A>T), GTT1636GGG (6035T>G 6036T>G), TAC1637TTT (6038A>T 6039C>T), ATC1641ATT (6051C>T), CTG1642CTT (6054G>T), GTC1643ATT (6055G>A 6057C>T)								

Proteins

polyprotein (YP_010797894.1)	1528	1645	5.9%	335	39.7%	118 (99.2%)	50 (42.0%)	1/0/0/0	0
Protein mutations:	T1528F (5710A>T 5711C>T), G1529V (5714G>T 5715A>T), E1530K (5716G>A 5718A>G), E1531K (5719G>A 5721G>A), K1532Q (5722A>C 5724G>A), K1533D (5725A>G 5727A>T), K1535S (5732A>G 5733G>T), E1536M (5734G>A 5735A>T), R1537K (5738G>A), M1538I (5742G>A), V1539C (5743G>T 5744T>G 5745C>T), F1540I (5746T>A 5748C>A), Y1542N (5752T>A), K1543R (5755A>C 5756A>G), T1544Q (5758A>C 5759C>A 5760C>A), D1547T (5767G>A 5768A>C 5769C>G), N1548V (5770A>G 5771A>T), F1550I (5776T>A 5778C>T), K1551R (5779A>C 5780A>G), D1552N (5782G>A 5784T>C), Q1553K (5785C>A), G1558R (5800G>C 5802C>T), N1560D (5806A>G 5808C>T), T1561D (5809A>G 5810C>A 5811A>T), I1562L (5812A>C), I1563F (5815A>T 5817A>T), Q1564D (5818C>G 5820G>C), K1565Q (5821A>C), V1566L (5824G>T 5826C>A), C1567Q (5827T>C 5828G>A 5829T>G), F1575I (5851T>A 5853C>T), D1576N (5854G>A), K1578R (5861A>G 5862G>A), F1581Y (5870T>A), I1584F (5878A>T), M1585K (5882T>A), M1586I (5886G>T), D1587K (5887G>A 5889C>G), E1588P (5890G>C 5891A>C 5892A>G), E1589K (5893G>A 5895A>G), S1590D (5896T>G 5897C>A 5898C>T), I1591V (5899A>G 5901C>G), W1593R (5905T>A), L1597R (5917C>A 5918T>G 5919G>A), P1599R (5924C>G 5925A>G), D1600Y (5926G>T), L1602H (5933T>A 5934T>C), W1605F (5942G>T 5943G>C), V1607M (5947G>A 5949T>G), P1609S (5953C>T 5955A>G), F1610I (5956T>A 5958C>T), K1613T (5966A>C 5967G>C), V1618A (5981T>C 5982G>C), Q1620M (5986C>A 5987A>T 5988A>G), R1621S (5989C>A 5991A>T), K1622L (5992A>T 5993A>T), D1624N (5998G>A 6000C>T), N1625G (6001A>G 6002A>G 6003C>G), C1626V (6004T>G 6005G>T 6006T>G), R1628K (6011G>A 6012A>G), D1629P (6013G>C 6014A>C 6015C>A), D1629_L1630insF (6015_6016insTTT), S1631D (6019T>G 6020C>A 6021A>T), G1632S (6022G>T 6023G>C 6024C>G), A1635I (6031G>A 6032C>T 6033A>T), V1636G (6035T>G 6036T>G), Y1637F (6038A>T 6039C>T), V1643I (6055G>A 6057C>T)								
Codon mutations:	GCA1527..G (5709A>G), ACT1528TTT (5710A>T 5711C>T), GGA1529GTT (5714G>T 5715A>T), GAA1530AAG (5716G>A 5718A>G), GAG1531AAA (5719G>A 5721G>A), AAG1532CAA (5722A>C 5724G>A), AAA1533GAT (5725A>G 5727A>T), GGA1534GGT (5730A>T), AAG1535AGT (5732A>G 5733G>T), GAG1536ATG (5734G>A 5735A>T), AGA1537AAA (5738G>A), ATG1538ATA (5742G>A), GTC1539TGT (5743G>T 5744T>G 5745C>T), TTC1540ATA (5746T>A 5748C>A), TAC1542AAC (5752T>A), AAA1543CGA (5755A>C 5756A>G), ACC1544CAA (5758A>C 5759C>A 5760C>A), TTG1545TTA (5763G>A), AAC1546AAT (5766C>T), GAC1547ACG (5767G>A 5768A>C 5769C>G), AAC1548GTC (5770A>G 5771A>T), ACG1549ACC (5775G>C), TTC1550ATT (5776T>A 5778C>T), AAA1551CGA (5779A>C 5780A>G), GAT1552AAC (5782G>A 5784T>C), CAG1553AAG (5785C>A), TCT1555TCA (5793T>A), CTA1556TTG (5794C>T 5796A>G), GGC1558CGT (5800G>C 5802C>T), ATT1559ATA (5805T>A), AAC1560GAT (5806A>G 5808C>T), ACA1561GAT (5809A>G 5810C>A 5811A>T), ATC1562CTC (5812A>C), ATA1563TTT (5815A>T 5817A>T), CAG1564GAC (5818C>G 5820G>C), AAG1565CAG (5821A>C), GTC1566TTA (5824G>T 5826C>A), TGT1567CAG (5827T>C 5828G>A 5829T>G), GCC1569GCA (5835C>A), AGT1570TCA (5836A>T 5837G>C 5838T>A), GTC1571GTG (5841C>G), TCC1573TCA (5847C>A), AAG1574AAA (5850G>A), TTC1575ATT (5851T>A 5853C>T), GAT1576AAT (5854G>A), CTG1577CTA (5859G>A), AAG1578AGA (5861A>G 5862G>A), AGC1579TCT (5863A>T 5864G>C 5865C>T), GGA1580GGT (5868A>T), TTC1581TAC (5870T>A), CAC1582CAT (5874C>T), ATT1584TTT (5878A>T), ATG1585AAG (5882T>A), ATG1586ATT (5886G>T), GAC1587AAG (5887G>A 5889C>G), GAA1588CCG (5890G>C 5891A>C 5892A>G), GAA1589AAG (5893G>A 5895A>G), TCC1590GAT (5896T>G 5897C>A 5898C>T), ATC1591GTG (5899A>G 5901C>G), TGG1593AGG (5905T>A), ACC1594ACT (5910C>T), GCC1595GCT (5913C>T), TTC1596TTT (5916C>T), CTG1597AGA (5917C>A 5918T>G 5919G>A), CCA1599CGG (5924C>G 5925A>G), GAT1600TAT (5926G>T), GGA1601GGG (5931A>G), CTT1602CAC (5933T>A 5934T>C), TGG1605TTC (5942G>T 5943G>C), TTA1606TTG (5946A>G), GTT1607ATG (5947G>A 5949T>G), CCA1609TCG (5953C>T 5955A>G), TTC1610ATT (5956T>A 5958C>T), GGA1611GGG (5961A>G), AAG1613ACC (5966A>C 5967G>C), GCC1615GCG (5973C>G), CCT1616CCA (5976T>A), GCT1617GCA (5979T>A), GTG1618GCC (5981T>C 5982G>C), CAA1620ATG (5986C>A 5987A>T 5988A>G), CGA1621AGT (5989C>A 5991A>T), AAG1622TTG (5992A>T 5993A>T), GAC1624AAT (5998G>A 6000C>T), AAC1625GGG (6001A>G 6002A>G 6003C>G), TGT1626GTG (6004T>G 6005G>T 6006T>G), TTC1627TTT (6009C>T), AGA1628AAG (6011G>A 6012A>G), GAC1629CCA (6013G>C 6014A>C 6015C>A), GAC1629_CTG1630insTTT (6015_6016insTTT), CTG1630TTG (6016C>T), TCA1631GAT (6019T>G 6020C>A 6021A>T), GGC1632TCG (6022G>T 6023G>C 6024C>G), GTA1634GTG (6030A>G), GCA1635ATT (6031G>A 6032C>T 6033A>T), GTT1636GGG (6035T>G 6036T>G), TAC1637TTT (6038A>T 6039C>T), ATC1641ATT (6051C>T), CTG1642CTT (6054G>T), GTC1643ATT (6055G>A 6057C>T)								

*: Inserts / Deletes / Misaligned / Frameshifts

Analysis details

This analysis was performed with panviral2.64

NGS Details (UN59): Badnavirus rutilanscamelliae

Assembly

**Coverage Length**	283 (1 contig(s))
**Depth Of Coverage**	9.1
**Number Of Reads**	21
**Reads Per Million**	0.46 rpm (after QC)
**Ambiguities**	0
**Assembly Method**	de novo + reference guided assembly
**Consensus Caller**	Bcf Tools

Coverage Map

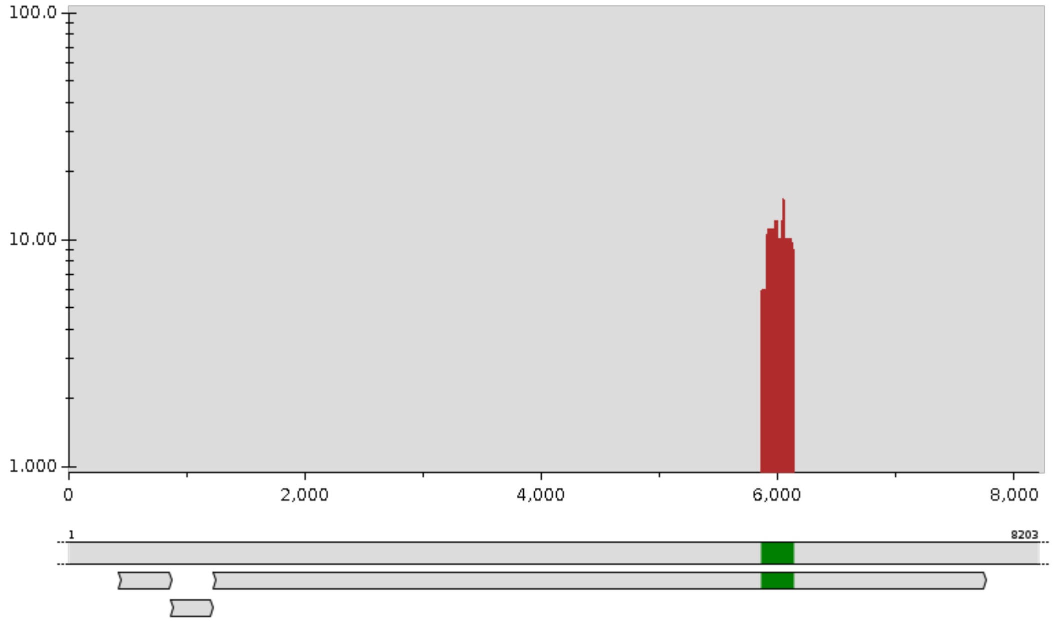

Assignment

**Type**	Badnavirus rutilanscamelliae (Taxonomy ID: 3047719)
**Reference Genome**	NC_055598.1
**NT Identity (%)**	52.4476
**AA Identity (%)**	38.5417
**Number Of Stop Codons**	0
**Number Of CDS**	3

Alignment

**Alignment Score**	22.0 (NT) + 272.0 (AA) = 294.0
**Concordance (%)**	23.0588

Alignment Method	Global, seeded, nucleotide + amino acids (AGA)

Genome Region

Sequence starts at position 5861 and ends at position 6143 relative to NC_055598.1 reference sequence.

Alignment Detailed Statistics

	Begin	End	Coverage	Score	Concordance	Matches	Identities	I/D/M/F*	Stop Codons
**NT**	**5861**	**6143**	**3.4%**	**22**	**3.9%**	**283 (99.0%)**	**150 (52.4%)**	**3/0**	
Mutations:	5861A>C, 5862G>A, 5864C>T, 5866T>G, 5869C>G, 5870G>A, 5872C>G, 5873A>G, 5874A>T, 5875C>G, 5877C>T, 5878T>A, 5879C>G, 5880A>T, 5884A>G, 5885G>A, 5888C>A, 5890A>G, 5894A>C, 5895G>C, 5896C>T, 5902A>T, 5903G>A, 5911C>T, 5912A>G, 5913C>A, 5915A>C, 5917T>G, 5918G>T, 5920G>T, 5922C>A, 5923G>C, 5924A>C, 5925G>A, 5927A>C, 5929A>T, 5930G>C, 5933C>G, 5934A>G, 5936A>G, 5937G>C, 5938C>A, 5939A>G, 5940A>C, 5941A>T, 5942A>G, 5944C>G, 5946A>T, 5947T>C, 5950G>C, 5954T>G, 5957G>A, 5959C>T, 5960C>T, 5964A>G, 5968A>T, 5971G>A, 5973T>A, 5980A>G, 5981G>T, 5983T>G, 5984G>A, 5985C>G, 5986T>A, 5989G>C, 5990G>A, 5991A>G, 5992T>G, 5993C>G, 5996C>T, 5997A>C, 5998A>T, 5999C>G, 6005G>C, 6006A>C, 6007A>T, 6008T>A, 6009G>A, 6012C>T, 6013C>A, 6014G>T, 6026C>A, 6027C>G, 6028A>G, 6029G>T, 6030T>A, 6031G>T, 6034C>A, 6035T>C, 6036T>A, 6037A>C, 6043A>G, 6045G>T, 6046G>C, 6049G>T, 6050G>A, 6052T>G, 6056C>T, 6058A>C, 6064G>A, 6067T>A, 6069A>T, 6070G>T, 6082A>C, 6084T>C, 6085A>G, 6089C>A, 6090A>T, 6091A>G, 6092A>G, 6093G>A, 6095A>T, 6096A>T, 6101G>A, 6103C>T, 6104C>G, 6106A>T, 6107A>G, 6109C>A, 6110T>C, 6113A>C, 6114A>G, 6115T>A, 6121C>T, 6121_6122insTTG, 6123C>A, 6125G>T, 6126G>T, 6127A>C, 6134G>A, 6135C>T, 6139G>C, 6141A>T, 6142T>C								
**CDS**									
KM754_gp3	1547	1641	4.4%	272	38.0%	95 (99.0%)	37 (38.5%)	1/0/0/0	0
Protein mutations:	R1547Q (5861A>C 5862G>A), N1549K (5869C>G), D1550K (5870G>A 5872C>G), N1551V (5873A>G 5874A>T 5875C>G), T1552I (5877C>T 5878T>A), H1553V (5879C>G 5880A>T), D1555N (5885G>A), Q1556K (5888C>A 5890A>G), S1558P (5894A>C 5895G>C 5896C>T), G1561R (5903G>A), T1564D (5912A>G 5913C>A), I1565L (5915A>C 5917T>G), V1566F (5918G>T 5920G>T), A1567D (5922C>A 5923G>C), R1568Q (5924A>C 5925G>A), I1569L (5927A>C 5929A>T), G1570R (5930G>C), H1571G (5933C>G 5934A>G), S1572A (5936A>G 5937G>C 5938C>A), K1573A (5939A>G 5940A>C 5941A>T), I1574V (5942A>G 5944C>G), Y1575F (5946A>T 5947T>C), F1578V (5954T>G), D1579N (5957G>A 5959C>T), K1581R (5964A>G), F1584Y (5973T>A), V1587L (5981G>T 5983T>G), A1588R (5984G>A 5985C>G 5986T>A), M1589I (5989G>C), D1590R (5990G>A 5991A>G 5992T>G), P1591A (5993C>G), Q1592S (5996C>T 5997A>C 5998A>T), H1593D (5999C>G), E1595P (6005G>C 6006A>C 6007A>T), W1596K (6008T>A 6009G>A), T1597I (6012C>T 6013C>A), A1598S (6014G>T), P1602R (6026C>A 6027C>G 6028A>G), V1603Y (6029G>T 6030T>A 6031G>T), L1605H (6035T>C 6036T>A 6037A>C), W1608F (6045G>T 6046G>C), V1610M (6050G>A 6052T>G), P1612S (6056C>T 6058A>C), K1616I (6069A>T 6070G>T), V1621A (6084T>C 6085A>G), Q1623M (6089C>A 6090A>T 6091A>G), R1624E (6092A>G 6093G>A), K1625L (6095A>T 6096A>T), D1627N (6101G>A 6103C>T), R1628G (6104C>G 6106A>T), I1629V (6107A>G 6109C>A), F1630L (6110T>C), N1631R (6113A>C 6114A>G 6115T>A), Y1633_A1634insL (6121_6122insTTG), A1634D (6123C>A), G1635F (6125G>T 6126G>T 6127A>C), A1638I (6134G>A 6135C>T), Y1640F (6141A>T 6142T>C)								
Codon mutations:	AGG1547CAG (5861A>C 5862G>A), CTT1548TTG (5864C>T 5866T>G), AAC1549AAG (5869C>G), GAC1550AAG (5870G>A 5872C>G), AAC1551GTG (5873A>G 5874A>T 5875C>G), ACT1552ATA (5877C>T 5878T>A), CAC1553GTC (5879C>G 5880A>T), AAA1554AAG (5884A>G), GAC1555AAC (5885G>A), CAA1556AAG (5888C>A 5890A>G), AGC1558CCT (5894A>C 5895G>C 5896C>T), CCA1560CCT (5902A>T), GGG1561AGG (5903G>A), GAC1563GAT (5911C>T), ACT1564GAT (5912A>G 5913C>A), ATT1565CTG (5915A>C 5917T>G), GTG1566TTT (5918G>T 5920G>T), GCG1567GAC (5922C>A 5923G>C), AGG1568CAG (5924A>C 5925G>A), ATA1569CTT (5927A>C 5929A>T), GGG1570CGG (5930G>C), CAT1571GGT (5933C>G 5934A>G), AGC1572GCA (5936A>G 5937G>C 5938C>A), AAA1573GCT (5939A>G 5940A>C 5941A>T), ATC1574GTG (5942A>G 5944C>G), TAT1575TTC (5946A>T 5947T>C), TCG1576TCC (5950G>C), TTC1578GTC (5954T>G), GAC1579AAT (5957G>A 5959C>T), CTG1580TTG (5960C>T), AAG1581AGG (5964A>G), TCA1582TCT (5968A>T), GGG1583GGA (5971G>A), TTT1584TAT (5973T>A), CAA1586CAG (5980A>G), GTT1587TTG (5981G>T 5983T>G), GCT1588AGA (5984G>A 5985C>G 5986T>A), ATG1589ATC (5989G>C), GAT1590AGG (5990G>A 5991A>G 5992T>G), CCT1591GCT (5993C>G), CAA1592TCT (5996C>T 5997A>C 5998A>T), CAC1593GAC (5999C>G), GAA1595CCT (6005G>C 6006A>C 6007A>T), TGG1596AAG (6008T>A 6009G>A), ACC1597ATA (6012C>T 6013C>A), GCT1598TCT (6014G>T), CCA1602AGG (6026C>A 6027C>G 6028A>G), GTG1603TAT (6029G>T 6030T>A 6031G>T), GGC1604GGA (6034C>A), TTA1605CAC (6035T>C 6036T>A 6037A>C), GAA1607GAG (6043A>G), TGG1608TTC (6045G>T 6046G>C), CTG1609CTT (6049G>T), GTT1610ATG (6050G>A 6052T>G), CCA1612TCC (6056C>T 6058A>C), GGG1614GGA (6064G>A), CTT1615CTA (6067T>A), AAG1616ATT (6069A>T 6070G>T), GCA1620GCC (6082A>C), GTA1621GCG (6084T>C 6085A>G), CAA1623ATG (6089C>A 6090A>T 6091A>G), AGG1624GAG (6092A>G 6093G>A), AAG1625TTG (6095A>T 6096A>T), GAC1627AAT (6101G>A 6103C>T), CGA1628GGT (6104C>G 6106A>T), ATC1629GTA (6107A>G 6109C>A), TTT1630CTT (6110T>C), AAT1631CGA (6113A>C 6114A>G 6115T>A), TAC1633TAT (6121C>T), TAC1633_GCT1634insTTG (6121_6122insTTG), GCT1634GAT (6123C>A), GGA1635TTC (6125G>T 6126G>T 6127A>C), GCT1638ATT (6134G>A 6135C>T), GTG1639GTC (6139G>C), TAT1640TTC (6141A>T 6142T>C)								
**Proteins**									
polyprotein (YP_010087856.1)	1547	1641	4.4%	272	38.0%	95 (99.0%)	37 (38.5%)	1/0/0/0	0
Protein mutations:	R1547Q (5861A>C 5862G>A), N1549K (5869C>G), D1550K (5870G>A 5872C>G), N1551V (5873A>G 5874A>T 5875C>G), T1552I (5877C>T 5878T>A), H1553V (5879C>G 5880A>T), D1555N (5885G>A), Q1556K (5888C>A 5890A>G), S1558P (5894A>C 5895G>C 5896C>T), G1561R (5903G>A), T1564D (5912A>G 5913C>A), I1565L (5915A>C 5917T>G), V1566F (5918G>T 5920G>T), A1567D (5922C>A 5923G>C), R1568Q (5924A>C 5925G>A), I1569L (5927A>C 5929A>T), G1570R (5930G>C), H1571G (5933C>G 5934A>G), S1572A (5936A>G 5937G>C 5938C>A), K1573A (5939A>G 5940A>C 5941A>T), I1574V (5942A>G 5944C>G), Y1575F (5946A>T 5947T>C), F1578V (5954T>G), D1579N (5957G>A 5959C>T), K1581R (5964A>G), F1584Y (5973T>A), V1587L (5981G>T 5983T>G), A1588R (5984G>A 5985C>G 5986T>A), M1589I (5989G>C), D1590R (5990G>A 5991A>G 5992T>G), P1591A (5993C>G), Q1592S (5996C>T 5997A>C 5998A>T), H1593D (5999C>G), E1595P (6005G>C 6006A>C 6007A>T), W1596K (6008T>A 6009G>A), T1597I (6012C>T 6013C>A), A1598S (6014G>T), P1602R (6026C>A 6027C>G 6028A>G), V1603Y (6029G>T 6030T>A 6031G>T), L1605H (6035T>C 6036T>A 6037A>C), W1608F (6045G>T 6046G>C), V1610M (6050G>A 6052T>G), P1612S (6056C>T 6058A>C), K1616I (6069A>T 6070G>T), V1621A (6084T>C 6085A>G), Q1623M (6089C>A 6090A>T 6091A>G), R1624E (6092A>G 6093G>A), K1625L (6095A>T 6096A>T), D1627N (6101G>A 6103C>T), R1628G (6104C>G 6106A>T), I1629V (6107A>G 6109C>A), F1630L (6110T>C), N1631R (6113A>C 6114A>G 6115T>A), Y1633_A1634insL (6121_6122insTTG), A1634D (6123C>A), G1635F (6125G>T 6126G>T 6127A>C), A1638I (6134G>A 6135C>T), Y1640F (6141A>T 6142T>C)								
Codon mutations:	AGG1547CAG (5861A>C 5862G>A), CTT1548TTG (5864C>T 5866T>G), AAC1549AAG (5869C>G), GAC1550AAG (5870G>A 5872C>G), AAC1551GTG (5873A>G 5874A>T 5875C>G), ACT1552ATA (5877C>T 5878T>A), CAC1553GTC (5879C>G 5880A>T), AAA1554AAG (5884A>G), GAC1555AAC (5885G>A), CAA1556AAG (5888C>A 5890A>G), AGC1558CCT (5894A>C 5895G>C 5896C>T), CCA1560CCT (5902A>T), GGG1561AGG (5903G>A), GAC1563GAT (5911C>T), ACT1564GAT (5912A>G 5913C>A), ATT1565CTG (5915A>C 5917T>G), GTG1566TTT (5918G>T 5920G>T), GCG1567GAC (5922C>A 5923G>C), AGG1568CAG (5924A>C 5925G>A), ATA1569CTT (5927A>C 5929A>T), GGG1570CGG (5930G>C), CAT1571GGT (5933C>G 5934A>G), AGC1572GCA (5936A>G 5937G>C 5938C>A), AAA1573GCT (5939A>G 5940A>C 5941A>T), ATC1574GTG (5942A>G 5944C>G), TAT1575TTC (5946A>T 5947T>C), TCG1576TCC (5950G>C), TTC1578GTC (5954T>G), GAC1579AAT (5957G>A 5959C>T), CTG1580TTG (5960C>T), AAG1581AGG (5964A>G), TCA1582TCT (5968A>T), GGG1583GGA (5971G>A), TTT1584TAT (5973T>A), CAA1586CAG (5980A>G), GTT1587TTG (5981G>T 5983T>G), GCT1588AGA (5984G>A 5985C>G 5986T>A), ATG1589ATC (5989G>C), GAT1590AGG (5990G>A 5991A>G 5992T>G), CCT1591GCT (5993C>G), CAA1592TCT (5996C>T 5997A>C 5998A>T), CAC1593GAC (5999C>G), GAA1595CCT (6005G>C 6006A>C 6007A>T), TGG1596AAG (6008T>A 6009G>A), ACC1597ATA (6012C>T 6013C>A), GCT1598TCT (6014G>T), CCA1602AGG (6026C>A 6027C>G 6028A>G), GTG1603TAT (6029G>T 6030T>A 6031G>T), GGC1604GGA (6034C>A), TTA1605CAC (6035T>C 6036T>A 6037A>C), GAA1607GAG (6043A>G), TGG1608TTC (6045G>T 6046G>C), CTG1609CTT (6049G>T), GTT1610ATG (6050G>A 6052T>G), CCA1612TCC (6056C>T 6058A>C), GGG1614GGA (6064G>A), CTT1615CTA (6067T>A), AAG1616ATT (6069A>T 6070G>T), GCA1620GCC (6082A>C), GTA1621GCG (6084T>C 6085A>G), CAA1623ATG (6089C>A 6090A>T 6091A>G), AGG1624GAG (6092A>G 6093G>A), AAG1625TTG (6095A>T 6096A>T), GAC1627AAT (6101G>A 6103C>T), CGA1628GGT (6104C>G 6106A>T), ATC1629GTA (6107A>G 6109C>A), TTT1630CTT (6110T>C), AAT1631CGA (6113A>C 6114A>G 6115T>A), TAC1633TAT (6121C>T), TAC1633_GCT1634insTTG (6121_6122insTTG), GCT1634GAT (6123C>A), GGA1635TTC (6125G>T 6126G>T 6127A>C), GCT1638ATT (6134G>A 6135C>T), GTG1639GTC (6139G>C), TAT1640TTC (6141A>T 6142T>C)								

*: Inserts / Deletes / Misaligned / Frameshifts

Analysis details

This analysis was performed with panviral2.64

NGS Details (UN59): Unknown

Assembly

**Coverage Length**	420 (1 contig(s))
**Depth Of Coverage**	5.6
**Number Of Reads**	20
**Reads Per Million**	0.43 rpm (after QC)
**Ambiguities**	0
**Assembly Method**	de novo + reference guided assembly
**Consensus Caller**	Bcf Tools

Coverage Map

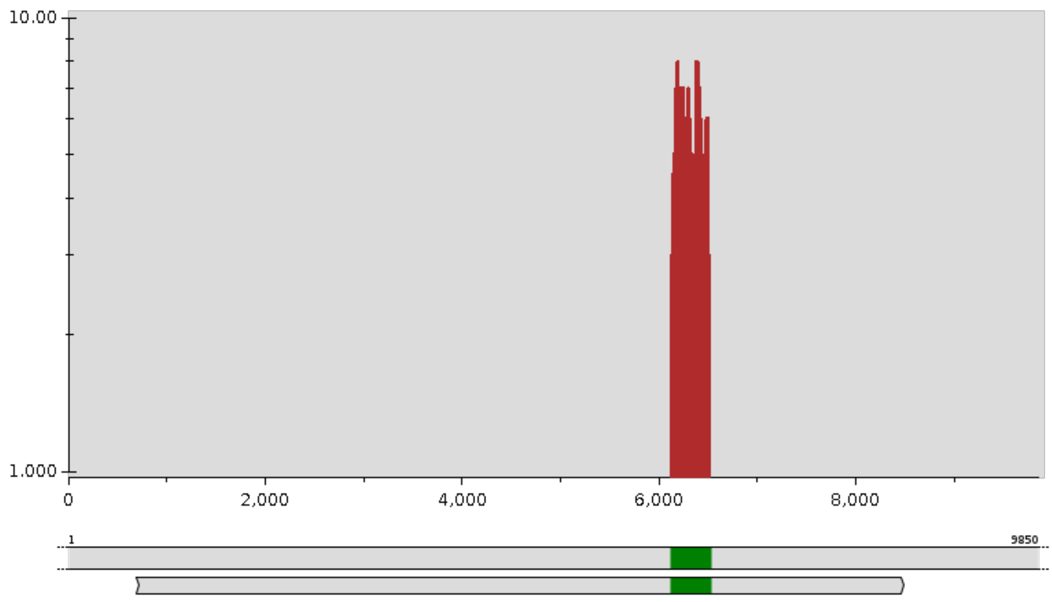

Assignment

**Type**	Unknown (Taxonomy ID: -1)
**Reference Genome**	NC_028462.1
**NT Identity (%)**	56.6986
**AA Identity (%)**	53.5714
**Number Of Stop Codons**	0
**Number Of CDS**	1

Alignment

**Alignment Score**	101.0 (NT) + 432.0 (AA) = 533.0
**Concordance (%)**	31.585

Alignment Method	Global, seeded, nucleotide + amino acids (AGA)

Genome Region

Sequence starts at position 6114 and ends at position 6533 relative to NC_028462.1 reference sequence.

Alignment Detailed Statistics

	Begin	End	Coverage	Score	Concordance	Matches	Identities	I/D/M/F*	Stop Codons
**NT**	**6114**	**6533**	**4.3%**	**101**	**12.2%**	**418 (99.5%)**	**237 (56.4%)**	**0/2**	
Mutations:	6116C>T, 6119A>T, 6122C>A, 6125T>A, 6126G>A, 6131C>T, 6134C>T, 6137C>T, 6140C>T, 6143G>T, 6146T>C, 6149G>T, 6159A>T, 6160A>T, 6161G>A, 6164C>A, 6165C>A, 6167T>G, 6168C>T, 6182G>A, 6192A>C, 6194C>A, 6197A>T, 6200C>T, 6203G>A, 6204C>A, 6208G>A, 6209G>A, 6213G>T, 6215C>T, 6221C>A, 6224C>T, 6225A>T, 6226A>C, 6227A>T, 6230T>A, 6231_6232delTG, 6233T>A, 6234T>A, 6236T>G, 6237C>A, 6239G>A, 6242C>T, 6244G>T, 6245T>G, 6246G>A, 6247G>T, 6248T>A, 6255G>A, 6257C>A, 6260C>T, 6263T>C, 6266A>T, 6267C>T, 6269C>G, 6272C>A, 6274T>G, 6278C>A, 6280A>C, 6282G>A, 6283A>C, 6284C>A, 6285A>T, 6287A>T, 6288G>C, 6290A>G, 6296C>T, 6299A>T, 6300C>A, 6301T>C, 6302G>A, 6308G>T, 6309G>A, 6311T>A, 6312A>G, 6313T>A, 6314C>A, 6315A>T, 6317C>T, 6318A>T, 6319T>C, 6320C>A, 6323C>A, 6324A>C, 6325A>T, 6326A>C, 6327A>T, 6328T>G, 6333C>A, 6335G>T, 6340G>A, 6341A>C, 6344C>A, 6345A>G, 6347C>A, 6348A>T, 6350C>A, 6351G>A, 6353A>T, 6354A>C, 6355G>A, 6356A>G, 6358C>A, 6359C>A, 6362G>A, 6363T>G, 6364A>C, 6365C>A, 6369T>A, 6371G>A, 6372G>A, 6373T>A, 6374C>T, 6376G>A, 6378G>A, 6380A>T, 6382G>A, 6383C>A, 6386T>A, 6389C>A, 6392T>C, 6393T>C, 6395A>T, 6399G>A, 6401C>G, 6403C>T, 6404C>T, 6407A>T, 6409G>C, 6410C>A, 6414G>A, 6415G>A, 6416A>T, 6417A>G, 6418A>G, 6419G>A, 6422C>A, 6423A>G, 6425G>A, 6428G>A, 6431G>A, 6432C>A, 6440C>A, 6441C>T, 6442T>C, 6443T>C, 6445C>A, 6446T>A, 6447C>A, 6449C>G, 6450C>A, 6452C>A, 6453A>T, 6455G>A, 6470A>G, 6471C>T, 6473C>A, 6475A>T, 6476G>A, 6477G>A, 6478A>C, 6479T>C, 6480C>A, 6481G>A, 6484T>A, 6485T>A, 6486C>G, 6488G>A, 6489G>A, 6491C>A, 6494G>A, 6495C>A, 6496A>G, 6497G>A, 6503C>T, 6509T>C, 6510G>T, 6512C>A, 6518C>T, 6521C>A, 6525A>G, 6527G>A								

CDS

	Begin	End	Coverage	Score	Concordance	Matches	Identities	I/D/M/F*	Stop Codons
ATL80_gp1	1808	1947	5.4%	432	42.8%	140 (100%)	75 (53.6%)	0/0/1/1	0
Protein mutations:	A1812T (6126G>A), K1817N (6143G>T), Q1819H (6149G>T), K1823L (6159A>T 6160A>T 6161G>A), L1825M (6165C>A 6167T>G), P1826S (6168C>T), N1834Q (6192A>C 6194C>A), R1839K (6208G>A 6209G>A), D1841Y (6213G>T 6215C>T), K1845S (6225A>T 6226A>C 6227A>T), D1846E (6230T>A), Y1848K (6234T>A 6236T>G), Q1849K (6237C>A 6239G>A), C1851L (6244G>T 6245T>G), G1852I (6246G>A 6247G>T 6248T>A), V1855I (6255G>A 6257C>A), F1861C (6274T>G), K1863T (6280A>C), D1864T (6282G>A 6283A>C 6284C>A), K1865Y (6285A>T 6287A>T), E1866Q (6288G>C 6290A>G), L1870T (6300C>A 6301T>C 6302G>A), D1873K (6309G>A 6311T>A), I1874E (6312A>G 6313T>A 6314C>A), I1875F (6315A>T 6317C>T), I1876S (6318A>T 6319T>C 6320C>A), N1877K (6323C>A), K1878L (6324A>C 6325A>T 6326A>C), I1879C (6327A>T 6328T>G), Q1881N (6333C>A 6335G>T), G1883D (6340G>A 6341A>C), I1885V (6345A>G 6347C>A), I1886L (6348A>T 6350C>A), G1887S (6351G>A 6353A>T), R1888Q (6354A>C 6355G>A 6356A>G), T1889K (6358C>A 6359C>A), Y1891A (6363T>G 6364A>C 6365C>A), L1893I (6369T>A 6371G>A), V1894N (6372G>A 6373T>A 6374C>T), R1895K (6376G>A), E1896N (6378G>A 6380A>T), S1897K (6382G>A 6383C>A), D1899E (6389C>A), V1903M (6399G>A 6401C>G), T1904I (6403C>T 6404C>T), C1906S (6409G>C 6410C>A), G1908N (6414G>A 6415G>A 6416A>T), K1909G (6417A>G 6418A>G 6419G>A), K1911E (6423A>G 6425G>A), P1914T (6432C>A), L1917S (6441C>T 6442T>C 6443T>C), T1918K (6445C>A 6446T>A), H1919K (6447C>A 6449C>G), L1920I (6450C>A 6452C>A), M1921L (6453A>T 6455G>A), K1928I (6475A>T 6476G>A), D1929T (6477G>A 6478A>C 6479T>C), R1930K (6480C>A 6481G>A), L1931Q (6484T>A 6485T>A), Q1932E (6486C>G 6488G>A), V1933I (6489G>A 6491C>A), Q1935R (6495C>A 6496A>G 6497G>A), V1940L (6510G>T 6512C>A), K1945E (6525A>G 6527G>A)								
Codon mutations:	ATC1808ATT (6116C>T), CCA1809CCT (6119A>T), CTC1810CTA (6122C>A), ACT1811ACA (6125T>A), GCA1812ACA (6126G>A), TTC1813TTT (6131C>T), AGC1814AGT (6134C>T), ACC1815ACT (6137C>T), CCC1816CCT (6140C>T), AAG1817AAT (6143G>T), GGT1818GGC (6146T>C), CAG1819CAT (6149G>T), AAG1823TTA (6159A>T 6160A>T 6161G>A), GTC1824GTA (6164C>A), CTT1825ATG (6165C>A 6167T>G), CCA1826TCA (6168C>T), AAG1830AAA (6182G>A), AAC1834CAA (6192A>C 6194C>A), ATA1835ATT (6197A>T), TTC1836TTT (6200C>T), CAG1837CAA (6203G>A), CGA1838AGA (6204C>A), AGG1839AAA (6208G>A 6209G>A), GAC1841TAT (6213G>T 6215C>T), ATC1843ATA (6221C>A), TTC1844TTT (6224C>T), AAA1845TCT (6225A>T 6226A>C 6227A>T), GAT1846GAA (6230T>A), TGT1847--A (6231_6232delTG 6233T>A), TAT1848AAG (6234T>A 6236T>G), CAG1849AAA (6237C>A 6239G>A), TTC1850TTT (6242C>T), TGT1851TTG (6244G>T 6245T>G), GGT1852ATA (6246G>A 6247G>T 6248T>A), GTC1855ATA (6255G>A 6257C>A), GAC1856GAT (6260C>T), GAT1857GAC (6263T>C), ATA1858ATT (6266A>T), CTC1859TTG (6267C>T 6269C>G), GTC1860GTA (6272C>A), TTC1861TGC (6274T>G), TCC1862TCA (6278C>A), AAA1863ACA (6280A>C), GAC1864ACA (6282G>A 6283A>C 6284C>A), AAA1865TAT (6285A>T 6287A>T), GAA1866CAG (6288G>C 6290A>G), CAC1868CAT (6296C>T), CTA1869CTT (6299A>T), CTG1870ACA (6300C>A 6301T>C 6302G>A), CTG1872CTT (6308G>T), GAT1873AAA (6309G>A 6311T>A), ATC1874GAA (6312A>G 6313T>A 6314C>A), ATC1875TTT (6315A>T 6317C>T), ATC1876TCA (6318A>T 6319T>C 6320C>A), AAC1877AAA (6323C>A), AAA1878CTC (6324A>C 6325A>T 6326A>C), ATT1879TGT (6327A>T 6328T>G), CAG1881AAT (6333C>A 6335G>T), GGA1883GAC (6340G>A 6341A>C), ATC1884ATA (6344C>A), ATC1885GTA (6345A>G 6347C>A), ATC1886TTA (6348A>T 6350C>A), GGA1887AGT (6351G>A 6353A>T), AGA1888CAG (6354A>C 6355G>A 6356A>G), ACC1889AAA (6358C>A 6359C>A), AAG1890AAA (6362G>A), TAC1891GCA (6363T>G 6364A>C 6365C>A), TTG1893ATA (6369T>A 6371G>A), GTC1894AAT (6372G>A 6373T>A 6374C>T), AGA1895AAA (6376G>A), GAA1896AAT (6378G>A 6380A>T), AGC1897AAA (6382G>A 6383C>A), ATT1898ATA (6386T>A), GAC1899GAA (6389C>A), TTT1900TTC (6392T>C), TTA1901CTT (6393T>C 6395A>T), GTC1903ATG (6399G>A 6401C>G), ACC1904ATT (6403C>T 6404C>T), ATA1905ATT (6407A>T), TGC1906TCA (6409G>C 6410C>A), GGA1908AAT (6414G>A 6415G>A 6416A>T), AAG1909GGA (6417A>G 6418A>G 6419G>A), ATC1910ATA (6422C>A), AAG1911GAA (6423A>G 6425G>A), TTG1912TTA (6428G>A), CAG1913CAA (6431G>A), CCT1914ACT (6432C>A), ATC1916ATA (6440C>A), CTT1917TCC (6441C>T 6442T>C 6443T>C), ACT1918AAA (6445C>A 6446T>A), CAC1919AAG (6447C>A 6449C>G), CTC1920ATA (6450C>A 6452C>A), ATG1921TTA (6453A>T 6455G>A), AAA1926AAG (6470A>G), CTC1927TTA (6471C>T 6473C>A), AAG1928ATA (6475A>T 6476G>A), GAT1929ACC (6477G>A 6478A>C 6479T>C), CGA1930AAA (6480C>A 6481G>A), CTT1931CAA (6484T>A 6485T>A), CAG1932GAA (6486C>G 6488G>A), GTC1933ATA (6489G>A 6491C>A), CAG1934CAA (6494G>A), CAG1935AGA (6495C>A 6496A>G 6497G>A), CTC1937CTT (6503C>T), TGT1939TGC (6509T>C), GTC1940TTA (6510G>T 6512C>A), TAC1942TAT (6518C>T), GCC1943GCA (6521C>A), AAG1945GAA (6525A>G 6527G>A)								

Proteins

	Begin	End	Coverage	Score	Concordance	Matches	Identities	I/D/M/F*	Stop Codons
polyprotein (YP_009182100.1)	1808	1947	5.4%	432	42.8%	140 (100%)	75 (53.6%)	0/0/1/1	0
Protein mutations:	A1812T (6126G>A), K1817N (6143G>T), Q1819H (6149G>T), K1823L (6159A>T 6160A>T 6161G>A), L1825M (6165C>A 6167T>G), P1826S (6168C>T), N1834Q (6192A>C 6194C>A), R1839K (6208G>A 6209G>A), D1841Y (6213G>T 6215C>T), K1845S (6225A>T 6226A>C 6227A>T), D1846E (6230T>A), Y1848K (6234T>A 6236T>G), Q1849K (6237C>A 6239G>A), C1851L (6244G>T 6245T>G), G1852I (6246G>A 6247G>T 6248T>A), V1855I (6255G>A 6257C>A), F1861C (6274T>G), K1863T (6280A>C), D1864T (6282G>A 6283A>C 6284C>A), K1865Y (6285A>T 6287A>T), E1866Q (6288G>C 6290A>G), L1870T (6300C>A 6301T>C 6302G>A), D1873K (6309G>A 6311T>A), I1874E (6312A>G 6313T>A 6314C>A), I1875F (6315A>T 6317C>T), I1876S (6318A>T 6319T>C 6320C>A), N1877K (6323C>A), K1878L (6324A>C 6325A>T 6326A>C), I1879C (6327A>T 6328T>G), Q1881N (6333C>A 6335G>T), G1883D (6340G>A 6341A>C), I1885V (6345A>G 6347C>A), I1886L (6348A>T 6350C>A), G1887S (6351G>A 6353A>T), R1888Q (6354A>C 6355G>A 6356A>G), T1889K (6358C>A 6359C>A), Y1891A (6363T>G 6364A>C 6365C>A), L1893I (6369T>A 6371G>A), V1894N (6372G>A 6373T>A 6374C>T), R1895K (6376G>A), E1896N (6378G>A 6380A>T), S1897K (6382G>A 6383C>A), D1899E (6389C>A), V1903M (6399G>A 6401C>G), T1904I (6403C>T 6404C>T), C1906S (6409G>C 6410C>A), G1908N (6414G>A 6415G>A 6416A>T), K1909G (6417A>G 6418A>G 6419G>A), K1911E (6423A>G 6425G>A), P1914T (6432C>A), L1917S (6441C>T 6442T>C 6443T>C), T1918K (6445C>A 6446T>A), H1919K (6447C>A 6449C>G), L1920I (6450C>A 6452C>A), M1921L (6453A>T 6455G>A), K1928I (6475A>T 6476G>A), D1929T (6477G>A 6478A>C 6479T>C), R1930K (6480C>A 6481G>A), L1931Q (6484T>A 6485T>A), Q1932E (6486C>G 6488G>A), V1933I (6489G>A 6491C>A), Q1935R (6495C>A 6496A>G 6497G>A), V1940L (6510G>T 6512C>A), K1945E (6525A>G 6527G>A)								
Codon mutations:	ATC1808ATT (6116C>T), CCA1809CCT (6119A>T), CTC1810CTA (6122C>A), ACT1811ACA (6125T>A), GCA1812ACA (6126G>A), TTC1813TTT (6131C>T), AGC1814AGT (6134C>T), ACC1815ACT (6137C>T), CCC1816CCT (6140C>T), AAG1817AAT (6143G>T), GGT1818GGC (6146T>C), CAG1819CAT (6149G>T), AAG1823TTA (6159A>T 6160A>T 6161G>A), GTC1824GTA (6164C>A), CTT1825ATG (6165C>A 6167T>G), CCA1826TCA (6168C>T), AAG1830AAA (6182G>A), AAC1834CAA (6192A>C 6194C>A), ATA1835ATT (6197A>T), TTC1836TTT (6200C>T), CAG1837CAA (6203G>A), CGA1838AGA (6204C>A), AGG1839AAA (6208G>A 6209G>A), GAC1841TAT (6213G>T 6215C>T), ATC1843ATA (6221C>A), TTC1844TTT (6224C>T), AAA1845TCT (6225A>T 6226A>C 6227A>T), GAT1846GAA (6230T>A), TGT1847--A (6231_6232delTG 6233T>A), TAT1848AAG (6234T>A 6236T>G), CAG1849AAA (6237C>A 6239G>A), TTC1850TTT (6242C>T), TGT1851TTG (6244G>T 6245T>G), GGT1852ATA (6246G>A 6247G>T 6248T>A), GTC1855ATA (6255G>A 6257C>A), GAC1856GAT (6260C>T), GAT1857GAC (6263T>C), ATA1858ATT (6266A>T), CTC1859TTG (6267C>T 6269C>G), GTC1860GTA (6272C>A), TTC1861TGC (6274T>G), TCC1862TCA (6278C>A), AAA1863ACA (6280A>C), GAC1864ACA (6282G>A 6283A>C 6284C>A), AAA1865TAT (6285A>T 6287A>T), GAA1866CAG (6288G>C 6290A>G), CAC1868CAT (6296C>T), CTA1869CTT (6299A>T), CTG1870ACA (6300C>A 6301T>C 6302G>A), CTG1872CTT (6308G>T), GAT1873AAA (6309G>A 6311T>A), ATC1874GAA (6312A>G 6313T>A 6314C>A), ATC1875TTT (6315A>T 6317C>T), ATC1876TCA (6318A>T 6319T>C 6320C>A), AAC1877AAA (6323C>A), AAA1878CTC (6324A>C 6325A>T 6326A>C), ATT1879TGT (6327A>T 6328T>G), CAG1881AAT (6333C>A 6335G>T), GGA1883GAC (6340G>A 6341A>C), ATC1884ATA (6344C>A), ATC1885GTA (6345A>G 6347C>A), ATC1886TTA (6348A>T 6350C>A), GGA1887AGT (6351G>A 6353A>T), AGA1888CAG (6354A>C 6355G>A 6356A>G), ACC1889AAA (6358C>A 6359C>A), AAG1890AAA (6362G>A), TAC1891GCA (6363T>G 6364A>C 6365C>A), TTG1893ATA (6369T>A 6371G>A), GTC1894AAT (6372G>A 6373T>A 6374C>T), AGA1895AAA (6376G>A), GAA1896AAT (6378G>A 6380A>T), AGC1897AAA (6382G>A 6383C>A), ATT1898ATA (6386T>A), GAC1899GAA (6389C>A), TTT1900TTC (6392T>C), TTA1901CTT (6393T>C 6395A>T), GTC1903ATG (6399G>A 6401C>G), ACC1904ATT (6403C>T 6404C>T), ATA1905ATT (6407A>T), TGC1906TCA (6409G>C 6410C>A), GGA1908AAT (6414G>A 6415G>A 6416A>T), AAG1909GGA (6417A>G 6418A>G 6419G>A), ATC1910ATA (6422C>A), AAG1911GAA (6423A>G 6425G>A), TTG1912TTA (6428G>A), CAG1913CAA (6431G>A), CCT1914ACT (6432C>A), ATC1916ATA (6440C>A), CTT1917TCC (6441C>T 6442T>C 6443T>C), ACT1918AAA (6445C>A 6446T>A), CAC1919AAG (6447C>A 6449C>G), CTC1920ATA (6450C>A 6452C>A), ATG1921TTA (6453A>T 6455G>A), AAA1926AAG (6470A>G), CTC1927TTA (6471C>T 6473C>A), AAG1928ATA (6475A>T 6476G>A), GAT1929ACC (6477G>A 6478A>C 6479T>C), CGA1930AAA (6480C>A 6481G>A), CTT1931CAA (6484T>A 6485T>A), CAG1932GAA (6486C>G 6488G>A), GTC1933ATA (6489G>A 6491C>A), CAG1934CAA (6494G>A), CAG1935AGA (6495C>A 6496A>G 6497G>A), CTC1937CTT (6503C>T), TGT1939TGC (6509T>C), GTC1940TTA (6510G>T 6512C>A), TAC1942TAT (6518C>T), GCC1943GCA (6521C>A), AAG1945GAA (6525A>G 6527G>A)								

*: Inserts / Deletes / Misaligned / Frameshifts

Analysis details

This analysis was performed with panviral2.64

NGS Details (UN59): Badnavirus venabougainvilleae

Assembly

**Coverage Length**	303 (1 contig(s))
**Depth Of Coverage**	6.9
**Number Of Reads**	18
**Reads Per Million**	0.39 rpm (after QC)
**Ambiguities**	0
**Assembly Method**	de novo + reference guided assembly
**Consensus Caller**	Bcf Tools

Coverage Map

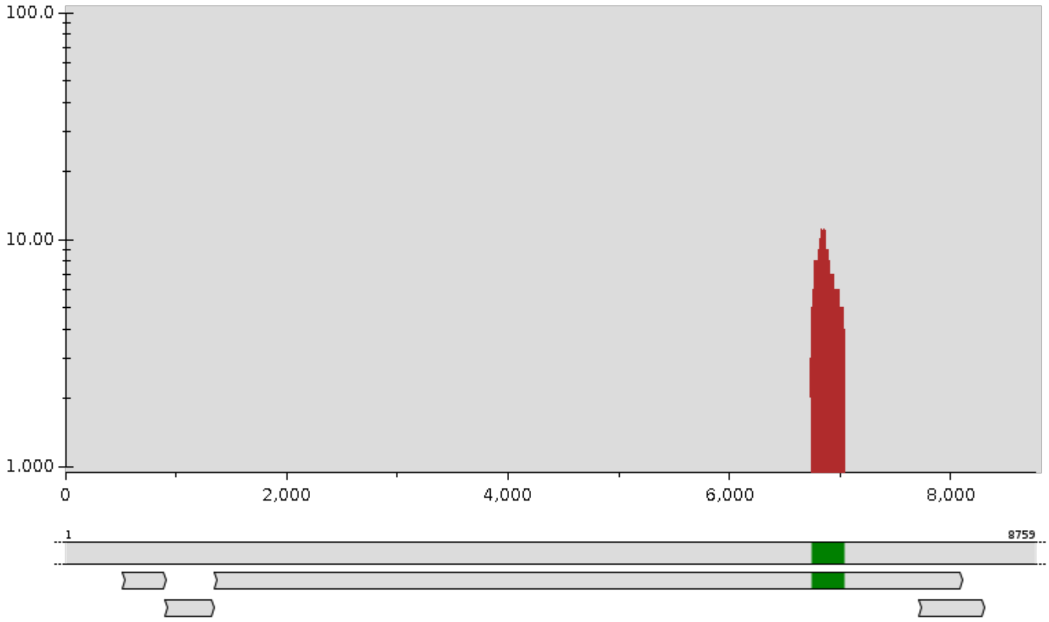

Assignment

**Type**	Badnavirus venabougainvilleae (Taxonomy ID: 3047697)
**Reference Genome**	NC_011592.1
**NT Identity (%)**	53.268
**AA Identity (%)**	46.0784
**Number Of Stop Codons**	0
**Number Of CDS**	4

Alignment

**Alignment Score**	34.0 (NT) + 315.0 (AA) = 349.0
**Concordance (%)**	26.5198

**Alignment Method**	Global, seeded, nucleotide + amino acids (AGA)

Genome Region

Sequence starts at position 6741 and ends at position 7043 relative to NC_011592.1 reference sequence.

Alignment Detailed Statistics

	Begin	End	Coverage	Score	Concordance	Matches	Identities	I/D/M/F*	Stop Codons
**NT**	**6741**	**7043**	**3.5%**	**34**	**5.6%**	**303 (99.0%)**	**163 (53.3%)**	**3/0**	
Mutations:	6741C>T, 6747A>C, 6748T>A, 6749G>A, 6759C>T, 6761T>G, 6762T>A, 6765T>A, 6767C>G, 6768A>G, 6769C>T, 6771A>T, 6774A>T, 6775T>C, 6776T>A, 6777G>T, 6783A>G, 6785G>T, 6786G>T, 6789A>G, 6792C>T, 6796C>T, 6804T>G, 6809A>C, 6810G>A, 6819A>G, 6822T>A, 6823A>G, 6824T>C, 6828C>T, 6829C>A, 6830A>T, 6831A>G, 6832A>G, 6833G>A, 6834G>T, 6835A>T, 6836A>T, 6837G>A, 6841G>A, 6843C>T, 6845A>G, 6846C>G, 6847T>G, 6848G>T, 6849C>G, 6853T>A, 6854C>A, 6855A>G, 6857G>C, 6860A>T, 6861C>T, 6862A>T, 6863G>T, 6864C>G, 6867_6868insGTG, 6870C>T, 6871A>G, 6873T>G, 6874G>A, 6875C>T, 6876T>A, 6881A>T, 6885T>A, 6894A>T, 6902T>A, 6906T>A, 6907G>A, 6908A>G, 6909G>A, 6910A>T, 6911G>C, 6912T>C, 6916A>G, 6917G>A, 6918A>G, 6926A>T, 6927G>T, 6930G>T, 6933T>C, 6934C>T, 6936C>G, 6937A>C, 6940G>C, 6941T>A, 6942T>A, 6943A>G, 6946C>T, 6948T>A, 6952G>A, 6955T>C, 6956G>T, 6959A>G, 6960A>T, 6963A>T, 6964A>C, 6965A>G, 6967G>A, 6968G>A, 6969C>G, 6973G>T, 6974T>A, 6975T>C, 6976C>G, 6977T>C, 6979T>A, 6980C>A, 6981C>A, 6982C>T, 6983C>T, 6985A>T, 6991A>T, 6992T>G, 6993G>T, 6994A>G, 6996G>T, 6997G>T, 7000G>T, 7001C>G, 7003G>T, 7005C>A, 7007A>G, 7009A>T, 7011T>A, 7012A>G, 7014T>G, 7016A>C, 7017G>A, 7021C>T, 7023T>A, 7026A>T, 7027G>C, 7028C>A, 7029A>T, 7032A>T, 7033C>G, 7036G>T, 7037G>C, 7038G>T, 7039A>G, 7042C>G								
**CDS**									
BSCVV_gp3	1800	1900	4.5%	315	43.6%	101 (99.0%)	47 (46.1%)	1/0/0/0	0
Protein mutations:	W1802K (6748T>A 6749G>A), L1806R (6761T>G 6762T>A), P1808R (6767C>G 6768A>G), Q1809Y (6769C>T 6771A>T), L1811H (6775T>C 6776T>A 6777G>T), W1814F (6785G>T 6786G>T), P1818S (6796C>T), K1822T (6809A>C 6810G>A), I1827A (6823A>G 6824T>C), Q1829M (6829C>A 6830A>T 6831A>G), R1830D (6832A>G 6833G>A 6834G>T), K1831L (6835A>T 6836A>T 6837G>A), D1833N (6841G>A 6843C>T), N1834R (6845A>G 6846C>G), C1835V (6847T>G 6848G>T 6849C>G), S1837K (6853T>A 6854C>A 6855A>G), R1838P (6857G>C), Y1839F (6860A>T 6861C>T), S1840L (6862A>T 6863G>T 6864C>G), D1841_F1842insV (6867_6868insGTG), I1843V (6871A>G 6873T>G), A1844I (6874G>A 6875C>T 6876T>A), Y1846F (6881A>T), F1853Y (6902T>A), E1855R (6907G>A 6908A>G 6909G>A), R1858E (6916A>G 6917G>A 6918A>G), E1861V (6926A>T 6927G>T), K1862N (6930G>T), V1866Q (6940G>C 6941T>A 6942T>A), M1867V (6943A>G), V1870I (6952G>A), C1871L (6955T>C 6956G>T), Q1872R (6959A>G 6960A>T), E1873D (6963A>T), N1874R (6964A>C 6965A>G), G1875K (6967G>A 6968G>A 6969C>G), V1877Y (6973G>T 6974T>A 6975T>C), L1878A (6976C>G 6977T>C), S1879K (6979T>A 6980C>A 6981C>A), P1880F (6982C>T 6983C>T), T1881S (6985A>T), M1883C (6991A>T 6992T>G 6993G>T), K1884D (6994A>G 6996G>T), V1885F (6997G>T), A1886W (7000G>T 7001C>G), V1887L (7003G>T 7005C>A), K1888R (7007A>G), T1889S (7009A>T 7011T>A), I1890V (7012A>G 7014T>G), E1891A (7016A>C 7017G>A), A1895H (7027G>C 7028C>A 7029A>T), L1897V (7033C>G), G1898S (7036G>T 7037G>C 7038G>T), N1899D (7039A>G)								
Codon mutations:	TCC1799..T (6741C>T), CCA1801CCC (6747A>C), TGG1802AAG (6748T>A 6749G>A), TTC1805TTT (6759C>T), CTT1806CGA (6761T>G 6762T>A), ACT1807ACA (6765T>A), CCA1808CGG (6767C>G 6768A>G), CAA1809TAT (6769C>T 6771A>T), GGA1810GGT (6774A>T), TTG1811CAT (6775T>C 6776T>A 6777G>T), GAA1813GAG (6783A>G), TGG1814TTT (6785G>T 6786G>T), CTA1815CTG (6789A>G), GTC1816GTT (6792C>T), CCA1818TCA (6796C>T), GGT1820GGG (6804T>G), AAG1822ACA (6809A>C 6810G>A), CCA1825CCG (6819A>G), GCT1826GCA (6822T>A), ATC1827GCC (6823A>G 6824T>C), TTC1828TTT (6828C>T), CAA1829ATG (6829C>A 6830A>T 6831A>G), AGG1830GAT (6832A>G 6833G>A 6834G>T), AAG1831TTA (6835A>T 6836A>T 6837G>A), GAC1833AAT (6841G>A 6843C>T), AAC1834AGG (6845A>G 6846C>G), TGC1835GTG (6847T>G 6848G>T 6849C>G), TCA1837AAG (6853T>A 6854C>A 6855A>G), CGA1838CCA (6857G>C), TAC1839TTT (6860A>T 6861C>T), AGC1840TTG (6862A>T 6863G>T 6864C>G), GAT1841_TTC1842insGTG (6867_6868insGTG), TTC1842TTT (6870C>T), ATT1843GTG (6871A>G 6873T>G), GCT1844ATA (6874G>A 6875C>T 6876T>A), TAC1846TTC (6881A>T), ATT1847ATA (6885T>A), ATA1850ATT (6894A>T), TTT1853TAT (6902T>A), TCT1854TCA (6906T>A), GAG1855AGA (6907G>A 6908A>G 6909G>A), AGT1856TCC (6910A>T 6911G>C 6912T>C), AGA1858GAG (6916A>G 6917G>A 6918A>G), GAG1861GTT (6926A>T 6927G>T), AAG1862AAT (6930G>T), CAT1863CAC (6933T>C), CTC1864TTG (6934C>T 6936C>G), AGA1865CGA (6937A>C), GTT1866CAA (6940G>C 6941T>A 6942T>A), ATG1867GTG (6943A>G), CTT1868TTA (6946C>T 6948T>A), GTT1870ATT (6952G>A), TGT1871CTT (6955T>C 6956G>T), CAA1872CGT (6959A>G 6960A>T), GAA1873GAT (6963A>T), AAT1874CGT (6964A>C 6965A>G), GGC1875AAG (6967G>A 6968G>A 6969C>G), GTT1877TAC (6973G>T 6974T>A 6975T>C), CTA1878GCA (6976C>G 6977T>C), TCC1879AAA (6979T>A 6980C>A 6981C>A), CCC1880TTC (6982C>T 6983C>T), ACT1881TCT (6985A>T), ATG1883TGT (6991A>T 6992T>G 6993G>T), AAG1884GAT (6994A>G 6996G>T), GTT1885TTT (6997G>T), GCG1886TGG (7000G>T 7001C>G), GTC1887TTA (7003G>T 7005C>A), AAA1888AGA (7007A>G), ACT1889TCA (7009A>T 7011T>A), ATT1890GTG (7012A>G 7014T>G), GAG1891GCA (7016A>C 7017G>A), CTT1893TTA (7021C>T 7023T>A), GGA1894GGT (7026A>T), GCA1895CAT (7027G>C 7028C>A 7029A>T), ATA1896ATT (7032A>T), CTA1897GTA (7033C>G), GGG1898TCT (7036G>T 7037G>C 7038G>T), AAT1899GAT (7039A>G), CAA1900GA. (7042C>G)								
**Proteins**									
polyprotein (YP_002321513.1)	1800	1900	4.5%	315	43.6%	101 (99.0%)	47 (46.1%)	1/0/0/0	0
Protein mutations:	W1802K (6748T>A 6749G>A), L1806R (6761T>G 6762T>A), P1808R (6767C>G 6768A>G), Q1809Y (6769C>T 6771A>T), L1811H (6775T>C 6776T>A 6777G>T), W1814F (6785G>T 6786G>T), P1818S (6796C>T), K1822T (6809A>C 6810G>A), I1827A (6823A>G 6824T>C), Q1829M (6829C>A 6830A>T 6831A>G), R1830D (6832A>G 6833G>A 6834G>T), K1831L (6835A>T 6836A>T 6837G>A), D1833N (6841G>A 6843C>T), N1834R (6845A>G 6846C>G), C1835V (6847T>G 6848G>T 6849C>G), S1837K (6853T>A 6854C>A 6855A>G), R1838P (6857G>C), Y1839F (6860A>T 6861C>T), S1840L (6862A>T 6863G>T 6864C>G), D1841_F1842insV (6867_6868insGTG), I1843V (6871A>G 6873T>G), A1844I (6874G>A 6875C>T 6876T>A), Y1846F (6881A>T), F1853Y (6902T>A), E1855R (6907G>A 6908A>G 6909G>A), R1858E (6916A>G 6917G>A 6918A>G), E1861V (6926A>T 6927G>T), K1862N (6930G>T), V1866Q (6940G>C 6941T>A 6942T>A), M1867V (6943A>G), V1870I (6952G>A), C1871L (6955T>C 6956G>T), Q1872R (6959A>G 6960A>T), E1873D (6963A>T), N1874R (6964A>C 6965A>G), G1875K (6967G>A 6968G>A 6969C>G), V1877Y (6973G>T 6974T>A 6975T>C), L1878A (6976C>G 6977T>C), S1879K (6979T>A 6980C>A 6981C>A), P1880F (6982C>T 6983C>T), T1881S (6985A>T), M1883C (6991A>T 6992T>G 6993G>T), K1884D (6994A>G 6996G>T), V1885F (6997G>T), A1886W (7000G>T 7001C>G), V1887L (7003G>T 7005C>A), K1888R (7007A>G), T1889S (7009A>T 7011T>A), I1890V (7012A>G 7014T>G), E1891A (7016A>C 7017G>A), A1895H (7027G>C 7028C>A 7029A>T), L1897V (7033C>G), G1898S (7036G>T 7037G>C 7038G>T), N1899D (7039A>G)								
Codon mutations:	TCC1799..T (6741C>T), CCA1801CCC (6747A>C), TGG1802AAG (6748T>A 6749G>A), TTC1805TTT (6759C>T), CTT1806CGA (6761T>G 6762T>A), ACT1807ACA (6765T>A), CCA1808CGG (6767C>G 6768A>G), CAA1809TAT (6769C>T 6771A>T), GGA1810GGT (6774A>T), TTG1811CAT (6775T>C 6776T>A 6777G>T), GAA1813GAG (6783A>G), TGG1814TTT (6785G>T 6786G>T), CTA1815CTG (6789A>G), GTC1816GTT (6792C>T), CCA1818TCA (6796C>T), GGT1820GGG (6804T>G), AAG1822ACA (6809A>C 6810G>A), CCA1825CCG (6819A>G), GCT1826GCA (6822T>A), ATC1827GCC (6823A>G 6824T>C), TTC1828TTT (6828C>T), CAA1829ATG (6829C>A 6830A>T 6831A>G), AGG1830GAT (6832A>G 6833G>A 6834G>T), AAG1831TTA (6835A>T 6836A>T 6837G>A), GAC1833AAT (6841G>A 6843C>T), AAC1834AGG (6845A>G 6846C>G), TGC1835GTG (6847T>G 6848G>T 6849C>G), TCA1837AAG (6853T>A 6854C>A 6855A>G), CGA1838CCA (6857G>C), TAC1839TTT (6860A>T 6861C>T), AGC1840TTG (6862A>T 6863G>T 6864C>G), GAT1841_TTC1842insGTG (6867_6868insGTG), TTC1842TTT (6870C>T), ATT1843GTG (6871A>G 6873T>G), GCT1844ATA (6874G>A 6875C>T 6876T>A), TAC1846TTC (6881A>T), ATT1847ATA (6885T>A), ATA1850ATT (6894A>T), TTT1853TAT (6902T>A), TCT1854TCA (6906T>A), GAG1855AGA (6907G>A 6908A>G 6909G>A), AGT1856TCC (6910A>T 6911G>C 6912T>C), AGA1858GAG (6916A>G 6917G>A 6918A>G), GAG1861GTT (6926A>T 6927G>T), AAG1862AAT (6930G>T), CAT1863CAC (6933T>C), CTC1864TTG (6934C>T 6936C>G), AGA1865CGA (6937A>C), GTT1866CAA (6940G>C 6941T>A 6942T>A), ATG1867GTG (6943A>G), CTT1868TTA (6946C>T 6948T>A), GTT1870ATT (6952G>A), TGT1871CTT (6955T>C 6956G>T), CAA1872CGT (6959A>G 6960A>T), GAA1873GAT (6963A>T), AAT1874CGT (6964A>C 6965A>G), GGC1875AAG (6967G>A 6968G>A 6969C>G), GTT1877TAC (6973G>T 6974T>A 6975T>C), CTA1878GCA (6976C>G 6977T>C), TCC1879AAA (6979T>A 6980C>A 6981C>A), CCC1880TTC (6982C>T 6983C>T), ACT1881TCT (6985A>T), ATG1883TGT (6991A>T 6992T>G 6993G>T), AAG1884GAT (6994A>G 6996G>T), GTT1885TTT (6997G>T), GCG1886TGG (7000G>T 7001C>G), GTC1887TTA (7003G>T 7005C>A), AAA1888AGA (7007A>G), ACT1889TCA (7009A>T 7011T>A), ATT1890GTG (7012A>G 7014T>G), GAG1891GCA (7016A>C 7017G>A), CTT1893TTA (7021C>T 7023T>A), GGA1894GGT (7026A>T), GCA1895CAT (7027G>C 7028C>A 7029A>T), ATA1896ATT (7032A>T), CTA1897GTA (7033C>G), GGG1898TCT (7036G>T 7037G>C 7038G>T), AAT1899GAT (7039A>G), CAA1900GA. (7042C>G)								

*: Inserts / Deletes / Misaligned / Frameshifts

Analysis details

This analysis was performed with panviral2.64

NGS Details (UN59): Epiphyllum badnavirus 1

Assembly

**Coverage Length**	275 (1 contig(s))
**Depth Of Coverage**	6.7
**Number Of Reads**	16
**Reads Per Million**	0.35 rpm (after QC)
**Ambiguities**	0
**Assembly Method**	de novo + reference guided assembly
**Consensus Caller**	Bcf Tools

Coverage Map

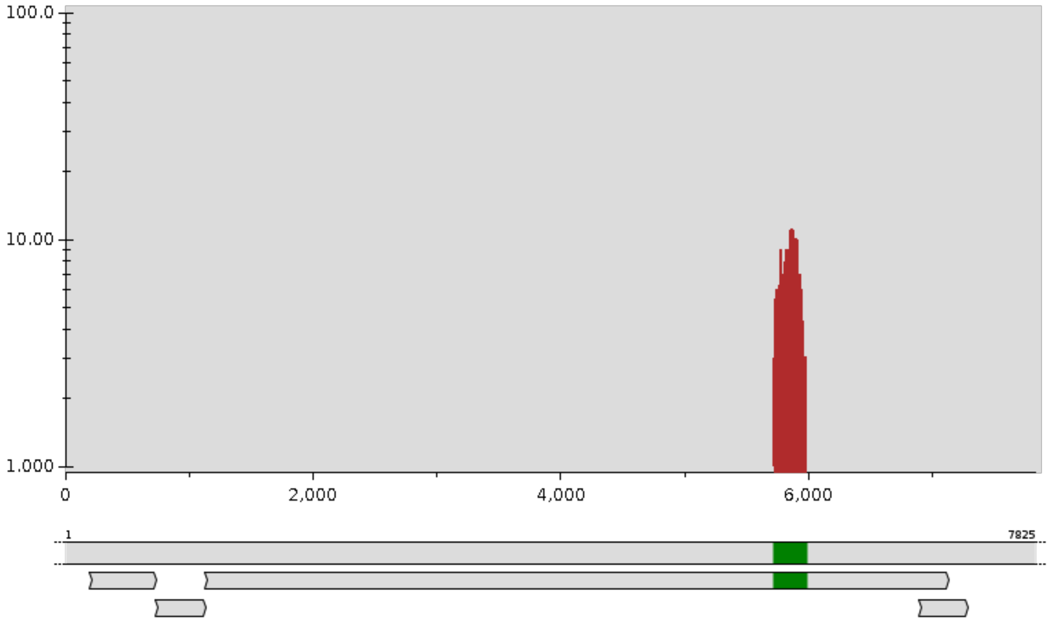

Assignment

**Type**	Epiphyllum badnavirus 1 (Taxonomy ID: 2518008)
**Reference Genome**	NC_076247.1
**NT Identity (%)**	57.971
**AA Identity (%)**	50.0
**Number Of Stop Codons**	0
**Number Of CDS**	4

Alignment

**Alignment Score**	80.0 (NT) + 187.0 (AA) = 267.0
**Concordance (%)**	24.4983

Alignment Method	Global, seeded, nucleotide + amino acids (AGA)

Genome Region

Sequence starts at position 5714 and ends at position 5988 relative to NC_076247.1 reference sequence.

Alignment Detailed Statistics

	Begin	End	Coverage	Score	Concordance	Matches	Identities	I/D/M/F*	Stop Codons
**NT**	**5714**	**5988**	**3.5%**	**80**	**14.5%**	**275 (99.6%)**	**160 (58.0%)**	**1/0**	
Mutations:	5719G>A, 5725A>C, 5727A>T, 5730A>T, 5732A>G, 5733G>C, 5734G>A, 5735A>T, 5743G>T, 5744T>G, 5745C>T, 5746T>A, 5748C>T, 5749A>G, 5754C>T, 5755A>C, 5756A>G, 5757A>T, 5758A>C, 5759C>A, 5760C>G, 5767G>A, 5769C>A, 5770A>G, 5771A>T, 5772C>G, 5775G>C, 5776T>A, 5781A>G, 5782G>A, 5784T>C, 5785C>A, 5790C>T, 5791T>A, 5794C>T, 5796A>G, 5800G>C, 5802C>T, 5806A>G, 5808C>T, 5809A>G, 5810C>A, 5811A>T, 5812A>T, 5814C>A, 5815A>T, 5817A>T, 5818C>G, 5820G>T, 5821A>C, 5824G>C, 5826C>T, 5827T>C, 5828G>A, 5829T>G, 5830A>G, 5835C>T, 5836A>T, 5837G>C, 5838T>A, 5841C>G, 5847C>T, 5850G>A, 5851T>A, 5853C>T, 5857C>T, 5861A>G, 5863A>T, 5864G>C, 5865C>T, 5868A>G, 5870T>A, 5871C>T, 5878A>T, 5880T>G, 5882T>A, 5884A>G, 5886G>T, 5887G>C, 5888A>G, 5889C>G, 5892A>G, 5892_5893insT, 5893G>C, 5896T>A, 5897C>A, 5904T>G, 5905T>A, 5906G>A, 5910C>T, 5913C>T, 5917C>A, 5918T>A, 5922C>A, 5924C>G, 5926G>T, 5933T>A, 5934T>C, 5940A>G, 5942G>T, 5943G>T, 5944T>A, 5946A>G, 5953C>T, 5955A>T, 5958C>T, 5960G>C, 5961A>G, 5962T>C, 5964G>T, 5966A>C, 5967G>T, 5970T>C, 5979T>C, 5981T>C, 5982G>T								
**CDS**									
QKM20_gp3	1530	1620	4.5%	187	28.6%	91 (98.9%)	46 (50.0%)	1/0/1/1	0
Protein mutations:	E1531K (5719G>A), K1533H (5725A>C 5727A>T), K1535S (5732A>G 5733G>C), E1536M (5734G>A 5735A>T), V1539C (5743G>T 5744T>G 5745C>T), F1540I (5746T>A 5748C>T), N1541D (5749A>G), K1543R (5755A>C 5756A>G 5757A>T), T1544Q (5758A>C 5759C>A 5760C>G), D1547K (5767G>A 5769C>A), N1548V (5770A>G 5771A>T 5772C>G), F1550I (5776T>A), D1552N (5782G>A 5784T>C), Q1553K (5785C>A), S1555T (5791T>A), G1558R (5800G>C 5802C>T), N1560D (5806A>G 5808C>T), T1561D (5809A>G 5810C>A 5811A>T), I1562L (5812A>T 5814C>A), I1563F (5815A>T 5817A>T), Q1564D (5818C>G 5820G>T), K1565Q (5821A>C), V1566L (5824G>C 5826C>T), C1567Q (5827T>C 5828G>A 5829T>G), N1568D (5830A>G), F1575I (5851T>A 5853C>T), K1578R (5861A>G), F1581Y (5870T>A 5871C>T), I1584L (5878A>T 5880T>G), M1585K (5882T>A), M1586V (5884A>G 5886G>T), D1587R (5887G>C 5888A>G 5889C>G), E1588_E1589insX (5892_5893insT), E1589Q (5893G>C), S1590N (5896T>A 5897C>A), W1593K (5905T>A 5906G>A), L1597K (5917C>A 5918T>A), P1599R (5924C>G), D1600Y (5926G>T), L1602H (5933T>A 5934T>C), W1605F (5942G>T 5943G>T), L1606M (5944T>A 5946A>G), P1609S (5953C>T 5955A>T), G1611A (5960G>C 5961A>G), K1613T (5966A>C 5967G>T), V1618A (5981T>C 5982G>T)								
Codon mutations:	GAG1531AAG (5719G>A), AAA1533CAT (5725A>C 5727A>T), GGA1534GGT (5730A>T), AAG1535AGC (5732A>G 5733G>C), GAG1536ATG (5734G>A 5735A>T), GTC1539TGT (5743G>T 5744T>G 5745C>T), TTC1540ATT (5746T>A 5748C>T), AAC1541GAC (5749A>G), TAC1542TAT (5754C>T), AAA1543CGT (5755A>C 5756A>G 5757A>T), ACC1544CAG (5758A>C 5759C>A 5760C>G), GAC1547AAA (5767G>A 5769C>A), AAC1548GTG (5770A>G 5771A>T 5772C>G), ACG1549ACC (5775G>C), TTC1550ATC (5776T>A), AAA1551AAG (5781A>G), GAT1552AAC (5782G>A 5784T>C), CAG1553AAG (5785C>A), TAC1554TAT (5790C>T), TCT1555ACT (5791T>A), CTA1556TTG (5794C>T 5796A>G), GGC1558CGT (5800G>C 5802C>T), AAC1560GAT (5806A>G 5808C>T), ACA1561GAT (5809A>G 5810C>A 5811A>T), ATC1562TTA (5812A>T 5814C>A), ATA1563TTT (5815A>T 5817A>T), CAG1564GAT (5818C>G 5820G>T), AAG1565CAG (5821A>C), GTC1566CTT (5824G>C 5826C>T), TGT1567CAG (5827T>C 5828G>A 5829T>G), AAT1568GAT (5830A>G), GCC1569GCT (5835C>T), AGT1570TCA (5836A>T 5837G>C 5838T>A), GTC1571GTG (5841C>G), TCC1573TCT (5847C>T), AAG1574AAA (5850G>A), TTC1575ATT (5851T>A 5853C>T), CTG1577TTG (5857C>T), AAG1578AGG (5861A>G), AGC1579TCT (5863A>T 5864G>C 5865C>T), GGA1580GGG (5868A>G), TTC1581TAT (5870T>A 5871C>T), ATT1584TTG (5878A>T 5880T>G), ATG1585AAG (5882T>A), ATG1586GTT (5884A>G 5886G>T), GAC1587CGG (5887G>C 5888A>G 5889C>G), GAA1588GAG (5892A>G), GAA1588_GAA1589insT-- (5892_5893insT), GAA1589CAA (5893G>C), TCC1590AAC (5896T>A 5897C>A), CCT1592CCG (5904T>G), TGG1593AAG (5905T>A 5906G>A), ACC1594ACT (5910C>T), GCC1595GCT (5913C>T), CTG1597AAG (5917C>A 5918T>A), ACC1598ACA (5922C>A), CCA1599CGA (5924C>G), GAT1600TAT (5926G>T), CTT1602CAC (5933T>A 5934T>C), GAA1604GAG (5940A>G), TGG1605TTT (5942G>T 5943G>T), TTA1606ATG (5944T>A 5946A>G), CCA1609TCT (5953C>T 5955A>T), TTC1610TTT (5958C>T), GGA1611GCG (5960G>C 5961A>G), TTG1612CTT (5962T>C 5964G>T), AAG1613ACT (5966A>C 5967G>T), AAT1614AAC (5970T>C), GCT1617GCC (5979T>C), GTG1618GCT (5981T>C 5982G>T)								
**Proteins**									
polyprotein (YP_010797894.1)	1530	1620	4.5%	187	28.6%	91 (98.9%)	46 (50.0%)	1/0/1/1	0
Protein mutations:	E1531K (5719G>A), K1533H (5725A>C 5727A>T), K1535S (5732A>G 5733G>C), E1536M (5734G>A 5735A>T), V1539C (5743G>T 5744T>G 5745C>T), F1540I (5746T>A 5748C>T), N1541D (5749A>G), K1543R (5755A>C 5756A>G 5757A>T), T1544Q (5758A>C 5759C>A 5760C>G), D1547K (5767G>A 5769C>A), N1548V (5770A>G 5771A>T 5772C>G), F1550I (5776T>A), D1552N (5782G>A 5784T>C), Q1553K (5785C>A), S1555T (5791T>A), G1558R (5800G>C 5802C>T), N1560D (5806A>G 5808C>T), T1561D (5809A>G 5810C>A 5811A>T), I1562L (5812A>T 5814C>A), I1563F (5815A>T 5817A>T), Q1564D (5818C>G 5820G>T), K1565Q (5821A>C), V1566L (5824G>C 5826C>T), C1567Q (5827T>C 5828G>A 5829T>G), N1568D (5830A>G), F1575I (5851T>A 5853C>T), K1578R (5861A>G), F1581Y (5870T>A 5871C>T), I1584L (5878A>T 5880T>G), M1585K (5882T>A), M1586V (5884A>G 5886G>T), D1587R (5887G>C 5888A>G 5889C>G), E1588_E1589insX (5892_5893insT), E1589Q (5893G>C), S1590N (5896T>A 5897C>A), W1593K (5905T>A 5906G>A), L1597K (5917C>A 5918T>A), P1599R (5924C>G), D1600Y (5926G>T), L1602H (5933T>A 5934T>C), W1605F (5942G>T 5943G>T), L1606M (5944T>A 5946A>G), P1609S (5953C>T 5955A>T), G1611A (5960G>C 5961A>G), K1613T (5966A>C 5967G>T), V1618A (5981T>C 5982G>T)								
Codon mutations:	GAG1531AAG (5719G>A), AAA1533CAT (5725A>C 5727A>T), GGA1534GGT (5730A>T), AAG1535AGC (5732A>G 5733G>C), GAG1536ATG (5734G>A 5735A>T), GTC1539TGT (5743G>T 5744T>G 5745C>T), TTC1540ATT (5746T>A 5748C>T), AAC1541GAC (5749A>G), TAC1542TAT (5754C>T), AAA1543CGT (5755A>C 5756A>G 5757A>T), ACC1544CAG (5758A>C 5759C>A 5760C>G), GAC1547AAA (5767G>A 5769C>A), AAC1548GTG (5770A>G 5771A>T 5772C>G), ACG1549ACC (5775G>C), TTC1550ATC (5776T>A), AAA1551AAG (5781A>G), GAT1552AAC (5782G>A 5784T>C), CAG1553AAG (5785C>A), TAC1554TAT (5790C>T), TCT1555ACT (5791T>A), CTA1556TTG (5794C>T 5796A>G), GGC1558CGT (5800G>C 5802C>T), AAC1560GAT (5806A>G 5808C>T), ACA1561GAT (5809A>G 5810C>A 5811A>T), ATC1562TTA (5812A>T 5814C>A), ATA1563TTT (5815A>T 5817A>T), CAG1564GAT (5818C>G 5820G>T), AAG1565CAG (5821A>C), GTC1566CTT (5824G>C 5826C>T), TGT1567CAG (5827T>C 5828G>A 5829T>G), AAT1568GAT (5830A>G), GCC1569GCT (5835C>T), AGT1570TCA (5836A>T 5837G>C 5838T>A), GTC1571GTG (5841C>G), TCC1573TCT (5847C>T), AAG1574AAA (5850G>A), TTC1575ATT (5851T>A 5853C>T), CTG1577TTG (5857C>T), AAG1578AGG (5861A>G), AGC1579TCT (5863A>T 5864G>C 5865C>T), GGA1580GGG (5868A>G), TTC1581TAT (5870T>A 5871C>T), ATT1584TTG (5878A>T 5880T>G), ATG1585AAG (5882T>A), ATG1586GTT (5884A>G 5886G>T), GAC1587CGG (5887G>C 5888A>G 5889C>G), GAA1588GAG (5892A>G), GAA1588_GAA1589insT-- (5892_5893insT), GAA1589CAA (5893G>C), TCC1590AAC (5896T>A 5897C>A), CCT1592CCG (5904T>G), TGG1593AAG (5905T>A 5906G>A), ACC1594ACT (5910C>T), GCC1595GCT (5913C>T), CTG1597AAG (5917C>A 5918T>A), ACC1598ACA (5922C>A), CCA1599CGA (5924C>G), GAT1600TAT (5926G>T), CTT1602CAC (5933T>A 5934T>C), GAA1604GAG (5940A>G), TGG1605TTT (5942G>T 5943G>T), TTA1606ATG (5944T>A 5946A>G), CCA1609TCT (5953C>T 5955A>T), TTC1610TTT (5958C>T), GGA1611GCG (5960G>C 5961A>G), TTG1612CTT (5962T>C 5964G>T), AAG1613ACT (5966A>C 5967G>T), AAT1614AAC (5970T>C), GCT1617GCC (5979T>C), GTG1618GCT (5981T>C 5982G>T)								

*: Inserts / Deletes / Misaligned / Frameshifts

Analysis details

This analysis was performed with panviral2.64

NGS Details (UN59): Cavemovirus deltaepiphylli

Assembly

**Coverage Length**	413 (1 contig(s))
**Depth Of Coverage**	4.4
**Number Of Reads**	15
**Reads Per Million**	0.33 rpm (after QC)
**Ambiguities**	0
**Assembly Method**	de novo + reference guided assembly
**Consensus Caller**	Bcf Tools

Coverage Map

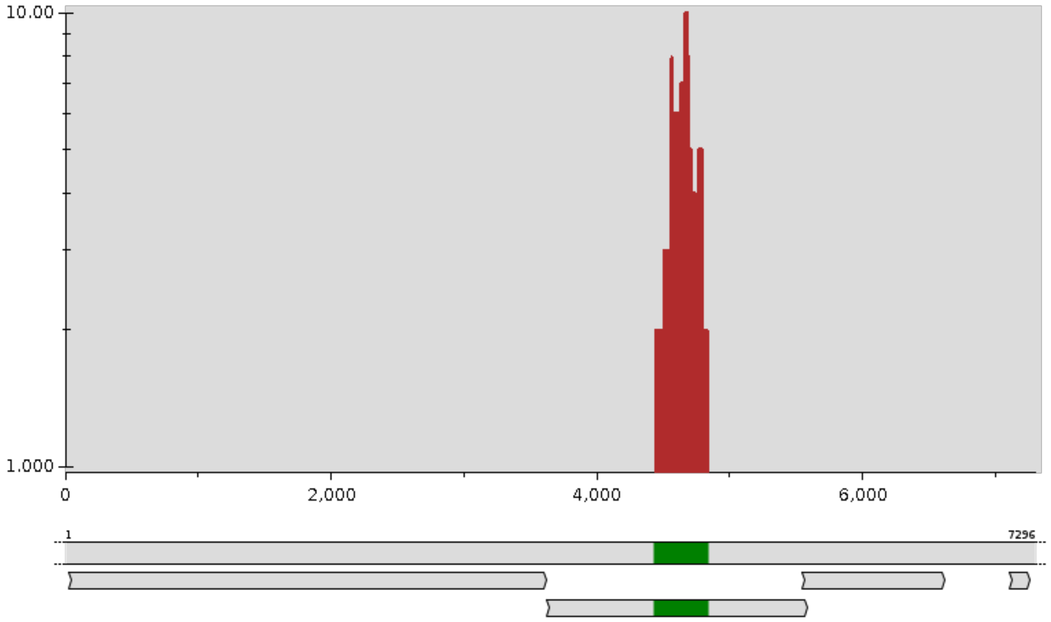

Assignment

**Type**	Cavemovirus deltaepiphylli (Taxonomy ID: 3047827)
**Reference Genome**	NC_055588.1
**NT Identity (%)**	60.0484
**AA Identity (%)**	52.8986
**Number Of Stop Codons**	0
**Number Of CDS**	4

Alignment

**Alignment Score**	166.0 (NT) + 526.0 (AA) = 692.0
**Concordance (%)**	38.1898

Alignment Method	Global, seeded, nucleotide + amino acids (AGA)

Genome Region

Sequence starts at position 4433 and ends at position 4845 relative to NC_055588.1 reference sequence.

Alignment Detailed Statistics

	Begin	End	Coverage	Score	Concordance	Matches	Identities	I/D/M/F*	Stop Codons
**NT**	**4433**	**4845**	**5.7%**	**166**	**20.1%**	**413 (100%)**	**248 (60.0%)**	**0/0**	
Mutations:	4439C>G, 4443A>T, 4444T>G, 4445A>G, 4450A>G, 4451A>G, 4452C>T, 4454C>T, 4457A>T, 4460A>T, 4463A>C, 4469A>G, 4470G>A, 4472T>G, 4473A>G, 4474T>A, 4475A>T, 4479A>T, 4481C>A, 4482A>T, 4483T>C, 4484A>C, 4488G>T, 4489G>T, 4491A>G, 4492T>A, 4493A>T, 4496A>T, 4499C>T, 4503T>A, 4504A>T, 4505T>A, 4509A>C, 4510G>C, 4511C>A, 4517C>T, 4520C>T, 4522G>T, 4523T>A, 4534T>A, 4537A>G, 4538C>G, 4541C>A, 4544C>A, 4545A>C, 4548C>A, 4551G>T, 4552A>T, 4559A>G, 4560A>C, 4561G>A, 4564A>C, 4565A>T, 4566A>T, 4568A>T, 4569T>A, 4570A>G, 4571T>A, 4574A>T, 4577A>T, 4582C>A, 4583G>T, 4586A>C, 4590C>T, 4591A>T, 4592A>T, 4596T>C, 4598C>A, 4601C>T, 4602C>G, 4610A>T, 4613A>C, 4614T>A, 4616A>G, 4619C>A, 4622C>T, 4626T>C, 4627A>T, 4628T>A, 4629C>A, 4631T>A, 4635T>G, 4637A>T, 4640A>T, 4641A>T, 4642G>C, 4644A>G, 4645T>A, 4649C>T, 4652A>G, 4653C>A, 4656T>A, 4658T>C, 4662G>A, 4665A>G, 4667A>T, 4670A>T, 4675G>A, 4676G>T, 4683T>A, 4684A>T, 4685T>G, 4688A>T, 4691T>C, 4695T>A, 4697A>T, 4700A>C, 4712T>C, 4719A>G, 4721C>T, 4723T>A, 4724C>T, 4728C>A, 4730T>A, 4733A>T, 4734G>T, 4735A>T, 4736G>T, 4740G>A, 4741A>T, 4742T>G, 4746T>A, 4748A>T, 4751A>G, 4755A>T, 4758A>G, 4760A>G, 4763A>C, 4769A>G, 4770G>A, 4773T>A, 4774G>T, 4775C>T, 4776A>G, 4778A>T, 4779T>A, 4781G>A, 4790A>T, 4791A>T, 4793A>G, 4794A>G, 4795C>T, 4796C>A, 4797T>A, 4800A>T, 4801G>C, 4805T>A, 4806A>C, 4807A>C, 4811G>A, 4813G>T, 4814C>G, 4815G>A, 4816A>G, 4817A>T, 4821G>T, 4822G>T, 4824A>C, 4829A>T, 4830A>G, 4831C>A, 4837A>G, 4838C>G, 4841C>T, 4842C>T								
**CDS**									
KM744_gp2	271	408	21.1%	526	52.7%	138 (100%)	73 (52.9%)	0/0/0/0	0
Protein mutations:	I274W (4443A>T 4444T>G 4445A>G), K276R (4450A>G 4451A>G), H277Y (4452C>T 4454C>T), D283K (4470G>A 4472T>G), I284D (4473A>G 4474T>A 4475A>T), I286L (4479A>T 4481C>A), I287S (4482A>T 4483T>C 4484A>C), G289L (4488G>T 4489G>T), I290D (4491A>G 4492T>A 4493A>T), K291N (4496A>T), Y294I (4503T>A 4504A>T 4505T>A), S296P (4509A>C 4510G>C 4511C>A), C300L (4522G>T 4523T>A), F304Y (4534T>A), Y305W (4537A>G 4538C>G), H306Q (4541C>A), K308Q (4545A>C), L309I (4548C>A), D310F (4551G>T 4552A>T), S313H (4560A>C 4561G>A), K314T (4564A>C 4565A>T), K315Y (4566A>T 4568A>T), Y316R (4569T>A 4570A>G 4571T>A), T320N (4582C>A 4583G>T), Q323F (4590C>T 4591A>T 4592A>T), Y325Q (4596T>C 4598C>A), Q327E (4602C>G), K329N (4610A>T), L331M (4614T>A 4616A>G), Y335L (4626T>C 4627A>T 4628T>A), H336K (4629C>A 4631T>A), S338A (4635T>G 4637A>T), I341E (4644A>G 4645T>A), Q344K (4653C>A), F345I (4656T>A 4658T>C), D347N (4662G>A), K348D (4665A>G 4667A>T), R351N (4675G>A 4676G>T), Y354M (4683T>A 4684A>T 4685T>G), E355D (4688A>T), L358I (4695T>A 4697A>T), I366V (4719A>G 4721C>T), F367Y (4723T>A 4724C>T), H369K (4728C>A 4730T>A), E371F (4734G>T 4735A>T 4736G>T), D373M (4740G>A 4741A>T 4742T>G), L375I (4746T>A 4748A>T), I378L (4755A>T), K379E (4758A>G 4760A>G), E383K (4770G>A), C384I (4773T>A 4774G>T 4775C>T), I385V (4776A>G 4778A>T), L386I (4779T>A 4781G>A), I390L (4791A>T 4793A>G), T391V (4794A>G 4795C>T 4796C>A), L392I (4797T>A), N394K (4805T>A), K395P (4806A>C 4807A>C), S397M (4813G>T 4814C>G), E398S (4815G>A 4816A>G 4817A>T), G400F (4821G>T 4822G>T), K401Q (4824A>C), T403D (4830A>G 4831C>A), H405R (4837A>G 4838C>G)								
Codon mutations:	GCC272GCG (4439C>G), ATA274TGG (4443A>T 4444T>G 4445A>G), AAA276AGG (4450A>G 4451A>G), CAC277TAT (4452C>T 4454C>T), CCA278CCT (4457A>T), ATA279ATT (4460A>T), CCA280CCC (4463A>C), AAA282AAG (4469A>G), GAT283AAG (4470G>A 4472T>G), ATA284GAT (4473A>G 4474T>A 4475A>T), ATC286TTA (4479A>T 4481C>A), ATA287TCC (4482A>T 4483T>C 4484A>C), GGA289TTA (4488G>T 4489G>T), ATA290GAT (4491A>G 4492T>A 4493A>T), AAA291AAT (4496A>T), GCC292GCT (4499C>T), TAT294ATA (4503T>A 4504A>T 4505T>A), AGC296CCA (4509A>C 4510G>C 4511C>A), TTC298TTT (4517C>T), GAC299GAT (4520C>T), TGT300TTA (4522G>T 4523T>A), TTT304TAT (4534T>A), TAC305TGG (4537A>G 4538C>G), CAC306CAA (4541C>A), ATC307ATA (4544C>A), AAG308CAG (4545A>C), CTA309ATA (4548C>A), GAT310TTT (4551G>T 4552A>T), GAA312GAG (4559A>G), AGT313CAT (4560A>C 4561G>A), AAA314ACT (4564A>C 4565A>T), AAA315TAT (4566A>T 4568A>T), TAT316AGA (4569T>A 4570A>G 4571T>A), ACA317ACT (4574A>T), GCA318GCT (4577A>T), ACG320AAT (4582C>A 4583G>T), GTA321GTC (4586A>C), CAA323TTT (4590C>T 4591A>T 4592A>T), TAC325CAA (4596T>C 4598C>A), TAC326TAT (4601C>T), CAA327GAA (4602C>G), AAA329AAT (4610A>T), GTA330GTC (4613A>C), TTA331ATG (4614T>A 4616A>G), CCC332CCA (4619C>A), TTC333TTT (4622C>T), TAT335CTA (4626T>C 4627A>T 4628T>A), CAT336AAA (4629C>A 4631T>A), TCA338GCT (4635T>G 4637A>T), CCA339CCT (4640A>T), AGT340TCT (4641A>T 4642G>C), ATA341GAA (4644A>G 4645T>A), TTC342TTT (4649C>T), CAA343CAG (4652A>G), CAA344AAA (4653C>A), TTT345ATC (4656T>A 4658T>C), GAT347AAT (4662G>A), AAA348GAT (4665A>G 4667A>T), ATA349ATT (4670A>T), AGG351AAT (4675G>A 4676G>T), TAT354ATG (4683T>A 4684A>T 4685T>G), GAA355GAT (4688A>T), TTT356TTC (4691T>C), TTA358ATT (4695T>A 4697A>T), GTA359GTC (4700A>C), GAT363GAC (4712T>C), ATC366GTT (4719A>G 4721C>T), TTC367TAT (4723T>A 4724C>T), CAT369AAA (4728C>A 4730T>A), ACA370ACT (4733A>T), GAG371TTT (4734G>T 4735A>T 4736G>T), GAT373ATG (4740G>A 4741A>T 4742T>G), TTA375ATT (4746T>A 4748A>T), AAA376AAG (4751A>G), ATA378TTA (4755A>T), AAA379GAG (4758A>G 4760A>G), ATA380ATC (4763A>C), AAA382AAG (4769A>G), GAA383AAA (4770G>A), TGC384ATT (4773T>A 4774G>T 4775C>T), ATA385GTT (4776A>G 4778A>T), TTG386ATA (4779T>A 4781G>A), GGA389GGT (4790A>T), ATA390TTG (4791A>T 4793A>G), ACC391GTA (4794A>G 4795C>T 4796C>A), TTA392ATA (4797T>A), AGT393TCT (4800A>T 4801G>C), AAT394AAA (4805T>A), AAA395CCA (4806A>C 4807A>C), AAG396AAA (4811G>A), AGC397ATG (4813G>T 4814C>G), GAA398AGT (4815G>A 4816A>G 4817A>T), GGC400TTC (4821G>T 4822G>T), AAA401CAA (4824A>C), ACA402ACT (4829A>T), ACT403GAT (4830A>G 4831C>A), CAC405CGG (4837A>G 4838C>G), TTC406TTT (4841C>T), CTA407TTA (4842C>T)								
**Proteins**									
replicase (YP_010087807.1)	271	408	21.1%	526	52.7%	138 (100%)	73 (52.9%)	0/0/0/0	0
Protein mutations:	I274W (4443A>T 4444T>G 4445A>G), K276R (4450A>G 4451A>G), H277Y (4452C>T 4454C>T), D283K (4470G>A 4472T>G), I284D (4473A>G 4474T>A 4475A>T), I286L (4479A>T 4481C>A), I287S (4482A>T 4483T>C 4484A>C), G289L (4488G>T 4489G>T), I290D (4491A>G 4492T>A 4493A>T), K291N (4496A>T), Y294I (4503T>A 4504A>T 4505T>A), S296P (4509A>C 4510G>C 4511C>A), C300L (4522G>T 4523T>A), F304Y (4534T>A), Y305W (4537A>G 4538C>G), H306Q (4541C>A), K308Q (4545A>C), L309I (4548C>A), D310F (4551G>T 4552A>T), S313H (4560A>C 4561G>A), K314T (4564A>C 4565A>T), K315Y (4566A>T 4568A>T), Y316R (4569T>A 4570A>G 4571T>A), T320N (4582C>A 4583G>T), Q323F (4590C>T 4591A>T 4592A>T), Y325Q (4596T>C 4598C>A), Q327E (4602C>G), K329N (4610A>T), L331M (4614T>A 4616A>G), Y335L (4626T>C 4627A>T 4628T>A), H336K (4629C>A 4631T>A), S338A (4635T>G 4637A>T), I341E (4644A>G 4645T>A), Q344K (4653C>A), F345I (4656T>A 4658T>C), D347N (4662G>A), K348D (4665A>G 4667A>T), R351N (4675G>A 4676G>T), Y354M (4683T>A 4684A>T 4685T>G), E355D (4688A>T), L358I (4695T>A 4697A>T), I366V (4719A>G 4721C>T), F367Y (4723T>A 4724C>T), H369K (4728C>A 4730T>A), E371F (4734G>T 4735A>T 4736G>T), D373M (4740G>A 4741A>T 4742T>G), L375I (4746T>A 4748A>T), I378L (4755A>T), K379E (4758A>G 4760A>G), E383K (4770G>A), C384I (4773T>A 4774G>T 4775C>T), I385V (4776A>G 4778A>T), L386I (4779T>A 4781G>A), I390L (4791A>T 4793A>G), T391V (4794A>G 4795C>T 4796C>A), L392I (4797T>A), N394K (4805T>A), K395P (4806A>C 4807A>C), S397M (4813G>T 4814C>G), E398S (4815G>A 4816A>G 4817A>T), G400F (4821G>T 4822G>T), K401Q (4824A>C), T403D (4830A>G 4831C>A), H405R (4837A>G 4838C>G)								
Codon mutations:	GCC272GCG (4439C>G), ATA274TGG (4443A>T 4444T>G 4445A>G), AAA276AGG (4450A>G 4451A>G), CAC277TAT (4452C>T 4454C>T), CCA278CCT (4457A>T), ATA279ATT (4460A>T), CCA280CCC (4463A>C), AAA282AAG (4469A>G), GAT283AAG (4470G>A 4472T>G), ATA284GAT (4473A>G 4474T>A 4475A>T), ATC286TTA (4479A>T 4481C>A), ATA287TCC (4482A>T 4483T>C 4484A>C), GGA289TTA (4488G>T 4489G>T), ATA290GAT (4491A>G 4492T>A 4493A>T), AAA291AAT (4496A>T), GCC292GCT (4499C>T), TAT294ATA (4503T>A 4504A>T 4505T>A), AGC296CCA (4509A>C 4510G>C 4511C>A), TTC298TTT (4517C>T), GAC299GAT (4520C>T), TGT300TTA (4522G>T 4523T>A), TTT304TAT (4534T>A), TAC305TGG (4537A>G 4538C>G), CAC306CAA (4541C>A), ATC307ATA (4544C>A), AAG308CAG (4545A>C), CTA309ATA (4548C>A), GAT310TTT (4551G>T 4552A>T), GAA312GAG (4559A>G), AGT313CAT (4560A>C 4561G>A), AAA314ACT (4564A>C 4565A>T), AAA315TAT (4566A>T 4568A>T), TAT316AGA (4569T>A 4570A>G 4571T>A), ACA317ACT (4574A>T), GCA318GCT (4577A>T), ACG320AAT (4582C>A 4583G>T), GTA321GTC (4586A>C), CAA323TTT (4590C>T 4591A>T 4592A>T), TAC325CAA (4596T>C 4598C>A), TAC326TAT (4601C>T), CAA327GAA (4602C>G), AAA329AAT (4610A>T), GTA330GTC (4613A>C), TTA331ATG (4614T>A 4616A>G), CCC332CCA (4619C>A), TTC333TTT (4622C>T), TAT335CTA (4626T>C 4627A>T 4628T>A), CAT336AAA (4629C>A 4631T>A), TCA338GCT (4635T>G 4637A>T), CCA339CCT (4640A>T), AGT340TCT (4641A>T 4642G>C), ATA341GAA (4644A>G 4645T>A), TTC342TTT (4649C>T), CAA343CAG (4652A>G), CAA344AAA (4653C>A), TTT345ATC (4656T>A 4658T>C), GAT347AAT (4662G>A), AAA348GAT (4665A>G 4667A>T), ATA349ATT (4670A>T), AGG351AAT (4675G>A 4676G>T), TAT354ATG (4683T>A 4684A>T 4685T>G), GAA355GAT (4688A>T), TTT356TTC (4691T>C), TTA358ATT (4695T>A 4697A>T), GTA359GTC (4700A>C), GAT363GAC (4712T>C), ATC366GTT (4719A>G 4721C>T), TTC367TAT (4723T>A 4724C>T), CAT369AAA (4728C>A 4730T>A), ACA370ACT (4733A>T), GAG371TTT (4734G>T 4735A>T 4736G>T), GAT373ATG (4740G>A 4741A>T 4742T>G), TTA375ATT (4746T>A 4748A>T), AAA376AAG (4751A>G), ATA378TTA (4755A>T), AAA379GAG (4758A>G 4760A>G), ATA380ATC (4763A>C), AAA382AAG (4769A>G), GAA383AAA (4770G>A), TGC384ATT (4773T>A 4774G>T 4775C>T), ATA385GTT (4776A>G 4778A>T), TTG386ATA (4779T>A 4781G>A), GGA389GGT (4790A>T), ATA390TTG (4791A>T 4793A>G), ACC391GTA (4794A>G 4795C>T 4796C>A), TTA392ATA (4797T>A), AGT393TCT (4800A>T 4801G>C), AAT394AAA (4805T>A), AAA395CCA (4806A>C 4807A>C), AAG396AAA (4811G>A), AGC397ATG (4813G>T 4814C>G), GAA398AGT (4815G>A 4816A>G 4817A>T), GGC400TTC (4821G>T 4822G>T), AAA401CAA (4824A>C), ACA402ACT (4829A>T), ACT403GAT (4830A>G 4831C>A), CAC405CGG (4837A>G 4838C>G), TTC406TTT (4841C>T), CTA407TTA (4842C>T)								

*: Inserts / Deletes / Misaligned / Frameshifts

Analysis details

This analysis was performed with panviral2.64

NGS Details (UN59): Dioscorea bacilliform virus

Assembly

**Coverage Length**	421 (1 contig(s))
**Depth Of Coverage**	3.5
**Number Of Reads**	11
**Reads Per Million**	0.24 rpm (after QC)
**Ambiguities**	0
**Assembly Method**	de novo + reference guided assembly
**Consensus Caller**	Bcf Tools

Coverage Map

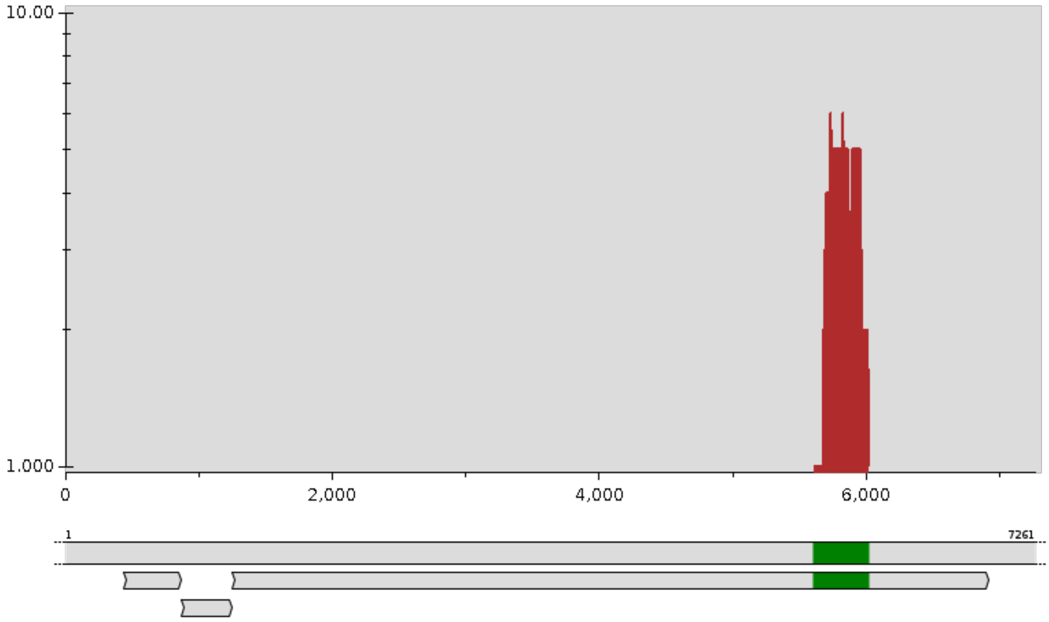

Assignment

**Type**	Dioscorea bacilliform virus (Taxonomy ID: 52996)
**Reference Genome**	NC_009010.1
**NT Identity (%)**	53.066
**AA Identity (%)**	39.7163
**Number Of Stop Codons**	1
**Number Of CDS**	3

Alignment

**Alignment Score**	46.0 (NT) + 394.0 (AA) = 440.0
**Concordance (%)**	24.3363

Alignment Method	Global, seeded, nucleotide + amino acids (AGA)

Genome Region

Sequence starts at position 5600 and ends at position 6020 relative to NC_009010.1 reference sequence.

Alignment Detailed Statistics

	Begin	End	Coverage	Score	Concordance	Matches	Identities	I/D/M/F*	Stop Codons
**NT**	**5600**	**6020**	**5.8%**	**46**	**5.5%**	**421 (99.3%)**	**225 (53.1%)**	**3/0**	
Mutations:	5601G>A, 5602G>A, 5607C>G, 5608A>G, 5610A>T, 5615A>T, 5616G>C, 5617G>C, 5618A>T, 5619G>A, 5620A>T, 5626G>T, 5627T>G, 5628C>T, 5629T>A, 5631C>T, 5632A>G, 5639A>G, 5640A>G, 5641A>C, 5642G>A, 5643A>G, 5650G>A, 5652C>G, 5653A>G, 5654A>T, 5655C>G, 5660A>T, 5665G>A, 5669A>G, 5670A>T, 5673T>C, 5674A>C, 5675G>C, 5677C>A, 5679T>G, 5680C>A, 5682A>T, 5683G>T, 5684G>A, 5685G>T, 5688C>T, 5689A>G, 5691C>T, 5692A>G, 5693C>A, 5694A>T, 5695A>T, 5697T>A, 5698C>T, 5700G>C, 5701A>C, 5703G>C, 5704A>C, 5705G>A, 5706A>G, 5707G>C, 5710G>T, 5711G>A, 5712T>G, 5713C>G, 5715A>T, 5716A>G, 5717G>C, 5720A>C, 5722A>G, 5724A>G, 5726A>T, 5727T>C, 5730A>T, 5733A>G, 5734T>G, 5742A>G, 5744A>G, 5746A>T, 5747G>C, 5751C>T, 5753T>A, 5757T>C, 5761G>T, 5764G>A, 5765C>G, 5766A>G, 5769G>T, 5770G>A, 5771C>G, 5772G>A, 5773C>G, 5774C>T, 5776C>G, 5777A>C, 5778A>T, 5779T>G, 5780C>A, 5781A>C, 5782G>A, 5784T>A, 5785G>C, 5786A>C, 5787A>T, 5788T>A, 5789G>A, 5793G>T, 5794G>A, 5795C>T, 5796G>T, 5801T>G, 5802A>C, 5803G>A, 5805G>C, 5807C>G, 5808A>T, 5809G>T, 5810G>A, 5811A>T, 5814A>C, 5816T>A, 5820C>T, 5823A>G, 5825G>T, 5826G>C, 5827T>C, 5829G>A, 5836C>T, 5838A>T, 5844A>G, 5847G>T, 5849A>G, 5850G>T, 5856A>C, 5858C>T, 5861C>T, 5864T>C, 5865G>T, 5868C>T, 5869C>A, 5870A>T, 5872A>G, 5873G>A, 5874G>C, 5875A>C, 5876A>T, 5877G>T, 5881G>A, 5882A>C, 5883C>T, 5884A>C, 5885A>G, 5886C>A, 5889C>A, 5893C>A, 5895A>G, 5896G>C, 5897G>C, 5898_5899insTAT, 5899A>C, 5900C>T, 5902G>A, 5904A>C, 5905G>T, 5906A>C, 5907A>T, 5910T>C, 5913T>G, 5914G>A, 5915C>T, 5919A>G, 5921A>T, 5931T>C, 5937G>T, 5939T>C, 5942T>A, 5943T>C, 5946T>A, 5947G>C, 5951C>G, 5952A>T, 5953G>A, 5954A>G, 5965T>C, 5968A>C, 5970G>A, 5974C>T, 5979G>A, 5980A>G, 5982A>T, 5983C>G, 5991A>G, 5993T>C, 5996G>T, 5997T>G, 5998C>A, 5999A>G, 6001C>G, 6003A>G, 6006C>T, 6007G>C								

CDS

	Begin	End	Coverage	Score	Concordance	Matches	Identities	I/D/M/F*	Stop Codons
DBV_gp3	1453	1592	7.4%	394	40.5%	140 (99.3%)	56 (39.7%)	1/0/0/0	1
Protein mutations:	E1453K (5602G>A), N1454K (5607C>G), K1455D (5608A>G 5610A>T), K1457I (5615A>T 5616G>C), E1458L (5617G>C 5618A>T 5619G>A), R1459W (5620A>T), V1461C (5626G>T 5627T>G 5628C>T), F1462I (5629T>A 5631C>T), N1463D (5632A>G), K1465R (5639A>G 5640A>G), R1466Q (5641A>C 5642G>A 5643A>G), D1469K (5650G>A 5652C>G), N1470V (5653A>G 5654A>T 5655C>G), E1472V (5660A>T), D1474N (5665G>A), Q1475R (5669A>G 5670A>T), S1477P (5674A>C 5675G>C), L1478M (5677C>A 5679T>G), P1479T (5680C>A 5682A>T), G1480Y (5683G>T 5684G>A 5685G>T), N1482D (5689A>G 5691C>T), T1483D (5692A>G 5693C>A 5694A>T), I1484L (5695A>T 5697T>A), L1485F (5698C>T 5700G>C), K1486H (5701A>C 5703G>C), R1487Q (5704A>C 5705G>A 5706A>G), V1488L (5707G>C), G1489* (5710G>T 5711G>A 5712T>G), Q1490D (5713C>G 5715A>T), S1491A (5716A>G 5717G>C), K1492T (5720A>C), I1493V (5722A>G 5724A>G), Y1494F (5726A>T 5727T>C), F1497V (5734T>G), K1500R (5744A>G), F1503Y (5753T>A), V1506L (5761G>T), A1507R (5764G>A 5765C>G 5766A>G), M1508I (5769G>T), A1509R (5770G>A 5771C>G 5772G>A), P1510V (5773C>G 5774C>T), Q1511A (5776C>G 5777A>C 5778A>T), S1512D (5779T>G 5780C>A 5781A>C), V1513I (5782G>A 5784T>A), E1514P (5785G>C 5786A>C 5787A>T), W1515K (5788T>A 5789G>A), A1517I (5794G>A 5795C>T 5796G>T), L1519R (5801T>G 5802A>C), A1520T (5803G>A 5805G>C), P1521R (5807C>G 5808A>T), G1522Y (5809G>T 5810G>A 5811A>T), L1524H (5816T>A), W1527F (5825G>T 5826G>C), P1531S (5836C>T 5838A>T), K1535S (5849A>G 5850G>T), P1538L (5858C>T), A1539V (5861C>T), V1540A (5864T>C 5865G>T), Q1542M (5869C>A 5870A>T), R1543D (5872A>G 5873G>A 5874G>C), K1544L (5875A>C 5876A>T 5877G>T), D1546T (5881G>A 5882A>C 5883C>T), N1547R (5884A>C 5885A>G 5886C>A), G1551P (5896G>C 5897G>C), G1551_T1552insY (5898_5899insTAT), T1552L (5899A>C 5900C>T), E1553N (5902G>A 5904A>C), E1554S (5905G>T 5906A>C 5907A>T), I1556M (5913T>G), A1557I (5914G>A 5915C>T), Y1559F (5921A>T), V1565A (5939T>C), F1566Y (5942T>A 5943T>C), E1568Q (5947G>C), T1569S (5951C>G 5952A>T), E1570R (5953G>A 5954A>G), K1575Q (5968A>C 5970G>A), I1579V (5980A>G 5982A>T), L1580V (5983C>G), I1583T (5993T>C), C1584L (5996G>T 5997T>G), Q1585R (5998C>A 5999A>G), Q1586E (6001C>G 6003A>G), G1588R (6007G>C)								
Codon mutations:	AAG1452.AA (5601G>A), GAG1453AAG (5602G>A), AAC1454AAG (5607C>G), AAA1455GAT (5608A>G 5610A>T), AAG1457ATC (5615A>T 5616G>C), GAG1458CTA (5617G>C 5618A>T 5619G>A), AGG1459TGG (5620A>T), GTC1461TGT (5626G>T 5627T>G 5628C>T), TTC1462ATT (5629T>A 5631C>T), AAC1463GAC (5632A>G), AAA1465AGG (5639A>G 5640A>G), AGA1466CAG (5641A>C 5642G>A 5643A>G), GAC1469AAG (5650G>A 5652C>G), AAC1470GTG (5653A>G 5654A>T 5655C>G), GAG1472GTG (5660A>T), GAC1474AAC (5665G>A), CAA1475CGT (5669A>G 5670A>T), TAT1476TAC (5673T>C), AGC1477CCC (5674A>C 5675G>C), CTT1478ATG (5677C>A 5679T>G), CCA1479ACT (5680C>A 5682A>T), GGG1480TAT (5683G>T 5684G>A 5685G>T), ATC1481ATT (5688C>T), AAC1482GAT (5689A>G 5691C>T), ACA1483GAT (5692A>G 5693C>A 5694A>T), ATT1484TTA (5695A>T 5697T>A), CTG1485TTC (5698C>T 5700G>C), AAG1486CAC (5701A>C 5703G>C), AGA1487CAG (5704A>C 5705G>A 5706A>G), GTC1488CTC (5707G>C), GGT1489TAG (5710G>T 5711G>A 5712T>G), CAA1490GAT (5713C>G 5715A>T), AGC1491GCC (5716A>G 5717G>C), AAA1492ACA (5720A>C), ATA1493GTG (5722A>G 5724A>G), TAT1494TTC (5726A>T 5727T>C), TCA1495TCT (5730A>T), AAA1496AAG (5733A>G), TTT1497GTT (5734T>G), TTA1499TTG (5742A>G), AAG1500AGG (5744A>G), AGC1501TCC (5746A>T 5747G>C), GGC1502GGT (5751C>T), TTT1503TAT (5753T>A), CAT1504CAC (5757T>C), GTG1506TTG (5761G>T), GCA1507AGG (5764G>A 5765C>G 5766A>G), ATG1508ATT (5769G>T), GCG1509AGA (5770G>A 5771C>G 5772G>A), CCA1510GTA (5773C>G 5774C>T), CAA1511GCT (5776C>G 5777A>C 5778A>T), TCA1512GAC (5779T>G 5780C>A 5781A>C), GTT1513ATA (5782G>A 5784T>A), GAA1514CCT (5785G>C 5786A>C 5787A>T), TGG1515AAG (5788T>A 5789G>A), ACG1516ACT (5793G>T), GCG1517ATT (5794G>A 5795C>T 5796G>T), CTA1519CGC (5801T>G 5802A>C), GCG1520ACC (5803G>A 5805G>C), CCA1521CGT (5807C>G 5808A>T), GGA1522TAT (5809G>T 5810G>A 5811A>T), GGA1523GGC (5814A>C), CTT1524CAT (5816T>A), TAC1525TAT (5820C>T), GAA1526GAG (5823A>G), TGG1527TTC (5825G>T 5826G>C), TTG1528CTA (5827T>C 5829G>A), CCA1531TCT (5836C>T 5838A>T), GGA1533GGG (5844A>G), CTG1534CTT (5847G>T), AAG1535AGT (5849A>G 5850G>T), GCA1537GCC (5856A>C), CCA1538CTA (5858C>T), GCA1539GTA (5861C>T), GTG1540GCT (5864T>C 5865G>T), TTC1541TTT (5868C>T), CAG1542ATG (5869C>A 5870A>T), AGG1543GAC (5872A>G 5873G>A 5874G>C), AAG1544CTT (5875A>C 5876A>T 5877G>T), GAC1546ACT (5881G>A 5882A>C 5883C>T), AAC1547CGA (5884A>C 5885A>G 5886C>A), GTC1548GTA (5889C>A), CGA1550AGG (5893C>A 5895A>G), GGT1551CCT (5896G>C 5897G>C), GGT1551_ACT1552insTAT (5898_5899insTAT), ACT1552CTT (5899A>C 5900C>T), GAA1553AAC (5902G>A 5904A>C), GAA1554TCT (5905G>T 5906A>C 5907A>T), TTT1555TTC (5910T>C), ATT1556ATG (5913T>G), GCT1557ATT (5914G>A 5915C>T), GTA1558GTG (5919A>G), TAC1559TTC (5921A>T), GAT1562GAC (5931T>C), CTG1564CTT (5937G>T), GTG1565GCG (5939T>C), TTT1566TAC (5942T>A 5943T>C), TCT1567TCA (5946T>A), GAA1568CAA (5947G>C), ACA1569AGT (5951C>G 5952A>T), GAA1570AGA (5953G>A 5954A>G), TTG1574CTG (5965T>C), AAG1575CAA (5968A>C 5970G>A), CTG1577TTG (5974C>T), AGG1578AGA (5979G>A), ATA1579GTT (5980A>G 5982A>T), CTG1580GTG (5983C>G), CAA1582CAG (5991A>G), ATC1583ACC (5993T>C), TGT1584TTG (5996G>T 5997T>G), CAA1585AGA (5998C>A 5999A>G), CAA1586GAG (6001C>G 6003A>G), CAC1587CAT (6006C>T), GGG1588CGG (6007G>C)								

Proteins

	Begin	End	Coverage	Score	Concordance	Matches	Identities	I/D/M/F*	Stop Codons
ORF3 protein (YP_001036293.1)	1453	1592	7.4%	394	40.5%	140 (99.3%)	56 (39.7%)	1/0/0/0	1
Protein mutations:	E1453K (5602G>A), N1454K (5607C>G), K1455D (5608A>G 5610A>T), K1457I (5615A>T 5616G>C), E1458L (5617G>C 5618A>T 5619G>A), R1459W (5620A>T), V1461C (5626G>T 5627T>G 5628C>T), F1462I (5629T>A 5631C>T), N1463D (5632A>G), K1465R (5639A>G 5640A>G), R1466Q (5641A>C 5642G>A 5643A>G), D1469K (5650G>A 5652C>G), N1470V (5653A>G 5654A>T 5655C>G), E1472V (5660A>T), D1474N (5665G>A), Q1475R (5669A>G 5670A>T), S1477P (5674A>C 5675G>C), L1478M (5677C>A 5679T>G), P1479T (5680C>A 5682A>T), G1480Y (5683G>T 5684G>A 5685G>T), N1482D (5689A>G 5691C>T), T1483D (5692A>G 5693C>A 5694A>T), I1484L (5695A>T 5697T>A), L1485F (5698C>T 5700G>C), K1486H (5701A>C 5703G>C), R1487Q (5704A>C 5705G>A 5706A>G), V1488L (5707G>C), G1489* (5710G>T 5711G>A 5712T>G), Q1490D (5713C>G 5715A>T), S1491A (5716A>G 5717G>C), K1492T (5720A>C), I1493V (5722A>G 5724A>G), Y1494F (5726A>T 5727T>C), F1497V (5734T>G), K1500R (5744A>G), F1503Y (5753T>A), V1506L (5761G>T), A1507R (5764G>A 5765C>G 5766A>G), M1508I (5769G>T), A1509R (5770G>A 5771C>G 5772G>A), P1510V (5773C>G 5774C>T), Q1511A (5776C>G 5777A>C 5778A>T), S1512D (5779T>G 5780C>A 5781A>C), V1513I (5782G>A 5784T>A), E1514P (5785G>C 5786A>C 5787A>T), W1515K (5788T>A 5789G>A), A1517I (5794G>A 5795C>T 5796G>T), L1519R (5801T>G 5802A>C), A1520T (5803G>A 5805G>C), P1521R (5807C>G 5808A>T), G1522Y (5809G>T 5810G>A 5811A>T), L1524H (5816T>A), W1527F (5825G>T 5826G>C), P1531S (5836C>T 5838A>T), K1535S (5849A>G 5850G>T), P1538L (5858C>T), A1539V (5861C>T), V1540A (5864T>C 5865G>T), Q1542M (5869C>A 5870A>T), R1543D (5872A>G 5873G>A 5874G>C), K1544L (5875A>C 5876A>T 5877G>T), D1546T (5881G>A 5882A>C 5883C>T), N1547R (5884A>C 5885A>G 5886C>A), G1551P (5896G>C 5897G>C), G1551_T1552insY (5898_5899insTAT), T1552L (5899A>C 5900C>T), E1553N (5902G>A 5904A>C), E1554S (5905G>T 5906A>C 5907A>T), I1556M (5913T>G), A1557I (5914G>A 5915C>T), Y1559F (5921A>T), V1565A (5939T>C), F1566Y (5942T>A 5943T>C), E1568Q (5947G>C), T1569S (5951C>G 5952A>T), E1570R (5953G>A 5954A>G), K1575Q (5968A>C 5970G>A), I1579V (5980A>G 5982A>T), L1580V (5983C>G), I1583T (5993T>C), C1584L (5996G>T 5997T>G), Q1585R (5998C>A 5999A>G), Q1586E (6001C>G 6003A>G), G1588R (6007G>C)								

	Begin	End	Coverage	Score	Concordance	Matches	Identities	I/D/M/F*	Stop Codons
**NT**	**5600**	**6020**	**5.8%**	**46**	**5.5%**	**421 (99.3%)**	**225 (53.1%)**	**3/0**	
Codon mutations:	AAG1452.AA (5601G>A), GAG1453AAG (5602G>A), AAC1454AAG (5607C>G), AAA1455GAT (5608A>G 5610A>T), AAG1457ATC (5615A>T 5616G>C), GAG1458CTA (5617G>C 5618A>T 5619G>A), AGG1459TGG (5620A>T), GTC1461TGT (5626G>T 5627T>G 5628C>T), TTC1462ATT (5629T>A 5631C>T), AAC1463GAC (5632A>G), AAA1465AGG (5639A>G 5640A>G), AGA1466CAG (5641A>C 5642G>A 5643A>G), GAC1469AAG (5650G>A 5652C>G), AAC1470GTG (5653A>G 5654A>T 5655C>G), GAG1472GTG (5660A>T), GAC1474AAC (5665G>A), CAA1475CGT (5669A>G 5670A>T), TAT1476TAC (5673T>C), AGC1477CCC (5674A>C 5675G>C), CTT1478ATG (5677C>A 5679T>G), CCA1479ACT (5680C>A 5682A>T), GGG1480TAT (5683G>T 5684G>A 5685G>T), ATC1481ATT (5688C>T), AAC1482GAT (5689A>G 5691C>T), ACA1483GAT (5692A>G 5693C>A 5694A>T), ATT1484TTA (5695A>T 5697T>A), CTG1485TTC (5698C>T 5700G>C), AAG1486CAC (5701A>C 5703G>C), AGA1487CAG (5704A>C 5705G>A 5706A>G), GTC1488CTC (5707G>C), GGT1489TAG (5710G>T 5711G>A 5712T>G), CAA1490GAT (5713C>G 5715A>T), AGC1491GCC (5716A>G 5717G>C), AAA1492ACA (5720A>C), ATA1493GTG (5722A>G 5724A>G), TAT1494TTC (5726A>T 5727T>C), TCA1495TCT (5730A>T), AAA1496AAG (5733A>G), TTT1497GTT (5734T>G), TTA1499TTG (5742A>G), AAG1500AGG (5744A>G), AGC1501TCC (5746A>T 5747G>C), GGC1502GGT (5751C>T), TTT1503TAT (5753T>A), CAT1504CAC (5757T>C), GTG1506TTG (5761G>T), GCA1507AGG (5764G>A 5765C>G 5766A>G), ATG1508ATT (5769G>T), GCG1509AGA (5770G>A 5771C>G 5772G>A), CCA1510GTA (5773C>G 5774C>T), CAA1511GCT (5776C>G 5777A>C 5778A>T), TCA1512GAC (5779T>G 5780C>A 5781A>C), GTT1513ATA (5782G>A 5784T>A), GAA1514CCT (5785G>C 5786A>C 5787A>T), TGG1515AAG (5788T>A 5789G>A), ACG1516ACT (5793G>T), GCG1517ATT (5794G>A 5795C>T 5796G>T), CTA1519CGC (5801T>G 5802A>C), GCG1520ACC (5803G>A 5805G>C), CCA1521CGT (5807C>G 5808A>T), GGA1522TAT (5809G>T 5810G>A 5811A>T), GGA1523GGC (5814A>C), CTT1524CAT (5816T>A), TAC1525TAT (5820C>T), GAA1526GAG (5823A>G), TGG1527TTC (5825G>T 5826G>C), TTG1528CTA (5827T>C 5829G>A), CCA1531TCT (5836C>T 5838A>T), GGA1533GGG (5844A>G), CTG1534CTT (5847G>T), AAG1535AGT (5849A>G 5850G>T), GCA1537GCC (5856A>C), CCA1538CTA (5858C>T), GCA1539GTA (5861C>T), GTG1540GCT (5864T>C 5865G>T), TTC1541TTT (5868C>T), CAG1542ATG (5869C>A 5870A>T), AGG1543GAC (5872A>G 5873G>A 5874G>C), AAG1544CTT (5875A>C 5876A>T 5877G>T), GAC1546ACT (5881G>A 5882A>C 5883C>T), AAC1547CGA (5884A>C 5885A>G 5886C>A), GTC1548GTA (5889C>A), CGA1550AGG (5893C>A 5895A>G), GGT1551CCT (5896G>C 5897G>C), GGT1551_ACT1552insTAT (5898_5899insTAT), ACT1552CTT (5899A>C 5900C>T), GAA1553AAC (5902G>A 5904A>C), GAA1554TCT (5905G>T 5906A>C 5907A>T), TTT1555TTC (5910T>C), ATT1556ATG (5913T>G), GCT1557ATT (5914G>A 5915C>T), GTA1558GTG (5919A>G), TAC1559TTC (5921A>T), GAT1562GAC (5931T>C), CTG1564CTT (5937G>T), GTG1565GCG (5939T>C), TTT1566TAC (5942T>A 5943T>C), TCT1567TCA (5946T>A), GAA1568CAA (5947G>C), ACA1569AGT (5951C>G 5952A>T), GAA1570AGA (5953G>A 5954A>G), TTG1574CTG (5965T>C), AAG1575CAA (5968A>C 5970G>A), CTG1577TTG (5974C>T), AGG1578AGA (5979G>A), ATA1579GTT (5980A>G 5982A>T), CTG1580GTG (5983C>G), CAA1582CAG (5991A>G), ATC1583ACC (5993T>C), TGT1584TTG (5996G>T 5997T>G), CAA1585AGA (5998C>A 5999A>G), CAA1586GAG (6001C>G 6003A>G), CAC1587CAT (6006C>T), GGG1588CGG (6007G>C)								

*: Inserts / Deletes / Misaligned / Frameshifts

Analysis details

This analysis was performed with panviral2.64

NGS Details (UN59): Alphabaculovirus lydisparis

Assembly

**Coverage Length**	192 (1 contig(s))
**Depth Of Coverage**	4.3
**Number Of Reads**	8
**Reads Per Million**	0.17 rpm (after QC)
**Ambiguities**	0
**Assembly Method**	de novo + reference guided assembly
**Consensus Caller**	Bcf Tools

Coverage Map

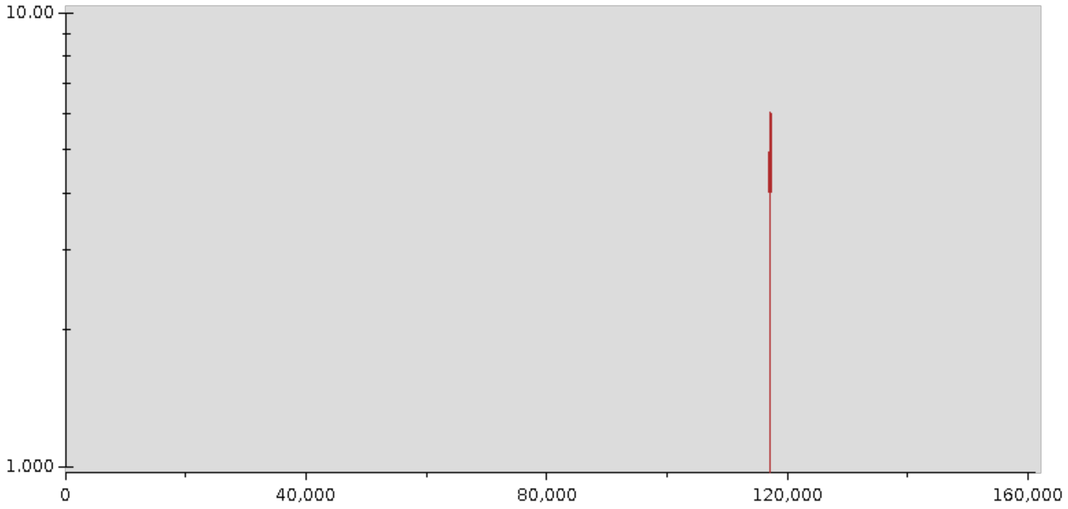

Assignment

**Type**	Alphabaculovirus lydisparis (Taxonomy ID: 3048188)
**Reference Genome**	NC_001973.1
**NT Identity (%)**	78.6458
**AA Identity (%)**	89.0625
**Number Of Stop Codons**	0
**Number Of CDS**	164

Alignment

**Alignment Score**	220.0 (NT) + 423.0 (AA) = 643.0
**Concordance (%)**	75.915
**Alignment Method**	Local, heuristic, nucleotide (BLASTN)

Genome Region

Sequence starts at position 117029 and ends at position 117220 relative to NC_001973.1 reference sequence.

Alignment Detailed Statistics

	Begin	End	Coverage	Score	Concordance	Matches	Identities	I/D/M/F*	Stop Codons
**NT**	**117029**	**117220**	**0.1%**	**220**	**57.3%**	**192 (100%)**	**151 (78.6%)**	**0/0**	

Mutations: 117044G>A, 117046G>T, 117049C>G, 117055G>C, 117068G>A, 117070C>T, 117076T>C, 117083A>T, 117084G>C, 117085T>G, 117088C>T, 117091C>G, 117092G>T, 117097A>C, 117112A>G, 117130C>A, 117131C>T, 117136T>G, 117139C>T, 117140A>T, 117141G>C, 117142C>G, 117157C>G, 117158A>C, 117160G>C, 117169T>G, 117175T>C, 117176T>A, 117177G>C, 117187C>G, 117190T>C, 117191T>C, 117199C>T, 117201A>C, 117205T>C, 117206T>C, 117209G>C, 117210T>G, 117212C>A, 117213A>G, 117217A>G

*: Inserts / Deletes / Misaligned / Frameshifts

Analysis details

This analysis was performed with panviral2.64

NGS Details (UN59): Trialeurodes vaporariorum mononega-like virus 2

Assembly

**Coverage Length**	268 (2 contig(s))
**Depth Of Coverage**	4.0
**Number Of Reads**	8
**Reads Per Million**	0.17 rpm (after QC)
**Ambiguities**	0
**Assembly Method**	de novo + reference guided assembly
**Consensus Caller**	Bcf Tools

Coverage Map

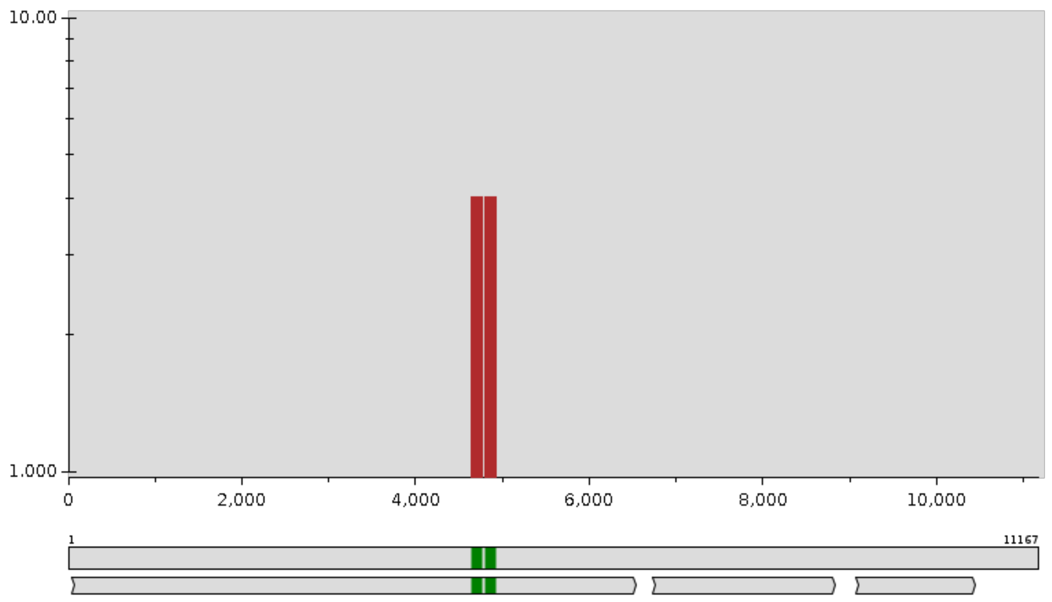

Assignment

**Type**	Trialeurodes vaporariorum mononega-like virus 2 (Taxonomy ID: 2973797)
**Reference Genome**	NC_076992.1
**NT Identity (%)**	99.6269
**AA Identity (%)**	97.7528
**Number Of Stop Codons**	0
**Number Of CDS**	3

Alignment

**Alignment Score**	532.0 (NT) + 602.0 (AA) = 1134.0
**Concordance (%)**	98.7805

Alignment Method	Global, seeded, nucleotide + amino acids (AGA)

Genome Region

Sequence starts at position 4641 and ends at position 4933 relative to NC_076992.1 reference sequence.

Alignment Detailed Statistics

	Begin	End	Coverage	Score	Concordance	Matches	Identities	I/D/M/F *	Stop Codons
**NT**	**4641**	**4933**	**2.4%**	**532**	**99.3%**	**268 (100%)**	**267 (99.6%)**	**0/0**	
Mutations:	4763C>T								
**CDS**									
QKT73_gp1	1537	1633	4.1%	602	96.6%	89 (100%)	87 (97.8%)	0/0/0/0	0
Protein mutations:	R1577C (4763C>T)								
Codon mutations:	CGT1577TGT (4763C>T)								
**Proteins**									
RNA-dependent RNA polymerase (YP_010802337.1)	1537	1633	4.1%	602	96.6%	89 (100%)	87 (97.8%)	0/0/0/0	0
Protein mutations:	R1577C (4763C>T)								
Codon mutations:	CGT1577TGT (4763C>T)								

*: Inserts / Deletes / Misaligned / Frameshifts

Analysis details

This analysis was performed with panviral2.64

NGS Details (UN59): Caulimovirus venafragariae

Assembly

**Coverage Length**	396 (2 contig(s))
**Depth Of Coverage**	2.2
**Number Of Reads**	7
**Reads Per Million**	0.15 rpm (after QC)
**Ambiguities**	0
**Assembly Method**	de novo + reference guided assembly
**Consensus Caller**	Bcf Tools

Coverage Map

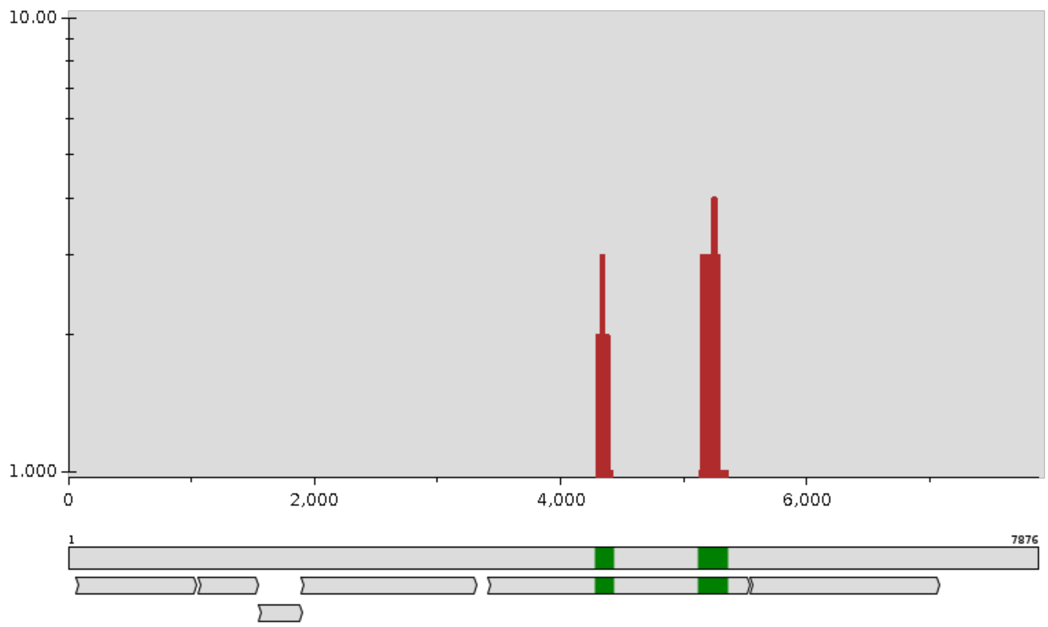

Assignment

**Type**	Caulimovirus venafragariae (Taxonomy ID: 3048344)
**Reference Genome**	NC_001725.1
**NT Identity (%)**	65.1042
**AA Identity (%)**	60.9375
**Number Of Stop Codons**	1
**Number Of CDS**	6

Alignment

**Alignment Score**	211.0 (NT) + 577.0 (AA) = 788.0
**Concordance (%)**	49.4354

**Alignment Method**	Global, seeded, nucleotide + amino acids (AGA)

Genome Region

Sequence starts at position 4281 and ends at position 5363 relative to NC_001725.1 reference sequence.

Alignment Detailed Statistics

	Begin	End	Coverage	Score	Concordance	Matches	Identities	I/D/M/F*	Stop Codons
**NT**	**4281**	**5363**	**5.0%**	**211**	**28.2%**	**384 (97.0%)**	**250 (63.1%)**	**0/12**	

Mutations: 4281A>T, 4284G>A, 4287T>C, 4289A>G, 4292C>T, 4299C>T, 4300G>C, 4302A>G, 4308A>T, 4314G>A, 4315C>A, 4317T>G, 4318G>A, 4319G>A, 4320T>G, 4321A>T, 4323C>A, 4330C>G, 4332T>A, 4337A>G, 4338G>A, 4347T>C, 4348T>A, 4349C>A, 4351T>A, 4352C>G, 4353A>T, 4356A>C, 4359C>G, 4371G>A, 4374T>C, 4380C>G, 4383G>A, 4384A>C, 4385T>A, 4386C>A, 4395C>T, 4398A>G, 4401A>T, 4402A>C, 4410A>C, 4413T>A, 4416C>T, 4423A>G, 4426T>C, 4428A>T, 5126C>A, 5133G>A, 5139G>C, 5141C>T, 5142T>G, 5143A>G, 5145C>A, 5146A>T, 5148C>A, 5149G>C, 5152T>A, 5153G>C, 5154C>A, 5160A>C, 5163A>C, 5166T>C, 5167G>T, 5182A>T, 5184C>G, 5185C>T, 5187C>A, 5188A>C, 5191G>A, 5194A>G, 5196A>T, 5197C>T, 5199T>G, 5200C>A, 5201C>A, 5202A>T, 5206_5217delGGTAAAGAGGTA, 5220A>T, 5223C>T, 5226G>A, 5230G>A, 5233T>A, 5234C>G, 5235A>T, 5241C>T, 5247A>T, 5248C>G, 5249C>A, 5253T>A, 5256G>A, 5258A>C, 5259G>T, 5262T>A, 5265C>T, 5266C>T, 5267A>C, 5270G>C, 5271T>C, 5284A>T, 5286C>A, 5287C>T, 5289T>A, 5290T>G, 5292C>A, 5296A>G, 5299A>C, 5300A>G, 5301G>A, 5303C>G, 5309A>G, 5311G>A, 5312C>A, 5313T>A, 5317A>T, 5318G>C, 5320G>T, 5324A>T, 5325T>C, 5326A>C, 5328C>A, 5334T>A, 5335T>A, 5337T>A, 5338A>C, 5343T>C, 5344C>A, 5346T>C, 5347G>A, 5349A>C, 5350A>C, 5351G>A, 5352G>A, 5353A>T, 5355T>A, 5358T>C

CDS

	Begin	End	Coverage	Score	Concordance	Matches	Identities	I/D/M/F*	Stop Codons
ORF_V	293	653	18.6%	577	65.9%	128 (97.0%)	78 (59.1%)	0/4/0/0	1

Protein mutations: K295R (4289A>G), T296I (4292C>T), E299Q (4300G>C 4302A>G), L304M (4315C>A 4317T>G), G305K (4318G>A 4319G>A 4320T>G), I306L (4321A>T 4323C>A), P309A (4330C>G 4332T>A), K311R (4337A>G 4338G>A), S315N (4348T>A 4349C>A), I327Q (4384A>C 4385T>A 4386C>A), K340E (4423A>G), S574* (5126C>A), K578N (5139G>C), P579L (5141C>T 5142T>G), I580V (5143A>G 5145C>A), I581L (5146A>T 5148C>A), E582Q (5149G>C), C583T (5152T>A 5153G>C 5154C>A), D588Y (5167G>T), I593L (5182A>T 5184C>G), K595Q (5188A>C), A596T (5191G>A), K597D (5194A>G 5196A>T), P599N (5200C>A 5201C>A 5202A>T), G601_V604del (5206_5217delGGTAAAGAGGTA), A609T (5230G>A), K614N (5247A>T), P615E (5248C>G 5249C>A), K618T (5258A>C 5259G>T), N619K (5262T>A), H621S (5266C>T 5267A>C), S622T (5270G>C 5271T>C), I627L (5284A>T 5286C>A), S629A (5290T>G 5292C>A), I631V (5296A>G), K632R (5299A>C 5300A>G 5301G>A), A633G (5303C>G), K635R (5309A>G), A636K (5311G>A 5312C>A 5313T>A), R638S (5317A>T 5318G>C), A639S (5320G>T), Y640F (5324A>T 5325T>C), I641L (5326A>C 5328C>A), Y644K (5335T>A 5337T>A), K645Q (5338A>C), L647I (5344C>A 5346T>C), V648I (5347G>A 5349A>C), R649Q (5350A>C 5351G>A 5352G>A), T650S (5353A>T 5355T>A)

Codon mutations: GAA292..T (4281A>T), GAG293GAA (4284G>A), TTT294TTC (4287T>C), AAA295AGA (4289A>G), ACT296ATT (4292C>T), ATC298ATT (4299C>T), GAA299CAG (4300G>C 4302A>G), CTA301CTT (4308A>T), AAG303AAA (4314G>A), CTT304ATG (4315C>A 4317T>G), GGT305AAG (4318G>A 4319G>A 4320T>G), ATC306TTA (4321A>T 4323C>A), CCT309GCA (4330C>G 4332T>A), AAG311AGA (4337A>G 4338G>A), CAT314CAC (4347T>C), TCC315AAC (4348T>A 4349C>A), TCA316AGT (4351T>A 4352C>G 4353A>T), CCA317CCC (4356A>C), GCC318GCG (4359C>G), AGG322AGA (4371G>A), AAT323AAC (4374T>C), GCC325GCG (4380C>G), GAG326GAA (4383G>A), ATC327CAA (4384A>C 4385T>A 4386C>A), GGC330GGT (4395C>T), AAA331AAG (4398A>G), GCA332GCT (4401A>T), AGA333CGA (4402A>C), GTA335GTC (4410A>C), ATT336ATA (4413T>A), AAC337AAT (4416C>T), AAG340GAG (4423A>G), TTA341CTT (4426T>C 4428A>T), TCA574TAA (5126C>A), GAG576GAA (5133G>A), AAG578AAC (5139G>C), CCT579CTG (5141C>T 5142T>G), ATC580GTA (5143A>G 5145C>A), ATC581TTA (5146A>T 5148C>A), GAA582CAA (5149G>C), TGC583ACA (5152T>A 5153G>C 5154C>A), GCA585GCC (5160A>C), TCA586TCC (5163A>C), GAT587GAC (5166T>C), GAC588TAC (5167G>T), ATC593TTG (5182A>T 5184C>G), CTC594TTA (5185C>T 5187C>A), AAA595CAA (5188A>C), GCA596ACA (5191G>A), AAA597GAT (5194A>G 5196A>T), CTT598TTG (5197C>T 5199T>G), CCA599AAT (5200C>A 5201C>A 5202A>T), GGT601_GTA604del (5206_5217delGGTAAAGAGGTA), ATA605ATT (5220A>T), TGC606TGT (5223C>T), AGG607AGA (5226G>A), GCT609ACT (5230G>A), TCA610AGT (5233T>A 5234C>G 5235A>T), ACC612ACT (5241C>T), AAA614AAT (5247A>T), CCA615GAA (5248C>G 5249C>A), GCT616GCA (5253T>A), GAG617GAA (5256G>A), AAG618ACT (5258A>C 5259G>T), AAT619AAA (5262T>A), TAC620TAT (5265C>T), CAT621TCT (5266C>T 5267A>C), AGT622ACC (5270G>C 5271T>C), ATC627TTA (5284A>T 5286C>A), CTT628TTA (5287C>T 5289T>A), TCC629GCA (5290T>G 5292C>A), ATT631GTT (5296A>G), AAG632CGA (5299A>C 5300A>G 5301G>A), GCA633GGA (5303C>G), AAA635AGA (5309A>G), GCT636AAA (5311G>A 5312C>A 5313T>A), AGA638TCA (5317A>T 5318G>C), GCT639TCT (5320G>T), TAT640TTC (5324A>T 5325T>C), ATC641CTA (5326A>C 5328C>A), CCT643CCA (5334T>A), TAT644AAA (5335T>A 5337T>A), AAA645CAA (5338A>C), TTT646TTC (5343T>C), CTT647ATC (5344C>A 5346T>C), GTA648ATC (5347G>A 5349A>C), AGG649CAA (5350A>C 5351G>A 5352G>A), ACT650TCA (5353A>T 5355T>A), GAT651GAC (5358T>C)

Proteins

	Begin	End	Coverage	Score	Concordance	Matches	Identities	I/D/M/F*	Stop Codons
hypothetical protein (NP_043933.1)	293	653	18.6%	577	65.9%	128 (97.0%)	78 (59.1%)	0/4/0/0	1

Protein mutations: K295R (4289A>G), T296I (4292C>T), E299Q (4300G>C 4302A>G), L304M (4315C>A 4317T>G), G305K (4318G>A 4319G>A 4320T>G), I306L (4321A>T 4323C>A), P309A (4330C>G 4332T>A), K311R (4337A>G 4338G>A), S315N (4348T>A 4349C>A), I327Q (4384A>C 4385T>A 4386C>A), K340E (4423A>G), S574* (5126C>A), K578N (5139G>C), P579L (5141C>T 5142T>G), I580V (5143A>G 5145C>A), I581L (5146A>T 5148C>A), E582Q (5149G>C), C583T (5152T>A 5153G>C 5154C>A), D588Y (5167G>T), I593L (5182A>T 5184C>G), K595Q (5188A>C), A596T (5191G>A), K597D (5194A>G 5196A>T), P599N (5200C>A 5201C>A 5202A>T), G601_V604del (5206_5217delGGTAAAGAGGTA), A609T (5230G>A), K614N (5247A>T), P615E (5248C>G 5249C>A), K618T (5258A>C 5259G>T), N619K (5262T>A), H621S (5266C>T 5267A>C), S622T (5270G>C 5271T>C), I627L (5284A>T 5286C>A), S629A (5290T>G 5292C>A), I631V (5296A>G), K632R (5299A>C 5300A>G 5301G>A), A633G (5303C>G), K635R (5309A>G), A636K (5311G>A 5312C>A 5313T>A), R638S (5317A>T 5318G>C), A639S (5320G>T), Y640F (5324A>T 5325T>C), I641L (5326A>C 5328C>A), Y644K (5335T>A 5337T>A), K645Q (5338A>C), L647I (5344C>A 5346T>C), V648I (5347G>A 5349A>C), R649Q (5350A>C 5351G>A 5352G>A), T650S (5353A>T 5355T>A)

Codon mutations: GAA292..T (4281A>T), GAG293GAA (4284G>A), TTT294TTC (4287T>C), AAA295AGA (4289A>G), ACT296ATT (4292C>T), ATC298ATT (4299C>T), GAA299CAG (4300G>C 4302A>G), CTA301CTT (4308A>T), AAG303AAA (4314G>A), CTT304ATG (4315C>A 4317T>G), GGT305AAG (4318G>A 4319G>A 4320T>G), ATC306TTA (4321A>T 4323C>A), CCT309GCA (4330C>G 4332T>A), AAG311AGA (4337A>G 4338G>A), CAT314CAC (4347T>C), TCC315AAC (4348T>A 4349C>A), TCA316AGT (4351T>A 4352C>G 4353A>T), CCA317CCC (4356A>C), GCC318GCG (4359C>G), AGG322AGA (4371G>A), AAT323AAC (4374T>C), GCC325GCG (4380C>G), GAG326GAA (4383G>A), ATC327CAA (4384A>C 4385T>A 4386C>A), GGC330GGT (4395C>T), AAA331AAG (4398A>G), GCA332GCT (4401A>T), AGA333CGA (4402A>C), GTA335GTC (4410A>C), ATT336ATA (4413T>A), AAC337AAT (4416C>T), AAG340GAG (4423A>G), TTA341CTT (4426T>C 4428A>T), TCA574TAA (5126C>A), GAG576GAA (5133G>A), AAG578AAC (5139G>C), CCT579CTG (5141C>T 5142T>G), ATC580GTA (5143A>G 5145C>A), ATC581TTA (5146A>T 5148C>A), GAA582CAA (5149G>C), TGC583ACA (5152T>A 5153G>C 5154C>A), GCA585GCC (5160A>C), TCA586TCC (5163A>C), GAT587GAC (5166T>C), GAC588TAC (5167G>T), ATC593TTG (5182A>T 5184C>G), CTC594TTA (5185C>T 5187C>A), AAA595CAA (5188A>C), GCA596ACA (5191G>A), AAA597GAT (5194A>G 5196A>T), CTT598TTG (5197C>T 5199T>G), CCA599AAT (5200C>A 5201C>A 5202A>T), GGT601_GTA604del (5206_5217delGGTAAAGAGGTA), ATA605ATT (5220A>T), TGC606TGT (5223C>T), AGG607AGA (5226G>A), GCT609ACT (5230G>A), TCA610AGT (5233T>A 5234C>G 5235A>T), ACC612ACT (5241C>T), AAA614AAT (5247A>T), CCA615GAA (5248C>G 5249C>A), GCT616GCA (5253T>A), GAG617GAA (5256G>A), AAG618ACT (5258A>C 5259G>T), AAT619AAA (5262T>A), TAC620TAT (5265C>T), CAT621TCT (5266C>T 5267A>C), AGT622ACC (5270G>C 5271T>C), ATC627TTA (5284A>T 5286C>A), CTT628TTA (5287C>T 5289T>A), TCC629GCA (5290T>G 5292C>A), ATT631GTT (5296A>G), AAG632CGA (5299A>C 5300A>G 5301G>A), GCA633GGA (5303C>G), AAA635AGA (5309A>G), GCT636AAA (5311G>A 5312C>A 5313T>A), AGA638TCA (5317A>T 5318G>C), GCT639TCT (5320G>T), TAT640TTC (5324A>T 5325T>C), ATC641CTA (5326A>C 5328C>A), CCT643CCA (5334T>A), TAT644AAA (5335T>A 5337T>A), AAA645CAA (5338A>C), TTT646TTC (5343T>C), CTT647ATC (5344C>A 5346T>C), GTA648ATC (5347G>A 5349A>C), AGG649CAA (5350A>C 5351G>A 5352G>A), ACT650TCA (5353A>T 5355T>A), GAT651GAC (5358T>C)

*: Inserts / Deletes / Misaligned / Frameshifts

Analysis details

This analysis was performed with panviral2.64

NGS Details (UN59): Burkholderia phage Magia

Assembly

**Coverage Length**	197 (1 contig(s))
**Depth Of Coverage**	2.6
**Number Of Reads**	4
**Reads Per Million**	0.09 rpm (after QC)
**Ambiguities**	0
**Assembly Method**	de novo + reference guided assembly
**Consensus Caller**	Bcf Tools

Coverage Map

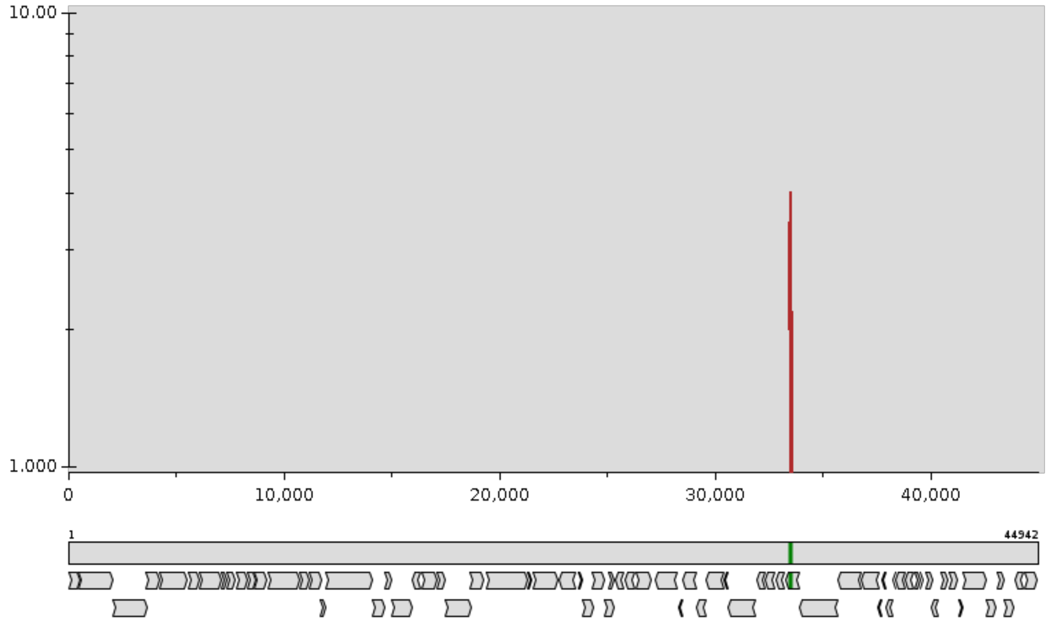

Assignment

**Type**	Burkholderia phage Magia (Taxonomy ID: 2767577)
**Reference Genome**	NC_070954.1
**NT Identity (%)**	86.802
**AA Identity (%)**	84.8485
**Number Of Stop Codons**	0
**Number Of CDS**	70

Alignment

**Alignment Score**	290.0 (NT) + 381.0 (AA) = 671.0
**Concordance (%)**	82.2304

Alignment Method	Local, heuristic, nucleotide (BLASTN)

Genome Region

Sequence starts at position 33387 and ends at position 33583 relative to NC_070954.1 reference sequence.

Alignment Detailed Statistics

	Begin	End	Coverage	Score	Concordance	Matches	Identities	I/D/M/F*	Stop Codons
**NT**	**33387**	**33583**	**0.4%**	**290**	**73.6%**	**197 (100%)**	**171 (86.8%)**	**0/0**	
Mutations:	33394T>G, 33398T>C, 33404T>G, 33407G>T, 33415T>C, 33416C>G, 33422A>G, 33434A>G, 33437G>T, 33464A>G, 33473G>C, 33476T>C, 33482C>T, 33483G>A, 33509C>T, 33518T>C, 33527G>A, 33536T>G, 33538C>T, 33544T>G, 33545C>G, 33551A>G, 33567T>C, 33569T>G, 33570G>C, 33579C>T								

CDS

	Begin	End	Coverage	Score	Concordance	Matches	Identities	I/D/M/F*	Stop Codons
PQC04_gp48	102	167	31.7%	381	87.2%	66 (100%)	56 (84.8%)	0/0/0/0	0
Protein mutations:	R103Q (33579C>T), A106G (33569T>G 33570G>C), D107G (33567T>C), I115L (33544T>G), A117T (33536T>G 33538C>T), T135I (33482C>T 33483G>A), I158V (33415T>C), K161N (33404T>G), K165Q (33394T>G)								
Codon mutations:	CGG103CAG (33579C>T), GCA106GGC (33569T>G 33570G>C), GAC107GGC (33567T>C), GTT112GTC (33551A>G), CGG114CGC (33545C>G), ATC115CTC (33544T>G), GCA117ACC (33536T>G 33538C>T), GAC120GAT (33527G>A), CAA123CAG (33518T>C), GCG126GCA (33509C>T), ACG135ATA (33482C>T 33483G>A), GAA137GAG (33476T>C), GCC138GCG (33473G>C), GAT141GAC (33464A>G), GCC150GCA (33437G>T), CGT151CGC (33434A>G), TTT155TTC (33422A>G), ACG157ACC (33416C>G), ATC158GTC (33415T>C), CGC160CGA (33407G>T), AAA161AAC (33404T>G), AAA163AAG (33398T>C), AAG165CAG (33394T>G)								

Proteins

	Begin	End	Coverage	Score	Concordance	Matches	Identities	I/D/M/F*	Stop Codons
transcriptional regulator (YP_010668144.1)	102	167	31.7%	381	87.2%	66 (100%)	56 (84.8%)	0/0/0/0	0
Protein mutations:	R103Q (33579C>T), A106G (33569T>G 33570G>C), D107G (33567T>C), I115L (33544T>G), A117T (33536T>G 33538C>T), T135I (33482C>T 33483G>A), I158V (33415T>C), K161N (33404T>G), K165Q (33394T>G)								
Codon mutations:	CGG103CAG (33579C>T), GCA106GGC (33569T>G 33570G>C), GAC107GGC (33567T>C), GTT112GTC (33551A>G), CGG114CGC (33545C>G), ATC115CTC (33544T>G), GCA117ACC (33536T>G 33538C>T), GAC120GAT (33527G>A), CAA123CAG (33518T>C), GCG126GCA (33509C>T), ACG135ATA (33482C>T 33483G>A), GAA137GAG (33476T>C), GCC138GCG (33473G>C), GAT141GAC (33464A>G), GCC150GCA (33437G>T), CGT151CGC (33434A>G), TTT155TTC (33422A>G), ACG157ACC (33416C>G), ATC158GTC (33415T>C), CGC160CGA (33407G>T), AAA161AAC (33404T>G), AAA163AAG (33398T>C), AAG165CAG (33394T>G)								

*: Inserts / Deletes / Misaligned / Frameshifts

Analysis details

This analysis was performed with panviral2.64

NGS Details (UN59): Sugarcane chlorotic streak virus

Assembly

**Coverage Length**	200 (1 contig(s))
**Depth Of Coverage**	1.6
**Number Of Reads**	3
**Reads Per Million**	0.07 rpm (after QC)
**Ambiguities**	0
**Assembly Method**	de novo + reference guided assembly
**Consensus Caller**	Bcf Tools

Coverage Map

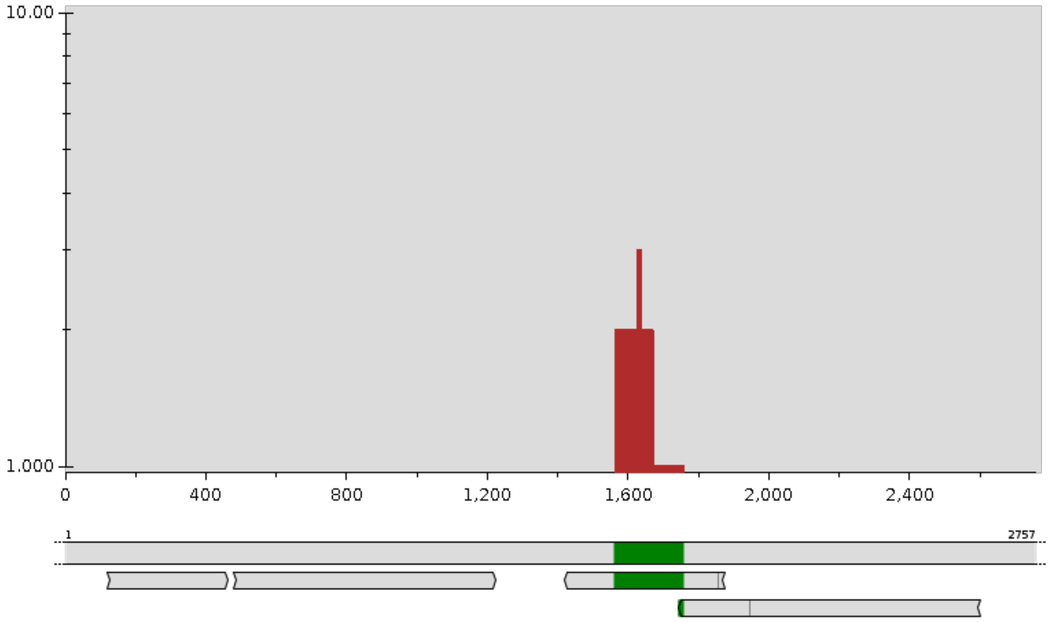

Assignment

**Type**	Sugarcane chlorotic streak virus (Taxonomy ID: 1919062)
**Reference Genome**	NC_032004.1
**NT Identity (%)**	59.8985
**AA Identity (%)**	56.2044
**Number Of Stop Codons**	1
**Number Of CDS**	5

Alignment

**Alignment Score**	66.0 (NT) + 560.0 (AA) = 626.0
**Concordance (%)**	45.3623

Alignment Method	Global, seeded, nucleotide + amino acids (AGA)

Genome Region

Sequence starts at position 1562 and ends at position 1761 relative to NC_032004.1 reference sequence.

Alignment Detailed Statistics

	Begin	End	Coverage	Score	Concordance	Matches	Identities	I/D/M/F*	Stop Codons
**NT**	**1562**	**1761**	**7.3%**	**66**	**17.3%**	**197 (98.5%)**	**118 (59.0%)**	**0/3**	
Mutations:	1562C>T, 1566C>A, 1567T>G, 1573G>C, 1574C>A, 1578C>A, 1584A>C, 1585G>C, 1587T>C, 1593G>T, 1595T>C, 1596T>C, 1598T>C, 1599T>C, 1600G>T, 1601A>T, 1602_1604delTGC, 1605G>A, 1607C>T, 1609T>C, 1611C>T, 1614C>T, 1615C>T, 1617T>C, 1620G>T, 1622C>T, 1626C>T, 1629C>T, 1630G>T, 1631G>C, 1632G>A, 1638A>T, 1639A>G, 1641G>A, 1644A>G, 1645T>C, 1647C>G, 1648T>A, 1649T>A, 1650C>T, 1656G>A, 1659A>T, 1661T>A, 1662T>C, 1664A>G, 1665C>T, 1667G>C, 1672C>T, 1673A>T, 1675C>T, 1676A>T, 1677A>T, 1680A>T, 1681C>A, 1683G>A, 1684T>A, 1689G>A, 1692A>C, 1695T>G, 1701A>G, 1704A>T, 1710G>A, 1713G>A, 1714A>T, 1715C>A, 1716G>C, 1718C>A, 1721C>T, 1722C>G, 1723T>A, 1724C>T, 1725T>A, 1734A>G, 1735G>T, 1736A>T, 1737G>A, 1738G>C, 1740C>G, 1741C>A, 1744G>T								

CDS

	Begin	End	Coverage	Score	Concordance	Matches	Identities	I/D/M/F*	Stop Codons
BS327_gp3	252	318	18.4%	268	53.3%	66 (98.5%)	36 (53.7%)	0/1/0/0	0
Protein mutations:	A257D (1744G>T), W258F (1740C>G 1741C>A), T259S (1737G>A 1738G>C), S260N (1734A>G 1735G>T 1736A>T), E263D (1725T>A), E264I (1722C>G 1723T>A 1724C>T), A265T (1721C>T), V266L (1716G>C 1718C>A), V267Y (1713G>A 1714A>T 1715C>A), Y277F (1683G>A 1684T>A), C278L (1680A>T 1681C>A), C280N (1675C>T 1676A>T), W281K (1672C>T 1673A>T), Q283E (1665C>T 1667G>C), I285L (1659A>T 1661T>A), K289F (1647C>G 1648T>A 1649T>A), D290G (1644A>G 1645T>C), I292T (1638A>T 1639A>G), P295E (1629C>T 1630G>T 1631G>C), G298R (1620G>T 1622C>T), R300K (1614C>T 1615C>T), K302R (1609T>C), V303I (1605G>A 1607C>T), A304del (1602_1604delTGC), S305K (1599T>C 1600G>T 1601A>T), R306G (1596T>C 1598T>C), S307G (1593G>T 1595T>C), T310R (1584A>C 1585G>C), A314C (1573G>C 1574C>A), E316A (1566C>A 1567T>G)								
Codon mutations:	GCC257GAC (1744G>T), TGG258TTC (1740C>G 1741C>A), ACC259AGT (1737G>A 1738G>C), TCT260AAC (1734A>G 1735G>T 1736A>T), GAA263GAT (1725T>A), GAG264ATC (1722C>G 1723T>A 1724C>T), GCA265ACA (1721C>T), GTC266TTG (1716G>C 1718C>A), GTC267TAT (1713G>A 1714A>T 1715C>A), AAC268AAT (1710G>A), ATT270ATA (1704A>T), GAT271GAC (1701A>G), ATA273ATC (1695T>G), CCT274CCG (1692A>C), TTC275TTT (1689G>A), TAC277TTT (1683G>A 1684T>A), TGT278TTA (1680A>T 1681C>A), CCT279CCA (1677A>T), TGC280AAC (1675C>T 1676A>T), TGG281AAG (1672C>T 1673A>T), CAG283GAA (1665C>T 1667G>C), TTA284CTG (1662T>C 1664A>G), ATT285TTA (1659A>T 1661T>A), GGC286GGT (1656G>A), CAG288CAA (1650C>T), AAG289TTC (1647C>G 1648T>A 1649T>A), GAT290GGC (1644A>G 1645T>C), TAC291TAT (1641G>A), ATT292ACA (1638A>T 1639A>G), AAC294AAT (1632G>A), CCG295GAA (1629C>T 1630G>T 1631G>C), AAG296AAA (1626C>T), GGC298AGA (1620G>T 1622C>T), AAA299AAG (1617T>C), AGG300AAA (1614C>T 1615C>T), AAG301AAA (1611C>T), AAG302AGG (1609T>C), GTC303ATT (1605G>A 1607C>T), GCA304del (1602_1604delTGC), TCA305AAG (1599T>C 1600G>T 1601A>T), AGA306GGG (1596T>C 1598T>C), AGC307GGA (1593G>T 1595T>C), TCA309TCG (1587T>C), ACT310AGG (1584A>C 1585G>C), GTG312GTT (1578C>A), GCC314TGC (1573G>C 1574C>A), GAG316GCT (1566C>A 1567T>G), GAG318A.. (1562C>T)								
BS327_gp4	40	106	44.1%	268	53.3%	66 (98.5%)	36 (53.7%)	0/1/0/0	0
Protein mutations:	A45D (1744G>T), W46F (1740C>G 1741C>A), T47S (1737G>A 1738G>C), S48N (1734A>G 1735G>T 1736A>T), E51D (1725T>A), E52I (1722C>G 1723T>A 1724C>T), A53T (1721C>T), V54L (1716G>C 1718C>A), V55Y (1713G>A 1714A>T 1715C>A), Y65F (1683G>A 1684T>A), C66L (1680A>T 1681C>A), C68N (1675C>T 1676A>T), W69K (1672C>T 1673A>T), Q71E (1665C>T 1667G>C), I73L (1659A>T 1661T>A), K77F (1647C>G 1648T>A 1649T>A), D78G (1644A>G 1645T>C), I80T (1638A>T 1639A>G), P83E (1629C>T 1630G>T 1631G>C), G86R (1620G>T 1622C>T), R88K (1614C>T 1615C>T), K90R (1609T>C), V91I (1605G>A 1607C>T), A92del (1602_1604delTGC), S93K (1599T>C 1600G>T 1601A>T), R94G (1596T>C 1598T>C), S95G (1593G>T 1595T>C), T98R (1584A>C 1585G>C), A102C (1573G>C 1574C>A), E104A (1566C>A 1567T>G)								
Codon mutations:	GCC45GAC (1744G>T), TGG46TTC (1740C>G 1741C>A), ACC47AGT (1737G>A 1738G>C), TCT48AAC (1734A>G 1735G>T 1736A>T), GAA51GAT (1725T>A), GAG52ATC (1722C>G 1723T>A 1724C>T), GCA53ACA (1721C>T), GTC54TTG (1716G>C 1718C>A), GTC55TAT (1713G>A 1714A>T 1715C>A), AAC56AAT (1710G>A), ATT58ATA (1704A>T), GAT59GAC (1701A>G), ATA61ATC (1695T>G), CCT62CCG (1692A>C), TTC63TTT (1689G>A), TAC65TTT (1683G>A 1684T>A), TGT66TTA (1680A>T 1681C>A), CCT67CCA (1677A>T), TGC68AAC (1675C>T 1676A>T), TGG69AAG (1672C>T 1673A>T), CAG71GAA (1665C>T 1667G>C), TTA72CTG (1662T>C 1664A>G), ATT73TTA (1659A>T 1661T>A), GGC74GGT (1656G>A), CAG76CAA (1650C>T), AAG77TTC (1647C>G 1648T>A 1649T>A), GAT78GGC (1644A>G 1645T>C), TAC79TAT (1641G>A), ATT80ACA (1638A>T 1639A>G), AAC82AAT (1632G>A), CCG83GAA (1629C>T 1630G>T 1631G>C), AAG84AAA (1626C>T), GGC86AGA (1620G>T 1622C>T), AAA87AAG (1617T>C), AGG88AAA (1614C>T 1615C>T), AAG89AAA (1611C>T), AAG90AGG (1609T>C), GTC91ATT (1605G>A 1607C>T), GCA92del (1602_1604delTGC), TCA93AAG (1599T>C 1600G>T 1601A>T), AGA94GGG (1596T>C 1598T>C), AGC95GGA (1593G>T 1595T>C), TCA97TCG (1587T>C), ACT98AGG (1584A>C 1585G>C), GTG100GTT (1578C>A), GCC102TGC (1573G>C 1574C>A), GAG104GCT (1566C>A 1567T>G), GAG106A.. (1562C>T)								
BS327_gp5	283	287	1.7%	24	82.8%	5 (100%)	5 (100%)	0/0/0/0	1

Proteins

	Begin	End	Coverage	Score	Concordance	Matches	Identities	I/D/M/F*	Stop Codons
Rep-associated protein (YP_009325925.1)	252	318	18.4%	268	53.3%	66 (98.5%)	36 (53.7%)	0/1/0/0	0
Protein mutations:	A257D (1744G>T), W258F (1740C>G 1741C>A), T259S (1737G>A 1738G>C), S260N (1734A>G 1735G>T 1736A>T), E263D (1725T>A), E264I (1722C>G 1723T>A 1724C>T), A265T (1721C>T), V266L (1716G>C 1718C>A), V267Y (1713G>A 1714A>T 1715C>A), Y277F (1683G>A 1684T>A), C278L (1680A>T 1681C>A), C280N (1675C>T 1676A>T), W281K (1672C>T 1673A>T), Q283E (1665C>T 1667G>C), I285L (1659A>T 1661T>A), K289F (1647C>G 1648T>A 1649T>A), D290G (1644A>G 1645T>C), I292T (1638A>T 1639A>G), P295E (1629C>T 1630G>T 1631G>C), G298R (1620G>T 1622C>T), R300K (1614C>T 1615C>T), K302R (1609T>C), V303I (1605G>A 1607C>T), A304del (1602_1604delTGC), S305K (1599T>C 1600G>T 1601A>T), R306G (1596T>C 1598T>C), S307G (1593G>T 1595T>C), T310R (1584A>C 1585G>C), A314C (1573G>C 1574C>A), E316A (1566C>A 1567T>G)								
Codon mutations:	GCC257GAC (1744G>T), TGG258TTC (1740C>G 1741C>A), ACC259AGT (1737G>A 1738G>C), TCT260AAC (1734A>G 1735G>T 1736A>T), GAA263GAT (1725T>A), GAG264ATC (1722C>G 1723T>A 1724C>T), GCA265ACA (1721C>T), GTC266TTG (1716G>C 1718C>A), GTC267TAT (1713G>A 1714A>T 1715C>A), AAC268AAT (1710G>A), ATT270ATA (1704A>T), GAT271GAC (1701A>G), ATA273ATC (1695T>G), CCT274CCG (1692A>C), TTC275TTT (1689G>A), TAC277TTT (1683G>A 1684T>A), TGT278TTA (1680A>T 1681C>A), CCT279CCA (1677A>T), TGC280AAC (1675C>T 1676A>T), TGG281AAG (1672C>T 1673A>T), CAG283GAA (1665C>T 1667G>C), TTA284CTG (1662T>C 1664A>G), ATT285TTA (1659A>T 1661T>A), GGC286GGT (1656G>A), CAG288CAA (1650C>T), AAG289TTC (1647C>G 1648T>A 1649T>A), GAT290GGC (1644A>G 1645T>C), TAC291TAT (1641G>A), ATT292ACA (1638A>T 1639A>G), AAC294AAT (1632G>A), CCG295GAA (1629C>T 1630G>T 1631G>C), AAG296AAA (1626C>T), GGC298AGA (1620G>T 1622C>T), AAA299AAG (1617T>C), AGG300AAA (1614C>T 1615C>T), AAG301AAA (1611C>T), AAG302AGG (1609T>C), GTC303ATT (1605G>A 1607C>T), GCA304del (1602_1604delTGC), TCA305AAG (1599T>C 1600G>T 1601A>T), AGA306GGG (1596T>C 1598T>C), AGC307GGA (1593G>T 1595T>C), TCA309TCG (1587T>C), ACT310AGG (1584A>C 1585G>C), GTG312GTT (1578C>A), GCC314TGC (1573G>C 1574C>A), GAG316GCT (1566C>A 1567T>G), GAG318A.. (1562C>T)								
RepB (YP_009325926.1)	40	106	44.1%	268	53.3%	66 (98.5%)	36 (53.7%)	0/1/0/0	0
Protein mutations:	A45D (1744G>T), W46F (1740C>G 1741C>A), T47S (1737G>A 1738G>C), S48N (1734A>G 1735G>T 1736A>T), E51D (1725T>A), E52I (1722C>G 1723T>A 1724C>T), A53T (1721C>T), V54L (1716G>C 1718C>A), V55Y (1713G>A 1714A>T 1715C>A), Y65F (1683G>A 1684T>A), C66L (1680A>T 1681C>A), C68N (1675C>T 1676A>T), W69K (1672C>T 1673A>T), Q71E (1665C>T 1667G>C), I73L (1659A>T 1661T>A), K77F (1647C>G 1648T>A 1649T>A), D78G (1644A>G 1645T>C), I80T (1638A>T 1639A>G), P83E (1629C>T 1630G>T 1631G>C), G86R (1620G>T 1622C>T), R88K (1614C>T 1615C>T), K90R (1609T>C), V91I (1605G>A 1607C>T), A92del (1602_1604delTGC), S93K (1599T>C 1600G>T 1601A>T), R94G (1596T>C 1598T>C), S95G (1593G>T 1595T>C), T98R (1584A>C 1585G>C), A102C (1573G>C 1574C>A), E104A (1566C>A 1567T>G)								
Codon mutations:	GCC45GAC (1744G>T), TGG46TTC (1740C>G 1741C>A), ACC47AGT (1737G>A 1738G>C), TCT48AAC (1734A>G 1735G>T 1736A>T), GAA51GAT (1725T>A), GAG52ATC (1722C>G 1723T>A 1724C>T), GCA53ACA (1721C>T), GTC54TTG (1716G>C 1718C>A), GTC55TAT (1713G>A 1714A>T 1715C>A), AAC56AAT (1710G>A), ATT58ATA (1704A>T), GAT59GAC (1701A>G), ATA61ATC (1695T>G), CCT62CCG (1692A>C), TTC63TTT (1689G>A), TAC65TTT (1683G>A 1684T>A), TGT66TTA (1680A>T 1681C>A), CCT67CCA (1677A>T), TGC68AAC (1675C>T 1676A>T), TGG69AAG (1672C>T 1673A>T), CAG71GAA (1665C>T 1667G>C), TTA72CTG (1662T>C 1664A>G), ATT73TTA (1659A>T 1661T>A), GGC74GGT (1656G>A), CAG76CAA (1650C>T), AAG77TTC (1647C>G 1648T>A 1649T>A), GAT78GGC (1644A>G 1645T>C), TAC79TAT (1641G>A), ATT80ACA (1638A>T 1639A>G), AAC82AAT (1632G>A), CCG83GAA (1629C>T 1630G>T 1631G>C), AAG84AAA (1626C>T), GGC86AGA (1620G>T 1622C>T), AAA87AAG (1617T>C), AGG88AAA (1614C>T 1615C>T), AAG89AAA (1611C>T), AAG90AGG (1609T>C), GTC91ATT (1605G>A 1607C>T), GCA92del (1602_1604delTGC), TCA93AAG (1599T>C 1600G>T 1601A>T), AGA94GGG (1596T>C 1598T>C), AGC95GGA (1593G>T 1595T>C), TCA97TCG (1587T>C), ACT98AGG (1584A>C 1585G>C), GTG100GTT (1578C>A), GCC102TGC (1573G>C 1574C>A), GAG104GCT (1566C>A 1567T>G), GAG106A.. (1562C>T)								
RepA (YP_009325927.1)	283	287	1.7%	24	82.8%	5 (100%)	5 (100%)	0/0/0/0	1
Protein mutations:	*none*								
Codon mutations:	*none*								

*: Inserts / Deletes / Misaligned / Frameshifts

Analysis details

This analysis was performed with panviral2.64

NGS Details (UN59): Alphafusarivirus pleosporae

Assembly

**Coverage Length**	254 (1 contig(s))
**Depth Of Coverage**	1.1
**Number Of Reads**	2
**Reads Per Million**	0.04 rpm (after QC)
**Ambiguities**	0
**Assembly Method**	de novo + reference guided assembly
**Consensus Caller**	Bcf Tools

Coverage Map

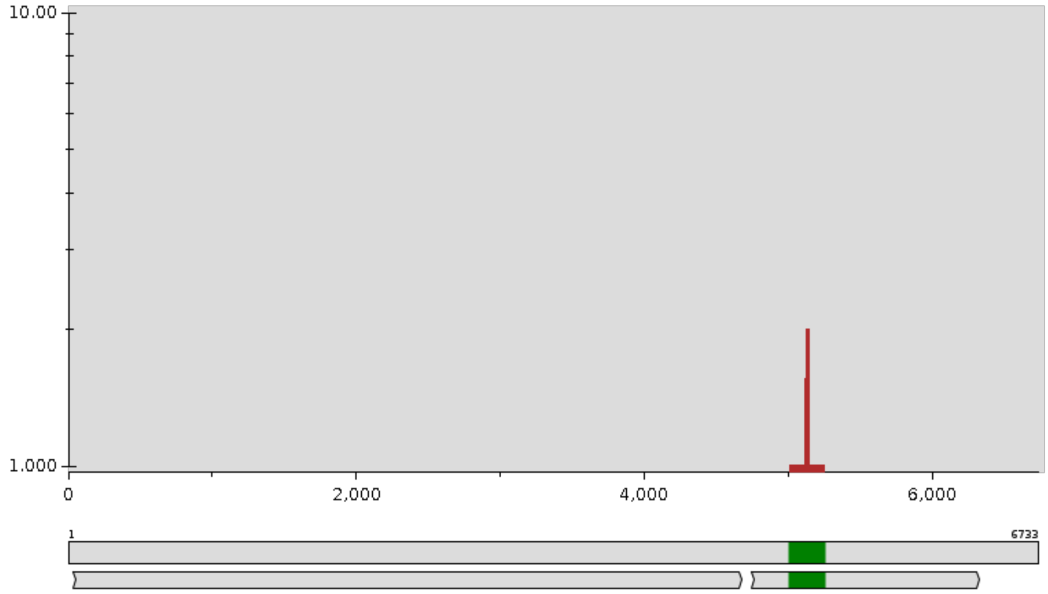

Assignment

**Type**	Alphafusarivirus pleosporae (Taxonomy ID: 2955271)
**Reference Genome**	NC_028470.1
**NT Identity (%)**	75.1969
**AA Identity (%)**	85.7143
**Number Of Stop Codons**	0
**Number Of CDS**	2

Alignment

**Alignment Score**	256.0 (NT) + 417.0 (AA) = 673.0
**Concordance (%)**	68.4639

Alignment Method	Global, seeded, nucleotide + amino acids (AGA)

Genome Region

Sequence starts at position 5007 and ends at position 5260 relative to NC_028470.1 reference sequence.

Alignment Detailed Statistics

	Begin	End	Coverage	Score	Concordance	Matches	Identities	I/D/M/F*	Stop Codons
**NT**	**5007**	**5260**	**3.8%**	**256**	**50.4%**	**254 (100%)**	**191 (75.2%)**	**0/0**	
Mutations:	5011C>T, 5014T>A, 5027T>C, 5029G>T, 5035T>A, 5039T>C, 5041G>T, 5048C>T, 5050T>G, 5051C>A, 5053T>G, 5059A>G, 5062A>C, 5074A>G, 5075A>T, 5077C>T, 5095G>A, 5101T>C, 5104G>A, 5105G>C, 5106C>A, 5107C>A, 5110C>T, 5116A>T, 5119G>A, 5123T>C, 5125G>C, 5128T>C, 5134A>C, 5140A>C, 5146A>G, 5154C>T, 5155A>T, 5158A>T, 5159A>G, 5165T>G, 5168C>G, 5173T>A, 5174G>A, 5176T>C, 5177C>T, 5179G>A, 5182C>T, 5188T>C, 5189A>T, 5190G>C, 5191C>T, 5192A>G, 5193A>G, 5197G>T, 5200A>G, 5206G>A, 5207C>T, 5209C>G, 5210A>C, 5211A>G, 5224A>T, 5225A>T, 5226A>C, 5230T>C, 5236T>C, 5239T>C, 5244G>A								

CDS

	Begin	End	Coverage	Score	Concordance	Matches	Identities	I/D/M/F*	Stop Codons
AT135_gp2	90	173	15.9%	417	85.8%	84 (100%)	72 (85.7%)	0/0/0/0	0
Protein mutations:	T112S (5075A>T 5077C>T), A122Q (5105G>C 5106C>A 5107C>A), A138V (5154C>T 5155A>T), I140V (5159A>G), S142A (5165T>G), Q143E (5168C>G), D144E (5173T>A), G145S (5174G>A 5176T>C), N151G (5192A>G 5193A>G), K157R (5210A>C 5211A>G), K162S (5225A>T 5226A>C), R168K (5244G>A)								
Codon mutations:	GTC90GTT (5011C>T), TCT91TCA (5014T>A), TTG96CTT (5027T>C 5029G>T), CGT98CGA (5035T>A), TTG100CTT (5039T>C 5041G>T), CTT103TTG (5048C>T 5050T>G), CGT104AGG (5051C>A 5053T>G), AAA106AAG (5059A>G), GCA107GCC (5062A>C), AAA111AAG (5074A>G), ACC112TCT (5075A>T 5077C>T), GAG118GAA (5095G>A), GCT120GCC (5101T>C), CAG121CAA (5104G>A), GCC122CAA (5105G>C 5106C>A 5107C>A), AAC123AAT (5110C>T), ACA125ACT (5116A>T), AAG126AAA (5119G>A), TTG128CTC (5123T>C 5125G>C), AAT129AAC (5128T>C), GCA131GCC (5134A>C), GCA133GCC (5140A>C), GAA135GAG (5146A>G), GCA138GTT (5154C>T 5155A>T), CGA139CGT (5158A>T), ATT140GTT (5159A>G), TCT142GCT (5165T>G), CAG143GAG (5168C>G), GAT144GAA (5173T>A), GGT145AGC (5174G>A 5176T>C), CTG146TTA (5177C>T 5179G>A), GCC147GCT (5182C>T), CTT149CTC (5188T>C), AGC150TCT (5189A>T 5190G>C 5191C>T), AAC151GGC (5192A>G 5193A>G), ACG152ACT (5197G>T), CAA153CAG (5200A>G), GAG155GAA (5206G>A), CTC156TTG (5207C>T 5209C>G), AAG157CGG (5210A>C 5211A>G), GCA161GCT (5224A>T), AAA162TCA (5225A>T 5226A>C), GTT163GTC (5230T>C), GCT165GCC (5236T>C), TCT166TCC (5239T>C), AGG168AAG (5244G>A)								

Proteins

	Begin	End	Coverage	Score	Concordance	Matches	Identities	I/D/M/F*	Stop Codons
59 kDa protein (YP_009182159.1)	90	173	15.9%	417	85.8%	84 (100%)	72 (85.7%)	0/0/0/0	0
Protein mutations:	T112S (5075A>T 5077C>T), A122Q (5105G>C 5106C>A 5107C>A), A138V (5154C>T 5155A>T), I140V (5159A>G), S142A (5165T>G), Q143E (5168C>G), D144E (5173T>A), G145S (5174G>A 5176T>C), N151G (5192A>G 5193A>G), K157R (5210A>C 5211A>G), K162S (5225A>T 5226A>C), R168K (5244G>A)								
Codon mutations:	GTC90GTT (5011C>T), TCT91TCA (5014T>A), TTG96CTT (5027T>C 5029G>T), CGT98CGA (5035T>A), TTG100CTT (5039T>C 5041G>T), CTT103TTG (5048C>T 5050T>G), CGT104AGG (5051C>A 5053T>G), AAA106AAG (5059A>G), GCA107GCC (5062A>C), AAA111AAG (5074A>G), ACC112TCT (5075A>T 5077C>T), GAG118GAA (5095G>A), GCT120GCC (5101T>C), CAG121CAA (5104G>A), GCC122CAA (5105G>C 5106C>A 5107C>A), AAC123AAT (5110C>T), ACA125ACT (5116A>T), AAG126AAA (5119G>A), TTG128CTC (5123T>C 5125G>C), AAT129AAC (5128T>C), GCA131GCC (5134A>C), GCA133GCC (5140A>C), GAA135GAG (5146A>G), GCA138GTT (5154C>T 5155A>T), CGA139CGT (5158A>T), ATT140GTT (5159A>G), TCT142GCT (5165T>G), CAG143GAG (5168C>G), GAT144GAA (5173T>A), GGT145AGC (5174G>A 5176T>C), CTG146TTA (5177C>T 5179G>A), GCC147GCT (5182C>T), CTT149CTC (5188T>C), AGC150TCT (5189A>T 5190G>C 5191C>T), AAC151GGC (5192A>G 5193A>G), ACG152ACT (5197G>T), CAA153CAG (5200A>G), GAG155GAA (5206G>A), CTC156TTG (5207C>T 5209C>G), AAG157CGG (5210A>C 5211A>G), GCA161GCT (5224A>T), AAA162TCA (5225A>T 5226A>C), GTT163GTC (5230T>C), GCT165GCC (5236T>C), TCT166TCC (5239T>C), AGG168AAG (5244G>A)								

*: Inserts / Deletes / Misaligned / Frameshifts

Analysis details

This analysis was performed with panviral2.64

NGS Details (UN59): Potato virus X

Assembly

**Coverage Length**	225 (1 contig(s))
**Depth Of Coverage**	1.2
**Number Of Reads**	2
**Reads Per Million**	0.04 rpm (after QC)
**Ambiguities**	0
**Assembly Method**	de novo + reference guided assembly
**Consensus Caller**	Bcf Tools

Coverage Map

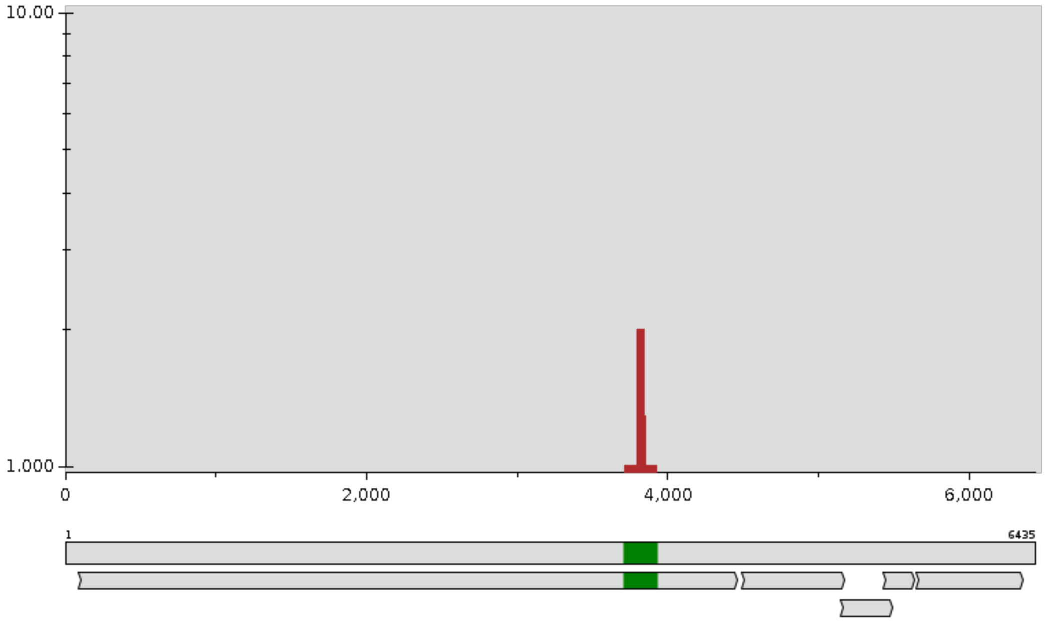

Assignment

**Type**	Potato virus X (Taxonomy ID: 12183)
**Reference Genome**	NC_011620.1
**NT Identity (%)**	79.1111
**AA Identity (%)**	97.3333
**Number Of Stop Codons**	0
**Number Of CDS**	5

Alignment

**Alignment Score**	262.0 (NT) + 544.0 (AA) = 806.0
**Concordance (%)**	80.7615

**Alignment Method**	Global, seeded, nucleotide + amino acids (AGA)

Genome Region

Sequence starts at position 3708 and ends at position 3932 relative to NC_011620.1 reference sequence.

Alignment Detailed Statistics

	Begin	End	Coverage	Score	Concordance	Matches	Identities	I/D/M/F*	Stop Codons
**NT**	**3708**	**3932**	**3.5%**	**262**	**58.2%**	**225 (100%)**	**178 (79.1%)**	**0/0**	
Mutations:	3711G>A, 3714A>C, 3717A>G, 3723C>G, 3729A>C, 3735T>C, 3738G>A, 3741T>A, 3747A>T, 3750A>G, 3761T>C, 3768C>T, 3771G>A, 3783T>C, 3787A>T, 3788G>C, 3795T>C, 3799T>C, 3801A>G, 3804T>C, 3816A>T, 3819T>A, 3822C>T, 3825C>T, 3831T>A, 3834G>A, 3840A>T, 3843T>C, 3847C>T, 3849G>A, 3861G>A, 3864C>A, 3867A>G, 3873G>A, 3876C>T, 3879C>T, 3885A>C, 3894A>G, 3900C>A, 3903A>G, 3906A>G, 3912A>T, 3918T>C, 3921G>A, 3924C>T, 3927T>C, 3930A>C								

CDS

	Begin	End	Coverage	Score	Concordance	Matches	Identities	I/D/M/F*	Stop Codons
PVX_gp1	1209	1283	5.1%	544	96.1%	75 (100%)	73 (97.3%)	0/0/0/0	0
Protein mutations:	V1226A (3761T>C)								
Codon mutations:	CAG1209CAA (3711G>A), CCA1210CCC (3714A>C), AAA1211AAG (3717A>G), GTC1213GTG (3723C>G), ATA1215ATC (3729A>C), TGT1217TGC (3735T>C), GAG1218GAA (3738G>A), ACT1219ACA (3741T>A), CCA1221CCT (3747A>T), GAA1222GAG (3750A>G), GTA1226GCA (3761T>C), GCC1228GCT (3768C>T), TTG1229TTA (3771G>A), AAT1233AAC (3783T>C), AGC1235TCC (3787A>T 3788G>C), CCT1237CCC (3795T>C), TTA1239CTG (3799T>C 3801A>G), GCT1240GCC (3804T>C), ACA1244ACT (3816A>T), GCT1245GCA (3819T>A), TTC1246TTT (3822C>T), GAC1247GAT (3825C>T), TCT1249TCA (3831T>A), CAG1250CAA (3834G>A), GGA1252GGT (3840A>T), GCT1253GCC (3843T>C), CTG1255TTA (3847C>T 3849G>A), GTG1259GTA (3861G>A), CTC1260CTA (3864C>A), AAA1261AAG (3867A>G), AAG1263AAA (3873G>A), CAC1264CAT (3876C>T), CAC1265CAT (3879C>T), ATA1267ATC (3885A>C), GAA1270GAG (3894A>G), ATC1272ATA (3900C>A), CAA1273CAG (3903A>G), GCA1274GCG (3906A>G), ATA1276ATT (3912A>T), ATT1278ATC (3918T>C), AAG1279AAA (3921G>A), ACC1280ACT (3924C>T), AAT1281AAC (3927T>C), GCA1282GCC (3930A>C)								

Proteins

	Begin	End	Coverage	Score	Concordance	Matches	Identities	I/D/M/F*	Stop Codons
replicase (YP_002332929.1)	1209	1283	5.1%	544	96.1%	75 (100%)	73 (97.3%)	0/0/0/0	0
Protein mutations:	V1226A (3761T>C)								
Codon mutations:	CAG1209CAA (3711G>A), CCA1210CCC (3714A>C), AAA1211AAG (3717A>G), GTC1213GTG (3723C>G), ATA1215ATC (3729A>C), TGT1217TGC (3735T>C), GAG1218GAA (3738G>A), ACT1219ACA (3741T>A), CCA1221CCT (3747A>T), GAA1222GAG (3750A>G), GTA1226GCA (3761T>C), GCC1228GCT (3768C>T), TTG1229TTA (3771G>A), AAT1233AAC (3783T>C), AGC1235TCC (3787A>T 3788G>C), CCT1237CCC (3795T>C), TTA1239CTG (3799T>C 3801A>G), GCT1240GCC (3804T>C), ACA1244ACT (3816A>T), GCT1245GCA (3819T>A), TTC1246TTT (3822C>T), GAC1247GAT (3825C>T), TCT1249TCA (3831T>A), CAG1250CAA (3834G>A), GGA1252GGT (3840A>T), GCT1253GCC (3843T>C), CTG1255TTA (3847C>T 3849G>A), GTG1259GTA (3861G>A), CTC1260CTA (3864C>A), AAA1261AAG (3867A>G), AAG1263AAA (3873G>A), CAC1264CAT (3876C>T), CAC1265CAT (3879C>T), ATA1267ATC (3885A>C), GAA1270GAG (3894A>G), ATC1272ATA (3900C>A), CAA1273CAG (3903A>G), GCA1274GCG (3906A>G), ATA1276ATT (3912A>T), ATT1278ATC (3918T>C), AAG1279AAA (3921G>A), ACC1280ACT (3924C>T), AAT1281AAC (3927T>C), GCA1282GCC (3930A>C)								

*: Inserts / Deletes / Misaligned / Frameshifts

Analysis details

This analysis was performed with panviral2.64

NGS Details (UN59): Colombian datura virus

Assembly

**Coverage Length**	269 (2 contig(s))
**Depth Of Coverage**	1.0
**Number Of Reads**	2
**Reads Per Million**	0.04 rpm (after QC)
**Ambiguities**	0
**Assembly Method**	de novo + reference guided assembly
**Consensus Caller**	Bcf Tools

Coverage Map

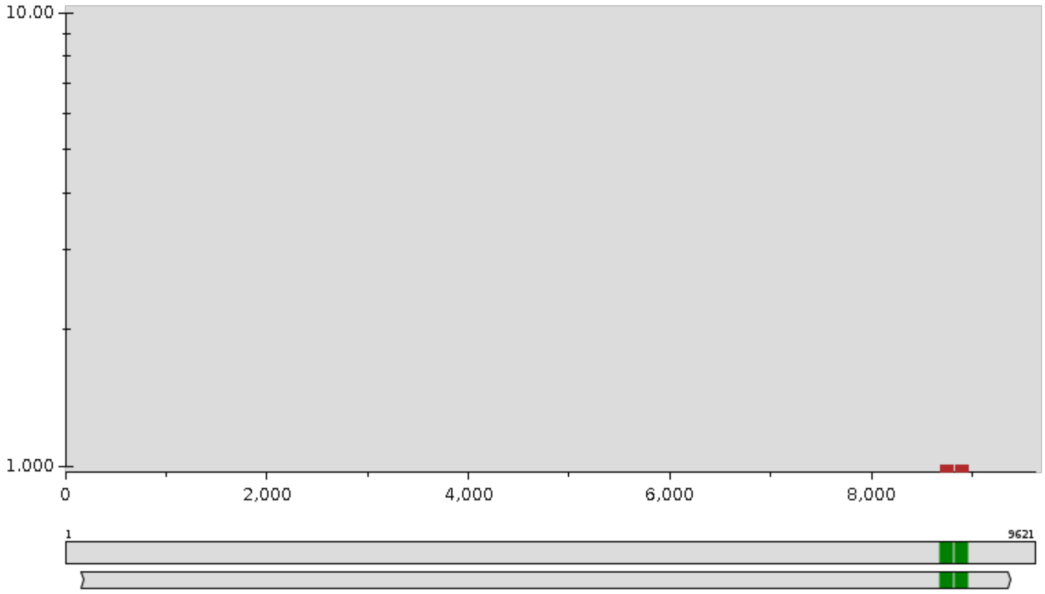

Assignment

**Type**	Colombian datura virus (Taxonomy ID: 91613)
**Reference Genome**	NC_020072.1
**NT Identity (%)**	98.1413
**AA Identity (%)**	97.7778
**Number Of Stop Codons**	0
**Number Of CDS**	1

Alignment

**Alignment Score**	518.0 (NT) + 640.0 (AA) = 1158.0
**Concordance (%)**	98.3022

Alignment Method	Global, seeded, nucleotide + amino acids (AGA)

Genome Region

Sequence starts at position 8680 and ends at position 8961 relative to NC_020072.1 reference sequence.

Alignment Detailed Statistics

	Begin	End	Coverage	Score	Concordance	Matches	Identities	I/D/M/F*	Stop Codons
**NT**	**8680**	**8961**	**2.8%**	**518**	**96.3%**	**269 (100%)**	**264 (98.1%)**	**0/0**	
Mutations:	8761T>C, 8767A>G, 8788G>T, 8893T>C, 8914G>A								
**CDS**									
G357_gp1	2843	2936	2.9%	640	96.1%	90 (100%)	88 (97.8%)	0/0/0/0	0
**Proteins**									
polyprotein (YP_007346986.1)	2843	2936	2.9%	640	96.1%	90 (100%)	88 (97.8%)	0/0/0/0	0
Protein mutations:	*none*								
Codon mutations:	AAT2869AAC (8761T>C), CAA2871CAG (8767A>G), CTG2878CTT (8788G>T), GAT2913GAC (8893T>C), TTG2920TTA (8914G>A)								

*: Inserts / Deletes / Misaligned / Frameshifts

Analysis details

This analysis was performed with panviral2.64

NGS Details (UN59): Rahariannevirus raharianne

Assembly

**Coverage Length**	251 (2 contig(s))
**Depth Of Coverage**	1.0
**Number Of Reads**	2
**Reads Per Million**	0.04 rpm (after QC)
**Ambiguities**	0
**Assembly Method**	de novo + reference guided assembly
**Consensus Caller**	Bcf Tools

Coverage Map

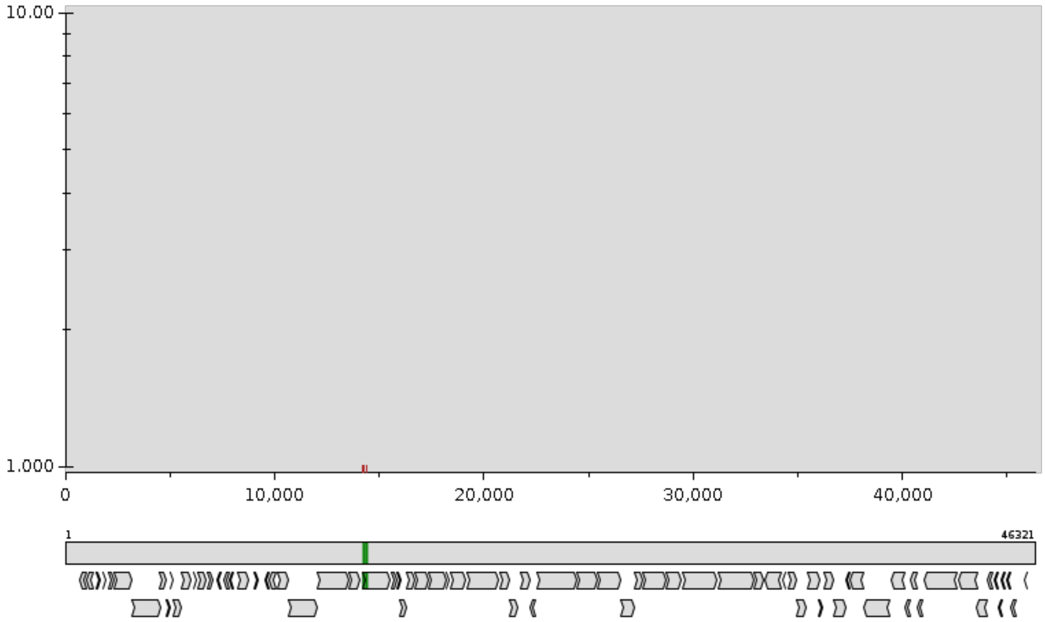

Assignment

**Type**	Rahariannevirus raharianne (Taxonomy ID: 2846050)
**Reference Genome**	NC_054955.1
**NT Identity (%)**	82.0717
**AA Identity (%)**	82.1429
**Number Of Stop Codons**	0
**Number Of CDS**	75

Alignment

**Alignment Score**	322.0 (NT) + 482.0 (AA) = 804.0
**Concordance (%)**	76.1364

Alignment Method	Local, heuristic, nucleotide (BLASTN)

Genome Region

Sequence starts at position 14196 and ends at position 14467 relative to NC_054955.1 reference sequence.

Alignment Detailed Statistics

	Begin	End	Coverage	Score	Concordance	Matches	Identities	I/D/M/F*	Stop Codons
**NT**	**14196**	**14467**	**0.5%**	**322**	**64.1%**	**251 (100%)**	**206 (82.1%)**	**0/0**	
Mutations:	14204C>T, 14205A>C, 14206G>A, 14207C>A, 14210T>C, 14219C>T, 14222T>C, 14231G>C, 14264T>C, 14270T>A, 14272C>G, 14285C>G, 14288C>G, 14289G>A, 14293T>A, 14294C>T, 14298C>T, 14299A>C, 14306A>C, 14309T>C, 14336T>C, 14339A>G, 14342C>A, 14349A>C, 14357T>C, 14363T>C, 14366G>C, 14372G>T, 14375G>C, 14378C>G, 14381C>G, 14394C>G, 14397T>G, 14399G>C, 14400G>A, 14401C>G, 14402A>C, 14427T>A, 14432G>C, 14433T>A, 14434C>G, 14435G>C, 14447G>C, 14457T>A, 14462C>G								
**CDS**									
KMC43_gp27	1	91	19.0%	482	83.8%	84 (100%)	69 (82.1%)	0/0/0/0	0
Protein mutations:	S4Q (14205A>C 14206G>A 14207C>A), D25E (14270T>A), A26G (14272C>G), I30M (14285C>G), G32S (14289G>A), F33Y (14293T>A 14294C>T), Q35S (14298C>T 14299A>C), K52Q (14349A>C), P67A (14394C>G), S68A (14397T>G 14399G>C), A69S (14400G>A 14401C>G 14402A>C), S78T (14427T>A), S88T (14457T>A)								
Codon mutations:	ATC3ATT (14204C>T), AGC4CAA (14205A>C 14206G>A 14207C>A), AAT5AAC (14210T>C), GCC8GCT (14219C>T), GCT9GCC (14222T>C), CCG12CCC (14231G>C), TTT23TTC (14264T>C), GAT25GAA (14270T>A), GCC26GGC (14272C>G), ATC30ATG (14285C>G), CTC31CTG (14288C>G), GGC32AGC (14289G>A), TTC33TAT (14293T>A 14294C>T), CAG35TCG (14298C>T 14299A>C), GCA37GCC (14306A>C), CGT38CGC (14309T>C), GGT47GGC (14336T>C), GAA48GAG (14339A>G), ACC49ACA (14342C>A), AAG52CAG (14349A>C), CGT54CGC (14357T>C), GGT56GGC (14363T>C), CTG57CTC (14366G>C), GCG59GCT (14372G>T), CCG60CCC (14375G>C), GTC61GTG (14378C>G), ACC62ACG (14381C>G), CCG67GCG (14394C>G), TCG68GCC (14397T>G 14399G>C), GCA69AGC (14400G>A 14401C>G 14402A>C), TCG78ACG (14427T>A), CCG79CCC (14432G>C), TCG80AGC (14433T>A 14434C>G 14435G>C), GTG84GTC (14447G>C), TCG88ACG (14457T>A), CTC89CTG (14462C>G)								
**Proteins**									
DUF4043 domain-containing protein (YP_010078151.1)	1	91	19.0%	482	83.8%	84 (100%)	69 (82.1%)	0/0/0/0	0
Protein mutations:	S4Q (14205A>C 14206G>A 14207C>A), D25E (14270T>A), A26G (14272C>G), I30M (14285C>G), G32S (14289G>A), F33Y (14293T>A 14294C>T), Q35S (14298C>T 14299A>C), K52Q (14349A>C), P67A (14394C>G), S68A (14397T>G 14399G>C), A69S (14400G>A 14401C>G 14402A>C), S78T (14427T>A), S88T (14457T>A)								
Codon mutations:	ATC3ATT (14204C>T), AGC4CAA (14205A>C 14206G>A 14207C>A), AAT5AAC (14210T>C), GCC8GCT (14219C>T), GCT9GCC (14222T>C), CCG12CCC (14231G>C), TTT23TTC (14264T>C), GAT25GAA (14270T>A), GCC26GGC (14272C>G), ATC30ATG (14285C>G), CTC31CTG (14288C>G), GGC32AGC (14289G>A), TTC33TAT (14293T>A 14294C>T), CAG35TCG (14298C>T 14299A>C), GCA37GCC (14306A>C), CGT38CGC (14309T>C), GGT47GGC (14336T>C), GAA48GAG (14339A>G), ACC49ACA (14342C>A), AAG52CAG (14349A>C), CGT54CGC (14357T>C), GGT56GGC (14363T>C), CTG57CTC (14366G>C), GCG59GCT (14372G>T), CCG60CCC (14375G>C), GTC61GTG (14378C>G), ACC62ACG (14381C>G), CCG67GCG (14394C>G), TCG68GCC (14397T>G 14399G>C), GCA69AGC (14400G>A 14401C>G 14402A>C), TCG78ACG (14427T>A), CCG79CCC (14432G>C), TCG80AGC (14433T>A 14434C>G 14435G>C), GTG84GTC (14447G>C), TCG88ACG (14457T>A), CTC89CTG (14462C>G)								

*: Inserts / Deletes / Misaligned / Frameshifts

Analysis details

This analysis was performed with panviral2.64

NGS Details (UN59): Euphorbia caput-medusae latent virus

Assembly

**Coverage Length**	123 (1 contig(s))
**Depth Of Coverage**	1.0
**Number Of Reads**	1
**Reads Per Million**	0.02 rpm (after QC)
**Ambiguities**	0
**Assembly Method**	de novo + reference guided assembly
**Consensus Caller**	Bcf Tools

Coverage Map

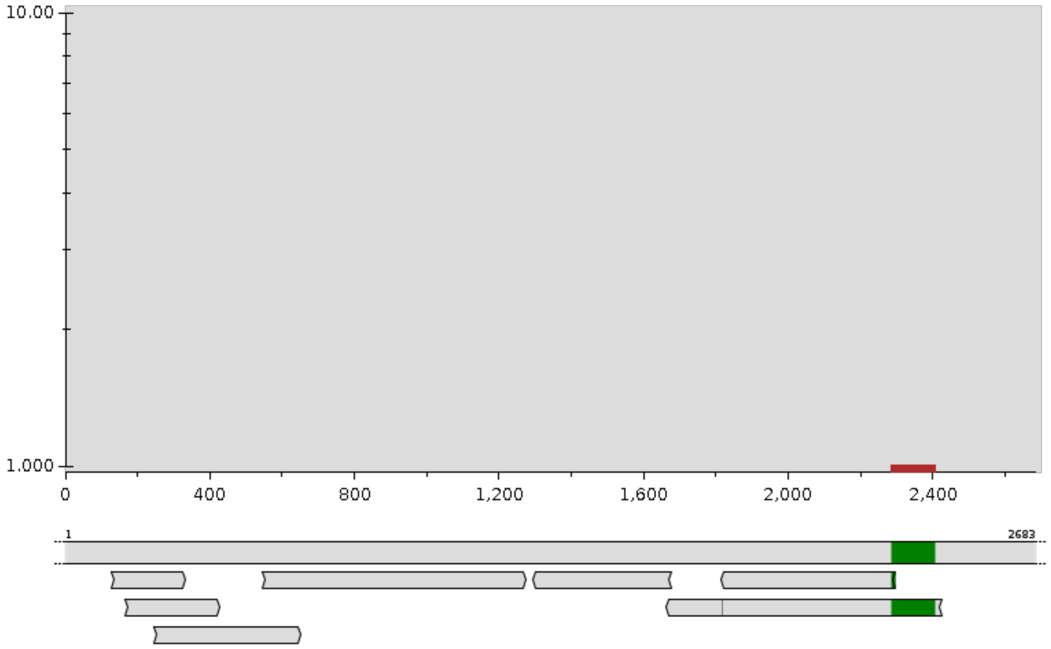

Assignment

**Type**	Euphorbia caput-medusae latent virus (Taxonomy ID: 1853865)
**Reference Genome**	NC_030238.1
**NT Identity (%)**	64.2276
**AA Identity (%)**	66.2791
**Number Of Stop Codons**	0
**Number Of CDS**	7

Alignment

**Alignment Score**	70.0 (NT) + 438.0 (AA) = 508.0
**Concordance (%)**	59.4152

**Alignment Method**	Global, seeded, nucleotide + amino acids (AGA)

Genome Region

Sequence starts at position 2286 and ends at position 2408 relative to NC_030238.1 reference sequence.

Alignment Detailed Statistics

	Begin	End	Coverage	Score	Concordance	Matches	Identities	I/D/M/F*	Stop Codons
**NT**	**2286**	**2408**	**4.6%**	**70**	**28.5%**	**123 (100%)**	**79 (64.2%)**	**0/0**	
Mutations:	2286T>A, 2289C>T, 2293G>A, 2294C>T, 2298T>A, 2299G>T, 2307A>G, 2308A>T, 2312T>C, 2313A>T, 2315T>G, 2318A>G, 2319T>A, 2321G>T, 2324G>T, 2325A>T, 2327A>T, 2328T>G, 2330A>G, 2331A>G, 2332T>A, 2334A>G, 2337A>T, 2340T>G, 2342A>C, 2343A>G, 2346A>T, 2349A>T, 2351G>C, 2352A>G, 2354G>T, 2355A>T, 2364T>C, 2367T>G, 2376C>G, 2382A>T, 2384T>G, 2385A>T, 2388C>T, 2391A>T, 2397T>A, 2399A>G, 2400G>T, 2402G>T								
**CDS**									
A9K86_gp5	8	48	12.3%	218	73.6%	41 (100%)	28 (68.3%)	0/0/0/0	0
Protein mutations:	I16L (2382A>T 2384T>G), L26I (2352A>G 2354G>T), P27A (2349A>T 2351G>C), L30V (2340T>G 2342A>C), Y33F (2331A>G 2332T>A), Y35K (2325A>T 2327A>T), Q36K (2324G>T), L37I (2319T>A 2321G>T), N39Q (2313A>T 2315T>G), N40D (2312T>C), F41Y (2307A>G 2308A>T), T44N (2298T>A 2299G>T), A46I (2293G>A 2294C>T)								
Codon mutations:	CGC10AGA (2400G>T 2402G>T), TTA11CTT (2397T>A 2399A>G), GGT13GGA (2391A>T), AAG14AAA (2388C>T), TCT15TCA (2385A>T), ATT16CTA (2382A>T 2384T>G), CTG18CTC (2376C>G), CCA21CCC (2367T>G), AAA22AAG (2364T>C), CTT25CTA (2355A>T), CTT26ATC (2352A>G 2354G>T), CCT27GCA (2349A>T 2351G>C), ATT28ATA (2346A>T), TTT29TTC (2343A>G), TTA30GTC (2340T>G 2342A>C), ATT31ATA (2337A>T), GAT32GAC (2334A>G), TAT33TTC (2331A>G 2332T>A), TTA34CTC (2328T>G 2330A>G), TAT35AAA (2325A>T 2327A>T), CAA36AAA (2324G>T), CTA37ATT (2319T>A 2321G>T), TTA38CTA (2318A>G), AAT39CAA (2313A>T 2315T>G), AAT40GAT (2312T>C), TTT41TAC (2307A>G 2308A>T), ACA44AAT (2298T>A 2299G>T), GCT46ATT (2293G>A 2294C>T), AGG47AGA (2289C>T), GTA48GTT (2286T>A)								
A9K86_gp6	8	48	16.1%	218	73.6%	41 (100%)	28 (68.3%)	0/0/0/0	0
Protein mutations:	I16L (2382A>T 2384T>G), L26I (2352A>G 2354G>T), P27A (2349A>T 2351G>C), L30V (2340T>G 2342A>C), Y33F (2331A>G 2332T>A), Y35K (2325A>T 2327A>T), Q36K (2324G>T), L37I (2319T>A 2321G>T), N39Q (2313A>T 2315T>G), N40D (2312T>C), F41Y (2307A>G 2308A>T), T44N (2298T>A 2299G>T), A46I (2293G>A 2294C>T)								
Codon mutations:	CGC10AGA (2400G>T 2402G>T), TTA11CTT (2397T>A 2399A>G), GGT13GGA (2391A>T), AAG14AAA (2388C>T), TCT15TCA (2385A>T), ATT16CTA (2382A>T 2384T>G), CTG18CTC (2376C>G), CCA21CCC (2367T>G), AAA22AAG (2364T>C), CTT25CTA (2355A>T), CTT26ATC (2352A>G 2354G>T), CCT27GCA (2349A>T 2351G>C), ATT28ATA (2346A>T), TTT29TTC (2343A>G), TTA30GTC (2340T>G 2342A>C), ATT31ATA (2337A>T), GAT32GAC (2334A>G), TAT33TTC (2331A>G 2332T>A), TTA34CTC (2328T>G 2330A>G), TAT35AAA (2325A>T 2327A>T), CAA36AAA (2324G>T), CTA37ATT (2319T>A 2321G>T), TTA38CTA (2318A>G), AAT39CAA (2313A>T 2315T>G), AAT40GAT (2312T>C), TTT41TAC (2307A>G 2308A>T), ACA44AAT (2298T>A 2299G>T), GCT46ATT (2293G>A 2294C>T), AGG47AGA (2289C>T), GTA48GTT (2286T>A)								
A9K86_gp7	1	4	2.5%	2	7.4%	4 (100%)	1 (25.0%)	0/0/0/0	0
Protein mutations:	M1I (2294C>T), G3E (2289C>T)								
Codon mutations:	ATG1ATA (2294C>T), CTA2TTA (2293G>A), GGG3GAG (2289C>T), TAT4TT. (2286T>A)								
**Proteins**									
replication-associated protein (YP_009255247.1)	8	48	12.3%	218	73.6%	41 (100%)	28 (68.3%)	0/0/0/0	0
Protein mutations:	I16L (2382A>T 2384T>G), L26I (2352A>G 2354G>T), P27A (2349A>T 2351G>C), L30V (2340T>G 2342A>C), Y33F (2331A>G 2332T>A), Y35K (2325A>T 2327A>T), Q36K (2324G>T), L37I (2319T>A 2321G>T), N39Q (2313A>T 2315T>G), N40D (2312T>C), F41Y (2307A>G 2308A>T), T44N (2298T>A 2299G>T), A46I (2293G>A 2294C>T)								
Codon mutations:	CGC10AGA (2400G>T 2402G>T), TTA11CTT (2397T>A 2399A>G), GGT13GGA (2391A>T), AAG14AAA (2388C>T), TCT15TCA (2385A>T), ATT16CTA (2382A>T 2384T>G), CTG18CTC (2376C>G), CCA21CCC (2367T>G), AAA22AAG (2364T>C), CTT25CTA (2355A>T), CTT26ATC (2352A>G 2354G>T), CCT27GCA (2349A>T 2351G>C), ATT28ATA (2346A>T), TTT29TTC (2343A>G), TTA30GTC (2340T>G 2342A>C), ATT31ATA (2337A>T), GAT32GAC (2334A>G), TAT33TTC (2331A>G 2332T>A), TTA34CTC (2328T>G 2330A>G), TAT35AAA (2325A>T 2327A>T), CAA36AAA (2324G>T), CTA37ATT (2319T>A 2321G>T), TTA38CTA (2318A>G), AAT39CAA (2313A>T 2315T>G), AAT40GAT (2312T>C), TTT41TAC (2307A>G 2308A>T), ACA44AAT (2298T>A 2299G>T), GCT46ATT (2293G>A 2294C>T), AGG47AGA (2289C>T), GTA48GTT (2286T>A)								
RepA (YP_009255248.1)	8	48	16.1%	218	73.6%	41 (100%)	28 (68.3%)	0/0/0/0	0
Protein mutations:	I16L (2382A>T 2384T>G), L26I (2352A>G 2354G>T), P27A (2349A>T 2351G>C), L30V (2340T>G 2342A>C), Y33F (2331A>G 2332T>A), Y35K (2325A>T 2327A>T), Q36K (2324G>T), L37I (2319T>A 2321G>T), N39Q (2313A>T 2315T>G), N40D (2312T>C), F41Y (2307A>G 2308A>T), T44N (2298T>A 2299G>T), A46I (2293G>A 2294C>T)								
Codon mutations:	CGC10AGA (2400G>T 2402G>T), TTA11CTT (2397T>A 2399A>G), GGT13GGA (2391A>T), AAG14AAA (2388C>T), TCT15TCA (2385A>T), ATT16CTA (2382A>T 2384T>G), CTG18CTC (2376C>G), CCA21CCC (2367T>G), AAA22AAG (2364T>C), CTT25CTA (2355A>T), CTT26ATC (2352A>G 2354G>T), CCT27GCA (2349A>T 2351G>C), ATT28ATA (2346A>T), TTT29TTC (2343A>G), TTA30GTC (2340T>G 2342A>C), ATT31ATA (2337A>T), GAT32GAC (2334A>G), TAT33TTC (2331A>G 2332T>A), TTA34CTC (2328T>G 2330A>G), TAT35AAA (2325A>T 2327A>T), CAA36AAA (2324G>T), CTA37ATT (2319T>A 2321G>T), TTA38CTA (2318A>G), AAT39CAA (2313A>T 2315T>G), AAT40GAT (2312T>C), TTT41TAC (2307A>G 2308A>T), ACA44AAT (2298T>A 2299G>T), GCT46ATT (2293G>A 2294C>T), AGG47AGA (2289C>T), GTA48GTT (2286T>A)								
C3 (YP_009255249.1)	1	4	2.5%	2	7.4%	4 (100%)	1 (25.0%)	0/0/0/0	0
Protein mutations:	M1I (2294C>T), G3E (2289C>T)								
Codon mutations:	ATG1ATA (2294C>T), CTA2TTA (2293G>A), GGG3GAG (2289C>T), TAT4TT. (2286T>A)								

*: Inserts / Deletes / Misaligned / Frameshifts

Analysis details

This analysis was performed with panviral2.64

NGS Details (UN59): Escherichia virus DE3

Assembly

**Coverage Length**	135 (1 contig(s))
**Depth Of Coverage**	1.0
**Number Of Reads**	1
**Reads Per Million**	0.02 rpm (after QC)
**Ambiguities**	0
**Assembly Method**	de novo + reference guided assembly
**Consensus Caller**	Bcf Tools

Coverage Map

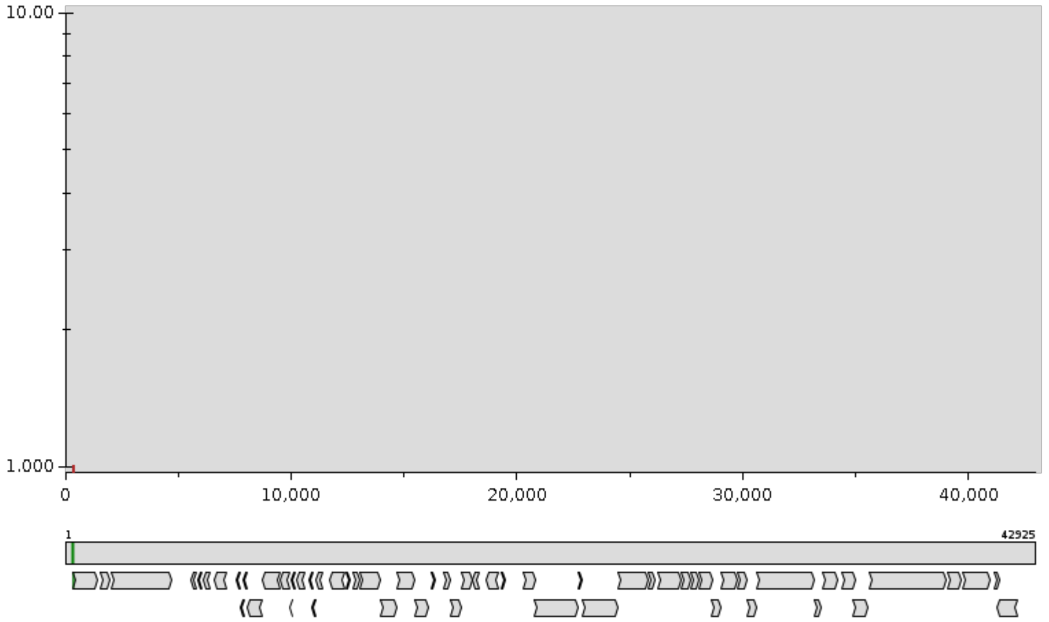

Assignment

**Type**	Escherichia virus DE3 (Taxonomy ID: 2169967)
**Reference Genome**	NC_042057.1
**NT Identity (%)**	100.0
**AA Identity (%)**	96.2963
**Number Of Stop Codons**	0
**Number Of CDS**	57

Alignment

**Alignment Score**	270.0 (NT) + 162.0 (AA) = 432.0
**Concordance (%)**	100.0

Alignment Method	Local, heuristic, nucleotide (BLASTN)

Genome Region

Sequence starts at position 285 and ends at position 419 relative to NC_042057.1 reference sequence.

Alignment Detailed Statistics

	Begin	End	Coverage	Score	Concordance	Matches	Identities	I/D/M/F *	Stop Codons
**NT**	**285**	**419**	**0.3%**	**270**	**100%**	**135 (100%)**	**135 (100%)**	**0/0**	
Mutations:	*none*								
**CDS**									
lacI	1	27	7.5%	162	97.6%	27 (100%)	26 (96.3%)	0/0/0/0	0
**Proteins**									
DNA-binding transcriptional repressor LacI (YP_009617199.1)	1	27	7.5%	162	97.6%	27 (100%)	26 (96.3%)	0/0/0/0	0
Protein mutations:	*none*								
Codon mutations:	*none*								

*: Inserts / Deletes / Misaligned / Frameshifts

Analysis details

This analysis was performed with panviral2.64
