# Supplementary material for: Nucleotide sequence analysis reveals the presence of PVY-Tam isolates affecting tamarillo in Colombia
Source: Virol J. 2026 Apr 20;23:145. doi: 10.1186/s12985-026-03166-6 (PMC13234967; doi:10.1186/s12985-026-03166-6)
Supplement: Supplementary file 6 — Additional file 6. [file 12985_2026_3166_MOESM6_ESM.pdf]

Analysis of UN60

**Technology**	Paired-end short reads
**Input Files**	UN60_R1.fq.gz (1.51 GB), UN60_R2.fq.gz (1.55 GB)
**Submitted On**	2023-09-26 13:17:18 UTC
**Duration**	3h 13m 35s
**Tool Version**	panviral2.64
**Location**	https://www.genomedetective.com/db/ui/analysis/0a18e193-0210-4915-bede-4b932408d4fe

Statistics

**Original Read Length**	20 - 150
**Trimmed Read Length**	50 - 135

	**# Reads**	**% of Reads**
**Input file**	47463978	100.0%
**After QC**	47148532	99.3%
**After filtering**	5923550	12.5%
**Mapped back to contigs**	3180271	6.7%

Assignments

**Assignment**	**No. of Reads**	**Depth of Coverage**	**Identity**		**Genome Coverage**	
			**NT**	**AA**		
Potato virus Y	1048341	14480.8	82.7%	90.0%	99.8%	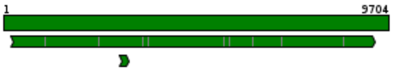
Torradovirus lycopersici (2 segments out of 2)	1041429	10584.1	88.8%	94.7%	99.3%	
Torradovirus lycopersici (segment RNA 2)	685969	16080.2	85.3%	89.6%	99.6%	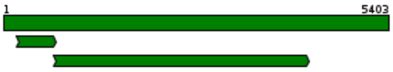
Torradovirus lycopersici (segment RNA 1)	355460	6761.0	91.2%	98.0%	99.1%	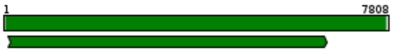
Tomato chocolate spot virus (2 segments out of 2)	367028	13358.9	67.5%	69.9%	28.2%	
Tomato chocolate spot virus (segment RNA2)	239197	15922.8	67.3%	70.6%	33.1%	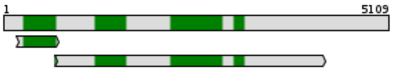
Tomato chocolate spot virus (segment RNA 1)	127831	11026.4	67.7%	68.8%	24.8%	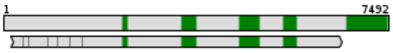
Torradovirus marchitezum (2 segments out of 2)	348393	5946.0	64.9%	66.8%	61.4%	

Assignment	No. of Reads	Depth of Coverage	Identity		Genome Coverage	
			NT	AA		
Torradovirus marchitezum (segment RNA 2)	209020	18973.4	67.3%	67.5%	27.8%	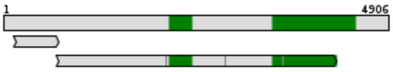
Torradovirus marchitezum (segment RNA 1)	139373	3023.2	64.3%	66.7%	84.2%	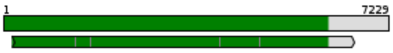
Tomato necrotic dwarf virus (segment RNA1)	244103	5251.7	64.2%	65.6%	85.3%	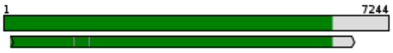
Diachasmimorpha longicaudata entomopoxvirus (segment NC_043455.1)	7189	803.2	60.5%	55.2%	83.9%	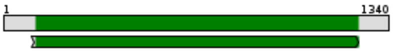
Bracoviriform glomeratae (segment NC_043292.1)	6143	2381.0	72.0%	83.5%	73.0%	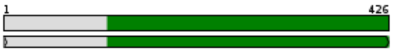
Potato leafroll virus	5542	126.6	97.5%	96.5%	97.3%	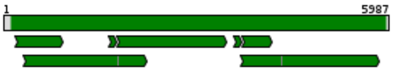
Duamitovirus soch1	351	22.1	67.5%	68.2%	70.6%	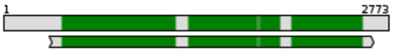
Solendovirus venanicotianae	130	11.4	77.9%	75.6%	17.7%	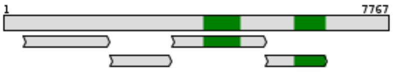
Macrosiphum euphorbiae virus 1	106	2.6	92.8%	96.5%	24.3%	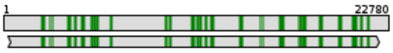
Duamitovirus peex1	26	4.4	68.7%	67.1%	27.0%	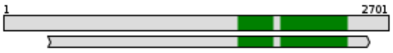

Discoveries

Similar to	No. of Reads	Depth of Coverage	Identity		Genome Coverage	
			NT	AA		
Brazilian marseillevirus	20377	8722.3	82.6%	93.1%	0.1%	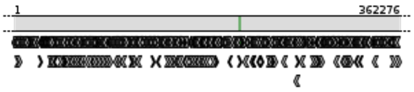
Brazilian marseillevirus	18320	8619.2	79.5%	95.6%	0.1%	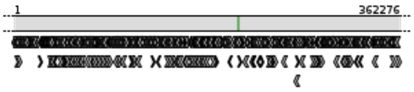
Brazilian marseillevirus	12747	5214.8	79.2%	94.7%	0.1%	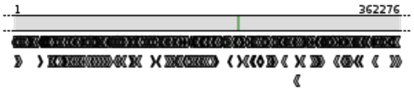
Brazilian marseillevirus	11487	6274.7	81.4%	92.6%	0.0%	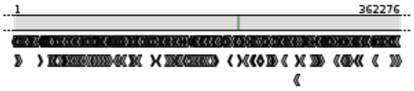
Makelovirus prm1	10355	1317.0	76.8%	87.5%	0.7%	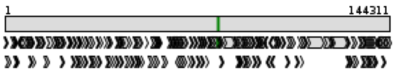

Similar to	No. of Reads	Depth of Coverage	Identity		Genome Coverage	
			NT	AA		
Brazilian marseillevirus	9741	4288.3	82.0%	97.1%	0.0%	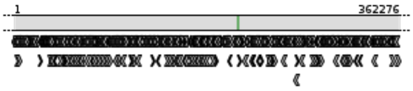
Yellowstone lake phycodnavirus 1	5848	3350.0	82.1%	93.1%	0.1%	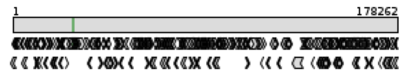
Brazilian marseillevirus	5218	2083.3	79.6%	94.7%	0.1%	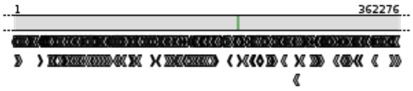
Yellowstone lake phycodnavirus 1	5137	2919.0	80.0%	90.4%	0.1%	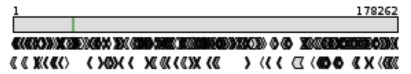
Brazilian marseillevirus	2553	1024.8	80.9%	94.7%	0.1%	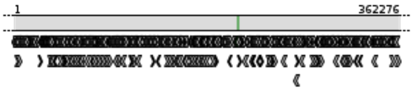
Noumeavirus	2495	1143.9	78.7%	95.9%	0.1%	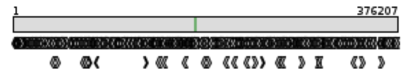
Betabaculovirus disaccharalis	2005	1183.5	82.0%	93.0%	0.1%	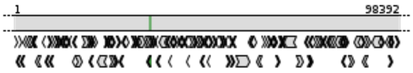
Betabaculovirus disaccharalis	1706	1065.4	84.7%	92.0%	0.2%	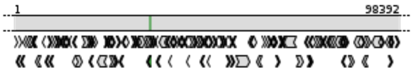
Cladosporium fulvum T-1 virus	605	46.5	52.2%	47.5%	20.9%	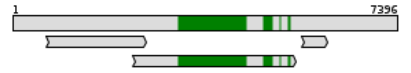
Vellamovirus rhodeisland44	604	154.1	75.0%	86.8%	0.2%	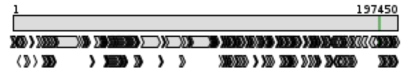
Cassava brown streak virus	356	82.0	60.3%	57.2%	5.8%	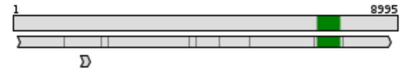
Lowelvirus tuscon4d	355	188.4	80.9%	90.6%	0.1%	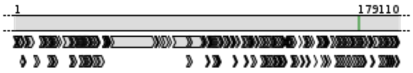
Badnavirus occultipomeae	353	112.5	51.7%	45.7%	4.3%	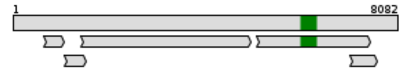
Errantivirus	227	29.2	55.2%	43.5%	8.5%	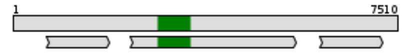
Ichnoviriform fugitivi (4 segments out of 56)	173	18.4	57.0%	47.2%	32.8%	
Ichnoviriform fugitivi (segment B17)	173	18.4	57.0%	47.2%	32.8%	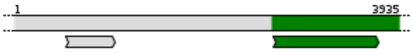
Ichnoviriform fugitivi (segment B17)	20	8.2	58.3%	47.9%	7.4%	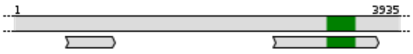

Similar to	No. of Reads	Depth of Coverage	Identity		Genome Coverage	
			NT	AA		
Ichnoviriform fugitivi (segment B17)	13	5.5	57.2%	54.3%	7.3%	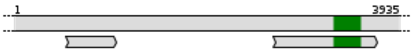
Ichnoviriform fugitivi (segment B17)	1	1.0	64.2%	62.2%	3.4%	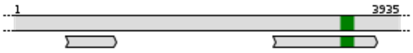
Badnavirus maculasmallanthi	110	43.1	56.5%	55.2%	3.4%	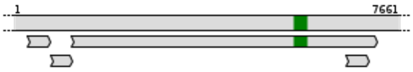
Caulimovirus venafragariae	77	9.9	56.3%	52.3%	13.2%	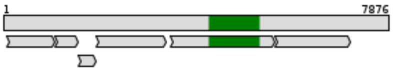
Caulimovirus tesselloscrophulariae	48	14.5	53.6%	37.9%	4.8%	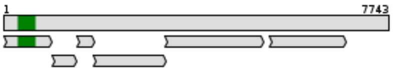
Brazilian marseillevirus	47	25.7	81.2%	95.7%	0.0%	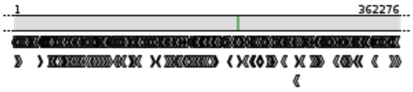
Errantivirus	46	16.8	53.0%	41.0%	4.0%	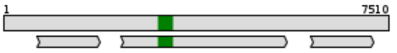
Caulimovirus venafragariae	45	6.3	58.5%	54.0%	10.8%	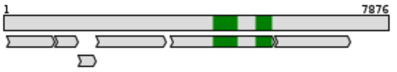
Badnavirus rutilanscamelliae	44	15.0	54.0%	45.0%	3.6%	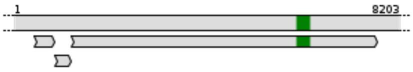
Synechococcus phage S-SRM01	43	25.2	76.8%	88.4%	0.1%	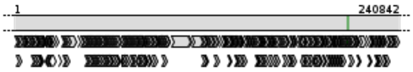
Marseillevirus marseillevirus	38	25.6	81.4%	94.7%	0.0%	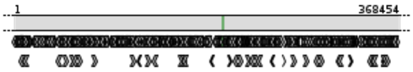
Petuvirus venapetuniae	36	5.1	58.6%	53.7%	13.0%	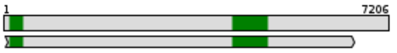
Cavemovirus venamanihotis	33	6.4	59.7%	48.3%	7.6%	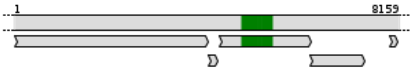
Cavemovirus collusipomeae	32	7.0	69.0%	57.8%	6.4%	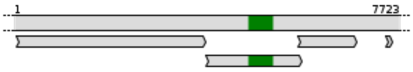
Dioscovirus dioscoreae	26	3.7	58.5%	48.6%	11.5%	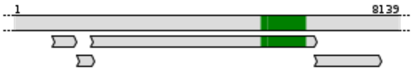
Rahariannevirus raharianne	26	4.0	79.5%	81.2%	1.9%	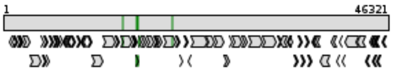
Badnavirus maculasmallanthi	21	9.4	56.5%	51.6%	3.5%	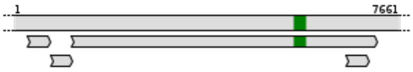

Similar to	No. of Reads	Depth of Coverage	Identity		Genome Coverage	
			NT	AA		
Blunervirus vaccinii (segment RNA 2)	14	6.4	61.7%	61.7%	4.6%	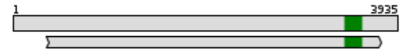
Alfalfa leaf curl virus	14	6.0	71.5%	58.2%	9.8%	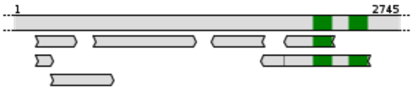
Duamitovirus peex1	13	4.7	70.8%	73.6%	10.1%	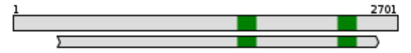
Pseudomonas phage YMC11/07/P54_PAE_BP	13	4.8	78.4%	83.3%	0.7%	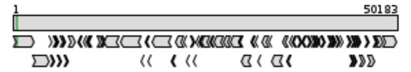
Pinus nigra virus 1	11	3.1	56.8%	45.9%	6.7%	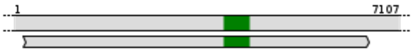
Badnavirus rutilanscamelliae	10	3.9	61.5%	56.2%	3.2%	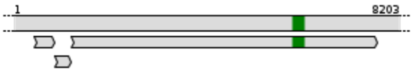
Duamitovirus dapi1	8	6.5	68.9%	66.7%	4.8%	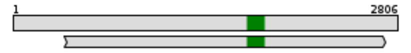
Alphabaculovirus lyxylinae	8	6.5	80.0%	83.8%	0.1%	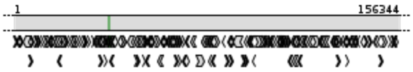
Caulimovirus tessellobrassicae	6	2.6	62.7%	52.6%	3.2%	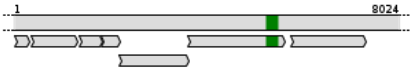
Badnavirus occultipomeae	6	3.2	54.3%	43.5%	3.1%	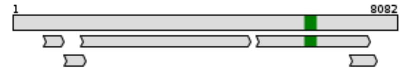
Moloney murine leukemia virus	6	3.0	75.6%	100.0%	3.2%	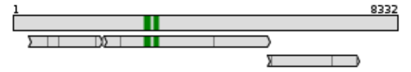
Marseillevirus marseillevirus	6	4.4	81.9%	88.6%	0.0%	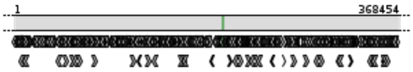
Yaba monkey tumor virus	4	3.3	81.3%	84.1%	0.1%	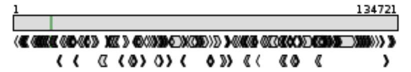
Alphabaculovirus agipsilonis	4	3.5	78.5%	77.5%	0.1%	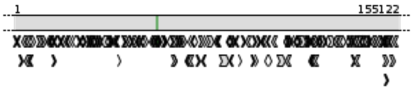
Badnavirus tessellocastaneae	4	2.1	59.4%	53.7%	3.4%	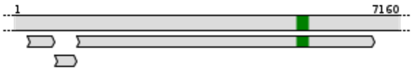
Escherichia virus DE3	4	2.5	100.0%	98.6%	0.5%	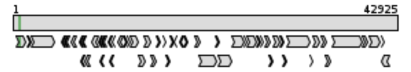
Epiphyllum badnavirus 1	4	2.0	54.8%	50.0%	3.4%	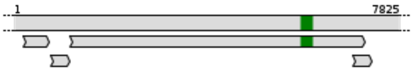

Similar to	No. of Reads	Depth of Coverage	Identity		Genome Coverage	
			NT	AA		
Goose dicistrovirus	4	2.2	85.0%	95.0%	2.6%	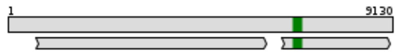
Melanoplus sanguinipes entomopoxvirus	2	1.4	81.7%	76.6%	0.1%	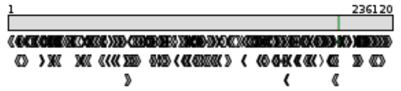
Cotia virus	2	1.8	80.0%	93.3%	0.1%	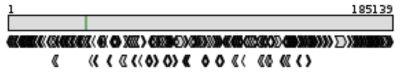
Scottvirus scott	2	1.1	82.0%	92.6%	0.6%	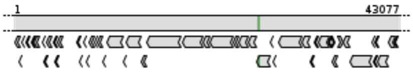
Punavirus P1	2	1.2	100.0%	98.3%	0.2%	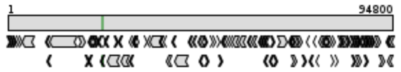
Harvey murine sarcoma virus	2	1.2	69.5%	85.1%	14.1%	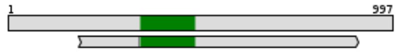
Ochrobactrum phage POA1180	2	2.0	74.8%	80.0%	0.3%	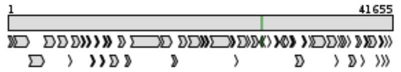
Pseudomonas phage PA8	2	2.0	83.0%	80.0%	0.2%	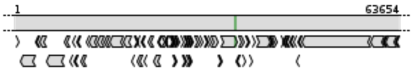
Tomato associated geminivirus 1	2	1.2	73.0%	75.3%	8.9%	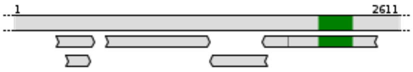
Euphorbia caput-medusae latent virus	1	1.0	47.6%	45.4%	5.5%	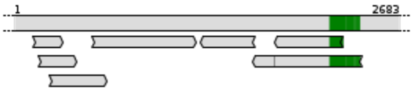

NGS Details (UN60): Potato virus Y

Assembly

**Coverage Length**	9683 (1 contig(s))
**Depth Of Coverage**	14480.8
**Number Of Reads**	1048341
**Reads Per Million**	22234.86 rpm (after QC)
**Ambiguities**	0
**Assembly Method**	de novo + reference guided assembly
**Consensus Caller**	Bcf Tools

Coverage Map

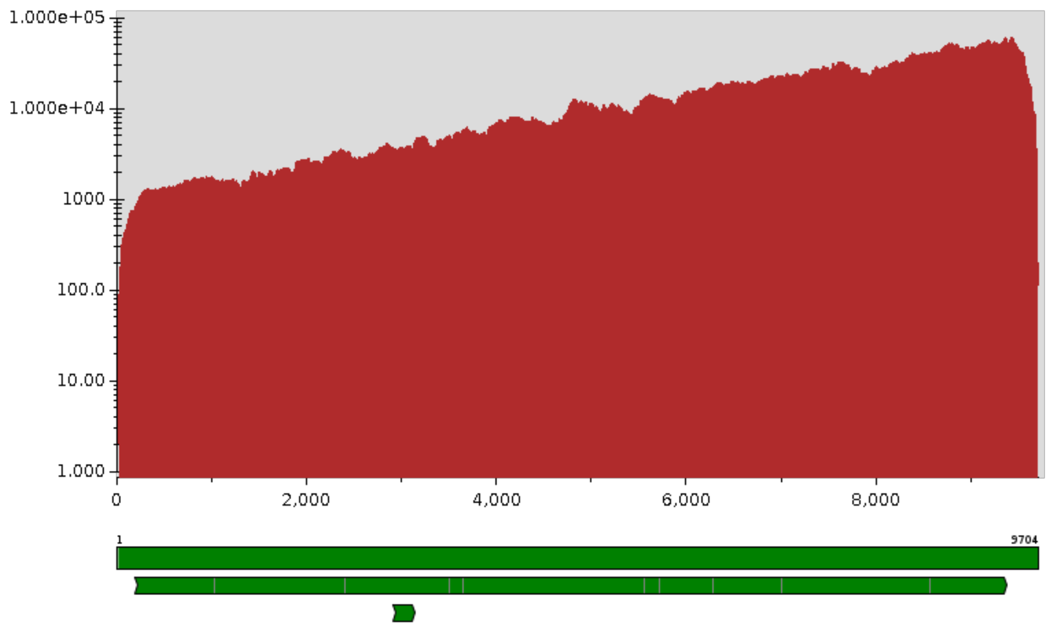

Assignment

**Type**	Potato virus Y (Taxonomy ID: 12216)
**Reference Genome**	NC_001616.1
**NT Identity (%)**	82.6671
**AA Identity (%)**	89.9682
**Number Of Stop Codons**	3
**Number Of CDS**	2

Alignment

**Alignment Score**	12522.0 (NT) + 19018.0 (AA) = 31540.0
**Concordance (%)**	78.1255

Alignment Method	Global, seeded, nucleotide + amino acids (AGA)

Genome Region

Sequence starts at position 22 and ends at position 9704 relative to NC_001616.1 reference sequence.

Alignment Detailed Statistics

	Begin	End	Coverage	Score	Concordance	Matches	Identities	I/D/M/F*	Stop Codons
**NT**	**22**	**9704**	**99.8%**	**12522**	**65.0%**	**9671 (99.8%)**	**8003 (82.6%)**	**10/12**	
Mutations:	31A>C, 32A>G, 36C>A, 37G>A, 38C>G, 42A>T, 44C>T, 50T>C, 52A>G, 54C>A, 56C>T, 58C>T, 59A>T, 60_61insAAA, 75C>T, 79C>T, 83G>A, 85T>C, 87C>T, 89G>A, 90T>C, 93A>T, 94A>G, 95A>T, 100T>C, 101T>A, 102C>T, 104T>C, 107C>T, 110T>G, 112C>T, 114C>T, 115T>C, 116A>T, 117G>C, 119A>G, 120C>T, 121A>T, 125T>G, 126T>C, 127G>A, 128G>T, 131A>G, 132C>T, 139A>T, 139_140insG, 141C>T, 143C>T, 146C>T, 150C>T, 150_151insT, 154T>C, 156C>A, 158T>A, 160A>C, 164C>T, 168C>A, 169C>G, 171A>C, 172T>C, 173T>A, 174T>G, 177G>C, 178A>G, 179T>C, 180C>T, 181C>A, 182T>G, 183C>T, 193T>C, 194T>C, 196C>A, 202A>G, 208C>T, 209T>C, 210G>T, 211T>G, 220G>A, 221T>A, 223T>G, 226A>G, 235A>T, 241C>T, 247A>T, 251T>C, 255G>T, 256C>T, 258A>G, 262T>C, 263A>G, 265T>G, 267T>C, 273A>T, 277A>T, 280A>G, 283G>T, 286G>A, 287G>A, 289T>C, 293G>A, 294T>C, 301T>C, 304C>T, 305G>A, 306C>T, 309A>G, 311C>T, 316A>T, 317A>G, 318C>T, 319A>G, 322A>G, 331A>T, 339T>A, 340C>A, 342A>G, 347A>G, 348A>C, 349A>G, 350T>C, 355T>A, 356A>G, 357C>T, 364T>C, 365G>A, 366T>C, 367G>A, 368C>T, 369T>C, 373A>G, 376C>T, 384T>G, 388G>A, 391C>T, 394A>G, 400G>A, 406T>C, 408C>T, 409C>T, 412G>A, 415A>T, 416A>G, 417T>A, 424T>C, 427G>A, 433A>G, 436G>C, 437G>A, 440A>G, 441G>A, 443A>C, 444A>G, 445G>A, 448T>A, 454A>G, 456A>C, 460T>C, 461C>A, 469A>G, 475C>G, 481T>A, 502T>C, 505A>T, 511C>T, 517T>A, 520T>A, 523A>T, 531C>T, 535G>A, 536C>T, 539C>T, 540A>G, 541A>G, 549G>A, 550A>G, 556C>T, 559T>C, 562T>C, 568T>C, 571A>G, 576T>C, 580T>C, 585T>C, 586C>G, 593C>T, 594G>A, 595C>T, 597C>G, 599A>G, 601A>G, 602A>C, 603T>C, 604A>G, 613A>G, 616A>G, 619C>G, 622G>A, 625G>A, 628T>C, 631C>T, 635A>G, 637T>C, 644A>G, 646A>G, 655T>C, 661G>A, 663A>C, 664G>C, 667A>G, 679C>T, 682C>A, 683T>C, 685A>G, 691T>C, 699C>G, 703T>A, 706T>A, 719A>G, 721G>A, 722A>G, 724A>G, 725C>T, 727T>G, 730T>A, 731G>T, 734T>C, 735A>C, 736C>T, 737T>C, 738C>G, 739C>T, 744C>T, 745G>C, 749C>T, 751A>C, 754T>C, 765T>A, 770T>C, 773C>A, 774G>A, 775A>G, 776C>A, 790C>T, 801T>A, 813G>C, 814A>G, 816T>A, 818T>C, 823A>G, 825G>A, 826T>A, 833C>A, 835G>A, 841C>T, 851A>G, 853T>C, 859C>T, 860C>T, 862C>T, 863A>G, 865T>C, 867T>C, 869A>G, 871C>T, 872A>T, 874A>G, 876G>A, 879G>A, 886T>C, 898C>T, 903A>G, 906C>A, 907A>C, 909A>C, 910A>C, 912G>A, 913C>T, 922C>A, 931T>A, 938T>C, 939C>T, 940A>T, 943A>G, 952C>T, 967A>G, 973A>G, 976G>C, 980T>A, 982G>C, 991A>G, 994T>C, 999G>A, 1003T>C, 1007C>A, 1008A>G, 1009G>A, 1010A>G, 1012T>G, 1013A>G, 1018A>G, 1019A>C, 1021C>G, 1030C>T, 1039G>A, 1042T>C, 1045C>A, 1048C>A, 1051T>C, 1060G>A, 1063T>A, 1064C>T, 1069C>T, 1083G>A, 1093T>C, 1097T>G, 1099G>A, 1105C>T, 1114A>G, 1117T>G, 1123A>G, 1126T>A, 1129C>T, 1143G>A, 1147A>T, 1150T>A, 1154T>A, 1156G>A, 1165C>T, 1168T>C, 1172C>T, 1174T>A, 1201C>T, 1221G>A, 1229G>A, 1230T>C, 1231T>C, 1233G>A, 1234C>T, 1240G>A, 1246G>A, 1250T>C, 1252G>A, 1255T>C, 1257A>C, 1258A>G, 1267A>G, 1273T>C, 1276G>A, 1278A>G, 1283T>C, 1290C>T, 1291G>A, 1298G>A, 1299A>C, 1300C>T, 1306T>C, 1307A>G, 1309A>G, 1312T>C, 1316A>G, 1318T>A, 1327G>A, 1329T>C, 1333G>T, 1336A>G, 1343C>T, 1345A>G, 1354G>A, 1357G>T, 1360C>T, 1367C>T, 1369C>G, 1373C>A, 1378C>T, 1384G>A, 1385A>G, 1393A>G, 1396C>T, 1405G>T, 1408A>G, 1411G>A, 1415G>T, 1417A>T, 1420G>C, 1429T>C, 1432A>G, 1436G>A, 1438C>T, 1439T>C, 1441A>G, 1444T>C, 1453C>T, 1456G>A, 1468A>G, 1492A>G, 1499T>C, 1501G>A, 1504T>C, 1507G>A, 1510C>T, 1513A>G, 1516A>G, 1519A>T, 1522G>A, 1525C>T, 1528G>A, 1534C>T, 1540T>A, 1546C>T, 1555A>G, 1558T>C, 1561T>C, 1564A>C, 1567T>A, 1573C>T, 1576A>G, 1583T>C, 1588T>A, 1594G>A, 1600C>T, 1609G>A, 1618G>A, 1621C>T, 1624C>T, 1631C>T, 1639A>G, 1651C>T, 1654C>G, 1675T>C, 1693A>G, 1702T>C, 1707A>G, 1718G>A, 1726A>G, 1729C>T, 1732A>C, 1738T>C, 1742A>G, 1744C>T, 1747C>T, 1753T>C, 1758G>A, 1768G>A, 1786C>T, 1789A>G, 1790G>A, 1795C>T, 1805T>C, 1807A>G, 1813G>A, 1819G>A, 1825G>A, 1837C>T, 1840T>C, 1849A>G, 1858C>G, 1864A>G, 1870C>T, 1876T>C, 1882A>G, 1888T>A, 1891T>C, 1906T>C, 1915A>T, 1930T>C, 1936C>T, 1937A>G, 1939T>C, 1945A>G, 1951C>T, 1960A>T, 1978A>G, 1981T>A, 1984C>T, 1987T>C, 1996C>T, 2004T>A, 2020A>G, 2023G>A, 2026T>C, 2029G>T, 2032G>A, 2059T>C, 2068T>G, 2071C>T, 2072G>A, 2074G>A, 2087C>T, 2089G>A, 2101C>T, 2104G>A, 2107G>A, 2116G>A, 2122C>T, 2137C>T, 2158T>C, 2164C>T, 2170A>G, 2182T>C, 2188G>A, 2191C>A, 2208G>A, 2218T>C, 2224C>T, 2227A>T, 2242G>A, 2245C>T, 2254G>A, 2257T>C, 2260C>T, 2266C>G, 2272A>G, 2275G>A, 2293A>G, 2299C>T, 2302G>A, 2305G>A, 2314A>T, 2317T>C, 2326A>G, 2341G>A, 2347A>G, 2350T>C, 2356G>A, 2365T>C, 2371A>G, 2374A>G, 2383T>C, 2386A>T, 2389A>G, 2392T>C, 2395T>C, 2414A>G, 2415A>G, 2416T>G, 2419T>A, 2420A>T, 2440A>T, 2467T>C, 2476T>C, 2482G>A, 2491G>A, 2497G>T, 2503G>A, 2518A>G, 2521T>C, 2523A>G, 2533A>G, 2539G>A, 2545A>G, 2551G>A, 2554T>A, 2558C>T, 2561T>C, 2563G>C, 2572A>G, 2584T>C, 2587C>T, 2593G>C, 2599T>C, 2608T>C, 2620T>C, 2629G>A, 2632G>A, 2634G>A, 2638G>A, 2644T>A, 2653A>G, 2665T>C, 2678C>T, 2683A>G, 2689T>C, 2698A>C, 2707A>G, 2710G>A, 2716A>G, 2719A>G, 2722C>T, 2723G>A, 2725C>T, 2728A>G, 2734G>A, 2737T>A, 2746T>C, 2747G>A, 2749T>A, 2752A>T, 2755T>A, 2761C>T, 2764C>T, 2779T>C, 2785G>T, 2791C>T, 2795C>A, 2806C>T, 2816T>C, 2818G>C, 2822G>A, 2824G>A, 2827G>A, 2836T>C, 2839G>A, 2842T>C, 2863C>A, 2864C>T, 2882C>A, 2885A>C, 2886G>A, 2893C>T, 2895C>T, 2902C>T, 2905A>G, 2911T>C, 2923T>C, 2931A>G, 2940A>G, 2947T>C, 2962T>C, 2971_2972insA, 2989T>C, 2991C>T, 2994A>G, 2999G>A, 3000A>G, 3016delC, 3021G>A, 3024A>T, 3025C>T, 3036C>T, 3039A>G, 3040A>C, 3042A>C, 3043G>A, 3052G>A, 3061A>G, 3082G>A, 3091C>T, 3099C>T, 3100C>A, 3104G>A, 3105T>G, 3106G>A, 3109C>T, 3112A>G, 3118T>C, 3121C>T, 3127A>G, 3132G>A, 3137C>A, 3138G>A, 3139A>G, 3151T>C, 3152T>A, 3154C>T, 3165G>A, 3169A>G, 3199C>T, 3203A>C, 3208T>C, 3214A>G, 3217C>T, 3218T>C, 3223C>A, 3225C>A, 3230G>A, 3235C>T, 3257T>C, 3262C>T, 3265C>T, 3266G>A, 3268A>G, 3271G>A, 3275G>A, 3284G>A, 3301G>A, 3315G>A, 3316A>G, 3318A>C, 3322C>T, 3325G>A, 3328G>A, 3337A>T, 3349G>A, 3355C>T, 3358C>T, 3359G>A, 3367A>G, 3370T>C, 3377T>C, 3379A>G, 3394A>G, 3397C>T, 3400T>G, 3406A>G, 3418C>T, 3421T>A, 3427T>C, 3433G>A, 3436A>T, 3439A>G, 3442C>T, 3445T>C, 3457G>A, 3460T>C, 3463T>A, 3469G>T, 3472G>A, 3496C>T, 3499G>A, 3505C>T, 3513T>G, 3520A>G, 3529A>G, 3532A>G, 3545A>G, 3548G>A, 3551T>C, 3554G>A, 3556C>T, 3583T>C, 3586T>C, 3589C>T, 3595C>T, 3607T>C, 3613T>C, 3616G>A, 3619C>T, 3623C>G, 3628C>T, 3632C>A, 3637T>C, 3638T>C, 3643A>G, 3661A>G, 3670G>A, 3682T>C, 3688G>A, 3697G>A, 3698A>G, 3706T>C, 3709T>C, 3713T>C, 3715G>A, 3717G>A, 3721G>A, 3731C>A, 3732G>A, 3736T>A, 3746C>T, 3748A>G, 3754A>G, 3763T>C, 3778A>G, 3781A>G, 3784C>T, 3796T>C, 3805A>T, 3811C>T, 3814A>G, 3829C>T, 3838T>C, 3844A>G, 3847A>C, 3856T>A, 3862G>T, 3871C>T, 3877C>A, 3883C>T, 3886A>G, 3892A>G, 3895C>T, 3898T>C, 3904A>G, 3905C>A, 3907G>A, 3913T>C, 3928G>A, 3931A>T, 3937G>A, 3955T>C, 3958G>A, 3961C>T, 3964A>G, 3967T>C, 3976A>G, 3979T>C, 3985A>T, 3997A>T, 4000G>A, 4006C>T, 4009T>G, 4012C>T, 4015A>G, 4024T>C, 4030A>G, 4033A>T, 4042G>A, 4052C>T, 4054G>A, 4057T>C, 4063T>A, 4066A>G, 4073A>G, 4078T>C, 4081C>T, 4084T>C, 4096C>T, 4108C>T, 4111A>T, 4117G>A, 4118C>T, 4120A>G, 4126C>T, 4128T>A, 4129C>T, 4132C>T, 4135T>C, 4147A>G, 4148T>C, 4151G>T, 4162C>T, 4166G>A, 4171A>C, 4180G>A, 4190C>T, 4198T>C, 4201C>T, 4210G>T, 4213C>T, 4219T>C, 4220C>T, 4223C>A, 4225G>A, 4231T>G, 4237T>C, 4252A>G, 4253T>C, 4255A>G, 4258A>G, 4270T>C, 4282A>G, 4285G>A, 4291A>G, 4303G>A, 4312C>T, 4319A>G, 4321A>G, 4324G>T, 4327G>A, 4336G>T, 4339T>C, 4348A>T, 4360A>G, 4366T>C, 4369T>A, 4375T>C, 4387T>C, 4395T>A, 4406G>A, 4411T>C, 4438T>C, 4444C>A, 4450C>T, 4456C>T, 4465C>T, 4475A>G, 4477G>T, 4480C>T, 4481A>T, 4483A>G, 4495C>T, 4507G>A, 4510C>T, 4513T>G, 4517C>T, 4528C>T, 4534A>G, 4540C>T, 4544G>T, 4545C>T, 4546G>A, 4548G>A, 4558T>C, 4564A>T, 4570C>T, 4576A>T, 4597G>A, 4600C>T, 4606C>T, 4609G>A, 4615G>A, 4618C>T, 4625T>C, 4627G>T, 4648C>T, 4651T>C, 4660G>A, 4663C>T, 4669T>A, 4679G>A, 4681G>C, 4684T>C, 4687T>G, 4699G>A, 4702A>G, 4708A>G, 4711G>A, 4712C>T, 4738A>G, 4744A>T, 4747A>G, 4748T>C, 4765T>C, 4796G>A, 4798C>T, 4803C>G, 4804T>C, 4807G>A, 4810G>T, 4813T>G, 4816T>C, 4831T>C, 4834C>T, 4840A>T, 4852A>G, 4855C>T, 4867T>C, 4870G>C, 4873T>A, 4882T>C, 4903A>T, 4909G>A, 4915T>C, 4918A>G, 4919T>C, 4921G>A, 4924T>C, 4927C>G, 4933T>C, 4936C>A, 4939G>A, 4942T>C, 4945C>T, 4963A>G, 4972T>C, 4990T>C, 5002A>G, 5023T>C, 5024T>C, 5029C>T, 5032T>C, 5038T>C, 5044A>T, 5053A>T, 5056G>A, 5057A>G, 5060A>G, 5071G>T, 5090C>A, 5095A>G, 5098G>T, 5100C>T, 5104A>G, 5107A>T, 5110T>C, 5113A>G, 5116G>A, 5119A>G, 5120G>T, 5122C>T, 5126A>G, 5137T>C, 5140C>T, 5143A>G, 5146G>A, 5161C>A, 5167A>G, 5173T>G, 5182G>A, 5183G>A, 5188C>T, 5191G>A, 5197A>G, 5200C>T, 5206C>T, 5209A>G, 5218C>T, 5230G>A, 5236C>T, 5239C>T, 5245C>T, 5263A>T, 5269C>T, 5272C>T, 5275C>A, 5278C>T, 5281A>C, 5284A>G, 5287T>C, 5288C>T, 5293A>T, 5294T>C, 5296G>A, 5302G>A, 5311T>A, 5314A>G, 5323A>T, 5347T>C, 5353C>T, 5365C>T, 5386T>C, 5395C>A, 5422A>G, 5425T>C, 5431C>T, 5437G>A, 5452T>C, 5458G>A, 5467T>C, 5470A>G, 5471C>T, 5473A>G, 5479A>G, 5482C>T, 5485A>C, 5488T>C, 5489T>C, 5491G>A, 5497T>C, 5506T>C, 5509G>A, 5512G>A, 5516T>C, 5530T>C, 5533G>A, 5536G>T, 5539G>A, 5548T>G, 5554C>T, 5560T>A, 5561G>A, 5563G>A, 5566G>C, 5569A>G, 5584C>G, 5593G>A, 5596G>A, 5597A>G, 5605C>A, 5617G>T, 5619C>T, 5620T>G, 5629G>A, 5630A>C, 5641C>T, 5650A>T, 5662A>T, 5665A>G, 5689A>G, 5693G>A, 5698G>A, 5713A>G, 5716G>C, 5719A>G, 5743C>A, 5755C>A, 5758T>C, 5764T>C, 5770A>G, 5779C>T, 5782T>C, 5791C>T, 5803C>T, 5836G>A, 5839A>G, 5845A>G, 5848A>G, 5853A>G, 5857T>C, 5860C>T, 5863A>T, 5869T>A, 5875T>C, 5884C>T, 5893C>T, 5894A>G, 5896C>G, 5899C>T, 5908G>A, 5911T>C, 5923G>A, 5938A>G, 5941C>T, 5944T>C, 5947T>C, 5959G>A, 5960C>G, 5961G>C, 5962G>C, 5974A>G, 5986T>A, 5989C>T, 5990A>G, 5998T>C, 6008A>C, 6010A>T, 6019A>T, 6022G>T, 6025A>T, 6030A>C, 6049C>A, 6058G>A, 6061A>G, 6064C>A, 6070T>C, 6072G>A, 6073T>C, 6074A>C, 6079G>A, 6081C>A, 6085A>T, 6091A>T, 6094C>T, 6111G>C, 6112T>C, 6118A>G, 6124G>A, 6127G>A, 6128A>G, 6130T>C, 6133T>C, 6136A>G, 6148C>T, 6157A>G, 6158G>A, 6163T>C, 6169A>G, 6170A>T, 6172A>G, 6178C>T, 6187A>G, 6190T>C, 6196G>A, 6203C>T, 6205C>T, 6208A>G, 6209C>T, 6211A>G, 6217G>A, 6229T>A, 6230G>A, 6238C>T, 6247G>A, 6253A>T, 6257G>A, 6258C>A, 6274T>C, 6282A>G, 6286G>A, 6298C>T, 6301G>A, 6304A>G, 6307C>T, 6322C>T, 6328A>T, 6331T>G, 6334T>C, 6337G>A, 6338C>T, 6358T>C, 6361G>A, 6362G>A, 6367A>T, 6370G>A, 6376C>T, 6379T>G, 6385A>C, 6391A>T, 6394A>G, 6397C>T, 6401G>A, 6405C>T, 6412C>T, 6418A>G, 6421T>C, 6429A>T, 6436T>A, 6445G>A, 6450A>G, 6454C>G, 6460C>T, 6463T>A, 6469C>T, 6472G>A, 6475G>A, 6481T>C, 6485C>A, 6487C>T, 6490T>C, 6496C>T, 6502G>A, 6514T>C, 6517G>A, 6527C>A, 6532C>A, 6541G>A, 6544A>G, 6556C>T, 6559T>C, 6565G>A, 6569T>C, 6571G>A, 6586T>A, 6588C>T, 6598A>G, 6601A>G, 6604T>C, 6607T>C, 6616A>G, 6622C>T, 6631G>A, 6634G>A, 6635T>C, 6640T>A, 6646G>T, 6647A>G, 6652C>T, 6664C>T, 6670T>G, 6677A>G, 6679A>G, 6685C>T, 6691A>T, 6694C>T, 6715A>G, 6716G>A, 6719A>G, 6724A>G, 6734C>T, 6742G>A, 6745G>A, 6754C>T, 6760A>G, 6764A>T, 6766A>G, 6775T>A, 6782C>T, 6787A>T, 6790C>T, 6795C>T, 6796A>G, 6799C>A, 6802C>A, 6808C>T, 6811C>T, 6814C>T, 6823C>T, 6829A>G, 6847C>T, 6853A>C, 6857A>G, 6862G>A, 6871A>G, 6875G>A, 6876T>C, 6877C>A, 6880G>A, 6883T>A, 6889T>G, 6901C>T, 6916C>T, 6919G>A, 6925A>G, 6928T>C, 6934C>A, 6949G>C, 6955C>T, 6958A>G, 6976A>G, 6980C>T, 6985C>T, 6997A>T, 7000G>T, 7001G>A, 7003G>A, 7012T>C, 7015G>A, 7018T>C, 7032T>A, 7039C>T, 7040T>C, 7042G>A, 7048A>G, 7060T>A, 7063C>A, 7078C>T, 7082T>C, 7084A>G, 7087A>G, 7090C>A, 7096T>C, 7099A>G, 7102T>C, 7111G>A, 7117A>G, 7125C>A, 7129A>G, 7132T>C, 7133C>T, 7135G>A, 7141G>A, 7144T>C, 7145G>T, 7147A>G, 7150G>A, 7159A>T, 7160_7165delGAGGCA, 7171C>T, 7189G>T, 7198A>G, 7201C>T, 7205C>T, 7210T>C, 7216T>A, 7219G>A, 7225C>A, 7228G>A, 7240G>A, 7243T>C, 7249A>G, 7252T>G, 7258T>A, 7261T>C, 7264T>A, 7267C>G, 7270G>A, 7273_7274insT, 7274C>G, 7275G>T, 7281A>T, 7284T>C, 7296C>T, 7311C>T, 7314C>T, 7317G>A, 7321T>A, 7322delG, 7327C>T, 7330C>T, 7333C>T, 7334A>C, 7342T>C, 7343G>A, 7344C>A, 7345A>C, 7354T>C, 7363A>G, 7369T>C, 7372C>T, 7378G>T, 7381C>T, 7396A>T, 7399C>T, 7405C>T, 7407G>T, 7408T>G, 7414G>T, 7415T>G, 7420A>G, 7429C>T, 7432T>C, 7441T>C, 7459G>A, 7462A>G, 7468A>T, 7471C>A, 7477A>G, 7480C>T, 7486G>T, 7495T>C, 7498A>G, 7501T>C, 7503T>C, 7504G>A, 7513T>A, 7519C>T, 7522A>G, 7531G>A, 7537G>A, 7540C>T, 7546T>C, 7570G>A, 7573G>A, 7579G>A, 7588T>C, 7595C>T, 7604T>C, 7606G>A, 7615T>A, 7624T>C, 7633C>T, 7636T>C, 7639T>C, 7648T>C, 7666A>G, 7672T>C, 7678A>T, 7684G>A, 7690T>C, 7696T>C, 7702T>G, 7709G>A, 7713A>G, 7714A>G, 7715C>T, 7726T>C, 7735G>A, 7744A>G, 7747C>T, 7762C>T, 7768G>A, 7771T>C, 7780A>G, 7783A>C, 7786T>C, 7793C>T, 7795A>G, 7798C>T, 7804T>C, 7812C>T, 7834A>G, 7837C>T, 7844G>T, 7846G>A, 7855G>A, 7861G>A, 7870A>G, 7873C>T, 7876T>A, 7879G>A, 7882T>A, 7883G>A, 7894A>T, 7897T>C, 7921C>T, 7927G>A, 7936T>C, 7939T>C, 7942T>C, 7966G>A, 7969T>C, 7975T>C, 7987C>T, 7990T>C, 8005T>C, 8032A>G, 8038C>T, 8041C>T, 8043G>A, 8080G>A, 8089G>A, 8095G>A, 8098G>A, 8107C>T, 8110T>C, 8116T>C, 8128A>G, 8146T>C, 8151A>G, 8152C>T, 8164G>A, 8178G>A, 8182G>A, 8191G>A, 8197C>T, 8203C>T, 8208G>A, 8213C>T, 8215G>A, 8218A>G, 8221C>T, 8224G>A, 8239A>G, 8254G>A, 8260T>C, 8263A>G, 8281T>C, 8284A>G, 8294C>G, 8296A>G, 8299G>A, 8302C>T, 8317T>C, 8320C>T, 8326T>A, 8335G>A, 8338C>T, 8349C>T, 8353A>G, 8362C>T, 8365A>G, 8368C>T, 8380C>T, 8383A>G, 8389A>G, 8404T>C, 8408G>T, 8413A>G, 8419G>T, 8434T>C, 8440T>C, 8452G>A, 8458A>G, 8460G>A, 8464A>G, 8467G>A, 8479G>A, 8480G>A, 8482T>A, 8497G>A, 8498C>T, 8500A>G, 8502G>A, 8503A>G, 8506C>T, 8515A>T, 8524C>T, 8527A>C, 8528T>C, 8530A>G, 8539G>C, 8546C>T, 8547T>G, 8552T>A, 8563A>G, 8574C>G, 8585A>G, 8598G>A, 8602C>T, 8603A>T, 8604A>C, 8605C>T, 8620A>G, 8622C>A, 8625A>G, 8626G>A, 8629G>A, 8632C>T, 8638C>T, 8641G>A, 8647C>T, 8648C>T, 8649C>T, 8650G>C, 8653C>T, 8656A>G, 8658G>A, 8664A>C, 8665T>G, 8674T>G, 8679C>T, 8680A>T, 8683C>A, 8692G>A, 8695A>T, 8707G>A, 8708A>C, 8716G>A, 8722C>T, 8725G>A, 8731A>G, 8737A>G, 8743C>T, 8745C>A, 8746A>G, 8752G>A, 8755A>T, 8770C>T, 8771T>C, 8776A>G, 8779T>C, 8782G>A, 8791T>C, 8797A>G, 8800A>G, 8803A>G, 8809T>C, 8812T>C, 8818T>C, 8824G>A, 8827A>G, 8848G>A, 8854T>C, 8856A>G, 8857G>A, 8863G>A, 8865G>A, 8867A>C, 8869G>T, 8872A>G, 8883G>A, 8888A>T, 8929C>T, 8954G>A, 8959C>T, 8962A>T, 8983G>A, 8984A>G, 8986T>C, 8988A>T, 8998G>A, 9004G>A, 9007G>A, 9016C>T, 9037C>A, 9085A>T, 9097C>T, 9131A>G, 9136A>T, 9145G>T, 9149A>G, 9150T>G, 9151G>A, 9152G>A, 9157A>G, 9160G>T, 9163T>C, 9184G>A, 9196A>G, 9214G>A, 9217A>G, 9220G>A, 9244A>T, 9247G>A, 9256C>T, 9260C>T, 9262T>C, 9274G>A, 9280C>T, 9289C>T, 9298A>C, 9304G>A, 9334C>T, 9364C>G, 9377_9378insT, 9387C>T, 9404G>A, 9411C>T, 9412A>G, 9414A>T, 9437C>T, 9454A>G, 9458T>C, 9467G>A, 9470_9471insT, 9482G>A, 9483C>T, 9498T>C, 9516T>G, 9517delA, 9533G>A, 9534G>T, 9548A>T, 9551C>T, 9553_9554delTA, 9556A>T, 9557T>A, 9558A>T, 9574T>A, 9581G>T, 9582T>G, 9583G>C, 9584_9585insG, 9590C>T, 9591T>C, 9607C>T, 9610T>C, 9615delC, 9620A>G, 9648A>G, 9674T>C, 9690C>T								

	Begin	End	Coverage	Score	Concordance	Matches	Identities	I/D/M/F*	Stop Codons
**NT**	**22**	**9704**	**99.8%**	**12522**	**65.0%**	**9671 (99.8%)**	**8003 (82.6%)**	**10/12**	
**CDS**									
PVYgp1	1	3064	100%	18923	89.7%	3062 (99.9%)	2771 (90.4%)	2/2/4/4	1
Protein mutations:	Y4Q (194T>C 196C>A), C9L (209T>C 210G>T 211T>G), F13M (221T>A 223T>G), S23P (251T>C), C24F (255G>T 256C>T), E25G (258A>G), I27V (263A>G 265T>G), V28A (267T>C), E30V (273A>T), A35T (287G>A 289T>C), V37T (293G>A 294T>C), A41I (305G>A 306C>T), D42G (309A>G), E44D (316A>T), T45V (317A>G 318C>T 319A>G), L52Q (339T>A 340C>A), K53R (342A>G), K55A (347A>G 348A>C 349A>G), Y56H (350T>C), T58V (356A>G 357C>T), V61T (365G>A 366T>C 367G>A), L62S (368C>T 369T>C), F67C (384T>G), A75V (408C>T 409C>T), M78E (416A>G 417T>A), E85K (437G>A), R86E (440A>G 441G>A), K87R (443A>C 444A>G 445G>A), D88E (448T>A), E91A (456A>C), H93N (461C>A), D111E (517T>A), S116F (531C>T), P118S (536C>T), Q119W (539C>T 540A>G 541A>G), R122K (549G>A 550A>G), M131T (576T>C), V134A (585T>C 586C>G), R137Y (593C>T 594G>A 595C>T), P138R (597C>G), I139V (599A>G 601A>G), I140P (602A>C 603T>C 604A>G), M147I (625G>A), I151V (635A>G 637T>C), I154V (644A>G 646A>G), E160A (663A>C 664G>C), H166Q (682C>A), T172S (699C>G), H174Q (706T>A), K179E (719A>G 721G>A), I180V (722A>G 724A>G), A183S (731G>T), Y184P (734T>C 735A>C 736C>T), S185R (737T>C 738C>G 739C>T), A187V (744C>T 745G>C), R189C (749C>T 751A>C), M194K (765T>A), R197K (773C>A 774G>A 775A>G), M206K (801T>A), G210A (813G>C 814A>G), L211H (816T>A), R214Q (825G>A 826T>A), N223D (851A>G 853T>C), R226C (860C>T 862C>T), T227A (863A>G 865T>C), I228T (867T>C), N229D (869A>G 871C>T), I230L (872A>T 874A>G), R231Q (876G>A), R232K (879G>A), N240S (903A>G), T241N (906C>A 907A>C), K242T (909A>C 910A>C), S243N (912G>A 913C>T), S252L (938T>C 939C>T 940A>T), L266I (980T>A 982G>C), R272K (999G>A), Q275R (1007C>A 1008A>G 1009G>A), S276G (1010A>G 1012T>G), I277V (1013A>G), N279Q (1019A>C 1021C>G), D288E (1048C>A), R300Q (1083G>A), S305A (1097T>G 1099G>A), R320K (1143G>A), L324I (1154T>A 1156G>A), S346N (1221G>A), V349T (1229G>A 1230T>C 1231T>C), S350N (1233G>A 1234C>T), K358T (1257A>C 1258A>G), N365S (1278A>G), A369V (1290C>T 1291G>A), D372T (1298G>A 1299A>C 1300C>T), I375V (1307A>G 1309A>G), N378E (1316A>G 1318T>A), I382T (1329T>C), L397I (1373C>A), I401V (1385A>G), E407D (1405G>T), A411S (1415G>T 1417A>T), V418I (1436G>A 1438C>T), E508G (1707A>G), A512T (1718G>A), I520V (1742A>G 1744C>T), S525N (1758G>A), V536I (1790G>A), I585V (1937A>G 1939T>C), F607Y (2004T>A), I628M (2068T>G), V630I (2072G>A 2074G>A), R675K (2208G>A), D694E (2266C>G), N744G (2414A>G 2415A>G 2416T>G), S746C (2420A>T), K780R (2523A>G), R817K (2634G>A), V847I (2723G>A 2725C>T), A855T (2747G>A 2749T>A), H871N (2795C>A), V880I (2822G>A 2824G>A), P900T (2882C>A), S901H (2885A>C 2886G>A), T904M (2895C>T), N916S (2931A>G), N919S (2940A>G), E929_N930insX (2971_2972insA), T936I (2991C>T), H937R (2994A>G), E939R (2999G>A 3000A>G), R946Q (3021G>A), Y947F (3024A>T 3025C>T), T951I (3036C>T), E952G (3039A>G 3040A>C), K953T (3042A>C 3043G>A), A972V (3099C>T 3100C>A), V974R (3104G>A 3105T>G 3106G>A), S983N (3132G>A), R985K (3137C>A 3138G>A 3139A>G), F990I (3152T>A 3154C>T), C994Y (3165G>A), F1012L (3218T>C), T1014N (3225C>A), V1016I (3230G>A), V1028M (3266G>A 3268A>G), V1031M (3275G>A), A1034T (3284G>A), R1044K (3315G>A 3316A>G), E1045A (3318A>C), M1059V (3359A>G), D1072E (3400T>G), V1110G (3513T>G), M1121V (3545A>G), A1122T (3548G>A), V1124I (3554G>A 3556C>T), L1147V (3623C>G), L1150M (3632C>A), Y1152H (3638T>C), I1172V (3698A>G), S1178N (3717G>A), R1183K (3731C>A 3732G>A), I1297V (4073A>G), F1315Y (4128T>A 4129C>T), A1323S (4151G>T), V1328I (4166G>A), L1347I (4223C>A 4225G>A), I1379V (4319A>G 4321A>G), F1404Y (4395T>A), V1408I (4406G>A), M1431V (4475A>G 4477G>T), T1433S (4481A>T 4483A>G), A1454L (4544G>T 4545C>T 4546G>A), R1455K (4548G>A), V1499I (4679G>A 4681G>C), V1538I (4796G>A 4798C>T), T1540S (4803C>G 4804T>C), S1625G (5057A>G), T1626A (5060A>G), L1636I (5090C>A), A1639V (5100C>T), E1641D (5107A>T), V1646F (5120G>T 5122C>T), I1648V (5126A>G), V1667I (5183G>A), A1793T (5561G>A 5563G>A), I1805V (5597A>G), N1807K (5605C>A), A1812V (5619C>T 5620T>G), I1816L (5630A>C), V1837I (5693G>A), K1890R (5853A>G), I1904V (5894A>G 5896C>G), R1926A (5960C>G 5961G>C 5962G>C), I1936V (5990A>G), E1945D (6019A>T), K1949T (6030A>C), D1955E (6049C>A), M1958I (6058G>A), S1963N (6072G>A 6073T>C), N1964H (6074A>C), T1966N (6081C>A), C1976S (6111G>C 6112T>C), I1982V (6128A>G 6130T>C), V1992I (6158G>A), T1996S (6170A>T 6172A>G), L2007F (6203C>T 6205C>T), V2016I (6230G>A), A2025K (6257G>A 6258C>A), K2033R (6282A>G), A2060T (6362G>A), V2073I (6401G>A), A2074V (6405C>T), Y2082F (6429A>T), Q2089R (6450A>G), H2101N (6485C>A 6487C>T), L2115I (6527C>A), T2135I (6588C>T), Y2151H (6635T>C), I2155V (6647A>G), I2165V (6677A>G 6679A>G), D2178N (6716G>A), N2179D (6719A>G), I2194L (6764A>T 6766A>G), A2204V (6795C>T 6796A>G), H2205Q (6799C>A), N2225D (6857A>G), V2231T (6875G>A 6876T>C 6877C>A), D2250E (6934C>A), V2273I (7001G>A 7003G>A), F2283Y (7032T>A), T2314K (7125C>A), A2321S (7145G>T 7147A>G), E2326_A2327del (7160_7165delGAGGCA), D2344E (7216T>A), D2358E (7258T>A), D2363_R2364insX (7273_7274insT), R2364V (7274C>G 7275G>T), H2366L (7281A>T), L2367S (7284T>C), S2371L (7296C>T), S2376F (7311C>T), T2377I (7314C>T), C2378Y (7317G>A), N2379K (7321T>A), K2384Q (7334A>C), A2387N (7343G>A 7344C>A 7345A>C), S2408M (7407G>T 7408T>G), C2411G (7415T>G), L2440S (7503T>C 7504G>A), D2509N (7709G>A), K2510R (7713A>G 7714A>G), T2543I (7812C>T), V2554L (7844G>T 7846G>A), V2567I (7883G>A), S2620N (8043G>A), N2656S (8151A>G 8152C>T), R2665K (8178G>A), R2675K (8208G>A), P2704A (8294C>G 8296A>G), S2722F (8349C>T), A2742S (8408G>T), M2756I (8452G>A), R2759K (8460G>A), A2766T (8480G>A 8482T>A), R2773K (8502G>A 8503A>G), E2777D (8515A>T), E2785D (8539G>C), L2788C (8546C>T 8547T>G), S2790T (8552T>A), A2797G (8574C>G), I2801V (8585A>G), G2805E (8598G>A), N2807S (8603A>T 8604A>C 8605C>T), P2813Q (8622C>A), E2814G (8625A>G 8626G>A), P2822F (8648C>T 8649C>T 8650G>C), G2825E (8658G>A), D2827A (8664A>C 8665T>G), A2832V (8679C>T 8680A>T), T2854K (8745C>A 8746A>G), E2891G (8856A>G 8857G>A), R2894Q (8865G>A), M2895L (8867A>C 8869G>T), G2900E (8883G>A), T2902S (8888A>T), V2924I (8954G>A), N2934D (8984A>G 8986T>C), E2935V (8988A>T), I2983V (9131A>G), M2989G (9149A>G 9150T>G 9151G>A), G2990S (9152G>A), P3026S (9260C>T 9262T>C), Q3038H (9298A>C)								

	Begin	End	Coverage	Score	Concordance	Matches	Identities	I/D/M/F*	Stop Codons
**NT**	**22**	**9704**	**99.8%**	**12522**	**65.0%**	**9671 (99.8%)**	**8003 (82.6%)**	**10/12**	

Codon mutations:

ACT3ACC (193T>C), TAC4CAA (194T>C 196C>A), TCA6TCG (202A>G), ATC8ATT (208C>T), TGT9CTG (209T>C 210G>T 211T>G), TCG12TCA (220G>A), TTT13ATG (221T>A 223T>G), GAA14GAG (226A>G), CTA17CTT (235A>T), TAC19TAT (241C>T), CCA21CCT (247A>T), TCT23CCT (251T>C), TGC24TTT (255G>T 256C>T), GAG25GGG (258A>G), CAT26CAC (262T>C), ATT27GTG (263A>G 265T>G), GTG28GCG (267T>C), GAA30GTA (273A>T), CGA31CGT (277A>T), GAA32GAG (280A>G), GTG33GTT (283G>T), CCG34CCA (286G>A), GCT35ACC (287G>A 289T>C), GTT37ACT (293G>A 294T>C), CCT39CCC (301T>C), TTC40TTT (304C>T), GCA41ATA (305G>A 306C>T), GAT42GGT (309A>G), CTG43TTG (311C>T), GAA44GAT (316A>T), ACA45GTG (317A>G 318C>T 319A>G), CAA46CAG (322A>G), GCA49GCT (331A>T), CTC52CAA (339T>A 340C>A), AAG53AGG (342A>G), AAA55GCG (347A>G 348A>C 349A>G), TAT56CAT (350T>C), GCT57GCA (355T>A), ACT58GTT (356A>G 357C>T), CGT60CGC (364T>C), GTG61ACA (365G>A 366T>C 367G>A), CTC62TCC (368C>T 369T>C), AAA63AAG (373A>G), AAC64AAT (376C>T), TTT67TGT (384T>G), ACG68ACA (388G>A), TAC69TAT (391C>T), CGA70CGG (394A>G), AAG72AAA (400G>A), GAT74GAC (406T>C), GCC75GTT (408C>T 409C>T), CAG76CAA (412G>A), ATA77ATT (415A>T), ATG78GAG (416A>G 417T>A), ATT80ATC (424T>C), CAG81CAA (427G>A), AAA83AAG (433A>G), CTG84CTC (436G>C), GAG85AAG (437G>A), AGG86GAG (440A>G 441G>A), AAG87CGA (443A>C 444A>G 445G>A), GAT88GAA (448T>A), GAA90GAG (454A>G), GAA91GCA (456A>C), TAT92TAC (460T>C), CAC93AAC (461C>A), CAA95CAG (469A>G), GCC97GCG (475C>G), CCT99CCA (481T>A), ACT106ACC (502T>C), ATA107ATT (505A>T), GGC109GGT (511C>T), GAT111GAA (517T>A), CCT112CCA (520T>A), CCA113CCT (523A>T), TCT116TTT (531C>T), GAG117GAA (535G>A), CCA118TCA (536C>T), CAA119TGG (539C>T 540A>G 541A>G), AGA122AAG (549G>A 550A>G), ATC124ATT (556C>T), ATT125ATC (559T>C), CAT126CAC (562T>C), ACT128ACC (568T>C), CCA129CCG (571A>G), ATG131ACG (576T>C), CGT132CGC (580T>C), GTC134GCG (585T>C 586C>G), CGC137TAT (593C>T 594G>A 595C>T), CCC138CGC (597C>G), ATA139GTG (599A>G 601A>G), ATA140CCG (602A>C 603T>C 604A>G), ACA143ACG (613A>G), GAA144GAG (616A>G), GGC145GGG (619C>G), CAG146CAA (622G>A), ATG147ATA (625G>A), AAT148AAC (628T>C), CAC149CAT (631C>T), ATT151GTC (635A>G 637T>C), ATA154GTG (644A>G 646A>G), ATT157ATC (655T>C), TCG159TCA (661G>A), GAG160GCC (663A>C 664G>C), AAA161AAG (667A>G), GTC165GTT (679C>T), CAC166CAA (682C>A), TTA167CTG (683T>C 685A>G), AGT169AGC (691T>C), ACC172AGC (699C>G), ACT173ACA (703T>A), CAT174CAA (706T>A), AAG179GAA (719A>G 721G>A), ATA180GTG (722A>G 724A>G), CTT181TTG (725C>T 727T>G), GGT182GGA (730T>A), GCA183TCA (731G>T), TAC184CCT (734T>C 735A>C 736C>T), TCC185CGT (737T>C 738C>G 739C>T), GCG187GTC (744C>T 745G>C), CGA189TGC (749C>T 751A>C), ACT190ACC (754T>C), ATG194AAG (765T>A), TTG196CTG (770T>C), CGA197AAG (773C>A 774G>A 775A>G), CGG198AGG (776C>A), TTC202TTT (790C>T), ATG206AAG (801T>A), GGA210GCG (813G>C 814A>G), CTT211CAT (816T>A), TTG212CTG (818T>C), CAA213CAG (823A>G), CGT214CAA (825G>A 826T>A), CGG217AGA (833C>A 835G>A), GAC219GAT (841C>T), AAT223GAC (851A>G 853T>C), GTC225GTT (859C>T), CGC226TGT (860C>T 862C>T), ACT227GCC (863A>G 865T>C), ATC228ACC (867T>C), AAC229GAT (869A>G 871C>T), ATA230TTG (872A>T 874A>G), CGA231CAA (876G>A), AGG232AAG (879G>A), GAT234GAC (886T>C), ATC238ATT (898C>T), AAC240AGC (903A>G), ACA241AAC (906C>A 907A>C), AAA242ACC (909A>C 910A>C), AGC243AAT (912G>A 913C>T), GGC246GGA (922C>A), GGT249GGA (931T>A), TCA252CTT (938T>C 939C>T 940A>T), GGA253GGG (943A>G), TTC256TTT (952C>T), TCA261TCG (967A>G), GAA263GAG (973A>G), GGG264GGC (976G>C), TTG266ATC (980T>A 982G>C), GCA269GCG (991A>G), CGT270CGC (994T>C), AGA272AAA (999G>A), GTT273GTC (1003T>C), CAG275AGA (1007C>A 1008A>G 1009G>A), AGT276GGG (1010A>G 1012T>G), ATT277GTT (1013A>G), TTA278TTG (1018A>G), AAC279CAG (1019A>C 1021C>G), ATC282ATT (1030C>T), TCG285TCA (1039G>A), AAT286AAC (1042T>C), GCC287GCA (1045C>A), GAC288GAA (1048C>A), AAT289AAC (1051T>C), AAG292AAA (1060G>A), GGT293GGA (1063T>A), CTG294TTG (1064C>T), GAC295GAT (1069C>T), CGA300CAA (1083G>A), TAT303TAC (1093T>C), TCG305GCA (1097T>G 1099G>A), CAC307CAT (1105C>T), GTA310GTG (1114A>G), GCT311GCG (1117T>G), TTA313TTG (1123A>G), CCT314CCA (1126T>A), GTC315GTT (1129C>T), AGG320AAG (1143G>A), GTA321GTT (1147A>T), GCT322GCA (1150T>A), TTG324ATA (1154T>A 1156G>A), CAC327CAT (1165C>T), AGT328AGC (1168T>C), CTT330TTA (1172C>T 1174T>A), ACC339ACT (1201C>T), AGC346AAC (1221G>A), GTT349ACC (1229G>A 1230T>C 1231T>C), AGC350AAT (1233G>A 1234C>T), CTG352CTA (1240G>A), AAG354AAA (1246G>A), TTG356CTA (1250T>C 1252G>A), CAT357CAC (1255T>C), AAA358ACG (1257A>C 1258A>G), AGA361AGG (1267A>G), GGT363GGC (1273T>C), TTG364TTA (1276G>A), AAT365AGT (1278A>G), TTG367CTG (1283T>C), GCG369GTA (1290C>T 1291G>A), GAC372ACT (1298G>A 1299A>C 1300C>T), TTT374TTC (1306T>C), ATA375GTG (1307A>G 1309A>G), CAT376CAC (1312T>C), AAT378GAA (1316A>G 1318T>A), TTG381TTA (1327G>A), ATA382ACA (1329T>C), GCG383GCT (1333G>T), TTA384TTG (1336A>G), CTA387TTG (1343C>T 1345A>G), CCG390CCA (1354G>A), GTG391GTT (1357G>T), GAC392GAT (1360C>T), CTC395TTG (1367C>T 1369C>G), CTT397ATT (1373C>A), TTC398TTT (1378C>T), GAG400GAA (1384G>A), ATA401GTA (1385A>G), AAA403AAG (1393A>G), TCC404TCT (1396C>T), GAG407GAT (1405G>T), AAA408AAG (1408A>G), CAG409CAA (1411G>A), GCA411TCT (1415G>T 1417A>T), CCG412CCC (1420G>C), AAT415AAC (1429T>C), TTA416TTG (1432A>G), GTC418ATT (1436G>A 1438C>T), TTA419CTG (1439T>C 1441A>G), AAT420AAC (1444T>C), TTC423TTT (1453C>T), CTG424CTA (1456G>A), GAA428GAG (1468A>G), GTA436GTG (1492A>G), TTG439CTA (1499T>C 1501G>A), AGT440AGC (1504T>C), TTG441TTA (1507G>A), CTC442CTT (1510C>T), GAA443GAG (1513A>G), TTA444TTG (1516A>G), GCA445GCT (1519A>T), AGG446AGA (1522G>A), TTC447TTT (1525C>T), CAG448CAA (1528G>A), AAC450AAT (1534C>T), ACT452ACA (1540T>A), AAC454AAT (1546C>T), AAA457AAG (1555A>G), GGT458GGC (1558T>C), GAT459GAC (1561T>C), ATA460ATC (1564A>C), TCT461TCA (1567T>A), TTC463TTT (1573C>T), AGA464AGG (1576A>G), TTA467CTA (1583T>C), TCT468TCA (1588T>A), AAG470AAA (1594G>A), AAC472AAT (1600C>T), CTG475CTA (1609G>A), TCG478TCA (1618G>A), TGC479TGT (1621C>T), GAC480GAT (1624C>T), CTG483TTG (1631C>T), AAA485AAG (1639A>G), TTC489TTT (1651C>T), CTC490CTG (1654C>G), CAT497CAC (1675T>C), TCA503TCG (1693A>G), TTT506TTC (1702T>C), GAA508GGA (1707A>G), GCA512ACA (1718G>A), GGA514GGG (1726A>G), TAC515TAT (1729C>T), TCA516TCC (1732A>C), TAT518TAC (1738T>C), ATC520GTT (1742A>G 1744C>T), CGC521CGT (1747C>T), CAT523CAC (1753T>C), AGT525AAT (1758G>A), AGG528AGA (1768G>A), AAC534AAT (1786C>T), TTA535TTG (1789A>G), GTT536ATT (1790G>A), GTC537GTT (1795C>T), TTA541CTG (1805T>C 1807A>G), GAG543GAA (1813G>A), AGG545AGA (1819G>A), AAG547AAA (1825G>A), GAC551GAT (1837C>T), TAT552TAC (1840T>C), CAA555CAG (1849A>G), GTC558GTG (1858C>G), AAA560AAG (1864A>G), TGC562TGT (1870C>T), AGT564AGC (1876T>C), AAA566AAG (1882A>G), GGT568GGA (1888T>A), AAT569AAC (1891T>C), TGT574TGC (1906T>C), ACA577ACT (1915A>T), GGT582GGC (1930T>C), GCC584GCT (1936C>T), ATT585GTC (1937A>G 1939T>C), TCA587TCG (1945A>G), TTC589TTT (1951C>T), CCA592CCT (1960A>T), GTA598GTG (1978A>G), ATT599ATA (1981T>A), GGC600GGT (1984C>T), AAT601AAC (1987T>C), GAC604GAT (1996C>T), TTT607TAT (2004T>A), AAA612AAG (2020A>G), GGG613GGA (2023G>A), GAT614GAC (2026T>C), TCG615TCT (2029G>T), GAG616GAA (2032G>A), TAT625TAC (2059T>C), ATT628ATG (2068T>G), AAC629AAT (2071C>T), GTG630ATA (2072G>A 2074G>A), CTG635TTA (2087C>T 2089G>A), AGC639AGT (2101C>T), GAG640GAA (2104G>A), GAG641GAA (2107G>A), AAG644AAA (2116G>A), TTC646TTT (2122C>T), CGC651CGT (2137C>T), CTT658CTC (2158T>C), ACC660ACT (2164C>T), CCA662CCG (2170A>G), GAT666GAC (2182T>C), GCG668GCA (2188G>A), ACC669ACA (2191C>A), AGA675AAA (2208G>A), TAT678TAC (2218T>C), GAC680GAT (2224C>T), GTA681GTT (2227A>T), TTG686TTA (2242G>A), CCC687CCT (2245C>T), TTG690TTA (2254G>A), GTT691GTC (2257T>C), GAC692GAT (2260C>T), GAC694GAG (2266C>G), CAA696CAG (2272A>G), ACG697ACA (2275G>A), TCA703TCG (2293A>G), GGC705GGT (2299C>T), TCG706TCA (2302G>A), CAG707CAA (2305G>A), GGA710GGT (2314A>T), TAT711TAC (2317T>C), CTA714CTG (2326A>G), GTG719GTA (2341G>A), CAA721CAG (2347A>G), CTT722CTC (2350T>C), TTG724TTA (2356G>A), AAT727AAC (2365T>C), GAA729GAG (2371A>G), TTA730TTG (2374A>G), GAT733GAC (2383T>C), ATA734ATT (2386A>T), AAA735AAG (2389A>G), CAT736CAC (2392T>C), TAT737TAC (2395T>C), AAT744GGG (2414A>G 2415A>G 2416T>G), GCT745GCA (2419T>A), AGC746TGC (2420A>T), ACA752ACT (2440A>T), GTT761GTC (2467T>C), TCT764TCC (2476T>C), TCG766TCA (2482G>A), CTG769CTA (2491G>A), CTG771CTT (2497G>T), TTG773TTA (2503G>A), AGA778AGG (2518A>G), CCT779CCC (2521T>C), AAG780AGG (2523A>G), AGA783AGG (2533A>G), TTG785TTA (2539G>A), TTA787TTG (2545A>G), GAG789GAA (2551G>A), CCT790CCA (2554T>A), CTG792TTG (2558C>T), TTG793CTC (2561T>C 2563G>C), TCA796TCG (2572A>G), CCT800CCC (2584T>C), GGC801GGT (2587C>T), CTG803CTC (2593G>C), GCT805GCC (2599T>C), AAT808AAC (2608T>C), TTT812TTC (2620T>C), GCG815GCA (2629G>A), GTG816GTA (2632G>A), AGG817AAG (2634G>A), TTG818TTA (2638G>A), ATT820ATA (2644T>A), AAA823AAG (2653A>G), GCT827GCC (2665T>C), CTA832TTA (2678C>T), CTA833CTG (2683A>G), GCT835GCC (2689T>C), CTA838CTC (2698A>C), TCA841TCG (2707A>G), GCG842GCA (2710G>A), GAA844GAG (2716A>G), ACA845ACG (2719A>G), CTC846CTT (2722C>T), GTC847ATT (2723G>A 2725C>T), GCA848GCG (2728A>G), AGG850AGA (2734G>A), ATT851ATA (2737T>A), GAT854GAC (2746T>C), GCT855ACA (2747G>A 2749T>A), GCA856GCT (2752A>T), GCT857GCA (2755T>A), GAC859GAT (2761C>T), CTC860CTT (2764C>T), TGT865TGC (2779T>C), GGG867GGT (2785G>T), AAC869AAT (2791C>T), CAT871AAT (2795C>A), TAC874TAT (2806C>T), TTG878CTC (2816T>C 2818G>C), GTG880ATA (2822G>A 2824G>A), TTG881TTA (2827G>A), GTT884GTC (2836T>C), AAG885AAA (2839G>A), AAT886AAC (2842T>C), ACC893ACA (2863C>A), CTA894TTA (2864C>T), CCA900ACA (2882C>A), AGT901CAT (2885A>C 2886G>A), AAC903AAT (2893C>T), ACG904ATG (2895C>T), GTC906GTT (2902C>T), GTA907GTG (2905A>G), ATT909ATC (2911T>C), AAT913AAC (2923T>C), AAT916AGT (2931A>G), AAC919AGC (2940A>G), GCT921GCC (2947T>C), ACT926ACC (2962T>C), GAA929_AAT930insA-- (2971_2972insA), GGT935GGC (2989T>C), ACT936ATT (2991C>T), CAT937CGT (2994A>G), GAG939AGG (2999G>A 3000A>G), TCC944TC- (3016delC), CGG946CAG (3021G>A), TAC947TTT (3024A>T 3025C>T), ACA951ATA (3036C>T), GAA952GGC (3039A>G 3040A>C), AAG953ACA (3042A>C 3043G>A), TTG956TTA (3052G>A), TTA959TTG (3061A>G), GCG966GCA (3082G>A), GGC969GGT (3091C>T), GCC972GTA (3099C>T 3100C>A), GTG974AGA (3104G>A 3105T>G 3106G>A), GTC975GTT (3109C>T), AAA976AAG (3112A>G), ACT978ACC (3118T>C), GCC979GCT (3121C>T), GGA981GGG (3127A>G), AGC983AAC (3132G>A), CGA985AAG (3137C>A 3138G>A 3139A>G), TAT989TAC (3151T>C), TTC990ATT (3152T>A 3154C>T), TGT994TAT (3165G>A), GTA995GTG (3169A>G), ATC1005ATT (3199C>T), AGG1007CGG (3203A>C), CGT1008CGC (3208T>C), CCA1010CCG (3214A>G), ACC1011ACT (3217C>T), TTT1012CTT (3218T>C), GTC1013GTA (3223C>A), ACT1014AAT (3225C>A), GTT1016ATT (3230G>A), AAC1017AAT (3235C>T), TTA1025CTA (3257T>C), ACC1026ACT (3262C>T), AGC1027AGT (3265C>T), GTA1028ATG (3266G>A 3268A>G), GTG1029GTA (3271G>A), GTG1031ATG (3275G>A), GCA1034ACA (3284G>A), CAG1039CAA (3301G>A), AGA1044AAG (3315G>A 3316A>G), GAA1045GCA (3318A>C), ATC1046ATT (3322C>T), GAG1047GAA (3325G>A), TTG1048TTA (3328G>A), ATA1051ATT (3337A>T), GAG1055GAA (3349G>A), GTC1057GTT (3355C>T), TGC1058TGT (3358C>T), ATG1059GTG (3359A>G), CTA1061CTG (3367A>G), TAT1062TAC (3370T>C), TTA1065CTG (3377T>C 3379A>G), GAA1070GAG (3394A>G), CGC1071CGT (3397C>T), GAT1072GAG (3400T>G), ACA1074ACG (3406A>G), TAC1078TAT (3418C>T), ATT1079ATA (3421T>A), TAT1081TAC (3427T>C), AAG1083AAA (3433G>A), TCA1084TCT (3436A>T), GTA1085GTG (3439A>G), AAC1086AAT (3442C>T), CCT1087CCC (3445T>C), CAG1091CAA (3457G>A), TTT1092TTC (3460T>C), GCT1093GCA (3463T>A), GCG1095GCT (3469G>T), CAG1096CAA (3472G>A), CAC1104CAT (3496C>T), CAG1105CAA (3499G>A), TCC1107TCT (3505C>T), GTT1110GGT (3513T>G), AAA1112AAG (3520A>G), GAA1115GAG (3529A>G), CAA1116CAG (3532A>G), ATG1121GTG (3545A>G), GCT1122ACT (3548G>A), TTA1123CTA (3551T>C), GTC1124ATT (3554G>A 3556C>T), AGT1133AGC (3583T>C), GAT1134GAC (3586T>C), TGC1135TGT (3589C>T), TTC1137TTT (3595C>T), AAT1141AAC (3607T>C), TTT1143TTC (3613T>C), AAG1144AAA (3616G>A), GGT1145GGC (3619T>C), CTT1147GTT (3623C>G), TCC1148TCT (3628C>T), CTG1150ATG (3632C>A), GAT1151GAC (3637T>C), TAT1152CAT (3638T>C), GAA1153GAG (3643A>G), TTA1159TTG (3661A>G), GTG1162GTA (3670G>A), TTT1166TTC (3682T>C), GAG1168GAA (3688G>A), GAG1171GAA (3697G>A), ATT1172GTT (3698A>G), GAT1174GAC (3706T>C), TTT1175TTC (3709T>C), TTG1177CTA (3713T>C 3715G>A), AGT1178AAT (3717G>A), GAG1179GAA (3721G>A), CGA1183AAA (3731C>A 3732G>A), ACT1184ACA (3736T>A), CTA1188TTG (3746C>T 3748A>G), ACA1190ACG (3754A>G), AGT1193AGC (3763T>C), CGA1198CGG (3778A>G), CAA1199CAG (3781A>G), ATC1200ATT (3784C>T), CAT1204CAC (3796T>C), CCA1207CCT (3805A>T), TAC1209TAT (3811C>T), AGA1210AGG (3814A>G), TTC1215TTT (3829C>T), TTT1218TTC (3838T>C), AGA1220AGG (3844A>G), GCA1221GCC (3847A>C), GTT1224GTA (3856T>A), GTG1226GTT (3862G>T), GAC1229GAT (3871C>T), GCC1231GCA (3877C>A), AGC1233AGT (3883C>T), GAA1234GAG (3886A>G), CTA1236CTG (3892A>G), GAC1237GAT (3895C>T), TTT1238TTC (3898T>C), GTA1240GTG (3904A>G), CGG1241AGA (3905C>A 3907G>A), GCT1243GCC (3913T>C), AAG1248AAA (3928G>A), TCA1249TCT (3931A>T), GGG1251GGA (3937G>A), AGT1257AGC (3955T>C), GTG1258GTA (3958G>A), GCC1259GCT (3961C>T), GGA1260GGG (3964A>G), TCT1261TCC (3967T>C), TTA1264TTG (3976A>G), ATT1265ATC (3979T>C), CCA1267CCT (3985A>T), CTA1271CTT (3997A>T), GCG1272GCA (4000G>A), AAC1274AAT (4006C>T), GTT1275GTG (4009T>G), TTC1276TTT (4012C>T), AAA1277AAG (4015A>G), TCT1280TCC (4024T>C), GAA1282GAG (4030A>G), CCA1283CCT (4033A>T), AAG1286AAA (4042G>A), CTG1290TTA (4052C>T 4054G>A), CGT1291CGC (4057T>C), CGT1293CGA (4063T>A), GGA1294GGG (4066A>G), ATA1297GTA (4073A>G), TTT1298TTC (4078T>C), GGC1299GGT (4081C>T), TCT1300TCC (4084T>C), TCC1304TCT (4096C>T), AGC1308AGT (4108C>T), GGA1309GGT (4111A>T), GCG1311GCA (4117G>A), CTA1312TTG (4118C>T 4120A>G), TAC1314TAT (4126C>T), TTC1315TAT (4128T>A 4129C>T), GCC1316GCT (4132C>T), AAT1317AAC (4135T>C), CAA1321CAG (4147A>G), TTA1322CTA (4148T>C), GCT1323TCT (4151G>T), AAC1326AAT (4162C>T), GTA1328ATA (4166G>A), ATA1329ATC (4171A>C), GAG1332GAA (4180G>A), CTG1336TTG (4190C>T), CCT1338CCC (4198T>C), TCC1339TCT (4201C>T), GCG1342GCT (4210G>T), TTC1343TTT (4213C>T), AGT1345AGC (4219T>C), CTG1346TTG (4220C>T), CTG1347ATA (4223C>A 4225G>A), GTT1349GTG (4231T>G), CAT1351CAC (4237T>C), GTA1356GTG (4252A>G), TTA1357CTG (4253T>C 4255A>G), AAA1358AAG (4258A>G), ACT1362ACC (4270T>C), AGA1366AGG (4282A>G), GAG1367GAA (4285G>A), GAA1369GAG (4291A>G), CAG1373CAA (4303G>A), GTC1376GTT (4312C>T), ATA1379GTG (4319A>G 4321A>G), GTG1380GTT (4324G>T), GAG1381GAA (4327G>A), CTG1384CTT (4336G>T), TCT1385TCC (4339T>C), TCA1388TCT (4348A>T), GCA1392GCG (4360A>G), GGT1394GGC (4366T>C), TCT1395TCA (4369T>A), ACT1397ACC (4375T>C), GTT1401GTC (4387T>C), TTT1404TAT (4395T>A), GTA1408ATA (4406G>A), CTT1409CTC (4411T>C), GTT1418GTC (4438T>C), ACC1420ACA (4444C>A), GCC1422GCT (4450C>T), CTC1424CTT (4456C>T), GAC1427GAT (4465C>T), ATG1431GTT (4475A>G 4477G>T), GTC1432GTT (4480C>T), ACA1433TCG (4481A>T 4483A>G), GGC1437GGT (4495C>T), AAG1441AAA (4507G>A), CAC1442CAT (4510C>T), GGT1443GGG (4513T>G), CTA1445TTA (4517C>T), GTC1448GTT (4528C>T), AAA1450AAG (4534A>G), ACC1452ACT (4540C>T), GCG1454TTA (4544G>T 4545C>T 4546G>A), AGA1455AAA (4548G>A), TTT1458TTC (4558T>C), GTA1460GTT (4564A>T), ACC1462ACT (4570C>T), ATA1464ATT (4576A>T), TTG1471TTA (4597G>A), GAC1472GAT (4600C>T), GAC1474GAT (4606C>T), GTG1475GTA (4609G>A), GTG1477GTA (4615G>A), GAC1478GAT (4618C>T), TTG1481CTT (4625T>C 4627G>T), GAC1488GAT (4648C>T), ATT1489ATC (4651T>C), AGG1492AGA (4660G>A), AGC1493AGT (4663C>T), GCT1495GCA (4669T>A), GTG1499ATC (4679G>A 4681G>C), AGT1500AGC (4684T>C), GTT1501GTG (4687T>G), GAG1505GAA (4699G>A), AGA1506AGG (4702A>G), CAA1508CAG (4708A>G), AGG1509AGA (4711G>A), CTG1510TTG (4712C>T), AAA1518AAG (4738A>G), GTA1520GTT (4744A>T), GCA1521GCG (4747A>G), TTG1522CTG (4748T>C), ACT1527ACC (4765T>C), GTC1538ATT (4796G>A 4798C>T), ACT1540AGC (4803C>G 4804T>C), GAG1541GAA (4807G>A), GCG1542GCT (4810G>T), GCT1543GCG (4813T>G), CTT1544CTC (4816T>C), TAT1549TAC

	(4831T>C), AAC1550AAT (4834C>T), CCA1552CCT (4840A>T), GGA1556GGG (4852A>G), GGC1557GGT (4855C>T), AGT1561AGC (4867T>C), CTG1562CTC (4870G>C), ATT1563ATA (4873T>A), TGT1566TGC (4882T>C), ACA1573ACT (4903A>T), CAG1575CAA (4909G>A), TTT1577TTC (4915T>C), GAA1578GAG (4918A>G), TTG1579CTA (4919T>C 4921G>A), AGT1580AGC (4924T>C), CCC1581CCG (4927C>G), TTT1583TTC (4933T>C), ATC1584ATA (4936C>A), CAG1585CAA (4939G>A), AAT1586AAC (4942T>C), TTC1587TTT (4945C>T), TCA1593TCG (4963A>G), CCT1596CCC (4972T>C), CTT1602CTC (4990T>C), AAA1606AAG (5002A>G), CCT1613CCC (5023T>C), TTG1614CTG (5024T>C), TGC1615TGT (5029C>T), GAT1616GAC (5032T>C), TCT1618TCC (5038T>C), CCA1620CCT (5044A>T), GCA1623GCT (5053A>T), TCG1624TCA (5056G>A), AGT1625GGT (5057A>G), ACT1626GCT (5060A>G), TCG1629TCT (5071G>T), CTT1636ATT (5090C>A), GGA1637GGG (5095A>G), GTG1638GTT (5098G>T), GCC1639GTC (5100C>T), TTA1640TTG (5104A>G), GAA1641GAT (5107A>T), ATT1642ATC (5110T>C), CCA1643CCG (5113A>G), AAG1644AAA (5116G>A), CAA1645CAG (5119A>G), GTC1646TTT (5120G>T 5122C>T), ATT1648GTT (5126A>G), CAT1651CAC (5137T>C), ATC1652ATT (5140C>T), AAA1653AAG (5143A>G), GAG1654GAA (5146G>A), CTC1659CTA (5161C>A), GAA1661GAG (5167A>G), CTT1663CTG (5173T>G), ACG1666ACA (5182G>A), GTT1667ATT (5183G>A), GTC1668GTT (5188C>T), AAG1669AAA (5191G>A), AAA1671AAG (5197A>G), GAC1672GAT (5200C>T), TGC1674TGT (5206C>T), TTA1675TTG (5209A>G), AGC1678AGT (5218C>T), TCG1682TCA (5230G>A), ATC1684ATT (5236C>T), AGC1685AGT (5239C>T), ATC1687ATT (5245C>T), ACA1693ACT (5263A>T), CTC1695CTT (5269C>T), TTC1696TTT (5272C>T), GCC1697GCA (5275C>A), ATC1698ATT (5278C>T), CCA1699CCC (5281A>C), AGA1700AGG (5284A>G), ACT1701ACC (5287T>C), CTA1702TTA (5288C>T), ATA1703ATT (5293A>T), TTG1704CTA (5294T>C 5296G>A), GAG1706GAA (5302G>A), CTT1709CTA (5311T>A), GAA1710GAG (5314A>G), CGA1713CGT (5323A>T), AGT1721AGC (5347T>C), ATC1723ATT (5353C>T), TGC1727TGT (5365C>T), GTT1734GTC (5386T>C), ACC1737ACA (5395C>A), AAA1746AAG (5422A>G), GAT1747GAC (5425T>C), ACC1749ACT (5431C>T), GAG1751GAA (5437G>A), CTT1756CTC (5452T>C), AAG1758AAA (5458G>A), AGT1761AGC (5467T>C), CAA1762CAG (5470A>G), CTA1763TTG (5471C>T 5473A>G), GAA1765GAG (5479A>G), TTC1766TTT (5482C>T), TCA1767TCC (5485A>C), AAT1768AAC (5488T>C), TTG1769CTA (5489T>C 5491G>A), GGT1771GGC (5497T>C), TGT1774TGC (5506T>C), GAG1775GAA (5509G>A), GAG1776GAA (5512G>A), TTA1778CTA (5516T>C), TAT1782TAC (5530T>C), GAG1783GAA (5533G>A), TCG1784TCT (5536G>T), TTG1785TTA (5539G>A), GTT1788GTG (5548T>G), CAC1790CAT (5554C>T), GCT1792GCA (5560T>A), GCG1793ACA (5561G>A 5563G>A), ACG1794ACC (5566G>C), TCA1795TCG (5569A>G), CTC1800CTG (5584C>G), AAG1803AAA (5593G>A), GGG1804GGA (5596G>A), ATT1805GTT (5597A>G), AAC1807AAA (5605C>A), GTG1811GTT (5617G>T), GCT1812GTG (5619C>T 5620T>G), TTG1815TTA (5629G>A), ATC1816CTC (5630A>C), GGC1819GGT (5641C>T), GCA1822GCT (5650A>T), ATA1826ATT (5662A>T), GGA1827GGG (5665A>G), CAA1835CAG (5689A>G), GTT1837ATT (5693G>A), GAG1838GAA (5698G>A), CAA1843CAG (5713A>G), GGG1844GGC (5716G>C), AAA1845AAG (5719A>G), GCC1853GCA (5743C>A), CGC1857CGA (5755C>A), CAT1858CAC (5758T>C), CGT1860CGC (5764T>C), AAA1862AAG (5770A>G), GGC1865GGT (5779C>T), TTT1866TTC (5782T>C), GAC1869GAT (5791C>T), GAC1873GAT (5803C>T), AGG1884AGA (5836G>A), AAA1885AAG (5839A>G), GGA1887GGG (5845A>G), AAA1888AAG (5848A>G), AAA1890AGA (5853A>G), GGT1891GGC (5857T>C), ACC1892ACT (5860C>T), ACA1893ACT (5863A>T), GGT1895GGA (5869T>A), GGT1897GGC (5875T>C), AGC1900AGT (5884C>T), TTC1903TTT (5893C>T), ATC1904GTG (5894A>G 5896C>G), AAC1905AAT (5899C>T), GGG1908GGA (5908G>A), TTT1909TTC (5911T>C), GAG1913GAA (5923G>A), CAA1918CAG (5938A>G), TTC1919TTT (5941C>T), GTT1920GTC (5944T>C), GAT1921GAC (5947T>C), GGG1925GGA (5959G>A), CGG1926GCC (5960C>G 5961G>C 5962G>C), GAA1930GAG (5974A>G), GCT1934GCA (5986T>A), GAC1935GAT (5989C>T), ATT1936GTT (5990A>G), GAT1938GAC (5998T>C), AGA1942CGT (6008A>C 6010A>T), GAA1945GAT (6019A>T), GTG1946GTT (6022G>T), CGA1947CGT (6025A>T), AAA1949ACA (6030A>C), GAC1955GAA (6049C>A), ATG1958ATA (6058G>A), CAA1959CAG (6061A>G), GCC1960GCA (6064C>A), GGT1962GGC (6070T>C), AGT1963AAC (6072G>A 6073T>C), AAC1964CAC (6074A>C), ACG1965ACA (6079G>A), ACC1966AAC (6081C>A), ATA1967ATT (6085A>T), GCA1969GCT (6091A>T), TAC1970TAT (6094C>T), TGT1976TCC (6111G>C 6112T>C), AAA1978AAG (6118A>G), TTG1980TTA (6124G>A), AAG1981AAA (6127G>A), ATT1982GTC (6128A>G 6130T>C), GAT1983GAC (6133T>C), TTA1984TTG (6136A>G), AAC1988AAT (6148C>T), AAA1991AAG (6157A>G), GTT1992ATT (6158G>A), TGT1993TGC (6163T>C), AAA1995AAG (6169A>G), ACA1996TCG (6170A>T 6172A>G), GGC1998GGT (6178C>T), AAA2001AAG (6187A>G), TTT2002TTC (6190T>C), GAG2004GAA (6196G>A), CTC2007TTT (6203C>T 6205C>T), GAA2008GAG (6208A>G), CTA2009TTG (6209C>T 6211A>G), CAG2011CAA (6217G>A), GCT2015GCA (6229T>A), GTA2016ATA (6230G>A), GTC2018GTT (6238C>T), AAG2021AAA (6247G>A), ATA2023ATT (6253A>T), GCA2025AAA (6257G>A 6258C>A), CAT2030CAC (6274T>C), AAA2033AGA (6282A>G), TCG2034TCA (6286G>A), GGC2038GGT (6298C>T), TTG2039TTA (6301G>A), AGA2040AGG (6304A>G), GAC2041GAT (6307C>T), GCC2046GCT (6322C>T), ACA2048ACT (6328A>T), GTT2049GTG (6331T>G), TGT2050TGC (6334T>C), AGG2051AGA (6337G>A), CTG2052TTG (6338C>T), TAT2058TAC (6358T>C), GGG2059GGA (6361G>A), GCA2060ACA (6362G>A), TCA2061TCT (6367A>T), GAG2062GAA (6370G>A), TAC2064TAT (6376C>T), GGT2065GGG (6379T>G), GGA2067GGC (6385A>C), GGA2069GGT (6391A>T), GCA2070GCG (6394A>G), TAC2071TAT (6397C>T), GTA2073ATA (6401G>A), GCG2074GTG (6405C>T), CAC2076CAT (6412C>T), TTA2078TTG (6418A>G), TTT2079TTC (6421T>C), TAC2082TTC (6429A>T), GGT2084GGA (6436T>A), GAG2087GAA (6445G>A), CAA2089CGA (6450A>G), TCC2090TCG (6454C>G), CAC2092CAT (6460C>T), GGT2093GGA (6463T>A), TTC2095TTT (6469C>T), AGG2096AGA (6472G>A), GTG2097GTA (6475G>A), AAT2099AAC (6481T>C), CAC2101AAT (6485C>A 6487C>T), AGT2102AGC (6490T>C), AGC2104AGT (6496C>T), CTG2106CTA (6502G>A), GGT2110GGC (6514T>C), AGG2111AGA (6517G>A), CTC2115ATC (6527C>A), ATC2116ATA (6532C>A), CCG2119CCA (6541G>A), AAA2120AAG (6544A>G), GTC2124GTT (6556C>T), TTT2125TTC (6559T>C), CAG2127CAA (6565G>A), TTG2129CTA (6569T>C 6571G>A), CCT2134CCA (6586T>A), ACA2135ATA (6588C>T), GAA2138GAG (6598A>G), AGA2139AGG (6601A>G), ATT2140ATC (6604T>C), TGT2141TGC (6607T>C), GGA2144GGG (6616A>G), AAC2146AAT (6622C>T), GAG2149GAA (6631G>A), AAG2150AAA (6634G>A), TAT2151CAT (6635T>C), GCT2152GCA (6640T>A), TCG2154TCT (6646G>T), ATC2155GTC (6647A>G), ATC2156ATT (6652C>T), AGC2160AGT (6664C>T), ACT2162ACG (6670T>G), ATA2165GTG (6677A>G 6679A>G), GGC2167GGT (6685C>T), ACA2169ACT (6691A>T), TTC2170TTT (6694C>T), ACA2177ACG (6715A>G), GAT2178AAT (6716G>A), AAT2179GAT (6719A>G), GGA2180GGG (6724A>G), CTA2184TTA (6734C>T), GTG2186GTA (6742G>A), GTG2187GTA (6745G>A), GCC2190GCT (6754C>T), GGA2192GGG (6760A>G), ATA2194TTG (6764A>T 6766A>G), ATT2197ATA (6775T>A), CTG2200TTG (6782C>T), GCA2201GCT (6787A>T), AAC2202AAT (6790C>T), GCA2204GTG (6795C>T 6796A>G), CAC2205CAA (6799C>A), ACC2206ACA (6802C>A), AAC2208AAT (6808C>T), TAC2209TAT (6811C>T), TAC2210TAT (6814C>T), TTC2213TTT (6823C>T), GAA2215GAG (6829A>G), TAC2221TAT (6847C>T), CGA2223CGC (6853A>C), AAT2225GAT (6857A>G), GAG2226GAA (6862G>A), GAA2229GAG (6871A>G), GTC2231ACA (6875G>A 6876T>C 6877C>A), AAG2232AAA (6880G>A), TCT2233TCA (6883T>A), GTT2235GTG (6889T>G), GAC2239GAT (6901C>T), GGC2244GGT (6916C>T), CCG2245CCA (6919G>A), AAA2247AAG (6925A>G), CTT2248CTC (6928T>C), GAC2250GAA (6934C>A), GGG2255GGC (6949G>C), TTC2257TTT (6955C>T), AAA2258AAG (6958A>G), CAA2264CAG (6976A>G), CTA2266TTA (6980C>T), ATC2267ATT (6985C>T), GTA2271GTT (6997A>T), GTG2272GTT (7000G>T), GTG2273ATA (7001G>A 7003G>A), GCT2276GCC (7012T>C), AAG2277AAA (7015G>A), CAT2278CAC (7018T>C), TTT2283TAT (7032T>A), GCC2285GCT (7039C>T), TTG2286CTA (7040T>C 7042G>A), GGA2288GGG (7048A>G), GCT2292GCA (7060T>A), GTC2293GTA (7063C>A), AGC2298AGT (7078C>T), TTA2300CTG (7082T>C 7084A>G), GTA2301GTG (7087A>G), ACC2302ACA (7090C>A), CAT2304CAC (7096T>C), GTA2305GTG (7099A>G), GTT2306GTC (7102T>C), GAG2309GAA (7111G>A), CGA2311CGG (7117A>G), ACA2314AAA (7125C>A), GAA2315GAG (7129A>G), TTT2316TTC (7132T>C), CTG2317TTA (7133C>T 7135G>A), GTG2319GTA (7141G>A), GAT2320GAC (7144T>C), GCA2321TCG (7145G>T 7147A>G), GAG2322GAA (7150G>A), GCA2325GCT (7159A>T), GAG2326_GCA2327del (7160_7165delGAGGCA), TTC2329TTT (7171C>T), GCG2335GCT (7189G>T), AAA2338AAG (7198A>G), AGC2339AGT (7201C>T), CTA2341TTA (7205C>T), AAT2342AAC (7210T>C), GAT2344GAA (7216T>A), GCG2345GCA (7219G>A), ATC2347ATA (7225C>A), AAG2348AAA (7228G>A), AAG2352AAA (7240G>A), TAT2353TAC (7243T>C), AAA2355AAG (7249A>G), CCT2356CCG (7252T>G), GAT2358GAA (7258T>A), GTT2359GTC (7261T>C), GGT2360GGA (7264T>A), GTC2361GTG (7267C>G), GTG2362GTA (7270G>A), GAT2363_CGG2364insT-- (7273_7274insT), CGG2364GTG (7274C>G 7275G>T), CAT2366CTT (7281A>T), TTG2367TCG (7284T>C), TCA2371TTA (7296C>T), TCT2376TTT (7311C>T), ACC2377ATC (7314C>T), TGC2378TAC (7317G>A), AAT2379AAA (7321T>A), GTG2380-TG (7322delG), CAC2381CAT (7327C>T), GGC2382GGT (7330C>T), TTC2383TTT (7333C>T), AAG2384CAG (7334A>C), TGT2386TGC (7342T>C), GCA2387AAC (7343G>A 7344C>A 7345A>C), ACT2390ACC (7354T>C), CAA2393CAG (7363A>G), ATT2395ATC (7369T>C), TTC2396TTT (7372C>T), GCG2398GCT (7378G>T), CTC2399CTT (7381C>T), GCA2404GCT (7396A>T), GTC2405GTT (7399C>T), GCC2407GCT (7405C>T), AGT2408ATG (7407G>T 7408T>G), GGG2410GGT (7414G>T), TGC2411GGC (7415T>G), AAA2412AAG (7420A>G), GAC2415GAT (7429C>T), TAT2416TAC (7432T>C), CAT2419CAC (7441T>C), AAG2425AAA (7459G>A), GAA2426GAG (7462A>G), ATA2428ATT (7468A>T), GTC2429GTA (7471C>A), CAA2431CAG (7477A>G), AGC2432AGT (7480C>T), CTG2434CTT (7486G>T), TAT2437TAC (7495T>C), AAA2438AAG (7498A>G), GGT2439GGC (7501T>C), TTG2440TCA (7503T>C 7504G>A), ATT2443ATA (7513T>A), AAC2445AAT (7519C>T), GGA2446GGG (7522A>G), AAG2449AAA (7531G>A), GAG2451GAA (7537G>A), CTC2452CTT (7540C>T), TGT2454TGC (7546T>C), AAG2462AAA (7570G>A), ACG2463ACA (7573G>A), ACG2465ACA (7579G>A), GCT2468GCC (7588T>C), CTA2471TTA (7595C>T), TTG2474CTA (7604T>C 7606G>A), GGT2477GGA (7615T>A), TGT2480TGC (7624T>C), GAC2483GAT (7633C>T), TTC2484TTT (7636C>T), AAT2485AAC (7639T>C), TTT2488TTC (7648T>C), GAA2494GAG (7666A>G), TGT2496TGC (7672T>C), ACA2498ACT (7678A>T), GGG2500GGA (7684G>A), ACT2502ACC (7690T>C), TTT2504TTC (7696T>C), GGT2506GGG (7702T>G), GAT2509AAT (7709G>A), AAA2510AGG (7713A>G 7714A>G), CTG2511TTG (7715C>T), CGT2514CGC (7726T>C), GAG2517GAA (7735G>A), GTA2520GTG (7744A>G), TAC2521TAT (7747C>T), GGC2526GGT (7762C>T), CAG2528CAA (7768G>A), TTT2529TTC (7771T>C), TCA2532TCG (7780A>G), CTA2533CTC (7783A>C), ACT2534ACC (7786T>C), CTA2537TTG (7793C>T 7795A>G), ATC2538ATT (7798C>T), GCT2540GCC (7804T>C), ACC2543ATC (7812C>T), GAA2550GAG (7834A>G), GAC2551GAT (7837C>T), GTG2554TTA (7844G>T 7846G>A), CAG2557CAA (7855G>A), CTG2559CTA (7861G>A), TTA2562TTG (7870A>G), TAC2563TAT (7873C>T), ACT2564ACA (7876T>A), GAG2565GAA (7879G>A), ATT2566ATA (7882T>A), GTT2567ATT (7883G>A), CCA2570CCT (7894A>T), ATT2571ATC (7897T>C), GTC2579GTT (7921C>T), AAG2581AAA (7927G>A), GGT2584GGC (7936T>C), AAT2585AAC (7939T>C), AAT2586AAC (7942T>C), GTG2594GTA (7966G>A), GAT2595GAC (7969T>C), TCT2597TCC (7975T>C), GTC2601GTT (7987C>T), CTT2602CTC (7990T>C), GCT2607GCC (8005T>C), GAA2616GAG (8032A>G), ATC2618ATT (8038C>T), GAC2619GAT (8041C>T), AGC2620AAC (8043G>A), TTG2632TTA (8080G>A), GTG2635GTA (8089G>A), CCG2637CCA (8095G>A), GAG2638GAA (8098G>A), AGC2641AGT (8107C>T), ATT2642ATC (8110T>C), GAT2644GAC (8116T>C), CAA2648CAG (8128A>G), GGT2654GGC (8146T>C), AAC2656AGT (8151A>G 8152C>T), TCG2660TCA (8164G>A), AGG2665AAG (8178G>A), AAG2666AAA (8182G>A), TTG2669TTA (8191G>A), TTC2671TTT (8197C>T), TCC2673TCT (8203C>T), AGA2675AAA (8208G>A), CTG2677TTA (8213C>T 8215G>A), CTA2678CTG (8218A>G), ATC2679ATT (8221C>T), GAG2680GAA (8224G>A), CCA2685CCG (8239A>G), GAG2690GAA (8254G>A), ATT2692ATC (8260T>C), GTA2693GTG (8263A>G), GAT2699GAC (8281T>C), AGA2700AGG (8284A>G), CCA2704GCG (8294C>G 8296A>G), GAG2705GAA (8299G>A), CAC2706CAT (8302C>T), ATT2711ATC (8317T>C), TGC2712TGT (8320C>T), GCT2714GCA (8326T>A), GAG2717GAA (8335G>A), TCC2718TCT (8338C>T), TCT2722TTT (8349C>T), GAA2723GAG (8353A>G), CAC2726CAT (8362C>T), CAA2727CAG (8365A>G), ATC2728ATT (8368C>T), TAC2732TAT (8380C>T), TCA2733TCG (8383A>G), TTA2735TTG (8389A>G), CCT2740CCC (8404T>C), GCA2742TCA (8408G>T), ACA2743ACG (8413A>G), GCG2745GCT (8419G>T), GCT2750GCC (8434T>C), TAT2752TAC (8440T>C), ATG2756ATA (8452G>A), CTA2758CTG (8458A>G), AGG2759AAG (8460G>A), AAA2760AAG (8464A>G), CTG2761CTA (8467G>A), AGG2765AGA (8479G>A), GCT2766ACA (8480G>A 8482T>A), GAG2771GAA (8497G>A), CTA2772TTG (8498C>T 8500A>G), AGA2773AAG (8502G>A 8503A>G), GCC2774GCT (8506C>T), GAA2777GAT (8515A>T), GTC2780GTT (8524C>T), GCA2781GCC (8527A>C), TTA2782CTG (8528T>C 8530A>G), GAG2785GAC (8539G>C), CTT2788TGT (8546C>T 8547T>G), TCT2790ACT (8552T>A), GTA2793GTG (8563A>G), GCA2797GGA (8574C>G), ATT2801GTT (8585A>G), GGA2805GAA (8598G>A), AGC2806AGT (8602C>T), AAC2807TCT (8603A>T 8604A>C 8605C>T), AAA2812AAG (8620A>G), CCA2813CAA (8622C>A), GAG2814GGA (8625A>G 8626G>A), CAG2815CAA (8629G>A), GGC2816GGT (8632C>T), ATC2818ATT (8638C>T), CAG2819CAA (8641G>A), AAC2821AAT (8647C>T), CCG2822TTC (8648C>T 8649C>T 8650G>C), AAC2823AAT (8653C>T), AAA2824AAG (8656A>G), GGA2825GAA (8658G>A), GAT2827GCG (8664A>C 8665T>G), GTT2830GTG (8674T>G), GCA2832GTT (8679C>T 8680A>T), GGC2833GGA (8683C>A), GGG2836GGA (8692G>A), ACA2837ACT (8695A>T), CCG2841CCA (8707G>A), AGA2842CGA (8708A>C), AAG2844AAA (8716G>A), ATC2846ATT (8722C>T), ACG2847ACA (8725G>A), AAA2849AAG (8731A>G), AGA2851AGG (8737A>G), CCC2853CCT (8743C>T), ACA2854AAG (8745C>A 8746A>G), AAG2856AAA (8752G>A), GGA2857GGT (8755A>T), AAC2862AAT (8770C>T), TTA2863CTA (8771T>C), GAA2864GAG (8776A>G), CAT2865CAC (8779T>C), TTG2866TTA (8782G>A), TAT2869TAC (8791T>C), CCA2871CCG (8797A>G), CAA2872CAG (8800A>G), CAA2873CAG (8803A>G), GAT2875GAC (8809T>C), ATT2876ATC (8812T>C), AAT2878AAC (8818T>C), CGG2880CGA (8824G>A), GCA2881GCG (8827A>G), ACG2888ACA (8848G>A), TAT2890TAC (8854T>C), GAG2891GGA (8856A>G 8857G>A), GTG2893GTA (8863G>A), CGG2894CAG (8865G>A), ATG2895CTT (8867A>C 8869G>T), GCA2896GCG (8872A>G), GGA2900GAA (8883G>A), ACT2902TCT (8888A>T), TGC2915TGT (8929C>T), GTC2924ATC (8954G>A), AAC2925AAT (8959C>T), GGA2926GGT (8962A>T), GGG2933GGA (8983G>A), AAT2934GAC (8984A>G 8986T>C), GAA2935GTA (8988A>T), GAG2938GAA (8998G>A), CCG2940CCA (9004G>A), TTG2941TTA (9007G>A), ATC2944ATT (9016C>T), ACC2951ACA (9037C>A), ATA2967ATT (9085A>T), AAC2971AAT (9097C>T), ATT2983GTT (9131A>G), CGA2984CGT (9136A>T), CGG2987CGT (9145G>T), ATG2989GGA (9149A>G 9150T>G 9151G>A), GGT2990AGT (9152G>A), TTA2991TTG (9157A>G), GCG2992GCT (9160G>T), CGT2993CGC (9163T>C), GAG3000GAA (9184G>A), CGA3004CGG (9196A>G), AGG3010AGA (9214G>A), GAA3011GAG (9217A>G), GCG3012GCA (9220G>A), GCA3020GCT (9244A>T), TTG3021TTA (9247G>A), GCC3024GCT (9256C>T), CCT3026TCC (9260C>T 9262T>C), GGG3030GGA (9274G>A), GAC3032GAT (9280C>T), ATC3035ATT (9289C>T), CAA3038CAC (9298A>C), GAG3040GAA (9304G>A), GTC3050GTT (9334C>T), GTC3060GTG (9364C>G)								
PVYgp2	1	75	100%	95	18.1%	75 (98.7%)	54 (71.1%)	1/0/3/2	2
Protein mutations:	R17_K17insX (2971_2972insA), Y23H (2989T>C 2991C>T), R26K (2999G>A 3000A>G), H35Y (3025C>T), K40H (3040A>C 3042A>C), G41S (3043G>A), E44K (3052G>A), I47V (3061A>G), V54I (3082G>A), P57S (3091C>T), P60T (3100C>A), G61E (3104G>A 3105T>G), G62S (3106G>A), Q63* (3109C>T), R64G (3112A>G), C66R (3118T>C), L67F (3121C>T), I69V (3127A>G), A72E (3137C>A 3138G>A), I73V (3139A>G)								
Codon mutations:	TTA2CTA (2923T>C), AAA4AAG (2931A>G), GAA7GAG (2940A>G), TTG10CTG (2947T>C), TTG15CTG (2962T>C), AGA17_AAA17insAA- (2971_2972insA), TAC23CAT (2989T>C 2991C>T), TCA24TCG (2994A>G), AGA26AAG (2999G>A 3000A>G), CAC32-AC (3016delC), TCG33TCA (3021G>A), GTA34GTT (3024A>T), CAT35TAT (3025C>T), CAC38CAT (3036C>T), AGA39AGG (3039A>G), AAA40CAC (3040A>C 3042A>C), GGC41AGC (3043G>A), GAA44AAA (3052G>A), ATA47GTA (3061A>G), GTT54ATT (3082G>A), CCG57TCG (3091C>T), CGC59CGT (3099C>T), CCA60ACA (3100C>A), GGT61GAG (3104G>A 3105T>G), GGT62AGT (3106G>A), CAA63TAA (3109C>T), AGG64GGG (3112A>G), TGC66CGC (3118T>C), CTC67TTC (3121C>T), ATT69GTT (3127A>G), GAG70GAA (3132G>A), GCG72GAA (3137C>A 3138G>A), ATT73GTT (3139A>G)								

	Begin	End	Coverage	Score	Concordance	Matches	Identities	I/D/M/F*	Stop Codons
**NT**	**22**	**9704**	**99.8%**	**12522**	**65.0%**	**9671 (99.8%)**	**8003 (82.6%)**	**10/12**	
**Proteins**									
polyprotein (NP_056759.1)	1	3064	100%	18923	89.7%	3062 (99.9%)	2771 (90.4%)	2/2/4/4	1
Protein mutations:	Y4Q (194T>C 196C>A), C9L (209T>C 210G>T 211T>G), F13M (221T>A 223T>G), S23P (251T>C), C24F (255G>T 256C>T), E25G (258A>G), I27V (263A>G 265T>G), V28A (267T>C), E30V (273A>T), A35T (287G>A 289T>C), V37T (293G>A 294T>C), A41I (305G>A 306C>T), D42G (309A>G), E44D (316A>T), T45V (317A>G 318C>T 319A>G), L52Q (339T>A 340C>A), K53R (342A>G), K55A (347A>G 348A>C 349A>G), Y56H (350T>C), T58V (356A>G 357C>T), V61T (365G>A 366T>C 367G>A), L62S (368C>T 369T>C), F67C (384T>G), A75V (408C>T 409C>T), M78E (416A>G 417T>A), E85K (437G>A), R86E (440A>G 441G>A), K87R (443A>C 444A>G 445G>A), D88E (448T>A), E91A (456A>C), H93N (461C>A), D111E (517T>A), S116F (531C>T), P118S (536C>T), Q119W (539C>T 540A>G 541A>G), R122K (549G>A 550A>G), M131T (576T>C), V134A (585T>C 586C>G), R137Y (593C>T 594G>A 595C>T), P138R (597C>G), I139V (599A>G 601A>G), I140P (602A>C 603T>C 604A>G), M147I (625G>A), I151V (635A>G 637T>C), I154V (644A>G 646A>G), E160A (663A>C 664G>C), H166Q (682C>A), T172S (699C>G), H174Q (706T>A), K179E (719A>G 721G>A), I180V (722A>G 724A>G), A183S (731G>T), Y184P (734T>C 735A>C 736C>T), S185R (737T>C 738C>G 739C>T), A187V (744C>T 745G>C), R189C (749C>T 751A>C), M194K (765T>A), R197K (773C>A 774G>A 775A>G), M206K (801T>A), G210A (813G>C 814A>G), L211H (816T>A), R214Q (825G>A 826T>A), N223D (851A>G 853T>C), R226C (860C>T 862C>T), T227A (863A>G 865T>C), I228T (867T>C), N229D (869A>G 871C>T), I230L (872A>T 874A>G), R231Q (876G>A), R232K (879G>A), N240S (903A>G), T241N (906C>A 907A>C), K242T (909A>C 910A>C), S243N (912G>A 913C>T), S252L (938T>C 939C>T 940A>T), L266I (980T>A 982G>C), R272K (999G>A), Q275R (1007C>A 1008A>G 1009G>A), S276G (1010A>G 1012T>G), I277V (1013A>G), N279Q (1019A>C 1021C>G), D288E (1048C>A), R300Q (1083G>A), S305A (1097T>G 1099G>A), R320K (1143G>A), L324I (1154T>A 1156G>A), S346N (1221G>A), V349T (1229G>A 1230T>C 1231T>C), S350N (1233G>A 1234C>T), K358T (1257A>C 1258A>G), N365S (1278A>G), A369V (1290C>T 1291G>A), D372T (1298G>A 1299A>C 1300C>T), I375V (1307A>G 1309A>G), N378E (1316A>G 1318T>A), I382T (1329T>C), L397I (1373C>A), I401V (1385A>G), E407D (1405G>T), A411S (1415G>T 1417A>T), V418I (1436G>A 1438C>T), E508G (1707A>G), A512T (1718G>A), I520V (1742A>G 1744C>T), S525N (1758G>A), V536I (1790G>A), I585V (1937A>G 1939T>C), F607Y (2004T>A), I628M (2068T>G), V630I (2072G>A 2074G>A), R675K (2208G>A), D694E (2266C>G), N744G (2414A>G 2415A>G 2416T>G), S746C (2420A>T), K780R (2523A>G), R817K (2634G>A), V847I (2723G>A 2725C>T), A855T (2747G>A 2749T>A), H871N (2795C>A), V880I (2822G>A 2824G>A), P900T (2882C>A), S901H (2885A>C 2886G>A), T904M (2895C>T), N916S (2931A>G), N919S (2940A>G), E929_N930insX (2971_2972insA), T936I (2991C>T), H937R (2994A>G), E939R (2999G>A 3000A>G), R946Q (3021G>A), Y947F (3024A>T 3025C>T), T951I (3036C>T), E952G (3039A>G 3040A>C), K953T (3042A>C 3043G>A), A972V (3099C>T 3100C>A), V974R (3104G>A 3105T>G 3106G>A), S983N (3132G>A), R985K (3137C>A 3138G>A 3139A>G), F990I (3152T>A 3154C>T), C994Y (3165G>A), F1012L (3218T>C), T1014N (3225C>A), V1016I (3230G>A), V1028M (3266G>A 3268A>G), V1031M (3275G>A), A1034T (3284G>A), R1044K (3315G>A 3316A>G), E1045A (3318A>C), M1059V (3359A>G), D1072E (3400T>G), V1110G (3513T>G), M1121V (3545A>G), A1122T (3548G>A), V1124I (3554G>A 3556C>T), L1147V (3623C>G), L1150M (3632C>A), Y1152H (3638T>C), I1172V (3698A>G), S1178N (3717G>A), R1183K (3731C>A 3732G>A), I1297V (4073A>G), F1315Y (4128T>A 4129C>T), A1323S (4151G>T), V1328I (4166G>A), L1347I (4223C>A 4225G>A), I1379V (4319A>G 4321A>G), F1404Y (4395T>A), V1408I (4406G>A), M1431V (4475A>G 4477G>T), T1433S (4481A>T 4483A>G), A1454L (4544G>T 4545C>T 4546G>A), R1455K (4548G>A), V1499I (4679G>A 4681G>C), V1538I (4796G>A 4798C>T), T1540S (4803C>G 4804T>C), S1625G (5057A>G), T1626A (5060A>G), L1636I (5090C>A), A1639V (5100C>T), E1641D (5107A>T), V1646F (5120G>T 5122C>T), I1648V (5126A>G), V1667I (5183G>A), A1793T (5561G>A 5563G>A), I1805V (5597A>G), N1807K (5605C>A), A1812V (5619C>T 5620T>G), I1816L (5630A>C), V1837I (5693G>A), K1890R (5853A>G), I1904V (5894A>G 5896C>G), R1926A (5960C>G 5961G>C 5962G>C), I1936V (5990A>G), E1945D (6019A>T), K1949T (6030A>C), D1955E (6049C>A), M1958I (6058G>A), S1963N (6072G>A 6073T>C), N1964H (6074A>C), T1966N (6081C>A), C1976S (6111G>C 6112T>C), I1982V (6128A>G 6130T>C), V1992I (6158G>A), T1996S (6170A>T 6172A>G), L2007F (6203C>T 6205C>T), V2016I (6230G>A), A2025K (6257G>A 6258C>A), K2033R (6282A>G), A2060T (6362G>A), V2073I (6401G>A), A2074V (6405C>T), Y2082F (6429A>T), Q2089R (6450A>G), H2101N (6485C>A 6487C>T), L2115I (6527C>A), T2135I (6588C>T), Y2151H (6635T>C), I2155V (6647A>G), I2165V (6677A>G 6679A>G), D2178N (6716G>A), N2179D (6719A>G), I2194L (6764A>T 6766A>G), A2204V (6795C>T 6796A>G), H2205Q (6799C>A), N2225D (6857A>G), V2231T (6875G>A 6876T>C 6877C>A), D2250E (6934C>A), V2273I (7001G>A 7003G>A), F2283Y (7032T>A), T2314K (7125C>A), A2321S (7145G>T 7147A>G), E2326_A2327del (7160_7165delGAGGCA), D2344E (7216T>A), D2358E (7258T>A), D2363_R2364insX (7273_7274insT), R2364V (7274C>G 7275G>T), H2366L (7281A>T), L2367S (7284T>C), S2371L (7296C>T), S2376F (7311C>T), T2377I (7314C>T), C2378Y (7317G>A), N2379K (7321T>A), K2384Q (7334A>C), A2387N (7343G>A 7344C>A 7345A>C), S2408M (7407G>T 7408T>G), C2411G (7415T>G), L2440S (7503T>C 7504G>A), D2509N (7709G>A), K2510R (7713A>G 7714A>G), T2543I (7812C>T), V2554L (7844G>T 7846G>A), V2567I (7883G>A), S2620N (8043G>A), N2656S (8151A>G 8152C>T), R2665K (8178G>A), R2675K (8208G>A), P2704A (8294C>G 8296A>G), S2722F (8349C>T), A2742S (8408G>T), M2756I (8452G>A), R2759K (8460G>A), A2766T (8480G>A 8482T>A), R2773K (8502G>A 8503A>G), E2777D (8515A>T), E2785D (8539G>C), L2788C (8546C>T 8547T>G), S2790T (8552T>A), A2797G (8574C>G), I2801V (8585A>G), G2805E (8598G>A), N2807S (8603A>T 8604A>C 8605C>T), P2813Q (8622C>A), E2814G (8625A>G 8626G>A), P2822F (8648C>T 8649C>T 8650G>C), G2825E (8658G>A), D2827A (8664A>C 8665T>G), A2832V (8679C>T 8680A>T), T2854K (8745C>A 8746A>G), E2891G (8856A>G 8857G>A), R2894Q (8865G>A), M2895L (8867A>C 8869G>T), G2900E (8883G>A), T2902S (8888A>T), V2924I (8954G>A), N2934D (8984A>G 8986T>C), E2935V (8988A>T), I2983V (9131A>G), M2989G (9149A>G 9150T>G 9151G>A), G2990S (9152G>A), P3026S (9260C>T 9262T>C), Q3038H (9298A>C)								

	Begin	End	Coverage	Score	Concordance	Matches	Identities	I/D/M/F*	Stop Codons
**NT**	**22**	**9704**	**99.8%**	**12522**	**65.0%**	**9671 (99.8%)**	**8003 (82.6%)**	**10/12**	

Codon mutations:

ACT3ACC (193T>C), TAC4CAA (194T>C 196C>A), TCA6TCG (202A>G), ATC8ATT (208C>T), TGT9CTG (209T>C 210G>T 211T>G), TCG12TCA (220G>A), TTT13ATG (221T>A 223T>G), GAA14GAG (226A>G), CTA17CTT (235A>T), TAC19TAT (241C>T), CCA21CCT (247A>T), TCT23CCT (251T>C), TGC24TTT (255G>T 256C>T), GAG25GGG (258A>G), CAT26CAC (262T>C), ATT27GTG (263A>G 265T>G), GTG28GCG (267T>C), GAA30GTA (273A>T), CGA31CGT (277A>T), GAA32GAG (280A>G), GTG33GTT (283G>T), CCG34CCA (286G>A), GCT35ACC (287G>A 289T>C), GTT37ACT (293G>A 294T>C), CCT39CCC (301T>C), TTC40TTT (304C>T), GCA41ATA (305G>A 306C>T), GAT42GGT (309A>G), CTG43TTG (311C>T), GAA44GAT (316A>T), ACA45GTG (317A>G 318C>T 319A>G), CAA46CAG (322A>G), GCA49GCT (331A>T), CTC52CAA (339T>A 340C>A), AAG53AGG (342A>G), AAA55GCG (347A>G 348A>C 349A>G), TAT56CAT (350T>C), GCT57GCA (355T>A), ACT58GTT (356A>G 357C>T), CGT60CGC (364T>C), GTG61ACA (365G>A 366T>C 367G>A), CTC62TCC (368C>T 369T>C), AAA63AAG (373A>G), AAC64AAT (376C>T), TTT67TGT (384T>G), ACG68ACA (388G>A), TAC69TAT (391C>T), CGA70CGG (394A>G), AAG72AAA (400G>A), GAT74GAC (406T>C), GCC75GTT (408C>T 409C>T), CAG76CAA (412G>A), ATA77ATT (415A>T), ATG78GAG (416A>G 417T>A), ATT80ATC (424T>C), CAG81CAA (427G>A), AAA83AAG (433A>G), CTG84CTC (436G>C), GAG85AAG (437G>A), AGG86GAG (440A>G 441G>A), AAG87CGA (443A>C 444A>G 445G>A), GAT88GAA (448T>A), GAA90GAG (454A>G), GAA91GCA (456A>C), TAT92TAC (460T>C), CAC93AAC (461C>A), CAA95CAG (469A>G), GCC97GCG (475C>G), CCT99CCA (481T>A), ACT106ACC (502T>C), ATA107ATT (505A>T), GGC109GGT (511C>T), GAT111GAA (517T>A), CCT112CCA (520T>A), CCA113CCT (523A>T), TCT116TTT (531C>T), GAG117GAA (535G>A), CCA118TCA (536C>T), CAA119TGG (539C>T 540A>G 541A>G), AGA122AAG (549G>A 550A>G), ATC124ATT (556C>T), ATT125ATC (559T>C), CAT126CAC (562T>C), ACT128ACC (568T>C), CCA129CCG (571A>G), ATG131ACG (576T>C), CGT132CGC (580T>C), GTC134GCG (585T>C 586C>G), CGC137TAT (593C>T 594G>A 595C>T), CCC138CGC (597C>G), ATA139GTG (599A>G 601A>G), ATA140CCG (602A>C 603T>C 604A>G), ACA143ACG (613A>G), GAA144GAG (616A>G), GGC145GGG (619C>G), CAG146CAA (622G>A), ATG147ATA (625G>A), AAT148AAC (628T>C), CAC149CAT (631C>T), ATT151GTC (635A>G 637T>C), ATA154GTG (644A>G 646A>G), ATT157ATC (655T>C), TCG159TCA (661G>A), GAG160GCC (663A>C 664G>C), AAA161AAG (667A>G), GTC165GTT (679C>T), CAC166CAA (682C>A), TTA167CTG (683T>C 685A>G), AGT169AGC (691T>C), ACC172AGC (699C>G), ACT173ACA (703T>A), CAT174CAA (706T>A), AAG179GAA (719A>G 721G>A), ATA180GTG (722A>G 724A>G), CTT181TTG (725C>T 727T>G), GGT182GGA (730T>A), GCA183TCA (731G>T), TAC184CCT (734T>C 735A>C 736C>T), TCC185CGT (737T>C 738C>G 739C>T), GCG187GTC (744C>T 745G>C), CGA189TGC (749C>T 751A>C), ACT190ACC (754T>C), ATG194AAG (765T>A), TTG196CTG (770T>C), CGA197AAG (773C>A 774G>A 775A>G), CGG198AGG (776C>A), TTC202TTT (790C>T), ATG206AAG (801T>A), GGA210GCG (813G>C 814A>G), CTT211CAT (816T>A), TTG212CTG (818T>C), CAA213CAG (823A>G), CGT214CAA (825G>A 826T>A), CGG217AGA (833C>A 835G>A), GAC219GAT (841C>T), AAT223GAC (851A>G 853T>C), GTC225GTT (859C>T), CGC226TGT (860C>T 862C>T), ACT227GCC (863A>G 865T>C), ATC228ACC (867T>C), AAC229GAT (869A>G 871C>T), ATA230TTG (872A>T 874A>G), CGA231CAA (876G>A), AGG232AAG (879G>A), GAT234GAC (886T>C), ATC238ATT (898C>T), AAC240AGC (903A>G), ACA241AAC (906C>A 907A>C), AAA242ACC (909A>C 910A>C), AGC243AAT (912G>A 913C>T), GGC246GGA (922C>A), GGT249GGA (931T>A), TCA252CTT (938T>C 939C>T 940A>T), GGA253GGG (943A>G), TTC256TTT (952C>T), TCA261TCG (967A>G), GAA263GAG (973A>G), GGG264GGC (976G>C), TTG266ATC (980T>A 982G>C), GCA269GCG (991A>G), CGT270CGC (994T>C), AGA272AAA (999G>A), GTT273GTC (1003T>C), CAG275AGA (1007C>A 1008A>G 1009G>A), AGT276GGG (1010A>G 1012T>G), ATT277GTT (1013A>G), TTA278TTG (1018A>G), AAC279CAG (1019A>C 1021C>G), ATC282ATT (1030C>T), TCG285TCA (1039G>A), AAT286AAC (1042T>C), GCC287GCA (1045C>A), GAC288GAA (1048C>A), AAT289AAC (1051T>C), AAG292AAA (1060G>A), GGT293GGA (1063T>A), CTG294TTG (1064C>T), GAC295GAT (1069C>T), CGA300CAA (1083G>A), TAT303TAC (1093T>C), TCG305GCA (1097T>G 1099G>A), CAC307CAT (1105C>T), GTA310GTG (1114A>G), GCT311GCG (1117T>G), TTA313TTG (1123A>G), CCT314CCA (1126T>A), GTC315GTT (1129C>T), AGG320AAG (1143G>A), GTA321GTT (1147A>T), GCT322GCA (1150T>A), TTG324ATA (1154T>A 1156G>A), CAC327CAT (1165C>T), AGT328AGC (1168T>C), CTT330TTA (1172C>T 1174T>A), ACC339ACT (1201C>T), AGC346AAC (1221G>A), GTT349ACC (1229G>A 1230T>C 1231T>C), AGC350AAT (1233G>A 1234C>T), CTG352CTA (1240G>A), AAG354AAA (1246G>A), TTG356CTA (1250T>C 1252G>A), CAT357CAC (1255T>C), AAA358ACG (1257A>C 1258A>G), AGA361AGG (1267A>G), GGT363GGC (1273T>C), TTG364TTA (1276G>A), AAT365AGT (1278A>G), TTG367CTG (1283T>C), GCG369GTA (1290C>T 1291G>A), GAC372ACT (1298G>A 1299A>C 1300C>T), TTT374TTC (1306T>C), ATA375GTG (1307A>G 1309A>G), CAT376CAC (1312T>C), AAT378GAA (1316A>G 1318T>A), TTG381TTA (1327G>A), ATA382ACA (1329T>C), GCG383GCT (1333G>T), TTA384TTG (1336A>G), CTA387TTG (1343C>T 1345A>G), CCG390CCA (1354G>A), GTG391GTT (1357G>T), GAC392GAT (1360C>T), CTC395TTG (1367C>T 1369C>G), CTT397ATT (1373C>A), TTC398TTT (1378C>T), GAG400GAA (1384G>A), ATA401GTA (1385A>G), AAA403AAG (1393A>G), TCC404TCT (1396C>T), GAG407GAT (1405G>T), AAA408AAG (1408A>G), CAG409CAA (1411G>A), GCA411TCT (1415G>T 1417A>T), CCG412CCC (1420G>C), AAT415AAC (1429T>C), TTA416TTG (1432A>G), GTC418ATT (1436G>A 1438C>T), TTA419CTG (1439T>C 1441A>G), AAT420AAC (1444T>C), TTC423TTT (1453C>T), CTG424CTA (1456G>A), GAA428GAG (1468A>G), GTA436GTG (1492A>G), TTG439CTA (1499T>C 1501G>A), AGT440AGC (1504T>C), TTG441TTA (1507G>A), CTC442CTT (1510C>T), GAA443GAG (1513A>G), TTA444TTG (1516A>G), GCA445GCT (1519A>T), AGG446AGA (1522G>A), TTC447TTT (1525C>T), CAG448CAA (1528G>A), AAC450AAT (1534C>T), ACT452ACA (1540T>A), AAC454AAT (1546C>T), AAA457AAG (1555A>G), GGT458GGC (1558T>C), GAT459GAC (1561T>C), ATA460ATC (1564A>C), TCT461TCA (1567T>A), TTC463TTT (1573C>T), AGA464AGG (1576A>G), TTA467CTA (1583T>C), TCT468TCA (1588T>A), AAG470AAA (1594G>A), AAC472AAT (1600C>T), CTG475CTA (1609G>A), TCG478TCA (1618G>A), TGC479TGT (1621C>T), GAC480GAT (1624C>T), CTG483TTG (1631C>T), AAA485AAG (1639A>G), TTC489TTT (1651C>T), CTC490CTG (1654C>G), CAT497CAC (1675T>C), TCA503TCG (1693A>G), TTT506TTC (1702T>C), GAA508GGA (1707A>G), GCA512ACA (1718G>A), GGA514GGG (1726A>G), TAC515TAT (1729C>T), TCA516TCC (1732A>C), TAT518TAC (1738T>C), ATC520GTT (1742A>G 1744C>T), CGC521CGT (1747C>T), CAT523CAC (1753T>C), AGT525AAT (1758G>A), AGG528AGA (1768G>A), AAC534AAT (1786C>T), TTA535TTG (1789A>G), GTT536ATT (1790G>A), GTC537GTT (1795C>T), TTA541CTG (1805T>C 1807A>G), GAG543GAA (1813G>A), AGG545AGA (1819G>A), AAG547AAA (1825G>A), GAC551GAT (1837C>T), TAT552TAC (1840T>C), CAA555CAG (1849A>G), GTC558GTG (1858C>G), AAA560AAG (1864A>G), TGC562TGT (1870C>T), AGT564AGC (1876T>C), AAA566AAG (1882A>G), GGT568GGA (1888T>A), AAT569AAC (1891T>C), TGT574TGC (1906T>C), ACA577ACT (1915A>T), GGT582GGC (1930T>C), GCC584GCT (1936C>T), ATT585GTC (1937A>G 1939T>C), TCA587TCG (1945A>G), TTC589TTT (1951C>T), CCA592CCT (1960A>T), GTA598GTG (1978A>G), ATT599ATA (1981T>A), GGC600GGT (1984C>T), AAT601AAC (1987T>C), GAC604GAT (1996C>T), TTT607TAT (2004T>A), AAA612AAG (2020A>G), GGG613GGA (2023G>A), GAT614GAC (2026T>C), TCG615TCT (2029G>T), GAG616GAA (2032G>A), TAT625TAC (2059T>C), ATT628ATG (2068T>G), AAC629AAT (2071C>T), GTG630ATA (2072G>A 2074G>A), CTG635TTA (2087C>T 2089G>A), AGC639AGT (2101C>T), GAG640GAA (2104G>A), GAG641GAA (2107G>A), AAG644AAA (2116G>A), TTC646TTT (2122C>T), CGC651CGT (2137C>T), CTT658CTC (2158T>C), ACC660ACT (2164C>T), CCA662CCG (2170A>G), GAT666GAC (2182T>C), GCG668GCA (2188G>A), ACC669ACA (2191C>A), AGA675AAA (2208G>A), TAT678TAC (2218T>C), GAC680GAT (2224C>T), GTA681GTT (2227A>T), TTG686TTA (2242G>A), CCC687CCT (2245C>T), TTG690TTA (2254G>A), GTT691GTC (2257T>C), GAC692GAT (2260C>T), GAC694GAG (2266C>G), CAA696CAG (2272A>G), ACG697ACA (2275G>A), TCA703TCG (2293A>G), GGC705GGT (2299C>T), TCG706TCA (2302G>A), CAG707CAA (2305G>A), GGA710GGT (2314A>T), TAT711TAC (2317T>C), CTA714CTG (2326A>G), GTG719GTA (2341G>A), CAA721CAG (2347A>G), CTT722CTC (2350T>C), TTG724TTA (2356G>A), AAT727AAC (2365T>C), GAA729GAG (2371A>G), TTA730TTG (2374A>G), GAT733GAC (2383T>C), ATA734ATT (2386A>T), AAA735AAG (2389A>G), CAT736CAC (2392T>C), TAT737TAC (2395T>C), AAT744GGG (2414A>G 2415A>G 2416T>G), GCT745GCA (2419T>A), AGC746TGC (2420A>T), ACA752ACT (2440A>T), GTT761GTC (2467T>C), TCT764TCC (2476T>C), TCG766TCA (2482G>A), CTG769CTA (2491G>A), CTG771CTT (2497G>T), TTG773TTA (2503G>A), AGA778AGG (2518A>G), CCT779CCC (2521T>C), AAG780AGG (2523A>G), AGA783AGG (2533A>G), TTG785TTA (2539G>A), TTA787TTG (2545A>G), GAG789GAA (2551G>A), CCT790CCA (2554T>A), CTG792TTG (2558C>T), TTG793CTC (2561T>C 2563G>C), TCA796TCG (2572A>G), CCT800CCC (2584T>C), GGC801GGT (2587C>T), CTG803CTC (2593G>C), GCT805GCC (2599T>C), AAT808AAC (2608T>C), TTT812TTC (2620T>C), GCG815GCA (2629G>A), GTG816GTA (2632G>A), AGG817AAG (2634G>A), TTG818TTA (2638G>A), ATT820ATA (2644T>A), AAA823AAG (2653A>G), GCT827GCC (2665T>C), CTA832TTA (2678C>T), CTA833CTG (2683A>G), GCT835GCC (2689T>C), CTA838CTC (2698A>C), TCA841TCG (2707A>G), GCG842GCA (2710G>A), GAA844GAG (2716A>G), ACA845ACG (2719A>G), CTC846CTT (2722C>T), GTC847ATT (2723G>A 2725C>T), GCA848GCG (2728A>G), AGG850AGA (2734G>A), ATT851ATA (2737T>A), GAT854GAC (2746T>C), GCT855ACA (2747G>A 2749T>A), GCA856GCT (2752A>T), GCT857GCA (2755T>A), GAC859GAT (2761C>T), CTC860CTT (2764C>T), TGT865TGC (2779T>C), GGG867GGT (2785G>T), AAC869AAT (2791C>T), CAT871AAT (2795C>A), TAC874TAT (2806C>T), TTG878CTC (2816T>C 2818G>C), GTG880ATA (2822G>A 2824G>A), TTG881TTA (2827G>A), GTT884GTC (2836T>C), AAG885AAA (2839G>A), AAT886AAC (2842T>C), ACC893ACA (2863C>A), CTA894TTA (2864C>T), CCA900ACA (2882C>A), AGT901CAT (2885A>C 2886G>A), AAC903AAT (2893C>T), ACG904ATG (2895C>T), GTC906GTT (2902C>T), GTA907GTG (2905A>G), ATT909ATC (2911T>C), AAT913AAC (2923T>C), AAT916AGT (2931A>G), AAC919AGC (2940A>G), GCT921GCC (2947T>C), ACT926ACC (2962T>C), GAA929_AAT930insA-- (2971_2972insA), GGT935GGC (2989T>C), ACT936ATT (2991C>T), CAT937CGT (2994A>G), GAG939AGG (2999G>A 3000A>G), TCC944TC- (3016delC), CGG946CAG (3021G>A), TAC947TTT (3024A>T 3025C>T), ACA951ATA (3036C>T), GAA952GGC (3039A>G 3040A>C), AAG953ACA (3042A>C 3043G>A), TTG956TTA (3052G>A), TTA959TTG (3061A>G), GCG966GCA (3082G>A), GGC969GGT (3091C>T), GCC972GTA (3099C>T 3100C>A), GTG974AGA (3104G>A 3105T>G 3106G>A), GTC975GTT (3109C>T), AAA976AAG (3112A>G), ACT978ACC (3118T>C), GCC979GCT (3121C>T), GGA981GGG (3127A>G), AGC983AAC (3132G>A), CGA985AAG (3137C>A 3138G>A 3139A>G), TAT989TAC (3151T>C), TTC990ATT (3152T>A 3154C>T), TGT994TAT (3165G>A), GTA995GTG (3169A>G), ATC1005ATT (3199C>T), AGG1007CGG (3203A>C), CGT1008CGC (3208T>C), CCA1010CCG (3214A>G), ACC1011ACT (3217C>T), TTT1012CTT (3218T>C), GTC1013GTA (3223C>A), ACT1014AAT (3225C>A), GTT1016ATT (3230G>A), AAC1017AAT (3235C>T), TTA1025CTA (3257T>C), ACC1026ACT (3262C>T), AGC1027AGT (3265C>T), GTA1028ATG (3266G>A 3268A>G), GTG1029GTA (3271G>A), GTG1031ATG (3275G>A), GCA1034ACA (3284G>A), CAG1039CAA (3301G>A), AGA1044AAG (3315G>A 3316A>G), GAA1045GCA (3318A>C), ATC1046ATT (3322C>T), GAG1047GAA (3325G>A), TTG1048TTA (3328G>A), ATA1051ATT (3337A>T), GAG1055GAA (3349G>A), GTC1057GTT (3355C>T), TGC1058TGT (3358C>T), ATG1059GTG (3359A>G), CTA1061CTG (3367A>G), TAT1062TAC (3370T>C), TTA1065CTG (3377T>C 3379A>G), GAA1070GAG (3394A>G), CGC1071CGT (3397C>T), GAT1072GAG (3400T>G), ACA1074ACG (3406A>G), TAC1078TAT (3418C>T), ATT1079ATA (3421T>A), TAT1081TAC (3427T>C), AAG1083AAA (3433G>A), TCA1084TCT (3436A>T), GTA1085GTG (3439A>G), AAC1086AAT (3442C>T), CCT1087CCC (3445T>C), CAG1091CAA (3457G>A), TTT1092TTC (3460T>C), GCT1093GCA (3463T>A), GCG1095GCT (3469G>T), CAG1096CAA (3472G>A), CAC1104CAT (3496C>T), CAG1105CAA (3499G>A), TCC1107TCT (3505C>T), GTT1110GGT (3513T>G), AAA1112AAG (3520A>G), GAA1115GAG (3529A>G), CAA1116CAG (3532A>G), ATG1121GTG (3545A>G), GCT1122ACT (3548G>A), TTA1123CTA (3551T>C), GTC1124ATT (3554G>A 3556C>T), AGT1133AGC (3583T>C), GAT1134GAC (3586T>C), TGC1135TGT (3589C>T), TTC1137TTT (3595C>T), AAT1141AAC (3607T>C), TTT1143TTC (3613T>C), AAG1144AAA (3616G>A), GGT1145GGC (3619T>C), CTT1147GTT (3623C>G), TCC1148TCT (3628C>T), CTG1150ATG (3632C>A), GAT1151GAC (3637T>C), TAT1152CAT (3638T>C), GAA1153GAG (3643A>G), TTA1159TTG (3661A>G), GTG1162GTA (3670G>A), TTT1166TTC (3682T>C), GAG1168GAA (3688G>A), GAG1171GAA (3697G>A), ATT1172GTT (3698A>G), GAT1174GAC (3706T>C), TTT1175TTC (3709T>C), TTG1177CTA (3713T>C 3715G>A), AGT1178AAT (3717G>A), GAG1179GAA (3721G>A), CGA1183AAA (3731C>A 3732G>A), ACT1184ACA (3736T>A), CTA1188TTG (3746C>T 3748A>G), ACA1190ACG (3754A>G), AGT1193AGC (3763T>C), CGA1198CGG (3778A>G), CAA1199CAG (3781A>G), ATC1200ATT (3784C>T), CAT1204CAC (3796T>C), CCA1207CCT (3805A>T), TAC1209TAT (3811C>T), AGA1210AGG (3814A>G), TTC1215TTT (3829C>T), TTT1218TTC (3838T>C), AGA1220AGG (3844A>G), GCA1221GCC (3847A>C), GTT1224GTA (3856T>A), GTG1226GTT (3862G>T), GAC1229GAT (3871C>T), GCC1231GCA (3877C>A), AGC1233AGT (3883C>T), GAA1234GAG (3886A>G), CTA1236CTG (3892A>G), GAC1237GAT (3895C>T), TTT1238TTC (3898T>C), GTA1240GTG (3904A>G), CGG1241AGA (3905C>A 3907G>A), GCT1243GCC (3913T>C), AAG1248AAA (3928G>A), TCA1249TCT (3931A>T), GGG1251GGA (3937G>A), AGT1257AGC (3955T>C), GTG1258GTA (3958G>A), GCC1259GCT (3961C>T), GGA1260GGG (3964A>G), TCT1261TCC (3967T>C), TTA1264TTG (3976A>G), ATT1265ATC (3979T>C), CCA1267CCT (3985A>T), CTA1271CTT (3997A>T), GCG1272GCA (4000G>A), AAC1274AAT (4006C>T), GTT1275GTG (4009T>G), TTC1276TTT (4012C>T), AAA1277AAG (4015A>G), TCT1280TCC (4024T>C), GAA1282GAG (4030A>G), CCA1283CCT (4033A>T), AAG1286AAA (4042G>A), CTG1290TTA (4052C>T 4054G>A), CGT1291CGC (4057T>C), CGT1293CGA (4063T>A), GGA1294GGG (4066A>G), ATA1297GTA (4073A>G), TTT1298TTC (4078T>C), GGC1299GGT (4081C>T), TCT1300TCC (4084T>C), TCC1304TCT (4096C>T), AGC1308AGT (4108C>T), GGA1309GGT (4111A>T), GCG1311GCA (4117G>A), CTA1312TTG (4118C>T 4120A>G), TAC1314TAT (4126C>T), TTC1315TAT (4128T>A 4129C>T), GCC1316GCT (4132C>T), AAT1317AAC (4135T>C), CAA1321CAG (4147A>G), TTA1322CTA (4148T>C), GCT1323TCT (4151G>T), AAC1326AAT (4162C>T), GTA1328ATA (4166G>A), ATA1329ATC (4171A>C), GAG1332GAA (4180G>A), CTG1336TTG (4190C>T), CCT1338CCC (4198T>C), TCC1339TCT (4201C>T), GCG1342GCT (4210G>T), TTC1343TTT (4213C>T), AGT1345AGC (4219T>C), CTG1346TTG (4220C>T), CTG1347ATA (4223C>A 4225G>A), GTT1349GTG (4231T>G), CAT1351CAC (4237T>C), GTA1356GTG (4252A>G), TTA1357CTG (4253T>C 4255A>G), AAA1358AAG (4258A>G), ACT1362ACC (4270T>C), AGA1366AGG (4282A>G), GAG1367GAA (4285G>A), GAA1369GAG (4291A>G), CAG1373CAA (4303G>A), GTC1376GTT (4312C>T), ATA1379GTG (4319A>G 4321A>G), GTG1380GTT (4324G>T), GAG1381GAA (4327G>A), CTG1384CTT (4336G>T), TCT1385TCC (4339T>C), TCA1388TCT (4348A>T), GCA1392GCG (4360A>G), GGT1394GGC (4366T>C), TCT1395TCA (4369T>A), ACT1397ACC (4375T>C), GTT1401GTC (4387T>C), TTT1404TAT (4395T>A), GTA1408ATA (4406G>A), CTT1409CTC (4411T>C), GTT1418GTC (4438T>C), ACC1420ACA (4444C>A), GCC1422GCT (4450C>T), CTC1424CTT (4456C>T), GAC1427GAT (4465C>T), ATG1431GTT (4475A>G 4477G>T), GTC1432GTT (4480C>T), ACA1433TCG (4481A>T 4483A>G), GGC1437GGT (4495C>T), AAG1441AAA (4507G>A), CAC1442CAT (4510C>T), GGT1443GGG (4513T>G), CTA1445TTA (4517C>T), GTC1448GTT (4528C>T), AAA1450AAG (4534A>G), ACC1452ACT (4540C>T), GCG1454TTA (4544G>T 4545C>T 4546G>A), AGA1455AAA (4548G>A), TTT1458TTC (4558T>C), GTA1460GTT (4564A>T), ACC1462ACT (4570C>T), ATA1464ATT (4576A>T), TTG1471TTA (4597G>A), GAC1472GAT (4600C>T), GAC1474GAT (4606C>T), GTG1475GTA (4609G>A), GTG1477GTA (4615G>A), GAC1478GAT (4618C>T), TTG1481CTT (4625T>C 4627G>T), GAC1488GAT (4648C>T), ATT1489ATC (4651T>C), AGG1492AGA (4660G>A), AGC1493AGT (4663C>T), GCT1495GCA (4669T>A), GTG1499ATC (4679G>A 4681G>C), AGT1500AGC (4684T>C), GTT1501GTG (4687T>G), GAG1505GAA (4699G>A), AGA1506AGG (4702A>G), CAA1508CAG (4708A>G), AGG1509AGA (4711G>A), CTG1510TTG (4712C>T), AAA1518AAG (4738A>G), GTA1520GTT (4744A>T), GCA1521GCG (4747A>G), TTG1522CTG (4748T>C), ACT1527ACC (4765T>C), GTC1538ATT (4796G>A 4798C>T), ACT1540AGC (4803C>G 4804T>C), GAG1541GAA (4807G>A), GCG1542GCT (4810G>T), GCT1543GCG (4813T>G), CTT1544CTC (4816T>C), TAT1549TAC

	(4831T>C), AAC1550AAT (4834C>T), CCA1552CCT (4840A>T), GGA1556GGG (4852A>G), GGC1557GGT (4855C>T), AGT1561AGC (4867T>C), CTG1562CTC (4870G>C), ATT1563ATA (4873T>A), TGT1566TGC (4882T>C), ACA1573ACT (4903A>T), CAG1575CAA (4909G>A), TTT1577TTC (4915T>C), GAA1578GAG (4918A>G), TTG1579CTA (4919T>C 4921G>A), AGT1580AGC (4924T>C), CCC1581CCG (4927C>G), TTT1583TTC (4933T>C), ATC1584ATA (4936C>A), CAG1585CAA (4939G>A), AAT1586AAC (4942T>C), TTC1587TTT (4945C>T), TCA1593TCG (4963A>G), CCT1596CCC (4972T>C), CTT1602CTC (4990T>C), AAA1606AAG (5002A>G), CCT1613CCC (5023T>C), TTG1614CTG (5024T>C), TGC1615TGT (5029C>T), GAT1616GAC (5032T>C), TCT1618TCC (5038T>C), CCA1620CCT (5044A>T), GCA1623GCT (5053A>T), TCG1624TCA (5056G>A), AGT1625GGT (5057A>G), ACT1626GCT (5060A>G), TCG1629TCT (5071G>T), CTT1636ATT (5090C>A), GGA1637GGG (5095A>G), GTG1638GTT (5098G>T), GCC1639GTC (5100C>T), TTA1640TTG (5104A>G), GAA1641GAT (5107A>T), ATT1642ATC (5110T>C), CCA1643CCG (5113A>G), AAG1644AAA (5116G>A), CAA1645CAG (5119A>G), GTC1646TTT (5120G>T 5122C>T), ATT1648GTT (5126A>G), CAT1651CAC (5137T>C), ATC1652ATT (5140C>T), AAA1653AAG (5143A>G), GAG1654GAA (5146G>A), CTC1659CTA (5161C>A), GAA1661GAG (5167A>G), CTT1663CTG (5173T>G), ACG1666ACA (5182G>A), GTT1667ATT (5183G>A), GTC1668GTT (5188C>T), AAG1669AAA (5191G>A), AAA1671AAG (5197A>G), GAC1672GAT (5200C>T), TGC1674TGT (5206C>T), TTA1675TTG (5209A>G), AGC1678AGT (5218C>T), TCG1682TCA (5230G>A), ATC1684ATT (5236C>T), AGC1685AGT (5239C>T), ATC1687ATT (5245C>T), ACA1693ACT (5263A>T), CTC1695CTT (5269C>T), TTC1696TTT (5272C>T), GCC1697GCA (5275C>A), ATC1698ATT (5278C>T), CCA1699CCC (5281A>C), AGA1700AGG (5284A>G), ACT1701ACC (5287T>C), CTA1702TTA (5288C>T), ATA1703ATT (5293A>T), TTG1704CTA (5294T>C 5296G>A), GAG1706GAA (5302G>A), CTT1709CTA (5311T>A), GAA1710GAG (5314A>G), CGA1713CGT (5323A>T), AGT1721AGC (5347T>C), ATC1723ATT (5353C>T), TGC1727TGT (5365C>T), GTT1734GTC (5386T>C), ACC1737ACA (5395C>A), AAA1746AAG (5422A>G), GAT1747GAC (5425T>C), ACC1749ACT (5431C>T), GAG1751GAA (5437G>A), CTT1756CTC (5452T>C), AAG1758AAA (5458G>A), AGT1761AGC (5467T>C), CAA1762CAG (5470A>G), CTA1763TTG (5471C>T 5473A>G), GAA1765GAG (5479A>G), TTC1766TTT (5482C>T), TCA1767TCC (5485A>C), AAT1768AAC (5488T>C), TTG1769CTA (5489T>C 5491G>A), GGT1771GGC (5497T>C), TGT1774TGC (5506T>C), GAG1775GAA (5509G>A), GAG1776GAA (5512G>A), TTA1778CTA (5516T>C), TAT1782TAC (5530T>C), GAG1783GAA (5533G>A), TCG1784TCT (5536G>T), TTG1785TTA (5539G>A), GTT1788GTG (5548T>G), CAC1790CAT (5554C>T), GCT1792GCA (5560T>A), GCG1793ACA (5561G>A 5563G>A), ACG1794ACC (5566G>C), TCA1795TCG (5569A>G), CTC1800CTG (5584C>G), AAG1803AAA (5593G>A), GGG1804GGA (5596G>A), ATT1805GTT (5597A>G), AAC1807AAA (5605C>A), GTG1811GTT (5617G>T), GCT1812GTG (5619C>T 5620T>G), TTG1815TTA (5629G>A), ATC1816CTC (5630A>C), GGC1819GGT (5641C>T), GCA1822GCT (5650A>T), ATA1826ATT (5662A>T), GGA1827GGG (5665A>G), CAA1835CAG (5689A>G), GTT1837ATT (5693G>A), GAG1838GAA (5698G>A), CAA1843CAG (5713A>G), GGG1844GGC (5716G>C), AAA1845AAG (5719A>G), GCC1853GCA (5743C>A), CGC1857CGA (5755C>A), CAT1858CAC (5758T>C), CGT1860CGC (5764T>C), AAA1862AAG (5770A>G), GGC1865GGT (5779C>T), TTT1866TTC (5782T>C), GAC1869GAT (5791C>T), GAC1873GAT (5803C>T), AGG1884AGA (5836G>A), AAA1885AAG (5839A>G), GGA1887GGG (5845A>G), AAA1888AAG (5848A>G), AAA1890AGA (5853A>G), GGT1891GGC (5857T>C), ACC1892ACT (5860C>T), ACA1893ACT (5863A>T), GGT1895GGA (5869T>A), GGT1897GGC (5875T>C), AGC1900AGT (5884C>T), TTC1903TTT (5893C>T), ATC1904GTG (5894A>G 5896C>G), AAC1905AAT (5899C>T), GGG1908GGA (5908G>A), TTT1909TTC (5911T>C), GAG1913GAA (5923G>A), CAA1918CAG (5938A>G), TTC1919TTT (5941C>T), GTT1920GTC (5944T>C), GAT1921GAC (5947T>C), GGG1925GGA (5959G>A), CGG1926GCC (5960C>G 5961G>C 5962G>C), GAA1930GAG (5974A>G), GCT1934GCA (5986T>A), GAC1935GAT (5989C>T), ATT1936GTT (5990A>G), GAT1938GAC (5998T>C), AGA1942CGT (6008A>C 6010A>T), GAA1945GAT (6019A>T), GTG1946GTT (6022G>T), CGA1947CGT (6025A>T), AAA1949ACA (6030A>C), GAC1955GAA (6049C>A), ATG1958ATA (6058G>A), CAA1959CAG (6061A>G), GCC1960GCA (6064C>A), GGT1962GGC (6070T>C), AGT1963AAC (6072G>A 6073T>C), AAC1964CAC (6074A>C), ACG1965ACA (6079G>A), ACC1966AAC (6081C>A), ATA1967ATT (6085A>T), GCA1969GCT (6091A>T), TAC1970TAT (6094C>T), TGT1976TCC (6111G>C 6112T>C), AAA1978AAG (6118A>G), TTG1980TTA (6124G>A), AAG1981AAA (6127G>A), ATT1982GTC (6128A>G 6130T>C), GAT1983GAC (6133T>C), TTA1984TTG (6136A>G), AAC1988AAT (6148C>T), AAA1991AAG (6157A>G), GTT1992ATT (6158G>A), TGT1993TGC (6163T>C), AAA1995AAG (6169A>G), ACA1996TCG (6170A>T 6172A>G), GGC1998GGT (6178C>T), AAA2001AAG (6187A>G), TTT2002TTC (6190T>C), GAG2004GAA (6196G>A), CTC2007TTT (6203C>T 6205C>T), GAA2008GAG (6208A>G), CTA2009TTG (6209C>T 6211A>G), CAG2011CAA (6217G>A), GCT2015GCA (6229T>A), GTA2016ATA (6230G>A), GTC2018GTT (6238C>T), AAG2021AAA (6247G>A), ATA2023ATT (6253A>T), GCA2025AAA (6257G>A 6258C>A), CAT2030CAC (6274T>C), AAA2033AGA (6282A>G), TCG2034TCA (6286G>A), GGC2038GGT (6298C>T), TTG2039TTA (6301G>A), AGA2040AGG (6304A>G), GAC2041GAT (6307C>T), GCC2046GCT (6322C>T), ACA2048ACT (6328A>T), GTT2049GTG (6331T>G), TGT2050TGC (6334T>C), AGG2051AGA (6337G>A), CTG2052TTG (6338C>T), TAT2058TAC (6358T>C), GGG2059GGA (6361G>A), GCA2060ACA (6362G>A), TCA2061TCT (6367A>T), GAG2062GAA (6370G>A), TAC2064TAT (6376C>T), GGT2065GGG (6379T>G), GGA2067GGC (6385A>C), GGA2069GGT (6391A>T), GCA2070GCG (6394A>G), TAC2071TAT (6397C>T), GTA2073ATA (6401G>A), GCG2074GTG (6405C>T), CAC2076CAT (6412C>T), TTA2078TTG (6418A>G), TTT2079TTC (6421T>C), TAC2082TTC (6429A>T), GGT2084GGA (6436T>A), GAG2087GAA (6445G>A), CAA2089CGA (6450A>G), TCC2090TCG (6454C>G), CAC2092CAT (6460C>T), GGT2093GGA (6463T>A), TTC2095TTT (6469C>T), AGG2096AGA (6472G>A), GTG2097GTA (6475G>A), AAT2099AAC (6481T>C), CAC2101AAT (6485C>A 6487C>T), AGT2102AGC (6490T>C), AGC2104AGT (6496C>T), CTG2106CTA (6502G>A), GGT2110GGC (6514T>C), AGG2111AGA (6517G>A), CTC2115ATC (6527C>A), ATC2116ATA (6532C>A), CCG2119CCA (6541G>A), AAA2120AAG (6544A>G), GTC2124GTT (6556C>T), TTT2125TTC (6559T>C), CAG2127CAA (6565G>A), TTG2129CTA (6569T>C 6571G>A), CCT2134CCA (6586T>A), ACA2135ATA (6588C>T), GAA2138GAG (6598A>G), AGA2139AGG (6601A>G), ATT2140ATC (6604T>C), TGT2141TGC (6607T>C), GGA2144GGG (6616A>G), AAC2146AAT (6622C>T), GAG2149GAA (6631G>A), AAG2150AAA (6634G>A), TAT2151CAT (6635T>C), GCT2152GCA (6640T>A), TCG2154TCT (6646G>T), ATC2155GTC (6647A>G), ATC2156ATT (6652C>T), AGC2160AGT (6664C>T), ACT2162ACG (6670T>G), ATA2165GTG (6677A>G 6679A>G), GGC2167GGT (6685C>T), ACA2169ACT (6691A>T), TTC2170TTT (6694C>T), ACA2177ACG (6715A>G), GAT2178AAT (6716G>A), AAT2179GAT (6719A>G), GGA2180GGG (6724A>G), CTA2184TTA (6734C>T), GTG2186GTA (6742G>A), GTG2187GTA (6745G>A), GCC2190GCT (6754C>T), GGA2192GGG (6760A>G), ATA2194TTG (6764A>T 6766A>G), ATT2197ATA (6775T>A), CTG2200TTG (6782C>T), GCA2201GCT (6787A>T), AAC2202AAT (6790C>T), GCA2204GTG (6795C>T 6796A>G), CAC2205CAA (6799C>A), ACC2206ACA (6802C>A), AAC2208AAT (6808C>T), TAC2209TAT (6811C>T), TAC2210TAT (6814C>T), TTC2213TTT (6823C>T), GAA2215GAG (6829A>G), TAC2221TAT (6847C>T), CGA2223CGC (6853A>C), AAT2225GAT (6857A>G), GAG2226GAA (6862G>A), GAA2229GAG (6871A>G), GTC2231ACA (6875G>A 6876T>C 6877C>A), AAG2232AAA (6880G>A), TCT2233TCA (6883T>A), GTT2235GTG (6889T>G), GAC2239GAT (6901C>T), GGC2244GGT (6916C>T), CCG2245CCA (6919G>A), AAA2247AAG (6925A>G), CTT2248CTC (6928T>C), GAC2250GAA (6934C>A), GGG2255GGC (6949G>C), TTC2257TTT (6955C>T), AAA2258AAG (6958A>G), CAA2264CAG (6976A>G), CTA2266TTA (6980C>T), ATC2267ATT (6985C>T), GTA2271GTT (6997A>T), GTG2272GTT (7000G>T), GTG2273ATA (7001G>A 7003G>A), GCT2276GCC (7012T>C), AAG2277AAA (7015G>A), CAT2278CAC (7018T>C), TTT2283TAT (7032T>A), GCC2285GCT (7039C>T), TTG2286CTA (7040T>C 7042G>A), GGA2288GGG (7048A>G), GCT2292GCA (7060T>A), GTC2293GTA (7063C>A), AGC2298AGT (7078C>T), TTA2300CTG (7082T>C 7084A>G), GTA2301GTG (7087A>G), ACC2302ACA (7090C>A), CAT2304CAC (7096T>C), GTA2305GTG (7099A>G), GTT2306GTC (7102T>C), GAG2309GAA (7111G>A), CGA2311CGG (7117A>G), ACA2314AAA (7125C>A), GAA2315GAG (7129A>G), TTT2316TTC (7132T>C), CTG2317TTA (7133C>T 7135G>A), GTG2319GTA (7141G>A), GAT2320GAC (7144T>C), GCA2321TCG (7145G>T 7147A>G), GAG2322GAA (7150G>A), GCA2325GCT (7159A>T), GAG2326_GCA2327del (7160_7165delGAGGCA), TTC2329TTT (7171C>T), GCG2335GCT (7189G>T), AAA2338AAG (7198A>G), AGC2339AGT (7201C>T), CTA2341TTA (7205C>T), AAT2342AAC (7210T>C), GAT2344GAA (7216T>A), GCG2345GCA (7219G>A), ATC2347ATA (7225C>A), AAG2348AAA (7228G>A), AAG2352AAA (7240G>A), TAT2353TAC (7243T>C), AAA2355AAG (7249A>G), CCT2356CCG (7252T>G), GAT2358GAA (7258T>A), GTT2359GTC (7261T>C), GGT2360GGA (7264T>A), GTC2361GTG (7267C>G), GTG2362GTA (7270G>A), GAT2363_CGG2364insT-- (7273_7274insT), CGG2364GTG (7274C>G 7275G>T), CAT2366CTT (7281A>T), TTG2367TCG (7284T>C), TCA2371TTA (7296C>T), TCT2376TTT (7311C>T), ACC2377ATC (7314C>T), TGC2378TAC (7317G>A), AAT2379AAA (7321T>A), GTG2380-TG (7322delG), CAC2381CAT (7327C>T), GGC2382GGT (7330C>T), TTC2383TTT (7333C>T), AAG2384CAG (7334A>C), TGT2386TGC (7342T>C), GCA2387AAC (7343G>A 7344C>A 7345A>C), ACT2390ACC (7354T>C), CAA2393CAG (7363A>G), ATT2395ATC (7369T>C), TTC2396TTT (7372C>T), GCG2398GCT (7378G>T), CTC2399CTT (7381C>T), GCA2404GCT (7396A>T), GTC2405GTT (7399C>T), GCC2407GCT (7405C>T), AGT2408ATG (7407G>T 7408T>G), GGG2410GGT (7414G>T), TGC2411GGC (7415T>G), AAA2412AAG (7420A>G), GAC2415GAT (7429C>T), TAT2416TAC (7432T>C), CAT2419CAC (7441T>C), AAG2425AAA (7459G>A), GAA2426GAG (7462A>G), ATA2428ATT (7468A>T), GTC2429GTA (7471C>A), CAA2431CAG (7477A>G), AGC2432AGT (7480C>T), CTG2434CTT (7486G>T), TAT2437TAC (7495T>C), AAA2438AAG (7498A>G), GGT2439GGC (7501T>C), TTG2440TCA (7503T>C 7504G>A), ATT2443ATA (7513T>A), AAC2445AAT (7519C>T), GGA2446GGG (7522A>G), AAG2449AAA (7531G>A), GAG2451GAA (7537G>A), CTC2452CTT (7540C>T), TGT2454TGC (7546T>C), AAG2462AAA (7570G>A), ACG2463ACA (7573G>A), ACG2465ACA (7579G>A), GCT2468GCC (7588T>C), CTA2471TTA (7595C>T), TTG2474CTA (7604T>C 7606G>A), GGT2477GGA (7615T>A), TGT2480TGC (7624T>C), GAC2483GAT (7633C>T), TTC2484TTT (7636C>T), AAT2485AAC (7639T>C), TTT2488TTC (7648T>C), GAA2494GAG (7666A>G), TGT2496TGC (7672T>C), ACA2498ACT (7678A>T), GGG2500GGA (7684G>A), ACT2502ACC (7690T>C), TTT2504TTC (7696T>C), GGT2506GGG (7702T>G), GAT2509AAT (7709G>A), AAA2510AGG (7713A>G 7714A>G), CTG2511TTG (7715C>T), CGT2514CGC (7726T>C), GAG2517GAA (7735G>A), GTA2520GTG (7744A>G), TAC2521TAT (7747C>T), GGC2526GGT (7762C>T), CAG2528CAA (7768G>A), TTT2529TTC (7771T>C), TCA2532TCG (7780A>G), CTA2533CTC (7783A>C), ACT2534ACC (7786T>C), CTA2537TTG (7793C>T 7795A>G), ATC2538ATT (7798C>T), GCT2540GCC (7804T>C), ACC2543ATC (7812C>T), GAA2550GAG (7834A>G), GAC2551GAT (7837C>T), GTG2554TTA (7844G>T 7846G>A), CAG2557CAA (7855G>A), CTG2559CTA (7861G>A), TTA2562TTG (7870A>G), TAC2563TAT (7873C>T), ACT2564ACA (7876T>A), GAG2565GAA (7879G>A), ATT2566ATA (7882T>A), GTT2567ATT (7883G>A), CCA2570CCT (7894A>T), ATT2571ATC (7897T>C), GTC2579GTT (7921C>T), AAG2581AAA (7927G>A), GGT2584GGC (7936T>C), AAT2585AAC (7939T>C), AAT2586AAC (7942T>C), GTG2594GTA (7966G>A), GAT2595GAC (7969T>C), TCT2597TCC (7975T>C), GTC2601GTT (7987C>T), CTT2602CTC (7990T>C), GCT2607GCC (8005T>C), GAA2616GAG (8032A>G), ATC2618ATT (8038C>T), GAC2619GAT (8041C>T), AGC2620AAC (8043G>A), TTG2632TTA (8080G>A), GTG2635GTA (8089G>A), CCG2637CCA (8095G>A), GAG2638GAA (8098G>A), AGC2641AGT (8107C>T), ATT2642ATC (8110T>C), GAT2644GAC (8116T>C), CAA2648CAG (8128A>G), GGT2654GGC (8146T>C), AAC2656AGT (8151A>G 8152C>T), TCG2660TCA (8164G>A), AGG2665AAG (8178G>A), AAG2666AAA (8182G>A), TTG2669TTA (8191G>A), TTC2671TTT (8197C>T), TCC2673TCT (8203C>T), AGA2675AAA (8208G>A), CTG2677TTA (8213C>T 8215G>A), CTA2678CTG (8218A>G), ATC2679ATT (8221C>T), GAG2680GAA (8224G>A), CCA2685CCG (8239A>G), GAG2690GAA (8254G>A), ATT2692ATC (8260T>C), GTA2693GTG (8263A>G), GAT2699GAC (8281T>C), AGA2700AGG (8284A>G), CCA2704GCG (8294C>G 8296A>G), GAG2705GAA (8299G>A), CAC2706CAT (8302C>T), ATT2711ATC (8317T>C), TGC2712TGT (8320C>T), GCT2714GCA (8326T>A), GAG2717GAA (8335G>A), TCC2718TCT (8338C>T), TCT2722TTT (8349C>T), GAA2723GAG (8353A>G), CAC2726CAT (8362C>T), CAA2727CAG (8365A>G), ATC2728ATT (8368C>T), TAC2732TAT (8380C>T), TCA2733TCG (8383A>G), TTA2735TTG (8389A>G), CCT2740CCC (8404T>C), GCA2742TCA (8408G>T), ACA2743ACG (8413A>G), GCG2745GCT (8419G>T), GCT2750GCC (8434T>C), TAT2752TAC (8440T>C), ATG2756ATA (8452G>A), CTA2758CTG (8458A>G), AGG2759AAG (8460G>A), AAA2760AAG (8464A>G), CTG2761CTA (8467G>A), AGG2765AGA (8479G>A), GCT2766ACA (8480G>A 8482T>A), GAG2771GAA (8497G>A), CTA2772TTG (8498C>T 8500A>G), AGA2773AAG (8502G>A 8503A>G), GCC2774GCT (8506C>T), GAA2777GAT (8515A>T), GTC2780GTT (8524C>T), GCA2781GCC (8527A>C), TTA2782CTG (8528T>C 8530A>G), GAG2785GAC (8539G>C), CTT2788TGT (8546C>T 8547T>G), TCT2790ACT (8552T>A), GTA2793GTG (8563A>G), GCA2797GGA (8574C>G), ATT2801GTT (8585A>G), GGA2805GAA (8598G>A), AGC2806AGT (8602C>T), AAC2807TCT (8603A>T 8604A>C 8605C>T), AAA2812AAG (8620A>G), CCA2813CAA (8622C>A), GAG2814GGA (8625A>G 8626G>A), CAG2815CAA (8629G>A), GGC2816GGT (8632C>T), ATC2818ATT (8638C>T), CAG2819CAA (8641G>A), AAC2821AAT (8647C>T), CCG2822TTC (8648C>T 8649C>T 8650G>C), AAC2823AAT (8653C>T), AAA2824AAG (8656A>G), GGA2825GAA (8658G>A), GAT2827GCG (8664A>C 8665T>G), GTT2830GTG (8674T>G), GCA2832GTT (8679C>T 8680A>T), GGC2833GGA (8683C>A), GGG2836GGA (8692G>A), ACA2837ACT (8695A>T), CCG2841CCA (8707G>A), AGA2842CGA (8708A>C), AAG2844AAA (8716G>A), ATC2846ATT (8722C>T), ACG2847ACA (8725G>A), AAA2849AAG (8731A>G), AGA2851AGG (8737A>G), CCC2853CCT (8743C>T), ACA2854AAG (8745C>A 8746A>G), AAG2856AAA (8752G>A), GGA2857GGT (8755A>T), AAC2862AAT (8770C>T), TTA2863CTA (8771T>C), GAA2864GAG (8776A>G), CAT2865CAC (8779T>C), TTG2866TTA (8782G>A), TAT2869TAC (8791T>C), CCA2871CCG (8797A>G), CAA2872CAG (8800A>G), CAA2873CAG (8803A>G), GAT2875GAC (8809T>C), ATT2876ATC (8812T>C), AAT2878AAC (8818T>C), CGG2880CGA (8824G>A), GCA2881GCG (8827A>G), ACG2888ACA (8848G>A), TAT2890TAC (8854T>C), GAG2891GGA (8856A>G 8857G>A), GTG2893GTA (8863G>A), CGG2894CAG (8865G>A), ATG2895CTT (8867A>C 8869G>T), GCA2896GCG (8872A>G), GGA2900GAA (8883G>A), ACT2902TCT (8888A>T), TGC2915TGT (8929C>T), GTC2924ATC (8954G>A), AAC2925AAT (8959C>T), GGA2926GGT (8962A>T), GGG2933GGA (8983G>A), AAT2934GAC (8984A>G 8986T>C), GAA2935GTA (8988A>T), GAG2938GAA (8998G>A), CCG2940CCA (9004G>A), TTG2941TTA (9007G>A), ATC2944ATT (9016C>T), ACC2951ACA (9037C>A), ATA2967ATT (9085A>T), AAC2971AAT (9097C>T), ATT2983GTT (9131A>G), CGA2984CGT (9136A>T), CGG2987CGT (9145G>T), ATG2989GGA (9149A>G 9150T>G 9151G>A), GGT2990AGT (9152G>A), TTA2991TTG (9157A>G), GCG2992GCT (9160G>T), CGT2993CGC (9163T>C), GAG3000GAA (9184G>A), CGA3004CGG (9196A>G), AGG3010AGA (9214G>A), GAA3011GAG (9217A>G), GCG3012GCA (9220G>A), GCA3020GCT (9244A>T), TTG3021TTA (9247G>A), GCC3024GCT (9256C>T), CCT3026TCC (9260C>T 9262T>C), GGG3030GGA (9274G>A), GAC3032GAT (9280C>T), ATC3035ATT (9289C>T), CAA3038CAC (9298A>C), GAG3040GAA (9304G>A), GTC3050GTT (9334C>T), GTC3060GTG (9364C>G)								
P1 protein (NP_734243.1)	1	284	100%	1394	72.7%	284 (100%)	203 (71.5%)	0/0/0/0	0

	Begin	End	Coverage	Score	Concordance	Matches	Identities	I/D/M/F*	Stop Codons
**NT**	**22**	**9704**	**99.8%**	**12522**	**65.0%**	**9671 (99.8%)**	**8003 (82.6%)**	**10/12**	
Protein mutations:	Y4Q (194T>C 196C>A), C9L (209T>C 210G>T 211T>G), F13M (221T>A 223T>G), S23P (251T>C), C24F (255G>T 256C>T), E25G (258A>G), I27V (263A>G 265T>G), V28A (267T>C), E30V (273A>T), A35T (287G>A 289T>C), V37T (293G>A 294T>C), A41I (305G>A 306C>T), D42G (309A>G), E44D (316A>T), T45V (317A>G 318C>T 319A>G), L52Q (339T>A 340C>A), K53R (342A>G), K55A (347A>G 348A>C 349A>G), Y56H (350T>C), T58V (356A>G 357C>T), V61T (365G>A 366T>C 367G>A), L62S (368C>T 369T>C), F67C (384T>G), A75V (408C>T 409C>T), M78E (416A>G 417T>A), E85K (437G>A), R86E (440A>G 441G>A), K87R (443A>C 444A>G 445G>A), D88E (448T>A), E91A (456A>C), H93N (461C>A), D111E (517T>A), S116F (531C>T), P118S (536C>T), Q119W (539C>T 540A>G 541A>G), R122K (549G>A 550A>G), M131T (576T>C), V134A (585T>C 586C>G), R137Y (593C>T 594G>A 595C>T), P138R (597C>G), I139V (599A>G 601A>G), I140P (602A>C 603T>C 604A>G), M147I (625G>A), I151V (635A>G 637T>C), I154V (644A>G 646A>G), E160A (663A>C 664G>C), H166Q (682C>A), T172S (699C>G), H174Q (706T>A), K179E (719A>G 721G>A), I180V (722A>G 724A>G), A183S (731G>T), Y184P (734T>C 735A>C 736C>T), S185R (737T>C 738C>G 739C>T), A187V (744C>T 745G>C), R189C (749C>T 751A>C), M194K (765T>A), R197K (773C>A 774G>A 775A>G), M206K (801T>A), G210A (813G>C 814A>G), L211H (816T>A), R214Q (825G>A 826T>A), N223D (851A>G 853T>C), R226C (860C>T 862C>T), T227A (863A>G 865T>C), I228T (867T>C), N229D (869A>G 871C>T), I230L (872A>T 874A>G), R231Q (876G>A), R232K (879G>A), N240S (903A>G), T241N (906C>A 907A>C), K242T (909A>C 910A>C), S243N (912G>A 913C>T), S252L (938T>C 939C>T 940A>T), L266I (980T>A 982G>C), R272K (999G>A), Q275R (1007C>A 1008A>G 1009G>A), S276G (1010A>G 1012T>G), I277V (1013A>G), N279Q (1019A>C 1021C>G)								
Codon mutations:	ACT3ACC (193T>C), TAC4CAA (194T>C 196C>A), TCA6TCG (202A>G), ATC8ATT (208C>T), TGT9CTG (209T>C 210G>T 211T>G), TCG12TCA (220G>A), TTT13ATG (221T>A 223T>G), GAA14GAG (226A>G), CTA17CTT (235A>T), TAC19TAT (241C>T), CCA21CCT (247A>T), TCT23CCT (251T>C), TGC24TTT (255G>T 256C>T), GAG25GGG (258A>G), CAT26CAC (262T>C), ATT27GTG (263A>G 265T>G), GTG28GCG (267T>C), GAA30GTA (273A>T), CGA31CGT (277A>T), GAA32GAG (280A>G), GTG33GTT (283G>T), CCG34CCA (286G>A), GCT35ACC (287G>A 289T>C), GTT37ACT (293G>A 294T>C), CCT39CCC (301T>C), TTC40TTT (304C>T), GCA41ATA (305G>A 306C>T), GAT42GGT (309A>G), CTG43TTG (311C>T), GAA44GAT (316A>T), ACA45GTG (317A>G 318C>T 319A>G), CAA46CAG (322A>G), GCA49GCT (331A>T), CTC52CAA (339T>A 340C>A), AAG53AGG (342A>G), AAA55GCG (347A>G 348A>C 349A>G), TAT56CAT (350T>C), GCT57GCA (355T>A), ACT58GTT (356A>G 357C>T), CGT60CGC (364T>C), GTG61ACA (365G>A 366T>C 367G>A), CTC62TCC (368C>T 369T>C), AAA63AAG (373A>G), AAC64AAT (376C>T), TTT67TGT (384T>G), ACG68ACA (388G>A), TAC69TAT (391C>T), CGA70CGG (394A>G), AAG72AAA (400G>A), GAT74GAC (406T>C), GCC75GTT (408C>T 409C>T), CAG76CAA (412G>A), ATA77ATT (415A>T), ATG78GAG (416A>G 417T>A), ATT80ATC (424T>C), CAG81CAA (427G>A), AAA83AAG (433A>G), CTG84CTC (436G>C), GAG85AAG (437G>A), AGG86GAG (440A>G 441G>A), AAG87CGA (443A>C 444A>G 445G>A), GAT88GAA (448T>A), GAA90GAG (454A>G), GAA91GCA (456A>C), TAT92TAC (460T>C), CAC93AAC (461C>A), CAA95CAG (469A>G), GCC97GCG (475C>G), CCT99CCA (481T>A), ACT106ACC (502T>C), ATA107ATT (505A>T), GGC109GGT (511C>T), GAT111GAA (517T>A), CCT112CCA (520T>A), CCA113CCT (523A>T), TCT116TTT (531C>T), GAG117GAA (535G>A), CCA118TCA (536C>T), CAA119TGG (539C>T 540A>G 541A>G), AGA122AAG (549G>A 550A>G), ATC124ATT (556C>T), ATT125ATC (559T>C), CAT126CAC (562T>C), ACT128ACC (568T>C), CCA129CCG (571A>G), ATG131ACG (576T>C), CGT132CGC (580T>C), GTC134GCG (585T>C 586C>G), CGC137TAT (593C>T 594G>A 595C>T), CCC138CGC (597C>G), ATA139GTG (599A>G 601A>G), ATA140CCG (602A>C 603T>C 604A>G), ACA143ACG (613A>G), GAA144GAG (616A>G), GGC145GGG (619C>G), CAG146CAA (622G>A), ATG147ATA (625G>A), AAT148AAC (628T>C), CAC149CAT (631C>T), ATT151GTC (635A>G 637T>C), ATA154GTG (644A>G 646A>G), ATT157ATC (655T>C), TCG159TCA (661G>A), GAG160GCC (663A>C 664G>C), AAA161AAG (667A>G), GTC165GTT (679C>T), CAC166CAA (682C>A), TTA167CTG (683T>C 685A>G), AGT169AGC (691T>C), ACC172AGC (699C>G), ACT173ACA (703T>A), CAT174CAA (706T>A), AAG179GAA (719A>G 721G>A), ATA180GTG (722A>G 724A>G), CTT181TTG (725C>T 727T>G), GGT182GGA (730T>A), GCA183TCA (731G>T), TAC184CCT (734T>C 735A>C 736C>T), TCC185CGT (737T>C 738C>G 739C>T), GCG187GTC (744C>T 745G>C), CGA189TGC (749C>T 751A>C), ACT190ACC (754T>C), ATG194AAG (765T>A), TTG196CTG (770T>C), CGA197AAG (773C>A 774G>A 775A>G), CGG198AGG (776C>A), TTC202TTT (790C>T), ATG206AAG (801T>A), GGA210GCG (813G>C 814A>G), CTT211CAT (816T>A), TTG212CTG (818T>C), CAA213CAG (823A>G), CGT214CAA (825G>A 826T>A), CGG217AGA (833C>A 835G>A), GAC219GAT (841C>T), AAT223GAC (851A>G 853T>C), GTC225GTT (859C>T), CGC226TGT (860C>T 862C>T), ACT227GCC (863A>G 865T>C), ATC228ACC (867T>C), AAC229GAT (869A>G 871C>T), ATA230TTG (872A>T 874A>G), CGA231CAA (876G>A), AGG232AAG (879G>A), GAT234GAC (886T>C), ATC238ATT (898C>T), AAC240AGC (903A>G), ACA241AAC (906C>A 907A>C), AAA242ACC (909A>C 910A>C), AGC243AAT (912G>A 913C>T), GGC246GGA (922C>A), GGT249GGA (931T>A), TCA252CTT (938T>C 939C>T 940A>T), GGA253GGG (943A>G), TTC256TTT (952C>T), TCA261TCG (967A>G), GAA263GAG (973A>G), GGG264GGC (976G>C), TTG266ATC (980T>A 982G>C), GCA269GCG (991A>G), CGT270CGC (994T>C), AGA272AAA (999G>A), GTT273GTC (1003T>C), CAG275AGA (1007C>A 1008A>G 1009G>A), AGT276GGG (1010A>G 1012T>G), ATT277GTT (1013A>G), TTA278TTG (1018A>G), AAC279CAG (1019A>C 1021C>G), ATC282ATT (1030C>T)								
HC-Pro protein (NP_734244.1)	1	456	100%	3144	95.7%	456 (100%)	425 (93.2%)	0/0/0/0	0
Protein mutations:	D4E (1048C>A), R16Q (1083G>A), S21A (1097T>G 1099G>A), R36K (1143G>A), L40I (1154T>A 1156G>A), S62N (1221G>A), V65T (1229G>A 1230T>C 1231T>C), S66N (1233G>A 1234C>T), K74T (1257A>C 1258A>G), N81S (1278A>G), A85V (1290C>T 1291G>A), D88T (1298G>A 1299A>C 1300C>T), I91V (1307A>G 1309A>G), N94E (1316A>G 1318T>A), I98T (1329T>C), L113I (1373C>A), I117V (1385A>G), E123D (1405G>T), A127S (1415G>T 1417A>T), V134I (1436G>A 1438C>T), E224G (1707A>G), A228T (1718G>A), I236V (1742A>G 1744C>T), S241N (1758G>A), V252I (1790G>A), I301V (1937A>G 1939T>C), F323Y (2004T>A), I344M (2068T>G), V346I (2072G>A 2074G>A), R391K (2208G>A), D410E (2266C>G)								
Codon mutations:	TCG1TCA (1039G>A), AAT2AAC (1042T>C), GCC3GCA (1045C>A), GAC4GAA (1048C>A), AAT5AAC (1051T>C), AAG8AAA (1060G>A), GGT9GGA (1063T>A), CTG10TTG (1064C>T), GAC11GAT (1069C>T), CGA16CAA (1083G>A), TAT19TAC (1093T>C), TCG21GCA (1097T>G 1099G>A), CAC23CAT (1105C>T), GTA26GTG (1114A>G), GCT27GCG (1117T>G), TTA29TTG (1123A>G), CCT30CCA (1126T>A), GTC31GTT (1129C>T), AGG36AAG (1143G>A), GTA37GTT (1147A>T), GCT38GCA (1150T>A), TTG40ATA (1154T>A 1156G>A), CAC43CAT (1165C>T), AGT44AGC (1168T>C), CTT46TTA (1172C>T 1174T>A), ACC55ACT (1201C>T), AGC62AAC (1221G>A), GTT65ACC (1229G>A 1230T>C 1231T>C), AGC66AAT (1233G>A 1234C>T), CTG68CTA (1240G>A), AAG70AAA (1246G>A), TTG72CTA (1250T>C 1252G>A), CAT73CAC (1255T>C), AAA74ACG (1257A>C 1258A>G), AGA77AGG (1267A>G), GGT79GGC (1273T>C), TTG80TTA (1276G>A), AAT81AGT (1278A>G), TTG83CTG (1283T>C), GCG85GTA (1290C>T 1291G>A), GAC88ACT (1298G>A 1299A>C 1300C>T), TTT90TTC (1306T>C), ATA91GTG (1307A>G 1309A>G), CAT92CAC (1312T>C), AAT94GAA (1316A>G 1318T>A), TTG97TTA (1327G>A), ATA98ACA (1329T>C), GCG99GCT (1333G>T), TTA100TTG (1336A>G), CTA103TTG (1343C>T 1345A>G), CCG106CCA (1354G>A), GTG107GTT (1357G>T), GAC108GAT (1360C>T), CTC111TTG (1367C>T 1369C>G), CTT113ATT (1373C>A), TTC114TTT (1378C>T), GAG116GAA (1384G>A), ATA117GTA (1385A>G), AAA119AAG (1393A>G), TCC120TCT (1396C>T), GAG123GAT (1405G>T), AAA124AAG (1408A>G), CAG125CAA (1411G>A), GCA127TCT (1415G>T 1417A>T), CCG128CCC (1420G>C), AAT131AAC (1429T>C), TTA132TTG (1432A>G), GTC134ATT (1436G>A 1438C>T), TTA135CTG (1439T>C 1441A>G), AAT136AAC (1444T>C), TTC139TTT (1453C>T), CTG140CTA (1456G>A), GAA144GAG (1468A>G), GTA152GTG (1492A>G), TTG155CTA (1499T>C 1501G>A), AGT156AGC (1504T>C), TTG157TTA (1507G>A), CTC158CTT (1510C>T), GAA159GAG (1513A>G), TTA160TTG (1516A>G), GCA161GCT (1519A>T), AGG162AGA (1522G>A), TTC163TTT (1525C>T), CAG164CAA (1528G>A), AAC166AAT (1534C>T), ACT168ACA (1540T>A), AAC170AAT (1546C>T), AAA173AAG (1555A>G), GGT174GGC (1558T>C), GAT175GAC (1561T>C), ATA176ATC (1564A>C), TCT177TCA (1567T>A), TTC179TTT (1573C>T), AGA180AGG (1576A>G), TTA183CTA (1583T>C), TCT184TCA (1588T>A), AAG186AAA (1594G>A), AAC188AAT (1600C>T), CTG191CTA (1609G>A), TCG194TCA (1618G>A), TGC195TGT (1621C>T), GAC196GAT (1624C>T), CTG199TTG (1631C>T), AAA201AAG (1639A>G), TTC205TTT (1651C>T), CTC206CTG (1654C>G), CAT213CAC (1675T>C), TCA219TCG (1693A>G), TTT222TTC (1702T>C), GAA224GGA (1707A>G), GCA228ACA (1718G>A), GGA230GGG (1726A>G), TAC231TAT (1729C>T), TCA232TCC (1732A>C), TAT234TAC (1738T>C), ATC236GTT (1742A>G 1744C>T), CGC237CGT (1747C>T), CAT239CAC (1753T>C), AGT241AAT (1758G>A), AGG244AGA (1768G>A), AAC250AAT (1786C>T), TTA251TTG (1789A>G), GTT252ATT (1790G>A), GTC253GTT (1795C>T), TTA257CTG (1805T>C 1807A>G), GAG259GAA (1813G>A), AGG261AGA (1819G>A), AAG263AAA (1825G>A), GAC267GAT (1837C>T), TAT268TAC (1840T>C), CAA271CAG (1849A>G), GTC274GTG (1858C>G), AAA276AAG (1864A>G), TGC278TGT (1870C>T), AGT280AGC (1876T>C), AAA282AAG (1882A>G), GGT284GGA (1888T>A), AAT285AAC (1891T>C), TGT290TGC (1906T>C), ACA293ACT (1915A>T), GGT298GGC (1930T>C), GCC300GCT (1936C>T), ATT301GTC (1937A>G 1939T>C), TCA303TCG (1945A>G), TTC305TTT (1951C>T), CCA308CCT (1960A>T), GTA314GTG (1978A>G), ATT315ATA (1981T>A), GGC316GGT (1984C>T), AAT317AAC (1987T>C), GAC320GAT (1996C>T), TTT323TAT (2004T>A), AAA328AAG (2020A>G), GGG329GGA (2023G>A), GAT330GAC (2026T>C), TCG331TCT (2029G>T), GAG332GAA (2032G>A), TAT341TAC (2059T>C), ATT344ATG (2068T>G), AAC345AAT (2071C>T), GTG346ATA (2072G>A 2074G>A), CTG351TTA (2087C>T 2089G>A), AGC355AGT (2101C>T), GAG356GAA (2104G>A), GAG357GAA (2107G>A), AAG360AAA (2116G>A), TTC362TTT (2122C>T), CGC367CGT (2137C>T), CTT374CTC (2158T>C), ACC376ACT (2164C>T), CCA378CCG (2170A>G), GAT382GAC (2182T>C), GCG384GCA (2188G>A), ACC385ACA (2191C>A), AGA391AAA (2208G>A), TAT394TAC (2218T>C), GAC396GAT (2224C>T), GTA397GTT (2227A>T), TTG402TTA (2242G>A), CCC403CCT (2245C>T), TTG406TTA (2254G>A), GTT407GTC (2257T>C), GAC408GAT (2260C>T), GAC410GAG (2266C>G), CAA412CAG (2272A>G), ACG413ACA (2275G>A), TCA419TCG (2293A>G), GGC421GGT (2299C>T), TCG422TCA (2302G>A), CAG423CAA (2305G>A), GGA426GGT (2314A>T), TAT427TAC (2317T>C), CTA430CTG (2326A>G), GTG435GTA (2341G>A), CAA437CAG (2347A>G), CTT438CTC (2350T>C), TTG440TTA (2356G>A), AAT443AAC (2365T>C), GAA445GAG (2371A>G), TTA446TTG (2374A>G), GAT449GAC (2383T>C), ATA450ATT (2386A>T), AAA451AAG (2389A>G), CAT452CAC (2392T>C), TAT453TAC (2395T>C)								
P3 protein (NP_734251.1)	1	365	100%	1924	79.6%	365 (99.7%)	327 (89.3%)	1/0/2/2	0
Protein mutations:	N4G (2414A>G 2415A>G 2416T>G), S6C (2420A>T), K40R (2523A>G), R77K (2634G>A), V107I (2723G>A 2725C>T), A115T (2747G>A 2749T>A), H131N (2795C>A), V140I (2822G>A 2824G>A), P160T (2882C>A), S161H (2885A>C 2886G>A), T164M (2895C>T), N176S (2931A>G), N179S (2940A>G), E189_N190insX (2971_2972insA), T196I (2991C>T), H197R (2994A>G), E199R (2999G>A 3000A>G), R206Q (3021G>A), Y207F (3024A>T 3025C>T), T211I (3036C>T), E212G (3039A>G 3040A>C), K213T (3042A>C 3043G>A), A232V (3099C>T 3100C>A), V234R (3104G>A 3105T>G 3106G>A), S243N (3132G>A), R245K (3137C>A 3138G>A 3139A>G), F250I (3152T>A 3154C>T), C254Y (3165G>A), F272L (3218T>C), T274N (3225C>A), V276I (3230G>A), V288M (3266G>A 3268A>G), V291M (3275G>A), A294T (3284G>A), R304K (3315G>A 3316A>G), E305A (3318A>C), M319V (3359A>G), D332E (3400T>G)								

	Begin	End	Coverage	Score	Concordance	Matches	Identities	I/D/M/F*	Stop Codons
**NT**	**22**	**9704**	**99.8%**	**12522**	**65.0%**	**9671 (99.8%)**	**8003 (82.6%)**	**10/12**	
Codon mutations:	AAT4GGG (2414A>G 2415A>G 2416T>G), GCT5GCA (2419T>A), AGC6TGC (2420A>T), ACA12ACT (2440A>T), GTT21GTC (2467T>C), TCT24TCC (2476T>C), TCG26TCA (2482G>A), CTG29CTA (2491G>A), CTG31CTT (2497G>T), TTG33TTA (2503G>A), AGA38AGG (2518A>G), CCT39CCC (2521T>C), AAG40AGG (2523A>G), AGA43AGG (2533A>G), TTG45TTA (2539G>A), TTA47TTG (2545A>G), GAG49GAA (2551G>A), CCT50CCA (2554T>A), CTG52TTG (2558C>T), TTG53CTC (2561T>C 2563G>C), TCA56TCG (2572A>G), CCT60CCC (2584T>C), GGC61GGT (2587C>T), CTG63CTC (2593G>C), GCT65GCC (2599T>C), AAT68AAC (2608T>C), TTT72TTC (2620T>C), GCG75GCA (2629G>A), GTG76GTA (2632G>A), AGG77AAG (2634G>A), TTG78TTA (2638G>A), ATT80ATA (2644T>A), AAA83AAG (2653A>G), GCT87GCC (2665T>C), CTA92TTA (2678C>T), CTA93CTG (2683A>G), GCT95GCC (2689T>C), CTA98CTC (2698A>C), TCA101TCG (2707A>G), GCG102GCA (2710G>A), GAA104GAG (2716A>G), ACA105ACG (2719A>G), CTC106CTT (2722C>T), GTC107ATT (2723G>A 2725C>T), GCA108GCG (2728A>G), AGG110AGA (2734G>A), ATT111ATA (2737T>A), GAT114GAC (2746T>C), GCT115ACA (2747G>A 2749T>A), GCA116GCT (2752A>T), GCT117GCA (2755T>A), GAC119GAT (2761C>T), CTC120CTT (2764C>T), TGT125TGC (2779T>C), GGG127GGT (2785G>T), AAC129AAT (2791C>T), CAT131AAT (2795C>A), TAC134TAT (2806C>T), TTG138CTC (2816T>C 2818G>C), GTG140ATA (2822G>A 2824G>A), TTG141TTA (2827G>A), GTT144GTC (2836T>C), AAG145AAA (2839G>A), AAT146AAC (2842T>C), ACC153ACA (2863C>A), CTA154TTA (2864C>T), CCA160ACA (2882C>A), AGT161CAT (2885A>C 2886G>A), AAC163AAT (2893C>T), ACG164ATG (2895C>T), GTC166GTT (2902C>T), GTA167GTG (2905A>G), ATT169ATC (2911T>C), AAT173AAC (2923T>C), AAT176AGT (2931A>G), AAC179AGC (2940A>G), GCT181GCC (2947T>C), ACT186ACC (2962T>C), GAA189_AAT190insA-- (2971_2972insA), GGT195GGC (2989T>C), ACT196ATT (2991C>T), CAT197CGT (2994A>G), GAG199AGG (2999G>A 3000A>G), TCC204TC- (3016delC), CGG206CAG (3021G>A), TAC207TTT (3024A>T 3025C>T), ACA211ATA (3036C>T), GAA212GGC (3039A>G 3040A>C), AAG213ACA (3042A>C 3043G>A), TTG216TTA (3052G>A), TTA219TTG (3061A>G), GCG226GCA (3082G>A), GGC229GGT (3091C>T), GCC232GTA (3099C>T 3100C>A), GTG234AGA (3104G>A 3105T>G 3106G>A), GTC235GTT (3109C>T), AAA236AAG (3112A>G), ACT238ACC (3118T>C), GCC239GCT (3121C>T), GGA241GGG (3127A>G), AGC243AAC (3132G>A), CGA245AAG (3137C>A 3138G>A 3139A>G), TAT249TAC (3151T>C), TTC250ATT (3152T>A 3154C>T), TGT254TAT (3165G>A), GTA255GTG (3169A>G), ATC265ATT (3199C>T), AGG267CGG (3203A>C), CGT268CGC (3208T>C), CCA270CCG (3214A>G), ACC271ACT (3217C>T), TTT272CTT (3218T>C), GTC273GTA (3223C>A), ACT274AAT (3225C>A), GTT276ATT (3230G>A), AAC277AAT (3235C>T), TTA285CTA (3257T>C), ACC286ACT (3262C>T), AGC287AGT (3265C>T), GTA288ATG (3266G>A 3268A>G), GTG289GTA (3271G>A), GTG291ATG (3275G>A), GCA294ACA (3284G>A), CAG299CAA (3301G>A), AGA304AAG (3315G>A 3316A>G), GAA305GCA (3318A>C), ATC306ATT (3322C>T), GAG307GAA (3325G>A), TTG308TTA (3328G>A), ATA311ATT (3337A>T), GAG315GAA (3349G>A), GTC317GTT (3355C>T), TGC318TGT (3358C>T), ATG319GTG (3359A>G), CTA321CTG (3367A>G), TAT322TAC (3370T>C), TTA325CTG (3377T>C 3379A>G), GAA330GAG (3394A>G), CGC331CGT (3397C>T), GAT332GAG (3400T>G), ACA334ACG (3406A>G), TAC338TAT (3418C>T), ATT339ATA (3421T>A), TAT341TAC (3427T>C), AAG343AAA (3433G>A), TCA344TCT (3436A>T), GTA345GTG (3439A>G), AAC346AAT (3442C>T), CCT347CCC (3445T>C), CAG351CAA (3457G>A), TTT352TTC (3460T>C), GCT353GCA (3463T>A), GCG355GCT (3469G>T), CAG356CAA (3472G>A), CAC364CAT (3496C>T), CAG365CAA (3499G>A)								
6K1 protein (NP_734245.1)	1	52	100%	305	90.5%	52 (100%)	45 (86.5%)	0/0/0/0	0
Protein mutations:	V5G (3513T>G), M16V (3545A>G), A17T (3548G>A), V19I (3554G>A 3556C>T), L42V (3623C>G), L45M (3632C>A), Y47H (3638T>C)								
Codon mutations:	TCC2TCT (3505C>T), GTT5GGT (3513T>G), AAA7AAG (3520A>G), GAA10GAG (3529A>G), CAA11CAG (3532A>G), ATG16GTG (3545A>G), GCT17ACT (3548G>A), TTA18CTA (3551T>C), GTC19ATT (3554G>A 3556C>T), AGT28AGC (3583T>C), GAT29GAC (3586T>C), TGC30TGT (3589C>T), TTC32TTT (3595C>T), AAT36AAC (3607T>C), TTT38TTC (3613T>C), AAG39AAA (3616G>A), GGT40GGC (3619T>C), CTT42GTT (3623C>G), TCC43TCT (3628C>T), CTG45ATG (3632C>A), GAT46GAC (3637T>C), TAT47CAT (3638T>C), GAA48GAG (3643A>G)								
CI protein (NP_734246.1)	1	634	100%	4190	97.9%	634 (100%)	608 (95.9%)	0/0/0/0	0
Protein mutations:	I15V (3698A>G), S21N (3717G>A), R26K (3731C>A 3732G>A), I140V (4073A>G), F158Y (4128T>A 4129C>T), A166S (4151G>T), V171I (4166G>A), L190I (4223C>A 4225G>A), I222V (4319A>G 4321A>G), F247Y (4395T>A), V251I (4406G>A), M274V (4475A>G 4477G>T), T276S (4481A>T 4483A>G), A297L (4544G>T 4545C>T 4546G>A), R298K (4548G>A), V342I (4679G>A 4681G>C), V381I (4796G>A 4798C>T), T383S (4803C>G 4804T>C), S468G (5057A>G), T469A (5060A>G), L479I (5090C>A), A482V (5100C>T), E484D (5107A>T), V489F (5120G>T 5122C>T), I491V (5126A>G), V510I (5183G>A)								
Codon mutations:	TTA2TTG (3661A>G), GTG5GTA (3670G>A), TTT9TTC (3682T>C), GAG11GAA (3688G>A), GAG14GAA (3697G>A), ATT15GTT (3698A>G), GAT17GAC (3706T>C), TTT18TTC (3709T>C), TTG20CTA (3713T>C 3715G>A), AGT21AAT (3717G>A), GAG22GAA (3721G>A), CGA26AAA (3731C>A 3732G>A), ACT27ACA (3736T>A), CTA31TTG (3746C>T 3748A>G), ACA33ACG (3754A>G), AGT36AGC (3763T>C), CGA41CGG (3778A>G), CAA42CAG (3781A>G), ATC43ATT (3784C>T), CAT47CAC (3796T>C), CCA50CCT (3805A>T), TAC52TAT (3811C>T), AGA53AGG (3814A>G), TTC58TTT (3829C>T), TTT61TTC (3838T>C), AGA63AGG (3844A>G), GCA64GCC (3847A>C), GTT67GTA (3856T>A), GTG69GTT (3862G>T), GAC72GAT (3871C>T), GCC74GCA (3877C>A), AGC76AGT (3883C>T), GAA77GAG (3886A>G), CTA79CTG (3892A>G), GAC80GAT (3895C>T), TTT81TTC (3898T>C), GTA83GTG (3904A>G), CGG84AGA (3905C>A 3907G>A), GCT86GCC (3913T>C), AAG91AAA (3928G>A), TCA92TCT (3931A>T), GGG94GGA (3937G>A), AGT100AGC (3955T>C), GTG101GTA (3958G>A), GCC102GCT (3961C>T), GGA103GGG (3964A>G), TCT104TCC (3967T>C), TTA107TTG (3976A>G), ATT108ATC (3979T>C), CCA110CCT (3985A>T), CTA114CTT (3997A>T), GCG115GCA (4000G>A), AAC117AAT (4006C>T), GTT118GTG (4009T>G), TTC119TTT (4012C>T), AAA120AAG (4015A>G), TCT123TCC (4024T>C), GAA125GAG (4030A>G), CCA126CCT (4033A>T), AAG129AAA (4042G>A), CTG133TTA (4052C>T 4054G>A), CGT134CGC (4057T>C), CGT136CGA (4063T>A), GGA137GGG (4066A>G), ATA140GTA (4073A>G), TTT141TTC (4078T>C), GGC142GGT (4081C>T), TCT143TCC (4084T>C), TCC147TCT (4096C>T), AGC151AGT (4108C>T), GGA152GGT (4111A>T), GCG154GCA (4117G>A), CTA155TTG (4118C>T 4120A>G), TAC157TAT (4126C>T), TTC158TAT (4128T>A 4129C>T), GCC159GCT (4132C>T), AAT160AAC (4135T>C), CAA164CAG (4147A>G), TTA165CTA (4148T>C), GCT166TCT (4151G>T), AAC169AAT (4162C>T), GTA171ATA (4166G>A), ATA172ATC (4171A>C), GAG175GAA (4180G>A), CTG179TTG (4190C>T), CCT181CCC (4198T>C), TCC182TCT (4201C>T), GCG185GCT (4210G>T), TTC186TTT (4213C>T), AGT188AGC (4219T>C), CTG189TTG (4220C>T), CTG190ATA (4223C>A 4225G>A), GTT192GTG (4231T>G), CAT194CAC (4237T>C), GTA199GTG (4252A>G), TTA200CTG (4253T>C 4255A>G), AAA201AAG (4258A>G), ACT205ACC (4270T>C), AGA209AGG (4282A>G), GAG210GAA (4285G>A), GAA212GAG (4291A>G), CAG216CAA (4303G>A), GTC219GTT (4312C>T), ATA222GTG (4319A>G 4321A>G), GTG223GTT (4324G>T), GAG224GAA (4327G>A), CTG227CTT (4336G>T), TCT228TCC (4339T>C), TCA231TCT (4348A>T), GCA235GCG (4360A>G), GGT237GGC (4366T>C), TCT238TCA (4369T>A), ACT240ACC (4375T>C), GTT244GTC (4387T>C), TTT247TAT (4395T>A), GTA251ATA (4406G>A), CTT252CTC (4411T>C), GTT261GTC (4438T>C), ACC263ACA (4444C>A), GCC265GCT (4450C>T), CTC267CTT (4456C>T), GAC270GAT (4465C>T), ATG274GTT (4475A>G 4477G>T), GTC275GTT (4480C>T), ACA276TCG (4481A>T 4483A>G), GGC280GGT (4495C>T), AAG284AAA (4507G>A), CAC285CAT (4510C>T), GGT286GGG (4513T>G), CTA288TTA (4517C>T), GTC291GTT (4528C>T), AAA293AAG (4534A>G), ACC295ACT (4540C>T), GCG297TTA (4544G>T 4545C>T 4546G>A), AGA298AAA (4548G>A), TTT301TTC (4558T>C), GTA303GTT (4564A>T), ACC305ACT (4570C>T), ATA307ATT (4576A>T), TTG314TTA (4597G>A), GAC315GAT (4600C>T), GAC317GAT (4606C>T), GTG318GTA (4609G>A), GTG320GTA (4615G>A), GAC321GAT (4618C>T), TTG324CTT (4625T>C 4627G>T), GAC331GAT (4648C>T), ATT332ATC (4651T>C), AGG335AGA (4660G>A), AGC336AGT (4663C>T), GCT338GCA (4669T>A), GTG342ATC (4679G>A 4681G>C), AGT343AGC (4684T>C), GTT344GTG (4687T>G), GAG348GAA (4699G>A), AGA349AGG (4702A>G), CAA351CAG (4708A>G), AGG352AGA (4711G>A), CTG353TTG (4712C>T), AAA361AAG (4738A>G), GTA363GTT (4744A>T), GCA364GCG (4747A>G), TTG365CTG (4748T>C), ACT370ACC (4765T>C), GTC381ATT (4796G>A 4798C>T), ACT383AGC (4803C>G 4804T>C), GAG384GAA (4807G>A), GCG385GCT (4810G>T), GCT386GCG (4813T>G), CTT387CTC (4816T>C), TAT392TAC (4831T>C), AAC393AAT (4834C>T), CCA395CCT (4840A>T), GGA399GGG (4852A>G), GGC400GGT (4855C>T), AGT404AGC (4867T>C), CTG405CTC (4870G>C), ATT406ATA (4873T>A), TGT409TGC (4882T>C), ACA416ACT (4903A>T), CAG418CAA (4909G>A), TTT420TTC (4915T>C), GAA421GAG (4918A>G), TTG422CTA (4919T>C 4921G>A), AGT423AGC (4924T>C), CCC424CCG (4927C>G), TTT426TTC (4933T>C), ATC427ATA (4936C>A), CAG428CAA (4939G>A), AAT429AAC (4942T>C), TTC430TTT (4945C>T), TCA436TCG (4963A>G), CCT439CCC (4972T>C), CTT445CTC (4990T>C), AAA449AAG (5002A>G), CCT456CCC (5023T>C), TTG457CTG (5024T>C), TGC458TGT (5029C>T), GAT459GAC (5032T>C), TCT461TCC (5038T>C), CCA463CCT (5044A>T), GCA466GCT (5053A>T), TCG467TCA (5056G>A), AGT468GGT (5057A>G), ACT469GCT (5060A>G), TCG472TCT (5071G>T), CTT479ATT (5090C>A), GGA480GGG (5095A>G), GTG481GTT (5098G>T), GCC482GTC (5100C>T), TTA483TTG (5104A>G), GAA484GAT (5107A>T), ATT485ATC (5110T>C), CCA486CCG (5113A>G), AAG487AAA (5116G>A), CAA488CAG (5119A>G), GTC489TTT (5120G>T 5122C>T), ATT491GTT (5126A>G), CAT494CAC (5137T>C), ATC495ATT (5140C>T), AAA496AAG (5143A>G), GAG497GAA (5146G>A), CTC502CTA (5161C>A), GAA504GAG (5167A>G), CTT506CTG (5173T>G), ACG509ACA (5182G>A), GTT510ATT (5183G>A), GTC511GTT (5188C>T), AAG512AAA (5191G>A), AAA514AAG (5197A>G), GAC515GAT (5200C>T), TGC517TGT (5206C>T), TTA518TTG (5209A>G), AGC521AGT (5218C>T), TCG525TCA (5230G>A), ATC527ATT (5236C>T), AGC528AGT (5239C>T), ATC530ATT (5245C>T), ACA536ACT (5263A>T), CTC538CTT (5269C>T), TTC539TTT (5272C>T), GCC540GCA (5275C>A), ATC541ATT (5278C>T), CCA542CCC (5281A>C), AGA543AGG (5284A>G), ACT544ACC (5287T>C), CTA545TTA (5288C>T), ATA546ATT (5293A>T), TTG547CTA (5294T>C 5296G>A), GAG549GAA (5302G>A), CTT552CTA (5311T>A), GAA553GAG (5314A>G), CGA556CGT (5323A>T), AGT564AGC (5347T>C), ATC566ATT (5353C>T), TGC570TGT (5365C>T), GTT577GTC (5386T>C), ACC580ACA (5395C>A), AAA589AAG (5422A>G), GAT590GAC (5425T>C), ACC592ACT (5431C>T), GAG594GAA (5437G>A), CTT599CTC (5452T>C), AAG601AAA (5458G>A), AGT604AGC (5467T>C), CAA605CAG (5470A>G), CTA606TTG (5471C>T 5473A>G), GAA608GAG (5479A>G), TTC609TTT (5482C>T), TCA610TCC (5485A>C), AAT611AAC (5488T>C), TTG612CTA (5489T>C 5491G>A), GGT614GGC (5497T>C), TGT617TGC (5506T>C), GAG618GAA (5509G>A), GAG619GAA (5512G>A), TTA621CTA (5516T>C), TAT625TAC (5530T>C), GAG626GAA (5533G>A), TCG627TCT (5536G>T), TTG628TTA (5539G>A), GTT631GTG (5548T>G), CAC633CAT (5554C>T)								
6K2 protein (NP_734247.1)	1	52	100%	303	93.5%	52 (100%)	46 (88.5%)	0/0/0/0	0
Protein mutations:	A2T (5561G>A 5563G>A), I14V (5597A>G), N16K (5605C>A), A21V (5619C>T 5620T>G), I25L (5630A>C), V46I (5693G>A)								
Codon mutations:	GCT1GCA (5560T>A), GCG2ACA (5561G>A 5563G>A), ACG3ACC (5566G>C), TCA4TCG (5569A>G), CTC9CTG (5584C>G), AAG12AAA (5593G>A), GGG13GGA (5596G>A), ATT14GTT (5597A>G), AAC16AAA (5605C>A), GTG20GTT (5617G>T), GCT21GTG (5619C>T 5620T>G), TTG24TTA (5629G>A), ATC25CTC (5630A>C), GGC28GGT (5641C>T), GCA31GCT (5650A>T), ATA35ATT (5662A>T), GGA36GGG (5665A>G), CAA44CAG (5689A>G), GTT46ATT (5693G>A), GAG47GAA (5698G>A), CAA52CAG (5713A>G)								
NIa-VPg protein (NP_734252.1)	1	188	100%	1227	93.3%	188 (100%)	170 (90.4%)	0/0/0/0	0
Protein mutations:	K47R (5853A>G), I61V (5894A>G 5896C>G), R83A (5960C>G 5961G>C 5962G>C), I93V (5990A>G), E102D (6019A>T), K106T (6030A>C), D112E (6049C>A), M115I (6058G>A), S120N (6072G>A 6073T>C), N121H (6074A>C), T123N (6081C>A), C133S (6111G>C 6112T>C), I139V (6128A>G 6130T>C), V149I (6158G>A), T153S (6170A>T 6172A>G), L164F (6203C>T 6205C>T), V173I (6230G>A), A182K (6257G>A 6258C>A)								

	Begin	End	Coverage	Score	Concordance	Matches	Identities	I/D/M/F*	Stop Codons
**NT**	**22**	**9704**	**99.8%**	**12522**	**65.0%**	**9671 (99.8%)**	**8003 (82.6%)**	**10/12**	
Codon mutations:	GGG1GGC (5716G>C), AAA2AAG (5719A>G), GCC10GCA (5743C>A), CGC14CGA (5755C>A), CAT15CAC (5758T>C), CGT17CGC (5764T>C), AAA19AAG (5770A>G), GGC22GGT (5779C>T), TTT23TTC (5782T>C), GAC26GAT (5791C>T), GAC30GAT (5803C>T), AGG41AGA (5836G>A), AAA42AAG (5839A>G), GGA44GGG (5845A>G), AAA45AAG (5848A>G), AAA47AGA (5853A>G), GGT48GGC (5857T>C), ACC49ACT (5860C>T), ACA50ACT (5863A>T), GGT52GGA (5869T>A), GGT54GGC (5875T>C), AGC57AGT (5884C>T), TTC60TTT (5893C>T), ATC61GTG (5894A>G 5896C>G), AAC62AAT (5899C>T), GGG65GGA (5908G>A), TTT66TTC (5911T>C), GAG70GAA (5923G>A), CAA75CAG (5938A>G), TTC76TTT (5941C>T), GTT77GTC (5944T>C), GAT78GAC (5947T>C), GGG82GGA (5959G>A), CGG83GCC (5960C>G 5961G>C 5962G>C), GAA87GAG (5974A>G), GCT91GCA (5986T>A), GAC92GAT (5989C>T), ATT93GTT (5990A>G), GAT95GAC (5998T>C), AGA99CGT (6008A>C 6010A>T), GAA102GAT (6019A>T), GTG103GTT (6022G>T), CGA104CGT (6025A>T), AAA106ACA (6030A>C), GAC112GAA (6049C>A), ATG115ATA (6058G>A), CAA116CAG (6061A>G), GCC117GCA (6064C>A), GGT119GGC (6070T>C), AGT120AAC (6072G>A 6073T>C), AAC121CAC (6074A>C), ACG122ACA (6079G>A), ACC123AAC (6081C>A), ATA124ATT (6085A>T), GCA126GCT (6091A>T), TAC127TAT (6094C>T), TGT133TCC (6111G>C 6112T>C), AAA135AAG (6118A>G), TTG137TTA (6124G>A), AAG138AAA (6127G>A), ATT139GTC (6128A>G 6130T>C), GAT140GAC (6133T>C), TTA141TTG (6136A>G), AAC145AAT (6148C>T), AAA148AAG (6157A>G), GTT149ATT (6158G>A), TGT150TGC (6163T>C), AAA152AAG (6169A>G), ACA153TCG (6170A>T 6172A>G), GGC155GGT (6178C>T), AAA158AAG (6187A>G), TTT159TTC (6190T>C), GAG161GAA (6196G>A), CTC164TTT (6203C>T 6205C>T), GAA165GAG (6208A>G), CTA166TTG (6209C>T 6211A>G), CAG168CAA (6217G>A), GCT172GCA (6229T>A), GTA173ATA (6230G>A), GTC175GTT (6238C>T), AAG178AAA (6247G>A), ATA180ATT (6253A>T), GCA182AAA (6257G>A 6258C>A), CAT187CAC (6274T>C)								
NIa-Pro protein (NP_734248.1)	1	244	100%	1654	93.7%	244 (100%)	223 (91.4%)	0/0/0/0	0
Protein mutations:	K2R (6282A>G), A29T (6362G>A), V42I (6401G>A), A43V (6405C>T), Y51F (6429A>T), Q58R (6450A>G), H70N (6485C>A 6487C>T), L84I (6527C>A), T104I (6588C>T), Y120H (6635T>C), I124V (6647A>G), I134V (6677A>G 6679A>G), D147N (6716G>A), N148D (6719A>G), I163L (6764A>T 6766A>G), A173V (6795C>T 6796A>G), H174Q (6799C>A), N194D (6857A>G), V200T (6875G>A 6876T>C 6877C>A), D219E (6934C>A), V242I (7001G>A 7003G>A)								
Codon mutations:	AAA2AGA (6282A>G), TCG3TCA (6286G>A), GGC7GGT (6298C>T), TTG8TTA (6301G>A), AGA9AGG (6304A>G), GAC10GAT (6307C>T), GCC15GCT (6322C>T), ACA17ACT (6328A>T), GTT18GTG (6331T>G), TGT19TGC (6334T>C), AGG20AGA (6337G>A), CTG21TTG (6338C>T), TAT27TAC (6358T>C), GGG28GGA (6361G>A), GCA29ACA (6362G>A), TCA30TCT (6367A>T), GAG31GAA (6370G>A), TAC33TAT (6376C>T), GGT34GGG (6379T>G), GGA36GGC (6385A>C), GGA38GGT (6391A>T), GCA39GCG (6394A>G), TAC40TAT (6397C>T), GTA42ATA (6401G>A), GCG43GTG (6405C>T), CAC45CAT (6412C>T), TTA47TTG (6418A>G), TTT48TTC (6421T>C), TAC51TTC (6429A>T), GGT53GGA (6436T>A), GAG56GAA (6445G>A), CAA58CGA (6450A>G), TCC59TCG (6454C>G), CAC61CAT (6460C>T), GGT62GGA (6463T>A), TTC64TTT (6469C>T), AGG65AGA (6472G>A), GTG66GTA (6475G>A), AAT68AAC (6481T>C), CAC70AAT (6485C>A 6487C>T), AGT71AGC (6490T>C), AGC73AGT (6496C>T), CTG75CTA (6502G>A), GGT79GGC (6514T>C), AGG80AGA (6517G>A), CTC84ATC (6527C>A), ATC85ATA (6532C>A), CCG88CCA (6541G>A), AAA89AAG (6544A>G), GTC93GTT (6556C>T), TTT94TTC (6559T>C), CAG96CAA (6565G>A), TTG98CTA (6569T>C 6571G>A), CCT103CCA (6586T>A), ACA104ATA (6588C>T), GAA107GAG (6598A>G), AGA108AGG (6601A>G), ATT109ATC (6604T>C), TGT110TGC (6607T>C), GGA113GGG (6616A>G), AAC115AAT (6622C>T), GAG118GAA (6631G>A), AAG119AAA (6634G>A), TAT120CAT (6635T>C), GCT121GCA (6640T>A), TCG123TCT (6646G>T), ATC124GTC (6647A>G), ATC125ATT (6652C>T), AGC129AGT (6664C>T), ACT131ACG (6670T>G), ATA134GTG (6677A>G 6679A>G), GGC136GGT (6685C>T), ACA138ACT (6691A>T), TTC139TTT (6694C>T), ACA146ACG (6715A>G), GAT147AAT (6716G>A), AAT148GAT (6719A>G), GGA149GGG (6724A>G), CTA153TTA (6734C>T), GTG155GTA (6742G>A), GTG156GTA (6745G>A), GCC159GCT (6754C>T), GGA161GGG (6760A>G), ATA163TTG (6764A>T 6766A>G), ATT166ATA (6775T>A), CTG169TTG (6782C>T), GCA170GCT (6787A>T), AAC171AAT (6790C>T), GCA173GTG (6795C>T 6796A>G), CAC174CAA (6799C>A), ACC175ACA (6802C>A), AAC177AAT (6808C>T), TAC178TAT (6811C>T), TAC179TAT (6814C>T), TTC182TTT (6823C>T), GAA184GAG (6829A>G), TAC190TAT (6847C>T), CGA192CGC (6853A>C), AAT194GAT (6857A>G), GAG195GAA (6862G>A), GAA198GAG (6871A>G), GTC200ACA (6875G>A 6876T>C 6877C>A), AAG201AAA (6880G>A), TCT202TCA (6883T>A), GTT204GTG (6889T>G), GAC208GAT (6901C>T), GGC213GGT (6916C>T), CCG214CCA (6919G>A), AAA216AAG (6925A>G), CTT217CTC (6928T>C), GAC219GAA (6934C>A), GGG224GGC (6949G>C), TTC226TTT (6955C>T), AAA227AAG (6958A>G), CAA233CAG (6976A>G), CTA235TTA (6980C>T), ATC236ATT (6985C>T), GTA240GTT (6997A>T), GTG241GTT (7000G>T), GTG242ATA (7001G>A 7003G>A)								
NIb protein (NP_734249.1)	1	521	100%	3129	86.1%	519 (99.4%)	480 (92.0%)	1/2/2/2	0
Protein mutations:	F8Y (7032T>A), T39K (7125C>A), A46S (7145G>T 7147A>G), E51_A52del (7160_7165delGAGGCA), D69E (7216T>A), D83E (7258T>A), D88_R89insX (7273_7274insT), R89V (7274C>G 7275G>T), H91L (7281A>T), L92S (7284T>C), S96L (7296C>T), S101F (7311C>T), T102I (7314C>T), C103Y (7317G>A), N104K (7321T>A), K109Q (7334A>C), A112N (7343G>A 7344C>A 7345A>C), S133M (7407G>T 7408T>G), C136G (7415T>G), L165S (7503T>C 7504G>A), D234N (7709G>A), K235R (7713A>G 7714A>G), T268I (7812C>T), V279L (7844G>T 7846G>A), V292I (7883G>A), S345N (8043G>A), N381S (8151A>G 8152C>T), R390K (8178G>A), R400K (8208G>A), P429A (8294C>G 8296A>G), S447F (8349C>T), A467S (8408G>T), M481I (8452G>A), R484K (8460G>A), A491T (8480G>A 8482T>A), R498K (8502G>A 8503A>G), E502D (8515A>T), E510D (8539G>C), L513C (8546C>T 8547T>G), S515T (8552T>A)								
Codon mutations:	GCT1GCC (7012T>C), AAG2AAA (7015G>A), CAT3CAC (7018T>C), TTT8TAT (7032T>A), GCC10GCT (7039C>T), TTG11CTA (7040T>C 7042G>A), GGA13GGG (7048A>G), GCT17GCA (7060T>A), GTC18GTA (7063C>A), AGC23AGT (7078C>T), TTA25CTG (7082T>C 7084A>G), GTA26GTG (7087A>G), ACC27ACA (7090C>A), CAT29CAC (7096T>C), GTA30GTG (7099A>G), GTT31GTC (7102T>C), GAG34GAA (7111G>A), CGA36CGG (7117A>G), ACA39AAA (7125C>A), GAA40GAG (7129A>G), TTT41TTC (7132T>C), CTG42TTA (7133C>T 7135G>A), GTG44GTA (7141G>A), GAT45GAC (7144T>C), GCA46TCG (7145G>T 7147A>G), GAG47GAA (7150G>A), GCA50GCT (7159A>T), GAG51_GCA52del (7160_7165delGAGGCA), TTC54TTT (7171C>T), GCG60GCT (7189G>T), AAA63AAG (7198A>G), AGC64AGT (7201C>T), CTA66TTA (7205C>T), AAT67AAC (7210T>C), GAT69GAA (7216T>A), GCG70GCA (7219G>A), ATC72ATA (7225C>A), AAG73AAA (7228G>A), AAG77AAA (7240G>A), TAT78TAC (7243T>C), AAA80AAG (7249A>G), CCT81CCG (7252T>G), GAT83GAA (7258T>A), GTT84GTC (7261T>C), GGT85GGA (7264T>A), GTC86GTG (7267C>G), GTG87GTA (7270G>A), GAT88_CGG89insT-- (7273_7274insT), CGG89GTG (7274C>G 7275G>T), CAT91CTT (7281A>T), TTG92TCG (7284T>C), TCA96TTA (7296C>T), TCT101TTT (7311C>T), ACC102ATC (7314C>T), TGC103TAC (7317G>A), AAT104AAA (7321T>A), GTG105-TG (7322delG), CAC106CAT (7327C>T), GGC107GGT (7330C>T), TTC108TTT (7333C>T), AAG109CAG (7334A>C), TGT111TGC (7342T>C), GCA112AAC (7343G>A 7344C>A 7345A>C), ACT115ACC (7354T>C), CAA118CAG (7363A>G), ATT120ATC (7369T>C), TTC121TTT (7372C>T), GCG123GCT (7378G>T), CTC124CTT (7381C>T), GCA129GCT (7396A>T), GTC130GTT (7399C>T), GCC132GCT (7405C>T), AGT133ATG (7407G>T 7408T>G), GGG135GGT (7414G>T), TGC136GGC (7415T>G), AAA137AAG (7420A>G), GAC140GAT (7429C>T), TAT141TAC (7432T>C), CAT144CAC (7441T>C), AAG150AAA (7459G>A), GAA151GAG (7462A>G), ATA153ATT (7468A>T), GTC154GTA (7471C>A), CAA156CAG (7477A>G), AGC157AGT (7480C>T), CTG159CTT (7486G>T), TAT162TAC (7495T>C), AAA163AAG (7498A>G), GGT164GGC (7501T>C), TTG165TCA (7503T>C 7504G>A), ATT168ATA (7513T>A), AAC170AAT (7519C>T), GGA171GGG (7522A>G), AAG174AAA (7531G>A), GAG176GAA (7537G>A), CTC177CTT (7540C>T), TGT179TGC (7546T>C), AAG187AAA (7570G>A), ACG188ACA (7573G>A), ACG190ACA (7579G>A), GCT193GCC (7588T>C), CTA196TTA (7595C>T), TTG199CTA (7604T>C 7606G>A), GGT202GGA (7615T>A), TGT205TGC (7624T>C), GAC208GAT (7633C>T), TTC209TTT (7636C>T), AAT210AAC (7639T>C), TTT213TTC (7648T>C), GAA219GAG (7666A>G), TGT221TGC (7672T>C), ACA223ACT (7678A>T), GGG225GGA (7684G>A), ACT227ACC (7690T>C), TTT229TTC (7696T>C), GGT231GGG (7702T>G), GAT234AAT (7709G>A), AAA235AGG (7713A>G 7714A>G), CTG236TTG (7715C>T), CGT239CGC (7726T>C), GAG242GAA (7735G>A), GTA245GTG (7744A>G), TAC246TAT (7747C>T), GGC251GGT (7762C>T), CAG253CAA (7768G>A), TTT254TTC (7771T>C), TCA257TCG (7780A>G), CTA258CTC (7783A>C), ACT259ACC (7786T>C), CTA262TTG (7793C>T 7795A>G), ATC263ATT (7798C>T), GCT265GCC (7804T>C), ACC268ATC (7812C>T), GAA275GAG (7834A>G), GAC276GAT (7837C>T), GTG279TTA (7844G>T 7846G>A), CAG282CAA (7855G>A), CTG284CTA (7861G>A), TTA287TTG (7870A>G), TAC288TAT (7873C>T), ACT289ACA (7876T>A), GAG290GAA (7879G>A), ATT291ATA (7882T>A), GTT292ATT (7883G>A), CCA295CCT (7894A>T), ATT296ATC (7897T>C), GTC304GTT (7921C>T), AAG306AAA (7927G>A), GGT309GGC (7936T>C), AAT310AAC (7939T>C), AAT311AAC (7942T>C), GTG319GTA (7966G>A), GAT320GAC (7969T>C), TCT322TCC (7975T>C), GTC326GTT (7987C>T), CTT327CTC (7990T>C), GCT332GCC (8005T>C), GAA341GAG (8032A>G), ATC343ATT (8038C>T), GAC344GAT (8041C>T), AGC345AAC (8043G>A), TTG357TTA (8080G>A), GTG360GTA (8089G>A), CCG362CCA (8095G>A), GAG363GAA (8098G>A), AGC366AGT (8107C>T), ATT367ATC (8110T>C), GAT369GAC (8116T>C), CAA373CAG (8128A>G), GGT379GGC (8146T>C), AAC381AGT (8151A>G 8152C>T), TCG385TCA (8164G>A), AGG390AAG (8178G>A), AAG391AAA (8182G>A), TTG394TTA (8191G>A), TTC396TTT (8197C>T), TCC398TCT (8203C>T), AGA400AAA (8208G>A), CTG402TTA (8213C>T 8215G>A), CTA403CTG (8218A>G), ATC404ATT (8221C>T), GAG405GAA (8224G>A), CCA410CCG (8239A>G), GAG415GAA (8254G>A), ATT417ATC (8260T>C), GTA418GTG (8263A>G), GAT424GAC (8281T>C), AGA425AGG (8284A>G), CCA429GCG (8294C>G 8296A>G), GAG430GAA (8299G>A), CAC431CAT (8302C>T), ATT436ATC (8317T>C), TGC437TGT (8320C>T), GCT439GCA (8326T>A), GAG442GAA (8335G>A), TCC443TCT (8338C>T), TCT447TTT (8349C>T), GAA448GAG (8353A>G), CAC451CAT (8362C>T), CAA452CAG (8365A>G), ATC453ATT (8368C>T), TAC457TAT (8380C>T), TCA458TCG (8383A>G), TTA460TTG (8389A>G), CCT465CCC (8404T>C), GCA467TCA (8408G>T), ACA468ACG (8413A>G), GCG470GCT (8419G>T), GCT475GCC (8434T>C), TAT477TAC (8440T>C), ATG481ATA (8452G>A), CTA483CTG (8458A>G), AGG484AAG (8460G>A), AAA485AAG (8464A>G), CTG486CTA (8467G>A), AGG490AGA (8479G>A), GCT491ACA (8480G>A 8482T>A), GAG496GAA (8497G>A), CTA497TTG (8498C>T 8500A>G), AGA498AAG (8502G>A 8503A>G), GCC499GCT (8506C>T), GAA502GAT (8515A>T), GTC505GTT (8524C>T), GCA506GCC (8527A>C), TTA507CTG (8528T>C 8530A>G), GAG510GAC (8539G>C), CTT513TGT (8546C>T 8547T>G), TCT515ACT (8552T>A), GTA518GTG (8563A>G)								
coat protein (NP_734250.1)	1	267	100%	1652	90.6%	267 (100%)	243 (91.0%)	0/0/0/0	0
Protein mutations:	A1G (8574C>G), I5V (8585A>G), G9E (8598G>A), N11S (8603A>T 8604A>C 8605C>T), P17Q (8622C>A), E18G (8625A>G 8626G>A), P26F (8648C>T 8649C>T 8650G>C), G29E (8658G>A), D31A (8664A>C 8665T>G), A36V (8679C>T 8680A>T), T58K (8745C>A 8746A>G), E95G (8856A>G 8857G>A), R98Q (8865G>A), M99L (8867A>C 8869G>T), G104E (8883G>A), T106S (8888A>T), V128I (8954G>A), N138D (8984A>G 8986T>C), E139V (8988A>T), I187V (9131A>G), M193G (9149A>G 9150T>G 9151G>A), G194S (9152G>A), P230S (9260C>T 9262T>C), Q242H (9298A>C)								
Codon mutations:	GCA1GGA (8574C>G), ATT5GTT (8585A>G), GGA9GAA (8598G>A), AGC10AGT (8602C>T), AAC11TCT (8603A>T 8604A>C 8605C>T), AAA16AAG (8620A>G), CCA17CAA (8622C>A), GAG18GGA (8625A>G 8626G>A), CAG19CAA (8629G>A), GGC20GGT (8632C>T), ATC22ATT (8638C>T), CAG23CAA (8641G>A), AAC25AAT (8647C>T), CCG26TTC (8648C>T 8649C>T 8650G>C), AAC27AAT (8653C>T), AAA28AAG (8656A>G), GGA29GAA (8658G>A), GAT31GCG (8664A>C 8665T>G), GTT34GTG (8674T>G), GCA36GTT (8679C>T 8680A>T), GGC37GGA (8683C>A), GGG40GGA (8692G>A), ACA41ACT (8695A>T), CCG45CCA (8707G>A), AGA46CGA (8708A>C), AAG48AAA (8716G>A), ATC50ATT (8722C>T), ACG51ACA (8725G>A), AAA53AAG (8731A>G), AGA55AGG (8737A>G), CCC57CCT (8743C>T), ACA58AAG (8745C>A 8746A>G), AAG60AAA (8752G>A), GGA61GGT (8755A>T), AAC66AAT (8770C>T), TTA67CTA (8771T>C), GAA68GAG (8776A>G), CAT69CAC (8779T>C), TTG70TTA (8782G>A), TAT73TAC (8791T>C), CCA75CCG (8797A>G), CAA76CAG (8800A>G), CAA77CAG (8803A>G), GAT79GAC (8809T>C), ATT80ATC (8812T>C), AAT82AAC (8818T>C), CGG84CGA (8824G>A), GCA85GCG (8827A>G), ACG92ACA (8848G>A), TAT94TAC (8854T>C), GAG95GGA (8856A>G 8857G>A), GTG97GTA (8863G>A), CGG98CAG (8865G>A), ATG99CTT (8867A>C 8869G>T), GCA100GCG (8872A>G), GGA104GAA (8883G>A), ACT106TCT (8888A>T), TGC119TGT (8929C>T), GTC128ATC (8954G>A), AAC129AAT (8959C>T), GGA130GGT (8962A>T), GGG137GGA (8983G>A), AAT138GAC (8984A>G 8986T>C), GAA139GTA (8988A>T), GAG142GAA (8998G>A), CCG144CCA (9004G>A), TTG145TTA (9007G>A), ATC148ATT (9016C>T), ACC155ACA (9037C>A), ATA171ATT (9085A>T), AAC175AAT (9097C>T), ATT187GTT (9131A>G), CGA188CGT (9136A>T), CGG191CGT (9145G>T), ATG193GGA (9149A>G 9150T>G 9151G>A), GGT194AGT (9152G>A), TTA195TTG (9157A>G), GCG196GCT (9160G>T), CGT197CGC (9163T>C), GAG204GAA (9184G>A), CGA208CGG (9196A>G), AGG214AGA (9214G>A), GAA215GAG (9217A>G), GCG216GCA (9220G>A), GCA224GCT (9244A>T), TTG225TTA (9247G>A), GCC228GCT (9256C>T), CCT230TCC (9260C>T 9262T>C), GGG234GGA (9274G>A), GAC236GAT (9280C>T), ATC239ATT (9289C>T), CAA242CAC (9298A>C), GAG244GAA (9304G>A), GTC254GTT (9334C>T), GTC264GTG (9364C>G)								

	Begin	End	Coverage	Score	Concordance	Matches	Identities	I/D/M/F*	Stop Codons
**NT**	**22**	**9704**	**99.8%**	**12522**	**65.0%**	**9671 (99.8%)**	**8003 (82.6%)**	**10/12**	
PIPO (YP_006393460.1)	1	75	100%	95	18.1%	75 (98.7%)	54 (71.1%)	1/0/3/2	2
Protein mutations:	R17_K17insX (2971_2972insA), Y23H (2989T>C 2991C>T), R26K (2999G>A 3000A>G), H35Y (3025C>T), K40H (3040A>C 3042A>C), G41S (3043G>A), E44K (3052G>A), I47V (3061A>G), V54I (3082G>A), P57S (3091C>T), P60T (3100C>A), G61E (3104G>A 3105T>G), G62S (3106G>A), Q63* (3109C>T), R64G (3112A>G), C66R (3118T>C), L67F (3121C>T), I69V (3127A>G), A72E (3137C>A 3138G>A), I73V (3139A>G)								
Codon mutations:	TTA2CTA (2923T>C), AAA4AAG (2931A>G), GAA7GAG (2940A>G), TTG10CTG (2947T>C), TTG15CTG (2962T>C), AGA17_AAA17insAA- (2971_2972insA), TAC23CAT (2989T>C 2991C>T), TCA24TCG (2994A>G), AGA26AAG (2999G>A 3000A>G), CAC32-AC (3016delC), TCG33TCA (3021G>A), GTA34GTT (3024A>T), CAT35TAT (3025C>T), CAC38CAT (3036C>T), AGA39AGG (3039A>G), AAA40CAC (3040A>C 3042A>C), GGC41AGC (3043G>A), GAA44AAA (3052G>A), ATA47GTA (3061A>G), GTT54ATT (3082G>A), CCG57TCG (3091C>T), CGC59CGT (3099C>T), CCA60ACA (3100C>A), GGT61GAG (3104G>A 3105T>G), GGT62AGT (3106G>A), CAA63TAA (3109C>T), AGG64GGG (3112A>G), TGC66CGC (3118T>C), CTC67TTC (3121C>T), ATT69GTT (3127A>G), GAG70GAA (3132G>A), GCG72GAA (3137C>A 3138G>A), ATT73GTT (3139A>G)								

*: Inserts / Deletes / Misaligned / Frameshifts

Analysis details

This analysis was performed with panviral2.64

NGS Details (UN60): Torradovirus lycopersici (segment RNA 1)

Assembly

**Coverage Length**	7740 (1 contig(s))
**Depth Of Coverage**	6761.0
**Number Of Reads**	355460
**Reads Per Million**	7539.15 rpm (after QC)
**Ambiguities**	0
**Assembly Method**	de novo + reference guided assembly
**Consensus Caller**	Bcf Tools

Coverage Map

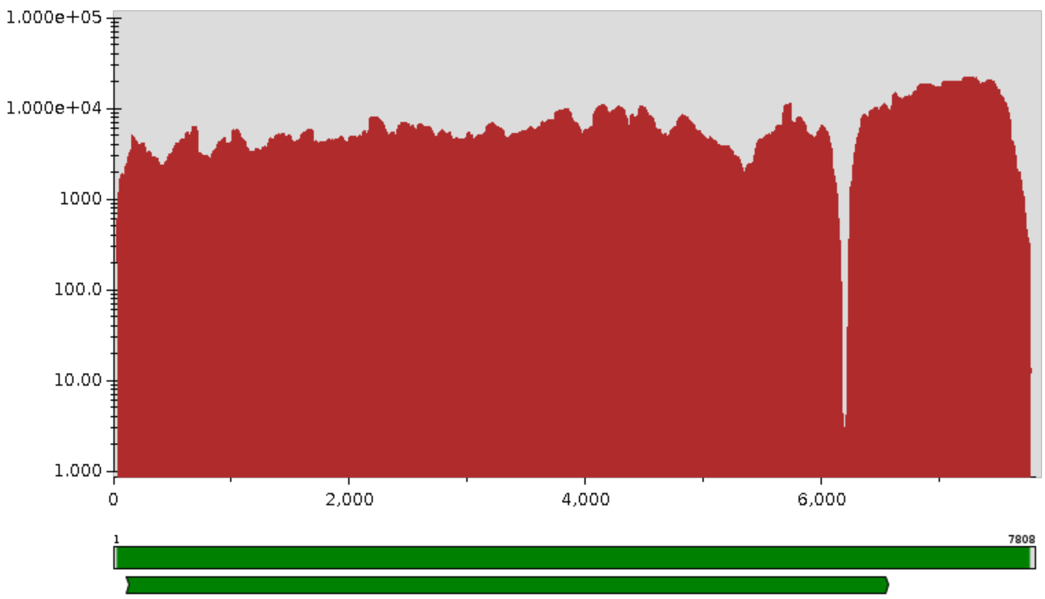

Assignment

**Type**	Torradovirus lycopersici (Taxonomy ID: 3048378)
**Reference Genome**	NC_009013.1
**NT Identity (%)**	91.2359
**AA Identity (%)**	97.962
**Number Of Stop Codons**	1
**Number Of CDS**	1

Alignment

**Alignment Score**	11591.0 (NT) + 14866.0 (AA) = 26457.0
**Concordance (%)**	90.408

**Alignment Method**	Global, seeded, nucleotide + amino acids (AGA)

Genome Region

Sequence starts at position 30 and ends at position 7769 relative to NC_009013.1 reference sequence.

Alignment Detailed Statistics

	Begin	End	Coverage	Score	Concordance	Matches	Identities	I/D/M/F*	Stop Codons
**NT**	**30**	**7769**	**99.1%**	**11591**	**81.7%**	**7347 (94.6%)**	**6725 (86.6%)**	**24/393**	
Mutations:	31C>A, 69C>T, 76_77insT, 83C>T, 83_84insT, 85T>C, 87C>T, 101G>A, 102G>C, 115T>C, 127C>T, 128C>T, 130C>T, 135T>C, 148T>C, 151A>G, 158G>T, 161A>G, 178T>G, 187A>G, 199T>A, 208T>C, 220C>T, 224C>T, 265C>T, 328T>C, 334T>C, 340G>A, 349G>A, 373C>T, 376C>T, 433G>A, 487C>T, 502G>A, 541T>C, 559C>T, 578C>T, 628C>T, 655C>T, 676A>G, 694A>G, 736A>G, 781C>A, 790C>T, 799A>G, 805T>C, 818C>T, 820A>G, 832A>T, 835A>C, 841T>C, 842C>T, 847C>T, 883G>A, 886T>C, 901T>C, 925C>T, 952A>G, 964T>G, 970G>A, 997C>T, 1006T>C, 1033G>A, 1060T>C, 1078A>T, 1081C>T, 1093T>C, 1105A>G, 1108T>C, 1109C>T, 1165C>T, 1180G>A, 1196A>G, 1231G>A, 1240C>T, 1255G>A, 1267T>C, 1276A>G, 1300G>A, 1351A>G, 1372C>T, 1408T>C, 1411T>C, 1417T>C, 1423T>C, 1426T>C, 1429C>T, 1430C>T, 1441C>T, 1450T>C, 1451C>T, 1480C>T, 1481A>G, 1489T>C, 1522C>T, 1552C>T, 1555C>T, 1570A>G, 1573T>C, 1582C>T, 1585A>T, 1591C>T, 1594T>C, 1607G>T, 1612G>A, 1618T>G, 1619C>T, 1624C>A, 1645A>G, 1669C>T, 1681T>A, 1684G>A, 1699T>A, 1705T>C, 1738A>G, 1771C>T, 1781T>C, 1789A>G, 1897A>G, 1910T>C, 1954T>C, 1960A>G, 1984C>T, 2014A>G, 2042G>A, 2056T>C, 2082T>A, 2092A>G, 2107A>G, 2128T>C, 2138C>T, 2143T>C, 2149T>C, 2155T>C, 2174C>T, 2179A>C, 2182G>A, 2194T>A, 2200T>C, 2227C>T, 2241A>G, 2250G>A, 2251C>T, 2257C>T, 2263G>A, 2275T>C, 2287G>A, 2290C>T, 2314C>T, 2323A>T, 2347C>T, 2350A>G, 2356C>T, 2359G>A, 2362C>T, 2377G>A, 2380T>C, 2392A>G, 2407T>C, 2409G>A, 2443T>C, 2446G>A, 2479G>A, 2485C>T, 2497G>A, 2500T>C, 2503G>A, 2509C>T, 2512T>C, 2542G>A, 2548C>T, 2557C>T, 2620G>A, 2626T>C, 2650G>A, 2665T>C, 2675T>C, 2683A>G, 2692G>A, 2698T>C, 2702C>T, 2710A>G, 2725T>C, 2731C>T, 2734C>T, 2743A>G, 2744T>C, 2752A>G, 2765T>C, 2773A>G, 2785C>T, 2788C>T, 2809C>T, 2821G>T, 2827A>G, 2842T>C, 2860C>T, 2866T>A, 2869C>T, 2872A>T, 2926G>A, 2929C>T, 2930T>C, 2935G>A, 2941A>G, 2950C>T, 2951C>T, 2998A>G, 3031T>A, 3034G>A, 3037C>T, 3040T>C, 3049A>G, 3064A>G, 3073G>A, 3079T>C, 3109T>C, 3115G>A, 3145A>G, 3151C>T, 3163T>C, 3169C>T, 3172T>C, 3202T>C, 3241T>C, 3253C>T, 3256G>A, 3265A>G, 3418C>T, 3463A>G, 3466G>A, 3481C>T, 3484A>T, 3508G>A, 3559G>A, 3580T>C, 3583C>T, 3610A>G, 3613T>C, 3625C>T, 3628G>A, 3643A>G, 3646C>T, 3661A>G, 3664T>C, 3673G>A, 3676C>T, 3686C>T, 3706T>C, 3716T>C, 3721T>A, 3739A>T, 3754G>A, 3758G>A, 3766G>A, 3784C>G, 3808T>C, 3814C>T, 3817G>C, 3824T>C, 3835T>C, 3844T>C, 3857T>C, 3859G>A, 3868C>T, 3871T>C, 3883G>A, 3898T>C, 3907A>T, 3916C>T, 3925A>G, 3928G>A, 3949C>T, 3955G>A, 3961G>A, 3970A>G, 3973T>C, 4034T>C, 4066T>C, 4075A>G, 4084C>T, 4100T>C, 4118C>T, 4129C>T, 4147C>A, 4159G>A, 4162A>T, 4168A>G, 4195A>G, 4207G>A, 4210T>C, 4219C>T, 4228C>T, 4294A>G, 4348C>T, 4354T>C, 4396T>C, 4408C>T, 4414T>G, 4417T>C, 4420G>A, 4459T>C, 4462C>T, 4465C>T, 4468C>T, 4471T>C, 4474T>C, 4486T>C, 4510T>A, 4511C>T, 4514T>C, 4555A>G, 4570C>T, 4576A>T, 4588C>A, 4591T>C, 4625T>C, 4636T>C, 4648T>A, 4660A>G, 4684A>C, 4696C>T, 4702G>A, 4705C>T, 4714T>C, 4735C>T, 4747A>G, 4756A>T, 4759C>T, 4768T>C, 4774G>A, 4777C>T, 4783A>G, 4786A>G, 4789C>T, 4813A>G, 4816T>C, 4843G>A, 4898G>A, 4906G>A, 4969G>A, 4988A>G, 4993A>T, 5005T>C, 5012T>C, 5023C>T, 5029G>T, 5044A>C, 5059G>T, 5074T>C, 5077T>C, 5079T>C, 5102T>C, 5119T>C, 5120G>A, 5122T>A, 5123C>T, 5132T>A, 5146T>C, 5148C>A, 5200G>A, 5218T>G, 5244G>A, 5266C>T, 5281C>T, 5329C>T, 5341T>A, 5344A>G, 5347C>T, 5369G>A, 5371G>A, 5386C>T, 5392G>A, 5407A>T, 5419A>T, 5437T>C, 5440C>T, 5461T>C, 5471T>C, 5482A>G, 5533T>A, 5575A>G, 5578T>C, 5591G>T, 5603A>C, 5608A>G, 5611A>T, 5617A>T, 5641T>C, 5647T>C, 5674A>T, 5677G>T, 5686G>A, 5690T>C, 5710T>A, 5716G>T, 5719A>G, 5731C>T, 5734T>C, 5737T>C, 5739G>A, 5746T>C, 5750T>C, 5752G>A, 5758C>T, 5773G>A, 5782A>G, 5784T>C, 5786T>C, 5797A>G, 5806C>T, 5836A>T, 5849T>C, 5850T>A, 5851G>A, 5869G>A, 5875A>G, 5878G>T, 5884T>C, 5887C>T, 5890C>T, 5902G>A, 5914T>C, 5923T>C, 5927C>T, 5932C>T, 5955A>G, 5965G>A, 5998A>G, 6004C>T, 6016A>G, 6028C>T, 6031A>T, 6033C>T, 6035G>T, 6055T>A, 6067C>A, 6070T>C, 6092C>T, 6094T>A, 6100T>G, 6103G>A, 6106G>A, 6112A>G, 6121A>T, 6139T>C, 6145T>C, 6148T>C, 6154C>G, 6163C>G, 6184A>G, 6187T>G, 6193C>T, 6196A>G, 6202G>C, 6208A>G, 6217G>T, 6223A>T, 6226G>A, 6230A>G, 6232A>G, 6235G>A, 6244A>G, 6253A>C, 6256C>A, 6262A>T, 6265G>A, 6268G>T, 6273G>T, 6274C>G, 6296G>C, 6298A>G, 6304A>G, 6308T>C, 6313T>C, 6319A>G, 6322G>A, 6323A>G, 6344C>T, 6358T>C, 6382C>T, 6385C>T, 6406T>C, 6412C>T, 6414G>A, 6427A>G, 6442C>G, 6445C>T, 6448G>A, 6451T>C, 6457C>A, 6466T>C, 6484A>C, 6490A>G, 6493T>C, 6500A>T, 6517C>T, 6532C>T, 6545C>T, 6571C>T, 6587G>A, 6643A>G, 6670C>T, 6685A>G, 6715C>T, 6721A>T, 6742A>T, 6766delC, 6786T>C, 6798_6799insA, 6807A>G, 6808A>G, 6809C>T, 6810G>C, 6811G>T, 6814A>T, 6815G>A, 6817A>G, 6818C>A, 6821A>G, 6822C>T, 6823T>G, 6825T>A, 6826A>T, 6828C>T, 6831_6897delGTTGGGAGTCCGGCTCCATTTGGAGTACCAATGAATTTACATTGGTTAAAGAGATTTGCACGCCTCT, 6905T>C, 6907T>A, 6908G>T, 6911T>C, 6912_6916delCGTGT, 6921T>G, 6923C>G, 6924T>G, 6927_6941delGTTAGAAAGTTCCTT, 6947A>C, 6948_6971delGGTTTACAAAGTACGGGGAGCACT, 6976G>A, 6979T>A, 6983_7024delATAGTGCAGGTGCTATCCCATGATAGTCCTTTAACTCAAGGG, 7026T>C, 7031T>A, 7033G>A, 7035T>G, 7038G>A, 7040_7072delATCTTTTGCCGTGATGAAAGATGAGGTAGCTTC, 7074C>A, 7077T>C, 7083G>T, 7085_7196delAGGCTGAAACTACACATATGTAGTGGGTTTGACTGAGTCCTATAATCAGTCCGTTTGAAATTCGATAATTTTCCGTAGCTTGCGTCAAGCTGCTCACGTTAGGGGTGTGAGT, 7198A>T, 7204_7273delCGCCGTACCACGTCTTCCCCGGCAATGCCAGTGGTTCAGAGCGGGCCCTCAGAATAGAGGTTAAAACTAG, 7292G>T, 7293A>T, 7297_7298insC, 7299G>A, 7311T>A, 7318G>T, 7320G>T, 7342T>G, 7343C>A, 7344C>A, 7346A>T, 7346_7347insG, 7354G>A, 7354_7355insCAGTAGTGCCATTTA, 7358A>C, 7360G>A, 7361A>C, 7362A>C, 7364C>T, 7368G>A, 7372A>T, 7375T>G, 7376T>G, 7377T>A, 7379A>T, 7383_7384delAA, 7388C>T, 7397G>A, 7398C>A, 7399G>A, 7400A>T, 7404C>T, 7407A>T, 7408G>T, 7423A>T, 7425G>A, 7435T>C, 7436G>A, 7444C>T, 7445A>G, 7457A>G, 7458T>C, 7460G>A, 7498C>G, 7503C>T, 7505A>G, 7506T>C, 7511_7512insC, 7514A>C, 7525_7526insC, 7526_7527insG, 7569G>A, 7577C>T, 7591G>A, 7596C>T, 7600T>G, 7601T>A, 7602T>A, 7603_7604insC, 7610C>T, 7612G>A, 7615A>G, 7616A>T, 7617A>T, 7618C>T, 7624T>A, 7625A>T, 7628_7633delGCTTTA, 7637G>A, 7642T>G, 7644G>T, 7647T>G, 7648G>T, 7649T>C, 7654C>A, 7657_7672delGTTTTATTGTGTGTTT, 7686T>A, 7687T>C, 7689G>T, 7698G>A, 7701T>C, 7702C>T, 7705T>A, 7717A>T, 7730G>A, 7731A>G, 7739T>A, 7747C>T, 7750T>C, 7752G>A, 7757G>T, 7759T>A									

CDS

	Begin	End	Coverage	Score	Concordance	Matches	Identities	I/D/M/F*	Stop Codons
ToTV_sRNA1gp1	1	2159	100%	14866	98.6%	2159 (100%)	2115 (98.0%)	0/0/0/0	1
Protein mutations:	P8S (128C>T 130C>T), F10S (135T>C), A18S (158G>T), T19A (161A>G), T364A (1196A>G), I459V (1481A>G), A501S (1607G>T), A646T (2042G>A), F659Y (2082T>A), N712S (2241A>G), S715N (2250G>A 2251C>T), R768K (2409G>A), V1218I (3758G>A), V1598I (4898G>A), T1628A (4988A>G), I1658T (5079T>C), V1672I (5120G>A 5122T>A), S1676T (5132T>A), T1681N (5148C>A), R1713K (5244G>A), V1755I (5369G>A 5371G>A), I1792M (5482A>G), A1829S (5591G>T), T1833P (5603A>C), Q1857H (5677G>T), R1878K (5739G>A), I1893T (5784T>C), L1915Q (5849T>C 5850T>A 5851G>A), N1950S (5955A>G), T1976I (6033C>T), A1977S (6035G>T), I2002M (6112A>G), M2032I (6202G>C), E2039D (6223A>T), I2042V (6230A>G 6232A>G), I2046M (6244A>G), L2049F (6253A>C), S2056M (6273G>T 6274C>G), V2064L (6296G>C 6298A>G), I2071M (6319A>G), I2073V (6323A>G), R2103Q (6414G>A), I2132F (6500A>T), L2147F (6545C>T)									

	Begin	End	Coverage	Score	Concordance	Matches	Identities	I/D/M/F*	Stop Codons
**NT**	**30**	**7769**	**99.1%**	**11591**	**81.7%**	**7347 (94.6%)**	**6725 (86.6%)**	**24/393**	
Codon mutations:	TTT3TTC (115T>C), TTC7TTT (127C>T), CCC8TCT (128C>T 130C>T), TTC10TCC (135T>C), ACT14ACC (148T>C), GAA15GAG (151A>G), GCT18TCT (158G>T), ACC19GCC (161A>G), TCT24TCG (178T>G), TCA27TCG (187A>G), GCT31GCA (199T>A), AGT34AGC (208T>C), CGC38CGT (220C>T), CTG40TTG (224C>T), CAC53CAT (265C>T), TCT74TCC (328T>C), ACT76ACC (334T>C), TTG78TTA (340G>A), AAG81AAA (349G>A), TTC89TTT (373C>T), ATC90ATT (376C>T), AAG109AAA (433G>A), GAC127GAT (487C>T), GTG132GTA (502G>A), TCT145TCC (541T>C), TTC151TTT (559C>T), CTA158TTA (578C>T), AAC174AAT (628C>T), GAC183GAT (655C>T), CAA190CAG (676A>G), GCA196GCG (694A>G), CCA210CCG (736A>G), ACC225ACA (781C>A), TTC228TTT (790C>T), GTA231GTG (799A>G), GGT233GGC (805T>C), CTA238TTG (818C>T 820A>G), CTA242CTT (832A>T), TCA243TCC (835A>C), ATT245ATC (841T>C), CTG246TTG (842C>T), TAC247TAT (847C>T), TTG259TTA (883G>A), TTT260TTC (886T>C), AAT265AAC (901T>C), GCC273GCT (925C>T), GTA282GTG (952A>G), GGT286GGG (964T>G), AAG288AAA (970G>A), GCC297GCT (997C>T), TCT300TCC (1006T>C), GAG309GAA (1033G>A), CAT318CAC (1060T>C), GCA324GCT (1078A>T), AAC325AAT (1081C>T), ACT329ACC (1093T>C), CAA333CAG (1105A>G), CAT334CAC (1108T>C), CTA335TTA (1109C>T), GCC353GCT (1165C>T), AGG358AGA (1180G>A), ACC364GCC (1196A>G), CAG375CAA (1231G>A), GGC378GGT (1240C>T), AGG383AGA (1255G>A), TTT387TTC (1267T>C), CAA390CAG (1276A>G), GGG398GGA (1300G>A), GAA415GAG (1351A>G), CTC422CTT (1372C>T), AAT434AAC (1408T>C), TAT435TAC (1411T>C), CAT437CAC (1417T>C), ACT439ACC (1423T>C), GCT440GCC (1426T>C), ATC441ATT (1429C>T), CTG442TTG (1430C>T), GAC445GAT (1441C>T), GCT448GCC (1450T>C), CTG449TTG (1451C>T), GAC458GAT (1480C>T), ATT459GTT (1481A>G), GAT461GAC (1489T>C), CCC472CCT (1522C>T), GGC482GGT (1552C>T), CGC483CGT (1555C>T), CGA488CGG (1570A>G), TAT489TAC (1573T>C), GCC492GCT (1582C>T), ACA493ACT (1585A>T), AAC495AAT (1591C>T), CTT496CTC (1594T>C), GCT501TCT (1607G>T), CAG502CAA (1612G>A), GGT504GGG (1618T>G), CTA505TTA (1619C>T), ACC506ACA (1624C>A), AGA513AGG (1645A>G), TGC521TGT (1669C>T), GGT525GGA (1681T>A), GAG526GAA (1684G>A), GCT531GCA (1699T>A), ACT533ACC (1705T>C), AAA544AAG (1738A>G), AGC555AGT (1771C>T), TTG559CTG (1781T>C), GAA561GAG (1789A>G), GCA597GCG (1897A>G), TTG602CTG (1910T>C), GTT616GTC (1954T>C), GTA618GTG (1960A>G), TAC626TAT (1984C>T), AAA636AAG (2014A>G), GCA646ACA (2042G>A), CCT650CCC (2056T>C), TTT659TAT (2082T>A), CTA662CTG (2092A>G), AAA667AAG (2107A>G), GTT674GTC (2128T>C), CTG678TTG (2138C>T), TAT679TAC (2143T>C), GGT681GGC (2149T>C), TGT683TGC (2155T>C), CTG690TTG (2174C>T), ATA691ATC (2179A>C), AAG692AAA (2182G>A), GCT696GCA (2194T>A), TAT698TAC (2200T>C), TAC707TAT (2227C>T), AAT712AGT (2241A>G), AGC715AAT (2250G>A 2251C>T), GAC717GAT (2257C>T), CAG719CAA (2263G>A), CCT723CCC (2275T>C), GCG727GCA (2287G>A), CAC728CAT (2290C>T), GAC736GAT (2314C>T), ATA739ATT (2323A>T), CAC747CAT (2347C>T), GTA748GTG (2350A>G), TTC750TTT (2356C>T), TTG751TTA (2359G>A), TAC752TAT (2362C>T), AGG757AGA (2377G>A), TAT758TAC (2380T>C), AGA762AGG (2392A>G), CCT767CCC (2407T>C), AGG768AAG (2409G>A), ATT779ATC (2443T>C), GTG780GTA (2446G>A), CCG791CCA (2479G>A), ATC793ATT (2485C>T), CTG797CTA (2497G>A), ATT798ATC (2500T>C), AAG799AAA (2503G>A), GGC801GGT (2509C>T), CTT802CTC (2512T>C), GGG812GGA (2542G>A), GTC814GTT (2548C>T), TTC817TTT (2557C>T), GAG838GAA (2620G>A), AAT840AAC (2626T>C), AAG848AAA (2650G>A), TTT853TTC (2665T>C), TTG857CTG (2675T>C), GCA859GCG (2683A>G), AAG862AAA (2692G>A), GAT864GAC (2698T>C), CTG866TTG (2702C>T), AAA868AAG (2710A>G), GAT873GAC (2725T>C), TTC875TTT (2731C>T), ATC876ATT (2734C>T), GGA879GGG (2743A>G), TTG880CTG (2744T>C), AAA882AAG (2752A>G), TTG887CTG (2765T>C), CTA889CTG (2773A>G), GTC893GTT (2785C>T), TAC894TAT (2788C>T), TGC901TGT (2809C>T), GTG905GTT (2821G>T), ACA907ACG (2827A>G), TTT912TTC (2842T>C), GGC918GGT (2860C>T), ACT920ACA (2866T>A), TTC921TTT (2869C>T), TCA922TCT (2872A>T), CGG940CGA (2926G>A), TTC941TTT (2929C>T), TTG942CTG (2930T>C), AAG943AAA (2935G>A), CAA945CAG (2941A>G), GTC948GTT (2950C>T), CTG949TTG (2951C>T), AAA964AAG (2998A>G), TCT975TCA (3031T>A), AAG976AAA (3034G>A), GGC977GGT (3037C>T), ACT978ACC (3040T>C), GTA981GTG (3049A>G), GTA986GTG (3064A>G), AAG989AAA (3073G>A), GCT991GCC (3079T>C), TTT1001TTC (3109T>C), GTG1003GTA (3115G>A), GTA1013GTG (3145A>G), TAC1015TAT (3151C>T), TCT1019TCC (3163T>C), TCC1021TCT (3169C>T), TTT1022TTC (3172T>C), TAT1032TAC (3202T>C), CTT1045CTC (3241T>C), AAC1049AAT (3253C>T), GTG1050GTA (3256G>A), AAA1053AAG (3265A>G), CTC1104CTT (3418C>T), AAA1119AAG (3463A>G), CAG1120CAA (3466G>A), GGC1125GGT (3481C>T), ACA1126ACT (3484A>T), ACG1134ACA (3508G>A), GGG1151GGA (3559G>A), TCT1158TCC (3580T>C), ATC1159ATT (3583C>T), GAA1168GAG (3610A>G), GGT1169GGC (3613T>C), TAC1173TAT (3625C>T), GTG1174GTA (3628G>A), AAA1179AAG (3643A>G), TAC1180TAT (3646C>T), CAA1185CAG (3661A>G), AAT1186AAC (3664T>C), GAG1189GAA (3673G>A), GAC1190GAT (3676C>T), CTA1194TTA (3686C>T), CGT1200CGC (3706T>C), TTG1204CTG (3716T>C), CCT1205CCA (3721T>A), CGA1211CGT (3739A>T), AAG1216AAA (3754G>A), GTC1218ATC (3758G>A), CAG1220CAA (3766G>A), CTC1226CTG (3784C>G), TAT1234TAC (3808T>C), GAC1236GAT (3814C>T), GGG1237GGC (3817G>C), TTG1240CTG (3824T>C), TCT1243TCC (3835T>C), TAT1246TAC (3844T>C), TTG1251CTA (3857T>C 3859G>A), GGC1254GGT (3868C>T), ACT1255ACC (3871T>C), GAG1259GAA (3883G>A), AGT1264AGC (3898T>C), GGA1267GGT (3907A>T), GTC1270GTT (3916C>T), AAA1273AAG (3925A>G), AGG1274AGA (3928G>A), GAC1281GAT (3949C>T), GAG1283GAA (3955G>A), TTG1285TTA (3961G>A), GAA1288GAG (3970A>G), ATT1289ATC (3973T>C), TTG1310CTG (4034T>C), CGT1320CGC (4066T>C), GAA1323GAG (4075A>G), CCC1326CCT (4084C>T), TTG1332CTG (4100T>C), CTG1338TTG (4118C>T), TCC1341TCT (4129C>T), TCC1347TCA (4147C>A), CTG1351CTA (4159G>A), CCA1352CCT (4162A>T), AAA1354AAG (4168A>G), GAA1363GAG (4195A>G), TTG1367TTA (4207G>A), GCT1368GCC (4210T>C), TTC1371TTT (4219C>T), GTC1374GTT (4228C>T), CAA1396CAG (4294A>G), GAC1414GAT (4348C>T), AAT1416AAC (4354T>C), ACT1430ACC (4396T>C), AAC1434AAT (4408C>T), GTT1436GTG (4414T>G), TTT1437TTC (4417T>C), AGG1438AGA (4420G>A), GTT1451GTC (4459T>C), ATC1452ATT (4462C>T), TTC1453TTT (4465C>T), AAC1454AAT (4468C>T), TCT1455TCC (4471T>C), CTT1456CTC (4474T>C), TTT1460TTC (4486T>C), TCT1468TCA (4510T>A), CTA1469TTA (4511C>T), TTG1470CTG (4514T>C), AAA1483AAG (4555A>G), AAC1488AAT (4570C>T), CTA1490CTT (4576A>T), GTC1494GTA (4588C>A), TAT1495TAC (4591T>C), TTG1507CTG (4625T>C), TAT1510TAC (4636T>C), GCT1514GCA (4648T>A), GAA1518GAG (4660A>G), ATA1526ATC (4684A>C), ATC1530ATT (4696C>T), AAG1532AAA (4702G>A), AAC1533AAT (4705C>T), GTT1536GTC (4714T>C), GAC1543GAT (4735C>T), CTA1547CTG (4747A>G), CTA1550CTT (4756A>T), AGC1551AGT (4759C>T), TTT1554TTC (4768T>C), AGG1556AGA (4774G>A), CAC1557CAT (4777C>T), CTA1559CTG (4783A>G), GGA1560GGG (4786A>G), TAC1561TAT (4789C>T), GTA1569GTG (4813A>G), TCT1570TCC (4816T>C), AGG1579AGA (4843G>A), GTT1598ATT (4898G>A), GAG1600GAA (4906G>A), AAG1621AAA (4969G>A), ACC1628GCC (4988A>G), ACA1629ACT (4993A>T), TAT1633TAC (5005T>C), TTG1636CTG (5012T>C), GAC1639GAT (5023C>T), CTG1641CTT (5029G>T), GGA1646GGC (5044A>C), GGG1651GGT (5059G>T), CTT1656CTC (5074T>C), CAT1657CAC (5077T>C), ATT1658ACT (5079T>C), TTG1666CTG (5102T>C), ACT1671ACC (5119T>C), GTT1672ATA (5120G>A 5122T>A), CTG1673TTG (5123C>T), TCT1676ACT (5132T>A), CAT1680CAC (5146T>C), ACT1681AAT (5148C>A), CAG1698CAA (5200G>A), GTT1704GTG (5218T>G), AGA1713AAA (5244G>A), CCC1720CCT (5266C>T), GAC1725GAT (5281C>T), CAC1741CAT (5329C>T), ATT1745ATA (5341T>A), GTA1746GTG (5344A>G), ACC1747ACT (5347C>T), GTG1755ATA (5369G>A 5371G>A), GGC1760GGT (5386C>T), GGG1762GGA (5392G>A), CCA1767CCT (5407A>T), GCA1771GCT (5419A>T), ACT1777ACC (5437T>C), GGC1778GGT (5440C>T), GTT1785GTC (5461T>C), TTG1789CTG (5471T>C), ATA1792ATG (5482A>G), CTT1809CTA (5533T>A), ACA1823ACG (5575A>G), TGT1824TGC (5578T>C), GCC1829TCC (5591G>T), ACA1833CCA (5603A>C), CCA1834CCG (5608A>G), GCA1835GCT (5611A>T), ATA1837ATT (5617A>T), GGT1845GGC (5641T>C), TTT1847TTC (5647T>C), CTA1856CTT (5674A>T), CAG1857CAT (5677G>T), CAG1860CAA (5686G>A), TTG1862CTG (5690T>C), CCT1868CCA (5710T>A), GGG1870GGT (5716G>T), GGA1871GGG (5719A>G), CAC1875CAT (5731C>T), ACT1876ACC (5734T>C), ACT1877ACC (5737T>C), AGA1878AAA (5739G>A), CTT1880CTC (5746T>C), TTG1882CTA (5750T>C 5752G>A), CTC1884CTT (5758C>T), AAG1889AAA (5773G>A), CAA1892CAG (5782A>G), ATT1893ACT (5784T>C), TTA1894CTA (5786T>C), AGA1897AGG (5797A>G), TTC1900TTT (5806C>T), TCA1910TCT (5836A>T), TTG1915CAA (5849T>C 5850T>A 5851G>A), AGG1921AGA (5869G>A), CAA1923CAG (5875A>G), GGG1924GGT (5878G>T), TTT1926TTC (5884T>C), CCC1927CCT (5887C>T), CGC1928CGT (5890C>T), GTG1932GTA (5902G>A), CTT1936CTC (5914T>C), TTT1939TTC (5923T>C), CTG1941TTG (5927C>T), AGC1942AGT (5932C>T), AAC1950AGC (5955A>G), ACG1953ACA (5965G>A), AAA1964AAG (5998A>G), ACC1966ACT (6004C>T), AAA1970AAG (6016A>G), TCC1974TCT (6028C>T), GCA1975GCT (6031A>T), ACA1976ATA (6033C>T), GCT1977TCT (6035G>T), GGT1983GGA (6055T>A), TCC1987TCA (6067C>A), GCT1988GCC (6070T>C), CTT1996TTA (6092C>T 6094T>A), CTT1998CTG (6100T>G), CAG1999CAA (6103G>A), AAG2000AAA (6106G>A), ATA2002ATG (6112A>G), TCA2005TCT (6121A>T), TTT2011TTC (6139T>C), ATT2013ATC (6145T>C), TAT2014TAC (6148T>C), GTC2016GTG (6154C>G), GGC2019GGG (6163C>G), GTA2026GTG (6184A>G), ACT2027ACG (6187T>G), TTC2029TTT (6193C>T), CAA2030CAG (6196A>G), ATG2032ATC (6202G>C), CAA2034CAG (6208A>G), CTG2037CTT (6217G>T), GAA2039GAT (6223A>T), GGG2040GGA (6226G>A), ATA2042GTG (6230A>G 6232A>G), GAG2043GAA (6235G>A), ATA2046ATG (6244A>G), TTA2049TTC (6253A>C), ACC2050ACA (6256C>A), ACA2052ACT (6262A>T), CTG2053CTA (6265G>A), TCG2054TCT (6268G>T), AGC2056ATG (6273G>T 6274C>G), GTA2064CTG (6296G>C 6298A>G), TTA2066TTG (6304A>G), TTG2068CTG (6308T>C), GAT2069GAC (6313T>C), ATA2071ATG (6319A>G), GAG2072GAA (6322G>A), ATC2073GTC (6323A>G), CTA2080TTA (6344C>T), AAT2084AAC (6358T>C), GCC2092GCT (6382C>T), ATC2093ATT (6385C>T), TGT2100TGC (6406T>C), AAC2102AAT (6412C>T), CGA2103CAA (6414G>A), AAA2107AAG (6427A>G), CGC2112CGG (6442C>G), TAC2113TAT (6445C>T), ACG2114ACA (6448G>A), ACT2115ACC (6451T>C), TCC2117TCA (6457C>A), GTT2120GTC (6466T>C), GCA2126GCC (6484A>C), AGA2128AGG (6490A>G), CAT2129CAC (6493T>C), ATT2132TTT (6500A>T), TCC2137TCT (6517C>T), CAC2142CAT (6532C>T), CTT2147TTT (6545C>T), TAC2155TAT (6571C>T)								

Proteins

	Begin	End	Coverage	Score	Concordance	Matches	Identities	I/D/M/F*	Stop Codons
polyprotein (YP_001039627.1)	1	2159	100%	14866	98.6%	2159 (100%)	2115 (98.0%)	0/0/0/0	1
Protein mutations:	P8S (128C>T 130C>T), F10S (135T>C), A18S (158G>T), T19A (161A>G), T364A (1196A>G), I459V (1481A>G), A501S (1607G>T), A646T (2042G>A), F659Y (2082T>A), N712S (2241A>G), S715N (2250G>A 2251C>T), R768K (2409G>A), V1218I (3758G>A), V1598I (4898G>A), T1628A (4988A>G), I1658T (5079T>C), V1672I (5120G>A 5122T>A), S1676T (5132T>A), T1681N (5148C>A), R1713K (5244G>A), V1755I (5369G>A 5371G>A), I1792M (5482A>G), A1829S (5591G>T), T1833P (5603A>C), Q1857H (5677G>T), R1878K (5739G>A), I1893T (5784T>C), L1915Q (5849T>C 5850T>A 5851G>A), N1950S (5955A>G), T1976I (6033C>T), A1977S (6035G>T), I2002M (6112A>G), M2032I (6202G>C), E2039D (6223A>T), I2042V (6230A>G 6232A>G), I2046M (6244A>G), L2049F (6253A>C), S2056M (6273G>T 6274C>G), V2064L (6296G>C 6298A>G), I2071M (6319A>G), I2073V (6323A>G), R2103Q (6414G>A), I2132F (6500A>T), L2147F (6545C>T)								

	Begin	End	Coverage	Score	Concordance	Matches	Identities	I/D/M/F*	Stop Codons
**NT**	**30**	**7769**	**99.1%**	**11591**	**81.7%**	**7347 (94.6%)**	**6725 (86.6%)**	**24/393**	
Codon mutations:	TTT3TTC (115T>C), TTC7TTT (127C>T), CCC8TCT (128C>T 130C>T), TTC10TCC (135T>C), ACT14ACC (148T>C), GAA15GAG (151A>G), GCT18TCT (158G>T), ACC19GCC (161A>G), TCT24TCG (178T>G), TCA27TCG (187A>G), GCT31GCA (199T>A), AGT34AGC (208T>C), CGC38CGT (220C>T), CTG40TTG (224C>T), CAC53CAT (265C>T), TCT74TCC (328T>C), ACT76ACC (334T>C), TTG78TTA (340G>A), AAG81AAA (349G>A), TTC89TTT (373C>T), ATC90ATT (376C>T), AAG109AAA (433G>A), GAC127GAT (487C>T), GTG132GTA (502G>A), TCT145TCC (541T>C), TTC151TTT (559C>T), CTA158TTA (578C>T), AAC174AAT (628C>T), GAC183GAT (655C>T), CAA190CAG (676A>G), GCA196GCG (694A>G), CCA210CCG (736A>G), ACC225ACA (781C>A), TTC228TTT (790C>T), GTA231GTG (799A>G), GGT233GGC (805T>C), CTA238TTG (818C>T 820A>G), CTA242CTT (832A>T), TCA243TCC (835A>C), ATT245ATC (841T>C), CTG246TTG (842C>T), TAC247TAT (847C>T), TTG259TTA (883G>A), TTT260TTC (886T>C), AAT265AAC (901T>C), GCC273GCT (925C>T), GTA282GTG (952A>G), GGT286GGG (964T>G), AAG288AAA (970G>A), GCC297GCT (997C>T), TCT300TCC (1006T>C), GAG309GAA (1033G>A), CAT318CAC (1060T>C), GCA324GCT (1078A>T), AAC325AAT (1081C>T), ACT329ACC (1093T>C), CAA333CAG (1105A>G), CAT334CAC (1108T>C), CTA335TTA (1109C>T), GCC353GCT (1165C>T), AGG358AGA (1180G>A), ACC364GCC (1196A>G), CAG375CAA (1231G>A), GGC378GGT (1240C>T), AGG383AGA (1255G>A), TTT387TTC (1267T>C), CAA390CAG (1276A>G), GGG398GGA (1300G>A), GAA415GAG (1351A>G), CTC422CTT (1372C>T), AAT434AAC (1408T>C), TAT435TAC (1411T>C), CAT437CAC (1417T>C), ACT439ACC (1423T>C), GCT440GCC (1426T>C), ATC441ATT (1429C>T), CTG442TTG (1430C>T), GAC445GAT (1441C>T), GCT448GCC (1450T>C), CTG449TTG (1451C>T), GAC458GAT (1480C>T), ATT459GTT (1481A>G), GAT461GAC (1489T>C), CCC472CCT (1522C>T), GGC482GGT (1552C>T), CGC483CGT (1555C>T), CGA488CGG (1570A>G), TAT489TAC (1573T>C), GCC492GCT (1582C>T), ACA493ACT (1585A>T), AAC495AAT (1591C>T), CTT496CTC (1594T>C), GCT501TCT (1607G>T), CAG502CAA (1612G>A), GGT504GGG (1618T>G), CTA505TTA (1619C>T), ACC506ACA (1624C>A), AGA513AGG (1645A>G), TGC521TGT (1669C>T), GGT525GGA (1681T>A), GAG526GAA (1684G>A), GCT531GCA (1699T>A), ACT533ACC (1705T>C), AAA544AAG (1738A>G), AGC555AGT (1771C>T), TTG559CTG (1781T>C), GAA561GAG (1789A>G), GCA597GCG (1897A>G), TTG602CTG (1910T>C), GTT616GTC (1954T>C), GTA618GTG (1960A>G), TAC626TAT (1984C>T), AAA636AAG (2014A>G), GCA646ACA (2042G>A), CCT650CCC (2056T>C), TTT659TAT (2082T>A), CTA662CTG (2092A>G), AAA667AAG (2107A>G), GTT674GTC (2128T>C), CTG678TTG (2138C>T), TAT679TAC (2143T>C), GGT681GGC (2149T>C), TGT683TGC (2155T>C), CTG690TTG (2174C>T), ATA691ATC (2179A>C), AAG692AAA (2182G>A), GCT696GCA (2194T>A), TAT698TAC (2200T>C), TAC707TAT (2227C>T), AAT712AGT (2241A>G), AGC715AAT (2250G>A 2251C>T), GAC717GAT (2257C>T), CAG719CAA (2263G>A), CCT723CCC (2275T>C), GCG727GCA (2287G>A), CAC728CAT (2290C>T), GAC736GAT (2314C>T), ATA739ATT (2323A>T), CAC747CAT (2347C>T), GTA748GTG (2350A>G), TTC750TTT (2356C>T), TTG751TTA (2359G>A), TAC752TAT (2362C>T), AGG757AGA (2377G>A), TAT758TAC (2380T>C), AGA762AGG (2392A>G), CCT767CCC (2407T>C), AGG768AAG (2409G>A), ATT779ATC (2443T>C), GTG780GTA (2446G>A), CCG791CCA (2479G>A), ATC793ATT (2485C>T), CTG797CTA (2497G>A), ATT798ATC (2500T>C), AAG799AAA (2503G>A), GGC801GGT (2509C>T), CTT802CTC (2512T>C), GGG812GGA (2542G>A), GTC814GTT (2548C>T), TTC817TTT (2557C>T), GAG838GAA (2620G>A), AAT840AAC (2626T>C), AAG848AAA (2650G>A), TTT853TTC (2665T>C), TTG857CTG (2675T>C), GCA859GCG (2683A>G), AAG862AAA (2692G>A), GAT864GAC (2698T>C), CTG866TTG (2702C>T), AAA868AAG (2710A>G), GAT873GAC (2725T>C), TTC875TTT (2731C>T), ATC876ATT (2734C>T), GGA879GGG (2743A>G), TTG880CTG (2744T>C), AAA882AAG (2752A>G), TTG887CTG (2765T>C), CTA889CTG (2773A>G), GTC893GTT (2785C>T), TAC894TAT (2788C>T), TGC901TGT (2809C>T), GTG905GTT (2821G>T), ACA907ACG (2827A>G), TTT912TTC (2842T>C), GGC918GGT (2860C>T), ACT920ACA (2866T>A), TTC921TTT (2869C>T), TCA922TCT (2872A>T), CGG940CGA (2926G>A), TTC941TTT (2929C>T), TTG942CTG (2930T>C), AAG943AAA (2935G>A), CAA945CAG (2941A>G), GTC948GTT (2950C>T), CTG949TTG (2951C>T), AAA964AAG (2998A>G), TCT975TCA (3031T>A), AAG976AAA (3034G>A), GGC977GGT (3037C>T), ACT978ACC (3040T>C), GTA981GTG (3049A>G), GTA986GTG (3064A>G), AAG989AAA (3073G>A), GCT991GCC (3079T>C), TTT1001TTC (3109T>C), GTG1003GTA (3115G>A), GTA1013GTG (3145A>G), TAC1015TAT (3151C>T), TCT1019TCC (3163T>C), TCC1021TCT (3169C>T), TTT1022TTC (3172T>C), TAT1032TAC (3202T>C), CTT1045CTC (3241T>C), AAC1049AAT (3253C>T), GTG1050GTA (3256G>A), AAA1053AAG (3265A>G), CTC1104CTT (3418C>T), AAA1119AAG (3463A>G), CAG1120CAA (3466G>A), GGC1125GGT (3481C>T), ACA1126ACT (3484A>T), ACG1134ACA (3508G>A), GGG1151GGA (3559G>A), TCT1158TCC (3580T>C), ATC1159ATT (3583C>T), GAA1168GAG (3610A>G), GGT1169GGC (3613T>C), TAC1173TAT (3625C>T), GTG1174GTA (3628G>A), AAA1179AAG (3643A>G), TAC1180TAT (3646C>T), CAA1185CAG (3661A>G), AAT1186AAC (3664T>C), GAG1189GAA (3673G>A), GAC1190GAT (3676C>T), CTA1194TTA (3686C>T), CGT1200CGC (3706T>C), TTG1204CTG (3716T>C), CCT1205CCA (3721T>A), CGA1211CGT (3739A>T), AAG1216AAA (3754G>A), GTC1218ATC (3758G>A), CAG1220CAA (3766G>A), CTC1226CTG (3784C>G), TAT1234TAC (3808T>C), GAC1236GAT (3814C>T), GGG1237GGC (3817G>C), TTG1240CTG (3824T>C), TCT1243TCC (3835T>C), TAT1246TAC (3844T>C), TTG1251CTA (3857T>C 3859G>A), GGC1254GGT (3868C>T), ACT1255ACC (3871T>C), GAG1259GAA (3883G>A), AGT1264AGC (3898T>C), GGA1267GGT (3907A>T), GTC1270GTT (3916C>T), AAA1273AAG (3925A>G), AGG1274AGA (3928G>A), GAC1281GAT (3949C>T), GAG1283GAA (3955G>A), TTG1285TTA (3961G>A), GAA1288GAG (3970A>G), ATT1289ATC (3973T>C), TTG1310CTG (4034T>C), CGT1320CGC (4066T>C), GAA1323GAG (4075A>G), CCC1326CCT (4084C>T), TTG1332CTG (4100T>C), CTG1338TTG (4118C>T), TCC1341TCT (4129C>T), TCC1347TCA (4147C>A), CTG1351CTA (4159G>A), CCA1352CCT (4162A>T), AAA1354AAG (4168A>G), GAA1363GAG (4195A>G), TTG1367TTA (4207G>A), GCT1368GCC (4210T>C), TTC1371TTT (4219C>T), GTC1374GTT (4228C>T), CAA1396CAG (4294A>G), GAC1414GAT (4348C>T), AAT1416AAC (4354T>C), ACT1430ACC (4396T>C), AAC1434AAT (4408C>T), GTT1436GTG (4414T>G), TTT1437TTC (4417T>C), AGG1438AGA (4420G>A), GTT1451GTC (4459T>C), ATC1452ATT (4462C>T), TTC1453TTT (4465C>T), AAC1454AAT (4468C>T), TCT1455TCC (4471T>C), CTT1456CTC (4474T>C), TTT1460TTC (4486T>C), TCT1468TCA (4510T>A), CTA1469TTA (4511C>T), TTG1470CTG (4514T>C), AAA1483AAG (4555A>G), AAC1488AAT (4570C>T), CTA1490CTT (4576A>T), GTC1494GTA (4588C>A), TAT1495TAC (4591T>C), TTG1507CTG (4625T>C), TAT1510TAC (4636T>C), GCT1514GCA (4648T>A), GAA1518GAG (4660A>G), ATA1526ATC (4684A>C), ATC1530ATT (4696C>T), AAG1532AAA (4702G>A), AAC1533AAT (4705C>T), GTT1536GTC (4714T>C), GAC1543GAT (4735C>T), CTA1547CTG (4747A>G), CTA1550CTT (4756A>T), AGC1551AGT (4759C>T), TTT1554TTC (4768T>C), AGG1556AGA (4774G>A), CAC1557CAT (4777C>T), CTA1559CTG (4783A>G), GGA1560GGG (4786A>G), TAC1561TAT (4789C>T), GTA1569GTG (4813A>G), TCT1570TCC (4816T>C), AGG1579AGA (4843G>A), GTT1598ATT (4898G>A), GAG1600GAA (4906G>A), AAG1621AAA (4969G>A), ACC1628GCC (4988A>G), ACA1629ACT (4993A>T), TAT1633TAC (5005T>C), TTG1636CTG (5012T>C), GAC1639GAT (5023C>T), CTG1641CTT (5029G>T), GGA1646GGC (5044A>C), GGG1651GGT (5059G>T), CTT1656CTC (5074T>C), CAT1657CAC (5077T>C), ATT1658ACT (5079T>C), TTG1666CTG (5102T>C), ACT1671ACC (5119T>C), GTT1672ATA (5120G>A 5122T>A), CTG1673TTG (5123C>T), TCT1676ACT (5132T>A), CAT1680CAC (5146T>C), ACT1681AAT (5148C>A), CAG1698CAA (5200G>A), GTT1704GTG (5218T>G), AGA1713AAA (5244G>A), CCC1720CCT (5266C>T), GAC1725GAT (5281C>T), CAC1741CAT (5329C>T), ATT1745ATA (5341T>A), GTA1746GTG (5344A>G), ACC1747ACT (5347C>T), GTG1755ATA (5369G>A 5371G>A), GGC1760GGT (5386C>T), GGG1762GGA (5392G>A), CCA1767CCT (5407A>T), GCA1771GCT (5419A>T), ACT1777ACC (5437T>C), GGC1778GGT (5440C>T), GTT1785GTC (5461T>C), TTG1789CTG (5471T>C), ATA1792ATG (5482A>G), CTT1809CTA (5533T>A), ACA1823ACG (5575A>G), TGT1824TGC (5578T>C), GCC1829TCC (5591G>T), ACA1833CCA (5603A>C), CCA1834CCG (5608A>G), GCA1835GCT (5611A>T), ATA1837ATT (5617A>T), GGT1845GGC (5641T>C), TTT1847TTC (5647T>C), CTA1856CTT (5674A>T), CAG1857CAT (5677G>T), CAG1860CAA (5686G>A), TTG1862CTG (5690T>C), CCT1868CCA (5710T>A), GGG1870GGT (5716G>T), GGA1871GGG (5719A>G), CAC1875CAT (5731C>T), ACT1876ACC (5734T>C), ACT1877ACC (5737T>C), AGA1878AAA (5739G>A), CTT1880CTC (5746T>C), TTG1882CTA (5750T>C 5752G>A), CTC1884CTT (5758C>T), AAG1889AAA (5773G>A), CAA1892CAG (5782A>G), ATT1893ACT (5784T>C), TTA1894CTA (5786T>C), AGA1897AGG (5797A>G), TTC1900TTT (5806C>T), TCA1910TCT (5836A>T), TTG1915CAA (5849T>C 5850T>A 5851G>A), AGG1921AGA (5869G>A), CAA1923CAG (5875A>G), GGG1924GGT (5878G>T), TTT1926TTC (5884T>C), CCC1927CCT (5887C>T), CGC1928CGT (5890C>T), GTG1932GTA (5902G>A), CTT1936CTC (5914T>C), TTT1939TTC (5923T>C), CTG1941TTG (5927C>T), AGC1942AGT (5932C>T), AAC1950AGC (5955A>G), ACG1953ACA (5965G>A), AAA1964AAG (5998A>G), ACC1966ACT (6004C>T), AAA1970AAG (6016A>G), TCC1974TCT (6028C>T), GCA1975GCT (6031A>T), ACA1976ATA (6033C>T), GCT1977TCT (6035G>T), GGT1983GGA (6055T>A), TCC1987TCA (6067C>A), GCT1988GCC (6070T>C), CTT1996TTA (6092C>T 6094T>A), CTT1998CTG (6100T>G), CAG1999CAA (6103G>A), AAG2000AAA (6106G>A), ATA2002ATG (6112A>G), TCA2005TCT (6121A>T), TTT2011TTC (6139T>C), ATT2013ATC (6145T>C), TAT2014TAC (6148T>C), GTC2016GTG (6154C>G), GGC2019GGG (6163C>G), GTA2026GTG (6184A>G), ACT2027ACG (6187T>G), TTC2029TTT (6193C>T), CAA2030CAG (6196A>G), ATG2032ATC (6202G>C), CAA2034CAG (6208A>G), CTG2037CTT (6217G>T), GAA2039GAT (6223A>T), GGG2040GGA (6226G>A), ATA2042GTG (6230A>G 6232A>G), GAG2043GAA (6235G>A), ATA2046ATG (6244A>G), TTA2049TTC (6253A>C), ACC2050ACA (6256C>A), ACA2052ACT (6262A>T), CTG2053CTA (6265G>A), TCG2054TCT (6268G>T), AGC2056ATG (6273G>T 6274C>G), GTA2064CTG (6296G>C 6298A>G), TTA2066TTG (6304A>G), TTG2068CTG (6308T>C), GAT2069GAC (6313T>C), ATA2071ATG (6319A>G), GAG2072GAA (6322G>A), ATC2073GTC (6323A>G), CTA2080TTA (6344C>T), AAT2084AAC (6358T>C), GCC2092GCT (6382C>T), ATC2093ATT (6385C>T), TGT2100TGC (6406T>C), AAC2102AAT (6412C>T), CGA2103CAA (6414G>A), AAA2107AAG (6427A>G), CGC2112CGG (6442C>G), TAC2113TAT (6445C>T), ACG2114ACA (6448G>A), ACT2115ACC (6451T>C), TCC2117TCA (6457C>A), GTT2120GTC (6466T>C), GCA2126GCC (6484A>C), AGA2128AGG (6490A>G), CAT2129CAC (6493T>C), ATT2132TTT (6500A>T), TCC2137TCT (6517C>T), CAC2142CAT (6532C>T), CTT2147TTT (6545C>T), TAC2155TAT (6571C>T)								

*: Inserts / Deletes / Misaligned / Frameshifts

Analysis details

This analysis was performed with panviral2.64

NGS Details (UN60): Torradovirus lycopersici (segment RNA 2)

Assembly

**Coverage Length**	5384 (1 contig(s))
**Depth Of Coverage**	16080.2
**Number Of Reads**	685969
**Reads Per Million**	14549.11 rpm (after QC)
**Ambiguities**	0
**Assembly Method**	de novo + reference guided assembly
**Consensus Caller**	Bcf Tools

Coverage Map

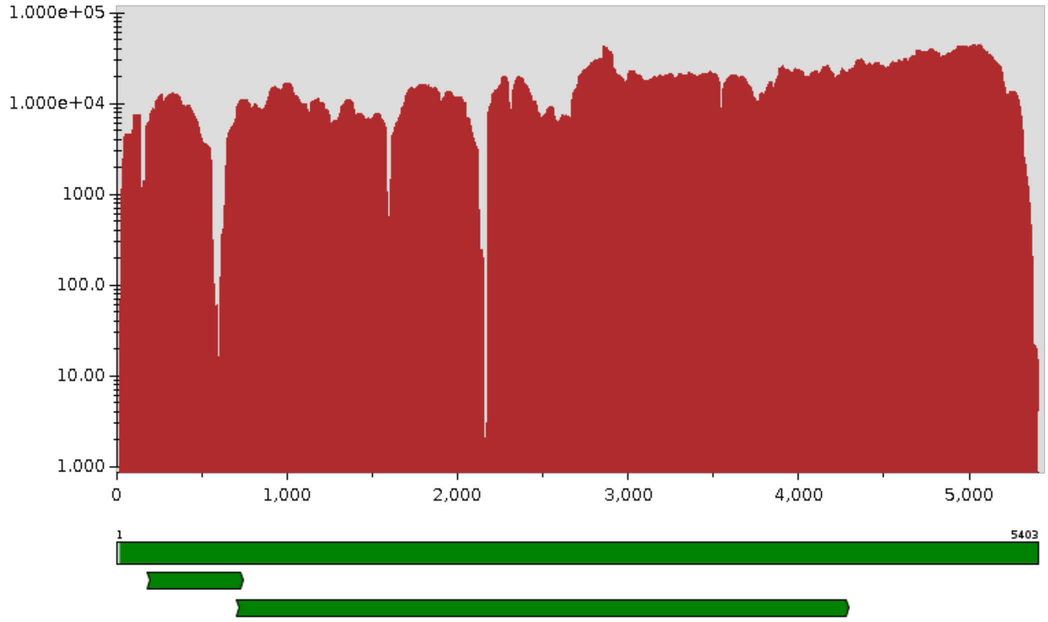

Assignment

**Type**	Torradovirus lycopersici (Taxonomy ID: 3048378)
**Reference Genome**	NC_009032.1
**NT Identity (%)**	85.2794
**AA Identity (%)**	89.6179
**Number Of Stop Codons**	5
**Number Of CDS**	2

Alignment

**Alignment Score**	7578.0 (NT) + 8662.0 (AA) = 16240.0
**Concordance (%)**	80.1777

Alignment Method	Global, seeded, nucleotide + amino acids (AGA)

Genome Region

Sequence starts at position 20 and ends at position 5403 relative to NC_009032.1 reference sequence.

Alignment Detailed Statistics

	Begin	End	Coverage	Score	Concordance	Matches	Identities	I/D/M/F*	Stop Codons
**NT**	**20**	**5403**	**99.6%**	**7578**	**70.4%**	**5384 (99.9%)**	**4594 (85.3%)**	**3/0**	
Mutations:	52C>T, 58T>C, 60G>A, 69A>G, 79A>G, 91T>A, 96C>T, 99T>G, 103G>C, 104A>G, 105G>T, 107G>A, 108C>G, 111C>A, 112G>A, 117C>T, 120A>G, 121A>C, 132C>T, 137T>C, 149G>T, 161A>T, 164T>A, 170G>T, 181T>C, 187A>C, 212C>G, 223G>A, 232C>T, 235G>A, 238A>G, 244A>T, 247C>T, 265G>A, 268T>A, 277C>T, 280T>C, 286A>C, 289A>C, 292C>T, 301A>T, 304T>C, 305C>T, 307A>G, 313C>T, 316G>A, 325C>T, 329A>G, 334T>A, 340A>T, 352A>T, 356G>A, 370C>T, 372A>C, 376C>T, 383C>T, 385G>A, 388T>A, 400T>C, 401T>C, 403A>C, 418T>G, 433C>T, 439G>A, 445T>A, 448C>A, 451A>T, 461A>C, 481C>T, 484C>A, 487A>G, 496A>G, 502T>A, 505G>C, 507G>A, 508G>A, 509A>G, 511T>A, 515G>A, 520C>T, 541C>T, 547T>G, 552C>A, 553T>G, 554G>A, 555C>A, 564A>G, 565A>T, 568A>T, 570A>G, 576C>A, 578C>G, 586A>G, 587T>C, 589A>T, 595G>T, 599C>T, 601T>G, 603A>C, 604C>T, 607G>A, 610C>T, 613A>G, 618G>A, 619C>A, 622A>G, 631A>G, 632G>T, 637A>G, 644C>G, 645T>C, 654T>A, 658C>A, 665T>G, 670A>G, 673A>G, 679G>A, 683T>A, 685G>A, 689A>T, 690A>C, 696T>C, 706T>C, 731G>A, 733A>G, 750A>G, 755A>T, 758T>A, 761A>G, 788A>T, 818G>A, 821T>C, 824C>G, 833C>T, 842A>T, 844A>T, 851T>C, 854C>A, 860C>G, 863C>T, 866A>G, 878C>T, 884A>G, 887G>T, 893C>T, 911T>C, 929T>A, 932A>T, 968A>G, 1010T>G, 1061T>C, 1085G>A, 1095G>A, 1100G>A, 1109C>T, 1115G>A, 1118A>C, 1130A>G, 1139G>A, 1142C>T, 1151A>G, 1163A>G, 1169C>G, 1178C>T, 1187A>T, 1205T>A, 1220T>C, 1235C>A, 1247A>G, 1262T>C, 1313T>C, 1316C>T, 1317C>A, 1319A>G, 1325A>G, 1328T>C, 1331C>T, 1343G>A, 1355G>A, 1361A>T, 1367A>T, 1370A>T, 1376T>A, 1383A>G, 1385C>T, 1388C>T, 1390C>G, 1394A>C, 1395A>T, 1397T>A, 1400T>G, 1403A>T, 1406G>A, 1407C>T, 1409T>A, 1412G>T, 1418G>A, 1426A>C, 1427G>T, 1434G>A, 1439A>C, 1451G>T, 1454A>T, 1456G>C, 1457C>T, 1460A>G, 1461G>T, 1465G>A, 1472C>G, 1475G>C, 1478C>G, 1481C>T, 1487C>G, 1490G>A, 1493G>A, 1496C>T, 1502C>T, 1514T>C, 1524T>C, 1526C>T, 1529A>T, 1538A>T, 1543C>T, 1545A>G, 1547C>T, 1553T>C, 1559C>A, 1560C>T, 1562C>A, 1568C>G, 1574G>A, 1575C>A, 1577T>G, 1589A>G, 1593C>G, 1595G>T, 1603G>A, 1605A>G, 1608T>C, 1610C>G, 1613A>G, 1616G>A, 1621G>C, 1622T>A, 1626G>T, 1627A>C, 1628T>C, 1631G>A, 1634T>A, 1643C>T, 1646T>C, 1651A>G, 1655T>A, 1661T>C, 1662C>T, 1663T>A, 1664G>T, 1667C>T, 1674C>T, 1676T>C, 1677G>A, 1679G>T, 1685T>C, 1688T>C, 1695A>G, 1700G>A, 1721G>C, 1724C>T, 1727A>G, 1730C>T, 1740C>T, 1745G>A, 1748G>A, 1749A>C, 1754G>A, 1757A>G, 1760T>G, 1763T>A, 1766A>G, 1769A>T, 1778A>C, 1784A>T, 1790G>A, 1793T>A, 1794G>A, 1797T>G, 1799C>G, 1805C>T, 1811A>C, 1814T>C, 1817A>T, 1819T>A, 1820C>G, 1823C>T, 1832G>C, 1841C>T, 1843A>G, 1845T>C, 1847G>T, 1856C>T, 1859C>T, 1863C>T, 1865C>T, 1875G>A, 1878A>G, 1892C>T, 1898T>G, 1899G>A, 1900T>C, 1901G>A, 1906C>T, 1907C>G, 1913T>A, 1928A>C, 1931C>T, 1938A>G, 1941C>T, 1943T>G, 1946C>A, 1947T>C, 1952A>C, 1955G>A, 1958A>G, 1967G>A, 1970A>G, 1979A>G, 1986G>A, 1994T>C, 2002T>A, 2012C>T, 2024T>C, 2027T>A, 2049T>A, 2051A>T, 2054G>T, 2059A>G, 2060A>C, 2066C>G, 2074T>A, 2083T>C, 2084G>A, 2087A>C, 2094C>G, 2096G>C, 2098C>A, 2099G>A, 2100C>A, 2101G>T, 2102C>G, 2106A>G, 2107G>A, 2111T>G, 2114C>T, 2115A>C, 2116A>G, 2119A>C, 2120G>A, 2123G>T, 2125C>T, 2126C>T, 2127A>G, 2128G>T, 2130G>A, 2132T>C, 2135G>A, 2136G>C, 2138G>T, 2144C>G, 2159T>A, 2162T>C, 2164T>G, 2170A>T, 2171A>T, 2174A>T, 2177A>T, 2178T>G, 2183C>T, 2186A>C, 2189C>T, 2195A>C, 2198G>A, 2199G>A, 2200T>C, 2204C>T, 2205A>G, 2210G>A, 2216C>G, 2219T>C, 2225T>A, 2228C>T, 2229A>G, 2230T>A, 2231G>T, 2240T>A, 2243T>C, 2252A>C, 2256G>A, 2259A>C, 2261T>A, 2262G>C, 2270A>G, 2285T>C, 2294A>C, 2303A>G, 2312T>C, 2315T>A, 2320G>C, 2321T>C, 2324G>C, 2326C>T, 2339A>G, 2345A>G, 2375C>T, 2393G>A, 2414T>C, 2420T>G, 2429G>A, 2432G>A, 2436T>G, 2444G>C, 2447C>A, 2453G>A, 2459C>G, 2469T>A, 2471C>G, 2474G>A, 2489A>C, 2492G>A, 2495A>G, 2501A>G, 2503G>A, 2507T>C, 2516C>T, 2523A>C, 2546T>C, 2552C>T, 2561T>G, 2567T>A, 2570G>A, 2573T>A, 2588T>A, 2609C>A, 2627C>T, 2630C>G, 2645G>C, 2660T>C, 2672A>T, 2693G>A, 2702T>A, 2708A>G, 2717T>C, 2720C>A, 2729C>T, 2732T>G, 2747C>T, 2753G>C, 2792C>T, 2795C>T, 2798G>T, 2819G>A, 2835A>G, 2837C>T, 2840T>C, 2841C>T, 2846G>T, 2852A>G, 2861G>C, 2864C>T, 2870C>A, 2879T>A, 2882T>G, 2885T>G, 2886T>C, 2894A>G, 2900A>T, 2909T>C, 2915A>G, 2921C>T, 2927A>G, 2936A>C, 2939A>G, 2948A>G, 2954G>A, 2957A>T, 2960G>A, 2966C>T, 2972T>C, 2981G>T, 2982G>A, 2996T>G, 2999T>A, 3003T>C, 3011C>T, 3012A>G, 3014C>T, 3020T>C, 3023G>C, 3029C>T, 3032A>T, 3044C>T, 3047T>A, 3050G>C, 3051C>T, 3059A>T, 3062C>T, 3063T>C, 3065G>A, 3071A>G, 3077A>T, 3083C>A, 3101T>G, 3104C>T, 3107T>G, 3113G>C, 3122T>G, 3128T>C, 3131G>A, 3143C>T, 3146T>C, 3152T>A, 3158G>A, 3167A>T, 3168C>T, 3176A>C, 3179G>T, 3182C>T, 3187G>A, 3197C>T, 3206C>T, 3227T>G, 3230T>A, 3236C>T, 3239T>G, 3242G>A, 3248A>C, 3251A>T, 3253A>G, 3265G>A, 3269T>C, 3270A>T, 3272G>T, 3278C>T, 3293A>G, 3299T>A, 3303A>T, 3305C>T, 3311T>A, 3324C>T, 3329G>T, 3350C>T, 3354A>C, 3359G>A, 3368A>T, 3371C>G, 3372T>C, 3374G>C, 3386A>T, 3395A>C, 3404A>G, 3410G>A, 3413C>T, 3428G>A, 3429C>T, 3431T>G, 3434A>G, 3437C>T, 3440A>T, 3447T>C, 3449G>A, 3452C>A, 3455T>A, 3458G>A, 3461C>T, 3470T>C, 3473T>C, 3479C>T, 3482T>C, 3485C>T, 3488A>G, 3491C>T, 3503A>G, 3508A>G, 3512T>C, 3515G>T, 3522C>T, 3524A>G, 3527C>T, 3533G>A, 3543T>A, 3551C>T, 3560T>C, 3564A>C, 3567T>A, 3569A>G, 3575G>A, 3578A>G, 3581C>T, 3590A>C, 3605A>G, 3611G>A, 3617A>T, 3620G>A, 3623A>T, 3629G>C, 3631C>A, 3634T>C, 3635C>T, 3638A>G, 3641C>T, 3650A>T, 3656G>T, 3659T>C, 3662A>G, 3674G>C, 3689C>T, 3698A>C, 3702T>C, 3704G>A, 3707T>G, 3708T>C, 3713A>C, 3716C>T, 3719A>G, 3722C>A, 3728C>G, 3742A>C, 3743G>C, 3746G>A, 3749A>T, 3752G>A, 3755A>G, 3767C>A, 3770A>C, 3773C>T, 3776A>T, 3779C>T, 3800C>T, 3815A>T, 3827C>T, 3833T>A, 3839A>G, 3845A>G, 3848G>A, 3851G>A, 3852A>G, 3854T>C, 3857C>A, 3866A>C, 3869A>T, 3872A>T, 3878G>C, 3881C>T, 3882A>G, 3884C>A, 3885A>G, 3890A>G, 3893T>C, 3896A>T, 3914T>A, 3920A>T, 3923C>T, 3927A>C, 3928A>G, 3929G>A, 3932C>A, 3933A>G, 3935C>T, 3941T>G, 3944C>A, 3947T>C, 3950T>A, 3953T>A, 3960G>A, 3971T>A, 3983T>A, 3984A>G, 3986G>T, 3988G>A, 3989C>T, 3990G>A, 3991A>G, 3995C>T, 3998C>T, 4001T>C, 4016T>G, 4025G>A, 4028T>C, 4031T>C, 4034T>G, 4035A>C, 4037A>T, 4040C>T, 4052C>T, 4059A>G, 4061A>T, 4067A>T, 4068A>G, 4069A>G, 4070T>C, 4072C>T, 4073T>G, 4077A>C, 4088T>A, 4091G>A, 4100A>C, 4103A>T, 4106A>C, 4112C>T, 4133A>G, 4142A>C, 4145C>T, 4149C>A, 4151G>A, 4154A>G, 4157C>A, 4166G>A, 4181C>T, 4187G>A, 4188C>G, 4196T>A, 4208A>T, 4211A>T, 4223G>A, 4224C>T, 4227T>C, 4229G>T, 4250C>T, 4256T>G, 4262T>C, 4265T>A, 4274A>T, 4281A>C, 4284A>G, 4286T>A, 4289T>C, 4300T>C, 4302T>G, 4316T>C, 4354G>A, 4355T>G, 4382A>T, 4383C>T, 4388A>T, 4391A>C, 4393A>G, 4424C>T, 4425T>C, 4429T>C, 4457A>G, 4468T>C, 4496T>A, 4506A>T, 4525T>C, 4567_4568insT, 4605T>C, 4633T>A, 4634G>A, 4643G>A, 4648G>A, 4650T>C, 4651G>A, 4675A>T, 4812C>T, 4823A>G, 4974_4975insT, 5089T>C, 5192C>T, 5206C>T, 5213A>G, 5214C>T, 5222C>T, 5224G>A, 5225C>A, 5226T>G, 5233G>T, 5236G>T, 5239T>A, 5242T>G, 5244G>A, 5249A>G, 5258A>G, 5259T>A, 5262A>T, 5282T>C, 5313A>C, 5315T>A, 5369G>T, 5371G>A, 5374T>A, 5378A>T, 5379A>C, 5380_5381insT, 5400A>C								

CDS

	Begin	End	Coverage	Score	Concordance	Matches	Identities	I/D/M/F*	Stop Codons
ToTV_sRNA2gp1	1	188	100%	1080	89.3%	188 (100%)	162 (86.2%)	0/0/0/0	2
Protein mutations:	L11V (212C>G), T50A (329A>G), V59I (356G>A), N64T (372A>C), L90F (451A>T), Q108H (505G>C), R109K (507G>A 508G>A), I110V (509A>G 511T>A), V112I (515G>A), A124E (552C>A 553T>G), A125K (554G>A 555C>A), Q128R (564A>G 565A>T), Q130R (570A>G), T132K (576C>A), L133V (578C>G), E138D (595G>T), D141A (603A>C 604C>T), R146Q (618G>A 619C>A), E151* (632G>T), L155A (644C>G 645T>C), L158Q (654T>A), S162A (665T>G), L168I (683T>A 685G>A), N170S (689A>T 690A>C), L172P (696T>C), E184K (731G>A 733A>G)								
Codon mutations:	TCA2TCC (187A>C), CTT11GTT (212C>G), GAG14GAA (223G>A), CAC17CAT (232C>T), AAG18AAA (235G>A), CAA19CAG (238A>G), GCA21GCT (244A>T), GAC22GAT (247C>T), TCG28TCA (265G>A), GTT29GTA (268T>A), GGC32GGT (277C>T), TCT33TCC (280T>C), ATA35ATC (286A>C), ATA36ATC (289A>C), AAC37AAT (292C>T), CCA40CCT (301A>T), ACT41ACC (304T>C), CTA42TTG (305C>T 307A>G), TTC44TTT (313C>T), AAG45AAA (316G>A), CCC48CCT (325C>T), ACA50GCA (329A>G), GGT51GGA (334T>A), GCA53GCT (340A>T), CTA57CTT (352A>T), GTT59ATT (356G>A), AAC63AAT (370C>T), AAT64ACT (372A>C), AGC65AGT (376C>T), CTG68TTA (383C>T 385G>A), GTT69GTA (388T>A), TAT73TAC (400T>C), TTA74CTC (401T>C 403A>C), ACT79ACG (418T>G), GGC84GGT (433C>T), AAG86AAA (439G>A), TCT88TCA (445T>A), GGC89GGA (448C>A), TTA90TTT (451A>T), AGA94CGA (461A>C), ACC100ACT (481C>T), ACC101ACA (484C>A), AGA102AGG (487A>G), CTA105CTG (496A>G), GCT107GCA (502T>A), CAG108CAC (505G>C), AGG109AAA (507G>A 508G>A), ATT110GTA (509A>G 511T>A), GTA112ATA (515G>A), TCC113TCT (520C>T), TCC120TCT (541C>T), GCT122GCG (547T>G), GCT124GAG (552C>A 553T>G), GCA125AAA (554G>A 555C>A), CAA128CGT (564A>G 565A>T), CCA129CCT (568A>T), CAA130CGA (570A>G), ACG132AAG (576C>A), CTT133GTT (578C>G), CAA135CAG (586A>G), TTA136CTT (587T>C 589A>T), GAG138GAT (595G>T), CTT140TTG (599C>T 601T>G), GAC141GCT (603A>C 604C>T), GAG142GAA (607G>A), GCC143GCT (610C>T), AAA144AAG (613A>G), CGC146CAA (618G>A 619C>A), TTA147TTG (622A>G), AAA150AAG (631A>G), GAG151TAG (632G>T), AAA152AAG (637A>G), CTT155GCT (644C>G 645T>C), CTA158CAA (654T>A), TCC159TCA (658C>A), TCC162GCC (665T>G), AAA163AAG (670A>G), TTA164TTG (673A>G), TTG166TTA (679G>A), TTG168ATA (683T>A 685G>A), AAC170TCC (689A>T 690A>C), CTA172CCA (696T>C), GAT175GAC (706T>C), GAA184AAG (731G>A 733A>G)								
ToTV_sRNA2gp2	1	1199	100%	7582	91.6%	1199 (100%)	1081 (90.2%)	0/0/0/0	3
Protein mutations:	M2T (706T>C), K11R (733A>G), S17G (750A>G), Y48F (844A>T), V132I (1095G>A), T228A (1383A>G 1385C>T), S230* (1390C>G), T232S (1395A>T 1397T>A), D233E (1400T>G), Q242P (1426A>C 1427G>T), D245N (1434G>A), S252T (1456G>C 1457C>T), A254S (1461G>T), R255K (1465G>A), I257M (1472C>G), S275P (1524T>C 1526C>T), S281F (1543C>T), T282A (1545A>G 1547C>T), H286Q (1559C>A), Q298D (1593C>G 1595G>T), S301N (1603G>A), R302G (1605A>G), Y303Q (1608T>C 1610C>G), G307A (1621G>C 1622T>A), D309S (1626G>T 1627A>C 1628T>C), K317R (1651A>G), L321Y (1662C>T 1663T>A 1664G>T), H325Y (1674C>T 1676T>C), V326I (1677G>A 1679G>T), I332V (1695A>G), V365I (1794G>A), C366G (1797T>G 1799C>G), K370N (1811A>C), I373K (1819T>A 1820C>G), K377N (1832G>C), H381R (1843A>G), H388Y (1863C>T 1865C>T), V392M (1875G>A), I393V (1878A>G), I399M (1898T>G), V400T (1899G>A 1900T>C 1901G>A), A402V (1906C>T 1907C>G), E409D (1928A>C), N413D (1938A>G), D429N (1986G>A), F434Y (2002T>A), L450I (2049T>A 2051A>T), K453S (2059A>G 2060A>C), L458Q (2074T>A), V461A (2083T>C 2084G>A), Q465D (2094C>G 2096G>C), A466E (2098C>A 2099G>A), R467M (2100C>A 2101G>T 2102C>G), R469E (2106A>G 2107G>A), F470L (2111T>G), K472R (2115A>C 2116A>G), E473A (2119A>C 2120G>A), A475V (2125C>T 2126C>T), R476V (2127A>G 2128G>T), A477T (2130G>A 2132T>C), V479L (2136G>C 2138G>T), N481K (2144C>G), V488G (2164T>G), E490V (2170A>T 2171A>T), S493A (2178T>G), V500T (2199G>A 2200T>C), M502V (2205A>G), D508E (2225T>A), M510D (2229A>G 2230T>A 2231G>T), E517D (2252A>C), A519T (2256G>A), N520Q (2259A>C 2261T>A), E521Q (2262G>C), E531D (2294A>C), N538K (2315T>A), G540A (2320G>C 2321T>C), T542I (2326C>T), S579A (2436T>G), S590T (2469T>A 2471C>G), R601K (2503G>A), I608L (2523A>C), I643M (2630C>G), C667* (2702T>A), L699F (2798G>T), I712V (2835A>G 2837C>T), Q715H (2846G>T), A761T (2982G>A), I771V (3012A>G 3014C>T), I807M (3122T>G), S829N (3187G>A), K851R (3253A>G), R855K (3265G>A), T857S (3270A>T 3272G>T), T868S (3303A>T 3305C>T), N936S (3508A>G), S948T (3543T>A), K955Q (3564A>C), L956M (3567T>A 3569A>G), P977Q (3631C>A), L978P (3634T>C 3635C>T), Q1014P (3742A>C 3743G>C), T1051A (3852A>G 3854T>C), I1061V (3882A>G 3884C>A), T1062A (3885A>G), K1076R (3927A>C 3928A>G 3929G>A), T1078A (3933A>G 3935C>T), A1087T (3960G>A), M1095V (3984A>G 3986G>T), S1096N (3988G>A 3989C>T), D1097S (3990G>A 3991A>G), H1105Q (4016T>G), I1120V (4059A>G 4061A>T), N1123G (4068A>G 4069A>G 4070T>C), A1124V (4072C>T 4073T>G), L1150I (4149C>A 4151G>A), Q1163E (4188C>G), H1175Y (4224C>T), S1195G (4284A>G 4286T>A)								

	Begin	End	Coverage	Score	Concordance	Matches	Identities	I/D/M/F*	Stop Codons
**NT**	**20**	**5403**	**99.6%**	**7578**	**70.4%**	**5384 (99.9%)**	**4594 (85.3%)**	**3/0**	
Codon mutations:	ATG2ACG (706T>C), AGG10AGA (731G>A), AAG11AGG (733A>G), AGT17GGT (750A>G), GCA18GCT (755A>T), GCT19GCA (758T>A), GTA20GTG (761A>G), ACA29ACT (788A>T), CTG39CTA (818G>A), CTT40CTC (821T>C), CGC41CGG (824C>G), GGC44GGT (833C>T), TCA47TCT (842A>T), TAT48TTT (844A>T), ATT50ATC (851T>C), CTC51CTA (854C>A), GGC53GGG (860C>G), GCC54GCT (863C>T), CAA55CAG (866A>G), GCC59GCT (878C>T), GAA61GAG (884A>G), CTG62CTT (887G>T), GAC64GAT (893C>T), AAT70AAC (911T>C), TCT76TCA (929T>A), TCA77TCT (932A>T), GGA89GGG (968A>G), GTT103GTG (1010T>G), AGT120AGC (1061T>C), CTG128CTA (1085G>A), GTT132ATT (1095G>A), TTG133TTA (1100G>A), TCC136TCT (1109C>T), GGG138GGA (1115G>A), GCA139GCC (1118A>C), TTA143TTG (1130A>G), CCG146CCA (1139G>A), ACC147ACT (1142C>T), CTA150CTG (1151A>G), AAA154AAG (1163A>G), GGC156GGG (1169C>G), TTC159TTT (1178C>T), ACA162ACT (1187A>T), ACT168ACA (1205T>A), CCT173CCC (1220T>C), GCC178GCA (1235C>A), CAA182CAG (1247A>G), CGT187CGC (1262T>C), CTT204CTC (1313T>C), CAC205CAT (1316C>T), CGA206AGG (1317C>A 1319A>G), CAA208CAG (1325A>G), TTT209TTC (1328T>C), TTC210TTT (1331C>T), CAG214CAA (1343G>A), CAG218CAA (1355G>A), ATA220ATT (1361A>T), TCA222TCT (1367A>T), GTA223GTT (1370A>T), TCT225TCA (1376T>A), ACC228GCT (1383A>G 1385C>T), TCC229TCT (1388C>T), TCA230TGA (1390C>G), GCA231GCC (1394A>C), ACT232TCA (1395A>T 1397T>A), GAT233GAG (1400T>G), TCA234TCT (1403A>T), GTG235GTA (1406G>A), CTT236TTA (1407C>T 1409T>A), CCG237CCT (1412G>T), GTG239GTA (1418G>A), CAG242CCT (1426A>C 1427G>T), GAC245AAC (1434G>A), ACA246ACC (1439A>C), GTG250GTT (1451G>T), CCA251CCT (1454A>T), AGC252ACT (1456G>C 1457C>T), GAA253GAG (1460A>G), GCA254TCA (1461G>T), AGA255AAA (1465G>A), ATC257ATG (1472C>G), CGG258CGC (1475G>C), TCC259TCG (1478C>G), TCC260TCT (1481C>T), CTC262CTG (1487C>G), CGG263CGA (1490G>A), GTG264GTA (1493G>A), CCC265CCT (1496C>T), CTC267CTT (1502C>T), AGT271AGC (1514T>C), TCC275CCT (1524T>C 1526C>T), TCA276TCT (1529A>T), ATA279ATT (1538A>T), TCT281TTT (1543C>T), ACC282GCT (1545A>G 1547C>T), ACT284ACC (1553T>C), CAC286CAA (1559C>A), CTC287TTA (1560C>T 1562C>A), GGC289GGG (1568C>G), ACG291ACA (1574G>A), CGT292AGG (1575C>A 1577T>G), GTA296GTG (1589A>G), CAG298GAT (1593C>G 1595G>T), AGT301AAT (1603G>A), AGG302GGG (1605A>G), TAC303CAG (1608T>C 1610C>G), GAA304GAG (1613A>G), GAG305GAA (1616G>A), GGT307GCA (1621G>C 1622T>A), GAT309TCC (1626G>T 1627A>C 1628T>C), GGG310GGA (1631G>A), GTT311GTA (1634T>A), CCC314CCT (1643C>T), GTT315GTC (1646T>C), AAG317AGG (1651A>G), GCT318GCA (1655T>A), GGT320GGC (1661T>C), CTG321TAT (1662C>T 1663T>A 1664G>T), AAC322AAT (1667C>T), CAT325TAC (1674C>T 1676T>C), GTG326ATT (1677G>A 1679G>T), GAT328GAC (1685T>C), AAT329AAC (1688T>C), ATT332GTT (1695A>G), GAG333GAA (1700G>A), CTG340CTC (1721G>C), CCC341CCT (1724C>T), GAA342GAG (1727A>G), AAC343AAT (1730C>T), CTG347TTG (1740C>T), TCG348TCA (1745G>A), GTG349GTA (1748G>A), AGA350CGA (1749A>C), CAG351CAA (1754G>A), CAA352CAG (1757A>G), GTT353GTG (1760T>G), GCT354GCA (1763T>A), GCA355GCG (1766A>G), GCA356GCT (1769A>T), GCA359GCC (1778A>C), GCA361GCT (1784A>T), AAG363AAA (1790G>A), GGT364GGA (1793T>A), GTT365ATT (1794G>A), TGC366GGG (1797T>G 1799C>G), GTC368GTT (1805C>T), AAA370AAC (1811A>C), CAT371CAC (1814T>C), ATA372ATT (1817A>T), ATC373AAG (1819T>A 1820C>G), AAC374AAT (1823C>T), AAG377AAC (1832G>C), ATC380ATT (1841C>T), CAC381CGC (1843A>G), TTG382CTT (1845T>C 1847G>T), ATC385ATT (1856C>T), ACC386ACT (1859C>T), CAC388TAT (1863C>T 1865C>T), GTG392ATG (1875G>A), ATA393GTA (1878A>G), TAC397TAT (1892C>T), ATT399ATG (1898T>G), GTG400ACA (1899G>A 1900T>C 1901G>A), GCC402GTG (1906C>T 1907C>G), TCT404TCA (1913T>A), GAA409GAC (1928A>C), AAC410AAT (1931C>T), AAT413GAT (1938A>G), CTT414TTG (1941C>T 1943T>G), ACC415ACA (1946C>A), TTG416CTG (1947T>C), GCA417GCC (1952A>C), CAG418CAA (1955G>A), AGA419AGG (1958A>G), GAG422GAA (1967G>A), CTA423CTG (1970A>G), CAA426CAG (1979A>G), GAT429AAT (1986G>A), TAT431TAC (1994T>C), TTT434TAT (2002T>A), AAC437AAT (2012C>T), TAT441TAC (2024T>C), CCT442CCA (2027T>A), TTA450ATT (2049T>A 2051A>T), ACG451ACT (2054G>T), AAA453AGC (2059A>G 2060A>C), CGC455CGG (2066C>G), CTA458CAA (2074T>A), GTG461GCA (2083T>C 2084G>A), GCA462GCC (2087A>C), CAG465GAC (2094C>G 2096G>C), GCG466GAA (2098C>A 2099G>A), CGC467ATG (2100C>A 2101G>T 2102C>G), AGA469GAA (2106A>G 2107G>A), TTT470TTG (2111T>G), TAC471TAT (2114C>T), AAG472CGG (2115A>C 2116A>G), GAG473GCA (2119A>C 2120G>A), GCG474GCT (2123G>T), GCC475GTT (2125C>T 2126C>T), AGG476GTG (2127A>G 2128G>T), GCT477ACC (2130G>A 2132T>C), CAG478CAA (2135G>A), GTG479CTT (2136G>C 2138G>T), AAC481AAG (2144C>G), ACT486ACA (2159T>A), AGT487AGC (2162T>C), GTG488GGG (2164T>G), GAA490GTT (2170A>T 2171A>T), ATA491ATT (2174A>T), CCA492CCT (2177A>T), TCC493GCC (2178T>G), ACC494ACT (2183C>T), TCA495TCC (2186A>C), TTC496TTT (2189C>T), ACA498ACC (2195A>C), AAG499AAA (2198G>A), GTT500ACT (2199G>A 2200T>C), GCC501GCT (2204C>T), ATG502GTG (2205A>G), GAG503GAA (2210G>A), GGC505GGG (2216C>G), AGT506AGC (2219T>C), GAT508GAA (2225T>A), AGC509AGT (2228C>T), ATG510GAT (2229A>G 2230T>A 2231G>T), GCT513GCA (2240T>A), ATT514ATC (2243T>C), GAA517GAC (2252A>C), GCC519ACC (2256G>A), AAT520CAA (2259A>C 2261T>A), GAA521CAA (2262G>C), GTA523GTG (2270A>G), GTT528GTC (2285T>C), GAA531GAC (2294A>C), CAA534CAG (2303A>G), AAT537AAC (2312T>C), AAT538AAA (2315T>A), GGT540GCC (2320G>C 2321T>C), GCG541GCC (2324G>C), ACA542ATA (2326C>T), TTA546TTG (2339A>G), CAA548CAG (2345A>G), TTC558TTT (2375C>T), GAG564GAA (2393G>A), AGT571AGC (2414T>C), GTT573GTG (2420T>G), GAG576GAA (2429G>A), GTG577GTA (2432G>A), TCA579GCA (2436T>G), GCG581GCC (2444G>C), CTC582CTA (2447C>A), GCG584GCA (2453G>A), CTC586CTG (2459C>G), TCC590ACG (2469T>A 2471C>G), AAG591AAA (2474G>A), GGA596GGC (2489A>C), TCG597TCA (2492G>A), CAA598CAG (2495A>G), CTA600CTG (2501A>G), AGG601AAG (2503G>A), TAT602TAC (2507T>C), GCC605GCT (2516C>T), ATT608CTT (2523A>C), TAT615TAC (2546T>C), TCC617TCT (2552C>T), GGT620GGG (2561T>G), ATT622ATA (2567T>A), TCG623TCA (2570G>A), GGT624GGA (2573T>A), GTT629GTA (2588T>A), CTC636CTA (2609C>A), TTC642TTT (2627C>T), ATC643ATG (2630C>G), CTG648CTC (2645G>C), CAT653CAC (2660T>C), TCA657TCT (2672A>T), GGG664GGA (2693G>A), TGT667TGA (2702T>A), ACA669ACG (2708A>G), GGT672GGC (2717T>C), ATC673ATA (2720C>A), TTC676TTT (2729C>T), GTT677GTG (2732T>G), GCC682GCT (2747C>T), GGG684GGC (2753G>C), CAC697CAT (2792C>T), CCC698CCT (2795C>T), TTG699TTT (2798G>T), GAG706GAA (2819G>A), ATC712GTT (2835A>G 2837C>T), CAT713CAC (2840T>C), CTG714TTG (2841C>T), CAG715CAT (2846G>T), AAA717AAG (2852A>G), TCG720TCC (2861G>C), ACC721ACT (2864C>T), ATC723ATA (2870C>A), CCT726CCA (2879T>A), CCT727CCG (2882T>G), CGT728CGG (2885T>G), TTG729CTG (2886T>C), GCA731GCG (2894A>G), GCA733GCT (2900A>T), GGT736GGC (2909T>C), AAA738AAG (2915A>G), GAC740GAT (2921C>T), ACA742ACG (2927A>G), CCA745CCC (2936A>C), CGA746CGG (2939A>G), ACA749ACG (2948A>G), CAG751CAA (2954G>A), GTA752GTT (2957A>T), CTG753CTA (2960G>A), CAC755CAT (2966C>T), AAT757AAC (2972T>C), GTG760GTT (2981G>T), GCT761ACT (2982G>A), GGT765GGG (2996T>G), ACT766ACA (2999T>A), TTA768CTA (3003T>C), AGC770AGT (3011C>T), ATC771GTT (3012A>G 3014C>T), TCT773TCC (3020T>C), CCG774CCC (3023G>C), GGC776GGT (3029C>T), ATA777ATT (3032A>T), GAC781GAT (3044C>T), GGT782GGA (3047T>A), ACG783ACC (3050G>C), CTG784TTG (3051C>T), CCA786CCT (3059A>T), TCC787TCT (3062C>T), TTG788CTA (3063T>C 3065G>A), GGA790GGG (3071A>G), ATA792ATT (3077A>T), CGC794CGA (3083C>A), ACT800ACG (3101T>G), GGC801GGT (3104C>T), ACT802ACG (3107T>G), GTG804GTC (3113G>C), ATT807ATG (3122T>G), ATT809ATC (3128T>C), GAG810GAA (3131G>A), TTC814TTT (3143C>T), CAT815CAC (3146T>C), GGT817GGA (3152T>A), TTG819TTA (3158G>A), GGA822GGT (3167A>T), CTG823TTG (3168C>T), ACA825ACC (3176A>C), CTG826CTT (3179G>T), AAC827AAT (3182C>T), AGC829AAC (3187G>A), ACC832ACT (3197C>T), GAC835GAT (3206C>T), GTT842GTG (3227T>G), ATT843ATA (3230T>A), AAC845AAT (3236C>T), CTT846CTG (3239T>G), GAG847GAA (3242G>A), GGA849GGC (3248A>C), CGA850CGT (3251A>T), AAG851AGG (3253A>G), AGG855AAG (3265G>A), TGT856TGC (3269T>C), ACG857TCT (3270A>T 3272G>T), ACC859ACT (3278C>T), AAA864AAG (3293A>G), CTT866CTA (3299T>A), ACC868TCT (3303A>T 3305C>T), GGT870GGA (3311T>A), CTA875TTA (3324C>T), CCG876CCT (3329G>T), CAC883CAT (3350C>T), AGG885CGG (3354A>C), TTG886TTA (3359G>A), ACA889ACT (3368A>T), GTC890GTG (3371C>G), TTG891CTC (3372T>C 3374G>C), GTA895GTT (3386A>T), TCA898TCC (3395A>C), CTA901CTG (3404A>G), ACG903ACA (3410G>A), GTC904GTT (3413C>T), CAG909CAA (3428G>A), CTT910TTG (3429C>T 3431T>G), AAA911AAG (3434A>G), TGC912TGT (3437C>T), ATA913ATT (3440A>T), TTG916CTA (3447T>C 3449G>A), GTC917GTA (3452C>A), CTT918CTA (3455T>A), GGG919GGA (3458G>A), GGC920GGT (3461C>T), TCT923TCC (3470T>C), GTT924GTC (3473T>C), CCC926CCT (3479C>T), ATT927ATC (3482T>C), TAC928TAT (3485C>T), GGA929GGG (3488A>G), CAC930CAT (3491C>T), GGA934GGG (3503A>G), AAT936AGT (3508A>G), GCT937GCC (3512T>C), GTG938GTT (3515G>T), CTA941TTG (3522C>T 3524A>G), TTC942TTT (3527C>T), GAG944GAA (3533G>A), TCT948ACT (3543T>A), CGC950CGT (3551C>T), ATT953ATC (3560T>C), AAA955CAA (3564A>C), TTA956ATG (3567T>A 3569A>G), AAG958AAA (3575G>A), GAA959GAG (3578A>G), AAC960AAT (3581C>T), ACA963ACC (3590A>C), GGA968GGG (3605A>G), AAG970AAA (3611G>A), CCA972CCT (3617A>T), CAG973CAA (3620G>A), GTA974GTT (3623A>T), GTG976GTC (3629G>C), CCG977CAG (3631C>A), CTC978CCT (3634T>C 3635C>T), AGA979AGG (3638A>G), GAC980GAT (3641C>T), TCA983TCT (3650A>T), GGG985GGT (3656G>T), GCT986GCC (3659T>C), GTA987GTG (3662A>G), GTG991GTC (3674G>C), GAC996GAT (3689C>T), CGA999CGC (3698A>C), TTG1001CTA (3702T>C 3704G>A), GTT1002GTG (3707T>G), TTA1003CTA (3708T>C), CCA1004CCC (3713A>C), TGC1005TGT (3716C>T), GCA1006GCG (3719A>G), CCC1007CCA (3722C>A), TCC1009TCG (3728C>G), CAG1014CCC (3742A>C 3743G>C), GGG1015GGA (3746G>A), GCA1016GCT (3749A>T), CTG1017CTA (3752G>A), GTA1018GTG (3755A>G), ATC1022ATA (3767C>A), ACA1023ACC (3770A>C), TGC1024TGT (3773C>T), CCA1025CCT (3776A>T), TTC1026TTT (3779C>T), TTC1033TTT (3800C>T), GGA1038GGT (3815A>T), TAC1042TAT (3827C>T), ATT1044ATA (3833T>A), GTA1046GTG (3839A>G), AGA1048AGG (3845A>G), GTG1049GTA (3848G>A), CAG1050CAA (3851G>A), ACT1051GCC (3852A>G 3854T>C), TCC1052TCA (3857C>A), ATA1055ATC (3866A>C), GGA1056GGT (3869A>T), GGA1057GGT (3872A>T), CTG1059CTC (3878G>C), AAC1060AAT (3881C>T), ATC1061GTA (3882A>G 3884C>A), ACC1062GCC (3885A>G), TTA1063TTG (3890A>G), GAT1064GAC (3893T>C), TCA1065TCT (3896A>T), CCT1071CCA (3914T>A), GGA1073GGT (3920A>T), ATC1074ATT (3923C>T), AAG1076CGA (3927A>C 3928A>G 3929G>A), GGC1077GGA (3932C>A), ACC1078GCT (3933A>G 3935C>T), GTT1080GTG (3941T>G), GTC1081GTA (3944C>A), TCT1082TCC (3947T>C), GCT1083GCA (3950T>A), GGT1084GGA (3953T>A), GCA1087ACA (3960G>A), GCT1090GCA (3971T>A), GGT1094GGA (3983T>A), ATG1095GTT (3984A>G 3986G>T), AGC1096AAT (3988G>A 3989C>T), GAC1097AGC (3990G>A 3991A>G), AAC1098AAT (3995C>T), ATC1099ATT (3998C>T), TTT1100TTC (4001T>C), CAT1105CAG (4016T>G), GAG1108GAA (4025G>A), TTT1109TTC (4028T>C), TTT1110TTC (4031T>C), CCT1111CCG (4034T>G), AGA1112CGT (4035A>C 4037A>T), CGC1113CGT (4040C>T), GCC1117GCT (4052C>T), ATA1120GTT (4059A>G 4061A>T), CCA1122CCT (4067A>T), AAT1123GGC (4068A>G 4069A>G 4070T>C), GCT1124GTG (4072C>T 4073T>G), AGA1126CGA (4077A>C), ACT1129ACA (4088T>A), CTG1130CTA (4091G>A), CGA1133CGC (4100A>C), CTA1134CTT (4103A>T), GGA1135GGC (4106A>C), CTC1137CTT (4112C>T), AAA1144AAG (4133A>G), ATA1147ATC (4142A>C), AGC1148AGT (4145C>T), CTG1150ATA (4149C>A 4151G>A), GAA1151GAG (4154A>G), ATC1152ATA (4157C>A), AAG1155AAA (4166G>A), TTC1160TTT (4181C>T), TTG1162TTA (4187G>A), CAA1163GAA (4188C>G), GCT1165GCA (4196T>A), TCA1169TCT (4208A>T), GCA1170GCT (4211A>T), AAG1174AAA (4223G>A), CAT1175TAT (4224C>T), TTG1176CTT (4227T>C 4229G>T), ACC1183ACT (4250C>T), CTT1185CTG (4256T>G), ACT1187ACC (4262T>C), CCT1188CCA (4265T>A), TCA1191TCT (4274A>T), AGG1194CGG (4281A>C), AGT1195GGA (4284A>G 4286T>A), TTT1196TTC (4289T>C)								

Proteins

	Begin	End	Coverage	Score	Concordance	Matches	Identities	I/D/M/F*	Stop Codons
Orf1 (YP_001040017.1)	1	188	100%	1080	89.3%	188 (100%)	162 (86.2%)	0/0/0/0	2
Protein mutations:	L11V (212C>G), T50A (329A>G), V59I (356G>A), N64T (372A>C), L90F (451A>T), Q108H (505G>C), R109K (507G>A 508G>A), I110V (509A>G 511T>A), V112I (515G>A), A124E (552C>A 553T>G), A125K (554G>A 555C>A), Q128R (564A>G 565A>T), Q130R (570A>G), T132K (576C>A), L133V (578C>G), E138D (595G>T), D141A (603A>C 604C>T), R146Q (618G>A 619C>A), E151* (632G>T), L155A (644C>G 645T>C), L158Q (654T>A), S162A (665T>G), L168I (683T>A 685G>A), N170S (689A>T 690A>C), L172P (696T>C), E184K (731G>A 733A>G)								
Codon mutations:	TCA2TCC (187A>C), CTT11GTT (212C>G), GAG14GAA (223G>A), CAC17CAT (232C>T), AAG18AAA (235G>A), CAA19CAG (238A>G), GCA21GCT (244A>T), GAC22GAT (247C>T), TCG28TCA (265G>A), GTT29GTA (268T>A), GGC32GGT (277C>T), TCT33TCC (280T>C), ATA35ATC (286A>C), ATA36ATC (289A>C), AAC37AAT (292C>T), CCA40CCT (301A>T), ACT41ACC (304T>C), CTA42TTG (305C>T 307A>G), TTC44TTT (313C>T), AAG45AAA (316G>A), CCC48CCT (325C>T), ACA50GCA (329A>G), GGT51GGA (334T>A), GCA53GCT (340A>T), CTA57CTT (352A>T), GTT59ATT (356G>A), AAC63AAT (370C>T), AAT64ACT (372A>C), AGC65AGT (376C>T), CTG68TTA (383C>T 385G>A), GTT69GTA (388T>A), TAT73TAC (400T>C), TTA74CTC (401T>C 403A>C), ACT79ACG (418T>G), GGC84GGT (433C>T), AAG86AAA (439G>A), TCT88TCA (445T>A), GGC89GGA (448C>A), TTA90TTT (451A>T), AGA94CGA (461A>C), ACC100ACT (481C>T), ACC101ACA (484C>A), AGA102AGG (487A>G), CTA105CTG (496A>G), GCT107GCA (502T>A), CAG108CAC (505G>C), AGG109AAA (507G>A 508G>A), ATT110GTA (509A>G 511T>A), GTA112ATA (515G>A), TCC113TCT (520C>T), TCC120TCT (541C>T), GCT122GCG (547T>G), GCT124GAG (552C>A 553T>G), GCA125AAA (554G>A 555C>A), CAA128CGT (564A>G 565A>T), CCA129CCT (568A>T), CAA130CGA (570A>G), ACG132AAG (576C>A), CTT133GTT (578C>G), CAA135CAG (586A>G), TTA136CTT (587T>C 589A>T), GAG138GAT (595G>T), CTT140TTG (599C>T 601T>G), GAC141GCT (603A>C 604C>T), GAG142GAA (607G>A), GCC143GCT (610C>T), AAA144AAG (613A>G), CGC146CAA (618G>A 619C>A), TTA147TTG (622A>G), AAA150AAG (631A>G), GAG151TAG (632G>T), AAA152AAG (637A>G), CTT155GCT (644C>G 645T>C), CTA158CAA (654T>A), TCC159TCA (658C>A), TCC162GCC (665T>G), AAA163AAG (670A>G), TTA164TTG (673A>G), TTG166TTA (679G>A), TTG168ATA (683T>A 685G>A), AAC170TCC (689A>T 690A>C), CTA172CCA (696T>C), GAT175GAC (706T>C), GAA184AAG (731G>A 733A>G)								
polyprotein (YP_001040018.1)	1	1199	100%	7582	91.6%	1199 (100%)	1081 (90.2%)	0/0/0/0	3

	Begin	End	Coverage	Score	Concordance	Matches	Identities	I/D/M/F*	Stop Codons
**NT**	**20**	**5403**	**99.6%**	**7578**	**70.4%**	**5384 (99.9%)**	**4594 (85.3%)**	**3/0**	
Protein mutations:	M2T (706T>C), K11R (733A>G), S17G (750A>G), Y48F (844A>T), V132I (1095G>A), T228A (1383A>G 1385C>T), S230* (1390C>G), T232S (1395A>T 1397T>A), D233E (1400T>G), Q242P (1426A>C 1427G>T), D245N (1434G>A), S252T (1456G>C 1457C>T), A254S (1461G>T), R255K (1465G>A), I257M (1472C>G), S275P (1524T>C 1526C>T), S281F (1543C>T), T282A (1545A>G 1547C>T), H286Q (1559C>A), Q298D (1593C>G 1595G>T), S301N (1603G>A), R302G (1605A>G), Y303Q (1608T>C 1610C>G), G307A (1621G>C 1622T>A), D309S (1626G>T 1627A>C 1628T>C), K317R (1651A>G), L321Y (1662C>T 1663T>A 1664G>T), H325Y (1674C>T 1676T>C), V326I (1677G>A 1679G>T), I332V (1695A>G), V365I (1794G>A), C366G (1797T>G 1799C>G), K370N (1811A>C), I373K (1819T>A 1820C>G), K377N (1832G>C), H381R (1843A>G), H388Y (1863C>T 1865C>T), V392M (1875G>A), I393V (1878A>G), I399M (1898T>G), V400T (1899G>A 1900T>C 1901G>A), A402V (1906C>T 1907C>G), E409D (1928A>C), N413D (1938A>G), D429N (1986G>A), F434Y (2002T>A), L450I (2049T>A 2051A>T), K453S (2059A>G 2060A>C), L458Q (2074T>A), V461A (2083T>C 2084G>A), Q465D (2094C>G 2096G>C), A466E (2098C>A 2099G>A), R467M (2100C>A 2101G>T 2102C>G), R469E (2106A>G 2107G>A), F470L (2111T>G), K472R (2115A>C 2116A>G), E473A (2119A>C 2120G>A), A475V (2125C>T 2126C>T), R476V (2127A>G 2128G>T), A477T (2130G>A 2132T>C), V479L (2136G>C 2138G>T), N481K (2144C>G), V488G (2164T>G), E490V (2170A>T 2171A>T), S493A (2178T>G), V500T (2199G>A 2200T>C), M502V (2205A>G), D508E (2225T>A), M510D (2229A>G 2230T>A 2231G>T), E517D (2252A>C), A519T (2256G>A), N520Q (2259A>C 2261T>A), E521Q (2262G>C), E531D (2294A>C), N538K (2315T>A), G540A (2320G>C 2321T>C), T542I (2326C>T), S579A (2436T>G), S590T (2469T>A 2471C>G), R601K (2503G>A), I608L (2523A>C), I643M (2630C>G), C667* (2702T>A), L699F (2798G>T), I712V (2835A>G 2837C>T), Q715H (2846G>T), A761T (2982G>A), I771V (3012A>G 3014C>T), I807M (3122T>G), S829N (3187G>A), K851R (3253A>G), R855K (3265G>A), T857S (3270A>T 3272G>T), T868S (3303A>T 3305C>T), N936S (3508A>G), S948T (3543T>A), K955Q (3564A>C), L956M (3567T>A 3569A>G), P977Q (3631C>A), L978P (3634T>C 3635C>T), Q1014P (3742A>C 3743G>C), T1051A (3852A>G 3854T>C), I1061V (3882A>G 3884C>A), T1062A (3885A>G), K1076R (3927A>C 3928A>G 3929G>A), T1078A (3933A>G 3935C>T), A1087T (3960G>A), M1095V (3984A>G 3986G>T), S1096N (3988G>A 3989C>T), D1097S (3990G>A 3991A>G), H1105Q (4016T>G), I1120V (4059A>G 4061A>T), N1123G (4068A>G 4069A>G 4070T>C), A1124V (4072C>T 4073T>G), L1150I (4149C>A 4151G>A), Q1163E (4188C>G), H1175Y (4224C>T), S1195G (4284A>G 4286T>A)								
Codon mutations:	ATG2ACG (706T>C), AGG10AGA (731G>A), AAG11AGG (733A>G), AGT17GGT (750A>G), GCA18GCT (755A>T), GCT19GCA (758T>A), GTA20GTG (761A>G), ACA29ACT (788A>T), CTG39CTA (818G>A), CTT40CTC (821T>C), CGC41CGG (824C>G), GGC44GGT (833C>T), TCA47TCT (842A>T), TAT48TTT (844A>T), ATT50ATC (851T>C), CTC51CTA (854C>A), GGC53GGG (860C>G), GCC54GCT (863C>T), CAA55CAG (866A>G), GCC59GCT (878C>T), GAA61GAG (884A>G), CTG62CTT (887G>T), GAC64GAT (893C>T), AAT70AAC (911T>C), TCT76TCA (929T>A), TCA77TCT (932A>T), GGA89GGG (968A>G), GTT103GTG (1010T>G), AGT120AGC (1061T>C), CTG128CTA (1085G>A), GTT132ATT (1095G>A), TTG133TTA (1100G>A), TCC136TCT (1109C>T), GGG138GGA (1115G>A), GCA139GCC (1118A>C), TTA143TTG (1130A>G), CCG146CCA (1139G>A), ACC147ACT (1142C>T), CTA150CTG (1151A>G), AAA154AAG (1163A>G), GGC156GGG (1169C>G), TTC159TTT (1178C>T), ACA162ACT (1187A>T), ACT168ACA (1205T>A), CCT173CCC (1220T>C), GCC178GCA (1235C>A), CAA182CAG (1247A>G), CGT187CGC (1262T>C), CTT204CTC (1313T>C), CAC205CAT (1316C>T), CGA206AGG (1317C>A 1319A>G), CAA208CAG (1325A>G), TTT209TTC (1328T>C), TTC210TTT (1331C>T), CAG214CAA (1343G>A), CAG218CAA (1355G>A), ATA220ATT (1361A>T), TCA222TCT (1367A>T), GTA223GTT (1370A>T), TCT225TCA (1376T>A), ACC228GCT (1383A>G 1385C>T), TCC229TCT (1388C>T), TCA230TGA (1390C>G), GCA231GCC (1394A>C), ACT232TCA (1395A>T 1397T>A), GAT233GAG (1400T>G), TCA234TCT (1403A>T), GTG235GTA (1406G>A), CTT236TTA (1407C>T 1409T>A), CCG237CCT (1412G>T), GTG239GTA (1418G>A), CAG242CCT (1426A>C 1427G>T), GAC245AAC (1434G>A), ACA246ACC (1439A>C), GTG250GTT (1451G>T), CCA251CCT (1454A>T), AGC252ACT (1456G>C 1457C>T), GAA253GAG (1460A>G), GCA254TCA (1461G>T), AGA255AAA (1465G>A), ATC257ATG (1472C>G), CGG258CGC (1475G>C), TCC259TCG (1478C>G), TCC260TCT (1481C>T), CTC262CTG (1487C>G), CGG263CGA (1490G>A), GTG264GTA (1493G>A), CCC265CCT (1496C>T), CTC267CTT (1502C>T), AGT271AGC (1514T>C), TCC275CCT (1524T>C 1526C>T), TCA276TCT (1529A>T), ATA279ATT (1538A>T), TCT281TTT (1543C>T), ACC282GCT (1545A>G 1547C>T), ACT284ACC (1553T>C), CAC286CAA (1559C>A), CTC287TTA (1560C>T 1562C>A), GGC289GGG (1568C>G), ACG291ACA (1574G>A), CGT292AGG (1575C>A 1577T>G), GTA296GTG (1589A>G), CAG298GAT (1593C>G 1595G>T), AGT301AAT (1603G>A), AGG302GGG (1605A>G), TAC303CAG (1608T>C 1610C>G), GAA304GAG (1613A>G), GAG305GAA (1616G>A), GGT307GCA (1621G>C 1622T>A), GAT309TCC (1626G>T 1627A>C 1628T>C), GGG310GGA (1631G>A), GTT311GTA (1634T>A), CCC314CCT (1643C>T), GTT315GTC (1646T>C), AAG317AGG (1651A>G), GCT318GCA (1655T>A), GGT320GGC (1661T>C), CTG321TAT (1662C>T 1663T>A 1664G>T), AAC322AAT (1667C>T), CAT325TAC (1674C>T 1676T>C), GTG326ATT (1677G>A 1679G>T), GAT328GAC (1685T>C), AAT329AAC (1688T>C), ATT332GTT (1695A>G), GAG333GAA (1700G>A), CTG340CTC (1721G>C), CCC341CCT (1724C>T), GAA342GAG (1727A>G), AAC343AAT (1730C>T), CTG347TTG (1740C>T), TCG348TCA (1745G>A), GTG349GTA (1748G>A), AGA350CGA (1749A>C), CAG351CAA (1754G>A), CAA352CAG (1757A>G), GTT353GTG (1760T>G), GCT354GCA (1763T>A), GCA355GCG (1766A>G), GCA356GCT (1769A>T), GCA359GCC (1778A>C), GCA361GCT (1784A>T), AAG363AAA (1790G>A), GGT364GGA (1793T>A), GTT365ATT (1794G>A), TGC366GGG (1797T>G 1799C>G), GTC368GTT (1805C>T), AAA370AAC (1811A>C), CAT371CAC (1814T>C), ATA372ATT (1817A>T), ATC373AAG (1819T>A 1820C>G), AAC374AAT (1823C>T), AAG377AAC (1832G>C), ATC380ATT (1841C>T), CAC381CGC (1843A>G), TTG382CTT (1845T>C 1847G>T), ATC385ATT (1856C>T), ACC386ACT (1859C>T), CAC388TAT (1863C>T 1865C>T), GTG392ATG (1875G>A), ATA393GTA (1878A>G), TAC397TAT (1892C>T), ATT399ATG (1898T>G), GTG400ACA (1899G>A 1900T>C 1901G>A), GCC402GTG (1906C>T 1907C>G), TCT404TCA (1913T>A), GAA409GAC (1928A>C), AAC410AAT (1931C>T), AAT413GAT (1938A>G), CTT414TTG (1941C>T 1943T>G), ACC415ACA (1946C>A), TTG416CTG (1947T>C), GCA417GCC (1952A>C), CAG418CAA (1955G>A), AGA419AGG (1958A>G), GAG422GAA (1967G>A), CTA423CTG (1970A>G), CAA426CAG (1979A>G), GAT429AAT (1986G>A), TAT431TAC (1994T>C), TTT434TAT (2002T>A), AAC437AAT (2012C>T), TAT441TAC (2024T>C), CCT442CCA (2027T>A), TTA450ATT (2049T>A 2051A>T), ACG451ACT (2054G>T), AAA453AGC (2059A>G 2060A>C), CGC455CGG (2066C>G), CTA458CAA (2074T>A), GTG461GCA (2083T>C 2084G>A), GCA462GCC (2087A>C), CAG465GAC (2094C>G 2096G>C), GCG466GAA (2098C>A 2099G>A), CGC467ATG (2100C>A 2101G>T 2102C>G), AGA469GAA (2106A>G 2107G>A), TTT470TTG (2111T>G), TAC471TAT (2114C>T), AAG472CGG (2115A>C 2116A>G), GAG473GCA (2119A>C 2120G>A), GCG474GCT (2123G>T), GCC475GTT (2125C>T 2126C>T), AGG476GTG (2127A>G 2128G>T), GCT477ACC (2130G>A 2132T>C), CAG478CAA (2135G>A), GTG479CTT (2136G>C 2138G>T), AAC481AAG (2144C>G), ACT486ACA (2159T>A), AGT487AGC (2162T>C), GTG488GGG (2164T>G), GAA490GTT (2170A>T 2171A>T), ATA491ATT (2174A>T), CCA492CCT (2177A>T), TCC493GCC (2178T>G), ACC494ACT (2183C>T), TCA495TCC (2186A>C), TTC496TTT (2189C>T), ACA498ACC (2195A>C), AAG499AAA (2198G>A), GTT500ACT (2199G>A 2200T>C), GCC501GCT (2204C>T), ATG502GTG (2205A>G), GAG503GAA (2210G>A), GGC505GGG (2216C>G), AGT506AGC (2219T>C), GAT508GAA (2225T>A), AGC509AGT (2228C>T), ATG510GAT (2229A>G 2230T>A 2231G>T), GCT513GCA (2240T>A), ATT514ATC (2243T>C), GAA517GAC (2252A>C), GCC519ACC (2256G>A), AAT520CAA (2259A>C 2261T>A), GAA521CAA (2262G>C), GTA523GTG (2270A>G), GTT528GTC (2285T>C), GAA531GAC (2294A>C), CAA534CAG (2303A>G), AAT537AAC (2312T>C), AAT538AAA (2315T>A), GGT540GCC (2320G>C 2321T>C), GCG541GCC (2324G>C), ACA542ATA (2326C>T), TTA546TTG (2339A>G), CAA548CAG (2345A>G), TTC558TTT (2375C>T), GAG564GAA (2393G>A), AGT571AGC (2414T>C), GTT573GTG (2420T>G), GAG576GAA (2429G>A), GTG577GTA (2432G>A), TCA579GCA (2436T>G), GCG581GCC (2444G>C), CTC582CTA (2447C>A), GCG584GCA (2453G>A), CTC586CTG (2459C>G), TCC590ACG (2469T>A 2471C>G), AAG591AAA (2474G>A), GGA596GGC (2489A>C), TCG597TCA (2492G>A), CAA598CAG (2495A>G), CTA600CTG (2501A>G), AGG601AAG (2503G>A), TAT602TAC (2507T>C), GCC605GCT (2516C>T), ATT608CTT (2523A>C), TAT615TAC (2546T>C), TCC617TCT (2552C>T), GGT620GGG (2561T>G), ATT622ATA (2567T>A), TCG623TCA (2570G>A), GGT624GGA (2573T>A), GTT629GTA (2588T>A), CTC636CTA (2609C>A), TTC642TTT (2627C>T), ATC643ATG (2630C>G), CTG648CTC (2645G>C), CAT653CAC (2660T>C), TCA657TCT (2672A>T), GGG664GGA (2693G>A), TGT667TGA (2702T>A), ACA669ACG (2708A>G), GGT672GGC (2717T>C), ATC673ATA (2720C>A), TTC676TTT (2729C>T), GTT677GTG (2732T>G), GCC682GCT (2747C>T), GGG684GGC (2753G>C), CAC697CAT (2792C>T), CCC698CCT (2795C>T), TTG699TTT (2798G>T), GAG706GAA (2819G>A), ATC712GTT (2835A>G 2837C>T), CAT713CAC (2840T>C), CTG714TTG (2841C>T), CAG715CAT (2846G>T), AAA717AAG (2852A>G), TCG720TCC (2861G>C), ACC721ACT (2864C>T), ATC723ATA (2870C>A), CCT726CCA (2879T>A), CCT727CCG (2882T>G), CGT728CGG (2885T>G), TTG729CTG (2886T>C), GCA731GCG (2894A>G), GCA733GCT (2900A>T), GGT736GGC (2909T>C), AAA738AAG (2915A>G), GAC740GAT (2921C>T), ACA742ACG (2927A>G), CCA745CCC (2936A>C), CGA746CGG (2939A>G), ACA749ACG (2948A>G), CAG751CAA (2954G>A), GTA752GTT (2957A>T), CTG753CTA (2960G>A), CAC755CAT (2966C>T), AAT757AAC (2972T>C), GTG760GTT (2981G>T), GCT761ACT (2982G>A), GGT765GGG (2996T>G), ACT766ACA (2999T>A), TTA768CTA (3003T>C), AGC770AGT (3011C>T), ATC771GTT (3012A>G 3014C>T), TCT773TCC (3020T>C), CCG774CCC (3023G>C), GGC776GGT (3029C>T), ATA777ATT (3032A>T), GAC781GAT (3044C>T), GGT782GGA (3047T>A), ACG783ACC (3050G>C), CTG784TTG (3051C>T), CCA786CCT (3059A>T), TCC787TCT (3062C>T), TTG788CTA (3063T>C 3065G>A), GGA790GGG (3071A>G), ATA792ATT (3077A>T), CGC794CGA (3083C>A), ACT800ACG (3101T>G), GGC801GGT (3104C>T), ACT802ACG (3107T>G), GTG804GTC (3113G>C), ATT807ATG (3122T>G), ATT809ATC (3128T>C), GAG810GAA (3131G>A), TTC814TTT (3143C>T), CAT815CAC (3146T>C), GGT817GGA (3152T>A), TTG819TTA (3158G>A), GGA822GGT (3167A>T), CTG823TTG (3168C>T), ACA825ACC (3176A>C), CTG826CTT (3179G>T), AAC827AAT (3182C>T), AGC829AAC (3187G>A), ACC832ACT (3197C>T), GAC835GAT (3206C>T), GTT842GTG (3227T>G), ATT843ATA (3230T>A), AAC845AAT (3236C>T), CTT846CTG (3239T>G), GAG847GAA (3242G>A), GGA849GGC (3248A>C), CGA850CGT (3251A>T), AAG851AGG (3253A>G), AGG855AAG (3265G>A), TGT856TGC (3269T>C), ACG857TCT (3270A>T 3272G>T), ACC859ACT (3278C>T), AAA864AAG (3293A>G), CTT866CTA (3299T>A), ACC868TCT (3303A>T 3305C>T), GGT870GGA (3311T>A), CTA875TTA (3324C>T), CCG876CCT (3329G>T), CAC883CAT (3350C>T), AGG885CGG (3354A>C), TTG886TTA (3359G>A), ACA889ACT (3368A>T), GTC890GTG (3371C>G), TTG891CTC (3372T>C 3374G>C), GTA895GTT (3386A>T), TCA898TCC (3395A>C), CTA901CTG (3404A>G), ACG903ACA (3410G>A), GTC904GTT (3413C>T), CAG909CAA (3428G>A), CTT910TTG (3429C>T 3431T>G), AAA911AAG (3434A>G), TGC912TGT (3437C>T), ATA913ATT (3440A>T), TTG916CTA (3447T>C 3449G>A), GTC917GTA (3452C>A), CTT918CTA (3455T>A), GGG919GGA (3458G>A), GGC920GGT (3461C>T), TCT923TCC (3470T>C), GTT924GTC (3473T>C), CCC926CCT (3479C>T), ATT927ATC (3482T>C), TAC928TAT (3485C>T), GGA929GGG (3488A>G), CAC930CAT (3491C>T), GGA934GGG (3503A>G), AAT936AGT (3508A>G), GCT937GCC (3512T>C), GTG938GTT (3515G>T), CTA941TTG (3522C>T 3524A>G), TTC942TTT (3527C>T), GAG944GAA (3533G>A), TCT948ACT (3543T>A), CGC950CGT (3551C>T), ATT953ATC (3560T>C), AAA955CAA (3564A>C), TTA956ATG (3567T>A 3569A>G), AAG958AAA (3575G>A), GAA959GAG (3578A>G), AAC960AAT (3581C>T), ACA963ACC (3590A>C), GGA968GGG (3605A>G), AAG970AAA (3611G>A), CCA972CCT (3617A>T), CAG973CAA (3620G>A), GTA974GTT (3623A>T), GTG976GTC (3629G>C), CCG977CAG (3631C>A), CTC978CCT (3634T>C 3635C>T), AGA979AGG (3638A>G), GAC980GAT (3641C>T), TCA983TCT (3650A>T), GGG985GGT (3656G>T), GCT986GCC (3659T>C), GTA987GTG (3662A>G), GTG991GTC (3674G>C), GAC996GAT (3689C>T), CGA999CGC (3698A>C), TTG1001CTA (3702T>C 3704G>A), GTT1002GTG (3707T>G), TTA1003CTA (3708T>C), CCA1004CCC (3713A>C), TGC1005TGT (3716C>T), GCA1006GCG (3719A>G), CCC1007CCA (3722C>A), TCC1009TCG (3728C>G), CAG1014CCC (3742A>C 3743G>C), GGG1015GGA (3746G>A), GCA1016GCT (3749A>T), CTG1017CTA (3752G>A), GTA1018GTG (3755A>G), ATC1022ATA (3767C>A), ACA1023ACC (3770A>C), TGC1024TGT (3773C>T), CCA1025CCT (3776A>T), TTC1026TTT (3779C>T), TTC1033TTT (3800C>T), GGA1038GGT (3815A>T), TAC1042TAT (3827C>T), ATT1044ATA (3833T>A), GTA1046GTG (3839A>G), AGA1048AGG (3845A>G), GTG1049GTA (3848G>A), CAG1050CAA (3851G>A), ACT1051GCC (3852A>G 3854T>C), TCC1052TCA (3857C>A), ATA1055ATC (3866A>C), GGA1056GGT (3869A>T), GGA1057GGT (3872A>T), CTG1059CTC (3878G>C), AAC1060AAT (3881C>T), ATC1061GTA (3882A>G 3884C>A), ACC1062GCC (3885A>G), TTA1063TTG (3890A>G), GAT1064GAC (3893T>C), TCA1065TCT (3896A>T), CCT1071CCA (3914T>A), GGA1073GGT (3920A>T), ATC1074ATT (3923C>T), AAG1076CGA (3927A>C 3928A>G 3929G>A), GGC1077GGA (3932C>A), ACC1078GCT (3933A>G 3935C>T), GTT1080GTG (3941T>G), GTC1081GTA (3944C>A), TCT1082TCC (3947T>C), GCT1083GCA (3950T>A), GGT1084GGA (3953T>A), GCA1087ACA (3960G>A), GCT1090GCA (3971T>A), GGT1094GGA (3983T>A), ATG1095GTT (3984A>G 3986G>T), AGC1096AAT (3988G>A 3989C>T), GAC1097AGC (3990G>A 3991A>G), AAC1098AAT (3995C>T), ATC1099ATT (3998C>T), TTT1100TTC (4001T>C), CAT1105CAG (4016T>G), GAG1108GAA (4025G>A), TTT1109TTC (4028T>C), TTT1110TTC (4031T>C), CCT1111CCG (4034T>G), AGA1112CGT (4035A>C 4037A>T), CGC1113CGT (4040C>T), GCC1117GCT (4052C>T), ATA1120GTT (4059A>G 4061A>T), CCA1122CCT (4067A>T), AAT1123GGC (4068A>G 4069A>G 4070T>C), GCT1124GTG (4072C>T 4073T>G), AGA1126CGA (4077A>C), ACT1129ACA (4088T>A), CTG1130CTA (4091G>A), CGA1133CGC (4100A>C), CTA1134CTT (4103A>T), GGA1135GGC (4106A>C), CTC1137CTT (4112C>T), AAA1144AAG (4133A>G), ATA1147ATC (4142A>C), AGC1148AGT (4145C>T), CTG1150ATA (4149C>A 4151G>A), GAA1151GAG (4154A>G), ATC1152ATA (4157C>A), AAG1155AAA (4166G>A), TTC1160TTT (4181C>T), TTG1162TTA (4187G>A), CAA1163GAA (4188C>G), GCT1165GCA (4196T>A), TCA1169TCT (4208A>T), GCA1170GCT (4211A>T), AAG1174AAA (4223G>A), CAT1175TAT (4224C>T), TTG1176CTT (4227T>C 4229G>T), ACC1183ACT (4250C>T), CTT1185CTG (4256T>G), ACT1187ACC (4262T>C), CCT1188CCA (4265T>A), TCA1191TCT (4274A>T), AGG1194CGG (4281A>C), AGT1195GGA (4284A>G 4286T>A), TTT1196TTC (4289T>C)								

*: Inserts / Deletes / Misaligned / Frameshifts

Analysis details

This analysis was performed with panviral2.64

NGS Details (UN60): Tomato necrotic dwarf virus (segment RNA1)

Assembly

**Coverage Length**	6176 (1 contig(s))
**Depth Of Coverage**	5251.7
**Number Of Reads**	244103
**Reads Per Million**	5177.32 rpm (after QC)
**Ambiguities**	0
**Assembly Method**	de novo + reference guided assembly
**Consensus Caller**	Bcf Tools

Coverage Map

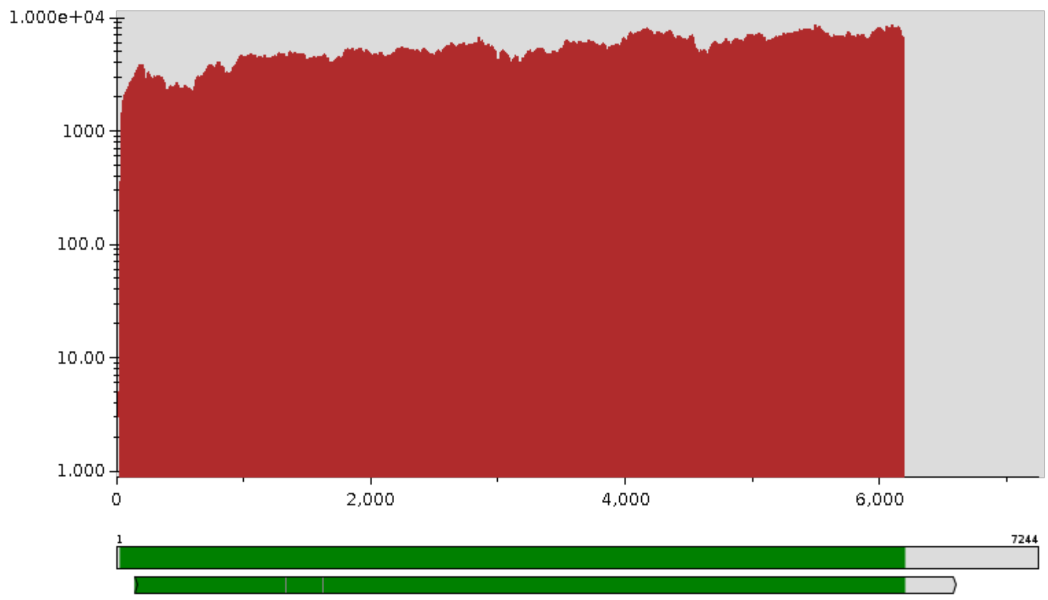

Assignment

**Type**	Tomato necrotic dwarf virus (Taxonomy ID: 1481465)
**Reference Genome**	NC_027926.1
**NT Identity (%)**	64.2066
**AA Identity (%)**	65.5893
**Number Of Stop Codons**	0
**Number Of CDS**	1

Alignment

**Alignment Score**	3315.0 (NT) + 9642.0 (AA) = 12957.0
**Concordance (%)**	49.8691

Alignment Method	Global, seeded, nucleotide + amino acids (AGA)

Genome Region

Sequence starts at position 25 and ends at position 6200 relative to NC_027926.1 reference sequence.

Alignment Detailed Statistics

	Begin	End	Coverage	Score	Concordance	Matches	Identities	I/D/M/F*	Stop Codons
NT	25	6200	85.3%	3315	27.4%	6117 (98.7%)	3941 (63.6%)	21/59	

	Begin	End	Coverage	Score	Concordance	Matches	Identities	I/D/M/F*	Stop Codons
**NT**	**25**	**6200**	**85.3%**	**3315**	**27.4%**	**6117 (98.7%)**	**3941 (63.6%)**	**21/59**	

Mutations:

35T>C, 40G>A, 42C>T, 43C>T, 49T>C, 50C>T, 52A>C, 54T>C, 62A>C, 64T>C, 65T>A, 66G>T, 67G>A, 69A>C, 70T>A, 71T>A, 74A>C, 77G>T, 79T>C, 82A>T, 84G>T,
85_93delCTAAACTTC, 99C>A, 101T>C, 109_119delGAAACTTTTAA, 121A>C, 122C>T, 123A>C, 125T>A, 129T>C, 130T>C, 131C>T, 134C>G, 135T>A, 136T>A, 137A>T, 138T>C,
142G>A, 143T>C, 144G>A, 145A>T, 146T>A, 148A>C, 156T>A, 157C>T, 165A>G, 170T>G, 171T>G, 172G>A, 175T>C, 178T>C, 180A>T, 181T>G,
184_201delGAGAAGACTGGATCAGTT, 202C>T, 204C>T, 207T>A, 208A>C, 213T>A, 221G>C, 222T>A, 226C>G, 228G>T, 232G>T, 233G>C, 238A>T, 240C>T, 243A>T, 244A>T,
245A>C, 246A>T, 247T>C, 249C>T, 255A>G, 256G>A, 259A>C, 261T>C, 262A>T, 263G>C, 267C>T, 268A>G, 270C>T, 272C>A, 273A>T, 274T>A, 275C>A, 279A>T, 287A>C,
288A>C, 292T>G, 293C>T, 294T>A, 300T>C, 301T>A, 306T>G, 310T>G, 312G>C, 315A>G, 318A>G, 319T>C, 321T>A, 324G>T, 326C>T, 327T>G, 329A>T, 330T>A, 334T>G,
336T>A, 337T>A, 339T>A, 342T>A, 347T>C, 348T>C, 357T>C, 358T>A, 359C>A, 360C>T, 363A>T, 364T>G, 366G>A, 367A>G, 369T>A, 371A>G, 375G>T, 376C>T, 378T>A,
387A>T, 389G>C, 390T>A, 391T>A, 392C>A, 393C>A, 394T>A, 396G>A, 399T>G, 405T>C, 408T>C, 409C>A, 411G>C, 414A>G, 416C>G, 417A>T, 420C>T, 421T>G, 422C>A,
427T>G, 429G>A, 430G>A, 431C>A, 435C>T, 445T>A, 450C>A, 451A>G, 452A>T, 453G>A, 457A>C, 459G>T, 462T>A, 463T>C, 465A>T, 466C>A, 467G>A, 469T>A, 472T>G,
474C>T, 475G>T, 476A>T, 481T>G, 483G>C, 484T>A, 485C>A, 486C>G, 487A>G, 493G>T, 495T>C, 497G>T, 498A>C, 499A>C, 506T>A, 507T>C, 508C>G, 510G>A, 514G>T,
516T>A, 522G>A, 525G>A, 526A>G, 527G>T, 532G>T, 533A>C, 534T>A, 535G>T, 543T>A, 546A>G, 547G>T, 550T>A, 551A>T, 552T>A, 554G>C, 561G>A, 570A>T, 571A>T,
573A>G, 574A>G, 576T>C, 577C>A, 580C>T, 582T>G, 583G>C, 585T>A, 586T>C, 588G>A, 589T>C, 593G>A, 597T>A, 604T>G, 605C>T, 609C>T, 612A>T, 613A>G, 615T>A,
616G>A, 617A>C, 618A>G, 619G>C, 620G>C, 621A>T, 622G>T, 624A>T, 625T>A, 626C>A, 629G>A, 631A>G, 632C>T, 633C>A, 634A>G, 637A>G, 638A>T, 639A>T, 641A>C,
643A>T, 645T>G, 648C>T, 650T>C, 652A>G, 654A>T, 657A>T, 660G>A, 664A>T, 666G>T, 669T>C, 673A>T, 674G>C, 675T>A, 684C>T, 687G>A, 690C>T, 699C>A, 701A>C,
702G>A, 706G>T, 710A>G, 717C>T, 718A>T, 720A>C, 721A>C, 722T>A, 724G>C, 726A>G, 729A>G, 736A>G, 737G>C, 738T>A, 739A>G, 741G>A, 744A>T, 753G>A, 754T>A,
755C>G, 756T>C, 757G>T, 759C>A, 760T>A, 762A>G, 765C>T, 768T>A, 774A>T, 775T>A, 776C>G, 780G>A, 781T>C, 783G>T, 784A>T, 786G>A, 789A>C, 790A>T, 792T>A,
793A>T, 795T>G, 796A>T, 801A>T, 810T>C, 813C>A, 816G>T, 817C>T, 819C>A, 822G>A, 826A>G, 828A>T, 831A>T, 834G>T, 837A>T, 840A>G, 846C>A, 853T>C, 855G>T,
856A>T, 857G>C, 858C>A, 861T>C, 864C>A, 867C>G, 876T>C, 885A>T, 886A>G, 891G>A, 897G>T, 900T>G, 915A>G, 916T>C, 921T>C, 922A>C, 923C>A, 924A>G, 933A>G,
936T>C, 942A>G, 948C>T, 951T>A, 954A>T, 960A>C, 963T>C, 966T>A, 972G>T, 978A>G, 981T>A, 982A>G, 984A>G, 988G>A, 990T>C, 991G>A, 992C>T, 1002C>T, 1005G>A,
1014G>A, 1015C>T, 1017T>G, 1020T>A, 1023A>G, 1026T>A, 1028G>A, 1029C>T, 1032A>T, 1035A>C, 1038G>A, 1041T>C, 1044T>C, 1048T>C, 1050G>T, 1054G>C, 1056A>G,
1063C>A, 1064A>C, 1071G>T, 1072G>T, 1073A>C, 1074T>G, 1076C>A, 1077T>G, 1081T>G, 1082G>C, 1086T>A, 1090G>T, 1094C>T, 1095C>G, 1096A>C, 1098G>C, 1099T>C,
1101G>T, 1104G>A, 1107C>T, 1113C>A, 1116T>C, 1117A>C, 1118A>C, 1119A>T, 1122C>A, 1125C>T, 1128T>A, 1129G>A, 1130A>T, 1131C>T, 1134A>G, 1138A>C, 1140T>A,
1143C>T, 1144C>T, 1146T>A, 1148G>A, 1149G>A, 1152G>A, 1158A>C, 1159C>G, 1161G>C, 1164G>A, 1167T>A, 1173A>T, 1185A>C, 1188C>T, 1194T>C, 1197C>A, 1204A>T,
1212T>C, 1213C>A, 1215A>G, 1218G>T, 1219A>T, 1220G>C, 1224A>T, 1227T>C, 1230C>A, 1233T>G, 1239T>A, 1242A>T, 1245G>A, 1246G>C, 1248C>G, 1249T>G, 1250A>T,
1254G>T, 1258A>G, 1259G>C, 1260T>C, 1261T>A, 1264A>C, 1265G>A, 1270G>T, 1271T>C, 1278T>G, 1281T>C, 1284G>A, 1290A>G, 1296A>G, 1299C>T, 1308T>G, 1316A>T,
1320T>A, 1323C>T, 1329G>A, 1344A>G, 1345G>T, 1346C>G, 1347A>T, 1353A>C, 1354T>C, 1356G>T, 1372T>C, 1374G>C, 1377T>C, 1378C>G, 1379G>C, 1380C>A, 1386G>T,
1389G>A, 1392T>A, 1398T>G, 1402A>C, 1404A>C, 1405C>T, 1407T>G, 1413T>G, 1416T>A, 1419C>T, 1422G>A, 1423C>A, 1425C>A, 1431C>A, 1440T>C, 1443T>C, 1449C>A,
1452C>T, 1455G>A, 1461T>A, 1465T>C, 1467G>T, 1476C>T, 1482A>T, 1485A>T, 1486T>C, 1488G>T, 1489A>C, 1491A>G, 1503A>G, 1506A>G, 1512A>G, 1518T>A, 1536T>C,
1542C>T, 1545A>C, 1551A>T, 1557A>T, 1560G>T, 1563C>A, 1569A>T, 1572T>A, 1575G>C, 1578A>G, 1581C>T, 1584G>A, 1587G>A, 1593T>C, 1599T>A, 1608T>C, 1617A>C,
1627C>T, 1629T>G, 1630G>A, 1635A>T, 1638A>T, 1641T>A, 1644C>T, 1656T>A, 1659C>G, 1671T>A, 1677G>A, 1678C>A, 1680T>G, 1683A>G, 1686G>A, 1692C>T, 1704T>C,
1706T>G, 1707C>A, 1712A>T, 1713A>G, 1716T>C, 1719A>T, 1723T>G, 1724C>A, 1726A>C, 1729G>A, 1731G>T, 1732G>A, 1733A>C, 1734C>T, 1740C>A, 1742G>C, 1743A>C,
1749G>A, 1752G>T, 1756A>G, 1758C>T, 1759A>G, 1761T>G, 1771C>A, 1773G>A, 1776G>A, 1788A>C, 1798C>G, 1799A>C, 1800C>T, 1804A>T, 1805G>C, 1806T>C, 1809C>T,
1810T>G, 1811C>A, 1812G>T, 1821A>G, 1822G>C, 1824C>A, 1836C>A, 1842T>C, 1844A>G, 1848T>G, 1851T>C, 1852G>T, 1853C>A, 1858A>G, 1860A>G, 1865A>G, 1866G>C,
1869G>A, 1872G>T, 1874T>A, 1875T>C, 1876G>A, 1878A>T, 1879G>A, 1881A>T, 1882G>A, 1883A>C, 1885A>T, 1887T>G, 1890C>A, 1891A>T, 1893T>G, 1894T>G, 1896A>T,
1897A>G, 1899T>C, 1903A>G, 1904C>T, 1905A>T, 1908C>T, 1914T>C, 1915G>A, 1916C>A, 1920A>T, 1921T>G, 1924T>G, 1925C>T, 1929A>T, 1932G>A, 1935T>G, 1936G>C,
1940C>A, 1941A>T, 1945G>T, 1947T>C, 1948G>C, 1950T>A, 1952G>A, 1953G>T, 1956T>C, 1957_1959delCGC, 1962C>A, 1963A>C, 1966A>G, 1967C>T, 1968A>T, 1969A>T,
1970A>T, 1971A>G, 1973A>C, 1974A>T, 1975G>C, 1976A>C, 1979T>A, 1981C>T, 1984A>G, 1985T>C, 1988T>C, 1989G>T, 1990A>T, 1991C>A, 1993C>G, 1994C>A, 1995C>G,
1996A>C, 1997C>A, 1998A>G, 1999G>A, 2000T>C, 2001T>A, 2004A>T, 2010G>A, 2012A>C, 2013A>T, 2016T>A, 2018G>A, 2020G>A, 2022G>T, 2023A>G, 2031T>C, 2032A>C,
2034A>G, 2038T>C, 2040A>C, 2046G>T, 2049A>G, 2055T>C, 2060T>A, 2067T>C, 2070G>A, 2073C>G, 2077T>A, 2080G>A, 2082C>T, 2083C>A, 2084C>A, 2085G>A, 2086G>T,
2087A>T, 2088G>T, 2089A>G, 2090G>A, 2091T>G, 2094G>T, 2097T>C, 2100T>A, 2107T>C, 2108A>T, 2109T>C, 2110C>T, 2111A>C, 2112A>C, 2115A>G, 2118A>G, 2122C>A,
2126T>A, 2131T>G, 2132C>T, 2133C>G, 2137G>T, 2138A>C, 2140G>T, 2142A>T, 2145G>T, 2146C>G, 2147A>C, 2149C>A, 2151G>A, 2154A>G, 2155G>A, 2156T>A, 2157C>T,
2160C>T, 2161A>G, 2162T>A, 2163G>A, 2165G>T, 2166A>G, 2170A>G, 2172G>A, 2174C>A, 2178C>T, 2179A>T, 2182A>T, 2184C>G, 2185T>G, 2186A>T, 2193T>A, 2196C>A,
2198A>G, 2200G>C, 2203G>C, 2205A>G, 2208A>G, 2211C>T, 2212A>G, 2213G>C, 2214G>T, 2225A>G, 2229T>C, 2245C>A, 2256T>C, 2259A>G, 2262C>T, 2268C>T, 2272G>A,
2273C>G, 2274T>C, 2275T>A, 2277C>A, 2283C>T, 2285G>C, 2290A>G, 2293A>C, 2294A>T, 2300C>A, 2304A>C, 2308A>C, 2309C>G, 2310A>C, 2311G>A, 2313G>T, 2314A>C,
2316G>T, 2317C>G, 2318G>A, 2319C>G, 2320A>C, 2321C>A, 2325A>G, 2326T>A, 2327T>A, 2328T>A, 2331A>G, 2332C>T, 2337T>G, 2338T>G, 2339G>C, 2340T>G, 2341A>C,
2344G>C, 2346T>G, 2354T>C, 2355T>A, 2362T>C, 2364G>T, 2366T>C, 2367T>G, 2370C>T, 2373C>T, 2374C>G, 2376G>A, 2389T>A, 2390T>G, 2391T>A, 2394G>A, 2400T>C,
2401T>C, 2409A>T, 2412C>T, 2421T>C, 2424T>C, 2432C>G, 2433C>G, 2440T>G, 2442C>A, 2442_2443insGGT, 2444A>C, 2445A>T, 2448G>C, 2449G>A, 2450C>A, 2451C>G,
2454A>G, 2455G>A, 2456A>G, 2457C>A, 2458T>A, 2459C>A, 2464G>A, 2466C>T, 2468A>T, 2469G>C, 2470A>G, 2471T>A, 2475T>C, 2476T>A, 2477A>T, 2478C>G, 2479C>A,
2480A>T, 2481C>T, 2482A>G, 2483A>G, 2484A>T, 2487T>C, 2488G>A, 2489T>A, 2493T>C, 2494T>A, 2496C>A, 2501A>T, 2502A>C, 2503G>C, 2505A>G, 2507C>T, 2508T>G,
2512T>A, 2513G>A, 2514G>T, 2515A>G, 2520A>G, 2523A>C, 2525A>T, 2526T>C, 2527G>A, 2528C>T, 2529A>C, 2530C>T, 2534G>A, 2553C>A, 2556A>G, 2560A>T, 2562C>G,
2565G>A, 2568G>A, 2577A>C, 2578T>A, 2579T>C, 2580G>C, 2581T>A, 2583T>G, 2584G>A, 2585G>A, 2586G>T, 2588G>C, 2589A>C, 2590A>G, 2592A>T, 2595A>T, 2601T>C,
2602C>A, 2605G>A, 2610T>C, 2611T>C, 2612G>T, 2613G>C, 2614C>A, 2616A>G, 2617A>G, 2618G>C, 2619C>A, 2620A>G, 2621A>C, 2622T>A, 2623T>A, 2627T>C, 2628T>C,
2631A>T, 2632A>T, 2633G>T, 2634T>A, 2638A>G, 2639G>A, 2643A>C, 2649T>G, 2652T>C, 2655C>A, 2658A>C, 2673C>T, 2679T>C, 2685A>G, 2688G>A, 2691C>T, 2694G>A,
2697C>G, 2698C>A, 2699C>A, 2700C>G, 2701T>A, 2705G>A, 2707C>T, 2709G>C, 2710A>G, 2711A>C, 2712G>A, 2715T>A, 2717T>C, 2718G>C, 2722G>C, 2724G>A, 2730G>A,
2731A>G, 2732G>C, 2733T>G, 2734A>G, 2735G>C, 2736C>A, 2738C>A, 2744T>A, 2745T>C, 2746A>C, 2747C>A, 2748A>T, 2749C>G, 2750A>T, 2751A>G, 2754A>C,
2754_2755insCGG, 2762C>A, 2763T>A, 2764G>C, 2766A>G, 2772G>A, 2778C>T, 2781C>T, 2782C>G, 2785G>A, 2786C>G, 2787A>T, 2790C>G, 2791A>C, 2793A>C, 2796G>A,
2799T>C, 2800A>T, 2803A>G, 2808A>T, 2809C>T, 2812C>A, 2814G>A, 2815C>A, 2819G>C, 2820A>T, 2823G>A, 2829G>C, 2832C>T, 2844A>G, 2850G>T, 2856A>T, 2859G>T,
2865A>T, 2866A>C, 2868G>C, 2883A>C, 2886C>T, 2887C>A, 2888A>G, 2889C>A, 2892T>C, 2893G>A, 2898A>G, 2904A>C, 2907A>T, 2914A>T, 2919T>C, 2922C>T, 2926C>G,
2927C>A, 2928T>G, 2929A>C, 2931C>T, 2934A>C, 2936G>T, 2937G>T, 2939C>G, 2940A>T, 2943G>T, 2951A>T, 2955C>T, 2959C>A, 2960G>C, 2961G>C, 2962G>C, 2967C>A,
2969A>G, 2971G>T, 2973C>G, 2976T>A, 2982A>T, 2989A>G, 2991C>G, 2995C>T, 2998C>T, 3000A>G, 3001C>A, 3003T>G, 3006G>A, 3009T>G, 3012C>T, 3015T>A, 3028C>T,
3030T>A, 3036T>A, 3037C>A, 3039C>A, 3042G>A, 3051A>T, 3063G>T, 3066C>G, 3069C>T, 3071T>A, 3073A>T, 3075C>T, 3078A>G, 3081T>C, 3084T>A, 3085C>A, 3086A>T,
3093A>G, 3096T>C, 3097T>G, 3098G>T, 3099T>G, 3102A>G, 3105C>T, 3108G>A, 3111G>T, 3116C>A, 3117C>G, 3123A>C, 3125G>A, 3126A>G, 3127C>A, 3128T>A, 3129G>A,
3130G>T, 3131G>C, 3135C>T, 3136C>T, 3138A>G, 3141A>G, 3142T>G, 3145T>C, 3147G>T, 3152T>A, 3168C>A, 3171A>G, 3177T>C, 3180A>C, 3182T>A, 3183C>A, 3186G>A,
3189G>C, 3190G>A, 3191A>C, 3192G>A, 3197A>G, 3198G>A, 3204C>T, 3207T>G, 3208G>A, 3209C>A, 3210C>T, 3211A>G, 3212G>C, 3213T>A, 3217A>G, 3218A>C, 3219A>T,
3220T>C, 3222G>T, 3225T>C, 3228G>A, 3231G>A, 3232C>G, 3233A>T, 3234A>T, 3235T>G, 3243A>G, 3246T>C, 3249T>C, 3252G>T, 3256G>A, 3258A>T, 3267T>A, 3276A>T,
3283T>C, 3285A>C, 3286C>T, 3288T>A, 3294C>A, 3296G>C, 3300T>G, 3309A>G, 3312A>G, 3315T>G, 3316G>T, 3317T>G, 3318T>C, 3324G>T, 3327C>A, 3330T>A, 3339T>A,
3342A>T, 3350G>A, 3353T>C, 3354T>C, 3357A>T, 3363A>G, 3369A>T, 3371T>A, 3373A>G, 3375T>G, 3376T>G, 3381C>A, 3384T>C, 3387C>A, 3390G>A, 3393C>A, 3394A>G,
3396C>G, 3399C>T, 3400C>A, 3402G>A, 3405C>T, 3408G>A, 3409C>T, 3411T>G, 3415A>G, 3417C>T, 3429G>A, 3432T>C, 3433G>A, 3440C>T, 3444A>T, 3453A>C, 3459C>A,
3460A>C, 3462T>G, 3463C>T, 3465C>G, 3468A>G, 3469G>C, 3470C>A, 3472A>C, 3474T>G, 3477G>A, 3478A>T, 3479G>C, 3480T>C, 3482G>A, 3483A>G, 3486A>G, 3487A>G,
3488C>A, 3489C>A, 3491C>G, 3492T>A, 3493C>T, 3495T>C, 3501A>G, 3507C>A, 3510A>G, 3513C>G, 3514T>G, 3516C>T, 3519T>C, 3520C>T, 3522T>G, 3531T>A, 3534T>G,
3535C>T, 3536A>T, 3537A>G, 3541T>A, 3546C>T, 3549A>T, 3555A>G, 3558C>G, 3561T>C, 3567T>A, 3570A>C, 3573C>T, 3575G>A, 3577A>C, 3578A>G, 3579G>C, 3584A>C,
3585A>C, 3586T>C, 3597A>T, 3603T>G, 3604C>G, 3605C>A, 3606T>C, 3610G>C, 3612A>G, 3615C>T, 3618T>C, 3621C>A, 3624A>C, 3627C>T, 3630C>A, 3631A>T, 3633C>T,
3635T>C, 3639T>A, 3646C>A, 3654G>A, 3657A>G, 3660C>T, 3666A>C, 3675A>T, 3681G>C, 3682A>G, 3683T>A, 3687A>G, 3690C>T, 3699T>A, 3703C>T, 3704A>C, 3705T>C,
3706G>A, 3707G>A, 3708T>C, 3714T>G, 3717T>G, 3718G>C, 3719A>C, 3720T>A, 3726T>A, 3729A>G, 3730T>C, 3733T>G, 3735C>T, 3741A>G, 3742A>G, 3743C>G, 3747C>T,
3751A>G, 3752G>C, 3753T>A, 3756A>C, 3757C>A, 3759G>T, 3762G>T, 3765A>T, 3766A>G, 3767C>T, 3768A>G, 3769A>C, 3771G>T, 3774A>G, 3776A>T, 3777C>G, 3780A>G,
3781A>C, 3782A>G, 3795C>G, 3801A>T, 3802C>G, 3803A>C, 3804G>A, 3805G>A, 3806A>T, 3807G>C, 3811G>A, 3819T>C, 3822G>A, 3828G>C, 3837T>A, 3845G>T, 3854A>T,
3860G>C, 3861C>A, 3868C>T, 3870C>G, 3876A>G, 3879C>A, 3888C>T, 3894C>T, 3901A>T, 3906C>A, 3907A>C, 3909G>T, 3912A>T, 3913A>T, 3914T>C, 3915G>T, 3921G>A,
3922C>A, 3927G>A, 3933C>G, 3935T>A, 3937C>G, 3939A>C, 3940A>C, 3941C>A, 3943G>C, 3944G>C, 3946G>A, 3948A>C, 3949G>T, 3950G>C, 3951T>G, 3952G>C, 3954T>G,
3956A>G, 3957A>G, 3960A>T, 3961G>C, 3962A>T, 3963G>T, 3966T>C, 3968G>T, 3973G>A, 3981A>T, 3984G>C, 3990A>T, 3993T>A, 3999G>A, 4002A>G, 4003C>T, 4009A>T,
4010T>C, 4011T>G, 4014A>G, 4015A>G, 4017T>A, 4023G>A, 4028T>C, 4030C>T, 4031A>T, 4035G>T, 4038T>C, 4041A>C, 4042A>G, 4047C>A, 4050A>C, 4053A>G, 4059C>G,
4062G>A, 4068G>A, 4071C>G, 4072C>T, 4074T>G, 4077T>A, 4083A>G, 4104A>G, 4107A>C, 4108A>C, 4110A>C, 4111T>C, 4113A>G, 4116T>C, 4119G>A, 4122A>C, 4137C>T,
4149C>G, 4150A>C, 4161T>C, 4173C>T, 4176C>T, 4177A>T, 4178C>G, 4179C>T, 4188A>T, 4189T>G, 4190T>C, 4197T>C, 4200C>A, 4201A>C, 4206A>T, 4209T>C, 4215T>A,
4218A>T, 4221A>C, 4222G>A, 4230C>G, 4233T>C, 4234T>C, 4235C>A, 4243T>A, 4245T>A, 4246C>G, 4248T>G, 4251T>G, 4254C>T, 4256A>G, 4257C>T, 4259A>G, 4263C>T,
4267G>T, 4269T>A, 4279A>G, 4280C>A, 4281T>G, 4284G>A, 4287T>C, 4293T>C, 4302A>G, 4305T>C, 4311C>T, 4314G>A, 4315C>A, 4317C>A, 4323A>C, 4324G>A, 4326G>C,
4329C>T, 4332T>A, 4335T>C, 4338G>A, 4351T>C, 4356T>G, 4361A>C, 4362G>T, 4364A>T, 4370A>G, 4372A>G, 4373C>A, 4374C>T, 4375A>G, 4383A>G, 4384G>A, 4385A>G,
4386G>T, 4391C>A, 4392T>G, 4395C>A, 4401A>T, 4404A>G, 4407T>C, 4408G>A, 4410T>A, 4411C>T, 4413T>A, 4414C>T, 4416C>A, 4425A>C, 4428A>T, 4434C>T, 4437C>T,
4441T>C, 4443A>C, 4450G>A, 4452T>C, 4455A>G, 4456G>T, 4458T>G, 4461C>T, 4464T>C, 4465T>C, 4467G>A, 4470T>G, 4476C>A, 4482G>C, 4494A>T, 4495C>T, 4497T>G,
4500C>T, 4509C>T, 4512C>T, 4516C>T, 4519T>C, 4521G>A, 4537C>A, 4542T>C, 4545T>G, 4548C>T, 4551C>A, 4557T>G, 4560A>G, 4563A>G, 4566G>A, 4569T>A, 4572C>T,
4573A>G, 4575T>C, 4576G>A, 4577C>A, 4578C>A, 4581T>C, 4583G>A, 4585G>T, 4588A>G, 4590A>G, 4593T>C, 4594G>A, 4596A>T, 4602T>A, 4603G>A, 4604G>A, 4608T>C,
4614C>T, 4618C>T, 4620T>G, 4638A>T, 4647T>C, 4648C>T, 4653G>T, 4656T>G, 4659G>T, 4662T>A, 4663C>A, 4669C>T, 4680T>C, 4681T>G, 4682C>A, 4683A>T, 4686G>A,
4689T>A, 4690A>T, 4691A>C, 4692T>C, 4698C>A, 4699C>A, 4700A>G, 4701G>A, 4707T>G, 4710C>A, 4711A>G, 4712T>C, 4713G>T, 4716A>T, 4719T>C, 4728C>T, 4731A>G,
4737G>T, 4738C>T, 4743T>C, 4749T>A, 4759A>G, 4761C>G, 4762C>A, 4765G>A, 4766T>C, 4767G>A, 4768C>A, 4770G>A, 4773C>T, 4775A>T, 4779C>T, 4785A>T, 4788A>G,
4794T>C, 4803C>T, 4809A>T, 4812T>C, 4815A>G, 4818T>A, 4824C>A, 4825C>T, 4827T>G, 4830G>A, 4836C>A, 4857G>T, 4861A>G, 4863A>C, 4866T>C, 4872T>C, 4875G>T,
4878C>T, 4884C>T, 4887A>G, 4890T>A, 4894C>G, 4895C>T, 4899T>A, 4909A>G, 4910C>T, 4911T>A, 4912G>C, 4913T>A, 4914G>A, 4917A>T, 4921A>T, 4922T>A, 4923G>T,
4926G>A, 4930G>A, 4932G>T, 4938T>A, 4942C>A, 4944T>C, 4945C>A, 4947T>A, 4950G>A, 4956T>C, 4966A>C, 4967G>A, 4968A>G, 4970T>C, 4971T>C, 4972G>A, 4974A>T,
4980G>A, 4982A>G, 4983G>T, 4986A>T, 4987A>G, 4988C>T, 4989T>A, 4990T>A, 4999C>T, 5000A>T, 5001G>T, 5002T>G, 5004T>A, 5007C>T, 5012G>A, 5013A>G, 5014G>C,
5017C>A, 5019T>A, 5020G>A, 5021G>A, 5022A>T, 5026G>C, 5027G>C, 5029G>A, 5030C>T, 5031A>C, 5033C>T, 5034A>G, 5038T>A, 5040T>G, 5043T>C, 5044T>A, 5049T>C,
5050A>T, 5051T>A, 5052G>T, 5056G>C, 5058G>T, 5061G>A, 5062C>A, 5063A>G, 5064G>T, 5065G>C, 5071A>T, 5073C>G, 5074G>A, 5077A>G, 5093C>A, 5094A>G, 5095T>A,
5096C>G, 5097A>C, 5098G>A, 5099C>T, 5100G>T, 5101T>G, 5102T>G, 5104A>G, 5105C>T, 5106A>T, 5107A>T, 5109C>G, 5110T>A, 5111G>T, 5112T>G, 5115A>G, 5116A>C,
5118G>A, 5119G>T, 5121C>A, 5122A>G, 5124A>T, 5128T>A, 5129C>A, 5130T>A, 5132T>A, 5133C>T, 5136T>A, 5138T>A, 5142A>C, 5148G>A, 5151A>T, 5152C>G, 5158A>G,
5160T>A, 5164G>A, 5167_5169delGAA, 5178A>G, 5179A>G, 5180G>A, 5181A>G, 5183A>G, 5185C>G, 5188A>C, 5191T>G, 5192G>A, 5193G>A, 5198C>T, 5199C>G, 5202A>T,
5203A>G, 5205C>T, 5206A>G, 5208T>A, 5209G>T, 5210G>T, 5217C>T, 5218T>A, 5219C>G, 5220A>T, 5223C>T, 5226T>G, 5233G>T, 5235C>A, 5238G>C, 5239A>C, 5240C>A,
5241T>A, 5242G>C, 5248G>T, 5249A>C, 5250G>A, 5251C>T, 5253T>A, 5256G>T, 5257T>A, 5258G>A, 5262A>C, 5263G>C, 5265A>G, 5271C>T, 5274T>C, 5277A>T, 5280G>A,
5283G>C, 5284A>C, 5285A>G, 5286G>C, 5288A>G, 5289G>A, 5291G>C, 5292C>A, 5294A>C, 5301T>G, 5302C>A, 5304C>A, 5307A>G, 5313T>C, 5315G>A, 5325T>C, 5340G>A,
5346A>C, 5349T>C, 5352C>T, 5353A>T, 5356T>G, 5364T>C, 5366T>A, 5371G>C, 5372C>A, 5377A>C, 5379A>C, 5380G>A, 5381G>A, 5382C>G, 5384A>G, 5385A>T, 5386A>C,
5389T>G, 5390C>A, 5393A>T, 5394G>T, 5396A>G, 5397C>T, 5398A>G, 5399T>A, 5402C>G, 5403T>G, 5410G>T, 5411A>C, 5415T>C, 5416G>A, 5417T>C, 5418G>C, 5424G>T,
5430G>T, 5442T>A, 5443G>A, 5445G>A, 5446A>G, 5447G>C, 5448C>T, 5463T>A, 5466A>G, 5467A>T, 5469G>T, 5472T>A, 5473G>T, 5483G>A, 5487G>A, 5490T>C, 5491T>A,
5493C>G, 5494A>G, 5495T>C, 5496T>C, 5497G>A, 5498A>C, 5499G>A, 5502T>C, 5503A>C, 5505C>T, 5512A>C, 5514G>C, 5515T>C, 5517G>A, 5520C>A, 5523T>C, 5526T>G,
5528G>C, 5529C>T, 5530A>G, 5531A>G, 5532T>G, 5536T>C, 5550A>C, 5553T>C, 5556C>T, 5567T>C, 5568T>G, 5571G>A, 5577T>G, 5581C>A, 5589G>T, 5590C>G, 5591C>A,
5592T>A, 5593C>G, 5595G>C, 5595_5596insACC, 5596G>T, 5597A>C, 5599G>C, 5600G>A, 5601G>C, 5604A>G, 5605A>T, 5609C>G, 5614G>A, 5615T>G, 5620T>A, 5622G>A,
5623_5625delTCT, 5627T>A, 5628G>T, 5629C>G, 5630A>G, 5631A>C, 5633G>C, 5634T>C, 5636G>C, 5642T>A, 5643G>C, 5649A>T, 5649_5650insCACAGT, 5651G>C, 5652T>A,

	5656G>T, 5657A>C, 5658G>A, 5659C>G, 5664C>A, 5666A>T, 5667A>T, 5668A>C, 5670T>A, 5671C>A, 5673T>G, 5674G>A, 5676C>A, 5677A>G, 5679C>T, 5681A>C, 5682C>T, 5683G>T, 5684G>C, 5688T>C, 5689T>C, 5691A>C, 5694A>T, 5695A>G, 5696T>A, 5697G>A, 5698G>A, 5700A>G, 5707T>G, 5709G>T, 5712C>T, 5713A>T, 5721T>C, 5722G>A, 5723T>C, 5724T>A, 5725G>A, 5727G>T, 5730C>T, 5733A>T, 5734C>G, 5736T>A, 5739T>A, 5742A>T, 5747G>C, 5749A>C, 5751C>G, 5753T>G, 5754T>C, 5755A>T, 5757C>A, 5760A>G, 5763A>G, 5766T>C, 5769T>G, 5770G>A, 5772T>G, 5775A>T, 5776T>G, 5777T>C, 5778G>T, 5781T>G, 5783C>A, 5784A>G, 5786G>C, 5787T>A, 5788C>T, 5790T>G, 5791G>T, 5792A>C, 5793A>T, 5794A>G, 5795A>T, 5796G>C, 5797T>A, 5806G>A, 5807A>C, 5808A>T, 5809T>C, 5811G>T, 5812C>G, 5813T>C, 5814T>C, 5819A>G, 5820G>A, 5821A>G, 5822A>G, 5823C>A, 5824A>G, 5825C>T, 5826G>C, 5830C>G, 5835C>A, 5845G>A, 5847G>A, 5848A>G, 5851A>T, 5853G>C, 5854A>T, 5856T>C, 5860A>C, 5862A>C, 5866G>T, 5867T>G, 5868G>T, 5870C>A, 5872C>A, 5873A>T, 5874A>G, 5878G>C, 5880C>T, 5881A>G, 5882G>A, 5883T>G, 5887G>C, 5889A>G, 5890A>G, 5893C>A, 5894A>G, 5895G>T, 5902T>G, 5904C>A, 5905T>G, 5906A>T, 5907T>A, 5908C>T, 5909C>T, 5912A>T, 5915C>G, 5916C>G, 5918C>G, 5919A>C, 5920A>G, 5921C>T, 5922C>T, 5923G>A, 5924T>A, 5926G>A, 5931G>A, 5932C>A, 5934C>G, 5935A>T, 5936A>T, 5938G>T, 5939T>C, 5940A>C, 5943T>C, 5944G>A, 5945C>T, 5946T>G, 5947G>A, 5950G>C, 5951C>A, 5952T>A, 5955T>G, 5955_5956insATG, 5956A>T, 5958C>A, 5960T>A, 5961T>A, 5962G>A, 5963T>C, 5965A>C, 5968A>G, 5969C>T, 5973T>C, 5975A>G, 5976A>C, 5979T>A, 5981T>G, 5982C>T, 5983C>T, 5985G>T, 5986_5988delGGA, 5991A>C, 5992G>A, 5993T>C, 5994T>A, 5997C>T, 5998C>A, 6000G>T, 6004A>G, 6005A>C, 6006T>A, 6008T>G, 6009T>G, 6012C>A, 6014G>C, 6016C>A, 6017C>A, 6018T>G, 6021G>C, 6022T>A, 6023G>T, 6024G>T, 6024_6025insGTC, 6026A>T, 6027A>G, 6029T>C, 6030T>A, 6031G>A, 6032G>A, 6037G>A, 6042C>G, 6043A>C, 6046_6048delATG, 6050C>G, 6052A>T, 6054A>T, 6055C>A, 6057C>T, 6058T>C, 6060A>T, 6066G>A, 6068G>C, 6069T>A, 6070C>G, 6072T>G, 6073C>T, 6077C>T, 6078A>T, 6086T>C, 6087G>T, 6090G>A, 6094T>C, 6095C>T, 6098C>T, 6099C>T, 6100A>G, 6104A>G, 6105G>A, 6109T>A, 6112C>T, 6114C>G, 6115A>C, 6119G>A, 6120A>T, 6121T>A, 6122A>G, 6123C>T, 6124A>G, 6125A>G, 6128G>A, 6129A>G, 6131A>G, 6133A>T, 6134T>A, 6135G>C, 6138T>A, 6142C>A, 6143A>C, 6144T>A, 6145T>A, 6146G>C, 6147C>A, 6148A>C, 6150A>G, 6151A>G, 6154T>C, 6156G>T, 6159A>T, 6160C>T, 6162C>T, 6164A>G, 6165G>A, 6168A>G, 6171T>C, 6172G>A, 6174T>G, 6175_6180delCTCAAC, 6182T>G, 6183C>T, 6186G>A, 6187A>T, 6188T>G, 6190C>G, 6192G>A, 6193A>C, 6195T>C, 6199G>C, 6200A>C

CDS

APL33_sRA11gp1	1	2017	93.8%	9642	69.4%	2004 (99.0%)	1319 (65.2%)	7/13/0/0	0

	Begin	End	Coverage	Score	Concordance	Matches	Identities	I/D/M/F*	Stop Codons
**NT**	**25**	**6200**	**85.3%**	**3315**	**27.4%**	**6117 (98.7%)**	**3941 (63.6%)**	**21/59**	

Protein mutations:

L3F (157C>T), F7W (170T>G 171T>G), G8S (172G>A), S9P (175T>C), S10P (178T>C 180A>T), S11A (181T>G), E12_V17del (184_201delGAGAAGACTGGATCAGTT), P18S (202C>T 204C>T), T20P (208A>C), G24A (221G>C 222T>A), L26V (226C>G 228G>T), G28S (232G>T 233G>C), I30F (238A>T 240C>T), K32S (244A>T 245A>C 246A>T), F33L (247T>C 249C>T), A36T (256G>A), I37L (259A>C 261T>C), I40V (268A>G 270C>T), T41N (272C>A 273A>T), S42K (274T>A 275C>A), K46T (287A>C 288A>C), S48V (292T>G 293C>T 294T>A), L51I (301T>A), D52E (306T>G), L54V (310T>G 312G>C), S57P (319T>C 321T>A), T59M (326C>T 327T>G), D60V (329A>T 330T>A), S62A (334T>G 336T>A), S63T (337T>A 339T>A), V66A (347T>C 348T>C), S70N (358T>A 359C>A 360C>T), L72V (364T>G 366G>A), N73E (367A>G 369T>A), K74R (371A>G), G80A (389G>C 390T>A), S81K (391T>A 392C>A 393C>A), L82I (394T>A 396G>A), I83M (399T>G), L87I (409C>A 411G>C), A89G (416C>G 417A>T), S91D (421T>G 422C>A), L93V (427T>G 429G>A), A94K (430G>A 431C>A), S99T (445T>A), K101V (451A>G 452A>T 453G>A), M103L (457A>C 459G>T), R106K (466C>A 467G>A), L107M (469T>A), F108V (472T>G 474C>T), E109L (475G>T 476A>T), L111V (481T>G 483G>C), S112K (484T>A 485C>A 486C>G), N113D (487A>G), V115F (493G>T 495T>C), R116I (497G>T 498A>C), I117L (499A>C), F119Y (506T>A 507T>C), Q120E (508C>G 510G>A), V122L (514G>T 516T>A), S126V (526A>G 527G>T), D128S (532G>T 533A>C 534T>A), V129L (535G>T), V133F (547G>T), Y134I (550T>A 551A>T 552T>A), G135A (554G>C), I141L (571A>T 573A>G), T142A (574A>G 576T>C), L143I (577C>A), V145L (583G>C 585T>A), C148Y (593G>A), S152V (604T>G 605C>T), I155V (613A>G 615T>A), E156T (616G>A 617A>C 618A>G), G157P (619G>C 620G>C 621A>T), V158F (622G>T 624A>T), S159N (625T>A 626C>A), S160N (629G>A), T161V (631A>G 632C>T 633C>A), I162V (634A>G), K163V (637A>G 638A>T 639A>T), N164T (641A>C), I165L (643A>T 645T>G), L167S (650T>C), I168V (652A>G 654A>T), M172F (664A>T 666G>T), K184T (701A>C 702G>A), A186S (706G>T), E187G (710A>G), T190S (718A>T 720A>C), M191Q (721A>C 722T>A), V192L (724G>C 726A>G), S196A (736A>G 737G>C 738T>A), M197V (739A>G 741G>A), V203L (757G>T 759C>A), S204T (760T>A 762A>G), M212L (784A>T 786G>A), T214S (790A>T 792T>A), I215L (793A>T 795T>G), M216L (796A>T), I226V (826A>G 828A>T), T246A (886A>G), T258Q (922A>C 923C>A 924A>G), I278V (982A>G 984A>G), G280S (988G>A 990T>C), A281I (991G>A 992C>T), S293N (1028G>A 1029C>T), E302Q (1054G>C 1056A>G), Q305T (1063C>A 1064A>C), D308S (1072G>T 1073A>C 1074T>G), A309E (1076C>A 1077T>G), C311A (1081T>G 1082G>C), D312E (1086T>A), A314S (1090G>T), T315M (1094C>T 1095C>G), K323P (1117A>C 1118A>C 1119A>T), D327I (1129G>A 1130A>T 1131C>T), N330Q (1138A>C 1140T>A), R333K (1148G>A 1149G>A), L337V (1159C>G 1161G>C), M352L (1204A>T), E364D (1242A>T), V366L (1246G>C 1248C>G), Y367V (1249T>G 1250A>T), S370A (1258A>G 1259G>C 1260T>C), C371S (1261T>A), R372Q (1264A>C 1265G>A), V374S (1270G>T 1271T>C), Y389F (1316A>T), A399C (1345G>T 1346C>G 1347A>T), R410A (1378C>G 1379G>C 1380C>A), V494I (1630G>A), I519R (1706T>G 1707C>A), E521V (1712A>T 1713A>G), E523D (1719A>T), S525D (1723T>G 1724C>A), T526P (1726A>C), E527N (1729G>A 1731G>T), D528T (1732G>A 1733A>C 1734C>T), G531A (1742G>C 1743A>C), M534I (1752G>T), N536D (1756A>G 1758C>T), I537V (1759A>G 1761T>G), H550A (1798C>G 1799A>C 1800C>T), S554D (1810T>G 1811C>A 1812G>T), D558Q (1822G>C 1824C>A), Q565R (1844A>G), A568Y (1852G>T 1853C>A), I570V (1858A>G 1860A>G), K572S (1865A>G 1866G>C), F575Y (1874T>A 1875T>C), E576N (1876G>A 1878A>T), A577T (1879G>A 1881A>T), E578T (1882G>A 1883A>C), T579S (1885A>T 1887T>G), I581L (1891A>T 1893T>G), S582A (1894T>G 1896A>T), N583D (1897A>G 1899T>C), T585V (1903A>G 1904C>T 1905A>T), A589N (1915G>A 1916C>A), S591A (1921T>G), S592V (1924T>G 1925C>T), E593D (1929A>T), V596L (1936G>C), A597D (1940C>A 1941A>T), V599F (1945G>T 1947T>C), D600Q (1948G>C 1950T>A), G601D (1952G>A 1953G>T), R603del (1957_1959delCGC), F604L (1962C>A), K605Q (1963A>C), T606V (1966A>G 1967C>T 1968A>T), K607L (1969A>T 1970A>T 1971A>G), Q608P (1973A>C 1974A>T), D609P (1975G>C 1976A>C), V610E (1979T>A), P611S (1981C>T), I612A (1984A>G 1985T>C), V613A (1988T>C 1989G>T), T614Y (1990A>T 1991C>A), P615E (1993C>G 1994C>A 1995C>G), T616Q (1996A>C 1997C>A 1998A>G), V617T (1999G>A 2000T>C 2001T>A), E621A (2012A>C 2013A>T), D622E (2016T>A), R623K (2018G>A), V624I (2020G>A 2022G>T), I625V (2023A>G), K628Q (2032A>C 2034A>G), F637Y (2060T>A), L643M (2077T>A), D644N (2080G>A 2082C>T), P645K (2083C>A 2084C>A 2085G>A), E646F (2086G>T 2087A>T 2088G>T), S647E (2089A>G 2090G>A 2091T>G), L648F (2094G>T), Y653L (2107T>C 2108A>T 2109T>C), Q654S (2110C>T 2111A>C 2112A>C), L658I (2122C>A), F659Y (2126T>A), S661V (2131T>G 2132C>T 2133C>G), D663S (2137G>T 2138A>C), A664S (2140G>T 2142A>T), E665D (2145G>T), H666A (2146C>G 2147A>C), Q667K (2149C>A 2151G>A), V669N (2155G>A 2156T>A 2157C>T), M671E (2161A>G 2162T>A 2163G>A), R672M (2165G>T 2166A>G), M674V (2170A>G 2172G>A), T675N (2174C>A), M677L (2179A>T), I678L (2182A>T 2184C>G), Y679V (2185T>G 2186A>T), N683S (2198A>G), A684P (2200G>C), E685Q (2203G>C 2205A>G), R688A (2212A>G 2213G>C 2214G>T), Q692R (2225A>G), L699I (2245C>A), A708S (2272G>A 2273C>G 2274T>C), S709T (2275T>A 2277C>A), C712S (2285G>C), K714E (2290A>G), K715L (2293A>C 2294A>T), A717D (2300C>A), T720R (2308A>C 2309C>G 2310A>C), E721N (2311G>A 2313G>T), M722L (2314A>C 2316G>T), R723E (2317C>G 2318G>A 2319C>G), T724Q (2320A>C 2321C>A), F726K (2326T>A 2327T>A 2328T>A), H728Y (2332C>T), N729K (2337T>G), C730A (2338T>G 2339G>C 2340T>G), I731L (2341A>C), D732Q (2344G>C 2346T>G), V735A (2354T>C 2355T>A), I739T (2366T>C 2367T>G), Q742E (2374C>G 2376G>A), F747R (2389T>A 2390T>G 2391T>A), T761R (2432C>G 2433C>G), S764A (2440T>G 2442C>A), S764_K765insG (2442_2443insGGT), K765T (2444A>C 2445A>T), E766D (2448G>C), A767K (2449G>A 2450C>A 2451C>G), D769R (2455G>A 2456A>G 2457C>A), S770N (2458T>A 2459C>A), V772I (2464G>A 2466C>T), K773I (2468A>T 2469G>C), I774E (2470A>G 2471T>A), Y776M (2476T>A 2477A>T 2478C>G), H777I (2479C>A 2480A>T 2481C>T), K778G (2482A>G 2483A>G 2484A>T), V780K (2488G>A 2489T>A), Y782K (2494T>A 2496C>A), K784I (2501A>T 2502A>C), E785Q (2503G>C 2505A>G), T786M (2507C>T 2508T>G), W788N (2512T>A 2513G>A 2514G>T), N789D (2515A>G), Y792F (2525A>T 2526T>C), A793I (2527G>A 2528C>T 2529A>C), R795K (2534G>A), I804L (2560A>T 2562C>G), M805I (2565G>A), L810T (2578T>A 2579T>C 2580G>C), F811M (2581T>A 2583T>G), G812N (2584G>A 2585G>A 2586G>T), G813A (2588G>C 2589A>C), I814V (2590A>G 2592A>T), L818I (2602C>A), A819T (2605G>A), W821L (2611T>C 2612G>T 2613G>C), Q822K (2614C>A 2616A>G), S823A (2617A>G 2618G>C 2619C>A), N824A (2620A>G 2621A>C 2622T>A), S825T (2623T>A), F826S (2627T>C 2628T>C), S828L (2632A>T 2633G>T 2634T>A), S830D (2638A>G 2639G>A), P850K (2698C>A 2699C>A 2700C>G), S851T (2701T>A), R852H (2705G>A), L853F (2707C>T 2709G>C), K854A (2710A>G 2711A>C 2712G>A), N855K (2715T>A), L856S (2717T>C 2718G>C), V858L (2722G>C 2724G>A), S861A (2731A>G 2732G>C 2733T>G), S862A (2734A>G 2735G>C 2736C>A), A863D (2738C>A), I865N (2744T>A 2745T>C), T866H (2746A>C 2747C>A 2748A>T), Q867V (2749C>G 2750A>T 2751A>G), E868D (2754A>C), E868_W869insR (2754_2755insCGG), A871E (2762C>A 2763T>A), E872Q (2764G>C 2766A>G), Q878E (2782C>G), A879S (2785G>A 2786C>G 2787A>T), K881H (2791A>C 2793A>C), M884L (2800A>T), I885V (2803A>G), L889I (2815C>A), G890A (2819G>C 2820A>T), M906L (2866A>C 2868G>C), H913R (2887C>A 2888A>G 2889C>A), V915M (2893G>A), T922S (2914A>T), P926E (2926C>G 2927C>A 2928T>G), N927H (2929A>C 2931C>T), R929I (2936G>T 2937G>T), S930C (2939C>G 2940A>T), K931N (2943G>T), Y934F (2951A>T), R937T (2959C>A 2960G>C 2961G>C), E938Q (2962G>C), K940R (2969A>G), V941L (2971G>T 2973C>G), I947V (2989A>G 2991C>G), P960S (3028C>T 3030T>A), E971D (3063G>T), F974Y (3071T>A), T975S (3073A>T 3075C>T), Q979M (3085C>A 3086A>T), C983V (3097T>G 3098G>T 3099T>G), T989K (3116C>A 3117C>G), R992K (3125G>A 3126A>G), L993K (3127C>A 3128T>A 3129G>A), G994S (3130G>T 3131G>C), F998V (3142T>G), F1001Y (3152T>A), V1011E (3182T>A 3183C>A), E1014T (3190G>A 3191A>C 3192G>A), E1016G (3197A>G 3198G>A), A1020N (3208G>A 3209C>A 3210C>T), S1021A (3211A>G 3212G>C 3213T>A), K1023A (3217A>G 3218A>C 3219A>T), Q1028V (3232C>G 3233A>T 3234A>T), S1029A (3235T>G), M1034I (3252G>T), G1036S (3256G>A 3258A>T), S1049T (3296G>C), V1056C (3316G>T 3317T>G 3318T>C), G1067D (3350G>A), I1068T (3353T>C 3354T>C), F1074Y (3371T>A), I1075V (3373A>G 3375T>G), S1076A (3376T>G), I1082V (3394A>G 3396C>G), N1089D (3415A>G 3417C>T), V1095I (3433G>A), A1097V (3440C>T), D1103E (3459C>A), I1104L (3460A>C 3462T>G), A1107H (3469G>C 3470C>A), I1108L (3472A>C 3474T>G), R1111K (3482G>A 3483A>G), T1113E (3487A>G 3488C>A 3489C>A), T1114R (3491C>G 3492T>A), L1115F (3493C>T 3495T>C), N1119K (3507C>A), S1122A (3514T>G 3516C>T), Q1129L (3535C>T 3536A>T 3537A>G), L1131M (3541T>A), R1142K (3575G>A), K1143R (3577A>C 3578A>G 3579G>C), Q1145P (3584A>C 3585A>C), P1152D (3604C>G 3605C>A 3606T>C), E1154Q (3610G>C 3612A>G), T1161S (3631A>T 3633C>T), V1162A (3635T>C), N1163K (3639T>A), M1178E (3682A>G 3683T>A), H1185S (3703C>T 3704A>C 3705T>C), G1186N (3706G>A 3707G>A 3708T>C), D1188E (3714T>G), D1189E (3717T>G), D1190P (3718G>C 3719A>C 3720T>A), S1195A (3733T>G 3735C>T), T1198G (3742A>G 3743C>G), S1201A (3751A>G 3752G>C 3753T>A), L1203I (3757C>A 3759G>T), T1206V (3766A>G 3767C>T 3768A>G), M1207L (3769A>C 3771G>T), N1209M (3776A>T 3777C>G), K1211R (3781A>C 3782A>G), Q1218A (3802C>G 3803A>C 3804G>A), E1219I (3805G>A 3806A>T 3807G>C), D1221N (3811G>A), C1232F (3845G>T), Y1235F (3854A>T), G1237A (3860G>C 3861C>A), M1251L (3901A>T), M1253L (3907A>C 3909G>T), M1255S (3913A>T 3914T>C 3915G>T), V1262E (3935T>A), P1263A (3937C>G 3939A>C), T1264Q (3940A>C 3941C>A), G1265P (3943G>C 3944G>C), E1266N (3946G>A 3948A>C), G1267S (3949G>T 3950G>C 3951T>G), D1268Q (3952G>C 3954T>G), K1269R (3956A>G 3957A>G), E1271L (3961G>C 3962A>T 3963G>T), R1273I (3968G>T), D1275N (3973G>A), E1280D (3990A>T), I1287S (4009A>T 4010T>C 4011T>G), I1289V (4015A>G 4017T>A), I1293T (4028T>C), H1294F (4030C>T 4031A>T), I1298V (4042A>G), T1343C (4177A>T 4178C>G 4179C>T), L1347A (4189T>G 4190T>C), I1351L (4201A>C), D1358N (4222G>A), S1362H (4234T>C 4235C>A), S1365T (4243T>A 4245T>A), L1366V (4246C>G 4248T>G), N1369S (4256A>G 4257C>T), N1370S (4259A>G), A1373S (4267G>T 4269T>A), T1377E (4279A>G 4280C>A 4281T>G), V1392I (4324G>A 4326G>C), E1404A (4361A>C 4362G>T), Y1405F (4364A>T), D1407G (4370A>G), T1408D (4372A>G 4373C>A 4374C>T), K1409E (4375A>G), E1412S (4384G>A 4385A>G 4386G>T), A1414E (4391C>A 4392T>G), A1420T (4408G>A 4410T>A), D1434N (4450G>A 4452T>C), V1436L (4456G>T 4458T>G), I1475V (4573A>G 4575T>C), A1476K (4576G>A 4577C>A 4578C>A), R1478K (4583G>A), A1479S (4585G>T), I1480V (4588A>G 4590A>G), V1482I (4594G>A 4596A>T), G1485N (4603G>A 4604G>A), L1505M (4663C>A), S1511D (4681T>G 4682C>A 4683A>T), N1514S (4690A>T 4691A>C 4692T>C), Q1517R (4699C>A 4700A>G 4701G>A), D1520E (4710C>A), M1521A (4711A>G 4712T>C 4713G>T), N1537E (4759A>G 4761C>G), Q1538K (4762C>A), V1539T (4765G>A 4766T>C 4767G>A), Q1540K (4768C>A 4770G>A), Q1542L (4775A>T), H1562Q (4836C>A), I1571V (4861A>G 4863A>C), M1575I (4875G>T), P1582V (4894C>G 4895C>T), T1587V (4909A>G 4910C>T 4911T>A), V1588Q (4912G>C 4913T>A 4914G>A), M1591Y (4921A>T 4922T>A 4923G>T), V1594I (4930G>A 4932G>T), L1598I (4942C>A 4944T>C), R1606Q (4966A>C 4967G>A 4968A>G), V1607A (4970T>C 4971T>C), A1608T (4972G>A 4974A>T), K1611S (4982A>G 4983G>T), L1612F (4986A>T), T1613V (4987A>G 4988C>T 4989T>A), Y1614N (4990T>A), Q1617F (4999C>T 5000A>T 5001G>T), C1618G (5002T>G 5004T>A), R1621K (5012G>A 5013A>G), E1622Q (5014G>C), G1624N (5020G>A 5021G>A 5022A>T), G1626P (5026G>C 5027G>C), A1627I (5029G>A 5030C>T 5031A>C), T1628M (5033C>T 5034A>G), F1630M (5038T>A 5040T>G), S1632T (5044T>A), M1634Y (5050A>T 5051T>A 5052G>T), V1636L (5056G>C 5058G>T), Q1638S (5062C>A 5063A>G 5064G>T), E1639Q (5065G>C), I1641L (5071A>T 5073C>G), A1642T (5074G>A), K1643E (5077A>G), A1648E (5093C>A 5094A>G), A1650I (5098G>A 5099C>T 5100G>T), L1651G (5101T>G 5102T>G), T1652V (5104A>G 5105C>T 5106A>T), I1653L (5107A>T 5109C>G), C1654M (5110T>A 5111G>T 5112T>G), M1656L (5116A>C 5118G>A), A1657S (5119G>T 5121C>A), K1658D (5122A>G 5124A>T), S1660K (5128T>A 5129C>A 5130T>A), I1661N (5132T>A 5133C>T), F1663Y (5138T>A), P1668A (5152C>G), N1670E (5158A>G 5160T>A), V1672I (5164G>A), E1673del (5167_5169delGAA), R1677E (5179A>G 5180G>A 5181A>G), D1678G (5183A>G), L1679V (5185C>G), N1680H (5188A>C), W1681E (5191T>G 5192G>A 5193G>A), A1683V (5198C>T 5199C>G), N1685D (5203A>G 5205C>T), I1686V (5206A>G 5208T>A), G1687F (5209G>T 5210G>T), A1695S (5233G>T 5235C>A), K1696N (5238G>C), T1697Q (5239A>C 5240C>A 5241T>A), E1698Q (5242G>C), E1700S (5248G>T 5249A>C 5250G>A), P1701S (5251C>T 5253T>A), C1703N (5257T>A 5258G>A), V1705L (5263G>C 5265A>G), K1712R (5284A>C 5285A>G 5286G>C), K1713R (5288A>G 5289G>A), S1714T (5291G>C 5292C>A), K1715T (5294A>C), S1722N (5315G>A), M1730I (5340G>A), M1735L (5353A>T), S1736A (5356T>G), L1739Q (5366T>A), A1741H (5371G>C 5372C>A), G1744K (5380G>A 5381G>A 5382C>G), K1745S (5384A>G 5385A>T), M1746L (5386A>C), S1747E (5389T>G 5390C>A), E1748V (5393A>T 5394G>T), D1749G (5396A>G 5397C>T), I1750D (5398A>G 5399T>A), A1751G (5402C>G 5403T>G), D1754S (5410G>T 5411A>C), V1756T (5416G>A 5417T>C 5418G>C), V1765I (5443G>A 5445G>A), S1766A (5446A>G 5447G>C 5448C>T), M1773F (5467A>T 5469G>T), V1775L (5473G>T), R1778H (5483G>A), C1781R (5491T>A 5493C>G), I1782A (5494A>G 5495T>C 5496T>C), E1783T (5497G>A 5498A>C 5499G>A), I1785L (5503A>C 5505C>T), I1792M (5526T>G), G1793A (5528G>C 5529C>T), N1794G (5530A>G 5531A>G 5532T>G), V1806A (5567T>C 5568T>G), F1809L (5577T>G), Q1811K (5581C>A), P1814E (5590C>G 5591C>A 5592T>A), Q1815D (5593C>G 5595G>C), Q1815_E1816insT (5595_5596insACC), E1816S (5596G>T 5597A>C), G1817H (5599G>C 5600G>A 5601G>C), M1819L (5605A>T), A1820G (5609C>G), V1822S (5614G>A 5615T>G), S1824T (5620T>A 5622G>A), S1825del (5623_5625delTCT), L1826Y (5627T>A 5628G>T), Q1827G (5629C>G 5630A>G 5631A>C), S1828T (5633G>C 5634T>C), S1829T (5636G>C), M1831N (5642T>A 5643G>C), Q1833H (5649A>T), Q1833_G1834insHS (5649_5650insCACAGT), G1834A (5651G>C 5652T>A), E1836S (5656G>T 5657A>C 5658G>A), L1837V (5659C>G), Q1839L (5666A>T 5667A>T), I1840L (5668A>C 5670T>A), V1842I (5674G>A 5676C>A), I1843V (5677A>G 5679C>T), H1844P (5681A>C 5682C>T), G1845S (5683G>T 5684G>C), M1849E (5695A>G 5696T>A 5697G>A), E1850K (5698G>A 5700A>G), L1853V (5707T>G 5709G>T), M1855L (5713A>T), V1858T (5722G>A 5723T>C 5724T>A), V1859I (5725G>A 5727G>T), H1862E (5734C>G 5736T>A), G1866A (5747G>C), N1867Q (5749A>C 5751C>G), F1868C (5753T>G 5754T>C), T1869S (5755A>T 5757C>A), D1874K (5770G>A 5772T>G), Q1875H (5775A>T), L1876A (5776T>G 5777T>C 5778G>T), F1877L (5781T>G), A1878E (5783C>A 5784A>G), S1879T (5786G>C 5787T>A), E1881S (5791G>T 5792A>C 5793A>T), K1882V (5794A>G 5795A>T 5796G>C), C1883S (5797T>A), E1886T (5806G>A 5807A>C 5808A>T), L1888A (5812C>G 5813T>C 5814T>C), K1890R (5819A>G 5820G>A), N1891G (5821A>G 5822A>G 5823C>A), T1892V (5824A>G 5825C>T 5826G>C), L1894V (5830C>G), E1899K (5845G>A 5847G>A), K1900E (5848A>G), M1901F (5851A>T 5853G>C), T1902S (5854A>T 5856T>C), K1904H (5860A>C 5862A>C), V1906C (5866G>T 5867T>G 5868G>T), A1907E (5870C>A), Q1908M (5872C>A 5873A>T 5874A>G), V1910L (5878G>C 5880C>T), S1911E (5881A>G 5882G>A 5883T>G), V1913L (5887G>C 5889A>G), K1914E (5890A>G), Q1915S (5893C>A 5894A>G 5895G>T), F1918V (5902T>G 5904C>A), Y1919V (5905T>G 5906A>T 5907T>A), P1920F (5908C>T 5909C>T), K1921M (5912A>T), A1922G (5915C>G 5916C>G), T1923S (5918C>G 5919A>C), T1924V (5920A>G 5921C>T 5922C>T), V1925K (5923G>A 5924T>A), E1926K (5926G>A), L1928M (5932C>A 5934C>G), K1929L (5935A>T 5936A>T), V1930S (5938G>T 5939T>C 5940A>C), A1932M (5944G>A 5945C>T 5946T>G), D1933N (5947G>A), A1934Q (5950G>C 5951C>A 5952T>A), H1935Q (5955T>G), H1935_T1936insM (5955_5956insATG), T1936S (5956A>T 5958C>A), I1937K (5960T>A 5961T>A), V1938T (5962G>A 5963T>C), K1939Q (5965A>C), T1940V (5968A>G 5969C>T), K1942S (5975A>G 5976A>C), F1944C (5981T>G 5982C>T), R1945C (5983C>T 5985G>T), G1946del (5986_5988delGGA), V1948T (5992G>A 5993T>C 5994T>A), R1950S (5998C>A 6000G>T), N1952A (6004A>G 6005A>C 6006T>A), F1953W (6008T>G 6009T>G), S1955T (6014G>C), P1956K (6016C>A 6017C>A 6018T>G), E1957D (6021G>C), W1958I (6022T>A 6023G>T 6024G>T), W1958_K1959insV (6024_6025insGTC), K1959M (6026A>T 6027A>G), I1960T (6029T>C 6030T>A), G1961K (6031G>A 6032G>A), E1963K (6037G>A), N1964K (6042C>G), T1965P (6043A>C), M1966del (6046_6048delATG), A1967G (6050C>G), T1968S (6052A>T 6054A>T), L1969I (6055C>A 6057C>T), S1970P (6058T>C 6060A>T), S1973T (6068G>C 6069T>A), L1974V (6070C>G 6072T>G), L1975F (6073C>T), P1976L (6077C>T 6078A>T), L1979P (6086T>C 6087G>T), S1982L (6094T>C 6095C>T), A1983V (6098C>T 6099C>T), M1984V (6100A>G), K1985R (6104A>G 6105G>A), L1987M (6109T>A), S1989R (6115A>C), R1990N (6119G>A 6120A>T), Y1991S (6121T>A 6122A>G 6123C>T), K1992G (6124A>G

	6125A>G), R1993K (6128G>A 6129A>G), N1994S (6131A>G), M1995Y (6133A>T 6134T>A 6135G>C), H1998T (6142C>A 6143A>C 6144T>A), C1999T (6145T>A 6146G>C 6147C>A), I2000L (6148A>C 6150A>G), K2001E (6151A>G), L2004F (6160C>T 6162C>T), K2005R (6164A>G 6165G>A), I2006M (6168A>G), V2008M (6172G>A 6174T>G), L2009_N2010del (6175_6180delCTCAAC), F2011C (6182T>G 6183C>T), M2013W (6187A>T 6188T>G), L2014V (6190C>G 6192G>A), N2015H (6193A>C 6195T>C)

	Begin	End	Coverage	Score	Concordance	Matches	Identities	I/D/M/F*	Stop Codons
**NT**	**25**	**6200**	**85.3%**	**3315**	**27.4%**	**6117 (98.7%)**	**3941 (63.6%)**	**21/59**	

GCT2GCA (156T>A), CTT3TTT (157C>T), AAA5AAG (165A>G), TTT7TGG (170T>G 171T>G), GGT8AGT (172G>A), TCT9CCT (175T>C), TCA10CCT (178T>C 180A>T), TCT11GCT (181T>G), GAG12_GTT17del (184_201delGAGAAGACTGGATCAGTT), CCC18TCT (202C>T 204C>T), TCT19TCA (207T>A), ACT20CCT (208A>C), TCT21TCA (213T>A), GGT24GCA (221G>C 222T>A), CTG26GTT (226C>G 228G>T), GGT28TCT (232G>T 233G>C), ATC30TTT (238A>T 240C>T), TCA31TCT (243A>T), AAA32TCT (244A>T 245A>C 246A>T), TTC33CTT (247T>C 249C>T), AAA35AAG (255A>G), GCT36ACT (256G>A), ATT37CTC (259A>C 261T>C), AGC38TCC (262A>T 263G>C), AAC39AAT (267C>T), ATC40GTT (268A>G 270C>T), ACA41AAT (272C>A 273A>T), TCA42AAA (274T>A 275C>A), GTA43GTT (279A>T), AAA46ACC (287A>C 288A>C), TCT48GTA (292T>G 293C>T 294T>A), CAT50CAC (300T>C), TTA51ATA (301T>A), GAT52GAG (306T>G), TTG54GTC (310T>G 312G>C), AAA55AAG (315A>G), CCA56CCG (318A>G), TCT57CCA (319T>C 321T>A), GTG58GTT (324G>T), ACT59ATG (326C>T 327T>G), GAT60GTA (329A>T 330T>A), TCT62GCA (334T>G 336T>A), TCT63ACA (337T>A 339T>A), TCT64TCA (342T>A), GTT66GCC (347T>C 348T>C), TGT69TGC (357T>C), TCC70AAT (358T>A 359C>A 360C>T), GCA71GCT (363A>T), TTG72GTA (364T>G 366G>A), AAT73GAA (367A>G 369T>A), AAA74AGA (371A>G), GTG75GTT (375G>T), CTT76TTA (376C>T 378T>A), ATA79ATT (387A>T), GGT80GCA (389G>C 390T>A), TCC81AAA (391T>A 392C>A 393C>A), TTG82ATA (394T>A 396G>A), ATT83ATG (399T>G), CCT85CCC (405T>C), TTT86TTC (408T>C), CTG87ATC (409C>A 411G>C), AAA88AAG (414A>G), GCA89GGT (416C>G 417A>T), TAC90TAT (420C>T), TCT91GAT (421T>G 422C>A), TTG93GTA (427T>G 429G>A), GCA94AAA (430G>A 431C>A), ACC95ACT (435C>T), TCT99ACT (445T>A), ATC100ATA (450C>A), AAG101GTA (451A>G 452A>T 453G>A), ATG103CTT (457A>C 459G>T), GTT104GTA (462T>A), TTA105CTT (463T>C 465A>T), CGG106AAG (466C>A 467G>A), TTG107ATG (469T>A), TTC108GTT (472T>G 474C>T), GAA109TTA (475G>T 476A>T), TTG111GTC (481T>G 483G>C), TCC112AAG (484T>A 485C>A 486C>G), AAT113GAT (487A>G), GTT115TTC (493G>T 495T>C), AGA116ATC (497G>T 498A>C), ATA117CTA (499A>C), TTT119TAC (506T>A 507T>C), CAG120GAA (508C>G 510G>A), GTT122TTA (514G>T 516T>A), GAG124GAA (522G>A), AAG125AAA (525G>A), AGT126GTT (526A>G 527G>T), GAT128TCA (532G>T 533A>C 534T>A), GTA129TTA (535G>T), ATT131ATA (543T>A), GTA132GTG (546A>G), GTT133TTT (547G>T), TAT134ATA (550T>A 551A>T 552T>A), GGT135GCT (554G>C), TTG137TTA (561G>A), GCA140GCT (570A>T), ATA141TTG (571A>T 573A>G), ACT142GCC (574A>G 576T>C), CTT143ATT (577C>A), CTT144TTG (580C>T 582T>G), GTT145CTA (583G>C 585T>A), TTG146CTA (586T>C 588G>A), TTG147CTG (589T>C), TGT148TAT (593G>A), GTT149GTA (597T>A), TCA152GTA (604T>G 605C>T), GAC153GAT (609C>T), ATA154ATT (612A>T), ATT155GTA (613A>G 615T>A), GAA156ACG (616G>A 617A>C 618A>G), GGA157CCT (619G>C 620G>C 621A>T), GTA158TTT (622G>T 624A>T), TCT159AAT (625T>A 626C>A), AGT160AAT (629G>A), ACC161GTA (631A>G 632C>T 633C>A), ATT162GTT (634A>G), AAA163GTT (637A>G 638A>T 639A>T), AAT164ACT (641A>C), ATT165TTG (643A>T 645T>G), TTC166TTT (648C>T), TTA167TCA (650T>C), ATA168GTT (652A>G 654A>T), GTA169GTT (657A>T), GGG170GGA (660G>A), ATG172TTT (664A>T 666G>T), TTT173TTC (669T>C), AGT175TCA (673A>T 674G>C 675T>A), AAC178AAT (684C>T), TTG179TTA (687G>A), GAC180GAT (690C>T), CCC183CCA (699C>A), AAG184ACA (701A>C 702G>A), GCT186TCT (706G>T), GAG187GGG (710A>G), TTC189TTT (717C>T), ACA190TCC (718A>T 720A>C), ATG191CAG (721A>C 722T>A), GTA192CTG (724G>C 726A>G), GCA193GCG (729A>G), AGT196GCA (736A>G 737G>C 738T>A), ATG197GTA (739A>G 741G>A), CTA198CTT (744A>T), GAG201GAA (753G>A), TCT202AGC (754T>A 755C>G 756T>C), GTC203TTA (757G>T 759C>A), TCA204ACG (760T>A 762A>G), CAC205CAT (765C>T), ACT206ACA (768T>A), ACA208ACT (774A>T), TCC209AGC (775T>A 776C>G), CAG210CAA (780G>A), TTG211CTT (781T>C 783G>T), ATG212TTA (784A>T 786G>A), TCA213TCC (789A>C), ACT214TCA (790A>T 792T>A), ATT215TTG (793A>T 795T>G), ATG216TTG (796A>T), GCA217GCT (801A>T), ATT220ATC (810T>C), TCC221TCA (813C>A), ACG222ACT (816G>T), CTC223TTA (817C>T 819C>A), GTG224GTA (822G>A), ATA226GTT (826A>G 828A>T), GCA227GCT (831A>T), GTG228GTT (834G>T), CCA229CCT (837A>T), GGA230GGG (840A>G), CCC232CCA (846C>A), TTG235CTT (853T>C 855G>T), AGC236TCA (856A>T 857G>C 858C>A), AAT237AAC (861T>C), CCC238CCA (864C>A), CTC239CTG (867C>G), ATT242ATC (876T>C), TCA245TCT (885A>T), ACT246GCT (886A>G), GGG247GGA (891G>A), GGG249GGT (897G>T), GCT250GCG (900T>G), CAA255CAG (915A>G), TTG256CTG (916T>C), TTT257TTC (921T>C), ACA258CAG (922A>C 923C>A 924A>G), AGA261AGG (933A>G), AAT262AAC (936T>C), AAA264AAG (942A>G), TGC266TGT (948C>T), ACT267ACA (951T>A), TCA268TCT (954A>T), GCA270GCC (960A>C), TTT271TTC (963T>C), TCT272TCA (966T>A), GTG274GTT (972G>T), GAA276GAG (978A>G), ATT277ATA (981T>A), ATA278GTG (982A>G 984A>G), GGT280AGC (988G>A 990T>C), GCT281ATT (991G>A 992C>T), TTC284TTT (1002C>T), AAG285AAA (1005G>A), GTG288GTA (1014G>A), CTT289TTG (1015C>T 1017T>G), TCT290TCA (1020T>A), GCA291GCG (1023A>G), ATT292ATA (1026T>A), AGC293AAT (1028G>A 1029C>T), GCA294GCT (1032A>T), ACA295ACC (1035A>C), TTG296TTA (1038G>A), TCT297TCC (1041T>C), ACT298ACC (1044T>C), TTG300CTT (1048T>C 1050G>T), GAA302CAG (1054G>C 1056A>G), CAG305ACG (1063C>A 1064A>C), GTG307GTT (1071G>T), GAT308TCG (1072G>T 1073A>C 1074T>G), GCT309GAG (1076C>A 1077T>G), TGT311GCT (1081T>G 1082G>C), GAT312GAA (1086T>A), GCT314TCT (1090G>T), ACC315ATG (1094C>T 1095C>G), AGG316CGC (1096A>C 1098G>C), TTG317CTT (1099T>C 1101G>T), GAG318GAA (1104G>A), AAC319AAT (1107C>T), GCC321GCA (1113C>A), AAT322AAC (1116T>C), AAA323CCT (1117A>C 1118A>C 1119A>T), GCC324GCA (1122C>A), TTC325TTT (1125C>T), CCT326CCA (1128T>A), GAC327ATT (1129G>A 1130A>T 1131C>T), AAA328AAG (1134A>G), AAT330CAA (1138A>C 1140T>A), CAC331CAT (1143C>T), CTT332TTA (1144C>T 1146T>A), AGG333AAA (1148G>A 1149G>A), GAG334GAA (1152G>A), GCA336GCC (1158A>C), CTG337GTC (1159C>G 1161G>C), AAG338AAA (1164G>A), ATT339ATA (1167T>A), GCA341GCT (1173A>T), ACA345ACC (1185A>C), CAC346CAT (1188C>T), GTT348GTC (1194T>C), GCC349GCA (1197C>A), ATG352TTG (1204A>T), CAT354CAC (1212T>C), CGA355AGG (1213C>A 1215A>G), GTG356GTT (1218G>T), AGT357TCT (1219A>T 1220G>C), GCA358GCT (1224A>T), GCT359GCC (1227T>C), ATC360ATA (1230C>A), ACT361ACG (1233T>G), CTT363CTA (1239T>A), GAA364GAT (1242A>T), AAG365AAA (1245G>A), GTC366CTG (1246G>C 1248C>G), TAT367GTT (1249T>G 1250A>T), GCG368GCT (1254G>T), AGT370GCC (1258A>G 1259G>C 1260T>C), TGC371AGC (1261T>A), AGA372CAA (1264A>C 1265G>A), GTT374TCT (1270G>T 1271T>C), GTT376GTG (1278T>G), GGT377GGC (1281T>C), CAG378CAA (1284G>A), AGA380AGG (1290A>G), GAA382GAG (1296A>G), CCC383CCT (1299C>T), GTT386GTG (1308T>G), TAT389TTT (1316A>T), GGT390GGA (1320T>A), GCC391GCT (1323C>T), GGG393GGA (1329G>A), ACA398ACG (1344A>G), GCA399TGT (1345G>T 1346C>G 1347A>T), CGA401CGC (1353A>C), TTG402CTT (1354T>C 1356G>T), TTG408CTC (1372T>C 1374G>C), GAT409GAC (1377T>C), CGC410GCA (1378C>G 1379G>C 1380C>A), GGG412GGT (1386G>T), GAG413GAA (1389G>A), CCT414CCA (1392T>A), CTT416CTG (1398T>G), AGA418CGC (1402A>C 1404A>C), CTT419TTG (1405C>T 1407T>G), GCT421GCG (1413T>G), GTT422GTA (1416T>A), AGC423AGT (1419C>T), AAG424AAA (1422G>A), CGC425AGA (1423C>A 1425C>A), GCC427GCA (1431C>A), TCT430TCC (1440T>C), AAT431AAC (1443T>C), GCC433GCA (1449C>A), CAC434CAT (1452C>T), CAG435CAA (1455G>A), GCT437GCA (1461T>A), TTG439CTT (1465T>C 1467G>T), GAC442GAT (1476C>T), GGA444GGT (1482A>T), GCA445GCT (1485A>T), TTG446CTT (1486T>C 1488G>T), AGA447CGG (1489A>C 1491A>G), GGA451GGG (1503A>G), CAA452CAG (1506A>G), CAA454CAG (1512A>G), ATT456ATA (1518T>A), ATT462ATC (1536T>C), TCC464TCT (1542C>T), ACA465ACC (1545A>C), CCA467CCT (1551A>T), CCA469CCT (1557A>T), CTG470CTT (1560G>T), CCC471CCA (1563C>A), GCA473GCT (1569A>T), GCT474GCA (1572T>A), GTG475GTC (1575G>C), GAA476GAG (1578A>G), GAC477GAT (1581C>T), AAG478AAA (1584G>A), GGG479GGA (1587G>A), CAT481CAC (1593T>C), ACT483ACA (1599T>A), TAT486TAC (1608T>C), GCA489GCC (1617A>C), CTT493TTG (1627C>T 1629T>G), GTA494ATA (1630G>A), TCA495TCT (1635A>T), GCA496GCT (1638A>T), CCT497CCA (1641T>A), GCC498GCT (1644C>T), CTT502CTA (1656T>A), ACC503ACG (1659C>G), GCT507GCA (1671T>A), GAG509GAA (1677G>A), CGT510AGG (1678C>A 1680T>G), AGA511AGG (1683A>G), AGG512AGA (1686G>A), ATC514ATT (1692C>T), TGT518TGC (1704T>C), ATC519AGA (1706T>G 1707C>A), GAA521GTG (1712A>T 1713A>G), GGT522GGC (1716T>C), GAA523GAT (1719A>T), TCT525GAT (1723T>G 1724C>A), ACA526CCA (1726A>C), GAG527AAT (1729G>A 1731G>T), GAC528ACT (1732G>A 1733A>C 1734C>T), ACC530ACA (1740C>A), GGA531GCC (1742G>C 1743A>C), CTG533CTA (1749G>A), ATG534ATT (1752G>T), AAC536GAT (1756A>G 1758C>T), ATT537GTG (1759A>G 1761T>G), CGG541AGA (1771C>A 1773G>A), AGG542AGA (1776G>A), ATA546ATC (1788A>C), CAC550GCT (1798C>G 1799A>C 1800C>T), AGT552TCC (1804A>T 1805G>C 1806T>C), TAC553TAT (1809C>T), TCG554GAT (1810T>G 1811C>A 1812G>T), TTA557TTG (1821A>G), GAC558CAA (1822G>C 1824C>A), GCC562GCA (1836C>A), TGT564TGC (1842T>C), CAG565CGG (1844A>G), GTT566GTG (1848T>G), CAT567CAC (1851T>C), GCT568TAT (1852G>T 1853C>A), ATA570GTG (1858A>G 1860A>G), AAG572AGC (1865A>G 1866G>C), CAG573CAA (1869G>A), CTG574CTT (1872G>T), TTT575TAC (1874T>A 1875T>C), GAA576AAT (1876G>A 1878A>T), GCA577ACT (1879G>A 1881A>T), GAA578ACA (1882G>A 1883A>C), ACT579TCG (1885A>T 1887T>G), GGC580GGA (1890C>A), ATT581TTG (1891A>T 1893T>G), TCA582GCT (1894T>G 1896A>T), AAT583GAC (1897A>G 1899T>C), ACA585GTT (1903A>G 1904C>T 1905A>T), GCC586GCT (1908C>T), GTT588GTC (1914T>C), GCT589AAT (1915G>A 1916C>A), GTA590GTT (1920A>T), TCT591GCT (1921T>G), TCT592GTT (1924T>G 1925C>T), GAA593GAT (1929A>T), GAG594GAA (1932G>A), GTT595GTG (1935T>G), GTG596CTG (1936G>C), GCA597GAT (1940C>A 1941A>T), GTT599TTC (1945G>T 1947T>C), GAT600CAA (1948G>C 1950T>A), GGG601GAT (1952G>A 1953G>T), GCT602GCC (1956T>C), CGC603del (1957_1959delCGC), TTC604TTA (1962C>A), AAA605CAA (1963A>C), ACA606GTT (1966A>G 1967C>T 1968A>T), AAA607TTG (1969A>T 1970A>T 1971A>G), CAA608CCT (1973A>C 1974A>T), GAT609CCT (1975G>C 1976A>C), GTG610GAG (1979T>A), CCA611TCA (1981C>T), ATA612GCA (1984A>G 1985T>C), GTG613GCT (1988T>C 1989G>T), ACT614TAT (1990A>T 1991C>A), CCC615GAG (1993C>G 1994C>A 1995C>G), ACA616CAG (1996A>C 1997C>A 1998A>G), GTT617ACA (1999G>A 2000T>C 2001T>A), ATA618ATT (2004A>T), GAG620GAA (2010G>A), GAA621GCT (2012A>C 2013A>T), GAT622GAA (2016T>A), AGA623AAA (2018G>A), GTG624ATT (2020G>A 2022G>T), ATT625GTT (2023A>G), TCT627TCC (2031T>C), AAA628CAG (2032A>C 2034A>G), TTA630CTC (2038T>C 2040A>C), GTG632GTT (2046G>T), GAA633GAG (2049A>G), CTT635CTC (2055T>C), TTT637TAT (2060T>A), TAT639TAC (2067T>C), CAG640CAA (2070G>A), GGC641GGG (2073C>G), TTG643ATG (2077T>A), GAC644AAT (2080G>A 2082C>T), CCG645AAA (2083C>A 2084C>A 2085G>A), GAG646TTT (2086G>T 2087A>T 2088G>T), AGT647GAG (2089A>G 2090G>A 2091T>G), TTG648TTT (2094G>T), TTT649TTC (2097T>C), CCT650CCA (2100T>A), TAT653CTC (2107T>C 2108A>T 2109T>C), CAA654TCC (2110C>T 2111A>C 2112A>C), AAA655AAG (2115A>G), CAA656CAG (2118A>G), CTC658ATC (2122C>A), TTT659TAT (2126T>A), TCC661GTG (2131T>G 2132C>T 2133C>G), GAT663TCT (2137G>T 2138A>C), GCA664TCT (2140G>T 2142A>T), GAG665GAT (2145G>T), CAC666GCC (2146C>G 2147A>C), CAG667AAA (2149C>A 2151G>A), CAA668CAG (2154A>G), GTC669AAT (2155G>A 2156T>A 2157C>T), TTC670TTT (2160C>T), ATG671GAA (2161A>G 2162T>A 2163G>A), AGA672ATG (2165G>T 2166A>G), ATG674GTA (2170A>G 2172G>A), ACT675AAT (2174C>A), GAC676GAT (2178C>T), ATG677TTG (2179A>T), ATC678TTG (2182A>T 2184C>G), TAC679GTC (2185T>G 2186A>T), GGT681GGA (2193T>A), GCC682GCA (2196C>A), AAT683AGT (2198A>G), GCT684CCT (2200G>C), GAA685CAG (2203G>C 2205A>G), CAA686CAG (2208A>G), TAC687TAT (2211C>T), AGG688GCT (2212A>G 2213G>C 2214G>T), CAA692CGA (2225A>G), AAT693AAC (2229T>C), CTC699ATC (2245C>A), TTT702TTC (2256T>C), AAA703AAG (2259A>G), AGC704AGT (2262C>T), ATC706ATT (2268C>T), GCT708AGC (2272G>A 2273C>G 2274T>C), TCC709ACA (2275T>A 2277C>A), ATC711ATT (2283C>T), TGT712TCT (2285G>C), AAG714GAG (2290A>G), AAG715CTG (2293A>C 2294A>T), GCT717GAT (2300C>A), GTA718GTC (2304A>C), ACA720CGC (2308A>C 2309C>G 2310A>C), GAG721AAT (2311G>A 2313G>T), ATG722CTT (2314A>C 2316G>T), CGC723GAG (2317C>G 2318G>A 2319C>G), ACA724CAA (2320A>C 2321C>A), GGA725GGG (2325A>G), TTT726AAA (2326T>A 2327T>A 2328T>A), GCA727GCG (2331A>G), CAT728TAT (2332C>T), AAT729AAG (2337T>G), TGT730GCG (2338T>G 2339G>C 2340T>G), ATT731CTT (2341A>C), GAT732CAG (2344G>C 2346T>G), GTT735GCA (2354T>C 2355T>A), TTG738CTT (2362T>C 2364G>T), ATT739ACG (2366T>C 2367T>G), TGC740TGT (2370C>T), ATC741ATT (2373C>T), CAG742GAA (2374C>G 2376G>A), TTT747AGA (2389T>A 2390T>G 2391T>A), GTG748GTA (2394G>A), TTT750TTC (2400T>C), TTG751CTG (2401T>C), ACA753ACT (2409A>T), GCC754GCT (2412C>T), CGT757CGC (2421T>C), TAT758TAC (2424T>C), ACC761AGG (2432C>G 2433C>G), TCC764GCA (2440T>G 2442C>A), TCC764_AAA765insGGT (2442_2443insGGT), AAA765ACT (2444A>C 2445A>T), GAG766GAC (2448G>C), GCC767AAG (2449G>A 2450C>A 2451C>G), AAA768AAG (2454A>G), GAC769AGA (2455G>A 2456A>G 2457C>A), TCT770AAT (2458T>A 2459C>A), GTC772ATT (2464G>A 2466C>T), AAG773ATC (2468A>T 2469G>C), ATA774GAA (2470A>G 2471T>A), TGT775TGC (2475T>C), TAC776ATG (2476T>A 2477A>T 2478C>G), CAC777ATT (2479C>A 2480A>T 2481C>T), AAA778GGT (2482A>G 2483A>G 2484A>T), ATT779ATC (2487T>C), GTG780AAG (2488G>A 2489T>A), GAT781GAC (2493T>C), TAC782AAA (2494T>A 2496C>A), AAA784ATC (2501A>T 2502A>C), GAA785CAG (2503G>C 2505A>G), ACT786ATG (2507C>T 2508T>G), TGG788AAT (2512T>A 2513G>A 2514G>T), AAC789GAC (2515A>G), TTA790TTG (2520A>G), CCA791CCC (2523A>C), TAT792TTC (2525A>T 2526T>C), GCA793ATC (2527G>A 2528C>T 2529A>C), CTG794TTG (2530C>T), AGG795AAG (2534G>A), GGC801GGA (2553C>A), CTA802CTG (2556A>G), ATC804TTG (2560A>T 2562C>G), ATG805ATA (2565G>A), GCG806GCA (2568G>A), GGA809GGC (2577A>C), TTG810ACC (2578T>A 2579T>C 2580G>C), TTT811ATG (2581T>A 2583T>G), GGG812AAT (2584G>A 2585G>A 2586G>T), GGA813GCC (2588G>C 2589A>C), ATA814GTT (2590A>G 2592A>T), ACA815ACT (2595A>T), TTT817TTC (2601T>C), CTT818ATT (2602C>A), GCT819ACT (2605G>A), TGT820TGC (2610T>C), TGG821CTC (2611T>C 2612G>T 2613G>C), CAA822AAG (2614C>A 2616A>G), AGC823GCA (2617A>G 2618G>C 2619C>A), AAT824GCA (2620A>G 2621A>C 2622T>A), TCT825ACT (2623T>A), TTT826TCC (2627T>C 2628T>C), CCA827CCT (2631A>T), AGT828TTA (2632A>T 2633G>T 2634T>A), AGT830GAT (2638A>G 2639G>A), GGA831GGC (2643A>C), GGT833GGG (2649T>G), GGT834GGC (2652T>C), GTC835GTA (2655C>A), ACA836ACC (2658A>C), AGC841AGT (2673C>T), TCT843TCC (2679T>C), AAA845AAG (2685A>G), AAG846AAA (2688G>A), AAC847AAT (2691C>T), AAG848AAA (2694G>A), GGC849GGG (2697C>G), CCC850AAG (2698C>A 2699C>A 2700C>G), TCT851ACT (2701T>A), CGT852CAT (2705G>A), CTG853TTC (2707C>T 2709G>C), AAG854GCA (2710A>G 2711A>C 2712G>A), AAT855AAA (2715T>A), TTG856TCC (2717T>C 2718G>C), GTG858CTA (2722G>C 2724G>A), CAG860CAA (2730G>A), AGT861GCG (2731A>G 2732G>C 2733T>G), AGC862GCA (2734A>G 2735G>C 2736C>A), GCT863GAT (2738C>A), ATT865AAC (2744T>A 2745T>C), ACA866CAT (2746A>C 2747C>A 2748A>T), CAA867GTG (2749C>G 2750A>T 2751A>G), GAA868GAC (2754A>C), GAA868_TGG869insCGG (2754_2755insCGG), GCT871GAA (2762C>A 2763T>A), GAA872CAG (2764G>C 2766A>G), GGG874GGA (2772G>A), GTC876GTT (2778C>T), AAC877AAT (2781C>T), CAA878GAA (2782C>G), GCA879AGT (2785G>A 2786C>G 2787A>T), CTC880CTG (2790C>G), AAA881CAC (2791A>C 2793A>C), AAG882AAA (2796G>A), AAT883AAC (2799T>C), ATG884TTG (2800A>T), ATT885GTT (2803A>G), GTA886GTT (2808A>T), CTG887TTG (2809C>T), CGG888AGA (2812C>A 2814G>A), CTT889ATT (2815C>A), GGA890GCT (2819G>C 2820A>T), GAG891GAA (2823G>A), GTG893GTC (2829G>C), TAC894TAT (2832C>T), ACA898ACG (2844A>G), GTG900GTT (2850G>T), TCA902TCT (2856A>T), GGG903GGT (2859G>T), ATA905ATT (2865A>T), ATG906CTC (2866A>C 2868G>C), GCA911GCC (2883A>C), TTC912TTT (2886C>T), CAC913AGA (2887C>A 2888A>G 2889C>A), AAT914AAC (2892T>C), GTG915ATG (2893G>A), AGA916AGG (2898A>G), GGA918GGC (2904A>C), ACA919ACT (2907A>T), ACC922TCC (2914A>T), ATT923ATC (2919T>C), ATC924ATT (2922C>T), CCT926GAG (2926C>G 2927C>A 2928T>G), AAC927CAT (2929A>C 2931C>T), TCA928TCC (2934A>C), AGG929ATT (2936G>T 2937G>T), TCA930TGT (2939C>G 2940A>T), AAG931AAT (2943G>T), TAC934TTC (2951A>T), AAC935AAT (2955C>T), CGG937ACC (2959C>A 2960G>C 2961G>C), GAA938CAA (2962G>C), TCC939TCA (2967C>A), AAG940AGG (2969A>G), GTC941TTG (2971G>T 2973C>G), ATT942ATA (2976T>A), GGA944GGT (2982A>T), ATC947GTG (2989A>G 2991C>G), CTG949TTG (2995C>T),

Codon mutations:

CTA950TTG (2998C>T 3000A>G), CGT951AGG (3001C>A 3003T>G), GTG952GTA (3006G>A), GGT953GGG (3009T>G), GAC954GAT (3012C>T), CCT955CCA (3015T>A), CCT960TCA (3028C>T 3030T>A), ATT962ATA (3036T>A), CGC963AGA (3037C>A 3039C>A), AAG964AAA (3042G>A), CCA967CCT (3051A>T), GAG971GAT (3063G>T), GTC972GTG (3066C>G), TGC973TGT (3069C>T), TTC974TAC (3071T>A), ACC975TCT (3073A>T 3075C>T), AAA976AAG (3078A>G), GGT977GGC (3081T>C), TCT978TCA (3084T>A), CAG979ATG (3085C>A 3086A>T), TTA981TTG (3093A>G), TGT982TGC (3096T>C), TGT983GTG (3097T>G 3098G>T 3099T>G), AGA984AGG (3102A>G), GCC985GCT (3105C>T), GTG986GTA (3108G>A), GCG987GCT (3111G>T), ACC989AAG (3116C>A 3117C>G), CCA991CCC (3123A>C), AGA992AAG (3125G>A 3126A>G), CTG993AAA (3127C>A 3128T>A 3129G>A), GGC994TCC (3130G>T 3131G>C), AAC995AAT (3135C>T), CTA996TTG (3136C>T 3138A>G), GAA997GAG (3141A>G), TTT998GTT (3142T>G), TTG999CTT (3145T>C 3147G>T), TTT1001TAT (3152T>A), TCC1006TCA (3168C>A), AAA1007AAG (3171A>G), TAT1009TAC (3177T>C), ACA1010ACC (3180A>C), GTC1011GAA (3182T>A 3183C>A), AAG1012AAA (3186G>A), GTG1013GTC (3189G>C), GAG1014ACA (3190G>A 3191A>C 3192G>A), GAG1016GGA (3197A>G 3198G>A), GAC1018GAT (3204C>T), TCT1019TCG (3207T>G), GCC1020AAT (3208G>A 3209C>A 3210C>T), AGT1021GCA (3211A>G 3212G>C 3213T>A), AAA1023GCT (3217A>G 3218A>C 3219A>T), TTG1024CTT (3220T>C 3222G>T), AGT1025AGC (3225T>C), TCG1026TCA (3228G>A), CAG1027CAA (3231G>A), CAA1028GTT (3232C>G 3233A>T 3234A>T), TCA1029GCA (3235T>G), GAA1031GAG (3243A>G), TAT1032TAC (3246T>C), CAT1033CAC (3249T>C), ATG1034ATT (3252G>T), GGA1036AGT (3256G>A 3258A>T), GGT1039GGA (3267T>A), GGA1042GGT (3276A>T), TTA1045CTC (3283T>C 3285A>C), CTT1046TTA (3286C>T 3288T>A), CCC1048CCA (3294C>A), AGT1049ACT (3296G>C), GTT1050GTG (3300T>G), AAA1053AAG (3309A>G), CAA1054CAG (3312A>G), CCT1055CCG (3315T>G), GTT1056TGC (3316G>T 3317T>G 3318T>C), GTG1058GTT (3324G>T), GGC1059GGA (3327C>A), ATT1060ATA (3330T>A), GCT1063GCA (3339T>A), TCA1064TCT (3342A>T), GGT1067GAT (3350G>A), ATT1068ACC (3353T>C 3354T>C), GCA1069GCT (3357A>T), GAA1071GAG (3363A>G), GGA1073GGT (3369A>T), TTC1074TAC (3371T>A), ATT1075GTG (3373A>G 3375T>G), TCT1076GCT (3376T>G), TCC1077TCA (3381C>A), AAT1078AAC (3384T>C), GCC1079GCA (3387C>A), ACG1080ACA (3390G>A), GCC1081GCA (3393C>A), ATC1082GTG (3394A>G 3396C>G), TTC1083TTT (3399C>T), CGG1084AGA (3400C>A 3402G>A), GAC1085GAT (3405C>T), CAG1086CAA (3408G>A), CTT1087TTG (3409C>T 3411T>G), AAC1089GAT (3415A>G 3417C>T), GGG1093GGA (3429G>A), CCT1094CCC (3432T>C), GTT1095ATT (3433G>A), GCG1097GTG (3440C>T), GCA1098GCT (3444A>T), CGA1101CGC (3453A>C), GAC1103GAA (3459C>A), ATT1104CTG (3460A>C 3462T>G), CTC1105TTG (3463C>T 3465C>G), AAA1106AAG (3468A>G), GCC1107CAC (3469G>C 3470C>A), ATT1108CTG (3472A>C 3474T>G), AGG1109AGA (3477G>A), AGT1110TCC (3478A>T 3479G>C 3480T>C), AGA1111AAG (3482G>A 3483A>G), GAA1112GAG (3486A>G), ACC1113GAA (3487A>G 3488C>A 3489C>A), ACT1114AGA (3491C>G 3492T>A), CTT1115TTC (3493C>T 3495T>C), GAA1117GAG (3501A>G), AAC1119AAA (3507C>A), CAA1120CAG (3510A>G), GTC1121GTG (3513C>G), TCC1122GCT (3514T>G 3516C>T), TTT1123TTC (3519T>C), CTT1124TTG (3520C>T 3522T>G), GTT1127GTA (3531T>A), CCT1128CCG (3534T>G), CAA1129TTG (3535C>T 3536A>T 3537A>G), TTG1131ATG (3541T>A), GCC1132GCT (3546C>T), GCA1133GCT (3549A>T), GTA1135GTG (3555A>G), CCC1136CCG (3558C>G), CAT1137CAC (3561T>C), ACT1139ACA (3567T>A), ACA1140ACC (3570A>C), CTC1141CTT (3573C>T), AGA1142AAA (3575G>A), AAG1143CGC (3577A>C 3578A>G 3579G>C), CAA1145CCC (3584A>C 3585A>C), TTG1146CTG (3586T>C), GCA1149GCT (3597A>T), GGT1151GGG (3603T>G), CCT1152GAC (3604C>G 3605C>A 3606T>C), GAA1154CAG (3610G>C 3612A>G), ACC1155ACT (3615C>T), GCT1156GCC (3618T>C), CCC1157CCA (3621C>A), TCA1158TCC (3624A>C), ATC1159ATT (3627C>T), CTC1160CTA (3630C>A), ACC1161TCT (3631A>T 3633C>T), GTC1162GCC (3635T>C), AAT1163AAA (3639T>A), CGG1166AGG (3646C>A), GAG1168GAA (3654G>A), GGA1169GGG (3657A>G), TTC1170TTT (3660C>T), CCA1172CCC (3666A>C), GCA1175GCT (3675A>T), GTG1177GTC (3681G>C), ATG1178GAG (3682A>G 3683T>A), AAA1179AAG (3687A>G), TAC1180TAT (3690C>T), ACT1183ACA (3699T>A), CAT1185TCC (3703C>T 3704A>C 3705T>C), GGT1186AAC (3706G>A 3707G>A 3708T>C), GAT1188GAG (3714T>G), GAT1189GAG (3717T>G), GAT1190CCA (3718G>C 3719A>C 3720T>A), GCT1192GCA (3726T>A), AAA1193AAG (3729A>G), TTG1194CTG (3730T>C), TCC1195GCT (3733T>G 3735C>T), GAA1197GAG (3741A>G), ACT1198GGT (3742A>G 3743C>G), CTC1199CTT (3747C>T), AGT1201GCA (3751A>G 3752G>C 3753T>A), TCA1202TCC (3756A>C), CTG1203ATT (3757C>A 3759G>T), CTG1204CTT (3762G>T), CCA1205CCT (3765A>T), ACA1206GTG (3766A>G 3767C>T 3768A>G), ATG1207CTT (3769A>C 3771G>T), AAA1208AAG (3774A>G), AAC1209ATG (3776A>T 3777C>G), CAA1210CAG (3780A>G), AAA1211CGA (3781A>C 3782A>G), GGC1215GGG (3795C>G), CCA1217CCT (3801A>T), CAG1218GCA (3802C>G 3803A>C 3804G>A), GAG1219ATC (3805G>A 3806A>T 3807G>C), GAT1221AAT (3811G>A), GAT1223GAC (3819T>C), GTG1224GTA (3822G>A), CTG1226CTC (3828G>C), ATT1229ATA (3837T>A), TGT1232TTT (3845G>T), TAT1235TTT (3854A>T), GGC1237GCA (3860G>C 3861C>A), CTC1240TTG (3868C>T 3870C>G), ACA1242ACG (3876A>G), TCC1243TCA (3879C>A), TAC1246TAT (3888C>T), TTC1248TTT (3894C>T), ATG1251TTG (3901A>T), GGC1252GGA (3906C>A), ATG1253CTT (3907A>C 3909G>T), GGA1254GGT (3912A>T), ATG1255TCT (3913A>T 3914T>C 3915G>T), AAG1257AAA (3921G>A), CGG1258AGG (3922C>A), GAG1259GAA (3927G>A), GTC1261GTG (3933C>G), GTA1262GAA (3935T>A), CCA1263GCC (3937C>G 3939A>C), ACA1264CAA (3940A>C 3941C>A), GGA1265CCA (3943G>C 3944G>C), GAA1266AAC (3946G>A 3948A>C), GGT1267TCG (3949G>T 3950G>C 3951T>G), GAT1268CAG (3952G>C 3954T>G), AAA1269AGG (3956A>G 3957A>G), GTA1270GTT (3960A>T), GAG1271CTT (3961G>C 3962A>T 3963G>T), CTT1272CTC (3966T>C), AGA1273ATA (3968G>T), GAC1275AAC (3973G>A), CCA1277CCT (3981A>T), GTG1278GTC (3984G>C), GAA1280GAT (3990A>T), GCT1281GCA (3993T>A), GAG1283GAA (3999G>A), GAA1284GAG (4002A>G), CTG1285TTG (4003C>T), ATT1287TCG (4009A>T 4010T>C 4011T>G), CAA1288CAG (4014A>G), ATT1289GTA (4015A>G 4017T>A), CAG1291CAA (4023G>A), ATA1293ACA (4028T>C), CAT1294TTT (4030C>T 4031A>T), GTG1295GTT (4035G>T), GAT1296GAC (4038T>C), CTA1297CTC (4041A>C), ATC1298GTC (4042A>G), ACC1299ACA (4047C>A), ACA1300ACC (4050A>C), CAA1301CAG (4053A>G), GCC1303GCG (4059C>G), AAG1304AAA (4062G>A), GAG1306GAA (4068G>A), CGC1307CGG (4071C>G), CTT1308TTG (4072C>T 4074T>G), CCT1309CCA (4077T>A), GAA1311GAG (4083A>G), AAA1318AAG (4104A>G), ACA1319ACC (4107A>C), AGA1320CGC (4108A>C 4110A>C), TTA1321CTG (4111T>C 4113A>G), TTT1322TTC (4116T>C), GAG1323GAA (4119G>A), ATA1324ATC (4122A>C), TAC1329TAT (4137C>T), GTC1333GTG (4149C>G), AGG1334CGG (4150A>C), TTT1337TTC (4161T>C), TCC1341TCT (4173C>T), GCC1342GCT (4176C>T), ACC1343TGT (4177A>T 4178C>G 4179C>T), GCA1346GCT (4188A>T), TTA1347GCA (4189T>G 4190T>C), AAT1349AAC (4197T>C), GTC1350GTA (4200C>A), ATC1351CTC (4201A>C), CCA1352CCT (4206A>T), TGT1353TGC (4209T>C), GTT1355GTA (4215T>A), GGA1356GGT (4218A>T), ATA1357ATC (4221A>C), GAT1358AAT (4222G>A), ACC1360ACG (4230C>G), AGT1361AGC (4233T>C), TCT1362CAT (4234T>C 4235C>A), TCT1365ACA (4243T>A 4245T>A), CTT1366GTG (4246C>G 4248T>G), CTT1367CTG (4251T>G), GCC1368GCT (4254C>T), AAC1369AGT (4256A>G 4257C>T), AAC1370AGC (4259A>G), TTC1371TTT (4263C>T), GCT1373TCA (4267G>T 4269T>A), ACT1377GAG (4279A>G 4280C>A 4281T>G), GGG1378GGA (4284G>A), TTT1379TTC (4287T>C), GCT1381GCC (4293T>C), TCA1384TCG (4302A>G), AGT1385AGC (4305T>C), GAC1387GAT (4311C>T), GGG1388GGA (4314G>A), CGC1389AGA (4315C>A 4317C>A), CCA1391CCC (4323A>C), GTG1392ATC (4324G>A 4326G>C), TTC1393TTT (4329C>T), GCT1394GCA (4332T>A), TTT1395TTC (4335T>C), CAG1396CAA (4338G>A), TTG1401CTG (4351T>C), GTT1402GTG (4356T>G), GAG1404GCT (4361A>C 4362G>T), TAT1405TTT (4364A>T), GAT1407GGT (4370A>G), ACC1408GAT (4372A>G 4373C>A 4374C>T), AAG1409GAG (4375A>G), GGA1411GGG (4383A>G), GAG1412AGT (4384G>A 4385A>G 4386G>T), GCT1414GAG (4391C>A 4392T>G), TCC1415TCA (4395C>A), GCA1417GCT (4401A>T), AGA1418AGG (4404A>G), CAT1419CAC (4407T>C), GCT1420ACA (4408G>A 4410T>A), CTT1421TTA (4411C>T 4413T>A), CTC1422TTA (4414C>T 4416C>A), GCA1425GCC (4425A>C), TCA1426TCT (4428A>T), CAC1428CAT (4434C>T), TAC1429TAT (4437C>T), TTA1431CTC (4441T>C 4443A>C), GAT1434AAC (4450G>A 4452T>C), AAA1435AAG (4455A>G), GTT1436TTG (4456G>T 4458T>G), TTC1437TTT (4461C>T), CGT1438CGC (4464T>C), TTG1439CTA (4465T>C 4467G>A), GTT1440GTG (4470T>G), GGC1442GGA (4476C>A), CCG1444CCC (4482G>C), GCA1448GCT (4494A>T), CTT1449TTG (4495C>T 4497T>G), ACC1450ACT (4500C>T), TTC1453TTT (4509C>T), AAC1454AAT (4512C>T), CTG1456TTG (4516C>T), TTG1457CTA (4519T>C 4521G>A), CGG1463AGG (4537C>A), TAT1464TAC (4542T>C), GCT1465GCG (4545T>G), TTC1466TTT (4548C>T), ATC1467ATA (4551C>A), CTT1469CTG (4557T>G), TTA1470TTG (4560A>G), AGA1471AGG (4563A>G), AGG1472AGA (4566G>A), CCT1473CCA (4569T>A), CAC1474CAT (4572C>T), ATT1475GTC (4573A>G 4575T>C), GCC1476AAA (4576G>A 4577C>A 4578C>A), GCT1477GCC (4581T>C), AGA1478AAA (4583G>A), GCT1479TCT (4585G>T), ATA1480GTG (4588A>G 4590A>G), GGT1481GGC (4593T>C), GTA1482ATT (4594G>A 4596A>T), CCT1484CCA (4602T>A), GGT1485AAT (4603G>A 4604G>A), GAT1486GAC (4608T>C), AAC1488AAT (4614C>T), CTT1490TTG (4618C>T 4620T>G), GGA1496GGT (4638A>T), AAT1499AAC (4647T>C), CTG1500TTG (4648C>T), GTG1501GTT (4653G>T), GCT1502GCG (4656T>G), GTG1503GTT (4659G>T), CCT1504CCA (4662T>A), CTG1505ATG (4663C>A), CTG1507TTG (4669C>T), TAT1510TAC (4680T>C), TCA1511GAT (4681T>G 4682C>A 4683A>T), CTG1512CTA (4686G>A), CCT1513CCA (4689T>A), AAT1514TCC (4690A>T 4691A>C 4692T>C), GCC1516GCA (4698C>A), CAG1517AGA (4699C>A 4700A>G 4701G>A), CTT1519CTG (4707T>G), GAC1520GAA (4710C>A), ATG1521GCT (4711A>G 4712T>C 4713G>T), GTA1522GTT (4716A>T), AAT1523AAC (4719T>C), ATC1526ATT (4728C>T), AAA1527AAG (4731A>G), GGG1529GGT (4737G>T), CTG1530TTG (4738C>T), GAT1531GAC (4743T>C), TCT1533TCA (4749T>A), AAC1537GAG (4759A>G 4761C>G), CAG1538AAG (4762C>A), GTG1539ACA (4765G>A 4766T>C 4767G>A), CAG1540AAA (4768C>A 4770G>A), TTC1541TTT (4773C>T), CAG1542CTG (4775A>T), GAC1543GAT (4779C>T), TCA1545TCT (4785A>T), GAA1546GAG (4788A>G), ACT1548ACC (4794T>C), AGC1551AGT (4803C>T), GGA1553GGT (4809A>T), TTT1554TTC (4812T>C), AAA1555AAG (4815A>G), CGT1556CGA (4818T>A), GCC1558GCA (4824C>A), CTT1559TTG (4825C>T 4827T>G), GGG1560GGA (4830G>A), CAC1562CAA (4836C>A), GTG1569GTT (4857G>T), ATA1571GTC (4861A>G 4863A>C), ATT1572ATC (4866T>C), CCT1574CCC (4872T>C), ATG1575ATT (4875G>T), TAC1576TAT (4878C>T), ATC1578ATT (4884C>T), AGA1579AGG (4887A>G), CCT1580CCA (4890T>A), CCG1582GTG (4894C>G 4895C>T), GGT1583GGA (4899T>A), ACT1587GTA (4909A>G 4910C>T 4911T>A), GTG1588CAA (4912G>C 4913T>A 4914G>A), GCA1589GCT (4917A>T), ATG1591TAT (4921A>T 4922T>A 4923G>T), GAG1592GAA (4926G>A), GTG1594ATT (4930G>A 4932G>T), ACT1596ACA (4938T>A), CTT1598ATC (4942C>A 4944T>C), CGT1599AGA (4945C>A 4947T>A), GAG1600GAA (4950G>A), TTT1602TTC (4956T>C), AGA1606CAG (4966A>C 4967G>A 4968A>G), GTT1607GCC (4970T>C 4971T>C), GCA1608ACT (4972G>A 4974A>T), GAG1610GAA (4980G>A), AAG1611AGT (4982A>G 4983G>T), TTA1612TTT (4986A>T), ACT1613GTA (4987A>G 4988C>T 4989T>A), TAT1614AAT (4990T>A), CAG1617TTT (4999C>T 5000A>T 5001G>T), TGT1618GGA (5002T>G 5004T>A), GCC1619GCT (5007C>T), AGA1621AAG (5012G>A 5013A>G), GAG1622CAG (5014G>C), CGT1623AGA (5017C>A 5019T>A), GGA1624AAT (5020G>A 5021G>A 5022A>T), GGC1626CCC (5026G>C 5027G>C), GCA1627ATC (5029G>A 5030C>T 5031A>C), ACA1628ATG (5033C>T 5034A>G), TTT1630ATG (5038T>A 5040T>G), CCT1631CCC (5043T>C), TCT1632ACT (5044T>A), TAT1633TAC (5049T>C), ATG1634TAT (5050A>T 5051T>A 5052G>T), GTG1636CTT (5056G>C 5058G>T), GAG1637GAA (5061G>A), CAG1638AGT (5062C>A 5063A>G 5064G>T), GAA1639CAA (5065G>C), ATC1641TTG (5071A>T 5073C>G), GCA1642ACA (5074G>A), AAG1643GAG (5077A>G), GCA1648GAG (5093C>A 5094A>G), TCA1649AGC (5095T>A 5096C>G 5097A>C), GCG1650ATT (5098G>A 5099C>T 5100G>T), TTG1651GGG (5101T>G 5102T>G), ACA1652GTT (5104A>G 5105C>T 5106A>T), ATC1653TTG (5107A>T 5109C>G), TGT1654ATG (5110T>A 5111G>T 5112T>G), GAA1655GAG (5115A>G), ATG1656CTA (5116A>C 5118G>A), GCC1657TCA (5119G>T 5121C>A), AAA1658GAT (5122A>G 5124A>T), TCT1660AAA (5128T>A 5129C>A 5130T>A), ATC1661AAT (5132T>A 5133C>T), ACT1662ACA (5136T>A), TTC1663TAC (5138T>A), ACA1664ACC (5142A>C), TTG1666TTA (5148G>A), CCA1667CCT (5151A>T), CCT1668GCT (5152C>G), AAT1670GAA (5158A>G 5160T>A), GTT1672ATT (5164G>A), GAA1673del (5167_5169delGAA), GAA1676GAG (5178A>G), AGA1677GAG (5179A>G 5180G>A 5181A>G), GAT1678GGT (5183A>G), CTC1679GTC (5185C>G), AAT1680CAT (5188A>C), TGG1681GAA (5191T>G 5192G>A 5193G>A), GCC1683GTG (5198C>T 5199C>G), CCA1684CCT (5202A>T), AAC1685GAT (5203A>G 5205C>T), ATT1686GTA (5206A>G 5208T>A), GGT1687TTT (5209G>T 5210G>T), TGC1689TGT (5217C>T), TCA1690AGT (5218T>A 5219C>G 5220A>T), GCC1691GCT (5223C>T), CGT1692CGG (5226T>G), GCC1695TCA (5233G>T 5235C>A), AAG1696AAC (5238G>C), ACT1697CAA (5239A>C 5240C>A 5241T>A), GAA1698CAA (5242G>C), GAG1700TCA (5248G>T 5249A>C 5250G>A), CCT1701TCA (5251C>T 5253T>A), GGG1702GGT (5256G>T), TGC1703AAC (5257T>A 5258G>A), ATA1704ATC (5262A>C), GTA1705CTG (5263G>C 5265A>G), AAC1707AAT (5271C>T), TGT1708TGC (5274T>C), ACA1709ACT (5277A>T), GGG1710GGA (5280G>A), GCG1711GCC (5283G>C), AAG1712CGC (5284A>C 5285A>G 5286G>C), AAG1713AGA (5288A>G 5289G>A), AGC1714ACA (5291G>C 5292C>A), AAA1715ACA (5294A>C), GTT1717GTG (5301T>G), CGC1718AGA (5302C>A 5304C>A), GGA1719GGG (5307A>G), GCT1721GCC (5313T>C), AGT1722AAT (5315G>A), GAT1725GAC (5325T>C), ATG1730ATA (5340G>A), CCA1732CCC (5346A>C), TAT1733TAC (5349T>C), ACC1734ACT (5352C>T), ATG1735TTG (5353A>T), TCA1736GCA (5356T>G), ATT1738ATC (5364T>C), CTG1739CAG (5366T>A), GCT1741CAT (5371G>C 5372C>A), AGA1743CGC (5377A>C 5379A>C), GGC1744AAG (5380G>A 5381G>A 5382C>G), AAA1745AGT (5384A>G 5385A>T), ATG1746CTG (5386A>C), TCA1747GAA (5389T>G 5390C>A), GAG1748GTT (5393A>T 5394G>T), GAC1749GGT (5396A>G 5397C>T), ATT1750GAT (5398A>G 5399T>A), GCT1751GGG (5402C>G 5403T>G), GAT1754TCT (5410G>T 5411A>C), GTT1755GTC (5415T>C), GTG1756ACC (5416G>A 5417T>C 5418G>C), GTG1758GTT (5424G>T), GGG1760GGT (5430G>T), GCT1764GCA (5442T>A), GTG1765ATA (5443G>A 5445G>A), AGC1766GCT (5446A>G 5447G>C 5448C>T), GCT1771GCA (5463T>A), TTA1772TTG (5466A>G), ATG1773TTT (5467A>T 5469G>T), GCT1774GCA (5472T>A), GTG1775TTG (5473G>T), CGT1778CAT (5483G>A), CAG1779CAA (5487G>A), TAT1780TAC (5490T>C), TGC1781AGG (5491T>A 5493C>G), ATT1782GCC (5494A>G 5495T>C 5496T>C), GAG1783ACA (5497G>A 5498A>C 5499G>A), GAT1784GAC (5502T>C), ATC1785CTT (5503A>C 5505C>T), AGG1788CGC (5512A>C 5514G>C), TTG1789CTA (5515T>C 5517G>A), CGC1790CGA (5520C>A), ACT1791ACC (5523T>C), ATT1792ATG (5526T>G), GGC1793GCT (5528G>C 5529C>T), AAT1794GGG (5530A>G 5531A>G 5532T>G), TTG1796CTG (5536T>C), ACA1800ACC (5550A>C), TAT1801TAC (5553T>C), CCC1802CCT (5556C>T), GTT1806GCG (5567T>C 5568T>G), CAG1807CAA (5571G>A), TTT1809TTG (5577T>G), CAA1811AAA (5581C>A), GTG1813GTT (5589G>T), CCT1814GAA (5590C>G 5591C>A 5592T>A), CAG1815GAC (5593C>G 5595G>C), CAG1815_GAA1816insACC (5595_5596insACC), GAA1816TCA (5596G>T 5597A>C), GGG1817CAC (5599G>C 5600G>A 5601G>C), AAA1818AAG (5604A>G), ATG1819TTG (5605A>T), GCA1820GGA (5609C>G), GTT1822AGT (5614G>A 5615T>G), TCG1824ACA (5620T>A 5622G>A), TCT1825del (5623_5625delTCT), TTG1826TAT (5627T>A 5628G>T), CAA1827GGC (5629C>G 5630A>G 5631A>C), AGT1828ACC (5633G>C 5634T>C), AGT1829ACT (5636G>C), ATG1831AAC (5642T>A 5643G>C), CAA1833CAT (5649A>T), CAA1833_GGT1834insCACAGT (5649_5650insCACAGT), GGT1834GCA (5651G>C 5652T>A), GAG1836TCA (5656G>T 5657A>C 5658G>A), CTT1837GTT (5659C>G), GGC1838GGA (5664C>A), CAA1839CTT (5666A>T 5667A>T), ATT1840CTA (5668A>C 5670T>A), CGT1841AGG (5671C>A 5673T>G), GTC1842ATA (5674G>A 5676C>A), ATC1843GTT (5677A>G 5679C>T), CAC1844CCT (5681A>C 5682C>T), GGT1845TCT (5683G>T 5684G>C), GAT1846GAC (5688T>C), TTA1847CTC (5689T>C 5691A>C), TCA1848TCT (5694A>T), ATG1849GAA (5695A>G 5696T>A 5697G>A), GAA1850AAG (5698G>A 5700A>G), TTG1853GTT (5707T>G 5709G>T), CGC1854CGT (5712C>T), ATG1855TTG (5713A>T), TAT1857TAC (5721T>C), GTT1858ACA (5722G>A 5723T>C 5724T>A), GTG1859ATT (5725G>A 5727G>T), GGC1860GGT (5730C>T), CCA1861CCT (5733A>T), CAT1862GAA (5734C>G 5736T>A), GGT1863GGA (5739T>A), GGA1864GGT (5742A>T), GGC1866GCC (5747G>C), AAC1867CAG (5749A>C 5751C>G), TTT1868TGC (5753T>G 5754T>C), ACC1869TCA (5755A>T 5757C>A), ACA1870ACG (5760A>G), AAA1871AAG (5763A>G), AAT1872AAC (5766T>C), CTT1873CTG (5769T>G), GAT1874AAG (5770G>A 5772T>G), CAA1875CAT (5775A>T), TTG1876GCT (5776T>G 5777T>C 5778G>T), TTT1877TTG (5781T>G), GCA1878GAG (5783C>A 5784A>G), AGT1879ACA (5786G>C 5787T>A), CTT1880TTG (5788C>T 5790T>G), GAA1881TCT (5791G>T 5792A>C 5793A>T), AAG1882GTC (5794A>G 5795A>T 5796G>C), TGT1883AGT (5797T>A), GAA1886ACT (5806G>A 5807A>C 5808A>T), TTG1887CTT (5809T>C 5811G>T), CTT1888GCC (5812C>G 5813T>C 5814T>C), AAG1890AGA (5819A>G 5820G>A), AAC1891GGA (5821A>G 5822A>G 5823C>A), ACG1892GTC (5824A>G 5825C>T 5826G>C), CTG1894GTG (5830C>G), ACC1895ACA (5835C>A), GAG1899AAA (5845G>A 5847G>A), AAA1900GAA (5848A>G), ATG1901TTC (5851A>T 5853G>C), ACT1902TCC (5854A>T 5856T>C), AAA1904CAC (5860A>C 5862A>C), GTG1906TGT (5866G>T 5867T>G 5868G>T), GCA1907GAA (5870C>A), CAA1908ATG (5872C>A 5873A>T 5874A>G), GTC1910CTT (5878G>C 5880C>T), AGT1911GAG (5881A>G 5882G>A 5883T>G), GTA1913CTG (5887G>C 5889A>G), AAA1914GAA (5890A>G),

	CAG1915AGT (5893C>A 5894A>G 5895G>T), TTC1918GTA (5902T>G 5904C>A), TAT1919GTA (5905T>G 5906A>T 5907T>A), CCC1920TTC (5908C>T 5909C>T), AAG1921ATG (5912A>T), GCC1922GGG (5915C>G 5916C>G), ACA1923AGC (5918C>G 5919A>C), ACC1924GTT (5920A>G 5921C>T 5922C>T), GTG1925AAG (5923G>A 5924T>A), GAA1926AAA (5926G>A), CAG1927CAA (5931G>A), CTC1928ATG (5932C>A 5934C>G), AAG1929TTG (5935A>T 5936A>T), GTA1930TCC (5938G>T 5939T>C 5940A>C), TTT1931TTC (5943T>C), GCT1932ATG (5944G>A 5945C>T 5946T>G), GAT1933AAT (5947G>A), GCT1934CAA (5950G>C 5951C>A 5952T>A), CAT1935CAG (5955T>G), CAT1935_ACC1936insATG (5955_5956insATG), ACC1936TCA (5956A>T 5958C>A), ATT1937AAA (5960T>A 5961T>A), GTC1938ACC (5962G>A 5963T>C), AAA1939CAA (5965A>C), ACT1940GTT (5968A>G 5969C>T), GCT1941GCC (5973T>C), AAA1942AGC (5975A>G 5976A>C), CCT1943CCA (5979T>A), TTC1944TGT (5981T>G 5982C>T), CGG1945TGT (5983C>T 5985G>T), GGA1946del (5986_5988delGGA), GTA1947GTC (5991A>C), GTT1948ACA (5992G>A 5993T>C 5994T>A), TTC1949TTT (5997C>T), CGG1950AGT (5998C>A 6000G>T), AAT1952GCA (6004A>G 6005A>C 6006T>A), TTT1953TGG (6008T>G 6009T>G), CTC1954CTA (6012C>A), AGT1955ACT (6014G>C), CCT1956AAG (6016C>A 6017C>A 6018T>G), GAG1957GAC (6021G>C), TGG1958ATT (6022T>A 6023G>T 6024G>T), TGG1958_AAA1959insGTC (6024_6025insGTC), AAA1959ATG (6026A>T 6027A>G), ATT1960ACA (6029T>C 6030T>A), GGA1961AAA (6031G>A 6032G>A), GAG1963AAG (6037G>A), AAC1964AAG (6042C>G), ACA1965CCA (6043A>C), ATG1966del (6046_6048delATG), GCA1967GGA (6050C>G), ACA1968TCT (6052A>T 6054A>T), CTC1969ATT (6055C>A 6057C>T), TCA1970CCT (6058T>C 6060A>T), GAG1972GAA (6066G>A), AGT1973ACA (6068G>C 6069T>A), CTT1974GTG (6070C>G 6072T>G), CTT1975TTT (6073C>T), CCA1976CTT (6077C>T 6078A>T), CTG1979CCT (6086T>C 6087G>T), TCG1980TCA (6090G>A), TCA1982CTA (6094T>C 6095C>T), GCC1983GTT (6098C>T 6099C>T), ATG1984GTG (6100A>G), AAG1985AGA (6104A>G 6105G>A), TTG1987ATG (6109T>A), CTC1988TTG (6112C>T 6114C>G), AGC1989CGC (6115A>C), AGA1990AAT (6119G>A 6120A>T), TAC1991AGT (6121T>A 6122A>G 6123C>T), AAA1992GGA (6124A>G 6125A>G), AGA1993AAG (6128G>A 6129A>G), AAC1994AGC (6131A>G), ATG1995TAC (6133A>T 6134T>A 6135G>C), TCT1996TCA (6138T>A), CAT1998ACA (6142C>A 6143A>C 6144T>A), TGC1999ACA (6145T>A 6146G>C 6147C>A), ATA2000CTG (6148A>C 6150A>G), AAA2001GAA (6151A>G), TTG2002CTT (6154T>C 6156G>T), GCA2003GCT (6159A>T), CTC2004TTT (6160C>T 6162C>T), AAG2005AGA (6164A>G 6165G>A), ATA2006ATG (6168A>G), TAT2007TAC (6171T>C), GTT2008ATG (6172G>A 6174T>G), CTC2009_AAC2010del (6175_6180delCTCAAC), TTC2011TGT (6182T>G 6183C>T), CAG2012CAA (6186G>A), ATG2013TGG (6187A>T 6188T>G), CTG2014GTA (6190C>G 6192G>A), AAT2015CAC (6193A>C 6195T>C), GAA2017CC. (6199G>C 6200A>C)

Proteins

polyprotein (YP_009165993.1)	1	2017	93.8%	9642	69.4%	2004 (99.0%)	1319 (65.2%)	7/13/0/0	0

	Begin	End	Coverage	Score	Concordance	Matches	Identities	I/D/M/F*	Stop Codons
**NT**	**25**	**6200**	**85.3%**	**3315**	**27.4%**	**6117 (98.7%)**	**3941 (63.6%)**	**21/59**	

Protein mutations:

L3F (157C>T), F7W (170T>G 171T>G), G8S (172G>A), S9P (175T>C), S10P (178T>C 180A>T), S11A (181T>G), E12_V17del (184_201delGAGAAGACTGGATCAGTT), P18S (202C>T 204C>T), T20P (208A>C), G24A (221G>C 222T>A), L26V (226C>G 228G>T), G28S (232G>T 233G>C), I30F (238A>T 240C>T), K32S (244A>T 245A>C 246A>T), F33L (247T>C 249C>T), A36T (256G>A), I37L (259A>C 261T>C), I40V (268A>G 270C>T), T41N (272C>A 273A>T), S42K (274T>A 275C>A), K46T (287A>C 288A>C), S48V (292T>G 293C>T 294T>A), L51I (301T>A), D52E (306T>G), L54V (310T>G 312G>C), S57P (319T>C 321T>A), T59M (326C>T 327T>G), D60V (329A>T 330T>A), S62A (334T>G 336T>A), S63T (337T>A 339T>A), V66A (347T>C 348T>C), S70N (358T>A 359C>A 360C>T), L72V (364T>G 366G>A), N73E (367A>G 369T>A), K74R (371A>G), G80A (389G>C 390T>A), S81K (391T>A 392C>A 393C>A), L82I (394T>A 396G>A), I83M (399T>G), L87I (409C>A 411G>C), A89G (416C>G 417A>T), S91D (421T>G 422C>A), L93V (427T>G 429G>A), A94K (430G>A 431C>A), S99T (445T>A), K101V (451A>G 452A>T 453G>A), M103L (457A>C 459G>T), R106K (466C>A 467G>A), L107M (469T>A), F108V (472T>G 474C>T), E109L (475G>T 476A>T), L111V (481T>G 483G>C), S112K (484T>A 485C>A 486C>G), N113D (487A>G), V115F (493G>T 495T>C), R116I (497G>T 498A>C), I117L (499A>C), F119Y (506T>A 507T>C), Q120E (508C>G 510G>A), V122L (514G>T 516T>A), S126V (526A>G 527G>T), D128S (532G>T 533A>C 534T>A), V129L (535G>T), V133F (547G>T), Y134I (550T>A 551A>T 552T>A), G135A (554G>C), I141L (571A>T 573A>G), T142A (574A>G 576T>C), L143I (577C>A), V145L (583G>C 585T>A), C148Y (593G>A), S152V (604T>G 605C>T), I155V (613A>G 615T>A), E156T (616G>A 617A>C 618A>G), G157P (619G>C 620G>C 621A>T), V158F (622G>T 624A>T), S159N (625T>A 626C>A), S160N (629G>A), T161V (631A>G 632C>T 633C>A), I162V (634A>G), K163V (637A>G 638A>T 639A>T), N164T (641A>C), I165L (643A>T 645T>G), L167S (650T>C), I168V (652A>G 654A>T), M172F (664A>T 666G>T), K184T (701A>C 702G>A), A186S (706G>T), E187G (710A>G), T190S (718A>T 720A>C), M191Q (721A>C 722T>A), V192L (724G>C 726A>G), S196A (736A>G 737G>C 738T>A), M197V (739A>G 741G>A), V203L (757G>T 759C>A), S204T (760T>A 762A>G), M212L (784A>T 786G>A), T214S (790A>T 792T>A), I215L (793A>T 795T>G), M216L (796A>T), I226V (826A>G 828A>T), T246A (886A>G), T258Q (922A>C 923C>A 924A>G), I278V (982A>G 984A>G), G280S (988G>A 990T>C), A281I (991G>A 992C>T), S293N (1028G>A 1029C>T), E302Q (1054G>C 1056A>G), Q305T (1063C>A 1064A>C), D308S (1072G>T 1073A>C 1074T>G), A309E (1076C>A 1077T>G), C311A (1081T>G 1082G>C), D312E (1086T>A), A314S (1090G>T), T315M (1094C>T 1095C>G), K323P (1117A>C 1118A>C 1119A>T), D327I (1129G>A 1130A>T 1131C>T), N330Q (1138A>C 1140T>A), R333K (1148G>A 1149G>A), L337V (1159C>G 1161G>C), M352L (1204A>T), E364D (1242A>T), V366L (1246G>C 1248C>G), Y367V (1249T>G 1250A>T), S370A (1258A>G 1259G>C 1260T>C), C371S (1261T>A), R372Q (1264A>C 1265G>A), V374S (1270G>T 1271T>C), Y389F (1316A>T), A399C (1345G>T 1346C>G 1347A>T), R410A (1378C>G 1379G>C 1380C>A), V494I (1630G>A), I519R (1706T>G 1707C>A), E521V (1712A>T 1713A>G), E523D (1719A>T), S525D (1723T>G 1724C>A), T526P (1726A>C), E527N (1729G>A 1731G>T), D528T (1732G>A 1733A>C 1734C>T), G531A (1742G>C 1743A>C), M534I (1752G>T), N536D (1756A>G 1758C>T), I537V (1759A>G 1761T>G), H550A (1798C>G 1799A>C 1800C>T), S554D (1810T>G 1811C>A 1812G>T), D558Q (1822G>C 1824C>A), Q565R (1844A>G), A568Y (1852G>T 1853C>A), I570V (1858A>G 1860A>G), K572S (1865A>G 1866G>C), F575Y (1874T>A 1875T>C), E576N (1876G>A 1878A>T), A577T (1879G>A 1881A>T), E578T (1882G>A 1883A>C), T579S (1885A>T 1887T>G), I581L (1891A>T 1893T>G), S582A (1894T>G 1896A>T), N583D (1897A>G 1899T>C), T585V (1903A>G 1904C>T 1905A>T), A589N (1915G>A 1916C>A), S591A (1921T>G), S592V (1924T>G 1925C>T), E593D (1929A>T), V596L (1936G>C), A597D (1940C>A 1941A>T), V599F (1945G>T 1947T>C), D600Q (1948G>C 1950T>A), G601D (1952G>A 1953G>T), R603del (1957_1959delCGC), F604L (1962C>A), K605Q (1963A>C), T606V (1966A>G 1967C>T 1968A>T), K607L (1969A>T 1970A>T 1971A>G), Q608P (1973A>C 1974A>T), D609P (1975G>C 1976A>C), V610E (1979T>A), P611S (1981C>T), I612A (1984A>G 1985T>C), V613A (1988T>C 1989G>T), T614Y (1990A>T 1991C>A), P615E (1993C>G 1994C>A 1995C>G), T616Q (1996A>C 1997C>A 1998A>G), V617T (1999G>A 2000T>C 2001T>A), E621A (2012A>C 2013A>T), D622E (2016T>A), R623K (2018G>A), V624I (2020G>A 2022G>T), I625V (2023A>G), K628Q (2032A>C 2034A>G), F637Y (2060T>A), L643M (2077T>A), D644N (2080G>A 2082C>T), P645K (2083C>A 2084C>A 2085G>A), E646F (2086G>T 2087A>T 2088G>T), S647E (2089A>G 2090G>A 2091T>G), L648F (2094G>T), Y653L (2107T>C 2108A>T 2109T>C), Q654S (2110C>T 2111A>C 2112A>C), L658I (2122C>A), F659Y (2126T>A), S661V (2131T>G 2132C>T 2133C>G), D663S (2137G>T 2138A>C), A664S (2140G>T 2142A>T), E665D (2145G>T), H666A (2146C>G 2147A>C), Q667K (2149C>A 2151G>A), V669N (2155G>A 2156T>A 2157C>T), M671E (2161A>G 2162T>A 2163G>A), R672M (2165G>T 2166A>G), M674V (2170A>G 2172G>A), T675N (2174C>A), M677L (2179A>T), I678L (2182A>T 2184C>G), Y679V (2185T>G 2186A>T), N683S (2198A>G), A684P (2200G>C), E685Q (2203G>C 2205A>G), R688A (2212A>G 2213G>C 2214G>T), Q692R (2225A>G), L699I (2245C>A), A708S (2272G>A 2273C>G 2274T>C), S709T (2275T>A 2277C>A), C712S (2285G>C), K714E (2290A>G), K715L (2293A>C 2294A>T), A717D (2300C>A), T720R (2308A>C 2309C>G 2310A>C), E721N (2311G>A 2313G>T), M722L (2314A>C 2316G>T), R723E (2317C>G 2318G>A 2319C>G), T724Q (2320A>C 2321C>A), F726K (2326T>A 2327T>A 2328T>A), H728Y (2332C>T), N729K (2337T>G), C730A (2338T>G 2339G>C 2340T>G), I731L (2341A>C), D732Q (2344G>C 2346T>G), V735A (2354T>C 2355T>A), I739T (2366T>C 2367T>G), Q742E (2374C>G 2376G>A), F747R (2389T>A 2390T>G 2391T>A), T761R (2432C>G 2433C>G), S764A (2440T>G 2442C>A), S764_K765insG (2442_2443insGGT), K765T (2444A>C 2445A>T), E766D (2448G>C), A767K (2449G>A 2450C>A 2451C>G), D769R (2455G>A 2456A>G 2457C>A), S770N (2458T>A 2459C>A), V772I (2464G>A 2466C>T), K773I (2468A>T 2469G>C), I774E (2470A>G 2471T>A), Y776M (2476T>A 2477A>T 2478C>G), H777I (2479C>A 2480A>T 2481C>T), K778G (2482A>G 2483A>G 2484A>T), V780K (2488G>A 2489T>A), Y782K (2494T>A 2496C>A), K784I (2501A>T 2502A>C), E785Q (2503G>C 2505A>G), T786M (2507C>T 2508T>G), W788N (2512T>A 2513G>A 2514G>T), N789D (2515A>G), Y792F (2525A>T 2526T>C), A793I (2527G>A 2528C>T 2529A>C), R795K (2534G>A), I804L (2560A>T 2562C>G), M805I (2565G>A), L810T (2578T>A 2579T>C 2580G>C), F811M (2581T>A 2583T>G), G812N (2584G>A 2585G>A 2586G>T), G813A (2588G>C 2589A>C), I814V (2590A>G 2592A>T), L818I (2602C>A), A819T (2605G>A), W821L (2611T>C 2612G>T 2613G>C), Q822K (2614C>A 2616A>G), S823A (2617A>G 2618G>C 2619C>A), N824A (2620A>G 2621A>C 2622T>A), S825T (2623T>A), F826S (2627T>C 2628T>C), S828L (2632A>T 2633G>T 2634T>A), S830D (2638A>G 2639G>A), P850K (2698C>A 2699C>A 2700C>G), S851T (2701T>A), R852H (2705G>A), L853F (2707C>T 2709G>C), K854A (2710A>G 2711A>C 2712G>A), N855K (2715T>A), L856S (2717T>C 2718G>C), V858L (2722G>C 2724G>A), S861A (2731A>G 2732G>C 2733T>G), S862A (2734A>G 2735G>C 2736C>A), A863D (2738C>A), I865N (2744T>A 2745T>C), T866H (2746A>C 2747C>A 2748A>T), Q867V (2749C>G 2750A>T 2751A>G), E868D (2754A>C), E868_W869insR (2754_2755insCGG), A871E (2762C>A 2763T>A), E872Q (2764G>C 2766A>G), Q878E (2782C>G), A879S (2785G>A 2786C>G 2787A>T), K881H (2791A>C 2793A>C), M884L (2800A>T), I885V (2803A>G), L889I (2815C>A), G890A (2819G>C 2820A>T), M906L (2866A>C 2868G>C), H913R (2887C>A 2888A>G 2889C>A), V915M (2893G>A), T922S (2914A>T), P926E (2926C>G 2927C>A 2928T>G), N927H (2929A>C 2931C>T), R929I (2936G>T 2937G>T), S930C (2939C>G 2940A>T), K931N (2943G>T), Y934F (2951A>T), R937T (2959C>A 2960G>C 2961G>C), E938Q (2962G>C), K940R (2969A>G), V941L (2971G>T 2973C>G), I947V (2989A>G 2991C>G), P960S (3028C>T 3030T>A), E971D (3063G>T), F974Y (3071T>A), T975S (3073A>T 3075C>T), Q979M (3085C>A 3086A>T), C983V (3097T>G 3098G>T 3099T>G), T989K (3116C>A 3117C>G), R992K (3125G>A 3126A>G), L993K (3127C>A 3128T>A 3129G>A), G994S (3130G>T 3131G>C), F998V (3142T>G), F1001Y (3152T>A), V1011E (3182T>A 3183C>A), E1014T (3190G>A 3191A>C 3192G>A), E1016G (3197A>G 3198G>A), A1020N (3208G>A 3209C>A 3210C>T), S1021A (3211A>G 3212G>C 3213T>A), K1023A (3217A>G 3218A>C 3219A>T), Q1028V (3232C>G 3233A>T 3234A>T), S1029A (3235T>G), M1034I (3252G>T), G1036S (3256G>A 3258A>T), S1049T (3296G>C), V1056C (3316G>T 3317T>G 3318T>C), G1067D (3350G>A), I1068T (3353T>C 3354T>C), F1074Y (3371T>A), I1075V (3373A>G 3375T>G), S1076A (3376T>G), I1082V (3394A>G 3396C>G), N1089D (3415A>G 3417C>T), V1095I (3433G>A), A1097V (3440C>T), D1103E (3459C>A), I1104L (3460A>C 3462T>G), A1107H (3469G>C 3470C>A), I1108L (3472A>C 3474T>G), R1111K (3482G>A 3483A>G), T1113E (3487A>G 3488C>A 3489C>A), T1114R (3491C>G 3492T>A), L1115F (3493C>T 3495T>C), N1119K (3507C>A), S1122A (3514T>G 3516C>T), Q1129L (3535C>T 3536A>T 3537A>G), L1131M (3541T>A), R1142K (3575G>A), K1143R (3577A>C 3578A>G 3579G>C), Q1145P (3584A>C 3585A>C), P1152D (3604C>G 3605C>A 3606T>C), E1154Q (3610G>C 3612A>G), T1161S (3631A>T 3633C>T), V1162A (3635T>C), N1163K (3639T>A), M1178E (3682A>G 3683T>A), H1185S (3703C>T 3704A>C 3705T>C), G1186N (3706G>A 3707G>A 3708T>C), D1188E (3714T>G), D1189E (3717T>G), D1190P (3718G>C 3719A>C 3720T>A), S1195A (3733T>G 3735C>T), T1198G (3742A>G 3743C>G), S1201A (3751A>G 3752G>C 3753T>A), L1203I (3757C>A 3759G>T), T1206V (3766A>G 3767C>T 3768A>G), M1207L (3769A>C 3771G>T), N1209M (3776A>T 3777C>G), K1211R (3781A>C 3782A>G), Q1218A (3802C>G 3803A>C 3804G>A), E1219I (3805G>A 3806A>T 3807G>C), D1221N (3811G>A), C1232F (3845G>T), Y1235F (3854A>T), G1237A (3860G>C 3861C>A), M1251L (3901A>T), M1253L (3907A>C 3909G>T), M1255S (3913A>T 3914T>C 3915G>T), V1262E (3935T>A), P1263A (3937C>G 3939A>C), T1264Q (3940A>C 3941C>A), G1265P (3943G>C 3944G>C), E1266N (3946G>A 3948A>C), G1267S (3949G>T 3950G>C 3951T>G), D1268Q (3952G>C 3954T>G), K1269R (3956A>G 3957A>G), E1271L (3961G>C 3962A>T 3963G>T), R1273I (3968G>T), D1275N (3973G>A), E1280D (3990A>T), I1287S (4009A>T 4010T>C 4011T>G), I1289V (4015A>G 4017T>A), I1293T (4028T>C), H1294F (4030C>T 4031A>T), I1298V (4042A>G), T1343C (4177A>T 4178C>G 4179C>T), L1347A (4189T>G 4190T>C), I1351L (4201A>C), D1358N (4222G>A), S1362H (4234T>C 4235C>A), S1365T (4243T>A 4245T>A), L1366V (4246C>G 4248T>G), N1369S (4256A>G 4257C>T), N1370S (4259A>G), A1373S (4267G>T 4269T>A), T1377E (4279A>G 4280C>A 4281T>G), V1392I (4324G>A 4326G>C), E1404A (4361A>C 4362G>T), Y1405F (4364A>T), D1407G (4370A>G), T1408D (4372A>G 4373C>A 4374C>T), K1409E (4375A>G), E1412S (4384G>A 4385A>G 4386G>T), A1414E (4391C>A 4392T>G), A1420T (4408G>A 4410T>A), D1434N (4450G>A 4452T>C), V1436L (4456G>T 4458T>G), I1475V (4573A>G 4575T>C), A1476K (4576G>A 4577C>A 4578C>A), R1478K (4583G>A), A1479S (4585G>T), I1480V (4588A>G 4590A>G), V1482I (4594G>A 4596A>T), G1485N (4603G>A 4604G>A), L1505M (4663C>A), S1511D (4681T>G 4682C>A 4683A>T), N1514S (4690A>T 4691A>C 4692T>C), Q1517R (4699C>A 4700A>G 4701G>A), D1520E (4710C>A), M1521A (4711A>G 4712T>C 4713G>T), N1537E (4759A>G 4761C>G), Q1538K (4762C>A), V1539T (4765G>A 4766T>C 4767G>A), Q1540K (4768C>A 4770G>A), Q1542L (4775A>T), H1562Q (4836C>A), I1571V (4861A>G 4863A>C), M1575I (4875G>T), P1582V (4894C>G 4895C>T), T1587V (4909A>G 4910C>T 4911T>A), V1588Q (4912G>C 4913T>A 4914G>A), M1591Y (4921A>T 4922T>A 4923G>T), V1594I (4930G>A 4932G>T), L1598I (4942C>A 4944T>C), R1606Q (4966A>C 4967G>A 4968A>G), V1607A (4970T>C 4971T>C), A1608T (4972G>A 4974A>T), K1611S (4982A>G 4983G>T), L1612F (4986A>T), T1613V (4987A>G 4988C>T 4989T>A), Y1614N (4990T>A), Q1617F (4999C>T 5000A>T 5001G>T), C1618G (5002T>G 5004T>A), R1621K (5012G>A 5013A>G), E1622Q (5014G>C), G1624N (5020G>A 5021G>A 5022A>T), G1626P (5026G>C 5027G>C), A1627I (5029G>A 5030C>T 5031A>C), T1628M (5033C>T 5034A>G), F1630M (5038T>A 5040T>G), S1632T (5044T>A), M1634Y (5050A>T 5051T>A 5052G>T), V1636L (5056G>C 5058G>T), Q1638S (5062C>A 5063A>G 5064G>T), E1639Q (5065G>C), I1641L (5071A>T 5073C>G), A1642T (5074G>A), K1643E (5077A>G), A1648E (5093C>A 5094A>G), A1650I (5098G>A 5099C>T 5100G>T), L1651G (5101T>G 5102T>G), T1652V (5104A>G 5105C>T 5106A>T), I1653L (5107A>T 5109C>G), C1654M (5110T>A 5111G>T 5112T>G), M1656L (5116A>C 5118G>A), A1657S (5119G>T 5121C>A), K1658D (5122A>G 5124A>T), S1660K (5128T>A 5129C>A 5130T>A), I1661N (5132T>A 5133C>T), F1663Y (5138T>A), P1668A (5152C>G), N1670E (5158A>G 5160T>A), V1672I (5164G>A), E1673del (5167_5169delGAA), R1677E (5179A>G 5180G>A 5181A>G), D1678G (5183A>G), L1679V (5185C>G), N1680H (5188A>C), W1681E (5191T>G 5192G>A 5193G>A), A1683V (5198C>T 5199C>G), N1685D (5203A>G 5205C>T), I1686V (5206A>G 5208T>A), G1687F (5209G>T 5210G>T), A1695S (5233G>T 5235C>A), K1696N (5238G>C), T1697Q (5239A>C 5240C>A 5241T>A), E1698Q (5242G>C), E1700S (5248G>T 5249A>C 5250G>A), P1701S (5251C>T 5253T>A), C1703N (5257T>A 5258G>A), V1705L (5263G>C 5265A>G), K1712R (5284A>C 5285A>G 5286G>C), K1713R (5288A>G 5289G>A), S1714T (5291G>C 5292C>A), K1715T (5294A>C), S1722N (5315G>A), M1730I (5340G>A), M1735L (5353A>T), S1736A (5356T>G), L1739Q (5366T>A), A1741H (5371G>C 5372C>A), G1744K (5380G>A 5381G>A 5382C>G), K1745S (5384A>G 5385A>T), M1746L (5386A>C), S1747E (5389T>G 5390C>A), E1748V (5393A>T 5394G>T), D1749G (5396A>G 5397C>T), I1750D (5398A>G 5399T>A), A1751G (5402C>G 5403T>G), D1754S (5410G>T 5411A>C), V1756T (5416G>A 5417T>C 5418G>C), V1765I (5443G>A 5445G>A), S1766A (5446A>G 5447G>C 5448C>T), M1773F (5467A>T 5469G>T), V1775L (5473G>T), R1778H (5483G>A), C1781R (5491T>A 5493C>G), I1782A (5494A>G 5495T>C 5496T>C), E1783T (5497G>A 5498A>C 5499G>A), I1785L (5503A>C 5505C>T), I1792M (5526T>G), G1793A (5528G>C 5529C>T), N1794G (5530A>G 5531A>G 5532T>G), V1806A (5567T>C 5568T>G), F1809L (5577T>G), Q1811K (5581C>A), P1814E (5590C>G 5591C>A 5592T>A), Q1815D (5593C>G 5595G>C), Q1815_E1816insT (5595_5596insACC), E1816S (5596G>T 5597A>C), G1817H (5599G>C 5600G>A 5601G>C), M1819L (5605A>T), A1820G (5609C>G), V1822S (5614G>A 5615T>G), S1824T (5620T>A 5622G>A), S1825del (5623_5625delTCT), L1826Y (5627T>A 5628G>T), Q1827G (5629C>G 5630A>G 5631A>C), S1828T (5633G>C 5634T>C), S1829T (5636G>C), M1831N (5642T>A 5643G>C), Q1833H (5649A>T), Q1833_G1834insHS (5649_5650insCACAGT), G1834A (5651G>C 5652T>A), E1836S (5656G>T 5657A>C 5658G>A), L1837V (5659C>G), Q1839L (5666A>T 5667A>T), I1840L (5668A>C 5670T>A), V1842I (5674G>A 5676C>A), I1843V (5677A>G 5679C>T), H1844P (5681A>C 5682C>T), G1845S (5683G>T 5684G>C), M1849E (5695A>G 5696T>A 5697G>A), E1850K (5698G>A 5700A>G), L1853V (5707T>G 5709G>T), M1855L (5713A>T), V1858T (5722G>A 5723T>C 5724T>A), V1859I (5725G>A 5727G>T), H1862E (5734C>G 5736T>A), G1866A (5747G>C), N1867Q (5749A>C 5751C>G), F1868C (5753T>G 5754T>C), T1869S (5755A>T 5757C>A), D1874K (5770G>A 5772T>G), Q1875H (5775A>T), L1876A (5776T>G 5777T>C 5778G>T), F1877L (5781T>G), A1878E (5783C>A 5784A>G), S1879T (5786G>C 5787T>A), E1881S (5791G>T 5792A>C 5793A>T), K1882V (5794A>G 5795A>T 5796G>C), C1883S (5797T>A), E1886T (5806G>A 5807A>C 5808A>T), L1888A (5812C>G 5813T>C 5814T>C), K1890R (5819A>G 5820G>A), N1891G (5821A>G 5822A>G 5823C>A), T1892V (5824A>G 5825C>T 5826G>C), L1894V (5830C>G), E1899K (5845G>A 5847G>A), K1900E (5848A>G), M1901F (5851A>T 5853G>C), T1902S (5854A>T 5856T>C), K1904H (5860A>C 5862A>C), V1906C (5866G>T 5867T>G 5868G>T), A1907E (5870C>A), Q1908M (5872C>A 5873A>T 5874A>G), V1910L (5878G>C 5880C>T), S1911E (5881A>G 5882G>A 5883T>G), V1913L (5887G>C 5889A>G), K1914E (5890A>G), Q1915S (5893C>A 5894A>G 5895G>T), F1918V (5902T>G 5904C>A), Y1919V (5905T>G 5906A>T 5907T>A), P1920F (5908C>T 5909C>T), K1921M (5912A>T), A1922G (5915C>G 5916C>G), T1923S (5918C>G 5919A>C), T1924V (5920A>G 5921C>T 5922C>T), V1925K (5923G>A 5924T>A), E1926K (5926G>A), L1928M (5932C>A 5934C>G), K1929L (5935A>T 5936A>T), V1930S (5938G>T 5939T>C 5940A>C), A1932M (5944G>A 5945C>T 5946T>G), D1933N (5947G>A), A1934Q (5950G>C 5951C>A 5952T>A), H1935Q (5955T>G), H1935_T1936insM (5955_5956insATG), T1936S (5956A>T 5958C>A), I1937K (5960T>A 5961T>A), V1938T (5962G>A 5963T>C), K1939Q (5965A>C), T1940V (5968A>G 5969C>T), K1942S (5975A>G 5976A>C), F1944C (5981T>G 5982C>T), R1945C (5983C>T 5985G>T), G1946del (5986_5988delGGA), V1948T (5992G>A 5993T>C 5994T>A), R1950S (5998C>A 6000G>T), N1952A (6004A>G 6005A>C 6006T>A), F1953W (6008T>G 6009T>G), S1955T (6014G>C), P1956K (6016C>A 6017C>A 6018T>G), E1957D (6021G>C), W1958I (6022T>A 6023G>T 6024G>T), W1958_K1959insV (6024_6025insGTC), K1959M (6026A>T 6027A>G), I1960T (6029T>C 6030T>A), G1961K (6031G>A 6032G>A), E1963K (6037G>A), N1964K (6042C>G), T1965P (6043A>C), M1966del (6046_6048delATG), A1967G (6050C>G), T1968S (6052A>T 6054A>T), L1969I (6055C>A 6057C>T), S1970P (6058T>C 6060A>T), S1973T (6068G>C 6069T>A), L1974V (6070C>G 6072T>G), L1975F (6073C>T), P1976L (6077C>T 6078A>T), L1979P (6086T>C 6087G>T), S1982L (6094T>C 6095C>T), A1983V (6098C>T 6099C>T), M1984V (6100A>G), K1985R (6104A>G 6105G>A), L1987M (6109T>A), S1989R (6115A>C), R1990N (6119G>A 6120A>T), Y1991S (6121T>A 6122A>G 6123C>T), K1992G (6124A>G

6125A>G), R1993K (6128G>A 6129A>G), N1994S (6131A>G), M1995Y (6133A>T 6134T>A 6135G>C), H1998T (6142C>A 6143A>C 6144T>A), C1999T (6145T>A 6146G>C 6147C>A), I2000L (6148A>C 6150A>G), K2001E (6151A>G), L2004F (6160C>T 6162C>T), K2005R (6164A>G 6165G>A), I2006M (6168A>G), V2008M (6172G>A 6174T>G), L2009_N2010del (6175_6180delCTCAAC), F2011C (6182T>G 6183C>T), M2013W (6187A>T 6188T>G), L2014V (6190C>G 6192G>A), N2015H (6193A>C 6195T>C)

	Begin	End	Coverage	Score	Concordance	Matches	Identities	I/D/M/F*	Stop Codons
**NT**	**25**	**6200**	**85.3%**	**3315**	**27.4%**	**6117 (98.7%)**	**3941 (63.6%)**	**21/59**	

GCT2GCA (156T>A), CTT3TTT (157C>T), AAA5AAG (165A>G), TTT7TGG (170T>G 171T>G), GGT8AGT (172G>A), TCT9CCT (175T>C), TCA10CCT (178T>C 180A>T), TCT11GCT (181T>G), GAG12_GTT17del (184_201delGAGAAGACTGGATCAGTT), CCC18TCT (202C>T 204C>T), TCT19TCA (207T>A), ACT20CCT (208A>C), TCT21TCA (213T>A), GGT24GCA (221G>C 222T>A), CTG26GTT (226C>G 228G>T), GGT28TCT (232G>T 233G>C), ATC30TTT (238A>T 240C>T), TCA31TCT (243A>T), AAA32TCT (244A>T 245A>C 246A>T), TTC33CTT (247T>C 249C>T), AAA35AAG (255A>G), GCT36ACT (256G>A), ATT37CTC (259A>C 261T>C), AGC38TCC (262A>T 263G>C), AAC39AAT (267C>T), ATC40GTT (268A>G 270C>T), ACA41AAT (272C>A 273A>T), TCA42AAA (274T>A 275C>A), GTA43GTT (279A>T), AAA46ACC (287A>C 288A>C), TCT48GTA (292T>G 293C>T 294T>A), CAT50CAC (300T>C), TTA51ATA (301T>A), GAT52GAG (306T>G), TTG54GTC (310T>G 312G>C), AAA55AAG (315A>G), CCA56CCG (318A>G), TCT57CCA (319T>C 321T>A), GTG58GTT (324G>T), ACT59ATG (326C>T 327T>G), GAT60GTA (329A>T 330T>A), TCT62GCA (334T>G 336T>A), TCT63ACA (337T>A 339T>A), TCT64TCA (342T>A), GTT66GCC (347T>C 348T>C), TGT69TGC (357T>C), TCC70AAT (358T>A 359C>A 360C>T), GCA71GCT (363A>T), TTG72GTA (364T>G 366G>A), AAT73GAA (367A>G 369T>A), AAA74AGA (371A>G), GTG75GTT (375G>T), CTT76TTA (376C>T 378T>A), ATA79ATT (387A>T), GGT80GCA (389G>C 390T>A), TCC81AAA (391T>A 392C>A 393C>A), TTG82ATA (394T>A 396G>A), ATT83ATG (399T>G), CCT85CCC (405T>C), TTT86TTC (408T>C), CTG87ATC (409C>A 411G>C), AAA88AAG (414A>G), GCA89GGT (416C>G 417A>T), TAC90TAT (420C>T), TCT91GAT (421T>G 422C>A), TTG93GTA (427T>G 429G>A), GCA94AAA (430G>A 431C>A), ACC95ACT (435C>T), TCT99ACT (445T>A), ATC100ATA (450C>A), AAG101GTA (451A>G 452A>T 453G>A), ATG103CTT (457A>C 459G>T), GTT104GTA (462T>A), TTA105CTT (463T>C 465A>T), CGG106AAG (466C>A 467G>A), TTG107ATG (469T>A), TTC108GTT (472T>G 474C>T), GAA109TTA (475G>T 476A>T), TTG111GTC (481T>G 483G>C), TCC112AAG (484T>A 485C>A 486C>G), AAT113GAT (487A>G), GTT115TTC (493G>T 495T>C), AGA116ATC (497G>T 498A>C), ATA117CTA (499A>C), TTT119TAC (506T>A 507T>C), CAG120GAA (508C>G 510G>A), GTT122TTA (514G>T 516T>A), GAG124GAA (522G>A), AAG125AAA (525G>A), AGT126GTT (526A>G 527G>T), GAT128TCA (532G>T 533A>C 534T>A), GTA129TTA (535G>T), ATT131ATA (543T>A), GTA132GTG (546A>G), GTT133TTT (547G>T), TAT134ATA (550T>A 551A>T 552T>A), GGT135GCT (554G>C), TTG137TTA (561G>A), GCA140GCT (570A>T), ATA141TTG (571A>T 573A>G), ACT142GCC (574A>G 576T>C), CTT143ATT (577C>A), CTT144TTG (580C>T 582T>G), GTT145CTA (583G>C 585T>A), TTG146CTA (586T>C 588G>A), TTG147CTG (589T>C), TGT148TAT (593G>A), GTT149GTA (597T>A), TCA152GTA (604T>G 605C>T), GAC153GAT (609C>T), ATA154ATT (612A>T), ATT155GTA (613A>G 615T>A), GAA156ACG (616G>A 617A>C 618A>G), GGA157CCT (619G>C 620G>C 621A>T), GTA158TTT (622G>T 624A>T), TCT159AAT (625T>A 626C>A), AGT160AAT (629G>A), ACC161GTA (631A>G 632C>T 633C>A), ATT162GTT (634A>G), AAA163GTT (637A>G 638A>T 639A>T), AAT164ACT (641A>C), ATT165TTG (643A>T 645T>G), TTC166TTT (648C>T), TTA167TCA (650T>C), ATA168GTT (652A>G 654A>T), GTA169GTT (657A>T), GGG170GGA (660G>A), ATG172TTT (664A>T 666G>T), TTT173TTC (669T>C), AGT175TCA (673A>T 674G>C 675T>A), AAC178AAT (684C>T), TTG179TTA (687G>A), GAC180GAT (690C>T), CCC183CCA (699C>A), AAG184ACA (701A>C 702G>A), GCT186TCT (706G>T), GAG187GGG (710A>G), TTC189TTT (717C>T), ACA190TCC (718A>T 720A>C), ATG191CAG (721A>C 722T>A), GTA192CTG (724G>C 726A>G), GCA193GCG (729A>G), AGT196GCA (736A>G 737G>C 738T>A), ATG197GTA (739A>G 741G>A), CTA198CTT (744A>T), GAG201GAA (753G>A), TCT202AGC (754T>A 755C>G 756T>C), GTC203TTA (757G>T 759C>A), TCA204ACG (760T>A 762A>G), CAC205CAT (765C>T), ACT206ACA (768T>A), ACA208ACT (774A>T), TCC209AGC (775T>A 776C>G), CAG210CAA (780G>A), TTG211CTT (781T>C 783G>T), ATG212TTA (784A>T 786G>A), TCA213TCC (789A>C), ACT214TCA (790A>T 792T>A), ATT215TTG (793A>T 795T>G), ATG216TTG (796A>T), GCA217GCT (801A>T), ATT220ATC (810T>C), TCC221TCA (813C>A), ACG222ACT (816G>T), CTC223TTA (817C>T 819C>A), GTG224GTA (822G>A), ATA226GTT (826A>G 828A>T), GCA227GCT (831A>T), GTG228GTT (834G>T), CCA229CCT (837A>T), GGA230GGG (840A>G), CCC232CCA (846C>A), TTG235CTT (853T>C 855G>T), AGC236TCA (856A>T 857G>C 858C>A), AAT237AAC (861T>C), CCC238CCA (864C>A), CTC239CTG (867C>G), ATT242ATC (876T>C), TCA245TCT (885A>T), ACT246GCT (886A>G), GGG247GGA (891G>A), GGG249GGT (897G>T), GCT250GCG (900T>G), CAA255CAG (915A>G), TTG256CTG (916T>C), TTT257TTC (921T>C), ACA258CAG (922A>C 923C>A 924A>G), AGA261AGG (933A>G), AAT262AAC (936T>C), AAA264AAG (942A>G), TGC266TGT (948C>T), ACT267ACA (951T>A), TCA268TCT (954A>T), GCA270GCC (960A>C), TTT271TTC (963T>C), TCT272TCA (966T>A), GTG274GTT (972G>T), GAA276GAG (978A>G), ATT277ATA (981T>A), ATA278GTG (982A>G 984A>G), GGT280AGC (988G>A 990T>C), GCT281ATT (991G>A 992C>T), TTC284TTT (1002C>T), AAG285AAA (1005G>A), GTG288GTA (1014G>A), CTT289TTG (1015C>T 1017T>G), TCT290TCA (1020T>A), GCA291GCG (1023A>G), ATT292ATA (1026T>A), AGC293AAT (1028G>A 1029C>T), GCA294GCT (1032A>T), ACA295ACC (1035A>C), TTG296TTA (1038G>A), TCT297TCC (1041T>C), ACT298ACC (1044T>C), TTG300CTT (1048T>C 1050G>T), GAA302CAG (1054G>C 1056A>G), CAG305ACG (1063C>A 1064A>C), GTG307GTT (1071G>T), GAT308TCG (1072G>T 1073A>C 1074T>G), GCT309GAG (1076C>A 1077T>G), TGT311GCT (1081T>G 1082G>C), GAT312GAA (1086T>A), GCT314TCT (1090G>T), ACC315ATG (1094C>T 1095C>G), AGG316CGC (1096A>C 1098G>C), TTG317CTT (1099T>C 1101G>T), GAG318GAA (1104G>A), AAC319AAT (1107C>T), GCC321GCA (1113C>A), AAT322AAC (1116T>C), AAA323CCT (1117A>C 1118A>C 1119A>T), GCC324GCA (1122C>A), TTC325TTT (1125C>T), CCT326CCA (1128T>A), GAC327ATT (1129G>A 1130A>T 1131C>T), AAA328AAG (1134A>G), AAT330CAA (1138A>C 1140T>A), CAC331CAT (1143C>T), CTT332TTA (1144C>T 1146T>A), AGG333AAA (1148G>A 1149G>A), GAG334GAA (1152G>A), GCA336GCC (1158A>C), CTG337GTC (1159C>G 1161G>C), AAG338AAA (1164G>A), ATT339ATA (1167T>A), GCA341GCT (1173A>T), ACA345ACC (1185A>C), CAC346CAT (1188C>T), GTT348GTC (1194T>C), GCC349GCA (1197C>A), ATG352TTG (1204A>T), CAT354CAC (1212T>C), CGA355AGG (1213C>A 1215A>G), GTG356GTT (1218G>T), AGT357TCT (1219A>T 1220G>C), GCA358GCT (1224A>T), GCT359GCC (1227T>C), ATC360ATA (1230C>A), ACT361ACG (1233T>G), CTT363CTA (1239T>A), GAA364GAT (1242A>T), AAG365AAA (1245G>A), GTC366CTG (1246G>C 1248C>G), TAT367GTT (1249T>G 1250A>T), GCG368GCT (1254G>T), AGT370GCC (1258A>G 1259G>C 1260T>C), TGC371AGC (1261T>A), AGA372CAA (1264A>C 1265G>A), GTT374TCT (1270G>T 1271T>C), GTT376GTG (1278T>G), GGT377GGC (1281T>C), CAG378CAA (1284G>A), AGA380AGG (1290A>G), GAA382GAG (1296A>G), CCC383CCT (1299C>T), GTT386GTG (1308T>G), TAT389TTT (1316A>T), GGT390GGA (1320T>A), GCC391GCT (1323C>T), GGG393GGA (1329G>A), ACA398ACG (1344A>G), GCA399TGT (1345G>T 1346C>G 1347A>T), CGA401CGC (1353A>C), TTG402CTT (1354T>C 1356G>T), TTG408CTC (1372T>C 1374G>C), GAT409GAC (1377T>C), CGC410GCA (1378C>G 1379G>C 1380C>A), GGG412GGT (1386G>T), GAG413GAA (1389G>A), CCT414CCA (1392T>A), CTT416CTG (1398T>G), AGA418CGC (1402A>C 1404A>C), CTT419TTG (1405C>T 1407T>G), GCT421GCG (1413T>G), GTT422GTA (1416T>A), AGC423AGT (1419C>T), AAG424AAA (1422G>A), CGC425AGA (1423C>A 1425C>A), GCC427GCA (1431C>A), TCT430TCC (1440T>C), AAT431AAC (1443T>C), GCC433GCA (1449C>A), CAC434CAT (1452C>T), CAG435CAA (1455G>A), GCT437GCA (1461T>A), TTG439CTT (1465T>C 1467G>T), GAC442GAT (1476C>T), GGA444GGT (1482A>T), GCA445GCT (1485A>T), TTG446CTT (1486T>C 1488G>T), AGA447CGG (1489A>C 1491A>G), GGA451GGG (1503A>G), CAA452CAG (1506A>G), CAA454CAG (1512A>G), ATT456ATA (1518T>A), ATT462ATC (1536T>C), TCC464TCT (1542C>T), ACA465ACC (1545A>C), CCA467CCT (1551A>T), CCA469CCT (1557A>T), CTG470CTT (1560G>T), CCC471CCA (1563C>A), GCA473GCT (1569A>T), GCT474GCA (1572T>A), GTG475GTC (1575G>C), GAA476GAG (1578A>G), GAC477GAT (1581C>T), AAG478AAA (1584G>A), GGG479GGA (1587G>A), CAT481CAC (1593T>C), ACT483ACA (1599T>A), TAT486TAC (1608T>C), GCA489GCC (1617A>C), CTT493TTG (1627C>T 1629T>G), GTA494ATA (1630G>A), TCA495TCT (1635A>T), GCA496GCT (1638A>T), CCT497CCA (1641T>A), GCC498GCT (1644C>T), CTT502CTA (1656T>A), ACC503ACG (1659C>G), GCT507GCA (1671T>A), GAG509GAA (1677G>A), CGT510AGG (1678C>A 1680T>G), AGA511AGG (1683A>G), AGG512AGA (1686G>A), ATC514ATT (1692C>T), TGT518TGC (1704T>C), ATC519AGA (1706T>G 1707C>A), GAA521GTG (1712A>T 1713A>G), GGT522GGC (1716T>C), GAA523GAT (1719A>T), TCT525GAT (1723T>G 1724C>A), ACA526CCA (1726A>C), GAG527AAT (1729G>A 1731G>T), GAC528ACT (1732G>A 1733A>C 1734C>T), ACC530ACA (1740C>A), GGA531GCC (1742G>C 1743A>C), CTG533CTA (1749G>A), ATG534ATT (1752G>T), AAC536GAT (1756A>G 1758C>T), ATT537GTG (1759A>G 1761T>G), CGG541AGA (1771C>A 1773G>A), AGG542AGA (1776G>A), ATA546ATC (1788A>C), CAC550GCT (1798C>G 1799A>C 1800C>T), AGT552TCC (1804A>T 1805G>C 1806T>C), TAC553TAT (1809C>T), TCG554GAT (1810T>G 1811C>A 1812G>T), TTA557TTG (1821A>G), GAC558CAA (1822G>C 1824C>A), GCC562GCA (1836C>A), TGT564TGC (1842T>C), CAG565CGG (1844A>G), GTT566GTG (1848T>G), CAT567CAC (1851T>C), GCT568TAT (1852G>T 1853C>A), ATA570GTG (1858A>G 1860A>G), AAG572AGC (1865A>G 1866G>C), CAG573CAA (1869G>A), CTG574CTT (1872G>T), TTT575TAC (1874T>A 1875T>C), GAA576AAT (1876G>A 1878A>T), GCA577ACT (1879G>A 1881A>T), GAA578ACA (1882G>A 1883A>C), ACT579TCG (1885A>T 1887T>G), GGC580GGA (1890C>A), ATT581TTG (1891A>T 1893T>G), TCA582GCT (1894T>G 1896A>T), AAT583GAC (1897A>G 1899T>C), ACA585GTT (1903A>G 1904C>T 1905A>T), GCC586GCT (1908C>T), GTT588GTC (1914T>C), GCT589AAT (1915G>A 1916C>A), GTA590GTT (1920A>T), TCT591GCT (1921T>G), TCT592GTT (1924T>G 1925C>T), GAA593GAT (1929A>T), GAG594GAA (1932G>A), GTT595GTG (1935T>G), GTG596CTG (1936G>C), GCA597GAT (1940C>A 1941A>T), GTT599TTC (1945G>T 1947T>C), GAT600CAA (1948G>C 1950T>A), GGG601GAT (1952G>A 1953G>T), GCT602GCC (1956T>C), CGC603del (1957_1959delCGC), TTC604TTA (1962C>A), AAA605CAA (1963A>C), ACA606GTT (1966A>G 1967C>T 1968A>T), AAA607TTG (1969A>T 1970A>T 1971A>G), CAA608CCT (1973A>C 1974A>T), GAT609CCT (1975G>C 1976A>C), GTG610GAG (1979T>A), CCA611TCA (1981C>T), ATA612GCA (1984A>G 1985T>C), GTG613GCT (1988T>C 1989G>T), ACT614TAT (1990A>T 1991C>A), CCC615GAG (1993C>G 1994C>A 1995C>G), ACA616CAG (1996A>C 1997C>A 1998A>G), GTT617ACA (1999G>A 2000T>C 2001T>A), ATA618ATT (2004A>T), GAG620GAA (2010G>A), GAA621GCT (2012A>C 2013A>T), GAT622GAA (2016T>A), AGA623AAA (2018G>A), GTG624ATT (2020G>A 2022G>T), ATT625GTT (2023A>G), TCT627TCC (2031T>C), AAA628CAG (2032A>C 2034A>G), TTA630CTC (2038T>C 2040A>C), GTG632GTT (2046G>T), GAA633GAG (2049A>G), CTT635CTC (2055T>C), TTT637TAT (2060T>A), TAT639TAC (2067T>C), CAG640CAA (2070G>A), GGC641GGG (2073C>G), TTG643ATG (2077T>A), GAC644AAT (2080G>A 2082C>T), CCG645AAA (2083C>A 2084C>A 2085G>A), GAG646TTT (2086G>T 2087A>T 2088G>T), AGT647GAG (2089A>G 2090G>A 2091T>G), TTG648TTT (2094G>T), TTT649TTC (2097T>C), CCT650CCA (2100T>A), TAT653CTC (2107T>C 2108A>T 2109T>C), CAA654TCC (2110C>T 2111A>C 2112A>C), AAA655AAG (2115A>G), CAA656CAG (2118A>G), CTC658ATC (2122C>A), TTT659TAT (2126T>A), TCC661GTG (2131T>G 2132C>T 2133C>G), GAT663TCT (2137G>T 2138A>C), GCA664TCT (2140G>T 2142A>T), GAG665GAT (2145G>T), CAC666GCC (2146C>G 2147A>C), CAG667AAA (2149C>A 2151G>A), CAA668CAG (2154A>G), GTC669AAT (2155G>A 2156T>A 2157C>T), TTC670TTT (2160C>T), ATG671GAA (2161A>G 2162T>A 2163G>A), AGA672ATG (2165G>T 2166A>G), ATG674GTA (2170A>G 2172G>A), ACT675AAT (2174C>A), GAC676GAT (2178C>T), ATG677TTG (2179A>T), ATC678TTG (2182A>T 2184C>G), TAC679GTC (2185T>G 2186A>T), GGT681GGA (2193T>A), GCC682GCA (2196C>A), AAT683AGT (2198A>G), GCT684CCT (2200G>C), GAA685CAG (2203G>C 2205A>G), CAA686CAG (2208A>G), TAC687TAT (2211C>T), AGG688GCT (2212A>G 2213G>C 2214G>T), CAA692CGA (2225A>G), AAT693AAC (2229T>C), CTC699ATC (2245C>A), TTT702TTC (2256T>C), AAA703AAG (2259A>G), AGC704AGT (2262C>T), ATC706ATT (2268C>T), GCT708AGC (2272G>A 2273C>G 2274T>C), TCC709ACA (2275T>A 2277C>A), ATC711ATT (2283C>T), TGT712TCT (2285G>C), AAG714GAG (2290A>G), AAG715CTG (2293A>C 2294A>T), GCT717GAT (2300C>A), GTA718GTC (2304A>C), ACA720CGC (2308A>C 2309C>G 2310A>C), GAG721AAT (2311G>A 2313G>T), ATG722CTT (2314A>C 2316G>T), CGC723GAG (2317C>G 2318G>A 2319C>G), ACA724CAA (2320A>C 2321C>A), GGA725GGG (2325A>G), TTT726AAA (2326T>A 2327T>A 2328T>A), GCA727GCG (2331A>G), CAT728TAT (2332C>T), AAT729AAG (2337T>G), TGT730GCG (2338T>G 2339G>C 2340T>G), ATT731CTT (2341A>C), GAT732CAG (2344G>C 2346T>G), GTT735GCA (2354T>C 2355T>A), TTG738CTT (2362T>C 2364G>T), ATT739ACG (2366T>C 2367T>G), TGC740TGT (2370C>T), ATC741ATT (2373C>T), CAG742GAA (2374C>G 2376G>A), TTT747AGA (2389T>A 2390T>G 2391T>A), GTG748GTA (2394G>A), TTT750TTC (2400T>C), TTG751CTG (2401T>C), ACA753ACT (2409A>T), GCC754GCT (2412C>T), CGT757CGC (2421T>C), TAT758TAC (2424T>C), ACC761AGG (2432C>G 2433C>G), TCC764GCA (2440T>G 2442C>A), TCC764_AAA765insGGT (2442_2443insGGT), AAA765ACT (2444A>C 2445A>T), GAG766GAC (2448G>C), GCC767AAG (2449G>A 2450C>A 2451C>G), AAA768AAG (2454A>G), GAC769AGA (2455G>A 2456A>G 2457C>A), TCT770AAT (2458T>A 2459C>A), GTC772ATT (2464G>A 2466C>T), AAG773ATC (2468A>T 2469G>C), ATA774GAA (2470A>G 2471T>A), TGT775TGC (2475T>C), TAC776ATG (2476T>A 2477A>T 2478C>G), CAC777ATT (2479C>A 2480A>T 2481C>T), AAA778GGT (2482A>G 2483A>G 2484A>T), ATT779ATC (2487T>C), GTG780AAG (2488G>A 2489T>A), GAT781GAC (2493T>C), TAC782AAA (2494T>A 2496C>A), AAA784ATC (2501A>T 2502A>C), GAA785CAG (2503G>C 2505A>G), ACT786ATG (2507C>T 2508T>G), TGG788AAT (2512T>A 2513G>A 2514G>T), AAC789GAC (2515A>G), TTA790TTG (2520A>G), CCA791CCC (2523A>C), TAT792TTC (2525A>T 2526T>C), GCA793ATC (2527G>A 2528C>T 2529A>C), CTG794TTG (2530C>T), AGG795AAG (2534G>A), GGC801GGA (2553C>A), CTA802CTG (2556A>G), ATC804TTG (2560A>T 2562C>G), ATG805ATA (2565G>A), GCG806GCA (2568G>A), GGA809GGC (2577A>C), TTG810ACC (2578T>A 2579T>C 2580G>C), TTT811ATG (2581T>A 2583T>G), GGG812AAT (2584G>A 2585G>A 2586G>T), GGA813GCC (2588G>C 2589A>C), ATA814GTT (2590A>G 2592A>T), ACA815ACT (2595A>T), TTT817TTC (2601T>C), CTT818ATT (2602C>A), GCT819ACT (2605G>A), TGT820TGC (2610T>C), TGG821CTC (2611T>C 2612G>T 2613G>C), CAA822AAG (2614C>A 2616A>G), AGC823GCA (2617A>G 2618G>C 2619C>A), AAT824GCA (2620A>G 2621A>C 2622T>A), TCT825ACT (2623T>A), TTT826TCC (2627T>C 2628T>C), CCA827CCT (2631A>T), AGT828TTA (2632A>T 2633G>T 2634T>A), AGT830GAT (2638A>G 2639G>A), GGA831GGC (2643A>C), GGT833GGG (2649T>G), GGT834GGC (2652T>C), GTC835GTA (2655C>A), ACA836ACC (2658A>C), AGC841AGT (2673C>T), TCT843TCC (2679T>C), AAA845AAG (2685A>G), AAG846AAA (2688G>A), AAC847AAT (2691C>T), AAG848AAA (2694G>A), GGC849GGG (2697C>G), CCC850AAG (2698C>A 2699C>A 2700C>G), TCT851ACT (2701T>A), CGT852CAT (2705G>A), CTG853TTC (2707C>T 2709G>C), AAG854GCA (2710A>G 2711A>C 2712G>A), AAT855AAA (2715T>A), TTG856TCC (2717T>C 2718G>C), GTG858CTA (2722G>C 2724G>A), CAG860CAA (2730G>A), AGT861GCG (2731A>G 2732G>C 2733T>G), AGC862GCA (2734A>G 2735G>C 2736C>A), GCT863GAT (2738C>A), ATT865AAC (2744T>A 2745T>C), ACA866CAT (2746A>C 2747C>A 2748A>T), CAA867GTG (2749C>G 2750A>T 2751A>G), GAA868GAC (2754A>C), GAA868_TGG869insCGG (2754_2755insCGG), GCT871GAA (2762C>A 2763T>A), GAA872CAG (2764G>C 2766A>G), GGG874GGA (2772G>A), GTC876GTT (2778C>T), AAC877AAT (2781C>T), CAA878GAA (2782C>G), GCA879AGT (2785G>A 2786C>G 2787A>T), CTC880CTG (2790C>G), AAA881CAC (2791A>C 2793A>C), AAG882AAA (2796G>A), AAT883AAC (2799T>C), ATG884TTG (2800A>T), ATT885GTT (2803A>G), GTA886GTT (2808A>T), CTG887TTG (2809C>T), CGG888AGA (2812C>A 2814G>A), CTT889ATT (2815C>A), GGA890GCT (2819G>C 2820A>T), GAG891GAA (2823G>A), GTG893GTC (2829G>C), TAC894TAT (2832C>T), ACA898ACG (2844A>G), GTG900GTT (2850G>T), TCA902TCT (2856A>T), GGG903GGT (2859G>T), ATA905ATT (2865A>T), ATG906CTC (2866A>C 2868G>C), GCA911GCC (2883A>C), TTC912TTT (2886C>T), CAC913AGA (2887C>A 2888A>G 2889C>A), AAT914AAC (2892T>C), GTG915ATG (2893G>A), AGA916AGG (2898A>G), GGA918GGC (2904A>C), ACA919ACT (2907A>T), ACC922TCC (2914A>T), ATT923ATC (2919T>C), ATC924ATT (2922C>T), CCT926GAG (2926C>G 2927C>A 2928T>G), AAC927CAT (2929A>C 2931C>T), TCA928TCC (2934A>C), AGG929ATT (2936G>T 2937G>T), TCA930TGT (2939C>G 2940A>T), AAG931AAT (2943G>T), TAC934TTC (2951A>T), AAC935AAT (2955C>T), CGG937ACC (2959C>A 2960G>C 2961G>C), GAA938CAA (2962G>C), TCC939TCA (2967C>A), AAG940AGG (2969A>G), GTC941TTG (2971G>T 2973C>G), ATT942ATA (2976T>A), GGA944GGT (2982A>T), ATC947GTG (2989A>G 2991C>G), CTG949TTG (2995C>T),

Codon mutations:

CTA950TTG (2998C>T 3000A>G), CGT951AGG (3001C>A 3003T>G), GTG952GTA (3006G>A), GGT953GGG (3009T>G), GAC954GAT (3012C>T), CCT955CCA (3015T>A), CCT960TCA (3028C>T 3030T>A), ATT962ATA (3036T>A), CGC963AGA (3037C>A 3039C>A), AAG964AAA (3042G>A), CCA967CCT (3051A>T), GAG971GAT (3063G>T), GTC972GTG (3066C>G), TGC973TGT (3069C>T), TTC974TAC (3071T>A), ACC975TCT (3073A>T 3075C>T), AAA976AAG (3078A>G), GGT977GGC (3081T>C), TCT978TCA (3084T>A), CAG979ATG (3085C>A 3086A>T), TTA981TTG (3093A>G), TGT982TGC (3096T>C), TGT983GTG (3097T>G 3098G>T 3099T>G), AGA984AGG (3102A>G), GCC985GCT (3105C>T), GTG986GTA (3108G>A), GCG987GCT (3111G>T), ACC989AAG (3116C>A 3117C>G), CCA991CCC (3123A>C), AGA992AAG (3125G>A 3126A>G), CTG993AAA (3127C>A 3128T>A 3129G>A), GGC994TCC (3130G>T 3131G>C), AAC995AAT (3135C>T), CTA996TTG (3136C>T 3138A>G), GAA997GAG (3141A>G), TTT998GTT (3142T>G), TTG999CTT (3145T>C 3147G>T), TTT1001TAT (3152T>A), TCC1006TCA (3168C>A), AAA1007AAG (3171A>G), TAT1009TAC (3177T>C), ACA1010ACC (3180A>C), GTC1011GAA (3182T>A 3183C>A), AAG1012AAA (3186G>A), GTG1013GTC (3189G>C), GAG1014ACA (3190G>A 3191A>C 3192G>A), GAG1016GGA (3197A>G 3198G>A), GAC1018GAT (3204C>T), TCT1019TCG (3207T>G), GCC1020AAT (3208G>A 3209C>A 3210C>T), AGT1021GCA (3211A>G 3212G>C 3213T>A), AAA1023GCT (3217A>G 3218A>C 3219A>T), TTG1024CTT (3220T>C 3222G>T), AGT1025AGC (3225T>C), TCG1026TCA (3228G>A), CAG1027CAA (3231G>A), CAA1028GTT (3232C>G 3233A>T 3234A>T), TCA1029GCA (3235T>G), GAA1031GAG (3243A>G), TAT1032TAC (3246T>C), CAT1033CAC (3249T>C), ATG1034ATT (3252G>T), GGA1036AGT (3256G>A 3258A>T), GGT1039GGA (3267T>A), GGA1042GGT (3276A>T), TTA1045CTC (3283T>C 3285A>C), CTT1046TTA (3286C>T 3288T>A), CCC1048CCA (3294C>A), AGT1049ACT (3296G>C), GTT1050GTG (3300T>G), AAA1053AAG (3309A>G), CAA1054CAG (3312A>G), CCT1055CCG (3315T>G), GTT1056TGC (3316G>T 3317T>G 3318T>C), GTG1058GTT (3324G>T), GGC1059GGA (3327C>A), ATT1060ATA (3330T>A), GCT1063GCA (3339T>A), TCA1064TCT (3342A>T), GGT1067GAT (3350G>A), ATT1068ACC (3353T>C 3354T>C), GCA1069GCT (3357A>T), GAA1071GAG (3363A>G), GGA1073GGT (3369A>T), TTC1074TAC (3371T>A), ATT1075GTG (3373A>G 3375T>G), TCT1076GCT (3376T>G), TCC1077TCA (3381C>A), AAT1078AAC (3384T>C), GCC1079GCA (3387C>A), ACG1080ACA (3390G>A), GCC1081GCA (3393C>A), ATC1082GTG (3394A>G 3396C>G), TTC1083TTT (3399C>T), CGG1084AGA (3400C>A 3402G>A), GAC1085GAT (3405C>T), CAG1086CAA (3408G>A), CTT1087TTG (3409C>T 3411T>G), AAC1089GAT (3415A>G 3417C>T), GGG1093GGA (3429G>A), CCT1094CCC (3432T>C), GTT1095ATT (3433G>A), GCG1097GTG (3440C>T), GCA1098GCT (3444A>T), CGA1101CGC (3453A>C), GAC1103GAA (3459C>A), ATT1104CTG (3460A>C 3462T>G), CTC1105TTG (3463C>T 3465C>G), AAA1106AAG (3468A>G), GCC1107CAC (3469G>C 3470C>A), ATT1108CTG (3472A>C 3474T>G), AGG1109AGA (3477G>A), AGT1110TCC (3478A>T 3479G>C 3480T>C), AGA1111AAG (3482G>A 3483A>G), GAA1112GAG (3486A>G), ACC1113GAA (3487A>G 3488C>A 3489C>A), ACT1114AGA (3491C>G 3492T>A), CTT1115TTC (3493C>T 3495T>C), GAA1117GAG (3501A>G), AAC1119AAA (3507C>A), CAA1120CAG (3510A>G), GTC1121GTG (3513C>G), TCC1122GCT (3514T>G 3516C>T), TTT1123TTC (3519T>C), CTT1124TTG (3520C>T 3522T>G), GTT1127GTA (3531T>A), CCT1128CCG (3534T>G), CAA1129TTG (3535C>T 3536A>T 3537A>G), TTG1131ATG (3541T>A), GCC1132GCT (3546C>T), GCA1133GCT (3549A>T), GTA1135GTG (3555A>G), CCC1136CCG (3558C>G), CAT1137CAC (3561T>C), ACT1139ACA (3567T>A), ACA1140ACC (3570A>C), CTC1141CTT (3573C>T), AGA1142AAA (3575G>A), AAG1143CGC (3577A>C 3578A>G 3579G>C), CAA1145CCC (3584A>C 3585A>C), TTG1146CTG (3586T>C), GCA1149GCT (3597A>T), GGT1151GGG (3603T>G), CCT1152GAC (3604C>G 3605C>A 3606T>C), GAA1154CAG (3610G>C 3612A>G), ACC1155ACT (3615C>T), GCT1156GCC (3618T>C), CCC1157CCA (3621C>A), TCA1158TCC (3624A>C), ATC1159ATT (3627C>T), CTC1160CTA (3630C>A), ACC1161TCT (3631A>T 3633C>T), GTC1162GCC (3635T>C), AAT1163AAA (3639T>A), CGG1166AGG (3646C>A), GAG1168GAA (3654G>A), GGA1169GGG (3657A>G), TTC1170TTT (3660C>T), CCA1172CCC (3666A>C), GCA1175GCT (3675A>T), GTG1177GTC (3681G>C), ATG1178GAG (3682A>G 3683T>A), AAA1179AAG (3687A>G), TAC1180TAT (3690C>T), ACT1183ACA (3699T>A), CAT1185TCC (3703C>T 3704A>C 3705T>C), GGT1186AAC (3706G>A 3707G>A 3708T>C), GAT1188GAG (3714T>G), GAT1189GAG (3717T>G), GAT1190CCA (3718G>C 3719A>C 3720T>A), GCT1192GCA (3726T>A), AAA1193AAG (3729A>G), TTG1194CTG (3730T>C), TCC1195GCT (3733T>G 3735C>T), GAA1197GAG (3741A>G), ACT1198GGT (3742A>G 3743C>G), CTC1199CTT (3747C>T), AGT1201GCA (3751A>G 3752G>C 3753T>A), TCA1202TCC (3756A>C), CTG1203ATT (3757C>A 3759G>T), CTG1204CTT (3762G>T), CCA1205CCT (3765A>T), ACA1206GTG (3766A>G 3767C>T 3768A>G), ATG1207CTT (3769A>C 3771G>T), AAA1208AAG (3774A>G), AAC1209ATG (3776A>T 3777C>G), CAA1210CAG (3780A>G), AAA1211CGA (3781A>C 3782A>G), GGC1215GGG (3795C>G), CCA1217CCT (3801A>T), CAG1218GCA (3802C>G 3803A>C 3804G>A), GAG1219ATC (3805G>A 3806A>T 3807G>C), GAT1221AAT (3811G>A), GAT1223GAC (3819T>C), GTG1224GTA (3822G>A), CTG1226CTC (3828G>C), ATT1229ATA (3837T>A), TGT1232TTT (3845G>T), TAT1235TTT (3854A>T), GGC1237GCA (3860G>C 3861C>A), CTC1240TTG (3868C>T 3870C>G), ACA1242ACG (3876A>G), TCC1243TCA (3879C>A), TAC1246TAT (3888C>T), TTC1248TTT (3894C>T), ATG1251TTG (3901A>T), GGC1252GGA (3906C>A), ATG1253CTT (3907A>C 3909G>T), GGA1254GGT (3912A>T), ATG1255TCT (3913A>T 3914T>C 3915G>T), AAG1257AAA (3921G>A), CGG1258AGG (3922C>A), GAG1259GAA (3927G>A), GTC1261GTG (3933C>G), GTA1262GAA (3935T>A), CCA1263GCC (3937C>G 3939A>C), ACA1264CAA (3940A>C 3941C>A), GGA1265CCA (3943G>C 3944G>C), GAA1266AAC (3946G>A 3948A>C), GGT1267TCG (3949G>T 3950G>C 3951T>G), GAT1268CAG (3952G>C 3954T>G), AAA1269AGG (3956A>G 3957A>G), GTA1270GTT (3960A>T), GAG1271CTT (3961G>C 3962A>T 3963G>T), CTT1272CTC (3966T>C), AGA1273ATA (3968G>T), GAC1275AAC (3973G>A), CCA1277CCT (3981A>T), GTG1278GTC (3984G>C), GAA1280GAT (3990A>T), GCT1281GCA (3993T>A), GAG1283GAA (3999G>A), GAA1284GAG (4002A>G), CTG1285TTG (4003C>T), ATT1287TCG (4009A>T 4010T>C 4011T>G), CAA1288CAG (4014A>G), ATT1289GTA (4015A>G 4017T>A), CAG1291CAA (4023G>A), ATA1293ACA (4028T>C), CAT1294TTT (4030C>T 4031A>T), GTG1295GTT (4035G>T), GAT1296GAC (4038T>C), CTA1297CTC (4041A>C), ATC1298GTC (4042A>G), ACC1299ACA (4047C>A), ACA1300ACC (4050A>C), CAA1301CAG (4053A>G), GCC1303GCG (4059C>G), AAG1304AAA (4062G>A), GAG1306GAA (4068G>A), CGC1307CGG (4071C>G), CTT1308TTG (4072C>T 4074T>G), CCT1309CCA (4077T>A), GAA1311GAG (4083A>G), AAA1318AAG (4104A>G), ACA1319ACC (4107A>C), AGA1320CGC (4108A>C 4110A>C), TTA1321CTG (4111T>C 4113A>G), TTT1322TTC (4116T>C), GAG1323GAA (4119G>A), ATA1324ATC (4122A>C), TAC1329TAT (4137C>T), GTC1333GTG (4149C>G), AGG1334CGG (4150A>C), TTT1337TTC (4161T>C), TCC1341TCT (4173C>T), GCC1342GCT (4176C>T), ACC1343TGT (4177A>T 4178C>G 4179C>T), GCA1346GCT (4188A>T), TTA1347GCA (4189T>G 4190T>C), AAT1349AAC (4197T>C), GTC1350GTA (4200C>A), ATC1351CTC (4201A>C), CCA1352CCT (4206A>T), TGT1353TGC (4209T>C), GTT1355GTA (4215T>A), GGA1356GGT (4218A>T), ATA1357ATC (4221A>C), GAT1358AAT (4222G>A), ACC1360ACG (4230C>G), AGT1361AGC (4233T>C), TCT1362CAT (4234T>C 4235C>A), TCT1365ACA (4243T>A 4245T>A), CTT1366GTG (4246C>G 4248T>G), CTT1367CTG (4251T>G), GCC1368GCT (4254C>T), AAC1369AGT (4256A>G 4257C>T), AAC1370AGC (4259A>G), TTC1371TTT (4263C>T), GCT1373TCA (4267G>T 4269T>A), ACT1377GAG (4279A>G 4280C>A 4281T>G), GGG1378GGA (4284G>A), TTT1379TTC (4287T>C), GCT1381GCC (4293T>C), TCA1384TCG (4302A>G), AGT1385AGC (4305T>C), GAC1387GAT (4311C>T), GGG1388GGA (4314G>A), CGC1389AGA (4315C>A 4317C>A), CCA1391CCC (4323A>C), GTG1392ATC (4324G>A 4326G>C), TTC1393TTT (4329C>T), GCT1394GCA (4332T>A), TTT1395TTC (4335T>C), CAG1396CAA (4338G>A), TTG1401CTG (4351T>C), GTT1402GTG (4356T>G), GAG1404GCT (4361A>C 4362G>T), TAT1405TTT (4364A>T), GAT1407GGT (4370A>G), ACC1408GAT (4372A>G 4373C>A 4374C>T), AAG1409GAG (4375A>G), GGA1411GGG (4383A>G), GAG1412AGT (4384G>A 4385A>G 4386G>T), GCT1414GAG (4391C>A 4392T>G), TCC1415TCA (4395C>A), GCA1417GCT (4401A>T), AGA1418AGG (4404A>G), CAT1419CAC (4407T>C), GCT1420ACA (4408G>A 4410T>A), CTT1421TTA (4411C>T 4413T>A), CTC1422TTA (4414C>T 4416C>A), GCA1425GCC (4425A>C), TCA1426TCT (4428A>T), CAC1428CAT (4434C>T), TAC1429TAT (4437C>T), TTA1431CTC (4441T>C 4443A>C), GAT1434AAC (4450G>A 4452T>C), AAA1435AAG (4455A>G), GTT1436TTG (4456G>T 4458T>G), TTC1437TTT (4461C>T), CGT1438CGC (4464T>C), TTG1439CTA (4465T>C 4467G>A), GTT1440GTG (4470T>G), GGC1442GGA (4476C>A), CCG1444CCC (4482G>C), GCA1448GCT (4494A>T), CTT1449TTG (4495C>T 4497T>G), ACC1450ACT (4500C>T), TTC1453TTT (4509C>T), AAC1454AAT (4512C>T), CTG1456TTG (4516C>T), TTG1457CTA (4519T>C 4521G>A), CGG1463AGG (4537C>A), TAT1464TAC (4542T>C), GCT1465GCG (4545T>G), TTC1466TTT (4548C>T), ATC1467ATA (4551C>A), CTT1469CTG (4557T>G), TTA1470TTG (4560A>G), AGA1471AGG (4563A>G), AGG1472AGA (4566G>A), CCT1473CCA (4569T>A), CAC1474CAT (4572C>T), ATT1475GTC (4573A>G 4575T>C), GCC1476AAA (4576G>A 4577C>A 4578C>A), GCT1477GCC (4581T>C), AGA1478AAA (4583G>A), GCT1479TCT (4585G>T), ATA1480GTG (4588A>G 4590A>G), GGT1481GGC (4593T>C), GTA1482ATT (4594G>A 4596A>T), CCT1484CCA (4602T>A), GGT1485AAT (4603G>A 4604G>A), GAT1486GAC (4608T>C), AAC1488AAT (4614C>T), CTT1490TTG (4618C>T 4620T>G), GGA1496GGT (4638A>T), AAT1499AAC (4647T>C), CTG1500TTG (4648C>T), GTG1501GTT (4653G>T), GCT1502GCG (4656T>G), GTG1503GTT (4659G>T), CCT1504CCA (4662T>A), CTG1505ATG (4663C>A), CTG1507TTG (4669C>T), TAT1510TAC (4680T>C), TCA1511GAT (4681T>G 4682C>A 4683A>T), CTG1512CTA (4686G>A), CCT1513CCA (4689T>A), AAT1514TCC (4690A>T 4691A>C 4692T>C), GCC1516GCA (4698C>A), CAG1517AGA (4699C>A 4700A>G 4701G>A), CTT1519CTG (4707T>G), GAC1520GAA (4710C>A), ATG1521GCT (4711A>G 4712T>C 4713G>T), GTA1522GTT (4716A>T), AAT1523AAC (4719T>C), ATC1526ATT (4728C>T), AAA1527AAG (4731A>G), GGG1529GGT (4737G>T), CTG1530TTG (4738C>T), GAT1531GAC (4743T>C), TCT1533TCA (4749T>A), AAC1537GAG (4759A>G 4761C>G), CAG1538AAG (4762C>A), GTG1539ACA (4765G>A 4766T>C 4767G>A), CAG1540AAA (4768C>A 4770G>A), TTC1541TTT (4773C>T), CAG1542CTG (4775A>T), GAC1543GAT (4779C>T), TCA1545TCT (4785A>T), GAA1546GAG (4788A>G), ACT1548ACC (4794T>C), AGC1551AGT (4803C>T), GGA1553GGT (4809A>T), TTT1554TTC (4812T>C), AAA1555AAG (4815A>G), CGT1556CGA (4818T>A), GCC1558GCA (4824C>A), CTT1559TTG (4825C>T 4827T>G), GGG1560GGA (4830G>A), CAC1562CAA (4836C>A), GTG1569GTT (4857G>T), ATA1571GTC (4861A>G 4863A>C), ATT1572ATC (4866T>C), CCT1574CCC (4872T>C), ATG1575ATT (4875G>T), TAC1576TAT (4878C>T), ATC1578ATT (4884C>T), AGA1579AGG (4887A>G), CCT1580CCA (4890T>A), CCG1582GTG (4894C>G 4895C>T), GGT1583GGA (4899T>A), ACT1587GTA (4909A>G 4910C>T 4911T>A), GTG1588CAA (4912G>C 4913T>A 4914G>A), GCA1589GCT (4917A>T), ATG1591TAT (4921A>T 4922T>A 4923G>T), GAG1592GAA (4926G>A), GTG1594ATT (4930G>A 4932G>T), ACT1596ACA (4938T>A), CTT1598ATC (4942C>A 4944T>C), CGT1599AGA (4945C>A 4947T>A), GAG1600GAA (4950G>A), TTT1602TTC (4956T>C), AGA1606CAG (4966A>C 4967G>A 4968A>G), GTT1607GCC (4970T>C 4971T>C), GCA1608ACT (4972G>A 4974A>T), GAG1610GAA (4980G>A), AAG1611AGT (4982A>G 4983G>T), TTA1612TTT (4986A>T), ACT1613GTA (4987A>G 4988C>T 4989T>A), TAT1614AAT (4990T>A), CAG1617TTT (4999C>T 5000A>T 5001G>T), TGT1618GGA (5002T>G 5004T>A), GCC1619GCT (5007C>T), AGA1621AAG (5012G>A 5013A>G), GAG1622CAG (5014G>C), CGT1623AGA (5017C>A 5019T>A), GGA1624AAT (5020G>A 5021G>A 5022A>T), GGC1626CCC (5026G>C 5027G>C), GCA1627ATC (5029G>A 5030C>T 5031A>C), ACA1628ATG (5033C>T 5034A>G), TTT1630ATG (5038T>A 5040T>G), CCT1631CCC (5043T>C), TCT1632ACT (5044T>A), TAT1633TAC (5049T>C), ATG1634TAT (5050A>T 5051T>A 5052G>T), GTG1636CTT (5056G>C 5058G>T), GAG1637GAA (5061G>A), CAG1638AGT (5062C>A 5063A>G 5064G>T), GAA1639CAA (5065G>C), ATC1641TTG (5071A>T 5073C>G), GCA1642ACA (5074G>A), AAG1643GAG (5077A>G), GCA1648GAG (5093C>A 5094A>G), TCA1649AGC (5095T>A 5096C>G 5097A>C), GCG1650ATT (5098G>A 5099C>T 5100G>T), TTG1651GGG (5101T>G 5102T>G), ACA1652GTT (5104A>G 5105C>T 5106A>T), ATC1653TTG (5107A>T 5109C>G), TGT1654ATG (5110T>A 5111G>T 5112T>G), GAA1655GAG (5115A>G), ATG1656CTA (5116A>C 5118G>A), GCC1657TCA (5119G>T 5121C>A), AAA1658GAT (5122A>G 5124A>T), TCT1660AAA (5128T>A 5129C>A 5130T>A), ATC1661AAT (5132T>A 5133C>T), ACT1662ACA (5136T>A), TTC1663TAC (5138T>A), ACA1664ACC (5142A>C), TTG1666TTA (5148G>A), CCA1667CCT (5151A>T), CCT1668GCT (5152C>G), AAT1670GAA (5158A>G 5160T>A), GTT1672ATT (5164G>A), GAA1673del (5167_5169delGAA), GAA1676GAG (5178A>G), AGA1677GAG (5179A>G 5180G>A 5181A>G), GAT1678GGT (5183A>G), CTC1679GTC (5185C>G), AAT1680CAT (5188A>C), TGG1681GAA (5191T>G 5192G>A 5193G>A), GCC1683GTG (5198C>T 5199C>G), CCA1684CCT (5202A>T), AAC1685GAT (5203A>G 5205C>T), ATT1686GTA (5206A>G 5208T>A), GGT1687TTT (5209G>T 5210G>T), TGC1689TGT (5217C>T), TCA1690AGT (5218T>A 5219C>G 5220A>T), GCC1691GCT (5223C>T), CGT1692CGG (5226T>G), GCC1695TCA (5233G>T 5235C>A), AAG1696AAC (5238G>C), ACT1697CAA (5239A>C 5240C>A 5241T>A), GAA1698CAA (5242G>C), GAG1700TCA (5248G>T 5249A>C 5250G>A), CCT1701TCA (5251C>T 5253T>A), GGG1702GGT (5256G>T), TGC1703AAC (5257T>A 5258G>A), ATA1704ATC (5262A>C), GTA1705CTG (5263G>C 5265A>G), AAC1707AAT (5271C>T), TGT1708TGC (5274T>C), ACA1709ACT (5277A>T), GGG1710GGA (5280G>A), GCG1711GCC (5283G>C), AAG1712CGC (5284A>C 5285A>G 5286G>C), AAG1713AGA (5288A>G 5289G>A), AGC1714ACA (5291G>C 5292C>A), AAA1715ACA (5294A>C), GTT1717GTG (5301T>G), CGC1718AGA (5302C>A 5304C>A), GGA1719GGG (5307A>G), GCT1721GCC (5313T>C), AGT1722AAT (5315G>A), GAT1725GAC (5325T>C), ATG1730ATA (5340G>A), CCA1732CCC (5346A>C), TAT1733TAC (5349T>C), ACC1734ACT (5352C>T), ATG1735TTG (5353A>T), TCA1736GCA (5356T>G), ATT1738ATC (5364T>C), CTG1739CAG (5366T>A), GCT1741CAT (5371G>C 5372C>A), AGA1743CGC (5377A>C 5379A>C), GGC1744AAG (5380G>A 5381G>A 5382C>G), AAA1745AGT (5384A>G 5385A>T), ATG1746CTG (5386A>C), TCA1747GAA (5389T>G 5390C>A), GAG1748GTT (5393A>T 5394G>T), GAC1749GGT (5396A>G 5397C>T), ATT1750GAT (5398A>G 5399T>A), GCT1751GGG (5402C>G 5403T>G), GAT1754TCT (5410G>T 5411A>C), GTT1755GTC (5415T>C), GTG1756ACC (5416G>A 5417T>C 5418G>C), GTG1758GTT (5424G>T), GGG1760GGT (5430G>T), GCT1764GCA (5442T>A), GTG1765ATA (5443G>A 5445G>A), AGC1766GCT (5446A>G 5447G>C 5448C>T), GCT1771GCA (5463T>A), TTA1772TTG (5466A>G), ATG1773TTT (5467A>T 5469G>T), GCT1774GCA (5472T>A), GTG1775TTG (5473G>T), CGT1778CAT (5483G>A), CAG1779CAA (5487G>A), TAT1780TAC (5490T>C), TGC1781AGG (5491T>A 5493C>G), ATT1782GCC (5494A>G 5495T>C 5496T>C), GAG1783ACA (5497G>A 5498A>C 5499G>A), GAT1784GAC (5502T>C), ATC1785CTT (5503A>C 5505C>T), AGG1788CGC (5512A>C 5514G>C), TTG1789CTA (5515T>C 5517G>A), CGC1790CGA (5520C>A), ACT1791ACC (5523T>C), ATT1792ATG (5526T>G), GGC1793GCT (5528G>C 5529C>T), AAT1794GGG (5530A>G 5531A>G 5532T>G), TTG1796CTG (5536T>C), ACA1800ACC (5550A>C), TAT1801TAC (5553T>C), CCC1802CCT (5556C>T), GTT1806GCG (5567T>C 5568T>G), CAG1807CAA (5571G>A), TTT1809TTG (5577T>G), CAA1811AAA (5581C>A), GTG1813GTT (5589G>T), CCT1814GAA (5590C>G 5591C>A 5592T>A), CAG1815GAC (5593C>G 5595G>C), CAG1815_GAA1816insACC (5595_5596insACC), GAA1816TCA (5596G>T 5597A>C), GGG1817CAC (5599G>C 5600G>A 5601G>C), AAA1818AAG (5604A>G), ATG1819TTG (5605A>T), GCA1820GGA (5609C>G), GTT1822AGT (5614G>A 5615T>G), TCG1824ACA (5620T>A 5622G>A), TCT1825del (5623_5625delTCT), TTG1826TAT (5627T>A 5628G>T), CAA1827GGC (5629C>G 5630A>G 5631A>C), AGT1828ACC (5633G>C 5634T>C), AGT1829ACT (5636G>C), ATG1831AAC (5642T>A 5643G>C), CAA1833CAT (5649A>T), CAA1833_GGT1834insCACAGT (5649_5650insCACAGT), GGT1834GCA (5651G>C 5652T>A), GAG1836TCA (5656G>T 5657A>C 5658G>A), CTT1837GTT (5659C>G), GGC1838GGA (5664C>A), CAA1839CTT (5666A>T 5667A>T), ATT1840CTA (5668A>C 5670T>A), CGT1841AGG (5671C>A 5673T>G), GTC1842ATA (5674G>A 5676C>A), ATC1843GTT (5677A>G 5679C>T), CAC1844CCT (5681A>C 5682C>T), GGT1845TCT (5683G>T 5684G>C), GAT1846GAC (5688T>C), TTA1847CTC (5689T>C 5691A>C), TCA1848TCT (5694A>T), ATG1849GAA (5695A>G 5696T>A 5697G>A), GAA1850AAG (5698G>A 5700A>G), TTG1853GTT (5707T>G 5709G>T), CGC1854CGT (5712C>T), ATG1855TTG (5713A>T), TAT1857TAC (5721T>C), GTT1858ACA (5722G>A 5723T>C 5724T>A), GTG1859ATT (5725G>A 5727G>T), GGC1860GGT (5730C>T), CCA1861CCT (5733A>T), CAT1862GAA (5734C>G 5736T>A), GGT1863GGA (5739T>A), GGA1864GGT (5742A>T), GGC1866GCC (5747G>C), AAC1867CAG (5749A>C 5751C>G), TTT1868TGC (5753T>G 5754T>C), ACC1869TCA (5755A>T 5757C>A), ACA1870ACG (5760A>G), AAA1871AAG (5763A>G), AAT1872AAC (5766T>C), CTT1873CTG (5769T>G), GAT1874AAG (5770G>A 5772T>G), CAA1875CAT (5775A>T), TTG1876GCT (5776T>G 5777T>C 5778G>T), TTT1877TTG (5781T>G), GCA1878GAG (5783C>A 5784A>G), AGT1879ACA (5786G>C 5787T>A), CTT1880TTG (5788C>T 5790T>G), GAA1881TCT (5791G>T 5792A>C 5793A>T), AAG1882GTC (5794A>G 5795A>T 5796G>C), TGT1883AGT (5797T>A), GAA1886ACT (5806G>A 5807A>C 5808A>T), TTG1887CTT (5809T>C 5811G>T), CTT1888GCC (5812C>G 5813T>C 5814T>C), AAG1890AGA (5819A>G 5820G>A), AAC1891GGA (5821A>G 5822A>G 5823C>A), ACG1892GTC (5824A>G 5825C>T 5826G>C), CTG1894GTG (5830C>G), ACC1895ACA (5835C>A), GAG1899AAA (5845G>A 5847G>A), AAA1900GAA (5848A>G), ATG1901TTC (5851A>T 5853G>C), ACT1902TCC (5854A>T 5856T>C), AAA1904CAC (5860A>C 5862A>C), GTG1906TGT (5866G>T 5867T>G 5868G>T), GCA1907GAA (5870C>A), CAA1908ATG (5872C>A 5873A>T 5874A>G), GTC1910CTT (5878G>C 5880C>T), AGT1911GAG (5881A>G 5882G>A 5883T>G), GTA1913CTG (5887G>C 5889A>G), AAA1914GAA (5890A>G),

	CAG1915AGT (5893C>A 5894A>G 5895G>T), TTC1918GTA (5902T>G 5904C>A), TAT1919GTA (5905T>G 5906A>T 5907T>A), CCC1920TTC (5908C>T 5909C>T), AAG1921ATG (5912A>T), GCC1922GGG (5915C>G 5916C>G), ACA1923AGC (5918C>G 5919A>C), ACC1924GTT (5920A>G 5921C>T 5922C>T), GTG1925AAG (5923G>A 5924T>A), GAA1926AAA (5926G>A), CAG1927CAA (5931G>A), CTC1928ATG (5932C>A 5934C>G), AAG1929TTG (5935A>T 5936A>T), GTA1930TCC (5938G>T 5939T>C 5940A>C), TTT1931TTC (5943T>C), GCT1932ATG (5944G>A 5945C>T 5946T>G), GAT1933AAT (5947G>A), GCT1934CAA (5950G>C 5951C>A 5952T>A), CAT1935CAG (5955T>G), CAT1935_ACC1936insATG (5955_5956insATG), ACC1936TCA (5956A>T 5958C>A), ATT1937AAA (5960T>A 5961T>A), GTC1938ACC (5962G>A 5963T>C), AAA1939CAA (5965A>C), ACT1940GTT (5968A>G 5969C>T), GCT1941GCC (5973T>C), AAA1942AGC (5975A>G 5976A>C), CCT1943CCA (5979T>A), TTC1944TGT (5981T>G 5982C>T), CGG1945TGT (5983C>T 5985G>T), GGA1946del (5986_5988delGGA), GTA1947GTC (5991A>C), GTT1948ACA (5992G>A 5993T>C 5994T>A), TTC1949TTT (5997C>T), CGG1950AGT (5998C>A 6000G>T), AAT1952GCA (6004A>G 6005A>C 6006T>A), TTT1953TGG (6008T>G 6009T>G), CTC1954CTA (6012C>A), AGT1955ACT (6014G>C), CCT1956AAG (6016C>A 6017C>A 6018T>G), GAG1957GAC (6021G>C), TGG1958ATT (6022T>A 6023G>T 6024G>T), TGG1958_AAA1959insGTC (6024_6025insGTC), AAA1959ATG (6026A>T 6027A>G), ATT1960ACA (6029T>C 6030T>A), GGA1961AAA (6031G>A 6032G>A), GAG1963AAG (6037G>A), AAC1964AAG (6042C>G), ACA1965CCA (6043A>C), ATG1966del (6046_6048delATG), GCA1967GGA (6050C>G), ACA1968TCT (6052A>T 6054A>T), CTC1969ATT (6055C>A 6057C>T), TCA1970CCT (6058T>C 6060A>T), GAG1972GAA (6066G>A), AGT1973ACA (6068G>C 6069T>A), CTT1974GTG (6070C>G 6072T>G), CTT1975TTT (6073C>T), CCA1976CTT (6077C>T 6078A>T), CTG1979CCT (6086T>C 6087G>T), TCG1980TCA (6090G>A), TCA1982CTA (6094T>C 6095C>T), GCC1983GTT (6098C>T 6099C>T), ATG1984GTG (6100A>G), AAG1985AGA (6104A>G 6105G>A), TTG1987ATG (6109T>A), CTC1988TTG (6112C>T 6114C>G), AGC1989CGC (6115A>C), AGA1990AAT (6119G>A 6120A>T), TAC1991AGT (6121T>A 6122A>G 6123C>T), AAA1992GGA (6124A>G 6125A>G), AGA1993AAG (6128G>A 6129A>G), AAC1994AGC (6131A>G), ATG1995TAC (6133A>T 6134T>A 6135G>C), TCT1996TCA (6138T>A), CAT1998ACA (6142C>A 6143A>C 6144T>A), TGC1999ACA (6145T>A 6146G>C 6147C>A), ATA2000CTG (6148A>C 6150A>G), AAA2001GAA (6151A>G), TTG2002CTT (6154T>C 6156G>T), GCA2003GCT (6159A>T), CTC2004TTT (6160C>T 6162C>T), AAG2005AGA (6164A>G 6165G>A), ATA2006ATG (6168A>G), TAT2007TAC (6171T>C), GTT2008ATG (6172G>A 6174T>G), CTC2009_AAC2010del (6175_6180delCTCAAC), TTC2011TGT (6182T>G 6183C>T), CAG2012CAA (6186G>A), ATG2013TGG (6187A>T 6188T>G), CTG2014GTA (6190C>G 6192G>A), AAT2015CAC (6193A>C 6195T>C), GAA2017CC. (6199G>C 6200A>C)								
helicase motif A-C (YP_009167369.1)	1	98	100%	654	97.6%	98 (100%)	96 (98.0%)	0/0/0/0	0
Protein mutations:	A5C (1345G>T 1346C>G 1347A>T), R16A (1378C>G 1379G>C 1380C>A)								
Codon mutations:	ACA4ACG (1344A>G), GCA5TGT (1345G>T 1346C>G 1347A>T), CGA7CGC (1353A>C), TTG8CTT (1354T>C 1356G>T), TTG14CTC (1372T>C 1374G>C), GAT15GAC (1377T>C), CGC16GCA (1378C>G 1379G>C 1380C>A), GGG18GGT (1386G>T), GAG19GAA (1389G>A), CCT20CCA (1392T>A), CTT22CTG (1398T>G), AGA24CGC (1402A>C 1404A>C), CTT25TTG (1405C>T 1407T>G), GCT27GCG (1413T>G), GTT28GTA (1416T>A), AGC29AGT (1419C>T), AAG30AAA (1422G>A), CGC31AGA (1423C>A 1425C>A), GCC33GCA (1431C>A), TCT36TCC (1440T>C), AAT37AAC (1443T>C), GCC39GCA (1449C>A), CAC40CAT (1452C>T), CAG41CAA (1455G>A), GCT43GCA (1461T>A), TTG45CTT (1465T>C 1467G>T), GAC48GAT (1476C>T), GGA50GGT (1482A>T), GCA51GCT (1485A>T), TTG52CTT (1486T>C 1488G>T), AGA53CGG (1489A>C 1491A>G), GGA57GGG (1503A>G), CAA58CAG (1506A>G), CAA60CAG (1512A>G), ATT62ATA (1518T>A), ATT68ATC (1536T>C), TCC70TCT (1542C>T), ACA71ACC (1545A>C), CCA73CCT (1551A>T), CCA75CCT (1557A>T), CTG76CTT (1560G>T), CCC77CCA (1563C>A), GCA79GCT (1569A>T), GCT80GCA (1572T>A), GTG81GTC (1575G>C), GAA82GAG (1578A>G), GAC83GAT (1581C>T), AAG84AAA (1584G>A), GGG85GGA (1587G>A), CAT87CAC (1593T>C), ACT89ACA (1599T>A), TAT92TAC (1608T>C), GCA95GCC (1617A>C)								

*: Inserts / Deletes / Misaligned / Frameshifts

Analysis details

This analysis was performed with panviral2.64

NGS Details (UN60): Tomato chocolate spot virus (segment RNA 1)

Assembly

**Coverage Length**	1861 (5 contig(s))
**Depth Of Coverage**	11026.4
**Number Of Reads**	127831
**Reads Per Million**	2711.24 rpm (after QC)
**Ambiguities**	0
**Assembly Method**	de novo + reference guided assembly
**Consensus Caller**	Bcf Tools

Coverage Map

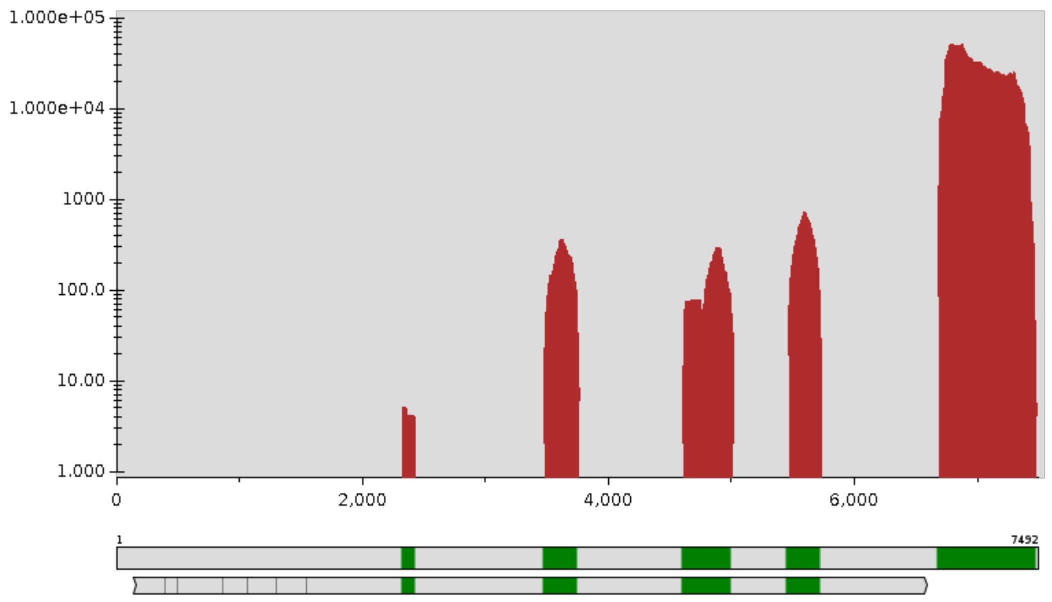

Assignment

**Type**	Tomato chocolate spot virus (Taxonomy ID: 661101)
**Reference Genome**	NC_013075.1
**NT Identity (%)**	67.7475
**AA Identity (%)**	68.8022
**Number Of Stop Codons**	0
**Number Of CDS**	1

Alignment

**Alignment Score**	777.0 (NT) + 1773.0 (AA) = 2550.0
**Concordance (%)**	46.3047

**Alignment Method**	Global, seeded, nucleotide + amino acids (AGA)

Genome Region

Sequence starts at position 2324 and ends at position 7477 relative to NC_013075.1 reference sequence.

Alignment Detailed Statistics

	Begin	End	Coverage	Score	Concordance	Matches	Identities	I/D/M/F*	Stop Codons
**NT**	**2324**	**7477**	**24.8%**	**777**	**26.0%**	**1698 (89.8%)**	**1170 (61.9%)**	**29/163**	
Mutations:	2325T>A, 2329C>T, 2331C>T, 2335T>G, 2336G>C, 2337C>G, 2338A>C, 2341G>C, 2343T>G, 2351T>C, 2352T>A, 2355C>T, 2361C>T, 2363T>C, 2364A>G, 2367C>T, 2382C>A, 2385T>C, 2386T>A, 2387T>G, 2388T>A, 2391C>A, 2394A>G, 2397T>C, 2400T>G, 2403C>T, 2409C>T, 2415T>G, 2416A>C, 2418A>C, 2429A>G, 2430C>G, 2437T>G, 3484G>A, 3489C>T, 3490T>C, 3492G>C, 3498G>C, 3501G>A, 3504T>G, 3511T>G, 3513A>T, 3517C>T, 3519T>G, 3531A>T, 3537A>G, 3538T>A, 3549C>G, 3552C>G, 3562T>A, 3564A>C, 3567A>C, 3573A>G, 3575G>A, 3579C>T, 3581A>C, 3582G>A, 3594T>A, 3600A>T, 3601C>G, 3602C>A, 3603T>A, 3607G>C, 3612A>T, 3615A>T, 3618A>T, 3622A>C, 3624A>G, 3628A>T, 3630C>T, 3633C>G, 3636T>A, 3639C>T, 3642G>A, 3645G>A, 3648T>G, 3651G>T, 3654C>A, 3663A>T, 3675G>C, 3676G>A, 3678C>T, 3679A>G, 3680T>A, 3699A>C, 3700C>T, 3701A>C, 3702C>A, 3711T>G, 3714A>C, 3715G>A, 3717A>C, 3723C>A, 3724A>C, 3725A>G, 3727A>G, 3728T>C, 3729G>T, 3732C>A, 3738G>A, 3739A>G, 3740C>G, 3741C>T, 3747A>G, 3749G>C, 3753T>C, 3754T>A, 3756G>T, 3759G>A, 4615C>T, 4617T>A, 4620C>T, 4621G>A, 4626T>A, 4635A>G, 4644C>T, 4650T>G, 4656T>G, 4659C>A, 4660T>A, 4666T>C, 4668G>T, 4671G>A, 4677T>C, 4678T>G, 4679C>A, 4680A>G, 4683G>A, 4686C>T, 4687A>G, 4688A>C, 4689C>A, 4692T>A, 4701A>G, 4704G>A, 4707T>A, 4708A>C, 4709T>A, 4710G>A, 4713A>G, 4716T>C, 4720G>A, 4722A>T, 4725T>C, 4728A>G, 4731C>T, 4734T>C, 4735T>C, 4737G>T, 4740C>T, 4744T>A, 4745C>A, 4746A>C, 4750G>A, 4755G>C, 4761G>T, 4764G>C, 4765C>A, 4767G>A, 4768T>C, 4770T>C, 4785A>G, 4786C>T, 4791T>G, 4795C>T, 4806G>C, 4813A>C, 4815A>C, 4822C>T, 4827C>T, 4833T>A, 4834A>C, 4836G>A, 4839A>C, 4842G>C, 4845T>C, 4854A>T, 4860A>T, 4861A>G, 4863A>G, 4881A>C, 4882C>A, 4891C>G, 4892C>T, 4893C>T, 4896T>G, 4902T>A, 4906G>A, 4907C>T, 4908A>C, 4909C>A, 4910A>C, 4911G>T, 4914A>C, 4918A>T, 4919T>A, 4920G>T, 4929T>G, 4935T>G, 4938C>T, 4939A>G, 4950C>T, 4953T>C, 4956T>G, 4962T>A, 4965A>G, 4968T>C, 4970C>T, 4971A>T, 4977G>C, 4979A>C, 4984G>A, 4985T>C, 4986G>A, 4988C>A, 4989A>C, 4990G>A, 4995G>C, 4999G>A, 5005C>A, 5007G>A, 5009A>T, 5010T>G, 5011G>C, 5013A>G, 5016T>A, 5019A>C, 5469T>A, 5472G>A, 5473G>T, 5475G>A, 5478T>C, 5480G>A, 5481G>T, 5486T>A, 5488T>A, 5490T>A, 5497G>C, 5499C>A, 5500A>C, 5505T>A, 5508A>C, 5511G>A, 5515C>A, 5517C>A, 5521A>T, 5523T>G, 5526T>G, 5527A>G, 5528A>G, 5529T>C, 5530G>T, 5544C>T, 5545A>C, 5546C>A, 5547A>G, 5550C>T, 5563G>A, 5564C>T, 5565A>T, 5568G>A, 5571C>T, 5577A>C, 5578C>A, 5579A>C, 5580G>A, 5583C>T, 5586_5587insGTG, 5588C>A, 5589C>G, 5590C>A, 5592T>A, 5593G>A, 5596G>T, 5597A>G, 5598T>C, 5601A>G, 5602G>A, 5603T>C, 5604G>A, 5607A>T, 5614G>A, 5617_5619delATT, 5620T>G, 5621C>G, 5624T>G, 5625G>C, 5626C>G, 5628C>T, 5629A>G, 5631C>A, 5633G>T, 5634T>G, 5637T>G, 5638G>A, 5639C>A, 5640A>C, 5643T>C, 5646A>T, 5646_5647insCATGCT, 5648G>C, 5651T>A, 5654A>C, 5655A>C, 5656T>C, 5658G>T, 5661C>A, 5664T>C, 5665T>C, 5667C>T, 5668A>C, 5674A>G, 5676C>G, 5677C>G, 5679T>C, 5680G>T, 5683G>T, 5684A>C, 5685C>A, 5688C>T, 5693A>G, 5694G>A, 5696C>A, 5697T>G, 5699C>T, 5700T>G, 5703C>A, 5704T>G, 5710A>T, 5715A>C, 5718T>C, 5719G>A, 5720T>C, 5721G>T, 5727A>G, 5729C>A, 5730C>G, 5733T>A, 5735G>T, 5736A>T, 6706A>G, 6707T>A, 6721G>C, 6722G>C, 6729T>C, 6731G>A, 6735G>A, 6736T>G, 6740C>A, 6741G>A, 6753G>T, 6757T>G, 6758_6759insT, 6759_6760insG, 6772C>T, 6777T>C, 6795A>G, 6806C>T, 6814C>T, 6815_6834delAATTGTTTGGTGTTGTCAAC, 6837G>T, 6840G>A, 6842C>A, 6843T>C, 6845T>G, 6847_6850delGCCA, 6853A>G, 6855A>T, 6857C>A, 6859C>T, 6860C>G, 6861C>G, 6862C>T, 6863T>C, 6867_6868delGC, 6870T>G, 6872A>G, 6873T>A, 6878A>G, 6879A>T, 6881G>A, 6885A>C, 6886G>T, 6887A>T, 6889_6894delGTGGAG, 6897G>A, 6903C>A, 6906G>C, 6908A>C, 6909A>G, 6911C>G, 6912A>G, 6914A>G, 6915A>T, 6916G>A, 6918A>T, 6919_6921delGAG, 6923C>T, 6924T>C, 6926T>C, 6930_6937delGGTCGGGC, 6942G>C, 6944_6946delTGG, 6949G>A, 6950C>G, 6951C>T, 6953G>C, 6955A>G, 6956T>G, 6958A>T, 6960A>T, 6961G>T, 6962C>T, 6964T>C, 6965T>C, 6967G>A, 6969G>A, 6970T>G, 6972T>A, 6973G>A, 6976T>A, 6978T>A, 6982_6985delCATC, 6987G>T, 6989T>A, 6991T>C, 6996A>C, 6998C>T, 7001_7012delCTCGTTTGGAGT, 7014T>G, 7016A>G, 7018C>G, 7020A>T, 7022delT, 7026G>T, 7030_7035delTGGTGT, 7040A>C, 7041C>G, 7042G>A, 7047_7067delGCAATGACACCCGGGGTGTGC, 7073C>G, 7077T>C, 7079A>T, 7080A>G, 7084_7088delAAGTA, 7091G>A, 7093T>C, 7097delT, 7100C>G, 7102T>A, 7103A>T, 7108_7116delAACTAGAGC, 7118T>G, 7121_7129delGTCTCAACT, 7132A>G, 7134G>T, 7135A>G, 7136G>A, 7140_7145delCCCGTT, 7148A>T, 7151A>C, 7152T>A, 7154G>A, 7155A>C, 7156T>C, 7158A>C, 7159A>T, 7161T>C, 7161_7162insC, 7165G>C, 7169C>G, 7172delG, 7177A>C, 7180_7183delCTGC, 7186T>G, 7188G>T, 7190T>C, 7193G>A, 7195_7218delGCAACAGTGAAGAATCATCACACC, 7220C>G, 7223C>A, 7224T>C, 7225T>C, 7227C>T, 7228C>G, 7230A>C, 7231G>T, 7234T>G, 7235C>G, 7238C>T, 7241G>C, 7244G>A, 7245T>A, 7247_7248delTC, 7251A>T, 7259_7260delCT, 7262C>G, 7264T>A, 7266G>A, 7270A>C, 7274G>C, 7288G>C, 7289T>C, 7291T>G, 7292T>G, 7293G>A, 7294C>T, 7296_7297insGCTTCTCCTAGTTAA, 7298G>A, 7299A>C, 7300C>G, 7302A>G, 7303T>C, 7304G>A, 7308C>T, 7310T>A, 7311A>T, 7314G>C, 7315T>C, 7316T>A, 7317T>G, 7318G>T, 7319G>A, 7321T>A, 7325C>A, 7327T>A, 7328A>T, 7330A>T, 7335_7336insT, 7342T>G, 7346A>T, 7348A>T, 7350G>C, 7351G>T, 7352_7353insC, 7356A>G, 7358C>G, 7367C>T, 7369G>A, 7372G>T, 7373delA, 7375T>C, 7376G>C, 7378A>C, 7381C>G, 7385C>T, 7387T>G, 7388T>C, 7389C>A, 7390A>T, 7393_7396delTATT, 7407G>T, 7410delG, 7425A>C, 7428delC, 7430T>C, 7431C>A, 7447A>C, 7453A>G, 7454G>A, 7455A>T, 7459T>C, 7460C>G								

CDS

ToChSV_s1_gp1	730	1866	16.6%	1773	69.6%	356 (98.9%)	247 (68.6%)	3/1/0/0	0
Protein mutations:	H731Y (2329C>T 2331C>T), C733A (2335T>G 2336G>C 2337C>G), I734L (2338A>C), D735Q (2341G>C 2343T>G), V738A (2351T>C 2352T>A), I742T (2363T>C 2364A>G), F750R (2386T>A 2387T>G 2388T>A), N764R (2429A>G 2430C>G), A1116T (3484G>A), E1120D (3498G>C), N1122K (3504T>G), S1125A (3511T>G 3513A>T), L1134M (3538T>A), S1142T (3562T>A 3564A>C), R1146K (3575G>A), Q1148P (3581A>C 3582G>A), P1155E (3601C>G 3602C>A 3603T>A), E1157Q (3607G>C), I1162L (3622A>C 3624A>G), T1164S (3628A>T 3630C>T), N1166K (3636T>A), E1171D (3651G>T), V1180I (3676G>A 3678C>T), M1181E (3679A>G 3680T>A), H1188S (3700C>T 3701A>C 3702C>A), D1191E (3711T>G), E1192D (3714A>C), G1193S (3715G>A 3717A>C), K1196R (3724A>C 3725A>G), M1197A (3727A>G 3728T>C 3729G>T), T1201G (3739A>G 3740C>G 3741C>T), C1204S (3749G>C), L1206I (3754T>A 3756G>T), V1495I (4621G>A), L1508M (4660T>A), S1514E (4678T>G 4679C>A 4680A>G), N1517A (4687A>G 4688A>C 4689C>A), D1523E (4707T>A), M1524Q (4708A>C 4709T>A 4710G>A), V1528I (4720G>A 4722A>T), S1536N (4744T>A 4745C>A 4746A>C), D1538N (4750G>A), E1541D (4761G>T), Q1543K (4765C>A 4767G>A), F1544L (4768T>C 4770T>C), H1565Q (4833T>A), M1566L (4834A>C 4836G>A), I1575V (4861A>G 4863A>G), P1585V (4891C>G 4892C>T 4893C>T), A1590I (4906G>A 4907C>T 4908A>C), Q1591T (4909C>A 4910A>C 4911G>T), M1594Y (4918A>T 4919T>A 4920G>T), I1601V (4939A>G), H1606Q (4956T>G), A1611V (4970C>T 4971A>T), E1613D (4977G>C), K1614T (4979A>C), V1616T (4984G>A 4985T>C 4986G>A), A1617D (4988C>A 4989A>C), D1618N (4990G>A), E1621K (4999G>A), L1623I (5005C>A 5007G>A), N1624M (5009A>T 5010T>G), E1625Q (5011G>C 5013A>G), A1779S (5473G>T 5475G>A), R1781N (5480G>A 5481G>T), F1783Y (5486T>A), C1784R (5488T>A 5490T>A), D1787Q (5497G>C 5499C>A), I1788L (5500A>C), I1795L (5521A>T 5523T>G), N1797G (5527A>G 5528A>G 5529T>C), V1798L (5530G>T), T1803Q (5545A>C 5546C>A 5547A>G), A1809I (5563G>A 5564C>T 5565A>T), Q1814T (5578C>A 5579A>C 5580G>A), V1816_P1817insV (5586_5587insGTG), P1817Q (5588C>A 5589C>G), H1818K (5590C>A 5592T>A), G1819S (5593G>A), D1820C (5596G>T 5597A>G 5598T>C), V1822T (5602G>A 5603T>C 5604G>A), G1826S (5614G>A), I1827del (5617_5619delATT), S1828G (5620T>G 5621C>G), L1829R (5624T>G 5625G>C), H1830D (5626C>G 5628C>T), S1831G (5629A>G 5631C>A), S1832M (5633G>T 5634T>G), F1833L (5637T>G), A1834N (5638G>A 5639C>A 5640A>C), Q1836H (5646A>T), Q1836_G1837insHA (5646_5647insCATGCT), G1837A (5648G>C), F1838Y (5651T>A), E1839A (5654A>C 5655A>C), F1843L (5665T>C 5667C>T), I1846V (5674A>G 5676C>G), R1847G (5677C>G 5679T>C), G1848C (5680G>T), D1849S (5683G>T 5684A>C 5685C>A), K1852R (5693A>G 5694G>A), T1853K (5696C>A 5697T>G), T1854M (5699C>T 5700T>G), L1856V (5704T>G), M1858L (5710A>T), V1861T (5719G>A 5720T>C 5721G>T), P1864Q (5729C>A 5730C>G), N1865K (5733T>A), G1866V (5735G>T 5736A>T)								

	Begin	End	Coverage	Score	Concordance	Matches	Identities	I/D/M/F*	Stop Codons
**NT**	**2324**	**7477**	**24.8%**	**777**	**26.0%**	**1698 (89.8%)**	**1170 (61.9%)**	**29/163**	
Codon mutations:	TTT729.TA (2325T>A), CAC731TAT (2329C>T 2331C>T), TGC733GCG (2335T>G 2336G>C 2337C>G), ATT734CTT (2338A>C), GAT735CAG (2341G>C 2343T>G), GTT738GCA (2351T>C 2352T>A), GAC739GAT (2355C>T), CTC741CTT (2361C>T), ATA742ACG (2363T>C 2364A>G), TGC743TGT (2367C>T), CCC748CCA (2382C>A), CCT749CCC (2385T>C), TTT750AGA (2386T>A 2387T>G 2388T>A), GTC751GTA (2391C>A), CAA752CAG (2394A>G), TTT753TTC (2397T>C), CTT754CTG (2400T>G), TAC755TAT (2403C>T), GCC757GCT (2409C>T), GTT759GTG (2415T>G), AGA760CGC (2416A>C 2418A>C), AAC764AGG (2429A>G 2430C>G), TCA767GC. (2437T>G), GCT1116ACT (3484G>A), GCC1117GCT (3489C>T), TTG1118CTC (3490T>C 3492G>C), GAG1120GAC (3498G>C), GAG1121GAA (3501G>A), AAT1122AAG (3504T>G), TCA1125GCT (3511T>G 3513A>T), CTT1127TTG (3517C>T 3519T>G), CCA1131CCT (3531A>T), GAA1133GAG (3537A>G), TTG1134ATG (3538T>A), ACC1137ACG (3549C>G), GTC1138GTG (3552C>G), TCA1142ACC (3562T>A 3564A>C), ACA1143ACC (3567A>C), AGA1145AGG (3573A>G), AGA1146AAA (3575G>A), AGC1147AGT (3579C>T), CAG1148CCA (3581A>C 3582G>A), GCT1152GCA (3594T>A), GGA1154GGT (3600A>T), CCT1155GAA (3601C>G 3602C>A 3603T>A), GAA1157CAA (3607G>C), ACA1158ACT (3612A>T), GCA1159GCT (3615A>T), CCA1160CCT (3618A>T), ATA1162CTG (3622A>C 3624A>G), ACC1164TCT (3628A>T 3630C>T), GTC1165GTG (3633C>G), AAT1166AAA (3636T>A), GAC1167GAT (3639C>T), AAG1168AAA (3642G>A), AGG1169AGA (3645G>A), GGT1170GGG (3648T>G), GAG1171GAT (3651G>T), GGC1172GGA (3654C>A), CCA1175CCT (3663A>T), GGG1179GGC (3675G>C), GTC1180ATT (3676G>A 3678C>T), ATG1181GAG (3679A>G 3680T>A), GCA1187GCC (3699A>C), CAC1188TCA (3700C>T 3701A>C 3702C>A), GAT1191GAG (3711T>G), GAA1192GAC (3714A>C), GGA1193AGC (3715G>A 3717A>C), GCC1195GCA (3723C>A), AAG1196CGG (3724A>C 3725A>G), ATG1197GCT (3727A>G 3728T>C 3729G>T), GCC1198GCA (3732C>A), GAG1200GAA (3738G>A), ACC1201GGT (3739A>G 3740C>G 3741C>T), AAA1203AAG (3747A>G), TGT1204TCT (3749G>C), GCT1205GCC (3753T>C), TTG1206ATT (3754T>A 3756G>T), TTG1207TTA (3759G>A), CTT1493TTA (4615C>T 4617T>A), TTC1494TTT (4620C>T), GTT1495ATT (4621G>A), GCT1496GCA (4626T>A), GGA1499GGG (4635A>G), AAC1502AAT (4644C>T), GTT1504GTG (4650T>G), GTT1506GTG (4656T>G), CCC1507CCA (4659C>A), TTG1508ATG (4660T>A), TTG1510CTT (4666T>C 4668G>T), CAG1511CAA (4671G>A), TAT1513TAC (4677T>C), TCA1514GAG (4678T>G 4679C>A 4680A>G), TTG1515TTA (4683G>A), CCC1516CCT (4686C>T), AAC1517GCA (4687A>G 4688A>C 4689C>A), ATT1518ATA (4692T>A), GAA1521GAG (4701A>G), CTG1522CTA (4704G>A), GAT1523GAA (4707T>A), ATG1524CAA (4708A>C 4709T>A 4710G>A), GTA1525GTG (4713A>G), AAT1526AAC (4716T>C), GTA1528ATT (4720G>A 4722A>T), ATT1529ATC (4725T>C), AAA1530AAG (4728A>G), AAC1531AAT (4731C>T), GGT1532GGC (4734T>C), TTG1533CTT (4735T>C 4737G>T), GAC1534GAT (4740C>T), TCA1536AAC (4744T>A 4745C>A 4746A>C), GAT1538AAT (4750G>A), GTG1539GTC (4755G>C), GAG1541GAT (4761G>T), GTG1542GTC (4764G>C), CAG1543AAA (4765C>A 4767G>A), TTT1544CTC (4768T>C 4770T>C), GAA1549GAG (4785A>G), CTG1550TTG (4786C>T), ACT1551ACG (4791T>G), CTG1553TTG (4795C>T), GGG1556GGC (4806G>C), AGA1559CGC (4813A>C 4815A>C), CTG1562TTG (4822C>T), GGC1563GGT (4827C>T), CAT1565CAA (4833T>A), ATG1566CTA (4834A>C 4836G>A), GCA1567GCC (4839A>C), CCG1568CCC (4842G>C), CTT1569CTC (4845T>C), GTA1572GTT (4854A>T), ATA1574ATT (4860A>T), ATA1575GTG (4861A>G 4863A>G), ATA1581ATC (4881A>C), CGA1582AGA (4882C>A), CCC1585GTT (4891C>G 4892C>T 4893C>T), GGT1586GGG (4896T>G), CCT1588CCA (4902T>A), GCA1590ATC (4906G>A 4907C>T 4908A>C), CAG1591ACT (4909C>A 4910A>C 4911G>T), GCA1592GCC (4914A>C), ATG1594TAT (4918A>T 4919T>A 4920G>T), GTT1597GTG (4929T>G), ACT1599ACG (4935T>G), GGC1600GGT (4938C>T), ATA1601GTA (4939A>G), GCC1604GCT (4950C>T), TTT1605TTC (4953T>C), CAT1606CAG (4956T>G), GGT1608GGA (4962T>A), CAA1609CAG (4965A>G), GTT1610GTC (4968T>C), GCA1611GTT (4970C>T 4971A>T), GAG1613GAC (4977G>C), AAA1614ACA (4979A>C), GTG1616ACA (4984G>A 4985T>C 4986G>A), GCA1617GAC (4988C>A 4989A>C), GAT1618AAT (4990G>A), GTG1619GTC (4995G>C), GAA1621AAA (4999G>A), CTG1623ATA (5005C>A 5007G>A), AAT1624ATG (5009A>T 5010T>G), GAA1625CAG (5011G>C 5013A>G), CGT1626CGA (5016T>A), GGA1627GGC (5019A>C), GCT1777..A (5469T>A), TTG1778TTA (5472G>A), GCG1779TCA (5473G>T 5475G>A), ACT1780ACC (5478T>C), AGG1781AAT (5480G>A 5481G>T), TTT1783TAT (5486T>A), TGT1784AGA (5488T>A 5490T>A), GAC1787CAA (5497G>C 5499C>A), ATT1788CTT (5500A>C), ATT1789ATA (5505T>A), GTA1790GTC (5508A>C), AGG1791AGA (5511G>A), CGC1793AGA (5515C>A 5517C>A), ATT1795TTG (5521A>T 5523T>G), GCT1796GCG (5526T>G), AAT1797GGC (5527A>G 5528A>G 5529T>C), GTG1798TTG (5530G>T), AAC1802AAT (5544C>T), ACA1803CAG (5545A>C 5546C>A 5547A>G), TAC1804TAT (5550C>T), GCA1809ATT (5563G>A 5564C>T 5565A>T), CAG1810CAA (5568G>A), TAC1811TAT (5571C>T), CTA1813CTC (5577A>C), CAG1814ACA (5578C>A 5579A>C 5580G>A), TGC1815TGT (5583C>T), GTC1816_CCC1817insGTG (5586_5587insGTG), CCC1817CAG (5588C>A 5589C>G), CAT1818AAA (5590C>A 5592T>A), GGT1819AGT (5593G>A), GAT1820TGC (5596G>T 5597A>G 5598T>C), AAA1821AAG (5601A>G), GTG1822ACA (5602G>A 5603T>C 5604G>A), GCA1823GCT (5607A>T), GGT1826AGT (5614G>A), ATT1827del (5617_5619delATT), TCG1828GGG (5620T>G 5621C>G), CTG1829CGC (5624T>G 5625G>C), CAC1830GAT (5626C>G 5628C>T), AGC1831GGA (5629A>G 5631C>A), AGT1832ATG (5633G>T 5634T>G), TTT1833TTG (5637T>G), GCA1834AAC (5638G>A 5639C>A 5640A>C), CAT1835CAC (5643T>C), CAA1836CAT (5646A>T), CAA1836_GGT1837insCATGCT (5646_5647insCATGCT), GGT1837GCT (5648G>C), TTT1838TAT (5651T>A), GAA1839GCC (5654A>C 5655A>C), TTG1840CTT (5656T>C 5658G>T), GGC1841GGA (5661C>A), AAT1842AAC (5664T>C), TTC1843CTT (5665T>C 5667C>T), AGA1844CGA (5668A>C), ATC1846GTG (5674A>G 5676C>G), CGT1847GGC (5677C>G 5679T>C), GGT1848TGT (5680G>T), GAC1849TCA (5683G>T 5684A>C 5685C>A), ATC1850ATT (5688C>T), AAG1852AGA (5693A>G 5694G>A), ACT1853AAG (5696C>A 5697T>G), ACT1854ATG (5699C>T 5700T>G), GCC1855GCA (5703C>A), TTG1856GTG (5704T>G), ATG1858TTG (5710A>T), CCA1859CCC (5715A>C), TAT1860TAC (5718T>C), GTG1861ACT (5719G>A 5720T>C 5721G>T), GGA1863GGG (5727A>G), CCC1864CAG (5729C>A 5730C>G), AAT1865AAA (5733T>A), GGA1866GTT (5735G>T 5736A>T)								

Proteins

	Begin	End	Coverage	Score	Concordance	Matches	Identities	I/D/M/F*	Stop Codons
polyprotein (YP_003097229.1)	730	1866	16.6%	1773	69.6%	356 (98.9%)	247 (68.6%)	3/1/0/0	0
Protein mutations:	H731Y (2329C>T 2331C>T), C733A (2335T>G 2336G>C 2337C>G), I734L (2338A>C), D735Q (2341G>C 2343T>G), V738A (2351T>C 2352T>A), I742T (2363T>C 2364A>G), F750R (2386T>A 2387T>G 2388T>A), N764R (2429A>G 2430C>G), A1116T (3484G>A), E1120D (3498G>C), N1122K (3504T>G), S1125A (3511T>G 3513A>T), L1134M (3538T>A), S1142T (3562T>A 3564A>C), R1146K (3575G>A), Q1148P (3581A>C 3582G>A), P1155E (3601C>G 3602C>A 3603T>A), E1157Q (3607G>C), I1162L (3622A>C 3624A>G), T1164S (3628A>T 3630C>T), N1166K (3636T>A), E1171D (3651G>T), V1180I (3676G>A 3678C>T), M1181E (3679A>G 3680T>A), H1188S (3700C>T 3701A>C 3702C>A), D1191E (3711T>G), E1192D (3714A>C), G1193S (3715G>A 3717A>C), K1196R (3724A>C 3725A>G), M1197A (3727A>G 3728T>C 3729G>T), T1201G (3739A>G 3740C>G 3741C>T), C1204S (3749G>C), L1206I (3754T>A 3756G>T), V1495I (4621G>A), L1508M (4660T>A), S1514E (4678T>G 4679C>A 4680A>G), N1517A (4687A>G 4688A>C 4689C>A), D1523E (4707T>A), M1524Q (4708A>C 4709T>A 4710G>A), V1528I (4720G>A 4722A>T), S1536N (4744T>A 4745C>A 4746A>C), D1538N (4750G>A), E1541D (4761G>T), Q1543K (4765C>A 4767G>A), F1544L (4768T>C 4770T>C), H1565Q (4833T>A), M1566L (4834A>C 4836G>A), I1575V (4861A>G 4863A>G), P1585V (4891C>G 4892C>T 4893C>T), A1590I (4906G>A 4907C>T 4908A>C), Q1591T (4909C>A 4910A>C 4911G>T), M1594Y (4918A>T 4919T>A 4920G>T), I1601V (4939A>G), H1606Q (4956T>G), A1611V (4970C>T 4971A>T), E1613D (4977G>C), K1614T (4979A>C), V1616T (4984G>A 4985T>C 4986G>A), A1617D (4988C>A 4989A>C), D1618N (4990G>A), E1621K (4999G>A), L1623I (5005C>A 5007G>A), N1624M (5009A>T 5010T>G), E1625Q (5011G>C 5013A>G), A1779S (5473G>T 5475G>A), R1781N (5480G>A 5481G>T), F1783Y (5486T>A), C1784R (5488T>A 5490T>A), D1787Q (5497G>C 5499C>A), I1788L (5500A>C), I1795L (5521A>T 5523T>G), N1797G (5527A>G 5528A>G 5529T>C), V1798L (5530G>T), T1803Q (5545A>C 5546C>A 5547A>G), A1809I (5563G>A 5564C>T 5565A>T), Q1814T (5578C>A 5579A>C 5580G>A), V1816_P1817insV (5586_5587insGTG), P1817Q (5588C>A 5589C>G), H1818K (5590C>A 5592T>A), G1819S (5593G>A), D1820C (5596G>T 5597A>G 5598T>C), V1822T (5602G>A 5603T>C 5604G>A), G1826S (5614G>A), I1827del (5617_5619delATT), S1828G (5620T>G 5621C>G), L1829R (5624T>G 5625G>C), H1830D (5626C>G 5628C>T), S1831G (5629A>G 5631C>A), S1832M (5633G>T 5634T>G), F1833L (5637T>G), A1834N (5638G>A 5639C>A 5640A>C), Q1836H (5646A>T), Q1836_G1837insHA (5646_5647insCATGCT), G1837A (5648G>C), F1838Y (5651T>A), E1839A (5654A>C 5655A>C), F1843L (5665T>C 5667C>T), I1846V (5674A>G 5676C>G), R1847G (5677C>G 5679T>C), G1848C (5680G>T), D1849S (5683G>T 5684A>C 5685C>A), K1852R (5693A>G 5694G>A), T1853K (5696C>A 5697T>G), T1854M (5699C>T 5700T>G), L1856V (5704T>G), M1858L (5710A>T), V1861T (5719G>A 5720T>C 5721G>T), P1864Q (5729C>A 5730C>G), N1865K (5733T>A), G1866V (5735G>T 5736A>T)								

	Begin	End	Coverage	Score	Concordance	Matches	Identities	I/D/M/F*	Stop Codons
**NT**	**2324**	**7477**	**24.8%**	**777**	**26.0%**	**1698 (89.8%)**	**1170 (61.9%)**	**29/163**	
Codon mutations:	TTT729.TA (2325T>A), CAC731TAT (2329C>T 2331C>T), TGC733GCG (2335T>G 2336G>C 2337C>G), ATT734CTT (2338A>C), GAT735CAG (2341G>C 2343T>G), GTT738GCA (2351T>C 2352T>A), GAC739GAT (2355C>T), CTC741CTT (2361C>T), ATA742ACG (2363T>C 2364A>G), TGC743TGT (2367C>T), CCC748CCA (2382C>A), CCT749CCC (2385T>C), TTT750AGA (2386T>A 2387T>G 2388T>A), GTC751GTA (2391C>A), CAA752CAG (2394A>G), TTT753TTC (2397T>C), CTT754CTG (2400T>G), TAC755TAT (2403C>T), GCC757GCT (2409C>T), GTT759GTG (2415T>G), AGA760CGC (2416A>C 2418A>C), AAC764AGG (2429A>G 2430C>G), TCA767GC. (2437T>G), GCT1116ACT (3484G>A), GCC1117GCT (3489C>T), TTG1118CTC (3490T>C 3492G>C), GAG1120GAC (3498G>C), GAG1121GAA (3501G>A), AAT1122AAG (3504T>G), TCA1125GCT (3511T>G 3513A>T), CTT1127TTG (3517C>T 3519T>G), CCA1131CCT (3531A>T), GAA1133GAG (3537A>G), TTG1134ATG (3538T>A), ACC1137ACG (3549C>G), GTC1138GTG (3552C>G), TCA1142ACC (3562T>A 3564A>C), ACA1143ACC (3567A>C), AGA1145AGG (3573A>G), AGA1146AAA (3575G>A), AGC1147AGT (3579C>T), CAG1148CCA (3581A>C 3582G>A), GCT1152GCA (3594T>A), GGA1154GGT (3600A>T), CCT1155GAA (3601C>G 3602C>A 3603T>A), GAA1157CAA (3607G>C), ACA1158ACT (3612A>T), GCA1159GCT (3615A>T), CCA1160CCT (3618A>T), ATA1162CTG (3622A>C 3624A>G), ACC1164TCT (3628A>T 3630C>T), GTC1165GTG (3633C>G), AAT1166AAA (3636T>A), GAC1167GAT (3639C>T), AAG1168AAA (3642G>A), AGG1169AGA (3645G>A), GGT1170GGG (3648T>G), GAG1171GAT (3651G>T), GGC1172GGA (3654C>A), CCA1175CCT (3663A>T), GGG1179GGC (3675G>C), GTC1180ATT (3676G>A 3678C>T), ATG1181GAG (3679A>G 3680T>A), GCA1187GCC (3699A>C), CAC1188TCA (3700C>T 3701A>C 3702C>A), GAT1191GAG (3711T>G), GAA1192GAC (3714A>C), GGA1193AGC (3715G>A 3717A>C), GCC1195GCA (3723C>A), AAG1196CGG (3724A>C 3725A>G), ATG1197GCT (3727A>G 3728T>C 3729G>T), GCC1198GCA (3732C>A), GAG1200GAA (3738G>A), ACC1201GGT (3739A>G 3740C>G 3741C>T), AAA1203AAG (3747A>G), TGT1204TCT (3749G>C), GCT1205GCC (3753T>C), TTG1206ATT (3754T>A 3756G>T), TTG1207TTA (3759G>A), CTT1493TTA (4615C>T 4617T>A), TTC1494TTT (4620C>T), GTT1495ATT (4621G>A), GCT1496GCA (4626T>A), GGA1499GGG (4635A>G), AAC1502AAT (4644C>T), GTT1504GTG (4650T>G), GTT1506GTG (4656T>G), CCC1507CCA (4659C>A), TTG1508ATG (4660T>A), TTG1510CTT (4666T>C 4668G>T), CAG1511CAA (4671G>A), TAT1513TAC (4677T>C), TCA1514GAG (4678T>G 4679C>A 4680A>G), TTG1515TTA (4683G>A), CCC1516CCT (4686C>T), AAC1517GCA (4687A>G 4688A>C 4689C>A), ATT1518ATA (4692T>A), GAA1521GAG (4701A>G), CTG1522CTA (4704G>A), GAT1523GAA (4707T>A), ATG1524CAA (4708A>C 4709T>A 4710G>A), GTA1525GTG (4713A>G), AAT1526AAC (4716T>C), GTA1528ATT (4720G>A 4722A>T), ATT1529ATC (4725T>C), AAA1530AAG (4728A>G), AAC1531AAT (4731C>T), GGT1532GGC (4734T>C), TTG1533CTT (4735T>C 4737G>T), GAC1534GAT (4740C>T), TCA1536AAC (4744T>A 4745C>A 4746A>C), GAT1538AAT (4750G>A), GTG1539GTC (4755G>C), GAG1541GAT (4761G>T), GTG1542GTC (4764G>C), CAG1543AAA (4765C>A 4767G>A), TTT1544CTC (4768T>C 4770T>C), GAA1549GAG (4785A>G), CTG1550TTG (4786C>T), ACT1551ACG (4791T>G), CTG1553TTG (4795C>T), GGG1556GGC (4806G>C), AGA1559CGC (4813A>C 4815A>C), CTG1562TTG (4822C>T), GGC1563GGT (4827C>T), CAT1565CAA (4833T>A), ATG1566CTA (4834A>C 4836G>A), GCA1567GCC (4839A>C), CCG1568CCC (4842G>C), CTT1569CTC (4845T>C), GTA1572GTT (4854A>T), ATA1574ATT (4860A>T), ATA1575GTG (4861A>G 4863A>G), ATA1581ATC (4881A>C), CGA1582AGA (4882C>A), CCC1585GTT (4891C>G 4892C>T 4893C>T), GGT1586GGG (4896T>G), CCT1588CCA (4902T>A), GCA1590ATC (4906G>A 4907C>T 4908A>C), CAG1591ACT (4909C>A 4910A>C 4911G>T), GCA1592GCC (4914A>C), ATG1594TAT (4918A>T 4919T>A 4920G>T), GTT1597GTG (4929T>G), ACT1599ACG (4935T>G), GGC1600GGT (4938C>T), ATA1601GTA (4939A>G), GCC1604GCT (4950C>T), TTT1605TTC (4953T>C), CAT1606CAG (4956T>G), GGT1608GGA (4962T>A), CAA1609CAG (4965A>G), GTT1610GTC (4968T>C), GCA1611GTT (4970C>T 4971A>T), GAG1613GAC (4977G>C), AAA1614ACA (4979A>C), GTG1616ACA (4984G>A 4985T>C 4986G>A), GCA1617GAC (4988C>A 4989A>C), GAT1618AAT (4990G>A), GTG1619GTC (4995G>C), GAA1621AAA (4999G>A), CTG1623ATA (5005C>A 5007G>A), AAT1624ATG (5009A>T 5010T>G), GAA1625CAG (5011G>C 5013A>G), CGT1626CGA (5016T>A), GGA1627GGC (5019A>C), GCT1777..A (5469T>A), TTG1778TTA (5472G>A), GCG1779TCA (5473G>T 5475G>A), ACT1780ACC (5478T>C), AGG1781AAT (5480G>A 5481G>T), TTT1783TAT (5486T>A), TGT1784AGA (5488T>A 5490T>A), GAC1787CAA (5497G>C 5499C>A), ATT1788CTT (5500A>C), ATT1789ATA (5505T>A), GTA1790GTC (5508A>C), AGG1791AGA (5511G>A), CGC1793AGA (5515C>A 5517C>A), ATT1795TTG (5521A>T 5523T>G), GCT1796GCG (5526T>G), AAT1797GGC (5527A>G 5528A>G 5529T>C), GTG1798TTG (5530G>T), AAC1802AAT (5544C>T), ACA1803CAG (5545A>C 5546C>A 5547A>G), TAC1804TAT (5550C>T), GCA1809ATT (5563G>A 5564C>T 5565A>T), CAG1810CAA (5568G>A), TAC1811TAT (5571C>T), CTA1813CTC (5577A>C), CAG1814ACA (5578C>A 5579A>C 5580G>A), TGC1815TGT (5583C>T), GTC1816_CCC1817insGTG (5586_5587insGTG), CCC1817CAG (5588C>A 5589C>G), CAT1818AAA (5590C>A 5592T>A), GGT1819AGT (5593G>A), GAT1820TGC (5596G>T 5597A>G 5598T>C), AAA1821AAG (5601A>G), GTG1822ACA (5602G>A 5603T>C 5604G>A), GCA1823GCT (5607A>T), GGT1826AGT (5614G>A), ATT1827del (5617_5619delATT), TCG1828GGG (5620T>G 5621C>G), CTG1829CGC (5624T>G 5625G>C), CAC1830GAT (5626C>G 5628C>T), AGC1831GGA (5629A>G 5631C>A), AGT1832ATG (5633G>T 5634T>G), TTT1833TTG (5637T>G), GCA1834AAC (5638G>A 5639C>A 5640A>C), CAT1835CAC (5643T>C), CAA1836CAT (5646A>T), CAA1836_GGT1837insCATGCT (5646_5647insCATGCT), GGT1837GCT (5648G>C), TTT1838TAT (5651T>A), GAA1839GCC (5654A>C 5655A>C), TTG1840CTT (5656T>C 5658G>T), GGC1841GGA (5661C>A), AAT1842AAC (5664T>C), TTC1843CTT (5665T>C 5667C>T), AGA1844CGA (5668A>C), ATC1846GTG (5674A>G 5676C>G), CGT1847GGC (5677C>G 5679T>C), GGT1848TGT (5680G>T), GAC1849TCA (5683G>T 5684A>C 5685C>A), ATC1850ATT (5688C>T), AAG1852AGA (5693A>G 5694G>A), ACT1853AAG (5696C>A 5697T>G), ACT1854ATG (5699C>T 5700T>G), GCC1855GCA (5703C>A), TTG1856GTG (5704T>G), ATG1858TTG (5710A>T), CCA1859CCC (5715A>C), TAT1860TAC (5718T>C), GTG1861ACT (5719G>A 5720T>C 5721G>T), GGA1863GGG (5727A>G), CCC1864CAG (5729C>A 5730C>G), AAT1865AAA (5733T>A), GGA1866GTT (5735G>T 5736A>T)								

*: Inserts / Deletes / Misaligned / Frameshifts

Analysis details

This analysis was performed with panviral2.64

NGS Details (UN60): Tomato chocolate spot virus (segment RNA2)

Assembly

**Coverage Length**	1693 (4 contig(s))
**Depth Of Coverage**	15922.8
**Number Of Reads**	239197
**Reads Per Million**	5073.27 rpm (after QC)
**Ambiguities**	0
**Assembly Method**	de novo + reference guided assembly
**Consensus Caller**	Bcf Tools

Coverage Map

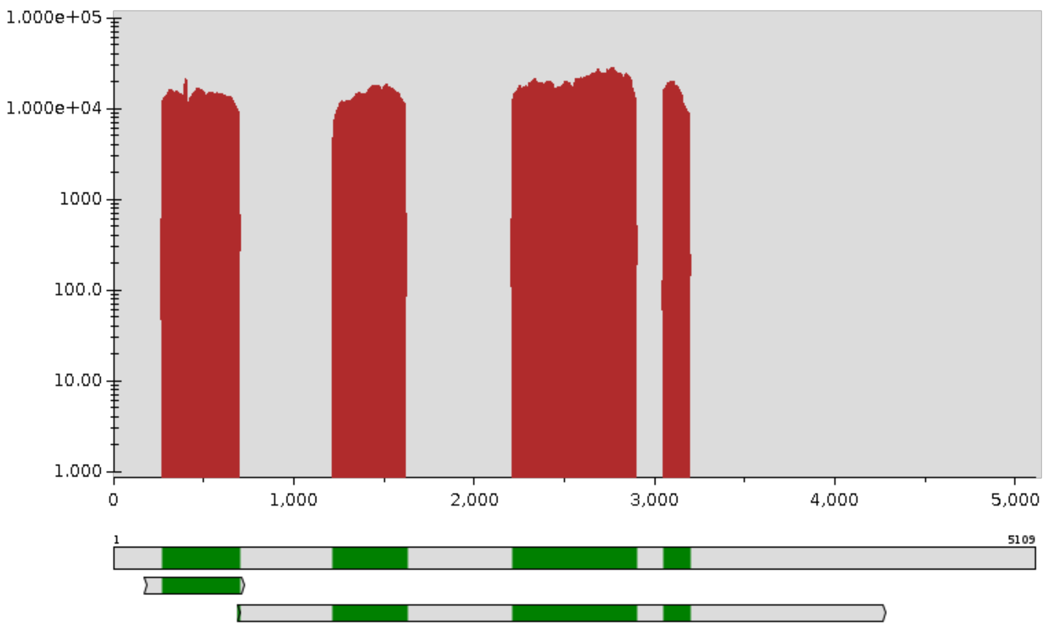

Assignment

**Type**	Tomato chocolate spot virus (Taxonomy ID: 661101)
**Reference Genome**	NC_013076.1
**NT Identity (%)**	67.2803
**AA Identity (%)**	70.5986
**Number Of Stop Codons**	0
**Number Of CDS**	2

Alignment

**Alignment Score**	1110.0 (NT) + 2795.0 (AA) = 3905.0
**Concordance (%)**	55.524

Alignment Method	Global, seeded, nucleotide + amino acids (AGA)

Genome Region

Sequence starts at position 268 and ends at position 3198 relative to NC_013076.1 reference sequence.

Alignment Detailed Statistics

	Begin	End	Coverage	Score	Concordance	Matches	Identities	I/D/M/F*	Stop Codons
**NT**	**268**	**3198**	**33.1%**	**1110**	**33.6%**	**1675 (98.4%)**	**1133 (66.6%)**	**9/18**	
Mutations:	277A>G, 288C>T, 291C>G, 294T>G, 297A>T, 303T>C, 307G>A, 309G>C, 310A>T, 311T>G, 312T>C, 315C>G, 321A>T, 331G>A, 336A>T, 339G>C, 340C>A, 342A>C, 343A>T, 345T>C, 346G>A, 348T>C, 351G>C, 357G>A, 363A>T, 366A>T, 369A>G, 372G>A, 373T>C, 378T>A, 381G>T, 384C>T, 390T>C, 402T>G, 404G>A, 406_414delACTTGGCCT, 415G>A, 417T>C, 421A>G, 422A>C, 423C>A, 426G>A, 430T>C, 432A>T, 435T>A, 438A>C, 439C>T, 441C>G, 442T>A, 444G>T, 449A>G, 450T>A, 456T>C, 457G>A, 460C>A, 461G>A, 462T>A, 471C>A, 474C>A, 475A>C, 480A>G, 481G>T, 483T>G, 484C>T, 486A>G, 490C>G, 491A>C, 492G>C, 501T>G, 502T>C, 503C>A, 504A>T, 507A>T, 508G>T, 511_516delCAACAA, 517A>T, 520G>A, 521A>C, 522A>C, 523A>T, 524A>C, 529G>T, 530T>C, 534T>C, 535G>A, 538A>C, 539A>C, 540A>C, 541G>T, 542G>C, 543G>T, 544A>T, 545A>C, 546A>T, 549_550insTTA, 550A>T, 552C>A, 558A>T, 561T>A, 562T>A, 564G>T, 565A>T, 566A>T, 567G>A, 568T>A, 569T>G, 571A>G, 572A>G, 577G>A, 579A>C, 581T>A, 582T>G, 585T>A, 586_588delCAT, 591C>T, 594C>G, 596C>A, 597T>A, 599A>C, 600G>C, 606G>A, 607C>G, 608G>T, 609A>T, 612A>C, 618G>A, 622A>C, 624A>G, 625G>C, 626C>A, 631A>G, 632A>C, 635A>G, 636A>G, 639A>T, 645A>T, 646T>G, 647C>T, 651G>A, 652T>C, 654G>C, 655C>A, 656A>G, 657A>G, 658C>T, 660T>G, 663G>A, 664C>A, 666C>T, 667T>C, 668C>A, 669C>A, 670A>G, 671A>G, 675G>A, 678T>A, 681T>C, 685A>G, 687T>G, 691A>G, 1219G>T, 1222A>T, 1228A>G, 1231A>G, 1238G>A, 1239A>G, 1240T>C, 1241A>C, 1246G>A, 1252C>T, 1255A>T, 1258C>T, 1259T>A, 1261T>G, 1262C>G, 1263A>C, 1267G>A, 1273T>A, 1277T>A, 1278C>G, 1285C>A, 1291A>G, 1294A>T, 1295T>C, 1297G>C, 1300C>T, 1306T>A, 1309A>G, 1312T>C, 1315C>T, 1316C>T, 1317A>C, 1318A>T, 1327G>A, 1333T>A, 1336C>A, 1351A>T, 1354A>C, 1360T>G, 1366G>A, 1367C>G, 1369A>G, 1372T>G, 1373T>G, 1375C>G, 1376C>T, 1377A>C, 1378G>T, 1379A>C, 1380T>A, 1381G>A, 1385T>C, 1387G>A, 1394C>A, 1396T>A, 1397G>C, 1405T>A, 1408A>T, 1410A>C, 1411G>A, 1412A>C, 1413G>T, 1414G>T, 1423T>C, 1426A>T, 1428G>A, 1429A>C, 1433G>A, 1438A>T, 1440T>C, 1441G>C, 1442A>C, 1443G>A, 1444T>G, 1448A>T, 1449G>C, 1450C>T, 1453C>A, 1454A>T, 1456G>A, 1458C>T, 1459T>G, 1463A>G, 1464G>C, 1465C>G, 1468T>A, 1471C>G, 1474G>T, 1477A>C, 1483G>A, 1489C>A, 1493G>A, 1495G>C, 1501T>C, 1503G>A, 1505C>A, 1513A>T, 1516C>G, 1522A>G, 1525A>G, 1531C>T, 1532G>T, 1534G>T, 1535C>T, 1537T>A, 1539A>G, 1540C>G, 1541A>G, 1547C>T, 1548G>C, 1551T>C, 1552A>G, 1555_1556insGTAGGT, 1556A>G, 1559C>A, 1572C>G, 1573T>G, 1574C>G, 1575A>T, 1576C>A, 1582A>T, 1585C>T, 1586A>T, 1588T>C, 1589G>A, 1590G>A, 1592G>T, 1593G>T, 1595T>C, 1603A>T, 1606T>C, 1610A>G, 2218C>G, 2221A>T, 2224G>A, 2225C>G, 2228A>C, 2229T>C, 2230T>G, 2235A>G, 2236T>G, 2237G>A, 2239T>A, 2243A>G, 2244C>A, 2245T>G, 2249G>A, 2250G>A, 2254T>C, 2255G>A, 2258A>T, 2260T>C, 2266C>T, 2269C>T, 2272T>G, 2273T>G, 2274C>A, 2275A>G, 2278C>A, 2282G>A, 2283A>T, 2284C>G, 2285T>G, 2286C>G, 2287A>T, 2288A>G, 2290C>T, 2291C>G, 2293A>C, 2294G>C, 2296C>G, 2297A>T, 2300G>A, 2303T>C, 2305A>G, 2309A>T, 2317A>G, 2322T>C, 2332A>T, 2335C>T, 2341A>G, 2356T>G, 2357G>A, 2359G>T, 2365G>A, 2368C>T, 2369G>C, 2375G>A, 2386A>C, 2392G>T, 2395C>T, 2401A>G, 2404C>T, 2405A>C, 2407G>T, 2408T>G, 2411T>C, 2413G>A, 2414G>T, 2416A>C, 2419A>C, 2423G>A, 2424C>T, 2425T>G, 2435G>A, 2436C>G, 2437A>C, 2446A>G, 2449G>A, 2450A>G, 2452A>T, 2462G>T, 2464A>C, 2479T>C, 2485T>C, 2491T>A, 2492C>A, 2493T>A, 2495A>C, 2497C>T, 2502A>T, 2503C>T, 2506G>A, 2518T>C, 2519G>A, 2521G>T, 2524C>T, 2528G>A, 2530T>G, 2533T>A, 2536C>G, 2537A>G, 2539A>G, 2542A>G, 2548A>G, 2549T>C, 2551G>A, 2554T>C, 2557A>G, 2560C>G, 2577T>C, 2578T>A, 2581C>T, 2589A>C, 2590G>C, 2593A>G, 2599T>C, 2602A>C, 2605C>T, 2608T>A, 2614T>C, 2615C>A, 2626C>T, 2629G>A, 2632T>C, 2633A>C, 2634C>A, 2635C>T, 2638T>G, 2641T>G, 2644T>A, 2647A>T, 2650C>A, 2651C>G, 2653G>A, 2656A>G, 2657C>T, 2658A>C, 2659G>C, 2664A>G, 2666G>A, 2668A>G, 2672T>G, 2673G>T, 2674T>G, 2677T>C, 2678A>T, 2683A>T, 2684A>C, 2685C>A, 2689G>T, 2695G>T, 2697A>C, 2698G>C, 2700A>T, 2704T>A, 2708C>T, 2710T>G, 2715A>C, 2716G>T, 2717G>T, 2718G>C, 2719T>A, 2720A>C, 2721C>T, 2722T>G, 2727C>T, 2728C>T, 2729A>C, 2731G>T, 2734C>A, 2735T>C, 2737A>T, 2740A>C, 2743C>T, 2744A>G, 2746A>T, 2747C>A, 2749T>A, 2758A>G, 2761A>C, 2768T>C, 2770A>G, 2771T>G, 2782A>T, 2785G>A, 2789T>G, 2790C>A, 2791A>G, 2792A>T, 2793A>C, 2794T>A, 2795G>A, 2797C>A, 2800A>T, 2803C>T, 2806T>C, 2809C>A, 2812T>C, 2813T>C, 2815G>A, 2816C>T, 2817A>C, 2818G>C, 2827T>G, 2833C>A, 2842C>A, 2843C>A, 2848G>A, 2854T>A, 2855C>A, 2858C>T, 2860A>G, 2862T>G, 2863T>A, 2865C>G, 2866A>G, 2870T>G, 2872G>T, 2885A>C, 2886A>G, 2890G>A, 3055G>A, 3072C>G, 3073T>A, 3076G>C, 3079G>C, 3085C>T, 3091G>A, 3092A>G, 3094T>G, 3098A>G, 3109G>T, 3112C>G, 3115C>T, 3118C>T, 3121A>T, 3124A>G, 3128T>C, 3130G>T, 3133C>T, 3139A>G, 3140C>T, 3142C>G, 3145G>C, 3148A>G, 3152A>G, 3153A>C, 3154C>A, 3158A>G, 3160A>G, 3166A>C, 3168A>C, 3169T>A, 3172A>G, 3181T>A, 3184T>G								

CDS

	Begin	End	Coverage	Score	Concordance	Matches	Identities	I/D/M/F*	Stop Codons
ToChV_s2_gp1	33	177	78.4%	565	66.8%	139 (95.2%)	90 (61.6%)	1/6/0/0	0
Protein mutations:	I36V (277A>G), V46I (307G>A 309G>C), I47C (310A>T 311T>G 312T>C), V54I (331G>A), L57I (340C>A 342A>C), T58S (343A>T 345T>C), V59I (346G>A 348T>C), S77R (402T>G), G78D (404G>A), T79_P81del (406_414delACTTGGCCT), V82I (415G>A 417T>C), N84A (421A>G 422A>C 423C>A), L91I (442T>A 444G>T), H93R (449A>G 450T>A), V96I (457G>A), R97K (460C>A 461G>A 462T>A), V104L (481G>T 483T>G), Q107A (490C>G 491A>C 492G>C), S111H (502T>C 503C>A 504A>T), A113S (508G>T), Q114_Q115del (511_516delCAACAA), T116S (517A>T), E117T (520G>A 521A>C 522A>C), N118S (523A>T 524A>C), V120S (529G>T 530T>C), D122N (535G>A), K123P (538A>C 539A>C 540A>C), G124S (541G>T 542G>C 543G>T), K125S (544A>T 545A>C 546A>T), T126_T127insL (549_550insTTA), T127S (550A>T 552C>A), W131S (562T>A 564G>T), K132L (565A>T 566A>T 567G>A), F133S (568T>A 569T>G), K134G (571A>G 572A>G), E136N (577G>A 579A>C), I137K (581T>A 582T>G), H139del (586_588delCAT), N141K (594C>G), A142E (596C>A 597T>A), E143A (599A>C 600G>C), R146V (607C>G 608G>T 609A>T), I151L (622A>C 624A>G), A152Q (625G>C 626C>A), K154A (631A>G 632A>C), Q155R (635A>G 636A>G), S159V (646T>G 647C>T), Q162R (655C>A 656A>G 657A>G), L165I (664C>A 666C>T), S166Q (667T>C 668C>A 669C>A), N167G (670A>G 671A>G), N172E (685A>G 687T>G), I174V (691A>G)								
Codon mutations:	ATT36GTT (277A>G), AAC39AAT (288C>T), CCC40CCG (291C>G), ACT41ACG (294T>G), CTA42CTT (297A>T), TTT44TTC (303T>C), GTG46ATC (307G>A 309G>C), ATT47TGC (310A>T 311T>G 312T>C), CCC48CCG (315C>G), ACA50ACT (321A>T), GTT54ATT (331G>A), TCA55TCT (336A>T), GTG56GTC (339G>C), CTA57ATC (340C>A 342A>C), ACT58TCC (343A>T 345T>C), GTT59ATC (346G>A 348T>C), TCG60TCC (351G>C), GAG62GAA (357G>A), TCA64TCT (363A>T), ACA65ACT (366A>T), CCA66CCG (369A>G), CAG67CAA (372G>A), TTG68CTG (373T>C), GTT69GTA (378T>A), CCG70CCT (381G>T), GGC71GGT (384C>T), TAT73TAC (390T>C), AGT77AGG (402T>G), GGT78GAT (404G>A), ACT79_CCT81del (406_414delACTTGGCCT), GTT82ATC (415G>A 417T>C), AAC84GCA (421A>G 422A>C 423C>A), GTG85GTA (426G>A), TTA87CTT (430T>C 432A>T), TCT88TCA (435T>A), GGA89GGC (438A>C), CTC90TTG (439C>T 441C>G), TTG91ATT (442T>A 444G>T), CAT93CGA (449A>G 450T>A), TCT95TCC (456T>C), GTT96ATT (457G>A), CGT97AAA (460C>A 461G>A 462T>A), ACC100ACA (471C>A), ACC101ACA (474C>A), AGG102CGG (475A>C), AAA103AAG (480A>G), GTT104TTG (481G>T 483T>G), CTA105TTG (484C>T 486A>G), CAG107GCC (490C>G 491A>C 492G>C), GTT110GTG (501T>G), TCA111CAT (502T>C 503C>A 504A>T), ATA112ATT (507A>T), GCT113TCT (508G>T), CAA114_CAA115del (511_516delCAACAA), ACT116TCT (517A>T), GAA117ACC (520G>A 521A>C 522A>C), AAT118TCT (523A>T 524A>C), GTA120TCA (529G>T 530T>C), TCT121TCC (534T>C), GAC122AAC (535G>A), AAA123CCC (538A>C 539A>C 540A>C), GGG124TCT (541G>T 542G>C 543G>T), AAA125TCT (544A>T 545A>C 546A>T), ACA126_ACC127insTTA (549_550insTTA), ACC127TCA (550A>T 552C>A), ACA129ACT (558A>T), GCT130GCA (561T>A), TGG131AGT (562T>A 564G>T), AAG132TTA (565A>T 566A>T 567G>A), TTC133AGC (568T>A 569T>G), AAA134GGA (571A>G 572A>G), GAA136AAC (577G>A 579A>C), ATT137AAG (581T>A 582T>G), GCT138GCA (585T>A), CAT139del (586_588delCAT), CTC140CTT (591C>T), AAC141AAG (594C>G), GCT142GAA (596C>A 597T>A), GAG143GCC (599A>C 600G>C), GAG145GAA (606G>A), CGA146GTT (607C>G 608G>T 609A>T), GCA147GCC (612A>C), AAG149AAA (618G>A), ATA151CTG (622A>C 624A>G), GCA152CAA (625G>C 626C>A), AAA154GCA (631A>G 632A>C), CAA155CGG (635A>G 636A>G), TCA156TCT (639A>T), ATA158ATT (645A>T), TCC159GTC (646T>G 647C>T), AAG160AAA (651G>A), TTG161CTC (652T>C 654G>C), CAA162AGG (655C>A 656A>G 657A>G), CTT163TTG (658C>T 660T>G), CAG164CAA (663G>A), CTC165ATT (664C>A 666C>T), TCC166CAA (667T>C 668C>A 669C>A), AAT167GGT (670A>G 671A>G), CAG168CAA (675G>A), CCT169CCA (678T>A), AGT170AGC (681T>C), AAT172GAG (685A>G 687T>G), ATC174GTC (691A>G)								
ToChV_s2_gp2	1	838	35.6%	2230	76.3%	426 (99.5%)	311 (72.7%)	2/0/0/0	0
Protein mutations:	M1R (687T>G), I2M (691A>G), D185S (1238G>A 1239A>G 1240T>C), T186P (1241A>C), S192T (1259T>A 1261T>G), Q193A (1262C>G 1263A>C), Q211S (1316C>T 1317A>C 1318A>T), Q228E (1367C>G 1369A>G), S229R (1372T>G), S230A (1373T>G 1375C>G), Q231S (1376C>T 1377A>C 1378G>T), M232Q (1379A>C 1380T>A 1381G>A), A238P (1397G>C), E242A (1410A>C 1411G>A), R243L (1412A>C 1413G>T 1414G>T), R248H (1428G>A 1429A>C), D250N (1433G>A), L252P (1440T>C 1441G>C), S253Q (1442A>C 1443G>A 1444T>G), M257L (1454A>T 1456G>A), T258M (1458C>T 1459T>G), S260A (1463A>G 1464G>C 1465C>G), V270I (1493G>A 1495G>C), R273K (1503G>A), V283F (1532G>T 1534G>T), P284S (1535C>T 1537T>A), N285R (1539A>G 1540C>G), T286A (1541A>G), R288S (1547C>T 1548G>C), L289S (1551T>C 1552A>G), T290_M291insVG (1555_1556insGTAGGT), M291V (1556A>G), A296G (1572C>G 1573T>G), H297V (1574C>G 1575A>T 1576C>A), E299D (1582A>T), T301S (1586A>T 1588T>C), G302N (1589G>A 1590G>A), G303L (1592G>T 1593G>T), K309E (1610A>G), H514D (2225C>G), I515P (2228A>C 2229T>C 2230T>G), D517G (2235A>G 2236T>G), D518K (2237G>A 2239T>A), T520E (2243A>G 2244C>A 2245T>G), G522N (2249G>A 2250G>A), V524I (2255G>A), I525F (2258A>T 2260T>C), S530E (2273T>G 2274C>A 2275A>G), D531E (2278C>A), D533M (2282G>A 2283A>T 2284C>G), S534G (2285T>G 2286C>G 2287A>T), N535D (2288A>G 2290C>T), P536A (2291C>G 2293A>C), A537P (2294G>C 2296C>G), I538L (2297A>T), D539N (2300G>A), M542L (2309A>T), V546A (2322T>C), V558I (2357G>A 2359G>T), V562L (2369G>C), E564K (2375G>A), K567N (2386A>C), M574L (2405A>C 2407G>T), S575A (2408T>G), A577S (2414G>T 2416A>C), A580M (2423G>A 2424C>T 2425T>G), A584S (2435G>A 2436C>G 2437A>C), T589A (2450A>G 2452A>T), A593S (2462G>T 2464A>C), L603N (2492C>A 2493T>A), I604L (2495A>C 2497C>T), Y606F (2502A>T 2503C>T), V612I (2519G>A 2521G>T), V615M (2528G>A 2530T>G), I618V (2537A>G 2539A>G), V631A (2577T>C 2578T>A), K635T (2589A>C 2590G>C), L644I (2615C>A), T650H (2633A>C 2634C>A 2635C>T), Q656E (2651C>G 2653G>A), Q658S (2657C>T 2658A>C 2659G>C), E660G (2664A>G), E661K (2666G>A 2668A>G), C663V (2672T>G 2673G>T 2674T>G), T665S (2678A>T), T667Q (2684A>C 2685C>A), K671T (2697A>C 2698G>C), Y672F (2700A>T), E677A (2715A>C 2716G>T), G678S (2717G>T 2718G>C 2719T>A), T679L (2720A>C 2721C>T 2722T>G), A681V (2727C>T 2728C>T), T682P (2729A>C 2731G>T), D683E (2734C>A), I687V (2744A>G 2746A>T), S696A (2771T>G), S702E (2789T>G 2790C>A 2791A>G), N703S (2792A>T 2793A>C 2794T>A), V704I (2795G>A 2797C>A), Q711S (2816C>T 2817A>C 2818G>C), L720M (2843C>A), I726R (2862T>G 2863T>A), A727G (2865C>G 2866A>G), S729A (2870T>G 2872G>T), K734R (2885A>C 2886A>G), T796R (3072C>G 3073T>A), I803V (3092A>G 3094T>G), I805V (3098A>G), N823A (3152A>G 3153A>C 3154C>A), K825E (3158A>G 3160A>G), N828T (3168A>C 3169T>A), F833L (3184T>G)								

	Begin	End	Coverage	Score	Concordance	Matches	Identities	I/D/M/F*	Stop Codons
**NT**	**268**	**3198**	**33.1%**	**1110**	**33.6%**	**1675 (98.4%)**	**1133 (66.6%)**	**9/18**	
Codon mutations:	ATG1AGG (687T>G), ATA2ATG (691A>G), GCG178GCT (1219G>T), ATA179ATT (1222A>T), AAA181AAG (1228A>G), CAA182CAG (1231A>G), GAT185AGC (1238G>A 1239A>G 1240T>C), ACA186CCA (1241A>C), AGG187AGA (1246G>A), GCC189GCT (1252C>T), GTA190GTT (1255A>T), TAC191TAT (1258C>T), TCT192ACG (1259T>A 1261T>G), CAA193GCA (1262C>G 1263A>C), CAG194CAA (1267G>A), ACT196ACA (1273T>A), TCT198AGT (1277T>A 1278C>G), GCC200GCA (1285C>A), GAA202GAG (1291A>G), GCA203GCT (1294A>T), TTG204CTC (1295T>C 1297G>C), AAC205AAT (1300C>T), CCT207CCA (1306T>A), CAA208CAG (1309A>G), TTT209TTC (1312T>C), TTC210TTT (1315C>T), CAA211TCT (1316C>T 1317A>C 1318A>T), CAG214CAA (1327G>A), GCT216GCA (1333T>A), GCC217GCA (1336C>A), TCA222TCT (1351A>T), GTA223GTC (1354A>C), TCT225TCG (1360T>G), GGG227GGA (1366G>A), CAA228GAG (1367C>G 1369A>G), AGT229AGG (1372T>G), TCC230GCG (1373T>G 1375C>G), CAG231TCT (1376C>T 1377A>C 1378G>T), ATG232CAA (1379A>C 1380T>A 1381G>A), TTG234CTA (1385T>C 1387G>A), CGT237AGA (1394C>A 1396T>A), GCT238CCT (1397G>C), GTT240GTA (1405T>A), GGA241GGT (1408A>T), GAG242GCA (1410A>C 1411G>A), AGG243CTT (1412A>C 1413G>T 1414G>T), AAT246AAC (1423T>C), ACA247ACT (1426A>T), CGA248CAC (1428G>A 1429A>C), GAT250AAT (1433G>A), GTA251GTT (1438A>T), CTG252CCC (1440T>C 1441G>C), AGT253CAG (1442A>C 1443G>A 1444T>G), AGC255TCT (1448A>T 1449G>C 1450C>T), TCC256TCA (1453C>A), ATG257TTA (1454A>T 1456G>A), ACT258ATG (1458C>T 1459T>G), AGC260GCG (1463A>G 1464G>C 1465C>G), TCT261TCA (1468T>A), TCC262TCG (1471C>G), CTG263CTT (1474G>T), CGA264CGC (1477A>C), CCG266CCA (1483G>A), CTC268CTA (1489C>A), GTG270ATC (1493G>A 1495G>C), AGT272AGC (1501T>C), AGG273AAG (1503G>A), CGG274AGG (1505C>A), CCA276CCT (1513A>T), TCC277TCG (1516C>G), GAA279GAG (1522A>G), CTA280CTG (1525A>G), GAC282GAT (1531C>T), GTG283TTT (1532G>T 1534G>T), CCT284TCA (1535C>T 1537T>A), AAC285AGG (1539A>G 1540C>G), ACC286GCC (1541A>G), CGA288TCA (1547C>T 1548G>C), TTA289TCG (1551T>C 1552A>G), ACA290_ATG291insGTAGGT (1555_1556insGTAGGT), ATG291GTG (1556A>G), CGA292AGA (1559C>A), GCT296GGG (1572C>G 1573T>G), CAC297GTA (1574C>G 1575A>T 1576C>A), GAA299GAT (1582A>T), GAC300GAT (1585C>T), ACT301TCC (1586A>T 1588T>C), GGT302AAT (1589G>A 1590G>A), GGA303TTA (1592G>T 1593G>T), TTG304CTG (1595T>C), CCA306CCT (1603A>T), GTT307GTC (1606T>C), AAG309GAG (1610A>G), ACC511ACG (2218C>G), GCA512GCT (2221A>T), GAG513GAA (2224G>A), CAT514GAT (2225C>G), ATT515CCG (2228A>C 2229T>C 2230T>G), GAT517GGG (2235A>G 2236T>G), GAT518AAA (2237G>A 2239T>A), ACT520GAG (2243A>G 2244C>A 2245T>G), GGT522AAT (2249G>A 2250G>A), GAT523GAC (2254T>C), GTT524ATT (2255G>A), ATT525TTC (2258A>T 2260T>C), GAC527GAT (2266C>T), TTC528TTT (2269C>T), GGT529GGG (2272T>G), TCA530GAG (2273T>G 2274C>A 2275A>G), GAC531GAA (2278C>A), GAC533ATG (2282G>A 2283A>T 2284C>G), TCA534GGT (2285T>G 2286C>G 2287A>T), AAC535GAT (2288A>G 2290C>T), CCA536GCC (2291C>G 2293A>C), GCC537CCG (2294G>C 2296C>G), ATA538TTA (2297A>T), GAT539AAT (2300G>A), TTA540CTG (2303T>C 2305A>G), ATG542TTG (2309A>T), CAA544CAG (2317A>G), GTA546GCA (2322T>C), GCA549GCT (2332A>T), AGC550AGT (2335C>T), GAA552GAG (2341A>G), GGT557GGG (2356T>G), GTG558ATT (2357G>A 2359G>T), GAG560GAA (2365G>A), TTC561TTT (2368C>T), GTG562CTG (2369G>C), GAA564AAA (2375G>A), AAA567AAC (2386A>C), GTG569GTT (2392G>T), GCC570GCT (2395C>T), CAA572CAG (2401A>G), GTC573GTT (2404C>T), ATG574CTT (2405A>C 2407G>T), TCA575GCA (2408T>G), TTG576CTA (2411T>C 2413G>A), GCA577TCC (2414G>T 2416A>C), CTA578CTC (2419A>C), GCT580ATG (2423G>A 2424C>T 2425T>G), GCA584AGC (2435G>A 2436C>G 2437A>C), AAA587AAG (2446A>G), GAG588GAA (2449G>A), ACA589GCT (2450A>G 2452A>T), GCA593TCC (2462G>T 2464A>C), TAT598TAC (2479T>C), GAT600GAC (2485T>C), GCT602GCA (2491T>A), CTC603AAC (2492C>A 2493T>A), ATC604CTT (2495A>C 2497C>T), TAC606TTT (2502A>T 2503C>T), AAG607AAA (2506G>A), TAT611TAC (2518T>C), GTG612ATT (2519G>A 2521G>T), TCC613TCT (2524C>T), GTT615ATG (2528G>A 2530T>G), GGT616GGA (2533T>A), GCC617GCG (2536C>G), ATA618GTG (2537A>G 2539A>G), TCA619TCG (2542A>G), CAA621CAG (2548A>G), TTG622CTA (2549T>C 2551G>A), GCT623GCC (2554T>C), TTA624TTG (2557A>G), GTC625GTG (2560C>G), GTT631GCA (2577T>C 2578T>A), CTC632CTT (2581C>T), AAG635ACC (2589A>C 2590G>C), AAA636AAG (2593A>G), TTT638TTC (2599T>C), ATA639ATC (2602A>C), AAC640AAT (2605C>T), ATT641ATA (2608T>A), ACT643ACC (2614T>C), CTT644ATT (2615C>A), AGC647AGT (2626C>T), AAG648AAA (2629G>A), CAT649CAC (2632T>C), ACC650CAT (2633A>C 2634C>A 2635C>T), CTT651CTG (2638T>G), GTT652GTG (2641T>G), TCT653TCA (2644T>A), GCA654GCT (2647A>T), TCC655TCA (2650C>A), CAG656GAA (2651C>G 2653G>A), CAA657CAG (2656A>G), CAG658TCC (2657C>T 2658A>C 2659G>C), GAG660GGG (2664A>G), GAA661AAG (2666G>A 2668A>G), TGT663GTG (2672T>G 2673G>T 2674T>G), TTT664TTC (2677T>C), ACA665TCA (2678A>T), CCA666CCT (2683A>T), ACA667CAA (2684A>C 2685C>A), GGG668GGT (2689G>T), GGG670GGT (2695G>T), AAG671ACC (2697A>C 2698G>C), TAT672TTT (2700A>T), GTT673GTA (2704T>A), CTT675TTG (2708C>T 2710T>G), GAG677GCT (2715A>C 2716G>T), GGT678TCA (2717G>T 2718G>C 2719T>A), ACT679CTG (2720A>C 2721C>T 2722T>G), GCC681GTT (2727C>T 2728C>T), ACG682CCT (2729A>C 2731G>T), GAC683GAA (2734C>A), TTA684CTT (2735T>C 2737A>T), GGA685GGC (2740A>C), AGC686AGT (2743C>T), ATA687GTT (2744A>G 2746A>T), CGT688AGA (2747C>A 2749T>A), GTA691GTG (2758A>G), ACA692ACC (2761A>C), TTA695CTG (2768T>C 2770A>G), TCA696GCA (2771T>G), ACA699ACT (2782A>T), GAG700GAA (2785G>A), TCA702GAG (2789T>G 2790C>A 2791A>G), AAT703TCA (2792A>T 2793A>C 2794T>A), GTC704ATA (2795G>A 2797C>A), CCA705CCT (2800A>T), TGC706TGT (2803C>T), CAT707CAC (2806T>C), ATC708ATA (2809C>A), CAT709CAC (2812T>C), TTG710CTA (2813T>C 2815G>A), CAG711TCC (2816C>T 2817A>C 2818G>C), GTT714GTG (2827T>G), TCC716TCA (2833C>A), ATC719ATA (2842C>A), CTG720ATG (2843C>A), CAG721CAA (2848G>A), CCT723CCA (2854T>A), CGG724AGG (2855C>A), CTA725TTG (2858C>T 2860A>G), ATT726AGA (2862T>G 2863T>A), GCA727GGG (2865C>G 2866A>G), TCG729GCT (2870T>G 2872G>T), AAG734CGG (2885A>C 2886A>G), CCG735CCA (2890G>A), CGG790CGA (3055G>A), ACT796AGA (3072C>G 3073T>A), GGG797GGC (3076G>C), ACG798ACC (3079G>C), GTC800GTT (3085C>T), GAG802GAA (3091G>A), ATT803GTG (3092A>G 3094T>G), ATT805GTT (3098A>G), ACG808ACT (3109G>T), CTC809CTG (3112C>G), TTC810TTT (3115C>T), CAC811CAT (3118C>T), TCA812TCT (3121A>T), GGA813GGG (3124A>G), TTG815CTT (3128T>C 3130G>T), GCC816GCT (3133C>T), GGA818GGG (3139A>G), CTC819TTG (3140C>T 3142C>G), GGG820GGC (3145G>C), ACA821ACG (3148A>G), AAC823GCA (3152A>G 3153A>C 3154C>A), AAA825GAG (3158A>G 3160A>G), TCA827TCC (3166A>C), AAT828ACA (3168A>C 3169T>A), GCA829GCG (3172A>G), ATT832ATA (3181T>A), TTT833TTG (3184T>G)								

Proteins

	Begin	End	Coverage	Score	Concordance	Matches	Identities	I/D/M/F*	Stop Codons
hypothetical protein (YP_003097230.1)	33	177	78.4%	565	66.8%	139 (95.2%)	90 (61.6%)	1/6/0/0	0
Protein mutations:	I36V (277A>G), V46I (307G>A 309G>C), I47C (310A>T 311T>G 312T>C), V54I (331G>A), L57I (340C>A 342A>C), T58S (343A>T 345T>C), V59I (346G>A 348T>C), S77R (402T>G), G78D (404G>A), T79_P81del (406_414delACTTGGCCT), V82I (415G>A 417T>C), N84A (421A>G 422A>C 423C>A), L91I (442T>A 444G>T), H93R (449A>G 450T>A), V96I (457G>A), R97K (460C>A 461G>A 462T>A), V104L (481G>T 483T>G), Q107A (490C>G 491A>C 492G>C), S111H (502T>C 503C>A 504A>T), A113S (508G>T), Q114_Q115del (511_516delCAACAA), T116S (517A>T), E117T (520G>A 521A>C 522A>C), N118S (523A>T 524A>C), V120S (529G>T 530T>C), D122N (535G>A), K123P (538A>C 539A>C 540A>C), G124S (541G>T 542G>C 543G>T), K125S (544A>T 545A>C 546A>T), T126_T127insL (549_550insTTA), T127S (550A>T 552C>A), W131S (562T>A 564G>T), K132L (565A>T 566A>T 567G>A), F133S (568T>A 569T>G), K134G (571A>G 572A>G), E136N (577G>A 579A>C), I137K (581T>A 582T>G), H139del (586_588delCAT), N141K (594C>G), A142E (596C>A 597T>A), E143A (599A>C 600G>C), R146V (607C>G 608G>T 609A>T), I151L (622A>C 624A>G), A152Q (625G>C 626C>A), K154A (631A>G 632A>C), Q155R (635A>G 636A>G), S159V (646T>G 647C>T), Q162R (655C>A 656A>G 657A>G), L165I (664C>A 666C>T), S166Q (667T>C 668C>A 669C>A), N167G (670A>G 671A>G), N172E (685A>G 687T>G), I174V (691A>G)								
Codon mutations:	ATT36GTT (277A>G), AAC39AAT (288C>T), CCC40CCG (291C>G), ACT41ACG (294T>G), CTA42CTT (297A>T), TTT44TTC (303T>C), GTG46ATC (307G>A 309G>C), ATT47TGC (310A>T 311T>G 312T>C), CCC48CCG (315C>G), ACA50ACT (321A>T), GTT54ATT (331G>A), TCA55TCT (336A>T), GTG56GTC (339G>C), CTA57ATC (340C>A 342A>C), ACT58TCC (343A>T 345T>C), GTT59ATC (346G>A 348T>C), TCG60TCC (351G>C), GAG62GAA (357G>A), TCA64TCT (363A>T), ACA65ACT (366A>T), CCA66CCG (369A>G), CAG67CAA (372G>A), TTG68CTG (373T>C), GTT69GTA (378T>A), CCG70CCT (381G>T), GGC71GGT (384C>T), TAT73TAC (390T>C), AGT77AGG (402T>G), GGT78GAT (404G>A), ACT79_CCT81del (406_414delACTTGGCCT), GTT82ATC (415G>A 417T>C), AAC84GCA (421A>G 422A>C 423C>A), GTG85GTA (426G>A), TTA87CTT (430T>C 432A>T), TCT88TCA (435T>A), GGA89GGC (438A>C), CTC90TTG (439C>T 441C>G), TTG91ATT (442T>A 444G>T), CAT93CGA (449A>G 450T>A), TCT95TCC (456T>C), GTT96ATT (457G>A), CGT97AAA (460C>A 461G>A 462T>A), ACC100ACA (471C>A), ACC101ACA (474C>A), AGG102CGG (475A>C), AAA103AAG (480A>G), GTT104TTG (481G>T 483T>G), CTA105TTG (484C>T 486A>G), CAG107GCC (490C>G 491A>C 492G>C), GTT110GTG (501T>G), TCA111CAT (502T>C 503C>A 504A>T), ATA112ATT (507A>T), GCT113TCT (508G>T), CAA114_CAA115del (511_516delCAACAA), ACT116TCT (517A>T), GAA117ACC (520G>A 521A>C 522A>C), AAT118TCT (523A>T 524A>C), GTA120TCA (529G>T 530T>C), TCT121TCC (534T>C), GAC122AAC (535G>A), AAA123CCC (538A>C 539A>C 540A>C), GGG124TCT (541G>T 542G>C 543G>T), AAA125TCT (544A>T 545A>C 546A>T), ACA126_ACC127insTTA (549_550insTTA), ACC127TCA (550A>T 552C>A), ACA129ACT (558A>T), GCT130GCA (561T>A), TGG131AGT (562T>A 564G>T), AAG132TTA (565A>T 566A>T 567G>A), TTC133AGC (568T>A 569T>G), AAA134GGA (571A>G 572A>G), GAA136AAC (577G>A 579A>C), ATT137AAG (581T>A 582T>G), GCT138GCA (585T>A), CAT139del (586_588delCAT), CTC140CTT (591C>T), AAC141AAG (594C>G), GCT142GAA (596C>A 597T>A), GAG143GCC (599A>C 600G>C), GAG145GAA (606G>A), CGA146GTT (607C>G 608G>T 609A>T), GCA147GCC (612A>C), AAG149AAA (618G>A), ATA151CTG (622A>C 624A>G), GCA152CAA (625G>C 626C>A), AAA154GCA (631A>G 632A>C), CAA155CGG (635A>G 636A>G), TCA156TCT (639A>T), ATA158ATT (645A>T), TCC159GTC (646T>G 647C>T), AAG160AAA (651G>A), TTG161CTC (652T>C 654G>C), CAA162AGG (655C>A 656A>G 657A>G), CTT163TTG (658C>T 660T>G), CAG164CAA (663G>A), CTC165ATT (664C>A 666C>T), TCC166CAA (667T>C 668C>A 669C>A), AAT167GGT (670A>G 671A>G), CAG168CAA (675G>A), CCT169CCA (678T>A), AGT170AGC (681T>C), AAT172GAG (685A>G 687T>G), ATC174GTC (691A>G)								
polyprotein (YP_003097231.1)	1	838	35.6%	2230	76.3%	426 (99.5%)	311 (72.7%)	2/0/0/0	0
Protein mutations:	M1R (687T>G), I2M (691A>G), D185S (1238G>A 1239A>G 1240T>C), T186P (1241A>C), S192T (1259T>A 1261T>G), Q193A (1262C>G 1263A>C), Q211S (1316C>T 1317A>C 1318A>T), Q228E (1367C>G 1369A>G), S229R (1372T>G), S230A (1373T>G 1375C>G), Q231S (1376C>T 1377A>C 1378G>T), M232Q (1379A>C 1380T>A 1381G>A), A238P (1397G>C), E242A (1410A>C 1411G>A), R243L (1412A>C 1413G>T 1414G>T), R248H (1428G>A 1429A>C), D250N (1433G>A), L252P (1440T>C 1441G>C), S253Q (1442A>C 1443G>A 1444T>G), M257L (1454A>T 1456G>A), T258M (1458C>T 1459T>G), S260A (1463A>G 1464G>C 1465C>G), V270I (1493G>A 1495G>C), R273K (1503G>A), V283F (1532G>T 1534G>T), P284S (1535C>T 1537T>A), N285R (1539A>G 1540C>G), T286A (1541A>G), R288S (1547C>T 1548G>C), L289S (1551T>C 1552A>G), T290_M291insVG (1555_1556insGTAGGT), M291V (1556A>G), A296G (1572C>G 1573T>G), H297V (1574C>G 1575A>T 1576C>A), E299D (1582A>T), T301S (1586A>T 1588T>C), G302N (1589G>A 1590G>A), G303L (1592G>T 1593G>T), K309E (1610A>G), H514D (2225C>G), I515P (2228A>C 2229T>C 2230T>G), D517G (2235A>G 2236T>G), D518K (2237G>A 2239T>A), T520E (2243A>G 2244C>A 2245T>G), G522N (2249G>A 2250G>A), V524I (2255G>A), I525F (2258A>T 2260T>C), S530E (2273T>G 2274C>A 2275A>G), D531E (2278C>A), D533M (2282G>A 2283A>T 2284C>G), S534G (2285T>G 2286C>G 2287A>T), N535D (2288A>G 2290C>T), P536A (2291C>G 2293A>C), A537P (2294G>C 2296C>G), I538L (2297A>T), D539N (2300G>A), M542L (2309A>T), V546A (2322T>C), V558I (2357G>A 2359G>T), V562L (2369G>C), E564K (2375G>A), K567N (2386A>C), M574L (2405A>C 2407G>T), S575A (2408T>G), A577S (2414G>T 2416A>C), A580M (2423G>A 2424C>T 2425T>G), A584S (2435G>A 2436C>G 2437A>C), T589A (2450A>G 2452A>T), A593S (2462G>T 2464A>C), L603N (2492C>A 2493T>A), I604L (2495A>C 2497C>T), Y606F (2502A>T 2503C>T), V612I (2519G>A 2521G>T), V615M (2528G>A 2530T>G), I618V (2537A>G 2539A>G), V631A (2577T>C 2578T>A), K635T (2589A>C 2590G>C), L644I (2615C>A), T650H (2633A>C 2634C>A 2635C>T), Q656E (2651C>G 2653G>A), Q658S (2657C>T 2658A>C 2659G>C), E660G (2664A>G), E661K (2666G>A 2668A>G), C663V (2672T>G 2673G>T 2674T>G), T665S (2678A>T), T667Q (2684A>C 2685C>A), K671T (2697A>C 2698G>C), Y672F (2700A>T), E677A (2715A>C 2716G>T), G678S (2717G>T 2718G>C 2719T>A), T679L (2720A>C 2721C>T 2722T>G), A681V (2727C>T 2728C>T), T682P (2729A>C 2731G>T), D683E (2734C>A), I687V (2744A>G 2746A>T), S696A (2771T>G), S702E (2789T>G 2790C>A 2791A>G), N703S (2792A>T 2793A>C 2794T>A), V704I (2795G>A 2797C>A), Q711S (2816C>T 2817A>C 2818G>C), L720M (2843C>A), I726R (2862T>G 2863T>A), A727G (2865C>G 2866A>G), S729A (2870T>G 2872G>T), K734R (2885A>C 2886A>G), T796R (3072C>G 3073T>A), I803V (3092A>G 3094T>G), I805V (3098A>G), N823A (3152A>G 3153A>C 3154C>A), K825E (3158A>G 3160A>G), N828T (3168A>C 3169T>A), F833L (3184T>G)								

	Begin	End	Coverage	Score	Concordance	Matches	Identities	I/D/M/F*	Stop Codons
NT	268	3198	33.1%	1110	33.6%	1675 (98.4%)	1133 (66.6%)	9/18	

Codon mutations: ATG1AGG (687T>G), ATA2ATG (691A>G), GCG178GCT (1219G>T), ATA179ATT (1222A>T), AAA181AAG (1228A>G), CAA182CAG (1231A>G), GAT185AGC (1238G>A 1239A>G 1240T>C), ACA186CCA (1241A>C), AGG187AGA (1246G>A), GCC189GCT (1252C>T), GTA190GTT (1255A>T), TAC191TAT (1258C>T), TCT192ACG (1259T>A 1261T>G), CAA193GCA (1262C>G 1263A>C), CAG194CAA (1267G>A), ACT196ACA (1273T>A), TCT198AGT (1277T>A 1278C>G), GCC200GCA (1285C>A), GAA202GAG (1291A>G), GCA203GCT (1294A>T), TTG204CTC (1295T>C 1297G>C), AAC205AAT (1300C>T), CCT207CCA (1306T>A), CAA208CAG (1309A>G), TTT209TTC (1312T>C), TTC210TTT (1315C>T), CAA211TCT (1316C>T 1317A>C 1318A>T), CAG214CAA (1327G>A), GCT216GCA (1333T>A), GCC217GCA (1336C>A), TCA222TCT (1351A>T), GTA223GTC (1354A>C), TCT225TCG (1360T>G), GGG227GGA (1366G>A), CAA228GAG (1367C>G 1369A>G), AGT229AGG (1372T>G), TCC230GCG (1373T>G 1375C>G), CAG231TCT (1376C>T 1377A>C 1378G>T), ATG232CAA (1379A>C 1380T>A 1381G>A), TTG234CTA (1385T>C 1387G>A), CGT237AGA (1394C>A 1396T>A), GCT238CCT (1397G>C), GTT240GTA (1405T>A), GGA241GGT (1408A>T), GAG242GCA (1410A>C 1411G>A), AGG243CTT (1412A>C 1413G>T 1414G>T), AAT246AAC (1423T>C), ACA247ACT (1426A>T), CGA248CAC (1428G>A 1429A>C), GAT250AAT (1433G>A), GTA251GTT (1438A>T), CTG252CCC (1440T>C 1441G>C), AGT253CAG (1442A>C 1443G>A 1444T>G), AGC255TCT (1448A>T 1449G>C 1450C>T), TCC256TCA (1453C>A), ATG257TTA (1454A>T 1456G>A), ACT258ATG (1458C>T 1459T>G), AGC260GCG (1463A>G 1464G>C 1465C>G), TCT261TCA (1468T>A), TCC262TCG (1471C>G), CTG263CTT (1474G>T), CGA264CGC (1477A>C), CCG266CCA (1483G>A), CTC268CTA (1489C>A), GTG270ATC (1493G>A 1495G>C), AGT272AGC (1501T>C), AGG273AAG (1503G>A), CGG274AGG (1505C>A), CCA276CCT (1513A>T), TCC277TCG (1516C>G), GAA279GAG (1522A>G), CTA280CTG (1525A>G), GAC282GAT (1531C>T), GTG283TTT (1532G>T 1534G>T), CCT284TCA (1535C>T 1537T>A), AAC285AGG (1539A>G 1540C>G), ACC286GCC (1541A>G), CGA288TCA (1547C>T 1548G>C), TTA289TCG (1551T>C 1552A>G), ACA290_ATG291insGTAGGT (1555_1556insGTAGGT), ATG291GTG (1556A>G), CGA292AGA (1559C>A), GCT296GGG (1572C>G 1573T>G), CAC297GTA (1574C>G 1575A>T 1576C>A), GAA299GAT (1582A>T), GAC300GAT (1585C>T), ACT301TCC (1586A>T 1588T>C), GGT302AAT (1589G>A 1590G>A), GGA303TTA (1592G>T 1593G>T), TTG304CTG (1595T>C), CCA306CCT (1603A>T), GTT307GTC (1606T>C), AAG309GAG (1610A>G), ACC511ACG (2218C>G), GCA512GCT (2221A>T), GAG513GAA (2224G>A), CAT514GAT (2225C>G), ATT515CCG (2228A>C 2229T>C 2230T>G), GAT517GGG (2235A>G 2236T>G), GAT518AAA (2237G>A 2239T>A), ACT520GAG (2243A>G 2244C>A 2245T>G), GGT522AAT (2249G>A 2250G>A), GAT523GAC (2254T>C), GTT524ATT (2255G>A), ATT525TTC (2258A>T 2260T>C), GAC527GAT (2266C>T), TTC528TTT (2269C>T), GGT529GGG (2272T>G), TCA530GAG (2273T>G 2274C>A 2275A>G), GAC531GAA (2278C>A), GAC533ATG (2282G>A 2283A>T 2284C>G), TCA534GGT (2285T>G 2286C>G 2287A>T), AAC535GAT (2288A>G 2290C>T), CCA536GCC (2291C>G 2293A>C), GCC537CCG (2294G>C 2296C>G), ATA538TTA (2297A>T), GAT539AAT (2300G>A), TTA540CTG (2303T>C 2305A>G), ATG542TTG (2309A>T), CAA544CAG (2317A>G), GTA546GCA (2322T>C), GCA549GCT (2332A>T), AGC550AGT (2335C>T), GAA552GAG (2341A>G), GGT557GGG (2356T>G), GTG558ATT (2357G>A 2359G>T), GAG560GAA (2365G>A), TTC561TTT (2368C>T), GTG562CTG (2369G>C), GAA564AAA (2375G>A), AAA567AAC (2386A>C), GTG569GTT (2392G>T), GCC570GCT (2395C>T), CAA572CAG (2401A>G), GTC573GTT (2404C>T), ATG574CTT (2405A>C 2407G>T), TCA575GCA (2408T>G), TTG576CTA (2411T>C 2413G>A), GCA577TCC (2414G>T 2416A>C), CTA578CTC (2419A>C), GCT580ATG (2423G>A 2424C>T 2425T>G), GCA584AGC (2435G>A 2436C>G 2437A>C), AAA587AAG (2446A>G), GAG588GAA (2449G>A), ACA589GCT (2450A>G 2452A>T), GCA593TCC (2462G>T 2464A>C), TAT598TAC (2479T>C), GAT600GAC (2485T>C), GCT602GCA (2491T>A), CTC603AAC (2492C>A 2493T>A), ATC604CTT (2495A>C 2497C>T), TAC606TTT (2502A>T 2503C>T), AAG607AAA (2506G>A), TAT611TAC (2518T>C), GTG612ATT (2519G>A 2521G>T), TCC613TCT (2524C>T), GTT615ATG (2528G>A 2530T>G), GGT616GGA (2533T>A), GCC617GCG (2536C>G), ATA618GTG (2537A>G 2539A>G), TCA619TCG (2542A>G), CAA621CAG (2548A>G), TTG622CTA (2549T>C 2551G>A), GCT623GCC (2554T>C), TTA624TTG (2557A>G), GTC625GTG (2560C>G), GTT631GCA (2577T>C 2578T>A), CTC632CTT (2581C>T), AAG635ACC (2589A>C 2590G>C), AAA636AAG (2593A>G), TTT638TTC (2599T>C), ATA639ATC (2602A>C), AAC640AAT (2605C>T), ATT641ATA (2608T>A), ACT643ACC (2614T>C), CTT644ATT (2615C>A), AGC647AGT (2626C>T), AAG648AAA (2629G>A), CAT649CAC (2632T>C), ACC650CAT (2633A>C 2634C>A 2635C>T), CTT651CTG (2638T>G), GTT652GTG (2641T>G), TCT653TCA (2644T>A), GCA654GCT (2647A>T), TCC655TCA (2650C>A), CAG656GAA (2651C>G 2653G>A), CAA657CAG (2656A>G), CAG658TCC (2657C>T 2658A>C 2659G>C), GAG660GGG (2664A>G), GAA661AAG (2666G>A 2668A>G), TGT663GTG (2672T>G 2673G>T 2674T>G), TTT664TTC (2677T>C), ACA665TCA (2678A>T), CCA666CCT (2683A>T), ACA667CAA (2684A>C 2685C>A), GGG668GGT (2689G>T), GGG670GGT (2695G>T), AAG671ACC (2697A>C 2698G>C), TAT672TTT (2700A>T), GTT673GTA (2704T>A), CTT675TTG (2708C>T 2710T>G), GAG677GCT (2715A>C 2716G>T), GGT678TCA (2717G>T 2718G>C 2719T>A), ACT679CTG (2720A>C 2721C>T 2722T>G), GCC681GTT (2727C>T 2728C>T), ACG682CCT (2729A>C 2731G>T), GAC683GAA (2734C>A), TTA684CTT (2735T>C 2737A>T), GGA685GGC (2740A>C), AGC686AGT (2743C>T), ATA687GTT (2744A>G 2746A>T), CGT688AGA (2747C>A 2749T>A), GTA691GTG (2758A>G), ACA692ACC (2761A>C), TTA695CTG (2768T>C 2770A>G), TCA696GCA (2771T>G), ACA699ACT (2782A>T), GAG700GAA (2785G>A), TCA702GAG (2789T>G 2790C>A 2791A>G), AAT703TCA (2792A>T 2793A>C 2794T>A), GTC704ATA (2795G>A 2797C>A), CCA705CCT (2800A>T), TGC706TGT (2803C>T), CAT707CAC (2806T>C), ATC708ATA (2809C>A), CAT709CAC (2812T>C), TTG710CTA (2813T>C 2815G>A), CAG711TCC (2816C>T 2817A>C 2818G>C), GTT714GTG (2827T>G), TCC716TCA (2833C>A), ATC719ATA (2842C>A), CTG720ATG (2843C>A), CAG721CAA (2848G>A), CCT723CCA (2854T>A), CGG724AGG (2855C>A), CTA725TTG (2858C>T 2860A>G), ATT726AGA (2862T>G 2863T>A), GCA727GGG (2865C>G 2866A>G), TCG729GCT (2870T>G 2872G>T), AAG734CGG (2885A>C 2886A>G), CCG735CCA (2890G>A), CGG790CGA (3055G>A), ACT796AGA (3072C>G 3073T>A), GGG797GGC (3076G>C), ACG798ACC (3079G>C), GTC800GTT (3085C>T), GAG802GAA (3091G>A), ATT803GTG (3092A>G 3094T>G), ATT805GTT (3098A>G), ACG808ACT (3109G>T), CTC809CTG (3112C>G), TTC810TTT (3115C>T), CAC811CAT (3118C>T), TCA812TCT (3121A>T), GGA813GGG (3124A>G), TTG815CTT (3128T>C 3130G>T), GCC816GCT (3133C>T), GGA818GGG (3139A>G), CTC819TTG (3140C>T 3142C>G), GGG820GGC (3145G>C), ACA821ACG (3148A>G), AAC823GCA (3152A>G 3153A>C 3154C>A), AAA825GAG (3158A>G 3160A>G), TCA827TCC (3166A>C), AAT828ACA (3168A>C 3169T>A), GCA829GCG (3172A>G), ATT832ATA (3181T>A), TTT833TTG (3184T>G)

*: Inserts / Deletes / Misaligned / Frameshifts

Analysis details

This analysis was performed with panviral2.64

NGS Details (UN60): Torradovirus marchitezum (segment RNA 1)

Assembly

**Coverage Length**	6084 (1 contig(s))
**Depth Of Coverage**	3023.2
**Number Of Reads**	139373
**Reads Per Million**	2956.04 rpm (after QC)
**Ambiguities**	0
**Assembly Method**	de novo + reference guided assembly
**Consensus Caller**	Bcf Tools

Coverage Map

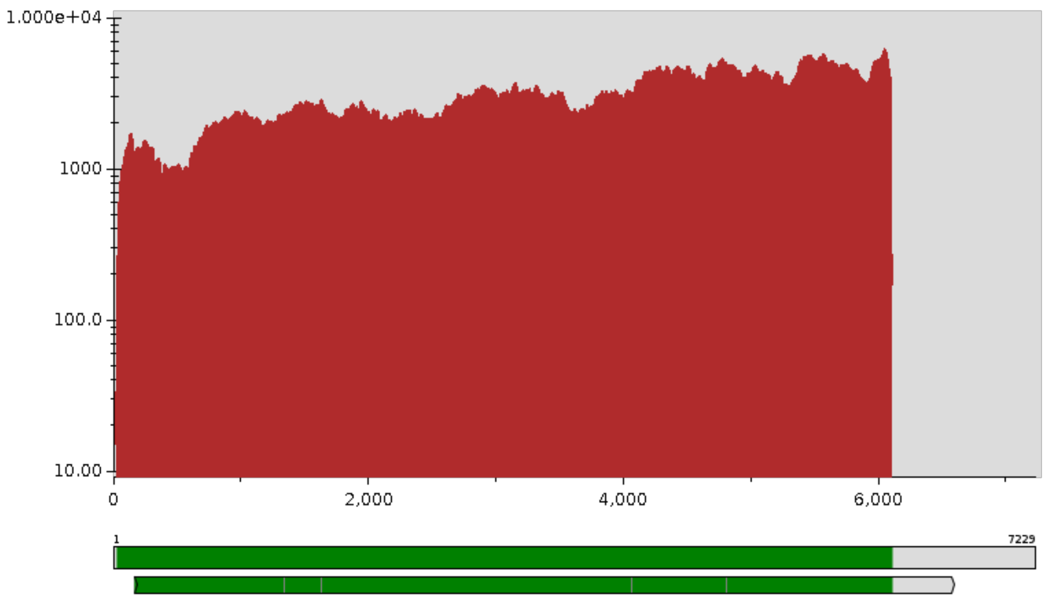

Assignment

**Type**	Torradovirus marchitezum (Taxonomy ID: 3048376)
**Reference Genome**	NC_010987.1
**NT Identity (%)**	64.3453
**AA Identity (%)**	66.7002
**Number Of Stop Codons**	0
**Number Of CDS**	1

Alignment

**Alignment Score**	3417.0 (NT) + 9624.0 (AA) = 13041.0
**Concordance (%)**	50.3981

**Alignment Method**	Global, seeded, nucleotide + amino acids (AGA)

Genome Region

Sequence starts at position 24 and ends at position 6107 relative to NC_010987.1 reference sequence.

Alignment Detailed Statistics

	Begin	End	Coverage	Score	Concordance	Matches	Identities	I/D/M/F*	Stop Codons
**NT**	**24**	**6107**	**84.2%**	**3417**	**28.3%**	**6063 (98.8%)**	**3936 (64.1%)**	**54/21**	

	Begin	End	Coverage	Score	Concordance	Matches	Identities	I/D/M/F*	Stop Codons
**NT**	**24**	**6107**	**84.2%**	**3417**	**28.3%**	**6063 (98.8%)**	**3936 (64.1%)**	**54/21**	
Mutations:	37G>A, 40C>T, 51C>T, 59A>C, 60_61insCAAGCTACCTT, 63G>A, 65T>A, 66A>T, 69T>C, 72T>G, 74C>T, 76T>A, 77G>C, 79A>T, 80A>G, 81A>T, 84T>C, 86T>C, 88_89insCATTCTACCTTTCAGA, 91C>A, 92A>T, 93T>C, 95T>A, 97C>T, 98T>G, 100C>T, 102C>T, 104T>A, 105C>A, 108A>T, 109C>G, 111C>G, 112G>T, 113A>T, 119A>T, 121T>C, 123A>G, 126C>T, 128C>T, 130T>C, 133A>T, 135T>C, 137T>C, 138C>T, 139C>T, 140A>T, 141A>C, 145C>T, 149T>C, 150T>C, 151C>A, 152A>C, 155G>T, 157T>C, 158G>C, 159T>A, 160T>C, 162T>A, 163C>T, 164C>T, 165A>C, 167G>A, 168G>A, 170T>G, 173T>A, 174_176delGGT, 177G>T, 180G>T, 181A>C, 182T>A, 183G>A, 185C>A, 186G>A, 187C>T, 189A>G, 190G>T, 198T>G, 200C>T, 203C>T, 204A>C, 209A>T, 212C>T, 213T>C, 216G>T, 217G>C, 221A>G, 222C>T, 224T>G, 227A>T, 228A>C, 230T>G, 231T>A, 233T>A, 234A>G, 236T>G, 239C>A, 240A>T, 241A>C, 243T>C, 245T>G, 248A>C, 251A>G, 252G>A, 254G>A, 255G>A, 257T>G, 259G>A, 260T>G, 262A>G, 266T>G, 268C>G, 269C>T, 271C>A, 272T>A, 275G>A, 278A>T, 279C>G, 284G>A, 285A>T, 287T>C, 292A>G, 293T>C, 297C>A, 302G>A, 305T>A, 306C>T, 308G>A, 314A>T, 317T>A, 318G>A, 320G>T, 321A>G, 322C>T, 324G>A, 326T>A, 327G>A, 328T>C, 329G>T, 330T>G, 332C>A, 333A>G, 334C>A, 335A>T, 338T>C, 342G>A, 343T>A, 344G>T, 345G>A, 346C>G, 347C>T, 348A>G, 349C>T, 359A>T, 360C>G, 362T>A, 363A>G, 365T>A, 369G>T, 371T>A, 374G>A, 376A>G, 377C>G, 385G>C, 386T>C, 387G>A, 389A>G, 390C>G, 395A>T, 398T>A, 401A>T, 405C>G, 412C>G, 413G>T, 414T>A, 415A>T, 417T>A, 418C>A, 425G>C, 426T>A, 427C>A, 428C>A, 437C>T, 439A>G, 441T>A, 443T>C, 446C>A, 448A>T, 449G>T, 459T>C, 461A>G, 465T>G, 467G>T, 468T>G, 471G>T, 472A>T, 474A>G, 476T>C, 477T>A, 479G>C, 480T>A, 481C>A, 482C>A, 488A>T, 489G>T, 493A>T, 494A>T, 495T>A, 496T>G, 502T>A, 504G>T, 506T>A, 510G>C, 515A>G, 516A>G, 518T>A, 520A>C, 523G>C, 524T>A, 526A>G, 527A>T, 530T>A, 531G>T, 532T>A, 533A>T, 537A>G, 539A>T, 542T>A, 545A>T, 546T>A, 547A>T, 548T>A, 550G>C, 557T>A, 560A>C, 564G>T, 566T>C, 567A>C, 569A>T, 570A>T, 573T>C, 575G>A, 578A>G, 579G>T, 581G>A, 582C>T, 584T>A, 586T>A, 588T>A, 590T>A, 591G>A, 593T>C, 600A>G, 601G>T, 602T>A, 611T>A, 614G>A, 615G>A, 616G>T, 617T>G, 621T>A, 622C>G, 623T>A, 625A>G, 626C>T, 627A>G, 628C>T, 629T>A, 632C>T, 633A>T, 634A>G, 635A>T, 641C>A, 645T>C, 647G>A, 648A>G, 650A>T, 653C>A, 656A>T, 660A>T, 662G>C, 665T>C, 668A>T, 669A>T, 670G>C, 674A>G, 677C>T, 681T>A, 683A>T, 684G>A, 685A>G, 695A>C, 696A>C, 697A>T, 698A>C, 702G>T, 704C>T, 707A>G, 708C>A, 709G>A, 713C>T, 714A>T, 716C>T, 717A>C, 718T>A, 720G>A, 725C>A, 728A>G, 732A>T, 733G>C, 735A>G, 740T>A, 746G>T, 749A>G, 750T>A, 751C>G, 752A>C, 753G>T, 755T>G, 756T>A, 757C>A, 758A>T, 770G>C, 771T>A, 772C>G, 776G>A, 780A>C, 785C>T, 786A>T, 788A>T, 789A>T, 791A>G, 792T>A, 794G>A, 797T>A, 803G>C, 806T>C, 809G>A, 818G>A, 821C>T, 822A>G, 824T>G, 830G>A, 839A>G, 849T>C, 851G>T, 853G>C, 854C>A, 860A>G, 861T>C, 863G>A, 873C>T, 875C>G, 881G>A, 882A>G, 884A>G, 887A>C, 893T>A, 899G>A, 914A>C, 918A>C, 919C>A, 920T>A, 921C>T, 923T>G, 926C>T, 927C>A, 928G>A, 929T>G, 932T>C, 938A>G, 947C>A, 962A>T, 968T>G, 969C>T, 971T>G, 977A>T, 978A>G, 980A>T, 982T>C, 983C>T, 985G>A, 986C>T, 988C>T, 989C>A, 992T>C, 995A>G, 998C>T, 1001A>G, 1004T>C, 1010G>T, 1011T>C, 1016A>T, 1019T>A, 1022A>T, 1031G>A, 1032C>A, 1034G>T, 1046G>C, 1049C>T, 1059C>A, 1060A>C, 1061G>T, 1064G>A, 1068G>T, 1069A>C, 1077T>G, 1078G>C, 1091G>A, 1092C>A, 1094C>G, 1100G>A, 1109C>T, 1113A>C, 1114A>C, 1115G>T, 1121C>T, 1122C>T, 1124T>A, 1126C>T, 1142G>T, 1144G>A, 1145G>A, 1148A>G, 1149G>C, 1151A>G, 1154T>A, 1163C>A, 1169T>C, 1172C>T, 1178A>T, 1187A>T, 1190T>G, 1199C>T, 1200A>T, 1205C>T, 1211G>A, 1215A>T, 1216G>C, 1221G>A, 1223C>A, 1228C>T, 1232G>A, 1235A>G, 1238A>T, 1242G>C, 1244T>C, 1245T>A, 1246A>T, 1247T>A, 1250T>A, 1255G>A, 1256T>C, 1260A>C, 1261G>A, 1262G>A, 1265C>T, 1267T>C, 1268G>A, 1271C>A, 1277C>T, 1283T>C, 1284C>A, 1286T>A, 1295T>C, 1304A>T, 1313C>T, 1316G>T, 1317T>G, 1318C>A, 1319C>G, 1325G>A, 1328T>C, 1331T>C, 1337T>C, 1340A>G, 1341T>A, 1342C>G, 1349C>A, 1350T>C, 1352A>G, 1355T>C, 1358T>C, 1367G>A, 1373C>T, 1374A>G, 1375G>C, 1376A>C, 1380G>C, 1381G>A, 1382T>A, 1388A>C, 1391A>G, 1394C>G, 1397T>C, 1398C>A, 1401C>T, 1403A>G, 1412A>T, 1419A>C, 1421G>C, 1427A>T, 1430T>C, 1436C>A, 1439C>T, 1463G>C, 1472C>T, 1478A>T, 1482T>C, 1487A>G, 1493G>A, 1499G>C, 1502G>A, 1508A>G, 1511T>C, 1517A>G, 1520T>C, 1523G>C, 1529T>C, 1538A>C, 1553C>A, 1556A>G, 1559A>C, 1565C>T, 1571T>A, 1574G>A, 1583C>G, 1584A>C, 1595C>A, 1598C>A, 1604T>C, 1616A>C, 1622T>C, 1623C>T, 1626A>G, 1628C>T, 1634T>A, 1637T>A, 1640C>A, 1646T>C, 1649T>G, 1652T>G, 1661A>T, 1673G>A, 1674C>A, 1679A>G, 1680A>C, 1688C>T, 1689C>T, 1697G>A, 1700T>C, 1701A>C, 1702T>G, 1706G>A, 1708A>T, 1712C>G, 1715G>A, 1718T>C, 1721C>A, 1724T>A, 1727A>G, 1728G>A, 1730T>C, 1733T>A, 1736G>A, 1738G>C, 1748A>C, 1751T>C, 1757A>C, 1758G>A, 1760G>T, 1762A>G, 1763G>A, 1769A>G, 1772A>G, 1781T>C, 1784C>A, 1787T>A, 1793G>A, 1794A>G, 1796T>C, 1797T>C, 1799G>C, 1802C>T, 1806A>T, 1807G>C, 1811T>C, 1812C>T, 1814T>G, 1820G>A, 1829A>G, 1835A>G, 1838T>C, 1839C>A, 1844A>C, 1847T>C, 1848G>C, 1849C>A, 1850A>C, 1854G>A, 1856A>T, 1857T>G, 1861A>C, 1862G>C, 1866T>C, 1868G>C, 1870T>A, 1871T>C, 1872G>A, 1874A>C, 1875G>C, 1876C>A, 1877T>G, 1878G>T, 1879A>C, 1881T>A, 1883T>A, 1886C>T, 1887A>T, 1888T>G, 1889A>T, 1890A>G, 1891G>A, 1892T>A, 1893C>G, 1894C>A, 1895T>G, 1898G>A, 1899A>G, 1901A>T, 1904T>C, 1910T>G, 1911C>G, 1921C>T, 1922A>T, 1925T>A, 1928T>A, 1934A>G, 1935G>A, 1937G>A, 1940T>C, 1941G>T, 1943G>T, 1944G>C, 1948G>A, 1949G>T, 1953A>C, 1954G>A, 1956T>A, 1958G>A, 1959C>A, 1962A>T, 1963C>T, 1964C>T, 1965A>G, 1967G>C, 1968C>G, 1972A>T, 1974G>A, 1975A>G, 1976A>C, 1979A>C, 1981T>G, 1982A>T, 1983T>G, 1985G>T, 1986_1991delGTGCCC, 1994T>A, 1998G>C, 2000G>C, 2001A>T, 2002G>C, 2006A>G, 2007A>G, 2019A>G, 2020T>G, 2021C>A, 2025G>T, 2028A>C, 2029G>A, 2030A>G, 2033G>A, 2036C>A, 2039G>T, 2042A>G, 2045G>A, 2049T>C, 2054A>G, 2060A>T, 2063C>T, 2066G>A, 2069C>A, 2070A>G, 2071G>C, 2072T>A, 2075G>A, 2076G>A, 2081T>A, 2082G>T, 2083A>T, 2084G>T, 2088C>T, 2089T>A, 2096T>C, 2102C>T, 2103C>G, 2104A>C, 2106C>T, 2107A>C, 2108T>A, 2117C>A, 2120G>T, 2122A>T, 2127T>G, 2128C>T, 2129T>C, 2132T>G, 2133G>T, 2134A>C, 2135T>A, 2136G>A, 2137A>G, 2142C>G, 2143A>C, 2144T>C, 2152T>G, 2153T>C, 2156C>T, 2158A>C, 2159C>T, 2160A>C, 2161A>G, 2162A>C, 2166A>G, 2167G>T, 2169G>A, 2170T>A, 2171C>T, 2172A>G, 2174C>G, 2178T>A, 2180G>T, 2183T>C, 2184A>G, 2185G>A, 2186A>G, 2189A>G, 2192A>T, 2193G>T, 2194A>C, 2195T>G, 2196G>A, 2200A>C, 2202C>G, 2203A>G, 2204G>A, 2208C>G, 2209G>C, 2210A>C, 2217G>A, 2219A>C, 2220C>A, 2222A>G, 2223A>G, 2224A>C, 2228T>C, 2231T>A, 2241A>G, 2243A>C, 2255G>A, 2258T>C, 2268G>A, 2269C>G, 2270A>T, 2271T>A, 2277A>G, 2279C>G, 2282T>A, 2285G>A, 2286A>G, 2287A>C, 2288G>T, 2289A>C, 2291A>C, 2292C>A, 2294A>G, 2296C>A, 2297A>T, 2300G>A, 2303G>A, 2305C>G, 2306A>G, 2307G>A, 2308A>T, 2310A>T, 2313A>T, 2314G>A, 2315G>C, 2317C>A, 2321A>C, 2322T>A, 2323T>G, 2324T>A, 2331T>A, 2332C>G, 2333T>A, 2334T>G, 2335G>C, 2336C>A, 2337A>C, 2339T>G, 2342T>A, 2345A>T, 2350T>C, 2357C>T, 2358C>T, 2360C>A, 2363A>G, 2369C>T, 2372G>A, 2375G>A, 2381A>T, 2385T>C, 2386T>A, 2390T>C, 2393G>A, 2396T>C, 2397T>C, 2402T>C, 2415A>C, 2417A>G, 2426_2427insAGAAGG, 2429C>G, 2431A>T, 2432A>G, 2435A>C, 2436T>A, 2438A>T, 2441A>G, 2444G>A, 2445C>A, 2448_2453delAAGGAA, 2456A>T, 2460A>G, 2462C>A, 2463A>C, 2466A>T, 2467T>C, 2468T>G, 2469T>C, 2470G>T, 2474C>T, 2475C>G, 2477T>C, 2478A>T, 2479A>C, 2480A>C, 2482T>C, 2483C>A, 2484G>A, 2488A>G, 2489A>G, 2490T>A, 2491A>G, 2492T>A, 2495C>G, 2496A>C, 2498G>A, 2501T>A, 2503C>T, 2504A>G, 2508T>A, 2509G>A, 2510G>T, 2511A>G, 2512G>A, 2513C>T, 2516T>G, 2522T>C, 2523G>A, 2524C>T, 2525A>T, 2526C>T, 2530G>A, 2537C>T, 2549T>G, 2556A>C, 2561G>A, 2567T>C, 2573A>G, 2574G>A, 2575T>C, 2577T>A, 2579T>G, 2582A>T, 2584G>C, 2586A>G, 2588T>A, 2591A>T, 2597T>C, 2598T>A, 2600G>T, 2601G>T, 2603G>T, 2610C>A, 2612G>A, 2615T>C, 2617A>G, 2620C>T, 2621T>G, 2622T>A, 2624C>T, 2629A>C, 2631G>T, 2633A>C, 2634T>C, 2635C>A, 2641G>A, 2651G>T, 2663C>G, 2669T>C, 2675C>A, 2678T>C, 2693T>A, 2695A>G, 2696T>A, 2696_2697insTCC, 2697A>C, 2699G>T, 2700C>G, 2702C>A, 2703A>T, 2704G>C, 2708T>G, 2709C>A, 2711T>G, 2714C>G, 2715G>T, 2718G>A, 2720T>C, 2729T>A, 2733T>C, 2736T>A, 2737T>A, 2738G>T, 2740C>A, 2741A>T, 2744A>G, 2744_2745insGGA, 2745G>A, 2747T>A, 2754G>T, 2756T>C, 2757G>A, 2758A>G, 2759A>G, 2765A>T, 2771A>G, 2774T>C, 2775C>G, 2777G>A, 2778A>T, 2779G>C, 2780C>T, 2783C>T, 2784A>G, 2785A>C, 2786G>C, 2798G>A, 2802T>C, 2807A>G, 2808C>G, 2810T>G, 2812G>C, 2813T>A, 2822G>T, 2829A>C, 2831A>T, 2834C>A, 2837C>T, 2846T>C, 2847T>A, 2848C>G, 2849T>C, 2851G>A, 2852T>A, 2864C>T, 2870T>A, 2873T>C, 2876A>T, 2880C>A, 2881A>C, 2882C>G, 2883A>C, 2885T>G, 2886G>C, 2887C>T, 2888T>C, 2891A>T, 2897T>A, 2900T>C, 2907A>T, 2915C>T, 2920C>A, 2921C>A, 2922A>C, 2928C>G, 2929G>T, 2930A>C, 2932C>G, 2933T>C, 2934A>C, 2936A>G, 2939T>G, 2948C>T, 2953G>A, 2955G>C, 2957A>T, 2958T>G, 2960A>C, 2962G>A, 2964A>T, 2967A>T, 2969T>G, 2970G>C, 2971A>C, 2972G>T, 2974G>A, 2975C>A, 2981C>T, 2982A>G, 2984C>T, 2985A>G, 2987T>A, 2988C>T, 2990G>A, 2991T>C, 2994C>A, 2996C>G, 3002T>C, 3003G>T, 3004A>G, 3008A>T, 3011T>C, 3014T>G, 3017A>T, 3020G>A, 3021C>G, 3022C>T, 3023T>C, 3030C>A, 3032T>A, 3035A>G, 3041C>T, 3042C>G, 3044A>T, 3045A>G, 3046G>A, 3055A>C, 3056G>A, 3071G>A, 3074C>A, 3077A>T, 3078C>G, 3083A>C, 3089C>T, 3090T>G, 3091G>T, 3092T>C, 3098T>A, 3101T>G, 3104G>A, 3107T>A, 3109C>A, 3116A>T, 3117C>G, 3118G>A, 3119T>G, 3120C>A, 3121T>A, 3122T>A, 3123G>C, 3124G>A, 3125C>G, 3128T>C, 3131A>G, 3134G>C, 3144A>T, 3145T>A, 3146G>T, 3147C>T, 3161A>C, 3164G>A, 3170C>T, 3175T>A, 3176T>G, 3182G>C, 3183G>A, 3184A>C, 3188T>C, 3189G>A, 3190A>G, 3191A>G, 3192C>A, 3196A>G, 3198T>A, 3200C>A, 3201T>A, 3202C>A, 3203C>T, 3204A>T, 3205G>C, 3206T>A, 3210A>C, 3212G>C, 3212_3213insTCA, 3215T>C, 3216T>A, 3218C>T, 3219T>G, 3225C>G, 3228T>G, 3230T>C, 3242C>T, 3243A>G, 3245A>C, 3249G>A, 3251G>C, 3263C>T, 3269C>A, 3272G>T, 3279C>T, 3281G>A, 3284A>G, 3288A>T, 3289G>C, 3291G>A, 3293T>C, 3296G>A, 3299T>C, 3308T>A, 3309G>T, 3310T>G, 3311G>C, 3314C>T, 3320C>T, 3323C>T, 3326C>T, 3332A>T, 3335A>C, 3343G>A, 3344C>A, 3346T>C, 3347A>T, 3351G>A, 3353T>A, 3356A>G, 3357C>T, 3358G>T, 3359T>G, 3366A>G, 3368C>A, 3369T>G, 3371T>A, 3380T>C, 3383A>T, 3387A>G, 3389C>A, 3398A>C, 3401A>G, 3404A>G, 3405G>C, 3407G>A, 3414C>G, 3416A>T, 3417A>C, 3418C>A, 3419T>A, 3425G>A, 3428C>T, 3432G>A, 3433C>T, 3434A>G, 3437A>C, 3443A>G, 3444C>A, 3446C>A, 3452T>C, 3453A>T, 3455T>G, 3458G>C, 3461G>A, 3462T>C, 3463C>A, 3464A>T, 3465A>G, 3468A>C, 3471A>T, 3472G>C, 3473C>A, 3482A>T, 3483C>G, 3484A>C, 3485G>T, 3488T>C, 3494G>C, 3500C>G, 3506G>T, 3507T>G, 3508A>C, 3509C>T, 3511A>T, 3512C>T, 3513C>T, 3515T>G, 3518A>C, 3521A>T, 3527A>T, 3530G>A, 3534T>A, 3539C>A, 3542C>A, 3545A>G, 3548T>G, 3551C>A, 3563T>C, 3566G>C, 3567C>A, 3575C>T, 3577A>C, 3579T>C, 3587A>G, 3593C>T, 3596A>T, 3597C>G, 3598C>A, 3599T>A, 3603G>C, 3605G>A, 3608A>T, 3611A>T, 3614A>T, 3617C>A, 3618A>C, 3620T>G, 3623A>C, 3624A>T, 3626A>T, 3629T>G, 3630C>A, 3632T>A, 3635C>T, 3644T>G, 3650T>A, 3656C>T, 3659C>T, 3665G>T, 3671G>C, 3672G>A, 3674A>T, 3675A>G, 3676T>A, 3695T>C, 3697G>C, 3698T>A, 3699G>A, 3700G>A, 3701A>T, 3707T>G, 3710T>C, 3711G>A, 3712A>G, 3719C>A, 3720A>C, 3721A>G, 3722A>G, 3723C>G, 3724T>C, 3725A>T, 3731C>T, 3735A>G, 3736A>G, 3745G>C, 3746C>T, 3747T>G, 3749G>C, 3750C>A, 3752G>T, 3753C>T, 3758T>A, 3759A>G, 3761C>T, 3762A>C, 3764G>C, 3765C>A, 3766G>A, 3767T>G, 3770C>T, 3773G>A, 3775A>G, 3777A>G, 3779C>G, 3782T>A, 3785G>T, 3788A>T, 3789C>A, 3790G>T, 3791T>C, 3794A>C, 3795T>A, 3796G>C, 3797T>A, 3798G>A, 3799A>C, 3801A>C, 3803G>T, 3804G>T, 3805A>G, 3812T>C, 3813G>A, 3815A>C, 3818G>T, 3821C>G, 3827A>T, 3828A>G, 3830A>T, 3836A>T, 3838G>T, 3842T>C, 3844A>T, 3847A>T, 3852G>T, 3853G>C, 3860G>A, 3863G>C, 3872T>A, 3875C>T, 3881T>C, 3884C>T, 3890C>T, 3893G>A, 3894A>C, 3899G>T, 3900A>T, 3905G>T, 3907T>C, 3908G>C, 3911C>T, 3914G>A, 3917A>G, 3923T>C, 3926G>T, 3927C>G, 3930T>G, 3932C>A, 3933A>C, 3934C>A, 3935T>G, 3937G>A, 3938C>G, 3939G>A, 3940A>G, 3941A>T, 3942G>A, 3943G>C, 3944A>C, 3945G>C, 3947G>A, 3950A>G, 3953G>A, 3954G>C, 3955A>T, 3962A>G, 3966G>A, 3968C>T, 3969A>T, 3977A>T, 3980T>C, 3981G>C, 3992A>G, 3995G>A, 3996C>T, 4005C>G, 4007G>A, 4010T>A, 4016A>G, 4019C>T, 4021T>A, 4023C>T, 4031T>A, 4032C>A, 4037C>T, 4043C>T, 4049C>T, 4052C>T, 4055A>G, 4058T>C, 4062C>A, 4064C>G, 4067C>A, 4070A>T, 4073T>G, 4076G>C, 4079A>G, 4082C>A, 4085T>C, 4088C>G, 4091G>A, 4093G>A, 4094A>G, 4097G>A, 4101A>C, 4103G>T, 4106C>T, 4115A>T, 4116C>T, 4118T>G, 4121T>A, 4130C>T, 4137T>C, 4142C>G, 4143A>C, 4145G>C, 4148G>A, 4154T>C, 4163C>T, 4166A>T, 4169C>A, 4170A>T, 4171C>G, 4172A>T, 4175G>A, 4182T>G, 4183T>C, 4184G>A, 4187C>T, 4192C>T, 4194A>C, 4196A>G, 4199A>C, 4208T>G, 4214T>C, 4215G>A, 4220T>C, 4222C>T, 4223A>T, 4224A>T, 4225G>C, 4226T>A, 4227T>C, 4228C>A, 4238A>C, 4239T>A, 4240T>C, 4241G>C, 4242T>C, 4250T>C, 4251G>C, 4252G>A, 4256T>C, 4257A>C, 4259A>G, 4260G>A, 4261C>G, 4268A>T, 4271C>T, 4274G>T, 4277A>T, 4283A>T, 4286T>A, 4289T>C, 4292T>C, 4310A>G, 4313A>G, 4316T>A, 4317G>A, 4319T>A, 4325T>A, 4331G>A, 4354A>C, 4361C>T, 4364A>G, 4368A>G, 4369A>C, 4370G>T, 4373T>A, 4376C>A, 4379T>C, 4380C>G, 4381C>A, 4382T>G, 4388C>A, 4394T>A, 4395C>A, 4403A>T, 4406T>G, 4409A>G, 4418A>T, 4424T>C, 4433A>T, 4436G>A, 4449G>C, 4451G>C, 4454T>C, 4460G>A, 4466G>T, 4469C>A, 4475A>T, 4478A>T, 4487A>T, 4490A>C, 4493G>C, 4499C>A, 4502C>T, 4505T>C, 4508T>C, 4511C>T, 4512C>T, 4514C>A, 4517T>C, 4532A>G, 4538C>T, 4541T>C, 4547A>G, 4548T>C, 4550G>C, 4551T>C, 4553A>C, 4559A>G, 4565T>C, 4568T>C, 4569G>A, 4570C>A, 4571T>A, 4574C>A, 4576G>A, 4580T>A, 4581A>T, 4583A>G, 4586A>T, 4589T>A, 4592A>G, 4593C>T, 4604C>T, 4608C>G, 4610G>C, 4611C>T, 4616C>T, 4619A>T, 4622T>A, 4625T>G, 4631A>G, 4646T>G, 4652A>G, 4656T>A, 4658A>G, 4661T>C, 4664C>T, 4667G>A, 4673T>C, 4674T>G, 4675C>A, 4676T>G, 4677C>T, 4679G>A, 4682A>T, 4683A>G, 4684A>C, 4685T>A, 4694T>A, 4698T>C, 4705T>A, 4706G>A, 4709C>G, 4712T>C, 4715A>T, 4718C>T, 4721T>C, 4730T>C, 4736C>T, 4740T>A, 4741C>A, 4742G>C, 4746G>A, 4751T>C, 4757G>T, 4760A>C, 4761C>A, 4766T>C, 4769A>G, 4782C>T, 4784A>G, 4787T>G, 4791C>T, 4796T>C, 4802T>C, 4805T>C, 4808A>G, 4811A>C, 4818C>T, 4820T>G, 4823A>T, 4829C>A, 4830A>C, 4832G>A, 4835T>C, 4838T>C, 4853A>C, 4856C>T, 4857A>G, 4859T>G, 4862G>A, 4865C>A, 4877A>C, 4883T>A, 4886C>A, 4887C>G, 4888C>T, 4889A>T, 4892T>G, 4898T>A, 4901C>T, 4903C>T, 4904T>C, 4905C>A, 4906A>C, 4907G>T, 4914A>T, 4915T>A, 4916G>T, 4931A>G, 4934A>T, 4935A>G, 4938C>A, 4940T>A, 4943A>G, 4952T>G, 4955C>T, 4958T>A, 4960G>A, 4961T>G, 4964G>C, 4966C>T, 4975A>C, 4976G>A, 4977C>T, 4979T>G, 4980G>A, 4981T>C, 4982C>A, 4983T>G, 4984T>A, 4985A>C, 4986G>A, 4991T>C, 4994G>A, 4996C>A, 4997G>A, 5000G>A, 5001T>A, 5003G>A, 5004G>A, 5005A>T, 5006T>G, 5007G>C, 5009A>G, 5010A>C, 5012G>A, 5015T>C, 5018C>G, 5019A>C, 5020G>C, 5022G>A, 5023C>T, 5024T>C, 5028A>C, 5029T>A, 5030C>T, 5036T>A, 5039C>T, 5043T>G, 5044T>C, 5045G>T, 5049G>C, 5050T>A, 5051G>A, 5055C>A, 5056A>G, 5057G>T, 5060A>G, 5064A>C, 5066T>A, 5067G>C, 5068C>A, 5070A>G, 5071A>C, 5072G>C, 5075A>T, 5078A>T, 5081G>A, 5084T>C, 5085T>A, 5086C>G, 5087A>T, 5089G>A, 5090T>C, 5091G>A, 5092C>T, 5093C>A, 5094C>G, 5095T>G, 5097A>G, 5099A>T, 5100A>C, 5102T>G, 5103T>C, 5104G>T, 5105T>G, 5109A>C, 5112G>A, 5113C>G, 5114A>C, 5117A>G, 5119C>A, 5120A>T, 5127T>A, 5129C>T, 5132T>C, 5133A>G, 5134C>T, 5138A>T, 5139T>C, 5141G>T, 5142G>C, 5143A>C, 5144C>A, 5146C>A, 5151G>A, 5153G>A, 5156G>A, 5159C>T, 5160A>G, 5161C>A, 5163A>G, 5169G>T, 5170A>C, 5171G>A, 5173G>A, 5174T>A, 5176A>G, 5178C>G, 5180G>C, 5181A>C, 5184T>G, 5185G>A, 5186G>A, 5190G>T, 5191C>G, 5192A>T, 5193C>T, 5195A>T, 5196A>G, 5197A>G, 5198C>A, 5199A>G, 5202G>T, 5203G>T, 5204T>C, 5207T>C, 5214G>T, 5216A>G, 5219T>A, 5222T>A, 5224C>A, 5227C>T, 5228C>T, 5229C>A, 5231C>T, 5232T>C, 5234C>T, 5236C>A, 5237G>A, 5246A>G, 5248G>A, 5249G>C, 5250T>A, 5251A>C, 5252C>A, 5256A>C, 5264C>T, 5267T>C, 5279G>A, 5282G>A, 5283T>A, 5285C>A, 5287A>C, 5288T>A, 5294T>G, 5300T>C, 5339G>C, 5342C>T, 5345T>A, 5349T>G, 5354T>C, 5357A>C, 5359T>A, 5363T>C, 5364G>A, 5365C>A, 5366C>T, 5370A>C, 5372G>A, 5374A>G, 5376G>C, 5377T>G, 5378G>C, 5382G>T, 5385G>A, 5386G>T, 5387A>G, 5390A>G, 5391C>A, 5392A>T, 5395T>G, 5397G>A, 5399A>C, 5400A>C, 5401C>A, 5402C>A, 5405T>A, 5408C>T, 5411T>C, 5415G>A, 5417G>T, 5423A>G, 5429A>T, 5438C>G, 5439G>A, 5441C>T, 5444A>C, 5453A>T, 5456G>T, 5460A>T, 5462G>T, 5465C>A, 5469G>T, 5474A>C, 5476G>A, 5477G>T, 5480A>G, 5484T>A, 5486T>A, 5488T>C, 5489G>T, 5493G>C, 5495C>A, 5496A>C, 5498A>T, 5504T>C, 5511C>A, 5513C>A, 5517A>T, 5519T>G, 5521G>C, 5522A>G, 5523A>G, 5524A>G, 5525T>C, 5526G>T, 5531T>G, 5534C>T, 5535C>T, 5537C>G, 5541A>C, 5542C>A, 5543C>G, 5555C>T, 5559G>A, 5561G>T, 5564G>A, 5570T>C, 5573T>C, 5574C>A, 5575A>C, 5579_5580insGTC, 5584C>A, 5585T>G, 5586C>A, 5588T>A, 5589G>A, 5591A>T, 5592G>T, 5593A>G, 5597A>G, 5598G>A, 5599T>C, 5600G>A, 5607A>G, 5608G>C, 5610G>A, 5614C>G, 5615A>G, 5616_5618delTCG, 5620T>G, 5622C>G, 5624C>T, 5625A>G, 5627T>A, 5629G>T, 5630T>G, 5633T>G, 5635T>A, 5636G>C,								

	5642A>T, 5642_5643insCATGCT, 5644G>C, 5645G>T, 5647T>A, 5650A>C, 5651A>C, 5652T>C, 5654G>T, 5657C>A, 5663C>T, 5666C>A, 5669T>A, 5670A>G, 5672A>G, 5673C>G, 5674A>G, 5675T>C, 5676G>T, 5679G>T, 5680A>C, 5681T>A, 5682T>A, 5684A>T, 5685G>T, 5689A>G, 5691C>A, 5695C>T, 5696A>G, 5700A>G, 5703C>A, 5705G>A, 5706A>T, 5711A>C, 5714T>C, 5715G>A, 5716T>C, 5717G>T, 5720A>T, 5723A>G, 5727C>A, 5728A>T, 5729T>G, 5732A>T, 5735A>T, 5740G>C, 5742A>G, 5746T>G, 5747T>C, 5750C>A, 5754C>A, 5755A>G, 5756G>A, 5757G>A, 5765G>T, 5766A>T, 5767G>C, 5769T>C, 5771G>C, 5772C>A, 5774A>C, 5775C>A, 5777C>A, 5778T>G, 5779A>C, 5780T>C, 5781T>C, 5783G>A, 5787C>T, 5788A>G, 5789G>C, 5790G>T, 5791G>C, 5792A>T, 5798A>T, 5800A>C, 5801G>A, 5803T>G, 5804A>T, 5805A>G, 5807T>G, 5808C>G, 5809A>C, 5810A>T, 5811A>C, 5812A>G, 5813G>T, 5814A>G, 5815A>G, 5816T>G, 5817A>T, 5818C>G, 5819A>T, 5823C>A, 5834T>C, 5837C>T, 5838A>G, 5840A>G, 5841G>A, 5843G>T, 5844C>T, 5846G>T, 5848G>A, 5850A>G, 5851T>C, 5852G>A, 5853G>C, 5857A>T, 5858T>A, 5860T>C, 5861G>T, 5862C>A, 5863A>C, 5864A>C, 5865C>A, 5866A>G, 5867A>G, 5868C>T, 5870G>A, 5871A>G, 5880G>C, 5885G>A, 5886C>A, 5887T>G, 5894G>T, 5895T>G, 5897T>G, 5900C>T, 5901C>A, 5902C>T, 5903A>C, 5905A>C, 5908A>C, 5909A>C, 5912T>A, 5913A>G, 5914C>G, 5916A>C, 5917T>A, 5918T>C, 5919C>A, 5921G>A, 5923A>T, 5925C>T, 5927C>G, 5928A>G, 5929A>T, 5930A>G, 5931A>C, 5933T>A, 5934T>A, 5935T>C, 5937G>C, 5939T>G, 5940G>A, 5941A>G, 5942T>C, 5943G>A, 5944C>A, 5946G>C, 5951T>G, 5953T>A, 5955A>T, 5957A>C, 5958T>C, 5959T>A, 5961A>G, 5964G>C, 5965C>A, 5969G>T, 5970A>T, 5972C>T, 5973T>G, 5975T>G, 5977G>A, 5979C>A, 5980A>G, 5981C>A, 5984A>C, 5985G>T, 5986T>A, 5993A>G, 5994A>C, 5995A>G, 5996G>A, 5999A>T, 6003T>C, 6005G>C, 6006A>T, 6007G>C, 6008T>A, 6009T>A, 6010C>A, 6012A>G, 6015T>A, 6016G>T, 6017G>A, 6019A>C, 6023_6024insTAC, 6024T>C, 6032A>T, 6033A>G, 6034G>C, 6035T>C, 6037T>A, 6038T>A, 6040T>A, 6045T>A, 6046C>T, 6048A>G, 6049G>T, 6050G>A, 6054_6056delGCA, 6063C>G, 6065A>G, 6069C>T, 6070C>T, 6071T>G, 6074C>T, 6078T>C, 6079T>C, 6080G>A, 6086T>C, 6087T>A, 6088C>T, 6089C>G, 6092A>T, 6097A>G, 6098A>G

CDS

ToMarV_RNA1gp1	1	1989	92.4%	9624	69.7%	1982 (99.2%)	1328 (66.5%)	9/7/0/0	0

	Begin	End	Coverage	Score	Concordance	Matches	Identities	I/D/M/F*	Stop Codons
**NT**	**24**	**6107**	**84.2%**	**3417**	**28.3%**	**6063 (98.8%)**	**3936 (64.1%)**	**54/21**	

Protein mutations:

M1L (141A>C), A2V (145C>T), S4H (150T>C 151C>A 152A>C), K5N (155G>T), M6T (157T>C 158G>C), F7T (159T>A 160T>C), S8I (162T>A 163C>T 164C>T), K9Q (165A>C 167G>A), V10M (168G>A 170T>G), G12del (174_176delGGT), V13F (177G>T), D14S (180G>T 181A>C 182T>A), D15K (183G>A 185C>A), A16I (186G>A 187C>T), S17V (189A>G 190G>T), S20A (198T>G 200C>T), T22P (204A>C), F25L (213T>C), G26S (216G>T 217G>C), N30Q (228A>C 230T>G), S31T (231T>A 233T>A), I32V (234A>G 236T>G), K34S (240A>T 241A>C), F35L (243T>C 245T>G), A38T (252G>A 254G>A), V39M (255G>A 257T>G), S40K (259G>A 260T>G), N41S (262A>G), I42M (266T>G), T43S (268C>G 269C>T), T44K (271C>A 272T>A), M45I (275G>A), Q47E (279C>G), I49F (285A>T 287T>C), D51G (292A>G 293T>C), L53I (297C>A), D55E (305T>A), V60I (318G>A 320G>T), T61V (321A>G 322C>T), D62K (324G>A 326T>A), V63T (327G>A 328T>C 329G>T), S64A (330T>G 332C>A), T65D (333A>G 334C>A 335A>T), V68N (342G>A 343T>A 344G>T), A69S (345G>A 346C>G 347C>T), T70V (348A>G 349C>T), L74V (360C>G 362T>A), N75E (363A>G 365T>A), V77L (369G>T 371T>A), D79G (376A>G 377C>G), G82A (385G>C 386T>C), A83T (387G>A 389A>G), L84V (390C>G), D86E (398T>A), L89V (405C>G), A91G (412C>G 413G>T), Y92I (414T>A 415A>T), S93N (417T>A 418C>A), L95F (425G>C), S96K (426T>A 427C>A 428C>A), K100R (439A>G), S101T (441T>A 443T>C), K103I (448A>T 449G>T), L109V (465T>G 467G>T), F110V (468T>G), E111L (471G>T 472A>T), S112G (474A>G 476T>C), L113I (477T>A 479G>C), S114K (480T>A 481C>A 482C>A), K116N (488A>T), V117F (489G>T), K118I (493A>T 494A>T), L119R (495T>A 496T>G), F121Y (502T>A), A122S (504G>T 506T>A), V124L (510G>C), N126E (516A>G 518T>A), K127T (520A>C), S128T (523G>C 524T>A), E129G (526A>G 527A>T), D130E (530T>A), V131Y (531G>T 532T>A 533A>T), I133V (537A>G 539A>T), Y136I (546T>A 547A>T 548T>A), G137A (550G>C), A142S (564G>T 566T>C), I143L (567A>C 569A>T), T144S (570A>T), V147L (579G>T 581G>A), L149Q (586T>A), F150I (588T>A 590T>A), V151I (591G>A 593T>C), S154V (600A>G 601G>T 602T>A), G159M (615G>A 616G>T 617T>G), S161R (621T>A 622C>G 623T>A), N162S (625A>G 626C>T), T163V (627A>G 628C>T 629T>A), K165C (633A>T 634A>G 635A>T), I170V (648A>G 650A>T), M174F (660A>T 662G>C), L181I (681T>A 683A>T), D182S (684G>A 685A>G), K186L (696A>C 697A>T 698A>C), A188S (702G>T 704C>T), R190K (708C>A 709G>A), T192S (714A>T 716C>T), M193Q (717A>C 718T>A), V194M (720G>A), M199V (735A>G), V205L (753G>T 755T>G), S206N (756T>A 757C>A 758A>T), M214L (780A>C), T216S (786A>T 788A>T), I217L (789A>T 791A>G), L218I (792T>A 794G>A), I228V (822A>G 824T>G), S238T (853G>C 854C>A), T248A (882A>G 884A>G), T260Q (918A>C 919C>A 920T>A), R263K (927C>A 928G>A 929T>G), I280V (978A>G 980A>T), V281A (982T>C 983C>T), G282D (985G>A 986C>T), T283I (988C>T 989C>A), L298I (1032C>A 1034G>T), Q307T (1059C>A 1060A>C 1061G>T), D310S (1068G>T 1069A>C), C313A (1077T>G 1078G>C), K325P (1113A>C 1114A>C 1115G>T), P328S (1122C>T 1124T>A), T329I (1126C>T), R335K (1144G>A 1145G>A), E337Q (1149G>C 1151A>G), M354L (1200A>T), A361T (1221G>A 1223C>A), A363V (1228C>T), E366D (1238A>T), V368L (1242G>C 1244T>C), Y369I (1245T>A 1246A>T 1247T>A), S372N (1255G>A 1256T>C), R374Q (1260A>C 1261G>A 1262G>A), M376T (1267T>C 1268G>A), S393E (1317T>G 1318C>A 1319C>G), R412A (1374A>G 1375G>C 1376A>C), G414Q (1380G>C 1381G>A 1382T>A), I496V (1626A>G 1628C>T), M521R (1701A>C 1702T>G), E523V (1708A>T), D530N (1728G>A 1730T>C), G533A (1738G>C), R536S (1748A>C), V540I (1758G>A 1760G>T), E541G (1762A>G 1763G>A), N552D (1794A>G 1796T>C), Q567K (1839C>A), A570H (1848G>C 1849C>A 1850A>C), V572I (1854G>A 1856A>T), S573A (1857T>G), K574T (1861A>C 1862G>C), F577Y (1870T>A 1871T>C), E578N (1872G>A 1874A>C), A579Q (1875G>C 1876C>A 1877T>G), E580S (1878G>T 1879A>C), S581T (1881T>A 1883T>A), I583C (1887A>T 1888T>G 1889A>T), S584E (1890A>G 1891G>A 1892T>A), P585E (1893C>G 1894C>A 1895T>G), I587V (1899A>G 1901A>T), Q591E (1911C>G), A594V (1921C>T 1922A>T), D595E (1925T>A), D596E (1928T>A), I598M (1934A>G), A599T (1935G>A 1937G>A), V601F (1941G>T 1943G>T), D602H (1944G>C), G603D (1948G>A 1949G>T), R605Q (1953A>C 1954G>A), L606I (1956T>A 1958G>A), R607S (1959C>A), T608F (1962A>T 1963C>T 1964C>T), K609D (1965A>G 1967G>C), Q610E (1968C>G), D611V (1972A>T), E612S (1974G>A 1975A>G 1976A>C), I614S (1981T>G 1982A>T), L615V (1983T>G 1985G>T), V616_P617del (1986_1991delGTGCCC), V620L (1998G>C 2000G>C), N623D (2007A>G), I627G (2019A>G 2020T>G 2021C>A), A629S (2025G>T), R630Q (2028A>C 2029G>A 2030A>G), S644A (2070A>G 2071G>C 2072T>A), D646N (2076G>A), E648F (2082G>T 2083A>T 2084G>T), L650Y (2088C>T 2089T>A), H655A (2103C>G 2104A>C), H656S (2106C>T 2107A>C 2108T>A), M660I (2120G>T), Y661F (2122A>T), S663V (2127T>G 2128C>T 2129T>C), D665S (2133G>T 2134A>C 2135T>A), D666S (2136G>A 2137A>G), H668A (2142C>G 2143A>C 2144T>C), I671S (2152T>G 2153T>C), N673T (2158A>C 2159C>T), K674R (2160A>C 2161A>G 2162A>C), R676V (2166A>G 2167G>T), V677N (2169G>A 2170T>A 2171C>T), N678E (2172A>G 2174C>G), L680I (2178T>A 2180G>T), R682E (2184A>G 2185G>A 2186A>G), D685S (2193G>T 2194A>C 2195T>G), A686T (2196G>A), E687A (2200A>C), Q688G (2202C>G 2203A>G 2204G>A), R690A (2208C>G 2209G>C 2210A>C), V693I (2217G>A 2219A>C), Q694K (2220C>A 2222A>G), N695A (2223A>G 2224A>C), I701V (2241A>G 2243A>C), A710S (2268G>A 2269C>G 2270A>T), S711T (2271T>A), I713V (2277A>G 2279C>G), K716A (2286A>G 2287A>C 2288G>T), K717H (2289A>C 2291A>C), L718M (2292C>A 2294A>G), A719D (2296C>A 2297A>T), T722R (2305C>G 2306A>G), E723M (2307G>A 2308A>T), M724L (2310A>T), R725Y (2313A>T 2314G>A 2315G>C), T726N (2317C>A), F728R (2322T>A 2323T>G 2324T>A), S731R (2331T>A 2332C>G 2333T>A), C732A (2334T>G 2335G>C 2336C>A), I733L (2337A>C 2339T>G), D734E (2342T>A), V737A (2350T>C), I741M (2363A>G), F749H (2385T>C 2386T>A), C762_N763insRR (2426_2427insAGAAGG), N763K (2429C>G), K764M (2431A>T 2432A>G), S766T (2436T>A 2438A>T), P769T (2445C>A), K770_E771del (2448_2453delAAGGAA), I774V (2460A>G 2462C>A), K775Q (2463A>C), I776S (2466A>T 2467T>C 2468T>G), C777L (2469T>C 2470G>T), H779D (2475C>G 2477T>C), K780S (2478A>T 2479A>C 2480A>C), I781T (2482T>C 2483C>A), V782I (2484G>A), E783G (2488A>G 2489A>G), Y784R (2490T>A 2491A>G 2492T>A), I785M (2495C>G), K786Q (2496A>C 2498G>A), D787E (2501T>A), T788M (2503C>T 2504A>G), W790N (2508T>A 2509G>A 2510G>T), S791D (2511A>G 2512G>A 2513C>T), A795I (2523G>A 2524C>T 2525A>T), R797K (2530G>A), I806L (2556A>C), M807I (2561G>A), V812T (2574G>A 2575T>C), F813M (2577T>A 2579T>G), G815A (2584G>C), I816V (2586A>G 2588T>A), L820I (2598T>A 2600G>T), A821S (2601G>T 2603G>T), Q824K (2610C>A 2612G>A), N826S (2617A>G), S827L (2620C>T 2621T>G), F828I (2622T>A 2624C>T), N830T (2629A>C), A831S (2631G>T 2633A>C), S832Q (2634T>C 2635C>A), R834K (2641G>A), N852R (2695A>G 2696T>A), N852_K853insS (2696_2697insTCC), K853H (2697A>C 2699G>T), L854V (2700C>G 2702C>A), R855S (2703A>T 2704G>C), N856K (2708T>G), L857M (2709C>A 2711T>G), V859F (2715G>T), G860S (2718G>A 2720T>C), S865P (2733T>C), L866N (2736T>A 2737T>A 2738G>T), A867D (2740C>A 2741A>T), Q868_D869insG (2744_2745insGGA), D869K (2745G>A 2747T>A), A872S (2754G>T 2756T>C), E873R (2757G>A 2758A>G 2759A>G), Q879E (2775C>G 2777G>A), K882A (2784A>G 2785A>C 2786G>C), L890V (2808C>G 2810T>G), G891A (2812G>C 2813T>A), G904E (2851G>A 2852T>A), H914T (2880C>A 2881A>C 2882C>G), N915Q (2883A>C 2885T>G), A916L (2886G>C 2887C>T 2888T>C), T923S (2907A>T), A927E (2920C>A 2921C>A), N928H (2922A>C), R930V (2928C>G 2929G>T 2930A>C), S931C (2932C>G 2933T>C), K932Q (2934A>C 2936A>G), R938K (2953G>A), E939H (2955G>C 2957A>T), S940A (2958T>G 2960A>C), R941K (2962G>A), I942F (2964A>T), I943L (2967A>T 2969T>G), E944P (2970G>C 2971A>C 2972G>T), G945E (2974G>A 2975C>A), I948V (2982A>G 2984C>T), I949V (2985A>G 2987T>A), D955C (3003G>T 3004A>G), P961V (3021C>G 3022C>T 3023T>C), P968A (3042C>G 3044A>T), R969E (3045A>G 3046G>A), E972A (3055A>C 3056G>A), Q980E (3078C>G), C984V (3090T>G 3091G>T 3092T>C), T990K (3109C>A), R993E (3117C>G 3118G>A 3119T>G), L994K (3120C>A 3121T>A 3122T>A), G995Q (3123G>C 3124G>A 3125C>G), E998D (3134G>C), M1002Y (3144A>T 3145T>A 3146G>T), P1003S (3147C>T), V1012E (3175T>A 3176T>G), E1015T (3183G>A 3184A>C), E1017R (3189G>A 3190A>G 3191A>G), L1018M (3192C>A), N1019S (3196A>G), S1020T (3198T>A 3200C>A), S1021N (3201T>A 3202C>A 3203C>T), K1024H (3210A>C 3212G>C), K1024_I1025insS (3212_3213insTCA), C1026S (3216T>A 3218C>T), S1027A (3219T>G), Q1029E (3225C>G), S1030A (3228T>G 3230T>C), I1035V (3243A>G 3245A>C), G1037S (3249G>A 3251G>C), V1051I (3291G>A 3293T>C), V1057C (3309G>T 3310T>G 3311G>C), G1068E (3343G>A 3344C>A), V1069A (3346T>C 3347A>T), A1071T (3351G>A 3353T>A), R1073L (3357C>T 3358G>T 3359T>G), I1076V (3366A>G 3368C>A), S1077A (3369T>G 3371T>A), I1083V (3387A>G 3389C>A), E1086D (3398A>C), E1089Q (3405G>C 3407G>A), P1092A (3414C>G 3416A>T), T1093Q (3417A>C 3418C>A 3419T>A), A1098M (3432G>A 3433C>T 3434A>G), I1105L (3453A>T 3455T>G), S1108H (3462T>C 3463C>A 3464A>T), I1109V (3465A>G), Q1115A (3483C>G 3484A>C 3485G>T), E1118D (3494G>C), N1120K (3500C>G), Y1123A (3507T>G 3508A>C 3509C>T), Y1124F (3511A>T 3512C>T), L1132M (3534T>A), Q1146P (3577A>C), P1153E (3597C>G 3598C>A 3599T>A), E1155Q (3603G>C 3605G>A), I1160L (3618A>C 3620T>G), T1162S (3624A>T 3626A>T), H1164K (3630C>A 3632T>A), V1178I (3672G>A 3674A>T), M1179E (3675A>G 3676T>A), C1186S (3697G>C 3698T>A), G1187N (3699G>A 3700G>A 3701A>T), D1189E (3707T>G), D1191S (3711G>A 3712A>G), K1194R (3720A>C 3721A>G 3722A>G), L1195A (3723C>G 3724T>C 3725A>T), N1199G (3735A>G 3736A>G), C1202S (3745G>C 3746C>T), S1203A (3747T>G 3749G>C), L1204I (3750C>A 3752G>T), I1207V (3759A>G 3761C>T), M1208L (3762A>C 3764G>C), R1209K (3765C>A 3766G>A 3767T>G), K1212R (3775A>G), I1213V (3777A>G 3779C>G), R1217I (3789C>A 3790G>T 3791T>C), C1219T (3795T>A 3796G>C 3797T>A), E1220T (3798G>A 3799A>C), D1222C (3804G>T 3805A>G), V1225I (3813G>A 3815A>C), I1230V (3828A>G 3830A>T), C1233F (3838G>T), Y1235F (3844A>T), Y1236F (3847A>T), G1238S (3852G>T 3853G>C), M1252L (3894A>C), M1254L (3900A>T), M1256T (3907T>C 3908G>C), Q1263E (3927C>G), S1264A (3930T>G 3932C>A), T1265Q (3933A>C 3934C>A 3935T>G), G1266E (3937G>A 3938C>G), E1267S (3939G>A 3940A>G 3941A>T), G1268T (3942G>A 3943G>C 3944A>C), E1269Q (3945G>C 3947G>A), E1272L (3954G>C 3955A>T), D1276N (3966G>A 3968C>T), T1277S (3969A>T), E1281Q (3981G>C), Q1289E (4005C>G 4007G>A), I1294N (4021T>A), H1295Y (4023C>T), D1297E (4031T>A), L1298M (4032C>A), E1312D (4076G>C), R1318K (4093G>A 4094A>G), T1344C (4170A>T 4171C>G 4172A>T), L1348A (4182T>G 4183T>C 4184G>A), A1351V (4192C>T), I1352L (4194A>C 4196A>G), D1359N (4215G>A), T1361I (4222C>T 4223A>T), S1363H (4227T>C 4228C>A), L1367T (4239T>A 4240T>C 4241G>C), G1371Q (4251G>C 4252G>A), A1374S (4260G>A 4261C>G), V1393I (4317G>A 4319T>A), E1405A (4354A>C), K1410A (4368A>G 4369A>C 4370G>T), P1414E (4380C>G 4381C>A 4382T>G), V1437L (4449G>C 4451G>C), A1477K (4569G>A 4570C>A 4571T>A), R1479K (4576G>A), I1481L (4581A>T 4583A>G), P1485S (4593C>T), Q1490D (4608C>G 4610G>C), L1506M (4656T>A 4658A>G), S1512E (4674T>G 4675C>A 4676T>G), N1515A (4683A>G 4684A>C 4685T>A), H1518Q (4694T>A), L1522Q (4705T>A 4706G>A), S1534N (4740T>A 4741C>A 4742G>C), D1536N (4746G>A), E1539D (4757G>T), Q1541K (4761C>A), H1563Q (4829C>A), M1564L (4830A>C 4832G>A), I1573V (4857A>G 4859T>G), P1583V (4887C>G 4888C>T 4889A>T), T1588I (4903C>T 4904T>C), Q1589T (4905C>A 4906A>C 4907G>T), M1592Y (4914A>T 4915T>A 4916G>T), I1599V (4935A>G), H1604Q (4952T>G), R1607Q (4960G>A 4961T>G), A1609V (4966C>T), K1612T (4975A>C 4976G>A), V1614T (4980G>A 4981T>C 4982C>A), L1615D (4983T>G 4984T>A 4985A>C), D1616N (4986G>A), T1619K (4996C>A 4997G>A), L1621I (5001T>A 5003G>A), D1622M (5004G>A 5005A>T 5006T>G), E1623Q (5007G>C 5009A>G), F1626L (5018C>G), R1627P (5019A>C 5020G>C), A1628I (5022G>A 5023C>T 5024T>C), I1630H (5028A>C 5029T>A 5030C>T), L1635A (5043T>G 5044T>C 5045G>T), V1637Q (5049G>C 5050T>A 5051G>A), Q1639S (5055C>A 5056A>G 5057G>T), I1642L (5064A>C 5066T>A), A1643Q (5067G>C 5068C>A), K1644A (5070A>G 5071A>C 5072G>C), S1650N (5089G>A 5090T>C), A1651I (5091G>A 5092C>T 5093C>A), L1652G (5094C>G 5095T>G), T1653A (5097A>G 5099A>T), I1654L (5100A>C 5102T>G), C1655L (5103T>C 5104G>T 5105T>G), M1657L (5109A>C), A1658S (5112G>A 5113C>G 5114A>C), A1660D (5119C>A 5120A>T), S1663T (5127T>A 5129C>T), T1665V (5133A>G 5134C>T), D1668P (5142G>C 5143A>C 5144C>A), A1669E (5146C>A), E1671K (5151G>A 5153G>A), T1674E (5160A>G 5161C>A), N1675D (5163A>G), E1677S (5169G>T 5170A>C 5171G>A), R1678Q (5173G>A 5174T>A), D1679G (5176A>G), L1680V (5178C>G 5180G>C), N1681H (5181A>C), W1682E (5184T>G 5185G>A 5186G>A), A1684C (5190G>T 5191C>G 5192A>T), P1685S (5193C>T 5195A>T), N1686G (5196A>G 5197A>G 5198C>A), I1687V (5199A>G), G1688F (5202G>T 5203G>T 5204T>C), A1692S (5214G>T 5216A>G), A1695E (5224C>A), A1696V (5227C>T 5228C>T), H1697N (5229C>A 5231C>T), Y1698H (5232T>C 5234C>T), T1699K (5236C>A 5237G>A), G1703D (5248G>A 5249G>C), Y1704T (5250T>A 5251A>C 5252C>A), I1706L (5256A>C), S1715T (5283T>A 5285C>A), N1716T (5287A>C 5288T>A), S1737A (5349T>G), M1740K (5359T>A), A1742N (5364G>A 5365C>A 5366C>T), K1744Q (5370A>C 5372G>A), N1745S (5374A>G), V1746R (5376G>C 5377T>G 5378G>C), A1748S (5382G>T), G1749M (5385G>A 5386G>T 5387A>G), H1751I (5391C>A 5392A>T), V1752G (5395T>G), A1753T (5397G>A 5399A>C), T1754Q (5400A>C 5401C>A 5402C>A), N1755K (5405T>A), V1759I (5415G>A 5417G>T), G1767S (5439G>A 5441C>T), M1774F (5460A>T 5462G>T), A1777S (5469G>T), R1779N (5476G>A 5477G>T), C1782R (5484T>A 5486T>A), V1783A (5488T>C 5489G>T), D1785Q (5493G>C 5495C>A), I1786L (5496A>C 5498A>T), I1793L (5517A>T 5519T>G), G1794A (5521G>C 5522A>G), N1795G (5523A>G 5524A>G 5525T>C), V1796L (5526G>T), T1801Q (5541A>C 5542C>A 5543C>G), V1807I (5559G>A 5561G>T), Q1812T (5574C>A 5575A>C), C1813_V1814insV (5579_5580insGTC), P1815Q (5584C>A 5585T>G), H1816K (5586C>A 5588T>A), G1817S (5589G>A 5591A>T), D1818C (5592G>T 5593A>G), V1820T (5598G>A 5599T>C 5600G>A), S1823A (5607A>G 5608G>C), G1824S (5610G>A), A1825G (5614C>G 5615A>G), S1826del (5616_5618delTCG), L1827R (5620T>G), H1828D (5622C>G 5624C>T), S1829G (5625A>G 5627T>A), S1830M (5629G>T 5630T>G), F1831L (5633T>G), M1832N (5635T>A 5636G>C), Q1834H (5642A>T), Q1834_G1835insHA (5642_5643insCATGCT), G1835A (5644G>C 5645G>T), F1836Y (5647T>A), E1837A (5650A>C 5651A>C), I1844V (5670A>G 5672A>G), H1845G (5673C>G 5674A>G 5675T>C), G1846C (5676G>T), D1847S (5679G>T 5680A>C 5681T>A), L1848I (5682T>A 5684A>T), A1849S (5685G>T), K1850R (5689A>G), Q1851K (5691C>A), T1852M (5695C>T 5696A>G), M1854V (5700A>G), M1856L (5706A>T), V1859T (5715G>A 5716T>C 5717G>T), H1863M (5727C>A 5728A>T 5729T>G), G1867A (5740G>C), N1868D (5742A>G), F1869C (5746T>G 5747T>C), Q1872R (5754C>A 5755A>G 5756G>A), D1873N (5757G>A), E1875D (5765G>T), L1878I (5772C>A 5774A>C), H1879K (5775C>A 5777C>A), Y1880A (5778T>G 5779A>C 5780T>C), Q1883C (5787C>T 5788A>G 5789G>C), G1884S (5790G>T 5791G>C 5792A>T), E1887A (5800A>C 5801G>A), L1888C (5803T>G 5804A>T), I1889V (5805A>G 5807T>G), Q1890A (5808C>G 5809A>C 5810A>T), K1891R (5811A>C 5812A>G 5813G>T), N1892G (5814A>G 5815A>G 5816T>G), T1893C (5817A>T 5818C>G 5819A>T), L1895I (5823C>A), K1900E (5838A>G 5840A>G), E1901N (5841G>A 5843G>T), L1902F (5844C>T 5846G>T), S1903N (5848G>A), M1904A (5850A>G 5851T>C 5852G>A), E1905Q (5853G>C), N1906I (5857A>T 5858T>A), V1907A (5860T>C 5861G>T), Q1908T (5862C>A 5863A>C 5864A>C), Q1909R (5865C>A 5866A>G 5867A>G), I1911V (5871A>G), V1914L (5880G>C), L1916S (5886C>A 5887T>G), F1919V (5895T>G 5897T>G), P1921I (5901C>A 5902C>T 5903A>C), K1922T (5905A>C), E1923A (5908A>C 5909A>C), T1925G (5913A>G 5914C>G), I1926H (5916A>C 5917T>A 5918T>C), Q1927K (5919C>A 5921G>A), K1928M (5923A>T), K1930V (5928A>G 5929A>T 5930A>G), N1931Q (5931A>C 5933T>A), F1932T (5934T>A 5935T>C), V1933L (5937G>C 5939T>G), D1934S (5940G>A 5941A>G 5942T>C), A1935N (5943G>A 5944C>A), E1936Q (5946G>C), C1937W (5951T>G), L1938Q (5953T>A), T1939S (5955A>T 5957A>C), F1940H (5958T>C 5959T>A), K1941E (5961A>G), A1942Q (5964G>C 5965C>A), R1943S (5969G>T), S1944C (5970A>T 5972C>T), F1945V (5973T>G 5975T>G), R1946K (5977G>A), H1947R (5979C>A 5980A>G 5981C>A), V1949Y (5985G>T 5986T>A), K1952R (5994A>C 5995A>G 5996G>A), K1953N (5999A>T), S1957N (6009T>A 6010C>A), T1958A (6012A>G), W1959I (6015T>A 6016G>T 6017G>A), K1960T (6019A>C), M1961_C1962insY (6023_6024insTAC), C1962R (6024T>C), E1964D (6032A>T), S1965A (6033A>G 6034G>C 6035T>C), I1966K (6037T>A 6038T>A), V1967D (6040T>A), S1969I (6045T>A 6046C>T), R1970V (6048A>G 6049G>T 6050G>A), A1972del (6054_6056delGCA), L1975V (6063C>G 6065A>G), P1977L (6069C>T 6070C>T 6071T>G), L1980P (6078T>C 6079T>C 6080G>A), S1983M (6087T>A 6088C>T 6089C>G), K1986R (6097A>G 6098A>G)

	Begin	End	Coverage	Score	Concordance	Matches	Identities	I/D/M/F*	Stop Codons
**NT**	**24**	**6107**	**84.2%**	**3417**	**28.3%**	**6063 (98.8%)**	**3936 (64.1%)**	**54/21**	

ATG1CTG (141A>C), GCT2GTT (145C>T), TTT3TTC (149T>C), TCA4CAC (150T>C 151C>A 152A>C), AAG5AAT (155G>T), ATG6ACC (157T>C 158G>C), TTC7ACC (159T>A 160T>C), TCC8ATT (162T>A 163C>T 164C>T), AAG9CAA (165A>C 167G>A), GTT10ATG (168G>A 170T>G), GCT11GCA (173T>A), GGT12del (174_176delGGT), GTT13TTT (177G>T), GAT14TCA (180G>T 181A>C 182T>A), GAC15AAA (183G>A 185C>A), GCC16ATC (186G>A 187C>T), AGT17GTT (189A>G 190G>T), TCC20GCT (198T>G 200C>T), ACC21ACT (203C>T), ACT22CCT (204A>C), TCA23TCT (209A>T), TTC24TTT (212C>T), TTT25CTT (213T>C), GGT26TCT (216G>T 217G>C), GAA27GAG (221A>G), CTT28TTG (222C>T 224T>G), TCA29TCT (227A>T), AAT30CAG (228A>C 230T>G), TCT31ACA (231T>A 233T>A), ATT32GTG (234A>G 236T>G), TCC33TCA (239C>A), AAA34TCA (240A>T 241A>C), TTT35CTG (243T>C 245T>G), TCA36TCC (248A>C), AAA37AAG (251A>G), GCG38ACA (252G>A 254G>A), GTT39ATG (255G>A 257T>G), AGT40AAG (259G>A 260T>G), AAT41AGT (262A>G), ATT42ATG (266T>G), ACC43AGT (268C>G 269C>T), ACT44AAA (271C>A 272T>A), ATG45ATA (275G>A), TCA46TCT (278A>T), CAG47GAG (279C>G), AAG48AAA (284G>A), ATT49TTC (285A>T 287T>C), GAT51GGC (292A>G 293T>C), CTT53ATT (297C>A), GAG54GAA (302G>A), GAT55GAA (305T>A), CTG56TTA (306C>T 308G>A), CCA58CCT (314A>T), TCT59TCA (317T>A), GTG60ATT (318G>A 320G>T), ACT61GTT (321A>G 322C>T), GAT62AAA (324G>A 326T>A), GTG63ACT (327G>A 328T>C 329G>T), TCC64GCA (330T>G 332C>A), ACA65GAT (333A>G 334C>A 335A>T), TCT66TCC (338T>C), GTG68AAT (342G>A 343T>A 344G>T), GCC69AGT (345G>A 346C>G 347C>T), ACT70GTT (348A>G 349C>T), TCA73TCT (359A>T), CTT74GTA (360C>G 362T>A), AAT75GAA (363A>G 365T>A), GTT77TTA (369G>T 371T>A), TTG78TTA (374G>A), GAC79GGG (376A>G 377C>G), GGT82GCC (385G>C 386T>C), GCA83ACG (387G>A 389A>G), CTT84GTT (390C>G), ATA85ATT (395A>T), GAT86GAA (398T>A), CCA87CCT (401A>T), CTT89GTT (405C>G), GCG91GGT (412C>G 413G>T), TAC92ATC (414T>A 415A>T), TCT93AAT (417T>A 418C>A), TTG95TTC (425G>C), TCC96AAA (426T>A 427C>A 428C>A), TAC99TAT (437C>T), AAA100AGA (439A>G), TCT101ACC (441T>A 443T>C), ATC102ATA (446C>A), AAG103ATT (448A>T 449G>T), TTA107CTG (459T>C 461A>G), TTG109GTT (465T>G 467G>T), TTT110GTT (468T>G), GAG111TTG (471G>T 472A>T), AGT112GGC (474A>G 476T>C), TTG113ATC (477T>A 479G>C), TCC114AAA (480T>A 481C>A 482C>A), AAA116AAT (488A>T), GTT117TTT (489G>T), AAA118ATT (493A>T 494A>T), TTA119AGA (495T>A 496T>G), TTT121TAT (502T>A), GCT122TCA (504G>T 506T>A), GTG124CTG (510G>C), TTA125TTG (515A>G), AAT126GAA (516A>G 518T>A), AAA127ACA (520A>C), AGT128ACA (523G>C 524T>A), GAA129GGT (526A>G 527A>T), GAT130GAA (530T>A), GTA131TAT (531G>T 532T>A 533A>T), ATA133GTT (537A>G 539A>T), GTT134GTA (542T>A), GTA135GTT (545A>T), TAT136ATA (546T>A 547A>T 548T>A), GGT137GCT (550G>C), CTT139CTA (557T>A), GTA140GTC (560A>C), GCT142TCC (564G>T 566T>C), ATA143CTT (567A>C 569A>T), ACA144TCA (570A>T), TTG145CTA (573T>C 575G>A), TTA146TTG (578A>G), GTG147TTA (579G>T 581G>A), CTT148TTA (582C>T 584T>A), CTA149CAA (586T>A), TTT150ATA (588T>A 590T>A), GTT151ATC (591G>A 593T>C), AGT154GTA (600A>G 601G>T 602T>A), GTT157GTA (611T>A), GAG158GAA (614G>A), GGT159ATG (615G>A 616G>T 617T>G), TCT161AGA (621T>A 622C>G 623T>A), AAC162AGT (625A>G 626C>T), ACT163GTA (627A>G 628C>T 629T>A), GTC164GTT (632C>T), AAA165TGT (633A>T 634A>G 635A>T), ATC167ATA (641C>A), TTG169CTA (645T>C 647G>A), ATA170GTT (648A>G 650A>T), GTC171GTA (653C>A), GGA172GGT (656A>T), ATG174TTC (660A>T 662G>C), TTT175TTC (665T>C), TCA176TCT (668A>T), AGT177TCT (669A>T 670G>C), CTA178CTG (674A>G), TAC179TAT (677C>T), TTA181ATT (681T>A 683A>T), GAT182AGT (684G>A 685A>G), CCA185CCC (695A>C), AAA186CTC (696A>C 697A>T 698A>C), GCC188TCT (702G>T 704C>T), GAA189GAG (707A>G), CGA190AAA (708C>A 709G>A), TTC191TTT (713C>T), ACC192TCT (714A>T 716C>T), ATG193CAG (717A>C 718T>A), GTG194ATG (720G>A), GCC195GCA (725C>A), CAA196CAG (728A>G), AGT198TCT (732A>T 733G>C), ATG199GTG (735A>G), CTT200CTA (740T>A), GGG202GGT (746G>T), GAA203GAG (749A>G), TCA204AGC (750T>A 751C>G 752A>C), GTT205TTG (753G>T 755T>G), TCA206AAT (756T>A 757C>A 758A>T), ACG210ACC (770G>C), TCT211AGT (771T>A 772C>G), CAG212CAA (776G>A), ATG214CTG (780A>C), TCC215TCT (785C>T), ACA216TCT (786A>T 788A>T), ATA217TTG (789A>T 791A>G), TTG218ATA (792T>A 794G>A), GCT219GCA (797T>A), GGG221GGC (803G>C), ATT222ATC (806T>C), TCG223TCA (809G>A), GTG226GTA (818G>A), TTC227TTT (821C>T), ATT228GTG (822A>G 824T>G), GTG230GTA (830G>A), AGA233AGG (839A>G), TTG237CTT (849T>C 851G>T), AGC238ACA (853G>C 854C>A), CCA240CCG (860A>G), TTG241CTA (861T>C 863G>A), CTC245TTG (873C>T 875C>G), TCG247TCA (881G>A), ACA248GCG (882A>G 884A>G), GGA249GGC (887A>C), GGT251GGA (893T>A), CAG253CAA (899G>A), CTA258CTC (914A>C), ACT260CAA (918A>C 919C>A 920T>A), CTT261TTG (921C>T 923T>G), TAC262TAT (926C>T), CGT263AAG (927C>A 928G>A 929T>G), AAT264AAC (932T>C), AAA266AAG (938A>G), ACC269ACA (947C>A), TCA274TCT (962A>T), GTT276GTG (968T>G), CTT277TTG (969C>T 971T>G), ATA279ATT (977A>T), ATA280GTT (978A>G 980A>T), GTC281GCT (982T>C 983C>T), GGC282GAT (985G>A 986C>T), ACC283ATA (988C>T 989C>A), TTT284TTC (992T>C), GGA285GGG (995A>G), TTC286TTT (998C>T), AAA287AAG (1001A>G), AAT288AAC (1004T>C), GTG290GTT (1010G>T), TTG291CTG (1011T>C), TCA292TCT (1016A>T), GCT293GCA (1019T>A), ATA294ATT (1022A>T), ACG297ACA (1031G>A), CTG298ATT (1032C>A 1034G>T), CTG302CTC (1046G>C), TTC303TTT (1049C>T), CAG307ACT (1059C>A 1060A>C 1061G>T), GAG308GAA (1064G>A), GAT310TCT (1068G>T 1069A>C), TGT313GCT (1077T>G 1078G>C), ACG317ACA (1091G>A), CGC318AGG (1092C>A 1094C>G), GAG320GAA (1100G>A), GCC323GCT (1109C>T), AAG325CCT (1113A>C 1114A>C 1115G>T), TTC327TTT (1121C>T), CCT328TCA (1122C>T 1124T>A), ACC329ATC (1126C>T), CTG334CTT (1142G>T), AGG335AAA (1144G>A 1145G>A), GAA336GAG (1148A>G), GAA337CAG (1149G>C 1151A>G), GCT338GCA (1154T>A), ATC341ATA (1163C>A), GCT343GCC (1169T>C), TAC344TAT (1172C>T), GCA346GCT (1178A>T), CCA349CCT (1187A>T), GTT350GTG (1190T>G), TTC353TTT (1199C>T), ATG354TTG (1200A>T), AGC355AGT (1205C>T), AGG357AGA (1211G>A), AGT359TCT (1215A>T 1216G>C), GCC361ACA (1221G>A 1223C>A), GCG363GTG (1228C>T), CAG364CAA (1232G>A), TTA365TTG (1235A>G), GAA366GAT (1238A>T), GTT368CTC (1242G>C 1244T>C), TAT369ATA (1245T>A 1246A>T 1247T>A), GCT370GCA (1250T>A), AGT372AAC (1255G>A 1256T>C), AGG374CAA (1260A>C 1261G>A 1262G>A), CAC375CAT (1265C>T), ATG376ACA (1267T>C 1268G>A), GGC377GGA (1271C>A), GGC379GGT (1277C>T), TAT381TAC (1283T>C), CGT382AGA (1284C>A 1286T>A), CCT385CCC (1295T>C), GTA388GTT (1304A>T), TTC391TTT (1313C>T), GGG392GGT (1316G>T), TCC393GAG (1317T>G 1318C>A 1319C>G), GGG395GGA (1325G>A), TGT396TGC (1328T>C), GGT397GGC (1331T>C), TCT399TCC (1337T>C), ACA400ACG (1340A>G), TCC401AGC (1341T>A 1342C>G), CGC403CGA (1349C>A), TTA404CTG (1350T>C 1352A>G), TTT405TTC (1355T>C), ATT406ATC (1358T>C), GTG409GTA (1367G>A), GAC411GAT (1373C>T), AGA412GCC (1374A>G 1375G>C 1376A>C), GGT414CAA (1380G>C 1381G>A 1382T>A), CCA416CCC (1388A>C), AAA417AAG (1391A>G), CTC418CTG (1394C>G), AAT419AAC (1397T>C), CGG420AGG (1398C>A), CTA421TTG (1401C>T 1403A>G), GTA424GTT (1412A>T), AGG427CGC (1419A>C 1421G>C), GCA429GCT (1427A>T), TAT430TAC (1430T>C), TCC432TCA (1436C>A), AAC433AAT (1439C>T), CTG441CTC (1463G>C), GAC444GAT (1472C>T), GGA446GGT (1478A>T), TTG448CTG (1482T>C), CGA449CGG (1487A>G), GGG451GGA (1493G>A), GGG453GGC (1499G>C), CAG454CAA (1502G>A), CAA456CAG (1508A>G), GAT457GAC (1511T>C), AAA459AAG (1517A>G), GAT460GAC (1520T>C), CTG461CTC (1523G>C), GAT463GAC (1529T>C), TCA466TCC (1538A>C), CCC471CCA (1553C>A), TTA472TTG (1556A>G), CCA473CCC (1559A>C), GCC475GCT (1565C>T), GTT477GTA (1571T>A), GAG478GAA (1574G>A), GGC481GGG (1583C>G), AGG482CGG (1584A>C), ACC485ACA (1595C>A), TCC486TCA (1598C>A), TAT488TAC (1604T>C), ACA492ACC (1616A>C), AAT494AAC (1622T>C), CTG495TTG (1623C>T), ATC496GTT (1626A>G 1628C>T), GCT498GCA (1634T>A), CCT499CCA (1637T>A), GCC500GCA (1640C>A), TGT502TGC (1646T>C), GGT503GGG (1649T>G), CTT504CTG (1652T>G), CCA507CCT (1661A>T), GAG511GAA (1673G>A), CGG512AGG (1674C>A), AGA513AGG (1679A>G), AGG514CGG (1680A>C), ATC516ATT (1688C>T), CTG517TTG (1689C>T), GAG519GAA (1697G>A), TGT520TGC (1700T>C), ATG521CGG (1701A>C 1702T>G), AAG522AAA (1706G>A), GAG523GTG (1708A>T), GGC524GGG (1712C>G), GAG525GAA (1715G>A), TTT526TTC (1718T>C), TCC527TCA (1721C>A), ACT528ACA (1724T>A), GAA529GAG (1727A>G), GAT530AAC (1728G>A 1730T>C), CCT531CCA (1733T>A), ACG532ACA (1736G>A), GGA533GCA (1738G>C), AGA536AGC (1748A>C), TTT537TTC (1751T>C), ATA539ATC (1757A>C), GTG540ATT (1758G>A 1760G>T), GAG541GGA (1762A>G 1763G>A), AGA543AGG (1769A>G), AGA544AGG (1772A>G), GCT547GCC (1781T>C), ATC548ATA (1784C>A), ACT549ACA (1787T>A), AGG551AGA (1793G>A), AAT552GAC (1794A>G 1796T>C), TTG553CTC (1797T>C 1799G>C), ACC554ACT (1802C>T), AGT556TCT (1806A>T 1807G>C), GAT557GAC (1811T>C), CTT558TTG (1812C>T 1814T>G), GAG560GAA (1820G>A), GTA563GTG (1829A>G), AAA565AAG (1835A>G), TGT566TGC (1838T>C), CAG567AAG (1839C>A), GTA568GTC (1844A>C), CAT569CAC (1847T>C), GCA570CAC (1848G>C 1849C>A 1850A>C), GTA572ATT (1854G>A 1856A>T), TCA573GCA (1857T>G), AAG574ACC (1861A>C 1862G>C), TTG576CTC (1866T>C 1868G>C), TTT577TAC (1870T>A 1871T>C), GAA578AAC (1872G>A 1874A>C), GCT579CAG (1875G>C 1876C>A 1877T>G), GAA580TCA (1878G>T 1879A>C), TCT581ACA (1881T>A 1883T>A), GGC582GGT (1886C>T), ATA583TGT (1887A>T 1888T>G 1889A>T), AGT584GAA (1890A>G 1891G>A 1892T>A), CCT585GAG (1893C>G 1894C>A 1895T>G), AAG586AAA (1898G>A), ATA587GTT (1899A>G 1901A>T), GCT588GCC (1904T>C), GTT590GTG (1910T>G), CAA591GAA (1911C>G), GCA594GTT (1921C>T 1922A>T), GAT595GAA (1925T>A), GAT596GAA (1928T>A), ATA598ATG (1934A>G), GCG599ACA (1935G>A 1937G>A), TCT600TCC (1940T>C), GTG601TTT (1941G>T 1943G>T), GAT602CAT (1944G>C), GGG603GAT (1948G>A 1949G>T), AGA605CAA (1953A>C 1954G>A), TTG606ATA (1956T>A 1958G>A), CGC607AGC (1959C>A), ACC608TTT (1962A>T 1963C>T 1964C>T), AAG609GAC (1965A>G 1967G>C), CAA610GAA (1968C>G), GAT611GTT (1972A>T), GAA612AGC (1974G>A 1975A>G 1976A>C), CCA613CCC (1979A>C), ATA614AGT (1981T>G 1982A>T), TTG615GTT (1983T>G 1985G>T), GTG616_CCC617del (1986_1991delGTGCCC), ACT618ACA (1994T>A), GTG620CTC (1998G>C 2000G>C), AGT621TCT (2001A>T 2002G>C), GAA622GAG (2006A>G), AAT623GAT (2007A>G), ATC627GGA (2019A>G 2020T>G 2021C>A), GCA629TCA (2025G>T), AGA630CAG (2028A>C 2029G>A 2030A>G), GAG631GAA (2033G>A), CTC632CTA (2036C>A), ACG633ACT (2039G>T), GTA634GTG (2042A>G), GAG635GAA (2045G>A), TTG637CTG (2049T>C), AAA638AAG (2054A>G), GCA640GCT (2060A>T), TAC641TAT (2063C>T), CAG642CAA (2066G>A), GGC643GGA (2069C>A), AGT644GCA (2070A>G 2071G>C 2072T>A), TTG645TTA (2075G>A), GAC646AAC (2076G>A), CCT647CCA (2081T>A), GAG648TTT (2082G>T 2083A>T 2084G>T), CTT650TAT (2088C>T 2089T>A), CCT652CCC (2096T>C), GAC654GAT (2102C>T), CAT655GCT (2103C>G 2104A>C), CAT656TCA (2106C>T 2107A>C 2108T>A), GCC659GCA (2117C>A), ATG660ATT (2120G>T), TAT661TTT (2122A>T), TCT663GTC (2127T>G 2128C>T 2129T>C), CTT664CTG (2132T>G), GAT665TCA (2133G>T 2134A>C 2135T>A), GAT666AGT (2136G>A 2137A>G), CAT668GCC (2142C>G 2143A>C 2144T>C), ATT671AGC (2152T>G 2153T>C), TTC672TTT (2156C>T), AAC673ACT (2158A>C 2159C>T), AAA674CGC (2160A>C 2161A>G 2162A>C), AGA676GTA (2166A>G 2167G>T), GTC677AAT (2169G>A 2170T>A 2171C>T), AAC678GAG (2172A>G 2174C>G), TTG680ATT (2178T>A 2180G>T), TAT681TAC (2183T>C), AGA682GAG (2184A>G 2185G>A 2186A>G), GGA683GGG (2189A>G), GCA684GCT (2192A>T), GAT685TCG (2193G>T 2194A>C 2195T>G), GCT686ACT (2196G>A), GAA687GCA (2200A>C), CAG688GGA (2202C>G 2203A>G 2204G>A), CGA690GCC (2208C>G 2209G>C 2210A>C), GTA693ATC (2217G>A 2219A>C), CAA694AAG (2220C>A 2222A>G), AAC695GCC (2223A>G 2224A>C), ATT696ATC (2228T>C), CCT697CCA (2231T>A), ATA701GTC (2241A>G 2243A>C), AAG705AAA (2255G>A), AGT706AGC (2258T>C), GCA710AGT (2268G>A 2269C>G 2270A>T), TCA711ACA (2271T>A), ATC713GTG (2277A>G 2279C>G), TCT714TCA (2282T>A), GAG715GAA (2285G>A), AAG716GCT (2286A>G 2287A>C 2288G>T), AAA717CAC (2289A>C 2291A>C), CTA718ATG (2292C>A 2294A>G), GCA719GAT (2296C>A 2297A>T), GTG720GTA (2300G>A), CAG721CAA (2303G>A), ACA722AGG (2305C>G 2306A>G), GAG723ATG (2307G>A 2308A>T), ATG724TTG (2310A>T), AGG725TAC (2313A>T 2314G>A 2315G>C), ACT726AAT (2317C>A), GGA727GGC (2321A>C), TTT728AGA (2322T>A 2323T>G 2324T>A), TCT731AGA (2331T>A 2332C>G 2333T>A), TGC732GCA (2334T>G 2335G>C 2336C>A), ATT733CTG (2337A>C 2339T>G), GAT734GAA (2342T>A), GCA735GCT (2345A>T), GTT737GCT (2350T>C), ACC739ACT (2357C>T), CTC740TTA (2358C>T 2360C>A), ATA741ATG (2363A>G), ATC743ATT (2369C>T), GAG744GAA (2372G>A), CAG745CAA (2375G>A), CCA747CCT (2381A>T), TTT749CAT (2385T>C 2386T>A), GTT750GTC (2390T>C), CAG751CAA (2393G>A), TTT752TTC (2396T>C), TTA753CTA (2397T>C), TAT754TAC (2402T>C), AGA759CGG (2415A>C 2417A>G), TGC762_AAC763insAGAAGG (2426_2427insAGAAGG), AAC763AAG (2429C>G), AAA764ATG (2431A>T 2432A>G), GTA765GTC (2435A>C), TCA766ACT (2436T>A 2438A>T), AAA767AAG (2441A>G), GAG768GAA (2444G>A), CCA769ACA (2445C>A), AAG770_GAA771del (2448_2453delAAGGAA), TCA772TCT (2456A>T), ATC774GTA (2460A>G 2462C>A), AAA775CAA (2463A>C), ATT776TCG (2466A>T 2467T>C 2468T>G), TGC777CTC (2469T>C 2470G>T), TAC778TAT (2474C>T), CAT779GAC (2475C>G 2477T>C), AAA780TCC (2478A>T 2479A>C 2480A>C), ATC781ACA (2482T>C 2483C>A), GTT782ATT (2484G>A), GAA783GGG (2488A>G 2489A>G), TAT784AGA (2490T>A 2491A>G 2492T>A), ATC785ATG (2495C>G), AAG786CAA (2496A>C 2498G>A), GAT787GAA (2501T>A), ACA788ATG (2503C>T 2504A>G), TGG790AAT (2508T>A 2509G>A 2510G>T), AGC791GAT (2511A>G 2512G>A 2513C>T), CTT792CTG (2516T>G), TAT794TAC (2522T>C), GCA795ATT (2523G>A 2524C>T 2525A>T), CTG796TTG (2526C>T), AGA797AAA (2530G>A), CTC799CTT (2537C>T), GGT803GGG (2549T>G), ATA806CTA (2556A>C), ATG807ATA (2561G>A), CTT809CTC (2567T>C), GGA811GGG (2573A>G), GTT812ACT (2574G>A 2575T>C), TTT813ATG (2577T>A 2579T>G), GGA814GGT (2582A>T), GGC815GCC (2584G>C), ATT816GTA (2586A>G 2588T>A), ACA817ACT (2591A>T), TTT819TTC (2597T>C), TTG820ATT (2598T>A 2600G>T), GCG821TCT (2601G>T 2603G>T), CAG824AAA (2610C>A 2612G>A), AGT825AGC (2615T>C), AAC826AGC (2617A>G), TCT827TTG (2620C>T 2621T>G), TTC828ATT (2622T>A 2624C>T), AAT830ACT (2629A>C), GCA831TCC (2631G>T 2633A>C), TCG832CAG (2634T>C 2635C>A), AGA834AAA (2641G>A), GTG837GTT (2651G>T), TCC841TCG (2663C>G), AGT843AGC (2669T>C), TCC845TCA (2675C>A), AGT846AGC (2678T>C), GGT851GGA (2693T>A), AAT852AGA (2695A>G 2696T>A), AAT852_AAG853insTCC (2696_2697insTCC), AAG853CAT (2697A>C 2699G>T), CTC854GTA (2700C>G 2702C>A), AGA855TCA (2703A>T 2704G>C), AAT856AAG (2708T>G), CTT857ATG (2709C>A 2711T>G), CTC858CTG (2714C>G), GTT859TTT (2715G>T), GGT860AGC (2718G>A 2720T>C), TCT863TCA (2729T>A), TCA865CCA (2733T>C), TTG866AAT (2736T>A 2737T>A 2738G>T), GCA867GAT (2740C>A 2741A>T), CAA868CAG (2744A>G), CAA868_GAT869insGGA (2744_2745insGGA), GAT869AAA (2745G>A 2747T>A), GCT872TCC (2754G>T 2756T>C), GAA873AGG (2757G>A 2758A>G 2759A>G), GGA875GGT (2765A>T), GTA877GTG (2771A>G), AAT878AAC (2774T>C), CAG879GAA (2775C>G 2777G>A), AGC880TCT (2778A>T 2779G>C 2780C>T), CTC881CTT (2783C>T), AAG882GCC (2784A>G 2785A>C 2786G>C), GTG886GTA (2798G>A), TTA888CTA (2802T>C), AGA889AGG (2807A>G), CTT890GTG (2808C>G 2810T>G), GGT891GCA (2812G>C 2813T>A), GTG894GTT (2822G>T), AGA897CGT (2829A>C 2831A>T), GGC898GGA (2834C>A), ACC899ACT (2837C>T), TGT902TGC (2846T>C), TCT903AGC (2847T>A 2848C>G 2849T>C), GGT904GAA (2851G>A 2852T>A), ACC908ACT (2864C>T), GCT910GCA (2870T>A), CAT911CAC (2873T>C), GCA912GCT (2876A>T), CAC914ACG (2880C>A 2881A>C 2882C>G), AAT915CAG (2883A>C 2885T>G), GCT916CTC (2886G>C 2887C>T 2888T>C), CGA917CGT (2891A>T), GGT919GGA (2897T>A), ACT920ACC (2900T>C), ACA923TCA (2907A>T), ATC925ATT (2915C>T), GCC927GAA (2920C>A 2921C>A), AAT928CAT (2922A>C), CGA930GTC (2928C>G 2929G>T 2930A>C), TCT931TGC (2932C>G 2933T>C), AAA932CAG (2934A>C 2936A>G), GTT933GTG (2939T>G), AAC936AAT (2948C>T), AGA938AAA (2953G>A), GAA939CAT (2955G>C 2957A>T), TCA940GCC (2958T>G 2960A>C), AGG941AAG (2962G>A), ATT942TTT (2964A>T), ATT943TTG (2967A>T 2969T>G), GAG944CCT (2970G>C 2971A>C 2972G>T), GGC945GAA (2974G>A 2975C>A), GAC947GAT (2981C>T), ATC948GTT (2982A>G 2984C>T), ATT949GTA (2985A>G 2987T>A), CTG950TTA (2988C>T 2990G>A), TTG951CTG (2991T>C), CGC952AGG (2994C>A 2996C>G), GGT954GGC (3002T>C), GAT955TGT (3003G>T 3004A>G), CCA956CCT (3008A>T), GAT957GAC (3011T>C),

Codon mutations:

GGT958GGG (3014T>G), CCA959CCT (3017A>T), AAG960AAA (3020G>A), CCT961GTC (3021C>G 3022C>T 3023T>C), CGT964AGA (3030C>A 3032T>A), AAA965AAG (3035A>G), TTC967TTT (3041C>T), CCA968GCT (3042C>G 3044A>T), AGA969GAA (3045A>G 3046G>A), GAG972GCA (3055A>C 3056G>A), AAG977AAA (3071G>A), GGC978GGA (3074C>A), TCA979TCT (3077A>T), CAA980GAA (3078C>G), GGA981GGC (3083A>C), TGC983TGT (3089C>T), TGT984GTC (3090T>G 3091G>T 3092T>C), GCT986GCA (3098T>A), GTT987GTG (3101T>G), GCG988GCA (3104G>A), TCT989TCA (3107T>A), ACA990AAA (3109C>A), CCA992CCT (3116A>T), CGT993GAG (3117C>G 3118G>A 3119T>G), CTT994AAA (3120C>A 3121T>A 3122T>A), GGC995CAG (3123G>C 3124G>A 3125C>G), AAT996AAC (3128T>C), TTA997TTG (3131A>G), GAG998GAC (3134G>C), ATG1002TAT (3144A>T 3145T>A 3146G>T), CCA1003TCA (3147C>T), TCA1007TCC (3161A>C), AAG1008AAA (3164G>A), TAC1010TAT (3170C>T), GTT1012GAG (3175T>A 3176T>G), GTG1014GTC (3182G>C), GAA1015ACA (3183G>A 3184A>C), TAT1016TAC (3188T>C), GAA1017AGG (3189G>A 3190A>G 3191A>G), CTG1018ATG (3192C>A), AAC1019AGC (3196A>G), TCC1020ACA (3198T>A 3200C>A), TCC1021AAT (3201T>A 3202C>A 3203C>T), AGT1022TCA (3204A>T 3205G>C 3206T>A), AAG1024CAC (3210A>C 3212G>C), AAG1024_ATT1025insTCA (3212_3213insTCA), ATT1025ATC (3215T>C), TGC1026AGT (3216T>A 3218C>T), TCT1027GCT (3219T>G), CAA1029GAA (3225C>G), TCT1030GCC (3228T>G 3230T>C), CAC1034CAT (3242C>T), ATA1035GTC (3243A>G 3245A>C), GGG1037AGC (3249G>A 3251G>C), GAC1041GAT (3263C>T), GGC1043GGA (3269C>A), ACG1044ACT (3272G>T), CTG1047TTA (3279C>T 3281G>A), TTA1048TTG (3284A>G), AGT1050TCT (3288A>T 3289G>C), GTT1051ATC (3291G>A 3293T>C), CAG1052CAA (3296G>A), AAT1053AAC (3299T>C), CCT1056CCA (3308T>A), GTG1057TGC (3309G>T 3310T>G 3311G>C), ATC1058ATT (3314C>T), GGC1060GGT (3320C>T), ATC1061ATT (3323C>T), CAC1062CAT (3326C>T), GCA1064GCT (3332A>T), TCA1065TCC (3335A>C), GGC1068GAA (3343G>A 3344C>A), GTA1069GCT (3346T>C 3347A>T), GCT1071ACA (3351G>A 3353T>A), GAA1072GAG (3356A>G), CGT1073TTG (3357C>T 3358G>T 3359T>G), ATC1076GTA (3366A>G 3368C>A), TCT1077GCA (3369T>G 3371T>A), GCT1080GCC (3380T>C), ACA1081ACT (3383A>T), ATC1083GTA (3387A>G 3389C>A), GAA1086GAC (3398A>C), CAA1087CAG (3401A>G), CTA1088CTG (3404A>G), GAG1089CAA (3405G>C 3407G>A), CCA1092GCT (3414C>G 3416A>T), ACT1093CAA (3417A>C 3418C>A 3419T>A), CCG1095CCA (3425G>A), GTC1096GTT (3428C>T), GCA1098ATG (3432G>A 3433C>T 3434A>G), GCA1099GCC (3437A>C), GTA1101GTG (3443A>G), CGC1102AGA (3444C>A 3446C>A), GAT1104GAC (3452T>C), ATT1105TTG (3453A>T 3455T>G), CTG1106CTC (3458G>C), AAG1107AAA (3461G>A), TCA1108CAT (3462T>C 3463C>A 3464A>T), ATT1109GTT (3465A>G), AGA1110CGA (3468A>C), AGC1111TCA (3471A>T 3472G>C 3473C>A), ACA1114ACT (3482A>T), CAG1115GCT (3483C>G 3484A>C 3485G>T), CTT1116CTC (3488T>C), GAG1118GAC (3494G>C), AAC1120AAG (3500C>G), GTG1122GTT (3506G>T), TAC1123GCT (3507T>G 3508A>C 3509C>T), TAC1124TTT (3511A>T 3512C>T), CTT1125TTG (3513C>T 3515T>G), GGA1126GGC (3518A>C), ACA1127ACT (3521A>T), CCA1129CCT (3527A>T), CAG1130CAA (3530G>A), TTG1132ATG (3534T>A), GCC1133GCA (3539C>A), GCC1134GCA (3542C>A), ACA1135ACG (3545A>G), GTT1136GTG (3548T>G), CCC1137CCA (3551C>A), ACT1141ACC (3563T>C), CTG1142CTC (3566G>C), CGG1143AGG (3567C>A), AGC1145AGT (3575C>T), CAA1146CCA (3577A>C), TTG1147CTG (3579T>C), GAA1149GAG (3587A>G), TTC1151TTT (3593C>T), GGA1152GGT (3596A>T), CCT1153GAA (3597C>G 3598C>A 3599T>A), GAG1155CAA (3603G>C 3605G>A), ACA1156ACT (3608A>T), GCA1157GCT (3611A>T), CCA1158CCT (3614A>T), TCC1159TCA (3617C>A), ATT1160CTG (3618A>C 3620T>G), CTA1161CTC (3623A>C), ACA1162TCT (3624A>T 3626A>T), GTT1163GTG (3629T>G), CAT1164AAA (3630C>A 3632T>A), GAC1165GAT (3635C>T), GGT1168GGG (3644T>G), GGT1170GGA (3650T>A), GAC1172GAT (3656C>T), CCC1173CCT (3659C>T), GTG1175GTT (3665G>T), GGG1177GGC (3671G>C), GTA1178ATT (3672G>A 3674A>T), ATG1179GAG (3675A>G 3676T>A), GCT1185GCC (3695T>C), TGT1186TCA (3697G>C 3698T>A), GGA1187AAT (3699G>A 3700G>A 3701A>T), GAT1189GAG (3707T>G), GAT1190GAC (3710T>C), GAC1191AGC (3711G>A 3712A>G), GCC1193GCA (3719C>A), AAA1194CGG (3720A>C 3721A>G 3722A>G), CTA1195GCT (3723C>G 3724T>C 3725A>T), TTC1197TTT (3731C>T), AAT1199GGT (3735A>G 3736A>G), TGC1202TCT (3745G>C 3746C>T), TCG1203GCC (3747T>G 3749G>C), CTG1204ATT (3750C>A 3752G>T), CTA1205TTA (3753C>T), CCT1206CCA (3758T>A), ATC1207GTT (3759A>G 3761C>T), ATG1208CTC (3762A>C 3764G>C), CGT1209AAG (3765C>A 3766G>A 3767T>G), AGC1210AGT (3770C>T), CAG1211CAA (3773G>A), AAG1212AGG (3775A>G), ATC1213GTG (3777A>G 3779C>G), CCT1214CCA (3782T>A), GGG1215GGT (3785G>T), GGA1216GGT (3788A>T), CGT1217ATC (3789C>A 3790G>T 3791T>C), CCA1218CCC (3794A>C), TGT1219ACA (3795T>A 3796G>C 3797T>A), GAA1220ACA (3798G>A 3799A>C), AGG1221CGT (3801A>C 3803G>T), GAT1222TGT (3804G>T 3805A>G), GAT1224GAC (3812T>C), GTA1225ATC (3813G>A 3815A>C), GTG1226GTT (3818G>T), CTC1227CTG (3821C>G), GGA1229GGT (3827A>T), ATA1230GTT (3828A>G 3830A>T), GGA1232GGT (3836A>T), TGT1233TTT (3838G>T), GAT1234GAC (3842T>C), TAC1235TTC (3844A>T), TAT1236TTT (3847A>T), GGC1238TCC (3852G>T 3853G>C), GAG1240GAA (3860G>A), CTG1241CTC (3863G>C), TCT1244TCA (3872T>A), TGC1245TGT (3875C>T), TAT1247TAC (3881T>C), CCC1248CCT (3884C>T), AAC1250AAT (3890C>T), AAG1251AAA (3893G>A), ATG1252CTG (3894A>C), GGG1253GGT (3899G>T), ATG1254TTG (3900A>T), GGG1255GGT (3905G>T), ATG1256ACC (3907T>C 3908G>C), AAC1257AAT (3911C>T), AAG1258AAA (3914G>A), AGA1259AGG (3917A>G), TTT1261TTC (3923T>C), GTG1262GTT (3926G>T), CAA1263GAA (3927C>G), TCC1264GCA (3930T>G 3932C>A), ACT1265CAG (3933A>C 3934C>A 3935T>G), GGC1266GAG (3937G>A 3938C>G), GAA1267AGT (3939G>A 3940A>G 3941A>T), GGA1268ACC (3942G>A 3943G>C 3944A>C), GAG1269CAA (3945G>C 3947G>A), AGA1270AGG (3950A>G), GTG1271GTA (3953G>A), GAA1272CTA (3954G>C 3955A>T), AAA1274AAG (3962A>G), GAC1276AAT (3966G>A 3968C>T), ACT1277TCT (3969A>T), GTA1279GTT (3977A>T), TTT1280TTC (3980T>C), GAA1281CAA (3981G>C), GAA1284GAG (3992A>G), GAG1285GAA (3995G>A), CTA1286TTA (3996C>T), CAG1289GAA (4005C>G 4007G>A), ATT1290ATA (4010T>A), AAA1292AAG (4016A>G), GGC1293GGT (4019C>T), ATC1294AAC (4021T>A), CAT1295TAT (4023C>T), GAT1297GAA (4031T>A), CTG1298ATG (4032C>A), GTC1299GTT (4037C>T), ACC1301ACT (4043C>T), TGC1303TGT (4049C>T), GCC1304GCT (4052C>T), AAA1305AAG (4055A>G), GAT1306GAC (4058T>C), CGC1308AGG (4062C>A 4064C>G), CTC1309CTA (4067C>A), CCA1310CCT (4070A>T), CTT1311CTG (4073T>G), GAG1312GAC (4076G>C), AAA1313AAG (4079A>G), ATC1314ATA (4082C>A), TAT1315TAC (4085T>C), GGC1316GGG (4088C>G), AAG1317AAA (4091G>A), AGA1318AAG (4093G>A 4094A>G), AAG1319AAA (4097G>A), AGG1321CGT (4101A>C 4103G>T), CTC1322CTT (4106C>T), ATA1325ATT (4115A>T), CTT1326TTG (4116C>T 4118T>G), CCT1327CCA (4121T>A), TAC1330TAT (4130C>T), TTG1333CTG (4137T>C), GTC1334GTG (4142C>G), AGG1335CGC (4143A>C 4145G>C), AAG1336AAA (4148G>A), TTT1338TTC (4154T>C), TTC1341TTT (4163C>T), TCA1342TCT (4166A>T), GCC1343GCA (4169C>A), ACA1344TGT (4170A>T 4171C>G 4172A>T), TTG1345TTA (4175G>A), TTG1348GCA (4182T>G 4183T>C 4184G>A), CAC1349CAT (4187C>T), GCT1351GTT (4192C>T), ATA1352CTG (4194A>C 4196A>G), CCA1353CCC (4199A>C), GTT1356GTG (4208T>G), ATT1358ATC (4214T>C), GAT1359AAT (4215G>A), CCT1360CCC (4220T>C), ACA1361ATT (4222C>T 4223A>T), AGT1362TCA (4224A>T 4225G>C 4226T>A), TCT1363CAT (4227T>C 4228C>A), ACA1366ACC (4238A>C), TTG1367ACC (4239T>A 4240T>C 4241G>C), TTG1368CTG (4242T>C), AAT1370AAC (4250T>C), GGG1371CAG (4251G>C 4252G>A), TTT1372TTC (4256T>C), AGA1373CGG (4257A>C 4259A>G), GCT1374AGT (4260G>A 4261C>G), TCA1376TCT (4268A>T), GAC1377GAT (4271C>T), GTG1378GTT (4274G>T), GGA1379GGT (4277A>T), TCA1381TCT (4283A>T), GCT1382GCA (4286T>A), GAT1383GAC (4289T>C), TAT1384TAC (4292T>C), AGA1390AGG (4310A>G), GCA1391GCG (4313A>G), CCT1392CCA (4316T>A), GTT1393ATA (4317G>A 4319T>A), GCT1395GCA (4325T>A), CAG1397CAA (4331G>A), GAA1405GCA (4354A>C), TAC1407TAT (4361C>T), GGA1408GGG (4364A>G), AAG1410GCT (4368A>G 4369A>C 4370G>T), CCT1411CCA (4373T>A), GGC1412GGA (4376C>A), AGT1413AGC (4379T>C), CCT1414GAG (4380C>G 4381C>A 4382T>G), TCC1416TCA (4388C>A), GCT1418GCA (4394T>A), CGA1419AGA (4395C>A), GCA1421GCT (4403A>T), CTT1422CTG (4406T>G), TTA1423TTG (4409A>G), GCA1426GCT (4418A>T), TGT1428TGC (4424T>C), ACA1431ACT (4433A>T), CTG1432CTA (4436G>A), GTG1437CTC (4449G>C 4451G>C), TTT1438TTC (4454T>C), TTG1440TTA (4460G>A), GGG1442GGT (4466G>T), GGC1443GGA (4469C>A), CCA1445CCT (4475A>T), TCA1446TCT (4478A>T), GCA1449GCT (4487A>T), CTA1450CTC (4490A>C), ACG1451ACC (4493G>C), ATC1453ATA (4499C>A), TTC1454TTT (4502C>T), AAT1455AAC (4505T>C), TCT1456TCC (4508T>C), CTC1457CTT (4511C>T), CTC1458TTA (4512C>T 4514C>A), AAT1459AAC (4517T>C), CGA1464CGG (4532A>G), GCC1466GCT (4538C>T), TTT1467TTC (4541T>C), TCA1469TCG (4547A>G), TTG1470CTC (4548T>C 4550G>C), TTA1471CTC (4551T>C 4553A>C), AGA1473AGG (4559A>G), CAT1475CAC (4565T>C), ATT1476ATC (4568T>C), GCT1477AAA (4569G>A 4570C>A 4571T>A), GCC1478GCA (4574C>A), AGG1479AAG (4576G>A), GCT1480GCA (4580T>A), ATA1481TTG (4581A>T 4583A>G), GGA1482GGT (4586A>T), GTT1483GTA (4589T>A), AAA1484AAG (4592A>G), CCA1485TCA (4593C>T), TTC1488TTT (4604C>T), CAG1490GAC (4608C>G 4610G>C), CTA1491TTA (4611C>T), TTC1492TTT (4616C>T), ATA1493ATT (4619A>T), GCT1494GCA (4622T>A), GTT1495GTG (4625T>G), GGA1497GGG (4631A>G), GTT1502GTG (4646T>G), GTA1504GTG (4652A>G), TTA1506ATG (4656T>A 4658A>G), CAT1507CAC (4661T>C), CTC1508CTT (4664C>T), CAG1509CAA (4667G>A), TAT1511TAC (4673T>C), TCT1512GAG (4674T>G 4675C>A 4676T>G), CTG1513TTA (4677C>T 4679G>A), CCA1514CCT (4682A>T), AAT1515GCA (4683A>G 4684A>C 4685T>A), CAT1518CAA (4694T>A), TTA1520CTA (4698T>C), CTG1522CAA (4705T>A 4706G>A), GTC1523GTG (4709C>G), AAT1524AAC (4712T>C), GTA1525GTT (4715A>T), ATC1526ATT (4718C>T), ATT1527ATC (4721T>C), GGT1530GGC (4730T>C), GAC1532GAT (4736C>T), TCG1534AAC (4740T>A 4741C>A 4742G>C), GAT1536AAT (4746G>A), GTT1537GTC (4751T>C), GAG1539GAT (4757G>T), GTA1540GTC (4760A>C), CAA1541AAA (4761C>A), TTT1542TTC (4766T>C), CAA1543CAG (4769A>G), CTA1548TTG (4782C>T 4784A>G), ACT1549ACG (4787T>G), CTG1551TTG (4791C>T), AGT1552AGC (4796T>C), GGT1554GGC (4802T>C), TTT1555TTC (4805T>C), AAA1556AAG (4808A>G), CGA1557CGC (4811A>C), CTT1560TTG (4818C>T 4820T>G), GGA1561GGT (4823A>T), CAC1563CAA (4829C>A), ATG1564CTA (4830A>C 4832G>A), GCT1565GCC (4835T>C), CCT1566CCC (4838T>C), TCA1571TCC (4853A>C), ATC1572ATT (4856C>T), ATT1573GTG (4857A>G 4859T>G), GAG1574GAA (4862G>A), CCC1575CCA (4865C>A), ATA1579ATC (4877A>C), CCT1581CCA (4883T>A), GCC1582GCA (4886C>A), CCA1583GTT (4887C>G 4888C>T 4889A>T), GGT1584GGG (4892T>G), CCT1586CCA (4898T>A), GAC1587GAT (4901C>T), ACT1588ATC (4903C>T 4904T>C), CAG1589ACT (4905C>A 4906A>C 4907G>T), ATG1592TAT (4914A>T 4915T>A 4916G>T), ACA1597ACG (4931A>G), GGA1598GGT (4934A>T), ATA1599GTA (4935A>G), CGT1600AGA (4938C>A 4940T>A), GAA1601GAG (4943A>G), CAT1604CAG (4952T>G), CAC1605CAT (4955C>T), GGT1606GGA (4958T>A), CGT1607CAG (4960G>A 4961T>G), GTG1608GTC (4964G>C), GCT1609GTT (4966C>T), AAG1612ACA (4975A>C 4976G>A), CTT1613TTG (4977C>T 4979T>G), GTC1614ACA (4980G>A 4981T>C 4982C>A), TTA1615GAC (4983T>G 4984T>A 4985A>C), GAT1616AAT (4986G>A), GTT1617GTC (4991T>C), CAG1618CAA (4994G>A), ACG1619AAA (4996C>A 4997G>A), GCG1620GCA (5000G>A), TTG1621ATA (5001T>A 5003G>A), GAT1622ATG (5004G>A 5005A>T 5006T>G), GAA1623CAG (5007G>C 5009A>G), AGG1624CGA (5010A>C 5012G>A), GGT1625GGC (5015T>C), TTC1626TTG (5018C>G), AGA1627CCA (5019A>C 5020G>C), GCT1628ATC (5022G>A 5023C>T 5024T>C), ATC1630CAT (5028A>C 5029T>A 5030C>T), CCT1632CCA (5036T>A), TCC1633TCT (5039C>T), TTG1635GCT (5043T>G 5044T>C 5045G>T), GTG1637CAA (5049G>C 5050T>A 5051G>A), CAG1639AGT (5055C>A 5056A>G 5057G>T), GAA1640GAG (5060A>G), ATT1642CTA (5064A>C 5066T>A), GCA1643CAA (5067G>C 5068C>A), AAG1644GCC (5070A>G 5071A>C 5072G>C), GTA1645GTT (5075A>T), ACA1646ACT (5078A>T), GGG1647GGA (5081G>A), GAT1648GAC (5084T>C), TCA1649AGT (5085T>A 5086C>G 5087A>T), AGT1650AAC (5089G>A 5090T>C), GCC1651ATA (5091G>A 5092C>T 5093C>A), CTG1652GGG (5094C>G 5095T>G), ACA1653GCT (5097A>G 5099A>T), ATT1654CTG (5100A>C 5102T>G), TGT1655CTG (5103T>C 5104G>T 5105T>G), ATG1657CTG (5109A>C), GCA1658AGC (5112G>A 5113C>G 5114A>C), AAA1659AAG (5117A>G), GCA1660GAT (5119C>A 5120A>T), TCC1663ACT (5127T>A 5129C>T), TAT1664TAC (5132T>C), ACG1665GTG (5133A>G 5134C>T), CCA1666CCT (5138A>T), TTG1667CTT (5139T>C 5141G>T), GAC1668CCA (5142G>C 5143A>C 5144C>A), GCA1669GAA (5146C>A), GAG1671AAA (5151G>A 5153G>A), AAG1672AAA (5156G>A), ATC1673ATT (5159C>T), ACA1674GAA (5160A>G 5161C>A), AAT1675GAT (5163A>G), GAG1677TCA (5169G>T 5170A>C 5171G>A), CGT1678CAA (5173G>A 5174T>A), GAT1679GGT (5176A>G), CTG1680GTC (5178C>G 5180G>C), AAT1681CAT (5181A>C), TGG1682GAA (5184T>G 5185G>A 5186G>A), GCA1684TGT (5190G>T 5191C>G 5192A>T), CCA1685TCT (5193C>T 5195A>T), AAC1686GGA (5196A>G 5197A>G 5198C>A), ATT1687GTT (5199A>G), GGT1688TTC (5202G>T 5203G>T 5204T>C), TTT1689TTC (5207T>C), GCA1692TCG (5214G>T 5216A>G), CGT1693CGA (5219T>A), ACT1694ACA (5222T>A), GCA1695GAA (5224C>A), GCC1696GTT (5227C>T 5228C>T), CAC1697AAT (5229C>A 5231C>T), TAC1698CAT (5232T>C 5234C>T), ACG1699AAA (5236C>A 5237G>A), GAA1702GAG (5246A>G), GGG1703GAC (5248G>A 5249G>C), TAC1704ACA (5250T>A 5251A>C 5252C>A), ATT1706CTT (5256A>C), AAC1708AAT (5264C>T), TGT1709TGC (5267T>C), AAG1713AAA (5279G>A), AAG1714AAA (5282G>A), TCC1715ACA (5283T>A 5285C>A), AAT1716ACA (5287A>C 5288T>A), GTT1718GTG (5294T>G), GGT1720GGC (5300T>C), CCG1733CCC (5339G>C), TAC1734TAT (5342C>T), ACT1735ACA (5345T>A), TCA1737GCA (5349T>G), GCT1738GCC (5354T>C), ATA1739ATC (5357A>C), ATG1740AAG (5359T>A), GAT1741GAC (5363T>C), GCC1742AAT (5364G>A 5365C>A 5366C>T), AAG1744CAA (5370A>C 5372G>A), AAT1745AGT (5374A>G), GTG1746CGC (5376G>C 5377T>G 5378G>C), GCA1748TCA (5382G>T), GGA1749ATG (5385G>A 5386G>T 5387A>G), GGA1750GGG (5390A>G), CAT1751ATT (5391C>A 5392A>T), GTA1752GGA (5395T>G), GCA1753ACC (5397G>A 5399A>C), ACC1754CAA (5400A>C 5401C>A 5402C>A), AAT1755AAA (5405T>A), GTC1756GTT (5408C>T), GTT1757GTC (5411T>C), GTG1759ATT (5415G>A 5417G>T), GGA1761GGG (5423A>G), GGA1763GGT (5429A>T), GTC1766GTG (5438C>G), GGC1767AGT (5439G>A 5441C>T), CCA1768CCC (5444A>C), GCA1771GCT (5453A>T), GCG1772GCT (5456G>T), ATG1774TTT (5460A>T 5462G>T), GCC1775GCA (5465C>A), GCA1777TCA (5469G>T), ACA1778ACC (5474A>C), AGG1779AAT (5476G>A 5477G>T), CAA1780CAG (5480A>G), TGT1782AGA (5484T>A 5486T>A), GTG1783GCT (5488T>C 5489G>T), GAC1785CAA (5493G>C 5495C>A), ATA1786CTT (5496A>C 5498A>T), GTT1788GTC (5504T>C), CGC1791AGA (5511C>A 5513C>A), ATT1793TTG (5517A>T 5519T>G), GGA1794GCG (5521G>C 5522A>G), AAT1795GGC (5523A>G 5524A>G 5525T>C), GTG1796TTG (5526G>T), CTT1797CTG (5531T>G), GAC1798GAT (5534C>T), CTC1799TTG (5535C>T 5537C>G), ACC1801CAG (5541A>C 5542C>A 5543C>G), GGC1805GGT (5555C>T), GTG1807ATT (5559G>A 5561G>T), CAG1808CAA (5564G>A), TTT1810TTC (5570T>C), CTT1811CTC (5573T>C), CAA1812ACA (5574C>A 5575A>C), TGT1813_GTG1814insGTC (5579_5580insGTC), CCT1815CAG (5584C>A 5585T>G), CAT1816AAA (5586C>A 5588T>A), GGA1817AGT (5589G>A 5591A>T), GAC1818TGC (5592G>T 5593A>G), AAA1819AAG (5597A>G), GTG1820ACA (5598G>A 5599T>C 5600G>A), AGT1823GCT (5607A>G 5608G>C), GGT1824AGT (5610G>A), GCA1825GGG (5614C>G 5615A>G), TCG1826del (5616_5618delTCG), CTC1827CGC (5620T>G), CAC1828GAT (5622C>G 5624C>T), AGT1829GGA (5625A>G 5627T>A), AGT1830ATG (5629G>T 5630T>G), TTT1831TTG (5633T>G), ATG1832AAC (5635T>A 5636G>C), CAA1834CAT (5642A>T), CAA1834_GGG1835insCATGCT (5642_5643insCATGCT), GGG1835GCT (5644G>C 5645G>T), TTT1836TAT (5647T>A), GAA1837GCC (5650A>C 5651A>C), TTG1838CTT (5652T>C 5654G>T), GGC1839GGA (5657C>A), CTC1841CTT (5663C>T), CGC1842CGA (5666C>A), ATT1843ATA (5669T>A), ATA1844GTG (5670A>G 5672A>G), CAT1845GGC (5673C>G 5674A>G 5675T>C), GGT1846TGT (5676G>T), GAT1847TCA (5679G>T 5680A>C 5681T>A), TTA1848ATT (5682T>A 5684A>T), GCA1849TCA (5685G>T), AAA1850AGA (5689A>G), CAG1851AAG (5691C>A), ACA1852ATG (5695C>T 5696A>G), ATG1854GTG (5700A>G), CGG1855AGA (5703C>A 5705G>A), ATG1856TTG (5706A>T), CCA1857CCC (5711A>C), TAT1858TAC (5714T>C), GTG1859ACT (5715G>A 5716T>C 5717G>T), GTA1860GTT (5720A>T), GGA1861GGG (5723A>G), CAT1863ATG (5727C>A 5728A>T 5729T>G), GGA1864GGT (5732A>T), GGA1865GGT (5735A>T), GGA1867GCA (5740G>C), AAT1868GAT (5742A>G), TTT1869TGC (5746T>G 5747T>C), TCC1870TCA (5750C>A), CAG1872AGA (5754C>A 5755A>G 5756G>A), GAT1873AAT (5757G>A), GAG1875GAT (5765G>T), AGT1876TCT (5766A>T 5767G>C), TTG1877CTC (5769T>C 5771G>C), CTA1878ATC (5772C>A 5774A>C), CAC1879AAA (5775C>A 5777C>A), TAT1880GCC (5778T>G 5779A>C 5780T>C), TTG1881CTA (5781T>C 5783G>A), CAG1883TGC (5787C>T 5788A>G 5789G>C), GGA1884TCT (5790G>T 5791G>C 5792A>T), GCA1886GCT (5798A>T), GAG1887GCA (5800A>C 5801G>A), TTA1888TGT (5803T>G 5804A>T), ATT1889GTG (5805A>G 5807T>G), CAA1890GCT (5808C>G 5809A>C 5810A>T), AAG1891CGT (5811A>C 5812A>G 5813G>T), AAT1892GGG (5814A>G 5815A>G 5816T>G), ACA1893TGT (5817A>T 5818C>G 5819A>T), CTA1895ATA (5823C>A), TAT1898TAC (5834T>C), TTC1899TTT (5837C>T), AAA1900GAG (5838A>G 5840A>G), GAG1901AAT (5841G>A 5843G>T), CTG1902TTT (5844C>T 5846G>T), AGT1903AAT (5848G>A), ATG1904GCA (5850A>G 5851T>C 5852G>A), GAA1905CAA (5853G>C), AAT1906ATA (5857A>T 5858T>A), GTG1907GCT (5860T>C 5861G>T), CAA1908ACC (5862C>A 5863A>C 5864A>C), CAA1909AGG (5865C>A 5866A>G 5867A>G), CTG1910TTA (5868C>T 5870G>A), ATA1911GTA (5871A>G), GTT1914CTT (5880G>C),

	AAG1915AAA (5885G>A), CTT1916AGT (5886C>A 5887T>G), GGG1918GGT (5894G>T), TTT1919GTG (5895T>G 5897T>G), TTC1920TTT (5900C>T), CCA1921ATC (5901C>A 5902C>T 5903A>C), AAA1922ACA (5905A>C), GAA1923GCC (5908A>C 5909A>C), ACT1924ACA (5912T>A), ACC1925GGC (5913A>G 5914C>G), ATT1926CAC (5916A>C 5917T>A 5918T>C), CAG1927AAA (5919C>A 5921G>A), AAG1928ATG (5923A>T), CTC1929TTG (5925C>T 5927C>G), AAA1930GTG (5928A>G 5929A>T 5930A>G), AAT1931CAA (5931A>C 5933T>A), TTT1932ACT (5934T>A 5935T>C), GTT1933CTG (5937G>C 5939T>G), GAT1934AGC (5940G>A 5941A>G 5942T>C), GCT1935AAT (5943G>A 5944C>A), GAA1936CAA (5946G>C), TGT1937TGG (5951T>G), CTA1938CAA (5953T>A), ACA1939TCC (5955A>T 5957A>C), TTC1940CAC (5958T>C 5959T>A), AAA1941GAA (5961A>G), GCA1942CAA (5964G>C 5965C>A), AGG1943AGT (5969G>T), AGC1944TGT (5970A>T 5972C>T), TTT1945GTG (5973T>G 5975T>G), AGG1946AAG (5977G>A), CAC1947AGA (5979C>A 5980A>G 5981C>A), GTA1948GTC (5984A>C), GTT1949TAT (5985G>T 5986T>A), AAA1951AAG (5993A>G), AAG1952CGA (5994A>C 5995A>G 5996G>A), AAA1953AAT (5999A>T), TTG1955CTC (6003T>C 6005G>C), AGT1956TCA (6006A>T 6007G>C 6008T>A), TCC1957AAC (6009T>A 6010C>A), ACA1958GCA (6012A>G), TGG1959ATA (6015T>A 6016G>T 6017G>A), AAA1960ACA (6019A>C), ATG1961_TGT1962insTAC (6023_6024insTAC), TGT1962CGT (6024T>C), GAA1964GAT (6032A>T), AGT1965GCC (6033A>G 6034G>C 6035T>C), ATT1966AAA (6037T>A 6038T>A), GTT1967GAT (6040T>A), TCA1969ATA (6045T>A 6046C>T), AGG1970GTA (6048A>G 6049G>T 6050G>A), GCA1972del (6054_6056delGCA), CTA1975GTG (6063C>G 6065A>G), CCT1977TTG (6069C>T 6070C>T 6071T>G), GGC1978GGT (6074C>T), TTG1980CCA (6078T>C 6079T>C 6080G>A), GCT1982GCC (6086T>C), TCC1983ATG (6087T>A 6088C>T 6089C>G), GTA1984GTT (6092A>T), AAA1986AGG (6097A>G 6098A>G)

Proteins

polyprotein (YP_001976147.1)	1	1989	92.4%	9624	69.7%	1982 (99.2%)	1328 (66.5%)	9/7/0/0	0

	Begin	End	Coverage	Score	Concordance	Matches	Identities	I/D/M/F*	Stop Codons
**NT**	**24**	**6107**	**84.2%**	**3417**	**28.3%**	**6063 (98.8%)**	**3936 (64.1%)**	**54/21**	

Protein mutations:

M1L (141A>C), A2V (145C>T), S4H (150T>C 151C>A 152A>C), K5N (155G>T), M6T (157T>C 158G>C), F7T (159T>A 160T>C), S8I (162T>A 163C>T 164C>T), K9Q (165A>C 167G>A), V10M (168G>A 170T>G), G12del (174_176delGGT), V13F (177G>T), D14S (180G>T 181A>C 182T>A), D15K (183G>A 185C>A), A16I (186G>A 187C>T), S17V (189A>G 190G>T), S20A (198T>G 200C>T), T22P (204A>C), F25L (213T>C), G26S (216G>T 217G>C), N30Q (228A>C 230T>G), S31T (231T>A 233T>A), I32V (234A>G 236T>G), K34S (240A>T 241A>C), F35L (243T>C 245T>G), A38T (252G>A 254G>A), V39M (255G>A 257T>G), S40K (259G>A 260T>G), N41S (262A>G), I42M (266T>G), T43S (268C>G 269C>T), T44K (271C>A 272T>A), M45I (275G>A), Q47E (279C>G), I49F (285A>T 287T>C), D51G (292A>G 293T>C), L53I (297C>A), D55E (305T>A), V60I (318G>A 320G>T), T61V (321A>G 322C>T), D62K (324G>A 326T>A), V63T (327G>A 328T>C 329G>T), S64A (330T>G 332C>A), T65D (333A>G 334C>A 335A>T), V68N (342G>A 343T>A 344G>T), A69S (345G>A 346C>G 347C>T), T70V (348A>G 349C>T), L74V (360C>G 362T>A), N75E (363A>G 365T>A), V77L (369G>T 371T>A), D79G (376A>G 377C>G), G82A (385G>C 386T>C), A83T (387G>A 389A>G), L84V (390C>G), D86E (398T>A), L89V (405C>G), A91G (412C>G 413G>T), Y92I (414T>A 415A>T), S93N (417T>A 418C>A), L95F (425G>C), S96K (426T>A 427C>A 428C>A), K100R (439A>G), S101T (441T>A 443T>C), K103I (448A>T 449G>T), L109V (465T>G 467G>T), F110V (468T>G), E111L (471G>T 472A>T), S112G (474A>G 476T>C), L113I (477T>A 479G>C), S114K (480T>A 481C>A 482C>A), K116N (488A>T), V117F (489G>T), K118I (493A>T 494A>T), L119R (495T>A 496T>G), F121Y (502T>A), A122S (504G>T 506T>A), V124L (510G>C), N126E (516A>G 518T>A), K127T (520A>C), S128T (523G>C 524T>A), E129G (526A>G 527A>T), D130E (530T>A), V131Y (531G>T 532T>A 533A>T), I133V (537A>G 539A>T), Y136I (546T>A 547A>T 548T>A), G137A (550G>C), A142S (564G>T 566T>C), I143L (567A>C 569A>T), T144S (570A>T), V147L (579G>T 581G>A), L149Q (586T>A), F150I (588T>A 590T>A), V151I (591G>A 593T>C), S154V (600A>G 601G>T 602T>A), G159M (615G>A 616G>T 617T>G), S161R (621T>A 622C>G 623T>A), N162S (625A>G 626C>T), T163V (627A>G 628C>T 629T>A), K165C (633A>T 634A>G 635A>T), I170V (648A>G 650A>T), M174F (660A>T 662G>C), L181I (681T>A 683A>T), D182S (684G>A 685A>G), K186L (696A>C 697A>T 698A>C), A188S (702G>T 704C>T), R190K (708C>A 709G>A), T192S (714A>T 716C>T), M193Q (717A>C 718T>A), V194M (720G>A), M199V (735A>G), V205L (753G>T 755T>G), S206N (756T>A 757C>A 758A>T), M214L (780A>C), T216S (786A>T 788A>T), I217L (789A>T 791A>G), L218I (792T>A 794G>A), I228V (822A>G 824T>G), S238T (853G>C 854C>A), T248A (882A>G 884A>G), T260Q (918A>C 919C>A 920T>A), R263K (927C>A 928G>A 929T>G), I280V (978A>G 980A>T), V281A (982T>C 983C>T), G282D (985G>A 986C>T), T283I (988C>T 989C>A), L298I (1032C>A 1034G>T), Q307T (1059C>A 1060A>C 1061G>T), D310S (1068G>T 1069A>C), C313A (1077T>G 1078G>C), K325P (1113A>C 1114A>C 1115G>T), P328S (1122C>T 1124T>A), T329I (1126C>T), R335K (1144G>A 1145G>A), E337Q (1149G>C 1151A>G), M354L (1200A>T), A361T (1221G>A 1223C>A), A363V (1228C>T), E366D (1238A>T), V368L (1242G>C 1244T>C), Y369I (1245T>A 1246A>T 1247T>A), S372N (1255G>A 1256T>C), R374Q (1260A>C 1261G>A 1262G>A), M376T (1267T>C 1268G>A), S393E (1317T>G 1318C>A 1319C>G), R412A (1374A>G 1375G>C 1376A>C), G414Q (1380G>C 1381G>A 1382T>A), I496V (1626A>G 1628C>T), M521R (1701A>C 1702T>G), E523V (1708A>T), D530N (1728G>A 1730T>C), G533A (1738G>C), R536S (1748A>C), V540I (1758G>A 1760G>T), E541G (1762A>G 1763G>A), N552D (1794A>G 1796T>C), Q567K (1839C>A), A570H (1848G>C 1849C>A 1850A>C), V572I (1854G>A 1856A>T), S573A (1857T>G), K574T (1861A>C 1862G>C), F577Y (1870T>A 1871T>C), E578N (1872G>A 1874A>C), A579Q (1875G>C 1876C>A 1877T>G), E580S (1878G>T 1879A>C), S581T (1881T>A 1883T>A), I583C (1887A>T 1888T>G 1889A>T), S584E (1890A>G 1891G>A 1892T>A), P585E (1893C>G 1894C>A 1895T>G), I587V (1899A>G 1901A>T), Q591E (1911C>G), A594V (1921C>T 1922A>T), D595E (1925T>A), D596E (1928T>A), I598M (1934A>G), A599T (1935G>A 1937G>A), V601F (1941G>T 1943G>T), D602H (1944G>C), G603D (1948G>A 1949G>T), R605Q (1953A>C 1954G>A), L606I (1956T>A 1958G>A), R607S (1959C>A), T608F (1962A>T 1963C>T 1964C>T), K609D (1965A>G 1967G>C), Q610E (1968C>G), D611V (1972A>T), E612S (1974G>A 1975A>G 1976A>C), I614S (1981T>G 1982A>T), L615V (1983T>G 1985G>T), V616_P617del (1986_1991delGTGCCC), V620L (1998G>C 2000G>C), N623D (2007A>G), I627G (2019A>G 2020T>G 2021C>A), A629S (2025G>T), R630Q (2028A>C 2029G>A 2030A>G), S644A (2070A>G 2071G>C 2072T>A), D646N (2076G>A), E648F (2082G>T 2083A>T 2084G>T), L650Y (2088C>T 2089T>A), H655A (2103C>G 2104A>C), H656S (2106C>T 2107A>C 2108T>A), M660I (2120G>T), Y661F (2122A>T), S663V (2127T>G 2128C>T 2129T>C), D665S (2133G>T 2134A>C 2135T>A), D666S (2136G>A 2137A>G), H668A (2142C>G 2143A>C 2144T>C), I671S (2152T>G 2153T>C), N673T (2158A>C 2159C>T), K674R (2160A>C 2161A>G 2162A>C), R676V (2166A>G 2167G>T), V677N (2169G>A 2170T>A 2171C>T), N678E (2172A>G 2174C>G), L680I (2178T>A 2180G>T), R682E (2184A>G 2185G>A 2186A>G), D685S (2193G>T 2194A>C 2195T>G), A686T (2196G>A), E687A (2200A>C), Q688G (2202C>G 2203A>G 2204G>A), R690A (2208C>G 2209G>C 2210A>C), V693I (2217G>A 2219A>C), Q694K (2220C>A 2222A>G), N695A (2223A>G 2224A>C), I701V (2241A>G 2243A>C), A710S (2268G>A 2269C>G 2270A>T), S711T (2271T>A), I713V (2277A>G 2279C>G), K716A (2286A>G 2287A>C 2288G>T), K717H (2289A>C 2291A>C), L718M (2292C>A 2294A>G), A719D (2296C>A 2297A>T), T722R (2305C>G 2306A>G), E723M (2307G>A 2308A>T), M724L (2310A>T), R725Y (2313A>T 2314G>A 2315G>C), T726N (2317C>A), F728R (2322T>A 2323T>G 2324T>A), S731R (2331T>A 2332C>G 2333T>A), C732A (2334T>G 2335G>C 2336C>A), I733L (2337A>C 2339T>G), D734E (2342T>A), V737A (2350T>C), I741M (2363A>G), F749H (2385T>C 2386T>A), C762_N763insRR (2426_2427insAGAAGG), N763K (2429C>G), K764M (2431A>T 2432A>G), S766T (2436T>A 2438A>T), P769T (2445C>A), K770_E771del (2448_2453delAAGGAA), I774V (2460A>G 2462C>A), K775Q (2463A>C), I776S (2466A>T 2467T>C 2468T>G), C777L (2469T>C 2470G>T), H779D (2475C>G 2477T>C), K780S (2478A>T 2479A>C 2480A>C), I781T (2482T>C 2483C>A), V782I (2484G>A), E783G (2488A>G 2489A>G), Y784R (2490T>A 2491A>G 2492T>A), I785M (2495C>G), K786Q (2496A>C 2498G>A), D787E (2501T>A), T788M (2503C>T 2504A>G), W790N (2508T>A 2509G>A 2510G>T), S791D (2511A>G 2512G>A 2513C>T), A795I (2523G>A 2524C>T 2525A>T), R797K (2530G>A), I806L (2556A>C), M807I (2561G>A), V812T (2574G>A 2575T>C), F813M (2577T>A 2579T>G), G815A (2584G>C), I816V (2586A>G 2588T>A), L820I (2598T>A 2600G>T), A821S (2601G>T 2603G>T), Q824K (2610C>A 2612G>A), N826S (2617A>G), S827L (2620C>T 2621T>G), F828I (2622T>A 2624C>T), N830T (2629A>C), A831S (2631G>T 2633A>C), S832Q (2634T>C 2635C>A), R834K (2641G>A), N852R (2695A>G 2696T>A), N852_K853insS (2696_2697insTCC), K853H (2697A>C 2699G>T), L854V (2700C>G 2702C>A), R855S (2703A>T 2704G>C), N856K (2708T>G), L857M (2709C>A 2711T>G), V859F (2715G>T), G860S (2718G>A 2720T>C), S865P (2733T>C), L866N (2736T>A 2737T>A 2738G>T), A867D (2740C>A 2741A>T), Q868_D869insG (2744_2745insGGA), D869K (2745G>A 2747T>A), A872S (2754G>T 2756T>C), E873R (2757G>A 2758A>G 2759A>G), Q879E (2775C>G 2777G>A), K882A (2784A>G 2785A>C 2786G>C), L890V (2808C>G 2810T>G), G891A (2812G>C 2813T>A), G904E (2851G>A 2852T>A), H914T (2880C>A 2881A>C 2882C>G), N915Q (2883A>C 2885T>G), A916L (2886G>C 2887C>T 2888T>C), T923S (2907A>T), A927E (2920C>A 2921C>A), N928H (2922A>C), R930V (2928C>G 2929G>T 2930A>C), S931C (2932C>G 2933T>C), K932Q (2934A>C 2936A>G), R938K (2953G>A), E939H (2955G>C 2957A>T), S940A (2958T>G 2960A>C), R941K (2962G>A), I942F (2964A>T), I943L (2967A>T 2969T>G), E944P (2970G>C 2971A>C 2972G>T), G945E (2974G>A 2975C>A), I948V (2982A>G 2984C>T), I949V (2985A>G 2987T>A), D955C (3003G>T 3004A>G), P961V (3021C>G 3022C>T 3023T>C), P968A (3042C>G 3044A>T), R969E (3045A>G 3046G>A), E972A (3055A>C 3056G>A), Q980E (3078C>G), C984V (3090T>G 3091G>T 3092T>C), T990K (3109C>A), R993E (3117C>G 3118G>A 3119T>G), L994K (3120C>A 3121T>A 3122T>A), G995Q (3123G>C 3124G>A 3125C>G), E998D (3134G>C), M1002Y (3144A>T 3145T>A 3146G>T), P1003S (3147C>T), V1012E (3175T>A 3176T>G), E1015T (3183G>A 3184A>C), E1017R (3189G>A 3190A>G 3191A>G), L1018M (3192C>A), N1019S (3196A>G), S1020T (3198T>A 3200C>A), S1021N (3201T>A 3202C>A 3203C>T), K1024H (3210A>C 3212G>C), K1024_I1025insS (3212_3213insTCA), C1026S (3216T>A 3218C>T), S1027A (3219T>G), Q1029E (3225C>G), S1030A (3228T>G 3230T>C), I1035V (3243A>G 3245A>C), G1037S (3249G>A 3251G>C), V1051I (3291G>A 3293T>C), V1057C (3309G>T 3310T>G 3311G>C), G1068E (3343G>A 3344C>A), V1069A (3346T>C 3347A>T), A1071T (3351G>A 3353T>A), R1073L (3357C>T 3358G>T 3359T>G), I1076V (3366A>G 3368C>A), S1077A (3369T>G 3371T>A), I1083V (3387A>G 3389C>A), E1086D (3398A>C), E1089Q (3405G>C 3407G>A), P1092A (3414C>G 3416A>T), T1093Q (3417A>C 3418C>A 3419T>A), A1098M (3432G>A 3433C>T 3434A>G), I1105L (3453A>T 3455T>G), S1108H (3462T>C 3463C>A 3464A>T), I1109V (3465A>G), Q1115A (3483C>G 3484A>C 3485G>T), E1118D (3494G>C), N1120K (3500C>G), Y1123A (3507T>G 3508A>C 3509C>T), Y1124F (3511A>T 3512C>T), L1132M (3534T>A), Q1146P (3577A>C), P1153E (3597C>G 3598C>A 3599T>A), E1155Q (3603G>C 3605G>A), I1160L (3618A>C 3620T>G), T1162S (3624A>T 3626A>T), H1164K (3630C>A 3632T>A), V1178I (3672G>A 3674A>T), M1179E (3675A>G 3676T>A), C1186S (3697G>C 3698T>A), G1187N (3699G>A 3700G>A 3701A>T), D1189E (3707T>G), D1191S (3711G>A 3712A>G), K1194R (3720A>C 3721A>G 3722A>G), L1195A (3723C>G 3724T>C 3725A>T), N1199G (3735A>G 3736A>G), C1202S (3745G>C 3746C>T), S1203A (3747T>G 3749G>C), L1204I (3750C>A 3752G>T), I1207V (3759A>G 3761C>T), M1208L (3762A>C 3764G>C), R1209K (3765C>A 3766G>A 3767T>G), K1212R (3775A>G), I1213V (3777A>G 3779C>G), R1217I (3789C>A 3790G>T 3791T>C), C1219T (3795T>A 3796G>C 3797T>A), E1220T (3798G>A 3799A>C), D1222C (3804G>T 3805A>G), V1225I (3813G>A 3815A>C), I1230V (3828A>G 3830A>T), C1233F (3838G>T), Y1235F (3844A>T), Y1236F (3847A>T), G1238S (3852G>T 3853G>C), M1252L (3894A>C), M1254L (3900A>T), M1256T (3907T>C 3908G>C), Q1263E (3927C>G), S1264A (3930T>G 3932C>A), T1265Q (3933A>C 3934C>A 3935T>G), G1266E (3937G>A 3938C>G), E1267S (3939G>A 3940A>G 3941A>T), G1268T (3942G>A 3943G>C 3944A>C), E1269Q (3945G>C 3947G>A), E1272L (3954G>C 3955A>T), D1276N (3966G>A 3968C>T), T1277S (3969A>T), E1281Q (3981G>C), Q1289E (4005C>G 4007G>A), I1294N (4021T>A), H1295Y (4023C>T), D1297E (4031T>A), L1298M (4032C>A), E1312D (4076G>C), R1318K (4093G>A 4094A>G), T1344C (4170A>T 4171C>G 4172A>T), L1348A (4182T>G 4183T>C 4184G>A), A1351V (4192C>T), I1352L (4194A>C 4196A>G), D1359N (4215G>A), T1361I (4222C>T 4223A>T), S1363H (4227T>C 4228C>A), L1367T (4239T>A 4240T>C 4241G>C), G1371Q (4251G>C 4252G>A), A1374S (4260G>A 4261C>G), V1393I (4317G>A 4319T>A), E1405A (4354A>C), K1410A (4368A>G 4369A>C 4370G>T), P1414E (4380C>G 4381C>A 4382T>G), V1437L (4449G>C 4451G>C), A1477K (4569G>A 4570C>A 4571T>A), R1479K (4576G>A), I1481L (4581A>T 4583A>G), P1485S (4593C>T), Q1490D (4608C>G 4610G>C), L1506M (4656T>A 4658A>G), S1512E (4674T>G 4675C>A 4676T>G), N1515A (4683A>G 4684A>C 4685T>A), H1518Q (4694T>A), L1522Q (4705T>A 4706G>A), S1534N (4740T>A 4741C>A 4742G>C), D1536N (4746G>A), E1539D (4757G>T), Q1541K (4761C>A), H1563Q (4829C>A), M1564L (4830A>C 4832G>A), I1573V (4857A>G 4859T>G), P1583V (4887C>G 4888C>T 4889A>T), T1588I (4903C>T 4904T>C), Q1589T (4905C>A 4906A>C 4907G>T), M1592Y (4914A>T 4915T>A 4916G>T), I1599V (4935A>G), H1604Q (4952T>G), R1607Q (4960G>A 4961T>G), A1609V (4966C>T), K1612T (4975A>C 4976G>A), V1614T (4980G>A 4981T>C 4982C>A), L1615D (4983T>G 4984T>A 4985A>C), D1616N (4986G>A), T1619K (4996C>A 4997G>A), L1621I (5001T>A 5003G>A), D1622M (5004G>A 5005A>T 5006T>G), E1623Q (5007G>C 5009A>G), F1626L (5018C>G), R1627P (5019A>C 5020G>C), A1628I (5022G>A 5023C>T 5024T>C), I1630H (5028A>C 5029T>A 5030C>T), L1635A (5043T>G 5044T>C 5045G>T), V1637Q (5049G>C 5050T>A 5051G>A), Q1639S (5055C>A 5056A>G 5057G>T), I1642L (5064A>C 5066T>A), A1643Q (5067G>C 5068C>A), K1644A (5070A>G 5071A>C 5072G>C), S1650N (5089G>A 5090T>C), A1651I (5091G>A 5092C>T 5093C>A), L1652G (5094C>G 5095T>G), T1653A (5097A>G 5099A>T), I1654L (5100A>C 5102T>G), C1655L (5103T>C 5104G>T 5105T>G), M1657L (5109A>C), A1658S (5112G>A 5113C>G 5114A>C), A1660D (5119C>A 5120A>T), S1663T (5127T>A 5129C>T), T1665V (5133A>G 5134C>T), D1668P (5142G>C 5143A>C 5144C>A), A1669E (5146C>A), E1671K (5151G>A 5153G>A), T1674E (5160A>G 5161C>A), N1675D (5163A>G), E1677S (5169G>T 5170A>C 5171G>A), R1678Q (5173G>A 5174T>A), D1679G (5176A>G), L1680V (5178C>G 5180G>C), N1681H (5181A>C), W1682E (5184T>G 5185G>A 5186G>A), A1684C (5190G>T 5191C>G 5192A>T), P1685S (5193C>T 5195A>T), N1686G (5196A>G 5197A>G 5198C>A), I1687V (5199A>G), G1688F (5202G>T 5203G>T 5204T>C), A1692S (5214G>T 5216A>G), A1695E (5224C>A), A1696V (5227C>T 5228C>T), H1697N (5229C>A 5231C>T), Y1698H (5232T>C 5234C>T), T1699K (5236C>A 5237G>A), G1703D (5248G>A 5249G>C), Y1704T (5250T>A 5251A>C 5252C>A), I1706L (5256A>C), S1715T (5283T>A 5285C>A), N1716T (5287A>C 5288T>A), S1737A (5349T>G), M1740K (5359T>A), A1742N (5364G>A 5365C>A 5366C>T), K1744Q (5370A>C 5372G>A), N1745S (5374A>G), V1746R (5376G>C 5377T>G 5378G>C), A1748S (5382G>T), G1749M (5385G>A 5386G>T 5387A>G), H1751I (5391C>A 5392A>T), V1752G (5395T>G), A1753T (5397G>A 5399A>C), T1754Q (5400A>C 5401C>A 5402C>A), N1755K (5405T>A), V1759I (5415G>A 5417G>T), G1767S (5439G>A 5441C>T), M1774F (5460A>T 5462G>T), A1777S (5469G>T), R1779N (5476G>A 5477G>T), C1782R (5484T>A 5486T>A), V1783A (5488T>C 5489G>T), D1785Q (5493G>C 5495C>A), I1786L (5496A>C 5498A>T), I1793L (5517A>T 5519T>G), G1794A (5521G>C 5522A>G), N1795G (5523A>G 5524A>G 5525T>C), V1796L (5526G>T), T1801Q (5541A>C 5542C>A 5543C>G), V1807I (5559G>A 5561G>T), Q1812T (5574C>A 5575A>C), C1813_V1814insV (5579_5580insGTC), P1815Q (5584C>A 5585T>G), H1816K (5586C>A 5588T>A), G1817S (5589G>A 5591A>T), D1818C (5592G>T 5593A>G), V1820T (5598G>A 5599T>C 5600G>A), S1823A (5607A>G 5608G>C), G1824S (5610G>A), A1825G (5614C>G 5615A>G), S1826del (5616_5618delTCG), L1827R (5620T>G), H1828D (5622C>G 5624C>T), S1829G (5625A>G 5627T>A), S1830M (5629G>T 5630T>G), F1831L (5633T>G), M1832N (5635T>A 5636G>C), Q1834H (5642A>T), Q1834_G1835insHA (5642_5643insCATGCT), G1835A (5644G>C 5645G>T), F1836Y (5647T>A), E1837A (5650A>C 5651A>C), I1844V (5670A>G 5672A>G), H1845G (5673C>G 5674A>G 5675T>C), G1846C (5676G>T), D1847S (5679G>T 5680A>C 5681T>A), L1848I (5682T>A 5684A>T), A1849S (5685G>T), K1850R (5689A>G), Q1851K (5691C>A), T1852M (5695C>T 5696A>G), M1854V (5700A>G), M1856L (5706A>T), V1859T (5715G>A 5716T>C 5717G>T), H1863M (5727C>A 5728A>T 5729T>G), G1867A (5740G>C), N1868D (5742A>G), F1869C (5746T>G 5747T>C), Q1872R (5754C>A 5755A>G 5756G>A), D1873N (5757G>A), E1875D (5765G>T), L1878I (5772C>A 5774A>C), H1879K (5775C>A 5777C>A), Y1880A (5778T>G 5779A>C 5780T>C), Q1883C (5787C>T 5788A>G 5789G>C), G1884S (5790G>T 5791G>C 5792A>T), E1887A (5800A>C 5801G>A), L1888C (5803T>G 5804A>T), I1889V (5805A>G 5807T>G), Q1890A (5808C>G 5809A>C 5810A>T), K1891R (5811A>C 5812A>G 5813G>T), N1892G (5814A>G 5815A>G 5816T>G), T1893C (5817A>T 5818C>G 5819A>T), L1895I (5823C>A), K1900E (5838A>G 5840A>G), E1901N (5841G>A 5843G>T), L1902F (5844C>T 5846G>T), S1903N (5848G>A), M1904A (5850A>G 5851T>C 5852G>A), E1905Q (5853G>C), N1906I (5857A>T 5858T>A), V1907A (5860T>C 5861G>T), Q1908T (5862C>A 5863A>C 5864A>C), Q1909R (5865C>A 5866A>G 5867A>G), I1911V (5871A>G), V1914L (5880G>C), L1916S (5886C>A 5887T>G), F1919V (5895T>G 5897T>G), P1921I (5901C>A 5902C>T 5903A>C), K1922T (5905A>C), E1923A (5908A>C 5909A>C), T1925G (5913A>G 5914C>G), I1926H (5916A>C 5917T>A 5918T>C), Q1927K (5919C>A 5921G>A), K1928M (5923A>T), K1930V (5928A>G 5929A>T 5930A>G), N1931Q (5931A>C 5933T>A), F1932T (5934T>A 5935T>C), V1933L (5937G>C 5939T>G), D1934S (5940G>A 5941A>G 5942T>C), A1935N (5943G>A 5944C>A), E1936Q (5946G>C), C1937W (5951T>G), L1938Q (5953T>A), T1939S (5955A>T 5957A>C), F1940H (5958T>C 5959T>A), K1941E (5961A>G), A1942Q (5964G>C 5965C>A), R1943S (5969G>T), S1944C (5970A>T 5972C>T), F1945V (5973T>G 5975T>G), R1946K (5977G>A), H1947R (5979C>A 5980A>G 5981C>A), V1949Y (5985G>T 5986T>A), K1952R (5994A>C 5995A>G 5996G>A), K1953N (5999A>T), S1957N (6009T>A 6010C>A), T1958A (6012A>G), W1959I (6015T>A 6016G>T 6017G>A), K1960T (6019A>C), M1961_C1962insY (6023_6024insTAC), C1962R (6024T>C), E1964D (6032A>T), S1965A (6033A>G 6034G>C 6035T>C), I1966K (6037T>A 6038T>A), V1967D (6040T>A), S1969I (6045T>A 6046C>T), R1970V (6048A>G 6049G>T 6050G>A), A1972del (6054_6056delGCA), L1975V (6063C>G 6065A>G), P1977L (6069C>T 6070C>T 6071T>G), L1980P (6078T>C 6079T>C 6080G>A), S1983M (6087T>A 6088C>T 6089C>G), K1986R (6097A>G 6098A>G)

	Begin	End	Coverage	Score	Concordance	Matches	Identities	I/D/M/F*	Stop Codons
**NT**	**24**	**6107**	**84.2%**	**3417**	**28.3%**	**6063 (98.8%)**	**3936 (64.1%)**	**54/21**	

ATG1CTG (141A>C), GCT2GTT (145C>T), TTT3TTC (149T>C), TCA4CAC (150T>C 151C>A 152A>C), AAG5AAT (155G>T), ATG6ACC (157T>C 158G>C), TTC7ACC (159T>A 160T>C), TCC8ATT (162T>A 163C>T 164C>T), AAG9CAA (165A>C 167G>A), GTT10ATG (168G>A 170T>G), GCT11GCA (173T>A), GGT12del (174_176delGGT), GTT13TTT (177G>T), GAT14TCA (180G>T 181A>C 182T>A), GAC15AAA (183G>A 185C>A), GCC16ATC (186G>A 187C>T), AGT17GTT (189A>G 190G>T), TCC20GCT (198T>G 200C>T), ACC21ACT (203C>T), ACT22CCT (204A>C), TCA23TCT (209A>T), TTC24TTT (212C>T), TTT25CTT (213T>C), GGT26TCT (216G>T 217G>C), GAA27GAG (221A>G), CTT28TTG (222C>T 224T>G), TCA29TCT (227A>T), AAT30CAG (228A>C 230T>G), TCT31ACA (231T>A 233T>A), ATT32GTG (234A>G 236T>G), TCC33TCA (239C>A), AAA34TCA (240A>T 241A>C), TTT35CTG (243T>C 245T>G), TCA36TCC (248A>C), AAA37AAG (251A>G), GCG38ACA (252G>A 254G>A), GTT39ATG (255G>A 257T>G), AGT40AAG (259G>A 260T>G), AAT41AGT (262A>G), ATT42ATG (266T>G), ACC43AGT (268C>G 269C>T), ACT44AAA (271C>A 272T>A), ATG45ATA (275G>A), TCA46TCT (278A>T), CAG47GAG (279C>G), AAG48AAA (284G>A), ATT49TTC (285A>T 287T>C), GAT51GGC (292A>G 293T>C), CTT53ATT (297C>A), GAG54GAA (302G>A), GAT55GAA (305T>A), CTG56TTA (306C>T 308G>A), CCA58CCT (314A>T), TCT59TCA (317T>A), GTG60ATT (318G>A 320G>T), ACT61GTT (321A>G 322C>T), GAT62AAA (324G>A 326T>A), GTG63ACT (327G>A 328T>C 329G>T), TCC64GCA (330T>G 332C>A), ACA65GAT (333A>G 334C>A 335A>T), TCT66TCC (338T>C), GTG68AAT (342G>A 343T>A 344G>T), GCC69AGT (345G>A 346C>G 347C>T), ACT70GTT (348A>G 349C>T), TCA73TCT (359A>T), CTT74GTA (360C>G 362T>A), AAT75GAA (363A>G 365T>A), GTT77TTA (369G>T 371T>A), TTG78TTA (374G>A), GAC79GGG (376A>G 377C>G), GGT82GCC (385G>C 386T>C), GCA83ACG (387G>A 389A>G), CTT84GTT (390C>G), ATA85ATT (395A>T), GAT86GAA (398T>A), CCA87CCT (401A>T), CTT89GTT (405C>G), GCG91GGT (412C>G 413G>T), TAC92ATC (414T>A 415A>T), TCT93AAT (417T>A 418C>A), TTG95TTC (425G>C), TCC96AAA (426T>A 427C>A 428C>A), TAC99TAT (437C>T), AAA100AGA (439A>G), TCT101ACC (441T>A 443T>C), ATC102ATA (446C>A), AAG103ATT (448A>T 449G>T), TTA107CTG (459T>C 461A>G), TTG109GTT (465T>G 467G>T), TTT110GTT (468T>G), GAG111TTG (471G>T 472A>T), AGT112GGC (474A>G 476T>C), TTG113ATC (477T>A 479G>C), TCC114AAA (480T>A 481C>A 482C>A), AAA116AAT (488A>T), GTT117TTT (489G>T), AAA118ATT (493A>T 494A>T), TTA119AGA (495T>A 496T>G), TTT121TAT (502T>A), GCT122TCA (504G>T 506T>A), GTG124CTG (510G>C), TTA125TTG (515A>G), AAT126GAA (516A>G 518T>A), AAA127ACA (520A>C), AGT128ACA (523G>C 524T>A), GAA129GGT (526A>G 527A>T), GAT130GAA (530T>A), GTA131TAT (531G>T 532T>A 533A>T), ATA133GTT (537A>G 539A>T), GTT134GTA (542T>A), GTA135GTT (545A>T), TAT136ATA (546T>A 547A>T 548T>A), GGT137GCT (550G>C), CTT139CTA (557T>A), GTA140GTC (560A>C), GCT142TCC (564G>T 566T>C), ATA143CTT (567A>C 569A>T), ACA144TCA (570A>T), TTG145CTA (573T>C 575G>A), TTA146TTG (578A>G), GTG147TTA (579G>T 581G>A), CTT148TTA (582C>T 584T>A), CTA149CAA (586T>A), TTT150ATA (588T>A 590T>A), GTT151ATC (591G>A 593T>C), AGT154GTA (600A>G 601G>T 602T>A), GTT157GTA (611T>A), GAG158GAA (614G>A), GGT159ATG (615G>A 616G>T 617T>G), TCT161AGA (621T>A 622C>G 623T>A), AAC162AGT (625A>G 626C>T), ACT163GTA (627A>G 628C>T 629T>A), GTC164GTT (632C>T), AAA165TGT (633A>T 634A>G 635A>T), ATC167ATA (641C>A), TTG169CTA (645T>C 647G>A), ATA170GTT (648A>G 650A>T), GTC171GTA (653C>A), GGA172GGT (656A>T), ATG174TTC (660A>T 662G>C), TTT175TTC (665T>C), TCA176TCT (668A>T), AGT177TCT (669A>T 670G>C), CTA178CTG (674A>G), TAC179TAT (677C>T), TTA181ATT (681T>A 683A>T), GAT182AGT (684G>A 685A>G), CCA185CCC (695A>C), AAA186CTC (696A>C 697A>T 698A>C), GCC188TCT (702G>T 704C>T), GAA189GAG (707A>G), CGA190AAA (708C>A 709G>A), TTC191TTT (713C>T), ACC192TCT (714A>T 716C>T), ATG193CAG (717A>C 718T>A), GTG194ATG (720G>A), GCC195GCA (725C>A), CAA196CAG (728A>G), AGT198TCT (732A>T 733G>C), ATG199GTG (735A>G), CTT200CTA (740T>A), GGG202GGT (746G>T), GAA203GAG (749A>G), TCA204AGC (750T>A 751C>G 752A>C), GTT205TTG (753G>T 755T>G), TCA206AAT (756T>A 757C>A 758A>T), ACG210ACC (770G>C), TCT211AGT (771T>A 772C>G), CAG212CAA (776G>A), ATG214CTG (780A>C), TCC215TCT (785C>T), ACA216TCT (786A>T 788A>T), ATA217TTG (789A>T 791A>G), TTG218ATA (792T>A 794G>A), GCT219GCA (797T>A), GGG221GGC (803G>C), ATT222ATC (806T>C), TCG223TCA (809G>A), GTG226GTA (818G>A), TTC227TTT (821C>T), ATT228GTG (822A>G 824T>G), GTG230GTA (830G>A), AGA233AGG (839A>G), TTG237CTT (849T>C 851G>T), AGC238ACA (853G>C 854C>A), CCA240CCG (860A>G), TTG241CTA (861T>C 863G>A), CTC245TTG (873C>T 875C>G), TCG247TCA (881G>A), ACA248GCG (882A>G 884A>G), GGA249GGC (887A>C), GGT251GGA (893T>A), CAG253CAA (899G>A), CTA258CTC (914A>C), ACT260CAA (918A>C 919C>A 920T>A), CTT261TTG (921C>T 923T>G), TAC262TAT (926C>T), CGT263AAG (927C>A 928G>A 929T>G), AAT264AAC (932T>C), AAA266AAG (938A>G), ACC269ACA (947C>A), TCA274TCT (962A>T), GTT276GTG (968T>G), CTT277TTG (969C>T 971T>G), ATA279ATT (977A>T), ATA280GTT (978A>G 980A>T), GTC281GCT (982T>C 983C>T), GGC282GAT (985G>A 986C>T), ACC283ATA (988C>T 989C>A), TTT284TTC (992T>C), GGA285GGG (995A>G), TTC286TTT (998C>T), AAA287AAG (1001A>G), AAT288AAC (1004T>C), GTG290GTT (1010G>T), TTG291CTG (1011T>C), TCA292TCT (1016A>T), GCT293GCA (1019T>A), ATA294ATT (1022A>T), ACG297ACA (1031G>A), CTG298ATT (1032C>A 1034G>T), CTG302CTC (1046G>C), TTC303TTT (1049C>T), CAG307ACT (1059C>A 1060A>C 1061G>T), GAG308GAA (1064G>A), GAT310TCT (1068G>T 1069A>C), TGT313GCT (1077T>G 1078G>C), ACG317ACA (1091G>A), CGC318AGG (1092C>A 1094C>G), GAG320GAA (1100G>A), GCC323GCT (1109C>T), AAG325CCT (1113A>C 1114A>C 1115G>T), TTC327TTT (1121C>T), CCT328TCA (1122C>T 1124T>A), ACC329ATC (1126C>T), CTG334CTT (1142G>T), AGG335AAA (1144G>A 1145G>A), GAA336GAG (1148A>G), GAA337CAG (1149G>C 1151A>G), GCT338GCA (1154T>A), ATC341ATA (1163C>A), GCT343GCC (1169T>C), TAC344TAT (1172C>T), GCA346GCT (1178A>T), CCA349CCT (1187A>T), GTT350GTG (1190T>G), TTC353TTT (1199C>T), ATG354TTG (1200A>T), AGC355AGT (1205C>T), AGG357AGA (1211G>A), AGT359TCT (1215A>T 1216G>C), GCC361ACA (1221G>A 1223C>A), GCG363GTG (1228C>T), CAG364CAA (1232G>A), TTA365TTG (1235A>G), GAA366GAT (1238A>T), GTT368CTC (1242G>C 1244T>C), TAT369ATA (1245T>A 1246A>T 1247T>A), GCT370GCA (1250T>A), AGT372AAC (1255G>A 1256T>C), AGG374CAA (1260A>C 1261G>A 1262G>A), CAC375CAT (1265C>T), ATG376ACA (1267T>C 1268G>A), GGC377GGA (1271C>A), GGC379GGT (1277C>T), TAT381TAC (1283T>C), CGT382AGA (1284C>A 1286T>A), CCT385CCC (1295T>C), GTA388GTT (1304A>T), TTC391TTT (1313C>T), GGG392GGT (1316G>T), TCC393GAG (1317T>G 1318C>A 1319C>G), GGG395GGA (1325G>A), TGT396TGC (1328T>C), GGT397GGC (1331T>C), TCT399TCC (1337T>C), ACA400ACG (1340A>G), TCC401AGC (1341T>A 1342C>G), CGC403CGA (1349C>A), TTA404CTG (1350T>C 1352A>G), TTT405TTC (1355T>C), ATT406ATC (1358T>C), GTG409GTA (1367G>A), GAC411GAT (1373C>T), AGA412GCC (1374A>G 1375G>C 1376A>C), GGT414CAA (1380G>C 1381G>A 1382T>A), CCA416CCC (1388A>C), AAA417AAG (1391A>G), CTC418CTG (1394C>G), AAT419AAC (1397T>C), CGG420AGG (1398C>A), CTA421TTG (1401C>T 1403A>G), GTA424GTT (1412A>T), AGG427CGC (1419A>C 1421G>C), GCA429GCT (1427A>T), TAT430TAC (1430T>C), TCC432TCA (1436C>A), AAC433AAT (1439C>T), CTG441CTC (1463G>C), GAC444GAT (1472C>T), GGA446GGT (1478A>T), TTG448CTG (1482T>C), CGA449CGG (1487A>G), GGG451GGA (1493G>A), GGG453GGC (1499G>C), CAG454CAA (1502G>A), CAA456CAG (1508A>G), GAT457GAC (1511T>C), AAA459AAG (1517A>G), GAT460GAC (1520T>C), CTG461CTC (1523G>C), GAT463GAC (1529T>C), TCA466TCC (1538A>C), CCC471CCA (1553C>A), TTA472TTG (1556A>G), CCA473CCC (1559A>C), GCC475GCT (1565C>T), GTT477GTA (1571T>A), GAG478GAA (1574G>A), GGC481GGG (1583C>G), AGG482CGG (1584A>C), ACC485ACA (1595C>A), TCC486TCA (1598C>A), TAT488TAC (1604T>C), ACA492ACC (1616A>C), AAT494AAC (1622T>C), CTG495TTG (1623C>T), ATC496GTT (1626A>G 1628C>T), GCT498GCA (1634T>A), CCT499CCA (1637T>A), GCC500GCA (1640C>A), TGT502TGC (1646T>C), GGT503GGG (1649T>G), CTT504CTG (1652T>G), CCA507CCT (1661A>T), GAG511GAA (1673G>A), CGG512AGG (1674C>A), AGA513AGG (1679A>G), AGG514CGG (1680A>C), ATC516ATT (1688C>T), CTG517TTG (1689C>T), GAG519GAA (1697G>A), TGT520TGC (1700T>C), ATG521CGG (1701A>C 1702T>G), AAG522AAA (1706G>A), GAG523GTG (1708A>T), GGC524GGG (1712C>G), GAG525GAA (1715G>A), TTT526TTC (1718T>C), TCC527TCA (1721C>A), ACT528ACA (1724T>A), GAA529GAG (1727A>G), GAT530AAC (1728G>A 1730T>C), CCT531CCA (1733T>A), ACG532ACA (1736G>A), GGA533GCA (1738G>C), AGA536AGC (1748A>C), TTT537TTC (1751T>C), ATA539ATC (1757A>C), GTG540ATT (1758G>A 1760G>T), GAG541GGA (1762A>G 1763G>A), AGA543AGG (1769A>G), AGA544AGG (1772A>G), GCT547GCC (1781T>C), ATC548ATA (1784C>A), ACT549ACA (1787T>A), AGG551AGA (1793G>A), AAT552GAC (1794A>G 1796T>C), TTG553CTC (1797T>C 1799G>C), ACC554ACT (1802C>T), AGT556TCT (1806A>T 1807G>C), GAT557GAC (1811T>C), CTT558TTG (1812C>T 1814T>G), GAG560GAA (1820G>A), GTA563GTG (1829A>G), AAA565AAG (1835A>G), TGT566TGC (1838T>C), CAG567AAG (1839C>A), GTA568GTC (1844A>C), CAT569CAC (1847T>C), GCA570CAC (1848G>C 1849C>A 1850A>C), GTA572ATT (1854G>A 1856A>T), TCA573GCA (1857T>G), AAG574ACC (1861A>C 1862G>C), TTG576CTC (1866T>C 1868G>C), TTT577TAC (1870T>A 1871T>C), GAA578AAC (1872G>A 1874A>C), GCT579CAG (1875G>C 1876C>A 1877T>G), GAA580TCA (1878G>T 1879A>C), TCT581ACA (1881T>A 1883T>A), GGC582GGT (1886C>T), ATA583TGT (1887A>T 1888T>G 1889A>T), AGT584GAA (1890A>G 1891G>A 1892T>A), CCT585GAG (1893C>G 1894C>A 1895T>G), AAG586AAA (1898G>A), ATA587GTT (1899A>G 1901A>T), GCT588GCC (1904T>C), GTT590GTG (1910T>G), CAA591GAA (1911C>G), GCA594GTT (1921C>T 1922A>T), GAT595GAA (1925T>A), GAT596GAA (1928T>A), ATA598ATG (1934A>G), GCG599ACA (1935G>A 1937G>A), TCT600TCC (1940T>C), GTG601TTT (1941G>T 1943G>T), GAT602CAT (1944G>C), GGG603GAT (1948G>A 1949G>T), AGA605CAA (1953A>C 1954G>A), TTG606ATA (1956T>A 1958G>A), CGC607AGC (1959C>A), ACC608TTT (1962A>T 1963C>T 1964C>T), AAG609GAC (1965A>G 1967G>C), CAA610GAA (1968C>G), GAT611GTT (1972A>T), GAA612AGC (1974G>A 1975A>G 1976A>C), CCA613CCC (1979A>C), ATA614AGT (1981T>G 1982A>T), TTG615GTT (1983T>G 1985G>T), GTG616_CCC617del (1986_1991delGTGCCC), ACT618ACA (1994T>A), GTG620CTC (1998G>C 2000G>C), AGT621TCT (2001A>T 2002G>C), GAA622GAG (2006A>G), AAT623GAT (2007A>G), ATC627GGA (2019A>G 2020T>G 2021C>A), GCA629TCA (2025G>T), AGA630CAG (2028A>C 2029G>A 2030A>G), GAG631GAA (2033G>A), CTC632CTA (2036C>A), ACG633ACT (2039G>T), GTA634GTG (2042A>G), GAG635GAA (2045G>A), TTG637CTG (2049T>C), AAA638AAG (2054A>G), GCA640GCT (2060A>T), TAC641TAT (2063C>T), CAG642CAA (2066G>A), GGC643GGA (2069C>A), AGT644GCA (2070A>G 2071G>C 2072T>A), TTG645TTA (2075G>A), GAC646AAC (2076G>A), CCT647CCA (2081T>A), GAG648TTT (2082G>T 2083A>T 2084G>T), CTT650TAT (2088C>T 2089T>A), CCT652CCC (2096T>C), GAC654GAT (2102C>T), CAT655GCT (2103C>G 2104A>C), CAT656TCA (2106C>T 2107A>C 2108T>A), GCC659GCA (2117C>A), ATG660ATT (2120G>T), TAT661TTT (2122A>T), TCT663GTC (2127T>G 2128C>T 2129T>C), CTT664CTG (2132T>G), GAT665TCA (2133G>T 2134A>C 2135T>A), GAT666AGT (2136G>A 2137A>G), CAT668GCC (2142C>G 2143A>C 2144T>C), ATT671AGC (2152T>G 2153T>C), TTC672TTT (2156C>T), AAC673ACT (2158A>C 2159C>T), AAA674CGC (2160A>C 2161A>G 2162A>C), AGA676GTA (2166A>G 2167G>T), GTC677AAT (2169G>A 2170T>A 2171C>T), AAC678GAG (2172A>G 2174C>G), TTG680ATT (2178T>A 2180G>T), TAT681TAC (2183T>C), AGA682GAG (2184A>G 2185G>A 2186A>G), GGA683GGG (2189A>G), GCA684GCT (2192A>T), GAT685TCG (2193G>T 2194A>C 2195T>G), GCT686ACT (2196G>A), GAA687GCA (2200A>C), CAG688GGA (2202C>G 2203A>G 2204G>A), CGA690GCC (2208C>G 2209G>C 2210A>C), GTA693ATC (2217G>A 2219A>C), CAA694AAG (2220C>A 2222A>G), AAC695GCC (2223A>G 2224A>C), ATT696ATC (2228T>C), CCT697CCA (2231T>A), ATA701GTC (2241A>G 2243A>C), AAG705AAA (2255G>A), AGT706AGC (2258T>C), GCA710AGT (2268G>A 2269C>G 2270A>T), TCA711ACA (2271T>A), ATC713GTG (2277A>G 2279C>G), TCT714TCA (2282T>A), GAG715GAA (2285G>A), AAG716GCT (2286A>G 2287A>C 2288G>T), AAA717CAC (2289A>C 2291A>C), CTA718ATG (2292C>A 2294A>G), GCA719GAT (2296C>A 2297A>T), GTG720GTA (2300G>A), CAG721CAA (2303G>A), ACA722AGG (2305C>G 2306A>G), GAG723ATG (2307G>A 2308A>T), ATG724TTG (2310A>T), AGG725TAC (2313A>T 2314G>A 2315G>C), ACT726AAT (2317C>A), GGA727GGC (2321A>C), TTT728AGA (2322T>A 2323T>G 2324T>A), TCT731AGA (2331T>A 2332C>G 2333T>A), TGC732GCA (2334T>G 2335G>C 2336C>A), ATT733CTG (2337A>C 2339T>G), GAT734GAA (2342T>A), GCA735GCT (2345A>T), GTT737GCT (2350T>C), ACC739ACT (2357C>T), CTC740TTA (2358C>T 2360C>A), ATA741ATG (2363A>G), ATC743ATT (2369C>T), GAG744GAA (2372G>A), CAG745CAA (2375G>A), CCA747CCT (2381A>T), TTT749CAT (2385T>C 2386T>A), GTT750GTC (2390T>C), CAG751CAA (2393G>A), TTT752TTC (2396T>C), TTA753CTA (2397T>C), TAT754TAC (2402T>C), AGA759CGG (2415A>C 2417A>G), TGC762_AAC763insAGAAGG (2426_2427insAGAAGG), AAC763AAG (2429C>G), AAA764ATG (2431A>T 2432A>G), GTA765GTC (2435A>C), TCA766ACT (2436T>A 2438A>T), AAA767AAG (2441A>G), GAG768GAA (2444G>A), CCA769ACA (2445C>A), AAG770_GAA771del (2448_2453delAAGGAA), TCA772TCT (2456A>T), ATC774GTA (2460A>G 2462C>A), AAA775CAA (2463A>C), ATT776TCG (2466A>T 2467T>C 2468T>G), TGC777CTC (2469T>C 2470G>T), TAC778TAT (2474C>T), CAT779GAC (2475C>G 2477T>C), AAA780TCC (2478A>T 2479A>C 2480A>C), ATC781ACA (2482T>C 2483C>A), GTT782ATT (2484G>A), GAA783GGG (2488A>G 2489A>G), TAT784AGA (2490T>A 2491A>G 2492T>A), ATC785ATG (2495C>G), AAG786CAA (2496A>C 2498G>A), GAT787GAA (2501T>A), ACA788ATG (2503C>T 2504A>G), TGG790AAT (2508T>A 2509G>A 2510G>T), AGC791GAT (2511A>G 2512G>A 2513C>T), CTT792CTG (2516T>G), TAT794TAC (2522T>C), GCA795ATT (2523G>A 2524C>T 2525A>T), CTG796TTG (2526C>T), AGA797AAA (2530G>A), CTC799CTT (2537C>T), GGT803GGG (2549T>G), ATA806CTA (2556A>C), ATG807ATA (2561G>A), CTT809CTC (2567T>C), GGA811GGG (2573A>G), GTT812ACT (2574G>A 2575T>C), TTT813ATG (2577T>A 2579T>G), GGA814GGT (2582A>T), GGC815GCC (2584G>C), ATT816GTA (2586A>G 2588T>A), ACA817ACT (2591A>T), TTT819TTC (2597T>C), TTG820ATT (2598T>A 2600G>T), GCG821TCT (2601G>T 2603G>T), CAG824AAA (2610C>A 2612G>A), AGT825AGC (2615T>C), AAC826AGC (2617A>G), TCT827TTG (2620C>T 2621T>G), TTC828ATT (2622T>A 2624C>T), AAT830ACT (2629A>C), GCA831TCC (2631G>T 2633A>C), TCG832CAG (2634T>C 2635C>A), AGA834AAA (2641G>A), GTG837GTT (2651G>T), TCC841TCG (2663C>G), AGT843AGC (2669T>C), TCC845TCA (2675C>A), AGT846AGC (2678T>C), GGT851GGA (2693T>A), AAT852AGA (2695A>G 2696T>A), AAT852_AAG853insTCC (2696_2697insTCC), AAG853CAT (2697A>C 2699G>T), CTC854GTA (2700C>G 2702C>A), AGA855TCA (2703A>T 2704G>C), AAT856AAG (2708T>G), CTT857ATG (2709C>A 2711T>G), CTC858CTG (2714C>G), GTT859TTT (2715G>T), GGT860AGC (2718G>A 2720T>C), TCT863TCA (2729T>A), TCA865CCA (2733T>C), TTG866AAT (2736T>A 2737T>A 2738G>T), GCA867GAT (2740C>A 2741A>T), CAA868CAG (2744A>G), CAA868_GAT869insGGA (2744_2745insGGA), GAT869AAA (2745G>A 2747T>A), GCT872TCC (2754G>T 2756T>C), GAA873AGG (2757G>A 2758A>G 2759A>G), GGA875GGT (2765A>T), GTA877GTG (2771A>G), AAT878AAC (2774T>C), CAG879GAA (2775C>G 2777G>A), AGC880TCT (2778A>T 2779G>C 2780C>T), CTC881CTT (2783C>T), AAG882GCC (2784A>G 2785A>C 2786G>C), GTG886GTA (2798G>A), TTA888CTA (2802T>C), AGA889AGG (2807A>G), CTT890GTG (2808C>G 2810T>G), GGT891GCA (2812G>C 2813T>A), GTG894GTT (2822G>T), AGA897CGT (2829A>C 2831A>T), GGC898GGA (2834C>A), ACC899ACT (2837C>T), TGT902TGC (2846T>C), TCT903AGC (2847T>A 2848C>G 2849T>C), GGT904GAA (2851G>A 2852T>A), ACC908ACT (2864C>T), GCT910GCA (2870T>A), CAT911CAC (2873T>C), GCA912GCT (2876A>T), CAC914ACG (2880C>A 2881A>C 2882C>G), AAT915CAG (2883A>C 2885T>G), GCT916CTC (2886G>C 2887C>T 2888T>C), CGA917CGT (2891A>T), GGT919GGA (2897T>A), ACT920ACC (2900T>C), ACA923TCA (2907A>T), ATC925ATT (2915C>T), GCC927GAA (2920C>A 2921C>A), AAT928CAT (2922A>C), CGA930GTC (2928C>G 2929G>T 2930A>C), TCT931TGC (2932C>G 2933T>C), AAA932CAG (2934A>C 2936A>G), GTT933GTG (2939T>G), AAC936AAT (2948C>T), AGA938AAA (2953G>A), GAA939CAT (2955G>C 2957A>T), TCA940GCC (2958T>G 2960A>C), AGG941AAG (2962G>A), ATT942TTT (2964A>T), ATT943TTG (2967A>T 2969T>G), GAG944CCT (2970G>C 2971A>C 2972G>T), GGC945GAA (2974G>A 2975C>A), GAC947GAT (2981C>T), ATC948GTT (2982A>G 2984C>T), ATT949GTA (2985A>G 2987T>A), CTG950TTA (2988C>T 2990G>A), TTG951CTG (2991T>C), CGC952AGG (2994C>A 2996C>G), GGT954GGC (3002T>C), GAT955TGT (3003G>T 3004A>G), CCA956CCT (3008A>T), GAT957GAC (3011T>C),

Codon mutations:

GGT958GGG (3014T>G), CCA959CCT (3017A>T), AAG960AAA (3020G>A), CCT961GTC (3021C>G 3022C>T 3023T>C), CGT964AGA (3030C>A 3032T>A), AAA965AAG (3035A>G), TTC967TTT (3041C>T), CCA968GCT (3042C>G 3044A>T), AGA969GAA (3045A>G 3046G>A), GAG972GCA (3055A>C 3056G>A), AAG977AAA (3071G>A), GGC978GGA (3074C>A), TCA979TCT (3077A>T), CAA980GAA (3078C>G), GGA981GGC (3083A>C), TGC983TGT (3089C>T), TGT984GTC (3090T>G 3091G>T 3092T>C), GCT986GCA (3098T>A), GTT987GTG (3101T>G), GCG988GCA (3104G>A), TCT989TCA (3107T>A), ACA990AAA (3109C>A), CCA992CCT (3116A>T), CGT993GAG (3117C>G 3118G>A 3119T>G), CTT994AAA (3120C>A 3121T>A 3122T>A), GGC995CAG (3123G>C 3124G>A 3125C>G), AAT996AAC (3128T>C), TTA997TTG (3131A>G), GAG998GAC (3134G>C), ATG1002TAT (3144A>T 3145T>A 3146G>T), CCA1003TCA (3147C>T), TCA1007TCC (3161A>C), AAG1008AAA (3164G>A), TAC1010TAT (3170C>T), GTT1012GAG (3175T>A 3176T>G), GTG1014GTC (3182G>C), GAA1015ACA (3183G>A 3184A>C), TAT1016TAC (3188T>C), GAA1017AGG (3189G>A 3190A>G 3191A>G), CTG1018ATG (3192C>A), AAC1019AGC (3196A>G), TCC1020ACA (3198T>A 3200C>A), TCC1021AAT (3201T>A 3202C>A 3203C>T), AGT1022TCA (3204A>T 3205G>C 3206T>A), AAG1024CAC (3210A>C 3212G>C), AAG1024_ATT1025insTCA (3212_3213insTCA), ATT1025ATC (3215T>C), TGC1026AGT (3216T>A 3218C>T), TCT1027GCT (3219T>G), CAA1029GAA (3225C>G), TCT1030GCC (3228T>G 3230T>C), CAC1034CAT (3242C>T), ATA1035GTC (3243A>G 3245A>C), GGG1037AGC (3249G>A 3251G>C), GAC1041GAT (3263C>T), GGC1043GGA (3269C>A), ACG1044ACT (3272G>T), CTG1047TTA (3279C>T 3281G>A), TTA1048TTG (3284A>G), AGT1050TCT (3288A>T 3289G>C), GTT1051ATC (3291G>A 3293T>C), CAG1052CAA (3296G>A), AAT1053AAC (3299T>C), CCT1056CCA (3308T>A), GTG1057TGC (3309G>T 3310T>G 3311G>C), ATC1058ATT (3314C>T), GGC1060GGT (3320C>T), ATC1061ATT (3323C>T), CAC1062CAT (3326C>T), GCA1064GCT (3332A>T), TCA1065TCC (3335A>C), GGC1068GAA (3343G>A 3344C>A), GTA1069GCT (3346T>C 3347A>T), GCT1071ACA (3351G>A 3353T>A), GAA1072GAG (3356A>G), CGT1073TTG (3357C>T 3358G>T 3359T>G), ATC1076GTA (3366A>G 3368C>A), TCT1077GCA (3369T>G 3371T>A), GCT1080GCC (3380T>C), ACA1081ACT (3383A>T), ATC1083GTA (3387A>G 3389C>A), GAA1086GAC (3398A>C), CAA1087CAG (3401A>G), CTA1088CTG (3404A>G), GAG1089CAA (3405G>C 3407G>A), CCA1092GCT (3414C>G 3416A>T), ACT1093CAA (3417A>C 3418C>A 3419T>A), CCG1095CCA (3425G>A), GTC1096GTT (3428C>T), GCA1098ATG (3432G>A 3433C>T 3434A>G), GCA1099GCC (3437A>C), GTA1101GTG (3443A>G), CGC1102AGA (3444C>A 3446C>A), GAT1104GAC (3452T>C), ATT1105TTG (3453A>T 3455T>G), CTG1106CTC (3458G>C), AAG1107AAA (3461G>A), TCA1108CAT (3462T>C 3463C>A 3464A>T), ATT1109GTT (3465A>G), AGA1110CGA (3468A>C), AGC1111TCA (3471A>T 3472G>C 3473C>A), ACA1114ACT (3482A>T), CAG1115GCT (3483C>G 3484A>C 3485G>T), CTT1116CTC (3488T>C), GAG1118GAC (3494G>C), AAC1120AAG (3500C>G), GTG1122GTT (3506G>T), TAC1123GCT (3507T>G 3508A>C 3509C>T), TAC1124TTT (3511A>T 3512C>T), CTT1125TTG (3513C>T 3515T>G), GGA1126GGC (3518A>C), ACA1127ACT (3521A>T), CCA1129CCT (3527A>T), CAG1130CAA (3530G>A), TTG1132ATG (3534T>A), GCC1133GCA (3539C>A), GCC1134GCA (3542C>A), ACA1135ACG (3545A>G), GTT1136GTG (3548T>G), CCC1137CCA (3551C>A), ACT1141ACC (3563T>C), CTG1142CTC (3566G>C), CGG1143AGG (3567C>A), AGC1145AGT (3575C>T), CAA1146CCA (3577A>C), TTG1147CTG (3579T>C), GAA1149GAG (3587A>G), TTC1151TTT (3593C>T), GGA1152GGT (3596A>T), CCT1153GAA (3597C>G 3598C>A 3599T>A), GAG1155CAA (3603G>C 3605G>A), ACA1156ACT (3608A>T), GCA1157GCT (3611A>T), CCA1158CCT (3614A>T), TCC1159TCA (3617C>A), ATT1160CTG (3618A>C 3620T>G), CTA1161CTC (3623A>C), ACA1162TCT (3624A>T 3626A>T), GTT1163GTG (3629T>G), CAT1164AAA (3630C>A 3632T>A), GAC1165GAT (3635C>T), GGT1168GGG (3644T>G), GGT1170GGA (3650T>A), GAC1172GAT (3656C>T), CCC1173CCT (3659C>T), GTG1175GTT (3665G>T), GGG1177GGC (3671G>C), GTA1178ATT (3672G>A 3674A>T), ATG1179GAG (3675A>G 3676T>A), GCT1185GCC (3695T>C), TGT1186TCA (3697G>C 3698T>A), GGA1187AAT (3699G>A 3700G>A 3701A>T), GAT1189GAG (3707T>G), GAT1190GAC (3710T>C), GAC1191AGC (3711G>A 3712A>G), GCC1193GCA (3719C>A), AAA1194CGG (3720A>C 3721A>G 3722A>G), CTA1195GCT (3723C>G 3724T>C 3725A>T), TTC1197TTT (3731C>T), AAT1199GGT (3735A>G 3736A>G), TGC1202TCT (3745G>C 3746C>T), TCG1203GCC (3747T>G 3749G>C), CTG1204ATT (3750C>A 3752G>T), CTA1205TTA (3753C>T), CCT1206CCA (3758T>A), ATC1207GTT (3759A>G 3761C>T), ATG1208CTC (3762A>C 3764G>C), CGT1209AAG (3765C>A 3766G>A 3767T>G), AGC1210AGT (3770C>T), CAG1211CAA (3773G>A), AAG1212AGG (3775A>G), ATC1213GTG (3777A>G 3779C>G), CCT1214CCA (3782T>A), GGG1215GGT (3785G>T), GGA1216GGT (3788A>T), CGT1217ATC (3789C>A 3790G>T 3791T>C), CCA1218CCC (3794A>C), TGT1219ACA (3795T>A 3796G>C 3797T>A), GAA1220ACA (3798G>A 3799A>C), AGG1221CGT (3801A>C 3803G>T), GAT1222TGT (3804G>T 3805A>G), GAT1224GAC (3812T>C), GTA1225ATC (3813G>A 3815A>C), GTG1226GTT (3818G>T), CTC1227CTG (3821C>G), GGA1229GGT (3827A>T), ATA1230GTT (3828A>G 3830A>T), GGA1232GGT (3836A>T), TGT1233TTT (3838G>T), GAT1234GAC (3842T>C), TAC1235TTC (3844A>T), TAT1236TTT (3847A>T), GGC1238TCC (3852G>T 3853G>C), GAG1240GAA (3860G>A), CTG1241CTC (3863G>C), TCT1244TCA (3872T>A), TGC1245TGT (3875C>T), TAT1247TAC (3881T>C), CCC1248CCT (3884C>T), AAC1250AAT (3890C>T), AAG1251AAA (3893G>A), ATG1252CTG (3894A>C), GGG1253GGT (3899G>T), ATG1254TTG (3900A>T), GGG1255GGT (3905G>T), ATG1256ACC (3907T>C 3908G>C), AAC1257AAT (3911C>T), AAG1258AAA (3914G>A), AGA1259AGG (3917A>G), TTT1261TTC (3923T>C), GTG1262GTT (3926G>T), CAA1263GAA (3927C>G), TCC1264GCA (3930T>G 3932C>A), ACT1265CAG (3933A>C 3934C>A 3935T>G), GGC1266GAG (3937G>A 3938C>G), GAA1267AGT (3939G>A 3940A>G 3941A>T), GGA1268ACC (3942G>A 3943G>C 3944A>C), GAG1269CAA (3945G>C 3947G>A), AGA1270AGG (3950A>G), GTG1271GTA (3953G>A), GAA1272CTA (3954G>C 3955A>T), AAA1274AAG (3962A>G), GAC1276AAT (3966G>A 3968C>T), ACT1277TCT (3969A>T), GTA1279GTT (3977A>T), TTT1280TTC (3980T>C), GAA1281CAA (3981G>C), GAA1284GAG (3992A>G), GAG1285GAA (3995G>A), CTA1286TTA (3996C>T), CAG1289GAA (4005C>G 4007G>A), ATT1290ATA (4010T>A), AAA1292AAG (4016A>G), GGC1293GGT (4019C>T), ATC1294AAC (4021T>A), CAT1295TAT (4023C>T), GAT1297GAA (4031T>A), CTG1298ATG (4032C>A), GTC1299GTT (4037C>T), ACC1301ACT (4043C>T), TGC1303TGT (4049C>T), GCC1304GCT (4052C>T), AAA1305AAG (4055A>G), GAT1306GAC (4058T>C), CGC1308AGG (4062C>A 4064C>G), CTC1309CTA (4067C>A), CCA1310CCT (4070A>T), CTT1311CTG (4073T>G), GAG1312GAC (4076G>C), AAA1313AAG (4079A>G), ATC1314ATA (4082C>A), TAT1315TAC (4085T>C), GGC1316GGG (4088C>G), AAG1317AAA (4091G>A), AGA1318AAG (4093G>A 4094A>G), AAG1319AAA (4097G>A), AGG1321CGT (4101A>C 4103G>T), CTC1322CTT (4106C>T), ATA1325ATT (4115A>T), CTT1326TTG (4116C>T 4118T>G), CCT1327CCA (4121T>A), TAC1330TAT (4130C>T), TTG1333CTG (4137T>C), GTC1334GTG (4142C>G), AGG1335CGC (4143A>C 4145G>C), AAG1336AAA (4148G>A), TTT1338TTC (4154T>C), TTC1341TTT (4163C>T), TCA1342TCT (4166A>T), GCC1343GCA (4169C>A), ACA1344TGT (4170A>T 4171C>G 4172A>T), TTG1345TTA (4175G>A), TTG1348GCA (4182T>G 4183T>C 4184G>A), CAC1349CAT (4187C>T), GCT1351GTT (4192C>T), ATA1352CTG (4194A>C 4196A>G), CCA1353CCC (4199A>C), GTT1356GTG (4208T>G), ATT1358ATC (4214T>C), GAT1359AAT (4215G>A), CCT1360CCC (4220T>C), ACA1361ATT (4222C>T 4223A>T), AGT1362TCA (4224A>T 4225G>C 4226T>A), TCT1363CAT (4227T>C 4228C>A), ACA1366ACC (4238A>C), TTG1367ACC (4239T>A 4240T>C 4241G>C), TTG1368CTG (4242T>C), AAT1370AAC (4250T>C), GGG1371CAG (4251G>C 4252G>A), TTT1372TTC (4256T>C), AGA1373CGG (4257A>C 4259A>G), GCT1374AGT (4260G>A 4261C>G), TCA1376TCT (4268A>T), GAC1377GAT (4271C>T), GTG1378GTT (4274G>T), GGA1379GGT (4277A>T), TCA1381TCT (4283A>T), GCT1382GCA (4286T>A), GAT1383GAC (4289T>C), TAT1384TAC (4292T>C), AGA1390AGG (4310A>G), GCA1391GCG (4313A>G), CCT1392CCA (4316T>A), GTT1393ATA (4317G>A 4319T>A), GCT1395GCA (4325T>A), CAG1397CAA (4331G>A), GAA1405GCA (4354A>C), TAC1407TAT (4361C>T), GGA1408GGG (4364A>G), AAG1410GCT (4368A>G 4369A>C 4370G>T), CCT1411CCA (4373T>A), GGC1412GGA (4376C>A), AGT1413AGC (4379T>C), CCT1414GAG (4380C>G 4381C>A 4382T>G), TCC1416TCA (4388C>A), GCT1418GCA (4394T>A), CGA1419AGA (4395C>A), GCA1421GCT (4403A>T), CTT1422CTG (4406T>G), TTA1423TTG (4409A>G), GCA1426GCT (4418A>T), TGT1428TGC (4424T>C), ACA1431ACT (4433A>T), CTG1432CTA (4436G>A), GTG1437CTC (4449G>C 4451G>C), TTT1438TTC (4454T>C), TTG1440TTA (4460G>A), GGG1442GGT (4466G>T), GGC1443GGA (4469C>A), CCA1445CCT (4475A>T), TCA1446TCT (4478A>T), GCA1449GCT (4487A>T), CTA1450CTC (4490A>C), ACG1451ACC (4493G>C), ATC1453ATA (4499C>A), TTC1454TTT (4502C>T), AAT1455AAC (4505T>C), TCT1456TCC (4508T>C), CTC1457CTT (4511C>T), CTC1458TTA (4512C>T 4514C>A), AAT1459AAC (4517T>C), CGA1464CGG (4532A>G), GCC1466GCT (4538C>T), TTT1467TTC (4541T>C), TCA1469TCG (4547A>G), TTG1470CTC (4548T>C 4550G>C), TTA1471CTC (4551T>C 4553A>C), AGA1473AGG (4559A>G), CAT1475CAC (4565T>C), ATT1476ATC (4568T>C), GCT1477AAA (4569G>A 4570C>A 4571T>A), GCC1478GCA (4574C>A), AGG1479AAG (4576G>A), GCT1480GCA (4580T>A), ATA1481TTG (4581A>T 4583A>G), GGA1482GGT (4586A>T), GTT1483GTA (4589T>A), AAA1484AAG (4592A>G), CCA1485TCA (4593C>T), TTC1488TTT (4604C>T), CAG1490GAC (4608C>G 4610G>C), CTA1491TTA (4611C>T), TTC1492TTT (4616C>T), ATA1493ATT (4619A>T), GCT1494GCA (4622T>A), GTT1495GTG (4625T>G), GGA1497GGG (4631A>G), GTT1502GTG (4646T>G), GTA1504GTG (4652A>G), TTA1506ATG (4656T>A 4658A>G), CAT1507CAC (4661T>C), CTC1508CTT (4664C>T), CAG1509CAA (4667G>A), TAT1511TAC (4673T>C), TCT1512GAG (4674T>G 4675C>A 4676T>G), CTG1513TTA (4677C>T 4679G>A), CCA1514CCT (4682A>T), AAT1515GCA (4683A>G 4684A>C 4685T>A), CAT1518CAA (4694T>A), TTA1520CTA (4698T>C), CTG1522CAA (4705T>A 4706G>A), GTC1523GTG (4709C>G), AAT1524AAC (4712T>C), GTA1525GTT (4715A>T), ATC1526ATT (4718C>T), ATT1527ATC (4721T>C), GGT1530GGC (4730T>C), GAC1532GAT (4736C>T), TCG1534AAC (4740T>A 4741C>A 4742G>C), GAT1536AAT (4746G>A), GTT1537GTC (4751T>C), GAG1539GAT (4757G>T), GTA1540GTC (4760A>C), CAA1541AAA (4761C>A), TTT1542TTC (4766T>C), CAA1543CAG (4769A>G), CTA1548TTG (4782C>T 4784A>G), ACT1549ACG (4787T>G), CTG1551TTG (4791C>T), AGT1552AGC (4796T>C), GGT1554GGC (4802T>C), TTT1555TTC (4805T>C), AAA1556AAG (4808A>G), CGA1557CGC (4811A>C), CTT1560TTG (4818C>T 4820T>G), GGA1561GGT (4823A>T), CAC1563CAA (4829C>A), ATG1564CTA (4830A>C 4832G>A), GCT1565GCC (4835T>C), CCT1566CCC (4838T>C), TCA1571TCC (4853A>C), ATC1572ATT (4856C>T), ATT1573GTG (4857A>G 4859T>G), GAG1574GAA (4862G>A), CCC1575CCA (4865C>A), ATA1579ATC (4877A>C), CCT1581CCA (4883T>A), GCC1582GCA (4886C>A), CCA1583GTT (4887C>G 4888C>T 4889A>T), GGT1584GGG (4892T>G), CCT1586CCA (4898T>A), GAC1587GAT (4901C>T), ACT1588ATC (4903C>T 4904T>C), CAG1589ACT (4905C>A 4906A>C 4907G>T), ATG1592TAT (4914A>T 4915T>A 4916G>T), ACA1597ACG (4931A>G), GGA1598GGT (4934A>T), ATA1599GTA (4935A>G), CGT1600AGA (4938C>A 4940T>A), GAA1601GAG (4943A>G), CAT1604CAG (4952T>G), CAC1605CAT (4955C>T), GGT1606GGA (4958T>A), CGT1607CAG (4960G>A 4961T>G), GTG1608GTC (4964G>C), GCT1609GTT (4966C>T), AAG1612ACA (4975A>C 4976G>A), CTT1613TTG (4977C>T 4979T>G), GTC1614ACA (4980G>A 4981T>C 4982C>A), TTA1615GAC (4983T>G 4984T>A 4985A>C), GAT1616AAT (4986G>A), GTT1617GTC (4991T>C), CAG1618CAA (4994G>A), ACG1619AAA (4996C>A 4997G>A), GCG1620GCA (5000G>A), TTG1621ATA (5001T>A 5003G>A), GAT1622ATG (5004G>A 5005A>T 5006T>G), GAA1623CAG (5007G>C 5009A>G), AGG1624CGA (5010A>C 5012G>A), GGT1625GGC (5015T>C), TTC1626TTG (5018C>G), AGA1627CCA (5019A>C 5020G>C), GCT1628ATC (5022G>A 5023C>T 5024T>C), ATC1630CAT (5028A>C 5029T>A 5030C>T), CCT1632CCA (5036T>A), TCC1633TCT (5039C>T), TTG1635GCT (5043T>G 5044T>C 5045G>T), GTG1637CAA (5049G>C 5050T>A 5051G>A), CAG1639AGT (5055C>A 5056A>G 5057G>T), GAA1640GAG (5060A>G), ATT1642CTA (5064A>C 5066T>A), GCA1643CAA (5067G>C 5068C>A), AAG1644GCC (5070A>G 5071A>C 5072G>C), GTA1645GTT (5075A>T), ACA1646ACT (5078A>T), GGG1647GGA (5081G>A), GAT1648GAC (5084T>C), TCA1649AGT (5085T>A 5086C>G 5087A>T), AGT1650AAC (5089G>A 5090T>C), GCC1651ATA (5091G>A 5092C>T 5093C>A), CTG1652GGG (5094C>G 5095T>G), ACA1653GCT (5097A>G 5099A>T), ATT1654CTG (5100A>C 5102T>G), TGT1655CTG (5103T>C 5104G>T 5105T>G), ATG1657CTG (5109A>C), GCA1658AGC (5112G>A 5113C>G 5114A>C), AAA1659AAG (5117A>G), GCA1660GAT (5119C>A 5120A>T), TCC1663ACT (5127T>A 5129C>T), TAT1664TAC (5132T>C), ACG1665GTG (5133A>G 5134C>T), CCA1666CCT (5138A>T), TTG1667CTT (5139T>C 5141G>T), GAC1668CCA (5142G>C 5143A>C 5144C>A), GCA1669GAA (5146C>A), GAG1671AAA (5151G>A 5153G>A), AAG1672AAA (5156G>A), ATC1673ATT (5159C>T), ACA1674GAA (5160A>G 5161C>A), AAT1675GAT (5163A>G), GAG1677TCA (5169G>T 5170A>C 5171G>A), CGT1678CAA (5173G>A 5174T>A), GAT1679GGT (5176A>G), CTG1680GTC (5178C>G 5180G>C), AAT1681CAT (5181A>C), TGG1682GAA (5184T>G 5185G>A 5186G>A), GCA1684TGT (5190G>T 5191C>G 5192A>T), CCA1685TCT (5193C>T 5195A>T), AAC1686GGA (5196A>G 5197A>G 5198C>A), ATT1687GTT (5199A>G), GGT1688TTC (5202G>T 5203G>T 5204T>C), TTT1689TTC (5207T>C), GCA1692TCG (5214G>T 5216A>G), CGT1693CGA (5219T>A), ACT1694ACA (5222T>A), GCA1695GAA (5224C>A), GCC1696GTT (5227C>T 5228C>T), CAC1697AAT (5229C>A 5231C>T), TAC1698CAT (5232T>C 5234C>T), ACG1699AAA (5236C>A 5237G>A), GAA1702GAG (5246A>G), GGG1703GAC (5248G>A 5249G>C), TAC1704ACA (5250T>A 5251A>C 5252C>A), ATT1706CTT (5256A>C), AAC1708AAT (5264C>T), TGT1709TGC (5267T>C), AAG1713AAA (5279G>A), AAG1714AAA (5282G>A), TCC1715ACA (5283T>A 5285C>A), AAT1716ACA (5287A>C 5288T>A), GTT1718GTG (5294T>G), GGT1720GGC (5300T>C), CCG1733CCC (5339G>C), TAC1734TAT (5342C>T), ACT1735ACA (5345T>A), TCA1737GCA (5349T>G), GCT1738GCC (5354T>C), ATA1739ATC (5357A>C), ATG1740AAG (5359T>A), GAT1741GAC (5363T>C), GCC1742AAT (5364G>A 5365C>A 5366C>T), AAG1744CAA (5370A>C 5372G>A), AAT1745AGT (5374A>G), GTG1746CGC (5376G>C 5377T>G 5378G>C), GCA1748TCA (5382G>T), GGA1749ATG (5385G>A 5386G>T 5387A>G), GGA1750GGG (5390A>G), CAT1751ATT (5391C>A 5392A>T), GTA1752GGA (5395T>G), GCA1753ACC (5397G>A 5399A>C), ACC1754CAA (5400A>C 5401C>A 5402C>A), AAT1755AAA (5405T>A), GTC1756GTT (5408C>T), GTT1757GTC (5411T>C), GTG1759ATT (5415G>A 5417G>T), GGA1761GGG (5423A>G), GGA1763GGT (5429A>T), GTC1766GTG (5438C>G), GGC1767AGT (5439G>A 5441C>T), CCA1768CCC (5444A>C), GCA1771GCT (5453A>T), GCG1772GCT (5456G>T), ATG1774TTT (5460A>T 5462G>T), GCC1775GCA (5465C>A), GCA1777TCA (5469G>T), ACA1778ACC (5474A>C), AGG1779AAT (5476G>A 5477G>T), CAA1780CAG (5480A>G), TGT1782AGA (5484T>A 5486T>A), GTG1783GCT (5488T>C 5489G>T), GAC1785CAA (5493G>C 5495C>A), ATA1786CTT (5496A>C 5498A>T), GTT1788GTC (5504T>C), CGC1791AGA (5511C>A 5513C>A), ATT1793TTG (5517A>T 5519T>G), GGA1794GCG (5521G>C 5522A>G), AAT1795GGC (5523A>G 5524A>G 5525T>C), GTG1796TTG (5526G>T), CTT1797CTG (5531T>G), GAC1798GAT (5534C>T), CTC1799TTG (5535C>T 5537C>G), ACC1801CAG (5541A>C 5542C>A 5543C>G), GGC1805GGT (5555C>T), GTG1807ATT (5559G>A 5561G>T), CAG1808CAA (5564G>A), TTT1810TTC (5570T>C), CTT1811CTC (5573T>C), CAA1812ACA (5574C>A 5575A>C), TGT1813_GTG1814insGTC (5579_5580insGTC), CCT1815CAG (5584C>A 5585T>G), CAT1816AAA (5586C>A 5588T>A), GGA1817AGT (5589G>A 5591A>T), GAC1818TGC (5592G>T 5593A>G), AAA1819AAG (5597A>G), GTG1820ACA (5598G>A 5599T>C 5600G>A), AGT1823GCT (5607A>G 5608G>C), GGT1824AGT (5610G>A), GCA1825GGG (5614C>G 5615A>G), TCG1826del (5616_5618delTCG), CTC1827CGC (5620T>G), CAC1828GAT (5622C>G 5624C>T), AGT1829GGA (5625A>G 5627T>A), AGT1830ATG (5629G>T 5630T>G), TTT1831TTG (5633T>G), ATG1832AAC (5635T>A 5636G>C), CAA1834CAT (5642A>T), CAA1834_GGG1835insCATGCT (5642_5643insCATGCT), GGG1835GCT (5644G>C 5645G>T), TTT1836TAT (5647T>A), GAA1837GCC (5650A>C 5651A>C), TTG1838CTT (5652T>C 5654G>T), GGC1839GGA (5657C>A), CTC1841CTT (5663C>T), CGC1842CGA (5666C>A), ATT1843ATA (5669T>A), ATA1844GTG (5670A>G 5672A>G), CAT1845GGC (5673C>G 5674A>G 5675T>C), GGT1846TGT (5676G>T), GAT1847TCA (5679G>T 5680A>C 5681T>A), TTA1848ATT (5682T>A 5684A>T), GCA1849TCA (5685G>T), AAA1850AGA (5689A>G), CAG1851AAG (5691C>A), ACA1852ATG (5695C>T 5696A>G), ATG1854GTG (5700A>G), CGG1855AGA (5703C>A 5705G>A), ATG1856TTG (5706A>T), CCA1857CCC (5711A>C), TAT1858TAC (5714T>C), GTG1859ACT (5715G>A 5716T>C 5717G>T), GTA1860GTT (5720A>T), GGA1861GGG (5723A>G), CAT1863ATG (5727C>A 5728A>T 5729T>G), GGA1864GGT (5732A>T), GGA1865GGT (5735A>T), GGA1867GCA (5740G>C), AAT1868GAT (5742A>G), TTT1869TGC (5746T>G 5747T>C), TCC1870TCA (5750C>A), CAG1872AGA (5754C>A 5755A>G 5756G>A), GAT1873AAT (5757G>A), GAG1875GAT (5765G>T), AGT1876TCT (5766A>T 5767G>C), TTG1877CTC (5769T>C 5771G>C), CTA1878ATC (5772C>A 5774A>C), CAC1879AAA (5775C>A 5777C>A), TAT1880GCC (5778T>G 5779A>C 5780T>C), TTG1881CTA (5781T>C 5783G>A), CAG1883TGC (5787C>T 5788A>G 5789G>C), GGA1884TCT (5790G>T 5791G>C 5792A>T), GCA1886GCT (5798A>T), GAG1887GCA (5800A>C 5801G>A), TTA1888TGT (5803T>G 5804A>T), ATT1889GTG (5805A>G 5807T>G), CAA1890GCT (5808C>G 5809A>C 5810A>T), AAG1891CGT (5811A>C 5812A>G 5813G>T), AAT1892GGG (5814A>G 5815A>G 5816T>G), ACA1893TGT (5817A>T 5818C>G 5819A>T), CTA1895ATA (5823C>A), TAT1898TAC (5834T>C), TTC1899TTT (5837C>T), AAA1900GAG (5838A>G 5840A>G), GAG1901AAT (5841G>A 5843G>T), CTG1902TTT (5844C>T 5846G>T), AGT1903AAT (5848G>A), ATG1904GCA (5850A>G 5851T>C 5852G>A), GAA1905CAA (5853G>C), AAT1906ATA (5857A>T 5858T>A), GTG1907GCT (5860T>C 5861G>T), CAA1908ACC (5862C>A 5863A>C 5864A>C), CAA1909AGG (5865C>A 5866A>G 5867A>G), CTG1910TTA (5868C>T 5870G>A), ATA1911GTA (5871A>G), GTT1914CTT (5880G>C),

	AAG1915AAA (5885G>A), CTT1916AGT (5886C>A 5887T>G), GGG1918GGT (5894G>T), TTT1919GTG (5895T>G 5897T>G), TTC1920TTT (5900C>T), CCA1921ATC (5901C>A 5902C>T 5903A>C), AAA1922ACA (5905A>C), GAA1923GCC (5908A>C 5909A>C), ACT1924ACA (5912T>A), ACC1925GGC (5913A>G 5914C>G), ATT1926CAC (5916A>C 5917T>A 5918T>C), CAG1927AAA (5919C>A 5921G>A), AAG1928ATG (5923A>T), CTC1929TTG (5925C>T 5927C>G), AAA1930GTG (5928A>G 5929A>T 5930A>G), AAT1931CAA (5931A>C 5933T>A), TTT1932ACT (5934T>A 5935T>C), GTT1933CTG (5937G>C 5939T>G), GAT1934AGC (5940G>A 5941A>G 5942T>C), GCT1935AAT (5943G>A 5944C>A), GAA1936CAA (5946G>C), TGT1937TGG (5951T>G), CTA1938CAA (5953T>A), ACA1939TCC (5955A>T 5957A>C), TTC1940CAC (5958T>C 5959T>A), AAA1941GAA (5961A>G), GCA1942CAA (5964G>C 5965C>A), AGG1943AGT (5969G>T), AGC1944TGT (5970A>T 5972C>T), TTT1945GTG (5973T>G 5975T>G), AGG1946AAG (5977G>A), CAC1947AGA (5979C>A 5980A>G 5981C>A), GTA1948GTC (5984A>C), GTT1949TAT (5985G>T 5986T>A), AAA1951AAG (5993A>G), AAG1952CGA (5994A>C 5995A>G 5996G>A), AAA1953AAT (5999A>T), TTG1955CTC (6003T>C 6005G>C), AGT1956TCA (6006A>T 6007G>C 6008T>A), TCC1957AAC (6009T>A 6010C>A), ACA1958GCA (6012A>G), TGG1959ATA (6015T>A 6016G>T 6017G>A), AAA1960ACA (6019A>C), ATG1961_TGT1962insTAC (6023_6024insTAC), TGT1962CGT (6024T>C), GAA1964GAT (6032A>T), AGT1965GCC (6033A>G 6034G>C 6035T>C), ATT1966AAA (6037T>A 6038T>A), GTT1967GAT (6040T>A), TCA1969ATA (6045T>A 6046C>T), AGG1970GTA (6048A>G 6049G>T 6050G>A), GCA1972del (6054_6056delGCA), CTA1975GTG (6063C>G 6065A>G), CCT1977TTG (6069C>T 6070C>T 6071T>G), GGC1978GGT (6074C>T), TTG1980CCA (6078T>C 6079T>C 6080G>A), GCT1982GCC (6086T>C), TCC1983ATG (6087T>A 6088C>T 6089C>G), GTA1984GTT (6092A>T), AAA1986AGG (6097A>G 6098A>G)									
helicase (YP_001976154.1)	1	98	100%	652	97.2%	98 (100%)	96 (98.0%)	0/0/0/0	0	
Protein mutations:	R16A (1374A>G 1375G>C 1376A>C), G18Q (1380G>C 1381G>A 1382T>A)									
Codon mutations:	GGT1GGC (1331T>C), TCT3TCC (1337T>C), ACA4ACG (1340A>G), TCC5AGC (1341T>A 1342C>G), CGC7CGA (1349C>A), TTA8CTG (1350T>C 1352A>G), TTT9TTC (1355T>C), ATT10ATC (1358T>C), GTG13GTA (1367G>A), GAC15GAT (1373C>T), AGA16GCC (1374A>G 1375G>C 1376A>C), GGT18CAA (1380G>C 1381G>A 1382T>A), CCA20CCC (1388A>C), AAA21AAG (1391A>G), CTC22CTG (1394C>G), AAT23AAC (1397T>C), CGG24AGG (1398C>A), CTA25TTG (1401C>T 1403A>G), GTA28GTT (1412A>T), AGG31CGC (1419A>C 1421G>C), GCA33GCT (1427A>T), TAT34TAC (1430T>C), TCC36TCA (1436C>A), AAC37AAT (1439C>T), CTG45CTC (1463G>C), GAC48GAT (1472C>T), GGA50GGT (1478A>T), TTG52CTG (1482T>C), CGA53CGG (1487A>G), GGG55GGA (1493G>A), GGG57GGC (1499G>C), CAG58CAA (1502G>A), CAA60CAG (1508A>G), GAT61GAC (1511T>C), AAA63AAG (1517A>G), GAT64GAC (1520T>C), CTG65CTC (1523G>C), GAT67GAC (1529T>C), TCA70TCC (1538A>C), CCC75CCA (1553C>A), TTA76TTG (1556A>G), CCA77CCC (1559A>C), GCC79GCT (1565C>T), GTT81GTA (1571T>A), GAG82GAA (1574G>A), GGC85GGG (1583C>G), AGG86CGG (1584A>C), ACC89ACA (1595C>A), TCC90TCA (1598C>A), TAT92TAC (1604T>C), ACA96ACC (1616A>C), AAT98AAC (1622T>C)									
RNA-dependent RNA polymerase (YP_001976153.1)	1	249	100%	1569	89.4%	249 (100%)	218 (87.6%)	0/0/0/0	0	
Protein mutations:	E8D (4076G>C), R14K (4093G>A 4094A>G), T40C (4170A>T 4171C>G 4172A>T), L44A (4182T>G 4183T>C 4184G>A), A47V (4192C>T), I48L (4194A>C 4196A>G), D55N (4215G>A), T57I (4222C>T 4223A>T), S59H (4227T>C 4228C>A), L63T (4239T>A 4240T>C 4241G>C), G67Q (4251G>C 4252G>A), A70S (4260G>A 4261C>G), V89I (4317G>A 4319T>A), E101A (4354A>C), K106A (4368A>G 4369A>C 4370G>T), P110E (4380C>G 4381C>A 4382T>G), V133L (4449G>C 4451G>C), A173K (4569G>A 4570C>A 4571T>A), R175K (4576G>A), I177L (4581A>T 4583A>G), P181S (4593C>T), Q186D (4608C>G 4610G>C), L202M (4656T>A 4658A>G), S208E (4674T>G 4675C>A 4676T>G), N211A (4683A>G 4684A>C 4685T>A), H214Q (4694T>A), L218Q (4705T>A 4706G>A), S230N (4740T>A 4741C>A 4742G>C), D232N (4746G>A), E235D (4757G>T), Q237K (4761C>A)									
Codon mutations:	AAA1AAG (4055A>G), GAT2GAC (4058T>C), CGC4AGG (4062C>A 4064C>G), CTC5CTA (4067C>A), CCA6CCT (4070A>T), CTT7CTG (4073T>G), GAG8GAC (4076G>C), AAA9AAG (4079A>G), ATC10ATA (4082C>A), TAT11TAC (4085T>C), GGC12GGG (4088C>G), AAG13AAA (4091G>A), AGA14AAG (4093G>A 4094A>G), AAG15AAA (4097G>A), AGG17CGT (4101A>C 4103G>T), CTC18CTT (4106C>T), ATA21ATT (4115A>T), CTT22TTG (4116C>T 4118T>G), CCT23CCA (4121T>A), TAC26TAT (4130C>T), TTG29CTG (4137T>C), GTC30GTG (4142C>G), AGG31CGC (4143A>C 4145G>C), AAG32AAA (4148G>A), TTT34TTC (4154T>C), TTC37TTT (4163C>T), TCA38TCT (4166A>T), GCC39GCA (4169C>A), ACA40TGT (4170A>T 4171C>G 4172A>T), TTG41TTA (4175G>A), TTG44GCA (4182T>G 4183T>C 4184G>A), CAC45CAT (4187C>T), GCT47GTT (4192C>T), ATA48CTG (4194A>C 4196A>G), CCA49CCC (4199A>C), GTT52GTG (4208T>G), ATT54ATC (4214T>C), GAT55AAT (4215G>A), CCT56CCC (4220T>C), ACA57ATT (4222C>T 4223A>T), AGT58TCA (4224A>T 4225G>C 4226T>A), TCT59CAT (4227T>C 4228C>A), ACA62ACC (4238A>C), TTG63ACC (4239T>A 4240T>C 4241G>C), TTG64CTG (4242T>C), AAT66AAC (4250T>C), GGG67CAG (4251G>C 4252G>A), TTT68TTC (4256T>C), AGA69CGG (4257A>C 4259A>G), GCT70AGT (4260G>A 4261C>G), TCA72TCT (4268A>T), GAC73GAT (4271C>T), GTG74GTT (4274G>T), GGA75GGT (4277A>T), TCA77TCT (4283A>T), GCT78GCA (4286T>A), GAT79GAC (4289T>C), TAT80TAC (4292T>C), AGA86AGG (4310A>G), GCA87GCG (4313A>G), CCT88CCA (4316T>A), GTT89ATA (4317G>A 4319T>A), GCT91GCA (4325T>A), CAG93CAA (4331G>A), GAA101GCA (4354A>C), TAC103TAT (4361C>T), GGA104GGG (4364A>G), AAG106GCT (4368A>G 4369A>C 4370G>T), CCT107CCA (4373T>A), GGC108GGA (4376C>A), AGT109AGC (4379T>C), CCT110GAG (4380C>G 4381C>A 4382T>G), TCC112TCA (4388C>A), GCT114GCA (4394T>A), CGA115AGA (4395C>A), GCA117GCT (4403A>T), CTT118CTG (4406T>G), TTA119TTG (4409A>G), GCA122GCT (4418A>T), TGT124TGC (4424T>C), ACA127ACT (4433A>T), CTG128CTA (4436G>A), GTG133CTC (4449G>C 4451G>C), TTT134TTC (4454T>C), TTG136TTA (4460G>A), GGG138GGT (4466G>T), GGC139GGA (4469C>A), CCA141CCT (4475A>T), TCA142TCT (4478A>T), GCA145GCT (4487A>T), CTA146CTC (4490A>C), ACG147ACC (4493G>C), ATC149ATA (4499C>A), TTC150TTT (4502C>T), AAT151AAC (4505T>C), TCT152TCC (4508T>C), CTC153CTT (4511C>T), CTC154TTA (4512C>T 4514C>A), AAT155AAC (4517T>C), CGA160CGG (4532A>G), GCC162GCT (4538C>T), TTT163TTC (4541T>C), TCA165TCG (4547A>G), TTG166CTC (4548T>C 4550G>C), TTA167CTC (4551T>C 4553A>C), AGA169AGG (4559A>G), CAT171CAC (4565T>C), ATT172ATC (4568T>C), GCT173AAA (4569G>A 4570C>A 4571T>A), GCC174GCA (4574C>A), AGG175AAG (4576G>A), GCT176GCA (4580T>A), ATA177TTG (4581A>T 4583A>G), GGA178GGT (4586A>T), GTT179GTA (4589T>A), AAA180AAG (4592A>G), CCA181TCA (4593C>T), TTC184TTT (4604C>T), CAG186GAC (4608C>G 4610G>C), CTA187TTA (4611C>T), TTC188TTT (4616C>T), ATA189ATT (4619A>T), GCT190GCA (4622T>A), GTT191GTG (4625T>G), GGA193GGG (4631A>G), GTT198GTG (4646T>G), GTA200GTG (4652A>G), TTA202ATG (4656T>A 4658A>G), CAT203CAC (4661T>C), CTC204CTT (4664C>T), CAG205CAA (4667G>A), TAT207TAC (4673T>C), TCT208GAG (4674T>G 4675C>A 4676T>G), CTG209TTA (4677C>T 4679G>A), CCA210CCT (4682A>T), AAT211GCA (4683A>G 4684A>C 4685T>A), CAT214CAA (4694T>A), TTA216CTA (4698T>C), CTG218CAA (4705T>A 4706G>A), GTC219GTG (4709C>G), AAT220AAC (4712T>C), GTA221GTT (4715A>T), ATC222ATT (4718C>T), ATT223ATC (4721T>C), GGT226GGC (4730T>C), GAC228GAT (4736C>T), TCG230AAC (4740T>A 4741C>A 4742G>C), GAT232AAT (4746G>A), GTT233GTC (4751T>C), GAG235GAT (4757G>T), GTA236GTC (4760A>C), CAA237AAA (4761C>A), TTT238TTC (4766T>C), CAA239CAG (4769A>G), CTA244TTG (4782C>T 4784A>G), ACT245ACG (4787T>G), CTG247TTG (4791C>T), AGT248AGC (4796T>C)									

*: Inserts / Deletes / Misaligned / Frameshifts

Analysis details

This analysis was performed with panviral2.64

NGS Details (UN60): Torradovirus marchitezum (segment RNA 2)

Assembly

**Coverage Length**	1365 (2 contig(s))
**Depth Of Coverage**	18973.4
**Number Of Reads**	209020
**Reads Per Million**	4433.22 rpm (after QC)
**Ambiguities**	0
**Assembly Method**	de novo + reference guided assembly
**Consensus Caller**	Bcf Tools

Coverage Map

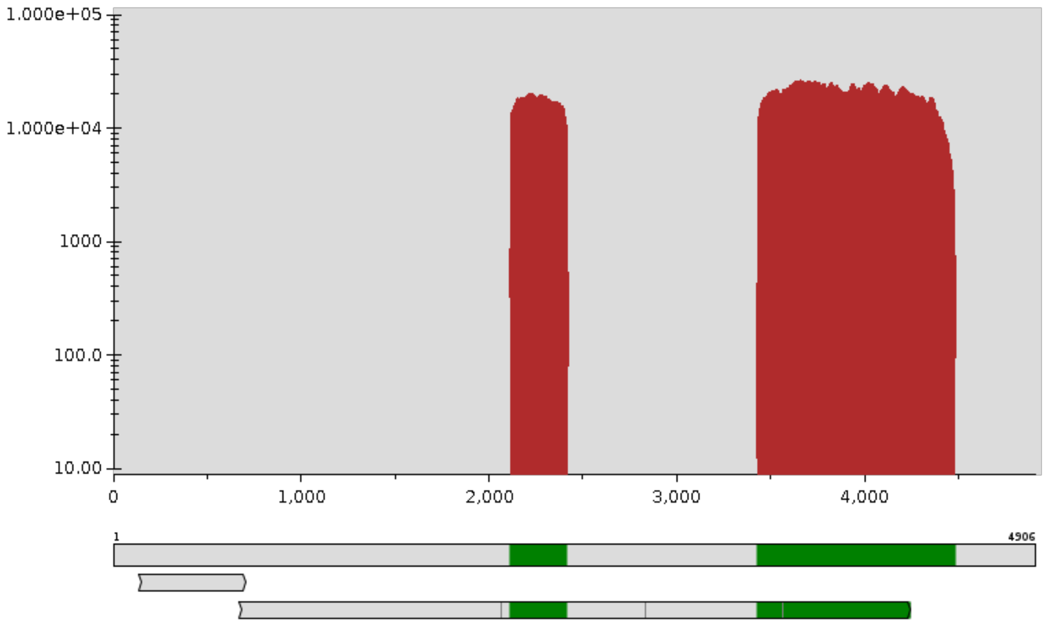

Assignment

**Type**	Torradovirus marchitezum (Taxonomy ID: 3048376)
**Reference Genome**	NC_010988.1
**NT Identity (%)**	67.2951
**AA Identity (%)**	67.5462
**Number Of Stop Codons**	1
**Number Of CDS**	2

Alignment

**Alignment Score**	905.0 (NT) + 1799.0 (AA) = 2704.0
**Concordance (%)**	50.9423

Alignment Method	Global, seeded, nucleotide + amino acids (AGA)

Genome Region

Sequence starts at position 2113 and ends at position 4485 relative to NC_010988.1 reference sequence.

Alignment Detailed Statistics

	Begin	End	Coverage	Score	Concordance	Matches	Identities	I/D/M/F*	Stop Codons
**NT**	**2113**	**4485**	**27.8%**	**905**	**33.2%**	**1365 (99.0%)**	**928 (67.3%)**	**14/0**	
Mutations:	2122T>G, 2133T>C, 2134G>A, 2135T>A, 2137A>T, 2140T>A, 2143C>T, 2144A>G, 2146G>A, 2150G>C, 2152_2153insAGC, 2155T>G, 2156C>G, 2157C>G, 2158C>A, 2160C>T, 2164G>C, 2165C>A, 2166C>A, 2167A>G, 2170T>C, 2171A>C, 2173G>A, 2176A>T, 2178A>T, 2182T>A, 2184T>A, 2185T>G, 2187C>T, 2188A>T, 2190A>C, 2191G>T, 2193G>A, 2194A>C, 2195G>C, 2200A>G, 2201G>C, 2203A>G, 2207A>G, 2208C>T, 2209T>C, 2210C>T, 2211A>C, 2217G>A, 2218T>C, 2224T>C, 2225A>T, 2227T>C, 2239A>G, 2240C>G, 2241C>A, 2242A>G, 2249G>A, 2250A>T, 2251C>G, 2252A>G, 2253C>A, 2255T>G, 2256C>A, 2257C>T, 2258A>G, 2259T>C, 2260G>C, 2261G>C, 2263A>C, 2264G>A, 2267G>C, 2270C>T, 2275T>A, 2276A>T, 2281G>A, 2284A>G, 2287G>A, 2289T>C, 2290G>C, 2293T>C, 2296A>T, 2299T>C, 2305T>G, 2308T>G, 2314C>T, 2315A>C, 2320T>C, 2323A>C, 2326T>C, 2335C>T, 2336G>A, 2337T>A, 2338T>C, 2342G>A, 2347G>A, 2350C>T, 2353T>C, 2356T>C, 2359T>G, 2363G>C, 2365G>A, 2366C>G, 2368A>G, 2369G>C, 2372A>C, 2374G>A, 2375A>G, 2376G>C, 2377C>T, 2378T>C, 2383T>C, 2386G>A, 2389C>T, 2392T>A, 2395C>A, 2398A>G, 2402T>A, 2403C>G, 2404A>T, 2409G>A, 2410C>G, 3442C>G, 3445A>G, 3448C>T, 3449T>G, 3451A>C, 3457T>C, 3460C>T, 3461C>T, 3463T>G, 3466C>T, 3476G>A, 3478T>C, 3481A>G, 3482T>A, 3484A>T, 3493G>A, 3497A>C, 3506T>C, 3514G>A, 3517C>A, 3520C>T, 3521A>G, 3528A>C, 3529G>C, 3532C>T, 3533C>G, 3535G>T, 3538G>T, 3544C>G, 3544_3545insAAGAAG, 3550T>C, 3551C>A, 3552C>A, 3554C>G, 3555C>A, 3556C>G, 3559G>A, 3560A>G, 3561A>T, 3564C>G, 3568A>G, 3569A>G, 3570G>T, 3572A>G, 3573T>C, 3574T>A, 3577G>T, 3578T>G, 3579C>G, 3580C>A, 3581A>C, 3583A>T, 3584A>C, 3586A>G, 3589G>A, 3595T>C, 3598C>T, 3604T>A, 3610A>T, 3619C>T, 3622C>T, 3623A>T, 3624T>C, 3625G>T, 3627G>C, 3628T>C, 3638G>A, 3639A>C, 3641G>A, 3642A>G, 3643A>C, 3644A>C, 3646A>C, 3647T>C, 3650T>G, 3655A>T, 3656A>C, 3661T>A, 3667A>T, 3670A>G, 3676C>T, 3677A>G, 3680A>C, 3682G>A, 3685T>C, 3686G>T, 3687A>C, 3689G>C, 3690G>C, 3691G>C, 3692C>A, 3693A>C, 3700T>C, 3712A>C, 3715C>A, 3718T>G, 3724A>C, 3730A>C, 3739T>C, 3740A>T, 3742A>T, 3751T>C, 3760A>T, 3763T>C, 3767T>C, 3769G>T, 3770A>G, 3772T>G, 3775T>C, 3776T>A, 3778A>C, 3779A>C, 3781T>C, 3782G>A, 3784G>C, 3787A>C, 3790C>T, 3793A>G, 3796A>C, 3800T>G, 3801C>G, 3803A>T, 3804G>C, 3806C>A, 3807C>A, 3811T>C, 3812G>A, 3817A>T, 3826A>T, 3832T>G, 3838T>G, 3841T>C, 3842G>A, 3860C>T, 3862A>T, 3863G>T, 3864C>A, 3868G>T, 3869C>G, 3871T>G, 3872A>G, 3873A>C, 3874T>A, 3875A>G, 3877A>G, 3880A>G, 3881A>G, 3882A>C, 3883T>A, 3884T>C, 3890G>C, 3892A>T, 3901T>C, 3904A>T, 3907C>T, 3908A>T, 3910A>C, 3917G>A, 3922T>C, 3925A>C, 3931T>A, 3934G>T, 3935G>A, 3937A>C, 3940A>T, 3943T>C, 3945C>T, 3946G>T, 3949C>T, 3952A>C, 3955C>T, 3956A>G, 3957C>T, 3958T>G, 3967T>C, 3971G>A, 3973G>A, 3982A>T, 3983A>C, 3986C>A, 3989C>T, 3994A>T, 3996G>A, 3998A>G, 3999T>C, 4000G>A, 4001A>C, 4003G>A, 4009C>T, 4010T>G, 4011C>A, 4012A>C, 4014G>A, 4015C>T, 4019C>A, 4020A>G, 4021A>T, 4024C>T, 4027C>T, 4030C>T, 4031A>G, 4033G>T, 4036A>T, 4037C>T, 4039A>G, 4045T>C, 4047G>A, 4048G>A, 4049C>T, 4051T>G, 4054A>T, 4055A>C, 4056A>T, 4060G>T, 4061A>T, 4063C>G, 4066A>C, 4070T>C, 4072G>A, 4075T>A, 4078T>C, 4079T>G, 4080C>T, 4081C>A, 4083C>A, 4085A>T, 4088G>A, 4090G>T, 4093T>C, 4102G>A, 4105A>C, 4106C>A, 4108T>G, 4109A>G, 4111A>T, 4112T>A, 4113C>A, 4114T>G, 4117T>A, 4120A>G, 4122T>C, 4123T>A, 4129C>T, 4131A>G, 4135G>A, 4138G>A, 4139T>C, 4141G>T, 4154T>C, 4155C>T, 4156T>G, 4159C>T, 4162C>T, 4165T>A, 4165_4166insT, 4180C>T, 4181A>G, 4192T>G, 4195C>T, 4198C>T, 4201T>C, 4204G>A, 4213A>T, 4219T>C, 4222T>A, 4225G>A, 4228A>G, 4229C>A, 4231T>A, 4234T>C, 4237T>C, 4238G>T, 4242T>A, 4243T>C, 4248A>C, 4252T>A, 4279A>T, 4279_4280insT, 4280A>T, 4280_4281insC, 4283A>G, 4286C>T, 4287T>A, 4288T>C, 4291C>A, 4294C>T, 4306G>A, 4309G>A, 4312G>T, 4313C>A, 4313_4314insG, 4315A>G, 4316A>T, 4318T>C, 4319G>T, 4320G>A, 4323T>G, 4324T>A, 4328T>C, 4330G>A, 4333A>G, 4335G>T, 4336A>C, 4338A>G, 4339A>G, 4340G>T, 4341G>C, 4342G>T, 4343A>T, 4345A>T, 4346G>C, 4349A>C, 4364C>T, 4365T>C, 4369A>G, 4372A>G, 4373G>A, 4392T>C, 4393C>T, 4419_4420insG, 4422A>G, 4426G>A, 4430G>A, 4437C>T, 4441T>C, 4449A>T, 4451T>G								
**CDS**									
ToMarV_RNA2gp2	482	1192	31.5%	1799	67.7%	375 (98.9%)	256 (67.5%)	4/0/1/1	1
Protein mutations:	V488A (2133T>C 2134G>A), S489T (2135T>A 2137A>T), T492A (2144A>G 2146G>A), E494Q (2150G>C), E494_D495insS (2152_2153insAGC), D495E (2155T>G), P496G (2156C>G 2157C>G 2158C>A), T497I (2160C>T), K498N (2164G>C), P499K (2165C>A 2166C>A 2167A>G), K501Q (2171A>C 2173G>A), E503V (2178A>T), V505E (2184T>A 2185T>G), A506V (2187C>T 2188A>T), E507A (2190A>C 2191G>T), G508D (2193G>A 2194A>C), A509P (2195G>C), E511Q (2201G>C 2203A>G), T513V (2207A>G 2208C>T 2209T>C), Q514S (2210C>T 2211A>C), G516D (2217G>A 2218T>C), I519F (2225A>T 2227T>C), P524E (2240C>G 2241C>A 2242A>G), D527M (2249G>A 2250A>T 2251C>G), T528E (2252A>G 2253C>A), S529D (2255T>G 2256C>A 2257C>T), M530A (2258A>G 2259T>C 2260G>C), A531P (2261G>C 2263A>C), V532I (2264G>A), E533Q (2267G>C), D535E (2275T>A), M536L (2276A>T), V540A (2289T>C 2290G>C), N545K (2305T>G), D546E (2308T>G), N549H (2315A>C), V556N (2336G>A 2337T>A 2338T>C), E558K (2342G>A), E565Q (2363G>C 2365G>A), Q566E (2366C>G 2368A>G), V567L (2369G>C), M568L (2372A>C 2374G>A), S569A (2375A>G 2376G>C 2377C>T), S580K (2409G>A 2410C>G), S927A (3449T>G 3451A>C), D936N (3476G>A 3478T>C), S938T (3482T>A 3484A>T), I943L (3497A>C), D949E (3517C>A), I951V (3521A>G), E953A (3528A>C 3529G>C), Q955D (3533C>G 3535G>T), E956D (3538G>T), G958_K959insKK (3544_3545insAAGAAG), P961K (3551C>A 3552C>A), P962E (3554C>G 3555C>A 3556C>G), K964V (3560A>G 3561A>T), A965G (3564C>G), S967V (3569A>G 3570G>T), I968A (3572A>G 3573T>C 3574T>A), S970G (3578T>G 3579C>G 3580C>A), I971L (3581A>C 3583A>T), M985S (3623A>T 3624T>C 3625G>T), G986A (3627G>C 3628T>C), D990T (3638G>A 3639A>C), E991S (3641G>A 3642A>G 3643A>C), L994V (3650T>G), I996L (3656A>C), I1003V (3677A>G), E1006S (3686G>T 3687A>C), G1007P (3689G>C 3690G>C 3691G>C), H1008T (3692C>A 3693A>C), T1024S (3740A>T 3742A>T), N1034E (3770A>G 3772T>G), S1036T (3776T>A 3778A>C), I1037L (3779A>C 3781T>C), V1038I (3782G>A 3784G>C), S1044G (3800T>G 3801C>G), P1046N (3806C>A 3807C>A), V1048I (3812G>A), F1056L (3838T>G), A1058T (3842G>A), P1064S (3860C>T 3862A>T), A1065Y (3863G>T 3864C>A), L1067V (3869C>G 3871T>G), N1068A (3872A>G 3873A>C 3874T>A), K1069E (3875A>G 3877A>G), N1071A (3881A>G 3882A>C 3883T>A), Y1072H (3884T>C), V1074L (3890G>C 3892A>T), T1080S (3908A>T 3910A>C), D1083N (3917G>A), A1089T (3935G>A 3937A>C), T1092I (3945C>T 3946G>T), T1096V (3956A>G 3957C>T 3958T>G), E1101K (3971G>A 3973G>A), H1107Y (3989C>T), R1109K (3996G>A), M1110A (3998A>G 3999T>C 4000G>A), S1114D (4010T>G 4011C>A 4012A>C), S1115N (4014G>A 4015C>T), Q1117S (4019C>A 4020A>G 4021A>T), M1121V (4031A>G 4033G>T), R1126K (4047G>A 4048G>A), N1129L (4055A>C 4056A>T), I1131L (4061A>T 4063C>G), S1137V (4079T>G 4080C>T 4081C>A), A1138D (4083C>A), I1139L (4085A>T), V1140I (4088G>A 4090G>T), L1146M (4106C>A 4108T>G), I1147V (4109A>G 4111A>T), S1148K (4112T>A 4113C>A 4114T>G), L1151P (4122T>C 4123T>A), K1154R (4131A>G), S1162L (4154T>C 4155C>T 4156T>G), H1165Q (4165T>A), H1165_E1166insX (4165_4166insT), N1171D (4181A>G), A1190S (4238G>T), I1191N (4242T>A 4243T>C)								

	Begin	End	Coverage	Score	Concordance	Matches	Identities	I/D/M/F*	Stop Codons
**NT**	**2113**	**4485**	**27.8%**	**905**	**33.2%**	**1365 (99.0%)**	**928 (67.3%)**	**14/0**	

Codon mutations: ACT484ACG (2122T>G), GTG488GCA (2133T>C 2134G>A), TCA489ACT (2135T>A 2137A>T), ACT490ACA (2140T>A), ACC491ACT (2143C>T), ACG492GCA (2144A>G 2146G>A), GAA494CAA (2150G>C), GAA494_GAT495insAGC (2152_2153insAGC), GAT495GAG (2155T>G), CCC496GGA (2156C>G 2157C>G 2158C>A), ACC497ATC (2160C>T), AAG498AAC (2164G>C), CCA499AAG (2165C>A 2166C>A 2167A>G), GAT500GAC (2170T>C), AAG501CAA (2171A>C 2173G>A), ATA502ATT (2176A>T), GAA503GTA (2178A>T), ATT504ATA (2182T>A), GTT505GAG (2184T>A 2185T>G), GCA506GTT (2187C>T 2188A>T), GAG507GCT (2190A>C 2191G>T), GGA508GAC (2193G>A 2194A>C), GCA509CCA (2195G>C), GAA510GAG (2200A>G), GAA511CAG (2201G>C 2203A>G), ACT513GTC (2207A>G 2208C>T 2209T>C), CAA514TCA (2210C>T 2211A>C), GGT516GAC (2217G>A 2218T>C), GTT518GTC (2224T>C), ATT519TTC (2225A>T 2227T>C), GGA523GGG (2239A>G), CCA524GAG (2240C>G 2241C>A 2242A>G), GAC527ATG (2249G>A 2250A>T 2251C>G), ACA528GAA (2252A>G 2253C>A), TCC529GAT (2255T>G 2256C>A 2257C>T), ATG530GCC (2258A>G 2259T>C 2260G>C), GCA531CCC (2261G>C 2263A>C), GTT532ATT (2264G>A), GAA533CAA (2267G>C), CTG534TTG (2270C>T), GAT535GAA (2275T>A), ATG536TTG (2276A>T), CAG537CAA (2281G>A), CAA538CAG (2284A>G), CCG539CCA (2287G>A), GTG540GCC (2289T>C 2290G>C), TGT541TGC (2293T>C), GTA542GTT (2296A>T), GCT543GCC (2299T>C), AAT545AAG (2305T>G), GAT546GAG (2308T>G), TTC548TTT (2314C>T), AAT549CAT (2315A>C), GTT550GTC (2320T>C), GGA551GGC (2323A>C), GTT552GTC (2326T>C), TTC555TTT (2335C>T), GTT556AAC (2336G>A 2337T>A 2338T>C), GAG558AAG (2342G>A), AAG559AAA (2347G>A), TCC560TCT (2350C>T), GCT561GCC (2353T>C), AAT562AAC (2356T>C), GTT563GTG (2359T>G), GAG565CAA (2363G>C 2365G>A), CAA566GAG (2366C>G 2368A>G), GTA567CTA (2369G>C), ATG568CTA (2372A>C 2374G>A), AGC569GCT (2375A>G 2376G>C 2377C>T), TTG570CTG (2378T>C), GCT571GCC (2383T>C), TTG572TTA (2386G>A), CCC573CCT (2389C>T), GCT574GCA (2392T>A), GCC575GCA (2395C>A), CTA576CTG (2398A>G), TCA578AGT (2402T>A 2403C>G 2404A>T), AGC580AAG (2409G>A 2410C>G), GGC924GGG (3442C>G), AAA925AAG (3445A>G), AGC926AGT (3448C>T), TCA927GCC (3449T>G 3451A>C), GAT929GAC (3457T>C), TTC930TTT (3460C>T), CTT931TTG (3461C>T 3463T>G), TTC932TTT (3466C>T), GAT936AAC (3476G>A 3478T>C), TTA937TTG (3481A>G), TCA938ACT (3482T>A 3484A>T), AAG941AAA (3493G>A), ATT943CTT (3497A>C), TTG946CTG (3506T>C), AAG948AAA (3514G>A), GAC949GAA (3517C>A), AAC950AAT (3520C>T), ATT951GTT (3521A>G), GAG953GCC (3528A>C 3529G>C), TAC954TAT (3532C>T), CAG955GAT (3533C>G 3535G>T), GAG956GAT (3538G>T), GGC958GGG (3544C>G), GGC958_AAA959insAAGAAG (3544_3545insAAGAAG), GAT960GAC (3550T>C), CCG961AAG (3551C>A 3552C>A), CCC962GAG (3554C>G 3555C>A 3556C>G), AAG963AAA (3559G>A), AAG964GTG (3560A>G 3561A>T), GCT965GGT (3564C>G), CAA966CAG (3568A>G), AGT967GTT (3569A>G 3570G>T), ATT968GCA (3572A>G 3573T>C 3574T>A), CTG969CTT (3577G>T), TCC970GGA (3578T>G 3579C>G 3580C>A), ATA971CTT (3581A>C 3583A>T), AGA972CGG (3584A>C 3586A>G), GAG973GAA (3589G>A), TTT975TTC (3595T>C), TCC976TCT (3598C>T), GGT978GGA (3604T>A), GTA980GTT (3610A>T), TTC983TTT (3619C>T), TGC984TGT (3622C>T), ATG985TCT (3623A>T 3624T>C 3625G>T), GGT986GCC (3627G>C 3628T>C), GAC990ACC (3638G>A 3639A>C), GAA991AGC (3641G>A 3642A>G 3643A>C), AGA992CGC (3644A>C 3646A>C), TTG993CTG (3647T>C), TTG994GTG (3650T>G), GTA995GTT (3655A>T), ATT996CTT (3656A>C), CCT997CCA (3661T>A), GCA999GCT (3667A>T), CCA1000CCG (3670A>G), TCC1002TCT (3676C>T), ATA1003GTA (3677A>G), AGG1004CGA (3680A>C 3682G>A), TTT1005TTC (3685T>C), GAA1006TCA (3686G>T 3687A>C), GGG1007CCC (3689G>C 3690G>C 3691G>C), CAC1008ACC (3692C>A 3693A>C), CCT1010CCC (3700T>C), GCA1014GCC (3712A>C), ATC1015ATA (3715C>A), ACT1016ACG (3718T>G), CCA1018CCC (3724A>C), ATA1020ATC (3730A>C), TGT1023TGC (3739T>C), ACA1024TCT (3740A>T 3742A>T), TGT1027TGC (3751T>C), TCA1030TCT (3760A>T), GGT1031GGC (3763T>C), TTG1033CTT (3767T>C 3769G>T), AAT1034GAG (3770A>G 3772T>G), TAT1035TAC (3775T>C), TCA1036ACC (3776T>A 3778A>C), ATT1037CTC (3779A>C 3781T>C), GTG1038ATC (3782G>A 3784G>C), ATA1039ATC (3787A>C), CAC1040CAT (3790C>T), AGA1041AGG (3793A>G), GTA1042GTC (3796A>C), TCC1044GGC (3800T>G 3801C>G), AGT1045TCT (3803A>T 3804G>C), CCT1046AAT (3806C>A 3807C>A), AAT1047AAC (3811T>C), GTT1048ATT (3812G>A), GGA1049GGT (3817A>T), CTA1052CTT (3826A>T), GTT1054GTG (3832T>G), TTT1056TTG (3838T>G), GAT1057GAC (3841T>C), GCC1058ACC (3842G>A), CCA1064TCT (3860C>T 3862A>T), GCT1065TAT (3863G>T 3864C>A), GGG1066GGT (3868G>T), CTT1067GTG (3869C>G 3871T>G), AAT1068GCA (3872A>G 3873A>C 3874T>A), AAA1069GAG (3875A>G 3877A>G), GGA1070GGG (3880A>G), AAT1071GCA (3881A>G 3882A>C 3883T>A), TAT1072CAT (3884T>C), GTA1074CTT (3890G>C 3892A>T), GGT1077GGC (3901T>C), GGA1078GGT (3904A>T), GGC1079GGT (3907C>T), ACA1080TCC (3908A>T 3910A>C), GAT1083AAT (3917G>A), TTT1084TTC (3922T>C), TCA1085TCC (3925A>C), GGT1087GGA (3931T>A), GTG1088GTT (3934G>T), GCA1089ACC (3935G>A 3937A>C), ACA1090ACT (3940A>T), AAT1091AAC (3943T>C), ACG1092ATT (3945C>T 3946G>T), TTC1093TTT (3949C>T), TCA1094TCC (3952A>C), TTC1095TTT (3955C>T), ACT1096GTG (3956A>G 3957C>T 3958T>G), GAT1099GAC (3967T>C), GAG1101AAA (3971G>A 3973G>A), CCA1104CCT (3982A>T), AGG1105CGG (3983A>C), CGG1106AGG (3986C>A), CAT1107TAT (3989C>T), ACA1108ACT (3994A>T), AGG1109AAG (3996G>A), ATG1110GCA (3998A>G 3999T>C 4000G>A), AGG1111CGA (4001A>C 4003G>A), TTC1113TTT (4009C>T), TCA1114GAC (4010T>G 4011C>A 4012A>C), AGC1115AAT (4014G>A 4015C>T), CAA1117AGT (4019C>A 4020A>G 4021A>T), TCC1118TCT (4024C>T), CGC1119CGT (4027C>T), ATC1120ATT (4030C>T), ATG1121GTT (4031A>G 4033G>T), TCA1122TCT (4036A>T), CTA1123TTG (4037C>T 4039A>G), GAT1125GAC (4045T>C), AGG1126AAA (4047G>A 4048G>A), CTT1127TTG (4049C>T 4051T>G), GGA1128GGT (4054A>T), AAT1129CTT (4055A>C 4056A>T), CTG1130CTT (4060G>T), ATC1131TTG (4061A>T 4063C>G), ATA1132ATC (4066A>C), TTG1134CTA (4070T>C 4072G>A), CCT1135CCA (4075T>A), CCT1136CCC (4078T>C), TCC1137GTA (4079T>G 4080C>T 4081C>A), GCC1138GAC (4083C>A), ATA1139TTA (4085A>T), GTG1140ATT (4088G>A 4090G>T), AGT1141AGC (4093T>C), GAG1144GAA (4102G>A), ATA1145ATC (4105A>C), CTT1146ATG (4106C>A 4108T>G), ATA1147GTT (4109A>G 4111A>T), TCT1148AAG (4112T>A 4113C>A 4114T>G), CCT1149CCA (4117T>A), GGA1150GGG (4120A>G), CTT1151CCA (4122T>C 4123T>A), TTC1153TTT (4129C>T), AAG1154AGG (4131A>G), TTG1155TTA (4135G>A), GAG1156GAA (4138G>A), TTG1157CTT (4139T>C 4141G>T), TCT1162CTG (4154T>C 4155C>T 4156T>G), GCC1163GCT (4159C>T), AAC1164AAT (4162C>T), CAT1165CAA (4165T>A), CAT1165_GAA1166insT-- (4165_4166insT), GGC1170GGT (4180C>T), AAT1171GAT (4181A>G), ACT1174ACG (4192T>G), CAC1175CAT (4195C>T), ACC1176ACT (4198C>T), TAT1177TAC (4201T>C), CAG1178CAA (4204G>A), TCA1181TCT (4213A>T), TTT1183TTC (4219T>C), TCT1184TCA (4222T>A), GAG1185GAA (4225G>A), CTA1186CTG (4228A>G), CGT1187AGA (4229C>A 4231T>A), GAT1188GAC (4234T>C), TTT1189TTC (4237T>C), GCG1190TCG (4238G>T), ATT1191AAC (4242T>A 4243T>C)

Proteins

polyprotein (YP_001976149.1)	482	1192	31.5%	1799	67.7%	375 (98.9%)	256 (67.5%)	4/0/1/1	1

Protein mutations: V488A (2133T>C 2134G>A), S489T (2135T>A 2137A>T), T492A (2144A>G 2146G>A), E494Q (2150G>C), E494_D495insS (2152_2153insAGC), D495E (2155T>G), P496G (2156C>G 2157C>G 2158C>A), T497I (2160C>T), K498N (2164G>C), P499K (2165C>A 2166C>A 2167A>G), K501Q (2171A>C 2173G>A), E503V (2178A>T), V505E (2184T>A 2185T>G), A506V (2187C>T 2188A>T), E507A (2190A>C 2191G>T), G508D (2193G>A 2194A>C), A509P (2195G>C), E511Q (2201G>C 2203A>G), T513V (2207A>G 2208C>T 2209T>C), Q514S (2210C>T 2211A>C), G516D (2217G>A 2218T>C), I519F (2225A>T 2227T>C), P524E (2240C>G 2241C>A 2242A>G), D527M (2249G>A 2250A>T 2251C>G), T528E (2252A>G 2253C>A), S529D (2255T>G 2256C>A 2257C>T), M530A (2258A>G 2259T>C 2260G>C), A531P (2261G>C 2263A>C), V532I (2264G>A), E533Q (2267G>C), D535E (2275T>A), M536L (2276A>T), V540A (2289T>C 2290G>C), N545K (2305T>G), D546E (2308T>G), N549H (2315A>C), V556N (2336G>A 2337T>A 2338T>C), E558K (2342G>A), E565Q (2363G>C 2365G>A), Q566E (2366C>G 2368A>G), V567L (2369G>C), M568L (2372A>C 2374G>A), S569A (2375A>G 2376G>C 2377C>T), S580K (2409G>A 2410C>G), S927A (3449T>G 3451A>C), D936N (3476G>A 3478T>C), S938T (3482T>A 3484A>T), I943L (3497A>C), D949E (3517C>A), I951V (3521A>G), E953A (3528A>C 3529G>C), Q955D (3533C>G 3535G>T), E956D (3538G>T), G958_K959insKK (3544_3545insAAGAAG), P961K (3551C>A 3552C>A), P962E (3554C>G 3555C>A 3556C>G), K964V (3560A>G 3561A>T), A965G (3564C>G), S967V (3569A>G 3570G>T), I968A (3572A>G 3573T>C 3574T>A), S970G (3578T>G 3579C>G 3580C>A), I971L (3581A>C 3583A>T), M985S (3623A>T 3624T>C 3625G>T), G986A (3627G>C 3628T>C), D990T (3638G>A 3639A>C), E991S (3641G>A 3642A>G 3643A>C), L994V (3650T>G), I996L (3656A>C), I1003V (3677A>G), E1006S (3686G>T 3687A>C), G1007P (3689G>C 3690G>C 3691G>C), H1008T (3692C>A 3693A>C), T1024S (3740A>T 3742A>T), N1034E (3770A>G 3772T>G), S1036T (3776T>A 3778A>C), I1037L (3779A>C 3781T>C), V1038I (3782G>A 3784G>C), S1044G (3800T>G 3801C>G), P1046N (3806C>A 3807C>A), V1048I (3812G>A), F1056L (3838T>G), A1058T (3842G>A), P1064S (3860C>T 3862A>T), A1065Y (3863G>T 3864C>A), L1067V (3869C>G 3871T>G), N1068A (3872A>G 3873A>C 3874T>A), K1069E (3875A>G 3877A>G), N1071A (3881A>G 3882A>C 3883T>A), Y1072H (3884T>C), V1074L (3890G>C 3892A>T), T1080S (3908A>T 3910A>C), D1083N (3917G>A), A1089T (3935G>A 3937A>C), T1092I (3945C>T 3946G>T), T1096V (3956A>G 3957C>T 3958T>G), E1101K (3971G>A 3973G>A), H1107Y (3989C>T), R1109K (3996G>A), M1110A (3998A>G 3999T>C 4000G>A), S1114D (4010T>G 4011C>A 4012A>C), S1115N (4014G>A 4015C>T), Q1117S (4019C>A 4020A>G 4021A>T), M1121V (4031A>G 4033G>T), R1126K (4047G>A 4048G>A), N1129L (4055A>C 4056A>T), I1131L (4061A>T 4063C>G), S1137V (4079T>G 4080C>T 4081C>A), A1138D (4083C>A), I1139L (4085A>T), V1140I (4088G>A 4090G>T), L1146M (4106C>A 4108T>G), I1147V (4109A>G 4111A>T), S1148K (4112T>A 4113C>A 4114T>G), L1151P (4122T>C 4123T>A), K1154R (4131A>G), S1162L (4154T>C 4155C>T 4156T>G), H1165Q (4165T>A), H1165_E1166insX (4165_4166insT), N1171D (4181A>G), A1190S (4238G>T), I1191N (4242T>A 4243T>C)

	Begin	End	Coverage	Score	Concordance	Matches	Identities	I/D/M/F*	Stop Codons
**NT**	**2113**	**4485**	**27.8%**	**905**	**33.2%**	**1365 (99.0%)**	**928 (67.3%)**	**14/0**	
Codon mutations:	ACT484ACG (2122T>G), GTG488GCA (2133T>C 2134G>A), TCA489ACT (2135T>A 2137A>T), ACT490ACA (2140T>A), ACC491ACT (2143C>T), ACG492GCA (2144A>G 2146G>A), GAA494CAA (2150G>C), GAA494_GAT495insAGC (2152_2153insAGC), GAT495GAG (2155T>G), CCC496GGA (2156C>G 2157C>G 2158C>A), ACC497ATC (2160C>T), AAG498AAC (2164G>C), CCA499AAG (2165C>A 2166C>A 2167A>G), GAT500GAC (2170T>C), AAG501CAA (2171A>C 2173G>A), ATA502ATT (2176A>T), GAA503GTA (2178A>T), ATT504ATA (2182T>A), GTT505GAG (2184T>A 2185T>G), GCA506GTT (2187C>T 2188A>T), GAG507GCT (2190A>C 2191G>T), GGA508GAC (2193G>A 2194A>C), GCA509CCA (2195G>C), GAA510GAG (2200A>G), GAA511CAG (2201G>C 2203A>G), ACT513GTC (2207A>G 2208C>T 2209T>C), CAA514TCA (2210C>T 2211A>C), GGT516GAC (2217G>A 2218T>C), GTT518GTC (2224T>C), ATT519TTC (2225A>T 2227T>C), GGA523GGG (2239A>G), CCA524GAG (2240C>G 2241C>A 2242A>G), GAC527ATG (2249G>A 2250A>T 2251C>G), ACA528GAA (2252A>G 2253C>A), TCC529GAT (2255T>G 2256C>A 2257C>T), ATG530GCC (2258A>G 2259T>C 2260G>C), GCA531CCC (2261G>C 2263A>C), GTT532ATT (2264G>A), GAA533CAA (2267G>C), CTG534TTG (2270C>T), GAT535GAA (2275T>A), ATG536TTG (2276A>T), CAG537CAA (2281G>A), CAA538CAG (2284A>G), CCG539CCA (2287G>A), GTG540GCC (2289T>C 2290G>C), TGT541TGC (2293T>C), GTA542GTT (2296A>T), GCT543GCC (2299T>C), AAT545AAG (2305T>G), GAT546GAG (2308T>G), TTC548TTT (2314C>T), AAT549CAT (2315A>C), GTT550GTC (2320T>C), GGA551GGC (2323A>C), GTT552GTC (2326T>C), TTC555TTT (2335C>T), GTT556AAC (2336G>A 2337T>A 2338T>C), GAG558AAG (2342G>A), AAG559AAA (2347G>A), TCC560TCT (2350C>T), GCT561GCC (2353T>C), AAT562AAC (2356T>C), GTT563GTG (2359T>G), GAG565CAA (2363G>C 2365G>A), CAA566GAG (2366C>G 2368A>G), GTA567CTA (2369G>C), ATG568CTA (2372A>C 2374G>A), AGC569GCT (2375A>G 2376G>C 2377C>T), TTG570CTG (2378T>C), GCT571GCC (2383T>C), TTG572TTA (2386G>A), CCC573CCT (2389C>T), GCT574GCA (2392T>A), GCC575GCA (2395C>A), CTA576CTG (2398A>G), TCA578AGT (2402T>A 2403C>G 2404A>T), AGC580AAG (2409G>A 2410C>G), GGC924GGG (3442C>G), AAA925AAG (3445A>G), AGC926AGT (3448C>T), TCA927GCC (3449T>G 3451A>C), GAT929GAC (3457T>C), TTC930TTT (3460C>T), CTT931TTG (3461C>T 3463T>G), TTC932TTT (3466C>T), GAT936AAC (3476G>A 3478T>C), TTA937TTG (3481A>G), TCA938ACT (3482T>A 3484A>T), AAG941AAA (3493G>A), ATT943CTT (3497A>C), TTG946CTG (3506T>C), AAG948AAA (3514G>A), GAC949GAA (3517C>A), AAC950AAT (3520C>T), ATT951GTT (3521A>G), GAG953GCC (3528A>C 3529G>C), TAC954TAT (3532C>T), CAG955GAT (3533C>G 3535G>T), GAG956GAT (3538G>T), GGC958GGG (3544C>G), GGC958_AAA959insAAGAAG (3544_3545insAAGAAG), GAT960GAC (3550T>C), CCG961AAG (3551C>A 3552C>A), CCC962GAG (3554C>G 3555C>A 3556C>G), AAG963AAA (3559G>A), AAG964GTG (3560A>G 3561A>T), GCT965GGT (3564C>G), CAA966CAG (3568A>G), AGT967GTT (3569A>G 3570G>T), ATT968GCA (3572A>G 3573T>C 3574T>A), CTG969CTT (3577G>T), TCC970GGA (3578T>G 3579C>G 3580C>A), ATA971CTT (3581A>C 3583A>T), AGA972CGG (3584A>C 3586A>G), GAG973GAA (3589G>A), TTT975TTC (3595T>C), TCC976TCT (3598C>T), GGT978GGA (3604T>A), GTA980GTT (3610A>T), TTC983TTT (3619C>T), TGC984TGT (3622C>T), ATG985TCT (3623A>T 3624T>C 3625G>T), GGT986GCC (3627G>C 3628T>C), GAC990ACC (3638G>A 3639A>C), GAA991AGC (3641G>A 3642A>G 3643A>C), AGA992CGC (3644A>C 3646A>C), TTG993CTG (3647T>C), TTG994GTG (3650T>G), GTA995GTT (3655A>T), ATT996CTT (3656A>C), CCT997CCA (3661T>A), GCA999GCT (3667A>T), CCA1000CCG (3670A>G), TCC1002TCT (3676C>T), ATA1003GTA (3677A>G), AGG1004CGA (3680A>C 3682G>A), TTT1005TTC (3685T>C), GAA1006TCA (3686G>T 3687A>C), GGG1007CCC (3689G>C 3690G>C 3691G>C), CAC1008ACC (3692C>A 3693A>C), CCT1010CCC (3700T>C), GCA1014GCC (3712A>C), ATC1015ATA (3715C>A), ACT1016ACG (3718T>G), CCA1018CCC (3724A>C), ATA1020ATC (3730A>C), TGT1023TGC (3739T>C), ACA1024TCT (3740A>T 3742A>T), TGT1027TGC (3751T>C), TCA1030TCT (3760A>T), GGT1031GGC (3763T>C), TTG1033CTT (3767T>C 3769G>T), AAT1034GAG (3770A>G 3772T>G), TAT1035TAC (3775T>C), TCA1036ACC (3776T>A 3778A>C), ATT1037CTC (3779A>C 3781T>C), GTG1038ATC (3782G>A 3784G>C), ATA1039ATC (3787A>C), CAC1040CAT (3790C>T), AGA1041AGG (3793A>G), GTA1042GTC (3796A>C), TCC1044GGC (3800T>G 3801C>G), AGT1045TCT (3803A>T 3804G>C), CCT1046AAT (3806C>A 3807C>A), AAT1047AAC (3811T>C), GTT1048ATT (3812G>A), GGA1049GGT (3817A>T), CTA1052CTT (3826A>T), GTT1054GTG (3832T>G), TTT1056TTG (3838T>G), GAT1057GAC (3841T>C), GCC1058ACC (3842G>A), CCA1064TCT (3860C>T 3862A>T), GCT1065TAT (3863G>T 3864C>A), GGG1066GGT (3868G>T), CTT1067GTG (3869C>G 3871T>G), AAT1068GCA (3872A>G 3873A>C 3874T>A), AAA1069GAG (3875A>G 3877A>G), GGA1070GGG (3880A>G), AAT1071GCA (3881A>G 3882A>C 3883T>A), TAT1072CAT (3884T>C), GTA1074CTT (3890G>C 3892A>T), GGT1077GGC (3901T>C), GGA1078GGT (3904A>T), GGC1079GGT (3907C>T), ACA1080TCC (3908A>T 3910A>C), GAT1083AAT (3917G>A), TTT1084TTC (3922T>C), TCA1085TCC (3925A>C), GGT1087GGA (3931T>A), GTG1088GTT (3934G>T), GCA1089ACC (3935G>A 3937A>C), ACA1090ACT (3940A>T), AAT1091AAC (3943T>C), ACG1092ATT (3945C>T 3946G>T), TTC1093TTT (3949C>T), TCA1094TCC (3952A>C), TTC1095TTT (3955C>T), ACT1096GTG (3956A>G 3957C>T 3958T>G), GAT1099GAC (3967T>C), GAG1101AAA (3971G>A 3973G>A), CCA1104CCT (3982A>T), AGG1105CGG (3983A>C), CGG1106AGG (3986C>A), CAT1107TAT (3989C>T), ACA1108ACT (3994A>T), AGG1109AAG (3996G>A), ATG1110GCA (3998A>G 3999T>C 4000G>A), AGG1111CGA (4001A>C 4003G>A), TTC1113TTT (4009C>T), TCA1114GAC (4010T>G 4011C>A 4012A>C), AGC1115AAT (4014G>A 4015C>T), CAA1117AGT (4019C>A 4020A>G 4021A>T), TCC1118TCT (4024C>T), CGC1119CGT (4027C>T), ATC1120ATT (4030C>T), ATG1121GTT (4031A>G 4033G>T), TCA1122TCT (4036A>T), CTA1123TTG (4037C>T 4039A>G), GAT1125GAC (4045T>C), AGG1126AAA (4047G>A 4048G>A), CTT1127TTG (4049C>T 4051T>G), GGA1128GGT (4054A>T), AAT1129CTT (4055A>C 4056A>T), CTG1130CTT (4060G>T), ATC1131TTG (4061A>T 4063C>G), ATA1132ATC (4066A>C), TTG1134CTA (4070T>C 4072G>A), CCT1135CCA (4075T>A), CCT1136CCC (4078T>C), TCC1137GTA (4079T>G 4080C>T 4081C>A), GCC1138GAC (4083C>A), ATA1139TTA (4085A>T), GTG1140ATT (4088G>A 4090G>T), AGT1141AGC (4093T>C), GAG1144GAA (4102G>A), ATA1145ATC (4105A>C), CTT1146ATG (4106C>A 4108T>G), ATA1147GTT (4109A>G 4111A>T), TCT1148AAG (4112T>A 4113C>A 4114T>G), CCT1149CCA (4117T>A), GGA1150GGG (4120A>G), CTT1151CCA (4122T>C 4123T>A), TTC1153TTT (4129C>T), AAG1154AGG (4131A>G), TTG1155TTA (4135G>A), GAG1156GAA (4138G>A), TTG1157CTT (4139T>C 4141G>T), TCT1162CTG (4154T>C 4155C>T 4156T>G), GCC1163GCT (4159C>T), AAC1164AAT (4162C>T), CAT1165CAA (4165T>A), CAT1165_GAA1166insT-- (4165_4166insT), GGC1170GGT (4180C>T), AAT1171GAT (4181A>G), ACT1174ACG (4192T>G), CAC1175CAT (4195C>T), ACC1176ACT (4198C>T), TAT1177TAC (4201T>C), CAG1178CAA (4204G>A), TCA1181TCT (4213A>T), TTT1183TTC (4219T>C), TCT1184TCA (4222T>A), GAG1185GAA (4225G>A), CTA1186CTG (4228A>G), CGT1187AGA (4229C>A 4231T>A), GAT1188GAC (4234T>C), TTT1189TTC (4237T>C), GCG1190TCG (4238G>T), ATT1191AAC (4242T>A 4243T>C)								
Coat protein A (YP_001976155.1)	14	116	40.6%	415	60.3%	103 (99.0%)	59 (56.7%)	1/0/0/0	0
Protein mutations:	V20A (2133T>C 2134G>A), S21T (2135T>A 2137A>T), T24A (2144A>G 2146G>A), E26Q (2150G>C), E26_D27insS (2152_2153insAGC), D27E (2155T>G), P28G (2156C>G 2157C>G 2158C>A), T29I (2160C>T), K30N (2164G>C), P31K (2165C>A 2166C>A 2167A>G), K33Q (2171A>C 2173G>A), E35V (2178A>T), V37E (2184T>A 2185T>G), A38V (2187C>T 2188A>T), E39A (2190A>C 2191G>T), G40D (2193G>A 2194A>C), A41P (2195G>C), E43Q (2201G>C 2203A>G), T45V (2207A>G 2208C>T 2209T>C), Q46S (2210C>T 2211A>C), G48D (2217G>A 2218T>C), I51F (2225A>T 2227T>C), P56E (2240C>G 2241C>A 2242A>G), D59M (2249G>A 2250A>T 2251C>G), T60E (2252A>G 2253C>A), S61D (2255T>G 2256C>A 2257C>T), M62A (2258A>G 2259T>C 2260G>C), A63P (2261G>C 2263A>C), V64I (2264G>A), E65Q (2267G>C), D67E (2275T>A), M68L (2276A>T), V72A (2289T>C 2290G>C), N77K (2305T>G), D78E (2308T>G), N81H (2315A>C), V88N (2336G>A 2337T>A 2338T>C), E90K (2342G>A), E97Q (2363G>C 2365G>A), Q98E (2366C>G 2368A>G), V99L (2369G>C), M100L (2372A>C 2374G>A), S101A (2375A>G 2376G>C 2377C>T), S112K (2409G>A 2410C>G)								
Codon mutations:	ACT16ACG (2122T>G), GTG20GCA (2133T>C 2134G>A), TCA21ACT (2135T>A 2137A>T), ACT22ACA (2140T>A), ACC23ACT (2143C>T), ACG24GCA (2144A>G 2146G>A), GAA26CAA (2150G>C), GAA26_GAT27insAGC (2152_2153insAGC), GAT27GAG (2155T>G), CCC28GGA (2156C>G 2157C>G 2158C>A), ACC29ATC (2160C>T), AAG30AAC (2164G>C), CCA31AAG (2165C>A 2166C>A 2167A>G), GAT32GAC (2170T>C), AAG33CAA (2171A>C 2173G>A), ATA34ATT (2176A>T), GAA35GTA (2178A>T), ATT36ATA (2182T>A), GTT37GAG (2184T>A 2185T>G), GCA38GTT (2187C>T 2188A>T), GAG39GCT (2190A>C 2191G>T), GGA40GAC (2193G>A 2194A>C), GCA41CCA (2195G>C), GAA42GAG (2200A>G), GAA43CAG (2201G>C 2203A>G), ACT45GTC (2207A>G 2208C>T 2209T>C), CAA46TCA (2210C>T 2211A>C), GGT48GAC (2217G>A 2218T>C), GTT50GTC (2224T>C), ATT51TTC (2225A>T 2227T>C), GGA55GGG (2239A>G), CCA56GAG (2240C>G 2241C>A 2242A>G), GAC59ATG (2249G>A 2250A>T 2251C>G), ACA60GAA (2252A>G 2253C>A), TCC61GAT (2255T>G 2256C>A 2257C>T), ATG62GCC (2258A>G 2259T>C 2260G>C), GCA63CCC (2261G>C 2263A>C), GTT64ATT (2264G>A), GAA65CAA (2267G>C), CTG66TTG (2270C>T), GAT67GAA (2275T>A), ATG68TTG (2276A>T), CAG69CAA (2281G>A), CAA70CAG (2284A>G), CCG71CCA (2287G>A), GTG72GCC (2289T>C 2290G>C), TGT73TGC (2293T>C), GTA74GTT (2296A>T), GCT75GCC (2299T>C), AAT77AAG (2305T>G), GAT78GAG (2308T>G), TTC80TTT (2314C>T), AAT81CAT (2315A>C), GTT82GTC (2320T>C), GGA83GGC (2323A>C), GTT84GTC (2326T>C), TTC87TTT (2335C>T), GTT88AAC (2336G>A 2337T>A 2338T>C), GAG90AAG (2342G>A), AAG91AAA (2347G>A), TCC92TCT (2350C>T), GCT93GCC (2353T>C), AAT94AAC (2356T>C), GTT95GTG (2359T>G), GAG97CAA (2363G>C 2365G>A), CAA98GAG (2366C>G 2368A>G), GTA99CTA (2369G>C), ATG100CTA (2372A>C 2374G>A), AGC101GCT (2375A>G 2376G>C 2377C>T), TTG102CTG (2378T>C), GCT103GCC (2383T>C), TTG104TTA (2386G>A), CCC105CCT (2389C>T), GCT106GCA (2392T>A), GCC107GCA (2395C>A), CTA108CTG (2398A>G), TCA110AGT (2402T>A 2403C>G 2404A>T), AGC112AAG (2409G>A 2410C>G)								
Coat protein B (YP_001976156.1)	199	244	18.9%	218	68.8%	46 (95.8%)	33 (68.8%)	2/0/0/0	0
Protein mutations:	S205A (3449T>G 3451A>C), D214N (3476G>A 3478T>C), S216T (3482T>A 3484A>T), I221L (3497A>C), D227E (3517C>A), I229V (3521A>G), E231A (3528A>C 3529G>C), Q233D (3533C>G 3535G>T), E234D (3538G>T), G236_K237insKK (3544_3545insAAGAAG), P239K (3551C>A 3552C>A), P240E (3554C>G 3555C>A 3556C>G), K242V (3560A>G 3561A>T), A243G (3564C>G)								
Codon mutations:	GGC202GGG (3442C>G), AAA203AAG (3445A>G), AGC204AGT (3448C>T), TCA205GCC (3449T>G 3451A>C), GAT207GAC (3457T>C), TTC208TTT (3460C>T), CTT209TTG (3461C>T 3463T>G), TTC210TTT (3466C>T), GAT214AAC (3476G>A 3478T>C), TTA215TTG (3481A>G), TCA216ACT (3482T>A 3484A>T), AAG219AAA (3493G>A), ATT221CTT (3497A>C), TTG224CTG (3506T>C), AAG226AAA (3514G>A), GAC227GAA (3517C>A), AAC228AAT (3520C>T), ATT229GTT (3521A>G), GAG231GCC (3528A>C 3529G>C), TAC232TAT (3532C>T), CAG233GAT (3533C>G 3535G>T), GAG234GAT (3538G>T), GGC236GGG (3544C>G), GGC236_AAA237insAAGAAG (3544_3545insAAGAAG), GAT238GAC (3550T>C), CCG239AAG (3551C>A 3552C>A), CCC240GAG (3554C>G 3555C>A 3556C>G), AAG241AAA (3559G>A), AAG242GTG (3560A>G 3561A>T), GCT243GGT (3564C>G), CAA244CAG (3568A>G)								
Coat protein C (YP_001976157.1)	1	225	100%	1165	70.6%	225 (99.6%)	163 (72.1%)	1/0/1/1	0
Protein mutations:	S1V (3569A>G 3570G>T), I2A (3572A>G 3573T>C 3574T>A), S4G (3578T>G 3579C>G 3580C>A), I5L (3581A>C 3583A>T), M19S (3623A>T 3624T>C 3625G>T), G20A (3627G>C 3628T>C), D24T (3638G>A 3639A>C), E25S (3641G>A 3642A>G 3643A>C), L28V (3650T>G), I30L (3656A>C), I37V (3677A>G), E40S (3686G>T 3687A>C), G41P (3689G>C 3690G>C 3691G>C), H42T (3692C>A 3693A>C), T58S (3740A>T 3742A>T), N68E (3770A>G 3772T>G), S70T (3776T>A 3778A>C), I71L (3779A>C 3781T>C), V72I (3782G>A 3784G>C), S78G (3800T>G 3801C>G), P80N (3806C>A 3807C>A), V82I (3812G>A), F90L (3838T>G), A92T (3842G>A), P98S (3860C>T 3862A>T), A99Y (3863G>T 3864C>A), L101V (3869C>G 3871T>G), N102A (3872A>G 3873A>C 3874T>A), K103E (3875A>G 3877A>G), N105A (3881A>G 3882A>C 3883T>A), Y106H (3884T>C), V108L (3890G>C 3892A>T), T114S (3908A>T 3910A>C), D117N (3917G>A), A123T (3935G>A 3937A>C), T126I (3945C>T 3946G>T), T130V (3956A>G 3957C>T 3958T>G), E135K (3971G>A 3973G>A), H141Y (3989C>T), R143K (3996G>A), M144A (3998A>G 3999T>C 4000G>A), S148D (4010T>G 4011C>A 4012A>C), S149N (4014G>A 4015C>T), Q151S (4019C>A 4020A>G 4021A>T), M155V (4031A>G 4033G>T), R160K (4047G>A 4048G>A), N163L (4055A>C 4056A>T), I165L (4061A>T 4063C>G), S171V (4079T>G 4080C>T 4081C>A), A172D (4083C>A), I173L (4085A>T), V174I (4088G>A 4090G>T), L180M (4106C>A 4108T>G), I181V (4109A>G 4111A>T), S182K (4112T>A 4113C>A 4114T>G), L185P (4122T>C 4123T>A), K188R (4131A>G), S196L (4154T>C 4155C>T 4156T>G), H199Q (4165T>A), H199_E200insX (4165_4166insT), N205D (4181A>G), A224S (4238G>T), I225N (4242T>A 4243T>C)								

	Begin	End	Coverage	Score	Concordance	Matches	Identities	I/D/M/F*	Stop Codons
NT	2113	4485	27.8%	905	33.2%	1365 (99.0%)	928 (67.3%)	14/0	
Codon mutations:	AGT1GTT (3569A>G 3570G>T), ATT2GCA (3572A>G 3573T>C 3574T>A), CTG3CTT (3577G>T), TCC4GGA (3578T>G 3579C>G 3580C>A), ATA5CTT (3581A>C 3583A>T), AGA6CGG (3584A>C 3586A>G), GAG7GAA (3589G>A), TTT9TTC (3595T>C), TCC10TCT (3598C>T), GGT12GGA (3604T>A), GTA14GTT (3610A>T), TTC17TTT (3619C>T), TGC18TGT (3622C>T), ATG19TCT (3623A>T 3624T>C 3625G>T), GGT20GCC (3627G>C 3628T>C), GAC24ACC (3638G>A 3639A>C), GAA25AGC (3641G>A 3642A>G 3643A>C), AGA26CGC (3644A>C 3646A>C), TTG27CTG (3647T>C), TTG28GTG (3650T>G), GTA29GTT (3655A>T), ATT30CTT (3656A>C), CCT31CCA (3661T>A), GCA33GCT (3667A>T), CCA34CCG (3670A>G), TCC36TCT (3676C>T), ATA37GTA (3677A>G), AGG38CGA (3680A>C 3682G>A), TTT39TTC (3685T>C), GAA40TCA (3686G>T 3687A>C), GGG41CCC (3689G>C 3690G>C 3691G>C), CAC42ACC (3692C>A 3693A>C), CCT44CCC (3700T>C), GCA48GCC (3712A>C), ATC49ATA (3715C>A), ACT50ACG (3718T>G), CCA52CCC (3724A>C), ATA54ATC (3730A>C), TGT57TGC (3739T>C), ACA58TCT (3740A>T 3742A>T), TGT61TGC (3751T>C), TCA64TCT (3760A>T), GGT65GGC (3763T>C), TTG67CTT (3767T>C 3769G>T), AAT68GAG (3770A>G 3772T>G), TAT69TAC (3775T>C), TCA70ACC (3776T>A 3778A>C), ATT71CTC (3779A>C 3781T>C), GTG72ATC (3782G>A 3784G>C), ATA73ATC (3787A>C), CAC74CAT (3790C>T), AGA75AGG (3793A>G), GTA76GTC (3796A>C), TCC78GGC (3800T>G 3801C>G), AGT79TCT (3803A>T 3804G>C), CCT80AAT (3806C>A 3807C>A), AAT81AAC (3811T>C), GTT82ATT (3812G>A), GGA83GGT (3817A>T), CTA86CTT (3826A>T), GTT88GTG (3832T>G), TTT90TTG (3838T>G), GAT91GAC (3841T>C), GCC92ACC (3842G>A), CCA98TCT (3860C>T 3862A>T), GCT99TAT (3863G>T 3864C>A), GGG100GGT (3868G>T), CTT101GTG (3869C>G 3871T>G), AAT102GCA (3872A>G 3873A>C 3874T>A), AAA103GAG (3875A>G 3877A>G), GGA104GGG (3880A>G), AAT105GCA (3881A>G 3882A>C 3883T>A), TAT106CAT (3884T>C), GTA108CTT (3890G>C 3892A>T), GGT111GGC (3901T>C), GGA112GGT (3904A>T), GGC113GGT (3907C>T), ACA114TCC (3908A>T 3910A>C), GAT117AAT (3917G>A), TTT118TTC (3922T>C), TCA119TCC (3925A>C), GGT121GGA (3931T>A), GTG122GTT (3934G>T), GCA123ACC (3935G>A 3937A>C), ACA124ACT (3940A>T), AAT125AAC (3943T>C), ACG126ATT (3945C>T 3946G>T), TTC127TTT (3949C>T), TCA128TCC (3952A>C), TTC129TTT (3955C>T), ACT130GTG (3956A>G 3957C>T 3958T>G), GAT133GAC (3967T>C), GAG135AAA (3971G>A 3973G>A), CCA138CCT (3982A>T), AGG139CGG (3983A>C), CGG140AGG (3986C>A), CAT141TAT (3989C>T), ACA142ACT (3994A>T), AGG143AAG (3996G>A), ATG144GCA (3998A>G 3999T>C 4000G>A), AGG145CGA (4001A>C 4003G>A), TTC147TTT (4009C>T), TCA148GAC (4010T>G 4011C>A 4012A>C), AGC149AAT (4014G>A 4015C>T), CAA151AGT (4019C>A 4020A>G 4021A>T), TCC152TCT (4024C>T), CGC153CGT (4027C>T), ATC154ATT (4030C>T), ATG155GTT (4031A>G 4033G>T), TCA156TCT (4036A>T), CTA157TTG (4037C>T 4039A>G), GAT159GAC (4045T>C), AGG160AAA (4047G>A 4048G>A), CTT161TTG (4049C>T 4051T>G), GGA162GGT (4054A>T), AAT163CTT (4055A>C 4056A>T), CTG164CTT (4060G>T), ATC165TTG (4061A>T 4063C>G), ATA166ATC (4066A>C), TTG168CTA (4070T>C 4072G>A), CCT169CCA (4075T>A), CCT170CCC (4078T>C), TCC171GTA (4079T>G 4080C>T 4081C>A), GCC172GAC (4083C>A), ATA173TTA (4085A>T), GTG174ATT (4088G>A 4090G>T), AGT175AGC (4093T>C), GAG178GAA (4102G>A), ATA179ATC (4105A>C), CTT180ATG (4106C>A 4108T>G), ATA181GTT (4109A>G 4111A>T), TCT182AAG (4112T>A 4113C>A 4114T>G), CCT183CCA (4117T>A), GGA184GGG (4120A>G), CTT185CCA (4122T>C 4123T>A), TTC187TTT (4129C>T), AAG188AGG (4131A>G), TTG189TTA (4135G>A), GAG190GAA (4138G>A), TTG191CTT (4139T>C 4141G>T), TCT196CTG (4154T>C 4155C>T 4156T>G), GCC197GCT (4159C>T), AAC198AAT (4162C>T), CAT199CAA (4165T>A), CAT199_GAA200insT-- (4165_4166insT), GGC204GGT (4180C>T), AAT205GAT (4181A>G), ACT208ACG (4192T>G), CAC209CAT (4195C>T), ACC210ACT (4198C>T), TAT211TAC (4201T>C), CAG212CAA (4204G>A), TCA215TCT (4213A>T), TTT217TTC (4219T>C), TCT218TCA (4222T>A), GAG219GAA (4225G>A), CTA220CTG (4228A>G), CGT221AGA (4229C>A 4231T>A), GAT222GAC (4234T>C), TTT223TTC (4237T>C), GCG224TCG (4238G>T), ATT225AAC (4242T>A 4243T>C)								

*: Inserts / Deletes / Misaligned / Frameshifts

Analysis details

This analysis was performed with panviral2.64

NGS Details (UN60): Diachasmimorpha longicaudata entomopoxvirus (segment NC_043455.1)

Assembly

**Coverage Length**	1124 (1 contig(s))
**Depth Of Coverage**	803.2
**Number Of Reads**	7189
**Reads Per Million**	152.48 rpm (after QC)
**Ambiguities**	0
**Assembly Method**	de novo + reference guided assembly
**Consensus Caller**	Bcf Tools

Coverage Map

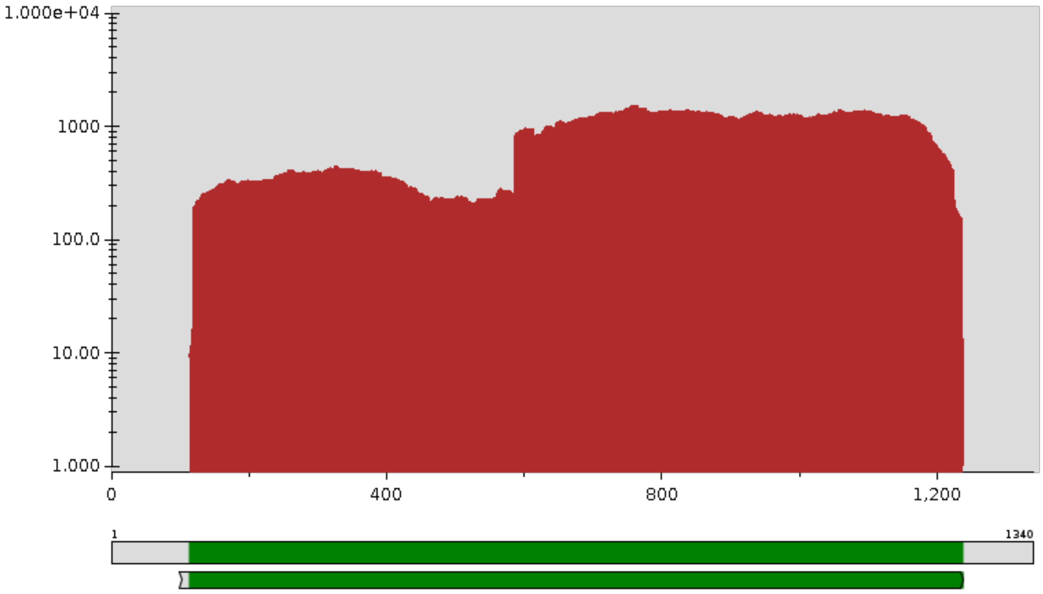

Assignment

**Type**	Diachasmimorpha longicaudata entomopoxvirus (Taxonomy ID: 109981)
**Reference Genome**	NC_043455.1
**NT Identity (%)**	60.4982
**AA Identity (%)**	55.2
**Number Of Stop Codons**	0
**Number Of CDS**	1

Alignment

**Alignment Score**	454.0 (NT) + 1463.0 (AA) = 1917.0
**Concordance (%)**	40.8394

Alignment Method	Global, seeded, nucleotide + amino acids (AGA)

Genome Region

Sequence starts at position 115 and ends at position 1238 relative to NC_043455.1 reference sequence.

Alignment Detailed Statistics

	Begin	End	Coverage	Score	Concordance	Matches	Identities	I/D/M/F*	Stop Codons
**NT**	**115**	**1238**	**83.9%**	**454**	**20.4%**	**1121 (99.5%)**	**680 (60.3%)**	**3/3**	
Mutations:	121A>T, 126A>C, 127C>T, 128C>G, 130T>G, 136C>G, 137C>A, 139C>T, 142G>A, 143A>G, 145A>T, 146A>G, 148T>C, 149A>C, 151C>T, 154A>T, 156A>G, 157G>A, 160T>G, 163A>T, 166T>C, 167T>C, 168C>A, 169T>G, 171G>A, 172T>C, 175A>G, 178T>C, 187T>A, 190A>T, 191A>G, 193T>C, 203A>C, 204A>G, 208C>T, 209A>G, 211T>A, 214T>G, 217A>T, 218T>A, 219G>T, 223T>A, 226T>G, 230A>C, 231A>G, 232A>T, 238G>C, 241A>T, 243T>C, 244C>T, 247A>G, 256G>C, 259A>T, 262A>C, 271A>C, 272G>T, 274A>C, 276C>T, 277A>G, 278T>A, 279A>T, 283A>T, 284A>C, 286C>T, 288G>C, 289T>C, 294T>G, 295A>C, 298G>A, 299C>G, 300A>T, 301A>T, 302A>G, 304A>G, 310T>C, 311T>A, 312C>A, 313T>A, 320A>G, 322T>G, 323A>G, 325C>A, 331T>A, 337C>A, 341A>T, 343C>T, 346A>T, 352T>G, 355A>G, 356T>C, 358G>T, 361T>A, 362C>G, 363T>C, 364C>T, 368G>A, 370T>G, 371C>G, 374C>A, 375G>A, 376T>G, 379A>G, 380T>A, 382G>T, 383C>T, 384A>T, 385A>G, 386A>G, 394G>A, 395A>G, 400T>C, 401C>A, 403A>T, 404_406delTAT, 409T>C, 410T>A, 412T>A, 413A>C, 415A>G, 416T>G, 417G>C, 418T>A, 421A>T, 423T>C, 424T>G, 438C>A, 442T>C, 443A>G, 445C>G, 446A>G, 447A>G, 448G>T, 452A>G, 453G>A, 454C>T, 455C>A, 456A>T, 457G>C, 458G>A, 459A>G, 462C>A, 463T>G, 467A>G, 469_470insCAT, 470A>G, 471A>G, 474C>T, 475C>A, 478G>A, 482T>G, 484G>C, 485A>T, 486T>C, 500C>A, 503A>G, 505G>C, 506A>T, 507T>G, 511T>C, 512C>A, 514A>G, 515C>A, 517C>T, 519C>A, 520C>A, 521C>A, 523A>G, 525A>G, 528G>C, 530A>T, 532C>A, 533G>C, 534A>G, 535T>A, 540A>G, 541A>G, 544A>C, 547T>A, 551A>T, 554G>T, 556T>G, 559A>T, 560A>C, 562A>T, 569G>T, 585T>G, 586A>C, 587G>C, 588A>G, 589T>A, 590A>G, 591A>G, 595T>C, 596T>A, 597T>A, 602A>C, 604A>G, 607A>T, 608C>T, 610A>T, 616T>A, 620G>C, 622A>G, 623T>C, 628T>G, 629G>C, 630A>C, 632A>C, 633G>C, 634T>A, 635C>A, 637T>A, 638G>A, 646T>G, 647A>G, 648T>G, 650T>G, 652A>T, 655A>C, 658A>T, 661A>C, 664C>T, 665G>A, 667T>G, 671T>C, 674A>G, 675G>A, 678T>C, 680A>C, 682C>T, 683A>G, 685T>G, 687C>T, 688A>T, 689T>A, 692C>A, 693A>G, 694G>A, 695G>A, 696T>A, 697C>G, 700T>C, 705G>A, 706A>C, 707G>A, 709T>G, 715A>G, 717A>G, 718A>G, 724G>T, 727A>G, 730A>G, 731A>C, 732A>G, 735C>A, 739A>G, 740T>C, 742G>C, 745A>T, 748C>T, 754C>T, 759G>A, 765A>T, 770A>G, 772A>C, 778A>C, 779A>G, 781G>T, 784A>G, 785A>G, 787T>A, 790T>G, 792T>G, 793T>G, 796A>G, 797G>C, 798C>T, 799A>T, 810T>G, 820T>C, 823C>G, 824C>A, 825A>C, 830A>G, 831G>C, 833C>A, 838T>C, 841A>G, 843C>G, 844A>T, 845C>G, 847A>C, 850A>C, 854T>G, 855G>T, 856T>G, 859T>C, 862A>C, 863C>A, 865T>G, 866A>C, 868A>T, 869C>A, 878G>T, 879T>G, 880A>G, 885T>C, 886G>T, 889A>T, 890T>A, 891G>A, 892T>G, 893T>A, 895A>G, 896A>C, 897C>G, 898A>C, 900A>G, 901T>C, 902A>C, 904A>T, 905A>G, 908T>C, 909T>A, 915C>T, 919A>T, 920A>G, 921G>C, 922C>A, 924T>C, 925C>T, 934T>C, 938T>G, 939C>A, 943A>G, 944C>A, 946A>C, 947G>A, 948A>C, 949A>T, 950C>A, 952T>A, 956T>A, 958T>C, 965A>C, 966A>G, 970A>G, 973T>C, 974A>C, 977G>T, 978A>C, 982T>G, 983A>T, 984A>C, 986A>T, 989A>C, 991A>T, 992A>G, 994A>G, 997T>C, 998C>A, 1000T>C, 1001T>A, 1003A>T, 1007A>G, 1012A>T, 1015A>G, 1018A>T, 1019A>C, 1021A>T, 1024C>T, 1033T>A, 1036A>G, 1040A>G, 1042A>C, 1045T>C, 1046T>C, 1048A>T, 1051T>C, 1054C>T, 1057T>C, 1063T>C, 1064T>C, 1070C>A, 1071A>C, 1073A>C, 1075T>A, 1076A>C, 1077G>C, 1087T>C, 1088A>C, 1090A>G, 1103A>C, 1105A>T, 1111T>A, 1112A>C, 1120C>A, 1121C>A, 1123A>G, 1126A>G, 1129G>T, 1130A>G, 1141T>C, 1145A>G, 1147A>C, 1150T>C, 1151G>A, 1153A>G, 1156C>T, 1159A>T, 1160T>G, 1161T>A, 1163C>A, 1164C>G, 1165A>G, 1167A>T, 1172G>T, 1173C>T, 1177A>C, 1178T>A, 1181G>C, 1183A>G, 1185C>A, 1186G>A, 1192T>C, 1195T>C, 1196A>G, 1197C>T, 1198C>T, 1199A>G, 1200A>T, 1204A>C, 1207T>G, 1210A>G, 1211A>C, 1218A>C, 1219G>C, 1223A>G, 1227T>C, 1232T>C, 1234G>C, 1235A>C, 1237A>G								

CDS

	Begin	End	Coverage	Score	Concordance	Matches	Identities	I/D/M/F*	Stop Codons
FLA14_p101	6	380	98.7%	1463	59.0%	374 (99.5%)	207 (55.1%)	1/1/0/0	0
Protein mutations:	E7D (121A>T), D9A (126A>C 127C>T), H10E (128C>G 130T>G), L13I (137C>A 139C>T), K15D (143A>G 145A>T), N16D (146A>G 148T>C), I17L (149A>C 151C>T), K19R (156A>G 157G>A), S23Q (167T>C 168C>A 169T>G), C24Y (171G>A 172T>C), T31A (191A>G 193T>C), K35R (203A>C 204A>G), I37V (209A>G 211T>A), F38L (214T>G), C40I (218T>A 219G>T), K44R (230A>C 231A>G 232A>T), V48A (243T>C 244C>T), A58S (272G>T 274A>C), T59M (276C>T 277A>G), Y60I (278T>A 279A>T), I62L (284A>C 286C>T), S63T (288G>C 289T>C), L65C (294T>G 295A>C), Q67V (299C>G 300A>T 301A>T), I68V (302A>G 304A>G), S71K (311T>A 312C>A 313T>A), N74E (320A>G 322T>G), I75V (323A>G 325C>A), T81S (341A>T 343C>T), L88A (362C>G 363T>C 364C>T), A90T (368G>A 370T>G), Q91E (371C>G), R92K (374C>A 375G>A 376T>G), L94I (380T>A 382G>T), Q95L (383C>T 384A>T 385A>G), T96A (386A>G), N99D (395A>G), L101I (401C>A 403A>T), Y102del (404_406delTAT), F104I (410T>A 412T>A), K105Q (413A>C 415A>G), C106A (416T>G 417G>C 418T>A), Q107H (421A>T), V108A (423T>C 424T>G), T113K (438C>A), I115V (443A>G 445C>G), K116G (446A>G 447A>G 448G>T), S118D (452A>G 453G>A 454C>T), Q119I (455C>A 456A>T 457G>C), E120R (458G>A 459A>G), T121K (462C>A 463T>G), K123E (467A>G), K123_K124insH (469_470insCAT), K124G (470A>G 471A>G), A125V (474C>T 475C>A), L128V (482T>G 484G>C), I129S (485A>T 486T>C), M135V (503A>G 505G>C), I136C (506A>T 507T>G), L138M (512C>A 514A>G), L139I (515C>A 517C>T), T140K (519C>A 520C>A), K142R (525A>G), S143T (528G>C), I144L (530A>T 532C>A), D145R (533G>C 534A>G 535T>A), K147R (540A>G 541A>G), I151L (551A>T), V152L (554G>T 556T>G), I154L (560A>C 562A>T), A157S (569G>T), I162S (585T>G 586A>C), D163R (587G>C 588A>G 589T>A), N164G (590A>G 591A>G), L166K (596T>A 597T>A), K168Q (602A>C 604A>G), Q170Y (608C>T 610A>T), E174Q (620G>C 622A>G), F175L (623T>C), F176L (628T>G), E177P (629G>C 630A>C), S178P (632A>C 633G>C 634T>A), H179K (635C>A 637T>A), V180I (638G>A), I183G (647A>G 648T>G), L184V (650T>G 652A>T), L185F (655A>C), V189M (665G>A 667T>G), S191P (671T>C), R192E (674A>G 675G>A), V193A (678T>C), I194L (680A>C 682C>T), N195E (683A>G 685T>G), T196I (687C>T 688A>T), S197T (689T>A), Q198R (692C>A 693A>G 694G>A), V199K (695G>A 696T>A 697C>G), R202N (705G>A 706A>C), D203K (707G>A 709T>G), K206R (717A>G 718A>G), N211R (731A>C 732A>G), A212D (735C>A), R220K (759G>A), Y222F (765A>T), I224V (770A>G 772A>C), K227D (779A>G 781G>T), N229E (785A>G 787T>A), D230E (790T>G), F231W (792T>G 793T>G), A233L (797G>C 798C>T 799A>T), F237C (810T>G), D241E (823C>G), H242T (824C>A 825A>C), S244A (830A>G 831G>C), L245I (833C>A), T248S (843C>G 844A>T), L249V (845C>G 847A>C), C252V (854T>G 855G>T 856T>G), Q257K (869C>A), V260W (878G>T 879T>G 880A>G), M262T (885T>C 886G>T), E263D (889A>T), C264K (890T>A 891G>A 892T>G), L265M (893T>A 895A>G), T266R (896A>C 897C>G 898A>C), N267S (900A>G 901T>C), N269D (905A>G), F270H (908T>C 909T>A), A272V (915C>T), S274A (920A>G 921G>C 922C>A), I275T (924T>C 925C>T), S280D (938T>G 939C>A), Q282N (944C>A 946A>C), E283T (947G>A 948A>C 949A>T), F286I (956T>A 958T>C), K289R (965A>C 966A>G), D293S (977G>T 978A>C), K295S (983A>T 984A>C), T296S (986A>T), I298V (992A>G 994A>G), L300I (998C>A 1000T>C), S301T (1001T>A 1003A>T), N303D (1007A>G), I314V (1040A>G 1042A>C), H324T (1070C>A 1071A>C), N325Q (1073A>C 1075T>A), R326P (1076A>C 1077G>C), I330L (1088A>C 1090A>G), I344V (1130A>G), I349V (1145A>G 1147A>C), E351K (1151G>A 1153A>G), E353D (1159A>T), L354E (1160T>G 1161T>A), P355R (1163C>A 1164C>G 1165A>G), K356M (1167A>T), A358F (1172G>T 1173C>T), E359D (1177A>C), L360I (1178T>A), E361Q (1181G>C 1183A>G), T362K (1185C>A 1186G>A), T366V (1196A>G 1197C>T 1198C>T), K367V (1199A>G 1200A>T), D369E (1207T>G), M371L (1211A>C), E373A (1218A>C 1219G>C), I375V (1223A>G), V376A (1227T>C), I379L (1235A>C 1237A>G)								

	Begin	End	Coverage	Score	Concordance	Matches	Identities	I/D/M/F*	Stop Codons
**NT**	**115**	**1238**	**83.9%**	**454**	**20.4%**	**1121 (99.5%)**	**680 (60.3%)**	**3/3**	
Codon mutations:	GAA7GAT (121A>T), GAC9GCT (126A>C 127C>T), CAT10GAG (128C>G 130T>G), GGC12GGG (136C>G), CTC13ATT (137C>A 139C>T), AAG14AAA (142G>A), AAA15GAT (143A>G 145A>T), AAT16GAC (146A>G 148T>C), ATC17CTT (149A>C 151C>T), CTA18CTT (154A>T), AAG19AGA (156A>G 157G>A), GGT20GGG (160T>G), ATA21ATT (163A>T), TAT22TAC (166T>C), TCT23CAG (167T>C 168C>A 169T>G), TGT24TAC (171G>A 172T>C), GGA25GGG (175A>G), TTT26TTC (178T>C), CCT29CCA (187T>A), TCA30TCT (190A>T), ACT31GCC (191A>G 193T>C), AAA35CGA (203A>C 204A>G), GCC36GCT (208C>T), ATT37GTA (209A>G 211T>A), TTT38TTG (214T>G), CCA39CCT (217A>T), TGT40ATT (218T>A 219G>T), ATT41ATA (223T>A), TCT42TCG (226T>G), AAA44CGT (230A>C 231A>G 232A>T), GTG46GTC (238G>C), ATA47ATT (241A>T), GTC48GCT (243T>C 244C>T), CAA49CAG (247A>G), TCG52TCC (256G>C), GGA53GGT (259A>T), ACA54ACC (262A>C), ACA57ACC (271A>C), GCA58TCC (272G>T 274A>C), ACA59ATG (276C>T 277A>G), TAT60ATT (278T>A 279A>T), GCA61GCT (283A>T), ATC62CTT (284A>C 286C>T), AGT63ACC (288G>C 289T>C), TTA65TGC (294T>G 295A>C), CAG66CAA (298G>A), CAA67GTT (299C>G 300A>T 301A>T), ATA68GTG (302A>G 304A>G), ACT70ACC (310T>C), TCT71AAA (311T>A 312C>A 313T>A), AAT74GAG (320A>G 322T>G), ATC75GTA (323A>G 325C>A), GCT77GCA (331T>A), ATC79ATA (337C>A), ACC81TCT (341A>T 343C>T), CCA82CCT (346A>T), CGT84CGG (352T>G), GAA85GAG (355A>G), TTG86CTT (356T>C 358G>T), GCT87GCA (361T>A), CTC88GCT (362C>G 363T>C 364C>T), GCT90ACG (368G>A 370T>G), CAA91GAA (371C>G), CGT92AAG (374C>A 375G>A 376T>G), GTA93GTG (379A>G), TTG94ATT (380T>A 382G>T), CAA95TTG (383C>T 384A>T 385A>G), ACA96GCA (386A>G), GGG98GGA (394G>A), AAT99GAT (395A>G), TAT100TAC (400T>C), CTA101ATT (401C>A 403A>T), TAT102del (404_406delTAT), AAT103AAC (409T>C), TTT104ATA (410T>A 412T>A), AAA105CAG (413A>C 415A>G), TGT106GCA (416T>G 417G>C 418T>A), CAA107CAT (421A>T), GTT108GCG (423T>C 424T>G), ACA113AAA (438C>A), AGT114AGC (442T>C), ATC115GTG (443A>G 445C>G), AAG116GGT (446A>G 447A>G 448G>T), AGC118GAT (452A>G 453G>A 454C>T), CAG119ATC (455C>A 456A>T 457G>C), GAA120AGA (458G>A 459A>G), ACT121AAG (462C>A 463T>G), AAG123GAG (467A>G), AAG123_AAA124insCAT (469_470insCAT), AAA124GGA (470A>G 471A>G), GCC125GTA (474C>T 475C>A), CAG126CAA (478G>A), TTG128GTC (482T>G 484G>C), ATT129TCT (485A>T 486T>C), CGA134AGA (500C>A), ATG135GTC (503A>G 505G>C), ATT136TGT (506A>T 507T>G), GAT137GAC (511T>C), CTA138ATG (512C>A 514A>G), CTC139ATT (515C>A 517C>T), ACC140AAA (519C>A 520C>A), CGA141AGG (521C>A 523A>G), AAA142AGA (525A>G), AGT143ACT (528G>C), ATC144TTA (530A>T 532C>A), GAT145CGA (533G>C 534A>G 535T>A), AAA147AGG (540A>G 541A>G), GCA148GCC (544A>C), ATT149ATA (547T>A), ATA151TTA (551A>T), GTT152TTG (554G>T 556T>G), GTA153GTT (559A>T), ATA154CTT (560A>C 562A>T), GCT157TCT (569G>T), ATA162AGC (585T>G 586A>C), GAT163CGA (587G>C 588A>G 589T>A), AAT164GGT (590A>G 591A>G), TTT165TTC (595T>C), TTG166AAG (596T>A 597T>A), AAA168CAG (602A>C 604A>G), ATA169ATT (607A>T), CAA170TAT (608C>T 610A>T), ATT172ATA (616T>A), GAA174CAG (620G>C 622A>G), TTT175CTT (623T>C), TTT176TTG (628T>G), GAA177CCA (629G>C 630A>C), AGT178CCA (632A>C 633G>C 634T>A), CAT179AAA (635C>A 637T>A), GTC180ATC (638G>A), GTT182GTG (646T>G), ATT183GGT (647A>G 648T>G), TTA184GTT (650T>G 652A>T), TTA185TTC (655A>C), TCA186TCT (658A>T), GCA187GCC (661A>C), ACC188ACT (664C>T), GTT189ATG (665G>A 667T>G), TCA191CCA (671T>C), AGG192GAG (674A>G 675G>A), GTT193GCT (678T>C), ATC194CTT (680A>C 682C>T), AAT195GAG (683A>G 685T>G), ACA196ATT (687C>T 688A>T), TCT197ACT (689T>A), CAG198AGA (692C>A 693A>G 694G>A), GTC199AAG (695G>A 696T>A 697C>G), TTT200TTC (700T>C), AGA202AAC (705G>A 706A>C), GAT203AAG (707G>A 709T>G), GTA205GTG (715A>G), AAA206AGG (717A>G 718A>G), CTG208CTT (724G>T), GTA209GTG (727A>G), AAA210AAG (730A>G), AAT211CGT (731A>C 732A>G), GCT212GAT (735C>A), GAA213GAG (739A>G), TTG214CTC (740T>C 742G>C), ACA215ACT (745A>T), CTC216CTT (748C>T), GGC218GGT (754C>T), AGG220AAG (759G>A), TAT222TTT (765A>T), ATA224GTC (770A>G 772A>C), GTA226GTC (778A>C), AAG227GAT (779A>G 781G>T), AAA228AAG (784A>G), AAT229GAA (785A>G 787T>A), GAT230GAG (790T>G), TTT231TGG (792T>G 793T>G), AAA232AAG (796A>G), GCA233CTT (797G>C 798C>T 799A>T), TTT237TGT (810T>G), TAT240TAC (820T>C), GAC241GAG (823C>G), CAC242ACC (824C>A 825A>C), AGC244GCC (830A>G 831G>C), CTC245ATC (833C>A), ACT246ACC (838T>C), CAA247CAG (841A>G), ACA248AGT (843C>G 844A>T), CTA249GTC (845C>G 847A>C), ATA250ATC (850A>C), TGT252GTG (854T>G 855G>T 856T>G), AAT253AAC (859T>C), ACA254ACC (862A>C), CGT255AGG (863C>A 865T>G), AGA256CGT (866A>C 868A>T), CAA257AAA (869C>A), GTA260TGG (878G>T 879T>G 880A>G), ATG262ACT (885T>C 886G>T), GAA263GAT (889A>T), TGT264AAG (890T>A 891G>A 892T>G), TTA265ATG (893T>A 895A>G), ACA266CGC (896A>C 897C>G 898A>C), AAT267AGC (900A>G 901T>C), AGA268CGT (902A>C 904A>T), AAT269GAT (905A>G), TTC270CAC (908T>C 909T>A), GCA272GTA (915C>T), TCA273TCT (919A>T), AGC274GCA (920A>G 921G>C 922C>A), ATC275ACT (924T>C 925C>T), GAT278GAC (934T>C), TCC280GAC (938T>G 939C>A), CAA281CAG (943A>G), CAA282AAC (944C>A 946A>C), GAA283ACT (947G>A 948A>C 949A>T), CGT284AGA (950C>A 952T>A), TTT286ATC (956T>A 958T>C), AAA289CGA (965A>C 966A>G), GAA290GAG (970A>G), TTT291TTC (973T>C), AGA292CGA (974A>C), GAT293TCT (977G>T 978A>C), GGT294GGG (982T>G), AAA295TCA (983A>T 984A>C), ACT296TCT (986A>T), AGA297CGT (989A>C 991A>T), ATA298GTG (992A>G 994A>G), CTT299CTC (997T>C), CTT300ATC (998C>A 1000T>C), TCA301ACT (1001T>A 1003A>T), AAT303GAT (1007A>G), CTA304CTT (1012A>T), TTA305TTG (1015A>G), GCA306GCT (1018A>T), AGA307CGT (1019A>C 1021A>T), GGC308GGT (1024C>T), GTT311GTA (1033T>A), CAA312CAG (1036A>G), ATA314GTC (1040A>G 1042A>C), TCT315TCC (1045T>C), TTA316CTT (1046T>C 1048A>T), GTT317GTC (1051T>C), ATC318ATT (1054C>T), AAT319AAC (1057T>C), GAT321GAC (1063T>C), TTG322CTG (1064T>C), CAT324ACT (1070C>A 1071A>C), AAT325CAA (1073A>C 1075T>A), AGA326CCA (1076A>C 1077G>C), TAT329TAC (1087T>C), ATA330CTG (1088A>C 1090A>G), AGA335CGT (1103A>C 1105A>T), GGT337GGA (1111T>A), AGA338CGA (1112A>C), GGC340GGA (1120C>A), CGA341AGG (1121C>A 1123A>G), AAA342AAG (1126A>G), GGG343GGT (1129G>T), ATT344GTT (1130A>G), AAT347AAC (1141T>C), ATA349GTC (1145A>G 1147A>C), ACT350ACC (1150T>C), GAA351AAG (1151G>A 1153A>G), GAC352GAT (1156C>T), GAA353GAT (1159A>T), TTA354GAA (1160T>G 1161T>A), CCA355AGG (1163C>A 1164C>G 1165A>G), AAG356ATG (1167A>T), GCT358TTT (1172G>T 1173C>T), GAA359GAC (1177A>C), TTA360ATA (1178T>A), GAA361CAG (1181G>C 1183A>G), ACG362AAA (1185C>A 1186G>A), TAT364TAC (1192T>C), AAT365AAC (1195T>C), ACC366GTT (1196A>G 1197C>T 1198C>T), AAA367GTA (1199A>G 1200A>T), ATA368ATC (1204A>C), GAT369GAG (1207T>G), GAA370GAG (1210A>G), ATG371CTG (1211A>C), GAG373GCC (1218A>C 1219G>C), ATT375GTT (1223A>G), GTT376GCT (1227T>C), TTG378CTC (1232T>C 1234G>C), ATA379CTG (1235A>C 1237A>G)								

Proteins

	Begin	End	Coverage	Score	Concordance	Matches	Identities	I/D/M/F*	Stop Codons
hypothetical protein (YP_009666148.1)	6	380	98.7%	1463	59.0%	374 (99.5%)	207 (55.1%)	1/1/0/0	0
Protein mutations:	E7D (121A>T), D9A (126A>C 127C>T), H10E (128C>G 130T>G), L13I (137C>A 139C>T), K15D (143A>G 145A>T), N16D (146A>G 148T>C), I17L (149A>C 151C>T), K19R (156A>G 157G>A), S23Q (167T>C 168C>A 169T>G), C24Y (171G>A 172T>C), T31A (191A>G 193T>C), K35R (203A>C 204A>G), I37V (209A>G 211T>A), F38L (214T>G), C40I (218T>A 219G>T), K44R (230A>C 231A>G 232A>T), V48A (243T>C 244C>T), A58S (272G>T 274A>C), T59M (276C>T 277A>G), Y60I (278T>A 279A>T), I62L (284A>C 286C>T), S63T (288G>C 289T>C), L65C (294T>G 295A>C), Q67V (299C>G 300A>T 301A>T), I68V (302A>G 304A>G), S71K (311T>A 312C>A 313T>A), N74E (320A>G 322T>G), I75V (323A>G 325C>A), T81S (341A>T 343C>T), L88A (362C>G 363T>C 364C>T), A90T (368G>A 370T>G), Q91E (371C>G), R92K (374C>A 375G>A 376T>G), L94I (380T>A 382G>T), Q95L (383C>T 384A>T 385A>G), T96A (386A>G), N99D (395A>G), L101I (401C>A 403A>T), Y102del (404_406delTAT), F104I (410T>A 412T>A), K105Q (413A>C 415A>G), C106A (416T>G 417G>C 418T>A), Q107H (421A>T), V108A (423T>C 424T>G), T113K (438C>A), I115V (443A>G 445C>G), K116G (446A>G 447A>G 448G>T), S118D (452A>G 453G>A 454C>T), Q119I (455C>A 456A>T 457G>C), E120R (458G>A 459A>G), T121K (462C>A 463T>G), K123E (467A>G), K123_K124insH (469_470insCAT), K124G (470A>G 471A>G), A125V (474C>T 475C>A), L128V (482T>G 484G>C), I129S (485A>T 486T>C), M135V (503A>G 505G>C), I136C (506A>T 507T>G), L138M (512C>A 514A>G), L139I (515C>A 517C>T), T140K (519C>A 520C>A), K142R (525A>G), S143T (528G>C), I144L (530A>T 532C>A), D145R (533G>C 534A>G 535T>A), K147R (540A>G 541A>G), I151L (551A>T), V152L (554G>T 556T>G), I154L (560A>C 562A>T), A157S (569G>T), I162S (585T>G 586A>C), D163R (587G>C 588A>G 589T>A), N164G (590A>G 591A>G), L166K (596T>A 597T>A), K168Q (602A>C 604A>G), Q170Y (608C>T 610A>T), E174Q (620G>C 622A>G), F175L (623T>C), F176L (628T>G), E177P (629G>C 630A>C), S178P (632A>C 633G>C 634T>A), H179K (635C>A 637T>A), V180I (638G>A), I183G (647A>G 648T>G), L184V (650T>G 652A>T), L185F (655A>C), V189M (665G>A 667T>G), S191P (671T>C), R192E (674A>G 675G>A), V193A (678T>C), I194L (680A>C 682C>T), N195E (683A>G 685T>G), T196I (687C>T 688A>T), S197T (689T>A), Q198R (692C>A 693A>G 694G>A), V199K (695G>A 696T>A 697C>G), R202N (705G>A 706A>C), D203K (707G>A 709T>G), K206R (717A>G 718A>G), N211R (731A>C 732A>G), A212D (735C>A), R220K (759G>A), Y222F (765A>T), I224V (770A>G 772A>C), K227D (779A>G 781G>T), N229E (785A>G 787T>A), D230E (790T>G), F231W (792T>G 793T>G), A233L (797G>C 798C>T 799A>T), F237C (810T>G), D241E (823C>G), H242T (824C>A 825A>C), S244A (830A>G 831G>C), L245I (833C>A), T248S (843C>G 844A>T), L249V (845C>G 847A>C), C252V (854T>G 855G>T 856T>G), Q257K (869C>A), V260W (878G>T 879T>G 880A>G), M262T (885T>C 886G>T), E263D (889A>T), C264K (890T>A 891G>A 892T>G), L265M (893T>A 895A>G), T266R (896A>C 897C>G 898A>C), N267S (900A>G 901T>C), N269D (905A>G), F270H (908T>C 909T>A), A272V (915C>T), S274A (920A>G 921G>C 922C>A), I275T (924T>C 925C>T), S280D (938T>G 939C>A), Q282N (944C>A 946A>C), E283T (947G>A 948A>C 949A>T), F286I (956T>A 958T>C), K289R (965A>C 966A>G), D293S (977G>T 978A>C), K295S (983A>T 984A>C), T296S (986A>T), I298V (992A>G 994A>G), L300I (998C>A 1000T>C), S301T (1001T>A 1003A>T), N303D (1007A>G), I314V (1040A>G 1042A>C), H324T (1070C>A 1071A>C), N325Q (1073A>C 1075T>A), R326P (1076A>C 1077G>C), I330L (1088A>C 1090A>G), I344V (1130A>G), I349V (1145A>G 1147A>C), E351K (1151G>A 1153A>G), E353D (1159A>T), L354E (1160T>G 1161T>A), P355R (1163C>A 1164C>G 1165A>G), K356M (1167A>T), A358F (1172G>T 1173C>T), E359D (1177A>C), L360I (1178T>A), E361Q (1181G>C 1183A>G), T362K (1185C>A 1186G>A), T366V (1196A>G 1197C>T 1198C>T), K367V (1199A>G 1200A>T), D369E (1207T>G), M371L (1211A>C), E373A (1218A>C 1219G>C), I375V (1223A>G), V376A (1227T>C), I379L (1235A>C 1237A>G)								

	Begin	End	Coverage	Score	Concordance	Matches	Identities	I/D/M/F*	Stop Codons
**NT**	**115**	**1238**	**83.9%**	**454**	**20.4%**	**1121 (99.5%)**	**680 (60.3%)**	**3/3**	

Codon mutations: GAA7GAT (121A>T), GAC9GCT (126A>C 127C>T), CAT10GAG (128C>G 130T>G), GGC12GGG (136C>G), CTC13ATT (137C>A 139C>T), AAG14AAA (142G>A), AAA15GAT (143A>G 145A>T), AAT16GAC (146A>G 148T>C), ATC17CTT (149A>C 151C>T), CTA18CTT (154A>T), AAG19AGA (156A>G 157G>A), GGT20GGG (160T>G), ATA21ATT (163A>T), TAT22TAC (166T>C), TCT23CAG (167T>C 168C>A 169T>G), TGT24TAC (171G>A 172T>C), GGA25GGG (175A>G), TTT26TTC (178T>C), CCT29CCA (187T>A), TCA30TCT (190A>T), ACT31GCC (191A>G 193T>C), AAA35CGA (203A>C 204A>G), GCC36GCT (208C>T), ATT37GTA (209A>G 211T>A), TTT38TTG (214T>G), CCA39CCT (217A>T), TGT40ATT (218T>A 219G>T), ATT41ATA (223T>A), TCT42TCG (226T>G), AAA44CGT (230A>C 231A>G 232A>T), GTG46GTC (238G>C), ATA47ATT (241A>T), GTC48GCT (243T>C 244C>T), CAA49CAG (247A>G), TCG52TCC (256G>C), GGA53GGT (259A>T), ACA54ACC (262A>C), ACA57ACC (271A>C), GCA58TCC (272G>T 274A>C), ACA59ATG (276C>T 277A>G), TAT60ATT (278T>A 279A>T), GCA61GCT (283A>T), ATC62CTT (284A>C 286C>T), AGT63ACC (288G>C 289T>C), TTA65TGC (294T>G 295A>C), CAG66CAA (298G>A), CAA67GTT (299C>G 300A>T 301A>T), ATA68GTG (302A>G 304A>G), ACT70ACC (310T>C), TCT71AAA (311T>A 312C>A 313T>A), AAT74GAG (320A>G 322T>G), ATC75GTA (323A>G 325C>A), GCT77GCA (331T>A), ATC79ATA (337C>A), ACC81TCT (341A>T 343C>T), CCA82CCT (346A>T), CGT84CGG (352T>G), GAA85GAG (355A>G), TTG86CTT (356T>C 358G>T), GCT87GCA (361T>A), CTC88GCT (362C>G 363T>C 364C>T), GCT90ACG (368G>A 370T>G), CAA91GAA (371C>G), CGT92AAG (374C>A 375G>A 376T>G), GTA93GTG (379A>G), TTG94ATT (380T>A 382G>T), CAA95TTG (383C>T 384A>T 385A>G), ACA96GCA (386A>G), GGG98GGA (394G>A), AAT99GAT (395A>G), TAT100TAC (400T>C), CTA101ATT (401C>A 403A>T), TAT102del (404_406delTAT), AAT103AAC (409T>C), TTT104ATA (410T>A 412T>A), AAA105CAG (413A>C 415A>G), TGT106GCA (416T>G 417G>C 418T>A), CAA107CAT (421A>T), GTT108GCG (423T>C 424T>G), ACA113AAA (438C>A), AGT114AGC (442T>C), ATC115GTG (443A>G 445C>G), AAG116GGT (446A>G 447A>G 448G>T), AGC118GAT (452A>G 453G>A 454C>T), CAG119ATC (455C>A 456A>T 457G>C), GAA120AGA (458G>A 459A>G), ACT121AAG (462C>A 463T>G), AAG123GAG (467A>G), AAG123_AAA124insCAT (469_470insCAT), AAA124GGA (470A>G 471A>G), GCC125GTA (474C>T 475C>A), CAG126CAA (478G>A), TTG128GTC (482T>G 484G>C), ATT129TCT (485A>T 486T>C), CGA134AGA (500C>A), ATG135GTC (503A>G 505G>C), ATT136TGT (506A>T 507T>G), GAT137GAC (511T>C), CTA138ATG (512C>A 514A>G), CTC139ATT (515C>A 517C>T), ACC140AAA (519C>A 520C>A), CGA141AGG (521C>A 523A>G), AAA142AGA (525A>G), AGT143ACT (528G>C), ATC144TTA (530A>T 532C>A), GAT145CGA (533G>C 534A>G 535T>A), AAA147AGG (540A>G 541A>G), GCA148GCC (544A>C), ATT149ATA (547T>A), ATA151TTA (551A>T), GTT152TTG (554G>T 556T>G), GTA153GTT (559A>T), ATA154CTT (560A>C 562A>T), GCT157TCT (569G>T), ATA162AGC (585T>G 586A>C), GAT163CGA (587G>C 588A>G 589T>A), AAT164GGT (590A>G 591A>G), TTT165TTC (595T>C), TTG166AAG (596T>A 597T>A), AAA168CAG (602A>C 604A>G), ATA169ATT (607A>T), CAA170TAT (608C>T 610A>T), ATT172ATA (616T>A), GAA174CAG (620G>C 622A>G), TTT175CTT (623T>C), TTT176TTG (628T>G), GAA177CCA (629G>C 630A>C), AGT178CCA (632A>C 633G>C 634T>A), CAT179AAA (635C>A 637T>A), GTC180ATC (638G>A), GTT182GTG (646T>G), ATT183GGT (647A>G 648T>G), TTA184GTT (650T>G 652A>T), TTA185TTC (655A>C), TCA186TCT (658A>T), GCA187GCC (661A>C), ACC188ACT (664C>T), GTT189ATG (665G>A 667T>G), TCA191CCA (671T>C), AGG192GAG (674A>G 675G>A), GTT193GCT (678T>C), ATC194CTT (680A>C 682C>T), AAT195GAG (683A>G 685T>G), ACA196ATT (687C>T 688A>T), TCT197ACT (689T>A), CAG198AGA (692C>A 693A>G 694G>A), GTC199AAG (695G>A 696T>A 697C>G), TTT200TTC (700T>C), AGA202AAC (705G>A 706A>C), GAT203AAG (707G>A 709T>G), GTA205GTG (715A>G), AAA206AGG (717A>G 718A>G), CTG208CTT (724G>T), GTA209GTG (727A>G), AAA210AAG (730A>G), AAT211CGT (731A>C 732A>G), GCT212GAT (735C>A), GAA213GAG (739A>G), TTG214CTC (740T>C 742G>C), ACA215ACT (745A>T), CTC216CTT (748C>T), GGC218GGT (754C>T), AGG220AAG (759G>A), TAT222TTT (765A>T), ATA224GTC (770A>G 772A>C), GTA226GTC (778A>C), AAG227GAT (779A>G 781G>T), AAA228AAG (784A>G), AAT229GAA (785A>G 787T>A), GAT230GAG (790T>G), TTT231TGG (792T>G 793T>G), AAA232AAG (796A>G), GCA233CTT (797G>C 798C>T 799A>T), TTT237TGT (810T>G), TAT240TAC (820T>C), GAC241GAG (823C>G), CAC242ACC (824C>A 825A>C), AGC244GCC (830A>G 831G>C), CTC245ATC (833C>A), ACT246ACC (838T>C), CAA247CAG (841A>G), ACA248AGT (843C>G 844A>T), CTA249GTC (845C>G 847A>C), ATA250ATC (850A>C), TGT252GTG (854T>G 855G>T 856T>G), AAT253AAC (859T>C), ACA254ACC (862A>C), CGT255AGG (863C>A 865T>G), AGA256CGT (866A>C 868A>T), CAA257AAA (869C>A), GTA260TGG (878G>T 879T>G 880A>G), ATG262ACT (885T>C 886G>T), GAA263GAT (889A>T), TGT264AAG (890T>A 891G>A 892T>G), TTA265ATG (893T>A 895A>G), ACA266CGC (896A>C 897C>G 898A>C), AAT267AGC (900A>G 901T>C), AGA268CGT (902A>C 904A>T), AAT269GAT (905A>G), TTC270CAC (908T>C 909T>A), GCA272GTA (915C>T), TCA273TCT (919A>T), AGC274GCA (920A>G 921G>C 922C>A), ATC275ACT (924T>C 925C>T), GAT278GAC (934T>C), TCC280GAC (938T>G 939C>A), CAA281CAG (943A>G), CAA282AAC (944C>A 946A>C), GAA283ACT (947G>A 948A>C 949A>T), CGT284AGA (950C>A 952T>A), TTT286ATC (956T>A 958T>C), AAA289CGA (965A>C 966A>G), GAA290GAG (970A>G), TTT291TTC (973T>C), AGA292CGA (974A>C), GAT293TCT (977G>T 978A>C), GGT294GGG (982T>G), AAA295TCA (983A>T 984A>C), ACT296TCT (986A>T), AGA297CGT (989A>C 991A>T), ATA298GTG (992A>G 994A>G), CTT299CTC (997T>C), CTT300ATC (998C>A 1000T>C), TCA301ACT (1001T>A 1003A>T), AAT303GAT (1007A>G), CTA304CTT (1012A>T), TTA305TTG (1015A>G), GCA306GCT (1018A>T), AGA307CGT (1019A>C 1021A>T), GGC308GGT (1024C>T), GTT311GTA (1033T>A), CAA312CAG (1036A>G), ATA314GTC (1040A>G 1042A>C), TCT315TCC (1045T>C), TTA316CTT (1046T>C 1048A>T), GTT317GTC (1051T>C), ATC318ATT (1054C>T), AAT319AAC (1057T>C), GAT321GAC (1063T>C), TTG322CTG (1064T>C), CAT324ACT (1070C>A 1071A>C), AAT325CAA (1073A>C 1075T>A), AGA326CCA (1076A>C 1077G>C), TAT329TAC (1087T>C), ATA330CTG (1088A>C 1090A>G), AGA335CGT (1103A>C 1105A>T), GGT337GGA (1111T>A), AGA338CGA (1112A>C), GGC340GGA (1120C>A), CGA341AGG (1121C>A 1123A>G), AAA342AAG (1126A>G), GGG343GGT (1129G>T), ATT344GTT (1130A>G), AAT347AAC (1141T>C), ATA349GTC (1145A>G 1147A>C), ACT350ACC (1150T>C), GAA351AAG (1151G>A 1153A>G), GAC352GAT (1156C>T), GAA353GAT (1159A>T), TTA354GAA (1160T>G 1161T>A), CCA355AGG (1163C>A 1164C>G 1165A>G), AAG356ATG (1167A>T), GCT358TTT (1172G>T 1173C>T), GAA359GAC (1177A>C), TTA360ATA (1178T>A), GAA361CAG (1181G>C 1183A>G), ACG362AAA (1185C>A 1186G>A), TAT364TAC (1192T>C), AAT365AAC (1195T>C), ACC366GTT (1196A>G 1197C>T 1198C>T), AAA367GTA (1199A>G 1200A>T), ATA368ATC (1204A>C), GAT369GAG (1207T>G), GAA370GAG (1210A>G), ATG371CTG (1211A>C), GAG373GCC (1218A>C 1219G>C), ATT375GTT (1223A>G), GTT376GCT (1227T>C), TTG378CTC (1232T>C 1234G>C), ATA379CTG (1235A>C 1237A>G)

*: Inserts / Deletes / Misaligned / Frameshifts

Analysis details

This analysis was performed with panviral2.64

NGS Details (UN60): Bracoviriform glomeratae (segment NC_043292.1)

Assembly

**Coverage Length**	311 (1 contig(s))
**Depth Of Coverage**	2381.0
**Number Of Reads**	6143
**Reads Per Million**	130.29 rpm (after QC)
**Ambiguities**	0
**Assembly Method**	de novo + reference guided assembly
**Consensus Caller**	Bcf Tools

Coverage Map

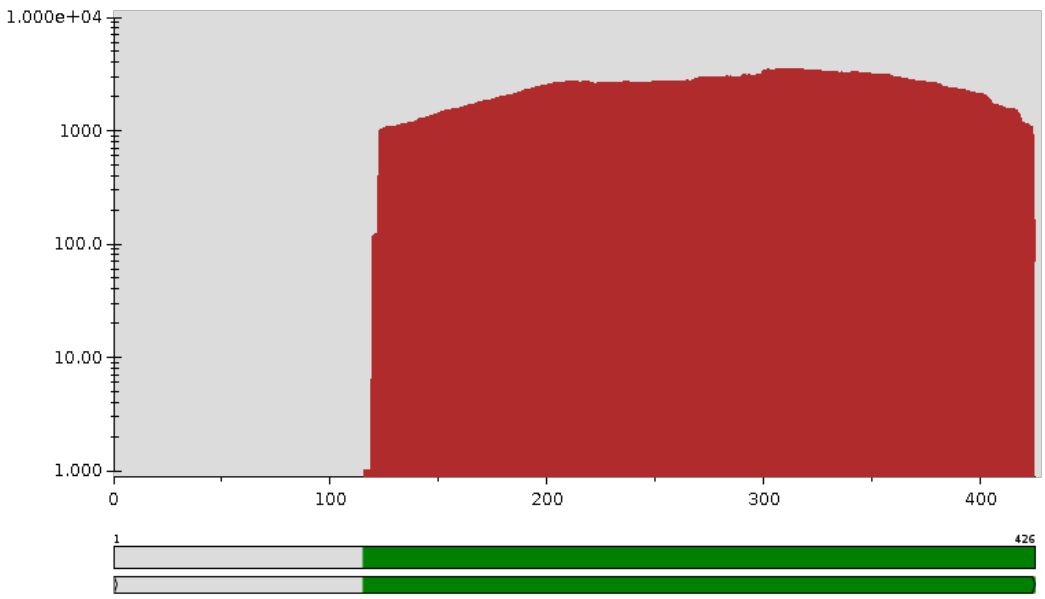

Assignment

**Type**	Bracoviriform glomeratae (Taxonomy ID: 257816)
**Reference Genome**	NC_043292.1
**NT Identity (%)**	72.0257
**AA Identity (%)**	83.4951
**Number Of Stop Codons**	1
**Number Of CDS**	1

Alignment

**Alignment Score**	274.0 (NT) + 552.0 (AA) = 826.0
**Concordance (%)**	64.5817

Alignment Method	Global, seeded, nucleotide + amino acids (AGA)

Genome Region

Sequence starts at position 116 and ends at position 426 relative to NC_043292.1 reference sequence.

Alignment Detailed Statistics

	Begin	End	Coverage	Score	Concordance	Matches	Identities	I/D/M/F*	Stop Codons
**NT**	**116**	**426**	**73.0%**	**274**	**44.1%**	**311 (100%)**	**224 (72.0%)**	**0/0**	
Mutations:	119G>C, 124T>C, 125T>G, 126G>T, 129C>A, 132A>G, 136A>G, 137C>G, 138G>C, 150A>C, 153A>G, 155C>G, 156G>A, 159C>A, 166C>A, 168T>G, 169T>C, 172C>A, 174C>G, 177A>G, 183T>A, 184C>A, 189T>C, 198A>G, 201A>C, 207C>T, 214A>G, 219C>T, 222T>C, 223C>A, 225T>G, 228G>T, 235C>A, 237T>G, 243A>T, 246C>G, 255C>T, 261A>G, 262T>C, 264A>G, 265G>A, 273A>G, 278T>C, 279T>C, 284A>G, 285T>A, 288T>G, 289T>C, 300C>T, 301C>T, 303C>G, 309C>T, 312T>G, 315C>T, 322G>T, 329T>C, 333T>C, 336C>T, 339A>G, 345A>C, 347A>G, 348A>G, 349C>A, 351T>A, 360C>T, 363C>T, 366C>T, 375C>T, 378C>T, 384A>C, 385T>C, 387G>C, 390A>G, 391C>A, 392A>G, 393T>A, 394A>C, 396A>G, 399C>A, 400C>A, 402T>G, 404T>C, 405G>T, 406A>C, 414T>G, 416A>T, 420A>T								

CDS

	Begin	End	Coverage	Score	Concordance	Matches	Identities	I/D/M/F*	Stop Codons
FK954_p501	40	142	72.5%	552	83.0%	103 (100%)	86 (83.5%)	0/0/0/0	1
Protein mutations:	R40T (119G>C), L42R (124T>C 125T>G 126G>T), T46G (136A>G 137C>G 138G>C), A52G (155C>G 156G>A), Y57H (169T>C), T72A (214A>G), V89I (265G>A), I93T (278T>C 279T>C), D95G (284A>G 285T>A), A108S (322G>T), I110T (329T>C), K116R (347A>G 348A>G), H131R (391C>A 392A>G 393T>A), K132Q (394A>C 396A>G), M135T (404T>C 405G>T), I136L (406A>C), Y139F (416A>T)								
Codon mutations:	AGA40ACA (119G>C), TTG42CGT (124T>C 125T>G 126G>T), GGC43GGA (129C>A), AAA44AAG (132A>G), ACG46GGC (136A>G 137C>G 138G>C), GGA50GGC (150A>C), AAA51AAG (153A>G), GCG52GGA (155C>G 156G>A), GGC53GGA (159C>A), CGT56AGG (166C>A 168T>G), TAT57CAT (169T>C), CGC58AGG (172C>A 174C>G), AAA59AAG (177A>G), CTT61CTA (183T>A), CGA62AGA (184C>A), GAT63GAC (189T>C), CAA66CAG (198A>G), GGA67GGC (201A>C), ACC69ACT (207C>T), ACT72GCT (214A>G), ATC73ATT (219C>T), CGT74CGC (222T>C), CGT75AGG (223C>A 225T>G), CTG76CTT (228G>T), CGT79AGG (235C>A 237T>G), GGA81GGT (243A>T), GTC82GTG (246C>G), ATC85ATT (255C>T), GGA87GGG (261A>G), TTA88CTG (262T>C 264A>G), GTC89ATC (265G>A), GAA91GAG (273A>G), ATT93ACC (278T>C 279T>C), GAT95GGA (284A>G 285T>A), GTT96GTG (288T>G), TTG97CTG (289T>C), TTC100TTT (300C>T), CTC101TTG (301C>T 303C>G), AAC103AAT (309C>T), GTT104GTG (312T>G), ATC105ATT (315C>T), GCT108TCT (322G>T), ATC110ACC (329T>C), TAT111TAC (333T>C), ACC112ACT (336C>T), GAA113GAG (339A>G), GCA115GCC (345A>C), AAA116AGG (347A>G 348A>G), CGT117AGA (349C>A 351T>A), GTC120GTT (360C>T), ACC121ACT (363C>T), GCC122GCT (366C>T), GTC125GTT (375C>T), GTC126GTT (378C>T), GCA128GCC (384A>C), TTG129CTC (385T>C 387G>C), AAA130AAG (390A>G), CAT131AGA (391C>A 392A>G 393T>A), AAA132CAG (394A>C 396A>G), GGC133GGA (399C>A), CGT134AGG (400C>A 402T>G), ATG135ACT (404T>C 405G>T), ATC136CTC (406A>C), GGT138GGG (414T>G), TAT139TTT (416A>T), GGA140GGT (420A>T)								

Proteins

	Begin	End	Coverage	Score	Concordance	Matches	Identities	I/D/M/F*	Stop Codons
putative histone 4 (YP_009665791.1)	40	142	72.5%	552	83.0%	103 (100%)	86 (83.5%)	0/0/0/0	1
Protein mutations:	R40T (119G>C), L42R (124T>C 125T>G 126G>T), T46G (136A>G 137C>G 138G>C), A52G (155C>G 156G>A), Y57H (169T>C), T72A (214A>G), V89I (265G>A), I93T (278T>C 279T>C), D95G (284A>G 285T>A), A108S (322G>T), I110T (329T>C), K116R (347A>G 348A>G), H131R (391C>A 392A>G 393T>A), K132Q (394A>C 396A>G), M135T (404T>C 405G>T), I136L (406A>C), Y139F (416A>T)								
Codon mutations:	AGA40ACA (119G>C), TTG42CGT (124T>C 125T>G 126G>T), GGC43GGA (129C>A), AAA44AAG (132A>G), ACG46GGC (136A>G 137C>G 138G>C), GGA50GGC (150A>C), AAA51AAG (153A>G), GCG52GGA (155C>G 156G>A), GGC53GGA (159C>A), CGT56AGG (166C>A 168T>G), TAT57CAT (169T>C), CGC58AGG (172C>A 174C>G), AAA59AAG (177A>G), CTT61CTA (183T>A), CGA62AGA (184C>A), GAT63GAC (189T>C), CAA66CAG (198A>G), GGA67GGC (201A>C), ACC69ACT (207C>T), ACT72GCT (214A>G), ATC73ATT (219C>T), CGT74CGC (222T>C), CGT75AGG (223C>A 225T>G), CTG76CTT (228G>T), CGT79AGG (235C>A 237T>G), GGA81GGT (243A>T), GTC82GTG (246C>G), ATC85ATT (255C>T), GGA87GGG (261A>G), TTA88CTG (262T>C 264A>G), GTC89ATC (265G>A), GAA91GAG (273A>G), ATT93ACC (278T>C 279T>C), GAT95GGA (284A>G 285T>A), GTT96GTG (288T>G), TTG97CTG (289T>C), TTC100TTT (300C>T), CTC101TTG (301C>T 303C>G), AAC103AAT (309C>T), GTT104GTG (312T>G), ATC105ATT (315C>T), GCT108TCT (322G>T), ATC110ACC (329T>C), TAT111TAC (333T>C), ACC112ACT (336C>T), GAA113GAG (339A>G), GCA115GCC (345A>C), AAA116AGG (347A>G 348A>G), CGT117AGA (349C>A 351T>A), GTC120GTT (360C>T), ACC121ACT (363C>T), GCC122GCT (366C>T), GTC125GTT (375C>T), GTC126GTT (378C>T), GCA128GCC (384A>C), TTG129CTC (385T>C 387G>C), AAA130AAG (390A>G), CAT131AGA (391C>A 392A>G 393T>A), AAA132CAG (394A>C 396A>G), GGC133GGA (399C>A), CGT134AGG (400C>A 402T>G), ATG135ACT (404T>C 405G>T), ATC136CTC (406A>C), GGT138GGG (414T>G), TAT139TTT (416A>T), GGA140GGT (420A>T)								

*: Inserts / Deletes / Misaligned / Frameshifts

Analysis details

This analysis was performed with panviral2.64

NGS Details (UN60): Potato leafroll virus

Assembly

**Coverage Length**	5826 (1 contig(s))
**Depth Of Coverage**	126.6
**Number Of Reads**	5542
**Reads Per Million**	117.54 rpm (after QC)
**Ambiguities**	0
**Assembly Method**	read mapping against reference + variant calling
**Consensus Caller**	Bcf Tools

Coverage Map

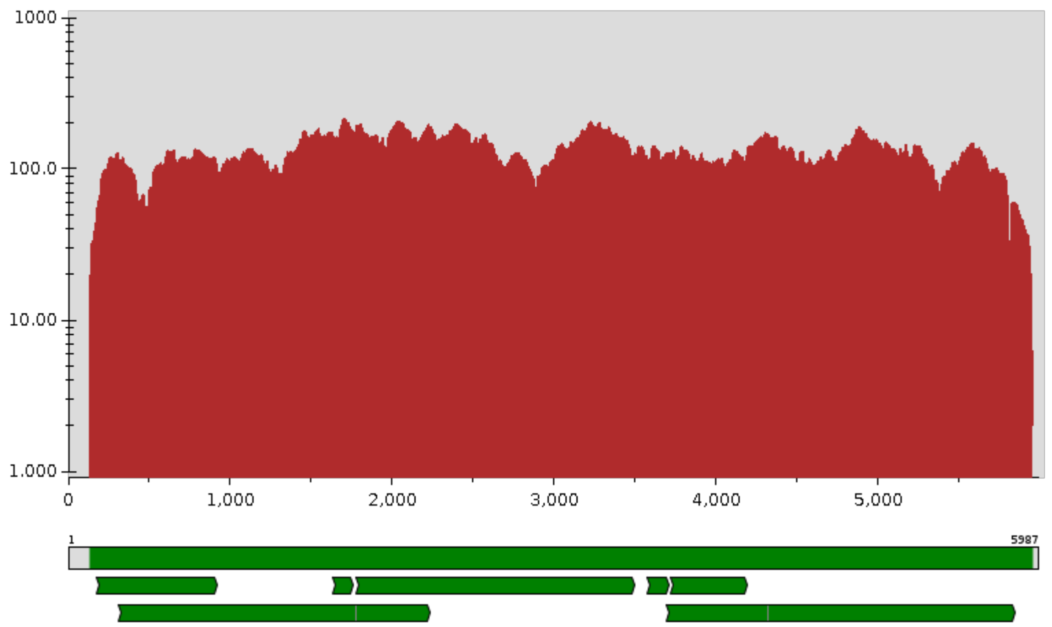

Assignment

**Type**	Potato leafroll virus (Taxonomy ID: 12045)
**Reference Genome**	NC_001747.1
**NT Identity (%)**	97.5455
**AA Identity (%)**	96.4823
**Number Of Stop Codons**	8
**Number Of CDS**	8

Alignment

**Alignment Score**	11044.0 (NT) + 20020.0 (AA) = 31064.0
**Concordance (%)**	95.2114

**Alignment Method**	Global, seeded, nucleotide + amino acids (AGA)

Genome Region

Sequence starts at position 129 and ends at position 5954 relative to NC_001747.1 reference sequence.

Alignment Detailed Statistics

	Begin	End	Coverage	Score	Concordance	Matches	Identities	I/D/M/F*	Stop Codons
**NT**	**129**	**5954**	**97.3%**	**11044**	**95.0%**	**5823 (99.9%)**	**5683 (97.5%)**	**3/3**	
Mutations:	191A>C, 221T>C, 228C>T, 269T>C, 276G>A, 300C>T, 343C>T, 462T>A, 476_477insTT, 479_480delTT, 492T>C, 525C>T, 561C>T, 600T>C, 665A>G, 697C>A, 699A>C, 822C>T, 825T>G, 922G>A, 931A>T, 958G>A, 1054C>T, 1132T>G, 1147C>T, 1171C>T, 1183T>C, 1204A>G, 1319G>A, 1356G>A, 1381T>C, 1411T>G, 1434A>G, 1465C>T, 1474G>A, 1534G>A, 1577G>T, 1593G>A, 1701A>G, 1716A>G, 1755T>C, 1757A>G, 1774T>C, 1814G>T, 1830C>T, 1894C>A, 1924G>A, 2007T>C, 2021A>G, 2047G>A, 2064A>G, 2079C>T, 2154C>T, 2187C>T, 2265A>G, 2283G>A, 2316T>G, 2400C>T, 2433T>C, 2470G>A, 2550G>A, 2574C>T, 2580A>G, 2586A>G, 2673C>T, 2704C>T, 2729A>G, 2850A>G, 2856G>A, 2869G>A, 2877C>T, 2898T>C, 2931T>C, 3000T>C, 3033C>T, 3036C>T, 3054T>C, 3069C>T, 3096C>T, 3105C>T, 3117C>T, 3195C>T, 3321C>T, 3379A>G, 3420A>G, 3437A>G, 3441C>T, 3572T>A, 3584_3585insT, 3591delT, 3619C>T, 3652T>A, 3712A>G, 3713A>G, 3721T>G, 3748T>G, 3750C>A, 3777G>T, 3788T>A, 3939A>T, 4013C>T, 4031A>G, 4034C>T, 4132A>T, 4151T>C, 4214T>G, 4265T>C, 4280C>T, 4295G>A, 4341A>G, 4379G>A, 4385C>T, 4421A>G, 4430G>A, 4433C>T, 4511T>C, 4520G>T, 4521T>G, 4661T>C, 4700C>T, 4733C>G, 4736A>G, 4817T>C, 4835C>T, 4883C>T, 4886T>C, 4949G>A, 4964A>G, 4979C>T, 5062C>T, 5072C>T, 5095G>A, 5129G>A, 5162C>T, 5199C>T, 5210G>A, 5215G>A, 5375T>G, 5385G>A, 5432T>C, 5493G>A, 5517A>G, 5530T>C, 5925T>C								
**CDS**									
PLRVgp1	1	248	100%	1428	82.4%	248 (99.6%)	241 (96.8%)	1/0/2/2	1
Protein mutations:	Q6P (191A>C), F16S (221T>C), L32P (269T>C), H57Y (343C>T), G101_L102insX (476_477insTT), Q164R (665A>G), Q175N (697C>A 699A>C)								
Codon mutations:	CAG6CCG (191A>C), TTT16TCT (221T>C), CTC18CTT (228C>T), CTT32CCT (269T>C), CTG34CTA (276G>A), GGC42GGT (300C>T), CAT57TAT (343C>T), GCT96GCA (462T>A), GGC101GGT (476_477insTT), GGC101_CTT102insT-C (476_477insTT), CTT102C-- (479_480delTT), ATT106ATC (492T>C), TAC117TAT (525C>T), TAC129TAT (561C>T), CAT142CAC (600T>C), CAA164CGA (665A>G), CAA175AAC (697C>A 699A>C), CGC216CGT (822C>T), GCT217GCG (825T>G)								
PLRVgp2	1	1063	100%	7021	94.1%	1063 (99.9%)	1031 (96.9%)	1/0/2/2	1
Protein mutations:	L52Q (462T>A), W56_A56insV (476_477insTT), L61S (492T>C), T72I (525C>T), T84I (561C>T), M97T (600T>C), K119E (665A>G), N130T (699A>C), A171V (822C>T), L172R (825T>G), E207D (931A>T), A337T (1319G>A), G349E (1356G>A), E375G (1434A>G), V423F (1577G>T), R428K (1593G>A), Q464R (1701A>G), E469G (1716A>G), L482P (1755T>C), T483A (1757A>G), W489R (1774T>C), R502L (1814G>T), Q529K (1894C>A), E539K (1924G>A), N571S (2021A>G), E580K (2047G>A), A721T (2470G>A), N807S (2729A>G), A854T (2869G>A), I1024V (3379A>G), E1043G (3437A>G)								
Codon mutations:	TTC12TTT (343C>T), CTA52CAA (462T>A), TGG56_GCC56insGTT (476_477insTT), TTA57--A (479_480delTT), TTA61TCA (492T>C), ACA72ATA (525C>T), ACA84ATA (561C>T), ATG97ACG (600T>C), AAA119GAA (665A>G), ATC129ATA (697C>A), AAC130ACC (699A>C), GCG171GTG (822C>T), CTA172CGA (825T>G), AGG204AGA (922G>A), GAA207GAT (931A>T), CAG216CAA (958G>A), GCC248GCT (1054C>T), ACT274ACG (1132T>G), TTC279TTT (1147C>T), TCC287TCT (1171C>T), GGT291GGC (1183T>C), CTA298CTG (1204A>G), GCC337ACC (1319G>A), GGA349GAA (1356G>A), TTT357TTC (1381T>C), CTT367CTG (1411T>G), GAG375GGG (1434A>G), CCC385CCT (1465C>T), CCG388CCA (1474G>A), TCG408TCA (1534G>A), GTC423TTC (1577G>T), AGA428AAA (1593G>A), CAA464CGA (1701A>G), GAG469GGG (1716A>G), CTA482CCA (1755T>C), ACA483GCA (1757A>G), AAT488AAC (1774T>C), TGG489CGG (1774T>C), CGA502CTA (1814G>T), CGC507CGT (1830C>T), CAA529AAA (1894C>A), GAG539AAG (1924G>A), TTT566TTC (2007T>C), AAC571AGC (2021A>G), GAA580AAA (2047G>A), CCA585CCG (2064A>G), TGC590TGT (2079C>T), CAC615CAT (2154C>T), GGC626GGT (2187C>T), TCA652TCG (2265A>G), GCG658GCA (2283G>A), GTT669GTG (2316T>G), ATC697ATT (2400C>T), GCT708GCC (2433T>C), GCA721ACA (2470G>A), TCG747TCA (2550G>A), AGC755AGT (2574C>T), GAA757GAG (2580A>G), CTA759CTG (2586A>G), CGC788CGT (2673C>T), CTG799TTG (2704C>T), AAT807AGT (2729A>G), CCA847CCG (2850A>G), GTG849GTA (2856G>A), GCA854ACA (2869G>A), CAC856CAT (2877C>T), ACT863ACC (2898T>C), TAT874TAC (2931T>C), CGT897CGC (3000T>C), AAC908AAT (3033C>T), TCC909TCT (3036C>T), GAT915GAC (3054T>C), GCC920GCT (3069C>T), AGC929AGT (3096C>T), TAC932TAT (3105C>T), TCC936TCT (3117C>T), CTC962CTT (3195C>T), AAC1004AAT (3321C>T), ATT1024GTT (3379A>G), GAA1037GAG (3420A>G), GAG1043GGG (3437A>G), CTC1044CTT (3441C>T)								
PLRVgp3	1	640	100%	3892	90.7%	640 (99.8%)	611 (95.3%)	1/0/2/2	1
Protein mutations:	L52Q (462T>A), W56_A56insV (476_477insTT), L61S (492T>C), T72I (525C>T), T84I (561C>T), M97T (600T>C), K119E (665A>G), N130T (699A>C), A171V (822C>T), L172R (825T>G), E207D (931A>T), A337T (1319G>A), G349E (1356G>A), E375G (1434A>G), V423F (1577G>T), R428K (1593G>A), Q464R (1701A>G), E469G (1716A>G), L482P (1755T>C), T483A (1757A>G), D502Y (1814G>T), A507V (1830C>T), L566S (2007T>C), T571A (2021A>G), Q585R (2064A>G), A590V (2079C>T), T615I (2154C>T), A626V (2187C>T)								
Codon mutations:	TTC12TTT (343C>T), CTA52CAA (462T>A), TGG56_GCC56insGTT (476_477insTT), TTA57--A (479_480delTT), TTA61TCA (492T>C), ACA72ATA (525C>T), ACA84ATA (561C>T), ATG97ACG (600T>C), AAA119GAA (665A>G), ATC129ATA (697C>A), AAC130ACC (699A>C), GCG171GTG (822C>T), CTA172CGA (825T>G), AGG204AGA (922G>A), GAA207GAT (931A>T), CAG216CAA (958G>A), GCC248GCT (1054C>T), ACT274ACG (1132T>G), TTC279TTT (1147C>T), TCC287TCT (1171C>T), GGT291GGC (1183T>C), CTA298CTG (1204A>G), GCC337ACC (1319G>A), GGA349GAA (1356G>A), TTT357TTC (1381T>C), CTT367CTG (1411T>G), GAG375GGG (1434A>G), CCC385CCT (1465C>T), CCG388CCA (1474G>A), TCG408TCA (1534G>A), GTC423TTC (1577G>T), AGA428AAA (1593G>A), CAA464CGA (1701A>G), GAG469GGG (1716A>G), CTA482CCA (1755T>C), ACA483GCA (1757A>G), AAT488AAC (1774T>C), GAC502TAC (1814G>T), GCT507GTT (1830C>T), ATC528ATA (1894C>A), GTG538GTA (1924G>A), TTA566TCA (2007T>C), ACG571GCG (2021A>G), AAG579AAA (2047G>A), CAG585CGG (2064A>G), GCG590GTG (2079C>T), ACC615ATC (2154C>T), GCT626GTT (2187C>T)								
Rap1	1	42	100%	267	93.0%	42 (100%)	39 (92.9%)	0/0/0/0	0
Protein mutations:	K24E (1701A>G), R29G (1716A>G), *42Q (1755T>C 1757A>G)								
Codon mutations:	AAA24GAA (1701A>G), AGA29GGA (1716A>G), TAA42CAG (1755T>C 1757A>G)								
ORF3a	1	46	100%	17	5.7%	46 (97.9%)	43 (91.5%)	1/0/3/2	1
Protein mutations:	Y3_K3insX (3584_3585insT), F25L (3652T>A)								
Codon mutations:	TAT3_AAA3insAT- (3584_3585insT), TTA5T-A (3591delT), TCC14TCT (3619C>T), TTT25TTA (3652T>A), TAA45TAG (3712A>G)								
PLRVgp4	1	718	100%	4940	98.1%	718 (100%)	700 (97.5%)	0/0/0/0	2
Protein mutations:	K7R (3712A>G 3713A>G), V10G (3721T>G), M19R (3748T>G), A29S (3777G>T), T83S (3939A>T), Y147F (4132A>T), S217G (4341A>G), K276N (4520G>T), F277V (4521T>G), A457V (5062C>T), G468E (5095G>A), P503S (5199C>T), R508Q (5215G>A), N561K (5375T>G), E565K (5385G>A), V601I (5493G>A), K609E (5517A>G), L613P (5530T>C)								
Codon mutations:	AAA7AGG (3712A>G 3713A>G), GTC10GGC (3721T>G), ATG19AGG (3748T>G), CGA20AGA (3750C>A), GCT29TCT (3777G>T), GTT32GTA (3788T>A), ACC83TCC (3939A>T), TAC107TAT (4013C>T), ACA113ACG (4031A>G), AGC114AGT (4034C>T), TAC147TTC (4132A>T), ATT153ATC (4151T>C), TCT174TCG (4214T>G), GAT191GAC (4265T>C), TTC196TTT (4280C>T), AAG201AAA (4295G>A), AGT217GGT (4341A>G), CAG229CAA (4379G>A), CAC231CAT (4385C>T), CTA243CTG (4421A>G), CAG246CAA (4430G>A), GCC247GCT (4433C>T), AAT273AAC (4511T>C), AAG276AAT (4520G>T), TTT277GTT (4521T>G), GGT323GGC (4661T>C), TCC336TCT (4700C>T), GTC347GTG (4733C>G), AAA348AAG (4736A>G), GGT375GGC (4817T>C), GAC381GAT (4835C>T), TTC397TTT (4883C>T), CTT398CTC (4886T>C), ACG419ACA (4949G>A), GAA424GAG (4964A>G), ACC429ACT (4979C>T), GCC457GTC (5062C>T), CAC460CAT (5072C>T), GGA468GAA (5095G>A), ACG479ACA (5129G>A), GAC490GAT (5162C>T), CCA503TCA (5199C>T), TCG506TCA (5210G>A), CGA508CAA (5215G>A), AAT561AAG (5375T>G), GAA565AAA (5385G>A), ACT580ACC (5432T>C), GTC601ATC (5493G>A), AAG609GAG (5517A>G), CTT613CCT (5530T>C)								
PLRVgp5	1	209	100%	1406	98.0%	209 (100%)	203 (97.1%)	0/0/0/0	1
Protein mutations:	K7R (3712A>G 3713A>G), V10G (3721T>G), M19R (3748T>G), A29S (3777G>T), T83S (3939A>T), Y147F (4132A>T)								
Codon mutations:	AAA7AGG (3712A>G 3713A>G), GTC10GGC (3721T>G), ATG19AGG (3748T>G), CGA20AGA (3750C>A), GCT29TCT (3777G>T), GTT32GTA (3788T>A), ACC83TCC (3939A>T), TAC107TAT (4013C>T), ACA113ACG (4031A>G), AGC114AGT (4034C>T), TAC147TTC (4132A>T), ATT153ATC (4151T>C), TCT174TCG (4214T>G), GAT191GAC (4265T>C), TTC196TTT (4280C>T), AAG201AAA (4295G>A)								
PLRVgp6	1	157	100%	1049	95.2%	157 (100%)	149 (94.9%)	0/0/0/0	1
Protein mutations:	S2A (3721T>G), C11G (3748T>G 3750C>A), F24Y (3788T>A), T99I (4013C>T), Q105R (4031A>G), A106V (4034C>T), T139S (4132A>T), L145S (4151T>C)								
Codon mutations:	TCA2GCA (3721T>G), TGC11GGA (3748T>G 3750C>A), GCG20GCT (3777G>T), TTC24TAC (3788T>A), ACA74ACT (3939A>T), ACC99ATC (4013C>T), CAA105CGA (4031A>G), GCA106GTA (4034C>T), ACG139TCG (4132A>T), TTA145TCA (4151T>C)								

	Begin	End	Coverage	Score	Concordance	Matches	Identities	I/D/M/F*	Stop Codons
**NT**	**129**	**5954**	**97.3%**	**11044**	**95.0%**	**5823 (99.9%)**	**5683 (97.5%)**	**3/3**	
**Proteins**									
P0 protein (NP_056746.1)	1	248	100%	1428	82.4%	248 (99.6%)	241 (96.8%)	1/0/2/2	1
Protein mutations:	Q6P (191A>C), F16S (221T>C), L32P (269T>C), H57Y (343C>T), G101_L102insX (476_477insTT), Q164R (665A>G), Q175N (697C>A 699A>C)								
Codon mutations:	CAG6CCG (191A>C), TTT16TCT (221T>C), CTC18CTT (228C>T), CTT32CCT (269T>C), CTG34CTA (276G>A), GGC42GGT (300C>T), CAT57TAT (343C>T), GCT96GCA (462T>A), GGC101GGT (476_477insTT), GGC101_CTT102insT-C (476_477insTT), CTT102C-- (479_480delTT), ATT106ATC (492T>C), TAC117TAT (525C>T), TAC129TAT (561C>T), CAT142CAC (600T>C), CAA164CGA (665A>G), CAA175AAC (697C>A 699A>C), CGC216CGT (822C>T), GCT217GCG (825T>G)								
RNA-dependent RNA polymerase (NP_056748.3)	1	1063	100%	7021	94.1%	1063 (99.9%)	1031 (96.9%)	1/0/2/2	1
Protein mutations:	L52Q (462T>A), W56_A56insV (476_477insTT), L61S (492T>C), T72I (525C>T), T84I (561C>T), M97T (600T>C), K119E (665A>G), N130T (699A>C), A171V (822C>T), L172R (825T>G), E207D (931A>T), A337T (1319G>A), G349E (1356G>A), E375G (1434A>G), V423F (1577G>T), R428K (1593G>A), Q464R (1701A>G), E469G (1716A>G), L482P (1755T>C), T483A (1757A>G), W489R (1774T>C), R502L (1814G>T), Q529K (1894C>A), E539K (1924G>A), N571S (2021A>G), E580K (2047G>A), A721T (2470G>A), N807S (2729A>G), A854T (2869G>A), I1024V (3379A>G), E1043G (3437A>G)								
Codon mutations:	TTC12TTT (343C>T), CTA52CAA (462T>A), TGG56_GCC56insGTT (476_477insTT), TTA57--A (479_480delTT), TTA61TCA (492T>C), ACA72ATA (525C>T), ACA84ATA (561C>T), ATG97ACG (600T>C), AAA119GAA (665A>G), ATC129ATA (697C>A), AAC130ACC (699A>C), GCG171GTG (822C>T), CTA172CGA (825T>G), AGG204AGA (922G>A), GAA207GAT (931A>T), CAG216CAA (958G>A), GCC248GCT (1054C>T), ACT274ACG (1132T>G), TTC279TTT (1147C>T), TCC287TCT (1171C>T), GGT291GGC (1183T>C), CTA298CTG (1204A>G), GCC337ACC (1319G>A), GGA349GAA (1356G>A), TTT357TTC (1381T>C), CTT367CTG (1411T>G), GAG375GGG (1434A>G), CCC385CCT (1465C>T), CCG388CCA (1474G>A), TCG408TCA (1534G>A), GTC423TTC (1577G>T), AGA428AAA (1593G>A), CAA464CGA (1701A>G), GAG469GGG (1716A>G), CTA482CCA (1755T>C), ACA483GCA (1757A>G), AAT488AAC (1774T>C), TGG489CGG (1774T>C), CGA502CTA (1814G>T), CGC507CGT (1830C>T), CAA529AAA (1894C>A), GAG539AAG (1924G>A), TTT566TTC (2007T>C), AAC571AGC (2021A>G), GAA580AAA (2047G>A), CCA585CCG (2064A>G), TGC590TGT (2079C>T), CAC615CAT (2154C>T), GGC626GGT (2187C>T), TCA652TCG (2265A>G), GCG658GCA (2283G>A), GTT669GTG (2316T>G), ATC697ATT (2400C>T), GCT708GCC (2433T>C), GCA721ACA (2470G>A), TCG747TCA (2550G>A), AGC755AGT (2574C>T), GAA757GAG (2580A>G), CTA759CTG (2586A>G), CGC788CGT (2673C>T), CTG799TTG (2704C>T), AAT807AGT (2729A>G), CCA847CCG (2850A>G), GTG849GTA (2856G>A), GCA854ACA (2869G>A), CAC856CAT (2877C>T), ACT863ACC (2898T>C), TAT874TAC (2931T>C), CGT897CGC (3000T>C), AAC908AAT (3033C>T), TCC909TCT (3036C>T), GAT915GAC (3054T>C), GCC920GCT (3069C>T), AGC929AGT (3096C>T), TAC932TAT (3105C>T), TCC936TCT (3117C>T), CTC962CTT (3195C>T), AAC1004AAT (3321C>T), ATT1024GTT (3379A>G), GAA1037GAG (3420A>G), GAG1043GGG (3437A>G), CTC1044CTT (3441C>T)								
P1 protein (NP_056747.1)	1	640	100%	3892	90.7%	640 (99.8%)	611 (95.3%)	1/0/2/2	1
Protein mutations:	L52Q (462T>A), W56_A56insV (476_477insTT), L61S (492T>C), T72I (525C>T), T84I (561C>T), M97T (600T>C), K119E (665A>G), N130T (699A>C), A171V (822C>T), L172R (825T>G), E207D (931A>T), A337T (1319G>A), G349E (1356G>A), E375G (1434A>G), V423F (1577G>T), R428K (1593G>A), Q464R (1701A>G), E469G (1716A>G), L482P (1755T>C), T483A (1757A>G), D502Y (1814G>T), A507V (1830C>T), L566S (2007T>C), T571A (2021A>G), Q585R (2064A>G), A590V (2079C>T), T615I (2154C>T), A626V (2187C>T)								
Codon mutations:	TTC12TTT (343C>T), CTA52CAA (462T>A), TGG56_GCC56insGTT (476_477insTT), TTA57--A (479_480delTT), TTA61TCA (492T>C), ACA72ATA (525C>T), ACA84ATA (561C>T), ATG97ACG (600T>C), AAA119GAA (665A>G), ATC129ATA (697C>A), AAC130ACC (699A>C), GCG171GTG (822C>T), CTA172CGA (825T>G), AGG204AGA (922G>A), GAA207GAT (931A>T), CAG216CAA (958G>A), GCC248GCT (1054C>T), ACT274ACG (1132T>G), TTC279TTT (1147C>T), TCC287TCT (1171C>T), GGT291GGC (1183T>C), CTA298CTG (1204A>G), GCC337ACC (1319G>A), GGA349GAA (1356G>A), TTT357TTC (1381T>C), CTT367CTG (1411T>G), GAG375GGG (1434A>G), CCC385CCT (1465C>T), CCG388CCA (1474G>A), TCG408TCA (1534G>A), GTC423TTC (1577G>T), AGA428AAA (1593G>A), CAA464CGA (1701A>G), GAG469GGG (1716A>G), CTA482CCA (1755T>C), ACA483GCA (1757A>G), AAT488AAC (1774T>C), GAC502TAC (1814G>T), GCT507GTT (1830C>T), ATC528ATA (1894C>A), GTG538GTA (1924G>A), TTA566TCA (2007T>C), ACG571GCG (2021A>G), AAG579AAA (2047G>A), CAG585CGG (2064A>G), GCG590GTG (2079C>T), ACC615ATC (2154C>T), GCT626GTT (2187C>T)								
Replication-associated protein (YP_006355442.1)	1	42	100%	267	93.0%	42 (100%)	39 (92.9%)	0/0/0/0	0
Protein mutations:	K24E (1701A>G), R29G (1716A>G), *42Q (1755T>C 1757A>G)								
Codon mutations:	AAA24GAA (1701A>G), AGA29GGA (1716A>G), TAA42CAG (1755T>C 1757A>G)								
protein 3a (YP_009179365.2)	1	46	100%	17	5.7%	46 (97.9%)	43 (91.5%)	1/0/3/2	1
Protein mutations:	Y3_K3insX (3584_3585insT), F25L (3652T>A)								
Codon mutations:	TAT3_AAA3insAT- (3584_3585insT), TTA5T-A (3591delT), TCC14TCT (3619C>T), TTT25TTA (3652T>A), TAA45TAG (3712A>G)								
CP read-through protein (NP_056751.2)	1	718	100%	4940	98.1%	718 (100%)	700 (97.5%)	0/0/0/0	2
Protein mutations:	K7R (3712A>G 3713A>G), V10G (3721T>G), M19R (3748T>G), A29S (3777G>T), T83S (3939A>T), Y147F (4132A>T), S217G (4341A>G), K276N (4520G>T), F277V (4521T>G), A457V (5062C>T), G468E (5095G>A), P503S (5199C>T), R508Q (5215G>A), N561K (5375T>G), E565K (5385G>A), V601I (5493G>A), K609E (5517A>G), L613P (5530T>C)								
Codon mutations:	AAA7AGG (3712A>G 3713A>G), GTC10GGC (3721T>G), ATG19AGG (3748T>G), CGA20AGA (3750C>A), GCT29TCT (3777G>T), GTT32GTA (3788T>A), ACC83TCC (3939A>T), TAC107TAT (4013C>T), ACA113ACG (4031A>G), AGC114AGT (4034C>T), TAC147TTC (4132A>T), ATT153ATC (4151T>C), TCT174TCG (4214T>G), GAT191GAC (4265T>C), TTC196TTT (4280C>T), AAG201AAA (4295G>A), AGT217GGT (4341A>G), CAG229CAA (4379G>A), CAC231CAT (4385C>T), CTA243CTG (4421A>G), CAG246CAA (4430G>A), GCC247GCT (4433C>T), AAT273AAC (4511T>C), AAG276AAT (4520G>T), TTT277GTT (4521T>G), GGT323GGC (4661T>C), TCC336TCT (4700C>T), GTC347GTG (4733C>G), AAA348AAG (4736A>G), GGT375GGC (4817T>C), GAC381GAT (4835C>T), TTC397TTT (4883C>T), CTT398CTC (4886T>C), ACG419ACA (4949G>A), GAA424GAG (4964A>G), ACC429ACT (4979C>T), GCC457GTC (5062C>T), CAC460CAT (5072C>T), GGA468GAA (5095G>A), ACG479ACA (5129G>A), GAC490GAT (5162C>T), CCA503TCA (5199C>T), TCG506TCA (5210G>A), CGA508CAA (5215G>A), AAT561AAG (5375T>G), GAA565AAA (5385G>A), ACT580ACC (5432T>C), GTC601ATC (5493G>A), AAG609GAG (5517A>G), CTT613CCT (5530T>C)								
coat protein (NP_056749.1)	1	209	100%	1406	98.0%	209 (100%)	203 (97.1%)	0/0/0/0	1
Protein mutations:	K7R (3712A>G 3713A>G), V10G (3721T>G), M19R (3748T>G), A29S (3777G>T), T83S (3939A>T), Y147F (4132A>T)								
Codon mutations:	AAA7AGG (3712A>G 3713A>G), GTC10GGC (3721T>G), ATG19AGG (3748T>G), CGA20AGA (3750C>A), GCT29TCT (3777G>T), GTT32GTA (3788T>A), ACC83TCC (3939A>T), TAC107TAT (4013C>T), ACA113ACG (4031A>G), AGC114AGT (4034C>T), TAC147TTC (4132A>T), ATT153ATC (4151T>C), TCT174TCG (4214T>G), GAT191GAC (4265T>C), TTC196TTT (4280C>T), AAG201AAA (4295G>A)								
movement protein (NP_056750.1)	1	157	100%	1049	95.2%	157 (100%)	149 (94.9%)	0/0/0/0	1
Protein mutations:	S2A (3721T>G), C11G (3748T>G 3750C>A), F24Y (3788T>A), T99I (4013C>T), Q105R (4031A>G), A106V (4034C>T), T139S (4132A>T), L145S (4151T>C)								
Codon mutations:	TCA2GCA (3721T>G), TGC11GGA (3748T>G 3750C>A), GCG20GCT (3777G>T), TTC24TAC (3788T>A), ACA74ACT (3939A>T), ACC99ATC (4013C>T), CAA105CGA (4031A>G), GCA106GTA (4034C>T), ACG139TCG (4132A>T), TTA145TCA (4151T>C)								

*: Inserts / Deletes / Misaligned / Frameshifts

Analysis details

This analysis was performed with panviral2.64

NGS Details (UN60): Duamitovirus soch1

Assembly

**Coverage Length**	1957 (6 contig(s))
**Depth Of Coverage**	22.1
**Number Of Reads**	351
**Reads Per Million**	7.44 rpm (after QC)
**Ambiguities**	0
**Assembly Method**	de novo + reference guided assembly
**Consensus Caller**	Bcf Tools

Coverage Map

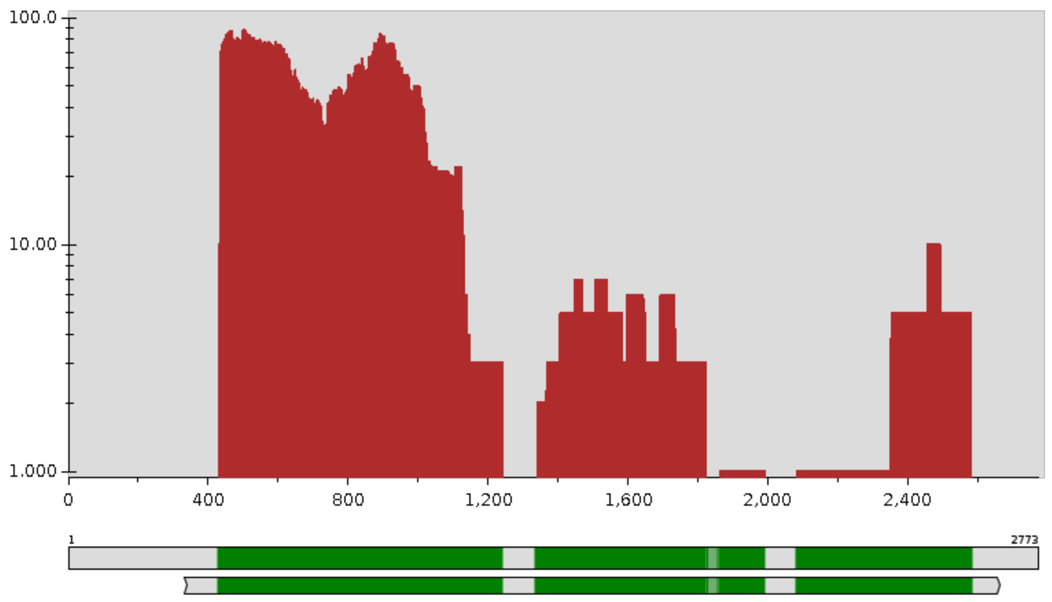

Assignment

**Type**	Duamitovirus soch1 (Taxonomy ID: 2955838)
**Reference Genome**	NC_076524.1
**NT Identity (%)**	67.4872
**AA Identity (%)**	68.2099
**Number Of Stop Codons**	4
**Number Of CDS**	1

Alignment

**Alignment Score**	1294.0 (NT) + 2702.0 (AA) = 3996.0
**Concordance (%)**	50.319

Alignment Method	Global, seeded, nucleotide + amino acids (AGA)

Genome Region

Sequence starts at position 428 and ends at position 2585 relative to NC_076524.1 reference sequence.

Alignment Detailed Statistics

	Begin	End	Coverage	Score	Concordance	Matches	Identities	I/D/M/F*	Stop Codons
**NT**	**428**	**2585**	**70.6%**	**1294**	**33.9%**	**1940 (98.6%)**	**1316 (66.9%)**	**10/17**	
Mutations:	437A>G, 441A>C, 443C>T, 447C>T, 459C>T, 466C>T, 470C>T, 471G>A, 472C>T, 473T>G, 477T>A, 480A>C, 481C>A, 482A>T, 485A>G, 489A>G, 494T>C, 497T>A, 500T>G, 503A>C, 506T>C, 509C>T, 512T>C, 513G>A, 516T>A, 517C>A, 518T>A, 520A>C, 524G>T, 525A>G, 526C>T, 527T>G, 529C>A, 530C>A, 531T>C, 533A>G, 534C>A, 535G>A, 536C>A, 539G>A, 540A>C, 541A>G, 542G>T, 545G>A, 548C>T, 550A>T, 556T>A, 557C>T, 560A>T, 569C>T, 575G>A, 581C>T, 582G>A, 587A>T, 589G>C, 591T>C, 593A>T, 599A>T, 602C>T, 612T>A, 613C>G, 614C>T, 616T>A, 618C>T, 619G>C, 620A>T, 624G>A, 626A>T, 629A>C, 630C>A, 632A>C, 633C>A, 635T>G, 638G>T, 641G>T, 644T>A, 647G>T, 651C>T, 653C>G, 656A>C, 657A>C, 661C>G, 662G>T, 665T>A, 668C>T, 672C>A, 673G>A, 674T>G, 680C>A, 683C>T, 684A>G, 685A>C, 686A>C, 692G>A, 695T>A, 699G>C, 700C>A, 701A>C, 702A>G, 705C>A, 707T>G, 708A>G, 709G>C, 710G>A, 713A>G, 714G>T, 715A>C, 719A>T, 722C>T, 737T>C, 746C>T, 752A>T, 761G>A, 765G>T, 767T>A, 770G>A, 771G>T, 772T>A, 773T>G, 774T>A, 776G>T, 777A>G, 778A>C, 779A>G, 782T>G, 796G>A, 797T>G, 803A>C, 809T>C, 810G>C, 815C>A, 818A>T, 821T>G, 824C>T, 827A>T, 831C>T, 833T>A, 841G>A, 842G>T, 850T>G, 851A>T, 857G>T, 860G>T, 862A>G, 869T>G, 875G>T, 880T>G, 881C>A, 885C>A, 887T>G, 892A>G, 905A>T, 908C>A, 909T>C, 910T>A, 916G>A, 918G>T, 919G>C, 923T>A, 926A>T, 927A>C, 929G>T, 934T>A, 935T>G, 938G>T, 942T>A, 943C>A, 948C>A, 971C>A, 972C>T, 975A>C, 976A>T, 977T>C, 983T>C, 990A>C, 992G>C, 993T>C, 994C>G, 995A>G, 997A>T, 998C>T, 999G>C, 1000G>A, 1001T>A, 1003T>A, 1004A>T, 1005G>T, 1007C>G, 1010A>G, 1016C>T, 1017A>G, 1018A>T, 1019A>T, 1022T>A, 1025T>C, 1026C>G, 1027A>G, 1030A>C, 1031G>A, 1034T>G, 1035A>G, 1036G>C, 1044T>G, 1046C>T, 1049G>A, 1050T>G, 1051T>A, 1052T>A, 1054C>A, 1055A>C, 1061T>C, 1067T>G, 1073G>A, 1076A>G, 1083C>A, 1088G>T, 1091C>G, 1099G>T, 1104A>G, 1105C>T, 1106A>G, 1115T>G, 1121A>C, 1122T>A, 1124C>T, 1127G>A, 1130C>T, 1133C>G, 1142A>C, 1145C>T, 1148G>T, 1151G>T, 1152T>C, 1154G>T, 1160C>T, 1161A>C, 1164T>A, 1165C>T, 1166T>G, 1169C>T, 1172C>A, 1174T>A, 1175T>C, 1178T>A, 1181A>T, 1184C>T, 1185A>T, 1186G>C, 1187G>T, 1188G>A, 1189C>A, 1191G>A, 1192C>A, 1193A>T, 1196T>C, 1197_1199delGAA, 1205A>T, 1211C>T, 1212T>G, 1213T>A, 1217T>C, 1220C>T, 1223A>T, 1224C>T, 1225A>T, 1226C>A, 1232G>A, 1236A>C, 1237G>A, 1238T>A, 1241C>A, 1340A>C, 1345T>C, 1349G>A, 1355C>T, 1359A>G, 1364_1365insAAAA, 1369G>A, 1370A>G, 1371C>T, 1372T>G, 1373T>A, 1376C>G, 1377C>T, 1378A>T, 1379C>T, 1385T>C, 1389G>C, 1391T>A, 1397T>A, 1402C>A, 1409G>A, 1413A>C, 1414A>G, 1415A>C, 1416A>C, 1418A>T, 1419A>C, 1420A>C, 1422A>G, 1424A>G, 1427C>T, 1430G>A, 1431A>G, 1433T>A, 1445G>A, 1446A>C, 1447A>G, 1448A>T, 1451T>G, 1455T>G, 1457G>A, 1458G>C, 1459C>G, 1460T>A, 1464A>C, 1465A>G, 1466G>A, 1467C>T, 1468A>C, 1469A>T, 1470A>C, 1472G>A, 1475C>G, 1476T>A, 1477T>A, 1478T>A, 1479A>G, 1480A>G, 1481A>T, 1486G>C, 1489A>T, 1490T>C, 1502A>C, 1505G>A, 1508A>G, 1526G>A, 1538G>A, 1541T>C, 1545C>T, 1547T>C, 1553T>C, 1554A>T, 1555T>G, 1556G>C, 1559A>G, 1566C>A, 1567C>G, 1571C>A, 1572T>C, 1580C>A, 1589T>C, 1593_1597delAGCTC, 1598T>A, 1607T>C, 1613G>T, 1614A>T, 1617C>T, 1619T>G, 1620G>A, 1622T>C, 1623A>G, 1624A>T, 1626A>C, 1627A>C, 1628A>T, 1632T>A, 1634A>G, 1635A>G, 1636C>T, 1637A>C, 1643A>G, 1647T>A, 1648A>G, 1664G>A, 1670G>A, 1676T>A, 1679G>A, 1682C>T, 1686C>T, 1691T>A, 1698G>T, 1700A>T, 1703T>C, 1707T>G, 1709G>T, 1710T>C, 1712G>A, 1724T>C, 1736A>G, 1738C>G, 1742G>A, 1744G>A, 1745C>G, 1759G>A, 1760A>C, 1761T>C, 1766C>A, 1767T>A, 1769T>C, 1772A>C, 1775T>C, 1776G>A, 1777G>A, 1778G>A, 1781T>C, 1784C>T, 1787T>C, 1790A>G, 1808A>G, 1814C>T, 1818A>G, 1819G>C, 1820T>A, 1823A>G, 1829C>G, 1830A>C, 1831A>G, 1832T>G, 1833G>C, 1840A>G, 1847A>G, 1848T>C, 1856A>G, 1859C>T, 1860A>G, 1861A>G, 1865C>T, 1867A>C, 1871T>C, 1874G>T, 1875delA, 1878C>G, 1880A>T, 1886T>G, 1887T>A, 1888T>G, 1889G>A, 1895T>C, 1898T>G, 1901T>C, 1910T>C, 1919G>T, 1925G>A, 1934T>C, 1937A>G, 1938T>G, 1939C>A, 1940A>C, 1950G>T, 1952T>C, 1953T>C, 1955A>T, 1964T>C, 1967C>T, 1976T>A, 1980T>A, 1981T>C, 1982A>T, 1985T>C, 1989C>A, 1991T>G, 1994T>A, 2084G>T, 2088T>C, 2090A>G, 2091T>A, 2093C>G, 2094A>T, 2099C>T, 2102A>G, 2103A>T, 2104G>C, 2105C>A, 2106A>C, 2107A>C, 2108G>A, 2111T>A, 2117G>A, 2118C>A, 2120T>A, 2126A>G, 2129T>G, 2133C>G, 2134A>C, 2136C>T, 2137T>G, 2141G>A, 2145G>A, 2146T>A, 2147T>C, 2148C>A, 2152C>T, 2156C>T, 2157T>C, 2160C>T, 2166T>C, 2167A>T, 2168C>T, 2173G>T, 2174G>T, 2187G>T, 2188G>T, 2190A>G, 2191T>G, 2192G>A, 2193C>A, 2204C>T, 2213A>G, 2215G>C, 2217_2219delAAG, 2223G>A, 2226A>G, 2227G>A, 2231C>T, 2234C>G, 2236T>G, 2238A>G, 2240A>G, 2242A>C, 2243A>T, 2244T>G, 2249A>G, 2250G>C, 2251T>C, 2256G>C, 2259T>A, 2264G>A, 2276G>A, 2280C>T, 2285G>A, 2288C>T, 2294T>A, 2298G>C, 2299T>G, 2300T>G, 2306A>C, 2309A>T, 2312A>G, 2315T>G, 2322C>A, 2324A>C, 2326G>T, 2327C>T, 2328C>A, 2330A>T, 2334G>A, 2336A>G, 2337G>A, 2348A>G, 2350A>C, 2351G>T, 2355_2359delCTCAA, 2360C>T, 2366C>T, 2372G>T, 2373A>G, 2375C>A, 2376T>G, 2377G>C, 2378T>A, 2379A>T, 2381G>A, 2382A>T, 2383A>C, 2384C>A, 2393A>G, 2394T>A, 2400A>G, 2402G>T, 2403A>C, 2404G>A, 2405T>A, 2408C>A, 2409A>T, 2411C>T, 2414C>T, 2415T>G, 2416C>T, 2417C>T, 2423A>G, 2426C>T, 2429A>C, 2432A>T, 2435C>T, 2442A>C, 2444G>A, 2445C>T, 2446T>C, 2447C>A, 2448G>A, 2450A>C, 2451A>G, 2452C>A, 2453T>A, 2458T>A, 2462T>A, 2474C>T, 2477C>G, 2478C>T, 2479T>A, 2480A>C, 2481A>G, 2483C>T, 2489A>G, 2504T>A, 2516T>C, 2518C>T, 2519C>T, 2525C>T, 2528T>A, 2531A>T, 2532C>A, 2533A>G, 2545A>G, 2550A>C, 2551A>C, 2552C>A, 2555_2556insCTCAGT, 2556G>T, 2561T>A, 2562C>T, 2563A>T, 2564A>G, 2565T>G, 2568A>G, 2571A>C, 2575G>A, 2577A>G, 2582A>T, 2585G>T								
**CDS**									
RdRp	32	750	83.4%	2702	58.7%	644 (98.8%)	442 (67.8%)	4/4/5/4	4
Protein mutations:	I36L (441A>C 443C>T), L38F (447C>T), A44V (466C>T), A46M (471G>A 472C>T 473T>G), L48M (477T>A), T49H (480A>C 481C>A 482A>T), I52V (489A>G), V60I (513G>A), S61K (516T>A 517C>A 518T>A), K62T (520A>C), T64V (525A>G 526C>T 527T>G), T65K (529C>A 530C>A), R67K (534C>A 535G>A 536C>A), K69R (540A>C 541A>G 542G>T), Y72F (550A>T), F74Y (556T>A 557C>T), A83T (582G>A), C85S (589G>C), F94Y (616T>A), R95S (618C>T 619G>C 620A>T), E97N (624G>A 626A>T), E98D (629A>C), P99T (630C>A 632A>C), L100M (633C>A 635T>G), S109C (661C>G 662G>T), R113K (672C>A 673G>A 674T>G), K117A (684A>G 685A>C 686A>C), A122H (699G>C 700C>A 701A>C), I123V (702A>G), R125A (708A>G 709G>C 710G>A), D127S (714G>T 715A>C), E128D (719A>T), A144S (765G>T 767T>A), V146* (771G>T 772T>A 773T>G), L147I (774T>A 776G>T), K148A (777A>G 778A>C 779A>G), S154K (796G>A 797T>G), E159Q (810G>C), P166S (831C>T 833T>A), G169D (841G>A 842G>T), L172R (850T>G 851A>T), E176G (862A>G), I178M (869T>G), F182* (880T>G 881C>A), H184K (885C>A 887T>G), Q186R (892A>G), F192H (909T>C 910T>A), R194Q (916G>A), G195S (918G>T 919G>C), M198L (927A>C 929G>T), I200K (934T>A 935T>G), S203N (942T>A 943C>A), Q205K (948C>A), N214L (975A>C 976A>T 977T>C), S220R (993T>C 994C>G 995A>G), Y221F (997A>T 998C>T), G222Q (999G>C 1000G>A 1001T>A), L223Y (1003T>A 1004A>T), A224S (1005G>T 1007C>G), K228V (1017A>G 1018A>T 1019A>T), Q231G (1026C>G 1027A>G), E232A (1030A>C 1031G>A), S234A (1035A>G 1036G>C), F237V (1044T>G 1046C>T), F239E (1050T>G 1051T>A 1052T>A), P240H (1054C>A 1055A>C), L250I (1083C>A), W255L (1099G>T), T257V (1104A>G 1105C>T 1106A>G), I260M (1115T>G), F263I (1122T>A 1124C>T), M272I (1151G>T), K276Q (1161A>C), S277M (1164T>A 1165C>T 1166T>G), F279L (1172C>A), F280Y (1174T>A 1175T>C), L282F (1181A>T), R284S (1185A>T 1186G>C 1187G>T), A285N (1188G>A 1189C>A), A286N (1191G>A 1192C>A 1193A>T), E288del (1197_1199delGAA), L290F (1205A>T), L293E (1212T>G 1213T>A), E296D (1223A>T), H297L (1224C>T 1225A>T 1226C>A), S301Q (1236A>C 1237G>A 1238T>A), V337A (1345T>C), M338I (1349G>A), I342V (1359A>G), K343_Q344insKX (1364_1365insAAAA), R345K (1369G>A 1370A>G), L346* (1371C>T 1372T>G 1373T>A), H348F (1377C>T 1378A>T 1379C>T), D352Q (1389G>C 1391T>A), T356K (1402C>A), K360R (1413A>C 1414A>G 1415A>C), I361L (1416A>C 1418A>T), K362P (1419A>C 1420A>C), T363A (1422A>G 1424A>G), T366A (1431A>G 1433T>A), K371R (1446A>C 1447A>G 1448A>T), L374V (1455T>G 1457G>A), A375R (1458G>C 1459C>G 1460T>A), K377R (1464A>C 1465A>G 1466G>A), Q378S (1467C>T 1468A>C 1469A>T), K379Q (1470A>C 1472G>A), N380K (1475C>G), F381K (1476T>A 1477T>A 1478T>A), K382G (1479A>G 1480A>G 1481A>T), C384S (1486G>C), Y385F (1489A>T 1490T>C), L389F (1502A>C), M401I (1538G>A), L404F (1545C>T 1547T>C), M407C (1554A>T 1555T>G 1556G>C), I408M (1559A>G), P411S (1566C>A 1567C>G), S420del (1593_1597delAGCTC), I427F (1614A>T), V429I (1620G>A 1622T>C), N430V (1623A>G 1624A>T), K431P (1626A>C 1627A>C 1628A>T), L433M (1632T>A 1634A>G), T434V (1635A>G 1636C>T 1637A>C), Y438S (1647T>A 1648A>G), H451Y (1686C>T), A455S (1698G>T 1700A>T), S458A (1707T>G 1709G>T), A468G (1738C>G), S470K (1744G>A 1745C>G), G475D (1759G>A 1760A>C), C476R (1761T>C), N477K (1766C>A), S478T (1767T>A 1769T>C), G481K (1776G>A 1777G>A 1778G>A), S495A (1818A>G 1819G>C 1820T>A), N499R (1830A>C 1831A>G 1832T>G), K511T (1867A>C), P515A (1878C>G 1880A>T), L518R (1887T>A 1888T>G 1889G>A), D521E (1898T>G), S535D (1938T>G 1939C>A 1940A>C), D539Y (1950G>T 1952T>C), L549T (1980T>A 1981T>C 1982A>T), F586M (2091T>A 2093C>G), T587S (2094A>T), K591P (2106A>C 2107A>C 2108G>A), H600A (2133C>G 2134A>C), L601C (2136C>T 2137T>G), V604N (2145G>A 2146T>A 2147T>C), S606L (2152C>T), Y611L (2166T>C 2167A>T 2168C>T), W613F (2173G>T 2174G>T), G618F (2187G>T 2188G>T), M619G (2190A>G 2191T>G 2192G>A), P620T (2193C>A), G627A (2215G>C), K628del (2217_2219delAAG), V630I (2223G>A), S631D (2226A>G 2227G>A), L634R (2236T>G), K635E (2238A>G 2240A>G), E636A (2242A>C 2243A>T), L637V (2244T>G), V639P (2250G>C 2251T>C), E641Q (2256G>C), L642I (2259T>A), V655R (2298G>C 2299T>G 2300T>G), L663I (2322C>A 2324A>C), R664L (2326G>T 2327C>T), Q665N (2328C>A 2330A>T), V667M (2334G>A 2336A>G), E668K (2337G>A), K672T (2350A>C 2351G>T), L674del (2355_2359delCTCAA), I680V (2373A>G 2375C>A), C681A (2376T>G 2377G>C 2378T>A), M682L (2379A>T 2381G>A), N683S (2382A>T 2383A>C 2384C>A), S687T (2394T>A), K689D (2400A>G 2402G>T), S690Q (2403A>C 2404G>A 2405T>A), I692F (2409A>T 2411C>T), S694V (2415T>G 2416C>T 2417C>T), I696M (2423A>G), L704S (2445C>T 2446T>C 2447C>A), E705N (2448G>A 2450A>C), T706E (2451A>G 2452C>A 2453T>A), F708Y (2458T>A), D709E (2462T>A), L715Y (2478C>T 2479T>A 2480A>C), I716V (2481A>G 2483C>T), P728L (2518C>T 2519C>T), Q733R (2532C>A 2533A>G), D737G (2545A>G), N739P (2550A>C 2551A>C 2552C>A), T740_V741insLS (2555_2556insCTCAGT), V741F (2556G>T), Q743L (2562C>T 2563A>T 2564A>G), F744V (2565T>G), N745D (2568A>G), K746Q (2571A>C), W747* (2575G>A), I748V (2577A>G), L749F (2582A>T)								

	Begin	End	Coverage	Score	Concordance	Matches	Identities	I/D/M/F*	Stop Codons
**NT**	**428**	**2585**	**70.6%**	**1294**	**33.9%**	**1940 (98.6%)**	**1316 (66.9%)**	**10/17**	
Codon mutations:	AAA34AAG (437A>G), ATC36CTT (441A>C 443C>T), CTT38TTT (447C>T), CTA42TTA (459C>T), GCC44GTC (466C>T), TCC45TCT (470C>T), GCT46ATG (471G>A 472C>T 473T>G), TTG48ATG (477T>A), ACA49CAT (480A>C 481C>A 482A>T), AAA50AAG (485A>G), ATC52GTC (489A>G), GCT53GCC (494T>C), CTT54CTA (497T>A), GCT55GCG (500T>G), GGA56GGC (503A>C), TGT57TGC (506T>C), GAC58GAT (509C>T), TTT59TTC (512T>C), GTA60ATA (513G>A), TCT61AAA (516T>A 517C>A 518T>A), AAA62ACA (520A>C), GTG63GTT (524G>T), ACT64GTG (525A>G 526C>T 527T>G), ACC65AAA (529C>A 530C>A), TTA66CTG (531T>C 533A>G), CGC67AAA (534C>A 535G>A 536C>A), CGG68CGA (539G>A), AAG69CGT (540A>C 541A>G 542G>T), TCG70TCA (545G>A), GGC71GGT (548C>T), TAT72TTT (550A>T), TTC74TAT (556T>A 557C>T), ACA75ACT (560A>T), TAC78TAT (569C>T), AAG80AAA (575G>A), TGC82TGT (581C>T), GCA83ACA (582G>A), GTA84GTT (587A>T), TGC85TCC (589G>C), TTA86CTT (591T>C 593A>T), CGA88CGT (599A>T), TAC89TAT (602C>T), TCC93AGT (612T>A 613C>G 614C>T), TTT94TAT (616T>A), CGA95TCT (618C>T 619G>C 620A>T), GAA97AAT (624G>A 626A>T), GAA98GAC (629A>C), CCA99ACC (630C>A 632A>C), CTT100ATG (633C>A 635T>G), TCG101TCT (638G>T), GTG102GTT (641G>T), CCT103CCA (644T>A), GTG104GTT (647G>T), CTC106TTG (651C>T 653C>G), ACA107ACC (656A>C), AGA108CGA (657A>C), TCG109TGT (661C>G 662G>T), GGT110GGA (665T>A), ATC111ATT (668C>T), CGT113AAG (672C>A 673G>A 674T>G), ATC115ATA (680C>A), CCC116CCT (683C>T), AAA117GCC (684A>G 685A>C 686A>C), TTG119TTA (692G>A), CGT120CGA (695T>A), GCA122CAC (699G>C 700C>A 701A>C), ATA123GTA (702A>G), CGT124AGG (705C>A 707T>G), AGG125GCA (708A>G 709G>C 710G>A), AAA126AAG (713A>G), GAT127TCT (714G>T 715A>C), GAA128GAT (719A>T), CAC129CAT (722C>T), GTT134GTC (737T>C), TAC137TAT (746C>T), TCA139TCT (752A>T), GGG142GGA (761G>A), GCT144TCA (765G>T 767T>A), AAG145AAA (770G>A), GTT146TAG (771G>T 772T>A 773T>G), TTG147ATT (774T>A 776G>T), AAA148GCG (777A>G 778A>C 779A>G), GTT149GTG (782T>G), AGT154AAG (796G>A 797T>G), GCA156GCC (803A>C), TTT158TTC (809T>C), GAA159CAA (810G>C), TCC160TCA (815C>A), ATA161ATT (818A>T), ACT162ACG (821T>G), ACC163ACT (824C>T), CCA164CCT (827A>T), CCT166TCA (831C>T 833T>A), GGG169GAT (841G>A 842G>T), CTA172CGT (850T>G 851A>T), GTG174GTT (857G>T), CTG175CTT (860G>T), GAA176GGA (862A>G), ATT178ATG (869T>G), ACG180ACT (875G>T), TTC182TGA (880T>G 881C>A), CAT184AAG (885C>A 887T>G), CAA186CGA (892A>G), CTA190CTT (905A>T), CCC191CCA (908C>A), TTT192CAT (909T>C 910T>A), CGA194CAA (916G>A), GGC195TCC (918G>T 919G>C), ATT196ATA (923T>A), CCA197CCT (926A>T), ATG198CTT (927A>C 929G>T), ATT200AAG (934T>A 935T>G), GGG201GGT (938G>T), TCT203AAT (942T>A 943C>A), CAA205AAA (948C>A), CCC212CCA (971C>A), CTA213TTA (972C>T), AAT214CTC (975A>C 976A>T 977T>C), AAT216AAC (983T>C), AGG219CGC (990A>C 992G>C), TCA220CGG (993T>C 994C>G 995A>G), TAC221TTT (997A>T 998C>T), GGT222CAA (999G>C 1000G>A 1001T>A), TTA223TAT (1003T>A 1004A>T), GCC224TCG (1005G>T 1007C>G), GTA225GTG (1010A>G), GAC227GAT (1016C>T), AAA228GTT (1017A>G 1018A>T 1019A>T), GTT229GTA (1022T>A), GAT230GAC (1025T>C), CAA231GGA (1026C>G 1027A>G), GAG232GCA (1030A>C 1031G>A), GCT233GCG (1034T>G), AGT234GCT (1035A>G 1036G>C), TTC237GTT (1044T>G 1046C>T), AAG238AAA (1049G>A), TTT239GAA (1050T>G 1051T>A 1052T>A), CCA240CAC (1054C>A 1055A>C), ATT242ATC (1061T>C), GTT244GTG (1067T>G), TTG246TTA (1073G>A), AAA247AAG (1076A>G), CTA250ATA (1083C>A), GCG251GCT (1088G>T), GCC252GCG (1091C>G), TGG255TTG (1099G>T), ACA257GTG (1104A>G 1105C>T 1106A>G), ATT260ATG (1115T>G), TCA262TCC (1121A>C), TTC263ATT (1122T>A 1124C>T), CAG264CAA (1127G>A), GAC265GAT (1130C>T), GGC266GGG (1133C>G), TCA269TCC (1142A>C), CCC270CCT (1145C>T), GGG271GGT (1148G>T), ATG272ATT (1151G>T), TTG273CTT (1152T>C 1154G>T), TCC275TCT (1160C>T), AAA276CAA (1161A>C), TCT277ATG (1164T>A 1165C>T 1166T>G), GTC278GTT (1169C>T), TTC279TTA (1172C>A), TTT280TAC (1174T>A 1175T>C), CCT281CCA (1178T>A), TTA282TTT (1181A>T), GAC283GAT (1184C>T), AGG284TCT (1185A>T 1186G>C 1187G>T), GCT285AAT (1188G>A 1189C>A), GCA286AAT (1191G>A 1192C>A 1193A>T), ACT287ACC (1196T>C), GAA288del (1197_1199delGAA), TTA290TTT (1205A>T), AAC292AAT (1211C>T), TTA293GAA (1212T>G 1213T>A), TCT294TCC (1217T>C), CTC295CTT (1220C>T), GAA296GAT (1223A>T), CAC297TTA (1224C>T 1225A>T 1226C>A), GAG299GAA (1232G>A), AGT301CAA (1236A>C 1237G>A 1238T>A), GTC302GTA (1241C>A), GTA335..C (1340A>C), GTA337GCA (1345T>C), ATG338ATA (1349G>A), AAC340AAT (1355C>T), ATA342GTA (1359A>G), AAA343_CAA344insAAAA-- (1364_1365insAAAA), AGA345AAG (1369G>A 1370A>G), CTT346TGA (1371C>T 1372T>G 1373T>A), CTC347CTG (1376C>G), CAC348TTT (1377C>T 1378A>T 1379C>T), GTT350GTC (1385T>C), GAT352CAA (1389G>C 1391T>A), GCT354GCA (1397T>A), ACA356AAA (1402C>A), CTG358CTA (1409G>A), AAA360CGC (1413A>C 1414A>G 1415A>C), ATA361CTT (1416A>C 1418A>T), AAG362CCG (1419A>C 1420A>C), ACA363GCG (1422A>G 1424A>G), GAC364GAT (1427C>T), GGG365GGA (1430G>A), ACT366GCA (1431A>G 1433T>A), GAG370GAA (1445G>A), AAA371CGT (1446A>C 1447A>G 1448A>T), CCT372CCG (1451T>G), TTG374GTA (1455T>G 1457G>A), GCT375CGA (1458G>C 1459C>G 1460T>A), AAG377CGA (1464A>C 1465A>G 1466G>A), CAA378TCT (1467C>T 1468A>C 1469A>T), AAG379CAA (1470A>C 1472G>A), AAC380AAG (1475C>G), TTT381AAA (1476T>A 1477T>A 1478T>A), AAA382GGT (1479A>G 1480A>G 1481A>T), TGT384TCT (1486G>C), TAT385TTC (1489A>T 1490T>C), TTA389TTC (1502A>C), AAG390AAA (1505G>A), TCA391TCG (1508A>G), CCG397CCA (1526G>A), ATG401ATA (1538G>A), TAT402TAC (1541T>C), CTT404TTC (1545C>T 1547T>C), TCT406TCC (1553T>C), ATG407TGC (1554A>T 1555T>G 1556G>C), ATA408ATG (1559A>G), CCT411AGT (1566C>A 1567C>G), ACC412ACA (1571C>A), TTG413CTG (1572T>C), TCC415TCA (1580C>A), GTT418GTC (1589T>C), AGC420del (1593_1597delAGCTC), TCT421--A (1593_1597delAGCTC 1598T>A), CTT424CTC (1607T>C), ACG426ACT (1613G>T), ATC427TTC (1614A>T), CTT428TTG (1617C>T 1619T>G), GTT429ATC (1620G>A 1622T>C), AAC430GTC (1623A>G 1624A>T), AAA431CCT (1626A>C 1627A>C 1628A>T), TTA433ATG (1632T>A 1634A>G), ACA434GTC (1635A>G 1636C>T 1637A>C), AGA436AGG (1643A>G), TAT438AGT (1647T>A 1648A>G), CTG443CTA (1664G>A), GGG445GGA (1670G>A), CCT447CCA (1676T>A), TTG448TTA (1679G>A), GGC449GGT (1682C>T), CAC451TAC (1686C>T), GGT452GGA (1691T>A), GCA455TCT (1698G>T 1700A>T), CTT456CTC (1703T>C), TCG458GCT (1707T>G 1709G>T), TTG459CTA (1710T>C 1712G>A), TAT463TAC (1724T>C), TTA467TTG (1736A>G), GCA468GGA (1738C>G), GCG469GCA (1742G>A), AGC470AAG (1744G>A 1745C>G), GGA475GAC (1759G>A 1760A>C), TGT476CGT (1761T>C), AAC477AAA (1766C>A), TCT478ACC (1767T>A 1769T>C), CCA479CCC (1772A>C), TTT480TTC (1775T>C), GGG481AAA (1776G>A 1777G>A 1778G>A), GAT482GAC (1781T>C), TAC483TAT (1784C>T), GCT484GCC (1787T>C), TTA485TTG (1790A>G), CTA491CTG (1808A>G), ACC493ACT (1814C>T), AGT495GCA (1818A>G 1819G>C 1820T>A), AAA496AAG (1823A>G), GCC498..G (1829C>G), AAT499CGG (1830A>C 1831A>G 1832T>G), GAG500C.. (1833G>C), AAG502.G. (1840A>G), TTA504..G (1847A>G), TTG505C.. (1848T>C), AGA507..G (1856A>G), CTC508..T (1859C>T), AAT509GG. (1860A>G 1861A>G), GTC510GTT (1865C>T), AAA511ACA (1867A>C), ATT512ATC (1871T>C), TCG513TCT (1874G>T), ATA514-TA (1875delA), CCA515GCT (1878C>G 1880A>T), TCT517TCG (1886T>G), TTG518AGA (1887T>A 1888T>G 1889G>A), TCT520TCC (1895T>C), GAT521GAG (1898T>G), AAT522AAC (1901T>C), ATT525ATC (1910T>C), GCG528GCT (1919G>T), AGG530AGA (1925G>A), ACT533ACC (1934T>C), AAA534AAG (1937A>G), TCA535GAC (1938T>G 1939C>A 1940A>C), GAT539TAC (1950G>T 1952T>C), TTA540CTT (1953T>C 1955A>T), ATT543ATC (1964T>C), TCC544TCT (1967C>T), GCT547GCA (1976T>A), TTA549ACT (1980T>A 1981T>C 1982A>T), TCT550TCC (1985T>C), CGT552AGG (1989C>A 1991T>G), ACT553ACA (1994T>A), GCG583.CT (2084G>T), TTA585CTG (2088T>C 2090A>G), TTC586ATG (2091T>A 2093C>G), ACT587TCT (2094A>T), ACC588ACT (2099C>T), CAA589CAG (2102A>G), AGC590TCA (2103A>T 2104G>C 2105C>A), AAG591CCA (2106A>C 2107A>C 2108G>A), CGT592CGA (2111T>A), GAG594GAA (2117G>A), CGT595AGA (2118C>A 2120T>A), AAA597AAG (2126A>G), GCT598GCG (2129T>G), CAT600GCT (2133C>G 2134A>C), CTT601TGT (2136C>T 2137T>G), AAG602AAA (2141G>A), GTT604AAC (2145G>A 2146T>A 2147T>C), CGG605AGG (2148C>A), TCA606TTA (2152C>T), CAC607CAT (2156C>T), TTA608CTA (2157T>C), CTA609TTA (2160C>T), TAC611CTT (2166T>C 2167A>T 2168C>T), TGG613TTT (2173G>T 2174G>T), GGT618TTT (2187G>T 2188G>T), ATG619GGA (2190A>G 2191T>G 2192G>A), CCC620ACC (2193C>A), CCC623CCT (2204C>T), AAA626AAG (2213A>G), GGG627GCG (2215G>C), AAG628del (2217_2219delAAG), GTA630ATA (2223G>A), AGT631GAT (2226A>G 2227G>A), TAC632TAT (2231C>T), CTC633CTG (2234C>G), CTT634CGT (2236T>G), AAA635GAG (2238A>G 2240A>G), GAA636GCT (2242A>C 2243A>T), TTA637GTA (2244T>G), AAA638AAG (2249A>G), GTT639CCT (2250G>C 2251T>C), GAG641CAG (2256G>C), TTA642ATA (2259T>A), CAG643CAA (2264G>A), AAG647AAA (2276G>A), CTG649TTG (2280C>T), GTG650GTA (2285G>A), TTC651TTT (2288C>T), GGT653GGA (2294T>A), GTT655CGG (2298G>C 2299T>G 2300T>G), ATA657ATC (2306A>C), CTA658CTT (2309A>T), GAA659GAG (2312A>G), CGT660CGG (2315T>G), CTA663ATC (2322C>A 2324A>C), CGC664CTT (2326G>T 2327C>T), CAA665AAT (2328C>A 2330A>T), GTA667ATG (2334G>A 2336A>G), GAA668AAA (2337G>A), TTA671TTG (2348A>G), AAG672ACT (2350A>C 2351G>T), CTC674del (2355_2359delCTCAA), AAC675--T (2355_2359delCTCAA 2360C>T), TAC677TAT (2366C>T), ACG679ACT (2372G>T), ATC680GTA (2373A>G 2375C>A), TGT681GCA (2376T>G 2377G>C 2378T>A), ATG682TTA (2379A>T 2381G>A), AAC683TCA (2382A>T 2383A>C 2384C>A), GTA686GTG (2393A>G), TCC687ACC (2394T>A), AAG689GAT (2400A>G 2402G>T), AGT690CAA (2403A>C 2404G>A 2405T>A), CTC691CTA (2408C>A), ATC692TTT (2409A>T 2411C>T), GAC693GAT (2414C>T), TCC694GTT (2415T>G 2416C>T 2417C>T), ATA696ATG (2423A>G), TGC697TGT (2426C>T), GCA698GCC (2429A>C), ACA699ACT (2432A>T), TCC700TCT (2435C>T), AGG703CGA (2442A>C 2444G>A), CTC704TCA (2445C>T 2446T>C 2447C>A), GAA705AAC (2448G>A 2450A>C), ACT706GAA (2451A>G 2452C>A 2453T>A), TTT708TAT (2458T>A), GAT709GAA (2462T>A), TTC713TTT (2474C>T), GGC714GGG (2477C>G), CTA715TAC (2478C>T 2479T>A 2480A>C), ATC716GTT (2481A>G 2483C>T), AAA718AAG (2489A>G), GGT723GGA (2504T>A), AAT727AAC (2516T>C), CCC728CTT (2518C>T 2519C>T), ACC730ACT (2525C>T), ACT731ACA (2528T>A), CCA732CCT (2531A>T), CAG733AGG (2532C>A 2533A>G), GAT737GGT (2545A>G), AAC739CCA (2550A>C 2551A>C 2552C>A), ACT740_GTT741insCTCAGT (2555_2556insCTCAGT), GTT741TTT (2556G>T), ATT742ATA (2561T>A), CAA743TTG (2562C>T 2563A>T 2564A>G), TTT744GTT (2565T>G), AAT745GAT (2568A>G), AAA746CAA (2571A>C), TGG747TAG (2575G>A), ATA748GTA (2577A>G), TTA749TTT (2582A>T), GGG750GGT (2585G>T)								

Proteins

	Begin	End	Coverage	Score	Concordance	Matches	Identities	I/D/M/F*	Stop Codons
RNA-dependent RNA polymerase (YP_010798874.1)	32	750	83.4%	2702	58.7%	644 (98.8%)	442 (67.8%)	4/4/5/4	4

	Begin	End	Coverage	Score	Concordance	Matches	Identities	I/D/M/F*	Stop Codons
NT	428	2585	70.6%	1294	33.9%	1940 (98.6%)	1316 (66.9%)	10/17	

Protein mutations:

I36L (441A>C 443C>T), L38F (447C>T), A44V (466C>T), A46M (471G>A 472C>T 473T>G), L48M (477T>A), T49H (480A>C 481C>A 482A>T), I52V (489A>G), V60I (513G>A), S61K (516T>A 517C>A 518T>A), K62T (520A>C), T64V (525A>G 526C>T 527T>G), T65K (529C>A 530C>A), R67K (534C>A 535G>A 536C>A), K69R (540A>C 541A>G 542G>T), Y72F (550A>T), F74Y (556T>A 557C>T), A83T (582G>A), C85S (589G>C), F94Y (616T>A), R95S (618C>T 619G>C 620A>T), E97N (624G>A 626A>T), E98D (629A>C), P99T (630C>A 632A>C), L100M (633C>A 635T>G), S109C (661C>G 662G>T), R113K (672C>A 673G>A 674T>G), K117A (684A>G 685A>C 686A>C), A122H (699G>C 700C>A 701A>C), I123V (702A>G), R125A (708A>G 709G>C 710G>A), D127S (714G>T 715A>C), E128D (719A>T), A144S (765G>T 767T>A), V146* (771G>T 772T>A 773T>G), L147I (774T>A 776G>T), K148A (777A>G 778A>C 779A>G), S154K (796G>A 797T>G), E159Q (810G>C), P166S (831C>T 833T>A), G169D (841G>A 842G>T), L172R (850T>G 851A>T), E176G (862A>G), I178M (869T>G), F182* (880T>G 881C>A), H184K (885C>A 887T>G), Q186R (892A>G), F192H (909T>C 910T>A), R194Q (916G>A), G195S (918G>T 919G>C), M198L (927A>C 929G>T), I200K (934T>A 935T>G), S203N (942T>A 943C>A), Q205K (948C>A), N214L (975A>C 976A>T 977T>C), S220R (993T>C 994C>G 995A>G), Y221F (997A>T 998C>T), G222Q (999G>C 1000G>A 1001T>A), L223Y (1003T>A 1004A>T), A224S (1005G>T 1007C>G), K228V (1017A>G 1018A>T 1019A>T), Q231G (1026C>G 1027A>G), E232A (1030A>C 1031G>A), S234A (1035A>G 1036G>C), F237V (1044T>G 1046C>T), F239E (1050T>G 1051T>A 1052T>A), P240H (1054C>A 1055A>C), L250I (1083C>A), W255L (1099G>T), T257V (1104A>G 1105C>T 1106A>G), I260M (1115T>G), F263I (1122T>A 1124C>T), M272I (1151G>T), K276Q (1161A>C), S277M (1164T>A 1165C>T 1166T>G), F279L (1172C>A), F280Y (1174T>A 1175T>C), L282F (1181A>T), R284S (1185A>T 1186G>C 1187G>T), A285N (1188G>A 1189C>A), A286N (1191G>A 1192C>A 1193A>T), E288del (1197_1199delGAA), L290F (1205A>T), L293E (1212T>G 1213T>A), E296D (1223A>T), H297L (1224C>T 1225A>T 1226C>A), S301Q (1236A>C 1237G>A 1238T>A), V337A (1345T>C), M338I (1349G>A), I342V (1359A>G), K343_Q344insKX (1364_1365insAAAA), R345K (1369G>A 1370A>G), L346* (1371C>T 1372T>G 1373T>A), H348F (1377C>T 1378A>T 1379C>T), D352Q (1389G>C 1391T>A), T356K (1402C>A), K360R (1413A>C 1414A>G 1415A>C), I361L (1416A>C 1418A>T), K362P (1419A>C 1420A>C), T363A (1422A>G 1424A>G), T366A (1431A>G 1433T>A), K371R (1446A>C 1447A>G 1448A>T), L374V (1455T>G 1457G>A), A375R (1458G>C 1459C>G 1460T>A), K377R (1464A>C 1465A>G 1466G>A), Q378S (1467C>T 1468A>C 1469A>T), K379Q (1470A>C 1472G>A), N380K (1475C>G), F381K (1476T>A 1477T>A 1478T>A), K382G (1479A>G 1480A>G 1481A>T), C384S (1486G>C), Y385F (1489A>T 1490T>C), L389F (1502A>C), M401I (1538G>A), L404F (1545C>T 1547T>C), M407C (1554A>T 1555T>G 1556G>C), I408M (1559A>G), P411S (1566C>A 1567C>G), S420del (1593_1597delAGCTC), I427F (1614A>T), V429I (1620G>A 1622T>C), N430V (1623A>G 1624A>T), K431P (1626A>C 1627A>C 1628A>T), L433M (1632T>A 1634A>G), T434V (1635A>G 1636C>T 1637A>C), Y438S (1647T>A 1648A>G), H451Y (1686C>T), A455S (1698G>T 1700A>T), S458A (1707T>G 1709G>T), A468G (1738C>G), S470K (1744G>A 1745C>G), G475D (1759G>A 1760A>C), C476R (1761T>C), N477K (1766C>A), S478T (1767T>A 1769T>C), G481K (1776G>A 1777G>A 1778G>A), S495A (1818A>G 1819G>C 1820T>A), N499R (1830A>C 1831A>G 1832T>G), K511T (1867A>C), P515A (1878C>G 1880A>T), L518R (1887T>A 1888T>G 1889G>A), D521E (1898T>G), S535D (1938T>G 1939C>A 1940A>C), D539Y (1950G>T 1952T>C), L549T (1980T>A 1981T>C 1982A>T), F586M (2091T>A 2093C>G), T587S (2094A>T), K591P (2106A>C 2107A>C 2108G>A), H600A (2133C>G 2134A>C), L601C (2136C>T 2137T>G), V604N (2145G>A 2146T>A 2147T>C), S606L (2152C>T), Y611L (2166T>C 2167A>T 2168C>T), W613F (2173G>T 2174G>T), G618F (2187G>T 2188G>T), M619G (2190A>G 2191T>G 2192G>A), P620T (2193C>A), G627A (2215G>C), K628del (2217_2219delAAG), V630I (2223G>A), S631D (2226A>G 2227G>A), L634R (2236T>G), K635E (2238A>G 2240A>G), E636A (2242A>C 2243A>T), L637V (2244T>G), V639P (2250G>C 2251T>C), E641Q (2256G>C), L642I (2259T>A), V655R (2298G>C 2299T>G 2300T>G), L663I (2322C>A 2324A>C), R664L (2326G>T 2327C>T), Q665N (2328C>A 2330A>T), V667M (2334G>A 2336A>G), E668K (2337G>A), K672T (2350A>C 2351G>T), L674del (2355_2359delCTCAA), I680V (2373A>G 2375C>A), C681A (2376T>G 2377G>C 2378T>A), M682L (2379A>T 2381G>A), N683S (2382A>T 2383A>C 2384C>A), S687T (2394T>A), K689D (2400A>G 2402G>T), S690Q (2403A>C 2404G>A 2405T>A), I692F (2409A>T 2411C>T), S694V (2415T>G 2416C>T 2417C>T), I696M (2423A>G), L704S (2445C>T 2446T>C 2447C>A), E705N (2448G>A 2450A>C), T706E (2451A>G 2452C>A 2453T>A), F708Y (2458T>A), D709E (2462T>A), L715Y (2478C>T 2479T>A 2480A>C), I716V (2481A>G 2483C>T), P728L (2518C>T 2519C>T), Q733R (2532C>A 2533A>G), D737G (2545A>G), N739P (2550A>C 2551A>C 2552C>A), T740_V741insLS (2555_2556insCTCAGT), V741F (2556G>T), Q743L (2562C>T 2563A>T 2564A>G), F744V (2565T>G), N745D (2568A>G), K746Q (2571A>C), W747* (2575G>A), I748V (2577A>G), L749F (2582A>T)

Codon mutations:

AAA34AAG (437A>G), ATC36CTT (441A>C 443C>T), CTT38TTT (447C>T), CTA42TTA (459C>T), GCC44GTC (466C>T), TCC45TCT (470C>T), GCT46ATG (471G>A 472C>T 473T>G), TTG48ATG (477T>A), ACA49CAT (480A>C 481C>A 482A>T), AAA50AAG (485A>G), ATC52GTC (489A>G), GCT53GCC (494T>C), CTT54CTA (497T>A), GCT55GCG (500T>G), GGA56GGC (503A>C), TGT57TGC (506T>C), GAC58GAT (509C>T), TTT59TTC (512T>C), GTA60ATA (513G>A), TCT61AAA (516T>A 517C>A 518T>A), AAA62ACA (520A>C), GTG63GTT (524G>T), ACT64GTG (525A>G 526C>T 527T>G), ACC65AAA (529C>A 530C>A), TTA66CTG (531T>C 533A>G), CGC67AAA (534C>A 535G>A 536C>A), CGG68CGA (539G>A), AAG69CGT (540A>C 541A>G 542G>T), TCG70TCA (545G>A), GGC71GGT (548C>T), TAT72TTT (550A>T), TTC74TAT (556T>A 557C>T), ACA75ACT (560A>T), TAC78TAT (569C>T), AAG80AAA (575G>A), TGC82TGT (581C>T), GCA83ACA (582G>A), GTA84GTT (587A>T), TGC85TCC (589G>C), TTA86CTT (591T>C 593A>T), CGA88CGT (599A>T), TAC89TAT (602C>T), TCC93AGT (612T>A 613C>G 614C>T), TTT94TAT (616T>A), CGA95TCT (618C>T 619G>C 620A>T), GAA97AAT (624G>A 626A>T), GAA98GAC (629A>C), CCA99ACC (630C>A 632A>C), CTT100ATG (633C>A 635T>G), TCG101TCT (638G>T), GTG102GTT (641G>T), CCT103CCA (644T>A), GTG104GTT (647G>T), CTC106TTG (651C>T 653C>G), ACA107ACC (656A>C), AGA108CGA (657A>C), TCG109TGT (661C>G 662G>T), GGT110GGA (665T>A), ATC111ATT (668C>T), CGT113AAG (672C>A 673G>A 674T>G), ATC115ATA (680C>A), CCC116CCT (683C>T), AAA117GCC (684A>G 685A>C 686A>C), TTG119TTA (692G>A), CGT120CGA (695T>A), GCA122CAC (699G>C 700C>A 701A>C), ATA123GTA (702A>G), CGT124AGG (705C>A 707T>G), AGG125GCA (708A>G 709G>C 710G>A), AAA126AAG (713A>G), GAT127TCT (714G>T 715A>C), GAA128GAT (719A>T), CAC129CAT (722C>T), GTT134GTC (737T>C), TAC137TAT (746C>T), TCA139TCT (752A>T), GGG142GGA (761G>A), GCT144TCA (765G>T 767T>A), AAG145AAA (770G>A), GTT146TAG (771G>T 772T>A 773T>G), TTG147ATT (774T>A 776G>T), AAA148GCG (777A>G 778A>C 779A>G), GTT149GTG (782T>G), AGT154AAG (796G>A 797T>G), GCA156GCC (803A>C), TTT158TTC (809T>C), GAA159CAA (810G>C), TCC160TCA (815C>A), ATA161ATT (818A>T), ACT162ACG (821T>G), ACC163ACT (824C>T), CCA164CCT (827A>T), CCT166TCA (831C>T 833T>A), GGG169GAT (841G>A 842G>T), CTA172CGT (850T>G 851A>T), GTG174GTT (857G>T), CTG175CTT (860G>T), GAA176GGA (862A>G), ATT178ATG (869T>G), ACG180ACT (875G>T), TTC182TGA (880T>G 881C>A), CAT184AAG (885C>A 887T>G), CAA186CGA (892A>G), CTA190CTT (905A>T), CCC191CCA (908C>A), TTT192CAT (909T>C 910T>A), CGA194CAA (916G>A), GGC195TCC (918G>T 919G>C), ATT196ATA (923T>A), CCA197CCT (926A>T), ATG198CTT (927A>C 929G>T), ATT200AAG (934T>A 935T>G), GGG201GGT (938G>T), TCT203AAT (942T>A 943C>A), CAA205AAA (948C>A), CCC212CCA (971C>A), CTA213TTA (972C>T), AAT214CTC (975A>C 976A>T 977T>C), AAT216AAC (983T>C), AGG219CGC (990A>C 992G>C), TCA220CGG (993T>C 994C>G 995A>G), TAC221TTT (997A>T 998C>T), GGT222CAA (999G>C 1000G>A 1001T>A), TTA223TAT (1003T>A 1004A>T), GCC224TCG (1005G>T 1007C>G), GTA225GTG (1010A>G), GAC227GAT (1016C>T), AAA228GTT (1017A>G 1018A>T 1019A>T), GTT229GTA (1022T>A), GAT230GAC (1025T>C), CAA231GGA (1026C>G 1027A>G), GAG232GCA (1030A>C 1031G>A), GCT233GCG (1034T>G), AGT234GCT (1035A>G 1036G>C), TTC237GTT (1044T>G 1046C>T), AAG238AAA (1049G>A), TTT239GAA (1050T>G 1051T>A 1052T>A), CCA240CAC (1054C>A 1055A>C), ATT242ATC (1061T>C), GTT244GTG (1067T>G), TTG246TTA (1073G>A), AAA247AAG (1076A>G), CTA250ATA (1083C>A), GCG251GCT (1088G>T), GCC252GCG (1091C>G), TGG255TTG (1099G>T), ACA257GTG (1104A>G 1105C>T 1106A>G), ATT260ATG (1115T>G), TCA262TCC (1121A>C), TTC263ATT (1122T>A 1124C>T), CAG264CAA (1127G>A), GAC265GAT (1130C>T), GGC266GGG (1133C>G), TCA269TCC (1142A>C), CCC270CCT (1145C>T), GGG271GGT (1148G>T), ATG272ATT (1151G>T), TTG273CTT (1152T>C 1154G>T), TCC275TCT (1160C>T), AAA276CAA (1161A>C), TCT277ATG (1164T>A 1165C>T 1166T>G), GTC278GTT (1169C>T), TTC279TTA (1172C>A), TTT280TAC (1174T>A 1175T>C), CCT281CCA (1178T>A), TTA282TTT (1181A>T), GAC283GAT (1184C>T), AGG284TCT (1185A>T 1186G>C 1187G>T), GCT285AAT (1188G>A 1189C>A), GCA286AAT (1191G>A 1192C>A 1193A>T), ACT287ACC (1196T>C), GAA288del (1197_1199delGAA), TTA290TTT (1205A>T), AAC292AAT (1211C>T), TTA293GAA (1212T>G 1213T>A), TCT294TCC (1217T>C), CTC295CTT (1220C>T), GAA296GAT (1223A>T), CAC297TTA (1224C>T 1225A>T 1226C>A), GAG299GAA (1232G>A), AGT301CAA (1236A>C 1237G>A 1238T>A), GTC302GTA (1241C>A), GTA335..C (1340A>C), GTA337GCA (1345T>C), ATG338ATA (1349G>A), AAC340AAT (1355C>T), ATA342GTA (1359A>G), AAA343_CAA344insAAAA-- (1364_1365insAAAA), AGA345AAG (1369G>A 1370A>G), CTT346TGA (1371C>T 1372T>G 1373T>A), CTC347CTG (1376C>G), CAC348TTT (1377C>T 1378A>T 1379C>T), GTT350GTC (1385T>C), GAT352CAA (1389G>C 1391T>A), GCT354GCA (1397T>A), ACA356AAA (1402C>A), CTG358CTA (1409G>A), AAA360CGC (1413A>C 1414A>G 1415A>C), ATA361CTT (1416A>C 1418A>T), AAG362CCG (1419A>C 1420A>C), ACA363GCG (1422A>G 1424A>G), GAC364GAT (1427C>T), GGG365GGA (1430G>A), ACT366GCA (1431A>G 1433T>A), GAG370GAA (1445G>A), AAA371CGT (1446A>C 1447A>G 1448A>T), CCT372CCG (1451T>G), TTG374GTA (1455T>G 1457G>A), GCT375CGA (1458G>C 1459C>G 1460T>A), AAG377CGA (1464A>C 1465A>G 1466G>A), CAA378TCT (1467C>T 1468A>C 1469A>T), AAG379CAA (1470A>C 1472G>A), AAC380AAG (1475C>G), TTT381AAA (1476T>A 1477T>A 1478T>A), AAA382GGT (1479A>G 1480A>G 1481A>T), TGT384TCT (1486G>C), TAT385TTC (1489A>T 1490T>C), TTA389TTC (1502A>C), AAG390AAA (1505G>A), TCA391TCG (1508A>G), CCG397CCA (1526G>A), ATG401ATA (1538G>A), TAT402TAC (1541T>C), CTT404TTC (1545C>T 1547T>C), TCT406TCC (1553T>C), ATG407TGC (1554A>T 1555T>G 1556G>C), ATA408ATG (1559A>G), CCT411AGT (1566C>A 1567C>G), ACC412ACA (1571C>A), TTG413CTG (1572T>C), TCC415TCA (1580C>A), GTT418GTC (1589T>C), AGC420del (1593_1597delAGCTC), TCT421--A (1593_1597delAGCTC 1598T>A), CTT424CTC (1607T>C), ACG426ACT (1613G>T), ATC427TTC (1614A>T), CTT428TTG (1617C>T 1619T>G), GTT429ATC (1620G>A 1622T>C), AAC430GTC (1623A>G 1624A>T), AAA431CCT (1626A>C 1627A>C 1628A>T), TTA433ATG (1632T>A 1634A>G), ACA434GTC (1635A>G 1636C>T 1637A>C), AGA436AGG (1643A>G), TAT438AGT (1647T>A 1648A>G), CTG443CTA (1664G>A), GGG445GGA (1670G>A), CCT447CCA (1676T>A), TTG448TTA (1679G>A), GGC449GGT (1682C>T), CAC451TAC (1686C>T), GGT452GGA (1691T>A), GCA455TCT (1698G>T 1700A>T), CTT456CTC (1703T>C), TCG458GCT (1707T>G 1709G>T), TTG459CTA (1710T>C 1712G>A), TAT463TAC (1724T>C), TTA467TTG (1736A>G), GCA468GGA (1738C>G), GCG469GCA (1742G>A), AGC470AAG (1744G>A 1745C>G), GGA475GAC (1759G>A 1760A>C), TGT476CGT (1761T>C), AAC477AAA (1766C>A), TCT478ACC (1767T>A 1769T>C), CCA479CCC (1772A>C), TTT480TTC (1775T>C), GGG481AAA (1776G>A 1777G>A 1778G>A), GAT482GAC (1781T>C), TAC483TAT (1784C>T), GCT484GCC (1787T>C), TTA485TTG (1790A>G), CTA491CTG (1808A>G), ACC493ACT (1814C>T), AGT495GCA (1818A>G 1819G>C 1820T>A), AAA496AAG (1823A>G), GCC498..G (1829C>G), AAT499CGG (1830A>C 1831A>G 1832T>G), GAG500C.. (1833G>C), AAG502.G. (1840A>G), TTA504..G (1847A>G), TTG505C.. (1848T>C), AGA507..G (1856A>G), CTC508..T (1859C>T), AAT509GG. (1860A>G 1861A>G), GTC510GTT (1865C>T), AAA511ACA (1867A>C), ATT512ATC (1871T>C), TCG513TCT (1874G>T), ATA514-TA (1875delA), CCA515GCT (1878C>G 1880A>T), TCT517TCG (1886T>G), TTG518AGA (1887T>A 1888T>G 1889G>A), TCT520TCC (1895T>C), GAT521GAG (1898T>G), AAT522AAC (1901T>C), ATT525ATC (1910T>C), GCG528GCT (1919G>T), AGG530AGA (1925G>A), ACT533ACC (1934T>C), AAA534AAG (1937A>G), TCA535GAC (1938T>G 1939C>A 1940A>C), GAT539TAC (1950G>T 1952T>C), TTA540CTT (1953T>C 1955A>T), ATT543ATC (1964T>C), TCC544TCT (1967C>T), GCT547GCA (1976T>A), TTA549ACT (1980T>A 1981T>C 1982A>T), TCT550TCC (1985T>C), CGT552AGG (1989C>A 1991T>G), ACT553ACA (1994T>A), GCG583.CT (2084G>T), TTA585CTG (2088T>C 2090A>G), TTC586ATG (2091T>A 2093C>G), ACT587TCT (2094A>T), ACC588ACT (2099C>T), CAA589CAG (2102A>G), AGC590TCA (2103A>T 2104G>C 2105C>A), AAG591CCA (2106A>C 2107A>C 2108G>A), CGT592CGA (2111T>A), GAG594GAA (2117G>A), CGT595AGA (2118C>A 2120T>A), AAA597AAG (2126A>G), GCT598GCG (2129T>G), CAT600GCT (2133C>G 2134A>C), CTT601TGT (2136C>T 2137T>G), AAG602AAA (2141G>A), GTT604AAC (2145G>A 2146T>A 2147T>C), CGG605AGG (2148C>A), TCA606TTA (2152C>T), CAC607CAT (2156C>T), TTA608CTA (2157T>C), CTA609TTA (2160C>T), TAC611CTT (2166T>C 2167A>T 2168C>T), TGG613TTT (2173G>T 2174G>T), GGT618TTT (2187G>T 2188G>T), ATG619GGA (2190A>G 2191T>G 2192G>A), CCC620ACC (2193C>A), CCC623CCT (2204C>T), AAA626AAG (2213A>G), GGG627GCG (2215G>C), AAG628del (2217_2219delAAG), GTA630ATA (2223G>A), AGT631GAT (2226A>G 2227G>A), TAC632TAT (2231C>T), CTC633CTG (2234C>G), CTT634CGT (2236T>G), AAA635GAG (2238A>G 2240A>G), GAA636GCT (2242A>C 2243A>T), TTA637GTA (2244T>G), AAA638AAG (2249A>G), GTT639CCT (2250G>C 2251T>C), GAG641CAG (2256G>C), TTA642ATA (2259T>A), CAG643CAA (2264G>A), AAG647AAA (2276G>A), CTG649TTG (2280C>T), GTG650GTA (2285G>A), TTC651TTT (2288C>T), GGT653GGA (2294T>A), GTT655CGG (2298G>C 2299T>G 2300T>G), ATA657ATC (2306A>C), CTA658CTT (2309A>T), GAA659GAG (2312A>G), CGT660CGG (2315T>G), CTA663ATC (2322C>A 2324A>C), CGC664CTT (2326G>T 2327C>T), CAA665AAT (2328C>A 2330A>T), GTA667ATG (2334G>A 2336A>G), GAA668AAA (2337G>A), TTA671TTG (2348A>G), AAG672ACT (2350A>C 2351G>T), CTC674del (2355_2359delCTCAA), AAC675--T (2355_2359delCTCAA 2360C>T), TAC677TAT (2366C>T), ACG679ACT (2372G>T), ATC680GTA (2373A>G 2375C>A), TGT681GCA (2376T>G 2377G>C 2378T>A), ATG682TTA (2379A>T 2381G>A), AAC683TCA (2382A>T 2383A>C 2384C>A), GTA686GTG (2393A>G), TCC687ACC (2394T>A), AAG689GAT (2400A>G 2402G>T), AGT690CAA (2403A>C 2404G>A 2405T>A), CTC691CTA (2408C>A), ATC692TTT (2409A>T 2411C>T), GAC693GAT (2414C>T), TCC694GTT (2415T>G 2416C>T 2417C>T), ATA696ATG (2423A>G), TGC697TGT (2426C>T), GCA698GCC (2429A>C), ACA699ACT (2432A>T), TCC700TCT (2435C>T), AGG703CGA (2442A>C 2444G>A), CTC704TCA (2445C>T 2446T>C 2447C>A), GAA705AAC (2448G>A 2450A>C), ACT706GAA (2451A>G 2452C>A 2453T>A), TTT708TAT (2458T>A), GAT709GAA (2462T>A), TTC713TTT (2474C>T), GGC714GGG (2477C>G), CTA715TAC (2478C>T 2479T>A 2480A>C), ATC716GTT (2481A>G 2483C>T), AAA718AAG (2489A>G), GGT723GGA (2504T>A), AAT727AAC (2516T>C), CCC728CTT (2518C>T 2519C>T), ACC730ACT (2525C>T), ACT731ACA (2528T>A), CCA732CCT (2531A>T), CAG733AGG (2532C>A 2533A>G), GAT737GGT (2545A>G), AAC739CCA (2550A>C 2551A>C 2552C>A), ACT740_GTT741insCTCAGT (2555_2556insCTCAGT), GTT741TTT (2556G>T), ATT742ATA (2561T>A), CAA743TTG (2562C>T 2563A>T 2564A>G), TTT744GTT (2565T>G), AAT745GAT (2568A>G), AAA746CAA (2571A>C), TGG747TAG (2575G>A), ATA748GTA (2577A>G), TTA749TTT (2582A>T), GGG750GGT (2585G>T)

*: Inserts / Deletes / Misaligned / Frameshifts

Analysis details

This analysis was performed with panviral2.64

NGS Details (UN60): Solendovirus venanicotianae

Assembly

**Coverage Length**	1373 (2 contig(s))
**Depth Of Coverage**	11.4
**Number Of Reads**	130
**Reads Per Million**	2.76 rpm (after QC)
**Ambiguities**	0
**Assembly Method**	de novo + reference guided assembly
**Consensus Caller**	Bcf Tools

Coverage Map

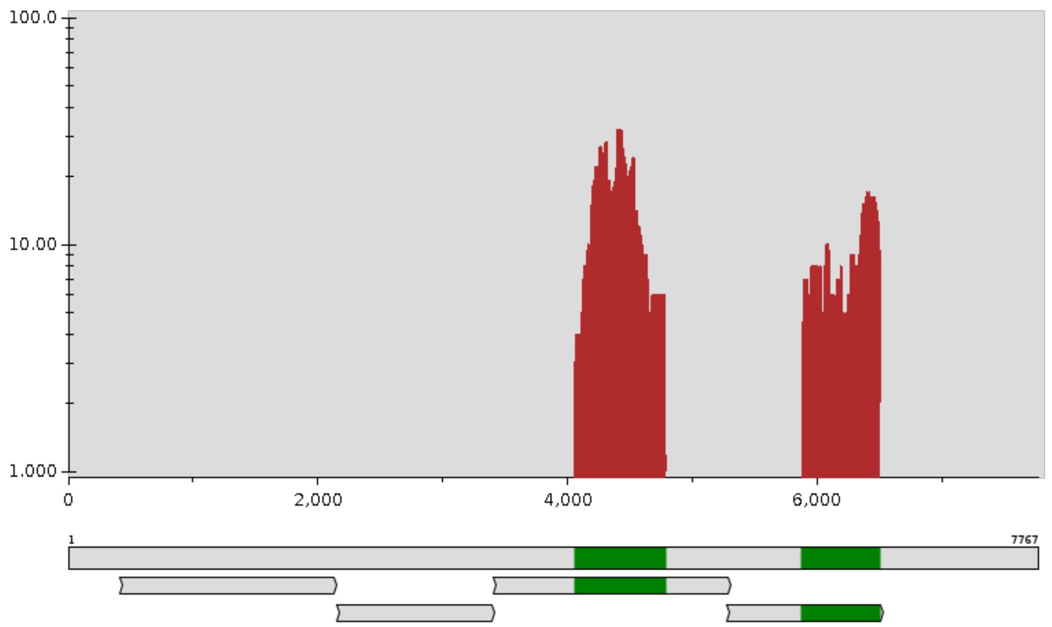

Assignment

**Type**	Solendovirus venanicotianae (Taxonomy ID: 3048371)
**Reference Genome**	NC_003378.1
**NT Identity (%)**	77.8592
**AA Identity (%)**	75.6044
**Number Of Stop Codons**	1
**Number Of CDS**	4

Alignment

**Alignment Score**	1493.0 (NT) + 2180.0 (AA) = 3673.0
**Concordance (%)**	66.1114

**Alignment Method**	Global, seeded, nucleotide + amino acids (AGA)

Genome Region

Sequence starts at position 4050 and ends at position 6507 relative to NC_003378.1 reference sequence.

Alignment Detailed Statistics

	**Begin**	**End**	**Coverage**	**Score**	**Concordance**	**Matches**	**Identities**	**I/D/M/F***	**Stop Codons**
**NT**	**4050**	**6507**	**17.7%**	**1493**	**55.3%**	**1364 (99.3%)**	**1062 (77.3%)**	**0/9**	
Mutations:	4052C>T, 4056A>G, 4061T>C, 4065A>G, 4066C>A, 4073T>C, 4079A>G, 4088A>C, 4091T>A, 4095T>C, 4097G>A, 4098G>A, 4100T>A, 4103T>G, 4104A>G, 4105A>G, 4115A>G, 4116G>A, 4121T>C, 4122A>G, 4124T>A, 4125A>G, 4127C>T, 4133T>C, 4161C>T, 4165G>A, 4181A>T, 4190G>A, 4196C>T, 4199A>T, 4202C>T, 4212T>C, 4235T>C, 4247A>C, 4250C>A, 4253T>C, 4260T>C, 4265G>A, 4292C>T, 4298T>C, 4310T>C, 4313G>A, 4325T>C, 4328T>C, 4329T>C, 4331G>A, 4340G>A, 4343T>A, 4355A>G, 4364T>A, 4376A>C, 4385T>C, 4400T>A, 4401T>C, 4406A>T, 4436C>T, 4440C>T, 4451C>T, 4453A>G, 4467C>T, 4470G>C, 4471A>C, 4475T>C, 4478T>C, 4482G>A, 4484A>T, 4497A>G, 4500C>T, 4503C>T, 4513G>A, 4515A>C, 4517A>T, 4518C>A, 4523T>A, 4530A>T, 4545A>C, 4550C>T, 4551G>A, 4552C>T, 4554C>A, 4559T>A, 4560A>G, 4568C>A, 4571T>A, 4574T>A, 4581T>C, 4591C>A, 4592C>G, 4598A>T, 4601A>G, 4604T>A, 4605A>T, 4607G>T, 4626T>C, 4643T>C, 4653A>G, 4667T>A, 4688C>T, 4691T>C, 4692C>T, 4693T>C, 4694T>A, 4703C>T, 4706T>A, 4730A>C, 4734C>T, 4740A>C, 4745A>T, 4748C>T, 4755A>C, 4757G>A, 4772A>G, 4784C>T, 4785T>C, 5872T>C, 5874G>A, 5880T>A, 5881T>C, 5882T>A, 5889T>A, 5892A>T, 5894G>A, 5898A>C, 5901A>T, 5907A>T, 5908G>A, 5909C>G, 5910C>A, 5913C>A, 5916C>T, 5919C>T, 5921A>G, 5932C>T, 5935G>A, 5940T>C, 5949A>C, 5950C>T, 5952G>T, 5953A>G, 5954C>A, 5955A>C, 5959C>G, 5960A>G, 5964G>A, 5968G>T, 5970C>A, 5971A>G, 5972A>C, 5979T>A, 5982G>A, 5983T>C, 5988C>T, 5991G>A, 5994A>C, 5997C>T, 6001C>A, 6002A>T, 6006T>C, 6009G>A, 6011G>A, 6012G>A, 6015A>T, 6018T>A, 6020A>G, 6023G>A, 6024G>A, 6026C>T, 6027A>T, 6033C>T, 6035A>T, 6036C>T, 6037G>A, 6040C>A, 6041G>A, 6044T>G, 6045C>T, 6049T>A, 6051G>A, 6054T>C, 6055A>C, 6067C>T, 6075A>T, 6078G>A, 6084G>A, 6087T>A, 6088A>G, 6090C>T, 6096A>G, 6099T>G, 6105A>G, 6108T>A, 6109G>T, 6112C>T, 6114C>G, 6120G>A, 6123A>T, 6129C>T, 6133C>G, 6139A>G, 6141G>T, 6144A>T, 6146A>G, 6148G>C, 6154G>C, 6155A>C, 6163G>C, 6164A>C, 6169G>A, 6171A>T, 6172A>G, 6174T>G, 6177T>A, 6181G>A, 6182A>G, 6183A>T, 6186C>T, 6189T>C, 6190G>A, 6192T>C, 6198A>C, 6201G>A, 6204A>T, 6211A>C, 6214T>G, 6219T>A, 6223C>A, 6225A>C, 6232C>T, 6236C>G, 6240C>T, 6246T>C, 6247C>A, 6248T>C, 6253C>G, 6254A>G, 6255G>A, 6258T>C, 6259G>C, 6260C>T, 6264T>G, 6269G>T, 6270C>T, 6277T>A, 6279A>C, 6282G>A, 6285A>T, 6288G>A, 6290C>T, 6308G>C, 6309C>A, 6315T>A, 6318A>G, 6319A>C, 6322G>A, 6325G>C, 6326C>A, 6334G>A, 6340C>G, 6341T>C, 6345A>G, 6352G>A, 6354C>A, 6355T>A, 6357A>G, 6359G>C, 6360T>A, 6363T>G, 6369A>T, 6371C>T, 6376C>A, 6377A>G, 6380C>A, 6381T>A, 6382A>C, 6384A>T, 6401G>A, 6402G>A, 6404G>T, 6406delA, 6407A>G, 6408T>A, 6411C>T, 6414C>T, 6417C>A, 6418C>G, 6419C>A, 6422A>G, 6423T>A, 6424C>T, 6426G>A, 6429A>T, 6444G>A, 6445C>G, 6446T>A, 6447A>T, 6451A>G, 6453T>A, 6456C>G, 6462T>C, 6464C>A, 6466_6473delGACCACCT, 6477C>T, 6480A>T, 6489A>C								

CDS

Tvvgp3	214	459	38.7%	1475	87.0%	246 (100%)	211 (85.8%)	0/0/0/0	0
Protein mutations:	N216D (4056A>G), T219E (4065A>G 4066C>A), I223M (4079A>G), K226N (4088A>C), D227E (4091T>A), D230K (4098G>A 4100T>A), N231K (4103T>G), K232G (4104A>G 4105A>G), E236K (4116G>A), N238E (4122A>G 4124T>A), S239G (4125A>G 4127C>T), H251Y (4161C>T), S252N (4165G>A), D311E (4343T>A), H344Y (4440C>T), N348S (4453A>G), E354P (4470G>C 4471A>C), V358I (4482G>A 4484A>T), I363V (4497A>G), R368K (4513G>A), T369P (4515A>C 4517A>T), Q370K (4518C>A), D371E (4523T>A), I374L (4530A>T), K379Q (4545A>C), A381I (4551G>A 4552C>T), H382N (4554C>A), I384V (4560A>G), N386K (4568C>A), T394K (4591C>A 4592C>G), M399F (4605A>T 4607G>T), I415V (4653A>G), L428S (4692C>T 4693T>C 4694T>A), I444L (4740A>C)								
Codon mutations:	AAC214AAT (4052C>T), AAT216GAT (4056A>G), GAT217GAC (4061T>C), ACA219GAA (4065A>G 4066C>A), TTT221TTC (4073T>C), ATA223ATG (4079A>G), AAA226AAC (4088A>C), GAT227GAA (4091T>A), TTG229CTA (4095T>C 4097G>A), GAT230AAA (4098G>A 4100T>A), AAT231AAG (4103T>G), AAA232GGA (4104A>G 4105A>G), CAA235CAG (4115A>G), GAA236AAA (4116G>A), AGT237AGC (4121T>C), AAT238GAA (4122A>G 4124T>A), AGC239GGT (4125A>G 4127C>T), CAT241CAC (4133T>C), CAT251TAT (4161C>T), AGT252AAT (4165G>A), GGA257GGT (4181A>T), AGG260AGA (4190G>A), GTC262GTT (4196C>T), ATA263ATT (4199A>T), GAC264GAT (4202C>T), TTA268CTA (4212T>C), TAT275TAC (4235T>C), ATA279ATC (4247A>C), CCC280CCA (4250C>A), AAT281AAC (4253T>C), TTA284CTA (4260T>C), AAG285AAA (4265G>A), TAC294TAT (4292C>T), AGT296AGC (4298T>C), TGT300TGC (4310T>C), AAG301AAA (4313G>A), TAT305TAC (4325T>C), CAT306CAC (4328T>C), TTG307CTA (4329T>C 4331G>A), GAG310GAA (4340G>A), GAT311GAA (4343T>A), AAA315AAG (4355A>G), GCT318GCA (4364T>A), CCA322CCC (4376A>C), TTT325TTC (4385T>C), GTT330GTA (4400T>A), TTA331CTA (4401T>C), CCA332CCT (4406A>T), TAC342TAT (4436C>T), CAT344TAT (4440C>T), GAC347GAT (4451C>T), AAC348AGC (4453A>G), CTA353TTA (4467C>T), GAA354CCA (4470G>C 4471A>C), AAT355AAC (4475T>C), TGT356TGC (4478T>C), GTA358ATT (4482G>A 4484A>T), ATA363GTA (4497A>G), CTA364TTA (4500C>T), CTA365TTA (4503C>T), AGA368AAA (4513G>A), ACA369CCT (4515A>C 4517A>T), CAA370AAA (4518C>A), GAT371GAA (4523T>A), ATA374TTA (4530A>T), AAA379CAA (4545A>C), TTC380TTT (4550C>T), GCA381ATA (4551G>A 4552C>T), CAT382AAT (4554C>A), ATT383ATA (4559T>A), ATA384GTA (4560A>G), AAC386AAA (4568C>A), TCT387TCA (4571T>A), GGT388GGA (4574T>A), TTA391CTA (4581T>C), ACC394AAG (4591C>A 4592C>G), GCA396GCT (4598A>T), GAA397GAG (4601A>G), ATT398ATA (4604T>A), ATG399TTT (4605A>T 4607G>T), TTA406CTA (4626T>C), GAT411GAC (4643T>C), ATA415GTA (4653A>G), ACT419ACA (4667T>A), ATC426ATT (4688C>T), AAT427AAC (4691T>C), CTT428TCA (4692C>T 4693T>C 4694T>A), AAC431AAT (4703C>T), ATT432ATA (4706T>A), TCA440TCC (4730A>C), CTA442TTA (4734C>T), ATA444CTA (4740A>C), GTA445GTT (4745A>T), AAC446AAT (4748C>T), AGG449CGA (4755A>C 4757G>A), AAA454AAG (4772A>G), AAC458AAT (4784C>T), TTA459CT. (4785T>C)								
Tvvgp4	200	411	50.5%	705	49.9%	209 (99.1%)	133 (63.0%)	0/2/2/2	1
Protein mutations:	L203Q (5881T>C 5882T>A), N205K (5889T>A), S207N (5894G>A), K209N (5901A>T), A212R (5908G>A 5909C>G 5910C>A), N216S (5921A>G), D221N (5935G>A), Q226Y (5950C>T 5952G>T), T227D (5953A>G 5954C>A 5955A>C), Q229G (5959C>G 5960A>G), A232S (5968G>T 5970C>A), N233A (5971A>G 5972A>C), Q243I (6001C>A 6002A>T), R246K (6011G>A 6012G>A), K249R (6020A>G), G250E (6023G>A 6024G>A), T251I (6026C>T 6027A>T), Y254F (6035A>T 6036C>T), V255I (6037G>A), R256K (6040C>A 6041G>A), F257C (6044T>G 6045C>T), S259T (6049T>A 6051G>A), T261P (6055A>C), E267D (6075A>T), I272V (6088A>G 6090C>T), G279* (6109G>T), E283D (6123A>T), P287A (6133C>G), K289D (6139A>G 6141G>T), E291G (6146A>G), E292Q (6148G>C), E294P (6154G>C 6155A>C), E297P (6163G>C 6164A>C), V299I (6169G>A 6171A>T), N300E (6172A>G 6174T>G), E303S (6181G>A 6182A>G 6183A>T), A306T (6190G>A 6192T>C), K308N (6198A>C), I313L (6211A>C), S314A (6214T>G), L317I (6223C>A 6225A>C), A321G (6236C>G), L325T (6247C>A 6248T>C), Q327G (6253C>G 6254A>G 6255G>A), A329L (6259G>C 6260C>T), I330M (6264T>G), S332I (6269G>T 6270C>T), S335T (6277T>A 6279A>C), E337D (6285A>T), T339I (6290C>T), C345S (6308G>C 6309C>A), D347E (6315T>A), I348M (6318A>G), E350K (6322G>A), A351Q (6325G>C 6326C>A), E354K (6334G>A), L356A (6340C>G 6341T>C), V360I (6352G>A 6354C>A), L361M (6355T>A 6357A>G), S362T (6359G>C 6360T>A), K365N (6369A>T), P366L (6371C>T), Q368R (6376C>A 6377A>G), P369Q (6380C>A 6381T>A), T370P (6382A>C 6384A>T), R376K (6401G>A 6402G>A), R377M (6404G>T), P382E (6418C>G 6419C>A), D383G (6422A>G 6423T>A), L385F (6429A>T), L391D (6445C>G 6446T>A 6447A>T), S393G (6451A>G 6453T>A), H394Q (6456C>G), P397Q (6464C>A), D398_H399del (6466_6473delGACCACCT), K405N (6489A>C)								
Codon mutations:	TTG200CTA (5872T>C 5874G>A), GCT202GCA (5880T>A), TTA203CAA (5881T>C 5882T>A), AAT205AAA (5889T>A), ACA206ACT (5892A>T), AGT207AAT (5894G>A), GCA208GCC (5898A>C), AAA209AAT (5901A>T), GTA211GTT (5907A>T), GCC212AGA (5908G>A 5909C>G 5910C>A), ACC213ACA (5913C>A), TGC214TGT (5916C>T), TAC215TAT (5919C>T), AAC216AGC (5921A>G), CTA220TTA (5932C>T), GAT221AAT (5935G>A), ACT222ACC (5940T>C), ACA225ACC (5949A>C), CAG226TAT (5950C>T 5952G>T), ACA227GAC (5953A>G 5954C>A 5955A>C), CAA229GGA (5959C>G 5960A>G), GAG230GAA (5964G>A), GCC232TCA (5968G>T 5970C>A), AAT233GCT (5971A>G 5972A>C), CCT235CCA (5979T>A), GAG236GAA (5982G>A), TTA237CTA (5983T>C), TAC238TAT (5988C>T), AAG239AAA (5991G>A), GCA240GCC (5994A>C), TTC241TTT (5997C>T), CAA243ATA (6001C>A 6002A>T), TAT244TAC (6006T>C), AAG245AAA (6009G>A), AGG246AAA (6011G>A 6012G>A), ATA247ATT (6015A>T), ACT248ACA (6018T>A), AAA249AGA (6020A>G), GGG250GAA (6023G>A 6024G>A), ACA251ATT (6026C>T 6027A>T), TTC253TTT (6033C>T), TAC254TTT (6035A>T 6036C>T), GTA255ATA (6037G>A), CGA256AAA (6040C>A 6041G>A), TTC257TGT (6044T>G 6045C>T), TCG259ACA (6049T>A 6051G>A), GCT260GCC (6054T>C), ACA261CCA (6055A>C), CTA265TTA (6067C>T), GAA267GAT (6075A>T), GAG268GAA (6078G>A), AAG270AAA (6084G>A), CCT271CCA (6087T>A), ATC272GTT (6088A>G 6090C>T), CAA274CAG (6096A>G), GTT275GTG (6099T>G), AAA277AAG (6105A>G), ATT278ATA (6108T>A), GGA279TGA (6109G>T), CTC280TTG (6112C>T 6114C>G), AGG282AGA (6120G>A), GAA283GAT (6123A>T), ATC285ATT (6129C>T), CCA287GCA (6133C>G), AAG289GAT (6139A>G 6141G>T), ATA290ATT (6144A>T), GAA291GGA (6146A>G), GAA292CAA (6148G>C), GAA294CCA (6154G>C 6155A>C), GAA297CCA (6163G>C 6164A>C), GTA299ATT (6169G>A 6171A>T), AAT300GAG (6172A>G 6174T>G), ATT301ATA (6177T>A), GAA303AGT (6181G>A 6182A>G 6183A>T), TTC304TTT (6186C>T), TAT305TAC (6189T>C), GCT306ACC (6190G>A 6192T>C), AAA308AAC (6198A>C), AGG309AGA (6201G>A), ATA310ATT (6204A>T), ATA313CTA (6211A>C), TCT314GCT (6214T>G), ACT315ACA (6219T>A), CTA317ATC (6223C>A 6225A>C), CTA320TTA (6232C>T), GCA321GGA (6236C>G), AAC322AAT (6240C>T), TAT324TAC (6246T>C), CTA325ACA (6247C>A 6248T>C), CAG327GGA (6253C>G 6254A>G 6255G>A), AAT328AAC (6258T>C), GCA329CTA (6259G>C 6260C>T), ATT330ATG (6264T>G), AGC332ATT (6269G>T 6270C>T), TCA335ACC (6277T>A 6279A>C), AGG336AGA (6282G>A), GAA337GAT (6285A>T), CAG338CAA (6288G>A), ACA339ATA (6290C>T), TGC345TCA (6308G>C 6309C>A), GAT347GAA (6315T>A), ATA348ATG (6318A>G), AGA349CGA (6319A>C), GAA350AAA (6322G>A), GCA351CAA (6325G>C 6326C>A), GAA354AAA (6334G>A), CTA356GCA (6340C>G 6341T>C), AGA357AGG (6345A>G), GTC360ATA (6352G>A 6354C>A), TTA361ATG (6355T>A 6357A>G), AGT362ACA (6359G>C 6360T>A), CTT363CTG (6363T>G), AAA365AAT (6369A>T), CCA366CTA (6371C>T), CAA368AGA (6376C>A 6377A>G), CCT369CAA (6380C>A 6381T>A), ACA370CCT (6382A>C 6384A>T), AGG376AAA (6401G>A 6402G>A), AGG377ATG (6404G>T), AAT378-GA (6406delA 6407A>G 6408T>A), TTC379TTT (6411C>T), ATC380ATT (6414C>T), TCC381TCA (6417C>A), CCA382GAA (6418C>G 6419C>A), GAT383GGA (6422A>G 6423T>A), CTG384TTA (6424C>T 6426G>A), TTA385TTT (6429A>T), AAG390AAA (6444G>A), CTA391GAT (6445C>G 6446T>A 6447A>T), AGT393GGA (6451A>G 6453T>A), CAC394CAG (6456C>G), TAT396TAC (6462T>C), CCA397CAA (6464C>A), GAC398_CAC399del (6466_6473delGACCACCT), CTA400--A (6466_6473delGACCACCT), TGC401TGT (6477C>T), TCA402TCT (6480A>T), AAA405AAC (6489A>C)								

Proteins

polyprotein (NP_569141.1)	214	459	38.7%	1475	87.0%	246 (100%)	211 (85.8%)	0/0/0/0	0
Protein mutations:	N216D (4056A>G), T219E (4065A>G 4066C>A), I223M (4079A>G), K226N (4088A>C), D227E (4091T>A), D230K (4098G>A 4100T>A), N231K (4103T>G), K232G (4104A>G 4105A>G), E236K (4116G>A), N238E (4122A>G 4124T>A), S239G (4125A>G 4127C>T), H251Y (4161C>T), S252N (4165G>A), D311E (4343T>A), H344Y (4440C>T), N348S (4453A>G), E354P (4470G>C 4471A>C), V358I (4482G>A 4484A>T), I363V (4497A>G), R368K (4513G>A), T369P (4515A>C 4517A>T), Q370K (4518C>A), D371E (4523T>A), I374L (4530A>T), K379Q (4545A>C), A381I (4551G>A 4552C>T), H382N (4554C>A), I384V (4560A>G), N386K (4568C>A), T394K (4591C>A 4592C>G), M399F (4605A>T 4607G>T), I415V (4653A>G), L428S (4692C>T 4693T>C 4694T>A), I444L (4740A>C)								

	Begin	End	Coverage	Score	Concordance	Matches	Identities	I/D/M/F*	Stop Codons
**NT**	**4050**	**6507**	**17.7%**	**1493**	**55.3%**	**1364 (99.3%)**	**1062 (77.3%)**	**0/9**	
Codon mutations:	AAC214AAT (4052C>T), AAT216GAT (4056A>G), GAT217GAC (4061T>C), ACA219GAA (4065A>G 4066C>A), TTT221TTC (4073T>C), ATA223ATG (4079A>G), AAA226AAC (4088A>C), GAT227GAA (4091T>A), TTG229CTA (4095T>C 4097G>A), GAT230AAA (4098G>A 4100T>A), AAT231AAG (4103T>G), AAA232GGA (4104A>G 4105A>G), CAA235CAG (4115A>G), GAA236AAA (4116G>A), AGT237AGC (4121T>C), AAT238GAA (4122A>G 4124T>A), AGC239GGT (4125A>G 4127C>T), CAT241CAC (4133T>C), CAT251TAT (4161C>T), AGT252AAT (4165G>A), GGA257GGT (4181A>T), AGG260AGA (4190G>A), GTC262GTT (4196C>T), ATA263ATT (4199A>T), GAC264GAT (4202C>T), TTA268CTA (4212T>C), TAT275TAC (4235T>C), ATA279ATC (4247A>C), CCC280CCA (4250C>A), AAT281AAC (4253T>C), TTA284CTA (4260T>C), AAG285AAA (4265G>A), TAC294TAT (4292C>T), AGT296AGC (4298T>C), TGT300TGC (4310T>C), AAG301AAA (4313G>A), TAT305TAC (4325T>C), CAT306CAC (4328T>C), TTG307CTA (4329T>C 4331G>A), GAG310GAA (4340G>A), GAT311GAA (4343T>A), AAA315AAG (4355A>G), GCT318GCA (4364T>A), CCA322CCC (4376A>C), TTT325TTC (4385T>C), GTT330GTA (4400T>A), TTA331CTA (4401T>C), CCA332CCT (4406A>T), TAC342TAT (4436C>T), CAT344TAT (4440C>T), GAC347GAT (4451C>T), AAC348AGC (4453A>G), CTA353TTA (4467C>T), GAA354CCA (4470G>C 4471A>C), AAT355AAC (4475T>C), TGT356TGC (4478T>C), GTA358ATT (4482G>A 4484A>T), ATA363GTA (4497A>G), CTA364TTA (4500C>T), CTA365TTA (4503C>T), AGA368AAA (4513G>A), ACA369CCT (4515A>C 4517A>T), CAA370AAA (4518C>A), GAT371GAA (4523T>A), ATA374TTA (4530A>T), AAA379CAA (4545A>C), TTC380TTT (4550C>T), GCA381ATA (4551G>A 4552C>T), CAT382AAT (4554C>A), ATT383ATA (4559T>A), ATA384GTA (4560A>G), AAC386AAA (4568C>A), TCT387TCA (4571T>A), GGT388GGA (4574T>A), TTA391CTA (4581T>C), ACC394AAG (4591C>A 4592C>G), GCA396GCT (4598A>T), GAA397GAG (4601A>G), ATT398ATA (4604T>A), ATG399TTT (4605A>T 4607G>T), TTA406CTA (4626T>C), GAT411GAC (4643T>C), ATA415GTA (4653A>G), ACT419ACA (4667T>A), ATC426ATT (4688C>T), AAT427AAC (4691T>C), CTT428TCA (4692C>T 4693T>C 4694T>A), AAC431AAT (4703C>T), ATT432ATA (4706T>A), TCA440TCC (4730A>C), CTA442TTA (4734C>T), ATA444CTA (4740A>C), GTA445GTT (4745A>T), AAC446AAT (4748C>T), AGG449CGA (4755A>C 4757G>A), AAA454AAG (4772A>G), AAC458AAT (4784C>T), TTA459CT. (4785T>C)								
putative transactivator factor (NP_569142.1)	200	411	50.5%	705	49.9%	209 (99.1%)	133 (63.0%)	0/2/2/2	1
Protein mutations:	L203Q (5881T>C 5882T>A), N205K (5889T>A), S207N (5894G>A), K209N (5901A>T), A212R (5908G>A 5909C>G 5910C>A), N216S (5921A>G), D221N (5935G>A), Q226Y (5950C>T 5952G>T), T227D (5953A>G 5954C>A 5955A>C), Q229G (5959C>G 5960A>G), A232S (5968G>T 5970C>A), N233A (5971A>G 5972A>C), Q243I (6001C>A 6002A>T), R246K (6011G>A 6012G>A), K249R (6020A>G), G250E (6023G>A 6024G>A), T251I (6026C>T 6027A>T), Y254F (6035A>T 6036C>T), V255I (6037G>A), R256K (6040C>A 6041G>A), F257C (6044T>G 6045C>T), S259T (6049T>A 6051G>A), T261P (6055A>C), E267D (6075A>T), I272V (6088A>G 6090C>T), G279* (6109G>T), E283D (6123A>T), P287A (6133C>G), K289D (6139A>G 6141G>T), E291G (6146A>G), E292Q (6148G>C), E294P (6154G>C 6155A>C), E297P (6163G>C 6164A>C), V299I (6169G>A 6171A>T), N300E (6172A>G 6174T>G), E303S (6181G>A 6182A>G 6183A>T), A306T (6190G>A 6192T>C), K308N (6198A>C), I313L (6211A>C), S314A (6214T>G), L317I (6223C>A 6225A>C), A321G (6236C>G), L325T (6247C>A 6248T>C), Q327G (6253C>G 6254A>G 6255G>A), A329L (6259G>C 6260C>T), I330M (6264T>G), S332I (6269G>T 6270C>T), S335T (6277T>A 6279A>C), E337D (6285A>T), T339I (6290C>T), C345S (6308G>C 6309C>A), D347E (6315T>A), I348M (6318A>G), E350K (6322G>A), A351Q (6325G>C 6326C>A), E354K (6334G>A), L356A (6340C>G 6341T>C), V360I (6352G>A 6354C>A), L361M (6355T>A 6357A>G), S362T (6359G>C 6360T>A), K365N (6369A>T), P366L (6371C>T), Q368R (6376C>A 6377A>G), P369Q (6380C>A 6381T>A), T370P (6382A>C 6384A>T), R376K (6401G>A 6402G>A), R377M (6404G>T), P382E (6418C>G 6419C>A), D383G (6422A>G 6423T>A), L385F (6429A>T), L391D (6445C>G 6446T>A 6447A>T), S393G (6451A>G 6453T>A), H394Q (6456C>G), P397Q (6464C>A), D398_H399del (6466_6473delGACCACCT), K405N (6489A>C)								
Codon mutations:	TTG200CTA (5872T>C 5874G>A), GCT202GCA (5880T>A), TTA203CAA (5881T>C 5882T>A), AAT205AAA (5889T>A), ACA206ACT (5892A>T), AGT207AAT (5894G>A), GCA208GCC (5898A>C), AAA209AAT (5901A>T), GTA211GTT (5907A>T), GCC212AGA (5908G>A 5909C>G 5910C>A), ACC213ACA (5913C>A), TGC214TGT (5916C>T), TAC215TAT (5919C>T), AAC216AGC (5921A>G), CTA220TTA (5932C>T), GAT221AAT (5935G>A), ACT222ACC (5940T>C), ACA225ACC (5949A>C), CAG226TAT (5950C>T 5952G>T), ACA227GAC (5953A>G 5954C>A 5955A>C), CAA229GGA (5959C>G 5960A>G), GAG230GAA (5964G>A), GCC232TCA (5968G>T 5970C>A), AAT233GCT (5971A>G 5972A>C), CCT235CCA (5979T>A), GAG236GAA (5982G>A), TTA237CTA (5983T>C), TAC238TAT (5988C>T), AAG239AAA (5991G>A), GCA240GCC (5994A>C), TTC241TTT (5997C>T), CAA243ATA (6001C>A 6002A>T), TAT244TAC (6006T>C), AAG245AAA (6009G>A), AGG246AAA (6011G>A 6012G>A), ATA247ATT (6015A>T), ACT248ACA (6018T>A), AAA249AGA (6020A>G), GGG250GAA (6023G>A 6024G>A), ACA251ATT (6026C>T 6027A>T), TTC253TTT (6033C>T), TAC254TTT (6035A>T 6036C>T), GTA255ATA (6037G>A), CGA256AAA (6040C>A 6041G>A), TTC257TGT (6044T>G 6045C>T), TCG259ACA (6049T>A 6051G>A), GCT260GCC (6054T>C), ACA261CCA (6055A>C), CTA265TTA (6067C>T), GAA267GAT (6075A>T), GAG268GAA (6078G>A), AAG270AAA (6084G>A), CCT271CCA (6087T>A), ATC272GTT (6088A>G 6090C>T), CAA274CAG (6096A>G), GTT275GTG (6099T>G), AAA277AAG (6105A>G), ATT278ATA (6108T>A), GGA279TGA (6109G>T), CTC280TTG (6112C>T 6114C>G), AGG282AGA (6120G>A), GAA283GAT (6123A>T), ATC285ATT (6129C>T), CCA287GCA (6133C>G), AAG289GAT (6139A>G 6141G>T), ATA290ATT (6144A>T), GAA291GGA (6146A>G), GAA292CAA (6148G>C), GAA294CCA (6154G>C 6155A>C), GAA297CCA (6163G>C 6164A>C), GTA299ATT (6169G>A 6171A>T), AAT300GAG (6172A>G 6174T>G), ATT301ATA (6177T>A), GAA303AGT (6181G>A 6182A>G 6183A>T), TTC304TTT (6186C>T), TAT305TAC (6189T>C), GCT306ACC (6190G>A 6192T>C), AAA308AAC (6198A>C), AGG309AGA (6201G>A), ATA310ATT (6204A>T), ATA313CTA (6211A>C), TCT314GCT (6214T>G), ACT315ACA (6219T>A), CTA317ATC (6223C>A 6225A>C), CTA320TTA (6232C>T), GCA321GGA (6236C>G), AAC322AAT (6240C>T), TAT324TAC (6246T>C), CTA325ACA (6247C>A 6248T>C), CAG327GGA (6253C>G 6254A>G 6255G>A), AAT328AAC (6258T>C), GCA329CTA (6259G>C 6260C>T), ATT330ATG (6264T>G), AGC332ATT (6269G>T 6270C>T), TCA335ACC (6277T>A 6279A>C), AGG336AGA (6282G>A), GAA337GAT (6285A>T), CAG338CAA (6288G>A), ACA339ATA (6290C>T), TGC345TCA (6308G>C 6309C>A), GAT347GAA (6315T>A), ATA348ATG (6318A>G), AGA349CGA (6319A>C), GAA350AAA (6322G>A), GCA351CAA (6325G>C 6326C>A), GAA354AAA (6334G>A), CTA356GCA (6340C>G 6341T>C), AGA357AGG (6345A>G), GTC360ATA (6352G>A 6354C>A), TTA361ATG (6355T>A 6357A>G), AGT362ACA (6359G>C 6360T>A), CTT363CTG (6363T>G), AAA365AAT (6369A>T), CCA366CTA (6371C>T), CAA368AGA (6376C>A 6377A>G), CCT369CAA (6380C>A 6381T>A), ACA370CCT (6382A>C 6384A>T), AGG376AAA (6401G>A 6402G>A), AGG377ATG (6404G>T), AAT378-GA (6406delA 6407A>G 6408T>A), TTC379TTT (6411C>T), ATC380ATT (6414C>T), TCC381TCA (6417C>A), CCA382GAA (6418C>G 6419C>A), GAT383GGA (6422A>G 6423T>A), CTG384TTA (6424C>T 6426G>A), TTA385TTT (6429A>T), AAG390AAA (6444G>A), CTA391GAT (6445C>G 6446T>A 6447A>T), AGT393GGA (6451A>G 6453T>A), CAC394CAG (6456C>G), TAT396TAC (6462T>C), CCA397CAA (6464C>A), GAC398_CAC399del (6466_6473delGACCACCT), CTA400--A (6466_6473delGACCACCT), TGC401TGT (6477C>T), TCA402TCT (6480A>T), AAA405AAC (6489A>C)								

*: Inserts / Deletes / Misaligned / Frameshifts

Analysis details

This analysis was performed with panviral2.64

NGS Details (UN60): Macrosiphum euphorbiae virus 1

Assembly

**Coverage Length**	5542 (29 contig(s))
**Depth Of Coverage**	2.6
**Number Of Reads**	106
**Reads Per Million**	2.25 rpm (after QC)
**Ambiguities**	3
**Assembly Method**	de novo + reference guided assembly
**Consensus Caller**	Bcf Tools

Coverage Map

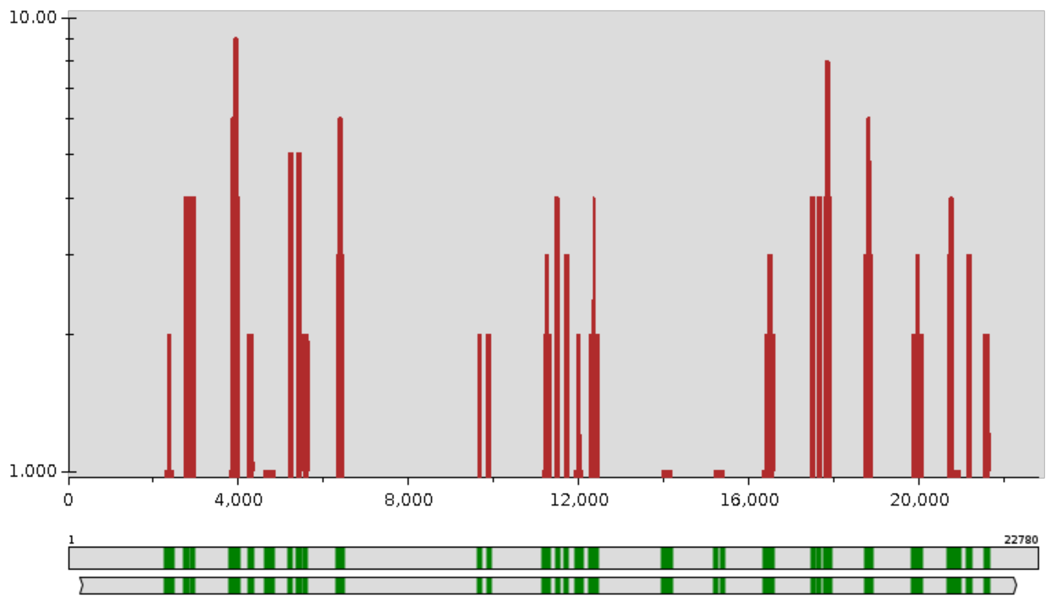

Assignment

**Type**	Macrosiphum euphorbiae virus 1 (Taxonomy ID: 1685953)
**Reference Genome**	NC_028137.1
**NT Identity (%)**	92.8378
**AA Identity (%)**	96.4655
**Number Of Stop Codons**	0
**Number Of CDS**	1

Alignment

**Alignment Score**	9449.0 (NT) + 12248.0 (AA) = 21697.0
**Concordance (%)**	91.9092

**Alignment Method**	Global, seeded, nucleotide + amino acids (AGA)

Genome Region

Sequence starts at position 2251 and ends at position 21653 relative to NC_028137.1 reference sequence.

Alignment Detailed Statistics

	Begin	End	Coverage	Score	Concordance	Matches	Identities	I/D/M/F*	Stop Codons
**NT**	**2251**	**21653**	**24.3%**	**9449**	**85.7%**	**5529 (99.8%)**	**5133 (92.6%)**	**0/13**	
Mutations:	2262C>T, 2277G>A, 2279G>T, 2282C>T, 2291T>C, 2303T>C, 2312C>A, 2318T>C, 2321A>G, 2324T>C, 2327C>T, 2336T>G, 2343A>G, 2344G>A, 2354T>C, 2363T>C, 2369T>C, 2381A>G, 2399T>C, 2405T>C, 2411C>T, 2420A>G, 2429C>G, 2432A>G, 2438A>G, 2456T>C, 2465A>G, 2466C>T, 2471C>T, 2474C>T, 2475C>T, 2480T>C, 2711A>G, 2717T>C, 2720G>T, 2735C>T, 2738T>A, 2747G>A, 2748G>A, 2759C>T, 2762G>A, 2765C>T, 2769T>G, 2771T>C, 2774G>A, 2777T>C, 2780A>T, 2783A>G, 2789A>G, 2798A>G, 2802C>T, 2804A>G, 2808A>G, 2810T>G, 2825G>A, 2831G>T, 2836G>A, 2837C>T, 2855C>A, 2867A>C, 2870A>T, 2871G>A, 2872T>C, 2876A>G, 2879T>C, 2881G>K, 2882G>C, 2883C>A, 2885C>G, 2888G>A, 2895C>G, 2900A>G, 2903C>A, 2904A>G, 2906C>T, 2912A>C, 2918A>G, 2930G>A, 2939A>G, 2942T>C, 2945A>G, 2948C>T, 2950T>C, 2951T>A, 2954G>T, 2957C>T, 2965A>T, 2972C>T, 3770G>A, 3776G>C, 3779A>T, 3782C>T, 3785T>C, 3788A>G, 3791A>G, 3794C>T, 3797T>C, 3806C>T, 3815T>C, 3821C>T, 3824C>T, 3827C>T, 3836C>T, 3842C>T, 3845G>A, 3860T>C, 3866C>T, 3872T>C, 3884C>T, 3885A>G, 3886C>T, 3887A>G, 3888C>T, 3890T>A, 3893C>T, 3896A>G, 3905A>G, 3911T>C, 3914G>A, 3916A>T, 3917G>A, 3920A>G, 3925A>G, 3929A>G, 3931T>G, 3932T>C, 3938C>T, 3947T>C, 3953T>C, 3956C>T, 3959T>C, 3962A>G, 3963C>T, 3971C>T, 3974G>T, 3983C>T, 3992G>C, 4004A>G, 4007A>G, 4008T>C, 4016T>C, 4022T>C, 4031T>C, 4034T>C, 4208T>C, 4212C>T, 4217T>C, 4223G>A, 4232T>C, 4237T>C, 4238C>T, 4247C>T, 4250A>G, 4259C>A, 4265C>T, 4268T>C, 4271C>T, 4289G>A, 4304T>C, 4316T>C, 4334T>C, 4337T>A, 4340C>T, 4343A>G, 4346G>A, 4355G>A, 4358G>A, 4598T>A, 4601A>G, 4607T>C, 4610A>T, 4620C>T, 4624A>G, 4625C>T, 4628T>C, 4631C>T, 4634G>A, 4638A>C, 4643A>G, 4661T>C, 4667A>G, 4673T>C, 4674C>T, 4676A>G, 4677A>G, 4685T>C, 4691C>T, 4694T>C, 4700T>C, 4705C>T, 4751T>C, 4784T>C, 4786C>T, 4807G>A, 4828A>G, 4838C>T, 4854T>C, 4855T>C, 5156C>T, 5165T>C, 5166G>A, 5174C>T, 5183T>C, 5203T>G, 5210A>G, 5217T>C, 5228C>T, 5246C>T, 5264C>T, 5285C>T, 5358G>A, 5363G>A, 5366G>A, 5390A>C, 5397T>C, 5411T>C, 5414T>C, 5417C>T, 5438A>G, 5439C>T, 5450C>T, 5454G>A, 5459T>C, 5462A>G, 5474T>C, 5489A>G, 5507C>T, 5528C>T, 5537C>T, 5540C>T, 5543G>A, 5546T>C, 5561C>T, 5579G>A, 5582A>G, 5591T>C, 5612A>T, 5621T>C, 5636T>C, 5640G>A, 5642C>T, 5645A>G, 6296C>T, 6302A>C, 6303A>G, 6309A>G, 6326C>T, 6344C>G, 6347T>C, 6356C>T, 6365T>C, 6371C>T, 6377T>C, 6401G>A, 6402C>T, 6449T>C, 6458G>A, 6488T>A, 9674T>C, 9701T>C, 9720G>K, 9823A>W, 9878G>A, 9910T>C, 11189T>C, 11231T>C, 11296T>G, 11513C>T, 11549T>C, 11555C>T, 11646T>C, 11681T>C, 11756G>C, 11915C>T, 11922A>G, 12257T>C, 12350C>T, 12383G>A, 12443T>C, 13928T>C, 13940T>C, 13946A>G, 13955T>A, 14058_14064delGAAGATA, 14084C>T, 15161T>C, 15183C>T, 15317T>C, 16307T>C, 16349C>T, 16369A>G, 16376C>T, 16430T>C, 16481C>T, 16523G>T, 16553T>C, 16577T>C, 17471T>C, 17516C>T, 17635G>A, 17684T>C, 17838T>G, 17912T>C, 17942C>T, 18701T>C, 18722C>T, 18743A>C, 18752A>G, 18755T>G, 18758C>T, 18761T>C, 18764G>A, 18794C>T, 18797G>A, 18833C>T, 18864T>C, 18878T>C, 18887T>C, 18890A>G, 18896G>T, 18905G>A, 18908C>T, 18920G>A, 19808A>G, 19818G>T, 19829C>T, 19835T>C, 19859C>T, 19904T>C, 19907G>T, 19926C>A, 19961C>T, 19982C>T, 20000T>C, 20036C>T, 20078T>C, 20082G>A, 20083T>C, 20084T>G, 20648T>C, 20654C>T, 20663G>A, 20666T>C, 20672G>A, 20675C>T, 20711T>C, 20716A>G, 20720A>G, 20732T>C, 20735T>C, 20756T>C, 20759T>C, 20771T>A, 20780C>T, 20783T>C, 20786C>T, 20807G>A, 20810T>C, 20816T>C, 20822C>T, 20828G>A, 20840T>C, 20844_20849delTCCATT, 20855G>A, 20867A>C, 20873C>T, 20876G>A, 20882A>G, 20891A>G, 20909C>T, 20912T>C, 20924T>C, 20939T>G, 21122T>C, 21137T>C, 21143T>C, 21156T>C, 21158G>T, 21164C>T, 21170C>T, 21179C>T, 21182C>T, 21185A>G, 21188T>C, 21191A>G, 21194C>T, 21200C>T, 21210A>G, 21218G>A, 21503T>C, 21518C>T, 21525A>G, 21526G>A, 21599C>T, 21617T>C, 21618G>A, 21635C>T, 21647G>A								
**CDS**									
ASK90_gp1	655	7121	25.1%	12248	94.7%	1839 (99.8%)	1774 (96.3%)	0/4/4/1	0
Protein mutations:	V663I (2277G>A 2279G>T), S685D (2343A>G 2344G>A), A820T (2748G>A), S827A (2769T>G 2771T>C), I840V (2808A>G 2810T>G), R849H (2836G>A 2837C>T), V861T (2871G>A 2872T>C), Q869E (2895C>G), I872V (2904A>G 2906C>T), V887A (2950T>C 2951T>A), N892I (2965A>T), T1199V (3885A>G 3886C>T 3887A>G), Q1209L (3916A>T 3917G>A), N1212S (3925A>G), V1214G (3931T>G 3932T>C), L1308F (4212C>T), V1316A (4237T>C 4238C>T), E1440D (4610A>T), H1444Y (4620C>T), D1445G (4624A>G 4625C>T), M1450L (4638A>C), K1463E (4677A>G), A1472V (4705C>T), T1499I (4786C>T), R1506Q (4807G>A), D1513G (4828A>G), L1522P (4854T>C 4855T>C), D1626N (5166G>A), V1638G (5203T>G), A1690T (5358G>A), V1722I (5454G>A), V1784I (5640G>A 5642C>T), I2005V (6303A>G), I2007V (6309A>G), V3207A (9910T>C), I3669R (11296T>G), C3786R (11646T>C), I3878V (11922A>G), E4590_D4591del (14058_14064delGAAGATA), D5360G (16369A>G), R5782K (17635G>A), S5850A (17838T>G), V6510L (19818G>T), K6539N (19907G>T), V6598T (20082G>A 20083T>C 20084T>G), K6809R (20716A>G), S6852_I6853del (20844_20849delTCCATT), I6974V (21210A>G), S7079D (21525A>G 21526G>A), D7110N (21618G>A)								

	Begin	End	Coverage	Score	Concordance	Matches	Identities	I/D/M/F*	Stop Codons
**NT**	**2251**	**21653**	**24.3%**	**9449**	**85.7%**	**5529 (99.8%)**	**5133 (92.6%)**	**0/13**	
Codon mutations:	CTG658TTG (2262C>T), GTG663ATT (2277G>A 2279G>T), TTC664TTT (2282C>T), GCT667GCC (2291T>C), GTT671GTC (2303T>C), TCC674TCA (2312C>A), GCT676GCC (2318T>C), AAA677AAG (2321A>G), TAT678TAC (2324T>C), CTC679CTT (2327C>T), ACT682ACG (2336T>G), AGC685GAC (2343A>G 2344G>A), CCT688CCC (2354T>C), ATT691ATC (2363T>C), GAT693GAC (2369T>C), GAA697GAG (2381A>G), CGT703CGC (2399T>C), CTT705CTC (2405T>C), TAC707TAT (2411C>T), GCA710GCG (2420A>G), TCC713TCG (2429C>G), AAA714AAG (2432A>G), AAA716AAG (2438A>G), ACT722ACC (2456T>C), CAA725CAG (2465A>G), CTG726TTG (2466C>T), ATC727ATT (2471C>T), GGC728GGT (2474C>T), CTA729TTA (2475C>T), GCT730GCC (2480T>C), GGA807GGG (2711A>G), TCT809TCC (2717T>C), TCG810TCT (2720G>T), TAC815TAT (2735C>T), GGT816GGA (2738T>A), CCG819CCA (2747G>A), GCC820ACC (2748G>A), CAC823CAT (2759C>T), GGG824GGA (2762G>A), TTC825TTT (2765C>T), TCT827GCC (2769T>G 2771T>C), TCG828TCA (2774G>A), CGT829CGC (2777T>C), GGA830GGT (2780A>T), GGA831GGG (2783A>G), AGA833AGG (2789A>G), GGA836GGG (2798A>G), CTA838TTG (2802C>T 2804A>G), ATT840GTG (2808A>G 2810T>G), GAG845GAA (2825G>A), GTG847GTT (2831G>T), CGC849CAT (2836G>A 2837C>T), GTC855..A (2855C>A), CCA859CCC (2867A>C), TCA860TCT (2870A>T), GTG861ACG (2871G>A 2872T>C), CCA862CCG (2876A>G), ATT863ATC (2879T>C), CGG864CKC (2881G>K 2882G>C), CGC865AGG (2883C>A 2885C>G), CTG866CTA (2888G>A), CAG869GAG (2895C>G), AAA870AAG (2900A>G), CCC871CCA (2903C>A), ATC872GTT (2904A>G 2906C>T), TCA874TCC (2912A>C), GAA876GAG (2918A>G), GAG880GAA (2930G>A), AAA883AAG (2939A>G), ACT884ACC (2942T>C), TTA885TTG (2945A>G), AAC886AAT (2948C>T), GTT887GCA (2950T>C 2951T>A), GGG888GGT (2954G>T), GTC889GTT (2957C>T), AAC892ATC (2965A>T), TCC894TCT (2972C>T), TTG1160TTA (3770G>A), GGG1162GGC (3776G>C), GCA1163GCT (3779A>T), GCC1164GCT (3782C>T), TTT1165TTC (3785T>C), GGA1166GGG (3788A>G), CAA1167CAG (3791A>G), TGC1168TGT (3794C>T), TTT1169TTC (3797T>C), TTC1172TTT (3806C>T), TCT1175TCC (3815T>C), TTC1177TTT (3821C>T), ATC1178ATT (3824C>T), AGC1179AGT (3827C>T), GTC1182GTT (3836C>T), TGC1184TGT (3842C>T), GGG1185GGA (3845G>A), CTT1190CTC (3860T>C), GAC1192GAT (3866C>T), AAT1194AAC (3872T>C), ACC1198ACT (3884C>T), ACA1199GTG (3885A>G 3886C>T 3887A>G), CTT1200TTA (3888C>T 3890T>A), AGC1201AGT (3893C>T), GAA1202GAG (3896A>G), AGA1205AGG (3905A>G), TGT1207TGC (3911T>C), TTG1208TTA (3914G>A), CAG1209CTA (3916A>T 3917G>A), ACA1210ACG (3920A>G), AAC1212AGC (3925A>G), ACA1213ACG (3929A>G), GTT1214GGC (3931T>G 3932T>C), CGC1216CGT (3938C>T), TAT1219TAC (3947T>C), GTT1221GTC (3953T>C), AGC1222AGT (3956C>T), AAT1223AAC (3959T>C), AAA1224AAG (3962A>G), CTG1225TTG (3963C>T), GAC1227GAT (3971C>T), GTG1228GTT (3974G>T), CCC1231CCT (3983C>T), GTG1234GTC (3992G>C), AAA1238AAG (4004A>G), GAA1239GAG (4007A>G), TTG1240CTG (4008T>C), ACT1242ACC (4016T>C), GCT1244GCC (4022T>C), GAT1247GAC (4031T>C), TAT1248TAC (4034T>C), GTT1306GTC (4208T>C), CTT1308TTT (4212C>T), TGT1309TGC (4217T>C), GTG1311GTA (4223G>A), AAT1314AAC (4232T>C), GTC1316GCT (4237T>C 4238C>T), GTC1319GTT (4247C>T), GAA1320GAG (4250A>G), CCC1323CCA (4259C>A), GAC1325GAT (4265C>T), ATT1326ATC (4268T>C), GTC1327GTT (4271C>T), GAG1333GAA (4289G>A), GCT1338GCC (4304T>C), GAT1342GAC (4316T>C), GCT1348GCC (4334T>C), GCT1349GCA (4337T>A), GCC1350GCT (4340C>T), TTA1351TTG (4343A>G), AAG1352AAA (4346G>A), GCG1355GCA (4355G>A), GTG1356GTA (4358G>A), TCT1436TCA (4598T>A), AGA1437AGG (4601A>G), GCT1439GCC (4607T>C), GAA1440GAT (4610A>T), CAT1444TAT (4620C>T), GAC1445GGT (4624A>G 4625C>T), GAT1446GAC (4628T>C), CCC1447CCT (4631C>T), GAG1448GAA (4634G>A), ATG1450CTG (4638A>C), TCA1451TCG (4643A>G), GCT1457GCC (4661T>C), CAA1459CAG (4667A>G), AAT1461AAC (4673T>C), CTA1462TTG (4674C>T 4676A>G), AAA1463GAA (4677A>G), CGT1465CGC (4685T>C), GAC1467GAT (4691C>T), GAT1468GAC (4694T>C), ATT1470ATC (4700T>C), GCT1472GTT (4705C>T), GCT1487GCC (4751T>C), GAT1498GAC (4784T>C), ACT1499ATT (4786C>T), CGA1506CAA (4807G>A), GAC1513GGC (4828A>G), ACC1516ACT (4838C>T), TTG1522CCG (4854T>C 4855T>C), TTC1622TTT (5156C>T), AAT1625AAC (5165T>C), GAC1626AAC (5166G>A), TAC1628TAT (5174C>T), GCT1631GCC (5183T>C), GTC1638GGC (5203T>G), TTA1640TTG (5210A>G), TTG1643CTG (5217T>C), GCC1646GCT (5228C>T), AAC1652AAT (5246C>T), ATC1658ATT (5264C>T), GGC1665GGT (5285C>T), GCC1690ACC (5358G>A), GAG1691GAA (5363G>A), AAG1692AAA (5366G>A), TCA1700TCC (5390A>C), TTA1703CTA (5397T>C), AAT1707AAC (5411T>C), AGT1708AGC (5414T>C), GAC1709GAT (5417C>T), CTA1716CTG (5438A>G), CTG1717TTG (5439C>T), GAC1720GAT (5450C>T), GTT1722ATT (5454G>A), TAT1723TAC (5459T>C), GAA1724GAG (5462A>G), GGT1728GGC (5474T>C), ACA1733ACG (5489A>G), TCC1739.CT (5507C>T), CTC1746CTT (5528C>T), GCC1749GCT (5537C>T), AAC1750AAT (5540C>T), TCG1751TCA (5543G>A), GCT1752GCC (5546T>C), CGC1757CGT (5561C>T), GTG1763GTA (5579G>A), GAA1764GAG (5582A>G), TCT1767TCC (5591T>C), GGA1774GGT (5612A>T), AGT1777AGC (5621T>C), GGT1782GGC (5636T>C), GTC1784ATT (5640G>A 5642C>T), GCA1785GCG (5645A>G), CGC2002CGT (6296C>T), CGA2004CGC (6302A>C), ATT2005GTT (6303A>G), ATT2007GTT (6309A>G), GCC2012GCT (6326C>T), TCC2018TCG (6344C>G), TAT2019TAC (6347T>C), AAC2022AAT (6356C>T), AAT2025AAC (6365T>C), TTC2027TTT (6371C>T), CAT2029CAC (6377T>C), AAG2037AAA (6401G>A), CTG2038TTG (6402C>T), GAT2053GAC (6449T>C), AAG2056AAA (6458G>A), ATT2066ATA (6488T>A), TCT3128TCC (9674T>C), CGT3137CGC (9701T>C), GCC3144KCC (9720G>K), AAA3178AWA (9823A>W), CAG3196CAA (9878G>A), GTT3207GCT (9910T>C), TTT3633TTC (11189T>C), AAT3647AAC (11231T>C), ATA3669AGA (11296T>G), GAC3741GAT (11513C>T), TTT3753TTC (11549T>C), AAC3755AAT (11555C>T), TGC3786CGC (11646T>C), AGT3797AGC (11681T>C), CCG3822CCC (11756G>C), TCC3875TCT (11915C>T), ATA3878GTA (11922A>G), GCT3989GCC (12257T>C), CAC4020CAT (12350C>T), AGG4031AGA (12383G>A), TTT4051TTC (12443T>C), AGT4546AGC (13928T>C), TTT4550TTC (13940T>C), GTA4552GTG (13946A>G), GTT4555GTA (13955T>A), GAA4590_GAT4591del (14058_14064delGAAGATA), ATG4592-TG (14058_14064delGAAGATA), TAC4598TAT (14084C>T), GTT4957GTC (15161T>C), CTG4965TTG (15183C>T), GAT5009GAC (15317T>C), GTT5339GTC (16307T>C), GAC5353GAT (16349C>T), GAT5360GGT (16369A>G), TAC5362TAT (16376C>T), AAT5380AAC (16430T>C), GTC5397GTT (16481C>T), GCG5411GCT (16523G>T), CTT5421CTC (16553T>C), GAT5429GAC (16577T>C), GAT5727GAC (17471T>C), AAC5742AAT (17516C>T), AGG5782AAG (17635G>A), CAT5798CAC (17684T>C), TCT5850GCT (17838T>G), GCT5874GCC (17912T>C), CAC5884CAT (17942C>T), AGT6137AGC (18701T>C), GAC6144GAT (18722C>T), ACA6151ACC (18743A>C), GAA6154GAG (18752A>G), GTT6155GTG (18755T>G), AGC6156AGT (18758C>T), GAT6157GAC (18761T>C), GAG6158GAA (18764G>A), AAC6168AAT (18794C>T), AAG6169AAA (18797G>A), TCC6181TCT (18833C>T), TTG6192CTG (18864T>C), GAT6196GAC (18878T>C), AAT6199AAC (18887T>C), GTA6200GTG (18890A>G), GCG6202GCT (18896G>T), GCG6205GCA (18905G>A), GCC6206GCT (18908C>T), CAG6210CAA (18920G>A), CCA6506CCG (19808A>G), GTG6510TTG (19818G>T), GAC6513GAT (19829C>T), GAT6515GAC (19835T>C), CGC6523CGT (19859C>T), TCT6538TCC (19904T>C), AAG6539AAT (19907G>T), CGA6546AGA (19926C>A), AGC6557AGT (19961C>T), GGC6564GGT (19982C>T), GTT6570GTC (20000T>C), AAC6582AAT (20036C>T), AAT6596AAC (20078T>C), GTT6598ACG (20082G>A 20083T>C 20084T>G), ATT6786.TC (20648T>C), GGC6788GGT (20654C>T), CCG6791CCA (20663G>A), CAT6792CAC (20666T>C), CAG6794CAA (20672G>A), GGC6795GGT (20675C>T), CCT6807CCC (20711T>C), AAG6809AGG (20716A>G), AAA6810AAG (20720A>G), CAT6814CAC (20732T>C), GTT6815GTC (20735T>C), GAT6822GAC (20756T>C), ATT6823ATC (20759T>C), CCT6827CCA (20771T>A), GGC6830GGT (20780C>T), TAT6831TAC (20783T>C), CTC6832CTT (20786C>T), AAG6839AAA (20807G>A), ATT6840ATC (20810T>C), TCT6842TCC (20816T>C), GAC6844GAT (20822C>T), GAG6846GAA (20828G>A), GGT6850GGC (20840T>C), TCC6852_ATT6853del (20844_20849delTCCATT), GAG6855GAA (20855G>A), ACA6859ACC (20867A>C), AAC6861AAT (20873C>T), GAG6862GAA (20876G>A), CAA6864CAG (20882A>G), TTA6867TTG (20891A>G), ATC6873ATT (20909C>T), GAT6874GAC (20912T>C), CTT6878CTC (20924T>C), ACT6883ACG (20939T>G), AAT6944AAC (21122T>C), ACT6949ACC (21137T>C), AAT6951AAC (21143T>C), TTG6956CTT (21156T>C 21158G>T), CAC6958CAT (21164C>T), CTC6960CTT (21170C>T), AGC6963AGT (21179C>T), ATC6964ATT (21182C>T), GAA6965GAG (21185A>G), GAT6966GAC (21188T>C), CTA6967CTG (21191A>G), ACC6968ACT (21194C>T), ACC6970ACT (21200C>T), ATC6974GTC (21210A>G), GTG6976GTA (21218G>A), TAT7071TAC (21503T>C), ATC7076ATT (21518C>T), AGC7079GAC (21525A>G 21526G>A), CTC7103CTT (21599C>T), AAT7109AAC (21617T>C), GAC7110AAC (21618G>A), TTC7115TTT (21635C>T), AGG7119AGA (21647G>A)								

Proteins

polyprotein (YP_009175071.1)	655	7121	25.1%	12248	94.7%	1839 (99.8%)	1774 (96.3%)	0/4/4/1	0
Protein mutations:	V663I (2277G>A 2279G>T), S685D (2343A>G 2344G>A), A820T (2748G>A), S827A (2769T>G 2771T>C), I840V (2808A>G 2810T>G), R849H (2836G>A 2837C>T), V861T (2871G>A 2872T>C), Q869E (2895C>G), I872V (2904A>G 2906C>T), V887A (2950T>C 2951T>A), N892I (2965A>T), T1199V (3885A>G 3886C>T 3887A>G), Q1209L (3916A>T 3917G>A), N1212S (3925A>G), V1214G (3931T>G 3932T>C), L1308F (4212C>T), V1316A (4237T>C 4238C>T), E1440D (4610A>T), H1444Y (4620C>T), D1445G (4624A>G 4625C>T), M1450L (4638A>C), K1463E (4677A>G), A1472V (4705C>T), T1499I (4786C>T), R1506Q (4807G>A), D1513G (4828A>G), L1522P (4854T>C 4855T>C), D1626N (5166G>A), V1638G (5203T>G), A1690T (5358G>A), V1722I (5454G>A), V1784I (5640G>A 5642C>T), I2005V (6303A>G), I2007V (6309A>G), V3207A (9910T>C), I3669R (11296T>G), C3786R (11646T>C), I3878V (11922A>G), E4590_D4591del (14058_14064delGAAGATA), D5360G (16369A>G), R5782K (17635G>A), S5850A (17838T>G), V6510L (19818G>T), K6539N (19907G>T), V6598T (20082G>A 20083T>C 20084T>G), K6809R (20716A>G), S6852_I6853del (20844_20849delTCCATT), I6974V (21210A>G), S7079D (21525A>G 21526G>A), D7110N (21618G>A)								

	Begin	End	Coverage	Score	Concordance	Matches	Identities	I/D/M/F*	Stop Codons
**NT**	**2251**	**21653**	**24.3%**	**9449**	**85.7%**	**5529 (99.8%)**	**5133 (92.6%)**	**0/13**	
Codon mutations:	CTG658TTG (2262C>T), GTG663ATT (2277G>A 2279G>T), TTC664TTT (2282C>T), GCT667GCC (2291T>C), GTT671GTC (2303T>C), TCC674TCA (2312C>A), GCT676GCC (2318T>C), AAA677AAG (2321A>G), TAT678TAC (2324T>C), CTC679CTT (2327C>T), ACT682ACG (2336T>G), AGC685GAC (2343A>G 2344G>A), CCT688CCC (2354T>C), ATT691ATC (2363T>C), GAT693GAC (2369T>C), GAA697GAG (2381A>G), CGT703CGC (2399T>C), CTT705CTC (2405T>C), TAC707TAT (2411C>T), GCA710GCG (2420A>G), TCC713TCG (2429C>G), AAA714AAG (2432A>G), AAA716AAG (2438A>G), ACT722ACC (2456T>C), CAA725CAG (2465A>G), CTG726TTG (2466C>T), ATC727ATT (2471C>T), GGC728GGT (2474C>T), CTA729TTA (2475C>T), GCT730GCC (2480T>C), GGA807GGG (2711A>G), TCT809TCC (2717T>C), TCG810TCT (2720G>T), TAC815TAT (2735C>T), GGT816GGA (2738T>A), CCG819CCA (2747G>A), GCC820ACC (2748G>A), CAC823CAT (2759C>T), GGG824GGA (2762G>A), TTC825TTT (2765C>T), TCT827GCC (2769T>G 2771T>C), TCG828TCA (2774G>A), CGT829CGC (2777T>C), GGA830GGT (2780A>T), GGA831GGG (2783A>G), AGA833AGG (2789A>G), GGA836GGG (2798A>G), CTA838TTG (2802C>T 2804A>G), ATT840GTG (2808A>G 2810T>G), GAG845GAA (2825G>A), GTG847GTT (2831G>T), CGC849CAT (2836G>A 2837C>T), GTC855..A (2855C>A), CCA859CCC (2867A>C), TCA860TCT (2870A>T), GTG861ACG (2871G>A 2872T>C), CCA862CCG (2876A>G), ATT863ATC (2879T>C), CGG864CKC (2881G>K 2882G>C), CGC865AGG (2883C>A 2885C>G), CTG866CTA (2888G>A), CAG869GAG (2895C>G), AAA870AAG (2900A>G), CCC871CCA (2903C>A), ATC872GTT (2904A>G 2906C>T), TCA874TCC (2912A>C), GAA876GAG (2918A>G), GAG880GAA (2930G>A), AAA883AAG (2939A>G), ACT884ACC (2942T>C), TTA885TTG (2945A>G), AAC886AAT (2948C>T), GTT887GCA (2950T>C 2951T>A), GGG888GGT (2954G>T), GTC889GTT (2957C>T), AAC892ATC (2965A>T), TCC894TCT (2972C>T), TTG1160TTA (3770G>A), GGG1162GGC (3776G>C), GCA1163GCT (3779A>T), GCC1164GCT (3782C>T), TTT1165TTC (3785T>C), GGA1166GGG (3788A>G), CAA1167CAG (3791A>G), TGC1168TGT (3794C>T), TTT1169TTC (3797T>C), TTC1172TTT (3806C>T), TCT1175TCC (3815T>C), TTC1177TTT (3821C>T), ATC1178ATT (3824C>T), AGC1179AGT (3827C>T), GTC1182GTT (3836C>T), TGC1184TGT (3842C>T), GGG1185GGA (3845G>A), CTT1190CTC (3860T>C), GAC1192GAT (3866C>T), AAT1194AAC (3872T>C), ACC1198ACT (3884C>T), ACA1199GTG (3885A>G 3886C>T 3887A>G), CTT1200TTA (3888C>T 3890T>A), AGC1201AGT (3893C>T), GAA1202GAG (3896A>G), AGA1205AGG (3905A>G), TGT1207TGC (3911T>C), TTG1208TTA (3914G>A), CAG1209CTA (3916A>T 3917G>A), ACA1210ACG (3920A>G), AAC1212AGC (3925A>G), ACA1213ACG (3929A>G), GTT1214GGC (3931T>G 3932T>C), CGC1216CGT (3938C>T), TAT1219TAC (3947T>C), GTT1221GTC (3953T>C), AGC1222AGT (3956C>T), AAT1223AAC (3959T>C), AAA1224AAG (3962A>G), CTG1225TTG (3963C>T), GAC1227GAT (3971C>T), GTG1228GTT (3974G>T), CCC1231CCT (3983C>T), GTG1234GTC (3992G>C), AAA1238AAG (4004A>G), GAA1239GAG (4007A>G), TTG1240CTG (4008T>C), ACT1242ACC (4016T>C), GCT1244GCC (4022T>C), GAT1247GAC (4031T>C), TAT1248TAC (4034T>C), GTT1306GTC (4208T>C), CTT1308TTT (4212C>T), TGT1309TGC (4217T>C), GTG1311GTA (4223G>A), AAT1314AAC (4232T>C), GTC1316GCT (4237T>C 4238C>T), GTC1319GTT (4247C>T), GAA1320GAG (4250A>G), CCC1323CCA (4259C>A), GAC1325GAT (4265C>T), ATT1326ATC (4268T>C), GTC1327GTT (4271C>T), GAG1333GAA (4289G>A), GCT1338GCC (4304T>C), GAT1342GAC (4316T>C), GCT1348GCC (4334T>C), GCT1349GCA (4337T>A), GCC1350GCT (4340C>T), TTA1351TTG (4343A>G), AAG1352AAA (4346G>A), GCG1355GCA (4355G>A), GTG1356GTA (4358G>A), TCT1436TCA (4598T>A), AGA1437AGG (4601A>G), GCT1439GCC (4607T>C), GAA1440GAT (4610A>T), CAT1444TAT (4620C>T), GAC1445GGT (4624A>G 4625C>T), GAT1446GAC (4628T>C), CCC1447CCT (4631C>T), GAG1448GAA (4634G>A), ATG1450CTG (4638A>C), TCA1451TCG (4643A>G), GCT1457GCC (4661T>C), CAA1459CAG (4667A>G), AAT1461AAC (4673T>C), CTA1462TTG (4674C>T 4676A>G), AAA1463GAA (4677A>G), CGT1465CGC (4685T>C), GAC1467GAT (4691C>T), GAT1468GAC (4694T>C), ATT1470ATC (4700T>C), GCT1472GTT (4705C>T), GCT1487GCC (4751T>C), GAT1498GAC (4784T>C), ACT1499ATT (4786C>T), CGA1506CAA (4807G>A), GAC1513GGC (4828A>G), ACC1516ACT (4838C>T), TTG1522CCG (4854T>C 4855T>C), TTC1622TTT (5156C>T), AAT1625AAC (5165T>C), GAC1626AAC (5166G>A), TAC1628TAT (5174C>T), GCT1631GCC (5183T>C), GTC1638GGC (5203T>G), TTA1640TTG (5210A>G), TTG1643CTG (5217T>C), GCC1646GCT (5228C>T), AAC1652AAT (5246C>T), ATC1658ATT (5264C>T), GGC1665GGT (5285C>T), GCC1690ACC (5358G>A), GAG1691GAA (5363G>A), AAG1692AAA (5366G>A), TCA1700TCC (5390A>C), TTA1703CTA (5397T>C), AAT1707AAC (5411T>C), AGT1708AGC (5414T>C), GAC1709GAT (5417C>T), CTA1716CTG (5438A>G), CTG1717TTG (5439C>T), GAC1720GAT (5450C>T), GTT1722ATT (5454G>A), TAT1723TAC (5459T>C), GAA1724GAG (5462A>G), GGT1728GGC (5474T>C), ACA1733ACG (5489A>G), TCC1739.CT (5507C>T), CTC1746CTT (5528C>T), GCC1749GCT (5537C>T), AAC1750AAT (5540C>T), TCG1751TCA (5543G>A), GCT1752GCC (5546T>C), CGC1757CGT (5561C>T), GTG1763GTA (5579G>A), GAA1764GAG (5582A>G), TCT1767TCC (5591T>C), GGA1774GGT (5612A>T), AGT1777AGC (5621T>C), GGT1782GGC (5636T>C), GTC1784ATT (5640G>A 5642C>T), GCA1785GCG (5645A>G), CGC2002CGT (6296C>T), CGA2004CGC (6302A>C), ATT2005GTT (6303A>G), ATT2007GTT (6309A>G), GCC2012GCT (6326C>T), TCC2018TCG (6344C>G), TAT2019TAC (6347T>C), AAC2022AAT (6356C>T), AAT2025AAC (6365T>C), TTC2027TTT (6371C>T), CAT2029CAC (6377T>C), AAG2037AAA (6401G>A), CTG2038TTG (6402C>T), GAT2053GAC (6449T>C), AAG2056AAA (6458G>A), ATT2066ATA (6488T>A), TCT3128TCC (9674T>C), CGT3137CGC (9701T>C), GCC3144KCC (9720G>K), AAA3178AWA (9823A>W), CAG3196CAA (9878G>A), GTT3207GCT (9910T>C), TTT3633TTC (11189T>C), AAT3647AAC (11231T>C), ATA3669AGA (11296T>G), GAC3741GAT (11513C>T), TTT3753TTC (11549T>C), AAC3755AAT (11555C>T), TGC3786CGC (11646T>C), AGT3797AGC (11681T>C), CCG3822CCC (11756G>C), TCC3875TCT (11915C>T), ATA3878GTA (11922A>G), GCT3989GCC (12257T>C), CAC4020CAT (12350C>T), AGG4031AGA (12383G>A), TTT4051TTC (12443T>C), AGT4546AGC (13928T>C), TTT4550TTC (13940T>C), GTA4552GTG (13946A>G), GTT4555GTA (13955T>A), GAA4590_GAT4591del (14058_14064delGAAGATA), ATG4592-TG (14058_14064delGAAGATA), TAC4598TAT (14084C>T), GTT4957GTC (15161T>C), CTG4965TTG (15183C>T), GAT5009GAC (15317T>C), GTT5339GTC (16307T>C), GAC5353GAT (16349C>T), GAT5360GGT (16369A>G), TAC5362TAT (16376C>T), AAT5380AAC (16430T>C), GTC5397GTT (16481C>T), GCG5411GCT (16523G>T), CTT5421CTC (16553T>C), GAT5429GAC (16577T>C), GAT5727GAC (17471T>C), AAC5742AAT (17516C>T), AGG5782AAG (17635G>A), CAT5798CAC (17684T>C), TCT5850GCT (17838T>G), GCT5874GCC (17912T>C), CAC5884CAT (17942C>T), AGT6137AGC (18701T>C), GAC6144GAT (18722C>T), ACA6151ACC (18743A>C), GAA6154GAG (18752A>G), GTT6155GTG (18755T>G), AGC6156AGT (18758C>T), GAT6157GAC (18761T>C), GAG6158GAA (18764G>A), AAC6168AAT (18794C>T), AAG6169AAA (18797G>A), TCC6181TCT (18833C>T), TTG6192CTG (18864T>C), GAT6196GAC (18878T>C), AAT6199AAC (18887T>C), GTA6200GTG (18890A>G), GCG6202GCT (18896G>T), GCG6205GCA (18905G>A), GCC6206GCT (18908C>T), CAG6210CAA (18920G>A), CCA6506CCG (19808A>G), GTG6510TTG (19818G>T), GAC6513GAT (19829C>T), GAT6515GAC (19835T>C), CGC6523CGT (19859C>T), TCT6538TCC (19904T>C), AAG6539AAT (19907G>T), CGA6546AGA (19926C>A), AGC6557AGT (19961C>T), GGC6564GGT (19982C>T), GTT6570GTC (20000T>C), AAC6582AAT (20036C>T), AAT6596AAC (20078T>C), GTT6598ACG (20082G>A 20083T>C 20084T>G), ATT6786.TC (20648T>C), GGC6788GGT (20654C>T), CCG6791CCA (20663G>A), CAT6792CAC (20666T>C), CAG6794CAA (20672G>A), GGC6795GGT (20675C>T), CCT6807CCC (20711T>C), AAG6809AGG (20716A>G), AAA6810AAG (20720A>G), CAT6814CAC (20732T>C), GTT6815GTC (20735T>C), GAT6822GAC (20756T>C), ATT6823ATC (20759T>C), CCT6827CCA (20771T>A), GGC6830GGT (20780C>T), TAT6831TAC (20783T>C), CTC6832CTT (20786C>T), AAG6839AAA (20807G>A), ATT6840ATC (20810T>C), TCT6842TCC (20816T>C), GAC6844GAT (20822C>T), GAG6846GAA (20828G>A), GGT6850GGC (20840T>C), TCC6852_ATT6853del (20844_20849delTCCATT), GAG6855GAA (20855G>A), ACA6859ACC (20867A>C), AAC6861AAT (20873C>T), GAG6862GAA (20876G>A), CAA6864CAG (20882A>G), TTA6867TTG (20891A>G), ATC6873ATT (20909C>T), GAT6874GAC (20912T>C), CTT6878CTC (20924T>C), ACT6883ACG (20939T>G), AAT6944AAC (21122T>C), ACT6949ACC (21137T>C), AAT6951AAC (21143T>C), TTG6956CTT (21156T>C 21158G>T), CAC6958CAT (21164C>T), CTC6960CTT (21170C>T), AGC6963AGT (21179C>T), ATC6964ATT (21182C>T), GAA6965GAG (21185A>G), GAT6966GAC (21188T>C), CTA6967CTG (21191A>G), ACC6968ACT (21194C>T), ACC6970ACT (21200C>T), ATC6974GTC (21210A>G), GTG6976GTA (21218G>A), TAT7071TAC (21503T>C), ATC7076ATT (21518C>T), AGC7079GAC (21525A>G 21526G>A), CTC7103CTT (21599C>T), AAT7109AAC (21617T>C), GAC7110AAC (21618G>A), TTC7115TTT (21635C>T), AGG7119AGA (21647G>A)								

*: Inserts / Deletes / Misaligned / Frameshifts

Analysis details

This analysis was performed with panviral2.64

NGS Details (UN60): Duamitovirus peex1

Assembly

**Coverage Length**	729 (2 contig(s))
**Depth Of Coverage**	4.4
**Number Of Reads**	26
**Reads Per Million**	0.55 rpm (after QC)
**Ambiguities**	0
**Assembly Method**	de novo + reference guided assembly
**Consensus Caller**	Bcf Tools

Coverage Map

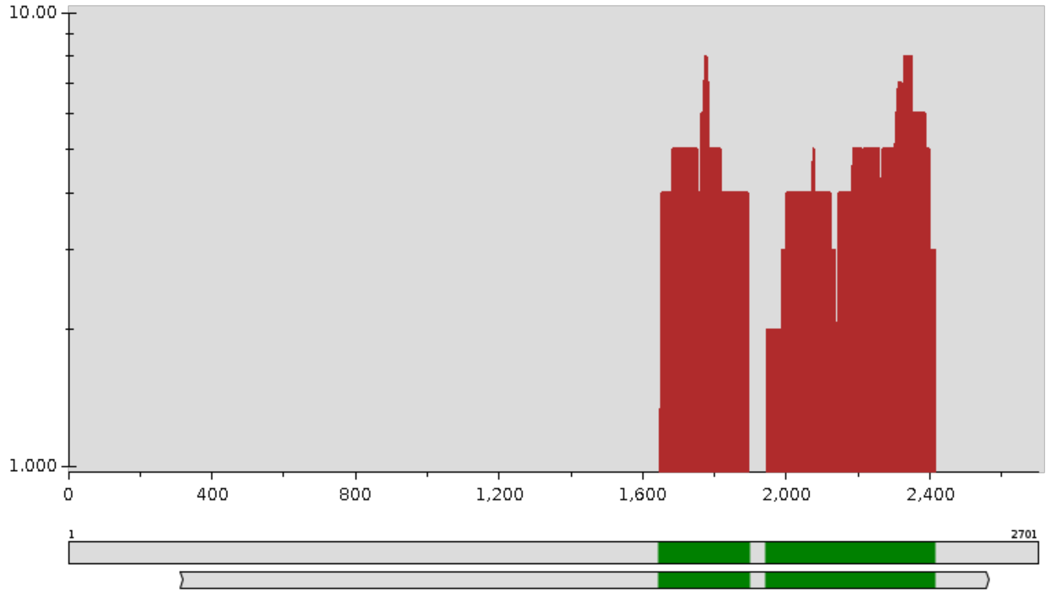

Assignment

**Type**	Duamitovirus peex1 (Taxonomy ID: 2955799)
**Reference Genome**	NC_076525.1
**NT Identity (%)**	68.6813
**AA Identity (%)**	67.0782
**Number Of Stop Codons**	1
**Number Of CDS**	1

Alignment

**Alignment Score**	534.0 (NT) + 1035.0 (AA) = 1569.0
**Concordance (%)**	51.9883

Alignment Method	Global, seeded, nucleotide + amino acids (AGA)

Genome Region

Sequence starts at position 1645 and ends at position 2417 relative to NC_076525.1 reference sequence.

Alignment Detailed Statistics

	Begin	End	Coverage	Score	Concordance	Matches	Identities	I/D/M/F*	Stop Codons
**NT**	**1645**	**2417**	**27.0%**	**534**	**36.9%**	**728 (99.9%)**	**500 (68.6%)**	**0/1**	
Mutations:	1655C>G, 1658G>T, 1661A>C, 1671T>A, 1676A>G, 1680T>C, 1682G>A, 1685G>T, 1689G>A, 1691G>A, 1692A>T, 1694G>T, 1696T>C, 1697C>A, 1703G>A, 1704G>A, 1705G>C, 1706C>G, 1707C>A, 1711G>C, 1713C>A, 1714G>C, 1715G>C, 1721C>T, 1722T>G, 1723G>C, 1724G>T, 1727A>T, 1730T>C, 1736T>G, 1737C>T, 1739C>A, 1751T>C, 1754C>A, 1757T>C, 1758G>A, 1764G>A, 1765A>C, 1766G>A, 1772C>G, 1774G>C, 1775C>G, 1776A>C, 1777A>G, 1781A>G, 1784T>C, 1785G>A, 1786C>G, 1788G>A, 1789A>T, 1790A>T, 1792A>T, 1793C>G, 1799G>T, 1800C>A, 1808A>T, 1814C>A, 1817T>C, 1820G>T, 1821delA, 1824C>G, 1825A>C, 1834T>G, 1841G>C, 1842A>G, 1844T>G, 1847T>C, 1850T>A, 1851T>A, 1853T>C, 1854C>A, 1856T>C, 1862C>T, 1865A>T, 1871G>A, 1880T>C, 1884T>G, 1885T>A, 1886A>C, 1893G>A, 1944A>G, 1946T>G, 1958G>A, 1959A>C, 1961T>C, 1963G>C, 1964G>A, 1966T>C, 1967G>C, 1974G>T, 1975G>C, 1976A>G, 1977C>A, 1980C>T, 1981A>C, 1984T>C, 1985G>A, 1988C>G, 1989G>A, 1990C>T, 1996A>C, 1997A>C, 1998C>A, 1999G>T, 2001C>T, 2003A>T, 2004G>T, 2005C>G, 2006T>G, 2009C>G, 2012A>C, 2015C>A, 2017A>T, 2018T>C, 2021G>A, 2027A>T, 2033C>T, 2034C>T, 2036T>G, 2040A>T, 2042G>T, 2048G>A, 2057C>T, 2066G>A, 2067A>C, 2069T>G, 2072G>A, 2075T>G, 2077T>C, 2081T>A, 2082T>A, 2084T>C, 2088T>C, 2090T>A, 2091T>C, 2095T>G, 2096A>G, 2102G>T, 2104G>T, 2105G>A, 2109T>C, 2114T>C, 2119G>A, 2120G>T, 2129A>G, 2132G>A, 2133G>T, 2134G>T, 2135A>T, 2138G>A, 2141C>T, 2145G>A, 2152A>T, 2156A>G, 2158G>A, 2159A>G, 2162T>A, 2171C>A, 2173C>A, 2180T>C, 2183T>G, 2189G>A, 2194A>C, 2209A>G, 2210G>A, 2211T>C, 2213G>C, 2214A>T, 2216A>T, 2222A>T, 2223C>G, 2225A>G, 2226C>T, 2234A>T, 2243A>G, 2244C>A, 2245A>G, 2246T>A, 2249G>A, 2255A>T, 2268G>T, 2272G>T, 2273T>C, 2274C>A, 2275A>G, 2276T>A, 2280A>G, 2283G>A, 2294T>G, 2295C>A, 2296G>A, 2301C>G, 2303T>C, 2306A>T, 2312T>C, 2315C>T, 2321G>T, 2325A>G, 2326A>C, 2327T>A, 2330C>A, 2331C>G, 2333G>A, 2339G>A, 2345A>T, 2348C>T, 2351A>G, 2352C>T, 2354T>G, 2363G>T, 2373G>A, 2374C>G, 2375G>T, 2377C>A, 2378T>A, 2381T>C, 2384G>A, 2388C>A, 2390T>A, 2393T>A, 2394C>A, 2396G>C, 2397A>G, 2398C>A, 2399T>A, 2406C>G, 2411A>G, 2412G>A, 2414G>C								

CDS

	Begin	End	Coverage	Score	Concordance	Matches	Identities	I/D/M/F*	Stop Codons
RdRp	446	702	32.4%	1035	59.8%	243 (100%)	163 (67.1%)	0/0/1/1	1
Protein mutations:	L454M (1671T>A), E460K (1689G>A 1691G>A), K461Y (1692A>T 1694G>T), V462A (1696T>C 1697C>A), G465T (1704G>A 1705G>C 1706C>G), R466S (1707C>A), S467T (1711G>C), R468T (1713C>A 1714G>C 1715G>C), W471A (1722T>G 1723G>C 1724G>T), E472D (1727A>T), A483T (1758G>A), E485T (1764G>A 1765A>C 1766G>A), G488A (1774G>C 1775C>G), K489R (1776A>C 1777A>G), A492R (1785G>A 1786C>G), E493I (1788G>A 1789A>T 1790A>T), Y494L (1792A>T 1793C>G), E496D (1799G>T), H505A (1824C>G 1825A>C), I508R (1834T>G), N511E (1842A>G 1844T>G), S514T (1851T>A 1853T>C), L515I (1854C>A 1856T>C), L525D (1884T>G 1885T>A 1886A>C), V528I (1893G>A), I545V (1944A>G 1946T>G), I550L (1959A>C 1961T>C), G551A (1963G>C 1964G>A), M552T (1966T>C 1967G>C), G555S (1974G>T 1975G>C 1976A>G), L556I (1977C>A), Q557S (1980C>T 1981A>C), M558T (1984T>C 1985G>A), A560I (1989G>A 1990C>T), Q562P (1996A>C 1997A>C), R563I (1998C>A 1999G>T), L564F (2001C>T 2003A>T), A565W (2004G>T 2005C>G 2006T>G), Y569F (2017A>T 2018T>C), T577S (2040A>T 2042G>T), I586L (2067A>C 2069T>G), V589A (2077T>C), C591S (2082T>A 2084T>C), S593P (2088T>C 2090T>A), Y594H (2091T>C), I595R (2095T>G 2096A>G), Q597H (2102G>T), R598L (2104G>T 2105G>A), S600P (2109T>C), W603Y (2119G>A 2120G>T), G608F (2133G>T 2134G>T 2135A>T), D612N (2145G>A), Y614F (2152A>T), R616K (2158G>A 2159A>G), A621D (2173C>A), K628T (2194A>C), Q633R (2209A>G 2210G>A), I635F (2214A>T 2216A>T), K637N (2222A>T), Q638E (2223C>G 2225A>G), L641F (2234A>T), H645R (2244C>A 2245A>G 2246T>A), V653L (2268G>T), R654L (2272G>T 2273T>C), H655R (2274C>A 2275A>G 2276T>A), M657V (2280A>G), E658K (2283G>A), R662K (2295C>A 2296G>A), L664V (2301C>G 2303T>C), N672A (2325A>G 2326A>C 2327T>A), P674A (2331C>G 2333G>A), A688S (2373G>A 2374C>G 2375G>T), T689K (2377C>A 2378T>A), W691* (2384G>A), Q695N (2394C>A 2396G>C), T696E (2397A>G 2398C>A 2399T>A), H699D (2406C>G), V701I (2412G>A 2414G>C)								
Codon mutations:	GCC448GCG (1655C>G), CTG449CTT (1658G>T), TCA450TCC (1661A>C), TTG454ATG (1671T>A), GTA455GTG (1676A>G), TTG457CTA (1680T>C 1682G>A), GCG458GCT (1685G>T), GAG460AAA (1689G>A 1691G>A), AAG461TAT (1692A>T 1694G>T), GTC462GCA (1696T>C 1697C>A), CCG464CCA (1703G>A), GGC465ACG (1704G>A 1705G>C 1706C>G), CGT466AGT (1707C>A), AGT467ACT (1711G>C), CGG468ACC (1713C>A 1714G>C 1715G>C), TTC470TTT (1721C>T), TGG471GCT (1722T>G 1723G>C 1724G>T), GAA472GAT (1727A>T), TAT473TAC (1730T>C), CTT475CTG (1736T>G), CTC476TTA (1737C>T 1739C>A), ATT480ATC (1751T>C), CTC481CTA (1754C>A), ATT482ATC (1757T>C), GCC483ACC (1758G>A), GAG485ACA (1764G>A 1765A>C 1766G>A), GTC487GTG (1772C>G), GGC488GCG (1774G>C 1775C>G), AAG489CGG (1776A>C 1777A>G), CAA490CAG (1781A>G), TAT491TAC (1784T>C), GCG492AGG (1785G>A 1786C>G), GAA493ATT (1788G>A 1789A>T 1790A>T), TAC494TTG (1792A>T 1793C>G), GAG496GAT (1799G>T), CGG497AGG (1800C>A), GGA499GGT (1808A>T), ACC501ACA (1814C>A), ATT502ATC (1817T>C), TCG503TCT (1820G>T), ATA504-TA (1821delA), CAT505GCT (1824C>G 1825A>C), ATA508AGA (1834T>G), TCG510TCC (1841G>C), AAT511GAG (1842A>G 1844T>G), AAT512AAC (1847T>C), GGT513GGA (1850T>A), TCT514ACC (1851T>A 1853T>C), CTT515ATC (1854C>A 1856T>C), TTC517TTT (1862C>T), GCA518GCT (1865A>T), AGG520AGA (1871G>A), ACT523ACC (1880T>C), TTA525GAC (1884T>G 1885T>A 1886A>C), GTT528ATT (1893G>A), ATT545GTG (1944A>G 1946T>G), CAG549CAA (1958G>A), ATT550CTC (1959A>C 1961T>C), GGG551GCA (1963G>C 1964G>A), ATG552ACC (1966T>C 1967G>C), GGA555TCG (1974G>T 1975G>C 1976A>G), CTT556ATT (1977C>A), CAA557TCA (1980C>T 1981A>C), ATG558ACA (1984T>C 1985G>A), TCC559TCG (1988C>G), GCT560ATT (1989G>A 1990C>T), CAA562CCC (1996A>C 1997A>C), CGT563ATT (1998C>A 1999G>T), CTA564TTT (2001C>T 2003A>T), GCT565TGG (2004G>T 2005C>G 2006T>G), GGC566GGG (2009C>G), GCA567GCC (2012A>C), GGC568GGA (2015C>A), TAT569TTC (2017A>T 2018T>C), AGG570AGA (2021G>A), CGA572CGT (2027A>T), CGC574CGT (2033C>T), CTT575TTG (2034C>T 2036T>G), ACG577TCT (2040A>T 2042G>T), CAG579CAA (2048G>A), CGC582CGT (2057C>T), AGG585AGA (2066G>A), ATT586CTG (2067A>C 2069T>G), AAG587AAA (2072G>A), GCT588GCG (2075T>G), GTT589GCT (2077T>C), GCT590GCA (2081T>A), TGT591AGC (2082T>A 2084T>C), TCT593CCA (2088T>C 2090T>A), TAC594CAC (2091T>C), ATA595AGG (2095T>G 2096A>G), CAG597CAT (2102G>T), CGG598CTA (2104G>T 2105G>A), TCT600CCT (2109T>C), CTT601CTC (2114T>C), TGG603TAT (2119G>A 2120G>T), GGA606GGG (2129A>G), AGG607AGA (2132G>A), GGA608TTT (2133G>T 2134G>T 2135A>T), AAG609AAA (2138G>A), CCC610CCT (2141C>T), GAT612AAT (2145G>A), TAT614TTT (2152A>T), TTA615TTG (2156A>G), AGA616AAG (2158G>A 2159A>G), GGT617GGA (2162T>A), ATC620ATA (2171C>A), GCT621GAT (2173C>A), CTT623CTC (2180T>C), CGT624CGG (2183T>G), GAG626GAA (2189G>A), AAA628ACA (2194A>C), CAG633CGA (2209A>G 2210G>A), TTG634CTC (2211T>C 2213G>C), ATA635TTT (2214A>T 2216A>T), AAA637AAT (2222A>T), CAA638GAG (2223C>G 2225A>G), CTG639TTG (2226C>T), TTA641TTT (2234A>T), GAA644GAG (2243A>G), CAT645AGA (2244C>A 2245A>G 2246T>A), GAG646GAA (2249G>A), CTA648CTT (2255A>T), GTA653TTA (2268G>T), CGT654CTC (2272G>T 2273T>C), CAT655AGA (2274C>A 2275A>G 2276T>A), ATG657GTG (2280A>G), GAA658AAA (2283G>A), CTT661CTG (2294T>G), CGA662AAA (2295C>A 2296G>A), CTT664GTC (2301C>G 2303T>C), TCA665TCT (2306A>T), TAT667TAC (2312T>C), CAC668CAT (2315C>T), GTG670GTT (2321G>T), AAT672GCA (2325A>G 2326A>C 2327T>A), TCC673TCA (2330C>A), CCG674GCA (2331C>G 2333G>A), GTG676GTA (2339G>A), ATA678ATT (2345A>T), GAC679GAT (2348C>T), CAA680CAG (2351A>G), CTT681TTG (2352C>T 2354T>G), GTG684GTT (2363G>T), GCG688AGT (2373G>A 2374C>G 2375G>T), ACT689AAA (2377C>A 2378T>A), TCT690TCC (2381T>C), TGG691TGA (2384G>A), CGT693AGA (2388C>A 2390T>A), ACT694ACA (2393T>A), CAG695AAC (2394C>A 2396G>C), ACT696GAA (2397A>G 2398C>A 2399T>A), CAT699GAT (2406C>G), TTA700TTG (2411A>G), GTG701ATC (2412G>A 2414G>C)								

Proteins

	Begin	End	Coverage	Score	Concordance	Matches	Identities	I/D/M/F*	Stop Codons
RNA-dependent RNA polymerase (YP_010798875.1)	446	702	32.4%	1035	59.8%	243 (100%)	163 (67.1%)	0/0/1/1	1
Protein mutations:	L454M (1671T>A), E460K (1689G>A 1691G>A), K461Y (1692A>T 1694G>T), V462A (1696T>C 1697C>A), G465T (1704G>A 1705G>C 1706C>G), R466S (1707C>A), S467T (1711G>C), R468T (1713C>A 1714G>C 1715G>C), W471A (1722T>G 1723G>C 1724G>T), E472D (1727A>T), A483T (1758G>A), E485T (1764G>A 1765A>C 1766G>A), G488A (1774G>C 1775C>G), K489R (1776A>C 1777A>G), A492R (1785G>A 1786C>G), E493I (1788G>A 1789A>T 1790A>T), Y494L (1792A>T 1793C>G), E496D (1799G>T), H505A (1824C>G 1825A>C), I508R (1834T>G), N511E (1842A>G 1844T>G), S514T (1851T>A 1853T>C), L515I (1854C>A 1856T>C), L525D (1884T>G 1885T>A 1886A>C), V528I (1893G>A), I545V (1944A>G 1946T>G), I550L (1959A>C 1961T>C), G551A (1963G>C 1964G>A), M552T (1966T>C 1967G>C), G555S (1974G>T 1975G>C 1976A>G), L556I (1977C>A), Q557S (1980C>T 1981A>C), M558T (1984T>C 1985G>A), A560I (1989G>A 1990C>T), Q562P (1996A>C 1997A>C), R563I (1998C>A 1999G>T), L564F (2001C>T 2003A>T), A565W (2004G>T 2005C>G 2006T>G), Y569F (2017A>T 2018T>C), T577S (2040A>T 2042G>T), I586L (2067A>C 2069T>G), V589A (2077T>C), C591S (2082T>A 2084T>C), S593P (2088T>C 2090T>A), Y594H (2091T>C), I595R (2095T>G 2096A>G), Q597H (2102G>T), R598L (2104G>T 2105G>A), S600P (2109T>C), W603Y (2119G>A 2120G>T), G608F (2133G>T 2134G>T 2135A>T), D612N (2145G>A), Y614F (2152A>T), R616K (2158G>A 2159A>G), A621D (2173C>A), K628T (2194A>C), Q633R (2209A>G 2210G>A), I635F (2214A>T 2216A>T), K637N (2222A>T), Q638E (2223C>G 2225A>G), L641F (2234A>T), H645R (2244C>A 2245A>G 2246T>A), V653L (2268G>T), R654L (2272G>T 2273T>C), H655R (2274C>A 2275A>G 2276T>A), M657V (2280A>G), E658K (2283G>A), R662K (2295C>A 2296G>A), L664V (2301C>G 2303T>C), N672A (2325A>G 2326A>C 2327T>A), P674A (2331C>G 2333G>A), A688S (2373G>A 2374C>G 2375G>T), T689K (2377C>A 2378T>A), W691* (2384G>A), Q695N (2394C>A 2396G>C), T696E (2397A>G 2398C>A 2399T>A), H699D (2406C>G), V701I (2412G>A 2414G>C)								

	Begin	End	Coverage	Score	Concordance	Matches	Identities	I/D/M/F*	Stop Codons
**NT**	**1645**	**2417**	**27.0%**	**534**	**36.9%**	**728 (99.9%)**	**500 (68.6%)**	**0/1**	
Codon mutations:	GCC448GCG (1655C>G), CTG449CTT (1658G>T), TCA450TCC (1661A>C), TTG454ATG (1671T>A), GTA455GTG (1676A>G), TTG457CTA (1680T>C 1682G>A), GCG458GCT (1685G>T), GAG460AAA (1689G>A 1691G>A), AAG461TAT (1692A>T 1694G>T), GTC462GCA (1696T>C 1697C>A), CCG464CCA (1703G>A), GGC465ACG (1704G>A 1705G>C 1706C>G), CGT466AGT (1707C>A), AGT467ACT (1711G>C), CGG468ACC (1713C>A 1714G>C 1715G>C), TTC470TTT (1721C>T), TGG471GCT (1722T>G 1723G>C 1724G>T), GAA472GAT (1727A>T), TAT473TAC (1730T>C), CTT475CTG (1736T>G), CTC476TTA (1737C>T 1739C>A), ATT480ATC (1751T>C), CTC481CTA (1754C>A), ATT482ATC (1757T>C), GCC483ACC (1758G>A), GAG485ACA (1764G>A 1765A>C 1766G>A), GTC487GTG (1772C>G), GGC488GCG (1774G>C 1775C>G), AAG489CGG (1776A>C 1777A>G), CAA490CAG (1781A>G), TAT491TAC (1784T>C), GCG492AGG (1785G>A 1786C>G), GAA493ATT (1788G>A 1789A>T 1790A>T), TAC494TTG (1792A>T 1793C>G), GAG496GAT (1799G>T), CGG497AGG (1800C>A), GGA499GGT (1808A>T), ACC501ACA (1814C>A), ATT502ATC (1817T>C), TCG503TCT (1820G>T), ATA504-TA (1821delA), CAT505GCT (1824C>G 1825A>C), ATA508AGA (1834T>G), TCG510TCC (1841G>C), AAT511GAG (1842A>G 1844T>G), AAT512AAC (1847T>C), GGT513GGA (1850T>A), TCT514ACC (1851T>A 1853T>C), CTT515ATC (1854C>A 1856T>C), TTC517TTT (1862C>T), GCA518GCT (1865A>T), AGG520AGA (1871G>A), ACT523ACC (1880T>C), TTA525GAC (1884T>G 1885T>A 1886A>C), GTT528ATT (1893G>A), ATT545GTG (1944A>G 1946T>G), CAG549CAA (1958G>A), ATT550CTC (1959A>C 1961T>C), GGG551GCA (1963G>C 1964G>A), ATG552ACC (1966T>C 1967G>C), GGA555TCG (1974G>T 1975G>C 1976A>G), CTT556ATT (1977C>A), CAA557TCA (1980C>T 1981A>C), ATG558ACA (1984T>C 1985G>A), TCC559TCG (1988C>G), GCT560ATT (1989G>A 1990C>T), CAA562CCC (1996A>C 1997A>C), CGT563ATT (1998C>A 1999G>T), CTA564TTT (2001C>T 2003A>T), GCT565TGG (2004G>T 2005C>G 2006T>G), GGC566GGG (2009C>G), GCA567GCC (2012A>C), GGC568GGA (2015C>A), TAT569TTC (2017A>T 2018T>C), AGG570AGA (2021G>A), CGA572CGT (2027A>T), CGC574CGT (2033C>T), CTT575TTG (2034C>T 2036T>G), ACG577TCT (2040A>T 2042G>T), CAG579CAA (2048G>A), CGC582CGT (2057C>T), AGG585AGA (2066G>A), ATT586CTG (2067A>C 2069T>G), AAG587AAA (2072G>A), GCT588GCG (2075T>G), GTT589GCT (2077T>C), GCT590GCA (2081T>A), TGT591AGC (2082T>A 2084T>C), TCT593CCA (2088T>C 2090T>A), TAC594CAC (2091T>C), ATA595AGG (2095T>G 2096A>G), CAG597CAT (2102G>T), CGG598CTA (2104G>T 2105G>A), TCT600CCT (2109T>C), CTT601CTC (2114T>C), TGG603TAT (2119G>A 2120G>T), GGA606GGG (2129A>G), AGG607AGA (2132G>A), GGA608TTT (2133G>T 2134G>T 2135A>T), AAG609AAA (2138G>A), CCC610CCT (2141C>T), GAT612AAT (2145G>A), TAT614TTT (2152A>T), TTA615TTG (2156A>G), AGA616AAG (2158G>A 2159A>G), GGT617GGA (2162T>A), ATC620ATA (2171C>A), GCT621GAT (2173C>A), CTT623CTC (2180T>C), CGT624CGG (2183T>G), GAG626GAA (2189G>A), AAA628ACA (2194A>C), CAG633CGA (2209A>G 2210G>A), TTG634CTC (2211T>C 2213G>C), ATA635TTT (2214A>T 2216A>T), AAA637AAT (2222A>T), CAA638GAG (2223C>G 2225A>G), CTG639TTG (2226C>T), TTA641TTT (2234A>T), GAA644GAG (2243A>G), CAT645AGA (2244C>A 2245A>G 2246T>A), GAG646GAA (2249G>A), CTA648CTT (2255A>T), GTA653TTA (2268G>T), CGT654CTC (2272G>T 2273T>C), CAT655AGA (2274C>A 2275A>G 2276T>A), ATG657GTG (2280A>G), GAA658AAA (2283G>A), CTT661CTG (2294T>G), CGA662AAA (2295C>A 2296G>A), CTT664GTC (2301C>G 2303T>C), TCA665TCT (2306A>T), TAT667TAC (2312T>C), CAC668CAT (2315C>T), GTG670GTT (2321G>T), AAT672GCA (2325A>G 2326A>C 2327T>A), TCC673TCA (2330C>A), CCG674GCA (2331C>G 2333G>A), GTG676GTA (2339G>A), ATA678ATT (2345A>T), GAC679GAT (2348C>T), CAA680CAG (2351A>G), CTT681TTG (2352C>T 2354T>G), GTG684GTT (2363G>T), GCG688AGT (2373G>A 2374C>G 2375G>T), ACT689AAA (2377C>A 2378T>A), TCT690TCC (2381T>C), TGG691TGA (2384G>A), CGT693AGA (2388C>A 2390T>A), ACT694ACA (2393T>A), CAG695AAC (2394C>A 2396G>C), ACT696GAA (2397A>G 2398C>A 2399T>A), CAT699GAT (2406C>G), TTA700TTG (2411A>G), GTG701ATC (2412G>A 2414G>C)								

*: Inserts / Deletes / Misaligned / Frameshifts

Analysis details

This analysis was performed with panviral2.64

NGS Details (UN60): Brazilian marseillevirus

Assembly

**Coverage Length**	224 (1 contig(s))
**Depth Of Coverage**	8722.3
**Number Of Reads**	20377
**Reads Per Million**	432.19 rpm (after QC)
**Ambiguities**	0
**Assembly Method**	de novo + reference guided assembly
**Consensus Caller**	Bcf Tools

Coverage Map

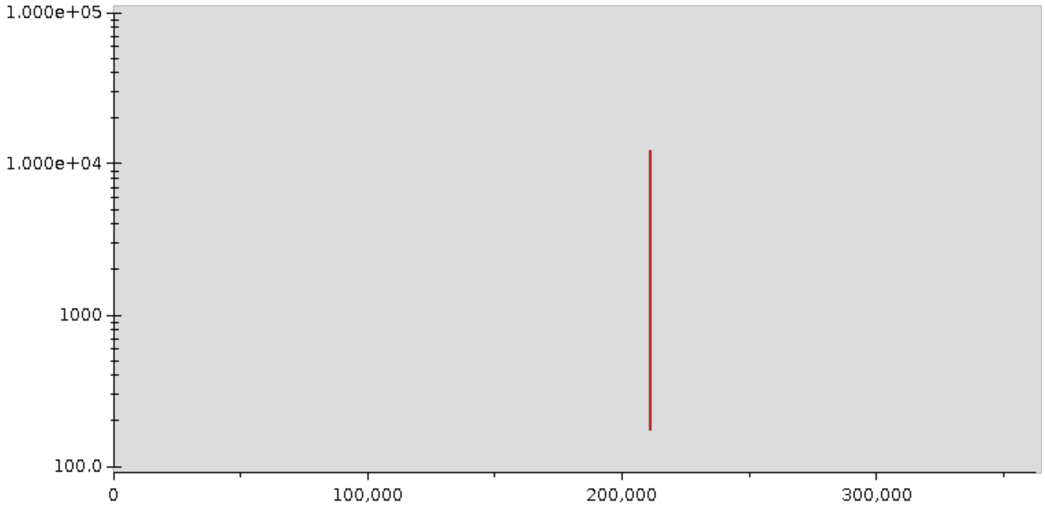

Assignment

**Type**	Brazilian marseillevirus (Taxonomy ID: 1813599)
**Reference Genome**	NC_029692.1
**NT Identity (%)**	82.5893
**AA Identity (%)**	93.0556
**Number Of Stop Codons**	0
**Number Of CDS**	491

Alignment

**Alignment Score**	292.0 (NT) + 432.0 (AA) = 724.0
**Concordance (%)**	80.9843
**Alignment Method**	Local, heuristic, nucleotide (BLASTN)

Genome Region

Sequence starts at position 210900 and ends at position 211123 relative to NC_029692.1 reference sequence.

Alignment Detailed Statistics

	Begin	End	Coverage	Score	Concordance	Matches	Identities	I/D/M/F*	Stop Codons
**NT**	**210900**	**211123**	**0.1%**	**292**	**65.2%**	**224 (100%)**	**185 (82.6%)**	**0/0**	

Mutations: 210914A>C, 210920G>T, 210926T>C, 210929T>C, 210932G>A, 210938A>G, 210953C>G, 210955T>G, 210956T>G, 210962T>C, 210965C>A, 210974G>A, 210977T>A, 210986A>C, 210988G>A, 210989G>C, 210995T>C, 211001A>T, 211004A>T, 211007A>G, 211009C>T, 211013T>C, 211031T>G, 211032T>G, 211033G>C, 211034T>C, 211037C>A, 211040G>A, 211043T>G, 211046G>A, 211061T>C, 211070G>C, 211079C>G, 211088C>G, 211091G>A, 211100T>A, 211103A>G, 211106C>G, 211108C>T

*: Inserts / Deletes / Misaligned / Frameshifts

Analysis details

This analysis was performed with panviral2.64

NGS Details (UN60): Brazilian marseillevirus

Assembly

**Coverage Length**	210 (1 contig(s))
**Depth Of Coverage**	8619.2
**Number Of Reads**	18320
**Reads Per Million**	388.56 rpm (after QC)
**Ambiguities**	0
**Assembly Method**	de novo + reference guided assembly
**Consensus Caller**	Bcf Tools

Coverage Map

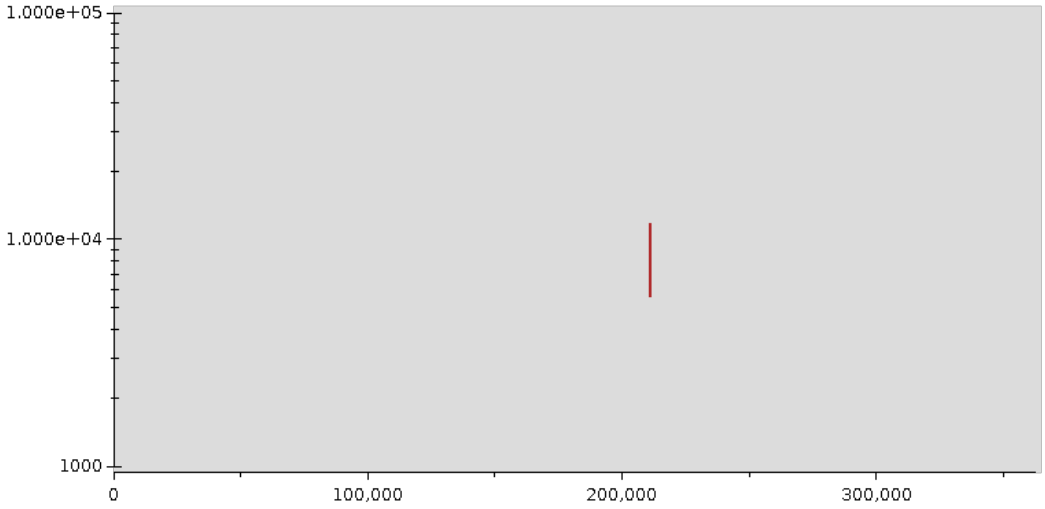

Assignment

**Type**	Brazilian marseillevirus (Taxonomy ID: 1813599)
**Reference Genome**	NC_029692.1
**NT Identity (%)**	79.5238
**AA Identity (%)**	95.5882
**Number Of Stop Codons**	1
**Number Of CDS**	491

Alignment

**Alignment Score**	248.0 (NT) + 412.0 (AA) = 660.0
**Concordance (%)**	78.1065
**Alignment Method**	Local, heuristic, nucleotide (BLASTN)

Genome Region

Sequence starts at position 210884 and ends at position 211093 relative to NC_029692.1 reference sequence.

Alignment Detailed Statistics

	Begin	End	Coverage	Score	Concordance	Matches	Identities	I/D/M/F*	Stop Codons
**NT**	**210884**	**211093**	**0.1%**	**248**	**59.0%**	**210 (100%)**	**167 (79.5%)**	**0/0**	

Mutations: 210896A>G, 210899T>A, 210901T>G, 210902T>G, 210914A>C, 210920G>T, 210926T>C, 210929T>C, 210935G>A, 210938A>G, 210941A>G, 210953C>G, 210955T>G, 210956T>G, 210965C>G, 210974G>A, 210983G>T, 210986A>C, 210989G>C, 210991G>T, 210992T>C, 210995T>C, 211001A>T, 211004A>T, 211007A>G, 211009C>T, 211010T>C, 211013T>C, 211025G>A, 211031T>A, 211032T>G, 211033G>C, 211040G>A, 211043T>G, 211046G>A, 211052G>A, 211058G>A, 211061T>C, 211064A>C, 211070G>C, 211072G>A, 211079C>G, 211082C>T

*: Inserts / Deletes / Misaligned / Frameshifts

Analysis details

This analysis was performed with panviral2.64

NGS Details (UN60): Brazilian marseillevirus

Assembly

**Coverage Length**	236 (1 contig(s))
**Depth Of Coverage**	5214.8
**Number Of Reads**	12747
**Reads Per Million**	270.36 rpm (after QC)
**Ambiguities**	0
**Assembly Method**	de novo + reference guided assembly
**Consensus Caller**	Bcf Tools

Coverage Map

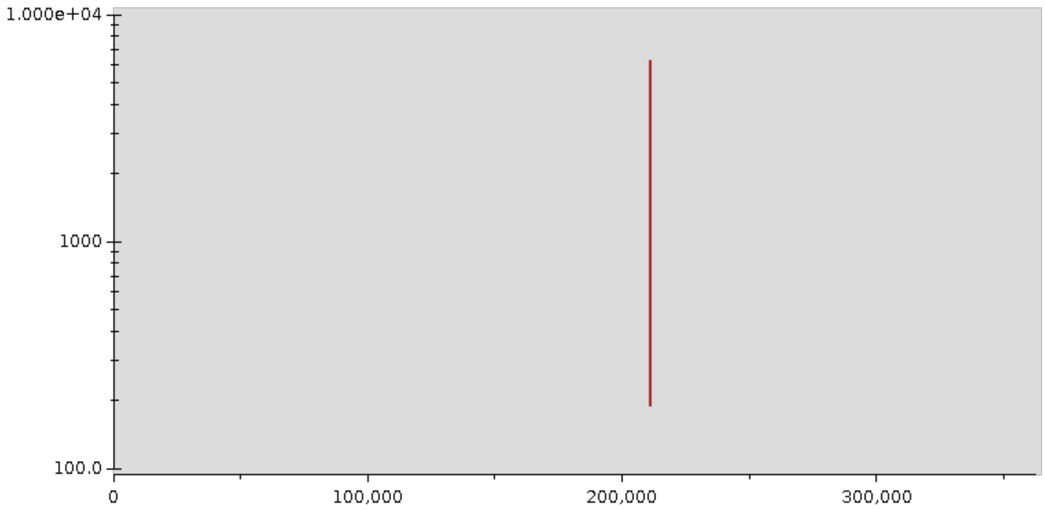

Assignment

**Type**	Brazilian marseillevirus (Taxonomy ID: 1813599)
**Reference Genome**	NC_029692.1
**NT Identity (%)**	79.2373
**AA Identity (%)**	94.6667
**Number Of Stop Codons**	1
**Number Of CDS**	491

Alignment

**Alignment Score**	276.0 (NT) + 454.0 (AA) = 730.0
**Concordance (%)**	77.6596
**Alignment Method**	Local, heuristic, nucleotide (BLASTN)

Genome Region

Sequence starts at position 210888 and ends at position 211123 relative to NC_029692.1 reference sequence.

Alignment Detailed Statistics

	Begin	End	Coverage	Score	Concordance	Matches	Identities	I/D/M/F*	Stop Codons
**NT**	**210888**	**211123**	**0.1%**	**276**	**58.5%**	**236 (100%)**	**187 (79.2%)**	**0/0**	

Mutations: 210899T>C, 210902T>C, 210905G>A, 210908A>C, 210910G>A, 210914A>G, 210917A>C, 210920G>C, 210926T>C, 210929T>C, 210932G>A, 210938A>G, 210947A>G, 210953C>G, 210955T>G, 210956T>G, 210962T>C, 210965C>T, 210974G>A, 210977T>A, 210980G>A, 210986A>C, 210988G>A, 210989G>C, 210991G>T, 210992T>C, 210995T>C, 211001A>T, 211004A>T, 211007A>G, 211009C>T, 211010T>C, 211013T>C, 211031T>G, 211032T>G, 211033G>C, 211034T>C, 211037C>G, 211040G>A, 211043T>G, 211046G>A, 211061T>C, 211070G>C, 211079C>G, 211088C>G, 211094A>G, 211100T>A, 211106C>A, 211108C>T

*: Inserts / Deletes / Misaligned / Frameshifts

Analysis details

This analysis was performed with panviral2.64

NGS Details (UN60): Brazilian marseillevirus

Assembly

**Coverage Length**	161 (1 contig(s))
**Depth Of Coverage**	6274.7
**Number Of Reads**	11487
**Reads Per Million**	243.63 rpm (after QC)
**Ambiguities**	0
**Assembly Method**	de novo + reference guided assembly
**Consensus Caller**	Bcf Tools

Coverage Map

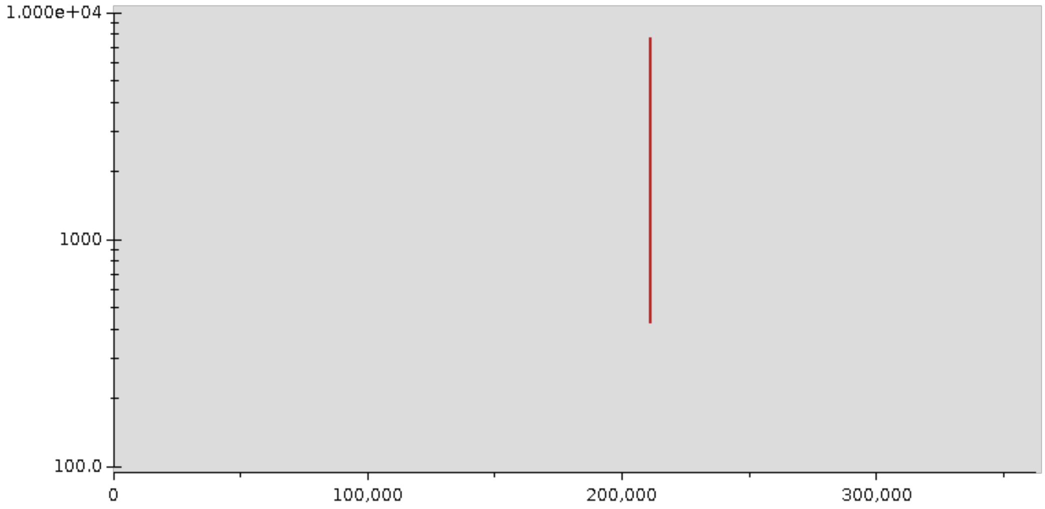

Assignment

**Type**	Brazilian marseillevirus (Taxonomy ID: 1813599)
**Reference Genome**	NC_029692.1
**NT Identity (%)**	81.3665
**AA Identity (%)**	92.5926
**Number Of Stop Codons**	0
**Number Of CDS**	491

Alignment

**Alignment Score**	202.0 (NT) + 335.0 (AA) = 537.0
**Concordance (%)**	80.0298
**Alignment Method**	Local, heuristic, nucleotide (BLASTN)

Genome Region

Sequence starts at position 210894 and ends at position 211054 relative to NC_029692.1 reference sequence.

Alignment Detailed Statistics

	Begin	End	Coverage	Score	Concordance	Matches	Identities	I/D/M/F*	Stop Codons
**NT**	**210894**	**211054**	**0.1%**	**202**	**62.7%**	**161 (100%)**	**131 (81.4%)**	**0/0**	

Mutations: 210908A>C, 210910G>A, 210917A>G, 210920G>A, 210926T>C, 210929T>C, 210938A>G, 210944C>A, 210956T>A, 210965C>G, 210971C>T, 210974G>C, 210977T>A, 210980G>A, 210986A>C, 210989G>T, 210991G>T, 210992T>C, 210995T>C, 210998G>A, 211001A>T, 211004A>G, 211009C>T, 211010T>C, 211031T>G, 211032T>G, 211033G>T, 211034T>C, 211037C>G, 211043T>G

*: Inserts / Deletes / Misaligned / Frameshifts

Analysis details

This analysis was performed with panviral2.64

NGS Details (UN60): Makelovirus prm1

Assembly

**Coverage Length**	1013 (1 contig(s))
**Depth Of Coverage**	1317.0
**Number Of Reads**	10355
**Reads Per Million**	219.63 rpm (after QC)
**Ambiguities**	0
**Assembly Method**	de novo + reference guided assembly
**Consensus Caller**	Bcf Tools

Coverage Map

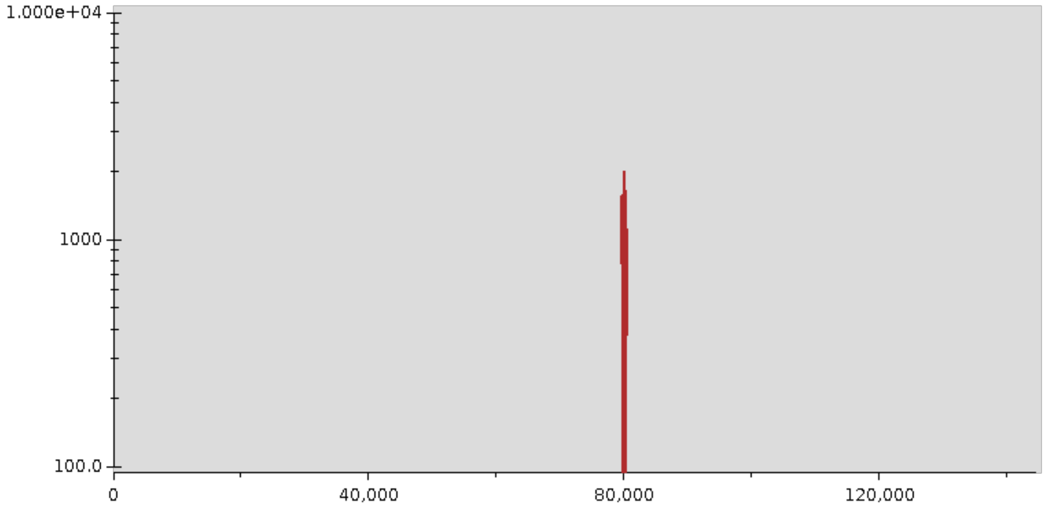

Assignment

**Type**	Makelovirus prm1 (Taxonomy ID: 2956181)
**Reference Genome**	NC_055761.1
**NT Identity (%)**	76.8016
**AA Identity (%)**	87.5371
**Number Of Stop Codons**	0
**Number Of CDS**	190

Alignment

**Alignment Score**	1086.0 (NT) + 2250.0 (AA) = 3336.0
**Concordance (%)**	74.3979
**Alignment Method**	Local, heuristic, nucleotide (BLASTN)

Genome Region

Sequence starts at position 79507 and ends at position 80519 relative to NC_055761.1 reference sequence.

Alignment Detailed Statistics

	Begin	End	Coverage	Score	Concordance	Matches	Identities	I/D/M/F*	Stop Codons
**NT**	**79507**	**80519**	**0.7%**	**1086**	**53.6%**	**1013 (100%)**	**778 (76.8%)**	**0/0**	
Mutations:	79519A>G, 79520A>T, 79522A>G, 79523A>C, 79529C>T, 79530G>A, 79532G>C, 79536G>A, 79541G>T, 79542T>A, 79543C>G, 79544T>C, 79547A>T, 79548A>G, 79550C>A, 79563G>A, 79565C>T, 79568T>A, 79574C>T, 79580G>T, 79581C>T, 79592A>T, 79595T>C, 79596C>T, 79598G>A, 79602G>A, 79604A>G, 79610C>T, 79611G>T, 79612T>C, 79613C>T, 79614T>G, 79615G>T, 79616C>A, 79619C>T, 79624C>T, 79628G>C, 79632G>A, 79643C>T, 79646T>A, 79649G>A, 79655C>T, 79658C>T, 79664C>T, 79673A>T, 79676C>T, 79677G>T, 79679T>A, 79682T>G, 79688G>A, 79689A>C, 79691G>T, 79694T>C, 79703C>T, 79712T>C, 79718T>C, 79719G>A, 79722G>A, 79724A>T, 79728T>A, 79733C>T, 79734A>G, 79735A>C, 79736C>A, 79739A>T, 79742T>A, 79746C>T, 79748T>A, 79751C>T, 79754C>T, 79760C>A, 79763T>C, 79772T>G, 79779C>G, 79791C>T, 79793C>A, 79799T>C, 79811C>T, 79812C>G, 79817G>A, 79818G>A, 79820C>T, 79823A>T, 79824T>C, 79826T>A, 79833C>T, 79835G>A, 79836A>C, 79838C>T, 79842A>G, 79844C>A, 79845T>G, 79846T>C, 79847C>T, 79850C>T, 79871A>G, 79874C>T, 79875T>A, 79876C>G, 79879A>T, 79880T>C, 79895T>A, 79898C>T, 79904C>T, 79905T>G, 79906G>C, 79907C>T, 79916C>T, 79919T>A, 79922A>T, 79931T>A, 79937A>T, 79938T>A, 79940T>C, 79946C>T, 79950C>T, 79953G>A, 79958T>C, 79961T>A, 79962T>A, 79973T>A, 79974T>A, 79975C>G, 79976G>T, 79979C>T, 79985C>T, 79994C>T, 79997C>A, 80000C>A, 80018C>T, 80025C>A, 80030C>A, 80039A>T, 80042A>G, 80054G>T, 80063C>A, 80066C>T, 80069T>C, 80073C>T, 80075T>A, 80078T>C, 80090C>A, 80096T>C, 80099T>C, 80105G>A, 80114A>T, 80120C>T, 80126T>C, 80127C>T, 80132T>A, 80139T>A, 80140C>G, 80141G>T, 80142C>T, 80145G>A, 80147T>C, 80148C>A, 80150T>G, 80159C>A, 80162G>A, 80165C>T, 80168G>A, 80171C>T, 80172C>G, 80173A>C, 80174G>T, 80177C>T, 80178T>G, 80180T>A, 80188A>G, 80189G>A, 80201A>G, 80207G>A, 80210C>T, 80213C>T, 80216C>T, 80222G>A, 80225T>C, 80237C>T, 80240C>T, 80243T>C, 80246C>A, 80247C>T, 80261C>T, 80265T>A, 80266C>G, 80267C>T, 80279C>T, 80286C>T, 80288G>A, 80291T>C, 80300T>A, 80306A>T, 80315T>A, 80318C>A, 80333T>C, 80336A>T, 80337C>T, 80339T>A, 80343G>A, 80346T>A, 80347C>G, 80351C>T, 80357A>T, 80366G>A, 80369C>T, 80378C>T, 80387G>A, 80390C>T, 80391A>G, 80393C>A, 80394A>G, 80396G>T, 80399T>C, 80400G>A, 80409A>C, 80410A>G, 80411A>T, 80414C>A, 80415C>A, 80417C>T, 80433G>A, 80435T>C, 80436C>A, 80438A>C, 80447C>T, 80453G>T, 80456G>T, 80462G>A, 80465A>T, 80471C>T, 80474G>A, 80486C>T, 80498G>A, 80504G>A								

*: Inserts / Deletes / Misaligned / Frameshifts

Analysis details

This analysis was performed with panviral2.64

NGS Details (UN60): Brazilian marseillevirus

Assembly

**Coverage Length**	111 (1 contig(s))
**Depth Of Coverage**	4288.3
**Number Of Reads**	9741
**Reads Per Million**	206.60 rpm (after QC)
**Ambiguities**	0
**Assembly Method**	de novo + reference guided assembly
**Consensus Caller**	Bcf Tools

Coverage Map

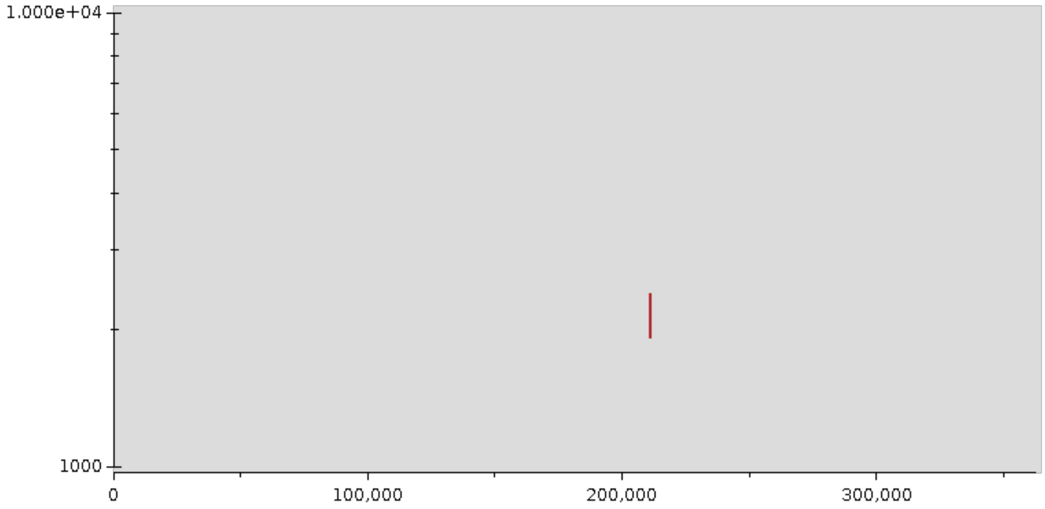

Assignment

**Type**	Brazilian marseillevirus (Taxonomy ID: 1813599)
**Reference Genome**	NC_029692.1
**NT Identity (%)**	81.982
**AA Identity (%)**	97.1429
**Number Of Stop Codons**	1
**Number Of CDS**	491

Alignment

**Alignment Score**	142.0 (NT) + 215.0 (AA) = 357.0
**Concordance (%)**	80.9524
**Alignment Method**	Local, heuristic, nucleotide (BLASTN)

Genome Region

Sequence starts at position 210884 and ends at position 210994 relative to NC_029692.1 reference sequence.

Alignment Detailed Statistics

	Begin	End	Coverage	Score	Concordance	Matches	Identities	I/D/M/F*	Stop Codons
**NT**	**210884**	**210994**	**0.1%**	**142**	**64.0%**	**111 (100%)**	**91 (82.0%)**	**0/0**	

Mutations: 210896A>G, 210899T>A, 210901T>G, 210902T>A, 210907C>A, 210914A>G, 210917A>G, 210920G>A, 210926T>C, 210929T>C, 210938A>G, 210941A>G, 210944C>A, 210947A>T, 210950G>T, 210953C>T, 210955T>G, 210962T>C, 210965C>A, 210983G>A

*: Inserts / Deletes / Misaligned / Frameshifts

Analysis details

This analysis was performed with panviral2.64

NGS Details (UN60): Yellowstone lake phycodnavirus 1

Assembly

**Coverage Length**	173 (1 contig(s))
**Depth Of Coverage**	3350.0
**Number Of Reads**	5848
**Reads Per Million**	124.03 rpm (after QC)
**Ambiguities**	0
**Assembly Method**	de novo + reference guided assembly
**Consensus Caller**	Bcf Tools

Coverage Map

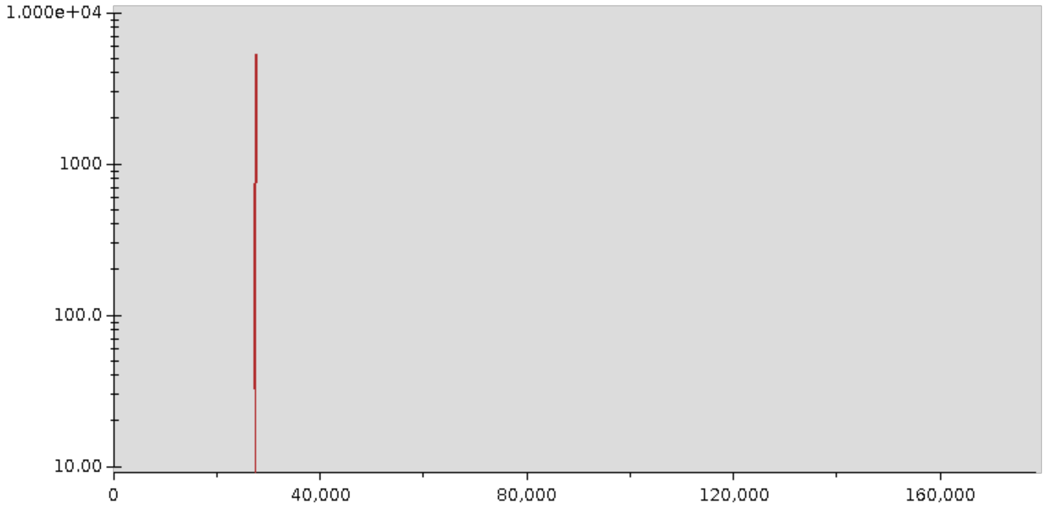

Assignment

**Type**	Yellowstone lake phycodnavirus 1 (Taxonomy ID: 1586713)
**Reference Genome**	NC_028112.1
**NT Identity (%)**	82.0809
**AA Identity (%)**	93.1034
**Number Of Stop Codons**	0
**Number Of CDS**	248

Alignment

**Alignment Score**	222.0 (NT) + 350.0 (AA) = 572.0
**Concordance (%)**	80.5634
**Alignment Method**	Local, heuristic, nucleotide (BLASTN)

Genome Region

Sequence starts at position 27469 and ends at position 27641 relative to NC_028112.1 reference sequence.

Alignment Detailed Statistics

	Begin	End	Coverage	Score	Concordance	Matches	Identities	I/D/M/F*	Stop Codons
**NT**	**27469**	**27641**	**0.1%**	**222**	**64.2%**	**173 (100%)**	**142 (82.1%)**	**0/0**	

Mutations: 27481T>G, 27486G>A, 27492A>G, 27510A>G, 27513T>C, 27516T>A, 27529C>A, 27531T>G, 27534A>G, 27537T>A, 27540T>C, 27543T>A, 27552A>G, 27558G>A, 27563A>G, 27564T>C, 27567T>C, 27573A>T, 27576A>G, 27582T>C, 27586G>A, 27591A>G, 27597A>G, 27600C>A, 27606T>G, 27609T>C, 27612A>T, 27616T>C, 27618G>C, 27624T>C, 27627C>T

*: Inserts / Deletes / Misaligned / Frameshifts

Analysis details

This analysis was performed with panviral2.64

NGS Details (UN60): Brazilian marseillevirus

Assembly

Coverage Length	240 (1 contig(s))
Depth Of Coverage	2083.3
Number Of Reads	5218
Reads Per Million	110.67 rpm (after QC)
Ambiguities	0
Assembly Method	de novo + reference guided assembly
Consensus Caller	Bcf Tools

Coverage Map

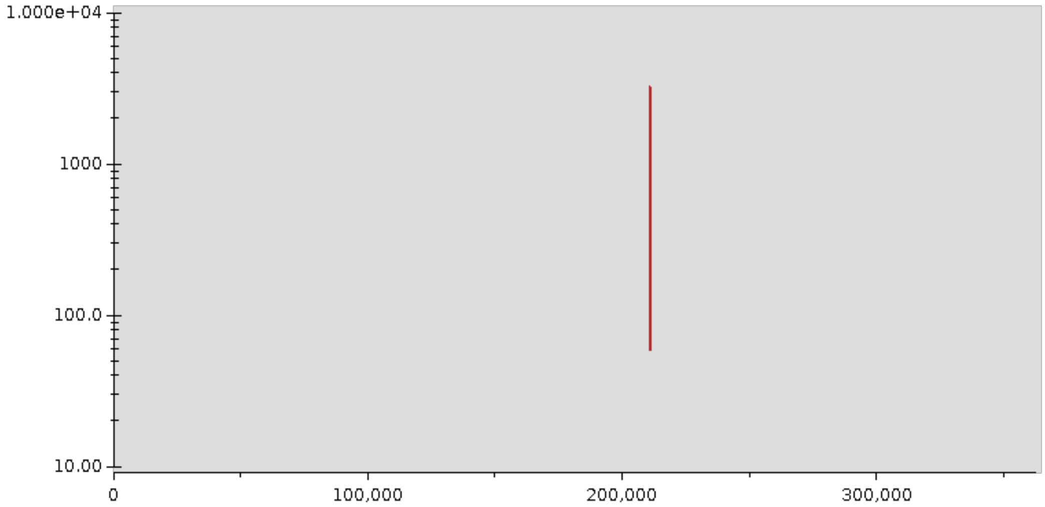

Assignment

Type	Brazilian marseillevirus (Taxonomy ID: 1813599)
Reference Genome	NC_029692.1
NT Identity (%)	79.5833
AA Identity (%)	94.6667
Number Of Stop Codons	1
Number Of CDS	491

Alignment

Alignment Score	284.0 (NT) + 454.0 (AA) = 738.0
Concordance (%)	77.8481
Alignment Method	Local, heuristic, nucleotide (BLASTN)

Genome Region

Sequence starts at position 210884 and ends at position 211123 relative to NC_029692.1 reference sequence.

Alignment Detailed Statistics

	Begin	End	Coverage	Score	Concordance	Matches	Identities	I/D/M/F*	Stop Codons
**NT**	**210884**	**211123**	**0.1%**	**284**	**59.2%**	**240 (100%)**	**191 (79.6%)**	**0/0**	

Mutations: 210896A>G, 210899T>A, 210901T>G, 210902T>G, 210914A>C, 210916G>A, 210920G>T, 210926T>C, 210938A>G, 210941A>G, 210944C>T, 210947A>C, 210950G>A, 210974G>C, 210980G>A, 210986A>C, 210989G>C, 210991G>T, 210992T>C, 210995T>C, 211001A>T, 211004A>G, 211009C>T, 211010T>G, 211025G>A, 211031T>A, 211032T>G, 211033G>C, 211034T>C, 211040G>A, 211043T>G, 211046G>A, 211049G>A, 211052G>A, 211058G>A, 211064A>C, 211070G>C, 211072G>A, 211076G>A, 211079C>G, 211082C>T, 211085T>C, 211088C>A, 211091G>C, 211093G>A, 211094A>T, 211100T>G, 211106C>G, 211108C>T

*: Inserts / Deletes / Misaligned / Frameshifts

Analysis details

This analysis was performed with panviral2.64

NGS Details (UN60): Yellowstone lake phycodnavirus 1

Assembly

**Coverage Length**	155 (1 contig(s))
**Depth Of Coverage**	2919.0
**Number Of Reads**	5137
**Reads Per Million**	108.95 rpm (after QC)
**Ambiguities**	0
**Assembly Method**	de novo + reference guided assembly
**Consensus Caller**	Bcf Tools

Coverage Map

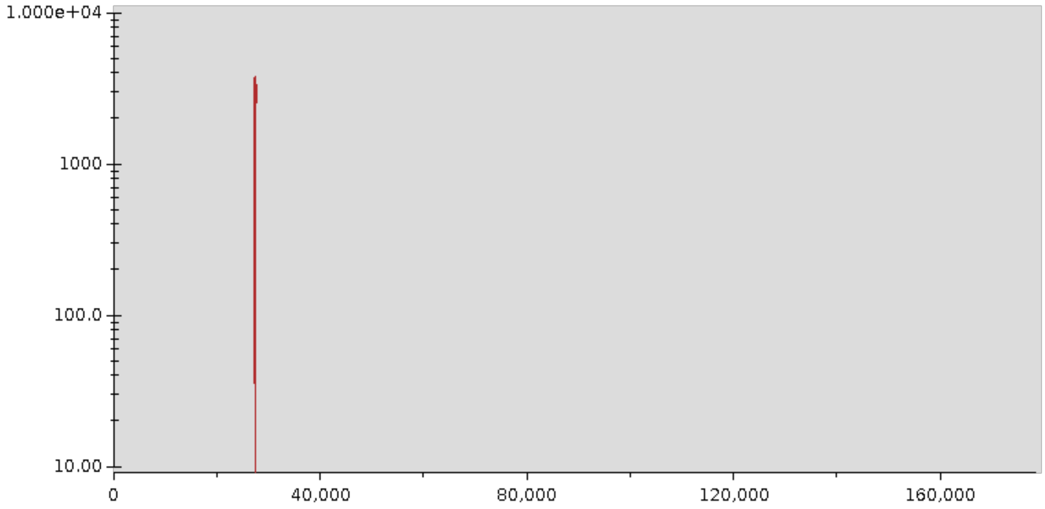

Assignment

**Type**	Yellowstone lake phycodnavirus 1 (Taxonomy ID: 1586713)
**Reference Genome**	NC_028112.1
**NT Identity (%)**	80.0
**AA Identity (%)**	90.3846
**Number Of Stop Codons**	0
**Number Of CDS**	248

Alignment

**Alignment Score**	186.0 (NT) + 299.0 (AA) = 485.0
**Concordance (%)**	77.476
**Alignment Method**	Local, heuristic, nucleotide (BLASTN)

Genome Region

Sequence starts at position 27397 and ends at position 27551 relative to NC_028112.1 reference sequence.

Alignment Detailed Statistics

	Begin	End	Coverage	Score	Concordance	Matches	Identities	I/D/M/F*	Stop Codons
NT	27397	27551	0.1%	186	60.0%	155 (100%)	124 (80.0%)	0/0	

Mutations: 27417T>C, 27420C>G, 27426G>C, 27427C>T, 27429T>G, 27432C>T, 27447T>C, 27456T>G, 27459G>A, 27460T>A, 27461C>G, 27462A>T, 27463G>T, 27464G>C, 27468T>C, 27470G>C, 27476C>A, 27477A>C, 27481T>G, 27483G>T, 27489T>C, 27492A>G, 27504G>A, 27510A>T, 27513T>C, 27516T>G, 27529C>A, 27531T>G, 27532C>T, 27534A>G, 27537T>A

*: Inserts / Deletes / Misaligned / Frameshifts

Analysis details

This analysis was performed with panviral2.64

NGS Details (UN60): Brazilian marseillevirus

Assembly

**Coverage Length**	241 (1 contig(s))
**Depth Of Coverage**	1024.8
**Number Of Reads**	2553
**Reads Per Million**	54.15 rpm (after QC)
**Ambiguities**	0
**Assembly Method**	de novo + reference guided assembly
**Consensus Caller**	Bcf Tools

Coverage Map

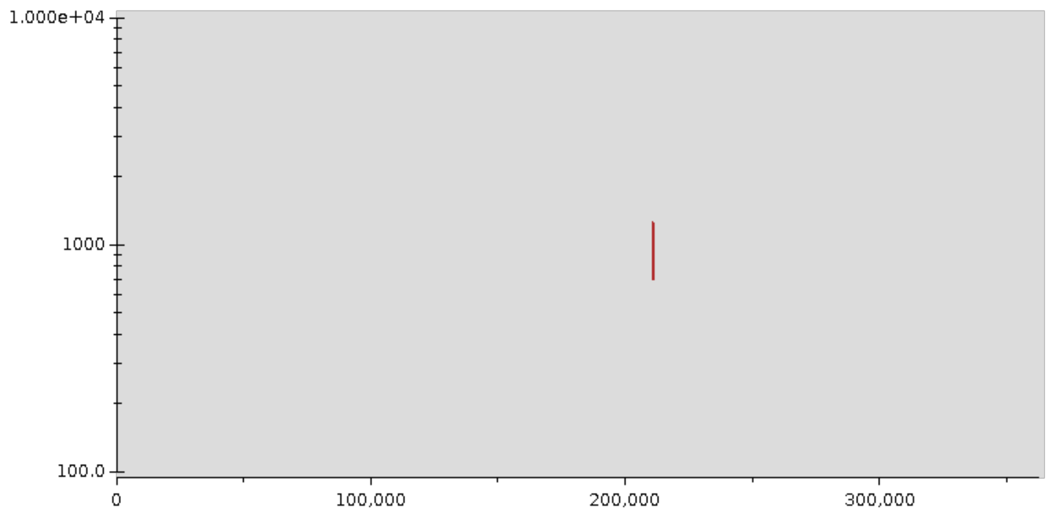

Assignment

**Type**	Brazilian marseillevirus (Taxonomy ID: 1813599)
**Reference Genome**	NC_029692.1
**NT Identity (%)**	80.9129
**AA Identity (%)**	94.6667
**Number Of Stop Codons**	1
**Number Of CDS**	491

Alignment

**Alignment Score**	298.0 (NT) + 454.0 (AA) = 752.0
**Concordance (%)**	79.1579
**Alignment Method**	Local, heuristic, nucleotide (BLASTN)

Genome Region

Sequence starts at position 210884 and ends at position 211124 relative to NC_029692.1 reference sequence.

Alignment Detailed Statistics

	Begin	End	Coverage	Score	Concordance	Matches	Identities	I/D/M/F*	Stop Codons
**NT**	**210884**	**211124**	**0.1%**	**298**	**61.8%**	**241 (100%)**	**195 (80.9%)**	**0/0**	

Mutations: 210899T>G, 210901T>G, 210902T>G, 210911G>A, 210917A>G, 210926T>C, 210938A>G, 210941A>G, 210944C>G, 210947A>T, 210950G>T, 210953C>T, 210955T>G, 210956T>G, 210965C>A, 210971C>T, 210974G>T, 210977T>G, 210980G>A, 210986A>C, 210989G>C, 210991G>T, 210992T>C, 210995T>C, 211004A>G, 211009C>T, 211010T>G, 211022C>T, 211025G>A, 211031T>A, 211032T>G, 211033G>C, 211037C>G, 211040G>A, 211043T>A, 211058G>A, 211061T>C, 211064A>G, 211070G>A, 211079C>G, 211088C>G, 211094A>C, 211100T>A, 211106C>G, 211108C>T, 211115T>C

*: Inserts / Deletes / Misaligned / Frameshifts

Analysis details

This analysis was performed with panviral2.64

NGS Details (UN60): Noumeavirus

Assembly

**Coverage Length**	225 (1 contig(s))
**Depth Of Coverage**	1143.9
**Number Of Reads**	2495
**Reads Per Million**	52.92 rpm (after QC)
**Ambiguities**	0
**Assembly Method**	de novo + reference guided assembly
**Consensus Caller**	Bcf Tools

Coverage Map

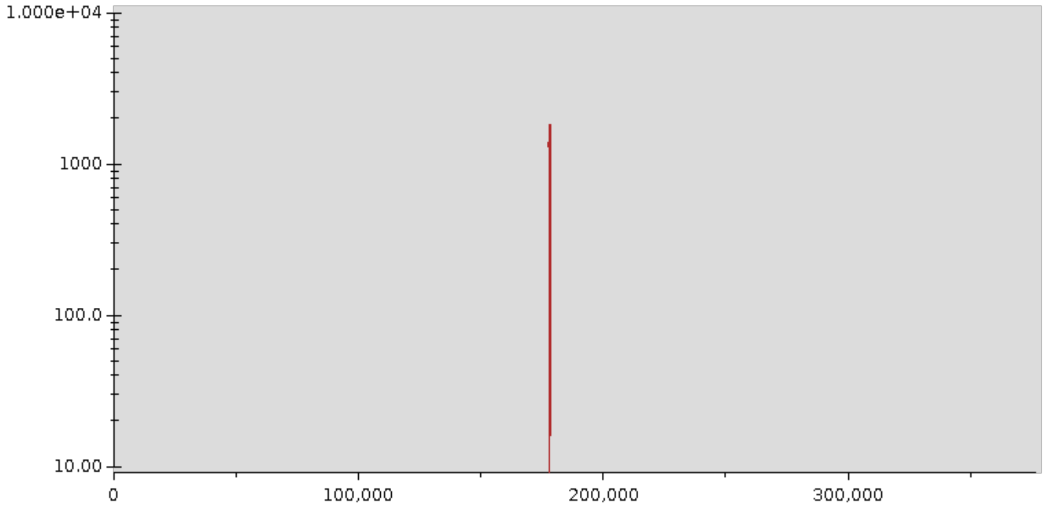

Assignment

**Type**	Noumeavirus (Taxonomy ID: 1955558)
**Reference Genome**	NC_033775.1
**NT Identity (%)**	78.6667
**AA Identity (%)**	95.9459
**Number Of Stop Codons**	1
**Number Of CDS**	452

Alignment

**Alignment Score**	258.0 (NT) + 450.0 (AA) = 708.0
**Concordance (%)**	77.5465
**Alignment Method**	Local, heuristic, nucleotide (BLASTN)

Genome Region

Sequence starts at position 177923 and ends at position 178147 relative to NC_033775.1 reference sequence.

Alignment Detailed Statistics

	Begin	End	Coverage	Score	Concordance	Matches	Identities	I/D/M/F*	Stop Codons
**NT**	**177923**	**178147**	**0.1%**	**258**	**57.3%**	**225 (100%)**	**177 (78.7%)**	**0/0**	

Mutations: 177947A>C, 177950A>T, 177956G>A, 177959C>T, 177962C>T, 177965C>G, 177968G>A, 177971G>T, 177975A>T, 177976G>C, 177978T>A, 177979C>G, 177980A>C, 177983C>T, 177986C>A, 177992G>C, 177995C>T, 178001A>G, 178002C>G, 178003A>C, 178004A>C, 178010T>A, 178019A>G, 178028C>T, 178031T>A, 178044C>A, 178049T>G, 178052C>T, 178055C>T, 178058C>T, 178061A>T, 178064A>G, 178079A>C, 178080A>C, 178082G>C, 178085T>C, 178086C>T, 178088T>G, 178091C>G, 178097T>C, 178101G>A, 178106A>G, 178118A>T, 178121G>A, 178127T>G, 178130T>G, 178131T>C, 178136A>G

*: Inserts / Deletes / Misaligned / Frameshifts

Analysis details

This analysis was performed with panviral2.64

NGS Details (UN60): Betabaculovirus disaccharalis

Assembly

**Coverage Length**	128 (1 contig(s))
**Depth Of Coverage**	1183.5
**Number Of Reads**	2005
**Reads Per Million**	42.53 rpm (after QC)
**Ambiguities**	0
**Assembly Method**	de novo + reference guided assembly
**Consensus Caller**	Bcf Tools

Coverage Map

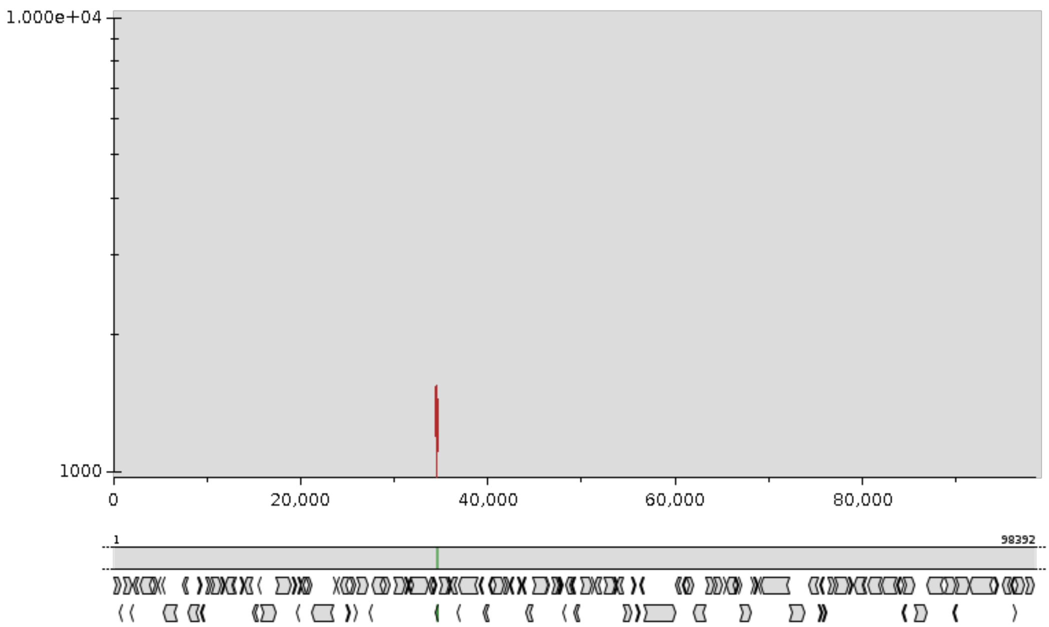

Assignment

**Type**	Betabaculovirus disaccharalis (Taxonomy ID: 3047795)
**Reference Genome**	NC_028491.1
**NT Identity (%)**	82.0312
**AA Identity (%)**	93.0233
**Number Of Stop Codons**	0
**Number Of CDS**	125

Alignment

**Alignment Score**	164.0 (NT) + 276.0 (AA) = 440.0
**Concordance (%)**	81.0313

**Alignment Method**	Local, heuristic, nucleotide (BLASTN)

Genome Region

Sequence starts at position 34471 and ends at position 34598 relative to NC_028491.1 reference sequence.

Alignment Detailed Statistics

	Begin	End	Coverage	Score	Concordance	Matches	Identities	I/D/M/F*	Stop Codons
**NT**	**34471**	**34598**	**0.1%**	**164**	**64.1%**	**128 (100%)**	**105 (82.0%)**	**0/0**	
Mutations:	34482G>T, 34485T>C, 34488A>C, 34490G>A, 34491A>C, 34512G>T, 34513C>G, 34514T>A, 34521T>C, 34524A>G, 34533A>G, 34538A>C, 34545T>C, 34547G>T, 34548C>G, 34549T>C, 34551G>A, 34554T>C, 34569G>A, 34575T>A, 34578T>C, 34580A>G, 34587T>C								

CDS

	Begin	End	Coverage	Score	Concordance	Matches	Identities	I/D/M/F*	Stop Codons
v-ubq	60	102	36.1%	276	89.3%	43 (100%)	40 (93.0%)	0/0/0/0	0
Protein mutations:	E76G (34548C>G 34549T>C), S80A (34538A>C)								
Codon mutations:	CAA63CAG (34587T>C), TTA66CTG (34578T>C 34580A>G), ATA67ATT (34575T>A), GCC69GCT (34569G>A), GAA74GAG (34554T>C), GAC75GAT (34551G>A), GAG76GGC (34548C>G 34549T>C), CGA77AGG (34545T>C 34547G>T), TCA80GCA (34538A>C), GAT81GAC (34533A>G), ATT84ATC (34524A>G), CAA85CAG (34521T>C), AGC88TCA (34512G>T 34513C>G 34514T>A), CGT95CGG (34491A>C), CTT96TTG (34488A>C 34490G>A), AGA97AGG (34485T>C), GGC98GGA (34482G>T)								

Proteins

	Begin	End	Coverage	Score	Concordance	Matches	Identities	I/D/M/F*	Stop Codons
ubiquitin-like protein (YP_009182246.1)	60	102	36.1%	276	89.3%	43 (100%)	40 (93.0%)	0/0/0/0	0
Protein mutations:	E76G (34548C>G 34549T>C), S80A (34538A>C)								
Codon mutations:	CAA63CAG (34587T>C), TTA66CTG (34578T>C 34580A>G), ATA67ATT (34575T>A), GCC69GCT (34569G>A), GAA74GAG (34554T>C), GAC75GAT (34551G>A), GAG76GGC (34548C>G 34549T>C), CGA77AGG (34545T>C 34547G>T), TCA80GCA (34538A>C), GAT81GAC (34533A>G), ATT84ATC (34524A>G), CAA85CAG (34521T>C), AGC88TCA (34512G>T 34513C>G 34514T>A), CGT95CGG (34491A>C), CTT96TTG (34488A>C 34490G>A), AGA97AGG (34485T>C), GGC98GGA (34482G>T)								

*: Inserts / Deletes / Misaligned / Frameshifts

Analysis details

This analysis was performed with panviral2.64

NGS Details (UN60): Betabaculovirus disaccharalis

Assembly

**Coverage Length**	150 (1 contig(s))
**Depth Of Coverage**	1065.4
**Number Of Reads**	1706
**Reads Per Million**	36.18 rpm (after QC)
**Ambiguities**	0
**Assembly Method**	de novo + reference guided assembly
**Consensus Caller**	Bcf Tools

Coverage Map

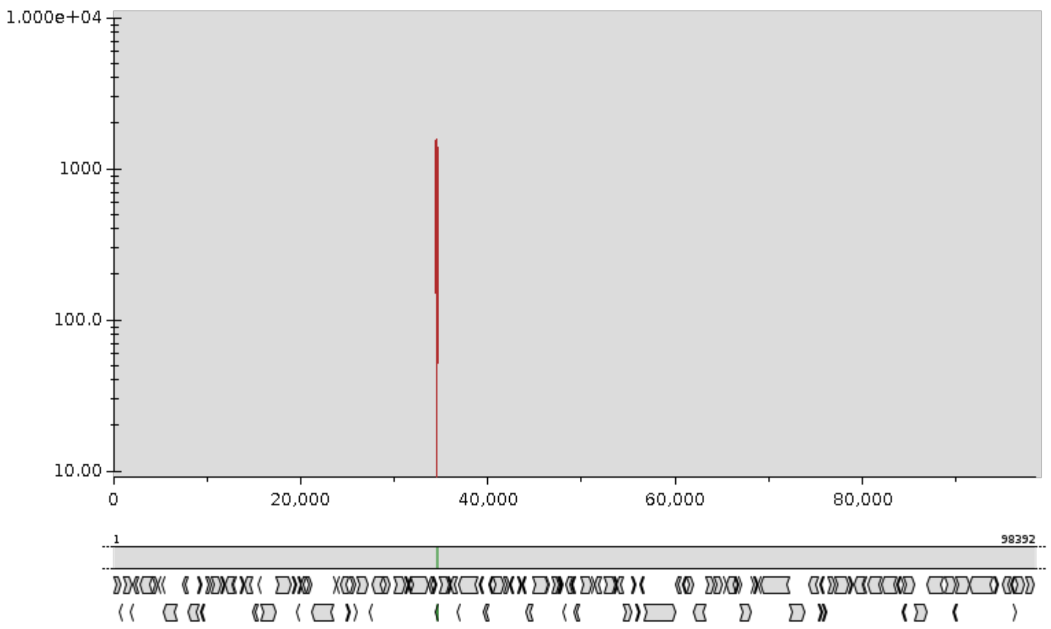

Assignment

**Type**	Betabaculovirus disaccharalis (Taxonomy ID: 3047795)
**Reference Genome**	NC_028491.1
**NT Identity (%)**	84.6667
**AA Identity (%)**	92.0
**Number Of Stop Codons**	0
**Number Of CDS**	125

Alignment

**Alignment Score**	208.0 (NT) + 314.0 (AA) = 522.0
**Concordance (%)**	83.3866

Alignment Method	Local, heuristic, nucleotide (BLASTN)

Genome Region

Sequence starts at position 34474 and ends at position 34623 relative to NC_028491.1 reference sequence.

Alignment Detailed Statistics

	Begin	End	Coverage	Score	Concordance	Matches	Identities	I/D/M/F*	Stop Codons
**NT**	**34474**	**34623**	**0.2%**	**208**	**69.3%**	**150 (100%)**	**127 (84.7%)**	**0/0**	
Mutations:	34485T>C, 34488A>C, 34490G>A, 34513C>G, 34514T>A, 34521T>C, 34524A>G, 34533A>G, 34538A>C, 34542T>A, 34545T>C, 34547G>T, 34549T>C, 34554T>C, 34569G>A, 34575T>G, 34578T>C, 34587T>C, 34593C>T, 34596C>G, 34599C>A, 34601C>T, 34602T>A								
**CDS**									
v-ubq	52	101	42.0%	314	91.3%	50 (100%)	46 (92.0%)	0/0/0/0	0
Protein mutations:	V59I (34599C>A 34601C>T), E76G (34549T>C), S80A (34538A>C)								
Codon mutations:	GGA58GGT (34602T>A), GTG59ATT (34599C>A 34601C>T), CCG60CCC (34596C>G), CCG61CCA (34593C>T), CAA63CAG (34587T>C), TTA66TTG (34578T>C), ATA67ATC (34575T>G), GCC69GCT (34569G>A), GAA74GAG (34554T>C), GAG76GGG (34549T>C), CGA77AGG (34545T>C 34547G>T), ACA78ACT (34542T>A), TCA80GCA (34538A>C), GAT81GAC (34533A>G), ATT84ATC (34524A>G), CAA85CAG (34521T>C), AGC88TCC (34513C>G 34514T>A), CTT96TTG (34488A>C 34490G>A), AGA97AGG (34485T>C)								
**Proteins**									
ubiquitin-like protein (YP_009182246.1)	52	101	42.0%	314	91.3%	50 (100%)	46 (92.0%)	0/0/0/0	0
Protein mutations:	V59I (34599C>A 34601C>T), E76G (34549T>C), S80A (34538A>C)								
Codon mutations:	GGA58GGT (34602T>A), GTG59ATT (34599C>A 34601C>T), CCG60CCC (34596C>G), CCG61CCA (34593C>T), CAA63CAG (34587T>C), TTA66TTG (34578T>C), ATA67ATC (34575T>G), GCC69GCT (34569G>A), GAA74GAG (34554T>C), GAG76GGG (34549T>C), CGA77AGG (34545T>C 34547G>T), ACA78ACT (34542T>A), TCA80GCA (34538A>C), GAT81GAC (34533A>G), ATT84ATC (34524A>G), CAA85CAG (34521T>C), AGC88TCC (34513C>G 34514T>A), CTT96TTG (34488A>C 34490G>A), AGA97AGG (34485T>C)								

*: Inserts / Deletes / Misaligned / Frameshifts

Analysis details

This analysis was performed with panviral2.64

NGS Details (UN60): Cladosporium fulvum T-1 virus

Assembly

**Coverage Length**	1543 (4 contig(s))
**Depth Of Coverage**	46.5
**Number Of Reads**	605
**Reads Per Million**	12.83 rpm (after QC)
**Ambiguities**	0
**Assembly Method**	de novo + reference guided assembly
**Consensus Caller**	Bcf Tools

Coverage Map

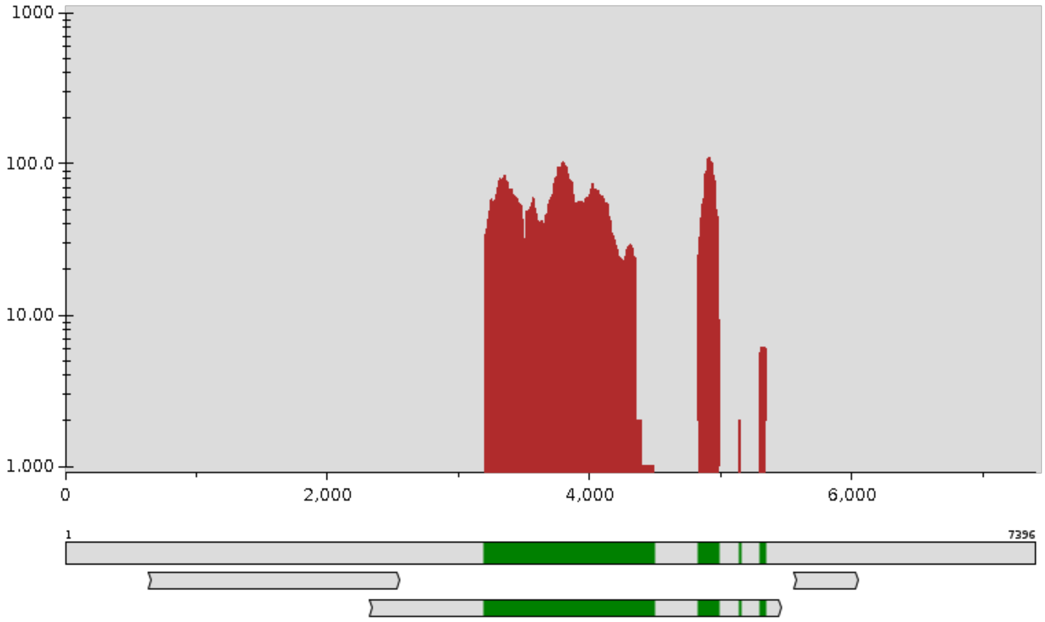

Assignment

**Type**	Cladosporium fulvum T-1 virus (Taxonomy ID: 2052899)
**Reference Genome**	NC_043491.1
**NT Identity (%)**	52.2078
**AA Identity (%)**	47.4708
**Number Of Stop Codons**	0
**Number Of CDS**	3

Alignment

**Alignment Score**	88.0 (NT) + 1723.0 (AA) = 1811.0
**Concordance (%)**	27.5563

Alignment Method	Global, seeded, nucleotide + amino acids (AGA)

Genome Region

Sequence starts at position 3196 and ends at position 5342 relative to NC_043491.1 reference sequence.

Alignment Detailed Statistics

	Begin	End	Coverage	Score	Concordance	Matches	Identities	I/D/M/F*	Stop Codons
**NT**	**3196**	**5342**	**20.9%**	**88**	**2.9%**	**1528 (98.3%)**	**804 (51.7%)**	**12/15**	
Mutations:	3204T>A, 3205C>G, 3207T>A, 3208T>C, 3210A>C, 3211A>T, 3214G>T, 3216A>T, 3217A>G, 3219G>T, 3220A>G, 3221G>C, 3222G>C, 3223C>A, 3224A>C, 3225A>C, 3226G>T, 3229C>G, 3230G>T, 3231C>T, 3232C>G, 3233T>C, 3234T>C, 3236C>A, 3237C>A, 3238T>G, 3243A>T, 3244C>G, 3246A>C, 3251T>A, 3253_3254insAGGGATATT, 3261A>G, 3262G>A, 3265A>T, 3266A>G, 3268C>G, 3269A>T, 3271T>G, 3272C>A, 3273A>T, 3280G>T, 3281A>T, 3282A>C, 3283A>T, 3284G>A, 3285A>C, 3289T>G, 3292A>T, 3293T>G, 3294G>C, 3295G>A, 3296G>C, 3297G>A, 3299C>G, 3301C>T, 3303T>C, 3304A>T, 3307T>C, 3308C>A, 3309A>G, 3314T>G, 3317G>C, 3318A>C, 3319G>T, 3326C>T, 3328A>G, 3329C>G, 3330A>C, 3331G>T, 3332A>G, 3333C>A, 3334C>A, 3337A>G, 3338C>A, 3340A>G, 3341G>A, 3343A>G, 3344T>C, 3345G>A, 3352G>C, 3355G>A, 3356A>T, 3357A>T, 3361A>T, 3363C>A, 3364C>T, 3365A>T, 3366A>C, 3371T>C, 3372G>T, 3376A>T, 3378G>A, 3379A>G, 3381G>C, 3382A>T, 3385C>T, 3387C>A, 3388C>G, 3389T>G, 3391A>T, 3392A>C, 3393G>C, 3394T>C, 3395G>T, 3396C>A, 3397A>T, 3400A>T, 3401A>G, 3403T>A, 3406A>T, 3407T>G, 3408G>T, 3409C>T, 3410A>T, 3412G>A, 3415C>T, 3416G>C, 3417T>A, 3418T>G, 3419C>A, 3420C>A, 3425G>C, 3426C>A, 3427A>G, 3428A>G, 3430C>T, 3434A>T, 3435A>C, 3437C>T, 3440C>A, 3442A>G, 3443C>A, 3445C>G, 3446G>T, 3447T>G, 3448A>C, 3449C>A, 3450A>T, 3451A>G, 3457C>T, 3460A>G, 3461A>G, 3462A>C, 3464T>C, 3470G>A, 3473A>G, 3478G>T, 3479A>G, 3481C>G, 3488C>A, 3489G>A, 3490A>G, 3496G>A, 3499A>T, 3502C>T, 3504A>G, 3505C>G, 3508C>T, 3511A>T, 3514A>T, 3515G>T, 3516C>T, 3517A>G, 3518C>A, 3519A>T, 3520A>G, 3526A>G, 3529A>G, 3531C>G, 3533G>A, 3534G>A, 3536T>G, 3538A>G, 3539G>T, 3540A>G, 3546A>T, 3550G>T, 3554A>C, 3556T>A, 3559C>T, 3562A>T, 3563C>A, 3565A>G, 3566G>T, 3567A>C, 3568C>A, 3570C>G, 3573T>A, 3574C>T, 3576A>G, 3577T>G, 3578G>C, 3579C>A, 3580T>G, 3581A>G, 3583C>T, 3584C>A, 3586A>G, 3589G>A, 3598A>G, 3601A>T, 3604A>G, 3605T>C, 3606G>C, 3607G>T, 3610A>G, 3613C>T, 3614G>A, 3618T>G, 3619C>T, 3620A>G, 3621G>T, 3622G>A, 3625A>T, 3628A>G, 3631C>T, 3634A>G, 3635C>T, 3636T>C, 3637C>A, 3640C>T, 3647T>C, 3649G>T, 3652C>A, 3658A>G, 3659A>T, 3661G>T, 3664A>G, 3670C>T, 3673C>T, 3676A>C, 3679C>G, 3682A>T, 3683T>A, 3687G>T, 3688C>T, 3689C>T, 3690A>G, 3691G>C, 3692G>A, 3694C>T, 3695C>T, 3697T>A, 3698G>A, 3700C>G, 3703C>T, 3706A>C, 3707A>G, 3708C>T, 3712T>G, 3713A>T, 3714G>T, 3715A>T, 3719C>T, 3720T>A, 3721A>T, 3724C>T, 3727C>T, 3729T>A, 3730G>T, 3732G>T, 3733C>T, 3736C>A, 3739T>A, 3741C>T, 3742T>C, 3745C>T, 3746A>C, 3748G>A, 3751C>T, 3757A>T, 3758C>G, 3760G>C, 3761G>A, 3763C>A, 3766C>T, 3768C>G, 3769A>T, 3770A>C, 3773_3775delGGA, 3776T>A, 3778C>G, 3779C>T, 3781C>G, 3782C>G, 3784G>T, 3791A>G, 3792C>T, 3793C>T, 3795A>G, 3796G>C, 3799A>T, 3800G>T, 3802T>G, 3803C>A, 3804A>G, 3805A>T, 3806G>T, 3807A>T, 3808T>G, 3811G>A, 3814C>A, 3815G>T, 3816A>C, 3817A>T, 3820A>T, 3821C>T, 3823C>A, 3825C>G, 3827A>C, 3829G>A, 3831C>A, 3833G>A, 3834G>C, 3835A>T, 3836T>C, 3839A>T, 3841G>T, 3842A>G, 3843C>T, 3844A>T, 3845G>A, 3846C>A, 3848C>A, 3849C>T, 3850C>G, 3856A>G, 3859C>T, 3862A>G, 3865C>T, 3866C>G, 3867A>C, 3869A>C, 3871G>A, 3877A>G, 3878G>A, 3880C>A, 3886T>C, 3887T>C, 3892C>G, 3893T>C, 3894T>A, 3896A>C, 3898C>A, 3899A>G, 3901C>T, 3905A>G, 3906C>A, 3909C>A, 3910A>C, 3911G>C, 3912G>A, 3913G>A, 3914A>G, 3918C>G, 3922C>G, 3925C>T, 3929G>A, 3930C>A, 3931A>G, 3934G>A, 3935A>G, 3936C>T, 3937A>G, 3940G>A, 3941T>G, 3943A>C, 3946C>T, 3947A>G, 3948G>T, 3949A>T, 3952A>T, 3957C>A, 3960A>C, 3964G>C, 3967G>A, 3970A>T, 3973C>G, 3980G>C, 3982A>G, 3983C>A, 3984A>G, 3988A>T, 3992C>T, 3994T>G, 3997A>G, 4000C>A, 4003C>T, 4010A>T, 4012C>T, 4025A>G, 4026A>C, 4027G>T, 4029A>G, 4030C>A, 4033T>C, 4041C>A, 4048A>T, 4049C>G, 4052A>C, 4057G>T, 4058A>G, 4059T>A, 4060G>T, 4061C>T, 4063T>G, 4064A>C, 4065C>T, 4066A>G, 4068G>A, 4069A>G, 4072A>G, 4075C>A, 4081C>G, 4085A>G, 4086A>T, 4091G>T, 4092G>C, 4093A>G, 4094A>G, 4095A>T, 4097G>A, 4098A>G, 4100C>T, 4101A>G, 4102G>C, 4103A>G, 4104C>A, 4105C>A, 4111G>T, 4114C>T, 4115A>C, 4117A>G, 4120A>T, 4121C>T, 4123C>G, 4130C>G, 4131A>C, 4132G>T, 4133T>A, 4134G>T, 4135C>T, 4138T>A, 4143C>A, 4144C>A, 4149C>T, 4150G>A, 4153T>G, 4154C>A, 4155G>A, 4156A>G, 4159A>G, 4160T>C, 4161T>C, 4162C>A, 4165T>C, 4166G>T, 4167G>T, 4169A>G, 4170G>A, 4171C>G, 4172A>C, 4173A>T, 4174G>A, 4175G>C, 4176A>C, 4177A>T, 4178G>T, 4180C>T, 4181C>G, 4183C>G, 4184A>G, 4186C>A, 4187G>C, 4189G>C, 4192C>T, 4195C>T, 4201T>G, 4204T>C, 4206T>A, 4207G>A, 4210A>T, 4211A>G, 4213A>G, 4216C>A, 4218C>G, 4219A>C, 4220T>G, 4221G>T, 4222T>A, 4223C>T, 4225A>G, 4226A>G, 4227C>T, 4228A>G, 4231G>A, 4232_4237delACACAC, 4240T>A, 4243G>T, 4244_4249delAAAAGA, 4252C>T, 4255A>G, 4263A>T, 4265T>G, 4267T>A, 4268T>A, 4269C>G, 4271C>A, 4277A>T, 4279G>A, 4281C>A, 4282C>T, 4283A>G, 4284C>A, 4285A>T, 4288G>T, 4291A>G, 4296A>G, 4297C>G, 4301G>T, 4302A>C, 4303C>T, 4305T>C, 4306C>T, 4312C>A, 4319C>A, 4321T>G, 4322C>G, 4324A>T, 4327C>A, 4328A>G, 4330T>G, 4333T>G, 4334G>C, 4335C>A, 4337G>T, 4338C>G, 4339C>T, 4340A>C, 4345A>G, 4348T>A, 4352A>C, 4354A>T, 4356T>C, 4357G>C, 4358T>G, 4360C>G, 4361G>C, 4363C>T, 4364G>C, 4366G>A, 4369C>A, 4369_4370insTCC, 4372A>T, 4374C>A, 4375G>C, 4378G>A, 4379T>A, 4381A>T, 4382A>G, 4383C>A, 4384G>A, 4390T>C, 4391T>A, 4396C>T, 4399C>T, 4402G>A, 4403A>G, 4404A>C, 4406C>T, 4408C>A, 4409A>G, 4410C>A, 4411G>A, 4417C>T, 4419C>T, 4421A>T, 4423G>A, 4426G>T, 4429G>A, 4430G>A, 4431A>C, 4432A>T, 4433C>T, 4435T>A, 4437C>A, 4439C>T, 4440G>C, 4441A>T, 4442A>C, 4444A>T, 4447A>G, 4450C>A, 4453C>T, 4457T>G, 4459G>A, 4462G>C, 4463C>T, 4465G>T, 4466C>T, 4468T>A, 4469G>T, 4470G>C, 4471G>C, 4472C>G, 4474G>A, 4477C>T, 4480G>C, 4483C>T, 4484G>A, 4485A>T, 4486A>T, 4832C>G, 4833G>A, 4834C>G, 4836A>T, 4837C>T, 4838A>G, 4840C>G, 4841A>G, 4842A>C, 4843G>C, 4846A>G, 4849C>T, 4850G>C, 4851T>C, 4853C>A, 4855C>T, 4865A>G, 4866A>T, 4867C>T, 4870A>G, 4872C>T, 4873T>A, 4875C>A, 4878G>A, 4879G>T, 4882C>A, 4883G>A, 4884C>G, 4886A>C, 4887A>C, 4889T>G, 4890A>G, 4894T>C, 4896A>T, 4897C>A, 4900A>T, 4904T>G, 4905T>A, 4906C>T, 4908G>T, 4909G>T, 4910A>G, 4911C>A, 4912A>G, 4913C>A, 4914C>T, 4915A>T, 4918A>T, 4921G>T, 4922A>T, 4923A>G, 4924A>G, 4925C>A, 4926C>A, 4927A>G, 4933C>A, 4939T>G, 4941C>A, 4942G>C, 4951C>T, 4952A>G, 4954T>C, 4955A>G, 4956C>T, 4958A>G, 4959A>G, 4961C>T, 4963C>A, 4966G>T, 4968G>A, 4970T>A, 4972A>T, 4974A>G, 4976G>A, 4977A>G, 4978T>G, 4980G>A, 5132G>T, 5133T>C, 5134A>C, 5135T>A, 5137C>T, 5141A>T, 5143A>T, 5146C>T, 5151A>G, 5152C>T, 5154A>C, 5155G>T, 5157T>A, 5294A>G, 5296C>G, 5298A>C, 5299C>G, 5300T>G, 5301A>C, 5302C>G, 5303G>A, 5304C>G, 5305A>C, 5308A>G, 5309G>A, 5310A>G, 5311C>A, 5314C>T, 5320T>G, 5321T>G, 5322C>A, 5323A>T, 5329G>T, 5330C>G, 5331C>A, 5332A>T, 5334T>C, 5335G>T, 5341G>A, 5342A>T									
**CDS**									
homologue_of_retroviral_POL_genes	291	1006	49.2%	1723	47.7%	510 (98.3%)	244 (47.0%)	4/5/0/0	0

	Begin	End	Coverage	Score	Concordance	Matches	Identities	I/D/M/F*	Stop Codons
**NT**	**3196**	**5342**	**20.9%**	**88**	**2.9%**	**1528 (98.3%)**	**804 (51.7%)**	**12/15**	
Protein mutations:	L293Q (3204T>A 3205C>G), F294Y (3207T>A 3208T>C), E295A (3210A>C 3211A>T), E296D (3214G>T), E297V (3216A>T 3217A>G), R298M (3219G>T 3220A>G), G299P (3221G>C 3222G>C 3223C>A), K300P (3224A>C 3225A>C 3226G>T), D301E (3229C>G), A302L (3230G>T 3231C>T 3232C>G), L303P (3233T>C 3234T>C), P304K (3236C>A 3237C>A 3238T>G), H306L (3243A>T 3244C>G), Q307P (3246A>C), W309R (3251T>A), W309_D310insRDI (3253_3254insAGGGATATT), K312R (3261A>G 3262G>A), N314E (3266A>G 3268C>G), I315L (3269A>T 3271T>G), Q316M (3272C>A 3273A>T), K319S (3281A>T 3282A>C 3283A>T), E320T (3284G>A 3285A>C), W323A (3293T>G 3294G>C 3295G>A), G324Q (3296G>C 3297G>A), P325A (3299C>G 3301C>T), L326P (3303T>C 3304A>T), Q328R (3308C>A 3309A>G), S330A (3314T>G), E331P (3317G>C 3318A>C 3319G>T), Q335A (3329C>G 3330A>C 3331G>T), T336E (3332A>G 3333C>A 3334C>A), E339K (3341G>A 3343A>G), W340Q (3344T>C 3345G>A), K342N (3352G>C), K344L (3356A>T 3357A>T), A346D (3363C>A 3364C>T), K347S (3365A>T 3366A>C), W349L (3371T>C 3372G>T), R351Q (3378G>A 3379A>G), R352P (3381G>C 3382A>T), T354K (3387C>A 3388C>G), S355A (3389T>G 3391A>T), S356P (3392A>C 3393G>C 3394T>C), A357Y (3395G>T 3396C>A 3397A>T), T359A (3401A>G 3403T>A), C361V (3407T>G 3408G>T 3409C>T), M362L (3410A>T 3412G>A), V364Q (3416G>C 3417T>A 3418T>G), P365K (3419C>A 3420C>A), A367Q (3425G>C 3426C>A 3427A>G), N368D (3428A>G 3430C>T), K370S (3434A>T 3435A>C), L373M (3443C>A 3445C>G), V374C (3446G>T 3447T>G 3448A>C), Q375M (3449C>A 3450A>T 3451A>G), K379A (3461A>G 3462A>C), E382K (3470G>A), I383V (3473A>G), I385V (3479A>G 3481C>G), R388K (3488C>A 3489G>A 3490A>G), N393R (3504A>G 3505C>G), E395D (3511A>T), E396D (3514A>T), A397L (3515G>T 3516C>T 3517A>G), Q398M (3518C>A 3519A>T 3520A>G), T402S (3531C>G), G403K (3533G>A 3534G>A), S404A (3536T>G 3538A>G), D405C (3539G>T 3540A>G), Y407F (3546A>T), I410L (3554A>C 3556T>A), D414S (3566G>T 3567A>C 3568C>A), A415G (3570C>G), F416Y (3573T>A 3574C>T), Y417W (3576A>G 3577T>G), A418Q (3578G>C 3579C>A 3580T>G), I419V (3581A>G 3583C>T), M421I (3589G>A), E425D (3601A>T), W427P (3605T>C 3606G>C 3607G>T), A430T (3614G>A), F431C (3618T>G 3619C>T), R432V (3620A>G 3621G>T 3622G>A), L437S (3635C>T 3636T>C 3637C>A), M445F (3659A>T 3661G>T), S453T (3683T>A), C454F (3687G>T 3688C>T), Q455C (3689C>T 3690A>G 3691G>C), D456N (3692G>A 3694C>T), V458M (3698G>A 3700C>G), E460D (3706A>C), T461V (3707A>G 3708C>T), R463F (3713A>T 3714G>T 3715A>T), L465Y (3719C>T 3720T>A 3721A>T), V468D (3729T>A 3730G>T), C469F (3732G>T 3733C>T), A472V (3741C>T 3742T>C), M474L (3746A>C 3748G>A), L478V (3758C>G 3760G>C), V479I (3761G>A 3763C>A), T481S (3768C>G 3769A>T), K482Q (3770A>C), G483del (3773_3775delGGA), S484T (3776T>A 3778C>G), Q486D (3782C>G 3784G>T), T489V (3791A>G 3792C>T 3793C>T), K490S (3795A>G 3796G>C), Q491H (3799A>T), V492L (3800G>T 3802T>G), Q493S (3803C>A 3804A>G 3805A>T), D494L (3806G>T 3807A>T 3808T>G), F496L (3814C>A), E497S (3815G>T 3816A>C 3817A>T), T500R (3825C>G), K501Q (3827A>C 3829G>A), S502Y (3831C>A), G503T (3833G>A 3834G>C 3835A>T), F504L (3836T>C), K505Y (3839A>T 3841G>T), T506V (3842A>G 3843C>T 3844A>T), A507K (3845G>A 3846C>A), P508M (3848C>A 3849C>T 3850C>G), H514A (3866C>G 3867A>C), K515Q (3869A>C 3871G>A), V518I (3878G>A 3880C>A), F523H (3893T>C 3894T>A), I524L (3896A>C 3898C>A), I525V (3899A>G 3901C>T), T527E (3905A>G 3906C>A), T528N (3909C>A 3910A>C), G529Q (3911G>C 3912G>A 3913G>A), I530V (3914A>G), T531R (3918C>G), I532M (3922C>G), A535K (3929G>A 3930C>A 3931A>G), T537V (3935A>G 3936C>T 3937A>G), S539A (3941T>G 3943A>C), R541V (3947A>G 3948G>T 3949A>T), E542D (3952A>T), P544Q (3957C>A), E545A (3960A>C), V552L (3980G>C 3982A>G), Q553R (3983C>A 3984A>G), N562Y (4010A>T 4012C>T), K567A (4025A>G 4026A>C 4027G>T), D568G (4029A>G 4030C>A), T572K (4041C>A), P575A (4049C>G), M576L (4052A>C), M578D (4058A>G 4059T>A 4060G>T), T580L (4064A>C 4065C>T 4066A>G), R581K (4068G>A 4069A>G), D583E (4075C>A), N585K (4081C>G), K587V (4085A>G 4086A>T), G589S (4091G>T 4092G>C 4093A>G), K590V (4094A>G 4095A>T), E591R (4097G>A 4098A>G), Q592C (4100C>T 4101A>G 4102G>C), T593E (4103A>G 4104C>A 4105C>A), K597Q (4115A>C 4117A>G), R598S (4120A>T), Q602A (4130C>G 4131A>C 4132G>T), C603I (4133T>A 4134G>T 4135C>T), A606E (4143C>A 4144C>A), T608I (4149C>T 4150G>A), R610K (4154C>A 4155G>A 4156A>G), F612P (4160T>C 4161T>C 4162C>A), G614F (4166G>T 4167G>T), S615E (4169A>G 4170G>A 4171C>G), K616L (4172A>C 4173A>T 4174G>A), E617P (4175G>C 4176A>C 4177A>T), V618F (4178G>T 4180C>T), H619E (4181C>G 4183C>G), I620V (4184A>G 4186C>A), E621H (4187G>C 4189G>C), M627K (4206T>A 4207G>A), I629V (4211A>G 4213A>G), A631G (4218C>G 4219A>C), C632V (4220T>G 4221G>T 4222T>A), T634V (4226A>G 4227C>T 4228A>G), T636_H637del (4232_4237delACACAC), D638E (4240T>A), K640_R641del (4244_4249delAAAAGA), Y646F (4263A>T), Y647E (4265T>G 4267T>A), M651L (4277A>T 4279G>A), T652N (4281C>A 4282C>T), T653D (4283A>G 4284C>A 4285A>T), N657R (4296A>G 4297C>G), D659S (4301G>T 4302A>C 4303C>T), I660T (4305T>C 4306C>T), D662E (4312C>A), L665M (4319C>A 4321T>G), L666V (4322C>G 4324A>T), I668V (4328A>G 4330T>G), A670H (4334G>C 4335C>A), A671C (4337G>T 4338C>G 4339C>T), M672L (4340A>C), H674Q (4348T>A), V677A (4356T>C 4357G>C), Y678E (4358T>G 4360C>G), V679L (4361G>C 4363C>T), E680Q (4364G>C 4366G>A), G681_P682insS (4369_4370insTCC), P683H (4374C>A 4375G>C), L685I (4379T>A 4381A>T), T686E (4382A>G 4383C>A 4384G>A), S689T (4391T>A), N693A (4403A>G 4404A>C), T695E (4409A>G 4410C>A 4411G>A), T698M (4419C>T), T699S (4421A>T 4423G>A), E702T (4430G>A 4431A>C 4432A>T), T704N (4437C>A), R705S (4439C>T 4440G>C 4441A>T), S711A (4457T>G 4459G>A), E712D (4462G>C), L713F (4463C>T 4465G>T), G715S (4469G>T 4470G>C 4471G>C), Q716E (4472C>G 4474G>A), K718N (4480G>C), E720I (4484G>A 4485A>T 4486A>T), R836E (4832C>G 4833G>A 4834C>G), Y837F (4836A>T 4837C>T), I838V (4838A>G 4840C>G), K839A (4841A>G 4842A>C 4843G>C), V842P (4850G>C 4851T>C), H843N (4853C>A 4855C>T), N847V (4865A>G 4866A>T 4867C>T), A849V (4872C>T 4873T>A), A850E (4875C>A), R851H (4878G>A 4879G>T), H852Q (4882C>A), A853R (4883G>A 4884C>G), K854P (4886A>C 4887A>C), Y855G (4889T>G 4890A>G), H857L (4896A>T 4897C>A), F860D (4904T>G 4905T>A 4906C>T), R861I (4908G>T 4909G>T), T862E (4910A>G 4911C>A 4912A>G), P863I (4913C>A 4914C>T 4915A>T), K866W (4922A>T 4923A>G 4924A>G), P867K (4925C>A 4926C>A 4927A>G), D869E (4933C>A), T872N (4941C>A 4942G>C), I876V (4952A>G 4954T>C), T877V (4955A>G 4956C>T), K878G (4958A>G 4959A>G), R881K (4968G>A), S882T (4970T>A 4972A>T), K883R (4974A>G), D884R (4976G>A 4977A>G 4978T>G), R885Q (4980G>A), V936S (5132G>T 5133T>C 5134A>C), F937I (5135T>A 5137C>T), T939S (5141A>T 5143A>T), D942G (5151A>G 5152C>T), K943T (5154A>C 5155G>T), I990V (5294A>G 5296C>G), N991T (5298A>C 5299C>G), Y992A (5300T>G 5301A>C 5302C>G), A993S (5303G>A 5304C>G 5305A>C), D995R (5309G>A 5310A>G 5311C>A), S999D (5321T>G 5322C>A 5323A>T), P1002D (5330C>G 5331C>A 5332A>T), M1003T (5334T>C 5335G>T)								

	Begin	End	Coverage	Score	Concordance	Matches	Identities	I/D/M/F*	Stop Codons
**NT**	**3196**	**5342**	**20.9%**	**88**	**2.9%**	**1528 (98.3%)**	**804 (51.7%)**	**12/15**	

Codon mutations:

CTC293CAG (3204T>A 3205C>G), TTT294TAC (3207T>A 3208T>C), GAA295GCT (3210A>C 3211A>T), GAG296GAT (3214G>T), GAA297GTG (3216A>T 3217A>G), AGA298ATG (3219G>T 3220A>G), GGC299CCA (3221G>C 3222G>C 3223C>A), AAG300CCT (3224A>C 3225A>C 3226G>T), GAC301GAG (3229C>G), GCC302TTG (3230G>T 3231C>T 3232C>G), TTA303CCA (3233T>C 3234T>C), CCT304AAG (3236C>A 3237C>A 3238T>G), CAC306CTG (3243A>T 3244C>G), CAA307CCA (3246A>C), TGG309AGG (3251T>A), TGG309_GAT310insAGGGATATT (3253_3254insAGGGATATT), AAG312AGA (3261A>G 3262G>A), ATA313ATT (3265A>T), AAC314GAG (3266A>G 3268C>G), ATT315TTG (3269A>T 3271T>G), CAG316ATG (3272C>A 3273A>T), GGG318GGT (3280G>T), AAA319TCT (3281A>T 3282A>C 3283A>T), GAG320ACG (3284G>A 3285A>C), CCT321CCG (3289T>G), CCA322CCT (3292A>T), TGG323GCA (3293T>G 3294G>C 3295G>A), GGA324CAA (3296G>C 3297G>A), CCC325GCT (3299C>G 3301C>T), CTA326CCT (3303T>C 3304A>T), TAT327TAC (3307T>C), CAA328AGA (3308C>A 3309A>G), TCT330GCT (3314T>G), GAG331CCT (3317G>C 3318A>C 3319G>T), CTA334TTG (3326C>T 3328A>G), CAG335GCT (3329C>G 3330A>C 3331G>T), ACC336GAA (3332A>G 3333C>A 3334C>A), CTA337CTG (3337A>G), CGA338AGG (3338C>A 3340A>G), GAA339AAG (3341G>A 3343A>G), TGG340CAG (3344T>C 3345G>A), AAG342AAC (3352G>C), GAG343GAA (3355G>A), AAG344TTG (3356A>T 3357A>T), CTA345CTT (3361A>T), GCC346GAT (3363C>A 3364C>T), AAA347TCA (3365A>T 3366A>C), TGG349CTG (3371T>C 3372G>T), ATA350ATT (3376A>T), CGA351CAG (3378G>A 3379A>G), CGA352CCT (3381G>C 3382A>T), TCC353TCT (3385C>T), ACC354AAG (3387C>A 3388C>G), TCA355GCT (3389T>G 3391A>T), AGT356CCC (3392A>C 3393G>C 3394T>C), GCA357TAT (3395G>T 3396C>A 3397A>T), GGA358GGT (3400A>T), ACT359GCA (3401A>G 3403T>A), CCA360CCT (3406A>T), TGC361GTT (3407T>G 3408G>T 3409C>T), ATG362TTA (3410A>T 3412G>A), TTC363TTT (3415C>T), GTT364CAG (3416G>C 3417T>A 3418T>G), CCA365AAA (3419C>A 3420C>A), GCA367CAG (3425G>C 3426C>A 3427A>G), AAC368GAT (3428A>G 3430C>T), AAA370TCA (3434A>T 3435A>C), CTA371TTA (3437C>T), CGA372AGG (3440C>A 3442A>G), CTC373ATG (3443C>A 3445C>G), GTA374TGC (3446G>T 3447T>G 3448A>C), CAA375ATG (3449C>A 3450A>T 3451A>G), TAC377TAT (3457C>T), CGA378CGG (3460A>G), AAG379GCG (3461A>G 3462A>C), TTG380CTG (3464T>C), GAG382AAG (3470G>A), ATC383GTC (3473A>G), ACG384ACT (3478G>T), ATC385GTG (3479A>G 3481C>G), CGA388AAG (3488C>A 3489G>A 3490A>G), CCG390CCA (3496G>A), CTA391CTT (3499A>T), CCC392CCT (3502C>T), AAC393AGG (3504A>G 3505C>G), ATC394ATT (3508C>T), GAA395GAT (3511A>T), GAA396GAT (3514A>T), GCA397TTG (3515G>T 3516C>T 3517A>G), CAA398ATG (3518C>A 3519A>T 3520A>G), AGA400AGG (3526A>G), TTA401TTG (3529A>G), ACC402AGC (3531C>G), GGA403AAA (3533G>A 3534G>A), TCA404GCG (3536T>G 3538A>G), GAC405TGC (3539G>T 3540A>G), TAC407TTC (3546A>T), ACG408ACT (3550G>T), ATT410CTA (3554A>C 3556T>A), GAC411GAT (3559C>T), CTA412CTT (3562A>T), CGA413AGG (3563C>A 3565A>G), GAC414TCA (3566G>T 3567A>C 3568C>A), GCC415GGC (3570C>G), TTC416TAT (3573T>A 3574C>T), TAT417TGG (3576A>G 3577T>G), GCT418CAG (3578G>C 3579C>A 3580T>G), ATC419GTT (3581A>G 3583C>T), CGA420AGG (3584C>A 3586A>G), ATG421ATA (3589G>A), GGA424GGG (3598A>G), GAA425GAT (3601A>T), GAA426GAG (3604A>G), TGG427CCT (3605T>C 3606G>C 3607G>T), AAA428AAG (3610A>G), ACC429ACT (3613C>T), GCT430ACT (3614G>A), TTC431TGT (3618T>G 3619C>T), AGG432GTA (3620A>G 3621G>T 3622G>A), ACA433ACT (3625A>T), AGA434AGG (3628A>G), TAC435TAT (3631C>T), GGA436GGG (3634A>G), CTC437TCA (3635C>T 3636T>C 3637C>A), TAC438TAT (3640C>T), TTG441CTT (3647T>C 3649G>T), GTC442GTA (3652C>A), CCA444CCG (3658A>G), ATG445TTT (3659A>T 3661G>T), GGA446GGG (3664A>G), ACC448ACT (3670C>T), AAC449AAT (3673C>T), GCA450GCC (3676A>C), CCC451CCG (3679C>G), GCA452GCT (3682A>T), TCC453ACC (3683T>A), TGC454TTT (3687G>T 3688C>T), CAG455TGC (3689C>T 3690A>G 3691G>C), GAC456AAT (3692G>A 3694C>T), CTT457TTA (3695C>T 3697T>A), GTC458ATG (3698G>A 3700C>G), AAC459AAT (3703C>T), GAA460GAC (3706A>C), ACA461GTA (3707A>G 3708C>T), CTT462CTG (3712T>G), AGA463TTT (3713A>T 3714G>T 3715A>T), CTA465TAT (3719C>T 3720T>A 3721A>T), CTC466CTT (3724C>T), GAC467GAT (3727C>T), GTG468GAT (3729T>A 3730G>T), TGC469TTT (3732G>T 3733C>T), GTC470GTA (3736C>A), GTT471GTA (3739T>A), GCT472GTC (3741C>T 3742T>C), TAC473TAT (3745C>T), ATG474CTA (3746A>C 3748G>A), GAC475GAT (3751C>T), ATA477ATT (3757A>T), CTG478GTC (3758C>G 3760G>C), GTC479ATA (3761G>A 3763C>A), TAC480TAT (3766C>T), ACA481AGT (3768C>G 3769A>T), AAA482CAA (3770A>C), GGA483del (3773_3775delGGA), TCC484ACG (3776T>A 3778C>G), CTC485TTG (3779C>T 3781C>G), CAG486GAT (3782C>G 3784G>T), ACC489GTT (3791A>G 3792C>T 3793C>T), AAG490AGC (3795A>G 3796G>C), CAA491CAT (3799A>T), GTT492TTG (3800G>T 3802T>G), CAA493AGT (3803C>A 3804A>G 3805A>T), GAT494TTG (3806G>T 3807A>T 3808T>G), GTG495GTA (3811G>A), TTC496TTA (3814C>A), GAA497TCT (3815G>T 3816A>C 3817A>T), CGA498CGT (3820A>T), CTC499TTA (3821C>T 3823C>A), ACG500AGG (3825C>G), AAG501CAA (3827A>C 3829G>A), TCC502TAC (3831C>A), GGA503ACT (3833G>A 3834G>C 3835A>T), TTC504CTC (3836T>C), AAG505TAT (3839A>T 3841G>T), ACA506GTT (3842A>G 3843C>T 3844A>T), GCA507AAA (3845G>A 3846C>A), CCC508ATG (3848C>A 3849C>T 3850C>G), AAA510AAG (3856A>G), TGC511TGT (3859C>T), GAA512GAG (3862A>G), TTC513TTT (3865C>T), CAC514GCC (3866C>G 3867A>C), AAG515CAA (3869A>C 3871G>A), GAA517GAG (3877A>G), GTC518ATA (3878G>A 3880C>A), TTT520TTC (3886T>C), TTA521CTA (3887T>C), GGC522GGG (3892C>G), TTT523CAT (3893T>C 3894T>A), ATC524CTA (3896A>C 3898C>A), ATC525GTT (3899A>G 3901C>T), ACA527GAA (3905A>G 3906C>A), ACA528AAC (3909C>A 3910A>C), GGG529CAA (3911G>C 3912G>A 3913G>A), ATA530GTA (3914A>G), ACG531AGG (3918C>G), ATC532ATG (3922C>G), GAC533GAT (3925C>T), GCA535AAG (3929G>A 3930C>A 3931A>G), AAG536AAA (3934G>A), ACA537GTG (3935A>G 3936C>T 3937A>G), CAG538CAA (3940G>A), TCA539GCC (3941T>G 3943A>C), ATC540ATT (3946C>T), AGA541GTT (3947A>G 3948G>T 3949A>T), GAA542GAT (3952A>T), CCA544CAA (3957C>A), GAA545GCA (3960A>C), CCG546CCC (3964G>C), AAG547AAA (3967G>A), ACA548ACT (3970A>T), GTC549GTG (3973C>G), GTA552CTG (3980G>C 3982A>G), CAG553AGG (3983C>A 3984A>G), TCA554TCT (3988A>T), CTT556TTG (3992C>T 3994T>G), GGA557GGG (3997A>G), CTC558CTA (4000C>A), GCC559GCT (4003C>T), AAC562TAT (4010A>T 4012C>T), AAG567GCT (4025A>G 4026A>C 4027G>T), GAC568GGA (4029A>G 4030C>A), TAT569TAC (4033T>C), ACA572AAA (4041C>A), GCA574GCT (4048A>T), CCA575GCA (4049C>G), ATG576CTG (4052A>C), ACG577ACT (4057G>T), ATG578GAT (4058A>G 4059T>A 4060G>T), CTT579TTG (4061C>T 4063T>G), ACA580CTG (4064A>C 4065C>T 4066A>G), AGA581AAG (4068G>A 4069A>G), AAA582AAG (4072A>G), GAC583GAA (4075C>A), AAC585AAG (4081C>G), AAA587GTA (4085A>G 4086A>T), GGA589TCG (4091G>T 4092G>C 4093A>G), AAA590GTA (4094A>G 4095A>T), GAA591AGA (4097G>A 4098A>G), CAG592TGC (4100C>T 4101A>G 4102G>C), ACC593GAA (4103A>G 4104C>A 4105C>A), GCG595GCT (4111G>T), TTC596TTT (4114C>T), AAA597CAG (4115A>C 4117A>G), AGA598AGT (4120A>T), CTC599TTG (4121C>T 4123C>G), CAG602GCT (4130C>G 4131A>C 4132G>T), TGC603ATT (4133T>A 4134G>T 4135C>T), GCT604GCA (4138T>A), GCC606GAA (4143C>A 4144C>A), ACG608ATA (4149C>T 4150G>A), CTT609CTG (4153T>G), CGA610AAG (4154C>A 4155G>A 4156A>G), CTA611CTG (4159A>G), TTC612CCA (4160T>C 4161T>C 4162C>A), GAT613GAC (4165T>C), GGT614TTT (4166G>T 4167G>T), AGC615GAG (4169A>G 4170G>A 4171C>G), AAG616CTA (4172A>C 4173A>T 4174G>A), GAA617CCT (4175G>C 4176A>C 4177A>T), GTC618TTT (4178G>T 4180C>T), CAC619GAG (4181C>G 4183C>G), ATC620GTA (4184A>G 4186C>A), GAG621CAC (4187G>C 4189G>C), ACC622ACT (4192C>T), GAC623GAT (4195C>T), TCT625TCG (4201T>G), GAT626GAC (4204T>C), ATG627AAA (4206T>A 4207G>A), GCA628GCT (4210A>T), ATA629GTG (4211A>G 4213A>G), GGC630GGA (4216C>A), GCA631GGC (4218C>G 4219A>C), TGT632GTA (4220T>G 4221G>T 4222T>A), CTA633TTG (4223C>T 4225A>G), ACA634GTG (4226A>G 4227C>T 4228A>G), CAG635CAA (4231G>A), ACA636_CAC637del (4232_4237delACACAC), GAT638GAA (4240T>A), GGG639GGT (4243G>T), AAA640_AGA641del (4244_4249delAAAAGA), CAC642CAT (4252C>T), CCA643CCG (4255A>G), TAT646TTT (4263A>T), TAT647GAA (4265T>G 4267T>A), TCC648AGC (4268T>A 4269C>G), CGG649AGG (4271C>A), ATG651TTA (4277A>T 4279G>A), ACC652AAT (4281C>A 4282C>T), ACA653GAT (4283A>G 4284C>A 4285A>T), GCG654GCT (4288G>T), GAA655GAG (4291A>G), AAC657AGG (4296A>G 4297C>G), GAC659TCT (4301G>T 4302A>C 4303C>T), ATC660ACT (4305T>C 4306C>T), GAC662GAA (4312C>A), CTT665ATG (4319C>A 4321T>G), CTA666GTT (4322C>G 4324A>T), GCC667GCA (4327C>A), ATT668GTG (4328A>G 4330T>G), GTT669GTG (4333T>G), GCC670CAC (4334G>C 4335C>A), GCC671TGT (4337G>T 4338C>G 4339C>T), ATG672CTG (4340A>C), CAA673CAG (4345A>G), CAT674CAA (4348T>A), AGA676CGT (4352A>C 4354A>T), GTG677GCC (4356T>C 4357G>C), TAC678GAG (4358T>G 4360C>G), GTC679CTT (4361G>C 4363C>T), GAG680CAA (4364G>C 4366G>A), GGC681GGA (4369C>A), GGC681_CCA682insTCC (4369_4370insTCC), CCA682CCT (4372A>T), CCG683CAC (4374C>A 4375G>C), AAG684AAA (4378G>A), TTA685ATT (4379T>A 4381A>T), ACG686GAA (4382A>G 4383C>A 4384G>A), CTT688CTC (4390T>C), TCA689ACA (4391T>A), GAC690GAT (4396C>T), CAC691CAT (4399C>T), AAG692AAA (4402G>A), AAT693GCT (4403A>G 4404A>C), CTC694TTA (4406C>T 4408C>A), ACG695GAA (4409A>G 4410C>A 4411G>A), TTC697TTT (4417C>T), ACG698ATG (4419C>T), ACG699TCA (4421A>T 4423G>A), ACG700ACT (4426G>T), AAG701AAA (4429G>A), GAA702ACT (4430G>A 4431A>C 4432A>T), CTT703TTA (4433C>T 4435T>A), ACC704AAC (4437C>A), CGA705TCT (4439C>T 4440G>C 4441A>T), AGA706CGT (4442A>C 4444A>T), CAA707CAG (4447A>G), GCC708GCA (4450C>A), CGC709CGT (4453C>T), TCG711GCA (4457T>G 4459G>A), GAG712GAC (4462G>C), CTG713TTT (4463C>T 4465G>T), CTT714TTA (4466C>T 4468T>A), GGG715TCC (4469G>T 4470G>C 4471G>C), CAG716GAA (4472C>G 4474G>A), TAC717TAT (4477C>T), AAG718AAC (4480G>C), TTC719TTT (4483C>T), GAA720ATT (4484G>A 4485A>T 4486A>T), CGC836GAG (4832C>G 4833G>A 4834C>G), TAC837TTT (4836A>T 4837C>T), ATC838GTG (4838A>G 4840C>G), AAG839GCC (4841A>G 4842A>C 4843G>C), AAA840AAG (4846A>G), TGC841TGT (4849C>T), GTA842CCA (4850G>C 4851T>C), CAC843AAT (4853C>A 4855C>T), AAC847GTT (4865A>G 4866A>T 4867C>T), AAA848AAG (4870A>G), GCT849GTA (4872C>T 4873T>A), GCA850GAA (4875C>A), CGG851CAT (4878G>A 4879G>T), CAC852CAA (4882C>A), GCG853AGG (4883G>A 4884C>G), AAA854CCA (4886A>C 4887A>C), TAC855GGC (4889T>G 4890A>G), GGT856GGC (4894T>C), CAC857CTA (4896A>T 4897C>A), CTA858CTT (4900A>T), TTC860GAT (4904T>G 4905T>A 4906C>T), AGG861ATT (4908G>T 4909G>T), ACA862GAG (4910A>G 4911C>A 4912A>G), CCA863ATT (4913C>A 4914C>T 4915A>T), CCA864CCT (4918A>T), ACG865ACT (4921G>T), AAA866TGG (4922A>T 4923A>G 4924A>G), CCA867AAG (4925C>A 4926C>A 4927A>G), GAC869GAA (4933C>A), GTT871GTG (4939T>G), ACG872AAC (4941C>A 4942G>C), TTC875TTT (4951C>T), ATT876GTC (4952A>G 4954T>C), ACG877GTG (4955A>G 4956C>T), AAA878GGA (4958A>G 4959A>G), CTC879TTA (4961C>T 4963C>A), CCG880CCT (4966G>T), AGG881AAG (4968G>A), TCA882ACT (4970T>A 4972A>T), AAG883AGG (4974A>G), GAT884AGG (4976G>A 4977A>G 4978T>G), CGA885CAA (4980G>A), GTA936TCC (5132G>T 5133T>C 5134A>C), TTC937ATT (5135T>A 5137C>T), ACA939TCT (5141A>T 5143A>T), GAC940GAT (5146C>T), GAC942GGT (5151A>G 5152C>T), AAG943ACT (5154A>C 5155G>T), CTC944CA. (5157T>A), ATC990GTG (5294A>G 5296C>G), AAC991ACG (5298A>C 5299C>G), TAC992GCG (5300T>G 5301A>C 5302C>G), GCA993AGC (5303G>A 5304C>G 5305A>C), CAA994CAG (5308A>G), GAC995AGA (5309G>A 5310A>G 5311C>A), AAC996AAT (5314C>T), GTT998GTG (5320T>G), TCA999GAT (5321T>G 5322C>A 5323A>T), CTG1001CTT (5329G>T), CCA1002GAT (5330C>G 5331C>A 5332A>T), ATG1003ACT (5334T>C 5335G>T), CAG1005CAA (5341G>A), ATC1006T.. (5342A>T)

Proteins

	Begin	End	Coverage	Score	Concordance	Matches	Identities	I/D/M/F*	Stop Codons
Reverse Transcriptase (YP_009666308.1)	291	1006	49.2%	1723	47.7%	510 (98.3%)	244 (47.0%)	4/5/0/0	0

	Begin	End	Coverage	Score	Concordance	Matches	Identities	I/D/M/F*	Stop Codons
**NT**	**3196**	**5342**	**20.9%**	**88**	**2.9%**	**1528 (98.3%)**	**804 (51.7%)**	**12/15**	
Protein mutations:	L293Q (3204T>A 3205C>G), F294Y (3207T>A 3208T>C), E295A (3210A>C 3211A>T), E296D (3214G>T), E297V (3216A>T 3217A>G), R298M (3219G>T 3220A>G), G299P (3221G>C 3222G>C 3223C>A), K300P (3224A>C 3225A>C 3226G>T), D301E (3229C>G), A302L (3230G>T 3231C>T 3232C>G), L303P (3233T>C 3234T>C), P304K (3236C>A 3237C>A 3238T>G), H306L (3243A>T 3244C>G), Q307P (3246A>C), W309R (3251T>A), W309_D310insRDI (3253_3254insAGGGATATT), K312R (3261A>G 3262G>A), N314E (3266A>G 3268C>G), I315L (3269A>T 3271T>G), Q316M (3272C>A 3273A>T), K319S (3281A>T 3282A>C 3283A>T), E320T (3284G>A 3285A>C), W323A (3293T>G 3294G>C 3295G>A), G324Q (3296G>C 3297G>A), P325A (3299C>G 3301C>T), L326P (3303T>C 3304A>T), Q328R (3308C>A 3309A>G), S330A (3314T>G), E331P (3317G>C 3318A>C 3319G>T), Q335A (3329C>G 3330A>C 3331G>T), T336E (3332A>G 3333C>A 3334C>A), E339K (3341G>A 3343A>G), W340Q (3344T>C 3345G>A), K342N (3352G>C), K344L (3356A>T 3357A>T), A346D (3363C>A 3364C>T), K347S (3365A>T 3366A>C), W349L (3371T>C 3372G>T), R351Q (3378G>A 3379A>G), R352P (3381G>C 3382A>T), T354K (3387C>A 3388C>G), S355A (3389T>G 3391A>T), S356P (3392A>C 3393G>C 3394T>C), A357Y (3395G>T 3396C>A 3397A>T), T359A (3401A>G 3403T>A), C361V (3407T>G 3408G>T 3409C>T), M362L (3410A>T 3412G>A), V364Q (3416G>C 3417T>A 3418T>G), P365K (3419C>A 3420C>A), A367Q (3425G>C 3426C>A 3427A>G), N368D (3428A>G 3430C>T), K370S (3434A>T 3435A>C), L373M (3443C>A 3445C>G), V374C (3446G>T 3447T>G 3448A>C), Q375M (3449C>A 3450A>T 3451A>G), K379A (3461A>G 3462A>C), E382K (3470G>A), I383V (3473A>G), I385V (3479A>G 3481C>G), R388K (3488C>A 3489G>A 3490A>G), N393R (3504A>G 3505C>G), E395D (3511A>T), E396D (3514A>T), A397L (3515G>T 3516C>T 3517A>G), Q398M (3518C>A 3519A>T 3520A>G), T402S (3531C>G), G403K (3533G>A 3534G>A), S404A (3536T>G 3538A>G), D405C (3539G>T 3540A>G), Y407F (3546A>T), I410L (3554A>C 3556T>A), D414S (3566G>T 3567A>C 3568C>A), A415G (3570C>G), F416Y (3573T>A 3574C>T), Y417W (3576A>G 3577T>G), A418Q (3578G>C 3579C>A 3580T>G), I419V (3581A>G 3583C>T), M421I (3589G>A), E425D (3601A>T), W427P (3605T>C 3606G>C 3607G>T), A430T (3614G>A), F431C (3618T>G 3619C>T), R432V (3620A>G 3621G>T 3622G>A), L437S (3635C>T 3636T>C 3637C>A), M445F (3659A>T 3661G>T), S453T (3683T>A), C454F (3687G>T 3688C>T), Q455C (3689C>T 3690A>G 3691G>C), D456N (3692G>A 3694C>T), V458M (3698G>A 3700C>G), E460D (3706A>C), T461V (3707A>G 3708C>T), R463F (3713A>T 3714G>T 3715A>T), L465Y (3719C>T 3720T>A 3721A>T), V468D (3729T>A 3730G>T), C469F (3732G>T 3733C>T), A472V (3741C>T 3742T>C), M474L (3746A>C 3748G>A), L478V (3758C>G 3760G>C), V479I (3761G>A 3763C>A), T481S (3768C>G 3769A>T), K482Q (3770A>C), G483del (3773_3775delGGA), S484T (3776T>A 3778C>G), Q486D (3782C>G 3784G>T), T489V (3791A>G 3792C>T 3793C>T), K490S (3795A>G 3796G>C), Q491H (3799A>T), V492L (3800G>T 3802T>G), Q493S (3803C>A 3804A>G 3805A>T), D494L (3806G>T 3807A>T 3808T>G), F496L (3814C>A), E497S (3815G>T 3816A>C 3817A>T), T500R (3825C>G), K501Q (3827A>C 3829G>A), S502Y (3831C>A), G503T (3833G>A 3834G>C 3835A>T), F504L (3836T>C), K505Y (3839A>T 3841G>T), T506V (3842A>G 3843C>T 3844A>T), A507K (3845G>A 3846C>A), P508M (3848C>A 3849C>T 3850C>G), H514A (3866C>G 3867A>C), K515Q (3869A>C 3871G>A), V518I (3878G>A 3880C>A), F523H (3893T>C 3894T>A), I524L (3896A>C 3898C>A), I525V (3899A>G 3901C>T), T527E (3905A>G 3906C>A), T528N (3909C>A 3910A>C), G529Q (3911G>C 3912G>A 3913G>A), I530V (3914A>G), T531R (3918C>G), I532M (3922C>G), A535K (3929G>A 3930C>A 3931A>G), T537V (3935A>G 3936C>T 3937A>G), S539A (3941T>G 3943A>C), R541V (3947A>G 3948G>T 3949A>T), E542D (3952A>T), P544Q (3957C>A), E545A (3960A>C), V552L (3980G>C 3982A>G), Q553R (3983C>A 3984A>G), N562Y (4010A>T 4012C>T), K567A (4025A>G 4026A>C 4027G>T), D568G (4029A>G 4030C>A), T572K (4041C>A), P575A (4049C>G), M576L (4052A>C), M578D (4058A>G 4059T>A 4060G>T), T580L (4064A>C 4065C>T 4066A>G), R581K (4068G>A 4069A>G), D583E (4075C>A), N585K (4081C>G), K587V (4085A>G 4086A>T), G589S (4091G>T 4092G>C 4093A>G), K590V (4094A>G 4095A>T), E591R (4097G>A 4098A>G), Q592C (4100C>T 4101A>G 4102G>C), T593E (4103A>G 4104C>A 4105C>A), K597Q (4115A>C 4117A>G), R598S (4120A>T), Q602A (4130C>G 4131A>C 4132G>T), C603I (4133T>A 4134G>T 4135C>T), A606E (4143C>A 4144C>A), T608I (4149C>T 4150G>A), R610K (4154C>A 4155G>A 4156A>G), F612P (4160T>C 4161T>C 4162C>A), G614F (4166G>T 4167G>T), S615E (4169A>G 4170G>A 4171C>G), K616L (4172A>C 4173A>T 4174G>A), E617P (4175G>C 4176A>C 4177A>T), V618F (4178G>T 4180C>T), H619E (4181C>G 4183C>G), I620V (4184A>G 4186C>A), E621H (4187G>C 4189G>C), M627K (4206T>A 4207G>A), I629V (4211A>G 4213A>G), A631G (4218C>G 4219A>C), C632V (4220T>G 4221G>T 4222T>A), T634V (4226A>G 4227C>T 4228A>G), T636_H637del (4232_4237delACACAC), D638E (4240T>A), K640_R641del (4244_4249delAAAAGA), Y646F (4263A>T), Y647E (4265T>G 4267T>A), M651L (4277A>T 4279G>A), T652N (4281C>A 4282C>T), T653D (4283A>G 4284C>A 4285A>T), N657R (4296A>G 4297C>G), D659S (4301G>T 4302A>C 4303C>T), I660T (4305T>C 4306C>T), D662E (4312C>A), L665M (4319C>A 4321T>G), L666V (4322C>G 4324A>T), I668V (4328A>G 4330T>G), A670H (4334G>C 4335C>A), A671C (4337G>T 4338C>G 4339C>T), M672L (4340A>C), H674Q (4348T>A), V677A (4356T>C 4357G>C), Y678E (4358T>G 4360C>G), V679L (4361G>C 4363C>T), E680Q (4364G>C 4366G>A), G681_P682insS (4369_4370insTCC), P683H (4374C>A 4375G>C), L685I (4379T>A 4381A>T), T686E (4382A>G 4383C>A 4384G>A), S689T (4391T>A), N693A (4403A>G 4404A>C), T695E (4409A>G 4410C>A 4411G>A), T698M (4419C>T), T699S (4421A>T 4423G>A), E702T (4430G>A 4431A>C 4432A>T), T704N (4437C>A), R705S (4439C>T 4440G>C 4441A>T), S711A (4457T>G 4459G>A), E712D (4462G>C), L713F (4463C>T 4465G>T), G715S (4469G>T 4470G>C 4471G>C), Q716E (4472C>G 4474G>A), K718N (4480G>C), E720I (4484G>A 4485A>T 4486A>T), R836E (4832C>G 4833G>A 4834C>G), Y837F (4836A>T 4837C>T), I838V (4838A>G 4840C>G), K839A (4841A>G 4842A>C 4843G>C), V842P (4850G>C 4851T>C), H843N (4853C>A 4855C>T), N847V (4865A>G 4866A>T 4867C>T), A849V (4872C>T 4873T>A), A850E (4875C>A), R851H (4878G>A 4879G>T), H852Q (4882C>A), A853R (4883G>A 4884C>G), K854P (4886A>C 4887A>C), Y855G (4889T>G 4890A>G), H857L (4896A>T 4897C>A), F860D (4904T>G 4905T>A 4906C>T), R861I (4908G>T 4909G>T), T862E (4910A>G 4911C>A 4912A>G), P863I (4913C>A 4914C>T 4915A>T), K866W (4922A>T 4923A>G 4924A>G), P867K (4925C>A 4926C>A 4927A>G), D869E (4933C>A), T872N (4941C>A 4942G>C), I876V (4952A>G 4954T>C), T877V (4955A>G 4956C>T), K878G (4958A>G 4959A>G), R881K (4968G>A), S882T (4970T>A 4972A>T), K883R (4974A>G), D884R (4976G>A 4977A>G 4978T>G), R885Q (4980G>A), V936S (5132G>T 5133T>C 5134A>C), F937I (5135T>A 5137C>T), T939S (5141A>T 5143A>T), D942G (5151A>G 5152C>T), K943T (5154A>C 5155G>T), I990V (5294A>G 5296C>G), N991T (5298A>C 5299C>G), Y992A (5300T>G 5301A>C 5302C>G), A993S (5303G>A 5304C>G 5305A>C), D995R (5309G>A 5310A>G 5311C>A), S999D (5321T>G 5322C>A 5323A>T), P1002D (5330C>G 5331C>A 5332A>T), M1003T (5334T>C 5335G>T)								

	Begin	End	Coverage	Score	Concordance	Matches	Identities	I/D/M/F*	Stop Codons
**NT**	**3196**	**5342**	**20.9%**	**88**	**2.9%**	**1528 (98.3%)**	**804 (51.7%)**	**12/15**	
Codon mutations:	CTC293CAG (3204T>A 3205C>G), TTT294TAC (3207T>A 3208T>C), GAA295GCT (3210A>C 3211A>T), GAG296GAT (3214G>T), GAA297GTG (3216A>T 3217A>G), AGA298ATG (3219G>T 3220A>G), GGC299CCA (3221G>C 3222G>C 3223C>A), AAG300CCT (3224A>C 3225A>C 3226G>T), GAC301GAG (3229C>G), GCC302TTG (3230G>T 3231C>T 3232C>G), TTA303CCA (3233T>C 3234T>C), CCT304AAG (3236C>A 3237C>A 3238T>G), CAC306CTG (3243A>T 3244C>G), CAA307CCA (3246A>C), TGG309AGG (3251T>A), TGG309_GAT310insAGGGATATT (3253_3254insAGGGATATT), AAG312AGA (3261A>G 3262G>A), ATA313ATT (3265A>T), AAC314GAG (3266A>G 3268C>G), ATT315TTG (3269A>T 3271T>G), CAG316ATG (3272C>A 3273A>T), GGG318GGT (3280G>T), AAA319TCT (3281A>T 3282A>C 3283A>T), GAG320ACG (3284G>A 3285A>C), CCT321CCG (3289T>G), CCA322CCT (3292A>T), TGG323GCA (3293T>G 3294G>C 3295G>A), GGA324CAA (3296G>C 3297G>A), CCC325GCT (3299C>G 3301C>T), CTA326CCT (3303T>C 3304A>T), TAT327TAC (3307T>C), CAA328AGA (3308C>A 3309A>G), TCT330GCT (3314T>G), GAG331CCT (3317G>C 3318A>C 3319G>T), CTA334TTG (3326C>T 3328A>G), CAG335GCT (3329C>G 3330A>C 3331G>T), ACC336GAA (3332A>G 3333C>A 3334C>A), CTA337CTG (3337A>G), CGA338AGG (3338C>A 3340A>G), GAA339AAG (3341G>A 3343A>G), TGG340CAG (3344T>C 3345G>A), AAG342AAC (3352G>C), GAG343GAA (3355G>A), AAG344TTG (3356A>T 3357A>T), CTA345CTT (3361A>T), GCC346GAT (3363C>A 3364C>T), AAA347TCA (3365A>T 3366A>C), TGG349CTG (3371T>C 3372G>T), ATA350ATT (3376A>T), CGA351CAG (3378G>A 3379A>G), CGA352CCT (3381G>C 3382A>T), TCC353TCT (3385C>T), ACC354AAG (3387C>A 3388C>G), TCA355GCT (3389T>G 3391A>T), AGT356CCC (3392A>C 3393G>C 3394T>C), GCA357TAT (3395G>T 3396C>A 3397A>T), GGA358GGT (3400A>T), ACT359GCA (3401A>G 3403T>A), CCA360CCT (3406A>T), TGC361GTT (3407T>G 3408G>T 3409C>T), ATG362TTA (3410A>T 3412G>A), TTC363TTT (3415C>T), GTT364CAG (3416G>C 3417T>A 3418T>G), CCA365AAA (3419C>A 3420C>A), GCA367CAG (3425G>C 3426C>A 3427A>G), AAC368GAT (3428A>G 3430C>T), AAA370TCA (3434A>T 3435A>C), CTA371TTA (3437C>T), CGA372AGG (3440C>A 3442A>G), CTC373ATG (3443C>A 3445C>G), GTA374TGC (3446G>T 3447T>G 3448A>C), CAA375ATG (3449C>A 3450A>T 3451A>G), TAC377TAT (3457C>T), CGA378CGG (3460A>G), AAG379GCG (3461A>G 3462A>C), TTG380CTG (3464T>C), GAG382AAG (3470G>A), ATC383GTC (3473A>G), ACG384ACT (3478G>T), ATC385GTG (3479A>G 3481C>G), CGA388AAG (3488C>A 3489G>A 3490A>G), CCG390CCA (3496G>A), CTA391CTT (3499A>T), CCC392CCT (3502C>T), AAC393AGG (3504A>G 3505C>G), ATC394ATT (3508C>T), GAA395GAT (3511A>T), GAA396GAT (3514A>T), GCA397TTG (3515G>T 3516C>T 3517A>G), CAA398ATG (3518C>A 3519A>T 3520A>G), AGA400AGG (3526A>G), TTA401TTG (3529A>G), ACC402AGC (3531C>G), GGA403AAA (3533G>A 3534G>A), TCA404GCG (3536T>G 3538A>G), GAC405TGC (3539G>T 3540A>G), TAC407TTC (3546A>T), ACG408ACT (3550G>T), ATT410CTA (3554A>C 3556T>A), GAC411GAT (3559C>T), CTA412CTT (3562A>T), CGA413AGG (3563C>A 3565A>G), GAC414TCA (3566G>T 3567A>C 3568C>A), GCC415GGC (3570C>G), TTC416TAT (3573T>A 3574C>T), TAT417TGG (3576A>G 3577T>G), GCT418CAG (3578G>C 3579C>A 3580T>G), ATC419GTT (3581A>G 3583C>T), CGA420AGG (3584C>A 3586A>G), ATG421ATA (3589G>A), GGA424GGG (3598A>G), GAA425GAT (3601A>T), GAA426GAG (3604A>G), TGG427CCT (3605T>C 3606G>C 3607G>T), AAA428AAG (3610A>G), ACC429ACT (3613C>T), GCT430ACT (3614G>A), TTC431TGT (3618T>G 3619C>T), AGG432GTA (3620A>G 3621G>T 3622G>A), ACA433ACT (3625A>T), AGA434AGG (3628A>G), TAC435TAT (3631C>T), GGA436GGG (3634A>G), CTC437TCA (3635C>T 3636T>C 3637C>A), TAC438TAT (3640C>T), TTG441CTT (3647T>C 3649G>T), GTC442GTA (3652C>A), CCA444CCG (3658A>G), ATG445TTT (3659A>T 3661G>T), GGA446GGG (3664A>G), ACC448ACT (3670C>T), AAC449AAT (3673C>T), GCA450GCC (3676A>C), CCC451CCG (3679C>G), GCA452GCT (3682A>T), TCC453ACC (3683T>A), TGC454TTT (3687G>T 3688C>T), CAG455TGC (3689C>T 3690A>G 3691G>C), GAC456AAT (3692G>A 3694C>T), CTT457TTA (3695C>T 3697T>A), GTC458ATG (3698G>A 3700C>G), AAC459AAT (3703C>T), GAA460GAC (3706A>C), ACA461GTA (3707A>G 3708C>T), CTT462CTG (3712T>G), AGA463TTT (3713A>T 3714G>T 3715A>T), CTA465TAT (3719C>T 3720T>A 3721A>T), CTC466CTT (3724C>T), GAC467GAT (3727C>T), GTG468GAT (3729T>A 3730G>T), TGC469TTT (3732G>T 3733C>T), GTC470GTA (3736C>A), GTT471GTA (3739T>A), GCT472GTC (3741C>T 3742T>C), TAC473TAT (3745C>T), ATG474CTA (3746A>C 3748G>A), GAC475GAT (3751C>T), ATA477ATT (3757A>T), CTG478GTC (3758C>G 3760G>C), GTC479ATA (3761G>A 3763C>A), TAC480TAT (3766C>T), ACA481AGT (3768C>G 3769A>T), AAA482CAA (3770A>C), GGA483del (3773_3775delGGA), TCC484ACG (3776T>A 3778C>G), CTC485TTG (3779C>T 3781C>G), CAG486GAT (3782C>G 3784G>T), ACC489GTT (3791A>G 3792C>T 3793C>T), AAG490AGC (3795A>G 3796G>C), CAA491CAT (3799A>T), GTT492TTG (3800G>T 3802T>G), CAA493AGT (3803C>A 3804A>G 3805A>T), GAT494TTG (3806G>T 3807A>T 3808T>G), GTG495GTA (3811G>A), TTC496TTA (3814C>A), GAA497TCT (3815G>T 3816A>C 3817A>T), CGA498CGT (3820A>T), CTC499TTA (3821C>T 3823C>A), ACG500AGG (3825C>G), AAG501CAA (3827A>C 3829G>A), TCC502TAC (3831C>A), GGA503ACT (3833G>A 3834G>C 3835A>T), TTC504CTC (3836T>C), AAG505TAT (3839A>T 3841G>T), ACA506GTT (3842A>G 3843C>T 3844A>T), GCA507AAA (3845G>A 3846C>A), CCC508ATG (3848C>A 3849C>T 3850C>G), AAA510AAG (3856A>G), TGC511TGT (3859C>T), GAA512GAG (3862A>G), TTC513TTT (3865C>T), CAC514GCC (3866C>G 3867A>C), AAG515CAA (3869A>C 3871G>A), GAA517GAG (3877A>G), GTC518ATA (3878G>A 3880C>A), TTT520TTC (3886T>C), TTA521CTA (3887T>C), GGC522GGG (3892C>G), TTT523CAT (3893T>C 3894T>A), ATC524CTA (3896A>C 3898C>A), ATC525GTT (3899A>G 3901C>T), ACA527GAA (3905A>G 3906C>A), ACA528AAC (3909C>A 3910A>C), GGG529CAA (3911G>C 3912G>A 3913G>A), ATA530GTA (3914A>G), ACG531AGG (3918C>G), ATC532ATG (3922C>G), GAC533GAT (3925C>T), GCA535AAG (3929G>A 3930C>A 3931A>G), AAG536AAA (3934G>A), ACA537GTG (3935A>G 3936C>T 3937A>G), CAG538CAA (3940G>A), TCA539GCC (3941T>G 3943A>C), ATC540ATT (3946C>T), AGA541GTT (3947A>G 3948G>T 3949A>T), GAA542GAT (3952A>T), CCA544CAA (3957C>A), GAA545GCA (3960A>C), CCG546CCC (3964G>C), AAG547AAA (3967G>A), ACA548ACT (3970A>T), GTC549GTG (3973C>G), GTA552CTG (3980G>C 3982A>G), CAG553AGG (3983C>A 3984A>G), TCA554TCT (3988A>T), CTT556TTG (3992C>T 3994T>G), GGA557GGG (3997A>G), CTC558CTA (4000C>A), GCC559GCT (4003C>T), AAC562TAT (4010A>T 4012C>T), AAG567GCT (4025A>G 4026A>C 4027G>T), GAC568GGA (4029A>G 4030C>A), TAT569TAC (4033T>C), ACA572AAA (4041C>A), GCA574GCT (4048A>T), CCA575GCA (4049C>G), ATG576CTG (4052A>C), ACG577ACT (4057G>T), ATG578GAT (4058A>G 4059T>A 4060G>T), CTT579TTG (4061C>T 4063T>G), ACA580CTG (4064A>C 4065C>T 4066A>G), AGA581AAG (4068G>A 4069A>G), AAA582AAG (4072A>G), GAC583GAA (4075C>A), AAC585AAG (4081C>G), AAA587GTA (4085A>G 4086A>T), GGA589TCG (4091G>T 4092G>C 4093A>G), AAA590GTA (4094A>G 4095A>T), GAA591AGA (4097G>A 4098A>G), CAG592TGC (4100C>T 4101A>G 4102G>C), ACC593GAA (4103A>G 4104C>A 4105C>A), GCG595GCT (4111G>T), TTC596TTT (4114C>T), AAA597CAG (4115A>C 4117A>G), AGA598AGT (4120A>T), CTC599TTG (4121C>T 4123C>G), CAG602GCT (4130C>G 4131A>C 4132G>T), TGC603ATT (4133T>A 4134G>T 4135C>T), GCT604GCA (4138T>A), GCC606GAA (4143C>A 4144C>A), ACG608ATA (4149C>T 4150G>A), CTT609CTG (4153T>G), CGA610AAG (4154C>A 4155G>A 4156A>G), CTA611CTG (4159A>G), TTC612CCA (4160T>C 4161T>C 4162C>A), GAT613GAC (4165T>C), GGT614TTT (4166G>T 4167G>T), AGC615GAG (4169A>G 4170G>A 4171C>G), AAG616CTA (4172A>C 4173A>T 4174G>A), GAA617CCT (4175G>C 4176A>C 4177A>T), GTC618TTT (4178G>T 4180C>T), CAC619GAG (4181C>G 4183C>G), ATC620GTA (4184A>G 4186C>A), GAG621CAC (4187G>C 4189G>C), ACC622ACT (4192C>T), GAC623GAT (4195C>T), TCT625TCG (4201T>G), GAT626GAC (4204T>C), ATG627AAA (4206T>A 4207G>A), GCA628GCT (4210A>T), ATA629GTG (4211A>G 4213A>G), GGC630GGA (4216C>A), GCA631GGC (4218C>G 4219A>C), TGT632GTA (4220T>G 4221G>T 4222T>A), CTA633TTG (4223C>T 4225A>G), ACA634GTG (4226A>G 4227C>T 4228A>G), CAG635CAA (4231G>A), ACA636_CAC637del (4232_4237delACACAC), GAT638GAA (4240T>A), GGG639GGT (4243G>T), AAA640_AGA641del (4244_4249delAAAAGA), CAC642CAT (4252C>T), CCA643CCG (4255A>G), TAT646TTT (4263A>T), TAT647GAA (4265T>G 4267T>A), TCC648AGC (4268T>A 4269C>G), CGG649AGG (4271C>A), ATG651TTA (4277A>T 4279G>A), ACC652AAT (4281C>A 4282C>T), ACA653GAT (4283A>G 4284C>A 4285A>T), GCG654GCT (4288G>T), GAA655GAG (4291A>G), AAC657AGG (4296A>G 4297C>G), GAC659TCT (4301G>T 4302A>C 4303C>T), ATC660ACT (4305T>C 4306C>T), GAC662GAA (4312C>A), CTT665ATG (4319C>A 4321T>G), CTA666GTT (4322C>G 4324A>T), GCC667GCA (4327C>A), ATT668GTG (4328A>G 4330T>G), GTT669GTG (4333T>G), GCC670CAC (4334G>C 4335C>A), GCC671TGT (4337G>T 4338C>G 4339C>T), ATG672CTG (4340A>C), CAA673CAG (4345A>G), CAT674CAA (4348T>A), AGA676CGT (4352A>C 4354A>T), GTG677GCC (4356T>C 4357G>C), TAC678GAG (4358T>G 4360C>G), GTC679CTT (4361G>C 4363C>T), GAG680CAA (4364G>C 4366G>A), GGC681GGA (4369C>A), GGC681_CCA682insTCC (4369_4370insTCC), CCA682CCT (4372A>T), CCG683CAC (4374C>A 4375G>C), AAG684AAA (4378G>A), TTA685ATT (4379T>A 4381A>T), ACG686GAA (4382A>G 4383C>A 4384G>A), CTT688CTC (4390T>C), TCA689ACA (4391T>A), GAC690GAT (4396C>T), CAC691CAT (4399C>T), AAG692AAA (4402G>A), AAT693GCT (4403A>G 4404A>C), CTC694TTA (4406C>T 4408C>A), ACG695GAA (4409A>G 4410C>A 4411G>A), TTC697TTT (4417C>T), ACG698ATG (4419C>T), ACG699TCA (4421A>T 4423G>A), ACG700ACT (4426G>T), AAG701AAA (4429G>A), GAA702ACT (4430G>A 4431A>C 4432A>T), CTT703TTA (4433C>T 4435T>A), ACC704AAC (4437C>A), CGA705TCT (4439C>T 4440G>C 4441A>T), AGA706CGT (4442A>C 4444A>T), CAA707CAG (4447A>G), GCC708GCA (4450C>A), CGC709CGT (4453C>T), TCG711GCA (4457T>G 4459G>A), GAG712GAC (4462G>C), CTG713TTT (4463C>T 4465G>T), CTT714TTA (4466C>T 4468T>A), GGG715TCC (4469G>T 4470G>C 4471G>C), CAG716GAA (4472C>G 4474G>A), TAC717TAT (4477C>T), AAG718AAC (4480G>C), TTC719TTT (4483C>T), GAA720ATT (4484G>A 4485A>T 4486A>T), CGC836GAG (4832C>G 4833G>A 4834C>G), TAC837TTT (4836A>T 4837C>T), ATC838GTG (4838A>G 4840C>G), AAG839GCC (4841A>G 4842A>C 4843G>C), AAA840AAG (4846A>G), TGC841TGT (4849C>T), GTA842CCA (4850G>C 4851T>C), CAC843AAT (4853C>A 4855C>T), AAC847GTT (4865A>G 4866A>T 4867C>T), AAA848AAG (4870A>G), GCT849GTA (4872C>T 4873T>A), GCA850GAA (4875C>A), CGG851CAT (4878G>A 4879G>T), CAC852CAA (4882C>A), GCG853AGG (4883G>A 4884C>G), AAA854CCA (4886A>C 4887A>C), TAC855GGC (4889T>G 4890A>G), GGT856GGC (4894T>C), CAC857CTA (4896A>T 4897C>A), CTA858CTT (4900A>T), TTC860GAT (4904T>G 4905T>A 4906C>T), AGG861ATT (4908G>T 4909G>T), ACA862GAG (4910A>G 4911C>A 4912A>G), CCA863ATT (4913C>A 4914C>T 4915A>T), CCA864CCT (4918A>T), ACG865ACT (4921G>T), AAA866TGG (4922A>T 4923A>G 4924A>G), CCA867AAG (4925C>A 4926C>A 4927A>G), GAC869GAA (4933C>A), GTT871GTG (4939T>G), ACG872AAC (4941C>A 4942G>C), TTC875TTT (4951C>T), ATT876GTC (4952A>G 4954T>C), ACG877GTG (4955A>G 4956C>T), AAA878GGA (4958A>G 4959A>G), CTC879TTA (4961C>T 4963C>A), CCG880CCT (4966G>T), AGG881AAG (4968G>A), TCA882ACT (4970T>A 4972A>T), AAG883AGG (4974A>G), GAT884AGG (4976G>A 4977A>G 4978T>G), CGA885CAA (4980G>A), GTA936TCC (5132G>T 5133T>C 5134A>C), TTC937ATT (5135T>A 5137C>T), ACA939TCT (5141A>T 5143A>T), GAC940GAT (5146C>T), GAC942GGT (5151A>G 5152C>T), AAG943ACT (5154A>C 5155G>T), CTC944CA. (5157T>A), ATC990GTG (5294A>G 5296C>G), AAC991ACG (5298A>C 5299C>G), TAC992GCG (5300T>G 5301A>C 5302C>G), GCA993AGC (5303G>A 5304C>G 5305A>C), CAA994CAG (5308A>G), GAC995AGA (5309G>A 5310A>G 5311C>A), AAC996AAT (5314C>T), GTT998GTG (5320T>G), TCA999GAT (5321T>G 5322C>A 5323A>T), CTG1001CTT (5329G>T), CCA1002GAT (5330C>G 5331C>A 5332A>T), ATG1003ACT (5334T>C 5335G>T), CAG1005CAA (5341G>A), ATC1006T.. (5342A>T)									

*: Inserts / Deletes / Misaligned / Frameshifts

Analysis details

This analysis was performed with panviral2.64

NGS Details (UN60): Vellamovirus rhodeisland44

Assembly

**Coverage Length**	432 (1 contig(s))
**Depth Of Coverage**	154.1
**Number Of Reads**	604
**Reads Per Million**	12.81 rpm (after QC)
**Ambiguities**	0
**Assembly Method**	de novo + reference guided assembly
**Consensus Caller**	Bcf Tools

Coverage Map

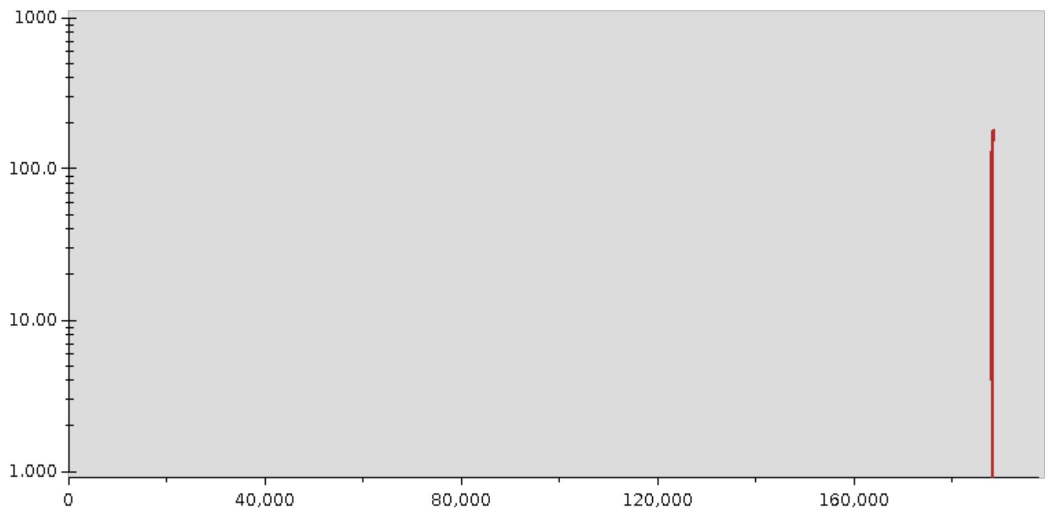

Assignment

**Type**	Vellamovirus rhodeisland44 (Taxonomy ID: 2734152)
**Reference Genome**	NC_047734.1
**NT Identity (%)**	75.0
**AA Identity (%)**	86.8056
**Number Of Stop Codons**	0
**Number Of CDS**	235

Alignment

**Alignment Score**	432.0 (NT) + 958.0 (AA) = 1390.0
**Concordance (%)**	72.6607
**Alignment Method**	Local, heuristic, nucleotide (BLASTN)

Genome Region

Sequence starts at position 188039 and ends at position 188470 relative to NC_047734.1 reference sequence.

Alignment Detailed Statistics

	Begin	End	Coverage	Score	Concordance	Matches	Identities	I/D/M/F*	Stop Codons
**NT**	**188039**	**188470**	**0.2%**	**432**	**50.0%**	**432 (100%)**	**324 (75.0%)**	**0/0**	
Mutations:	188048A>C, 188051T>G, 188054C>T, 188057G>T, 188060G>T, 188067G>A, 188069C>T, 188070G>A, 188071C>G, 188073A>T, 188075G>A, 188077G>C, 188078T>A, 188084T>G, 188085C>T, 188087T>G, 188088C>T, 188096T>A, 188108G>A, 188109T>G, 188111T>A, 188117G>A, 188120C>T, 188124C>A, 188125A>C, 188126A>T, 188129C>T, 188135C>T, 188139C>T, 188141T>G, 188147A>T, 188150C>G, 188155A>C, 188162G>T, 188165A>T, 188171C>T, 188174T>A, 188177C>T, 188182C>G, 188183T>C, 188186C>A, 188189T>A, 188199C>T, 188201T>A, 188204A>T, 188207G>A, 188210T>C, 188213A>G, 188222C>A, 188224T>C, 188225G>T, 188226A>G, 188229G>C, 188230G>A, 188231T>A, 188232A>T, 188234C>G, 188235C>A, 188237T>A, 188243C>T, 188249T>A, 188252T>C, 188255G>A, 188267T>A, 188282C>T, 188283A>T, 188284G>C, 188291C>T, 188294T>G, 188297C>T, 188306G>A, 188316T>G, 188317C>G, 188318A>T, 188339C>T, 188342G>T, 188351A>T, 188354C>A, 188358A>C, 188364C>A, 188366A>C, 188367T>C, 188369G>A, 188372C>T, 188373C>T, 188378G>A, 188381T>G, 188384C>T, 188387C>T, 188390C>T, 188397C>T, 188399C>G, 188408C>T, 188426T>C, 188430A>G, 188433C>T, 188435A>G, 188438A>C, 188440G>C, 188441A>T, 188444A>T, 188445C>T, 188447A>G, 188450C>A, 188451A>T, 188453T>C, 188456T>C, 188459C>T								

*: Inserts / Deletes / Misaligned / Frameshifts

Analysis details

This analysis was performed with panviral2.64

NGS Details (UN60): Cassava brown streak virus

Assembly

**Coverage Length**	519 (1 contig(s))
**Depth Of Coverage**	82.0
**Number Of Reads**	356
**Reads Per Million**	7.55 rpm (after QC)
**Ambiguities**	0
**Assembly Method**	de novo + reference guided assembly
**Consensus Caller**	Bcf Tools

Coverage Map

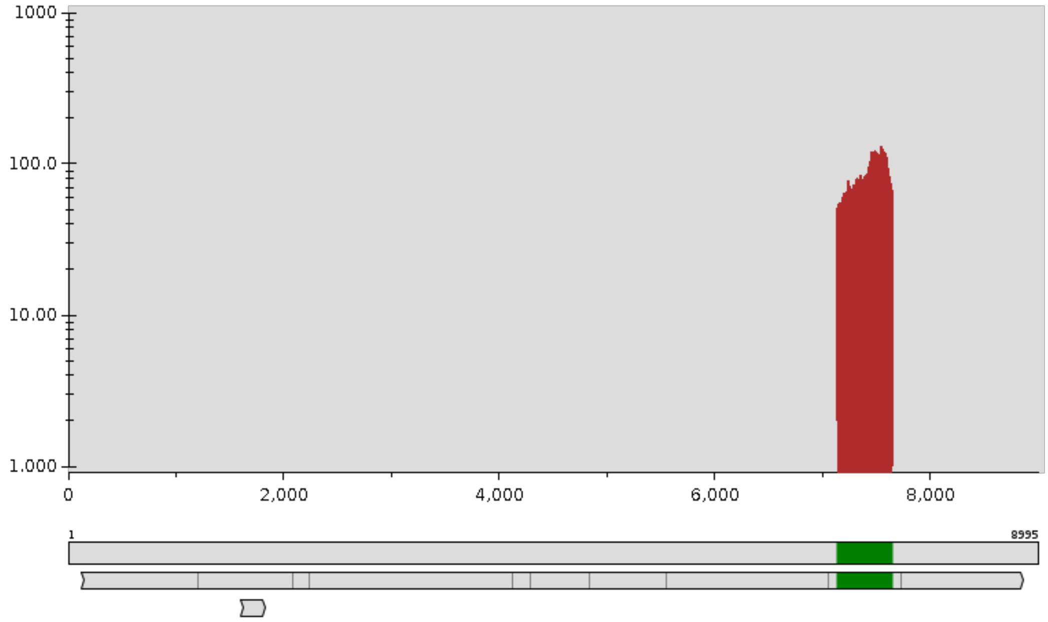

Assignment

**Type**	Cassava brown streak virus (Taxonomy ID: 137758)
**Reference Genome**	NC_012698.2
**NT Identity (%)**	60.3083
**AA Identity (%)**	57.2254
**Number Of Stop Codons**	0
**Number Of CDS**	2

Alignment

**Alignment Score**	214.0 (NT) + 728.0 (AA) = 942.0
**Concordance (%)**	41.9599

Alignment Method	Global, seeded, nucleotide + amino acids (AGA)

Genome Region

Sequence starts at position 7129 and ends at position 7647 relative to NC_012698.2 reference sequence.

Alignment Detailed Statistics

	Begin	End	Coverage	Score	Concordance	Matches	Identities	I/D/M/F*	Stop Codons
**NT**	**7129**	**7647**	**5.8%**	**214**	**20.6%**	**519 (100%)**	**313 (60.3%)**	**0/0**	
Mutations:	7130A>G, 7133A>G, 7134A>G, 7136T>G, 7142C>T, 7145A>T, 7148A>C, 7151G>A, 7157A>T, 7158C>A, 7163A>G, 7167A>G, 7175A>T, 7177A>G, 7178A>G, 7179C>G, 7180A>C, 7184A>T, 7185T>C, 7190T>C, 7192C>A, 7193C>G, 7194A>T, 7202A>C, 7203A>T, 7205C>T, 7206A>C, 7207T>A, 7208C>G, 7211T>C, 7213G>T, 7214G>T, 7217A>G, 7218G>C, 7229A>T, 7234C>T, 7235A>T, 7241A>G, 7242A>G, 7243C>A, 7244A>G, 7245G>C, 7246T>C, 7250G>A, 7253A>T, 7256T>A, 7257A>T, 7258T>C, 7270T>C, 7271A>T, 7272C>A, 7273G>A, 7274T>A, 7275G>A, 7277A>T, 7280G>T, 7281T>G, 7283T>C, 7284G>A, 7286G>A, 7287T>G, 7288T>A, 7290A>G, 7292T>G, 7293G>A, 7294G>A, 7295A>T, 7298G>A, 7304T>G, 7307C>A, 7310A>T, 7313A>G, 7319A>T, 7320A>T, 7323T>C, 7325G>C, 7331T>C, 7332G>A, 7334T>C, 7337T>C, 7340C>T, 7343T>G, 7355A>G, 7358A>T, 7361C>T, 7364T>C, 7374A>T, 7377G>C, 7380G>A, 7381G>A, 7385T>C, 7388A>T, 7390T>A, 7391A>T, 7397A>T, 7398T>C, 7400A>T, 7401T>A, 7403T>C, 7406G>C, 7409G>A, 7410G>T, 7413G>A, 7414A>T, 7416C>G, 7418G>T, 7422C>G, 7424A>G, 7425A>G, 7432T>C, 7433G>A, 7436T>A, 7437A>T, 7438G>A, 7442T>C, 7443C>A, 7445C>G, 7448C>T, 7449G>A, 7451G>C, 7455G>T, 7457T>A, 7458T>C, 7462T>C, 7463A>T, 7464A>C, 7465A>T, 7467A>G, 7468A>G, 7469A>G, 7470G>C, 7471T>C, 7472T>A, 7473G>A, 7474G>A, 7476G>A, 7477A>C, 7481T>G, 7484T>G, 7487A>G, 7489T>C, 7490C>T, 7494A>G, 7495A>T, 7496G>T, 7499T>A, 7500G>A, 7501T>A, 7502G>A, 7503C>A, 7504T>C, 7505A>G, 7506A>C, 7507G>T, 7508A>G, 7511T>A, 7512G>A, 7517T>A, 7520A>G, 7521A>C, 7522T>C, 7523G>A, 7524C>G, 7526A>T, 7527C>A, 7529C>G, 7535A>C, 7539T>A, 7540C>A, 7541A>T, 7544T>C, 7547G>A, 7553C>T, 7556A>G, 7559T>A, 7562C>T, 7568A>T, 7570T>A, 7572A>G, 7573A>G, 7576G>A, 7577G>T, 7578A>G, 7579G>A, 7580A>C, 7581A>C, 7586A>T, 7588T>A, 7589T>C, 7599A>C, 7600T>C, 7601G>C, 7602G>A, 7603C>A, 7604C>G, 7607G>A, 7610G>A, 7616T>C, 7618T>A, 7622A>T, 7628T>C, 7631A>C, 7632T>G, 7633T>G, 7635C>A, 7636G>A								

CDS

	Begin	End	Coverage	Score	Concordance	Matches	Identities	I/D/M/F*	Stop Codons
CBSV_gp1	2336	2508	5.9%	728	59.9%	173 (100%)	99 (57.2%)	0/0/0/0	0
Protein mutations:	I2337V (7134A>G 7136T>G), Q2345K (7158C>A), K2348E (7167A>G), K2351R (7177A>G 7178A>G), Q2352A (7179C>G 7180A>C), F2354L (7185T>C), P2356Q (7192C>A 7193C>G), T2357S (7194A>T), I2360F (7203A>T 7205C>T), I2361Q (7206A>C 7207T>A 7208C>G), R2363L (7213G>T 7214G>T), V2365L (7218G>C), P2370L (7234C>T 7235A>T), T2373E (7242A>G 7243C>A 7244A>G), V2374P (7245G>C 7246T>C), E2376D (7253A>T), I2378S (7257A>T 7258T>C), V2382A (7270T>C 7271A>T), R2383K (7272C>A 7273G>A 7274T>A), V2384I (7275G>A 7277A>T), S2386A (7281T>G 7283T>C), E2387K (7284G>A 7286G>A), L2388E (7287T>G 7288T>A), I2389V (7290A>G 7292T>G), G2390N (7293G>A 7294G>A 7295A>T), S2399C (7320A>T), D2403N (7332G>A 7334T>C), N2406K (7343T>G), M2417L (7374A>T), E2418Q (7377G>C), G2419K (7380G>A 7381G>A), L2422H (7390T>A 7391A>T), Y2426N (7401T>A 7403T>C), K2427N (7406G>C), V2429L (7410G>T), E2430M (7413G>A 7414A>T), P2431A (7416C>G 7418G>T), Q2433E (7422C>G 7424A>G), N2434D (7425A>G), M2436T (7432T>C 7433G>A), S2438Y (7437A>T 7438G>A), L2440M (7443C>A 7445C>G), V2442I (7449G>A 7451G>C), A2444S (7455G>T 7457T>A), F2445L (7458T>C), V2446A (7462T>C 7463A>T), N2447L (7464A>C 7465A>T), K2448G (7467A>G 7468A>G 7469A>G), V2449P (7470G>C 7471T>C 7472T>A), G2450N (7473G>A 7474G>A), D2451T (7476G>A 7477A>C), D2452E (7481T>G), I2454M (7487A>G), I2455T (7489T>C 7490C>T), K2457V (7494A>G 7495A>T 7496G>T), V2459K (7500G>A 7501T>A 7502G>A), L2460T (7503C>A 7504T>C 7505A>G), R2461L (7506A>C 7507G>T 7508A>G), E2463K (7512G>A), M2466P (7521A>C 7522T>C 7523G>A), P2467A (7524C>G 7526A>T), S2472N (7539T>A 7540C>A 7541A>T), L2482H (7570T>A), N2483G (7572A>G 7573A>G), W2484Y (7576G>A 7577G>T), R2485D (7578A>G 7579G>A 7580A>C), K2486Q (7581A>C), F2488Y (7588T>A 7589T>C), M2492P (7599A>C 7600T>C 7601G>C), A2493K (7602G>A 7603C>A 7604C>G), M2498K (7618T>A), F2503G (7632T>G 7633T>G), R2504K (7635C>A 7636G>A)								
Codon mutations:	GCA2335.CG (7130A>G), CCA2336CCG (7133A>G), ATT2337GTG (7134A>G 7136T>G), TTC2339TTT (7142C>T), GTA2340GTT (7145A>T), ACA2341ACC (7148A>C), GGG2342GGA (7151G>A), GCA2344GCT (7157A>T), CAA2345AAA (7158C>A), AAA2346AAG (7163A>G), AAG2348GAG (7167A>G), GTA2350GTT (7175A>T), AAA2351AGG (7177A>G 7178A>G), CAA2352GCA (7179C>G 7180A>C), ATA2353ATT (7184A>T), TTT2354CTT (7185T>C), GGT2355GGC (7190T>C), CCC2356CAG (7192C>A 7193C>G), ACT2357TCT (7194A>T), CCA2359CCC (7202A>C), ATC2360TTT (7203A>T 7205C>T), ATC2361CAG (7206A>C 7207T>A 7208C>G), TCT2362TCC (7211T>C), CGG2363CTT (7213G>T 7214G>T), AAA2364AAG (7217A>G), GTT2365CTT (7218G>C), CCA2368CCT (7229A>T), CCA2370CTT (7234C>T 7235A>T), GGA2372GGG (7241A>G), ACA2373GAG (7242A>G 7243C>A 7244A>G), GTT2374CCT (7245G>C 7246T>C), GAG2375GAA (7250G>A), GAA2376GAT (7253A>T), ATT2377ATA (7256T>A), ATC2378TCC (7257A>T 7258T>C), GTA2382GCT (7270T>C 7271A>T), CGT2383AAA (7272C>A 7273G>A 7274T>A), GTA2384ATT (7275G>A 7277A>T), GCG2385GCT (7280G>T), TCT2386GCC (7281T>G 7283T>C), GAG2387AAA (7284G>A 7286G>A), TTG2388GAG (7287T>G 7288T>A), ATT2389GTG (7290A>G 7292T>G), GGA2390AAT (7293G>A 7294G>A 7295A>T), GGG2391GGA (7298G>A), GTT2393GTG (7304T>G), CTC2394CTA (7307C>A), GTA2395GTT (7310A>T), GAA2396GAG (7313A>G), ACA2398ACT (7319A>T), AGT2399TGT (7320A>T), TTG2400CTC (7323T>C 7325G>C), TTT2402TTC (7331T>C), GAT2403AAC (7332G>A 7334T>C), GCT2404GCC (7337T>C), CTC2405CTT (7340C>T), AAT2406AAG (7343T>G), GGA2410GGG (7355A>G), CCA2411CCT (7358A>T), TAC2412TAT (7361C>T), ATT2413ATC (7364T>C), ATG2417TTG (7374A>T), GAA2418CAA (7377G>C), GGG2419AAG (7380G>A 7381G>A), ATT2420ATC (7385T>C), GGA2421GGT (7388A>T), CTA2422CAT (7390T>A 7391A>T), GGA2424GGT (7397A>T), TTA2425CTT (7398T>C 7400A>T), TAT2426AAC (7401T>A 7403T>C), AAG2427AAC (7406G>C), TTG2428TTA (7409G>A), GTG2429TTG (7410G>T), GAG2430ATG (7413G>A 7414A>T), CCG2431GCT (7416C>G 7418G>T), CAA2433GAG (7422C>G 7424A>G), AAT2434GAT (7425A>G), ATG2436ACA (7432T>C 7433G>A), GCT2437GCA (7436T>A), AGT2438TAT (7437A>T 7438G>A), GCT2439GCC (7442T>C), CTC2440ATG (7443C>A 7445C>G), TGC2441TGT (7448C>T), GTG2442ATC (7449G>A 7451G>C), GCT2444TCA (7455G>T 7457T>A), TTT2445CTT (7458T>C), GTA2446GCT (7462T>C 7463A>T), AAT2447CTT (7464A>C 7465A>T), AAA2448GGG (7467A>G 7468A>G 7469A>G), GTT2449CCA (7470G>C 7471T>C 7472T>A), GGT2450AAT (7473G>A 7474G>A), GAT2451ACT (7476G>A 7477A>C), GAT2452GAG (7481T>G), CCT2453CCG (7484T>G), ATA2454ATG (7487A>G), ATC2455ACT (7489T>C 7490C>T), AAG2457GTT (7494A>G 7495A>T 7496G>T), GGT2458GGA (7499T>A), GTG2459AAA (7500G>A 7501T>A 7502G>A), CTA2460ACG (7503C>A 7504T>C 7505A>G), AGA2461CTG (7506A>C 7507G>T 7508A>G), GGT2462GGA (7511T>A), GAG2463AAG (7512G>A), ATT2464ATA (7517T>A), GTA2465GTG (7520A>G), ATG2466CCA (7521A>C 7522T>C 7523G>A), CCA2467GCT (7524C>G 7526A>T), CGC2468AGG (7527C>A 7529C>G), CCA2470CCC (7535A>C), TCA2472AAT (7539T>A 7540C>A 7541A>T), TTT2473TTC (7544T>C), GGG2474GGA (7547G>A), GAC2476GAT (7553C>T), CCA2477CCG (7556A>G), ATT2478ATA (7559T>A), TTC2479TTT (7562C>T), CCA2481CCT (7568A>T), CTT2482CAT (7570T>A), AAC2483GGC (7572A>G 7573A>G), TGG2484TAT (7576G>A 7577G>T), AGA2485GAC (7578A>G 7579G>A 7580A>C), AAG2486CAG (7581A>C), ACA2487ACT (7586A>T), TTT2488TAC (7588T>A 7589T>C), ATG2492CCC (7599A>C 7600T>C 7601G>C), GCC2493AAG (7602G>A 7603C>A 7604C>G), GAG2494GAA (7607G>A), GAG2495GAA (7610G>A), AAT2497AAC (7616T>C), ATG2498AAG (7618T>A), ATA2499ATT (7622A>T), CAT2501CAC (7628T>C), CGA2502CGC (7631A>C), TTT2503GGT (7632T>G 7633T>G), CGA2504AAA (7635C>A 7636G>A)								

Proteins

	Begin	End	Coverage	Score	Concordance	Matches	Identities	I/D/M/F*	Stop Codons
polyprotein (YP_007027011.1)	2336	2508	5.9%	728	59.9%	173 (100%)	99 (57.2%)	0/0/0/0	0
Protein mutations:	I2337V (7134A>G 7136T>G), Q2345K (7158C>A), K2348E (7167A>G), K2351R (7177A>G 7178A>G), Q2352A (7179C>G 7180A>C), F2354L (7185T>C), P2356Q (7192C>A 7193C>G), T2357S (7194A>T), I2360F (7203A>T 7205C>T), I2361Q (7206A>C 7207T>A 7208C>G), R2363L (7213G>T 7214G>T), V2365L (7218G>C), P2370L (7234C>T 7235A>T), T2373E (7242A>G 7243C>A 7244A>G), V2374P (7245G>C 7246T>C), E2376D (7253A>T), I2378S (7257A>T 7258T>C), V2382A (7270T>C 7271A>T), R2383K (7272C>A 7273G>A 7274T>A), V2384I (7275G>A 7277A>T), S2386A (7281T>G 7283T>C), E2387K (7284G>A 7286G>A), L2388E (7287T>G 7288T>A), I2389V (7290A>G 7292T>G), G2390N (7293G>A 7294G>A 7295A>T), S2399C (7320A>T), D2403N (7332G>A 7334T>C), N2406K (7343T>G), M2417L (7374A>T), E2418Q (7377G>C), G2419K (7380G>A 7381G>A), L2422H (7390T>A 7391A>T), Y2426N (7401T>A 7403T>C), K2427N (7406G>C), V2429L (7410G>T), E2430M (7413G>A 7414A>T), P2431A (7416C>G 7418G>T), Q2433E (7422C>G 7424A>G), N2434D (7425A>G), M2436T (7432T>C 7433G>A), S2438Y (7437A>T 7438G>A), L2440M (7443C>A 7445C>G), V2442I (7449G>A 7451G>C), A2444S (7455G>T 7457T>A), F2445L (7458T>C), V2446A (7462T>C 7463A>T), N2447L (7464A>C 7465A>T), K2448G (7467A>G 7468A>G 7469A>G), V2449P (7470G>C 7471T>C 7472T>A), G2450N (7473G>A 7474G>A), D2451T (7476G>A 7477A>C), D2452E (7481T>G), I2454M (7487A>G), I2455T (7489T>C 7490C>T), K2457V (7494A>G 7495A>T 7496G>T), V2459K (7500G>A 7501T>A 7502G>A), L2460T (7503C>A 7504T>C 7505A>G), R2461L (7506A>C 7507G>T 7508A>G), E2463K (7512G>A), M2466P (7521A>C 7522T>C 7523G>A), P2467A (7524C>G 7526A>T), S2472N (7539T>A 7540C>A 7541A>T), L2482H (7570T>A), N2483G (7572A>G 7573A>G), W2484Y (7576G>A 7577G>T), R2485D (7578A>G 7579G>A 7580A>C), K2486Q (7581A>C), F2488Y (7588T>A 7589T>C), M2492P (7599A>C 7600T>C 7601G>C), A2493K (7602G>A 7603C>A 7604C>G), M2498K (7618T>A), F2503G (7632T>G 7633T>G), R2504K (7635C>A 7636G>A)								

	Begin	End	Coverage	Score	Concordance	Matches	Identities	I/D/M/F*	Stop Codons
**NT**	**7129**	**7647**	**5.8%**	**214**	**20.6%**	**519 (100%)**	**313 (60.3%)**	**0/0**	
Codon mutations:	GCA2335.CG (7130A>G), CCA2336CCG (7133A>G), ATT2337GTG (7134A>G 7136T>G), TTC2339TTT (7142C>T), GTA2340GTT (7145A>T), ACA2341ACC (7148A>C), GGG2342GGA (7151G>A), GCA2344GCT (7157A>T), CAA2345AAA (7158C>A), AAA2346AAG (7163A>G), AAG2348GAG (7167A>G), GTA2350GTT (7175A>T), AAA2351AGG (7177A>G 7178A>G), CAA2352GCA (7179C>G 7180A>C), ATA2353ATT (7184A>T), TTT2354CTT (7185T>C), GGT2355GGC (7190T>C), CCC2356CAG (7192C>A 7193C>G), ACT2357TCT (7194A>T), CCA2359CCC (7202A>C), ATC2360TTT (7203A>T 7205C>T), ATC2361CAG (7206A>C 7207T>A 7208C>G), TCT2362TCC (7211T>C), CGG2363CTT (7213G>T 7214G>T), AAA2364AAG (7217A>G), GTT2365CTT (7218G>C), CCA2368CCT (7229A>T), CCA2370CTT (7234C>T 7235A>T), GGA2372GGG (7241A>G), ACA2373GAG (7242A>G 7243C>A 7244A>G), GTT2374CCT (7245G>C 7246T>C), GAG2375GAA (7250G>A), GAA2376GAT (7253A>T), ATT2377ATA (7256T>A), ATC2378TCC (7257A>T 7258T>C), GTA2382GCT (7270T>C 7271A>T), CGT2383AAA (7272C>A 7273G>A 7274T>A), GTA2384ATT (7275G>A 7277A>T), GCG2385GCT (7280G>T), TCT2386GCC (7281T>G 7283T>C), GAG2387AAA (7284G>A 7286G>A), TTG2388GAG (7287T>G 7288T>A), ATT2389GTG (7290A>G 7292T>G), GGA2390AAT (7293G>A 7294G>A 7295A>T), GGG2391GGA (7298G>A), GTT2393GTG (7304T>G), CTC2394CTA (7307C>A), GTA2395GTT (7310A>T), GAA2396GAG (7313A>G), ACA2398ACT (7319A>T), AGT2399TGT (7320A>T), TTG2400CTC (7323T>C 7325G>C), TTT2402TTC (7331T>C), GAT2403AAC (7332G>A 7334T>C), GCT2404GCC (7337T>C), CTC2405CTT (7340C>T), AAT2406AAG (7343T>G), GGA2410GGG (7355A>G), CCA2411CCT (7358A>T), TAC2412TAT (7361C>T), ATT2413ATC (7364T>C), ATG2417TTG (7374A>T), GAA2418CAA (7377G>C), GGG2419AAG (7380G>A 7381G>A), ATT2420ATC (7385T>C), GGA2421GGT (7388A>T), CTA2422CAT (7390T>A 7391A>T), GGA2424GGT (7397A>T), TTA2425CTT (7398T>C 7400A>T), TAT2426AAC (7401T>A 7403T>C), AAG2427AAC (7406G>C), TTG2428TTA (7409G>A), GTG2429TTG (7410G>T), GAG2430ATG (7413G>A 7414A>T), CCG2431GCT (7416C>G 7418G>T), CAA2433GAG (7422C>G 7424A>G), AAT2434GAT (7425A>G), ATG2436ACA (7432T>C 7433G>A), GCT2437GCA (7436T>A), AGT2438TAT (7437A>T 7438G>A), GCT2439GCC (7442T>C), CTC2440ATG (7443C>A 7445C>G), TGC2441TGT (7448C>T), GTG2442ATC (7449G>A 7451G>C), GCT2444TCA (7455G>T 7457T>A), TTT2445CTT (7458T>C), GTA2446GCT (7462T>C 7463A>T), AAT2447CTT (7464A>C 7465A>T), AAA2448GGG (7467A>G 7468A>G 7469A>G), GTT2449CCA (7470G>C 7471T>C 7472T>A), GGT2450AAT (7473G>A 7474G>A), GAT2451ACT (7476G>A 7477A>C), GAT2452GAG (7481T>G), CCT2453CCG (7484T>G), ATA2454ATG (7487A>G), ATC2455ACT (7489T>C 7490C>T), AAG2457GTT (7494A>G 7495A>T 7496G>T), GGT2458GGA (7499T>A), GTG2459AAA (7500G>A 7501T>A 7502G>A), CTA2460ACG (7503C>A 7504T>C 7505A>G), AGA2461CTG (7506A>C 7507G>T 7508A>G), GGT2462GGA (7511T>A), GAG2463AAG (7512G>A), ATT2464ATA (7517T>A), GTA2465GTG (7520A>G), ATG2466CCA (7521A>C 7522T>C 7523G>A), CCA2467GCT (7524C>G 7526A>T), CGC2468AGG (7527C>A 7529C>G), CCA2470CCC (7535A>C), TCA2472AAT (7539T>A 7540C>A 7541A>T), TTT2473TTC (7544T>C), GGG2474GGA (7547G>A), GAC2476GAT (7553C>T), CCA2477CCG (7556A>G), ATT2478ATA (7559T>A), TTC2479TTT (7562C>T), CCA2481CCT (7568A>T), CTT2482CAT (7570T>A), AAC2483GGC (7572A>G 7573A>G), TGG2484TAT (7576G>A 7577G>T), AGA2485GAC (7578A>G 7579G>A 7580A>C), AAG2486CAG (7581A>C), ACA2487ACT (7586A>T), TTT2488TAC (7588T>A 7589T>C), ATG2492CCC (7599A>C 7600T>C 7601G>C), GCC2493AAG (7602G>A 7603C>A 7604C>G), GAG2494GAA (7607G>A), GAG2495GAA (7610G>A), AAT2497AAC (7616T>C), ATG2498AAG (7618T>A), ATA2499ATT (7622A>T), CAT2501CAC (7628T>C), CGA2502CGC (7631A>C), TTT2503GGT (7632T>G 7633T>G), CGA2504AAA (7635C>A 7636G>A)								
HAM1-like protein (YP_007032446.1)	28	200	76.5%	728	59.9%	173 (100%)	99 (57.2%)	0/0/0/0	0
Protein mutations:	I29V (7134A>G 7136T>G), Q37K (7158C>A), K40E (7167A>G), K43R (7177A>G 7178A>G), Q44A (7179C>G 7180A>C), F46L (7185T>C), P48Q (7192C>A 7193C>G), T49S (7194A>T), I52F (7203A>T 7205C>T), I53Q (7206A>C 7207T>A 7208C>G), R55L (7213G>T 7214G>T), V57L (7218G>C), P62L (7234C>T 7235A>T), T65E (7242A>G 7243C>A 7244A>G), V66P (7245G>C 7246T>C), E68D (7253A>T), I70S (7257A>T 7258T>C), V74A (7270T>C 7271A>T), R75K (7272C>A 7273G>A 7274T>A), V76I (7275G>A 7277A>T), S78A (7281T>G 7283T>C), E79K (7284G>A 7286G>A), L80E (7287T>G 7288T>A), I81V (7290A>G 7292T>G), G82N (7293G>A 7294G>A 7295A>T), S91C (7320A>T), D95N (7332G>A 7334T>C), N98K (7343T>G), M109L (7374A>T), E110Q (7377G>C), G111K (7380G>A 7381G>A), L114H (7390T>A 7391A>T), Y118N (7401T>A 7403T>C), K119N (7406G>C), V121L (7410G>T), E122M (7413G>A 7414A>T), P123A (7416C>G 7418G>T), Q125E (7422C>G 7424A>G), N126D (7425A>G), M128T (7432T>C 7433G>A), S130Y (7437A>T 7438G>A), L132M (7443C>A 7445C>G), V134I (7449G>A 7451G>C), A136S (7455G>T 7457T>A), F137L (7458T>C), V138A (7462T>C 7463A>T), N139L (7464A>C 7465A>T), K140G (7467A>G 7468A>G 7469A>G), V141P (7470G>C 7471T>C 7472T>A), G142N (7473G>A 7474G>A), D143T (7476G>A 7477A>C), D144E (7481T>G), I146M (7487A>G), I147T (7489T>C 7490C>T), K149V (7494A>G 7495A>T 7496G>T), V151K (7500G>A 7501T>A 7502G>A), L152T (7503C>A 7504T>C 7505A>G), R153L (7506A>C 7507G>T 7508A>G), E155K (7512G>A), M158P (7521A>C 7522T>C 7523G>A), P159A (7524C>G 7526A>T), S164N (7539T>A 7540C>A 7541A>T), L174H (7570T>A), N175G (7572A>G 7573A>G), W176Y (7576G>A 7577G>T), R177D (7578A>G 7579G>A 7580A>C), K178Q (7581A>C), F180Y (7588T>A 7589T>C), M184P (7599A>C 7600T>C 7601G>C), A185K (7602G>A 7603C>A 7604C>G), M190K (7618T>A), F195G (7632T>G 7633T>G), R196K (7635C>A 7636G>A)								
Codon mutations:	GCA27.CG (7130A>G), CCA28CCG (7133A>G), ATT29GTG (7134A>G 7136T>G), TTC31TTT (7142C>T), GTA32GTT (7145A>T), ACA33ACC (7148A>C), GGG34GGA (7151G>A), GCA36GCT (7157A>T), CAA37AAA (7158C>A), AAA38AAG (7163A>G), AAG40GAG (7167A>G), GTA42GTT (7175A>T), AAA43AGG (7177A>G 7178A>G), CAA44GCA (7179C>G 7180A>C), ATA45ATT (7184A>T), TTT46CTT (7185T>C), GGT47GGC (7190T>C), CCC48CAG (7192C>A 7193C>G), ACT49TCT (7194A>T), CCA51CCC (7202A>C), ATC52TTT (7203A>T 7205C>T), ATC53CAG (7206A>C 7207T>A 7208C>G), TCT54TCC (7211T>C), CGG55CTT (7213G>T 7214G>T), AAA56AAG (7217A>G), GTT57CTT (7218G>C), CCA60CCT (7229A>T), CCA62CTT (7234C>T 7235A>T), GGA64GGG (7241A>G), ACA65GAG (7242A>G 7243C>A 7244A>G), GTT66CCT (7245G>C 7246T>C), GAG67GAA (7250G>A), GAA68GAT (7253A>T), ATT69ATA (7256T>A), ATC70TCC (7257A>T 7258T>C), GTA74GCT (7270T>C 7271A>T), CGT75AAA (7272C>A 7273G>A 7274T>A), GTA76ATT (7275G>A 7277A>T), GCG77GCT (7280G>T), TCT78GCC (7281T>G 7283T>C), GAG79AAA (7284G>A 7286G>A), TTG80GAG (7287T>G 7288T>A), ATT81GTG (7290A>G 7292T>G), GGA82AAT (7293G>A 7294G>A 7295A>T), GGG83GGA (7298G>A), GTT85GTG (7304T>G), CTC86CTA (7307C>A), GTA87GTT (7310A>T), GAA88GAG (7313A>G), ACA90ACT (7319A>T), AGT91TGT (7320A>T), TTG92CTC (7323T>C 7325G>C), TTT94TTC (7331T>C), GAT95AAC (7332G>A 7334T>C), GCT96GCC (7337T>C), CTC97CTT (7340C>T), AAT98AAG (7343T>G), GGA102GGG (7355A>G), CCA103CCT (7358A>T), TAC104TAT (7361C>T), ATT105ATC (7364T>C), ATG109TTG (7374A>T), GAA110CAA (7377G>C), GGG111AAG (7380G>A 7381G>A), ATT112ATC (7385T>C), GGA113GGT (7388A>T), CTA114CAT (7390T>A 7391A>T), GGA116GGT (7397A>T), TTA117CTT (7398T>C 7400A>T), TAT118AAC (7401T>A 7403T>C), AAG119AAC (7406G>C), TTG120TTA (7409G>A), GTG121TTG (7410G>T), GAG122ATG (7413G>A 7414A>T), CCG123GCT (7416C>G 7418G>T), CAA125GAG (7422C>G 7424A>G), AAT126GAT (7425A>G), ATG128ACA (7432T>C 7433G>A), GCT129GCA (7436T>A), AGT130TAT (7437A>T 7438G>A), GCT131GCC (7442T>C), CTC132ATG (7443C>A 7445C>G), TGC133TGT (7448C>T), GTG134ATC (7449G>A 7451G>C), GCT136TCA (7455G>T 7457T>A), TTT137CTT (7458T>C), GTA138GCT (7462T>C 7463A>T), AAT139CTT (7464A>C 7465A>T), AAA140GGG (7467A>G 7468A>G 7469A>G), GTT141CCA (7470G>C 7471T>C 7472T>A), GGT142AAT (7473G>A 7474G>A), GAT143ACT (7476G>A 7477A>C), GAT144GAG (7481T>G), CCT145CCG (7484T>G), ATA146ATG (7487A>G), ATC147ACT (7489T>C 7490C>T), AAG149GTT (7494A>G 7495A>T 7496G>T), GGT150GGA (7499T>A), GTG151AAA (7500G>A 7501T>A 7502G>A), CTA152ACG (7503C>A 7504T>C 7505A>G), AGA153CTG (7506A>C 7507G>T 7508A>G), GGT154GGA (7511T>A), GAG155AAG (7512G>A), ATT156ATA (7517T>A), GTA157GTG (7520A>G), ATG158CCA (7521A>C 7522T>C 7523G>A), CCA159GCT (7524C>G 7526A>T), CGC160AGG (7527C>A 7529C>G), CCA162CCC (7535A>C), TCA164AAT (7539T>A 7540C>A 7541A>T), TTT165TTC (7544T>C), GGG166GGA (7547G>A), GAC168GAT (7553C>T), CCA169CCG (7556A>G), ATT170ATA (7559T>A), TTC171TTT (7562C>T), CCA173CCT (7568A>T), CTT174CAT (7570T>A), AAC175GGC (7572A>G 7573A>G), TGG176TAT (7576G>A 7577G>T), AGA177GAC (7578A>G 7579G>A 7580A>C), AAG178CAG (7581A>C), ACA179ACT (7586A>T), TTT180TAC (7588T>A 7589T>C), ATG184CCC (7599A>C 7600T>C 7601G>C), GCC185AAG (7602G>A 7603C>A 7604C>G), GAG186GAA (7607G>A), GAG187GAA (7610G>A), AAT189AAC (7616T>C), ATG190AAG (7618T>A), ATA191ATT (7622A>T), CAT193CAC (7628T>C), CGA194CGC (7631A>C), TTT195GGT (7632T>G 7633T>G), CGA196AAA (7635C>A 7636G>A)								

*: Inserts / Deletes / Misaligned / Frameshifts

Analysis details

This analysis was performed with panviral2.64

NGS Details (UN60): Lowelvirus tuscon4d

Assembly

**Coverage Length**	164 (1 contig(s))
**Depth Of Coverage**	188.4
**Number Of Reads**	355
**Reads Per Million**	7.53 rpm (after QC)
**Ambiguities**	0
**Assembly Method**	de novo + reference guided assembly
**Consensus Caller**	Bcf Tools

Coverage Map

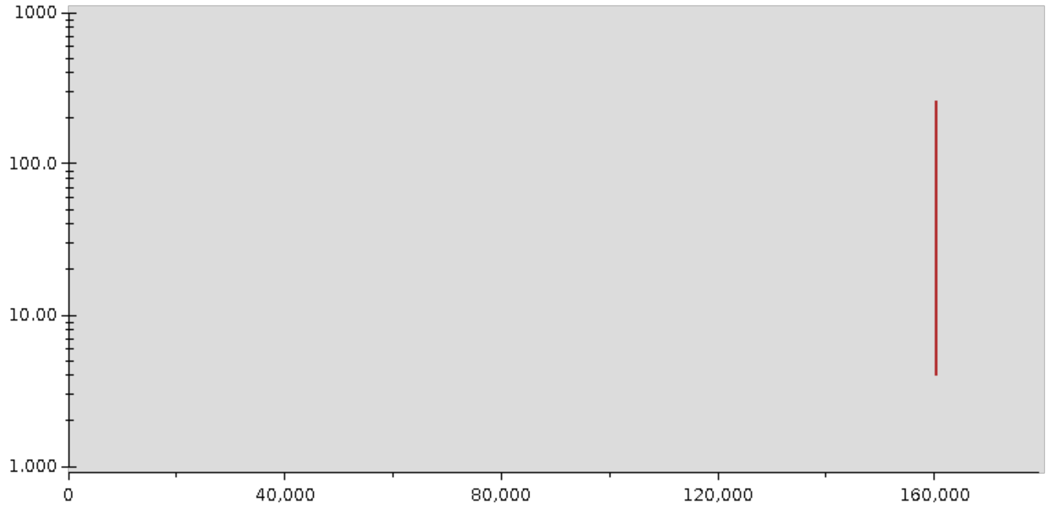

Assignment

**Type**	Lowelvirus tuscon4d (Taxonomy ID: 2956131)
**Reference Genome**	NC_026923.1
**NT Identity (%)**	80.8642
**AA Identity (%)**	90.566
**Number Of Stop Codons**	1
**Number Of CDS**	218

Alignment

**Alignment Score**	189.0 (NT) + 233.0 (AA) = 422.0
**Concordance (%)**	75.5481
**Alignment Method**	Local, heuristic, nucleotide (BLASTN)

Genome Region

Sequence starts at position 160235 and ends at position 160398 relative to NC_026923.1 reference sequence.

Alignment Detailed Statistics

	Begin	End	Coverage	Score	Concordance	Matches	Identities	I/D/M/F*	Stop Codons
**NT**	**160235**	**160398**	**0.1%**	**189**	**60.4%**	**162 (98.8%)**	**131 (79.9%)**	**0/2**	
Mutations:	160246A>C, 160248A>C, 160254A>G, 160270G>A, 160275C>T, 160287G>A, 160290C>T, 160291_160292delAT, 160293C>A, 160296A>C, 160299G>A, 160302T>C, 160308A>T, 160309C>A, 160326A>G, 160327C>G, 160330G>C, 160331C>A, 160332T>G, 160338A>G, 160341A>T, 160350C>T, 160356C>A, 160359C>T, 160368A>G, 160371A>T, 160377T>A, 160378A>C, 160380A>T, 160383T>A, 160389A>T, 160392G>T								

*: Inserts / Deletes / Misaligned / Frameshifts

Analysis details

This analysis was performed with panviral2.64

NGS Details (UN60): Badnavirus occultipomeae

Assembly

**Coverage Length**	345 (1 contig(s))
**Depth Of Coverage**	112.5
**Number Of Reads**	353
**Reads Per Million**	7.49 rpm (after QC)
**Ambiguities**	0
**Assembly Method**	de novo + reference guided assembly
**Consensus Caller**	Bcf Tools

Coverage Map

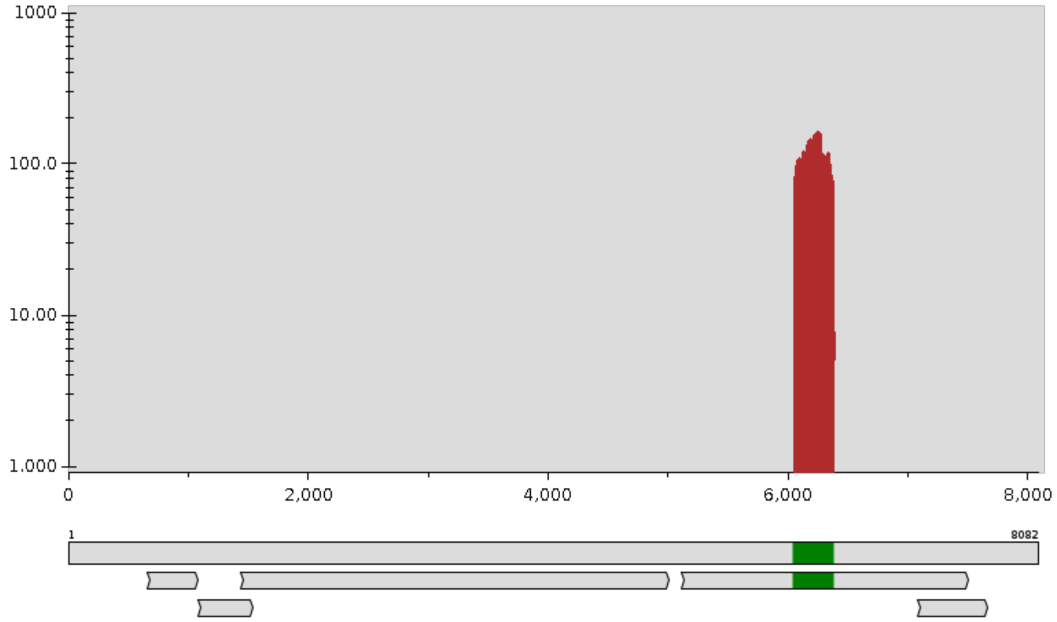

Assignment

**Type**	Badnavirus occultipomeae (Taxonomy ID: 3048353)
**Reference Genome**	NC_015655.1
**NT Identity (%)**	51.7241
**AA Identity (%)**	45.6897
**Number Of Stop Codons**	0
**Number Of CDS**	5

Alignment

**Alignment Score**	18.0 (NT) + 370.0 (AA) = 388.0
**Concordance (%)**	25.9532

Alignment Method	Global, seeded, nucleotide + amino acids (AGA)

Genome Region

Sequence starts at position 6040 and ends at position 6384 relative to NC_015655.1 reference sequence.

Alignment Detailed Statistics

	Begin	End	Coverage	Score	Concordance	Matches	Identities	I/D/M/F*	Stop Codons
**NT**	**6040**	**6384**	**4.3%**	**18**	**2.6%**	**345 (99.1%)**	**180 (51.7%)**	**3/0**	
Mutations:	6042A>G, 6043T>A, 6051C>T, 6052A>C, 6053A>G, 6055G>T, 6057A>G, 6060C>T, 6062T>A, 6063T>C, 6070A>T, 6072A>G, 6076A>G, 6078G>T, 6079G>C, 6080A>G, 6084A>G, 6085A>T, 6086A>C, 6087G>A, 6088T>G, 6089C>A, 6090C>T, 6092A>T, 6093A>T, 6096A>G, 6097T>A, 6098G>A, 6105T>G, 6108T>C, 6109T>A, 6114T>C, 6116C>G, 6117A>C, 6118G>T, 6120A>T, 6123G>T, 6125T>A, 6126C>T, 6132A>G, 6137A>T, 6138A>G, 6141C>T, 6145C>T, 6147C>T, 6153A>G, 6154C>T, 6158T>C, 6159G>T, 6165A>C, 6168A>T, 6171A>T, 6173A>C, 6177T>C, 6178C>A, 6179A>T, 6181A>G, 6182G>A, 6183A>G, 6184A>T, 6185A>T, 6190G>A, 6192T>C, 6193A>C, 6194A>G, 6195T>A, 6197C>T, 6198A>G, 6201T>C, 6203G>A, 6204G>A, 6205G>T, 6206G>C, 6207A>T, 6207_6208insTAT, 6208A>C, 6209C>T, 6210A>T, 6214G>C, 6215C>A, 6216A>G, 6220A>G, 6222C>G, 6223G>A, 6224C>T, 6225A>T, 6228A>G, 6230A>T, 6231T>C, 6234T>A, 6240C>T, 6243A>C, 6249A>T, 6251T>A, 6255T>C, 6256G>A, 6258A>G, 6260A>G, 6264A>G, 6265G>A, 6266A>G, 6267A>T, 6270G>T, 6275A>T, 6277G>A, 6279T>A, 6282T>C, 6285C>G, 6286T>A, 6287T>G, 6289A>T, 6290A>T, 6292T>G, 6294G>A, 6295G>T, 6296C>T, 6297C>A, 6300A>G, 6302T>G, 6303T>G, 6304G>T, 6309A>G, 6310A>G, 6311G>A, 6312A>T, 6315A>G, 6316G>A, 6317G>A, 6319C>T, 6321T>G, 6322A>T, 6323T>A, 6324T>C, 6325C>G, 6326T>C, 6327A>C, 6329G>A, 6330C>G, 6331C>T, 6332C>T, 6333A>C, 6334A>T, 6339A>G, 6340A>T, 6341T>G, 6342G>T, 6343A>G, 6345A>G, 6346A>T, 6349G>T, 6351A>G, 6352G>C, 6354C>A, 6355A>C, 6357A>G, 6358G>T, 6359A>C, 6363G>A, 6364G>T, 6365A>C, 6369C>T, 6375A>T, 6376A>C, 6377T>A, 6378A>T, 6379A>G								
**CDS**									
SPBVa_gp4	309	423	14.5%	370	46.0%	115 (99.1%)	53 (45.7%)	1/0/0/0	0
Protein mutations:	F310I (6043T>A), K313R (6052A>C 6053A>G), A314S (6055G>T 6057A>G), F316Y (6062T>A 6063T>C), I319L (6070A>T 6072A>G), M321V (6076A>G 6078G>T), E322R (6079G>C 6080A>G), K324S (6085A>T 6086A>C 6087G>A), S325D (6088T>G 6089C>A 6090C>T), K326I (6092A>T 6093A>T), W328K (6097T>A 6098G>A), W332R (6109T>A), P334R (6116C>G 6117A>C), E335Y (6118G>T 6120A>T), L337H (6125T>A 6126C>T), E341V (6137A>T 6138A>G), P344S (6145C>T 6147C>T), M348T (6158T>C 6159G>T), D353A (6173A>C), Q355M (6178C>A 6179A>T), R356E (6181A>G 6182G>A 6183A>G), K357L (6184A>T 6185A>T), D359N (6190G>A 6192T>C), N360R (6193A>C 6194A>G 6195T>A), A361V (6197C>T 6198A>G), R363K (6203G>A 6204G>A), G364S (6205G>T 6206G>C 6207A>T), G364_T365insY (6207_6208insTAT), T365L (6208A>C 6209C>T 6210A>T), A367Q (6214G>C 6215C>A 6216A>G), I369V (6220A>G 6222C>G), A370I (6223G>A 6224C>T 6225A>T), Y372F (6230A>T 6231T>C), F379Y (6251T>A), E381K (6256G>A 6258A>G), N382S (6260A>G), E384S (6265G>A 6266A>G 6267A>T), E385D (6270G>T), E387V (6275A>T), D388K (6277G>A 6279T>A), L391R (6286T>A 6287T>G), N392F (6289A>T 6290A>T), L393V (6292T>G 6294G>A), A394L (6295G>T 6296C>T 6297C>A), I396R (6302T>G 6303T>G), V397L (6304G>T), R399D (6310A>G 6311G>A 6312A>T), G401K (6316G>A 6317G>A), I403Y (6322A>T 6323T>A 6324T>C), L404A (6325C>G 6326T>C 6327A>C), S405K (6329G>A 6330C>G), P406F (6331C>T 6332C>T 6333A>C), T407S (6334A>T), M409C (6340A>T 6341T>G 6342G>T), K410E (6343A>G 6345A>G), I411F (6346A>T), G412W (6349G>T 6351A>G), V413L (6352G>C 6354C>A), K414Q (6355A>C 6357A>G), E415S (6358G>T 6359A>C), D417S (6364G>T 6365A>C), I421H (6376A>C 6377T>A 6378A>T), K422E (6379A>G)								
Codon mutations:	AAA309AAG (6042A>G), TTT310ATT (6043T>A), CTC312CTT (6051C>T), AAA313CGA (6052A>C 6053A>G), GCA314TCG (6055G>T 6057A>G), GGC315GGT (6060C>T), TTT316TAC (6062T>A 6063T>C), ATA319TTG (6070A>T 6072A>G), ATG321GTT (6076A>G 6078G>T), GAG322CGG (6079G>C 6080A>G), GAA323GAG (6084A>G), AAG324TCA (6085A>T 6086A>C 6087G>A), TCC325GAT (6088T>G 6089C>A 6090C>T), AAA326ATT (6092A>T 6093A>T), CCA327CCG (6096A>G), TGG328AAG (6097T>A 6098G>A), GCT330GCG (6105T>G), TTT331TTC (6108T>C), TGG332AGG (6109T>A), ACT333ACC (6114T>C), CCA334CGC (6116C>G 6117A>C), GAA335TAT (6118G>T 6120A>T), GGG336GGT (6123G>T), CTC337CAT (6125T>A 6126C>T), GAA339GAG (6132A>G), GAA341GTG (6137A>T 6138A>G), GTC342GTT (6141C>T), CCC344TCT (6145C>T 6147C>T), GGA346GGG (6153A>G), CTG347TTG (6154C>T), ATG348ACT (6158T>C 6159G>T), GCA350GCC (6165A>C), CCA351CCT (6168A>T), GCA352GCT (6171A>T), GAT353GCT (6173A>C), TTT354TTC (6177T>C), CAG355ATG (6178C>A 6179A>T), AGA356GAG (6181A>G 6182G>A 6183A>G), AAG357TTG (6184A>T 6185A>T), GAT359AAC (6190G>A 6192T>C), AAT360CGA (6193A>C 6194A>G 6195T>A), GCA361GTG (6197C>T 6198A>G), TTT362TTC (6201T>C), AGG363AAA (6203G>A 6204G>A), GGA364TCT (6205G>T 6206G>C 6207A>T), GGA364_ACA365insTAT (6207_6208insTAT), ACA365CTT (6208A>C 6209C>T 6210A>T), GCA367CAG (6214G>C 6215C>A 6216A>G), ATC369GTG (6220A>G 6222C>G), GCA370ATT (6223G>A 6224C>T 6225A>T), GTA371GTG (6228A>G), TAT372TTC (6230A>T 6231T>C), ATT373ATA (6234T>A), GAC375GAT (6240C>T), ATA376ATC (6243A>C), GTA378GTT (6249A>T), TTC379TAC (6251T>A), TCT380TCC (6255T>C), GAA381AAG (6256G>A 6258A>G), AAT382AGT (6260A>G), GAA383GAG (6264A>G), GAA384AGT (6265G>A 6266A>G 6267A>T), GAG385GAT (6270G>T), GAG387GTG (6275A>T), GAT388AAA (6277G>A 6279T>A), CAT389CAC (6282T>C), CTC390CTG (6285C>G), TTA391AGA (6286T>A 6287T>G), AAT392TTT (6289A>T 6290A>T), TTG393GTA (6292T>G 6294G>A), GCC394TTA (6295G>T 6296C>T 6297C>A), CAA395CAG (6300A>G), ATT396AGG (6302T>G 6303T>G), GTG397TTG (6304G>T), AGA398AGG (6309A>G), AGA399GAT (6310A>G 6311G>A 6312A>T), GAA400GAG (6315A>G), GGG401AAG (6316G>A 6317G>A), CTT402TTG (6319C>T 6321T>G), ATT403TAC (6322A>T 6323T>A 6324T>C), CTA404GCC (6325C>G 6326T>C 6327A>C), AGC405AAG (6329G>A 6330C>G), CCA406TTC (6331C>T 6332C>T 6333A>C), ACA407TCA (6334A>T), AAA408AAG (6339A>G), ATG409TGT (6340A>T 6341T>G 6342G>T), AAA410GAG (6343A>G 6345A>G), ATT411TTT (6346A>T), GGA412TGG (6349G>T 6351A>G), GTC413CTA (6352G>C 6354C>A), AAA414CAG (6355A>C 6357A>G), GAA415TCA (6358G>T 6359A>C), GTG416GTA (6363G>A), GAT417TCT (6364G>T 6365A>C), TTC418TTT (6369C>T), GGA420GGT (6375A>T), ATA421CAT (6376A>C 6377T>A 6378A>T), AAG422GAG (6379A>G)								
**Proteins**									
RNaseH/reverse transcriptase (YP_004581513.1)	309	423	14.5%	370	46.0%	115 (99.1%)	53 (45.7%)	1/0/0/0	0
Protein mutations:	F310I (6043T>A), K313R (6052A>C 6053A>G), A314S (6055G>T 6057A>G), F316Y (6062T>A 6063T>C), I319L (6070A>T 6072A>G), M321V (6076A>G 6078G>T), E322R (6079G>C 6080A>G), K324S (6085A>T 6086A>C 6087G>A), S325D (6088T>G 6089C>A 6090C>T), K326I (6092A>T 6093A>T), W328K (6097T>A 6098G>A), W332R (6109T>A), P334R (6116C>G 6117A>C), E335Y (6118G>T 6120A>T), L337H (6125T>A 6126C>T), E341V (6137A>T 6138A>G), P344S (6145C>T 6147C>T), M348T (6158T>C 6159G>T), D353A (6173A>C), Q355M (6178C>A 6179A>T), R356E (6181A>G 6182G>A 6183A>G), K357L (6184A>T 6185A>T), D359N (6190G>A 6192T>C), N360R (6193A>C 6194A>G 6195T>A), A361V (6197C>T 6198A>G), R363K (6203G>A 6204G>A), G364S (6205G>T 6206G>C 6207A>T), G364_T365insY (6207_6208insTAT), T365L (6208A>C 6209C>T 6210A>T), A367Q (6214G>C 6215C>A 6216A>G), I369V (6220A>G 6222C>G), A370I (6223G>A 6224C>T 6225A>T), Y372F (6230A>T 6231T>C), F379Y (6251T>A), E381K (6256G>A 6258A>G), N382S (6260A>G), E384S (6265G>A 6266A>G 6267A>T), E385D (6270G>T), E387V (6275A>T), D388K (6277G>A 6279T>A), L391R (6286T>A 6287T>G), N392F (6289A>T 6290A>T), L393V (6292T>G 6294G>A), A394L (6295G>T 6296C>T 6297C>A), I396R (6302T>G 6303T>G), V397L (6304G>T), R399D (6310A>G 6311G>A 6312A>T), G401K (6316G>A 6317G>A), I403Y (6322A>T 6323T>A 6324T>C), L404A (6325C>G 6326T>C 6327A>C), S405K (6329G>A 6330C>G), P406F (6331C>T 6332C>T 6333A>C), T407S (6334A>T), M409C (6340A>T 6341T>G 6342G>T), K410E (6343A>G 6345A>G), I411F (6346A>T), G412W (6349G>T 6351A>G), V413L (6352G>C 6354C>A), K414Q (6355A>C 6357A>G), E415S (6358G>T 6359A>C), D417S (6364G>T 6365A>C), I421H (6376A>C 6377T>A 6378A>T), K422E (6379A>G)								
Codon mutations:	AAA309AAG (6042A>G), TTT310ATT (6043T>A), CTC312CTT (6051C>T), AAA313CGA (6052A>C 6053A>G), GCA314TCG (6055G>T 6057A>G), GGC315GGT (6060C>T), TTT316TAC (6062T>A 6063T>C), ATA319TTG (6070A>T 6072A>G), ATG321GTT (6076A>G 6078G>T), GAG322CGG (6079G>C 6080A>G), GAA323GAG (6084A>G), AAG324TCA (6085A>T 6086A>C 6087G>A), TCC325GAT (6088T>G 6089C>A 6090C>T), AAA326ATT (6092A>T 6093A>T), CCA327CCG (6096A>G), TGG328AAG (6097T>A 6098G>A), GCT330GCG (6105T>G), TTT331TTC (6108T>C), TGG332AGG (6109T>A), ACT333ACC (6114T>C), CCA334CGC (6116C>G 6117A>C), GAA335TAT (6118G>T 6120A>T), GGG336GGT (6123G>T), CTC337CAT (6125T>A 6126C>T), GAA339GAG (6132A>G), GAA341GTG (6137A>T 6138A>G), GTC342GTT (6141C>T), CCC344TCT (6145C>T 6147C>T), GGA346GGG (6153A>G), CTG347TTG (6154C>T), ATG348ACT (6158T>C 6159G>T), GCA350GCC (6165A>C), CCA351CCT (6168A>T), GCA352GCT (6171A>T), GAT353GCT (6173A>C), TTT354TTC (6177T>C), CAG355ATG (6178C>A 6179A>T), AGA356GAG (6181A>G 6182G>A 6183A>G), AAG357TTG (6184A>T 6185A>T), GAT359AAC (6190G>A 6192T>C), AAT360CGA (6193A>C 6194A>G 6195T>A), GCA361GTG (6197C>T 6198A>G), TTT362TTC (6201T>C), AGG363AAA (6203G>A 6204G>A), GGA364TCT (6205G>T 6206G>C 6207A>T), GGA364_ACA365insTAT (6207_6208insTAT), ACA365CTT (6208A>C 6209C>T 6210A>T), GCA367CAG (6214G>C 6215C>A 6216A>G), ATC369GTG (6220A>G 6222C>G), GCA370ATT (6223G>A 6224C>T 6225A>T), GTA371GTG (6228A>G), TAT372TTC (6230A>T 6231T>C), ATT373ATA (6234T>A), GAC375GAT (6240C>T), ATA376ATC (6243A>C), GTA378GTT (6249A>T), TTC379TAC (6251T>A), TCT380TCC (6255T>C), GAA381AAG (6256G>A 6258A>G), AAT382AGT (6260A>G), GAA383GAG (6264A>G), GAA384AGT (6265G>A 6266A>G 6267A>T), GAG385GAT (6270G>T), GAG387GTG (6275A>T), GAT388AAA (6277G>A 6279T>A), CAT389CAC (6282T>C), CTC390CTG (6285C>G), TTA391AGA (6286T>A 6287T>G), AAT392TTT (6289A>T 6290A>T), TTG393GTA (6292T>G 6294G>A), GCC394TTA (6295G>T 6296C>T 6297C>A), CAA395CAG (6300A>G), ATT396AGG (6302T>G 6303T>G), GTG397TTG (6304G>T), AGA398AGG (6309A>G), AGA399GAT (6310A>G 6311G>A 6312A>T), GAA400GAG (6315A>G), GGG401AAG (6316G>A 6317G>A), CTT402TTG (6319C>T 6321T>G), ATT403TAC (6322A>T 6323T>A 6324T>C), CTA404GCC (6325C>G 6326T>C 6327A>C), AGC405AAG (6329G>A 6330C>G), CCA406TTC (6331C>T 6332C>T 6333A>C), ACA407TCA (6334A>T), AAA408AAG (6339A>G), ATG409TGT (6340A>T 6341T>G 6342G>T), AAA410GAG (6343A>G 6345A>G), ATT411TTT (6346A>T), GGA412TGG (6349G>T 6351A>G), GTC413CTA (6352G>C 6354C>A), AAA414CAG (6355A>C 6357A>G), GAA415TCA (6358G>T 6359A>C), GTG416GTA (6363G>A), GAT417TCT (6364G>T 6365A>C), TTC418TTT (6369C>T), GGA420GGT (6375A>T), ATA421CAT (6376A>C 6377T>A 6378A>T), AAG422GAG (6379A>G)								

*: Inserts / Deletes / Misaligned / Frameshifts

Analysis details

This analysis was performed with panviral2.64

NGS Details (UN60): Errantivirus

Assembly

**Coverage Length**	638 (1 contig(s))
**Depth Of Coverage**	29.2
**Number Of Reads**	227
**Reads Per Million**	4.81 rpm (after QC)
**Ambiguities**	0
**Assembly Method**	de novo + reference guided assembly
**Consensus Caller**	Bcf Tools

Coverage Map

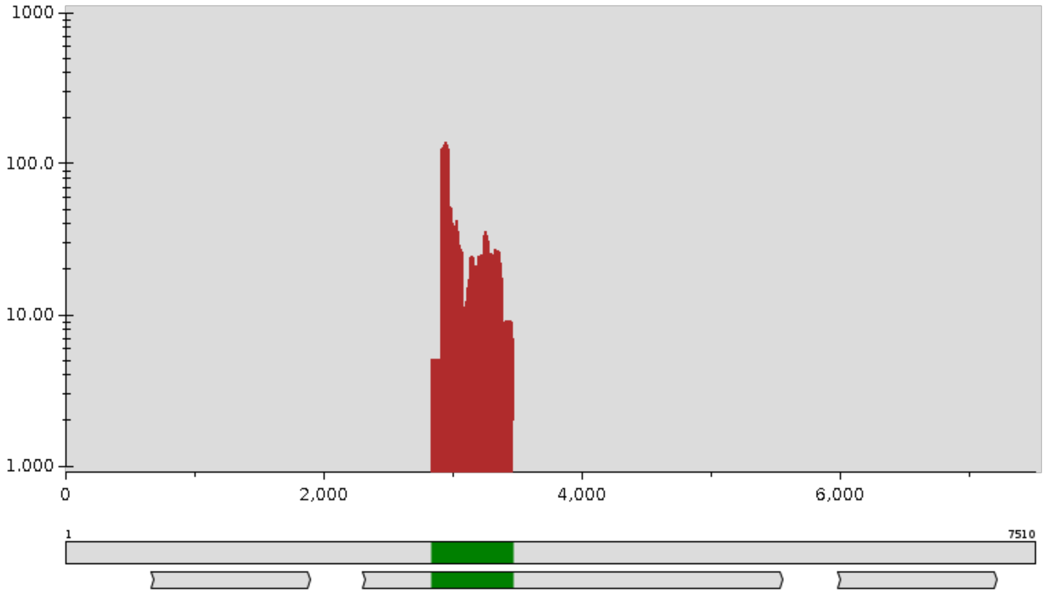

Assignment

**Type**	Errantivirus (Taxonomy ID: 186666)
**Reference Genome**	NC_038512.1
**NT Identity (%)**	55.1834
**AA Identity (%)**	43.5407
**Number Of Stop Codons**	0
**Number Of CDS**	3

Alignment

**Alignment Score**	101.0 (NT) + 551.0 (AA) = 652.0
**Concordance (%)**	25.7402

Alignment Method	Global, seeded, nucleotide + amino acids (AGA)

Genome Region

Sequence starts at position 2834 and ends at position 3471 relative to NC_038512.1 reference sequence.

Alignment Detailed Statistics

	Begin	End	Coverage	Score	Concordance	Matches	Identities	I/D/M/F*	Stop Codons
**NT**	**2834**	**3471**	**8.5%**	**101**	**8.2%**	**627 (98.3%)**	**346 (54.2%)**	**0/11**	
Mutations:	2836C>A, 2837A>G, 2839A>G, 2841G>T, 2843G>T, 2845T>A, 2849_2851delCCC, 2859T>A, 2860C>A, 2863C>T, 2865C>G, 2866T>G, 2867T>A, 2870_2877delGGGAAACA, 2880A>C, 2881G>A, 2885C>A, 2887T>A, 2888C>T, 2891G>T, 2892T>A, 2893A>T, 2894G>A, 2901T>A, 2903C>A, 2905T>A, 2906A>G, 2909T>C, 2914C>T, 2915G>A, 2917G>C, 2919A>T, 2920G>C, 2924A>G, 2926C>G, 2927G>A, 2928A>G, 2929T>G, 2932C>T, 2934A>G, 2935A>G, 2937A>T, 2941G>A, 2944A>C, 2949A>C, 2950C>T, 2953A>T, 2954A>G, 2955G>A, 2959C>G, 2960G>C, 2963C>T, 2968C>T, 2969A>G, 2972T>G, 2975G>A, 2976G>A, 2978A>G, 2979A>G, 2980G>C, 2981T>G, 2982G>C, 2983C>T, 2984C>G, 2985A>T, 2986A>T, 2989C>T, 2993A>T, 2995C>A, 2997C>A, 2998C>G, 2999T>C, 3001A>T, 3008G>T, 3009C>T, 3011A>T, 3012G>C, 3016G>A, 3018T>A, 3019T>C, 3020T>G, 3025G>A, 3026G>A, 3028G>T, 3029G>C, 3030A>G, 3032A>G, 3034G>T, 3035G>A, 3037C>G, 3038C>G, 3039C>T, 3040T>G, 3041C>G, 3042A>C, 3050T>C, 3052G>C, 3058C>T, 3061G>T, 3066A>G, 3067C>G, 3068G>A, 3069T>C, 3071G>C, 3072A>G, 3074C>T, 3076C>T, 3080C>A, 3081A>G, 3084T>A, 3095C>G, 3096G>T, 3103T>G, 3104A>T, 3106G>T, 3109A>G, 3110T>C, 3114A>C, 3115A>T, 3118C>T, 3119T>G, 3121A>C, 3124A>G, 3125T>G, 3133T>C, 3134C>T, 3135A>G, 3136A>T, 3138G>A, 3139A>C, 3140G>T, 3142T>A, 3146G>A, 3157A>T, 3158A>T, 3159G>A, 3160A>T, 3162G>A, 3164C>T, 3165T>A, 3166C>T, 3168A>T, 3169A>T, 3170A>G, 3173A>G, 3176A>T, 3179T>G, 3180G>T, 3181T>A, 3182C>G, 3184C>A, 3191C>T, 3193T>A, 3196C>T, 3199T>C, 3202T>A, 3203A>G, 3205T>C, 3206G>A, 3208C>A, 3216C>G, 3217T>A, 3218T>A, 3220C>T, 3221C>T, 3223A>G, 3224C>G, 3226G>A, 3229A>C, 3232C>T, 3233C>T, 3236G>C, 3237A>T, 3239A>C, 3241C>T, 3242C>T, 3245G>A, 3246A>G, 3247A>G, 3248C>A, 3249G>A, 3250A>G, 3253T>G, 3256C>G, 3272A>T, 3273G>A, 3274T>C, 3275A>C, 3277C>A, 3278T>C, 3281A>T, 3283A>T, 3284A>G, 3286T>C, 3287C>A, 3289A>G, 3295C>G, 3300C>G, 3301C>T, 3307C>T, 3309T>C, 3310G>T, 3312A>G, 3313G>T, 3315T>G, 3316C>T, 3319A>G, 3321C>T, 3322T>C, 3323G>A, 3324C>A, 3325T>G, 3327A>T, 3329C>T, 3331T>A, 3334T>C, 3337C>T, 3338A>T, 3340C>G, 3341A>G, 3343A>T, 3346C>T, 3347A>G, 3348G>A, 3349G>A, 3350G>A, 3353G>C, 3354G>A, 3355T>A, 3356A>G, 3358C>G, 3359A>C, 3360A>G, 3361G>C, 3362C>A, 3363C>T, 3364T>G, 3365A>G, 3371G>A, 3373T>G, 3376G>A, 3377A>G, 3379T>G, 3380T>C, 3381C>A, 3382C>G, 3385T>A, 3386A>G, 3388T>A, 3389C>G, 3390A>T, 3392A>G, 3394A>T, 3396A>G, 3397T>G, 3399T>A, 3400G>A, 3401A>G, 3402T>C, 3403T>A, 3406A>C, 3408A>C, 3409G>T, 3410A>G, 3411C>G, 3412C>G, 3413C>G, 3414C>T, 3415T>C, 3422A>C, 3426A>G, 3428C>T, 3429A>C, 3430A>C, 3431T>A, 3439C>G, 3442T>G, 3443C>G, 3445C>A, 3446G>A, 3447G>A, 3448T>C, 3454C>T								

CDS

	Begin	End	Coverage	Score	Concordance	Matches	Identities	I/D/M/F*	Stop Codons
D1R33_gp2	177	389	19.5%	551	38.1%	209 (98.6%)	91 (42.9%)	0/3/1/1	0
Protein mutations:	I178V (2837A>G 2839A>G), W179L (2841G>T), V180L (2843G>T 2845T>A), P182del (2849_2851delCCC), I185K (2859T>A 2860C>A), A187G (2865C>G 2866T>G), S188T (2867T>A), G189_K190del (2870_2877delGGGAAACA), K192T (2880A>C 2881G>A), L195F (2888C>T), V196Y (2891G>T 2892T>A 2893A>T), V197I (2894G>A), F199Y (2901T>A), K201E (2906A>G), E204N (2915G>A 2917G>C), K205I (2919A>T 2920G>C), I207V (2924A>G 2926C>G), D208R (2927G>A 2928A>G 2929T>G), K210R (2934A>G 2935A>G), Y211F (2937A>T), N215T (2949A>C 2950C>T), S217D (2954A>G 2955G>A), D218E (2959C>G), V219L (2960G>C), L220F (2963C>T), K222E (2969A>G), L223V (2972T>G), G224N (2975G>A 2976G>A), K225G (2978A>G 2979A>G 2980G>C), C226A (2981T>G 2982G>C 2983C>T), Q227V (2984C>G 2985A>T 2986A>T), T230S (2993A>T 2995C>A), T231K (2997C>A 2998C>G), A235L (3008G>T 3009C>T), F238Y (3018T>A 3019T>C), Y239D (3020T>G), V241I (3026G>A 3028G>T), E242R (3029G>C 3030A>G), M243V (3032A>G 3034G>T), D244K (3035G>A 3037C>G), P245V (3038C>G 3039C>T 3040T>G), Q246A (3041C>G 3042A>C), S249P (3050T>C 3052G>C), N254R (3066A>G 3067C>G), V255T (3068G>A 3069T>C), E256R (3071G>C 3072A>G), H257Y (3074C>T 3076C>T), H259S (3080C>A 3081A>G), F260Y (3084T>A), R264V (3095C>G 3096G>T), M267F (3104A>T 3106G>T), K270T (3114A>C 3115A>T), S272A (3119T>G 3121A>C), S274A (3125T>G), Q277C (3134C>T 3135A>G 3136A>T), R278N (3138G>A 3139A>C), V279L (3140G>T 3142T>A), D281N (3146G>A), R285Y (3158A>T 3159G>A 3160A>T), G286D (3162G>A), L287Y (3164C>T 3165T>A 3166C>T), Q288L (3168A>T 3169A>T), N289D (3170A>G), N290D (3173A>G), I291F (3176A>T), C292V (3179T>G 3180G>T 3181T>A), L293V (3182C>G 3184C>A), I300V (3203A>G 3205T>C), V301I (3206G>A 3208C>A), T304R (3216C>G 3217T>A), S305T (3218T>A 3220C>T), Q307E (3224C>G 3226G>A), E308D (3229A>C), E311L (3236G>C 3237A>T), N312H (3239A>C 3241C>T), E314R (3245G>A 3246A>G 3247A>G), R315K (3248C>A 3249G>A 3250A>G), F317L (3256C>G), S323Y (3272A>T 3273G>A 3274T>C), N324Q (3275A>C 3277C>A), F325L (3278T>C), K326Y (3281A>T 3283A>T), I327V (3284A>G 3286T>C), Q328K (3287C>A 3289A>G), D330E (3295C>G), S332C (3300C>G 3301C>T), L335S (3309T>C 3310G>T), K336S (3312A>G 3313G>T), L337R (3315T>G 3316C>T), T339I (3321C>T 3322T>C), A340K (3323G>A 3324C>A 3325T>G), Y341F (3327A>T), I345L (3338A>T 3340C>G), I346V (3341A>G 3343A>T), R348E (3347A>G 3348G>A 3349G>A), D349N (3350G>A), G350Q (3353G>C 3354G>A 3355T>A), I351V (3356A>G 3358C>G), K352R (3359A>C 3360A>G 3361G>C), P353M (3362C>A 3363C>T 3364T>G), N354D (3365A>G), D356K (3371G>A 3373T>G), I358V (3377A>G 3379T>G), S359Q (3380T>C 3381C>A 3382C>G), I361V (3386A>G 3388T>A), Q362V (3389C>G 3390A>T), K363D (3392A>G 3394A>T), Y364W (3396A>G 3397T>G), L365Q (3399T>A 3400G>A), I366A (3401A>G 3402T>C 3403T>A), K368T (3408A>C 3409G>T), T369G (3410A>G 3411C>G 3412C>G), P370V (3413C>G 3414C>T 3415T>C), I373L (3422A>C), K374R (3426A>G), Q375S (3428C>T 3429A>C 3430A>C), F376I (3431T>A), L380V (3443C>G 3445C>A), G381N (3446G>A 3447G>A 3448T>C)								
Codon mutations:	CCC177CCA (2836C>A), ATA178GTG (2837A>G 2839A>G), TGG179TTG (2841G>T), GTT180TTA (2843G>T 2845T>A), CCC182del (2849_2851delCCC), ATC185AAA (2859T>A 2860C>A), GAC186GAT (2863C>T), GCT187GGG (2865C>G 2866T>G), TCT188ACT (2867T>A), GGG189_AAA190del (2870_2877delGGGAAACA), CAA191--A (2870_2877delGGGAAACA), AAG192ACA (2880A>C 2881G>A), CGT194AGA (2885C>A 2887T>A), CTC195TTC (2888C>T), GTA196TAT (2891G>T 2892T>A 2893A>T), GTT197ATT (2894G>A), TTC199TAC (2901T>A), CGT200AGA (2903C>A 2905T>A), AAG201GAG (2906A>G), TTG202CTG (2909T>C), AAC203AAT (2914C>T), GAG204AAC (2915G>A 2917G>C), AAG205ATC (2919A>T 2920G>C), ATC207GTG (2924A>G 2926C>G), GAT208AGG (2927G>A 2928A>G 2929T>G), GAC209GAT (2932C>T), AAA210AGG (2934A>G 2935A>G), TAC211TTC (2937A>T), CCG212CCA (2941G>A), ATA213ATC (2944A>C), AAC215ACT (2949A>C 2950C>T), ATA216ATT (2953A>T), AGT217GAT (2954A>G 2955G>A), GAC218GAG (2959C>G), GTA219CTA (2960G>C), CTT220TTT (2963C>T), GAC221GAT (2968C>T), AAG222GAG (2969A>G), TTA223GTA (2972T>G), GGT224AAT (2975G>A 2976G>A), AAG225GGC (2978A>G 2979A>G 2980G>C), TGC226GCT (2981T>G 2982G>C 2983C>T), CAA227GTT (2984C>G 2985A>T 2986A>T), TAC228TAT (2989C>T), ACC230TCA (2993A>T 2995C>A), ACC231AAG (2997C>A 2998C>G), TTA232CTT (2999T>C 3001A>T), GCA235TTA (3008G>T 3009C>T), AGT236TCT (3011A>T 3012G>C), GGG237GGA (3016G>A), TTT238TAC (3018T>A 3019T>C), TAT239GAT (3020T>G), CAG240CAA (3025G>A), GTG241ATT (3026G>A 3028G>T), GAG242CGG (3029G>C 3030A>G), ATG243GTT (3032A>G 3034G>T), GAC244AAG (3035G>A 3037C>G), CCT245GTG (3038C>G 3039C>T 3040T>G), CAA246GCA (3041C>G 3042A>C), TCG249CCC (3050T>C 3052G>C), ACC251ACT (3058C>T), GCG252GCT (3061G>T), AAC254AGG (3066A>G 3067C>G), GTA255ACA (3068G>A 3069T>C), GAA256CGA (3071G>C 3072A>G), CAC257TAT (3074C>T 3076C>T), CAT259AGT (3080C>A 3081A>G), TTT260TAT (3084T>A), CGA264GTA (3095C>G 3096G>T), CCT266CCG (3103T>G), ATG267TTT (3104A>T 3106G>T), GGA268GGG (3109A>G), TTA269CTA (3110T>C), AAA270ACT (3114A>C 3115A>T), AAC271AAT (3118C>T), TCA272GCC (3119T>G 3121A>C), CCA273CCG (3124A>G), TCT274GCT (3125T>G), TTT276TTC (3133T>C), CAA277TGT (3134C>T 3135A>G 3136A>T), AGA278AAC (3138G>A 3139A>C), GTT279TTA (3140G>T 3142T>A), GAC281AAC (3146G>A), CTA284CTT (3157A>T), AGA285TAT (3158A>T 3159G>A 3160A>T), GGT286GAT (3162G>A), CTC287TAT (3164C>T 3165T>A 3166C>T), CAA288CTT (3168A>T 3169A>T), AAT289GAT (3170A>G), AAC290GAC (3173A>G), ATC291TTC (3176A>T), TGT292GTA (3179T>G 3180G>T 3181T>A), CTC293GTA (3182C>G 3184C>A), CTT296TTA (3191C>T 3193T>A), GAC297GAT (3196C>T), GAT298GAC (3199T>C), ATT299ATA (3202T>A), ATT300GTC (3203A>G 3205T>C), GTC301ATA (3206G>A 3208C>A), ACT304AGA (3216C>G 3217T>A), TCC305ACT (3218T>A 3220C>T), CTA306TTG (3221C>T 3223A>G), CAG307GAA (3224C>G 3226G>A), GAA308GAC (3229A>C), CAC309CAT (3232C>T), CTG310TTG (3233C>T), GAG311CTG (3236G>C 3237A>T), AAC312CAT (3239A>C 3241C>T), CTG313TTG (3242C>T), GAA314AGG (3245G>A 3246A>G 3247A>G), CGA315AAG (3248C>A 3249G>A 3250A>G), GTT316GTG (3253T>G), TTC317TTG (3256C>G), AGT323TAC (3272A>T 3273G>A 3274T>C), AAC324CAA (3275A>C 3277C>A), TTC325CTC (3278T>C), AAA326TAT (3281A>T 3283A>T), ATT327GTC (3284A>G 3286T>C), CAA328AAG (3287C>A 3289A>G), GAC330GAG (3295C>G), TCC332TGT (3300C>G 3301C>T), TTC334TTT (3307C>T), TTG335TCT (3309T>C 3310G>T), AAG336AGT (3312A>G 3313G>T), CTC337CGT (3315T>G 3316C>T), GAA338GAG (3319A>G), ACT339ATC (3321C>T 3322T>C), GCT340AAG (3323G>A 3324C>A 3325T>G), TAT341TTT (3327A>T), CTT342TTA (3329C>T 3331T>A), GGT343GGC (3334T>C), CAC344CAT (3337C>T), ATC345TTG (3338A>T 3340C>G), ATA346GTT (3341A>G 3343A>T), AGC347AGT (3346C>T), AGG348GAA (3347A>G 3348G>A 3349G>A), GAC349AAC (3350G>A), GGT350CAA (3353G>C 3354G>A 3355T>A), ATC351GTG (3356A>G 3358C>G), AAG352CGC (3359A>C 3360A>G 3361G>C), CCT353ATG (3362C>A 3363C>T 3364T>G), AAC354GAC (3365A>G), GAT356AAG (3371G>A 3373T>G), AAG357AAA (3376G>A), ATT358GTG (3377A>G 3379T>G), TCC359CAG (3380T>C 3381C>A 3382C>G), GCT360GCA (3385T>A), ATT361GTA (3386A>G 3388T>A), CAA362GTA (3389C>G 3390A>T), AAA363GAT (3392A>G 3394A>T), TAT364TGG (3396A>G 3397T>G), CTG365CAA (3399T>A 3400G>A), ATT366GCA (3401A>G 3402T>C 3403T>A), CCA367CCC (3406A>C), AAG368ACT (3408A>C 3409G>T), ACC369GGG (3410A>G 3411C>G 3412C>G), CCT370GTC (3413C>G 3414C>T 3415T>C), ATA373CTA (3422A>C), AAA374AGA (3426A>G), CAA375TCC (3428C>T 3429A>C 3430A>C), TTT376ATT (3431T>A), GGC378GGG (3439C>G), CTT379CTG (3442T>G), CTC380GTA (3443C>G 3445C>A), GGT381AAC (3446G>A 3447G>A 3448T>C), TAC383TAT (3454C>T)								

Proteins

	Begin	End	Coverage	Score	Concordance	Matches	Identities	I/D/M/F*	Stop Codons
ORF B (YP_009507248.1)	177	389	19.5%	551	38.1%	209 (98.6%)	91 (42.9%)	0/3/1/1	0

	Begin	End	Coverage	Score	Concordance	Matches	Identities	I/D/M/F*	Stop Codons
**NT**	**2834**	**3471**	**8.5%**	**101**	**8.2%**	**627 (98.3%)**	**346 (54.2%)**	**0/11**	

Protein mutations:	I178V (2837A>G 2839A>G), W179L (2841G>T), V180L (2843G>T 2845T>A), P182del (2849_2851delCCC), I185K (2859T>A 2860C>A), A187G (2865C>G 2866T>G), S188T (2867T>A), G189_K190del (2870_2877delGGGAAACA), K192T (2880A>C 2881G>A), L195F (2888C>T), V196Y (2891G>T 2892T>A 2893A>T), V197I (2894G>A), F199Y (2901T>A), K201E (2906A>G), E204N (2915G>A 2917G>C), K205I (2919A>T 2920G>C), I207V (2924A>G 2926C>G), D208R (2927G>A 2928A>G 2929T>G), K210R (2934A>G 2935A>G), Y211F (2937A>T), N215T (2949A>C 2950C>T), S217D (2954A>G 2955G>A), D218E (2959C>G), V219L (2960G>C), L220F (2963C>T), K222E (2969A>G), L223V (2972T>G), G224N (2975G>A 2976G>A), K225G (2978A>G 2979A>G 2980G>C), C226A (2981T>G 2982G>C 2983C>T), Q227V (2984C>G 2985A>T 2986A>T), T230S (2993A>T 2995C>A), T231K (2997C>A 2998C>G), A235L (3008G>T 3009C>T), F238Y (3018T>A 3019T>C), Y239D (3020T>G), V241I (3026G>A 3028G>T), E242R (3029G>C 3030A>G), M243V (3032A>G 3034G>T), D244K (3035G>A 3037C>G), P245V (3038C>G 3039C>T 3040T>G), Q246A (3041C>G 3042A>C), S249P (3050T>C 3052G>C), N254R (3066A>G 3067C>G), V255T (3068G>A 3069T>C), E256R (3071G>C 3072A>G), H257Y (3074C>T 3076C>T), H259S (3080C>A 3081A>G), F260Y (3084T>A), R264V (3095C>G 3096G>T), M267F (3104A>T 3106G>T), K270T (3114A>C 3115A>T), S272A (3119T>G 3121A>C), S274A (3125T>G), Q277C (3134C>T 3135A>G 3136A>T), R278N (3138G>A 3139A>C), V279L (3140G>T 3142T>A), D281N (3146G>A), R285Y (3158A>T 3159G>A 3160A>T), G286D (3162G>A), L287Y (3164C>T 3165T>A 3166C>T), Q288L (3168A>T 3169A>T), N289D (3170A>G), N290D (3173A>G), I291F (3176A>T), C292V (3179T>G 3180G>T 3181T>A), L293V (3182C>G 3184C>A), I300V (3203A>G 3205T>C), V301I (3206G>A 3208C>A), T304R (3216C>G 3217T>A), S305T (3218T>A 3220C>T), Q307E (3224C>G 3226G>A), E308D (3229A>C), E311L (3236G>C 3237A>T), N312H (3239A>C 3241C>T), E314R (3245G>A 3246A>G 3247A>G), R315K (3248C>A 3249G>A 3250A>G), F317L (3256C>G), S323Y (3272A>T 3273G>A 3274T>C), N324Q (3275A>C 3277C>A), F325L (3278T>C), K326Y (3281A>T 3283A>T), I327V (3284A>G 3286T>C), Q328K (3287C>A 3289A>G), D330E (3295C>G), S332C (3300C>G 3301C>T), L335S (3309T>C 3310G>T), K336S (3312A>G 3313G>T), L337R (3315T>G 3316C>T), T339I (3321C>T 3322T>C), A340K (3323G>A 3324C>A 3325T>G), Y341F (3327A>T), I345L (3338A>T 3340C>G), I346V (3341A>G 3343A>T), R348E (3347A>G 3348G>A 3349G>A), D349N (3350G>A), G350Q (3353G>C 3354G>A 3355T>A), I351V (3356A>G 3358C>G), K352R (3359A>C 3360A>G 3361G>C), P353M (3362C>A 3363C>T 3364T>G), N354D (3365A>G), D356K (3371G>A 3373T>G), I358V (3377A>G 3379T>G), S359Q (3380T>C 3381C>A 3382C>G), I361V (3386A>G 3388T>A), Q362V (3389C>G 3390A>T), K363D (3392A>G 3394A>T), Y364W (3396A>G 3397T>G), L365Q (3399T>A 3400G>A), I366A (3401A>G 3402T>C 3403T>A), K368T (3408A>C 3409G>T), T369G (3410A>G 3411C>G 3412C>G), P370V (3413C>G 3414C>T 3415T>C), I373L (3422A>C), K374R (3426A>G), Q375S (3428C>T 3429A>C 3430A>C), F376I (3431T>A), L380V (3443C>G 3445C>A), G381N (3446G>A 3447G>A 3448T>C)
Codon mutations:	CCC177CCA (2836C>A), ATA178GTG (2837A>G 2839A>G), TGG179TTG (2841G>T), GTT180TTA (2843G>T 2845T>A), CCC182del (2849_2851delCCC), ATC185AAA (2859T>A 2860C>A), GAC186GAT (2863C>T), GCT187GGG (2865C>G 2866T>G), TCT188ACT (2867T>A), GGG189_AAA190del (2870_2877delGGGAAACA), CAA191--A (2870_2877delGGGAAACA), AAG192ACA (2880A>C 2881G>A), CGT194AGA (2885C>A 2887T>A), CTC195TTC (2888C>T), GTA196TAT (2891G>T 2892T>A 2893A>T), GTT197ATT (2894G>A), TTC199TAC (2901T>A), CGT200AGA (2903C>A 2905T>A), AAG201GAG (2906A>G), TTG202CTG (2909T>C), AAC203AAT (2914C>T), GAG204AAC (2915G>A 2917G>C), AAG205ATC (2919A>T 2920G>C), ATC207GTG (2924A>G 2926C>G), GAT208AGG (2927G>A 2928A>G 2929T>G), GAC209GAT (2932C>T), AAA210AGG (2934A>G 2935A>G), TAC211TTC (2937A>T), CCG212CCA (2941G>A), ATA213ATC (2944A>C), AAC215ACT (2949A>C 2950C>T), ATA216ATT (2953A>T), AGT217GAT (2954A>G 2955G>A), GAC218GAG (2959C>G), GTA219CTA (2960G>C), CTT220TTT (2963C>T), GAC221GAT (2968C>T), AAG222GAG (2969A>G), TTA223GTA (2972T>G), GGT224AAT (2975G>A 2976G>A), AAG225GGC (2978A>G 2979A>G 2980G>C), TGC226GCT (2981T>G 2982G>C 2983C>T), CAA227GTT (2984C>G 2985A>T 2986A>T), TAC228TAT (2989C>T), ACC230TCA (2993A>T 2995C>A), ACC231AAG (2997C>A 2998C>G), TTA232CTT (2999T>C 3001A>T), GCA235TTA (3008G>T 3009C>T), AGT236TCT (3011A>T 3012G>C), GGG237GGA (3016G>A), TTT238TAC (3018T>A 3019T>C), TAT239GAT (3020T>G), CAG240CAA (3025G>A), GTG241ATT (3026G>A 3028G>T), GAG242CGG (3029G>C 3030A>G), ATG243GTT (3032A>G 3034G>T), GAC244AAG (3035G>A 3037C>G), CCT245GTG (3038C>G 3039C>T 3040T>G), CAA246GCA (3041C>G 3042A>C), TCG249CCC (3050T>C 3052G>C), ACC251ACT (3058C>T), GCG252GCT (3061G>T), AAC254AGG (3066A>G 3067C>G), GTA255ACA (3068G>A 3069T>C), GAA256CGA (3071G>C 3072A>G), CAC257TAT (3074C>T 3076C>T), CAT259AGT (3080C>A 3081A>G), TTT260TAT (3084T>A), CGA264GTA (3095C>G 3096G>T), CCT266CCG (3103T>G), ATG267TTT (3104A>T 3106G>T), GGA268GGG (3109A>G), TTA269CTA (3110T>C), AAA270ACT (3114A>C 3115A>T), AAC271AAT (3118C>T), TCA272GCC (3119T>G 3121A>C), CCA273CCG (3124A>G), TCT274GCT (3125T>G), TTT276TTC (3133T>C), CAA277TGT (3134C>T 3135A>G 3136A>T), AGA278AAC (3138G>A 3139A>C), GTT279TTA (3140G>T 3142T>A), GAC281AAC (3146G>A), CTA284CTT (3157A>T), AGA285TAT (3158A>T 3159G>A 3160A>T), GGT286GAT (3162G>A), CTC287TAT (3164C>T 3165T>A 3166C>T), CAA288CTT (3168A>T 3169A>T), AAT289GAT (3170A>G), AAC290GAC (3173A>G), ATC291TTC (3176A>T), TGT292GTA (3179T>G 3180G>T 3181T>A), CTC293GTA (3182C>G 3184C>A), CTT296TTA (3191C>T 3193T>A), GAC297GAT (3196C>T), GAT298GAC (3199T>C), ATT299ATA (3202T>A), ATT300GTC (3203A>G 3205T>C), GTC301ATA (3206G>A 3208C>A), ACT304AGA (3216C>G 3217T>A), TCC305ACT (3218T>A 3220C>T), CTA306TTG (3221C>T 3223A>G), CAG307GAA (3224C>G 3226G>A), GAA308GAC (3229A>C), CAC309CAT (3232C>T), CTG310TTG (3233C>T), GAG311CTG (3236G>C 3237A>T), AAC312CAT (3239A>C 3241C>T), CTG313TTG (3242C>T), GAA314AGG (3245G>A 3246A>G 3247A>G), CGA315AAG (3248C>A 3249G>A 3250A>G), GTT316GTG (3253T>G), TTC317TTG (3256C>G), AGT323TAC (3272A>T 3273G>A 3274T>C), AAC324CAA (3275A>C 3277C>A), TTC325CTC (3278T>C), AAA326TAT (3281A>T 3283A>T), ATT327GTC (3284A>G 3286T>C), CAA328AAG (3287C>A 3289A>G), GAC330GAG (3295C>G), TCC332TGT (3300C>G 3301C>T), TTC334TTT (3307C>T), TTG335TCT (3309T>C 3310G>T), AAG336AGT (3312A>G 3313G>T), CTC337CGT (3315T>G 3316C>T), GAA338GAG (3319A>G), ACT339ATC (3321C>T 3322T>C), GCT340AAG (3323G>A 3324C>A 3325T>G), TAT341TTT (3327A>T), CTT342TTA (3329C>T 3331T>A), GGT343GGC (3334T>C), CAC344CAT (3337C>T), ATC345TTG (3338A>T 3340C>G), ATA346GTT (3341A>G 3343A>T), AGC347AGT (3346C>T), AGG348GAA (3347A>G 3348G>A 3349G>A), GAC349AAC (3350G>A), GGT350CAA (3353G>C 3354G>A 3355T>A), ATC351GTG (3356A>G 3358C>G), AAG352CGC (3359A>C 3360A>G 3361G>C), CCT353ATG (3362C>A 3363C>T 3364T>G), AAC354GAC (3365A>G), GAT356AAG (3371G>A 3373T>G), AAG357AAA (3376G>A), ATT358GTG (3377A>G 3379T>G), TCC359CAG (3380T>C 3381C>A 3382C>G), GCT360GCA (3385T>A), ATT361GTA (3386A>G 3388T>A), CAA362GTA (3389C>G 3390A>T), AAA363GAT (3392A>G 3394A>T), TAT364TGG (3396A>G 3397T>G), CTG365CAA (3399T>A 3400G>A), ATT366GCA (3401A>G 3402T>C 3403T>A), CCA367CCC (3406A>C), AAG368ACT (3408A>C 3409G>T), ACC369GGG (3410A>G 3411C>G 3412C>G), CCT370GTC (3413C>G 3414C>T 3415T>C), ATA373CTA (3422A>C), AAA374AGA (3426A>G), CAA375TCC (3428C>T 3429A>C 3430A>C), TTT376ATT (3431T>A), GGC378GGG (3439C>G), CTT379CTG (3442T>G), CTC380GTA (3443C>G 3445C>A), GGT381AAC (3446G>A 3447G>A 3448T>C), TAC383TAT (3454C>T)

*: Inserts / Deletes / Misaligned / Frameshifts

Analysis details

This analysis was performed with panviral2.64

NGS Details (UN60): Ichnoviriform fugitivi (segment B17)

Assembly

**Coverage Length**	134 (1 contig(s))
**Depth Of Coverage**	1.0
**Number Of Reads**	1
**Reads Per Million**	0.02 rpm (after QC)
**Ambiguities**	0
**Assembly Method**	de novo + reference guided assembly
**Consensus Caller**	Bcf Tools

Coverage Map

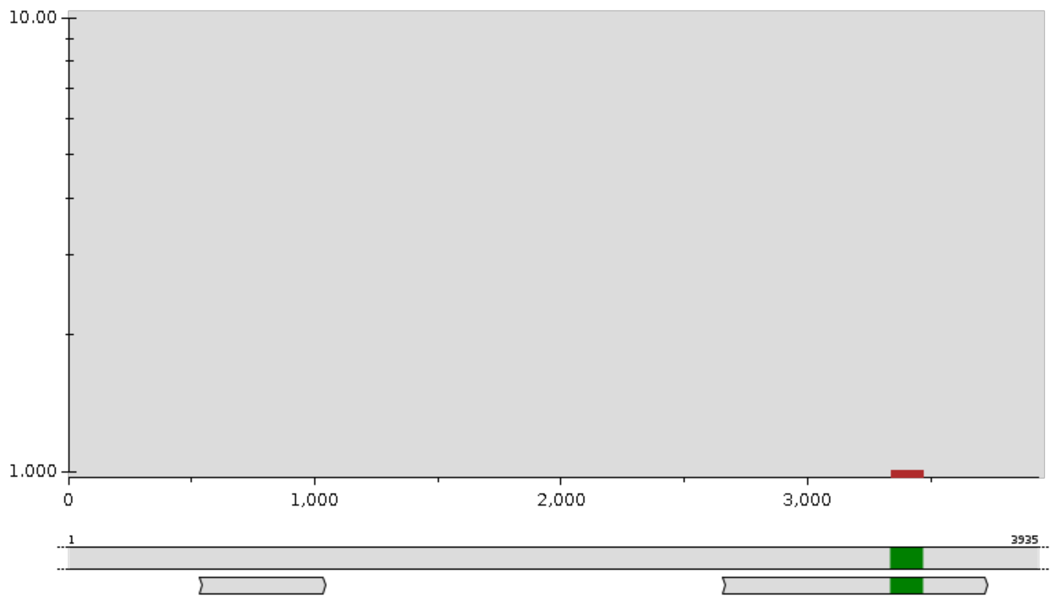

Assignment

**Type**	Ichnoviriform fugitivi (Taxonomy ID: 265522)
**Reference Genome**	NC_008953.1
**NT Identity (%)**	64.1791
**AA Identity (%)**	62.2222
**Number Of Stop Codons**	0
**Number Of CDS**	2

Alignment

**Alignment Score**	76.0 (NT) + 243.0 (AA) = 319.0
**Concordance (%)**	52.9022

Alignment Method	Global, seeded, nucleotide + amino acids (AGA)

Genome Region

Sequence starts at position 3339 and ends at position 3472 relative to NC_008953.1 reference sequence.

Alignment Detailed Statistics

	Begin	End	Coverage	Score	Concordance	Matches	Identities	I/D/M/F*	Stop Codons
**NT**	**3339**	**3472**	**3.4%**	**76**	**28.4%**	**134 (100%)**	**86 (64.2%)**	**0/0**	
Mutations:	3344G>A, 3348C>A, 3354A>T, 3355A>C, 3356C>G, 3357A>T, 3360C>G, 3363T>A, 3364C>A, 3365G>A, 3375T>C, 3378C>T, 3379A>C, 3380A>G, 3384C>T, 3388C>T, 3390T>A, 3393G>A, 3396C>T, 3397T>G, 3398C>A, 3400C>G, 3402G>A, 3403G>C, 3405A>G, 3407A>G, 3408C>A, 3409T>C, 3411C>T, 3414G>T, 3416G>C, 3420C>G, 3423C>T, 3429C>T, 3432G>A, 3433G>T, 3434A>T, 3439G>A, 3441G>T, 3442G>A, 3447C>T, 3450G>A, 3453G>A, 3456C>T, 3459C>T, 3462T>C, 3466C>T, 3468C>G								
**CDS**									
HfIV_sB17gp2	229	273	12.6%	243	69.0%	45 (100%)	28 (62.2%)	0/0/0/0	0
Protein mutations:	R230Q (3344G>A), T234R (3355A>C 3356C>G 3357A>T), I235M (3360C>G), R237K (3364C>A 3365G>A), K242R (3379A>C 3380A>G), P245S (3388C>T 3390T>A), S248D (3397T>G 3398C>A), L249V (3400C>G 3402G>A), E250Q (3403G>C 3405A>G), N251R (3407A>G 3408C>A), Y252H (3409T>C 3411C>T), E253D (3414G>T), G254A (3416G>C), E260L (3433G>T 3434A>T), V262I (3439G>A 3441G>T), V263I (3442G>A)								
Codon mutations:	CGA230CAA (3344G>A), GTC231GTA (3348C>A), CCA233CCT (3354A>T), ACA234CGT (3355A>C 3356C>G 3357A>T), ATC235ATG (3360C>G), ACT236ACA (3363T>A), CGA237AAA (3364C>A 3365G>A), TTT240TTC (3375T>C), CAC241CAT (3378C>T), AAA242CGA (3379A>C 3380A>G), TAC243TAT (3384C>T), CCT245TCA (3388C>T 3390T>A), TCG246TCA (3393G>A), GGC247GGT (3396C>T), TCC248GAC (3397T>G 3398C>A), CTG249GTA (3400C>G 3402G>A), GAA250CAG (3403G>C 3405A>G), AAC251AGA (3407A>G 3408C>A), TAC252CAT (3409T>C 3411C>T), GAG253GAT (3414G>T), GGA254GCA (3416G>C), CTC255CTG (3420C>G), TGC256TGT (3423C>T), CTC258CTT (3429C>T), CCG259CCA (3432G>A), GAG260TTG (3433G>T 3434A>T), GTG262ATT (3439G>A 3441G>T), GTC263ATC (3442G>A), AAC264AAT (3447C>T), GAG265GAA (3450G>A), AAG266AAA (3453G>A), ATC267ATT (3456C>T), TAC268TAT (3459C>T), ATT269ATC (3462T>C), CTC271TTG (3466C>T 3468C>G)								
**Proteins**									
viral inexin-like protein (YP_001031233.1)	229	273	12.6%	243	69.0%	45 (100%)	28 (62.2%)	0/0/0/0	0
Protein mutations:	R230Q (3344G>A), T234R (3355A>C 3356C>G 3357A>T), I235M (3360C>G), R237K (3364C>A 3365G>A), K242R (3379A>C 3380A>G), P245S (3388C>T 3390T>A), S248D (3397T>G 3398C>A), L249V (3400C>G 3402G>A), E250Q (3403G>C 3405A>G), N251R (3407A>G 3408C>A), Y252H (3409T>C 3411C>T), E253D (3414G>T), G254A (3416G>C), E260L (3433G>T 3434A>T), V262I (3439G>A 3441G>T), V263I (3442G>A)								
Codon mutations:	CGA230CAA (3344G>A), GTC231GTA (3348C>A), CCA233CCT (3354A>T), ACA234CGT (3355A>C 3356C>G 3357A>T), ATC235ATG (3360C>G), ACT236ACA (3363T>A), CGA237AAA (3364C>A 3365G>A), TTT240TTC (3375T>C), CAC241CAT (3378C>T), AAA242CGA (3379A>C 3380A>G), TAC243TAT (3384C>T), CCT245TCA (3388C>T 3390T>A), TCG246TCA (3393G>A), GGC247GGT (3396C>T), TCC248GAC (3397T>G 3398C>A), CTG249GTA (3400C>G 3402G>A), GAA250CAG (3403G>C 3405A>G), AAC251AGA (3407A>G 3408C>A), TAC252CAT (3409T>C 3411C>T), GAG253GAT (3414G>T), GGA254GCA (3416G>C), CTC255CTG (3420C>G), TGC256TGT (3423C>T), CTC258CTT (3429C>T), CCG259CCA (3432G>A), GAG260TTG (3433G>T 3434A>T), GTG262ATT (3439G>A 3441G>T), GTC263ATC (3442G>A), AAC264AAT (3447C>T), GAG265GAA (3450G>A), AAG266AAA (3453G>A), ATC267ATT (3456C>T), TAC268TAT (3459C>T), ATT269ATC (3462T>C), CTC271TTG (3466C>T 3468C>G)								

*: Inserts / Deletes / Misaligned / Frameshifts

Analysis details

This analysis was performed with panviral2.64

NGS Details (UN60): Ichnoviriform fugitivi (segment B17)

Assembly

**Coverage Length**	286 (1 contig(s))
**Depth Of Coverage**	5.5
**Number Of Reads**	13
**Reads Per Million**	0.28 rpm (after QC)
**Ambiguities**	0
**Assembly Method**	de novo + reference guided assembly
**Consensus Caller**	Bcf Tools

Coverage Map

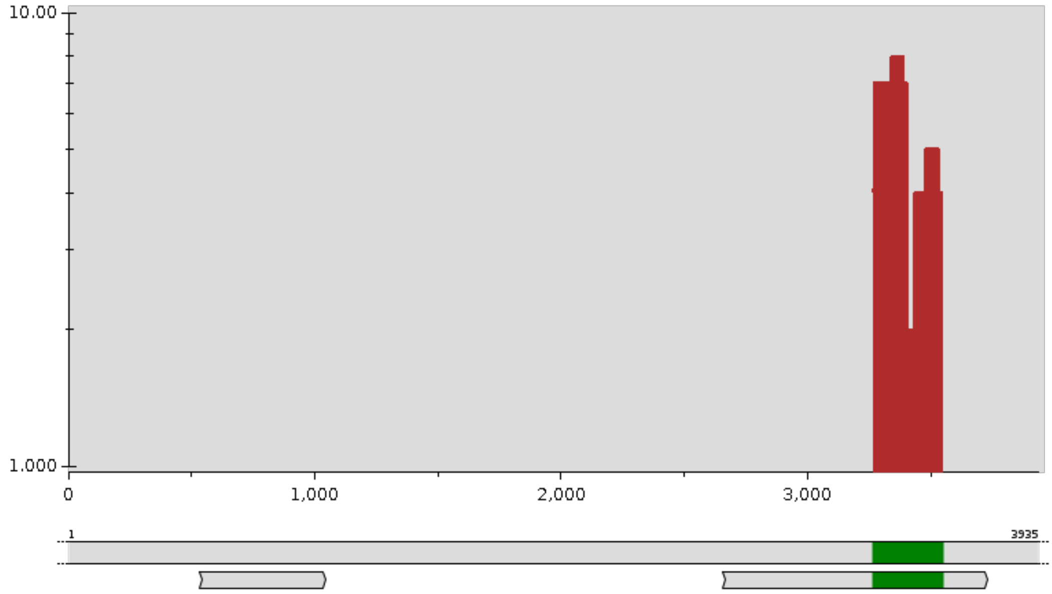

Assignment

**Type**	Ichnoviriform fugitivi (Taxonomy ID: 265522)
**Reference Genome**	NC_008953.1
**NT Identity (%)**	57.2438
**AA Identity (%)**	54.2553
**Number Of Stop Codons**	0
**Number Of CDS**	2

Alignment

**Alignment Score**	70.0 (NT) + 421.0 (AA) = 491.0
**Concordance (%)**	40.0489

Alignment Method	Global, seeded, nucleotide + amino acids (AGA)

Genome Region

Sequence starts at position 3266 and ends at position 3551 relative to NC_008953.1 reference sequence.

Alignment Detailed Statistics

	Begin	End	Coverage	Score	Concordance	Matches	Identities	I/D/M/F*	Stop Codons
**NT**	**3266**	**3551**	**7.3%**	**70**	**12.6%**	**283 (99.0%)**	**162 (56.6%)**	**0/3**	
Mutations:	3267C>A, 3273G>C, 3274T>A, 3275T>C, 3276G>A, 3282A>T, 3285C>T, 3286T>A, 3288T>A, 3291T>G, 3292A>T, 3293C>T, 3294C>A, 3295T>G, 3296T>A, 3297C>A, 3298T>C, 3302A>G, 3303C>T, 3306G>A, 3309A>G, 3311C>A, 3312C>T, 3313_3315delGCC, 3318T>A, 3319C>G, 3320C>A, 3321G>A, 3326T>G, 3327G>A, 3329C>T, 3330G>T, 3331A>G, 3333C>T, 3340A>G, 3341A>T, 3342G>A, 3343C>T, 3344G>C, 3345A>T, 3351T>C, 3356C>G, 3358A>G, 3360C>A, 3363T>A, 3364C>A, 3365G>A, 3366A>G, 3369T>C, 3372T>A, 3383A>T, 3390T>A, 3393G>T, 3396C>A, 3399C>A, 3400C>T, 3403G>C, 3405A>G, 3407A>C, 3408C>A, 3409T>C, 3411C>T, 3414G>T, 3416G>C, 3420C>T, 3423C>T, 3424A>G, 3426C>A, 3427C>T, 3429C>G, 3430C>G, 3432G>A, 3433G>T, 3434A>T, 3435G>A, 3438C>T, 3439G>A, 3441G>C, 3442G>T, 3444C>G, 3447C>T, 3450G>A, 3453G>A, 3462T>A, 3465C>T, 3466C>T, 3468C>G, 3474C>T, 3481T>A, 3482A>T, 3483C>T, 3484G>A, 3486G>A, 3487C>T, 3489A>G, 3492C>A, 3498C>A, 3499A>T, 3500G>C, 3501C>T, 3504T>A, 3507C>G, 3509T>C, 3510T>G, 3511G>T, 3513T>G, 3514C>G, 3515T>C, 3516G>C, 3520C>A, 3522C>T, 3529C>G, 3531G>T, 3532C>A, 3535G>A, 3537G>A, 3539C>T, 3540C>G, 3544G>T, 3546G>A, 3547C>A, 3549C>T								
**CDS**									
HfIV_sB17gp2	205	299	26.5%	421	61.0%	94 (98.9%)	51 (53.7%)	0/1/0/0	0
Protein mutations:	L206F (3273G>C), L207T (3274T>A 3275T>C 3276G>A), Y211K (3286T>A 3288T>A), T213L (3292A>T 3293C>T 3294C>A), F214E (3295T>G 3296T>A 3297C>A), F215L (3298T>C), N216S (3302A>G 3303C>T), A219D (3311C>A 3312C>T), A220del (3313_3315delGCC), H221Q (3318T>A), P222E (3319C>G 3320C>A 3321G>A), M224R (3326T>G 3327G>A), T225I (3329C>T 3330G>T), N226D (3331A>G 3333C>T), K229V (3340A>G 3341A>T 3342G>A), R230S (3343C>T 3344G>C 3345A>T), T234R (3356C>G), I235V (3358A>G 3360C>A), R237K (3364C>A 3365G>A 3366A>G), Y243F (3383A>T), E250Q (3403G>C 3405A>G), N251T (3407A>C 3408C>A), Y252H (3409T>C 3411C>T), E253D (3414G>T), G254A (3416G>C), I257V (3424A>G 3426C>A), P259A (3430C>G 3432G>A), E260L (3433G>T 3434A>T 3435G>A), V262I (3439G>A 3441G>C), V263L (3442G>T 3444C>G), Y276I (3481T>A 3482A>T 3483C>T), V277I (3484G>A 3486G>A), I284M (3507C>G), V285A (3509T>C 3510T>G), V286L (3511G>T 3513T>G), L287A (3514C>G 3515T>C 3516G>C), R289S (3520C>A 3522C>T), L292V (3529C>G 3531G>T), L293I (3532C>A), A294T (3535G>A 3537G>A), S295L (3539C>T 3540C>G), A297S (3544G>T 3546G>A), L298I (3547C>A 3549C>T)								
Codon mutations:	GAC204.AA (3267C>A), TTG206TTC (3273G>C), TTG207ACA (3274T>A 3275T>C 3276G>A), GGA209GGT (3282A>T), ATC210ATT (3285C>T), TAT211AAA (3286T>A 3288T>A), GTT212GTG (3291T>G), ACC213TTA (3292A>T 3293C>T 3294C>A), TTC214GAA (3295T>G 3296T>A 3297C>A), TTC215CTC (3298T>C), AAC216AGT (3302A>G 3303C>T), CAG217CAA (3306G>A), GAA218GAG (3309A>G), GCC219GAT (3311C>A 3312C>T), GCC220del (3313_3315delGCC), CAT221CAA (3318T>A), CCG222GAA (3319C>G 3320C>A 3321G>A), ATG224AGA (3326T>G 3327G>A), ACG225ATT (3329C>T 3330G>T), AAC226GAT (3331A>G 3333C>T), AAG229GTA (3340A>G 3341A>T 3342G>A), CGA230TCT (3343C>T 3344G>C 3345A>T), TTT232TTC (3351T>C), ACA234AGA (3356C>G), ATC235GTA (3358A>G 3360C>A), ACT236ACA (3363T>A), CGA237AAG (3364C>A 3365G>A 3366A>G), TGT238TGC (3369T>C), ACT239ACA (3372T>A), TAC243TTC (3383A>T), CCT245CCA (3390T>A), TCG246TCT (3393G>T), GGC247GGA (3396C>A), TCC248TCA (3399C>A), CTG249TTG (3400C>T), GAA250CAG (3403G>C 3405A>G), AAC251ACA (3407A>C 3408C>A), TAC252CAT (3409T>C 3411C>T), GAG253GAT (3414G>T), GGA254GCA (3416G>C), CTC255CTT (3420C>T), TGC256TGT (3423C>T), ATC257GTA (3424A>G 3426C>A), CTC258TTG (3427C>T 3429C>G), CCG259GCA (3430C>G 3432G>A), GAG260TTA (3433G>T 3434A>T 3435G>A), AAC261AAT (3438C>T), GTG262ATC (3439G>A 3441G>C), GTC263TTG (3442G>T 3444C>G), AAC264AAT (3447C>T), GAG265GAA (3450G>A), AAG266AAA (3453G>A), ATT269ATA (3462T>A), TTC270TTT (3465C>T), CTC271TTG (3466C>T 3468C>G), TTC273TTT (3474C>T), TAC276ATT (3481T>A 3482A>T 3483C>T), GTG277ATA (3484G>A 3486G>A), CTA278TTG (3487C>T 3489A>G), GCC279GCA (3492C>A), ATC281ATA (3498C>A), AGC282TCT (3499A>T 3500G>C 3501C>T), GGT283GGA (3504T>A), ATC284ATG (3507C>G), GTT285GCG (3509T>C 3510T>G), GTT286TTG (3511G>T 3513T>G), CTG287GCC (3514C>G 3515T>C 3516G>C), CGC289AGT (3520C>A 3522C>T), CTG292GTT (3529C>G 3531G>T), CTA293ATA (3532C>A), GCG294ACA (3535G>A 3537G>A), TCC295TTG (3539C>T 3540C>G), GCG297TCA (3544G>T 3546G>A), CTC298ATT (3547C>A 3549C>T)								
**Proteins**									
viral inexin-like protein (YP_001031233.1)	205	299	26.5%	421	61.0%	94 (98.9%)	51 (53.7%)	0/1/0/0	0
Protein mutations:	L206F (3273G>C), L207T (3274T>A 3275T>C 3276G>A), Y211K (3286T>A 3288T>A), T213L (3292A>T 3293C>T 3294C>A), F214E (3295T>G 3296T>A 3297C>A), F215L (3298T>C), N216S (3302A>G 3303C>T), A219D (3311C>A 3312C>T), A220del (3313_3315delGCC), H221Q (3318T>A), P222E (3319C>G 3320C>A 3321G>A), M224R (3326T>G 3327G>A), T225I (3329C>T 3330G>T), N226D (3331A>G 3333C>T), K229V (3340A>G 3341A>T 3342G>A), R230S (3343C>T 3344G>C 3345A>T), T234R (3356C>G), I235V (3358A>G 3360C>A), R237K (3364C>A 3365G>A 3366A>G), Y243F (3383A>T), E250Q (3403G>C 3405A>G), N251T (3407A>C 3408C>A), Y252H (3409T>C 3411C>T), E253D (3414G>T), G254A (3416G>C), I257V (3424A>G 3426C>A), P259A (3430C>G 3432G>A), E260L (3433G>T 3434A>T 3435G>A), V262I (3439G>A 3441G>C), V263L (3442G>T 3444C>G), Y276I (3481T>A 3482A>T 3483C>T), V277I (3484G>A 3486G>A), I284M (3507C>G), V285A (3509T>C 3510T>G), V286L (3511G>T 3513T>G), L287A (3514C>G 3515T>C 3516G>C), R289S (3520C>A 3522C>T), L292V (3529C>G 3531G>T), L293I (3532C>A), A294T (3535G>A 3537G>A), S295L (3539C>T 3540C>G), A297S (3544G>T 3546G>A), L298I (3547C>A 3549C>T)								
Codon mutations:	GAC204.AA (3267C>A), TTG206TTC (3273G>C), TTG207ACA (3274T>A 3275T>C 3276G>A), GGA209GGT (3282A>T), ATC210ATT (3285C>T), TAT211AAA (3286T>A 3288T>A), GTT212GTG (3291T>G), ACC213TTA (3292A>T 3293C>T 3294C>A), TTC214GAA (3295T>G 3296T>A 3297C>A), TTC215CTC (3298T>C), AAC216AGT (3302A>G 3303C>T), CAG217CAA (3306G>A), GAA218GAG (3309A>G), GCC219GAT (3311C>A 3312C>T), GCC220del (3313_3315delGCC), CAT221CAA (3318T>A), CCG222GAA (3319C>G 3320C>A 3321G>A), ATG224AGA (3326T>G 3327G>A), ACG225ATT (3329C>T 3330G>T), AAC226GAT (3331A>G 3333C>T), AAG229GTA (3340A>G 3341A>T 3342G>A), CGA230TCT (3343C>T 3344G>C 3345A>T), TTT232TTC (3351T>C), ACA234AGA (3356C>G), ATC235GTA (3358A>G 3360C>A), ACT236ACA (3363T>A), CGA237AAG (3364C>A 3365G>A 3366A>G), TGT238TGC (3369T>C), ACT239ACA (3372T>A), TAC243TTC (3383A>T), CCT245CCA (3390T>A), TCG246TCT (3393G>T), GGC247GGA (3396C>A), TCC248TCA (3399C>A), CTG249TTG (3400C>T), GAA250CAG (3403G>C 3405A>G), AAC251ACA (3407A>C 3408C>A), TAC252CAT (3409T>C 3411C>T), GAG253GAT (3414G>T), GGA254GCA (3416G>C), CTC255CTT (3420C>T), TGC256TGT (3423C>T), ATC257GTA (3424A>G 3426C>A), CTC258TTG (3427C>T 3429C>G), CCG259GCA (3430C>G 3432G>A), GAG260TTA (3433G>T 3434A>T 3435G>A), AAC261AAT (3438C>T), GTG262ATC (3439G>A 3441G>C), GTC263TTG (3442G>T 3444C>G), AAC264AAT (3447C>T), GAG265GAA (3450G>A), AAG266AAA (3453G>A), ATT269ATA (3462T>A), TTC270TTT (3465C>T), CTC271TTG (3466C>T 3468C>G), TTC273TTT (3474C>T), TAC276ATT (3481T>A 3482A>T 3483C>T), GTG277ATA (3484G>A 3486G>A), CTA278TTG (3487C>T 3489A>G), GCC279GCA (3492C>A), ATC281ATA (3498C>A), AGC282TCT (3499A>T 3500G>C 3501C>T), GGT283GGA (3504T>A), ATC284ATG (3507C>G), GTT285GCG (3509T>C 3510T>G), GTT286TTG (3511G>T 3513T>G), CTG287GCC (3514C>G 3515T>C 3516G>C), CGC289AGT (3520C>A 3522C>T), CTG292GTT (3529C>G 3531G>T), CTA293ATA (3532C>A), GCG294ACA (3535G>A 3537G>A), TCC295TTG (3539C>T 3540C>G), GCG297TCA (3544G>T 3546G>A), CTC298ATT (3547C>A 3549C>T)								

*: Inserts / Deletes / Misaligned / Frameshifts

Analysis details

This analysis was performed with panviral2.64

NGS Details (UN60): Ichnoviriform fugitivi (segment B17)

Assembly

**Coverage Length**	291 (1 contig(s))
**Depth Of Coverage**	8.2
**Number Of Reads**	20
**Reads Per Million**	0.42 rpm (after QC)
**Ambiguities**	0
**Assembly Method**	de novo + reference guided assembly
**Consensus Caller**	Bcf Tools

Coverage Map

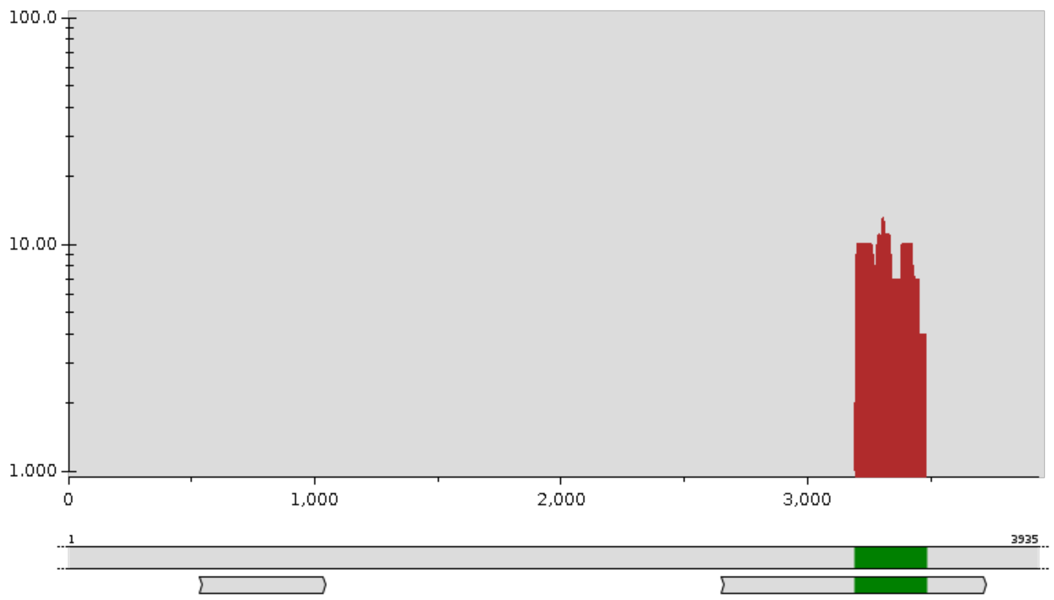

Assignment

**Type**	Ichnoviriform fugitivi (Taxonomy ID: 265522)
**Reference Genome**	NC_008953.1
**NT Identity (%)**	58.3333
**AA Identity (%)**	47.9167
**Number Of Stop Codons**	0
**Number Of CDS**	2

Alignment

**Alignment Score**	75.0 (NT) + 415.0 (AA) = 490.0
**Concordance (%)**	38.6435

Alignment Method	Global, seeded, nucleotide + amino acids (AGA)

Genome Region

Sequence starts at position 3195 and ends at position 3485 relative to NC_008953.1 reference sequence.

Alignment Detailed Statistics

	Begin	End	Coverage	Score	Concordance	Matches	Identities	I/D/M/F*	Stop Codons
**NT**	**3195**	**3485**	**7.4%**	**75**	**13.5%**	**285 (96.9%)**	**168 (57.1%)**	**3/6**	
Mutations:	3201C>T, 3204G>A, 3205C>A, 3207G>T, 3208C>A, 3210A>C, 3211A>T, 3213C>T, 3217G>A, 3219C>T, 3220A>C, 3222C>T, 3223G>C, 3226G>A, 3228C>T, 3229G>T, 3230C>G, 3231A>C, 3234G>A, 3240C>G, 3245T>C, 3246G>C, 3250G>C, 3251C>T, 3252C>G, 3255T>C, 3256A>C, 3258A>T, 3261C>T, 3263A>G, 3265G>T, 3270T>C, 3273G>T, 3275T>C, 3276G>A, 3278A>T, 3279C>G, 3282A>C, 3285_3286insGAG, 3287A>G, 3288T>G, 3289G>A, 3291T>G, 3292A>T, 3296T>A, 3297C>T, 3299T>C, 3300C>A, 3301A>C, 3303C>T, 3304C>A, 3305A>T, 3311C>A, 3312C>G, 3314C>A, 3315C>T, 3316_3321delCATCCG, 3324C>T, 3325A>C, 3326T>G, 3327G>T, 3328A>C, 3329C>A, 3330G>T, 3331A>G, 3333C>T, 3336C>T, 3337A>T, 3341A>T, 3342G>C, 3346G>A, 3347T>C, 3348C>T, 3354A>T, 3355A>C, 3356C>G, 3357A>T, 3358A>T, 3360C>A, 3363T>C, 3364C>A, 3365G>A, 3369T>C, 3370A>T, 3372T>A, 3375T>C, 3378C>T, 3384C>T, 3388C>T, 3389C>A, 3396C>T, 3397T>A, 3399C>A, 3400C>A, 3402G>T, 3405A>G, 3406A>G, 3407A>C, 3408C>T, 3409T>C, 3414G>T, 3416G>T, 3417A>T, 3423C>T, 3424A>T, 3426C>T, 3429C>T, 3432G>T, 3433G>C, 3434A>T, 3435G>C, 3439G>A, 3441G>T, 3450G>A, 3459C>T, 3460A>G, 3462T>C, 3463T>A, 3468C>T								

CDS

	Begin	End	Coverage	Score	Concordance	Matches	Identities	I/D/M/F*	Stop Codons
HfIV_sB17gp2	181	277	27.1%	415	57.0%	95 (96.9%)	46 (46.9%)	1/2/0/0	0
Protein mutations:	L184I (3205C>A 3207G>T), L185I (3208C>A 3210A>C), N186Y (3211A>T 3213C>T), V188I (3217G>A 3219C>T), N189H (3220A>C 3222C>T), V190L (3223G>C), V191I (3226G>A 3228C>T), A192C (3229G>T 3230C>G 3231A>C), C195W (3240C>G), M197T (3245T>C 3246G>C), A199L (3250G>C 3251C>T 3252C>G), I201L (3256A>C 3258A>T), E203G (3263A>G), D204Y (3265G>T), L206F (3273G>T), L207S (3275T>C 3276G>A), Y208L (3278A>T 3279C>G), I210_Y211insE (3285_3286insGAG), Y211W (3287A>G 3288T>G), V212M (3289G>A 3291T>G), T213S (3292A>T), F214Y (3296T>A 3297C>T), F215S (3299T>C 3300C>A), N216H (3301A>C 3303C>T), Q217M (3304C>A 3305A>T), A219E (3311C>A 3312C>G), A220D (3314C>A 3315C>T), H221_P222del (3316_3321delCATCCG), M224R (3325A>C 3326T>G 3327G>T), T225H (3328A>C 3329C>A 3330G>T), N226D (3331A>G 3333C>T), M228L (3337A>T), K229I (3341A>T 3342G>C), V231T (3346G>A 3347T>C 3348C>T), T234R (3355A>C 3356C>G 3357A>T), I235L (3358A>T 3360C>A), R237K (3364C>A 3365G>A), T239S (3370A>T 3372T>A), P245Y (3388C>T 3389C>A), S248T (3397T>A 3399C>A), L249I (3400C>A 3402G>T), N251A (3406A>G 3407A>C 3408C>T), Y252H (3409T>C), E253D (3414G>T), G254V (3416G>T 3417A>T), I257F (3424A>T 3426C>T), E260L (3433G>C 3434A>T 3435G>C), V262I (3439G>A 3441G>T), I269V (3460A>G 3462T>C), F270I (3463T>A)								
Codon mutations:	TGC182TGT (3201C>T), GAG183GAA (3204G>A), CTG184ATT (3205C>A 3207G>T), CTA185ATC (3208C>A 3210A>C), AAC186TAT (3211A>T 3213C>T), GTC188ATT (3217G>A 3219C>T), AAC189CAT (3220A>C 3222C>T), GTT190CTT (3223G>C), GTC191ATT (3226G>A 3228C>T), GCA192TGC (3229G>T 3230C>G 3231A>C), CAG193CAA (3234G>A), TGC195TGG (3240C>G), ATG197ACC (3245T>C 3246G>C), GCC199CTG (3250G>C 3251C>T 3252C>G), TTT200TTC (3255T>C), ATA201CTT (3256A>C 3258A>T), GGC202GGT (3261C>T), GAA203GGA (3263A>G), GAC204TAC (3265G>T), TTT205TTC (3270T>C), TTG206TTT (3273G>T), TTG207TCA (3275T>C 3276G>A), TAC208TTG (3278A>T 3279C>G), GGA209GGC (3282A>C), ATC210_TAT211insGAG (3285_3286insGAG), TAT211TGG (3287A>G 3288T>G), GTT212ATG (3289G>A 3291T>G), ACC213TCC (3292A>T), TTC214TAT (3296T>A 3297C>T), TTC215TCA (3299T>C 3300C>A), AAC216CAT (3301A>C 3303C>T), CAG217ATG (3304C>A 3305A>T), GCC219GAG (3311C>A 3312C>G), GCC220GAT (3314C>A 3315C>T), CAT221_CCG222del (3316_3321delCATCCG), AAC223AAT (3324C>T), ATG224CGT (3325A>C 3326T>G 3327G>T), ACG225CAT (3328A>C 3329C>A 3330G>T), AAC226GAT (3331A>G 3333C>T), CCC227CCT (3336C>T), ATG228TTG (3337A>T), AAG229ATC (3341A>T 3342G>C), GTC231ACT (3346G>A 3347T>C 3348C>T), CCA233CCT (3354A>T), ACA234CGT (3355A>C 3356C>G 3357A>T), ATC235TTA (3358A>T 3360C>A), ACT236ACC (3363T>C), CGA237AAA (3364C>A 3365G>A), TGT238TGC (3369T>C), ACT239TCA (3370A>T 3372T>A), TTT240TTC (3375T>C), CAC241CAT (3378C>T), TAC243TAT (3384C>T), CCT245TAT (3388C>T 3389C>A), GGC247GGT (3396C>T), TCC248ACA (3397T>A 3399C>A), CTG249ATT (3400C>A 3402G>T), GAA250GAG (3405A>G), AAC251GCT (3406A>G 3407A>C 3408C>T), TAC252CAC (3409T>C), GAG253GAT (3414G>T), GGA254GTT (3416G>T 3417A>T), TGC256TGT (3423C>T), ATC257TTT (3424A>T 3426C>T), CTC258CTT (3429C>T), CCG259CCT (3432G>T), GAG260CTC (3433G>C 3434A>T 3435G>C), GTG262ATT (3439G>A 3441G>T), GAG265GAA (3450G>A), TAC268TAT (3459C>T), ATT269GTC (3460A>G 3462T>C), TTC270ATC (3463T>A), CTC271CTT (3468C>T)								

Proteins

	Begin	End	Coverage	Score	Concordance	Matches	Identities	I/D/M/F*	Stop Codons
viral inexin-like protein (YP_001031233.1)	181	277	27.1%	415	57.0%	95 (96.9%)	46 (46.9%)	1/2/0/0	0
Protein mutations:	L184I (3205C>A 3207G>T), L185I (3208C>A 3210A>C), N186Y (3211A>T 3213C>T), V188I (3217G>A 3219C>T), N189H (3220A>C 3222C>T), V190L (3223G>C), V191I (3226G>A 3228C>T), A192C (3229G>T 3230C>G 3231A>C), C195W (3240C>G), M197T (3245T>C 3246G>C), A199L (3250G>C 3251C>T 3252C>G), I201L (3256A>C 3258A>T), E203G (3263A>G), D204Y (3265G>T), L206F (3273G>T), L207S (3275T>C 3276G>A), Y208L (3278A>T 3279C>G), I210_Y211insE (3285_3286insGAG), Y211W (3287A>G 3288T>G), V212M (3289G>A 3291T>G), T213S (3292A>T), F214Y (3296T>A 3297C>T), F215S (3299T>C 3300C>A), N216H (3301A>C 3303C>T), Q217M (3304C>A 3305A>T), A219E (3311C>A 3312C>G), A220D (3314C>A 3315C>T), H221_P222del (3316_3321delCATCCG), M224R (3325A>C 3326T>G 3327G>T), T225H (3328A>C 3329C>A 3330G>T), N226D (3331A>G 3333C>T), M228L (3337A>T), K229I (3341A>T 3342G>C), V231T (3346G>A 3347T>C 3348C>T), T234R (3355A>C 3356C>G 3357A>T), I235L (3358A>T 3360C>A), R237K (3364C>A 3365G>A), T239S (3370A>T 3372T>A), P245Y (3388C>T 3389C>A), S248T (3397T>A 3399C>A), L249I (3400C>A 3402G>T), N251A (3406A>G 3407A>C 3408C>T), Y252H (3409T>C), E253D (3414G>T), G254V (3416G>T 3417A>T), I257F (3424A>T 3426C>T), E260L (3433G>C 3434A>T 3435G>C), V262I (3439G>A 3441G>T), I269V (3460A>G 3462T>C), F270I (3463T>A)								
Codon mutations:	TGC182TGT (3201C>T), GAG183GAA (3204G>A), CTG184ATT (3205C>A 3207G>T), CTA185ATC (3208C>A 3210A>C), AAC186TAT (3211A>T 3213C>T), GTC188ATT (3217G>A 3219C>T), AAC189CAT (3220A>C 3222C>T), GTT190CTT (3223G>C), GTC191ATT (3226G>A 3228C>T), GCA192TGC (3229G>T 3230C>G 3231A>C), CAG193CAA (3234G>A), TGC195TGG (3240C>G), ATG197ACC (3245T>C 3246G>C), GCC199CTG (3250G>C 3251C>T 3252C>G), TTT200TTC (3255T>C), ATA201CTT (3256A>C 3258A>T), GGC202GGT (3261C>T), GAA203GGA (3263A>G), GAC204TAC (3265G>T), TTT205TTC (3270T>C), TTG206TTT (3273G>T), TTG207TCA (3275T>C 3276G>A), TAC208TTG (3278A>T 3279C>G), GGA209GGC (3282A>C), ATC210_TAT211insGAG (3285_3286insGAG), TAT211TGG (3287A>G 3288T>G), GTT212ATG (3289G>A 3291T>G), ACC213TCC (3292A>T), TTC214TAT (3296T>A 3297C>T), TTC215TCA (3299T>C 3300C>A), AAC216CAT (3301A>C 3303C>T), CAG217ATG (3304C>A 3305A>T), GCC219GAG (3311C>A 3312C>G), GCC220GAT (3314C>A 3315C>T), CAT221_CCG222del (3316_3321delCATCCG), AAC223AAT (3324C>T), ATG224CGT (3325A>C 3326T>G 3327G>T), ACG225CAT (3328A>C 3329C>A 3330G>T), AAC226GAT (3331A>G 3333C>T), CCC227CCT (3336C>T), ATG228TTG (3337A>T), AAG229ATC (3341A>T 3342G>C), GTC231ACT (3346G>A 3347T>C 3348C>T), CCA233CCT (3354A>T), ACA234CGT (3355A>C 3356C>G 3357A>T), ATC235TTA (3358A>T 3360C>A), ACT236ACC (3363T>C), CGA237AAA (3364C>A 3365G>A), TGT238TGC (3369T>C), ACT239TCA (3370A>T 3372T>A), TTT240TTC (3375T>C), CAC241CAT (3378C>T), TAC243TAT (3384C>T), CCT245TAT (3388C>T 3389C>A), GGC247GGT (3396C>T), TCC248ACA (3397T>A 3399C>A), CTG249ATT (3400C>A 3402G>T), GAA250GAG (3405A>G), AAC251GCT (3406A>G 3407A>C 3408C>T), TAC252CAC (3409T>C), GAG253GAT (3414G>T), GGA254GTT (3416G>T 3417A>T), TGC256TGT (3423C>T), ATC257TTT (3424A>T 3426C>T), CTC258CTT (3429C>T), CCG259CCT (3432G>T), GAG260CTC (3433G>C 3434A>T 3435G>C), GTG262ATT (3439G>A 3441G>T), GAG265GAA (3450G>A), TAC268TAT (3459C>T), ATT269GTC (3460A>G 3462T>C), TTC270ATC (3463T>A), CTC271CTT (3468C>T)								

*: Inserts / Deletes / Misaligned / Frameshifts

Analysis details

This analysis was performed with panviral2.64

NGS Details (UN60): Ichnoviriform fugitivi (segment B17)

Assembly

**Coverage Length**	1290 (1 contig(s))
**Depth Of Coverage**	18.4
**Number Of Reads**	173
**Reads Per Million**	3.67 rpm (after QC)
**Ambiguities**	0
**Assembly Method**	de novo + reference guided assembly
**Consensus Caller**	Bcf Tools

Coverage Map

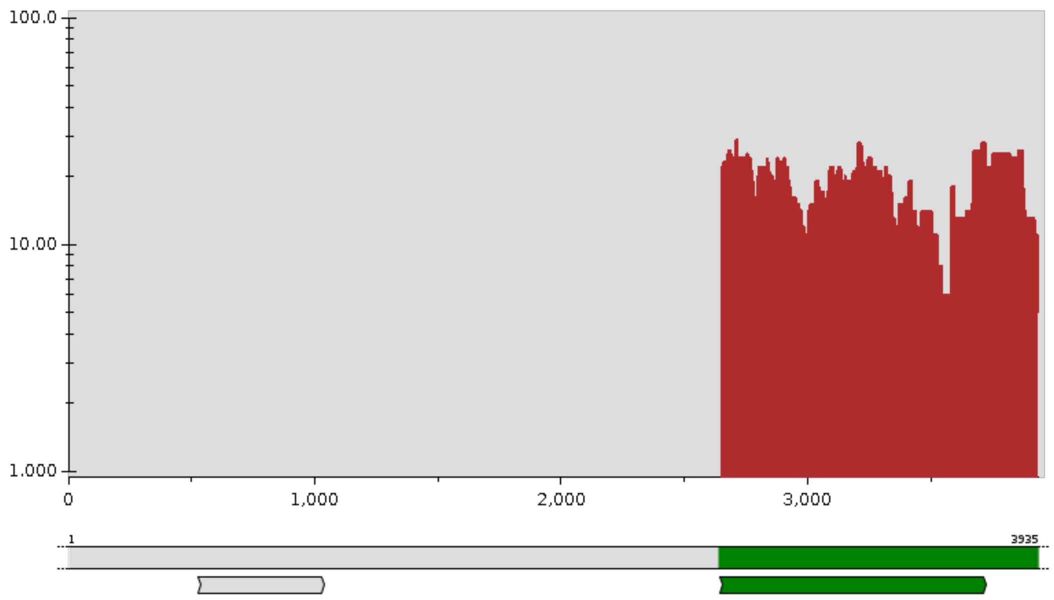

Assignment

**Type**	Ichnoviriform fugitivi (Taxonomy ID: 265522)
**Reference Genome**	NC_008953.1
**NT Identity (%)**	56.9547
**AA Identity (%)**	47.191
**Number Of Stop Codons**	0
**Number Of CDS**	2

Alignment

**Alignment Score**	164.0 (NT) + 1334.0 (AA) = 1498.0
**Concordance (%)**	31.124

**Alignment Method**	Global, seeded, nucleotide + amino acids (AGA)

Genome Region

Sequence starts at position 2646 and ends at position 3935 relative to NC_008953.1 reference sequence.

Alignment Detailed Statistics

	Begin	End	Coverage	Score	Concordance	Matches	Identities	I/D/M/F*	Stop Codons
**NT**	**2646**	**3935**	**32.8%**	**164**	**7.3%**	**1203 (92.4%)**	**692 (53.1%)**	**12/87**	
Mutations:	2659C>T, 2660G>T, 2661G>C, 2662A>G, 2665C>G, 2667A>G, 2668A>T, 2671A>G, 2672A>G, 2674G>T, 2679G>C, 2685C>G, 2686T>C, 2688A>G, 2689A>C, 2691A>G, 2694G>A, 2695C>A, 2697T>C, 2698C>G, 2699C>A, 2700A>C, 2702C>G, 2703A>C, 2706C>G, 2708C>G, 2718T>C, 2719G>A, 2720T>A, 2721A>C, 2722T>A, 2724C>A, 2736T>C, 2740C>A, 2743T>G, 2745C>G, 2751G>C, 2760C>G, 2761A>G, 2763C>G, 2764G>T, 2769T>C, 2772A>G, 2778G>C, 2781G>C, 2784T>G, 2787G>C, 2790G>C, 2797T>A, 2802A>C, 2803A>G, 2805G>C, 2807T>C, 2808C>G, 2811A>C, 2818C>A, 2819A>T, 2820T>C, 2821T>G, 2823T>C, 2824C>G, 2825C>A, 2826G>C, 2829C>T, 2830T>G, 2831A>T, 2832T>G, 2836T>C, 2837A>T, 2839G>A, 2840G>A, 2842T>A, 2843C>T, 2845C>A, 2847C>G, 2848A>G, 2850T>C, 2851G>A, 2852A>C, 2853C>G, 2855T>A, 2861C>G, 2862C>G, 2863G>A, 2865G>A, 2866C>T, 2868A>C, 2869C>T, 2874C>G, 2876A>T, 2877_2878insACCATA, 2879T>C, 2880A>C, 2881G>A, 2883A>C, 2884G>C, 2885T>G, 2886G>T, 2887A>C, 2892_2893insGGCCGC, 2894C>T, 2896A>G, 2897C>G, 2898A>C, 2899C>A, 2901T>A, 2904T>C, 2907T>G, 2908A>G, 2910A>C, 2911A>C, 2912G>A, 2913C>G, 2916C>G, 2917A>G, 2918T>G, 2920T>G, 2921C>T, 2922G>C, 2923C>G, 2925T>G, 2926C>T, 2927A>C, 2928T>C, 2931A>C, 2934T>G, 2935_2943delCGTACATCG, 2944A>G, 2946T>C, 2947C>G, 2948A>G, 2949G>C, 2950C>G, 2952A>G, 2953C>G, 2954G>A, 2955A>C, 2958A>G, 2959A>G, 2967T>C, 2968T>C, 2970T>C, 2971G>A, 2972G>A, 2973C>G, 2976T>C, 2982A>G, 2988T>G, 2990T>G, 2992A>T, 2997A>G, 2998T>C, 3000G>C, 3004A>T, 3012G>A, 3013G>A, 3023C>A, 3025A>C, 3027T>G, 3030G>C, 3031C>A, 3032A>G, 3039C>G, 3045G>A, 3046G>A, 3047T>C, 3048A>G, 3051T>G, 3054A>G, 3057A>T, 3060G>C, 3061A>C, 3062A>G, 3063A>C, 3066G>C, 3071C>T, 3072A>G, 3073T>C, 3075G>C, 3077C>T, 3078T>G, 3080A>T, 3081T>C, 3084T>C, 3087G>C, 3089C>A, 3090T>C, 3092C>G, 3093A>C, 3097T>A, 3100C>A, 3102G>C, 3106A>G, 3108A>G, 3116T>A, 3117C>G, 3119C>A, 3120A>C, 3123G>C, 3124A>C, 3125A>G, 3126G>C, 3127G>A, 3128T>A, 3130G>A, 3132T>G, 3134A>T, 3135T>G, 3136T>C, 3141G>C, 3144T>C, 3147T>C, 3151T>G, 3152T>C, 3155T>C, 3156G>C, 3157C>A, 3159G>T, 3166G>A, 3171A>G, 3176C>T, 3177T>C, 3184T>A, 3185A>T, 3186C>A, 3187A>C, 3188A>G, 3189A>C, 3191A>T, 3195T>C, 3196G>C, 3197G>T, 3198C>G, 3205C>T, 3207G>C, 3210A>C, 3216T>C, 3225T>C, 3226G>A, 3231A>G, 3237A>T, 3239G>T, 3247A>G, 3249T>C, 3250G>T, 3251C>A, 3255T>C, 3256A>C, 3258A>C, 3260G>A, 3261C>A, 3263A>G, 3264A>T, 3267C>G, 3270T>C, 3271T>A, 3272T>G, 3273G>T, 3274T>A, 3275T>C, 3282A>C, 3283A>T, 3284T>C, 3285C>G, 3286T>G, 3291T>G, 3292A>C, 3293C>T, 3294C>G, 3295T>C, 3296T>G, 3297C>G, 3302A>C, 3304_3306delCAG, 3310G>A, 3311C>T, 3312C>G, 3314C>A, 3315C>G, 3317A>C, 3318T>A, 3319C>G, 3320C>A, 3321G>A, 3322A>G, 3324C>A, 3325A>C, 3326T>G, 3327G>T, 3328A>G, 3329C>A, 3330G>A, 3331A>G, 3333C>T, 3340A>G, 3341A>C, 3342G>C, 3345A>G, 3348C>G, 3351T>C, 3354A>G, 3356C>A, 3358A>G, 3360C>T, 3363T>A, 3364C>A, 3365G>A, 3366A>G, 3372T>A, 3375T>C, 3384C>T, 3390T>A, 3393G>T, 3396C>A, 3400C>A, 3402G>C, 3403G>C, 3405A>G, 3408C>G, 3410A>T, 3411C>A, 3414G>C, 3417A>T, 3420C>T, 3423C>T, 3424A>G, 3426C>G, 3427C>T, 3429C>G, 3432G>A, 3433G>C, 3434A>T, 3435G>C, 3439G>A, 3441G>T, 3450G>A, 3453G>A, 3459C>T, 3460A>G, 3462T>G, 3468C>G, 3482A>T, 3483C>G, 3484G>T, 3486G>C, 3487C>A, 3489A>T, 3493A>G, 3495C>A, 3496A>C, 3501C>T, 3505A>C, 3508G>A, 3509T>A, 3510T>C, 3511G>T, 3513T>G, 3514C>G, 3524T>C, 3525T>A, 3528T>C, 3529C>G, 3531G>T, 3532C>G, 3536C>T, 3538T>A, 3539C>T, 3540C>G, 3543T>C, 3544G>A, 3545C>A, 3546G>A, 3547C>T, 3550A>C, 3552A>T, 3556T>C, 3557A>T, 3558C>G, 3559A>T, 3564C>G, 3565C>A, 3568A>G, 3569A>C, 3572C>G, 3573G>A, 3575G>C, 3576C>T, 3578T>G, 3579C>T, 3580A>T, 3583A>G, 3584A>C, 3585C>T, 3586T>C, 3587T>C, 3588C>T, 3590C>A, 3591C>G, 3594T>C, 3597C>A, 3600C>T, 3601C>G, 3604C>A, 3605T>C, 3606G>C, 3607G>A, 3609A>T, 3610C>A, 3611A>C, 3613G>A, 3615A>G, 3616C>A, 3619C>T, 3620T>G, 3621A>C, 3624A>G, 3627A>C, 3630A>T, 3637C>T, 3640C>G, 3642G>A, 3643G>T, 3645A>G, 3646C>T, 3648T>C, 3649G>C, 3650G>A, 3651T>G, 3652C>T, 3654T>G, 3655T>G, 3657G>A, 3660A>G, 3665C>T, 3666G>T, 3667A>G, 3672A>C, 3673A>T, 3675G>A, 3680A>T, 3684A>G, 3687A>G, 3690C>T, 3691A>G, 3694A>T, 3696G>A, 3697C>G, 3698G>A, 3700A>T, 3702T>A, 3705A>C, 3706C>A, 3708T>G, 3709_3714delCGTATC, 3720C>G, 3723T>C, 3725T>G, 3727T>A, 3730T>C, 3731delT, 3733C>G, 3734T>A, 3735T>G, 3736T>C, 3741T>A, 3742G>A, 3743T>A, 3745delC, 3748T>G, 3749T>A, 3753T>G, 3754T>A, 3756C>T, 3757T>C, 3758T>A, 3760T>C, 3762G>T, 3763T>C, 3767C>T, 3768G>T, 3769A>T, 3774_3809delACTCCCGTACCAAGAGTGCGGGTGTGTGTGTGTGTG, 3813T>A, 3814T>A, 3816G>A, 3817A>C, 3820T>A, 3822C>T, 3824C>A, 3826C>A, 3830T>A, 3833G>A, 3836T>G, 3838_3853delCCGTCATGTACCCTAG, 3860T>A, 3862T>G, 3863_3869delAATTAAT, 3875G>C, 3877G>A, 3878T>A, 3880G>A, 3883T>G, 3885T>A, 3886A>T, 3889C>G, 3890G>C, 3891_3898delTGTTCATT, 3901A>T, 3905A>T, 3906C>T, 3909A>G, 3910C>A, 3911A>C, 3913T>A, 3914A>T, 3915A>T, 3916A>T, 3917T>G, 3922T>A, 3930G>A, 3932C>T, 3935C>A								

CDS

	Begin	End	Coverage	Score	Concordance	Matches	Identities	I/D/M/F*	Stop Codons
HfIV_sB17gp2	1	358	100%	1334	51.8%	352 (97.2%)	168 (46.4%)	4/6/0/0	0
Protein mutations:	R2F (2659C>T 2660G>T 2661G>C), N3D (2662A>G), L4V (2665C>G 2667A>G), I5F (2668A>T), N6G (2671A>G 2672A>G), A7S (2674G>T), I12L (2689A>C 2691A>G), L14I (2695C>A 2697T>C), P15D (2698C>G 2699C>A 2700A>C), T16S (2702C>G 2703A>C), S18C (2708C>G), V22N (2719G>A 2720T>A 2721A>C), F23I (2722T>A 2724C>A), Q29K (2740C>A), F30V (2743T>G 2745C>G), I36V (2761A>G 2763C>G), A37S (2764G>T), F48I (2797T>A), K50D (2803A>G 2805G>C), L51P (2807T>C 2808C>G), H55I (2818C>A 2819A>T 2820T>C), F56V (2821T>G 2823T>C), P57D (2824C>G 2825C>A 2826G>C), Y59V (2830T>G 2831A>T 2832T>G), Y61L (2836T>C 2837A>T), G62N (2839G>A 2840G>A), S63I (2842T>A 2843C>T), L64M (2845C>A 2847C>G), N65D (2848A>G 2850T>C), D66T (2851G>A 2852A>C 2853C>G), F67Y (2855T>A), S69W (2861C>G 2862C>G), V70I (2863G>A 2865G>A), Q71Y (2866C>T 2868A>C), P72S (2869C>T), Y74F (2876A>T), Y74_L75insTI (2877_2878insACCATA), L75P (2879T>C 2880A>C), E76N (2881G>A 2883A>C), V77R (2884G>C 2885T>G 2886G>T), I78L (2887A>C), G79_T80insGR (2892_2893insGGCCGC), T80I (2894C>T), T81G (2896A>G 2897C>G 2898A>C), H82K (2899C>A 2901T>A), I85V (2908A>G 2910A>C), S86Q (2911A>C 2912G>A 2913C>G), I88G (2917A>G 2918T>G), S89V (2920T>G 2921C>T 2922G>C), P90A (2923C>G 2925T>G), H91S (2926C>T 2927A>C 2928T>C), Q92H (2931A>C), R94_S96del (2935_2943delCGTACATCG), N97D (2944A>G 2946T>C), Q98G (2947C>G 2948A>G 2949G>C), Q99E (2950C>G 2952A>G), R100D (2953C>G 2954G>A 2955A>C), I102V (2959A>G), Y105H (2968T>C 2970T>C), G106K (2971G>A 2972G>A 2973C>G), F112C (2990T>G), I113F (2992A>T), I117F (3004A>T), V120M (3013G>A), S123Y (3023C>A), I124L (3025A>C 3027T>G), Q126R (3031C>A 3032A>G), I128M (3039C>G), V131T (3046G>A 3047T>C 3048A>G), C132W (3051T>G), K136R (3061A>C 3062A>G 3063A>C), M137I (3066G>C), T139M (3071C>T 3072A>G), A141V (3077C>T 3078T>G), H142L (3080A>T 3081T>C), T145N (3089C>A 3090T>C), S146C (3092C>G 3093A>C), F148I (3097T>A), L149I (3100C>A 3102G>C), K151E (3106A>G 3108A>G), I154K (3116T>A 3117C>G), T155N (3119C>A 3120A>C), E156D (3123G>C), K157R (3124A>C 3125A>G 3126G>C), V158K (3127G>A 3128T>A), D159K (3130G>A 3132T>G), H160L (3134A>T 3135T>G), M162I (3141G>C), F166A (3151T>G 3152T>C), M167T (3155T>C 3156G>C), Q168N (3157C>A 3159G>T), A171T (3166G>A), S174F (3176C>T 3177T>C), Y177I (3184T>A 3185A>T 3186C>A), K178R (3187A>C 3188A>G 3189A>C), Y179F (3191A>T), G181L (3196G>C 3197G>T 3198C>G), L184F (3205C>T 3207G>C), V191I (3226G>A), C195F (3239G>T), N198D (3247A>G 3249T>C), A199Y (3250G>T 3251C>A), I201L (3256A>C 3258A>C), G202E (3260G>A 3261C>A), E203G (3263A>G 3264A>T), D204E (3267C>G), L206S (3271T>A 3272T>G 3273G>T), L207T (3274T>A 3275T>C), I210S (3283A>T 3284T>C 3285C>G), Y211D (3286T>G), T213L (3292A>C 3293C>T 3294C>G), F214R (3295T>C 3296T>G 3297C>G), N216T (3302A>C), Q217del (3304_3306delCAG), A219M (3310G>A 3311C>T 3312C>G), A220E (3314C>A 3315C>G), H221P (3317A>C 3318T>A), P222E (3319C>G 3320C>A 3321G>A), N223E (3322A>G 3324C>A), M224R (3325A>C 3326T>G 3327G>T), T225E (3328A>G 3329C>A 3330G>A), N226D (3331A>G 3333C>T), K229A (3340A>G 3341A>C 3342G>C), T234K (3356C>A), I235V (3358A>G 3360C>T), R237K (3364C>A 3365G>A 3366A>G), L249I (3400C>A 3402G>C), E250Q (3403G>C 3405A>G), N251K (3408C>G), Y252L (3410A>T 3411C>A), E253D (3414G>C), I257V (3424A>G 3426C>G), E260L (3433G>C 3434A>T 3435G>C), V262I (3439G>A 3441G>T), I269V (3460A>G 3462T>G), Y276L (3482A>T 3483C>G), V277F (3484G>T 3486G>C), L278I (3487C>A 3489A>T), I280V (3493A>G 3495C>A), I281L (3496A>C), I284L (3505A>C), V285N (3508G>A 3509T>A 3510T>C), V286L (3511G>T 3513T>G), L287V (3514C>G), I290T (3524T>C 3525T>A), L292V (3529C>G 3531G>T), L293V (3532C>G), A294V (3536C>T), S295M (3538T>A 3539C>T 3540C>G), A297K (3544G>A 3545C>A 3546G>A), L298F (3547C>T), Y301L (3556T>C 3557A>T 3558C>G), M302L (3559A>T), F303L (3564C>G), K305A (3568A>G 3569A>C), T306R (3572C>G 3573G>A), C307S (3575G>C 3576C>T), L308R (3578T>G 3579C>T), M309L (3580A>T), N310A (3583A>G 3584A>C 3585C>T), F311P (3586T>C 3587T>C 3588C>T), P312Q (3590C>A 3591C>G), D314E (3597C>A), Q316E (3601C>G), L317T (3604C>A 3605T>C 3606G>C), V318I (3607G>A 3609A>T), H319T (3610C>A 3611A>C), E320K (3613G>A 3615A>G), Q321K (3616C>A), L322C (3619C>T 3620T>G 3621A>C), L328F (3637C>T), L329V (3640C>G 3642G>A), V330L (3643G>T 3645A>G), H331Y (3646C>T 3648T>C), G332Q (3649G>C 3650G>A 3651T>G), W334G (3655T>G 3657G>A), T337I (3665C>T 3666G>T), N338D (3667A>G), M340L (3673A>T 3675G>A), Y342F (3680A>T), I346V (3691A>G), T347S (3694A>T 3696G>A), R348D (3697C>G 3698G>A), I349L (3700A>T 3702T>A), H351K (3706C>A 3708T>G), R352_I353del (3709_3714delCGTATC), F355L (3720C>G), V357G (3725T>G), *358K (3727T>A)								

	Begin	End	Coverage	Score	Concordance	Matches	Identities	I/D/M/F*	Stop Codons
**NT**	**2646**	**3935**	**32.8%**	**164**	**7.3%**	**1203 (92.4%)**	**692 (53.1%)**	**12/87**	

Codon mutations: CGG2TTC (2659C>T 2660G>T 2661G>C), AAC3GAC (2662A>G), CTA4GTG (2665C>G 2667A>G), ATC5TTC (2668A>T), AAC6GGC (2671A>G 2672A>G), GCC7TCC (2674G>T), GTG8GTC (2679G>C), GGC10GGG (2685C>G), TTA11CTG (2686T>C 2688A>G), ATA12CTG (2689A>C 2691A>G), AAG13AAA (2694G>A), CTT14ATC (2695C>A 2697T>C), CCA15GAC (2698C>G 2699C>A 2700A>C), ACA16AGC (2702C>G 2703A>C), GTC17GTG (2706C>G), TCC18TGC (2708C>G), AAT21AAC (2718T>C), GTA22AAC (2719G>A 2720T>A 2721A>C), TTC23ATA (2722T>A 2724C>A), CAT27CAC (2736T>C), CAG29AAG (2740C>A), TTC30GTG (2743T>G 2745C>G), GTG32GTC (2751G>C), CTC35CTG (2760C>G), ATC36GTG (2761A>G 2763C>G), GCG37TCG (2764G>T), TTT38TTC (2769T>C), TCA39TCG (2772A>G), CTG41CTC (2778G>C), GTG42GTC (2781G>C), ACT43ACG (2784T>G), TCG44TCC (2787G>C), CGG45CGC (2790G>C), TTC48ATC (2797T>A), GGA49GGC (2802A>C), AAG50GAC (2803A>G 2805G>C), CTC51CCG (2807T>C 2808C>G), ATA52ATC (2811A>C), CAT55ATC (2818C>A 2819A>T 2820T>C), TTT56GTC (2821T>G 2823T>C), CCG57GAC (2824C>G 2825C>A 2826G>C), GAC58GAT (2829C>T), TAT59GTG (2830T>G 2831A>T 2832T>G), TAC61CTC (2836T>C 2837A>T), GGC62AAC (2839G>A 2840G>A), TCC63ATC (2842T>A 2843C>T), CTC64ATG (2845C>A 2847C>G), AAT65GAC (2848A>G 2850T>C), GAC66ACG (2851G>A 2852A>C 2853C>G), TTC67TAC (2855T>A), TCC69TGG (2861C>G 2862C>G), GTG70ATA (2863G>A 2865G>A), CAA71TAC (2866C>T 2868A>C), CCG72TCG (2869C>T), ACC73ACG (2874C>G), TAC74TTC (2876A>T), TAC74_CTA75insACCATA (2877_2878insACCATA), CTA75CCC (2879T>C 2880A>C), GAA76AAC (2881G>A 2883A>C), GTG77CGT (2884G>C 2885T>G 2886G>T), ATC78CTC (2887A>C), GGT79_ACC80insGGCCGC (2892_2893insGGCCGC), ACC80ATC (2894C>T), ACA81GGC (2896A>G 2897C>G 2898A>C), CAT82AAA (2899C>A 2901T>A), GAT83GAC (2904T>C), GTT84GTG (2907T>G), ATA85GTC (2908A>G 2910A>C), AGC86CAG (2911A>C 2912G>A 2913C>G), CCC87CCG (2916C>G), ATC88GGC (2917A>G 2918T>G), TCG89GTC (2920T>G 2921C>T 2922G>C), CCT90GCG (2923C>G 2925T>G), CAT91TCC (2926C>T 2927A>C 2928T>C), CAA92CAC (2931A>C), GTT93GTG (2934T>G), CGT94_TCG96del (2935_2943delCGTACATCG), AAT97GAC (2944A>G 2946T>C), CAG98GGC (2947C>G 2948A>G 2949G>C), CAA99GAG (2950C>G 2952A>G), CGA100GAC (2953C>G 2954G>A 2955A>C), GAA101GAG (2958A>G), ATC102GTC (2959A>G), TAT104TAC (2967T>C), TAT105CAC (2968T>C 2970T>C), GGC106AAG (2971G>A 2972G>A 2973C>G), TAT107TAC (2976T>C), CAA109CAG (2982A>G), GTT111GTG (2988T>G), TTC112TGC (2990T>G), ATC113TTC (2992A>T), GTA114GTG (2997A>G), TTG115CTC (2998T>C 3000G>C), ATC117TTC (3004A>T), GCG119GCA (3012G>A), GTG120ATG (3013G>A), TCC123TAC (3023C>A), ATT124CTG (3025A>C 3027T>G), CCG125CCC (3030G>C), CAG126AGG (3031C>A 3032A>G), ATC128ATG (3039C>G), AAG130AAA (3045G>A), GTA131ACG (3046G>A 3047T>C 3048A>G), TGT132TGG (3051T>G), GAA133GAG (3054A>G), GGA134GGT (3057A>T), GGG135GGC (3060G>C), AAA136CGC (3061A>C 3062A>G 3063A>C), ATG137ATC (3066G>C), ACA139ATG (3071C>T 3072A>G), TTG140CTC (3073T>C 3075G>C), GCT141GTG (3077C>T 3078T>G), CAT142CTC (3080A>T 3081T>C), GAT143GAC (3084T>C), CTG144CTC (3087G>C), ACT145AAC (3089C>A 3090T>C), TCA146TGC (3092C>G 3093A>C), TTC148ATC (3097T>A), CTG149ATC (3100C>A 3102G>C), AAA151GAG (3106A>G 3108A>G), ATC154AAG (3116T>A 3117C>G), ACA155AAC (3119C>A 3120A>C), GAG156GAC (3123G>C), AAG157CGC (3124A>C 3125A>G 3126G>C), GTG158AAG (3127G>A 3128T>A), GAT159AAG (3130G>A 3132T>G), CAT160CTG (3134A>T 3135T>G), TTG161CTG (3136T>C), ATG162ATC (3141G>C), GAT163GAC (3144T>C), TAT164TAC (3147T>C), TTC166GCC (3151T>G 3152T>C), ATG167ACC (3155T>C 3156G>C), CAG168AAT (3157C>A 3159G>T), GCG171ACG (3166G>A), CAA172CAG (3171A>G), TCT174TTC (3176C>T 3177T>C), TAC177ATA (3184T>A 3185A>T 3186C>A), AAA178CGC (3187A>C 3188A>G 3189A>C), TAC179TTC (3191A>T), TTT180TTC (3195T>C), GGC181CTG (3196G>C 3197G>T 3198C>G), CTG184TTC (3205C>T 3207G>C), CTA185CTC (3210A>C), TTT187TTC (3216T>C), GTT190GTC (3225T>C), GTC191ATC (3226G>A), GCA192GCG (3231A>G), ATA194ATT (3237A>T), TGC195TTC (3239G>T), AAT198GAC (3247A>G 3249T>C), GCC199TAC (3250G>T 3251C>A), TTT200TTC (3255T>C), ATA201CTC (3256A>C 3258A>C), GGC202GAA (3260G>A 3261C>A), GAA203GGT (3263A>G 3264A>T), GAC204GAG (3267C>G), TTT205TTC (3270T>C), TTG206AGT (3271T>A 3272T>G 3273G>T), TTG207ACG (3274T>A 3275T>C), GGA209GGC (3282A>C), ATC210TCG (3283A>T 3284T>C 3285C>G), TAT211GAT (3286T>G), GTT212GTG (3291T>G), ACC213CTG (3292A>C 3293C>T 3294C>G), TTC214CGG (3295T>C 3296T>G 3297C>G), AAC216ACC (3302A>C), CAG217del (3304_3306delCAG), GCC219ATG (3310G>A 3311C>T 3312C>G), GCC220GAG (3314C>A 3315C>G), CAT221CCA (3317A>C 3318T>A), CCG222GAA (3319C>G 3320C>A 3321G>A), AAC223GAA (3322A>G 3324C>A), ATG224CGT (3325A>C 3326T>G 3327G>T), ACG225GAA (3328A>G 3329C>A 3330G>A), AAC226GAT (3331A>G 3333C>T), AAG229GCC (3340A>G 3341A>C 3342G>C), CGA230CGG (3345A>G), GTC231GTG (3348C>G), TTT232TTC (3351T>C), CCA233CCG (3354A>G), ACA234AAA (3356C>A), ATC235GTT (3358A>G 3360C>T), ACT236ACA (3363T>A), CGA237AAG (3364C>A 3365G>A 3366A>G), ACT239ACA (3372T>A), TTT240TTC (3375T>C), TAC243TAT (3384C>T), CCT245CCA (3390T>A), TCG246TCT (3393G>T), GGC247GGA (3396C>A), CTG249ATC (3400C>A 3402G>C), GAA250CAG (3403G>C 3405A>G), AAC251AAG (3408C>G), TAC252TTA (3410A>T 3411C>A), GAG253GAC (3414G>C), GGA254GGT (3417A>T), CTC255CTT (3420C>T), TGC256TGT (3423C>T), ATC257GTG (3424A>G 3426C>G), CTC258TTG (3427C>T 3429C>G), CCG259CCA (3432G>A), GAG260CTC (3433G>C 3434A>T 3435G>C), GTG262ATT (3439G>A 3441G>T), GAG265GAA (3450G>A), AAG266AAA (3453G>A), TAC268TAT (3459C>T), ATT269GTG (3460A>G 3462T>G), CTC271CTG (3468C>G), TAC276TTG (3482A>T 3483C>G), GTG277TTC (3484G>T 3486G>C), CTA278ATT (3487C>A 3489A>T), ATC280GTA (3493A>G 3495C>A), ATC281CTC (3496A>C), AGC282AGT (3501C>T), ATC284CTC (3505A>C), GTT285AAC (3508G>A 3509T>A 3510T>C), GTT286TTG (3511G>T 3513T>G), CTG287GTG (3514C>G), ATT290ACA (3524T>C 3525T>A), GCT291GCC (3528T>C), CTG292GTT (3529C>G 3531G>T), CTA293GTA (3532C>G), GCG294GTG (3536C>T), TCC295ATG (3538T>A 3539C>T 3540C>G), CCT296CCC (3543T>C), GCG297AAA (3544G>A 3545C>A 3546G>A), CTC298TTC (3547C>T), AGA299CGT (3550A>C 3552A>T), TAC301CTG (3556T>C 3557A>T 3558C>G), ATG302TTG (3559A>T), TTC303TTG (3564C>G), CGA304AGA (3565C>A), AAA305GCA (3568A>G 3569A>C), ACG306AGA (3572C>G 3573G>A), TGC307TCT (3575G>C 3576C>T), CTC308CGT (3578T>G 3579C>T), ATG309TTG (3580A>T), AAC310GCT (3583A>G 3584A>C 3585C>T), TTC311CCT (3586T>C 3587T>C 3588C>T), CCC312CAG (3590C>A 3591C>G), GAT313GAC (3594T>C), GAC314GAA (3597C>A), GTC315GTT (3600C>T), CAA316GAA (3601C>G), CTG317ACC (3604C>A 3605T>C 3606G>C), GTA318ATT (3607G>A 3609A>T), CAC319ACC (3610C>A 3611A>C), GAA320AAG (3613G>A 3615A>G), CAG321AAG (3616C>A), CTA322TGC (3619C>T 3620T>G 3621A>C), CAA323CAG (3624A>G), ATA324ATC (3627A>C), GGA325GGT (3630A>T), CTT328TTT (3637C>T), CTG329GTA (3640C>G 3642G>A), GTA330TTG (3643G>T 3645A>G), CAT331TAC (3646C>T 3648T>C), GGT332CAG (3649G>C 3650G>A 3651T>G), CTT333TTG (3652C>T 3654T>G), TGG334GGA (3655T>G 3657G>A), AAA335AAG (3660A>G), ACG337ATT (3665C>T 3666G>T), AAC338GAC (3667A>G), CCA339CCC (3672A>C), ATG340TTA (3673A>T 3675G>A), TAC342TTC (3680A>T), AAA343AAG (3684A>G), GAA344GAG (3687A>G), CTC345CTT (3690C>T), ATA346GTA (3691A>G), ACG347TCA (3694A>T 3696G>A), CGC348GAC (3697C>G 3698G>A), ATT349TTA (3700A>T 3702T>A), GCA350GCC (3705A>C), CAT351AAG (3706C>A 3708T>G), CGT352_ATC353del (3709_3714delCGTATC), TTC355TTG (3720C>G), GAT356GAC (3723T>C), GTT357GGT (3725T>G), TAG358AAG (3727T>A)

Proteins

	Begin	End	Coverage	Score	Concordance	Matches	Identities	I/D/M/F*	Stop Codons
viral inexin-like protein (YP_001031233.1)	1	358	100%	1334	51.8%	352 (97.2%)	168 (46.4%)	4/6/0/0	0

Protein mutations: R2F (2659C>T 2660G>T 2661G>C), N3D (2662A>G), L4V (2665C>G 2667A>G), I5F (2668A>T), N6G (2671A>G 2672A>G), A7S (2674G>T), I12L (2689A>C 2691A>G), L14I (2695C>A 2697T>C), P15D (2698C>G 2699C>A 2700A>C), T16S (2702C>G 2703A>C), S18C (2708C>G), V22N (2719G>A 2720T>A 2721A>C), F23I (2722T>A 2724C>A), Q29K (2740C>A), F30V (2743T>G 2745C>G), I36V (2761A>G 2763C>G), A37S (2764G>T), F48I (2797T>A), K50D (2803A>G 2805G>C), L51P (2807T>C 2808C>G), H55I (2818C>A 2819A>T 2820T>C), F56V (2821T>G 2823T>C), P57D (2824C>G 2825C>A 2826G>C), Y59V (2830T>G 2831A>T 2832T>G), Y61L (2836T>C 2837A>T), G62N (2839G>A 2840G>A), S63I (2842T>A 2843C>T), L64M (2845C>A 2847C>G), N65D (2848A>G 2850T>C), D66T (2851G>A 2852A>C 2853C>G), F67Y (2855T>A), S69W (2861C>G 2862C>G), V70I (2863G>A 2865G>A), Q71Y (2866C>T 2868A>C), P72S (2869C>T), Y74F (2876A>T), Y74_L75insTI (2877_2878insACCATA), L75P (2879T>C 2880A>C), E76N (2881G>A 2883A>C), V77R (2884G>C 2885T>G 2886G>T), I78L (2887A>C), G79_T80insGR (2892_2893insGGCCGC), T80I (2894C>T), T81G (2896A>G 2897C>G 2898A>C), H82K (2899C>A 2901T>A), I85V (2908A>G 2910A>C), S86Q (2911A>C 2912G>A 2913C>G), I88G (2917A>G 2918T>G), S89V (2920T>G 2921C>T 2922G>C), P90A (2923C>G 2925T>G), H91S (2926C>T 2927A>C 2928T>C), Q92H (2931A>C), R94_S96del (2935_2943delCGTACATCG), N97D (2944A>G 2946T>C), Q98G (2947C>G 2948A>G 2949G>C), Q99E (2950C>G 2952A>G), R100D (2953C>G 2954G>A 2955A>C), I102V (2959A>G), Y105H (2968T>C 2970T>C), G106K (2971G>A 2972G>A 2973C>G), F112C (2990T>G), I113F (2992A>T), I117F (3004A>T), V120M (3013G>A), S123Y (3023C>A), I124L (3025A>C 3027T>G), Q126R (3031C>A 3032A>G), I128M (3039C>G), V131T (3046G>A 3047T>C 3048A>G), C132W (3051T>G), K136R (3061A>C 3062A>G 3063A>C), M137I (3066G>C), T139M (3071C>T 3072A>G), A141V (3077C>T 3078T>G), H142L (3080A>T 3081T>C), T145N (3089C>A 3090T>C), S146C (3092C>G 3093A>C), F148I (3097T>A), L149I (3100C>A 3102G>C), K151E (3106A>G 3108A>G), I154K (3116T>A 3117C>G), T155N (3119C>A 3120A>C), E156D (3123G>C), K157R (3124A>C 3125A>G 3126G>C), V158K (3127G>A 3128T>A), D159K (3130G>A 3132T>G), H160L (3134A>T 3135T>G), M162I (3141G>C), F166A (3151T>G 3152T>C), M167T (3155T>C 3156G>C), Q168N (3157C>A 3159G>T), A171T (3166G>A), S174F (3176C>T 3177T>C), Y177I (3184T>A 3185A>T 3186C>A), K178R (3187A>C 3188A>G 3189A>C), Y179F (3191A>T), G181L (3196G>C 3197G>T 3198C>G), L184F (3205C>T 3207G>C), V191I (3226G>A), C195F (3239G>T), N198D (3247A>G 3249T>C), A199Y (3250G>T 3251C>A), I201L (3256A>C 3258A>C), G202E (3260G>A 3261C>A), E203G (3263A>G 3264A>T), D204E (3267C>G), L206S (3271T>A 3272T>G 3273G>T), L207T (3274T>A 3275T>C), I210S (3283A>T 3284T>C 3285C>G), Y211D (3286T>G), T213L (3292A>C 3293C>T 3294C>G), F214R (3295T>C 3296T>G 3297C>G), N216T (3302A>C), Q217del (3304_3306delCAG), A219M (3310G>A 3311C>T 3312C>G), A220E (3314C>A 3315C>G), H221P (3317A>C 3318T>A), P222E (3319C>G 3320C>A 3321G>A), N223E (3322A>G 3324C>A), M224R (3325A>C 3326T>G 3327G>T), T225E (3328A>G 3329C>A 3330G>A), N226D (3331A>G 3333C>T), K229A (3340A>G 3341A>C 3342G>C), T234K (3356C>A), I235V (3358A>G 3360C>T), R237K (3364C>A 3365G>A 3366A>G), L249I (3400C>A 3402G>C), E250Q (3403G>C 3405A>G), N251K (3408C>G), Y252L (3410A>T 3411C>A), E253D (3414G>C), I257V (3424A>G 3426C>G), E260L (3433G>C 3434A>T 3435G>C), V262I (3439G>A 3441G>T), I269V (3460A>G 3462T>G), Y276L (3482A>T 3483C>G), V277F (3484G>T 3486G>C), L278I (3487C>A 3489A>T), I280V (3493A>G 3495C>A), I281L (3496A>C), I284L (3505A>C), V285N (3508G>A 3509T>A 3510T>C), V286L (3511G>T 3513T>G), L287V (3514C>G), I290T (3524T>C 3525T>A), L292V (3529C>G 3531G>T), L293V (3532C>G), A294V (3536C>T), S295M (3538T>A 3539C>T 3540C>G), A297K (3544G>A 3545C>A 3546G>A), L298F (3547C>T), Y301L (3556T>C 3557A>T 3558C>G), M302L (3559A>T), F303L (3564C>G), K305A (3568A>G 3569A>C), T306R (3572C>G 3573G>A), C307S (3575G>C 3576C>T), L308R (3578T>G 3579C>T), M309L (3580A>T), N310A (3583A>G 3584A>C 3585C>T), F311P (3586T>C 3587T>C 3588C>T), P312Q (3590C>A 3591C>G), D314E (3597C>A), Q316E (3601C>G), L317T (3604C>A 3605T>C 3606G>C), V318I (3607G>A 3609A>T), H319T (3610C>A 3611A>C), E320K (3613G>A 3615A>G), Q321K (3616C>A), L322C (3619C>T 3620T>G 3621A>C), L328F (3637C>T), L329V (3640C>G 3642G>A), V330L (3643G>T 3645A>G), H331Y (3646C>T 3648T>C), G332Q (3649G>C 3650G>A 3651T>G), W334G (3655T>G 3657G>A), T337I (3665C>T 3666G>T), N338D (3667A>G), M340L (3673A>T 3675G>A), Y342F (3680A>T), I346V (3691A>G), T347S (3694A>T 3696G>A), R348D (3697C>G 3698G>A), I349L (3700A>T 3702T>A), H351K (3706C>A 3708T>G), R352_I353del (3709_3714delCGTATC), F355L (3720C>G), V357G (3725T>G), *358K (3727T>A)

	Begin	End	Coverage	Score	Concordance	Matches	Identities	I/D/M/F*	Stop Codons
**NT**	**2646**	**3935**	**32.8%**	**164**	**7.3%**	**1203 (92.4%)**	**692 (53.1%)**	**12/87**	

Codon mutations: CGG2TTC (2659C>T 2660G>T 2661G>C), AAC3GAC (2662A>G), CTA4GTG (2665C>G 2667A>G), ATC5TTC (2668A>T), AAC6GGC (2671A>G 2672A>G), GCC7TCC (2674G>T), GTG8GTC (2679G>C), GGC10GGG (2685C>G), TTA11CTG (2686T>C 2688A>G), ATA12CTG (2689A>C 2691A>G), AAG13AAA (2694G>A), CTT14ATC (2695C>A 2697T>C), CCA15GAC (2698C>G 2699C>A 2700A>C), ACA16AGC (2702C>G 2703A>C), GTC17GTG (2706C>G), TCC18TGC (2708C>G), AAT21AAC (2718T>C), GTA22AAC (2719G>A 2720T>A 2721A>C), TTC23ATA (2722T>A 2724C>A), CAT27CAC (2736T>C), CAG29AAG (2740C>A), TTC30GTG (2743T>G 2745C>G), GTG32GTC (2751G>C), CTC35CTG (2760C>G), ATC36GTG (2761A>G 2763C>G), GCG37TCG (2764G>T), TTT38TTC (2769T>C), TCA39TCG (2772A>G), CTG41CTC (2778G>C), GTG42GTC (2781G>C), ACT43ACG (2784T>G), TCG44TCC (2787G>C), CGG45CGC (2790G>C), TTC48ATC (2797T>A), GGA49GGC (2802A>C), AAG50GAC (2803A>G 2805G>C), CTC51CCG (2807T>C 2808C>G), ATA52ATC (2811A>C), CAT55ATC (2818C>A 2819A>T 2820T>C), TTT56GTC (2821T>G 2823T>C), CCG57GAC (2824C>G 2825C>A 2826G>C), GAC58GAT (2829C>T), TAT59GTG (2830T>G 2831A>T 2832T>G), TAC61CTC (2836T>C 2837A>T), GGC62AAC (2839G>A 2840G>A), TCC63ATC (2842T>A 2843C>T), CTC64ATG (2845C>A 2847C>G), AAT65GAC (2848A>G 2850T>C), GAC66ACG (2851G>A 2852A>C 2853C>G), TTC67TAC (2855T>A), TCC69TGG (2861C>G 2862C>G), GTG70ATA (2863G>A 2865G>A), CAA71TAC (2866C>T 2868A>C), CCG72TCG (2869C>T), ACC73ACG (2874C>G), TAC74TTC (2876A>T), TAC74_CTA75insACCATA (2877_2878insACCATA), CTA75CCC (2879T>C 2880A>C), GAA76AAC (2881G>A 2883A>C), GTG77CGT (2884G>C 2885T>G 2886G>T), ATC78CTC (2887A>C), GGT79_ACC80insGGCCGC (2892_2893insGGCCGC), ACC80ATC (2894C>T), ACA81GGC (2896A>G 2897C>G 2898A>C), CAT82AAA (2899C>A 2901T>A), GAT83GAC (2904T>C), GTT84GTG (2907T>G), ATA85GTC (2908A>G 2910A>C), AGC86CAG (2911A>C 2912G>A 2913C>G), CCC87CCG (2916C>G), ATC88GGC (2917A>G 2918T>G), TCG89GTC (2920T>G 2921C>T 2922G>C), CCT90GCG (2923C>G 2925T>G), CAT91TCC (2926C>T 2927A>C 2928T>C), CAA92CAC (2931A>C), GTT93GTG (2934T>G), CGT94_TCG96del (2935_2943delCGTACATCG), AAT97GAC (2944A>G 2946T>C), CAG98GGC (2947C>G 2948A>G 2949G>C), CAA99GAG (2950C>G 2952A>G), CGA100GAC (2953C>G 2954G>A 2955A>C), GAA101GAG (2958A>G), ATC102GTC (2959A>G), TAT104TAC (2967T>C), TAT105CAC (2968T>C 2970T>C), GGC106AAG (2971G>A 2972G>A 2973C>G), TAT107TAC (2976T>C), CAA109CAG (2982A>G), GTT111GTG (2988T>G), TTC112TGC (2990T>G), ATC113TTC (2992A>T), GTA114GTG (2997A>G), TTG115CTC (2998T>C 3000G>C), ATC117TTC (3004A>T), GCG119GCA (3012G>A), GTG120ATG (3013G>A), TCC123TAC (3023C>A), ATT124CTG (3025A>C 3027T>G), CCG125CCC (3030G>C), CAG126AGG (3031C>A 3032A>G), ATC128ATG (3039C>G), AAG130AAA (3045G>A), GTA131ACG (3046G>A 3047T>C 3048A>G), TGT132TGG (3051T>G), GAA133GAG (3054A>G), GGA134GGT (3057A>T), GGG135GGC (3060G>C), AAA136CGC (3061A>C 3062A>G 3063A>C), ATG137ATC (3066G>C), ACA139ATG (3071C>T 3072A>G), TTG140CTC (3073T>C 3075G>C), GCT141GTG (3077C>T 3078T>G), CAT142CTC (3080A>T 3081T>C), GAT143GAC (3084T>C), CTG144CTC (3087G>C), ACT145AAC (3089C>A 3090T>C), TCA146TGC (3092C>G 3093A>C), TTC148ATC (3097T>A), CTG149ATC (3100C>A 3102G>C), AAA151GAG (3106A>G 3108A>G), ATC154AAG (3116T>A 3117C>G), ACA155AAC (3119C>A 3120A>C), GAG156GAC (3123G>C), AAG157CGC (3124A>C 3125A>G 3126G>C), GTG158AAG (3127G>A 3128T>A), GAT159AAG (3130G>A 3132T>G), CAT160CTG (3134A>T 3135T>G), TTG161CTG (3136T>C), ATG162ATC (3141G>C), GAT163GAC (3144T>C), TAT164TAC (3147T>C), TTC166GCC (3151T>G 3152T>C), ATG167ACC (3155T>C 3156G>C), CAG168AAT (3157C>A 3159G>T), GCG171ACG (3166G>A), CAA172CAG (3171A>G), TCT174TTC (3176C>T 3177T>C), TAC177ATA (3184T>A 3185A>T 3186C>A), AAA178CGC (3187A>C 3188A>G 3189A>C), TAC179TTC (3191A>T), TTT180TTC (3195T>C), GGC181CTG (3196G>C 3197G>T 3198C>G), CTG184TTC (3205C>T 3207G>C), CTA185CTC (3210A>C), TTT187TTC (3216T>C), GTT190GTC (3225T>C), GTC191ATC (3226G>A), GCA192GCG (3231A>G), ATA194ATT (3237A>T), TGC195TTC (3239G>T), AAT198GAC (3247A>G 3249T>C), GCC199TAC (3250G>T 3251C>A), TTT200TTC (3255T>C), ATA201CTC (3256A>C 3258A>C), GGC202GAA (3260G>A 3261C>A), GAA203GGT (3263A>G 3264A>T), GAC204GAG (3267C>G), TTT205TTC (3270T>C), TTG206AGT (3271T>A 3272T>G 3273G>T), TTG207ACG (3274T>A 3275T>C), GGA209GGC (3282A>C), ATC210TCG (3283A>T 3284T>C 3285C>G), TAT211GAT (3286T>G), GTT212GTG (3291T>G), ACC213CTG (3292A>C 3293C>T 3294C>G), TTC214CGG (3295T>C 3296T>G 3297C>G), AAC216ACC (3302A>C), CAG217del (3304_3306delCAG), GCC219ATG (3310G>A 3311C>T 3312C>G), GCC220GAG (3314C>A 3315C>G), CAT221CCA (3317A>C 3318T>A), CCG222GAA (3319C>G 3320C>A 3321G>A), AAC223GAA (3322A>G 3324C>A), ATG224CGT (3325A>C 3326T>G 3327G>T), ACG225GAA (3328A>G 3329C>A 3330G>A), AAC226GAT (3331A>G 3333C>T), AAG229GCC (3340A>G 3341A>C 3342G>C), CGA230CGG (3345A>G), GTC231GTG (3348C>G), TTT232TTC (3351T>C), CCA233CCG (3354A>G), ACA234AAA (3356C>A), ATC235GTT (3358A>G 3360C>T), ACT236ACA (3363T>A), CGA237AAG (3364C>A 3365G>A 3366A>G), ACT239ACA (3372T>A), TTT240TTC (3375T>C), TAC243TAT (3384C>T), CCT245CCA (3390T>A), TCG246TCT (3393G>T), GGC247GGA (3396C>A), CTG249ATC (3400C>A 3402G>C), GAA250CAG (3403G>C 3405A>G), AAC251AAG (3408C>G), TAC252TTA (3410A>T 3411C>A), GAG253GAC (3414G>C), GGA254GGT (3417A>T), CTC255CTT (3420C>T), TGC256TGT (3423C>T), ATC257GTG (3424A>G 3426C>G), CTC258TTG (3427C>T 3429C>G), CCG259CCA (3432G>A), GAG260CTC (3433G>C 3434A>T 3435G>C), GTG262ATT (3439G>A 3441G>T), GAG265GAA (3450G>A), AAG266AAA (3453G>A), TAC268TAT (3459C>T), ATT269GTG (3460A>G 3462T>G), CTC271CTG (3468C>G), TAC276TTG (3482A>T 3483C>G), GTG277TTC (3484G>T 3486G>C), CTA278ATT (3487C>A 3489A>T), ATC280GTA (3493A>G 3495C>A), ATC281CTC (3496A>C), AGC282AGT (3501C>T), ATC284CTC (3505A>C), GTT285AAC (3508G>A 3509T>A 3510T>C), GTT286TTG (3511G>T 3513T>G), CTG287GTG (3514C>G), ATT290ACA (3524T>C 3525T>A), GCT291GCC (3528T>C), CTG292GTT (3529C>G 3531G>T), CTA293GTA (3532C>G), GCG294GTG (3536C>T), TCC295ATG (3538T>A 3539C>T 3540C>G), CCT296CCC (3543T>C), GCG297AAA (3544G>A 3545C>A 3546G>A), CTC298TTC (3547C>T), AGA299CGT (3550A>C 3552A>T), TAC301CTG (3556T>C 3557A>T 3558C>G), ATG302TTG (3559A>T), TTC303TTG (3564C>G), CGA304AGA (3565C>A), AAA305GCA (3568A>G 3569A>C), ACG306AGA (3572C>G 3573G>A), TGC307TCT (3575G>C 3576C>T), CTC308CGT (3578T>G 3579C>T), ATG309TTG (3580A>T), AAC310GCT (3583A>G 3584A>C 3585C>T), TTC311CCT (3586T>C 3587T>C 3588C>T), CCC312CAG (3590C>A 3591C>G), GAT313GAC (3594T>C), GAC314GAA (3597C>A), GTC315GTT (3600C>T), CAA316GAA (3601C>G), CTG317ACC (3604C>A 3605T>C 3606G>C), GTA318ATT (3607G>A 3609A>T), CAC319ACC (3610C>A 3611A>C), GAA320AAG (3613G>A 3615A>G), CAG321AAG (3616C>A), CTA322TGC (3619C>T 3620T>G 3621A>C), CAA323CAG (3624A>G), ATA324ATC (3627A>C), GGA325GGT (3630A>T), CTT328TTT (3637C>T), CTG329GTA (3640C>G 3642G>A), GTA330TTG (3643G>T 3645A>G), CAT331TAC (3646C>T 3648T>C), GGT332CAG (3649G>C 3650G>A 3651T>G), CTT333TTG (3652C>T 3654T>G), TGG334GGA (3655T>G 3657G>A), AAA335AAG (3660A>G), ACG337ATT (3665C>T 3666G>T), AAC338GAC (3667A>G), CCA339CCC (3672A>C), ATG340TTA (3673A>T 3675G>A), TAC342TTC (3680A>T), AAA343AAG (3684A>G), GAA344GAG (3687A>G), CTC345CTT (3690C>T), ATA346GTA (3691A>G), ACG347TCA (3694A>T 3696G>A), CGC348GAC (3697C>G 3698G>A), ATT349TTA (3700A>T 3702T>A), GCA350GCC (3705A>C), CAT351AAG (3706C>A 3708T>G), CGT352_ATC353del (3709_3714delCGTATC), TTC355TTG (3720C>G), GAT356GAC (3723T>C), GTT357GGT (3725T>G), TAG358AAG (3727T>A)

*: Inserts / Deletes / Misaligned / Frameshifts

Analysis details

This analysis was performed with panviral2.64

NGS Details (UN60): Badnavirus maculasmallanthi

Assembly

**Coverage Length**	257 (1 contig(s))
**Depth Of Coverage**	43.1
**Number Of Reads**	110
**Reads Per Million**	2.33 rpm (after QC)
**Ambiguities**	0
**Assembly Method**	de novo + reference guided assembly
**Consensus Caller**	Bcf Tools

Coverage Map

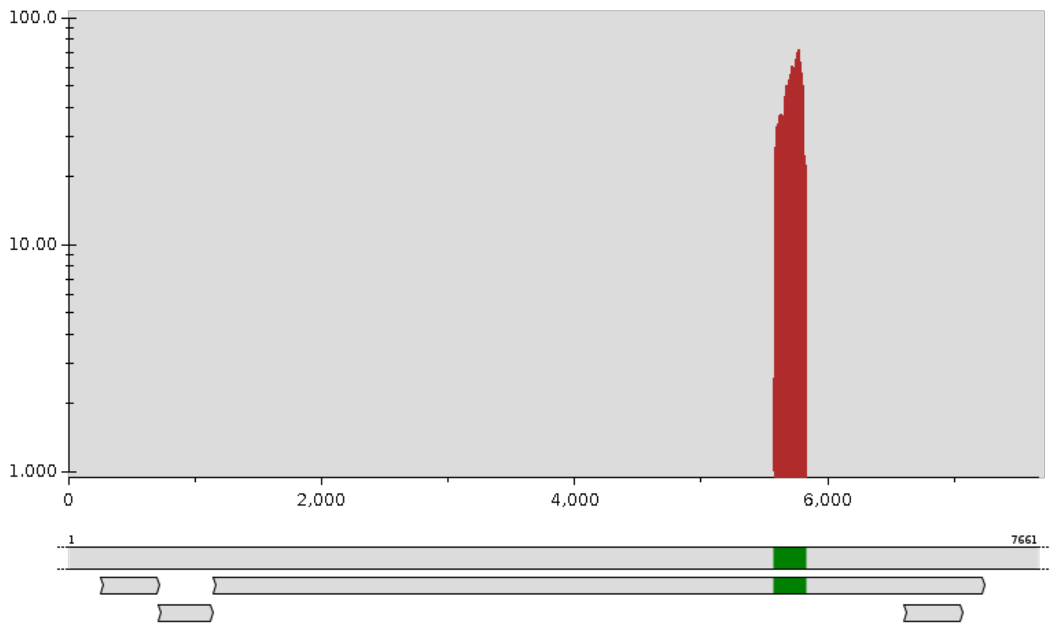

Assignment

**Type**	Badnavirus maculasmallanthi (Taxonomy ID: 3048453)
**Reference Genome**	NC_026472.1
**NT Identity (%)**	56.5385
**AA Identity (%)**	55.1724
**Number Of Stop Codons**	0
**Number Of CDS**	4

Alignment

**Alignment Score**	62.0 (NT) + 334.0 (AA) = 396.0
**Concordance (%)**	35.1375

Alignment Method	Global, seeded, nucleotide + amino acids (AGA)

Genome Region

Sequence starts at position 5577 and ends at position 5833 relative to NC_026472.1 reference sequence.

Alignment Detailed Statistics

	Begin	End	Coverage	Score	Concordance	Matches	Identities	I/D/M/F*	Stop Codons
**NT**	**5577**	**5833**	**3.4%**	**62**	**12.1%**	**257 (98.8%)**	**147 (56.5%)**	**3/0**	
Mutations:	5588A>G, 5589G>A, 5592T>G, 5595A>T, 5597T>A, 5604A>G, 5605G>T, 5607T>G, 5608G>A, 5609C>G, 5610C>A, 5611A>G, 5613G>T, 5614G>C, 5615A>G, 5616A>G, 5619A>G, 5620G>T, 5621A>C, 5623T>G, 5624C>A, 5625C>T, 5628A>C, 5631C>G, 5632T>A, 5633G>A, 5637G>T, 5640C>T, 5644T>C, 5645T>G, 5646A>G, 5648T>C, 5649C>A, 5650C>T, 5651C>G, 5652A>C, 5653G>T, 5654G>A, 5655A>T, 5658A>T, 5659T>C, 5660T>A, 5661A>T, 5664C>T, 5667A>G, 5669G>T, 5670G>T, 5671C>T, 5673T>G, 5676C>T, 5680C>T, 5682A>T, 5685C>T, 5688A>G, 5691C>T, 5693A>C, 5700A>C, 5703C>T, 5707A>G, 5708T>C, 5712T>C, 5713C>A, 5714A>T, 5716A>G, 5717G>A, 5718A>G, 5719A>T, 5720A>T, 5721A>G, 5725G>A, 5728A>C, 5729A>G, 5730A>G, 5731T>G, 5732G>T, 5733C>G, 5736C>T, 5739A>G, 5740G>T, 5741A>C, 5742_5743insTAT, 5743A>T, 5744C>T, 5745A>G, 5748A>T, 5749G>C, 5755A>G, 5757C>G, 5758G>A, 5759C>T, 5765A>T, 5766C>T, 5769C>A, 5772C>T, 5775C>T, 5779C>T, 5784A>T, 5787C>T, 5793C>G, 5799A>G, 5800G>A, 5801C>G, 5802G>T, 5810A>T, 5813G>A, 5814G>A, 5817T>C, 5820A>G, 5822A>G, 5823G>A, 5827A>G								

CDS

	Begin	End	Coverage	Score	Concordance	Matches	Identities	I/D/M/F*	Stop Codons
UF61_gp3	1479	1564	4.2%	334	53.4%	86 (98.9%)	48 (55.2%)	1/0/0/0	0
Protein mutations:	K1482R (5588A>G 5589G>A), F1485Y (5597T>A), V1488L (5605G>T 5607T>G), A1489R (5608G>A 5609C>G 5610C>A), M1490V (5611A>G 5613G>T), E1491R (5614G>C 5615A>G 5616A>G), E1493S (5620G>T 5621A>C), S1494D (5623T>G 5624C>A 5625C>T), W1497K (5632T>A 5633G>A), L1501R (5644T>C 5645T>G 5646A>G), I1502T (5648T>C 5649C>A), P1503C (5650C>T 5651C>G 5652A>C), G1504Y (5653G>T 5654G>A 5655A>T), L1506H (5659T>C 5660T>A 5661A>T), W1509F (5669G>T 5670G>T), P1513S (5680C>T 5682A>T), K1517T (5693A>C), I1522A (5707A>G 5708T>C), Q1524M (5713C>A 5714A>T), R1525E (5716A>G 5717G>A 5718A>G), K1526L (5719A>T 5720A>T 5721A>G), D1528N (5725G>A), K1529R (5728A>C 5729A>G 5730A>G), C1530V (5731T>G 5732G>T 5733C>G), D1533S (5740G>T 5741A>C), D1533_T1534insY (5742_5743insTAT), T1534L (5743A>T 5744C>T 5745A>G), E1535D (5748A>T), E1536Q (5749G>C), I1538V (5755A>G 5757C>G), A1539I (5758G>A 5759C>T), Y1541F (5765A>T 5766C>T), N1550K (5793C>G), A1553S (5800G>A 5801C>G 5802G>T), E1556V (5810A>T), R1557K (5813G>A 5814G>A), K1560R (5822A>G 5823G>A), M1562V (5827A>G)								
Codon mutations:	AAG1482AGA (5588A>G 5589G>A), TCT1483TCG (5592T>G), GGA1484GGT (5595A>T), TTT1485TAT (5597T>A), CAA1487CAG (5604A>G), GTT1488TTG (5605G>T 5607T>G), GCC1489AGA (5608G>A 5609C>G 5610C>A), ATG1490GTT (5611A>G 5613G>T), GAA1491CGG (5614G>C 5615A>G 5616A>G), GAA1492GAG (5619A>G), GAA1493TCA (5620G>T 5621A>C), TCC1494GAT (5623T>G 5624C>A 5625C>T), ATA1495ATC (5628A>C), CCC1496CCG (5631C>G), TGG1497AAG (5632T>A 5633G>A), ACG1498ACT (5637G>T), GCC1499GCT (5640C>T), TTA1501CGG (5644T>C 5645T>G 5646A>G), ATC1502ACA (5648T>C 5649C>A), CCA1503TGC (5650C>T 5651C>G 5652A>C), GGA1504TAT (5653G>T 5654G>A 5655A>T), GGA1505GGT (5658A>T), TTA1506CAT (5659T>C 5660T>A 5661A>T), TAC1507TAT (5664C>T), GAA1508GAG (5667A>G), TGG1509TTT (5669G>T 5670G>T), CTT1510TTG (5671C>T 5673T>G), GTC1511GTT (5676C>T), CCA1513TCT (5680C>T 5682A>T), TTC1514TTT (5685C>T), GGA1515GGG (5688A>G), CTC1516CTT (5691C>T), AAA1517ACA (5693A>C), GCA1519GCC (5700A>C), CCC1520CCT (5703C>T), ATT1522GCT (5707A>G 5708T>C), TTT1523TTC (5712T>C), CAG1524ATG (5713C>A 5714A>T), AGA1525GAG (5716A>G 5717G>A 5718A>G), AAA1526TTG (5719A>T 5720A>T 5721A>G), GAT1528AAT (5725G>A), AAA1529CGG (5728A>C 5729A>G 5730A>G), TGC1530GTG (5731T>G 5732G>T 5733C>G), TTC1531TTT (5736C>T), AAA1532AAG (5739A>G), GAC1533TCC (5740G>T 5741A>C), GAC1533_ACA1534insTAT (5742_5743insTAT), ACA1534TTG (5743A>T 5744C>T 5745A>G), GAA1535GAT (5748A>T), GAG1536CAG (5749G>C), ATC1538GTG (5755A>G 5757C>G), GCC1539ATC (5758G>A 5759C>T), TAC1541TTT (5765A>T 5766C>T), ATC1542ATA (5769C>A), GAC1543GAT (5772C>T), GAC1544GAT (5775C>T), CTG1546TTG (5779C>T), GTA1547GTT (5784A>T), TAC1548TAT (5787C>T), AAC1550AAG (5793C>G), GAA1552GAG (5799A>G), GCG1553AGT (5800G>A 5801C>G 5802G>T), GAA1556GTA (5810A>T), AGG1557AAA (5813G>A 5814G>A), CAT1558CAC (5817T>C), TTA1559TTG (5820A>G), AAG1560AGA (5822A>G 5823G>A), ATG1562GTG (5827A>G)								

Proteins

	Begin	End	Coverage	Score	Concordance	Matches	Identities	I/D/M/F*	Stop Codons
ORF3 (YP_009121747.1)	1479	1564	4.2%	334	53.4%	86 (98.9%)	48 (55.2%)	1/0/0/0	0
Protein mutations:	K1482R (5588A>G 5589G>A), F1485Y (5597T>A), V1488L (5605G>T 5607T>G), A1489R (5608G>A 5609C>G 5610C>A), M1490V (5611A>G 5613G>T), E1491R (5614G>C 5615A>G 5616A>G), E1493S (5620G>T 5621A>C), S1494D (5623T>G 5624C>A 5625C>T), W1497K (5632T>A 5633G>A), L1501R (5644T>C 5645T>G 5646A>G), I1502T (5648T>C 5649C>A), P1503C (5650C>T 5651C>G 5652A>C), G1504Y (5653G>T 5654G>A 5655A>T), L1506H (5659T>C 5660T>A 5661A>T), W1509F (5669G>T 5670G>T), P1513S (5680C>T 5682A>T), K1517T (5693A>C), I1522A (5707A>G 5708T>C), Q1524M (5713C>A 5714A>T), R1525E (5716A>G 5717G>A 5718A>G), K1526L (5719A>T 5720A>T 5721A>G), D1528N (5725G>A), K1529R (5728A>C 5729A>G 5730A>G), C1530V (5731T>G 5732G>T 5733C>G), D1533S (5740G>T 5741A>C), D1533_T1534insY (5742_5743insTAT), T1534L (5743A>T 5744C>T 5745A>G), E1535D (5748A>T), E1536Q (5749G>C), I1538V (5755A>G 5757C>G), A1539I (5758G>A 5759C>T), Y1541F (5765A>T 5766C>T), N1550K (5793C>G), A1553S (5800G>A 5801C>G 5802G>T), E1556V (5810A>T), R1557K (5813G>A 5814G>A), K1560R (5822A>G 5823G>A), M1562V (5827A>G)								
Codon mutations:	AAG1482AGA (5588A>G 5589G>A), TCT1483TCG (5592T>G), GGA1484GGT (5595A>T), TTT1485TAT (5597T>A), CAA1487CAG (5604A>G), GTT1488TTG (5605G>T 5607T>G), GCC1489AGA (5608G>A 5609C>G 5610C>A), ATG1490GTT (5611A>G 5613G>T), GAA1491CGG (5614G>C 5615A>G 5616A>G), GAA1492GAG (5619A>G), GAA1493TCA (5620G>T 5621A>C), TCC1494GAT (5623T>G 5624C>A 5625C>T), ATA1495ATC (5628A>C), CCC1496CCG (5631C>G), TGG1497AAG (5632T>A 5633G>A), ACG1498ACT (5637G>T), GCC1499GCT (5640C>T), TTA1501CGG (5644T>C 5645T>G 5646A>G), ATC1502ACA (5648T>C 5649C>A), CCA1503TGC (5650C>T 5651C>G 5652A>C), GGA1504TAT (5653G>T 5654G>A 5655A>T), GGA1505GGT (5658A>T), TTA1506CAT (5659T>C 5660T>A 5661A>T), TAC1507TAT (5664C>T), GAA1508GAG (5667A>G), TGG1509TTT (5669G>T 5670G>T), CTT1510TTG (5671C>T 5673T>G), GTC1511GTT (5676C>T), CCA1513TCT (5680C>T 5682A>T), TTC1514TTT (5685C>T), GGA1515GGG (5688A>G), CTC1516CTT (5691C>T), AAA1517ACA (5693A>C), GCA1519GCC (5700A>C), CCC1520CCT (5703C>T), ATT1522GCT (5707A>G 5708T>C), TTT1523TTC (5712T>C), CAG1524ATG (5713C>A 5714A>T), AGA1525GAG (5716A>G 5717G>A 5718A>G), AAA1526TTG (5719A>T 5720A>T 5721A>G), GAT1528AAT (5725G>A), AAA1529CGG (5728A>C 5729A>G 5730A>G), TGC1530GTG (5731T>G 5732G>T 5733C>G), TTC1531TTT (5736C>T), AAA1532AAG (5739A>G), GAC1533TCC (5740G>T 5741A>C), GAC1533_ACA1534insTAT (5742_5743insTAT), ACA1534TTG (5743A>T 5744C>T 5745A>G), GAA1535GAT (5748A>T), GAG1536CAG (5749G>C), ATC1538GTG (5755A>G 5757C>G), GCC1539ATC (5758G>A 5759C>T), TAC1541TTT (5765A>T 5766C>T), ATC1542ATA (5769C>A), GAC1543GAT (5772C>T), GAC1544GAT (5775C>T), CTG1546TTG (5779C>T), GTA1547GTT (5784A>T), TAC1548TAT (5787C>T), AAC1550AAG (5793C>G), GAA1552GAG (5799A>G), GCG1553AGT (5800G>A 5801C>G 5802G>T), GAA1556GTA (5810A>T), AGG1557AAA (5813G>A 5814G>A), CAT1558CAC (5817T>C), TTA1559TTG (5820A>G), AAG1560AGA (5822A>G 5823G>A), ATG1562GTG (5827A>G)								

*: Inserts / Deletes / Misaligned / Frameshifts

Analysis details

This analysis was performed with panviral2.64

NGS Details (UN60): Caulimovirus venafragariae

Assembly

Coverage Length	1041 (1 contig(s))
Depth Of Coverage	9.9
Number Of Reads	77
Reads Per Million	1.63 rpm (after QC)
Ambiguities	0
Assembly Method	de novo + reference guided assembly
Consensus Caller	Bcf Tools

Coverage Map

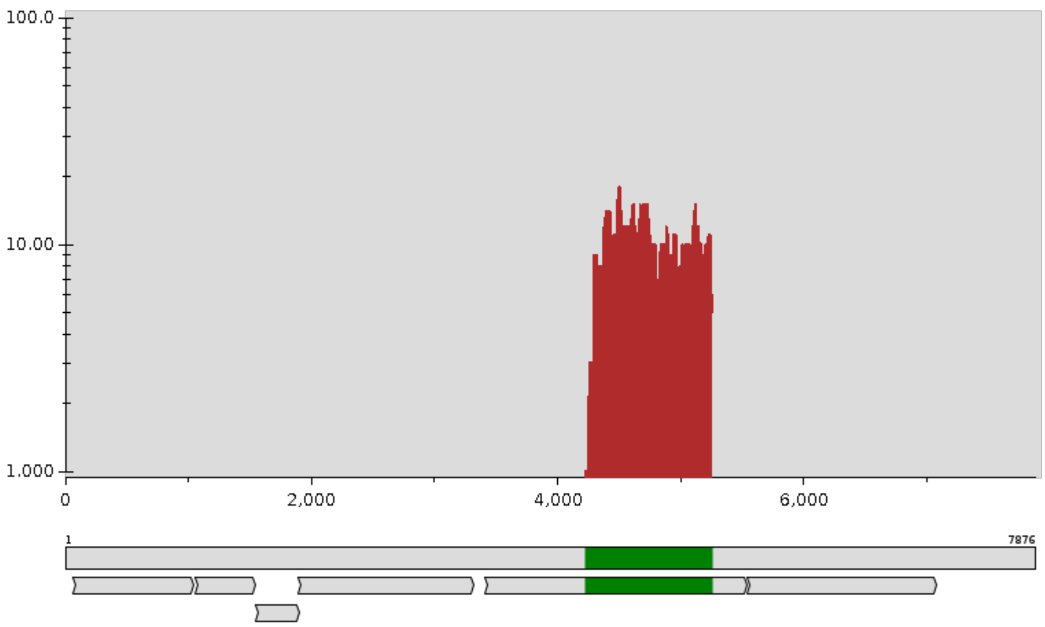

Assignment

Type	Caulimovirus venafragariae (Taxonomy ID: 3048344)
Reference Genome	NC_001725.1
NT Identity (%)	56.3477
AA Identity (%)	52.3392
Number Of Stop Codons	0
Number Of CDS	6

Alignment

Alignment Score	216.0 (NT) + 1173.0 (AA) = 1389.0
Concordance (%)	32.3873

Alignment Method	Global, seeded, nucleotide + amino acids (AGA)

Genome Region

Sequence starts at position 4219 and ends at position 5259 relative to NC_001725.1 reference sequence.

Alignment Detailed Statistics

	Begin	End	Coverage	Score	Concordance	Matches	Identities	I/D/M/F*	Stop Codons
**NT**	**4219**	**5259**	**13.2%**	**216**	**10.7%**	**1023 (98.2%)**	**577 (55.4%)**	**1/18**	
Mutations:	4219T>C, 4222T>A, 4223T>A, 4227C>T, 4228A>C, 4230T>C, 4232A>G, 4235A>T, 4236A>C, 4239T>A, 4242T>A, 4244A>G, 4245G>A, 4246T>G, 4247G>T, 4254A>T, 4259A>T, 4260G>A, 4263T>C, 4264T>A, 4265C>A, 4266A>T, 4267C>G, 4268C>A, 4269T>A, 4275C>T, 4276C>A, 4277G>A, 4279G>A, 4280A>C, 4281A>T, 4284G>A, 4292C>T, 4299C>T, 4300G>C, 4306C>T, 4309C>T, 4311C>A, 4314G>T, 4315C>A, 4317T>G, 4318G>A, 4319G>A, 4320T>A, 4321A>T, 4323C>A, 4326C>A, 4331C>A, 4335C>T, 4338G>A, 4348T>A, 4349C>G, 4350C>T, 4351T>A, 4352C>G, 4353A>C, 4356A>C, 4359C>A, 4362C>T, 4366G>A, 4371G>A, 4374T>C, 4379C>G, 4383G>A, 4384A>C, 4385T>A, 4386C>A, 4392A>T, 4395C>A, 4401A>T, 4402A>C, 4413T>A, 4423A>G, 4426T>C, 4428A>T, 4432G>A, 4434C>A, 4435C>A, 4438A>T, 4439C>G, 4440A>T, 4441A>G, 4442A>T, 4443G>C, 4444G>T, 4445G>T, 4446A>C, 4449T>C, 4452C>A, 4455C>T, 4456C>T, 4458A>C, 4459C>A, 4461T>C, 4464T>A, 4467C>T, 4470G>A, 4471G>A, 4473G>T, 4474C>A, 4476A>C, 4479G>T, 4480C>A, 4482T>A, 4483C>A, 4485A>T, 4486A>C, 4487G>T, 4488A>T, 4489A>G, 4490T>C, 4492G>A, 4493G>A, 4497T>A, 4500G>A, 4505T>A, 4506T>C, 4508A>T, 4509C>T, 4512T>A, 4513T>A, 4514C>A, 4515C>A, 4521C>T, 4524T>C, 4528T>A, 4529C>G, 4542G>A, 4543G>A, 4546C>A, 4547G>A, 4548C>A, 4551T>A, 4553C>A, 4554T>A, 4555C>G, 4556C>A, 4558G>A, 4560A>T, 4561A>T, 4563A>T, 4566T>A, 4567C>G, 4568A>C, 4569G>T, 4570C>T, 4575C>A, 4578T>G, 4584C>T, 4585T>A, 4586G>C, 4587T>G, 4590C>A, 4591C>A, 4593A>T, 4599C>T, 4614C>G, 4633C>A, 4635A>T, 4642G>C, 4643C>A, 4644T>A, 4647T>C, 4650C>T, 4653A>G, 4657C>A, 4659C>A, 4665C>T, 4666G>A, 4670G>T, 4671T>A, 4672C>T, 4674C>T, 4675A>T, 4676G>C, 4678A>C, 4680C>A, 4682T>A, 4684T>A, 4687_4692delCCACAG, 4696T>C, 4697G>T, 4698T>A, 4699G>A, 4700C>T, 4701T>C, 4704A>C, 4707T>C, 4708G>A, 4710T>A, 4716C>T, 4719C>A, 4720A>T, 4722C>A, 4723G>A, 4725C>A, 4727T>G, 4728C>T, 4729A>T, 4730G>C, 4734A>C, 4735A>T, 4737T>A, 4738G>T, 4740A>T, 4741G>C, 4753G>A, 4754G>C, 4755A>T, 4759G>C, 4761C>T, 4765A>C, 4766T>A, 4767T>A, 4768G>T, 4771C>T, 4772T>C, 4773A>T, 4774A>C, 4776C>A, 4778G>T, 4782C>T, 4783A>T, 4784A>G, 4785A>C, 4787C>A, 4788C>A, 4789C>A, 4790T>A, 4791A>T, 4797A>T, 4801C>T, 4803T>A, 4807A>C, 4809A>G, 4812G>A, 4815A>G, 4818C>A, 4819C>G, 4821G>C, 4822T>A, 4824G>A, 4825T>A, 4826G>A, 4827C>T, 4832C>A, 4833A>T, 4834A>G, 4835C>A, 4836T>A, 4839C>T, 4840A>G, 4842T>A, 4845C>T, 4848T>C, 4851T>A, 4852C>A, 4854A>G, 4855G>A, 4860A>T, 4861G>T, 4862A>C, 4865G>A, 4867G>A, 4868G>A, 4869A>T, 4870A>G, 4871A>G, 4872T>C, 4873C>A, 4875C>T, 4876A>G, 4879G>T, 4881C>A, 4884G>A, 4886G>C, 4890T>C, 4893C>A, 4894G>T, 4895G>C, 4897C>A, 4898T>A, 4900C>A, 4902C>A, 4902_4903insG, 4903T>A, 4905A>C, 4906G>T, 4907T>G, 4910C>A, 4911T>A, 4915C>T, 4921C>A, 4926T>A, 4928G>A, 4929T>C, 4930G>A, 4931A>C, 4932T>A, 4934G>A, 4935G>A, 4936A>C, 4938T>A, 4940C>A, 4941C>A, 4942T>A, 4944A>C, 4950A>G, 4954T>C, 4956A>T, 4959C>A, 4960C>T, 4961T>G, 4962C>T, 4963C>T, 4971T>C, 4972A>G, 4973T>C, 4974C>A, 4975T>A, 4976C>G, 4977C>T, 4979C>A, 4980T>A, 4982A>T, 4984T>A, 4986T>A, 4990A>G, 4992A>C, 4993A>C, 5001C>A, 5002C>A, 5003T>A, 5004T>G, 5007G>A, 5008T>A, 5009C>A, 5010A>T, 5012C>T, 5019G>A, 5020G>A, 5021T>G, 5022T>G, 5023A>C, 5024A>T, 5025A>T, 5026C>A, 5029A>C, 5030A>G, 5031G>A, 5035G>T, 5036A>C, 5037G>C, 5039T>A, 5040A>C, 5041A>C, 5042C>G, 5043T>A, 5044T>A, 5045G>T, 5047T>G, 5048C>G, 5049T>A, 5055T>A, 5058G>T, 5059A>G, 5062G>C, 5064C>T, 5067G>T, 5068G>A, 5071A>T, 5072C>G, 5076C>T, 5077A>C, 5078G>A, 5081A>G, 5082A>C, 5083A>C, 5088G>A, 5089A>C, 5090G>A, 5091T>A, 5092C>G, 5093T>A, 5095G>T, 5096T>G, 5097A>T, 5098A>T, 5099A>C, 5100A>C, 5102C>A, 5103T>A, 5110G>A, 5112C>G, 5115T>A, 5116T>A, 5117A>G, 5118C>A, 5119A>C, 5120A>T, 5124T>G, 5125T>G, 5126C>A, 5133G>A, 5136T>C, 5139G>C, 5140C>T, 5141C>T, 5142T>A, 5143A>G, 5145C>T, 5146A>T, 5148C>A, 5149G>C, 5152T>A, 5153G>C, 5154C>A, 5160A>T, 5167G>T, 5182A>T, 5184C>A, 5185C>T, 5187C>A, 5188A>C, 5191G>A, 5193A>T, 5194A>G, 5196A>C, 5200C>A, 5201C>A, 5202A>T, 5206_5217delGGTAAAGAGGTA, 5220A>T, 5223C>T, 5230G>A, 5232T>A, 5233T>A, 5234C>G, 5235A>T, 5238A>T, 5241C>A, 5247A>T, 5248C>G, 5249C>A, 5251G>A, 5253T>C, 5256G>A, 5258A>C, 5259G>T								

CDS

	Begin	End	Coverage	Score	Concordance	Matches	Identities	I/D/M/F*	Stop Codons
ORF_V	272	618	48.9%	1173	49.7%	341 (98.0%)	179 (51.4%)	1/6/1/1	0
Protein mutations:	L273K (4222T>A 4223T>A), T275P (4228A>C 4230T>C), N276S (4232A>G), K277I (4235A>T 4236A>C), K280R (4244A>G 4245G>A), C281V (4246T>G 4247G>T), Q285L (4259A>T 4260G>A), S287N (4264T>A 4265C>A 4266A>T), P288E (4267C>G 4268C>A 4269T>A), R291K (4276C>A 4277G>A), E292T (4279G>A 4280A>C 4281A>T), T296I (4292C>T), E299Q (4300G>C), K303N (4314G>T), L304M (4315C>A 4317T>G), G305K (4318G>A 4319G>A 4320T>A), I306L (4321A>T 4323C>A), P309H (4331C>A), V321I (4366G>A), A325G (4379C>G), I327Q (4384A>C 4385T>A 4386C>A), K340E (4423A>G), D343K (4432G>A 4434C>A), H344N (4435C>A), T345C (4438A>T 4439C>G 4440A>T), K346V (4441A>G 4442A>T 4443G>C), G347F (4444G>T 4445G>T 4446A>C), L351F (4456C>T 4458A>C), L352I (4459C>A 4461T>C), E356N (4471G>A 4473G>T), Q357N (4474C>A 4476A>C), L359I (4480C>A 4482T>A), Q360N (4483C>A 4485A>T), R361L (4486A>C 4487G>T 4488A>T), I362A (4489A>G 4490T>C), G363K (4492G>A 4493G>A), F367Y (4505T>A 4506T>C), Y368F (4508A>T 4509C>T), S370K (4513T>A 4514C>A 4515C>A), V380I (4543G>A), R381K (4546C>A 4547G>A 4548C>A), A383E (4553C>A 4554T>A), P384E (4555C>G 4556C>A), E385N (4558G>A 4560A>T), T386S (4561A>T 4563A>T), Q388A (4567C>G 4568A>C 4569G>T), C394T (4585T>A 4586G>C 4587T>G), Q396N (4591C>A 4593A>T), Q410N (4633C>A 4635A>T), A413Q (4642G>C 4643C>A 4644T>A), H418K (4657C>A 4659C>A), E421K (4666G>A), S422I (4670G>T 4671T>A), L423F (4672C>T 4674C>T), N425Q (4678A>C 4680C>A), M426K (4682T>A), Y427N (4684T>A), P428_Q429del (4687_4692delCCACAG), C431L (4696T>C 4697G>T 4698T>A), A432I (4699G>A 4700C>T 4701T>C), V435I (4708G>A 4710T>A), I439L (4720A>T 4722C>A), V440I (4723G>A 4725C>A), F441C (4727T>G 4728C>T), K443N (4734A>C), T444S (4735A>T 4737T>A), E445Y (4738G>T 4740A>T), E446Q (4741G>C), G450T (4753G>A 4754G>C 4755A>T), V452L (4759G>C 4761C>T), I454Q (4765A>C 4766T>A 4767T>A), V455F (4768G>T), L456S (4771C>T 4772T>C 4773A>T), N457Q (4774A>C 4776C>A), R458I (4778G>T), K460C (4783A>T 4784A>G 4785A>C), A461E (4787C>A 4788C>A), L462N (4789C>A 4790T>A 4791A>T), K468Q (4807A>C 4809A>G), Q472D (4819C>G 4821G>C), L473I (4822T>A 4824G>A), C474N (4825T>A 4826G>A 4827C>T), T476N (4832C>A 4833A>T), T477E (4834A>G 4835C>A 4836T>A), N479E (4840A>G 4842T>A), L483M (4852C>A 4854A>G), V484I (4855G>A), E486S (4861G>T 4862A>C), R487K (4865G>A), G488N (4867G>A 4868G>A 4869A>T), N489G (4870A>G 4871A>G 4872T>C), L490I (4873C>A 4875C>T), K491E (4876A>G), V492L (4879G>T 4881C>A), S494T (4886G>C), G497S (4894G>T 4895G>C), L498K (4897C>A 4898T>A), H499K (4900C>A 4902C>A), H499_L500insX (4902_4903insG), L500I (4903T>A 4905A>C), V501C (4906G>T 4907T>G), A502E (4910C>A 4911T>A), P504S (4915C>T), Q506K (4921C>A), S508N (4928G>A 4929T>C), D509T (4930G>A 4931A>C 4932T>A), R510K (4934G>A 4935G>A), N511Q (4936A>C 4938T>A), A512E (4940C>A 4941C>A), L513I (4942T>A 4944A>C), L519C (4960C>T 4961T>G 4962C>T), I523A (4972A>G 4973T>C 4974C>A), A525E (4979C>A 4980T>A), Y526F (4982A>T), F527I (4984T>A 4986T>A), K529D (4990A>G 4992A>C), I530L (4993A>C), N532K (5001C>A), L533K (5002C>A 5003T>A 5004T>G), S535N (5008T>A 5009C>A 5010A>T), P536L (5012C>T), V539R (5020G>A 5021T>G 5022T>G), K540L (5023A>C 5024A>T 5025A>T), L541I (5026C>A), K542R (5029A>C 5030A>G 5031G>A), E544S (5035G>T 5036A>C 5037G>C), I545N (5039T>A 5040A>C), T546R (5041A>C 5042C>G 5043T>A), W547M (5044T>A 5045G>T), S548G (5047T>G 5048C>G 5049T>A), E551D (5058G>T), K552E (5059A>G), D553H (5062G>C 5064C>T), E555K (5068G>A), T556C (5071A>T 5072C>G), R558Q (5077A>C 5078G>A), K559S (5081A>G 5082A>C), I560L (5083A>C), S562Q (5089A>C 5090G>A 5091T>A), L563E (5092C>G 5093T>A), V564C (5095G>T 5096T>G 5097A>T), K565S (5098A>T 5099A>C 5100A>C), T566K (5102C>A 5103T>A), D569K (5110G>A 5112C>G), Y571R (5116T>A 5117A>G 5118C>A), N572L (5119A>C 5120A>T), S574E (5125T>G 5126C>A), K578N (5139G>C), P579L (5140C>T 5141C>T 5142T>A), I580V (5143A>G 5145C>T), I581L (5146A>T 5148C>A), E582Q (5149G>C), C583T (5152T>A 5153G>C 5154C>A), D588Y (5167G>T), I593L (5182A>T 5184C>A), K595Q (5188A>C), A596T (5191G>A 5193A>T), K597D (5194A>G 5196A>C), P599N (5200C>A 5201C>A 5202A>T), G601_V604del (5206_5217delGGTAAAGAGGTA), A609T (5230G>A 5232T>A), K614N (5247A>T), P615E (5248C>G 5249C>A), A616T (5251G>A 5253T>C), K618T (5258A>C 5259G>T)								

	Begin	End	Coverage	Score	Concordance	Matches	Identities	I/D/M/F*	Stop Codons
**NT**	**4219**	**5259**	**13.2%**	**216**	**10.7%**	**1023 (98.2%)**	**577 (55.4%)**	**1/18**	
Codon mutations:	TTA272CTA (4219T>C), TTA273AAA (4222T>A 4223T>A), GAC274GAT (4227C>T), ACT275CCC (4228A>C 4230T>C), AAC276AGC (4232A>G), AAA277ATC (4235A>T 4236A>C), ATT278ATA (4239T>A), ATT279ATA (4242T>A), AAG280AGA (4244A>G 4245G>A), TGT281GTT (4246T>G 4247G>T), CCA283CCT (4254A>T), CAG285CTA (4259A>T 4260G>A), TAT286TAC (4263T>C), TCA287AAT (4264T>A 4265C>A 4266A>T), CCT288GAA (4267C>G 4268C>A 4269T>A), GAC290GAT (4275C>T), CGA291AAA (4276C>A 4277G>A), GAA292ACT (4279G>A 4280A>C 4281A>T), GAG293GAA (4284G>A), ACT296ATT (4292C>T), ATC298ATT (4299C>T), GAA299CAA (4300G>C), CTA301TTA (4306C>T), CTC302TTA (4309C>T 4311C>A), AAG303AAT (4314G>T), CTT304ATG (4315C>A 4317T>G), GGT305AAA (4318G>A 4319G>A 4320T>A), ATC306TTA (4321A>T 4323C>A), ATC307ATA (4326C>A), CCT309CAT (4331C>A), AGC310AGT (4335C>T), AAG311AAA (4338G>A), TCC315AGT (4348T>A 4349C>G 4350C>T), TCA316AGC (4351T>A 4352C>G 4353A>C), CCA317CCC (4356A>C), GCC318GCA (4359C>A), TTC319TTT (4362C>T), GTA321ATA (4366G>A), AGG322AGA (4371G>A), AAT323AAC (4374T>C), GCC325GGC (4379C>G), GAG326GAA (4383G>A), ATC327CAA (4384A>C 4385T>A 4386C>A), CGA329CGT (4392A>T), GGC330GGA (4395C>A), GCA332GCT (4401A>T), AGA333CGA (4402A>C), ATT336ATA (4413T>A), AAG340GAG (4423A>G), TTA341CTT (4426T>C 4428A>T), GAC343AAA (4432G>A 4434C>A), CAT344AAT (4435C>A), ACA345TGT (4438A>T 4439C>G 4440A>T), AAG346GTC (4441A>G 4442A>T 4443G>C), GGA347TTC (4444G>T 4445G>T 4446A>C), GAT348GAC (4449T>C), GGC349GGA (4452C>A), TAC350TAT (4455C>T), CTA351TTC (4456C>T 4458A>C), CTT352ATC (4459C>A 4461T>C), CCT353CCA (4464T>A), AAC354AAT (4467C>T), AAG355AAA (4470G>A), GAG356AAT (4471G>A 4473G>T), CAA357AAC (4474C>A 4476A>C), CTG358CTT (4479G>T), CTT359ATA (4480C>A 4482T>A), CAA360AAT (4483C>A 4485A>T), AGA361CTT (4486A>C 4487G>T 4488A>T), ATC362GCC (4489A>G 4490T>C), GGA363AAA (4492G>A 4493G>A), GGT364GGA (4497T>A), AAG365AAA (4500G>A), TTT367TAC (4505T>A 4506T>C), TAC368TTT (4508A>T 4509C>T), TCT369TCA (4512T>A), TCC370AAA (4513T>A 4514C>A 4515C>A), GAC372GAT (4521C>T), TGT373TGC (4524T>C), TCT375AGT (4528T>A 4529C>G), CAG379CAA (4542G>A), GTA380ATA (4543G>A), CGC381AAA (4546C>A 4547G>A 4548C>A), CTT382CTA (4551T>A), GCT383GAA (4553C>A 4554T>A), CCA384GAA (4555C>G 4556C>A), GAA385AAT (4558G>A 4560A>T), ACA386TCT (4561A>T 4563A>T), ATT387ATA (4566T>A), CAG388GCT (4567C>G 4568A>C 4569G>T), CTA389TTA (4570C>T), ACC390ACA (4575C>A), GCT391GCG (4578T>G), AGC393AGT (4584C>T), TGT394ACG (4585T>A 4586G>C 4587T>G), CCC395CCA (4590C>A), CAA396AAT (4591C>A 4593A>T), CAC398CAT (4599C>T), GTC403GTG (4614C>G), CAA410AAT (4633C>A 4635A>T), GCT413CAA (4642G>C 4643C>A 4644T>A), ATT414ATC (4647T>C), TTC415TTT (4650C>T), CAA416CAG (4653A>G), CAC418AAA (4657C>A 4659C>A), GAC420GAT (4665C>T), GAA421AAA (4666G>A), AGT422ATA (4670G>T 4671T>A), CTC423TTT (4672C>T 4674C>T), AGC424TCC (4675A>T 4676G>C), AAC425CAA (4678A>C 4680C>A), ATG426AAG (4682T>A), TAT427AAT (4684T>A), CCA428_CAG429del (4687_4692delCCACAG), TGT431CTA (4696T>C 4697G>T 4698T>A), GCT432ATC (4699G>A 4700C>T 4701T>C), GTA433GTC (4704A>C), TAT434TAC (4707T>C), GTT435ATA (4708G>A 4710T>A), GAC437GAT (4716C>T), ATC438ATA (4719C>A), ATC439TTA (4720A>T 4722C>A), GTC440ATA (4723G>A 4725C>A), TTC441TGT (4727T>G 4728C>T), AGC442TCC (4729A>T 4730G>C), AAA443AAC (4734A>C), ACT444TCA (4735A>T 4737T>A), GAA445TAT (4738G>T 4740A>T), GAA446CAA (4741G>C), GGA450ACT (4753G>A 4754G>C 4755A>T), GTC452CTT (4759G>C 4761C>T), ATT454CAA (4765A>C 4766T>A 4767T>A), GTT455TTT (4768G>T), CTA456TCT (4771C>T 4772T>C 4773A>T), AAC457CAA (4774A>C 4776C>A), AGA458ATA (4778G>T), TGC459TGT (4782C>T), AAA460TGC (4783A>T 4784A>G 4785A>C), GCC461GAA (4787C>A 4788C>A), CTA462AAT (4789C>A 4790T>A 4791A>T), ATA464ATT (4797A>T), CTT466TTA (4801C>T 4803T>A), AAA468CAG (4807A>C 4809A>G), AAG469AAA (4812G>A), AAA470AAG (4815A>G), GCC471GCA (4818C>A), CAG472GAC (4819C>G 4821G>C), TTG473ATA (4822T>A 4824G>A), TGC474AAT (4825T>A 4826G>A 4827C>T), ACA476AAT (4832C>A 4833A>T), ACT477GAA (4834A>G 4835C>A 4836T>A), ATC478ATT (4839C>T), AAT479GAA (4840A>G 4842T>A), TTC480TTT (4845C>T), CTT481CTC (4848T>C), GGT482GGA (4851T>A), CTA483ATG (4852C>A 4854A>G), GTA484ATA (4855G>A), ATA485ATT (4860A>T), GAA486TCA (4861G>T 4862A>C), AGA487AAA (4865G>A), GGA488AAT (4867G>A 4868G>A 4869A>T), AAT489GGC (4870A>G 4871A>G 4872T>C), CTC490ATT (4873C>A 4875C>T), AAA491GAA (4876A>G), GTC492TTA (4879G>T 4881C>A), CAG493CAA (4884G>A), AGT494ACT (4886G>C), CAT495CAC (4890T>C), ATC496ATA (4893C>A), GGA497TCA (4894G>T 4895G>C), CTA498AAA (4897C>A 4898T>A), CAC499AAA (4900C>A 4902C>A), CAC499_TTA500insG-- (4902_4903insG), TTA500ATC (4903T>A 4905A>C), GTT501TGT (4906G>T 4907T>G), GCT502GAA (4910C>A 4911T>A), CCA504TCA (4915C>T), CAA506AAA (4921C>A), CTT507CTA (4926T>A), AGT508AAC (4928G>A 4929T>C), GAT509ACA (4930G>A 4931A>C 4932T>A), AGG510AAA (4934G>A 4935G>A), AAT511CAA (4936A>C 4938T>A), GCC512GAA (4940C>A 4941C>A), TTA513ATC (4942T>A 4944A>C), AGA515AGG (4950A>G), TTA517CTT (4954T>C 4956A>T), GGC518GGA (4959C>A), CTC519TGT (4960C>T 4961T>G 4962C>T), CTA520TTA (4963C>T), TAT522TAC (4971T>C), ATC523GCA (4972A>G 4973T>C 4974C>A), TCC524AGT (4975T>A 4976C>G 4977C>T), GCT525GAA (4979C>A 4980T>A), TAT526TTT (4982A>T), TTT527ATA (4984T>A 4986T>A), AAA529GAC (4990A>G 4992A>C), ATA530CTA (4993A>C), AAC532AAA (5001C>A), CTT533AAG (5002C>A 5003T>A 5004T>G), AGG534AGA (5007G>A), TCA535AAT (5008T>A 5009C>A 5010A>T), CCT536CTT (5012C>T), CAG538CAA (5019G>A), GTT539AGG (5020G>A 5021T>G 5022T>G), AAA540CTT (5023A>C 5024A>T 5025A>T), CTT541ATT (5026C>A), AAG542CGA (5029A>C 5030A>G 5031G>A), GAG544TCC (5035G>T 5036A>C 5037G>C), ATA545AAC (5039T>A 5040A>C), ACT546CGA (5041A>C 5042C>G 5043T>A), TGG547ATG (5044T>A 5045G>T), TCT548GGA (5047T>G 5048C>G 5049T>A), ACT550ACA (5055T>A), GAG551GAT (5058G>T), AAA552GAA (5059A>G), GAC553CAT (5062G>C 5064C>T), ACG554ACT (5067G>T), GAA555AAA (5068G>A), ACT556TGT (5071A>T 5072C>G), GTC557GTT (5076C>T), AGA558CAA (5077A>C 5078G>A), AAA559AGC (5081A>G 5082A>C), ATA560CTA (5083A>C), AAG561AAA (5088G>A), AGT562CAA (5089A>C 5090G>A 5091T>A), CTA563GAA (5092C>G 5093T>A), GTA564TGT (5095G>T 5096T>G 5097A>T), AAA565TCC (5098A>T 5099A>C 5100A>C), ACT566AAA (5102C>A 5103T>A), GAC569AAG (5110G>A 5112C>G), CTT570CTA (5115T>A), TAC571AGA (5116T>A 5117A>G 5118C>A), AAT572CTT (5119A>C 5120A>T), CCT573CCG (5124T>G), TCA574GAA (5125T>G 5126C>A), GAG576GAA (5133G>A), GAT577GAC (5136T>C), AAG578AAC (5139G>C), CCT579TTA (5140C>T 5141C>T 5142T>A), ATC580GTT (5143A>G 5145C>T), ATC581TTA (5146A>T 5148C>A), GAA582CAA (5149G>C), TGC583ACA (5152T>A 5153G>C 5154C>A), GCA585GCT (5160A>T), GAC588TAC (5167G>T), ATC593TTA (5182A>T 5184C>A), CTC594TTA (5185C>T 5187C>A), AAA595CAA (5188A>C), GCA596ACT (5191G>A 5193A>T), AAA597GAC (5194A>G 5196A>C), CCA599AAT (5200C>A 5201C>A 5202A>T), GGT601_GTA604del (5206_5217delGGTAAAGAGGTA), ATA605ATT (5220A>T), TGC606TGT (5223C>T), GCT609ACA (5230G>A 5232T>A), TCA610AGT (5233T>A 5234C>G 5235A>T), GGA611GGT (5238A>T), ACC612ACA (5241C>A), AAA614AAT (5247A>T), CCA615GAA (5248C>G 5249C>A), GCT616ACC (5251G>A 5253T>C), GAG617GAA (5256G>A), AAG618ACT (5258A>C 5259G>T)								

Proteins

	Begin	End	Coverage	Score	Concordance	Matches	Identities	I/D/M/F*	Stop Codons
hypothetical protein (NP_043933.1)	272	618	48.9%	1173	49.7%	341 (98.0%)	179 (51.4%)	1/6/1/1	0
Protein mutations:	L273K (4222T>A 4223T>A), T275P (4228A>C 4230T>C), N276S (4232A>G), K277I (4235A>T 4236A>C), K280R (4244A>G 4245G>A), C281V (4246T>G 4247G>T), Q285L (4259A>T 4260G>A), S287N (4264T>A 4265C>A 4266A>T), P288E (4267C>G 4268C>A 4269T>A), R291K (4276C>A 4277G>A), E292T (4279G>A 4280A>C 4281A>T), T296I (4292C>T), E299Q (4300G>C), K303N (4314G>T), L304M (4315C>A 4317T>G), G305K (4318G>A 4319G>A 4320T>A), I306L (4321A>T 4323C>A), P309H (4331C>A), V321I (4366G>A), A325G (4379C>G), I327Q (4384A>C 4385T>A 4386C>A), K340E (4423A>G), D343K (4432G>A 4434C>A), H344N (4435C>A), T345C (4438A>T 4439C>G 4440A>T), K346V (4441A>G 4442A>T 4443G>C), G347F (4444G>T 4445G>T 4446A>C), L351F (4456C>T 4458A>C), L352I (4459C>A 4461T>C), E356N (4471G>A 4473G>T), Q357N (4474C>A 4476A>C), L359I (4480C>A 4482T>A), Q360N (4483C>A 4485A>T), R361L (4486A>C 4487G>T 4488A>T), I362A (4489A>G 4490T>C), G363K (4492G>A 4493G>A), F367Y (4505T>A 4506T>C), Y368F (4508A>T 4509C>T), S370K (4513T>A 4514C>A 4515C>A), V380I (4543G>A), R381K (4546C>A 4547G>A 4548C>A), A383E (4553C>A 4554T>A), P384E (4555C>G 4556C>A), E385N (4558G>A 4560A>T), T386S (4561A>T 4563A>T), Q388A (4567C>G 4568A>C 4569G>T), C394T (4585T>A 4586G>C 4587T>G), Q396N (4591C>A 4593A>T), Q410N (4633C>A 4635A>T), A413Q (4642G>C 4643C>A 4644T>A), H418K (4657C>A 4659C>A), E421K (4666G>A), S422I (4670G>T 4671T>A), L423F (4672C>T 4674C>T), N425Q (4678A>C 4680C>A), M426K (4682T>A), Y427N (4684T>A), P428_Q429del (4687_4692delCCACAG), C431L (4696T>C 4697G>T 4698T>A), A432I (4699G>A 4700C>T 4701T>C), V435I (4708G>A 4710T>A), I439L (4720A>T 4722C>A), V440I (4723G>A 4725C>A), F441C (4727T>G 4728C>T), K443N (4734A>C), T444S (4735A>T 4737T>A), E445Y (4738G>T 4740A>T), E446Q (4741G>C), G450T (4753G>A 4754G>C 4755A>T), V452L (4759G>C 4761C>T), I454Q (4765A>C 4766T>A 4767T>A), V455F (4768G>T), L456S (4771C>T 4772T>C 4773A>T), N457Q (4774A>C 4776C>A), R458I (4778G>T), K460C (4783A>T 4784A>G 4785A>C), A461E (4787C>A 4788C>A), L462N (4789C>A 4790T>A 4791A>T), K468Q (4807A>C 4809A>G), Q472D (4819C>G 4821G>C), L473I (4822T>A 4824G>A), C474N (4825T>A 4826G>A 4827C>T), T476N (4832C>A 4833A>T), T477E (4834A>G 4835C>A 4836T>A), N479E (4840A>G 4842T>A), L483M (4852C>A 4854A>G), V484I (4855G>A), E486S (4861G>T 4862A>C), R487K (4865G>A), G488N (4867G>A 4868G>A 4869A>T), N489G (4870A>G 4871A>G 4872T>C), L490I (4873C>A 4875C>T), K491E (4876A>G), V492L (4879G>T 4881C>A), S494T (4886G>C), G497S (4894G>T 4895G>C), L498K (4897C>A 4898T>A), H499K (4900C>A 4902C>A), H499_L500insX (4902_4903insG), L500I (4903T>A 4905A>C), V501C (4906G>T 4907T>G), A502E (4910C>A 4911T>A), P504S (4915C>T), Q506K (4921C>A), S508N (4928G>A 4929T>C), D509T (4930G>A 4931A>C 4932T>A), R510K (4934G>A 4935G>A), N511Q (4936A>C 4938T>A), A512E (4940C>A 4941C>A), L513I (4942T>A 4944A>C), L519C (4960C>T 4961T>G 4962C>T), I523A (4972A>G 4973T>C 4974C>A), A525E (4979C>A 4980T>A), Y526F (4982A>T), F527I (4984T>A 4986T>A), K529D (4990A>G 4992A>C), I530L (4993A>C), N532K (5001C>A), L533K (5002C>A 5003T>A 5004T>G), S535N (5008T>A 5009C>A 5010A>T), P536L (5012C>T), V539R (5020G>A 5021T>G 5022T>G), K540L (5023A>C 5024A>T 5025A>T), L541I (5026C>A), K542R (5029A>C 5030A>G 5031G>A), E544S (5035G>T 5036A>C 5037G>C), I545N (5039T>A 5040A>C), T546R (5041A>C 5042C>G 5043T>A), W547M (5044T>A 5045G>T), S548G (5047T>G 5048C>G 5049T>A), E551D (5058G>T), K552E (5059A>G), D553H (5062G>C 5064C>T), E555K (5068G>A), T556C (5071A>T 5072C>G), R558Q (5077A>C 5078G>A), K559S (5081A>G 5082A>C), I560L (5083A>C), S562Q (5089A>C 5090G>A 5091T>A), L563E (5092C>G 5093T>A), V564C (5095G>T 5096T>G 5097A>T), K565S (5098A>T 5099A>C 5100A>C), T566K (5102C>A 5103T>A), D569K (5110G>A 5112C>G), Y571R (5116T>A 5117A>G 5118C>A), N572L (5119A>C 5120A>T), S574E (5125T>G 5126C>A), K578N (5139G>C), P579L (5140C>T 5141C>T 5142T>A), I580V (5143A>G 5145C>T), I581L (5146A>T 5148C>A), E582Q (5149G>C), C583T (5152T>A 5153G>C 5154C>A), D588Y (5167G>T), I593L (5182A>T 5184C>A), K595Q (5188A>C), A596T (5191G>A 5193A>T), K597D (5194A>G 5196A>C), P599N (5200C>A 5201C>A 5202A>T), G601_V604del (5206_5217delGGTAAAGAGGTA), A609T (5230G>A 5232T>A), K614N (5247A>T), P615E (5248C>G 5249C>A), A616T (5251G>A 5253T>C), K618T (5258A>C 5259G>T)								

	Begin	End	Coverage	Score	Concordance	Matches	Identities	I/D/M/F*	Stop Codons
**NT**	**4219**	**5259**	**13.2%**	**216**	**10.7%**	**1023 (98.2%)**	**577 (55.4%)**	**1/18**	
Codon mutations:	TTA272CTA (4219T>C), TTA273AAA (4222T>A 4223T>A), GAC274GAT (4227C>T), ACT275CCC (4228A>C 4230T>C), AAC276AGC (4232A>G), AAA277ATC (4235A>T 4236A>C), ATT278ATA (4239T>A), ATT279ATA (4242T>A), AAG280AGA (4244A>G 4245G>A), TGT281GTT (4246T>G 4247G>T), CCA283CCT (4254A>T), CAG285CTA (4259A>T 4260G>A), TAT286TAC (4263T>C), TCA287AAT (4264T>A 4265C>A 4266A>T), CCT288GAA (4267C>G 4268C>A 4269T>A), GAC290GAT (4275C>T), CGA291AAA (4276C>A 4277G>A), GAA292ACT (4279G>A 4280A>C 4281A>T), GAG293GAA (4284G>A), ACT296ATT (4292C>T), ATC298ATT (4299C>T), GAA299CAA (4300G>C), CTA301TTA (4306C>T), CTC302TTA (4309C>T 4311C>A), AAG303AAT (4314G>T), CTT304ATG (4315C>A 4317T>G), GGT305AAA (4318G>A 4319G>A 4320T>A), ATC306TTA (4321A>T 4323C>A), ATC307ATA (4326C>A), CCT309CAT (4331C>A), AGC310AGT (4335C>T), AAG311AAA (4338G>A), TCC315AGT (4348T>A 4349C>G 4350C>T), TCA316AGC (4351T>A 4352C>G 4353A>C), CCA317CCC (4356A>C), GCC318GCA (4359C>A), TTC319TTT (4362C>T), GTA321ATA (4366G>A), AGG322AGA (4371G>A), AAT323AAC (4374T>C), GCC325GGC (4379C>G), GAG326GAA (4383G>A), ATC327CAA (4384A>C 4385T>A 4386C>A), CGA329CGT (4392A>T), GGC330GGA (4395C>A), GCA332GCT (4401A>T), AGA333CGA (4402A>C), ATT336ATA (4413T>A), AAG340GAG (4423A>G), TTA341CTT (4426T>C 4428A>T), GAC343AAA (4432G>A 4434C>A), CAT344AAT (4435C>A), ACA345TGT (4438A>T 4439C>G 4440A>T), AAG346GTC (4441A>G 4442A>T 4443G>C), GGA347TTC (4444G>T 4445G>T 4446A>C), GAT348GAC (4449T>C), GGC349GGA (4452C>A), TAC350TAT (4455C>T), CTA351TTC (4456C>T 4458A>C), CTT352ATC (4459C>A 4461T>C), CCT353CCA (4464T>A), AAC354AAT (4467C>T), AAG355AAA (4470G>A), GAG356AAT (4471G>A 4473G>T), CAA357AAC (4474C>A 4476A>C), CTG358CTT (4479G>T), CTT359ATA (4480C>A 4482T>A), CAA360AAT (4483C>A 4485A>T), AGA361CTT (4486A>C 4487G>T 4488A>T), ATC362GCC (4489A>G 4490T>C), GGA363AAA (4492G>A 4493G>A), GGT364GGA (4497T>A), AAG365AAA (4500G>A), TTT367TAC (4505T>A 4506T>C), TAC368TTT (4508A>T 4509C>T), TCT369TCA (4512T>A), TCC370AAA (4513T>A 4514C>A 4515C>A), GAC372GAT (4521C>T), TGT373TGC (4524T>C), TCT375AGT (4528T>A 4529C>G), CAG379CAA (4542G>A), GTA380ATA (4543G>A), CGC381AAA (4546C>A 4547G>A 4548C>A), CTT382CTA (4551T>A), GCT383GAA (4553C>A 4554T>A), CCA384GAA (4555C>G 4556C>A), GAA385AAT (4558G>A 4560A>T), ACA386TCT (4561A>T 4563A>T), ATT387ATA (4566T>A), CAG388GCT (4567C>G 4568A>C 4569G>T), CTA389TTA (4570C>T), ACC390ACA (4575C>A), GCT391GCG (4578T>G), AGC393AGT (4584C>T), TGT394ACG (4585T>A 4586G>C 4587T>G), CCC395CCA (4590C>A), CAA396AAT (4591C>A 4593A>T), CAC398CAT (4599C>T), GTC403GTG (4614C>G), CAA410AAT (4633C>A 4635A>T), GCT413CAA (4642G>C 4643C>A 4644T>A), ATT414ATC (4647T>C), TTC415TTT (4650C>T), CAA416CAG (4653A>G), CAC418AAA (4657C>A 4659C>A), GAC420GAT (4665C>T), GAA421AAA (4666G>A), AGT422ATA (4670G>T 4671T>A), CTC423TTT (4672C>T 4674C>T), AGC424TCC (4675A>T 4676G>C), AAC425CAA (4678A>C 4680C>A), ATG426AAG (4682T>A), TAT427AAT (4684T>A), CCA428_CAG429del (4687_4692delCCACAG), TGT431CTA (4696T>C 4697G>T 4698T>A), GCT432ATC (4699G>A 4700C>T 4701T>C), GTA433GTC (4704A>C), TAT434TAC (4707T>C), GTT435ATA (4708G>A 4710T>A), GAC437GAT (4716C>T), ATC438ATA (4719C>A), ATC439TTA (4720A>T 4722C>A), GTC440ATA (4723G>A 4725C>A), TTC441TGT (4727T>G 4728C>T), AGC442TCC (4729A>T 4730G>C), AAA443AAC (4734A>C), ACT444TCA (4735A>T 4737T>A), GAA445TAT (4738G>T 4740A>T), GAA446CAA (4741G>C), GGA450ACT (4753G>A 4754G>C 4755A>T), GTC452CTT (4759G>C 4761C>T), ATT454CAA (4765A>C 4766T>A 4767T>A), GTT455TTT (4768G>T), CTA456TCT (4771C>T 4772T>C 4773A>T), AAC457CAA (4774A>C 4776C>A), AGA458ATA (4778G>T), TGC459TGT (4782C>T), AAA460TGC (4783A>T 4784A>G 4785A>C), GCC461GAA (4787C>A 4788C>A), CTA462AAT (4789C>A 4790T>A 4791A>T), ATA464ATT (4797A>T), CTT466TTA (4801C>T 4803T>A), AAA468CAG (4807A>C 4809A>G), AAG469AAA (4812G>A), AAA470AAG (4815A>G), GCC471GCA (4818C>A), CAG472GAC (4819C>G 4821G>C), TTG473ATA (4822T>A 4824G>A), TGC474AAT (4825T>A 4826G>A 4827C>T), ACA476AAT (4832C>A 4833A>T), ACT477GAA (4834A>G 4835C>A 4836T>A), ATC478ATT (4839C>T), AAT479GAA (4840A>G 4842T>A), TTC480TTT (4845C>T), CTT481CTC (4848T>C), GGT482GGA (4851T>A), CTA483ATG (4852C>A 4854A>G), GTA484ATA (4855G>A), ATA485ATT (4860A>T), GAA486TCA (4861G>T 4862A>C), AGA487AAA (4865G>A), GGA488AAT (4867G>A 4868G>A 4869A>T), AAT489GGC (4870A>G 4871A>G 4872T>C), CTC490ATT (4873C>A 4875C>T), AAA491GAA (4876A>G), GTC492TTA (4879G>T 4881C>A), CAG493CAA (4884G>A), AGT494ACT (4886G>C), CAT495CAC (4890T>C), ATC496ATA (4893C>A), GGA497TCA (4894G>T 4895G>C), CTA498AAA (4897C>A 4898T>A), CAC499AAA (4900C>A 4902C>A), CAC499_TTA500insG-- (4902_4903insG), TTA500ATC (4903T>A 4905A>C), GTT501TGT (4906G>T 4907T>G), GCT502GAA (4910C>A 4911T>A), CCA504TCA (4915C>T), CAA506AAA (4921C>A), CTT507CTA (4926T>A), AGT508AAC (4928G>A 4929T>C), GAT509ACA (4930G>A 4931A>C 4932T>A), AGG510AAA (4934G>A 4935G>A), AAT511CAA (4936A>C 4938T>A), GCC512GAA (4940C>A 4941C>A), TTA513ATC (4942T>A 4944A>C), AGA515AGG (4950A>G), TTA517CTT (4954T>C 4956A>T), GGC518GGA (4959C>A), CTC519TGT (4960C>T 4961T>G 4962C>T), CTA520TTA (4963C>T), TAT522TAC (4971T>C), ATC523GCA (4972A>G 4973T>C 4974C>A), TCC524AGT (4975T>A 4976C>G 4977C>T), GCT525GAA (4979C>A 4980T>A), TAT526TTT (4982A>T), TTT527ATA (4984T>A 4986T>A), AAA529GAC (4990A>G 4992A>C), ATA530CTA (4993A>C), AAC532AAA (5001C>A), CTT533AAG (5002C>A 5003T>A 5004T>G), AGG534AGA (5007G>A), TCA535AAT (5008T>A 5009C>A 5010A>T), CCT536CTT (5012C>T), CAG538CAA (5019G>A), GTT539AGG (5020G>A 5021T>G 5022T>G), AAA540CTT (5023A>C 5024A>T 5025A>T), CTT541ATT (5026C>A), AAG542CGA (5029A>C 5030A>G 5031G>A), GAG544TCC (5035G>T 5036A>C 5037G>C), ATA545AAC (5039T>A 5040A>C), ACT546CGA (5041A>C 5042C>G 5043T>A), TGG547ATG (5044T>A 5045G>T), TCT548GGA (5047T>G 5048C>G 5049T>A), ACT550ACA (5055T>A), GAG551GAT (5058G>T), AAA552GAA (5059A>G), GAC553CAT (5062G>C 5064C>T), ACG554ACT (5067G>T), GAA555AAA (5068G>A), ACT556TGT (5071A>T 5072C>G), GTC557GTT (5076C>T), AGA558CAA (5077A>C 5078G>A), AAA559AGC (5081A>G 5082A>C), ATA560CTA (5083A>C), AAG561AAA (5088G>A), AGT562CAA (5089A>C 5090G>A 5091T>A), CTA563GAA (5092C>G 5093T>A), GTA564TGT (5095G>T 5096T>G 5097A>T), AAA565TCC (5098A>T 5099A>C 5100A>C), ACT566AAA (5102C>A 5103T>A), GAC569AAG (5110G>A 5112C>G), CTT570CTA (5115T>A), TAC571AGA (5116T>A 5117A>G 5118C>A), AAT572CTT (5119A>C 5120A>T), CCT573CCG (5124T>G), TCA574GAA (5125T>G 5126C>A), GAG576GAA (5133G>A), GAT577GAC (5136T>C), AAG578AAC (5139G>C), CCT579TTA (5140C>T 5141C>T 5142T>A), ATC580GTT (5143A>G 5145C>T), ATC581TTA (5146A>T 5148C>A), GAA582CAA (5149G>C), TGC583ACA (5152T>A 5153G>C 5154C>A), GCA585GCT (5160A>T), GAC588TAC (5167G>T), ATC593TTA (5182A>T 5184C>A), CTC594TTA (5185C>T 5187C>A), AAA595CAA (5188A>C), GCA596ACT (5191G>A 5193A>T), AAA597GAC (5194A>G 5196A>C), CCA599AAT (5200C>A 5201C>A 5202A>T), GGT601_GTA604del (5206_5217delGGTAAAGAGGTA), ATA605ATT (5220A>T), TGC606TGT (5223C>T), GCT609ACA (5230G>A 5232T>A), TCA610AGT (5233T>A 5234C>G 5235A>T), GGA611GGT (5238A>T), ACC612ACA (5241C>A), AAA614AAT (5247A>T), CCA615GAA (5248C>G 5249C>A), GCT616ACC (5251G>A 5253T>C), GAG617GAA (5256G>A), AAG618ACT (5258A>C 5259G>T)								

*: Inserts / Deletes / Misaligned / Frameshifts

Analysis details

This analysis was performed with panviral2.64

NGS Details (UN60): Caulimovirus tesselloscrophulariae

Assembly

**Coverage Length**	373 (1 contig(s))
**Depth Of Coverage**	14.5
**Number Of Reads**	48
**Reads Per Million**	1.02 rpm (after QC)
**Ambiguities**	0
**Assembly Method**	de novo + reference guided assembly
**Consensus Caller**	Bcf Tools

Coverage Map

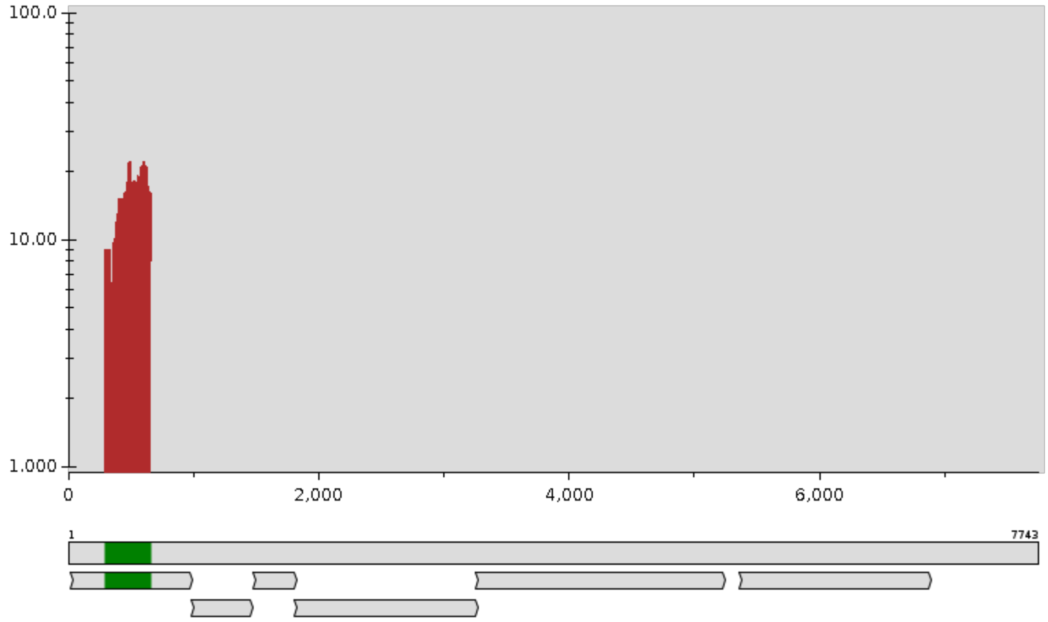

Assignment

**Type**	Caulimovirus tesselloscrophulariae (Taxonomy ID: 3047834)
**Reference Genome**	NC_003554.1
**NT Identity (%)**	53.6193
**AA Identity (%)**	37.9032
**Number Of Stop Codons**	0
**Number Of CDS**	6

Alignment

**Alignment Score**	54.0 (NT) + 317.0 (AA) = 371.0
**Concordance (%)**	24.4079

Alignment Method	Global, seeded, nucleotide + amino acids (AGA)

Genome Region

Sequence starts at position 288 and ends at position 660 relative to NC_003554.1 reference sequence.

Alignment Detailed Statistics

	Begin	End	Coverage	Score	Concordance	Matches	Identities	I/D/M/F*	Stop Codons
**NT**	**288**	**660**	**4.8%**	**54**	**7.2%**	**373 (100%)**	**200 (53.6%)**	**0/0**	
Mutations:	288T>C, 289C>T, 290T>A, 296C>A, 299G>C, 300T>A, 302T>A, 304T>A, 305C>T, 307T>A, 308C>A, 309C>A, 311C>T, 313T>A, 314A>T, 322G>A, 324A>C, 325G>A, 326G>A, 327A>T, 328A>G, 331A>T, 332C>A, 334A>T, 339A>T, 340G>C, 343G>A, 344A>C, 345T>A, 346G>A, 348A>G, 353G>C, 354A>C, 355T>A, 356C>G, 358A>C, 359T>A, 360C>G, 361A>C, 362G>A, 363T>A, 368A>C, 369G>A, 373A>G, 376C>G, 377T>A, 378C>A, 379A>T, 380A>T, 381T>A, 382G>T, 383A>G, 385C>T, 388C>T, 389C>T, 391T>G, 396C>G, 398G>A, 401A>C, 403G>A, 406T>C, 407C>A, 409A>G, 410C>G, 412T>G, 414C>A, 416G>T, 417C>T, 419C>A, 420A>C, 421A>T, 425C>A, 426G>A, 427G>A, 428C>G, 433A>T, 436T>A, 437G>A, 440A>T, 441C>G, 442G>T, 443T>C, 445A>T, 446G>A, 448T>A, 450A>T, 454G>A, 455G>A, 456C>A, 457A>C, 461A>T, 462T>C, 463C>T, 469T>A, 473A>T, 475C>T, 476G>A, 478C>T, 482C>A, 483G>C, 484A>T, 486A>G, 490T>A, 491A>G, 493T>A, 494C>A, 495T>A, 496G>T, 499C>T, 503G>A, 504C>T, 507C>T, 508A>T, 509A>G, 510G>A, 518C>T, 520C>A, 526T>C, 527G>A, 528G>C, 535T>A, 536A>C, 538G>T, 544C>A, 545G>T, 546T>A, 550C>T, 553T>C, 555A>G, 558T>A, 560G>T, 561C>G, 562A>T, 563T>A, 565G>A, 572C>A, 575A>G, 576G>A, 577C>T, 578A>G, 579A>C, 580G>T, 583T>C, 586A>T, 587G>A, 590A>G, 593A>G, 598A>G, 601C>T, 604T>G, 605A>C, 606T>A, 608C>T, 609A>T, 613A>G, 614T>G, 616T>C, 617G>A, 628C>T, 632A>T, 634G>C, 637A>G, 638A>C, 643C>T, 644G>A, 647A>C, 649A>T, 650G>A, 653T>A, 655T>G, 657C>G, 658G>T, 659G>A								
**CDS**									
FMVgp1	93	216	38.3%	317	40.4%	124 (100%)	47 (37.9%)	0/0/0/0	0
Protein mutations:	S93T (290T>A), Q95K (296C>A), V96H (299G>C 300T>A), Y97K (302T>A 304T>A), P99N (308C>A 309C>A), I101L (314A>T), Q104P (324A>C 325G>A), E105M (326G>A 327A>T 328A>G), Q107N (332C>A 334A>T), K109I (339A>T 340G>C), M111Q (344A>C 345T>A 346G>A), K112R (348A>G), D114P (353G>C 354A>C 355T>A), P115A (356C>G 358A>C), V117K (362G>A 363T>A), S119H (368A>C 369G>A), I121M (376C>G), S122N (377T>A 378C>A 379A>T), M123Y (380A>T 381T>A 382G>T), I124V (383A>G 385C>T), A128G (396C>G), V129I (398G>A), K130Q (401A>C 403G>A), L132M (407C>A 409A>G), L133V (410C>G 412T>G), T134K (414C>A), A135F (416G>T 417C>T), Q136T (419C>A 420A>C 421A>T), R138K (425C>A 426G>A 427G>A), Q139E (428C>G), D142N (437G>A), T143C (440A>T 441C>G 442G>T), S144P (443T>C 445A>T), V145I (446G>A 448T>A), K146I (450A>T), M147I (454G>A), A148N (455G>A 456C>A 457A>C), I150S (461A>T 462T>C 463C>T), D152E (469T>A), I154F (473A>T 475C>T), V155I (476G>A 478C>T), R157T (482C>A 483G>C 484A>T), K158R (486A>G), D159E (490T>A), S160G (491A>G 493T>A), L161N (494C>A 495T>A 496G>T), A164I (503G>A 504C>T), A165V (507C>T 508A>T), R166E (509A>G 510G>A), G172T (527G>A 528G>C), F174L (535T>A), M175L (536A>C 538G>T), V178Y (545G>T 546T>A), K181R (555A>G), F182Y (558T>A), A183C (560G>T 561C>G 562A>T), L184I (563T>A 565G>A), Q187K (572C>A), S188D (575A>G 576G>A 577C>T), K189A (578A>G 579A>C 580G>T), L191F (586A>T), D192N (587G>A), K193E (590A>G), T194A (593A>G), F197L (604T>G), I198H (605A>C 606T>A), H199F (608C>T 609A>T), F201V (614T>G 616T>C), E202K (617G>A), M207F (632A>T 634G>C), T209P (638A>C), D211N (644G>A), K212H (647A>C 649A>T), V213I (650G>A), F214M (653T>A 655T>G), T215S (657C>G 658G>T)								
Codon mutations:	GTC92.CT (288T>C 289C>T), TCT93ACT (290T>A), CAA95AAA (296C>A), GTT96CAT (299G>C 300T>A), TAT97AAA (302T>A 304T>A), CTT98TTA (305C>T 307T>A), CCT99AAT (308C>A 309C>A), CTT100TTA (311C>T 313T>A), ATA101TTA (314A>T), AAG103AAA (322G>A), CAG104CCA (324A>C 325G>A), GAA105ATG (326G>A 327A>T 328A>G), ATA106ATT (331A>T), CAA107AAT (332C>A 334A>T), AAG109ATC (339A>T 340G>C), CTG110CTA (343G>A), ATG111CAA (344A>C 345T>A 346G>A), AAA112AGA (348A>G), GAT114CCA (353G>C 354A>C 355T>A), CCA115GCC (356C>G 358A>C), TCA116AGC (359T>A 360C>G 361A>C), GTA117AAA (362G>A 363T>A), AGT119CAT (368A>C 369G>A), AAA120AAG (373A>G), ATC121ATG (376C>G), TCA122AAT (377T>A 378C>A 379A>T), ATG123TAT (380A>T 381T>A 382G>T), ATC124GTT (383A>G 385C>T), CAC125CAT (388C>T), CTT126TTG (389C>T 391T>G), GCA128GGA (396C>G), GTA129ATA (398G>A), AAG130CAA (401A>C 403G>A), ATT131ATC (406T>C), CTA132ATG (407C>A 409A>G), CTT133GTG (410C>G 412T>G), ACA134AAA (414C>A), GCC135TTC (416G>T 417C>T), CAA136ACT (419C>A 420A>C 421A>T), CGG138AAA (425C>A 426G>A 427G>A), CAA139GAA (428C>G), GGA140GGT (433A>T), ATT141ATA (436T>A), GAT142AAT (437G>A), ACG143TGT (440A>T 441C>G 442G>T), TCA144CCT (443T>C 445A>T), GTT145ATA (446G>A 448T>A), AAA146ATA (450A>T), ATG147ATA (454G>A), GCA148AAC (455G>A 456C>A 457A>C), ATC150TCT (461A>T 462T>C 463C>T), GAT152GAA (469T>A), ATC154TTT (473A>T 475C>T), GTC155ATT (476G>A 478C>T), CGA157ACT (482C>A 483G>C 484A>T), AAA158AGA (486A>G), GAT159GAA (490T>A), AGT160GGA (491A>G 493T>A), CTG161AAT (494C>A 495T>A 496G>T), CTC162CTT (499C>T), GCA164ATA (503G>A 504C>T), GCA165GTT (507C>T 508A>T), AGA166GAA (509A>G 510G>A), CTC169TTA (518C>T 520C>A), TAT171TAC (526T>C), GGT172ACT (527G>A 528G>C), TTT174TTA (535T>A), ATG175CTT (536A>C 538G>T), ACC177ACA (544C>A), GTT178TAT (545G>T 546T>A), TAC179TAT (550C>T), CCT180CCC (553T>C), AAA181AGA (555A>G), TTT182TAT (558T>A), GCA183TGT (560G>T 561C>G 562A>T), TTG184ATA (563T>A 565G>A), CAA187AAA (572C>A), AGC188GAT (575A>G 576G>A 577C>T), AAG189GCT (578A>G 579A>C 580G>T), AAT190AAC (583T>C), TTA191TTT (586A>T), GAT192AAT (587G>A), AAA193GAA (590A>G), ACA194GCA (593A>G), TTA195TTG (598A>G), AGC196AGT (601C>T), TTT197TTG (604T>G), ATT198CAT (605A>C 606T>A), CAT199TTT (608C>T 609A>T), CAA200CAG (613A>G), TTT201GTC (614T>G 616T>C), GAA202AAA (617G>A), GAC205GAT (628C>T), ATG207TTC (632A>T 634G>C), AAA208AAG (637A>G), ACA209CCA (638A>C), GGC210GGT (643C>T), GAT211AAT (644G>A), AAA212CAT (647A>C 649A>T), GTT213ATT (650G>A), TTT214ATG (653T>A 655T>G), ACG215AGT (657C>G 658G>T), GTC216AT. (659G>A)								
**Proteins**									
hypothetical protein (NP_619544.1)	93	216	38.3%	317	40.4%	124 (100%)	47 (37.9%)	0/0/0/0	0
Protein mutations:	S93T (290T>A), Q95K (296C>A), V96H (299G>C 300T>A), Y97K (302T>A 304T>A), P99N (308C>A 309C>A), I101L (314A>T), Q104P (324A>C 325G>A), E105M (326G>A 327A>T 328A>G), Q107N (332C>A 334A>T), K109I (339A>T 340G>C), M111Q (344A>C 345T>A 346G>A), K112R (348A>G), D114P (353G>C 354A>C 355T>A), P115A (356C>G 358A>C), V117K (362G>A 363T>A), S119H (368A>C 369G>A), I121M (376C>G), S122N (377T>A 378C>A 379A>T), M123Y (380A>T 381T>A 382G>T), I124V (383A>G 385C>T), A128G (396C>G), V129I (398G>A), K130Q (401A>C 403G>A), L132M (407C>A 409A>G), L133V (410C>G 412T>G), T134K (414C>A), A135F (416G>T 417C>T), Q136T (419C>A 420A>C 421A>T), R138K (425C>A 426G>A 427G>A), Q139E (428C>G), D142N (437G>A), T143C (440A>T 441C>G 442G>T), S144P (443T>C 445A>T), V145I (446G>A 448T>A), K146I (450A>T), M147I (454G>A), A148N (455G>A 456C>A 457A>C), I150S (461A>T 462T>C 463C>T), D152E (469T>A), I154F (473A>T 475C>T), V155I (476G>A 478C>T), R157T (482C>A 483G>C 484A>T), K158R (486A>G), D159E (490T>A), S160G (491A>G 493T>A), L161N (494C>A 495T>A 496G>T), A164I (503G>A 504C>T), A165V (507C>T 508A>T), R166E (509A>G 510G>A), G172T (527G>A 528G>C), F174L (535T>A), M175L (536A>C 538G>T), V178Y (545G>T 546T>A), K181R (555A>G), F182Y (558T>A), A183C (560G>T 561C>G 562A>T), L184I (563T>A 565G>A), Q187K (572C>A), S188D (575A>G 576G>A 577C>T), K189A (578A>G 579A>C 580G>T), L191F (586A>T), D192N (587G>A), K193E (590A>G), T194A (593A>G), F197L (604T>G), I198H (605A>C 606T>A), H199F (608C>T 609A>T), F201V (614T>G 616T>C), E202K (617G>A), M207F (632A>T 634G>C), T209P (638A>C), D211N (644G>A), K212H (647A>C 649A>T), V213I (650G>A), F214M (653T>A 655T>G), T215S (657C>G 658G>T)								
Codon mutations:	GTC92.CT (288T>C 289C>T), TCT93ACT (290T>A), CAA95AAA (296C>A), GTT96CAT (299G>C 300T>A), TAT97AAA (302T>A 304T>A), CTT98TTA (305C>T 307T>A), CCT99AAT (308C>A 309C>A), CTT100TTA (311C>T 313T>A), ATA101TTA (314A>T), AAG103AAA (322G>A), CAG104CCA (324A>C 325G>A), GAA105ATG (326G>A 327A>T 328A>G), ATA106ATT (331A>T), CAA107AAT (332C>A 334A>T), AAG109ATC (339A>T 340G>C), CTG110CTA (343G>A), ATG111CAA (344A>C 345T>A 346G>A), AAA112AGA (348A>G), GAT114CCA (353G>C 354A>C 355T>A), CCA115GCC (356C>G 358A>C), TCA116AGC (359T>A 360C>G 361A>C), GTA117AAA (362G>A 363T>A), AGT119CAT (368A>C 369G>A), AAA120AAG (373A>G), ATC121ATG (376C>G), TCA122AAT (377T>A 378C>A 379A>T), ATG123TAT (380A>T 381T>A 382G>T), ATC124GTT (383A>G 385C>T), CAC125CAT (388C>T), CTT126TTG (389C>T 391T>G), GCA128GGA (396C>G), GTA129ATA (398G>A), AAG130CAA (401A>C 403G>A), ATT131ATC (406T>C), CTA132ATG (407C>A 409A>G), CTT133GTG (410C>G 412T>G), ACA134AAA (414C>A), GCC135TTC (416G>T 417C>T), CAA136ACT (419C>A 420A>C 421A>T), CGG138AAA (425C>A 426G>A 427G>A), CAA139GAA (428C>G), GGA140GGT (433A>T), ATT141ATA (436T>A), GAT142AAT (437G>A), ACG143TGT (440A>T 441C>G 442G>T), TCA144CCT (443T>C 445A>T), GTT145ATA (446G>A 448T>A), AAA146ATA (450A>T), ATG147ATA (454G>A), GCA148AAC (455G>A 456C>A 457A>C), ATC150TCT (461A>T 462T>C 463C>T), GAT152GAA (469T>A), ATC154TTT (473A>T 475C>T), GTC155ATT (476G>A 478C>T), CGA157ACT (482C>A 483G>C 484A>T), AAA158AGA (486A>G), GAT159GAA (490T>A), AGT160GGA (491A>G 493T>A), CTG161AAT (494C>A 495T>A 496G>T), CTC162CTT (499C>T), GCA164ATA (503G>A 504C>T), GCA165GTT (507C>T 508A>T), AGA166GAA (509A>G 510G>A), CTC169TTA (518C>T 520C>A), TAT171TAC (526T>C), GGT172ACT (527G>A 528G>C), TTT174TTA (535T>A), ATG175CTT (536A>C 538G>T), ACC177ACA (544C>A), GTT178TAT (545G>T 546T>A), TAC179TAT (550C>T), CCT180CCC (553T>C), AAA181AGA (555A>G), TTT182TAT (558T>A), GCA183TGT (560G>T 561C>G 562A>T), TTG184ATA (563T>A 565G>A), CAA187AAA (572C>A), AGC188GAT (575A>G 576G>A 577C>T), AAG189GCT (578A>G 579A>C 580G>T), AAT190AAC (583T>C), TTA191TTT (586A>T), GAT192AAT (587G>A), AAA193GAA (590A>G), ACA194GCA (593A>G), TTA195TTG (598A>G), AGC196AGT (601C>T), TTT197TTG (604T>G), ATT198CAT (605A>C 606T>A), CAT199TTT (608C>T 609A>T), CAA200CAG (613A>G), TTT201GTC (614T>G 616T>C), GAA202AAA (617G>A), GAC205GAT (628C>T), ATG207TTC (632A>T 634G>C), AAA208AAG (637A>G), ACA209CCA (638A>C), GGC210GGT (643C>T), GAT211AAT (644G>A), AAA212CAT (647A>C 649A>T), GTT213ATT (650G>A), TTT214ATG (653T>A 655T>G), ACG215AGT (657C>G 658G>T), GTC216AT. (659G>A)								

*: Inserts / Deletes / Misaligned / Frameshifts

Analysis details

This analysis was performed with panviral2.64

NGS Details (UN60): Brazilian marseillevirus

Assembly

**Coverage Length**	138 (1 contig(s))
**Depth Of Coverage**	25.7
**Number Of Reads**	47
**Reads Per Million**	1.00 rpm (after QC)
**Ambiguities**	0
**Assembly Method**	de novo + reference guided assembly
**Consensus Caller**	Bcf Tools

Coverage Map

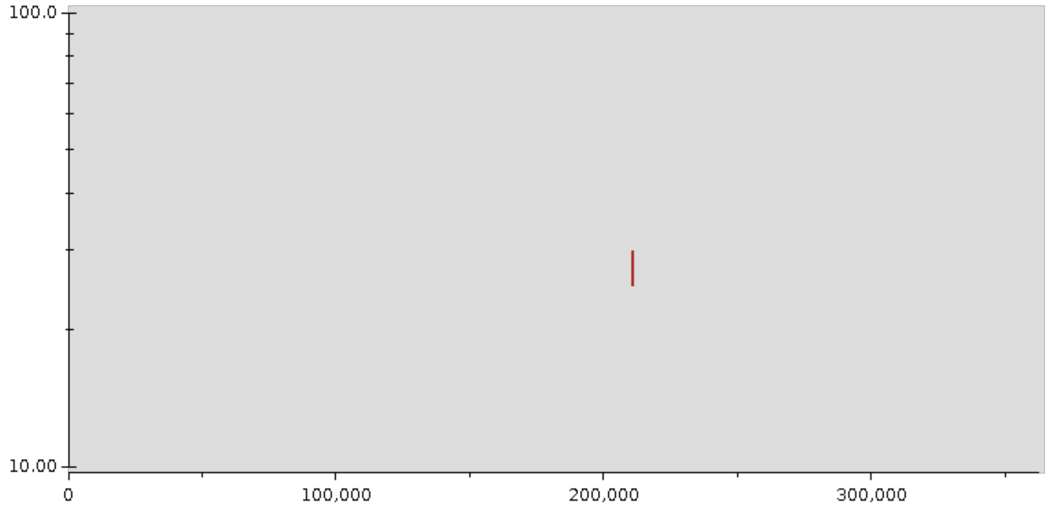

Assignment

**Type**	Brazilian marseillevirus (Taxonomy ID: 1813599)
**Reference Genome**	NC_029692.1
**NT Identity (%)**	81.1594
**AA Identity (%)**	95.6522
**Number Of Stop Codons**	0
**Number Of CDS**	491

Alignment

**Alignment Score**	172.0 (NT) + 303.0 (AA) = 475.0
**Concordance (%)**	81.4751
**Alignment Method**	Local, heuristic, nucleotide (BLASTN)

Genome Region

Sequence starts at position 210893 and ends at position 211030 relative to NC_029692.1 reference sequence.

Alignment Detailed Statistics

	Begin	End	Coverage	Score	Concordance	Matches	Identities	I/D/M/F*	Stop Codons
**NT**	**210893**	**211030**	**0.1%**	**172**	**62.3%**	**138 (100%)**	**112 (81.2%)**	**0/0**	

Mutations: 210901T>G, 210904G>A, 210908A>C, 210910G>A, 210920G>A, 210932G>A, 210938A>G, 210944C>G, 210945G>C, 210946C>T, 210947A>C, 210949G>A, 210950G>A, 210953C>A, 210955T>G, 210965C>A, 210971C>T, 210974G>A, 210977T>A, 210989G>A, 210992T>C, 211001A>G, 211004A>T, 211007A>G, 211009C>T, 211010T>A

*: Inserts / Deletes / Misaligned / Frameshifts

Analysis details

This analysis was performed with panviral2.64

NGS Details (UN60): Errantivirus

Assembly

**Coverage Length**	300 (1 contig(s))
**Depth Of Coverage**	16.8
**Number Of Reads**	46
**Reads Per Million**	0.98 rpm (after QC)
**Ambiguities**	0
**Assembly Method**	de novo + reference guided assembly
**Consensus Caller**	Bcf Tools

Coverage Map

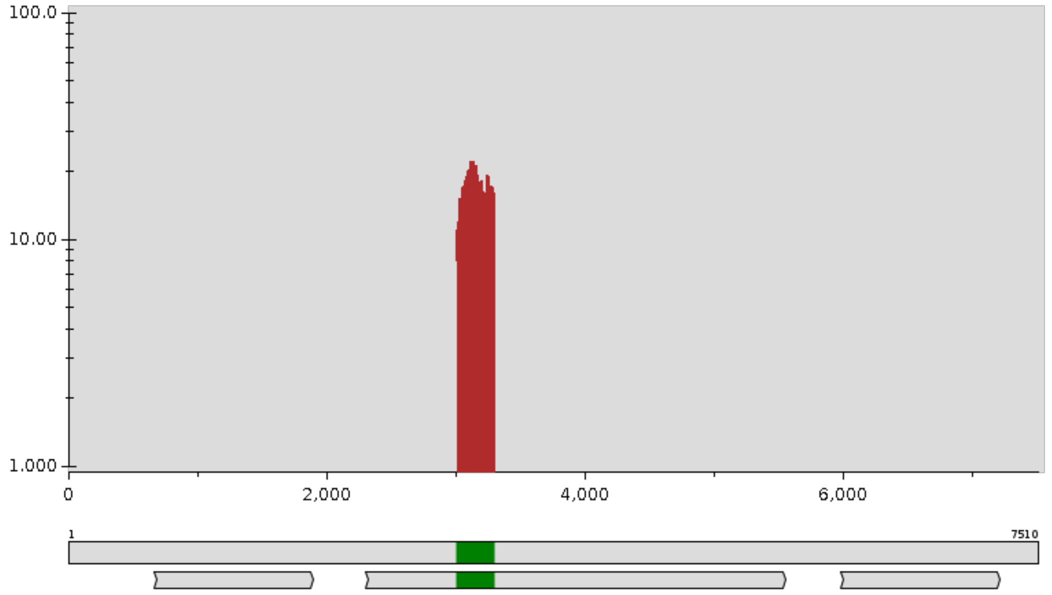

Assignment

**Type**	Errantivirus (Taxonomy ID: 186666)
**Reference Genome**	NC_038512.1
**NT Identity (%)**	53.0
**AA Identity (%)**	41.0
**Number Of Stop Codons**	0
**Number Of CDS**	3

Alignment

**Alignment Score**	36.0 (NT) + 301.0 (AA) = 337.0
**Concordance (%)**	26.1646

Alignment Method	Global, seeded, nucleotide + amino acids (AGA)

Genome Region

Sequence starts at position 3007 and ends at position 3306 relative to NC_038512.1 reference sequence.

Alignment Detailed Statistics

	Begin	End	Coverage	Score	Concordance	Matches	Identities	I/D/M/F*	Stop Codons
**NT**	**3007**	**3306**	**4.0%**	**36**	**6.0%**	**300 (100%)**	**159 (53.0%)**	**0/0**	
Mutations:	3007G>T, 3008G>A, 3009C>G, 3011A>G, 3012G>C, 3016G>A, 3018T>A, 3020T>C, 3022T>C, 3026G>T, 3029G>A, 3030A>G, 3031G>A, 3035G>T, 3037C>T, 3038C>G, 3039C>A, 3040T>A, 3041C>A, 3043A>G, 3047A>G, 3048T>C, 3049A>T, 3050T>C, 3051C>A, 3052G>C, 3055A>G, 3058C>T, 3061G>C, 3067C>A, 3068G>A, 3069T>C, 3070A>T, 3071G>C, 3073A>T, 3074C>G, 3076C>A, 3082T>C, 3084T>A, 3088A>G, 3091C>T, 3094T>G, 3095C>G, 3096G>T, 3103T>C, 3104A>T, 3106G>T, 3109A>G, 3114A>C, 3115A>C, 3118C>T, 3119T>G, 3121A>C, 3124A>T, 3128A>T, 3133T>C, 3138G>A, 3139A>C, 3140G>C, 3142T>C, 3146G>A, 3149A>T, 3150A>C, 3151T>A, 3154C>A, 3155C>T, 3157A>T, 3161G>C, 3162G>C, 3163T>A, 3164C>T, 3166C>A, 3167C>T, 3168A>T, 3171A>G, 3172T>G, 3175C>A, 3178C>T, 3179T>G, 3180G>T, 3181T>C, 3182C>T, 3184C>G, 3187C>T, 3189A>T, 3191C>T, 3196C>T, 3199T>C, 3202T>C, 3203A>C, 3206G>A, 3211T>C, 3214T>C, 3216C>A, 3217T>G, 3221C>A, 3223A>G, 3224C>A, 3225A>C, 3226G>T, 3229A>T, 3233C>A, 3235G>T, 3238G>A, 3239A>C, 3241C>T, 3242C>G, 3244G>T, 3245G>A, 3248C>G, 3249G>C, 3253T>G, 3256C>T, 3257C>G, 3259A>T, 3260A>C, 3261G>T, 3262A>G, 3263C>A, 3265T>G, 3266A>T, 3267G>T, 3269G>A, 3271A>G, 3272A>T, 3273G>T, 3275A>C, 3277C>G, 3280C>A, 3281A>T, 3283A>T, 3284A>G, 3285T>C, 3287C>A, 3293G>T, 3294A>C, 3295C>T, 3298G>A, 3300C>G, 3301C>T, 3303A>C								

CDS

	Begin	End	Coverage	Score	Concordance	Matches	Identities	I/D/M/F*	Stop Codons
D1R33_gp2	235	334	9.2%	301	42.8%	100 (100%)	41 (41.0%)	0/0/0/0	0
Protein mutations:	A235R (3008G>A 3009C>G), S236A (3011A>G 3012G>C), F238Y (3018T>A), Y239H (3020T>C 3022T>C), V241L (3026G>T), E242R (3029G>A 3030A>G 3031G>A), D244Y (3035G>T 3037C>T), P245E (3038C>G 3039C>A 3040T>A), Q246K (3041C>A 3043A>G), I248A (3047A>G 3048T>C 3049A>T), S249H (3050T>C 3051C>A 3052G>C), N254K (3067C>A), V255T (3068G>A 3069T>C 3070A>T), E256H (3071G>C 3073A>T), H257E (3074C>G 3076C>A), F260Y (3084T>A), R264V (3095C>G 3096G>T), M267F (3104A>T 3106G>T), K270T (3114A>C 3115A>C), S272A (3119T>G 3121A>C), T275S (3128A>T), R278N (3138G>A 3139A>C), V279L (3140G>C 3142T>C), D281N (3146G>A), N282S (3149A>T 3150A>C 3151T>A), L284F (3155C>T 3157A>T), G286P (3161G>C 3162G>C 3163T>A), Q288L (3167C>T 3168A>T), N289R (3171A>G 3172T>G), N290K (3175C>A), C292V (3179T>G 3180G>T 3181T>C), Y295F (3189A>T), L296F (3191C>T), I300L (3203A>C), V301I (3206G>A), T304K (3216C>A 3217T>G), L306M (3221C>A 3223A>G), Q307T (3224C>A 3225A>C 3226G>T), E308D (3229A>T), L310I (3233C>A 3235G>T), N312H (3239A>C 3241C>T), L313V (3242C>G 3244G>T), E314K (3245G>A), R315A (3248C>G 3249G>C), Q318D (3257C>G 3259A>T), R319L (3260A>C 3261G>T 3262A>G), L320M (3263C>A 3265T>G), R321L (3266A>T 3267G>T), E322K (3269G>A 3271A>G), S323F (3272A>T 3273G>T), N324Q (3275A>C 3277C>G), F325L (3280C>A), K326Y (3281A>T 3283A>T), I327A (3284A>G 3285T>C), Q328K (3287C>A), D330S (3293G>T 3294A>C 3295C>T), S332C (3300C>G 3301C>T), E333A (3303A>C)								
Codon mutations:	TTG234..T (3007G>T), GCA235AGA (3008G>A 3009C>G), AGT236GCT (3011A>G 3012G>C), GGG237GGA (3016G>A), TTT238TAT (3018T>A), TAT239CAC (3020T>C 3022T>C), GTG241TTG (3026G>T), GAG242AGA (3029G>A 3030A>G 3031G>A), GAC244TAT (3035G>T 3037C>T), CCT245GAA (3038C>G 3039C>A 3040T>A), CAA246AAG (3041C>A 3043A>G), ATA248GCT (3047A>G 3048T>C 3049A>T), TCG249CAC (3050T>C 3051C>A 3052G>C), AAA250AAG (3055A>G), ACC251ACT (3058C>T), GCG252GCC (3061G>C), AAC254AAA (3067C>A), GTA255ACT (3068G>A 3069T>C 3070A>T), GAA256CAT (3071G>C 3073A>T), CAC257GAA (3074C>G 3076C>A), CAT259CAC (3082T>C), TTT260TAT (3084T>A), GAA261GAG (3088A>G), TTC262TTT (3091C>T), CTT263CTG (3094T>G), CGA264GTA (3095C>G 3096G>T), CCT266CCC (3103T>C), ATG267TTT (3104A>T 3106G>T), GGA268GGG (3109A>G), AAA270ACC (3114A>C 3115A>C), AAC271AAT (3118C>T), TCA272GCC (3119T>G 3121A>C), CCA273CCT (3124A>T), ACT275TCT (3128A>T), TTT276TTC (3133T>C), AGA278AAC (3138G>A 3139A>C), GTT279CTC (3140G>C 3142T>C), GAC281AAC (3146G>A), AAT282TCA (3149A>T 3150A>C 3151T>A), GTC283GTA (3154C>A), CTA284TTT (3155C>T 3157A>T), GGT286CCA (3161G>C 3162G>C 3163T>A), CTC287TTA (3164C>T 3166C>A), CAA288TTA (3167C>T 3168A>T), AAT289AGG (3171A>G 3172T>G), AAC290AAA (3175C>A), ATC291ATT (3178C>T), TGT292GTC (3179T>G 3180G>T 3181T>C), CTC293TTG (3182C>T 3184C>G), GTC294GTT (3187C>T), TAC295TTC (3189A>T), CTT296TTT (3191C>T), GAC297GAT (3196C>T), GAT298GAC (3199T>C), ATT299ATC (3202T>C), ATT300CTT (3203A>C), GTC301ATC (3206G>A), TAT302TAC (3211T>C), AGT303AGC (3214T>C), ACT304AAG (3216C>A 3217T>G), CTA306ATG (3221C>A 3223A>G), CAG307ACT (3224C>A 3225A>C 3226G>T), GAA308GAT (3229A>T), CTG310ATT (3233C>A 3235G>T), GAG311GAA (3238G>A), AAC312CAT (3239A>C 3241C>T), CTG313GTT (3242C>G 3244G>T), GAA314AAA (3245G>A), CGA315GCA (3248C>G 3249G>C), GTT316GTG (3253T>G), TTC317TTT (3256C>T), CAA318GAT (3257C>G 3259A>T), AGA319CTG (3260A>C 3261G>T 3262A>G), CTT320ATG (3263C>A 3265T>G), AGA321TTA (3266A>T 3267G>T), GAA322AAG (3269G>A 3271A>G), AGT323TTT (3272A>T 3273G>T), AAC324CAG (3275A>C 3277C>G), TTC325TTA (3280C>A), AAA326TAT (3281A>T 3283A>T), ATT327GCT (3284A>G 3285T>C), CAA328AAA (3287C>A), GAC330TCT (3293G>T 3294A>C 3295C>T), AAG331AAA (3298G>A), TCC332TGT (3300C>G 3301C>T), GAA333GCA (3303A>C)								

Proteins

	Begin	End	Coverage	Score	Concordance	Matches	Identities	I/D/M/F*	Stop Codons
ORF B (YP_009507248.1)	235	334	9.2%	301	42.8%	100 (100%)	41 (41.0%)	0/0/0/0	0
Protein mutations:	A235R (3008G>A 3009C>G), S236A (3011A>G 3012G>C), F238Y (3018T>A), Y239H (3020T>C 3022T>C), V241L (3026G>T), E242R (3029G>A 3030A>G 3031G>A), D244Y (3035G>T 3037C>T), P245E (3038C>G 3039C>A 3040T>A), Q246K (3041C>A 3043A>G), I248A (3047A>G 3048T>C 3049A>T), S249H (3050T>C 3051C>A 3052G>C), N254K (3067C>A), V255T (3068G>A 3069T>C 3070A>T), E256H (3071G>C 3073A>T), H257E (3074C>G 3076C>A), F260Y (3084T>A), R264V (3095C>G 3096G>T), M267F (3104A>T 3106G>T), K270T (3114A>C 3115A>C), S272A (3119T>G 3121A>C), T275S (3128A>T), R278N (3138G>A 3139A>C), V279L (3140G>C 3142T>C), D281N (3146G>A), N282S (3149A>T 3150A>C 3151T>A), L284F (3155C>T 3157A>T), G286P (3161G>C 3162G>C 3163T>A), Q288L (3167C>T 3168A>T), N289R (3171A>G 3172T>G), N290K (3175C>A), C292V (3179T>G 3180G>T 3181T>C), Y295F (3189A>T), L296F (3191C>T), I300L (3203A>C), V301I (3206G>A), T304K (3216C>A 3217T>G), L306M (3221C>A 3223A>G), Q307T (3224C>A 3225A>C 3226G>T), E308D (3229A>T), L310I (3233C>A 3235G>T), N312H (3239A>C 3241C>T), L313V (3242C>G 3244G>T), E314K (3245G>A), R315A (3248C>G 3249G>C), Q318D (3257C>G 3259A>T), R319L (3260A>C 3261G>T 3262A>G), L320M (3263C>A 3265T>G), R321L (3266A>T 3267G>T), E322K (3269G>A 3271A>G), S323F (3272A>T 3273G>T), N324Q (3275A>C 3277C>G), F325L (3280C>A), K326Y (3281A>T 3283A>T), I327A (3284A>G 3285T>C), Q328K (3287C>A), D330S (3293G>T 3294A>C 3295C>T), S332C (3300C>G 3301C>T), E333A (3303A>C)								
Codon mutations:	TTG234..T (3007G>T), GCA235AGA (3008G>A 3009C>G), AGT236GCT (3011A>G 3012G>C), GGG237GGA (3016G>A), TTT238TAT (3018T>A), TAT239CAC (3020T>C 3022T>C), GTG241TTG (3026G>T), GAG242AGA (3029G>A 3030A>G 3031G>A), GAC244TAT (3035G>T 3037C>T), CCT245GAA (3038C>G 3039C>A 3040T>A), CAA246AAG (3041C>A 3043A>G), ATA248GCT (3047A>G 3048T>C 3049A>T), TCG249CAC (3050T>C 3051C>A 3052G>C), AAA250AAG (3055A>G), ACC251ACT (3058C>T), GCG252GCC (3061G>C), AAC254AAA (3067C>A), GTA255ACT (3068G>A 3069T>C 3070A>T), GAA256CAT (3071G>C 3073A>T), CAC257GAA (3074C>G 3076C>A), CAT259CAC (3082T>C), TTT260TAT (3084T>A), GAA261GAG (3088A>G), TTC262TTT (3091C>T), CTT263CTG (3094T>G), CGA264GTA (3095C>G 3096G>T), CCT266CCC (3103T>C), ATG267TTT (3104A>T 3106G>T), GGA268GGG (3109A>G), AAA270ACC (3114A>C 3115A>C), AAC271AAT (3118C>T), TCA272GCC (3119T>G 3121A>C), CCA273CCT (3124A>T), ACT275TCT (3128A>T), TTT276TTC (3133T>C), AGA278AAC (3138G>A 3139A>C), GTT279CTC (3140G>C 3142T>C), GAC281AAC (3146G>A), AAT282TCA (3149A>T 3150A>C 3151T>A), GTC283GTA (3154C>A), CTA284TTT (3155C>T 3157A>T), GGT286CCA (3161G>C 3162G>C 3163T>A), CTC287TTA (3164C>T 3166C>A), CAA288TTA (3167C>T 3168A>T), AAT289AGG (3171A>G 3172T>G), AAC290AAA (3175C>A), ATC291ATT (3178C>T), TGT292GTC (3179T>G 3180G>T 3181T>C), CTC293TTG (3182C>T 3184C>G), GTC294GTT (3187C>T), TAC295TTC (3189A>T), CTT296TTT (3191C>T), GAC297GAT (3196C>T), GAT298GAC (3199T>C), ATT299ATC (3202T>C), ATT300CTT (3203A>C), GTC301ATC (3206G>A), TAT302TAC (3211T>C), AGT303AGC (3214T>C), ACT304AAG (3216C>A 3217T>G), CTA306ATG (3221C>A 3223A>G), CAG307ACT (3224C>A 3225A>C 3226G>T), GAA308GAT (3229A>T), CTG310ATT (3233C>A 3235G>T), GAG311GAA (3238G>A), AAC312CAT (3239A>C 3241C>T), CTG313GTT (3242C>G 3244G>T), GAA314AAA (3245G>A), CGA315GCA (3248C>G 3249G>C), GTT316GTG (3253T>G), TTC317TTT (3256C>T), CAA318GAT (3257C>G 3259A>T), AGA319CTG (3260A>C 3261G>T 3262A>G), CTT320ATG (3263C>A 3265T>G), AGA321TTA (3266A>T 3267G>T), GAA322AAG (3269G>A 3271A>G), AGT323TTT (3272A>T 3273G>T), AAC324CAG (3275A>C 3277C>G), TTC325TTA (3280C>A), AAA326TAT (3281A>T 3283A>T), ATT327GCT (3284A>G 3285T>C), CAA328AAA (3287C>A), GAC330TCT (3293G>T 3294A>C 3295C>T), AAG331AAA (3298G>A), TCC332TGT (3300C>G 3301C>T), GAA333GCA (3303A>C)								

*: Inserts / Deletes / Misaligned / Frameshifts

Analysis details

This analysis was performed with panviral2.64

NGS Details (UN60): Caulimovirus venafragariae

Assembly

Coverage Length	847 (2 contig(s))
Depth Of Coverage	6.3
Number Of Reads	45
Reads Per Million	0.95 rpm (after QC)
Ambiguities	0
Assembly Method	de novo + reference guided assembly
Consensus Caller	Bcf Tools

Coverage Map

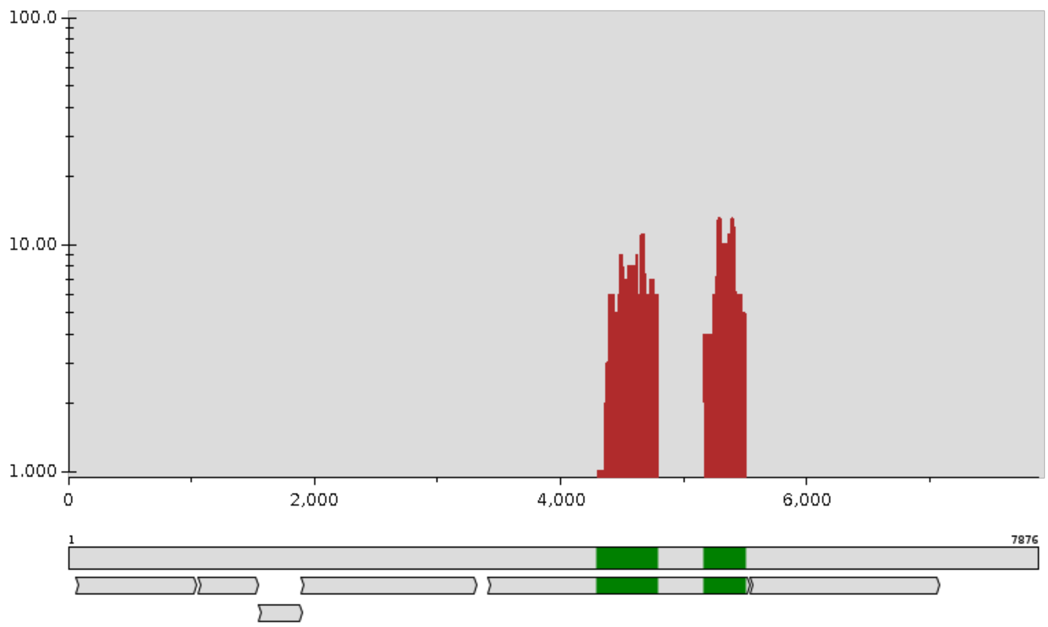

Assignment

Type	Caulimovirus venafragariae (Taxonomy ID: 3048344)
Reference Genome	NC_001725.1
NT Identity (%)	58.5042
AA Identity (%)	53.9855
Number Of Stop Codons	0
Number Of CDS	6

Alignment

Alignment Score	246.0 (NT) + 1127.0 (AA) = 1373.0
Concordance (%)	38.8182

Alignment Method	Global, seeded, nucleotide + amino acids (AGA)

Genome Region

Sequence starts at position 4295 and ends at position 5508 relative to NC_001725.1 reference sequence.

Alignment Detailed Statistics

	Begin	End	Coverage	Score	Concordance	Matches	Identities	I/D/M/F*	Stop Codons
**NT**	**4295**	**5508**	**10.8%**	**246**	**15.2%**	**829 (97.9%)**	**485 (57.3%)**	**0/18**	
Mutations:	4299C>T, 4300G>C, 4302A>G, 4308A>T, 4314G>A, 4315C>A, 4317T>G, 4318G>A, 4319G>A, 4320T>G, 4321A>T, 4323C>A, 4330C>G, 4332T>A, 4337A>G, 4338G>A, 4347T>C, 4348T>A, 4349C>A, 4351T>A, 4352C>G, 4353A>T, 4356A>C, 4359C>G, 4371G>A, 4374T>C, 4380C>G, 4381G>A, 4383G>A, 4384A>C, 4385T>A, 4386C>A, 4395C>A, 4401A>T, 4402A>C, 4413T>A, 4423A>G, 4426T>C, 4428A>T, 4432G>A, 4434C>A, 4435C>A, 4438A>T, 4439C>G, 4440A>T, 4441A>G, 4442A>T, 4443G>C, 4444G>T, 4445G>T, 4446A>T, 4452C>A, 4455C>T, 4456C>T, 4458A>T, 4459C>A, 4464T>A, 4470G>A, 4471G>A, 4473G>T, 4474C>A, 4476A>T, 4479G>T, 4480C>A, 4482T>A, 4483C>A, 4485A>T, 4486A>C, 4487G>T, 4489A>G, 4490T>C, 4492G>A, 4493G>A, 4497T>A, 4500G>A, 4505T>A, 4506T>C, 4508A>T, 4509C>T, 4512T>A, 4513T>A, 4514C>A, 4515C>A, 4528T>A, 4529C>G, 4542G>A, 4543G>A, 4546C>A, 4547G>A, 4548C>A, 4551T>A, 4552G>A, 4553C>A, 4554T>A, 4555C>G, 4556C>A, 4558G>A, 4560A>T, 4561A>T, 4563A>T, 4566T>A, 4567C>G, 4568A>C, 4569G>T, 4570C>T, 4575C>A, 4578T>A, 4584C>T, 4585T>A, 4586G>C, 4587T>A, 4590C>A, 4591C>A, 4593A>T, 4599C>T, 4602C>T, 4614C>A, 4633C>A, 4635A>T, 4642G>C, 4643C>A, 4644T>A, 4647T>C, 4650C>T, 4656G>A, 4657C>A, 4659C>A, 4665C>T, 4670G>T, 4671T>A, 4672C>T, 4674C>T, 4675A>T, 4676G>C, 4677C>T, 4678A>G, 4680C>A, 4682T>A, 4684T>A, 4687_4692delCCACAG, 4696T>C, 4697G>T, 4698T>A, 4699G>A, 4700C>T, 4701T>C, 4704A>C, 4708G>A, 4710T>A, 4716C>T, 4719C>A, 4720A>C, 4722C>A, 4723G>A, 4725C>A, 4727T>G, 4728C>T, 4729A>T, 4730G>C, 4731C>T, 4734A>T, 4735A>T, 4737T>A, 4738G>T, 4740A>T, 4741G>C, 4746A>G, 4753G>A, 4754G>C, 4758T>C, 4759G>C, 4761C>T, 4765A>C, 4766T>A, 4767T>A, 4768G>T, 4771C>T, 4772T>C, 4773A>T, 4774A>C, 4776C>A, 4777A>T, 4778G>T, 4782C>T, 4783A>G, 4784A>T, 4785A>T, 4787C>A, 4788C>A, 4789C>A, 4790T>A, 4791A>T, 5163A>C, 5167G>T, 5169C>T, 5182A>T, 5184C>A, 5185C>T, 5187C>A, 5188A>C, 5191G>A, 5193A>T, 5194A>G, 5196A>T, 5199T>A, 5200C>A, 5201C>A, 5202A>T, 5206_5217delGGTAAAGAGGTA, 5220A>T, 5223C>T, 5226G>A, 5230G>A, 5233T>A, 5234C>G, 5235A>T, 5247A>T, 5248C>G, 5249C>A, 5251G>A, 5253T>A, 5256G>A, 5258A>C, 5259G>T, 5261A>G, 5262T>G, 5266C>T, 5267A>T, 5268T>A, 5270G>C, 5271T>A, 5280G>A, 5284A>T, 5286C>A, 5289T>A, 5290T>G, 5292C>T, 5295T>A, 5296A>G, 5298T>A, 5300A>G, 5301G>A, 5303C>G, 5304A>C, 5307C>A, 5308A>C, 5309A>G, 5311G>A, 5312C>A, 5313T>G, 5317A>T, 5318G>C, 5319A>T, 5322T>A, 5324A>T, 5325T>C, 5326A>T, 5328C>A, 5331T>A, 5334T>A, 5335T>A, 5337T>A, 5338A>C, 5343T>C, 5344C>A, 5346T>C, 5347G>A, 5349A>C, 5352G>A, 5353A>T, 5355T>G, 5358T>C, 5364T>G, 5365A>C, 5367T>G, 5369C>T, 5370T>A, 5371G>T, 5373T>A, 5374T>G, 5375A>G, 5377T>C, 5379T>A, 5380G>A, 5382G>A, 5383A>T, 5384G>T, 5385A>C, 5387C>A, 5388A>T, 5391T>A, 5392A>C, 5395G>C, 5398G>T, 5399G>C, 5403T>A, 5404T>C, 5405A>C, 5407A>C, 5409G>A, 5410C>T, 5412A>T, 5415C>A, 5416C>A, 5418G>A, 5419C>T, 5421T>A, 5422G>C, 5423T>A, 5424G>T, 5434A>G, 5436G>A, 5437G>T, 5438C>T, 5439T>G, 5443A>T, 5444G>C, 5445A>T, 5446G>T, 5447A>T, 5448A>T, 5452T>A, 5454T>C, 5457T>C, 5458G>A, 5460T>A, 5463A>T, 5467C>A, 5470G>A, 5473T>A, 5474C>A, 5475T>A, 5478T>A, 5479C>T, 5480A>C, 5481A>T, 5482A>G, 5484A>T, 5491C>T, 5493A>T, 5496T>A, 5500A>T, 5503A>T, 5505G>A, 5506A>T, 5508T>A								

CDS

	Begin	End	Coverage	Score	Concordance	Matches	Identities	I/D/M/F*	Stop Codons
ORF_V	298	701	39.8%	1127	58.3%	276 (97.9%)	149 (52.8%)	0/6/0/0	0
Protein mutations:	E299Q (4300G>C 4302A>G), L304M (4315C>A 4317T>G), G305K (4318G>A 4319G>A 4320T>G), I306L (4321A>T 4323C>A), P309A (4330C>G 4332T>A), K311R (4337A>G 4338G>A), S315N (4348T>A 4349C>A), E326K (4381G>A 4383G>A), I327Q (4384A>C 4385T>A 4386C>A), K340E (4423A>G), D343K (4432G>A 4434C>A), H344N (4435C>A), T345C (4438A>T 4439C>G 4440A>T), K346V (4441A>G 4442A>T 4443G>C), G347F (4444G>T 4445G>T 4446A>T), L351F (4456C>T 4458A>T), L352I (4459C>A), E356N (4471G>A 4473G>T), Q357N (4474C>A 4476A>T), L359I (4480C>A 4482T>A), Q360N (4483C>A 4485A>T), R361L (4486A>C 4487G>T), I362A (4489A>G 4490T>C), G363K (4492G>A 4493G>A), F367Y (4505T>A 4506T>C), Y368F (4508A>T 4509C>T), S370K (4513T>A 4514C>A 4515C>A), V380I (4543G>A), R381K (4546C>A 4547G>A 4548C>A), A383K (4552G>A 4553C>A 4554T>A), P384E (4555C>G 4556C>A), E385N (4558G>A 4560A>T), T386S (4561A>T 4563A>T), Q388A (4567C>G 4568A>C 4569G>T), C394T (4585T>A 4586G>C 4587T>A), Q396N (4591C>A 4593A>T), Q410N (4633C>A 4635A>T), A413Q (4642G>C 4643C>A 4644T>A), H418K (4657C>A 4659C>A), S422I (4670G>T 4671T>A), L423F (4672C>T 4674C>T), N425E (4678A>G 4680C>A), M426K (4682T>A), Y427N (4684T>A), P428_Q429del (4687_4692delCCACAG), C431L (4696T>C 4697G>T 4698T>A), A432I (4699G>A 4700C>T 4701T>C), V435I (4708G>A 4710T>A), I439L (4720A>C 4722C>A), V440I (4723G>A 4725C>A), F441C (4727T>G 4728C>T), K443N (4734A>T), T444S (4735A>T 4737T>A), E445Y (4738G>T 4740A>T), E446Q (4741G>C), G450T (4753G>A 4754G>C), V452L (4759G>C 4761C>T), I454Q (4765A>C 4766T>A 4767T>A), V455F (4768G>T), L456S (4771C>T 4772T>C 4773A>T), N457Q (4774A>C 4776C>A), R458L (4777A>T 4778G>T), K460V (4783A>G 4784A>T 4785A>T), A461E (4787C>A 4788C>A), L462N (4789C>A 4790T>A 4791A>T), D588Y (5167G>T 5169C>T), I593L (5182A>T 5184C>A), K595Q (5188A>C), A596T (5191G>A 5193A>T), K597D (5194A>G 5196A>T), P599N (5200C>A 5201C>A 5202A>T), G601_V604del (5206_5217delGGTAAAGAGGTA), A609T (5230G>A), K614N (5247A>T), P615E (5248C>G 5249C>A), A616T (5251G>A 5253T>A), K618T (5258A>C 5259G>T), N619R (5261A>G 5262T>G), H621L (5266C>T 5267A>T 5268T>A), S622T (5270G>C 5271T>A), I627L (5284A>T 5286C>A), S629A (5290T>G 5292C>T), I631V (5296A>G 5298T>A), K632R (5300A>G 5301G>A), A633G (5303C>G 5304A>C), K635R (5308A>C 5309A>G), A636K (5311G>A 5312C>A 5313T>G), R638S (5317A>T 5318G>C 5319A>T), Y640F (5324A>T 5325T>C), I641L (5326A>T 5328C>A), Y644K (5335T>A 5337T>A), K645Q (5338A>C), L647I (5344C>A 5346T>C), V648I (5347G>A 5349A>C), T650S (5353A>T 5355T>G), N654Q (5365A>C 5367T>G), A655V (5369C>T 5370T>A), A656S (5371G>T 5373T>A), Y657G (5374T>G 5375A>G), F658L (5377T>C 5379T>A), V659I (5380G>A 5382G>A), R660F (5383A>T 5384G>T 5385A>C), T661N (5387C>A 5388A>T), N662K (5391T>A), I663L (5392A>C), A664P (5395G>C), G665S (5398G>T 5399G>C), D666E (5403T>A), Y667P (5404T>C 5405A>C), K668Q (5407A>C 5409G>A), Q669Y (5410C>T 5412A>T), S670R (5415C>A), V673H (5422G>C 5423T>A 5424G>T), M677V (5434A>G 5436G>A), A678L (5437G>T 5438C>T 5439T>G), R680S (5443A>T 5444G>C 5445A>T), E681F (5446G>T 5447A>T 5448A>T), S683T (5452T>A 5454T>C), D685K (5458G>A 5460T>A), H688N (5467C>A), V689I (5470G>A), S690K (5473T>A 5474C>A 5475T>A), Q692S (5479C>T 5480A>C 5481A>T), K693D (5482A>G 5484A>T), L696F (5491C>T 5493A>T), I699F (5500A>T), M700L (5503A>T 5505G>A), T701S (5506A>T 5508T>A)								
Codon mutations:	ATC298ATT (4299C>T), GAA299CAG (4300G>C 4302A>G), CTA301CTT (4308A>T), AAG303AAA (4314G>A), CTT304ATG (4315C>A 4317T>G), GGT305AAG (4318G>A 4319G>A 4320T>G), ATC306TTA (4321A>T 4323C>A), CCT309GCA (4330C>G 4332T>A), AAG311AGA (4337A>G 4338G>A), CAT314CAC (4347T>C), TCC315AAC (4348T>A 4349C>A), TCA316AGT (4351T>A 4352C>G 4353A>T), CCA317CCC (4356A>C), GCC318GCG (4359C>G), AGG322AGA (4371G>A), AAT323AAC (4374T>C), GCC325GCG (4380C>G), GAG326AAA (4381G>A 4383G>A), ATC327CAA (4384A>C 4385T>A 4386C>A), GGC330GGA (4395C>A), GCA332GCT (4401A>T), AGA333CGA (4402A>C), ATT336ATA (4413T>A), AAG340GAG (4423A>G), TTA341CTT (4426T>C 4428A>T), GAC343AAA (4432G>A 4434C>A), CAT344AAT (4435C>A), ACA345TGT (4438A>T 4439C>G 4440A>T), AAG346GTC (4441A>G 4442A>T 4443G>C), GGA347TTT (4444G>T 4445G>T 4446A>T), GGC349GGA (4452C>A), TAC350TAT (4455C>T), CTA351TTT (4456C>T 4458A>T), CTT352ATT (4459C>A), CCT353CCA (4464T>A), AAG355AAA (4470G>A), GAG356AAT (4471G>A 4473G>T), CAA357AAT (4474C>A 4476A>T), CTG358CTT (4479G>T), CTT359ATA (4480C>A 4482T>A), CAA360AAT (4483C>A 4485A>T), AGA361CTA (4486A>C 4487G>T), ATC362GCC (4489A>G 4490T>C), GGA363AAA (4492G>A 4493G>A), GGT364GGA (4497T>A), AAG365AAA (4500G>A), TTT367TAC (4505T>A 4506T>C), TAC368TTT (4508A>T 4509C>T), TCT369TCA (4512T>A), TCC370AAA (4513T>A 4514C>A 4515C>A), TCT375AGT (4528T>A 4529C>G), CAG379CAA (4542G>A), GTA380ATA (4543G>A), CGC381AAA (4546C>A 4547G>A 4548C>A), CTT382CTA (4551T>A), GCT383AAA (4552G>A 4553C>A 4554T>A), CCA384GAA (4555C>G 4556C>A), GAA385AAT (4558G>A 4560A>T), ACA386TCT (4561A>T 4563A>T), ATT387ATA (4566T>A), CAG388GCT (4567C>G 4568A>C 4569G>T), CTA389TTA (4570C>T), ACC390ACA (4575C>A), GCT391GCA (4578T>A), AGC393AGT (4584C>T), TGT394ACA (4585T>A 4586G>C 4587T>A), CCC395CCA (4590C>A), CAA396AAT (4591C>A 4593A>T), CAC398CAT (4599C>T), TAC399TAT (4602C>T), GTC403GTA (4614C>A), CAA410AAT (4633C>A 4635A>T), GCT413CAA (4642G>C 4643C>A 4644T>A), ATT414ATC (4647T>C), TTC415TTT (4650C>T), AGG417AGA (4656G>A), CAC418AAA (4657C>A 4659C>A), GAC420GAT (4665C>T), AGT422ATA (4670G>T 4671T>A), CTC423TTT (4672C>T 4674C>T), AGC424TCT (4675A>T 4676G>C 4677C>T), AAC425GAA (4678A>G 4680C>A), ATG426AAG (4682T>A), TAT427AAT (4684T>A), CCA428_CAG429del (4687_4692delCCACAG), TGT431CTA (4696T>C 4697G>T 4698T>A), GCT432ATC (4699G>A 4700C>T 4701T>C), GTA433GTC (4704A>C), GTT435ATA (4708G>A 4710T>A), GAC437GAT (4716C>T), ATC438ATA (4719C>A), ATC439CTA (4720A>C 4722C>A), GTC440ATA (4723G>A 4725C>A), TTC441TGT (4727T>G 4728C>T), AGC442TCT (4729A>T 4730G>C 4731C>T), AAA443AAT (4734A>T), ACT444TCA (4735A>T 4737T>A), GAA445TAT (4738G>T 4740A>T), GAA446CAA (4741G>C), GAA447GAG (4746A>G), GGA450ACA (4753G>A 4754G>C), CAT451CAC (4758T>C), GTC452CTT (4759G>C 4761C>T), ATT454CAA (4765A>C 4766T>A 4767T>A), GTT455TTT (4768G>T), CTA456TCT (4771C>T 4772T>C 4773A>T), AAC457CAA (4774A>C 4776C>A), AGA458TTA (4777A>T 4778G>T), TGC459TGT (4782C>T), AAA460GTT (4783A>G 4784A>T 4785A>T), GCC461GAA (4787C>A 4788C>A), CTA462AAT (4789C>A 4790T>A 4791A>T), TCA586..C (5163A>C), GAC588TAT (5167G>T 5169C>T), ATC593TTA (5182A>T 5184C>A), CTC594TTA (5185C>T 5187C>A), AAA595CAA (5188A>C), GCA596ACT (5191G>A 5193A>T), AAA597GAT (5194A>G 5196A>T), CTT598CTA (5199T>A), CCA599AAT (5200C>A 5201C>A 5202A>T), GGT601_GTA604del (5206_5217delGGTAAAGAGGTA), ATA605ATT (5220A>T), TGC606TGT (5223C>T), AGG607AGA (5226G>A), GCT609ACT (5230G>A), TCA610AGT (5233T>A 5234C>G 5235A>T), AAA614AAT (5247A>T), CCA615GAA (5248C>G 5249C>A), GCT616ACA (5251G>A 5253T>A), GAG617GAA (5256G>A), AAG618ACT (5258A>C 5259G>T), AAT619AGG (5261A>G 5262T>G), CAT621TTA (5266C>T 5267A>T 5268T>A), AGT622ACA (5270G>C 5271T>A), AAG625AAA (5280G>A), ATC627TTA (5284A>T 5286C>A), CTT628CTA (5289T>A), TCC629GCT (5290T>G 5292C>T), ATT630ATA (5295T>A), ATT631GTA (5296A>G 5298T>A), AAG632AGA (5300A>G 5301G>A), GCA633GGC (5303C>G 5304A>C), ATC634ATA (5307C>A), AAA635CGA (5308A>C 5309A>G), GCT636AAG (5311G>A 5312C>A 5313T>G), AGA638TCT (5317A>T 5318G>C 5319A>T), GCT639GCA (5322T>A), TAT640TTC (5324A>T 5325T>C), ATC641TTA (5326A>T 5328C>A), CTT642CTA (5331T>A), CCT643CCA (5334T>A), TAT644AAA (5335T>A 5337T>A), AAA645CAA (5338A>C), TTT646TTC (5343T>C), CTT647ATC (5344C>A 5346T>C), GTA648ATC (5347G>A 5349A>C), AGG649AGA (5352G>A), ACT650TCG (5353A>T 5355T>G), GAT651GAC (5358T>C), ACT653ACG (5364T>G), AAT654CAG (5365A>C 5367T>G), GCT655GTA (5369C>T 5370T>A), GCT656TCA (5371G>T 5373T>A), TAT657GGT (5374T>G 5375A>G), TTT658CTA (5377T>C 5379T>A), GTG659ATA (5380G>A 5382G>A), AGA660TTC (5383A>T 5384G>T 5385A>C), ACA661AAT (5387C>A 5388A>T), AAT662AAA (5391T>A), ATT663CTT (5392A>C), GCA664CCA (5395G>C), GGT665TCT (5398G>T 5399G>C), GAT666GAA (5403T>A), TAT667CCT (5404T>C 5405A>C), AAG668CAA (5407A>C 5409G>A), CAA669TAT (5410C>T 5412A>T), AGC670AGA (5415C>A), CGG671AGA (5416C>A 5418G>A), CTT672TTA (5419C>T 5421T>A), GTG673CAT (5422G>C 5423T>A 5424G>T), ATG677GTA (5434A>G 5436G>A), GCT678TTG (5437G>T 5438C>T 5439T>G), AGA680TCT (5443A>T 5444G>C 5445A>T), GAA681TTT (5446G>T 5447A>T 5448A>T), TCT683ACC (5452T>A 5454T>C), TTT684TTC (5457T>C), GAT685AAA (5458G>A 5460T>A), ATA686ATT (5463A>T), CAT688AAT (5467C>A), GTT689ATT (5470G>A), TCT690AAA (5473T>A 5474C>A 5475T>A), GGT691GGA (5478T>A), CAA692TCT (5479C>T 5480A>C 5481A>T), AAA693GAT (5482A>G 5484A>T), CTA696TTT (5491C>T 5493A>T), GCT697GCA (5496T>A), ATC699TTC (5500A>T), ATG700TTA (5503A>T 5505G>A), ACT701TCA (5506A>T 5508T>A)								

Proteins

	Begin	End	Coverage	Score	Concordance	Matches	Identities	I/D/M/F*	Stop Codons
hypothetical protein (NP_043933.1)	298	701	39.8%	1127	58.3%	276 (97.9%)	149 (52.8%)	0/6/0/0	0

	Begin	End	Coverage	Score	Concordance	Matches	Identities	I/D/M/F*	Stop Codons
**NT**	**4295**	**5508**	**10.8%**	**246**	**15.2%**	**829 (97.9%)**	**485 (57.3%)**	**0/18**	
Protein mutations:	E299Q (4300G>C 4302A>G), L304M (4315C>A 4317T>G), G305K (4318G>A 4319G>A 4320T>G), I306L (4321A>T 4323C>A), P309A (4330C>G 4332T>A), K311R (4337A>G 4338G>A), S315N (4348T>A 4349C>A), E326K (4381G>A 4383G>A), I327Q (4384A>C 4385T>A 4386C>A), K340E (4423A>G), D343K (4432G>A 4434C>A), H344N (4435C>A), T345C (4438A>T 4439C>G 4440A>T), K346V (4441A>G 4442A>T 4443G>C), G347F (4444G>T 4445G>T 4446A>T), L351F (4456C>T 4458A>T), L352I (4459C>A), E356N (4471G>A 4473G>T), Q357N (4474C>A 4476A>T), L359I (4480C>A 4482T>A), Q360N (4483C>A 4485A>T), R361L (4486A>C 4487G>T), I362A (4489A>G 4490T>C), G363K (4492G>A 4493G>A), F367Y (4505T>A 4506T>C), Y368F (4508A>T 4509C>T), S370K (4513T>A 4514C>A 4515C>A), V380I (4543G>A), R381K (4546C>A 4547G>A 4548C>A), A383K (4552G>A 4553C>A 4554T>A), P384E (4555C>G 4556C>A), E385N (4558G>A 4560A>T), T386S (4561A>T 4563A>T), Q388A (4567C>G 4568A>C 4569G>T), C394T (4585T>A 4586G>C 4587T>A), Q396N (4591C>A 4593A>T), Q410N (4633C>A 4635A>T), A413Q (4642G>C 4643C>A 4644T>A), H418K (4657C>A 4659C>A), S422I (4670G>T 4671T>A), L423F (4672C>T 4674C>T), N425E (4678A>G 4680C>A), M426K (4682T>A), Y427N (4684T>A), P428_Q429del (4687_4692delCCACAG), C431L (4696T>C 4697G>T 4698T>A), A432I (4699G>A 4700C>T 4701T>C), V435I (4708G>A 4710T>A), I439L (4720A>C 4722C>A), V440I (4723G>A 4725C>A), F441C (4727T>G 4728C>T), K443N (4734A>T), T444S (4735A>T 4737T>A), E445Y (4738G>T 4740A>T), E446Q (4741G>C), G450T (4753G>A 4754G>C), V452L (4759G>C 4761C>T), I454Q (4765A>C 4766T>A 4767T>A), V455F (4768G>T), L456S (4771C>T 4772T>C 4773A>T), N457Q (4774A>C 4776C>A), R458L (4777A>T 4778G>T), K460V (4783A>G 4784A>T 4785A>T), A461E (4787C>A 4788C>A), L462N (4789C>A 4790T>A 4791A>T), D588Y (5167G>T 5169C>T), I593L (5182A>T 5184C>A), K595Q (5188A>C), A596T (5191G>A 5193A>T), K597D (5194A>G 5196A>T), P599N (5200C>A 5201C>A 5202A>T), G601_V604del (5206_5217delGGTAAAGAGGTA), A609T (5230G>A), K614N (5247A>T), P615E (5248C>G 5249C>A), A616T (5251G>A 5253T>A), K618T (5258A>C 5259G>T), N619R (5261A>G 5262T>G), H621L (5266C>T 5267A>T 5268T>A), S622T (5270G>C 5271T>A), I627L (5284A>T 5286C>A), S629A (5290T>G 5292C>T), I631V (5296A>G 5298T>A), K632R (5300A>G 5301G>A), A633G (5303C>G 5304A>C), K635R (5308A>C 5309A>G), A636K (5311G>A 5312C>A 5313T>G), R638S (5317A>T 5318G>C 5319A>T), Y640F (5324A>T 5325T>C), I641L (5326A>T 5328C>A), Y644K (5335T>A 5337T>A), K645Q (5338A>C), L647I (5344C>A 5346T>C), V648I (5347G>A 5349A>C), T650S (5353A>T 5355T>G), N654Q (5365A>C 5367T>G), A655V (5369C>T 5370T>A), A656S (5371G>T 5373T>A), Y657G (5374T>G 5375A>G), F658L (5377T>C 5379T>A), V659I (5380G>A 5382G>A), R660F (5383A>T 5384G>T 5385A>C), T661N (5387C>A 5388A>T), N662K (5391T>A), I663L (5392A>C), A664P (5395G>C), G665S (5398G>T 5399G>C), D666E (5403T>A), Y667P (5404T>C 5405A>C), K668Q (5407A>C 5409G>A), Q669Y (5410C>T 5412A>T), S670R (5415C>A), V673H (5422G>C 5423T>A 5424G>T), M677V (5434A>G 5436G>A), A678L (5437G>T 5438C>T 5439T>G), R680S (5443A>T 5444G>C 5445A>T), E681F (5446G>T 5447A>T 5448A>T), S683T (5452T>A 5454T>C), D685K (5458G>A 5460T>A), H688N (5467C>A), V689I (5470G>A), S690K (5473T>A 5474C>A 5475T>A), Q692S (5479C>T 5480A>C 5481A>T), K693D (5482A>G 5484A>T), L696F (5491C>T 5493A>T), I699F (5500A>T), M700L (5503A>T 5505G>A), T701S (5506A>T 5508T>A)								
Codon mutations:	ATC298ATT (4299C>T), GAA299CAG (4300G>C 4302A>G), CTA301CTT (4308A>T), AAG303AAA (4314G>A), CTT304ATG (4315C>A 4317T>G), GGT305AAG (4318G>A 4319G>A 4320T>G), ATC306TTA (4321A>T 4323C>A), CCT309GCA (4330C>G 4332T>A), AAG311AGA (4337A>G 4338G>A), CAT314CAC (4347T>C), TCC315AAC (4348T>A 4349C>A), TCA316AGT (4351T>A 4352C>G 4353A>T), CCA317CCC (4356A>C), GCC318GCG (4359C>G), AGG322AGA (4371G>A), AAT323AAC (4374T>C), GCC325GCG (4380C>G), GAG326AAA (4381G>A 4383G>A), ATC327CAA (4384A>C 4385T>A 4386C>A), GGC330GGA (4395C>A), GCA332GCT (4401A>T), AGA333CGA (4402A>C), ATT336ATA (4413T>A), AAG340GAG (4423A>G), TTA341CTT (4426T>C 4428A>T), GAC343AAA (4432G>A 4434C>A), CAT344AAT (4435C>A), ACA345TGT (4438A>T 4439C>G 4440A>T), AAG346GTC (4441A>G 4442A>T 4443G>C), GGA347TTT (4444G>T 4445G>T 4446A>T), GGC349GGA (4452C>A), TAC350TAT (4455C>T), CTA351TTT (4456C>T 4458A>T), CTT352ATT (4459C>A), CCT353CCA (4464T>A), AAG355AAA (4470G>A), GAG356AAT (4471G>A 4473G>T), CAA357AAT (4474C>A 4476A>T), CTG358CTT (4479G>T), CTT359ATA (4480C>A 4482T>A), CAA360AAT (4483C>A 4485A>T), AGA361CTA (4486A>C 4487G>T), ATC362GCC (4489A>G 4490T>C), GGA363AAA (4492G>A 4493G>A), GGT364GGA (4497T>A), AAG365AAA (4500G>A), TTT367TAC (4505T>A 4506T>C), TAC368TTT (4508A>T 4509C>T), TCT369TCA (4512T>A), TCC370AAA (4513T>A 4514C>A 4515C>A), TCT375AGT (4528T>A 4529C>G), CAG379CAA (4542G>A), GTA380ATA (4543G>A), CGC381AAA (4546C>A 4547G>A 4548C>A), CTT382CTA (4551T>A), GCT383AAA (4552G>A 4553C>A 4554T>A), CCA384GAA (4555C>G 4556C>A), GAA385AAT (4558G>A 4560A>T), ACA386TCT (4561A>T 4563A>T), ATT387ATA (4566T>A), CAG388GCT (4567C>G 4568A>C 4569G>T), CTA389TTA (4570C>T), ACC390ACA (4575C>A), GCT391GCA (4578T>A), AGC393AGT (4584C>T), TGT394ACA (4585T>A 4586G>C 4587T>A), CCC395CCA (4590C>A), CAA396AAT (4591C>A 4593A>T), CAC398CAT (4599C>T), TAC399TAT (4602C>T), GTC403GTA (4614C>A), CAA410AAT (4633C>A 4635A>T), GCT413CAA (4642G>C 4643C>A 4644T>A), ATT414ATC (4647T>C), TTC415TTT (4650C>T), AGG417AGA (4656G>A), CAC418AAA (4657C>A 4659C>A), GAC420GAT (4665C>T), AGT422ATA (4670G>T 4671T>A), CTC423TTT (4672C>T 4674C>T), AGC424TCT (4675A>T 4676G>C 4677C>T), AAC425GAA (4678A>G 4680C>A), ATG426AAG (4682T>A), TAT427AAT (4684T>A), CCA428_CAG429del (4687_4692delCCACAG), TGT431CTA (4696T>C 4697G>T 4698T>A), GCT432ATC (4699G>A 4700C>T 4701T>C), GTA433GTC (4704A>C), GTT435ATA (4708G>A 4710T>A), GAC437GAT (4716C>T), ATC438ATA (4719C>A), ATC439CTA (4720A>C 4722C>A), GTC440ATA (4723G>A 4725C>A), TTC441TGT (4727T>G 4728C>T), AGC442TCT (4729A>T 4730G>C 4731C>T), AAA443AAT (4734A>T), ACT444TCA (4735A>T 4737T>A), GAA445TAT (4738G>T 4740A>T), GAA446CAA (4741G>C), GAA447GAG (4746A>G), GGA450ACA (4753G>A 4754G>C), CAT451CAC (4758T>C), GTC452CTT (4759G>C 4761C>T), ATT454CAA (4765A>C 4766T>A 4767T>A), GTT455TTT (4768G>T), CTA456TCT (4771C>T 4772T>C 4773A>T), AAC457CAA (4774A>C 4776C>A), AGA458TTA (4777A>T 4778G>T), TGC459TGT (4782C>T), AAA460GTT (4783A>G 4784A>T 4785A>T), GCC461GAA (4787C>A 4788C>A), CTA462AAT (4789C>A 4790T>A 4791A>T), TCA586..C (5163A>C), GAC588TAT (5167G>T 5169C>T), ATC593TTA (5182A>T 5184C>A), CTC594TTA (5185C>T 5187C>A), AAA595CAA (5188A>C), GCA596ACT (5191G>A 5193A>T), AAA597GAT (5194A>G 5196A>T), CTT598CTA (5199T>A), CCA599AAT (5200C>A 5201C>A 5202A>T), GGT601_GTA604del (5206_5217delGGTAAAGAGGTA), ATA605ATT (5220A>T), TGC606TGT (5223C>T), AGG607AGA (5226G>A), GCT609ACT (5230G>A), TCA610AGT (5233T>A 5234C>G 5235A>T), AAA614AAT (5247A>T), CCA615GAA (5248C>G 5249C>A), GCT616ACA (5251G>A 5253T>A), GAG617GAA (5256G>A), AAG618ACT (5258A>C 5259G>T), AAT619AGG (5261A>G 5262T>G), CAT621TTA (5266C>T 5267A>T 5268T>A), AGT622ACA (5270G>C 5271T>A), AAG625AAA (5280G>A), ATC627TTA (5284A>T 5286C>A), CTT628CTA (5289T>A), TCC629GCT (5290T>G 5292C>T), ATT630ATA (5295T>A), ATT631GTA (5296A>G 5298T>A), AAG632AGA (5300A>G 5301G>A), GCA633GGC (5303C>G 5304A>C), ATC634ATA (5307C>A), AAA635CGA (5308A>C 5309A>G), GCT636AAG (5311G>A 5312C>A 5313T>G), AGA638TCT (5317A>T 5318G>C 5319A>T), GCT639GCA (5322T>A), TAT640TTC (5324A>T 5325T>C), ATC641TTA (5326A>T 5328C>A), CTT642CTA (5331T>A), CCT643CCA (5334T>A), TAT644AAA (5335T>A 5337T>A), AAA645CAA (5338A>C), TTT646TTC (5343T>C), CTT647ATC (5344C>A 5346T>C), GTA648ATC (5347G>A 5349A>C), AGG649AGA (5352G>A), ACT650TCG (5353A>T 5355T>G), GAT651GAC (5358T>C), ACT653ACG (5364T>G), AAT654CAG (5365A>C 5367T>G), GCT655GTA (5369C>T 5370T>A), GCT656TCA (5371G>T 5373T>A), TAT657GGT (5374T>G 5375A>G), TTT658CTA (5377T>C 5379T>A), GTG659ATA (5380G>A 5382G>A), AGA660TTC (5383A>T 5384G>T 5385A>C), ACA661AAT (5387C>A 5388A>T), AAT662AAA (5391T>A), ATT663CTT (5392A>C), GCA664CCA (5395G>C), GGT665TCT (5398G>T 5399G>C), GAT666GAA (5403T>A), TAT667CCT (5404T>C 5405A>C), AAG668CAA (5407A>C 5409G>A), CAA669TAT (5410C>T 5412A>T), AGC670AGA (5415C>A), CGG671AGA (5416C>A 5418G>A), CTT672TTA (5419C>T 5421T>A), GTG673CAT (5422G>C 5423T>A 5424G>T), ATG677GTA (5434A>G 5436G>A), GCT678TTG (5437G>T 5438C>T 5439T>G), AGA680TCT (5443A>T 5444G>C 5445A>T), GAA681TTT (5446G>T 5447A>T 5448A>T), TCT683ACC (5452T>A 5454T>C), TTT684TTC (5457T>C), GAT685AAA (5458G>A 5460T>A), ATA686ATT (5463A>T), CAT688AAT (5467C>A), GTT689ATT (5470G>A), TCT690AAA (5473T>A 5474C>A 5475T>A), GGT691GGA (5478T>A), CAA692TCT (5479C>T 5480A>C 5481A>T), AAA693GAT (5482A>G 5484A>T), CTA696TTT (5491C>T 5493A>T), GCT697GCA (5496T>A), ATC699TTC (5500A>T), ATG700TTA (5503A>T 5505G>A), ACT701TCA (5506A>T 5508T>A)								

*: Inserts / Deletes / Misaligned / Frameshifts

Analysis details

This analysis was performed with panviral2.64

NGS Details (UN60): Badnavirus rutilanscamelliae

Assembly

**Coverage Length**	297 (1 contig(s))
**Depth Of Coverage**	15.0
**Number Of Reads**	44
**Reads Per Million**	0.93 rpm (after QC)
**Ambiguities**	0
**Assembly Method**	de novo + reference guided assembly
**Consensus Caller**	Bcf Tools

Coverage Map

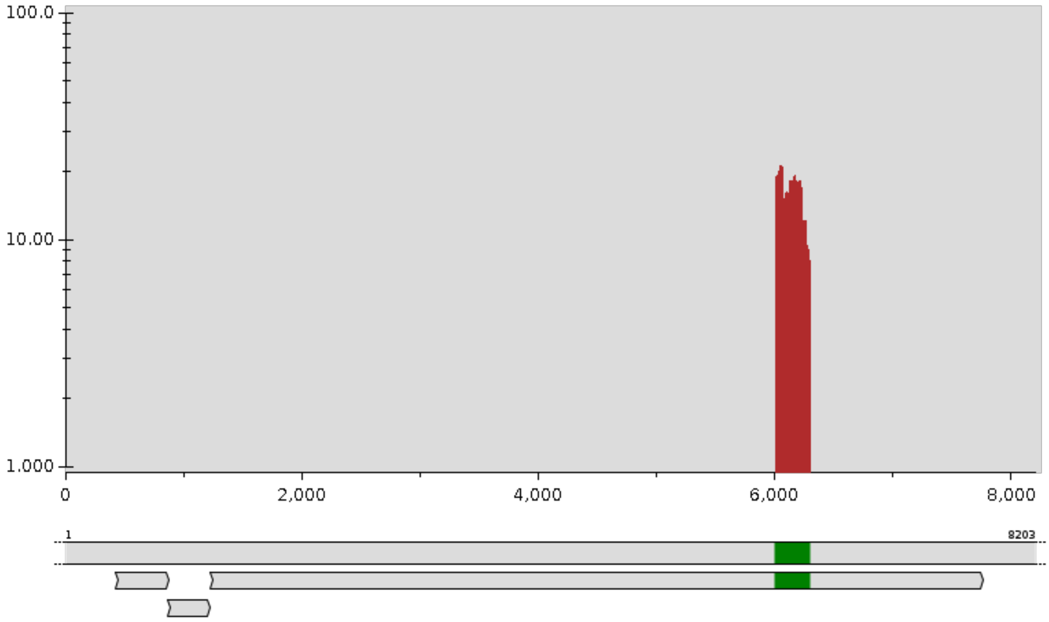

Assignment

**Type**	Badnavirus rutilanscamelliae (Taxonomy ID: 3047719)
**Reference Genome**	NC_055598.1
**NT Identity (%)**	54.0
**AA Identity (%)**	45.0
**Number Of Stop Codons**	0
**Number Of CDS**	3

Alignment

**Alignment Score**	42.0 (NT) + 327.0 (AA) = 369.0
**Concordance (%)**	28.4942

Alignment Method	Global, seeded, nucleotide + amino acids (AGA)

Genome Region

Sequence starts at position 6010 and ends at position 6306 relative to NC_055598.1 reference sequence.

Alignment Detailed Statistics

	Begin	End	Coverage	Score	Concordance	Matches	Identities	I/D/M/F*	Stop Codons
**NT**	**6010**	**6306**	**3.6%**	**42**	**7.1%**	**297 (99.0%)**	**162 (54.0%)**	**3/0**	

Mutations: 6013C>T, 6019T>C, 6020T>A, 6022G>A, 6027C>A, 6028A>T, 6031G>A, 6034C>G, 6035T>C, 6036T>A, 6037A>T, 6040T>C, 6043A>G, 6045G>T, 6046G>C, 6047C>A, 6048T>A, 6052T>G, 6058A>G, 6064G>C, 6069A>C, 6070G>A, 6082A>T, 6083G>A, 6084T>C, 6085A>T, 6088T>C, 6092A>G, 6093G>C, 6094G>C, 6095A>T, 6096A>T, 6097G>C, 6101G>A, 6103C>T, 6105G>A, 6106A>G, 6107A>G, 6109C>T, 6112T>C, 6113A>G, 6115T>A, 6118A>T, 6120A>T, 6121C>T, 6122G>C, 6123C>T, 6124T>G, 6125G>A, 6127_6128insAAG, 6131A>G, 6134G>T, 6135C>T, 6136T>G, 6139G>A, 6141A>T, 6143A>T, 6154C>A, 6157C>T, 6158A>G, 6160A>T, 6162T>A, 6166C>T, 6167C>T, 6168C>T, 6169A>G, 6173C>G, 6174C>T, 6175T>A, 6176G>A, 6177A>G, 6178A>T, 6180C>A, 6181C>T, 6185A>G, 6186A>T, 6187A>T, 6188A>C, 6196G>A, 6197G>A, 6198C>G, 6199C>G, 6200C>G, 6201A>T, 6202A>T, 6203T>G, 6206G>T, 6208G>T, 6213T>C, 6214A>T, 6215T>C, 6216G>T, 6221G>T, 6222A>G, 6223A>T, 6227G>T, 6228G>C, 6229G>A, 6232T>C, 6233G>T, 6234T>A, 6235C>T, 6236T>G, 6237T>C, 6238G>T, 6240G>A, 6241T>G, 6242C>A, 6243C>G, 6244A>G, 6245A>T, 6251A>T, 6252T>G, 6253G>T, 6254A>T, 6255A>C, 6256G>C, 6257G>T, 6259G>T, 6261C>G, 6266G>C, 6268C>A, 6272A>G, 6277C>G, 6279T>A, 6280T>C, 6283G>A, 6286C>T, 6287T>C, 6288C>A, 6289A>C, 6291C>T, 6298A>C, 6301T>A, 6303G>A, 6304G>A

CDS

	Begin	End	Coverage	Score	Concordance	Matches	Identities	I/D/M/F*	Stop Codons
KM754_gp3	1597	1695	4.5%	327	44.8%	99 (99.0%)	45 (45.0%)	1/0/0/0	0

Protein mutations: W1600R (6020T>A 6022G>A), P1602H (6027C>A 6028A>T), L1605H (6035T>C 6036T>A 6037A>T), W1608F (6045G>T 6046G>C), L1609K (6047C>A 6048T>A), K1616T (6069A>C 6070G>A), V1621T (6083G>A 6084T>C 6085A>T), R1624A (6092A>G 6093G>C 6094G>C), K1625F (6095A>T 6096A>T 6097G>C), D1627N (6101G>A 6103C>T), R1628Q (6105G>A 6106A>G), I1629V (6107A>G 6109C>T), N1631E (6113A>G 6115T>A), Y1633F (6120A>T 6121C>T), A1634L (6122G>C 6123C>T 6124T>G), G1635R (6125G>A), G1635_F1636insK (6127_6128insAAG), I1637V (6131A>G), A1638L (6134G>T 6135C>T 6136T>G), Y1640F (6141A>T), I1641F (6143A>T), I1646V (6158A>G 6160A>T), F1647Y (6162T>A), P1649L (6167C>T 6168C>T 6169A>G), P1651V (6173C>G 6174C>T 6175T>A), E1652S (6176G>A 6177A>G 6178A>T), A1653D (6180C>A 6181C>T), K1655V (6185A>G 6186A>T 6187A>T), K1656Q (6188A>C), A1659R (6197G>A 6198C>G 6199C>G), Q1660V (6200C>G 6201A>T 6202A>T), F1661V (6203T>G), V1662F (6206G>T 6208G>T), I1664T (6213T>C 6214A>T), C1665L (6215T>C 6216G>T), E1667C (6221G>T 6222A>G 6223A>T), G1669S (6227G>T 6228G>C 6229G>A), V1671Y (6233G>T 6234T>A 6235C>T), L1672A (6236T>G 6237T>C 6238G>T), S1673K (6240G>A 6241T>G), P1674R (6242C>A 6243C>G 6244A>G), T1675S (6245A>T), M1677C (6251A>T 6252T>G 6253G>T), K1678S (6254A>T 6255A>C 6256G>C), V1679F (6257G>T 6259G>T), A1680G (6261C>G), A1682P (6266G>C 6268C>A), I1684V (6272A>G), D1685E (6277C>G), F1686Y (6279T>A 6280T>C), S1689H (6287T>C 6288C>A 6289A>C), T1690I (6291C>T), N1693K (6301T>A), G1694E (6303G>A 6304G>A)

Codon mutations: ACC1597ACT (6013C>T), TTT1599TTC (6019T>C), TGG1600AGA (6020T>A 6022G>A), CCA1602CAT (6027C>A 6028A>T), GTG1603GTA (6031G>A), GGC1604GGG (6034C>G), TTA1605CAT (6035T>C 6036T>A 6037A>T), TAT1606TAC (6040T>C), GAA1607GAG (6043A>G), TGG1608TTC (6045G>T 6046G>C), CTG1609AAG (6047C>A 6048T>A), GTT1610GTG (6052T>G), CCA1612CCG (6058A>G), GGG1614GGC (6064G>C), AAG1616ACA (6069A>C 6070G>A), GCA1620GCT (6082A>T), GTA1621ACT (6083G>A 6084T>C 6085A>T), TTT1622TTC (6088T>C), AGG1624GCC (6092A>G 6093G>C 6094G>C), AAG1625TTC (6095A>T 6096A>T 6097G>C), GAC1627AAT (6101G>A 6103C>T), CGA1628CAG (6105G>A 6106A>G), ATC1629GTT (6107A>G 6109C>T), TTT1630TTC (6112T>C), AAT1631GAA (6113A>G 6115T>A), CCA1632CCT (6118A>T), TAC1633TTT (6120A>T 6121C>T), GCT1634CTG (6122G>C 6123C>T 6124T>G), GGA1635AGA (6125G>A), GGA1635_TTT1636insAAG (6127_6128insAAG), ATT1637GTT (6131A>G), GCT1638TTG (6134G>T 6135C>T 6136T>G), GTG1639GTA (6139G>A), TAT1640TTT (6141A>T), ATT1641TTT (6143A>T), ATC1644ATA (6154C>A), CTC1645CTT (6157C>T), ATA1646GTT (6158A>G 6160A>T), TTC1647TAC (6162T>A), AGC1648AGT (6166C>T), CCA1649TTG (6167C>T 6168C>T 6169A>G), CCT1651GTA (6173C>G 6174C>T 6175T>A), GAA1652AGT (6176G>A 6177A>G 6178A>T), GCC1653GAT (6180C>A 6181C>T), AAA1655GTT (6185A>G 6186A>T 6187A>T), AAG1656CAG (6188A>C), TTG1658TTA (6196G>A), GCC1659AGG (6197G>A 6198C>G 6199C>G), CAA1660GTT (6200C>G 6201A>T 6202A>T), TTC1661GTC (6203T>G), GTG1662TTT (6206G>T 6208G>T), ATA1664ACT (6213T>C 6214A>T), TGC1665CTC (6215T>C 6216G>T), GAA1667TGT (6221G>T 6222A>G 6223A>T), GGG1669TCA (6227G>T 6228G>C 6229G>A), CTT1670CTC (6232T>C), GTC1671TAT (6233G>T 6234T>A 6235C>T), TTG1672GCT (6236T>G 6237T>C 6238G>T), AGT1673AAG (6240G>A 6241T>G), CCA1674AGG (6242C>A 6243C>G 6244A>G), ACA1675TCA (6245A>T), ATG1677TGT (6251A>T 6252T>G 6253G>T), AAG1678TCC (6254A>T 6255A>C 6256G>C), GTG1679TTT (6257G>T 6259G>T), GCC1680GGC (6261C>G), GCC1682CCA (6266G>C 6268C>A), ATT1684GTT (6272A>G), GAC1685GAG (6277C>G), TTT1686TAC (6279T>A 6280T>C), TTG1687TTA (6283G>A), GGC1688GGT (6286C>T), TCA1689CAC (6287T>C 6288C>A 6289A>C), ACA1690ATA (6291C>T), ACA1692ACC (6298A>C), AAT1693AAA (6301T>A), GGG1694GAA (6303G>A 6304G>A)

Proteins

	Begin	End	Coverage	Score	Concordance	Matches	Identities	I/D/M/F*	Stop Codons
polyprotein (YP_010087856.1)	1597	1695	4.5%	327	44.8%	99 (99.0%)	45 (45.0%)	1/0/0/0	0

Protein mutations: W1600R (6020T>A 6022G>A), P1602H (6027C>A 6028A>T), L1605H (6035T>C 6036T>A 6037A>T), W1608F (6045G>T 6046G>C), L1609K (6047C>A 6048T>A), K1616T (6069A>C 6070G>A), V1621T (6083G>A 6084T>C 6085A>T), R1624A (6092A>G 6093G>C 6094G>C), K1625F (6095A>T 6096A>T 6097G>C), D1627N (6101G>A 6103C>T), R1628Q (6105G>A 6106A>G), I1629V (6107A>G 6109C>T), N1631E (6113A>G 6115T>A), Y1633F (6120A>T 6121C>T), A1634L (6122G>C 6123C>T 6124T>G), G1635R (6125G>A), G1635_F1636insK (6127_6128insAAG), I1637V (6131A>G), A1638L (6134G>T 6135C>T 6136T>G), Y1640F (6141A>T), I1641F (6143A>T), I1646V (6158A>G 6160A>T), F1647Y (6162T>A), P1649L (6167C>T 6168C>T 6169A>G), P1651V (6173C>G 6174C>T 6175T>A), E1652S (6176G>A 6177A>G 6178A>T), A1653D (6180C>A 6181C>T), K1655V (6185A>G 6186A>T 6187A>T), K1656Q (6188A>C), A1659R (6197G>A 6198C>G 6199C>G), Q1660V (6200C>G 6201A>T 6202A>T), F1661V (6203T>G), V1662F (6206G>T 6208G>T), I1664T (6213T>C 6214A>T), C1665L (6215T>C 6216G>T), E1667C (6221G>T 6222A>G 6223A>T), G1669S (6227G>T 6228G>C 6229G>A), V1671Y (6233G>T 6234T>A 6235C>T), L1672A (6236T>G 6237T>C 6238G>T), S1673K (6240G>A 6241T>G), P1674R (6242C>A 6243C>G 6244A>G), T1675S (6245A>T), M1677C (6251A>T 6252T>G 6253G>T), K1678S (6254A>T 6255A>C 6256G>C), V1679F (6257G>T 6259G>T), A1680G (6261C>G), A1682P (6266G>C 6268C>A), I1684V (6272A>G), D1685E (6277C>G), F1686Y (6279T>A 6280T>C), S1689H (6287T>C 6288C>A 6289A>C), T1690I (6291C>T), N1693K (6301T>A), G1694E (6303G>A 6304G>A)

Codon mutations: ACC1597ACT (6013C>T), TTT1599TTC (6019T>C), TGG1600AGA (6020T>A 6022G>A), CCA1602CAT (6027C>A 6028A>T), GTG1603GTA (6031G>A), GGC1604GGG (6034C>G), TTA1605CAT (6035T>C 6036T>A 6037A>T), TAT1606TAC (6040T>C), GAA1607GAG (6043A>G), TGG1608TTC (6045G>T 6046G>C), CTG1609AAG (6047C>A 6048T>A), GTT1610GTG (6052T>G), CCA1612CCG (6058A>G), GGG1614GGC (6064G>C), AAG1616ACA (6069A>C 6070G>A), GCA1620GCT (6082A>T), GTA1621ACT (6083G>A 6084T>C 6085A>T), TTT1622TTC (6088T>C), AGG1624GCC (6092A>G 6093G>C 6094G>C), AAG1625TTC (6095A>T 6096A>T 6097G>C), GAC1627AAT (6101G>A 6103C>T), CGA1628CAG (6105G>A 6106A>G), ATC1629GTT (6107A>G 6109C>T), TTT1630TTC (6112T>C), AAT1631GAA (6113A>G 6115T>A), CCA1632CCT (6118A>T), TAC1633TTT (6120A>T 6121C>T), GCT1634CTG (6122G>C 6123C>T 6124T>G), GGA1635AGA (6125G>A), GGA1635_TTT1636insAAG (6127_6128insAAG), ATT1637GTT (6131A>G), GCT1638TTG (6134G>T 6135C>T 6136T>G), GTG1639GTA (6139G>A), TAT1640TTT (6141A>T), ATT1641TTT (6143A>T), ATC1644ATA (6154C>A), CTC1645CTT (6157C>T), ATA1646GTT (6158A>G 6160A>T), TTC1647TAC (6162T>A), AGC1648AGT (6166C>T), CCA1649TTG (6167C>T 6168C>T 6169A>G), CCT1651GTA (6173C>G 6174C>T 6175T>A), GAA1652AGT (6176G>A 6177A>G 6178A>T), GCC1653GAT (6180C>A 6181C>T), AAA1655GTT (6185A>G 6186A>T 6187A>T), AAG1656CAG (6188A>C), TTG1658TTA (6196G>A), GCC1659AGG (6197G>A 6198C>G 6199C>G), CAA1660GTT (6200C>G 6201A>T 6202A>T), TTC1661GTC (6203T>G), GTG1662TTT (6206G>T 6208G>T), ATA1664ACT (6213T>C 6214A>T), TGC1665CTC (6215T>C 6216G>T), GAA1667TGT (6221G>T 6222A>G 6223A>T), GGG1669TCA (6227G>T 6228G>C 6229G>A), CTT1670CTC (6232T>C), GTC1671TAT (6233G>T 6234T>A 6235C>T), TTG1672GCT (6236T>G 6237T>C 6238G>T), AGT1673AAG (6240G>A 6241T>G), CCA1674AGG (6242C>A 6243C>G 6244A>G), ACA1675TCA (6245A>T), ATG1677TGT (6251A>T 6252T>G 6253G>T), AAG1678TCC (6254A>T 6255A>C 6256G>C), GTG1679TTT (6257G>T 6259G>T), GCC1680GGC (6261C>G), GCC1682CCA (6266G>C 6268C>A), ATT1684GTT (6272A>G), GAC1685GAG (6277C>G), TTT1686TAC (6279T>A 6280T>C), TTG1687TTA (6283G>A), GGC1688GGT (6286C>T), TCA1689CAC (6287T>C 6288C>A 6289A>C), ACA1690ATA (6291C>T), ACA1692ACC (6298A>C), AAT1693AAA (6301T>A), GGG1694GAA (6303G>A 6304G>A)

*: Inserts / Deletes / Misaligned / Frameshifts

Analysis details

This analysis was performed with panviral2.64

NGS Details (UN60): Synechococcus phage S-SRM01

Assembly

**Coverage Length**	207 (1 contig(s))
**Depth Of Coverage**	25.2
**Number Of Reads**	43
**Reads Per Million**	0.91 rpm (after QC)
**Ambiguities**	0
**Assembly Method**	de novo + reference guided assembly
**Consensus Caller**	Bcf Tools

Coverage Map

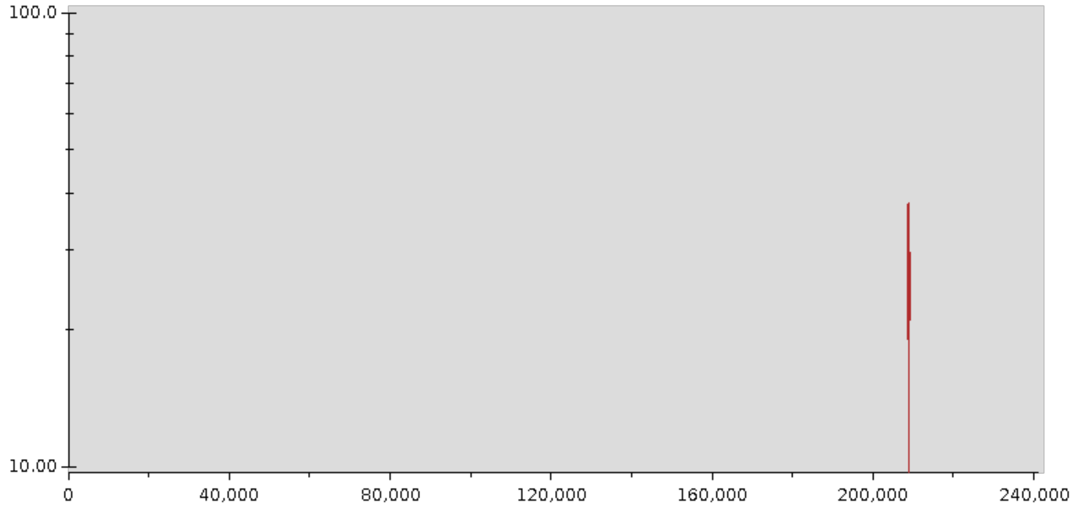

Assignment

**Type**	Synechococcus phage S-SRM01 (Taxonomy ID: 2781608)
**Reference Genome**	NC_070963.1
**NT Identity (%)**	76.8116
**AA Identity (%)**	88.4058
**Number Of Stop Codons**	0
**Number Of CDS**	353

Alignment

**Alignment Score**	222.0 (NT) + 433.0 (AA) = 655.0
**Concordance (%)**	74.3473
**Alignment Method**	Local, heuristic, nucleotide (BLASTN)

Genome Region

Sequence starts at position 208802 and ends at position 209008 relative to NC_070963.1 reference sequence.

Alignment Detailed Statistics

	Begin	End	Coverage	Score	Concordance	Matches	Identities	I/D/M/F*	Stop Codons
NT	208802	209008	0.1%	222	53.6%	207 (100%)	159 (76.8%)	0/0	

Mutations: 208807T>C, 208809G>A, 208812T>A, 208816A>T, 208817G>C, 208821A>T, 208830C>T, 208832A>T, 208837C>A, 208842G>A, 208851A>T, 208866G>T, 208875C>A, 208878C>T, 208881T>C, 208885C>T, 208887T>A, 208890A>C, 208893T>G, 208902C>A, 208908T>C, 208911T>C, 208914T>C, 208917G>A, 208932C>T, 208938T>C, 208939C>T, 208944T>A, 208950G>T, 208951T>A, 208952C>G, 208953A>T, 208954C>T, 208957G>A, 208959T>C, 208960C>A, 208962T>G, 208971T>A, 208976C>A, 208983A>T, 208984C>G, 208985A>C, 208986G>T, 208990T>G, 208992T>A, 209000A>G, 209001G>A, 209004T>C

*: Inserts / Deletes / Misaligned / Frameshifts

Analysis details

This analysis was performed with panviral2.64

NGS Details (UN60): Marseillevirus marseillevirus

Assembly

**Coverage Length**	113 (1 contig(s))
**Depth Of Coverage**	25.6
**Number Of Reads**	38
**Reads Per Million**	0.81 rpm (after QC)
**Ambiguities**	0
**Assembly Method**	de novo + reference guided assembly
**Consensus Caller**	Bcf Tools

Coverage Map

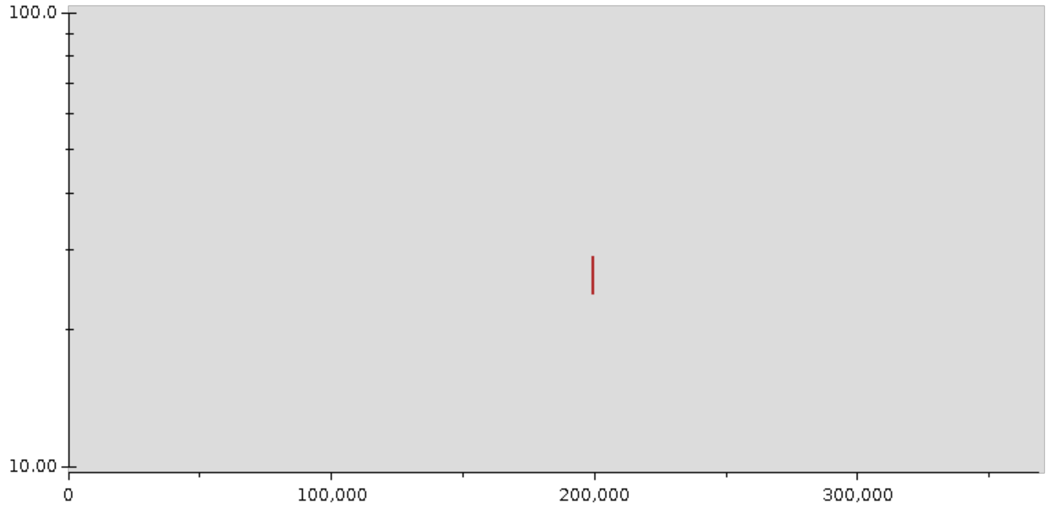

Assignment

**Type**	Marseillevirus marseillevirus (Taxonomy ID: 694581)
**Reference Genome**	NC_013756.1
**NT Identity (%)**	81.4159
**AA Identity (%)**	94.7368
**Number Of Stop Codons**	0
**Number Of CDS**	428

Alignment

**Alignment Score**	142.0 (NT) + 245.0 (AA) = 387.0
**Concordance (%)**	81.9915
**Alignment Method**	Local, heuristic, nucleotide (BLASTN)

Genome Region

Sequence starts at position 199409 and ends at position 199521 relative to NC_013756.1 reference sequence.

Alignment Detailed Statistics

	Begin	End	Coverage	Score	Concordance	Matches	Identities	I/D/M/F*	Stop Codons
**NT**	**199409**	**199521**	**0.1%**	**142**	**62.8%**	**113 (100%)**	**92 (81.4%)**	**0/0**	

Mutations: 199411A>C, 199414T>A, 199416T>G, 199423A>G, 199432A>G, 199444T>C, 199449C>T, 199459A>C, 199462C>A, 199464A>G, 199465A>G, 199470T>G, 199471T>G, 199474A>G, 199477T>C, 199480C>G, 199489T>G, 199495G>A, 199501G>C, 199503G>A, 199516C>T

*: Inserts / Deletes / Misaligned / Frameshifts

Analysis details

This analysis was performed with panviral2.64

NGS Details (UN60): Petuvirus venapetuniae

Assembly

**Coverage Length**	939 (2 contig(s))
**Depth Of Coverage**	5.1
**Number Of Reads**	36
**Reads Per Million**	0.76 rpm (after QC)
**Ambiguities**	0
**Assembly Method**	de novo + reference guided assembly
**Consensus Caller**	Bcf Tools

Coverage Map

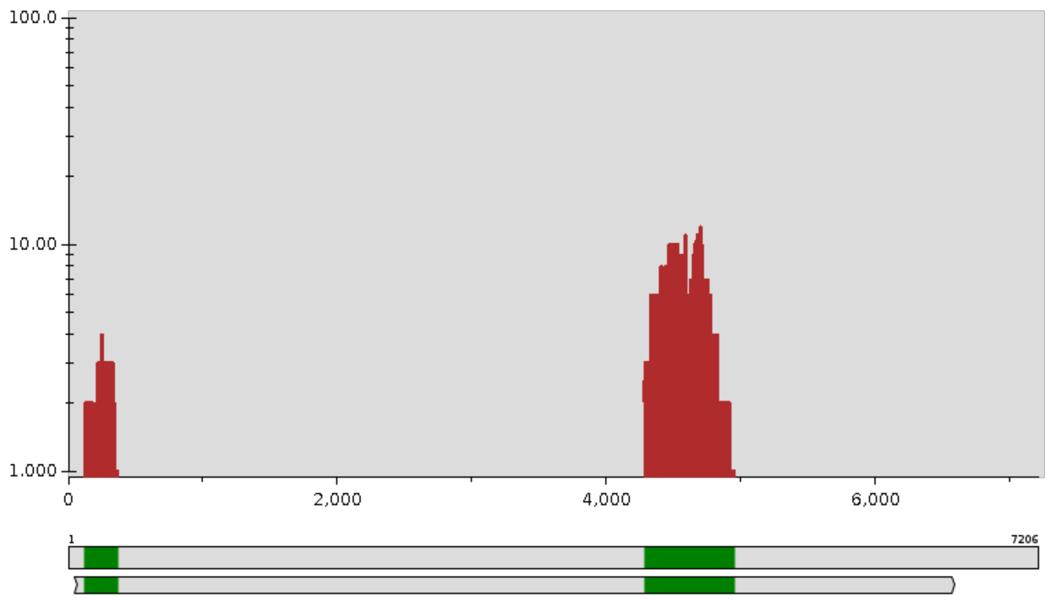

Assignment

**Type**	Petuvirus venapetuniae (Taxonomy ID: 3048265)
**Reference Genome**	NC_001839.2
**NT Identity (%)**	58.5729
**AA Identity (%)**	53.6741
**Number Of Stop Codons**	3
**Number Of CDS**	1

Alignment

**Alignment Score**	304.0 (NT) + 1191.0 (AA) = 1495.0
**Concordance (%)**	37.4218

Alignment Method	Global, seeded, nucleotide + amino acids (AGA)

Genome Region

Sequence starts at position 116 and ends at position 4957 relative to NC_001839.2 reference sequence.

Alignment Detailed Statistics

	Begin	End	Coverage	Score	Concordance	Matches	Identities	I/D/M/F*	Stop Codons
**NT**	**116**	**4957**	**13.0%**	**304**	**16.3%**	**936 (99.4%)**	**550 (58.4%)**	**3/3**	
Mutations:	116A>T, 119C>T, 121C>G, 122T>A, 126G>A, 128T>C, 129T>C, 131G>T, 134T>A, 136A>G, 137T>A, 149C>A, 150C>T, 152T>G, 159G>A, 161A>T, 165A>G, 170T>A, 171A>C, 172A>C, 173A>C, 177A>G, 179C>G, 186A>G, 187T>C, 192G>T, 193A>C, 194T>C, 197C>G, 199A>C, 200T>C, 201C>T, 203T>A, 207T>C, 209G>T, 210T>A, 221C>T, 233A>C, 238G>A, 239A>T, 241C>A, 242C>A, 243G>T, 245T>A, 246A>T, 248C>G, 251T>A, 255T>A, 256C>T, 257T>C, 260G>T, 263C>T, 265G>A, 269A>C, 278G>A, 279T>A, 280C>A, 281T>C, 286C>T, 287T>C, 288C>A, 289G>T, 290C>A, 291C>G, 292A>G, 293T>A, 296G>A, 298A>G, 310C>T, 317C>A, 321T>C, 323A>C, 324G>A, 329G>A, 333C>T, 336G>A, 338C>A, 339T>C, 344T>A, 347A>T, 348G>C, 349G>C, 359G>A, 362T>C, 363G>T, 366A>T, 4287C>A, 4289A>T, 4293G>A, 4294A>G, 4298C>T, 4302G>A, 4304T>A, 4307A>T, 4309A>C, 4310A>T, 4314G>A, 4315C>G, 4316A>C, 4320G>T, 4321A>C, 4325T>C, 4328T>C, 4334C>T, 4337C>T, 4340G>A, 4342G>C, 4343G>T, 4344T>G, 4346A>T, 4349G>A, 4350C>A, 4354T>A, 4355G>A, 4361A>C, 4362A>T, 4363A>C, 4364A>C, 4365T>C, 4366T>C, 4367A>T, 4376C>T, 4379A>T, 4386C>A, 4388A>G, 4391A>T, 4397C>T, 4398C>T, 4399A>C, 4401T>G, 4403C>T, 4404C>T, 4406C>A, 4407C>A, 4410G>T, 4411A>G, 4412T>G, 4413G>A, 4414A>T, 4415C>T, 4417A>G, 4418G>A, 4420T>A, 4421T>C, 4424C>G, 4430A>T, 4436G>A, 4437C>A, 4439C>G, 4440A>G, 4441C>A, 4442A>T, 4445C>T, 4448C>G, 4449T>A, 4450C>A, 4451T>A, 4452C>A, 4453A>G, 4454C>A, 4455T>A, 4456T>C, 4457A>T, 4459C>T, 4460C>T, 4464G>A, 4466A>C, 4469G>T, 4470C>A, 4472C>T, 4474T>A, 4475T>C, 4476T>A, 4477C>G, 4478A>T, 4481G>A, 4488C>A, 4490T>G, 4493G>A, 4499A>T, 4502T>C, 4509T>A, 4511A>T, 4512G>C, 4513G>A, 4517C>T, 4518C>T, 4519A>C, 4520T>G, 4521C>A, 4522C>A, 4523T>A, 4526T>A, 4529G>T, 4531G>A, 4533C>T, 4534C>A, 4535A>T, 4541A>T, 4543G>C, 4544G>T, 4547T>C, 4548T>A, 4549G>A, 4551A>G, 4553T>C, 4556T>C, 4557G>T, 4558A>T, 4559T>C, 4560A>G, 4565T>A, 4567T>A, 4569C>G, 4577G>T, 4580C>T, 4586A>C, 4589C>T, 4592T>A, 4593C>T, 4595T>A, 4600C>A, 4601A>T, 4611T>G, 4612T>A, 4613G>A, 4616C>T, 4622A>C, 4624C>T, 4625C>T, 4630T>A, 4631C>T, 4632A>T, 4633A>C, 4634A>T, 4637C>T, 4640C>T, 4641C>A, 4643G>T, 4645C>A, 4650C>A, 4651T>G, 4652T>C, 4653T>A, 4654T>A, 4655T>C, 4656T>A, 4657C>T, 4658A>C, 4659G>T, 4662T>A, 4664G>T, 4667C>G, 4670C>T, 4680A>G, 4682C>T, 4683C>T, 4685T>G, 4686C>A, 4688T>A, 4697A>G, 4698A>G, 4699C>A, 4700A>T, 4701C>A, 4703G>T, 4706A>T, 4707G>T, 4708A>C, 4712C>T, 4713A>T, 4715A>T, 4719T>C, 4720T>A, 4721G>T, 4722C>T, 4724T>A, 4727C>T, 4728C>A, 4729A>C, 4730G>C, 4734A>T, 4736A>C, 4738G>A, 4739T>A, 4740T>A, 4742G>T, 4745T>C, 4750A>G, 4752T>A, 4753T>A, 4757A>T, 4758G>C, 4760C>T, 4761A>G, 4763G>C, 4764T>A, 4766A>C, 4767T>A, 4768C>G, 4769G>T, 4772A>T, 4775A>G, 4778G>A, 4783T>A, 4784C>A, 4785C>T, 4787T>G, 4788G>T, 4789C>T, 4795A>C, 4796T>A, 4802T>C, 4803C>A, 4804A>G, 4808C>T, 4809T>C, 4811A>T, 4814A>G, 4815A>C, 4816T>A, 4817G>T, 4823T>A, 4824G>T, 4825C>A, 4827G>C, 4829T>A, 4835T>A, 4837T>A, 4839T>A, 4840C>A, 4844T>C, 4845G>A, 4846C>T, 4847T>C, 4848G>T, 4850T>C, 4851_4853delCAC, 4855T>G, 4856C>A, 4857T>G, 4859T>C, 4860T>A, 4862G>C, 4866C>T, 4868C>T, 4869C>T, 4870A>C, 4871A>T, 4871_4872insGGT, 4874G>A, 4877T>C, 4881G>A, 4884A>G, 4885C>A, 4888A>T, 4889T>C, 4890C>A, 4891T>C, 4892C>T, 4893T>G, 4894C>A, 4895G>C, 4896G>A, 4897T>A, 4900A>C, 4901G>T, 4905A>T, 4910A>G, 4911C>A, 4912A>G, 4916T>C, 4917C>T, 4919T>G, 4922G>A, 4924T>G, 4925T>C, 4926G>C, 4928T>C, 4935A>G, 4937C>T, 4938A>G, 4939G>C, 4943T>C, 4946T>C, 4952A>T, 4953G>A, 4955G>A								
**CDS**									
PVCVgp1	23	1636	14.4%	1191	55.2%	312 (99.4%)	168 (53.5%)	1/1/0/0	3
Protein mutations:	S24* (121C>G 122T>A), A26T (126G>A 128T>C), N29R (136A>G 137T>A), Y33* (149C>A), V37I (159G>A 161A>T), I39V (165A>G), K41P (171A>C 172A>C 173A>C), N43E (177A>G 179C>G), I46A (186A>G 187T>C), D48S (192G>T 193A>C 194T>C), Y50S (199A>C 200T>C), L54I (210T>A), R63N (238G>A 239A>T), S64* (241C>A 242C>A), V65L (243G>T 245T>A), T66S (246A>T 248C>G), S69I (255T>A 256C>T 257T>C), Q70H (260G>T), R72H (265G>A), S77N (279T>A 280C>A 281T>C), P79L (286C>T 287T>C), R80I (288C>A 289G>T 290C>A), H81G (291C>G 292A>G 293T>A), K83R (298A>G), A87V (310C>T), D92N (324G>A), P95S (333C>T), V96I (336G>A 338C>A), F97L (339T>C), G100P (348G>C 349G>C), V105F (363G>T), T106S (366A>T), L1413I (4287C>A 4289A>T), E1415R (4293G>A 4294A>G), D1418K (4302G>A 4304T>A), Q1420P (4309A>C 4310A>T), A1422S (4314G>A 4315C>G 4316A>C), E1424S (4320G>T 4321A>C), R1431T (4342G>C 4343G>T), S1432A (4344T>G 4346A>T), Q1434K (4350C>A), V1435E (4354T>A 4355G>A), K1438S (4362A>T 4363A>C 4364A>C), L1439P (4365T>C 4366T>C 4367A>T), Q1446K (4386C>A 4388A>G), H1450S (4398C>T 4399A>C), F1451V (4401T>G 4403C>T), Q1453K (4407C>A), D1454W (4410G>T 4411A>G 4412T>G), D1455I (4413G>A 4414A>T 4415C>T), K1456R (4417A>G 4418G>A), F1457Y (4420T>A 4421T>C), L1463M (4437C>A 4439C>G), T1464D (4440A>G 4441C>A 4442A>T), F1466L (4448C>G), S1467K (4449T>A 4450C>A 4451T>A), H1468R (4452C>A 4453A>G 4454C>A), L1469T (4455T>A 4456T>C 4457A>T), S1470F (4459C>T 4460C>T), A1472T (4464G>A 4466A>C), K1473N (4469G>T), L1474I (4470C>A 4472C>T), F1475Y (4474T>A 4475T>C), L1480M (4488C>A 4490T>G), L1487I (4509T>A 4511A>T), G1488Q (4512G>C 4513G>A), H1490S (4518C>T 4519A>C 4520T>G), P1491K (4521C>A 4522C>A 4523T>A), N1492K (4526T>A), E1493D (4529G>T), R1494K (4531G>A), P1495Y (4533C>T 4534C>A 4535A>T), G1498A (4543G>C 4544G>T), C1500N (4548T>A 4549G>A), I1501V (4551A>G 4553T>C), D1503F (4557G>T 4558A>T 4559T>C), R1504G (4560A>G), H1505Q (4565T>A), F1506Y (4567T>A), Q1507E (4569C>G), K1509N (4577G>T), T1517N (4600C>A 4601A>T), L1521E (4611T>G 4612T>A 4613G>A), K1524N (4622A>C), A1525V (4624C>T 4625C>T), I1527N (4630T>A 4631C>T), K1528S (4632A>T 4633A>C 4634A>T), Q1531N (4641C>A 4643G>T), P1532H (4645C>A), L1534S (4650C>A 4651T>G 4652T>C), F1535N (4653T>A 4654T>A 4655T>C), S1536I (4656T>A 4657C>T 4658A>C), A1537S (4659G>T), L1538I (4662T>A 4664G>T), I1544V (4680A>G 4682C>T), L1546I (4686C>A 4688T>A), T1550D (4698A>G 4699C>A 4700A>T), L1551I (4701C>A 4703G>T), E1552D (4706A>T), D1553S (4707G>T 4708A>C), I1555F (4713A>T 4715A>T), L1557H (4719T>C 4720T>A 4721G>T), Q1560T (4728C>A 4729A>C 4730G>C), I1562F (4734A>T 4736A>C), S1563K (4738G>A 4739T>A), L1564I (4740T>A 4742G>T), K1567R (4750A>G), F1568N (4752T>A 4753T>A), V1570L (4758G>C 4760C>T), M1571V (4761A>G 4763G>C), L1572I (4764T>A 4766A>C), I1578K (4783T>A 4784C>A), A1580F (4788G>T 4789C>T), N1582T (4795A>C 4796T>A), Q1585R (4803C>A 4804A>G), M1589H (4815A>C 4816T>A 4817G>T), F1591L (4823T>A), A1592Y (4824G>T 4825C>A), D1593Q (4827G>C 4829T>A), F1596Y (4837T>A), S1597K (4839T>A 4840C>A), A1599I (4845G>A 4846C>T 4847T>C), G1600C (4848G>T 4850T>C), H1601del (4851_4853delCAC), I1602R (4855T>G 4856C>A), S1603A (4857T>G 4859T>C), L1604I (4860T>A 4862G>C), L1606F (4866C>T 4868C>T), Q1607S (4869C>T 4870A>C 4871A>T), Q1607_K1608insG (4871_4872insGGT), D1611N (4881G>A), T1612E (4884A>G 4885C>A), N1613I (4888A>T 4889T>C), L1614T (4890C>A 4891T>C 4892C>T), S1615D (4893T>G 4894C>A 4895G>C), V1616K (4896G>A 4897T>A), K1617T (4900A>C 4901G>T), I1619L (4905A>T), Q1621R (4911C>A 4912A>G), I1625S (4924T>G 4925T>C), V1626L (4926G>C 4928T>C), I1629V (4935A>G 4937C>T), R1630A (4938A>G 4939G>C), E1635K (4953G>A 4955G>A)								
Codon mutations:	AAA22..T (116A>T), CTC23CTT (119C>T), TCT24TGA (121C>G 122T>A), GCT26ACC (126G>A 128T>C), TTG27CTT (129T>C 131G>T), TCT28TCA (134T>A), AAT29AGA (136A>G 137T>A), TAC33TAA (149C>A), CTT34TTG (150C>T 152T>G), GTA37ATT (159G>A 161A>T), ATC39GTC (165A>G), CTT40CTA (170T>A), AAA41CCC (171A>C 172A>C 173A>C), AAC43GAG (177A>G 179C>G), ATA46GCA (186A>G 187T>C), GAT48TCC (192G>T 193A>C 194T>C), ACC49ACG (197C>G), TAT50TCC (199A>C 200T>C), CTT51TTA (201C>T 203T>A), TTG53CTT (207T>C 209G>T), TTA54ATA (210T>A), TAC57TAT (221C>T), GCA61GCC (233A>C), AGA63AAT (238G>A 239A>T), TCC64TAA (241C>A 242C>A), GTT65TTA (243G>T 245T>A), ACC66TCG (246A>T 248C>G), CCT67CCA (251T>A), TCT69ATC (255T>A 256C>T 257T>C), CAG70CAT (260G>T), ATC71ATT (263C>T), CGT72CAT (265G>A), TCA73TCC (269A>C), CAG76CAA (278G>A), TCT77AAC (279T>A 280C>A 281T>C), CCT79CTC (286C>T 287T>C), CGC80ATA (288C>A 289G>T 290C>A), CAT81GGA (291C>G 292A>G 293T>A), GTG82GTA (296G>A), AAA83AGA (298A>G), GCT87GTT (310C>T), TCC89TCA (317C>A), TTA91CTC (321T>C 323A>C), GAT92AAT (324G>A), CAG93CAA (329G>A), CCT95TCT (333C>T), GTC96ATA (336G>A 338C>A), TTT97CTT (339T>C), GCT98GCA (344T>A), ACA99ACT (347A>T), GGA100CCA (348G>C 349G>C), CAG103CAA (359G>A), TTT104TTC (362T>C), GTT105TTT (363G>T), ACT106TCT (366A>T), CTA1413ATT (4287C>A 4289A>T), GAA1415AGA (4293G>A 4294A>G), CCC1416CCT (4298C>T), GAT1418AAA (4302G>A 4304T>A), TCA1419TCT (4307A>T), CAA1420CCT (4309A>C 4310A>T), GCA1422AGC (4314G>A 4315C>G 4316A>C), GAA1424TCA (4320G>T 4321A>C), GCT1425GCC (4325T>C), TTT1426TTC (4328T>C), GTC1428GTT (4334C>T), AAC1429AAT (4337C>T), AAG1430AAA (4340G>A), AGG1431ACT (4342G>C 4343G>T), TCA1432GCT (4344T>G 4346A>T), GAG1433GAA (4349G>A), CAA1434AAA (4350C>A), GTG1435GAA (4354T>A 4355G>A), GGA1437GGC (4361A>C), AAA1438TCC (4362A>T 4363A>C 4364A>C), TTA1439CCT (4365T>C 4366T>C 4367A>T), GTC1442GTT (4376C>T), ATA1443ATT (4379A>T), CAA1446AAG (4386C>A 4388A>G), CCA1447CCT (4391A>T), AAC1449AAT (4397C>T), CAT1450TCT (4398C>T 4399A>C), TTC1451GTT (4401T>G 4403C>T), CTC1452TTA (4404C>T 4406C>A), CAA1453AAA (4407C>A), GAT1454TGG (4410G>T 4411A>G 4412T>G), GAC1455ATT (4413G>A 4414A>T 4415C>T), AAG1456AGA (4417A>G 4418G>A), TTT1457TAC (4420T>A 4421T>C), CCC1458CCG (4424C>G), CCA1460CCT (4430A>T), AAG1462AAA (4436G>A), CTC1463ATG (4437C>A 4439C>G), ACA1464GAT (4440A>G 4441C>A 4442A>T), CTC1465CTT (4445C>T), TTC1466TTG (4448C>G), TCT1467AAA (4449T>A 4450C>A 4451T>A), CAC1468AGA (4452C>A 4453A>G 4454C>A), TTA1469ACT (4455T>A 4456T>C 4457A>T), TCC1470TTT (4459C>T 4460C>T), GCA1472ACC (4464G>A 4466A>C), AAG1473AAT (4469G>T), CTC1474ATT (4470C>A 4472C>T), TTT1475TAC (4474T>A 4475T>C), TCA1476AGT (4476T>A 4477C>G 4478A>T), AAG1477AAA (4481G>A), CTT1480ATG (4488C>A 4490T>G), AAG1481AAA (4493G>A), GGA1483GGT (4499A>T), TTT1484TTC (4502T>C), TTA1487ATT (4509T>A 4511A>T), GGA1488CAA (4512G>C 4513G>A), ATC1489ATT (4517C>T), CAT1490TCG (4518C>T 4519A>C 4520T>G), CCT1491AAA (4521C>A 4522C>A 4523T>A), AAT1492AAA (4526T>A), GAG1493GAT (4529G>T), AGA1494AAA (4531G>A), CCA1495TAT (4533C>T 4534C>A 4535A>T), ACA1497ACT (4541A>T), GGG1498GCT (4543G>C 4544G>T), TTT1499TTC (4547T>C), TGC1500AAC (4548T>A 4549G>A), ATT1501GTC (4551A>G 4553T>C), CCT1502CCC (4556T>C), GAT1503TTC (4557G>T 4558A>T 4559T>C), AGA1504GGA (4560A>G), CAT1505CAA (4565T>A), TTT1506TAT (4567T>A), CAA1507GAA (4569C>G), AAG1509AAT (4577G>T), GTC1510GTT (4580C>T), CCA1512CCC (4586A>C), TTC1513TTT (4589C>T), GGT1514GGA (4592T>A), CTT1515TTA (4593C>T 4595T>A), ACA1517AAT (4600C>A 4601A>T), TTG1521GAA (4611T>G 4612T>A 4613G>A), TTC1522TTT (4616C>T), AAA1524AAC (4622A>C), GCC1525GTT (4624C>T 4625C>T), ATC1527AAT (4630T>A 4631C>T), AAA1528TCT (4632A>T 4633A>C 4634A>T), ATC1529ATT (4637C>T), TTC1530TTT (4640C>T), CAG1531AAT (4641C>A 4643G>T), CCC1532CAC (4645C>A), CTT1534AGC (4650C>A 4651T>G 4652T>C), TTT1535AAC (4653T>A 4654T>A 4655T>C), TCA1536ATC (4656T>A 4657C>T 4658A>C), GCT1537TCT (4659G>T), TTG1538ATT (4662T>A 4664G>T), GTC1539GTG (4667C>G), TAC1540TAT (4670C>T), ATC1544GTT (4680A>G 4682C>T), CTT1545TTG (4683C>T 4685T>G), CTT1546ATA (4686C>A 4688T>A), GAA1549GAG (4697A>G), ACA1550GAT (4698A>G 4699C>A 4700A>T), CTG1551ATT (4701C>A 4703G>T), GAA1552GAT (4706A>T), GAT1553TCT (4707G>T 4708A>C), CAC1554CAT (4712C>T), ATA1555TTT (4713A>T 4715A>T), TTG1557CAT (4719T>C 4720T>A 4721G>T), CTT1558TTA (4722C>T 4724T>A), AAC1559AAT (4727C>T), CAG1560ACC (4728C>A 4729A>C 4730G>C), ATA1562TTC (4734A>T 4736A>C), AGT1563AAA (4738G>A 4739T>A), TTG1564ATT (4740T>A 4742G>T), GTT1565GTC (4745T>C), AAA1567AGA (4750A>G), TTT1568AAT (4752T>A 4753T>A), GGA1569GGT (4757A>T), GTC1570CTT (4758G>C 4760C>T), ATG1571GTC (4761A>G 4763G>C), TTA1572ATC (4764T>A 4766A>C), TCG1573AGT (4767T>A 4768C>G 4769G>T), GCA1574GCT (4772A>T), AAA1575AAG (4775A>G), AAG1576AAA (4778G>A), ATC1578AAA (4783T>A 4784C>A), CTT1579TTG (4785C>T 4787T>G), GCT1580TTT (4788G>T 4789C>T), AAT1582ACA (4795A>C 4796T>A), ATT1584ATC (4802T>C), CAA1585AGA (4803C>A 4804A>G), TTC1586TTT (4808C>T), TTA1587CTT (4809T>C 4811A>T), GGA1588GGG (4814A>G), ATG1589CAT (4815A>C 4816T>A 4817G>T), TTT1591TTA (4823T>A), GCT1592TAT (4824G>T 4825C>A), GAT1593CAA (4827G>C 4829T>A), ACT1595ACA (4835T>A), TTT1596TAT (4837T>A), TCA1597AAA (4839T>A 4840C>A), CCT1598CCC (4844T>C), GCT1599ATC (4845G>A 4846C>T 4847T>C), GGT1600TGC (4848G>T 4850T>C), CAC1601del (4851_4853delCAC), ATC1602AGA (4855T>G 4856C>A), TCT1603GCC (4857T>G 4859T>C), TTG1604ATC (4860T>A 4862G>C), CTC1606TTT (4866C>T 4868C>T), CAA1607TCT (4869C>T 4870A>C 4871A>T), CAA1607_AAG1608insGGT (4871_4872insGGT), AAG1608AAA (4874G>A), TTT1609TTC (4877T>C), GAT1611AAT (4881G>A), ACA1612GAA (4884A>G 4885C>A), AAT1613ATC (4888A>T 4889T>C), CTC1614ACT (4890C>A 4891T>C 4892C>T), TCG1615GAC (4893T>G 4894C>A 4895G>C), GTA1616AAA (4896G>A 4897T>A), AAG1617ACT (4900A>C 4901G>T), ATA1619TTA (4905A>T), CAA1620CAG (4910A>G), CAA1621AGA (4911C>A 4912A>G), TTT1622TTC (4916T>C), CTT1623TTG (4917C>T 4919T>G), GGG1624GGA (4922G>A), ATT1625AGC (4924T>G 4925T>C), GTT1626CTC (4926G>C 4928T>C), ATC1629GTT (4935A>G 4937C>T), AGA1630GCA (4938A>G 4939G>C), GAT1631GAC (4943T>C), TTT1632TTC (4946T>C), CCA1634CCT (4952A>T), GAG1635AAA (4953G>A 4955G>A)								

	Begin	End	Coverage	Score	Concordance	Matches	Identities	I/D/M/F*	Stop Codons
**NT**	**116**	**4957**	**13.0%**	**304**	**16.3%**	**936 (99.4%)**	**550 (58.4%)**	**3/3**	

Proteins

	Begin	End	Coverage	Score	Concordance	Matches	Identities	I/D/M/F*	Stop Codons
ORF I polyprotein (NP_127504.1)	23	1636	14.4%	1191	55.2%	312 (99.4%)	168 (53.5%)	1/1/0/0	3

Protein mutations: S24* (121C>G 122T>A), A26T (126G>A 128T>C), N29R (136A>G 137T>A), Y33* (149C>A), V37I (159G>A 161A>T), I39V (165A>G), K41P (171A>C 172A>C 173A>C), N43E (177A>G 179C>G), I46A (186A>G 187T>C), D48S (192G>T 193A>C 194T>C), Y50S (199A>C 200T>C), L54I (210T>A), R63N (238G>A 239A>T), S64* (241C>A 242C>A), V65L (243G>T 245T>A), T66S (246A>T 248C>G), S69I (255T>A 256C>T 257T>C), Q70H (260G>T), R72H (265G>A), S77N (279T>A 280C>A 281T>C), P79L (286C>T 287T>C), R80I (288C>A 289G>T 290C>A), H81G (291C>G 292A>G 293T>A), K83R (298A>G), A87V (310C>T), D92N (324G>A), P95S (333C>T), V96I (336G>A 338C>A), F97L (339T>C), G100P (348G>C 349G>C), V105F (363G>T), T106S (366A>T), L1413I (4287C>A 4289A>T), E1415R (4293G>A 4294A>G), D1418K (4302G>A 4304T>A), Q1420P (4309A>C 4310A>T), A1422S (4314G>A 4315C>G 4316A>C), E1424S (4320G>T 4321A>C), R1431T (4342G>C 4343G>T), S1432A (4344T>G 4346A>T), Q1434K (4350C>A), V1435E (4354T>A 4355G>A), K1438S (4362A>T 4363A>C 4364A>C), L1439P (4365T>C 4366T>C 4367A>T), Q1446K (4386C>A 4388A>G), H1450S (4398C>T 4399A>C), F1451V (4401T>G 4403C>T), Q1453K (4407C>A), D1454W (4410G>T 4411A>G 4412T>G), D1455I (4413G>A 4414A>T 4415C>T), K1456R (4417A>G 4418G>A), F1457Y (4420T>A 4421T>C), L1463M (4437C>A 4439C>G), T1464D (4440A>G 4441C>A 4442A>T), F1466L (4448C>G), S1467K (4449T>A 4450C>A 4451T>A), H1468R (4452C>A 4453A>G 4454C>A), L1469T (4455T>A 4456T>C 4457A>T), S1470F (4459C>T 4460C>T), A1472T (4464G>A 4466A>C), K1473N (4469G>T), L1474I (4470C>A 4472C>T), F1475Y (4474T>A 4475T>C), L1480M (4488C>A 4490T>G), L1487I (4509T>A 4511A>T), G1488Q (4512G>C 4513G>A), H1490S (4518C>T 4519A>C 4520T>G), P1491K (4521C>A 4522C>A 4523T>A), N1492K (4526T>A), E1493D (4529G>T), R1494K (4531G>A), P1495Y (4533C>T 4534C>A 4535A>T), G1498A (4543G>C 4544G>T), C1500N (4548T>A 4549G>A), I1501V (4551A>G 4553T>C), D1503F (4557G>T 4558A>T 4559T>C), R1504G (4560A>G), H1505Q (4565T>A), F1506Y (4567T>A), Q1507E (4569C>G), K1509N (4577G>T), T1517N (4600C>A 4601A>T), L1521E (4611T>G 4612T>A 4613G>A), K1524N (4622A>C), A1525V (4624C>T 4625C>T), I1527N (4630T>A 4631C>T), K1528S (4632A>T 4633A>C 4634A>T), Q1531N (4641C>A 4643G>T), P1532H (4645C>A), L1534S (4650C>A 4651T>G 4652T>C), F1535N (4653T>A 4654T>A 4655T>C), S1536I (4656T>A 4657C>T 4658A>C), A1537S (4659G>T), L1538I (4662T>A 4664G>T), I1544V (4680A>G 4682C>T), L1546I (4686C>A 4688T>A), T1550D (4698A>G 4699C>A 4700A>T), L1551I (4701C>A 4703G>T), E1552D (4706A>T), D1553S (4707G>T 4708A>C), I1555F (4713A>T 4715A>T), L1557H (4719T>C 4720T>A 4721G>T), Q1560T (4728C>A 4729A>C 4730G>C), I1562F (4734A>T 4736A>C), S1563K (4738G>A 4739T>A), L1564I (4740T>A 4742G>T), K1567R (4750A>G), F1568N (4752T>A 4753T>A), V1570L (4758G>C 4760C>T), M1571V (4761A>G 4763G>C), L1572I (4764T>A 4766A>C), I1578K (4783T>A 4784C>A), A1580F (4788G>T 4789C>T), N1582T (4795A>C 4796T>A), Q1585R (4803C>A 4804A>G), M1589H (4815A>C 4816T>A 4817G>T), F1591L (4823T>A), A1592Y (4824G>T 4825C>A), D1593Q (4827G>C 4829T>A), F1596Y (4837T>A), S1597K (4839T>A 4840C>A), A1599I (4845G>A 4846C>T 4847T>C), G1600C (4848G>T 4850T>C), H1601del (4851_4853delCAC), I1602R (4855T>G 4856C>A), S1603A (4857T>G 4859T>C), L1604I (4860T>A 4862G>C), L1606F (4866C>T 4868C>T), Q1607S (4869C>T 4870A>C 4871A>T), Q1607_K1608insG (4871_4872insGGT), D1611N (4881G>A), T1612E (4884A>G 4885C>A), N1613I (4888A>T 4889T>C), L1614T (4890C>A 4891T>C 4892C>T), S1615D (4893T>G 4894C>A 4895G>C), V1616K (4896G>A 4897T>A), K1617T (4900A>C 4901G>T), I1619L (4905A>T), Q1621R (4911C>A 4912A>G), I1625S (4924T>G 4925T>C), V1626L (4926G>C 4928T>C), I1629V (4935A>G 4937C>T), R1630A (4938A>G 4939G>C), E1635K (4953G>A 4955G>A)

Codon mutations: AAA22..T (116A>T), CTC23CTT (119C>T), TCT24TGA (121C>G 122T>A), GCT26ACC (126G>A 128T>C), TTG27CTT (129T>C 131G>T), TCT28TCA (134T>A), AAT29AGA (136A>G 137T>A), TAC33TAA (149C>A), CTT34TTG (150C>T 152T>G), GTA37ATT (159G>A 161A>T), ATC39GTC (165A>G), CTT40CTA (170T>A), AAA41CCC (171A>C 172A>C 173A>C), AAC43GAG (177A>G 179C>G), ATA46GCA (186A>G 187T>C), GAT48TCC (192G>T 193A>C 194T>C), ACC49ACG (197C>G), TAT50TCC (199A>C 200T>C), CTT51TTA (201C>T 203T>A), TTG53CTT (207T>C 209G>T), TTA54ATA (210T>A), TAC57TAT (221C>T), GCA61GCC (233A>C), AGA63AAT (238G>A 239A>T), TCC64TAA (241C>A 242C>A), GTT65TTA (243G>T 245T>A), ACC66TCG (246A>T 248C>G), CCT67CCA (251T>A), TCT69ATC (255T>A 256C>T 257T>C), CAG70CAT (260G>T), ATC71ATT (263C>T), CGT72CAT (265G>A), TCA73TCC (269A>C), CAG76CAA (278G>A), TCT77AAC (279T>A 280C>A 281T>C), CCT79CTC (286C>T 287T>C), CGC80ATA (288C>A 289G>T 290C>A), CAT81GGA (291C>G 292A>G 293T>A), GTG82GTA (296G>A), AAA83AGA (298A>G), GCT87GTT (310C>T), TCC89TCA (317C>A), TTA91CTC (321T>C 323A>C), GAT92AAT (324G>A), CAG93CAA (329G>A), CCT95TCT (333C>T), GTC96ATA (336G>A 338C>A), TTT97CTT (339T>C), GCT98GCA (344T>A), ACA99ACT (347A>T), GGA100CCA (348G>C 349G>C), CAG103CAA (359G>A), TTT104TTC (362T>C), GTT105TTT (363G>T), ACT106TCT (366A>T), CTA1413ATT (4287C>A 4289A>T), GAA1415AGA (4293G>A 4294A>G), CCC1416CCT (4298C>T), GAT1418AAA (4302G>A 4304T>A), TCA1419TCT (4307A>T), CAA1420CCT (4309A>C 4310A>T), GCA1422AGC (4314G>A 4315C>G 4316A>C), GAA1424TCA (4320G>T 4321A>C), GCT1425GCC (4325T>C), TTT1426TTC (4328T>C), GTC1428GTT (4334C>T), AAC1429AAT (4337C>T), AAG1430AAA (4340G>A), AGG1431ACT (4342G>C 4343G>T), TCA1432GCT (4344T>G 4346A>T), GAG1433GAA (4349G>A), CAA1434AAA (4350C>A), GTG1435GAA (4354T>A 4355G>A), GGA1437GGC (4361A>C), AAA1438TCC (4362A>T 4363A>C 4364A>C), TTA1439CCT (4365T>C 4366T>C 4367A>T), GTC1442GTT (4376C>T), ATA1443ATT (4379A>T), CAA1446AAG (4386C>A 4388A>G), CCA1447CCT (4391A>T), AAC1449AAT (4397C>T), CAT1450TCT (4398C>T 4399A>C), TTC1451GTT (4401T>G 4403C>T), CTC1452TTA (4404C>T 4406C>A), CAA1453AAA (4407C>A), GAT1454TGG (4410G>T 4411A>G 4412T>G), GAC1455ATT (4413G>A 4414A>T 4415C>T), AAG1456AGA (4417A>G 4418G>A), TTT1457TAC (4420T>A 4421T>C), CCC1458CCG (4424C>G), CCA1460CCT (4430A>T), AAG1462AAA (4436G>A), CTC1463ATG (4437C>A 4439C>G), ACA1464GAT (4440A>G 4441C>A 4442A>T), CTC1465CTT (4445C>T), TTC1466TTG (4448C>G), TCT1467AAA (4449T>A 4450C>A 4451T>A), CAC1468AGA (4452C>A 4453A>G 4454C>A), TTA1469ACT (4455T>A 4456T>C 4457A>T), TCC1470TTT (4459C>T 4460C>T), GCA1472ACC (4464G>A 4466A>C), AAG1473AAT (4469G>T), CTC1474ATT (4470C>A 4472C>T), TTT1475TAC (4474T>A 4475T>C), TCA1476AGT (4476T>A 4477C>G 4478A>T), AAG1477AAA (4481G>A), CTT1480ATG (4488C>A 4490T>G), AAG1481AAA (4493G>A), GGA1483GGT (4499A>T), TTT1484TTC (4502T>C), TTA1487ATT (4509T>A 4511A>T), GGA1488CAA (4512G>C 4513G>A), ATC1489ATT (4517C>T), CAT1490TCG (4518C>T 4519A>C 4520T>G), CCT1491AAA (4521C>A 4522C>A 4523T>A), AAT1492AAA (4526T>A), GAG1493GAT (4529G>T), AGA1494AAA (4531G>A), CCA1495TAT (4533C>T 4534C>A 4535A>T), ACA1497ACT (4541A>T), GGG1498GCT (4543G>C 4544G>T), TTT1499TTC (4547T>C), TGC1500AAC (4548T>A 4549G>A), ATT1501GTC (4551A>G 4553T>C), CCT1502CCC (4556T>C), GAT1503TTC (4557G>T 4558A>T 4559T>C), AGA1504GGA (4560A>G), CAT1505CAA (4565T>A), TTT1506TAT (4567T>A), CAA1507GAA (4569C>G), AAG1509AAT (4577G>T), GTC1510GTT (4580C>T), CCA1512CCC (4586A>C), TTC1513TTT (4589C>T), GGT1514GGA (4592T>A), CTT1515TTA (4593C>T 4595T>A), ACA1517AAT (4600C>A 4601A>T), TTG1521GAA (4611T>G 4612T>A 4613G>A), TTC1522TTT (4616C>T), AAA1524AAC (4622A>C), GCC1525GTT (4624C>T 4625C>T), ATC1527AAT (4630T>A 4631C>T), AAA1528TCT (4632A>T 4633A>C 4634A>T), ATC1529ATT (4637C>T), TTC1530TTT (4640C>T), CAG1531AAT (4641C>A 4643G>T), CCC1532CAC (4645C>A), CTT1534AGC (4650C>A 4651T>G 4652T>C), TTT1535AAC (4653T>A 4654T>A 4655T>C), TCA1536ATC (4656T>A 4657C>T 4658A>C), GCT1537TCT (4659G>T), TTG1538ATT (4662T>A 4664G>T), GTC1539GTG (4667C>G), TAC1540TAT (4670C>T), ATC1544GTT (4680A>G 4682C>T), CTT1545TTG (4683C>T 4685T>G), CTT1546ATA (4686C>A 4688T>A), GAA1549GAG (4697A>G), ACA1550GAT (4698A>G 4699C>A 4700A>T), CTG1551ATT (4701C>A 4703G>T), GAA1552GAT (4706A>T), GAT1553TCT (4707G>T 4708A>C), CAC1554CAT (4712C>T), ATA1555TTT (4713A>T 4715A>T), TTG1557CAT (4719T>C 4720T>A 4721G>T), CTT1558TTA (4722C>T 4724T>A), AAC1559AAT (4727C>T), CAG1560ACC (4728C>A 4729A>C 4730G>C), ATA1562TTC (4734A>T 4736A>C), AGT1563AAA (4738G>A 4739T>A), TTG1564ATT (4740T>A 4742G>T), GTT1565GTC (4745T>C), AAA1567AGA (4750A>G), TTT1568AAT (4752T>A 4753T>A), GGA1569GGT (4757A>T), GTC1570CTT (4758G>C 4760C>T), ATG1571GTC (4761A>G 4763G>C), TTA1572ATC (4764T>A 4766A>C), TCG1573AGT (4767T>A 4768C>G 4769G>T), GCA1574GCT (4772A>T), AAA1575AAG (4775A>G), AAG1576AAA (4778G>A), ATC1578AAA (4783T>A 4784C>A), CTT1579TTG (4785C>T 4787T>G), GCT1580TTT (4788G>T 4789C>T), AAT1582ACA (4795A>C 4796T>A), ATT1584ATC (4802T>C), CAA1585AGA (4803C>A 4804A>G), TTC1586TTT (4808C>T), TTA1587CTT (4809T>C 4811A>T), GGA1588GGG (4814A>G), ATG1589CAT (4815A>C 4816T>A 4817G>T), TTT1591TTA (4823T>A), GCT1592TAT (4824G>T 4825C>A), GAT1593CAA (4827G>C 4829T>A), ACT1595ACA (4835T>A), TTT1596TAT (4837T>A), TCA1597AAA (4839T>A 4840C>A), CCT1598CCC (4844T>C), GCT1599ATC (4845G>A 4846C>T 4847T>C), GGT1600TGC (4848G>T 4850T>C), CAC1601del (4851_4853delCAC), ATC1602AGA (4855T>G 4856C>A), TCT1603GCC (4857T>G 4859T>C), TTG1604ATC (4860T>A 4862G>C), CTC1606TTT (4866C>T 4868C>T), CAA1607TCT (4869C>T 4870A>C 4871A>T), CAA1607_AAG1608insGGT (4871_4872insGGT), AAG1608AAA (4874G>A), TTT1609TTC (4877T>C), GAT1611AAT (4881G>A), ACA1612GAA (4884A>G 4885C>A), AAT1613ATC (4888A>T 4889T>C), CTC1614ACT (4890C>A 4891T>C 4892C>T), TCG1615GAC (4893T>G 4894C>A 4895G>C), GTA1616AAA (4896G>A 4897T>A), AAG1617ACT (4900A>C 4901G>T), ATA1619TTA (4905A>T), CAA1620CAG (4910A>G), CAA1621AGA (4911C>A 4912A>G), TTT1622TTC (4916T>C), CTT1623TTG (4917C>T 4919T>G), GGG1624GGA (4922G>A), ATT1625AGC (4924T>G 4925T>C), GTT1626CTC (4926G>C 4928T>C), ATC1629GTT (4935A>G 4937C>T), AGA1630GCA (4938A>G 4939G>C), GAT1631GAC (4943T>C), TTT1632TTC (4946T>C), CCA1634CCT (4952A>T), GAG1635AAA (4953G>A 4955G>A)

*: Inserts / Deletes / Misaligned / Frameshifts

Analysis details

This analysis was performed with panviral2.64

NGS Details (UN60): Cavemovirus venamanihotis

Assembly

**Coverage Length**	620 (2 contig(s))
**Depth Of Coverage**	6.4
**Number Of Reads**	33
**Reads Per Million**	0.70 rpm (after QC)
**Ambiguities**	0
**Assembly Method**	de novo + reference guided assembly
**Consensus Caller**	Bcf Tools

Coverage Map

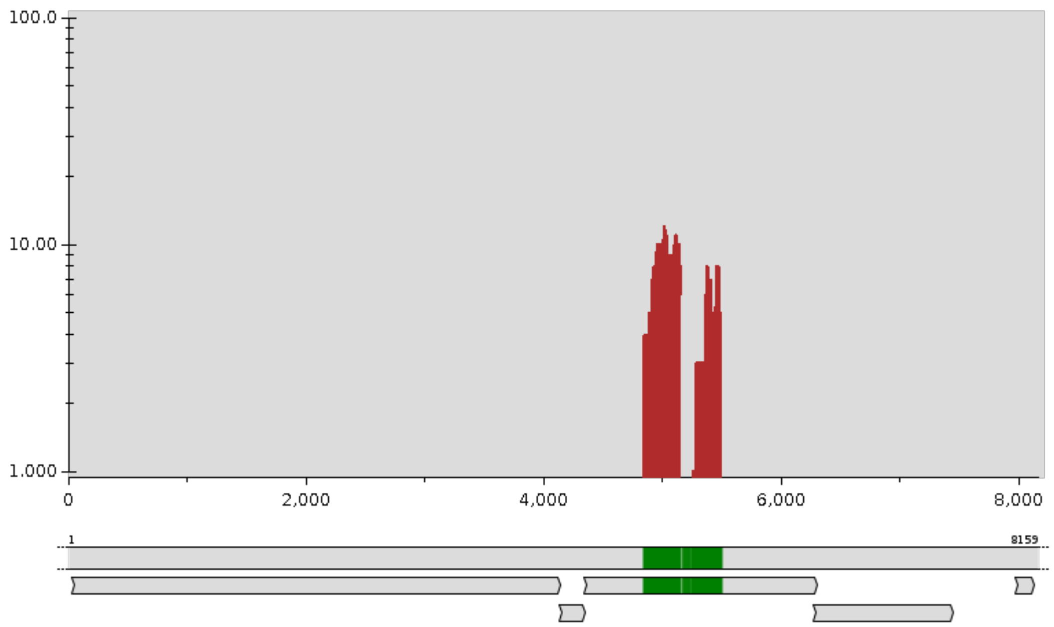

Assignment

**Type**	Cavemovirus venamanihotis (Taxonomy ID: 3047736)
**Reference Genome**	NC_001648.1
**NT Identity (%)**	59.6694
**AA Identity (%)**	48.2587
**Number Of Stop Codons**	1
**Number Of CDS**	5

Alignment

**Alignment Score**	201.0 (NT) + 585.0 (AA) = 786.0
**Concordance (%)**	32.0685

Alignment Method	Global, seeded, nucleotide + amino acids (AGA)

Genome Region

Sequence starts at position 4833 and ends at position 5501 relative to NC_001648.1 reference sequence.

Alignment Detailed Statistics

	Begin	End	Coverage	Score	Concordance	Matches	Identities	I/D/M/F*	Stop Codons
**NT**	**4833**	**5501**	**7.6%**	**201**	**17.1%**	**605 (97.6%)**	**361 (58.2%)**	**0/15**	
Mutations:	4842G>A, 4845G>A, 4853T>A, 4857A>G, 4858T>A, 4859A>T, 4863G>C, 4864G>A, 4867C>A, 4868A>G, 4869G>T, 4870A>C, 4872_4874delATA, 4880C>A, 4889A>C, 4890T>C, 4904A>T, 4908C>A, 4909A>G, 4911A>C, 4913A>C, 4914A>G, 4915C>A, 4917T>A, 4918T>A, 4921C>G, 4924A>T, 4925A>T, 4926A>T, 4929G>A, 4930A>T, 4931A>C, 4932T>C, 4934A>T, 4938A>G, 4941G>C, 4942A>T, 4943A>T, 4946A>G, 4947G>A, 4948A>T, 4951A>T, 4952T>A, 4955T>A, 4956T>A, 4957A>G, 4958T>G, 4959A>G, 4960A>T, 4962C>A, 4963C>G, 4977C>A, 4979A>T, 4984C>A, 4989T>A, 4990T>A, 4992C>T, 4994A>T, 4995G>A, 5000T>C, 5002A>G, 5006G>T, 5009T>G, 5012A>T, 5013G>C, 5019A>C, 5021G>T, 5022A>C, 5024T>C, 5028G>T, 5029A>T, 5031G>A, 5032G>A, 5033T>A, 5035T>A, 5036T>A, 5040G>A, 5041A>G, 5047A>G, 5048A>T, 5050C>A, 5051T>G, 5053A>G, 5055_5066delTTTGAAGATAAA, 5067A>C, 5068A>C, 5070T>C, 5073T>A, 5074C>G, 5075T>C, 5078C>T, 5081A>T, 5084T>A, 5090A>G, 5091G>A, 5095A>G, 5096T>A, 5099A>T, 5103T>G, 5105A>G, 5123A>G, 5124A>G, 5127A>C, 5135A>T, 5138T>A, 5139G>A, 5141T>C, 5144T>C, 5150T>G, 5151T>C, 5153A>T, 5160A>G, 5161A>C, 5162A>T, 5163G>T, 5164C>T, 5165T>A, 5169G>T, 5170T>G, 5171A>G, 5172G>A, 5174A>T, 5176A>G, 5180T>C, 5186C>T, 5189A>T, 5192T>C, 5196G>A, 5198T>G, 5199A>G, 5200C>A, 5201A>T, 5202T>C, 5204A>T, 5205A>C, 5208C>A, 5210T>A, 5215G>C, 5216T>C, 5217A>T, 5219A>T, 5220C>A, 5222G>A, 5227G>A, 5228A>C, 5229T>A, 5230A>T, 5231C>T, 5234T>C, 5247T>A, 5248G>T, 5249T>G, 5258A>T, 5260T>A, 5263A>G, 5264T>G, 5267T>A, 5271A>C, 5274T>A, 5276A>C, 5277G>T, 5278A>T, 5279A>T, 5280G>A, 5285T>G, 5286A>C, 5287G>A, 5290A>C, 5291A>T, 5292A>T, 5294A>T, 5295T>A, 5296A>G, 5297T>A, 5300A>T, 5308C>A, 5309A>T, 5315G>T, 5316C>T, 5317A>T, 5318G>T, 5322T>C, 5324T>A, 5328C>G, 5330G>A, 5336A>C, 5339A>T, 5340T>A, 5342A>G, 5345A>T, 5349G>T, 5351A>T, 5353A>T, 5354T>G, 5355C>A, 5357T>A, 5360C>T, 5361T>G, 5362C>T, 5363A>C, 5366A>T, 5367A>T, 5368G>C, 5370A>G, 5371T>A, 5372T>A, 5375C>T, 5378A>G, 5379C>A, 5382T>A, 5384T>C, 5388G>A, 5391A>G, 5392G>A, 5393A>T, 5396A>T, 5401G>A, 5402A>T, 5410A>T, 5411T>A, 5420T>C, 5426G>T, 5438T>C, 5441A>T, 5444A>G, 5445G>A, 5447C>T, 5449T>A, 5456G>A, 5459T>G, 5460A>T, 5466G>A, 5467A>T, 5473A>T, 5474A>C, 5476T>A, 5481A>C, 5485C>A, 5486A>T, 5488A>T, 5489A>C, 5492T>C, 5494G>A, 5496G>A, 5498T>A, 5501A>T								

CDS

	Begin	End	Coverage	Score	Concordance	Matches	Identities	I/D/M/F*	Stop Codons
CsVMVgp3	164	386	31.5%	585	39.9%	201 (97.6%)	97 (47.1%)	0/5/4/0	1
Protein mutations:	E167K (4842G>A), E168K (4845G>A), N170K (4853T>A), I172D (4857A>G 4858T>A 4859A>T), G174Q (4863G>C 4864G>A), T175K (4867C>A 4868A>G), D176S (4869G>T 4870A>C), I177del (4872_4874delATA), E187D (4904A>T), H189S (4908C>A 4909A>G), K190H (4911A>C 4913A>C), T191E (4914A>G 4915C>A), L192K (4917T>A 4918T>A), A193G (4921C>G), K194I (4924A>T 4925A>T), I195L (4926A>T), E196I (4929G>A 4930A>T 4931A>C), N199D (4938A>G), E200L (4941G>C 4942A>T 4943A>T), D202I (4947G>A 4948A>T), N203I (4951A>T 4952T>A), Y205R (4956T>A 4957A>G 4958T>G), K206V (4959A>G 4960A>T), P207R (4962C>A 4963C>G), Q212N (4977C>A 4979A>T), T214K (4984C>A), L216K (4989T>A 4990T>A), P217S (4992C>T 4994A>T), E218K (4995G>A), K220R (5002A>G), M221I (5006G>T), H222Q (5009T>G), E224Q (5013G>C), M226L (5019A>C 5021G>T), I227L (5022A>C 5024T>C), E229L (5028G>T 5029A>T), G230K (5031G>A 5032G>A 5033T>A), F231* (5035T>A 5036T>A), E233R (5040G>A 5041A>G), K235S (5047A>G 5048A>T), T236K (5050C>A 5051T>G), N237S (5053A>G), F238_K241del (5055_5066delTTTGAAGATAAA), K242P (5067A>C 5068A>C), Y243H (5070T>C), I249M (5090A>G), V250I (5091G>A), N251R (5095A>G 5096T>A), K252N (5099A>T), S254A (5103T>G 5105A>G), T261A (5124A>G), D266N (5139G>A 5141T>C), D269E (5150T>G), K273A (5160A>G 5161A>C 5162A>T), A274L (5163G>T 5164C>T 5165T>A), V276W (5169G>T 5170T>G 5171A>G), T286D (5199A>G 5200C>A 5201A>T), Y296I (5229T>A 5230A>T 5231C>T), C302M (5247T>A 5248G>T 5249T>G), F306Y (5260T>A), Y307W (5263A>G 5264T>G), H308Q (5267T>A), K310Q (5271A>C), L311I (5274T>A 5276A>C), E312F (5277G>T 5278A>T 5279A>T), E313K (5280G>A), D314E (5285T>G), S315H (5286A>C 5287G>A), K316T (5290A>C 5291A>T), K317Y (5292A>T 5294A>T), Y318R (5295T>A 5296A>G 5297T>A), T322N (5308C>A 5309A>T), Q325F (5316C>T 5317A>T 5318G>T), Y327Q (5322T>C 5324T>A), Q329E (5328C>G 5330G>A), K331N (5336A>C), L333M (5340T>A 5342A>G), G336C (5349G>T 5351A>T), Y337L (5353A>T 5354T>G), H338K (5355C>A 5357T>A), S340V (5361T>G 5362C>T 5363A>C), I343E (5370A>G 5371T>A 5372T>A), Q346K (5379C>A), F347I (5382T>A 5384T>C), D349N (5388G>A), R350D (5391A>G 5392G>A 5393A>T), R353N (5401G>A 5402A>T), Y356L (5410A>T 5411T>A), V368I (5445G>A 5447C>T), F369Y (5449T>A), I373F (5460A>T), E375M (5466G>A 5467A>T), K377I (5473A>T 5474A>C), I378K (5476T>A), I380L (5481A>C), A381D (5485C>A 5486A>T), K382I (5488A>T 5489A>C), R384K (5494G>A), D385K (5496G>A 5498T>A)								
Codon mutations:	GAA167AAA (4842G>A), GAA168AAA (4845G>A), AAT170AAA (4853T>A), ATA172GAT (4857A>G 4858T>A 4859A>T), GGA174CAA (4863G>C 4864G>A), ACA175AAG (4867C>A 4868A>G), GAT176TCT (4869G>T 4870A>C), ATA177del (4872_4874delATA), GGC179GGA (4880C>A), CCA182CCC (4889A>C), TTA183CTA (4890T>C), GAA187GAT (4904A>T), CAT189AGT (4908C>A 4909A>G), AAA190CAC (4911A>C 4913A>C), ACA191GAA (4914A>G 4915C>A), TTA192AAA (4917T>A 4918T>A), GCA193GGA (4921C>G), AAA194ATT (4924A>T 4925A>T), ATA195TTA (4926A>T), GAA196ATC (4929G>A 4930A>T 4931A>C), TTA197CTT (4932T>C 4934A>T), AAT199GAT (4938A>G), GAA200CTT (4941G>C 4942A>T 4943A>T), ACA201ACG (4946A>G), GAT202ATT (4947G>A 4948A>T), AAT203ATA (4951A>T 4952T>A), ATT204ATA (4955T>A), TAT205AGG (4956T>A 4957A>G 4958T>G), AAA206GTA (4959A>G 4960A>T), CCA207AGA (4962C>A 4963C>G), CAA212AAT (4977C>A 4979A>T), ACA214AAA (4984C>A), TTA216AAA (4989T>A 4990T>A), CCA217TCT (4992C>T 4994A>T), GAA218AAA (4995G>A), TTT219TTC (5000T>C), AAA220AGA (5002A>G), ATG221ATT (5006G>T), CAT222CAG (5009T>G), ATA223ATT (5012A>T), GAA224CAA (5013G>C), ATG226CTT (5019A>C 5021G>T), ATT227CTC (5022A>C 5024T>C), GAA229TTA (5028G>T 5029A>T), GGT230AAA (5031G>A 5032G>A 5033T>A), TTT231TAA (5035T>A 5036T>A), GAA233AGA (5040G>A 5041A>G), AAA235AGT (5047A>G 5048A>T), ACT236AAG (5050C>A 5051T>G), AAT237AGT (5053A>G), TTT238_AAA241del (5055_5066delTTTGAAGATAAA), AAA242CCA (5067A>C 5068A>C), TAT243CAT (5070T>C), TCT244AGC (5073T>A 5074C>G 5075T>C), AGC245AGT (5078C>T), CCA246CCT (5081A>T), GCT247GCA (5084T>A), ATA249ATG (5090A>G), GTA250ATA (5091G>A), AAT251AGA (5095A>G 5096T>A), AAA252AAT (5099A>T), TCA254GCG (5103T>G 5105A>G), AAA260AAG (5123A>G), ACT261GCT (5124A>G), AGA262CGA (5127A>C), GTA264GTT (5135A>T), ATT265ATA (5138T>A), GAT266AAC (5139G>A 5141T>C), TAT267TAC (5144T>C), GAT269GAG (5150T>G), TTA270CTT (5151T>C 5153A>T), AAA273GCT (5160A>G 5161A>C 5162A>T), GCT274TTA (5163G>T 5164C>T 5165T>A), GTA276TGG (5169G>T 5170T>G 5171A>G), GTA277A.T (5172G>A 5174A>T), AAA278.G. (5176A>G), TAT279..C (5180T>C), ATC281..T (5186C>T), CCA282..T (5189A>T), AAT283..C (5192T>C), GAT285A.G (5196G>A 5198T>G), ACA286GAT (5199A>G 5200C>A 5201A>T), TTA287C.T (5202T>C 5204A>T), ATA288C.. (5205A>C), CAT289A.A (5208C>A 5210T>A), AGT291.CC (5215G>C 5216T>C), ATA292T.T (5217A>T 5219A>T), CAG293A.A (5220C>A 5222G>A), AGA295.AC (5227G>A 5228A>C), TAC296ATT (5229T>A 5230A>T 5231C>T), TAT297..C (5234T>C), TGT302ATG (5247T>A 5248G>T 5249T>G), GGA305GGT (5258A>T), TTT306TAT (5260T>A), TAT307TGG (5263A>G 5264T>G), CAT308CAA (5267T>A), AAG310CAG (5271A>C), TTA311ATC (5274T>A 5276A>C), GAA312TTT (5277G>T 5278A>T 5279A>T), GAA313AAA (5280G>A), GAT314GAG (5285T>G), AGT315CAT (5286A>C 5287G>A), AAA316ACT (5290A>C 5291A>T), AAA317TAT (5292A>T 5294A>T), TAT318AGA (5295T>A 5296A>G 5297T>A), ACA319ACT (5300A>T), ACA322AAT (5308C>A 5309A>T), CCG324CCT (5315G>T), CAG325TTT (5316C>T 5317A>T 5318G>T), TAT327CAA (5322T>C 5324T>A), CAG329GAA (5328C>G 5330G>A), AAA331AAC (5336A>C), GTA332GTT (5339A>T), TTA333ATG (5340T>A 5342A>G), CCA334CCT (5345A>T), GGA336TGT (5349G>T 5351A>T), TAT337TTG (5353A>T 5354T>G), CAT338AAA (5355C>A 5357T>A), AAC339AAT (5360C>T), TCA340GTC (5361T>G 5362C>T 5363A>C), CCA341CCT (5366A>T), AGT342TCT (5367A>T 5368G>C), ATT343GAA (5370A>G 5371T>A 5372T>A), TTC344TTT (5375C>T), CAA345CAG (5378A>G), CAA346AAA (5379C>A), TTT347ATC (5382T>A 5384T>C), GAT349AAT (5388G>A), AGA350GAT (5391A>G 5392G>A 5393A>T), ATA351ATT (5396A>T), AGA353AAT (5401G>A 5402A>T), TAT356TTA (5410A>T 5411T>A), ATT359ATC (5420T>C), GTG361GTT (5426G>T), GAT365GAC (5438T>C), ATA366ATT (5441A>T), TTA367TTG (5444A>G), GTC368ATT (5445G>A 5447C>T), TTT369TAT (5449T>A), AAG371AAA (5456G>A), ACT372ACG (5459T>G), ATT373TTT (5460A>T), GAG375ATG (5466G>A 5467A>T), AAA377ATC (5473A>T 5474A>C), ATA378AAA (5476T>A), ATT380CTT (5481A>C), GCA381GAT (5485C>A 5486A>T), AAA382ATC (5488A>T 5489A>C), TTT383TTC (5492T>C), AGA384AAA (5494G>A), GAT385AAA (5496G>A 5498T>A), ATA386ATT (5501A>T)								

Proteins

	Begin	End	Coverage	Score	Concordance	Matches	Identities	I/D/M/F*	Stop Codons
aspartic protease/reverse transcriptase (NP_056848.1)	164	386	31.5%	585	39.9%	201 (97.6%)	97 (47.1%)	0/5/4/0	1
Protein mutations:	E167K (4842G>A), E168K (4845G>A), N170K (4853T>A), I172D (4857A>G 4858T>A 4859A>T), G174Q (4863G>C 4864G>A), T175K (4867C>A 4868A>G), D176S (4869G>T 4870A>C), I177del (4872_4874delATA), E187D (4904A>T), H189S (4908C>A 4909A>G), K190H (4911A>C 4913A>C), T191E (4914A>G 4915C>A), L192K (4917T>A 4918T>A), A193G (4921C>G), K194I (4924A>T 4925A>T), I195L (4926A>T), E196I (4929G>A 4930A>T 4931A>C), N199D (4938A>G), E200L (4941G>C 4942A>T 4943A>T), D202I (4947G>A 4948A>T), N203I (4951A>T 4952T>A), Y205R (4956T>A 4957A>G 4958T>G), K206V (4959A>G 4960A>T), P207R (4962C>A 4963C>G), Q212N (4977C>A 4979A>T), T214K (4984C>A), L216K (4989T>A 4990T>A), P217S (4992C>T 4994A>T), E218K (4995G>A), K220R (5002A>G), M221I (5006G>T), H222Q (5009T>G), E224Q (5013G>C), M226L (5019A>C 5021G>T), I227L (5022A>C 5024T>C), E229L (5028G>T 5029A>T), G230K (5031G>A 5032G>A 5033T>A), F231* (5035T>A 5036T>A), E233R (5040G>A 5041A>G), K235S (5047A>G 5048A>T), T236K (5050C>A 5051T>G), N237S (5053A>G), F238_K241del (5055_5066delTTTGAAGATAAA), K242P (5067A>C 5068A>C), Y243H (5070T>C), I249M (5090A>G), V250I (5091G>A), N251R (5095A>G 5096T>A), K252N (5099A>T), S254A (5103T>G 5105A>G), T261A (5124A>G), D266N (5139G>A 5141T>C), D269E (5150T>G), K273A (5160A>G 5161A>C 5162A>T), A274L (5163G>T 5164C>T 5165T>A), V276W (5169G>T 5170T>G 5171A>G), T286D (5199A>G 5200C>A 5201A>T), Y296I (5229T>A 5230A>T 5231C>T), C302M (5247T>A 5248G>T 5249T>G), F306Y (5260T>A), Y307W (5263A>G 5264T>G), H308Q (5267T>A), K310Q (5271A>C), L311I (5274T>A 5276A>C), E312F (5277G>T 5278A>T 5279A>T), E313K (5280G>A), D314E (5285T>G), S315H (5286A>C 5287G>A), K316T (5290A>C 5291A>T), K317Y (5292A>T 5294A>T), Y318R (5295T>A 5296A>G 5297T>A), T322N (5308C>A 5309A>T), Q325F (5316C>T 5317A>T 5318G>T), Y327Q (5322T>C 5324T>A), Q329E (5328C>G 5330G>A), K331N (5336A>C), L333M (5340T>A 5342A>G), G336C (5349G>T 5351A>T), Y337L (5353A>T 5354T>G), H338K (5355C>A 5357T>A), S340V (5361T>G 5362C>T 5363A>C), I343E (5370A>G 5371T>A 5372T>A), Q346K (5379C>A), F347I (5382T>A 5384T>C), D349N (5388G>A), R350D (5391A>G 5392G>A 5393A>T), R353N (5401G>A 5402A>T), Y356L (5410A>T 5411T>A), V368I (5445G>A 5447C>T), F369Y (5449T>A), I373F (5460A>T), E375M (5466G>A 5467A>T), K377I (5473A>T 5474A>C), I378K (5476T>A), I380L (5481A>C), A381D (5485C>A 5486A>T), K382I (5488A>T 5489A>C), R384K (5494G>A), D385K (5496G>A 5498T>A)								

	Begin	End	Coverage	Score	Concordance	Matches	Identities	I/D/M/F*	Stop Codons
**NT**	**4833**	**5501**	**7.6%**	**201**	**17.1%**	**605 (97.6%)**	**361 (58.2%)**	**0/15**	
Codon mutations:	GAA167AAA (4842G>A), GAA168AAA (4845G>A), AAT170AAA (4853T>A), ATA172GAT (4857A>G 4858T>A 4859A>T), GGA174CAA (4863G>C 4864G>A), ACA175AAG (4867C>A 4868A>G), GAT176TCT (4869G>T 4870A>C), ATA177del (4872_4874delATA), GGC179GGA (4880C>A), CCA182CCC (4889A>C), TTA183CTA (4890T>C), GAA187GAT (4904A>T), CAT189AGT (4908C>A 4909A>G), AAA190CAC (4911A>C 4913A>C), ACA191GAA (4914A>G 4915C>A), TTA192AAA (4917T>A 4918T>A), GCA193GGA (4921C>G), AAA194ATT (4924A>T 4925A>T), ATA195TTA (4926A>T), GAA196ATC (4929G>A 4930A>T 4931A>C), TTA197CTT (4932T>C 4934A>T), AAT199GAT (4938A>G), GAA200CTT (4941G>C 4942A>T 4943A>T), ACA201ACG (4946A>G), GAT202ATT (4947G>A 4948A>T), AAT203ATA (4951A>T 4952T>A), ATT204ATA (4955T>A), TAT205AGG (4956T>A 4957A>G 4958T>G), AAA206GTA (4959A>G 4960A>T), CCA207AGA (4962C>A 4963C>G), CAA212AAT (4977C>A 4979A>T), ACA214AAA (4984C>A), TTA216AAA (4989T>A 4990T>A), CCA217TCT (4992C>T 4994A>T), GAA218AAA (4995G>A), TTT219TTC (5000T>C), AAA220AGA (5002A>G), ATG221ATT (5006G>T), CAT222CAG (5009T>G), ATA223ATT (5012A>T), GAA224CAA (5013G>C), ATG226CTT (5019A>C 5021G>T), ATT227CTC (5022A>C 5024T>C), GAA229TTA (5028G>T 5029A>T), GGT230AAA (5031G>A 5032G>A 5033T>A), TTT231TAA (5035T>A 5036T>A), GAA233AGA (5040G>A 5041A>G), AAA235AGT (5047A>G 5048A>T), ACT236AAG (5050C>A 5051T>G), AAT237AGT (5053A>G), TTT238_AAA241del (5055_5066delTTTGAAGATAAA), AAA242CCA (5067A>C 5068A>C), TAT243CAT (5070T>C), TCT244AGC (5073T>A 5074C>G 5075T>C), AGC245AGT (5078C>T), CCA246CCT (5081A>T), GCT247GCA (5084T>A), ATA249ATG (5090A>G), GTA250ATA (5091G>A), AAT251AGA (5095A>G 5096T>A), AAA252AAT (5099A>T), TCA254GCG (5103T>G 5105A>G), AAA260AAG (5123A>G), ACT261GCT (5124A>G), AGA262CGA (5127A>C), GTA264GTT (5135A>T), ATT265ATA (5138T>A), GAT266AAC (5139G>A 5141T>C), TAT267TAC (5144T>C), GAT269GAG (5150T>G), TTA270CTT (5151T>C 5153A>T), AAA273GCT (5160A>G 5161A>C 5162A>T), GCT274TTA (5163G>T 5164C>T 5165T>A), GTA276TGG (5169G>T 5170T>G 5171A>G), GTA277A.T (5172G>A 5174A>T), AAA278.G. (5176A>G), TAT279..C (5180T>C), ATC281..T (5186C>T), CCA282..T (5189A>T), AAT283..C (5192T>C), GAT285A.G (5196G>A 5198T>G), ACA286GAT (5199A>G 5200C>A 5201A>T), TTA287C.T (5202T>C 5204A>T), ATA288C.. (5205A>C), CAT289A.A (5208C>A 5210T>A), AGT291.CC (5215G>C 5216T>C), ATA292T.T (5217A>T 5219A>T), CAG293A.A (5220C>A 5222G>A), AGA295.AC (5227G>A 5228A>C), TAC296ATT (5229T>A 5230A>T 5231C>T), TAT297..C (5234T>C), TGT302ATG (5247T>A 5248G>T 5249T>G), GGA305GGT (5258A>T), TTT306TAT (5260T>A), TAT307TGG (5263A>G 5264T>G), CAT308CAA (5267T>A), AAG310CAG (5271A>C), TTA311ATC (5274T>A 5276A>C), GAA312TTT (5277G>T 5278A>T 5279A>T), GAA313AAA (5280G>A), GAT314GAG (5285T>G), AGT315CAT (5286A>C 5287G>A), AAA316ACT (5290A>C 5291A>T), AAA317TAT (5292A>T 5294A>T), TAT318AGA (5295T>A 5296A>G 5297T>A), ACA319ACT (5300A>T), ACA322AAT (5308C>A 5309A>T), CCG324CCT (5315G>T), CAG325TTT (5316C>T 5317A>T 5318G>T), TAT327CAA (5322T>C 5324T>A), CAG329GAA (5328C>G 5330G>A), AAA331AAC (5336A>C), GTA332GTT (5339A>T), TTA333ATG (5340T>A 5342A>G), CCA334CCT (5345A>T), GGA336TGT (5349G>T 5351A>T), TAT337TTG (5353A>T 5354T>G), CAT338AAA (5355C>A 5357T>A), AAC339AAT (5360C>T), TCA340GTC (5361T>G 5362C>T 5363A>C), CCA341CCT (5366A>T), AGT342TCT (5367A>T 5368G>C), ATT343GAA (5370A>G 5371T>A 5372T>A), TTC344TTT (5375C>T), CAA345CAG (5378A>G), CAA346AAA (5379C>A), TTT347ATC (5382T>A 5384T>C), GAT349AAT (5388G>A), AGA350GAT (5391A>G 5392G>A 5393A>T), ATA351ATT (5396A>T), AGA353AAT (5401G>A 5402A>T), TAT356TTA (5410A>T 5411T>A), ATT359ATC (5420T>C), GTG361GTT (5426G>T), GAT365GAC (5438T>C), ATA366ATT (5441A>T), TTA367TTG (5444A>G), GTC368ATT (5445G>A 5447C>T), TTT369TAT (5449T>A), AAG371AAA (5456G>A), ACT372ACG (5459T>G), ATT373TTT (5460A>T), GAG375ATG (5466G>A 5467A>T), AAA377ATC (5473A>T 5474A>C), ATA378AAA (5476T>A), ATT380CTT (5481A>C), GCA381GAT (5485C>A 5486A>T), AAA382ATC (5488A>T 5489A>C), TTT383TTC (5492T>C), AGA384AAA (5494G>A), GAT385AAA (5496G>A 5498T>A), ATA386ATT (5501A>T)								

*: Inserts / Deletes / Misaligned / Frameshifts

Analysis details

This analysis was performed with panviral2.64

NGS Details (UN60): Cavemovirus collusipomeae

Assembly

**Coverage Length**	497 (1 contig(s))
**Depth Of Coverage**	7.0
**Number Of Reads**	32
**Reads Per Million**	0.68 rpm (after QC)
**Ambiguities**	0
**Assembly Method**	de novo + reference guided assembly
**Consensus Caller**	Bcf Tools

Coverage Map

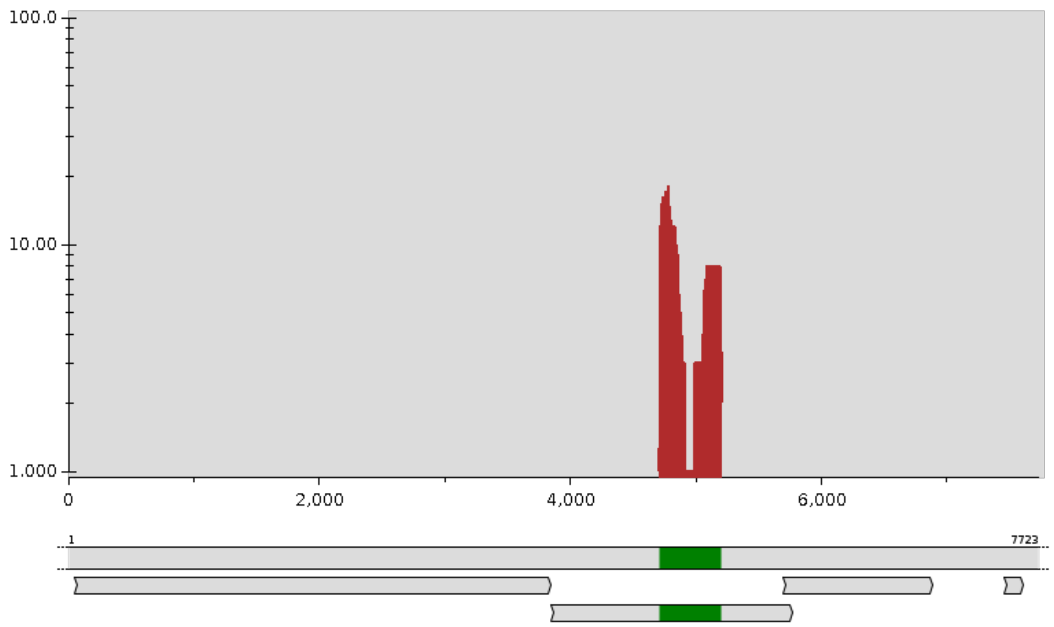

Assignment

**Type**	Cavemovirus collusipomeae (Taxonomy ID: 3048352)
**Reference Genome**	NC_015328.1
**NT Identity (%)**	69.0141
**AA Identity (%)**	57.8313
**Number Of Stop Codons**	1
**Number Of CDS**	4

Alignment

**Alignment Score**	378.0 (NT) + 640.0 (AA) = 1018.0
**Concordance (%)**	47.3709

**Alignment Method**	Global, seeded, nucleotide + amino acids (AGA)

Genome Region

Sequence starts at position 4709 and ends at position 5205 relative to NC_015328.1 reference sequence.

Alignment Detailed Statistics

	Begin	End	Coverage	Score	Concordance	Matches	Identities	I/D/M/F*	Stop Codons
**NT**	**4709**	**5205**	**6.4%**	**378**	**38.0%**	**497 (100%)**	**343 (69.0%)**	**0/0**	
Mutations:	4723T>C, 4729C>T, 4732C>T, 4734G>A, 4750T>C, 4753T>C, 4754A>C, 4756T>A, 4759A>G, 4760C>T, 4763G>A, 4764A>C, 4771T>A, 4772A>T, 4773G>C, 4778A>C, 4780A>G, 4782A>T, 4783T>A, 4809A>T, 4813T>C, 4815T>A, 4816A>G, 4818G>A, 4821T>A, 4828A>G, 4831T>A, 4841C>G, 4843T>A, 4846T>C, 4847T>A, 4853A>G, 4855T>A, 4857T>G, 4865C>T, 4867A>T, 4873G>T, 4877G>A, 4879A>C, 4880A>T, 4881T>G, 4882A>T, 4887G>A, 4888A>T, 4890C>A, 4893A>T, 4894T>A, 4895T>C, 4896A>C, 4897T>A, 4898G>A, 4900T>C, 4902T>G, 4903T>C, 4907C>A, 4909T>A, 4912A>G, 4924T>C, 4927A>T, 4930A>G, 4931A>G, 4935T>G, 4936T>C, 4939G>C, 4941A>C, 4948C>T, 4949G>C, 4951A>G, 4958A>T, 4959A>T, 4960A>T, 4967T>C, 4969G>T, 4970G>A, 4973A>G, 4974T>A, 4979A>T, 4980G>T, 4984T>A, 4985A>T, 4987A>T, 4988A>T, 4989T>G, 4990A>T, 4991A>G, 4993A>C, 4996A>T, 4997C>A, 5001G>A, 5002A>C, 5015A>C, 5029G>A, 5033G>A, 5034G>A, 5035A>T, 5039C>A, 5041A>T, 5053T>C, 5056A>T, 5060G>A, 5062T>G, 5064A>T, 5065G>T, 5068A>T, 5069G>T, 5070A>C, 5072C>A, 5075G>A, 5076G>A, 5077A>T, 5092G>A, 5093C>A, 5098T>C, 5102A>T, 5103T>C, 5104A>C, 5105G>A, 5107T>A, 5110A>G, 5119A>G, 5120A>T, 5121A>T, 5122A>T, 5124A>C, 5126A>G, 5127T>A, 5128A>C, 5131A>G, 5132A>T, 5136A>T, 5139G>C, 5140T>C, 5144A>C, 5150T>A, 5156T>A, 5157C>G, 5159A>T, 5161A>T, 5162T>C, 5164A>T, 5169T>G, 5170A>C, 5171G>T, 5177C>T, 5179A>T, 5180A>G, 5181T>C, 5182T>A, 5183A>G, 5186A>G, 5188T>A, 5192T>A, 5198A>G, 5200T>A								
**CDS**									
SPCV_gp2	290	455	25.9%	640	55.2%	166 (100%)	96 (57.8%)	0/0/0/0	1
Protein mutations:	C298Y (4734G>A), I305L (4754A>C 4756T>A), E308T (4763G>A 4764A>C), D310E (4771T>A), K313Q (4778A>C 4780A>G), Y314L (4782A>T 4783T>A), Y323F (4809A>T), V325E (4815T>A 4816A>G), W326* (4818G>A), I327N (4821T>A), H334E (4841C>G 4843T>A), S336T (4847T>A), S338G (4853A>G 4855T>A), I339R (4857T>G), Q342Y (4865C>T 4867A>T), M344I (4873G>T), G346S (4877G>A 4879A>C), I347C (4880A>T 4881T>G 4882A>T), R349N (4887G>A 4888A>T), P350Q (4890C>A), Y351L (4893A>T 4894T>A), Y352P (4895T>C 4896A>C 4897T>A), D353N (4898G>A 4900T>C), F354C (4902T>G 4903T>C), L356I (4907C>A 4909T>A), I364V (4931A>G), F365C (4935T>G 4936T>C), K367T (4941A>C), E370Q (4949G>C 4951A>G), K373F (4958A>T 4959A>T 4960A>T), E377K (4970G>A), I378E (4973A>G 4974T>A), R380L (4979A>T 4980G>T), N381K (4984T>A), I382F (4985A>T 4987A>T), I383C (4988A>T 4989T>G 4990A>T), I384V (4991A>G 4993A>C), K385N (4996A>T), H386N (4997C>A), G387D (5001G>A 5002A>C), K392Q (5015A>C), G398N (5033G>A 5034G>A 5035A>T), Q400N (5039C>A 5041A>T), V407M (5060G>A 5062T>G), K408I (5064A>T 5065G>T), E410S (5069G>T 5070A>C), Q411K (5072C>A), G412N (5075G>A 5076G>A 5077A>T), P418T (5093C>A), I421S (5102A>T 5103T>C 5104A>C), D422K (5105G>A 5107T>A), K427F (5120A>T 5121A>T 5122A>T), H428P (5124A>C), I429D (5126A>G 5127T>A 5128A>C), I431L (5132A>T), K432I (5136A>T), S433T (5139G>C 5140T>C), K435Q (5144A>C), L437I (5150T>A), S439R (5156T>A 5157C>G), I440F (5159A>T 5161A>T), L443C (5169T>G 5170A>C), V444L (5171G>T), Q446Y (5177C>T 5179A>T), I447A (5180A>G 5181T>C 5182T>A), R448G (5183A>G), N449E (5186A>G 5188T>A), L451I (5192T>A), N453E (5198A>G 5200T>A)								
Codon mutations:	AGT294AGC (4723T>C), TTC296TTT (4729C>T), GAC297GAT (4732C>T), TGT298TAT (4734G>A), TAT303TAC (4750T>C), CAT304CAC (4753T>C), ATT305CTA (4754A>C 4756T>A), AAA306AAG (4759A>G), CTA307TTA (4760C>T), GAA308ACA (4763G>A 4764A>C), GAT310GAA (4771T>A), AGT311TCT (4772A>T 4773G>C), AAA313CAG (4778A>C 4780A>G), TAT314TTA (4782A>T 4783T>A), TAT323TTT (4809A>T), TAT324TAC (4813T>C), GTA325GAG (4815T>A 4816A>G), TGG326TAG (4818G>A), ATT327AAT (4821T>A), TTA329TTG (4828A>G), CCT330CCA (4831T>A), CAT334GAA (4841C>G 4843T>A), AAT335AAC (4846T>C), TCA336ACA (4847T>A), AGT338GGA (4853A>G 4855T>A), ATA339AGA (4857T>G), CAA342TAT (4865C>T 4867A>T), ATG344ATT (4873G>T), GGA346AGC (4877G>A 4879A>C), ATA347TGT (4880A>T 4881T>G 4882A>T), AGA349AAT (4887G>A 4888A>T), CCA350CAA (4890C>A), TAT351TTA (4893A>T 4894T>A), TAT352CCA (4895T>C 4896A>C 4897T>A), GAT353AAC (4898G>A 4900T>C), TTT354TGC (4902T>G 4903T>C), CTT356ATA (4907C>A 4909T>A), GTA357GTG (4912A>G), GAT361GAC (4924T>C), ATA362ATT (4927A>T), TTA363TTG (4930A>G), ATA364GTA (4931A>G), TTT365TGC (4935T>G 4936T>C), TCG366TCC (4939G>C), AAA367ACA (4941A>C), TAC369TAT (4948C>T), GAA370CAG (4949G>C 4951A>G), AAA373TTT (4958A>T 4959A>T 4960A>T), TTG376CTT (4967T>C 4969G>T), GAA377AAA (4970G>A), ATA378GAA (4973A>G 4974T>A), AGA380TTA (4979A>T 4980G>T), AAT381AAA (4984T>A), ATA382TTT (4985A>T 4987A>T), ATA383TGT (4988A>T 4989T>G 4990A>T), ATA384GTC (4991A>G 4993A>C), AAA385AAT (4996A>T), CAT386AAT (4997C>A), GGA387GAC (5001G>A 5002A>C), AAG392CAG (5015A>C), GAG396GAA (5029G>A), GGA398AAT (5033G>A 5034G>A 5035A>T), CAA400AAT (5039C>A 5041A>T), TTT404TTC (5053T>C), CTA405CTT (5056A>T), GTT407ATG (5060G>A 5062T>G), AAG408ATT (5064A>T 5065G>T), ATA409ATT (5068A>T), GAA410TCA (5069G>T 5070A>C), CAA411AAA (5072C>A), GGA412AAT (5075G>A 5076G>A 5077A>T), CAG417CAA (5092G>A), CCT418ACT (5093C>A), CAT419CAC (5098T>C), ATA421TCC (5102A>T 5103T>C 5104A>C), GAT422AAA (5105G>A 5107T>A), AAA423AAG (5110A>G), GAA426GAG (5119A>G), AAA427TTT (5120A>T 5121A>T 5122A>T), CAT428CCT (5124A>C), ATA429GAC (5126A>G 5127T>A 5128A>C), AAA430AAG (5131A>G), ATA431TTA (5132A>T), AAA432ATA (5136A>T), AGT433ACC (5139G>C 5140T>C), AAA435CAA (5144A>C), TTA437ATA (5150T>A), TCA439AGA (5156T>A 5157C>G), ATA440TTT (5159A>T 5161A>T), TTA441CTT (5162T>C 5164A>T), TTA443TGC (5169T>G 5170A>C), GTA444TTA (5171G>T), CAA446TAT (5177C>T 5179A>T), ATT447GCA (5180A>G 5181T>C 5182T>A), AGA448GGA (5183A>G), AAT449GAA (5186A>G 5188T>A), TTA451ATA (5192T>A), AAT453GAA (5198A>G 5200T>A)								
**Proteins**									
replicase (YP_004347415.1)	290	455	25.9%	640	55.2%	166 (100%)	96 (57.8%)	0/0/0/0	1
Protein mutations:	C298Y (4734G>A), I305L (4754A>C 4756T>A), E308T (4763G>A 4764A>C), D310E (4771T>A), K313Q (4778A>C 4780A>G), Y314L (4782A>T 4783T>A), Y323F (4809A>T), V325E (4815T>A 4816A>G), W326* (4818G>A), I327N (4821T>A), H334E (4841C>G 4843T>A), S336T (4847T>A), S338G (4853A>G 4855T>A), I339R (4857T>G), Q342Y (4865C>T 4867A>T), M344I (4873G>T), G346S (4877G>A 4879A>C), I347C (4880A>T 4881T>G 4882A>T), R349N (4887G>A 4888A>T), P350Q (4890C>A), Y351L (4893A>T 4894T>A), Y352P (4895T>C 4896A>C 4897T>A), D353N (4898G>A 4900T>C), F354C (4902T>G 4903T>C), L356I (4907C>A 4909T>A), I364V (4931A>G), F365C (4935T>G 4936T>C), K367T (4941A>C), E370Q (4949G>C 4951A>G), K373F (4958A>T 4959A>T 4960A>T), E377K (4970G>A), I378E (4973A>G 4974T>A), R380L (4979A>T 4980G>T), N381K (4984T>A), I382F (4985A>T 4987A>T), I383C (4988A>T 4989T>G 4990A>T), I384V (4991A>G 4993A>C), K385N (4996A>T), H386N (4997C>A), G387D (5001G>A 5002A>C), K392Q (5015A>C), G398N (5033G>A 5034G>A 5035A>T), Q400N (5039C>A 5041A>T), V407M (5060G>A 5062T>G), K408I (5064A>T 5065G>T), E410S (5069G>T 5070A>C), Q411K (5072C>A), G412N (5075G>A 5076G>A 5077A>T), P418T (5093C>A), I421S (5102A>T 5103T>C 5104A>C), D422K (5105G>A 5107T>A), K427F (5120A>T 5121A>T 5122A>T), H428P (5124A>C), I429D (5126A>G 5127T>A 5128A>C), I431L (5132A>T), K432I (5136A>T), S433T (5139G>C 5140T>C), K435Q (5144A>C), L437I (5150T>A), S439R (5156T>A 5157C>G), I440F (5159A>T 5161A>T), L443C (5169T>G 5170A>C), V444L (5171G>T), Q446Y (5177C>T 5179A>T), I447A (5180A>G 5181T>C 5182T>A), R448G (5183A>G), N449E (5186A>G 5188T>A), L451I (5192T>A), N453E (5198A>G 5200T>A)								
Codon mutations:	AGT294AGC (4723T>C), TTC296TTT (4729C>T), GAC297GAT (4732C>T), TGT298TAT (4734G>A), TAT303TAC (4750T>C), CAT304CAC (4753T>C), ATT305CTA (4754A>C 4756T>A), AAA306AAG (4759A>G), CTA307TTA (4760C>T), GAA308ACA (4763G>A 4764A>C), GAT310GAA (4771T>A), AGT311TCT (4772A>T 4773G>C), AAA313CAG (4778A>C 4780A>G), TAT314TTA (4782A>T 4783T>A), TAT323TTT (4809A>T), TAT324TAC (4813T>C), GTA325GAG (4815T>A 4816A>G), TGG326TAG (4818G>A), ATT327AAT (4821T>A), TTA329TTG (4828A>G), CCT330CCA (4831T>A), CAT334GAA (4841C>G 4843T>A), AAT335AAC (4846T>C), TCA336ACA (4847T>A), AGT338GGA (4853A>G 4855T>A), ATA339AGA (4857T>G), CAA342TAT (4865C>T 4867A>T), ATG344ATT (4873G>T), GGA346AGC (4877G>A 4879A>C), ATA347TGT (4880A>T 4881T>G 4882A>T), AGA349AAT (4887G>A 4888A>T), CCA350CAA (4890C>A), TAT351TTA (4893A>T 4894T>A), TAT352CCA (4895T>C 4896A>C 4897T>A), GAT353AAC (4898G>A 4900T>C), TTT354TGC (4902T>G 4903T>C), CTT356ATA (4907C>A 4909T>A), GTA357GTG (4912A>G), GAT361GAC (4924T>C), ATA362ATT (4927A>T), TTA363TTG (4930A>G), ATA364GTA (4931A>G), TTT365TGC (4935T>G 4936T>C), TCG366TCC (4939G>C), AAA367ACA (4941A>C), TAC369TAT (4948C>T), GAA370CAG (4949G>C 4951A>G), AAA373TTT (4958A>T 4959A>T 4960A>T), TTG376CTT (4967T>C 4969G>T), GAA377AAA (4970G>A), ATA378GAA (4973A>G 4974T>A), AGA380TTA (4979A>T 4980G>T), AAT381AAA (4984T>A), ATA382TTT (4985A>T 4987A>T), ATA383TGT (4988A>T 4989T>G 4990A>T), ATA384GTC (4991A>G 4993A>C), AAA385AAT (4996A>T), CAT386AAT (4997C>A), GGA387GAC (5001G>A 5002A>C), AAG392CAG (5015A>C), GAG396GAA (5029G>A), GGA398AAT (5033G>A 5034G>A 5035A>T), CAA400AAT (5039C>A 5041A>T), TTT404TTC (5053T>C), CTA405CTT (5056A>T), GTT407ATG (5060G>A 5062T>G), AAG408ATT (5064A>T 5065G>T), ATA409ATT (5068A>T), GAA410TCA (5069G>T 5070A>C), CAA411AAA (5072C>A), GGA412AAT (5075G>A 5076G>A 5077A>T), CAG417CAA (5092G>A), CCT418ACT (5093C>A), CAT419CAC (5098T>C), ATA421TCC (5102A>T 5103T>C 5104A>C), GAT422AAA (5105G>A 5107T>A), AAA423AAG (5110A>G), GAA426GAG (5119A>G), AAA427TTT (5120A>T 5121A>T 5122A>T), CAT428CCT (5124A>C), ATA429GAC (5126A>G 5127T>A 5128A>C), AAA430AAG (5131A>G), ATA431TTA (5132A>T), AAA432ATA (5136A>T), AGT433ACC (5139G>C 5140T>C), AAA435CAA (5144A>C), TTA437ATA (5150T>A), TCA439AGA (5156T>A 5157C>G), ATA440TTT (5159A>T 5161A>T), TTA441CTT (5162T>C 5164A>T), TTA443TGC (5169T>G 5170A>C), GTA444TTA (5171G>T), CAA446TAT (5177C>T 5179A>T), ATT447GCA (5180A>G 5181T>C 5182T>A), AGA448GGA (5183A>G), AAT449GAA (5186A>G 5188T>A), TTA451ATA (5192T>A), AAT453GAA (5198A>G 5200T>A)								

*: Inserts / Deletes / Misaligned / Frameshifts

Analysis details

This analysis was performed with panviral2.64

NGS Details (UN60): Dioscovirus dioscoreae

Assembly

**Coverage Length**	932 (1 contig(s))
**Depth Of Coverage**	3.7
**Number Of Reads**	26
**Reads Per Million**	0.55 rpm (after QC)
**Ambiguities**	0
**Assembly Method**	de novo + reference guided assembly
**Consensus Caller**	Bcf Tools

Coverage Map

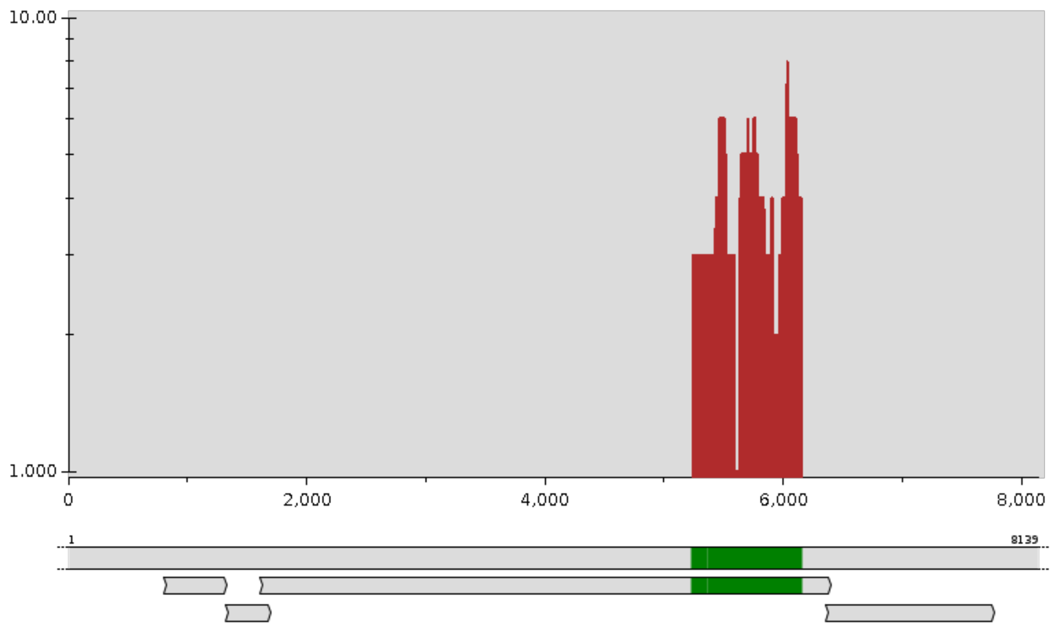

Assignment

**Type**	Dioscovirus dioscoreae (Taxonomy ID: 3052184)
**Reference Genome**	NC_040712.1
**NT Identity (%)**	58.5288
**AA Identity (%)**	48.5623
**Number Of Stop Codons**	0
**Number Of CDS**	4

Alignment

**Alignment Score**	299.0 (NT) + 1031.0 (AA) = 1330.0
**Concordance (%)**	33.4087

Alignment Method	Global, seeded, nucleotide + amino acids (AGA)

Genome Region

Sequence starts at position 5232 and ends at position 6164 relative to NC_040712.1 reference sequence.

Alignment Detailed Statistics

	Begin	End	Coverage	Score	Concordance	Matches	Identities	I/D/M/F*	Stop Codons
**NT**	**5232**	**6164**	**11.5%**	**299**	**16.2%**	**929 (98.7%)**	**549 (58.3%)**	**9/3**	
Mutations:	5233A>T, 5246A>G, 5247A>G, 5248G>C, 5249T>A, 5252T>A, 5259T>A, 5260A>T, 5261C>A, 5264T>C, 5267T>C, 5270G>A, 5271C>A, 5276A>T, 5285T>C, 5288A>T, 5289T>A, 5290A>G, 5291C>A, 5293A>C, 5294A>G, 5295G>A, 5297C>T, 5298C>G, 5299A>C, 5300A>C, 5303C>T, 5305C>A, 5306A>G, 5309C>A, 5310C>T, 5313A>G, 5314G>A, 5317T>A, 5318T>A, 5319G>A, 5320A>G, 5321C>T, 5322T>G, 5324T>A, 5325C>T, 5327C>A, 5328C>A, 5331C>A, 5332T>A, 5335A>G, 5339T>A, 5340A>C, 5344A>G, 5345C>T, 5350T>A, 5351T>A, 5352G>A, 5354C>A, 5356A>T, 5360C>T, 5370C>T, 5371T>G, 5372A>T, 5374A>G, 5378A>T, 5383T>A, 5384C>T, 5387C>T, 5390G>A, 5391A>C, 5395T>G, 5396G>A, 5401A>C, 5402T>C, 5403C>G, 5406C>G, 5409A>G, 5410G>A, 5412A>G, 5414A>G, 5415G>C, 5416A>C, 5418T>A, 5419G>A, 5420G>A, 5423A>T, 5426A>C, 5430G>A, 5431T>G, 5432C>A, 5433T>A, 5434G>C, 5435C>T, 5437C>A, 5438A>C, 5439C>T, 5440A>C, 5441G>T, 5444T>G, 5453A>G, 5455G>A, 5456G>C, 5457A>T, 5462A>C, 5468A>C, 5471C>T, 5477C>G, 5478A>T, 5479A>C, 5485C>T, 5486A>C, 5489T>A, 5490T>G, 5492A>C, 5493G>A, 5494T>C, 5498C>T, 5502C>G, 5505A>T, 5506A>T, 5511G>A, 5514A>T, 5515A>C, 5517A>G, 5519A>G, 5523A>C, 5525A>G, 5530A>T, 5532T>C, 5533C>T, 5534_5535insAGG, 5535G>A, 5537A>G, 5538T>C, 5539T>A, 5543C>G, 5545G>T, 5546T>G, 5549T>A, 5551A>T, 5552C>T, 5553A>T, 5555A>T, 5558C>T, 5564T>A, 5565T>C, 5567G>T, 5572T>A, 5574T>A, 5575C>G, 5576T>C, 5577G>A, 5578A>G, 5581G>C, 5582T>C, 5586C>G, 5589C>G, 5591A>T, 5595G>C, 5598C>G, 5599A>G, 5600A>T, 5603T>C, 5604T>C, 5607T>C, 5608T>A, 5609A>G, 5610C>A, 5611A>C, 5612G>A, 5613T>A, 5615C>A, 5618C>T, 5619C>A, 5620A>G, 5622G>C, 5626G>T, 5628A>G, 5629A>T, 5630G>A, 5631G>A, 5632A>G, 5633A>T, 5634G>A, 5636A>C, 5639G>C, 5640C>A, 5642T>A, 5645T>A, 5646T>G, 5648G>A, 5649T>A, 5650C>G, 5651A>C, 5656C>A, 5657A>G, 5661T>A, 5667A>T, 5670G>T, 5672A>T, 5673G>A, 5674T>A, 5676G>A, 5678A>C, 5679A>C, 5685G>A, 5687A>T, 5691C>T, 5693T>A, 5696C>G, 5697C>A, 5699A>T, 5700G>A, 5701A>T, 5703A>T, 5705A>G, 5711A>T, 5714A>G, 5717A>G, 5718G>A, 5720T>A, 5721C>A, 5726C>A, 5739T>G, 5740T>C, 5741A>T, 5748A>G, 5756A>T, 5757T>A, 5759T>G, 5762T>A, 5763_5765delGAA, 5768C>T, 5769C>A, 5777A>T, 5779C>A, 5781T>A, 5782T>C, 5786G>A, 5788G>A, 5790C>T, 5795G>A, 5798G>A, 5807G>A, 5808A>T, 5811T>G, 5813A>G, 5816C>T, 5822C>T, 5828T>C, 5830A>T, 5834C>A, 5836C>A, 5843G>A, 5844A>G, 5846T>A, 5850T>A, 5851A>T, 5853A>G, 5855T>A, 5856A>G, 5859A>C, 5860T>C, 5864T>A, 5868A>G, 5869A>C, 5870T>A, 5873G>A, 5874T>A, 5875G>T, 5876T>A, 5877T>G, 5878C>G, 5879C>A, 5882C>T, 5889G>C, 5893G>A, 5895A>C, 5897A>C, 5903T>C, 5904T>A, 5906T>A, 5907C>G, 5910G>A, 5912C>T, 5913T>A, 5914G>T, 5915G>T, 5918G>A, 5919A>C, 5924T>A, 5926G>A, 5928A>C, 5933C>T, 5940G>A, 5941T>A, 5945T>A, 5947C>A, 5948C>A, 5951A>C, 5952T>A, 5957C>A, 5958C>G, 5959C>A, 5960T>C, 5963G>A, 5965T>G, 5966T>C, 5967T>A, 5973A>C, 5974A>T, 5976C>G, 5977C>A, 5979C>T, 5980A>C, 5982A>G, 5983G>A, 5988A>C, 5990T>A, 5999G>A, 6005T>C, 6009T>A, 6010C>G, 6011T>C, 6014A>C, 6015G>C, 6026G>A, 6028G>C, 6029A>T, 6030A>G, 6032T>A, 6039T>G, 6040G>C, 6041G>A, 6047C>A, 6048T>A, 6049C>A, 6050T>C, 6051C>A, 6054C>T, 6056A>C, 6057G>A, 6058A>G, 6060T>C, 6062A>T, 6063C>A, 6064C>A, 6065T>A, 6066T>A, 6067C>A, 6069A>G, 6070C>A, 6082C>G, 6083A>T, 6084C>G, 6085G>C, 6086G>T, 6089T>C, 6090T>C, 6091G>A, 6092T>A, 6100G>A, 6101C>A, 6104T>C, 6107_6108insGAAAAG, 6108T>G, 6109C>G, 6111G>A, 6112C>A, 6116C>G, 6117T>A, 6118C>G, 6120G>A, 6121C>G, 6122A>T, 6130C>T, 6131T>C, 6135A>G, 6138A>T, 6140G>A, 6144T>A, 6145G>T, 6146T>A, 6154T>G, 6155A>G, 6156T>C, 6159G>A								
**CDS**									
EXK67_gp3	1208	1518	19.5%	1031	47.8%	310 (98.7%)	152 (48.4%)	3/1/1/0	0
Protein mutations:	Q1208L (5233A>T), S1213A (5247A>G 5248G>C 5249T>A), Y1217I (5259T>A 5260A>T 5261C>A), Y1227R (5289T>A 5290A>G 5291C>A), K1228T (5293A>C 5294A>G), D1229N (5295G>A 5297C>T), Q1230A (5298C>G 5299A>C 5300A>C), T1232K (5305C>A 5306A>G), P1234S (5310C>T), S1235D (5313A>G 5314G>A), I1236K (5317T>A 5318T>A), D1237S (5319G>A 5320A>G 5321C>T), Y1238E (5322T>G 5324T>A), L1240I (5328C>A), L1241N (5331C>A 5332T>A), K1242R (5335A>G), K1244Q (5340A>C), D1245G (5344A>G 5345C>T), I1247K (5350T>A 5351T>A), V1248I (5352G>A 5354C>A), Y1249F (5356A>T), L1254C (5370C>T 5371T>G 5372A>T), F1258Y (5383T>A 5384C>T), I1261L (5391A>C), M1262R (5395T>G 5396G>A), D1264A (5401A>C 5402T>C), P1265A (5403C>G), Q1266E (5406C>G), S1267D (5409A>G 5410G>A), I1268V (5412A>G 5414A>G), E1269P (5415G>C 5416A>C), W1270K (5418T>A 5419G>A 5420G>A), V1274R (5430G>A 5431T>G 5432C>A), C1275T (5433T>A 5434G>C 5435C>T), P1276H (5437C>A 5438A>C), Q1277S (5439C>T 5440A>C 5441G>T), W1282Y (5455G>A 5456G>C), I1283L (5457A>T), K1290S (5478A>T 5479A>C), A1292V (5485C>T 5486A>C), S1294A (5490T>G 5492A>C), V1295T (5493G>A 5494T>C), R1298G (5502C>G), K1299L (5505A>T 5506A>T), D1301N (5511G>A), N1302S (5514A>T 5515A>C), I1303V (5517A>G 5519A>G), K1305Q (5523A>C 5525A>G), Y1307F (5530A>T), S1308L (5532T>C 5533C>T), S1308_E1309insR (5534_5535insAGG), E1309K (5535G>A 5537A>G), F1310H (5538T>C 5539T>A), C1312L (5545G>T 5546T>G), Y1314F (5551A>T 5552C>T), I1315F (5553A>T 5555A>T), F1321Y (5572T>A), E1323R (5577G>A 5578A>G), S1324T (5581G>C 5582T>C), Q1326E (5586C>G), Q1327D (5589C>G 5591A>T), V1329L (5595G>C), Q1330G (5598C>G 5599A>G 5600A>T), L1333Q (5607T>C 5608T>A 5609A>G), Q1334T (5610C>A 5611A>C 5612G>A), F1335I (5613T>A 5615C>A), Q1337R (5619C>A 5620A>G), V1338L (5622G>C), C1339F (5626G>T), K1340V (5628A>G 5629A>T 5630G>A), E1341S (5631G>A 5632A>G 5633A>T), E1342N (5634G>A 5636A>C), L1344I (5640C>A 5642T>A), L1346V (5646T>G 5648G>A), T1349K (5656C>A 5657A>G), L1351M (5661T>A), I1353L (5667A>T), G1354C (5670G>T 5672A>T), V1355K (5673G>A 5674T>A), A1356T (5676G>A 5678A>C), N1357H (5679A>C), E1359N (5685G>A 5687A>T), L1363I (5697C>A 5699A>T), E1364I (5700G>A 5701A>T), I1365L (5703A>T 5705A>G), E1367D (5711A>T), V1370I (5718G>A 5720T>A), Q1371K (5721C>A), L1377A (5739T>G 5740T>C 5741A>T), I1380V (5748A>G), E1382D (5756A>T), F1383M (5757T>A 5759T>G), E1385del (5763_5765delGAA), Q1387K (5769C>A), E1389D (5777A>T), T1390K (5779C>A), L1391T (5781T>A 5782T>C), G1393D (5788G>A), I1400L (5808A>T), L1401V (5811T>G 5813A>G), Y1407F (5830A>T), P1409Q (5836C>A), S1412G (5844A>G 5846T>A), Y1414I (5850T>A 5851A>T), T1415A (5853A>G 5855T>A), R1416G (5856A>G), I1417P (5859A>C 5860T>C), F1418L (5864T>A), N1420A (5868A>G 5869A>C 5870T>A), C1422I (5874T>A 5875G>T 5876T>A), S1423G (5877T>G 5878C>G 5879C>A), E1427Q (5889G>C), R1428K (5893G>A), K1429H (5895A>C 5897A>C), S1432T (5904T>A 5906T>A), Q1433E (5907C>G), D1434N (5910G>A 5912C>T), W1435I (5913T>A 5914G>T 5915G>T), M1437L (5919A>C), R1439K (5926G>A), K1440Q (5928A>C), V1444K (5940G>A 5941T>A), T1446K (5947C>A 5948C>A), K1447N (5951A>C), L1448I (5952T>A), P1450D (5958C>G 5959C>A 5960T>C), I1452S (5965T>G 5966T>C), L1453I (5967T>A), K1455L (5973A>C 5974A>T), P1456E (5976C>G 5977C>A), Q1457S (5979C>T 5980A>C), S1458D (5982A>G 5983G>A), I1460L (5988A>C 5990T>A), E1469Q (6015G>C), G1473A (6028G>C 6029A>T), I1474V (6030A>G 6032T>A), W1477A (6039T>G 6040G>C 6041G>A), S1480N (6048T>A 6049C>A 6050T>C), Q1481K (6051C>A), Q1482Y (6054C>T 6056A>C), D1483S (6057G>A 6058A>G), S1484P (6060T>C 6062A>T), P1485K (6063C>A 6064C>A 6065T>A), S1486K (6066T>A 6067C>A), T1487E (6069A>G 6070C>A), S1491C (6082C>G 6083A>T), R1492A (6084C>G 6085G>C 6086G>T), C1494Q (6090T>C 6091G>A 6092T>A), S1497K (6100G>A 6101C>A), K1499_S1500insEK (6107_6108insGAAAAG), S1500G (6108T>G 6109C>G), A1501N (6111G>A 6112C>A), I1502M (6116C>G), A1504S (6120G>A 6121C>G 6122A>T), A1507V (6130C>T 6131T>C), I1509V (6135A>G), M1510L (6138A>T 6140G>A), C1512I (6144T>A 6145G>T 6146T>A), V1515G (6154T>G 6155A>G), D1517N (6159G>A)								

	Begin	End	Coverage	Score	Concordance	Matches	Identities	I/D/M/F*	Stop Codons
**NT**	**5232**	**6164**	**11.5%**	**299**	**16.2%**	**929 (98.7%)**	**549 (58.3%)**	**9/3**	
Codon mutations:	CAA1208CTA (5233A>T), AAA1212AAG (5246A>G), AGT1213GCA (5247A>G 5248G>C 5249T>A), CGT1214CGA (5252T>A), TAC1217ATA (5259T>A 5260A>T 5261C>A), AAT1218AAC (5264T>C), TAT1219TAC (5267T>C), AAG1220AAA (5270G>A), CGA1221AGA (5271C>A), CTA1222CTT (5276A>T), AAT1225AAC (5285T>C), ACA1226ACT (5288A>T), TAC1227AGA (5289T>A 5290A>G 5291C>A), AAA1228ACG (5293A>C 5294A>G), GAC1229AAT (5295G>A 5297C>T), CAA1230GCC (5298C>G 5299A>C 5300A>C), TAC1231TAT (5303C>T), ACA1232AAG (5305C>A 5306A>G), CTC1233CTA (5309C>A), CCA1234TCA (5310C>T), AGT1235GAT (5313A>G 5314G>A), ATT1236AAA (5317T>A 5318T>A), GAC1237AGT (5319G>A 5320A>G 5321C>T), TAT1238GAA (5322T>G 5324T>A), CTC1239TTA (5325C>T 5327C>A), CTA1240ATA (5328C>A), CTT1241AAT (5331C>A 5332T>A), AAA1242AGA (5335A>G), ATT1243ATA (5339T>A), AAA1244CAA (5340A>C), GAC1245GGT (5344A>G 5345C>T), ATT1247AAA (5350T>A 5351T>A), GTC1248ATA (5352G>A 5354C>A), TAC1249TTC (5356A>T), AGC1250AGT (5360C>T), CTA1254TGT (5370C>T 5371T>G 5372A>T), AAG1255.GG (5374A>G), TCA1256TCT (5378A>T), TTC1258TAT (5383T>A 5384C>T), CAC1259CAT (5387C>T), CAG1260CAA (5390G>A), ATT1261CTT (5391A>C), ATG1262AGA (5395T>G 5396G>A), GAT1264GCC (5401A>C 5402T>C), CCA1265GCA (5403C>G), CAG1266GAG (5406C>G), AGT1267GAT (5409A>G 5410G>A), ATA1268GTG (5412A>G 5414A>G), GAA1269CCA (5415G>C 5416A>C), TGG1270AAA (5418T>A 5419G>A 5420G>A), ACA1271ACT (5423A>T), GCA1272GCC (5426A>C), GTC1274AGA (5430G>A 5431T>G 5432C>A), TGC1275ACT (5433T>A 5434G>C 5435C>T), CCA1276CAC (5437C>A 5438A>C), CAG1277TCT (5439C>T 5440A>C 5441G>T), GGT1278GGG (5444T>G), GAA1281GAG (5453A>G), TGG1282TAC (5455G>A 5456G>C), ATA1283TTA (5457A>T), GTA1284GTC (5462A>C), CCA1286CCC (5468A>C), TTC1287TTT (5471C>T), CTC1289CTG (5477C>G), AAA1290TCA (5478A>T 5479A>C), GCA1292GTC (5485C>T 5486A>C), CCT1293CCA (5489T>A), TCA1294GCC (5490T>G 5492A>C), GTA1295ACA (5493G>A 5494T>C), TTC1296TTT (5498C>T), CGA1298GGA (5502C>G), AAA1299TTA (5505A>T 5506A>T), GAT1301AAT (5511G>A), AAT1302TCT (5514A>T 5515A>C), ATA1303GTG (5517A>G 5519A>G), AAA1305CAG (5523A>C 5525A>G), TAT1307TTT (5530A>T), TCA1308CTA (5532T>C 5533C>T), TCA1308_GAA1309insAGG (5534_5535insAGG), GAA1309AAG (5535G>A 5537A>G), TTT1310CAT (5538T>C 5539T>A), GTC1311GTG (5543C>G), TGT1312TTG (5545G>T 5546T>G), GTT1313GTA (5549T>A), TAC1314TTT (5551A>T 5552C>T), ATA1315TTT (5553A>T 5555A>T), GAC1316GAT (5558C>T), ATT1318ATA (5564T>A), TTG1319CTT (5565T>C 5567G>T), TTT1321TAT (5572T>A), TCT1322AGC (5574T>A 5575C>G 5576T>C), GAA1323AGA (5577G>A 5578A>G), AGT1324ACC (5581G>C 5582T>C), CAA1326GAA (5586C>G), CAA1327GAT (5589C>G 5591A>T), GTA1329CTA (5595G>C), CAA1330GGT (5598C>G 5599A>G 5600A>T), CAT1331CAC (5603T>C), TTA1332CTA (5604T>C), TTA1333CAG (5607T>C 5608T>A 5609A>G), CAG1334ACA (5610C>A 5611A>C 5612G>A), TTC1335ATA (5613T>A 5615C>A), TTC1336TTT (5618C>T), CAA1337AGA (5619C>A 5620A>G), GTA1338CTA (5622G>C), TGT1339TTT (5626G>T), AAG1340GTA (5628A>G 5629A>T 5630G>A), GAA1341AGT (5631G>A 5632A>G 5633A>T), GAA1342AAC (5634G>A 5636A>C), GGG1343GGC (5639G>C), CTT1344ATA (5640C>A 5642T>A), ATT1345ATA (5645T>A), TTG1346GTA (5646T>G 5648G>A), TCA1347AGC (5649T>A 5650C>G 5651A>C), ACA1349AAG (5656C>A 5657A>G), TTG1351ATG (5661T>A), ATA1353TTA (5667A>T), GGA1354TGT (5670G>T 5672A>T), GTA1355AAA (5673G>A 5674T>A), GCA1356ACC (5676G>A 5678A>C), AAT1357CAT (5679A>C), GAA1359AAT (5685G>A 5687A>T), CTT1361TTA (5691C>T 5693T>A), GGC1362GGG (5696C>G), CTA1363ATT (5697C>A 5699A>T), GAA1364ATA (5700G>A 5701A>T), ATA1365TTG (5703A>T 5705A>G), GAA1367GAT (5711A>T), GGA1368GGG (5714A>G), AAA1369AAG (5717A>G), GTT1370ATA (5718G>A 5720T>A), CAA1371AAA (5721C>A), CTC1372CTA (5726C>A), TTA1377GCT (5739T>G 5740T>C 5741A>T), ATA1380GTA (5748A>G), GAA1382GAT (5756A>T), TTT1383ATG (5757T>A 5759T>G), CCT1384CCA (5762T>A), GAA1385del (5763_5765delGAA), GAC1386GAT (5768C>T), CAA1387AAA (5769C>A), GAA1389GAT (5777A>T), ACA1390AAA (5779C>A), TTA1391ACA (5781T>A 5782T>C), AAG1392AAA (5786G>A), GGT1393GAT (5788G>A), CTA1394TTA (5790C>T), CAG1395CAA (5795G>A), AAG1396AAA (5798G>A), GGG1399GGA (5807G>A), ATA1400TTA (5808A>T), TTA1401GTG (5811T>G 5813A>G), AAC1402AAT (5816C>T), GCC1404GCT (5822C>T), AAT1406AAC (5828T>C), TAT1407TTT (5830A>T), ATC1408ATA (5834C>A), CCA1409CAA (5836C>A), TTG1411TTA (5843G>A), AGT1412GGA (5844A>G 5846T>A), TAT1414ATT (5850T>A 5851A>T), ACT1415GCA (5853A>G 5855T>A), AGA1416GGA (5856A>G), ATA1417CCA (5859A>C 5860T>C), TTT1418TTA (5864T>A), AAT1420GCA (5868A>G 5869A>C 5870T>A), AAG1421AAA (5873G>A), TGT1422ATA (5874T>A 5875G>T 5876T>A), TCC1423GGA (5877T>G 5878C>G 5879C>A), AGC1424AGT (5882C>T), GAA1427CAA (5889G>C), AGA1428AAA (5893G>A), AAA1429CAC (5895A>C 5897A>C), AAT1431AAC (5903T>C), TCT1432ACA (5904T>A 5906T>A), CAA1433GAA (5907C>G), GAC1434AAT (5910G>A 5912C>T), TGG1435ATT (5913T>A 5914G>T 5915G>T), AAG1436AAA (5918G>A), ATG1437CTG (5919A>C), GTT1438GTA (5924T>A), AGA1439AAA (5926G>A), AAA1440CAA (5928A>C), ATC1441ATT (5933C>T), GTA1444AAA (5940G>A 5941T>A), ATT1445ATA (5945T>A), ACC1446AAA (5947C>A 5948C>A), AAA1447AAC (5951A>C), TTA1448ATA (5952T>A), CCC1449CCA (5957C>A), CCT1450GAC (5958C>G 5959C>A 5960T>C), TTG1451TTA (5963G>A), ATT1452AGC (5965T>G 5966T>C), TTA1453ATA (5967T>A), AAA1455CTA (5973A>C 5974A>T), CCA1456GAA (5976C>G 5977C>A), CAA1457TCA (5979C>T 5980A>C), AGT1458GAT (5982A>G 5983G>A), ATT1460CTA (5988A>C 5990T>A), GAG1463GAA (5999G>A), GAT1465GAC (6005T>C), TCT1467AGC (6009T>A 6010C>G 6011T>C), CTA1468CTC (6014A>C), GAA1469CAA (6015G>C), GGG1472GGA (6026G>A), GGA1473GCT (6028G>C 6029A>T), ATT1474GTA (6030A>G 6032T>A), TGG1477GCA (6039T>G 6040G>C 6041G>A), CCC1479CCA (6047C>A), TCT1480AAC (6048T>A 6049C>A 6050T>C), CAA1481AAA (6051C>A), CAA1482TAC (6054C>T 6056A>C), GAT1483AGT (6057G>A 6058A>G), TCA1484CCT (6060T>C 6062A>T), CCT1485AAA (6063C>A 6064C>A 6065T>A), TCA1486AAA (6066T>A 6067C>A), ACA1487GAA (6069A>G 6070C>A), TCA1491TGT (6082C>G 6083A>T), CGG1492GCT (6084C>G 6085G>C 6086G>T), TAT1493TAC (6089T>C), TGT1494CAA (6090T>C 6091G>A 6092T>A), AGC1497AAA (6100G>A 6101C>A), TAT1498TAC (6104T>C), AAA1499_TCT1500insGAAAAG (6107_6108insGAAAAG), TCT1500GGT (6108T>G 6109C>G), GCC1501AAC (6111G>A 6112C>A), ATC1502ATG (6116C>G), TCT1503AGT (6117T>A 6118C>G), GCA1504AGT (6120G>A 6121C>G 6122A>T), GCT1507GTC (6130C>T 6131T>C), ATA1509GTA (6135A>G), ATG1510TTA (6138A>T 6140G>A), TGT1512ATA (6144T>A 6145G>T 6146T>A), GTA1515GGG (6154T>G 6155A>G), TTA1516CTA (6156T>C), GAT1517AAT (6159G>A)								
**Proteins**									
ORF3 (YP_009553219.1)	1208	1518	19.5%	1031	47.8%	310 (98.7%)	152 (48.4%)	3/1/1/0	0
Protein mutations:	Q1208L (5233A>T), S1213A (5247A>G 5248G>C 5249T>A), Y1217I (5259T>A 5260A>T 5261C>A), Y1227R (5289T>A 5290A>G 5291C>A), K1228T (5293A>C 5294A>G), D1229N (5295G>A 5297C>T), Q1230A (5298C>G 5299A>C 5300A>C), T1232K (5305C>A 5306A>G), P1234S (5310C>T), S1235D (5313A>G 5314G>A), I1236K (5317T>A 5318T>A), D1237S (5319G>A 5320A>G 5321C>T), Y1238E (5322T>G 5324T>A), L1240I (5328C>A), L1241N (5331C>A 5332T>A), K1242R (5335A>G), K1244Q (5340A>C), D1245G (5344A>G 5345C>T), I1247K (5350T>A 5351T>A), V1248I (5352G>A 5354C>A), Y1249F (5356A>T), L1254C (5370C>T 5371T>G 5372A>T), F1258Y (5383T>A 5384C>T), I1261L (5391A>C), M1262R (5395T>G 5396G>A), D1264A (5401A>C 5402T>C), P1265A (5403C>G), Q1266E (5406C>G), S1267D (5409A>G 5410G>A), I1268V (5412A>G 5414A>G), E1269P (5415G>C 5416A>C), W1270K (5418T>A 5419G>A 5420G>A), V1274R (5430G>A 5431T>G 5432C>A), C1275T (5433T>A 5434G>C 5435C>T), P1276H (5437C>A 5438A>C), Q1277S (5439C>T 5440A>C 5441G>T), W1282Y (5455G>A 5456G>C), I1283L (5457A>T), K1290S (5478A>T 5479A>C), A1292V (5485C>T 5486A>C), S1294A (5490T>G 5492A>C), V1295T (5493G>A 5494T>C), R1298G (5502C>G), K1299L (5505A>T 5506A>T), D1301N (5511G>A), N1302S (5514A>T 5515A>C), I1303V (5517A>G 5519A>G), K1305Q (5523A>C 5525A>G), Y1307F (5530A>T), S1308L (5532T>C 5533C>T), S1308_E1309insR (5534_5535insAGG), E1309K (5535G>A 5537A>G), F1310H (5538T>C 5539T>A), C1312L (5545G>T 5546T>G), Y1314F (5551A>T 5552C>T), I1315F (5553A>T 5555A>T), F1321Y (5572T>A), E1323R (5577G>A 5578A>G), S1324T (5581G>C 5582T>C), Q1326E (5586C>G), Q1327D (5589C>G 5591A>T), V1329L (5595G>C), Q1330G (5598C>G 5599A>G 5600A>T), L1333Q (5607T>C 5608T>A 5609A>G), Q1334T (5610C>A 5611A>C 5612G>A), F1335I (5613T>A 5615C>A), Q1337R (5619C>A 5620A>G), V1338L (5622G>C), C1339F (5626G>T), K1340V (5628A>G 5629A>T 5630G>A), E1341S (5631G>A 5632A>G 5633A>T), E1342N (5634G>A 5636A>C), L1344I (5640C>A 5642T>A), L1346V (5646T>G 5648G>A), T1349K (5656C>A 5657A>G), L1351M (5661T>A), I1353L (5667A>T), G1354C (5670G>T 5672A>T), V1355K (5673G>A 5674T>A), A1356T (5676G>A 5678A>C), N1357H (5679A>C), E1359N (5685G>A 5687A>T), L1363I (5697C>A 5699A>T), E1364I (5700G>A 5701A>T), I1365L (5703A>T 5705A>G), E1367D (5711A>T), V1370I (5718G>A 5720T>A), Q1371K (5721C>A), L1377A (5739T>G 5740T>C 5741A>T), I1380V (5748A>G), E1382D (5756A>T), F1383M (5757T>A 5759T>G), E1385del (5763_5765delGAA), Q1387K (5769C>A), E1389D (5777A>T), T1390K (5779C>A), L1391T (5781T>A 5782T>C), G1393D (5788G>A), I1400L (5808A>T), L1401V (5811T>G 5813A>G), Y1407F (5830A>T), P1409Q (5836C>A), S1412G (5844A>G 5846T>A), Y1414I (5850T>A 5851A>T), T1415A (5853A>G 5855T>A), R1416G (5856A>G), I1417P (5859A>C 5860T>C), F1418L (5864T>A), N1420A (5868A>G 5869A>C 5870T>A), C1422I (5874T>A 5875G>T 5876T>A), S1423G (5877T>G 5878C>G 5879C>A), E1427Q (5889G>C), R1428K (5893G>A), K1429H (5895A>C 5897A>C), S1432T (5904T>A 5906T>A), Q1433E (5907C>G), D1434N (5910G>A 5912C>T), W1435I (5913T>A 5914G>T 5915G>T), M1437L (5919A>C), R1439K (5926G>A), K1440Q (5928A>C), V1444K (5940G>A 5941T>A), T1446K (5947C>A 5948C>A), K1447N (5951A>C), L1448I (5952T>A), P1450D (5958C>G 5959C>A 5960T>C), I1452S (5965T>G 5966T>C), L1453I (5967T>A), K1455L (5973A>C 5974A>T), P1456E (5976C>G 5977C>A), Q1457S (5979C>T 5980A>C), S1458D (5982A>G 5983G>A), I1460L (5988A>C 5990T>A), E1469Q (6015G>C), G1473A (6028G>C 6029A>T), I1474V (6030A>G 6032T>A), W1477A (6039T>G 6040G>C 6041G>A), S1480N (6048T>A 6049C>A 6050T>C), Q1481K (6051C>A), Q1482Y (6054C>T 6056A>C), D1483S (6057G>A 6058A>G), S1484P (6060T>C 6062A>T), P1485K (6063C>A 6064C>A 6065T>A), S1486K (6066T>A 6067C>A), T1487E (6069A>G 6070C>A), S1491C (6082C>G 6083A>T), R1492A (6084C>G 6085G>C 6086G>T), C1494Q (6090T>C 6091G>A 6092T>A), S1497K (6100G>A 6101C>A), K1499_S1500insEK (6107_6108insGAAAAG), S1500G (6108T>G 6109C>G), A1501N (6111G>A 6112C>A), I1502M (6116C>G), A1504S (6120G>A 6121C>G 6122A>T), A1507V (6130C>T 6131T>C), I1509V (6135A>G), M1510L (6138A>T 6140G>A), C1512I (6144T>A 6145G>T 6146T>A), V1515G (6154T>G 6155A>G), D1517N (6159G>A)								

	Begin	End	Coverage	Score	Concordance	Matches	Identities	I/D/M/F*	Stop Codons
**NT**	**5232**	**6164**	**11.5%**	**299**	**16.2%**	**929 (98.7%)**	**549 (58.3%)**	**9/3**	
Codon mutations:	CAA1208CTA (5233A>T), AAA1212AAG (5246A>G), AGT1213GCA (5247A>G 5248G>C 5249T>A), CGT1214CGA (5252T>A), TAC1217ATA (5259T>A 5260A>T 5261C>A), AAT1218AAC (5264T>C), TAT1219TAC (5267T>C), AAG1220AAA (5270G>A), CGA1221AGA (5271C>A), CTA1222CTT (5276A>T), AAT1225AAC (5285T>C), ACA1226ACT (5288A>T), TAC1227AGA (5289T>A 5290A>G 5291C>A), AAA1228ACG (5293A>C 5294A>G), GAC1229AAT (5295G>A 5297C>T), CAA1230GCC (5298C>G 5299A>C 5300A>C), TAC1231TAT (5303C>T), ACA1232AAG (5305C>A 5306A>G), CTC1233CTA (5309C>A), CCA1234TCA (5310C>T), AGT1235GAT (5313A>G 5314G>A), ATT1236AAA (5317T>A 5318T>A), GAC1237AGT (5319G>A 5320A>G 5321C>T), TAT1238GAA (5322T>G 5324T>A), CTC1239TTA (5325C>T 5327C>A), CTA1240ATA (5328C>A), CTT1241AAT (5331C>A 5332T>A), AAA1242AGA (5335A>G), ATT1243ATA (5339T>A), AAA1244CAA (5340A>C), GAC1245GGT (5344A>G 5345C>T), ATT1247AAA (5350T>A 5351T>A), GTC1248ATA (5352G>A 5354C>A), TAC1249TTC (5356A>T), AGC1250AGT (5360C>T), CTA1254TGT (5370C>T 5371T>G 5372A>T), AAG1255.GG (5374A>G), TCA1256TCT (5378A>T), TTC1258TAT (5383T>A 5384C>T), CAC1259CAT (5387C>T), CAG1260CAA (5390G>A), ATT1261CTT (5391A>C), ATG1262AGA (5395T>G 5396G>A), GAT1264GCC (5401A>C 5402T>C), CCA1265GCA (5403C>G), CAG1266GAG (5406C>G), AGT1267GAT (5409A>G 5410G>A), ATA1268GTG (5412A>G 5414A>G), GAA1269CCA (5415G>C 5416A>C), TGG1270AAA (5418T>A 5419G>A 5420G>A), ACA1271ACT (5423A>T), GCA1272GCC (5426A>C), GTC1274AGA (5430G>A 5431T>G 5432C>A), TGC1275ACT (5433T>A 5434G>C 5435C>T), CCA1276CAC (5437C>A 5438A>C), CAG1277TCT (5439C>T 5440A>C 5441G>T), GGT1278GGG (5444T>G), GAA1281GAG (5453A>G), TGG1282TAC (5455G>A 5456G>C), ATA1283TTA (5457A>T), GTA1284GTC (5462A>C), CCA1286CCC (5468A>C), TTC1287TTT (5471C>T), CTC1289CTG (5477C>G), AAA1290TCA (5478A>T 5479A>C), GCA1292GTC (5485C>T 5486A>C), CCT1293CCA (5489T>A), TCA1294GCC (5490T>G 5492A>C), GTA1295ACA (5493G>A 5494T>C), TTC1296TTT (5498C>T), CGA1298GGA (5502C>G), AAA1299TTA (5505A>T 5506A>T), GAT1301AAT (5511G>A), AAT1302TCT (5514A>T 5515A>C), ATA1303GTG (5517A>G 5519A>G), AAA1305CAG (5523A>C 5525A>G), TAT1307TTT (5530A>T), TCA1308CTA (5532T>C 5533C>T), TCA1308_GAA1309insAGG (5534_5535insAGG), GAA1309AAG (5535G>A 5537A>G), TTT1310CAT (5538T>C 5539T>A), GTC1311GTG (5543C>G), TGT1312TTG (5545G>T 5546T>G), GTT1313GTA (5549T>A), TAC1314TTT (5551A>T 5552C>T), ATA1315TTT (5553A>T 5555A>T), GAC1316GAT (5558C>T), ATT1318ATA (5564T>A), TTG1319CTT (5565T>C 5567G>T), TTT1321TAT (5572T>A), TCT1322AGC (5574T>A 5575C>G 5576T>C), GAA1323AGA (5577G>A 5578A>G), AGT1324ACC (5581G>C 5582T>C), CAA1326GAA (5586C>G), CAA1327GAT (5589C>G 5591A>T), GTA1329CTA (5595G>C), CAA1330GGT (5598C>G 5599A>G 5600A>T), CAT1331CAC (5603T>C), TTA1332CTA (5604T>C), TTA1333CAG (5607T>C 5608T>A 5609A>G), CAG1334ACA (5610C>A 5611A>C 5612G>A), TTC1335ATA (5613T>A 5615C>A), TTC1336TTT (5618C>T), CAA1337AGA (5619C>A 5620A>G), GTA1338CTA (5622G>C), TGT1339TTT (5626G>T), AAG1340GTA (5628A>G 5629A>T 5630G>A), GAA1341AGT (5631G>A 5632A>G 5633A>T), GAA1342AAC (5634G>A 5636A>C), GGG1343GGC (5639G>C), CTT1344ATA (5640C>A 5642T>A), ATT1345ATA (5645T>A), TTG1346GTA (5646T>G 5648G>A), TCA1347AGC (5649T>A 5650C>G 5651A>C), ACA1349AAG (5656C>A 5657A>G), TTG1351ATG (5661T>A), ATA1353TTA (5667A>T), GGA1354TGT (5670G>T 5672A>T), GTA1355AAA (5673G>A 5674T>A), GCA1356ACC (5676G>A 5678A>C), AAT1357CAT (5679A>C), GAA1359AAT (5685G>A 5687A>T), CTT1361TTA (5691C>T 5693T>A), GGC1362GGG (5696C>G), CTA1363ATT (5697C>A 5699A>T), GAA1364ATA (5700G>A 5701A>T), ATA1365TTG (5703A>T 5705A>G), GAA1367GAT (5711A>T), GGA1368GGG (5714A>G), AAA1369AAG (5717A>G), GTT1370ATA (5718G>A 5720T>A), CAA1371AAA (5721C>A), CTC1372CTA (5726C>A), TTA1377GCT (5739T>G 5740T>C 5741A>T), ATA1380GTA (5748A>G), GAA1382GAT (5756A>T), TTT1383ATG (5757T>A 5759T>G), CCT1384CCA (5762T>A), GAA1385del (5763_5765delGAA), GAC1386GAT (5768C>T), CAA1387AAA (5769C>A), GAA1389GAT (5777A>T), ACA1390AAA (5779C>A), TTA1391ACA (5781T>A 5782T>C), AAG1392AAA (5786G>A), GGT1393GAT (5788G>A), CTA1394TTA (5790C>T), CAG1395CAA (5795G>A), AAG1396AAA (5798G>A), GGG1399GGA (5807G>A), ATA1400TTA (5808A>T), TTA1401GTG (5811T>G 5813A>G), AAC1402AAT (5816C>T), GCC1404GCT (5822C>T), AAT1406AAC (5828T>C), TAT1407TTT (5830A>T), ATC1408ATA (5834C>A), CCA1409CAA (5836C>A), TTG1411TTA (5843G>A), AGT1412GGA (5844A>G 5846T>A), TAT1414ATT (5850T>A 5851A>T), ACT1415GCA (5853A>G 5855T>A), AGA1416GGA (5856A>G), ATA1417CCA (5859A>C 5860T>C), TTT1418TTA (5864T>A), AAT1420GCA (5868A>G 5869A>C 5870T>A), AAG1421AAA (5873G>A), TGT1422ATA (5874T>A 5875G>T 5876T>A), TCC1423GGA (5877T>G 5878C>G 5879C>A), AGC1424AGT (5882C>T), GAA1427CAA (5889G>C), AGA1428AAA (5893G>A), AAA1429CAC (5895A>C 5897A>C), AAT1431AAC (5903T>C), TCT1432ACA (5904T>A 5906T>A), CAA1433GAA (5907C>G), GAC1434AAT (5910G>A 5912C>T), TGG1435ATT (5913T>A 5914G>T 5915G>T), AAG1436AAA (5918G>A), ATG1437CTG (5919A>C), GTT1438GTA (5924T>A), AGA1439AAA (5926G>A), AAA1440CAA (5928A>C), ATC1441ATT (5933C>T), GTA1444AAA (5940G>A 5941T>A), ATT1445ATA (5945T>A), ACC1446AAA (5947C>A 5948C>A), AAA1447AAC (5951A>C), TTA1448ATA (5952T>A), CCC1449CCA (5957C>A), CCT1450GAC (5958C>G 5959C>A 5960T>C), TTG1451TTA (5963G>A), ATT1452AGC (5965T>G 5966T>C), TTA1453ATA (5967T>A), AAA1455CTA (5973A>C 5974A>T), CCA1456GAA (5976C>G 5977C>A), CAA1457TCA (5979C>T 5980A>C), AGT1458GAT (5982A>G 5983G>A), ATT1460CTA (5988A>C 5990T>A), GAG1463GAA (5999G>A), GAT1465GAC (6005T>C), TCT1467AGC (6009T>A 6010C>G 6011T>C), CTA1468CTC (6014A>C), GAA1469CAA (6015G>C), GGG1472GGA (6026G>A), GGA1473GCT (6028G>C 6029A>T), ATT1474GTA (6030A>G 6032T>A), TGG1477GCA (6039T>G 6040G>C 6041G>A), CCC1479CCA (6047C>A), TCT1480AAC (6048T>A 6049C>A 6050T>C), CAA1481AAA (6051C>A), CAA1482TAC (6054C>T 6056A>C), GAT1483AGT (6057G>A 6058A>G), TCA1484CCT (6060T>C 6062A>T), CCT1485AAA (6063C>A 6064C>A 6065T>A), TCA1486AAA (6066T>A 6067C>A), ACA1487GAA (6069A>G 6070C>A), TCA1491TGT (6082C>G 6083A>T), CGG1492GCT (6084C>G 6085G>C 6086G>T), TAT1493TAC (6089T>C), TGT1494CAA (6090T>C 6091G>A 6092T>A), AGC1497AAA (6100G>A 6101C>A), TAT1498TAC (6104T>C), AAA1499_TCT1500insGAAAAG (6107_6108insGAAAAG), TCT1500GGT (6108T>G 6109C>G), GCC1501AAC (6111G>A 6112C>A), ATC1502ATG (6116C>G), TCT1503AGT (6117T>A 6118C>G), GCA1504AGT (6120G>A 6121C>G 6122A>T), GCT1507GTC (6130C>T 6131T>C), ATA1509GTA (6135A>G), ATG1510TTA (6138A>T 6140G>A), TGT1512ATA (6144T>A 6145G>T 6146T>A), GTA1515GGG (6154T>G 6155A>G), TTA1516CTA (6156T>C), GAT1517AAT (6159G>A)								

*: Inserts / Deletes / Misaligned / Frameshifts

Analysis details

This analysis was performed with panviral2.64

NGS Details (UN60): Rahariannevirus raharianne

Assembly

**Coverage Length**	885 (3 contig(s))
**Depth Of Coverage**	4.0
**Number Of Reads**	26
**Reads Per Million**	0.55 rpm (after QC)
**Ambiguities**	0
**Assembly Method**	de novo + reference guided assembly
**Consensus Caller**	Bcf Tools

Coverage Map

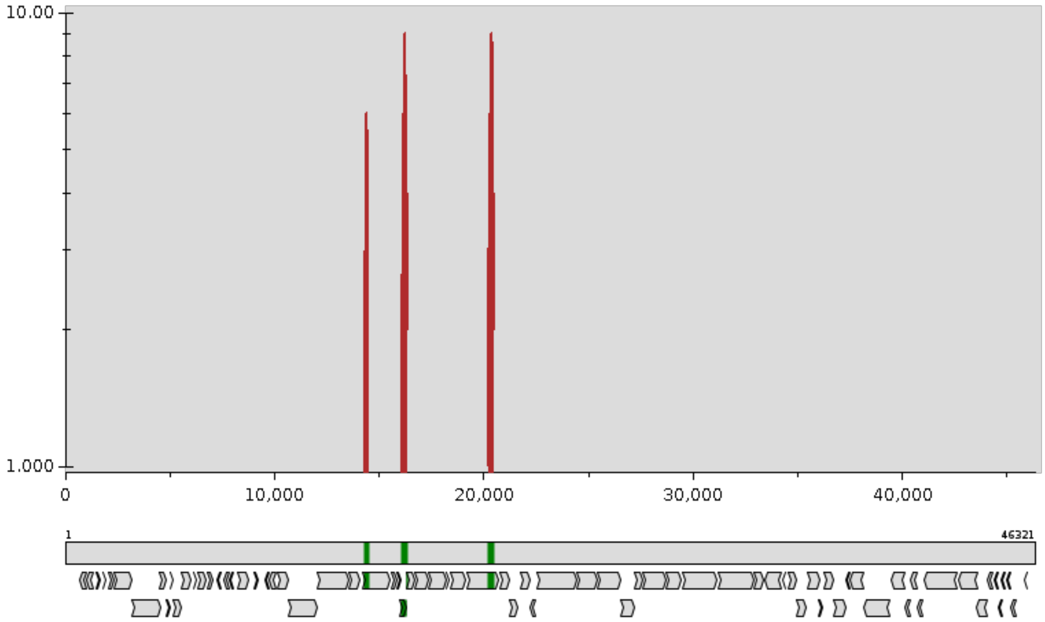

Assignment

**Type**	Rahariannevirus raharianne (Taxonomy ID: 2846050)
**Reference Genome**	NC_054955.1
**NT Identity (%)**	79.5249
**AA Identity (%)**	81.1644
**Number Of Stop Codons**	1
**Number Of CDS**	75

Alignment

**Alignment Score**	1034.0 (NT) + 1598.0 (AA) = 2632.0
**Concordance (%)**	72.1887

Alignment Method	Local, heuristic, nucleotide (BLASTN)

Genome Region

Sequence starts at position 14270 and ends at position 20492 relative to NC_054955.1 reference sequence.

Alignment Detailed Statistics

	Begin	End	Coverage	Score	Concordance	Matches	Identities	I/D/M/F*	Stop Codons
**NT**	**14270**	**20492**	**1.9%**	**1034**	**58.8%**	**884 (99.9%)**	**703 (79.4%)**	**0/1**	
Mutations:	14285C>G, 14288C>G, 14289G>A, 14293T>A, 14294C>T, 14298C>T, 14299A>C, 14306A>C, 14309T>C, 14317G>A, 14318T>G, 14336T>C, 14339A>G, 14342C>A, 14357T>C, 14363T>C, 14366G>C, 14372G>T, 14375G>C, 14378C>G, 14381C>G, 14394C>G, 14397T>G, 14399G>C, 14400G>A, 14401C>G, 14402A>C, 14427T>A, 14432G>C, 14433T>A, 14434C>G, 14435G>C, 14447G>C, 14457T>A, 14462C>G, 14486G>C, 14500C>T, 14504G>C, 14516C>G, 16035C>G, 16036A>C, 16037A>G, 16038A>C, 16040G>A, 16045A>G, 16047C>G, 16053T>C, 16057A>G, 16059G>T, 16061C>G, 16062C>G, 16065G>C, 16066C>A, 16067A>C, 16071G>C, 16074T>C, 16078T>A, 16086C>A, 16087A>G, 16088A>C, 16089G>C, 16091A>G, 16095T>C, 16099A>C, 16101C>G, 16113A>C, 16126A>C, 16128T>G, 16131T>G, 16134C>G, 16137T>G, 16140G>C, 16143G>C, 16146T>C, 16147G>A, 16149G>T, 16150G>C, 16151G>C, 16158C>G, 16161G>C, 16164C>T, 16170G>A, 16176G>C, 16177A>G, 16178C>T, 16179G>C, 16182C>T, 16183A>G, 16184C>G, 16190C>T, 16191T>C, 16198A>G, 16199A>T, 16204A>G, 16208G>C, 16209C>G, 16212G>C, 16218G>C, 16227G>C, 16231T>A, 16233A>C, 16239C>G, 16245T>C, 16251G>C, 16255C>T, 16258A>G, 16259A>C, 16260C>A, 16263C>G, 16270A>G, 16278T>C, 16282G>A, 16284T>C, 16293C>G, 16296T>C, 16297T>G, 16302T>C, 16305T>C, 16317C>T, 16319G>C, 16321G>C, 16323delG, 16335A>C, 16338C>G, 16339G>C, 16340C>A, 16341C>G, 16345A>C, 16346A>G, 20211A>T, 20229T>C, 20231T>G, 20235G>A, 20237G>C, 20240G>C, 20253T>A, 20254C>G, 20255G>C, 20268A>C, 20269C>A, 20279C>G, 20282C>T, 20286T>C, 20292T>C, 20293C>A, 20298A>G, 20299T>G, 20301C>A, 20302A>T, 20303G>C, 20306G>C, 20309T>C, 20336C>G, 20343T>A, 20344C>G, 20345G>C, 20355T>G, 20356G>C, 20357C>G, 20360G>C, 20362C>T, 20363G>C, 20369T>C, 20385G>C, 20388C>G, 20394C>A, 20396G>C, 20411C>G, 20433A>T, 20434G>A, 20439G>C, 20444T>C, 20461A>T, 20463G>A, 20468G>T, 20477A>G, 20478T>A, 20479C>G, 20480G>C, 20481G>T, 20483C>G, 20489G>C								

CDS

	Begin	End	Coverage	Score	Concordance	Matches	Identities	I/D/M/F*	Stop Codons
KMC43_gp27	26	109	19.0%	489	86.5%	84 (100%)	72 (85.7%)	0/0/0/0	0
Protein mutations:	I30M (14285C>G), G32S (14289G>A), F33Y (14293T>A 14294C>T), Q35S (14298C>T 14299A>C), R41Q (14317G>A 14318T>G), P67A (14394C>G), S68A (14397T>G 14399G>C), A69S (14400G>A 14401C>G 14402A>C), S78T (14427T>A), S88T (14457T>A), T102M (14500C>T)								
Codon mutations:	ATC30ATG (14285C>G), CTC31CTG (14288C>G), GGC32AGC (14289G>A), TTC33TAT (14293T>A 14294C>T), CAG35TCG (14298C>T 14299A>C), GCA37GCC (14306A>C), CGT38CGC (14309T>C), CGT41CAG (14317G>A 14318T>G), GGT47GGC (14336T>C), GAA48GAG (14339A>G), ACC49ACA (14342C>A), CGT54CGC (14357T>C), GGT56GGC (14363T>C), CTG57CTC (14366G>C), GCG59GCT (14372G>T), CCG60CCC (14375G>C), GTC61GTG (14378C>G), ACC62ACG (14381C>G), CCG67GCG (14394C>G), TCG68GCC (14397T>G 14399G>C), GCA69AGC (14400G>A 14401C>G 14402A>C), TCG78ACG (14427T>A), CCG79CCC (14432G>C), TCG80AGC (14433T>A 14434C>G 14435G>C), GTG84GTC (14447G>C), TCG88ACG (14457T>A), CTC89CTG (14462C>G), ACG97ACC (14486G>C), ACG102ATG (14500C>T), GTG103GTC (14504G>C), GTC107GTG (14516C>G)								
KMC43_gp30	7	103	94.2%	504	79.6%	97 (100%)	73 (75.3%)	0/0/0/0	1
Protein mutations:	K11R (16036A>C 16037A>G 16038A>C), S12N (16040G>A), T14A (16045A>G 16047C>G), T18A (16057A>G 16059G>T), A19G (16061C>G 16062C>G), Q21T (16066C>A 16067A>C), S25T (16078T>A), K28A (16087A>G 16088A>C 16089G>C), D29G (16091A>G), I32L (16099A>C 16101C>G), N41Q (16126A>C 16128T>G), A48T (16147G>A 16149G>T), G49P (16150G>C 16151G>C), T58V (16177A>G 16178C>T 16179G>C), T60G (16183A>G 16184C>G), A62V (16190C>T 16191T>C), N65V (16198A>G 16199A>T), I67V (16204A>G), G68A (16208G>C 16209C>G), S76T (16231T>A 16233A>C), N85A (16258A>G 16259A>C 16260C>A), I89V (16270A>G), A93T (16282G>A 16284T>C), S98A (16297T>G)								
Codon mutations:	GGC10GGG (16035C>G), AAA11CGC (16036A>C 16037A>G 16038A>C), AGC12AAC (16040G>A), ACC14GCG (16045A>G 16047C>G), AAT16AAC (16053T>C), ACG18GCT (16057A>G 16059G>T), GCC19GGG (16061C>G 16062C>G), GCG20GCC (16065G>C), CAG21ACG (16066C>A 16067A>C), GTG22GTC (16071G>C), GTT23GTC (16074T>C), TCG25ACG (16078T>A), CCC27CCA (16086C>A), AAG28GCC (16087A>G 16088A>C 16089G>C), GAC29GGC (16091A>G), TTT30TTC (16095T>C), ATC32CTG (16099A>C 16101C>G), CGA36CGC (16113A>C), AAT41CAG (16126A>C 16128T>G), GTT42GTG (16131T>G), CTC43CTG (16134C>G), GTT44GTG (16137T>G), GCG45GCC (16140G>C), GGG46GGC (16143G>C), ACT47ACC (16146T>C), GCG48ACT (16147G>A 16149G>T), GGG49CCG (16150G>C 16151G>C), GCC51GCG (16158C>G), GCG52GCC (16161G>C), TAC53TAT (16164C>T), GCG55GCA (16170G>A), ACG57ACC (16176G>C), ACG58GTC (16177A>G 16178C>T 16179G>C), GCC59GCT (16182C>T), ACC60GGC (16183A>G 16184C>G), GCT62GTC (16190C>T 16191T>C), AAC65GTC (16198A>G 16199A>T), ATC67GTC (16204A>G), GGC68GCG (16208G>C 16209C>G), GCG69GCC (16212G>C), CCG71CCC (16218G>C), GTG74GTC (16227G>C), TCA76ACC (16231T>A 16233A>C), CTC78CTG (16239C>G), GAT80GAC (16245T>C), CCG82CCC (16251G>C), CTG84TTG (16255C>T), AAC85GCA (16258A>G 16259A>C 16260C>A), GGC86GGG (16263C>G), ATC89GTC (16270A>G), CCT91CCC (16278T>C), GCT93ACC (16282G>A 16284T>C), ACC96ACG (16293C>G), GTT97GTC (16296T>C), TCT98GCT (16297T>G), GTT99GTC (16302T>C), TCT100TCC (16305T>C)								
KMC43_gp31	1	11	8.6%	58	90.6%	11 (100%)	9 (81.8%)	0/0/0/0	0
Protein mutations:	A6Q (16339G>C 16340C>A 16341C>G), K8R (16345A>C 16346A>G)								
Codon mutations:	ACA4ACC (16335A>C), ACC5ACG (16338C>G), GCC6CAG (16339G>C 16340C>A 16341C>G), AAG8CGG (16345A>C 16346A>G)								
KMC43_gp36	329	428	19.6%	547	84.2%	100 (100%)	83 (83.0%)	0/0/0/0	0
Protein mutations:	T335S (20211A>T), Y341Q (20229T>C 20231T>G), V343I (20235G>A 20237G>C), T354Q (20268A>C 20269C>A), S362Q (20292T>C 20293C>A), I364G (20298A>G 20299T>G), Q365I (20301C>A 20302A>T 20303G>C), C383A (20355T>G 20356G>C 20357C>G), S385F (20362C>T 20363G>C), A393P (20385G>C), L394V (20388C>G), Q396N (20394C>A 20396G>C), S409Y (20433A>T 20434G>A), V411L (20439G>C), Y418F (20461A>T), V419I (20463G>A), A425S (20481G>T 20483C>G)								
Codon mutations:	ACG335TCG (20211A>T), TAT341CAG (20229T>C 20231T>G), GTG343ATC (20235G>A 20237G>C), GTG344GTC (20240G>C), TCG349AGC (20253T>A 20254C>G 20255G>C), ACG354CAG (20268A>C 20269C>A), TCC357TCG (20279C>G), GCC358GCT (20282C>T), TTG360CTG (20286T>C), TCG362CAG (20292T>C 20293C>A), ATC364GGC (20298A>G 20299T>G), CAG365ATC (20301C>A 20302A>T 20303G>C), GCG366GCC (20306G>C), GGT367GGC (20309T>C), CCC376CCG (20336C>G), TCG379AGC (20343T>A 20344C>G 20345G>C), TGC383GCG (20355T>G 20356G>C 20357C>G), CGG384CGC (20360G>C), TCG385TTC (20362C>T 20363G>C), CAT387CAC (20369T>C), GCG393CCG (20385G>C), CTG394GTG (20388C>G), CAG396AAC (20394C>A 20396G>C), ACC401ACG (20411C>G), AGC409TAC (20433A>T 20434G>A), GTC411CTC (20439G>C), AAT412AAC (20444T>C), TAC418TTC (20461A>T), GTC419ATC (20463G>A), GGG420GGT (20468G>T), CAA423CAG (20477A>G), TCG424AGC (20478T>A 20479C>G 20480G>C), GCC425TCG (20481G>T 20483C>G), GTG427GTC (20489G>C)								

Proteins

	Begin	End	Coverage	Score	Concordance	Matches	Identities	I/D/M/F*	Stop Codons
DUF4043 domain-containing protein (YP_010078151.1)	26	109	19.0%	489	86.5%	84 (100%)	72 (85.7%)	0/0/0/0	0
Protein mutations:	I30M (14285C>G), G32S (14289G>A), F33Y (14293T>A 14294C>T), Q35S (14298C>T 14299A>C), R41Q (14317G>A 14318T>G), P67A (14394C>G), S68A (14397T>G 14399G>C), A69S (14400G>A 14401C>G 14402A>C), S78T (14427T>A), S88T (14457T>A), T102M (14500C>T)								
Codon mutations:	ATC30ATG (14285C>G), CTC31CTG (14288C>G), GGC32AGC (14289G>A), TTC33TAT (14293T>A 14294C>T), CAG35TCG (14298C>T 14299A>C), GCA37GCC (14306A>C), CGT38CGC (14309T>C), CGT41CAG (14317G>A 14318T>G), GGT47GGC (14336T>C), GAA48GAG (14339A>G), ACC49ACA (14342C>A), CGT54CGC (14357T>C), GGT56GGC (14363T>C), CTG57CTC (14366G>C), GCG59GCT (14372G>T), CCG60CCC (14375G>C), GTC61GTG (14378C>G), ACC62ACG (14381C>G), CCG67GCG (14394C>G), TCG68GCC (14397T>G 14399G>C), GCA69AGC (14400G>A 14401C>G 14402A>C), TCG78ACG (14427T>A), CCG79CCC (14432G>C), TCG80AGC (14433T>A 14434C>G 14435G>C), GTG84GTC (14447G>C), TCG88ACG (14457T>A), CTC89CTG (14462C>G), ACG97ACC (14486G>C), ACG102ATG (14500C>T), GTG103GTC (14504G>C), GTC107GTG (14516C>G)								
hypothetical protein (YP_010078154.1)	7	103	94.2%	504	79.6%	97 (100%)	73 (75.3%)	0/0/0/0	1
Protein mutations:	K11R (16036A>C 16037A>G 16038A>C), S12N (16040G>A), T14A (16045A>G 16047C>G), T18A (16057A>G 16059G>T), A19G (16061C>G 16062C>G), Q21T (16066C>A 16067A>C), S25T (16078T>A), K28A (16087A>G 16088A>C 16089G>C), D29G (16091A>G), I32L (16099A>C 16101C>G), N41Q (16126A>C 16128T>G), A48T (16147G>A 16149G>T), G49P (16150G>C 16151G>C), T58V (16177A>G 16178C>T 16179G>C), T60G (16183A>G 16184C>G), A62V (16190C>T 16191T>C), N65V (16198A>G 16199A>T), I67V (16204A>G), G68A (16208G>C 16209C>G), S76T (16231T>A 16233A>C), N85A (16258A>G 16259A>C 16260C>A), I89V (16270A>G), A93T (16282G>A 16284T>C), S98A (16297T>G)								

	Begin	End	Coverage	Score	Concordance	Matches	Identities	I/D/M/F*	Stop Codons
**NT**	**14270**	**20492**	**1.9%**	**1034**	**58.8%**	**884 (99.9%)**	**703 (79.4%)**	**0/1**	
Codon mutations:	GGC10GGG (16035C>G), AAA11CGC (16036A>C 16037A>G 16038A>C), AGC12AAC (16040G>A), ACC14GCG (16045A>G 16047C>G), AAT16AAC (16053T>C), ACG18GCT (16057A>G 16059G>T), GCC19GGG (16061C>G 16062C>G), GCG20GCC (16065G>C), CAG21ACG (16066C>A 16067A>C), GTG22GTC (16071G>C), GTT23GTC (16074T>C), TCG25ACG (16078T>A), CCC27CCA (16086C>A), AAG28GCC (16087A>G 16088A>C 16089G>C), GAC29GGC (16091A>G), TTT30TTC (16095T>C), ATC32CTG (16099A>C 16101C>G), CGA36CGC (16113A>C), AAT41CAG (16126A>C 16128T>G), GTT42GTG (16131T>G), CTC43CTG (16134C>G), GTT44GTG (16137T>G), GCG45GCC (16140G>C), GGG46GGC (16143G>C), ACT47ACC (16146T>C), GCG48ACT (16147G>A 16149G>T), GGG49CCG (16150G>C 16151G>C), GCC51GCG (16158C>G), GCG52GCC (16161G>C), TAC53TAT (16164C>T), GCG55GCA (16170G>A), ACG57ACC (16176G>C), ACG58GTC (16177A>G 16178C>T 16179G>C), GCC59GCT (16182C>T), ACC60GGC (16183A>G 16184C>G), GCT62GTC (16190C>T 16191T>C), AAC65GTC (16198A>G 16199A>T), ATC67GTC (16204A>G), GGC68GCG (16208G>C 16209C>G), GCG69GCC (16212G>C), CCG71CCC (16218G>C), GTG74GTC (16227G>C), TCA76ACC (16231T>A 16233A>C), CTC78CTG (16239C>G), GAT80GAC (16245T>C), CCG82CCC (16251G>C), CTG84TTG (16255C>T), AAC85GCA (16258A>G 16259A>C 16260C>A), GGC86GGG (16263C>G), ATC89GTC (16270A>G), CCT91CCC (16278T>C), GCT93ACC (16282G>A 16284T>C), ACC96ACG (16293C>G), GTT97GTC (16296T>C), TCT98GCT (16297T>G), GTT99GTC (16302T>C), TCT100TCC (16305T>C)								
hypothetical protein (YP_010078155.1)	1	11	8.6%	58	90.6%	11 (100%)	9 (81.8%)	0/0/0/0	0
Protein mutations:	A6Q (16339G>C 16340C>A 16341C>G), K8R (16345A>C 16346A>G)								
Codon mutations:	ACA4ACC (16335A>C), ACC5ACG (16338C>G), GCC6CAG (16339G>C 16340C>A 16341C>G), AAG8CGG (16345A>C 16346A>G)								
tail protein (YP_010078160.1)	329	428	19.6%	547	84.2%	100 (100%)	83 (83.0%)	0/0/0/0	0
Protein mutations:	T335S (20211A>T), Y341Q (20229T>C 20231T>G), V343I (20235G>A 20237G>C), T354Q (20268A>C 20269C>A), S362Q (20292T>C 20293C>A), I364G (20298A>G 20299T>G), Q365I (20301C>A 20302A>T 20303G>C), C383A (20355T>G 20356G>C 20357C>G), S385F (20362C>T 20363G>C), A393P (20385G>C), L394V (20388C>G), Q396N (20394C>A 20396G>C), S409Y (20433A>T 20434G>A), V411L (20439G>C), Y418F (20461A>T), V419I (20463G>A), A425S (20481G>T 20483C>G)								
Codon mutations:	ACG335TCG (20211A>T), TAT341CAG (20229T>C 20231T>G), GTG343ATC (20235G>A 20237G>C), GTG344GTC (20240G>C), TCG349AGC (20253T>A 20254C>G 20255G>C), ACG354CAG (20268A>C 20269C>A), TCC357TCG (20279C>G), GCC358GCT (20282C>T), TTG360CTG (20286T>C), TCG362CAG (20292T>C 20293C>A), ATC364GGC (20298A>G 20299T>G), CAG365ATC (20301C>A 20302A>T 20303G>C), GCG366GCC (20306G>C), GGT367GGC (20309T>C), CCC376CCG (20336C>G), TCG379AGC (20343T>A 20344C>G 20345G>C), TGC383GCG (20355T>G 20356G>C 20357C>G), CGG384CGC (20360G>C), TCG385TTC (20362C>T 20363G>C), CAT387CAC (20369T>C), GCG393CCG (20385G>C), CTG394GTG (20388C>G), CAG396AAC (20394C>A 20396G>C), ACC401ACG (20411C>G), AGC409TAC (20433A>T 20434G>A), GTC411CTC (20439G>C), AAT412AAC (20444T>C), TAC418TTC (20461A>T), GTC419ATC (20463G>A), GGG420GGT (20468G>T), CAA423CAG (20477A>G), TCG424AGC (20478T>A 20479C>G 20480G>C), GCC425TCG (20481G>T 20483C>G), GTG427GTC (20489G>C)								

*: Inserts / Deletes / Misaligned / Frameshifts

Analysis details

This analysis was performed with panviral2.64

NGS Details (UN60): Badnavirus maculasmallanthi

Assembly

**Coverage Length**	268 (1 contig(s))
**Depth Of Coverage**	9.4
**Number Of Reads**	21
**Reads Per Million**	0.45 rpm (after QC)
**Ambiguities**	0
**Assembly Method**	de novo + reference guided assembly
**Consensus Caller**	Bcf Tools

Coverage Map

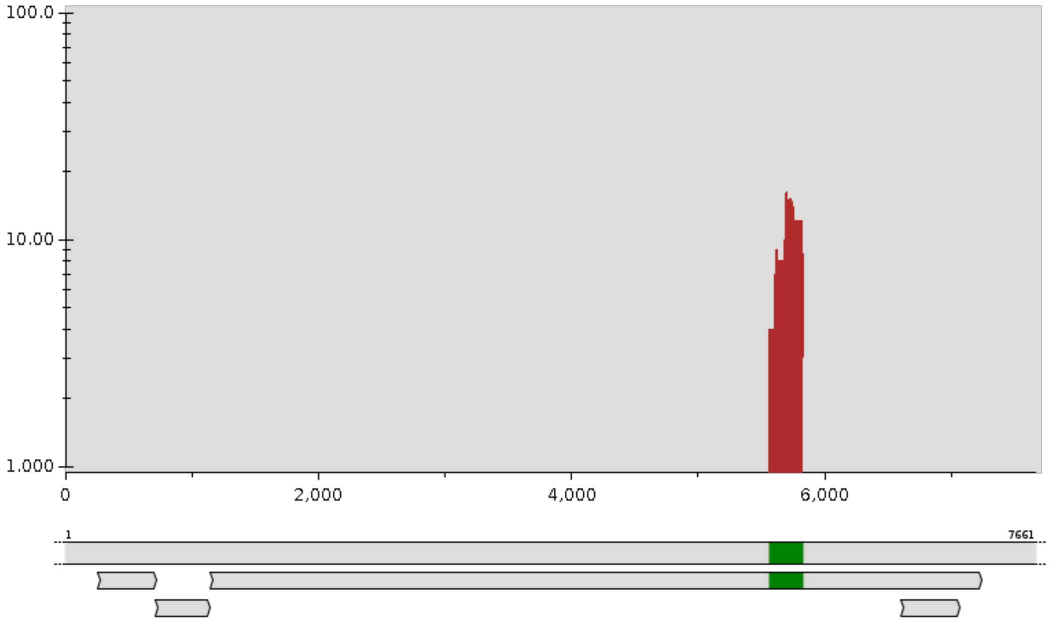

Assignment

**Type**	Badnavirus maculasmallanthi (Taxonomy ID: 3048453)
**Reference Genome**	NC_026472.1
**NT Identity (%)**	56.4576
**AA Identity (%)**	51.6484
**Number Of Stop Codons**	0
**Number Of CDS**	4

Alignment

**Alignment Score**	64.0 (NT) + 333.0 (AA) = 397.0
**Concordance (%)**	33.8737

**Alignment Method**	Global, seeded, nucleotide + amino acids (AGA)

Genome Region

Sequence starts at position 5560 and ends at position 5827 relative to NC_026472.1 reference sequence.

Alignment Detailed Statistics

	Begin	End	Coverage	Score	Concordance	Matches	Identities	I/D/M/F*	Stop Codons
**NT**	**5560**	**5827**	**3.5%**	**64**	**11.9%**	**268 (98.9%)**	**153 (56.5%)**	**3/0**	
Mutations:	5560G>T, 5562A>C, 5574A>T, 5577G>A, 5578T>A, 5580T>A, 5583T>C, 5584C>T, 5586G>A, 5588A>G, 5589G>A, 5597T>A, 5598T>C, 5605G>C, 5608G>A, 5609C>G, 5610C>A, 5615A>C, 5616A>C, 5617G>A, 5618A>C, 5622A>G, 5623T>G, 5624C>A, 5625C>T, 5626A>G, 5628A>G, 5631C>A, 5632T>A, 5633G>A, 5634G>A, 5637G>T, 5644T>A, 5645T>G, 5648T>C, 5649C>T, 5651C>A, 5652A>C, 5653G>T, 5654G>C, 5655A>T, 5658A>G, 5659T>C, 5660T>A, 5661A>T, 5663A>T, 5664C>T, 5667A>G, 5669G>A, 5670G>C, 5671C>T, 5673T>A, 5682A>C, 5685C>T, 5691C>G, 5692A>T, 5693A>C, 5699C>T, 5700A>C, 5703C>A, 5706T>C, 5708T>C, 5709T>A, 5715G>A, 5716A>G, 5719A>T, 5720A>T, 5725G>A, 5728A>T, 5729A>C, 5730A>T, 5731T>G, 5732G>T, 5733C>G, 5736_5737insCAG, 5740G>T, 5741A>T, 5742C>T, 5743A>C, 5744C>T, 5746G>A, 5747A>G, 5748A>G, 5749G>A, 5752T>C, 5753T>A, 5755A>G, 5757C>G, 5758G>T, 5759C>T, 5760C>G, 5765A>T, 5766C>T, 5767A>T, 5769C>T, 5772C>T, 5775C>T, 5778C>A, 5781G>T, 5782G>A, 5787C>T, 5788T>A, 5789C>G, 5792A>G, 5793C>A, 5795G>C, 5796T>C, 5797G>A, 5798A>T, 5801C>A, 5802G>A, 5809G>A, 5810A>T, 5812A>T, 5813G>T, 5820A>G								

CDS

	Begin	End	Coverage	Score	Concordance	Matches	Identities	I/D/M/F*	Stop Codons
UF61_gp3	1473	1562	4.4%	333	51.9%	90 (98.9%)	47 (51.6%)	1/0/0/0	0
Protein mutations:	A1473S (5560G>T 5562A>C), F1479I (5578T>A 5580T>A), K1482R (5588A>G 5589G>A), F1485Y (5597T>A 5598T>C), V1488L (5605G>C), A1489R (5608G>A 5609C>G 5610C>A), E1491A (5615A>C 5616A>C), E1492T (5617G>A 5618A>C), S1494D (5623T>G 5624C>A 5625C>T), I1495V (5626A>G 5628A>G), W1497K (5632T>A 5633G>A 5634G>A), L1501R (5644T>A 5645T>G), I1502T (5648T>C 5649C>T), P1503H (5651C>A 5652A>C), G1504S (5653G>T 5654G>C 5655A>T), L1506H (5659T>C 5660T>A 5661A>T), Y1507F (5663A>T 5664C>T), W1509Y (5669G>A 5670G>C), K1517S (5692A>T 5693A>C), A1519V (5699C>T 5700A>C), I1522T (5708T>C 5709T>A), R1525G (5716A>G), K1526L (5719A>T 5720A>T), D1528N (5725G>A), K1529S (5728A>T 5729A>C 5730A>T), C1530V (5731T>G 5732G>T 5733C>G), F1531_K1532insQ (5736_5737insCAG), D1533F (5740G>T 5741A>T 5742C>T), T1534L (5743A>C 5744C>T), E1535R (5746G>A 5747A>G 5748A>G), E1536K (5749G>A), F1537H (5752T>C 5753T>A), I1538V (5755A>G 5757C>G), A1539L (5758G>T 5759C>T 5760C>G), Y1541F (5765A>T 5766C>T), I1542F (5767A>T 5769C>T), V1547I (5782G>A), N1550R (5792A>G 5793C>A), S1551T (5795G>C 5796T>C), E1552I (5797G>A 5798A>T), A1553E (5801C>A 5802G>A), E1556I (5809G>A 5810A>T), R1557L (5812A>T 5813G>T)								
Codon mutations:	GCA1473TCC (5560G>T 5562A>C), TCA1477TCT (5574A>T), AAG1478AAA (5577G>A), TTT1479ATA (5578T>A 5580T>A), GAT1480GAC (5583T>C), CTG1481TTA (5584C>T 5586G>A), AAG1482AGA (5588A>G 5589G>A), TTT1485TAC (5597T>A 5598T>C), GTT1488CTT (5605G>C), GCC1489AGA (5608G>A 5609C>G 5610C>A), GAA1491GCC (5615A>C 5616A>C), GAA1492ACA (5617G>A 5618A>C), GAA1493GAG (5622A>G), TCC1494GAT (5623T>G 5624C>A 5625C>T), ATA1495GTG (5626A>G 5628A>G), CCC1496CCA (5631C>A), TGG1497AAA (5632T>A 5633G>A 5634G>A), ACG1498ACT (5637G>T), TTA1501AGA (5644T>A 5645T>G), ATC1502ACT (5648T>C 5649C>T), CCA1503CAC (5651C>A 5652A>C), GGA1504TCT (5653G>T 5654G>C 5655A>T), GGA1505GGG (5658A>G), TTA1506CAT (5659T>C 5660T>A 5661A>T), TAC1507TTT (5663A>T 5664C>T), GAA1508GAG (5667A>G), TGG1509TAC (5669G>A 5670G>C), CTT1510TTA (5671C>T 5673T>A), CCA1513CCC (5682A>C), TTC1514TTT (5685C>T), CTC1516CTG (5691C>G), AAA1517TCA (5692A>T 5693A>C), GCA1519GTC (5699C>T 5700A>C), CCC1520CCA (5703C>A), GCT1521GCC (5706T>C), ATT1522ACA (5708T>C 5709T>A), CAG1524CAA (5715G>A), AGA1525GGA (5716A>G), AAA1526TTA (5719A>T 5720A>T), GAT1528AAT (5725G>A), AAA1529TCT (5728A>T 5729A>C 5730A>T), TGC1530GTG (5731T>G 5732G>T 5733C>G), TTC1531_AAA1532insCAG (5736_5737insCAG), GAC1533TTT (5740G>T 5741A>T 5742C>T), ACA1534CTA (5743A>C 5744C>T), GAA1535AGG (5746G>A 5747A>G 5748A>G), GAG1536AAG (5749G>A), TTT1537CAT (5752T>C 5753T>A), ATC1538GTG (5755A>G 5757C>G), GCC1539TTG (5758G>T 5759C>T 5760C>G), TAC1541TTT (5765A>T 5766C>T), ATC1542TTT (5767A>T 5769C>T), GAC1543GAT (5772C>T), GAC1544GAT (5775C>T), ATC1545ATA (5778C>A), CTG1546CTT (5781G>T), GTA1547ATA (5782G>A), TAC1548TAT (5787C>T), TCC1549AGC (5788T>A 5789C>G), AAC1550AGA (5792A>G 5793C>A), AGT1551ACC (5795G>C 5796T>C), GAA1552ATA (5797G>A 5798A>T), GCG1553GAA (5801C>A 5802G>A), GAA1556ATA (5809G>A 5810A>T), AGG1557TTG (5812A>T 5813G>T), TTA1559TTG (5820A>G)								

Proteins

	Begin	End	Coverage	Score	Concordance	Matches	Identities	I/D/M/F*	Stop Codons
ORF3 (YP_009121747.1)	1473	1562	4.4%	333	51.9%	90 (98.9%)	47 (51.6%)	1/0/0/0	0
Protein mutations:	A1473S (5560G>T 5562A>C), F1479I (5578T>A 5580T>A), K1482R (5588A>G 5589G>A), F1485Y (5597T>A 5598T>C), V1488L (5605G>C), A1489R (5608G>A 5609C>G 5610C>A), E1491A (5615A>C 5616A>C), E1492T (5617G>A 5618A>C), S1494D (5623T>G 5624C>A 5625C>T), I1495V (5626A>G 5628A>G), W1497K (5632T>A 5633G>A 5634G>A), L1501R (5644T>A 5645T>G), I1502T (5648T>C 5649C>T), P1503H (5651C>A 5652A>C), G1504S (5653G>T 5654G>C 5655A>T), L1506H (5659T>C 5660T>A 5661A>T), Y1507F (5663A>T 5664C>T), W1509Y (5669G>A 5670G>C), K1517S (5692A>T 5693A>C), A1519V (5699C>T 5700A>C), I1522T (5708T>C 5709T>A), R1525G (5716A>G), K1526L (5719A>T 5720A>T), D1528N (5725G>A), K1529S (5728A>T 5729A>C 5730A>T), C1530V (5731T>G 5732G>T 5733C>G), F1531_K1532insQ (5736_5737insCAG), D1533F (5740G>T 5741A>T 5742C>T), T1534L (5743A>C 5744C>T), E1535R (5746G>A 5747A>G 5748A>G), E1536K (5749G>A), F1537H (5752T>C 5753T>A), I1538V (5755A>G 5757C>G), A1539L (5758G>T 5759C>T 5760C>G), Y1541F (5765A>T 5766C>T), I1542F (5767A>T 5769C>T), V1547I (5782G>A), N1550R (5792A>G 5793C>A), S1551T (5795G>C 5796T>C), E1552I (5797G>A 5798A>T), A1553E (5801C>A 5802G>A), E1556I (5809G>A 5810A>T), R1557L (5812A>T 5813G>T)								
Codon mutations:	GCA1473TCC (5560G>T 5562A>C), TCA1477TCT (5574A>T), AAG1478AAA (5577G>A), TTT1479ATA (5578T>A 5580T>A), GAT1480GAC (5583T>C), CTG1481TTA (5584C>T 5586G>A), AAG1482AGA (5588A>G 5589G>A), TTT1485TAC (5597T>A 5598T>C), GTT1488CTT (5605G>C), GCC1489AGA (5608G>A 5609C>G 5610C>A), GAA1491GCC (5615A>C 5616A>C), GAA1492ACA (5617G>A 5618A>C), GAA1493GAG (5622A>G), TCC1494GAT (5623T>G 5624C>A 5625C>T), ATA1495GTG (5626A>G 5628A>G), CCC1496CCA (5631C>A), TGG1497AAA (5632T>A 5633G>A 5634G>A), ACG1498ACT (5637G>T), TTA1501AGA (5644T>A 5645T>G), ATC1502ACT (5648T>C 5649C>T), CCA1503CAC (5651C>A 5652A>C), GGA1504TCT (5653G>T 5654G>C 5655A>T), GGA1505GGG (5658A>G), TTA1506CAT (5659T>C 5660T>A 5661A>T), TAC1507TTT (5663A>T 5664C>T), GAA1508GAG (5667A>G), TGG1509TAC (5669G>A 5670G>C), CTT1510TTA (5671C>T 5673T>A), CCA1513CCC (5682A>C), TTC1514TTT (5685C>T), CTC1516CTG (5691C>G), AAA1517TCA (5692A>T 5693A>C), GCA1519GTC (5699C>T 5700A>C), CCC1520CCA (5703C>A), GCT1521GCC (5706T>C), ATT1522ACA (5708T>C 5709T>A), CAG1524CAA (5715G>A), AGA1525GGA (5716A>G), AAA1526TTA (5719A>T 5720A>T), GAT1528AAT (5725G>A), AAA1529TCT (5728A>T 5729A>C 5730A>T), TGC1530GTG (5731T>G 5732G>T 5733C>G), TTC1531_AAA1532insCAG (5736_5737insCAG), GAC1533TTT (5740G>T 5741A>T 5742C>T), ACA1534CTA (5743A>C 5744C>T), GAA1535AGG (5746G>A 5747A>G 5748A>G), GAG1536AAG (5749G>A), TTT1537CAT (5752T>C 5753T>A), ATC1538GTG (5755A>G 5757C>G), GCC1539TTG (5758G>T 5759C>T 5760C>G), TAC1541TTT (5765A>T 5766C>T), ATC1542TTT (5767A>T 5769C>T), GAC1543GAT (5772C>T), GAC1544GAT (5775C>T), ATC1545ATA (5778C>A), CTG1546CTT (5781G>T), GTA1547ATA (5782G>A), TAC1548TAT (5787C>T), TCC1549AGC (5788T>A 5789C>G), AAC1550AGA (5792A>G 5793C>A), AGT1551ACC (5795G>C 5796T>C), GAA1552ATA (5797G>A 5798A>T), GCG1553GAA (5801C>A 5802G>A), GAA1556ATA (5809G>A 5810A>T), AGG1557TTG (5812A>T 5813G>T), TTA1559TTG (5820A>G)								

*: Inserts / Deletes / Misaligned / Frameshifts

Analysis details

This analysis was performed with panviral2.64

NGS Details (UN60): Blunervirus vaccinii (segment RNA 2)

Assembly

**Coverage Length**	180 (1 contig(s))
**Depth Of Coverage**	6.4
**Number Of Reads**	14
**Reads Per Million**	0.30 rpm (after QC)
**Ambiguities**	0
**Assembly Method**	de novo + reference guided assembly
**Consensus Caller**	Bcf Tools

Coverage Map

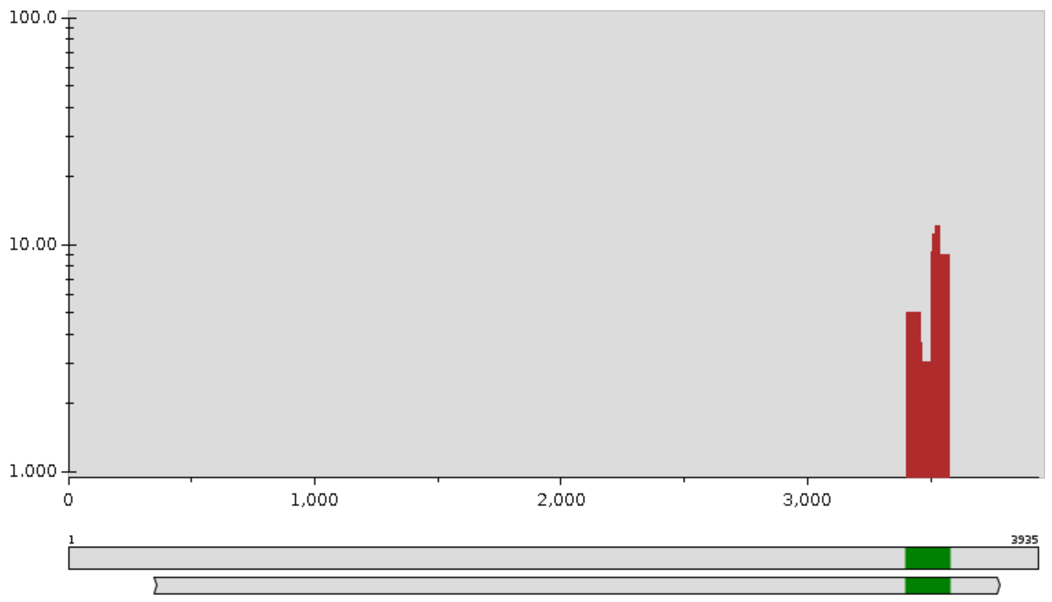

Assignment

**Type**	Blunervirus vaccinii (Taxonomy ID: 3047693)
**Reference Genome**	NC_016085.1
**NT Identity (%)**	61.6667
**AA Identity (%)**	61.6667
**Number Of Stop Codons**	0
**Number Of CDS**	1

Alignment

**Alignment Score**	84.0 (NT) + 266.0 (AA) = 350.0
**Concordance (%)**	43.4783

Alignment Method	Global, seeded, nucleotide + amino acids (AGA)

Genome Region

Sequence starts at position 3400 and ends at position 3579 relative to NC_016085.1 reference sequence.

Alignment Detailed Statistics

	Begin	End	Coverage	Score	Concordance	Matches	Identities	I/D/M/F*	Stop Codons
**NT**	**3400**	**3579**	**4.6%**	**84**	**23.3%**	**180 (100%)**	**111 (61.7%)**	**0/0**	
Mutations:	3405A>T, 3406T>G, 3409C>T, 3412A>T, 3418G>A, 3421G>A, 3423T>G, 3424T>C, 3425G>A, 3426A>T, 3427C>G, 3430C>T, 3431C>A, 3433G>C, 3435A>C, 3437C>A, 3439C>G, 3445C>T, 3448C>T, 3451G>A, 3457T>C, 3458G>T, 3460C>A, 3461C>T, 3463T>G, 3469G>T, 3470A>G, 3472T>A, 3476C>A, 3477G>T, 3478T>C, 3480G>T, 3481G>A, 3482G>A, 3483T>G, 3484T>A, 3485G>T, 3487C>A, 3488A>G, 3489C>T, 3490A>G, 3493C>T, 3499G>T, 3500C>G, 3505G>A, 3506C>G, 3507G>T, 3509G>T, 3510C>T, 3512G>A, 3514C>T, 3517A>G, 3518C>T, 3520C>G, 3523A>C, 3524C>A, 3526T>G, 3527G>C, 3528C>A, 3533C>T, 3535A>G, 3542T>G, 3553G>T, 3556G>A, 3565G>A, 3568G>A, 3574C>G, 3575A>T, 3577T>G								
**CDS**									
BNRBV_s2gp1	1019	1078	5.2%	266	57.5%	60 (100%)	37 (61.7%)	0/0/0/0	0
Protein mutations:	N1020M (3405A>T 3406T>G), E1022D (3412A>T), F1026C (3423T>G 3424T>C), D1027M (3425G>A 3426A>T 3427C>G), Q1029N (3431C>A 3433G>C), Y1030S (3435A>C), H1031K (3437C>A 3439C>G), V1038L (3458G>T 3460C>A), N1042E (3470A>G 3472T>A), R1044I (3476C>A 3477G>T 3478T>C), W1045L (3480G>T 3481G>A), V1046R (3482G>A 3483T>G 3484T>A), V1047L (3485G>T 3487C>A), T1048V (3488A>G 3489C>T 3490A>G), L1052V (3500C>G), R1054V (3506C>G 3507G>T), A1055L (3509G>T 3510C>T), V1056I (3512G>A 3514C>T), A1061H (3527G>C 3528C>A), F1066V (3542T>G), F1076L (3574C>G), I1077L (3575A>T 3577T>G)								
Codon mutations:	AAT1020ATG (3405A>T 3406T>G), TTC1021TTT (3409C>T), GAA1022GAT (3412A>T), AAG1024AAA (3418G>A), TTG1025TTA (3421G>A), TTT1026TGC (3423T>G 3424T>C), GAC1027ATG (3425G>A 3426A>T 3427C>G), TTC1028TTT (3430C>T), CAG1029AAC (3431C>A 3433G>C), TAT1030TCT (3435A>C), CAC1031AAG (3437C>A 3439C>G), TTC1033TTT (3445C>T), TGC1034TGT (3448C>T), TCG1035TCA (3451G>A), TTT1037TTC (3457T>C), GTC1038TTA (3458G>T 3460C>A), CTT1039TTG (3461C>T 3463T>G), GTG1041GTT (3469G>T), AAT1042GAA (3470A>G 3472T>A), CGT1044ATC (3476C>A 3477G>T 3478T>C), TGG1045TTA (3480G>T 3481G>A), GTT1046AGA (3482G>A 3483T>G 3484T>A), GTC1047TTA (3485G>T 3487C>A), ACA1048GTG (3488A>G 3489C>T 3490A>G), CCC1049CCT (3493C>T), CCG1051CCT (3499G>T), CTG1052GTG (3500C>G), AAG1053AAA (3505G>A), CGT1054GTT (3506C>G 3507G>T), GCG1055TTG (3509G>T 3510C>T), GTC1056ATT (3512G>A 3514C>T), AAA1057AAG (3517A>G), CTC1058TTG (3518C>T 3520C>G), GGA1059GGC (3523A>C), CGT1060AGG (3524C>A 3526T>G), GCT1061CAT (3527G>C 3528C>A), CTA1063TTG (3533C>T 3535A>G), TTT1066GTT (3542T>G), GTG1069GTT (3553G>T), GAG1070GAA (3556G>A), AGG1073AGA (3565G>A), GTG1074GTA (3568G>A), TTC1076TTG (3574C>G), ATT1077TTG (3575A>T 3577T>G)								
**Proteins**									
polymerase (YP_004901701.1)	1019	1078	5.2%	266	57.5%	60 (100%)	37 (61.7%)	0/0/0/0	0
Protein mutations:	N1020M (3405A>T 3406T>G), E1022D (3412A>T), F1026C (3423T>G 3424T>C), D1027M (3425G>A 3426A>T 3427C>G), Q1029N (3431C>A 3433G>C), Y1030S (3435A>C), H1031K (3437C>A 3439C>G), V1038L (3458G>T 3460C>A), N1042E (3470A>G 3472T>A), R1044I (3476C>A 3477G>T 3478T>C), W1045L (3480G>T 3481G>A), V1046R (3482G>A 3483T>G 3484T>A), V1047L (3485G>T 3487C>A), T1048V (3488A>G 3489C>T 3490A>G), L1052V (3500C>G), R1054V (3506C>G 3507G>T), A1055L (3509G>T 3510C>T), V1056I (3512G>A 3514C>T), A1061H (3527G>C 3528C>A), F1066V (3542T>G), F1076L (3574C>G), I1077L (3575A>T 3577T>G)								
Codon mutations:	AAT1020ATG (3405A>T 3406T>G), TTC1021TTT (3409C>T), GAA1022GAT (3412A>T), AAG1024AAA (3418G>A), TTG1025TTA (3421G>A), TTT1026TGC (3423T>G 3424T>C), GAC1027ATG (3425G>A 3426A>T 3427C>G), TTC1028TTT (3430C>T), CAG1029AAC (3431C>A 3433G>C), TAT1030TCT (3435A>C), CAC1031AAG (3437C>A 3439C>G), TTC1033TTT (3445C>T), TGC1034TGT (3448C>T), TCG1035TCA (3451G>A), TTT1037TTC (3457T>C), GTC1038TTA (3458G>T 3460C>A), CTT1039TTG (3461C>T 3463T>G), GTG1041GTT (3469G>T), AAT1042GAA (3470A>G 3472T>A), CGT1044ATC (3476C>A 3477G>T 3478T>C), TGG1045TTA (3480G>T 3481G>A), GTT1046AGA (3482G>A 3483T>G 3484T>A), GTC1047TTA (3485G>T 3487C>A), ACA1048GTG (3488A>G 3489C>T 3490A>G), CCC1049CCT (3493C>T), CCG1051CCT (3499G>T), CTG1052GTG (3500C>G), AAG1053AAA (3505G>A), CGT1054GTT (3506C>G 3507G>T), GCG1055TTG (3509G>T 3510C>T), GTC1056ATT (3512G>A 3514C>T), AAA1057AAG (3517A>G), CTC1058TTG (3518C>T 3520C>G), GGA1059GGC (3523A>C), CGT1060AGG (3524C>A 3526T>G), GCT1061CAT (3527G>C 3528C>A), CTA1063TTG (3533C>T 3535A>G), TTT1066GTT (3542T>G), GTG1069GTT (3553G>T), GAG1070GAA (3556G>A), AGG1073AGA (3565G>A), GTG1074GTA (3568G>A), TTC1076TTG (3574C>G), ATT1077TTG (3575A>T 3577T>G)								

*: Inserts / Deletes / Misaligned / Frameshifts

Analysis details

This analysis was performed with panviral2.64

NGS Details (UN60): Alfalfa leaf curl virus

Assembly

**Coverage Length**	270 (2 contig(s))
**Depth Of Coverage**	6.0
**Number Of Reads**	14
**Reads Per Million**	0.30 rpm (after QC)
**Ambiguities**	0
**Assembly Method**	de novo + reference guided assembly
**Consensus Caller**	Bcf Tools

Coverage Map

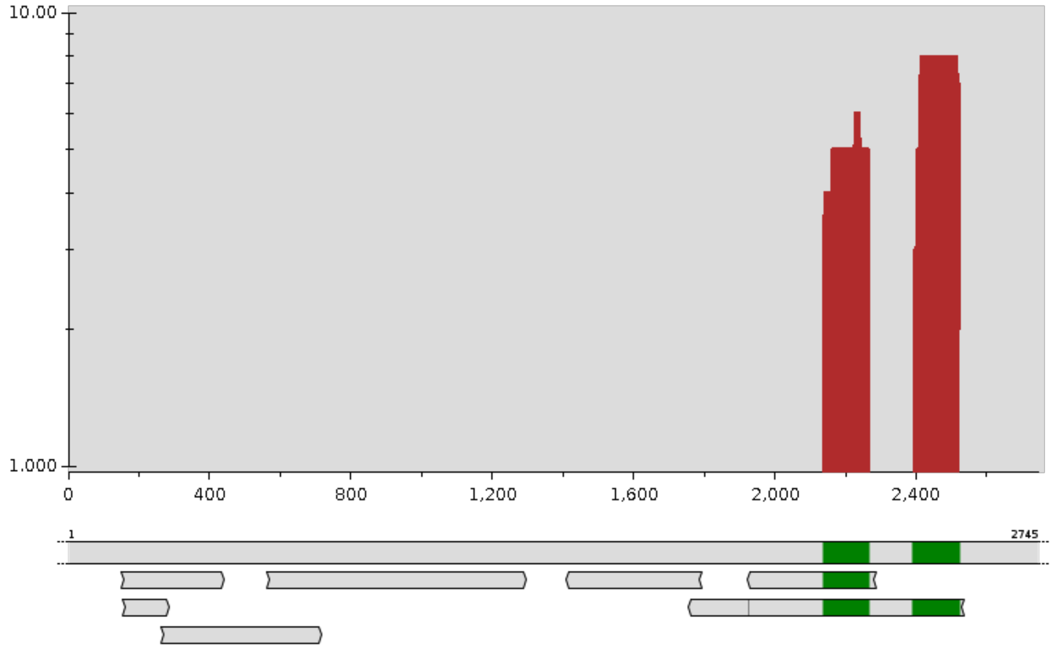

Assignment

**Type**	Alfalfa leaf curl virus (Taxonomy ID: 1306546)
**Reference Genome**	NC_027714.1
**NT Identity (%)**	71.4815
**AA Identity (%)**	58.2222
**Number Of Stop Codons**	0
**Number Of CDS**	7

Alignment

**Alignment Score**	232.0 (NT) + 989.0 (AA) = 1221.0
**Concordance (%)**	57.297

Alignment Method	Global, seeded, nucleotide + amino acids (AGA)

Genome Region

Sequence starts at position 2136 and ends at position 2525 relative to NC_027714.1 reference sequence.

Alignment Detailed Statistics

	Begin	End	Coverage	Score	Concordance	Matches	Identities	I/D/M/F*	Stop Codons
**NT**	**2136**	**2525**	**9.8%**	**232**	**43.0%**	**270 (100%)**	**193 (71.5%)**	**0/0**	
Mutations:	2141C>G, 2144G>T, 2147G>A, 2152C>T, 2158A>T, 2161T>G, 2162C>T, 2170T>C, 2173T>G, 2175C>T, 2179A>T, 2181G>T, 2182C>T, 2183T>C, 2184C>T, 2188G>A, 2191A>C, 2195T>A, 2197G>T, 2209C>T, 2210C>T, 2219G>T, 2225G>A, 2228G>A, 2233C>A, 2237A>G, 2239C>G, 2241G>T, 2242C>T, 2243A>G, 2247C>G, 2248T>A, 2251G>T, 2252G>C, 2255A>T, 2261G>A, 2264A>G, 2267T>G, 2396G>A, 2402T>A, 2403G>A, 2404C>T, 2405G>A, 2409G>T, 2414A>G, 2416C>T, 2417C>G, 2418C>T, 2420G>A, 2422T>C, 2423C>T, 2425T>G, 2428A>G, 2429T>A, 2431A>T, 2434G>T, 2435A>T, 2437A>T, 2442T>A, 2444A>G, 2447G>T, 2450T>G, 2452A>C, 2453A>G, 2456C>T, 2461G>C, 2467A>G, 2476G>T, 2477C>G, 2486T>G, 2488A>G, 2492A>T, 2494T>G, 2495A>T, 2498C>T, 2501A>T, 2504C>T								

CDS

	Begin	End	Coverage	Score	Concordance	Matches	Identities	I/D/M/F*	Stop Codons
AL349_gp5	5	134	27.2%	440	64.9%	90 (100%)	56 (62.2%)	0/0/0/0	0
Protein mutations:	I15L (2492A>T 2494T>G), Q21K (2476G>T), P26A (2461G>C), M27I (2456C>T), L29V (2450T>G 2452A>C), Y32F (2442T>A), Y34K (2435A>T 2437A>T), Q35K (2434G>T), L36I (2429T>A 2431A>T), K38Q (2423C>T 2425T>G), N39D (2420G>A 2422T>C), W40Y (2417C>G 2418C>T), D41N (2414A>G 2416C>T), T43N (2409G>T), A45I (2402T>A 2403G>A 2404C>T), R97S (2247C>G 2248T>A), A99K (2241G>T 2242C>T), D100H (2237A>G 2239C>G), A102S (2233C>A), V110I (2209C>T), R114S (2195T>A 2197G>T), L116V (2191A>C), L117F (2188G>A), R118K (2183T>C 2184C>T), A119N (2181G>T 2182C>T), S120T (2179A>T), R121K (2175C>T), S123G (2170T>C), K126Q (2161T>G), S127T (2158A>T), D129N (2152C>T), W132C (2141C>G)								
Codon mutations:	CAG11CAA (2504C>T), GGT12GGA (2501A>T), AAG13AAA (2498C>T), TCT14TCA (2495A>T), ATT15CTA (2492A>T 2494T>G), TTA17CTC (2486T>G 2488A>G), CCG20CCC (2477C>G), CAG21AAG (2476G>T), TTA24CTA (2467A>G), CCA26GCA (2461G>C), ATG27ATA (2456C>T), TTT28TTC (2453A>G), TTA29GTC (2450T>G 2452A>C), ATC30ATA (2447G>T), GAT31GAC (2444A>G), TAC32TTC (2442T>A), TAT34AAA (2435A>T 2437A>T), CAA35AAA (2434G>T), TTA36ATT (2429T>A 2431A>T), TTA37CTA (2428A>G), AAG38CAA (2423C>T 2425T>G), AAC39GAT (2420G>A 2422T>C), TGG40TAC (2417C>G 2418C>T), GAT41AAC (2414A>G 2416C>T), ACT43AAT (2409G>T), TAC44TAT (2405G>A), GCA45ATT (2402T>A 2403G>A 2404C>T), GTC47GTT (2396G>A), CCA90CCC (2267T>G), AAT91AAC (2264A>G), ACC92ACT (2261G>A), ATT94ATA (2255A>T), CCC95CCG (2252G>C), CGA96AGA (2251G>T), AGG97TCG (2247C>G 2248T>A), GAT98GAC (2243A>G), GCA99AAA (2241G>T 2242C>T), GAT100CAC (2237A>G 2239C>G), GCC102TCC (2233C>A), GAC103GAT (2228G>A), TAC104TAT (2225G>A), TCC106TCA (2219G>T), GGG109GGA (2210C>T), GTA110ATA (2209C>T), CGA114AGT (2195T>A 2197G>T), TTA116GTA (2191A>C), CTT117TTT (2188G>A), AGA118AAG (2183T>C 2184C>T), GCT119AAT (2181G>T 2182C>T), TCA120ACA (2179A>T), AGA121AAA (2175C>T), AGA122CGA (2173T>G), AGT123GGT (2170T>C), AAG125AAA (2162C>T), AAG126CAG (2161T>G), TCT127ACT (2158A>T), GAT129AAT (2152C>T), TCC130TCT (2147G>A), ATC131ATA (2144G>T), TGG132TGC (2141C>G)								
repA	5	134	34.6%	440	64.9%	90 (100%)	56 (62.2%)	0/0/0/0	0
Protein mutations:	I15L (2492A>T 2494T>G), Q21K (2476G>T), P26A (2461G>C), M27I (2456C>T), L29V (2450T>G 2452A>C), Y32F (2442T>A), Y34K (2435A>T 2437A>T), Q35K (2434G>T), L36I (2429T>A 2431A>T), K38Q (2423C>T 2425T>G), N39D (2420G>A 2422T>C), W40Y (2417C>G 2418C>T), D41N (2414A>G 2416C>T), T43N (2409G>T), A45I (2402T>A 2403G>A 2404C>T), R97S (2247C>G 2248T>A), A99K (2241G>T 2242C>T), D100H (2237A>G 2239C>G), A102S (2233C>A), V110I (2209C>T), R114S (2195T>A 2197G>T), L116V (2191A>C), L117F (2188G>A), R118K (2183T>C 2184C>T), A119N (2181G>T 2182C>T), S120T (2179A>T), R121K (2175C>T), S123G (2170T>C), K126Q (2161T>G), S127T (2158A>T), D129N (2152C>T), W132C (2141C>G)								
Codon mutations:	CAG11CAA (2504C>T), GGT12GGA (2501A>T), AAG13AAA (2498C>T), TCT14TCA (2495A>T), ATT15CTA (2492A>T 2494T>G), TTA17CTC (2486T>G 2488A>G), CCG20CCC (2477C>G), CAG21AAG (2476G>T), TTA24CTA (2467A>G), CCA26GCA (2461G>C), ATG27ATA (2456C>T), TTT28TTC (2453A>G), TTA29GTC (2450T>G 2452A>C), ATC30ATA (2447G>T), GAT31GAC (2444A>G), TAC32TTC (2442T>A), TAT34AAA (2435A>T 2437A>T), CAA35AAA (2434G>T), TTA36ATT (2429T>A 2431A>T), TTA37CTA (2428A>G), AAG38CAA (2423C>T 2425T>G), AAC39GAT (2420G>A 2422T>C), TGG40TAC (2417C>G 2418C>T), GAT41AAC (2414A>G 2416C>T), ACT43AAT (2409G>T), TAC44TAT (2405G>A), GCA45ATT (2402T>A 2403G>A 2404C>T), GTC47GTT (2396G>A), CCA90CCC (2267T>G), AAT91AAC (2264A>G), ACC92ACT (2261G>A), ATT94ATA (2255A>T), CCC95CCG (2252G>C), CGA96AGA (2251G>T), AGG97TCG (2247C>G 2248T>A), GAT98GAC (2243A>G), GCA99AAA (2241G>T 2242C>T), GAT100CAC (2237A>G 2239C>G), GCC102TCC (2233C>A), GAC103GAT (2228G>A), TAC104TAT (2225G>A), TCC106TCA (2219G>T), GGG109GGA (2210C>T), GTA110ATA (2209C>T), CGA114AGT (2195T>A 2197G>T), TTA116GTA (2191A>C), CTT117TTT (2188G>A), AGA118AAG (2183T>C 2184C>T), GCT119AAT (2181G>T 2182C>T), TCA120ACA (2179A>T), AGA121AAA (2175C>T), AGA122CGA (2173T>G), AGT123GGT (2170T>C), AAG125AAA (2162C>T), AAG126CAG (2161T>G), TCT127ACT (2158A>T), GAT129AAT (2152C>T), TCC130TCT (2147G>A), ATC131ATA (2144G>T), TGG132TGC (2141C>G)								
C3	8	52	36.9%	109	34.1%	45 (100%)	19 (42.2%)	0/0/0/0	0
Protein mutations:	Q8P (2267T>G), I9T (2264A>G), P10L (2261G>A), F12Y (2255A>T), P13R (2251G>T 2252G>C), E14D (2248T>A), G15R (2247C>G), M16T (2242C>T 2243A>G), Q17N (2239C>G 2241G>T), M18T (2237A>G), T21I (2228G>A), T22I (2225G>A), P24Q (2219G>T), G27E (2209C>T 2210C>T), N31K (2197G>T), E32V (2195T>A), D33E (2191A>C), E36R (2182C>T 2183T>C 2184C>T), L37I (2179A>T 2181G>T), E39N (2173T>G 2175C>T), R43N (2161T>G 2162C>T), S44R (2158A>T), P48L (2147G>A), S49Y (2144G>T), G50A (2141C>G)								
Codon mutations:	CAA8CCA (2267T>G), ATA9ACA (2264A>G), CCC10CTC (2261G>A), TTC12TAC (2255A>T), CCC13CGA (2251G>T 2252G>C), GAA14GAT (2248T>A), GGG15CGG (2247C>G), ATG16ACA (2242C>T 2243A>G), CAG17AAC (2239C>G 2241G>T), ATG18ACG (2237A>G), TCG19TCT (2233C>A), ACT21ATT (2228G>A), ACA22ATA (2225G>A), CCA24CAA (2219G>T), GGG27GAA (2209C>T 2210C>T), AAC31AAA (2197G>T), GAG32GTG (2195T>A), GAT33GAG (2191A>C), TAC34TAT (2188G>A), GAG36AGA (2182C>T 2183T>C 2184C>T), CTT37ATA (2179A>T 2181G>T), GAA39AAC (2173T>G 2175C>T), GAA40GAG (2170T>C), AGA43AAC (2161T>G 2162C>T), AGT44AGA (2158A>T), GGG46GGA (2152C>T), CCA48CTA (2147G>A), TCT49TAT (2144G>T), GGA50GCA (2141C>G)								

Proteins

	Begin	End	Coverage	Score	Concordance	Matches	Identities	I/D/M/F*	Stop Codons
replication associated protein (YP_009162635.2)	5	134	27.2%	440	64.9%	90 (100%)	56 (62.2%)	0/0/0/0	0
Protein mutations:	I15L (2492A>T 2494T>G), Q21K (2476G>T), P26A (2461G>C), M27I (2456C>T), L29V (2450T>G 2452A>C), Y32F (2442T>A), Y34K (2435A>T 2437A>T), Q35K (2434G>T), L36I (2429T>A 2431A>T), K38Q (2423C>T 2425T>G), N39D (2420G>A 2422T>C), W40Y (2417C>G 2418C>T), D41N (2414A>G 2416C>T), T43N (2409G>T), A45I (2402T>A 2403G>A 2404C>T), R97S (2247C>G 2248T>A), A99K (2241G>T 2242C>T), D100H (2237A>G 2239C>G), A102S (2233C>A), V110I (2209C>T), R114S (2195T>A 2197G>T), L116V (2191A>C), L117F (2188G>A), R118K (2183T>C 2184C>T), A119N (2181G>T 2182C>T), S120T (2179A>T), R121K (2175C>T), S123G (2170T>C), K126Q (2161T>G), S127T (2158A>T), D129N (2152C>T), W132C (2141C>G)								
Codon mutations:	CAG11CAA (2504C>T), GGT12GGA (2501A>T), AAG13AAA (2498C>T), TCT14TCA (2495A>T), ATT15CTA (2492A>T 2494T>G), TTA17CTC (2486T>G 2488A>G), CCG20CCC (2477C>G), CAG21AAG (2476G>T), TTA24CTA (2467A>G), CCA26GCA (2461G>C), ATG27ATA (2456C>T), TTT28TTC (2453A>G), TTA29GTC (2450T>G 2452A>C), ATC30ATA (2447G>T), GAT31GAC (2444A>G), TAC32TTC (2442T>A), TAT34AAA (2435A>T 2437A>T), CAA35AAA (2434G>T), TTA36ATT (2429T>A 2431A>T), TTA37CTA (2428A>G), AAG38CAA (2423C>T 2425T>G), AAC39GAT (2420G>A 2422T>C), TGG40TAC (2417C>G 2418C>T), GAT41AAC (2414A>G 2416C>T), ACT43AAT (2409G>T), TAC44TAT (2405G>A), GCA45ATT (2402T>A 2403G>A 2404C>T), GTC47GTT (2396G>A), CCA90CCC (2267T>G), AAT91AAC (2264A>G), ACC92ACT (2261G>A), ATT94ATA (2255A>T), CCC95CCG (2252G>C), CGA96AGA (2251G>T), AGG97TCG (2247C>G 2248T>A), GAT98GAC (2243A>G), GCA99AAA (2241G>T 2242C>T), GAT100CAC (2237A>G 2239C>G), GCC102TCC (2233C>A), GAC103GAT (2228G>A), TAC104TAT (2225G>A), TCC106TCA (2219G>T), GGG109GGA (2210C>T), GTA110ATA (2209C>T), CGA114AGT (2195T>A 2197G>T), TTA116GTA (2191A>C), CTT117TTT (2188G>A), AGA118AAG (2183T>C 2184C>T), GCT119AAT (2181G>T 2182C>T), TCA120ACA (2179A>T), AGA121AAA (2175C>T), AGA122CGA (2173T>G), AGT123GGT (2170T>C), AAG125AAA (2162C>T), AAG126CAG (2161T>G), TCT127ACT (2158A>T), GAT129AAT (2152C>T), TCC130TCT (2147G>A), ATC131ATA (2144G>T), TGG132TGC (2141C>G)								
RepA (YP_009162636.1)	5	134	34.6%	440	64.9%	90 (100%)	56 (62.2%)	0/0/0/0	0
Protein mutations:	I15L (2492A>T 2494T>G), Q21K (2476G>T), P26A (2461G>C), M27I (2456C>T), L29V (2450T>G 2452A>C), Y32F (2442T>A), Y34K (2435A>T 2437A>T), Q35K (2434G>T), L36I (2429T>A 2431A>T), K38Q (2423C>T 2425T>G), N39D (2420G>A 2422T>C), W40Y (2417C>G 2418C>T), D41N (2414A>G 2416C>T), T43N (2409G>T), A45I (2402T>A 2403G>A 2404C>T), R97S (2247C>G 2248T>A), A99K (2241G>T 2242C>T), D100H (2237A>G 2239C>G), A102S (2233C>A), V110I (2209C>T), R114S (2195T>A 2197G>T), L116V (2191A>C), L117F (2188G>A), R118K (2183T>C 2184C>T), A119N (2181G>T 2182C>T), S120T (2179A>T), R121K (2175C>T), S123G (2170T>C), K126Q (2161T>G), S127T (2158A>T), D129N (2152C>T), W132C (2141C>G)								

	Begin	End	Coverage	Score	Concordance	Matches	Identities	I/D/M/F*	Stop Codons
**NT**	**2136**	**2525**	**9.8%**	**232**	**43.0%**	**270 (100%)**	**193 (71.5%)**	**0/0**	
Codon mutations:	CAG11CAA (2504C>T), GGT12GGA (2501A>T), AAG13AAA (2498C>T), TCT14TCA (2495A>T), ATT15CTA (2492A>T 2494T>G), TTA17CTC (2486T>G 2488A>G), CCG20CCC (2477C>G), CAG21AAG (2476G>T), TTA24CTA (2467A>G), CCA26GCA (2461G>C), ATG27ATA (2456C>T), TTT28TTC (2453A>G), TTA29GTC (2450T>G 2452A>C), ATC30ATA (2447G>T), GAT31GAC (2444A>G), TAC32TTC (2442T>A), TAT34AAA (2435A>T 2437A>T), CAA35AAA (2434G>T), TTA36ATT (2429T>A 2431A>T), TTA37CTA (2428A>G), AAG38CAA (2423C>T 2425T>G), AAC39GAT (2420G>A 2422T>C), TGG40TAC (2417C>G 2418C>T), GAT41AAC (2414A>G 2416C>T), ACT43AAT (2409G>T), TAC44TAT (2405G>A), GCA45ATT (2402T>A 2403G>A 2404C>T), GTC47GTT (2396G>A), CCA90CCC (2267T>G), AAT91AAC (2264A>G), ACC92ACT (2261G>A), ATT94ATA (2255A>T), CCC95CCG (2252G>C), CGA96AGA (2251G>T), AGG97TCG (2247C>G 2248T>A), GAT98GAC (2243A>G), GCA99AAA (2241G>T 2242C>T), GAT100CAC (2237A>G 2239C>G), GCC102TCC (2233C>A), GAC103GAT (2228G>A), TAC104TAT (2225G>A), TCC106TCA (2219G>T), GGG109GGA (2210C>T), GTA110ATA (2209C>T), CGA114AGT (2195T>A 2197G>T), TTA116GTA (2191A>C), CTT117TTT (2188G>A), AGA118AAG (2183T>C 2184C>T), GCT119AAT (2181G>T 2182C>T), TCA120ACA (2179A>T), AGA121AAA (2175C>T), AGA122CGA (2173T>G), AGT123GGT (2170T>C), AAG125AAA (2162C>T), AAG126CAG (2161T>G), TCT127ACT (2158A>T), GAT129AAT (2152C>T), TCC130TCT (2147G>A), ATC131ATA (2144G>T), TGG132TGC (2141C>G)								
C3 (YP_009162637.1)	8	52	36.9%	109	34.1%	45 (100%)	19 (42.2%)	0/0/0/0	0
Protein mutations:	Q8P (2267T>G), I9T (2264A>G), P10L (2261G>A), F12Y (2255A>T), P13R (2251G>T 2252G>C), E14D (2248T>A), G15R (2247C>G), M16T (2242C>T 2243A>G), Q17N (2239C>G 2241G>T), M18T (2237A>G), T21I (2228G>A), T22I (2225G>A), P24Q (2219G>T), G27E (2209C>T 2210C>T), N31K (2197G>T), E32V (2195T>A), D33E (2191A>C), E36R (2182C>T 2183T>C 2184C>T), L37I (2179A>T 2181G>T), E39N (2173T>G 2175C>T), R43N (2161T>G 2162C>T), S44R (2158A>T), P48L (2147G>A), S49Y (2144G>T), G50A (2141C>G)								
Codon mutations:	CAA8CCA (2267T>G), ATA9ACA (2264A>G), CCC10CTC (2261G>A), TTC12TAC (2255A>T), CCC13CGA (2251G>T 2252G>C), GAA14GAT (2248T>A), GGG15CGG (2247C>G), ATG16ACA (2242C>T 2243A>G), CAG17AAC (2239C>G 2241G>T), ATG18ACG (2237A>G), TCG19TCT (2233C>A), ACT21ATT (2228G>A), ACA22ATA (2225G>A), CCA24CAA (2219G>T), GGG27GAA (2209C>T 2210C>T), AAC31AAA (2197G>T), GAG32GTG (2195T>A), GAT33GAG (2191A>C), TAC34TAT (2188G>A), GAG36AGA (2182C>T 2183T>C 2184C>T), CTT37ATA (2179A>T 2181G>T), GAA39AAC (2173T>G 2175C>T), GAA40GAG (2170T>C), AGA43AAC (2161T>G 2162C>T), AGT44AGA (2158A>T), GGG46GGA (2152C>T), CCA48CTA (2147G>A), TCT49TAT (2144G>T), GGA50GCA (2141C>G)								

*: Inserts / Deletes / Misaligned / Frameshifts

Analysis details

This analysis was performed with panviral2.64

NGS Details (UN60): Duamitovirus peex1

Assembly

**Coverage Length**	274 (2 contig(s))
**Depth Of Coverage**	4.7
**Number Of Reads**	13
**Reads Per Million**	0.28 rpm (after QC)
**Ambiguities**	0
**Assembly Method**	de novo + reference guided assembly
**Consensus Caller**	Bcf Tools

Coverage Map

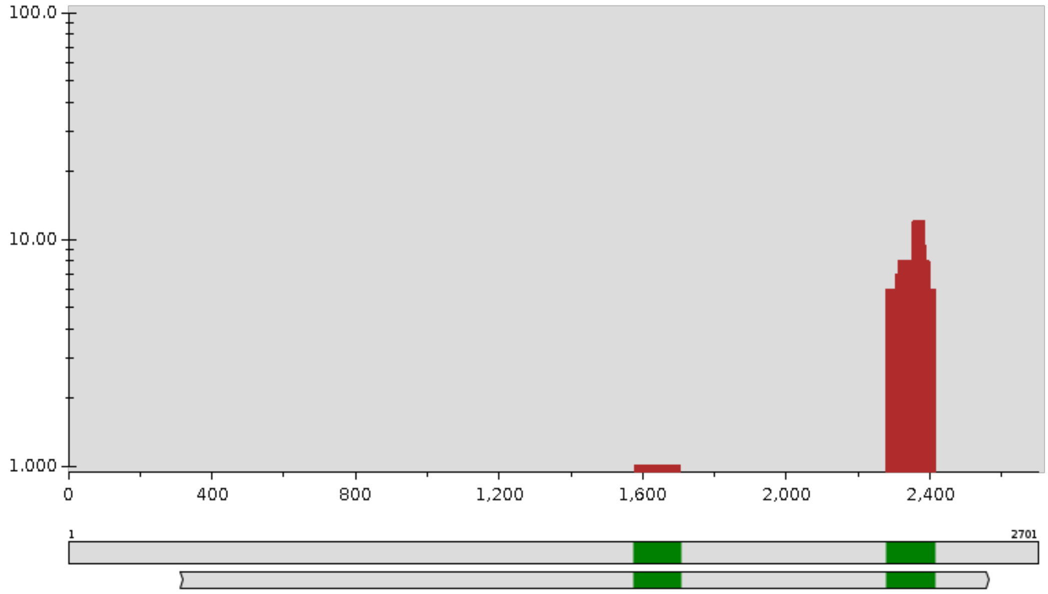

Assignment

**Type**	Duamitovirus peex1 (Taxonomy ID: 2955799)
**Reference Genome**	NC_076525.1
**NT Identity (%)**	70.8029
**AA Identity (%)**	73.6264
**Number Of Stop Codons**	0
**Number Of CDS**	1

Alignment

**Alignment Score**	228.0 (NT) + 541.0 (AA) = 769.0
**Concordance (%)**	63.5537

Alignment Method	Global, seeded, nucleotide + amino acids (AGA)

Genome Region

Sequence starts at position 1575 and ends at position 2417 relative to NC_076525.1 reference sequence.

Alignment Detailed Statistics

	Begin	End	Coverage	Score	Concordance	Matches	Identities	I/D/M/F*	Stop Codons
**NT**	**1575**	**2417**	**10.1%**	**228**	**41.6%**	**274 (100%)**	**194 (70.8%)**	**0/0**	
Mutations:	1583C>G, 1590G>A, 1592G>T, 1593T>A, 1594C>G, 1598G>A, 1599A>G, 1601C>A, 1604T>C, 1610G>T, 1611G>A, 1616G>A, 1619G>A, 1622T>G, 1623C>T, 1628C>T, 1632C>T, 1633T>A, 1634G>C, 1637A>T, 1646C>A, 1649A>G, 1652T>C, 1653G>T, 1655C>G, 1661A>C, 1664C>T, 1667T>C, 1671T>A, 1673G>A, 1676A>G, 1688G>T, 1689G>T, 1690A>T, 1696T>C, 1697C>T, 1700C>T, 2278G>A, 2281T>A, 2283G>A, 2294T>G, 2295C>A, 2296G>A, 2301C>G, 2303T>C, 2306A>T, 2311A>G, 2312T>C, 2315C>T, 2321G>T, 2325A>G, 2326A>C, 2327T>A, 2330C>A, 2331C>G, 2333G>T, 2339G>A, 2345A>T, 2348C>T, 2351A>G, 2352C>T, 2354T>C, 2363G>T, 2373G>A, 2374C>G, 2375G>T, 2377C>A, 2378T>A, 2379T>A, 2388C>A, 2390T>A, 2393T>A, 2394C>A, 2396G>C, 2398C>A, 2399T>A, 2406C>G, 2411A>G, 2412G>A, 2414G>C								

CDS

	Begin	End	Coverage	Score	Concordance	Matches	Identities	I/D/M/F*	Stop Codons
RdRp	422	702	12.1%	541	78.3%	91 (100%)	67 (73.6%)	0/0/0/0	0
Protein mutations:	V427I (1590G>A 1592G>T), I430V (1599A>G 1601C>A), A434T (1611G>A), L441Y (1632C>T 1633T>A 1634G>C), A448S (1653G>T 1655C>G), L454I (1671T>A 1673G>A), E460L (1689G>T 1690A>T), V462A (1696T>C 1697C>T), M657K (2281T>A), E658K (2283G>A), R662K (2295C>A 2296G>A), L664V (2301C>G 2303T>C), Y667C (2311A>G 2312T>C), N672A (2325A>G 2326A>C 2327T>A), P674A (2331C>G 2333G>T), L681F (2352C>T 2354T>C), A688S (2373G>A 2374C>G 2375G>T), T689K (2377C>A 2378T>A), S690T (2379T>A), Q695N (2394C>A 2396G>C), T696K (2398C>A 2399T>A), H699D (2406C>G), V701I (2412G>A 2414G>C)								
Codon mutations:	ACC424ACG (1583C>G), GTG427ATT (1590G>A 1592G>T), TCT428AGT (1593T>A 1594C>G), GAG429GAA (1598G>A), ATC430GTA (1599A>G 1601C>A), GCT431GCC (1604T>C), CTG433CTT (1610G>T), GCT434ACT (1611G>A), GGG435GGA (1616G>A), CAG436CAA (1619G>A), CCT437CCG (1622T>G), CTA438TTA (1623C>T), GGC439GGT (1628C>T), CTG441TAC (1632C>T 1633T>A 1634G>C), GGA442GGT (1637A>T), TCC445TCA (1646C>A), CTA446CTG (1649A>G), TTT447TTC (1652T>C), GCC448TCG (1653G>T 1655C>G), TCA450TCC (1661A>C), CAC451CAT (1664C>T), CAT452CAC (1667T>C), TTG454ATA (1671T>A 1673G>A), GTA455GTG (1676A>G), GCG459GCT (1688G>T), GAG460TTG (1689G>T 1690A>T), GTC462GCT (1696T>C 1697C>T), TAC463TAT (1700C>T), TGG656.AG (2278G>A), ATG657AAG (2281T>A), GAA658AAA (2283G>A), CTT661CTG (2294T>G), CGA662AAA (2295C>A 2296G>A), CTT664GTC (2301C>G 2303T>C), TCA665TCT (2306A>T), TAT667TGC (2311A>G 2312T>C), CAC668CAT (2315C>T), GTG670GTT (2321G>T), AAT672GCA (2325A>G 2326A>C 2327T>A), TCC673TCA (2330C>A), CCG674GCT (2331C>G 2333G>T), GTG676GTA (2339G>A), ATA678ATT (2345A>T), GAC679GAT (2348C>T), CAA680CAG (2351A>G), CTT681TTC (2352C>T 2354T>C), GTG684GTT (2363G>T), GCG688AGT (2373G>A 2374C>G 2375G>T), ACT689AAA (2377C>A 2378T>A), TCT690ACT (2379T>A), CGT693AGA (2388C>A 2390T>A), ACT694ACA (2393T>A), CAG695AAC (2394C>A 2396G>C), ACT696AAA (2398C>A 2399T>A), CAT699GAT (2406C>G), TTA700TTG (2411A>G), GTG701ATC (2412G>A 2414G>C)								

Proteins

	Begin	End	Coverage	Score	Concordance	Matches	Identities	I/D/M/F*	Stop Codons
RNA-dependent RNA polymerase (YP_010798875.1)	422	702	12.1%	541	78.3%	91 (100%)	67 (73.6%)	0/0/0/0	0
Protein mutations:	V427I (1590G>A 1592G>T), I430V (1599A>G 1601C>A), A434T (1611G>A), L441Y (1632C>T 1633T>A 1634G>C), A448S (1653G>T 1655C>G), L454I (1671T>A 1673G>A), E460L (1689G>T 1690A>T), V462A (1696T>C 1697C>T), M657K (2281T>A), E658K (2283G>A), R662K (2295C>A 2296G>A), L664V (2301C>G 2303T>C), Y667C (2311A>G 2312T>C), N672A (2325A>G 2326A>C 2327T>A), P674A (2331C>G 2333G>T), L681F (2352C>T 2354T>C), A688S (2373G>A 2374C>G 2375G>T), T689K (2377C>A 2378T>A), S690T (2379T>A), Q695N (2394C>A 2396G>C), T696K (2398C>A 2399T>A), H699D (2406C>G), V701I (2412G>A 2414G>C)								
Codon mutations:	ACC424ACG (1583C>G), GTG427ATT (1590G>A 1592G>T), TCT428AGT (1593T>A 1594C>G), GAG429GAA (1598G>A), ATC430GTA (1599A>G 1601C>A), GCT431GCC (1604T>C), CTG433CTT (1610G>T), GCT434ACT (1611G>A), GGG435GGA (1616G>A), CAG436CAA (1619G>A), CCT437CCG (1622T>G), CTA438TTA (1623C>T), GGC439GGT (1628C>T), CTG441TAC (1632C>T 1633T>A 1634G>C), GGA442GGT (1637A>T), TCC445TCA (1646C>A), CTA446CTG (1649A>G), TTT447TTC (1652T>C), GCC448TCG (1653G>T 1655C>G), TCA450TCC (1661A>C), CAC451CAT (1664C>T), CAT452CAC (1667T>C), TTG454ATA (1671T>A 1673G>A), GTA455GTG (1676A>G), GCG459GCT (1688G>T), GAG460TTG (1689G>T 1690A>T), GTC462GCT (1696T>C 1697C>T), TAC463TAT (1700C>T), TGG656.AG (2278G>A), ATG657AAG (2281T>A), GAA658AAA (2283G>A), CTT661CTG (2294T>G), CGA662AAA (2295C>A 2296G>A), CTT664GTC (2301C>G 2303T>C), TCA665TCT (2306A>T), TAT667TGC (2311A>G 2312T>C), CAC668CAT (2315C>T), GTG670GTT (2321G>T), AAT672GCA (2325A>G 2326A>C 2327T>A), TCC673TCA (2330C>A), CCG674GCT (2331C>G 2333G>T), GTG676GTA (2339G>A), ATA678ATT (2345A>T), GAC679GAT (2348C>T), CAA680CAG (2351A>G), CTT681TTC (2352C>T 2354T>C), GTG684GTT (2363G>T), GCG688AGT (2373G>A 2374C>G 2375G>T), ACT689AAA (2377C>A 2378T>A), TCT690ACT (2379T>A), CGT693AGA (2388C>A 2390T>A), ACT694ACA (2393T>A), CAG695AAC (2394C>A 2396G>C), ACT696AAA (2398C>A 2399T>A), CAT699GAT (2406C>G), TTA700TTG (2411A>G), GTG701ATC (2412G>A 2414G>C)								

*: Inserts / Deletes / Misaligned / Frameshifts

Analysis details

This analysis was performed with panviral2.64

NGS Details (UN60): Pseudomonas phage YMC11/07/P54_PAE_BP

Assembly

**Coverage Length**	334 (1 contig(s))
**Depth Of Coverage**	4.8
**Number Of Reads**	13
**Reads Per Million**	0.28 rpm (after QC)
**Ambiguities**	0
**Assembly Method**	de novo + reference guided assembly
**Consensus Caller**	Bcf Tools

Coverage Map

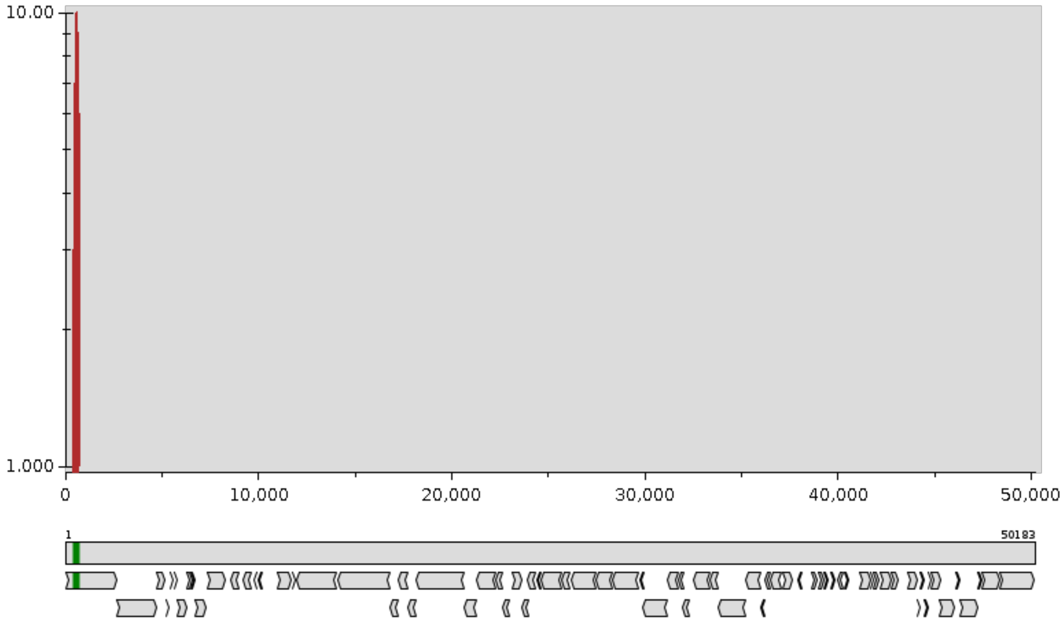

Assignment

**Type**	Pseudomonas phage YMC11/07/P54_PAE_BP (Taxonomy ID: 1777064)
**Reference Genome**	NC_030909.1
**NT Identity (%)**	78.4257
**AA Identity (%)**	83.3333
**Number Of Stop Codons**	0
**Number Of CDS**	73

Alignment

**Alignment Score**	390.0 (NT) + 622.0 (AA) = 1012.0
**Concordance (%)**	71.9772

Alignment Method	Local, heuristic, nucleotide (BLASTN)

Genome Region

Sequence starts at position 373 and ends at position 706 relative to NC_030909.1 reference sequence.

Alignment Detailed Statistics

	Begin	End	Coverage	Score	Concordance	Matches	Identities	I/D/M/F*	Stop Codons
**NT**	**373**	**706**	**0.7%**	**390**	**58.4%**	**334 (97.4%)**	**269 (78.4%)**	**9/0**	
Mutations:	385G>C, 391T>C, 397G>A, 400C>G, 401G>A, 410G>C, 418C>G, 424T>G, 426G>T, 427C>G, 429G>T, 442A>C, 454A>G, 457C>G, 458A>G, 459G>C, 460T>A, 466T>G, 490G>C, 508T>G, 517T>C, 520T>C, 526T>C, 529C>G, 529_530insCGCGACATG, 533G>A, 534C>A, 536G>C, 539A>G, 541C>G, 542C>A, 543G>A, 544C>G, 545G>A, 549A>C, 556C>G, 558G>C, 562C>T, 565G>C, 567A>T, 577T>G, 586A>C, 589C>T, 592G>A, 598A>G, 600A>T, 601C>T, 602A>G, 610A>G, 616T>C, 620T>A, 621C>G, 622G>C, 631C>G, 643T>C, 646C>T, 661C>G, 662A>C, 664G>T, 673A>G, 680T>A, 681C>G, 682T>C, 695C>A, 697A>C, 700C>G								
**CDS**									
BH775_gp01	114	224	12.7%	622	83.7%	111 (97.4%)	95 (83.3%)	3/0/0/0	0
Protein mutations:	V123I (401G>A), G126R (410G>C), R131L (426G>T 427C>G), R132L (429G>T), S142A (458A>G 459G>C 460T>A), R165_A166insRDM (529_530insCGCGACATG), A167N (533G>A 534C>A), G168R (536G>C), I169V (539A>G 541C>G), R170K (542C>A 543G>A 544C>G), G171S (545G>A), D172A (549A>C), R175P (558G>C), Y178F (567A>T), Y189F (600A>T 601C>T), I190V (602A>G), Q221N (695C>A 697A>C)								
Codon mutations:	CTG117CTC (385G>C), AAT119AAC (391T>C), GAG121GAA (397G>A), ACC122ACG (400C>G), GTC123ATC (401G>A), GGC126CGC (410G>C), CTC128CTG (418C>G), CCT130CCG (424T>G), CGC131CTG (426G>T 427C>G), CGG132CTG (429G>T), GCA136GCC (442A>C), GAA140GAG (454A>G), GCC141GCG (457C>G), AGT142GCA (458A>G 459G>C 460T>A), CTT144CTG (466T>G), ACG152ACC (490G>C), GCT158GCG (508T>G), GCT161GCC (517T>C), GGT162GGC (520T>C), GAT164GAC (526T>C), CGC165CGG (529C>G), CGC165_GCC166insCGCGACATG (529_530insCGCGACATG), GCC167AAC (533G>A 534C>A), GGC168CGC (536G>C), ATC169GTG (539A>G 541C>G), CGC170AAG (542C>A 543G>A 544C>G), GGC171AGC (545G>A), GAT172GCT (549A>C), GTC174GTG (556C>G), CGG175CCG (558G>C), TAC176TAT (562C>T), CGG177CGC (565G>C), TAC178TTC (567A>T), ACT181ACG (577T>G), CCA184CCC (586A>C), AGC185AGT (589C>T), CCG186CCA (592G>A), GAA188GAG (598A>G), TAC189TTT (600A>T 601C>T), ATC190GTC (602A>G), CTA192CTG (610A>G), GCT194GCC (616T>C), TCG196AGC (620T>A 621C>G 622G>C), CTC199CTG (631C>G), GAT203GAC (643T>C), GTC204GTT (646C>T), ACC209ACG (661C>G), AGG210CGT (662A>C 664G>T), AAA213AAG (673A>G), TCT216AGC (680T>A 681C>G 682T>C), CAA221AAC (695C>A 697A>C), CTC222CTG (700C>G)								
**Proteins**									
DNA methylase N-4 (YP_009273683.1)	114	224	12.7%	622	83.7%	111 (97.4%)	95 (83.3%)	3/0/0/0	0
Protein mutations:	V123I (401G>A), G126R (410G>C), R131L (426G>T 427C>G), R132L (429G>T), S142A (458A>G 459G>C 460T>A), R165_A166insRDM (529_530insCGCGACATG), A167N (533G>A 534C>A), G168R (536G>C), I169V (539A>G 541C>G), R170K (542C>A 543G>A 544C>G), G171S (545G>A), D172A (549A>C), R175P (558G>C), Y178F (567A>T), Y189F (600A>T 601C>T), I190V (602A>G), Q221N (695C>A 697A>C)								
Codon mutations:	CTG117CTC (385G>C), AAT119AAC (391T>C), GAG121GAA (397G>A), ACC122ACG (400C>G), GTC123ATC (401G>A), GGC126CGC (410G>C), CTC128CTG (418C>G), CCT130CCG (424T>G), CGC131CTG (426G>T 427C>G), CGG132CTG (429G>T), GCA136GCC (442A>C), GAA140GAG (454A>G), GCC141GCG (457C>G), AGT142GCA (458A>G 459G>C 460T>A), CTT144CTG (466T>G), ACG152ACC (490G>C), GCT158GCG (508T>G), GCT161GCC (517T>C), GGT162GGC (520T>C), GAT164GAC (526T>C), CGC165CGG (529C>G), CGC165_GCC166insCGCGACATG (529_530insCGCGACATG), GCC167AAC (533G>A 534C>A), GGC168CGC (536G>C), ATC169GTG (539A>G 541C>G), CGC170AAG (542C>A 543G>A 544C>G), GGC171AGC (545G>A), GAT172GCT (549A>C), GTC174GTG (556C>G), CGG175CCG (558G>C), TAC176TAT (562C>T), CGG177CGC (565G>C), TAC178TTC (567A>T), ACT181ACG (577T>G), CCA184CCC (586A>C), AGC185AGT (589C>T), CCG186CCA (592G>A), GAA188GAG (598A>G), TAC189TTT (600A>T 601C>T), ATC190GTC (602A>G), CTA192CTG (610A>G), GCT194GCC (616T>C), TCG196AGC (620T>A 621C>G 622G>C), CTC199CTG (631C>G), GAT203GAC (643T>C), GTC204GTT (646C>T), ACC209ACG (661C>G), AGG210CGT (662A>C 664G>T), AAA213AAG (673A>G), TCT216AGC (680T>A 681C>G 682T>C), CAA221AAC (695C>A 697A>C), CTC222CTG (700C>G)								

*: Inserts / Deletes / Misaligned / Frameshifts

Analysis details

This analysis was performed with panviral2.64

NGS Details (UN60): Pinus nigra virus 1

Assembly

**Coverage Length**	474 (1 contig(s))
**Depth Of Coverage**	3.1
**Number Of Reads**	11
**Reads Per Million**	0.23 rpm (after QC)
**Ambiguities**	0
**Assembly Method**	de novo + reference guided assembly
**Consensus Caller**	Bcf Tools

Coverage Map

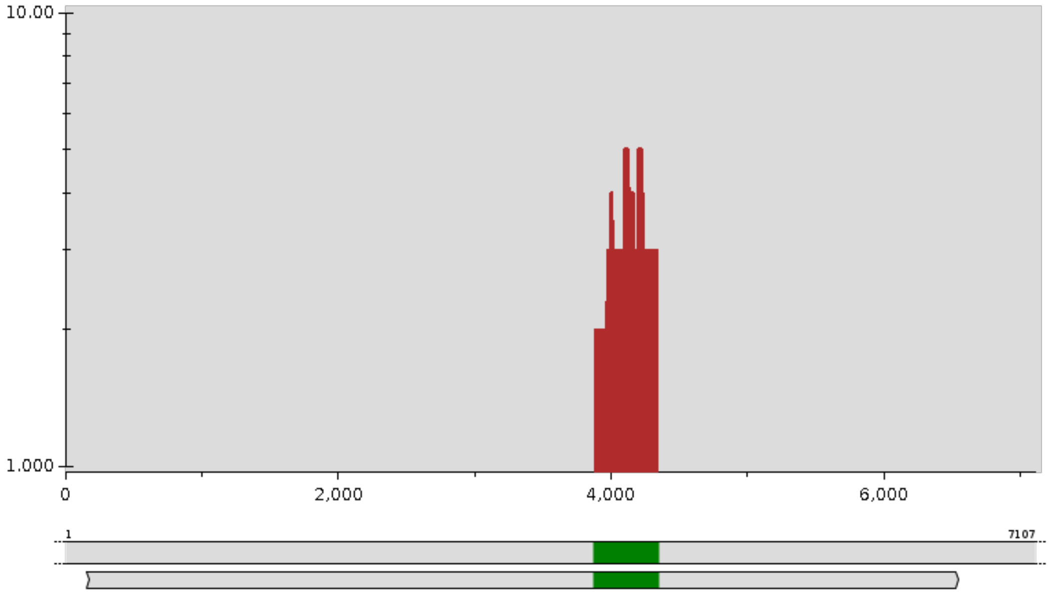

Assignment

**Type**	Pinus nigra virus 1 (Taxonomy ID: 2267679)
**Reference Genome**	NC_040841.1
**NT Identity (%)**	56.8134
**AA Identity (%)**	45.9119
**Number Of Stop Codons**	1
**Number Of CDS**	1

Alignment

**Alignment Score**	115.0 (NT) + 496.0 (AA) = 611.0
**Concordance (%)**	29.5597

Alignment Method	Global, seeded, nucleotide + amino acids (AGA)

Genome Region

Sequence starts at position 3879 and ends at position 4352 relative to NC_040841.1 reference sequence.

Alignment Detailed Statistics

	Begin	End	Coverage	Score	Concordance	Matches	Identities	I/D/M/F*	Stop Codons
**NT**	**3879**	**4352**	**6.7%**	**115**	**12.4%**	**471 (98.1%)**	**271 (56.5%)**	**6/3**	
Mutations:	3880G>T, 3884C>G, 3885A>T, 3887T>G, 3892G>A, 3894T>A, 3897T>A, 3899A>T, 3901A>C, 3903G>A, 3904T>G, 3905A>T, 3906A>G, 3907A>T, 3914A>C, 3916G>T, 3919A>T, 3922A>C, 3935C>T, 3937T>G, 3941G>A, 3943C>T, 3944A>G, 3945T>C, 3946C>T, 3947A>T, 3948C>T, 3949T>A, 3950C>A, 3952G>A, 3953A>T, 3954A>G, 3955T>G, 3956T>A, 3960A>G, 3961T>A, 3967A>T, 3968T>A, 3970G>T, 3973A>T, 3975G>A, 3976A>C, 3977C>A, 3978C>A, 3980G>A, 3982A>G, 3984T>A, 3985G>T, 3986A>C, 3989A>C, 3991G>A, 3992C>A, 3996A>G, 3999T>C, 4000T>C, 4001C>T, 4002A>T, 4003G>T, 4004C>A, 4006T>A, 4007A>G, 4008G>C, 4009T>A, 4012G>C, 4013G>A, 4015G>T, 4017T>A, 4021C>T, 4024G>A, 4030C>T, 4036G>A, 4044A>T, 4047A>G, 4048T>G, 4051G>A, 4054A>T, 4057A>G, 4061C>T, 4062A>C, 4063G>T, 4064C>G, 4065C>A, 4067G>A, 4072T>C, 4074G>A, 4076C>T, 4078C>G, 4081A>G, 4082A>T, 4084A>C, 4090C>T, 4092T>A, 4093A>T, 4094T>G, 4095G>T, 4099A>G, 4100G>T, 4101C>T, 4102A>T, 4105A>T, 4106T>C, 4107T>A, 4108C>A, 4109T>C, 4112C>G, 4114G>A, 4120A>C, 4124G>A, 4126T>G, 4128C>T, 4129A>T, 4135T>G, 4136T>C, 4138G>T, 4141G>A, 4147G>C, 4150A>C, 4151G>T, 4153C>A, 4154T>G, 4155T>A, 4156C>A, 4164G>A, 4167G>T, 4168G>T, 4172G>A, 4175T>G, 4181T>A, 4184G>A, 4185C>A, 4187A>C, 4189A>T, 4191A>T, 4192T>G, 4193G>A, 4194A>G, 4195T>C, 4195_4196insCATATG, 4197T>C, 4198T>A, 4205_4207delACA, 4210C>T, 4213A>T, 4217G>A, 4220A>G, 4228T>C, 4229C>T, 4230A>T, 4231T>C, 4232A>T, 4233G>C, 4235C>G, 4236C>A, 4237A>G, 4240T>C, 4241G>A, 4243A>C, 4244C>G, 4246G>T, 4247A>T, 4248A>C, 4258G>A, 4262T>C, 4265G>A, 4267A>T, 4273T>A, 4274C>T, 4277G>A, 4279A>C, 4280G>A, 4281A>C, 4282A>C, 4288G>A, 4290A>G, 4292C>A, 4297A>T, 4298A>T, 4300T>G, 4303T>A, 4304T>G, 4306G>A, 4307T>A, 4308C>G, 4309A>C, 4310G>A, 4311A>C, 4312A>T, 4314G>A, 4321G>T, 4322T>A, 4323C>A, 4324C>A, 4325T>C, 4327A>T, 4333G>A, 4334G>A, 4335A>C, 4336T>A, 4339T>A, 4343G>A, 4344A>G, 4345T>A								

CDS

	Begin	End	Coverage	Score	Concordance	Matches	Identities	I/D/M/F*	Stop Codons
EXL67_gp1	1243	1400	7.4%	496	43.0%	157 (98.1%)	73 (45.6%)	2/1/0/0	1
Protein mutations:	H1244V (3884C>G 3885A>T), S1245A (3887T>G), I1247K (3894T>A), V1248E (3897T>A), R1249C (3899A>T 3901A>C), G1250E (3903G>A 3904T>G), K1251C (3905A>T 3906A>G 3907A>T), M1254L (3914A>C 3916G>T), A1263T (3941G>A 3943C>T), I1264A (3944A>G 3945T>C 3946C>T), T1265L (3947A>T 3948C>T 3949T>A), Q1266K (3950C>A 3952G>A), N1267W (3953A>T 3954A>G 3955T>G), F1268I (3956T>A), N1269R (3960A>G 3961T>A), L1272I (3968T>A 3970G>T), R1274N (3975G>A 3976A>C), P1275K (3977C>A 3978C>A), E1276K (3980G>A 3982A>G), V1277D (3984T>A 3985G>T), I1278L (3986A>C), M1279L (3989A>C 3991G>A), Q1280K (3992C>A), K1281R (3996A>G), I1282T (3999T>C 4000T>C), Q1283F (4001C>T 4002A>T 4003G>T), H1284K (4004C>A 4006T>A), S1285A (4007A>G 4008G>C 4009T>A), K1286N (4012G>C), V1287I (4013G>A 4015G>T), F1288Y (4017T>A), Y1297F (4044A>T), Y1298W (4047A>G 4048T>G), Q1303S (4061C>T 4062A>C 4063G>T), P1304E (4064C>G 4065C>A), E1305K (4067G>A), R1307K (4074G>A), H1308* (4076C>T 4078C>G), T1310S (4082A>T 4084A>C), I1313N (4092T>A 4093A>T), C1314V (4094T>G 4095G>T), A1316F (4100G>T 4101C>T 4102A>T), F1318Q (4106T>C 4107T>A 4108C>A), Y1319H (4109T>C), Q1320E (4112C>G 4114G>A), K1322N (4120A>C), V1324M (4124G>A 4126T>G), P1325L (4128C>T 4129A>T), A1333S (4151G>T 4153C>A), F1334E (4154T>G 4155T>A 4156C>A), R1337K (4164G>A), R1338I (4167G>T 4168G>T), D1340N (4172G>A), Y1341D (4175T>G), F1343I (4181T>A), A1344N (4184G>A 4185C>A), K1345H (4187A>C 4189A>T), Y1346L (4191A>T 4192T>G), D1347S (4193G>A 4194A>G 4195T>C), D1347_F1348insHM (4195_4196insCATATG), F1348S (4197T>C 4198T>A), T1351del (4205_4207delACA), D1355N (4217G>A), I1356V (4220A>G), H1359F (4229C>T 4230A>T 4231T>C), P1361E (4235C>G 4236C>A 4237A>G), V1363I (4241G>A 4243A>C), Q1364D (4244C>G 4246G>T), N1365S (4247A>T 4248A>C), E1371N (4265G>A 4267A>T), F1373L (4273T>A), L1374F (4274C>T), E1375N (4277G>A 4279A>C), E1376T (4280G>A 4281A>C 4282A>C), K1379R (4290A>G), H1380N (4292C>A), I1382L (4298A>T 4300T>G), L1384V (4304T>G 4306G>A), E1386T (4310G>A 4311A>C 4312A>T), R1387K (4314G>A), M1389I (4321G>T), S1390K (4322T>A 4323C>A 4324C>A), D1394T (4334G>A 4335A>C 4336T>A), N1395K (4339T>A), D1397R (4343G>A 4344A>G 4345T>A)								
Codon mutations:	AGG1242.GT (3880G>T), CAT1244GTT (3884C>G 3885A>T), TCT1245GCT (3887T>G), GAG1246GAA (3892G>A), ATA1247AAA (3894T>A), GTA1248GAA (3897T>A), AGA1249TGC (3899A>T 3901A>C), GGT1250GAG (3903G>A 3904T>G), AAA1251TGT (3905A>T 3906A>G 3907A>T), ATG1254CTT (3914A>C 3916G>T), GTA1255GTT (3919A>T), ATA1256ATC (3922A>C), CTT1261TTG (3935C>T 3937T>G), GCC1263ACT (3941G>A 3943C>T), ATC1264GCT (3944A>G 3945T>C 3946C>T), ACT1265TTA (3947A>T 3948C>T 3949T>A), CAG1266AAA (3950C>A 3952G>A), AAT1267TGG (3953A>T 3954A>G 3955T>G), TTT1268ATT (3956T>A), AAT1269AGA (3960A>G 3961T>A), CCA1271CCT (3967A>T), TTG1272ATT (3968T>A 3970G>T), CCA1273CCT (3973A>T), AGA1274AAC (3975G>A 3976A>C), CCA1275AAA (3977C>A 3978C>A), GAA1276AAG (3980G>A 3982A>G), GTG1277GAT (3984T>A 3985G>T), ATT1278CTT (3986A>C), ATG1279CTA (3989A>C 3991G>A), CAA1280AAA (3992C>A), AAA1281AGA (3996A>G), ATT1282ACC (3999T>C 4000T>C), CAG1283TTT (4001C>T 4002A>T 4003G>T), CAT1284AAA (4004C>A 4006T>A), AGT1285GCA (4007A>G 4008G>C 4009T>A), AAG1286AAC (4012G>C), GTG1287ATT (4013G>A 4015G>T), TTC1288TAC (4017T>A), AGC1289AGT (4021C>T), AAG1290AAA (4024G>A), GAC1292GAT (4030C>T), AAG1294AAA (4036G>A), TAT1297TTT (4044A>T), TAT1298TGG (4047A>G 4048T>G), CAG1299CAA (4051G>A), ATA1300ATT (4054A>T), CAA1301CAG (4057A>G), CAG1303TCT (4061C>T 4062A>C 4063G>T), CCA1304GAA (4064C>G 4065C>A), GAA1305AAA (4067G>A), GAT1306GAC (4072T>C), AGA1307AAA (4074G>A), CAC1308TAG (4076C>T 4078C>G), AAA1309AAG (4081A>G), ACA1310TCC (4082A>T 4084A>C), TTC1312TTT (4090C>T), ATA1313AAT (4092T>A 4093A>T), TGT1314GTT (4094T>G 4095G>T), CCA1315CCG (4099A>G), GCA1316TTT (4100G>T 4101C>T 4102A>T), GGA1317GGT (4105A>T), TTC1318CAA (4106T>C 4107T>A 4108C>A), TAT1319CAT (4109T>C), CAG1320GAA (4112C>G 4114G>A), AAA1322AAC (4120A>C), GTT1324ATG (4124G>A 4126T>G), CCA1325CTT (4128C>T 4129A>T), GGT1327GGG (4135T>G), TTG1328CTT (4136T>C 4138G>T), AAG1329AAA (4141G>A), GCG1331GCC (4147G>C), CCA1332CCC (4150A>C), GCC1333TCA (4151G>T 4153C>A), TTC1334GAA (4154T>G 4155T>A 4156C>A), AGA1337AAA (4164G>A), AGG1338ATT (4167G>T 4168G>T), GAT1340AAT (4172G>A), TAT1341GAT (4175T>G), TTT1343ATT (4181T>A), GCT1344AAT (4184G>A 4185C>A), AAA1345CAT (4187A>C 4189A>T), TAT1346TTG (4191A>T 4192T>G), GAT1347AGC (4193G>A 4194A>G 4195T>C), GAT1347_TTT1348insCATATG (4195_4196insCATATG), TTT1348TCA (4197T>C 4198T>A), ACA1351del (4205_4207delACA), TAC1352TAT (4210C>T), ATA1353ATT (4213A>T), GAT1355AAT (4217G>A), ATT1356GTT (4220A>G), ATT1358ATC (4228T>C), CAT1359TTC (4229C>T 4230A>T 4231T>C), AGT1360TCT (4232A>T 4233G>C), CCA1361GAG (4235C>G 4236C>A 4237A>G), GAT1362GAC (4240T>C), GTA1363ATC (4241G>A 4243A>C), CAG1364GAT (4244C>G 4246G>T), AAT1365TCT (4247A>T 4248A>C), AAG1368AAA (4258G>A), TTA1370CTA (4262T>C), GAA1371AAT (4265G>A 4267A>T), TTT1373TTA (4273T>A), CTT1374TTT (4274C>T), GAA1375AAC (4277G>A 4279A>C), GAA1376ACC (4280G>A 4281A>C 4282A>C), AAG1378AAA (4288G>A), AAA1379AGA (4290A>G), CAT1380AAT (4292C>A), GGA1381GGT (4297A>T), ATT1382TTG (4298A>T 4300T>G), GTT1383GTA (4303T>A), TTG1384GTA (4304T>G 4306G>A), TCA1385AGC (4307T>A 4308C>G 4309A>C), GAA1386ACT (4310G>A 4311A>C 4312A>T), AGA1387AAA (4314G>A), ATG1389ATT (4321G>T), TCC1390AAA (4322T>A 4323C>A 4324C>A), TTA1391CTT (4325T>C 4327A>T), CAG1393CAA (4333G>A), GAT1394ACA (4334G>A 4335A>C 4336T>A), AAT1395AAA (4339T>A), GAT1397AGA (4343G>A 4344A>G 4345T>A)								

Proteins

	Begin	End	Coverage	Score	Concordance	Matches	Identities	I/D/M/F*	Stop Codons
polyprotein (YP_009553669.1)	1243	1400	7.4%	496	43.0%	157 (98.1%)	73 (45.6%)	2/1/0/0	1
Protein mutations:	H1244V (3884C>G 3885A>T), S1245A (3887T>G), I1247K (3894T>A), V1248E (3897T>A), R1249C (3899A>T 3901A>C), G1250E (3903G>A 3904T>G), K1251C (3905A>T 3906A>G 3907A>T), M1254L (3914A>C 3916G>T), A1263T (3941G>A 3943C>T), I1264A (3944A>G 3945T>C 3946C>T), T1265L (3947A>T 3948C>T 3949T>A), Q1266K (3950C>A 3952G>A), N1267W (3953A>T 3954A>G 3955T>G), F1268I (3956T>A), N1269R (3960A>G 3961T>A), L1272I (3968T>A 3970G>T), R1274N (3975G>A 3976A>C), P1275K (3977C>A 3978C>A), E1276K (3980G>A 3982A>G), V1277D (3984T>A 3985G>T), I1278L (3986A>C), M1279L (3989A>C 3991G>A), Q1280K (3992C>A), K1281R (3996A>G), I1282T (3999T>C 4000T>C), Q1283F (4001C>T 4002A>T 4003G>T), H1284K (4004C>A 4006T>A), S1285A (4007A>G 4008G>C 4009T>A), K1286N (4012G>C), V1287I (4013G>A 4015G>T), F1288Y (4017T>A), Y1297F (4044A>T), Y1298W (4047A>G 4048T>G), Q1303S (4061C>T 4062A>C 4063G>T), P1304E (4064C>G 4065C>A), E1305K (4067G>A), R1307K (4074G>A), H1308* (4076C>T 4078C>G), T1310S (4082A>T 4084A>C), I1313N (4092T>A 4093A>T), C1314V (4094T>G 4095G>T), A1316F (4100G>T 4101C>T 4102A>T), F1318Q (4106T>C 4107T>A 4108C>A), Y1319H (4109T>C), Q1320E (4112C>G 4114G>A), K1322N (4120A>C), V1324M (4124G>A 4126T>G), P1325L (4128C>T 4129A>T), A1333S (4151G>T 4153C>A), F1334E (4154T>G 4155T>A 4156C>A), R1337K (4164G>A), R1338I (4167G>T 4168G>T), D1340N (4172G>A), Y1341D (4175T>G), F1343I (4181T>A), A1344N (4184G>A 4185C>A), K1345H (4187A>C 4189A>T), Y1346L (4191A>T 4192T>G), D1347S (4193G>A 4194A>G 4195T>C), D1347_F1348insHM (4195_4196insCATATG), F1348S (4197T>C 4198T>A), T1351del (4205_4207delACA), D1355N (4217G>A), I1356V (4220A>G), H1359F (4229C>T 4230A>T 4231T>C), P1361E (4235C>G 4236C>A 4237A>G), V1363I (4241G>A 4243A>C), Q1364D (4244C>G 4246G>T), N1365S (4247A>T 4248A>C), E1371N (4265G>A 4267A>T), F1373L (4273T>A), L1374F (4274C>T), E1375N (4277G>A 4279A>C), E1376T (4280G>A 4281A>C 4282A>C), K1379R (4290A>G), H1380N (4292C>A), I1382L (4298A>T 4300T>G), L1384V (4304T>G 4306G>A), E1386T (4310G>A 4311A>C 4312A>T), R1387K (4314G>A), M1389I (4321G>T), S1390K (4322T>A 4323C>A 4324C>A), D1394T (4334G>A 4335A>C 4336T>A), N1395K (4339T>A), D1397R (4343G>A 4344A>G 4345T>A)								

	Begin	End	Coverage	Score	Concordance	Matches	Identities	I/D/M/F*	Stop Codons
**NT**	**3879**	**4352**	**6.7%**	**115**	**12.4%**	**471 (98.1%)**	**271 (56.5%)**	**6/3**	
Codon mutations:	AGG1242.GT (3880G>T), CAT1244GTT (3884C>G 3885A>T), TCT1245GCT (3887T>G), GAG1246GAA (3892G>A), ATA1247AAA (3894T>A), GTA1248GAA (3897T>A), AGA1249TGC (3899A>T 3901A>C), GGT1250GAG (3903G>A 3904T>G), AAA1251TGT (3905A>T 3906A>G 3907A>T), ATG1254CTT (3914A>C 3916G>T), GTA1255GTT (3919A>T), ATA1256ATC (3922A>C), CTT1261TTG (3935C>T 3937T>G), GCC1263ACT (3941G>A 3943C>T), ATC1264GCT (3944A>G 3945T>C 3946C>T), ACT1265TTA (3947A>T 3948C>T 3949T>A), CAG1266AAA (3950C>A 3952G>A), AAT1267TGG (3953A>T 3954A>G 3955T>G), TTT1268ATT (3956T>A), AAT1269AGA (3960A>G 3961T>A), CCA1271CCT (3967A>T), TTG1272ATT (3968T>A 3970G>T), CCA1273CCT (3973A>T), AGA1274AAC (3975G>A 3976A>C), CCA1275AAA (3977C>A 3978C>A), GAA1276AAG (3980G>A 3982A>G), GTG1277GAT (3984T>A 3985G>T), ATT1278CTT (3986A>C), ATG1279CTA (3989A>C 3991G>A), CAA1280AAA (3992C>A), AAA1281AGA (3996A>G), ATT1282ACC (3999T>C 4000T>C), CAG1283TTT (4001C>T 4002A>T 4003G>T), CAT1284AAA (4004C>A 4006T>A), AGT1285GCA (4007A>G 4008G>C 4009T>A), AAG1286AAC (4012G>C), GTG1287ATT (4013G>A 4015G>T), TTC1288TAC (4017T>A), AGC1289AGT (4021C>T), AAG1290AAA (4024G>A), GAC1292GAT (4030C>T), AAG1294AAA (4036G>A), TAT1297TTT (4044A>T), TAT1298TGG (4047A>G 4048T>G), CAG1299CAA (4051G>A), ATA1300ATT (4054A>T), CAA1301CAG (4057A>G), CAG1303TCT (4061C>T 4062A>C 4063G>T), CCA1304GAA (4064C>G 4065C>A), GAA1305AAA (4067G>A), GAT1306GAC (4072T>C), AGA1307AAA (4074G>A), CAC1308TAG (4076C>T 4078C>G), AAA1309AAG (4081A>G), ACA1310TCC (4082A>T 4084A>C), TTC1312TTT (4090C>T), ATA1313AAT (4092T>A 4093A>T), TGT1314GTT (4094T>G 4095G>T), CCA1315CCG (4099A>G), GCA1316TTT (4100G>T 4101C>T 4102A>T), GGA1317GGT (4105A>T), TTC1318CAA (4106T>C 4107T>A 4108C>A), TAT1319CAT (4109T>C), CAG1320GAA (4112C>G 4114G>A), AAA1322AAC (4120A>C), GTT1324ATG (4124G>A 4126T>G), CCA1325CTT (4128C>T 4129A>T), GGT1327GGG (4135T>G), TTG1328CTT (4136T>C 4138G>T), AAG1329AAA (4141G>A), GCG1331GCC (4147G>C), CCA1332CCC (4150A>C), GCC1333TCA (4151G>T 4153C>A), TTC1334GAA (4154T>G 4155T>A 4156C>A), AGA1337AAA (4164G>A), AGG1338ATT (4167G>T 4168G>T), GAT1340AAT (4172G>A), TAT1341GAT (4175T>G), TTT1343ATT (4181T>A), GCT1344AAT (4184G>A 4185C>A), AAA1345CAT (4187A>C 4189A>T), TAT1346TTG (4191A>T 4192T>G), GAT1347AGC (4193G>A 4194A>G 4195T>C), GAT1347_TTT1348insCATATG (4195_4196insCATATG), TTT1348TCA (4197T>C 4198T>A), ACA1351del (4205_4207delACA), TAC1352TAT (4210C>T), ATA1353ATT (4213A>T), GAT1355AAT (4217G>A), ATT1356GTT (4220A>G), ATT1358ATC (4228T>C), CAT1359TTC (4229C>T 4230A>T 4231T>C), AGT1360TCT (4232A>T 4233G>C), CCA1361GAG (4235C>G 4236C>A 4237A>G), GAT1362GAC (4240T>C), GTA1363ATC (4241G>A 4243A>C), CAG1364GAT (4244C>G 4246G>T), AAT1365TCT (4247A>T 4248A>C), AAG1368AAA (4258G>A), TTA1370CTA (4262T>C), GAA1371AAT (4265G>A 4267A>T), TTT1373TTA (4273T>A), CTT1374TTT (4274C>T), GAA1375AAC (4277G>A 4279A>C), GAA1376ACC (4280G>A 4281A>C 4282A>C), AAG1378AAA (4288G>A), AAA1379AGA (4290A>G), CAT1380AAT (4292C>A), GGA1381GGT (4297A>T), ATT1382TTG (4298A>T 4300T>G), GTT1383GTA (4303T>A), TTG1384GTA (4304T>G 4306G>A), TCA1385AGC (4307T>A 4308C>G 4309A>C), GAA1386ACT (4310G>A 4311A>C 4312A>T), AGA1387AAA (4314G>A), ATG1389ATT (4321G>T), TCC1390AAA (4322T>A 4323C>A 4324C>A), TTA1391CTT (4325T>C 4327A>T), CAG1393CAA (4333G>A), GAT1394ACA (4334G>A 4335A>C 4336T>A), AAT1395AAA (4339T>A), GAT1397AGA (4343G>A 4344A>G 4345T>A)								

*: Inserts / Deletes / Misaligned / Frameshifts

Analysis details

This analysis was performed with panviral2.64

NGS Details (UN60): Badnavirus rutilanscamelliae

Assembly

**Coverage Length**	262 (1 contig(s))
**Depth Of Coverage**	3.9
**Number Of Reads**	10
**Reads Per Million**	0.21 rpm (after QC)
**Ambiguities**	0
**Assembly Method**	de novo + reference guided assembly
**Consensus Caller**	Bcf Tools

Coverage Map

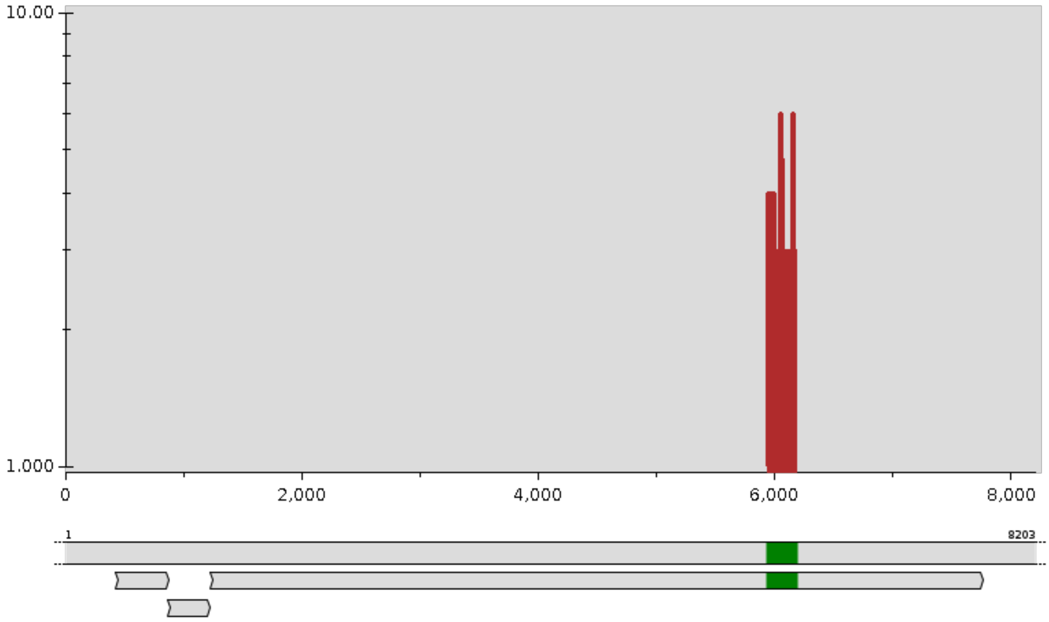

Assignment

**Type**	Badnavirus rutilanscamelliae (Taxonomy ID: 3047719)
**Reference Genome**	NC_055598.1
**NT Identity (%)**	61.5094
**AA Identity (%)**	56.1798
**Number Of Stop Codons**	0
**Number Of CDS**	3

Alignment

**Alignment Score**	98.0 (NT) + 398.0 (AA) = 496.0
**Concordance (%)**	42.7586

Alignment Method	Global, seeded, nucleotide + amino acids (AGA)

Genome Region

Sequence starts at position 5933 and ends at position 6194 relative to NC_055598.1 reference sequence.

Alignment Detailed Statistics

	Begin	End	Coverage	Score	Concordance	Matches	Identities	I/D/M/F*	Stop Codons
**NT**	**5933**	**6194**	**3.2%**	**98**	**19.4%**	**259 (96.6%)**	**163 (60.8%)**	**6/3**	
Mutations:	5937G>C, 5946A>T, 5950G>T, 5953G>A, 5954T>A, 5956C>T, 5959C>T, 5965G>A, 5966T>A, 5967C>G, 5968A>T, 5971G>T, 5973T>A, 5974T>C, 5978C>G, 5980A>G, 5981G>A, 5984G>C, 5985C>G, 5986T>A, 5990G>A, 5996C>G, 5997A>G, 5999C>G, 6001C>T, 6002_6004delATT, 6010_6011insAAA, 6013C>T, 6020T>A, 6021G>A, 6025T>C, 6026C>A, 6027C>A, 6029G>T, 6030T>A, 6031G>T, 6034C>A, 6035T>C, 6037A>T, 6043A>G, 6047C>A, 6058A>C, 6064G>C, 6065C>T, 6067T>A, 6069A>C, 6070G>T, 6076T>A, 6080G>A, 6081C>G, 6082A>C, 6083G>A, 6084T>C, 6085A>T, 6089C>A, 6090A>T, 6091A>G, 6094G>A, 6095A>C, 6096A>T, 6097G>A, 6101G>A, 6103C>T, 6105G>A, 6106A>T, 6107A>G, 6109C>T, 6115T>A, 6116C>G, 6117C>A, 6118A>T, 6120A>T, 6122G>C, 6123C>A, 6127_6128insAAA, 6131A>G, 6135C>T, 6136T>G, 6143A>T, 6151T>C, 6155C>T, 6157C>G, 6160A>C, 6163C>T, 6164A>T, 6165G>C, 6166C>T, 6168C>A, 6171C>A, 6172A>T, 6174C>T, 6175T>A, 6178A>T, 6180C>A, 6181C>A, 6184T>C, 6185A>C, 6186A>T, 6188A>G								

CDS

	Begin	End	Coverage	Score	Concordance	Matches	Identities	I/D/M/F*	Stop Codons
KM754_gp3	1571	1658	4.0%	398	60.5%	87 (96.7%)	50 (55.6%)	2/1/0/0	0
Protein mutations:	S1572T (5937G>C), Y1575F (5946A>T), F1578I (5954T>A 5956C>T), F1584Y (5973T>A 5974T>C), Q1586E (5978C>G 5980A>G), V1587I (5981G>A), A1588R (5984G>C 5985C>G 5986T>A), D1590N (5990G>A), Q1592G (5996C>G 5997A>G), H1593D (5999C>G 6001C>T), I1594del (6002_6004delATT), W1596_T1597insK (6010_6011insAAA), W1600K (6020T>A 6021G>A), P1602K (6026C>A 6027C>A), V1603Y (6029G>T 6030T>A 6031G>T), L1609M (6047C>A), K1616T (6069A>C 6070G>T), A1620S (6080G>A 6081C>G 6082A>C), V1621T (6083G>A 6084T>C 6085A>T), Q1623M (6089C>A 6090A>T 6091A>G), K1625L (6095A>C 6096A>T 6097G>A), D1627N (6101G>A 6103C>T), R1628H (6105G>A 6106A>T), I1629V (6107A>G 6109C>T), N1631K (6115T>A), P1632D (6116C>G 6117C>A 6118A>T), Y1633F (6120A>T), A1634H (6122G>C 6123C>A), G1635_F1636insK (6127_6128insAAA), I1637V (6131A>G), A1638V (6135C>T 6136T>G), I1641F (6143A>T), P1649Q (6168C>A), T1650N (6171C>A 6172A>T), P1651L (6174C>T 6175T>A), E1652D (6178A>T), A1653E (6180C>A 6181C>A), K1655L (6185A>C 6186A>T), K1656E (6188A>G)								
Codon mutations:	AGC1572ACC (5937G>C), TAT1575TTT (5946A>T), TCG1576TCT (5950G>T), AAG1577AAA (5953G>A), TTC1578ATT (5954T>A 5956C>T), GAC1579GAT (5959C>T), AAG1581AAA (5965G>A), TCA1582AGT (5966T>A 5967C>G 5968A>T), GGG1583GGT (5971G>T), TTT1584TAC (5973T>A 5974T>C), CAA1586GAG (5978C>G 5980A>G), GTT1587ATT (5981G>A), GCT1588CGA (5984G>C 5985C>G 5986T>A), GAT1590AAT (5990G>A), CAA1592GGA (5996C>G 5997A>G), CAC1593GAT (5999C>G 6001C>T), ATT1594del (6002_6004delATT), TGG1596_ACC1597insAAA (6010_6011insAAA), ACC1597ACT (6013C>T), TGG1600AAG (6020T>A 6021G>A), ACT1601ACC (6025T>C), CCA1602AAA (6026C>A 6027C>A), GTG1603TAT (6029G>T 6030T>A 6031G>T), GGC1604GGA (6034C>A), TTA1605CTT (6035T>C 6037A>T), GAA1607GAG (6043A>G), CTG1609ATG (6047C>A), CCA1612CCC (6058A>C), GGG1614GGC (6064G>C), CTT1615TTA (6065C>T 6067T>A), AAG1616ACT (6069A>C 6070G>T), GCT1618GCA (6076T>A), GCA1620AGC (6080G>A 6081C>G 6082A>C), GTA1621ACT (6083G>A 6084T>C 6085A>T), CAA1623ATG (6089C>A 6090A>T 6091A>G), AGG1624AGA (6094G>A), AAG1625CTA (6095A>C 6096A>T 6097G>A), GAC1627AAT (6101G>A 6103C>T), CGA1628CAT (6105G>A 6106A>T), ATC1629GTT (6107A>G 6109C>T), AAT1631AAA (6115T>A), CCA1632GAT (6116C>G 6117C>A 6118A>T), TAC1633TTC (6120A>T), GCT1634CAT (6122G>C 6123C>A), GGA1635_TTT1636insAAA (6127_6128insAAA), ATT1637GTT (6131A>G), GCT1638GTG (6135C>T 6136T>G), ATT1641TTT (6143A>T), GAT1643GAC (6151T>C), CTC1645TTG (6155C>T 6157C>G), ATA1646ATC (6160A>C), TTC1647TTT (6163C>T), AGC1648TCT (6164A>T 6165G>C 6166C>T), CCA1649CAA (6168C>A), ACA1650AAT (6171C>A 6172A>T), CCT1651CTA (6174C>T 6175T>A), GAA1652GAT (6178A>T), GCC1653GAA (6180C>A 6181C>A), CAT1654CAC (6184T>C), AAA1655CTA (6185A>C 6186A>T), AAG1656GAG (6188A>G)								

Proteins

	Begin	End	Coverage	Score	Concordance	Matches	Identities	I/D/M/F*	Stop Codons
polyprotein (YP_010087856.1)	1571	1658	4.0%	398	60.5%	87 (96.7%)	50 (55.6%)	2/1/0/0	0
Protein mutations:	S1572T (5937G>C), Y1575F (5946A>T), F1578I (5954T>A 5956C>T), F1584Y (5973T>A 5974T>C), Q1586E (5978C>G 5980A>G), V1587I (5981G>A), A1588R (5984G>C 5985C>G 5986T>A), D1590N (5990G>A), Q1592G (5996C>G 5997A>G), H1593D (5999C>G 6001C>T), I1594del (6002_6004delATT), W1596_T1597insK (6010_6011insAAA), W1600K (6020T>A 6021G>A), P1602K (6026C>A 6027C>A), V1603Y (6029G>T 6030T>A 6031G>T), L1609M (6047C>A), K1616T (6069A>C 6070G>T), A1620S (6080G>A 6081C>G 6082A>C), V1621T (6083G>A 6084T>C 6085A>T), Q1623M (6089C>A 6090A>T 6091A>G), K1625L (6095A>C 6096A>T 6097G>A), D1627N (6101G>A 6103C>T), R1628H (6105G>A 6106A>T), I1629V (6107A>G 6109C>T), N1631K (6115T>A), P1632D (6116C>G 6117C>A 6118A>T), Y1633F (6120A>T), A1634H (6122G>C 6123C>A), G1635_F1636insK (6127_6128insAAA), I1637V (6131A>G), A1638V (6135C>T 6136T>G), I1641F (6143A>T), P1649Q (6168C>A), T1650N (6171C>A 6172A>T), P1651L (6174C>T 6175T>A), E1652D (6178A>T), A1653E (6180C>A 6181C>A), K1655L (6185A>C 6186A>T), K1656E (6188A>G)								
Codon mutations:	AGC1572ACC (5937G>C), TAT1575TTT (5946A>T), TCG1576TCT (5950G>T), AAG1577AAA (5953G>A), TTC1578ATT (5954T>A 5956C>T), GAC1579GAT (5959C>T), AAG1581AAA (5965G>A), TCA1582AGT (5966T>A 5967C>G 5968A>T), GGG1583GGT (5971G>T), TTT1584TAC (5973T>A 5974T>C), CAA1586GAG (5978C>G 5980A>G), GTT1587ATT (5981G>A), GCT1588CGA (5984G>C 5985C>G 5986T>A), GAT1590AAT (5990G>A), CAA1592GGA (5996C>G 5997A>G), CAC1593GAT (5999C>G 6001C>T), ATT1594del (6002_6004delATT), TGG1596_ACC1597insAAA (6010_6011insAAA), ACC1597ACT (6013C>T), TGG1600AAG (6020T>A 6021G>A), ACT1601ACC (6025T>C), CCA1602AAA (6026C>A 6027C>A), GTG1603TAT (6029G>T 6030T>A 6031G>T), GGC1604GGA (6034C>A), TTA1605CTT (6035T>C 6037A>T), GAA1607GAG (6043A>G), CTG1609ATG (6047C>A), CCA1612CCC (6058A>C), GGG1614GGC (6064G>C), CTT1615TTA (6065C>T 6067T>A), AAG1616ACT (6069A>C 6070G>T), GCT1618GCA (6076T>A), GCA1620AGC (6080G>A 6081C>G 6082A>C), GTA1621ACT (6083G>A 6084T>C 6085A>T), CAA1623ATG (6089C>A 6090A>T 6091A>G), AGG1624AGA (6094G>A), AAG1625CTA (6095A>C 6096A>T 6097G>A), GAC1627AAT (6101G>A 6103C>T), CGA1628CAT (6105G>A 6106A>T), ATC1629GTT (6107A>G 6109C>T), AAT1631AAA (6115T>A), CCA1632GAT (6116C>G 6117C>A 6118A>T), TAC1633TTC (6120A>T), GCT1634CAT (6122G>C 6123C>A), GGA1635_TTT1636insAAA (6127_6128insAAA), ATT1637GTT (6131A>G), GCT1638GTG (6135C>T 6136T>G), ATT1641TTT (6143A>T), GAT1643GAC (6151T>C), CTC1645TTG (6155C>T 6157C>G), ATA1646ATC (6160A>C), TTC1647TTT (6163C>T), AGC1648TCT (6164A>T 6165G>C 6166C>T), CCA1649CAA (6168C>A), ACA1650AAT (6171C>A 6172A>T), CCT1651CTA (6174C>T 6175T>A), GAA1652GAT (6178A>T), GCC1653GAA (6180C>A 6181C>A), CAT1654CAC (6184T>C), AAA1655CTA (6185A>C 6186A>T), AAG1656GAG (6188A>G)								

*: Inserts / Deletes / Misaligned / Frameshifts

Analysis details

This analysis was performed with panviral2.64

NGS Details (UN60): Duamitovirus dapi1

Assembly

**Coverage Length**	135 (1 contig(s))
**Depth Of Coverage**	6.5
**Number Of Reads**	8
**Reads Per Million**	0.17 rpm (after QC)
**Ambiguities**	0
**Assembly Method**	de novo + reference guided assembly
**Consensus Caller**	Bcf Tools

Coverage Map

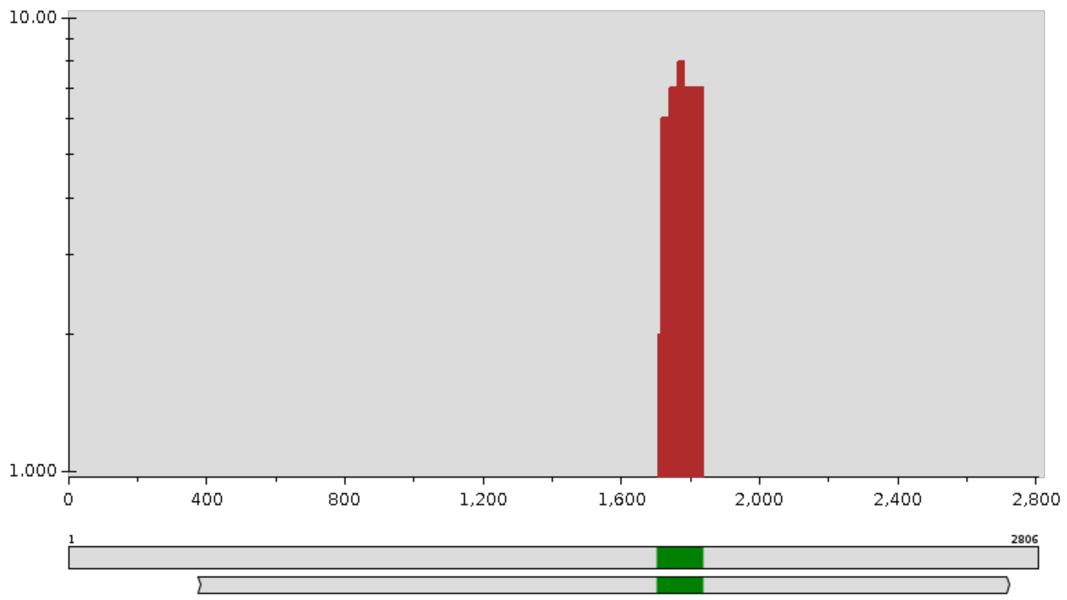

Assignment

**Type**	Duamitovirus dapi1 (Taxonomy ID: 2955778)
**Reference Genome**	NC_076528.1
**NT Identity (%)**	68.8889
**AA Identity (%)**	66.6667
**Number Of Stop Codons**	0
**Number Of CDS**	1

Alignment

**Alignment Score**	102.0 (NT) + 249.0 (AA) = 351.0
**Concordance (%)**	57.1661

Alignment Method	Global, seeded, nucleotide + amino acids (AGA)

Genome Region

Sequence starts at position 1706 and ends at position 1840 relative to NC_076528.1 reference sequence.

Alignment Detailed Statistics

	Begin	End	Coverage	Score	Concordance	Matches	Identities	I/D/M/F*	Stop Codons
**NT**	**1706**	**1840**	**4.8%**	**102**	**37.8%**	**135 (100%)**	**93 (68.9%)**	**0/0**	
Mutations:	1706G>C, 1710C>T, 1713A>G, 1715G>A, 1725T>C, 1728G>C, 1734C>T, 1736A>T, 1737C>G, 1738T>A, 1739C>G, 1743C>T, 1749G>T, 1750T>C, 1751C>T, 1756G>A, 1763C>A, 1774G>C, 1776A>T, 1783C>A, 1785G>A, 1788G>T, 1789G>A, 1791C>T, 1792T>G, 1793G>A, 1794G>A, 1800A>G, 1804C>G, 1805C>G, 1806T>C, 1809T>G, 1810C>A, 1811A>C, 1816T>C, 1819A>T, 1820A>G, 1821G>C, 1827C>T, 1830T>C, 1831C>A, 1839T>C								
**CDS**									
RdRp	445	489	5.7%	249	68.8%	45 (100%)	30 (66.7%)	0/0/0/0	0
Protein mutations:	R447K (1715G>A), Y454L (1736A>T 1737C>G), S459L (1750T>C 1751C>T), A461T (1756G>A), T463K (1763C>A), V467L (1774G>C 1776A>T), L470I (1783C>A 1785G>A), A472T (1789G>A 1791C>T), W473E (1792T>G 1793G>A 1794G>A), P477G (1804C>G 1805C>G 1806T>C), Q479T (1810C>A 1811A>C), F481L (1816T>C), K482C (1819A>T 1820A>G 1821G>C), L486I (1831C>A)								
Codon mutations:	CGG444.CG (1706G>C), TTC445TTT (1710C>T), ACA446ACG (1713A>G), AGG447AAG (1715G>A), CCT450CCC (1725T>C), CTG451CTC (1728G>C), TTC453TTT (1734C>T), TAC454TTG (1736A>T 1737C>G), TCT455AGT (1738T>A 1739C>G), TCC456TCT (1743C>T), CCG458CCT (1749G>T), TCA459CTA (1750T>C 1751C>T), GCA461ACA (1756G>A), ACA463AAA (1763C>A), GTA467CTT (1774G>C 1776A>T), CTG470ATA (1783C>A 1785G>A), GCG471GCT (1788G>T), GCC472ACT (1789G>A 1791C>T), TGG473GAA (1792T>G 1793G>A 1794G>A), GTA475GTG (1800A>G), CCT477GGC (1804C>G 1805C>G 1806T>C), ACT478ACG (1809T>G), CAG479ACG (1810C>A 1811A>C), TTT481CTT (1816T>C), AAG482TGC (1819A>T 1820A>G 1821G>C), TAC484TAT (1827C>T), GCT485GCC (1830T>C), CTT486ATT (1831C>A), GGT488GGC (1839T>C)								
**Proteins**									
RNA-dependent RNA polymerase (YP_010798878.1)	445	489	5.7%	249	68.8%	45 (100%)	30 (66.7%)	0/0/0/0	0
Protein mutations:	R447K (1715G>A), Y454L (1736A>T 1737C>G), S459L (1750T>C 1751C>T), A461T (1756G>A), T463K (1763C>A), V467L (1774G>C 1776A>T), L470I (1783C>A 1785G>A), A472T (1789G>A 1791C>T), W473E (1792T>G 1793G>A 1794G>A), P477G (1804C>G 1805C>G 1806T>C), Q479T (1810C>A 1811A>C), F481L (1816T>C), K482C (1819A>T 1820A>G 1821G>C), L486I (1831C>A)								
Codon mutations:	CGG444.CG (1706G>C), TTC445TTT (1710C>T), ACA446ACG (1713A>G), AGG447AAG (1715G>A), CCT450CCC (1725T>C), CTG451CTC (1728G>C), TTC453TTT (1734C>T), TAC454TTG (1736A>T 1737C>G), TCT455AGT (1738T>A 1739C>G), TCC456TCT (1743C>T), CCG458CCT (1749G>T), TCA459CTA (1750T>C 1751C>T), GCA461ACA (1756G>A), ACA463AAA (1763C>A), GTA467CTT (1774G>C 1776A>T), CTG470ATA (1783C>A 1785G>A), GCG471GCT (1788G>T), GCC472ACT (1789G>A 1791C>T), TGG473GAA (1792T>G 1793G>A 1794G>A), GTA475GTG (1800A>G), CCT477GGC (1804C>G 1805C>G 1806T>C), ACT478ACG (1809T>G), CAG479ACG (1810C>A 1811A>C), TTT481CTT (1816T>C), AAG482TGC (1819A>T 1820A>G 1821G>C), TAC484TAT (1827C>T), GCT485GCC (1830T>C), CTT486ATT (1831C>A), GGT488GGC (1839T>C)								

*: Inserts / Deletes / Misaligned / Frameshifts

Analysis details

This analysis was performed with panviral2.64

NGS Details (UN60): Alphabaculovirus lyxylinae

Assembly

**Coverage Length**	110 (1 contig(s))
**Depth Of Coverage**	6.5
**Number Of Reads**	8
**Reads Per Million**	0.17 rpm (after QC)
**Ambiguities**	0
**Assembly Method**	de novo + reference guided assembly
**Consensus Caller**	Bcf Tools

Coverage Map

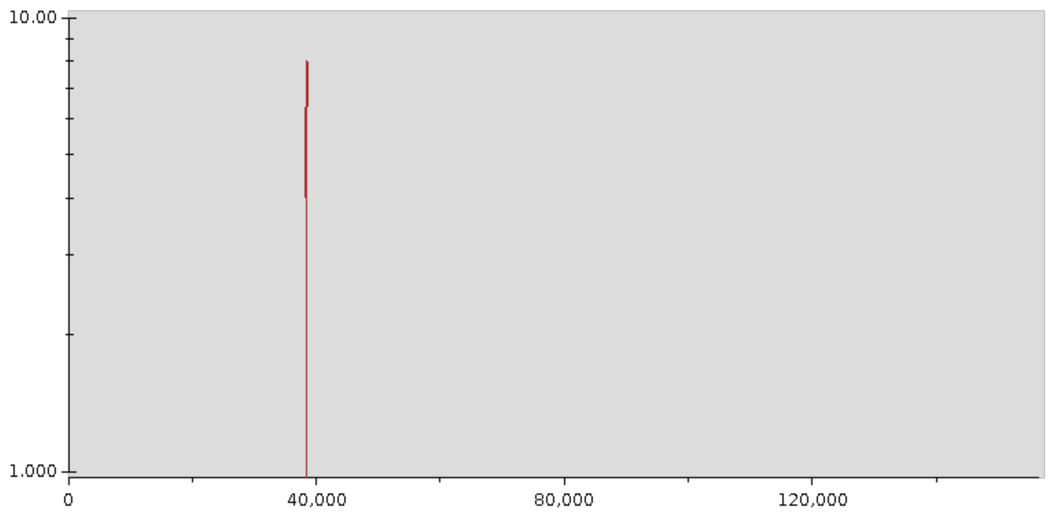

Assignment

**Type**	Alphabaculovirus lyxylinae (Taxonomy ID: 3051419)
**Reference Genome**	NC_013953.1
**NT Identity (%)**	80.0
**AA Identity (%)**	83.7838
**Number Of Stop Codons**	0
**Number Of CDS**	157

Alignment

**Alignment Score**	132.0 (NT) + 204.0 (AA) = 336.0
**Concordance (%)**	73.523
**Alignment Method**	Local, heuristic, nucleotide (BLASTN)

Genome Region

Sequence starts at position 38361 and ends at position 38470 relative to NC_013953.1 reference sequence.

Alignment Detailed Statistics

	Begin	End	Coverage	Score	Concordance	Matches	Identities	I/D/M/F*	Stop Codons
**NT**	**38361**	**38470**	**0.1%**	**132**	**60.0%**	**110 (100%)**	**88 (80.0%)**	**0/0**	

Mutations: 38363A>G, 38369G>C, 38377A>T, 38387A>G, 38393G>C, 38396C>A, 38401A>G, 38402G>C, 38408C>G, 38412A>G, 38413A>C, 38426C>T, 38432G>A, 38435A>G, 38436G>T, 38447T>C, 38448T>C, 38450G>C, 38453G>C, 38463A>C, 38465A>C, 38468C>T

*: Inserts / Deletes / Misaligned / Frameshifts

Analysis details

This analysis was performed with panviral2.64

NGS Details (UN60): Caulimovirus tessellobrassicae

Assembly

**Coverage Length**	254 (1 contig(s))
**Depth Of Coverage**	2.6
**Number Of Reads**	6
**Reads Per Million**	0.13 rpm (after QC)
**Ambiguities**	0
**Assembly Method**	de novo + reference guided assembly
**Consensus Caller**	Bcf Tools

Coverage Map

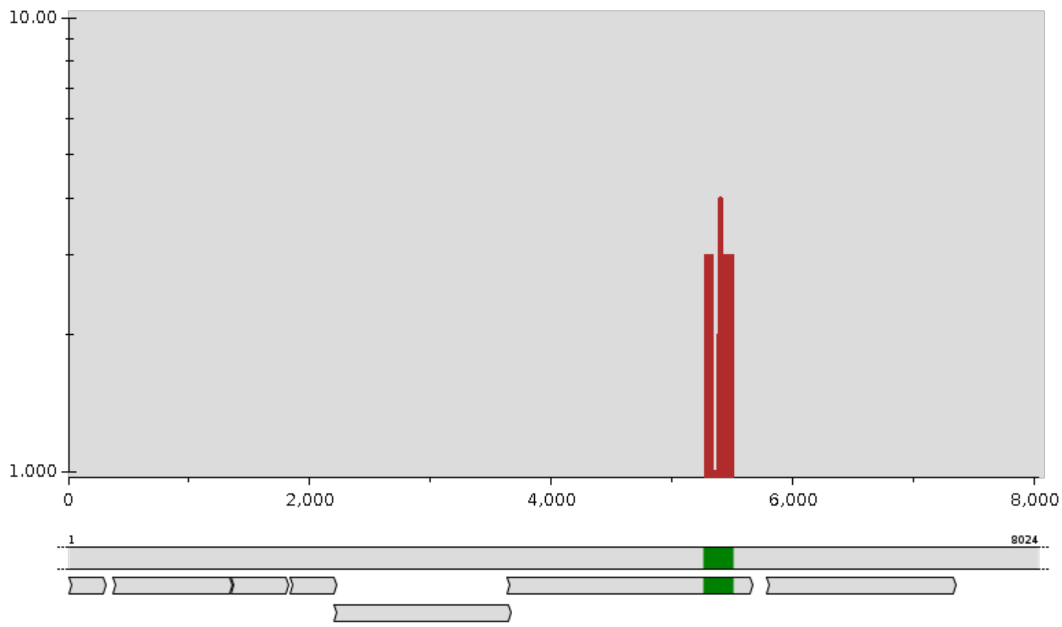

Assignment

**Type**	Caulimovirus tessellobrassicae (Taxonomy ID: 3047738)
**Reference Genome**	NC_001497.2
**NT Identity (%)**	62.6609
**AA Identity (%)**	52.5641
**Number Of Stop Codons**	0
**Number Of CDS**	7

Alignment

**Alignment Score**	79.0 (NT) + 296.0 (AA) = 375.0
**Concordance (%)**	40.1929

**Alignment Method**	Global, seeded, nucleotide + amino acids (AGA)

Genome Region

Sequence starts at position 5256 and ends at position 5509 relative to NC_001497.2 reference sequence.

Alignment Detailed Statistics

	Begin	End	Coverage	Score	Concordance	Matches	Identities	I/D/M/F*	Stop Codons
**NT**	**5256**	**5509**	**3.2%**	**79**	**18.5%**	**233 (91.7%)**	**146 (57.5%)**	**0/21**	
Mutations:	5256A>T, 5262G>A, 5265G>C, 5268G>T, 5272A>G, 5274C>A, 5275A>T, 5277C>A, 5278G>C, 5280G>A, 5283C>A, 5284G>A, 5288C>T, 5289A>C, 5292A>C, 5295C>T, 5296G>T, 5298C>T, 5299T>C, 5301C>T, 5309G>C, 5310T>A, 5311A>T, 5314T>C, 5317A>C, 5320G>A, 5322T>A, 5323_5325delATC, 5326A>G, 5328A>C, 5329A>C, 5331T>A, 5338_5355delGGTACTAATACTGAGTTA, 5361C>T, 5364A>G, 5367C>T, 5368G>A, 5371T>A, 5372C>G, 5378G>C, 5379C>T, 5385A>C, 5387C>A, 5388T>A, 5392G>A, 5395A>G, 5396A>T, 5397G>T, 5403C>T, 5404C>T, 5405A>C, 5408G>C, 5409C>A, 5415C>A, 5421G>A, 5422A>T, 5423C>T, 5424A>G, 5427G>A, 5434A>G, 5438A>G, 5439T>A, 5440A>G, 5441C>G, 5442T>A, 5446A>C, 5448G>A, 5455A>T, 5456G>C, 5457T>G, 5458A>G, 5459T>C, 5462A>G, 5463T>C, 5464C>T, 5467A>T, 5468C>T, 5469T>A, 5472T>A, 5473G>A, 5474T>A, 5475T>A, 5476C>T, 5477A>C, 5478T>A, 5482C>G, 5484G>T, 5489G>A, 5490G>A								

CDS

CaMVgp6	542	626	12.5%	296	57.6%	78 (91.8%)	41 (48.2%)	0/7/0/0	0
Protein mutations:	E544D (5265G>C), K545N (5268G>T), I547V (5272A>G 5274C>A), I548L (5275A>T 5277C>A), E549Q (5278G>C 5280G>A), D551N (5284G>A), A552V (5288C>T 5289A>C), D555Y (5296G>T 5298C>T), Y556H (5299T>C 5301C>T), G559A (5309G>C 5310T>A), M560L (5311A>T), K562Q (5317A>C), A563T (5320G>A 5322T>A), I564del (5323_5325delATC), K565D (5326A>G 5328A>C), I566L (5329A>C 5331T>A), G569_L574del (5338_5355delGGTACTAATACTGAGTTA), A579T (5368G>A), S582T (5378G>C 5379C>T), K584N (5385A>C), A585E (5387C>A 5388T>A), E587K (5392G>A), K588V (5395A>G 5396A>T 5397G>T), H591S (5404C>T 5405A>C), S592T (5408G>C 5409C>A), D594E (5415C>A), T597L (5422A>T 5423C>T 5424A>G), I601V (5434A>G), N602R (5438A>G 5439T>A), T603G (5440A>G 5441C>G 5442T>A), K605Q (5446A>C 5448G>A), I609A (5458A>G 5459T>C), Y610C (5462A>G 5463T>C), T612L (5467A>T 5468C>T 5469T>A), V614K (5473G>A 5474T>A 5475T>A), H615S (5476C>T 5477A>C 5478T>A), L617V (5482C>G 5484G>T), R619K (5489G>A 5490G>A)								
Codon mutations:	TTA541..T (5256A>T), GAG543GAA (5262G>A), GAG544GAC (5265G>C), AAG545AAT (5268G>T), ATC547GTA (5272A>G 5274C>A), ATC548TTA (5275A>T 5277C>A), GAG549CAA (5278G>C 5280G>A), ACC550ACA (5283C>A), GAT551AAT (5284G>A), GCA552GTC (5288C>T 5289A>C), TCA553TCC (5292A>C), GAC554GAT (5295C>T), GAC555TAT (5296G>T 5298C>T), TAC556CAT (5299T>C 5301C>T), GGT559GCA (5309G>C 5310T>A), ATG560TTG (5311A>T), TTA561CTA (5314T>C), AAA562CAA (5317A>C), GCT563ACA (5320G>A 5322T>A), ATC564del (5323_5325delATC), AAA565GAC (5326A>G 5328A>C), ATT566CTA (5329A>C 5331T>A), GGT569_TTA574del (5338_5355delGGTACTAATACTGAGTTA), TGC576TGT (5361C>T), AGA577AGG (5364A>G), TAC578TAT (5367C>T), GCA579ACA (5368G>A), TCT580AGT (5371T>A 5372C>G), AGC582ACT (5378G>C 5379C>T), AAA584AAC (5385A>C), GCT585GAA (5387C>A 5388T>A), GAA587AAA (5392G>A), AAG588GTT (5395A>G 5396A>T 5397G>T), TAC590TAT (5403C>T), CAC591TCC (5404C>T 5405A>C), AGC592ACA (5408G>C 5409C>A), GAC594GAA (5415C>A), GAG596GAA (5421G>A), ACA597TTG (5422A>T 5423C>T 5424A>G), TTG598TTA (5427G>A), ATA601GTA (5434A>G), AAT602AGA (5438A>G 5439T>A), ACT603GGA (5440A>G 5441C>G 5442T>A), AAG605CAA (5446A>C 5448G>A), AGT608TCG (5455A>T 5456G>C 5457T>G), ATT609GCT (5458A>G 5459T>C), TAT610TGC (5462A>G 5463T>C), CTA611TTA (5464C>T), ACT612TTA (5467A>T 5468C>T 5469T>A), CCT613CCA (5472T>A), GTT614AAA (5473G>A 5474T>A 5475T>A), CAT615TCA (5476C>T 5477A>C 5478T>A), CTG617GTT (5482C>G 5484G>T), AGG619AAA (5489G>A 5490G>A)								

Proteins

reverse transcriptase (NP_056728.1)	542	626	12.5%	296	57.6%	78 (91.8%)	41 (48.2%)	0/7/0/0	0
Protein mutations:	E544D (5265G>C), K545N (5268G>T), I547V (5272A>G 5274C>A), I548L (5275A>T 5277C>A), E549Q (5278G>C 5280G>A), D551N (5284G>A), A552V (5288C>T 5289A>C), D555Y (5296G>T 5298C>T), Y556H (5299T>C 5301C>T), G559A (5309G>C 5310T>A), M560L (5311A>T), K562Q (5317A>C), A563T (5320G>A 5322T>A), I564del (5323_5325delATC), K565D (5326A>G 5328A>C), I566L (5329A>C 5331T>A), G569_L574del (5338_5355delGGTACTAATACTGAGTTA), A579T (5368G>A), S582T (5378G>C 5379C>T), K584N (5385A>C), A585E (5387C>A 5388T>A), E587K (5392G>A), K588V (5395A>G 5396A>T 5397G>T), H591S (5404C>T 5405A>C), S592T (5408G>C 5409C>A), D594E (5415C>A), T597L (5422A>T 5423C>T 5424A>G), I601V (5434A>G), N602R (5438A>G 5439T>A), T603G (5440A>G 5441C>G 5442T>A), K605Q (5446A>C 5448G>A), I609A (5458A>G 5459T>C), Y610C (5462A>G 5463T>C), T612L (5467A>T 5468C>T 5469T>A), V614K (5473G>A 5474T>A 5475T>A), H615S (5476C>T 5477A>C 5478T>A), L617V (5482C>G 5484G>T), R619K (5489G>A 5490G>A)								
Codon mutations:	TTA541..T (5256A>T), GAG543GAA (5262G>A), GAG544GAC (5265G>C), AAG545AAT (5268G>T), ATC547GTA (5272A>G 5274C>A), ATC548TTA (5275A>T 5277C>A), GAG549CAA (5278G>C 5280G>A), ACC550ACA (5283C>A), GAT551AAT (5284G>A), GCA552GTC (5288C>T 5289A>C), TCA553TCC (5292A>C), GAC554GAT (5295C>T), GAC555TAT (5296G>T 5298C>T), TAC556CAT (5299T>C 5301C>T), GGT559GCA (5309G>C 5310T>A), ATG560TTG (5311A>T), TTA561CTA (5314T>C), AAA562CAA (5317A>C), GCT563ACA (5320G>A 5322T>A), ATC564del (5323_5325delATC), AAA565GAC (5326A>G 5328A>C), ATT566CTA (5329A>C 5331T>A), GGT569_TTA574del (5338_5355delGGTACTAATACTGAGTTA), TGC576TGT (5361C>T), AGA577AGG (5364A>G), TAC578TAT (5367C>T), GCA579ACA (5368G>A), TCT580AGT (5371T>A 5372C>G), AGC582ACT (5378G>C 5379C>T), AAA584AAC (5385A>C), GCT585GAA (5387C>A 5388T>A), GAA587AAA (5392G>A), AAG588GTT (5395A>G 5396A>T 5397G>T), TAC590TAT (5403C>T), CAC591TCC (5404C>T 5405A>C), AGC592ACA (5408G>C 5409C>A), GAC594GAA (5415C>A), GAG596GAA (5421G>A), ACA597TTG (5422A>T 5423C>T 5424A>G), TTG598TTA (5427G>A), ATA601GTA (5434A>G), AAT602AGA (5438A>G 5439T>A), ACT603GGA (5440A>G 5441C>G 5442T>A), AAG605CAA (5446A>C 5448G>A), AGT608TCG (5455A>T 5456G>C 5457T>G), ATT609GCT (5458A>G 5459T>C), TAT610TGC (5462A>G 5463T>C), CTA611TTA (5464C>T), ACT612TTA (5467A>T 5468C>T 5469T>A), CCT613CCA (5472T>A), GTT614AAA (5473G>A 5474T>A 5475T>A), CAT615TCA (5476C>T 5477A>C 5478T>A), CTG617GTT (5482C>G 5484G>T), AGG619AAA (5489G>A 5490G>A)								

*: Inserts / Deletes / Misaligned / Frameshifts

Analysis details

This analysis was performed with panviral2.64

NGS Details (UN60): Badnavirus occultipomeae

Assembly

**Coverage Length**	251 (1 contig(s))
**Depth Of Coverage**	3.2
**Number Of Reads**	6
**Reads Per Million**	0.13 rpm (after QC)
**Ambiguities**	0
**Assembly Method**	de novo + reference guided assembly
**Consensus Caller**	Bcf Tools

Coverage Map

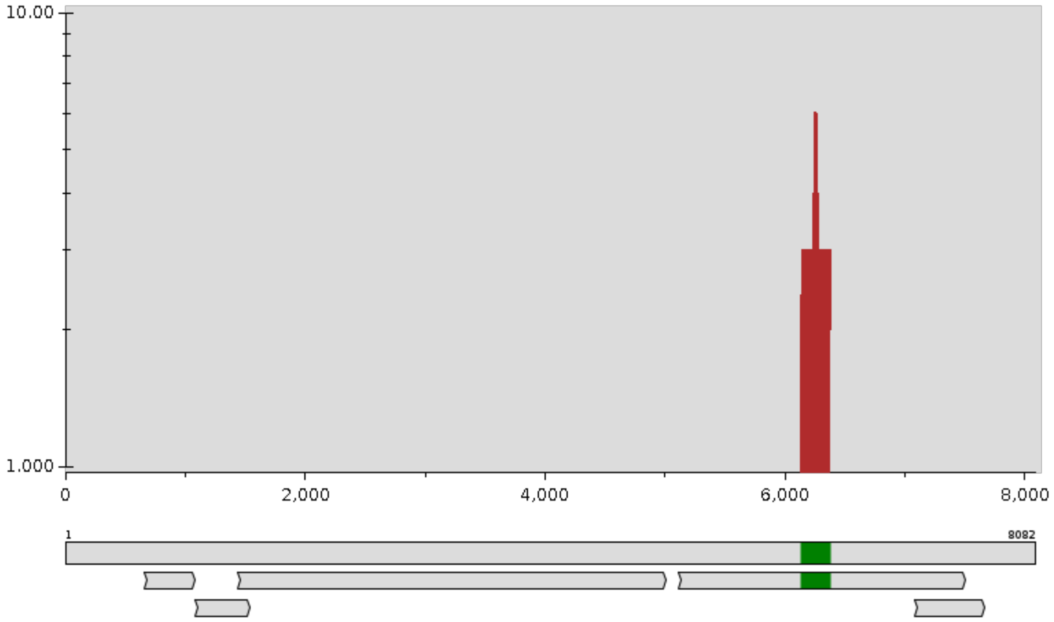

Assignment

**Type**	Badnavirus occultipomeae (Taxonomy ID: 3048353)
**Reference Genome**	NC_015655.1
**NT Identity (%)**	54.3307
**AA Identity (%)**	43.5294
**Number Of Stop Codons**	0
**Number Of CDS**	5

Alignment

**Alignment Score**	38.0 (NT) + 264.0 (AA) = 302.0
**Concordance (%)**	28.3568

**Alignment Method**	Global, seeded, nucleotide + amino acids (AGA)

Genome Region

Sequence starts at position 6129 and ends at position 6379 relative to NC_015655.1 reference sequence.

Alignment Detailed Statistics

	Begin	End	Coverage	Score	Concordance	Matches	Identities	I/D/M/F*	Stop Codons
**NT**	**6129**	**6379**	**3.1%**	**38**	**7.6%**	**251 (98.8%)**	**138 (54.3%)**	**3/0**	
Mutations:	6129T>C, 6132A>G, 6137A>T, 6138A>G, 6141C>A, 6145C>T, 6147C>T, 6153A>G, 6154C>T, 6165A>G, 6169G>A, 6171A>T, 6172G>A, 6173A>T, 6174T>A, 6178C>A, 6179A>T, 6181A>G, 6182G>A, 6183A>T, 6184A>C, 6185A>T, 6186G>T, 6190G>A, 6194A>G, 6195T>G, 6197C>T, 6198A>T, 6201T>C, 6203G>A, 6204G>A, 6205G>C, 6206G>A, 6207A>C, 6207_6208insTAC, 6208A>C, 6209C>T, 6210A>T, 6214G>C, 6215C>T, 6216A>T, 6220A>G, 6222C>T, 6224C>T, 6225A>G, 6226G>T, 6230A>T, 6241A>G, 6244T>C, 6246G>A, 6247G>A, 6249A>C, 6251T>A, 6255T>C, 6256G>C, 6260A>G, 6264A>G, 6265G>A, 6267A>G, 6270G>A, 6276G>A, 6279T>A, 6283C>T, 6285C>G, 6286T>A, 6287T>G, 6290A>T, 6291T>A, 6292T>G, 6294G>C, 6295G>C, 6296C>T, 6297C>T, 6302T>C, 6304G>T, 6310A>G, 6311G>A, 6312A>T, 6313G>A, 6315A>G, 6316G>A, 6317G>A, 6319C>T, 6321T>G, 6322A>T, 6323T>A, 6325C>G, 6326T>C, 6327A>C, 6329G>A, 6330C>A, 6331C>T, 6332C>T, 6333A>C, 6335C>A, 6336A>C, 6340A>T, 6341T>G, 6342G>T, 6343A>G, 6346A>T, 6349G>T, 6351A>G, 6352G>A, 6354C>G, 6363G>T, 6364G>T, 6365A>C, 6372A>T, 6375A>C, 6376A>C, 6377T>A, 6378A>T, 6379A>G								

CDS

	Begin	End	Coverage	Score	Concordance	Matches	Identities	I/D/M/F*	Stop Codons
SPBVa_gp4	339	422	10.6%	264	45.8%	84 (98.8%)	37 (43.5%)	1/0/0/0	0
Protein mutations:	E341V (6137A>T 6138A>G), P344S (6145C>T 6147C>T), A352T (6169G>A 6171A>T), D353I (6172G>A 6173A>T 6174T>A), Q355M (6178C>A 6179A>T), R356D (6181A>G 6182G>A 6183A>T), K357L (6184A>C 6185A>T 6186G>T), D359N (6190G>A), N360R (6194A>G 6195T>G), A361V (6197C>T 6198A>T), R363K (6203G>A 6204G>A), G364H (6205G>C 6206G>A 6207A>C), G364_T365insY (6207_6208insTAC), T365L (6208A>C 6209C>T 6210A>T), A367L (6214G>C 6215C>T 6216A>T), I369V (6220A>G 6222C>T), A370V (6224C>T 6225A>G), V371L (6226G>T), Y372F (6230A>T), I376V (6241A>G), V378I (6247G>A 6249A>C), F379Y (6251T>A), E381Q (6256G>C), N382S (6260A>G), E384K (6265G>A 6267A>G), D388E (6279T>A), L391R (6286T>A 6287T>G), N392I (6290A>T 6291T>A), L393V (6292T>G 6294G>C), A394L (6295G>C 6296C>T 6297C>T), I396T (6302T>C), V397L (6304G>T), R399D (6310A>G 6311G>A 6312A>T), E400K (6313G>A 6315A>G), G401K (6316G>A 6317G>A), I403Y (6322A>T 6323T>A), L404A (6325C>G 6326T>C 6327A>C), S405K (6329G>A 6330C>A), P406F (6331C>T 6332C>T 6333A>C), T407N (6335C>A 6336A>C), M409C (6340A>T 6341T>G 6342G>T), K410E (6343A>G), I411F (6346A>T), G412W (6349G>T 6351A>G), V413M (6352G>A 6354C>G), D417S (6364G>T 6365A>C), I421H (6376A>C 6377T>A 6378A>T)								
Codon mutations:	TAT338..C (6129T>C), GAA339GAG (6132A>G), GAA341GTG (6137A>T 6138A>G), GTC342GTA (6141C>A), CCC344TCT (6145C>T 6147C>T), GGA346GGG (6153A>G), CTG347TTG (6154C>T), GCA350GCG (6165A>G), GCA352ACT (6169G>A 6171A>T), GAT353ATA (6172G>A 6173A>T 6174T>A), CAG355ATG (6178C>A 6179A>T), AGA356GAT (6181A>G 6182G>A 6183A>T), AAG357CTT (6184A>C 6185A>T 6186G>T), GAT359AAT (6190G>A), AAT360AGG (6194A>G 6195T>G), GCA361GTT (6197C>T 6198A>T), TTT362TTC (6201T>C), AGG363AAA (6203G>A 6204G>A), GGA364CAC (6205G>C 6206G>A 6207A>C), GGA364_ACA365insTAC (6207_6208insTAC), ACA365CTT (6208A>C 6209C>T 6210A>T), GCA367CTT (6214G>C 6215C>T 6216A>T), ATC369GTT (6220A>G 6222C>T), GCA370GTG (6224C>T 6225A>G), GTA371TTA (6226G>T), TAT372TTT (6230A>T), ATA376GTA (6241A>G), TTG377CTA (6244T>C 6246G>A), GTA378ATC (6247G>A 6249A>C), TTC379TAC (6251T>A), TCT380TCC (6255T>C), GAA381CAA (6256G>C), AAT382AGT (6260A>G), GAA383GAG (6264A>G), GAA384AAG (6265G>A 6267A>G), GAG385GAA (6270G>A), GAG387GAA (6276G>A), GAT388GAA (6279T>A), CTC390TTG (6283C>T 6285C>G), TTA391AGA (6286T>A 6287T>G), AAT392ATA (6290A>T 6291T>A), TTG393GTC (6292T>G 6294G>C), GCC394CTT (6295G>C 6296C>T 6297C>T), ATT396ACT (6302T>C), GTG397TTG (6304G>T), AGA399GAT (6310A>G 6311G>A 6312A>T), GAA400AAG (6313G>A 6315A>G), GGG401AAG (6316G>A 6317G>A), CTT402TTG (6319C>T 6321T>G), ATT403TAT (6322A>T 6323T>A), CTA404GCC (6325C>G 6326T>C 6327A>C), AGC405AAA (6329G>A 6330C>A), CCA406TTC (6331C>T 6332C>T 6333A>C), ACA407AAC (6335C>A 6336A>C), ATG409TGT (6340A>T 6341T>G 6342G>T), AAA410GAA (6343A>G), ATT411TTT (6346A>T), GGA412TGG (6349G>T 6351A>G), GTC413ATG (6352G>A 6354C>G), GTG416GTT (6363G>T), GAT417TCT (6364G>T 6365A>C), CTA419CTT (6372A>T), GGA420GGC (6375A>C), ATA421CAT (6376A>C 6377T>A 6378A>T), AAG422G.. (6379A>G)								

Proteins

	Begin	End	Coverage	Score	Concordance	Matches	Identities	I/D/M/F*	Stop Codons
RNaseH/reverse transcriptase (YP_004581513.1)	339	422	10.6%	264	45.8%	84 (98.8%)	37 (43.5%)	1/0/0/0	0
Protein mutations:	E341V (6137A>T 6138A>G), P344S (6145C>T 6147C>T), A352T (6169G>A 6171A>T), D353I (6172G>A 6173A>T 6174T>A), Q355M (6178C>A 6179A>T), R356D (6181A>G 6182G>A 6183A>T), K357L (6184A>C 6185A>T 6186G>T), D359N (6190G>A), N360R (6194A>G 6195T>G), A361V (6197C>T 6198A>T), R363K (6203G>A 6204G>A), G364H (6205G>C 6206G>A 6207A>C), G364_T365insY (6207_6208insTAC), T365L (6208A>C 6209C>T 6210A>T), A367L (6214G>C 6215C>T 6216A>T), I369V (6220A>G 6222C>T), A370V (6224C>T 6225A>G), V371L (6226G>T), Y372F (6230A>T), I376V (6241A>G), V378I (6247G>A 6249A>C), F379Y (6251T>A), E381Q (6256G>C), N382S (6260A>G), E384K (6265G>A 6267A>G), D388E (6279T>A), L391R (6286T>A 6287T>G), N392I (6290A>T 6291T>A), L393V (6292T>G 6294G>C), A394L (6295G>C 6296C>T 6297C>T), I396T (6302T>C), V397L (6304G>T), R399D (6310A>G 6311G>A 6312A>T), E400K (6313G>A 6315A>G), G401K (6316G>A 6317G>A), I403Y (6322A>T 6323T>A), L404A (6325C>G 6326T>C 6327A>C), S405K (6329G>A 6330C>A), P406F (6331C>T 6332C>T 6333A>C), T407N (6335C>A 6336A>C), M409C (6340A>T 6341T>G 6342G>T), K410E (6343A>G), I411F (6346A>T), G412W (6349G>T 6351A>G), V413M (6352G>A 6354C>G), D417S (6364G>T 6365A>C), I421H (6376A>C 6377T>A 6378A>T)								
Codon mutations:	TAT338..C (6129T>C), GAA339GAG (6132A>G), GAA341GTG (6137A>T 6138A>G), GTC342GTA (6141C>A), CCC344TCT (6145C>T 6147C>T), GGA346GGG (6153A>G), CTG347TTG (6154C>T), GCA350GCG (6165A>G), GCA352ACT (6169G>A 6171A>T), GAT353ATA (6172G>A 6173A>T 6174T>A), CAG355ATG (6178C>A 6179A>T), AGA356GAT (6181A>G 6182G>A 6183A>T), AAG357CTT (6184A>C 6185A>T 6186G>T), GAT359AAT (6190G>A), AAT360AGG (6194A>G 6195T>G), GCA361GTT (6197C>T 6198A>T), TTT362TTC (6201T>C), AGG363AAA (6203G>A 6204G>A), GGA364CAC (6205G>C 6206G>A 6207A>C), GGA364_ACA365insTAC (6207_6208insTAC), ACA365CTT (6208A>C 6209C>T 6210A>T), GCA367CTT (6214G>C 6215C>T 6216A>T), ATC369GTT (6220A>G 6222C>T), GCA370GTG (6224C>T 6225A>G), GTA371TTA (6226G>T), TAT372TTT (6230A>T), ATA376GTA (6241A>G), TTG377CTA (6244T>C 6246G>A), GTA378ATC (6247G>A 6249A>C), TTC379TAC (6251T>A), TCT380TCC (6255T>C), GAA381CAA (6256G>C), AAT382AGT (6260A>G), GAA383GAG (6264A>G), GAA384AAG (6265G>A 6267A>G), GAG385GAA (6270G>A), GAG387GAA (6276G>A), GAT388GAA (6279T>A), CTC390TTG (6283C>T 6285C>G), TTA391AGA (6286T>A 6287T>G), AAT392ATA (6290A>T 6291T>A), TTG393GTC (6292T>G 6294G>C), GCC394CTT (6295G>C 6296C>T 6297C>T), ATT396ACT (6302T>C), GTG397TTG (6304G>T), AGA399GAT (6310A>G 6311G>A 6312A>T), GAA400AAG (6313G>A 6315A>G), GGG401AAG (6316G>A 6317G>A), CTT402TTG (6319C>T 6321T>G), ATT403TAT (6322A>T 6323T>A), CTA404GCC (6325C>G 6326T>C 6327A>C), AGC405AAA (6329G>A 6330C>A), CCA406TTC (6331C>T 6332C>T 6333A>C), ACA407AAC (6335C>A 6336A>C), ATG409TGT (6340A>T 6341T>G 6342G>T), AAA410GAA (6343A>G), ATT411TTT (6346A>T), GGA412TGG (6349G>T 6351A>G), GTC413ATG (6352G>A 6354C>G), GTG416GTT (6363G>T), GAT417TCT (6364G>T 6365A>C), CTA419CTT (6372A>T), GGA420GGC (6375A>C), ATA421CAT (6376A>C 6377T>A 6378A>T), AAG422G.. (6379A>G)								

*: Inserts / Deletes / Misaligned / Frameshifts

Analysis details

This analysis was performed with panviral2.64

NGS Details (UN60): Moloney murine leukemia virus

Assembly

**Coverage Length**	270 (2 contig(s))
**Depth Of Coverage**	3.0
**Number Of Reads**	6
**Reads Per Million**	0.13 rpm (after QC)
**Ambiguities**	0
**Assembly Method**	de novo + reference guided assembly
**Consensus Caller**	Bcf Tools

Coverage Map

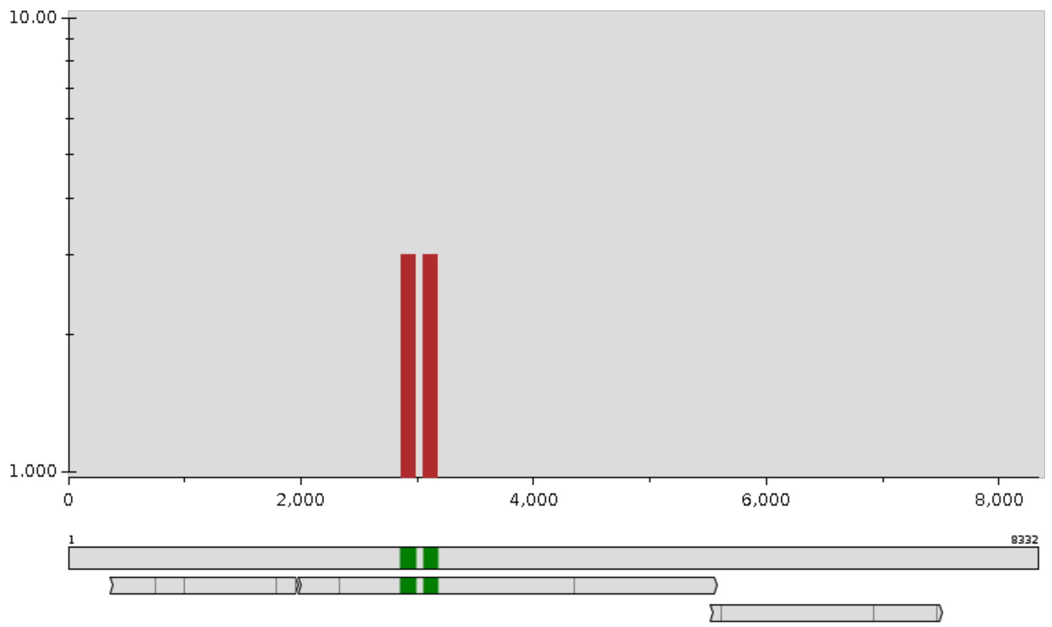

Assignment

**Type**	Moloney murine leukemia virus (Taxonomy ID: 11801)
**Reference Genome**	NC_001501.1
**NT Identity (%)**	75.5556
**AA Identity (%)**	100.0
**Number Of Stop Codons**	0
**Number Of CDS**	3

Alignment

**Alignment Score**	276.0 (NT) + 627.0 (AA) = 903.0
**Concordance (%)**	77.3779

Alignment Method	Global, seeded, nucleotide + amino acids (AGA)

Genome Region

Sequence starts at position 2856 and ends at position 3182 relative to NC_001501.1 reference sequence.

Alignment Detailed Statistics

	Begin	End	Coverage	Score	Concordance	Matches	Identities	I/D/M/F*	Stop Codons
**NT**	**2856**	**3182**	**3.2%**	**276**	**51.1%**	**270 (100%)**	**204 (75.6%)**	**0/0**	
Mutations:	2858A>T, 2861G>A, 2867A>C, 2870C>T, 2871T>A, 2872C>G, 2873A>C, 2876A>C, 2879A>G, 2880T>C, 2892A>C, 2894A>C, 2897C>G, 2900A>G, 2906T>C, 2909C>T, 2918T>C, 2921C>G, 2936G>A, 2939A>G, 2945C>T, 2946A>C, 2948A>C, 2951C>T, 2954A>G, 2957A>G, 2960C>T, 2963C>T, 2966G>C, 2969C>T, 2975C>T, 2978A>G, 2981C>T, 2982T>C, 2987C>T, 3050T>C, 3053T>C, 3056G>C, 3059C>G, 3063T>C, 3065A>G, 3068A>G, 3074A>G, 3077G>C, 3083C>G, 3086G>C, 3092G>C, 3095C>G, 3096T>A, 3097C>G, 3098G>C, 3101C>G, 3104G>A, 3110C>G, 3113A>G, 3131C>G, 3134G>A, 3143G>C, 3149T>G, 3152A>G, 3158G>A, 3161T>C, 3165A>C, 3167A>C, 3176T>C, 3179G>A								
**CDS**									
gag-pol	833	941	5.2%	627	100%	90 (100%)	90 (100%)	0/0/0/0	0
**Proteins**									
Pr180 (NP_057933.2)	833	941	5.2%	627	100%	90 (100%)	90 (100%)	0/0/0/0	0
Protein mutations:	*none*								
Codon mutations:	CCA833CCT (2858A>T), GAG834GAA (2861G>A), GGA836GGC (2867A>C), ATC837ATT (2870C>T), TCA838AGC (2871T>A 2872C>G 2873A>C), GGA839GGC (2876A>C), CAA840CAG (2879A>G), TTG841CTG (2880T>C), AGA845CGC (2892A>C 2894A>C), CTC846CTG (2897C>G), CCA847CCG (2900A>G), GGT849GGC (2906T>C), TTC850TTT (2909C>T), AGT853AGC (2918T>C), CCC854CCG (2921C>G), GAG859GAA (2936G>A), GCA860GCG (2939A>G), CAC862CAT (2945C>T), AGA863CGC (2946A>C 2948A>C), GAC864GAT (2951C>T), CTA865CTG (2954A>G), GCA866GCG (2957A>G), GAC867GAT (2960C>T), TTC868TTT (2963C>T), CGG869CGC (2966G>C), ATC870ATT (2969C>T), CAC872CAT (2975C>T), CCA873CCG (2978A>G), GAC874GAT (2981C>T), TTG875CTG (2982T>C), ATC876ATT (2987C>T), GGT897GGC (3050T>C), ACT898ACC (3053T>C), CGG899CGC (3056G>C), GCC900GCG (3059C>G), TTA902CTG (3063T>C 3065A>G), CAA903CAG (3068A>G), CTA905CTG (3074A>G), GGG906GGC (3077G>C), CTC908CTG (3083C>G), GGG909GGC (3086G>C), CGG911CGC (3092G>C), GCC912GCG (3095C>G), TCG913AGC (3096T>A 3097C>G 3098G>C), GCC914GCG (3101C>G), AAG915AAA (3104G>A), GCC917GCG (3110C>G), CAA918CAG (3113A>G), GTC924GTG (3131C>G), AAG925AAA (3134G>A), GGG928GGC (3143G>C), CTT930CTG (3149T>G), CTA931CTG (3152A>G), GAG933GAA (3158G>A), GGT934GGC (3161T>C), AGA936CGC (3165A>C 3167A>C), ACT939ACC (3176T>C), GAG940GAA (3179G>A)								
p80 RT (NP_955591.1)	174	282	13.4%	627	100%	90 (100%)	90 (100%)	0/0/0/0	0
Protein mutations:	*none*								
Codon mutations:	CCA174CCT (2858A>T), GAG175GAA (2861G>A), GGA177GGC (2867A>C), ATC178ATT (2870C>T), TCA179AGC (2871T>A 2872C>G 2873A>C), GGA180GGC (2876A>C), CAA181CAG (2879A>G), TTG182CTG (2880T>C), AGA186CGC (2892A>C 2894A>C), CTC187CTG (2897C>G), CCA188CCG (2900A>G), GGT190GGC (2906T>C), TTC191TTT (2909C>T), AGT194AGC (2918T>C), CCC195CCG (2921C>G), GAG200GAA (2936G>A), GCA201GCG (2939A>G), CAC203CAT (2945C>T), AGA204CGC (2946A>C 2948A>C), GAC205GAT (2951C>T), CTA206CTG (2954A>G), GCA207GCG (2957A>G), GAC208GAT (2960C>T), TTC209TTT (2963C>T), CGG210CGC (2966G>C), ATC211ATT (2969C>T), CAC213CAT (2975C>T), CCA214CCG (2978A>G), GAC215GAT (2981C>T), TTG216CTG (2982T>C), ATC217ATT (2987C>T), GGT238GGC (3050T>C), ACT239ACC (3053T>C), CGG240CGC (3056G>C), GCC241GCG (3059C>G), TTA243CTG (3063T>C 3065A>G), CAA244CAG (3068A>G), CTA246CTG (3074A>G), GGG247GGC (3077G>C), CTC249CTG (3083C>G), GGG250GGC (3086G>C), CGG252CGC (3092G>C), GCC253GCG (3095C>G), TCG254AGC (3096T>A 3097C>G 3098G>C), GCC255GCG (3101C>G), AAG256AAA (3104G>A), GCC258GCG (3110C>G), CAA259CAG (3113A>G), GTC265GTG (3131C>G), AAG266AAA (3134G>A), GGG269GGC (3143G>C), CTT271CTG (3149T>G), CTA272CTG (3152A>G), GAG274GAA (3158G>A), GGT275GGC (3161T>C), AGA277CGC (3165A>C 3167A>C), ACT280ACC (3176T>C), GAG281GAA (3179G>A)								

*: Inserts / Deletes / Misaligned / Frameshifts

Analysis details

This analysis was performed with panviral2.64

NGS Details (UN60): Marseillevirus marseillevirus

Assembly

**Coverage Length**	105 (1 contig(s))
**Depth Of Coverage**	4.4
**Number Of Reads**	6
**Reads Per Million**	0.13 rpm (after QC)
**Ambiguities**	1
**Assembly Method**	de novo + reference guided assembly
**Consensus Caller**	Bcf Tools

Coverage Map

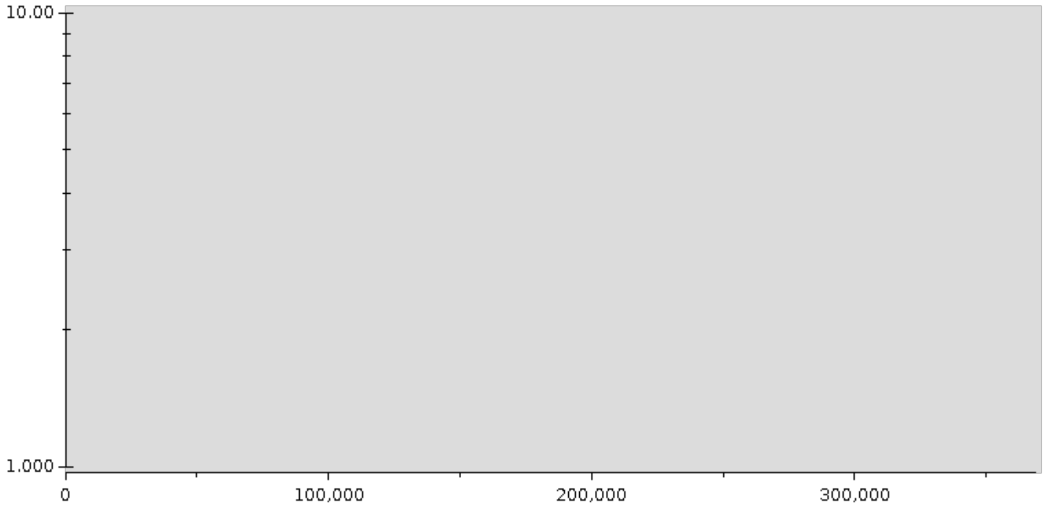

Assignment

**Type**	Marseillevirus marseillevirus (Taxonomy ID: 694581)
**Reference Genome**	NC_013756.1
**NT Identity (%)**	81.9048
**AA Identity (%)**	88.5714
**Number Of Stop Codons**	0
**Number Of CDS**	428

Alignment

**Alignment Score**	136.0 (NT) + 183.0 (AA) = 319.0
**Concordance (%)**	73.8426
**Alignment Method**	Local, heuristic, nucleotide (BLASTN)

Genome Region

Sequence starts at position 199412 and ends at position 199516 relative to NC_013756.1 reference sequence.

Alignment Detailed Statistics

	Begin	End	Coverage	Score	Concordance	Matches	Identities	I/D/M/F*	Stop Codons
NT	199412	199516	0.1%	136	64.8%	105 (100%)	86 (81.9%)	0/0	

Mutations: 199414T>C, 199420G>A, 199423A>C, 199425G>A, 199426G>M, 199429G>C, 199432A>G, 199444T>C, 199449C>T, 199450G>A, 199461C>A, 199462C>G, 199464A>G, 199465A>G, 199477T>C, 199480C>G, 199492G>A, 199495G>A, 199513G>A

*: Inserts / Deletes / Misaligned / Frameshifts

Analysis details

This analysis was performed with panviral2.64

NGS Details (UN60): Yaba monkey tumor virus

Assembly

**Coverage Length**	134 (1 contig(s))
**Depth Of Coverage**	3.3
**Number Of Reads**	4
**Reads Per Million**	0.08 rpm (after QC)
**Ambiguities**	0
**Assembly Method**	de novo + reference guided assembly
**Consensus Caller**	Bcf Tools

Coverage Map

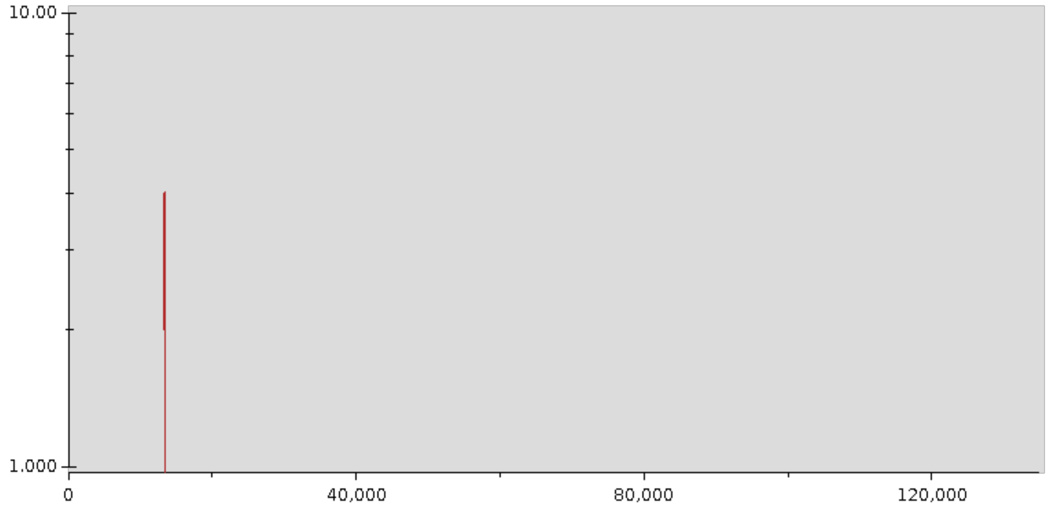

Assignment

**Type**	Yaba monkey tumor virus (Taxonomy ID: 38804)
**Reference Genome**	NC_005179.1
**NT Identity (%)**	81.3433
**AA Identity (%)**	84.0909
**Number Of Stop Codons**	0
**Number Of CDS**	140

Alignment

**Alignment Score**	168.0 (NT) + 273.0 (AA) = 441.0
**Concordance (%)**	75.7732
**Alignment Method**	Local, heuristic, nucleotide (BLASTN)

Genome Region

Sequence starts at position 13363 and ends at position 13496 relative to NC_005179.1 reference sequence.

Alignment Detailed Statistics

	Begin	End	Coverage	Score	Concordance	Matches	Identities	I/D/M/F*	Stop Codons
**NT**	**13363**	**13496**	**0.1%**	**168**	**62.7%**	**134 (100%)**	**109 (81.3%)**	**0/0**	

Mutations: 13366G>A, 13369G>C, 13370T>G, 13376T>G, 13381T>A, 13384T>A, 13387G>A, 13397T>A, 13399A>G, 13401T>C, 13408C>T, 13411A>T, 13412C>G, 13413T>G, 13418A>T, 13419A>G, 13426A>T, 13429A>G, 13432T>A, 13435C>T, 13453T>C, 13459A>G, 13472G>A, 13473C>T, 13483A>C

*: Inserts / Deletes / Misaligned / Frameshifts

Analysis details

This analysis was performed with panviral2.64

NGS Details (UN60): Alphabaculovirus agipsilonis

Assembly

**Coverage Length**	121 (1 contig(s))
**Depth Of Coverage**	3.5
**Number Of Reads**	4
**Reads Per Million**	0.08 rpm (after QC)
**Ambiguities**	0
**Assembly Method**	de novo + reference guided assembly
**Consensus Caller**	Bcf Tools

Coverage Map

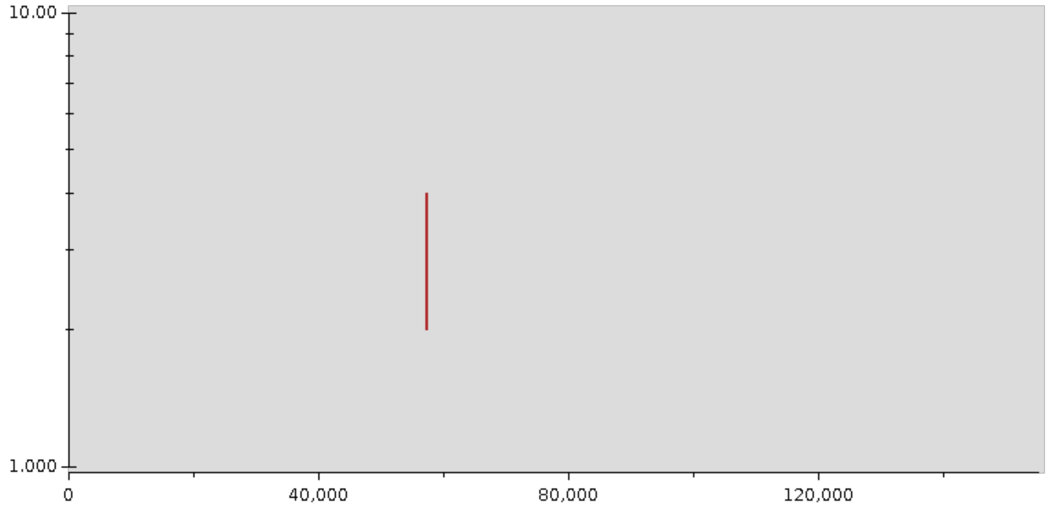

Assignment

**Type**	Alphabaculovirus agipsilonis (Taxonomy ID: 3047352)
**Reference Genome**	NC_011345.1
**NT Identity (%)**	78.5124
**AA Identity (%)**	77.5
**Number Of Stop Codons**	0
**Number Of CDS**	163

Alignment

**Alignment Score**	138.0 (NT) + 272.0 (AA) = 410.0
**Concordance (%)**	72.1831
**Alignment Method**	Local, heuristic, nucleotide (BLASTN)

Genome Region

Sequence starts at position 57321 and ends at position 57441 relative to NC_011345.1 reference sequence.

Alignment Detailed Statistics

	Begin	End	Coverage	Score	Concordance	Matches	Identities	I/D/M/F*	Stop Codons
**NT**	**57321**	**57441**	**0.1%**	**138**	**57.0%**	**121 (100%)**	**95 (78.5%)**	**0/0**	

Mutations: 57330C>T, 57334A>T, 57336G>A, 57339C>G, 57344T>C, 57357T>C, 57359C>T, 57361C>G, 57362T>C, 57363G>T, 57368T>C, 57375A>G, 57381C>G, 57382T>G, 57383C>G, 57384G>T, 57387C>A, 57390C>A, 57393C>T, 57399G>C, 57409G>T, 57414G>A, 57422C>G, 57432A>G, 57435T>G, 57439T>C

*: Inserts / Deletes / Misaligned / Frameshifts

Analysis details

This analysis was performed with panviral2.64

NGS Details (UN60): Badnavirus tessellocastaneae

Assembly

**Coverage Length**	245 (1 contig(s))
**Depth Of Coverage**	2.1
**Number Of Reads**	4
**Reads Per Million**	0.08 rpm (after QC)
**Ambiguities**	0
**Assembly Method**	de novo + reference guided assembly
**Consensus Caller**	Bcf Tools

Coverage Map

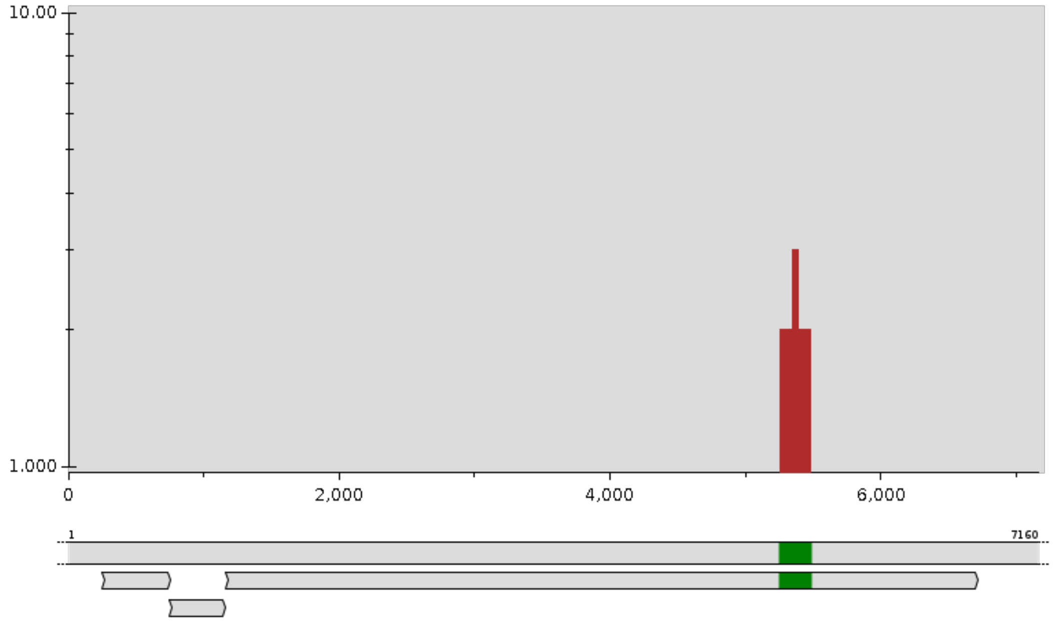

Assignment

**Type**	Badnavirus tessellocastaneae (Taxonomy ID: 3051987)
**Reference Genome**	NC_076885.1
**NT Identity (%)**	59.4262
**AA Identity (%)**	53.6585
**Number Of Stop Codons**	0
**Number Of CDS**	3

Alignment

**Alignment Score**	82.0 (NT) + 195.0 (AA) = 277.0
**Concordance (%)**	30.9948

Alignment Method	Global, seeded, nucleotide + amino acids (AGA)

Genome Region

Sequence starts at position 5248 and ends at position 5492 relative to NC_076885.1 reference sequence.

Alignment Detailed Statistics

	Begin	End	Coverage	Score	Concordance	Matches	Identities	I/D/M/F*	Stop Codons
**NT**	**5248**	**5492**	**3.4%**	**82**	**17.2%**	**244 (99.6%)**	**145 (59.2%)**	**0/1**	
Mutations:	5248G>A, 5254G>T, 5258G>A, 5259T>A, 5260G>T, 5263A>C, 5266A>C, 5267G>T, 5268G>T, 5269A>T, 5273T>C, 5274T>A, 5281G>A, 5285C>A, 5286T>A, 5287G>T, 5296A>T, 5305A>T, 5307G>A, 5314A>C, 5321A>G, 5322C>A, 5323C>A, 5326C>T, 5332G>T, 5334A>T, 5335G>T, 5339G>A, 5341C>T, 5342C>T, 5343A>C, 5345delT, 5346G>T, 5352G>A, 5353G>T, 5354G>C, 5355G>A, 5356G>T, 5357A>C, 5358C>T, 5359A>T, 5360G>A, 5361A>G, 5362A>C, 5363G>C, 5365A>T, 5369A>T, 5370T>C, 5371A>T, 5372T>A, 5373C>T, 5374A>T, 5386C>T, 5389T>C, 5390A>G, 5392A>T, 5393C>T, 5395C>A, 5396G>A, 5398C>A, 5404C>A, 5405C>G, 5406C>A, 5411C>A, 5416A>T, 5417A>T, 5423G>T, 5424C>T, 5425A>C, 5432T>C, 5434G>C, 5435G>A, 5437G>T, 5438A>G, 5439A>C, 5440A>T, 5441A>T, 5443G>C, 5444C>T, 5446G>C, 5447G>A, 5448C>A, 5451T>C, 5452C>T, 5453T>A, 5454G>T, 5455C>T, 5459G>A, 5460A>T, 5464T>C, 5470A>T, 5474C>A, 5476G>T, 5477T>A, 5478C>G, 5479A>T, 5480C>G, 5484C>A, 5485A>G, 5491G>A								

CDS

	Begin	End	Coverage	Score	Concordance	Matches	Identities	I/D/M/F*	Stop Codons
ORF3	1363	1444	4.4%	195	32.4%	82 (100%)	44 (53.7%)	0/0/1/1	0
Protein mutations:	V1366N (5258G>A 5259T>A 5260G>T), G1369F (5267G>T 5268G>T 5269A>T), L1371Q (5273T>C 5274T>A), L1375N (5285C>A 5286T>A 5287G>T), R1382K (5307G>A), T1387E (5321A>G 5322C>A 5323C>A), R1390S (5332G>T), K1391I (5334A>T 5335G>T), D1393N (5339G>A 5341C>T), Q1394S (5342C>T 5343A>C), R1397N (5352G>A 5353G>T), G1398H (5354G>C 5355G>A 5356G>T), T1399L (5357A>C 5358C>T 5359A>T), E1400S (5360G>A 5361A>G 5362A>C), E1401H (5363G>C 5365A>T), I1403S (5369A>T 5370T>C 5371A>T), S1404I (5372T>A 5373C>T 5374A>T), I1410V (5390A>G 5392A>T), V1412I (5396G>A 5398C>A), P1415E (5405C>G 5406C>A), L1417I (5411C>A), E1418D (5416A>T), T1419S (5417A>T), A1421F (5423G>T 5424C>T 5425A>C), E1425N (5435G>A 5437G>T), K1426A (5438A>G 5439A>C 5440A>T), M1427F (5441A>T 5443G>C), L1428F (5444C>T 5446G>C), A1429K (5447G>A 5448C>A), I1430T (5451T>C 5452C>T), C1431I (5453T>A 5454G>T 5455C>T), D1433I (5459G>A 5460A>T), L1438I (5474C>A 5476G>T), P1440A (5480C>G), T1441K (5484C>A 5485A>G), M1443I (5491G>A)								
Codon mutations:	TGG1362..A (5248G>A), GCG1364GCT (5254G>T), GTG1366AAT (5258G>A 5259T>A 5260G>T), GTA1367GTC (5263A>C), CCA1368CCC (5266A>C), GGA1369TTT (5267G>T 5268G>T 5269A>T), TTA1371CAA (5273T>C 5274T>A), GAG1373GAA (5281G>A), CTG1375AAT (5285C>A 5286T>A 5287G>T), CCA1378CCT (5296A>T), CTA1381CTT (5305A>T), AGA1382AAA (5307G>A), GCA1384GCC (5314A>C), ACC1387GAA (5321A>G 5322C>A 5323C>A), TTC1388TTT (5326C>T), AGG1390AGT (5332G>T), AAG1391ATT (5334A>T 5335G>T), GAC1393AAT (5339G>A 5341C>T), CAA1394TCA (5342C>T 5343A>C), TGT1395-TT (5345delT 5346G>T), AGG1397AAT (5352G>A 5353G>T), GGG1398CAT (5354G>C 5355G>A 5356G>T), ACA1399CTT (5357A>C 5358C>T 5359A>T), GAA1400AGC (5360G>A 5361A>G 5362A>C), GAA1401CAT (5363G>C 5365A>T), ATA1403TCT (5369A>T 5370T>C 5371A>T), TCA1404ATT (5372T>A 5373C>T 5374A>T), GAC1408GAT (5386C>T), GAT1409GAC (5389T>C), ATA1410GTT (5390A>G 5392A>T), CTC1411TTA (5393C>T 5395C>A), GTC1412ATA (5396G>A 5398C>A), TCC1414TCA (5404C>A), CCA1415GAA (5405C>G 5406C>A), CTT1417ATT (5411C>A), GAA1418GAT (5416A>T), ACT1419TCT (5417A>T), GCA1421TTC (5423G>T 5424C>T 5425A>C), TTG1424CTC (5432T>C 5434G>C), GAG1425AAT (5435G>A 5437G>T), AAA1426GCT (5438A>G 5439A>C 5440A>T), ATG1427TTC (5441A>T 5443G>C), CTG1428TTC (5444C>T 5446G>C), GCA1429AAA (5447G>A 5448C>A), ATC1430ACT (5451T>C 5452C>T), TGC1431ATT (5453T>A 5454G>T 5455C>T), GAC1433ATC (5459G>A 5460A>T), AAT1434AAC (5464T>C), CTA1436CTT (5470A>T), CTG1438ATT (5474C>A 5476G>T), TCA1439AGT (5477T>A 5478C>G 5479A>T), CCA1440GCA (5480C>G), ACA1441AAG (5484C>A 5485A>G), ATG1443ATA (5491G>A)								

Proteins

	Begin	End	Coverage	Score	Concordance	Matches	Identities	I/D/M/F*	Stop Codons
polyprotein (YP_010800602.1)	1363	1444	4.4%	195	32.4%	82 (100%)	44 (53.7%)	0/0/1/1	0
Protein mutations:	V1366N (5258G>A 5259T>A 5260G>T), G1369F (5267G>T 5268G>T 5269A>T), L1371Q (5273T>C 5274T>A), L1375N (5285C>A 5286T>A 5287G>T), R1382K (5307G>A), T1387E (5321A>G 5322C>A 5323C>A), R1390S (5332G>T), K1391I (5334A>T 5335G>T), D1393N (5339G>A 5341C>T), Q1394S (5342C>T 5343A>C), R1397N (5352G>A 5353G>T), G1398H (5354G>C 5355G>A 5356G>T), T1399L (5357A>C 5358C>T 5359A>T), E1400S (5360G>A 5361A>G 5362A>C), E1401H (5363G>C 5365A>T), I1403S (5369A>T 5370T>C 5371A>T), S1404I (5372T>A 5373C>T 5374A>T), I1410V (5390A>G 5392A>T), V1412I (5396G>A 5398C>A), P1415E (5405C>G 5406C>A), L1417I (5411C>A), E1418D (5416A>T), T1419S (5417A>T), A1421F (5423G>T 5424C>T 5425A>C), E1425N (5435G>A 5437G>T), K1426A (5438A>G 5439A>C 5440A>T), M1427F (5441A>T 5443G>C), L1428F (5444C>T 5446G>C), A1429K (5447G>A 5448C>A), I1430T (5451T>C 5452C>T), C1431I (5453T>A 5454G>T 5455C>T), D1433I (5459G>A 5460A>T), L1438I (5474C>A 5476G>T), P1440A (5480C>G), T1441K (5484C>A 5485A>G), M1443I (5491G>A)								
Codon mutations:	TGG1362..A (5248G>A), GCG1364GCT (5254G>T), GTG1366AAT (5258G>A 5259T>A 5260G>T), GTA1367GTC (5263A>C), CCA1368CCC (5266A>C), GGA1369TTT (5267G>T 5268G>T 5269A>T), TTA1371CAA (5273T>C 5274T>A), GAG1373GAA (5281G>A), CTG1375AAT (5285C>A 5286T>A 5287G>T), CCA1378CCT (5296A>T), CTA1381CTT (5305A>T), AGA1382AAA (5307G>A), GCA1384GCC (5314A>C), ACC1387GAA (5321A>G 5322C>A 5323C>A), TTC1388TTT (5326C>T), AGG1390AGT (5332G>T), AAG1391ATT (5334A>T 5335G>T), GAC1393AAT (5339G>A 5341C>T), CAA1394TCA (5342C>T 5343A>C), TGT1395-TT (5345delT 5346G>T), AGG1397AAT (5352G>A 5353G>T), GGG1398CAT (5354G>C 5355G>A 5356G>T), ACA1399CTT (5357A>C 5358C>T 5359A>T), GAA1400AGC (5360G>A 5361A>G 5362A>C), GAA1401CAT (5363G>C 5365A>T), ATA1403TCT (5369A>T 5370T>C 5371A>T), TCA1404ATT (5372T>A 5373C>T 5374A>T), GAC1408GAT (5386C>T), GAT1409GAC (5389T>C), ATA1410GTT (5390A>G 5392A>T), CTC1411TTA (5393C>T 5395C>A), GTC1412ATA (5396G>A 5398C>A), TCC1414TCA (5404C>A), CCA1415GAA (5405C>G 5406C>A), CTT1417ATT (5411C>A), GAA1418GAT (5416A>T), ACT1419TCT (5417A>T), GCA1421TTC (5423G>T 5424C>T 5425A>C), TTG1424CTC (5432T>C 5434G>C), GAG1425AAT (5435G>A 5437G>T), AAA1426GCT (5438A>G 5439A>C 5440A>T), ATG1427TTC (5441A>T 5443G>C), CTG1428TTC (5444C>T 5446G>C), GCA1429AAA (5447G>A 5448C>A), ATC1430ACT (5451T>C 5452C>T), TGC1431ATT (5453T>A 5454G>T 5455C>T), GAC1433ATC (5459G>A 5460A>T), AAT1434AAC (5464T>C), CTA1436CTT (5470A>T), CTG1438ATT (5474C>A 5476G>T), TCA1439AGT (5477T>A 5478C>G 5479A>T), CCA1440GCA (5480C>G), ACA1441AAG (5484C>A 5485A>G), ATG1443ATA (5491G>A)								

*: Inserts / Deletes / Misaligned / Frameshifts

Analysis details

This analysis was performed with panviral2.64

NGS Details (UN60): Escherichia virus DE3

Assembly

Coverage Length	219 (1 contig(s))
Depth Of Coverage	2.5
Number Of Reads	4
Reads Per Million	0.08 rpm (after QC)
Ambiguities	0
Assembly Method	de novo + reference guided assembly
Consensus Caller	Bcf Tools

Coverage Map

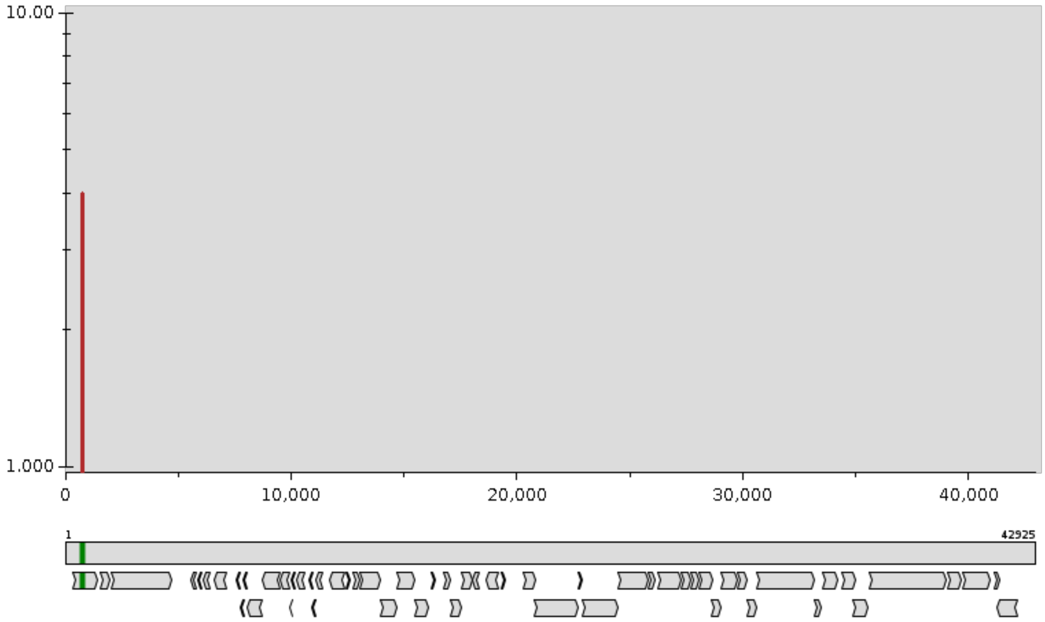

Assignment

Type	Escherichia virus DE3 (Taxonomy ID: 2169967)
Reference Genome	NC_042057.1
NT Identity (%)	100.0
AA Identity (%)	98.6301
Number Of Stop Codons	0
Number Of CDS	57

Alignment

Alignment Score	438.0 (NT) + 491.0 (AA) = 929.0
Concordance (%)	100.0

Alignment Method	Local, heuristic, nucleotide (BLASTN)

Genome Region

Sequence starts at position 657 and ends at position 875 relative to NC_042057.1 reference sequence.

Alignment Detailed Statistics

	Begin	End	Coverage	Score	Concordance	Matches	Identities	I/D/M/F *	Stop Codons
**NT**	**657**	**875**	**0.5%**	**438**	**100%**	**219 (100%)**	**219 (100%)**	**0/0**	
Mutations:	*none*								
**CDS**									
lacI	107	179	20.2%	491	96.3%	73 (100%)	72 (98.6%)	0/0/0/0	0
**Proteins**									
DNA-binding transcriptional repressor LacI (YP_009617199.1)	107	179	20.2%	491	96.3%	73 (100%)	72 (98.6%)	0/0/0/0	0
Protein mutations:	*none*								
Codon mutations:	*none*								

*: Inserts / Deletes / Misaligned / Frameshifts

Analysis details

This analysis was performed with panviral2.64

NGS Details (UN60): Epiphyllum badnavirus 1

Assembly

**Coverage Length**	267 (1 contig(s))
**Depth Of Coverage**	2.0
**Number Of Reads**	4
**Reads Per Million**	0.08 rpm (after QC)
**Ambiguities**	0
**Assembly Method**	de novo + reference guided assembly
**Consensus Caller**	Bcf Tools

Coverage Map

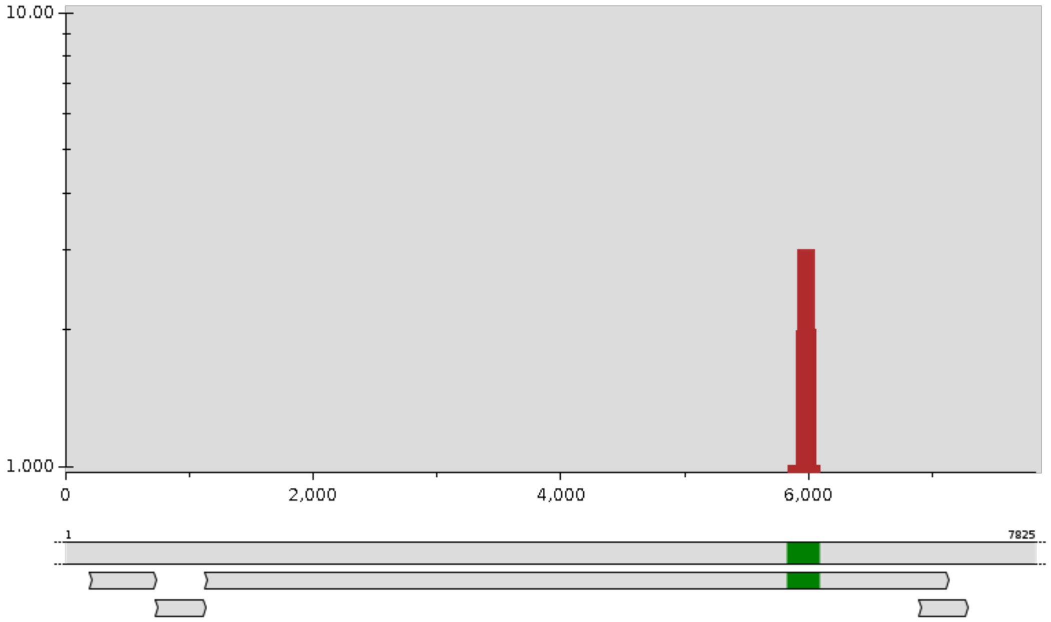

Assignment

**Type**	Epiphyllum badnavirus 1 (Taxonomy ID: 2518008)
**Reference Genome**	NC_076247.1
**NT Identity (%)**	54.8148
**AA Identity (%)**	50.0
**Number Of Stop Codons**	0
**Number Of CDS**	4

Alignment

**Alignment Score**	46.0 (NT) + 315.0 (AA) = 361.0
**Concordance (%)**	30.1839

Alignment Method	Global, seeded, nucleotide + amino acids (AGA)

Genome Region

Sequence starts at position 5825 and ends at position 6091 relative to NC_076247.1 reference sequence.

Alignment Detailed Statistics

	Begin	End	Coverage	Score	Concordance	Matches	Identities	I/D/M/F*	Stop Codons
**NT**	**5825**	**6091**	**3.4%**	**46**	**8.6%**	**267 (98.9%)**	**148 (54.8%)**	**3/0**	
Mutations:	5826C>G, 5830A>G, 5831A>G, 5832T>A, 5835C>T, 5836A>T, 5837G>C, 5841C>G, 5851T>A, 5853C>A, 5859G>T, 5861A>G, 5862G>A, 5863A>G, 5864G>C, 5865C>T, 5868A>C, 5870T>A, 5871C>T, 5877G>A, 5878A>C, 5880T>A, 5882T>G, 5883G>A, 5887G>A, 5888A>T, 5892A>G, 5893G>T, 5894A>G, 5895A>T, 5896T>G, 5897C>A, 5900T>C, 5901C>A, 5903C>A, 5905T>A, 5906G>A, 5907G>A, 5910C>G, 5913C>T, 5917C>A, 5918T>A, 5919G>A, 5922C>T, 5924C>A, 5925A>T, 5928T>G, 5931A>T, 5933T>A, 5937C>T, 5942G>T, 5943G>T, 5955A>T, 5958C>T, 5961A>C, 5966A>C, 5967G>A, 5976T>A, 5977G>T, 5980G>T, 5981T>C, 5982G>A, 5985C>T, 5988A>G, 5989C>A, 5991A>C, 5992A>C, 5993A>T, 5994G>C, 5998G>A, 6000C>T, 6001A>G, 6002A>T, 6003C>T, 6004T>G, 6005G>T, 6011G>A, 6013G>C, 6014A>C, 6015C>A, 6016C>T, 6018G>A, 6020C>T, 6021A>G, 6022G>A, 6024C>G, 6024_6025insAAA, 6026T>C, 6027T>A, 6030A>G, 6031G>C, 6032C>T, 6033A>G, 6038A>T, 6039C>T, 6040A>T, 6043G>T, 6052C>T, 6055G>A, 6057C>T, 6059T>A, 6060C>T, 6063T>C, 6066C>G, 6069T>C, 6070G>A, 6071A>T, 6072A>G, 6073G>T, 6074A>C, 6076G>A, 6077A>C, 6078C>T, 6082G>A, 6083C>T, 6084A>T, 6085C>G, 6087G>T, 6090C>T, 6091C>G								
**CDS**									
QKM20_gp3	1567	1655	4.4%	315	46.9%	89 (98.9%)	45 (50.0%)	1/0/0/0	0
Protein mutations:	N1568G (5830A>G 5831A>G 5832T>A), F1575I (5851T>A 5853C>A), K1578R (5861A>G 5862G>A), S1579A (5863A>G 5864G>C 5865C>T), F1581Y (5870T>A 5871C>T), I1584L (5878A>C 5880T>A), M1585R (5882T>G 5883G>A), D1587I (5887G>A 5888A>T), E1589C (5893G>T 5894A>G 5895A>T), S1590D (5896T>G 5897C>A), I1591T (5900T>C 5901C>A), P1592H (5903C>A), W1593K (5905T>A 5906G>A 5907G>A), L1597K (5917C>A 5918T>A 5919G>A), P1599H (5924C>A 5925A>T), D1600E (5928T>G), L1602H (5933T>A), W1605F (5942G>T 5943G>T), K1613T (5966A>C 5967G>A), A1617S (5977G>T), V1618S (5980G>T 5981T>C 5982G>A), R1621S (5989C>A 5991A>C), K1622L (5992A>C 5993A>T 5994G>C), D1624N (5998G>A 6000C>T), N1625V (6001A>G 6002A>T 6003C>T), C1626V (6004T>G 6005G>T), R1628K (6011G>A), D1629P (6013G>C 6014A>C 6015C>A), S1631L (6020C>T 6021A>G), G1632R (6022G>A 6024C>G), G1632_F1633insK (6024_6025insAAA), F1633S (6026T>C 6027T>A), A1635L (6031G>C 6032C>T 6033A>G), Y1637F (6038A>T 6039C>T), I1638F (6040A>T), D1639Y (6043G>T), V1643I (6055G>A 6057C>T), F1644Y (6059T>A 6060C>T), N1646K (6066C>G), E1648M (6070G>A 6071A>T 6072A>G), D1649S (6073G>T 6074A>C), D1650T (6076G>A 6077A>C 6078C>T), A1652I (6082G>A 6083C>T 6084A>T), Q1653D (6085C>G 6087G>T)								
Codon mutations:	GTC1566.TG (5826C>G), AAT1568GGA (5830A>G 5831A>G 5832T>A), GCC1569GCT (5835C>T), AGT1570TCT (5836A>T 5837G>C), GTC1571GTG (5841C>G), TTC1575ATA (5851T>A 5853C>A), CTG1577CTT (5859G>T), AAG1578AGA (5861A>G 5862G>A), AGC1579GCT (5863A>G 5864G>C 5865C>T), GGA1580GGC (5868A>C), TTC1581TAT (5870T>A 5871C>T), CAG1583CAA (5877G>A), ATT1584CTA (5878A>C 5880T>A), ATG1585AGA (5882T>G 5883G>A), GAC1587ATC (5887G>A 5888A>T), GAA1588GAG (5892A>G), GAA1589TGT (5893G>T 5894A>G 5895A>T), TCC1590GAC (5896T>G 5897C>A), ATC1591ACA (5900T>C 5901C>A), CCT1592CAT (5903C>A), TGG1593AAA (5905T>A 5906G>A 5907G>A), ACC1594ACG (5910C>G), GCC1595GCT (5913C>T), CTG1597AAA (5917C>A 5918T>A 5919G>A), ACC1598ACT (5922C>T), CCA1599CAT (5924C>A 5925A>T), GAT1600GAG (5928T>G), GGA1601GGT (5931A>T), CTT1602CAT (5933T>A), TAC1603TAT (5937C>T), TGG1605TTT (5942G>T 5943G>T), CCA1609CCT (5955A>T), TTC1610TTT (5958C>T), GGA1611GGC (5961A>C), AAG1613ACA (5966A>C 5967G>A), CCT1616CCA (5976T>A), GCT1617TCT (5977G>T), GTG1618TCA (5980G>T 5981T>C 5982G>A), TTC1619TTT (5985C>T), CAA1620CAG (5988A>G), CGA1621AGC (5989C>A 5991A>C), AAG1622CTC (5992A>C 5993A>T 5994G>C), GAC1624AAT (5998G>A 6000C>T), AAC1625GTT (6001A>G 6002A>T 6003C>T), TGT1626GTT (6004T>G 6005G>T), AGA1628AAA (6011G>A), GAC1629CCA (6013G>C 6014A>C 6015C>A), CTG1630TTA (6016C>T 6018G>A), TCA1631TTG (6020C>T 6021A>G), GGC1632AGG (6022G>A 6024C>G), GGC1632_TTT1633insAAA (6024_6025insAAA), TTT1633TCA (6026T>C 6027T>A), GTA1634GTG (6030A>G), GCA1635CTG (6031G>C 6032C>T 6033A>G), TAC1637TTT (6038A>T 6039C>T), ATT1638TTT (6040A>T), GAT1639TAT (6043G>T), CTG1642TTG (6052C>T), GTC1643ATT (6055G>A 6057C>T), TTC1644TAT (6059T>A 6060C>T), AGT1645AGC (6063T>C), AAC1646AAG (6066C>G), ACT1647ACC (6069T>C), GAA1648ATG (6070G>A 6071A>T 6072A>G), GAT1649TCT (6073G>T 6074A>C), GAC1650ACT (6076G>A 6077A>C 6078C>T), GCA1652ATT (6082G>A 6083C>T 6084A>T), CAG1653GAT (6085C>G 6087G>T), CAC1654CAT (6090C>T), CTG1655G.. (6091C>G)								
**Proteins**									
polyprotein (YP_010797894.1)	1567	1655	4.4%	315	46.9%	89 (98.9%)	45 (50.0%)	1/0/0/0	0
Protein mutations:	N1568G (5830A>G 5831A>G 5832T>A), F1575I (5851T>A 5853C>A), K1578R (5861A>G 5862G>A), S1579A (5863A>G 5864G>C 5865C>T), F1581Y (5870T>A 5871C>T), I1584L (5878A>C 5880T>A), M1585R (5882T>G 5883G>A), D1587I (5887G>A 5888A>T), E1589C (5893G>T 5894A>G 5895A>T), S1590D (5896T>G 5897C>A), I1591T (5900T>C 5901C>A), P1592H (5903C>A), W1593K (5905T>A 5906G>A 5907G>A), L1597K (5917C>A 5918T>A 5919G>A), P1599H (5924C>A 5925A>T), D1600E (5928T>G), L1602H (5933T>A), W1605F (5942G>T 5943G>T), K1613T (5966A>C 5967G>A), A1617S (5977G>T), V1618S (5980G>T 5981T>C 5982G>A), R1621S (5989C>A 5991A>C), K1622L (5992A>C 5993A>T 5994G>C), D1624N (5998G>A 6000C>T), N1625V (6001A>G 6002A>T 6003C>T), C1626V (6004T>G 6005G>T), R1628K (6011G>A), D1629P (6013G>C 6014A>C 6015C>A), S1631L (6020C>T 6021A>G), G1632R (6022G>A 6024C>G), G1632_F1633insK (6024_6025insAAA), F1633S (6026T>C 6027T>A), A1635L (6031G>C 6032C>T 6033A>G), Y1637F (6038A>T 6039C>T), I1638F (6040A>T), D1639Y (6043G>T), V1643I (6055G>A 6057C>T), F1644Y (6059T>A 6060C>T), N1646K (6066C>G), E1648M (6070G>A 6071A>T 6072A>G), D1649S (6073G>T 6074A>C), D1650T (6076G>A 6077A>C 6078C>T), A1652I (6082G>A 6083C>T 6084A>T), Q1653D (6085C>G 6087G>T)								
Codon mutations:	GTC1566.TG (5826C>G), AAT1568GGA (5830A>G 5831A>G 5832T>A), GCC1569GCT (5835C>T), AGT1570TCT (5836A>T 5837G>C), GTC1571GTG (5841C>G), TTC1575ATA (5851T>A 5853C>A), CTG1577CTT (5859G>T), AAG1578AGA (5861A>G 5862G>A), AGC1579GCT (5863A>G 5864G>C 5865C>T), GGA1580GGC (5868A>C), TTC1581TAT (5870T>A 5871C>T), CAG1583CAA (5877G>A), ATT1584CTA (5878A>C 5880T>A), ATG1585AGA (5882T>G 5883G>A), GAC1587ATC (5887G>A 5888A>T), GAA1588GAG (5892A>G), GAA1589TGT (5893G>T 5894A>G 5895A>T), TCC1590GAC (5896T>G 5897C>A), ATC1591ACA (5900T>C 5901C>A), CCT1592CAT (5903C>A), TGG1593AAA (5905T>A 5906G>A 5907G>A), ACC1594ACG (5910C>G), GCC1595GCT (5913C>T), CTG1597AAA (5917C>A 5918T>A 5919G>A), ACC1598ACT (5922C>T), CCA1599CAT (5924C>A 5925A>T), GAT1600GAG (5928T>G), GGA1601GGT (5931A>T), CTT1602CAT (5933T>A), TAC1603TAT (5937C>T), TGG1605TTT (5942G>T 5943G>T), CCA1609CCT (5955A>T), TTC1610TTT (5958C>T), GGA1611GGC (5961A>C), AAG1613ACA (5966A>C 5967G>A), CCT1616CCA (5976T>A), GCT1617TCT (5977G>T), GTG1618TCA (5980G>T 5981T>C 5982G>A), TTC1619TTT (5985C>T), CAA1620CAG (5988A>G), CGA1621AGC (5989C>A 5991A>C), AAG1622CTC (5992A>C 5993A>T 5994G>C), GAC1624AAT (5998G>A 6000C>T), AAC1625GTT (6001A>G 6002A>T 6003C>T), TGT1626GTT (6004T>G 6005G>T), AGA1628AAA (6011G>A), GAC1629CCA (6013G>C 6014A>C 6015C>A), CTG1630TTA (6016C>T 6018G>A), TCA1631TTG (6020C>T 6021A>G), GGC1632AGG (6022G>A 6024C>G), GGC1632_TTT1633insAAA (6024_6025insAAA), TTT1633TCA (6026T>C 6027T>A), GTA1634GTG (6030A>G), GCA1635CTG (6031G>C 6032C>T 6033A>G), TAC1637TTT (6038A>T 6039C>T), ATT1638TTT (6040A>T), GAT1639TAT (6043G>T), CTG1642TTG (6052C>T), GTC1643ATT (6055G>A 6057C>T), TTC1644TAT (6059T>A 6060C>T), AGT1645AGC (6063T>C), AAC1646AAG (6066C>G), ACT1647ACC (6069T>C), GAA1648ATG (6070G>A 6071A>T 6072A>G), GAT1649TCT (6073G>T 6074A>C), GAC1650ACT (6076G>A 6077A>C 6078C>T), GCA1652ATT (6082G>A 6083C>T 6084A>T), CAG1653GAT (6085C>G 6087G>T), CAC1654CAT (6090C>T), CTG1655G.. (6091C>G)								

*: Inserts / Deletes / Misaligned / Frameshifts

Analysis details

This analysis was performed with panviral2.64

NGS Details (UN60): Goose dicistrovirus

Assembly

**Coverage Length**	240 (1 contig(s))
**Depth Of Coverage**	2.2
**Number Of Reads**	4
**Reads Per Million**	0.08 rpm (after QC)
**Ambiguities**	0
**Assembly Method**	de novo + reference guided assembly
**Consensus Caller**	Bcf Tools

Coverage Map

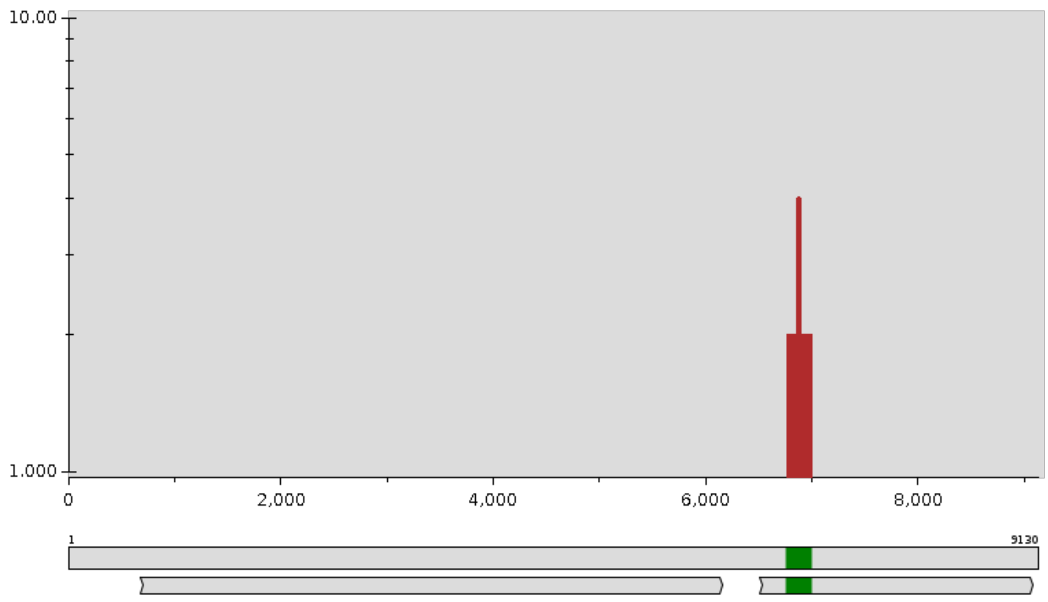

Assignment

**Type**	Goose dicistrovirus (Taxonomy ID: 1776109)
**Reference Genome**	NC_029052.1
**NT Identity (%)**	85.0
**AA Identity (%)**	95.0
**Number Of Stop Codons**	0
**Number Of CDS**	2

Alignment

**Alignment Score**	336.0 (NT) + 540.0 (AA) = 876.0
**Concordance (%)**	84.8015

Alignment Method	Global, seeded, nucleotide + amino acids (AGA)

Genome Region

Sequence starts at position 6764 and ends at position 7003 relative to NC_029052.1 reference sequence.

Alignment Detailed Statistics

	Begin	End	Coverage	Score	Concordance	Matches	Identities	I/D/M/F*	Stop Codons
**NT**	**6764**	**7003**	**2.6%**	**336**	**70.0%**	**240 (100%)**	**204 (85.0%)**	**0/0**	
Mutations:	6774C>T, 6780G>A, 6792T>C, 6795T>C, 6801A>G, 6802G>A, 6805C>T, 6807T>A, 6819T>A, 6828G>A, 6837G>T, 6846A>C, 6852T>C, 6855A>T, 6864T>C, 6870T>G, 6873A>G, 6879G>A, 6882G>A, 6885A>T, 6886A>C, 6900C>G, 6903G>C, 6907G>A, 6912T>A, 6924G>A, 6927T>A, 6943T>C, 6945G>A, 6955T>C, 6960T>G, 6972A>G, 6978T>C, 6993C>T, 7000C>T, 7002T>A								
**CDS**									
AXF40_gp2	87	166	9.3%	540	95.6%	80 (100%)	76 (95.0%)	0/0/0/0	0
Protein mutations:	A99T (6802G>A), V134I (6907G>A), Y150H (6955T>C)								
Codon mutations:	CCC89CCT (6774C>T), CAG91CAA (6780G>A), GGT95GGC (6792T>C), AAT96AAC (6795T>C), ACA98ACG (6801A>G), GCA99ACA (6802G>A), CTT100TTA (6805C>T 6807T>A), ACT104ACA (6819T>A), GGG107GGA (6828G>A), TCG110TCT (6837G>T), GGA113GGC (6846A>C), GAT115GAC (6852T>C), ATA116ATT (6855A>T), TGT119TGC (6864T>C), GGT121GGG (6870T>G), TCA122TCG (6873A>G), CCG124CCA (6879G>A), CAG125CAA (6882G>A), TCA126TCT (6885A>T), AGA127CGA (6886A>C), GCC131GCG (6900C>G), ACG132ACC (6903G>C), GTT134ATT (6907G>A), ATT135ATA (6912T>A), TCG139TCA (6924G>A), CCT140CCA (6927T>A), TTG146CTA (6943T>C 6945G>A), TAT150CAT (6955T>C), GGT151GGG (6960T>G), CAA155CAG (6972A>G), AAT157AAC (6978T>C), AGC162AGT (6993C>T), CTT165TTA (7000C>T 7002T>A)								
**Proteins**									
capsid protein precursor (YP_009221982.1)	87	166	9.3%	540	95.6%	80 (100%)	76 (95.0%)	0/0/0/0	0
Protein mutations:	A99T (6802G>A), V134I (6907G>A), Y150H (6955T>C)								
Codon mutations:	CCC89CCT (6774C>T), CAG91CAA (6780G>A), GGT95GGC (6792T>C), AAT96AAC (6795T>C), ACA98ACG (6801A>G), GCA99ACA (6802G>A), CTT100TTA (6805C>T 6807T>A), ACT104ACA (6819T>A), GGG107GGA (6828G>A), TCG110TCT (6837G>T), GGA113GGC (6846A>C), GAT115GAC (6852T>C), ATA116ATT (6855A>T), TGT119TGC (6864T>C), GGT121GGG (6870T>G), TCA122TCG (6873A>G), CCG124CCA (6879G>A), CAG125CAA (6882G>A), TCA126TCT (6885A>T), AGA127CGA (6886A>C), GCC131GCG (6900C>G), ACG132ACC (6903G>C), GTT134ATT (6907G>A), ATT135ATA (6912T>A), TCG139TCA (6924G>A), CCT140CCA (6927T>A), TTG146CTA (6943T>C 6945G>A), TAT150CAT (6955T>C), GGT151GGG (6960T>G), CAA155CAG (6972A>G), AAT157AAC (6978T>C), AGC162AGT (6993C>T), CTT165TTA (7000C>T 7002T>A)								

*: Inserts / Deletes / Misaligned / Frameshifts

Analysis details

This analysis was performed with panviral2.64

NGS Details (UN60): Melanoplus sanguinipes entomopoxvirus

Assembly

**Coverage Length**	142 (1 contig(s))
**Depth Of Coverage**	1.4
**Number Of Reads**	2
**Reads Per Million**	0.04 rpm (after QC)
**Ambiguities**	0
**Assembly Method**	de novo + reference guided assembly
**Consensus Caller**	Bcf Tools

Coverage Map

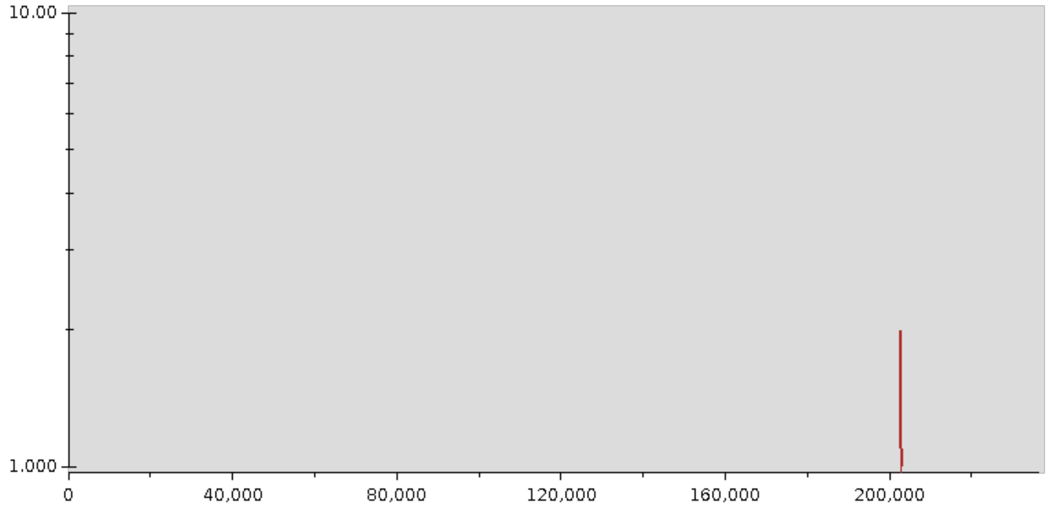

Assignment

**Type**	Melanoplus sanguinipes entomopoxvirus (Taxonomy ID: 83191)
**Reference Genome**	NC_001993.1
**NT Identity (%)**	81.6901
**AA Identity (%)**	76.5957
**Number Of Stop Codons**	0
**Number Of CDS**	267

Alignment

**Alignment Score**	180.0 (NT) + 336.0 (AA) = 516.0
**Concordance (%)**	75.7709
**Alignment Method**	Local, heuristic, nucleotide (BLASTN)

Genome Region

Sequence starts at position 202763 and ends at position 202904 relative to NC_001993.1 reference sequence.

Alignment Detailed Statistics

	Begin	End	Coverage	Score	Concordance	Matches	Identities	I/D/M/F*	Stop Codons
**NT**	**202763**	**202904**	**0.1%**	**180**	**63.4%**	**142 (100%)**	**116 (81.7%)**	**0/0**	

Mutations: 202768T>C, 202771A>T, 202773G>T, 202774T>C, 202775C>T, 202777A>G, 202784T>G, 202789A>G, 202790C>T, 202807A>T, 202813T>A, 202817G>T, 202818G>C, 202831T>C, 202832G>C, 202834T>C, 202835C>T, 202836T>G, 202852A>T, 202862A>G, 202874T>A, 202876T>A, 202880T>C, 202885A>G, 202891T>A, 202897T>C

*: Inserts / Deletes / Misaligned / Frameshifts

Analysis details

This analysis was performed with panviral2.64

NGS Details (UN60): Cotia virus

Assembly

**Coverage Length**	135 (1 contig(s))
**Depth Of Coverage**	1.8
**Number Of Reads**	2
**Reads Per Million**	0.04 rpm (after QC)
**Ambiguities**	0
**Assembly Method**	de novo + reference guided assembly
**Consensus Caller**	Bcf Tools

Coverage Map

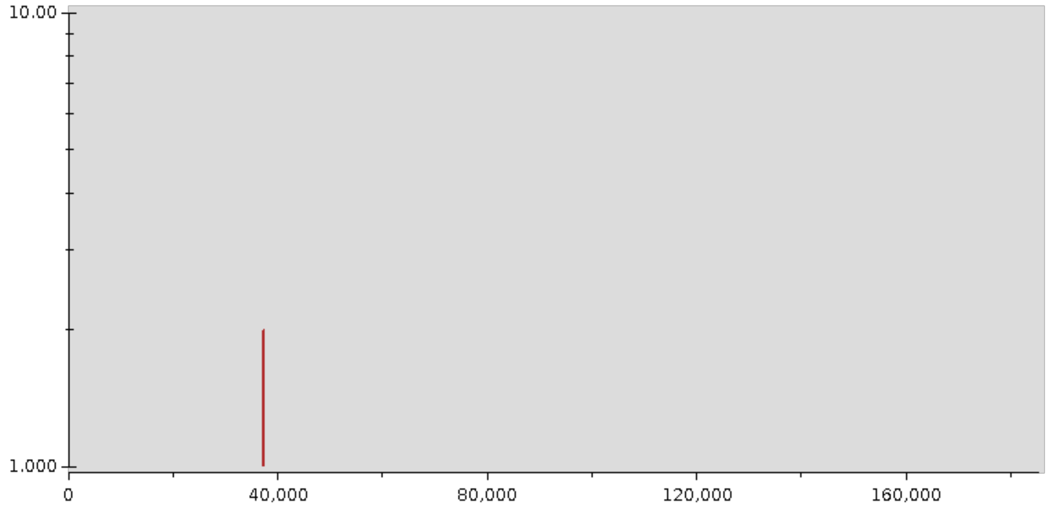

Assignment

**Type**	Cotia virus (Taxonomy ID: 39444)
**Reference Genome**	NC_016924.1
**NT Identity (%)**	80.0
**AA Identity (%)**	93.3333
**Number Of Stop Codons**	0
**Number Of CDS**	185

Alignment

**Alignment Score**	162.0 (NT) + 285.0 (AA) = 447.0
**Concordance (%)**	78.4211
**Alignment Method**	Local, heuristic, nucleotide (BLASTN)

Genome Region

Sequence starts at position 37199 and ends at position 37333 relative to NC_016924.1 reference sequence.

Alignment Detailed Statistics

	Begin	End	Coverage	Score	Concordance	Matches	Identities	I/D/M/F*	Stop Codons
**NT**	**37199**	**37333**	**0.1%**	**162**	**60.0%**	**135 (100%)**	**108 (80.0%)**	**0/0**	

Mutations: 37209C>G, 37210T>A, 37211T>A, 37216A>G, 37223A>G, 37224G>C, 37225A>T, 37229G>A, 37235T>A, 37247A>T, 37256T>C, 37259A>C, 37261T>A, 37270T>A, 37274T>A, 37281G>C, 37282A>T, 37289A>G, 37292G>A, 37295A>T, 37307T>A, 37310T>A, 37319T>A, 37322A>T, 37325A>C, 37329G>T, 37330T>C

*: Inserts / Deletes / Misaligned / Frameshifts

Analysis details

This analysis was performed with panviral2.64

NGS Details (UN60): Scottvirus scott

Assembly

**Coverage Length**	244 (1 contig(s))
**Depth Of Coverage**	1.1
**Number Of Reads**	2
**Reads Per Million**	0.04 rpm (after QC)
**Ambiguities**	0
**Assembly Method**	de novo + reference guided assembly
**Consensus Caller**	Bcf Tools

Coverage Map

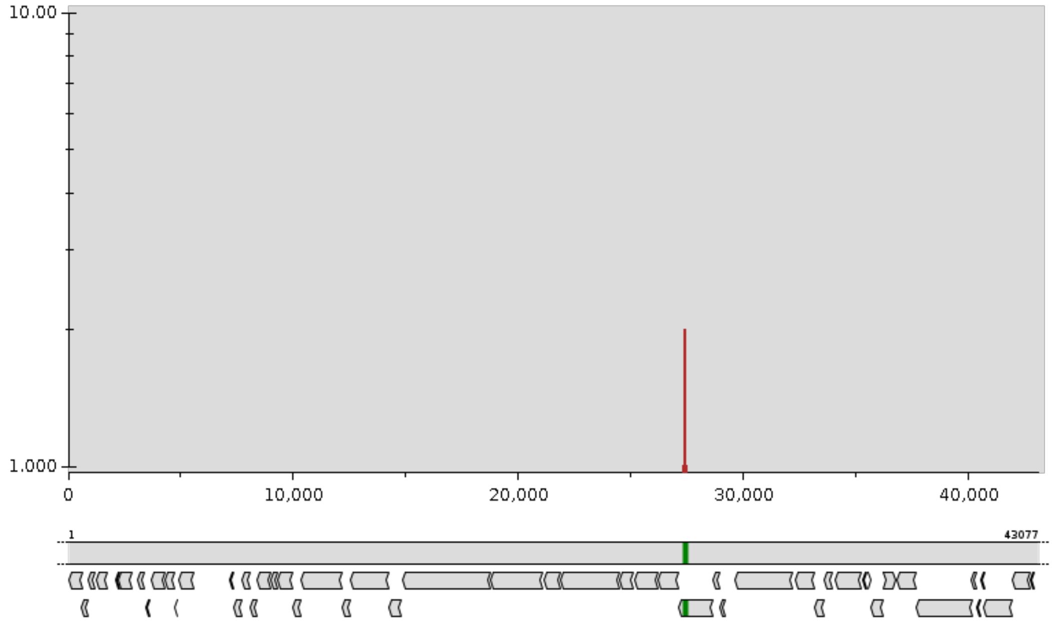

Assignment

**Type**	Scottvirus scott (Taxonomy ID: 2733954)
**Reference Genome**	NC_048029.1
**NT Identity (%)**	81.9672
**AA Identity (%)**	92.5926
**Number Of Stop Codons**	0
**Number Of CDS**	51

Alignment

**Alignment Score**	312.0 (NT) + 506.0 (AA) = 818.0
**Concordance (%)**	80.1961

Alignment Method	Local, heuristic, nucleotide (BLASTN)

Genome Region

Sequence starts at position 27285 and ends at position 27528 relative to NC_048029.1 reference sequence.

Alignment Detailed Statistics

	Begin	End	Coverage	Score	Concordance	Matches	Identities	I/D/M/F*	Stop Codons
**NT**	**27285**	**27528**	**0.6%**	**312**	**63.9%**	**244 (100%)**	**200 (82.0%)**	**0/0**	
Mutations:	27287T>C, 27291T>C, 27296G>C, 27299T>G, 27317T>A, 27318T>G, 27319G>C, 27323A>T, 27326C>T, 27329T>C, 27344A>G, 27347C>T, 27350A>G, 27353C>T, 27362G>C, 27368C>G, 27380G>A, 27392T>A, 27398G>C, 27401G>T, 27404A>T, 27410C>G, 27412A>G, 27416G>C, 27419A>T, 27428A>T, 27434G>A, 27440C>T, 27443A>G, 27445T>C, 27446C>G, 27461C>G, 27462C>T, 27463G>T, 27464C>A, 27476A>T, 27482A>C, 27485A>C, 27487G>T, 27491G>A, 27509A>G, 27515T>C, 27521G>A, 27524A>G								
**CDS**									
HOT83_gp32	373	453	15.9%	506	93.7%	81 (100%)	75 (92.6%)	0/0/0/0	0
Protein mutations:	L386M (27485A>C 27487G>T), R394N (27461C>G 27462C>T 27463G>T), T400A (27443A>G 27445T>C), Q442A (27317T>A 27318T>G 27319G>C), K451R (27291T>C)								
Codon mutations:	CTT373CTC (27524A>G), GGC374GGT (27521G>A), GCA376GCG (27515T>C), TCT378TCC (27509A>G), TTC384TTT (27491G>A), CTT386ATG (27485A>C 27487G>T), CCT387CCG (27482A>C), GCT389GCA (27476A>T), CTG393CTT (27464C>A), CGG394AAC (27461C>G 27462C>T 27463G>T), CCG399CCC (27446C>G), ACT400GCC (27443A>G 27445T>C), AAG401AAA (27440C>T), TTC403TTT (27434G>A), CCT405CCA (27428A>T), GTT408GTA (27419A>T), ACC409ACG (27416G>C), TTG411CTC (27410C>G 27412A>G), GCT413GCA (27404A>T), CTC414CTA (27401G>T), TCC415TCG (27398G>C), GGA417GGT (27392T>A), GAC421GAT (27380G>A), CTG425CTC (27368C>G), CTC427CTG (27362G>C), GTG430GTA (27353C>T), GCT431GCC (27350A>G), GCG432GCA (27347C>T), CTT433CTC (27344A>G), GAA438GAG (27329T>C), CCG439CCA (27326C>T), GCT440GCA (27323A>T), CAA442GCT (27317T>A 27318T>G 27319G>C), GCA448GCC (27299T>G), CTC449CTG (27296G>C), AAG451AGG (27291T>C), GCA452GCG (27287T>C)								
**Proteins**									
head-to-tail joining protein (YP_009807875.1)	373	453	15.9%	506	93.7%	81 (100%)	75 (92.6%)	0/0/0/0	0
Protein mutations:	L386M (27485A>C 27487G>T), R394N (27461C>G 27462C>T 27463G>T), T400A (27443A>G 27445T>C), Q442A (27317T>A 27318T>G 27319G>C), K451R (27291T>C)								
Codon mutations:	CTT373CTC (27524A>G), GGC374GGT (27521G>A), GCA376GCG (27515T>C), TCT378TCC (27509A>G), TTC384TTT (27491G>A), CTT386ATG (27485A>C 27487G>T), CCT387CCG (27482A>C), GCT389GCA (27476A>T), CTG393CTT (27464C>A), CGG394AAC (27461C>G 27462C>T 27463G>T), CCG399CCC (27446C>G), ACT400GCC (27443A>G 27445T>C), AAG401AAA (27440C>T), TTC403TTT (27434G>A), CCT405CCA (27428A>T), GTT408GTA (27419A>T), ACC409ACG (27416G>C), TTG411CTC (27410C>G 27412A>G), GCT413GCA (27404A>T), CTC414CTA (27401G>T), TCC415TCG (27398G>C), GGA417GGT (27392T>A), GAC421GAT (27380G>A), CTG425CTC (27368C>G), CTC427CTG (27362G>C), GTG430GTA (27353C>T), GCT431GCC (27350A>G), GCG432GCA (27347C>T), CTT433CTC (27344A>G), GAA438GAG (27329T>C), CCG439CCA (27326C>T), GCT440GCA (27323A>T), CAA442GCT (27317T>A 27318T>G 27319G>C), GCA448GCC (27299T>G), CTC449CTG (27296G>C), AAG451AGG (27291T>C), GCA452GCG (27287T>C)								

*: Inserts / Deletes / Misaligned / Frameshifts

Analysis details

This analysis was performed with panviral2.64

NGS Details (UN60): Punavirus P1

Assembly

**Coverage Length**	224 (1 contig(s))
**Depth Of Coverage**	1.2
**Number Of Reads**	2
**Reads Per Million**	0.04 rpm (after QC)
**Ambiguities**	0
**Assembly Method**	de novo + reference guided assembly
**Consensus Caller**	Bcf Tools

Coverage Map

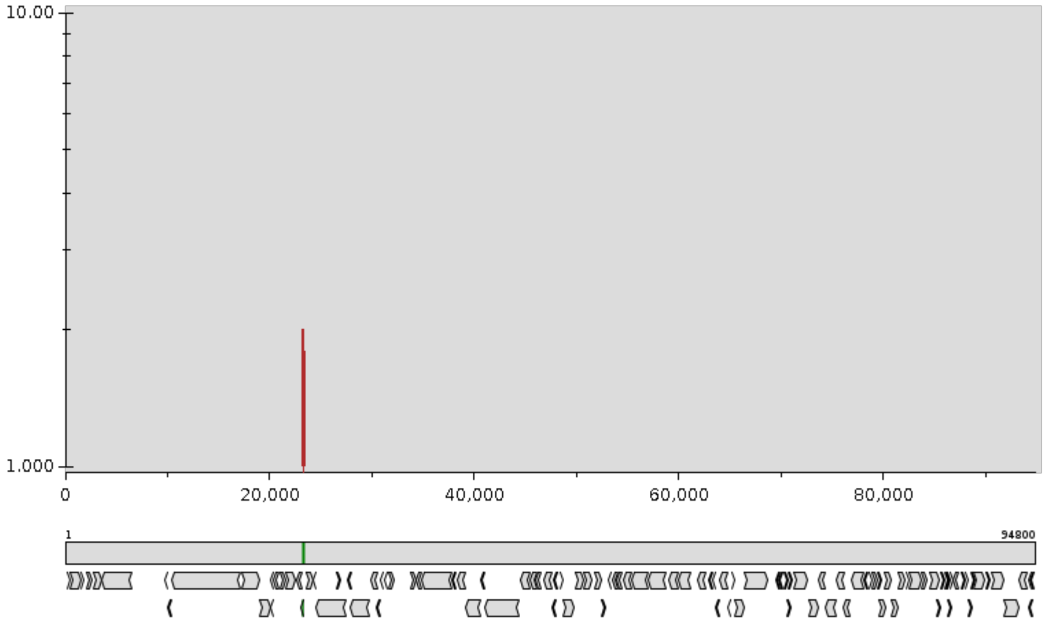

Assignment

**Type**	Punavirus P1 (Taxonomy ID: 10678)
**Reference Genome**	NC_005856.1
**NT Identity (%)**	100.0
**AA Identity (%)**	98.3051
**Number Of Stop Codons**	0
**Number Of CDS**	110

Alignment

**Alignment Score**	448.0 (NT) + 438.0 (AA) = 886.0
**Concordance (%)**	100.0

Alignment Method	Local, heuristic, nucleotide (BLASTN)

Genome Region

Sequence starts at position 23164 and ends at position 23387 relative to NC_005856.1 reference sequence.

Alignment Detailed Statistics

	Begin	End	Coverage	Score	Concordance	Matches	Identities	I/D/M/F*	Stop Codons
**NT**	**23164**	**23387**	**0.2%**	**448**	**100%**	**224 (100%)**	**224 (100%)**	**0/0**	
Mutations:	*none*								
**CDS**									
insA	1	59	64.1%	438	98.6%	59 (100%)	58 (98.3%)	0/0/0/0	0
**Proteins**									
IS1 family transposase (YP_006488.1)	1	59	64.1%	438	98.6%	59 (100%)	58 (98.3%)	0/0/0/0	0
Protein mutations:	*none*								
Codon mutations:	*none*								

*: Inserts / Deletes / Misaligned / Frameshifts

Analysis details

This analysis was performed with panviral2.64

NGS Details (UN60): Harvey murine sarcoma virus

Assembly

**Coverage Length**	141 (1 contig(s))
**Depth Of Coverage**	1.2
**Number Of Reads**	2
**Reads Per Million**	0.04 rpm (after QC)
**Ambiguities**	0
**Assembly Method**	de novo + reference guided assembly
**Consensus Caller**	Bcf Tools

Coverage Map

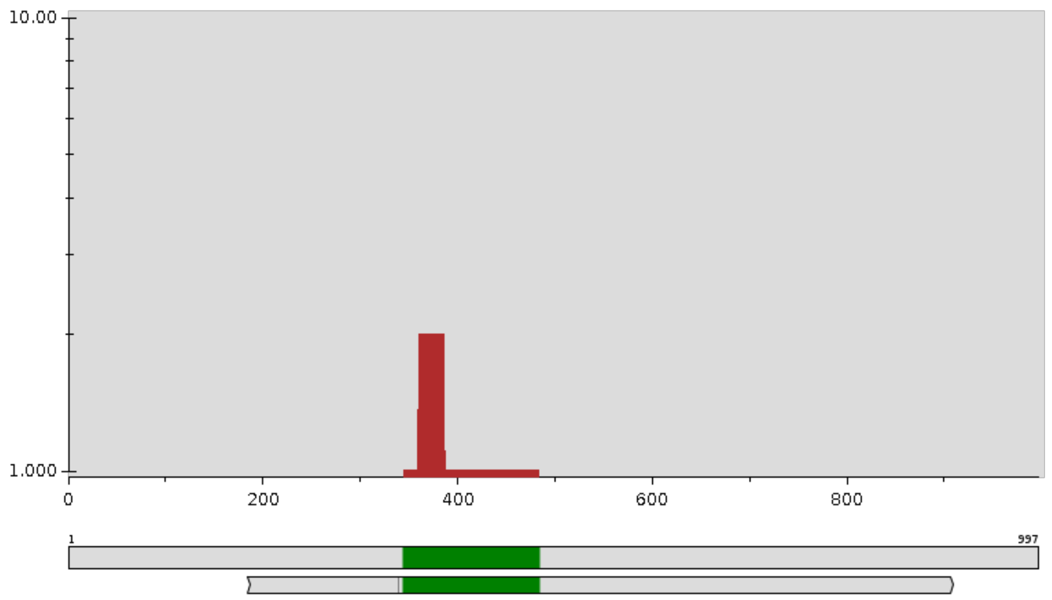

Assignment

**Type**	Harvey murine sarcoma virus (Taxonomy ID: 11807)
**Reference Genome**	NC_038668.1
**NT Identity (%)**	69.5035
**AA Identity (%)**	85.1064
**Number Of Stop Codons**	0
**Number Of CDS**	2

Alignment

**Alignment Score**	110.0 (NT) + 516.0 (AA) = 626.0
**Concordance (%)**	70.3371

**Alignment Method**	Global, seeded, nucleotide + amino acids (AGA)

Genome Region

Sequence starts at position 345 and ends at position 485 relative to NC_038668.1 reference sequence.

Alignment Detailed Statistics

	Begin	End	Coverage	Score	Concordance	Matches	Identities	I/D/M/F*	Stop Codons
**NT**	**345**	**485**	**14.1%**	**110**	**39.0%**	**141 (100%)**	**98 (69.5%)**	**0/0**	
Mutations:	345C>G, 349A>G, 358T>C, 361G>T, 364G>C, 367G>C, 370C>A, 372C>G, 373T>C, 374A>G, 379C>G, 389A>T, 390G>C, 391T>A, 392G>T, 393C>G, 394C>T, 397G>T, 400C>G, 406G>A, 407C>T, 409G>A, 412C>T, 415G>A, 417A>G, 418C>T, 424T>C, 427G>A, 430C>T, 433G>A, 442C>G, 445T>A, 448A>C, 451G>A, 457C>A, 463G>T, 469G>A, 470G>T, 471T>G, 472A>C, 475C>G, 481T>C, 483G>A								
**CDS**									
D1R95_gp1	55	101	19.4%	258	81.6%	47 (100%)	40 (85.1%)	0/0/0/0	0
Protein mutations:	A63G (372C>G 373T>C), R64G (374A>G), A70C (392G>T 393C>G 394C>T), N78S (417A>G 418C>T), V96C (470G>T 471T>G 472A>C), G100E (483G>A)								
Codon mutations:	ACA54.GA (345C>G), GAA55GAG (349A>G), CTT58CTC (358T>C), GTG59GTT (361G>T), GTG60GTC (364G>C), GTG61GTC (367G>C), GGC62GGA (370C>A), GCT63GGC (372C>G 373T>C), AGA64GGA (374A>G), GGC65GGG (379C>G), AGT69TCA (389A>T 390G>C 391T>A), GCC70TGT (392G>T 393C>G 394C>T), CTG71CTT (397G>T), ACC72ACG (400C>G), CAG74CAA (406G>A), CTG75TTA (407C>T 409G>A), ATC76ATT (412C>T), CAG77CAA (415G>A), AAC78AGT (417A>G 418C>T), TTT80TTC (424T>C), GTG81GTA (427G>A), GAC82GAT (430C>T), GAG83GAA (433G>A), CCC86CCG (442C>G), ACT87ACA (445T>A), ATA88ATC (448A>C), GAG89GAA (451G>A), TCC91TCA (457C>A), CGG93CGT (463G>T), CAG95CAA (469G>A), GTA96TGC (470G>T 471T>G 472A>C), GTC97GTG (475C>G), GAT99GAC (481T>C), GGG100GAG (483G>A)								
D1R95_gp2	3	49	24.7%	258	81.6%	47 (100%)	40 (85.1%)	0/0/0/0	0
Protein mutations:	A11G (372C>G 373T>C), R12G (374A>G), A18C (392G>T 393C>G 394C>T), N26S (417A>G 418C>T), V44C (470G>T 471T>G 472A>C), G48E (483G>A)								
Codon mutations:	ACA2.GA (345C>G), GAA3GAG (349A>G), CTT6CTC (358T>C), GTG7GTT (361G>T), GTG8GTC (364G>C), GTG9GTC (367G>C), GGC10GGA (370C>A), GCT11GGC (372C>G 373T>C), AGA12GGA (374A>G), GGC13GGG (379C>G), AGT17TCA (389A>T 390G>C 391T>A), GCC18TGT (392G>T 393C>G 394C>T), CTG19CTT (397G>T), ACC20ACG (400C>G), CAG22CAA (406G>A), CTG23TTA (407C>T 409G>A), ATC24ATT (412C>T), CAG25CAA (415G>A), AAC26AGT (417A>G 418C>T), TTT28TTC (424T>C), GTG29GTA (427G>A), GAC30GAT (430C>T), GAG31GAA (433G>A), CCC34CCG (442C>G), ACT35ACA (445T>A), ATA36ATC (448A>C), GAG37GAA (451G>A), TCC39TCA (457C>A), CGG41CGT (463G>T), CAG43CAA (469G>A), GTA44TGC (470G>T 471T>G 472A>C), GTC45GTG (475C>G), GAT47GAC (481T>C), GGG48GAG (483G>A)								
**Proteins**									
hypothetical protein (YP_009507788.1)	55	101	19.4%	258	81.6%	47 (100%)	40 (85.1%)	0/0/0/0	0
Protein mutations:	A63G (372C>G 373T>C), R64G (374A>G), A70C (392G>T 393C>G 394C>T), N78S (417A>G 418C>T), V96C (470G>T 471T>G 472A>C), G100E (483G>A)								
Codon mutations:	ACA54.GA (345C>G), GAA55GAG (349A>G), CTT58CTC (358T>C), GTG59GTT (361G>T), GTG60GTC (364G>C), GTG61GTC (367G>C), GGC62GGA (370C>A), GCT63GGC (372C>G 373T>C), AGA64GGA (374A>G), GGC65GGG (379C>G), AGT69TCA (389A>T 390G>C 391T>A), GCC70TGT (392G>T 393C>G 394C>T), CTG71CTT (397G>T), ACC72ACG (400C>G), CAG74CAA (406G>A), CTG75TTA (407C>T 409G>A), ATC76ATT (412C>T), CAG77CAA (415G>A), AAC78AGT (417A>G 418C>T), TTT80TTC (424T>C), GTG81GTA (427G>A), GAC82GAT (430C>T), GAG83GAA (433G>A), CCC86CCG (442C>G), ACT87ACA (445T>A), ATA88ATC (448A>C), GAG89GAA (451G>A), TCC91TCA (457C>A), CGG93CGT (463G>T), CAG95CAA (469G>A), GTA96TGC (470G>T 471T>G 472A>C), GTC97GTG (475C>G), GAT99GAC (481T>C), GGG100GAG (483G>A)								
hypothetical protein (YP_009507789.1)	3	49	24.7%	258	81.6%	47 (100%)	40 (85.1%)	0/0/0/0	0
Protein mutations:	A11G (372C>G 373T>C), R12G (374A>G), A18C (392G>T 393C>G 394C>T), N26S (417A>G 418C>T), V44C (470G>T 471T>G 472A>C), G48E (483G>A)								
Codon mutations:	ACA2.GA (345C>G), GAA3GAG (349A>G), CTT6CTC (358T>C), GTG7GTT (361G>T), GTG8GTC (364G>C), GTG9GTC (367G>C), GGC10GGA (370C>A), GCT11GGC (372C>G 373T>C), AGA12GGA (374A>G), GGC13GGG (379C>G), AGT17TCA (389A>T 390G>C 391T>A), GCC18TGT (392G>T 393C>G 394C>T), CTG19CTT (397G>T), ACC20ACG (400C>G), CAG22CAA (406G>A), CTG23TTA (407C>T 409G>A), ATC24ATT (412C>T), CAG25CAA (415G>A), AAC26AGT (417A>G 418C>T), TTT28TTC (424T>C), GTG29GTA (427G>A), GAC30GAT (430C>T), GAG31GAA (433G>A), CCC34CCG (442C>G), ACT35ACA (445T>A), ATA36ATC (448A>C), GAG37GAA (451G>A), TCC39TCA (457C>A), CGG41CGT (463G>T), CAG43CAA (469G>A), GTA44TGC (470G>T 471T>G 472A>C), GTC45GTG (475C>G), GAT47GAC (481T>C), GGG48GAG (483G>A)								

*: Inserts / Deletes / Misaligned / Frameshifts

Analysis details

This analysis was performed with panviral2.64

NGS Details (UN60): Ochrobactrum phage POA1180

Assembly

**Coverage Length**	119 (1 contig(s))
**Depth Of Coverage**	2.0
**Number Of Reads**	2
**Reads Per Million**	0.04 rpm (after QC)
**Ambiguities**	0
**Assembly Method**	de novo + reference guided assembly
**Consensus Caller**	Bcf Tools

Coverage Map

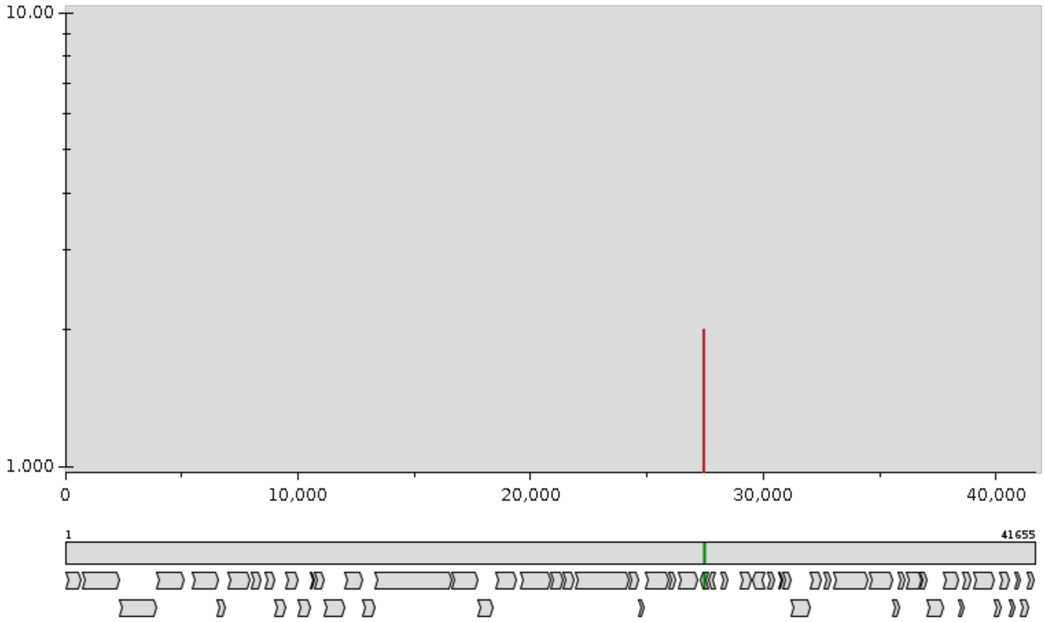

Assignment

**Type**	Ochrobactrum phage POA1180 (Taxonomy ID: 1897640)
**Reference Genome**	NC_070931.1
**NT Identity (%)**	74.7899
**AA Identity (%)**	80.0
**Number Of Stop Codons**	0
**Number Of CDS**	58

Alignment

**Alignment Score**	118.0 (NT) + 250.0 (AA) = 368.0
**Concordance (%)**	70.0952

Alignment Method	Local, heuristic, nucleotide (BLASTN)

Genome Region

Sequence starts at position 27400 and ends at position 27518 relative to NC_070931.1 reference sequence.

Alignment Detailed Statistics

	Begin	End	Coverage	Score	Concordance	Matches	Identities	I/D/M/F*	Stop Codons
**NT**	**27400**	**27518**	**0.3%**	**118**	**49.6%**	**119 (100%)**	**89 (74.8%)**	**0/0**	
Mutations:	27417C>G, 27418G>C, 27419T>C, 27420G>C, 27423G>C, 27424C>G, 27425C>A, 27426G>C, 27428T>C, 27435T>C, 27436T>C, 27437T>G, 27447G>C, 27449T>G, 27453C>G, 27465G>C, 27468A>G, 27474G>C, 27477G>C, 27480G>C, 27492G>C, 27493C>G, 27494T>A, 27495C>G, 27498G>C, 27501C>G, 27507C>G, 27510C>A, 27511G>A, 27512C>G								
**CDS**									
PQB33_gp31	30	69	36.7%	250	85.9%	40 (100%)	32 (80.0%)	0/0/0/0	0
Protein mutations:	A32L (27510C>A 27511G>A 27512C>G), I53L (27447G>C 27449T>G), K57R (27435T>C 27436T>C 27437T>G), I60V (27426G>C 27428T>C), G61S (27423G>C 27424C>G 27425C>A), D62E (27420G>C), T63G (27417C>G 27418G>C 27419T>C)								
Codon mutations:	GCG32CTT (27510C>A 27511G>A 27512C>G), CGG33CGC (27507C>G), GCG35GCC (27501C>G), GCC36GCG (27498G>C), CTG37CTC (27495C>G), AGC38TCG (27492G>C 27493C>G 27494T>A), CCC42CCG (27480G>C), GCC43GCG (27477G>C), GGC44GGG (27474G>C), ATT46ATC (27468A>G), GCC47GCG (27465G>C), CGG51CGC (27453C>G), ATC53CTG (27447G>C 27449T>G), AAA57CGG (27435T>C 27436T>C 27437T>G), ATC60GTG (27426G>C 27428T>C), GGC61TCG (27423G>C 27424C>G 27425C>A), GAC62GAG (27420G>C), ACG63GGC (27417C>G 27418G>C 27419T>C)								
**Proteins**									
chromate transport protein (YP_010665114.1)	30	69	36.7%	250	85.9%	40 (100%)	32 (80.0%)	0/0/0/0	0
Protein mutations:	A32L (27510C>A 27511G>A 27512C>G), I53L (27447G>C 27449T>G), K57R (27435T>C 27436T>C 27437T>G), I60V (27426G>C 27428T>C), G61S (27423G>C 27424C>G 27425C>A), D62E (27420G>C), T63G (27417C>G 27418G>C 27419T>C)								
Codon mutations:	GCG32CTT (27510C>A 27511G>A 27512C>G), CGG33CGC (27507C>G), GCG35GCC (27501C>G), GCC36GCG (27498G>C), CTG37CTC (27495C>G), AGC38TCG (27492G>C 27493C>G 27494T>A), CCC42CCG (27480G>C), GCC43GCG (27477G>C), GGC44GGG (27474G>C), ATT46ATC (27468A>G), GCC47GCG (27465G>C), CGG51CGC (27453C>G), ATC53CTG (27447G>C 27449T>G), AAA57CGG (27435T>C 27436T>C 27437T>G), ATC60GTG (27426G>C 27428T>C), GGC61TCG (27423G>C 27424C>G 27425C>A), GAC62GAG (27420G>C), ACG63GGC (27417C>G 27418G>C 27419T>C)								

*: Inserts / Deletes / Misaligned / Frameshifts

Analysis details

This analysis was performed with panviral2.64

NGS Details (UN60): Pseudomonas phage PA8

Assembly

**Coverage Length**	135 (1 contig(s))
**Depth Of Coverage**	2.0
**Number Of Reads**	2
**Reads Per Million**	0.04 rpm (after QC)
**Ambiguities**	0
**Assembly Method**	de novo + reference guided assembly
**Consensus Caller**	Bcf Tools

Coverage Map

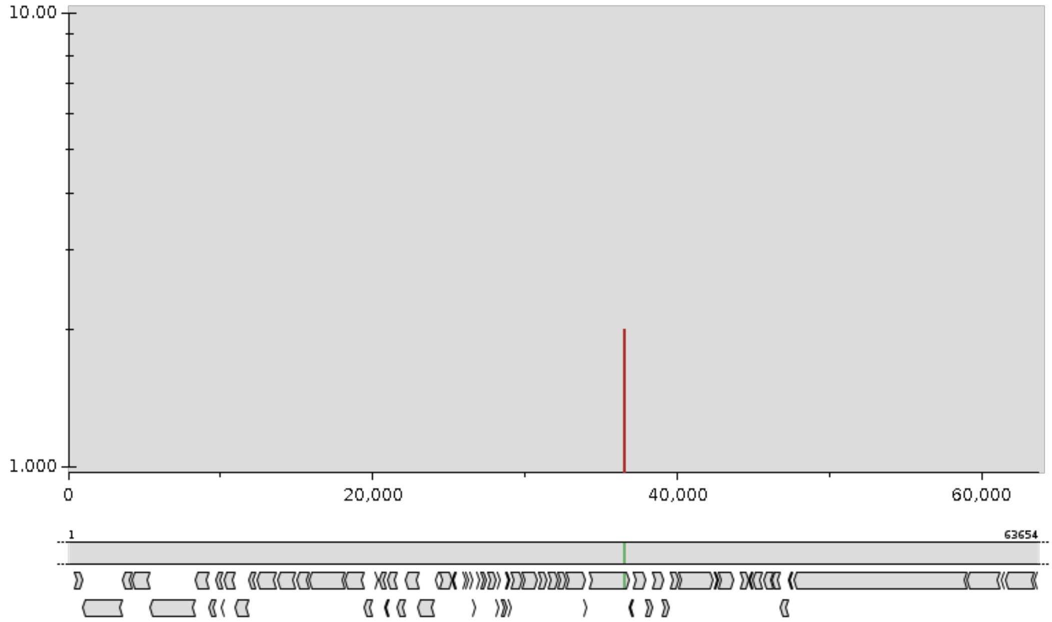

Assignment

**Type**	Pseudomonas phage PA8 (Taxonomy ID: 2860997)
**Reference Genome**	NC_073672.1
**NT Identity (%)**	82.963
**AA Identity (%)**	80.0
**Number Of Stop Codons**	0
**Number Of CDS**	69

Alignment

**Alignment Score**	178.0 (NT) + 275.0 (AA) = 453.0
**Concordance (%)**	74.0196

Alignment Method	Local, heuristic, nucleotide (BLASTN)

Genome Region

Sequence starts at position 36438 and ends at position 36572 relative to NC_073672.1 reference sequence.

Alignment Detailed Statistics

	Begin	End	Coverage	Score	Concordance	Matches	Identities	I/D/M/F*	Stop Codons
**NT**	**36438**	**36572**	**0.2%**	**178**	**65.9%**	**135 (100%)**	**112 (83.0%)**	**0/0**	
Mutations:	36444C>G, 36449C>G, 36461A>G, 36462T>G, 36469A>C, 36470G>C, 36472T>A, 36473C>G, 36474A>C, 36475C>G, 36479G>C, 36481A>T, 36492G>A, 36493G>C, 36508C>A, 36516T>A, 36517C>G, 36524C>T, 36533A>C, 36545C>G, 36546G>A, 36548C>G, 36557G>C								

CDS

	Begin	End	Coverage	Score	Concordance	Matches	Identities	I/D/M/F*	Stop Codons
QGM55_gp48	733	777	5.1%	275	80.4%	45 (100%)	36 (80.0%)	0/0/0/0	0
Protein mutations:	H735D (36444C>G), S741A (36462T>G), E743A (36469A>C 36470G>C), V744E (36472T>A 36473C>G), T745R (36474A>C 36475C>G), K747M (36481A>T), G751T (36492G>A 36493G>C), P756Q (36508C>A), A769T (36546G>A 36548C>G)								
Codon mutations:	CAC735GAC (36444C>G), GTC736GTG (36449C>G), GAA740GAG (36461A>G), TCG741GCG (36462T>G), GAG743GCC (36469A>C 36470G>C), GTC744GAG (36472T>A 36473C>G), ACC745CGC (36474A>C 36475C>G), GGG746GGC (36479G>C), AAG747ATG (36481A>T), GGC751ACC (36492G>A 36493G>C), CCG756CAG (36508C>A), TCC759AGC (36516T>A 36517C>G), CAC761CAT (36524C>T), GTA764GTC (36533A>C), GTC768GTG (36545C>G), GCC769ACG (36546G>A 36548C>G), CGG772CGC (36557G>C)								

Proteins

	Begin	End	Coverage	Score	Concordance	Matches	Identities	I/D/M/F*	Stop Codons
hypothetical protein (YP_010765738.1)	733	777	5.1%	275	80.4%	45 (100%)	36 (80.0%)	0/0/0/0	0
Protein mutations:	H735D (36444C>G), S741A (36462T>G), E743A (36469A>C 36470G>C), V744E (36472T>A 36473C>G), T745R (36474A>C 36475C>G), K747M (36481A>T), G751T (36492G>A 36493G>C), P756Q (36508C>A), A769T (36546G>A 36548C>G)								
Codon mutations:	CAC735GAC (36444C>G), GTC736GTG (36449C>G), GAA740GAG (36461A>G), TCG741GCG (36462T>G), GAG743GCC (36469A>C 36470G>C), GTC744GAG (36472T>A 36473C>G), ACC745CGC (36474A>C 36475C>G), GGG746GGC (36479G>C), AAG747ATG (36481A>T), GGC751ACC (36492G>A 36493G>C), CCG756CAG (36508C>A), TCC759AGC (36516T>A 36517C>G), CAC761CAT (36524C>T), GTA764GTC (36533A>C), GTC768GTG (36545C>G), GCC769ACG (36546G>A 36548C>G), CGG772CGC (36557G>C)								

*: Inserts / Deletes / Misaligned / Frameshifts

Analysis details

This analysis was performed with panviral2.64

NGS Details (UN60): Tomato associated geminivirus 1

Assembly

**Coverage Length**	233 (1 contig(s))
**Depth Of Coverage**	1.2
**Number Of Reads**	2
**Reads Per Million**	0.04 rpm (after QC)
**Ambiguities**	0
**Assembly Method**	de novo + reference guided assembly
**Consensus Caller**	Bcf Tools

Coverage Map

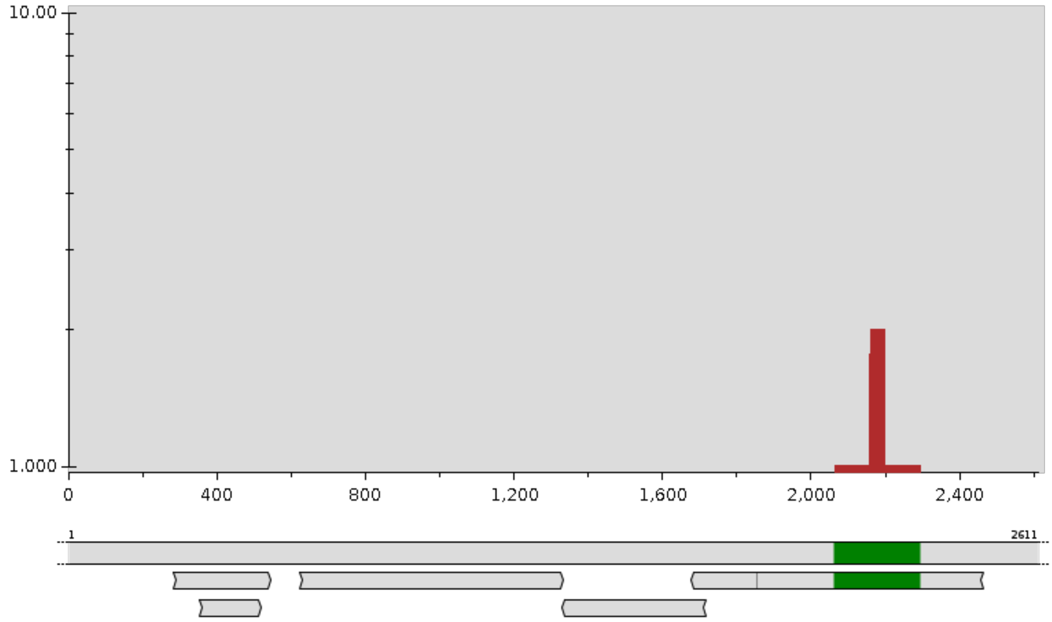

Assignment

**Type**	Tomato associated geminivirus 1 (Taxonomy ID: 2027645)
**Reference Genome**	NC_035676.1
**NT Identity (%)**	72.9614
**AA Identity (%)**	75.3247
**Number Of Stop Codons**	4
**Number Of CDS**	5

Alignment

**Alignment Score**	214.0 (NT) + 844.0 (AA) = 1058.0
**Concordance (%)**	64.7491

Alignment Method	Global, seeded, nucleotide + amino acids (AGA)

Genome Region

Sequence starts at position 2064 and ends at position 2296 relative to NC_035676.1 reference sequence.

Alignment Detailed Statistics

	Begin	End	Coverage	Score	Concordance	Matches	Identities	I/D/M/F*	Stop Codons
**NT**	**2064**	**2296**	**8.9%**	**214**	**45.9%**	**233 (100%)**	**170 (73.0%)**	**0/0**	
Mutations:	2065C>G, 2067T>A, 2070C>T, 2073G>T, 2076A>T, 2078C>A, 2079G>A, 2082T>C, 2084T>G, 2086T>G, 2087T>A, 2088C>T, 2094C>A, 2102T>A, 2105G>T, 2106G>T, 2118A>T, 2120T>A, 2121A>T, 2126T>G, 2136G>C, 2137G>T, 2138T>G, 2150A>C, 2151A>G, 2157G>A, 2160C>G, 2163A>G, 2175C>T, 2177T>G, 2190G>A, 2191A>C, 2192T>A, 2196T>G, 2201G>C, 2205A>G, 2209C>G, 2210C>T, 2216C>T, 2220A>T, 2223G>A, 2227G>A, 2228T>C, 2229G>A, 2241C>A, 2243T>A, 2244C>T, 2247G>A, 2251T>C, 2252G>T, 2253C>G, 2256C>G, 2258G>T, 2263C>T, 2264T>G, 2268C>T, 2271A>C, 2272G>A, 2277T>G, 2280C>A, 2289G>A, 2291T>G, 2295T>C								
**CDS**									
CK833_gp4	58	134	23.1%	422	70.9%	77 (100%)	58 (75.3%)	0/0/0/0	2
Protein mutations:	I59L (2289G>A 2291T>G), T65M (2271A>C 2272G>A), R68Q (2263C>T 2264T>G), Q70N (2256C>G 2258G>T), Q72R (2251T>C 2252G>T), R75C (2241C>A 2243T>A), T80V (2227G>A 2228T>C), G84R (2216C>T), G86T (2209C>G 2210C>T), H89D (2201G>C), I92C (2190G>A 2191A>C 2192T>A), S106A (2150A>C), T110Q (2136G>C 2137G>T 2138T>G), I116L (2118A>T 2120T>A), R122* (2102T>A), N127S (2086T>G 2087T>A), A130S (2076A>T 2078C>A), W132* (2070C>T), R134T (2065C>G)								
Codon mutations:	CCA57.CG (2295T>C), ATC59CTT (2289G>A 2291T>G), CTG62CTT (2280C>A), GTA63GTC (2277T>G), ACT65ATG (2271A>C 2272G>A), GAG66GAA (2268C>T), AGG68CAG (2263C>T 2264T>G), CAG70AAC (2256C>G 2258G>T), ACG71ACC (2253C>G), CAG72AGG (2251T>C 2252G>T), AAC73AAT (2247G>A), CAG74CAA (2244C>T), AGG75TGT (2241C>A 2243T>A), ATC79ATT (2229G>A), ACC80GTC (2227G>A 2228T>C), GAC81GAT (2223G>A), CCT82CCA (2220A>T), GGA84AGA (2216C>T), GGA86ACA (2209C>G 2210C>T), ATT87ATC (2205A>G), CAT89GAT (2201G>C), CCA90CCC (2196T>G), ATC92TGT (2190G>A 2191A>C 2192T>A), AGG97CGA (2175C>T 2177T>G), GTT101GTC (2163A>G), GCG102GCC (2160C>G), GAC103GAT (2157G>A), ATT105ATC (2151A>G), TCC106GCC (2150A>C), ACC110CAG (2136G>C 2137G>T 2138T>G), AGA114CGA (2126T>G), GGT115GGA (2121A>T), ATT116TTA (2118A>T 2120T>A), TCC120TCA (2106G>T), CGA121AGA (2105G>T), AGA122TGA (2102T>A), CCG124CCT (2094C>A), AAG126AAA (2088C>T), AAT127TCT (2086T>G 2087T>A), AGA128CGG (2082T>C 2084T>G), GAC129GAT (2079G>A), GCT130TCA (2076A>T 2078C>A), ATC131ATA (2073G>T), TGG132TGA (2070C>T), ACA133ACT (2067T>A), AGA134ACA (2065C>G)								
CK833_gp5	58	134	29.4%	422	70.9%	77 (100%)	58 (75.3%)	0/0/0/0	2
Protein mutations:	I59L (2289G>A 2291T>G), T65M (2271A>C 2272G>A), R68Q (2263C>T 2264T>G), Q70N (2256C>G 2258G>T), Q72R (2251T>C 2252G>T), R75C (2241C>A 2243T>A), T80V (2227G>A 2228T>C), G84R (2216C>T), G86T (2209C>G 2210C>T), H89D (2201G>C), I92C (2190G>A 2191A>C 2192T>A), S106A (2150A>C), T110Q (2136G>C 2137G>T 2138T>G), I116L (2118A>T 2120T>A), R122* (2102T>A), N127S (2086T>G 2087T>A), A130S (2076A>T 2078C>A), W132* (2070C>T), R134T (2065C>G)								
Codon mutations:	CCA57.CG (2295T>C), ATC59CTT (2289G>A 2291T>G), CTG62CTT (2280C>A), GTA63GTC (2277T>G), ACT65ATG (2271A>C 2272G>A), GAG66GAA (2268C>T), AGG68CAG (2263C>T 2264T>G), CAG70AAC (2256C>G 2258G>T), ACG71ACC (2253C>G), CAG72AGG (2251T>C 2252G>T), AAC73AAT (2247G>A), CAG74CAA (2244C>T), AGG75TGT (2241C>A 2243T>A), ATC79ATT (2229G>A), ACC80GTC (2227G>A 2228T>C), GAC81GAT (2223G>A), CCT82CCA (2220A>T), GGA84AGA (2216C>T), GGA86ACA (2209C>G 2210C>T), ATT87ATC (2205A>G), CAT89GAT (2201G>C), CCA90CCC (2196T>G), ATC92TGT (2190G>A 2191A>C 2192T>A), AGG97CGA (2175C>T 2177T>G), GTT101GTC (2163A>G), GCG102GCC (2160C>G), GAC103GAT (2157G>A), ATT105ATC (2151A>G), TCC106GCC (2150A>C), ACC110CAG (2136G>C 2137G>T 2138T>G), AGA114CGA (2126T>G), GGT115GGA (2121A>T), ATT116TTA (2118A>T 2120T>A), TCC120TCA (2106G>T), CGA121AGA (2105G>T), AGA122TGA (2102T>A), CCG124CCT (2094C>A), AAG126AAA (2088C>T), AAT127TCT (2086T>G 2087T>A), AGA128CGG (2082T>C 2084T>G), GAC129GAT (2079G>A), GCT130TCA (2076A>T 2078C>A), ATC131ATA (2073G>T), TGG132TGA (2070C>T), ACA133ACT (2067T>A), AGA134ACA (2065C>G)								
**Proteins**									
replication-associated protein (YP_009417307.1)	58	134	23.1%	422	70.9%	77 (100%)	58 (75.3%)	0/0/0/0	2
Protein mutations:	I59L (2289G>A 2291T>G), T65M (2271A>C 2272G>A), R68Q (2263C>T 2264T>G), Q70N (2256C>G 2258G>T), Q72R (2251T>C 2252G>T), R75C (2241C>A 2243T>A), T80V (2227G>A 2228T>C), G84R (2216C>T), G86T (2209C>G 2210C>T), H89D (2201G>C), I92C (2190G>A 2191A>C 2192T>A), S106A (2150A>C), T110Q (2136G>C 2137G>T 2138T>G), I116L (2118A>T 2120T>A), R122* (2102T>A), N127S (2086T>G 2087T>A), A130S (2076A>T 2078C>A), W132* (2070C>T), R134T (2065C>G)								
Codon mutations:	CCA57.CG (2295T>C), ATC59CTT (2289G>A 2291T>G), CTG62CTT (2280C>A), GTA63GTC (2277T>G), ACT65ATG (2271A>C 2272G>A), GAG66GAA (2268C>T), AGG68CAG (2263C>T 2264T>G), CAG70AAC (2256C>G 2258G>T), ACG71ACC (2253C>G), CAG72AGG (2251T>C 2252G>T), AAC73AAT (2247G>A), CAG74CAA (2244C>T), AGG75TGT (2241C>A 2243T>A), ATC79ATT (2229G>A), ACC80GTC (2227G>A 2228T>C), GAC81GAT (2223G>A), CCT82CCA (2220A>T), GGA84AGA (2216C>T), GGA86ACA (2209C>G 2210C>T), ATT87ATC (2205A>G), CAT89GAT (2201G>C), CCA90CCC (2196T>G), ATC92TGT (2190G>A 2191A>C 2192T>A), AGG97CGA (2175C>T 2177T>G), GTT101GTC (2163A>G), GCG102GCC (2160C>G), GAC103GAT (2157G>A), ATT105ATC (2151A>G), TCC106GCC (2150A>C), ACC110CAG (2136G>C 2137G>T 2138T>G), AGA114CGA (2126T>G), GGT115GGA (2121A>T), ATT116TTA (2118A>T 2120T>A), TCC120TCA (2106G>T), CGA121AGA (2105G>T), AGA122TGA (2102T>A), CCG124CCT (2094C>A), AAG126AAA (2088C>T), AAT127TCT (2086T>G 2087T>A), AGA128CGG (2082T>C 2084T>G), GAC129GAT (2079G>A), GCT130TCA (2076A>T 2078C>A), ATC131ATA (2073G>T), TGG132TGA (2070C>T), ACA133ACT (2067T>A), AGA134ACA (2065C>G)								
RepA (YP_009417308.1)	58	134	29.4%	422	70.9%	77 (100%)	58 (75.3%)	0/0/0/0	2
Protein mutations:	I59L (2289G>A 2291T>G), T65M (2271A>C 2272G>A), R68Q (2263C>T 2264T>G), Q70N (2256C>G 2258G>T), Q72R (2251T>C 2252G>T), R75C (2241C>A 2243T>A), T80V (2227G>A 2228T>C), G84R (2216C>T), G86T (2209C>G 2210C>T), H89D (2201G>C), I92C (2190G>A 2191A>C 2192T>A), S106A (2150A>C), T110Q (2136G>C 2137G>T 2138T>G), I116L (2118A>T 2120T>A), R122* (2102T>A), N127S (2086T>G 2087T>A), A130S (2076A>T 2078C>A), W132* (2070C>T), R134T (2065C>G)								
Codon mutations:	CCA57.CG (2295T>C), ATC59CTT (2289G>A 2291T>G), CTG62CTT (2280C>A), GTA63GTC (2277T>G), ACT65ATG (2271A>C 2272G>A), GAG66GAA (2268C>T), AGG68CAG (2263C>T 2264T>G), CAG70AAC (2256C>G 2258G>T), ACG71ACC (2253C>G), CAG72AGG (2251T>C 2252G>T), AAC73AAT (2247G>A), CAG74CAA (2244C>T), AGG75TGT (2241C>A 2243T>A), ATC79ATT (2229G>A), ACC80GTC (2227G>A 2228T>C), GAC81GAT (2223G>A), CCT82CCA (2220A>T), GGA84AGA (2216C>T), GGA86ACA (2209C>G 2210C>T), ATT87ATC (2205A>G), CAT89GAT (2201G>C), CCA90CCC (2196T>G), ATC92TGT (2190G>A 2191A>C 2192T>A), AGG97CGA (2175C>T 2177T>G), GTT101GTC (2163A>G), GCG102GCC (2160C>G), GAC103GAT (2157G>A), ATT105ATC (2151A>G), TCC106GCC (2150A>C), ACC110CAG (2136G>C 2137G>T 2138T>G), AGA114CGA (2126T>G), GGT115GGA (2121A>T), ATT116TTA (2118A>T 2120T>A), TCC120TCA (2106G>T), CGA121AGA (2105G>T), AGA122TGA (2102T>A), CCG124CCT (2094C>A), AAG126AAA (2088C>T), AAT127TCT (2086T>G 2087T>A), AGA128CGG (2082T>C 2084T>G), GAC129GAT (2079G>A), GCT130TCA (2076A>T 2078C>A), ATC131ATA (2073G>T), TGG132TGA (2070C>T), ACA133ACT (2067T>A), AGA134ACA (2065C>G)								

*: Inserts / Deletes / Misaligned / Frameshifts

Analysis details

This analysis was performed with panviral2.64

NGS Details (UN60): Euphorbia caput-medusae latent virus

Assembly

**Coverage Length**	147 (3 contig(s))
**Depth Of Coverage**	1.0
**Number Of Reads**	1
**Reads Per Million**	0.02 rpm (after QC)
**Ambiguities**	0
**Assembly Method**	de novo + reference guided assembly
**Consensus Caller**	Bcf Tools

Coverage Map

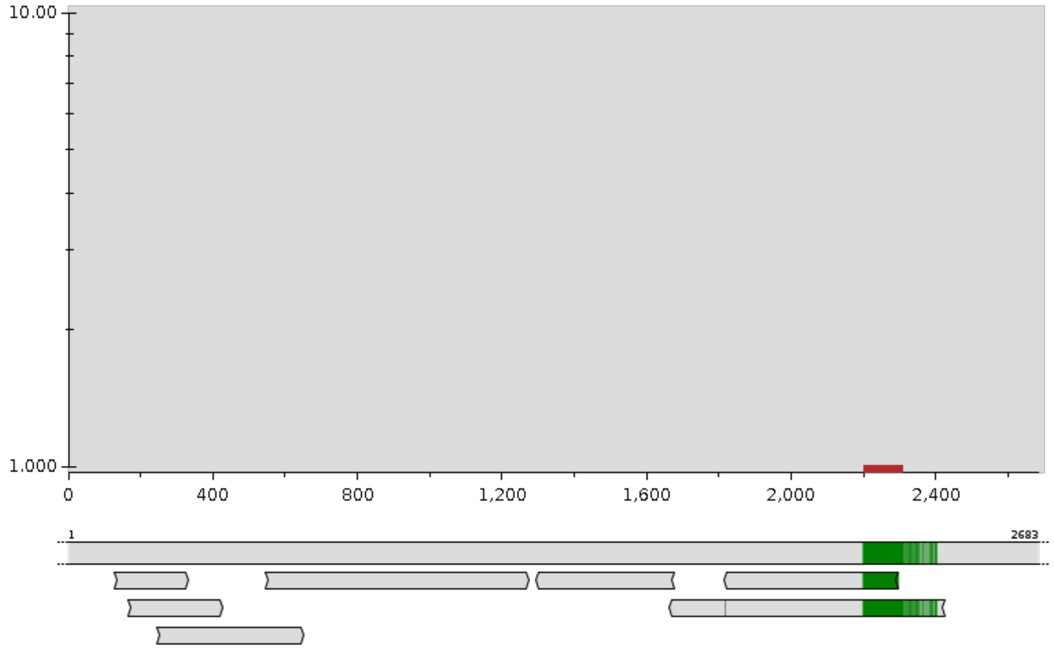

Assignment

**Type**	Euphorbia caput-medusae latent virus (Taxonomy ID: 1853865)
**Reference Genome**	NC_030238.1
**NT Identity (%)**	47.619
**AA Identity (%)**	45.3704
**Number Of Stop Codons**	6
**Number Of CDS**	7

Alignment

**Alignment Score**	-14.0 (NT) + 366.0 (AA) = 352.0
**Concordance (%)**	31.6832

**Alignment Method**	Global, seeded, nucleotide + amino acids (AGA)

Genome Region

Sequence starts at position 2201 and ends at position 2402 relative to NC_030238.1 reference sequence.

Alignment Detailed Statistics

	Begin	End	Coverage	Score	Concordance	Matches	Identities	I/D/M/F*	Stop Codons
**NT**	**2201**	**2402**	**5.5%**	**-14**	**-4.8%**	**147 (100%)**	**70 (47.6%)**	**0/0**	
Mutations:	2201A>G, 2204G>T, 2206T>A, 2211G>T, 2212G>A, 2214T>A, 2215G>A, 2217G>A, 2219T>G, 2222G>A, 2223C>T, 2224C>T, 2229G>C, 2231C>A, 2232C>G, 2233A>G, 2238G>A, 2239A>C, 2240C>A, 2241A>T, 2243G>T, 2253G>A, 2259C>T, 2262A>C, 2265G>T, 2271G>A, 2274A>T, 2275T>G, 2277C>T, 2279C>A, 2280C>T, 2283A>G, 2284C>G, 2286T>A, 2289C>T, 2293G>A, 2294C>T, 2298T>A, 2299G>T, 2307A>G, 2308A>T, 2312T>C, 2313A>T, 2315T>G, 2318A>G, 2319T>A, 2321G>T, 2324G>T, 2325A>T, 2327A>T, 2328T>G, 2330A>G, 2331A>G, 2332T>A, 2334A>G, 2337A>T, 2340T>G, 2342A>C, 2343A>G, 2346A>T, 2349A>T, 2351G>C, 2352A>G, 2354G>T, 2355A>T, 2364T>C, 2367T>G, 2376C>G, 2382A>T, 2384T>G, 2385A>T, 2388C>T, 2391A>T, 2397T>A, 2399A>G, 2400G>T, 2402G>T								

CDS

	Begin	End	Coverage	Score	Concordance	Matches	Identities	I/D/M/F*	Stop Codons
A9K86_gp5	40	77	11.4%	160	34.6%	38 (100%)	19 (50.0%)	0/0/0/0	2
Protein mutations:	N40D (2312T>C), F41Y (2307A>G 2308A>T), T44N (2298T>A 2299G>T), A46I (2293G>A 2294C>T), C49S (2283A>G 2284C>G), E51* (2277C>T 2279C>A), N52T (2274A>T 2275T>G), D55E (2265G>T), L63I (2241A>T 2243G>T), V64C (2238G>A 2239A>C 2240C>A), M66T (2232C>G 2233A>G), D67* (2229G>C 2231C>A), R69K (2223C>T 2224C>T), L70F (2222G>A), N71H (2217G>A 2219T>G), T72I (2214T>A 2215G>A), T73I (2211G>T 2212G>A), Q75L (2206T>A)								
Codon mutations:	CGC10A.A (2400G>T 2402G>T), TTA11C.T (2397T>A 2399A>G), GGT13..A (2391A>T), AAG14..A (2388C>T), TCT15..A (2385A>T), ATT16C.A (2382A>T 2384T>G), CTG18..C (2376C>G), CCA21..C (2367T>G), AAA22..G (2364T>C), CTT25..A (2355A>T), CTT26A.C (2352A>G 2354G>T), CCT27G.A (2349A>T 2351G>C), ATT28..A (2346A>T), TTT29..C (2343A>G), TTA30G.C (2340T>G 2342A>C), ATT31..A (2337A>T), GAT32..C (2334A>G), TAT33.TC (2331A>G 2332T>A), TTA34C.C (2328T>G 2330A>G), TAT35A.A (2325A>T 2327A>T), CAA36A.. (2324G>T), CTA37A.T (2319T>A 2321G>T), TTA38C.. (2318A>G), AAT39C.A (2313A>T 2315T>G), AAT40GAT (2312T>C), TTT41TAC (2307A>G 2308A>T), ACA44AAT (2298T>A 2299G>T), GCT46ATT (2293G>A 2294C>T), AGG47AGA (2289C>T), GTA48GTT (2286T>A), TGT49TCC (2283A>G 2284C>G), ACG50ACA (2280C>T), GAG51TAA (2277C>T 2279C>A), AAT52ACA (2274A>T 2275T>G), CAC53CAT (2271G>A), GAC55GAA (2265G>T), GGT56GGG (2262A>C), GAG57GAA (2259C>T), CAC59CAT (2253G>A), CTT63ATA (2241A>T 2243G>T), GTC64TGT (2238G>A 2239A>C 2240C>A), ATG66ACC (2232C>G 2233A>G), GAC67TAG (2229G>C 2231C>A), AGG69AAA (2223C>T 2224C>T), CTC70TTC (2222G>A), AAC71CAT (2217G>A 2219T>G), ACA72ATT (2214T>A 2215G>A), ACC73ATA (2211G>T 2212G>A), CAA75CTA (2206T>A), CGG76AGG (2204G>T), TTC77C.. (2201A>G)								
A9K86_gp6	40	77	14.9%	160	34.6%	38 (100%)	19 (50.0%)	0/0/0/0	2
Protein mutations:	N40D (2312T>C), F41Y (2307A>G 2308A>T), T44N (2298T>A 2299G>T), A46I (2293G>A 2294C>T), C49S (2283A>G 2284C>G), E51* (2277C>T 2279C>A), N52T (2274A>T 2275T>G), D55E (2265G>T), L63I (2241A>T 2243G>T), V64C (2238G>A 2239A>C 2240C>A), M66T (2232C>G 2233A>G), D67* (2229G>C 2231C>A), R69K (2223C>T 2224C>T), L70F (2222G>A), N71H (2217G>A 2219T>G), T72I (2214T>A 2215G>A), T73I (2211G>T 2212G>A), Q75L (2206T>A)								
Codon mutations:	CGC10A.A (2400G>T 2402G>T), TTA11C.T (2397T>A 2399A>G), GGT13..A (2391A>T), AAG14..A (2388C>T), TCT15..A (2385A>T), ATT16C.A (2382A>T 2384T>G), CTG18..C (2376C>G), CCA21..C (2367T>G), AAA22..G (2364T>C), CTT25..A (2355A>T), CTT26A.C (2352A>G 2354G>T), CCT27G.A (2349A>T 2351G>C), ATT28..A (2346A>T), TTT29..C (2343A>G), TTA30G.C (2340T>G 2342A>C), ATT31..A (2337A>T), GAT32..C (2334A>G), TAT33.TC (2331A>G 2332T>A), TTA34C.C (2328T>G 2330A>G), TAT35A.A (2325A>T 2327A>T), CAA36A.. (2324G>T), CTA37A.T (2319T>A 2321G>T), TTA38C.. (2318A>G), AAT39C.A (2313A>T 2315T>G), AAT40GAT (2312T>C), TTT41TAC (2307A>G 2308A>T), ACA44AAT (2298T>A 2299G>T), GCT46ATT (2293G>A 2294C>T), AGG47AGA (2289C>T), GTA48GTT (2286T>A), TGT49TCC (2283A>G 2284C>G), ACG50ACA (2280C>T), GAG51TAA (2277C>T 2279C>A), AAT52ACA (2274A>T 2275T>G), CAC53CAT (2271G>A), GAC55GAA (2265G>T), GGT56GGG (2262A>C), GAG57GAA (2259C>T), CAC59CAT (2253G>A), CTT63ATA (2241A>T 2243G>T), GTC64TGT (2238G>A 2239A>C 2240C>A), ATG66ACC (2232C>G 2233A>G), GAC67TAG (2229G>C 2231C>A), AGG69AAA (2223C>T 2224C>T), CTC70TTC (2222G>A), AAC71CAT (2217G>A 2219T>G), ACA72ATT (2214T>A 2215G>A), ACC73ATA (2211G>T 2212G>A), CAA75CTA (2206T>A), CGG76AGG (2204G>T), TTC77C.. (2201A>G)								
A9K86_gp7	1	32	19.9%	46	20.8%	32 (100%)	11 (34.4%)	0/0/0/0	2
Protein mutations:	M1I (2294C>T), G3E (2289C>T), Y4F (2286T>A), V5P (2283A>G 2284C>G), R6H (2279C>A 2280C>T), R7K (2277C>T), I8H (2274A>T 2275T>G), T9I (2271G>A), T11K (2265G>T), V12G (2262A>C), S13N (2259C>T), T15I (2253G>A), L19Y (2240C>A 2241A>T), S20V (2238G>A 2239A>C), W22P (2231C>A 2232C>G 2233A>G), T23R (2229G>C), G25N (2222G>A 2223C>T 2224C>T), T27I (2217G>A), Q28L (2214T>A 2215G>A), P29* (2211G>T 2212G>A), N31* (2204G>T 2206T>A)								
Codon mutations:	ATG1ATA (2294C>T), CTA2TTA (2293G>A), GGG3GAG (2289C>T), TAT4TTT (2286T>A), GTA5CCA (2283A>G 2284C>G), CGG6CAT (2279C>A 2280C>T), AGA7AAA (2277C>T), ATC8CAC (2274A>T 2275T>G), ACC9ATC (2271G>A), ACG11AAG (2265G>T), GTG12GGG (2262A>C), AGC13AAC (2259C>T), ACC15ATC (2253G>A), GCC18GCA (2243G>T), TTG19TAT (2240C>A 2241A>T), TCC20GTC (2238G>A 2239A>C), TGG22CCT (2231C>A 2232C>G 2233A>G), ACA23AGA (2229G>C), GGC25AAT (2222G>A 2223C>T 2224C>T), TCA26TCC (2219T>G), ACA27ATA (2217G>A), CAA28TTA (2214T>A 2215G>A), CCA29TAA (2211G>T 2212G>A), AAC31TAA (2204G>T 2206T>A), GGT32GGC (2201A>G)								

Proteins

	Begin	End	Coverage	Score	Concordance	Matches	Identities	I/D/M/F*	Stop Codons
replication-associated protein (YP_009255247.1)	40	77	11.4%	160	34.6%	38 (100%)	19 (50.0%)	0/0/0/0	2
Protein mutations:	N40D (2312T>C), F41Y (2307A>G 2308A>T), T44N (2298T>A 2299G>T), A46I (2293G>A 2294C>T), C49S (2283A>G 2284C>G), E51* (2277C>T 2279C>A), N52T (2274A>T 2275T>G), D55E (2265G>T), L63I (2241A>T 2243G>T), V64C (2238G>A 2239A>C 2240C>A), M66T (2232C>G 2233A>G), D67* (2229G>C 2231C>A), R69K (2223C>T 2224C>T), L70F (2222G>A), N71H (2217G>A 2219T>G), T72I (2214T>A 2215G>A), T73I (2211G>T 2212G>A), Q75L (2206T>A)								
Codon mutations:	CGC10A.A (2400G>T 2402G>T), TTA11C.T (2397T>A 2399A>G), GGT13..A (2391A>T), AAG14..A (2388C>T), TCT15..A (2385A>T), ATT16C.A (2382A>T 2384T>G), CTG18..C (2376C>G), CCA21..C (2367T>G), AAA22..G (2364T>C), CTT25..A (2355A>T), CTT26A.C (2352A>G 2354G>T), CCT27G.A (2349A>T 2351G>C), ATT28..A (2346A>T), TTT29..C (2343A>G), TTA30G.C (2340T>G 2342A>C), ATT31..A (2337A>T), GAT32..C (2334A>G), TAT33.TC (2331A>G 2332T>A), TTA34C.C (2328T>G 2330A>G), TAT35A.A (2325A>T 2327A>T), CAA36A.. (2324G>T), CTA37A.T (2319T>A 2321G>T), TTA38C.. (2318A>G), AAT39C.A (2313A>T 2315T>G), AAT40GAT (2312T>C), TTT41TAC (2307A>G 2308A>T), ACA44AAT (2298T>A 2299G>T), GCT46ATT (2293G>A 2294C>T), AGG47AGA (2289C>T), GTA48GTT (2286T>A), TGT49TCC (2283A>G 2284C>G), ACG50ACA (2280C>T), GAG51TAA (2277C>T 2279C>A), AAT52ACA (2274A>T 2275T>G), CAC53CAT (2271G>A), GAC55GAA (2265G>T), GGT56GGG (2262A>C), GAG57GAA (2259C>T), CAC59CAT (2253G>A), CTT63ATA (2241A>T 2243G>T), GTC64TGT (2238G>A 2239A>C 2240C>A), ATG66ACC (2232C>G 2233A>G), GAC67TAG (2229G>C 2231C>A), AGG69AAA (2223C>T 2224C>T), CTC70TTC (2222G>A), AAC71CAT (2217G>A 2219T>G), ACA72ATT (2214T>A 2215G>A), ACC73ATA (2211G>T 2212G>A), CAA75CTA (2206T>A), CGG76AGG (2204G>T), TTC77C.. (2201A>G)								
RepA (YP_009255248.1)	40	77	14.9%	160	34.6%	38 (100%)	19 (50.0%)	0/0/0/0	2
Protein mutations:	N40D (2312T>C), F41Y (2307A>G 2308A>T), T44N (2298T>A 2299G>T), A46I (2293G>A 2294C>T), C49S (2283A>G 2284C>G), E51* (2277C>T 2279C>A), N52T (2274A>T 2275T>G), D55E (2265G>T), L63I (2241A>T 2243G>T), V64C (2238G>A 2239A>C 2240C>A), M66T (2232C>G 2233A>G), D67* (2229G>C 2231C>A), R69K (2223C>T 2224C>T), L70F (2222G>A), N71H (2217G>A 2219T>G), T72I (2214T>A 2215G>A), T73I (2211G>T 2212G>A), Q75L (2206T>A)								
Codon mutations:	CGC10A.A (2400G>T 2402G>T), TTA11C.T (2397T>A 2399A>G), GGT13..A (2391A>T), AAG14..A (2388C>T), TCT15..A (2385A>T), ATT16C.A (2382A>T 2384T>G), CTG18..C (2376C>G), CCA21..C (2367T>G), AAA22..G (2364T>C), CTT25..A (2355A>T), CTT26A.C (2352A>G 2354G>T), CCT27G.A (2349A>T 2351G>C), ATT28..A (2346A>T), TTT29..C (2343A>G), TTA30G.C (2340T>G 2342A>C), ATT31..A (2337A>T), GAT32..C (2334A>G), TAT33.TC (2331A>G 2332T>A), TTA34C.C (2328T>G 2330A>G), TAT35A.A (2325A>T 2327A>T), CAA36A.. (2324G>T), CTA37A.T (2319T>A 2321G>T), TTA38C.. (2318A>G), AAT39C.A (2313A>T 2315T>G), AAT40GAT (2312T>C), TTT41TAC (2307A>G 2308A>T), ACA44AAT (2298T>A 2299G>T), GCT46ATT (2293G>A 2294C>T), AGG47AGA (2289C>T), GTA48GTT (2286T>A), TGT49TCC (2283A>G 2284C>G), ACG50ACA (2280C>T), GAG51TAA (2277C>T 2279C>A), AAT52ACA (2274A>T 2275T>G), CAC53CAT (2271G>A), GAC55GAA (2265G>T), GGT56GGG (2262A>C), GAG57GAA (2259C>T), CAC59CAT (2253G>A), CTT63ATA (2241A>T 2243G>T), GTC64TGT (2238G>A 2239A>C 2240C>A), ATG66ACC (2232C>G 2233A>G), GAC67TAG (2229G>C 2231C>A), AGG69AAA (2223C>T 2224C>T), CTC70TTC (2222G>A), AAC71CAT (2217G>A 2219T>G), ACA72ATT (2214T>A 2215G>A), ACC73ATA (2211G>T 2212G>A), CAA75CTA (2206T>A), CGG76AGG (2204G>T), TTC77C.. (2201A>G)								
C3 (YP_009255249.1)	1	32	19.9%	46	20.8%	32 (100%)	11 (34.4%)	0/0/0/0	2
Protein mutations:	M1I (2294C>T), G3E (2289C>T), Y4F (2286T>A), V5P (2283A>G 2284C>G), R6H (2279C>A 2280C>T), R7K (2277C>T), I8H (2274A>T 2275T>G), T9I (2271G>A), T11K (2265G>T), V12G (2262A>C), S13N (2259C>T), T15I (2253G>A), L19Y (2240C>A 2241A>T), S20V (2238G>A 2239A>C), W22P (2231C>A 2232C>G 2233A>G), T23R (2229G>C), G25N (2222G>A 2223C>T 2224C>T), T27I (2217G>A), Q28L (2214T>A 2215G>A), P29* (2211G>T 2212G>A), N31* (2204G>T 2206T>A)								

	Begin	End	Coverage	Score	Concordance	Matches	Identities	I/D/M/F*	Stop Codons
**NT**	**2201**	**2402**	**5.5%**	**-14**	**-4.8%**	**147 (100%)**	**70 (47.6%)**	**0/0**	
Codon mutations:	ATG1ATA (2294C>T), CTA2TTA (2293G>A), GGG3GAG (2289C>T), TAT4TTT (2286T>A), GTA5CCA (2283A>G 2284C>G), CGG6CAT (2279C>A 2280C>T), AGA7AAA (2277C>T), ATC8CAC (2274A>T 2275T>G), ACC9ATC (2271G>A), ACG11AAG (2265G>T), GTG12GGG (2262A>C), AGC13AAC (2259C>T), ACC15ATC (2253G>A), GCC18GCA (2243G>T), TTG19TAT (2240C>A 2241A>T), TCC20GTC (2238G>A 2239A>C), TGG22CCT (2231C>A 2232C>G 2233A>G), ACA23AGA (2229G>C), GGC25AAT (2222G>A 2223C>T 2224C>T), TCA26TCC (2219T>G), ACA27ATA (2217G>A), CAA28TTA (2214T>A 2215G>A), CCA29TAA (2211G>T 2212G>A), AAC31TAA (2204G>T 2206T>A), GGT32GGC (2201A>G)								

*: Inserts / Deletes / Misaligned / Frameshifts

Analysis details

This analysis was performed with panviral2.64
