# Supplementary material for: Nucleotide sequence analysis reveals the presence of PVY-Tam isolates affecting tamarillo in Colombia
Source: Virol J. 2026 Apr 20;23:145. doi: 10.1186/s12985-026-03166-6 (PMC13234967; doi:10.1186/s12985-026-03166-6)
Supplement: Supplementary file 7 — Additional file 7. [file 12985_2026_3166_MOESM7_ESM.pdf]

Analysis of UN62

**Technology**	Paired-end short reads
**Input Files**	UN62_R1.fq.gz (1.38 GB), UN62_R2.fq.gz (1.41 GB)
**Submitted On**	2023-09-26 14:21:19 UTC
**Duration**	2h 33m 10s
**Tool Version**	panviral2.64
**Location**	https://www.genomedetective.com/db/ui/analysis/1abb2807-2d0c-4c65-b599-46af9f9dd8ba

Statistics

**Original Read Length**	20 - 150
**Trimmed Read Length**	50 - 135

	# Reads	% of Reads
**Input file**	43419312	100.0%
**After QC**	43139000	99.4%
**After filtering**	3469850	8.0%
**Mapped back to contigs**	1265033	2.9%

Assignments

Assignment	No. of Reads	Depth of Coverage	Identity		Genome Coverage	
			NT	AA		
Torradovirus lycopersici (2 segments out of 2)	464494	4623.0	88.8%	93.7%	99.8%	
Torradovirus lycopersici (segment RNA 2)	333725	7488.0	91.5%	94.3%	99.6%	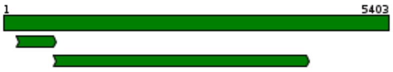
Torradovirus lycopersici (segment RNA 1)	130769	2646.1	87.0%	93.2%	99.9%	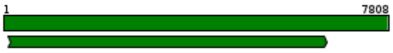
Potato virus Y	431062	5971.0	82.8%	90.0%	99.5%	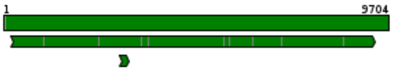
Torradovirus marchitezum (2 segments out of 2)	240631	4344.5	67.6%	71.0%	58.2%	
Torradovirus marchitezum (segment RNA 2)	143680	14475.3	69.5%	76.0%	25.4%	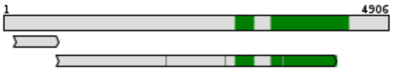
Torradovirus marchitezum (segment RNA 1)	96951	2179.8	67.2%	70.0%	80.5%	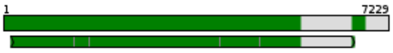
Diachasmimorpha longicaudata entomopoxvirus (segment NC_043455.1)	8833	993.3	60.0%	55.5%	84.0%	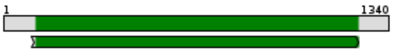

Assignment	No. of Reads	Depth of Coverage	Identity		Genome Coverage	
			NT	AA		
Bracoviriform glomeratae (segment NC_043292.1)	8084	3029.1	72.6%	84.5%	72.8%	
Potato leafroll virus	3283	75.3	98.1%	97.2%	97.2%	
Duamitovirus soch1	543	32.4	68.3%	69.2%	75.7%	
Solendovirus venanicotianae	124	8.5	77.0%	73.5%	20.1%	

Discoveries

Similar to	No. of Reads	Depth of Coverage	Identity		Genome Coverage	
			NT	AA		
Tomato chocolate spot virus (2 segments out of 2)	79686	12924.2	73.1%	70.5%	5.5%	
Tomato chocolate spot virus (segment RNA 1)	61095	24274.9	82.1%	0.0%	3.7%	
Tomato chocolate spot virus (segment RNA2)	18591	5209.1	67.0%	70.5%	8.0%	
Noumeavirus	24245	10898.8	82.0%	94.4%	0.1%	
Tomato necrotic dwarf virus (segment RNA1)	2020	356.8	64.8%	63.2%	10.3%	
Cladosporium fulvum T-1 virus	511	39.2	53.0%	47.2%	18.5%	
Bracoviriform congregatae (3 segments out of 30)	408	206.7	78.9%	90.0%	0.7%	
Bracoviriform congregatae (segment Circle 7)	408	206.7	78.9%	90.0%	0.7%	
Bracoviriform congregatae (segment Circle 7)	277	198.1	78.6%	86.8%	0.5%	
Bracoviriform congregatae (segment Circle 7)	5	3.1	75.5%	85.4%	0.6%	
Cassava brown streak virus	391	85.7	60.8%	57.7%	5.8%	
Human gammaherpesvirus 8 (subtype: Could not assign)	322	200.8	83.7%	84.4%	0.1%	

Similar to	No. of Reads	Depth of Coverage	Identity		Genome Coverage	
			NT	AA		
Hibiscus bacilliform virus GD1	53	11.3	51.6%	42.9%	4.5%	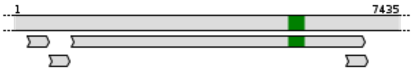
Caulimovirus venafragariae	51	6.8	57.6%	51.7%	11.1%	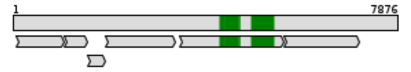
Badnavirus occultipomeae	46	8.1	49.5%	38.4%	5.3%	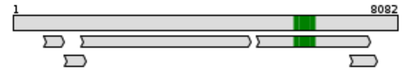
Errantivirus	38	8.9	37.9%	33.3%	7.7%	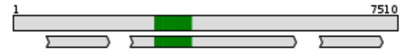
Badnavirus volubetulae	30	10.1	52.6%	42.1%	4.3%	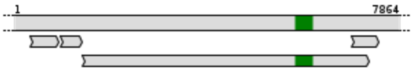
Badnavirus zetavirgamusae	29	8.4	53.9%	40.4%	6.3%	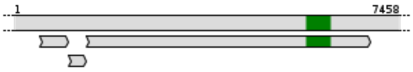
Badnavirus maculasmallanthi	15	6.2	57.1%	48.8%	3.2%	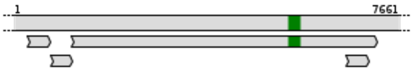
Chinaberry tree badnavirus 1	13	6.5	55.4%	45.8%	3.5%	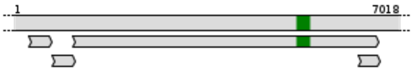
Pinus nigra virus 1	12	4.5	55.3%	47.4%	4.1%	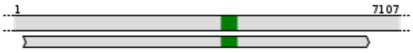
Dioscovirus dioscoreae	11	2.7	61.1%	54.9%	5.3%	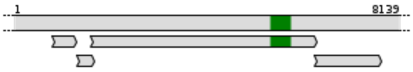
Duamitovirus peex1	10	5.1	73.4%	64.4%	9.7%	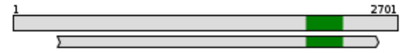
Cacao swollen shoot Ghana J virus	10	4.1	58.0%	49.5%	4.3%	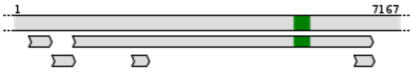
Hibiscus bacilliform virus GD1	10	5.5	56.4%	46.3%	3.2%	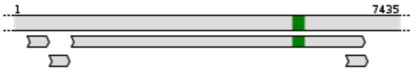
Badnavirus maculasmallanthi	9	2.8	51.8%	45.0%	5.5%	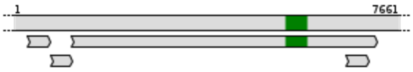
Badnavirus venazanthoxyli	8	3.8	58.4%	53.2%	3.5%	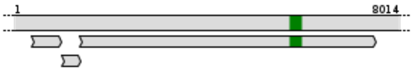
Dioscovirus dioscoreae	8	3.1	63.4%	48.7%	4.2%	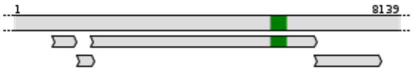
Duamitovirus soch1	7	2.5	69.2%	73.9%	12.9%	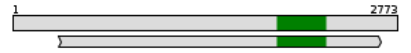

Similar to	No. of Reads	Depth of Coverage	Identity		Genome Coverage	
			NT	AA		
Escherichia virus DE3	6	3.6	100.0%	100.0%	0.5%	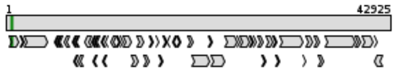
Potato virus X	4	2.4	73.7%	88.0%	3.5%	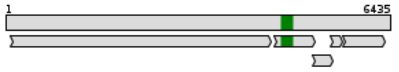
Pandoravirus salinus	4	2.7	75.4%	80.5%	0.0%	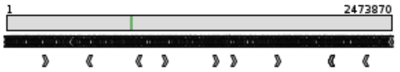
Dioscorea bacilliform virus	4	1.7	52.9%	47.1%	4.3%	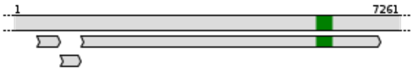
Rhizobium phage RHEph10	3	2.9	80.0%	82.2%	0.1%	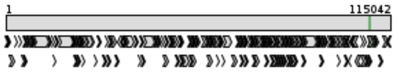
Melanoplus sanguinipes entomopoxvirus	2	2.0	80.0%	80.0%	0.1%	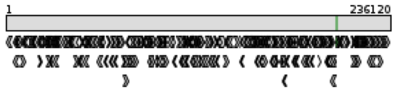
Wadgaonvirus wv5004651	2	1.4	100.0%	100.0%	0.5%	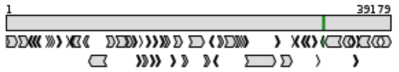
Enterobacteria phage P7	2	1.6	100.0%	100.0%	0.2%	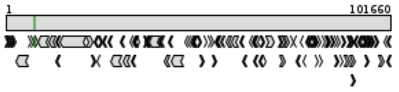
Orthomarburgvirus marburgense	2	1.0	70.4%	98.9%	1.4%	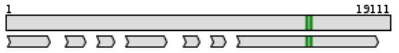
Sclerotinia sclerotiorum deltaflexivirus 2	2	1.2	60.7%	65.8%	3.2%	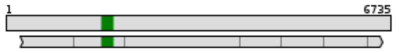
Friend murine leukemia virus	1	1.0	77.0%	97.8%	1.6%	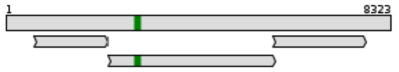
Moloney murine leukemia virus	1	1.0	76.3%	100.0%	1.6%	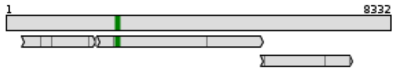
Ochrobactrum phage POA1180	1	1.0	79.3%	71.1%	0.3%	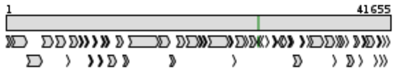

NGS Details (UN62): Potato virus Y

Assembly

**Coverage Length**	9651 (1 contig(s))
**Depth Of Coverage**	5971.0
**Number Of Reads**	431062
**Reads Per Million**	9992.40 rpm (after QC)
**Ambiguities**	0
**Assembly Method**	de novo + reference guided assembly
**Consensus Caller**	Bcf Tools

Coverage Map

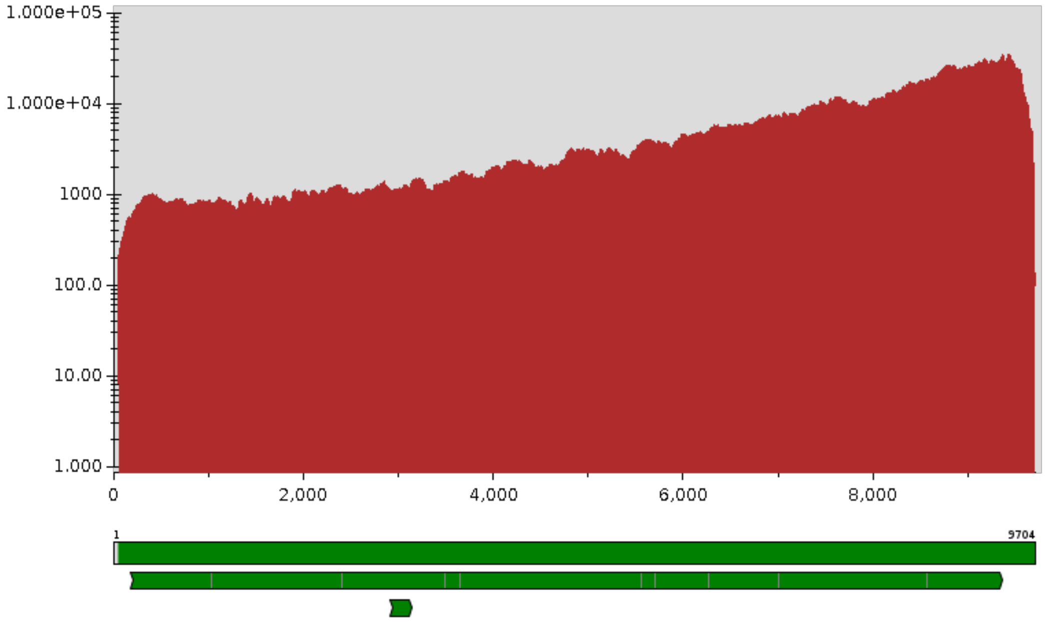

Assignment

**Type**	Potato virus Y (Taxonomy ID: 12216)
**Reference Genome**	NC_001616.1
**NT Identity (%)**	82.7701
**AA Identity (%)**	89.9682
**Number Of Stop Codons**	3
**Number Of CDS**	2

Alignment

**Alignment Score**	12522.0 (NT) + 19019.0 (AA) = 31541.0
**Concordance (%)**	78.2514

Alignment Method	Global, seeded, nucleotide + amino acids (AGA)

Genome Region

Sequence starts at position 54 and ends at position 9704 relative to NC_001616.1 reference sequence.

Alignment Detailed Statistics

	Begin	End	Coverage	Score	Concordance	Matches	Identities	I/D/M/F*	Stop Codons
NT	54	9704	99.5%	12522	65.2%	9639 (99.8%)	7984 (82.7%)	7/12	
Mutations:	75C>T, 79C>T, 83G>A, 85T>C, 87C>T, 89G>A, 90T>C, 93A>T, 94A>G, 95A>T, 100T>C, 101T>A, 102C>T, 104T>C, 107C>T, 110T>G, 112C>T, 114C>T, 115T>C, 116A>T, 117G>C, 119A>G, 120C>T, 121A>T, 125T>G, 126T>C, 127G>A, 128G>T, 131A>G, 132C>T, 139A>T, 139_140insG, 141C>T, 143C>T, 146C>T, 150C>T, 150_151insT, 154T>C, 156C>A, 158T>A, 160A>C, 164C>T, 168C>A, 169C>G, 171A>C, 172T>C, 173T>A, 174T>G, 177G>C, 178A>G, 179T>C, 180C>T, 181C>A, 182T>G, 183C>T, 193T>C, 194T>C, 196C>A, 202A>G, 208C>T, 209T>C, 210G>T, 211T>G, 220G>A, 221T>A, 223T>G, 226A>G, 235A>T, 241C>T, 247A>T, 251T>C, 255G>T, 256C>T, 258A>G, 262T>C, 263A>G, 265T>G, 267T>C, 273A>T, 277A>T, 280A>G, 283G>T, 286G>A, 287G>A, 289T>C, 293G>A, 294T>C, 301T>C, 304C>T, 305G>A, 306C>T, 309A>G, 311C>T, 316A>T, 317A>G, 318C>T, 319A>G, 322A>G, 331A>T, 339T>A, 340C>A, 342A>G, 347A>G, 348A>C, 349A>G, 350T>C, 355T>A, 356A>G, 357C>T, 364T>C, 365G>A, 366T>C, 367G>A, 368C>T, 369T>C, 373A>G, 376C>T, 384T>G, 388G>A, 391C>T, 394A>G, 400G>A, 406T>C, 408C>T, 409C>T, 412G>A, 415A>T, 416A>G, 417T>A, 424T>C, 427G>A, 433A>G, 436G>C, 437G>A, 440A>G, 441G>A, 443A>C, 444A>G, 445G>A, 448T>A, 454A>G, 456A>C, 460T>C, 461C>A, 469A>G, 475C>G, 481T>A, 502T>C, 505A>T, 511C>T, 517T>A, 520T>A, 523A>T, 531C>T, 535G>A, 536C>T, 539C>T, 540A>G, 541A>G, 549G>A, 550A>G, 556C>T, 559T>C, 562T>C, 568T>C, 571A>G, 576T>C, 580T>C, 585T>C, 586C>G, 593C>T, 594G>A, 595C>T, 597C>G, 599A>G, 601A>G, 602A>C, 603T>C, 604A>G, 613A>G, 616A>G, 619C>G, 622G>A, 625G>A, 628T>C, 631C>T, 635A>G, 637T>C, 644A>G, 646A>G, 655T>C, 661G>A, 663A>C, 664G>C, 667A>G, 679C>T, 682C>A, 683T>C, 685A>G, 691T>C, 699C>G, 703T>A, 706T>A, 719A>G, 721G>A, 722A>G, 724A>G, 725C>T, 727T>G, 730T>A, 731G>T, 734T>C, 735A>C, 736C>T, 737T>C, 738C>G, 739C>T, 744C>T, 745G>C, 749C>T, 751A>C, 754T>C, 765T>A, 770T>C, 773C>A, 774G>A, 775A>G, 776C>A, 790C>T, 801T>A, 813G>C, 814A>G, 816T>A, 818T>C, 823A>G, 825G>A, 826T>A, 833C>A, 835G>A, 841C>T, 851A>G, 853T>C, 859C>T, 860C>T, 862C>T, 863A>G, 865T>C, 867T>C, 869A>G, 871C>T, 872A>T, 874A>G, 876G>A, 879G>A, 886T>C, 898C>T, 903A>G, 906C>A, 907A>C, 909A>C, 910A>C, 912G>A, 913C>T, 922C>A, 931T>A, 938T>C, 939C>T, 940A>T, 943A>G, 952C>T, 967A>G, 973A>G, 976G>C, 980T>A, 982G>C, 991A>G, 994T>C, 999G>A, 1003T>C, 1007C>A, 1008A>G, 1009G>A, 1010A>G, 1012T>G, 1013A>G, 1018A>G, 1019A>C, 1021C>G, 1030C>T, 1039G>A, 1042T>C, 1045C>A, 1048C>A, 1051T>C, 1060G>A, 1063T>A, 1064C>T, 1069C>T, 1083G>A, 1093T>C, 1097T>G, 1099G>A, 1105C>T, 1114A>G, 1117T>G, 1123A>G, 1126T>A, 1129C>T, 1143G>A, 1147A>T, 1150T>A, 1154T>A, 1156G>A, 1165C>T, 1168T>C, 1172C>T, 1174T>A, 1201C>T, 1221G>A, 1229G>A, 1230T>C, 1231T>C, 1233G>A, 1234C>T, 1240G>A, 1246G>A, 1250T>C, 1252G>A, 1255T>C, 1257A>C, 1258A>G, 1267A>G, 1273T>C, 1276G>A, 1278A>G, 1283T>C, 1290C>T, 1291G>A, 1298G>A, 1299A>C, 1300C>T, 1306T>C, 1307A>G, 1309A>G, 1312T>C, 1316A>G, 1318T>A, 1327G>A, 1329T>C, 1333G>T, 1336A>G, 1343C>T, 1345A>G, 1354G>A, 1357G>T, 1360C>T, 1367C>T, 1369C>G, 1373C>A, 1378C>T, 1384G>A, 1385A>G, 1393A>G, 1396C>T, 1405G>T, 1408A>G, 1411G>A, 1415G>T, 1417A>T, 1420G>C, 1429T>C, 1432A>G, 1436G>A, 1438C>T, 1439T>C, 1441A>G, 1444T>C, 1453C>T, 1456G>A, 1468A>G, 1492A>G, 1499T>C, 1501G>A, 1504T>C, 1507G>A, 1510C>T, 1513A>G, 1516A>G, 1519A>T, 1522G>A, 1525C>T, 1528G>A, 1534C>T, 1540T>A, 1546C>T, 1555A>G, 1558T>C, 1561T>C, 1564A>C, 1567T>A, 1573C>T, 1576A>G, 1583T>C, 1588T>A, 1594G>A, 1600C>T, 1609G>A, 1618G>A, 1621C>T, 1624C>T, 1631C>T, 1639A>G, 1651C>T, 1654C>G, 1675T>C, 1693A>G, 1702T>C, 1706G>A, 1707A>G, 1718G>A, 1726A>G, 1729C>T, 1732A>C, 1738T>C, 1742A>G, 1744C>T, 1747C>T, 1753T>C, 1758G>A, 1768G>A, 1786C>T, 1789A>G, 1790G>A, 1795C>T, 1805T>C, 1807A>G, 1813G>A, 1819G>A, 1825G>A, 1837C>T, 1840T>C, 1849A>G, 1858C>G, 1864A>G, 1870C>T, 1876T>C, 1882A>G, 1888T>A, 1891T>C, 1906T>C, 1915A>T, 1930T>C, 1936C>T, 1937A>G, 1939T>C, 1945A>G, 1951C>T, 1960A>T, 1978A>G, 1981T>A, 1984C>T, 1987T>C, 1996C>T, 2004T>A, 2020A>G, 2023G>A, 2026T>C, 2029G>T, 2032G>A, 2059T>C, 2068T>G, 2071C>T, 2072G>A, 2074G>A, 2087C>T, 2089G>A, 2101C>T, 2104G>A, 2107G>A, 2116G>A, 2122C>T, 2137C>T, 2158T>C, 2164C>T, 2170A>G, 2182T>C, 2188G>A, 2191C>A, 2208G>A, 2218T>C, 2224C>T, 2227A>T, 2242G>A, 2245C>T, 2254G>A, 2257T>C, 2260C>T, 2266C>G, 2272A>G, 2275G>A, 2293A>G, 2299C>T, 2302G>A, 2305G>A, 2314A>T, 2317T>C, 2326A>G, 2341G>A, 2347A>G, 2350T>C, 2356G>A, 2365T>C, 2371A>G, 2374A>G, 2383T>C, 2386A>T, 2389A>G, 2392T>C, 2395T>C, 2414A>G, 2415A>G, 2416T>G, 2419T>A, 2420A>T, 2440A>T, 2467T>C, 2476T>C, 2482G>A, 2491G>A, 2497G>T, 2503G>A, 2518A>G, 2521T>C, 2523A>G, 2533A>G, 2539G>A, 2545A>G, 2551G>A, 2554T>A, 2558C>T, 2561T>C, 2563G>C, 2572A>G, 2584T>C, 2587C>T, 2593G>C, 2599T>C, 2608T>C, 2620T>C, 2629G>A, 2632G>A, 2634G>A, 2638G>A, 2644T>A, 2653A>G, 2665T>C, 2678C>T, 2683A>G, 2689T>C, 2698A>C, 2707A>G, 2710G>A, 2716A>G, 2719A>G, 2722C>T, 2723G>A, 2725C>T, 2728A>G, 2734G>A, 2737T>A, 2746T>C, 2747G>A, 2749T>A, 2752A>T, 2755T>A, 2761C>T, 2764C>T, 2779T>C, 2785G>T, 2791C>T, 2795C>A, 2806C>T, 2816T>C, 2818G>C, 2822G>A, 2824G>A, 2827G>A, 2836T>C, 2839G>A, 2842T>C, 2863C>A, 2864C>T, 2882C>A, 2885A>C, 2886G>A, 2893C>T, 2895C>T, 2902C>T, 2905A>G, 2911T>C, 2923T>C, 2931A>G, 2940A>G, 2947T>C, 2962T>C, 2971_2972insA, 2989T>C, 2991C>T, 2994A>G, 2999G>A, 3000A>G, 3016delC, 3021G>A, 3024A>T, 3025C>T, 3036C>T, 3039A>G, 3040A>C, 3042A>C, 3043G>A, 3052G>A, 3061A>G, 3082G>A, 3091C>T, 3099C>T, 3100C>A, 3104G>A, 3105T>G, 3106G>A, 3109C>T, 3112A>G, 3118T>C, 3121C>T, 3127A>G, 3132G>A, 3137C>A, 3138G>A, 3139A>G, 3151T>C, 3152T>A, 3154C>T, 3165G>A, 3169A>G, 3199C>T, 3203A>C, 3208T>C, 3214A>G, 3217C>T, 3218T>C, 3223C>A, 3225C>A, 3230G>A, 3235C>T, 3257T>C, 3262C>T, 3265C>T, 3266G>A, 3268A>G, 3271G>A, 3275G>A, 3284G>A, 3301G>A, 3315G>A, 3316A>G, 3318A>C, 3322C>T, 3325G>A, 3328G>A, 3337A>T, 3349G>A, 3355C>T, 3358C>T, 3359A>G, 3367A>G, 3370T>C, 3377T>C, 3379A>G, 3394A>G, 3397C>T, 3400T>G, 3406A>G, 3418C>T, 3421T>A, 3427T>C, 3433G>A, 3436A>T, 3439A>G, 3442C>T, 3445T>C, 3457G>A, 3460T>C, 3463T>A, 3469G>T, 3472G>A, 3496C>T, 3499G>A, 3505C>T, 3513T>G, 3520A>G, 3529A>G, 3532A>G, 3545A>G, 3548G>A, 3551T>C, 3554G>A, 3556C>T, 3583T>C, 3586T>C, 3589C>T, 3595C>T, 3607T>C, 3613T>C, 3616G>A, 3619T>C, 3623C>G, 3628C>T, 3632C>A, 3637T>C, 3638T>C, 3643A>G, 3661A>G, 3670G>A, 3682T>C, 3688G>A, 3697G>A, 3698A>G, 3706T>C, 3709T>C, 3713T>C, 3715G>A, 3717G>A, 3721G>A, 3731C>A, 3732G>A, 3736T>A, 3746C>T, 3748A>G, 3754A>G, 3763T>C, 3778A>G, 3781A>G, 3784C>T, 3796T>C, 3805A>T, 3811C>T, 3814A>G, 3829C>T, 3838T>C, 3844A>G, 3847A>C, 3856T>A, 3862G>T, 3871C>T, 3877C>A, 3883C>T, 3886A>G, 3892A>G, 3895C>T, 3898T>C, 3904A>G, 3905C>A, 3907G>A, 3913T>C, 3928G>A, 3931A>T, 3937G>A, 3955T>C, 3958G>A, 3961C>T, 3964A>G, 3967T>C, 3976A>G, 3979T>C, 3985A>T, 3997A>T, 4000G>A, 4006C>T, 4009T>G, 4012C>T, 4015A>G, 4024T>C, 4030A>G, 4033A>T, 4042G>A, 4052C>T, 4054G>A, 4057T>C, 4063T>A, 4066A>G, 4073A>G, 4078T>C, 4081C>T, 4084T>C, 4096C>T, 4108C>T, 4111A>T, 4117G>A, 4118C>T, 4120A>G, 4126C>T, 4128T>A, 4129C>T, 4132C>T, 4135T>C, 4147A>G, 4148T>C, 4151G>T, 4162C>T, 4166G>A, 4171A>C, 4180G>A, 4190C>T, 4198T>C, 4201C>T, 4210G>T, 4213C>T, 4219T>C, 4220C>T, 4223C>A, 4225G>A, 4231T>G, 4237T>C, 4252A>G, 4253T>C, 4255A>G, 4258A>G, 4270T>C, 4282A>G, 4285G>A, 4291A>G, 4303G>A, 4312C>T, 4319A>G, 4321A>G, 4324G>T, 4327G>A, 4336G>T, 4339T>C, 4348A>T, 4360A>G, 4366T>C, 4369T>A, 4375T>C, 4387T>C, 4395T>A, 4406G>A, 4411T>C, 4438T>C, 4444C>A, 4450C>T, 4456C>T, 4465C>T, 4475A>G, 4477G>T, 4480C>T, 4481A>T, 4483A>G, 4495C>T, 4507G>A, 4510C>T, 4513T>G, 4517C>T, 4528C>T, 4534A>G, 4540C>T, 4544G>T, 4545C>T, 4546G>A, 4548G>A, 4558T>C, 4564A>T, 4570C>T, 4576A>T, 4597G>A, 4600C>T, 4606C>T, 4609G>A, 4615G>A, 4618C>T, 4625T>C, 4627G>T, 4648C>T, 4651T>C, 4660G>A, 4663C>T, 4669T>A, 4679G>A, 4681G>C, 4684T>C, 4687T>G, 4699G>A, 4702A>G, 4708A>G, 4711G>A, 4712C>T, 4738A>G, 4744A>T, 4747A>G, 4748T>C, 4765T>C, 4796G>A, 4798C>T, 4803C>G, 4804T>C, 4807G>A, 4810G>T, 4813T>G, 4816T>C, 4831T>C, 4834C>T, 4840A>T, 4852A>G, 4855C>T, 4867T>C, 4870G>C, 4873T>A, 4882T>C, 4903A>T, 4909G>A, 4915T>C, 4918A>G, 4919T>C, 4921G>A, 4924T>C, 4927C>G, 4933T>C, 4936C>A, 4939G>A, 4942T>C, 4945C>T, 4963A>G, 4972T>C, 4990T>C, 5002A>G, 5023T>C, 5024T>C, 5029C>T, 5032T>C, 5038T>C, 5044A>T, 5053A>T, 5056G>A, 5057A>G, 5060A>G, 5071G>T, 5090C>A, 5095A>G, 5098G>T, 5100C>T, 5104A>G, 5107A>T, 5110T>C, 5113A>G, 5116G>A, 5119A>G, 5120G>T, 5122C>T, 5126A>G, 5137T>C, 5140C>T, 5143A>G, 5146G>A, 5161C>A, 5167A>G, 5173T>G, 5182G>A, 5183G>A, 5188C>T, 5191G>A, 5197A>G, 5200C>T, 5206C>T, 5209A>G, 5218C>T, 5230G>A, 5236C>T, 5239C>T, 5245C>T, 5263A>T, 5269C>T, 5272C>T, 5275C>A, 5278C>T, 5281A>C, 5284A>G, 5287T>C, 5288C>T, 5293A>T, 5294T>C, 5296G>A, 5302G>A, 5311T>A, 5314A>G, 5323A>T, 5347T>C, 5353C>T, 5365C>T, 5386T>C, 5395C>A, 5422A>G, 5425T>C, 5431C>T, 5437G>A, 5452T>C, 5458G>A, 5467T>C, 5470A>G, 5471C>T, 5473A>G, 5479A>G, 5482C>T, 5485A>C, 5488T>C, 5489T>C, 5491G>A, 5497T>C, 5506T>C, 5509G>A, 5512G>A, 5516T>C, 5530T>C, 5533G>A, 5536G>T, 5539G>A, 5548T>G, 5554C>T, 5560T>A, 5561G>A, 5563G>A, 5566G>C, 5569A>G, 5584C>G, 5593G>A, 5596G>A, 5597A>G, 5605C>A, 5617G>T, 5619C>T, 5620T>G, 5629G>A, 5630A>C, 5641C>T, 5650A>T, 5662A>T, 5665A>G, 5689A>G, 5693G>A, 5698G>A, 5713A>G, 5716G>C, 5719A>G, 5743C>A, 5755C>A, 5758T>C, 5764T>C, 5770A>G, 5779C>T, 5782T>C, 5791C>T, 5803C>T, 5836G>A, 5839A>G, 5845A>G, 5848A>G, 5853A>G, 5857T>C, 5860C>T, 5863A>T, 5869T>A, 5875T>C, 5884C>T, 5893C>T, 5894A>G, 5896C>G, 5899C>T, 5908G>A, 5911T>C, 5923G>A, 5938A>G, 5941C>T, 5944T>C, 5947T>C, 5959G>A, 5960C>G, 5961G>C, 5962G>C, 5974A>G, 5986T>A, 5989C>T, 5990A>G, 5998T>C, 6008A>C, 6010A>T, 6019A>T, 6022G>T, 6025A>T, 6030A>C, 6049C>A, 6058G>A, 6061A>G, 6064C>A, 6070T>C, 6072G>A, 6073T>C, 6074A>C, 6079G>A, 6081C>A, 6085A>T, 6091A>T, 6094C>T, 6111G>C, 6112T>C, 6118A>G, 6124G>A, 6127G>A, 6128A>G, 6130T>C, 6133T>C, 6136A>G, 6148C>T, 6157A>G, 6158G>A, 6163T>C, 6169A>G, 6170A>T, 6172A>G, 6178C>T, 6187A>G, 6190T>C, 6196G>A, 6203C>T, 6205C>T, 6208A>G, 6209C>T, 6211A>G, 6217G>A, 6229T>A, 6230G>A, 6238C>T, 6247G>A, 6253A>T, 6257G>A, 6258C>A, 6274T>C, 6282A>G, 6286G>A, 6298C>T, 6301G>A, 6304A>G, 6307C>T, 6322C>T, 6328A>T, 6331T>G, 6334T>C, 6337G>A, 6338C>T, 6358T>C, 6361G>A, 6362G>A, 6367A>T, 6370G>A, 6376C>T, 6379T>G, 6385A>C, 6391A>T, 6394A>G, 6397C>T, 6401G>A, 6405C>T, 6412C>T, 6418A>G, 6421T>C, 6429A>T, 6436T>A, 6445G>A, 6450A>G, 6454C>G, 6460C>T, 6463T>A, 6469C>T, 6472G>A, 6475G>A, 6481T>C, 6485C>A, 6487C>T, 6490T>C, 6496C>T, 6502G>A, 6514T>C, 6517G>A, 6527C>A, 6532C>A, 6541G>A, 6544A>G, 6556C>T, 6559T>C, 6565G>A, 6569T>C, 6571G>A, 6586T>A, 6588C>T, 6598A>G, 6601A>G, 6604T>C, 6607T>C, 6616A>G, 6622C>T, 6631G>A, 6634G>A, 6635T>C, 6640T>A, 6646G>T, 6647A>G, 6652C>T, 6664C>T, 6670T>G, 6677A>G, 6679A>G, 6685C>T, 6691A>T, 6694C>T, 6715A>G, 6716G>A, 6719A>G, 6724A>G, 6734C>T, 6742G>A, 6745G>A, 6754C>T, 6760A>G, 6764A>T, 6766A>G, 6775T>A, 6782C>T, 6787A>T, 6790C>T, 6795C>T, 6796A>G, 6799C>A, 6802C>A, 6808C>T, 6811C>T, 6814C>T, 6823C>T, 6829A>G, 6847C>T, 6853A>C, 6857A>G, 6862G>A, 6871A>G, 6875G>A, 6876T>C, 6877C>A, 6880G>A, 6883T>A, 6889T>G, 6901C>T, 6916C>T, 6919G>A, 6925A>G, 6928T>C, 6934C>A, 6949G>C, 6955C>T, 6958A>G, 6976A>G, 6980C>T, 6985C>T, 6997A>T, 7000G>T, 7001G>A, 7003G>A, 7012T>C, 7015G>A, 7018T>C, 7032T>A, 7039C>T, 7040T>C, 7042G>A, 7048A>G, 7060T>A, 7063C>A, 7078C>T, 7082T>C, 7084A>G, 7087A>G, 7090C>A, 7096T>C, 7099A>G, 7102T>C, 7111G>A, 7117A>G, 7125C>A, 7129A>G, 7132T>C, 7133C>T, 7135G>A, 7141G>A, 7144T>C, 7145G>T, 7147A>G, 7150G>A, 7159A>T, 7160_7165delGAGGCA, 7171C>T, 7189G>T, 7198A>G, 7201C>T, 7205C>T, 7210T>C, 7216T>A, 7219G>A, 7225C>A, 7228G>A, 7240G>A, 7243T>C, 7249A>G, 7252T>G, 7258T>A, 7261T>C, 7264T>A, 7267C>G, 7270G>A, 7273_7274insT, 7274C>G, 7275G>T, 7281A>T, 7284T>C, 7296C>T, 7311C>T, 7314C>T, 7317G>A, 7321T>A, 7322delG, 7327C>T, 7330C>T, 7333C>T, 7334A>C, 7342T>C, 7343G>A, 7344C>A, 7345A>C, 7354T>C, 7363A>G, 7369T>C, 7372C>T, 7378G>T, 7381C>T, 7396A>T, 7399C>T, 7405C>T, 7407G>T, 7408T>G, 7414G>T, 7415T>G, 7420A>G, 7429C>T, 7432T>C, 7441T>C, 7459G>A, 7462A>G, 7468A>T, 7471C>A, 7477A>G, 7480C>T, 7486G>T, 7495T>C, 7498A>G, 7501T>C, 7503T>C, 7504G>A, 7513T>A, 7519C>T, 7522A>G, 7531G>A, 7537G>A, 7540C>T, 7546T>C, 7570G>A, 7573G>A, 7579G>A, 7588T>C, 7595C>T, 7604T>C, 7606G>A, 7615T>A, 7624T>C, 7633C>T, 7636C>T, 7639T>C, 7648T>C, 7666A>G, 7672T>C, 7678A>T, 7684G>A, 7690T>C, 7696T>C, 7702T>G, 7709G>A, 7713A>G, 7714A>G, 7715C>T, 7726T>C, 7735G>A, 7744A>G, 7747C>T, 7762C>T, 7768G>A, 7771T>C, 7780A>G, 7783A>C, 7786T>C, 7793C>T, 7795A>G, 7798C>T, 7804T>C, 7812C>T, 7834A>G, 7837C>T, 7844G>T, 7846G>A, 7855G>A, 7861G>A, 7870A>G, 7873C>T, 7876T>A, 7879G>A, 7882T>A, 7883G>A, 7894A>T, 7897T>C, 7921C>T, 7927G>A, 7936T>C, 7939T>C, 7942T>C, 7966G>A, 7969T>C, 7975T>C, 7987C>T, 7990T>C, 8005T>C, 8032A>G, 8038C>T, 8041C>T, 8043G>A, 8080G>A, 8089G>A, 8095G>A, 8098G>A, 8107C>T, 8110T>C, 8116T>C, 8128A>G, 8146T>C, 8151A>G, 8152C>T, 8164G>A, 8178G>A, 8182G>A, 8191G>A, 8197C>T, 8203C>T, 8208G>A, 8213C>T, 8215G>A, 8218A>G, 8221C>T, 8224G>A, 8239A>G, 8254G>A, 8260T>C, 8263A>G, 8281T>C, 8284A>G, 8294C>G, 8296A>G, 8299G>A, 8302C>T, 8317T>C, 8320C>T, 8326T>A, 8335G>A, 8338C>T, 8349C>T, 8353A>G, 8362C>T, 8365A>G, 8368C>T, 8380C>T, 8383A>G, 8389A>G, 8404T>C, 8408G>T, 8413A>G, 8419G>T, 8434T>C, 8440T>C, 8452G>A, 8458A>G, 8460G>A, 8464A>G, 8467G>A, 8479G>A, 8480G>A, 8482T>A, 8497G>A, 8498C>T, 8500A>G, 8502G>A, 8503A>G, 8506C>T, 8515A>T, 8524C>T, 8527A>C, 8528T>C, 8530A>G, 8539G>C, 8546C>T, 8547T>G, 8552T>A, 8563A>G, 8574C>G, 8585A>G, 8598G>A, 8602C>T, 8603A>T, 8604A>C, 8605C>T, 8620A>G, 8622C>A, 8625A>G, 8626G>A, 8629G>A, 8632C>T, 8638C>T, 8641G>A, 8647C>T, 8648C>T, 8649C>T, 8650G>C, 8653C>T, 8656A>G, 8658G>A, 8664A>C, 8665T>G, 8674T>G, 8679C>T, 8680A>T, 8683C>A, 8692G>A, 8695A>T, 8707G>A, 8708A>C, 8716G>A, 8722C>T, 8725G>A, 8731A>G, 8737A>G, 8743C>T, 8745C>A, 8752G>A, 8755A>T, 8770C>T, 8771T>C, 8776A>G, 8779T>C, 8782G>A, 8791T>C, 8797A>G, 8800A>G, 8803A>G, 8809T>C, 8812T>C, 8818T>C, 8824G>A, 8827A>G, 8848G>A, 8854T>C, 8856A>G, 8857G>A, 8863G>A, 8865G>A, 8867A>C, 8869G>T, 8872A>G, 8883G>A, 8888A>T, 8929C>T, 8954G>A, 8959C>T, 8962A>T, 8983G>A, 8984A>G, 8986T>C, 8988A>T, 8998G>A, 9004G>A, 9007G>A, 9016C>T, 9037C>A, 9085A>T, 9097C>T, 9131A>G, 9136A>T, 9145G>T, 9149A>G, 9150T>G, 9151G>A, 9152G>A, 9157A>G, 9160G>T, 9163T>C, 9184G>A, 9196A>G, 9214G>A, 9217A>G, 9220G>A, 9244A>T, 9247G>A, 9256C>T, 9260C>T, 9262T>C, 9274G>A, 9280C>T, 9289C>T, 9298A>C, 9304G>A, 9334C>T, 9364C>G, 9377_9378insT, 9387C>T, 9404G>A, 9411C>T, 9412A>G, 9414A>T, 9437C>T, 9454A>G, 9458T>C, 9467G>A, 9470_9471insT, 9482G>A, 9483C>T, 9498T>C, 9516T>G, 9517delA, 9533G>A, 9534G>T, 9548A>T, 9551C>T, 9553_9554delTA, 9556A>T, 9557T>A, 9558A>T, 9574T>A, 9581G>T, 9582T>G, 9583G>C, 9584_9585insG, 9590C>T, 9591T>C, 9607C>T, 9610T>C, 9615delC, 9620A>G, 9648A>G, 9674T>C, 9690C>T								

	Begin	End	Coverage	Score	Concordance	Matches	Identities	I/D/M/F*	Stop Codons
**NT**	**54**	**9704**	**99.5%**	**12522**	**65.2%**	**9639 (99.8%)**	**7984 (82.7%)**	**7/12**	
**CDS**									
PVYgp1	1	3064	100%	18924	89.7%	3062 (99.9%)	2771 (90.4%)	2/2/4/4	1
Protein mutations:	Y4Q (194T>C 196C>A), C9L (209T>C 210G>T 211T>G), F13M (221T>A 223T>G), S23P (251T>C), C24F (255G>T 256C>T), E25G (258A>G), I27V (263A>G 265T>G), V28A (267T>C), E30V (273A>T), A35T (287G>A 289T>C), V37T (293G>A 294T>C), A41I (305G>A 306C>T), D42G (309A>G), E44D (316A>T), T45V (317A>G 318C>T 319A>G), L52Q (339T>A 340C>A), K53R (342A>G), K55A (347A>G 348A>C 349A>G), Y56H (350T>C), T58V (356A>G 357C>T), V61T (365G>A 366T>C 367G>A), L62S (368C>T 369T>C), F67C (384T>G), A75V (408C>T 409C>T), M78E (416A>G 417T>A), E85K (437G>A), R86E (440A>G 441G>A), K87R (443A>C 444A>G 445G>A), D88E (448T>A), E91A (456A>C), H93N (461C>A), D111E (517T>A), S116F (531C>T), P118S (536C>T), Q119W (539C>T 540A>G 541A>G), R122K (549G>A 550A>G), M131T (576T>C), V134A (585T>C 586C>G), R137Y (593C>T 594G>A 595C>T), P138R (597C>G), I139V (599A>G 601A>G), I140P (602A>C 603T>C 604A>G), M147I (625G>A), I151V (635A>G 637T>C), I154V (644A>G 646A>G), E160A (663A>C 664G>C), H166Q (682C>A), T172S (699C>G), H174Q (706T>A), K179E (719A>G 721G>A), I180V (722A>G 724A>G), A183S (731G>T), Y184P (734T>C 735A>C 736C>T), S185R (737T>C 738C>G 739C>T), A187V (744C>T 745G>C), R189C (749C>T 751A>C), M194K (765T>A), R197K (773C>A 774G>A 775A>G), M206K (801T>A), G210A (813G>C 814A>G), L211H (816T>A), R214Q (825G>A 826T>A), N223D (851A>G 853T>C), R226C (860C>T 862C>T), T227A (863A>G 865T>C), I228T (867T>C), N229D (869A>G 871C>T), I230L (872A>T 874A>G), R231Q (876G>A), R232K (879G>A), N240S (903A>G), T241N (906C>A 907A>C), K242T (909A>C 910A>C), S243N (912G>A 913C>T), S252L (938T>C 939C>T 940A>T), L266I (980T>A 982G>C), R272K (999G>A), Q275R (1007C>A 1008A>G 1009G>A), S276G (1010A>G 1012T>G), I277V (1013A>G), N279Q (1019A>C 1021C>G), D288E (1048C>A), R300Q (1083G>A), S305A (1097T>G 1099G>A), R320K (1143G>A), L324I (1154T>A 1156G>A), S346N (1221G>A), V349T (1229G>A 1230T>C 1231T>C), S350N (1233G>A 1234C>T), K358T (1257A>C 1258A>G), N365S (1278A>G), A369V (1290C>T 1291G>A), D372T (1298G>A 1299A>C 1300C>T), I375V (1307A>G 1309A>G), N378E (1316A>G 1318T>A), I382T (1329T>C), L397I (1373C>A), I401V (1385A>G), E407D (1405G>T), A411S (1415G>T 1417A>T), V418I (1436G>A 1438C>T), E508R (1706G>A 1707A>G), A512T (1718G>A), I520V (1742A>G 1744C>T), S525N (1758G>A), V536I (1790G>A), I585V (1937A>G 1939T>C), F607Y (2004T>A), I628M (2068T>G), V630I (2072G>A 2074G>A), R675K (2208G>A), D694E (2266C>G), N744G (2414A>G 2415A>G 2416T>G), S746C (2420A>T), K780R (2523A>G), R817K (2634G>A), V847I (2723G>A 2725C>T), A855T (2747G>A 2749T>A), H871N (2795C>A), V880I (2822G>A 2824G>A), P900T (2882C>A), S901H (2885A>C 2886G>A), T904M (2895C>T), N916S (2931A>G), N919S (2940A>G), E929_N930insX (2971_2972insA), T936I (2991C>T), H937R (2994A>G), E939R (2999G>A 3000A>G), R946Q (3021G>A), Y947F (3024A>T 3025C>T), T951I (3036C>T), E952G (3039A>G 3040A>C), K953T (3042A>C 3043G>A), A972V (3099C>T 3100C>A), V974R (3104G>A 3105T>G 3106G>A), S983N (3132G>A), R985K (3137C>A 3138G>A 3139A>G), F990I (3152T>A 3154C>T), C994Y (3165G>A), F1012L (3218T>C), T1014N (3225C>A), V1016I (3230G>A), V1028M (3266G>A 3268A>G), V1031M (3275G>A), A1034T (3284G>A), R1044K (3315G>A 3316A>G), E1045A (3318A>C), M1059V (3359A>G), D1072E (3400T>G), V1110G (3513T>G), M1121V (3545A>G), A1122T (3548G>A), V1124I (3554G>A 3556C>T), L1147V (3623C>G), L1150M (3632C>A), Y1152H (3638T>C), I1172V (3698A>G), S1178N (3717G>A), R1183K (3731C>A 3732G>A), I1297V (4073A>G), F1315Y (4128T>A 4129C>T), A1323S (4151G>T), V1328I (4166G>A), L1347I (4223C>A 4225G>A), I1379V (4319A>G 4321A>G), F1404Y (4395T>A), V1408I (4406G>A), M1431V (4475A>G 4477G>T), T1433S (4481A>T 4483A>G), A1454L (4544G>T 4545C>T 4546G>A), R1455K (4548G>A), V1499I (4679G>A 4681G>C), V1538I (4796G>A 4798C>T), T1540S (4803C>G 4804T>C), S1625G (5057A>G), T1626A (5060A>G), L1636I (5090C>A), A1639V (5100C>T), E1641D (5107A>T), V1646F (5120G>T 5122C>T), I1648V (5126A>G), V1667I (5183G>A), A1793T (5561G>A 5563G>A), I1805V (5597A>G), N1807K (5605C>A), A1812V (5619C>T 5620T>G), I1816L (5630A>C), V1837I (5693G>A), K1890R (5853A>G), I1904V (5894A>G 5896C>G), R1926A (5960C>G 5961G>C 5962G>C), I1936V (5990A>G), E1945D (6019A>T), K1949T (6030A>C), D1955E (6049C>A), M1958I (6058G>A), S1963N (6072G>A 6073T>C), N1964H (6074A>C), T1966N (6081C>A), C1976S (6111G>C 6112T>C), I1982V (6128A>G 6130T>C), V1992I (6158G>A), T1996S (6170A>T 6172A>G), L2007F (6203C>T 6205C>T), V2016I (6230G>A), A2025K (6257G>A 6258C>A), K2033R (6282A>G), A2060T (6362G>A), V2073I (6401G>A), A2074V (6405C>T), Y2082F (6429A>T), Q2089R (6450A>G), H2101N (6485C>A 6487C>T), L2115I (6527C>A), T2135I (6588C>T), Y2151H (6635T>C), I2155V (6647A>G), I2165V (6677A>G 6679A>G), D2178N (6716G>A), N2179D (6719A>G), I2194L (6764A>T 6766A>G), A2204V (6795C>T 6796A>G), H2205Q (6799C>A), N2225D (6857A>G), V2231T (6875G>A 6876T>C 6877C>A), D2250E (6934C>A), V2273I (7001G>A 7003G>A), F2283Y (7032T>A), T2314K (7125C>A), A2321S (7145G>T 7147A>G), E2326_A2327del (7160_7165delGAGGCA), D2344E (7216T>A), D2358E (7258T>A), D2363_R2364insX (7273_7274insT), R2364V (7274C>G 7275G>T), H2366L (7281A>T), L2367S (7284T>C), S2371L (7296C>T), S2376F (7311C>T), T2377I (7314C>T), C2378Y (7317G>A), N2379K (7321T>A), K2384Q (7334A>C), A2387N (7343G>A 7344C>A 7345A>C), S2408M (7407G>T 7408T>G), C2411G (7415T>G), L2440S (7503T>C 7504G>A), D2509N (7709G>A), K2510R (7713A>G 7714A>G), T2543I (7812C>T), V2554L (7844G>T 7846G>A), V2567I (7883G>A), S2620N (8043G>A), N2656S (8151A>G 8152C>T), R2665K (8178G>A), R2675K (8208G>A), P2704A (8294C>G 8296A>G), S2722F (8349C>T), A2742S (8408G>T), M2756I (8452G>A), R2759K (8460G>A), A2766T (8480G>A 8482T>A), R2773K (8502G>A 8503A>G), E2777D (8515A>T), E2785D (8539G>C), L2788C (8546C>T 8547T>G), S2790T (8552T>A), A2797G (8574C>G), I2801V (8585A>G), G2805E (8598G>A), N2807S (8603A>T 8604A>C 8605C>T), P2813Q (8622C>A), E2814G (8625A>G 8626G>A), P2822F (8648C>T 8649C>T 8650G>C), G2825E (8658G>A), D2827A (8664A>C 8665T>G), A2832V (8679C>T 8680A>T), T2854K (8745C>A), E2891G (8856A>G 8857G>A), R2894Q (8865G>A), M2895L (8867A>C 8869G>T), G2900E (8883G>A), T2902S (8888A>T), V2924I (8954G>A), N2934D (8984A>G 8986T>C), E2935V (8988A>T), I2983V (9131A>G), M2989G (9149A>G 9150T>G 9151G>A), G2990S (9152G>A), P3026S (9260C>T 9262T>C), Q3038H (9298A>C)								

	Begin	End	Coverage	Score	Concordance	Matches	Identities	I/D/M/F*	Stop Codons
**NT**	**54**	**9704**	**99.5%**	**12522**	**65.2%**	**9639 (99.8%)**	**7984 (82.7%)**	**7/12**	

Codon mutations:

ACT3ACC (193T>C), TAC4CAA (194T>C 196C>A), TCA6TCG (202A>G), ATC8ATT (208C>T), TGT9CTG (209T>C 210G>T 211T>G), TCG12TCA (220G>A), TTT13ATG (221T>A 223T>G), GAA14GAG (226A>G), CTA17CTT (235A>T), TAC19TAT (241C>T), CCA21CCT (247A>T), TCT23CCT (251T>C), TGC24TTT (255G>T 256C>T), GAG25GGG (258A>G), CAT26CAC (262T>C), ATT27GTG (263A>G 265T>G), GTG28GCG (267T>C), GAA30GTA (273A>T), CGA31CGT (277A>T), GAA32GAG (280A>G), GTG33GTT (283G>T), CCG34CCA (286G>A), GCT35ACC (287G>A 289T>C), GTT37ACT (293G>A 294T>C), CCT39CCC (301T>C), TTC40TTT (304C>T), GCA41ATA (305G>A 306C>T), GAT42GGT (309A>G), CTG43TTG (311C>T), GAA44GAT (316A>T), ACA45GTG (317A>G 318C>T 319A>G), CAA46CAG (322A>G), GCA49GCT (331A>T), CTC52CAA (339T>A 340C>A), AAG53AGG (342A>G), AAA55GCG (347A>G 348A>C 349A>G), TAT56CAT (350T>C), GCT57GCA (355T>A), ACT58GTT (356A>G 357C>T), CGT60CGC (364T>C), GTG61ACA (365G>A 366T>C 367G>A), CTC62TCC (368C>T 369T>C), AAA63AAG (373A>G), AAC64AAT (376C>T), TTT67TGT (384T>G), ACG68ACA (388G>A), TAC69TAT (391C>T), CGA70CGG (394A>G), AAG72AAA (400G>A), GAT74GAC (406T>C), GCC75GTT (408C>T 409C>T), CAG76CAA (412G>A), ATA77ATT (415A>T), ATG78GAG (416A>G 417T>A), ATT80ATC (424T>C), CAG81CAA (427G>A), AAA83AAG (433A>G), CTG84CTC (436G>C), GAG85AAG (437G>A), AGG86GAG (440A>G 441G>A), AAG87CGA (443A>C 444A>G 445G>A), GAT88GAA (448T>A), GAA90GAG (454A>G), GAA91GCA (456A>C), TAT92TAC (460T>C), CAC93AAC (461C>A), CAA95CAG (469A>G), GCC97GCG (475C>G), CCT99CCA (481T>A), ACT106ACC (502T>C), ATA107ATT (505A>T), GGC109GGT (511C>T), GAT111GAA (517T>A), CCT112CCA (520T>A), CCA113CCT (523A>T), TCT116TTT (531C>T), GAG117GAA (535G>A), CCA118TCA (536C>T), CAA119TGG (539C>T 540A>G 541A>G), AGA122AAG (549G>A 550A>G), ATC124ATT (556C>T), ATT125ATC (559T>C), CAT126CAC (562T>C), ACT128ACC (568T>C), CCA129CCG (571A>G), ATG131ACG (576T>C), CGT132CGC (580T>C), GTC134GCG (585T>C 586C>G), CGC137TAT (593C>T 594G>A 595C>T), CCC138CGC (597C>G), ATA139GTG (599A>G 601A>G), ATA140CCG (602A>C 603T>C 604A>G), ACA143ACG (613A>G), GAA144GAG (616A>G), GGC145GGG (619C>G), CAG146CAA (622G>A), ATG147ATA (625G>A), AAT148AAC (628T>C), CAC149CAT (631C>T), ATT151GTC (635A>G 637T>C), ATA154GTG (644A>G 646A>G), ATT157ATC (655T>C), TCG159TCA (661G>A), GAG160GCC (663A>C 664G>C), AAA161AAG (667A>G), GTC165GTT (679C>T), CAC166CAA (682C>A), TTA167CTG (683T>C 685A>G), AGT169AGC (691T>C), ACC172AGC (699C>G), ACT173ACA (703T>A), CAT174CAA (706T>A), AAG179GAA (719A>G 721G>A), ATA180GTG (722A>G 724A>G), CTT181TTG (725C>T 727T>G), GGT182GGA (730T>A), GCA183TCA (731G>T), TAC184CCT (734T>C 735A>C 736C>T), TCC185CGT (737T>C 738C>G 739C>T), GCG187GTC (744C>T 745G>C), CGA189TGC (749C>T 751A>C), ACT190ACC (754T>C), ATG194AAG (765T>A), TTG196CTG (770T>C), CGA197AAG (773C>A 774G>A 775A>G), CGG198AGG (776C>A), TTC202TTT (790C>T), ATG206AAG (801T>A), GGA210GCG (813G>C 814A>G), CTT211CAT (816T>A), TTG212CTG (818T>C), CAA213CAG (823A>G), CGT214CAA (825G>A 826T>A), CGG217AGA (833C>A 835G>A), GAC219GAT (841C>T), AAT223GAC (851A>G 853T>C), GTC225GTT (859C>T), CGC226TGT (860C>T 862C>T), ACT227GCC (863A>G 865T>C), ATC228ACC (867T>C), AAC229GAT (869A>G 871C>T), ATA230TTG (872A>T 874A>G), CGA231CAA (876G>A), AGG232AAG (879G>A), GAT234GAC (886T>C), ATC238ATT (898C>T), AAC240AGC (903A>G), ACA241AAC (906C>A 907A>C), AAA242ACC (909A>C 910A>C), AGC243AAT (912G>A 913C>T), GGC246GGA (922C>A), GGT249GGA (931T>A), TCA252CTT (938T>C 939C>T 940A>T), GGA253GGG (943A>G), TTC256TTT (952C>T), TCA261TCG (967A>G), GAA263GAG (973A>G), GGG264GGC (976G>C), TTG266ATC (980T>A 982G>C), GCA269GCG (991A>G), CGT270CGC (994T>C), AGA272AAA (999G>A), GTT273GTC (1003T>C), CAG275AGA (1007C>A 1008A>G 1009G>A), AGT276GGG (1010A>G 1012T>G), ATT277GTT (1013A>G), TTA278TTG (1018A>G), AAC279CAG (1019A>C 1021C>G), ATC282ATT (1030C>T), TCG285TCA (1039G>A), AAT286AAC (1042T>C), GCC287GCA (1045C>A), GAC288GAA (1048C>A), AAT289AAC (1051T>C), AAG292AAA (1060G>A), GGT293GGA (1063T>A), CTG294TTG (1064C>T), GAC295GAT (1069C>T), CGA300CAA (1083G>A), TAT303TAC (1093T>C), TCG305GCA (1097T>G 1099G>A), CAC307CAT (1105C>T), GTA310GTG (1114A>G), GCT311GCG (1117T>G), TTA313TTG (1123A>G), CCT314CCA (1126T>A), GTC315GTT (1129C>T), AGG320AAG (1143G>A), GTA321GTT (1147A>T), GCT322GCA (1150T>A), TTG324ATA (1154T>A 1156G>A), CAC327CAT (1165C>T), AGT328AGC (1168T>C), CTT330TTA (1172C>T 1174T>A), ACC339ACT (1201C>T), AGC346AAC (1221G>A), GTT349ACC (1229G>A 1230T>C 1231T>C), AGC350AAT (1233G>A 1234C>T), CTG352CTA (1240G>A), AAG354AAA (1246G>A), TTG356CTA (1250T>C 1252G>A), CAT357CAC (1255T>C), AAA358ACG (1257A>C 1258A>G), AGA361AGG (1267A>G), GGT363GGC (1273T>C), TTG364TTA (1276G>A), AAT365AGT (1278A>G), TTG367CTG (1283T>C), GCG369GTA (1290C>T 1291G>A), GAC372ACT (1298G>A 1299A>C 1300C>T), TTT374TTC (1306T>C), ATA375GTG (1307A>G 1309A>G), CAT376CAC (1312T>C), AAT378GAA (1316A>G 1318T>A), TTG381TTA (1327G>A), ATA382ACA (1329T>C), GCG383GCT (1333G>T), TTA384TTG (1336A>G), CTA387TTG (1343C>T 1345A>G), CCG390CCA (1354G>A), GTG391GTT (1357G>T), GAC392GAT (1360C>T), CTC395TTG (1367C>T 1369C>G), CTT397ATT (1373C>A), TTC398TTT (1378C>T), GAG400GAA (1384G>A), ATA401GTA (1385A>G), AAA403AAG (1393A>G), TCC404TCT (1396C>T), GAG407GAT (1405G>T), AAA408AAG (1408A>G), CAG409CAA (1411G>A), GCA411TCT (1415G>T 1417A>T), CCG412CCC (1420G>C), AAT415AAC (1429T>C), TTA416TTG (1432A>G), GTC418ATT (1436G>A 1438C>T), TTA419CTG (1439T>C 1441A>G), AAT420AAC (1444T>C), TTC423TTT (1453C>T), CTG424CTA (1456G>A), GAA428GAG (1468A>G), GTA436GTG (1492A>G), TTG439CTA (1499T>C 1501G>A), AGT440AGC (1504T>C), TTG441TTA (1507G>A), CTC442CTT (1510C>T), GAA443GAG (1513A>G), TTA444TTG (1516A>G), GCA445GCT (1519A>T), AGG446AGA (1522G>A), TTC447TTT (1525C>T), CAG448CAA (1528G>A), AAC450AAT (1534C>T), ACT452ACA (1540T>A), AAC454AAT (1546C>T), AAA457AAG (1555A>G), GGT458GGC (1558T>C), GAT459GAC (1561T>C), ATA460ATC (1564A>C), TCT461TCA (1567T>A), TTC463TTT (1573C>T), AGA464AGG (1576A>G), TTA467CTA (1583T>C), TCT468TCA (1588T>A), AAG470AAA (1594G>A), AAC472AAT (1600C>T), CTG475CTA (1609G>A), TCG478TCA (1618G>A), TGC479TGT (1621C>T), GAC480GAT (1624C>T), CTG483TTG (1631C>T), AAA485AAG (1639A>G), TTC489TTT (1651C>T), CTC490CTG (1654C>G), CAT497CAC (1675T>C), TCA503TCG (1693A>G), TTT506TTC (1702T>C), GAA508AGA (1706G>A 1707A>G), GCA512ACA (1718G>A), GGA514GGG (1726A>G), TAC515TAT (1729C>T), TCA516TCC (1732A>C), TAT518TAC (1738T>C), ATC520GTT (1742A>G 1744C>T), CGC521CGT (1747C>T), CAT523CAC (1753T>C), AGT525AAT (1758G>A), AGG528AGA (1768G>A), AAC534AAT (1786C>T), TTA535TTG (1789A>G), GTT536ATT (1790G>A), GTC537GTT (1795C>T), TTA541CTG (1805T>C 1807A>G), GAG543GAA (1813G>A), AGG545AGA (1819G>A), AAG547AAA (1825G>A), GAC551GAT (1837C>T), TAT552TAC (1840T>C), CAA555CAG (1849A>G), GTC558GTG (1858C>G), AAA560AAG (1864A>G), TGC562TGT (1870C>T), AGT564AGC (1876T>C), AAA566AAG (1882A>G), GGT568GGA (1888T>A), AAT569AAC (1891T>C), TGT574TGC (1906T>C), ACA577ACT (1915A>T), GGT582GGC (1930T>C), GCC584GCT (1936C>T), ATT585GTC (1937A>G 1939T>C), TCA587TCG (1945A>G), TTC589TTT (1951C>T), CCA592CCT (1960A>T), GTA598GTG (1978A>G), ATT599ATA (1981T>A), GGC600GGT (1984C>T), AAT601AAC (1987T>C), GAC604GAT (1996C>T), TTT607TAT (2004T>A), AAA612AAG (2020A>G), GGG613GGA (2023G>A), GAT614GAC (2026T>C), TCG615TCT (2029G>T), GAG616GAA (2032G>A), TAT625TAC (2059T>C), ATT628ATG (2068T>G), AAC629AAT (2071C>T), GTG630ATA (2072G>A 2074G>A), CTG635TTA (2087C>T 2089G>A), AGC639AGT (2101C>T), GAG640GAA (2104G>A), GAG641GAA (2107G>A), AAG644AAA (2116G>A), TTC646TTT (2122C>T), CGC651CGT (2137C>T), CTT658CTC (2158T>C), ACC660ACT (2164C>T), CCA662CCG (2170A>G), GAT666GAC (2182T>C), GCG668GCA (2188G>A), ACC669ACA (2191C>A), AGA675AAA (2208G>A), TAT678TAC (2218T>C), GAC680GAT (2224C>T), GTA681GTT (2227A>T), TTG686TTA (2242G>A), CCC687CCT (2245C>T), TTG690TTA (2254G>A), GTT691GTC (2257T>C), GAC692GAT (2260C>T), GAC694GAG (2266C>G), CAA696CAG (2272A>G), ACG697ACA (2275G>A), TCA703TCG (2293A>G), GGC705GGT (2299C>T), TCG706TCA (2302G>A), CAG707CAA (2305G>A), GGA710GGT (2314A>T), TAT711TAC (2317T>C), CTA714CTG (2326A>G), GTG719GTA (2341G>A), CAA721CAG (2347A>G), CTT722CTC (2350T>C), TTG724TTA (2356G>A), AAT727AAC (2365T>C), GAA729GAG (2371A>G), TTA730TTG (2374A>G), GAT733GAC (2383T>C), ATA734ATT (2386A>T), AAA735AAG (2389A>G), CAT736CAC (2392T>C), TAT737TAC (2395T>C), AAT744GGG (2414A>G 2415A>G 2416T>G), GCT745GCA (2419T>A), AGC746TGC (2420A>T), ACA752ACT (2440A>T), GTT761GTC (2467T>C), TCT764TCC (2476T>C), TCG766TCA (2482G>A), CTG769CTA (2491G>A), CTG771CTT (2497G>T), TTG773TTA (2503G>A), AGA778AGG (2518A>G), CCT779CCC (2521T>C), AAG780AGG (2523A>G), AGA783AGG (2533A>G), TTG785TTA (2539G>A), TTA787TTG (2545A>G), GAG789GAA (2551G>A), CCT790CCA (2554T>A), CTG792TTG (2558C>T), TTG793CTC (2561T>C 2563G>C), TCA796TCG (2572A>G), CCT800CCC (2584T>C), GGC801GGT (2587C>T), CTG803CTC (2593G>C), GCT805GCC (2599T>C), AAT808AAC (2608T>C), TTT812TTC (2620T>C), GCG815GCA (2629G>A), GTG816GTA (2632G>A), AGG817AAG (2634G>A), TTG818TTA (2638G>A), ATT820ATA (2644T>A), AAA823AAG (2653A>G), GCT827GCC (2665T>C), CTA832TTA (2678C>T), CTA833CTG (2683A>G), GCT835GCC (2689T>C), CTA838CTC (2698A>C), TCA841TCG (2707A>G), GCG842GCA (2710G>A), GAA844GAG (2716A>G), ACA845ACG (2719A>G), CTC846CTT (2722C>T), GTC847ATT (2723G>A 2725C>T), GCA848GCG (2728A>G), AGG850AGA (2734G>A), ATT851ATA (2737T>A), GAT854GAC (2746T>C), GCT855ACA (2747G>A 2749T>A), GCA856GCT (2752A>T), GCT857GCA (2755T>A), GAC859GAT (2761C>T), CTC860CTT (2764C>T), TGT865TGC (2779T>C), GGG867GGT (2785G>T), AAC869AAT (2791C>T), CAT871AAT (2795C>A), TAC874TAT (2806C>T), TTG878CTC (2816T>C 2818G>C), GTG880ATA (2822G>A 2824G>A), TTG881TTA (2827G>A), GTT884GTC (2836T>C), AAG885AAA (2839G>A), AAT886AAC (2842T>C), ACC893ACA (2863C>A), CTA894TTA (2864C>T), CCA900ACA (2882C>A), AGT901CAT (2885A>C 2886G>A), AAC903AAT (2893C>T), ACG904ATG (2895C>T), GTC906GTT (2902C>T), GTA907GTG (2905A>G), ATT909ATC (2911T>C), AAT913AAC (2923T>C), AAT916AGT (2931A>G), AAC919AGC (2940A>G), GCT921GCC (2947T>C), ACT926ACC (2962T>C), GAA929_AAT930insA-- (2971_2972insA), GGT935GGC (2989T>C), ACT936ATT (2991C>T), CAT937CGT (2994A>G), GAG939AGG (2999G>A 3000A>G), TCC944TC- (3016delC), CGG946CAG (3021G>A), TAC947TTT (3024A>T 3025C>T), ACA951ATA (3036C>T), GAA952GGC (3039A>G 3040A>C), AAG953ACA (3042A>C 3043G>A), TTG956TTA (3052G>A), TTA959TTG (3061A>G), GCG966GCA (3082G>A), GGC969GGT (3091C>T), GCC972GTA (3099C>T 3100C>A), GTG974AGA (3104G>A 3105T>G 3106G>A), GTC975GTT (3109C>T), AAA976AAG (3112A>G), ACT978ACC (3118T>C), GCC979GCT (3121C>T), GGA981GGG (3127A>G), AGC983AAC (3132G>A), CGA985AAG (3137C>A 3138G>A 3139A>G), TAT989TAC (3151T>C), TTC990ATT (3152T>A 3154C>T), TGT994TAT (3165G>A), GTA995GTG (3169A>G), ATC1005ATT (3199C>T), AGG1007CGG (3203A>C), CGT1008CGC (3208T>C), CCA1010CCG (3214A>G), ACC1011ACT (3217C>T), TTT1012CTT (3218T>C), GTC1013GTA (3223C>A), ACT1014AAT (3225C>A), GTT1016ATT (3230G>A), AAC1017AAT (3235C>T), TTA1025CTA (3257T>C), ACC1026ACT (3262C>T), AGC1027AGT (3265C>T), GTA1028ATG (3266G>A 3268A>G), GTG1029GTA (3271G>A), GTG1031ATG (3275G>A), GCA1034ACA (3284G>A), CAG1039CAA (3301G>A), AGA1044AAG (3315G>A 3316A>G), GAA1045GCA (3318A>C), ATC1046ATT (3322C>T), GAG1047GAA (3325G>A), TTG1048TTA (3328G>A), ATA1051ATT (3337A>T), GAG1055GAA (3349G>A), GTC1057GTT (3355C>T), TGC1058TGT (3358C>T), ATG1059GTG (3359A>G), CTA1061CTG (3367A>G), TAT1062TAC (3370T>C), TTA1065CTG (3377T>C 3379A>G), GAA1070GAG (3394A>G), CGC1071CGT (3397C>T), GAT1072GAG (3400T>G), ACA1074ACG (3406A>G), TAC1078TAT (3418C>T), ATT1079ATA (3421T>A), TAT1081TAC (3427T>C), AAG1083AAA (3433G>A), TCA1084TCT (3436A>T), GTA1085GTG (3439A>G), AAC1086AAT (3442C>T), CCT1087CCC (3445T>C), CAG1091CAA (3457G>A), TTT1092TTC (3460T>C), GCT1093GCA (3463T>A), GCG1095GCT (3469G>T), CAG1096CAA (3472G>A), CAC1104CAT (3496C>T), CAG1105CAA (3499G>A), TCC1107TCT (3505C>T), GTT1110GGT (3513T>G), AAA1112AAG (3520A>G), GAA1115GAG (3529A>G), CAA1116CAG (3532A>G), ATG1121GTG (3545A>G), GCT1122ACT (3548G>A), TTA1123CTA (3551T>C), GTC1124ATT (3554G>A 3556C>T), AGT1133AGC (3583T>C), GAT1134GAC (3586T>C), TGC1135TGT (3589C>T), TTC1137TTT (3595C>T), AAT1141AAC (3607T>C), TTT1143TTC (3613T>C), AAG1144AAA (3616G>A), GGT1145GGC (3619T>C), CTT1147GTT (3623C>G), TCC1148TCT (3628C>T), CTG1150ATG (3632C>A), GAT1151GAC (3637T>C), TAT1152CAT (3638T>C), GAA1153GAG (3643A>G), TTA1159TTG (3661A>G), GTG1162GTA (3670G>A), TTT1166TTC (3682T>C), GAG1168GAA (3688G>A), GAG1171GAA (3697G>A), ATT1172GTT (3698A>G), GAT1174GAC (3706T>C), TTT1175TTC (3709T>C), TTG1177CTA (3713T>C 3715G>A), AGT1178AAT (3717G>A), GAG1179GAA (3721G>A), CGA1183AAA (3731C>A 3732G>A), ACT1184ACA (3736T>A), CTA1188TTG (3746C>T 3748A>G), ACA1190ACG (3754A>G), AGT1193AGC (3763T>C), CGA1198CGG (3778A>G), CAA1199CAG (3781A>G), ATC1200ATT (3784C>T), CAT1204CAC (3796T>C), CCA1207CCT (3805A>T), TAC1209TAT (3811C>T), AGA1210AGG (3814A>G), TTC1215TTT (3829C>T), TTT1218TTC (3838T>C), AGA1220AGG (3844A>G), GCA1221GCC (3847A>C), GTT1224GTA (3856T>A), GTG1226GTT (3862G>T), GAC1229GAT (3871C>T), GCC1231GCA (3877C>A), AGC1233AGT (3883C>T), GAA1234GAG (3886A>G), CTA1236CTG (3892A>G), GAC1237GAT (3895C>T), TTT1238TTC (3898T>C), GTA1240GTG (3904A>G), CGG1241AGA (3905C>A 3907G>A), GCT1243GCC (3913T>C), AAG1248AAA (3928G>A), TCA1249TCT (3931A>T), GGG1251GGA (3937G>A), AGT1257AGC (3955T>C), GTG1258GTA (3958G>A), GCC1259GCT (3961C>T), GGA1260GGG (3964A>G), TCT1261TCC (3967T>C), TTA1264TTG (3976A>G), ATT1265ATC (3979T>C), CCA1267CCT (3985A>T), CTA1271CTT (3997A>T), GCG1272GCA (4000G>A), AAC1274AAT (4006C>T), GTT1275GTG (4009T>G), TTC1276TTT (4012C>T), AAA1277AAG (4015A>G), TCT1280TCC (4024T>C), GAA1282GAG (4030A>G), CCA1283CCT (4033A>T), AAG1286AAA (4042G>A), CTG1290TTA (4052C>T 4054G>A), CGT1291CGC (4057T>C), CGT1293CGA (4063T>A), GGA1294GGG (4066A>G), ATA1297GTA (4073A>G), TTT1298TTC (4078T>C), GGC1299GGT (4081C>T), TCT1300TCC (4084T>C), TCC1304TCT (4096C>T), AGC1308AGT (4108C>T), GGA1309GGT (4111A>T), GCG1311GCA (4117G>A), CTA1312TTG (4118C>T 4120A>G), TAC1314TAT (4126C>T), TTC1315TAT (4128T>A 4129C>T), GCC1316GCT (4132C>T), AAT1317AAC (4135T>C), CAA1321CAG (4147A>G), TTA1322CTA (4148T>C), GCT1323TCT (4151G>T), AAC1326AAT (4162C>T), GTA1328ATA (4166G>A), ATA1329ATC (4171A>C), GAG1332GAA (4180G>A), CTG1336TTG (4190C>T), CCT1338CCC (4198T>C), TCC1339TCT (4201C>T), GCG1342GCT (4210G>T), TTC1343TTT (4213C>T), AGT1345AGC (4219T>C), CTG1346TTG (4220C>T), CTG1347ATA (4223C>A 4225G>A), GTT1349GTG (4231T>G), CAT1351CAC (4237T>C), GTA1356GTG (4252A>G), TTA1357CTG (4253T>C 4255A>G), AAA1358AAG (4258A>G), ACT1362ACC (4270T>C), AGA1366AGG (4282A>G), GAG1367GAA (4285G>A), GAA1369GAG (4291A>G), CAG1373CAA (4303G>A), GTC1376GTT (4312C>T), ATA1379GTG (4319A>G 4321A>G), GTG1380GTT (4324G>T), GAG1381GAA (4327G>A), CTG1384CTT (4336G>T), TCT1385TCC (4339T>C), TCA1388TCT (4348A>T), GCA1392GCG (4360A>G), GGT1394GGC (4366T>C), TCT1395TCA (4369T>A), ACT1397ACC (4375T>C), GTT1401GTC (4387T>C), TTT1404TAT (4395T>A), GTA1408ATA (4406G>A), CTT1409CTC (4411T>C), GTT1418GTC (4438T>C), ACC1420ACA (4444C>A), GCC1422GCT (4450C>T), CTC1424CTT (4456C>T), GAC1427GAT (4465C>T), ATG1431GTT (4475A>G 4477G>T), GTC1432GTT (4480C>T), ACA1433TCG (4481A>T 4483A>G), GGC1437GGT (4495C>T), AAG1441AAA (4507G>A), CAC1442CAT (4510C>T), GGT1443GGG (4513T>G), CTA1445TTA (4517C>T), GTC1448GTT (4528C>T), AAA1450AAG (4534A>G), ACC1452ACT (4540C>T), GCG1454TTA (4544G>T 4545C>T 4546G>A), AGA1455AAA (4548G>A), TTT1458TTC (4558T>C), GTA1460GTT (4564A>T), ACC1462ACT (4570C>T), ATA1464ATT (4576A>T), TTG1471TTA (4597G>A), GAC1472GAT (4600C>T), GAC1474GAT (4606C>T), GTG1475GTA (4609G>A), GTG1477GTA (4615G>A), GAC1478GAT (4618C>T), TTG1481CTT (4625T>C 4627G>T), GAC1488GAT (4648C>T), ATT1489ATC (4651T>C), AGG1492AGA (4660G>A), AGC1493AGT (4663C>T), GCT1495GCA (4669T>A), GTG1499ATC (4679G>A 4681G>C), AGT1500AGC (4684T>C), GTT1501GTG (4687T>G), GAG1505GAA (4699G>A), AGA1506AGG (4702A>G), CAA1508CAG (4708A>G), AGG1509AGA (4711G>A), CTG1510TTG (4712C>T), AAA1518AAG (4738A>G), GTA1520GTT (4744A>T), GCA1521GCG (4747A>G), TTG1522CTG (4748T>C), ACT1527ACC (4765T>C), GTC1538ATT (4796G>A 4798C>T), ACT1540AGC (4803C>G 4804T>C), GAG1541GAA (4807G>A), GCG1542GCT (4810G>T), GCT1543GCG (4813T>G), CTT1544CTC

	(4816T>C), TAT1549TAC (4831T>C), AAC1550AAT (4834C>T), CCA1552CCT (4840A>T), GGA1556GGG (4852A>G), GGC1557GGT (4855C>T), AGT1561AGC (4867T>C), CTG1562CTC (4870G>C), ATT1563ATA (4873T>A), TGT1566TGC (4882T>C), ACA1573ACT (4903A>T), CAG1575CAA (4909G>A), TTT1577TTC (4915T>C), GAA1578GAG (4918A>G), TTG1579CTA (4919T>C 4921G>A), AGT1580AGC (4924T>C), CCC1581CCG (4927C>G), TTT1583TTC (4933T>C), ATC1584ATA (4936C>A), CAG1585CAA (4939G>A), AAT1586AAC (4942T>C), TTC1587TTT (4945C>T), TCA1593TCG (4963A>G), CCT1596CCC (4972T>C), CTT1602CTC (4990T>C), AAA1606AAG (5002A>G), CCT1613CCC (5023T>C), TTG1614CTG (5024T>C), TGC1615TGT (5029C>T), GAT1616GAC (5032T>C), TCT1618TCC (5038T>C), CCA1620CCT (5044A>T), GCA1623GCT (5053A>T), TCG1624TCA (5056G>A), AGT1625GGT (5057A>G), ACT1626GCT (5060A>G), TCG1629TCT (5071G>T), CTT1636ATT (5090C>A), GGA1637GGG (5095A>G), GTG1638GTT (5098G>T), GCC1639GTC (5100C>T), TTA1640TTG (5104A>G), GAA1641GAT (5107A>T), ATT1642ATC (5110T>C), CCA1643CCG (5113A>G), AAG1644AAA (5116G>A), CAA1645CAG (5119A>G), GTC1646TTT (5120G>T 5122C>T), ATT1648GTT (5126A>G), CAT1651CAC (5137T>C), ATC1652ATT (5140C>T), AAA1653AAG (5143A>G), GAG1654GAA (5146G>A), CTC1659CTA (5161C>A), GAA1661GAG (5167A>G), CTT1663CTG (5173T>G), ACG1666ACA (5182G>A), GTT1667ATT (5183G>A), GTC1668GTT (5188C>T), AAG1669AAA (5191G>A), AAA1671AAG (5197A>G), GAC1672GAT (5200C>T), TGC1674TGT (5206C>T), TTA1675TTG (5209A>G), AGC1678AGT (5218C>T), TCG1682TCA (5230G>A), ATC1684ATT (5236C>T), AGC1685AGT (5239C>T), ATC1687ATT (5245C>T), ACA1693ACT (5263A>T), CTC1695CTT (5269C>T), TTC1696TTT (5272C>T), GCC1697GCA (5275C>A), ATC1698ATT (5278C>T), CCA1699CCC (5281A>C), AGA1700AGG (5284A>G), ACT1701ACC (5287T>C), CTA1702TTA (5288C>T), ATA1703ATT (5293A>T), TTG1704CTA (5294T>C 5296G>A), GAG1706GAA (5302G>A), CTT1709CTA (5311T>A), GAA1710GAG (5314A>G), CGA1713CGT (5323A>T), AGT1721AGC (5347T>C), ATC1723ATT (5353C>T), TGC1727TGT (5365C>T), GTT1734GTC (5386T>C), ACC1737ACA (5395C>A), AAA1746AAG (5422A>G), GAT1747GAC (5425T>C), ACC1749ACT (5431C>T), GAG1751GAA (5437G>A), CTT1756CTC (5452T>C), AAG1758AAA (5458G>A), AGT1761AGC (5467T>C), CAA1762CAG (5470A>G), CTA1763TTG (5471C>T 5473A>G), GAA1765GAG (5479A>G), TTC1766TTT (5482C>T), TCA1767TCC (5485A>C), AAT1768AAC (5488T>C), TTG1769CTA (5489T>C 5491G>A), GGT1771GGC (5497T>C), TGT1774TGC (5506T>C), GAG1775GAA (5509G>A), GAG1776GAA (5512G>A), TTA1778CTA (5516T>C), TAT1782TAC (5530T>C), GAG1783GAA (5533G>A), TCG1784TCT (5536G>T), TTG1785TTA (5539G>A), GTT1788GTG (5548T>G), CAC1790CAT (5554C>T), GCT1792GCA (5560T>A), GCG1793ACA (5561G>A 5563G>A), ACG1794ACC (5566G>C), TCA1795TCG (5569A>G), CTC1800CTG (5584C>G), AAG1803AAA (5593G>A), GGG1804GGA (5596G>A), ATT1805GTT (5597A>G), AAC1807AAA (5605C>A), GTG1811GTT (5617G>T), GCT1812GTG (5619C>T 5620T>G), TTG1815TTA (5629G>A), ATC1816CTC (5630A>C), GGC1819GGT (5641C>T), GCA1822GCT (5650A>T), ATA1826ATT (5662A>T), GGA1827GGG (5665A>G), CAA1835CAG (5689A>G), GTT1837ATT (5693G>A), GAG1838GAA (5698G>A), CAA1843CAG (5713A>G), GGG1844GGC (5716G>C), AAA1845AAG (5719A>G), GCC1853GCA (5743C>A), CGC1857CGA (5755C>A), CAT1858CAC (5758T>C), CGT1860CGC (5764T>C), AAA1862AAG (5770A>G), GGC1865GGT (5779C>T), TTT1866TTC (5782T>C), GAC1869GAT (5791C>T), GAC1873GAT (5803C>T), AGG1884AGA (5836G>A), AAA1885AAG (5839A>G), GGA1887GGG (5845A>G), AAA1888AAG (5848A>G), AAA1890AGA (5853A>G), GGT1891GGC (5857T>C), ACC1892ACT (5860C>T), ACA1893ACT (5863A>T), GGT1895GGA (5869T>A), GGT1897GGC (5875T>C), AGC1900AGT (5884C>T), TTC1903TTT (5893C>T), ATC1904GTG (5894A>G 5896C>G), AAC1905AAT (5899C>T), GGG1908GGA (5908G>A), TTT1909TTC (5911T>C), GAG1913GAA (5923G>A), CAA1918CAG (5938A>G), TTC1919TTT (5941C>T), GTT1920GTC (5944T>C), GAT1921GAC (5947T>C), GGG1925GGA (5959G>A), CGG1926GCC (5960C>G 5961G>C 5962G>C), GAA1930GAG (5974A>G), GCT1934GCA (5986T>A), GAC1935GAT (5989C>T), ATT1936GTT (5990A>G), GAT1938GAC (5998T>C), AGA1942CGT (6008A>C 6010A>T), GAA1945GAT (6019A>T), GTG1946GTT (6022G>T), CGA1947CGT (6025A>T), AAA1949ACA (6030A>C), GAC1955GAA (6049C>A), ATG1958ATA (6058G>A), CAA1959CAG (6061A>G), GCC1960GCA (6064C>A), GGT1962GGC (6070T>C), AGT1963AAC (6072G>A 6073T>C), AAC1964CAC (6074A>C), ACG1965ACA (6079G>A), ACC1966AAC (6081C>A), ATA1967ATT (6085A>T), GCA1969GCT (6091A>T), TAC1970TAT (6094C>T), TGT1976TCC (6111G>C 6112T>C), AAA1978AAG (6118A>G), TTG1980TTA (6124G>A), AAG1981AAA (6127G>A), ATT1982GTC (6128A>G 6130T>C), GAT1983GAC (6133T>C), TTA1984TTG (6136A>G), AAC1988AAT (6148C>T), AAA1991AAG (6157A>G), GTT1992ATT (6158G>A), TGT1993TGC (6163T>C), AAA1995AAG (6169A>G), ACA1996TCG (6170A>T 6172A>G), GGC1998GGT (6178C>T), AAA2001AAG (6187A>G), TTT2002TTC (6190T>C), GAG2004GAA (6196G>A), CTC2007TTT (6203C>T 6205C>T), GAA2008GAG (6208A>G), CTA2009TTG (6209C>T 6211A>G), CAG2011CAA (6217G>A), GCT2015GCA (6229T>A), GTA2016ATA (6230G>A), GTC2018GTT (6238C>T), AAG2021AAA (6247G>A), ATA2023ATT (6253A>T), GCA2025AAA (6257G>A 6258C>A), CAT2030CAC (6274T>C), AAA2033AGA (6282A>G), TCG2034TCA (6286G>A), GGC2038GGT (6298C>T), TTG2039TTA (6301G>A), AGA2040AGG (6304A>G), GAC2041GAT (6307C>T), GCC2046GCT (6322C>T), ACA2048ACT (6328A>T), GTT2049GTG (6331T>G), TGT2050TGC (6334T>C), AGG2051AGA (6337G>A), CTG2052TTG (6338C>T), TAT2058TAC (6358T>C), GGG2059GGA (6361G>A), GCA2060ACA (6362G>A), TCA2061TCT (6367A>T), GAG2062GAA (6370G>A), TAC2064TAT (6376C>T), GGT2065GGG (6379T>G), GGA2067GGC (6385A>C), GGA2069GGT (6391A>T), GCA2070GCG (6394A>G), TAC2071TAT (6397C>T), GTA2073ATA (6401G>A), GCG2074GTG (6405C>T), CAC2076CAT (6412C>T), TTA2078TTG (6418A>G), TTT2079TTC (6421T>C), TAC2082TTC (6429A>T), GGT2084GGA (6436T>A), GAG2087GAA (6445G>A), CAA2089CGA (6450A>G), TCC2090TCG (6454C>G), CAC2092CAT (6460C>T), GGT2093GGA (6463T>A), TTC2095TTT (6469C>T), AGG2096AGA (6472G>A), GTG2097GTA (6475G>A), AAT2099AAC (6481T>C), CAC2101AAT (6485C>A 6487C>T), AGT2102AGC (6490T>C), AGC2104AGT (6496C>T), CTG2106CTA (6502G>A), GGT2110GGC (6514T>C), AGG2111AGA (6517G>A), CTC2115ATC (6527C>A), ATC2116ATA (6532C>A), CCG2119CCA (6541G>A), AAA2120AAG (6544A>G), GTC2124GTT (6556C>T), TTT2125TTC (6559T>C), CAG2127CAA (6565G>A), TTG2129CTA (6569T>C 6571G>A), CCT2134CCA (6586T>A), ACA2135ATA (6588C>T), GAA2138GAG (6598A>G), AGA2139AGG (6601A>G), ATT2140ATC (6604T>C), TGT2141TGC (6607T>C), GGA2144GGG (6616A>G), AAC2146AAT (6622C>T), GAG2149GAA (6631G>A), AAG2150AAA (6634G>A), TAT2151CAT (6635T>C), GCT2152GCA (6640T>A), TCG2154TCT (6646G>T), ATC2155GTC (6647A>G), ATC2156ATT (6652C>T), AGC2160AGT (6664C>T), ACT2162ACG (6670T>G), ATA2165GTG (6677A>G 6679A>G), GGC2167GGT (6685C>T), ACA2169ACT (6691A>T), TTC2170TTT (6694C>T), ACA2177ACG (6715A>G), GAT2178AAT (6716G>A), AAT2179GAT (6719A>G), GGA2180GGG (6724A>G), CTA2184TTA (6734C>T), GTG2186GTA (6742G>A), GTG2187GTA (6745G>A), GCC2190GCT (6754C>T), GGA2192GGG (6760A>G), ATA2194TTG (6764A>T 6766A>G), ATT2197ATA (6775T>A), CTG2200TTG (6782C>T), GCA2201GCT (6787A>T), AAC2202AAT (6790C>T), GCA2204GTG (6795C>T 6796A>G), CAC2205CAA (6799C>A), ACC2206ACA (6802C>A), AAC2208AAT (6808C>T), TAC2209TAT (6811C>T), TAC2210TAT (6814C>T), TTC2213TTT (6823C>T), GAA2215GAG (6829A>G), TAC2221TAT (6847C>T), CGA2223CGC (6853A>C), AAT2225GAT (6857A>G), GAG2226GAA (6862G>A), GAA2229GAG (6871A>G), GTC2231ACA (6875G>A 6876T>C 6877C>A), AAG2232AAA (6880G>A), TCT2233TCA (6883T>A), GTT2235GTG (6889T>G), GAC2239GAT (6901C>T), GGC2244GGT (6916C>T), CCG2245CCA (6919G>A), AAA2247AAG (6925A>G), CTT2248CTC (6928T>C), GAC2250GAA (6934C>A), GGG2255GGC (6949G>C), TTC2257TTT (6955C>T), AAA2258AAG (6958A>G), CAA2264CAG (6976A>G), CTA2266TTA (6980C>T), ATC2267ATT (6985C>T), GTA2271GTT (6997A>T), GTG2272GTT (7000G>T), GTG2273ATA (7001G>A 7003G>A), GCT2276GCC (7012T>C), AAG2277AAA (7015G>A), CAT2278CAC (7018T>C), TTT2283TAT (7032T>A), GCC2285GCT (7039C>T), TTG2286CTA (7040T>C 7042G>A), GGA2288GGG (7048A>G), GCT2292GCA (7060T>A), GTC2293GTA (7063C>A), AGC2298AGT (7078C>T), TTA2300CTG (7082T>C 7084A>G), GTA2301GTG (7087A>G), ACC2302ACA (7090C>A), CAT2304CAC (7096T>C), GTA2305GTG (7099A>G), GTT2306GTC (7102T>C), GAG2309GAA (7111G>A), CGA2311CGG (7117A>G), ACA2314AAA (7125C>A), GAA2315GAG (7129A>G), TTT2316TTC (7132T>C), CTG2317TTA (7133C>T 7135G>A), GTG2319GTA (7141G>A), GAT2320GAC (7144T>C), GCA2321TCG (7145G>T 7147A>G), GAG2322GAA (7150G>A), GCA2325GCT (7159A>T), GAG2326_GCA2327del (7160_7165delGAGGCA), TTC2329TTT (7171C>T), GCG2335GCT (7189G>T), AAA2338AAG (7198A>G), AGC2339AGT (7201C>T), CTA2341TTA (7205C>T), AAT2342AAC (7210T>C), GAT2344GAA (7216T>A), GCG2345GCA (7219G>A), ATC2347ATA (7225C>A), AAG2348AAA (7228G>A), AAG2352AAA (7240G>A), TAT2353TAC (7243T>C), AAA2355AAG (7249A>G), CCT2356CCG (7252T>G), GAT2358GAA (7258T>A), GTT2359GTC (7261T>C), GGT2360GGA (7264T>A), GTC2361GTG (7267C>G), GTG2362GTA (7270G>A), GAT2363_CGG2364insT-- (7273_7274insT), CGG2364GTG (7274C>G 7275G>T), CAT2366CTT (7281A>T), TTG2367TCG (7284T>C), TCA2371TTA (7296C>T), TCT2376TTT (7311C>T), ACC2377ATC (7314C>T), TGC2378TAC (7317G>A), AAT2379AAA (7321T>A), GTG2380-TG (7322delG), CAC2381CAT (7327C>T), GGC2382GGT (7330C>T), TTC2383TTT (7333C>T), AAG2384CAG (7334A>C), TGT2386TGC (7342T>C), GCA2387AAC (7343G>A 7344C>A 7345A>C), ACT2390ACC (7354T>C), CAA2393CAG (7363A>G), ATT2395ATC (7369T>C), TTC2396TTT (7372C>T), GCG2398GCT (7378G>T), CTC2399CTT (7381C>T), GCA2404GCT (7396A>T), GTC2405GTT (7399C>T), GCC2407GCT (7405C>T), AGT2408ATG (7407G>T 7408T>G), GGG2410GGT (7414G>T), TGC2411GGC (7415T>G), AAA2412AAG (7420A>G), GAC2415GAT (7429C>T), TAT2416TAC (7432T>C), CAT2419CAC (7441T>C), AAG2425AAA (7459G>A), GAA2426GAG (7462A>G), ATA2428ATT (7468A>T), GTC2429GTA (7471C>A), CAA2431CAG (7477A>G), AGC2432AGT (7480C>T), CTG2434CTT (7486G>T), TAT2437TAC (7495T>C), AAA2438AAG (7498A>G), GGT2439GGC (7501T>C), TTG2440TCA (7503T>C 7504G>A), ATT2443ATA (7513T>A), AAC2445AAT (7519C>T), GGA2446GGG (7522A>G), AAG2449AAA (7531G>A), GAG2451GAA (7537G>A), CTC2452CTT (7540C>T), TGT2454TGC (7546T>C), AAG2462AAA (7570G>A), ACG2463ACA (7573G>A), ACG2465ACA (7579G>A), GCT2468GCC (7588T>C), CTA2471TTA (7595C>T), TTG2474CTA (7604T>C 7606G>A), GGT2477GGA (7615T>A), TGT2480TGC (7624T>C), GAC2483GAT (7633C>T), TTC2484TTT (7636C>T), AAT2485AAC (7639T>C), TTT2488TTC (7648T>C), GAA2494GAG (7666A>G), TGT2496TGC (7672T>C), ACA2498ACT (7678A>T), GGG2500GGA (7684G>A), ACT2502ACC (7690T>C), TTT2504TTC (7696T>C), GGT2506GGG (7702T>G), GAT2509AAT (7709G>A), AAA2510AGG (7713A>G 7714A>G), CTG2511TTG (7715C>T), CGT2514CGC (7726T>C), GAG2517GAA (7735G>A), GTA2520GTG (7744A>G), TAC2521TAT (7747C>T), GGC2526GGT (7762C>T), CAG2528CAA (7768G>A), TTT2529TTC (7771T>C), TCA2532TCG (7780A>G), CTA2533CTC (7783A>C), ACT2534ACC (7786T>C), CTA2537TTG (7793C>T 7795A>G), ATC2538ATT (7798C>T), GCT2540GCC (7804T>C), ACC2543ATC (7812C>T), GAA2550GAG (7834A>G), GAC2551GAT (7837C>T), GTG2554TTA (7844G>T 7846G>A), CAG2557CAA (7855G>A), CTG2559CTA (7861G>A), TTA2562TTG (7870A>G), TAC2563TAT (7873C>T), ACT2564ACA (7876T>A), GAG2565GAA (7879G>A), ATT2566ATA (7882T>A), GTT2567ATT (7883G>A), CCA2570CCT (7894A>T), ATT2571ATC (7897T>C), GTC2579GTT (7921C>T), AAG2581AAA (7927G>A), GGT2584GGC (7936T>C), AAT2585AAC (7939T>C), AAT2586AAC (7942T>C), GTG2594GTA (7966G>A), GAT2595GAC (7969T>C), TCT2597TCC (7975T>C), GTC2601GTT (7987C>T), CTT2602CTC (7990T>C), GCT2607GCC (8005T>C), GAA2616GAG (8032A>G), ATC2618ATT (8038C>T), GAC2619GAT (8041C>T), AGC2620AAC (8043G>A), TTG2632TTA (8080G>A), GTG2635GTA (8089G>A), CCG2637CCA (8095G>A), GAG2638GAA (8098G>A), AGC2641AGT (8107C>T), ATT2642ATC (8110T>C), GAT2644GAC (8116T>C), CAA2648CAG (8128A>G), GGT2654GGC (8146T>C), AAC2656AGT (8151A>G 8152C>T), TCG2660TCA (8164G>A), AGG2665AAG (8178G>A), AAG2666AAA (8182G>A), TTG2669TTA (8191G>A), TTC2671TTT (8197C>T), TCC2673TCT (8203C>T), AGA2675AAA (8208G>A), CTG2677TTA (8213C>T 8215G>A), CTA2678CTG (8218A>G), ATC2679ATT (8221C>T), GAG2680GAA (8224G>A), CCA2685CCG (8239A>G), GAG2690GAA (8254G>A), ATT2692ATC (8260T>C), GTA2693GTG (8263A>G), GAT2699GAC (8281T>C), AGA2700AGG (8284A>G), CCA2704GCG (8294C>G 8296A>G), GAG2705GAA (8299G>A), CAC2706CAT (8302C>T), ATT2711ATC (8317T>C), TGC2712TGT (8320C>T), GCT2714GCA (8326T>A), GAG2717GAA (8335G>A), TCC2718TCT (8338C>T), TCT2722TTT (8349C>T), GAA2723GAG (8353A>G), CAC2726CAT (8362C>T), CAA2727CAG (8365A>G), ATC2728ATT (8368C>T), TAC2732TAT (8380C>T), TCA2733TCG (8383A>G), TTA2735TTG (8389A>G), CCT2740CCC (8404T>C), GCA2742TCA (8408G>T), ACA2743ACG (8413A>G), GCG2745GCT (8419G>T), GCT2750GCC (8434T>C), TAT2752TAC (8440T>C), ATG2756ATA (8452G>A), CTA2758CTG (8458A>G), AGG2759AAG (8460G>A), AAA2760AAG (8464A>G), CTG2761CTA (8467G>A), AGG2765AGA (8479G>A), GCT2766ACA (8480G>A 8482T>A), GAG2771GAA (8497G>A), CTA2772TTG (8498C>T 8500A>G), AGA2773AAG (8502G>A 8503A>G), GCC2774GCT (8506C>T), GAA2777GAT (8515A>T), GTC2780GTT (8524C>T), GCA2781GCC (8527A>C), TTA2782CTG (8528T>C 8530A>G), GAG2785GAC (8539G>C), CTT2788TGT (8546C>T 8547T>G), TCT2790ACT (8552T>A), GTA2793GTG (8563A>G), GCA2797GGA (8574C>G), ATT2801GTT (8585A>G), GGA2805GAA (8598G>A), AGC2806AGT (8602C>T), AAC2807TCT (8603A>T 8604A>C 8605C>T), AAA2812AAG (8620A>G), CCA2813CAA (8622C>A), GAG2814GGA (8625A>G 8626G>A), CAG2815CAA (8629G>A), GGC2816GGT (8632C>T), ATC2818ATT (8638C>T), CAG2819CAA (8641G>A), AAC2821AAT (8647C>T), CCG2822TTC (8648C>T 8649C>T 8650G>C), AAC2823AAT (8653C>T), AAA2824AAG (8656A>G), GGA2825GAA (8658G>A), GAT2827GCG (8664A>C 8665T>G), GTT2830GTG (8674T>G), GCA2832GTT (8679C>T 8680A>T), GGC2833GGA (8683C>A), GGG2836GGA (8692G>A), ACA2837ACT (8695A>T), CCG2841CCA (8707G>A), AGA2842CGA (8708A>C), AAG2844AAA (8716G>A), ATC2846ATT (8722C>T), ACG2847ACA (8725G>A), AAA2849AAG (8731A>G), AGA2851AGG (8737A>G), CCC2853CCT (8743C>T), ACA2854AAA (8745C>A), AAG2856AAA (8752G>A), GGA2857GGT (8755A>T), AAC2862AAT (8770C>T), TTA2863CTA (8771T>C), GAA2864GAG (8776A>G), CAT2865CAC (8779T>C), TTG2866TTA (8782G>A), TAT2869TAC (8791T>C), CCA2871CCG (8797A>G), CAA2872CAG (8800A>G), CAA2873CAG (8803A>G), GAT2875GAC (8809T>C), ATT2876ATC (8812T>C), AAT2878AAC (8818T>C), CGG2880CGA (8824G>A), GCA2881GCG (8827A>G), ACG2888ACA (8848G>A), TAT2890TAC (8854T>C), GAG2891GGA (8856A>G 8857G>A), GTG2893GTA (8863G>A), CGG2894CAG (8865G>A), ATG2895CTT (8867A>C 8869G>T), GCA2896GCG (8872A>G), GGA2900GAA (8883G>A), ACT2902TCT (8888A>T), TGC2915TGT (8929C>T), GTC2924ATC (8954G>A), AAC2925AAT (8959C>T), GGA2926GGT (8962A>T), GGG2933GGA (8983G>A), AAT2934GAC (8984A>G 8986T>C), GAA2935GTA (8988A>T), GAG2938GAA (8998G>A), CCG2940CCA (9004G>A), TTG2941TTA (9007G>A), ATC2944ATT (9016C>T), ACC2951ACA (9037C>A), ATA2967ATT (9085A>T), AAC2971AAT (9097C>T), ATT2983GTT (9131A>G), CGA2984CGT (9136A>T), CGG2987CGT (9145G>T), ATG2989GGA (9149A>G 9150T>G 9151G>A), GGT2990AGT (9152G>A), TTA2991TTG (9157A>G), GCG2992GCT (9160G>T), CGT2993CGC (9163T>C), GAG3000GAA (9184G>A), CGA3004CGG (9196A>G), AGG3010AGA (9214G>A), GAA3011GAG (9217A>G), GCG3012GCA (9220G>A), GCA3020GCT (9244A>T), TTG3021TTA (9247G>A), GCC3024GCT (9256C>T), CCT3026TCC (9260C>T 9262T>C), GGG3030GGA (9274G>A), GAC3032GAT (9280C>T), ATC3035ATT (9289C>T), CAA3038CAC (9298A>C), GAG3040GAA (9304G>A), GTC3050GTT (9334C>T), GTC3060GTG (9364C>G)								
PVYgp2	1	75	100%	95	18.1%	75 (98.7%)	54 (71.1%)	1/0/3/2	2
Protein mutations:	R17_K17insX (2971_2972insA), Y23H (2989T>C 2991C>T), R26K (2999G>A 3000A>G), H35Y (3025C>T), K40H (3040A>C 3042A>C), G41S (3043G>A), E44K (3052G>A), I47V (3061A>G), V54I (3082G>A), P57S (3091C>T), P60T (3100C>A), G61E (3104G>A 3105T>G), G62S (3106G>A), Q63* (3109C>T), R64G (3112A>G), C66R (3118T>C), L67F (3121C>T), I69V (3127A>G), A72E (3137C>A 3138G>A), I73V (3139A>G)								
Codon mutations:	TTA2CTA (2923T>C), AAA4AAG (2931A>G), GAA7GAG (2940A>G), TTG10CTG (2947T>C), TTG15CTG (2962T>C), AGA17_AAA17insAA- (2971_2972insA), TAC23CAT (2989T>C 2991C>T), TCA24TCG (2994A>G), AGA26AAG (2999G>A 3000A>G), CAC32-AC (3016delC), TCG33TCA (3021G>A), GTA34GTT (3024A>T), CAT35TAT (3025C>T), CAC38CAT (3036C>T), AGA39AGG (3039A>G), AAA40CAC (3040A>C 3042A>C), GGC41AGC (3043G>A), GAA44AAA (3052G>A), ATA47GTA (3061A>G), GTT54ATT (3082G>A), CCG57TCG (3091C>T), CGC59CGT (3099C>T), CCA60ACA (3100C>A), GGT61GAG (3104G>A 3105T>G), GGT62AGT (3106G>A), CAA63TAA (3109C>T), AGG64GGG (3112A>G), TGC66CGC (3118T>C), CTC67TTC (3121C>T), ATT69GTT (3127A>G), GAG70GAA (3132G>A), GCG72GAA (3137C>A 3138G>A), ATT73GTT (3139A>G)								

	Begin	End	Coverage	Score	Concordance	Matches	Identities	I/D/M/F*	Stop Codons
**NT**	**54**	**9704**	**99.5%**	**12522**	**65.2%**	**9639 (99.8%)**	**7984 (82.7%)**	**7/12**	
**Proteins**									
polyprotein (NP_056759.1)	1	3064	100%	18924	89.7%	3062 (99.9%)	2771 (90.4%)	2/2/4/4	1
Protein mutations:	Y4Q (194T>C 196C>A), C9L (209T>C 210G>T 211T>G), F13M (221T>A 223T>G), S23P (251T>C), C24F (255G>T 256C>T), E25G (258A>G), I27V (263A>G 265T>G), V28A (267T>C), E30V (273A>T), A35T (287G>A 289T>C), V37T (293G>A 294T>C), A41I (305G>A 306C>T), D42G (309A>G), E44D (316A>T), T45V (317A>G 318C>T 319A>G), L52Q (339T>A 340C>A), K53R (342A>G), K55A (347A>G 348A>C 349A>G), Y56H (350T>C), T58V (356A>G 357C>T), V61T (365G>A 366T>C 367G>A), L62S (368C>T 369T>C), F67C (384T>G), A75V (408C>T 409C>T), M78E (416A>G 417T>A), E85K (437G>A), R86E (440A>G 441G>A), K87R (443A>C 444A>G 445G>A), D88E (448T>A), E91A (456A>C), H93N (461C>A), D111E (517T>A), S116F (531C>T), P118S (536C>T), Q119W (539C>T 540A>G 541A>G), R122K (549G>A 550A>G), M131T (576T>C), V134A (585T>C 586C>G), R137Y (593C>T 594G>A 595C>T), P138R (597C>G), I139V (599A>G 601A>G), I140P (602A>C 603T>C 604A>G), M147I (625G>A), I151V (635A>G 637T>C), I154V (644A>G 646A>G), E160A (663A>C 664G>C), H166Q (682C>A), T172S (699C>G), H174Q (706T>A), K179E (719A>G 721G>A), I180V (722A>G 724A>G), A183S (731G>T), Y184P (734T>C 735A>C 736C>T), S185R (737T>C 738C>G 739C>T), A187V (744C>T 745G>C), R189C (749C>T 751A>C), M194K (765T>A), R197K (773C>A 774G>A 775A>G), M206K (801T>A), G210A (813G>C 814A>G), L211H (816T>A), R214Q (825G>A 826T>A), N223D (851A>G 853T>C), R226C (860C>T 862C>T), T227A (863A>G 865T>C), I228T (867T>C), N229D (869A>G 871C>T), I230L (872A>T 874A>G), R231Q (876G>A), R232K (879G>A), N240S (903A>G), T241N (906C>A 907A>C), K242T (909A>C 910A>C), S243N (912G>A 913C>T), S252L (938T>C 939C>T 940A>T), L266I (980T>A 982G>C), R272K (999G>A), Q275R (1007C>A 1008A>G 1009G>A), S276G (1010A>G 1012T>G), I277V (1013A>G), N279Q (1019A>C 1021C>G), D288E (1048C>A), R300Q (1083G>A), S305A (1097T>G 1099G>A), R320K (1143G>A), L324I (1154T>A 1156G>A), S346N (1221G>A), V349T (1229G>A 1230T>C 1231T>C), S350N (1233G>A 1234C>T), K358T (1257A>C 1258A>G), N365S (1278A>G), A369V (1290C>T 1291G>A), D372T (1298G>A 1299A>C 1300C>T), I375V (1307A>G 1309A>G), N378E (1316A>G 1318T>A), I382T (1329T>C), L397I (1373C>A), I401V (1385A>G), E407D (1405G>T), A411S (1415G>T 1417A>T), V418I (1436G>A 1438C>T), E508R (1706G>A 1707A>G), A512T (1718G>A), I520V (1742A>G 1744C>T), S525N (1758G>A), V536I (1790G>A), I585V (1937A>G 1939T>C), F607Y (2004T>A), I628M (2068T>G), V630I (2072G>A 2074G>A), R675K (2208G>A), D694E (2266C>G), N744G (2414A>G 2415A>G 2416T>G), S746C (2420A>T), K780R (2523A>G), R817K (2634G>A), V847I (2723G>A 2725C>T), A855T (2747G>A 2749T>A), H871N (2795C>A), V880I (2822G>A 2824G>A), P900T (2882C>A), S901H (2885A>C 2886G>A), T904M (2895C>T), N916S (2931A>G), N919S (2940A>G), E929_N930insX (2971_2972insA), T936I (2991C>T), H937R (2994A>G), E939R (2999G>A 3000A>G), R946Q (3021G>A), Y947F (3024A>T 3025C>T), T951I (3036C>T), E952G (3039A>G 3040A>C), K953T (3042A>C 3043G>A), A972V (3099C>T 3100C>A), V974R (3104G>A 3105T>G 3106G>A), S983N (3132G>A), R985K (3137C>A 3138G>A 3139A>G), F990I (3152T>A 3154C>T), C994Y (3165G>A), F1012L (3218T>C), T1014N (3225C>A), V1016I (3230G>A), V1028M (3266G>A 3268A>G), V1031M (3275G>A), A1034T (3284G>A), R1044K (3315G>A 3316A>G), E1045A (3318A>C), M1059V (3359A>G), D1072E (3400T>G), V1110G (3513T>G), M1121V (3545A>G), A1122T (3548G>A), V1124I (3554G>A 3556C>T), L1147V (3623C>G), L1150M (3632C>A), Y1152H (3638T>C), I1172V (3698A>G), S1178N (3717G>A), R1183K (3731C>A 3732G>A), I1297V (4073A>G), F1315Y (4128T>A 4129C>T), A1323S (4151G>T), V1328I (4166G>A), L1347I (4223C>A 4225G>A), I1379V (4319A>G 4321A>G), F1404Y (4395T>A), V1408I (4406G>A), M1431V (4475A>G 4477G>T), T1433S (4481A>T 4483A>G), A1454L (4544G>T 4545C>T 4546G>A), R1455K (4548G>A), V1499I (4679G>A 4681G>C), V1538I (4796G>A 4798C>T), T1540S (4803C>G 4804T>C), S1625G (5057A>G), T1626A (5060A>G), L1636I (5090C>A), A1639V (5100C>T), E1641D (5107A>T), V1646F (5120G>T 5122C>T), I1648V (5126A>G), V1667I (5183G>A), A1793T (5561G>A 5563G>A), I1805V (5597A>G), N1807K (5605C>A), A1812V (5619C>T 5620T>G), I1816L (5630A>C), V1837I (5693G>A), K1890R (5853A>G), I1904V (5894A>G 5896C>G), R1926A (5960C>G 5961G>C 5962G>C), I1936V (5990A>G), E1945D (6019A>T), K1949T (6030A>C), D1955E (6049C>A), M1958I (6058G>A), S1963N (6072G>A 6073T>C), N1964H (6074A>C), T1966N (6081C>A), C1976S (6111G>C 6112T>C), I1982V (6128A>G 6130T>C), V1992I (6158G>A), T1996S (6170A>T 6172A>G), L2007F (6203C>T 6205C>T), V2016I (6230G>A), A2025K (6257G>A 6258C>A), K2033R (6282A>G), A2060T (6362G>A), V2073I (6401G>A), A2074V (6405C>T), Y2082F (6429A>T), Q2089R (6450A>G), H2101N (6485C>A 6487C>T), L2115I (6527C>A), T2135I (6588C>T), Y2151H (6635T>C), I2155V (6647A>G), I2165V (6677A>G 6679A>G), D2178N (6716G>A), N2179D (6719A>G), I2194L (6764A>T 6766A>G), A2204V (6795C>T 6796A>G), H2205Q (6799C>A), N2225D (6857A>G), V2231T (6875G>A 6876T>C 6877C>A), D2250E (6934C>A), V2273I (7001G>A 7003G>A), F2283Y (7032T>A), T2314K (7125C>A), A2321S (7145G>T 7147A>G), E2326_A2327del (7160_7165delGAGGCA), D2344E (7216T>A), D2358E (7258T>A), D2363_R2364insX (7273_7274insT), R2364V (7274C>G 7275G>T), H2366L (7281A>T), L2367S (7284T>C), S2371L (7296C>T), S2376F (7311C>T), T2377I (7314C>T), C2378Y (7317G>A), N2379K (7321T>A), K2384Q (7334A>C), A2387N (7343G>A 7344C>A 7345A>C), S2408M (7407G>T 7408T>G), C2411G (7415T>G), L2440S (7503T>C 7504G>A), D2509N (7709G>A), K2510R (7713A>G 7714A>G), T2543I (7812C>T), V2554L (7844G>T 7846G>A), V2567I (7883G>A), S2620N (8043G>A), N2656S (8151A>G 8152C>T), R2665K (8178G>A), R2675K (8208G>A), P2704A (8294C>G 8296A>G), S2722F (8349C>T), A2742S (8408G>T), M2756I (8452G>A), R2759K (8460G>A), A2766T (8480G>A 8482T>A), R2773K (8502G>A 8503A>G), E2777D (8515A>T), E2785D (8539G>C), L2788C (8546C>T 8547T>G), S2790T (8552T>A), A2797G (8574C>G), I2801V (8585A>G), G2805E (8598G>A), N2807S (8603A>T 8604A>C 8605C>T), P2813Q (8622C>A), E2814G (8625A>G 8626G>A), P2822F (8648C>T 8649C>T 8650G>C), G2825E (8658G>A), D2827A (8664A>C 8665T>G), A2832V (8679C>T 8680A>T), T2854K (8745C>A), E2891G (8856A>G 8857G>A), R2894Q (8865G>A), M2895L (8867A>C 8869G>T), G2900E (8883G>A), T2902S (8888A>T), V2924I (8954G>A), N2934D (8984A>G 8986T>C), E2935V (8988A>T), I2983V (9131A>G), M2989G (9149A>G 9150T>G 9151G>A), G2990S (9152G>A), P3026S (9260C>T 9262T>C), Q3038H (9298A>C)								

	Begin	End	Coverage	Score	Concordance	Matches	Identities	I/D/M/F*	Stop Codons
NT	54	9704	99.5%	12522	65.2%	9639 (99.8%)	7984 (82.7%)	7/12	

Codon mutations:

ACT3ACC (193T>C), TAC4CAA (194T>C 196C>A), TCA6TCG (202A>G), ATC8ATT (208C>T), TGT9CTG (209T>C 210G>T 211T>G), TCG12TCA (220G>A), TTT13ATG (221T>A 223T>G), GAA14GAG (226A>G), CTA17CTT (235A>T), TAC19TAT (241C>T), CCA21CCT (247A>T), TCT23CCT (251T>C), TGC24TTT (255G>T 256C>T), GAG25GGG (258A>G), CAT26CAC (262T>C), ATT27GTG (263A>G 265T>G), GTG28GCG (267T>C), GAA30GTA (273A>T), CGA31CGT (277A>T), GAA32GAG (280A>G), GTG33GTT (283G>T), CCG34CCA (286G>A), GCT35ACC (287G>A 289T>C), GTT37ACT (293G>A 294T>C), CCT39CCC (301T>C), TTC40TTT (304C>T), GCA41ATA (305G>A 306C>T), GAT42GGT (309A>G), CTG43TTG (311C>T), GAA44GAT (316A>T), ACA45GTG (317A>G 318C>T 319A>G), CAA46CAG (322A>G), GCA49GCT (331A>T), CTC52CAA (339T>A 340C>A), AAG53AGG (342A>G), AAA55GCG (347A>G 348A>C 349A>G), TAT56CAT (350T>C), GCT57GCA (355T>A), ACT58GTT (356A>G 357C>T), CGT60CGC (364T>C), GTG61ACA (365G>A 366T>C 367G>A), CTC62TCC (368C>T 369T>C), AAA63AAG (373A>G), AAC64AAT (376C>T), TTT67TGT (384T>G), ACG68ACA (388G>A), TAC69TAT (391C>T), CGA70CGG (394A>G), AAG72AAA (400G>A), GAT74GAC (406T>C), GCC75GTT (408C>T 409C>T), CAG76CAA (412G>A), ATA77ATT (415A>T), ATG78GAG (416A>G 417T>A), ATT80ATC (424T>C), CAG81CAA (427G>A), AAA83AAG (433A>G), CTG84CTC (436G>C), GAG85AAG (437G>A), AGG86GAG (440A>G 441G>A), AAG87CGA (443A>C 444A>G 445G>A), GAT88GAA (448T>A), GAA90GAG (454A>G), GAA91GCA (456A>C), TAT92TAC (460T>C), CAC93AAC (461C>A), CAA95CAG (469A>G), GCC97GCG (475C>G), CCT99CCA (481T>A), ACT106ACC (502T>C), ATA107ATT (505A>T), GGC109GGT (511C>T), GAT111GAA (517T>A), CCT112CCA (520T>A), CCA113CCT (523A>T), TCT116TTT (531C>T), GAG117GAA (535G>A), CCA118TCA (536C>T), CAA119TGG (539C>T 540A>G 541A>G), AGA122AAG (549G>A 550A>G), ATC124ATT (556C>T), ATT125ATC (559T>C), CAT126CAC (562T>C), ACT128ACC (568T>C), CCA129CCG (571A>G), ATG131ACG (576T>C), CGT132CGC (580T>C), GTC134GCG (585T>C 586C>G), CGC137TAT (593C>T 594G>A 595C>T), CCC138CGC (597C>G), ATA139GTG (599A>G 601A>G), ATA140CCG (602A>C 603T>C 604A>G), ACA143ACG (613A>G), GAA144GAG (616A>G), GGC145GGG (619C>G), CAG146CAA (622G>A), ATG147ATA (625G>A), AAT148AAC (628T>C), CAC149CAT (631C>T), ATT151GTC (635A>G 637T>C), ATA154GTG (644A>G 646A>G), ATT157ATC (655T>C), TCG159TCA (661G>A), GAG160GCC (663A>C 664G>C), AAA161AAG (667A>G), GTC165GTT (679C>T), CAC166CAA (682C>A), TTA167CTG (683T>C 685A>G), AGT169AGC (691T>C), ACC172AGC (699C>G), ACT173ACA (703T>A), CAT174CAA (706T>A), AAG179GAA (719A>G 721G>A), ATA180GTG (722A>G 724A>G), CTT181TTG (725C>T 727T>G), GGT182GGA (730T>A), GCA183TCA (731G>T), TAC184CCT (734T>C 735A>C 736C>T), TCC185CGT (737T>C 738C>G 739C>T), GCG187GTC (744C>T 745G>C), CGA189TGC (749C>T 751A>C), ACT190ACC (754T>C), ATG194AAG (765T>A), TTG196CTG (770T>C), CGA197AAG (773C>A 774G>A 775A>G), CGG198AGG (776C>A), TTC202TTT (790C>T), ATG206AAG (801T>A), GGA210GCG (813G>C 814A>G), CTT211CAT (816T>A), TTG212CTG (818T>C), CAA213CAG (823A>G), CGT214CAA (825G>A 826T>A), CGG217AGA (833C>A 835G>A), GAC219GAT (841C>T), AAT223GAC (851A>G 853T>C), GTC225GTT (859C>T), CGC226TGT (860C>T 862C>T), ACT227GCC (863A>G 865T>C), ATC228ACC (867T>C), AAC229GAT (869A>G 871C>T), ATA230TTG (872A>T 874A>G), CGA231CAA (876G>A), AGG232AAG (879G>A), GAT234GAC (886T>C), ATC238ATT (898C>T), AAC240AGC (903A>G), ACA241AAC (906C>A 907A>C), AAA242ACC (909A>C 910A>C), AGC243AAT (912G>A 913C>T), GGC246GGA (922C>A), GGT249GGA (931T>A), TCA252CTT (938T>C 939C>T 940A>T), GGA253GGG (943A>G), TTC256TTT (952C>T), TCA261TCG (967A>G), GAA263GAG (973A>G), GGG264GGC (976G>C), TTG266ATC (980T>A 982G>C), GCA269GCG (991A>G), CGT270CGC (994T>C), AGA272AAA (999G>A), GTT273GTC (1003T>C), CAG275AGA (1007C>A 1008A>G 1009G>A), AGT276GGG (1010A>G 1012T>G), ATT277GTT (1013A>G), TTA278TTG (1018A>G), AAC279CAG (1019A>C 1021C>G), ATC282ATT (1030C>T), TCG285TCA (1039G>A), AAT286AAC (1042T>C), GCC287GCA (1045C>A), GAC288GAA (1048C>A), AAT289AAC (1051T>C), AAG292AAA (1060G>A), GGT293GGA (1063T>A), CTG294TTG (1064C>T), GAC295GAT (1069C>T), CGA300CAA (1083G>A), TAT303TAC (1093T>C), TCG305GCA (1097T>G 1099G>A), CAC307CAT (1105C>T), GTA310GTG (1114A>G), GCT311GCG (1117T>G), TTA313TTG (1123A>G), CCT314CCA (1126T>A), GTC315GTT (1129C>T), AGG320AAG (1143G>A), GTA321GTT (1147A>T), GCT322GCA (1150T>A), TTG324ATA (1154T>A 1156G>A), CAC327CAT (1165C>T), AGT328AGC (1168T>C), CTT330TTA (1172C>T 1174T>A), ACC339ACT (1201C>T), AGC346AAC (1221G>A), GTT349ACC (1229G>A 1230T>C 1231T>C), AGC350AAT (1233G>A 1234C>T), CTG352CTA (1240G>A), AAG354AAA (1246G>A), TTG356CTA (1250T>C 1252G>A), CAT357CAC (1255T>C), AAA358ACG (1257A>C 1258A>G), AGA361AGG (1267A>G), GGT363GGC (1273T>C), TTG364TTA (1276G>A), AAT365AGT (1278A>G), TTG367CTG (1283T>C), GCG369GTA (1290C>T 1291G>A), GAC372ACT (1298G>A 1299A>C 1300C>T), TTT374TTC (1306T>C), ATA375GTG (1307A>G 1309A>G), CAT376CAC (1312T>C), AAT378GAA (1316A>G 1318T>A), TTG381TTA (1327G>A), ATA382ACA (1329T>C), GCG383GCT (1333G>T), TTA384TTG (1336A>G), CTA387TTG (1343C>T 1345A>G), CCG390CCA (1354G>A), GTG391GTT (1357G>T), GAC392GAT (1360C>T), CTC395TTG (1367C>T 1369C>G), CTT397ATT (1373C>A), TTC398TTT (1378C>T), GAG400GAA (1384G>A), ATA401GTA (1385A>G), AAA403AAG (1393A>G), TCC404TCT (1396C>T), GAG407GAT (1405G>T), AAA408AAG (1408A>G), CAG409CAA (1411G>A), GCA411TCT (1415G>T 1417A>T), CCG412CCC (1420G>C), AAT415AAC (1429T>C), TTA416TTG (1432A>G), GTC418ATT (1436G>A 1438C>T), TTA419CTG (1439T>C 1441A>G), AAT420AAC (1444T>C), TTC423TTT (1453C>T), CTG424CTA (1456G>A), GAA428GAG (1468A>G), GTA436GTG (1492A>G), TTG439CTA (1499T>C 1501G>A), AGT440AGC (1504T>C), TTG441TTA (1507G>A), CTC442CTT (1510C>T), GAA443GAG (1513A>G), TTA444TTG (1516A>G), GCA445GCT (1519A>T), AGG446AGA (1522G>A), TTC447TTT (1525C>T), CAG448CAA (1528G>A), AAC450AAT (1534C>T), ACT452ACA (1540T>A), AAC454AAT (1546C>T), AAA457AAG (1555A>G), GGT458GGC (1558T>C), GAT459GAC (1561T>C), ATA460ATC (1564A>C), TCT461TCA (1567T>A), TTC463TTT (1573C>T), AGA464AGG (1576A>G), TTA467CTA (1583T>C), TCT468TCA (1588T>A), AAG470AAA (1594G>A), AAC472AAT (1600C>T), CTG475CTA (1609G>A), TCG478TCA (1618G>A), TGC479TGT (1621C>T), GAC480GAT (1624C>T), CTG483TTG (1631C>T), AAA485AAG (1639A>G), TTC489TTT (1651C>T), CTC490CTG (1654C>G), CAT497CAC (1675T>C), TCA503TCG (1693A>G), TTT506TTC (1702T>C), GAA508AGA (1706G>A 1707A>G), GCA512ACA (1718G>A), GGA514GGG (1726A>G), TAC515TAT (1729C>T), TCA516TCC (1732A>C), TAT518TAC (1738T>C), ATC520GTT (1742A>G 1744C>T), CGC521CGT (1747C>T), CAT523CAC (1753T>C), AGT525AAT (1758G>A), AGG528AGA (1768G>A), AAC534AAT (1786C>T), TTA535TTG (1789A>G), GTT536ATT (1790G>A), GTC537GTT (1795C>T), TTA541CTG (1805T>C 1807A>G), GAG543GAA (1813G>A), AGG545AGA (1819G>A), AAG547AAA (1825G>A), GAC551GAT (1837C>T), TAT552TAC (1840T>C), CAA555CAG (1849A>G), GTC558GTG (1858C>G), AAA560AAG (1864A>G), TGC562TGT (1870C>T), AGT564AGC (1876T>C), AAA566AAG (1882A>G), GGT568GGA (1888T>A), AAT569AAC (1891T>C), TGT574TGC (1906T>C), ACA577ACT (1915A>T), GGT582GGC (1930T>C), GCC584GCT (1936C>T), ATT585GTC (1937A>G 1939T>C), TCA587TCG (1945A>G), TTC589TTT (1951C>T), CCA592CCT (1960A>T), GTA598GTG (1978A>G), ATT599ATA (1981T>A), GGC600GGT (1984C>T), AAT601AAC (1987T>C), GAC604GAT (1996C>T), TTT607TAT (2004T>A), AAA612AAG (2020A>G), GGG613GGA (2023G>A), GAT614GAC (2026T>C), TCG615TCT (2029G>T), GAG616GAA (2032G>A), TAT625TAC (2059T>C), ATT628ATG (2068T>G), AAC629AAT (2071C>T), GTG630ATA (2072G>A 2074G>A), CTG635TTA (2087C>T 2089G>A), AGC639AGT (2101C>T), GAG640GAA (2104G>A), GAG641GAA (2107G>A), AAG644AAA (2116G>A), TTC646TTT (2122C>T), CGC651CGT (2137C>T), CTT658CTC (2158T>C), ACC660ACT (2164C>T), CCA662CCG (2170A>G), GAT666GAC (2182T>C), GCG668GCA (2188G>A), ACC669ACA (2191C>A), AGA675AAA (2208G>A), TAT678TAC (2218T>C), GAC680GAT (2224C>T), GTA681GTT (2227A>T), TTG686TTA (2242G>A), CCC687CCT (2245C>T), TTG690TTA (2254G>A), GTT691GTC (2257T>C), GAC692GAT (2260C>T), GAC694GAG (2266C>G), CAA696CAG (2272A>G), ACG697ACA (2275G>A), TCA703TCG (2293A>G), GGC705GGT (2299C>T), TCG706TCA (2302G>A), CAG707CAA (2305G>A), GGA710GGT (2314A>T), TAT711TAC (2317T>C), CTA714CTG (2326A>G), GTG719GTA (2341G>A), CAA721CAG (2347A>G), CTT722CTC (2350T>C), TTG724TTA (2356G>A), AAT727AAC (2365T>C), GAA729GAG (2371A>G), TTA730TTG (2374A>G), GAT733GAC (2383T>C), ATA734ATT (2386A>T), AAA735AAG (2389A>G), CAT736CAC (2392T>C), TAT737TAC (2395T>C), AAT744GGG (2414A>G 2415A>G 2416T>G), GCT745GCA (2419T>A), AGC746TGC (2420A>T), ACA752ACT (2440A>T), GTT761GTC (2467T>C), TCT764TCC (2476T>C), TCG766TCA (2482G>A), CTG769CTA (2491G>A), CTG771CTT (2497G>T), TTG773TTA (2503G>A), AGA778AGG (2518A>G), CCT779CCC (2521T>C), AAG780AGG (2523A>G), AGA783AGG (2533A>G), TTG785TTA (2539G>A), TTA787TTG (2545A>G), GAG789GAA (2551G>A), CCT790CCA (2554T>A), CTG792TTG (2558C>T), TTG793CTC (2561T>C 2563G>C), TCA796TCG (2572A>G), CCT800CCC (2584T>C), GGC801GGT (2587C>T), CTG803CTC (2593G>C), GCT805GCC (2599T>C), AAT808AAC (2608T>C), TTT812TTC (2620T>C), GCG815GCA (2629G>A), GTG816GTA (2632G>A), AGG817AAG (2634G>A), TTG818TTA (2638G>A), ATT820ATA (2644T>A), AAA823AAG (2653A>G), GCT827GCC (2665T>C), CTA832TTA (2678C>T), CTA833CTG (2683A>G), GCT835GCC (2689T>C), CTA838CTC (2698A>C), TCA841TCG (2707A>G), GCG842GCA (2710G>A), GAA844GAG (2716A>G), ACA845ACG (2719A>G), CTC846CTT (2722C>T), GTC847ATT (2723G>A 2725C>T), GCA848GCG (2728A>G), AGG850AGA (2734G>A), ATT851ATA (2737T>A), GAT854GAC (2746T>C), GCT855ACA (2747G>A 2749T>A), GCA856GCT (2752A>T), GCT857GCA (2755T>A), GAC859GAT (2761C>T), CTC860CTT (2764C>T), TGT865TGC (2779T>C), GGG867GGT (2785G>T), AAC869AAT (2791C>T), CAT871AAT (2795C>A), TAC874TAT (2806C>T), TTG878CTC (2816T>C 2818G>C), GTG880ATA (2822G>A 2824G>A), TTG881TTA (2827G>A), GTT884GTC (2836T>C), AAG885AAA (2839G>A), AAT886AAC (2842T>C), ACC893ACA (2863C>A), CTA894TTA (2864C>T), CCA900ACA (2882C>A), AGT901CAT (2885A>C 2886G>A), AAC903AAT (2893C>T), ACG904ATG (2895C>T), GTC906GTT (2902C>T), GTA907GTG (2905A>G), ATT909ATC (2911T>C), AAT913AAC (2923T>C), AAT916AGT (2931A>G), AAC919AGC (2940A>G), GCT921GCC (2947T>C), ACT926ACC (2962T>C), GAA929_AAT930insA-- (2971_2972insA), GGT935GGC (2989T>C), ACT936ATT (2991C>T), CAT937CGT (2994A>G), GAG939AGG (2999G>A 3000A>G), TCC944TC- (3016delC), CGG946CAG (3021G>A), TAC947TTT (3024A>T 3025C>T), ACA951ATA (3036C>T), GAA952GGC (3039A>G 3040A>C), AAG953ACA (3042A>C 3043G>A), TTG956TTA (3052G>A), TTA959TTG (3061A>G), GCG966GCA (3082G>A), GGC969GGT (3091C>T), GCC972GTA (3099C>T 3100C>A), GTG974AGA (3104G>A 3105T>G 3106G>A), GTC975GTT (3109C>T), AAA976AAG (3112A>G), ACT978ACC (3118T>C), GCC979GCT (3121C>T), GGA981GGG (3127A>G), AGC983AAC (3132G>A), CGA985AAG (3137C>A 3138G>A 3139A>G), TAT989TAC (3151T>C), TTC990ATT (3152T>A 3154C>T), TGT994TAT (3165G>A), GTA995GTG (3169A>G), ATC1005ATT (3199C>T), AGG1007CGG (3203A>C), CGT1008CGC (3208T>C), CCA1010CCG (3214A>G), ACC1011ACT (3217C>T), TTT1012CTT (3218T>C), GTC1013GTA (3223C>A), ACT1014AAT (3225C>A), GTT1016ATT (3230G>A), AAC1017AAT (3235C>T), TTA1025CTA (3257T>C), ACC1026ACT (3262C>T), AGC1027AGT (3265C>T), GTA1028ATG (3266G>A 3268A>G), GTG1029GTA (3271G>A), GTG1031ATG (3275G>A), GCA1034ACA (3284G>A), CAG1039CAA (3301G>A), AGA1044AAG (3315G>A 3316A>G), GAA1045GCA (3318A>C), ATC1046ATT (3322C>T), GAG1047GAA (3325G>A), TTG1048TTA (3328G>A), ATA1051ATT (3337A>T), GAG1055GAA (3349G>A), GTC1057GTT (3355C>T), TGC1058TGT (3358C>T), ATG1059GTG (3359A>G), CTA1061CTG (3367A>G), TAT1062TAC (3370T>C), TTA1065CTG (3377T>C 3379A>G), GAA1070GAG (3394A>G), CGC1071CGT (3397C>T), GAT1072GAG (3400T>G), ACA1074ACG (3406A>G), TAC1078TAT (3418C>T), ATT1079ATA (3421T>A), TAT1081TAC (3427T>C), AAG1083AAA (3433G>A), TCA1084TCT (3436A>T), GTA1085GTG (3439A>G), AAC1086AAT (3442C>T), CCT1087CCC (3445T>C), CAG1091CAA (3457G>A), TTT1092TTC (3460T>C), GCT1093GCA (3463T>A), GCG1095GCT (3469G>T), CAG1096CAA (3472G>A), CAC1104CAT (3496C>T), CAG1105CAA (3499G>A), TCC1107TCT (3505C>T), GTT1110GGT (3513T>G), AAA1112AAG (3520A>G), GAA1115GAG (3529A>G), CAA1116CAG (3532A>G), ATG1121GTG (3545A>G), GCT1122ACT (3548G>A), TTA1123CTA (3551T>C), GTC1124ATT (3554G>A 3556C>T), AGT1133AGC (3583T>C), GAT1134GAC (3586T>C), TGC1135TGT (3589C>T), TTC1137TTT (3595C>T), AAT1141AAC (3607T>C), TTT1143TTC (3613T>C), AAG1144AAA (3616G>A), GGT1145GGC (3619T>C), CTT1147GTT (3623C>G), TCC1148TCT (3628C>T), CTG1150ATG (3632C>A), GAT1151GAC (3637T>C), TAT1152CAT (3638T>C), GAA1153GAG (3643A>G), TTA1159TTG (3661A>G), GTG1162GTA (3670G>A), TTT1166TTC (3682T>C), GAG1168GAA (3688G>A), GAG1171GAA (3697G>A), ATT1172GTT (3698A>G), GAT1174GAC (3706T>C), TTT1175TTC (3709T>C), TTG1177CTA (3713T>C 3715G>A), AGT1178AAT (3717G>A), GAG1179GAA (3721G>A), CGA1183AAA (3731C>A 3732G>A), ACT1184ACA (3736T>A), CTA1188TTG (3746C>T 3748A>G), ACA1190ACG (3754A>G), AGT1193AGC (3763T>C), CGA1198CGG (3778A>G), CAA1199CAG (3781A>G), ATC1200ATT (3784C>T), CAT1204CAC (3796T>C), CCA1207CCT (3805A>T), TAC1209TAT (3811C>T), AGA1210AGG (3814A>G), TTC1215TTT (3829C>T), TTT1218TTC (3838T>C), AGA1220AGG (3844A>G), GCA1221GCC (3847A>C), GTT1224GTA (3856T>A), GTG1226GTT (3862G>T), GAC1229GAT (3871C>T), GCC1231GCA (3877C>A), AGC1233AGT (3883C>T), GAA1234GAG (3886A>G), CTA1236CTG (3892A>G), GAC1237GAT (3895C>T), TTT1238TTC (3898T>C), GTA1240GTG (3904A>G), CGG1241AGA (3905C>A 3907G>A), GCT1243GCC (3913T>C), AAG1248AAA (3928G>A), TCA1249TCT (3931A>T), GGG1251GGA (3937G>A), AGT1257AGC (3955T>C), GTG1258GTA (3958G>A), GCC1259GCT (3961C>T), GGA1260GGG (3964A>G), TCT1261TCC (3967T>C), TTA1264TTG (3976A>G), ATT1265ATC (3979T>C), CCA1267CCT (3985A>T), CTA1271CTT (3997A>T), GCG1272GCA (4000G>A), AAC1274AAT (4006C>T), GTT1275GTG (4009T>G), TTC1276TTT (4012C>T), AAA1277AAG (4015A>G), TCT1280TCC (4024T>C), GAA1282GAG (4030A>G), CCA1283CCT (4033A>T), AAG1286AAA (4042G>A), CTG1290TTA (4052C>T 4054G>A), CGT1291CGC (4057T>C), CGT1293CGA (4063T>A), GGA1294GGG (4066A>G), ATA1297GTA (4073A>G), TTT1298TTC (4078T>C), GGC1299GGT (4081C>T), TCT1300TCC (4084T>C), TCC1304TCT (4096C>T), AGC1308AGT (4108C>T), GGA1309GGT (4111A>T), GCG1311GCA (4117G>A), CTA1312TTG (4118C>T 4120A>G), TAC1314TAT (4126C>T), TTC1315TAT (4128T>A 4129C>T), GCC1316GCT (4132C>T), AAT1317AAC (4135T>C), CAA1321CAG (4147A>G), TTA1322CTA (4148T>C), GCT1323TCT (4151G>T), AAC1326AAT (4162C>T), GTA1328ATA (4166G>A), ATA1329ATC (4171A>C), GAG1332GAA (4180G>A), CTG1336TTG (4190C>T), CCT1338CCC (4198T>C), TCC1339TCT (4201C>T), GCG1342GCT (4210G>T), TTC1343TTT (4213C>T), AGT1345AGC (4219T>C), CTG1346TTG (4220C>T), CTG1347ATA (4223C>A 4225G>A), GTT1349GTG (4231T>G), CAT1351CAC (4237T>C), GTA1356GTG (4252A>G), TTA1357CTG (4253T>C 4255A>G), AAA1358AAG (4258A>G), ACT1362ACC (4270T>C), AGA1366AGG (4282A>G), GAG1367GAA (4285G>A), GAA1369GAG (4291A>G), CAG1373CAA (4303G>A), GTC1376GTT (4312C>T), ATA1379GTG (4319A>G 4321A>G), GTG1380GTT (4324G>T), GAG1381GAA (4327G>A), CTG1384CTT (4336G>T), TCT1385TCC (4339T>C), TCA1388TCT (4348A>T), GCA1392GCG (4360A>G), GGT1394GGC (4366T>C), TCT1395TCA (4369T>A), ACT1397ACC (4375T>C), GTT1401GTC (4387T>C), TTT1404TAT (4395T>A), GTA1408ATA (4406G>A), CTT1409CTC (4411T>C), GTT1418GTC (4438T>C), ACC1420ACA (4444C>A), GCC1422GCT (4450C>T), CTC1424CTT (4456C>T), GAC1427GAT (4465C>T), ATG1431GTT (4475A>G 4477G>T), GTC1432GTT (4480C>T), ACA1433TCG (4481A>T 4483A>G), GGC1437GGT (4495C>T), AAG1441AAA (4507G>A), CAC1442CAT (4510C>T), GGT1443GGG (4513T>G), CTA1445TTA (4517C>T), GTC1448GTT (4528C>T), AAA1450AAG (4534A>G), ACC1452ACT (4540C>T), GCG1454TTA (4544G>T 4545C>T 4546G>A), AGA1455AAA (4548G>A), TTT1458TTC (4558T>C), GTA1460GTT (4564A>T), ACC1462ACT (4570C>T), ATA1464ATT (4576A>T), TTG1471TTA (4597G>A), GAC1472GAT (4600C>T), GAC1474GAT (4606C>T), GTG1475GTA (4609G>A), GTG1477GTA (4615G>A), GAC1478GAT (4618C>T), TTG1481CTT (4625T>C 4627G>T), GAC1488GAT (4648C>T), ATT1489ATC (4651T>C), AGG1492AGA (4660G>A), AGC1493AGT (4663C>T), GCT1495GCA (4669T>A), GTG1499ATC (4679G>A 4681G>C), AGT1500AGC (4684T>C), GTT1501GTG (4687T>G), GAG1505GAA (4699G>A), AGA1506AGG (4702A>G), CAA1508CAG (4708A>G), AGG1509AGA (4711G>A), CTG1510TTG (4712C>T), AAA1518AAG (4738A>G), GTA1520GTT (4744A>T), GCA1521GCG (4747A>G), TTG1522CTG (4748T>C), ACT1527ACC (4765T>C), GTC1538ATT (4796G>A 4798C>T), ACT1540AGC (4803C>G 4804T>C), GAG1541GAA (4807G>A), GCG1542GCT (4810G>T), GCT1543GCG (4813T>G), CTT1544CTC

	(4816T>C), TAT1549TAC (4831T>C), AAC1550AAT (4834C>T), CCA1552CCT (4840A>T), GGA1556GGG (4852A>G), GGC1557GGT (4855C>T), AGT1561AGC (4867T>C), CTG1562CTC (4870G>C), ATT1563ATA (4873T>A), TGT1566TGC (4882T>C), ACA1573ACT (4903A>T), CAG1575CAA (4909G>A), TTT1577TTC (4915T>C), GAA1578GAG (4918A>G), TTG1579CTA (4919T>C 4921G>A), AGT1580AGC (4924T>C), CCC1581CCG (4927C>G), TTT1583TTC (4933T>C), ATC1584ATA (4936C>A), CAG1585CAA (4939G>A), AAT1586AAC (4942T>C), TTC1587TTT (4945C>T), TCA1593TCG (4963A>G), CCT1596CCC (4972T>C), CTT1602CTC (4990T>C), AAA1606AAG (5002A>G), CCT1613CCC (5023T>C), TTG1614CTG (5024T>C), TGC1615TGT (5029C>T), GAT1616GAC (5032T>C), TCT1618TCC (5038T>C), CCA1620CCT (5044A>T), GCA1623GCT (5053A>T), TCG1624TCA (5056G>A), AGT1625GGT (5057A>G), ACT1626GCT (5060A>G), TCG1629TCT (5071G>T), CTT1636ATT (5090C>A), GGA1637GGG (5095A>G), GTG1638GTT (5098G>T), GCC1639GTC (5100C>T), TTA1640TTG (5104A>G), GAA1641GAT (5107A>T), ATT1642ATC (5110T>C), CCA1643CCG (5113A>G), AAG1644AAA (5116G>A), CAA1645CAG (5119A>G), GTC1646TTT (5120G>T 5122C>T), ATT1648GTT (5126A>G), CAT1651CAC (5137T>C), ATC1652ATT (5140C>T), AAA1653AAG (5143A>G), GAG1654GAA (5146G>A), CTC1659CTA (5161C>A), GAA1661GAG (5167A>G), CTT1663CTG (5173T>G), ACG1666ACA (5182G>A), GTT1667ATT (5183G>A), GTC1668GTT (5188C>T), AAG1669AAA (5191G>A), AAA1671AAG (5197A>G), GAC1672GAT (5200C>T), TGC1674TGT (5206C>T), TTA1675TTG (5209A>G), AGC1678AGT (5218C>T), TCG1682TCA (5230G>A), ATC1684ATT (5236C>T), AGC1685AGT (5239C>T), ATC1687ATT (5245C>T), ACA1693ACT (5263A>T), CTC1695CTT (5269C>T), TTC1696TTT (5272C>T), GCC1697GCA (5275C>A), ATC1698ATT (5278C>T), CCA1699CCC (5281A>C), AGA1700AGG (5284A>G), ACT1701ACC (5287T>C), CTA1702TTA (5288C>T), ATA1703ATT (5293A>T), TTG1704CTA (5294T>C 5296G>A), GAG1706GAA (5302G>A), CTT1709CTA (5311T>A), GAA1710GAG (5314A>G), CGA1713CGT (5323A>T), AGT1721AGC (5347T>C), ATC1723ATT (5353C>T), TGC1727TGT (5365C>T), GTT1734GTC (5386T>C), ACC1737ACA (5395C>A), AAA1746AAG (5422A>G), GAT1747GAC (5425T>C), ACC1749ACT (5431C>T), GAG1751GAA (5437G>A), CTT1756CTC (5452T>C), AAG1758AAA (5458G>A), AGT1761AGC (5467T>C), CAA1762CAG (5470A>G), CTA1763TTG (5471C>T 5473A>G), GAA1765GAG (5479A>G), TTC1766TTT (5482C>T), TCA1767TCC (5485A>C), AAT1768AAC (5488T>C), TTG1769CTA (5489T>C 5491G>A), GGT1771GGC (5497T>C), TGT1774TGC (5506T>C), GAG1775GAA (5509G>A), GAG1776GAA (5512G>A), TTA1778CTA (5516T>C), TAT1782TAC (5530T>C), GAG1783GAA (5533G>A), TCG1784TCT (5536G>T), TTG1785TTA (5539G>A), GTT1788GTG (5548T>G), CAC1790CAT (5554C>T), GCT1792GCA (5560T>A), GCG1793ACA (5561G>A 5563G>A), ACG1794ACC (5566G>C), TCA1795TCG (5569A>G), CTC1800CTG (5584C>G), AAG1803AAA (5593G>A), GGG1804GGA (5596G>A), ATT1805GTT (5597A>G), AAC1807AAA (5605C>A), GTG1811GTT (5617G>T), GCT1812GTG (5619C>T 5620T>G), TTG1815TTA (5629G>A), ATC1816CTC (5630A>C), GGC1819GGT (5641C>T), GCA1822GCT (5650A>T), ATA1826ATT (5662A>T), GGA1827GGG (5665A>G), CAA1835CAG (5689A>G), GTT1837ATT (5693G>A), GAG1838GAA (5698G>A), CAA1843CAG (5713A>G), GGG1844GGC (5716G>C), AAA1845AAG (5719A>G), GCC1853GCA (5743C>A), CGC1857CGA (5755C>A), CAT1858CAC (5758T>C), CGT1860CGC (5764T>C), AAA1862AAG (5770A>G), GGC1865GGT (5779C>T), TTT1866TTC (5782T>C), GAC1869GAT (5791C>T), GAC1873GAT (5803C>T), AGG1884AGA (5836G>A), AAA1885AAG (5839A>G), GGA1887GGG (5845A>G), AAA1888AAG (5848A>G), AAA1890AGA (5853A>G), GGT1891GGC (5857T>C), ACC1892ACT (5860C>T), ACA1893ACT (5863A>T), GGT1895GGA (5869T>A), GGT1897GGC (5875T>C), AGC1900AGT (5884C>T), TTC1903TTT (5893C>T), ATC1904GTG (5894A>G 5896C>G), AAC1905AAT (5899C>T), GGG1908GGA (5908G>A), TTT1909TTC (5911T>C), GAG1913GAA (5923G>A), CAA1918CAG (5938A>G), TTC1919TTT (5941C>T), GTT1920GTC (5944T>C), GAT1921GAC (5947T>C), GGG1925GGA (5959G>A), CGG1926GCC (5960C>G 5961G>C 5962G>C), GAA1930GAG (5974A>G), GCT1934GCA (5986T>A), GAC1935GAT (5989C>T), ATT1936GTT (5990A>G), GAT1938GAC (5998T>C), AGA1942CGT (6008A>C 6010A>T), GAA1945GAT (6019A>T), GTG1946GTT (6022G>T), CGA1947CGT (6025A>T), AAA1949ACA (6030A>C), GAC1955GAA (6049C>A), ATG1958ATA (6058G>A), CAA1959CAG (6061A>G), GCC1960GCA (6064C>A), GGT1962GGC (6070T>C), AGT1963AAC (6072G>A 6073T>C), AAC1964CAC (6074A>C), ACG1965ACA (6079G>A), ACC1966AAC (6081C>A), ATA1967ATT (6085A>T), GCA1969GCT (6091A>T), TAC1970TAT (6094C>T), TGT1976TCC (6111G>C 6112T>C), AAA1978AAG (6118A>G), TTG1980TTA (6124G>A), AAG1981AAA (6127G>A), ATT1982GTC (6128A>G 6130T>C), GAT1983GAC (6133T>C), TTA1984TTG (6136A>G), AAC1988AAT (6148C>T), AAA1991AAG (6157A>G), GTT1992ATT (6158G>A), TGT1993TGC (6163T>C), AAA1995AAG (6169A>G), ACA1996TCG (6170A>T 6172A>G), GGC1998GGT (6178C>T), AAA2001AAG (6187A>G), TTT2002TTC (6190T>C), GAG2004GAA (6196G>A), CTC2007TTT (6203C>T 6205C>T), GAA2008GAG (6208A>G), CTA2009TTG (6209C>T 6211A>G), CAG2011CAA (6217G>A), GCT2015GCA (6229T>A), GTA2016ATA (6230G>A), GTC2018GTT (6238C>T), AAG2021AAA (6247G>A), ATA2023ATT (6253A>T), GCA2025AAA (6257G>A 6258C>A), CAT2030CAC (6274T>C), AAA2033AGA (6282A>G), TCG2034TCA (6286G>A), GGC2038GGT (6298C>T), TTG2039TTA (6301G>A), AGA2040AGG (6304A>G), GAC2041GAT (6307C>T), GCC2046GCT (6322C>T), ACA2048ACT (6328A>T), GTT2049GTG (6331T>G), TGT2050TGC (6334T>C), AGG2051AGA (6337G>A), CTG2052TTG (6338C>T), TAT2058TAC (6358T>C), GGG2059GGA (6361G>A), GCA2060ACA (6362G>A), TCA2061TCT (6367A>T), GAG2062GAA (6370G>A), TAC2064TAT (6376C>T), GGT2065GGG (6379T>G), GGA2067GGC (6385A>C), GGA2069GGT (6391A>T), GCA2070GCG (6394A>G), TAC2071TAT (6397C>T), GTA2073ATA (6401G>A), GCG2074GTG (6405C>T), CAC2076CAT (6412C>T), TTA2078TTG (6418A>G), TTT2079TTC (6421T>C), TAC2082TTC (6429A>T), GGT2084GGA (6436T>A), GAG2087GAA (6445G>A), CAA2089CGA (6450A>G), TCC2090TCG (6454C>G), CAC2092CAT (6460C>T), GGT2093GGA (6463T>A), TTC2095TTT (6469C>T), AGG2096AGA (6472G>A), GTG2097GTA (6475G>A), AAT2099AAC (6481T>C), CAC2101AAT (6485C>A 6487C>T), AGT2102AGC (6490T>C), AGC2104AGT (6496C>T), CTG2106CTA (6502G>A), GGT2110GGC (6514T>C), AGG2111AGA (6517G>A), CTC2115ATC (6527C>A), ATC2116ATA (6532C>A), CCG2119CCA (6541G>A), AAA2120AAG (6544A>G), GTC2124GTT (6556C>T), TTT2125TTC (6559T>C), CAG2127CAA (6565G>A), TTG2129CTA (6569T>C 6571G>A), CCT2134CCA (6586T>A), ACA2135ATA (6588C>T), GAA2138GAG (6598A>G), AGA2139AGG (6601A>G), ATT2140ATC (6604T>C), TGT2141TGC (6607T>C), GGA2144GGG (6616A>G), AAC2146AAT (6622C>T), GAG2149GAA (6631G>A), AAG2150AAA (6634G>A), TAT2151CAT (6635T>C), GCT2152GCA (6640T>A), TCG2154TCT (6646G>T), ATC2155GTC (6647A>G), ATC2156ATT (6652C>T), AGC2160AGT (6664C>T), ACT2162ACG (6670T>G), ATA2165GTG (6677A>G 6679A>G), GGC2167GGT (6685C>T), ACA2169ACT (6691A>T), TTC2170TTT (6694C>T), ACA2177ACG (6715A>G), GAT2178AAT (6716G>A), AAT2179GAT (6719A>G), GGA2180GGG (6724A>G), CTA2184TTA (6734C>T), GTG2186GTA (6742G>A), GTG2187GTA (6745G>A), GCC2190GCT (6754C>T), GGA2192GGG (6760A>G), ATA2194TTG (6764A>T 6766A>G), ATT2197ATA (6775T>A), CTG2200TTG (6782C>T), GCA2201GCT (6787A>T), AAC2202AAT (6790C>T), GCA2204GTG (6795C>T 6796A>G), CAC2205CAA (6799C>A), ACC2206ACA (6802C>A), AAC2208AAT (6808C>T), TAC2209TAT (6811C>T), TAC2210TAT (6814C>T), TTC2213TTT (6823C>T), GAA2215GAG (6829A>G), TAC2221TAT (6847C>T), CGA2223CGC (6853A>C), AAT2225GAT (6857A>G), GAG2226GAA (6862G>A), GAA2229GAG (6871A>G), GTC2231ACA (6875G>A 6876T>C 6877C>A), AAG2232AAA (6880G>A), TCT2233TCA (6883T>A), GTT2235GTG (6889T>G), GAC2239GAT (6901C>T), GGC2244GGT (6916C>T), CCG2245CCA (6919G>A), AAA2247AAG (6925A>G), CTT2248CTC (6928T>C), GAC2250GAA (6934C>A), GGG2255GGC (6949G>C), TTC2257TTT (6955C>T), AAA2258AAG (6958A>G), CAA2264CAG (6976A>G), CTA2266TTA (6980C>T), ATC2267ATT (6985C>T), GTA2271GTT (6997A>T), GTG2272GTT (7000G>T), GTG2273ATA (7001G>A 7003G>A), GCT2276GCC (7012T>C), AAG2277AAA (7015G>A), CAT2278CAC (7018T>C), TTT2283TAT (7032T>A), GCC2285GCT (7039C>T), TTG2286CTA (7040T>C 7042G>A), GGA2288GGG (7048A>G), GCT2292GCA (7060T>A), GTC2293GTA (7063C>A), AGC2298AGT (7078C>T), TTA2300CTG (7082T>C 7084A>G), GTA2301GTG (7087A>G), ACC2302ACA (7090C>A), CAT2304CAC (7096T>C), GTA2305GTG (7099A>G), GTT2306GTC (7102T>C), GAG2309GAA (7111G>A), CGA2311CGG (7117A>G), ACA2314AAA (7125C>A), GAA2315GAG (7129A>G), TTT2316TTC (7132T>C), CTG2317TTA (7133C>T 7135G>A), GTG2319GTA (7141G>A), GAT2320GAC (7144T>C), GCA2321TCG (7145G>T 7147A>G), GAG2322GAA (7150G>A), GCA2325GCT (7159A>T), GAG2326_GCA2327del (7160_7165delGAGGCA), TTC2329TTT (7171C>T), GCG2335GCT (7189G>T), AAA2338AAG (7198A>G), AGC2339AGT (7201C>T), CTA2341TTA (7205C>T), AAT2342AAC (7210T>C), GAT2344GAA (7216T>A), GCG2345GCA (7219G>A), ATC2347ATA (7225C>A), AAG2348AAA (7228G>A), AAG2352AAA (7240G>A), TAT2353TAC (7243T>C), AAA2355AAG (7249A>G), CCT2356CCG (7252T>G), GAT2358GAA (7258T>A), GTT2359GTC (7261T>C), GGT2360GGA (7264T>A), GTC2361GTG (7267C>G), GTG2362GTA (7270G>A), GAT2363_CGG2364insT-- (7273_7274insT), CGG2364GTG (7274C>G 7275G>T), CAT2366CTT (7281A>T), TTG2367TCG (7284T>C), TCA2371TTA (7296C>T), TCT2376TTT (7311C>T), ACC2377ATC (7314C>T), TGC2378TAC (7317G>A), AAT2379AAA (7321T>A), GTG2380-TG (7322delG), CAC2381CAT (7327C>T), GGC2382GGT (7330C>T), TTC2383TTT (7333C>T), AAG2384CAG (7334A>C), TGT2386TGC (7342T>C), GCA2387AAC (7343G>A 7344C>A 7345A>C), ACT2390ACC (7354T>C), CAA2393CAG (7363A>G), ATT2395ATC (7369T>C), TTC2396TTT (7372C>T), GCG2398GCT (7378G>T), CTC2399CTT (7381C>T), GCA2404GCT (7396A>T), GTC2405GTT (7399C>T), GCC2407GCT (7405C>T), AGT2408ATG (7407G>T 7408T>G), GGG2410GGT (7414G>T), TGC2411GGC (7415T>G), AAA2412AAG (7420A>G), GAC2415GAT (7429C>T), TAT2416TAC (7432T>C), CAT2419CAC (7441T>C), AAG2425AAA (7459G>A), GAA2426GAG (7462A>G), ATA2428ATT (7468A>T), GTC2429GTA (7471C>A), CAA2431CAG (7477A>G), AGC2432AGT (7480C>T), CTG2434CTT (7486G>T), TAT2437TAC (7495T>C), AAA2438AAG (7498A>G), GGT2439GGC (7501T>C), TTG2440TCA (7503T>C 7504G>A), ATT2443ATA (7513T>A), AAC2445AAT (7519C>T), GGA2446GGG (7522A>G), AAG2449AAA (7531G>A), GAG2451GAA (7537G>A), CTC2452CTT (7540C>T), TGT2454TGC (7546T>C), AAG2462AAA (7570G>A), ACG2463ACA (7573G>A), ACG2465ACA (7579G>A), GCT2468GCC (7588T>C), CTA2471TTA (7595C>T), TTG2474CTA (7604T>C 7606G>A), GGT2477GGA (7615T>A), TGT2480TGC (7624T>C), GAC2483GAT (7633C>T), TTC2484TTT (7636C>T), AAT2485AAC (7639T>C), TTT2488TTC (7648T>C), GAA2494GAG (7666A>G), TGT2496TGC (7672T>C), ACA2498ACT (7678A>T), GGG2500GGA (7684G>A), ACT2502ACC (7690T>C), TTT2504TTC (7696T>C), GGT2506GGG (7702T>G), GAT2509AAT (7709G>A), AAA2510AGG (7713A>G 7714A>G), CTG2511TTG (7715C>T), CGT2514CGC (7726T>C), GAG2517GAA (7735G>A), GTA2520GTG (7744A>G), TAC2521TAT (7747C>T), GGC2526GGT (7762C>T), CAG2528CAA (7768G>A), TTT2529TTC (7771T>C), TCA2532TCG (7780A>G), CTA2533CTC (7783A>C), ACT2534ACC (7786T>C), CTA2537TTG (7793C>T 7795A>G), ATC2538ATT (7798C>T), GCT2540GCC (7804T>C), ACC2543ATC (7812C>T), GAA2550GAG (7834A>G), GAC2551GAT (7837C>T), GTG2554TTA (7844G>T 7846G>A), CAG2557CAA (7855G>A), CTG2559CTA (7861G>A), TTA2562TTG (7870A>G), TAC2563TAT (7873C>T), ACT2564ACA (7876T>A), GAG2565GAA (7879G>A), ATT2566ATA (7882T>A), GTT2567ATT (7883G>A), CCA2570CCT (7894A>T), ATT2571ATC (7897T>C), GTC2579GTT (7921C>T), AAG2581AAA (7927G>A), GGT2584GGC (7936T>C), AAT2585AAC (7939T>C), AAT2586AAC (7942T>C), GTG2594GTA (7966G>A), GAT2595GAC (7969T>C), TCT2597TCC (7975T>C), GTC2601GTT (7987C>T), CTT2602CTC (7990T>C), GCT2607GCC (8005T>C), GAA2616GAG (8032A>G), ATC2618ATT (8038C>T), GAC2619GAT (8041C>T), AGC2620AAC (8043G>A), TTG2632TTA (8080G>A), GTG2635GTA (8089G>A), CCG2637CCA (8095G>A), GAG2638GAA (8098G>A), AGC2641AGT (8107C>T), ATT2642ATC (8110T>C), GAT2644GAC (8116T>C), CAA2648CAG (8128A>G), GGT2654GGC (8146T>C), AAC2656AGT (8151A>G 8152C>T), TCG2660TCA (8164G>A), AGG2665AAG (8178G>A), AAG2666AAA (8182G>A), TTG2669TTA (8191G>A), TTC2671TTT (8197C>T), TCC2673TCT (8203C>T), AGA2675AAA (8208G>A), CTG2677TTA (8213C>T 8215G>A), CTA2678CTG (8218A>G), ATC2679ATT (8221C>T), GAG2680GAA (8224G>A), CCA2685CCG (8239A>G), GAG2690GAA (8254G>A), ATT2692ATC (8260T>C), GTA2693GTG (8263A>G), GAT2699GAC (8281T>C), AGA2700AGG (8284A>G), CCA2704GCG (8294C>G 8296A>G), GAG2705GAA (8299G>A), CAC2706CAT (8302C>T), ATT2711ATC (8317T>C), TGC2712TGT (8320C>T), GCT2714GCA (8326T>A), GAG2717GAA (8335G>A), TCC2718TCT (8338C>T), TCT2722TTT (8349C>T), GAA2723GAG (8353A>G), CAC2726CAT (8362C>T), CAA2727CAG (8365A>G), ATC2728ATT (8368C>T), TAC2732TAT (8380C>T), TCA2733TCG (8383A>G), TTA2735TTG (8389A>G), CCT2740CCC (8404T>C), GCA2742TCA (8408G>T), ACA2743ACG (8413A>G), GCG2745GCT (8419G>T), GCT2750GCC (8434T>C), TAT2752TAC (8440T>C), ATG2756ATA (8452G>A), CTA2758CTG (8458A>G), AGG2759AAG (8460G>A), AAA2760AAG (8464A>G), CTG2761CTA (8467G>A), AGG2765AGA (8479G>A), GCT2766ACA (8480G>A 8482T>A), GAG2771GAA (8497G>A), CTA2772TTG (8498C>T 8500A>G), AGA2773AAG (8502G>A 8503A>G), GCC2774GCT (8506C>T), GAA2777GAT (8515A>T), GTC2780GTT (8524C>T), GCA2781GCC (8527A>C), TTA2782CTG (8528T>C 8530A>G), GAG2785GAC (8539G>C), CTT2788TGT (8546C>T 8547T>G), TCT2790ACT (8552T>A), GTA2793GTG (8563A>G), GCA2797GGA (8574C>G), ATT2801GTT (8585A>G), GGA2805GAA (8598G>A), AGC2806AGT (8602C>T), AAC2807TCT (8603A>T 8604A>C 8605C>T), AAA2812AAG (8620A>G), CCA2813CAA (8622C>A), GAG2814GGA (8625A>G 8626G>A), CAG2815CAA (8629G>A), GGC2816GGT (8632C>T), ATC2818ATT (8638C>T), CAG2819CAA (8641G>A), AAC2821AAT (8647C>T), CCG2822TTC (8648C>T 8649C>T 8650G>C), AAC2823AAT (8653C>T), AAA2824AAG (8656A>G), GGA2825GAA (8658G>A), GAT2827GCG (8664A>C 8665T>G), GTT2830GTG (8674T>G), GCA2832GTT (8679C>T 8680A>T), GGC2833GGA (8683C>A), GGG2836GGA (8692G>A), ACA2837ACT (8695A>T), CCG2841CCA (8707G>A), AGA2842CGA (8708A>C), AAG2844AAA (8716G>A), ATC2846ATT (8722C>T), ACG2847ACA (8725G>A), AAA2849AAG (8731A>G), AGA2851AGG (8737A>G), CCC2853CCT (8743C>T), ACA2854AAA (8745C>A), AAG2856AAA (8752G>A), GGA2857GGT (8755A>T), AAC2862AAT (8770C>T), TTA2863CTA (8771T>C), GAA2864GAG (8776A>G), CAT2865CAC (8779T>C), TTG2866TTA (8782G>A), TAT2869TAC (8791T>C), CCA2871CCG (8797A>G), CAA2872CAG (8800A>G), CAA2873CAG (8803A>G), GAT2875GAC (8809T>C), ATT2876ATC (8812T>C), AAT2878AAC (8818T>C), CGG2880CGA (8824G>A), GCA2881GCG (8827A>G), ACG2888ACA (8848G>A), TAT2890TAC (8854T>C), GAG2891GGA (8856A>G 8857G>A), GTG2893GTA (8863G>A), CGG2894CAG (8865G>A), ATG2895CTT (8867A>C 8869G>T), GCA2896GCG (8872A>G), GGA2900GAA (8883G>A), ACT2902TCT (8888A>T), TGC2915TGT (8929C>T), GTC2924ATC (8954G>A), AAC2925AAT (8959C>T), GGA2926GGT (8962A>T), GGG2933GGA (8983G>A), AAT2934GAC (8984A>G 8986T>C), GAA2935GTA (8988A>T), GAG2938GAA (8998G>A), CCG2940CCA (9004G>A), TTG2941TTA (9007G>A), ATC2944ATT (9016C>T), ACC2951ACA (9037C>A), ATA2967ATT (9085A>T), AAC2971AAT (9097C>T), ATT2983GTT (9131A>G), CGA2984CGT (9136A>T), CGG2987CGT (9145G>T), ATG2989GGA (9149A>G 9150T>G 9151G>A), GGT2990AGT (9152G>A), TTA2991TTG (9157A>G), GCG2992GCT (9160G>T), CGT2993CGC (9163T>C), GAG3000GAA (9184G>A), CGA3004CGG (9196A>G), AGG3010AGA (9214G>A), GAA3011GAG (9217A>G), GCG3012GCA (9220G>A), GCA3020GCT (9244A>T), TTG3021TTA (9247G>A), GCC3024GCT (9256C>T), CCT3026TCC (9260C>T 9262T>C), GGG3030GGA (9274G>A), GAC3032GAT (9280C>T), ATC3035ATT (9289C>T), CAA3038CAC (9298A>C), GAG3040GAA (9304G>A), GTC3050GTT (9334C>T), GTC3060GTG (9364C>G)								
P1 protein (NP_734243.1)	1	284	100%	1394	72.7%	284 (100%)	203 (71.5%)	0/0/0/0	0

	Begin	End	Coverage	Score	Concordance	Matches	Identities	I/D/M/F*	Stop Codons
**NT**	**54**	**9704**	**99.5%**	**12522**	**65.2%**	**9639 (99.8%)**	**7984 (82.7%)**	**7/12**	
Protein mutations:	Y4Q (194T>C 196C>A), C9L (209T>C 210G>T 211T>G), F13M (221T>A 223T>G), S23P (251T>C), C24F (255G>T 256C>T), E25G (258A>G), I27V (263A>G 265T>G), V28A (267T>C), E30V (273A>T), A35T (287G>A 289T>C), V37T (293G>A 294T>C), A41I (305G>A 306C>T), D42G (309A>G), E44D (316A>T), T45V (317A>G 318C>T 319A>G), L52Q (339T>A 340C>A), K53R (342A>G), K55A (347A>G 348A>C 349A>G), Y56H (350T>C), T58V (356A>G 357C>T), V61T (365G>A 366T>C 367G>A), L62S (368C>T 369T>C), F67C (384T>G), A75V (408C>T 409C>T), M78E (416A>G 417T>A), E85K (437G>A), R86E (440A>G 441G>A), K87R (443A>C 444A>G 445G>A), D88E (448T>A), E91A (456A>C), H93N (461C>A), D111E (517T>A), S116F (531C>T), P118S (536C>T), Q119W (539C>T 540A>G 541A>G), R122K (549G>A 550A>G), M131T (576T>C), V134A (585T>C 586C>G), R137Y (593C>T 594G>A 595C>T), P138R (597C>G), I139V (599A>G 601A>G), I140P (602A>C 603T>C 604A>G), M147I (625G>A), I151V (635A>G 637T>C), I154V (644A>G 646A>G), E160A (663A>C 664G>C), H166Q (682C>A), T172S (699C>G), H174Q (706T>A), K179E (719A>G 721G>A), I180V (722A>G 724A>G), A183S (731G>T), Y184P (734T>C 735A>C 736C>T), S185R (737T>C 738C>G 739C>T), A187V (744C>T 745G>C), R189C (749C>T 751A>C), M194K (765T>A), R197K (773C>A 774G>A 775A>G), M206K (801T>A), G210A (813G>C 814A>G), L211H (816T>A), R214Q (825G>A 826T>A), N223D (851A>G 853T>C), R226C (860C>T 862C>T), T227A (863A>G 865T>C), I228T (867T>C), N229D (869A>G 871C>T), I230L (872A>T 874A>G), R231Q (876G>A), R232K (879G>A), N240S (903A>G), T241N (906C>A 907A>C), K242T (909A>C 910A>C), S243N (912G>A 913C>T), S252L (938T>C 939C>T 940A>T), L266I (980T>A 982G>C), R272K (999G>A), Q275R (1007C>A 1008A>G 1009G>A), S276G (1010A>G 1012T>G), I277V (1013A>G), N279Q (1019A>C 1021C>G)								
Codon mutations:	ACT3ACC (193T>C), TAC4CAA (194T>C 196C>A), TCA6TCG (202A>G), ATC8ATT (208C>T), TGT9CTG (209T>C 210G>T 211T>G), TCG12TCA (220G>A), TTT13ATG (221T>A 223T>G), GAA14GAG (226A>G), CTA17CTT (235A>T), TAC19TAT (241C>T), CCA21CCT (247A>T), TCT23CCT (251T>C), TGC24TTT (255G>T 256C>T), GAG25GGG (258A>G), CAT26CAC (262T>C), ATT27GTG (263A>G 265T>G), GTG28GCG (267T>C), GAA30GTA (273A>T), CGA31CGT (277A>T), GAA32GAG (280A>G), GTG33GTT (283G>T), CCG34CCA (286G>A), GCT35ACC (287G>A 289T>C), GTT37ACT (293G>A 294T>C), CCT39CCC (301T>C), TTC40TTT (304C>T), GCA41ATA (305G>A 306C>T), GAT42GGT (309A>G), CTG43TTG (311C>T), GAA44GAT (316A>T), ACA45GTG (317A>G 318C>T 319A>G), CAA46CAG (322A>G), GCA49GCT (331A>T), CTC52CAA (339T>A 340C>A), AAG53AGG (342A>G), AAA55GCG (347A>G 348A>C 349A>G), TAT56CAT (350T>C), GCT57GCA (355T>A), ACT58GTT (356A>G 357C>T), CGT60CGC (364T>C), GTG61ACA (365G>A 366T>C 367G>A), CTC62TCC (368C>T 369T>C), AAA63AAG (373A>G), AAC64AAT (376C>T), TTT67TGT (384T>G), ACG68ACA (388G>A), TAC69TAT (391C>T), CGA70CGG (394A>G), AAG72AAA (400G>A), GAT74GAC (406T>C), GCC75GTT (408C>T 409C>T), CAG76CAA (412G>A), ATA77ATT (415A>T), ATG78GAG (416A>G 417T>A), ATT80ATC (424T>C), CAG81CAA (427G>A), AAA83AAG (433A>G), CTG84CTC (436G>C), GAG85AAG (437G>A), AGG86GAG (440A>G 441G>A), AAG87CGA (443A>C 444A>G 445G>A), GAT88GAA (448T>A), GAA90GAG (454A>G), GAA91GCA (456A>C), TAT92TAC (460T>C), CAC93AAC (461C>A), CAA95CAG (469A>G), GCC97GCG (475C>G), CCT99CCA (481T>A), ACT106ACC (502T>C), ATA107ATT (505A>T), GGC109GGT (511C>T), GAT111GAA (517T>A), CCT112CCA (520T>A), CCA113CCT (523A>T), TCT116TTT (531C>T), GAG117GAA (535G>A), CCA118TCA (536C>T), CAA119TGG (539C>T 540A>G 541A>G), AGA122AAG (549G>A 550A>G), ATC124ATT (556C>T), ATT125ATC (559T>C), CAT126CAC (562T>C), ACT128ACC (568T>C), CCA129CCG (571A>G), ATG131ACG (576T>C), CGT132CGC (580T>C), GTC134GCG (585T>C 586C>G), CGC137TAT (593C>T 594G>A 595C>T), CCC138CGC (597C>G), ATA139GTG (599A>G 601A>G), ATA140CCG (602A>C 603T>C 604A>G), ACA143ACG (613A>G), GAA144GAG (616A>G), GGC145GGG (619C>G), CAG146CAA (622G>A), ATG147ATA (625G>A), AAT148AAC (628T>C), CAC149CAT (631C>T), ATT151GTC (635A>G 637T>C), ATA154GTG (644A>G 646A>G), ATT157ATC (655T>C), TCG159TCA (661G>A), GAG160GCC (663A>C 664G>C), AAA161AAG (667A>G), GTC165GTT (679C>T), CAC166CAA (682C>A), TTA167CTG (683T>C 685A>G), AGT169AGC (691T>C), ACC172AGC (699C>G), ACT173ACA (703T>A), CAT174CAA (706T>A), AAG179GAA (719A>G 721G>A), ATA180GTG (722A>G 724A>G), CTT181TTG (725C>T 727T>G), GGT182GGA (730T>A), GCA183TCA (731G>T), TAC184CCT (734T>C 735A>C 736C>T), TCC185CGT (737T>C 738C>G 739C>T), GCG187GTC (744C>T 745G>C), CGA189TGC (749C>T 751A>C), ACT190ACC (754T>C), ATG194AAG (765T>A), TTG196CTG (770T>C), CGA197AAG (773C>A 774G>A 775A>G), CGG198AGG (776C>A), TTC202TTT (790C>T), ATG206AAG (801T>A), GGA210GCG (813G>C 814A>G), CTT211CAT (816T>A), TTG212CTG (818T>C), CAA213CAG (823A>G), CGT214CAA (825G>A 826T>A), CGG217AGA (833C>A 835G>A), GAC219GAT (841C>T), AAT223GAC (851A>G 853T>C), GTC225GTT (859C>T), CGC226TGT (860C>T 862C>T), ACT227GCC (863A>G 865T>C), ATC228ACC (867T>C), AAC229GAT (869A>G 871C>T), ATA230TTG (872A>T 874A>G), CGA231CAA (876G>A), AGG232AAG (879G>A), GAT234GAC (886T>C), ATC238ATT (898C>T), AAC240AGC (903A>G), ACA241AAC (906C>A 907A>C), AAA242ACC (909A>C 910A>C), AGC243AAT (912G>A 913C>T), GGC246GGA (922C>A), GGT249GGA (931T>A), TCA252CTT (938T>C 939C>T 940A>T), GGA253GGG (943A>G), TTC256TTT (952C>T), TCA261TCG (967A>G), GAA263GAG (973A>G), GGG264GGC (976G>C), TTG266ATC (980T>A 982G>C), GCA269GCG (991A>G), CGT270CGC (994T>C), AGA272AAA (999G>A), GTT273GTC (1003T>C), CAG275AGA (1007C>A 1008A>G 1009G>A), AGT276GGG (1010A>G 1012T>G), ATT277GTT (1013A>G), TTA278TTG (1018A>G), AAC279CAG (1019A>C 1021C>G), ATC282ATT (1030C>T)								
HC-Pro protein (NP_734244.1)	1	456	100%	3145	95.7%	456 (100%)	425 (93.2%)	0/0/0/0	0
Protein mutations:	D4E (1048C>A), R16Q (1083G>A), S21A (1097T>G 1099G>A), R36K (1143G>A), L40I (1154T>A 1156G>A), S62N (1221G>A), V65T (1229G>A 1230T>C 1231T>C), S66N (1233G>A 1234C>T), K74T (1257A>C 1258A>G), N81S (1278A>G), A85V (1290C>T 1291G>A), D88T (1298G>A 1299A>C 1300C>T), I91V (1307A>G 1309A>G), N94E (1316A>G 1318T>A), I98T (1329T>C), L113I (1373C>A), I117V (1385A>G), E123D (1405G>T), A127S (1415G>T 1417A>T), V134I (1436G>A 1438C>T), E224R (1706G>A 1707A>G), A228T (1718G>A), I236V (1742A>G 1744C>T), S241N (1758G>A), V252I (1790G>A), I301V (1937A>G 1939T>C), F323Y (2004T>A), I344M (2068T>G), V346I (2072G>A 2074G>A), R391K (2208G>A), D410E (2266C>G)								
Codon mutations:	TCG1TCA (1039G>A), AAT2AAC (1042T>C), GCC3GCA (1045C>A), GAC4GAA (1048C>A), AAT5AAC (1051T>C), AAG8AAA (1060G>A), GGT9GGA (1063T>A), CTG10TTG (1064C>T), GAC11GAT (1069C>T), CGA16CAA (1083G>A), TAT19TAC (1093T>C), TCG21GCA (1097T>G 1099G>A), CAC23CAT (1105C>T), GTA26GTG (1114A>G), GCT27GCG (1117T>G), TTA29TTG (1123A>G), CCT30CCA (1126T>A), GTC31GTT (1129C>T), AGG36AAG (1143G>A), GTA37GTT (1147A>T), GCT38GCA (1150T>A), TTG40ATA (1154T>A 1156G>A), CAC43CAT (1165C>T), AGT44AGC (1168T>C), CTT46TTA (1172C>T 1174T>A), ACC55ACT (1201C>T), AGC62AAC (1221G>A), GTT65ACC (1229G>A 1230T>C 1231T>C), AGC66AAT (1233G>A 1234C>T), CTG68CTA (1240G>A), AAG70AAA (1246G>A), TTG72CTA (1250T>C 1252G>A), CAT73CAC (1255T>C), AAA74ACG (1257A>C 1258A>G), AGA77AGG (1267A>G), GGT79GGC (1273T>C), TTG80TTA (1276G>A), AAT81AGT (1278A>G), TTG83CTG (1283T>C), GCG85GTA (1290C>T 1291G>A), GAC88ACT (1298G>A 1299A>C 1300C>T), TTT90TTC (1306T>C), ATA91GTG (1307A>G 1309A>G), CAT92CAC (1312T>C), AAT94GAA (1316A>G 1318T>A), TTG97TTA (1327G>A), ATA98ACA (1329T>C), GCG99GCT (1333G>T), TTA100TTG (1336A>G), CTA103TTG (1343C>T 1345A>G), CCG106CCA (1354G>A), GTG107GTT (1357G>T), GAC108GAT (1360C>T), CTC111TTG (1367C>T 1369C>G), CTT113ATT (1373C>A), TTC114TTT (1378C>T), GAG116GAA (1384G>A), ATA117GTA (1385A>G), AAA119AAG (1393A>G), TCC120TCT (1396C>T), GAG123GAT (1405G>T), AAA124AAG (1408A>G), CAG125CAA (1411G>A), GCA127TCT (1415G>T 1417A>T), CCG128CCC (1420G>C), AAT131AAC (1429T>C), TTA132TTG (1432A>G), GTC134ATT (1436G>A 1438C>T), TTA135CTG (1439T>C 1441A>G), AAT136AAC (1444T>C), TTC139TTT (1453C>T), CTG140CTA (1456G>A), GAA144GAG (1468A>G), GTA152GTG (1492A>G), TTG155CTA (1499T>C 1501G>A), AGT156AGC (1504T>C), TTG157TTA (1507G>A), CTC158CTT (1510C>T), GAA159GAG (1513A>G), TTA160TTG (1516A>G), GCA161GCT (1519A>T), AGG162AGA (1522G>A), TTC163TTT (1525C>T), CAG164CAA (1528G>A), AAC166AAT (1534C>T), ACT168ACA (1540T>A), AAC170AAT (1546C>T), AAA173AAG (1555A>G), GGT174GGC (1558T>C), GAT175GAC (1561T>C), ATA176ATC (1564A>C), TCT177TCA (1567T>A), TTC179TTT (1573C>T), AGA180AGG (1576A>G), TTA183CTA (1583T>C), TCT184TCA (1588T>A), AAG186AAA (1594G>A), AAC188AAT (1600C>T), CTG191CTA (1609G>A), TCG194TCA (1618G>A), TGC195TGT (1621C>T), GAC196GAT (1624C>T), CTG199TTG (1631C>T), AAA201AAG (1639A>G), TTC205TTT (1651C>T), CTC206CTG (1654C>G), CAT213CAC (1675T>C), TCA219TCG (1693A>G), TTT222TTC (1702T>C), GAA224AGA (1706G>A 1707A>G), GCA228ACA (1718G>A), GGA230GGG (1726A>G), TAC231TAT (1729C>T), TCA232TCC (1732A>C), TAT234TAC (1738T>C), ATC236GTT (1742A>G 1744C>T), CGC237CGT (1747C>T), CAT239CAC (1753T>C), AGT241AAT (1758G>A), AGG244AGA (1768G>A), AAC250AAT (1786C>T), TTA251TTG (1789A>G), GTT252ATT (1790G>A), GTC253GTT (1795C>T), TTA257CTG (1805T>C 1807A>G), GAG259GAA (1813G>A), AGG261AGA (1819G>A), AAG263AAA (1825G>A), GAC267GAT (1837C>T), TAT268TAC (1840T>C), CAA271CAG (1849A>G), GTC274GTG (1858C>G), AAA276AAG (1864A>G), TGC278TGT (1870C>T), AGT280AGC (1876T>C), AAA282AAG (1882A>G), GGT284GGA (1888T>A), AAT285AAC (1891T>C), TGT290TGC (1906T>C), ACA293ACT (1915A>T), GGT298GGC (1930T>C), GCC300GCT (1936C>T), ATT301GTC (1937A>G 1939T>C), TCA303TCG (1945A>G), TTC305TTT (1951C>T), CCA308CCT (1960A>T), GTA314GTG (1978A>G), ATT315ATA (1981T>A), GGC316GGT (1984C>T), AAT317AAC (1987T>C), GAC320GAT (1996C>T), TTT323TAT (2004T>A), AAA328AAG (2020A>G), GGG329GGA (2023G>A), GAT330GAC (2026T>C), TCG331TCT (2029G>T), GAG332GAA (2032G>A), TAT341TAC (2059T>C), ATT344ATG (2068T>G), AAC345AAT (2071C>T), GTG346ATA (2072G>A 2074G>A), CTG351TTA (2087C>T 2089G>A), AGC355AGT (2101C>T), GAG356GAA (2104G>A), GAG357GAA (2107G>A), AAG360AAA (2116G>A), TTC362TTT (2122C>T), CGC367CGT (2137C>T), CTT374CTC (2158T>C), ACC376ACT (2164C>T), CCA378CCG (2170A>G), GAT382GAC (2182T>C), GCG384GCA (2188G>A), ACC385ACA (2191C>A), AGA391AAA (2208G>A), TAT394TAC (2218T>C), GAC396GAT (2224C>T), GTA397GTT (2227A>T), TTG402TTA (2242G>A), CCC403CCT (2245C>T), TTG406TTA (2254G>A), GTT407GTC (2257T>C), GAC408GAT (2260C>T), GAC410GAG (2266C>G), CAA412CAG (2272A>G), ACG413ACA (2275G>A), TCA419TCG (2293A>G), GGC421GGT (2299C>T), TCG422TCA (2302G>A), CAG423CAA (2305G>A), GGA426GGT (2314A>T), TAT427TAC (2317T>C), CTA430CTG (2326A>G), GTG435GTA (2341G>A), CAA437CAG (2347A>G), CTT438CTC (2350T>C), TTG440TTA (2356G>A), AAT443AAC (2365T>C), GAA445GAG (2371A>G), TTA446TTG (2374A>G), GAT449GAC (2383T>C), ATA450ATT (2386A>T), AAA451AAG (2389A>G), CAT452CAC (2392T>C), TAT453TAC (2395T>C)								
P3 protein (NP_734251.1)	1	365	100%	1924	79.6%	365 (99.7%)	327 (89.3%)	1/0/2/2	0
Protein mutations:	N4G (2414A>G 2415A>G 2416T>G), S6C (2420A>T), K40R (2523A>G), R77K (2634G>A), V107I (2723G>A 2725C>T), A115T (2747G>A 2749T>A), H131N (2795C>A), V140I (2822G>A 2824G>A), P160T (2882C>A), S161H (2885A>C 2886G>A), T164M (2895C>T), N176S (2931A>G), N179S (2940A>G), E189_N190insX (2971_2972insA), T196I (2991C>T), H197R (2994A>G), E199R (2999G>A 3000A>G), R206Q (3021G>A), Y207F (3024A>T 3025C>T), T211I (3036C>T), E212G (3039A>G 3040A>C), K213T (3042A>C 3043G>A), A232V (3099C>T 3100C>A), V234R (3104G>A 3105T>G 3106G>A), S243N (3132G>A), R245K (3137C>A 3138G>A 3139A>G), F250I (3152T>A 3154C>T), C254Y (3165G>A), F272L (3218T>C), T274N (3225C>A), V276I (3230G>A), V288M (3266G>A 3268A>G), V291M (3275G>A), A294T (3284G>A), R304K (3315G>A 3316A>G), E305A (3318A>C), M319V (3359A>G), D332E (3400T>G)								

	Begin	End	Coverage	Score	Concordance	Matches	Identities	I/D/M/F*	Stop Codons
**NT**	**54**	**9704**	**99.5%**	**12522**	**65.2%**	**9639 (99.8%)**	**7984 (82.7%)**	**7/12**	
Codon mutations:	AAT4GGG (2414A>G 2415A>G 2416T>G), GCT5GCA (2419T>A), AGC6TGC (2420A>T), ACA12ACT (2440A>T), GTT21GTC (2467T>C), TCT24TCC (2476T>C), TCG26TCA (2482G>A), CTG29CTA (2491G>A), CTG31CTT (2497G>T), TTG33TTA (2503G>A), AGA38AGG (2518A>G), CCT39CCC (2521T>C), AAG40AGG (2523A>G), AGA43AGG (2533A>G), TTG45TTA (2539G>A), TTA47TTG (2545A>G), GAG49GAA (2551G>A), CCT50CCA (2554T>A), CTG52TTG (2558C>T), TTG53CTC (2561T>C 2563G>C), TCA56TCG (2572A>G), CCT60CCC (2584T>C), GGC61GGT (2587C>T), CTG63CTC (2593G>C), GCT65GCC (2599T>C), AAT68AAC (2608T>C), TTT72TTC (2620T>C), GCG75GCA (2629G>A), GTG76GTA (2632G>A), AGG77AAG (2634G>A), TTG78TTA (2638G>A), ATT80ATA (2644T>A), AAA83AAG (2653A>G), GCT87GCC (2665T>C), CTA92TTA (2678C>T), CTA93CTG (2683A>G), GCT95GCC (2689T>C), CTA98CTC (2698A>C), TCA101TCG (2707A>G), GCG102GCA (2710G>A), GAA104GAG (2716A>G), ACA105ACG (2719A>G), CTC106CTT (2722C>T), GTC107ATT (2723G>A 2725C>T), GCA108GCG (2728A>G), AGG110AGA (2734G>A), ATT111ATA (2737T>A), GAT114GAC (2746T>C), GCT115ACA (2747G>A 2749T>A), GCA116GCT (2752A>T), GCT117GCA (2755T>A), GAC119GAT (2761C>T), CTC120CTT (2764C>T), TGT125TGC (2779T>C), GGG127GGT (2785G>T), AAC129AAT (2791C>T), CAT131AAT (2795C>A), TAC134TAT (2806C>T), TTG138CTC (2816T>C 2818G>C), GTG140ATA (2822G>A 2824G>A), TTG141TTA (2827G>A), GTT144GTC (2836T>C), AAG145AAA (2839G>A), AAT146AAC (2842T>C), ACC153ACA (2863C>A), CTA154TTA (2864C>T), CCA160ACA (2882C>A), AGT161CAT (2885A>C 2886G>A), AAC163AAT (2893C>T), ACG164ATG (2895C>T), GTC166GTT (2902C>T), GTA167GTG (2905A>G), ATT169ATC (2911T>C), AAT173AAC (2923T>C), AAT176AGT (2931A>G), AAC179AGC (2940A>G), GCT181GCC (2947T>C), ACT186ACC (2962T>C), GAA189_AAT190insA-- (2971_2972insA), GGT195GGC (2989T>C), ACT196ATT (2991C>T), CAT197CGT (2994A>G), GAG199AGG (2999G>A 3000A>G), TCC204TC- (3016delC), CGG206CAG (3021G>A), TAC207TTT (3024A>T 3025C>T), ACA211ATA (3036C>T), GAA212GGC (3039A>G 3040A>C), AAG213ACA (3042A>C 3043G>A), TTG216TTA (3052G>A), TTA219TTG (3061A>G), GCG226GCA (3082G>A), GGC229GGT (3091C>T), GCC232GTA (3099C>T 3100C>A), GTG234AGA (3104G>A 3105T>G 3106G>A), GTC235GTT (3109C>T), AAA236AAG (3112A>G), ACT238ACC (3118T>C), GCC239GCT (3121C>T), GGA241GGG (3127A>G), AGC243AAC (3132G>A), CGA245AAG (3137C>A 3138G>A 3139A>G), TAT249TAC (3151T>C), TTC250ATT (3152T>A 3154C>T), TGT254TAT (3165G>A), GTA255GTG (3169A>G), ATC265ATT (3199C>T), AGG267CGG (3203A>C), CGT268CGC (3208T>C), CCA270CCG (3214A>G), ACC271ACT (3217C>T), TTT272CTT (3218T>C), GTC273GTA (3223C>A), ACT274AAT (3225C>A), GTT276ATT (3230G>A), AAC277AAT (3235C>T), TTA285CTA (3257T>C), ACC286ACT (3262C>T), AGC287AGT (3265C>T), GTA288ATG (3266G>A 3268A>G), GTG289GTA (3271G>A), GTG291ATG (3275G>A), GCA294ACA (3284G>A), CAG299CAA (3301G>A), AGA304AAG (3315G>A 3316A>G), GAA305GCA (3318A>C), ATC306ATT (3322C>T), GAG307GAA (3325G>A), TTG308TTA (3328G>A), ATA311ATT (3337A>T), GAG315GAA (3349G>A), GTC317GTT (3355C>T), TGC318TGT (3358C>T), ATG319GTG (3359A>G), CTA321CTG (3367A>G), TAT322TAC (3370T>C), TTA325CTG (3377T>C 3379A>G), GAA330GAG (3394A>G), CGC331CGT (3397C>T), GAT332GAG (3400T>G), ACA334ACG (3406A>G), TAC338TAT (3418C>T), ATT339ATA (3421T>A), TAT341TAC (3427T>C), AAG343AAA (3433G>A), TCA344TCT (3436A>T), GTA345GTG (3439A>G), AAC346AAT (3442C>T), CCT347CCC (3445T>C), CAG351CAA (3457G>A), TTT352TTC (3460T>C), GCT353GCA (3463T>A), GCG355GCT (3469G>T), CAG356CAA (3472G>A), CAC364CAT (3496C>T), CAG365CAA (3499G>A)								
6K1 protein (NP_734245.1)	1	52	100%	305	90.5%	52 (100%)	45 (86.5%)	0/0/0/0	0
Protein mutations:	V5G (3513T>G), M16V (3545A>G), A17T (3548G>A), V19I (3554G>A 3556C>T), L42V (3623C>G), L45M (3632C>A), Y47H (3638T>C)								
Codon mutations:	TCC2TCT (3505C>T), GTT5GGT (3513T>G), AAA7AAG (3520A>G), GAA10GAG (3529A>G), CAA11CAG (3532A>G), ATG16GTG (3545A>G), GCT17ACT (3548G>A), TTA18CTA (3551T>C), GTC19ATT (3554G>A 3556C>T), AGT28AGC (3583T>C), GAT29GAC (3586T>C), TGC30TGT (3589C>T), TTC32TTT (3595C>T), AAT36AAC (3607T>C), TTT38TTC (3613T>C), AAG39AAA (3616G>A), GGT40GGC (3619T>C), CTT42GTT (3623C>G), TCC43TCT (3628C>T), CTG45ATG (3632C>A), GAT46GAC (3637T>C), TAT47CAT (3638T>C), GAA48GAG (3643A>G)								
CI protein (NP_734246.1)	1	634	100%	4190	97.9%	634 (100%)	608 (95.9%)	0/0/0/0	0
Protein mutations:	I15V (3698A>G), S21N (3717G>A), R26K (3731C>A 3732G>A), I140V (4073A>G), F158Y (4128T>A 4129C>T), A166S (4151G>T), V171I (4166G>A), L190I (4223C>A 4225G>A), I222V (4319A>G 4321A>G), F247Y (4395T>A), V251I (4406G>A), M274V (4475A>G 4477G>T), T276S (4481A>T 4483A>G), A297L (4544G>T 4545C>T 4546G>A), R298K (4548G>A), V342I (4679G>A 4681G>C), V381I (4796G>A 4798C>T), T383S (4803C>G 4804T>C), S468G (5057A>G), T469A (5060A>G), L479I (5090C>A), A482V (5100C>T), E484D (5107A>T), V489F (5120G>T 5122C>T), I491V (5126A>G), V510I (5183G>A)								
Codon mutations:	TTA2TTG (3661A>G), GTG5GTA (3670G>A), TTT9TTC (3682T>C), GAG11GAA (3688G>A), GAG14GAA (3697G>A), ATT15GTT (3698A>G), GAT17GAC (3706T>C), TTT18TTC (3709T>C), TTG20CTA (3713T>C 3715G>A), AGT21AAT (3717G>A), GAG22GAA (3721G>A), CGA26AAA (3731C>A 3732G>A), ACT27ACA (3736T>A), CTA31TTG (3746C>T 3748A>G), ACA33ACG (3754A>G), AGT36AGC (3763T>C), CGA41CGG (3778A>G), CAA42CAG (3781A>G), ATC43ATT (3784C>T), CAT47CAC (3796T>C), CCA50CCT (3805A>T), TAC52TAT (3811C>T), AGA53AGG (3814A>G), TTC58TTT (3829C>T), TTT61TTC (3838T>C), AGA63AGG (3844A>G), GCA64GCC (3847A>C), GTT67GTA (3856T>A), GTG69GTT (3862G>T), GAC72GAT (3871C>T), GCC74GCA (3877C>A), AGC76AGT (3883C>T), GAA77GAG (3886A>G), CTA79CTG (3892A>G), GAC80GAT (3895C>T), TTT81TTC (3898T>C), GTA83GTG (3904A>G), CGG84AGA (3905C>A 3907G>A), GCT86GCC (3913T>C), AAG91AAA (3928G>A), TCA92TCT (3931A>T), GGG94GGA (3937G>A), AGT100AGC (3955T>C), GTG101GTA (3958G>A), GCC102GCT (3961C>T), GGA103GGG (3964A>G), TCT104TCC (3967T>C), TTA107TTG (3976A>G), ATT108ATC (3979T>C), CCA110CCT (3985A>T), CTA114CTT (3997A>T), GCG115GCA (4000G>A), AAC117AAT (4006C>T), GTT118GTG (4009T>G), TTC119TTT (4012C>T), AAA120AAG (4015A>G), TCT123TCC (4024T>C), GAA125GAG (4030A>G), CCA126CCT (4033A>T), AAG129AAA (4042G>A), CTG133TTA (4052C>T 4054G>A), CGT134CGC (4057T>C), CGT136CGA (4063T>A), GGA137GGG (4066A>G), ATA140GTA (4073A>G), TTT141TTC (4078T>C), GGC142GGT (4081C>T), TCT143TCC (4084T>C), TCC147TCT (4096C>T), AGC151AGT (4108C>T), GGA152GGT (4111A>T), GCG154GCA (4117G>A), CTA155TTG (4118C>T 4120A>G), TAC157TAT (4126C>T), TTC158TAT (4128T>A 4129C>T), GCC159GCT (4132C>T), AAT160AAC (4135T>C), CAA164CAG (4147A>G), TTA165CTA (4148T>C), GCT166TCT (4151G>T), AAC169AAT (4162C>T), GTA171ATA (4166G>A), ATA172ATC (4171A>C), GAG175GAA (4180G>A), CTG179TTG (4190C>T), CCT181CCC (4198T>C), TCC182TCT (4201C>T), GCG185GCT (4210G>T), TTC186TTT (4213C>T), AGT188AGC (4219T>C), CTG189TTG (4220C>T), CTG190ATA (4223C>A 4225G>A), GTT192GTG (4231T>G), CAT194CAC (4237T>C), GTA199GTG (4252A>G), TTA200CTG (4253T>C 4255A>G), AAA201AAG (4258A>G), ACT205ACC (4270T>C), AGA209AGG (4282A>G), GAG210GAA (4285G>A), GAA212GAG (4291A>G), CAG216CAA (4303G>A), GTC219GTT (4312C>T), ATA222GTG (4319A>G 4321A>G), GTG223GTT (4324G>T), GAG224GAA (4327G>A), CTG227CTT (4336G>T), TCT228TCC (4339T>C), TCA231TCT (4348A>T), GCA235GCG (4360A>G), GGT237GGC (4366T>C), TCT238TCA (4369T>A), ACT240ACC (4375T>C), GTT244GTC (4387T>C), TTT247TAT (4395T>A), GTA251ATA (4406G>A), CTT252CTC (4411T>C), GTT261GTC (4438T>C), ACC263ACA (4444C>A), GCC265GCT (4450C>T), CTC267CTT (4456C>T), GAC270GAT (4465C>T), ATG274GTT (4475A>G 4477G>T), GTC275GTT (4480C>T), ACA276TCG (4481A>T 4483A>G), GGC280GGT (4495C>T), AAG284AAA (4507G>A), CAC285CAT (4510C>T), GGT286GGG (4513T>G), CTA288TTA (4517C>T), GTC291GTT (4528C>T), AAA293AAG (4534A>G), ACC295ACT (4540C>T), GCG297TTA (4544G>T 4545C>T 4546G>A), AGA298AAA (4548G>A), TTT301TTC (4558T>C), GTA303GTT (4564A>T), ACC305ACT (4570C>T), ATA307ATT (4576A>T), TTG314TTA (4597G>A), GAC315GAT (4600C>T), GAC317GAT (4606C>T), GTG318GTA (4609G>A), GTG320GTA (4615G>A), GAC321GAT (4618C>T), TTG324CTT (4625T>C 4627G>T), GAC331GAT (4648C>T), ATT332ATC (4651T>C), AGG335AGA (4660G>A), AGC336AGT (4663C>T), GCT338GCA (4669T>A), GTG342ATC (4679G>A 4681G>C), AGT343AGC (4684T>C), GTT344GTG (4687T>G), GAG348GAA (4699G>A), AGA349AGG (4702A>G), CAA351CAG (4708A>G), AGG352AGA (4711G>A), CTG353TTG (4712C>T), AAA361AAG (4738A>G), GTA363GTT (4744A>T), GCA364GCG (4747A>G), TTG365CTG (4748T>C), ACT370ACC (4765T>C), GTC381ATT (4796G>A 4798C>T), ACT383AGC (4803C>G 4804T>C), GAG384GAA (4807G>A), GCG385GCT (4810G>T), GCT386GCG (4813T>G), CTT387CTC (4816T>C), TAT392TAC (4831T>C), AAC393AAT (4834C>T), CCA395CCT (4840A>T), GGA399GGG (4852A>G), GGC400GGT (4855C>T), AGT404AGC (4867T>C), CTG405CTC (4870G>C), ATT406ATA (4873T>A), TGT409TGC (4882T>C), ACA416ACT (4903A>T), CAG418CAA (4909G>A), TTT420TTC (4915T>C), GAA421GAG (4918A>G), TTG422CTA (4919T>C 4921G>A), AGT423AGC (4924T>C), CCC424CCG (4927C>G), TTT426TTC (4933T>C), ATC427ATA (4936C>A), CAG428CAA (4939G>A), AAT429AAC (4942T>C), TTC430TTT (4945C>T), TCA436TCG (4963A>G), CCT439CCC (4972T>C), CTT445CTC (4990T>C), AAA449AAG (5002A>G), CCT456CCC (5023T>C), TTG457CTG (5024T>C), TGC458TGT (5029C>T), GAT459GAC (5032T>C), TCT461TCC (5038T>C), CCA463CCT (5044A>T), GCA466GCT (5053A>T), TCG467TCA (5056G>A), AGT468GGT (5057A>G), ACT469GCT (5060A>G), TCG472TCT (5071G>T), CTT479ATT (5090C>A), GGA480GGG (5095A>G), GTG481GTT (5098G>T), GCC482GTC (5100C>T), TTA483TTG (5104A>G), GAA484GAT (5107A>T), ATT485ATC (5110T>C), CCA486CCG (5113A>G), AAG487AAA (5116G>A), CAA488CAG (5119A>G), GTC489TTT (5120G>T 5122C>T), ATT491GTT (5126A>G), CAT494CAC (5137T>C), ATC495ATT (5140C>T), AAA496AAG (5143A>G), GAG497GAA (5146G>A), CTC502CTA (5161C>A), GAA504GAG (5167A>G), CTT506CTG (5173T>G), ACG509ACA (5182G>A), GTT510ATT (5183G>A), GTC511GTT (5188C>T), AAG512AAA (5191G>A), AAA514AAG (5197A>G), GAC515GAT (5200C>T), TGC517TGT (5206C>T), TTA518TTG (5209A>G), AGC521AGT (5218C>T), TCG525TCA (5230G>A), ATC527ATT (5236C>T), AGC528AGT (5239C>T), ATC530ATT (5245C>T), ACA536ACT (5263A>T), CTC538CTT (5269C>T), TTC539TTT (5272C>T), GCC540GCA (5275C>A), ATC541ATT (5278C>T), CCA542CCC (5281A>C), AGA543AGG (5284A>G), ACT544ACC (5287T>C), CTA545TTA (5288C>T), ATA546ATT (5293A>T), TTG547CTA (5294T>C 5296G>A), GAG549GAA (5302G>A), CTT552CTA (5311T>A), GAA553GAG (5314A>G), CGA556CGT (5323A>T), AGT564AGC (5347T>C), ATC566ATT (5353C>T), TGC570TGT (5365C>T), GTT577GTC (5386T>C), ACC580ACA (5395C>A), AAA589AAG (5422A>G), GAT590GAC (5425T>C), ACC592ACT (5431C>T), GAG594GAA (5437G>A), CTT599CTC (5452T>C), AAG601AAA (5458G>A), AGT604AGC (5467T>C), CAA605CAG (5470A>G), CTA606TTG (5471C>T 5473A>G), GAA608GAG (5479A>G), TTC609TTT (5482C>T), TCA610TCC (5485A>C), AAT611AAC (5488T>C), TTG612CTA (5489T>C 5491G>A), GGT614GGC (5497T>C), TGT617TGC (5506T>C), GAG618GAA (5509G>A), GAG619GAA (5512G>A), TTA621CTA (5516T>C), TAT625TAC (5530T>C), GAG626GAA (5533G>A), TCG627TCT (5536G>T), TTG628TTA (5539G>A), GTT631GTG (5548T>G), CAC633CAT (5554C>T)								
6K2 protein (NP_734247.1)	1	52	100%	303	93.5%	52 (100%)	46 (88.5%)	0/0/0/0	0
Protein mutations:	A2T (5561G>A 5563G>A), I14V (5597A>G), N16K (5605C>A), A21V (5619C>T 5620T>G), I25L (5630A>C), V46I (5693G>A)								
Codon mutations:	GCT1GCA (5560T>A), GCG2ACA (5561G>A 5563G>A), ACG3ACC (5566G>C), TCA4TCG (5569A>G), CTC9CTG (5584C>G), AAG12AAA (5593G>A), GGG13GGA (5596G>A), ATT14GTT (5597A>G), AAC16AAA (5605C>A), GTG20GTT (5617G>T), GCT21GTG (5619C>T 5620T>G), TTG24TTA (5629G>A), ATC25CTC (5630A>C), GGC28GGT (5641C>T), GCA31GCT (5650A>T), ATA35ATT (5662A>T), GGA36GGG (5665A>G), CAA44CAG (5689A>G), GTT46ATT (5693G>A), GAG47GAA (5698G>A), CAA52CAG (5713A>G)								
NIa-VPg protein (NP_734252.1)	1	188	100%	1227	93.3%	188 (100%)	170 (90.4%)	0/0/0/0	0
Protein mutations:	K47R (5853A>G), I61V (5894A>G 5896C>G), R83A (5960C>G 5961G>C 5962G>C), I93V (5990A>G), E102D (6019A>T), K106T (6030A>C), D112E (6049C>A), M115I (6058G>A), S120N (6072G>A 6073T>C), N121H (6074A>C), T123N (6081C>A), C133S (6111G>C 6112T>C), I139V (6128A>G 6130T>C), V149I (6158G>A), T153S (6170A>T 6172A>G), L164F (6203C>T 6205C>T), V173I (6230G>A), A182K (6257G>A 6258C>A)								

	Begin	End	Coverage	Score	Concordance	Matches	Identities	I/D/M/F*	Stop Codons
**NT**	**54**	**9704**	**99.5%**	**12522**	**65.2%**	**9639 (99.8%)**	**7984 (82.7%)**	**7/12**	
Codon mutations:	GGG1GGC (5716G>C), AAA2AAG (5719A>G), GCC10GCA (5743C>A), CGC14CGA (5755C>A), CAT15CAC (5758T>C), CGT17CGC (5764T>C), AAA19AAG (5770A>G), GGC22GGT (5779C>T), TTT23TTC (5782T>C), GAC26GAT (5791C>T), GAC30GAT (5803C>T), AGG41AGA (5836G>A), AAA42AAG (5839A>G), GGA44GGG (5845A>G), AAA45AAG (5848A>G), AAA47AGA (5853A>G), GGT48GGC (5857T>C), ACC49ACT (5860C>T), ACA50ACT (5863A>T), GGT52GGA (5869T>A), GGT54GGC (5875T>C), AGC57AGT (5884C>T), TTC60TTT (5893C>T), ATC61GTG (5894A>G 5896C>G), AAC62AAT (5899C>T), GGG65GGA (5908G>A), TTT66TTC (5911T>C), GAG70GAA (5923G>A), CAA75CAG (5938A>G), TTC76TTT (5941C>T), GTT77GTC (5944T>C), GAT78GAC (5947T>C), GGG82GGA (5959G>A), CGG83GCC (5960C>G 5961G>C 5962G>C), GAA87GAG (5974A>G), GCT91GCA (5986T>A), GAC92GAT (5989C>T), ATT93GTT (5990A>G), GAT95GAC (5998T>C), AGA99CGT (6008A>C 6010A>T), GAA102GAT (6019A>T), GTG103GTT (6022G>T), CGA104CGT (6025A>T), AAA106ACA (6030A>C), GAC112GAA (6049C>A), ATG115ATA (6058G>A), CAA116CAG (6061A>G), GCC117GCA (6064C>A), GGT119GGC (6070T>C), AGT120AAC (6072G>A 6073T>C), AAC121CAC (6074A>C), ACG122ACA (6079G>A), ACC123AAC (6081C>A), ATA124ATT (6085A>T), GCA126GCT (6091A>T), TAC127TAT (6094C>T), TGT133TCC (6111G>C 6112T>C), AAA135AAG (6118A>G), TTG137TTA (6124G>A), AAG138AAA (6127G>A), ATT139GTC (6128A>G 6130T>C), GAT140GAC (6133T>C), TTA141TTG (6136A>G), AAC145AAT (6148C>T), AAA148AAG (6157A>G), GTT149ATT (6158G>A), TGT150TGC (6163T>C), AAA152AAG (6169A>G), ACA153TCG (6170A>T 6172A>G), GGC155GGT (6178C>T), AAA158AAG (6187A>G), TTT159TTC (6190T>C), GAG161GAA (6196G>A), CTC164TTT (6203C>T 6205C>T), GAA165GAG (6208A>G), CTA166TTG (6209C>T 6211A>G), CAG168CAA (6217G>A), GCT172GCA (6229T>A), GTA173ATA (6230G>A), GTC175GTT (6238C>T), AAG178AAA (6247G>A), ATA180ATT (6253A>T), GCA182AAA (6257G>A 6258C>A), CAT187CAC (6274T>C)								
NIa-Pro protein (NP_734248.1)	1	244	100%	1654	93.7%	244 (100%)	223 (91.4%)	0/0/0/0	0
Protein mutations:	K2R (6282A>G), A29T (6362G>A), V42I (6401G>A), A43V (6405C>T), Y51F (6429A>T), Q58R (6450A>G), H70N (6485C>A 6487C>T), L84I (6527C>A), T104I (6588C>T), Y120H (6635T>C), I124V (6647A>G), I134V (6677A>G 6679A>G), D147N (6716G>A), N148D (6719A>G), I163L (6764A>T 6766A>G), A173V (6795C>T 6796A>G), H174Q (6799C>A), N194D (6857A>G), V200T (6875G>A 6876T>C 6877C>A), D219E (6934C>A), V242I (7001G>A 7003G>A)								
Codon mutations:	AAA2AGA (6282A>G), TCG3TCA (6286G>A), GGC7GGT (6298C>T), TTG8TTA (6301G>A), AGA9AGG (6304A>G), GAC10GAT (6307C>T), GCC15GCT (6322C>T), ACA17ACT (6328A>T), GTT18GTG (6331T>G), TGT19TGC (6334T>C), AGG20AGA (6337G>A), CTG21TTG (6338C>T), TAT27TAC (6358T>C), GGG28GGA (6361G>A), GCA29ACA (6362G>A), TCA30TCT (6367A>T), GAG31GAA (6370G>A), TAC33TAT (6376C>T), GGT34GGG (6379T>G), GGA36GGC (6385A>C), GGA38GGT (6391A>T), GCA39GCG (6394A>G), TAC40TAT (6397C>T), GTA42ATA (6401G>A), GCG43GTG (6405C>T), CAC45CAT (6412C>T), TTA47TTG (6418A>G), TTT48TTC (6421T>C), TAC51TTC (6429A>T), GGT53GGA (6436T>A), GAG56GAA (6445G>A), CAA58CGA (6450A>G), TCC59TCG (6454C>G), CAC61CAT (6460C>T), GGT62GGA (6463T>A), TTC64TTT (6469C>T), AGG65AGA (6472G>A), GTG66GTA (6475G>A), AAT68AAC (6481T>C), CAC70AAT (6485C>A 6487C>T), AGT71AGC (6490T>C), AGC73AGT (6496C>T), CTG75CTA (6502G>A), GGT79GGC (6514T>C), AGG80AGA (6517G>A), CTC84ATC (6527C>A), ATC85ATA (6532C>A), CCG88CCA (6541G>A), AAA89AAG (6544A>G), GTC93GTT (6556C>T), TTT94TTC (6559T>C), CAG96CAA (6565G>A), TTG98CTA (6569T>C 6571G>A), CCT103CCA (6586T>A), ACA104ATA (6588C>T), GAA107GAG (6598A>G), AGA108AGG (6601A>G), ATT109ATC (6604T>C), TGT110TGC (6607T>C), GGA113GGG (6616A>G), AAC115AAT (6622C>T), GAG118GAA (6631G>A), AAG119AAA (6634G>A), TAT120CAT (6635T>C), GCT121GCA (6640T>A), TCG123TCT (6646G>T), ATC124GTC (6647A>G), ATC125ATT (6652C>T), AGC129AGT (6664C>T), ACT131ACG (6670T>G), ATA134GTG (6677A>G 6679A>G), GGC136GGT (6685C>T), ACA138ACT (6691A>T), TTC139TTT (6694C>T), ACA146ACG (6715A>G), GAT147AAT (6716G>A), AAT148GAT (6719A>G), GGA149GGG (6724A>G), CTA153TTA (6734C>T), GTG155GTA (6742G>A), GTG156GTA (6745G>A), GCC159GCT (6754C>T), GGA161GGG (6760A>G), ATA163TTG (6764A>T 6766A>G), ATT166ATA (6775T>A), CTG169TTG (6782C>T), GCA170GCT (6787A>T), AAC171AAT (6790C>T), GCA173GTG (6795C>T 6796A>G), CAC174CAA (6799C>A), ACC175ACA (6802C>A), AAC177AAT (6808C>T), TAC178TAT (6811C>T), TAC179TAT (6814C>T), TTC182TTT (6823C>T), GAA184GAG (6829A>G), TAC190TAT (6847C>T), CGA192CGC (6853A>C), AAT194GAT (6857A>G), GAG195GAA (6862G>A), GAA198GAG (6871A>G), GTC200ACA (6875G>A 6876T>C 6877C>A), AAG201AAA (6880G>A), TCT202TCA (6883T>A), GTT204GTG (6889T>G), GAC208GAT (6901C>T), GGC213GGT (6916C>T), CCG214CCA (6919G>A), AAA216AAG (6925A>G), CTT217CTC (6928T>C), GAC219GAA (6934C>A), GGG224GGC (6949G>C), TTC226TTT (6955C>T), AAA227AAG (6958A>G), CAA233CAG (6976A>G), CTA235TTA (6980C>T), ATC236ATT (6985C>T), GTA240GTT (6997A>T), GTG241GTT (7000G>T), GTG242ATA (7001G>A 7003G>A)								
NIb protein (NP_734249.1)	1	521	100%	3129	86.1%	519 (99.4%)	480 (92.0%)	1/2/2/2	0
Protein mutations:	F8Y (7032T>A), T39K (7125C>A), A46S (7145G>T 7147A>G), E51_A52del (7160_7165delGAGGCA), D69E (7216T>A), D83E (7258T>A), D88_R89insX (7273_7274insT), R89V (7274C>G 7275G>T), H91L (7281A>T), L92S (7284T>C), S96L (7296C>T), S101F (7311C>T), T102I (7314C>T), C103Y (7317G>A), N104K (7321T>A), K109Q (7334A>C), A112N (7343G>A 7344C>A 7345A>C), S133M (7407G>T 7408T>G), C136G (7415T>G), L165S (7503T>C 7504G>A), D234N (7709G>A), K235R (7713A>G 7714A>G), T268I (7812C>T), V279L (7844G>T 7846G>A), V292I (7883G>A), S345N (8043G>A), N381S (8151A>G 8152C>T), R390K (8178G>A), R400K (8208G>A), P429A (8294C>G 8296A>G), S447F (8349C>T), A467S (8408G>T), M481I (8452G>A), R484K (8460G>A), A491T (8480G>A 8482T>A), R498K (8502G>A 8503A>G), E502D (8515A>T), E510D (8539G>C), L513C (8546C>T 8547T>G), S515T (8552T>A)								
Codon mutations:	GCT1GCC (7012T>C), AAG2AAA (7015G>A), CAT3CAC (7018T>C), TTT8TAT (7032T>A), GCC10GCT (7039C>T), TTG11CTA (7040T>C 7042G>A), GGA13GGG (7048A>G), GCT17GCA (7060T>A), GTC18GTA (7063C>A), AGC23AGT (7078C>T), TTA25CTG (7082T>C 7084A>G), GTA26GTG (7087A>G), ACC27ACA (7090C>A), CAT29CAC (7096T>C), GTA30GTG (7099A>G), GTT31GTC (7102T>C), GAG34GAA (7111G>A), CGA36CGG (7117A>G), ACA39AAA (7125C>A), GAA40GAG (7129A>G), TTT41TTC (7132T>C), CTG42TTA (7133C>T 7135G>A), GTG44GTA (7141G>A), GAT45GAC (7144T>C), GCA46TCG (7145G>T 7147A>G), GAG47GAA (7150G>A), GCA50GCT (7159A>T), GAG51_GCA52del (7160_7165delGAGGCA), TTC54TTT (7171C>T), GCG60GCT (7189G>T), AAA63AAG (7198A>G), AGC64AGT (7201C>T), CTA66TTA (7205C>T), AAT67AAC (7210T>C), GAT69GAA (7216T>A), GCG70GCA (7219G>A), ATC72ATA (7225C>A), AAG73AAA (7228G>A), AAG77AAA (7240G>A), TAT78TAC (7243T>C), AAA80AAG (7249A>G), CCT81CCG (7252T>G), GAT83GAA (7258T>A), GTT84GTC (7261T>C), GGT85GGA (7264T>A), GTC86GTG (7267C>G), GTG87GTA (7270G>A), GAT88_CGG89insT-- (7273_7274insT), CGG89GTG (7274C>G 7275G>T), CAT91CTT (7281A>T), TTG92TCG (7284T>C), TCA96TTA (7296C>T), TCT101TTT (7311C>T), ACC102ATC (7314C>T), TGC103TAC (7317G>A), AAT104AAA (7321T>A), GTG105-TG (7322delG), CAC106CAT (7327C>T), GGC107GGT (7330C>T), TTC108TTT (7333C>T), AAG109CAG (7334A>C), TGT111TGC (7342T>C), GCA112AAC (7343G>A 7344C>A 7345A>C), ACT115ACC (7354T>C), CAA118CAG (7363A>G), ATT120ATC (7369T>C), TTC121TTT (7372C>T), GCG123GCT (7378G>T), CTC124CTT (7381C>T), GCA129GCT (7396A>T), GTC130GTT (7399C>T), GCC132GCT (7405C>T), AGT133ATG (7407G>T 7408T>G), GGG135GGT (7414G>T), TGC136GGC (7415T>G), AAA137AAG (7420A>G), GAC140GAT (7429C>T), TAT141TAC (7432T>C), CAT144CAC (7441T>C), AAG150AAA (7459G>A), GAA151GAG (7462A>G), ATA153ATT (7468A>T), GTC154GTA (7471C>A), CAA156CAG (7477A>G), AGC157AGT (7480C>T), CTG159CTT (7486G>T), TAT162TAC (7495T>C), AAA163AAG (7498A>G), GGT164GGC (7501T>C), TTG165TCA (7503T>C 7504G>A), ATT168ATA (7513T>A), AAC170AAT (7519C>T), GGA171GGG (7522A>G), AAG174AAA (7531G>A), GAG176GAA (7537G>A), CTC177CTT (7540C>T), TGT179TGC (7546T>C), AAG187AAA (7570G>A), ACG188ACA (7573G>A), ACG190ACA (7579G>A), GCT193GCC (7588T>C), CTA196TTA (7595C>T), TTG199CTA (7604T>C 7606G>A), GGT202GGA (7615T>A), TGT205TGC (7624T>C), GAC208GAT (7633C>T), TTC209TTT (7636C>T), AAT210AAC (7639T>C), TTT213TTC (7648T>C), GAA219GAG (7666A>G), TGT221TGC (7672T>C), ACA223ACT (7678A>T), GGG225GGA (7684G>A), ACT227ACC (7690T>C), TTT229TTC (7696T>C), GGT231GGG (7702T>G), GAT234AAT (7709G>A), AAA235AGG (7713A>G 7714A>G), CTG236TTG (7715C>T), CGT239CGC (7726T>C), GAG242GAA (7735G>A), GTA245GTG (7744A>G), TAC246TAT (7747C>T), GGC251GGT (7762C>T), CAG253CAA (7768G>A), TTT254TTC (7771T>C), TCA257TCG (7780A>G), CTA258CTC (7783A>C), ACT259ACC (7786T>C), CTA262TTG (7793C>T 7795A>G), ATC263ATT (7798C>T), GCT265GCC (7804T>C), ACC268ATC (7812C>T), GAA275GAG (7834A>G), GAC276GAT (7837C>T), GTG279TTA (7844G>T 7846G>A), CAG282CAA (7855G>A), CTG284CTA (7861G>A), TTA287TTG (7870A>G), TAC288TAT (7873C>T), ACT289ACA (7876T>A), GAG290GAA (7879G>A), ATT291ATA (7882T>A), GTT292ATT (7883G>A), CCA295CCT (7894A>T), ATT296ATC (7897T>C), GTC304GTT (7921C>T), AAG306AAA (7927G>A), GGT309GGC (7936T>C), AAT310AAC (7939T>C), AAT311AAC (7942T>C), GTG319GTA (7966G>A), GAT320GAC (7969T>C), TCT322TCC (7975T>C), GTC326GTT (7987C>T), CTT327CTC (7990T>C), GCT332GCC (8005T>C), GAA341GAG (8032A>G), ATC343ATT (8038C>T), GAC344GAT (8041C>T), AGC345AAC (8043G>A), TTG357TTA (8080G>A), GTG360GTA (8089G>A), CCG362CCA (8095G>A), GAG363GAA (8098G>A), AGC366AGT (8107C>T), ATT367ATC (8110T>C), GAT369GAC (8116T>C), CAA373CAG (8128A>G), GGT379GGC (8146T>C), AAC381AGT (8151A>G 8152C>T), TCG385TCA (8164G>A), AGG390AAG (8178G>A), AAG391AAA (8182G>A), TTG394TTA (8191G>A), TTC396TTT (8197C>T), TCC398TCT (8203C>T), AGA400AAA (8208G>A), CTG402TTA (8213C>T 8215G>A), CTA403CTG (8218A>G), ATC404ATT (8221C>T), GAG405GAA (8224G>A), CCA410CCG (8239A>G), GAG415GAA (8254G>A), ATT417ATC (8260T>C), GTA418GTG (8263A>G), GAT424GAC (8281T>C), AGA425AGG (8284A>G), CCA429GCG (8294C>G 8296A>G), GAG430GAA (8299G>A), CAC431CAT (8302C>T), ATT436ATC (8317T>C), TGC437TGT (8320C>T), GCT439GCA (8326T>A), GAG442GAA (8335G>A), TCC443TCT (8338C>T), TCT447TTT (8349C>T), GAA448GAG (8353A>G), CAC451CAT (8362C>T), CAA452CAG (8365A>G), ATC453ATT (8368C>T), TAC457TAT (8380C>T), TCA458TCG (8383A>G), TTA460TTG (8389A>G), CCT465CCC (8404T>C), GCA467TCA (8408G>T), ACA468ACG (8413A>G), GCG470GCT (8419G>T), GCT475GCC (8434T>C), TAT477TAC (8440T>C), ATG481ATA (8452G>A), CTA483CTG (8458A>G), AGG484AAG (8460G>A), AAA485AAG (8464A>G), CTG486CTA (8467G>A), AGG490AGA (8479G>A), GCT491ACA (8480G>A 8482T>A), GAG496GAA (8497G>A), CTA497TTG (8498C>T 8500A>G), AGA498AAG (8502G>A 8503A>G), GCC499GCT (8506C>T), GAA502GAT (8515A>T), GTC505GTT (8524C>T), GCA506GCC (8527A>C), TTA507CTG (8528T>C 8530A>G), GAG510GAC (8539G>C), CTT513TGT (8546C>T 8547T>G), TCT515ACT (8552T>A), GTA518GTG (8563A>G)								
coat protein (NP_734250.1)	1	267	100%	1652	90.6%	267 (100%)	243 (91.0%)	0/0/0/0	0
Protein mutations:	A1G (8574C>G), I5V (8585A>G), G9E (8598G>A), N11S (8603A>T 8604A>C 8605C>T), P17Q (8622C>A), E18G (8625A>G 8626G>A), P26F (8648C>T 8649C>T 8650G>C), G29E (8658G>A), D31A (8664A>C 8665T>G), A36V (8679C>T 8680A>T), T58K (8745C>A), E95G (8856A>G 8857G>A), R98Q (8865G>A), M99L (8867A>C 8869G>T), G104E (8883G>A), T106S (8888A>T), V128I (8954G>A), N138D (8984A>G 8986T>C), E139V (8988A>T), I187V (9131A>G), M193G (9149A>G 9150T>G 9151G>A), G194S (9152G>A), P230S (9260C>T 9262T>C), Q242H (9298A>C)								
Codon mutations:	GCA1GGA (8574C>G), ATT5GTT (8585A>G), GGA9GAA (8598G>A), AGC10AGT (8602C>T), AAC11TCT (8603A>T 8604A>C 8605C>T), AAA16AAG (8620A>G), CCA17CAA (8622C>A), GAG18GGA (8625A>G 8626G>A), CAG19CAA (8629G>A), GGC20GGT (8632C>T), ATC22ATT (8638C>T), CAG23CAA (8641G>A), AAC25AAT (8647C>T), CCG26TTC (8648C>T 8649C>T 8650G>C), AAC27AAT (8653C>T), AAA28AAG (8656A>G), GGA29GAA (8658G>A), GAT31GCG (8664A>C 8665T>G), GTT34GTG (8674T>G), GCA36GTT (8679C>T 8680A>T), GGC37GGA (8683C>A), GGG40GGA (8692G>A), ACA41ACT (8695A>T), CCG45CCA (8707G>A), AGA46CGA (8708A>C), AAG48AAA (8716G>A), ATC50ATT (8722C>T), ACG51ACA (8725G>A), AAA53AAG (8731A>G), AGA55AGG (8737A>G), CCC57CCT (8743C>T), ACA58AAA (8745C>A), AAG60AAA (8752G>A), GGA61GGT (8755A>T), AAC66AAT (8770C>T), TTA67CTA (8771T>C), GAA68GAG (8776A>G), CAT69CAC (8779T>C), TTG70TTA (8782G>A), TAT73TAC (8791T>C), CCA75CCG (8797A>G), CAA76CAG (8800A>G), CAA77CAG (8803A>G), GAT79GAC (8809T>C), ATT80ATC (8812T>C), AAT82AAC (8818T>C), CGG84CGA (8824G>A), GCA85GCG (8827A>G), ACG92ACA (8848G>A), TAT94TAC (8854T>C), GAG95GGA (8856A>G 8857G>A), GTG97GTA (8863G>A), CGG98CAG (8865G>A), ATG99CTT (8867A>C 8869G>T), GCA100GCG (8872A>G), GGA104GAA (8883G>A), ACT106TCT (8888A>T), TGC119TGT (8929C>T), GTC128ATC (8954G>A), AAC129AAT (8959C>T), GGA130GGT (8962A>T), GGG137GGA (8983G>A), AAT138GAC (8984A>G 8986T>C), GAA139GTA (8988A>T), GAG142GAA (8998G>A), CCG144CCA (9004G>A), TTG145TTA (9007G>A), ATC148ATT (9016C>T), ACC155ACA (9037C>A), ATA171ATT (9085A>T), AAC175AAT (9097C>T), ATT187GTT (9131A>G), CGA188CGT (9136A>T), CGG191CGT (9145G>T), ATG193GGA (9149A>G 9150T>G 9151G>A), GGT194AGT (9152G>A), TTA195TTG (9157A>G), GCG196GCT (9160G>T), CGT197CGC (9163T>C), GAG204GAA (9184G>A), CGA208CGG (9196A>G), AGG214AGA (9214G>A), GAA215GAG (9217A>G), GCG216GCA (9220G>A), GCA224GCT (9244A>T), TTG225TTA (9247G>A), GCC228GCT (9256C>T), CCT230TCC (9260C>T 9262T>C), GGG234GGA (9274G>A), GAC236GAT (9280C>T), ATC239ATT (9289C>T), CAA242CAC (9298A>C), GAG244GAA (9304G>A), GTC254GTT (9334C>T), GTC264GTG (9364C>G)								

	Begin	End	Coverage	Score	Concordance	Matches	Identities	I/D/M/F*	Stop Codons
**NT**	**54**	**9704**	**99.5%**	**12522**	**65.2%**	**9639 (99.8%)**	**7984 (82.7%)**	**7/12**	
PIPO (YP_006393460.1)	1	75	100%	95	18.1%	75 (98.7%)	54 (71.1%)	1/0/3/2	2
Protein mutations:	R17_K17insX (2971_2972insA), Y23H (2989T>C 2991C>T), R26K (2999G>A 3000A>G), H35Y (3025C>T), K40H (3040A>C 3042A>C), G41S (3043G>A), E44K (3052G>A), I47V (3061A>G), V54I (3082G>A), P57S (3091C>T), P60T (3100C>A), G61E (3104G>A 3105T>G), G62S (3106G>A), Q63* (3109C>T), R64G (3112A>G), C66R (3118T>C), L67F (3121C>T), I69V (3127A>G), A72E (3137C>A 3138G>A), I73V (3139A>G)								
Codon mutations:	TTA2CTA (2923T>C), AAA4AAG (2931A>G), GAA7GAG (2940A>G), TTG10CTG (2947T>C), TTG15CTG (2962T>C), AGA17_AAA17insAA- (2971_2972insA), TAC23CAT (2989T>C 2991C>T), TCA24TCG (2994A>G), AGA26AAG (2999G>A 3000A>G), CAC32-AC (3016delC), TCG33TCA (3021G>A), GTA34GTT (3024A>T), CAT35TAT (3025C>T), CAC38CAT (3036C>T), AGA39AGG (3039A>G), AAA40CAC (3040A>C 3042A>C), GGC41AGC (3043G>A), GAA44AAA (3052G>A), ATA47GTA (3061A>G), GTT54ATT (3082G>A), CCG57TCG (3091C>T), CGC59CGT (3099C>T), CCA60ACA (3100C>A), GGT61GAG (3104G>A 3105T>G), GGT62AGT (3106G>A), CAA63TAA (3109C>T), AGG64GGG (3112A>G), TGC66CGC (3118T>C), CTC67TTC (3121C>T), ATT69GTT (3127A>G), GAG70GAA (3132G>A), GCG72GAA (3137C>A 3138G>A), ATT73GTT (3139A>G)								

*: Inserts / Deletes / Misaligned / Frameshifts

Analysis details

This analysis was performed with panviral2.64

NGS Details (UN62): Torradovirus lycopersici (segment RNA 1)

Assembly

**Coverage Length**	7803 (1 contig(s))
**Depth Of Coverage**	2646.1
**Number Of Reads**	130769
**Reads Per Million**	3031.34 rpm (after QC)
**Ambiguities**	0
**Assembly Method**	de novo + reference guided assembly
**Consensus Caller**	Bcf Tools

Coverage Map

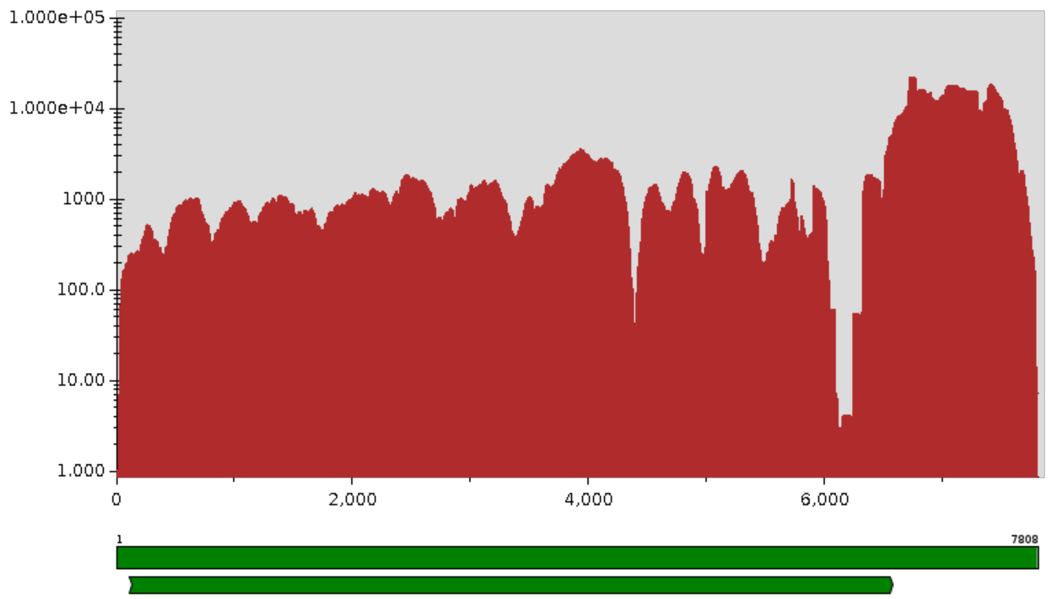

Assignment

**Type**	Torradovirus lycopersici (Taxonomy ID: 3048378)
**Reference Genome**	NC_009013.1
**NT Identity (%)**	87.0179
**AA Identity (%)**	93.2376
**Number Of Stop Codons**	3
**Number Of CDS**	1

Alignment

**Alignment Score**	10368.0 (NT) + 14226.0 (AA) = 24594.0
**Concordance (%)**	83.7585

Alignment Method	Global, seeded, nucleotide + amino acids (AGA)

Genome Region

Sequence starts at position 1 and ends at position 7803 relative to NC_009013.1 reference sequence.

Alignment Detailed Statistics

	Begin	End	Coverage	Score	Concordance	Matches	Identities	I/D/M/F*	Stop Codons
**NT**	**1**	**7803**	**99.9%**	**10368**	**72.5%**	**7431 (95.1%)**	**6475 (82.9%)**	**10/372**	
Mutations:	1T>G, 69C>T, 72C>T, 77A>T, 87C>T, 101G>T, 115T>C, 127C>T, 128C>T, 130C>T, 135T>C, 138A>G, 144T>C, 147C>T, 155T>A, 157C>T, 159C>T, 161A>G, 178T>A, 187A>T, 197G>C, 198C>A, 199T>A, 220C>T, 262T>A, 265C>T, 289T>G, 292A>T, 301T>A, 302T>G, 307C>T, 310T>C, 311A>T, 313C>T, 322T>C, 324A>G, 325C>T, 328T>A, 340G>A, 349G>A, 358T>A, 359T>C, 361A>T, 367A>G, 370C>T, 373C>T, 376C>T, 382T>A, 388T>C, 394C>T, 409A>G, 412A>C, 427T>G, 433G>A, 443A>G, 451A>G, 487C>T, 502G>A, 541T>C, 559C>T, 578C>T, 599A>T, 606T>C, 615A>G, 618C>T, 619A>T, 655C>T, 661A>G, 676A>G, 694A>G, 707C>T, 715G>A, 718A>G, 719T>G, 727A>T, 748G>T, 751A>T, 760C>T, 766C>T, 778T>A, 781C>T, 790C>T, 799A>T, 802T>A, 806A>C, 821T>A, 829G>A, 832A>T, 835A>C, 842C>T, 847C>T, 883G>A, 886T>C, 889C>A, 901T>C, 913T>C, 925C>T, 938C>T, 952A>G, 964T>A, 970G>A, 977G>A, 991T>C, 997C>T, 1001T>C, 1006T>A, 1018C>T, 1019A>G, 1020C>T, 1033G>A, 1060T>C, 1063C>A, 1093T>C, 1109C>T, 1129A>G, 1135T>A, 1165C>A, 1180G>A, 1186G>A, 1192A>C, 1195A>T, 1196A>T, 1198C>T, 1204T>G, 1207T>G, 1219G>C, 1240C>T, 1249A>G, 1255G>A, 1276A>G, 1300G>A, 1309C>A, 1318G>A, 1319C>T, 1324C>T, 1336T>G, 1337T>C, 1339G>T, 1357A>C, 1361T>C, 1411T>C, 1429C>T, 1489T>C, 1522C>A, 1534A>T, 1552C>T, 1553C>A, 1555C>G, 1568C>A, 1570A>G, 1576A>C, 1582C>T, 1585A>C, 1591C>T, 1607G>T, 1612G>A, 1618T>G, 1619C>T, 1624C>A, 1630C>T, 1643A>C, 1669C>T, 1681T>A, 1684G>A, 1692C>T, 1699T>G, 1706T>G, 1708T>C, 1714T>G, 1737A>G, 1738A>G, 1739A>C, 1755C>T, 1756A>C, 1770G>A, 1771C>T, 1781T>C, 1789A>G, 1813T>G, 1825C>A, 1828A>T, 1834A>G, 1839A>T, 1844G>A, 1849A>C, 1850T>A, 1858G>A, 1867G>A, 1876G>C, 1877T>A, 1879A>T, 1885G>A, 1889A>G, 1891A>G, 1894A>T, 1906T>A, 1907A>C, 1908T>C, 1909G>A, 1911T>G, 1927C>A, 1938C>T, 1942G>A, 1957T>G, 1960A>C, 1966A>G, 1978G>A, 1984C>T, 2014A>G, 2092A>G, 2120A>C, 2128T>C, 2130A>C, 2134C>T, 2138C>T, 2141T>C, 2143T>A, 2146A>G, 2149T>A, 2155T>C, 2174C>T, 2179A>C, 2200T>C, 2224C>T, 2227C>T, 2230T>G, 2240A>T, 2241A>G, 2250G>A, 2251C>A, 2257C>T, 2263G>A, 2270C>T, 2281A>T, 2282A>T, 2287G>A, 2290C>T, 2299A>T, 2318C>T, 2323A>T, 2344C>A, 2347C>T, 2350A>G, 2356C>T, 2359G>A, 2365A>T, 2368A>T, 2375A>C, 2380T>C, 2387C>A, 2389C>A, 2392A>G, 2407T>C, 2409G>A, 2446G>A, 2479G>A, 2485C>T, 2488G>A, 2491A>G, 2494T>G, 2497G>C, 2500T>C, 2503G>A, 2506T>A, 2509C>T, 2512T>C, 2516T>C, 2524A>C, 2542G>T, 2605A>T, 2614C>A, 2620G>A, 2626T>C, 2639C>A, 2650G>A, 2662C>T, 2692G>A, 2716A>C, 2726G>A, 2731C>T, 2734C>T, 2743A>C, 2744T>C, 2765T>C, 2767G>C, 2768A>C, 2773A>G, 2776T>A, 2806C>T, 2809C>T, 2812G>A, 2821G>A, 2827A>T, 2848C>T, 2852C>A, 2854T>A, 2860C>G, 2866T>A, 2869C>T, 2875A>T, 2878A>C, 2881T>C, 2882G>T, 2890A>C, 2914C>T, 2926G>A, 2929C>T, 2932G>A, 2947T>A, 2950C>T, 2992T>C, 2998A>G, 3031T>A, 3049A>C, 3064A>T, 3079T>C, 3083C>A, 3085A>G, 3101T>C, 3103G>T, 3109T>C, 3124C>A, 3136T>G, 3146A>T, 3151C>T, 3166T>C, 3169C>T, 3172T>C, 3175A>G, 3176C>T, 3184T>A, 3202T>C, 3238T>G, 3241T>A, 3242C>T, 3253C>T, 3265A>G, 3277T>A, 3289T>C, 3298T>A, 3331A>T, 3349T>A, 3365T>C, 3376T>G, 3379G>A, 3382T>A, 3388G>T, 3400A>C, 3406A>G, 3413C>G, 3418C>A, 3421T>C, 3430A>G, 3431C>A, 3445G>T, 3451T>C, 3466G>A, 3476G>C, 3481C>T, 3484A>T, 3508G>T, 3520A>G, 3529G>C, 3530C>A, 3532A>G, 3534G>A, 3541C>T, 3542T>C, 3544G>T, 3553T>A, 3559G>A, 3580T>C, 3583C>A, 3589A>G, 3591C>T, 3592T>C, 3593T>C, 3594C>A, 3610A>G, 3613T>C, 3622G>A, 3625C>T, 3628G>A, 3629G>T, 3646C>T, 3658A>C, 3661A>G, 3676C>T, 3679T>A, 3682G>C, 3688A>G, 3691C>T, 3693A>T, 3701C>T, 3710G>T, 3713A>G, 3716T>C, 3739A>T, 3742T>G, 3754G>A, 3758G>A, 3766G>A, 3776G>A, 3778G>T, 3781G>T, 3784C>G, 3790T>A, 3814C>T, 3824T>C, 3847G>A, 3856G>A, 3868C>T, 3871T>C, 3883G>A, 3895A>C, 3907A>T, 3910T>C, 3911C>A, 3949C>T, 3955G>A, 3958T>A, 3959T>C, 3961G>A, 3973T>C, 3982A>C, 4018A>G, 4028T>C, 4033G>T, 4034T>C, 4055C>A, 4067T>C, 4078T>A, 4084C>T, 4093C>T, 4118C>T, 4129C>T, 4147C>A, 4157C>T, 4159G>A, 4160C>G, 4162A>T, 4189T>A, 4192T>G, 4207G>A, 4210T>C, 4225A>T, 4228C>T, 4282C>A, 4288C>A, 4291T>C, 4294A>G, 4309T>G, 4318C>T, 4327A>T, 4339T>C, 4348C>T, 4358A>C, 4360A>C, 4381T>C, 4384A>C, 4393T>C, 4414T>G, 4417T>C, 4421T>C, 4426G>T, 4429A>G, 4441A>T, 4459T>C, 4468C>T, 4471T>C, 4501A>T, 4510T>A, 4537T>A, 4549G>C, 4555A>G, 4588C>T, 4606A>T, 4618C>A, 4625T>C, 4636T>C, 4648T>A, 4651T>A, 4654C>T, 4661T>C, 4663G>A, 4678T>C, 4684A>T, 4696C>T, 4699C>T, 4714T>C, 4716G>A, 4719G>A, 4735C>T, 4736T>C, 4747A>G, 4753T>C, 4756A>T, 4759C>T, 4765T>C, 4768T>C, 4774G>A, 4780T>C, 4783A>G, 4786A>G, 4789C>T, 4801T>C, 4802C>T, 4813A>G, 4816T>C, 4828A>C, 4834C>T, 4843G>A, 4861C>T, 4870C>G, 4885C>T, 4894G>T, 4897A>C, 4898G>A, 4906G>A, 4909A>C, 4921G>T, 4947C>A, 4954T>G, 4958A>C, 4960C>T, 4969G>A, 4975G>A, 4981C>T, 4987C>T, 4988A>G, 4990C>A, 4999T>C, 5002A>T, 5005T>C, 5012T>C, 5017G>A, 5020G>A, 5023C>T, 5030G>A, 5035G>A, 5041A>T, 5044A>C, 5059G>T, 5065C>T, 5074T>C, 5077T>C, 5079T>C, 5083A>T, 5090G>T, 5102T>C, 5105C>G, 5113C>T, 5116T>C, 5119T>C, 5120G>A, 5123C>T, 5131T>C, 5132T>A, 5143G>T, 5146T>C, 5158C>T, 5167T>C, 5173C>T, 5176C>T, 5182A>G, 5194A>C, 5200G>A, 5206C>T, 5209C>A, 5218T>G, 5227C>T, 5239A>C, 5266C>A, 5269C>A, 5278G>A, 5290G>T, 5293T>C, 5302T>A, 5329C>T, 5344A>G, 5347C>A, 5357A>T, 5360A>C, 5366C>A, 5369G>A, 5371G>T, 5374T>A, 5386C>G, 5392G>A, 5399A>G, 5407A>T, 5410G>T, 5413C>T, 5419A>T, 5429C>T, 5437T>C, 5440C>G, 5459G>A, 5461T>C, 5462G>A, 5471T>C, 5482A>T, 5485A>T, 5486G>A, 5491T>C, 5492A>C, 5494A>T, 5498C>T, 5500C>G, 5512A>T, 5515A>T, 5531C>T, 5533T>G, 5534C>G, 5537A>C, 5543G>T, 5558G>C, 5566T>G, 5569A>T, 5572T>C, 5578T>C, 5582A>G, 5591G>T, 5602G>A, 5604C>T, 5611A>T, 5613A>T, 5617A>T, 5620T>A, 5626A>T, 5638T>G, 5641T>C, 5647T>C, 5664G>A, 5674A>T, 5677G>T, 5686G>A, 5689C>A, 5707A>T, 5710T>A, 5711T>C, 5716G>T, 5719A>T, 5731C>T, 5734T>C, 5737T>C, 5739G>A, 5746T>C, 5751T>A, 5752G>A, 5767G>T, 5773G>A, 5774A>G, 5776A>G, 5779T>C, 5786T>C, 5791C>A, 5797A>G, 5804T>A, 5805T>C, 5806C>T, 5815C>G, 5818C>T, 5826A>T, 5845G>A, 5849T>C, 5850T>A, 5857T>G, 5860C>A, 5866T>G, 5869G>A, 5872C>G, 5878G>T, 5881T>C, 5887C>T, 5890C>T, 5894A>C, 5907A>T, 5914T>C, 5923T>C, 5927C>T, 5942C>T, 5944C>T, 5955A>G, 5965G>A, 5968T>G, 5974T>C, 5975A>C, 5980G>A, 5983T>C, 5986T>C, 5998A>G, 6002A>G, 6003C>T, 6004C>T, 6009G>A, 6016A>G, 6028C>T, 6031A>T, 6033C>T, 6035G>T, 6040T>A, 6055T>G, 6070T>C, 6079C>G, 6085T>C, 6089C>T, 6091A>G, 6092C>T, 6097T>A, 6100T>G, 6103G>A, 6106G>A, 6112A>G, 6118T>C, 6121A>T, 6130G>T, 6139T>C, 6154C>A, 6163C>G, 6166C>T, 6184A>G, 6185A>G, 6186C>A, 6187T>C, 6189A>G, 6193C>T, 6196A>G, 6199C>T, 6202G>C, 6214T>G, 6215C>G, 6217G>T, 6218A>C, 6221G>C, 6222A>C, 6225G>A, 6227A>G, 6230A>G, 6232A>G, 6235G>A, 6239G>A, 6240T>C, 6251T>C, 6253A>T, 6256C>A, 6262A>T, 6263C>T, 6265G>A, 6266T>A, 6268G>T, 6273G>T, 6274C>G, 6291T>A, 6292T>A, 6296G>C, 6298A>T, 6301T>A, 6302T>A, 6304A>G, 6308T>C, 6311G>T, 6313T>A, 6317A>G, 6319A>T, 6322G>A, 6337T>C, 6344C>T, 6371G>A, 6382C>T, 6397C>G, 6404T>C, 6405G>A, 6414G>T, 6415A>G, 6416T>C, 6417C>A, 6418A>C, 6433G>T, 6436C>T, 6437T>A, 6445C>T, 6448G>A, 6457C>A, 6458T>A, 6461C>T, 6463T>A, 6481T>A, 6485C>A, 6490A>C, 6491C>A, 6493T>C, 6496G>A, 6497A>C, 6499A>T, 6500A>G, 6501T>A, 6502T>G, 6514G>T, 6515T>C, 6517C>A, 6520C>T, 6532C>T, 6545C>T, 6556A>C, 6562A>T, 6566G>A, 6567G>A, 6568C>T, 6571C>T, 6574A>G, 6587G>A, 6643A>G, 6670C>T, 6685A>G, 6721A>T, 6742A>T, 6766delC, 6786T>C, 6798_6799insA, 6806T>G, 6807_6832delAACGGAGAGTACTGACTTTAACTAGT, 6835G>T, 6838_6850delGTCCGGCTCCATT, 6854A>T, 6858_6867delCCAATGAATT, 6871_6895delATTGGTTAAAGAGATTTGCACGCCT, 6897T>A, 6905T>G, 6907T>A, 6908G>T, 6911T>C, 6912_6916delCGTGT, 6921T>G, 6923C>G, 6924T>A, 6927_6941delGTTAGAAAGTTCCTT, 6947_6970delAGGTTTACAAAGTACGGGGAGCAC, 6972C>T, 6976G>A, 6984T>C, 6984_6985insG, 6988G>A, 6994G>T, 6995C>A, 6997A>C, 6998T>A, 7002A>T, 7003_7023delTGATAGTCCTTTAACTCAAGG, 7028A>T, 7030T>G, 7032C>A, 7034G>A, 7036T>G, 7039_7050delCATCTTTTGCCG, 7056_7106delAAAGATGAGGTAGCTTCCCCCTTATTGGGAGGCTGAAACTACACATATGTA, 7110G>T, 7111G>A, 7113T>A, 7115G>C, 7119_7121delGAG, 7123C>T, 7124C>A, 7125T>A, 7131C>G, 7133G>C, 7134T>A, 7137G>C, 7138_7185delTTTGAAATTCGATAATTTTCCGTAGCTTGCGTCAAGCTGCTCACGTTA, 7189G>A, 7194_7216delAGTGAAGATGCGCCGTACCACGT, 7221C>T, 7224_7231delGGCAATGC, 7236_7285delGGTTCAGAGCGGGCCCTCAGAATAGAGGTTAAAACTAGTGTGATGGTGTA, 7288T>A, 7294T>G, 7295A>C, 7297A>C, 7300T>G, 7303C>A, 7304_7315delACCCGGGTTGTG, 7320G>C, 7322C>T, 7323_7328delTAGTTA, 7332C>A, 7333G>C, 7336C>T, 7338C>A, 7339A>T, 7341_7347delGTCCCAC, 7350T>A, 7351A>C, 7353A>T, 7354G>A, 7358A>C, 7360G>A, 7361A>C, 7362A>C, 7363A>G, 7364C>T, 7368G>A, 7372A>T, 7374T>C, 7375T>G, 7376T>G, 7377T>A, 7379A>T, 7383_7384delAA, 7388C>T, 7397G>A, 7398C>A, 7399G>A, 7400A>T, 7404C>T, 7407A>T, 7408G>T, 7423A>T, 7425G>A, 7435T>C, 7436G>A, 7444C>T, 7445A>G, 7457A>G, 7458T>C, 7460G>A, 7503C>T, 7505A>G, 7506T>C, 7511_7512insT, 7513A>C, 7514A>T, 7515A>C, 7525_7526insG, 7526T>A, 7526_7527insG, 7545T>A, 7554A>G, 7569G>A, 7585T>C, 7589T>A, 7600T>G, 7601T>A, 7602T>G, 7604A>C, 7604_7605insG, 7610C>T, 7612G>A, 7615A>G, 7616A>C, 7617A>T, 7618C>T, 7622C>T, 7625delA, 7627T>A, 7629C>A, 7634A>G, 7637G>T, 7644_7652delGTTTGTTTT, 7657G>C, 7660T>A, 7665G>T, 7666T>C, 7667G>T, 7678C>T, 7686T>G, 7687T>A, 7689G>T, 7698G>A, 7717A>C, 7719T>A, 7723T>C, 7739T>A, 7742_7743insC, 7749T>C, 7751T>C, 7752G>A, 7754_7755insG, 7756_7757insT, 7762C>T, 7763A>C, 7764A>T, 7767C>T, 7773G>T, 7775G>A, 7778T>A, 7782A>T, 7783A>C, 7784_7785insT								

CDS

ToTV_sRNA1gp1	1	2159	100%	14226	94.4%	2159 (100%)	2013 (93.2%)	0/0/0/0	3
Protein mutations:	P8S (128C>T 130C>T), F10S (135T>C), N11S (138A>G), V13A (144T>C), T14I (147C>T), C17S (155T>A 157C>T), A18V (159C>T), T19A (161A>G), A31Q (197G>C 198C>A 199T>A), S66A (302T>G), T69S (311A>T 313C>T), N73S (324A>G 325C>T), S113G (443A>G), T165S (599A>T), I167T (606T>C), N170S (615A>G), T171I (618C>T 619A>T), S205A (719T>G), S239T (821T>A), V291I (977G>A), T305V (1019A>G 1020C>T), T364S (1196A>T 1198C>T), D367E (1207T>G), A501S (1607G>T), T529I (1692C>T), S534A (1706T>G 1708T>C), K544R (1737A>G 1738A>G), T550I (1755C>T 1756A>C), S555N (1770G>A 1771C>T), Y578F (1839A>T), A580T (1844G>A), S582T (1850T>A), S591T (1877T>A 1879A>T), I595V (1889A>G 1891A>G), D600E (1906T>A), M601P (1907A>C 1908T>C 1909G>A), L602W (1911T>G), A611V (1938C>T), N675T (2130A>C), Y679Q (2141T>C 2143T>A), N712C (2240A>T 2241A>G), S715K (2250G>A 2251C>A), M726L (2282A>T), R768K (2409G>A), G874S (2726G>A), A926S (2882G>T), Q993K (3083C>A 3085A>G), T1014S (3146A>T), L1046F (3242C>T), Q1103E (3413C>G), V1124L (3476G>C), R1143K (3534G>A), A1162V (3591C>T 3592T>C), S1163Q (3593T>C 3594C>A), A1175S (3629G>T), Y1196F (3693A>T), A1202S (3710G>T), I1203V (3713A>G), V1218I (3758G>A), V1224I (3776G>A 3778G>T), P1352A (4160C>G 4162A>T), I1362M (4192T>G), S1537N (4716G>A), S1538N (4719G>A), V1598I (4898G>A), T1614K (4947C>A), N1618H (4958A>C 4960C>T), T1628A (4988A>G 4990C>A), V1642M (5030G>A), I1658T (5079T>C), A1662S (5090G>T), P1667A (5105C>G), V1672I (5120G>A), S1676T (5132T>A), T1751S (5357A>T), K1752Q (5360A>C), H1754N (5366C>A), V1755I (5369G>A 5371G>T), I1765V (5399A>G), V1785I (5459G>A 5461T>C), V1786I (5462G>A), D1794N (5486G>A), T1796P (5492A>C 5494A>T), L1810V (5534C>G), K1811Q (5537A>C), A1813S (5543G>T), E1818Q (5558G>C), I1826V (5582A>G), A1829S (5591G>T), T1833I (5604C>T), Y1836F (5613A>T), R1853K (5664G>A), Q1857H (5677G>T), Y1869H (5711T>C), R1878K (5739G>A), L1882* (5751T>A 5752G>A), M1887I (5767G>T), I1890V (5774A>G 5776A>G), F1900T (5804T>A 5805T>C 5806C>T), Y1907F (5826A>T), L1915Q (5849T>C 5850T>A), D1918E (5860C>A), N1930H (5894A>C), Y1934F (5907A>T), H1946Y (5942C>T 5944C>T), N1950S (5955A>G), T1966V (6002A>G 6003C>T 6004C>T), G1968E (6009G>A), T1976I (6033C>T), A1977S (6035G>T), L1996F (6092C>T), H1997Q (6097T>A), I2002M (6112A>G), E2008D (6130G>T), T2027D (6185A>G 6186C>A 6187T>C), H2028R (6189A>G), M2032I (6202G>C), D2036E (6214T>G), L2037V (6215C>G 6217G>T), T2038P (6218A>C), E2039P (6221G>C 6222A>C), G2040E (6225G>A), I2041V (6227A>G), I2042V (6230A>G 6232A>G), V2045T (6239G>A 6240T>C), S2054T (6266T>A 6268G>T), S2056M (6273G>T 6274C>G), V2062E (6291T>A 6292T>A), V2064L (6296G>C 6298A>T), L2066M (6302T>A 6304A>G), D2069* (6311G>T 6313T>A), I2071V (6317A>G 6319A>T), A2089T (6371G>A), C2100H (6404T>C 6405G>A), R2103L (6414G>T 6415A>G), S2104H (6416T>C 6417C>A 6418A>C), C2111S (6437T>A), S2118T (6458T>A), H2127N (6485C>A), R2128S (6490A>C), H2129N (6491C>A 6493T>C), T2131P (6497A>C 6499A>T), I2132E (6500A>G 6501T>A 6502T>G), S2137P (6515T>C 6517C>A), L2147F (6545C>T), G2154N (6566G>A 6567G>A 6568C>T)								

	Begin	End	Coverage	Score	Concordance	Matches	Identities	I/D/M/F*	Stop Codons
**NT**	**1**	**7803**	**99.9%**	**10368**	**72.5%**	**7431 (95.1%)**	**6475 (82.9%)**	**10/372**	

Codon mutations:

TTT3TTC (115T>C), TTC7TTT (127C>T), CCC8TCT (128C>T 130C>T), TTC10TCC (135T>C), AAC11AGC (138A>G), GTT13GCT (144T>C), ACT14ATT (147C>T), TGC17AGT (155T>A 157C>T), GCT18GTT (159C>T), ACC19GCC (161A>G), TCT24TCA (178T>A), TCA27TCT (187A>T), GCT31CAA (197G>C 198C>A 199T>A), CGC38CGT (220C>T), TCT52TCA (262T>A), CAC53CAT (265C>T), GTT61GTG (289T>G), ACA62ACT (292A>T), GCT65GCA (301T>A), TCT66GCT (302T>G), TCC67TCT (307C>T), TTT68TTC (310T>C), ACC69TCT (311A>T 313C>T), TGT72TGC (322T>C), AAC73AGT (324A>G 325C>T), TCT74TCA (328T>A), TTG78TTA (340G>A), AAG81AAA (349G>A), ACT84ACA (358T>A), TTA85CTT (359T>C 361A>T), GAA87GAG (367A>G), CCC88CCT (370C>T), TTC89TTT (373C>T), ATC90ATT (376C>T), GCT92GCA (382T>A), TCT94TCC (388T>C), GTC96GTT (394C>T), AAA101AAG (409A>G), TCA102TCC (412A>C), GTT107GTG (427T>G), AAG109AAA (433G>A), AGC113GGC (443A>G), AAA115AAG (451A>G), GAC127GAT (487C>T), GTG132GTA (502G>A), TCT145TCC (541T>C), TTC151TTT (559C>T), CTA158TTA (578C>T), ACT165TCT (599A>T), ATA167ACA (606T>C), AAT170AGT (615A>G), ACA171ATT (618C>T 619A>T), GAC183GAT (655C>T), TTA185TTG (661A>G), CAA190CAG (676A>G), GCA196GCG (694A>G), CTA201TTA (707C>T), GGG203GGA (715G>A), GAA204GAG (718A>G), TCC205GCC (719T>G), TCA207TCT (727A>T), GTG214GTT (748G>T), GTA215GTT (751A>T), CTC218CTT (760C>T), GCC220GCT (766C>T), TCT224TCA (778T>A), ACC225ACT (781C>T), TTC228TTT (790C>T), GTA231GTT (799A>T), CCT232CCA (802T>A), AGA234CGA (806A>C), TCC239ACC (821T>A), CCG241CCA (829G>A), CTA242CTT (832A>T), TCA243TCC (835A>C), CTG246TTG (842C>T), TAC247TAT (847C>T), TTG259TTA (883G>A), TTT260TTC (886T>C), ACC261ACA (889C>A), AAT265AAC (901T>C), TGT269TGC (913T>C), GCC273GCT (925C>T), CTG278TTG (938C>T), GTA282GTG (952A>G), GGT286GGA (964T>A), AAG288AAA (970G>A), GTT291ATT (977G>A), ATT295ATC (991T>C), GCC297GCT (997C>T), TTG299CTG (1001T>C), TCT300TCA (1006T>A), TTC304TTT (1018C>T), ACG305GTG (1019A>G 1020C>T), GAG309GAA (1033G>A), CAT318CAC (1060T>C), CGC319CGA (1063C>A), ACT329ACC (1093T>C), CTA335TTA (1109C>T), AAA341AAG (1129A>G), TCT343TCA (1135T>A), GCC353GCA (1165C>A), AGG358AGA (1180G>A), ACG360ACA (1186G>A), GCA362GCC (1192A>C), ATA363ATT (1195A>T), ACC364TCT (1196A>T 1198C>T), CTT366CTG (1204T>G), GAT367GAG (1207T>G), GGG371GGC (1219G>C), GGC378GGT (1240C>T), CAA381CAG (1249A>G), AGG383AGA (1255G>A), CAA390CAG (1276A>G), GGG398GGA (1300G>A), ACC401ACA (1309C>A), CGG404CGA (1318G>A), CTG405TTG (1319C>T), TTC406TTT (1324C>T), GTT410GTG (1336T>G), TTG411CTT (1337T>C 1339G>T), CCA417CCC (1357A>C), TTG419CTG (1361T>C), TAT435TAC (1411T>C), ATC441ATT (1429C>T), GAT461GAC (1489T>C), CCC472CCA (1522C>A), GCA476GCT (1534A>T), GGC482GGT (1552C>T), CGC483AGG (1553C>A 1555C>G), CGA488AGG (1568C>A 1570A>G), ATA490ATC (1576A>C), GCC492GCT (1582C>T), ACA493ACC (1585A>C), AAC495AAT (1591C>T), GCT501TCT (1607G>T), CAG502CAA (1612G>A), GGT504GGG (1618T>G), CTA505TTA (1619C>T), ACC506ACA (1624C>A), CCC508CCT (1630C>T), AGA513CGA (1643A>C), TGC521TGT (1669C>T), GGT525GGA (1681T>A), GAG526GAA (1684G>A), ACT529ATT (1692C>T), GCT531GCG (1699T>G), TCT534GCC (1706T>G 1708T>C), CTT536CTG (1714T>G), AAA544AGG (1737A>G 1738A>G), AGA545CGA (1739A>C), ACA550ATC (1755C>T 1756A>C), AGC555AAT (1770G>A 1771C>T), TTG559CTG (1781T>C), GAA561GAG (1789A>G), GTT569GTG (1813T>G), ATC573ATA (1825C>A), TCA574TCT (1828A>T), AAA576AAG (1834A>G), TAT578TTT (1839A>T), GCC580ACC (1844G>A), ACA581ACC (1849A>C), TCA582ACA (1850T>A), AAG584AAA (1858G>A), CAG587CAA (1867G>A), GTG590GTC (1876G>C), TCA591ACT (1877T>A 1879A>T), GAG593GAA (1885G>A), ATA595GTG (1889A>G 1891A>G), ATA596ATT (1894A>T), GAT600GAA (1906T>A), ATG601CCA (1907A>C 1908T>C 1909G>A), TTG602TGG (1911T>G), ACC607ACA (1927C>A), GCT611GTT (1938C>T), AAG612AAA (1942G>A), GTT617GTG (1957T>G), GTA618GTC (1960A>C), GAA620GAG (1966A>G), GTG624GTA (1978G>A), TAC626TAT (1984C>T), AAA636AAG (2014A>G), CTA662CTG (2092A>G), AGG672CGG (2120A>C), GTT674GTC (2128T>C), AAT675ACT (2130A>C), GAC676GAT (2134C>T), CTG678TTG (2138C>T), TAT679CAA (2141T>C 2143T>A), CAA680CAG (2146A>G), GGT681GGA (2149T>A), TGT683TGC (2155T>C), CTG690TTG (2174C>T), ATA691ATC (2179A>C), TAT698TAC (2200T>C), ATC706ATT (2224C>T), TAC707TAT (2227C>T), GCT708GCG (2230T>G), AAT712TGT (2240A>T 2241A>G), AGC715AAA (2250G>A 2251C>A), GAC717GAT (2257C>T), CAG719CAA (2263G>A), CTG722TTG (2270C>T), GGA725GGT (2281A>T), ATG726TTG (2282A>T), GCG727GCA (2287G>A), CAC728CAT (2290C>T), ATA731ATT (2299A>T), CTG738TTG (2318C>T), ATA739ATT (2323A>T), GCC746GCA (2344C>A), CAC747CAT (2347C>T), GTA748GTG (2350A>G), TTC750TTT (2356C>T), TTG751TTA (2359G>A), ACA753ACT (2365A>T), GCA754GCT (2368A>T), AGG757CGG (2375A>C), TAT758TAC (2380T>C), CGC761AGA (2387C>A 2389C>A), AGA762AGG (2392A>G), CCT767CCC (2407T>C), AGG768AAG (2409G>A), GTG780GTA (2446G>A), CCG791CCA (2479G>A), ATC793ATT (2485C>T), TTG794TTA (2488G>A), AGA795AGG (2491A>G), GTT796GTG (2494T>G), CTG797CTC (2497G>C), ATT798ATC (2500T>C), AAG799AAA (2503G>A), GCT800GCA (2506T>A), GGC801GGT (2509C>T), CTT802CTC (2512T>C), TTA804CTA (2516T>C), GCA806GCC (2524A>C), GGG812GGT (2542G>T), GGA833GGT (2605A>T), ACC836ACA (2614C>A), GAG838GAA (2620G>A), AAT840AAC (2626T>C), CGG845AGG (2639C>A), AAG848AAA (2650G>A), ATC852ATT (2662C>T), AAG862AAA (2692G>A), GCA870GCC (2716A>C), GGC874AGC (2726G>A), TTC875TTT (2731C>T), ATC876ATT (2734C>T), GGA879GGC (2743A>C), TTG880CTG (2744T>C), TTG887CTC (2765T>C 2767G>C), AGA888CGA (2768A>C), CTA889CTG (2773A>G), GGT890GGA (2776T>A), GTC900GTT (2806C>T), TGC901TGT (2809C>T), TCG902TCA (2812G>A), GTG905GTA (2821G>A), ACA907ACT (2827A>T), AGC914AGT (2848C>T), CGT916AGA (2852C>A 2854T>A), GGC918GGG (2860C>G), ACT920ACA (2866T>A), TTC921TTT (2869C>T), ATA923ATT (2875A>T), ATA924ATC (2878A>C), CAT925CAC (2881T>C), GCC926TCC (2882G>T), TCA928TCC (2890A>C), GCC936GCT (2914C>T), CGG940CGA (2926G>A), TTC941TTT (2929C>T), TTG942TTA (2932G>A), ATT947ATA (2947T>A), GTC948GTT (2950C>T), ATT962ATC (2992T>C), AAA964AAG (2998A>G), TCT975TCA (3031T>A), GTA981GTC (3049A>C), GTA986GTT (3064A>T), GCT991GCC (3079T>C), CAA993AAG (3083C>A 3085A>G), TTG999CTT (3101T>C 3103G>T), TTT1001TTC (3109T>C), TCC1006TCA (3124C>A), CTT1010CTG (3136T>G), ACG1014TCG (3146A>T), TAC1015TAT (3151C>T), AGT1020AGC (3166T>C), TCC1021TCT (3169C>T), TTT1022TTC (3172T>C), AAA1023AAG (3175A>G), CTG1024TTG (3176C>T), TCT1026TCA (3184T>A), TAT1032TAC (3202T>C), CTT1044CTG (3238T>G), CTT1045CTA (3241T>A), CTT1046TTT (3242C>T), AAC1049AAT (3253C>T), AAA1053AAG (3265A>G), ATT1057ATA (3277T>A), CAT1061CAC (3289T>C), TCT1064TCA (3298T>A), GTA1075GTT (3331A>T), GCT1081GCA (3349T>A), TTG1087CTG (3365T>C), CTT1090CTG (3376T>G), CCG1091CCA (3379G>A), ACT1092ACA (3382T>A), CCG1094CCT (3388G>T), GCA1098GCC (3400A>C), GTA1100GTG (3406A>G), CAG1103GAG (3413C>G), CTC1104CTA (3418C>A), CTT1105CTC (3421T>C), CTA1108CTG (3430A>G), CGA1109AGA (3431C>A), GCG1113GCT (3445G>T), CTT1115CTC (3451T>C), CAG1120CAA (3466G>A), GTT1124CTT (3476G>C), GGC1125GGT (3481C>T), ACA1126ACT (3484A>T), ACG1134ACT (3508G>T), AAA1138AAG (3520A>G), CTG1141CTC (3529G>C), CGA1142AGG (3530C>A 3532A>G), AGG1143AAG (3534G>A), GGC1145GGT (3541C>T), TTG1146CTT (3542T>C 3544G>T), GCT1149GCA (3553T>A), GGG1151GGA (3559G>A), TCT1158TCC (3580T>C), ATC1159ATA (3583C>A), TCA1161TCG (3589A>G), GCT1162GTC (3591C>T 3592T>C), TCA1163CAA (3593T>C 3594C>A), GAA1168GAG (3610A>G), GGT1169GGC (3613T>C), CCG1172CCA (3622G>A), TAC1173TAT (3625C>T), GTG1174GTA (3628G>A), GCT1175TCT (3629G>T), TAC1180TAT (3646C>T), GCA1184GCC (3658A>C), CAA1185CAG (3661A>G), GAC1190GAT (3676C>T), ATT1191ATA (3679T>A), GCG1192GCC (3682G>C), CTA1194CTG (3688A>G), GCC1195GCT (3691C>T), TAT1196TTT (3693A>T), CTA1199TTA (3701C>T), GCA1202TCA (3710G>T), ATT1203GTT (3713A>G), TTG1204CTG (3716T>C), CGA1211CGT (3739A>T), GTT1212GTG (3742T>G), AAG1216AAA (3754G>A), GTC1218ATC (3758G>A), CAG1220CAA (3766G>A), GTG1224ATT (3776G>A 3778G>T), GTG1225GTT (3781G>T), CTC1226CTG (3784C>G), GGT1228GGA (3790T>A), GAC1236GAT (3814C>T), TTG1240CTG (3824T>C), CCG1247CCA (3847G>A), AAG1250AAA (3856G>A), GGC1254GGT (3868C>T), ACT1255ACC (3871T>C), GAG1259GAA (3883G>A), CCA1263CCC (3895A>C), GGA1267GGT (3907A>T), GAT1268GAC (3910T>C), CGA1269AGA (3911C>A), GAC1281GAT (3949C>T), GAG1283GAA (3955G>A), GCT1284GCA (3958T>A), TTG1285CTA (3959T>C 3961G>A), ATT1289ATC (3973T>C), GGA1292GGC (3982A>C), AAA1304AAG (4018A>G), TTG1308CTG (4028T>C), CCG1309CCT (4033G>T), TTG1310CTG (4034T>C), CGG1317AGG (4055C>A), TTG1321CTG (4067T>C), ATT1324ATA (4078T>A), CCC1326CCT (4084C>T), TAC1329TAT (4093C>T), CTG1338TTG (4118C>T), TCC1341TCT (4129C>T), TCC1347TCA (4147C>A), CTG1351TTA (4157C>T 4159G>A), CCA1352GCT (4160C>G 4162A>T), GGT1361GGA (4189T>A), ATT1362ATG (4192T>G), TTG1367TTA (4207G>A), GCT1368GCC (4210T>C), GCA1373GCT (4225A>T), GTC1374GTT (4228C>T), ATC1392ATA (4282C>A), GCC1394GCA (4288C>A), TTT1395TTC (4291T>C), CAA1396CAG (4294A>G), CTT1401CTG (4309T>G), GAC1404GAT (4318C>T), GGA1407GGT (4327A>T), GGT1411GGC (4339T>C), GAC1414GAT (4348C>T), AGA1418CGC (4358A>C 4360A>C), GCT1425GCC (4381T>C), TCA1426TCC (4384A>C), TAT1429TAC (4393T>C), GTT1436GTG (4414T>G), TTT1437TTC (4417T>C), TTG1439CTG (4421T>C), GTG1440GTT (4426G>T), GGA1441GGG (4429A>G), TCA1445TCT (4441A>T), GTT1451GTC (4459T>C), AAC1454AAT (4468C>T), TCT1455TCC (4471T>C), GCA1465GCT (4501A>T), TCT1468TCA (4510T>A), GCT1477GCA (4537T>A), GGG1481GGC (4549G>C), AAA1483AAG (4555A>G), GTC1494GTT (4588C>T), CTA1500CTT (4606A>T), CCC1504CCA (4618C>A), TTG1507CTG (4625T>C), TAT1510TAC (4636T>C), GCT1514GCA (4648T>A), ATT1515ATA (4651T>A), GCC1516GCT (4654C>T), TTG1519CTA (4661T>C 4663G>A), GTT1524GTC (4678T>C), ATA1526ATT (4684A>T), ATC1530ATT (4696C>T), GAC1531GAT (4699C>T), GTT1536GTC (4714T>C), AGC1537AAC (4716G>A), AGT1538AAT (4719G>A), GAC1543GAT (4735C>T), TTG1544CTG (4736T>C), CTA1547CTG (4747A>G), TTT1549TTC (4753T>C), CTA1550CTT (4756A>T), AGC1551AGT (4759C>T), GGT1553GGC (4765T>C), TTT1554TTC (4768T>C), AGG1556AGA (4774G>A), CGT1558CGC (4780T>C), CTA1559CTG (4783A>G), GGA1560GGG (4786A>G), TAC1561TAT (4789C>T), CCT1565CCC (4801T>C), CTG1566TTG (4802C>T), GTA1569GTG (4813A>G), TCT1570TCC (4816T>C), CCA1574CCC (4828A>C), TAC1576TAT (4834C>T), AGG1579AGA (4843G>A), CCC1585CCT (4861C>T), CTC1588CTG (4870C>G), AAC1593AAT (4885C>T), ACG1596ACT (4894G>T), GGA1597GGC (4897A>C), GTT1598ATT (4898G>A), GAG1600GAA (4906G>A), GCA1601GCC (4909A>C), GGG1605GGT (4921G>T), ACA1614AAA (4947C>A), GTT1616GTG (4954T>G), AAC1618CAT (4958A>C 4960C>T), AAG1621AAA (4969G>A), CGG1623CGA (4975G>A), TTC1625TTT (4981C>T), GCC1627GCT (4987C>T), ACC1628GCA (4988A>G 4990C>A), CCT1631CCC (4999T>C), ACA1632ACT (5002A>T), TAT1633TAC (5005T>C), TTG1636CTG (5012T>C), GAG1637GAA (5017G>A), CAG1638CAA (5020G>A), GAC1639GAT (5023C>T), GTG1642ATG (5030G>A), GAG1643GAA (5035G>A), ACA1645ACT (5041A>T), GGA1646GGC (5044A>C), GGG1651GGT (5059G>T), ATC1653ATT (5065C>T), CTT1656CTC (5074T>C), CAT1657CAC (5077T>C), ATT1658ACT (5079T>C), GCA1659GCT (5083A>T), GCT1662TCT (5090G>T), TTG1666CTG (5102T>C), CCC1667GCC (5105C>G), GGC1669GGT (5113C>T), AAT1670AAC (5116T>C), ACT1671ACC (5119T>C), GTT1672ATT (5120G>A), CTG1673TTG (5123C>T), TTT1675TTC (5131T>C), TCT1676ACT (5132T>A), GTG1679GTT (5143G>T), CAT1680CAC (5146T>C), GAC1684GAT (5158C>T), AGT1687AGC (5167T>C), TGC1689TGT (5173C>T), TCC1690TCT (5176C>T), CGA1692CGG (5182A>G), GCA1696GCC (5194A>C), CAG1698CAA (5200G>A), GAC1700GAT (5206C>T), ACC1701ACA (5209C>A), GTT1704GTG (5218T>G), AAC1707AAT (5227C>T), GCA1711GCC (5239A>C), CCC1720CCA (5266C>A), ACC1721ACA (5269C>A), AGG1724AGA (5278G>A), GGG1728GGT (5290G>T), CTT1729CTC (5293T>C), CCT1732CCA (5302T>A), CAC1741CAT (5329C>T), GTA1746GTG (5344A>G), ACC1747ACA (5347C>A), ACC1751TCC (5357A>T), AAA1752CAA (5360A>C), CAT1754AAT (5366C>A), GTG1755ATT (5369G>A 5371G>T), GTT1756GTA (5374T>A), GGC1760GGG (5386C>G), GGG1762GGA (5392G>A), ATT1765GTT (5399A>G), CCA1767CCT (5407A>T), GTG1768GTT (5410G>T), TGC1769TGT (5413C>T), GCA1771GCT (5419A>T), CTG1775TTG (5429C>T), ACT1777ACC (5437T>C), GGC1778GGG (5440C>G), GTT1785ATC (5459G>A 5461T>C), GTT1786ATT (5462G>A), TTG1789CTG (5471T>C), ATA1792ATT (5482A>T), GCA1793GCT (5485A>T), GAT1794AAT (5486G>A), GTT1795GTC (5491T>C), ACA1796CCT (5492A>C 5494A>T), CTC1798TTG (5498C>T 5500C>G), CCA1802CCT (5512A>T), GGA1803GGT (5515A>T), CTT1809TTG (5531C>T 5533T>G), CTG1810GTG (5534C>G), AAA1811CAA (5537A>C), GCT1813TCT (5543G>T), GAA1818CAA (5558G>C), CTT1820CTG (5566T>G), GCA1821GCT (5569A>T), GAT1822GAC (5572T>C), TGT1824TGC (5578T>C), ATT1826GTT (5582A>G), GCC1829TCC (5591G>T), GAG1832GAA (5602G>A), ACA1833ATA (5604C>T), GCA1835GCT (5611A>T), TAC1836TTC (5613A>T), ATA1837ATT (5617A>T), CCT1838CCA (5620T>A), GGA1840GGT (5626A>T), CTT1844CTG (5638T>G), GGT1845GGC (5641T>C), TTT1847TTC (5647T>C), AGA1853AAA (5664G>A), CTA1856CTT (5674A>T), CAG1857CAT (5677G>T), CAG1860CAA (5686G>A), CGC1861CGA (5689C>A), GGA1867GGT (5707A>T), CCT1868CCA (5710T>A), TAT1869CAT (5711T>C), GGG1870GGT (5716G>T), GGA1871GGT (5719A>T), CAC1875CAT (5731C>T), ACT1876ACC (5734T>C), ACT1877ACC (5737T>C), AGA1878AAA (5739G>A), CTT1880CTC (5746T>C), TTG1882TAA (5751T>A 5752G>A), ATG1887ATT (5767G>T), AAG1889AAA (5773G>A), ATA1890GTG (5774A>G 5776A>G), TAT1891TAC (5779T>C), TTA1894CTA (5786T>C), GTC1895GTA (5791C>A), AGA1897AGG (5797A>G), TTC1900ACT (5804T>A 5805T>C 5806C>T), CTC1903CTG (5815C>G), TAC1904TAT (5818C>T), TAT1907TTT (5826A>T), GTG1913GTA (5845G>A), TTG1915CAG (5849T>C 5850T>A), GTT1917GTG (5857T>G), GAC1918GAA (5860C>A), CTT1920CTG (5866T>G), AGG1921AGA (5869G>A), CTC1922CTG (5872C>G), GGG1924GGT (5878G>T), TTT1925TTC (5881T>C), CCC1927CCT (5887C>T), CGC1928CGT (5890C>T), AAT1930CAT (5894A>C), TAC1934TTC (5907A>T), CTT1936CTC (5914T>C), TTT1939TTC (5923T>C), CTG1941TTG (5927C>T), CAC1946TAT (5942C>T 5944C>T), AAC1950AGC (5955A>G), ACG1953ACA (5965G>A), GTT1954GTG (5968T>G), TTT1956TTC (5974T>C), AGA1957CGA (5975A>C), AAG1958AAA (5980G>A), CCT1959CCC (5983T>C), TTT1960TTC (5986T>C), AAA1964AAG (5998A>G), ACC1966GTT (6002A>G 6003C>T 6004C>T), GGG1968GAG (6009G>A), AAA1970AAG (6016A>G), TCC1974TCT (6028C>T), GCA1975GCT (6031A>T), ACA1976ATA (6033C>T), GCT1977TCT (6035G>T), GCT1978GCA (6040T>A), GGT1983GGG (6055T>G), GCT1988GCC (6070T>C), CTC1991CTG (6079C>G), AGT1993AGC (6085T>C), CTA1995TTG (6089C>T 6091A>G), CTT1996TTT (6092C>T), CAT1997CAA (6097T>A), CTT1998CTG (6100T>G), CAG1999CAA (6103G>A), AAG2000AAA (6106G>A), ATA2002ATG (6112A>G), TGT2004TGC (6118T>C), TCA2005TCT (6121A>T), GAG2008GAT (6130G>T), TTT2011TTC (6139T>C), GTC2016GTA (6154C>A), GGC2019GGG (6163C>G), CAC2020CAT (6166C>T), GTA2026GTG (6184A>G), ACT2027GAC (6185A>G 6186C>A 6187T>C), CAC2028CGC (6189A>G), TTC2029TTT (6193C>T), CAA2030CAG (6196A>G), CGC2031CGT (6199C>T), ATG2032ATC (6202G>C), GAT2036GAG (6214T>G), CTG2037GTT (6215C>G 6217G>T), ACA2038CCA (6218A>C), GAA2039CCA (6221G>C 6222A>C), GGG2040GAG (6225G>A), ATC2041GTC (6227A>G), ATA2042GTG (6230A>G 6232A>G), GAG2043GAA (6235G>A), GTG2045ACG (6239G>A 6240T>C), TTA2049CTT (6251T>C 6253A>T), ACC2050ACA (6256C>A), ACA2052ACT (6262A>T), CTG2053TTA (6263C>T 6265G>A), TCG2054ACT (6266T>A 6268G>T), AGC2056ATG (6273G>T 6274C>G), GTT2062GAA (6291T>A 6292T>A), GTA2064CTT (6296G>C 6298A>T), CCT2065CCA (6301T>A), TTA2066ATG (6302T>A 6304A>G), TTG2068CTG (6308T>C), GAT2069TAA (6311G>T 6313T>A), ATA2071GTT (6317A>G 6319A>T), GAG2072GAA (6322G>A), GGT2077GGC (6337T>C), CTA2080TTA (6344C>T), GCA2089ACA (6371G>A), GCC2092GCT (6382C>T), CTC2097CTG (6397C>G), TGT2100CAT (6404T>C 6405G>A), CGA2103CTG (6414G>T 6415A>G), TCA2104CAC (6416T>C 6417C>A 6418A>C), GTG2109GTT (6433G>T), TTC2110TTT (6436C>T), TGT2111AGT (6437T>A), TAC2113TAT (6445C>T), ACG2114ACA (6448G>A), TCC2117TCA (6457C>A), TCT2118ACT (6458T>A), CTT2119TTA (6461C>T 6463T>A), CTT2125CTA (6481T>A), CAT2127AAT (6485C>A), AGA2128AGC (6490A>C), CAT2129AAC (6491C>A 6493T>C), CAG2130CAA (6496G>A), ACA2131CCT (6497A>C 6499A>T), ATT2132GAG (6500A>G 6501T>A 6502T>G), CCG2136CCT (6514G>T), TCC2137CCA (6515T>C 6517C>A), TTC2138TTT (6520C>T), CAC2142CAT (6532C>T), CTT2147TTT (6545C>T), CCA2150CCC (6556A>C), CTA2152CTT (6562A>T), GGC2154AAT (6566G>A 6567G>A 6568C>T), TAC2155TAT (6571C>T), AAA2156AAG (6574A>G)

	Begin	End	Coverage	Score	Concordance	Matches	Identities	I/D/M/F*	Stop Codons
**NT**	**1**	**7803**	**99.9%**	**10368**	**72.5%**	**7431 (95.1%)**	**6475 (82.9%)**	**10/372**	
**Proteins**									
polyprotein (YP_001039627.1)	1	2159	100%	14226	94.4%	2159 (100%)	2013 (93.2%)	0/0/0/0	3
Protein mutations:	P8S (128C>T 130C>T), F10S (135T>C), N11S (138A>G), V13A (144T>C), T14I (147C>T), C17S (155T>A 157C>T), A18V (159C>T), T19A (161A>G), A31Q (197G>C 198C>A 199T>A), S66A (302T>G), T69S (311A>T 313C>T), N73S (324A>G 325C>T), S113G (443A>G), T165S (599A>T), I167T (606T>C), N170S (615A>G), T171I (618C>T 619A>T), S205A (719T>G), S239T (821T>A), V291I (977G>A), T305V (1019A>G 1020C>T), T364S (1196A>T 1198C>T), D367E (1207T>G), A501S (1607G>T), T529I (1692C>T), S534A (1706T>G 1708T>C), K544R (1737A>G 1738A>G), T550I (1755C>T 1756A>C), S555N (1770G>A 1771C>T), Y578F (1839A>T), A580T (1844G>A), S582T (1850T>A), S591T (1877T>A 1879A>T), I595V (1889A>G 1891A>G), D600E (1906T>A), M601P (1907A>C 1908T>C 1909G>A), L602W (1911T>G), A611V (1938C>T), N675T (2130A>C), Y679Q (2141T>C 2143T>A), N712C (2240A>T 2241A>G), S715K (2250G>A 2251C>A), M726L (2282A>T), R768K (2409G>A), G874S (2726G>A), A926S (2882G>T), Q993K (3083C>A 3085A>G), T1014S (3146A>T), L1046F (3242C>T), Q1103E (3413C>G), V1124L (3476G>C), R1143K (3534G>A), A1162V (3591C>T 3592T>C), S1163Q (3593T>C 3594C>A), A1175S (3629G>T), Y1196F (3693A>T), A1202S (3710G>T), I1203V (3713A>G), V1218I (3758G>A), V1224I (3776G>A 3778G>T), P1352A (4160C>G 4162A>T), I1362M (4192T>G), S1537N (4716G>A), S1538N (4719G>A), V1598I (4898G>A), T1614K (4947C>A), N1618H (4958A>C 4960C>T), T1628A (4988A>G 4990C>A), V1642M (5030G>A), I1658T (5079T>C), A1662S (5090G>T), P1667A (5105C>G), V1672I (5120G>A), S1676T (5132T>A), T1751S (5357A>T), K1752Q (5360A>C), H1754N (5366C>A), V1755I (5369G>A 5371G>T), I1765V (5399A>G), V1785I (5459G>A 5461T>C), V1786I (5462G>A), D1794N (5486G>A), T1796P (5492A>C 5494A>T), L1810V (5534C>G), K1811Q (5537A>C), A1813S (5543G>T), E1818Q (5558G>C), I1826V (5582A>G), A1829S (5591G>T), T1833I (5604C>T), Y1836F (5613A>T), R1853K (5664G>A), Q1857H (5677G>T), Y1869H (5711T>C), R1878K (5739G>A), L1882* (5751T>A 5752G>A), M1887I (5767G>T), I1890V (5774A>G 5776A>G), F1900T (5804T>A 5805T>C 5806C>T), Y1907F (5826A>T), L1915Q (5849T>C 5850T>A), D1918E (5860C>A), N1930H (5894A>C), Y1934F (5907A>T), H1946Y (5942C>T 5944C>T), N1950S (5955A>G), T1966V (6002A>G 6003C>T 6004C>T), G1968E (6009G>A), T1976I (6033C>T), A1977S (6035G>T), L1996F (6092C>T), H1997Q (6097T>A), I2002M (6112A>G), E2008D (6130G>T), T2027D (6185A>G 6186C>A 6187T>C), H2028R (6189A>G), M2032I (6202G>C), D2036E (6214T>G), L2037V (6215C>G 6217G>T), T2038P (6218A>C), E2039P (6221G>C 6222A>C), G2040E (6225G>A), I2041V (6227A>G), I2042V (6230A>G 6232A>G), V2045T (6239G>A 6240T>C), S2054T (6266T>A 6268G>T), S2056M (6273G>T 6274C>G), V2062E (6291T>A 6292T>A), V2064L (6296G>C 6298A>T), L2066M (6302T>A 6304A>G), D2069* (6311G>T 6313T>A), I2071V (6317A>G 6319A>T), A2089T (6371G>A), C2100H (6404T>C 6405G>A), R2103L (6414G>T 6415A>G), S2104H (6416T>C 6417C>A 6418A>C), C2111S (6437T>A), S2118T (6458T>A), H2127N (6485C>A), R2128S (6490A>C), H2129N (6491C>A 6493T>C), T2131P (6497A>C 6499A>T), I2132E (6500A>G 6501T>A 6502T>G), S2137P (6515T>C 6517C>A), L2147F (6545C>T), G2154N (6566G>A 6567G>A 6568C>T)								

	Begin	End	Coverage	Score	Concordance	Matches	Identities	I/D/M/F*	Stop Codons
**NT**	**1**	**7803**	**99.9%**	**10368**	**72.5%**	**7431 (95.1%)**	**6475 (82.9%)**	**10/372**	

Codon mutations:

TTT3TTC (115T>C), TTC7TTT (127C>T), CCC8TCT (128C>T 130C>T), TTC10TCC (135T>C), AAC11AGC (138A>G), GTT13GCT (144T>C), ACT14ATT (147C>T), TGC17AGT (155T>A 157C>T), GCT18GTT (159C>T), ACC19GCC (161A>G), TCT24TCA (178T>A), TCA27TCT (187A>T), GCT31CAA (197G>C 198C>A 199T>A), CGC38CGT (220C>T), TCT52TCA (262T>A), CAC53CAT (265C>T), GTT61GTG (289T>G), ACA62ACT (292A>T), GCT65GCA (301T>A), TCT66GCT (302T>G), TCC67TCT (307C>T), TTT68TTC (310T>C), ACC69TCT (311A>T 313C>T), TGT72TGC (322T>C), AAC73AGT (324A>G 325C>T), TCT74TCA (328T>A), TTG78TTA (340G>A), AAG81AAA (349G>A), ACT84ACA (358T>A), TTA85CTT (359T>C 361A>T), GAA87GAG (367A>G), CCC88CCT (370C>T), TTC89TTT (373C>T), ATC90ATT (376C>T), GCT92GCA (382T>A), TCT94TCC (388T>C), GTC96GTT (394C>T), AAA101AAG (409A>G), TCA102TCC (412A>C), GTT107GTG (427T>G), AAG109AAA (433G>A), AGC113GGC (443A>G), AAA115AAG (451A>G), GAC127GAT (487C>T), GTG132GTA (502G>A), TCT145TCC (541T>C), TTC151TTT (559C>T), CTA158TTA (578C>T), ACT165TCT (599A>T), ATA167ACA (606T>C), AAT170AGT (615A>G), ACA171ATT (618C>T 619A>T), GAC183GAT (655C>T), TTA185TTG (661A>G), CAA190CAG (676A>G), GCA196GCG (694A>G), CTA201TTA (707C>T), GGG203GGA (715G>A), GAA204GAG (718A>G), TCC205GCC (719T>G), TCA207TCT (727A>T), GTG214GTT (748G>T), GTA215GTT (751A>T), CTC218CTT (760C>T), GCC220GCT (766C>T), TCT224TCA (778T>A), ACC225ACT (781C>T), TTC228TTT (790C>T), GTA231GTT (799A>T), CCT232CCA (802T>A), AGA234CGA (806A>C), TCC239ACC (821T>A), CCG241CCA (829G>A), CTA242CTT (832A>T), TCA243TCC (835A>C), CTG246TTG (842C>T), TAC247TAT (847C>T), TTG259TTA (883G>A), TTT260TTC (886T>C), ACC261ACA (889C>A), AAT265AAC (901T>C), TGT269TGC (913T>C), GCC273GCT (925C>T), CTG278TTG (938C>T), GTA282GTG (952A>G), GGT286GGA (964T>A), AAG288AAA (970G>A), GTT291ATT (977G>A), ATT295ATC (991T>C), GCC297GCT (997C>T), TTG299CTG (1001T>C), TCT300TCA (1006T>A), TTC304TTT (1018C>T), ACG305GTG (1019A>G 1020C>T), GAG309GAA (1033G>A), CAT318CAC (1060T>C), CGC319CGA (1063C>A), ACT329ACC (1093T>C), CTA335TTA (1109C>T), AAA341AAG (1129A>G), TCT343TCA (1135T>A), GCC353GCA (1165C>A), AGG358AGA (1180G>A), ACG360ACA (1186G>A), GCA362GCC (1192A>C), ATA363ATT (1195A>T), ACC364TCT (1196A>T 1198C>T), CTT366CTG (1204T>G), GAT367GAG (1207T>G), GGG371GGC (1219G>C), GGC378GGT (1240C>T), CAA381CAG (1249A>G), AGG383AGA (1255G>A), CAA390CAG (1276A>G), GGG398GGA (1300G>A), ACC401ACA (1309C>A), CGG404CGA (1318G>A), CTG405TTG (1319C>T), TTC406TTT (1324C>T), GTT410GTG (1336T>G), TTG411CTT (1337T>C 1339G>T), CCA417CCC (1357A>C), TTG419CTG (1361T>C), TAT435TAC (1411T>C), ATC441ATT (1429C>T), GAT461GAC (1489T>C), CCC472CCA (1522C>A), GCA476GCT (1534A>T), GGC482GGT (1552C>T), CGC483AGG (1553C>A 1555C>G), CGA488AGG (1568C>A 1570A>G), ATA490ATC (1576A>C), GCC492GCT (1582C>T), ACA493ACC (1585A>C), AAC495AAT (1591C>T), GCT501TCT (1607G>T), CAG502CAA (1612G>A), GGT504GGG (1618T>G), CTA505TTA (1619C>T), ACC506ACA (1624C>A), CCC508CCT (1630C>T), AGA513CGA (1643A>C), TGC521TGT (1669C>T), GGT525GGA (1681T>A), GAG526GAA (1684G>A), ACT529ATT (1692C>T), GCT531GCG (1699T>G), TCT534GCC (1706T>G 1708T>C), CTT536CTG (1714T>G), AAA544AGG (1737A>G 1738A>G), AGA545CGA (1739A>C), ACA550ATC (1755C>T 1756A>C), AGC555AAT (1770G>A 1771C>T), TTG559CTG (1781T>C), GAA561GAG (1789A>G), GTT569GTG (1813T>G), ATC573ATA (1825C>A), TCA574TCT (1828A>T), AAA576AAG (1834A>G), TAT578TTT (1839A>T), GCC580ACC (1844G>A), ACA581ACC (1849A>C), TCA582ACA (1850T>A), AAG584AAA (1858G>A), CAG587CAA (1867G>A), GTG590GTC (1876G>C), TCA591ACT (1877T>A 1879A>T), GAG593GAA (1885G>A), ATA595GTG (1889A>G 1891A>G), ATA596ATT (1894A>T), GAT600GAA (1906T>A), ATG601CCA (1907A>C 1908T>C 1909G>A), TTG602TGG (1911T>G), ACC607ACA (1927C>A), GCT611GTT (1938C>T), AAG612AAA (1942G>A), GTT617GTG (1957T>G), GTA618GTC (1960A>C), GAA620GAG (1966A>G), GTG624GTA (1978G>A), TAC626TAT (1984C>T), AAA636AAG (2014A>G), CTA662CTG (2092A>G), AGG672CGG (2120A>C), GTT674GTC (2128T>C), AAT675ACT (2130A>C), GAC676GAT (2134C>T), CTG678TTG (2138C>T), TAT679CAA (2141T>C 2143T>A), CAA680CAG (2146A>G), GGT681GGA (2149T>A), TGT683TGC (2155T>C), CTG690TTG (2174C>T), ATA691ATC (2179A>C), TAT698TAC (2200T>C), ATC706ATT (2224C>T), TAC707TAT (2227C>T), GCT708GCG (2230T>G), AAT712TGT (2240A>T 2241A>G), AGC715AAA (2250G>A 2251C>A), GAC717GAT (2257C>T), CAG719CAA (2263G>A), CTG722TTG (2270C>T), GGA725GGT (2281A>T), ATG726TTG (2282A>T), GCG727GCA (2287G>A), CAC728CAT (2290C>T), ATA731ATT (2299A>T), CTG738TTG (2318C>T), ATA739ATT (2323A>T), GCC746GCA (2344C>A), CAC747CAT (2347C>T), GTA748GTG (2350A>G), TTC750TTT (2356C>T), TTG751TTA (2359G>A), ACA753ACT (2365A>T), GCA754GCT (2368A>T), AGG757CGG (2375A>C), TAT758TAC (2380T>C), CGC761AGA (2387C>A 2389C>A), AGA762AGG (2392A>G), CCT767CCC (2407T>C), AGG768AAG (2409G>A), GTG780GTA (2446G>A), CCG791CCA (2479G>A), ATC793ATT (2485C>T), TTG794TTA (2488G>A), AGA795AGG (2491A>G), GTT796GTG (2494T>G), CTG797CTC (2497G>C), ATT798ATC (2500T>C), AAG799AAA (2503G>A), GCT800GCA (2506T>A), GGC801GGT (2509C>T), CTT802CTC (2512T>C), TTA804CTA (2516T>C), GCA806GCC (2524A>C), GGG812GGT (2542G>T), GGA833GGT (2605A>T), ACC836ACA (2614C>A), GAG838GAA (2620G>A), AAT840AAC (2626T>C), CGG845AGG (2639C>A), AAG848AAA (2650G>A), ATC852ATT (2662C>T), AAG862AAA (2692G>A), GCA870GCC (2716A>C), GGC874AGC (2726G>A), TTC875TTT (2731C>T), ATC876ATT (2734C>T), GGA879GGC (2743A>C), TTG880CTG (2744T>C), TTG887CTC (2765T>C 2767G>C), AGA888CGA (2768A>C), CTA889CTG (2773A>G), GGT890GGA (2776T>A), GTC900GTT (2806C>T), TGC901TGT (2809C>T), TCG902TCA (2812G>A), GTG905GTA (2821G>A), ACA907ACT (2827A>T), AGC914AGT (2848C>T), CGT916AGA (2852C>A 2854T>A), GGC918GGG (2860C>G), ACT920ACA (2866T>A), TTC921TTT (2869C>T), ATA923ATT (2875A>T), ATA924ATC (2878A>C), CAT925CAC (2881T>C), GCC926TCC (2882G>T), TCA928TCC (2890A>C), GCC936GCT (2914C>T), CGG940CGA (2926G>A), TTC941TTT (2929C>T), TTG942TTA (2932G>A), ATT947ATA (2947T>A), GTC948GTT (2950C>T), ATT962ATC (2992T>C), AAA964AAG (2998A>G), TCT975TCA (3031T>A), GTA981GTC (3049A>C), GTA986GTT (3064A>T), GCT991GCC (3079T>C), CAA993AAG (3083C>A 3085A>G), TTG999CTT (3101T>C 3103G>T), TTT1001TTC (3109T>C), TCC1006TCA (3124C>A), CTT1010CTG (3136T>G), ACG1014TCG (3146A>T), TAC1015TAT (3151C>T), AGT1020AGC (3166T>C), TCC1021TCT (3169C>T), TTT1022TTC (3172T>C), AAA1023AAG (3175A>G), CTG1024TTG (3176C>T), TCT1026TCA (3184T>A), TAT1032TAC (3202T>C), CTT1044CTG (3238T>G), CTT1045CTA (3241T>A), CTT1046TTT (3242C>T), AAC1049AAT (3253C>T), AAA1053AAG (3265A>G), ATT1057ATA (3277T>A), CAT1061CAC (3289T>C), TCT1064TCA (3298T>A), GTA1075GTT (3331A>T), GCT1081GCA (3349T>A), TTG1087CTG (3365T>C), CTT1090CTG (3376T>G), CCG1091CCA (3379G>A), ACT1092ACA (3382T>A), CCG1094CCT (3388G>T), GCA1098GCC (3400A>C), GTA1100GTG (3406A>G), CAG1103GAG (3413C>G), CTC1104CTA (3418C>A), CTT1105CTC (3421T>C), CTA1108CTG (3430A>G), CGA1109AGA (3431C>A), GCG1113GCT (3445G>T), CTT1115CTC (3451T>C), CAG1120CAA (3466G>A), GTT1124CTT (3476G>C), GGC1125GGT (3481C>T), ACA1126ACT (3484A>T), ACG1134ACT (3508G>T), AAA1138AAG (3520A>G), CTG1141CTC (3529G>C), CGA1142AGG (3530C>A 3532A>G), AGG1143AAG (3534G>A), GGC1145GGT (3541C>T), TTG1146CTT (3542T>C 3544G>T), GCT1149GCA (3553T>A), GGG1151GGA (3559G>A), TCT1158TCC (3580T>C), ATC1159ATA (3583C>A), TCA1161TCG (3589A>G), GCT1162GTC (3591C>T 3592T>C), TCA1163CAA (3593T>C 3594C>A), GAA1168GAG (3610A>G), GGT1169GGC (3613T>C), CCG1172CCA (3622G>A), TAC1173TAT (3625C>T), GTG1174GTA (3628G>A), GCT1175TCT (3629G>T), TAC1180TAT (3646C>T), GCA1184GCC (3658A>C), CAA1185CAG (3661A>G), GAC1190GAT (3676C>T), ATT1191ATA (3679T>A), GCG1192GCC (3682G>C), CTA1194CTG (3688A>G), GCC1195GCT (3691C>T), TAT1196TTT (3693A>T), CTA1199TTA (3701C>T), GCA1202TCA (3710G>T), ATT1203GTT (3713A>G), TTG1204CTG (3716T>C), CGA1211CGT (3739A>T), GTT1212GTG (3742T>G), AAG1216AAA (3754G>A), GTC1218ATC (3758G>A), CAG1220CAA (3766G>A), GTG1224ATT (3776G>A 3778G>T), GTG1225GTT (3781G>T), CTC1226CTG (3784C>G), GGT1228GGA (3790T>A), GAC1236GAT (3814C>T), TTG1240CTG (3824T>C), CCG1247CCA (3847G>A), AAG1250AAA (3856G>A), GGC1254GGT (3868C>T), ACT1255ACC (3871T>C), GAG1259GAA (3883G>A), CCA1263CCC (3895A>C), GGA1267GGT (3907A>T), GAT1268GAC (3910T>C), CGA1269AGA (3911C>A), GAC1281GAT (3949C>T), GAG1283GAA (3955G>A), GCT1284GCA (3958T>A), TTG1285CTA (3959T>C 3961G>A), ATT1289ATC (3973T>C), GGA1292GGC (3982A>C), AAA1304AAG (4018A>G), TTG1308CTG (4028T>C), CCG1309CCT (4033G>T), TTG1310CTG (4034T>C), CGG1317AGG (4055C>A), TTG1321CTG (4067T>C), ATT1324ATA (4078T>A), CCC1326CCT (4084C>T), TAC1329TAT (4093C>T), CTG1338TTG (4118C>T), TCC1341TCT (4129C>T), TCC1347TCA (4147C>A), CTG1351TTA (4157C>T 4159G>A), CCA1352GCT (4160C>G 4162A>T), GGT1361GGA (4189T>A), ATT1362ATG (4192T>G), TTG1367TTA (4207G>A), GCT1368GCC (4210T>C), GCA1373GCT (4225A>T), GTC1374GTT (4228C>T), ATC1392ATA (4282C>A), GCC1394GCA (4288C>A), TTT1395TTC (4291T>C), CAA1396CAG (4294A>G), CTT1401CTG (4309T>G), GAC1404GAT (4318C>T), GGA1407GGT (4327A>T), GGT1411GGC (4339T>C), GAC1414GAT (4348C>T), AGA1418CGC (4358A>C 4360A>C), GCT1425GCC (4381T>C), TCA1426TCC (4384A>C), TAT1429TAC (4393T>C), GTT1436GTG (4414T>G), TTT1437TTC (4417T>C), TTG1439CTG (4421T>C), GTG1440GTT (4426G>T), GGA1441GGG (4429A>G), TCA1445TCT (4441A>T), GTT1451GTC (4459T>C), AAC1454AAT (4468C>T), TCT1455TCC (4471T>C), GCA1465GCT (4501A>T), TCT1468TCA (4510T>A), GCT1477GCA (4537T>A), GGG1481GGC (4549G>C), AAA1483AAG (4555A>G), GTC1494GTT (4588C>T), CTA1500CTT (4606A>T), CCC1504CCA (4618C>A), TTG1507CTG (4625T>C), TAT1510TAC (4636T>C), GCT1514GCA (4648T>A), ATT1515ATA (4651T>A), GCC1516GCT (4654C>T), TTG1519CTA (4661T>C 4663G>A), GTT1524GTC (4678T>C), ATA1526ATT (4684A>T), ATC1530ATT (4696C>T), GAC1531GAT (4699C>T), GTT1536GTC (4714T>C), AGC1537AAC (4716G>A), AGT1538AAT (4719G>A), GAC1543GAT (4735C>T), TTG1544CTG (4736T>C), CTA1547CTG (4747A>G), TTT1549TTC (4753T>C), CTA1550CTT (4756A>T), AGC1551AGT (4759C>T), GGT1553GGC (4765T>C), TTT1554TTC (4768T>C), AGG1556AGA (4774G>A), CGT1558CGC (4780T>C), CTA1559CTG (4783A>G), GGA1560GGG (4786A>G), TAC1561TAT (4789C>T), CCT1565CCC (4801T>C), CTG1566TTG (4802C>T), GTA1569GTG (4813A>G), TCT1570TCC (4816T>C), CCA1574CCC (4828A>C), TAC1576TAT (4834C>T), AGG1579AGA (4843G>A), CCC1585CCT (4861C>T), CTC1588CTG (4870C>G), AAC1593AAT (4885C>T), ACG1596ACT (4894G>T), GGA1597GGC (4897A>C), GTT1598ATT (4898G>A), GAG1600GAA (4906G>A), GCA1601GCC (4909A>C), GGG1605GGT (4921G>T), ACA1614AAA (4947C>A), GTT1616GTG (4954T>G), AAC1618CAT (4958A>C 4960C>T), AAG1621AAA (4969G>A), CGG1623CGA (4975G>A), TTC1625TTT (4981C>T), GCC1627GCT (4987C>T), ACC1628GCA (4988A>G 4990C>A), CCT1631CCC (4999T>C), ACA1632ACT (5002A>T), TAT1633TAC (5005T>C), TTG1636CTG (5012T>C), GAG1637GAA (5017G>A), CAG1638CAA (5020G>A), GAC1639GAT (5023C>T), GTG1642ATG (5030G>A), GAG1643GAA (5035G>A), ACA1645ACT (5041A>T), GGA1646GGC (5044A>C), GGG1651GGT (5059G>T), ATC1653ATT (5065C>T), CTT1656CTC (5074T>C), CAT1657CAC (5077T>C), ATT1658ACT (5079T>C), GCA1659GCT (5083A>T), GCT1662TCT (5090G>T), TTG1666CTG (5102T>C), CCC1667GCC (5105C>G), GGC1669GGT (5113C>T), AAT1670AAC (5116T>C), ACT1671ACC (5119T>C), GTT1672ATT (5120G>A), CTG1673TTG (5123C>T), TTT1675TTC (5131T>C), TCT1676ACT (5132T>A), GTG1679GTT (5143G>T), CAT1680CAC (5146T>C), GAC1684GAT (5158C>T), AGT1687AGC (5167T>C), TGC1689TGT (5173C>T), TCC1690TCT (5176C>T), CGA1692CGG (5182A>G), GCA1696GCC (5194A>C), CAG1698CAA (5200G>A), GAC1700GAT (5206C>T), ACC1701ACA (5209C>A), GTT1704GTG (5218T>G), AAC1707AAT (5227C>T), GCA1711GCC (5239A>C), CCC1720CCA (5266C>A), ACC1721ACA (5269C>A), AGG1724AGA (5278G>A), GGG1728GGT (5290G>T), CTT1729CTC (5293T>C), CCT1732CCA (5302T>A), CAC1741CAT (5329C>T), GTA1746GTG (5344A>G), ACC1747ACA (5347C>A), ACC1751TCC (5357A>T), AAA1752CAA (5360A>C), CAT1754AAT (5366C>A), GTG1755ATT (5369G>A 5371G>T), GTT1756GTA (5374T>A), GGC1760GGG (5386C>G), GGG1762GGA (5392G>A), ATT1765GTT (5399A>G), CCA1767CCT (5407A>T), GTG1768GTT (5410G>T), TGC1769TGT (5413C>T), GCA1771GCT (5419A>T), CTG1775TTG (5429C>T), ACT1777ACC (5437T>C), GGC1778GGG (5440C>G), GTT1785ATC (5459G>A 5461T>C), GTT1786ATT (5462G>A), TTG1789CTG (5471T>C), ATA1792ATT (5482A>T), GCA1793GCT (5485A>T), GAT1794AAT (5486G>A), GTT1795GTC (5491T>C), ACA1796CCT (5492A>C 5494A>T), CTC1798TTG (5498C>T 5500C>G), CCA1802CCT (5512A>T), GGA1803GGT (5515A>T), CTT1809TTG (5531C>T 5533T>G), CTG1810GTG (5534C>G), AAA1811CAA (5537A>C), GCT1813TCT (5543G>T), GAA1818CAA (5558G>C), CTT1820CTG (5566T>G), GCA1821GCT (5569A>T), GAT1822GAC (5572T>C), TGT1824TGC (5578T>C), ATT1826GTT (5582A>G), GCC1829TCC (5591G>T), GAG1832GAA (5602G>A), ACA1833ATA (5604C>T), GCA1835GCT (5611A>T), TAC1836TTC (5613A>T), ATA1837ATT (5617A>T), CCT1838CCA (5620T>A), GGA1840GGT (5626A>T), CTT1844CTG (5638T>G), GGT1845GGC (5641T>C), TTT1847TTC (5647T>C), AGA1853AAA (5664G>A), CTA1856CTT (5674A>T), CAG1857CAT (5677G>T), CAG1860CAA (5686G>A), CGC1861CGA (5689C>A), GGA1867GGT (5707A>T), CCT1868CCA (5710T>A), TAT1869CAT (5711T>C), GGG1870GGT (5716G>T), GGA1871GGT (5719A>T), CAC1875CAT (5731C>T), ACT1876ACC (5734T>C), ACT1877ACC (5737T>C), AGA1878AAA (5739G>A), CTT1880CTC (5746T>C), TTG1882TAA (5751T>A 5752G>A), ATG1887ATT (5767G>T), AAG1889AAA (5773G>A), ATA1890GTG (5774A>G 5776A>G), TAT1891TAC (5779T>C), TTA1894CTA (5786T>C), GTC1895GTA (5791C>A), AGA1897AGG (5797A>G), TTC1900ACT (5804T>A 5805T>C 5806C>T), CTC1903CTG (5815C>G), TAC1904TAT (5818C>T), TAT1907TTT (5826A>T), GTG1913GTA (5845G>A), TTG1915CAG (5849T>C 5850T>A), GTT1917GTG (5857T>G), GAC1918GAA (5860C>A), CTT1920CTG (5866T>G), AGG1921AGA (5869G>A), CTC1922CTG (5872C>G), GGG1924GGT (5878G>T), TTT1925TTC (5881T>C), CCC1927CCT (5887C>T), CGC1928CGT (5890C>T), AAT1930CAT (5894A>C), TAC1934TTC (5907A>T), CTT1936CTC (5914T>C), TTT1939TTC (5923T>C), CTG1941TTG (5927C>T), CAC1946TAT (5942C>T 5944C>T), AAC1950AGC (5955A>G), ACG1953ACA (5965G>A), GTT1954GTG (5968T>G), TTT1956TTC (5974T>C), AGA1957CGA (5975A>C), AAG1958AAA (5980G>A), CCT1959CCC (5983T>C), TTT1960TTC (5986T>C), AAA1964AAG (5998A>G), ACC1966GTT (6002A>G 6003C>T 6004C>T), GGG1968GAG (6009G>A), AAA1970AAG (6016A>G), TCC1974TCT (6028C>T), GCA1975GCT (6031A>T), ACA1976ATA (6033C>T), GCT1977TCT (6035G>T), GCT1978GCA (6040T>A), GGT1983GGG (6055T>G), GCT1988GCC (6070T>C), CTC1991CTG (6079C>G), AGT1993AGC (6085T>C), CTA1995TTG (6089C>T 6091A>G), CTT1996TTT (6092C>T), CAT1997CAA (6097T>A), CTT1998CTG (6100T>G), CAG1999CAA (6103G>A), AAG2000AAA (6106G>A), ATA2002ATG (6112A>G), TGT2004TGC (6118T>C), TCA2005TCT (6121A>T), GAG2008GAT (6130G>T), TTT2011TTC (6139T>C), GTC2016GTA (6154C>A), GGC2019GGG (6163C>G), CAC2020CAT (6166C>T), GTA2026GTG (6184A>G), ACT2027GAC (6185A>G 6186C>A 6187T>C), CAC2028CGC (6189A>G), TTC2029TTT (6193C>T), CAA2030CAG (6196A>G), CGC2031CGT (6199C>T), ATG2032ATC (6202G>C), GAT2036GAG (6214T>G), CTG2037GTT (6215C>G 6217G>T), ACA2038CCA (6218A>C), GAA2039CCA (6221G>C 6222A>C), GGG2040GAG (6225G>A), ATC2041GTC (6227A>G), ATA2042GTG (6230A>G 6232A>G), GAG2043GAA (6235G>A), GTG2045ACG (6239G>A 6240T>C), TTA2049CTT (6251T>C 6253A>T), ACC2050ACA (6256C>A), ACA2052ACT (6262A>T), CTG2053TTA (6263C>T 6265G>A), TCG2054ACT (6266T>A 6268G>T), AGC2056ATG (6273G>T 6274C>G), GTT2062GAA (6291T>A 6292T>A), GTA2064CTT (6296G>C 6298A>T), CCT2065CCA (6301T>A), TTA2066ATG (6302T>A 6304A>G), TTG2068CTG (6308T>C), GAT2069TAA (6311G>T 6313T>A), ATA2071GTT (6317A>G 6319A>T), GAG2072GAA (6322G>A), GGT2077GGC (6337T>C), CTA2080TTA (6344C>T), GCA2089ACA (6371G>A), GCC2092GCT (6382C>T), CTC2097CTG (6397C>G), TGT2100CAT (6404T>C 6405G>A), CGA2103CTG (6414G>T 6415A>G), TCA2104CAC (6416T>C 6417C>A 6418A>C), GTG2109GTT (6433G>T), TTC2110TTT (6436C>T), TGT2111AGT (6437T>A), TAC2113TAT (6445C>T), ACG2114ACA (6448G>A), TCC2117TCA (6457C>A), TCT2118ACT (6458T>A), CTT2119TTA (6461C>T 6463T>A), CTT2125CTA (6481T>A), CAT2127AAT (6485C>A), AGA2128AGC (6490A>C), CAT2129AAC (6491C>A 6493T>C), CAG2130CAA (6496G>A), ACA2131CCT (6497A>C 6499A>T), ATT2132GAG (6500A>G 6501T>A 6502T>G), CCG2136CCT (6514G>T), TCC2137CCA (6515T>C 6517C>A), TTC2138TTT (6520C>T), CAC2142CAT (6532C>T), CTT2147TTT (6545C>T), CCA2150CCC (6556A>C), CTA2152CTT (6562A>T), GGC2154AAT (6566G>A 6567G>A 6568C>T), TAC2155TAT (6571C>T), AAA2156AAG (6574A>G)

*: Inserts / Deletes / Misaligned / Frameshifts

Analysis details

This analysis was performed with panviral2.64

NGS Details (UN62): Torradovirus lycopersici (segment RNA 2)

Assembly

**Coverage Length**	5384 (1 contig(s))
**Depth Of Coverage**	7488.0
**Number Of Reads**	333725
**Reads Per Million**	7736.04 rpm (after QC)
**Ambiguities**	0
**Assembly Method**	de novo + reference guided assembly
**Consensus Caller**	Bcf Tools

Coverage Map

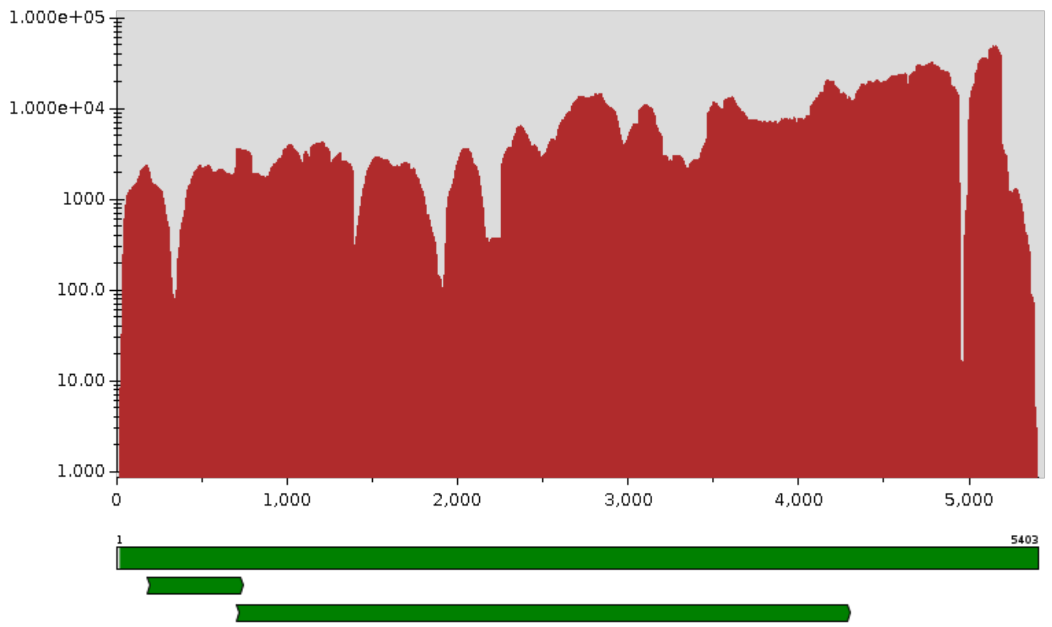

Assignment

**Type**	Torradovirus lycopersici (Taxonomy ID: 3048378)
**Reference Genome**	NC_009032.1
**NT Identity (%)**	91.4763
**AA Identity (%)**	94.3043
**Number Of Stop Codons**	2
**Number Of CDS**	2

Alignment

**Alignment Score**	8926.0 (NT) + 9075.0 (AA) = 18001.0
**Concordance (%)**	88.8719

Alignment Method	Global, seeded, nucleotide + amino acids (AGA)

Genome Region

Sequence starts at position 20 and ends at position 5403 relative to NC_009032.1 reference sequence.

Alignment Detailed Statistics

	Begin	End	Coverage	Score	Concordance	Matches	Identities	I/D/M/F*	Stop Codons
**NT**	**20**	**5403**	**99.6%**	**8926**	**82.9%**	**5384 (99.9%)**	**4926 (91.5%)**	**1/0**	
Mutations:	89G>A, 90C>T, 97C>T, 107G>A, 111C>A, 116A>G, 137T>C, 144A>G, 161A>G, 179T>C, 180C>T, 271T>C, 277C>T, 280T>C, 283C>T, 295T>C, 301A>T, 310C>T, 313C>T, 316G>A, 340A>T, 356G>A, 370C>T, 372A>C, 376C>T, 388T>A, 400T>C, 401T>C, 418T>G, 433C>T, 439G>A, 445T>A, 448C>A, 460T>C, 461A>C, 481C>A, 487A>G, 502T>A, 507G>A, 508G>A, 509A>G, 511T>A, 515G>A, 552C>T, 565A>T, 568A>T, 576C>A, 583A>T, 586A>G, 587T>C, 589A>T, 603A>C, 607G>A, 613A>G, 622A>G, 634G>A, 637A>G, 644C>G, 645T>C, 654T>A, 670A>G, 679G>A, 683T>A, 690A>C, 696T>C, 704G>A, 721C>T, 733A>G, 748A>G, 760T>C, 797T>C, 806C>T, 827A>G, 844A>T, 968A>G, 1043A>G, 1061T>C, 1083C>T, 1100G>A, 1106T>C, 1109C>T, 1139G>A, 1151A>G, 1163A>G, 1169C>G, 1178C>T, 1187A>T, 1205T>A, 1226A>G, 1235C>A, 1247A>G, 1277A>G, 1313T>C, 1317C>A, 1319A>G, 1325A>G, 1328T>C, 1331C>T, 1343G>A, 1355G>A, 1361A>T, 1367A>T, 1383A>G, 1388C>T, 1397T>A, 1403A>T, 1406G>A, 1412G>T, 1418G>A, 1421A>G, 1427G>T, 1434G>A, 1451G>T, 1457C>T, 1460A>G, 1461G>T, 1472C>G, 1490G>A, 1502C>T, 1514T>C, 1524T>C, 1547C>T, 1550A>C, 1589A>G, 1608T>C, 1619G>A, 1634T>A, 1646T>C, 1651A>G, 1655T>A, 1661T>C, 1662C>T, 1664G>T, 1667C>T, 1677G>A, 1685T>C, 1721G>C, 1724C>T, 1740C>T, 1748G>A, 1757A>G, 1778A>C, 1794G>A, 1796T>A, 1814T>C, 1817A>T, 1819T>A, 1820C>G, 1832G>C, 1845T>C, 1856C>T, 1859C>T, 1863C>T, 1865C>T, 1892C>T, 1899G>A, 1900T>C, 1901G>A, 1906C>T, 1907C>G, 1928A>C, 1931C>T, 1937C>T, 1941C>T, 1947T>C, 1955G>A, 1958A>G, 1967G>A, 1970A>G, 1979A>G, 1985C>T, 1994T>C, 2072G>A, 2084G>A, 2094C>G, 2099G>A, 2106A>G, 2108A>G, 2126C>T, 2128G>T, 2135G>A, 2136G>C, 2140A>C, 2144C>G, 2159T>A, 2164T>G, 2183C>T, 2186A>C, 2189C>T, 2198G>A, 2199G>A, 2200T>C, 2205A>G, 2210G>A, 2216C>G, 2229A>G, 2230T>A, 2232A>G, 2240T>A, 2243T>A, 2252A>T, 2256G>C, 2259A>G, 2261T>G, 2262G>C, 2270A>G, 2285T>C, 2294A>C, 2303A>G, 2312T>C, 2315T>A, 2320G>C, 2321T>C, 2326C>T, 2339A>G, 2345A>G, 2375C>T, 2393G>A, 2414T>C, 2420T>G, 2432G>A, 2436T>G, 2444G>C, 2447C>A, 2453G>A, 2459C>G, 2469T>A, 2471C>G, 2474G>A, 2489A>C, 2492G>A, 2495A>G, 2501A>G, 2503G>A, 2507T>C, 2516C>T, 2523A>C, 2546T>C, 2552C>T, 2561T>G, 2567T>A, 2570G>A, 2573T>A, 2630C>T, 2645G>A, 2660T>C, 2693G>A, 2708A>G, 2720C>A, 2732T>C, 2765G>A, 2792C>T, 2819G>A, 2840T>C, 2841C>T, 2852A>G, 2870C>A, 2879T>A, 2885T>C, 2886T>C, 2888G>A, 2900A>T, 2915A>G, 2939A>G, 2957A>T, 2958C>T, 2966C>T, 2972T>C, 2982G>A, 2999T>A, 3003T>C, 3011C>T, 3012A>G, 3020T>C, 3032A>T, 3050G>A, 3063T>C, 3122T>C, 3131G>A, 3146T>C, 3152T>A, 3167A>T, 3187G>A, 3206C>T, 3227T>G, 3230T>A, 3236C>T, 3242G>A, 3248A>C, 3253A>G, 3265G>A, 3270A>T, 3303A>T, 3305C>T, 3324C>T, 3335C>T, 3350C>T, 3354A>C, 3359G>A, 3368A>G, 3371C>G, 3386A>T, 3404A>G, 3413C>T, 3422A>G, 3428G>A, 3431T>C, 3437C>T, 3446T>C, 3449G>A, 3452C>T, 3455T>A, 3470T>C, 3479C>T, 3482T>C, 3485C>T, 3491C>T, 3503A>G, 3508A>G, 3512T>C, 3515G>T, 3522C>T, 3524A>G, 3527C>T, 3533G>A, 3543T>A, 3551C>T, 3560T>A, 3569A>G, 3575G>A, 3581C>T, 3590A>C, 3626A>G, 3635C>T, 3638A>G, 3650A>T, 3662A>T, 3674G>A, 3722C>T, 3743G>A, 3755A>G, 3767C>T, 3773C>T, 3800C>T, 3827C>T, 3831A>G, 3881C>T, 3885A>G, 3890A>G, 3893T>C, 3933A>G, 3941T>G, 3950T>A, 3983T>A, 3984A>G, 3986G>T, 3990G>A, 3995C>T, 3998C>T, 4013G>C, 4031T>C, 4040C>T, 4059A>G, 4061A>G, 4069A>G, 4100A>T, 4112C>T, 4130C>T, 4223G>A, 4224C>T, 4250C>T, 4336T>A, 4457A>G, 4590G>A, 4635C>T, 4812C>T, 4814T>C, 4823A>G, 4940C>A, 4941C>A, 4943A>T, 4949T>C, 4955A>C, 4959A>C, 4960A>G, 4961C>T, 4965G>A, 4969A>T, 4971T>C, 4972T>G, 4974T>A, 4980A>G, 4984C>T, 4993G>A, 4994C>A, 4995G>A, 4996A>T, 5000C>T, 5003A>T, 5004G>T, 5019A>T, 5031T>C, 5032G>A, 5040C>T, 5041A>G, 5053A>G, 5054T>C, 5056G>A, 5099C>T, 5101A>G, 5107A>T, 5110A>C, 5112A>G, 5118T>A, 5153A>G, 5173C>T, 5184C>T, 5185T>G, 5187G>A, 5196T>G, 5197T>A, 5198T>A, 5199_5200insC, 5206C>T, 5208G>A, 5211A>G, 5212A>T, 5213A>T, 5214C>T, 5220T>A, 5221A>T, 5236G>A, 5239T>A, 5242T>A, 5250C>T, 5262A>T, 5265G>A, 5266T>A, 5268T>C, 5282T>A, 5285G>T, 5294G>A, 5297T>C, 5298C>T, 5301T>A, 5313A>C, 5326G>A, 5327A>G, 5335T>A, 5346T>C, 5348G>A, 5360A>T, 5363C>A, 5364A>T, 5369G>T, 5371G>T, 5373G>A, 5374T>G, 5377G>T								
**CDS**									
ToTV_sRNA2gp1	1	188	100%	1130	93.5%	188 (100%)	173 (92.0%)	0/0/0/0	1
Protein mutations:	V59I (356G>A), N64T (372A>C), R109K (507G>A 508G>A), I110V (509A>G 511T>A), V112I (515G>A), A124V (552C>T), Q128H (565A>T), T132K (576C>A), D141A (603A>C), L155A (644C>G 645T>C), L158Q (654T>A), L168M (683T>A), N170T (690A>C), L172P (696T>C), D175N (704G>A)								
Codon mutations:	GAT30GAC (271T>C), GGC32GGT (277C>T), TCT33TCC (280T>C), GGC34GGT (283C>T), AGT38AGC (295T>C), CCA40CCT (301A>T), GAC43GAT (310C>T), TTC44TTT (313C>T), AAG45AAA (316G>A), GCA53GCT (340A>T), GTT59ATT (356G>A), AAC63AAT (370C>T), AAT64ACT (372A>C), AGC65AGT (376C>T), GTT69GTA (388T>A), TAT73TAC (400T>C), TTA74CTA (401T>C), ACT79ACG (418T>G), GGC84GGT (433C>T), AAG86AAA (439G>A), TCT88TCA (445T>A), GGC89GGA (448C>A), CAT93CAC (460T>C), AGA94CGA (461A>C), ACC100ACA (481C>A), AGA102AGG (487A>G), GCT107GCA (502T>A), AGG109AAA (507G>A 508G>A), ATT110GTA (509A>G 511T>A), GTA112ATA (515G>A), GCT124GTT (552C>T), CAA128CAT (565A>T), CCA129CCT (568A>T), ACG132AAG (576C>A), ACA134ACT (583A>T), CAA135CAG (586A>G), TTA136CTT (587T>C 589A>T), GAC141GCC (603A>C), GAG142GAA (607G>A), AAA144AAG (613A>G), TTA147TTG (622A>G), GAG151GAA (634G>A), AAA152AAG (637A>G), CTT155GCT (644C>G 645T>C), CTA158CAA (654T>A), AAA163AAG (670A>G), TTG166TTA (679G>A), TTG168ATG (683T>A), AAC170ACC (690A>C), CTA172CCA (696T>C), GAT175AAT (704G>A), GGC180GGT (721C>T), GAA184GAG (733A>G)								
ToTV_sRNA2gp2	1	1199	100%	7945	96.0%	1199 (100%)	1135 (94.7%)	0/0/0/0	1
Protein mutations:	M1I (704G>A), A7V (721C>T), K11R (733A>G), K16R (748A>G), V20A (760T>C), Y48F (844A>T), T228A (1383A>G), Q242H (1427G>T), D245N (1434G>A), A254S (1461G>T), I257M (1472C>G), S275P (1524T>C), Y303H (1608T>C), K317R (1651A>G), L321F (1662C>T 1664G>T), V326M (1677G>A), V365I (1794G>A 1796T>A), I373K (1819T>A 1820C>G), K377N (1832G>C), H388Y (1863C>T 1865C>T), V400T (1899G>A 1900T>C 1901G>A), A402V (1906C>T 1907C>G), E409D (1928A>C), L414F (1941C>T), Q465E (2094C>G), R469G (2106A>G 2108A>G), R476M (2128G>T), V479L (2136G>C), K480T (2140A>C), N481K (2144C>G), V488G (2164T>G), V500T (2199G>A 2200T>C), M502V (2205A>G), M510E (2229A>G 2230T>A), K511E (2232A>G), E517D (2252A>T), A519P (2256G>C), N520E (2259A>G 2261T>G), E521Q (2262G>C), E531D (2294A>C), N538K (2315T>A), G540A (2320G>C 2321T>C), T542I (2326C>T), S579A (2436T>G), S590T (2469T>A 2471C>G), R601K (2503G>A), I608L (2523A>C), A761T (2982G>A), I771V (3012A>G), S829N (3187G>A), K851R (3253A>G), R855K (3265G>A), T857S (3270A>T), T868S (3303A>T 3305C>T), N936S (3508A>G), S948T (3543T>A), I1044V (3831A>G), T1062A (3885A>G), T1078A (3933A>G), M1095V (3984A>G 3986G>T), D1097N (3990G>A), I1120V (4059A>G 4061A>G), N1123S (4069A>G), H1175Y (4224C>T)								

	Begin	End	Coverage	Score	Concordance	Matches	Identities	I/D/M/F*	Stop Codons
**NT**	**20**	**5403**	**99.6%**	**8926**	**82.9%**	**5384 (99.9%)**	**4926 (91.5%)**	**1/0**	
Codon mutations:	ATG1ATA (704G>A), GCT7GTT (721C>T), AAG11AGG (733A>G), AAA16AGA (748A>G), GTA20GCA (760T>C), GTT32GTC (797T>C), GGC35GGT (806C>T), AAA42AAG (827A>G), TAT48TTT (844A>T), GGA89GGG (968A>G), AAA114AAG (1043A>G), AGT120AGC (1061T>C), CTG128TTG (1083C>T), TTG133TTA (1100G>A), TTT135TTC (1106T>C), TCC136TCT (1109C>T), CCG146CCA (1139G>A), CTA150CTG (1151A>G), AAA154AAG (1163A>G), GGC156GGG (1169C>G), TTC159TTT (1178C>T), ACA162ACT (1187A>T), ACT168ACA (1205T>A), TCA175TCG (1226A>G), GCC178GCA (1235C>A), CAA182CAG (1247A>G), TCA192TCG (1277A>G), CTT204CTC (1313T>C), CGA206AGG (1317C>A 1319A>G), CAA208CAG (1325A>G), TTT209TTC (1328T>C), TTC210TTT (1331C>T), CAG214CAA (1343G>A), CAG218CAA (1355G>A), ATA220ATT (1361A>T), TCA222TCT (1367A>T), ACC228GCC (1383A>G), TCC229TCT (1388C>T), ACT232ACA (1397T>A), TCA234TCT (1403A>T), GTG235GTA (1406G>A), CCG237CCT (1412G>T), GTG239GTA (1418G>A), GGA240GGG (1421A>G), CAG242CAT (1427G>T), GAC245AAC (1434G>A), GTG250GTT (1451G>T), AGC252AGT (1457C>T), GAA253GAG (1460A>G), GCA254TCA (1461G>T), ATC257ATG (1472C>G), CGG263CGA (1490G>A), CTC267CTT (1502C>T), AGT271AGC (1514T>C), TCC275CCC (1524T>C), ACC282ACT (1547C>T), TCA283TCC (1550A>C), GTA296GTG (1589A>G), TAC303CAC (1608T>C), GAG306GAA (1619G>A), GTT311GTA (1634T>A), GTT315GTC (1646T>C), AAG317AGG (1651A>G), GCT318GCA (1655T>A), GGT320GGC (1661T>C), CTG321TTT (1662C>T 1664G>T), AAC322AAT (1667C>T), GTG326ATG (1677G>A), GAT328GAC (1685T>C), CTG340CTC (1721G>C), CCC341CCT (1724C>T), CTG347TTG (1740C>T), GTG349GTA (1748G>A), CAA352CAG (1757A>G), GCA359GCC (1778A>C), GTT365ATA (1794G>A 1796T>A), CAT371CAC (1814T>C), ATA372ATT (1817A>T), ATC373AAG (1819T>A 1820C>G), AAG377AAC (1832G>C), TTG382CTG (1845T>C), ATC385ATT (1856C>T), ACC386ACT (1859C>T), CAC388TAT (1863C>T 1865C>T), TAC397TAT (1892C>T), GTG400ACA (1899G>A 1900T>C 1901G>A), GCC402GTG (1906C>T 1907C>G), GAA409GAC (1928A>C), AAC410AAT (1931C>T), TAC412TAT (1937C>T), CTT414TTT (1941C>T), TTG416CTG (1947T>C), CAG418CAA (1955G>A), AGA419AGG (1958A>G), GAG422GAA (1967G>A), CTA423CTG (1970A>G), CAA426CAG (1979A>G), GAC428GAT (1985C>T), TAT431TAC (1994T>C), GAG457GAA (2072G>A), GTG461GTA (2084G>A), CAG465GAG (2094C>G), GCG466GCA (2099G>A), AGA469GGG (2106A>G 2108A>G), GCC475GCT (2126C>T), AGG476ATG (2128G>T), CAG478CAA (2135G>A), GTG479CTG (2136G>C), AAA480ACA (2140A>C), AAC481AAG (2144C>G), ACT486ACA (2159T>A), GTG488GGG (2164T>G), ACC494ACT (2183C>T), TCA495TCC (2186A>C), TTC496TTT (2189C>T), AAG499AAA (2198G>A), GTT500ACT (2199G>A 2200T>C), ATG502GTG (2205A>G), GAG503GAA (2210G>A), GGC505GGG (2216C>G), ATG510GAG (2229A>G 2230T>A), AAA511GAA (2232A>G), GCT513GCA (2240T>A), ATT514ATA (2243T>A), GAA517GAT (2252A>T), GCC519CCC (2256G>C), AAT520GAG (2259A>G 2261T>G), GAA521CAA (2262G>C), GTA523GTG (2270A>G), GTT528GTC (2285T>C), GAA531GAC (2294A>C), CAA534CAG (2303A>G), AAT537AAC (2312T>C), AAT538AAA (2315T>A), GGT540GCC (2320G>C 2321T>C), ACA542ATA (2326C>T), TTA546TTG (2339A>G), CAA548CAG (2345A>G), TTC558TTT (2375C>T), GAG564GAA (2393G>A), AGT571AGC (2414T>C), GTT573GTG (2420T>G), GTG577GTA (2432G>A), TCA579GCA (2436T>G), GCG581GCC (2444G>C), CTC582CTA (2447C>A), GCG584GCA (2453G>A), CTC586CTG (2459C>G), TCC590ACG (2469T>A 2471C>G), AAG591AAA (2474G>A), GGA596GGC (2489A>C), TCG597TCA (2492G>A), CAA598CAG (2495A>G), CTA600CTG (2501A>G), AGG601AAG (2503G>A), TAT602TAC (2507T>C), GCC605GCT (2516C>T), ATT608CTT (2523A>C), TAT615TAC (2546T>C), TCC617TCT (2552C>T), GGT620GGG (2561T>G), ATT622ATA (2567T>A), TCG623TCA (2570G>A), GGT624GGA (2573T>A), ATC643ATT (2630C>T), CTG648CTA (2645G>A), CAT653CAC (2660T>C), GGG664GGA (2693G>A), ACA669ACG (2708A>G), ATC673ATA (2720C>A), GTT677GTC (2732T>C), CTG688CTA (2765G>A), CAC697CAT (2792C>T), GAG706GAA (2819G>A), CAT713CAC (2840T>C), CTG714TTG (2841C>T), AAA717AAG (2852A>G), ATC723ATA (2870C>A), CCT726CCA (2879T>A), CGT728CGC (2885T>C), TTG729CTA (2886T>C 2888G>A), GCA733GCT (2900A>T), AAA738AAG (2915A>G), CGA746CGG (2939A>G), GTA752GTT (2957A>T), CTG753TTG (2958C>T), CAC755CAT (2966C>T), AAT757AAC (2972T>C), GCT761ACT (2982G>A), ACT766ACA (2999T>A), TTA768CTA (3003T>C), AGC770AGT (3011C>T), ATC771GTC (3012A>G), TCT773TCC (3020T>C), ATA777ATT (3032A>T), ACG783ACA (3050G>A), TTG788CTG (3063T>C), ATT807ATC (3122T>C), GAG810GAA (3131G>A), CAT815CAC (3146T>C), GGT817GGA (3152T>A), GGA822GGT (3167A>T), AGC829AAC (3187G>A), GAC835GAT (3206C>T), GTT842GTG (3227T>G), ATT843ATA (3230T>A), AAC845AAT (3236C>T), GAG847GAA (3242G>A), GGA849GGC (3248A>C), AAG851AGG (3253A>G), AGG855AAG (3265G>A), ACG857TCG (3270A>T), ACC868TCT (3303A>T 3305C>T), CTA875TTA (3324C>T), CCC878CCT (3335C>T), CAC883CAT (3350C>T), AGG885CGG (3354A>C), TTG886TTA (3359G>A), ACA889ACG (3368A>G), GTC890GTG (3371C>G), GTA895GTT (3386A>T), CTA901CTG (3404A>G), GTC904GTT (3413C>T), ACA907ACG (3422A>G), CAG909CAA (3428G>A), CTT910CTC (3431T>C), TGC912TGT (3437C>T), GAT915GAC (3446T>C), TTG916TTA (3449G>A), GTC917GTT (3452C>T), CTT918CTA (3455T>A), TCT923TCC (3470T>C), CCC926CCT (3479C>T), ATT927ATC (3482T>C), TAC928TAT (3485C>T), CAC930CAT (3491C>T), GGA934GGG (3503A>G), AAT936AGT (3508A>G), GCT937GCC (3512T>C), GTG938GTT (3515G>T), CTA941TTG (3522C>T 3524A>G), TTC942TTT (3527C>T), GAG944GAA (3533G>A), TCT948ACT (3543T>A), CGC950CGT (3551C>T), ATT953ATA (3560T>A), TTA956TTG (3569A>G), AAG958AAA (3575G>A), AAC960AAT (3581C>T), ACA963ACC (3590A>C), CAA975CAG (3626A>G), CTC978CTT (3635C>T), AGA979AGG (3638A>G), TCA983TCT (3650A>T), GTA987GTT (3662A>T), GTG991GTA (3674G>A), CCC1007CCT (3722C>T), CAG1014CAA (3743G>A), GTA1018GTG (3755A>G), ATC1022ATT (3767C>T), TGC1024TGT (3773C>T), TTC1033TTT (3800C>T), TAC1042TAT (3827C>T), ATT1044GTT (3831A>G), AAC1060AAT (3881C>T), ACC1062GCC (3885A>G), TTA1063TTG (3890A>G), GAT1064GAC (3893T>C), ACC1078GCC (3933A>G), GTT1080GTG (3941T>G), GCT1083GCA (3950T>A), GGT1094GGA (3983T>A), ATG1095GTT (3984A>G 3986G>T), GAC1097AAC (3990G>A), AAC1098AAT (3995C>T), ATC1099ATT (3998C>T), GTG1104GTC (4013G>C), TTT1110TTC (4031T>C), CGC1113CGT (4040C>T), ATA1120GTG (4059A>G 4061A>G), AAT1123AGT (4069A>G), CGA1133CGT (4100A>T), CTC1137CTT (4112C>T), GCC1143GCT (4130C>T), AAG1174AAA (4223G>A), CAT1175TAT (4224C>T), ACC1183ACT (4250C>T)								

Proteins

	Begin	End	Coverage	Score	Concordance	Matches	Identities	I/D/M/F*	Stop Codons
Orf1 (YP_001040017.1)	1	188	100%	1130	93.5%	188 (100%)	173 (92.0%)	0/0/0/0	1
Protein mutations:	V59I (356G>A), N64T (372A>C), R109K (507G>A 508G>A), I110V (509A>G 511T>A), V112I (515G>A), A124V (552C>T), Q128H (565A>T), T132K (576C>A), D141A (603A>C), L155A (644C>G 645T>C), L158Q (654T>A), L168M (683T>A), N170T (690A>C), L172P (696T>C), D175N (704G>A)								
Codon mutations:	GAT30GAC (271T>C), GGC32GGT (277C>T), TCT33TCC (280T>C), GGC34GGT (283C>T), AGT38AGC (295T>C), CCA40CCT (301A>T), GAC43GAT (310C>T), TTC44TTT (313C>T), AAG45AAA (316G>A), GCA53GCT (340A>T), GTT59ATT (356G>A), AAC63AAT (370C>T), AAT64ACT (372A>C), AGC65AGT (376C>T), GTT69GTA (388T>A), TAT73TAC (400T>C), TTA74CTA (401T>C), ACT79ACG (418T>G), GGC84GGT (433C>T), AAG86AAA (439G>A), TCT88TCA (445T>A), GGC89GGA (448C>A), CAT93CAC (460T>C), AGA94CGA (461A>C), ACC100ACA (481C>A), AGA102AGG (487A>G), GCT107GCA (502T>A), AGG109AAA (507G>A 508G>A), ATT110GTA (509A>G 511T>A), GTA112ATA (515G>A), GCT124GTT (552C>T), CAA128CAT (565A>T), CCA129CCT (568A>T), ACG132AAG (576C>A), ACA134ACT (583A>T), CAA135CAG (586A>G), TTA136CTT (587T>C 589A>T), GAC141GCC (603A>C), GAG142GAA (607G>A), AAA144AAG (613A>G), TTA147TTG (622A>G), GAG151GAA (634G>A), AAA152AAG (637A>G), CTT155GCT (644C>G 645T>C), CTA158CAA (654T>A), AAA163AAG (670A>G), TTG166TTA (679G>A), TTG168ATG (683T>A), AAC170ACC (690A>C), CTA172CCA (696T>C), GAT175AAT (704G>A), GGC180GGT (721C>T), GAA184GAG (733A>G)								
polyprotein (YP_001040018.1)	1	1199	100%	7945	96.0%	1199 (100%)	1135 (94.7%)	0/0/0/0	1
Protein mutations:	M1I (704G>A), A7V (721C>T), K11R (733A>G), K16R (748A>G), V20A (760T>C), Y48F (844A>T), T228A (1383A>G), Q242H (1427G>T), D245N (1434G>A), A254S (1461G>T), I257M (1472C>G), S275P (1524T>C), Y303H (1608T>C), K317R (1651A>G), L321F (1662C>T 1664G>T), V326M (1677G>A), V365I (1794G>A 1796T>A), I373K (1819T>A 1820C>G), K377N (1832G>C), H388Y (1863C>T 1865C>T), V400T (1899G>A 1900T>C 1901G>A), A402V (1906C>T 1907C>G), E409D (1928A>C), L414F (1941C>T), Q465E (2094C>G), R469G (2106A>G 2108A>G), R476M (2128G>T), V479L (2136G>C), K480T (2140A>C), N481K (2144C>G), V488G (2164T>G), V500T (2199G>A 2200T>C), M502V (2205A>G), M510E (2229A>G 2230T>A), K511E (2232A>G), E517D (2252A>T), A519P (2256G>C), N520E (2259A>G 2261T>G), E521Q (2262G>C), E531D (2294A>C), N538K (2315T>A), G540A (2320G>C 2321T>C), T542I (2326C>T), S579A (2436T>G), S590T (2469T>A 2471C>G), R601K (2503G>A), I608L (2523A>C), A761T (2982G>A), I771V (3012A>G), S829N (3187G>A), K851R (3253A>G), R855K (3265G>A), T857S (3270A>T), T868S (3303A>T 3305C>T), N936S (3508A>G), S948T (3543T>A), I1044V (3831A>G), T1062A (3885A>G), T1078A (3933A>G), M1095V (3984A>G 3986G>T), D1097N (3990G>A), I1120V (4059A>G 4061A>G), N1123S (4069A>G), H1175Y (4224C>T)								

	Begin	End	Coverage	Score	Concordance	Matches	Identities	I/D/M/F*	Stop Codons
**NT**	**20**	**5403**	**99.6%**	**8926**	**82.9%**	**5384 (99.9%)**	**4926 (91.5%)**	**1/0**	
Codon mutations:	ATG1ATA (704G>A), GCT7GTT (721C>T), AAG11AGG (733A>G), AAA16AGA (748A>G), GTA20GCA (760T>C), GTT32GTC (797T>C), GGC35GGT (806C>T), AAA42AAG (827A>G), TAT48TTT (844A>T), GGA89GGG (968A>G), AAA114AAG (1043A>G), AGT120AGC (1061T>C), CTG128TTG (1083C>T), TTG133TTA (1100G>A), TTT135TTC (1106T>C), TCC136TCT (1109C>T), CCG146CCA (1139G>A), CTA150CTG (1151A>G), AAA154AAG (1163A>G), GGC156GGG (1169C>G), TTC159TTT (1178C>T), ACA162ACT (1187A>T), ACT168ACA (1205T>A), TCA175TCG (1226A>G), GCC178GCA (1235C>A), CAA182CAG (1247A>G), TCA192TCG (1277A>G), CTT204CTC (1313T>C), CGA206AGG (1317C>A 1319A>G), CAA208CAG (1325A>G), TTT209TTC (1328T>C), TTC210TTT (1331C>T), CAG214CAA (1343G>A), CAG218CAA (1355G>A), ATA220ATT (1361A>T), TCA222TCT (1367A>T), ACC228GCC (1383A>G), TCC229TCT (1388C>T), ACT232ACA (1397T>A), TCA234TCT (1403A>T), GTG235GTA (1406G>A), CCG237CCT (1412G>T), GTG239GTA (1418G>A), GGA240GGG (1421A>G), CAG242CAT (1427G>T), GAC245AAC (1434G>A), GTG250GTT (1451G>T), AGC252AGT (1457C>T), GAA253GAG (1460A>G), GCA254TCA (1461G>T), ATC257ATG (1472C>G), CGG263CGA (1490G>A), CTC267CTT (1502C>T), AGT271AGC (1514T>C), TCC275CCC (1524T>C), ACC282ACT (1547C>T), TCA283TCC (1550A>C), GTA296GTG (1589A>G), TAC303CAC (1608T>C), GAG306GAA (1619G>A), GTT311GTA (1634T>A), GTT315GTC (1646T>C), AAG317AGG (1651A>G), GCT318GCA (1655T>A), GGT320GGC (1661T>C), CTG321TTT (1662C>T 1664G>T), AAC322AAT (1667C>T), GTG326ATG (1677G>A), GAT328GAC (1685T>C), CTG340CTC (1721G>C), CCC341CCT (1724C>T), CTG347TTG (1740C>T), GTG349GTA (1748G>A), CAA352CAG (1757A>G), GCA359GCC (1778A>C), GTT365ATA (1794G>A 1796T>A), CAT371CAC (1814T>C), ATA372ATT (1817A>T), ATC373AAG (1819T>A 1820C>G), AAG377AAC (1832G>C), TTG382CTG (1845T>C), ATC385ATT (1856C>T), ACC386ACT (1859C>T), CAC388TAT (1863C>T 1865C>T), TAC397TAT (1892C>T), GTG400ACA (1899G>A 1900T>C 1901G>A), GCC402GTG (1906C>T 1907C>G), GAA409GAC (1928A>C), AAC410AAT (1931C>T), TAC412TAT (1937C>T), CTT414TTT (1941C>T), TTG416CTG (1947T>C), CAG418CAA (1955G>A), AGA419AGG (1958A>G), GAG422GAA (1967G>A), CTA423CTG (1970A>G), CAA426CAG (1979A>G), GAC428GAT (1985C>T), TAT431TAC (1994T>C), GAG457GAA (2072G>A), GTG461GTA (2084G>A), CAG465GAG (2094C>G), GCG466GCA (2099G>A), AGA469GGG (2106A>G 2108A>G), GCC475GCT (2126C>T), AGG476ATG (2128G>T), CAG478CAA (2135G>A), GTG479CTG (2136G>C), AAA480ACA (2140A>C), AAC481AAG (2144C>G), ACT486ACA (2159T>A), GTG488GGG (2164T>G), ACC494ACT (2183C>T), TCA495TCC (2186A>C), TTC496TTT (2189C>T), AAG499AAA (2198G>A), GTT500ACT (2199G>A 2200T>C), ATG502GTG (2205A>G), GAG503GAA (2210G>A), GGC505GGG (2216C>G), ATG510GAG (2229A>G 2230T>A), AAA511GAA (2232A>G), GCT513GCA (2240T>A), ATT514ATA (2243T>A), GAA517GAT (2252A>T), GCC519CCC (2256G>C), AAT520GAG (2259A>G 2261T>G), GAA521CAA (2262G>C), GTA523GTG (2270A>G), GTT528GTC (2285T>C), GAA531GAC (2294A>C), CAA534CAG (2303A>G), AAT537AAC (2312T>C), AAT538AAA (2315T>A), GGT540GCC (2320G>C 2321T>C), ACA542ATA (2326C>T), TTA546TTG (2339A>G), CAA548CAG (2345A>G), TTC558TTT (2375C>T), GAG564GAA (2393G>A), AGT571AGC (2414T>C), GTT573GTG (2420T>G), GTG577GTA (2432G>A), TCA579GCA (2436T>G), GCG581GCC (2444G>C), CTC582CTA (2447C>A), GCG584GCA (2453G>A), CTC586CTG (2459C>G), TCC590ACG (2469T>A 2471C>G), AAG591AAA (2474G>A), GGA596GGC (2489A>C), TCG597TCA (2492G>A), CAA598CAG (2495A>G), CTA600CTG (2501A>G), AGG601AAG (2503G>A), TAT602TAC (2507T>C), GCC605GCT (2516C>T), ATT608CTT (2523A>C), TAT615TAC (2546T>C), TCC617TCT (2552C>T), GGT620GGG (2561T>G), ATT622ATA (2567T>A), TCG623TCA (2570G>A), GGT624GGA (2573T>A), ATC643ATT (2630C>T), CTG648CTA (2645G>A), CAT653CAC (2660T>C), GGG664GGA (2693G>A), ACA669ACG (2708A>G), ATC673ATA (2720C>A), GTT677GTC (2732T>C), CTG688CTA (2765G>A), CAC697CAT (2792C>T), GAG706GAA (2819G>A), CAT713CAC (2840T>C), CTG714TTG (2841C>T), AAA717AAG (2852A>G), ATC723ATA (2870C>A), CCT726CCA (2879T>A), CGT728CGC (2885T>C), TTG729CTA (2886T>C 2888G>A), GCA733GCT (2900A>T), AAA738AAG (2915A>G), CGA746CGG (2939A>G), GTA752GTT (2957A>T), CTG753TTG (2958C>T), CAC755CAT (2966C>T), AAT757AAC (2972T>C), GCT761ACT (2982G>A), ACT766ACA (2999T>A), TTA768CTA (3003T>C), AGC770AGT (3011C>T), ATC771GTC (3012A>G), TCT773TCC (3020T>C), ATA777ATT (3032A>T), ACG783ACA (3050G>A), TTG788CTG (3063T>C), ATT807ATC (3122T>C), GAG810GAA (3131G>A), CAT815CAC (3146T>C), GGT817GGA (3152T>A), GGA822GGT (3167A>T), AGC829AAC (3187G>A), GAC835GAT (3206C>T), GTT842GTG (3227T>G), ATT843ATA (3230T>A), AAC845AAT (3236C>T), GAG847GAA (3242G>A), GGA849GGC (3248A>C), AAG851AGG (3253A>G), AGG855AAG (3265G>A), ACG857TCG (3270A>T), ACC868TCT (3303A>T 3305C>T), CTA875TTA (3324C>T), CCC878CCT (3335C>T), CAC883CAT (3350C>T), AGG885CGG (3354A>C), TTG886TTA (3359G>A), ACA889ACG (3368A>G), GTC890GTG (3371C>G), GTA895GTT (3386A>T), CTA901CTG (3404A>G), GTC904GTT (3413C>T), ACA907ACG (3422A>G), CAG909CAA (3428G>A), CTT910CTC (3431T>C), TGC912TGT (3437C>T), GAT915GAC (3446T>C), TTG916TTA (3449G>A), GTC917GTT (3452C>T), CTT918CTA (3455T>A), TCT923TCC (3470T>C), CCC926CCT (3479C>T), ATT927ATC (3482T>C), TAC928TAT (3485C>T), CAC930CAT (3491C>T), GGA934GGG (3503A>G), AAT936AGT (3508A>G), GCT937GCC (3512T>C), GTG938GTT (3515G>T), CTA941TTG (3522C>T 3524A>G), TTC942TTT (3527C>T), GAG944GAA (3533G>A), TCT948ACT (3543T>A), CGC950CGT (3551C>T), ATT953ATA (3560T>A), TTA956TTG (3569A>G), AAG958AAA (3575G>A), AAC960AAT (3581C>T), ACA963ACC (3590A>C), CAA975CAG (3626A>G), CTC978CTT (3635C>T), AGA979AGG (3638A>G), TCA983TCT (3650A>T), GTA987GTT (3662A>T), GTG991GTA (3674G>A), CCC1007CCT (3722C>T), CAG1014CAA (3743G>A), GTA1018GTG (3755A>G), ATC1022ATT (3767C>T), TGC1024TGT (3773C>T), TTC1033TTT (3800C>T), TAC1042TAT (3827C>T), ATT1044GTT (3831A>G), AAC1060AAT (3881C>T), ACC1062GCC (3885A>G), TTA1063TTG (3890A>G), GAT1064GAC (3893T>C), ACC1078GCC (3933A>G), GTT1080GTG (3941T>G), GCT1083GCA (3950T>A), GGT1094GGA (3983T>A), ATG1095GTT (3984A>G 3986G>T), GAC1097AAC (3990G>A), AAC1098AAT (3995C>T), ATC1099ATT (3998C>T), GTG1104GTC (4013G>C), TTT1110TTC (4031T>C), CGC1113CGT (4040C>T), ATA1120GTG (4059A>G 4061A>G), AAT1123AGT (4069A>G), CGA1133CGT (4100A>T), CTC1137CTT (4112C>T), GCC1143GCT (4130C>T), AAG1174AAA (4223G>A), CAT1175TAT (4224C>T), ACC1183ACT (4250C>T)								

*: Inserts / Deletes / Misaligned / Frameshifts

Analysis details

This analysis was performed with panviral2.64

NGS Details (UN62): Torradovirus marchitezum (segment RNA 1)

Assembly

**Coverage Length**	5822 (2 contig(s))
**Depth Of Coverage**	2179.8
**Number Of Reads**	96951
**Reads Per Million**	2247.41 rpm (after QC)
**Ambiguities**	0
**Assembly Method**	de novo + reference guided assembly
**Consensus Caller**	Bcf Tools

Coverage Map

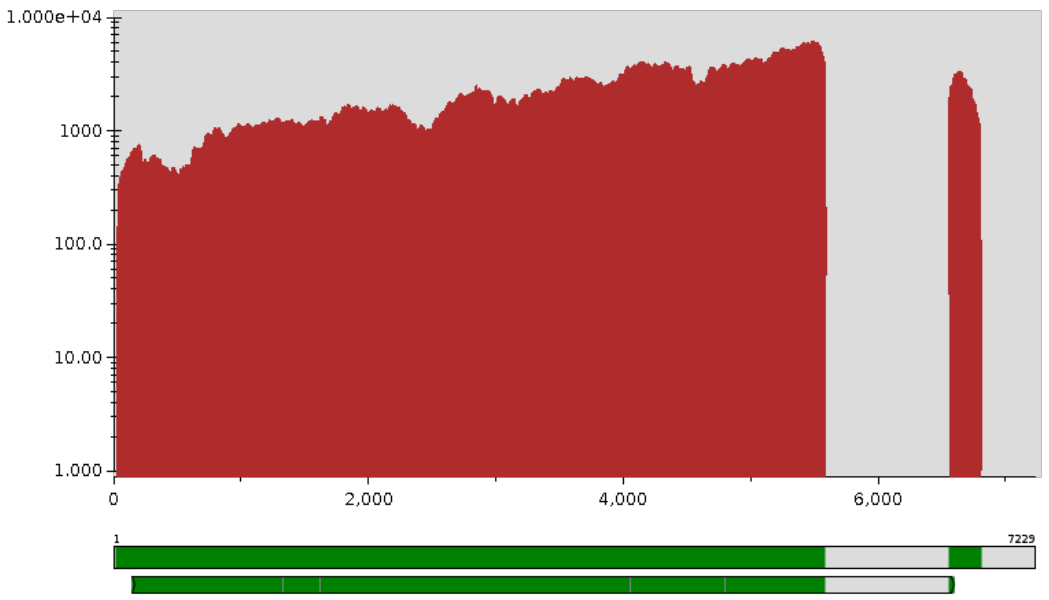

Assignment

**Type**	Torradovirus marchitezum (Taxonomy ID: 3048376)
**Reference Genome**	NC_010987.1
**NT Identity (%)**	67.1567
**AA Identity (%)**	69.9945
**Number Of Stop Codons**	1
**Number Of CDS**	1

Alignment

**Alignment Score**	3829.0 (NT) + 9356.0 (AA) = 13185.0
**Concordance (%)**	54.5579

**Alignment Method**	Global, seeded, nucleotide + amino acids (AGA)

Genome Region

Sequence starts at position 22 and ends at position 6809 relative to NC_010987.1 reference sequence.

Alignment Detailed Statistics

	Begin	End	Coverage	Score	Concordance	Matches	Identities	I/D/M/F*	Stop Codons
**NT**	**22**	**6809**	**80.5%**	**3829**	**33.5%**	**5772 (99.0%)**	**3883 (66.6%)**	**10/50**	

	Begin	End	Coverage	Score	Concordance	Matches	Identities	I/D/M/F*	Stop Codons
**NT**	**22**	**6809**	**80.5%**	**3829**	**33.5%**	**5772 (99.0%)**	**3883 (66.6%)**	**10/50**	
Mutations:	32T>C, 37G>A, 40C>T, 59A>C, 61T>C, 62T>A, 63G>T, 66A>C, 67_78delTTTGATTCTTGT, 81A>T, 82C>A, 83T>C, 87C>G, 89A>T, 90A>T, 91C>T, 94C>T, 99T>A, 100_101insC, 108A>T, 112G>C, 114C>T, 115C>T, 116A>C, 117T>C, 118T>C, 119A>T, 122T>G, 125A>T, 128C>A, 129C>A, 130T>A, 133_134delAT, 136T>A, 146T>A, 160T>G, 161C>G, 162T>A, 163C>G, 164C>T, 165_188delAAGGTTGCTGGTGTTGATGACGCC, 189A>C, 190G>C, 192T>C, 195T>G, 200C>T, 201A>T, 203C>A, 204A>C, 217G>C, 218T>A, 222C>G, 227A>T, 228A>T, 229A>C, 234A>T, 239C>T, 240A>T, 241A>C, 242A>T, 243T>C, 251A>G, 252G>A, 254G>T, 255G>C, 257T>C, 258A>T, 259G>C, 260T>C, 264A>G, 268C>A, 269C>T, 271C>A, 272T>A, 273A>G, 275G>T, 283A>C, 284G>C, 288T>G, 289C>T, 296T>C, 297C>A, 299T>A, 306C>G, 308G>C, 311A>G, 314A>G, 315T>C, 317T>A, 320G>T, 322C>T, 323T>G, 325A>C, 326T>A, 328T>C, 329G>A, 330T>G, 332C>A, 338T>A, 343T>C, 344G>C, 345G>T, 347C>T, 350T>A, 353T>C, 355G>A, 357T>G, 359A>T, 360C>G, 362T>A, 363A>G, 365T>A, 367A>G, 374G>A, 377C>T, 380A>G, 385G>C, 386T>A, 387G>A, 388C>A, 390C>A, 392T>A, 395A>G, 398T>A, 401A>C, 404T>C, 405C>A, 407T>C, 410A>G, 412C>G, 413G>T, 416C>T, 417T>G, 418C>A, 422C>T, 423T>G, 425G>A, 426T>A, 427C>A, 428C>A, 431A>T, 437C>T, 441T>A, 446C>A, 447A>G, 448A>T, 449G>A, 453A>C, 455G>T, 458T>A, 459T>C, 461A>T, 464A>G, 465T>A, 468T>G, 471G>T, 472A>T, 473G>A, 477T>G, 479G>C, 480T>A, 481C>A, 482C>G, 483A>G, 489G>T, 491T>C, 493A>T, 494A>C, 495T>C, 502T>A, 503T>C, 505C>A, 506T>A, 510G>T, 512G>A, 516A>G, 518T>A, 522A>G, 523G>T, 527A>G, 528G>T, 529A>C, 530T>A, 531G>T, 542T>G, 543G>T, 545A>T, 546T>A, 547A>T, 548T>A, 550G>C, 555C>T, 557T>A, 560A>T, 567A>T, 569A>G, 570A>G, 572A>C, 573T>A, 575G>T, 578A>G, 579G>C, 581G>A, 584T>A, 587A>G, 589T>A, 593T>A, 596T>C, 600A>G, 601G>T, 602T>A, 611T>A, 612G>A, 613A>C, 615G>C, 616G>C, 618G>T, 621T>A, 622C>A, 626C>T, 627A>G, 628C>T, 629T>A, 632C>T, 633A>G, 634A>T, 635A>T, 637A>C, 639A>T, 641C>G, 646T>C, 647G>A, 648A>G, 650A>T, 653C>T, 659C>T, 660A>T, 662G>T, 665T>C, 668A>T, 669A>T, 670G>C, 671T>A, 672C>T, 674A>G, 677C>T, 697A>C, 702G>T, 704C>T, 706A>G, 707A>G, 708C>A, 709G>A, 710A>G, 713C>T, 714A>T, 717A>C, 718T>A, 720G>C, 725C>G, 731T>A, 732A>G, 733G>C, 734T>A, 735A>G, 737G>A, 746G>T, 750T>A, 751C>G, 752A>C, 753G>T, 755T>A, 756T>A, 758A>G, 770G>T, 771T>A, 772C>G, 773T>C, 776G>A, 777T>C, 779G>T, 780A>T, 782G>A, 786A>T, 789A>T, 791A>G, 803G>T, 806T>C, 809G>A, 812G>T, 815G>A, 818G>A, 821C>T, 822A>G, 827A>T, 830G>T, 836T>G, 848C>T, 849T>C, 851G>T, 852A>T, 853G>C, 854C>A, 861T>C, 866C>A, 872T>C, 875C>G, 878T>C, 881G>T, 882A>G, 884A>T, 896T>G, 899G>A, 911A>G, 914A>G, 918A>C, 919C>A, 920T>G, 921C>T, 923T>G, 927C>A, 929T>G, 932T>C, 938A>G, 944C>T, 947C>A, 950C>T, 953G>A, 956T>C, 974A>G, 978A>G, 980A>G, 983C>T, 984G>A, 988C>T, 989C>T, 995A>T, 998C>T, 1010G>A, 1019T>G, 1024G>A, 1028A>T, 1031G>C, 1032C>T, 1034G>A, 1040T>C, 1046G>T, 1050G>C, 1059C>A, 1060A>C, 1064G>A, 1068G>T, 1069A>C, 1070T>G, 1072C>A, 1073A>G, 1076G>T, 1077T>G, 1078G>C, 1082T>A, 1085T>A, 1086G>T, 1088A>T, 1090C>T, 1100G>A, 1103C>T, 1109C>A, 1112T>C, 1113A>C, 1114A>C, 1115G>T, 1118T>A, 1121C>T, 1124T>A, 1126C>T, 1127C>T, 1130A>G, 1134A>C, 1136C>A, 1140C>T, 1142G>A, 1144G>A, 1145G>A, 1148A>G, 1149G>C, 1151A>G, 1154T>C, 1155C>G, 1160G>A, 1163C>A, 1178A>C, 1190T>C, 1193G>A, 1199C>T, 1200A>T, 1215A>T, 1216G>C, 1226T>A, 1227G>A, 1233T>C, 1238A>T, 1242G>C, 1244T>G, 1245T>G, 1246A>T, 1253A>G, 1254A>G, 1255G>C, 1256T>C, 1257T>A, 1259T>C, 1260A>C, 1261G>A, 1262G>A, 1265C>T, 1266A>T, 1267T>C, 1268G>T, 1271C>T, 1280G>A, 1284C>A, 1286T>G, 1292A>G, 1298T>C, 1304A>G, 1313C>T, 1316G>A, 1317T>G, 1319C>T, 1325G>A, 1337T>A, 1340A>G, 1342C>G, 1343C>T, 1350T>C, 1352A>T, 1355T>C, 1358T>C, 1367G>T, 1368T>C, 1370G>C, 1374A>G, 1375G>C, 1385G>A, 1391A>G, 1394C>G, 1397T>C, 1400G>C, 1401C>T, 1403A>G, 1409A>G, 1418G>A, 1421G>A, 1445T>A, 1448C>T, 1457T>A, 1458A>G, 1460C>G, 1463G>T, 1472C>T, 1478A>T, 1481A>T, 1482T>C, 1484G>T, 1487A>G, 1493G>A, 1496T>A, 1505C>T, 1508A>G, 1511T>C, 1514T>A, 1517A>G, 1529T>C, 1538A>T, 1541A>C, 1544A>G, 1547A>T, 1553C>T, 1554T>C, 1556A>T, 1565C>T, 1571T>C, 1577C>T, 1583C>A, 1589T>C, 1592C>T, 1595C>A, 1598C>A, 1604T>C, 1607A>C, 1616A>C, 1622T>C, 1623C>T, 1628C>A, 1631A>T, 1637T>A, 1640C>T, 1643A>G, 1649T>G, 1652T>A, 1655A>G, 1661A>T, 1667T>A, 1673G>A, 1674C>A, 1679A>G, 1682G>A, 1685C>T, 1688C>T, 1689C>T, 1691G>A, 1694G>T, 1700T>C, 1702T>G, 1703G>A, 1708A>T, 1715G>T, 1719T>G, 1720C>A, 1721C>T, 1722A>C, 1724T>A, 1725G>A, 1727A>T, 1728G>A, 1729A>C, 1733T>A, 1736G>A, 1738G>C, 1739A>C, 1745C>A, 1747G>T, 1748A>T, 1752A>G, 1754C>T, 1755A>G, 1757A>G, 1763G>A, 1775T>C, 1781T>C, 1787T>A, 1790C>T, 1793G>A, 1794A>G, 1795A>C, 1799G>A, 1800A>T, 1806A>G, 1807G>A, 1811T>C, 1814T>C, 1818G>C, 1820G>A, 1829A>G, 1832C>A, 1835A>G, 1838T>C, 1840A>G, 1844A>G, 1847T>C, 1848G>T, 1849C>A, 1850A>T, 1853A>G, 1856A>G, 1861A>G, 1862G>C, 1866T>C, 1868G>T, 1870T>A, 1871T>C, 1872G>A, 1874A>T, 1875G>A, 1878G>A, 1879A>C, 1883T>G, 1886C>A, 1887A>T, 1889A>G, 1890A>G, 1891G>C, 1893C>G, 1894C>A, 1895T>C, 1899A>G, 1901A>T, 1910T>C, 1911C>A, 1913A>T, 1917T>G, 1921C>T, 1922A>T, 1928T>A, 1932A>C, 1934A>G, 1936C>A, 1937G>T, 1941G>T, 1943G>C, 1944G>C, 1946T>A, 1948G>A, 1949G>T, 1952T>C, 1953_1955delAGA, 1958G>A, 1960G>A, 1961C>A, 1962A>G, 1963C>T, 1964C>T, 1965A>T, 1966A>T, 1969A>C, 1970A>T, 1971G>C, 1972A>C, 1976A>G, 1980A>G, 1981T>C, 1983T>G, 1984T>C, 1985G>T, 1986G>T, 1987T>A, 1988G>T, 1989C>G, 1990C>A, 1991C>G, 1992A>C, 1993C>A, 1994T>G, 1995G>A, 1996T>C, 1997T>A, 1998G>A, 2000G>T, 2002G>C, 2003T>A, 2007A>G, 2008A>C, 2012T>A, 2014G>A, 2016G>A, 2018G>T, 2019A>G, 2021C>T, 2024T>C, 2025G>T, 2027A>C, 2028A>C, 2029G>A, 2030A>G, 2033G>A, 2039G>A, 2042A>T, 2048A>C, 2049T>C, 2051G>C, 2060A>T, 2066G>A, 2069C>G, 2073T>A, 2076G>A, 2079C>A, 2080C>A, 2081T>A, 2082G>T, 2083A>T, 2084G>T, 2087A>G, 2088C>T, 2093T>C, 2096T>A, 2104A>T, 2105T>C, 2106C>T, 2107A>C, 2108T>C, 2117C>T, 2120G>C, 2127T>G, 2128C>T, 2129T>G, 2132T>A, 2133G>T, 2134A>C, 2136G>T, 2137A>C, 2141A>T, 2142C>G, 2143A>C, 2144T>C, 2148G>C, 2152T>A, 2157A>G, 2159C>A, 2161A>T, 2162A>G, 2166A>G, 2167G>T, 2169G>A, 2170T>A, 2171C>T, 2172A>G, 2174C>T, 2175A>T, 2181T>G, 2182A>T, 2183T>C, 2185G>A, 2193G>A, 2194A>G, 2196G>C, 2199G>C, 2201A>G, 2208C>G, 2209G>C, 2210A>T, 2219A>G, 2221A>G, 2237C>T, 2243A>C, 2249C>T, 2252T>C, 2268G>A, 2269C>G, 2270A>C, 2271T>A, 2279C>T, 2285G>A, 2286A>G, 2289A>C, 2290A>T, 2291A>G, 2294A>G, 2296C>A, 2297A>T, 2300G>C, 2303G>A, 2304A>C, 2305C>G, 2306A>C, 2307G>A, 2309G>T, 2310A>C, 2312G>T, 2313A>G, 2314G>A, 2316A>C, 2317C>A, 2318T>A, 2321A>G, 2322T>A, 2323T>A, 2324T>A, 2327A>G, 2328C>T, 2331T>A, 2332C>A, 2333T>G, 2334T>G, 2335G>C, 2336C>G, 2337A>C, 2340G>C, 2342T>G, 2345A>T, 2350T>C, 2351T>A, 2354C>T, 2357C>A, 2360C>T, 2362T>C, 2363A>G, 2369C>T, 2372G>A, 2385T>A, 2386T>G, 2387T>A, 2390T>A, 2396T>C, 2397T>C, 2399A>G, 2405A>T, 2408A>T, 2415A>C, 2417A>C, 2426C>T, 2428A>G, 2429C>G, 2435A>T, 2436T>G, 2438_2439insGGT, 2440A>C, 2441A>T, 2444G>C, 2445C>A, 2446C>A, 2447A>G, 2451G>A, 2452A>G, 2454T>A, 2455C>A, 2456A>T, 2462C>T, 2464A>T, 2465A>C, 2466A>G, 2467T>A, 2468T>A, 2472T>A, 2473A>T, 2474C>G, 2475C>A, 2476A>T, 2478A>G, 2479A>G, 2480A>T, 2484G>A, 2485T>A, 2486T>G, 2489A>C, 2490T>A, 2492T>A, 2495C>G, 2497A>T, 2498G>C, 2499G>C, 2501T>G, 2503C>T, 2504A>G, 2508T>A, 2509G>A, 2510G>T, 2511A>G, 2512G>A, 2514C>T, 2516T>G, 2521A>T, 2522T>C, 2523G>A, 2524C>T, 2525A>C, 2526C>T, 2530G>A, 2531A>G, 2537C>T, 2546A>C, 2549T>A, 2556A>T, 2558A>G, 2561G>A, 2564T>A, 2573A>C, 2574G>A, 2575T>C, 2576T>C, 2577T>A, 2579T>G, 2580G>A, 2581G>A, 2582A>T, 2584G>C, 2586A>G, 2591A>T, 2594A>T, 2597T>C, 2598T>A, 2600G>T, 2601G>A, 2603G>T, 2607T>C, 2608G>T, 2609G>C, 2610C>A, 2613A>G, 2614G>C, 2615T>A, 2616A>G, 2617A>C, 2618C>A, 2619T>A, 2623T>C, 2627C>T, 2628A>T, 2629A>T, 2630T>A, 2633A>T, 2634T>G, 2635C>A, 2636G>T, 2645A>G, 2648T>C, 2651G>A, 2660A>G, 2672A>C, 2678T>C, 2684G>A, 2687C>T, 2690G>A, 2693T>G, 2696T>G, 2698A>C, 2699G>T, 2699_2700insCAT, 2700C>T, 2703A>G, 2704G>C, 2708T>A, 2709C>T, 2710T>C, 2711T>C, 2712C>T, 2714C>G, 2715G>C, 2717T>A, 2720T>G, 2724A>G, 2725G>C, 2726T>G, 2727T>G, 2729T>A, 2730C>G, 2732A>T, 2733T>A, 2734C>A, 2735A>C, 2736T>A, 2737T>A, 2738G>C, 2739G>C, 2740C>A, 2741A>T, 2742C>G, 2743A>T, 2744A>G, 2747T>C, 2747_2748insCGG, 2753T>A, 2755C>A, 2756T>A, 2757G>C, 2759A>G, 2771A>T, 2775C>G, 2777G>A, 2780C>T, 2783C>G, 2784A>C, 2786G>C, 2792T>C, 2798G>T, 2801G>T, 2804A>G, 2808C>A, 2812G>C, 2822G>C, 2825C>T, 2828T>C, 2834C>T, 2837C>G, 2840C>T, 2843G>T, 2858A>T, 2859A>C, 2861G>C, 2864C>T, 2867A>G, 2870T>A, 2876A>C, 2879C>T, 2880C>A, 2881A>G, 2882C>A, 2885T>C, 2886G>A, 2887C>T, 2888T>G, 2889C>A, 2891A>G, 2897T>C, 2903A>T, 2907A>T, 2909A>C, 2915C>T, 2920C>A, 2921C>G, 2922A>C, 2927T>C, 2928C>A, 2929G>T, 2930A>T, 2932C>G, 2936A>T, 2939T>G, 2942A>G, 2944A>T, 2948C>T, 2953G>C, 2954A>C, 2955G>C, 2964A>T, 2966T>G, 2969T>A, 2975C>T, 2981C>T, 2982A>G, 2984C>G, 2988C>T, 2994C>A, 2996C>G, 2999T>A, 3002T>G, 3017A>C, 3020G>A, 3021C>T, 3023T>A, 3026C>T, 3029C>A, 3030C>A, 3032T>A, 3038C>T, 3044A>T, 3045A>C, 3047A>C, 3050G>A, 3056G>T, 3062C>T, 3064T>A, 3066A>T, 3068A>T, 3078C>A, 3079A>T, 3080A>G, 3083A>C, 3090T>G, 3091G>T, 3092T>G, 3095A>G, 3101T>A, 3104G>T, 3107T>A, 3109C>A, 3110A>G, 3116A>C, 3117C>A, 3118G>A, 3119T>G, 3120C>A, 3121T>A, 3122T>A, 3123G>T, 3124G>C, 3131A>G, 3135T>G, 3140C>T, 3143G>A, 3144A>T, 3145T>A, 3146G>T, 3167A>C, 3173A>C, 3175T>A, 3176T>A, 3182G>C, 3183G>A, 3184A>C, 3190A>G, 3192C>T, 3194G>A, 3195A>G, 3197C>T, 3200C>G, 3201T>A, 3202C>A, 3203C>T, 3204A>G, 3205G>C, 3206T>A, 3210A>G, 3211A>C, 3212G>T, 3213A>C, 3216T>A, 3221T>A, 3225C>G, 3226A>T, 3227A>T, 3228T>G, 3230T>A, 3236A>G, 3245A>T, 3249G>A, 3251G>T, 3254A>G, 3260T>A, 3263C>T, 3269C>T, 3272G>T, 3273T>C, 3275G>T, 3278A>C, 3279C>T, 3281G>A, 3284A>G, 3289G>C, 3293T>G, 3299T>C, 3305A>G, 3308T>G, 3309G>T, 3310T>G, 3311G>C, 3320C>A, 3323C>A, 3326C>T, 3335A>T, 3343G>A, 3344C>T, 3345G>A, 3346T>C, 3347A>C, 3350A>T, 3353T>A, 3356A>G, 3357C>A, 3358G>A, 3359T>G, 3362A>T, 3364T>A, 3365T>C, 3366A>G, 3368C>G, 3369T>G, 3377T>C, 3380T>A, 3386T>A, 3387A>G, 3389C>G, 3391A>T, 3392C>T, 3395G>A, 3398A>T, 3402C>T, 3404A>G, 3407G>A, 3416A>C, 3419T>G, 3422G>A, 3425G>C, 3426G>A, 3428C>T, 3433C>T, 3434A>G, 3437A>T, 3443A>G, 3452T>A, 3453A>C, 3455T>G, 3456C>T, 3462T>C, 3463C>A, 3464A>C, 3465A>C, 3467T>G, 3471A>T, 3472G>C, 3475G>A, 3476A>G, 3479A>G, 3480A>G, 3481C>A, 3483C>A, 3484A>G, 3485G>A, 3486C>T, 3488T>C, 3497A>G, 3500C>A, 3503A>G, 3507T>G, 3508A>C, 3509C>T, 3511A>T, 3513C>T, 3515T>G, 3518A>C, 3521A>T, 3524T>A, 3527A>G, 3528C>T, 3529A>T, 3533G>A, 3534T>A, 3539C>T, 3542C>T, 3548T>G, 3551C>G, 3557G>A, 3560C>A, 3563T>C, 3566G>T, 3567C>A, 3568G>A, 3569G>A, 3570A>C, 3571A>G, 3572A>C, 3575C>T, 3577A>C, 3578A>C, 3579T>C, 3587A>G, 3590A>T, 3593C>T, 3596A>G, 3597C>G, 3598C>A, 3599T>C, 3602A>T, 3603G>C, 3608A>T, 3611A>C, 3624A>T, 3626A>T, 3628T>C, 3629T>C, 3630C>A, 3632T>A, 3641A>G, 3644T>A, 3647T>A, 3650T>G, 3656C>T, 3671G>T, 3674A>C, 3675A>G, 3676T>A, 3680A>G, 3683C>T, 3697G>C, 3698T>C, 3699G>A, 3700G>A, 3701A>C, 3707T>G, 3710T>G, 3711G>C, 3712A>C, 3713C>A, 3719C>A, 3722A>G, 3725A>G, 3728A>T, 3731C>T, 3734A>G, 3735A>G, 3736A>G, 3740C>T, 3743G>A, 3744T>G, 3745G>C, 3746C>A, 3749G>C, 3750C>A, 3752G>T, 3755A>T, 3759A>G, 3761C>G, 3762A>C, 3764G>T, 3765C>A, 3766G>A, 3767T>G, 3769G>T, 3770C>G, 3774A>C, 3775A>G, 3776G>A, 3777A>G, 3779C>G, 3782T>C, 3788A>G, 3789C>A, 3790G>A, 3791T>A, 3794A>T, 3795T>G, 3796G>C, 3797T>A, 3798G>A, 3799A>T, 3800A>C, 3803G>A, 3804G>A, 3827A>G, 3838G>T, 3845C>T, 3847A>T, 3853G>C, 3854C>A, 3860G>A, 3861C>T, 3869A>G, 3872T>A, 3875C>T, 3878A>T, 3884C>T, 3887C>T, 3894A>T, 3899G>A, 3900A>C, 3902G>T, 3905G>T, 3906A>T, 3907T>C, 3908G>T, 3914G>A, 3917A>G, 3927C>G, 3930T>G, 3933A>C, 3934C>A, 3935T>A, 3936G>C, 3937G>C, 3938C>A, 3939G>A, 3941A>C, 3942G>T, 3943G>C, 3944A>G, 3945G>C, 3950A>G, 3953G>T, 3954G>C, 3955A>T, 3956A>T, 3961A>T, 3965A>G, 3966G>A, 3971T>A, 3977A>C, 3983A>T, 3996C>T, 3998A>G, 4001T>C, 4002G>T, 4003T>C, 4008A>G, 4010T>A, 4014A>C, 4021T>C, 4022C>A, 4023C>T, 4024A>T, 4028G>T, 4031T>C, 4034G>C, 4040C>A, 4046A>G, 4049C>T, 4052C>G, 4064C>G, 4065C>T, 4067C>G, 4079A>G, 4082C>T, 4088C>G, 4094A>G, 4101A>C, 4103G>C, 4106C>G, 4109T>C, 4112G>A, 4115A>C, 4116C>T, 4118T>G, 4130C>T, 4137T>C, 4142C>G, 4143A>C, 4148G>A, 4151T>C, 4154T>C, 4160T>C, 4163C>T, 4166A>T, 4169C>T, 4170A>T, 4171C>G, 4172A>T, 4182T>G, 4183T>C, 4184G>A, 4190T>C, 4192C>T, 4193T>A, 4194A>C, 4196A>C, 4199A>T, 4205A>G, 4208T>A, 4214T>C, 4215G>A, 4220T>A, 4223A>G, 4226T>C, 4227T>C, 4228C>A, 4239T>G, 4242T>C, 4247A>T, 4249A>G, 4251G>A, 4253G>C, 4259A>G, 4260G>T, 4262T>A, 4265G>A, 4268A>T, 4271C>T, 4273T>A, 4280T>C, 4286T>C, 4292T>C, 4295C>G, 4316T>C, 4317G>A, 4319T>C, 4325T>A, 4328T>C, 4331G>A, 4344T>C, 4354A>C, 4355A>T, 4357A>T, 4358C>T, 4361C>T, 4364A>T, 4365T>G, 4366C>A, 4367A>T, 4368A>G, 4376C>G, 4385T>G, 4388C>A, 4395C>A, 4397A>G, 4400T>C, 4401G>A, 4404C>T, 4406T>A, 4418A>C, 4424T>C, 4430C>T, 4433A>C, 4436G>C, 4439C>T, 4443G>A, 4445T>C, 4448A>G, 4449G>T, 4455A>C, 4457G>C, 4458T>C, 4460G>A, 4463T>G, 4466G>T, 4469C>A, 4475A>C, 4478A>T, 4481A>T, 4487A>T, 4488C>T, 4490A>G, 4493G>T, 4499C>A, 4502C>T, 4508T>A, 4509C>T, 4511C>G, 4514C>A, 4530C>A, 4532A>G, 4535T>C, 4538C>G, 4548T>C, 4553A>G, 4556A>G, 4562C>A, 4566A>G, 4568T>C, 4569G>A, 4570C>A, 4571T>A, 4576G>A, 4577G>A, 4578G>T, 4581A>G, 4583A>G, 4586A>C, 4587G>A, 4592A>G, 4597G>A, 4601T>C, 4611C>T, 4613A>G, 4622T>A, 4625T>A, 4631A>T, 4637C>T, 4640T>C, 4641C>T, 4643T>G, 4649T>G, 4652A>T, 4656T>A, 4658A>G, 4662C>T, 4664C>G, 4665C>A, 4666A>G, 4673T>C, 4674T>G, 4675C>A, 4679G>A, 4683A>T, 4684A>C, 4685T>C, 4688A>T, 4692C>A, 4693A>G, 4694T>A, 4697G>A, 4698T>C, 4700A>G, 4704C>G, 4705T>C, 4706G>T, 4709C>T, 4712T>C, 4715A>C, 4716A>G, 4731C>T, 4733T>G, 4739A>G, 4742G>A, 4751T>A, 4752A>G, 4754T>G, 4755G>A, 4758G>A, 4759T>C, 4761C>A, 4768A>T, 4769A>G, 4784A>C, 4787T>C, 4793G>C, 4799A>G, 4805T>C, 4808A>G, 4817T>A, 4818C>T, 4820T>G, 4826C>T, 4829C>A, 4835T>A, 4838T>A, 4844G>A, 4854A>G, 4859T>C, 4868G>T, 4877A>T, 4880A>G, 4883T>A, 4886C>A, 4887C>G, 4888C>T, 4889A>G, 4892T>A, 4901C>T, 4902A>G, 4903C>T, 4904T>A, 4907G>A, 4910C>T, 4914A>T, 4915T>A, 4916G>T, 4923G>A, 4925G>T, 4934A>C, 4937A>C, 4938C>A, 4940T>A, 4946T>C, 4955C>T, 4958T>G, 4960G>A, 4961T>G, 4963T>C, 4964G>C, 4965G>A, 4973C>A, 4975A>G, 4976G>T, 4977C>T, 4982C>A, 4983T>A, 4984T>A, 4985A>T, 4992C>T, 4993A>T, 4994G>T, 4995A>G, 4996C>G, 4997G>A, 5000G>T, 5001T>C, 5003G>C, 5004G>A, 5006T>G, 5007G>C, 5009A>G, 5012G>A, 5013G>A, 5014G>A, 5016T>C, 5019A>C, 5020G>C, 5021A>C, 5022G>A, 5023C>T, 5024T>C, 5025G>A, 5031T>A, 5033T>G, 5036T>C, 5037T>A, 5039C>T, 5042T>C, 5044T>A, 5045G>T, 5049G>C, 5051G>T, 5055C>A, 5056A>G, 5057G>T, 5058G>C, 5064A>T, 5066T>G, 5067G>A, 5070A>G, 5075A>C, 5081G>A, 5085T>G, 5086C>A, 5087A>G, 5090T>C, 5091G>A, 5092C>T, 5093C>T, 5094C>G, 5095T>G, 5097A>G, 5098C>T, 5099A>T, 5100A>T, 5102T>G, 5103T>A, 5104G>T, 5105T>G, 5108A>G, 5109A>C, 5111G>A, 5112G>T, 5115A>G, 5117A>T, 5120A>T, 5121G>A, 5122C>A, 5123T>A, 5125T>A, 5127T>A, 5129C>A, 5132T>C, 5135G>C, 5138A>T, 5141G>A, 5142G>C, 5143A>C, 5144C>T, 5147A>T, 5150T>G, 5153G>A, 5159C>T, 5160_5162delACA, 5172C>G, 5173G>A, 5174T>G, 5176A>G, 5178C>G, 5180G>C, 5181A>C, 5184T>G, 5185G>A, 5186G>A, 5191C>T, 5192A>G, 5195A>T, 5196A>G, 5198C>T, 5199A>G, 5201T>A, 5202G>T, 5203G>T, 5207T>C, 5211T>A, 5212C>G, 5213A>T, 5216A>T, 5219T>G, 5226G>T, 5228C>A, 5229C>A, 5232T>C, 5234C>A, 5235A>C, 5236C>A, 5237G>A, 5241G>T, 5242A>C, 5243T>A, 5244G>T, 5245A>C, 5249G>T, 5250T>A, 5255T>C, 5256A>C, 5258T>G, 5264C>T, 5267T>C, 5270A>T, 5273T>A, 5276A>C, 5277A>C, 5278A>G, 5279G>C, 5281A>G, 5282G>A, 5283T>A, 5285C>A, 5287A>C, 5288T>A, 5294T>G, 5300T>G, 5303A>T, 5309C>T, 5333G>A, 5339G>C, 5346A>T, 5349T>G, 5354T>A, 5357A>C, 5358A>C, 5359T>A, 5364G>C, 5365C>A, 5366C>T, 5370A>C, 5371A>G, 5372G>C, 5375T>G, 5376G>A, 5377T>G, 5378G>T, 5379T>C, 5383C>A, 5386G>T, 5387A>T, 5390A>T, 5391C>G, 5395T>G, 5396A>G, 5399A>T, 5403A>T, 5404A>C, 5409G>A, 5410T>C, 5411T>C, 5417G>T, 5423A>T, 5429A>T, 5435T>A, 5436G>A, 5438C>A, 5440G>C, 5441C>T, 5447T>C, 5456G>A, 5457C>T, 5460A>T, 5462G>T, 5465C>A, 5468A>G, 5471A>C, 5474A>T, 5475A>C, 5476G>A, 5477G>T, 5483T>C, 5484T>A, 5486T>G, 5488T>C, 5489G>C, 5490G>A, 5491A>C, 5492G>A, 5496A>C, 5498A>T, 5501A>T, 5504T>G, 5505A>C, 5507A>C, 5508T>C, 5510G>A, 5513C>A, 5516A>C, 5519T>G, 5521G>C, 5522A>T, 5523A>G, 5524A>G, 5525T>G, 5531T>G, 5534C>T, 5537C>G, 5540T>C, 5546T>C, 5555C>T, 5560T>C, 5564G>A, 5567T>C, 5570T>G, 5573T>C, 5574C>A, 6565A>C, 6567A>C, 6572T>C, 6584C>T, 6589A>C, 6621G>A, 6623G>A, 6624A>T, 6625T>G, 6626A>C, 6627T>A, 6628G>A, 6641T>G, 6643G>A, 6650A>T, 6651T>G, 6652_6653delAT, 6655G>A, 6657T>G, 6664G>T, 6668_6671delTGTT, 6674G>C, 6686T>G, 6693A>T, 6697A>T, 6698T>C, 6707C>G, 6708C>G, 6709T>C, 6713C>T, 6716T>C, 6721A>G, 6722G>T, 6726A>T, 6727A>G, 6733C>T, 6737A>G, 6738T>C, 6739T>A, 6741G>T, 6743A>T, 6745A>G, 6749G>A, 6750T>C, 6759T>C, 6760C>T, 6764T>C, 6772A>T, 6774T>G, 6775T>A								

	Begin	End	Coverage	Score	Concordance	Matches	Identities	I/D/M/F*	Stop Codons
**NT**	**22**	**6809**	**80.5%**	**3829**	**33.5%**	**5772 (99.0%)**	**3883 (66.6%)**	**10/50**	
**CDS**									
ToMarV_RNA1gp1	1	2152	85.0%	9356	73.4%	1820 (99.3%)	1276 (69.6%)	3/10/0/0	1
Protein mutations:	F7W (160T>G 161C>G), K9_A16del (165_188delAAGGTTGCTGGTGTTGATGACGCC), S17P (189A>C 190G>C), S18P (192T>C), S19A (195T>G), T21S (201A>T 203C>A), T22P (204A>C), G26A (217G>C 218T>A), L28V (222C>G), N30S (228A>T 229A>C), I32F (234A>T), K34S (240A>T 241A>C 242A>T), F35L (243T>C), A38T (252G>A 254G>T), V39L (255G>C 257T>C), I42V (264A>G), T43N (268C>A 269C>T), T44K (271C>A 272T>A), M45V (273A>G 275G>T), K48T (283A>C 284G>C), S50V (288T>G 289C>T), L53I (297C>A 299T>A), L56V (306C>G 308G>C), S59P (315T>C 317T>A), T61M (322C>T 323T>G), D62A (325A>C 326T>A), V63A (328T>C 329G>A), S64A (330T>G 332C>A), V68A (343T>C 344G>C), A69S (345G>T 347C>T), S72N (355G>A), S73A (357T>G 359A>T), L74V (360C>G 362T>A), N75E (363A>G 365T>A), K76R (367A>G), G82A (385G>C 386T>A), A83K (387G>A 388C>A), L84I (390C>A 392T>A), I85M (395A>G), D86E (398T>A), L89I (405C>A 407T>C), A91G (412C>G 413G>T), S93D (417T>G 418C>A), L95V (423T>G 425G>A), S96K (426T>A 427C>A 428C>A), S101T (441T>A), K103V (447A>G 448A>T 449G>A), M105L (453A>C 455G>T), L109M (465T>A), F110V (468T>G), E111L (471G>T 472A>T 473G>A), L113V (477T>G 479G>C), S114K (480T>A 481C>A 482C>G), N115D (483A>G), V117F (489G>T 491T>C), K118I (493A>T 494A>C), F121Y (502T>A 503T>C), A122E (505C>A 506T>A), V124L (510G>T 512G>A), N126E (516A>G 518T>A), S128V (522A>G 523G>T), D130S (528G>T 529A>C 530T>A), V131L (531G>T), V135F (543G>T 545A>T), Y136I (546T>A 547A>T 548T>A), G137A (550G>C), I143L (567A>T 569A>G), T144A (570A>G 572A>C), L145I (573T>A 575G>T), V147L (579G>C 581G>A), F150Y (589T>A), S154V (600A>G 601G>T 602T>A), E158T (612G>A 613A>C), G159P (615G>C 616G>C), V160F (618G>T), S161N (621T>A 622C>A), T163V (627A>G 628C>T 629T>A), K165V (633A>G 634A>T 635A>T), N166T (637A>C), I167L (639A>T 641C>G), L169S (646T>C 647G>A), I170V (648A>G 650A>T), M174F (660A>T 662G>T), K186T (697A>C), A188S (702G>T 704C>T), E189G (706A>G 707A>G), R190K (708C>A 709G>A 710A>G), T192S (714A>T), M193Q (717A>C 718T>A), V194L (720G>C), S198A (732A>G 733G>C 734T>A), M199V (735A>G 737G>A), V205L (753G>T 755T>A), S206T (756T>A 758A>G), M214L (780A>T 782G>A), T216S (786A>T), I217L (789A>T 791A>G), I228V (822A>G), T248A (882A>G 884A>T), T260Q (918A>C 919C>A 920T>G), I280V (978A>G 980A>G), G282S (984G>A), T283I (988C>T 989C>T), S295N (1024G>A), E304Q (1050G>C), Q307T (1059C>A 1060A>C), D310S (1068G>T 1069A>C 1070T>G), A311E (1072C>A 1073A>G), C313A (1077T>G 1078G>C), D314E (1082T>A), A316S (1086G>T 1088A>T), T317M (1090C>T), K325P (1113A>C 1114A>C 1115G>T), T329I (1126C>T 1127C>T), N332Q (1134A>C 1136C>A), R335K (1144G>A 1145G>A), E337Q (1149G>C 1151A>G), L339V (1155C>G), M354L (1200A>T), A363T (1227G>A), E366D (1238A>T), V368L (1242G>C 1244T>G), Y369V (1245T>G 1246A>T), S372A (1254A>G 1255G>C 1256T>C), C373S (1257T>A 1259T>C), R374Q (1260A>C 1261G>A 1262G>A), M376S (1266A>T 1267T>C 1268G>T), S393A (1317T>G 1319C>T), S401C (1342C>G 1343C>T), R412A (1374A>G 1375G>C), I440V (1458A>G 1460C>G), M521R (1702T>G 1703G>A), E523V (1708A>T), E525D (1715G>T), S527D (1719T>G 1720C>A 1721C>T), T528P (1722A>C 1724T>A), E529N (1725G>A 1727A>T), D530T (1728G>A 1729A>C), G533A (1738G>C 1739A>C), R536I (1747G>T 1748A>T), N538D (1752A>G 1754C>T), I539V (1755A>G 1757A>G), N552A (1794A>G 1795A>C), T554S (1800A>T), S556D (1806A>G 1807G>A), E560Q (1818G>C 1820G>A), Q567R (1840A>G), A570Y (1848G>T 1849C>A 1850A>T), K574S (1861A>G 1862G>C), F577Y (1870T>A 1871T>C), E578N (1872G>A 1874A>T), A579T (1875G>A), E580T (1878G>A 1879A>C), I583L (1887A>T 1889A>G), S584A (1890A>G 1891G>C), P585D (1893C>G 1894C>A 1895T>C), I587V (1899A>G 1901A>T), Q591N (1911C>A 1913A>T), S593A (1917T>G), A594V (1921C>T 1922A>T), D596E (1928T>A), I598L (1932A>C 1934A>G), A599D (1936C>A 1937G>T), V601F (1941G>T 1943G>C), D602Q (1944G>C 1946T>A), G603D (1948G>A 1949G>T), R605del (1953_1955delAGA), R607Q (1960G>A 1961C>A), T608V (1962A>G 1963C>T 1964C>T), K609L (1965A>T 1966A>T), Q610P (1969A>C 1970A>T), D611P (1971G>C 1972A>C), I614A (1980A>G 1981T>C), L615A (1983T>G 1984T>C 1985G>T), V616Y (1986G>T 1987T>A 1988G>T), P617E (1989C>G 1990C>A 1991C>G), T618Q (1992A>C 1993C>A 1994T>G), V619T (1995G>A 1996T>C 1997T>A), V620I (1998G>A 2000G>T), S621T (2002G>C 2003T>A), N623A (2007A>G 2008A>C), D624E (2012T>A), R625K (2014G>A), V626I (2016G>A 2018G>T), I627V (2019A>G 2021C>T), A629S (2025G>T 2027A>C), R630Q (2028A>C 2029G>A 2030A>G), L645M (2073T>A), D646N (2076G>A), P647K (2079C>A 2080C>A 2081T>A), E648F (2082G>T 2083A>T 2084G>T), L650F (2088C>T), H655L (2104A>T 2105T>C), H656S (2106C>T 2107A>C 2108T>C), M660I (2120G>C), S663V (2127T>G 2128C>T 2129T>G), D665S (2133G>T 2134A>C), D666S (2136G>T 2137A>C), E667D (2141A>T), H668A (2142C>G 2143A>C 2144T>C), E670Q (2148G>C), I671N (2152T>A), N673E (2157A>G 2159C>A), K674M (2161A>T 2162A>G), R676V (2166A>G 2167G>T), V677N (2169G>A 2170T>A 2171C>T), N678D (2172A>G 2174C>T), M679L (2175A>T), Y681V (2181T>G 2182A>T 2183T>C), R682K (2185G>A), D685S (2193G>A 2194A>G), A686P (2196G>C), E687Q (2199G>C 2201A>G), R690A (2208C>G 2209G>C 2210A>T), Q694R (2221A>G), A710S (2268G>A 2269C>G 2270A>C), S711T (2271T>A), K716E (2286A>G), K717L (2289A>C 2290A>T 2291A>G), A719D (2296C>A 2297A>T), T722R (2304A>C 2305C>G 2306A>C), E723N (2307G>A 2309G>T), M724L (2310A>C 2312G>T), R725E (2313A>G 2314G>A), T726Q (2316A>C 2317C>A 2318T>A), F728K (2322T>A 2323T>A 2324T>A), H730Y (2328C>T), S731K (2331T>A 2332C>A 2333T>G), C732A (2334T>G 2335G>C 2336C>G), I733L (2337A>C), D734Q (2340G>C 2342T>G), V737A (2350T>C 2351T>A), I741T (2362T>C 2363A>G), F749R (2385T>A 2386T>G 2387T>A), N763R (2428A>G 2429C>G), S766A (2436T>G), S766_K767insG (2438_2439insGGT), K767T (2440A>C 2441A>T), E768D (2444G>C), P769K (2445C>A 2446C>A 2447A>G), E771R (2451G>A 2452A>G), S772N (2454T>A 2455C>A 2456A>T), K775I (2464A>T 2465A>C), I776E (2466A>G 2467T>A 2468T>A), Y778M (2472T>A 2473A>T 2474C>G), H779I (2475C>A 2476A>T), K780G (2478A>G 2479A>G 2480A>T), V782K (2484G>A 2485T>A 2486T>G), E783D (2489A>C), Y784K (2490T>A 2492T>A), I785M (2495C>G), K786I (2497A>T 2498G>C), D787Q (2499G>C 2501T>G), T788M (2503C>T 2504A>G), W790N (2508T>A 2509G>A 2510G>T), S791D (2511A>G 2512G>A), Y794F (2521A>T 2522T>C), A795I (2523G>A 2524C>T 2525A>C), R797K (2530G>A 2531A>G), I806L (2556A>T 2558A>G), M807I (2561G>A), V812T (2574G>A 2575T>C 2576T>C), F813M (2577T>A 2579T>G), G814N (2580G>A 2581G>A 2582A>T), G815A (2584G>C), I816V (2586A>G), L820I (2598T>A 2600G>T), A821T (2601G>A 2603G>T), W823L (2607T>C 2608G>T 2609G>C), Q824K (2610C>A), S825A (2613A>G 2614G>C 2615T>A), N826A (2616A>G 2617A>C 2618C>A), S827T (2619T>A), F828S (2623T>C), N830L (2628A>T 2629A>T 2630T>A), S832D (2634T>G 2635C>A 2636G>T), N852K (2696T>G), K853T (2698A>C 2699G>T), K853_L854insH (2699_2700insCAT), L854F (2700C>T), R855A (2703A>G 2704G>C), N856K (2708T>A), L857S (2709C>T 2710T>C 2711T>C), V859L (2715G>C 2717T>A), S862A (2724A>G 2725G>C 2726T>G), S863A (2727T>G 2729T>A), Q864D (2730C>G 2732A>T), S865N (2733T>A 2734C>A 2735A>C), L866N (2736T>A 2737T>A 2738G>C), A867H (2739G>C 2740C>A 2741A>T), Q868V (2742C>G 2743A>T 2744A>G), D869_W870insR (2747_2748insCGG), A872E (2755C>A 2756T>A), E873Q (2757G>C 2759A>G), Q879E (2775C>G 2777G>A), K882H (2784A>C 2786G>C), L890I (2808C>A), G891A (2812G>C), M907L (2859A>C 2861G>C), H914R (2880C>A 2881A>G 2882C>A), A916M (2886G>A 2887C>T 2888T>G), T923S (2907A>T 2909A>C), A927E (2920C>A 2921C>G), N928H (2922A>C), R930I (2928C>A 2929G>T 2930A>T), S931C (2932C>G), K932N (2936A>T), Y935F (2944A>T), R938T (2953G>C 2954A>C), E939Q (2955G>C), I942L (2964A>T 2966T>G), I948V (2982A>G 2984C>G), P961S (3021C>T 3023T>A), E972D (3056G>T), F975Y (3064T>A), T976S (3066A>T 3068A>T), Q980M (3078C>A 3079A>T 3080A>G), C984V (3090T>G 3091G>T 3092T>G), T990K (3109C>A 3110A>G), R993K (3117C>A 3118G>A 3119T>G), L994K (3120C>A 3121T>A 3122T>A), G995S (3123G>T 3124G>C), F999V (3135T>G), M1002Y (3144A>T 3145T>A 3146G>T), V1012E (3175T>A 3176T>A), E1015T (3183G>A 3184A>C), E1017G (3190A>G), N1019D (3195A>G 3197C>T), S1021N (3201T>A 3202C>A 3203C>T), S1022A (3204A>G 3205G>C 3206T>A), K1024A (3210A>G 3211A>C 3212G>T), I1025L (3213A>C), C1026S (3216T>A), Q1029V (3225C>G 3226A>T 3227A>T), S1030A (3228T>G 3230T>A), G1037S (3249G>A 3251G>T), S1050T (3289G>C), V1057C (3309G>T 3310T>G 3311G>C), G1068D (3343G>A 3344C>T), V1069T (3345G>A 3346T>C 3347A>C), R1073K (3357C>A 3358G>A 3359T>G), F1075Y (3364T>A 3365T>C), I1076V (3366A>G 3368C>G), S1077A (3369T>G), I1083V (3387A>G 3389C>G), Y1084F (3391A>T 3392C>T), E1086D (3398A>T), V1096I (3426G>A 3428C>T), A1098V (3433C>T 3434A>G), D1104E (3452T>A), I1105L (3453A>C 3455T>G), S1108H (3462T>C 3463C>A 3464A>C), I1109L (3465A>C 3467T>G), R1112K (3475G>A 3476A>G), T1114E (3480A>G 3481C>A), Q1115R (3483C>A 3484A>G 3485G>A), L1116F (3486C>T 3488T>C), N1120K (3500C>A), Y1123A (3507T>G 3508A>C 3509C>T), Y1124F (3511A>T), Q1130L (3528C>T 3529A>T), L1132M (3534T>A), R1143K (3567C>A 3568G>A 3569G>A), K1144R (3570A>C 3571A>G 3572A>C), Q1146P (3577A>C 3578A>C), P1153D (3597C>G 3598C>A 3599T>C), E1155Q (3603G>C), T1162S (3624A>T 3626A>T), V1163A (3628T>C 3629T>C), H1164K (3630C>A 3632T>A), D1169E (3647T>A), M1179E (3675A>G 3676T>A), C1186S (3697G>C 3698T>C), G1187N (3699G>A 3700G>A 3701A>C), D1189E (3707T>G), D1190E (3710T>G), D1191P (3711G>C 3712A>C 3713C>A), N1199G (3735A>G 3736A>G), C1202A (3744T>G 3745G>C 3746C>A), L1204I (3750C>A 3752G>T), I1207V (3759A>G 3761C>G), M1208L (3762A>C 3764G>T), R1209K (3765C>A 3766G>A 3767T>G), S1210M (3769G>T 3770C>G), K1212R (3774A>C 3775A>G 3776G>A), I1213V (3777A>G 3779C>G), R1217K (3789C>A 3790G>A 3791T>A), C1219A (3795T>G 3796G>C 3797T>A), E1220I (3798G>A 3799A>T 3800A>C), D1222N (3804G>A), C1233F (3838G>T), Y1236F (3847A>T), G1238A (3853G>C 3854C>A), M1252L (3894A>T), M1254L (3900A>C 3902G>T), M1256S (3906A>T 3907T>C 3908G>T), Q1263E (3927C>G), S1264A (3930T>G), T1265Q (3933A>C 3934C>A 3935T>A), G1266P (3936G>C 3937G>C 3938C>A), E1267N (3939G>A 3941A>C), G1268S (3942G>T 3943G>C 3944A>G), E1269Q (3945G>C), E1272L (3954G>C 3955A>T 3956A>T), K1274I (3961A>T), D1276N (3966G>A), E1281D (3983A>T), V1288S (4002G>T 4003T>C), I1290V (4008A>G 4010T>A), K1292Q (4014A>C), I1294T (4021T>C 4022C>A), H1295F (4023C>T 4024A>T), T1344C (4170A>T 4171C>G 4172A>T), L1348A (4182T>G 4183T>C 4184G>A), A1351V (4192C>T 4193T>A), I1352L (4194A>C 4196A>C), D1359N (4215G>A), S1363H (4227T>C 4228C>A), L1367V (4239T>G), N1370S (4249A>G), G1371S (4251G>A 4253G>C), A1374S (4260G>T 4262T>A), V1378E (4273T>A), V1393I (4317G>A 4319T>C), E1405A (4354A>C 4355A>T), Y1406F (4357A>T 4358C>T), S1409D (4365T>G 4366C>A 4367A>T), K1410E (4368A>G), D1415E (4385T>G), A1421T (4401G>A), D1435N (4443G>A 4445T>C), V1437L (4449G>T), I1476V (4566A>G 4568T>C), A1477K (4569G>A 4570C>A 4571T>A), R1479K (4576G>A 4577G>A), A1480S (4578G>T), I1481V (4581A>G 4583A>G), V1483I (4587G>A), S1486N (4597G>A), L1506M (4656T>A 4658A>G), Q1509R (4665C>A 4666A>G), S1512D (4674T>G 4675C>A), N1515S (4683A>T 4684A>C 4685T>C), H1518R (4692C>A 4693A>G 4694T>A), L1522A (4704C>G 4705T>C 4706G>T), I1526V (4716A>G), N1538E (4752A>G 4754T>G), E1539K (4755G>A), V1540T (4758G>A 4759T>C), Q1541K (4761C>A), Q1543L (4768A>T 4769A>G), H1563Q (4829C>A), I1572V (4854A>G), M1576I (4868G>T), P1583V (4887C>G 4888C>T 4889A>G), T1588V (4902A>G 4903C>T 4904T>A), M1592Y (4914A>T 4915T>A 4916G>T), V1595I (4923G>A 4925G>T), R1607Q (4960G>A 4961T>G), V1608A (4963T>C 4964G>C), A1609T (4965G>A), D1611E (4973C>A), K1612S (4975A>G 4976G>T), L1613F (4977C>T), L1615N (4983T>A 4984T>A 4985A>T), Q1618F (4992C>T 4993A>T 4994G>T), T1619G (4995A>G 4996C>G 4997G>A), D1622K (5004G>A 5006T>G), E1623Q (5007G>C 5009A>G), G1625N (5013G>A 5014G>A), F1626L (5016T>C), R1627P (5019A>C 5020G>C 5021A>C), A1628I (5022G>A 5023C>T 5024T>C), V1629M (5025G>A), F1631M (5031T>A 5033T>G), S1633T (5037T>A 5039C>T), L1635Y (5044T>A 5045G>T), V1637L (5049G>C 5051G>T), Q1639S (5055C>A 5056A>G 5057G>T), E1640Q (5058G>C), I1642L (5064A>T 5066T>G), A1643T (5067G>A), K1644E (5070A>G), S1649E (5085T>G 5086C>A 5087A>G), A1651I (5091G>A 5092C>T 5093C>T), L1652G (5094C>G 5095T>G), T1653V (5097A>G 5098C>T 5099A>T), I1654L (5100A>T 5102T>G), C1655M (5103T>A 5104G>T 5105T>G), M1657L (5109A>C 5111G>A), A1658S (5112G>T), K1659D (5115A>G 5117A>T), A1661K (5121G>A 5122C>A 5123T>A), I1662N (5125T>A), S1663T (5127T>A 5129C>A), D1668P (5142G>C 5143A>C 5144C>T), T1674del (5160_5162delACA), R1678E (5172C>G 5173G>A 5174T>G), D1679G (5176A>G), L1680V (5178C>G 5180G>C), N1681H (5181A>C), W1682E (5184T>G 5185G>A 5186G>A), A1684V (5191C>T 5192A>G), N1686D (5196A>G 5198C>T), I1687V (5199A>G 5201T>A), G1688F (5202G>T 5203G>T), A1696S (5226G>T 5228C>A), H1697N (5229C>A), Y1698Q (5232T>C 5234C>A), T1699Q (5235A>C 5236C>A 5237G>A), D1701S (5241G>T 5242A>C 5243T>A), E1702S (5244G>T 5245A>C), Y1704N (5250T>A), I1706L (5256A>C 5258T>G), K1713R (5277A>C 5278A>G 5279G>C), K1714R (5281A>G 5282G>A), S1715T (5283T>A 5285C>A), N1716T (5287A>C 5288T>A), M1731I (5333G>A), M1736L (5346A>T), S1737A (5349T>G), M1740Q (5358A>C 5359T>A), A1742H (5364G>C 5365C>A 5366C>T), K1744R (5370A>C 5371A>G 5372G>C), N1745K (5375T>G), V1746S (5376G>A 5377T>G 5378G>T), A1748E (5383C>A), G1749V (5386G>T 5387A>T), H1751D (5391C>G), V1752G (5395T>G 5396A>G), N1755S (5403A>T 5404A>C), V1757T (5409G>A 5410T>C 5411T>C), V1766I (5436G>A 5438C>A), G1767A (5440G>C 5441C>T), M1774F (5460A>T 5462G>T), R1779H (5475A>C 5476G>A 5477G>T), C1782R (5484T>A 5486T>G), V1783A (5488T>C 5489G>C), E1784T (5490G>A 5491A>C 5492G>A), I1786L (5496A>C 5498A>T), I1793M (5519T>G), G1794A (5521G>C 5522A>T), N1795G (5523A>G 5524A>G 5525T>G), V1807A (5560T>C), F1810L (5570T>G), Q1812K (5574C>A), E2142A (6565A>C), T2143P (6567A>C), E2150A (6589A>C)								

	Begin	End	Coverage	Score	Concordance	Matches	Identities	I/D/M/F*	Stop Codons
**NT**	**22**	**6809**	**80.5%**	**3829**	**33.5%**	**5772 (99.0%)**	**3883 (66.6%)**	**10/50**	

Codon mutations:

GCT2GCA (146T>A), TTC7TGG (160T>G 161C>G), TCC8AGT (162T>A 163C>G 164C>T), AAG9_GCC16del (165_188delAAGGTTGCTGGTGTTGATGACGCC), AGT17CCT (189A>C 190G>C), TCT18CCT (192T>C), TCT19GCT (195T>G), TCC20TCT (200C>T), ACC21TCA (201A>T 203C>A), ACT22CCT (204A>C), GGT26GCA (217G>C 218T>A), CTT28GTT (222C>G), TCA29TCT (227A>T), AAT30TCT (228A>T 229A>C), ATT32TTT (234A>T), TCC33TCT (239C>T), AAA34TCT (240A>T 241A>C 242A>T), TTT35CTT (243T>C), AAA37AAG (251A>G), GCG38ACT (252G>A 254G>T), GTT39CTC (255G>C 257T>C), AGT40TCC (258A>T 259G>C 260T>C), ATT42GTT (264A>G), ACC43AAT (268C>A 269C>T), ACT44AAA (271C>A 272T>A), ATG45GTT (273A>G 275G>T), AAG48ACC (283A>C 284G>C), TCA50GTA (288T>G 289C>T), CAT52CAC (296T>C), CTT53ATA (297C>A 299T>A), CTG56GTC (306C>G 308G>C), AAA57AAG (311A>G), CCA58CCG (314A>G), TCT59CCA (315T>C 317T>A), GTG60GTT (320G>T), ACT61ATG (322C>T 323T>G), GAT62GCA (325A>C 326T>A), GTG63GCA (328T>C 329G>A), TCC64GCA (330T>G 332C>A), TCT66TCA (338T>A), GTG68GCC (343T>C 344G>C), GCC69TCT (345G>T 347C>T), ACT70ACA (350T>A), TGT71TGC (353T>C), AGT72AAT (355G>A), TCA73GCT (357T>G 359A>T), CTT74GTA (360C>G 362T>A), AAT75GAA (363A>G 365T>A), AAA76AGA (367A>G), TTG78TTA (374G>A), GAC79GAT (377C>T), AAA80AAG (380A>G), GGT82GCA (385G>C 386T>A), GCA83AAA (387G>A 388C>A), CTT84ATA (390C>A 392T>A), ATA85ATG (395A>G), GAT86GAA (398T>A), CCA87CCC (401A>C), TTT88TTC (404T>C), CTT89ATC (405C>A 407T>C), AAA90AAG (410A>G), GCG91GGT (412C>G 413G>T), TAC92TAT (416C>T), TCT93GAT (417T>G 418C>A), TTC94TTT (422C>T), TTG95GTA (423T>G 425G>A), TCC96AAA (426T>A 427C>A 428C>A), ACA97ACT (431A>T), TAC99TAT (437C>T), TCT101ACT (441T>A), ATC102ATA (446C>A), AAG103GTA (447A>G 448A>T 449G>A), ATG105CTT (453A>C 455G>T), GTT106GTA (458T>A), TTA107CTT (459T>C 461A>T), AAA108AAG (464A>G), TTG109ATG (465T>A), TTT110GTT (468T>G), GAG111TTA (471G>T 472A>T 473G>A), TTG113GTC (477T>G 479G>C), TCC114AAG (480T>A 481C>A 482C>G), AAT115GAT (483A>G), GTT117TTC (489G>T 491T>C), AAA118ATC (493A>T 494A>C), TTA119CTA (495T>C), TTT121TAC (502T>A 503T>C), GCT122GAA (505C>A 506T>A), GTG124TTA (510G>T 512G>A), AAT126GAA (516A>G 518T>A), AGT128GTT (522A>G 523G>T), GAA129GAG (527A>G), GAT130TCA (528G>T 529A>C 530T>A), GTA131TTA (531G>T), GTT134GTG (542T>G), GTA135TTT (543G>T 545A>T), TAT136ATA (546T>A 547A>T 548T>A), GGT137GCT (550G>C), CTT139TTA (555C>T 557T>A), GTA140GTT (560A>T), ATA143TTG (567A>T 569A>G), ACA144GCC (570A>G 572A>C), TTG145ATT (573T>A 575G>T), TTA146TTG (578A>G), GTG147CTA (579G>C 581G>A), CTT148CTA (584T>A), CTA149CTG (587A>G), TTT150TAT (589T>A), GTT151GTA (593T>A), TGT152TGC (596T>C), AGT154GTA (600A>G 601G>T 602T>A), GTT157GTA (611T>A), GAG158ACG (612G>A 613A>C), GGT159CCT (615G>C 616G>C), GTT160TTT (618G>T), TCT161AAT (621T>A 622C>A), AAC162AAT (626C>T), ACT163GTA (627A>G 628C>T 629T>A), GTC164GTT (632C>T), AAA165GTT (633A>G 634A>T 635A>T), AAT166ACT (637A>C), ATC167TTG (639A>T 641C>G), TTG169TCA (646T>C 647G>A), ATA170GTT (648A>G 650A>T), GTC171GTT (653C>T), AAC173AAT (659C>T), ATG174TTT (660A>T 662G>T), TTT175TTC (665T>C), TCA176TCT (668A>T), AGT177TCA (669A>T 670G>C 671T>A), CTA178TTG (672C>T 674A>G), TAC179TAT (677C>T), AAA186ACA (697A>C), GCC188TCT (702G>T 704C>T), GAA189GGG (706A>G 707A>G), CGA190AAG (708C>A 709G>A 710A>G), TTC191TTT (713C>T), ACC192TCC (714A>T), ATG193CAG (717A>C 718T>A), GTG194CTG (720G>C), GCC195GCG (725C>G), GCT197GCA (731T>A), AGT198GCA (732A>G 733G>C 734T>A), ATG199GTA (735A>G 737G>A), GGG202GGT (746G>T), TCA204AGC (750T>A 751C>G 752A>C), GTT205TTA (753G>T 755T>A), TCA206ACG (756T>A 758A>G), ACG210ACT (770G>T), TCT211AGC (771T>A 772C>G 773T>C), CAG212CAA (776G>A), TTG213CTT (777T>C 779G>T), ATG214TTA (780A>T 782G>A), ACA216TCA (786A>T), ATA217TTG (789A>T 791A>G), GGG221GGT (803G>T), ATT222ATC (806T>C), TCG223TCA (809G>A), ACG224ACT (812G>T), TTG225TTA (815G>A), GTG226GTA (818G>A), TTC227TTT (821C>T), ATT228GTT (822A>G), GCA229GCT (827A>T), GTG230GTT (830G>T), GGT232GGG (836T>G), GGC236GGT (848C>T), TTG237CTT (849T>C 851G>T), AGC238TCA (852A>T 853G>C 854C>A), TTG241CTG (861T>C), TCC242TCA (866C>A), ATT244ATC (872T>C), CTC245CTG (875C>G), TAT246TAC (878T>C), TCG247TCT (881G>T), ACA248GCT (882A>G 884A>T), GCT252GCG (896T>G), CAG253CAA (899G>A), CAA257CAG (911A>G), CTA258CTG (914A>G), ACT260CAG (918A>C 919C>A 920T>G), CTT261TTG (921C>T 923T>G), CGT263AGG (927C>A 929T>G), AAT264AAC (932T>C), AAA266AAG (938A>G), TGC268TGT (944C>T), ACC269ACA (947C>A), TCC270TCT (950C>T), CAG271CAA (953G>A), GCT272GCC (956T>C), GAA278GAG (974A>G), ATA280GTG (978A>G 980A>G), GTC281GTT (983C>T), GGC282AGC (984G>A), ACC283ATT (988C>T 989C>T), GGA285GGT (995A>T), TTC286TTT (998C>T), GTG290GTA (1010G>A), GCT293GCG (1019T>G), AGT295AAT (1024G>A), GCA296GCT (1028A>T), ACG297ACC (1031G>C), CTG298TTA (1032C>T 1034G>A), ACT300ACC (1040T>C), CTG302CTT (1046G>T), GAG304CAG (1050G>C), CAG307ACG (1059C>A 1060A>C), GAG308GAA (1064G>A), GAT310TCG (1068G>T 1069A>C 1070T>G), GCA311GAG (1072C>A 1073A>G), GTG312GTT (1076G>T), TGT313GCT (1077T>G 1078G>C), GAT314GAA (1082T>A), CCT315CCA (1085T>A), GCA316TCT (1086G>T 1088A>T), ACG317ATG (1090C>T), GAG320GAA (1100G>A), AAC321AAT (1103C>T), GCC323GCA (1109C>A), AAT324AAC (1112T>C), AAG325CCT (1113A>C 1114A>C 1115G>T), GCT326GCA (1118T>A), TTC327TTT (1121C>T), CCT328CCA (1124T>A), ACC329ATT (1126C>T 1127C>T), AAA330AAG (1130A>G), AAC332CAA (1134A>C 1136C>A), CTG334TTA (1140C>T 1142G>A), AGG335AAA (1144G>A 1145G>A), GAA336GAG (1148A>G), GAA337CAG (1149G>C 1151A>G), GCT338GCC (1154T>C), CTC339GTC (1155C>G), AAG340AAA (1160G>A), ATC341ATA (1163C>A), GCA346GCC (1178A>C), GTT350GTC (1190T>C), GCG351GCA (1193G>A), TTC353TTT (1199C>T), ATG354TTG (1200A>T), AGT359TCT (1215A>T 1216G>C), ATT362ATA (1226T>A), GCG363ACG (1227G>A), TTA365CTA (1233T>C), GAA366GAT (1238A>T), GTT368CTG (1242G>C 1244T>G), TAT369GTT (1245T>G 1246A>T), GAA371GAG (1253A>G), AGT372GCC (1254A>G 1255G>C 1256T>C), TGT373AGC (1257T>A 1259T>C), AGG374CAA (1260A>C 1261G>A 1262G>A), CAC375CAT (1265C>T), ATG376TCT (1266A>T 1267T>C 1268G>T), GGC377GGT (1271C>T), CAG380CAA (1280G>A), CGT382AGG (1284C>A 1286T>G), GAA384GAG (1292A>G), TTT386TTC (1298T>C), GTA388GTG (1304A>G), TTC391TTT (1313C>T), GGG392GGA (1316G>A), TCC393GCT (1317T>G 1319C>T), GGG395GGA (1325G>A), TCT399TCA (1337T>A), ACA400ACG (1340A>G), TCC401TGT (1342C>G 1343C>T), TTA404CTT (1350T>C 1352A>T), TTT405TTC (1355T>C), ATT406ATC (1358T>C), GTG409GTT (1367G>T), TTG410CTC (1368T>C 1370G>C), AGA412GCA (1374A>G 1375G>C), GAG415GAA (1385G>A), AAA417AAG (1391A>G), CTC418CTG (1394C>G), AAT419AAC (1397T>C), CGG420CGC (1400G>C), CTA421TTG (1401C>T 1403A>G), GCA423GCG (1409A>G), AAG426AAA (1418G>A), AGG427AGA (1421G>A), GCT435GCA (1445T>A), CAC436CAT (1448C>T), GCT439GCA (1457T>A), ATC440GTG (1458A>G 1460C>G), CTG441CTT (1463G>T), GAC444GAT (1472C>T), GGA446GGT (1478A>T), GCA447GCT (1481A>T), TTG448CTT (1482T>C 1484G>T), CGA449CGG (1487A>G), GGG451GGA (1493G>A), GCT452GCA (1496T>A), TGC455TGT (1505C>T), CAA456CAG (1508A>G), GAT457GAC (1511T>C), ATT458ATA (1514T>A), AAA459AAG (1517A>G), GAT463GAC (1529T>C), TCA466TCT (1538A>T), ACA467ACC (1541A>C), CAA468CAG (1544A>G), CCA469CCT (1547A>T), CCC471CCT (1553C>T), TTA472CTT (1554T>C 1556A>T), GCC475GCT (1565C>T), GTT477GTC (1571T>C), GAC479GAT (1577C>T), GGC481GGA (1583C>A), CAT483CAC (1589T>C), TTC484TTT (1592C>T), ACC485ACA (1595C>A), TCC486TCA (1598C>A), TAT488TAC (1604T>C), ATA489ATC (1607A>C), ACA492ACC (1616A>C), AAT494AAC (1622T>C), CTG495TTG (1623C>T), ATC496ATA (1628C>A), TCA497TCT (1631A>T), CCT499CCA (1637T>A), GCC500GCT (1640C>T), CAA501CAG (1643A>G), GGT503GGG (1649T>G), CTT504CTA (1652T>A), ACA505ACG (1655A>G), CCA507CCT (1661A>T), GCT509GCA (1667T>A), GAG511GAA (1673G>A), CGG512AGG (1674C>A), AGA513AGG (1679A>G), AGG514AGA (1682G>A), GAC515GAT (1685C>T), ATC516ATT (1688C>T), CTG517TTA (1689C>T 1691G>A), GTG518GTT (1694G>T), TGT520TGC (1700T>C), ATG521AGA (1702T>G 1703G>A), GAG523GTG (1708A>T), GAG525GAT (1715G>T), TCC527GAT (1719T>G 1720C>A 1721C>T), ACT528CCA (1722A>C 1724T>A), GAA529AAT (1725G>A 1727A>T), GAT530ACT (1728G>A 1729A>C), CCT531CCA (1733T>A), ACG532ACA (1736G>A), GGA533GCC (1738G>C 1739A>C), CTC535CTA (1745C>A), AGA536ATT (1747G>T 1748A>T), AAC538GAT (1752A>G 1754C>T), ATA539GTG (1755A>G 1757A>G), GAG541GAA (1763G>A), CCT545CCC (1775T>C), GCT547GCC (1781T>C), ACT549ACA (1787T>A), CAC550CAT (1790C>T), AGG551AGA (1793G>A), AAT552GCT (1794A>G 1795A>C), TTG553TTA (1799G>A), ACC554TCC (1800A>T), AGT556GAT (1806A>G 1807G>A), GAT557GAC (1811T>C), CTT558CTC (1814T>C), GAG560CAA (1818G>C 1820G>A), GTA563GTG (1829A>G), GCC564GCA (1832C>A), AAA565AAG (1835A>G), TGT566TGC (1838T>C), CAG567CGG (1840A>G), GTA568GTG (1844A>G), CAT569CAC (1847T>C), GCA570TAT (1848G>T 1849C>A 1850A>T), GAA571GAG (1853A>G), GTA572GTG (1856A>G), AAG574AGC (1861A>G 1862G>C), TTG576CTT (1866T>C 1868G>T), TTT577TAC (1870T>A 1871T>C), GAA578AAT (1872G>A 1874A>T), GCT579ACT (1875G>A), GAA580ACA (1878G>A 1879A>C), TCT581TCG (1883T>G), GGC582GGA (1886C>A), ATA583TTG (1887A>T 1889A>G), AGT584GCT (1890A>G 1891G>C), CCT585GAC (1893C>G 1894C>A 1895T>C), ATA587GTT (1899A>G 1901A>T), GTT590GTC (1910T>C), CAA591AAT (1911C>A 1913A>T), TCT593GCT (1917T>G), GCA594GTT (1921C>T 1922A>T), GAT596GAA (1928T>A), ATA598CTG (1932A>C 1934A>G), GCG599GAT (1936C>A 1937G>T), GTG601TTC (1941G>T 1943G>C), GAT602CAA (1944G>C 1946T>A), GGG603GAT (1948G>A 1949G>T), GCT604GCC (1952T>C), AGA605del (1953_1955delAGA), TTG606TTA (1958G>A), CGC607CAA (1960G>A 1961C>A), ACC608GTT (1962A>G 1963C>T 1964C>T), AAG609TTG (1965A>T 1966A>T), CAA610CCT (1969A>C 1970A>T), GAT611CCT (1971G>C 1972A>C), GAA612GAG (1976A>G), ATA614GCA (1980A>G 1981T>C), TTG615GCT (1983T>G 1984T>C 1985G>T), GTG616TAT (1986G>T 1987T>A 1988G>T), CCC617GAG (1989C>G 1990C>A 1991C>G), ACT618CAG (1992A>C 1993C>A 1994T>G), GTT619ACA (1995G>A 1996T>C 1997T>A), GTG620ATT (1998G>A 2000G>T), AGT621ACA (2002G>C 2003T>A), AAT623GCT (2007A>G 2008A>C), GAT624GAA (2012T>A), AGA625AAA (2014G>A), GTG626ATT (2016G>A 2018G>T), ATC627GTT (2019A>G 2021C>T), TAT628TAC (2024T>C), GCA629TCC (2025G>T 2027A>C), AGA630CAG (2028A>C 2029G>A 2030A>G), GAG631GAA (2033G>A), ACG633ACA (2039G>A), GTA634GTT (2042A>T), GCA636GCC (2048A>C), TTG637CTC (2049T>C 2051G>C), GCA640GCT (2060A>T), CAG642CAA (2066G>A), GGC643GGG (2069C>G), TTG645ATG (2073T>A), GAC646AAC (2076G>A), CCT647AAA (2079C>A 2080C>A 2081T>A), GAG648TTT (2082G>T 2083A>T 2084G>T), GAA649GAG (2087A>G), CTT650TTT (2088C>T), TTT651TTC (2093T>C), CCT652CCA (2096T>A), CAT655CTC (2104A>T 2105T>C), CAT656TCC (2106C>T 2107A>C 2108T>C), GCC659GCT (2117C>T), ATG660ATC (2120G>C), TCT663GTG (2127T>G 2128C>T 2129T>G), CTT664CTA (2132T>A), GAT665TCT (2133G>T 2134A>C), GAT666TCT (2136G>T 2137A>C), GAA667GAT (2141A>T), CAT668GCC (2142C>G 2143A>C 2144T>C), GAG670CAG (2148G>C), ATT671AAT (2152T>A), AAC673GAA (2157A>G 2159C>A), AAA674ATG (2161A>T 2162A>G), AGA676GTA (2166A>G 2167G>T), GTC677AAT (2169G>A 2170T>A 2171C>T), AAC678GAT (2172A>G 2174C>T), ATG679TTG (2175A>T), TAT681GTC (2181T>G 2182A>T 2183T>C), AGA682AAA (2185G>A), GAT685AGT (2193G>A 2194A>G), GCT686CCT (2196G>C), GAA687CAG (2199G>C 2201A>G), CGA690GCT (2208C>G 2209G>C 2210A>T), GTA693GTG (2219A>G), CAA694CGA (2221A>G), GAC699GAT (2237C>T), ATA701ATC (2243A>C), CAC703CAT (2249C>T), TTT704TTC (2252T>C), GCA710AGC (2268G>A 2269C>G 2270A>C), TCA711ACA (2271T>A), ATC713ATT (2279C>T), GAG715GAA (2285G>A), AAG716GAG (2286A>G), AAA717CTG (2289A>C 2290A>T 2291A>G), CTA718CTG (2294A>G), GCA719GAT (2296C>A 2297A>T), GTG720GTC (2300G>C), CAG721CAA (2303G>A), ACA722CGC (2304A>C 2305C>G 2306A>C), GAG723AAT (2307G>A 2309G>T), ATG724CTT (2310A>C 2312G>T), AGG725GAG (2313A>G 2314G>A), ACT726CAA (2316A>C 2317C>A 2318T>A), GGA727GGG (2321A>G), TTT728AAA (2322T>A 2323T>A 2324T>A), GCA729GCG (2327A>G), CAT730TAT (2328C>T), TCT731AAG (2331T>A 2332C>A 2333T>G), TGC732GCG (2334T>G 2335G>C 2336C>G), ATT733CTT (2337A>C), GAT734CAG (2340G>C 2342T>G), GCA735GCT (2345A>T), GTT737GCA (2350T>C 2351T>A), GAC738GAT (2354C>T), ACC739ACA (2357C>A), CTC740CTT (2360C>T), ATA741ACG (2362T>C 2363A>G), ATC743ATT (2369C>T), GAG744GAA (2372G>A), TTT749AGA (2385T>A 2386T>G 2387T>A), GTT750GTA (2390T>A), TTT752TTC (2396T>C), TTA753CTG (2397T>C 2399A>G), ACA755ACT (2405A>T), GCA756GCT (2408A>T), AGA759CGC (2415A>C 2417A>C), TGC762TGT (2426C>T), AAC763AGG (2428A>G 2429C>G), GTA765GTT (2435A>T), TCA766GCA (2436T>G), TCA766_AAA767insGGT (2438_2439insGGT), AAA767ACT (2440A>C 2441A>T), GAG768GAC (2444G>C), CCA769AAG (2445C>A 2446C>A 2447A>G), GAA771AGA (2451G>A 2452A>G), TCA772AAT (2454T>A 2455C>A 2456A>T), ATC774ATT (2462C>T), AAA775ATC (2464A>T 2465A>C), ATT776GAA (2466A>G 2467T>A 2468T>A), TAC778ATG (2472T>A 2473A>T 2474C>G), CAT779ATT (2475C>A 2476A>T), AAA780GGT (2478A>G 2479A>G 2480A>T), GTT782AAG (2484G>A 2485T>A 2486T>G), GAA783GAC (2489A>C), TAT784AAA (2490T>A 2492T>A), ATC785ATG (2495C>G), AAG786ATC (2497A>T 2498G>C), GAT787CAG (2499G>C 2501T>G), ACA788ATG (2503C>T 2504A>G), TGG790AAT (2508T>A 2509G>A 2510G>T), AGC791GAC (2511A>G 2512G>A), CTT792TTG (2514C>T 2516T>G), TAT794TTC (2521A>T 2522T>C), GCA795ATC (2523G>A 2524C>T 2525A>C), CTG796TTG (2526C>T), AGA797AAG (2530G>A 2531A>G), CTC799CTT (2537C>T), GCA802GCC (2546A>C), GGT803GGA (2549T>A), ATA806TTG (2556A>T 2558A>G), ATG807ATA (2561G>A), GCT808GCA (2564T>A), GGA811GGC (2573A>C), GTT812ACC (2574G>A 2575T>C 2576T>C), TTT813ATG (2577T>A 2579T>G), GGA814AAT (2580G>A 2581G>A 2582A>T), GGC815GCC (2584G>C), ATT816GTT (2586A>G), ACA817ACT (2591A>T), GCA818GCT (2594A>T), TTT819TTC (2597T>C), TTG820ATT (2598T>A 2600G>T), GCG821ACT (2601G>A 2603G>T), TGG823CTC (2607T>C 2608G>T 2609G>C), CAG824AAG (2610C>A), AGT825GCA (2613A>G 2614G>C 2615T>A), AAC826GCA (2616A>G 2617A>C 2618C>A), TCT827ACT (2619T>A), TTC828TCC (2623T>C), CCC829CCT (2627C>T), AAT830TTA (2628A>T 2629A>T 2630T>A), GCA831GCT (2633A>T), TCG832GAT (2634T>G 2635C>A 2636G>T), GGA835GGG (2645A>G), GGT836GGC (2648T>C), GTG837GTA (2651G>A), GAA840GAG (2660A>G), ATA844ATC (2672A>C), AGT846AGC (2678T>C), AAG848AAA (2684G>A), AAC849AAT (2687C>T), AAG850AAA (2690G>A), GGT851GGG (2693T>G), AAT852AAG (2696T>G), AAG853ACT (2698A>C 2699G>T), AAG853_CTC854insCAT (2699_2700insCAT), CTC854TTC (2700C>T), AGA855GCA (2703A>G 2704G>C), AAT856AAA (2708T>A), CTT857TCC (2709C>T 2710T>C 2711T>C), CTC858TTG (2712C>T 2714C>G), GTT859CTA (2715G>C 2717T>A), GGT860GGG (2720T>G), AGT862GCG (2724A>G 2725G>C 2726T>G), TCT863GCA (2727T>G 2729T>A), CAA864GAT (2730C>G 2732A>T), TCA865AAC (2733T>A 2734C>A 2735A>C), TTG866AAC (2736T>A 2737T>A 2738G>C), GCA867CAT (2739G>C 2740C>A 2741A>T), CAA868GTG (2742C>G 2743A>T 2744A>G), GAT869GAC (2747T>C), GAT869_TGG870insCGG (2747_2748insCGG), GCT871GCA (2753T>A), GCT872GAA (2755C>A 2756T>A), GAA873CAG (2757G>C 2759A>G), GTA877GTT (2771A>T), CAG879GAA (2775C>G 2777G>A), AGC880AGT (2780C>T), CTC881CTG (2783C>G), AAG882CAC (2784A>C 2786G>C), AAT884AAC (2792T>C), GTG886GTT (2798G>T), GTG887GTT (2801G>T), TTA888TTG (2804A>G), CTT890ATT (2808C>A), GGT891GCT (2812G>C), GTG894GTC (2822G>C), TAC895TAT (2825C>T), TTT896TTC (2828T>C), GGC898GGT (2834C>T), ACC899ACG (2837C>G), TAC900TAT (2840C>T), GTG901GTT (2843G>T), ATA906ATT (2858A>T), ATG907CTC (2859A>C 2861G>C), ACC908ACT (2864C>T), GTA909GTG (2867A>G), GCT910GCA (2870T>A), GCA912GCC (2876A>C), TTC913TTT (2879C>T), CAC914AGA (2880C>A 2881A>G 2882C>A), AAT915AAC (2885T>C), GCT916ATG (2886G>A 2887C>T 2888T>G), CGA917AGG (2889C>A 2891A>G), GGT919GGC (2897T>C), CCA921CCT (2903A>T), ACA923TCC (2907A>T 2909A>C), ATC925ATT (2915C>T), GCC927GAG (2920C>A 2921C>G), AAT928CAT (2922A>C), TCT929TCC (2927T>C), CGA930ATT (2928C>A 2929G>T 2930A>T), TCT931TGT (2932C>G), AAA932AAT (2936A>T), GTT933GTG (2939T>G), CAA934CAG (2942A>G), TAC935TTC (2944A>T), AAC936AAT (2948C>T), AGA938ACC (2953G>C 2954A>C), GAA939CAA (2955G>C), ATT942TTG (2964A>T 2966T>G), ATT943ATA (2969T>A), GGC945GGT (2975C>T), GAC947GAT (2981C>T), ATC948GTG (2982A>G 2984C>G), CTG950TTG (2988C>T), CGC952AGG (2994C>A 2996C>G), GTT953GTA (2999T>A), GGT954GGG (3002T>G), CCA959CCC (3017A>C), AAG960AAA (3020G>A), CCT961TCA (3021C>T 3023T>A), GAC962GAT (3026C>T), ATC963ATA (3029C>A), CGT964AGA (3030C>A 3032T>A), CAC966CAT (3038C>T), CCA968CCT (3044A>T), AGA969CGC (3045A>C 3047A>C), AGG970AGA (3050G>A),

GAG972GAT (3056G>T), TGC974TGT (3062C>T), TTC975TAC (3064T>A), ACA976TCT (3066A>T 3068A>T), CAA980ATG (3078C>A 3079A>T 3080A>G), GGA981GGC (3083A>C), TGT984GTG (3090T>G 3091G>T 3092T>G), AGA985AGG (3095A>G), GTT987GTA (3101T>A), GCG988GCT (3104G>T), TCT989TCA (3107T>A), ACA990AAG (3109C>A 3110A>G), CCA992CCC (3116A>C), CGT993AAG (3117C>A 3118G>A 3119T>G), CTT994AAA (3120C>A 3121T>A 3122T>A), GGC995TCC (3123G>T 3124G>C), TTA997TTG (3131A>G), TTT999GTT (3135T>G), CTC1000CTT (3140C>T), AAG1001AAA (3143G>A), ATG1002TAT (3144A>T 3145T>A 3146G>T), GGA1009GGC (3167A>C), ACA1011ACC (3173A>C), GTT1012GAA (3175T>A 3176T>A), GTG1014GTC (3182G>C), GAA1015ACA (3183G>A 3184A>C), GAA1017GGA (3190A>G), CTG1018TTA (3192C>T 3194G>A), AAC1019GAT (3195A>G 3197C>T), TCC1020TCG (3200C>G), TCC1021AAT (3201T>A 3202C>A 3203C>T), AGT1022GCA (3204A>G 3205G>C 3206T>A), AAG1024GCT (3210A>G 3211A>C 3212G>T), ATT1025CTT (3213A>C), TGC1026AGC (3216T>A), TCT1027TCA (3221T>A), CAA1029GTT (3225C>G 3226A>T 3227A>T), TCT1030GCA (3228T>G 3230T>A), GAA1032GAG (3236A>G), ATA1035ATT (3245A>T), GGG1037AGT (3249G>A 3251G>T), GAA1038GAG (3254A>G), GGT1040GGA (3260T>A), GAC1041GAT (3263C>T), GGC1043GGT (3269C>T), ACG1044ACT (3272G>T), TTG1045CTT (3273T>C 3275G>T), CTA1046CTC (3278A>C), CTG1047TTA (3279C>T 3281G>A), TTA1048TTG (3284A>G), AGT1050ACT (3289G>C), GTT1051GTG (3293T>G), AAT1053AAC (3299T>C), CAA1055CAG (3305A>G), CCT1056CCG (3308T>G), GTG1057TGC (3309G>T 3310T>G 3311G>C), GGC1060GGA (3320C>A), ATC1061ATA (3323C>A), CAC1062CAT (3326C>T), TCA1065TCT (3335A>T), GGC1068GAT (3343G>A 3344C>T), GTA1069ACC (3345G>A 3346T>C 3347A>C), GCA1070GCT (3350A>T), GCT1071GCA (3353T>A), GAA1072GAG (3356A>G), CGT1073AAG (3357C>A 3358G>A 3359T>G), GGA1074GGT (3362A>T), TTT1075TAC (3364T>A 3365T>C), ATC1076GTG (3366A>G 3368C>G), TCT1077GCT (3369T>G), AAT1079AAC (3377T>C), GCT1080GCA (3380T>A), GCT1082GCA (3386T>A), ATC1083GTG (3387A>G 3389C>G), TAC1084TTT (3391A>T 3392C>T), AGG1085AGA (3395G>A), GAA1086GAT (3398A>T), CTA1088TTG (3402C>T 3404A>G), GAG1089GAA (3407G>A), CCA1092CCC (3416A>C), ACT1093ACG (3419T>G), GGG1094GGA (3422G>A), CCG1095CCC (3425G>C), GTC1096ATT (3426G>A 3428C>T), GCA1098GTG (3433C>T 3434A>G), GCA1099GCT (3437A>T), GTA1101GTG (3443A>G), GAT1104GAA (3452T>A), ATT1105CTG (3453A>C 3455T>G), CTG1106TTG (3456C>T), TCA1108CAC (3462T>C 3463C>A 3464A>C), ATT1109CTG (3465A>C 3467T>G), AGC1111TCC (3471A>T 3472G>C), AGA1112AAG (3475G>A 3476A>G), GAA1113GAG (3479A>G), ACA1114GAA (3480A>G 3481C>A), CAG1115AGA (3483C>A 3484A>G 3485G>A), CTT1116TTC (3486C>T 3488T>C), GAA1119GAG (3497A>G), AAC1120AAA (3500C>A), CAA1121CAG (3503A>G), TAC1123GCT (3507T>G 3508A>C 3509C>T), TAC1124TTC (3511A>T), CTT1125TTG (3513C>T 3515T>G), GGA1126GGC (3518A>C), ACA1127ACT (3521A>T), GTT1128GTA (3524T>A), CCA1129CCG (3527A>G), CAG1130TTG (3528C>T 3529A>T), GAG1131GAA (3533G>A), TTG1132ATG (3534T>A), GCC1133GCT (3539C>T), GCC1134GCT (3542C>T), GTT1136GTG (3548T>G), CCC1137CCG (3551C>G), AAG1139AAA (3557G>A), ACC1140ACA (3560C>A), ACT1141ACC (3563T>C), CTG1142CTT (3566G>T), CGG1143AAA (3567C>A 3568G>A 3569G>A), AAA1144CGC (3570A>C 3571A>G 3572A>C), AGC1145AGT (3575C>T), CAA1146CCC (3577A>C 3578A>C), TTG1147CTG (3579T>C), GAA1149GAG (3587A>G), GCA1150GCT (3590A>T), TTC1151TTT (3593C>T), GGA1152GGG (3596A>G), CCT1153GAC (3597C>G 3598C>A 3599T>C), GCA1154GCT (3602A>T), GAG1155CAG (3603G>C), ACA1156ACT (3608A>T), GCA1157GCC (3611A>C), ACA1162TCT (3624A>T 3626A>T), GTT1163GCC (3628T>C 3629T>C), CAT1164AAA (3630C>A 3632T>A), AGA1167AGG (3641A>G), GGT1168GGA (3644T>A), GAT1169GAA (3647T>A), GGT1170GGG (3650T>G), GAC1172GAT (3656C>T), GGG1177GGT (3671G>T), GTA1178GTC (3674A>C), ATG1179GAG (3675A>G 3676T>A), AAA1180AAG (3680A>G), TAC1181TAT (3683C>T), TGT1186TCC (3697G>C 3698T>C), GGA1187AAC (3699G>A 3700G>A 3701A>C), GAT1189GAG (3707T>G), GAT1190GAG (3710T>G), GAC1191CCA (3711G>C 3712A>C 3713C>A), GCC1193GCA (3719C>A), AAA1194AAG (3722A>G), CTA1195CTG (3725A>G), GCA1196GCT (3728A>T), TTC1197TTT (3731C>T), GAA1198GAG (3734A>G), AAT1199GGT (3735A>G 3736A>G), CTC1200CTT (3740C>T), AAG1201AAA (3743G>A), TGC1202GCA (3744T>G 3745G>C 3746C>A), TCG1203TCC (3749G>C), CTG1204ATT (3750C>A 3752G>T), CTA1205CTT (3755A>T), ATC1207GTG (3759A>G 3761C>G), ATG1208CTT (3762A>C 3764G>T), CGT1209AAG (3765C>A 3766G>A 3767T>G), AGC1210ATG (3769G>T 3770C>G), AAG1212CGA (3774A>C 3775A>G 3776G>A), ATC1213GTG (3777A>G 3779C>G), CCT1214CCC (3782T>C), GGA1216GGG (3788A>G), CGT1217AAA (3789C>A 3790G>A 3791T>A), CCA1218CCT (3794A>T), TGT1219GCA (3795T>G 3796G>C 3797T>A), GAA1220ATC (3798G>A 3799A>T 3800A>C), AGG1221AGA (3803G>A), GAT1222AAT (3804G>A), GGA1229GGG (3827A>G), TGT1233TTT (3838G>T), TAC1235TAT (3845C>T), TAT1236TTT (3847A>T), GGC1238GCA (3853G>C 3854C>A), GAG1240GAA (3860G>A), CTG1241TTG (3861C>T), ACA1243ACG (3869A>G), TCT1244TCA (3872T>A), TGC1245TGT (3875C>T), GGA1246GGT (3878A>T), CCC1248CCT (3884C>T), TTC1249TTT (3887C>T), ATG1252TTG (3894A>T), GGG1253GGA (3899G>A), ATG1254CTT (3900A>C 3902G>T), GGG1255GGT (3905G>T), ATG1256TCT (3906A>T 3907T>C 3908G>T), AAG1258AAA (3914G>A), AGA1259AGG (3917A>G), CAA1263GAA (3927C>G), TCC1264GCC (3930T>G), ACT1265CAA (3933A>C 3934C>A 3935T>A), GGC1266CCA (3936G>C 3937G>C 3938C>A), GAA1267AAC (3939G>A 3941A>C), GGA1268TCG (3942G>T 3943G>C 3944A>G), GAG1269CAG (3945G>C), AGA1270AGG (3950A>G), GTG1271GTT (3953G>T), GAA1272CTT (3954G>C 3955A>T 3956A>T), AAA1274ATA (3961A>T), AGA1275AGG (3965A>G), GAC1276AAC (3966G>A), ACT1277ACA (3971T>A), GTA1279GTC (3977A>C), GAA1281GAT (3983A>T), CTA1286TTG (3996C>T 3998A>G), GAT1287GAC (4001T>C), GTG1288TCG (4002G>T 4003T>C), ATT1290GTA (4008A>G 4010T>A), AAA1292CAA (4014A>C), ATC1294ACA (4021T>C 4022C>A), CAT1295TTT (4023C>T 4024A>T), GTG1296GTT (4028G>T), GAT1297GAC (4031T>C), CTG1298CTC (4034G>C), ACC1300ACA (4040C>A), CAA1302CAG (4046A>G), TGC1303TGT (4049C>T), GCC1304GCG (4052C>G), CGC1308CGG (4064C>G), CTC1309TTG (4065C>T 4067C>G), AAA1313AAG (4079A>G), ATC1314ATT (4082C>T), GGC1316GGG (4088C>G), AGA1318AGG (4094A>G), AGG1321CGC (4101A>C 4103G>C), CTC1322CTG (4106C>G), TTT1323TTC (4109T>C), GAG1324GAA (4112G>A), ATA1325ATC (4115A>C), CTT1326TTG (4116C>T 4118T>G), TAC1330TAT (4130C>T), TTG1333CTG (4137T>C), GTC1334GTG (4142C>G), AGG1335CGG (4143A>C), AAG1336AAA (4148G>A), TAT1337TAC (4151T>C), TTT1338TTC (4154T>C), GAT1340GAC (4160T>C), TTC1341TTT (4163C>T), TCA1342TCT (4166A>T), GCC1343GCT (4169C>T), ACA1344TGT (4170A>T 4171C>G 4172A>T), TTG1348GCA (4182T>G 4183T>C 4184G>A), AAT1350AAC (4190T>C), GCT1351GTA (4192C>T 4193T>A), ATA1352CTC (4194A>C 4196A>C), CCA1353CCT (4199A>T), AAA1355AAG (4205A>G), GTT1356GTA (4208T>A), ATT1358ATC (4214T>C), GAT1359AAT (4215G>A), CCT1360CCA (4220T>A), ACA1361ACG (4223A>G), AGT1362AGC (4226T>C), TCT1363CAT (4227T>C 4228C>A), TTG1367GTG (4239T>G), TTG1368CTG (4242T>C), GCA1369GCT (4247A>T), AAT1370AGT (4249A>G), GGG1371AGC (4251G>A 4253G>C), AGA1373AGG (4259A>G), GCT1374TCA (4260G>T 4262T>A), GTG1375GTA (4265G>A), TCA1376TCT (4268A>T), GAC1377GAT (4271C>T), GTG1378GAG (4273T>A), TTT1380TTC (4280T>C), GCT1382GCC (4286T>C), TAT1384TAC (4292T>C), TCC1385TCG (4295C>G), CCT1392CCC (4316T>C), GTT1393ATC (4317G>A 4319T>C), GCT1395GCA (4325T>A), TTT1396TTC (4328T>C), CAG1397CAA (4331G>A), TTG1402CTG (4344T>C), GAA1405GCT (4354A>C 4355A>T), TAC1406TTT (4357A>T 4358C>T), TAC1407TAT (4361C>T), GGA1408GGT (4364A>T), TCA1409GAT (4365T>G 4366C>A 4367A>T), AAG1410GAG (4368A>G), GGC1412GGG (4376C>G), GAT1415GAG (4385T>G), TCC1416TCA (4388C>A), CGA1419AGG (4395C>A 4397A>G), CAT1420CAC (4400T>C), GCA1421ACA (4401G>A), CTT1422TTA (4404C>T 4406T>A), GCA1426GCC (4418A>C), TGT1428TGC (4424T>C), TAC1430TAT (4430C>T), ACA1431ACC (4433A>C), CTG1432CTC (4436G>C), TGC1433TGT (4439C>T), GAT1435AAC (4443G>A 4445T>C), AAA1436AAG (4448A>G), GTG1437TTG (4449G>T), AGG1439CGC (4455A>C 4457G>C), TTG1440CTA (4458T>C 4460G>A), GTT1441GTG (4463T>G), GGG1442GGT (4466G>T), GGC1443GGA (4469C>A), CCA1445CCC (4475A>C), TCA1446TCT (4478A>T), GGA1447GGT (4481A>T), GCA1449GCT (4487A>T), CTA1450TTG (4488C>T 4490A>G), ACG1451ACT (4493G>T), ATC1453ATA (4499C>A), TTC1454TTT (4502C>T), TCT1456TCA (4508T>A), CTC1457TTG (4509C>T 4511C>G), CTC1458CTA (4514C>A), CGA1464AGG (4530C>A 4532A>G), TAT1465TAC (4535T>C), GCC1466GCG (4538C>G), TTG1470CTG (4548T>C), TTA1471TTG (4553A>G), AGA1472AGG (4556A>G), CCC1474CCA (4562C>A), ATT1476GTC (4566A>G 4568T>C), GCT1477AAA (4569G>A 4570C>A 4571T>A), AGG1479AAA (4576G>A 4577G>A), GCT1480TCT (4578G>T), ATA1481GTG (4581A>G 4583A>G), GGA1482GGC (4586A>C), GTT1483ATT (4587G>A), AAA1484AAG (4592A>G), AGT1486AAT (4597G>A), GAT1487GAC (4601T>C), CTA1491TTG (4611C>T 4613A>G), GCT1494GCA (4622T>A), GTT1495GTA (4625T>A), GGA1497GGT (4631A>T), GAC1499GAT (4637C>T), AAT1500AAC (4640T>C), CTT1501TTG (4641C>T 4643T>G), GCT1503GCG (4649T>G), GTA1504GTT (4652A>T), TTA1506ATG (4656T>A 4658A>G), CTC1508TTG (4662C>T 4664C>G), CAG1509AGG (4665C>A 4666A>G), TAT1511TAC (4673T>C), TCT1512GAT (4674T>G 4675C>A), CTG1513CTA (4679G>A), AAT1515TCC (4683A>T 4684A>C 4685T>C), ATA1516ATT (4688A>T), CAT1518AGA (4692C>A 4693A>G 4694T>A), GAG1519GAA (4697G>A), TTA1520CTG (4698T>C 4700A>G), CTG1522GCT (4704C>G 4705T>C 4706G>T), GTC1523GTT (4709C>T), AAT1524AAC (4712T>C), GTA1525GTC (4715A>C), ATC1526GTC (4716A>G), CTT1531TTG (4731C>T 4733T>G), AAA1533AAG (4739A>G), TCG1534TCA (4742G>A), GTT1537GTA (4751T>A), AAT1538GAG (4752A>G 4754T>G), GAG1539AAG (4755G>A), GTA1540ACA (4758G>A 4759T>C), CAA1541AAA (4761C>A), CAA1543CTG (4768A>T 4769A>G), CTA1548CTC (4784A>C), ACT1549ACC (4787T>C), CTG1551CTC (4793G>C), AGA1553AGG (4799A>G), TTT1555TTC (4805T>C), AAA1556AAG (4808A>G), GCT1559GCA (4817T>A), CTT1560TTG (4818C>T 4820T>G), TAC1562TAT (4826C>T), CAC1563CAA (4829C>A), GCT1565GCA (4835T>A), CCT1566CCA (4838T>A), AAG1568AAA (4844G>A), ATC1572GTC (4854A>G), ATT1573ATC (4859T>C), ATG1576ATT (4868G>T), ATA1579ATT (4877A>T), AGA1580AGG (4880A>G), CCT1581CCA (4883T>A), GCC1582GCA (4886C>A), CCA1583GTG (4887C>G 4888C>T 4889A>G), GGT1584GGA (4892T>A), GAC1587GAT (4901C>T), ACT1588GTA (4902A>G 4903C>T 4904T>A), CAG1589CAA (4907G>A), GCC1590GCT (4910C>T), ATG1592TAT (4914A>T 4915T>A 4916G>T), GTG1595ATT (4923G>A 4925G>T), GGA1598GGC (4934A>C), ATA1599ATC (4937A>C), CGT1600AGA (4938C>A 4940T>A), GCT1602GCC (4946T>C), CAC1605CAT (4955C>T), GGT1606GGG (4958T>G), CGT1607CAG (4960G>A 4961T>G), GTG1608GCC (4963T>C 4964G>C), GCT1609ACT (4965G>A), GAC1611GAA (4973C>A), AAG1612AGT (4975A>G 4976G>T), CTT1613TTT (4977C>T), GTC1614GTA (4982C>A), TTA1615AAT (4983T>A 4984T>A 4985A>T), CAG1618TTT (4992C>T 4993A>T 4994G>T), ACG1619GGA (4995A>G 4996C>G 4997G>A), GCG1620GCT (5000G>T), TTG1621CTC (5001T>C 5003G>C), GAT1622AAG (5004G>A 5006T>G), GAA1623CAG (5007G>C 5009A>G), AGG1624AGA (5012G>A), GGT1625AAT (5013G>A 5014G>A), TTC1626CTC (5016T>C), AGA1627CCC (5019A>C 5020G>C 5021A>C), GCT1628ATC (5022G>A 5023C>T 5024T>C), GTG1629ATG (5025G>A), TTT1631ATG (5031T>A 5033T>G), CCT1632CCC (5036T>C), TCC1633ACT (5037T>A 5039C>T), TAT1634TAC (5042T>C), TTG1635TAT (5044T>A 5045G>T), GTG1637CTT (5049G>C 5051G>T), CAG1639AGT (5055C>A 5056A>G 5057G>T), GAA1640CAA (5058G>C), ATT1642TTG (5064A>T 5066T>G), GCA1643ACA (5067G>A), AAG1644GAG (5070A>G), GTA1645GTC (5075A>C), GGG1647GGA (5081G>A), TCA1649GAG (5085T>G 5086C>A 5087A>G), AGT1650AGC (5090T>C), GCC1651ATT (5091G>A 5092C>T 5093C>T), CTG1652GGG (5094C>G 5095T>G), ACA1653GTT (5097A>G 5098C>T 5099A>T), ATT1654TTG (5100A>T 5102T>G), TGT1655ATG (5103T>A 5104G>T 5105T>G), GAA1656GAG (5108A>G), ATG1657CTA (5109A>C 5111G>A), GCA1658TCA (5112G>T), AAA1659GAT (5115A>G 5117A>T), GCA1660GCT (5120A>T), GCT1661AAA (5121G>A 5122C>A 5123T>A), ATT1662AAT (5125T>A), TCC1663ACA (5127T>A 5129C>A), TAT1664TAC (5132T>C), ACG1665ACC (5135G>C), CCA1666CCT (5138A>T), TTG1667TTA (5141G>A), GAC1668CCT (5142G>C 5143A>C 5144C>T), GCA1669GCT (5147A>T), GGT1670GGG (5150T>G), GAG1671GAA (5153G>A), ATC1673ATT (5159C>T), ACA1674del (5160_5162delACA), CGT1678GAG (5172C>G 5173G>A 5174T>G), GAT1679GGT (5176A>G), CTG1680GTC (5178C>G 5180G>C), AAT1681CAT (5181A>C), TGG1682GAA (5184T>G 5185G>A 5186G>A), GCA1684GTG (5191C>T 5192A>G), CCA1685CCT (5195A>T), AAC1686GAT (5196A>G 5198C>T), ATT1687GTA (5199A>G 5201T>A), GGT1688TTT (5202G>T 5203G>T), TTT1689TTC (5207T>C), TCA1691AGT (5211T>A 5212C>G 5213A>T), GCA1692GCT (5216A>T), CGT1693CGG (5219T>G), GCC1696TCA (5226G>T 5228C>A), CAC1697AAC (5229C>A), TAC1698CAA (5232T>C 5234C>A), ACG1699CAA (5235A>C 5236C>A 5237G>A), GAT1701TCA (5241G>T 5242A>C 5243T>A), GAA1702TCA (5244G>T 5245A>C), GGG1703GGT (5249G>T), TAC1704AAC (5250T>A), ATT1705ATC (5255T>C), ATT1706CTG (5256A>C 5258T>G), AAC1708AAT (5264C>T), TGT1709TGC (5267T>C), ACA1710ACT (5270A>T), GGT1711GGA (5273T>A), GCA1712GCC (5276A>C), AAG1713CGC (5277A>C 5278A>G 5279G>C), AAG1714AGA (5281A>G 5282G>A), TCC1715ACA (5283T>A 5285C>A), AAT1716ACA (5287A>C 5288T>A), GTT1718GTG (5294T>G), GGT1720GGG (5300T>G), CCA1721CCT (5303A>T), AAC1723AAT (5309C>T), ATG1731ATA (5333G>A), CCG1733CCC (5339G>C), ATG1736TTG (5346A>T), TCA1737GCA (5349T>G), GCT1738GCA (5354T>A), ATA1739ATC (5357A>C), ATG1740CAG (5358A>C 5359T>A), GCC1742CAT (5364G>C 5365C>A 5366C>T), AAG1744CGC (5370A>C 5371A>G 5372G>C), AAT1745AAG (5375T>G), GTG1746AGT (5376G>A 5377T>G 5378G>T), TTG1747CTG (5379T>C), GCA1748GAA (5383C>A), GGA1749GTT (5386G>T 5387A>T), GGA1750GGT (5390A>T), CAT1751GAT (5391C>G), GTA1752GGG (5395T>G 5396A>G), GCA1753GCT (5399A>T), AAT1755TCT (5403A>T 5404A>C), GTT1757ACC (5409G>A 5410T>C 5411T>C), GTG1759GTT (5417G>T), GGA1761GGT (5423A>T), GGA1763GGT (5429A>T), GCT1765GCA (5435T>A), GTC1766ATA (5436G>A 5438C>A), GGC1767GCT (5440G>C 5441C>T), ATT1769ATC (5447T>C), GCG1772GCA (5456G>A), CTG1773TTG (5457C>T), ATG1774TTT (5460A>T 5462G>T), GCC1775GCA (5465C>A), TTA1776TTG (5468A>G), GCA1777GCC (5471A>C), ACA1778ACT (5474A>T), AGG1779CAT (5475A>C 5476G>A 5477G>T), TAT1781TAC (5483T>C), TGT1782AGG (5484T>A 5486T>G), GTG1783GCC (5488T>C 5489G>C), GAG1784ACA (5490G>A 5491A>C 5492G>A), ATA1786CTT (5496A>C 5498A>T), ATA1787ATT (5501A>T), GTT1788GTG (5504T>G), AGA1789CGC (5505A>C 5507A>C), TTG1790CTA (5508T>C 5510G>A), CGC1791CGA (5513C>A), ACA1792ACC (5516A>C), ATT1793ATG (5519T>G), GGA1794GCT (5521G>C 5522A>T), AAT1795GGG (5523A>G 5524A>G 5525T>G), CTT1797CTG (5531T>G), GAC1798GAT (5534C>T), CTC1799CTG (5537C>G), AAT1800AAC (5540T>C), TAT1802TAC (5546T>C), GGC1805GGT (5555C>T), GTG1807GCG (5560T>C), CAG1808CAA (5564G>A), TAT1809TAC (5567T>C), TTT1810TTG (5570T>G), CTT1811CTC (5573T>C), CAA1812AAA (5574C>A), GAA2142GCA (6565A>C), ACC2143CCC (6567A>C), ATT2144ATC (6572T>C), TAC2148TAT (6584C>T), GAG2150GCG (6589A>C)

Proteins

polyprotein (YP_001976147.1)	1	2152	85.0%	9356	73.4%	1820 (99.3%)	1276 (69.6%)	3/10/0/0	1

	Begin	End	Coverage	Score	Concordance	Matches	Identities	I/D/M/F*	Stop Codons
**NT**	**22**	**6809**	**80.5%**	**3829**	**33.5%**	**5772 (99.0%)**	**3883 (66.6%)**	**10/50**	

Protein mutations:

F7W (160T>G 161C>G), K9_A16del (165_188delAAGGTTGCTGGTGTTGATGACGCC), S17P (189A>C 190G>C), S18P (192T>C), S19A (195T>G), T21S (201A>T 203C>A), T22P (204A>C), G26A (217G>C 218T>A), L28V (222C>G), N30S (228A>T 229A>C), I32F (234A>T), K34S (240A>T 241A>C 242A>T), F35L (243T>C), A38T (252G>A 254G>T), V39L (255G>C 257T>C), I42V (264A>G), T43N (268C>A 269C>T), T44K (271C>A 272T>A), M45V (273A>G 275G>T), K48T (283A>C 284G>C), S50V (288T>G 289C>T), L53I (297C>A 299T>A), L56V (306C>G 308G>C), S59P (315T>C 317T>A), T61M (322C>T 323T>G), D62A (325A>C 326T>A), V63A (328T>C 329G>A), S64A (330T>G 332C>A), V68A (343T>C 344G>C), A69S (345G>T 347C>T), S72N (355G>A), S73A (357T>G 359A>T), L74V (360C>G 362T>A), N75E (363A>G 365T>A), K76R (367A>G), G82A (385G>C 386T>A), A83K (387G>A 388C>A), L84I (390C>A 392T>A), I85M (395A>G), D86E (398T>A), L89I (405C>A 407T>C), A91G (412C>G 413G>T), S93D (417T>G 418C>A), L95V (423T>G 425G>A), S96K (426T>A 427C>A 428C>A), S101T (441T>A), K103V (447A>G 448A>T 449G>A), M105L (453A>C 455G>T), L109M (465T>A), F110V (468T>G), E111L (471G>T 472A>T 473G>A), L113V (477T>G 479G>C), S114K (480T>A 481C>A 482C>G), N115D (483A>G), V117F (489G>T 491T>C), K118I (493A>T 494A>C), F121Y (502T>A 503T>C), A122E (505C>A 506T>A), V124L (510G>T 512G>A), N126E (516A>G 518T>A), S128V (522A>G 523G>T), D130S (528G>T 529A>C 530T>A), V131L (531G>T), V135F (543G>T 545A>T), Y136I (546T>A 547A>T 548T>A), G137A (550G>C), I143L (567A>T 569A>G), T144A (570A>G 572A>C), L145I (573T>A 575G>T), V147L (579G>C 581G>A), F150Y (589T>A), S154V (600A>G 601G>T 602T>A), E158T (612G>A 613A>C), G159P (615G>C 616G>C), V160F (618G>T), S161N (621T>A 622C>A), T163V (627A>G 628C>T 629T>A), K165V (633A>G 634A>T 635A>T), N166T (637A>C), I167L (639A>T 641C>G), L169S (646T>C 647G>A), I170V (648A>G 650A>T), M174F (660A>T 662G>T), K186T (697A>C), A188S (702G>T 704C>T), E189G (706A>G 707A>G), R190K (708C>A 709G>A 710A>G), T192S (714A>T), M193Q (717A>C 718T>A), V194L (720G>C), S198A (732A>G 733G>C 734T>A), M199V (735A>G 737G>A), V205L (753G>T 755T>A), S206T (756T>A 758A>G), M214L (780A>T 782G>A), T216S (786A>T), I217L (789A>T 791A>G), I228V (822A>G), T248A (882A>G 884A>T), T260Q (918A>C 919C>A 920T>G), I280V (978A>G 980A>G), G282S (984G>A), T283I (988C>T 989C>T), S295N (1024G>A), E304Q (1050G>C), Q307T (1059C>A 1060A>C), D310S (1068G>T 1069A>C 1070T>G), A311E (1072C>A 1073A>G), C313A (1077T>G 1078G>C), D314E (1082T>A), A316S (1086G>T 1088A>T), T317M (1090C>T), K325P (1113A>C 1114A>C 1115G>T), T329I (1126C>T 1127C>T), N332Q (1134A>C 1136C>A), R335K (1144G>A 1145G>A), E337Q (1149G>C 1151A>G), L339V (1155C>G), M354L (1200A>T), A363T (1227G>A), E366D (1238A>T), V368L (1242G>C 1244T>G), Y369V (1245T>G 1246A>T), S372A (1254A>G 1255G>C 1256T>C), C373S (1257T>A 1259T>C), R374Q (1260A>C 1261G>A 1262G>A), M376S (1266A>T 1267T>C 1268G>T), S393A (1317T>G 1319C>T), S401C (1342C>G 1343C>T), R412A (1374A>G 1375G>C), I440V (1458A>G 1460C>G), M521R (1702T>G 1703G>A), E523V (1708A>T), E525D (1715G>T), S527D (1719T>G 1720C>A 1721C>T), T528P (1722A>C 1724T>A), E529N (1725G>A 1727A>T), D530T (1728G>A 1729A>C), G533A (1738G>C 1739A>C), R536I (1747G>T 1748A>T), N538D (1752A>G 1754C>T), I539V (1755A>G 1757A>G), N552A (1794A>G 1795A>C), T554S (1800A>T), S556D (1806A>G 1807G>A), E560Q (1818G>C 1820G>A), Q567R (1840A>G), A570Y (1848G>T 1849C>A 1850A>T), K574S (1861A>G 1862G>C), F577Y (1870T>A 1871T>C), E578N (1872G>A 1874A>T), A579T (1875G>A), E580T (1878G>A 1879A>C), I583L (1887A>T 1889A>G), S584A (1890A>G 1891G>C), P585D (1893C>G 1894C>A 1895T>C), I587V (1899A>G 1901A>T), Q591N (1911C>A 1913A>T), S593A (1917T>G), A594V (1921C>T 1922A>T), D596E (1928T>A), I598L (1932A>C 1934A>G), A599D (1936C>A 1937G>T), V601F (1941G>T 1943G>C), D602Q (1944G>C 1946T>A), G603D (1948G>A 1949G>T), R605del (1953_1955delAGA), R607Q (1960G>A 1961C>A), T608V (1962A>G 1963C>T 1964C>T), K609L (1965A>T 1966A>T), Q610P (1969A>C 1970A>T), D611P (1971G>C 1972A>C), I614A (1980A>G 1981T>C), L615A (1983T>G 1984T>C 1985G>T), V616Y (1986G>T 1987T>A 1988G>T), P617E (1989C>G 1990C>A 1991C>G), T618Q (1992A>C 1993C>A 1994T>G), V619T (1995G>A 1996T>C 1997T>A), V620I (1998G>A 2000G>T), S621T (2002G>C 2003T>A), N623A (2007A>G 2008A>C), D624E (2012T>A), R625K (2014G>A), V626I (2016G>A 2018G>T), I627V (2019A>G 2021C>T), A629S (2025G>T 2027A>C), R630Q (2028A>C 2029G>A 2030A>G), L645M (2073T>A), D646N (2076G>A), P647K (2079C>A 2080C>A 2081T>A), E648F (2082G>T 2083A>T 2084G>T), L650F (2088C>T), H655L (2104A>T 2105T>C), H656S (2106C>T 2107A>C 2108T>C), M660I (2120G>C), S663V (2127T>G 2128C>T 2129T>G), D665S (2133G>T 2134A>C), D666S (2136G>T 2137A>C), E667D (2141A>T), H668A (2142C>G 2143A>C 2144T>C), E670Q (2148G>C), I671N (2152T>A), N673E (2157A>G 2159C>A), K674M (2161A>T 2162A>G), R676V (2166A>G 2167G>T), V677N (2169G>A 2170T>A 2171C>T), N678D (2172A>G 2174C>T), M679L (2175A>T), Y681V (2181T>G 2182A>T 2183T>C), R682K (2185G>A), D685S (2193G>A 2194A>G), A686P (2196G>C), E687Q (2199G>C 2201A>G), R690A (2208C>G 2209G>C 2210A>T), Q694R (2221A>G), A710S (2268G>A 2269C>G 2270A>C), S711T (2271T>A), K716E (2286A>G), K717L (2289A>C 2290A>T 2291A>G), A719D (2296C>A 2297A>T), T722R (2304A>C 2305C>G 2306A>C), E723N (2307G>A 2309G>T), M724L (2310A>C 2312G>T), R725E (2313A>G 2314G>A), T726Q (2316A>C 2317C>A 2318T>A), F728K (2322T>A 2323T>A 2324T>A), H730Y (2328C>T), S731K (2331T>A 2332C>A 2333T>G), C732A (2334T>G 2335G>C 2336C>G), I733L (2337A>C), D734Q (2340G>C 2342T>G), V737A (2350T>C 2351T>A), I741T (2362T>C 2363A>G), F749R (2385T>A 2386T>G 2387T>A), N763R (2428A>G 2429C>G), S766A (2436T>G), S766_K767insG (2438_2439insGGT), K767T (2440A>C 2441A>T), E768D (2444G>C), P769K (2445C>A 2446C>A 2447A>G), E771R (2451G>A 2452A>G), S772N (2454T>A 2455C>A 2456A>T), K775I (2464A>T 2465A>C), I776E (2466A>G 2467T>A 2468T>A), Y778M (2472T>A 2473A>T 2474C>G), H779I (2475C>A 2476A>T), K780G (2478A>G 2479A>G 2480A>T), V782K (2484G>A 2485T>A 2486T>G), E783D (2489A>C), Y784K (2490T>A 2492T>A), I785M (2495C>G), K786I (2497A>T 2498G>C), D787Q (2499G>C 2501T>G), T788M (2503C>T 2504A>G), W790N (2508T>A 2509G>A 2510G>T), S791D (2511A>G 2512G>A), Y794F (2521A>T 2522T>C), A795I (2523G>A 2524C>T 2525A>C), R797K (2530G>A 2531A>G), I806L (2556A>T 2558A>G), M807I (2561G>A), V812T (2574G>A 2575T>C 2576T>C), F813M (2577T>A 2579T>G), G814N (2580G>A 2581G>A 2582A>T), G815A (2584G>C), I816V (2586A>G), L820I (2598T>A 2600G>T), A821T (2601G>A 2603G>T), W823L (2607T>C 2608G>T 2609G>C), Q824K (2610C>A), S825A (2613A>G 2614G>C 2615T>A), N826A (2616A>G 2617A>C 2618C>A), S827T (2619T>A), F828S (2623T>C), N830L (2628A>T 2629A>T 2630T>A), S832D (2634T>G 2635C>A 2636G>T), N852K (2696T>G), K853T (2698A>C 2699G>T), K853_L854insH (2699_2700insCAT), L854F (2700C>T), R855A (2703A>G 2704G>C), N856K (2708T>A), L857S (2709C>T 2710T>C 2711T>C), V859L (2715G>C 2717T>A), S862A (2724A>G 2725G>C 2726T>G), S863A (2727T>G 2729T>A), Q864D (2730C>G 2732A>T), S865N (2733T>A 2734C>A 2735A>C), L866N (2736T>A 2737T>A 2738G>C), A867H (2739G>C 2740C>A 2741A>T), Q868V (2742C>G 2743A>T 2744A>G), D869_W870insR (2747_2748insCGG), A872E (2755C>A 2756T>A), E873Q (2757G>C 2759A>G), Q879E (2775C>G 2777G>A), K882H (2784A>C 2786G>C), L890I (2808C>A), G891A (2812G>C), M907L (2859A>C 2861G>C), H914R (2880C>A 2881A>G 2882C>A), A916M (2886G>A 2887C>T 2888T>G), T923S (2907A>T 2909A>C), A927E (2920C>A 2921C>G), N928H (2922A>C), R930I (2928C>A 2929G>T 2930A>T), S931C (2932C>G), K932N (2936A>T), Y935F (2944A>T), R938T (2953G>C 2954A>C), E939Q (2955G>C), I942L (2964A>T 2966T>G), I948V (2982A>G 2984C>G), P961S (3021C>T 3023T>A), E972D (3056G>T), F975Y (3064T>A), T976S (3066A>T 3068A>T), Q980M (3078C>A 3079A>T 3080A>G), C984V (3090T>G 3091G>T 3092T>G), T990K (3109C>A 3110A>G), R993K (3117C>A 3118G>A 3119T>G), L994K (3120C>A 3121T>A 3122T>A), G995S (3123G>T 3124G>C), F999V (3135T>G), M1002Y (3144A>T 3145T>A 3146G>T), V1012E (3175T>A 3176T>A), E1015T (3183G>A 3184A>C), E1017G (3190A>G), N1019D (3195A>G 3197C>T), S1021N (3201T>A 3202C>A 3203C>T), S1022A (3204A>G 3205G>C 3206T>A), K1024A (3210A>G 3211A>C 3212G>T), I1025L (3213A>C), C1026S (3216T>A), Q1029V (3225C>G 3226A>T 3227A>T), S1030A (3228T>G 3230T>A), G1037S (3249G>A 3251G>T), S1050T (3289G>C), V1057C (3309G>T 3310T>G 3311G>C), G1068D (3343G>A 3344C>T), V1069T (3345G>A 3346T>C 3347A>C), R1073K (3357C>A 3358G>A 3359T>G), F1075Y (3364T>A 3365T>C), I1076V (3366A>G 3368C>G), S1077A (3369T>G), I1083V (3387A>G 3389C>G), Y1084F (3391A>T 3392C>T), E1086D (3398A>T), V1096I (3426G>A 3428C>T), A1098V (3433C>T 3434A>G), D1104E (3452T>A), I1105L (3453A>C 3455T>G), S1108H (3462T>C 3463C>A 3464A>C), I1109L (3465A>C 3467T>G), R1112K (3475G>A 3476A>G), T1114E (3480A>G 3481C>A), Q1115R (3483C>A 3484A>G 3485G>A), L1116F (3486C>T 3488T>C), N1120K (3500C>A), Y1123A (3507T>G 3508A>C 3509C>T), Y1124F (3511A>T), Q1130L (3528C>T 3529A>T), L1132M (3534T>A), R1143K (3567C>A 3568G>A 3569G>A), K1144R (3570A>C 3571A>G 3572A>C), Q1146P (3577A>C 3578A>C), P1153D (3597C>G 3598C>A 3599T>C), E1155Q (3603G>C), T1162S (3624A>T 3626A>T), V1163A (3628T>C 3629T>C), H1164K (3630C>A 3632T>A), D1169E (3647T>A), M1179E (3675A>G 3676T>A), C1186S (3697G>C 3698T>C), G1187N (3699G>A 3700G>A 3701A>C), D1189E (3707T>G), D1190E (3710T>G), D1191P (3711G>C 3712A>C 3713C>A), N1199G (3735A>G 3736A>G), C1202A (3744T>G 3745G>C 3746C>A), L1204I (3750C>A 3752G>T), I1207V (3759A>G 3761C>G), M1208L (3762A>C 3764G>T), R1209K (3765C>A 3766G>A 3767T>G), S1210M (3769G>T 3770C>G), K1212R (3774A>C 3775A>G 3776G>A), I1213V (3777A>G 3779C>G), R1217K (3789C>A 3790G>A 3791T>A), C1219A (3795T>G 3796G>C 3797T>A), E1220I (3798G>A 3799A>T 3800A>C), D1222N (3804G>A), C1233F (3838G>T), Y1236F (3847A>T), G1238A (3853G>C 3854C>A), M1252L (3894A>T), M1254L (3900A>C 3902G>T), M1256S (3906A>T 3907T>C 3908G>T), Q1263E (3927C>G), S1264A (3930T>G), T1265Q (3933A>C 3934C>A 3935T>A), G1266P (3936G>C 3937G>C 3938C>A), E1267N (3939G>A 3941A>C), G1268S (3942G>T 3943G>C 3944A>G), E1269Q (3945G>C), E1272L (3954G>C 3955A>T 3956A>T), K1274I (3961A>T), D1276N (3966G>A), E1281D (3983A>T), V1288S (4002G>T 4003T>C), I1290V (4008A>G 4010T>A), K1292Q (4014A>C), I1294T (4021T>C 4022C>A), H1295F (4023C>T 4024A>T), T1344C (4170A>T 4171C>G 4172A>T), L1348A (4182T>G 4183T>C 4184G>A), A1351V (4192C>T 4193T>A), I1352L (4194A>C 4196A>C), D1359N (4215G>A), S1363H (4227T>C 4228C>A), L1367V (4239T>G), N1370S (4249A>G), G1371S (4251G>A 4253G>C), A1374S (4260G>T 4262T>A), V1378E (4273T>A), V1393I (4317G>A 4319T>C), E1405A (4354A>C 4355A>T), Y1406F (4357A>T 4358C>T), S1409D (4365T>G 4366C>A 4367A>T), K1410E (4368A>G), D1415E (4385T>G), A1421T (4401G>A), D1435N (4443G>A 4445T>C), V1437L (4449G>T), I1476V (4566A>G 4568T>C), A1477K (4569G>A 4570C>A 4571T>A), R1479K (4576G>A 4577G>A), A1480S (4578G>T), I1481V (4581A>G 4583A>G), V1483I (4587G>A), S1486N (4597G>A), L1506M (4656T>A 4658A>G), Q1509R (4665C>A 4666A>G), S1512D (4674T>G 4675C>A), N1515S (4683A>T 4684A>C 4685T>C), H1518R (4692C>A 4693A>G 4694T>A), L1522A (4704C>G 4705T>C 4706G>T), I1526V (4716A>G), N1538E (4752A>G 4754T>G), E1539K (4755G>A), V1540T (4758G>A 4759T>C), Q1541K (4761C>A), Q1543L (4768A>T 4769A>G), H1563Q (4829C>A), I1572V (4854A>G), M1576I (4868G>T), P1583V (4887C>G 4888C>T 4889A>G), T1588V (4902A>G 4903C>T 4904T>A), M1592Y (4914A>T 4915T>A 4916G>T), V1595I (4923G>A 4925G>T), R1607Q (4960G>A 4961T>G), V1608A (4963T>C 4964G>C), A1609T (4965G>A), D1611E (4973C>A), K1612S (4975A>G 4976G>T), L1613F (4977C>T), L1615N (4983T>A 4984T>A 4985A>T), Q1618F (4992C>T 4993A>T 4994G>T), T1619G (4995A>G 4996C>G 4997G>A), D1622K (5004G>A 5006T>G), E1623Q (5007G>C 5009A>G), G1625N (5013G>A 5014G>A), F1626L (5016T>C), R1627P (5019A>C 5020G>C 5021A>C), A1628I (5022G>A 5023C>T 5024T>C), V1629M (5025G>A), F1631M (5031T>A 5033T>G), S1633T (5037T>A 5039C>T), L1635Y (5044T>A 5045G>T), V1637L (5049G>C 5051G>T), Q1639S (5055C>A 5056A>G 5057G>T), E1640Q (5058G>C), I1642L (5064A>T 5066T>G), A1643T (5067G>A), K1644E (5070A>G), S1649E (5085T>G 5086C>A 5087A>G), A1651I (5091G>A 5092C>T 5093C>T), L1652G (5094C>G 5095T>G), T1653V (5097A>G 5098C>T 5099A>T), I1654L (5100A>T 5102T>G), C1655M (5103T>A 5104G>T 5105T>G), M1657L (5109A>C 5111G>A), A1658S (5112G>T), K1659D (5115A>G 5117A>T), A1661K (5121G>A 5122C>A 5123T>A), I1662N (5125T>A), S1663T (5127T>A 5129C>A), D1668P (5142G>C 5143A>C 5144C>T), T1674del (5160_5162delACA), R1678E (5172C>G 5173G>A 5174T>G), D1679G (5176A>G), L1680V (5178C>G 5180G>C), N1681H (5181A>C), W1682E (5184T>G 5185G>A 5186G>A), A1684V (5191C>T 5192A>G), N1686D (5196A>G 5198C>T), I1687V (5199A>G 5201T>A), G1688F (5202G>T 5203G>T), A1696S (5226G>T 5228C>A), H1697N (5229C>A), Y1698Q (5232T>C 5234C>A), T1699Q (5235A>C 5236C>A 5237G>A), D1701S (5241G>T 5242A>C 5243T>A), E1702S (5244G>T 5245A>C), Y1704N (5250T>A), I1706L (5256A>C 5258T>G), K1713R (5277A>C 5278A>G 5279G>C), K1714R (5281A>G 5282G>A), S1715T (5283T>A 5285C>A), N1716T (5287A>C 5288T>A), M1731I (5333G>A), M1736L (5346A>T), S1737A (5349T>G), M1740Q (5358A>C 5359T>A), A1742H (5364G>C 5365C>A 5366C>T), K1744R (5370A>C 5371A>G 5372G>C), N1745K (5375T>G), V1746S (5376G>A 5377T>G 5378G>T), A1748E (5383C>A), G1749V (5386G>T 5387A>T), H1751D (5391C>G), V1752G (5395T>G 5396A>G), N1755S (5403A>T 5404A>C), V1757T (5409G>A 5410T>C 5411T>C), V1766I (5436G>A 5438C>A), G1767A (5440G>C 5441C>T), M1774F (5460A>T 5462G>T), R1779H (5475A>C 5476G>A 5477G>T), C1782R (5484T>A 5486T>G), V1783A (5488T>C 5489G>C), E1784T (5490G>A 5491A>C 5492G>A), I1786L (5496A>C 5498A>T), I1793M (5519T>G), G1794A (5521G>C 5522A>T), N1795G (5523A>G 5524A>G 5525T>G), V1807A (5560T>C), F1810L (5570T>G), Q1812K (5574C>A), E2142A (6565A>C), T2143P (6567A>C), E2150A (6589A>C)

	Begin	End	Coverage	Score	Concordance	Matches	Identities	I/D/M/F*	Stop Codons
NT	22	6809	80.5%	3829	33.5%	5772 (99.0%)	3883 (66.6%)	10/50	

Codon mutations:

GCT2GCA (146T>A), TTC7TGG (160T>G 161C>G), TCC8AGT (162T>A 163C>G 164C>T), AAG9_GCC16del (165_188delAAGGTTGCTGGTGTTGATGACGCC), AGT17CCT (189A>C 190G>C), TCT18CCT (192T>C), TCT19GCT (195T>G), TCC20TCT (200C>T), ACC21TCA (201A>T 203C>A), ACT22CCT (204A>C), GGT26GCA (217G>C 218T>A), CTT28GTT (222C>G), TCA29TCT (227A>T), AAT30TCT (228A>T 229A>C), ATT32TTT (234A>T), TCC33TCT (239C>T), AAA34TCT (240A>T 241A>C 242A>T), TTT35CTT (243T>C), AAA37AAG (251A>G), GCG38ACT (252G>A 254G>T), GTT39CTC (255G>C 257T>C), AGT40TCC (258A>T 259G>C 260T>C), ATT42GTT (264A>G), ACC43AAT (268C>A 269C>T), ACT44AAA (271C>A 272T>A), ATG45GTT (273A>G 275G>T), AAG48ACC (283A>C 284G>C), TCA50GTA (288T>G 289C>T), CAT52CAC (296T>C), CTT53ATA (297C>A 299T>A), CTG56GTC (306C>G 308G>C), AAA57AAG (311A>G), CCA58CCG (314A>G), TCT59CCA (315T>C 317T>A), GTG60GTT (320G>T), ACT61ATG (322C>T 323T>G), GAT62GCA (325A>C 326T>A), GTG63GCA (328T>C 329G>A), TCC64GCA (330T>G 332C>A), TCT66TCA (338T>A), GTG68GCC (343T>C 344G>C), GCC69TCT (345G>T 347C>T), ACT70ACA (350T>A), TGT71TGC (353T>C), AGT72AAT (355G>A), TCA73GCT (357T>G 359A>T), CTT74GTA (360C>G 362T>A), AAT75GAA (363A>G 365T>A), AAA76AGA (367A>G), TTG78TTA (374G>A), GAC79GAT (377C>T), AAA80AAG (380A>G), GGT82GCA (385G>C 386T>A), GCA83AAA (387G>A 388C>A), CTT84ATA (390C>A 392T>A), ATA85ATG (395A>G), GAT86GAA (398T>A), CCA87CCC (401A>C), TTT88TTC (404T>C), CTT89ATC (405C>A 407T>C), AAA90AAG (410A>G), GCG91GGT (412C>G 413G>T), TAC92TAT (416C>T), TCT93GAT (417T>G 418C>A), TTC94TTT (422C>T), TTG95GTA (423T>G 425G>A), TCC96AAA (426T>A 427C>A 428C>A), ACA97ACT (431A>T), TAC99TAT (437C>T), TCT101ACT (441T>A), ATC102ATA (446C>A), AAG103GTA (447A>G 448A>T 449G>A), ATG105CTT (453A>C 455G>T), GTT106GTA (458T>A), TTA107CTT (459T>C 461A>T), AAA108AAG (464A>G), TTG109ATG (465T>A), TTT110GTT (468T>G), GAG111TTA (471G>T 472A>T 473G>A), TTG113GTC (477T>G 479G>C), TCC114AAG (480T>A 481C>A 482C>G), AAT115GAT (483A>G), GTT117TTC (489G>T 491T>C), AAA118ATC (493A>T 494A>C), TTA119CTA (495T>C), TTT121TAC (502T>A 503T>C), GCT122GAA (505C>A 506T>A), GTG124TTA (510G>T 512G>A), AAT126GAA (516A>G 518T>A), AGT128GTT (522A>G 523G>T), GAA129GAG (527A>G), GAT130TCA (528G>T 529A>C 530T>A), GTA131TTA (531G>T), GTT134GTG (542T>G), GTA135TTT (543G>T 545A>T), TAT136ATA (546T>A 547A>T 548T>A), GGT137GCT (550G>C), CTT139TTA (555C>T 557T>A), GTA140GTT (560A>T), ATA143TTG (567A>T 569A>G), ACA144GCC (570A>G 572A>C), TTG145ATT (573T>A 575G>T), TTA146TTG (578A>G), GTG147CTA (579G>C 581G>A), CTT148CTA (584T>A), CTA149CTG (587A>G), TTT150TAT (589T>A), GTT151GTA (593T>A), TGT152TGC (596T>C), AGT154GTA (600A>G 601G>T 602T>A), GTT157GTA (611T>A), GAG158ACG (612G>A 613A>C), GGT159CCT (615G>C 616G>C), GTT160TTT (618G>T), TCT161AAT (621T>A 622C>A), AAC162AAT (626C>T), ACT163GTA (627A>G 628C>T 629T>A), GTC164GTT (632C>T), AAA165GTT (633A>G 634A>T 635A>T), AAT166ACT (637A>C), ATC167TTG (639A>T 641C>G), TTG169TCA (646T>C 647G>A), ATA170GTT (648A>G 650A>T), GTC171GTT (653C>T), AAC173AAT (659C>T), ATG174TTT (660A>T 662G>T), TTT175TTC (665T>C), TCA176TCT (668A>T), AGT177TCA (669A>T 670G>C 671T>A), CTA178TTG (672C>T 674A>G), TAC179TAT (677C>T), AAA186ACA (697A>C), GCC188TCT (702G>T 704C>T), GAA189GGG (706A>G 707A>G), CGA190AAG (708C>A 709G>A 710A>G), TTC191TTT (713C>T), ACC192TCC (714A>T), ATG193CAG (717A>C 718T>A), GTG194CTG (720G>C), GCC195GCG (725C>G), GCT197GCA (731T>A), AGT198GCA (732A>G 733G>C 734T>A), ATG199GTA (735A>G 737G>A), GGG202GGT (746G>T), TCA204AGC (750T>A 751C>G 752A>C), GTT205TTA (753G>T 755T>A), TCA206ACG (756T>A 758A>G), ACG210ACT (770G>T), TCT211AGC (771T>A 772C>G 773T>C), CAG212CAA (776G>A), TTG213CTT (777T>C 779G>T), ATG214TTA (780A>T 782G>A), ACA216TCA (786A>T), ATA217TTG (789A>T 791A>G), GGG221GGT (803G>T), ATT222ATC (806T>C), TCG223TCA (809G>A), ACG224ACT (812G>T), TTG225TTA (815G>A), GTG226GTA (818G>A), TTC227TTT (821C>T), ATT228GTT (822A>G), GCA229GCT (827A>T), GTG230GTT (830G>T), GGT232GGG (836T>G), GGC236GGT (848C>T), TTG237CTT (849T>C 851G>T), AGC238TCA (852A>T 853G>C 854C>A), TTG241CTG (861T>C), TCC242TCA (866C>A), ATT244ATC (872T>C), CTC245CTG (875C>G), TAT246TAC (878T>C), TCG247TCT (881G>T), ACA248GCT (882A>G 884A>T), GCT252GCG (896T>G), CAG253CAA (899G>A), CAA257CAG (911A>G), CTA258CTG (914A>G), ACT260CAG (918A>C 919C>A 920T>G), CTT261TTG (921C>T 923T>G), CGT263AGG (927C>A 929T>G), AAT264AAC (932T>C), AAA266AAG (938A>G), TGC268TGT (944C>T), ACC269ACA (947C>A), TCC270TCT (950C>T), CAG271CAA (953G>A), GCT272GCC (956T>C), GAA278GAG (974A>G), ATA280GTG (978A>G 980A>G), GTC281GTT (983C>T), GGC282AGC (984G>A), ACC283ATT (988C>T 989C>T), GGA285GGT (995A>T), TTC286TTT (998C>T), GTG290GTA (1010G>A), GCT293GCG (1019T>G), AGT295AAT (1024G>A), GCA296GCT (1028A>T), ACG297ACC (1031G>C), CTG298TTA (1032C>T 1034G>A), ACT300ACC (1040T>C), CTG302CTT (1046G>T), GAG304CAG (1050G>C), CAG307ACG (1059C>A 1060A>C), GAG308GAA (1064G>A), GAT310TCG (1068G>T 1069A>C 1070T>G), GCA311GAG (1072C>A 1073A>G), GTG312GTT (1076G>T), TGT313GCT (1077T>G 1078G>C), GAT314GAA (1082T>A), CCT315CCA (1085T>A), GCA316TCT (1086G>T 1088A>T), ACG317ATG (1090C>T), GAG320GAA (1100G>A), AAC321AAT (1103C>T), GCC323GCA (1109C>A), AAT324AAC (1112T>C), AAG325CCT (1113A>C 1114A>C 1115G>T), GCT326GCA (1118T>A), TTC327TTT (1121C>T), CCT328CCA (1124T>A), ACC329ATT (1126C>T 1127C>T), AAA330AAG (1130A>G), AAC332CAA (1134A>C 1136C>A), CTG334TTA (1140C>T 1142G>A), AGG335AAA (1144G>A 1145G>A), GAA336GAG (1148A>G), GAA337CAG (1149G>C 1151A>G), GCT338GCC (1154T>C), CTC339GTC (1155C>G), AAG340AAA (1160G>A), ATC341ATA (1163C>A), GCA346GCC (1178A>C), GTT350GTC (1190T>C), GCG351GCA (1193G>A), TTC353TTT (1199C>T), ATG354TTG (1200A>T), AGT359TCT (1215A>T 1216G>C), ATT362ATA (1226T>A), GCG363ACG (1227G>A), TTA365CTA (1233T>C), GAA366GAT (1238A>T), GTT368CTG (1242G>C 1244T>G), TAT369GTT (1245T>G 1246A>T), GAA371GAG (1253A>G), AGT372GCC (1254A>G 1255G>C 1256T>C), TGT373AGC (1257T>A 1259T>C), AGG374CAA (1260A>C 1261G>A 1262G>A), CAC375CAT (1265C>T), ATG376TCT (1266A>T 1267T>C 1268G>T), GGC377GGT (1271C>T), CAG380CAA (1280G>A), CGT382AGG (1284C>A 1286T>G), GAA384GAG (1292A>G), TTT386TTC (1298T>C), GTA388GTG (1304A>G), TTC391TTT (1313C>T), GGG392GGA (1316G>A), TCC393GCT (1317T>G 1319C>T), GGG395GGA (1325G>A), TCT399TCA (1337T>A), ACA400ACG (1340A>G), TCC401TGT (1342C>G 1343C>T), TTA404CTT (1350T>C 1352A>T), TTT405TTC (1355T>C), ATT406ATC (1358T>C), GTG409GTT (1367G>T), TTG410CTC (1368T>C 1370G>C), AGA412GCA (1374A>G 1375G>C), GAG415GAA (1385G>A), AAA417AAG (1391A>G), CTC418CTG (1394C>G), AAT419AAC (1397T>C), CGG420CGC (1400G>C), CTA421TTG (1401C>T 1403A>G), GCA423GCG (1409A>G), AAG426AAA (1418G>A), AGG427AGA (1421G>A), GCT435GCA (1445T>A), CAC436CAT (1448C>T), GCT439GCA (1457T>A), ATC440GTG (1458A>G 1460C>G), CTG441CTT (1463G>T), GAC444GAT (1472C>T), GGA446GGT (1478A>T), GCA447GCT (1481A>T), TTG448CTT (1482T>C 1484G>T), CGA449CGG (1487A>G), GGG451GGA (1493G>A), GCT452GCA (1496T>A), TGC455TGT (1505C>T), CAA456CAG (1508A>G), GAT457GAC (1511T>C), ATT458ATA (1514T>A), AAA459AAG (1517A>G), GAT463GAC (1529T>C), TCA466TCT (1538A>T), ACA467ACC (1541A>C), CAA468CAG (1544A>G), CCA469CCT (1547A>T), CCC471CCT (1553C>T), TTA472CTT (1554T>C 1556A>T), GCC475GCT (1565C>T), GTT477GTC (1571T>C), GAC479GAT (1577C>T), GGC481GGA (1583C>A), CAT483CAC (1589T>C), TTC484TTT (1592C>T), ACC485ACA (1595C>A), TCC486TCA (1598C>A), TAT488TAC (1604T>C), ATA489ATC (1607A>C), ACA492ACC (1616A>C), AAT494AAC (1622T>C), CTG495TTG (1623C>T), ATC496ATA (1628C>A), TCA497TCT (1631A>T), CCT499CCA (1637T>A), GCC500GCT (1640C>T), CAA501CAG (1643A>G), GGT503GGG (1649T>G), CTT504CTA (1652T>A), ACA505ACG (1655A>G), CCA507CCT (1661A>T), GCT509GCA (1667T>A), GAG511GAA (1673G>A), CGG512AGG (1674C>A), AGA513AGG (1679A>G), AGG514AGA (1682G>A), GAC515GAT (1685C>T), ATC516ATT (1688C>T), CTG517TTA (1689C>T 1691G>A), GTG518GTT (1694G>T), TGT520TGC (1700T>C), ATG521AGA (1702T>G 1703G>A), GAG523GTG (1708A>T), GAG525GAT (1715G>T), TCC527GAT (1719T>G 1720C>A 1721C>T), ACT528CCA (1722A>C 1724T>A), GAA529AAT (1725G>A 1727A>T), GAT530ACT (1728G>A 1729A>C), CCT531CCA (1733T>A), ACG532ACA (1736G>A), GGA533GCC (1738G>C 1739A>C), CTC535CTA (1745C>A), AGA536ATT (1747G>T 1748A>T), AAC538GAT (1752A>G 1754C>T), ATA539GTG (1755A>G 1757A>G), GAG541GAA (1763G>A), CCT545CCC (1775T>C), GCT547GCC (1781T>C), ACT549ACA (1787T>A), CAC550CAT (1790C>T), AGG551AGA (1793G>A), AAT552GCT (1794A>G 1795A>C), TTG553TTA (1799G>A), ACC554TCC (1800A>T), AGT556GAT (1806A>G 1807G>A), GAT557GAC (1811T>C), CTT558CTC (1814T>C), GAG560CAA (1818G>C 1820G>A), GTA563GTG (1829A>G), GCC564GCA (1832C>A), AAA565AAG (1835A>G), TGT566TGC (1838T>C), CAG567CGG (1840A>G), GTA568GTG (1844A>G), CAT569CAC (1847T>C), GCA570TAT (1848G>T 1849C>A 1850A>T), GAA571GAG (1853A>G), GTA572GTG (1856A>G), AAG574AGC (1861A>G 1862G>C), TTG576CTT (1866T>C 1868G>T), TTT577TAC (1870T>A 1871T>C), GAA578AAT (1872G>A 1874A>T), GCT579ACT (1875G>A), GAA580ACA (1878G>A 1879A>C), TCT581TCG (1883T>G), GGC582GGA (1886C>A), ATA583TTG (1887A>T 1889A>G), AGT584GCT (1890A>G 1891G>C), CCT585GAC (1893C>G 1894C>A 1895T>C), ATA587GTT (1899A>G 1901A>T), GTT590GTC (1910T>C), CAA591AAT (1911C>A 1913A>T), TCT593GCT (1917T>G), GCA594GTT (1921C>T 1922A>T), GAT596GAA (1928T>A), ATA598CTG (1932A>C 1934A>G), GCG599GAT (1936C>A 1937G>T), GTG601TTC (1941G>T 1943G>C), GAT602CAA (1944G>C 1946T>A), GGG603GAT (1948G>A 1949G>T), GCT604GCC (1952T>C), AGA605del (1953_1955delAGA), TTG606TTA (1958G>A), CGC607CAA (1960G>A 1961C>A), ACC608GTT (1962A>G 1963C>T 1964C>T), AAG609TTG (1965A>T 1966A>T), CAA610CCT (1969A>C 1970A>T), GAT611CCT (1971G>C 1972A>C), GAA612GAG (1976A>G), ATA614GCA (1980A>G 1981T>C), TTG615GCT (1983T>G 1984T>C 1985G>T), GTG616TAT (1986G>T 1987T>A 1988G>T), CCC617GAG (1989C>G 1990C>A 1991C>G), ACT618CAG (1992A>C 1993C>A 1994T>G), GTT619ACA (1995G>A 1996T>C 1997T>A), GTG620ATT (1998G>A 2000G>T), AGT621ACA (2002G>C 2003T>A), AAT623GCT (2007A>G 2008A>C), GAT624GAA (2012T>A), AGA625AAA (2014G>A), GTG626ATT (2016G>A 2018G>T), ATC627GTT (2019A>G 2021C>T), TAT628TAC (2024T>C), GCA629TCC (2025G>T 2027A>C), AGA630CAG (2028A>C 2029G>A 2030A>G), GAG631GAA (2033G>A), ACG633ACA (2039G>A), GTA634GTT (2042A>T), GCA636GCC (2048A>C), TTG637CTC (2049T>C 2051G>C), GCA640GCT (2060A>T), CAG642CAA (2066G>A), GGC643GGG (2069C>G), TTG645ATG (2073T>A), GAC646AAC (2076G>A), CCT647AAA (2079C>A 2080C>A 2081T>A), GAG648TTT (2082G>T 2083A>T 2084G>T), GAA649GAG (2087A>G), CTT650TTT (2088C>T), TTT651TTC (2093T>C), CCT652CCA (2096T>A), CAT655CTC (2104A>T 2105T>C), CAT656TCC (2106C>T 2107A>C 2108T>C), GCC659GCT (2117C>T), ATG660ATC (2120G>C), TCT663GTG (2127T>G 2128C>T 2129T>G), CTT664CTA (2132T>A), GAT665TCT (2133G>T 2134A>C), GAT666TCT (2136G>T 2137A>C), GAA667GAT (2141A>T), CAT668GCC (2142C>G 2143A>C 2144T>C), GAG670CAG (2148G>C), ATT671AAT (2152T>A), AAC673GAA (2157A>G 2159C>A), AAA674ATG (2161A>T 2162A>G), AGA676GTA (2166A>G 2167G>T), GTC677AAT (2169G>A 2170T>A 2171C>T), AAC678GAT (2172A>G 2174C>T), ATG679TTG (2175A>T), TAT681GTC (2181T>G 2182A>T 2183T>C), AGA682AAA (2185G>A), GAT685AGT (2193G>A 2194A>G), GCT686CCT (2196G>C), GAA687CAG (2199G>C 2201A>G), CGA690GCT (2208C>G 2209G>C 2210A>T), GTA693GTG (2219A>G), CAA694CGA (2221A>G), GAC699GAT (2237C>T), ATA701ATC (2243A>C), CAC703CAT (2249C>T), TTT704TTC (2252T>C), GCA710AGC (2268G>A 2269C>G 2270A>C), TCA711ACA (2271T>A), ATC713ATT (2279C>T), GAG715GAA (2285G>A), AAG716GAG (2286A>G), AAA717CTG (2289A>C 2290A>T 2291A>G), CTA718CTG (2294A>G), GCA719GAT (2296C>A 2297A>T), GTG720GTC (2300G>C), CAG721CAA (2303G>A), ACA722CGC (2304A>C 2305C>G 2306A>C), GAG723AAT (2307G>A 2309G>T), ATG724CTT (2310A>C 2312G>T), AGG725GAG (2313A>G 2314G>A), ACT726CAA (2316A>C 2317C>A 2318T>A), GGA727GGG (2321A>G), TTT728AAA (2322T>A 2323T>A 2324T>A), GCA729GCG (2327A>G), CAT730TAT (2328C>T), TCT731AAG (2331T>A 2332C>A 2333T>G), TGC732GCG (2334T>G 2335G>C 2336C>G), ATT733CTT (2337A>C), GAT734CAG (2340G>C 2342T>G), GCA735GCT (2345A>T), GTT737GCA (2350T>C 2351T>A), GAC738GAT (2354C>T), ACC739ACA (2357C>A), CTC740CTT (2360C>T), ATA741ACG (2362T>C 2363A>G), ATC743ATT (2369C>T), GAG744GAA (2372G>A), TTT749AGA (2385T>A 2386T>G 2387T>A), GTT750GTA (2390T>A), TTT752TTC (2396T>C), TTA753CTG (2397T>C 2399A>G), ACA755ACT (2405A>T), GCA756GCT (2408A>T), AGA759CGC (2415A>C 2417A>C), TGC762TGT (2426C>T), AAC763AGG (2428A>G 2429C>G), GTA765GTT (2435A>T), TCA766GCA (2436T>G), TCA766_AAA767insGGT (2438_2439insGGT), AAA767ACT (2440A>C 2441A>T), GAG768GAC (2444G>C), CCA769AAG (2445C>A 2446C>A 2447A>G), GAA771AGA (2451G>A 2452A>G), TCA772AAT (2454T>A 2455C>A 2456A>T), ATC774ATT (2462C>T), AAA775ATC (2464A>T 2465A>C), ATT776GAA (2466A>G 2467T>A 2468T>A), TAC778ATG (2472T>A 2473A>T 2474C>G), CAT779ATT (2475C>A 2476A>T), AAA780GGT (2478A>G 2479A>G 2480A>T), GTT782AAG (2484G>A 2485T>A 2486T>G), GAA783GAC (2489A>C), TAT784AAA (2490T>A 2492T>A), ATC785ATG (2495C>G), AAG786ATC (2497A>T 2498G>C), GAT787CAG (2499G>C 2501T>G), ACA788ATG (2503C>T 2504A>G), TGG790AAT (2508T>A 2509G>A 2510G>T), AGC791GAC (2511A>G 2512G>A), CTT792TTG (2514C>T 2516T>G), TAT794TTC (2521A>T 2522T>C), GCA795ATC (2523G>A 2524C>T 2525A>C), CTG796TTG (2526C>T), AGA797AAG (2530G>A 2531A>G), CTC799CTT (2537C>T), GCA802GCC (2546A>C), GGT803GGA (2549T>A), ATA806TTG (2556A>T 2558A>G), ATG807ATA (2561G>A), GCT808GCA (2564T>A), GGA811GGC (2573A>C), GTT812ACC (2574G>A 2575T>C 2576T>C), TTT813ATG (2577T>A 2579T>G), GGA814AAT (2580G>A 2581G>A 2582A>T), GGC815GCC (2584G>C), ATT816GTT (2586A>G), ACA817ACT (2591A>T), GCA818GCT (2594A>T), TTT819TTC (2597T>C), TTG820ATT (2598T>A 2600G>T), GCG821ACT (2601G>A 2603G>T), TGG823CTC (2607T>C 2608G>T 2609G>C), CAG824AAG (2610C>A), AGT825GCA (2613A>G 2614G>C 2615T>A), AAC826GCA (2616A>G 2617A>C 2618C>A), TCT827ACT (2619T>A), TTC828TCC (2623T>C), CCC829CCT (2627C>T), AAT830TTA (2628A>T 2629A>T 2630T>A), GCA831GCT (2633A>T), TCG832GAT (2634T>G 2635C>A 2636G>T), GGA835GGG (2645A>G), GGT836GGC (2648T>C), GTG837GTA (2651G>A), GAA840GAG (2660A>G), ATA844ATC (2672A>C), AGT846AGC (2678T>C), AAG848AAA (2684G>A), AAC849AAT (2687C>T), AAG850AAA (2690G>A), GGT851GGG (2693T>G), AAT852AAG (2696T>G), AAG853ACT (2698A>C 2699G>T), AAG853_CTC854insCAT (2699_2700insCAT), CTC854TTC (2700C>T), AGA855GCA (2703A>G 2704G>C), AAT856AAA (2708T>A), CTT857TCC (2709C>T 2710T>C 2711T>C), CTC858TTG (2712C>T 2714C>G), GTT859CTA (2715G>C 2717T>A), GGT860GGG (2720T>G), AGT862GCG (2724A>G 2725G>C 2726T>G), TCT863GCA (2727T>G 2729T>A), CAA864GAT (2730C>G 2732A>T), TCA865AAC (2733T>A 2734C>A 2735A>C), TTG866AAC (2736T>A 2737T>A 2738G>C), GCA867CAT (2739G>C 2740C>A 2741A>T), CAA868GTG (2742C>G 2743A>T 2744A>G), GAT869GAC (2747T>C), GAT869_TGG870insCGG (2747_2748insCGG), GCT871GCA (2753T>A), GCT872GAA (2755C>A 2756T>A), GAA873CAG (2757G>C 2759A>G), GTA877GTT (2771A>T), CAG879GAA (2775C>G 2777G>A), AGC880AGT (2780C>T), CTC881CTG (2783C>G), AAG882CAC (2784A>C 2786G>C), AAT884AAC (2792T>C), GTG886GTT (2798G>T), GTG887GTT (2801G>T), TTA888TTG (2804A>G), CTT890ATT (2808C>A), GGT891GCT (2812G>C), GTG894GTC (2822G>C), TAC895TAT (2825C>T), TTT896TTC (2828T>C), GGC898GGT (2834C>T), ACC899ACG (2837C>G), TAC900TAT (2840C>T), GTG901GTT (2843G>T), ATA906ATT (2858A>T), ATG907CTC (2859A>C 2861G>C), ACC908ACT (2864C>T), GTA909GTG (2867A>G), GCT910GCA (2870T>A), GCA912GCC (2876A>C), TTC913TTT (2879C>T), CAC914AGA (2880C>A 2881A>G 2882C>A), AAT915AAC (2885T>C), GCT916ATG (2886G>A 2887C>T 2888T>G), CGA917AGG (2889C>A 2891A>G), GGT919GGC (2897T>C), CCA921CCT (2903A>T), ACA923TCC (2907A>T 2909A>C), ATC925ATT (2915C>T), GCC927GAG (2920C>A 2921C>G), AAT928CAT (2922A>C), TCT929TCC (2927T>C), CGA930ATT (2928C>A 2929G>T 2930A>T), TCT931TGT (2932C>G), AAA932AAT (2936A>T), GTT933GTG (2939T>G), CAA934CAG (2942A>G), TAC935TTC (2944A>T), AAC936AAT (2948C>T), AGA938ACC (2953G>C 2954A>C), GAA939CAA (2955G>C), ATT942TTG (2964A>T 2966T>G), ATT943ATA (2969T>A), GGC945GGT (2975C>T), GAC947GAT (2981C>T), ATC948GTG (2982A>G 2984C>G), CTG950TTG (2988C>T), CGC952AGG (2994C>A 2996C>G), GTT953GTA (2999T>A), GGT954GGG (3002T>G), CCA959CCC (3017A>C), AAG960AAA (3020G>A), CCT961TCA (3021C>T 3023T>A), GAC962GAT (3026C>T), ATC963ATA (3029C>A), CGT964AGA (3030C>A 3032T>A), CAC966CAT (3038C>T), CCA968CCT (3044A>T), AGA969CGC (3045A>C 3047A>C), AGG970AGA (3050G>A),

	GAG972GAT (3056G>T), TGC974TGT (3062C>T), TTC975TAC (3064T>A), ACA976TCT (3066A>T 3068A>T), CAA980ATG (3078C>A 3079A>T 3080A>G), GGA981GGC (3083A>C), TGT984GTG (3090T>G 3091G>T 3092T>G), AGA985AGG (3095A>G), GTT987GTA (3101T>A), GCG988GCT (3104G>T), TCT989TCA (3107T>A), ACA990AAG (3109C>A 3110A>G), CCA992CCC (3116A>C), CGT993AAG (3117C>A 3118G>A 3119T>G), CTT994AAA (3120C>A 3121T>A 3122T>A), GGC995TCC (3123G>T 3124G>C), TTA997TTG (3131A>G), TTT999GTT (3135T>G), CTC1000CTT (3140C>T), AAG1001AAA (3143G>A), ATG1002TAT (3144A>T 3145T>A 3146G>T), GGA1009GGC (3167A>C), ACA1011ACC (3173A>C), GTT1012GAA (3175T>A 3176T>A), GTG1014GTC (3182G>C), GAA1015ACA (3183G>A 3184A>C), GAA1017GGA (3190A>G), CTG1018TTA (3192C>T 3194G>A), AAC1019GAT (3195A>G 3197C>T), TCC1020TCG (3200C>G), TCC1021AAT (3201T>A 3202C>A 3203C>T), AGT1022GCA (3204A>G 3205G>C 3206T>A), AAG1024GCT (3210A>G 3211A>C 3212G>T), ATT1025CTT (3213A>C), TGC1026AGC (3216T>A), TCT1027TCA (3221T>A), CAA1029GTT (3225C>G 3226A>T 3227A>T), TCT1030GCA (3228T>G 3230T>A), GAA1032GAG (3236A>G), ATA1035ATT (3245A>T), GGG1037AGT (3249G>A 3251G>T), GAA1038GAG (3254A>G), GGT1040GGA (3260T>A), GAC1041GAT (3263C>T), GGC1043GGT (3269C>T), ACG1044ACT (3272G>T), TTG1045CTT (3273T>C 3275G>T), CTA1046CTC (3278A>C), CTG1047TTA (3279C>T 3281G>A), TTA1048TTG (3284A>G), AGT1050ACT (3289G>C), GTT1051GTG (3293T>G), AAT1053AAC (3299T>C), CAA1055CAG (3305A>G), CCT1056CCG (3308T>G), GTG1057TGC (3309G>T 3310T>G 3311G>C), GGC1060GGA (3320C>A), ATC1061ATA (3323C>A), CAC1062CAT (3326C>T), TCA1065TCT (3335A>T), GGC1068GAT (3343G>A 3344C>T), GTA1069ACC (3345G>A 3346T>C 3347A>C), GCA1070GCT (3350A>T), GCT1071GCA (3353T>A), GAA1072GAG (3356A>G), CGT1073AAG (3357C>A 3358G>A 3359T>G), GGA1074GGT (3362A>T), TTT1075TAC (3364T>A 3365T>C), ATC1076GTG (3366A>G 3368C>G), TCT1077GCT (3369T>G), AAT1079AAC (3377T>C), GCT1080GCA (3380T>A), GCT1082GCA (3386T>A), ATC1083GTG (3387A>G 3389C>G), TAC1084TTT (3391A>T 3392C>T), AGG1085AGA (3395G>A), GAA1086GAT (3398A>T), CTA1088TTG (3402C>T 3404A>G), GAG1089GAA (3407G>A), CCA1092CCC (3416A>C), ACT1093ACG (3419T>G), GGG1094GGA (3422G>A), CCG1095CCC (3425G>C), GTC1096ATT (3426G>A 3428C>T), GCA1098GTG (3433C>T 3434A>G), GCA1099GCT (3437A>T), GTA1101GTG (3443A>G), GAT1104GAA (3452T>A), ATT1105CTG (3453A>C 3455T>G), CTG1106TTG (3456C>T), TCA1108CAC (3462T>C 3463C>A 3464A>C), ATT1109CTG (3465A>C 3467T>G), AGC1111TCC (3471A>T 3472G>C), AGA1112AAG (3475G>A 3476A>G), GAA1113GAG (3479A>G), ACA1114GAA (3480A>G 3481C>A), CAG1115AGA (3483C>A 3484A>G 3485G>A), CTT1116TTC (3486C>T 3488T>C), GAA1119GAG (3497A>G), AAC1120AAA (3500C>A), CAA1121CAG (3503A>G), TAC1123GCT (3507T>G 3508A>C 3509C>T), TAC1124TTC (3511A>T), CTT1125TTG (3513C>T 3515T>G), GGA1126GGC (3518A>C), ACA1127ACT (3521A>T), GTT1128GTA (3524T>A), CCA1129CCG (3527A>G), CAG1130TTG (3528C>T 3529A>T), GAG1131GAA (3533G>A), TTG1132ATG (3534T>A), GCC1133GCT (3539C>T), GCC1134GCT (3542C>T), GTT1136GTG (3548T>G), CCC1137CCG (3551C>G), AAG1139AAA (3557G>A), ACC1140ACA (3560C>A), ACT1141ACC (3563T>C), CTG1142CTT (3566G>T), CGG1143AAA (3567C>A 3568G>A 3569G>A), AAA1144CGC (3570A>C 3571A>G 3572A>C), AGC1145AGT (3575C>T), CAA1146CCC (3577A>C 3578A>C), TTG1147CTG (3579T>C), GAA1149GAG (3587A>G), GCA1150GCT (3590A>T), TTC1151TTT (3593C>T), GGA1152GGG (3596A>G), CCT1153GAC (3597C>G 3598C>A 3599T>C), GCA1154GCT (3602A>T), GAG1155CAG (3603G>C), ACA1156ACT (3608A>T), GCA1157GCC (3611A>C), ACA1162TCT (3624A>T 3626A>T), GTT1163GCC (3628T>C 3629T>C), CAT1164AAA (3630C>A 3632T>A), AGA1167AGG (3641A>G), GGT1168GGA (3644T>A), GAT1169GAA (3647T>A), GGT1170GGG (3650T>G), GAC1172GAT (3656C>T), GGG1177GGT (3671G>T), GTA1178GTC (3674A>C), ATG1179GAG (3675A>G 3676T>A), AAA1180AAG (3680A>G), TAC1181TAT (3683C>T), TGT1186TCC (3697G>C 3698T>C), GGA1187AAC (3699G>A 3700G>A 3701A>C), GAT1189GAG (3707T>G), GAT1190GAG (3710T>G), GAC1191CCA (3711G>C 3712A>C 3713C>A), GCC1193GCA (3719C>A), AAA1194AAG (3722A>G), CTA1195CTG (3725A>G), GCA1196GCT (3728A>T), TTC1197TTT (3731C>T), GAA1198GAG (3734A>G), AAT1199GGT (3735A>G 3736A>G), CTC1200CTT (3740C>T), AAG1201AAA (3743G>A), TGC1202GCA (3744T>G 3745G>C 3746C>A), TCG1203TCC (3749G>C), CTG1204ATT (3750C>A 3752G>T), CTA1205CTT (3755A>T), ATC1207GTG (3759A>G 3761C>G), ATG1208CTT (3762A>C 3764G>T), CGT1209AAG (3765C>A 3766G>A 3767T>G), AGC1210ATG (3769G>T 3770C>G), AAG1212CGA (3774A>C 3775A>G 3776G>A), ATC1213GTG (3777A>G 3779C>G), CCT1214CCC (3782T>C), GGA1216GGG (3788A>G), CGT1217AAA (3789C>A 3790G>A 3791T>A), CCA1218CCT (3794A>T), TGT1219GCA (3795T>G 3796G>C 3797T>A), GAA1220ATC (3798G>A 3799A>T 3800A>C), AGG1221AGA (3803G>A), GAT1222AAT (3804G>A), GGA1229GGG (3827A>G), TGT1233TTT (3838G>T), TAC1235TAT (3845C>T), TAT1236TTT (3847A>T), GGC1238GCA (3853G>C 3854C>A), GAG1240GAA (3860G>A), CTG1241TTG (3861C>T), ACA1243ACG (3869A>G), TCT1244TCA (3872T>A), TGC1245TGT (3875C>T), GGA1246GGT (3878A>T), CCC1248CCT (3884C>T), TTC1249TTT (3887C>T), ATG1252TTG (3894A>T), GGG1253GGA (3899G>A), ATG1254CTT (3900A>C 3902G>T), GGG1255GGT (3905G>T), ATG1256TCT (3906A>T 3907T>C 3908G>T), AAG1258AAA (3914G>A), AGA1259AGG (3917A>G), CAA1263GAA (3927C>G), TCC1264GCC (3930T>G), ACT1265CAA (3933A>C 3934C>A 3935T>A), GGC1266CCA (3936G>C 3937G>C 3938C>A), GAA1267AAC (3939G>A 3941A>C), GGA1268TCG (3942G>T 3943G>C 3944A>G), GAG1269CAG (3945G>C), AGA1270AGG (3950A>G), GTG1271GTT (3953G>T), GAA1272CTT (3954G>C 3955A>T 3956A>T), AAA1274ATA (3961A>T), AGA1275AGG (3965A>G), GAC1276AAC (3966G>A), ACT1277ACA (3971T>A), GTA1279GTC (3977A>C), GAA1281GAT (3983A>T), CTA1286TTG (3996C>T 3998A>G), GAT1287GAC (4001T>C), GTG1288TCG (4002G>T 4003T>C), ATT1290GTA (4008A>G 4010T>A), AAA1292CAA (4014A>C), ATC1294ACA (4021T>C 4022C>A), CAT1295TTT (4023C>T 4024A>T), GTG1296GTT (4028G>T), GAT1297GAC (4031T>C), CTG1298CTC (4034G>C), ACC1300ACA (4040C>A), CAA1302CAG (4046A>G), TGC1303TGT (4049C>T), GCC1304GCG (4052C>G), CGC1308CGG (4064C>G), CTC1309TTG (4065C>T 4067C>G), AAA1313AAG (4079A>G), ATC1314ATT (4082C>T), GGC1316GGG (4088C>G), AGA1318AGG (4094A>G), AGG1321CGC (4101A>C 4103G>C), CTC1322CTG (4106C>G), TTT1323TTC (4109T>C), GAG1324GAA (4112G>A), ATA1325ATC (4115A>C), CTT1326TTG (4116C>T 4118T>G), TAC1330TAT (4130C>T), TTG1333CTG (4137T>C), GTC1334GTG (4142C>G), AGG1335CGG (4143A>C), AAG1336AAA (4148G>A), TAT1337TAC (4151T>C), TTT1338TTC (4154T>C), GAT1340GAC (4160T>C), TTC1341TTT (4163C>T), TCA1342TCT (4166A>T), GCC1343GCT (4169C>T), ACA1344TGT (4170A>T 4171C>G 4172A>T), TTG1348GCA (4182T>G 4183T>C 4184G>A), AAT1350AAC (4190T>C), GCT1351GTA (4192C>T 4193T>A), ATA1352CTC (4194A>C 4196A>C), CCA1353CCT (4199A>T), AAA1355AAG (4205A>G), GTT1356GTA (4208T>A), ATT1358ATC (4214T>C), GAT1359AAT (4215G>A), CCT1360CCA (4220T>A), ACA1361ACG (4223A>G), AGT1362AGC (4226T>C), TCT1363CAT (4227T>C 4228C>A), TTG1367GTG (4239T>G), TTG1368CTG (4242T>C), GCA1369GCT (4247A>T), AAT1370AGT (4249A>G), GGG1371AGC (4251G>A 4253G>C), AGA1373AGG (4259A>G), GCT1374TCA (4260G>T 4262T>A), GTG1375GTA (4265G>A), TCA1376TCT (4268A>T), GAC1377GAT (4271C>T), GTG1378GAG (4273T>A), TTT1380TTC (4280T>C), GCT1382GCC (4286T>C), TAT1384TAC (4292T>C), TCC1385TCG (4295C>G), CCT1392CCC (4316T>C), GTT1393ATC (4317G>A 4319T>C), GCT1395GCA (4325T>A), TTT1396TTC (4328T>C), CAG1397CAA (4331G>A), TTG1402CTG (4344T>C), GAA1405GCT (4354A>C 4355A>T), TAC1406TTT (4357A>T 4358C>T), TAC1407TAT (4361C>T), GGA1408GGT (4364A>T), TCA1409GAT (4365T>G 4366C>A 4367A>T), AAG1410GAG (4368A>G), GGC1412GGG (4376C>G), GAT1415GAG (4385T>G), TCC1416TCA (4388C>A), CGA1419AGG (4395C>A 4397A>G), CAT1420CAC (4400T>C), GCA1421ACA (4401G>A), CTT1422TTA (4404C>T 4406T>A), GCA1426GCC (4418A>C), TGT1428TGC (4424T>C), TAC1430TAT (4430C>T), ACA1431ACC (4433A>C), CTG1432CTC (4436G>C), TGC1433TGT (4439C>T), GAT1435AAC (4443G>A 4445T>C), AAA1436AAG (4448A>G), GTG1437TTG (4449G>T), AGG1439CGC (4455A>C 4457G>C), TTG1440CTA (4458T>C 4460G>A), GTT1441GTG (4463T>G), GGG1442GGT (4466G>T), GGC1443GGA (4469C>A), CCA1445CCC (4475A>C), TCA1446TCT (4478A>T), GGA1447GGT (4481A>T), GCA1449GCT (4487A>T), CTA1450TTG (4488C>T 4490A>G), ACG1451ACT (4493G>T), ATC1453ATA (4499C>A), TTC1454TTT (4502C>T), TCT1456TCA (4508T>A), CTC1457TTG (4509C>T 4511C>G), CTC1458CTA (4514C>A), CGA1464AGG (4530C>A 4532A>G), TAT1465TAC (4535T>C), GCC1466GCG (4538C>G), TTG1470CTG (4548T>C), TTA1471TTG (4553A>G), AGA1472AGG (4556A>G), CCC1474CCA (4562C>A), ATT1476GTC (4566A>G 4568T>C), GCT1477AAA (4569G>A 4570C>A 4571T>A), AGG1479AAA (4576G>A 4577G>A), GCT1480TCT (4578G>T), ATA1481GTG (4581A>G 4583A>G), GGA1482GGC (4586A>C), GTT1483ATT (4587G>A), AAA1484AAG (4592A>G), AGT1486AAT (4597G>A), GAT1487GAC (4601T>C), CTA1491TTG (4611C>T 4613A>G), GCT1494GCA (4622T>A), GTT1495GTA (4625T>A), GGA1497GGT (4631A>T), GAC1499GAT (4637C>T), AAT1500AAC (4640T>C), CTT1501TTG (4641C>T 4643T>G), GCT1503GCG (4649T>G), GTA1504GTT (4652A>T), TTA1506ATG (4656T>A 4658A>G), CTC1508TTG (4662C>T 4664C>G), CAG1509AGG (4665C>A 4666A>G), TAT1511TAC (4673T>C), TCT1512GAT (4674T>G 4675C>A), CTG1513CTA (4679G>A), AAT1515TCC (4683A>T 4684A>C 4685T>C), ATA1516ATT (4688A>T), CAT1518AGA (4692C>A 4693A>G 4694T>A), GAG1519GAA (4697G>A), TTA1520CTG (4698T>C 4700A>G), CTG1522GCT (4704C>G 4705T>C 4706G>T), GTC1523GTT (4709C>T), AAT1524AAC (4712T>C), GTA1525GTC (4715A>C), ATC1526GTC (4716A>G), CTT1531TTG (4731C>T 4733T>G), AAA1533AAG (4739A>G), TCG1534TCA (4742G>A), GTT1537GTA (4751T>A), AAT1538GAG (4752A>G 4754T>G), GAG1539AAG (4755G>A), GTA1540ACA (4758G>A 4759T>C), CAA1541AAA (4761C>A), CAA1543CTG (4768A>T 4769A>G), CTA1548CTC (4784A>C), ACT1549ACC (4787T>C), CTG1551CTC (4793G>C), AGA1553AGG (4799A>G), TTT1555TTC (4805T>C), AAA1556AAG (4808A>G), GCT1559GCA (4817T>A), CTT1560TTG (4818C>T 4820T>G), TAC1562TAT (4826C>T), CAC1563CAA (4829C>A), GCT1565GCA (4835T>A), CCT1566CCA (4838T>A), AAG1568AAA (4844G>A), ATC1572GTC (4854A>G), ATT1573ATC (4859T>C), ATG1576ATT (4868G>T), ATA1579ATT (4877A>T), AGA1580AGG (4880A>G), CCT1581CCA (4883T>A), GCC1582GCA (4886C>A), CCA1583GTG (4887C>G 4888C>T 4889A>G), GGT1584GGA (4892T>A), GAC1587GAT (4901C>T), ACT1588GTA (4902A>G 4903C>T 4904T>A), CAG1589CAA (4907G>A), GCC1590GCT (4910C>T), ATG1592TAT (4914A>T 4915T>A 4916G>T), GTG1595ATT (4923G>A 4925G>T), GGA1598GGC (4934A>C), ATA1599ATC (4937A>C), CGT1600AGA (4938C>A 4940T>A), GCT1602GCC (4946T>C), CAC1605CAT (4955C>T), GGT1606GGG (4958T>G), CGT1607CAG (4960G>A 4961T>G), GTG1608GCC (4963T>C 4964G>C), GCT1609ACT (4965G>A), GAC1611GAA (4973C>A), AAG1612AGT (4975A>G 4976G>T), CTT1613TTT (4977C>T), GTC1614GTA (4982C>A), TTA1615AAT (4983T>A 4984T>A 4985A>T), CAG1618TTT (4992C>T 4993A>T 4994G>T), ACG1619GGA (4995A>G 4996C>G 4997G>A), GCG1620GCT (5000G>T), TTG1621CTC (5001T>C 5003G>C), GAT1622AAG (5004G>A 5006T>G), GAA1623CAG (5007G>C 5009A>G), AGG1624AGA (5012G>A), GGT1625AAT (5013G>A 5014G>A), TTC1626CTC (5016T>C), AGA1627CCC (5019A>C 5020G>C 5021A>C), GCT1628ATC (5022G>A 5023C>T 5024T>C), GTG1629ATG (5025G>A), TTT1631ATG (5031T>A 5033T>G), CCT1632CCC (5036T>C), TCC1633ACT (5037T>A 5039C>T), TAT1634TAC (5042T>C), TTG1635TAT (5044T>A 5045G>T), GTG1637CTT (5049G>C 5051G>T), CAG1639AGT (5055C>A 5056A>G 5057G>T), GAA1640CAA (5058G>C), ATT1642TTG (5064A>T 5066T>G), GCA1643ACA (5067G>A), AAG1644GAG (5070A>G), GTA1645GTC (5075A>C), GGG1647GGA (5081G>A), TCA1649GAG (5085T>G 5086C>A 5087A>G), AGT1650AGC (5090T>C), GCC1651ATT (5091G>A 5092C>T 5093C>T), CTG1652GGG (5094C>G 5095T>G), ACA1653GTT (5097A>G 5098C>T 5099A>T), ATT1654TTG (5100A>T 5102T>G), TGT1655ATG (5103T>A 5104G>T 5105T>G), GAA1656GAG (5108A>G), ATG1657CTA (5109A>C 5111G>A), GCA1658TCA (5112G>T), AAA1659GAT (5115A>G 5117A>T), GCA1660GCT (5120A>T), GCT1661AAA (5121G>A 5122C>A 5123T>A), ATT1662AAT (5125T>A), TCC1663ACA (5127T>A 5129C>A), TAT1664TAC (5132T>C), ACG1665ACC (5135G>C), CCA1666CCT (5138A>T), TTG1667TTA (5141G>A), GAC1668CCT (5142G>C 5143A>C 5144C>T), GCA1669GCT (5147A>T), GGT1670GGG (5150T>G), GAG1671GAA (5153G>A), ATC1673ATT (5159C>T), ACA1674del (5160_5162delACA), CGT1678GAG (5172C>G 5173G>A 5174T>G), GAT1679GGT (5176A>G), CTG1680GTC (5178C>G 5180G>C), AAT1681CAT (5181A>C), TGG1682GAA (5184T>G 5185G>A 5186G>A), GCA1684GTG (5191C>T 5192A>G), CCA1685CCT (5195A>T), AAC1686GAT (5196A>G 5198C>T), ATT1687GTA (5199A>G 5201T>A), GGT1688TTT (5202G>T 5203G>T), TTT1689TTC (5207T>C), TCA1691AGT (5211T>A 5212C>G 5213A>T), GCA1692GCT (5216A>T), CGT1693CGG (5219T>G), GCC1696TCA (5226G>T 5228C>A), CAC1697AAC (5229C>A), TAC1698CAA (5232T>C 5234C>A), ACG1699CAA (5235A>C 5236C>A 5237G>A), GAT1701TCA (5241G>T 5242A>C 5243T>A), GAA1702TCA (5244G>T 5245A>C), GGG1703GGT (5249G>T), TAC1704AAC (5250T>A), ATT1705ATC (5255T>C), ATT1706CTG (5256A>C 5258T>G), AAC1708AAT (5264C>T), TGT1709TGC (5267T>C), ACA1710ACT (5270A>T), GGT1711GGA (5273T>A), GCA1712GCC (5276A>C), AAG1713CGC (5277A>C 5278A>G 5279G>C), AAG1714AGA (5281A>G 5282G>A), TCC1715ACA (5283T>A 5285C>A), AAT1716ACA (5287A>C 5288T>A), GTT1718GTG (5294T>G), GGT1720GGG (5300T>G), CCA1721CCT (5303A>T), AAC1723AAT (5309C>T), ATG1731ATA (5333G>A), CCG1733CCC (5339G>C), ATG1736TTG (5346A>T), TCA1737GCA (5349T>G), GCT1738GCA (5354T>A), ATA1739ATC (5357A>C), ATG1740CAG (5358A>C 5359T>A), GCC1742CAT (5364G>C 5365C>A 5366C>T), AAG1744CGC (5370A>C 5371A>G 5372G>C), AAT1745AAG (5375T>G), GTG1746AGT (5376G>A 5377T>G 5378G>T), TTG1747CTG (5379T>C), GCA1748GAA (5383C>A), GGA1749GTT (5386G>T 5387A>T), GGA1750GGT (5390A>T), CAT1751GAT (5391C>G), GTA1752GGG (5395T>G 5396A>G), GCA1753GCT (5399A>T), AAT1755TCT (5403A>T 5404A>C), GTT1757ACC (5409G>A 5410T>C 5411T>C), GTG1759GTT (5417G>T), GGA1761GGT (5423A>T), GGA1763GGT (5429A>T), GCT1765GCA (5435T>A), GTC1766ATA (5436G>A 5438C>A), GGC1767GCT (5440G>C 5441C>T), ATT1769ATC (5447T>C), GCG1772GCA (5456G>A), CTG1773TTG (5457C>T), ATG1774TTT (5460A>T 5462G>T), GCC1775GCA (5465C>A), TTA1776TTG (5468A>G), GCA1777GCC (5471A>C), ACA1778ACT (5474A>T), AGG1779CAT (5475A>C 5476G>A 5477G>T), TAT1781TAC (5483T>C), TGT1782AGG (5484T>A 5486T>G), GTG1783GCC (5488T>C 5489G>C), GAG1784ACA (5490G>A 5491A>C 5492G>A), ATA1786CTT (5496A>C 5498A>T), ATA1787ATT (5501A>T), GTT1788GTG (5504T>G), AGA1789CGC (5505A>C 5507A>C), TTG1790CTA (5508T>C 5510G>A), CGC1791CGA (5513C>A), ACA1792ACC (5516A>C), ATT1793ATG (5519T>G), GGA1794GCT (5521G>C 5522A>T), AAT1795GGG (5523A>G 5524A>G 5525T>G), CTT1797CTG (5531T>G), GAC1798GAT (5534C>T), CTC1799CTG (5537C>G), AAT1800AAC (5540T>C), TAT1802TAC (5546T>C), GGC1805GGT (5555C>T), GTG1807GCG (5560T>C), CAG1808CAA (5564G>A), TAT1809TAC (5567T>C), TTT1810TTG (5570T>G), CTT1811CTC (5573T>C), CAA1812AAA (5574C>A), GAA2142GCA (6565A>C), ACC2143CCC (6567A>C), ATT2144ATC (6572T>C), TAC2148TAT (6584C>T), GAG2150GCG (6589A>C)								
helicase (YP_001976154.1)	1	98	100%	654	97.5%	98 (100%)	95 (96.9%)	0/0/0/0	0
Protein mutations:	S5C (1342C>G 1343C>T), R16A (1374A>G 1375G>C), I44V (1458A>G 1460C>G)								
Codon mutations:	TCT3TCA (1337T>A), ACA4ACG (1340A>G), TCC5TGT (1342C>G 1343C>T), TTA8CTT (1350T>C 1352A>T), TTT9TTC (1355T>C), ATT10ATC (1358T>C), GTG13GTT (1367G>T), TTG14CTC (1368T>C 1370G>C), AGA16GCA (1374A>G 1375G>C), GAG19GAA (1385G>A), AAA21AAG (1391A>G), CTC22CTG (1394C>G), AAT23AAC (1397T>C), CGG24CGC (1400G>C), CTA25TTG (1401C>T 1403A>G), GCA27GCG (1409A>G), AAG30AAA (1418G>A), AGG31AGA (1421G>A), GCT39GCA (1445T>A), CAC40CAT (1448C>T), GCT43GCA (1457T>A), ATC44GTG (1458A>G 1460C>G), CTG45CTT (1463G>T), GAC48GAT (1472C>T), GGA50GGT (1478A>T), GCA51GCT (1481A>T), TTG52CTT (1482T>C 1484G>T), CGA53CGG (1487A>G), GGG55GGA (1493G>A), GCT56GCA (1496T>A), TGC59TGT (1505C>T), CAA60CAG (1508A>G), GAT61GAC (1511T>C), ATT62ATA (1514T>A), AAA63AAG (1517A>G), GAT67GAC (1529T>C), TCA70TCT (1538A>T), ACA71ACC (1541A>C), CAA72CAG (1544A>G), CCA73CCT (1547A>T), CCC75CCT (1553C>T), TTA76CTT (1554T>C 1556A>T), GCC79GCT (1565C>T), GTT81GTC (1571T>C), GAC83GAT (1577C>T), GGC85GGA (1583C>A), CAT87CAC (1589T>C), TTC88TTT (1592C>T), ACC89ACA (1595C>A), TCC90TCA (1598C>A), TAT92TAC (1604T>C), ATA93ATC (1607A>C), ACA96ACC (1616A>C), AAT98AAC (1622T>C)								

	Begin	End	Coverage	Score	Concordance	Matches	Identities	I/D/M/F*	Stop Codons
**NT**	**22**	**6809**	**80.5%**	**3829**	**33.5%**	**5772 (99.0%)**	**3883 (66.6%)**	**10/50**	
RNA-dependent RNA polymerase (YP_001976153.1)	1	249	100%	1552	88.4%	249 (100%)	210 (84.3%)	0/0/0/0	0
Protein mutations:	T40C (4170A>T 4171C>G 4172A>T), L44A (4182T>G 4183T>C 4184G>A), A47V (4192C>T 4193T>A), I48L (4194A>C 4196A>C), D55N (4215G>A), S59H (4227T>C 4228C>A), L63V (4239T>G), N66S (4249A>G), G67S (4251G>A 4253G>C), A70S (4260G>T 4262T>A), V74E (4273T>A), V89I (4317G>A 4319T>C), E101A (4354A>C 4355A>T), Y102F (4357A>T 4358C>T), S105D (4365T>G 4366C>A 4367A>T), K106E (4368A>G), D111E (4385T>G), A117T (4401G>A), D131N (4443G>A 4445T>C), V133L (4449G>T), I172V (4566A>G 4568T>C), A173K (4569G>A 4570C>A 4571T>A), R175K (4576G>A 4577G>A), A176S (4578G>T), I177V (4581A>G 4583A>G), V179I (4587G>A), S182N (4597G>A), L202M (4656T>A 4658A>G), Q205R (4665C>A 4666A>G), S208D (4674T>G 4675C>A), N211S (4683A>T 4684A>C 4685T>C), H214R (4692C>A 4693A>G 4694T>A), L218A (4704C>G 4705T>C 4706G>T), I222V (4716A>G), N234E (4752A>G 4754T>G), E235K (4755G>A), V236T (4758G>A 4759T>C), Q237K (4761C>A), Q239L (4768A>T 4769A>G)								
Codon mutations:	CGC4CGG (4064C>G), CTC5TTG (4065C>T 4067C>G), AAA9AAG (4079A>G), ATC10ATT (4082C>T), GGC12GGG (4088C>G), AGA14AGG (4094A>G), AGG17CGC (4101A>C 4103G>C), CTC18CTG (4106C>G), TTT19TTC (4109T>C), GAG20GAA (4112G>A), ATA21ATC (4115A>C), CTT22TTG (4116C>T 4118T>G), TAC26TAT (4130C>T), TTG29CTG (4137T>C), GTC30GTG (4142C>G), AGG31CGG (4143A>C), AAG32AAA (4148G>A), TAT33TAC (4151T>C), TTT34TTC (4154T>C), GAT36GAC (4160T>C), TTC37TTT (4163C>T), TCA38TCT (4166A>T), GCC39GCT (4169C>T), ACA40TGT (4170A>T 4171C>G 4172A>T), TTG44GCA (4182T>G 4183T>C 4184G>A), AAT46AAC (4190T>C), GCT47GTA (4192C>T 4193T>A), ATA48CTC (4194A>C 4196A>C), CCA49CCT (4199A>T), AAA51AAG (4205A>G), GTT52GTA (4208T>A), ATT54ATC (4214T>C), GAT55AAT (4215G>A), CCT56CCA (4220T>A), ACA57ACG (4223A>G), AGT58AGC (4226T>C), TCT59CAT (4227T>C 4228C>A), TTG63GTG (4239T>G), TTG64CTG (4242T>C), GCA65GCT (4247A>T), AAT66AGT (4249A>G), GGG67AGC (4251G>A 4253G>C), AGA69AGG (4259A>G), GCT70TCA (4260G>T 4262T>A), GTG71GTA (4265G>A), TCA72TCT (4268A>T), GAC73GAT (4271C>T), GTG74GAG (4273T>A), TTT76TTC (4280T>C), GCT78GCC (4286T>C), TAT80TAC (4292T>C), TCC81TCG (4295C>G), CCT88CCC (4316T>C), GTT89ATC (4317G>A 4319T>C), GCT91GCA (4325T>A), TTT92TTC (4328T>C), CAG93CAA (4331G>A), TTG98CTG (4344T>C), GAA101GCT (4354A>C 4355A>T), TAC102TTT (4357A>T 4358C>T), TAC103TAT (4361C>T), GGA104GGT (4364A>T), TCA105GAT (4365T>G 4366C>A 4367A>T), AAG106GAG (4368A>G), GGC108GGG (4376C>G), GAT111GAG (4385T>G), TCC112TCA (4388C>A), CGA115AGG (4395C>A 4397A>G), CAT116CAC (4400T>C), GCA117ACA (4401G>A), CTT118TTA (4404C>T 4406T>A), GCA122GCC (4418A>C), TGT124TGC (4424T>C), TAC126TAT (4430C>T), ACA127ACC (4433A>C), CTG128CTC (4436G>C), TGC129TGT (4439C>T), GAT131AAC (4443G>A 4445T>C), AAA132AAG (4448A>G), GTG133TTG (4449G>T), AGG135CGC (4455A>C 4457G>C), TTG136CTA (4458T>C 4460G>A), GTT137GTG (4463T>G), GGG138GGT (4466G>T), GGC139GGA (4469C>A), CCA141CCC (4475A>C), TCA142TCT (4478A>T), GGA143GGT (4481A>T), GCA145GCT (4487A>T), CTA146TTG (4488C>T 4490A>G), ACG147ACT (4493G>T), ATC149ATA (4499C>A), TTC150TTT (4502C>T), TCT152TCA (4508T>A), CTC153TTG (4509C>T 4511C>G), CTC154CTA (4514C>A), CGA160AGG (4530C>A 4532A>G), TAT161TAC (4535T>C), GCC162GCG (4538C>G), TTG166CTG (4548T>C), TTA167TTG (4553A>G), AGA168AGG (4556A>G), CCC170CCA (4562C>A), ATT172GTC (4566A>G 4568T>C), GCT173AAA (4569G>A 4570C>A 4571T>A), AGG175AAA (4576G>A 4577G>A), GCT176TCT (4578G>T), ATA177GTG (4581A>G 4583A>G), GGA178GGC (4586A>C), GTT179ATT (4587G>A), AAA180AAG (4592A>G), AGT182AAT (4597G>A), GAT183GAC (4601T>C), CTA187TTG (4611C>T 4613A>G), GCT190GCA (4622T>A), GTT191GTA (4625T>A), GGA193GGT (4631A>T), GAC195GAT (4637C>T), AAT196AAC (4640T>C), CTT197TTG (4641C>T 4643T>G), GCT199GCG (4649T>G), GTA200GTT (4652A>T), TTA202ATG (4656T>A 4658A>G), CTC204TTG (4662C>T 4664C>G), CAG205AGG (4665C>A 4666A>G), TAT207TAC (4673T>C), TCT208GAT (4674T>G 4675C>A), CTG209CTA (4679G>A), AAT211TCC (4683A>T 4684A>C 4685T>C), ATA212ATT (4688A>T), CAT214AGA (4692C>A 4693A>G 4694T>A), GAG215GAA (4697G>A), TTA216CTG (4698T>C 4700A>G), CTG218GCT (4704C>G 4705T>C 4706G>T), GTC219GTT (4709C>T), AAT220AAC (4712T>C), GTA221GTC (4715A>C), ATC222GTC (4716A>G), CTT227TTG (4731C>T 4733T>G), AAA229AAG (4739A>G), TCG230TCA (4742G>A), GTT233GTA (4751T>A), AAT234GAG (4752A>G 4754T>G), GAG235AAG (4755G>A), GTA236ACA (4758G>A 4759T>C), CAA237AAA (4761C>A), CAA239CTG (4768A>T 4769A>G), CTA244CTC (4784A>C), ACT245ACC (4787T>C), CTG247CTC (4793G>C), AGA249AGG (4799A>G)								

*: Inserts / Deletes / Misaligned / Frameshifts

Analysis details

This analysis was performed with panviral2.64

NGS Details (UN62): Torradovirus marchitezum (segment RNA 2)

Assembly

**Coverage Length**	1244 (2 contig(s))
**Depth Of Coverage**	14475.3
**Number Of Reads**	143680
**Reads Per Million**	3330.63 rpm (after QC)
**Ambiguities**	0
**Assembly Method**	de novo + reference guided assembly
**Consensus Caller**	Bcf Tools

Coverage Map

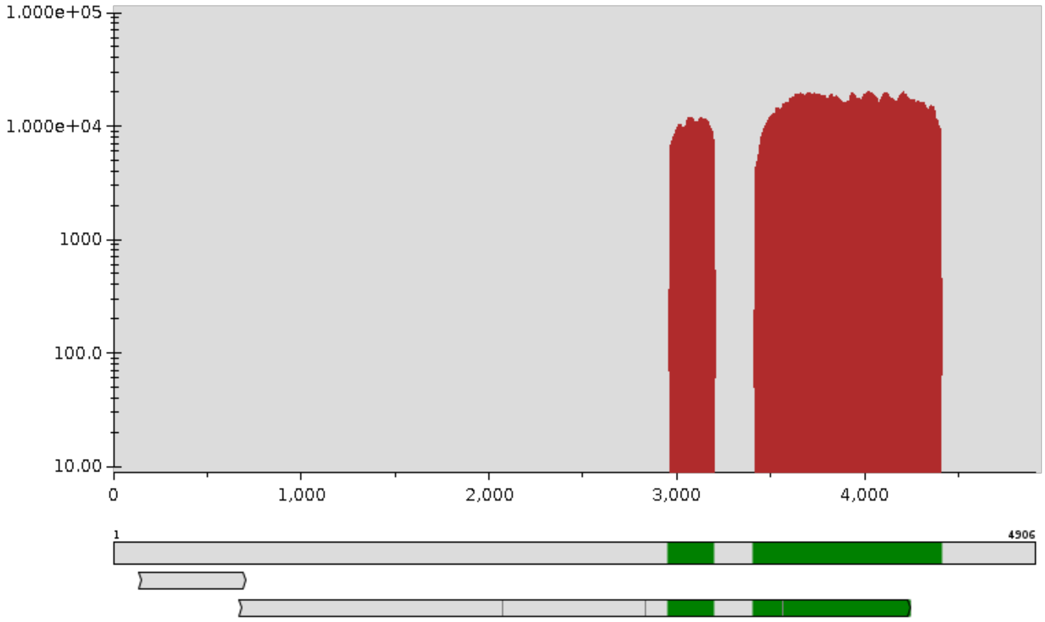

Assignment

**Type**	Torradovirus marchitezum (Taxonomy ID: 3048376)
**Reference Genome**	NC_010988.1
**NT Identity (%)**	69.4712
**AA Identity (%)**	75.9669
**Number Of Stop Codons**	1
**Number Of CDS**	2

Alignment

**Alignment Score**	931.0 (NT) + 2123.0 (AA) = 3054.0
**Concordance (%)**	60.0708

Alignment Method	Global, seeded, nucleotide + amino acids (AGA)

Genome Region

Sequence starts at position 2960 and ends at position 4410 relative to NC_010988.1 reference sequence.

Alignment Detailed Statistics

	Begin	End	Coverage	Score	Concordance	Matches	Identities	I/D/M/F*	Stop Codons
**NT**	**2960**	**4410**	**25.4%**	**931**	**37.8%**	**1239 (98.9%)**	**867 (69.2%)**	**9/5**	

Mutations: 2971A>T, 2977G>A, 2989C>A, 2990T>C, 2998G>A, 3001T>A, 3002T>C, 3005C>T, 3007T>G, 3010T>A, 3013C>T, 3016A>T, 3019C>T, 3022G>A, 3028C>T, 3038A>C, 3039C>G, 3040T>G, 3043A>C, 3046T>A, 3049T>C, 3058A>G, 3061T>A, 3064C>T, 3065A>G, 3067T>C, 3070G>A, 3073A>G, 3079G>A, 3085T>C, 3088A>T, 3091T>A, 3097G>T, 3100A>C, 3106A>T, 3107C>T, 3109T>G, 3112A>T, 3118G>A, 3119A>G, 3120A>C, 3121C>A, 3124C>A, 3125A>G, 3131A>G, 3134A>G, 3135A>C, 3136T>A, 3148A>T, 3149T>C, 3151T>A, 3154C>T, 3160A>C, 3163T>C, 3178T>A, 3182A>G, 3187T>C, 3190A>C, 3191A>C, 3192A>G, 3193A>T, 3427G>T, 3429A>T, 3436A>T, 3442C>G, 3445A>G, 3448C>T, 3449T>G, 3451A>C, 3457T>C, 3460C>T, 3461C>T, 3463T>G, 3466C>T, 3476G>A, 3478T>C, 3481A>G, 3482T>A, 3484A>T, 3493G>A, 3497A>C, 3506T>C, 3514G>A, 3517C>A, 3520C>T, 3521A>G, 3528A>T, 3529G>C, 3532C>T, 3533C>G, 3535G>T, 3538G>T, 3544C>G, 3544_3545insAAGAAG, 3550T>C, 3551C>A, 3552C>A, 3554C>G, 3555C>A, 3556C>G, 3559G>A, 3560A>G, 3561A>T, 3564C>G, 3568A>G, 3569A>G, 3570G>T, 3572A>G, 3573T>C, 3574T>A, 3577G>T, 3578T>G, 3579C>G, 3580C>A, 3581A>C, 3583A>T, 3584A>C, 3586A>G, 3589G>A, 3595T>C, 3598C>T, 3604T>A, 3610A>T, 3619C>T, 3622C>T, 3623A>T, 3624T>C, 3625G>T, 3627G>C, 3628T>C, 3638G>A, 3639A>C, 3641G>A, 3642A>G, 3643A>C, 3644A>C, 3646A>C, 3647T>C, 3650T>G, 3655A>T, 3656A>C, 3661T>A, 3667A>T, 3670A>G, 3676C>T, 3677A>G, 3680A>C, 3682G>A, 3685T>C, 3686G>T, 3687A>C, 3689G>C, 3690G>C, 3691G>C, 3692C>A, 3693A>C, 3700T>C, 3712A>C, 3715C>A, 3718T>G, 3724A>C, 3730A>C, 3739T>C, 3740A>T, 3742A>T, 3751T>C, 3760A>T, 3763T>C, 3767T>C, 3769G>T, 3770A>G, 3772T>G, 3775T>C, 3776T>A, 3778A>T, 3779A>C, 3781T>C, 3782G>A, 3784G>C, 3787A>C, 3790C>T, 3793A>G, 3796A>C, 3800T>G, 3801C>G, 3803A>T, 3804G>C, 3806C>A, 3807C>A, 3811T>C, 3812G>A, 3817A>T, 3826A>T, 3832T>G, 3838T>G, 3841T>C, 3842G>A, 3860C>T, 3862A>T, 3863G>T, 3864C>A, 3868G>T, 3869C>G, 3871T>G, 3872A>G, 3873A>C, 3874T>A, 3875A>G, 3877A>G, 3880A>G, 3881A>G, 3882A>C, 3883T>A, 3884T>C, 3890G>C, 3892A>T, 3901T>C, 3904A>T, 3907C>T, 3908A>T, 3910A>C, 3917G>A, 3922T>C, 3925A>C, 3931T>A, 3934G>T, 3935G>A, 3937A>C, 3940A>T, 3943T>C, 3945C>T, 3946G>T, 3949C>T, 3952A>C, 3955C>T, 3956A>G, 3957C>T, 3958T>G, 3967T>C, 3971G>A, 3973G>A, 3982A>T, 3983A>C, 3986C>A, 3989C>T, 3994A>T, 3996G>A, 3998A>G, 3999T>C, 4000G>A, 4001A>C, 4003G>A, 4009C>T, 4010T>G, 4011C>A, 4012A>C, 4014G>A, 4015C>T, 4019C>A, 4020A>G, 4021A>T, 4024C>T, 4027C>T, 4030C>T, 4031A>G, 4033G>T, 4036A>T, 4037C>T, 4039A>G, 4045T>C, 4047G>A, 4048G>A, 4049C>T, 4051T>G, 4054A>T, 4055A>C, 4056A>T, 4060G>T, 4061A>T, 4063C>G, 4066A>C, 4070T>C, 4072G>A, 4075T>A, 4078T>C, 4079T>G, 4080C>T, 4081C>A, 4083C>A, 4085A>T, 4088G>A, 4090G>T, 4093T>C, 4102G>A, 4105A>C, 4106C>A, 4108T>G, 4109A>G, 4111A>T, 4112T>A, 4113C>A, 4114T>G, 4117T>A, 4120A>G, 4122T>C, 4123T>A, 4129C>T, 4131A>G, 4135G>A, 4138G>A, 4139T>C, 4141G>T, 4154T>C, 4155C>T, 4156T>G, 4159C>T, 4162C>T, 4180C>T, 4181A>G, 4192T>G, 4195C>T, 4198C>T, 4201T>C, 4204G>A, 4213A>T, 4219T>C, 4222T>A, 4225G>A, 4228A>G, 4229C>A, 4231T>A, 4234T>C, 4237T>C, 4238G>T, 4242T>A, 4243T>C, 4248A>C, 4252T>A, 4279A>T, 4279_4280insT, 4280A>T, 4280_4281insC, 4283A>G, 4286C>T, 4287T>A, 4288T>C, 4291C>A, 4294C>T, 4306G>A, 4309G>A, 4312G>T, 4313C>A, 4313_4314insG, 4315A>G, 4316A>T, 4318T>C, 4319G>T, 4320G>A, 4323T>G, 4324T>A, 4328T>C, 4330G>A, 4333A>G, 4335G>T, 4336A>C, 4338A>G, 4339_4343delAGGGA, 4345A>T, 4346G>C, 4349A>C, 4364C>T, 4365T>C, 4369A>G, 4372A>G, 4373G>A, 4392T>C, 4393C>T

CDS

	Begin	End	Coverage	Score	Concordance	Matches	Identities	I/D/M/F*	Stop Codons
ToMarV_RNA2gp2	764	1192	30.2%	2123	80.3%	360 (99.4%)	275 (76.0%)	2/0/0/0	1

Protein mutations: T790R (3038A>C 3039C>G 3040T>G), I799V (3065A>G 3067T>C), N817A (3119A>G 3120A>C 3121C>A), K819E (3125A>G), T821A (3131A>G), N822A (3134A>G 3135A>C 3136T>A), F827L (3149T>C 3151T>A), M838V (3182A>G), K841R (3191A>C 3192A>G 3193A>T), H920L (3429A>T), S927A (3449T>G 3451A>C), D936N (3476G>A 3478T>C), S938T (3482T>A 3484A>T), I943L (3497A>C), D949E (3517C>A), I951V (3521A>G), E953V (3528A>T 3529G>C), Q955D (3533C>G 3535G>T), E956D (3538G>T), G958_K959insKK (3544_3545insAAGAAG), P961K (3551C>A 3552C>A), P962E (3554C>G 3555C>A 3556C>G), K964V (3560A>G 3561A>T), A965G (3564C>G), S967V (3569A>G 3570G>T), I968A (3572A>G 3573T>C 3574T>A), S970G (3578T>G 3579C>G 3580C>A), I971L (3581A>C 3583A>T), M985S (3623A>T 3624T>C 3625G>T), G986A (3627G>C 3628T>C), D990T (3638G>A 3639A>C), E991S (3641G>A 3642A>G 3643A>C), L994V (3650T>G), I996L (3656A>C), I1003V (3677A>G), E1006S (3686G>T 3687A>C), G1007P (3689G>C 3690G>C 3691G>C), H1008T (3692C>A 3693A>C), T1024S (3740A>T 3742A>T), N1034E (3770A>G 3772T>G), S1036T (3776T>A 3778A>T), I1037L (3779A>C 3781T>C), V1038I (3782G>A 3784G>C), S1044G (3800T>G 3801C>G), P1046N (3806C>A 3807C>A), V1048I (3812G>A), F1056L (3838T>G), A1058T (3842G>A), P1064S (3860C>T 3862A>T), A1065Y (3863G>T 3864C>A), L1067V (3869C>G 3871T>G), N1068A (3872A>G 3873A>C 3874T>A), K1069E (3875A>G 3877A>G), N1071A (3881A>G 3882A>C 3883T>A), Y1072H (3884T>C), V1074L (3890G>C 3892A>T), T1080S (3908A>T 3910A>C), D1083N (3917G>A), A1089T (3935G>A 3937A>C), T1092I (3945C>T 3946G>T), T1096V (3956A>G 3957C>T 3958T>G), E1101K (3971G>A 3973G>A), H1107Y (3989C>T), R1109K (3996G>A), M1110A (3998A>G 3999T>C 4000G>A), S1114D (4010T>G 4011C>A 4012A>C), S1115N (4014G>A 4015C>T), Q1117S (4019C>A 4020A>G 4021A>T), M1121V (4031A>G 4033G>T), R1126K (4047G>A 4048G>A), N1129L (4055A>C 4056A>T), I1131L (4061A>T 4063C>G), S1137V (4079T>G 4080C>T 4081C>A), A1138D (4083C>A), I1139L (4085A>T), V1140I (4088G>A 4090G>T), L1146M (4106C>A 4108T>G), I1147V (4109A>G 4111A>T), S1148K (4112T>A 4113C>A 4114T>G), L1151P (4122T>C 4123T>A), K1154R (4131A>G), S1162L (4154T>C 4155C>T 4156T>G), N1171D (4181A>G), A1190S (4238G>T), I1191N (4242T>A 4243T>C)

Codon mutations: ATA767ATT (2971A>T), GAG769GAA (2977G>A), ACC773ACA (2989C>A), TTG774CTG (2990T>C), CCG776CCA (2998G>A), TCT777TCA (3001T>A), TTG778CTG (3002T>C), CTT779TTG (3005C>T 3007T>G), GGT780GGA (3010T>A), AAC781AAT (3013C>T), ATA782ATT (3016A>T), GCC783GCT (3019C>T), AGG784AGA (3022G>A), TGC786TGT (3028C>T), ACT790CGG (3038A>C 3039C>G 3040T>G), GGA791GGC (3043A>C), ACT792ACA (3046T>A), TGT793TGC (3049T>C), GAA796GAG (3058A>G), ATT797ATA (3061T>A), TGC798TGT (3064C>T), ATT799GTC (3065A>G 3067T>C), GAG800GAA (3070G>A), AAA801AAG (3073A>G), TTG803TTA (3079G>A), CAT805CAC (3085T>C), TCA806TCT (3088A>T), GGT807GGA (3091T>A), CTG809CTT (3097G>T), GCA810GCC (3100A>C), GGA812GGT (3106A>T), CTT813TTG (3107C>T 3109T>G), GGA814GGT (3112A>T), CTG816CTA (3118G>A), AAC817GCA (3119A>G 3120A>C 3121C>A), ACC818ACA (3124C>A), AAA819GAA (3125A>G), ACC821GCC (3131A>G), AAT822GCA (3134A>G 3135A>C 3136T>A), ATA826ATT (3148A>T), TTT827CTA (3149T>C 3151T>A), AAC828AAT (3154C>T), CCA830CCC (3160A>C), CAT831CAC (3163T>C), CTT836CTA (3178T>A), ATG838GTG (3182A>G), GGT839GGC (3187T>C), CGA840CGC (3190A>C), AAA841CGT (3191A>C 3192A>G 3193A>T), GGG919GGT (3427G>T), CAT920CTT (3429A>T), ACA922ACT (3436A>T), GGC924GGG (3442C>G), AAA925AAG (3445A>G), AGC926AGT (3448C>T), TCA927GCC (3449T>G 3451A>C), GAT929GAC (3457T>C), TTC930TTT (3460C>T), CTT931TTG (3461C>T 3463T>G), TTC932TTT (3466C>T), GAT936AAC (3476G>A 3478T>C), TTA937TTG (3481A>G), TCA938ACT (3482T>A 3484A>T), AAG941AAA (3493G>A), ATT943CTT (3497A>C), TTG946CTG (3506T>C), AAG948AAA (3514G>A), GAC949GAA (3517C>A), AAC950AAT (3520C>T), ATT951GTT (3521A>G), GAG953GTC (3528A>T 3529G>C), TAC954TAT (3532C>T), CAG955GAT (3533C>G 3535G>T), GAG956GAT (3538G>T), GGC958GGG (3544C>G), GGC958_AAA959insAAGAAG (3544_3545insAAGAAG), GAT960GAC (3550T>C), CCG961AAG (3551C>A 3552C>A), CCC962GAG (3554C>G 3555C>A 3556C>G), AAG963AAA (3559G>A), AAG964GTG (3560A>G 3561A>T), GCT965GGT (3564C>G), CAA966CAG (3568A>G), AGT967GTT (3569A>G 3570G>T), ATT968GCA (3572A>G 3573T>C 3574T>A), CTG969CTT (3577G>T), TCC970GGA (3578T>G 3579C>G 3580C>A), ATA971CTT (3581A>C 3583A>T), AGA972CGG (3584A>C 3586A>G), GAG973GAA (3589G>A), TTT975TTC (3595T>C), TCC976TCT (3598C>T), GGT978GGA (3604T>A), GTA980GTT (3610A>T), TTC983TTT (3619C>T), TGC984TGT (3622C>T), ATG985TCT (3623A>T 3624T>C 3625G>T), GGT986GCC (3627G>C 3628T>C), GAC990ACC (3638G>A 3639A>C), GAA991AGC (3641G>A 3642A>G 3643A>C), AGA992CGC (3644A>C 3646A>C), TTG993CTG (3647T>C), TTG994GTG (3650T>G), GTA995GTT (3655A>T), ATT996CTT (3656A>C), CCT997CCA (3661T>A), GCA999GCT (3667A>T), CCA1000CCG (3670A>G), TCC1002TCT (3676C>T), ATA1003GTA (3677A>G), AGG1004CGA (3680A>C 3682G>A), TTT1005TTC (3685T>C), GAA1006TCA (3686G>T 3687A>C), GGG1007CCC (3689G>C 3690G>C 3691G>C), CAC1008ACC (3692C>A 3693A>C), CCT1010CCC (3700T>C), GCA1014GCC (3712A>C), ATC1015ATA (3715C>A), ACT1016ACG (3718T>G), CCA1018CCC (3724A>C), ATA1020ATC (3730A>C), TGT1023TGC (3739T>C), ACA1024TCT (3740A>T 3742A>T), TGT1027TGC (3751T>C), TCA1030TCT (3760A>T), GGT1031GGC (3763T>C), TTG1033CTT (3767T>C 3769G>T), AAT1034GAG (3770A>G 3772T>G), TAT1035TAC (3775T>C), TCA1036ACT (3776T>A 3778A>T), ATT1037CTC (3779A>C 3781T>C), GTG1038ATC (3782G>A 3784G>C), ATA1039ATC (3787A>C), CAC1040CAT (3790C>T), AGA1041AGG (3793A>G), GTA1042GTC (3796A>C), TCC1044GGC (3800T>G 3801C>G), AGT1045TCT (3803A>T 3804G>C), CCT1046AAT (3806C>A 3807C>A), AAT1047AAC (3811T>C), GTT1048ATT (3812G>A), GGA1049GGT (3817A>T), CTA1052CTT (3826A>T), GTT1054GTG (3832T>G), TTT1056TTG (3838T>G), GAT1057GAC (3841T>C), GCC1058ACC (3842G>A), CCA1064TCT (3860C>T 3862A>T), GCT1065TAT (3863G>T 3864C>A), GGG1066GGT (3868G>T), CTT1067GTG (3869C>G 3871T>G), AAT1068GCA (3872A>G 3873A>C 3874T>A), AAA1069GAG (3875A>G 3877A>G), GGA1070GGG (3880A>G), AAT1071GCA (3881A>G 3882A>C 3883T>A), TAT1072CAT (3884T>C), GTA1074CTT (3890G>C 3892A>T), GGT1077GGC (3901T>C), GGA1078GGT (3904A>T), GGC1079GGT (3907C>T), ACA1080TCC (3908A>T 3910A>C), GAT1083AAT (3917G>A), TTT1084TTC (3922T>C), TCA1085TCC (3925A>C), GGT1087GGA (3931T>A), GTG1088GTT (3934G>T), GCA1089ACC (3935G>A 3937A>C), ACA1090ACT (3940A>T), AAT1091AAC (3943T>C), ACG1092ATT (3945C>T 3946G>T), TTC1093TTT (3949C>T), TCA1094TCC (3952A>C), TTC1095TTT (3955C>T), ACT1096GTG (3956A>G 3957C>T 3958T>G), GAT1099GAC (3967T>C), GAG1101AAA (3971G>A 3973G>A), CCA1104CCT (3982A>T), AGG1105CGG (3983A>C), CGG1106AGG (3986C>A), CAT1107TAT (3989C>T), ACA1108ACT (3994A>T), AGG1109AAG (3996G>A), ATG1110GCA (3998A>G 3999T>C 4000G>A), AGG1111CGA (4001A>C 4003G>A), TTC1113TTT (4009C>T), TCA1114GAC (4010T>G 4011C>A 4012A>C), AGC1115AAT (4014G>A 4015C>T), CAA1117AGT (4019C>A 4020A>G 4021A>T), TCC1118TCT (4024C>T), CGC1119CGT (4027C>T), ATC1120ATT (4030C>T), ATG1121GTT (4031A>G 4033G>T), TCA1122TCT (4036A>T), CTA1123TTG (4037C>T 4039A>G), GAT1125GAC (4045T>C), AGG1126AAA (4047G>A 4048G>A), CTT1127TTG (4049C>T 4051T>G), GGA1128GGT (4054A>T), AAT1129CTT (4055A>C 4056A>T), CTG1130CTT (4060G>T), ATC1131TTG (4061A>T 4063C>G), ATA1132ATC (4066A>C), TTG1134CTA (4070T>C 4072G>A), CCT1135CCA (4075T>A), CCT1136CCC (4078T>C), TCC1137GTA (4079T>G 4080C>T 4081C>A), GCC1138GAC (4083C>A), ATA1139TTA (4085A>T), GTG1140ATT (4088G>A 4090G>T), AGT1141AGC (4093T>C), GAG1144GAA (4102G>A), ATA1145ATC (4105A>C), CTT1146ATG (4106C>A 4108T>G), ATA1147GTT (4109A>G 4111A>T), TCT1148AAG (4112T>A 4113C>A 4114T>G), CCT1149CCA (4117T>A), GGA1150GGG (4120A>G), CTT1151CCA (4122T>C 4123T>A), TTC1153TTT (4129C>T), AAG1154AGG (4131A>G), TTG1155TTA (4135G>A), GAG1156GAA (4138G>A), TTG1157CTT (4139T>C 4141G>T), TCT1162CTG (4154T>C 4155C>T 4156T>G), GCC1163GCT (4159C>T), AAC1164AAT (4162C>T), GGC1170GGT (4180C>T), AAT1171GAT (4181A>G), ACT1174ACG (4192T>G), CAC1175CAT (4195C>T), ACC1176ACT (4198C>T), TAT1177TAC (4201T>C), CAG1178CAA (4204G>A), TCA1181TCT (4213A>T), TTT1183TTC (4219T>C), TCT1184TCA (4222T>A), GAG1185GAA (4225G>A), CTA1186CTG (4228A>G), CGT1187AGA (4229C>A 4231T>A), GAT1188GAC (4234T>C), TTT1189TTC (4237T>C), GCG1190TCG (4238G>T), ATT1191AAC (4242T>A 4243T>C)

Proteins

	Begin	End	Coverage	Score	Concordance	Matches	Identities	I/D/M/F*	Stop Codons
polyprotein (YP_001976149.1)	764	1192	30.2%	2123	80.3%	360 (99.4%)	275 (76.0%)	2/0/0/0	1

	Begin	End	Coverage	Score	Concordance	Matches	Identities	I/D/M/F*	Stop Codons
**NT**	**2960**	**4410**	**25.4%**	**931**	**37.8%**	**1239 (98.9%)**	**867 (69.2%)**	**9/5**	
Protein mutations:	T790R (3038A>C 3039C>G 3040T>G), I799V (3065A>G 3067T>C), N817A (3119A>G 3120A>C 3121C>A), K819E (3125A>G), T821A (3131A>G), N822A (3134A>G 3135A>C 3136T>A), F827L (3149T>C 3151T>A), M838V (3182A>G), K841R (3191A>C 3192A>G 3193A>T), H920L (3429A>T), S927A (3449T>G 3451A>C), D936N (3476G>A 3478T>C), S938T (3482T>A 3484A>T), I943L (3497A>C), D949E (3517C>A), I951V (3521A>G), E953V (3528A>T 3529G>C), Q955D (3533C>G 3535G>T), E956D (3538G>T), G958_K959insKK (3544_3545insAAGAAG), P961K (3551C>A 3552C>A), P962E (3554C>G 3555C>A 3556C>G), K964V (3560A>G 3561A>T), A965G (3564C>G), S967V (3569A>G 3570G>T), I968A (3572A>G 3573T>C 3574T>A), S970G (3578T>G 3579C>G 3580C>A), I971L (3581A>C 3583A>T), M985S (3623A>T 3624T>C 3625G>T), G986A (3627G>C 3628T>C), D990T (3638G>A 3639A>C), E991S (3641G>A 3642A>G 3643A>C), L994V (3650T>G), I996L (3656A>C), I1003V (3677A>G), E1006S (3686G>T 3687A>C), G1007P (3689G>C 3690G>C 3691G>C), H1008T (3692C>A 3693A>C), T1024S (3740A>T 3742A>T), N1034E (3770A>G 3772T>G), S1036T (3776T>A 3778A>T), I1037L (3779A>C 3781T>C), V1038I (3782G>A 3784G>C), S1044G (3800T>G 3801C>G), P1046N (3806C>A 3807C>A), V1048I (3812G>A), F1056L (3838T>G), A1058T (3842G>A), P1064S (3860C>T 3862A>T), A1065Y (3863G>T 3864C>A), L1067V (3869C>G 3871T>G), N1068A (3872A>G 3873A>C 3874T>A), K1069E (3875A>G 3877A>G), N1071A (3881A>G 3882A>C 3883T>A), Y1072H (3884T>C), V1074L (3890G>C 3892A>T), T1080S (3908A>T 3910A>C), D1083N (3917G>A), A1089T (3935G>A 3937A>C), T1092I (3945C>T 3946G>T), T1096V (3956A>G 3957C>T 3958T>G), E1101K (3971G>A 3973G>A), H1107Y (3989C>T), R1109K (3996G>A), M1110A (3998A>G 3999T>C 4000G>A), S1114D (4010T>G 4011C>A 4012A>C), S1115N (4014G>A 4015C>T), Q1117S (4019C>A 4020A>G 4021A>T), M1121V (4031A>G 4033G>T), R1126K (4047G>A 4048G>A), N1129L (4055A>C 4056A>T), I1131L (4061A>T 4063C>G), S1137V (4079T>G 4080C>T 4081C>A), A1138D (4083C>A), I1139L (4085A>T), V1140I (4088G>A 4090G>T), L1146M (4106C>A 4108T>G), I1147V (4109A>G 4111A>T), S1148K (4112T>A 4113C>A 4114T>G), L1151P (4122T>C 4123T>A), K1154R (4131A>G), S1162L (4154T>C 4155C>T 4156T>G), N1171D (4181A>G), A1190S (4238G>T), I1191N (4242T>A 4243T>C)								
Codon mutations:	ATA767ATT (2971A>T), GAG769GAA (2977G>A), ACC773ACA (2989C>A), TTG774CTG (2990T>C), CCG776CCA (2998G>A), TCT777TCA (3001T>A), TTG778CTG (3002T>C), CTT779TTG (3005C>T 3007T>G), GGT780GGA (3010T>A), AAC781AAT (3013C>T), ATA782ATT (3016A>T), GCC783GCT (3019C>T), AGG784AGA (3022G>A), TGC786TGT (3028C>T), ACT790CGG (3038A>C 3039C>G 3040T>G), GGA791GGC (3043A>C), ACT792ACA (3046T>A), TGT793TGC (3049T>C), GAA796GAG (3058A>G), ATT797ATA (3061T>A), TGC798TGT (3064C>T), ATT799GTC (3065A>G 3067T>C), GAG800GAA (3070G>A), AAA801AAG (3073A>G), TTG803TTA (3079G>A), CAT805CAC (3085T>C), TCA806TCT (3088A>T), GGT807GGA (3091T>A), CTG809CTT (3097G>T), GCA810GCC (3100A>C), GGA812GGT (3106A>T), CTT813TTG (3107C>T 3109T>G), GGA814GGT (3112A>T), CTG816CTA (3118G>A), AAC817GCA (3119A>G 3120A>C 3121C>A), ACC818ACA (3124C>A), AAA819GAA (3125A>G), ACC821GCC (3131A>G), AAT822GCA (3134A>G 3135A>C 3136T>A), ATA826ATT (3148A>T), TTT827CTA (3149T>C 3151T>A), AAC828AAT (3154C>T), CCA830CCC (3160A>C), CAT831CAC (3163T>C), CTT836CTA (3178T>A), ATG838GTG (3182A>G), GGT839GGC (3187T>C), CGA840CGC (3190A>C), AAA841CGT (3191A>C 3192A>G 3193A>T), GGG919GGT (3427G>T), CAT920CTT (3429A>T), ACA922ACT (3436A>T), GGC924GGG (3442C>G), AAA925AAG (3445A>G), AGC926AGT (3448C>T), TCA927GCC (3449T>G 3451A>C), GAT929GAC (3457T>C), TTC930TTT (3460C>T), CTT931TTG (3461C>T 3463T>G), TTC932TTT (3466C>T), GAT936AAC (3476G>A 3478T>C), TTA937TTG (3481A>G), TCA938ACT (3482T>A 3484A>T), AAG941AAA (3493G>A), ATT943CTT (3497A>C), TTG946CTG (3506T>C), AAG948AAA (3514G>A), GAC949GAA (3517C>A), AAC950AAT (3520C>T), ATT951GTT (3521A>G), GAG953GTC (3528A>T 3529G>C), TAC954TAT (3532C>T), CAG955GAT (3533C>G 3535G>T), GAG956GAT (3538G>T), GGC958GGG (3544C>G), GGC958_AAA959insAAGAAG (3544_3545insAAGAAG), GAT960GAC (3550T>C), CCG961AAG (3551C>A 3552C>A), CCC962GAG (3554C>G 3555C>A 3556C>G), AAG963AAA (3559G>A), AAG964GTG (3560A>G 3561A>T), GCT965GGT (3564C>G), CAA966CAG (3568A>G), AGT967GTT (3569A>G 3570G>T), ATT968GCA (3572A>G 3573T>C 3574T>A), CTG969CTT (3577G>T), TCC970GGA (3578T>G 3579C>G 3580C>A), ATA971CTT (3581A>C 3583A>T), AGA972CGG (3584A>C 3586A>G), GAG973GAA (3589G>A), TTT975TTC (3595T>C), TCC976TCT (3598C>T), GGT978GGA (3604T>A), GTA980GTT (3610A>T), TTC983TTT (3619C>T), TGC984TGT (3622C>T), ATG985TCT (3623A>T 3624T>C 3625G>T), GGT986GCC (3627G>C 3628T>C), GAC990ACC (3638G>A 3639A>C), GAA991AGC (3641G>A 3642A>G 3643A>C), AGA992CGC (3644A>C 3646A>C), TTG993CTG (3647T>C), TTG994GTG (3650T>G), GTA995GTT (3655A>T), ATT996CTT (3656A>C), CCT997CCA (3661T>A), GCA999GCT (3667A>T), CCA1000CCG (3670A>G), TCC1002TCT (3676C>T), ATA1003GTA (3677A>G), AGG1004CGA (3680A>C 3682G>A), TTT1005TTC (3685T>C), GAA1006TCA (3686G>T 3687A>C), GGG1007CCC (3689G>C 3690G>C 3691G>C), CAC1008ACC (3692C>A 3693A>C), CCT1010CCC (3700T>C), GCA1014GCC (3712A>C), ATC1015ATA (3715C>A), ACT1016ACG (3718T>G), CCA1018CCC (3724A>C), ATA1020ATC (3730A>C), TGT1023TGC (3739T>C), ACA1024TCT (3740A>T 3742A>T), TGT1027TGC (3751T>C), TCA1030TCT (3760A>T), GGT1031GGC (3763T>C), TTG1033CTT (3767T>C 3769G>T), AAT1034GAG (3770A>G 3772T>G), TAT1035TAC (3775T>C), TCA1036ACT (3776T>A 3778A>T), ATT1037CTC (3779A>C 3781T>C), GTG1038ATC (3782G>A 3784G>C), ATA1039ATC (3787A>C), CAC1040CAT (3790C>T), AGA1041AGG (3793A>G), GTA1042GTC (3796A>C), TCC1044GGC (3800T>G 3801C>G), AGT1045TCT (3803A>T 3804G>C), CCT1046AAT (3806C>A 3807C>A), AAT1047AAC (3811T>C), GTT1048ATT (3812G>A), GGA1049GGT (3817A>T), CTA1052CTT (3826A>T), GTT1054GTG (3832T>G), TTT1056TTG (3838T>G), GAT1057GAC (3841T>C), GCC1058ACC (3842G>A), CCA1064TCT (3860C>T 3862A>T), GCT1065TAT (3863G>T 3864C>A), GGG1066GGT (3868G>T), CTT1067GTG (3869C>G 3871T>G), AAT1068GCA (3872A>G 3873A>C 3874T>A), AAA1069GAG (3875A>G 3877A>G), GGA1070GGG (3880A>G), AAT1071GCA (3881A>G 3882A>C 3883T>A), TAT1072CAT (3884T>C), GTA1074CTT (3890G>C 3892A>T), GGT1077GGC (3901T>C), GGA1078GGT (3904A>T), GGC1079GGT (3907C>T), ACA1080TCC (3908A>T 3910A>C), GAT1083AAT (3917G>A), TTT1084TTC (3922T>C), TCA1085TCC (3925A>C), GGT1087GGA (3931T>A), GTG1088GTT (3934G>T), GCA1089ACC (3935G>A 3937A>C), ACA1090ACT (3940A>T), AAT1091AAC (3943T>C), ACG1092ATT (3945C>T 3946G>T), TTC1093TTT (3949C>T), TCA1094TCC (3952A>C), TTC1095TTT (3955C>T), ACT1096GTG (3956A>G 3957C>T 3958T>G), GAT1099GAC (3967T>C), GAG1101AAA (3971G>A 3973G>A), CCA1104CCT (3982A>T), AGG1105CGG (3983A>C), CGG1106AGG (3986C>A), CAT1107TAT (3989C>T), ACA1108ACT (3994A>T), AGG1109AAG (3996G>A), ATG1110GCA (3998A>G 3999T>C 4000G>A), AGG1111CGA (4001A>C 4003G>A), TTC1113TTT (4009C>T), TCA1114GAC (4010T>G 4011C>A 4012A>C), AGC1115AAT (4014G>A 4015C>T), CAA1117AGT (4019C>A 4020A>G 4021A>T), TCC1118TCT (4024C>T), CGC1119CGT (4027C>T), ATC1120ATT (4030C>T), ATG1121GTT (4031A>G 4033G>T), TCA1122TCT (4036A>T), CTA1123TTG (4037C>T 4039A>G), GAT1125GAC (4045T>C), AGG1126AAA (4047G>A 4048G>A), CTT1127TTG (4049C>T 4051T>G), GGA1128GGT (4054A>T), AAT1129CTT (4055A>C 4056A>T), CTG1130CTT (4060G>T), ATC1131TTG (4061A>T 4063C>G), ATA1132ATC (4066A>C), TTG1134CTA (4070T>C 4072G>A), CCT1135CCA (4075T>A), CCT1136CCC (4078T>C), TCC1137GTA (4079T>G 4080C>T 4081C>A), GCC1138GAC (4083C>A), ATA1139TTA (4085A>T), GTG1140ATT (4088G>A 4090G>T), AGT1141AGC (4093T>C), GAG1144GAA (4102G>A), ATA1145ATC (4105A>C), CTT1146ATG (4106C>A 4108T>G), ATA1147GTT (4109A>G 4111A>T), TCT1148AAG (4112T>A 4113C>A 4114T>G), CCT1149CCA (4117T>A), GGA1150GGG (4120A>G), CTT1151CCA (4122T>C 4123T>A), TTC1153TTT (4129C>T), AAG1154AGG (4131A>G), TTG1155TTA (4135G>A), GAG1156GAA (4138G>A), TTG1157CTT (4139T>C 4141G>T), TCT1162CTG (4154T>C 4155C>T 4156T>G), GCC1163GCT (4159C>T), AAC1164AAT (4162C>T), GGC1170GGT (4180C>T), AAT1171GAT (4181A>G), ACT1174ACG (4192T>G), CAC1175CAT (4195C>T), ACC1176ACT (4198C>T), TAT1177TAC (4201T>C), CAG1178CAA (4204G>A), TCA1181TCT (4213A>T), TTT1183TTC (4219T>C), TCT1184TCA (4222T>A), GAG1185GAA (4225G>A), CTA1186CTG (4228A>G), CGT1187AGA (4229C>A 4231T>A), GAT1188GAC (4234T>C), TTT1189TTC (4237T>C), GCG1190TCG (4238G>T), ATT1191AAC (4242T>A 4243T>C)								
Coat protein B (YP_001976156.1)	42	244	54.9%	817	82.4%	134 (98.5%)	110 (80.9%)	2/0/0/0	0
Protein mutations:	T68R (3038A>C 3039C>G 3040T>G), I77V (3065A>G 3067T>C), N95A (3119A>G 3120A>C 3121C>A), K97E (3125A>G), T99A (3131A>G), N100A (3134A>G 3135A>C 3136T>A), F105L (3149T>C 3151T>A), M116V (3182A>G), K119R (3191A>C 3192A>G 3193A>T), H198L (3429A>T), S205A (3449T>G 3451A>C), D214N (3476G>A 3478T>C), S216T (3482T>A 3484A>T), I221L (3497A>C), D227E (3517C>A), I229V (3521A>G), E231V (3528A>T 3529G>C), Q233D (3533C>G 3535G>T), E234D (3538G>T), G236_K237insKK (3544_3545insAAGAAG), P239K (3551C>A 3552C>A), P240E (3554C>G 3555C>A 3556C>G), K242V (3560A>G 3561A>T), A243G (3564C>G)								
Codon mutations:	ATA45ATT (2971A>T), GAG47GAA (2977G>A), ACC51ACA (2989C>A), TTG52CTG (2990T>C), CCG54CCA (2998G>A), TCT55TCA (3001T>A), TTG56CTG (3002T>C), CTT57TTG (3005C>T 3007T>G), GGT58GGA (3010T>A), AAC59AAT (3013C>T), ATA60ATT (3016A>T), GCC61GCT (3019C>T), AGG62AGA (3022G>A), TGC64TGT (3028C>T), ACT68CGG (3038A>C 3039C>G 3040T>G), GGA69GGC (3043A>C), ACT70ACA (3046T>A), TGT71TGC (3049T>C), GAA74GAG (3058A>G), ATT75ATA (3061T>A), TGC76TGT (3064C>T), ATT77GTC (3065A>G 3067T>C), GAG78GAA (3070G>A), AAA79AAG (3073A>G), TTG81TTA (3079G>A), CAT83CAC (3085T>C), TCA84TCT (3088A>T), GGT85GGA (3091T>A), CTG87CTT (3097G>T), GCA88GCC (3100A>C), GGA90GGT (3106A>T), CTT91TTG (3107C>T 3109T>G), GGA92GGT (3112A>T), CTG94CTA (3118G>A), AAC95GCA (3119A>G 3120A>C 3121C>A), ACC96ACA (3124C>A), AAA97GAA (3125A>G), ACC99GCC (3131A>G), AAT100GCA (3134A>G 3135A>C 3136T>A), ATA104ATT (3148A>T), TTT105CTA (3149T>C 3151T>A), AAC106AAT (3154C>T), CCA108CCC (3160A>C), CAT109CAC (3163T>C), CTT114CTA (3178T>A), ATG116GTG (3182A>G), GGT117GGC (3187T>C), CGA118CGC (3190A>C), AAA119CGT (3191A>C 3192A>G 3193A>T), GGG197GGT (3427G>T), CAT198CTT (3429A>T), ACA200ACT (3436A>T), GGC202GGG (3442C>G), AAA203AAG (3445A>G), AGC204AGT (3448C>T), TCA205GCC (3449T>G 3451A>C), GAT207GAC (3457T>C), TTC208TTT (3460C>T), CTT209TTG (3461C>T 3463T>G), TTC210TTT (3466C>T), GAT214AAC (3476G>A 3478T>C), TTA215TTG (3481A>G), TCA216ACT (3482T>A 3484A>T), AAG219AAA (3493G>A), ATT221CTT (3497A>C), TTG224CTG (3506T>C), AAG226AAA (3514G>A), GAC227GAA (3517C>A), AAC228AAT (3520C>T), ATT229GTT (3521A>G), GAG231GTC (3528A>T 3529G>C), TAC232TAT (3532C>T), CAG233GAT (3533C>G 3535G>T), GAG234GAT (3538G>T), GGC236GGG (3544C>G), GGC236_AAA237insAAGAAG (3544_3545insAAGAAG), GAT238GAC (3550T>C), CCG239AAG (3551C>A 3552C>A), CCC240GAG (3554C>G 3555C>A 3556C>G), AAG241AAA (3559G>A), AAG242GTG (3560A>G 3561A>T), GCT243GGT (3564C>G), CAA244CAG (3568A>G)								
Coat protein C (YP_001976157.1)	1	225	100%	1305	79.1%	225 (100%)	164 (72.9%)	0/0/0/0	0
Protein mutations:	S1V (3569A>G 3570G>T), I2A (3572A>G 3573T>C 3574T>A), S4G (3578T>G 3579C>G 3580C>A), I5L (3581A>C 3583A>T), M19S (3623A>T 3624T>C 3625G>T), G20A (3627G>C 3628T>C), D24T (3638G>A 3639A>C), E25S (3641G>A 3642A>G 3643A>C), L28V (3650T>G), I30L (3656A>C), I37V (3677A>G), E40S (3686G>T 3687A>C), G41P (3689G>C 3690G>C 3691G>C), H42T (3692C>A 3693A>C), T58S (3740A>T 3742A>T), N68E (3770A>G 3772T>G), S70T (3776T>A 3778A>T), I71L (3779A>C 3781T>C), V72I (3782G>A 3784G>C), S78G (3800T>G 3801C>G), P80N (3806C>A 3807C>A), V82I (3812G>A), F90L (3838T>G), A92T (3842G>A), P98S (3860C>T 3862A>T), A99Y (3863G>T 3864C>A), L101V (3869C>G 3871T>G), N102A (3872A>G 3873A>C 3874T>A), K103E (3875A>G 3877A>G), N105A (3881A>G 3882A>C 3883T>A), Y106H (3884T>C), V108L (3890G>C 3892A>T), T114S (3908A>T 3910A>C), D117N (3917G>A), A123T (3935G>A 3937A>C), T126I (3945C>T 3946G>T), T130V (3956A>G 3957C>T 3958T>G), E135K (3971G>A 3973G>A), H141Y (3989C>T), R143K (3996G>A), M144A (3998A>G 3999T>C 4000G>A), S148D (4010T>G 4011C>A 4012A>C), S149N (4014G>A 4015C>T), Q151S (4019C>A 4020A>G 4021A>T), M155V (4031A>G 4033G>T), R160K (4047G>A 4048G>A), N163L (4055A>C 4056A>T), I165L (4061A>T 4063C>G), S171V (4079T>G 4080C>T 4081C>A), A172D (4083C>A), I173L (4085A>T), V174I (4088G>A 4090G>T), L180M (4106C>A 4108T>G), I181V (4109A>G 4111A>T), S182K (4112T>A 4113C>A 4114T>G), L185P (4122T>C 4123T>A), K188R (4131A>G), S196L (4154T>C 4155C>T 4156T>G), N205D (4181A>G), A224S (4238G>T), I225N (4242T>A 4243T>C)								

	Begin	End	Coverage	Score	Concordance	Matches	Identities	I/D/M/F*	Stop Codons
**NT**	**2960**	**4410**	**25.4%**	**931**	**37.8%**	**1239 (98.9%)**	**867 (69.2%)**	**9/5**	
Codon mutations:	AGT1GTT (3569A>G 3570G>T), ATT2GCA (3572A>G 3573T>C 3574T>A), CTG3CTT (3577G>T), TCC4GGA (3578T>G 3579C>G 3580C>A), ATA5CTT (3581A>C 3583A>T), AGA6CGG (3584A>C 3586A>G), GAG7GAA (3589G>A), TTT9TTC (3595T>C), TCC10TCT (3598C>T), GGT12GGA (3604T>A), GTA14GTT (3610A>T), TTC17TTT (3619C>T), TGC18TGT (3622C>T), ATG19TCT (3623A>T 3624T>C 3625G>T), GGT20GCC (3627G>C 3628T>C), GAC24ACC (3638G>A 3639A>C), GAA25AGC (3641G>A 3642A>G 3643A>C), AGA26CGC (3644A>C 3646A>C), TTG27CTG (3647T>C), TTG28GTG (3650T>G), GTA29GTT (3655A>T), ATT30CTT (3656A>C), CCT31CCA (3661T>A), GCA33GCT (3667A>T), CCA34CCG (3670A>G), TCC36TCT (3676C>T), ATA37GTA (3677A>G), AGG38CGA (3680A>C 3682G>A), TTT39TTC (3685T>C), GAA40TCA (3686G>T 3687A>C), GGG41CCC (3689G>C 3690G>C 3691G>C), CAC42ACC (3692C>A 3693A>C), CCT44CCC (3700T>C), GCA48GCC (3712A>C), ATC49ATA (3715C>A), ACT50ACG (3718T>G), CCA52CCC (3724A>C), ATA54ATC (3730A>C), TGT57TGC (3739T>C), ACA58TCT (3740A>T 3742A>T), TGT61TGC (3751T>C), TCA64TCT (3760A>T), GGT65GGC (3763T>C), TTG67CTT (3767T>C 3769G>T), AAT68GAG (3770A>G 3772T>G), TAT69TAC (3775T>C), TCA70ACT (3776T>A 3778A>T), ATT71CTC (3779A>C 3781T>C), GTG72ATC (3782G>A 3784G>C), ATA73ATC (3787A>C), CAC74CAT (3790C>T), AGA75AGG (3793A>G), GTA76GTC (3796A>C), TCC78GGC (3800T>G 3801C>G), AGT79TCT (3803A>T 3804G>C), CCT80AAT (3806C>A 3807C>A), AAT81AAC (3811T>C), GTT82ATT (3812G>A), GGA83GGT (3817A>T), CTA86CTT (3826A>T), GTT88GTG (3832T>G), TTT90TTG (3838T>G), GAT91GAC (3841T>C), GCC92ACC (3842G>A), CCA98TCT (3860C>T 3862A>T), GCT99TAT (3863G>T 3864C>A), GGG100GGT (3868G>T), CTT101GTG (3869C>G 3871T>G), AAT102GCA (3872A>G 3873A>C 3874T>A), AAA103GAG (3875A>G 3877A>G), GGA104GGG (3880A>G), AAT105GCA (3881A>G 3882A>C 3883T>A), TAT106CAT (3884T>C), GTA108CTT (3890G>C 3892A>T), GGT111GGC (3901T>C), GGA112GGT (3904A>T), GGC113GGT (3907C>T), ACA114TCC (3908A>T 3910A>C), GAT117AAT (3917G>A), TTT118TTC (3922T>C), TCA119TCC (3925A>C), GGT121GGA (3931T>A), GTG122GTT (3934G>T), GCA123ACC (3935G>A 3937A>C), ACA124ACT (3940A>T), AAT125AAC (3943T>C), ACG126ATT (3945C>T 3946G>T), TTC127TTT (3949C>T), TCA128TCC (3952A>C), TTC129TTT (3955C>T), ACT130GTG (3956A>G 3957C>T 3958T>G), GAT133GAC (3967T>C), GAG135AAA (3971G>A 3973G>A), CCA138CCT (3982A>T), AGG139CGG (3983A>C), CGG140AGG (3986C>A), CAT141TAT (3989C>T), ACA142ACT (3994A>T), AGG143AAG (3996G>A), ATG144GCA (3998A>G 3999T>C 4000G>A), AGG145CGA (4001A>C 4003G>A), TTC147TTT (4009C>T), TCA148GAC (4010T>G 4011C>A 4012A>C), AGC149AAT (4014G>A 4015C>T), CAA151AGT (4019C>A 4020A>G 4021A>T), TCC152TCT (4024C>T), CGC153CGT (4027C>T), ATC154ATT (4030C>T), ATG155GTT (4031A>G 4033G>T), TCA156TCT (4036A>T), CTA157TTG (4037C>T 4039A>G), GAT159GAC (4045T>C), AGG160AAA (4047G>A 4048G>A), CTT161TTG (4049C>T 4051T>G), GGA162GGT (4054A>T), AAT163CTT (4055A>C 4056A>T), CTG164CTT (4060G>T), ATC165TTG (4061A>T 4063C>G), ATA166ATC (4066A>C), TTG168CTA (4070T>C 4072G>A), CCT169CCA (4075T>A), CCT170CCC (4078T>C), TCC171GTA (4079T>G 4080C>T 4081C>A), GCC172GAC (4083C>A), ATA173TTA (4085A>T), GTG174ATT (4088G>A 4090G>T), AGT175AGC (4093T>C), GAG178GAA (4102G>A), ATA179ATC (4105A>C), CTT180ATG (4106C>A 4108T>G), ATA181GTT (4109A>G 4111A>T), TCT182AAG (4112T>A 4113C>A 4114T>G), CCT183CCA (4117T>A), GGA184GGG (4120A>G), CTT185CCA (4122T>C 4123T>A), TTC187TTT (4129C>T), AAG188AGG (4131A>G), TTG189TTA (4135G>A), GAG190GAA (4138G>A), TTG191CTT (4139T>C 4141G>T), TCT196CTG (4154T>C 4155C>T 4156T>G), GCC197GCT (4159C>T), AAC198AAT (4162C>T), GGC204GGT (4180C>T), AAT205GAT (4181A>G), ACT208ACG (4192T>G), CAC209CAT (4195C>T), ACC210ACT (4198C>T), TAT211TAC (4201T>C), CAG212CAA (4204G>A), TCA215TCT (4213A>T), TTT217TTC (4219T>C), TCT218TCA (4222T>A), GAG219GAA (4225G>A), CTA220CTG (4228A>G), CGT221AGA (4229C>A 4231T>A), GAT222GAC (4234T>C), TTT223TTC (4237T>C), GCG224TCG (4238G>T), ATT225AAC (4242T>A 4243T>C)								

*: Inserts / Deletes / Misaligned / Frameshifts

Analysis details

This analysis was performed with panviral2.64

NGS Details (UN62): Diachasmimorpha longicaudata entomopoxvirus (segment NC_043455.1)

Assembly

**Coverage Length**	1126 (1 contig(s))
**Depth Of Coverage**	993.3
**Number Of Reads**	8833
**Reads Per Million**	204.76 rpm (after QC)
**Ambiguities**	0
**Assembly Method**	de novo + reference guided assembly
**Consensus Caller**	Bcf Tools

Coverage Map

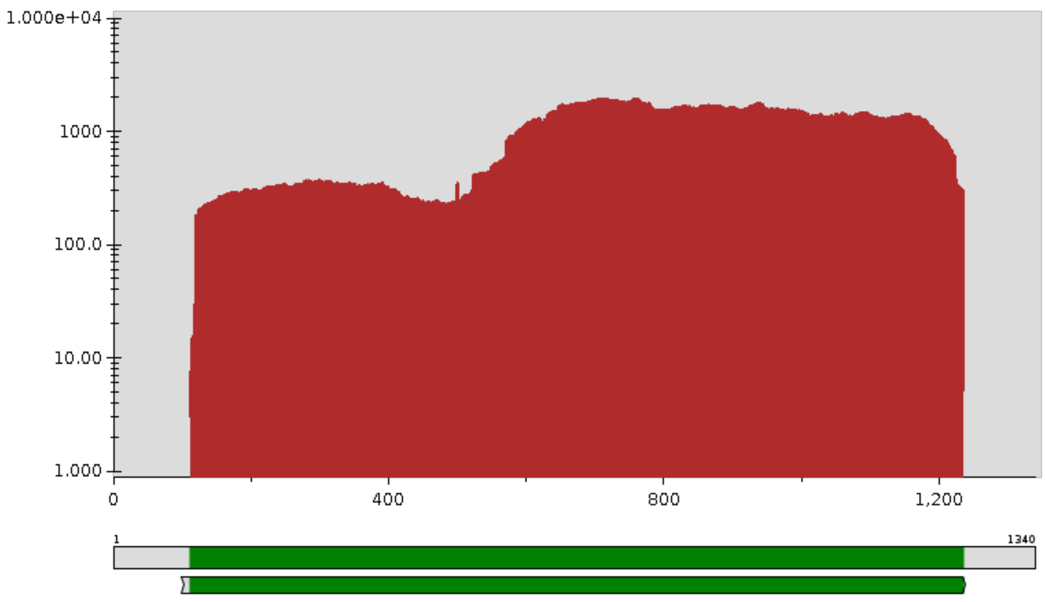

Assignment

**Type**	Diachasmimorpha longicaudata entomopoxvirus (Taxonomy ID: 109981)
**Reference Genome**	NC_043455.1
**NT Identity (%)**	60.0355
**AA Identity (%)**	55.4667
**Number Of Stop Codons**	0
**Number Of CDS**	1

Alignment

**Alignment Score**	434.0 (NT) + 1463.0 (AA) = 1897.0
**Concordance (%)**	40.2418

Alignment Method	Global, seeded, nucleotide + amino acids (AGA)

Genome Region

Sequence starts at position 112 and ends at position 1237 relative to NC_043455.1 reference sequence.

Alignment Detailed Statistics

	Begin	End	Coverage	Score	Concordance	Matches	Identities	I/D/M/F*	Stop Codons
**NT**	**112**	**1237**	**84.0%**	**434**	**19.4%**	**1123 (99.5%)**	**676 (59.9%)**	**3/3**	
Mutations:	121A>T, 126A>C, 127C>T, 128C>G, 130T>G, 136C>G, 137C>A, 139C>T, 142G>A, 143A>G, 145A>T, 146A>G, 148T>C, 149A>C, 151C>T, 154A>T, 156A>G, 157G>A, 160T>G, 163A>T, 166T>C, 167T>C, 168C>A, 169T>G, 171G>A, 172T>C, 175A>G, 178T>C, 187T>A, 190A>T, 191A>G, 193T>C, 203A>C, 204A>G, 208C>T, 209A>G, 211T>A, 214T>G, 217A>T, 218T>A, 219G>T, 223T>A, 226T>G, 230A>C, 231A>G, 232A>T, 238G>C, 241A>T, 243T>C, 244C>T, 247A>G, 256G>C, 259A>T, 262A>C, 271A>C, 272G>T, 274A>C, 276C>T, 277A>G, 278T>A, 279A>T, 283A>T, 284A>C, 286C>T, 288G>C, 289T>C, 294T>G, 295A>C, 298G>A, 299C>G, 300A>T, 301A>T, 302A>G, 304A>G, 310T>C, 311T>A, 312C>A, 313T>A, 320A>G, 322T>G, 323A>G, 325C>A, 331T>A, 337C>A, 341A>T, 343C>T, 346A>T, 352T>G, 355A>G, 356T>C, 358G>T, 361T>A, 362C>G, 363T>C, 364C>T, 368G>A, 370T>G, 371C>G, 374C>A, 375G>A, 376T>G, 379A>G, 380T>A, 382G>T, 383C>T, 384A>T, 385A>G, 386A>G, 394G>A, 395A>G, 400T>C, 401C>A, 403A>T, 404_406delTAT, 409T>C, 410T>A, 412T>A, 413A>C, 415A>G, 416T>G, 417G>C, 418T>A, 421A>T, 423T>C, 424T>G, 438C>A, 442T>C, 443A>G, 445C>G, 446A>G, 447A>G, 448G>T, 452A>G, 453G>A, 454C>T, 455C>A, 456A>T, 457G>C, 458G>A, 459A>G, 462C>A, 463T>G, 467A>G, 469_470insCAT, 470A>G, 471A>G, 474C>T, 475C>A, 478G>A, 482T>G, 484G>C, 485A>T, 486T>C, 500C>A, 503A>G, 505G>C, 506A>T, 507T>G, 511T>C, 512C>A, 514A>G, 515C>T, 517C>G, 519C>G, 520C>A, 521C>A, 524A>C, 526A>G, 527A>T, 528G>C, 530A>C, 533G>C, 534A>G, 535T>C, 536A>C, 538C>T, 539A>G, 541A>T, 542G>T, 543C>A, 544A>C, 549A>C, 550A>G, 553A>G, 554G>T, 560A>T, 562A>G, 565T>C, 568A>G, 577G>A, 581T>C, 583G>C, 584A>T, 585T>C, 586A>C, 587G>A, 588A>G, 589T>A, 590A>G, 591A>G, 595T>C, 596T>A, 597T>A, 602A>C, 604A>G, 607A>T, 608C>T, 610A>T, 620G>C, 622A>G, 623T>C, 628T>G, 629G>C, 630A>C, 632A>C, 633G>C, 634T>A, 635C>A, 637T>G, 638G>A, 646T>G, 647A>G, 648T>G, 650T>G, 652A>T, 655A>C, 658A>T, 661A>C, 664C>T, 665G>A, 667T>G, 671T>C, 674A>G, 675G>A, 678T>C, 680A>C, 682C>T, 683A>G, 685T>G, 687C>T, 688A>T, 689T>A, 692C>A, 693A>G, 694G>A, 695G>A, 696T>A, 697C>G, 700T>C, 705G>A, 706A>C, 707G>A, 709T>G, 715A>G, 717A>G, 718A>G, 724G>T, 727A>G, 730A>G, 731A>C, 732A>G, 735C>A, 739A>G, 740T>C, 742G>C, 745A>T, 748C>T, 754C>T, 759G>A, 765A>T, 770A>G, 772A>C, 778A>C, 779A>G, 781G>T, 784A>G, 785A>G, 787T>A, 790T>A, 792T>G, 793T>G, 796A>G, 797G>C, 798C>T, 799A>T, 810T>G, 823C>G, 824C>A, 825A>C, 826C>T, 830A>G, 831G>C, 833C>A, 838T>C, 841A>G, 843C>G, 844A>T, 845C>G, 847A>C, 850A>C, 854T>G, 855G>T, 856T>G, 859T>C, 862A>C, 863C>A, 865T>G, 866A>C, 868A>T, 869C>A, 878G>T, 879T>G, 880A>G, 885T>C, 886G>T, 889A>T, 890T>A, 891G>A, 892T>G, 893T>A, 895A>G, 896A>C, 897C>G, 898A>C, 900A>G, 901T>C, 902A>C, 904A>T, 905A>G, 908T>C, 909T>A, 915C>T, 919A>T, 920A>G, 921G>C, 922C>A, 924T>C, 925C>T, 934T>C, 938T>G, 939C>A, 943A>G, 944C>A, 946A>C, 947G>A, 948A>C, 949A>T, 950C>A, 952T>A, 956T>A, 958T>C, 965A>C, 966A>G, 970A>G, 973T>C, 974A>C, 977G>T, 978A>C, 982T>G, 983A>T, 984A>C, 986A>T, 989A>C, 991A>T, 992A>G, 994A>G, 997T>C, 998C>A, 1000T>C, 1001T>A, 1003A>T, 1007A>G, 1012A>T, 1015A>G, 1018A>T, 1019A>C, 1021A>T, 1024C>T, 1033T>A, 1036A>G, 1040A>G, 1042A>C, 1045T>C, 1046T>C, 1048A>T, 1051T>C, 1054C>T, 1057T>C, 1063T>C, 1064T>C, 1070C>A, 1071A>C, 1073A>C, 1075T>A, 1076A>C, 1077G>C, 1087T>C, 1088A>C, 1090A>G, 1103A>C, 1105A>T, 1111T>A, 1112A>C, 1120C>A, 1121C>A, 1123A>G, 1126A>G, 1129G>T, 1130A>G, 1141T>C, 1145A>G, 1147A>C, 1150T>C, 1151G>A, 1153A>G, 1156C>T, 1159A>T, 1160T>G, 1161T>A, 1163C>A, 1164C>G, 1165A>G, 1167A>T, 1172G>T, 1173C>T, 1177A>C, 1178T>A, 1181G>C, 1183A>G, 1185C>A, 1186G>A, 1192T>C, 1195T>C, 1196A>G, 1197C>T, 1199A>G, 1200A>T, 1204A>C, 1207T>G, 1210A>G, 1211A>C, 1218A>C, 1219G>C, 1223A>G, 1227T>C, 1232T>C, 1234G>C, 1235A>C, 1237A>G								
**CDS**									
FLA14_p101	5	379	98.7%	1463	58.8%	374 (99.5%)	208 (55.3%)	1/1/0/0	0
Protein mutations:	E7D (121A>T), D9A (126A>C 127C>T), H10E (128C>G 130T>G), L13I (137C>A 139C>T), K15D (143A>G 145A>T), N16D (146A>G 148T>C), I17L (149A>C 151C>T), K19R (156A>G 157G>A), S23Q (167T>C 168C>A 169T>G), C24Y (171G>A 172T>C), T31A (191A>G 193T>C), K35R (203A>C 204A>G), I37V (209A>G 211T>A), F38L (214T>G), C40I (218T>A 219G>T), K44R (230A>C 231A>G 232A>T), V48A (243T>C 244C>T), A58S (272G>T 274A>C), T59M (276C>T 277A>G), Y60I (278T>A 279A>T), I62L (284A>C 286C>T), S63T (288G>C 289T>C), L65C (294T>G 295A>C), Q67V (299C>G 300A>T 301A>T), I68V (302A>G 304A>G), S71K (311T>A 312C>A 313T>A), N74E (320A>G 322T>G), I75V (323A>G 325C>A), T81S (341A>T 343C>T), L88A (362C>G 363T>C 364C>T), A90T (368G>A 370T>G), Q91E (371C>G), R92K (374C>A 375G>A 376T>G), L94I (380T>A 382G>T), Q95L (383C>T 384A>T 385A>G), T96A (386A>G), N99D (395A>G), L101I (401C>A 403A>T), Y102del (404_406delTAT), F104I (410T>A 412T>A), K105Q (413A>C 415A>G), C106A (416T>G 417G>C 418T>A), Q107H (421A>T), V108A (423T>C 424T>G), T113K (438C>A), I115V (443A>G 445C>G), K116G (446A>G 447A>G 448G>T), S118D (452A>G 453G>A 454C>T), Q119I (455C>A 456A>T 457G>C), E120R (458G>A 459A>G), T121K (462C>A 463T>G), K123E (467A>G), K123_K124insH (469_470insCAT), K124G (470A>G 471A>G), A125V (474C>T 475C>A), L128V (482T>G 484G>C), I129S (485A>T 486T>C), M135V (503A>G 505G>C), I136C (506A>T 507T>G), L138M (512C>A 514A>G), T140R (519C>G 520C>A), K142Q (524A>C 526A>G), I144L (530A>C), D145R (533G>C 534A>G 535T>C), T146P (536A>C 538C>T), K147D (539A>G 541A>T), A148Y (542G>T 543C>A 544A>C), K150T (549A>C 550A>G), I151M (553A>G), V152F (554G>T), I154L (560A>T 562A>G), I162S (584A>T 585T>C 586A>C), D163R (587G>A 588A>G 589T>A), N164G (590A>G 591A>G), L166K (596T>A 597T>A), K168Q (602A>C 604A>G), Q170Y (608C>T 610A>T), E174Q (620G>C 622A>G), F175L (623T>C), F176L (628T>G), E177P (629G>C 630A>C), S178P (632A>C 633G>C 634T>A), H179K (635C>A 637T>G), V180I (638G>A), I183G (647A>G 648T>G), L184V (650T>G 652A>T), L185F (655A>C), V189M (665G>A 667T>G), S191P (671T>C), R192E (674A>G 675G>A), V193A (678T>C), I194L (680A>C 682C>T), N195E (683A>G 685T>G), T196I (687C>T 688A>T), S197T (689T>A), Q198R (692C>A 693A>G 694G>A), V199K (695G>A 696T>A 697C>G), R202N (705G>A 706A>C), D203K (707G>A 709T>G), K206R (717A>G 718A>G), N211R (731A>C 732A>G), A212D (735C>A), R220K (759G>A), Y222F (765A>T), I224V (770A>G 772A>C), K227D (779A>G 781G>T), N229E (785A>G 787T>A), D230E (790T>A), F231W (792T>G 793T>G), A233L (797G>C 798C>T 799A>T), F237C (810T>G), D241E (823C>G), H242T (824C>A 825A>C 826C>T), S244A (830A>G 831G>C), L245I (833C>A), T248S (843C>G 844A>T), L249V (845C>G 847A>C), C252V (854T>G 855G>T 856T>G), Q257K (869C>A), V260W (878G>T 879T>G 880A>G), M262T (885T>C 886G>T), E263D (889A>T), C264K (890T>A 891G>A 892T>G), L265M (893T>A 895A>G), T266R (896A>C 897C>G 898A>C), N267S (900A>G 901T>C), N269D (905A>G), F270H (908T>C 909T>A), A272V (915C>T), S274A (920A>G 921G>C 922C>A), I275T (924T>C 925C>T), S280D (938T>G 939C>A), Q282N (944C>A 946A>C), E283T (947G>A 948A>C 949A>T), F286I (956T>A 958T>C), K289R (965A>C 966A>G), D293S (977G>T 978A>C), K295S (983A>T 984A>C), T296S (986A>T), I298V (992A>G 994A>G), L300I (998C>A 1000T>C), S301T (1001T>A 1003A>T), N303D (1007A>G), I314V (1040A>G 1042A>C), H324T (1070C>A 1071A>C), N325Q (1073A>C 1075T>A), R326P (1076A>C 1077G>C), I330L (1088A>C 1090A>G), I344V (1130A>G), I349V (1145A>G 1147A>C), E351K (1151G>A 1153A>G), E353D (1159A>T), L354E (1160T>G 1161T>A), P355R (1163C>A 1164C>G 1165A>G), K356M (1167A>T), A358F (1172G>T 1173C>T), E359D (1177A>C), L360I (1178T>A), E361Q (1181G>C 1183A>G), T362K (1185C>A 1186G>A), T366V (1196A>G 1197C>T), K367V (1199A>G 1200A>T), D369E (1207T>G), M371L (1211A>C), E373A (1218A>C 1219G>C), I375V (1223A>G), V376A (1227T>C), I379L (1235A>C 1237A>G)								

	Begin	End	Coverage	Score	Concordance	Matches	Identities	I/D/M/F*	Stop Codons
**NT**	**112**	**1237**	**84.0%**	**434**	**19.4%**	**1123 (99.5%)**	**676 (59.9%)**	**3/3**	

Codon mutations:

GAA7GAT (121A>T), GAC9GCT (126A>C 127C>T), CAT10GAG (128C>G 130T>G), GGC12GGG (136C>G), CTC13ATT (137C>A 139C>T), AAG14AAA (142G>A), AAA15GAT (143A>G 145A>T), AAT16GAC (146A>G 148T>C), ATC17CTT (149A>C 151C>T), CTA18CTT (154A>T), AAG19AGA (156A>G 157G>A), GGT20GGG (160T>G), ATA21ATT (163A>T), TAT22TAC (166T>C), TCT23CAG (167T>C 168C>A 169T>G), TGT24TAC (171G>A 172T>C), GGA25GGG (175A>G), TTT26TTC (178T>C), CCT29CCA (187T>A), TCA30TCT (190A>T), ACT31GCC (191A>G 193T>C), AAA35CGA (203A>C 204A>G), GCC36GCT (208C>T), ATT37GTA (209A>G 211T>A), TTT38TTG (214T>G), CCA39CCT (217A>T), TGT40ATT (218T>A 219G>T), ATT41ATA (223T>A), TCT42TCG (226T>G), AAA44CGT (230A>C 231A>G 232A>T), GTG46GTC (238G>C), ATA47ATT (241A>T), GTC48GCT (243T>C 244C>T), CAA49CAG (247A>G), TCG52TCC (256G>C), GGA53GGT (259A>T), ACA54ACC (262A>C), ACA57ACC (271A>C), GCA58TCC (272G>T 274A>C), ACA59ATG (276C>T 277A>G), TAT60ATT (278T>A 279A>T), GCA61GCT (283A>T), ATC62CTT (284A>C 286C>T), AGT63ACC (288G>C 289T>C), TTA65TGC (294T>G 295A>C), CAG66CAA (298G>A), CAA67GTT (299C>G 300A>T 301A>T), ATA68GTG (302A>G 304A>G), ACT70ACC (310T>C), TCT71AAA (311T>A 312C>A 313T>A), AAT74GAG (320A>G 322T>G), ATC75GTA (323A>G 325C>A), GCT77GCA (331T>A), ATC79ATA (337C>A), ACC81TCT (341A>T 343C>T), CCA82CCT (346A>T), CGT84CGG (352T>G), GAA85GAG (355A>G), TTG86CTT (356T>C 358G>T), GCT87GCA (361T>A), CTC88GCT (362C>G 363T>C 364C>T), GCT90ACG (368G>A 370T>G), CAA91GAA (371C>G), CGT92AAG (374C>A 375G>A 376T>G), GTA93GTG (379A>G), TTG94ATT (380T>A 382G>T), CAA95TTG (383C>T 384A>T 385A>G), ACA96GCA (386A>G), GGG98GGA (394G>A), AAT99GAT (395A>G), TAT100TAC (400T>C), CTA101ATT (401C>A 403A>T), TAT102del (404_406delTAT), AAT103AAC (409T>C), TTT104ATA (410T>A 412T>A), AAA105CAG (413A>C 415A>G), TGT106GCA (416T>G 417G>C 418T>A), CAA107CAT (421A>T), GTT108GCG (423T>C 424T>G), ACA113AAA (438C>A), AGT114AGC (442T>C), ATC115GTG (443A>G 445C>G), AAG116GGT (446A>G 447A>G 448G>T), AGC118GAT (452A>G 453G>A 454C>T), CAG119ATC (455C>A 456A>T 457G>C), GAA120AGA (458G>A 459A>G), ACT121AAG (462C>A 463T>G), AAG123GAG (467A>G), AAG123_AAA124insCAT (469_470insCAT), AAA124GGA (470A>G 471A>G), GCC125GTA (474C>T 475C>A), CAG126CAA (478G>A), TTG128GTC (482T>G 484G>C), ATT129TCT (485A>T 486T>C), CGA134AGA (500C>A), ATG135GTC (503A>G 505G>C), ATT136TGT (506A>T 507T>G), GAT137GAC (511T>C), CTA138ATG (512C>A 514A>G), CTC139TTG (515C>T 517C>G), ACC140AGA (519C>G 520C>A), CGA141AGA (521C>A), AAA142CAG (524A>C 526A>G), AGT143TCT (527A>T 528G>C), ATC144CTC (530A>C), GAT145CGC (533G>C 534A>G 535T>C), ACC146CCT (536A>C 538C>T), AAA147GAT (539A>G 541A>T), GCA148TAC (542G>T 543C>A 544A>C), AAA150ACG (549A>C 550A>G), ATA151ATG (553A>G), GTT152TTT (554G>T), ATA154TTG (560A>T 562A>G), GAT155GAC (565T>C), GAA156GAG (568A>G), GAG159GAA (577G>A), TTG161CTC (581T>C 583G>C), ATA162TCC (584A>T 585T>C 586A>C), GAT163AGA (587G>A 588A>G 589T>A), AAT164GGT (590A>G 591A>G), TTT165TTC (595T>C), TTG166AAG (596T>A 597T>A), AAA168CAG (602A>C 604A>G), ATA169ATT (607A>T), CAA170TAT (608C>T 610A>T), GAA174CAG (620G>C 622A>G), TTT175CTT (623T>C), TTT176TTG (628T>G), GAA177CCA (629G>C 630A>C), AGT178CCA (632A>C 633G>C 634T>A), CAT179AAG (635C>A 637T>G), GTC180ATC (638G>A), GTT182GTG (646T>G), ATT183GGT (647A>G 648T>G), TTA184GTT (650T>G 652A>T), TTA185TTC (655A>C), TCA186TCT (658A>T), GCA187GCC (661A>C), ACC188ACT (664C>T), GTT189ATG (665G>A 667T>G), TCA191CCA (671T>C), AGG192GAG (674A>G 675G>A), GTT193GCT (678T>C), ATC194CTT (680A>C 682C>T), AAT195GAG (683A>G 685T>G), ACA196ATT (687C>T 688A>T), TCT197ACT (689T>A), CAG198AGA (692C>A 693A>G 694G>A), GTC199AAG (695G>A 696T>A 697C>G), TTT200TTC (700T>C), AGA202AAC (705G>A 706A>C), GAT203AAG (707G>A 709T>G), GTA205GTG (715A>G), AAA206AGG (717A>G 718A>G), CTG208CTT (724G>T), GTA209GTG (727A>G), AAA210AAG (730A>G), AAT211CGT (731A>C 732A>G), GCT212GAT (735C>A), GAA213GAG (739A>G), TTG214CTC (740T>C 742G>C), ACA215ACT (745A>T), CTC216CTT (748C>T), GGC218GGT (754C>T), AGG220AAG (759G>A), TAT222TTT (765A>T), ATA224GTC (770A>G 772A>C), GTA226GTC (778A>C), AAG227GAT (779A>G 781G>T), AAA228AAG (784A>G), AAT229GAA (785A>G 787T>A), GAT230GAA (790T>A), TTT231TGG (792T>G 793T>G), AAA232AAG (796A>G), GCA233CTT (797G>C 798C>T 799A>T), TTT237TGT (810T>G), GAC241GAG (823C>G), CAC242ACT (824C>A 825A>C 826C>T), AGC244GCC (830A>G 831G>C), CTC245ATC (833C>A), ACT246ACC (838T>C), CAA247CAG (841A>G), ACA248AGT (843C>G 844A>T), CTA249GTC (845C>G 847A>C), ATA250ATC (850A>C), TGT252GTG (854T>G 855G>T 856T>G), AAT253AAC (859T>C), ACA254ACC (862A>C), CGT255AGG (863C>A 865T>G), AGA256CGT (866A>C 868A>T), CAA257AAA (869C>A), GTA260TGG (878G>T 879T>G 880A>G), ATG262ACT (885T>C 886G>T), GAA263GAT (889A>T), TGT264AAG (890T>A 891G>A 892T>G), TTA265ATG (893T>A 895A>G), ACA266CGC (896A>C 897C>G 898A>C), AAT267AGC (900A>G 901T>C), AGA268CGT (902A>C 904A>T), AAT269GAT (905A>G), TTC270CAC (908T>C 909T>A), GCA272GTA (915C>T), TCA273TCT (919A>T), AGC274GCA (920A>G 921G>C 922C>A), ATC275ACT (924T>C 925C>T), GAT278GAC (934T>C), TCC280GAC (938T>G 939C>A), CAA281CAG (943A>G), CAA282AAC (944C>A 946A>C), GAA283ACT (947G>A 948A>C 949A>T), CGT284AGA (950C>A 952T>A), TTT286ATC (956T>A 958T>C), AAA289CGA (965A>C 966A>G), GAA290GAG (970A>G), TTT291TTC (973T>C), AGA292CGA (974A>C), GAT293TCT (977G>T 978A>C), GGT294GGG (982T>G), AAA295TCA (983A>T 984A>C), ACT296TCT (986A>T), AGA297CGT (989A>C 991A>T), ATA298GTG (992A>G 994A>G), CTT299CTC (997T>C), CTT300ATC (998C>A 1000T>C), TCA301ACT (1001T>A 1003A>T), AAT303GAT (1007A>G), CTA304CTT (1012A>T), TTA305TTG (1015A>G), GCA306GCT (1018A>T), AGA307CGT (1019A>C 1021A>T), GGC308GGT (1024C>T), GTT311GTA (1033T>A), CAA312CAG (1036A>G), ATA314GTC (1040A>G 1042A>C), TCT315TCC (1045T>C), TTA316CTT (1046T>C 1048A>T), GTT317GTC (1051T>C), ATC318ATT (1054C>T), AAT319AAC (1057T>C), GAT321GAC (1063T>C), TTG322CTG (1064T>C), CAT324ACT (1070C>A 1071A>C), AAT325CAA (1073A>C 1075T>A), AGA326CCA (1076A>C 1077G>C), TAT329TAC (1087T>C), ATA330CTG (1088A>C 1090A>G), AGA335CGT (1103A>C 1105A>T), GGT337GGA (1111T>A), AGA338CGA (1112A>C), GGC340GGA (1120C>A), CGA341AGG (1121C>A 1123A>G), AAA342AAG (1126A>G), GGG343GGT (1129G>T), ATT344GTT (1130A>G), AAT347AAC (1141T>C), ATA349GTC (1145A>G 1147A>C), ACT350ACC (1150T>C), GAA351AAG (1151G>A 1153A>G), GAC352GAT (1156C>T), GAA353GAT (1159A>T), TTA354GAA (1160T>G 1161T>A), CCA355AGG (1163C>A 1164C>G 1165A>G), AAG356ATG (1167A>T), GCT358TTT (1172G>T 1173C>T), GAA359GAC (1177A>C), TTA360ATA (1178T>A), GAA361CAG (1181G>C 1183A>G), ACG362AAA (1185C>A 1186G>A), TAT364TAC (1192T>C), AAT365AAC (1195T>C), ACC366GTC (1196A>G 1197C>T), AAA367GTA (1199A>G 1200A>T), ATA368ATC (1204A>C), GAT369GAG (1207T>G), GAA370GAG (1210A>G), ATG371CTG (1211A>C), GAG373GCC (1218A>C 1219G>C), ATT375GTT (1223A>G), GTT376GCT (1227T>C), TTG378CTC (1232T>C 1234G>C), ATA379CTG (1235A>C 1237A>G)

Proteins

	Begin	End	Coverage	Score	Concordance	Matches	Identities	I/D/M/F*	Stop Codons
hypothetical protein (YP_009666148.1)	5	379	98.7%	1463	58.8%	374 (99.5%)	208 (55.3%)	1/1/0/0	0

Protein mutations:

E7D (121A>T), D9A (126A>C 127C>T), H10E (128C>G 130T>G), L13I (137C>A 139C>T), K15D (143A>G 145A>T), N16D (146A>G 148T>C), I17L (149A>C 151C>T), K19R (156A>G 157G>A), S23Q (167T>C 168C>A 169T>G), C24Y (171G>A 172T>C), T31A (191A>G 193T>C), K35R (203A>C 204A>G), I37V (209A>G 211T>A), F38L (214T>G), C40I (218T>A 219G>T), K44R (230A>C 231A>G 232A>T), V48A (243T>C 244C>T), A58S (272G>T 274A>C), T59M (276C>T 277A>G), Y60I (278T>A 279A>T), I62L (284A>C 286C>T), S63T (288G>C 289T>C), L65C (294T>G 295A>C), Q67V (299C>G 300A>T 301A>T), I68V (302A>G 304A>G), S71K (311T>A 312C>A 313T>A), N74E (320A>G 322T>G), I75V (323A>G 325C>A), T81S (341A>T 343C>T), L88A (362C>G 363T>C 364C>T), A90T (368G>A 370T>G), Q91E (371C>G), R92K (374C>A 375G>A 376T>G), L94I (380T>A 382G>T), Q95L (383C>T 384A>T 385A>G), T96A (386A>G), N99D (395A>G), L101I (401C>A 403A>T), Y102del (404_406delTAT), F104I (410T>A 412T>A), K105Q (413A>C 415A>G), C106A (416T>G 417G>C 418T>A), Q107H (421A>T), V108A (423T>C 424T>G), T113K (438C>A), I115V (443A>G 445C>G), K116G (446A>G 447A>G 448G>T), S118D (452A>G 453G>A 454C>T), Q119I (455C>A 456A>T 457G>C), E120R (458G>A 459A>G), T121K (462C>A 463T>G), K123E (467A>G), K123_K124insH (469_470insCAT), K124G (470A>G 471A>G), A125V (474C>T 475C>A), L128V (482T>G 484G>C), I129S (485A>T 486T>C), M135V (503A>G 505G>C), I136C (506A>T 507T>G), L138M (512C>A 514A>G), T140R (519C>G 520C>A), K142Q (524A>C 526A>G), I144L (530A>C), D145R (533G>C 534A>G 535T>C), T146P (536A>C 538C>T), K147D (539A>G 541A>T), A148Y (542G>T 543C>A 544A>C), K150T (549A>C 550A>G), I151M (553A>G), V152F (554G>T), I154L (560A>T 562A>G), I162S (584A>T 585T>C 586A>C), D163R (587G>A 588A>G 589T>A), N164G (590A>G 591A>G), L166K (596T>A 597T>A), K168Q (602A>C 604A>G), Q170Y (608C>T 610A>T), E174Q (620G>C 622A>G), F175L (623T>C), F176L (628T>G), E177P (629G>C 630A>C), S178P (632A>C 633G>C 634T>A), H179K (635C>A 637T>G), V180I (638G>A), I183G (647A>G 648T>G), L184V (650T>G 652A>T), L185F (655A>C), V189M (665G>A 667T>G), S191P (671T>C), R192E (674A>G 675G>A), V193A (678T>C), I194L (680A>C 682C>T), N195E (683A>G 685T>G), T196I (687C>T 688A>T), S197T (689T>A), Q198R (692C>A 693A>G 694G>A), V199K (695G>A 696T>A 697C>G), R202N (705G>A 706A>C), D203K (707G>A 709T>G), K206R (717A>G 718A>G), N211R (731A>C 732A>G), A212D (735C>A), R220K (759G>A), Y222F (765A>T), I224V (770A>G 772A>C), K227D (779A>G 781G>T), N229E (785A>G 787T>A), D230E (790T>A), F231W (792T>G 793T>G), A233L (797G>C 798C>T 799A>T), F237C (810T>G), D241E (823C>G), H242T (824C>A 825A>C 826C>T), S244A (830A>G 831G>C), L245I (833C>A), T248S (843C>G 844A>T), L249V (845C>G 847A>C), C252V (854T>G 855G>T 856T>G), Q257K (869C>A), V260W (878G>T 879T>G 880A>G), M262T (885T>C 886G>T), E263D (889A>T), C264K (890T>A 891G>A 892T>G), L265M (893T>A 895A>G), T266R (896A>C 897C>G 898A>C), N267S (900A>G 901T>C), N269D (905A>G), F270H (908T>C 909T>A), A272V (915C>T), S274A (920A>G 921G>C 922C>A), I275T (924T>C 925C>T), S280D (938T>G 939C>A), Q282N (944C>A 946A>C), E283T (947G>A 948A>C 949A>T), F286I (956T>A 958T>C), K289R (965A>C 966A>G), D293S (977G>T 978A>C), K295S (983A>T 984A>C), T296S (986A>T), I298V (992A>G 994A>G), L300I (998C>A 1000T>C), S301T (1001T>A 1003A>T), N303D (1007A>G), I314V (1040A>G 1042A>C), H324T (1070C>A 1071A>C), N325Q (1073A>C 1075T>A), R326P (1076A>C 1077G>C), I330L (1088A>C 1090A>G), I344V (1130A>G), I349V (1145A>G 1147A>C), E351K (1151G>A 1153A>G), E353D (1159A>T), L354E (1160T>G 1161T>A), P355R (1163C>A 1164C>G 1165A>G), K356M (1167A>T), A358F (1172G>T 1173C>T), E359D (1177A>C), L360I (1178T>A), E361Q (1181G>C 1183A>G), T362K (1185C>A 1186G>A), T366V (1196A>G 1197C>T), K367V (1199A>G 1200A>T), D369E (1207T>G), M371L (1211A>C), E373A (1218A>C 1219G>C), I375V (1223A>G), V376A (1227T>C), I379L (1235A>C 1237A>G)

	Begin	End	Coverage	Score	Concordance	Matches	Identities	I/D/M/F*	Stop Codons
**NT**	**112**	**1237**	**84.0%**	**434**	**19.4%**	**1123 (99.5%)**	**676 (59.9%)**	**3/3**	
Codon mutations:	GAA7GAT (121A>T), GAC9GCT (126A>C 127C>T), CAT10GAG (128C>G 130T>G), GGC12GGG (136C>G), CTC13ATT (137C>A 139C>T), AAG14AAA (142G>A), AAA15GAT (143A>G 145A>T), AAT16GAC (146A>G 148T>C), ATC17CTT (149A>C 151C>T), CTA18CTT (154A>T), AAG19AGA (156A>G 157G>A), GGT20GGG (160T>G), ATA21ATT (163A>T), TAT22TAC (166T>C), TCT23CAG (167T>C 168C>A 169T>G), TGT24TAC (171G>A 172T>C), GGA25GGG (175A>G), TTT26TTC (178T>C), CCT29CCA (187T>A), TCA30TCT (190A>T), ACT31GCC (191A>G 193T>C), AAA35CGA (203A>C 204A>G), GCC36GCT (208C>T), ATT37GTA (209A>G 211T>A), TTT38TTG (214T>G), CCA39CCT (217A>T), TGT40ATT (218T>A 219G>T), ATT41ATA (223T>A), TCT42TCG (226T>G), AAA44CGT (230A>C 231A>G 232A>T), GTG46GTC (238G>C), ATA47ATT (241A>T), GTC48GCT (243T>C 244C>T), CAA49CAG (247A>G), TCG52TCC (256G>C), GGA53GGT (259A>T), ACA54ACC (262A>C), ACA57ACC (271A>C), GCA58TCC (272G>T 274A>C), ACA59ATG (276C>T 277A>G), TAT60ATT (278T>A 279A>T), GCA61GCT (283A>T), ATC62CTT (284A>C 286C>T), AGT63ACC (288G>C 289T>C), TTA65TGC (294T>G 295A>C), CAG66CAA (298G>A), CAA67GTT (299C>G 300A>T 301A>T), ATA68GTG (302A>G 304A>G), ACT70ACC (310T>C), TCT71AAA (311T>A 312C>A 313T>A), AAT74GAG (320A>G 322T>G), ATC75GTA (323A>G 325C>A), GCT77GCA (331T>A), ATC79ATA (337C>A), ACC81TCT (341A>T 343C>T), CCA82CCT (346A>T), CGT84CGG (352T>G), GAA85GAG (355A>G), TTG86CTT (356T>C 358G>T), GCT87GCA (361T>A), CTC88GCT (362C>G 363T>C 364C>T), GCT90ACG (368G>A 370T>G), CAA91GAA (371C>G), CGT92AAG (374C>A 375G>A 376T>G), GTA93GTG (379A>G), TTG94ATT (380T>A 382G>T), CAA95TTG (383C>T 384A>T 385A>G), ACA96GCA (386A>G), GGG98GGA (394G>A), AAT99GAT (395A>G), TAT100TAC (400T>C), CTA101ATT (401C>A 403A>T), TAT102del (404_406delTAT), AAT103AAC (409T>C), TTT104ATA (410T>A 412T>A), AAA105CAG (413A>C 415A>G), TGT106GCA (416T>G 417G>C 418T>A), CAA107CAT (421A>T), GTT108GCG (423T>C 424T>G), ACA113AAA (438C>A), AGT114AGC (442T>C), ATC115GTG (443A>G 445C>G), AAG116GGT (446A>G 447A>G 448G>T), AGC118GAT (452A>G 453G>A 454C>T), CAG119ATC (455C>A 456A>T 457G>C), GAA120AGA (458G>A 459A>G), ACT121AAG (462C>A 463T>G), AAG123GAG (467A>G), AAG123_AAA124insCAT (469_470insCAT), AAA124GGA (470A>G 471A>G), GCC125GTA (474C>T 475C>A), CAG126CAA (478G>A), TTG128GTC (482T>G 484G>C), ATT129TCT (485A>T 486T>C), CGA134AGA (500C>A), ATG135GTC (503A>G 505G>C), ATT136TGT (506A>T 507T>G), GAT137GAC (511T>C), CTA138ATG (512C>A 514A>G), CTC139TTG (515C>T 517C>G), ACC140AGA (519C>G 520C>A), CGA141AGA (521C>A), AAA142CAG (524A>C 526A>G), AGT143TCT (527A>T 528G>C), ATC144CTC (530A>C), GAT145CGC (533G>C 534A>G 535T>C), ACC146CCT (536A>C 538C>T), AAA147GAT (539A>G 541A>T), GCA148TAC (542G>T 543C>A 544A>C), AAA150ACG (549A>C 550A>G), ATA151ATG (553A>G), GTT152TTT (554G>T), ATA154TTG (560A>T 562A>G), GAT155GAC (565T>C), GAA156GAG (568A>G), GAG159GAA (577G>A), TTG161CTC (581T>C 583G>C), ATA162TCC (584A>T 585T>C 586A>C), GAT163AGA (587G>A 588A>G 589T>A), AAT164GGT (590A>G 591A>G), TTT165TTC (595T>C), TTG166AAG (596T>A 597T>A), AAA168CAG (602A>C 604A>G), ATA169ATT (607A>T), CAA170TAT (608C>T 610A>T), GAA174CAG (620G>C 622A>G), TTT175CTT (623T>C), TTT176TTG (628T>G), GAA177CCA (629G>C 630A>C), AGT178CCA (632A>C 633G>C 634T>A), CAT179AAG (635C>A 637T>G), GTC180ATC (638G>A), GTT182GTG (646T>G), ATT183GGT (647A>G 648T>G), TTA184GTT (650T>G 652A>T), TTA185TTC (655A>C), TCA186TCT (658A>T), GCA187GCC (661A>C), ACC188ACT (664C>T), GTT189ATG (665G>A 667T>G), TCA191CCA (671T>C), AGG192GAG (674A>G 675G>A), GTT193GCT (678T>C), ATC194CTT (680A>C 682C>T), AAT195GAG (683A>G 685T>G), ACA196ATT (687C>T 688A>T), TCT197ACT (689T>A), CAG198AGA (692C>A 693A>G 694G>A), GTC199AAG (695G>A 696T>A 697C>G), TTT200TTC (700T>C), AGA202AAC (705G>A 706A>C), GAT203AAG (707G>A 709T>G), GTA205GTG (715A>G), AAA206AGG (717A>G 718A>G), CTG208CTT (724G>T), GTA209GTG (727A>G), AAA210AAG (730A>G), AAT211CGT (731A>C 732A>G), GCT212GAT (735C>A), GAA213GAG (739A>G), TTG214CTC (740T>C 742G>C), ACA215ACT (745A>T), CTC216CTT (748C>T), GGC218GGT (754C>T), AGG220AAG (759G>A), TAT222TTT (765A>T), ATA224GTC (770A>G 772A>C), GTA226GTC (778A>C), AAG227GAT (779A>G 781G>T), AAA228AAG (784A>G), AAT229GAA (785A>G 787T>A), GAT230GAA (790T>A), TTT231TGG (792T>G 793T>G), AAA232AAG (796A>G), GCA233CTT (797G>C 798C>T 799A>T), TTT237TGT (810T>G), GAC241GAG (823C>G), CAC242ACT (824C>A 825A>C 826C>T), AGC244GCC (830A>G 831G>C), CTC245ATC (833C>A), ACT246ACC (838T>C), CAA247CAG (841A>G), ACA248AGT (843C>G 844A>T), CTA249GTC (845C>G 847A>C), ATA250ATC (850A>C), TGT252GTG (854T>G 855G>T 856T>G), AAT253AAC (859T>C), ACA254ACC (862A>C), CGT255AGG (863C>A 865T>G), AGA256CGT (866A>C 868A>T), CAA257AAA (869C>A), GTA260TGG (878G>T 879T>G 880A>G), ATG262ACT (885T>C 886G>T), GAA263GAT (889A>T), TGT264AAG (890T>A 891G>A 892T>G), TTA265ATG (893T>A 895A>G), ACA266CGC (896A>C 897C>G 898A>C), AAT267AGC (900A>G 901T>C), AGA268CGT (902A>C 904A>T), AAT269GAT (905A>G), TTC270CAC (908T>C 909T>A), GCA272GTA (915C>T), TCA273TCT (919A>T), AGC274GCA (920A>G 921G>C 922C>A), ATC275ACT (924T>C 925C>T), GAT278GAC (934T>C), TCC280GAC (938T>G 939C>A), CAA281CAG (943A>G), CAA282AAC (944C>A 946A>C), GAA283ACT (947G>A 948A>C 949A>T), CGT284AGA (950C>A 952T>A), TTT286ATC (956T>A 958T>C), AAA289CGA (965A>C 966A>G), GAA290GAG (970A>G), TTT291TTC (973T>C), AGA292CGA (974A>C), GAT293TCT (977G>T 978A>C), GGT294GGG (982T>G), AAA295TCA (983A>T 984A>C), ACT296TCT (986A>T), AGA297CGT (989A>C 991A>T), ATA298GTG (992A>G 994A>G), CTT299CTC (997T>C), CTT300ATC (998C>A 1000T>C), TCA301ACT (1001T>A 1003A>T), AAT303GAT (1007A>G), CTA304CTT (1012A>T), TTA305TTG (1015A>G), GCA306GCT (1018A>T), AGA307CGT (1019A>C 1021A>T), GGC308GGT (1024C>T), GTT311GTA (1033T>A), CAA312CAG (1036A>G), ATA314GTC (1040A>G 1042A>C), TCT315TCC (1045T>C), TTA316CTT (1046T>C 1048A>T), GTT317GTC (1051T>C), ATC318ATT (1054C>T), AAT319AAC (1057T>C), GAT321GAC (1063T>C), TTG322CTG (1064T>C), CAT324ACT (1070C>A 1071A>C), AAT325CAA (1073A>C 1075T>A), AGA326CCA (1076A>C 1077G>C), TAT329TAC (1087T>C), ATA330CTG (1088A>C 1090A>G), AGA335CGT (1103A>C 1105A>T), GGT337GGA (1111T>A), AGA338CGA (1112A>C), GGC340GGA (1120C>A), CGA341AGG (1121C>A 1123A>G), AAA342AAG (1126A>G), GGG343GGT (1129G>T), ATT344GTT (1130A>G), AAT347AAC (1141T>C), ATA349GTC (1145A>G 1147A>C), ACT350ACC (1150T>C), GAA351AAG (1151G>A 1153A>G), GAC352GAT (1156C>T), GAA353GAT (1159A>T), TTA354GAA (1160T>G 1161T>A), CCA355AGG (1163C>A 1164C>G 1165A>G), AAG356ATG (1167A>T), GCT358TTT (1172G>T 1173C>T), GAA359GAC (1177A>C), TTA360ATA (1178T>A), GAA361CAG (1181G>C 1183A>G), ACG362AAA (1185C>A 1186G>A), TAT364TAC (1192T>C), AAT365AAC (1195T>C), ACC366GTC (1196A>G 1197C>T), AAA367GTA (1199A>G 1200A>T), ATA368ATC (1204A>C), GAT369GAG (1207T>G), GAA370GAG (1210A>G), ATG371CTG (1211A>C), GAG373GCC (1218A>C 1219G>C), ATT375GTT (1223A>G), GTT376GCT (1227T>C), TTG378CTC (1232T>C 1234G>C), ATA379CTG (1235A>C 1237A>G)								

*: Inserts / Deletes / Misaligned / Frameshifts

Analysis details

This analysis was performed with panviral2.64

NGS Details (UN62): Bracoviriform glomeratae (segment NC_043292.1)

Assembly

**Coverage Length**	310 (1 contig(s))
**Depth Of Coverage**	3029.1
**Number Of Reads**	8084
**Reads Per Million**	187.39 rpm (after QC)
**Ambiguities**	0
**Assembly Method**	de novo + reference guided assembly
**Consensus Caller**	Bcf Tools

Coverage Map

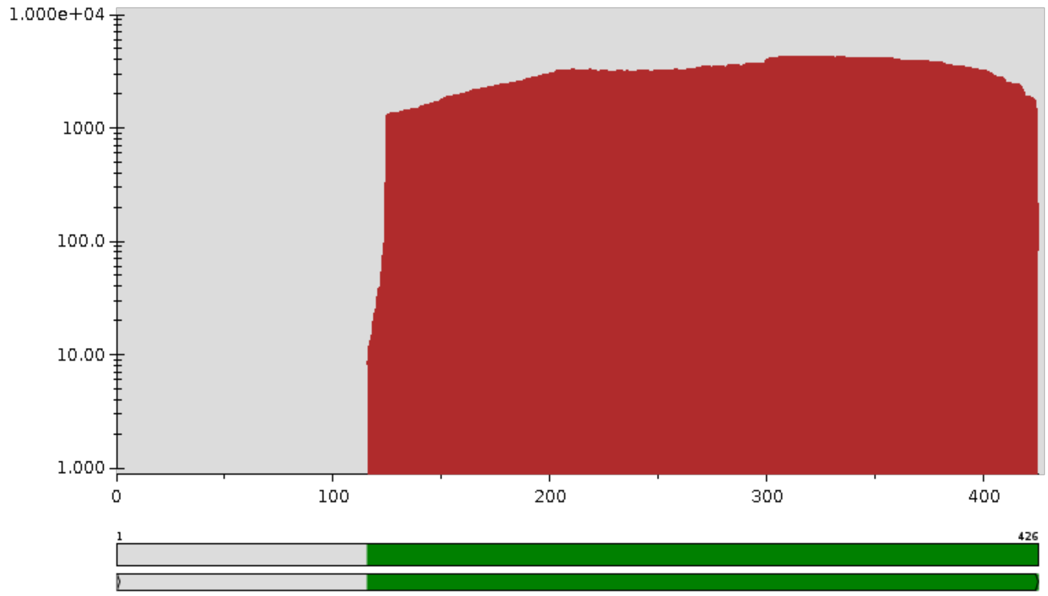

Assignment

**Type**	Bracoviriform glomeratae (Taxonomy ID: 257816)
**Reference Genome**	NC_043292.1
**NT Identity (%)**	72.5806
**AA Identity (%)**	84.466
**Number Of Stop Codons**	1
**Number Of CDS**	1

Alignment

**Alignment Score**	280.0 (NT) + 567.0 (AA) = 847.0
**Concordance (%)**	66.3273

Alignment Method	Global, seeded, nucleotide + amino acids (AGA)

Genome Region

Sequence starts at position 117 and ends at position 426 relative to NC_043292.1 reference sequence.

Alignment Detailed Statistics

	Begin	End	Coverage	Score	Concordance	Matches	Identities	I/D/M/F*	Stop Codons
**NT**	**117**	**426**	**72.8%**	**280**	**45.2%**	**310 (100%)**	**225 (72.6%)**	**0/0**	
Mutations:	126G>T, 129C>A, 132A>G, 136A>G, 137C>G, 138G>C, 150A>C, 153A>G, 155C>G, 156G>A, 159C>A, 166C>A, 168T>G, 169T>C, 172C>A, 174C>G, 177A>G, 183T>A, 184C>A, 189T>C, 198A>G, 201A>C, 207C>T, 214A>G, 219C>T, 222T>C, 223C>A, 225T>G, 228G>T, 235C>A, 237T>G, 243A>T, 246C>G, 255C>T, 261A>G, 262T>C, 264A>G, 265G>A, 273A>G, 278T>C, 279T>C, 284A>G, 285T>A, 288T>G, 289T>C, 300C>T, 301C>T, 303C>G, 309C>T, 312T>G, 315C>T, 322G>T, 324T>C, 329T>C, 333T>C, 336C>T, 339A>G, 345A>C, 347A>G, 348A>G, 349C>A, 351T>A, 360C>T, 363C>T, 366C>T, 375C>T, 378C>T, 384A>C, 385T>C, 387G>C, 390A>G, 391C>A, 392A>G, 393T>A, 394A>C, 396A>G, 399C>A, 400C>A, 402T>G, 404T>C, 405G>T, 406A>C, 414T>G, 416A>T, 420A>T								
**CDS**									
FK954_p501	40	142	72.5%	567	85.3%	103 (100%)	87 (84.5%)	0/0/0/0	1
Protein mutations:	L42F (126G>T), T46G (136A>G 137C>G 138G>C), A52G (155C>G 156G>A), Y57H (169T>C), T72A (214A>G), V89I (265G>A), I93T (278T>C 279T>C), D95G (284A>G 285T>A), A108S (322G>T 324T>C), I110T (329T>C), K116R (347A>G 348A>G), H131R (391C>A 392A>G 393T>A), K132Q (394A>C 396A>G), M135T (404T>C 405G>T), I136L (406A>C), Y139F (416A>T)								
Codon mutations:	TTG42TTT (126G>T), GGC43GGA (129C>A), AAA44AAG (132A>G), ACG46GGC (136A>G 137C>G 138G>C), GGA50GGC (150A>C), AAA51AAG (153A>G), GCG52GGA (155C>G 156G>A), GGC53GGA (159C>A), CGT56AGG (166C>A 168T>G), TAT57CAT (169T>C), CGC58AGG (172C>A 174C>G), AAA59AAG (177A>G), CTT61CTA (183T>A), CGA62AGA (184C>A), GAT63GAC (189T>C), CAA66CAG (198A>G), GGA67GGC (201A>C), ACC69ACT (207C>T), ACT72GCT (214A>G), ATC73ATT (219C>T), CGT74CGC (222T>C), CGT75AGG (223C>A 225T>G), CTG76CTT (228G>T), CGT79AGG (235C>A 237T>G), GGA81GGT (243A>T), GTC82GTG (246C>G), ATC85ATT (255C>T), GGA87GGG (261A>G), TTA88CTG (262T>C 264A>G), GTC89ATC (265G>A), GAA91GAG (273A>G), ATT93ACC (278T>C 279T>C), GAT95GGA (284A>G 285T>A), GTT96GTG (288T>G), TTG97CTG (289T>C), TTC100TTT (300C>T), CTC101TTG (301C>T 303C>G), AAC103AAT (309C>T), GTT104GTG (312T>G), ATC105ATT (315C>T), GCT108TCC (322G>T 324T>C), ATC110ACC (329T>C), TAT111TAC (333T>C), ACC112ACT (336C>T), GAA113GAG (339A>G), GCA115GCC (345A>C), AAA116AGG (347A>G 348A>G), CGT117AGA (349C>A 351T>A), GTC120GTT (360C>T), ACC121ACT (363C>T), GCC122GCT (366C>T), GTC125GTT (375C>T), GTC126GTT (378C>T), GCA128GCC (384A>C), TTG129CTC (385T>C 387G>C), AAA130AAG (390A>G), CAT131AGA (391C>A 392A>G 393T>A), AAA132CAG (394A>C 396A>G), GGC133GGA (399C>A), CGT134AGG (400C>A 402T>G), ATG135ACT (404T>C 405G>T), ATC136CTC (406A>C), GGT138GGG (414T>G), TAT139TTT (416A>T), GGA140GGT (420A>T)								
**Proteins**									
putative histone 4 (YP_009665791.1)	40	142	72.5%	567	85.3%	103 (100%)	87 (84.5%)	0/0/0/0	1
Protein mutations:	L42F (126G>T), T46G (136A>G 137C>G 138G>C), A52G (155C>G 156G>A), Y57H (169T>C), T72A (214A>G), V89I (265G>A), I93T (278T>C 279T>C), D95G (284A>G 285T>A), A108S (322G>T 324T>C), I110T (329T>C), K116R (347A>G 348A>G), H131R (391C>A 392A>G 393T>A), K132Q (394A>C 396A>G), M135T (404T>C 405G>T), I136L (406A>C), Y139F (416A>T)								
Codon mutations:	TTG42TTT (126G>T), GGC43GGA (129C>A), AAA44AAG (132A>G), ACG46GGC (136A>G 137C>G 138G>C), GGA50GGC (150A>C), AAA51AAG (153A>G), GCG52GGA (155C>G 156G>A), GGC53GGA (159C>A), CGT56AGG (166C>A 168T>G), TAT57CAT (169T>C), CGC58AGG (172C>A 174C>G), AAA59AAG (177A>G), CTT61CTA (183T>A), CGA62AGA (184C>A), GAT63GAC (189T>C), CAA66CAG (198A>G), GGA67GGC (201A>C), ACC69ACT (207C>T), ACT72GCT (214A>G), ATC73ATT (219C>T), CGT74CGC (222T>C), CGT75AGG (223C>A 225T>G), CTG76CTT (228G>T), CGT79AGG (235C>A 237T>G), GGA81GGT (243A>T), GTC82GTG (246C>G), ATC85ATT (255C>T), GGA87GGG (261A>G), TTA88CTG (262T>C 264A>G), GTC89ATC (265G>A), GAA91GAG (273A>G), ATT93ACC (278T>C 279T>C), GAT95GGA (284A>G 285T>A), GTT96GTG (288T>G), TTG97CTG (289T>C), TTC100TTT (300C>T), CTC101TTG (301C>T 303C>G), AAC103AAT (309C>T), GTT104GTG (312T>G), ATC105ATT (315C>T), GCT108TCC (322G>T 324T>C), ATC110ACC (329T>C), TAT111TAC (333T>C), ACC112ACT (336C>T), GAA113GAG (339A>G), GCA115GCC (345A>C), AAA116AGG (347A>G 348A>G), CGT117AGA (349C>A 351T>A), GTC120GTT (360C>T), ACC121ACT (363C>T), GCC122GCT (366C>T), GTC125GTT (375C>T), GTC126GTT (378C>T), GCA128GCC (384A>C), TTG129CTC (385T>C 387G>C), AAA130AAG (390A>G), CAT131AGA (391C>A 392A>G 393T>A), AAA132CAG (394A>C 396A>G), GGC133GGA (399C>A), CGT134AGG (400C>A 402T>G), ATG135ACT (404T>C 405G>T), ATC136CTC (406A>C), GGT138GGG (414T>G), TAT139TTT (416A>T), GGA140GGT (420A>T)								

*: Inserts / Deletes / Misaligned / Frameshifts

Analysis details

This analysis was performed with panviral2.64

NGS Details (UN62): Potato leafroll virus

Assembly

**Coverage Length**	5822 (1 contig(s))
**Depth Of Coverage**	75.3
**Number Of Reads**	3283
**Reads Per Million**	76.10 rpm (after QC)
**Ambiguities**	0
**Assembly Method**	read mapping against reference + variant calling
**Consensus Caller**	Bcf Tools

Coverage Map

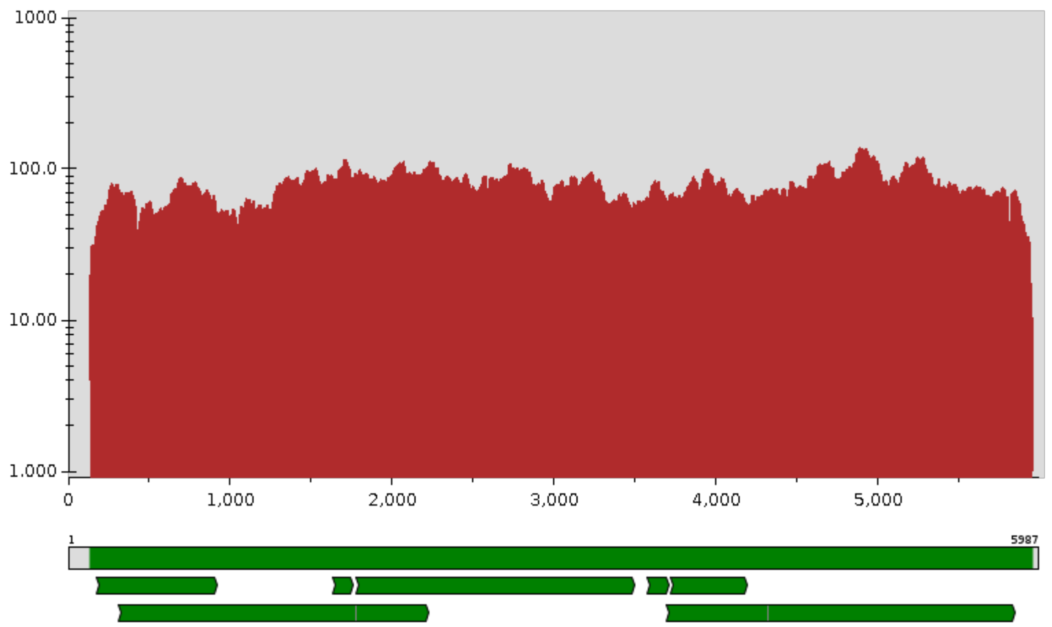

Assignment

**Type**	Potato leafroll virus (Taxonomy ID: 12045)
**Reference Genome**	NC_001747.1
**NT Identity (%)**	98.1106
**AA Identity (%)**	97.2178
**Number Of Stop Codons**	9
**Number Of CDS**	8

Alignment

**Alignment Score**	11168.0 (NT) + 20158.0 (AA) = 31326.0
**Concordance (%)**	95.9828

**Alignment Method**	Global, seeded, nucleotide + amino acids (AGA)

Genome Region

Sequence starts at position 135 and ends at position 5956 relative to NC_001747.1 reference sequence.

Alignment Detailed Statistics

	Begin	End	Coverage	Score	Concordance	Matches	Identities	I/D/M/F*	Stop Codons
**NT**	**135**	**5956**	**97.2%**	**11168**	**96.2%**	**5818 (99.9%)**	**5712 (98.0%)**	**4/4**	
Mutations:	191A>C, 221T>C, 228C>T, 269T>C, 276G>A, 300C>T, 343C>T, 462T>A, 476_477insTT, 479_480delTT, 492T>C, 525C>T, 561C>T, 600T>C, 665A>G, 699A>C, 822C>T, 825T>G, 931A>G, 958G>A, 1054C>T, 1132T>G, 1147C>T, 1171C>T, 1204A>G, 1231T>C, 1319G>A, 1356G>A, 1465C>T, 1474G>A, 1534G>A, 1577G>T, 1593G>A, 1611A>G, 1716A>G, 1754C>T, 1779A>G, 1894C>A, 2007T>C, 2051G>A, 2154C>T, 2265A>G, 2283G>A, 2316T>G, 2470G>A, 2555C>T, 2586A>G, 2640C>T, 2649C>T, 2729A>G, 2856G>A, 2877C>T, 2898T>C, 2931T>C, 3000T>C, 3033C>T, 3036C>T, 3069C>T, 3117C>T, 3195C>T, 3321C>T, 3379A>G, 3420A>G, 3437A>G, 3441C>T, 3619C>T, 3652T>A, 3721T>G, 3748T>G, 3750C>A, 3777G>T, 3788T>A, 3939A>T, 4031A>G, 4132A>T, 4214T>G, 4433C>T, 4511T>C, 4520G>T, 4521T>G, 4661T>C, 4700C>T, 4733C>G, 4817T>C, 4883C>T, 4886T>C, 4895T>C, 4898C>T, 4949G>A, 4964A>G, 4979C>T, 5062C>T, 5072C>T, 5095G>A, 5129G>A, 5162C>T, 5199C>T, 5215G>A, 5375T>G, 5385G>A, 5432T>C, 5474G>A, 5493G>A, 5517A>G, 5689_5690insGA, 5692_5693delGT, 5741T>G, 5748A>G, 5781G>A, 5925T>C								

CDS

	Begin	End	Coverage	Score	Concordance	Matches	Identities	I/D/M/F*	Stop Codons
PLRVgp1	1	248	100%	1429	82.5%	248 (99.6%)	241 (96.8%)	1/0/2/2	1
Protein mutations:	Q6P (191A>C), F16S (221T>C), L32P (269T>C), H57Y (343C>T), G101_L102insX (476_477insTT), Q164R (665A>G), Q175H (699A>C)								
Codon mutations:	CAG6CCG (191A>C), TTT16TCT (221T>C), CTC18CTT (228C>T), CTT32CCT (269T>C), CTG34CTA (276G>A), GGC42GGT (300C>T), CAT57TAT (343C>T), GCT96GCA (462T>A), GGC101GGT (476_477insTT), GGC101_CTT102insT-C (476_477insTT), CTT102C-- (479_480delTT), ATT106ATC (492T>C), TAC117TAT (525C>T), TAC129TAT (561C>T), CAT142CAC (600T>C), CAA164CGA (665A>G), CAA175CAC (699A>C), CGC216CGT (822C>T), GCT217GCG (825T>G)								
PLRVgp2	1	1063	100%	7076	94.9%	1063 (99.9%)	1039 (97.7%)	1/0/2/2	1
Protein mutations:	L52Q (462T>A), W56_A56insV (476_477insTT), L61S (492T>C), T72I (525C>T), T84I (561C>T), M97T (600T>C), K119E (665A>G), N130T (699A>C), A171V (822C>T), L172R (825T>G), A337T (1319G>A), G349E (1356G>A), V423F (1577G>T), R428K (1593G>A), D434G (1611A>G), E469G (1716A>G), Q529K (1894C>A), G581E (2051G>A), A721T (2470G>A), A749V (2555C>T), N807S (2729A>G), I1024V (3379A>G), E1043G (3437A>G)								
Codon mutations:	TTC12TTT (343C>T), CTA52CAA (462T>A), TGG56_GCC56insGTT (476_477insTT), TTA57--A (479_480delTT), TTA61TCA (492T>C), ACA72ATA (525C>T), ACA84ATA (561C>T), ATG97ACG (600T>C), AAA119GAA (665A>G), AAC130ACC (699A>C), GCG171GTG (822C>T), CTA172CGA (825T>G), GAA207GAG (931A>G), CAG216CAA (958G>A), GCC248GCT (1054C>T), ACT274ACG (1132T>G), TTC279TTT (1147C>T), TCC287TCT (1171C>T), CTA298CTG (1204A>G), ATT307ATC (1231T>C), GCC337ACC (1319G>A), GGA349GAA (1356G>A), CCC385CCT (1465C>T), CCG388CCA (1474G>A), TCG408TCA (1534G>A), GTC423TTC (1577G>T), AGA428AAA (1593G>A), GAC434GGC (1611A>G), GAG469GGG (1716A>G), CTA482TTA (1754C>T), GCA490GCG (1779A>G), CAA529AAA (1894C>A), TTT566TTC (2007T>C), GGA581GAA (2051G>A), CAC615CAT (2154C>T), TCA652TCG (2265A>G), GCG658GCA (2283G>A), GTT669GTG (2316T>G), GCA721ACA (2470G>A), GCC749GTC (2555C>T), CTA759CTG (2586A>G), CAC777CAT (2640C>T), AGC780AGT (2649C>T), AAT807AGT (2729A>G), GTG849GTA (2856G>A), CAC856CAT (2877C>T), ACT863ACC (2898T>C), TAT874TAC (2931T>C), CGT897CGC (3000T>C), AAC908AAT (3033C>T), TCC909TCT (3036C>T), GCC920GCT (3069C>T), TCC936TCT (3117C>T), CTC962CTT (3195C>T), AAC1004AAT (3321C>T), ATT1024GTT (3379A>G), GAA1037GAG (3420A>G), GAG1043GGG (3437A>G), CTC1044CTT (3441C>T)								
PLRVgp3	1	640	100%	3930	91.6%	640 (99.8%)	619 (96.6%)	1/0/2/2	1
Protein mutations:	L52Q (462T>A), W56_A56insV (476_477insTT), L61S (492T>C), T72I (525C>T), T84I (561C>T), M97T (600T>C), K119E (665A>G), N130T (699A>C), A171V (822C>T), L172R (825T>G), A337T (1319G>A), G349E (1356G>A), V423F (1577G>T), R428K (1593G>A), D434G (1611A>G), E469G (1716A>G), Q490R (1779A>G), L566S (2007T>C), E581K (2051G>A), T615I (2154C>T)								
Codon mutations:	TTC12TTT (343C>T), CTA52CAA (462T>A), TGG56_GCC56insGTT (476_477insTT), TTA57--A (479_480delTT), TTA61TCA (492T>C), ACA72ATA (525C>T), ACA84ATA (561C>T), ATG97ACG (600T>C), AAA119GAA (665A>G), AAC130ACC (699A>C), GCG171GTG (822C>T), CTA172CGA (825T>G), GAA207GAG (931A>G), CAG216CAA (958G>A), GCC248GCT (1054C>T), ACT274ACG (1132T>G), TTC279TTT (1147C>T), TCC287TCT (1171C>T), CTA298CTG (1204A>G), ATT307ATC (1231T>C), GCC337ACC (1319G>A), GGA349GAA (1356G>A), CCC385CCT (1465C>T), CCG388CCA (1474G>A), TCG408TCA (1534G>A), GTC423TTC (1577G>T), AGA428AAA (1593G>A), GAC434GGC (1611A>G), GAG469GGG (1716A>G), CTA482TTA (1754C>T), CAA490CGA (1779A>G), ATC528ATA (1894C>A), TTA566TCA (2007T>C), GAG581AAG (2051G>A), ACC615ATC (2154C>T)								
Rap1	1	42	100%	277	96.5%	42 (100%)	41 (97.6%)	0/0/0/0	1
Protein mutations:	R29G (1716A>G)								
Codon mutations:	AGA29GGA (1716A>G), CTC41CTT (1754C>T)								
ORF3a	1	46	100%	291	97.3%	46 (100%)	45 (97.8%)	0/0/0/0	1
Protein mutations:	F26L (3652T>A)								
Codon mutations:	TCC15TCT (3619C>T), TTT26TTA (3652T>A)								
PLRVgp4	1	718	100%	4683	93.0%	718 (99.9%)	699 (97.2%)	1/0/2/2	2
Protein mutations:	V10G (3721T>G), M19R (3748T>G), A29S (3777G>T), T83S (3939A>T), Y147F (4132A>T), K276N (4520G>T), F277V (4521T>G), A457V (5062C>T), G468E (5095G>A), P503S (5199C>T), R508Q (5215G>A), N561K (5375T>G), E565K (5385G>A), V601I (5493G>A), K609E (5517A>G), E666_R667insX (5689_5690insGA), F683L (5741T>G), T686A (5748A>G), E697K (5781G>A)								
Codon mutations:	GTC10GGC (3721T>G), ATG19AGG (3748T>G), CGA20AGA (3750C>A), GCT29TCT (3777G>T), GTT32GTA (3788T>A), ACC83TCC (3939A>T), ACA113ACG (4031A>G), TAC147TTC (4132A>T), TCT174TCG (4214T>G), GCC247GCT (4433C>T), AAT273AAC (4511T>C), AAG276AAT (4520G>T), TTT277GTT (4521T>G), GGT323GGC (4661T>C), TCC336TCT (4700C>T), GTC347GTG (4733C>G), GGT375GGC (4817T>C), TTC397TTT (4883C>T), CTT398CTC (4886T>C), CCT401CCC (4895T>C), GCC402GCT (4898C>T), ACG419ACA (4949G>A), GAA424GAG (4964A>G), ACC429ACT (4979C>T), GCC457GTC (5062C>T), CAC460CAT (5072C>T), GGA468GAA (5095G>A), ACG479ACA (5129G>A), GAC490GAT (5162C>T), CCA503TCA (5199C>T), CGA508CAA (5215G>A), AAT561AAG (5375T>G), GAA565AAA (5385G>A), ACT580ACC (5432T>C), TTG594TTA (5474G>A), GTC601ATC (5493G>A), AAG609GAG (5517A>G), GAA666GAG (5689_5690insGA), GAA666_CGT667insA-A (5689_5690insGA), CGT667C-- (5692_5693delGT), TTT683TTG (5741T>G), ACT686GCT (5748A>G), GAA697AAA (5781G>A)								
PLRVgp5	1	209	100%	1409	98.2%	209 (100%)	204 (97.6%)	0/0/0/0	1
Protein mutations:	V10G (3721T>G), M19R (3748T>G), A29S (3777G>T), T83S (3939A>T), Y147F (4132A>T)								
Codon mutations:	GTC10GGC (3721T>G), ATG19AGG (3748T>G), CGA20AGA (3750C>A), GCT29TCT (3777G>T), GTT32GTA (3788T>A), ACC83TCC (3939A>T), ACA113ACG (4031A>G), TAC147TTC (4132A>T), TCT174TCG (4214T>G)								
PLRVgp6	1	157	100%	1063	96.5%	157 (100%)	152 (96.8%)	0/0/0/0	1
Protein mutations:	S2A (3721T>G), C11G (3748T>G 3750C>A), F24Y (3788T>A), Q105R (4031A>G), T139S (4132A>T)								
Codon mutations:	TCA2GCA (3721T>G), TGC11GGA (3748T>G 3750C>A), GCG20GCT (3777G>T), TTC24TAC (3788T>A), ACA74ACT (3939A>T), CAA105CGA (4031A>G), ACG139TCG (4132A>T)								

Proteins

	Begin	End	Coverage	Score	Concordance	Matches	Identities	I/D/M/F*	Stop Codons
P0 protein (NP_056746.1)	1	248	100%	1429	82.5%	248 (99.6%)	241 (96.8%)	1/0/2/2	1
Protein mutations:	Q6P (191A>C), F16S (221T>C), L32P (269T>C), H57Y (343C>T), G101_L102insX (476_477insTT), Q164R (665A>G), Q175H (699A>C)								

	Begin	End	Coverage	Score	Concordance	Matches	Identities	I/D/M/F*	Stop Codons
**NT**	**135**	**5956**	**97.2%**	**11168**	**96.2%**	**5818 (99.9%)**	**5712 (98.0%)**	**4/4**	
Codon mutations:	CAG6CCG (191A>C), TTT16TCT (221T>C), CTC18CTT (228C>T), CTT32CCT (269T>C), CTG34CTA (276G>A), GGC42GGT (300C>T), CAT57TAT (343C>T), GCT96GCA (462T>A), GGC101GGT (476_477insTT), GGC101_CTT102insT-C (476_477insTT), CTT102C-- (479_480delTT), ATT106ATC (492T>C), TAC117TAT (525C>T), TAC129TAT (561C>T), CAT142CAC (600T>C), CAA164CGA (665A>G), CAA175CAC (699A>C), CGC216CGT (822C>T), GCT217GCG (825T>G)								
RNA-dependent RNA polymerase (NP_056748.3)	1	1063	100%	7076	94.9%	1063 (99.9%)	1039 (97.7%)	1/0/2/2	1
Protein mutations:	L52Q (462T>A), W56_A56insV (476_477insTT), L61S (492T>C), T72I (525C>T), T84I (561C>T), M97T (600T>C), K119E (665A>G), N130T (699A>C), A171V (822C>T), L172R (825T>G), A337T (1319G>A), G349E (1356G>A), V423F (1577G>T), R428K (1593G>A), D434G (1611A>G), E469G (1716A>G), Q529K (1894C>A), G581E (2051G>A), A721T (2470G>A), A749V (2555C>T), N807S (2729A>G), I1024V (3379A>G), E1043G (3437A>G)								
Codon mutations:	TTC12TTT (343C>T), CTA52CAA (462T>A), TGG56_GCC56insGTT (476_477insTT), TTA57--A (479_480delTT), TTA61TCA (492T>C), ACA72ATA (525C>T), ACA84ATA (561C>T), ATG97ACG (600T>C), AAA119GAA (665A>G), AAC130ACC (699A>C), GCG171GTG (822C>T), CTA172CGA (825T>G), GAA207GAG (931A>G), CAG216CAA (958G>A), GCC248GCT (1054C>T), ACT274ACG (1132T>G), TTC279TTT (1147C>T), TCC287TCT (1171C>T), CTA298CTG (1204A>G), ATT307ATC (1231T>C), GCC337ACC (1319G>A), GGA349GAA (1356G>A), CCC385CCT (1465C>T), CCG388CCA (1474G>A), TCG408TCA (1534G>A), GTC423TTC (1577G>T), AGA428AAA (1593G>A), GAC434GGC (1611A>G), GAG469GGG (1716A>G), CTA482TTA (1754C>T), GCA490GCG (1779A>G), CAA529AAA (1894C>A), TTT566TTC (2007T>C), GGA581GAA (2051G>A), CAC615CAT (2154C>T), TCA652TCG (2265A>G), GCG658GCA (2283G>A), GTT669GTG (2316T>G), GCA721ACA (2470G>A), GCC749GTC (2555C>T), CTA759CTG (2586A>G), CAC777CAT (2640C>T), AGC780AGT (2649C>T), AAT807AGT (2729A>G), GTG849GTA (2856G>A), CAC856CAT (2877C>T), ACT863ACC (2898T>C), TAT874TAC (2931T>C), CGT897CGC (3000T>C), AAC908AAT (3033C>T), TCC909TCT (3036C>T), GCC920GCT (3069C>T), TCC936TCT (3117C>T), CTC962CTT (3195C>T), AAC1004AAT (3321C>T), ATT1024GTT (3379A>G), GAA1037GAG (3420A>G), GAG1043GGG (3437A>G), CTC1044CTT (3441C>T)								
P1 protein (NP_056747.1)	1	640	100%	3930	91.6%	640 (99.8%)	619 (96.6%)	1/0/2/2	1
Protein mutations:	L52Q (462T>A), W56_A56insV (476_477insTT), L61S (492T>C), T72I (525C>T), T84I (561C>T), M97T (600T>C), K119E (665A>G), N130T (699A>C), A171V (822C>T), L172R (825T>G), A337T (1319G>A), G349E (1356G>A), V423F (1577G>T), R428K (1593G>A), D434G (1611A>G), E469G (1716A>G), Q490R (1779A>G), L566S (2007T>C), E581K (2051G>A), T615I (2154C>T)								
Codon mutations:	TTC12TTT (343C>T), CTA52CAA (462T>A), TGG56_GCC56insGTT (476_477insTT), TTA57--A (479_480delTT), TTA61TCA (492T>C), ACA72ATA (525C>T), ACA84ATA (561C>T), ATG97ACG (600T>C), AAA119GAA (665A>G), AAC130ACC (699A>C), GCG171GTG (822C>T), CTA172CGA (825T>G), GAA207GAG (931A>G), CAG216CAA (958G>A), GCC248GCT (1054C>T), ACT274ACG (1132T>G), TTC279TTT (1147C>T), TCC287TCT (1171C>T), CTA298CTG (1204A>G), ATT307ATC (1231T>C), GCC337ACC (1319G>A), GGA349GAA (1356G>A), CCC385CCT (1465C>T), CCG388CCA (1474G>A), TCG408TCA (1534G>A), GTC423TTC (1577G>T), AGA428AAA (1593G>A), GAC434GGC (1611A>G), GAG469GGG (1716A>G), CTA482TTA (1754C>T), CAA490CGA (1779A>G), ATC528ATA (1894C>A), TTA566TCA (2007T>C), GAG581AAG (2051G>A), ACC615ATC (2154C>T)								
Replication-associated protein (YP_006355442.1)	1	42	100%	277	96.5%	42 (100%)	41 (97.6%)	0/0/0/0	1
Protein mutations:	R29G (1716A>G)								
Codon mutations:	AGA29GGA (1716A>G), CTC41CTT (1754C>T)								
protein 3a (YP_009179365.2)	1	46	100%	291	97.3%	46 (100%)	45 (97.8%)	0/0/0/0	1
Protein mutations:	F26L (3652T>A)								
Codon mutations:	TCC15TCT (3619C>T), TTT26TTA (3652T>A)								
CP read-through protein (NP_056751.2)	1	718	100%	4683	93.0%	718 (99.9%)	699 (97.2%)	1/0/2/2	2
Protein mutations:	V10G (3721T>G), M19R (3748T>G), A29S (3777G>T), T83S (3939A>T), Y147F (4132A>T), K276N (4520G>T), F277V (4521T>G), A457V (5062C>T), G468E (5095G>A), P503S (5199C>T), R508Q (5215G>A), N561K (5375T>G), E565K (5385G>A), V601I (5493G>A), K609E (5517A>G), E666_R667insX (5689_5690insGA), F683L (5741T>G), T686A (5748A>G), E697K (5781G>A)								
Codon mutations:	GTC10GGC (3721T>G), ATG19AGG (3748T>G), CGA20AGA (3750C>A), GCT29TCT (3777G>T), GTT32GTA (3788T>A), ACC83TCC (3939A>T), ACA113ACG (4031A>G), TAC147TTC (4132A>T), TCT174TCG (4214T>G), GCC247GCT (4433C>T), AAT273AAC (4511T>C), AAG276AAT (4520G>T), TTT277GTT (4521T>G), GGT323GGC (4661T>C), TCC336TCT (4700C>T), GTC347GTG (4733C>G), GGT375GGC (4817T>C), TTC397TTT (4883C>T), CTT398CTC (4886T>C), CCT401CCC (4895T>C), GCC402GCT (4898C>T), ACG419ACA (4949G>A), GAA424GAG (4964A>G), ACC429ACT (4979C>T), GCC457GTC (5062C>T), CAC460CAT (5072C>T), GGA468GAA (5095G>A), ACG479ACA (5129G>A), GAC490GAT (5162C>T), CCA503TCA (5199C>T), CGA508CAA (5215G>A), AAT561AAG (5375T>G), GAA565AAA (5385G>A), ACT580ACC (5432T>C), TTG594TTA (5474G>A), GTC601ATC (5493G>A), AAG609GAG (5517A>G), GAA666GAG (5689_5690insGA), GAA666_CGT667insA-A (5689_5690insGA), CGT667C-- (5692_5693delGT), TTT683TTG (5741T>G), ACT686GCT (5748A>G), GAA697AAA (5781G>A)								
coat protein (NP_056749.1)	1	209	100%	1409	98.2%	209 (100%)	204 (97.6%)	0/0/0/0	1
Protein mutations:	V10G (3721T>G), M19R (3748T>G), A29S (3777G>T), T83S (3939A>T), Y147F (4132A>T)								
Codon mutations:	GTC10GGC (3721T>G), ATG19AGG (3748T>G), CGA20AGA (3750C>A), GCT29TCT (3777G>T), GTT32GTA (3788T>A), ACC83TCC (3939A>T), ACA113ACG (4031A>G), TAC147TTC (4132A>T), TCT174TCG (4214T>G)								
movement protein (NP_056750.1)	1	157	100%	1063	96.5%	157 (100%)	152 (96.8%)	0/0/0/0	1
Protein mutations:	S2A (3721T>G), C11G (3748T>G 3750C>A), F24Y (3788T>A), Q105R (4031A>G), T139S (4132A>T)								
Codon mutations:	TCA2GCA (3721T>G), TGC11GGA (3748T>G 3750C>A), GCG20GCT (3777G>T), TTC24TAC (3788T>A), ACA74ACT (3939A>T), CAA105CGA (4031A>G), ACG139TCG (4132A>T)								

*: Inserts / Deletes / Misaligned / Frameshifts

Analysis details

This analysis was performed with panviral2.64

NGS Details (UN62): Duamitovirus soch1

Assembly

**Coverage Length**	2098 (2 contig(s))
**Depth Of Coverage**	32.4
**Number Of Reads**	543
**Reads Per Million**	12.59 rpm (after QC)
**Ambiguities**	0
**Assembly Method**	de novo + reference guided assembly
**Consensus Caller**	Bcf Tools

Coverage Map

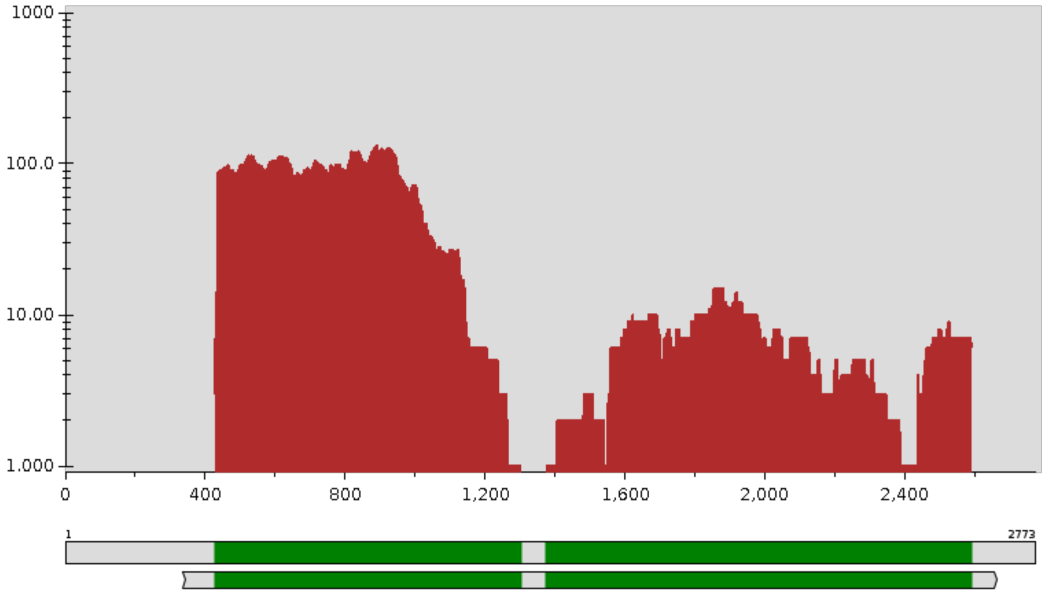

Assignment

**Type**	Duamitovirus soch1 (Taxonomy ID: 2955838)
**Reference Genome**	NC_076524.1
**NT Identity (%)**	68.3102
**AA Identity (%)**	69.1535
**Number Of Stop Codons**	3
**Number Of CDS**	1

Alignment

**Alignment Score**	1494.0 (NT) + 3210.0 (AA) = 4704.0
**Concordance (%)**	53.7195

Alignment Method	Global, seeded, nucleotide + amino acids (AGA)

Genome Region

Sequence starts at position 428 and ends at position 2593 relative to NC_076524.1 reference sequence.

Alignment Detailed Statistics

	Begin	End	Coverage	Score	Concordance	Matches	Identities	I/D/M/F*	Stop Codons
**NT**	**428**	**2593**	**75.7%**	**1494**	**36.1%**	**2089 (99.6%)**	**1427 (68.0%)**	**0/9**	
Mutations:	437A>G, 441A>C, 443C>T, 447C>T, 459C>T, 466C>T, 470C>T, 471G>A, 472C>T, 473T>G, 477T>A, 480A>C, 481C>A, 482A>T, 485A>G, 489A>G, 494T>C, 497T>A, 500T>G, 503A>C, 506T>C, 509C>T, 512T>C, 513G>A, 516T>A, 517C>A, 518T>A, 520A>C, 524G>T, 525A>G, 526C>T, 527T>G, 529C>A, 530C>A, 531T>C, 533A>G, 534C>A, 535G>A, 536C>A, 539G>A, 540A>C, 541A>G, 542G>T, 545G>A, 548C>T, 550A>T, 556T>A, 557C>T, 560A>T, 569C>T, 575G>A, 581C>T, 582G>A, 587A>T, 589G>C, 591T>C, 593A>T, 599A>T, 602C>T, 612T>A, 613C>G, 614C>T, 616T>A, 618C>T, 619G>C, 620A>T, 624G>A, 626A>T, 629A>C, 630C>A, 632A>C, 633C>A, 635T>G, 638G>T, 641G>T, 644T>A, 647G>T, 651C>T, 653C>G, 656A>C, 657A>C, 661C>G, 662G>T, 665T>A, 668C>T, 672C>A, 673G>A, 674T>G, 680C>A, 683C>T, 684A>G, 685A>C, 686A>C, 692G>A, 695T>A, 699G>C, 700C>A, 701A>C, 702A>G, 705C>A, 707T>G, 708A>G, 709G>C, 710G>A, 713A>G, 714G>T, 715A>C, 719A>T, 722C>T, 737T>C, 746C>T, 752A>T, 761G>A, 765G>T, 767T>A, 770G>A, 771G>T, 772T>A, 773T>G, 774T>A, 776G>T, 777A>G, 778A>C, 779A>G, 782T>G, 796G>A, 797T>G, 803A>C, 809T>C, 810G>C, 815C>A, 818A>T, 821T>G, 824C>T, 827A>T, 831C>T, 833T>A, 841G>A, 842G>T, 850T>G, 851A>T, 857G>T, 860G>T, 862A>G, 869T>G, 875G>T, 880T>G, 881C>A, 885C>A, 887T>G, 892A>G, 905A>T, 908C>A, 909T>C, 910T>A, 916G>A, 918G>T, 919G>C, 923T>A, 926A>T, 927A>C, 929G>T, 934T>A, 935T>G, 938G>T, 942T>A, 943C>A, 948C>A, 971C>A, 972C>T, 975A>C, 976A>T, 977T>C, 983T>C, 990A>C, 992G>C, 993T>C, 994C>G, 995A>G, 997A>T, 998C>T, 999G>C, 1000G>A, 1001T>A, 1003T>A, 1004A>T, 1005G>T, 1007C>G, 1010A>G, 1016C>T, 1017A>G, 1018A>T, 1019A>T, 1022T>A, 1025T>C, 1026C>G, 1027A>G, 1030A>C, 1031G>A, 1034T>G, 1035A>G, 1036G>C, 1044T>G, 1046C>T, 1049G>A, 1050T>G, 1051T>A, 1052T>A, 1054C>A, 1055A>C, 1061T>C, 1067T>G, 1073G>A, 1076A>G, 1083C>A, 1088G>T, 1091C>G, 1099G>T, 1104A>G, 1105C>T, 1106A>G, 1115T>G, 1121A>C, 1122T>A, 1124C>T, 1127G>A, 1130C>T, 1133C>G, 1142A>C, 1145C>T, 1148G>T, 1151G>T, 1152T>C, 1154G>T, 1160C>T, 1161A>C, 1164T>A, 1165C>T, 1166T>G, 1169C>T, 1172C>A, 1174T>A, 1175T>C, 1178T>A, 1181A>T, 1184C>T, 1185A>T, 1186G>C, 1187G>T, 1188G>A, 1189C>A, 1191G>A, 1192C>A, 1193A>T, 1196T>C, 1197_1199delGAA, 1205A>T, 1211C>T, 1212T>G, 1213T>A, 1217T>C, 1220C>T, 1223A>T, 1224C>T, 1225A>T, 1226C>A, 1232G>A, 1236A>C, 1237G>A, 1238T>A, 1241C>A, 1247A>C, 1248T>C, 1249G>T, 1250G>T, 1253T>C, 1256A>T, 1258C>G, 1265G>A, 1266A>G, 1268T>G, 1269C>G, 1271T>C, 1274T>C, 1276G>A, 1280G>A, 1283G>T, 1292T>G, 1376C>G, 1379C>T, 1382T>C, 1385T>C, 1388C>T, 1389G>A, 1390A>T, 1403A>T, 1406C>A, 1409G>A, 1412T>A, 1414A>G, 1415A>T, 1416A>C, 1418A>T, 1424A>T, 1427C>T, 1430G>T, 1433T>A, 1437G>A, 1445G>A, 1446A>C, 1447A>G, 1448A>T, 1451T>G, 1455T>C, 1456T>A, 1458G>T, 1459C>A, 1461T>C, 1465A>G, 1466G>A, 1468A>T, 1469A>T, 1472G>A, 1474A>G, 1475C>A, 1477T>C, 1478T>G, 1481A>G, 1482A>T, 1483A>C, 1486G>C, 1489A>T, 1490T>C, 1502A>C, 1505G>A, 1508A>G, 1526G>A, 1538G>A, 1541T>C, 1545C>T, 1547T>C, 1553T>C, 1554A>T, 1555T>G, 1556G>C, 1559A>G, 1566C>A, 1567C>G, 1571C>A, 1572T>C, 1580C>A, 1589T>C, 1593_1597delAGCTC, 1598T>A, 1607T>C, 1613G>T, 1614A>T, 1617C>T, 1619T>G, 1620G>A, 1622T>C, 1623A>G, 1624A>T, 1626A>C, 1627A>C, 1628A>T, 1632T>A, 1634A>G, 1635A>G, 1636C>T, 1637A>C, 1643A>G, 1647T>A, 1648A>G, 1655A>T, 1661T>C, 1662C>T, 1664G>A, 1667T>A, 1670G>A, 1676T>G, 1679G>A, 1682C>T, 1684T>A, 1686C>T, 1691T>A, 1694T>A, 1698G>T, 1700A>C, 1703T>A, 1706C>T, 1707T>G, 1710T>C, 1712G>T, 1715T>C, 1721C>T, 1727A>G, 1734T>C, 1739A>T, 1744G>A, 1745C>A, 1746A>T, 1748A>T, 1754T>C, 1758G>A, 1759G>C, 1760A>G, 1761T>A, 1765A>C, 1766C>T, 1767T>A, 1769T>C, 1772A>T, 1777G>C, 1778G>T, 1787T>C, 1788T>C, 1790A>G, 1791C>T, 1819G>C, 1820T>A, 1821A>G, 1823A>G, 1829C>G, 1830A>C, 1831A>G, 1832T>G, 1833G>C, 1840A>G, 1847A>G, 1848T>C, 1856A>G, 1859C>T, 1860A>G, 1861A>G, 1865C>T, 1867A>C, 1871T>C, 1874G>T, 1875delA, 1878C>G, 1880A>T, 1886T>G, 1887T>A, 1888T>G, 1889G>A, 1895T>C, 1898T>G, 1901T>C, 1910T>C, 1919G>T, 1925G>A, 1934T>C, 1937A>G, 1938T>G, 1939C>A, 1940A>C, 1950G>T, 1952T>C, 1953T>C, 1955A>T, 1964T>C, 1967C>T, 1976T>A, 1980T>A, 1981T>C, 1982A>T, 1985T>C, 1989C>A, 1991T>G, 1994T>A, 2000T>G, 2003A>T, 2006T>G, 2009C>T, 2010G>C, 2011C>A, 2012C>A, 2020T>C, 2028G>T, 2029A>C, 2030T>G, 2034A>T, 2035A>C, 2036C>A, 2038T>C, 2042A>G, 2043G>A, 2050A>C, 2051A>C, 2053G>T, 2054A>T, 2057G>T, 2058G>T, 2063T>G, 2069G>A, 2071A>T, 2074A>G, 2075G>A, 2078A>T, 2082G>T, 2084G>T, 2087C>T, 2090A>G, 2091T>A, 2093C>G, 2094A>T, 2099C>T, 2103A>T, 2104G>C, 2105C>T, 2108G>A, 2117G>A, 2118C>A, 2120T>A, 2123A>G, 2129T>G, 2133C>G, 2134A>C, 2135T>A, 2136C>A, 2137T>G, 2138T>C, 2141G>A, 2145G>C, 2146T>A, 2147T>C, 2148C>A, 2156C>T, 2157T>C, 2160C>T, 2166T>C, 2167A>T, 2173G>A, 2174G>T, 2183A>G, 2186G>A, 2187G>T, 2188G>T, 2191T>A, 2192G>A, 2195C>T, 2204C>T, 2206A>T, 2213A>G, 2216G>A, 2223G>A, 2226A>G, 2227G>A, 2231C>T, 2236T>G, 2237T>G, 2240A>G, 2248A>C, 2250G>C, 2251T>C, 2259T>A, 2263A>G, 2264G>A, 2265A>C, 2276G>T, 2279T>G, 2280C>T, 2288C>T, 2297A>G, 2298G>A, 2299T>G, 2300T>A, 2306A>C, 2309A>T, 2315T>C, 2322C>T, 2326G>T, 2328C>A, 2329A>G, 2336A>G, 2337G>A, 2346T>C, 2348A>G, 2351G>A, 2355C>G, 2358A>T, 2359A>C, 2360C>T, 2372G>T, 2373A>G, 2375C>T, 2376T>G, 2377G>C, 2378T>A, 2379A>G, 2380T>C, 2381G>A, 2382A>T, 2383A>C, 2384C>A, 2385C>G, 2390T>A, 2394T>A, 2400A>G, 2402G>T, 2403A>C, 2404G>A, 2405T>G, 2406C>T, 2408C>G, 2411C>G, 2414C>A, 2415T>G, 2416C>T, 2417C>T, 2423A>G, 2426C>T, 2427G>A, 2428C>G, 2429A>T, 2431C>A, 2438G>A, 2441A>G, 2442A>C, 2444G>A, 2445C>T, 2446T>C, 2447C>G, 2448G>A, 2450A>T, 2451A>G, 2452C>A, 2453T>A, 2456T>C, 2457T>C, 2459T>G, 2460G>A, 2462T>A, 2465A>G, 2466A>G, 2477C>T, 2478C>T, 2489A>G, 2492T>C, 2495C>T, 2504T>A, 2510T>C, 2514A>G, 2518C>T, 2519C>G, 2525C>T, 2528T>A, 2532C>A, 2533A>G, 2536G>A, 2537G>T, 2541T>C, 2543T>G, 2551A>G, 2552C>T, 2555T>C, 2556G>T, 2557T>C, 2561T>C, 2568A>G, 2571A>C, 2572A>G, 2582A>G, 2585G>A									
**CDS**									
RdRp	32	753	90.0%	3210	65.4%	697 (99.7%)	482 (69.0%)	0/2/2/2	3
Protein mutations:	I36L (441A>C 443C>T), L38F (447C>T), A44V (466C>T), A46M (471G>A 472C>T 473T>G), L48M (477T>A), T49H (480A>C 481C>A 482A>T), I52V (489A>G), V60I (513G>A), S61K (516T>A 517C>A 518T>A), K62T (520A>C), T64V (525A>G 526C>T 527T>G), T65K (529C>A 530C>A), R67K (534C>A 535G>A 536C>A), K69R (540A>C 541A>G 542G>T), Y72F (550A>T), F74Y (556T>A 557C>T), A83T (582G>A), C85S (589G>C), F94Y (616T>A), R95S (618C>T 619G>C 620A>T), E97N (624G>A 626A>T), E98D (629A>C), P99T (630C>A 632A>C), L100M (633C>A 635T>G), S109C (661C>G 662G>T), R113K (672C>A 673G>A 674T>G), K117A (684A>G 685A>C 686A>C), A122H (699G>C 700C>A 701A>C), I123V (702A>G), R125A (708A>G 709G>C 710G>A), D127S (714G>T 715A>C), E128D (719A>T), A144S (765G>T 767T>A), V146* (771G>T 772T>A 773T>G), L147I (774T>A 776G>T), K148A (777A>G 778A>C 779A>G), S154K (796G>A 797T>G), E159Q (810G>C), P166S (831C>T 833T>A), G169D (841G>A 842G>T), L172R (850T>G 851A>T), E176G (862A>G), I178M (869T>G), F182* (880T>G 881C>A), H184K (885C>A 887T>G), Q186R (892A>G), F192H (909T>C 910T>A), R194Q (916G>A), G195S (918G>T 919G>C), M198L (927A>C 929G>T), I200K (934T>A 935T>G), S203N (942T>A 943C>A), Q205K (948C>A), N214L (975A>C 976A>T 977T>C), S220R (993T>C 994C>G 995A>G), Y221F (997A>T 998C>T), G222Q (999G>C 1000G>A 1001T>A), L223Y (1003T>A 1004A>T), A224S (1005G>T 1007C>G), K228V (1017A>G 1018A>T 1019A>T), Q231G (1026C>G 1027A>G), E232A (1030A>C 1031G>A), S234A (1035A>G 1036G>C), F237V (1044T>G 1046C>T), F239E (1050T>G 1051T>A 1052T>A), P240H (1054C>A 1055A>C), L250I (1083C>A), W255L (1099G>T), T257V (1104A>G 1105C>T 1106A>G), I260M (1115T>G), F263I (1122T>A 1124C>T), M272I (1151G>T), K276Q (1161A>C), S277M (1164T>A 1165C>T 1166T>G), F279L (1172C>A), F280Y (1174T>A 1175T>C), L282F (1181A>T), R284S (1185A>T 1186G>C 1187G>T), A285N (1188G>A 1189C>A), A286N (1191G>A 1192C>A 1193A>T), E288del (1197_1199delGAA), L290F (1205A>T), L293E (1212T>G 1213T>A), E296D (1223A>T), H297L (1224C>T 1225A>T 1226C>A), S301Q (1236A>C 1237G>A 1238T>A), W305L (1248T>C 1249G>T 1250G>T), T308S (1258C>G), S311G (1266A>G 1268T>G), P312A (1269C>G 1271T>C), R314Q (1276G>A), D352I (1389G>A 1390A>T), K360S (1414A>G 1415A>T), I361L (1416A>C 1418A>T), D368N (1437G>A), K371R (1446A>C 1447A>G 1448A>T), L374Q (1455T>C 1456T>A), A375Y (1458G>T 1459C>A), K377R (1465A>G 1466G>A), Q378L (1468A>T 1469A>T), N380R (1474A>G 1475C>A), F381S (1477T>C 1478T>G), N383S (1482A>T 1483A>C), C384S (1486G>C), Y385F (1489A>T 1490T>C), L389F (1502A>C), M401I (1538G>A), L404F (1545C>T 1547T>C), M407C (1554A>T 1555T>G 1556G>C), I408M (1559A>G), P411S (1566C>A 1567C>G), S420del (1593_1597delAGCTC), I427F (1614A>T), V429I (1620G>A 1622T>C), N430V (1623A>G 1624A>T), K431P (1626A>C 1627A>C 1628A>T), L433M (1632T>A 1634A>G), T434V (1635A>G 1636C>T 1637A>C), Y438S (1647T>A 1648A>G), F450Y (1684T>A), H451Y (1686C>T), A455S (1698G>T 1700A>C), S458A (1707T>G), I464M (1727A>G), S470K (1744G>A 1745C>A), K471Y (1746A>T 1748A>T), G475T (1758G>A 1759G>C 1760A>G), C476S (1761T>A), N477T (1765A>C 1766C>T), S478T (1767T>A 1769T>C), G481A (1777G>C 1778G>T), S495T (1819G>C 1820T>A), K496E (1821A>G 1823A>G), N499R (1830A>C 1831A>G 1832T>G), E500Q (1833G>C), K502R (1840A>G), N509G (1860A>G 1861A>G), K511T (1867A>C), P515A (1878C>G 1880A>T), L518R (1887T>A 1888T>G 1889G>A), D521E (1898T>G), S535D (1938T>G 1939C>A 1940A>C), D539Y (1950G>T 1952T>C), L549T (1980T>A 1981T>C 1982A>T), A559Q (2010G>C 2011C>A 2012C>A), I562T (2020T>C), D565S (2028G>T 2029A>C 2030T>G), N567S (2034A>T 2035A>C 2036C>A), I568T (2038T>C), V570I (2043G>A), Q572P (2050A>C 2051A>C), R573I (2053G>T 2054A>T), L574F (2057G>T), G575W (2058G>T), Y579F (2071A>T), K580R (2074A>G 2075G>A), A583S (2082G>T 2084G>T), F586M (2091T>A 2093C>G), T587S (2094A>T), H600A (2133C>G 2134A>C 2135T>A), L601S (2136C>A 2137T>G 2138T>C), V604H (2145G>C 2146T>A 2147T>C), Y611L (2166T>C 2167A>T), W613Y (2173G>A 2174G>T), G618F (2187G>T 2188G>T), M619K (2191T>A 2192G>A), Y624F (2206A>T), V630I (2223G>A), S631D (2226A>G 2227G>A), L634R (2236T>G 2237T>G), K638T (2248A>C), V639P (2250G>C 2251T>C), L642I (2259T>A), Q643R (2263A>G 2264G>A), I644L (2265A>C), K647N (2276G>T), D648E (2279T>G), V655R (2298G>A 2299T>G 2300T>A), R664L (2326G>T), Q665R (2328C>A 2329A>G), E668K (2337G>A), L674V (2355C>G), N675S (2358A>T 2359A>C 2360C>T), I680V (2373A>G 2375C>T), C681A (2376T>G 2377G>C 2378T>A), M682A (2379A>G 2380T>C 2381G>A), N683S (2382A>T 2383A>C 2384C>A), P684A (2385C>G), D685E (2390T>A), S687T (2394T>A), K689D (2400A>G 2402G>T), S690Q (2403A>C 2404G>A 2405T>G), I692M (2411C>G), D693E (2414C>A), S694V (2415T>G 2416C>T 2417C>T), I696M (2423A>G), A698S (2427G>A 2428C>G 2429A>T), T699K (2431C>A), W701* (2438G>A), L704S (2445C>T 2446T>C 2447C>G), E705N (2448G>A 2450A>T), T706E (2451A>G 2452C>A 2453T>A), F708L (2457T>C 2459T>G), D709K (2460G>A 2462T>A), I711V (2466A>G), N727D (2514A>G), P728L (2518C>T 2519C>G), Q733R (2532C>A 2533A>G), W734Y (2536G>A 2537G>T), F736L (2541T>C 2543T>G), N739S (2551A>G 2552C>T), V741S (2556G>T 2557T>C), N745D (2568A>G), K746R (2571A>C 2572A>G)									

	Begin	End	Coverage	Score	Concordance	Matches	Identities	I/D/M/F*	Stop Codons
**NT**	**428**	**2593**	**75.7%**	**1494**	**36.1%**	**2089 (99.6%)**	**1427 (68.0%)**	**0/9**	
Codon mutations:	AAA34AAG (437A>G), ATC36CTT (441A>C 443C>T), CTT38TTT (447C>T), CTA42TTA (459C>T), GCC44GTC (466C>T), TCC45TCT (470C>T), GCT46ATG (471G>A 472C>T 473T>G), TTG48ATG (477T>A), ACA49CAT (480A>C 481C>A 482A>T), AAA50AAG (485A>G), ATC52GTC (489A>G), GCT53GCC (494T>C), CTT54CTA (497T>A), GCT55GCG (500T>G), GGA56GGC (503A>C), TGT57TGC (506T>C), GAC58GAT (509C>T), TTT59TTC (512T>C), GTA60ATA (513G>A), TCT61AAA (516T>A 517C>A 518T>A), AAA62ACA (520A>C), GTG63GTT (524G>T), ACT64GTG (525A>G 526C>T 527T>G), ACC65AAA (529C>A 530C>A), TTA66CTG (531T>C 533A>G), CGC67AAA (534C>A 535G>A 536C>A), CGG68CGA (539G>A), AAG69CGT (540A>C 541A>G 542G>T), TCG70TCA (545G>A), GGC71GGT (548C>T), TAT72TTT (550A>T), TTC74TAT (556T>A 557C>T), ACA75ACT (560A>T), TAC78TAT (569C>T), AAG80AAA (575G>A), TGC82TGT (581C>T), GCA83ACA (582G>A), GTA84GTT (587A>T), TGC85TCC (589G>C), TTA86CTT (591T>C 593A>T), CGA88CGT (599A>T), TAC89TAT (602C>T), TCC93AGT (612T>A 613C>G 614C>T), TTT94TAT (616T>A), CGA95TCT (618C>T 619G>C 620A>T), GAA97AAT (624G>A 626A>T), GAA98GAC (629A>C), CCA99ACC (630C>A 632A>C), CTT100ATG (633C>A 635T>G), TCG101TCT (638G>T), GTG102GTT (641G>T), CCT103CCA (644T>A), GTG104GTT (647G>T), CTC106TTG (651C>T 653C>G), ACA107ACC (656A>C), AGA108CGA (657A>C), TCG109TGT (661C>G 662G>T), GGT110GGA (665T>A), ATC111ATT (668C>T), CGT113AAG (672C>A 673G>A 674T>G), ATC115ATA (680C>A), CCC116CCT (683C>T), AAA117GCC (684A>G 685A>C 686A>C), TTG119TTA (692G>A), CGT120CGA (695T>A), GCA122CAC (699G>C 700C>A 701A>C), ATA123GTA (702A>G), CGT124AGG (705C>A 707T>G), AGG125GCA (708A>G 709G>C 710G>A), AAA126AAG (713A>G), GAT127TCT (714G>T 715A>C), GAA128GAT (719A>T), CAC129CAT (722C>T), GTT134GTC (737T>C), TAC137TAT (746C>T), TCA139TCT (752A>T), GGG142GGA (761G>A), GCT144TCA (765G>T 767T>A), AAG145AAA (770G>A), GTT146TAG (771G>T 772T>A 773T>G), TTG147ATT (774T>A 776G>T), AAA148GCG (777A>G 778A>C 779A>G), GTT149GTG (782T>G), AGT154AAG (796G>A 797T>G), GCA156GCC (803A>C), TTT158TTC (809T>C), GAA159CAA (810G>C), TCC160TCA (815C>A), ATA161ATT (818A>T), ACT162ACG (821T>G), ACC163ACT (824C>T), CCA164CCT (827A>T), CCT166TCA (831C>T 833T>A), GGG169GAT (841G>A 842G>T), CTA172CGT (850T>G 851A>T), GTG174GTT (857G>T), CTG175CTT (860G>T), GAA176GGA (862A>G), ATT178ATG (869T>G), ACG180ACT (875G>T), TTC182TGA (880T>G 881C>A), CAT184AAG (885C>A 887T>G), CAA186CGA (892A>G), CTA190CTT (905A>T), CCC191CCA (908C>A), TTT192CAT (909T>C 910T>A), CGA194CAA (916G>A), GGC195TCC (918G>T 919G>C), ATT196ATA (923T>A), CCA197CCT (926A>T), ATG198CTT (927A>C 929G>T), ATT200AAG (934T>A 935T>G), GGG201GGT (938G>T), TCT203AAT (942T>A 943C>A), CAA205AAA (948C>A), CCC212CCA (971C>A), CTA213TTA (972C>T), AAT214CTC (975A>C 976A>T 977T>C), AAT216AAC (983T>C), AGG219CGC (990A>C 992G>C), TCA220CGG (993T>C 994C>G 995A>G), TAC221TTT (997A>T 998C>T), GGT222CAA (999G>C 1000G>A 1001T>A), TTA223TAT (1003T>A 1004A>T), GCC224TCG (1005G>T 1007C>G), GTA225GTG (1010A>G), GAC227GAT (1016C>T), AAA228GTT (1017A>G 1018A>T 1019A>T), GTT229GTA (1022T>A), GAT230GAC (1025T>C), CAA231GGA (1026C>G 1027A>G), GAG232GCA (1030A>C 1031G>A), GCT233GCG (1034T>G), AGT234GCT (1035A>G 1036G>C), TTC237GTT (1044T>G 1046C>T), AAG238AAA (1049G>A), TTT239GAA (1050T>G 1051T>A 1052T>A), CCA240CAC (1054C>A 1055A>C), ATT242ATC (1061T>C), GTT244GTG (1067T>G), TTG246TTA (1073G>A), AAA247AAG (1076A>G), CTA250ATA (1083C>A), GCG251GCT (1088G>T), GCC252GCG (1091C>G), TGG255TTG (1099G>T), ACA257GTG (1104A>G 1105C>T 1106A>G), ATT260ATG (1115T>G), TCA262TCC (1121A>C), TTC263ATT (1122T>A 1124C>T), CAG264CAA (1127G>A), GAC265GAT (1130C>T), GGC266GGG (1133C>G), TCA269TCC (1142A>C), CCC270CCT (1145C>T), GGG271GGT (1148G>T), ATG272ATT (1151G>T), TTG273CTT (1152T>C 1154G>T), TCC275TCT (1160C>T), AAA276CAA (1161A>C), TCT277ATG (1164T>A 1165C>T 1166T>G), GTC278GTT (1169C>T), TTC279TTA (1172C>A), TTT280TAC (1174T>A 1175T>C), CCT281CCA (1178T>A), TTA282TTT (1181A>T), GAC283GAT (1184C>T), AGG284TCT (1185A>T 1186G>C 1187G>T), GCT285AAT (1188G>A 1189C>A), GCA286AAT (1191G>A 1192C>A 1193A>T), ACT287ACC (1196T>C), GAA288del (1197_1199delGAA), TTA290TTT (1205A>T), AAC292AAT (1211C>T), TTA293GAA (1212T>G 1213T>A), TCT294TCC (1217T>C), CTC295CTT (1220C>T), GAA296GAT (1223A>T), CAC297TTA (1224C>T 1225A>T 1226C>A), GAG299GAA (1232G>A), AGT301CAA (1236A>C 1237G>A 1238T>A), GTC302GTA (1241C>A), CCA304CCC (1247A>C), TGG305CTT (1248T>C 1249G>T 1250G>T), TTT306TTC (1253T>C), TCA307TCT (1256A>T), ACC308AGC (1258C>G), TTG310TTA (1265G>A), AGT311GGG (1266A>G 1268T>G), CCT312GCC (1269C>G 1271T>C), TAT313TAC (1274T>C), CGA314CAA (1276G>A), GGG315GGA (1280G>A), GTG316GTT (1283G>T), GTT319GTG (1292T>G), CTC347CTG (1376C>G), CAC348CAT (1379C>T), CCT349CCC (1382T>C), GTT350GTC (1385T>C), CAC351CAT (1388C>T), GAT352ATT (1389G>A 1390A>T), ACA356ACT (1403A>T), GTC357GTA (1406C>A), CTG358CTA (1409G>A), TCT359TCA (1412T>A), AAA360AGT (1414A>G 1415A>T), ATA361CTT (1416A>C 1418A>T), ACA363ACT (1424A>T), GAC364GAT (1427C>T), GGG365GGT (1430G>T), ACT366ACA (1433T>A), GAC368AAC (1437G>A), GAG370GAA (1445G>A), AAA371CGT (1446A>C 1447A>G 1448A>T), CCT372CCG (1451T>G), TTG374CAG (1455T>C 1456T>A), GCT375TAT (1458G>T 1459C>A), TTA376CTA (1461T>C), AAG377AGA (1465A>G 1466G>A), CAA378CTT (1468A>T 1469A>T), AAG379AAA (1472G>A), AAC380AGA (1474A>G 1475C>A), TTT381TCG (1477T>C 1478T>G), AAA382AAG (1481A>G), AAT383TCT (1482A>T 1483A>C), TGT384TCT (1486G>C), TAT385TTC (1489A>T 1490T>C), TTA389TTC (1502A>C), AAG390AAA (1505G>A), TCA391TCG (1508A>G), CCG397CCA (1526G>A), ATG401ATA (1538G>A), TAT402TAC (1541T>C), CTT404TTC (1545C>T 1547T>C), TCT406TCC (1553T>C), ATG407TGC (1554A>T 1555T>G 1556G>C), ATA408ATG (1559A>G), CCT411AGT (1566C>A 1567C>G), ACC412ACA (1571C>A), TTG413CTG (1572T>C), TCC415TCA (1580C>A), GTT418GTC (1589T>C), AGC420del (1593_1597delAGCTC), TCT421--A (1593_1597delAGCTC 1598T>A), CTT424CTC (1607T>C), ACG426ACT (1613G>T), ATC427TTC (1614A>T), CTT428TTG (1617C>T 1619T>G), GTT429ATC (1620G>A 1622T>C), AAC430GTC (1623A>G 1624A>T), AAA431CCT (1626A>C 1627A>C 1628A>T), TTA433ATG (1632T>A 1634A>G), ACA434GTC (1635A>G 1636C>T 1637A>C), AGA436AGG (1643A>G), TAT438AGT (1647T>A 1648A>G), ATA440ATT (1655A>T), TTT442TTC (1661T>C), CTG443TTA (1662C>T 1664G>A), ACT444ACA (1667T>A), GGG445GGA (1670G>A), CCT447CCG (1676T>G), TTG448TTA (1679G>A), GGC449GGT (1682C>T), TTC450TAC (1684T>A), CAC451TAC (1686C>T), GGT452GGA (1691T>A), TCT453TCA (1694T>A), GCA455TCC (1698G>T 1700A>C), CTT456CTA (1703T>A), TTC457TTT (1706C>T), TCG458GCG (1707T>G), TTG459CTT (1710T>C 1712G>T), TCT460TCC (1715T>C), CAC462CAT (1721C>T), ATA464ATG (1727A>G), TTA467CTA (1734T>C), GCA468GCT (1739A>T), AGC470AAA (1744G>A 1745C>A), AAA471TAT (1746A>T 1748A>T), TAT473TAC (1754T>C), GGA475ACG (1758G>A 1759G>C 1760A>G), TGT476AGT (1761T>A), AAC477ACT (1765A>C 1766C>T), TCT478ACC (1767T>A 1769T>C), CCA479CCT (1772A>T), GGG481GCT (1777G>C 1778G>T), GCT484GCC (1787T>C), TTA485CTG (1788T>C 1790A>G), CTA486TTA (1791C>T), AGT495ACA (1819G>C 1820T>A), AAA496GAG (1821A>G 1823A>G), GCC498GCG (1829C>G), AAT499CGG (1830A>C 1831A>G 1832T>G), GAG500CAG (1833G>C), AAG502AGG (1840A>G), TTA504TTG (1847A>G), TTG505CTG (1848T>C), AGA507AGG (1856A>G), CTC508CTT (1859C>T), AAT509GGT (1860A>G 1861A>G), GTC510GTT (1865C>T), AAA511ACA (1867A>C), ATT512ATC (1871T>C), TCG513TCT (1874G>T), ATA514-TA (1875delA), CCA515GCT (1878C>G 1880A>T), TCT517TCG (1886T>G), TTG518AGA (1887T>A 1888T>G 1889G>A), TCT520TCC (1895T>C), GAT521GAG (1898T>G), AAT522AAC (1901T>C), ATT525ATC (1910T>C), GCG528GCT (1919G>T), AGG530AGA (1925G>A), ACT533ACC (1934T>C), AAA534AAG (1937A>G), TCA535GAC (1938T>G 1939C>A 1940A>C), GAT539TAC (1950G>T 1952T>C), TTA540CTT (1953T>C 1955A>T), ATT543ATC (1964T>C), TCC544TCT (1967C>T), GCT547GCA (1976T>A), TTA549ACT (1980T>A 1981T>C 1982A>T), TCT550TCC (1985T>C), CGT552AGG (1989C>A 1991T>G), ACT553ACA (1994T>A), GTT555GTG (2000T>G), GGA556GGT (2003A>T), CTT557CTG (2006T>G), TGC558TGT (2009C>T), GCC559CAA (2010G>C 2011C>A 2012C>A), ATC562ACC (2020T>C), GAT565TCG (2028G>T 2029A>C 2030T>G), AAC567TCA (2034A>T 2035A>C 2036C>A), ATA568ACA (2038T>C), TCA569TCG (2042A>G), GTT570ATT (2043G>A), CAA572CCC (2050A>C 2051A>C), AGA573ATT (2053G>T 2054A>T), TTG574TTT (2057G>T), GGG575TGG (2058G>T), GGT576GGG (2063T>G), GGG578GGA (2069G>A), TAC579TTC (2071A>T), AAG580AGA (2074A>G 2075G>A), GTA581GTT (2078A>T), GCG583TCT (2082G>T 2084G>T), CGC584CGT (2087C>T), TTA585TTG (2090A>G), TTC586ATG (2091T>A 2093C>G), ACT587TCT (2094A>T), ACC588ACT (2099C>T), AGC590TCT (2103A>T 2104G>C 2105C>T), AAG591AAA (2108G>A), GAG594GAA (2117G>A), CGT595AGA (2118C>A 2120T>A), CTA596CTG (2123A>G), GCT598GCG (2129T>G), CAT600GCA (2133C>G 2134A>C 2135T>A), CTT601AGC (2136C>A 2137T>G 2138T>C), AAG602AAA (2141G>A), GTT604CAC (2145G>C 2146T>A 2147T>C), CGG605AGG (2148C>A), CAC607CAT (2156C>T), TTA608CTA (2157T>C), CTA609TTA (2160C>T), TAC611CTC (2166T>C 2167A>T), TGG613TAT (2173G>A 2174G>T), GGA616GGG (2183A>G), AGG617AGA (2186G>A), GGT618TTT (2187G>T 2188G>T), ATG619AAA (2191T>A 2192G>A), CCC620CCT (2195C>T), CCC623CCT (2204C>T), TAT624TTT (2206A>T), AAA626AAG (2213A>G), GGG627GGA (2216G>A), GTA630ATA (2223G>A), AGT631GAT (2226A>G 2227G>A), TAC632TAT (2231C>T), CTT634CGG (2236T>G 2237T>G), AAA635AAG (2240A>G), AAA638ACA (2248A>C), GTT639CCT (2250G>C 2251T>C), TTA642ATA (2259T>A), CAG643CGA (2263A>G 2264G>A), ATC644CTC (2265A>C), AAG647AAT (2276G>T), GAT648GAG (2279T>G), CTG649TTG (2280C>T), TTC651TTT (2288C>T), GAA654GAG (2297A>G), GTT655AGA (2298G>A 2299T>G 2300T>A), ATA657ATC (2306A>C), CTA658CTT (2309A>T), CGT660CGC (2315T>C), CTA663TTA (2322C>T), CGC664CTC (2326G>T), CAA665AGA (2328C>A 2329A>G), GTA667GTG (2336A>G), GAA668AAA (2337G>A), TTA671CTG (2346T>C 2348A>G), AAG672AAA (2351G>A), CTC674GTC (2355C>G), AAC675TCT (2358A>T 2359A>C 2360C>T), ACG679ACT (2372G>T), ATC680GTT (2373A>G 2375C>T), TGT681GCA (2376T>G 2377G>C 2378T>A), ATG682GCA (2379A>G 2380T>C 2381G>A), AAC683TCA (2382A>T 2383A>C 2384C>A), CCA684GCA (2385C>G), GAT685GAA (2390T>A), TCC687ACC (2394T>A), AAG689GAT (2400A>G 2402G>T), AGT690CAG (2403A>C 2404G>A 2405T>G), CTC691TTG (2406C>T 2408C>G), ATC692ATG (2411C>G), GAC693GAA (2414C>A), TCC694GTT (2415T>G 2416C>T 2417C>T), ATA696ATG (2423A>G), TGC697TGT (2426C>T), GCA698AGT (2427G>A 2428C>G 2429A>T), ACA699AAA (2431C>A), TGG701TGA (2438G>A), AAA702AAG (2441A>G), AGG703CGA (2442A>C 2444G>A), CTC704TCG (2445C>T 2446T>C 2447C>G), GAA705AAT (2448G>A 2450A>T), ACT706GAA (2451A>G 2452C>A 2453T>A), GAT707GAC (2456T>C), TTT708CTG (2457T>C 2459T>G), GAT709AAA (2460G>A 2462T>A), TTA710TTG (2465A>G), ATC711GTC (2466A>G), GGC714GGT (2477C>T), CTA715TTA (2478C>T), AAA718AAG (2489A>G), TGT719TGC (2492T>C), TAC720TAT (2495C>T), GGT723GGA (2504T>A), GGT725GGC (2510T>C), AAT727GAT (2514A>G), CCC728CTG (2518C>T 2519C>G), ACC730ACT (2525C>T), ACT731ACA (2528T>A), CAG733AGG (2532C>A 2533A>G), TGG734TAT (2536G>A 2537G>T), TTT736CTG (2541T>C 2543T>G), AAC739AGT (2551A>G 2552C>T), ACT740ACC (2555T>C), GTT741TCT (2556G>T 2557T>C), ATT742ATC (2561T>C), AAT745GAT (2568A>G), AAA746CGA (2571A>C 2572A>G), TTA749TTG (2582A>G), GGG750GGA (2585G>A)								

Proteins

RNA-dependent RNA polymerase (YP_010798874.1)	32	753	90.0%	3210	65.4%	697 (99.7%)	482 (69.0%)	0/2/2/2	3

	Begin	End	Coverage	Score	Concordance	Matches	Identities	I/D/M/F*	Stop Codons
**NT**	**428**	**2593**	**75.7%**	**1494**	**36.1%**	**2089 (99.6%)**	**1427 (68.0%)**	**0/9**	

Protein mutations:

I36L (441A>C 443C>T), L38F (447C>T), A44V (466C>T), A46M (471G>A 472C>T 473T>G), L48M (477T>A), T49H (480A>C 481C>A 482A>T), I52V (489A>G), V60I (513G>A), S61K (516T>A 517C>A 518T>A), K62T (520A>C), T64V (525A>G 526C>T 527T>G), T65K (529C>A 530C>A), R67K (534C>A 535G>A 536C>A), K69R (540A>C 541A>G 542G>T), Y72F (550A>T), F74Y (556T>A 557C>T), A83T (582G>A), C85S (589G>C), F94Y (616T>A), R95S (618C>T 619G>C 620A>T), E97N (624G>A 626A>T), E98D (629A>C), P99T (630C>A 632A>C), L100M (633C>A 635T>G), S109C (661C>G 662G>T), R113K (672C>A 673G>A 674T>G), K117A (684A>G 685A>C 686A>C), A122H (699G>C 700C>A 701A>C), I123V (702A>G), R125A (708A>G 709G>C 710G>A), D127S (714G>T 715A>C), E128D (719A>T), A144S (765G>T 767T>A), V146* (771G>T 772T>A 773T>G), L147I (774T>A 776G>T), K148A (777A>G 778A>C 779A>G), S154K (796G>A 797T>G), E159Q (810G>C), P166S (831C>T 833T>A), G169D (841G>A 842G>T), L172R (850T>G 851A>T), E176G (862A>G), I178M (869T>G), F182* (880T>G 881C>A), H184K (885C>A 887T>G), Q186R (892A>G), F192H (909T>C 910T>A), R194Q (916G>A), G195S (918G>T 919G>C), M198L (927A>C 929G>T), I200K (934T>A 935T>G), S203N (942T>A 943C>A), Q205K (948C>A), N214L (975A>C 976A>T 977T>C), S220R (993T>C 994C>G 995A>G), Y221F (997A>T 998C>T), G222Q (999G>C 1000G>A 1001T>A), L223Y (1003T>A 1004A>T), A224S (1005G>T 1007C>G), K228V (1017A>G 1018A>T 1019A>T), Q231G (1026C>G 1027A>G), E232A (1030A>C 1031G>A), S234A (1035A>G 1036G>C), F237V (1044T>G 1046C>T), F239E (1050T>G 1051T>A 1052T>A), P240H (1054C>A 1055A>C), L250I (1083C>A), W255L (1099G>T), T257V (1104A>G 1105C>T 1106A>G), I260M (1115T>G), F263I (1122T>A 1124C>T), M272I (1151G>T), K276Q (1161A>C), S277M (1164T>A 1165C>T 1166T>G), F279L (1172C>A), F280Y (1174T>A 1175T>C), L282F (1181A>T), R284S (1185A>T 1186G>C 1187G>T), A285N (1188G>A 1189C>A), A286N (1191G>A 1192C>A 1193A>T), E288del (1197_1199delGAA), L290F (1205A>T), L293E (1212T>G 1213T>A), E296D (1223A>T), H297L (1224C>T 1225A>T 1226C>A), S301Q (1236A>C 1237G>A 1238T>A), W305L (1248T>C 1249G>T 1250G>T), T308S (1258C>G), S311G (1266A>G 1268T>G), P312A (1269C>G 1271T>C), R314Q (1276G>A), D352I (1389G>A 1390A>T), K360S (1414A>G 1415A>T), I361L (1416A>C 1418A>T), D368N (1437G>A), K371R (1446A>C 1447A>G 1448A>T), L374Q (1455T>C 1456T>A), A375Y (1458G>T 1459C>A), K377R (1465A>G 1466G>A), Q378L (1468A>T 1469A>T), N380R (1474A>G 1475C>A), F381S (1477T>C 1478T>G), N383S (1482A>T 1483A>C), C384S (1486G>C), Y385F (1489A>T 1490T>C), L389F (1502A>C), M401I (1538G>A), L404F (1545C>T 1547T>C), M407C (1554A>T 1555T>G 1556G>C), I408M (1559A>G), P411S (1566C>A 1567C>G), S420del (1593_1597delAGCTC), I427F (1614A>T), V429I (1620G>A 1622T>C), N430V (1623A>G 1624A>T), K431P (1626A>C 1627A>C 1628A>T), L433M (1632T>A 1634A>G), T434V (1635A>G 1636C>T 1637A>C), Y438S (1647T>A 1648A>G), F450Y (1684T>A), H451Y (1686C>T), A455S (1698G>T 1700A>C), S458A (1707T>G), I464M (1727A>G), S470K (1744G>A 1745C>A), K471Y (1746A>T 1748A>T), G475T (1758G>A 1759G>C 1760A>G), C476S (1761T>A), N477T (1765A>C 1766C>T), S478T (1767T>A 1769T>C), G481A (1777G>C 1778G>T), S495T (1819G>C 1820T>A), K496E (1821A>G 1823A>G), N499R (1830A>C 1831A>G 1832T>G), E500Q (1833G>C), K502R (1840A>G), N509G (1860A>G 1861A>G), K511T (1867A>C), P515A (1878C>G 1880A>T), L518R (1887T>A 1888T>G 1889G>A), D521E (1898T>G), S535D (1938T>G 1939C>A 1940A>C), D539Y (1950G>T 1952T>C), L549T (1980T>A 1981T>C 1982A>T), A559Q (2010G>C 2011C>A 2012C>A), I562T (2020T>C), D565S (2028G>T 2029A>C 2030T>G), N567S (2034A>T 2035A>C 2036C>A), I568T (2038T>C), V570I (2043G>A), Q572P (2050A>C 2051A>C), R573I (2053G>T 2054A>T), L574F (2057G>T), G575W (2058G>T), Y579F (2071A>T), K580R (2074A>G 2075G>A), A583S (2082G>T 2084G>T), F586M (2091T>A 2093C>G), T587S (2094A>T), H600A (2133C>G 2134A>C 2135T>A), L601S (2136C>A 2137T>G 2138T>C), V604H (2145G>C 2146T>A 2147T>C), Y611L (2166T>C 2167A>T), W613Y (2173G>A 2174G>T), G618F (2187G>T 2188G>T), M619K (2191T>A 2192G>A), Y624F (2206A>T), V630I (2223G>A), S631D (2226A>G 2227G>A), L634R (2236T>G 2237T>G), K638T (2248A>C), V639P (2250G>C 2251T>C), L642I (2259T>A), Q643R (2263A>G 2264G>A), I644L (2265A>C), K647N (2276G>T), D648E (2279T>G), V655R (2298G>A 2299T>G 2300T>A), R664L (2326G>T), Q665R (2328C>A 2329A>G), E668K (2337G>A), L674V (2355C>G), N675S (2358A>T 2359A>C 2360C>T), I680V (2373A>G 2375C>T), C681A (2376T>G 2377G>C 2378T>A), M682A (2379A>G 2380T>C 2381G>A), N683S (2382A>T 2383A>C 2384C>A), P684A (2385C>G), D685E (2390T>A), S687T (2394T>A), K689D (2400A>G 2402G>T), S690Q (2403A>C 2404G>A 2405T>G), I692M (2411C>G), D693E (2414C>A), S694V (2415T>G 2416C>T 2417C>T), I696M (2423A>G), A698S (2427G>A 2428C>G 2429A>T), T699K (2431C>A), W701* (2438G>A), L704S (2445C>T 2446T>C 2447C>G), E705N (2448G>A 2450A>T), T706E (2451A>G 2452C>A 2453T>A), F708L (2457T>C 2459T>G), D709K (2460G>A 2462T>A), I711V (2466A>G), N727D (2514A>G), P728L (2518C>T 2519C>G), Q733R (2532C>A 2533A>G), W734Y (2536G>A 2537G>T), F736L (2541T>C 2543T>G), N739S (2551A>G 2552C>T), V741S (2556G>T 2557T>C), N745D (2568A>G), K746R (2571A>C 2572A>G)

Codon mutations:

AAA34AAG (437A>G), ATC36CTT (441A>C 443C>T), CTT38TTT (447C>T), CTA42TTA (459C>T), GCC44GTC (466C>T), TCC45TCT (470C>T), GCT46ATG (471G>A 472C>T 473T>G), TTG48ATG (477T>A), ACA49CAT (480A>C 481C>A 482A>T), AAA50AAG (485A>G), ATC52GTC (489A>G), GCT53GCC (494T>C), CTT54CTA (497T>A), GCT55GCG (500T>G), GGA56GGC (503A>C), TGT57TGC (506T>C), GAC58GAT (509C>T), TTT59TTC (512T>C), GTA60ATA (513G>A), TCT61AAA (516T>A 517C>A 518T>A), AAA62ACA (520A>C), GTG63GTT (524G>T), ACT64GTG (525A>G 526C>T 527T>G), ACC65AAA (529C>A 530C>A), TTA66CTG (531T>C 533A>G), CGC67AAA (534C>A 535G>A 536C>A), CGG68CGA (539G>A), AAG69CGT (540A>C 541A>G 542G>T), TCG70TCA (545G>A), GGC71GGT (548C>T), TAT72TTT (550A>T), TTC74TAT (556T>A 557C>T), ACA75ACT (560A>T), TAC78TAT (569C>T), AAG80AAA (575G>A), TGC82TGT (581C>T), GCA83ACA (582G>A), GTA84GTT (587A>T), TGC85TCC (589G>C), TTA86CTT (591T>C 593A>T), CGA88CGT (599A>T), TAC89TAT (602C>T), TCC93AGT (612T>A 613C>G 614C>T), TTT94TAT (616T>A), CGA95TCT (618C>T 619G>C 620A>T), GAA97AAT (624G>A 626A>T), GAA98GAC (629A>C), CCA99ACC (630C>A 632A>C), CTT100ATG (633C>A 635T>G), TCG101TCT (638G>T), GTG102GTT (641G>T), CCT103CCA (644T>A), GTG104GTT (647G>T), CTC106TTG (651C>T 653C>G), ACA107ACC (656A>C), AGA108CGA (657A>C), TCG109TGT (661C>G 662G>T), GGT110GGA (665T>A), ATC111ATT (668C>T), CGT113AAG (672C>A 673G>A 674T>G), ATC115ATA (680C>A), CCC116CCT (683C>T), AAA117GCC (684A>G 685A>C 686A>C), TTG119TTA (692G>A), CGT120CGA (695T>A), GCA122CAC (699G>C 700C>A 701A>C), ATA123GTA (702A>G), CGT124AGG (705C>A 707T>G), AGG125GCA (708A>G 709G>C 710G>A), AAA126AAG (713A>G), GAT127TCT (714G>T 715A>C), GAA128GAT (719A>T), CAC129CAT (722C>T), GTT134GTC (737T>C), TAC137TAT (746C>T), TCA139TCT (752A>T), GGG142GGA (761G>A), GCT144TCA (765G>T 767T>A), AAG145AAA (770G>A), GTT146TAG (771G>T 772T>A 773T>G), TTG147ATT (774T>A 776G>T), AAA148GCG (777A>G 778A>C 779A>G), GTT149GTG (782T>G), AGT154AAG (796G>A 797T>G), GCA156GCC (803A>C), TTT158TTC (809T>C), GAA159CAA (810G>C), TCC160TCA (815C>A), ATA161ATT (818A>T), ACT162ACG (821T>G), ACC163ACT (824C>T), CCA164CCT (827A>T), CCT166TCA (831C>T 833T>A), GGG169GAT (841G>A 842G>T), CTA172CGT (850T>G 851A>T), GTG174GTT (857G>T), CTG175CTT (860G>T), GAA176GGA (862A>G), ATT178ATG (869T>G), ACG180ACT (875G>T), TTC182TGA (880T>G 881C>A), CAT184AAG (885C>A 887T>G), CAA186CGA (892A>G), CTA190CTT (905A>T), CCC191CCA (908C>A), TTT192CAT (909T>C 910T>A), CGA194CAA (916G>A), GGC195TCC (918G>T 919G>C), ATT196ATA (923T>A), CCA197CCT (926A>T), ATG198CTT (927A>C 929G>T), ATT200AAG (934T>A 935T>G), GGG201GGT (938G>T), TCT203AAT (942T>A 943C>A), CAA205AAA (948C>A), CCC212CCA (971C>A), CTA213TTA (972C>T), AAT214CTC (975A>C 976A>T 977T>C), AAT216AAC (983T>C), AGG219CGC (990A>C 992G>C), TCA220CGG (993T>C 994C>G 995A>G), TAC221TTT (997A>T 998C>T), GGT222CAA (999G>C 1000G>A 1001T>A), TTA223TAT (1003T>A 1004A>T), GCC224TCG (1005G>T 1007C>G), GTA225GTG (1010A>G), GAC227GAT (1016C>T), AAA228GTT (1017A>G 1018A>T 1019A>T), GTT229GTA (1022T>A), GAT230GAC (1025T>C), CAA231GGA (1026C>G 1027A>G), GAG232GCA (1030A>C 1031G>A), GCT233GCG (1034T>G), AGT234GCT (1035A>G 1036G>C), TTC237GTT (1044T>G 1046C>T), AAG238AAA (1049G>A), TTT239GAA (1050T>G 1051T>A 1052T>A), CCA240CAC (1054C>A 1055A>C), ATT242ATC (1061T>C), GTT244GTG (1067T>G), TTG246TTA (1073G>A), AAA247AAG (1076A>G), CTA250ATA (1083C>A), GCG251GCT (1088G>T), GCC252GCG (1091C>G), TGG255TTG (1099G>T), ACA257GTG (1104A>G 1105C>T 1106A>G), ATT260ATG (1115T>G), TCA262TCC (1121A>C), TTC263ATT (1122T>A 1124C>T), CAG264CAA (1127G>A), GAC265GAT (1130C>T), GGC266GGG (1133C>G), TCA269TCC (1142A>C), CCC270CCT (1145C>T), GGG271GGT (1148G>T), ATG272ATT (1151G>T), TTG273CTT (1152T>C 1154G>T), TCC275TCT (1160C>T), AAA276CAA (1161A>C), TCT277ATG (1164T>A 1165C>T 1166T>G), GTC278GTT (1169C>T), TTC279TTA (1172C>A), TTT280TAC (1174T>A 1175T>C), CCT281CCA (1178T>A), TTA282TTT (1181A>T), GAC283GAT (1184C>T), AGG284TCT (1185A>T 1186G>C 1187G>T), GCT285AAT (1188G>A 1189C>A), GCA286AAT (1191G>A 1192C>A 1193A>T), ACT287ACC (1196T>C), GAA288del (1197_1199delGAA), TTA290TTT (1205A>T), AAC292AAT (1211C>T), TTA293GAA (1212T>G 1213T>A), TCT294TCC (1217T>C), CTC295CTT (1220C>T), GAA296GAT (1223A>T), CAC297TTA (1224C>T 1225A>T 1226C>A), GAG299GAA (1232G>A), AGT301CAA (1236A>C 1237G>A 1238T>A), GTC302GTA (1241C>A), CCA304CCC (1247A>C), TGG305CTT (1248T>C 1249G>T 1250G>T), TTT306TTC (1253T>C), TCA307TCT (1256A>T), ACC308AGC (1258C>G), TTG310TTA (1265G>A), AGT311GGG (1266A>G 1268T>G), CCT312GCC (1269C>G 1271T>C), TAT313TAC (1274T>C), CGA314CAA (1276G>A), GGG315GGA (1280G>A), GTG316GTT (1283G>T), GTT319GTG (1292T>G), CTC347CTG (1376C>G), CAC348CAT (1379C>T), CCT349CCC (1382T>C), GTT350GTC (1385T>C), CAC351CAT (1388C>T), GAT352ATT (1389G>A 1390A>T), ACA356ACT (1403A>T), GTC357GTA (1406C>A), CTG358CTA (1409G>A), TCT359TCA (1412T>A), AAA360AGT (1414A>G 1415A>T), ATA361CTT (1416A>C 1418A>T), ACA363ACT (1424A>T), GAC364GAT (1427C>T), GGG365GGT (1430G>T), ACT366ACA (1433T>A), GAC368AAC (1437G>A), GAG370GAA (1445G>A), AAA371CGT (1446A>C 1447A>G 1448A>T), CCT372CCG (1451T>G), TTG374CAG (1455T>C 1456T>A), GCT375TAT (1458G>T 1459C>A), TTA376CTA (1461T>C), AAG377AGA (1465A>G 1466G>A), CAA378CTT (1468A>T 1469A>T), AAG379AAA (1472G>A), AAC380AGA (1474A>G 1475C>A), TTT381TCG (1477T>C 1478T>G), AAA382AAG (1481A>G), AAT383TCT (1482A>T 1483A>C), TGT384TCT (1486G>C), TAT385TTC (1489A>T 1490T>C), TTA389TTC (1502A>C), AAG390AAA (1505G>A), TCA391TCG (1508A>G), CCG397CCA (1526G>A), ATG401ATA (1538G>A), TAT402TAC (1541T>C), CTT404TTC (1545C>T 1547T>C), TCT406TCC (1553T>C), ATG407TGC (1554A>T 1555T>G 1556G>C), ATA408ATG (1559A>G), CCT411AGT (1566C>A 1567C>G), ACC412ACA (1571C>A), TTG413CTG (1572T>C), TCC415TCA (1580C>A), GTT418GTC (1589T>C), AGC420del (1593_1597delAGCTC), TCT421--A (1593_1597delAGCTC 1598T>A), CTT424CTC (1607T>C), ACG426ACT (1613G>T), ATC427TTC (1614A>T), CTT428TTG (1617C>T 1619T>G), GTT429ATC (1620G>A 1622T>C), AAC430GTC (1623A>G 1624A>T), AAA431CCT (1626A>C 1627A>C 1628A>T), TTA433ATG (1632T>A 1634A>G), ACA434GTC (1635A>G 1636C>T 1637A>C), AGA436AGG (1643A>G), TAT438AGT (1647T>A 1648A>G), ATA440ATT (1655A>T), TTT442TTC (1661T>C), CTG443TTA (1662C>T 1664G>A), ACT444ACA (1667T>A), GGG445GGA (1670G>A), CCT447CCG (1676T>G), TTG448TTA (1679G>A), GGC449GGT (1682C>T), TTC450TAC (1684T>A), CAC451TAC (1686C>T), GGT452GGA (1691T>A), TCT453TCA (1694T>A), GCA455TCC (1698G>T 1700A>C), CTT456CTA (1703T>A), TTC457TTT (1706C>T), TCG458GCG (1707T>G), TTG459CTT (1710T>C 1712G>T), TCT460TCC (1715T>C), CAC462CAT (1721C>T), ATA464ATG (1727A>G), TTA467CTA (1734T>C), GCA468GCT (1739A>T), AGC470AAA (1744G>A 1745C>A), AAA471TAT (1746A>T 1748A>T), TAT473TAC (1754T>C), GGA475ACG (1758G>A 1759G>C 1760A>G), TGT476AGT (1761T>A), AAC477ACT (1765A>C 1766C>T), TCT478ACC (1767T>A 1769T>C), CCA479CCT (1772A>T), GGG481GCT (1777G>C 1778G>T), GCT484GCC (1787T>C), TTA485CTG (1788T>C 1790A>G), CTA486TTA (1791C>T), AGT495ACA (1819G>C 1820T>A), AAA496GAG (1821A>G 1823A>G), GCC498GCG (1829C>G), AAT499CGG (1830A>C 1831A>G 1832T>G), GAG500CAG (1833G>C), AAG502AGG (1840A>G), TTA504TTG (1847A>G), TTG505CTG (1848T>C), AGA507AGG (1856A>G), CTC508CTT (1859C>T), AAT509GGT (1860A>G 1861A>G), GTC510GTT (1865C>T), AAA511ACA (1867A>C), ATT512ATC (1871T>C), TCG513TCT (1874G>T), ATA514-TA (1875delA), CCA515GCT (1878C>G 1880A>T), TCT517TCG (1886T>G), TTG518AGA (1887T>A 1888T>G 1889G>A), TCT520TCC (1895T>C), GAT521GAG (1898T>G), AAT522AAC (1901T>C), ATT525ATC (1910T>C), GCG528GCT (1919G>T), AGG530AGA (1925G>A), ACT533ACC (1934T>C), AAA534AAG (1937A>G), TCA535GAC (1938T>G 1939C>A 1940A>C), GAT539TAC (1950G>T 1952T>C), TTA540CTT (1953T>C 1955A>T), ATT543ATC (1964T>C), TCC544TCT (1967C>T), GCT547GCA (1976T>A), TTA549ACT (1980T>A 1981T>C 1982A>T), TCT550TCC (1985T>C), CGT552AGG (1989C>A 1991T>G), ACT553ACA (1994T>A), GTT555GTG (2000T>G), GGA556GGT (2003A>T), CTT557CTG (2006T>G), TGC558TGT (2009C>T), GCC559CAA (2010G>C 2011C>A 2012C>A), ATC562ACC (2020T>C), GAT565TCG (2028G>T 2029A>C 2030T>G), AAC567TCA (2034A>T 2035A>C 2036C>A), ATA568ACA (2038T>C), TCA569TCG (2042A>G), GTT570ATT (2043G>A), CAA572CCC (2050A>C 2051A>C), AGA573ATT (2053G>T 2054A>T), TTG574TTT (2057G>T), GGG575TGG (2058G>T), GGT576GGG (2063T>G), GGG578GGA (2069G>A), TAC579TTC (2071A>T), AAG580AGA (2074A>G 2075G>A), GTA581GTT (2078A>T), GCG583TCT (2082G>T 2084G>T), CGC584CGT (2087C>T), TTA585TTG (2090A>G), TTC586ATG (2091T>A 2093C>G), ACT587TCT (2094A>T), ACC588ACT (2099C>T), AGC590TCT (2103A>T 2104G>C 2105C>T), AAG591AAA (2108G>A), GAG594GAA (2117G>A), CGT595AGA (2118C>A 2120T>A), CTA596CTG (2123A>G), GCT598GCG (2129T>G), CAT600GCA (2133C>G 2134A>C 2135T>A), CTT601AGC (2136C>A 2137T>G 2138T>C), AAG602AAA (2141G>A), GTT604CAC (2145G>C 2146T>A 2147T>C), CGG605AGG (2148C>A), CAC607CAT (2156C>T), TTA608CTA (2157T>C), CTA609TTA (2160C>T), TAC611CTC (2166T>C 2167A>T), TGG613TAT (2173G>A 2174G>T), GGA616GGG (2183A>G), AGG617AGA (2186G>A), GGT618TTT (2187G>T 2188G>T), ATG619AAA (2191T>A 2192G>A), CCC620CCT (2195C>T), CCC623CCT (2204C>T), TAT624TTT (2206A>T), AAA626AAG (2213A>G), GGG627GGA (2216G>A), GTA630ATA (2223G>A), AGT631GAT (2226A>G 2227G>A), TAC632TAT (2231C>T), CTT634CGG (2236T>G 2237T>G), AAA635AAG (2240A>G), AAA638ACA (2248A>C), GTT639CCT (2250G>C 2251T>C), TTA642ATA (2259T>A), CAG643CGA (2263A>G 2264G>A), ATC644CTC (2265A>C), AAG647AAT (2276G>T), GAT648GAG (2279T>G), CTG649TTG (2280C>T), TTC651TTT (2288C>T), GAA654GAG (2297A>G), GTT655AGA (2298G>A 2299T>G 2300T>A), ATA657ATC (2306A>C), CTA658CTT (2309A>T), CGT660CGC (2315T>C), CTA663TTA (2322C>T), CGC664CTC (2326G>T), CAA665AGA (2328C>A 2329A>G), GTA667GTG (2336A>G), GAA668AAA (2337G>A), TTA671CTG (2346T>C 2348A>G), AAG672AAA (2351G>A), CTC674GTC (2355C>G), AAC675TCT (2358A>T 2359A>C 2360C>T), ACG679ACT (2372G>T), ATC680GTT (2373A>G 2375C>T), TGT681GCA (2376T>G 2377G>C 2378T>A), ATG682GCA (2379A>G 2380T>C 2381G>A), AAC683TCA (2382A>T 2383A>C 2384C>A), CCA684GCA (2385C>G), GAT685GAA (2390T>A), TCC687ACC (2394T>A), AAG689GAT (2400A>G 2402G>T), AGT690CAG (2403A>C 2404G>A 2405T>G), CTC691TTG (2406C>T 2408C>G), ATC692ATG (2411C>G), GAC693GAA (2414C>A), TCC694GTT (2415T>G 2416C>T 2417C>T), ATA696ATG (2423A>G), TGC697TGT (2426C>T), GCA698AGT (2427G>A 2428C>G 2429A>T), ACA699AAA (2431C>A), TGG701TGA (2438G>A), AAA702AAG (2441A>G), AGG703CGA (2442A>C 2444G>A), CTC704TCG (2445C>T 2446T>C 2447C>G), GAA705AAT (2448G>A 2450A>T), ACT706GAA (2451A>G 2452C>A 2453T>A), GAT707GAC (2456T>C), TTT708CTG (2457T>C 2459T>G), GAT709AAA (2460G>A 2462T>A), TTA710TTG (2465A>G), ATC711GTC (2466A>G), GGC714GGT (2477C>T), CTA715TTA (2478C>T), AAA718AAG (2489A>G), TGT719TGC (2492T>C), TAC720TAT (2495C>T), GGT723GGA (2504T>A), GGT725GGC (2510T>C), AAT727GAT (2514A>G), CCC728CTG (2518C>T 2519C>G), ACC730ACT (2525C>T), ACT731ACA (2528T>A), CAG733AGG (2532C>A 2533A>G), TGG734TAT (2536G>A 2537G>T), TTT736CTG (2541T>C 2543T>G), AAC739AGT (2551A>G 2552C>T), ACT740ACC (2555T>C), GTT741TCT (2556G>T 2557T>C), ATT742ATC (2561T>C), AAT745GAT (2568A>G), AAA746CGA (2571A>C 2572A>G), TTA749TTG (2582A>G), GGG750GGA (2585G>A)

*: Inserts / Deletes / Misaligned / Frameshifts

Analysis details

This analysis was performed with panviral2.64

NGS Details (UN62): Solendovirus venanicotianae

Assembly

**Coverage Length**	1561 (5 contig(s))
**Depth Of Coverage**	8.5
**Number Of Reads**	124
**Reads Per Million**	2.87 rpm (after QC)
**Ambiguities**	10
**Assembly Method**	de novo + reference guided assembly
**Consensus Caller**	Bcf Tools

Coverage Map

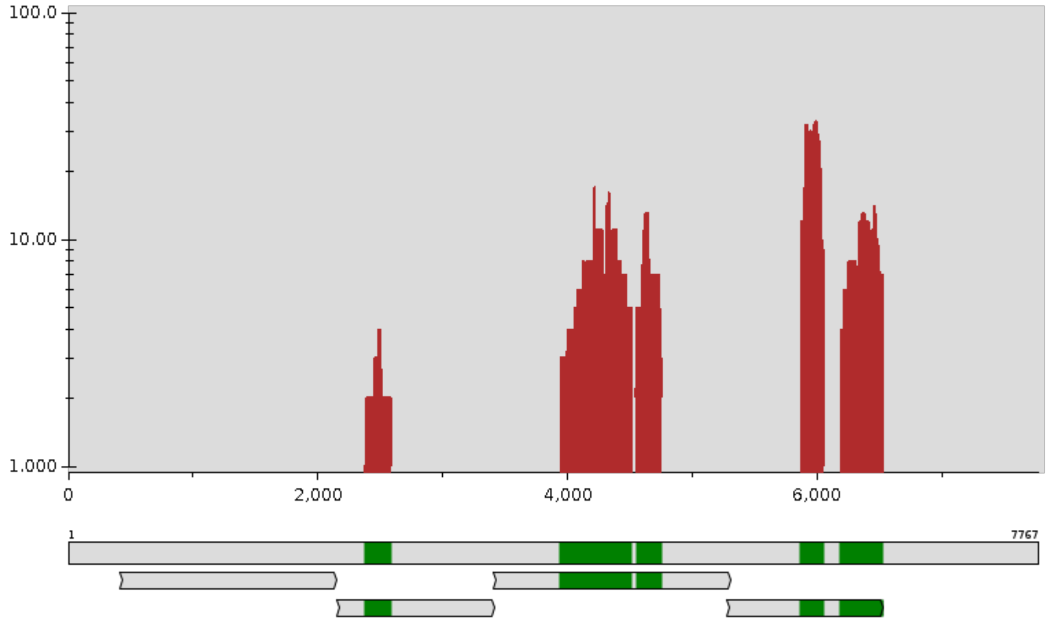

Assignment

**Type**	Solendovirus venanicotianae (Taxonomy ID: 3048371)
**Reference Genome**	NC_003378.1
**NT Identity (%)**	76.9529
**AA Identity (%)**	73.5409
**Number Of Stop Codons**	2
**Number Of CDS**	4

Alignment

**Alignment Score**	1669.0 (NT) + 2601.0 (AA) = 4270.0
**Concordance (%)**	64.6284

Alignment Method	Global, seeded, nucleotide + amino acids (AGA)

Genome Region

Sequence starts at position 2371 and ends at position 6532 relative to NC_003378.1 reference sequence.

Alignment Detailed Statistics

	Begin	End	Coverage	Score	Concordance	Matches	Identities	I/D/M/F*	Stop Codons
**NT**	**2371**	**6532**	**20.1%**	**1669**	**54.2%**	**1549 (99.2%)**	**1192 (76.4%)**	**0/12**	
Mutations:	2373T>A, 2376T>C, 2379C>T, 2388A>T, 2389G>A, 2390G>A, 2394T>A, 2397G>A, 2400C>T, 2426T>C, 2438A>G, 2442A>T, 2454G>A, 2472G>A, 2475T>C, 2482T>C, 2493T>A, 2511A>T, 2514T>C, 2535C>A, 2551C>T, 2564G>C, 2565A>G, 2571C>A, 3935A>T, 3939G>C, 3944A>G, 3946T>C, 3947A>T, 3951A>G, 3952A>G, 3953T>A, 3956T>A, 3959C>T, 3962T>C, 3966C>G, 3969G>A, 3970G>A, 3977G>T, 3981C>A, 3982A>G, 3984A>C, 3986A>C, 3987A>G, 3988C>A, 3994T>G, 3997A>T, 3998A>T, 3999A>T, 4002G>A, 4003A>T, 4004A>C, 4007A>T, 4009T>A, 4010T>A, 4013A>T, 4014G>C, 4015A>T, 4016G>T, 4018A>C, 4019T>G, 4021G>T, 4030C>G, 4031A>G, 4032C>G, 4033A>T, 4036A>G, 4040T>A, 4041C>A, 4043A>G, 4044A>T, 4045A>T, 4052C>T, 4053T>G, 4054T>A, 4055T>A, 4058T>A, 4062T>A, 4063T>A, 4065A>T, 4067A>T, 4068G>A, 4073T>C, 4075A>G, 4079A>T, 4082T>G, 4085A>T, 4086A>C, 4091T>A, 4092T>C, 4094A>T, 4095T>C, 4097G>C, 4098G>A, 4100T>A, 4101A>T, 4102A>T, 4103T>A, 4109T>A, 4113C>A, 4114A>G, 4124T>G, 4127C>T, 4128A>C, 4129A>C, 4135C>G, 4136A>C, 4142A>T, 4151A>G, 4152G>A, 4156A>G, 4157T>A, 4160A>T, 4164A>G, 4165G>C, 4166T>G, 4184A>G, 4185A>G, 4186G>C, 4187C>T, 4188A>C, 4190G>A, 4196C>T, 4200G>A, 4207G>A, 4209A>G, 4211T>G, 4212T>C, 4214A>T, 4228A>T, 4235T>C, 4238T>A, 4241T>C, 4250C>T, 4253T>C, 4260T>C, 4262A>T, 4265G>A, 4274A>G, 4313G>A, 4325T>C, 4328T>C, 4329T>C, 4331G>A, 4335C>T, 4340G>A, 4343T>A, 4364T>A, 4376A>C, 4400T>A, 4401T>C, 4406A>T, 4430A>T, 4444T>G, 4451C>T, 4462A>G, 4463T>C, 4484A>T, 4500C>T, 4503C>T, 4513G>A, 4550C>T, 4552C>T, 4553A>G, 4554C>A, 4559T>A, 4560A>T, 4562A>T, 4566A>T, 4567A>C, 4568C>T, 4571T>A, 4573G>A, 4574T>A, 4580C>T, 4586C>T, 4592C>A, 4595A>G, 4596G>T, 4604T>A, 4612A>C, 4613T>A, 4616G>A, 4648A>G, 4649T>C, 4662C>T, 4665A>T, 4676A>G, 4688C>T, 4691T>C, 4692C>T, 4693T>C, 4694T>A, 4695G>A, 4697T>C, 4701A>C, 4703C>A, 4706T>A, 4715A>G, 4718G>A, 4738G>T, 4740A>T, 4748C>T, 5865C>T, 5867A>G, 5868T>C, 5871A>G, 5872T>C, 5874G>A, 5877A>T, 5880T>A, 5881T>C, 5882T>A, 5886A>T, 5889T>A, 5892A>C, 5894G>A, 5901A>C, 5902T>C, 5908G>A, 5909C>A, 5910C>A, 5913C>A, 5916C>T, 5921A>G, 5922C>Y, 5925C>T, 5928A>W, 5929C>Y, 5931A>R, 5932C>Y, 5935G>A, 5936A>R, 5937T>Y, 5940T>C, 5950C>T, 5952G>T, 5953A>G, 5954C>A, 5955A>Y, 5959C>G, 5964G>A, 5968G>T, 5970C>R, 5972A>C, 5973T>Y, 5979T>A, 5991G>A, 5997C>T, 5999T>C, 6000G>A, 6001C>A, 6002A>C, 6009G>A, 6012G>A, 6015A>T, 6017C>T, 6018T>A, 6023G>A, 6024G>C, 6026C>A, 6027A>C, 6033C>T, 6036C>T, 6037G>A, 6040C>A, 6041G>A, 6042A>G, 6045C>T, 6181G>A, 6183A>T, 6186C>T, 6195C>A, 6201G>A, 6204A>T, 6209G>A, 6211A>C, 6214T>G, 6215C>T, 6219T>G, 6223C>A, 6225A>C, 6232C>T, 6246T>C, 6247C>A, 6248T>C, 6253C>G, 6254A>G, 6258T>C, 6259G>C, 6264T>G, 6269G>A, 6270C>T, 6271T>C, 6277T>A, 6279A>C, 6282G>A, 6285A>C, 6290C>T, 6300T>C, 6304A>C, 6307T>A, 6308G>T, 6310_6321delAGAGATATAAGA, 6325G>A, 6326C>G, 6340C>G, 6341T>C, 6344G>T, 6345A>G, 6352G>A, 6354C>A, 6355T>A, 6357A>G, 6359G>C, 6360T>A, 6361C>T, 6363T>G, 6369A>T, 6375A>G, 6376C>A, 6380C>A, 6381T>A, 6382A>C, 6384A>T, 6386C>T, 6387C>T, 6401G>A, 6402G>A, 6404G>A, 6407A>G, 6408T>A, 6411C>T, 6414C>T, 6417C>G, 6418C>A, 6419C>A, 6423T>A, 6424C>T, 6426G>A, 6427T>C, 6444G>A, 6446T>A, 6451A>G, 6453T>G, 6456C>A, 6462T>C, 6471C>T, 6477C>T, 6489A>C, 6502A>G, 6504T>A, 6505G>A, 6507T>C, 6513C>T, 6522G>A, 6525A>C, 6526T>G									

CDS

	Begin	End	Coverage	Score	Concordance	Matches	Identities	I/D/M/F*	Stop Codons
Tvvgp2	73	146	17.6%	474	93.3%	74 (100%)	70 (94.6%)	0/0/0/0	0
Protein mutations:	G79K (2389G>A 2390G>A), I91T (2426T>C), E95G (2438A>G), R137T (2564G>C 2565A>G)								
Codon mutations:	ATT73ATA (2373T>A), GAT74GAC (2376T>C), TGC75TGT (2379C>T), ACA78ACT (2388A>T), GGA79AAA (2389G>A 2390G>A), GGT80GGA (2394T>A), AAG81AAA (2397G>A), GCC82GCT (2400C>T), ATA91ACA (2426T>C), GAA95GGA (2438A>G), ATA96ATT (2442A>T), AAG100AAA (2454G>A), AAG106AAA (2472G>A), TAT107TAC (2475T>C), TTA110CTA (2482T>C), ACT113ACA (2493T>A), GCA119GCT (2511A>T), TGT120TGC (2514T>C), ACC127ACA (2535C>A), CTA133TTA (2551C>T), AGA137ACG (2564G>C 2565A>G), ATC139ATA (2571C>A)								
Tvvgp3	176	449	41.7%	1324	72.1%	265 (100%)	194 (73.2%)	0/0/0/0	2
Protein mutations:	E177Q (3939G>C), L179S (3946T>C 3947A>T), N181G (3951A>G 3952A>G 3953T>A), D182E (3956T>A), Q186E (3966C>G), G187K (3969G>A 3970G>A), E189D (3977G>T), H191S (3981C>A 3982A>G), K192H (3984A>C 3986A>C), T193E (3987A>G 3988C>A), V195G (3994T>G), K196I (3997A>T 3998A>T), I197L (3999A>T), E198I (4002G>A 4003A>T 4004A>C), I200K (4009T>A 4010T>A), E201D (4013A>T), E202L (4014G>C 4015A>T 4016G>T), N203T (4018A>C 4019T>G), S204I (4021G>T), T207R (4030C>G 4031A>G), Q208V (4032C>G 4033A>T), K209R (4036A>G), L211M (4041C>A 4043A>G), K212L (4044A>T 4045A>T), F215E (4053T>G 4054T>A 4055T>A), N216K (4058T>A), L218K (4062T>A 4063T>A), T219S (4065A>T 4067A>T), E220K (4068G>A), K222R (4075A>G), H224Q (4082T>G), K226Q (4086A>C), D227E (4091T>A), D230K (4098G>A 4100T>A), N231L (4101A>T 4102A>T 4103T>A), Y233* (4109T>A), Q235R (4113C>A 4114A>G), N238K (4124T>G), K240P (4128A>C 4129A>C), T242S (4135C>G 4136A>C), I247M (4151A>G), V248I (4152G>A), N249R (4156A>G 4157T>A), K250N (4160A>T), S252A (4164A>G 4165G>C 4166T>G), S259A (4185A>G 4186G>C 4187C>T), D264N (4200G>A), R266K (4207G>A), N267E (4209A>G 4211T>G), K273I (4228A>T), N276K (4238T>A), D311E (4343T>A), F345C (4444T>G), N351S (4462A>G 4463T>C), R368K (4513G>A), A381V (4552C>T 4553A>G), H382N (4554C>A), I384F (4560A>T 4562A>T), N386S (4566A>T 4567A>C 4568C>T), G388E (4573G>A 4574T>A), A396S (4596G>T), N401T (4612A>C 4613T>A), N413S (4648A>G 4649T>C), Q418* (4662C>T), T419S (4665A>T), L428S (4692C>T 4693T>C 4694T>A), D429N (4695G>A 4697T>C), N431Q (4701A>C 4703C>A), G443V (4738G>T), I444L (4740A>T)								
Codon mutations:	AAA175.AT (3935A>T), GAA177CAA (3939G>C), AAA178AAG (3944A>G), TTA179TCT (3946T>C 3947A>T), AAT181GGA (3951A>G 3952A>G 3953T>A), GAT182GAA (3956T>A), AAC183AAT (3959C>T), CCT184CCC (3962T>C), CAA186GAA (3966C>G), GGA187AAA (3969G>A 3970G>A), GAG189GAT (3977G>T), CAT191AGT (3981C>A 3982A>G), AAA192CAC (3984A>C 3986A>C), ACA193GAA (3987A>G 3988C>A), GTA195GGA (3994T>G), AAA196ATT (3997A>T 3998A>T), ATA197TTA (3999A>T), GAA198ATC (4002G>A 4003A>T 4004A>C), CTA199CTT (4007A>T), ATT200AAA (4009T>A 4010T>A), GAA201GAT (4013A>T), GAG202CTT (4014G>C 4015A>T 4016G>T), AAT203ACG (4018A>C 4019T>G), AGT204ATT (4021G>T), ACA207AGG (4030C>G 4031A>G), CAA208GTA (4032C>G 4033A>T), AAA209AGA (4036A>G), CCT210CCA (4040T>A), CTA211ATG (4041C>A 4043A>G), AAA212TTA (4044A>T 4045A>T), AAC214AAT (4052C>T), TTT215GAA (4053T>G 4054T>A 4055T>A), AAT216AAA (4058T>A), TTA218AAA (4062T>A 4063T>A), ACA219TCT (4065A>T 4067A>T), GAA220AAA (4068G>A), TTT221TTC (4073T>C), AAA222AGA (4075A>G), ATA223ATT (4079A>T), CAT224CAG (4082T>G), ATA225ATT (4085A>T), AAA226CAA (4086A>C), GAT227GAA (4091T>A), TTA228CTT (4092T>C 4094A>T), TTG229CTC (4095T>C 4097G>C), GAT230AAA (4098G>A 4100T>A), AAT231TTA (4101A>T 4102A>T 4103T>A), TAT233TAA (4109T>A), CAA235AGA (4113C>A 4114A>G), AAT238AAG (4124T>G), AGC239AGT (4127C>T), AAA240CCA (4128A>C 4129A>C), ACA242AGC (4135C>G 4136A>C), CCA244CCT (4142A>T), ATA247ATG (4151A>G), GTA248ATA (4152G>A), AAT249AGA (4156A>G 4157T>A), AAA250AAT (4160A>T), AGT252GCG (4164A>G 4165G>C 4166T>G), AAA258AAG (4184A>G), AGC259GCT (4185A>G 4186G>C 4187C>T), AGG260CGA (4188A>C 4190G>A), GTC262GTT (4196C>T), GAC264AAC (4200G>A), AGA266AAA (4207G>A), AAT267GAG (4209A>G 4211T>G), TTA268CTT (4212T>C 4214A>T), AAA273ATA (4228A>T), TAT275TAC (4235T>C), AAT276AAA (4238T>A), TAT277TAC (4241T>C), CCC280CCT (4250C>T), AAT281AAC (4253T>C), TTA284CTT (4260T>C 4262A>T), AAG285AAA (4265G>A), CAA288CAG (4274A>G), AAG301AAA (4313G>A), TAT305TAC (4325T>C), CAT306CAC (4328T>C), TTG307CTA (4329T>C 4331G>A), CTA309TTA (4335C>T), GAG310GAA (4340G>A), GAT311GAA (4343T>A), GCT318GCA (4364T>A), CCA322CCC (4376A>C), GTT330GTA (4400T>A), TTA331CTA (4401T>C), CCA332CCT (4406A>T), GGA340GGT (4430A>T), TTT345TGT (4444T>G), GAC347GAT (4451C>T), AAT351AGC (4462A>G 4463T>C), GTA358GTT (4484A>T), CTA364TTA (4500C>T), CTA365TTA (4503C>T), AGA368AAA (4513G>A), TTC380TTT (4550C>T), GCA381GTG (4552C>T 4553A>G), CAT382AAT (4554C>A), ATT383ATA (4559T>A), ATA384TTT (4560A>T 4562A>T), AAC386TCT (4566A>T 4567A>C 4568C>T), TCT387TCA (4571T>A), GGT388GAA (4573G>A 4574T>A), AGC390AGT (4580C>T), AGC392AGT (4586C>T), ACC394ACA (4592C>A), AAA395AAG (4595A>G), GCA396TCA (4596G>T), ATT398ATA (4604T>A), AAT401ACA (4612A>C 4613T>A), CAG402CAA (4616G>A), AAT413AGC (4648A>G 4649T>C), CAA418TAA (4662C>T), ACT419TCT (4665A>T), GTA422GTG (4676A>G), ATC426ATT (4688C>T), AAT427AAC (4691T>C), CTT428TCA (4692C>T 4693T>C 4694T>A), GAT429AAC (4695G>A 4697T>C), AAC431CAA (4701A>C 4703C>A), ATT432ATA (4706T>A), AAA435AAG (4715A>G), AAG436AAA (4718G>A), GGA443GTA (4738G>T), ATA444TTA (4740A>T), AAC446AAT (4748C>T)								
Tvvgp4	197	418	42.8%	803	65.8%	175 (97.8%)	114 (63.7%)	0/4/1/0	0
Protein mutations:	N198S (5867A>G 5868T>C), L203Q (5881T>C 5882T>A), N205K (5889T>A), S207N (5894G>A), K209N (5901A>C), A212K (5908G>A 5909C>A 5910C>A), N216S (5921A>G 5922C>Y), Q226Y (5950C>T 5952G>T), T227D (5953A>G 5954C>A 5955A>Y), Q229E (5959C>G), A232S (5968G>T 5970C>R), N233T (5972A>C 5973T>Y), M242T (5999T>C 6000G>A), Q243T (6001C>A 6002A>C), T248I (6017C>T 6018T>A), G250D (6023G>A 6024G>C), T251N (6026C>A 6027A>C), V255I (6037G>A), R256K (6040C>A 6041G>A 6042A>G), E303N (6181G>A 6183A>T), N307K (6195C>A), G312E (6209G>A), I313L (6211A>C), S314V (6214T>G 6215C>T), L317I (6223C>A 6225A>C), L325T (6247C>A 6248T>C), Q327G (6253C>G 6254A>G), A329P (6259G>C), I330M (6264T>G), S332N (6269G>A 6270C>T), Y333H (6271T>C), S335T (6277T>A 6279A>C), E337D (6285A>C), T339I (6290C>T), N344H (6304A>C), C345I (6307T>A 6308G>T), R346_R349del (6310_6321delAGAGATATAAGA), A351R (6325G>A 6326C>G), L356A (6340C>G 6341T>C), R357M (6344G>T 6345A>G), V360I (6352G>A 6354C>A), L361M (6355T>A 6357A>G), S362T (6359G>C 6360T>A), K365N (6369A>T), Q368K (6376C>A), P369Q (6380C>A 6381T>A), T370P (6382A>C 6384A>T), T371I (6386C>T 6387C>T), R376K (6401G>A 6402G>A), R377K (6404G>A), N378R (6407A>G 6408T>A), P382K (6418C>A 6419C>A), D383E (6423T>A), L391Q (6446T>A), S393G (6451A>G 6453T>G), H394Q (6456C>A), K405N (6489A>C), I410V (6502A>G 6504T>A), V411I (6505G>A 6507T>C), E417D (6525A>C), *418E (6526T>G)								

	Begin	End	Coverage	Score	Concordance	Matches	Identities	I/D/M/F*	Stop Codons
**NT**	**2371**	**6532**	**20.1%**	**1669**	**54.2%**	**1549 (99.2%)**	**1192 (76.4%)**	**0/12**	
Codon mutations:	TAC197TAT (5865C>T), AAT198AGC (5867A>G 5868T>C), AAA199AAG (5871A>G), TTG200CTA (5872T>C 5874G>A), ATA201ATT (5877A>T), GCT202GCA (5880T>A), TTA203CAA (5881T>C 5882T>A), CCA204CCT (5886A>T), AAT205AAA (5889T>A), ACA206ACC (5892A>C), AGT207AAT (5894G>A), AAA209AAC (5901A>C), TTA210CTA (5902T>C), GCC212AAA (5908G>A 5909C>A 5910C>A), ACC213ACA (5913C>A), TGC214TGT (5916C>T), AAC216AGY (5921A>G 5922C>Y), TAC217TAT (5925C>T), GGA218GGW (5928A>W), CTA219YTR (5929C>Y 5931A>R), CTA220YTA (5932C>Y), GAT221ARY (5935G>A 5936A>R 5937T>Y), ACT222ACC (5940T>C), CAG226TAT (5950C>T 5952G>T), ACA227GAY (5953A>G 5954C>A 5955A>Y), CAA229GAA (5959C>G), GAG230GAA (5964G>A), GCC232TCR (5968G>T 5970C>R), AAT233ACY (5972A>C 5973T>Y), CCT235CCA (5979T>A), AAG239AAA (5991G>A), TTC241TTT (5997C>T), ATG242ACA (5999T>C 6000G>A), CAA243ACA (6001C>A 6002A>C), AAG245AAA (6009G>A), AGG246AGA (6012G>A), ATA247ATT (6015A>T), ACT248ATA (6017C>T 6018T>A), GGG250GAC (6023G>A 6024G>C), ACA251AAC (6026C>A 6027A>C), TTC253TTT (6033C>T), TAC254TAT (6036C>T), GTA255ATA (6037G>A), CGA256AAG (6040C>A 6041G>A 6042A>G), TTC257TTT (6045C>T), GAA303AAT (6181G>A 6183A>T), TTC304TTT (6186C>T), AAC307AAA (6195C>A), AGG309AGA (6201G>A), ATA310ATT (6204A>T), GGA312GAA (6209G>A), ATA313CTA (6211A>C), TCT314GTT (6214T>G 6215C>T), ACT315ACG (6219T>G), CTA317ATC (6223C>A 6225A>C), CTA320TTA (6232C>T), TAT324TAC (6246T>C), CTA325ACA (6247C>A 6248T>C), CAG327GGG (6253C>G 6254A>G), AAT328AAC (6258T>C), GCA329CCA (6259G>C), ATT330ATG (6264T>G), AGC332AAT (6269G>A 6270C>T), TAT333CAT (6271T>C), TCA335ACC (6277T>A 6279A>C), AGG336AGA (6282G>A), GAA337GAC (6285A>C), ACA339ATA (6290C>T), TAT342TAC (6300T>C), AAT344CAT (6304A>C), TGC345ATC (6307T>A 6308G>T), AGA346_AGA349del (6310_6321delAGAGATATAAGA), GCA351AGA (6325G>A 6326C>G), CTA356GCA (6340C>G 6341T>C), AGA357ATG (6344G>T 6345A>G), GTC360ATA (6352G>A 6354C>A), TTA361ATG (6355T>A 6357A>G), AGT362ACA (6359G>C 6360T>A), CTT363TTG (6361C>T 6363T>G), AAA365AAT (6369A>T), GAA367GAG (6375A>G), CAA368AAA (6376C>A), CCT369CAA (6380C>A 6381T>A), ACA370CCT (6382A>C 6384A>T), ACC371ATT (6386C>T 6387C>T), AGG376AAA (6401G>A 6402G>A), AGG377AAG (6404G>A), AAT378AGA (6407A>G 6408T>A), TTC379TTT (6411C>T), ATC380ATT (6414C>T), TCC381TCG (6417C>G), CCA382AAA (6418C>A 6419C>A), GAT383GAA (6423T>A), CTG384TTA (6424C>T 6426G>A), TTA385CTA (6427T>C), AAG390AAA (6444G>A), CTA391CAA (6446T>A), AGT393GGG (6451A>G 6453T>G), CAC394CAA (6456C>A), TAT396TAC (6462T>C), CAC399CAT (6471C>T), TGC401TGT (6477C>T), AAA405AAC (6489A>C), ATT410GTA (6502A>G 6504T>A), GTT411ATC (6505G>A 6507T>C), GAC413GAT (6513C>T), CTG416CTA (6522G>A), GAA417GAC (6525A>C), TAA418GAA (6526T>G)								

Proteins

	Begin	End	Coverage	Score	Concordance	Matches	Identities	I/D/M/F*	Stop Codons
putative cell-to-cell movement protein (NP_569140.1)	73	146	17.6%	474	93.3%	74 (100%)	70 (94.6%)	0/0/0/0	0
Protein mutations:	G79K (2389G>A 2390G>A), I91T (2426T>C), E95G (2438A>G), R137T (2564G>C 2565A>G)								
Codon mutations:	ATT73ATA (2373T>A), GAT74GAC (2376T>C), TGC75TGT (2379C>T), ACA78ACT (2388A>T), GGA79AAA (2389G>A 2390G>A), GGT80GGA (2394T>A), AAG81AAA (2397G>A), GCC82GCT (2400C>T), ATA91ACA (2426T>C), GAA95GGA (2438A>G), ATA96ATT (2442A>T), AAG100AAA (2454G>A), AAG106AAA (2472G>A), TAT107TAC (2475T>C), TTA110CTA (2482T>C), ACT113ACA (2493T>A), GCA119GCT (2511A>T), TGT120TGC (2514T>C), ACC127ACA (2535C>A), CTA133TTA (2551C>T), AGA137ACG (2564G>C 2565A>G), ATC139ATA (2571C>A)								
polyprotein (NP_569141.1)	176	449	41.7%	1324	72.1%	265 (100%)	194 (73.2%)	0/0/0/0	2
Protein mutations:	E177Q (3939G>C), L179S (3946T>C 3947A>T), N181G (3951A>G 3952A>G 3953T>A), D182E (3956T>A), Q186E (3966C>G), G187K (3969G>A 3970G>A), E189D (3977G>T), H191S (3981C>A 3982A>G), K192H (3984A>C 3986A>C), T193E (3987A>G 3988C>A), V195G (3994T>G), K196I (3997A>T 3998A>T), I197L (3999A>T), E198I (4002G>A 4003A>T 4004A>C), I200K (4009T>A 4010T>A), E201D (4013A>T), E202L (4014G>C 4015A>T 4016G>T), N203T (4018A>C 4019T>G), S204I (4021G>T), T207R (4030C>G 4031A>G), Q208V (4032C>G 4033A>T), K209R (4036A>G), L211M (4041C>A 4043A>G), K212L (4044A>T 4045A>T), F215E (4053T>G 4054T>A 4055T>A), N216K (4058T>A), L218K (4062T>A 4063T>A), T219S (4065A>T 4067A>T), E220K (4068G>A), K222R (4075A>G), H224Q (4082T>G), K226Q (4086A>C), D227E (4091T>A), D230K (4098G>A 4100T>A), N231L (4101A>T 4102A>T 4103T>A), Y233* (4109T>A), Q235R (4113C>A 4114A>G), N238K (4124T>G), K240P (4128A>C 4129A>C), T242S (4135C>G 4136A>C), I247M (4151A>G), V248I (4152G>A), N249R (4156A>G 4157T>A), K250N (4160A>T), S252A (4164A>G 4165G>C 4166T>G), S259A (4185A>G 4186G>C 4187C>T), D264N (4200G>A), R266K (4207G>A), N267E (4209A>G 4211T>G), K273I (4228A>T), N276K (4238T>A), D311E (4343T>A), F345C (4444T>G), N351S (4462A>G 4463T>C), R368K (4513G>A), A381V (4552C>T 4553A>G), H382N (4554C>A), I384F (4560A>T 4562A>T), N386S (4566A>T 4567A>C 4568C>T), G388E (4573G>A 4574T>A), A396S (4596G>T), N401T (4612A>C 4613T>A), N413S (4648A>G 4649T>C), Q418* (4662C>T), T419S (4665A>T), L428S (4692C>T 4693T>C 4694T>A), D429N (4695G>A 4697T>C), N431Q (4701A>C 4703C>A), G443V (4738G>T), I444L (4740A>T)								
Codon mutations:	AAA175.AT (3935A>T), GAA177CAA (3939G>C), AAA178AAG (3944A>G), TTA179TCT (3946T>C 3947A>T), AAT181GGA (3951A>G 3952A>G 3953T>A), GAT182GAA (3956T>A), AAC183AAT (3959C>T), CCT184CCC (3962T>C), CAA186GAA (3966C>G), GGA187AAA (3969G>A 3970G>A), GAG189GAT (3977G>T), CAT191AGT (3981C>A 3982A>G), AAA192CAC (3984A>C 3986A>C), ACA193GAA (3987A>G 3988C>A), GTA195GGA (3994T>G), AAA196ATT (3997A>T 3998A>T), ATA197TTA (3999A>T), GAA198ATC (4002G>A 4003A>T 4004A>C), CTA199CTT (4007A>T), ATT200AAA (4009T>A 4010T>A), GAA201GAT (4013A>T), GAG202CTT (4014G>C 4015A>T 4016G>T), AAT203ACG (4018A>C 4019T>G), AGT204ATT (4021G>T), ACA207AGG (4030C>G 4031A>G), CAA208GTA (4032C>G 4033A>T), AAA209AGA (4036A>G), CCT210CCA (4040T>A), CTA211ATG (4041C>A 4043A>G), AAA212TTA (4044A>T 4045A>T), AAC214AAT (4052C>T), TTT215GAA (4053T>G 4054T>A 4055T>A), AAT216AAA (4058T>A), TTA218AAA (4062T>A 4063T>A), ACA219TCT (4065A>T 4067A>T), GAA220AAA (4068G>A), TTT221TTC (4073T>C), AAA222AGA (4075A>G), ATA223ATT (4079A>T), CAT224CAG (4082T>G), ATA225ATT (4085A>T), AAA226CAA (4086A>C), GAT227GAA (4091T>A), TTA228CTT (4092T>C 4094A>T), TTG229CTC (4095T>C 4097G>C), GAT230AAA (4098G>A 4100T>A), AAT231TTA (4101A>T 4102A>T 4103T>A), TAT233TAA (4109T>A), CAA235AGA (4113C>A 4114A>G), AAT238AAG (4124T>G), AGC239AGT (4127C>T), AAA240CCA (4128A>C 4129A>C), ACA242AGC (4135C>G 4136A>C), CCA244CCT (4142A>T), ATA247ATG (4151A>G), GTA248ATA (4152G>A), AAT249AGA (4156A>G 4157T>A), AAA250AAT (4160A>T), AGT252GCG (4164A>G 4165G>C 4166T>G), AAA258AAG (4184A>G), AGC259GCT (4185A>G 4186G>C 4187C>T), AGG260CGA (4188A>C 4190G>A), GTC262GTT (4196C>T), GAC264AAC (4200G>A), AGA266AAA (4207G>A), AAT267GAG (4209A>G 4211T>G), TTA268CTT (4212T>C 4214A>T), AAA273ATA (4228A>T), TAT275TAC (4235T>C), AAT276AAA (4238T>A), TAT277TAC (4241T>C), CCC280CCT (4250C>T), AAT281AAC (4253T>C), TTA284CTT (4260T>C 4262A>T), AAG285AAA (4265G>A), CAA288CAG (4274A>G), AAG301AAA (4313G>A), TAT305TAC (4325T>C), CAT306CAC (4328T>C), TTG307CTA (4329T>C 4331G>A), CTA309TTA (4335C>T), GAG310GAA (4340G>A), GAT311GAA (4343T>A), GCT318GCA (4364T>A), CCA322CCC (4376A>C), GTT330GTA (4400T>A), TTA331CTA (4401T>C), CCA332CCT (4406A>T), GGA340GGT (4430A>T), TTT345TGT (4444T>G), GAC347GAT (4451C>T), AAT351AGC (4462A>G 4463T>C), GTA358GTT (4484A>T), CTA364TTA (4500C>T), CTA365TTA (4503C>T), AGA368AAA (4513G>A), TTC380TTT (4550C>T), GCA381GTG (4552C>T 4553A>G), CAT382AAT (4554C>A), ATT383ATA (4559T>A), ATA384TTT (4560A>T 4562A>T), AAC386TCT (4566A>T 4567A>C 4568C>T), TCT387TCA (4571T>A), GGT388GAA (4573G>A 4574T>A), AGC390AGT (4580C>T), AGC392AGT (4586C>T), ACC394ACA (4592C>A), AAA395AAG (4595A>G), GCA396TCA (4596G>T), ATT398ATA (4604T>A), AAT401ACA (4612A>C 4613T>A), CAG402CAA (4616G>A), AAT413AGC (4648A>G 4649T>C), CAA418TAA (4662C>T), ACT419TCT (4665A>T), GTA422GTG (4676A>G), ATC426ATT (4688C>T), AAT427AAC (4691T>C), CTT428TCA (4692C>T 4693T>C 4694T>A), GAT429AAC (4695G>A 4697T>C), AAC431CAA (4701A>C 4703C>A), ATT432ATA (4706T>A), AAA435AAG (4715A>G), AAG436AAA (4718G>A), GGA443GTA (4738G>T), ATA444TTA (4740A>T), AAC446AAT (4748C>T)								
putative transactivator factor (NP_569142.1)	197	418	42.8%	803	65.8%	175 (97.8%)	114 (63.7%)	0/4/1/0	0
Protein mutations:	N198S (5867A>G 5868T>C), L203Q (5881T>C 5882T>A), N205K (5889T>A), S207N (5894G>A), K209N (5901A>C), A212K (5908G>A 5909C>A 5910C>A), N216S (5921A>G 5922C>Y), Q226Y (5950C>T 5952G>T), T227D (5953A>G 5954C>A 5955A>Y), Q229E (5959C>G), A232S (5968G>T 5970C>R), N233T (5972A>C 5973T>Y), M242T (5999T>C 6000G>A), Q243T (6001C>A 6002A>C), T248I (6017C>T 6018T>A), G250D (6023G>A 6024G>C), T251N (6026C>A 6027A>C), V255I (6037G>A), R256K (6040C>A 6041G>A 6042A>G), E303N (6181G>A 6183A>T), N307K (6195C>A), G312E (6209G>A), I313L (6211A>C), S314V (6214T>G 6215C>T), L317I (6223C>A 6225A>C), L325T (6247C>A 6248T>C), Q327G (6253C>G 6254A>G), A329P (6259G>C), I330M (6264T>G), S332N (6269G>A 6270C>T), Y333H (6271T>C), S335T (6277T>A 6279A>C), E337D (6285A>C), T339I (6290C>T), N344H (6304A>C), C345I (6307T>A 6308G>T), R346_R349del (6310_6321delAGAGATATAAGA), A351R (6325G>A 6326C>G), L356A (6340C>G 6341T>C), R357M (6344G>T 6345A>G), V360I (6352G>A 6354C>A), L361M (6355T>A 6357A>G), S362T (6359G>C 6360T>A), K365N (6369A>T), Q368K (6376C>A), P369Q (6380C>A 6381T>A), T370P (6382A>C 6384A>T), T371I (6386C>T 6387C>T), R376K (6401G>A 6402G>A), R377K (6404G>A), N378R (6407A>G 6408T>A), P382K (6418C>A 6419C>A), D383E (6423T>A), L391Q (6446T>A), S393G (6451A>G 6453T>G), H394Q (6456C>A), K405N (6489A>C), I410V (6502A>G 6504T>A), V411I (6505G>A 6507T>C), E417D (6525A>C), *418E (6526T>G)								
Codon mutations:	TAC197TAT (5865C>T), AAT198AGC (5867A>G 5868T>C), AAA199AAG (5871A>G), TTG200CTA (5872T>C 5874G>A), ATA201ATT (5877A>T), GCT202GCA (5880T>A), TTA203CAA (5881T>C 5882T>A), CCA204CCT (5886A>T), AAT205AAA (5889T>A), ACA206ACC (5892A>C), AGT207AAT (5894G>A), AAA209AAC (5901A>C), TTA210CTA (5902T>C), GCC212AAA (5908G>A 5909C>A 5910C>A), ACC213ACA (5913C>A), TGC214TGT (5916C>T), AAC216AGY (5921A>G 5922C>Y), TAC217TAT (5925C>T), GGA218GGW (5928A>W), CTA219YTR (5929C>Y 5931A>R), CTA220YTA (5932C>Y), GAT221ARY (5935G>A 5936A>R 5937T>Y), ACT222ACC (5940T>C), CAG226TAT (5950C>T 5952G>T), ACA227GAY (5953A>G 5954C>A 5955A>Y), CAA229GAA (5959C>G), GAG230GAA (5964G>A), GCC232TCR (5968G>T 5970C>R), AAT233ACY (5972A>C 5973T>Y), CCT235CCA (5979T>A), AAG239AAA (5991G>A), TTC241TTT (5997C>T), ATG242ACA (5999T>C 6000G>A), CAA243ACA (6001C>A 6002A>C), AAG245AAA (6009G>A), AGG246AGA (6012G>A), ATA247ATT (6015A>T), ACT248ATA (6017C>T 6018T>A), GGG250GAC (6023G>A 6024G>C), ACA251AAC (6026C>A 6027A>C), TTC253TTT (6033C>T), TAC254TAT (6036C>T), GTA255ATA (6037G>A), CGA256AAG (6040C>A 6041G>A 6042A>G), TTC257TTT (6045C>T), GAA303AAT (6181G>A 6183A>T), TTC304TTT (6186C>T), AAC307AAA (6195C>A), AGG309AGA (6201G>A), ATA310ATT (6204A>T), GGA312GAA (6209G>A), ATA313CTA (6211A>C), TCT314GTT (6214T>G 6215C>T), ACT315ACG (6219T>G), CTA317ATC (6223C>A 6225A>C), CTA320TTA (6232C>T), TAT324TAC (6246T>C), CTA325ACA (6247C>A 6248T>C), CAG327GGG (6253C>G 6254A>G), AAT328AAC (6258T>C), GCA329CCA (6259G>C), ATT330ATG (6264T>G), AGC332AAT (6269G>A 6270C>T), TAT333CAT (6271T>C), TCA335ACC (6277T>A 6279A>C), AGG336AGA (6282G>A), GAA337GAC (6285A>C), ACA339ATA (6290C>T), TAT342TAC (6300T>C), AAT344CAT (6304A>C), TGC345ATC (6307T>A 6308G>T), AGA346_AGA349del (6310_6321delAGAGATATAAGA), GCA351AGA (6325G>A 6326C>G), CTA356GCA (6340C>G 6341T>C), AGA357ATG (6344G>T 6345A>G), GTC360ATA (6352G>A 6354C>A), TTA361ATG (6355T>A 6357A>G), AGT362ACA (6359G>C 6360T>A), CTT363TTG (6361C>T 6363T>G), AAA365AAT (6369A>T), GAA367GAG (6375A>G), CAA368AAA (6376C>A), CCT369CAA (6380C>A 6381T>A), ACA370CCT (6382A>C 6384A>T), ACC371ATT (6386C>T 6387C>T), AGG376AAA (6401G>A 6402G>A), AGG377AAG (6404G>A), AAT378AGA (6407A>G 6408T>A), TTC379TTT (6411C>T), ATC380ATT (6414C>T), TCC381TCG (6417C>G), CCA382AAA (6418C>A 6419C>A), GAT383GAA (6423T>A), CTG384TTA (6424C>T 6426G>A), TTA385CTA (6427T>C), AAG390AAA (6444G>A), CTA391CAA (6446T>A), AGT393GGG (6451A>G 6453T>G), CAC394CAA (6456C>A), TAT396TAC (6462T>C), CAC399CAT (6471C>T), TGC401TGT (6477C>T), AAA405AAC (6489A>C), ATT410GTA (6502A>G 6504T>A), GTT411ATC (6505G>A 6507T>C), GAC413GAT (6513C>T), CTG416CTA (6522G>A), GAA417GAC (6525A>C), TAA418GAA (6526T>G)								

*: Inserts / Deletes / Misaligned / Frameshifts

Analysis details

This analysis was performed with panviral2.64

NGS Details (UN62): Tomato chocolate spot virus (segment RNA2)

Assembly

**Coverage Length**	409 (1 contig(s))
**Depth Of Coverage**	5209.1
**Number Of Reads**	18591
**Reads Per Million**	430.96 rpm (after QC)
**Ambiguities**	0
**Assembly Method**	de novo + reference guided assembly
**Consensus Caller**	Bcf Tools

Coverage Map

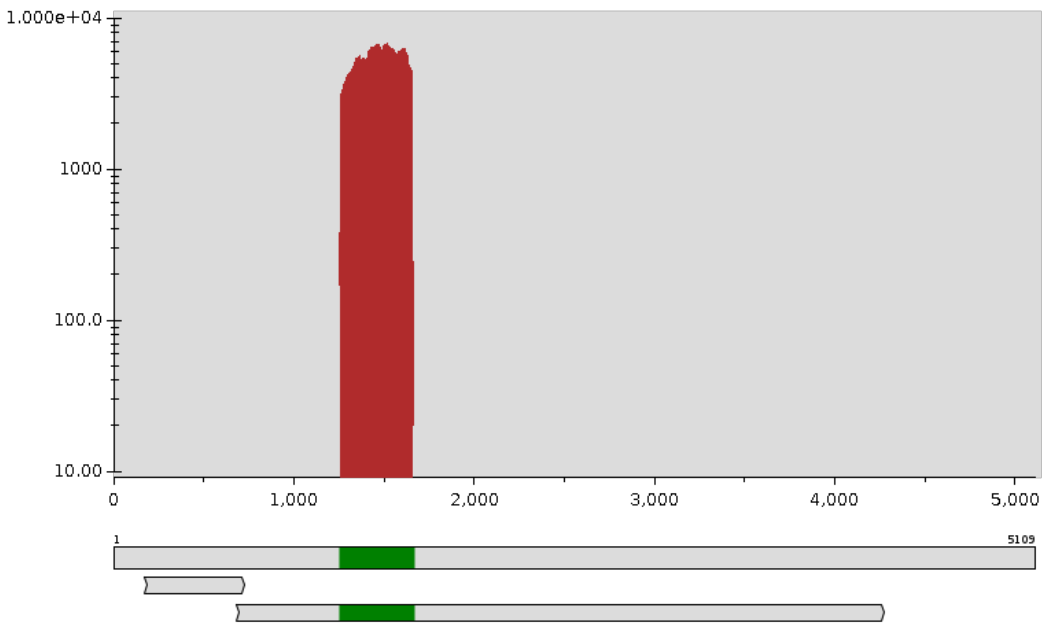

Assignment

**Type**	Tomato chocolate spot virus (Taxonomy ID: 661101)
**Reference Genome**	NC_013076.1
**NT Identity (%)**	66.988
**AA Identity (%)**	70.5036
**Number Of Stop Codons**	0
**Number Of CDS**	2

Alignment

**Alignment Score**	279.0 (NT) + 653.0 (AA) = 932.0
**Concordance (%)**	53.8728

**Alignment Method**	Global, seeded, nucleotide + amino acids (AGA)

Genome Region

Sequence starts at position 1256 and ends at position 1664 relative to NC_013076.1 reference sequence.

Alignment Detailed Statistics

	Begin	End	Coverage	Score	Concordance	Matches	Identities	I/D/M/F*	Stop Codons
**NT**	**1256**	**1664**	**8.0%**	**279**	**34.1%**	**409 (98.6%)**	**278 (67.0%)**	**6/0**	
Mutations:	1267G>A, 1273T>A, 1277T>A, 1278C>G, 1285C>A, 1291A>G, 1294A>T, 1295T>C, 1297G>C, 1300C>T, 1306T>A, 1309A>G, 1312T>C, 1315C>T, 1316C>T, 1317A>C, 1318A>T, 1327G>A, 1333T>A, 1336C>A, 1351A>T, 1354A>C, 1360T>G, 1366G>A, 1367C>G, 1369A>G, 1372T>G, 1373T>G, 1375C>G, 1376C>T, 1377A>C, 1378G>T, 1379A>C, 1380T>A, 1381G>A, 1385T>C, 1387G>A, 1394C>A, 1396T>A, 1397G>C, 1405T>A, 1408A>T, 1410A>C, 1411G>A, 1412A>C, 1413G>T, 1414G>T, 1423T>C, 1426A>T, 1428G>A, 1429A>C, 1433G>A, 1438A>T, 1440T>C, 1441G>C, 1442A>C, 1443G>A, 1444T>G, 1448A>T, 1449G>C, 1450C>T, 1453C>A, 1454A>T, 1456G>A, 1458C>T, 1459T>G, 1463A>G, 1464G>C, 1465C>G, 1468T>A, 1471C>G, 1474G>T, 1477A>C, 1483G>A, 1489C>A, 1493G>A, 1495G>C, 1501T>C, 1503G>A, 1505C>A, 1513A>T, 1516C>G, 1522A>G, 1525A>G, 1531C>T, 1532G>T, 1534G>T, 1535C>T, 1537T>A, 1539A>G, 1540C>G, 1541A>G, 1547C>T, 1548G>C, 1551T>C, 1552A>G, 1555_1556insGTAGGT, 1556A>G, 1559C>A, 1572C>G, 1573T>G, 1574C>G, 1575A>T, 1576C>A, 1582A>T, 1585C>T, 1586A>T, 1588T>C, 1589G>A, 1590G>A, 1592G>T, 1593G>T, 1595T>C, 1603A>C, 1606T>C, 1610A>G, 1618A>G, 1620G>C, 1622T>A, 1624C>T, 1625A>G, 1627C>T, 1628A>T, 1630G>T, 1633T>C, 1634G>C, 1635T>A, 1636T>G, 1645T>C, 1649C>T, 1650T>A, 1651G>T								

CDS

	Begin	End	Coverage	Score	Concordance	Matches	Identities	I/D/M/F*	Stop Codons
ToChV_s2_gp2	191	327	11.4%	653	70.7%	137 (98.6%)	98 (70.5%)	2/0/0/0	0
Protein mutations:	Q211S (1316C>T 1317A>C 1318A>T), Q228E (1367C>G 1369A>G), S229R (1372T>G), S230A (1373T>G 1375C>G), Q231S (1376C>T 1377A>C 1378G>T), M232Q (1379A>C 1380T>A 1381G>A), A238P (1397G>C), E242A (1410A>C 1411G>A), R243L (1412A>C 1413G>T 1414G>T), R248H (1428G>A 1429A>C), D250N (1433G>A), L252P (1440T>C 1441G>C), S253Q (1442A>C 1443G>A 1444T>G), M257L (1454A>T 1456G>A), T258M (1458C>T 1459T>G), S260A (1463A>G 1464G>C 1465C>G), V270I (1493G>A 1495G>C), R273K (1503G>A), V283F (1532G>T 1534G>T), P284S (1535C>T 1537T>A), N285R (1539A>G 1540C>G), T286A (1541A>G), R288S (1547C>T 1548G>C), L289S (1551T>C 1552A>G), T290_M291insVG (1555_1556insGTAGGT), M291V (1556A>G), A296G (1572C>G 1573T>G), H297V (1574C>G 1575A>T 1576C>A), E299D (1582A>T), T301S (1586A>T 1588T>C), G302N (1589G>A 1590G>A), G303L (1592G>T 1593G>T), K309E (1610A>G), G312A (1620G>C), F313I (1622T>A 1624C>T), N314D (1625A>G 1627C>T), M315F (1628A>T 1630G>T), V317Q (1634G>C 1635T>A 1636T>G), L322Y (1649C>T 1650T>A 1651G>T)								
Codon mutations:	CAG194CAA (1267G>A), ACT196ACA (1273T>A), TCT198AGT (1277T>A 1278C>G), GCC200GCA (1285C>A), GAA202GAG (1291A>G), GCA203GCT (1294A>T), TTG204CTC (1295T>C 1297G>C), AAC205AAT (1300C>T), CCT207CCA (1306T>A), CAA208CAG (1309A>G), TTT209TTC (1312T>C), TTC210TTT (1315C>T), CAA211TCT (1316C>T 1317A>C 1318A>T), CAG214CAA (1327G>A), GCT216GCA (1333T>A), GCC217GCA (1336C>A), TCA222TCT (1351A>T), GTA223GTC (1354A>C), TCT225TCG (1360T>G), GGG227GGA (1366G>A), CAA228GAG (1367C>G 1369A>G), AGT229AGG (1372T>G), TCC230GCG (1373T>G 1375C>G), CAG231TCT (1376C>T 1377A>C 1378G>T), ATG232CAA (1379A>C 1380T>A 1381G>A), TTG234CTA (1385T>C 1387G>A), CGT237AGA (1394C>A 1396T>A), GCT238CCT (1397G>C), GTT240GTA (1405T>A), GGA241GGT (1408A>T), GAG242GCA (1410A>C 1411G>A), AGG243CTT (1412A>C 1413G>T 1414G>T), AAT246AAC (1423T>C), ACA247ACT (1426A>T), CGA248CAC (1428G>A 1429A>C), GAT250AAT (1433G>A), GTA251GTT (1438A>T), CTG252CCC (1440T>C 1441G>C), AGT253CAG (1442A>C 1443G>A 1444T>G), AGC255TCT (1448A>T 1449G>C 1450C>T), TCC256TCA (1453C>A), ATG257TTA (1454A>T 1456G>A), ACT258ATG (1458C>T 1459T>G), AGC260GCG (1463A>G 1464G>C 1465C>G), TCT261TCA (1468T>A), TCC262TCG (1471C>G), CTG263CTT (1474G>T), CGA264CGC (1477A>C), CCG266CCA (1483G>A), CTC268CTA (1489C>A), GTG270ATC (1493G>A 1495G>C), AGT272AGC (1501T>C), AGG273AAG (1503G>A), CGG274AGG (1505C>A), CCA276CCT (1513A>T), TCC277TCG (1516C>G), GAA279GAG (1522A>G), CTA280CTG (1525A>G), GAC282GAT (1531C>T), GTG283TTT (1532G>T 1534G>T), CCT284TCA (1535C>T 1537T>A), AAC285AGG (1539A>G 1540C>G), ACC286GCC (1541A>G), CGA288TCA (1547C>T 1548G>C), TTA289TCG (1551T>C 1552A>G), ACA290_ATG291insGTAGGT (1555_1556insGTAGGT), ATG291GTG (1556A>G), CGA292AGA (1559C>A), GCT296GGG (1572C>G 1573T>G), CAC297GTA (1574C>G 1575A>T 1576C>A), GAA299GAT (1582A>T), GAC300GAT (1585C>T), ACT301TCC (1586A>T 1588T>C), GGT302AAT (1589G>A 1590G>A), GGA303TTA (1592G>T 1593G>T), TTG304CTG (1595T>C), CCA306CCC (1603A>C), GTT307GTC (1606T>C), AAG309GAG (1610A>G), CAA311CAG (1618A>G), GGC312GCC (1620G>C), TTC313ATT (1622T>A 1624C>T), AAC314GAT (1625A>G 1627C>T), ATG315TTT (1628A>T 1630G>T), GGT316GGC (1633T>C), GTT317CAG (1634G>C 1635T>A 1636T>G), GAT320GAC (1645T>C), CTG322TAT (1649C>T 1650T>A 1651G>T)								

Proteins

	Begin	End	Coverage	Score	Concordance	Matches	Identities	I/D/M/F*	Stop Codons
polyprotein (YP_003097231.1)	191	327	11.4%	653	70.7%	137 (98.6%)	98 (70.5%)	2/0/0/0	0
Protein mutations:	Q211S (1316C>T 1317A>C 1318A>T), Q228E (1367C>G 1369A>G), S229R (1372T>G), S230A (1373T>G 1375C>G), Q231S (1376C>T 1377A>C 1378G>T), M232Q (1379A>C 1380T>A 1381G>A), A238P (1397G>C), E242A (1410A>C 1411G>A), R243L (1412A>C 1413G>T 1414G>T), R248H (1428G>A 1429A>C), D250N (1433G>A), L252P (1440T>C 1441G>C), S253Q (1442A>C 1443G>A 1444T>G), M257L (1454A>T 1456G>A), T258M (1458C>T 1459T>G), S260A (1463A>G 1464G>C 1465C>G), V270I (1493G>A 1495G>C), R273K (1503G>A), V283F (1532G>T 1534G>T), P284S (1535C>T 1537T>A), N285R (1539A>G 1540C>G), T286A (1541A>G), R288S (1547C>T 1548G>C), L289S (1551T>C 1552A>G), T290_M291insVG (1555_1556insGTAGGT), M291V (1556A>G), A296G (1572C>G 1573T>G), H297V (1574C>G 1575A>T 1576C>A), E299D (1582A>T), T301S (1586A>T 1588T>C), G302N (1589G>A 1590G>A), G303L (1592G>T 1593G>T), K309E (1610A>G), G312A (1620G>C), F313I (1622T>A 1624C>T), N314D (1625A>G 1627C>T), M315F (1628A>T 1630G>T), V317Q (1634G>C 1635T>A 1636T>G), L322Y (1649C>T 1650T>A 1651G>T)								
Codon mutations:	CAG194CAA (1267G>A), ACT196ACA (1273T>A), TCT198AGT (1277T>A 1278C>G), GCC200GCA (1285C>A), GAA202GAG (1291A>G), GCA203GCT (1294A>T), TTG204CTC (1295T>C 1297G>C), AAC205AAT (1300C>T), CCT207CCA (1306T>A), CAA208CAG (1309A>G), TTT209TTC (1312T>C), TTC210TTT (1315C>T), CAA211TCT (1316C>T 1317A>C 1318A>T), CAG214CAA (1327G>A), GCT216GCA (1333T>A), GCC217GCA (1336C>A), TCA222TCT (1351A>T), GTA223GTC (1354A>C), TCT225TCG (1360T>G), GGG227GGA (1366G>A), CAA228GAG (1367C>G 1369A>G), AGT229AGG (1372T>G), TCC230GCG (1373T>G 1375C>G), CAG231TCT (1376C>T 1377A>C 1378G>T), ATG232CAA (1379A>C 1380T>A 1381G>A), TTG234CTA (1385T>C 1387G>A), CGT237AGA (1394C>A 1396T>A), GCT238CCT (1397G>C), GTT240GTA (1405T>A), GGA241GGT (1408A>T), GAG242GCA (1410A>C 1411G>A), AGG243CTT (1412A>C 1413G>T 1414G>T), AAT246AAC (1423T>C), ACA247ACT (1426A>T), CGA248CAC (1428G>A 1429A>C), GAT250AAT (1433G>A), GTA251GTT (1438A>T), CTG252CCC (1440T>C 1441G>C), AGT253CAG (1442A>C 1443G>A 1444T>G), AGC255TCT (1448A>T 1449G>C 1450C>T), TCC256TCA (1453C>A), ATG257TTA (1454A>T 1456G>A), ACT258ATG (1458C>T 1459T>G), AGC260GCG (1463A>G 1464G>C 1465C>G), TCT261TCA (1468T>A), TCC262TCG (1471C>G), CTG263CTT (1474G>T), CGA264CGC (1477A>C), CCG266CCA (1483G>A), CTC268CTA (1489C>A), GTG270ATC (1493G>A 1495G>C), AGT272AGC (1501T>C), AGG273AAG (1503G>A), CGG274AGG (1505C>A), CCA276CCT (1513A>T), TCC277TCG (1516C>G), GAA279GAG (1522A>G), CTA280CTG (1525A>G), GAC282GAT (1531C>T), GTG283TTT (1532G>T 1534G>T), CCT284TCA (1535C>T 1537T>A), AAC285AGG (1539A>G 1540C>G), ACC286GCC (1541A>G), CGA288TCA (1547C>T 1548G>C), TTA289TCG (1551T>C 1552A>G), ACA290_ATG291insGTAGGT (1555_1556insGTAGGT), ATG291GTG (1556A>G), CGA292AGA (1559C>A), GCT296GGG (1572C>G 1573T>G), CAC297GTA (1574C>G 1575A>T 1576C>A), GAA299GAT (1582A>T), GAC300GAT (1585C>T), ACT301TCC (1586A>T 1588T>C), GGT302AAT (1589G>A 1590G>A), GGA303TTA (1592G>T 1593G>T), TTG304CTG (1595T>C), CCA306CCC (1603A>C), GTT307GTC (1606T>C), AAG309GAG (1610A>G), CAA311CAG (1618A>G), GGC312GCC (1620G>C), TTC313ATT (1622T>A 1624C>T), AAC314GAT (1625A>G 1627C>T), ATG315TTT (1628A>T 1630G>T), GGT316GGC (1633T>C), GTT317CAG (1634G>C 1635T>A 1636T>G), GAT320GAC (1645T>C), CTG322TAT (1649C>T 1650T>A 1651G>T)								

*: Inserts / Deletes / Misaligned / Frameshifts

Analysis details

This analysis was performed with panviral2.64

NGS Details (UN62): Tomato chocolate spot virus (segment RNA 1)

Assembly

**Coverage Length**	278 (1 contig(s))
**Depth Of Coverage**	24274.9
**Number Of Reads**	61095
**Reads Per Million**	1416.24 rpm (after QC)
**Ambiguities**	0
**Assembly Method**	de novo + reference guided assembly
**Consensus Caller**	Bcf Tools

Coverage Map

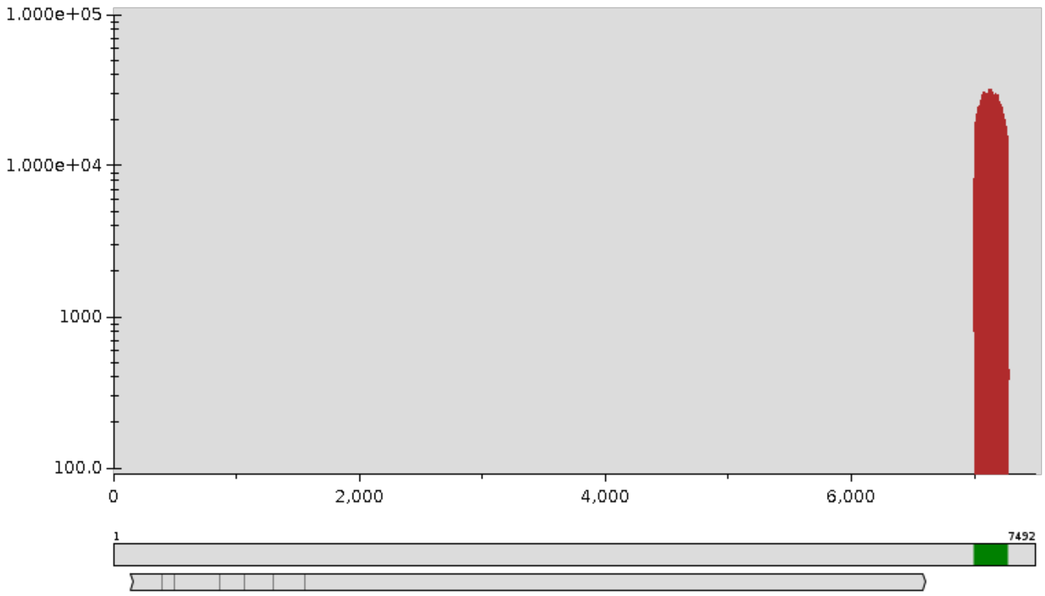

Assignment

**Type**	Tomato chocolate spot virus (Taxonomy ID: 661101)
**Reference Genome**	NC_013075.1
**NT Identity (%)**	82.0789
**AA Identity (%)**	0.0
**Number Of Stop Codons**	0
**Number Of CDS**	1

Alignment

**Alignment Score**	350.0 (NT) + 0.0 (AA) = 350.0
**Concordance (%)**	62.9496

Alignment Method	Global, seeded, nucleotide + amino acids (AGA)

Genome Region

Sequence starts at position 6998 and ends at position 7275 relative to NC_013075.1 reference sequence.

Alignment Detailed Statistics

	Begin	End	Coverage	Score	Concordance	Matches	Identities	I/D/M/F*	Stop Codons
NT	6998	7275	3.7%	350	62.9%	278 (99.6%)	229 (82.1%)	1/0	
Mutations:	7007T>C, 7012T>G, 7018C>T, 7019A>G, 7020A>G, 7020_7021insT, 7023C>A, 7042G>T, 7044A>T, 7047G>A, 7048C>G, 7066G>A, 7082G>A, 7083T>C, 7098C>T, 7099T>C, 7104T>A, 7107G>A, 7116C>G, 7121G>A, 7127A>C, 7140C>T, 7151A>T, 7152T>C, 7183C>T, 7184T>C, 7185G>T, 7186T>G, 7187T>C, 7188G>A, 7196C>T, 7197A>G, 7198A>C, 7199C>A, 7200A>G, 7201G>A, 7207A>T, 7208A>T, 7209T>G, 7210C>T, 7212T>A, 7219T>A, 7224T>C, 7242A>T, 7255A>T, 7257G>A, 7258G>C, 7260T>C, 7262C>G, 7264T>C								

CDS

Proteins

*: Inserts / Deletes / Misaligned / Frameshifts

Analysis details

This analysis was performed with panviral2.64

NGS Details (UN62): Noumeavirus

Assembly

**Coverage Length**	217 (1 contig(s))
**Depth Of Coverage**	10898.8
**Number Of Reads**	24245
**Reads Per Million**	562.02 rpm (after QC)
**Ambiguities**	0
**Assembly Method**	de novo + reference guided assembly
**Consensus Caller**	Bcf Tools

Coverage Map

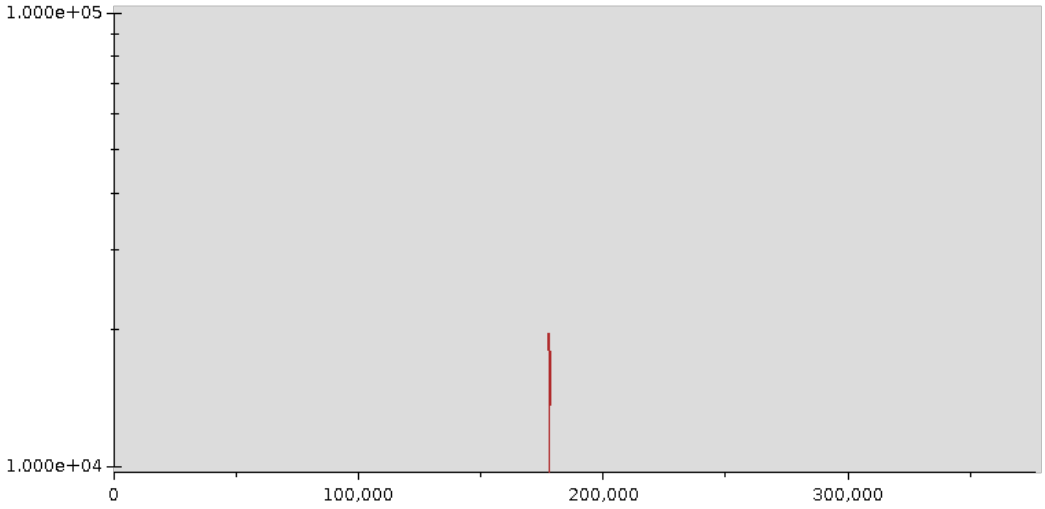

Assignment

**Type**	Noumeavirus (Taxonomy ID: 1955558)
**Reference Genome**	NC_033775.1
**NT Identity (%)**	82.0276
**AA Identity (%)**	94.4444
**Number Of Stop Codons**	0
**Number Of CDS**	452

Alignment

**Alignment Score**	278.0 (NT) + 437.0 (AA) = 715.0
**Concordance (%)**	80.8824
**Alignment Method**	Local, heuristic, nucleotide (BLASTN)

Genome Region

Sequence starts at position 177922 and ends at position 178138 relative to NC_033775.1 reference sequence.

Alignment Detailed Statistics

	Begin	End	Coverage	Score	Concordance	Matches	Identities	I/D/M/F*	Stop Codons
**NT**	**177922**	**178138**	**0.1%**	**278**	**64.1%**	**217 (100%)**	**178 (82.0%)**	**0/0**	

Mutations: 177944C>G, 177956G>C, 177965C>T, 177974A>G, 177977C>T, 177980A>T, 177992G>C, 177995C>T, 177998C>G, 178002C>G, 178003A>C, 178004A>T, 178019A>G, 178025A>G, 178034G>A, 178043A>G, 178044C>A, 178046A>G, 178049T>G, 178058C>T, 178061A>T, 178064A>G, 178073A>G, 178079A>C, 178080A>C, 178082G>C, 178091C>G, 178094T>C, 178097T>C, 178100C>T, 178101G>A, 178106A>G, 178109A>G, 178112A>G, 178115A>G, 178118A>C, 178119C>T, 178130T>G, 178136A>G

*: Inserts / Deletes / Misaligned / Frameshifts

Analysis details

This analysis was performed with panviral2.64

NGS Details (UN62): Tomato necrotic dwarf virus (segment RNA1)

Assembly

**Coverage Length**	749 (2 contig(s))
**Depth Of Coverage**	356.8
**Number Of Reads**	2020
**Reads Per Million**	46.83 rpm (after QC)
**Ambiguities**	0
**Assembly Method**	de novo + reference guided assembly
**Consensus Caller**	Bcf Tools

Coverage Map

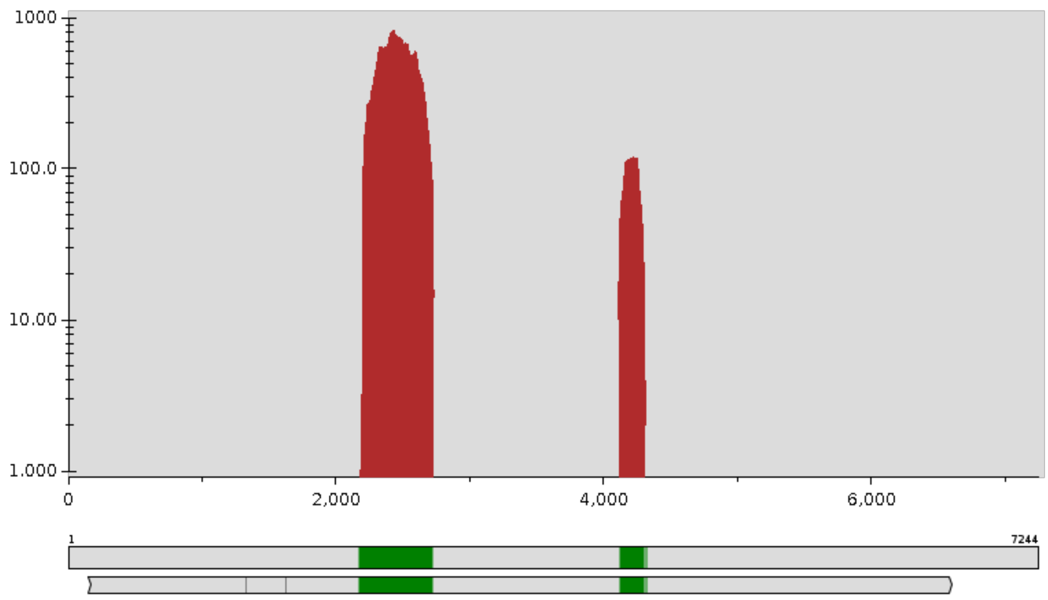

Assignment

**Type**	Tomato necrotic dwarf virus (Taxonomy ID: 1481465)
**Reference Genome**	NC_027926.1
**NT Identity (%)**	64.7606
**AA Identity (%)**	63.2
**Number Of Stop Codons**	0
**Number Of CDS**	1

Alignment

**Alignment Score**	438.0 (NT) + 1167.0 (AA) = 1605.0
**Concordance (%)**	49.0076

Alignment Method	Global, seeded, nucleotide + amino acids (AGA)

Genome Region

Sequence starts at position 2174 and ends at position 4311 relative to NC_027926.1 reference sequence.

Alignment Detailed Statistics

	Begin	End	Coverage	Score	Concordance	Matches	Identities	I/D/M/F*	Stop Codons
**NT**	**2174**	**4311**	**10.3%**	**438**	**29.2%**	**749 (99.6%)**	**487 (64.8%)**	**3/0**	

Mutations:	2174C>A, 2178C>T, 2179A>T, 2182A>T, 2184C>G, 2185T>G, 2186A>T, 2193T>A, 2194G>T, 2196C>A, 2198A>G, 2199T>A, 2200G>C, 2201C>A, 2203G>C, 2205A>G, 2208A>G, 2211C>T, 2212A>G, 2213G>C, 2214G>T, 2225A>G, 2229T>C, 2245C>A, 2256T>C, 2259A>G, 2262C>T, 2268C>T, 2272G>A, 2273C>G, 2274T>C, 2275T>A, 2277C>A, 2283C>T, 2285G>C, 2290A>G, 2293A>C, 2294A>T, 2300C>A, 2304A>C, 2308A>C, 2309C>G, 2310A>C, 2311G>A, 2313G>T, 2314A>C, 2316G>T, 2317C>G, 2318G>A, 2319C>G, 2320A>C, 2321C>A, 2325A>G, 2326T>A, 2327T>A, 2328T>A, 2331A>G, 2332C>T, 2337T>G, 2338T>G, 2339G>C, 2340T>G, 2341A>C, 2344G>C, 2346T>G, 2354T>C, 2355T>A, 2362T>C, 2364G>T, 2366T>C, 2367T>G, 2370C>T, 2373C>T, 2374C>G, 2376G>A, 2389T>A, 2390T>G, 2391T>A, 2394G>A, 2400T>C, 2401T>C, 2409A>T, 2412C>T, 2421T>C, 2424T>C, 2432C>G, 2433C>G, 2440T>G, 2442C>A, 2442_2443insGGT, 2444A>C, 2445A>T, 2448G>C, 2449G>A, 2450C>A, 2451C>G, 2454A>G, 2455G>A, 2456A>G, 2457C>A, 2458T>A, 2459C>A, 2464G>A, 2466C>T, 2468A>T, 2469G>C, 2470A>G, 2471T>A, 2475T>C, 2476T>A, 2477A>T, 2478C>G, 2479C>A, 2480A>T, 2481C>T, 2482A>G, 2483A>G, 2484A>T, 2487T>C, 2488G>A, 2489T>A, 2493T>C, 2494T>A, 2496C>A, 2501A>T, 2502A>C, 2503G>C, 2505A>G, 2507C>T, 2508T>G, 2512T>A, 2513G>A, 2514G>T, 2515A>G, 2520A>G, 2523A>C, 2525A>T, 2526T>C, 2527G>A, 2528C>T, 2529A>C, 2530C>T, 2534G>A, 2553C>A, 2556A>G, 2560A>T, 2562C>G, 2565G>A, 2568G>A, 2577A>C, 2578T>A, 2579T>C, 2580G>C, 2581T>A, 2583T>G, 2584G>A, 2585G>A, 2586G>T, 2588G>C, 2589A>C, 2590A>G, 2592A>T, 2595A>T, 2601T>C, 2602C>A, 2605G>A, 2610T>C, 2611T>C, 2612G>T, 2613G>C, 2614C>A, 2616A>G, 2617A>G, 2618G>C, 2619C>A, 2620A>G, 2621A>C, 2622T>A, 2623T>A, 2627T>C, 2628T>C, 2631A>T, 2632A>T, 2633G>T, 2634T>A, 2638A>G, 2639G>A, 2643A>C, 2649T>G, 2652T>C, 2655C>A, 2658A>C, 2673C>T, 2679T>C, 2685A>G, 2688G>A, 2691C>T, 2694G>A, 2697C>G, 2698C>A, 2699C>A, 2700C>G, 2701T>A, 2705G>A, 2707C>T, 2709G>C, 2710A>G, 2711A>C, 2712G>A, 2715T>A, 2717T>C, 2718G>C, 2722G>C, 2724G>A, 4116T>C, 4119G>A, 4122A>C, 4137C>T, 4149C>G, 4150A>C, 4161T>C, 4173C>T, 4176C>T, 4177A>T, 4178C>G, 4179C>T, 4188A>T, 4189T>G, 4190T>C, 4197T>C, 4200C>A, 4201A>C, 4206A>T, 4209T>C, 4215T>A, 4218A>T, 4221A>C, 4222G>A, 4230C>G, 4233T>C, 4234T>C, 4235C>A, 4243T>A, 4245T>A, 4246C>G, 4248T>G, 4251T>G, 4254C>T, 4256A>G, 4257C>T, 4259A>G, 4263C>T, 4267G>T, 4269T>A, 4279A>G, 4280C>A, 4281T>G, 4284G>A, 4287T>C, 4293T>C, 4302A>G, 4305T>C, 4311C>T

CDS

	Begin	End	Coverage	Score	Concordance	Matches	Identities	I/D/M/F*	Stop Codons
APL33_sRA11gp1	676	1387	11.6%	1167	64.8%	249 (99.6%)	158 (63.2%)	1/0/0/0	0

Protein mutations:	M677L (2179A>T), I678L (2182A>T 2184C>G), Y679V (2185T>G 2186A>T), A682S (2194G>T 2196C>A), N683R (2198A>G 2199T>A), A684H (2200G>C 2201C>A), E685Q (2203G>C 2205A>G), R688A (2212A>G 2213G>C 2214G>T), Q692R (2225A>G), L699I (2245C>A), A708S (2272G>A 2273C>G 2274T>C), S709T (2275T>A 2277C>A), C712S (2285G>C), K714E (2290A>G), K715L (2293A>C 2294A>T), A717D (2300C>A), T720R (2308A>C 2309C>G 2310A>C), E721N (2311G>A 2313G>T), M722L (2314A>C 2316G>T), R723E (2317C>G 2318G>A 2319C>G), T724Q (2320A>C 2321C>A), F726K (2326T>A 2327T>A 2328T>A), H728Y (2332C>T), N729K (2337T>G), C730A (2338T>G 2339G>C 2340T>G), I731L (2341A>C), D732Q (2344G>C 2346T>G), V735A (2354T>C 2355T>A), I739T (2366T>C 2367T>G), Q742E (2374C>G 2376G>A), F747R (2389T>A 2390T>G 2391T>A), T761R (2432C>G 2433C>G), S764A (2440T>G 2442C>A), S764_K765insG (2442_2443insGGT), K765T (2444A>C 2445A>T), E766D (2448G>C), A767K (2449G>A 2450C>A 2451C>G), D769R (2455G>A 2456A>G 2457C>A), S770N (2458T>A 2459C>A), V772I (2464G>A 2466C>T), K773I (2468A>T 2469G>C), I774E (2470A>G 2471T>A), Y776M (2476T>A 2477A>T 2478C>G), H777I (2479C>A 2480A>T 2481C>T), K778G (2482A>G 2483A>G 2484A>T), V780K (2488G>A 2489T>A), Y782K (2494T>A 2496C>A), K784I (2501A>T 2502A>C), E785Q (2503G>C 2505A>G), T786M (2507C>T 2508T>G), W788N (2512T>A 2513G>A 2514G>T), N789D (2515A>G), Y792F (2525A>T 2526T>C), A793I (2527G>A 2528C>T 2529A>C), R795K (2534G>A), I804L (2560A>T 2562C>G), M805I (2565G>A), L810T (2578T>A 2579T>C 2580G>C), F811M (2581T>A 2583T>G), G812N (2584G>A 2585G>A 2586G>T), G813A (2588G>C 2589A>C), I814V (2590A>G 2592A>T), L818I (2602C>A), A819T (2605G>A), W821L (2611T>C 2612G>T 2613G>C), Q822K (2614C>A 2616A>G), S823A (2617A>G 2618G>C 2619C>A), N824A (2620A>G 2621A>C 2622T>A), S825T (2623T>A), F826S (2627T>C 2628T>C), S828L (2632A>T 2633G>T 2634T>A), S830D (2638A>G 2639G>A), P850K (2698C>A 2699C>A 2700C>G), S851T (2701T>A), R852H (2705G>A), L853F (2707C>T 2709G>C), K854A (2710A>G 2711A>C 2712G>A), N855K (2715T>A), L856S (2717T>C 2718G>C), V858L (2722G>C 2724G>A), T1343C (4177A>T 4178C>G 4179C>T), L1347A (4189T>G 4190T>C), I1351L (4201A>C), D1358N (4222G>A), S1362H (4234T>C 4235C>A), S1365T (4243T>A 4245T>A), L1366V (4246C>G 4248T>G), N1369S (4256A>G 4257C>T), N1370S (4259A>G), A1373S (4267G>T 4269T>A), T1377E (4279A>G 4280C>A 4281T>G)
Codon mutations:	ACT675.AT (2174C>A), GAC676GAT (2178C>T), ATG677TTG (2179A>T), ATC678TTG (2182A>T 2184C>G), TAC679GTC (2185T>G 2186A>T), GGT681GGA (2193T>A), GCC682TCA (2194G>T 2196C>A), AAT683AGA (2198A>G 2199T>A), GCT684CAT (2200G>C 2201C>A), GAA685CAG (2203G>C 2205A>G), CAA686CAG (2208A>G), TAC687TAT (2211C>T), AGG688GCT (2212A>G 2213G>C 2214G>T), CAA692CGA (2225A>G), AAT693AAC (2229T>C), CTC699ATC (2245C>A), TTT702TTC (2256T>C), AAA703AAG (2259A>G), AGC704AGT (2262C>T), ATC706ATT (2268C>T), GCT708AGC (2272G>A 2273C>G 2274T>C), TCC709ACA (2275T>A 2277C>A), ATC711ATT (2283C>T), TGT712TCT (2285G>C), AAG714GAG (2290A>G), AAG715CTG (2293A>C 2294A>T), GCT717GAT (2300C>A), GTA718GTC (2304A>C), ACA720CGC (2308A>C 2309C>G 2310A>C), GAG721AAT (2311G>A 2313G>T), ATG722CTT (2314A>C 2316G>T), CGC723GAG (2317C>G 2318G>A 2319C>G), ACA724CAA (2320A>C 2321C>A), GGA725GGG (2325A>G), TTT726AAA (2326T>A 2327T>A 2328T>A), GCA727GCG (2331A>G), CAT728TAT (2332C>T), AAT729AAG (2337T>G), TGT730GCG (2338T>G 2339G>C 2340T>G), ATT731CTT (2341A>C), GAT732CAG (2344G>C 2346T>G), GTT735GCA (2354T>C 2355T>A), TTG738CTT (2362T>C 2364G>T), ATT739ACG (2366T>C 2367T>G), TGC740TGT (2370C>T), ATC741ATT (2373C>T), CAG742GAA (2374C>G 2376G>A), TTT747AGA (2389T>A 2390T>G 2391T>A), GTG748GTA (2394G>A), TTT750TTC (2400T>C), TTG751CTG (2401T>C), ACA753ACT (2409A>T), GCC754GCT (2412C>T), CGT757CGC (2421T>C), TAT758TAC (2424T>C), ACC761AGG (2432C>G 2433C>G), TCC764GCA (2440T>G 2442C>A), TCC764_AAA765insGGT (2442_2443insGGT), AAA765ACT (2444A>C 2445A>T), GAG766GAC (2448G>C), GCC767AAG (2449G>A 2450C>A 2451C>G), AAA768AAG (2454A>G), GAC769AGA (2455G>A 2456A>G 2457C>A), TCT770AAT (2458T>A 2459C>A), GTC772ATT (2464G>A 2466C>T), AAG773ATC (2468A>T 2469G>C), ATA774GAA (2470A>G 2471T>A), TGT775TGC (2475T>C), TAC776ATG (2476T>A 2477A>T 2478C>G), CAC777ATT (2479C>A 2480A>T 2481C>T), AAA778GGT (2482A>G 2483A>G 2484A>T), ATT779ATC (2487T>C), GTG780AAG (2488G>A 2489T>A), GAT781GAC (2493T>C), TAC782AAA (2494T>A 2496C>A), AAA784ATC (2501A>T 2502A>C), GAA785CAG (2503G>C 2505A>G), ACT786ATG (2507C>T 2508T>G), TGG788AAT (2512T>A 2513G>A 2514G>T), AAC789GAC (2515A>G), TTA790TTG (2520A>G), CCA791CCC (2523A>C), TAT792TTC (2525A>T 2526T>C), GCA793ATC (2527G>A 2528C>T 2529A>C), CTG794TTG (2530C>T), AGG795AAG (2534G>A), GGC801GGA (2553C>A), CTA802CTG (2556A>G), ATC804TTG (2560A>T 2562C>G), ATG805ATA (2565G>A), GCG806GCA (2568G>A), GGA809GGC (2577A>C), TTG810ACC (2578T>A 2579T>C 2580G>C), TTT811ATG (2581T>A 2583T>G), GGG812AAT (2584G>A 2585G>A 2586G>T), GGA813GCC (2588G>C 2589A>C), ATA814GTT (2590A>G 2592A>T), ACA815ACT (2595A>T), TTT817TTC (2601T>C), CTT818ATT (2602C>A), GCT819ACT (2605G>A), TGT820TGC (2610T>C), TGG821CTC (2611T>C 2612G>T 2613G>C), CAA822AAG (2614C>A 2616A>G), AGC823GCA (2617A>G 2618G>C 2619C>A), AAT824GCA (2620A>G 2621A>C 2622T>A), TCT825ACT (2623T>A), TTT826TCC (2627T>C 2628T>C), CCA827CCT (2631A>T), AGT828TTA (2632A>T 2633G>T 2634T>A), AGT830GAT (2638A>G 2639G>A), GGA831GGC (2643A>C), GGT833GGG (2649T>G), GGT834GGC (2652T>C), GTC835GTA (2655C>A), ACA836ACC (2658A>C), AGC841AGT (2673C>T), TCT843TCC (2679T>C), AAA845AAG (2685A>G), AAG846AAA (2688G>A), AAC847AAT (2691C>T), AAG848AAA (2694G>A), GGC849GGG (2697C>G), CCC850AAG (2698C>A 2699C>A 2700C>G), TCT851ACT (2701T>A), CGT852CAT (2705G>A), CTG853TTC (2707C>T 2709G>C), AAG854GCA (2710A>G 2711A>C 2712G>A), AAT855AAA (2715T>A), TTG856TCC (2717T>C 2718G>C), GTG858CTA (2722G>C 2724G>A), TTT1322.TC (4116T>C), GAG1323GAA (4119G>A), ATA1324ATC (4122A>C), TAC1329TAT (4137C>T), GTC1333GTG (4149C>G), AGG1334CGG (4150A>C), TTT1337TTC (4161T>C), TCC1341TCT (4173C>T), GCC1342GCT (4176C>T), ACC1343TGT (4177A>T 4178C>G 4179C>T), GCA1346GCT (4188A>T), TTA1347GCA (4189T>G 4190T>C), AAT1349AAC (4197T>C), GTC1350GTA (4200C>A), ATC1351CTC (4201A>C), CCA1352CCT (4206A>T), TGT1353TGC (4209T>C), GTT1355GTA (4215T>A), GGA1356GGT (4218A>T), ATA1357ATC (4221A>C), GAT1358AAT (4222G>A), ACC1360ACG (4230C>G), AGT1361AGC (4233T>C), TCT1362CAT (4234T>C 4235C>A), TCT1365ACA (4243T>A 4245T>A), CTT1366GTG (4246C>G 4248T>G), CTT1367CTG (4251T>G), GCC1368GCT (4254C>T), AAC1369AGT (4256A>G 4257C>T), AAC1370AGC (4259A>G), TTC1371TTT (4263C>T), GCT1373TCA (4267G>T 4269T>A), ACT1377GAG (4279A>G 4280C>A 4281T>G), GGG1378GGA (4284G>A), TTT1379TTC (4287T>C), GCT1381GCC (4293T>C), TCA1384TCG (4302A>G), AGT1385AGC (4305T>C), GAC1387..T (4311C>T)

Proteins

	Begin	End	Coverage	Score	Concordance	Matches	Identities	I/D/M/F*	Stop Codons
polyprotein (YP_009165993.1)	676	1387	11.6%	1167	64.8%	249 (99.6%)	158 (63.2%)	1/0/0/0	0

Protein mutations:	M677L (2179A>T), I678L (2182A>T 2184C>G), Y679V (2185T>G 2186A>T), A682S (2194G>T 2196C>A), N683R (2198A>G 2199T>A), A684H (2200G>C 2201C>A), E685Q (2203G>C 2205A>G), R688A (2212A>G 2213G>C 2214G>T), Q692R (2225A>G), L699I (2245C>A), A708S (2272G>A 2273C>G 2274T>C), S709T (2275T>A 2277C>A), C712S (2285G>C), K714E (2290A>G), K715L (2293A>C 2294A>T), A717D (2300C>A), T720R (2308A>C 2309C>G 2310A>C), E721N (2311G>A 2313G>T), M722L (2314A>C 2316G>T), R723E (2317C>G 2318G>A 2319C>G), T724Q (2320A>C 2321C>A), F726K (2326T>A 2327T>A 2328T>A), H728Y (2332C>T), N729K (2337T>G), C730A (2338T>G 2339G>C 2340T>G), I731L (2341A>C), D732Q (2344G>C 2346T>G), V735A (2354T>C 2355T>A), I739T (2366T>C 2367T>G), Q742E (2374C>G 2376G>A), F747R (2389T>A 2390T>G 2391T>A), T761R (2432C>G 2433C>G), S764A (2440T>G 2442C>A), S764_K765insG (2442_2443insGGT), K765T (2444A>C 2445A>T), E766D (2448G>C), A767K (2449G>A 2450C>A 2451C>G), D769R (2455G>A 2456A>G 2457C>A), S770N (2458T>A 2459C>A), V772I (2464G>A 2466C>T), K773I (2468A>T 2469G>C), I774E (2470A>G 2471T>A), Y776M (2476T>A 2477A>T 2478C>G), H777I (2479C>A 2480A>T 2481C>T), K778G (2482A>G 2483A>G 2484A>T), V780K (2488G>A 2489T>A), Y782K (2494T>A 2496C>A), K784I (2501A>T 2502A>C), E785Q (2503G>C 2505A>G), T786M (2507C>T 2508T>G), W788N (2512T>A 2513G>A 2514G>T), N789D (2515A>G), Y792F (2525A>T 2526T>C), A793I (2527G>A 2528C>T 2529A>C), R795K (2534G>A), I804L (2560A>T 2562C>G), M805I (2565G>A), L810T (2578T>A 2579T>C 2580G>C), F811M (2581T>A 2583T>G), G812N (2584G>A 2585G>A 2586G>T), G813A (2588G>C 2589A>C), I814V (2590A>G 2592A>T), L818I (2602C>A), A819T (2605G>A), W821L (2611T>C 2612G>T 2613G>C), Q822K (2614C>A 2616A>G), S823A (2617A>G 2618G>C 2619C>A), N824A (2620A>G 2621A>C 2622T>A), S825T (2623T>A), F826S (2627T>C 2628T>C), S828L (2632A>T 2633G>T 2634T>A), S830D (2638A>G 2639G>A), P850K (2698C>A 2699C>A 2700C>G), S851T (2701T>A), R852H (2705G>A), L853F (2707C>T 2709G>C), K854A (2710A>G 2711A>C 2712G>A), N855K (2715T>A), L856S (2717T>C 2718G>C), V858L (2722G>C 2724G>A), T1343C (4177A>T 4178C>G 4179C>T), L1347A (4189T>G 4190T>C), I1351L (4201A>C), D1358N (4222G>A), S1362H (4234T>C 4235C>A), S1365T (4243T>A 4245T>A), L1366V (4246C>G 4248T>G), N1369S (4256A>G 4257C>T), N1370S (4259A>G), A1373S (4267G>T 4269T>A), T1377E (4279A>G 4280C>A 4281T>G)

	Begin	End	Coverage	Score	Concordance	Matches	Identities	I/D/M/F*	Stop Codons
**NT**	**2174**	**4311**	**10.3%**	**438**	**29.2%**	**749 (99.6%)**	**487 (64.8%)**	**3/0**	
Codon mutations:	ACT675.AT (2174C>A), GAC676GAT (2178C>T), ATG677TTG (2179A>T), ATC678TTG (2182A>T 2184C>G), TAC679GTC (2185T>G 2186A>T), GGT681GGA (2193T>A), GCC682TCA (2194G>T 2196C>A), AAT683AGA (2198A>G 2199T>A), GCT684CAT (2200G>C 2201C>A), GAA685CAG (2203G>C 2205A>G), CAA686CAG (2208A>G), TAC687TAT (2211C>T), AGG688GCT (2212A>G 2213G>C 2214G>T), CAA692CGA (2225A>G), AAT693AAC (2229T>C), CTC699ATC (2245C>A), TTT702TTC (2256T>C), AAA703AAG (2259A>G), AGC704AGT (2262C>T), ATC706ATT (2268C>T), GCT708AGC (2272G>A 2273C>G 2274T>C), TCC709ACA (2275T>A 2277C>A), ATC711ATT (2283C>T), TGT712TCT (2285G>C), AAG714GAG (2290A>G), AAG715CTG (2293A>C 2294A>T), GCT717GAT (2300C>A), GTA718GTC (2304A>C), ACA720CGC (2308A>C 2309C>G 2310A>C), GAG721AAT (2311G>A 2313G>T), ATG722CTT (2314A>C 2316G>T), CGC723GAG (2317C>G 2318G>A 2319C>G), ACA724CAA (2320A>C 2321C>A), GGA725GGG (2325A>G), TTT726AAA (2326T>A 2327T>A 2328T>A), GCA727GCG (2331A>G), CAT728TAT (2332C>T), AAT729AAG (2337T>G), TGT730GCG (2338T>G 2339G>C 2340T>G), ATT731CTT (2341A>C), GAT732CAG (2344G>C 2346T>G), GTT735GCA (2354T>C 2355T>A), TTG738CTT (2362T>C 2364G>T), ATT739ACG (2366T>C 2367T>G), TGC740TGT (2370C>T), ATC741ATT (2373C>T), CAG742GAA (2374C>G 2376G>A), TTT747AGA (2389T>A 2390T>G 2391T>A), GTG748GTA (2394G>A), TTT750TTC (2400T>C), TTG751CTG (2401T>C), ACA753ACT (2409A>T), GCC754GCT (2412C>T), CGT757CGC (2421T>C), TAT758TAC (2424T>C), ACC761AGG (2432C>G 2433C>G), TCC764GCA (2440T>G 2442C>A), TCC764_AAA765insGGT (2442_2443insGGT), AAA765ACT (2444A>C 2445A>T), GAG766GAC (2448G>C), GCC767AAG (2449G>A 2450C>A 2451C>G), AAA768AAG (2454A>G), GAC769AGA (2455G>A 2456A>G 2457C>A), TCT770AAT (2458T>A 2459C>A), GTC772ATT (2464G>A 2466C>T), AAG773ATC (2468A>T 2469G>C), ATA774GAA (2470A>G 2471T>A), TGT775TGC (2475T>C), TAC776ATG (2476T>A 2477A>T 2478C>G), CAC777ATT (2479C>A 2480A>T 2481C>T), AAA778GGT (2482A>G 2483A>G 2484A>T), ATT779ATC (2487T>C), GTG780AAG (2488G>A 2489T>A), GAT781GAC (2493T>C), TAC782AAA (2494T>A 2496C>A), AAA784ATC (2501A>T 2502A>C), GAA785CAG (2503G>C 2505A>G), ACT786ATG (2507C>T 2508T>G), TGG788AAT (2512T>A 2513G>A 2514G>T), AAC789GAC (2515A>G), TTA790TTG (2520A>G), CCA791CCC (2523A>C), TAT792TTC (2525A>T 2526T>C), GCA793ATC (2527G>A 2528C>T 2529A>C), CTG794TTG (2530C>T), AGG795AAG (2534G>A), GGC801GGA (2553C>A), CTA802CTG (2556A>G), ATC804TTG (2560A>T 2562C>G), ATG805ATA (2565G>A), GCG806GCA (2568G>A), GGA809GGC (2577A>C), TTG810ACC (2578T>A 2579T>C 2580G>C), TTT811ATG (2581T>A 2583T>G), GGG812AAT (2584G>A 2585G>A 2586G>T), GGA813GCC (2588G>C 2589A>C), ATA814GTT (2590A>G 2592A>T), ACA815ACT (2595A>T), TTT817TTC (2601T>C), CTT818ATT (2602C>A), GCT819ACT (2605G>A), TGT820TGC (2610T>C), TGG821CTC (2611T>C 2612G>T 2613G>C), CAA822AAG (2614C>A 2616A>G), AGC823GCA (2617A>G 2618G>C 2619C>A), AAT824GCA (2620A>G 2621A>C 2622T>A), TCT825ACT (2623T>A), TTT826TCC (2627T>C 2628T>C), CCA827CCT (2631A>T), AGT828TTA (2632A>T 2633G>T 2634T>A), AGT830GAT (2638A>G 2639G>A), GGA831GGC (2643A>C), GGT833GGG (2649T>G), GGT834GGC (2652T>C), GTC835GTA (2655C>A), ACA836ACC (2658A>C), AGC841AGT (2673C>T), TCT843TCC (2679T>C), AAA845AAG (2685A>G), AAG846AAA (2688G>A), AAC847AAT (2691C>T), AAG848AAA (2694G>A), GGC849GGG (2697C>G), CCC850AAG (2698C>A 2699C>A 2700C>G), TCT851ACT (2701T>A), CGT852CAT (2705G>A), CTG853TTC (2707C>T 2709G>C), AAG854GCA (2710A>G 2711A>C 2712G>A), AAT855AAA (2715T>A), TTG856TCC (2717T>C 2718G>C), GTG858CTA (2722G>C 2724G>A), TTT1322.TC (4116T>C), GAG1323GAA (4119G>A), ATA1324ATC (4122A>C), TAC1329TAT (4137C>T), GTC1333GTG (4149C>G), AGG1334CGG (4150A>C), TTT1337TTC (4161T>C), TCC1341TCT (4173C>T), GCC1342GCT (4176C>T), ACC1343TGT (4177A>T 4178C>G 4179C>T), GCA1346GCT (4188A>T), TTA1347GCA (4189T>G 4190T>C), AAT1349AAC (4197T>C), GTC1350GTA (4200C>A), ATC1351CTC (4201A>C), CCA1352CCT (4206A>T), TGT1353TGC (4209T>C), GTT1355GTA (4215T>A), GGA1356GGT (4218A>T), ATA1357ATC (4221A>C), GAT1358AAT (4222G>A), ACC1360ACG (4230C>G), AGT1361AGC (4233T>C), TCT1362CAT (4234T>C 4235C>A), TCT1365ACA (4243T>A 4245T>A), CTT1366GTG (4246C>G 4248T>G), CTT1367CTG (4251T>G), GCC1368GCT (4254C>T), AAC1369AGT (4256A>G 4257C>T), AAC1370AGC (4259A>G), TTC1371TTT (4263C>T), GCT1373TCA (4267G>T 4269T>A), ACT1377GAG (4279A>G 4280C>A 4281T>G), GGG1378GGA (4284G>A), TTT1379TTC (4287T>C), GCT1381GCC (4293T>C), TCA1384TCG (4302A>G), AGT1385AGC (4305T>C), GAC1387..T (4311C>T)								

*: Inserts / Deletes / Misaligned / Frameshifts

Analysis details

This analysis was performed with panviral2.64

NGS Details (UN62): Cladosporium fulvum T-1 virus

Assembly

**Coverage Length**	1370 (3 contig(s))
**Depth Of Coverage**	39.2
**Number Of Reads**	511
**Reads Per Million**	11.85 rpm (after QC)
**Ambiguities**	0
**Assembly Method**	de novo + reference guided assembly
**Consensus Caller**	Bcf Tools

Coverage Map

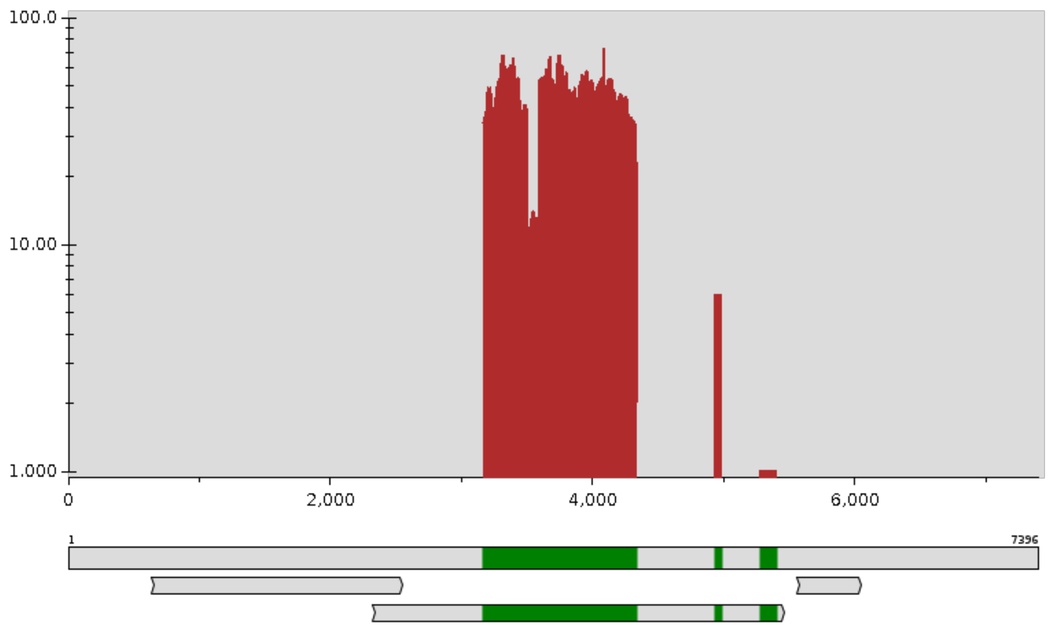

Assignment

**Type**	Cladosporium fulvum T-1 virus (Taxonomy ID: 2052899)
**Reference Genome**	NC_043491.1
**NT Identity (%)**	52.9758
**AA Identity (%)**	47.2406
**Number Of Stop Codons**	0
**Number Of CDS**	3

Alignment

**Alignment Score**	117.0 (NT) + 1494.0 (AA) = 1611.0
**Concordance (%)**	27.8431

Alignment Method	Global, seeded, nucleotide + amino acids (AGA)

Genome Region

Sequence starts at position 3165 and ends at position 5404 relative to NC_043491.1 reference sequence.

Alignment Detailed Statistics

	Begin	End	Coverage	Score	Concordance	Matches	Identities	I/D/M/F*	Stop Codons
**NT**	**3165**	**5404**	**18.5%**	**117**	**4.4%**	**1352 (98.0%)**	**721 (52.3%)**	**9/18**	
Mutations:	3165A>G, 3167T>G, 3170A>G, 3171G>A, 3172T>A, 3175T>A, 3178C>A, 3179A>G, 3181G>C, 3182G>T, 3183A>G, 3184G>T, 3185T>G, 3186A>T, 3187T>G, 3188C>G, 3189G>C, 3190C>A, 3191A>G, 3194T>C, 3195G>T, 3197T>C, 3198C>T, 3200C>A, 3201G>A, 3204T>A, 3205C>G, 3207T>A, 3208T>C, 3210A>C, 3211A>T, 3214G>T, 3216A>T, 3217A>G, 3219G>T, 3220A>G, 3221G>C, 3222G>C, 3223C>A, 3224A>C, 3225A>C, 3226G>T, 3229C>G, 3230G>T, 3231C>T, 3232C>G, 3233T>C, 3234T>C, 3236C>A, 3237C>A, 3238T>G, 3243A>T, 3244C>G, 3246A>C, 3251T>A, 3253_3254insAGGGATATT, 3261A>G, 3262G>A, 3265A>T, 3266A>G, 3268C>G, 3269A>T, 3271T>G, 3272C>A, 3273A>T, 3280G>T, 3281A>T, 3282A>C, 3283A>T, 3284G>A, 3285A>C, 3289T>G, 3292A>T, 3293T>G, 3294G>C, 3295G>A, 3296G>C, 3297G>A, 3299C>G, 3301C>T, 3303T>C, 3304A>T, 3307T>C, 3308C>A, 3309A>G, 3314T>G, 3317G>C, 3318A>C, 3319G>T, 3326C>T, 3328A>G, 3329C>G, 3330A>C, 3331G>T, 3332A>G, 3333C>A, 3334C>A, 3337A>G, 3338C>A, 3340A>G, 3341G>A, 3343A>G, 3344T>C, 3345G>A, 3352G>C, 3355G>A, 3356A>T, 3357A>T, 3361A>T, 3363C>A, 3364C>T, 3365A>T, 3366A>C, 3371T>C, 3372G>T, 3376A>T, 3378G>A, 3379A>G, 3381G>C, 3382A>T, 3385C>T, 3387C>A, 3388C>G, 3389T>G, 3391A>T, 3392A>C, 3393G>C, 3394T>C, 3395G>T, 3396C>A, 3397A>T, 3400A>T, 3401A>G, 3403T>A, 3406A>T, 3407T>G, 3408G>T, 3409C>T, 3410A>T, 3412G>A, 3415C>T, 3416G>C, 3417T>A, 3418T>G, 3419C>A, 3420C>A, 3425G>C, 3426C>A, 3427A>G, 3428A>G, 3430C>T, 3434A>T, 3435A>C, 3437C>T, 3440C>A, 3442A>G, 3443C>A, 3445C>G, 3446G>T, 3447T>G, 3448A>C, 3449C>A, 3450A>T, 3451A>G, 3454C>T, 3457C>T, 3460A>G, 3461A>G, 3462A>C, 3464T>C, 3470G>A, 3473A>G, 3479A>G, 3481C>T, 3487C>T, 3488C>A, 3489G>A, 3490A>G, 3493T>C, 3497C>A, 3499A>C, 3502C>A, 3503A>C, 3504A>T, 3508C>T, 3510A>C, 3511A>T, 3514A>T, 3515G>C, 3516C>T, 3517A>C, 3518C>T, 3519A>T, 3520A>T, 3523C>T, 3524A>C, 3525G>A, 3527T>C, 3529A>T, 3530A>G, 3531C>G, 3533G>C, 3534G>A, 3536T>G, 3539G>C, 3540A>G, 3541C>T, 3546A>T, 3550G>C, 3554A>T, 3556T>A, 3559C>T, 3562A>T, 3565A>G, 3566G>T, 3567A>C, 3568C>T, 3570C>G, 3571C>A, 3573T>A, 3578G>C, 3579C>A, 3580T>A, 3581A>G, 3583C>G, 3584C>A, 3589G>C, 3598A>G, 3601A>T, 3604A>G, 3605T>C, 3606G>C, 3607G>T, 3610A>G, 3613C>T, 3614G>A, 3618T>G, 3619C>T, 3620A>G, 3621G>T, 3622G>A, 3625A>T, 3628A>G, 3631C>T, 3634A>G, 3635C>T, 3636T>C, 3637C>A, 3640C>T, 3647T>C, 3649G>T, 3652C>A, 3658A>G, 3659A>T, 3661G>T, 3664A>G, 3670C>T, 3673C>T, 3676A>C, 3679C>G, 3682A>T, 3683T>A, 3687G>T, 3688C>T, 3689C>T, 3690A>G, 3691G>C, 3692G>A, 3694C>T, 3697T>A, 3698G>A, 3700C>G, 3703C>T, 3705A>G, 3706A>C, 3707A>G, 3708C>T, 3712T>G, 3713A>T, 3714G>A, 3715A>T, 3716G>C, 3717A>C, 3719C>T, 3720T>A, 3721A>C, 3724C>T, 3728G>A, 3729T>A, 3730G>T, 3732G>T, 3733C>T, 3736C>A, 3739T>A, 3741C>T, 3742T>C, 3745C>T, 3746A>C, 3748G>A, 3751C>T, 3757A>T, 3758C>G, 3760G>C, 3761G>A, 3763C>A, 3766C>T, 3768C>G, 3769A>T, 3770A>C, 3774G>A, 3775A>T, 3776T>A, 3778C>G, 3779C>T, 3781C>G, 3782C>G, 3784G>T, 3791A>G, 3792C>T, 3793C>T, 3795A>G, 3796G>C, 3799A>T, 3800G>T, 3802T>G, 3803C>A, 3804A>G, 3805A>T, 3806G>T, 3807A>T, 3808T>G, 3811G>A, 3814C>A, 3815G>T, 3816A>C, 3817A>T, 3820A>T, 3821C>T, 3823C>A, 3825C>G, 3827A>C, 3829G>A, 3831C>A, 3833G>A, 3834G>C, 3835A>T, 3836T>C, 3839A>T, 3841G>T, 3842A>G, 3843C>T, 3844A>T, 3845G>A, 3846C>A, 3848C>A, 3849C>T, 3850C>G, 3856A>G, 3859C>T, 3862A>G, 3865C>T, 3866C>G, 3867A>C, 3869A>C, 3871G>A, 3877A>G, 3878G>A, 3880C>A, 3886T>C, 3887T>C, 3892C>G, 3893T>C, 3894T>A, 3896A>C, 3898C>A, 3899A>G, 3901C>T, 3905A>G, 3906C>A, 3909C>A, 3910A>C, 3911G>C, 3912G>A, 3913G>A, 3914A>G, 3918C>G, 3922C>G, 3925C>T, 3929G>A, 3930C>A, 3931A>G, 3934G>A, 3935A>G, 3936C>T, 3937A>G, 3940G>A, 3941T>G, 3943A>C, 3946C>T, 3947A>G, 3948G>T, 3949A>T, 3952A>T, 3957C>A, 3960A>C, 3964G>C, 3967G>A, 3970A>T, 3973C>G, 3980G>C, 3982A>G, 3983C>A, 3984A>G, 3988A>T, 3992C>T, 3994T>G, 3997A>G, 4000C>A, 4003C>T, 4010A>T, 4012C>T, 4025A>G, 4026A>C, 4027G>T, 4029A>G, 4030C>A, 4033T>C, 4041C>A, 4048A>T, 4049C>G, 4052A>C, 4057G>T, 4058A>G, 4059T>A, 4060G>T, 4061C>T, 4063T>G, 4064A>C, 4065C>T, 4066A>G, 4068G>A, 4069A>G, 4072A>G, 4075C>A, 4081C>G, 4085A>G, 4086A>T, 4091G>A, 4092G>C, 4093A>C, 4094A>G, 4099A>G, 4100C>T, 4101A>G, 4102G>T, 4103A>G, 4104C>A, 4105C>G, 4107A>C, 4108A>G, 4109G>A, 4110C>G, 4111G>C, 4114C>T, 4115A>C, 4119G>A, 4120A>G, 4129A>C, 4130C>T, 4131A>T, 4132G>A, 4134G>T, 4135C>G, 4136G>A, 4147A>T, 4149C>T, 4150G>T, 4153T>C, 4154C>G, 4155G>C, 4157C>T, 4160T>C, 4161T>C, 4165T>G, 4167G>A, 4168T>G, 4169A>C, 4170G>C, 4171C>T, 4172A>T, 4173A>T, 4174G>T, 4177A>G, 4180C>A, 4184A>G, 4186C>G, 4187G>C, 4192C>T, 4195C>T, 4201T>G, 4204T>C, 4206T>A, 4207G>A, 4210A>T, 4211A>G, 4213A>G, 4216C>A, 4218C>G, 4219A>C, 4220T>G, 4221G>T, 4222T>A, 4223C>T, 4225A>G, 4226A>G, 4227C>T, 4228A>G, 4231G>A, 4232_4237delACACAC, 4240T>A, 4243G>T, 4244_4249delAAAAGA, 4252C>T, 4255A>G, 4263A>T, 4265T>G, 4267T>A, 4268T>A, 4269C>G, 4271C>A, 4277A>T, 4279G>A, 4281C>A, 4282C>T, 4283A>G, 4284C>A, 4285A>T, 4288G>T, 4291A>G, 4296A>G, 4297C>G, 4301G>T, 4302A>C, 4303C>T, 4305T>C, 4306C>T, 4312C>A, 4319C>A, 4321T>G, 4322C>G, 4324A>T, 4327C>A, 4328A>G, 4330T>G, 4333T>G, 4334G>C, 4923A>G, 4924A>T, 4933C>G, 4934G>A, 4935A>G, 4936G>T, 4942G>T, 4954T>A, 4957G>T, 4958A>T, 4959A>G, 4960A>C, 4963C>G, 4966G>A, 4968G>A, 4972A>C, 4975G>C, 4976G>A, 4978T>G, 5276C>T, 5277A>G, 5278A>C, 5284A>T, 5285C>A, 5292A>T, 5294A>G, 5296C>G, 5298A>G, 5301A>C, 5302C>T, 5303G>C, 5304C>A, 5305A>G, 5309G>A, 5311C>G, 5312A>G, 5314C>T, 5319T>C, 5321T>A, 5322C>A, 5323A>G, 5326A>G, 5329G>A, 5330C>G, 5331C>A, 5332A>T, 5333A>T, 5338G>A, 5339C>G, 5341G>A, 5342A>T, 5345G>C, 5348C>T, 5349T>A, 5350G>T, 5354A>T, 5355A>T, 5356C>A, 5359C>G, 5360A>T, 5361A>G, 5362A>G, 5363T>A, 5364C>G, 5365A>T, 5368G>A, 5369A>G, 5370C>T, 5371A>C, 5374T>C, 5375T>G, 5376C>G, 5377G>A, 5379C>G, 5383G>A, 5390A>G, 5391T>A, 5393_5398delCGAACT, 5404A>C								
**CDS**									
homologue_of_retroviral_POL_genes	281	1026	43.6%	1494	47.5%	450 (98.0%)	214 (46.6%)	3/6/0/0	0

	Begin	End	Coverage	Score	Concordance	Matches	Identities	I/D/M/F*	Stop Codons
**NT**	**3165**	**5404**	**18.5%**	**117**	**4.4%**	**1352 (98.0%)**	**721 (52.3%)**	**9/18**	

Protein mutations:

L281V (3167T>G), S282E (3170A>G 3171G>A 3172T>A), K285D (3179A>G 3181G>C), E286C (3182G>T 3183A>G 3184G>T), Y287V (3185T>G 3186A>T 3187T>G), R288A (3188C>G 3189G>C 3190C>A), K289E (3191A>G), W290L (3194T>C 3195G>T), S291L (3197T>C 3198C>T), R292K (3200C>A 3201G>A), L293Q (3204T>A 3205C>G), F294Y (3207T>A 3208T>C), E295A (3210A>C 3211A>T), E296D (3214G>T), E297V (3216A>T 3217A>G), R298M (3219G>T 3220A>G), G299P (3221G>C 3222G>C 3223C>A), K300P (3224A>C 3225A>C 3226G>T), D301E (3229C>G), A302L (3230G>T 3231C>T 3232C>G), L303P (3233T>C 3234T>C), P304K (3236C>A 3237C>A 3238T>G), H306L (3243A>T 3244C>G), Q307P (3246A>C), W309R (3251T>A), W309_D310insRDI (3253_3254insAGGGATATT), K312R (3261A>G 3262G>A), N314E (3266A>G 3268C>G), I315L (3269A>T 3271T>G), Q316M (3272C>A 3273A>T), K319S (3281A>T 3282A>C 3283A>T), E320T (3284G>A 3285A>C), W323A (3293T>G 3294G>C 3295G>A), G324Q (3296G>C 3297G>A), P325A (3299C>G 3301C>T), L326P (3303T>C 3304A>T), Q328R (3308C>A 3309A>G), S330A (3314T>G), E331P (3317G>C 3318A>C 3319G>T), Q335A (3329C>G 3330A>C 3331G>T), T336E (3332A>G 3333C>A 3334C>A), E339K (3341G>A 3343A>G), W340Q (3344T>C 3345G>A), K342N (3352G>C), K344L (3356A>T 3357A>T), A346D (3363C>A 3364C>T), K347S (3365A>T 3366A>C), W349L (3371T>C 3372G>T), R351Q (3378G>A 3379A>G), R352P (3381G>C 3382A>T), T354K (3387C>A 3388C>G), S355A (3389T>G 3391A>T), S356P (3392A>C 3393G>C 3394T>C), A357Y (3395G>T 3396C>A 3397A>T), T359A (3401A>G 3403T>A), C361V (3407T>G 3408G>T 3409C>T), M362L (3410A>T 3412G>A), V364Q (3416G>C 3417T>A 3418T>G), P365K (3419C>A 3420C>A), A367Q (3425G>C 3426C>A 3427A>G), N368D (3428A>G 3430C>T), K370S (3434A>T 3435A>C), L373M (3443C>A 3445C>G), V374C (3446G>T 3447T>G 3448A>C), Q375M (3449C>A 3450A>T 3451A>G), K379A (3461A>G 3462A>C), E382K (3470G>A), I383V (3473A>G), I385V (3479A>G 3481C>T), R388K (3488C>A 3489G>A 3490A>G), L391I (3497C>A 3499A>C), N393L (3503A>C 3504A>T), E395A (3510A>C 3511A>T), E396D (3514A>T), A397L (3515G>C 3516C>T 3517A>C), Q398F (3518C>T 3519A>T 3520A>T), R400Q (3524A>C 3525G>A), T402G (3530A>G 3531C>G), G403Q (3533G>C 3534G>A), S404A (3536T>G), D405R (3539G>C 3540A>G 3541C>T), Y407F (3546A>T), I410L (3554A>T 3556T>A), D414S (3566G>T 3567A>C 3568C>T), A415G (3570C>G 3571C>A), F416Y (3573T>A), A418Q (3578G>C 3579C>A 3580T>A), I419V (3581A>G 3583C>G), M421I (3589G>C), E425D (3601A>T), W427P (3605T>C 3606G>C 3607G>T), A430T (3614G>A), F431C (3618T>G 3619C>T), R432V (3620A>G 3621G>T 3622G>A), L437S (3635C>T 3636T>C 3637C>A), M445F (3659A>T 3661G>T), S453T (3683T>A), C454F (3687G>T 3688C>T), Q455C (3689C>T 3690A>G 3691G>C), D456N (3692G>A 3694C>T), V458M (3698G>A 3700C>G), E460G (3705A>G 3706A>C), T461V (3707A>G 3708C>T), R463Y (3713A>T 3714G>A 3715A>T), D464P (3716G>C 3717A>C), L465Y (3719C>T 3720T>A 3721A>C), V468N (3728G>A 3729T>A 3730G>T), C469F (3732G>T 3733C>T), A472V (3741C>T 3742T>C), M474L (3746A>C 3748G>A), L478V (3758C>G 3760G>C), V479I (3761G>A 3763C>A), T481S (3768C>G 3769A>T), K482Q (3770A>C), G483D (3774G>A 3775A>T), S484T (3776T>A 3778C>G), Q486D (3782C>G 3784G>T), T489V (3791A>G 3792C>T 3793C>T), K490S (3795A>G 3796G>C), Q491H (3799A>T), V492L (3800G>T 3802T>G), Q493S (3803C>A 3804A>G 3805A>T), D494L (3806G>T 3807A>T 3808T>G), F496L (3814C>A), E497S (3815G>T 3816A>C 3817A>T), T500R (3825C>G), K501Q (3827A>C 3829G>A), S502Y (3831C>A), G503T (3833G>A 3834G>C 3835A>T), F504L (3836T>C), K505Y (3839A>T 3841G>T), T506V (3842A>G 3843C>T 3844A>T), A507K (3845G>A 3846C>A), P508M (3848C>A 3849C>T 3850C>G), H514A (3866C>G 3867A>C), K515Q (3869A>C 3871G>A), V518I (3878G>A 3880C>A), F523H (3893T>C 3894T>A), I524L (3896A>C 3898C>A), I525V (3899A>G 3901C>T), T527E (3905A>G 3906C>A), T528N (3909C>A 3910A>C), G529Q (3911G>C 3912G>A 3913G>A), I530V (3914A>G), T531R (3918C>G), I532M (3922C>G), A535K (3929G>A 3930C>A 3931A>G), T537V (3935A>G 3936C>T 3937A>G), S539A (3941T>G 3943A>C), R541V (3947A>G 3948G>T 3949A>T), E542D (3952A>T), P544Q (3957C>A), E545A (3960A>C), V552L (3980G>C 3982A>G), Q553R (3983C>A 3984A>G), N562Y (4010A>T 4012C>T), K567A (4025A>G 4026A>C 4027G>T), D568G (4029A>G 4030C>A), T572K (4041C>A), P575A (4049C>G), M576L (4052A>C), M578D (4058A>G 4059T>A 4060G>T), T580L (4064A>C 4065C>T 4066A>G), R581K (4068G>A 4069A>G), D583E (4075C>A), N585K (4081C>G), K587V (4085A>G 4086A>T), G589T (4091G>A 4092G>C 4093A>C), K590E (4094A>G), Q592C (4100C>T 4101A>G 4102G>T), T593E (4103A>G 4104C>A 4105C>G), E594A (4107A>C 4108A>G), A595S (4109G>A 4110C>G 4111G>C), K597Q (4115A>C), R598K (4119G>A 4120A>G), E601D (4129A>C), Q602L (4130C>T 4131A>T 4132G>A), C603L (4134G>T 4135C>G), A604T (4136G>A), T608I (4149C>T 4150G>T), R610A (4154C>G 4155G>C), F612P (4160T>C 4161T>C), D613E (4165T>G), G614E (4167G>A 4168T>G), S615P (4169A>C 4170G>C 4171C>T), K616F (4172A>T 4173A>T 4174G>T), I620V (4184A>G 4186C>G), E621Q (4187G>C), M627K (4206T>A 4207G>A), I629V (4211A>G 4213A>G), A631G (4218C>G 4219A>C), C632V (4220T>G 4221G>T 4222T>A), T634V (4226A>G 4227C>T 4228A>G), T636_H637del (4232_4237delACACAC), D638E (4240T>A), K640_R641del (4244_4249delAAAAGA), Y646F (4263A>T), Y647E (4265T>G 4267T>A), M651L (4277A>T 4279G>A), T652N (4281C>A 4282C>T), T653D (4283A>G 4284C>A 4285A>T), N657R (4296A>G 4297C>G), D659S (4301G>T 4302A>C 4303C>T), I660T (4305T>C 4306C>T), D662E (4312C>A), L665M (4319C>A 4321T>G), L666V (4322C>G 4324A>T), I668V (4328A>G 4330T>G), A670P (4334G>C), D869E (4933C>G), E870S (4934G>A 4935A>G 4936G>T), K878C (4958A>T 4959A>G 4960A>C), R881K (4968G>A), K883N (4975G>C), D884K (4976G>A 4978T>G), Q984C (5276C>T 5277A>G 5278A>C), Y989F (5292A>T), I990V (5294A>G 5296C>G), N991S (5298A>G), Y992S (5301A>C 5302C>T), A993Q (5303G>C 5304C>A 5305A>G), D995K (5309G>A 5311C>G), N996D (5312A>G 5314C>T), V998A (5319T>C), S999K (5321T>A 5322C>A 5323A>G), P1002D (5330C>G 5331C>A 5332A>T), M1003L (5333A>T), Q1005E (5339C>G 5341G>A), I1006F (5342A>T), A1007P (5345G>C), L1008Y (5348C>T 5349T>A 5350G>T), N1010L (5354A>T 5355A>T 5356C>A), H1011Q (5359C>G), K1012W (5360A>T 5361A>G 5362A>G), T1015V (5369A>G 5370C>T 5371A>C), S1017G (5375T>G 5376C>G 5377G>A), T1018R (5379C>G), M1022E (5390A>G 5391T>A), R1023_T1024del (5393_5398delCGAACT)

	Begin	End	Coverage	Score	Concordance	Matches	Identities	I/D/M/F*	Stop Codons
**NT**	**3165**	**5404**	**18.5%**	**117**	**4.4%**	**1352 (98.0%)**	**721 (52.3%)**	**9/18**	
Codon mutations:	GAG280.GG (3165A>G), TTG281GTG (3167T>G), AGT282GAA (3170A>G 3171G>A 3172T>A), ATT283ATA (3175T>A), CCC284CCA (3178C>A), AAG285GAC (3179A>G 3181G>C), GAG286TGT (3182G>T 3183A>G 3184G>T), TAT287GTG (3185T>G 3186A>T 3187T>G), CGC288GCA (3188C>G 3189G>C 3190C>A), AAG289GAG (3191A>G), TGG290CTG (3194T>C 3195G>T), TCG291CTG (3197T>C 3198C>T), CGA292AAA (3200C>A 3201G>A), CTC293CAG (3204T>A 3205C>G), TTT294TAC (3207T>A 3208T>C), GAA295GCT (3210A>C 3211A>T), GAG296GAT (3214G>T), GAA297GTG (3216A>T 3217A>G), AGA298ATG (3219G>T 3220A>G), GGC299CCA (3221G>C 3222G>C 3223C>A), AAG300CCT (3224A>C 3225A>C 3226G>T), GAC301GAG (3229C>G), GCC302TTG (3230G>T 3231C>T 3232C>G), TTA303CCA (3233T>C 3234T>C), CCT304AAG (3236C>A 3237C>A 3238T>G), CAC306CTG (3243A>T 3244C>G), CAA307CCA (3246A>C), TGG309AGG (3251T>A), TGG309_GAT310insAGGGATATT (3253_3254insAGGGATATT), AAG312AGA (3261A>G 3262G>A), ATA313ATT (3265A>T), AAC314GAG (3266A>G 3268C>G), ATT315TTG (3269A>T 3271T>G), CAG316ATG (3272C>A 3273A>T), GGG318GGT (3280G>T), AAA319TCT (3281A>T 3282A>C 3283A>T), GAG320ACG (3284G>A 3285A>C), CCT321CCG (3289T>G), CCA322CCT (3292A>T), TGG323GCA (3293T>G 3294G>C 3295G>A), GGA324CAA (3296G>C 3297G>A), CCC325GCT (3299C>G 3301C>T), CTA326CCT (3303T>C 3304A>T), TAT327TAC (3307T>C), CAA328AGA (3308C>A 3309A>G), TCT330GCT (3314T>G), GAG331CCT (3317G>C 3318A>C 3319G>T), CTA334TTG (3326C>T 3328A>G), CAG335GCT (3329C>G 3330A>C 3331G>T), ACC336GAA (3332A>G 3333C>A 3334C>A), CTA337CTG (3337A>G), CGA338AGG (3338C>A 3340A>G), GAA339AAG (3341G>A 3343A>G), TGG340CAG (3344T>C 3345G>A), AAG342AAC (3352G>C), GAG343GAA (3355G>A), AAG344TTG (3356A>T 3357A>T), CTA345CTT (3361A>T), GCC346GAT (3363C>A 3364C>T), AAA347TCA (3365A>T 3366A>C), TGG349CTG (3371T>C 3372G>T), ATA350ATT (3376A>T), CGA351CAG (3378G>A 3379A>G), CGA352CCT (3381G>C 3382A>T), TCC353TCT (3385C>T), ACC354AAG (3387C>A 3388C>G), TCA355GCT (3389T>G 3391A>T), AGT356CCC (3392A>C 3393G>C 3394T>C), GCA357TAT (3395G>T 3396C>A 3397A>T), GGA358GGT (3400A>T), ACT359GCA (3401A>G 3403T>A), CCA360CCT (3406A>T), TGC361GTT (3407T>G 3408G>T 3409C>T), ATG362TTA (3410A>T 3412G>A), TTC363TTT (3415C>T), GTT364CAG (3416G>C 3417T>A 3418T>G), CCA365AAA (3419C>A 3420C>A), GCA367CAG (3425G>C 3426C>A 3427A>G), AAC368GAT (3428A>G 3430C>T), AAA370TCA (3434A>T 3435A>C), CTA371TTA (3437C>T), CGA372AGG (3440C>A 3442A>G), CTC373ATG (3443C>A 3445C>G), GTA374TGC (3446G>T 3447T>G 3448A>C), CAA375ATG (3449C>A 3450A>T 3451A>G), GAC376GAT (3454C>T), TAC377TAT (3457C>T), CGA378CGG (3460A>G), AAG379GCG (3461A>G 3462A>C), TTG380CTG (3464T>C), GAG382AAG (3470G>A), ATC383GTC (3473A>G), ATC385GTT (3479A>G 3481C>T), AAC387AAT (3487C>T), CGA388AAG (3488C>A 3489G>A 3490A>G), TAT389TAC (3493T>C), CTA391ATC (3497C>A 3499A>C), CCC392CCA (3502C>A), AAC393CTC (3503A>C 3504A>T), ATC394ATT (3508C>T), GAA395GCT (3510A>C 3511A>T), GAA396GAT (3514A>T), GCA397CTC (3515G>C 3516C>T 3517A>C), CAA398TTT (3518C>T 3519A>T 3520A>T), GAC399GAT (3523C>T), AGA400CAA (3524A>C 3525G>A), TTA401CTT (3527T>C 3529A>T), ACC402GGC (3530A>G 3531C>G), GGA403CAA (3533G>C 3534G>A), TCA404GCA (3536T>G), GAC405CGT (3539G>C 3540A>G 3541C>T), TAC407TTC (3546A>T), ACG408ACC (3550G>C), ATT410TTA (3554A>T 3556T>A), GAC411GAT (3559C>T), CTA412CTT (3562A>T), CGA413CGG (3565A>G), GAC414TCT (3566G>T 3567A>C 3568C>T), GCC415GGA (3570C>G 3571C>A), TTC416TAC (3573T>A), GCT418CAA (3578G>C 3579C>A 3580T>A), ATC419GTG (3581A>G 3583C>G), CGA420AGA (3584C>A), ATG421ATC (3589G>C), GGA424GGG (3598A>G), GAA425GAT (3601A>T), GAA426GAG (3604A>G), TGG427CCT (3605T>C 3606G>C 3607G>T), AAA428AAG (3610A>G), ACC429ACT (3613C>T), GCT430ACT (3614G>A), TTC431TGT (3618T>G 3619C>T), AGG432GTA (3620A>G 3621G>T 3622G>A), ACA433ACT (3625A>T), AGA434AGG (3628A>G), TAC435TAT (3631C>T), GGA436GGG (3634A>G), CTC437TCA (3635C>T 3636T>C 3637C>A), TAC438TAT (3640C>T), TTG441CTT (3647T>C 3649G>T), GTC442GTA (3652C>A), CCA444CCG (3658A>G), ATG445TTT (3659A>T 3661G>T), GGA446GGG (3664A>G), ACC448ACT (3670C>T), AAC449AAT (3673C>T), GCA450GCC (3676A>C), CCC451CCG (3679C>G), GCA452GCT (3682A>T), TCC453ACC (3683T>A), TGC454TTT (3687G>T 3688C>T), CAG455TGC (3689C>T 3690A>G 3691G>C), GAC456AAT (3692G>A 3694C>T), CTT457CTA (3697T>A), GTC458ATG (3698G>A 3700C>G), AAC459AAT (3703C>T), GAA460GGC (3705A>G 3706A>C), ACA461GTA (3707A>G 3708C>T), CTT462CTG (3712T>G), AGA463TAT (3713A>T 3714G>A 3715A>T), GAC464CCC (3716G>C 3717A>C), CTA465TAC (3719C>T 3720T>A 3721A>C), CTC466CTT (3724C>T), GTG468AAT (3728G>A 3729T>A 3730G>T), TGC469TTT (3732G>T 3733C>T), GTC470GTA (3736C>A), GTT471GTA (3739T>A), GCT472GTC (3741C>T 3742T>C), TAC473TAT (3745C>T), ATG474CTA (3746A>C 3748G>A), GAC475GAT (3751C>T), ATA477ATT (3757A>T), CTG478GTC (3758C>G 3760G>C), GTC479ATA (3761G>A 3763C>A), TAC480TAT (3766C>T), ACA481AGT (3768C>G 3769A>T), AAA482CAA (3770A>C), GGA483GAT (3774G>A 3775A>T), TCC484ACG (3776T>A 3778C>G), CTC485TTG (3779C>T 3781C>G), CAG486GAT (3782C>G 3784G>T), ACC489GTT (3791A>G 3792C>T 3793C>T), AAG490AGC (3795A>G 3796G>C), CAA491CAT (3799A>T), GTT492TTG (3800G>T 3802T>G), CAA493AGT (3803C>A 3804A>G 3805A>T), GAT494TTG (3806G>T 3807A>T 3808T>G), GTG495GTA (3811G>A), TTC496TTA (3814C>A), GAA497TCT (3815G>T 3816A>C 3817A>T), CGA498CGT (3820A>T), CTC499TTA (3821C>T 3823C>A), ACG500AGG (3825C>G), AAG501CAA (3827A>C 3829G>A), TCC502TAC (3831C>A), GGA503ACT (3833G>A 3834G>C 3835A>T), TTC504CTC (3836T>C), AAG505TAT (3839A>T 3841G>T), ACA506GTT (3842A>G 3843C>T 3844A>T), GCA507AAA (3845G>A 3846C>A), CCC508ATG (3848C>A 3849C>T 3850C>G), AAA510AAG (3856A>G), TGC511TGT (3859C>T), GAA512GAG (3862A>G), TTC513TTT (3865C>T), CAC514GCC (3866C>G 3867A>C), AAG515CAA (3869A>C 3871G>A), GAA517GAG (3877A>G), GTC518ATA (3878G>A 3880C>A), TTT520TTC (3886T>C), TTA521CTA (3887T>C), GGC522GGG (3892C>G), TTT523CAT (3893T>C 3894T>A), ATC524CTA (3896A>C 3898C>A), ATC525GTT (3899A>G 3901C>T), ACA527GAA (3905A>G 3906C>A), ACA528AAC (3909C>A 3910A>C), GGG529CAA (3911G>C 3912G>A 3913G>A), ATA530GTA (3914A>G), ACG531AGG (3918C>G), ATC532ATG (3922C>G), GAC533GAT (3925C>T), GCA535AAG (3929G>A 3930C>A 3931A>G), AAG536AAA (3934G>A), ACA537GTG (3935A>G 3936C>T 3937A>G), CAG538CAA (3940G>A), TCA539GCC (3941T>G 3943A>C), ATC540ATT (3946C>T), AGA541GTT (3947A>G 3948G>T 3949A>T), GAA542GAT (3952A>T), CCA544CAA (3957C>A), GAA545GCA (3960A>C), CCG546CCC (3964G>C), AAG547AAA (3967G>A), ACA548ACT (3970A>T), GTC549GTG (3973C>G), GTA552CTG (3980G>C 3982A>G), CAG553AGG (3983C>A 3984A>G), TCA554TCT (3988A>T), CTT556TTG (3992C>T 3994T>G), GGA557GGG (3997A>G), CTC558CTA (4000C>A), GCC559GCT (4003C>T), AAC562TAT (4010A>T 4012C>T), AAG567GCT (4025A>G 4026A>C 4027G>T), GAC568GGA (4029A>G 4030C>A), TAT569TAC (4033T>C), ACA572AAA (4041C>A), GCA574GCT (4048A>T), CCA575GCA (4049C>G), ATG576CTG (4052A>C), ACG577ACT (4057G>T), ATG578GAT (4058A>G 4059T>A 4060G>T), CTT579TTG (4061C>T 4063T>G), ACA580CTG (4064A>C 4065C>T 4066A>G), AGA581AAG (4068G>A 4069A>G), AAA582AAG (4072A>G), GAC583GAA (4075C>A), AAC585AAG (4081C>G), AAA587GTA (4085A>G 4086A>T), GGA589ACC (4091G>A 4092G>C 4093A>C), AAA590GAA (4094A>G), GAA591GAG (4099A>G), CAG592TGT (4100C>T 4101A>G 4102G>T), ACC593GAG (4103A>G 4104C>A 4105C>G), GAA594GCG (4107A>C 4108A>G), GCG595AGC (4109G>A 4110C>G 4111G>C), TTC596TTT (4114C>T), AAA597CAA (4115A>C), AGA598AAG (4119G>A 4120A>G), GAA601GAC (4129A>C), CAG602TTA (4130C>T 4131A>T 4132G>A), TGC603TTG (4134G>T 4135C>G), GCT604ACT (4136G>A), CCA607CCT (4147A>T), ACG608ATT (4149C>T 4150G>T), CTT609CTC (4153T>C), CGA610GCA (4154C>G 4155G>C), CTA611TTA (4157C>T), TTC612CCC (4160T>C 4161T>C), GAT613GAG (4165T>G), GGT614GAG (4167G>A 4168T>G), AGC615CCT (4169A>C 4170G>C 4171C>T), AAG616TTT (4172A>T 4173A>T 4174G>T), GAA617GAG (4177A>G), GTC618GTA (4180C>A), ATC620GTG (4184A>G 4186C>G), GAG621CAG (4187G>C), ACC622ACT (4192C>T), GAC623GAT (4195C>T), TCT625TCG (4201T>G), GAT626GAC (4204T>C), ATG627AAA (4206T>A 4207G>A), GCA628GCT (4210A>T), ATA629GTG (4211A>G 4213A>G), GGC630GGA (4216C>A), GCA631GGC (4218C>G 4219A>C), TGT632GTA (4220T>G 4221G>T 4222T>A), CTA633TTG (4223C>T 4225A>G), ACA634GTG (4226A>G 4227C>T 4228A>G), CAG635CAA (4231G>A), ACA636_CAC637del (4232_4237delACACAC), GAT638GAA (4240T>A), GGG639GGT (4243G>T), AAA640_AGA641del (4244_4249delAAAAGA), CAC642CAT (4252C>T), CCA643CCG (4255A>G), TAT646TTT (4263A>T), TAT647GAA (4265T>G 4267T>A), TCC648AGC (4268T>A 4269C>G), CGG649AGG (4271C>A), ATG651TTA (4277A>T 4279G>A), ACC652AAT (4281C>A 4282C>T), ACA653GAT (4283A>G 4284C>A 4285A>T), GCG654GCT (4288G>T), GAA655GAG (4291A>G), AAC657AGG (4296A>G 4297C>G), GAC659TCT (4301G>T 4302A>C 4303C>T), ATC660ACT (4305T>C 4306C>T), GAC662GAA (4312C>A), CTT665ATG (4319C>A 4321T>G), CTA666GTT (4322C>G 4324A>T), GCC667GCA (4327C>A), ATT668GTG (4328A>G 4330T>G), GTT669GTG (4333T>G), GCC670CCC (4334G>C), AAA866.GT (4923A>G 4924A>T), GAC869GAG (4933C>G), GAG870AGT (4934G>A 4935A>G 4936G>T), ACG872ACT (4942G>T), ATT876ATA (4954T>A), ACG877ACT (4957G>T), AAA878TGC (4958A>T 4959A>G 4960A>C), CTC879CTG (4963C>G), CCG880CCA (4966G>A), AGG881AAG (4968G>A), TCA882TCC (4972A>C), AAG883AAC (4975G>C), GAT884AAG (4976G>A 4978T>G), CAA984TGC (5276C>T 5277A>G 5278A>C), CTA986CTT (5284A>T), CGG987AGG (5285C>A), TAC989TTC (5292A>T), ATC990GTG (5294A>G 5296C>G), AAC991AGC (5298A>G), TAC992TCT (5301A>C 5302C>T), GCA993CAG (5303G>C 5304C>A 5305A>G), GAC995AAG (5309G>A 5311C>G), AAC996GAT (5312A>G 5314C>T), GTT998GCT (5319T>C), TCA999AAG (5321T>A 5322C>A 5323A>G), TTA1000TTG (5326A>G), CTG1001CTA (5329G>A), CCA1002GAT (5330C>G 5331C>A 5332A>T), ATG1003TTG (5333A>T), GCG1004GCA (5338G>A), CAG1005GAA (5339C>G 5341G>A), ATC1006TTC (5342A>T), GCA1007CCA (5345G>C), CTG1008TAT (5348C>T 5349T>A 5350G>T), AAC1010TTA (5354A>T 5355A>T 5356C>A), CAC1011CAG (5359C>G), AAA1012TGG (5360A>T 5361A>G 5362A>G), TCA1013AGT (5363T>A 5364C>G 5365A>T), GAG1014GAA (5368G>A), ACA1015GTC (5369A>G 5370C>T 5371A>C), ACT1016ACC (5374T>C), TCG1017GGA (5375T>G 5376C>G 5377G>A), ACG1018AGG (5379C>G), ACG1019ACA (5383G>A), ATG1022GAG (5390A>G 5391T>A), CGA1023_ACT1024del (5393_5398delCGAACT), GCA1026GCC (5404A>C)								
**Proteins**									
Reverse Transcriptase (YP_009666308.1)	281	1026	43.6%	1494	47.5%	450 (98.0%)	214 (46.6%)	3/6/0/0	0

	Begin	End	Coverage	Score	Concordance	Matches	Identities	I/D/M/F*	Stop Codons
**NT**	**3165**	**5404**	**18.5%**	**117**	**4.4%**	**1352 (98.0%)**	**721 (52.3%)**	**9/18**	
Protein mutations:	L281V (3167T>G), S282E (3170A>G 3171G>A 3172T>A), K285D (3179A>G 3181G>C), E286C (3182G>T 3183A>G 3184G>T), Y287V (3185T>G 3186A>T 3187T>G), R288A (3188C>G 3189G>C 3190C>A), K289E (3191A>G), W290L (3194T>C 3195G>T), S291L (3197T>C 3198C>T), R292K (3200C>A 3201G>A), L293Q (3204T>A 3205C>G), F294Y (3207T>A 3208T>C), E295A (3210A>C 3211A>T), E296D (3214G>T), E297V (3216A>T 3217A>G), R298M (3219G>T 3220A>G), G299P (3221G>C 3222G>C 3223C>A), K300P (3224A>C 3225A>C 3226G>T), D301E (3229C>G), A302L (3230G>T 3231C>T 3232C>G), L303P (3233T>C 3234T>C), P304K (3236C>A 3237C>A 3238T>G), H306L (3243A>T 3244C>G), Q307P (3246A>C), W309R (3251T>A), W309_D310insRDI (3253_3254insAGGGATATT), K312R (3261A>G 3262G>A), N314E (3266A>G 3268C>G), I315L (3269A>T 3271T>G), Q316M (3272C>A 3273A>T), K319S (3281A>T 3282A>C 3283A>T), E320T (3284G>A 3285A>C), W323A (3293T>G 3294G>C 3295G>A), G324Q (3296G>C 3297G>A), P325A (3299C>G 3301C>T), L326P (3303T>C 3304A>T), Q328R (3308C>A 3309A>G), S330A (3314T>G), E331P (3317G>C 3318A>C 3319G>T), Q335A (3329C>G 3330A>C 3331G>T), T336E (3332A>G 3333C>A 3334C>A), E339K (3341G>A 3343A>G), W340Q (3344T>C 3345G>A), K342N (3352G>C), K344L (3356A>T 3357A>T), A346D (3363C>A 3364C>T), K347S (3365A>T 3366A>C), W349L (3371T>C 3372G>T), R351Q (3378G>A 3379A>G), R352P (3381G>C 3382A>T), T354K (3387C>A 3388C>G), S355A (3389T>G 3391A>T), S356P (3392A>C 3393G>C 3394T>C), A357Y (3395G>T 3396C>A 3397A>T), T359A (3401A>G 3403T>A), C361V (3407T>G 3408G>T 3409C>T), M362L (3410A>T 3412G>A), V364Q (3416G>C 3417T>A 3418T>G), P365K (3419C>A 3420C>A), A367Q (3425G>C 3426C>A 3427A>G), N368D (3428A>G 3430C>T), K370S (3434A>T 3435A>C), L373M (3443C>A 3445C>G), V374C (3446G>T 3447T>G 3448A>C), Q375M (3449C>A 3450A>T 3451A>G), K379A (3461A>G 3462A>C), E382K (3470G>A), I383V (3473A>G), I385V (3479A>G 3481C>T), R388K (3488C>A 3489G>A 3490A>G), L391I (3497C>A 3499A>C), N393L (3503A>C 3504A>T), E395A (3510A>C 3511A>T), E396D (3514A>T), A397L (3515G>C 3516C>T 3517A>C), Q398F (3518C>T 3519A>T 3520A>T), R400Q (3524A>C 3525G>A), T402G (3530A>G 3531C>G), G403Q (3533G>C 3534G>A), S404A (3536T>G), D405R (3539G>C 3540A>G 3541C>T), Y407F (3546A>T), I410L (3554A>T 3556T>A), D414S (3566G>T 3567A>C 3568C>T), A415G (3570C>G 3571C>A), F416Y (3573T>A), A418Q (3578G>C 3579C>A 3580T>A), I419V (3581A>G 3583C>G), M421I (3589G>C), E425D (3601A>T), W427P (3605T>C 3606G>C 3607G>T), A430T (3614G>A), F431C (3618T>G 3619C>T), R432V (3620A>G 3621G>T 3622G>A), L437S (3635C>T 3636T>C 3637C>A), M445F (3659A>T 3661G>T), S453T (3683T>A), C454F (3687G>T 3688C>T), Q455C (3689C>T 3690A>G 3691G>C), D456N (3692G>A 3694C>T), V458M (3698G>A 3700C>G), E460G (3705A>G 3706A>C), T461V (3707A>G 3708C>T), R463Y (3713A>T 3714G>A 3715A>T), D464P (3716G>C 3717A>C), L465Y (3719C>T 3720T>A 3721A>C), V468N (3728G>A 3729T>A 3730G>T), C469F (3732G>T 3733C>T), A472V (3741C>T 3742T>C), M474L (3746A>C 3748G>A), L478V (3758C>G 3760G>C), V479I (3761G>A 3763C>A), T481S (3768C>G 3769A>T), K482Q (3770A>C), G483D (3774G>A 3775A>T), S484T (3776T>A 3778C>G), Q486D (3782C>G 3784G>T), T489V (3791A>G 3792C>T 3793C>T), K490S (3795A>G 3796G>C), Q491H (3799A>T), V492L (3800G>T 3802T>G), Q493S (3803C>A 3804A>G 3805A>T), D494L (3806G>T 3807A>T 3808T>G), F496L (3814C>A), E497S (3815G>T 3816A>C 3817A>T), T500R (3825C>G), K501Q (3827A>C 3829G>A), S502Y (3831C>A), G503T (3833G>A 3834G>C 3835A>T), F504L (3836T>C), K505Y (3839A>T 3841G>T), T506V (3842A>G 3843C>T 3844A>T), A507K (3845G>A 3846C>A), P508M (3848C>A 3849C>T 3850C>G), H514A (3866C>G 3867A>C), K515Q (3869A>C 3871G>A), V518I (3878G>A 3880C>A), F523H (3893T>C 3894T>A), I524L (3896A>C 3898C>A), I525V (3899A>G 3901C>T), T527E (3905A>G 3906C>A), T528N (3909C>A 3910A>C), G529Q (3911G>C 3912G>A 3913G>A), I530V (3914A>G), T531R (3918C>G), I532M (3922C>G), A535K (3929G>A 3930C>A 3931A>G), T537V (3935A>G 3936C>T 3937A>G), S539A (3941T>G 3943A>C), R541V (3947A>G 3948G>T 3949A>T), E542D (3952A>T), P544Q (3957C>A), E545A (3960A>C), V552L (3980G>C 3982A>G), Q553R (3983C>A 3984A>G), N562Y (4010A>T 4012C>T), K567A (4025A>G 4026A>C 4027G>T), D568G (4029A>G 4030C>A), T572K (4041C>A), P575A (4049C>G), M576L (4052A>C), M578D (4058A>G 4059T>A 4060G>T), T580L (4064A>C 4065C>T 4066A>G), R581K (4068G>A 4069A>G), D583E (4075C>A), N585K (4081C>G), K587V (4085A>G 4086A>T), G589T (4091G>A 4092G>C 4093A>C), K590E (4094A>G), Q592C (4100C>T 4101A>G 4102G>T), T593E (4103A>G 4104C>A 4105C>G), E594A (4107A>C 4108A>G), A595S (4109G>A 4110C>G 4111G>C), K597Q (4115A>C), R598K (4119G>A 4120A>G), E601D (4129A>C), Q602L (4130C>T 4131A>T 4132G>A), C603L (4134G>T 4135C>G), A604T (4136G>A), T608I (4149C>T 4150G>T), R610A (4154C>G 4155G>C), F612P (4160T>C 4161T>C), D613E (4165T>G), G614E (4167G>A 4168T>G), S615P (4169A>C 4170G>C 4171C>T), K616F (4172A>T 4173A>T 4174G>T), I620V (4184A>G 4186C>G), E621Q (4187G>C), M627K (4206T>A 4207G>A), I629V (4211A>G 4213A>G), A631G (4218C>G 4219A>C), C632V (4220T>G 4221G>T 4222T>A), T634V (4226A>G 4227C>T 4228A>G), T636_H637del (4232_4237delACACAC), D638E (4240T>A), K640_R641del (4244_4249delAAAAGA), Y646F (4263A>T), Y647E (4265T>G 4267T>A), M651L (4277A>T 4279G>A), T652N (4281C>A 4282C>T), T653D (4283A>G 4284C>A 4285A>T), N657R (4296A>G 4297C>G), D659S (4301G>T 4302A>C 4303C>T), I660T (4305T>C 4306C>T), D662E (4312C>A), L665M (4319C>A 4321T>G), L666V (4322C>G 4324A>T), I668V (4328A>G 4330T>G), A670P (4334G>C), D869E (4933C>G), E870S (4934G>A 4935A>G 4936G>T), K878C (4958A>T 4959A>G 4960A>C), R881K (4968G>A), K883N (4975G>C), D884K (4976G>A 4978T>G), Q984C (5276C>T 5277A>G 5278A>C), Y989F (5292A>T), I990V (5294A>G 5296C>G), N991S (5298A>G), Y992S (5301A>C 5302C>T), A993Q (5303G>C 5304C>A 5305A>G), D995K (5309G>A 5311C>G), N996D (5312A>G 5314C>T), V998A (5319T>C), S999K (5321T>A 5322C>A 5323A>G), P1002D (5330C>G 5331C>A 5332A>T), M1003L (5333A>T), Q1005E (5339C>G 5341G>A), I1006F (5342A>T), A1007P (5345G>C), L1008Y (5348C>T 5349T>A 5350G>T), N1010L (5354A>T 5355A>T 5356C>A), H1011Q (5359C>G), K1012W (5360A>T 5361A>G 5362A>G), T1015V (5369A>G 5370C>T 5371A>C), S1017G (5375T>G 5376C>G 5377G>A), T1018R (5379C>G), M1022E (5390A>G 5391T>A), R1023_T1024del (5393_5398delCGAACT)								

	Begin	End	Coverage	Score	Concordance	Matches	Identities	I/D/M/F*	Stop Codons
**NT**	**3165**	**5404**	**18.5%**	**117**	**4.4%**	**1352 (98.0%)**	**721 (52.3%)**	**9/18**	
Codon mutations:	GAG280.GG (3165A>G), TTG281GTG (3167T>G), AGT282GAA (3170A>G 3171G>A 3172T>A), ATT283ATA (3175T>A), CCC284CCA (3178C>A), AAG285GAC (3179A>G 3181G>C), GAG286TGT (3182G>T 3183A>G 3184G>T), TAT287GTG (3185T>G 3186A>T 3187T>G), CGC288GCA (3188C>G 3189G>C 3190C>A), AAG289GAG (3191A>G), TGG290CTG (3194T>C 3195G>T), TCG291CTG (3197T>C 3198C>T), CGA292AAA (3200C>A 3201G>A), CTC293CAG (3204T>A 3205C>G), TTT294TAC (3207T>A 3208T>C), GAA295GCT (3210A>C 3211A>T), GAG296GAT (3214G>T), GAA297GTG (3216A>T 3217A>G), AGA298ATG (3219G>T 3220A>G), GGC299CCA (3221G>C 3222G>C 3223C>A), AAG300CCT (3224A>C 3225A>C 3226G>T), GAC301GAG (3229C>G), GCC302TTG (3230G>T 3231C>T 3232C>G), TTA303CCA (3233T>C 3234T>C), CCT304AAG (3236C>A 3237C>A 3238T>G), CAC306CTG (3243A>T 3244C>G), CAA307CCA (3246A>C), TGG309AGG (3251T>A), TGG309_GAT310insAGGGATATT (3253_3254insAGGGATATT), AAG312AGA (3261A>G 3262G>A), ATA313ATT (3265A>T), AAC314GAG (3266A>G 3268C>G), ATT315TTG (3269A>T 3271T>G), CAG316ATG (3272C>A 3273A>T), GGG318GGT (3280G>T), AAA319TCT (3281A>T 3282A>C 3283A>T), GAG320ACG (3284G>A 3285A>C), CCT321CCG (3289T>G), CCA322CCT (3292A>T), TGG323GCA (3293T>G 3294G>C 3295G>A), GGA324CAA (3296G>C 3297G>A), CCC325GCT (3299C>G 3301C>T), CTA326CCT (3303T>C 3304A>T), TAT327TAC (3307T>C), CAA328AGA (3308C>A 3309A>G), TCT330GCT (3314T>G), GAG331CCT (3317G>C 3318A>C 3319G>T), CTA334TTG (3326C>T 3328A>G), CAG335GCT (3329C>G 3330A>C 3331G>T), ACC336GAA (3332A>G 3333C>A 3334C>A), CTA337CTG (3337A>G), CGA338AGG (3338C>A 3340A>G), GAA339AAG (3341G>A 3343A>G), TGG340CAG (3344T>C 3345G>A), AAG342AAC (3352G>C), GAG343GAA (3355G>A), AAG344TTG (3356A>T 3357A>T), CTA345CTT (3361A>T), GCC346GAT (3363C>A 3364C>T), AAA347TCA (3365A>T 3366A>C), TGG349CTG (3371T>C 3372G>T), ATA350ATT (3376A>T), CGA351CAG (3378G>A 3379A>G), CGA352CCT (3381G>C 3382A>T), TCC353TCT (3385C>T), ACC354AAG (3387C>A 3388C>G), TCA355GCT (3389T>G 3391A>T), AGT356CCC (3392A>C 3393G>C 3394T>C), GCA357TAT (3395G>T 3396C>A 3397A>T), GGA358GGT (3400A>T), ACT359GCA (3401A>G 3403T>A), CCA360CCT (3406A>T), TGC361GTT (3407T>G 3408G>T 3409C>T), ATG362TTA (3410A>T 3412G>A), TTC363TTT (3415C>T), GTT364CAG (3416G>C 3417T>A 3418T>G), CCA365AAA (3419C>A 3420C>A), GCA367CAG (3425G>C 3426C>A 3427A>G), AAC368GAT (3428A>G 3430C>T), AAA370TCA (3434A>T 3435A>C), CTA371TTA (3437C>T), CGA372AGG (3440C>A 3442A>G), CTC373ATG (3443C>A 3445C>G), GTA374TGC (3446G>T 3447T>G 3448A>C), CAA375ATG (3449C>A 3450A>T 3451A>G), GAC376GAT (3454C>T), TAC377TAT (3457C>T), CGA378CGG (3460A>G), AAG379GCG (3461A>G 3462A>C), TTG380CTG (3464T>C), GAG382AAG (3470G>A), ATC383GTC (3473A>G), ATC385GTT (3479A>G 3481C>T), AAC387AAT (3487C>T), CGA388AAG (3488C>A 3489G>A 3490A>G), TAT389TAC (3493T>C), CTA391ATC (3497C>A 3499A>C), CCC392CCA (3502C>A), AAC393CTC (3503A>C 3504A>T), ATC394ATT (3508C>T), GAA395GCT (3510A>C 3511A>T), GAA396GAT (3514A>T), GCA397CTC (3515G>C 3516C>T 3517A>C), CAA398TTT (3518C>T 3519A>T 3520A>T), GAC399GAT (3523C>T), AGA400CAA (3524A>C 3525G>A), TTA401CTT (3527T>C 3529A>T), ACC402GGC (3530A>G 3531C>G), GGA403CAA (3533G>C 3534G>A), TCA404GCA (3536T>G), GAC405CGT (3539G>C 3540A>G 3541C>T), TAC407TTC (3546A>T), ACG408ACC (3550G>C), ATT410TTA (3554A>T 3556T>A), GAC411GAT (3559C>T), CTA412CTT (3562A>T), CGA413CGG (3565A>G), GAC414TCT (3566G>T 3567A>C 3568C>T), GCC415GGA (3570C>G 3571C>A), TTC416TAC (3573T>A), GCT418CAA (3578G>C 3579C>A 3580T>A), ATC419GTG (3581A>G 3583C>G), CGA420AGA (3584C>A), ATG421ATC (3589G>C), GGA424GGG (3598A>G), GAA425GAT (3601A>T), GAA426GAG (3604A>G), TGG427CCT (3605T>C 3606G>C 3607G>T), AAA428AAG (3610A>G), ACC429ACT (3613C>T), GCT430ACT (3614G>A), TTC431TGT (3618T>G 3619C>T), AGG432GTA (3620A>G 3621G>T 3622G>A), ACA433ACT (3625A>T), AGA434AGG (3628A>G), TAC435TAT (3631C>T), GGA436GGG (3634A>G), CTC437TCA (3635C>T 3636T>C 3637C>A), TAC438TAT (3640C>T), TTG441CTT (3647T>C 3649G>T), GTC442GTA (3652C>A), CCA444CCG (3658A>G), ATG445TTT (3659A>T 3661G>T), GGA446GGG (3664A>G), ACC448ACT (3670C>T), AAC449AAT (3673C>T), GCA450GCC (3676A>C), CCC451CCG (3679C>G), GCA452GCT (3682A>T), TCC453ACC (3683T>A), TGC454TTT (3687G>T 3688C>T), CAG455TGC (3689C>T 3690A>G 3691G>C), GAC456AAT (3692G>A 3694C>T), CTT457CTA (3697T>A), GTC458ATG (3698G>A 3700C>G), AAC459AAT (3703C>T), GAA460GGC (3705A>G 3706A>C), ACA461GTA (3707A>G 3708C>T), CTT462CTG (3712T>G), AGA463TAT (3713A>T 3714G>A 3715A>T), GAC464CCC (3716G>C 3717A>C), CTA465TAC (3719C>T 3720T>A 3721A>C), CTC466CTT (3724C>T), GTG468AAT (3728G>A 3729T>A 3730G>T), TGC469TTT (3732G>T 3733C>T), GTC470GTA (3736C>A), GTT471GTA (3739T>A), GCT472GTC (3741C>T 3742T>C), TAC473TAT (3745C>T), ATG474CTA (3746A>C 3748G>A), GAC475GAT (3751C>T), ATA477ATT (3757A>T), CTG478GTC (3758C>G 3760G>C), GTC479ATA (3761G>A 3763C>A), TAC480TAT (3766C>T), ACA481AGT (3768C>G 3769A>T), AAA482CAA (3770A>C), GGA483GAT (3774G>A 3775A>T), TCC484ACG (3776T>A 3778C>G), CTC485TTG (3779C>T 3781C>G), CAG486GAT (3782C>G 3784G>T), ACC489GTT (3791A>G 3792C>T 3793C>T), AAG490AGC (3795A>G 3796G>C), CAA491CAT (3799A>T), GTT492TTG (3800G>T 3802T>G), CAA493AGT (3803C>A 3804A>G 3805A>T), GAT494TTG (3806G>T 3807A>T 3808T>G), GTG495GTA (3811G>A), TTC496TTA (3814C>A), GAA497TCT (3815G>T 3816A>C 3817A>T), CGA498CGT (3820A>T), CTC499TTA (3821C>T 3823C>A), ACG500AGG (3825C>G), AAG501CAA (3827A>C 3829G>A), TCC502TAC (3831C>A), GGA503ACT (3833G>A 3834G>C 3835A>T), TTC504CTC (3836T>C), AAG505TAT (3839A>T 3841G>T), ACA506GTT (3842A>G 3843C>T 3844A>T), GCA507AAA (3845G>A 3846C>A), CCC508ATG (3848C>A 3849C>T 3850C>G), AAA510AAG (3856A>G), TGC511TGT (3859C>T), GAA512GAG (3862A>G), TTC513TTT (3865C>T), CAC514GCC (3866C>G 3867A>C), AAG515CAA (3869A>C 3871G>A), GAA517GAG (3877A>G), GTC518ATA (3878G>A 3880C>A), TTT520TTC (3886T>C), TTA521CTA (3887T>C), GGC522GGG (3892C>G), TTT523CAT (3893T>C 3894T>A), ATC524CTA (3896A>C 3898C>A), ATC525GTT (3899A>G 3901C>T), ACA527GAA (3905A>G 3906C>A), ACA528AAC (3909C>A 3910A>C), GGG529CAA (3911G>C 3912G>A 3913G>A), ATA530GTA (3914A>G), ACG531AGG (3918C>G), ATC532ATG (3922C>G), GAC533GAT (3925C>T), GCA535AAG (3929G>A 3930C>A 3931A>G), AAG536AAA (3934G>A), ACA537GTG (3935A>G 3936C>T 3937A>G), CAG538CAA (3940G>A), TCA539GCC (3941T>G 3943A>C), ATC540ATT (3946C>T), AGA541GTT (3947A>G 3948G>T 3949A>T), GAA542GAT (3952A>T), CCA544CAA (3957C>A), GAA545GCA (3960A>C), CCG546CCC (3964G>C), AAG547AAA (3967G>A), ACA548ACT (3970A>T), GTC549GTG (3973C>G), GTA552CTG (3980G>C 3982A>G), CAG553AGG (3983C>A 3984A>G), TCA554TCT (3988A>T), CTT556TTG (3992C>T 3994T>G), GGA557GGG (3997A>G), CTC558CTA (4000C>A), GCC559GCT (4003C>T), AAC562TAT (4010A>T 4012C>T), AAG567GCT (4025A>G 4026A>C 4027G>T), GAC568GGA (4029A>G 4030C>A), TAT569TAC (4033T>C), ACA572AAA (4041C>A), GCA574GCT (4048A>T), CCA575GCA (4049C>G), ATG576CTG (4052A>C), ACG577ACT (4057G>T), ATG578GAT (4058A>G 4059T>A 4060G>T), CTT579TTG (4061C>T 4063T>G), ACA580CTG (4064A>C 4065C>T 4066A>G), AGA581AAG (4068G>A 4069A>G), AAA582AAG (4072A>G), GAC583GAA (4075C>A), AAC585AAG (4081C>G), AAA587GTA (4085A>G 4086A>T), GGA589ACC (4091G>A 4092G>C 4093A>C), AAA590GAA (4094A>G), GAA591GAG (4099A>G), CAG592TGT (4100C>T 4101A>G 4102G>T), ACC593GAG (4103A>G 4104C>A 4105C>G), GAA594GCG (4107A>C 4108A>G), GCG595AGC (4109G>A 4110C>G 4111G>C), TTC596TTT (4114C>T), AAA597CAA (4115A>C), AGA598AAG (4119G>A 4120A>G), GAA601GAC (4129A>C), CAG602TTA (4130C>T 4131A>T 4132G>A), TGC603TTG (4134G>T 4135C>G), GCT604ACT (4136G>A), CCA607CCT (4147A>T), ACG608ATT (4149C>T 4150G>T), CTT609CTC (4153T>C), CGA610GCA (4154C>G 4155G>C), CTA611TTA (4157C>T), TTC612CCC (4160T>C 4161T>C), GAT613GAG (4165T>G), GGT614GAG (4167G>A 4168T>G), AGC615CCT (4169A>C 4170G>C 4171C>T), AAG616TTT (4172A>T 4173A>T 4174G>T), GAA617GAG (4177A>G), GTC618GTA (4180C>A), ATC620GTG (4184A>G 4186C>G), GAG621CAG (4187G>C), ACC622ACT (4192C>T), GAC623GAT (4195C>T), TCT625TCG (4201T>G), GAT626GAC (4204T>C), ATG627AAA (4206T>A 4207G>A), GCA628GCT (4210A>T), ATA629GTG (4211A>G 4213A>G), GGC630GGA (4216C>A), GCA631GGC (4218C>G 4219A>C), TGT632GTA (4220T>G 4221G>T 4222T>A), CTA633TTG (4223C>T 4225A>G), ACA634GTG (4226A>G 4227C>T 4228A>G), CAG635CAA (4231G>A), ACA636_CAC637del (4232_4237delACACAC), GAT638GAA (4240T>A), GGG639GGT (4243G>T), AAA640_AGA641del (4244_4249delAAAAGA), CAC642CAT (4252C>T), CCA643CCG (4255A>G), TAT646TTT (4263A>T), TAT647GAA (4265T>G 4267T>A), TCC648AGC (4268T>A 4269C>G), CGG649AGG (4271C>A), ATG651TTA (4277A>T 4279G>A), ACC652AAT (4281C>A 4282C>T), ACA653GAT (4283A>G 4284C>A 4285A>T), GCG654GCT (4288G>T), GAA655GAG (4291A>G), AAC657AGG (4296A>G 4297C>G), GAC659TCT (4301G>T 4302A>C 4303C>T), ATC660ACT (4305T>C 4306C>T), GAC662GAA (4312C>A), CTT665ATG (4319C>A 4321T>G), CTA666GTT (4322C>G 4324A>T), GCC667GCA (4327C>A), ATT668GTG (4328A>G 4330T>G), GTT669GTG (4333T>G), GCC670CCC (4334G>C), AAA866.GT (4923A>G 4924A>T), GAC869GAG (4933C>G), GAG870AGT (4934G>A 4935A>G 4936G>T), ACG872ACT (4942G>T), ATT876ATA (4954T>A), ACG877ACT (4957G>T), AAA878TGC (4958A>T 4959A>G 4960A>C), CTC879CTG (4963C>G), CCG880CCA (4966G>A), AGG881AAG (4968G>A), TCA882TCC (4972A>C), AAG883AAC (4975G>C), GAT884AAG (4976G>A 4978T>G), CAA984TGC (5276C>T 5277A>G 5278A>C), CTA986CTT (5284A>T), CGG987AGG (5285C>A), TAC989TTC (5292A>T), ATC990GTG (5294A>G 5296C>G), AAC991AGC (5298A>G), TAC992TCT (5301A>C 5302C>T), GCA993CAG (5303G>C 5304C>A 5305A>G), GAC995AAG (5309G>A 5311C>G), AAC996GAT (5312A>G 5314C>T), GTT998GCT (5319T>C), TCA999AAG (5321T>A 5322C>A 5323A>G), TTA1000TTG (5326A>G), CTG1001CTA (5329G>A), CCA1002GAT (5330C>G 5331C>A 5332A>T), ATG1003TTG (5333A>T), GCG1004GCA (5338G>A), CAG1005GAA (5339C>G 5341G>A), ATC1006TTC (5342A>T), GCA1007CCA (5345G>C), CTG1008TAT (5348C>T 5349T>A 5350G>T), AAC1010TTA (5354A>T 5355A>T 5356C>A), CAC1011CAG (5359C>G), AAA1012TGG (5360A>T 5361A>G 5362A>G), TCA1013AGT (5363T>A 5364C>G 5365A>T), GAG1014GAA (5368G>A), ACA1015GTC (5369A>G 5370C>T 5371A>C), ACT1016ACC (5374T>C), TCG1017GGA (5375T>G 5376C>G 5377G>A), ACG1018AGG (5379C>G), ACG1019ACA (5383G>A), ATG1022GAG (5390A>G 5391T>A), CGA1023_ACT1024del (5393_5398delCGAACT), GCA1026GCC (5404A>C)								

*: Inserts / Deletes / Misaligned / Frameshifts

Analysis details

This analysis was performed with panviral2.64

NGS Details (UN62): Bracoviriform congregatae (segment Circle 7)

Assembly

**Coverage Length**	143 (1 contig(s))
**Depth Of Coverage**	3.1
**Number Of Reads**	5
**Reads Per Million**	0.12 rpm (after QC)
**Ambiguities**	0
**Assembly Method**	de novo + reference guided assembly
**Consensus Caller**	Bcf Tools

Coverage Map

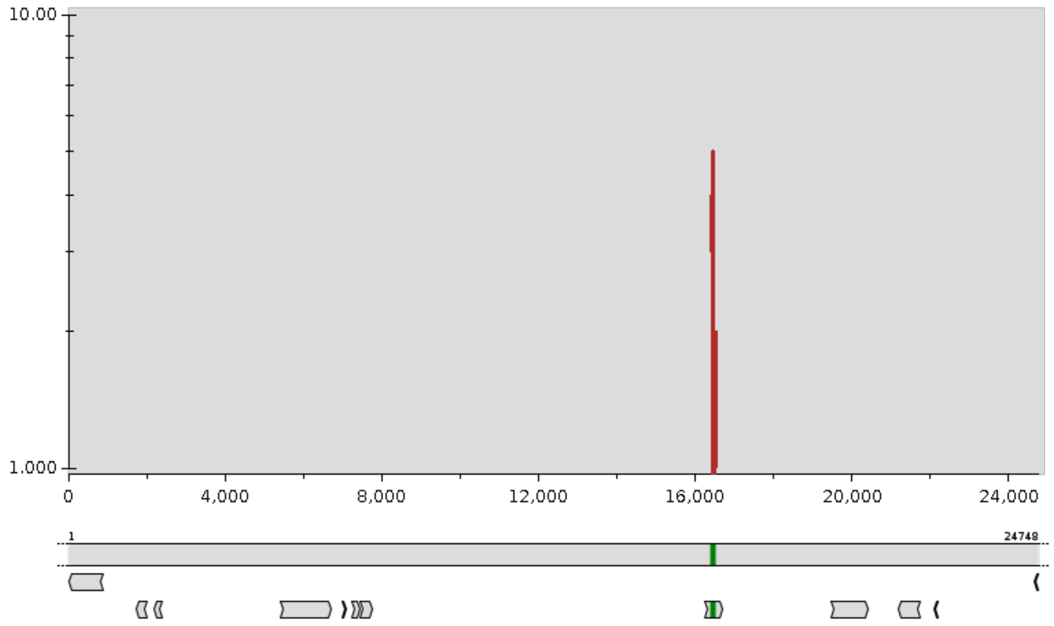

Assignment

**Type**	Bracoviriform congregatae (Taxonomy ID: 39640)
**Reference Genome**	NC_006639.1
**NT Identity (%)**	75.5245
**AA Identity (%)**	85.4167
**Number Of Stop Codons**	0
**Number Of CDS**	5

Alignment

**Alignment Score**	146.0 (NT) + 284.0 (AA) = 430.0
**Concordance (%)**	72.3906

Alignment Method	Global, seeded, nucleotide + amino acids (AGA)

Genome Region

Sequence starts at position 16402 and ends at position 16544 relative to NC_006639.1 reference sequence.

Alignment Detailed Statistics

	Begin	End	Coverage	Score	Concordance	Matches	Identities	I/D/M/F*	Stop Codons
**NT**	**16402**	**16544**	**0.6%**	**146**	**51.0%**	**143 (100%)**	**108 (75.5%)**	**0/0**	
Mutations:	16402A>C, 16408A>T, 16411G>C, 16414A>G, 16417A>C, 16418T>C, 16420G>C, 16423A>C, 16426A>G, 16429G>C, 16432C>A, 16435T>C, 16444T>C, 16449G>A, 16450A>G, 16451T>A, 16452C>T, 16459A>C, 16462T>C, 16471A>G, 16474A>T, 16486A>C, 16489T>C, 16495T>C, 16501G>C, 16504T>C, 16516A>T, 16526G>A, 16531T>A, 16533G>C, 16534A>C, 16535T>A, 16538G>A, 16540C>T, 16543T>C								
**CDS**									
Histone	58	105	30.8%	284	88.8%	48 (100%)	41 (85.4%)	0/0/0/0	0
Protein mutations:	R73K (16449G>A 16450A>G), S74I (16451T>A 16452C>T), V99I (16526G>A), G101A (16533G>C 16534A>C), L102M (16535T>A), V103I (16538G>A 16540C>T)								
Codon mutations:	GTA57..C (16402A>C), GGA59GGT (16408A>T), GGG60GGC (16411G>C), AAA61AAG (16414A>G), GGA62GGC (16417A>C), TTG63CTC (16418T>C 16420G>C), GGA64GGC (16423A>C), AAA65AAG (16426A>G), GGG66GGC (16429G>C), GGC67GGA (16432C>A), GCT68GCC (16435T>C), CAT71CAC (16444T>C), AGA73AAG (16449G>A 16450A>G), TCT74ATT (16451T>A 16452C>T), CGA76CGC (16459A>C), GAT77GAC (16462T>C), CAA80CAG (16471A>G), GGA81GGT (16474A>T), CCA85CCC (16486A>C), GCT86GCC (16489T>C), CGT88CGC (16495T>C), CTG90CTC (16501G>C), GCT91GCC (16504T>C), GGA95GGT (16516A>T), GTC99ATC (16526G>A), TCT100TCA (16531T>A), GGA101GCC (16533G>C 16534A>C), TTG102ATG (16535T>A), GTC103ATT (16538G>A 16540C>T), TAT104TAC (16543T>C)								
**Proteins**									
hypothetical protein (YP_184795.1)	58	105	30.8%	284	88.8%	48 (100%)	41 (85.4%)	0/0/0/0	0
Protein mutations:	R73K (16449G>A 16450A>G), S74I (16451T>A 16452C>T), V99I (16526G>A), G101A (16533G>C 16534A>C), L102M (16535T>A), V103I (16538G>A 16540C>T)								
Codon mutations:	GTA57..C (16402A>C), GGA59GGT (16408A>T), GGG60GGC (16411G>C), AAA61AAG (16414A>G), GGA62GGC (16417A>C), TTG63CTC (16418T>C 16420G>C), GGA64GGC (16423A>C), AAA65AAG (16426A>G), GGG66GGC (16429G>C), GGC67GGA (16432C>A), GCT68GCC (16435T>C), CAT71CAC (16444T>C), AGA73AAG (16449G>A 16450A>G), TCT74ATT (16451T>A 16452C>T), CGA76CGC (16459A>C), GAT77GAC (16462T>C), CAA80CAG (16471A>G), GGA81GGT (16474A>T), CCA85CCC (16486A>C), GCT86GCC (16489T>C), CGT88CGC (16495T>C), CTG90CTC (16501G>C), GCT91GCC (16504T>C), GGA95GGT (16516A>T), GTC99ATC (16526G>A), TCT100TCA (16531T>A), GGA101GCC (16533G>C 16534A>C), TTG102ATG (16535T>A), GTC103ATT (16538G>A 16540C>T), TAT104TAC (16543T>C)								

*: Inserts / Deletes / Misaligned / Frameshifts

Analysis details

This analysis was performed with panviral2.64

NGS Details (UN62): Bracoviriform congregatae (segment Circle 7)

Assembly

**Coverage Length**	112 (1 contig(s))
**Depth Of Coverage**	198.1
**Number Of Reads**	277
**Reads Per Million**	6.42 rpm (after QC)
**Ambiguities**	0
**Assembly Method**	de novo + reference guided assembly
**Consensus Caller**	Bcf Tools

Coverage Map

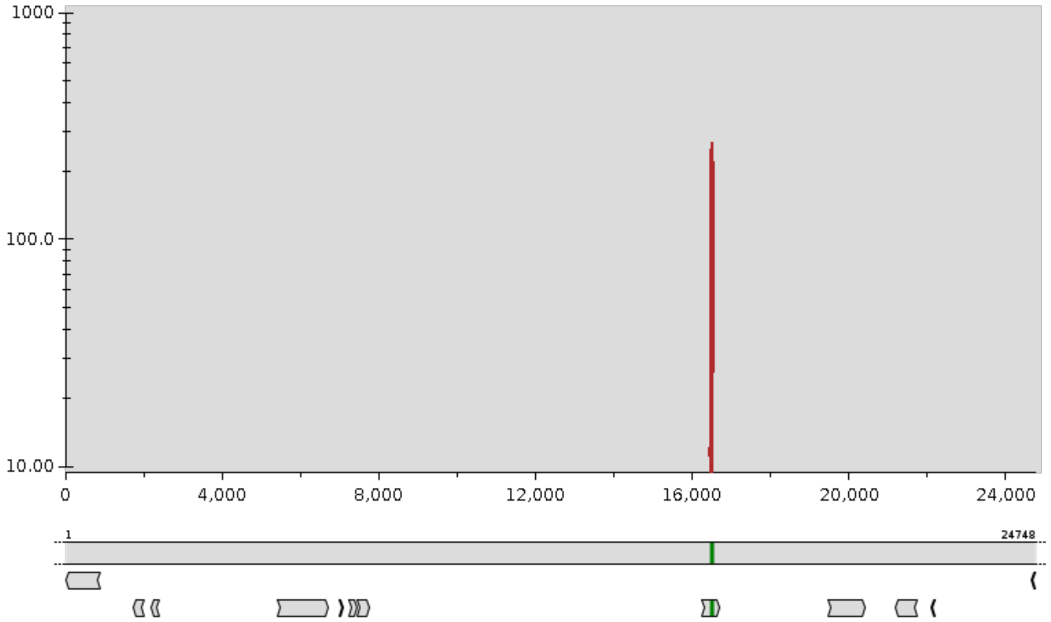

Assignment

**Type**	Bracoviriform congregatae (Taxonomy ID: 39640)
**Reference Genome**	NC_006639.1
**NT Identity (%)**	78.5714
**AA Identity (%)**	86.8421
**Number Of Stop Codons**	0
**Number Of CDS**	5

Alignment

**Alignment Score**	128.0 (NT) + 221.0 (AA) = 349.0
**Concordance (%)**	75.378

Alignment Method	Global, seeded, nucleotide + amino acids (AGA)

Genome Region

Sequence starts at position 16454 and ends at position 16565 relative to NC_006639.1 reference sequence.

Alignment Detailed Statistics

	Begin	End	Coverage	Score	Concordance	Matches	Identities	I/D/M/F*	Stop Codons
**NT**	**16454**	**16565**	**0.5%**	**128**	**57.1%**	**112 (100%)**	**88 (78.6%)**	**0/0**	
Mutations:	16468C>T, 16474A>T, 16480C>A, 16486A>G, 16492C>T, 16493C>A, 16495T>G, 16499C>T, 16508C>A, 16510T>A, 16513T>A, 16519C>G, 16526G>A, 16528C>T, 16529T>A, 16530C>G, 16534A>T, 16535T>C, 16537G>T, 16538G>A, 16546A>G, 16551T>C, 16557A>G, 16558T>G								
**CDS**									
Histone	75	112	24.4%	221	90.9%	38 (100%)	33 (86.8%)	0/0/0/0	0
Protein mutations:	V99I (16526G>A 16528C>T), V103I (16538G>A), I107T (16551T>C), D109G (16557A>G 16558T>G)								
Codon mutations:	ATC79ATT (16468C>T), GGA81GGT (16474A>T), ACC83ACA (16480C>A), CCA85CCG (16486A>G), ATC87ATT (16492C>T), CGT88AGG (16493C>A 16495T>G), CTG90TTG (16499C>T), CGT93AGA (16508C>A 16510T>A), GGT94GGA (16513T>A), GTC96GTG (16519C>G), GTC99ATT (16526G>A 16528C>T), TCT100AGT (16529T>A 16530C>G), GGA101GGT (16534A>T), TTG102CTT (16535T>C 16537G>T), GTC103ATC (16538G>A), GAA105GAG (16546A>G), ATT107ACT (16551T>C), GAT109GGG (16557A>G 16558T>G)								
**Proteins**									
hypothetical protein (YP_184795.1)	75	112	24.4%	221	90.9%	38 (100%)	33 (86.8%)	0/0/0/0	0
Protein mutations:	V99I (16526G>A 16528C>T), V103I (16538G>A), I107T (16551T>C), D109G (16557A>G 16558T>G)								
Codon mutations:	ATC79ATT (16468C>T), GGA81GGT (16474A>T), ACC83ACA (16480C>A), CCA85CCG (16486A>G), ATC87ATT (16492C>T), CGT88AGG (16493C>A 16495T>G), CTG90TTG (16499C>T), CGT93AGA (16508C>A 16510T>A), GGT94GGA (16513T>A), GTC96GTG (16519C>G), GTC99ATT (16526G>A 16528C>T), TCT100AGT (16529T>A 16530C>G), GGA101GGT (16534A>T), TTG102CTT (16535T>C 16537G>T), GTC103ATC (16538G>A), GAA105GAG (16546A>G), ATT107ACT (16551T>C), GAT109GGG (16557A>G 16558T>G)								

*: Inserts / Deletes / Misaligned / Frameshifts

Analysis details

This analysis was performed with panviral2.64

NGS Details (UN62): Bracoviriform congregatae (segment Circle 7)

Assembly

**Coverage Length**	180 (1 contig(s))
**Depth Of Coverage**	206.7
**Number Of Reads**	408
**Reads Per Million**	9.46 rpm (after QC)
**Ambiguities**	0
**Assembly Method**	de novo + reference guided assembly
**Consensus Caller**	Bcf Tools

Coverage Map

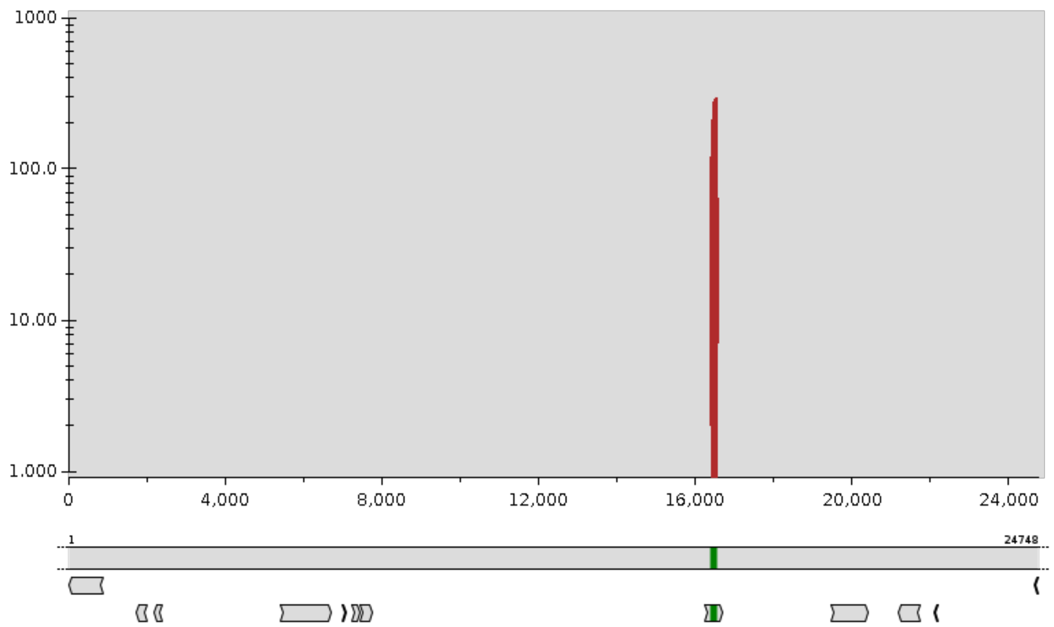

Assignment

**Type**	Bracoviriform congregatae (Taxonomy ID: 39640)
**Reference Genome**	NC_006639.1
**NT Identity (%)**	78.8889
**AA Identity (%)**	90.0
**Number Of Stop Codons**	0
**Number Of CDS**	5

Alignment

**Alignment Score**	208.0 (NT) + 360.0 (AA) = 568.0
**Concordance (%)**	75.7333

Alignment Method	Global, seeded, nucleotide + amino acids (AGA)

Genome Region

Sequence starts at position 16394 and ends at position 16573 relative to NC_006639.1 reference sequence.

Alignment Detailed Statistics

	Begin	End	Coverage	Score	Concordance	Matches	Identities	I/D/M/F*	Stop Codons
**NT**	**16394**	**16573**	**0.7%**	**208**	**57.8%**	**180 (100%)**	**142 (78.9%)**	**0/0**	
Mutations:	16405A>G, 16411G>A, 16414A>G, 16417A>G, 16426A>G, 16429G>A, 16432C>A, 16444T>C, 16447C>T, 16449G>A, 16450A>G, 16451T>G, 16452C>T, 16456T>C, 16459A>C, 16471A>G, 16486A>T, 16492C>T, 16493C>A, 16495T>G, 16504T>G, 16508C>A, 16510T>A, 16513T>A, 16519C>G, 16526G>A, 16528C>T, 16529T>A, 16530C>G, 16534A>T, 16535T>C, 16537G>T, 16538G>A, 16546A>G, 16551T>C, 16557A>G, 16558T>G, 16562T>C								
**CDS**									
Histone	55	114	38.5%	360	92.3%	60 (100%)	54 (90.0%)	0/0/0/0	0
Protein mutations:	R73K (16449G>A 16450A>G), S74V (16451T>G 16452C>T), V99I (16526G>A 16528C>T), V103I (16538G>A), I107T (16551T>C), D109G (16557A>G 16558T>G)								
Codon mutations:	AAA58AAG (16405A>G), GGG60GGA (16411G>A), AAA61AAG (16414A>G), GGA62GGG (16417A>G), AAA65AAG (16426A>G), GGG66GGA (16429G>A), GGC67GGA (16432C>A), CAT71CAC (16444T>C), CGC72CGT (16447C>T), AGA73AAG (16449G>A 16450A>G), TCT74GTT (16451T>G 16452C>T), CTT75CTC (16456T>C), CGA76CGC (16459A>C), CAA80CAG (16471A>G), CCA85CCT (16486A>T), ATC87ATT (16492C>T), CGT88AGG (16493C>A 16495T>G), GCT91GCG (16504T>G), CGT93AGA (16508C>A 16510T>A), GGT94GGA (16513T>A), GTC96GTG (16519C>G), GTC99ATT (16526G>A 16528C>T), TCT100AGT (16529T>A 16530C>G), GGA101GGT (16534A>T), TTG102CTT (16535T>C 16537G>T), GTC103ATC (16538G>A), GAA105GAG (16546A>G), ATT107ACT (16551T>C), GAT109GGG (16557A>G 16558T>G), TTG111CTG (16562T>C)								
**Proteins**									
hypothetical protein (YP_184795.1)	55	114	38.5%	360	92.3%	60 (100%)	54 (90.0%)	0/0/0/0	0
Protein mutations:	R73K (16449G>A 16450A>G), S74V (16451T>G 16452C>T), V99I (16526G>A 16528C>T), V103I (16538G>A), I107T (16551T>C), D109G (16557A>G 16558T>G)								
Codon mutations:	AAA58AAG (16405A>G), GGG60GGA (16411G>A), AAA61AAG (16414A>G), GGA62GGG (16417A>G), AAA65AAG (16426A>G), GGG66GGA (16429G>A), GGC67GGA (16432C>A), CAT71CAC (16444T>C), CGC72CGT (16447C>T), AGA73AAG (16449G>A 16450A>G), TCT74GTT (16451T>G 16452C>T), CTT75CTC (16456T>C), CGA76CGC (16459A>C), CAA80CAG (16471A>G), CCA85CCT (16486A>T), ATC87ATT (16492C>T), CGT88AGG (16493C>A 16495T>G), GCT91GCG (16504T>G), CGT93AGA (16508C>A 16510T>A), GGT94GGA (16513T>A), GTC96GTG (16519C>G), GTC99ATT (16526G>A 16528C>T), TCT100AGT (16529T>A 16530C>G), GGA101GGT (16534A>T), TTG102CTT (16535T>C 16537G>T), GTC103ATC (16538G>A), GAA105GAG (16546A>G), ATT107ACT (16551T>C), GAT109GGG (16557A>G 16558T>G), TTG111CTG (16562T>C)								

*: Inserts / Deletes / Misaligned / Frameshifts

Analysis details

This analysis was performed with panviral2.64

NGS Details (UN62): Cassava brown streak virus

Assembly

**Coverage Length**	526 (1 contig(s))
**Depth Of Coverage**	85.7
**Number Of Reads**	391
**Reads Per Million**	9.06 rpm (after QC)
**Ambiguities**	0
**Assembly Method**	de novo + reference guided assembly
**Consensus Caller**	Bcf Tools

Coverage Map

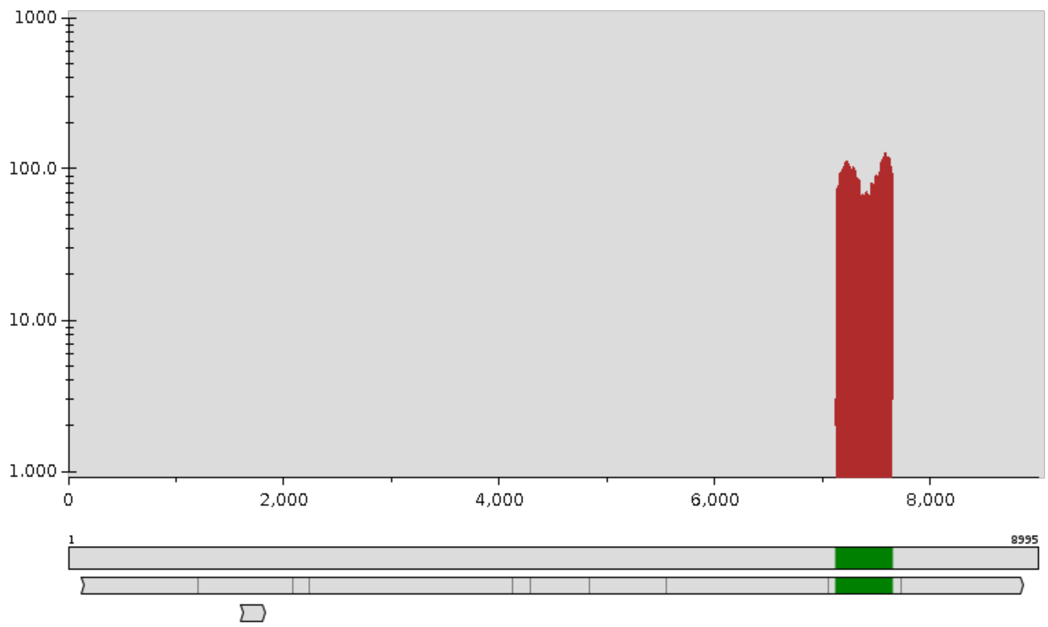

Assignment

**Type**	Cassava brown streak virus (Taxonomy ID: 137758)
**Reference Genome**	NC_012698.2
**NT Identity (%)**	60.8365
**AA Identity (%)**	57.7143
**Number Of Stop Codons**	0
**Number Of CDS**	2

Alignment

**Alignment Score**	228.0 (NT) + 738.0 (AA) = 966.0
**Concordance (%)**	42.5738

Alignment Method	Global, seeded, nucleotide + amino acids (AGA)

Genome Region

Sequence starts at position 7123 and ends at position 7648 relative to NC_012698.2 reference sequence.

Alignment Detailed Statistics

	Begin	End	Coverage	Score	Concordance	Matches	Identities	I/D/M/F*	Stop Codons
**NT**	**7123**	**7648**	**5.8%**	**228**	**21.7%**	**526 (100%)**	**320 (60.8%)**	**0/0**	
Mutations:	7130A>G, 7133A>G, 7134A>G, 7136T>G, 7142C>T, 7145A>T, 7148A>C, 7151G>A, 7157A>T, 7158C>A, 7163A>G, 7167A>G, 7175A>T, 7177A>G, 7178A>G, 7179C>G, 7180A>C, 7184A>T, 7185T>C, 7190T>C, 7192C>A, 7193C>G, 7194A>T, 7202A>C, 7203A>T, 7205C>T, 7206A>C, 7207T>A, 7208C>G, 7211T>C, 7213G>T, 7214G>T, 7217A>G, 7218G>C, 7229A>T, 7234C>T, 7235A>T, 7241A>G, 7242A>G, 7243C>A, 7244A>G, 7245G>C, 7246T>C, 7250G>A, 7253A>T, 7256T>A, 7257A>T, 7258T>C, 7270T>C, 7271A>T, 7272C>A, 7273G>A, 7274T>A, 7275G>A, 7277A>T, 7280G>T, 7281T>G, 7283T>C, 7284G>A, 7286G>A, 7287T>G, 7288T>A, 7290A>G, 7292T>G, 7293G>A, 7294G>A, 7295A>T, 7298G>A, 7304T>G, 7307C>A, 7310A>T, 7313A>G, 7319A>T, 7320A>T, 7323T>C, 7325G>C, 7331T>C, 7332G>A, 7334T>C, 7337T>C, 7340C>T, 7343T>G, 7355A>G, 7358A>T, 7361C>T, 7364T>C, 7374A>T, 7377G>C, 7380G>A, 7381G>A, 7385T>C, 7388A>T, 7390T>A, 7391A>T, 7397A>T, 7398T>C, 7400A>T, 7401T>A, 7403T>C, 7406G>C, 7409G>A, 7410G>T, 7413G>A, 7414A>T, 7416C>G, 7418G>T, 7422C>G, 7424A>G, 7425A>G, 7432T>C, 7433G>A, 7436T>A, 7437A>T, 7438G>A, 7442T>C, 7443C>A, 7445C>G, 7448C>T, 7449G>A, 7451G>C, 7455G>T, 7457T>A, 7458T>C, 7462T>C, 7463A>T, 7464A>C, 7465A>T, 7467A>G, 7468A>G, 7469A>G, 7470G>C, 7471T>C, 7472T>A, 7473G>A, 7474G>A, 7476G>A, 7477A>C, 7481T>G, 7484T>G, 7487A>G, 7489T>C, 7490C>T, 7494A>G, 7495A>T, 7496G>T, 7499T>A, 7500G>A, 7501T>A, 7502G>A, 7503C>A, 7504T>C, 7505A>G, 7506A>C, 7507G>T, 7508A>G, 7511T>A, 7512G>A, 7517T>A, 7520A>G, 7521A>C, 7522T>C, 7523G>A, 7524C>G, 7526A>T, 7527C>A, 7529C>G, 7535A>C, 7539T>A, 7540C>A, 7541A>T, 7544T>C, 7547G>A, 7553C>T, 7556A>G, 7559T>A, 7562C>T, 7568A>T, 7570T>A, 7572A>G, 7573A>G, 7576G>A, 7577G>T, 7578A>G, 7579G>A, 7580A>C, 7581A>C, 7586A>T, 7588T>A, 7589T>C, 7599A>C, 7600T>C, 7601G>C, 7602G>A, 7603C>A, 7604C>G, 7607G>A, 7610G>A, 7616T>C, 7618T>A, 7622A>T, 7628T>C, 7631A>C, 7632T>G, 7633T>G, 7635C>A, 7636G>A								

CDS

CBSV_gp1	2334	2508	6.0%	738	60.1%	175 (100%)	101 (57.7%)	0/0/0/0	0
Protein mutations:	I2337V (7134A>G 7136T>G), Q2345K (7158C>A), K2348E (7167A>G), K2351R (7177A>G 7178A>G), Q2352A (7179C>G 7180A>C), F2354L (7185T>C), P2356Q (7192C>A 7193C>G), T2357S (7194A>T), I2360F (7203A>T 7205C>T), I2361Q (7206A>C 7207T>A 7208C>G), R2363L (7213G>T 7214G>T), V2365L (7218G>C), P2370L (7234C>T 7235A>T), T2373E (7242A>G 7243C>A 7244A>G), V2374P (7245G>C 7246T>C), E2376D (7253A>T), I2378S (7257A>T 7258T>C), V2382A (7270T>C 7271A>T), R2383K (7272C>A 7273G>A 7274T>A), V2384I (7275G>A 7277A>T), S2386A (7281T>G 7283T>C), E2387K (7284G>A 7286G>A), L2388E (7287T>G 7288T>A), I2389V (7290A>G 7292T>G), G2390N (7293G>A 7294G>A 7295A>T), S2399C (7320A>T), D2403N (7332G>A 7334T>C), N2406K (7343T>G), M2417L (7374A>T), E2418Q (7377G>C), G2419K (7380G>A 7381G>A), L2422H (7390T>A 7391A>T), Y2426N (7401T>A 7403T>C), K2427N (7406G>C), V2429L (7410G>T), E2430M (7413G>A 7414A>T), P2431A (7416C>G 7418G>T), Q2433E (7422C>G 7424A>G), N2434D (7425A>G), M2436T (7432T>C 7433G>A), S2438Y (7437A>T 7438G>A), L2440M (7443C>A 7445C>G), V2442I (7449G>A 7451G>C), A2444S (7455G>T 7457T>A), F2445L (7458T>C), V2446A (7462T>C 7463A>T), N2447L (7464A>C 7465A>T), K2448G (7467A>G 7468A>G 7469A>G), V2449P (7470G>C 7471T>C 7472T>A), G2450N (7473G>A 7474G>A), D2451T (7476G>A 7477A>C), D2452E (7481T>G), I2454M (7487A>G), I2455T (7489T>C 7490C>T), K2457V (7494A>G 7495A>T 7496G>T), V2459K (7500G>A 7501T>A 7502G>A), L2460T (7503C>A 7504T>C 7505A>G), R2461L (7506A>C 7507G>T 7508A>G), E2463K (7512G>A), M2466P (7521A>C 7522T>C 7523G>A), P2467A (7524C>G 7526A>T), S2472N (7539T>A 7540C>A 7541A>T), L2482H (7570T>A), N2483G (7572A>G 7573A>G), W2484Y (7576G>A 7577G>T), R2485D (7578A>G 7579G>A 7580A>C), K2486Q (7581A>C), F2488Y (7588T>A 7589T>C), M2492P (7599A>C 7600T>C 7601G>C), A2493K (7602G>A 7603C>A 7604C>G), M2498K (7618T>A), F2503G (7632T>G 7633T>G), R2504K (7635C>A 7636G>A)								
Codon mutations:	GCA2335GCG (7130A>G), CCA2336CCG (7133A>G), ATT2337GTG (7134A>G 7136T>G), TTC2339TTT (7142C>T), GTA2340GTT (7145A>T), ACA2341ACC (7148A>C), GGG2342GGA (7151G>A), GCA2344GCT (7157A>T), CAA2345AAA (7158C>A), AAA2346AAG (7163A>G), AAG2348GAG (7167A>G), GTA2350GTT (7175A>T), AAA2351AGG (7177A>G 7178A>G), CAA2352GCA (7179C>G 7180A>C), ATA2353ATT (7184A>T), TTT2354CTT (7185T>C), GGT2355GGC (7190T>C), CCC2356CAG (7192C>A 7193C>G), ACT2357TCT (7194A>T), CCA2359CCC (7202A>C), ATC2360TTT (7203A>T 7205C>T), ATC2361CAG (7206A>C 7207T>A 7208C>G), TCT2362TCC (7211T>C), CGG2363CTT (7213G>T 7214G>T), AAA2364AAG (7217A>G), GTT2365CTT (7218G>C), CCA2368CCT (7229A>T), CCA2370CTT (7234C>T 7235A>T), GGA2372GGG (7241A>G), ACA2373GAG (7242A>G 7243C>A 7244A>G), GTT2374CCT (7245G>C 7246T>C), GAG2375GAA (7250G>A), GAA2376GAT (7253A>T), ATT2377ATA (7256T>A), ATC2378TCC (7257A>T 7258T>C), GTA2382GCT (7270T>C 7271A>T), CGT2383AAA (7272C>A 7273G>A 7274T>A), GTA2384ATT (7275G>A 7277A>T), GCG2385GCT (7280G>T), TCT2386GCC (7281T>G 7283T>C), GAG2387AAA (7284G>A 7286G>A), TTG2388GAG (7287T>G 7288T>A), ATT2389GTG (7290A>G 7292T>G), GGA2390AAT (7293G>A 7294G>A 7295A>T), GGG2391GGA (7298G>A), GTT2393GTG (7304T>G), CTC2394CTA (7307C>A), GTA2395GTT (7310A>T), GAA2396GAG (7313A>G), ACA2398ACT (7319A>T), AGT2399TGT (7320A>T), TTG2400CTC (7323T>C 7325G>C), TTT2402TTC (7331T>C), GAT2403AAC (7332G>A 7334T>C), GCT2404GCC (7337T>C), CTC2405CTT (7340C>T), AAT2406AAG (7343T>G), GGA2410GGG (7355A>G), CCA2411CCT (7358A>T), TAC2412TAT (7361C>T), ATT2413ATC (7364T>C), ATG2417TTG (7374A>T), GAA2418CAA (7377G>C), GGG2419AAG (7380G>A 7381G>A), ATT2420ATC (7385T>C), GGA2421GGT (7388A>T), CTA2422CAT (7390T>A 7391A>T), GGA2424GGT (7397A>T), TTA2425CTT (7398T>C 7400A>T), TAT2426AAC (7401T>A 7403T>C), AAG2427AAC (7406G>C), TTG2428TTA (7409G>A), GTG2429TTG (7410G>T), GAG2430ATG (7413G>A 7414A>T), CCG2431GCT (7416C>G 7418G>T), CAA2433GAG (7422C>G 7424A>G), AAT2434GAT (7425A>G), ATG2436ACA (7432T>C 7433G>A), GCT2437GCA (7436T>A), AGT2438TAT (7437A>T 7438G>A), GCT2439GCC (7442T>C), CTC2440ATG (7443C>A 7445C>G), TGC2441TGT (7448C>T), GTG2442ATC (7449G>A 7451G>C), GCT2444TCA (7455G>T 7457T>A), TTT2445CTT (7458T>C), GTA2446GCT (7462T>C 7463A>T), AAT2447CTT (7464A>C 7465A>T), AAA2448GGG (7467A>G 7468A>G 7469A>G), GTT2449CCA (7470G>C 7471T>C 7472T>A), GGT2450AAT (7473G>A 7474G>A), GAT2451ACT (7476G>A 7477A>C), GAT2452GAG (7481T>G), CCT2453CCG (7484T>G), ATA2454ATG (7487A>G), ATC2455ACT (7489T>C 7490C>T), AAG2457GTT (7494A>G 7495A>T 7496G>T), GGT2458GGA (7499T>A), GTG2459AAA (7500G>A 7501T>A 7502G>A), CTA2460ACG (7503C>A 7504T>C 7505A>G), AGA2461CTG (7506A>C 7507G>T 7508A>G), GGT2462GGA (7511T>A), GAG2463AAG (7512G>A), ATT2464ATA (7517T>A), GTA2465GTG (7520A>G), ATG2466CCA (7521A>C 7522T>C 7523G>A), CCA2467GCT (7524C>G 7526A>T), CGC2468AGG (7527C>A 7529C>G), CCA2470CCC (7535A>C), TCA2472AAT (7539T>A 7540C>A 7541A>T), TTT2473TTC (7544T>C), GGG2474GGA (7547G>A), GAC2476GAT (7553C>T), CCA2477CCG (7556A>G), ATT2478ATA (7559T>A), TTC2479TTT (7562C>T), CCA2481CCT (7568A>T), CTT2482CAT (7570T>A), AAC2483GGC (7572A>G 7573A>G), TGG2484TAT (7576G>A 7577G>T), AGA2485GAC (7578A>G 7579G>A 7580A>C), AAG2486CAG (7581A>C), ACA2487ACT (7586A>T), TTT2488TAC (7588T>A 7589T>C), ATG2492CCC (7599A>C 7600T>C 7601G>C), GCC2493AAG (7602G>A 7603C>A 7604C>G), GAG2494GAA (7607G>A), GAG2495GAA (7610G>A), AAT2497AAC (7616T>C), ATG2498AAG (7618T>A), ATA2499ATT (7622A>T), CAT2501CAC (7628T>C), CGA2502CGC (7631A>C), TTT2503GGT (7632T>G 7633T>G), CGA2504AAA (7635C>A 7636G>A)								

Proteins

polyprotein (YP_007027011.1)	2334	2508	6.0%	738	60.1%	175 (100%)	101 (57.7%)	0/0/0/0	0
Protein mutations:	I2337V (7134A>G 7136T>G), Q2345K (7158C>A), K2348E (7167A>G), K2351R (7177A>G 7178A>G), Q2352A (7179C>G 7180A>C), F2354L (7185T>C), P2356Q (7192C>A 7193C>G), T2357S (7194A>T), I2360F (7203A>T 7205C>T), I2361Q (7206A>C 7207T>A 7208C>G), R2363L (7213G>T 7214G>T), V2365L (7218G>C), P2370L (7234C>T 7235A>T), T2373E (7242A>G 7243C>A 7244A>G), V2374P (7245G>C 7246T>C), E2376D (7253A>T), I2378S (7257A>T 7258T>C), V2382A (7270T>C 7271A>T), R2383K (7272C>A 7273G>A 7274T>A), V2384I (7275G>A 7277A>T), S2386A (7281T>G 7283T>C), E2387K (7284G>A 7286G>A), L2388E (7287T>G 7288T>A), I2389V (7290A>G 7292T>G), G2390N (7293G>A 7294G>A 7295A>T), S2399C (7320A>T), D2403N (7332G>A 7334T>C), N2406K (7343T>G), M2417L (7374A>T), E2418Q (7377G>C), G2419K (7380G>A 7381G>A), L2422H (7390T>A 7391A>T), Y2426N (7401T>A 7403T>C), K2427N (7406G>C), V2429L (7410G>T), E2430M (7413G>A 7414A>T), P2431A (7416C>G 7418G>T), Q2433E (7422C>G 7424A>G), N2434D (7425A>G), M2436T (7432T>C 7433G>A), S2438Y (7437A>T 7438G>A), L2440M (7443C>A 7445C>G), V2442I (7449G>A 7451G>C), A2444S (7455G>T 7457T>A), F2445L (7458T>C), V2446A (7462T>C 7463A>T), N2447L (7464A>C 7465A>T), K2448G (7467A>G 7468A>G 7469A>G), V2449P (7470G>C 7471T>C 7472T>A), G2450N (7473G>A 7474G>A), D2451T (7476G>A 7477A>C), D2452E (7481T>G), I2454M (7487A>G), I2455T (7489T>C 7490C>T), K2457V (7494A>G 7495A>T 7496G>T), V2459K (7500G>A 7501T>A 7502G>A), L2460T (7503C>A 7504T>C 7505A>G), R2461L (7506A>C 7507G>T 7508A>G), E2463K (7512G>A), M2466P (7521A>C 7522T>C 7523G>A), P2467A (7524C>G 7526A>T), S2472N (7539T>A 7540C>A 7541A>T), L2482H (7570T>A), N2483G (7572A>G 7573A>G), W2484Y (7576G>A 7577G>T), R2485D (7578A>G 7579G>A 7580A>C), K2486Q (7581A>C), F2488Y (7588T>A 7589T>C), M2492P (7599A>C 7600T>C 7601G>C), A2493K (7602G>A 7603C>A 7604C>G), M2498K (7618T>A), F2503G (7632T>G 7633T>G), R2504K (7635C>A 7636G>A)								

	Begin	End	Coverage	Score	Concordance	Matches	Identities	I/D/M/F*	Stop Codons
**NT**	**7123**	**7648**	**5.8%**	**228**	**21.7%**	**526 (100%)**	**320 (60.8%)**	**0/0**	
Codon mutations:	GCA2335GCG (7130A>G), CCA2336CCG (7133A>G), ATT2337GTG (7134A>G 7136T>G), TTC2339TTT (7142C>T), GTA2340GTT (7145A>T), ACA2341ACC (7148A>C), GGG2342GGA (7151G>A), GCA2344GCT (7157A>T), CAA2345AAA (7158C>A), AAA2346AAG (7163A>G), AAG2348GAG (7167A>G), GTA2350GTT (7175A>T), AAA2351AGG (7177A>G 7178A>G), CAA2352GCA (7179C>G 7180A>C), ATA2353ATT (7184A>T), TTT2354CTT (7185T>C), GGT2355GGC (7190T>C), CCC2356CAG (7192C>A 7193C>G), ACT2357TCT (7194A>T), CCA2359CCC (7202A>C), ATC2360TTT (7203A>T 7205C>T), ATC2361CAG (7206A>C 7207T>A 7208C>G), TCT2362TCC (7211T>C), CGG2363CTT (7213G>T 7214G>T), AAA2364AAG (7217A>G), GTT2365CTT (7218G>C), CCA2368CCT (7229A>T), CCA2370CTT (7234C>T 7235A>T), GGA2372GGG (7241A>G), ACA2373GAG (7242A>G 7243C>A 7244A>G), GTT2374CCT (7245G>C 7246T>C), GAG2375GAA (7250G>A), GAA2376GAT (7253A>T), ATT2377ATA (7256T>A), ATC2378TCC (7257A>T 7258T>C), GTA2382GCT (7270T>C 7271A>T), CGT2383AAA (7272C>A 7273G>A 7274T>A), GTA2384ATT (7275G>A 7277A>T), GCG2385GCT (7280G>T), TCT2386GCC (7281T>G 7283T>C), GAG2387AAA (7284G>A 7286G>A), TTG2388GAG (7287T>G 7288T>A), ATT2389GTG (7290A>G 7292T>G), GGA2390AAT (7293G>A 7294G>A 7295A>T), GGG2391GGA (7298G>A), GTT2393GTG (7304T>G), CTC2394CTA (7307C>A), GTA2395GTT (7310A>T), GAA2396GAG (7313A>G), ACA2398ACT (7319A>T), AGT2399TGT (7320A>T), TTG2400CTC (7323T>C 7325G>C), TTT2402TTC (7331T>C), GAT2403AAC (7332G>A 7334T>C), GCT2404GCC (7337T>C), CTC2405CTT (7340C>T), AAT2406AAG (7343T>G), GGA2410GGG (7355A>G), CCA2411CCT (7358A>T), TAC2412TAT (7361C>T), ATT2413ATC (7364T>C), ATG2417TTG (7374A>T), GAA2418CAA (7377G>C), GGG2419AAG (7380G>A 7381G>A), ATT2420ATC (7385T>C), GGA2421GGT (7388A>T), CTA2422CAT (7390T>A 7391A>T), GGA2424GGT (7397A>T), TTA2425CTT (7398T>C 7400A>T), TAT2426AAC (7401T>A 7403T>C), AAG2427AAC (7406G>C), TTG2428TTA (7409G>A), GTG2429TTG (7410G>T), GAG2430ATG (7413G>A 7414A>T), CCG2431GCT (7416C>G 7418G>T), CAA2433GAG (7422C>G 7424A>G), AAT2434GAT (7425A>G), ATG2436ACA (7432T>C 7433G>A), GCT2437GCA (7436T>A), AGT2438TAT (7437A>T 7438G>A), GCT2439GCC (7442T>C), CTC2440ATG (7443C>A 7445C>G), TGC2441TGT (7448C>T), GTG2442ATC (7449G>A 7451G>C), GCT2444TCA (7455G>T 7457T>A), TTT2445CTT (7458T>C), GTA2446GCT (7462T>C 7463A>T), AAT2447CTT (7464A>C 7465A>T), AAA2448GGG (7467A>G 7468A>G 7469A>G), GTT2449CCA (7470G>C 7471T>C 7472T>A), GGT2450AAT (7473G>A 7474G>A), GAT2451ACT (7476G>A 7477A>C), GAT2452GAG (7481T>G), CCT2453CCG (7484T>G), ATA2454ATG (7487A>G), ATC2455ACT (7489T>C 7490C>T), AAG2457GTT (7494A>G 7495A>T 7496G>T), GGT2458GGA (7499T>A), GTG2459AAA (7500G>A 7501T>A 7502G>A), CTA2460ACG (7503C>A 7504T>C 7505A>G), AGA2461CTG (7506A>C 7507G>T 7508A>G), GGT2462GGA (7511T>A), GAG2463AAG (7512G>A), ATT2464ATA (7517T>A), GTA2465GTG (7520A>G), ATG2466CCA (7521A>C 7522T>C 7523G>A), CCA2467GCT (7524C>G 7526A>T), CGC2468AGG (7527C>A 7529C>G), CCA2470CCC (7535A>C), TCA2472AAT (7539T>A 7540C>A 7541A>T), TTT2473TTC (7544T>C), GGG2474GGA (7547G>A), GAC2476GAT (7553C>T), CCA2477CCG (7556A>G), ATT2478ATA (7559T>A), TTC2479TTT (7562C>T), CCA2481CCT (7568A>T), CTT2482CAT (7570T>A), AAC2483GGC (7572A>G 7573A>G), TGG2484TAT (7576G>A 7577G>T), AGA2485GAC (7578A>G 7579G>A 7580A>C), AAG2486CAG (7581A>C), ACA2487ACT (7586A>T), TTT2488TAC (7588T>A 7589T>C), ATG2492CCC (7599A>C 7600T>C 7601G>C), GCC2493AAG (7602G>A 7603C>A 7604C>G), GAG2494GAA (7607G>A), GAG2495GAA (7610G>A), AAT2497AAC (7616T>C), ATG2498AAG (7618T>A), ATA2499ATT (7622A>T), CAT2501CAC (7628T>C), CGA2502CGC (7631A>C), TTT2503GGT (7632T>G 7633T>G), CGA2504AAA (7635C>A 7636G>A)								
HAM1-like protein (YP_007032446.1)	26	200	77.4%	738	60.1%	175 (100%)	101 (57.7%)	0/0/0/0	0
Protein mutations:	I29V (7134A>G 7136T>G), Q37K (7158C>A), K40E (7167A>G), K43R (7177A>G 7178A>G), Q44A (7179C>G 7180A>C), F46L (7185T>C), P48Q (7192C>A 7193C>G), T49S (7194A>T), I52F (7203A>T 7205C>T), I53Q (7206A>C 7207T>A 7208C>G), R55L (7213G>T 7214G>T), V57L (7218G>C), P62L (7234C>T 7235A>T), T65E (7242A>G 7243C>A 7244A>G), V66P (7245G>C 7246T>C), E68D (7253A>T), I70S (7257A>T 7258T>C), V74A (7270T>C 7271A>T), R75K (7272C>A 7273G>A 7274T>A), V76I (7275G>A 7277A>T), S78A (7281T>G 7283T>C), E79K (7284G>A 7286G>A), L80E (7287T>G 7288T>A), I81V (7290A>G 7292T>G), G82N (7293G>A 7294G>A 7295A>T), S91C (7320A>T), D95N (7332G>A 7334T>C), N98K (7343T>G), M109L (7374A>T), E110Q (7377G>C), G111K (7380G>A 7381G>A), L114H (7390T>A 7391A>T), Y118N (7401T>A 7403T>C), K119N (7406G>C), V121L (7410G>T), E122M (7413G>A 7414A>T), P123A (7416C>G 7418G>T), Q125E (7422C>G 7424A>G), N126D (7425A>G), M128T (7432T>C 7433G>A), S130Y (7437A>T 7438G>A), L132M (7443C>A 7445C>G), V134I (7449G>A 7451G>C), A136S (7455G>T 7457T>A), F137L (7458T>C), V138A (7462T>C 7463A>T), N139L (7464A>C 7465A>T), K140G (7467A>G 7468A>G 7469A>G), V141P (7470G>C 7471T>C 7472T>A), G142N (7473G>A 7474G>A), D143T (7476G>A 7477A>C), D144E (7481T>G), I146M (7487A>G), I147T (7489T>C 7490C>T), K149V (7494A>G 7495A>T 7496G>T), V151K (7500G>A 7501T>A 7502G>A), L152T (7503C>A 7504T>C 7505A>G), R153L (7506A>C 7507G>T 7508A>G), E155K (7512G>A), M158P (7521A>C 7522T>C 7523G>A), P159A (7524C>G 7526A>T), S164N (7539T>A 7540C>A 7541A>T), L174H (7570T>A), N175G (7572A>G 7573A>G), W176Y (7576G>A 7577G>T), R177D (7578A>G 7579G>A 7580A>C), K178Q (7581A>C), F180Y (7588T>A 7589T>C), M184P (7599A>C 7600T>C 7601G>C), A185K (7602G>A 7603C>A 7604C>G), M190K (7618T>A), F195G (7632T>G 7633T>G), R196K (7635C>A 7636G>A)								
Codon mutations:	GCA27GCG (7130A>G), CCA28CCG (7133A>G), ATT29GTG (7134A>G 7136T>G), TTC31TTT (7142C>T), GTA32GTT (7145A>T), ACA33ACC (7148A>C), GGG34GGA (7151G>A), GCA36GCT (7157A>T), CAA37AAA (7158C>A), AAA38AAG (7163A>G), AAG40GAG (7167A>G), GTA42GTT (7175A>T), AAA43AGG (7177A>G 7178A>G), CAA44GCA (7179C>G 7180A>C), ATA45ATT (7184A>T), TTT46CTT (7185T>C), GGT47GGC (7190T>C), CCC48CAG (7192C>A 7193C>G), ACT49TCT (7194A>T), CCA51CCC (7202A>C), ATC52TTT (7203A>T 7205C>T), ATC53CAG (7206A>C 7207T>A 7208C>G), TCT54TCC (7211T>C), CGG55CTT (7213G>T 7214G>T), AAA56AAG (7217A>G), GTT57CTT (7218G>C), CCA60CCT (7229A>T), CCA62CTT (7234C>T 7235A>T), GGA64GGG (7241A>G), ACA65GAG (7242A>G 7243C>A 7244A>G), GTT66CCT (7245G>C 7246T>C), GAG67GAA (7250G>A), GAA68GAT (7253A>T), ATT69ATA (7256T>A), ATC70TCC (7257A>T 7258T>C), GTA74GCT (7270T>C 7271A>T), CGT75AAA (7272C>A 7273G>A 7274T>A), GTA76ATT (7275G>A 7277A>T), GCG77GCT (7280G>T), TCT78GCC (7281T>G 7283T>C), GAG79AAA (7284G>A 7286G>A), TTG80GAG (7287T>G 7288T>A), ATT81GTG (7290A>G 7292T>G), GGA82AAT (7293G>A 7294G>A 7295A>T), GGG83GGA (7298G>A), GTT85GTG (7304T>G), CTC86CTA (7307C>A), GTA87GTT (7310A>T), GAA88GAG (7313A>G), ACA90ACT (7319A>T), AGT91TGT (7320A>T), TTG92CTC (7323T>C 7325G>C), TTT94TTC (7331T>C), GAT95AAC (7332G>A 7334T>C), GCT96GCC (7337T>C), CTC97CTT (7340C>T), AAT98AAG (7343T>G), GGA102GGG (7355A>G), CCA103CCT (7358A>T), TAC104TAT (7361C>T), ATT105ATC (7364T>C), ATG109TTG (7374A>T), GAA110CAA (7377G>C), GGG111AAG (7380G>A 7381G>A), ATT112ATC (7385T>C), GGA113GGT (7388A>T), CTA114CAT (7390T>A 7391A>T), GGA116GGT (7397A>T), TTA117CTT (7398T>C 7400A>T), TAT118AAC (7401T>A 7403T>C), AAG119AAC (7406G>C), TTG120TTA (7409G>A), GTG121TTG (7410G>T), GAG122ATG (7413G>A 7414A>T), CCG123GCT (7416C>G 7418G>T), CAA125GAG (7422C>G 7424A>G), AAT126GAT (7425A>G), ATG128ACA (7432T>C 7433G>A), GCT129GCA (7436T>A), AGT130TAT (7437A>T 7438G>A), GCT131GCC (7442T>C), CTC132ATG (7443C>A 7445C>G), TGC133TGT (7448C>T), GTG134ATC (7449G>A 7451G>C), GCT136TCA (7455G>T 7457T>A), TTT137CTT (7458T>C), GTA138GCT (7462T>C 7463A>T), AAT139CTT (7464A>C 7465A>T), AAA140GGG (7467A>G 7468A>G 7469A>G), GTT141CCA (7470G>C 7471T>C 7472T>A), GGT142AAT (7473G>A 7474G>A), GAT143ACT (7476G>A 7477A>C), GAT144GAG (7481T>G), CCT145CCG (7484T>G), ATA146ATG (7487A>G), ATC147ACT (7489T>C 7490C>T), AAG149GTT (7494A>G 7495A>T 7496G>T), GGT150GGA (7499T>A), GTG151AAA (7500G>A 7501T>A 7502G>A), CTA152ACG (7503C>A 7504T>C 7505A>G), AGA153CTG (7506A>C 7507G>T 7508A>G), GGT154GGA (7511T>A), GAG155AAG (7512G>A), ATT156ATA (7517T>A), GTA157GTG (7520A>G), ATG158CCA (7521A>C 7522T>C 7523G>A), CCA159GCT (7524C>G 7526A>T), CGC160AGG (7527C>A 7529C>G), CCA162CCC (7535A>C), TCA164AAT (7539T>A 7540C>A 7541A>T), TTT165TTC (7544T>C), GGG166GGA (7547G>A), GAC168GAT (7553C>T), CCA169CCG (7556A>G), ATT170ATA (7559T>A), TTC171TTT (7562C>T), CCA173CCT (7568A>T), CTT174CAT (7570T>A), AAC175GGC (7572A>G 7573A>G), TGG176TAT (7576G>A 7577G>T), AGA177GAC (7578A>G 7579G>A 7580A>C), AAG178CAG (7581A>C), ACA179ACT (7586A>T), TTT180TAC (7588T>A 7589T>C), ATG184CCC (7599A>C 7600T>C 7601G>C), GCC185AAG (7602G>A 7603C>A 7604C>G), GAG186GAA (7607G>A), GAG187GAA (7610G>A), AAT189AAC (7616T>C), ATG190AAG (7618T>A), ATA191ATT (7622A>T), CAT193CAC (7628T>C), CGA194CGC (7631A>C), TTT195GGT (7632T>G 7633T>G), CGA196AAA (7635C>A 7636G>A)								

*: Inserts / Deletes / Misaligned / Frameshifts

Analysis details

This analysis was performed with panviral2.64

NGS Details (UN62): Human gammaherpesvirus 8

Assembly

**Coverage Length**	135 (1 contig(s))
**Depth Of Coverage**	200.8
**Number Of Reads**	322
**Reads Per Million**	7.46 rpm (after QC)
**Ambiguities**	0
**Assembly Method**	de novo + reference guided assembly
**Consensus Caller**	Bcf Tools

Coverage Map

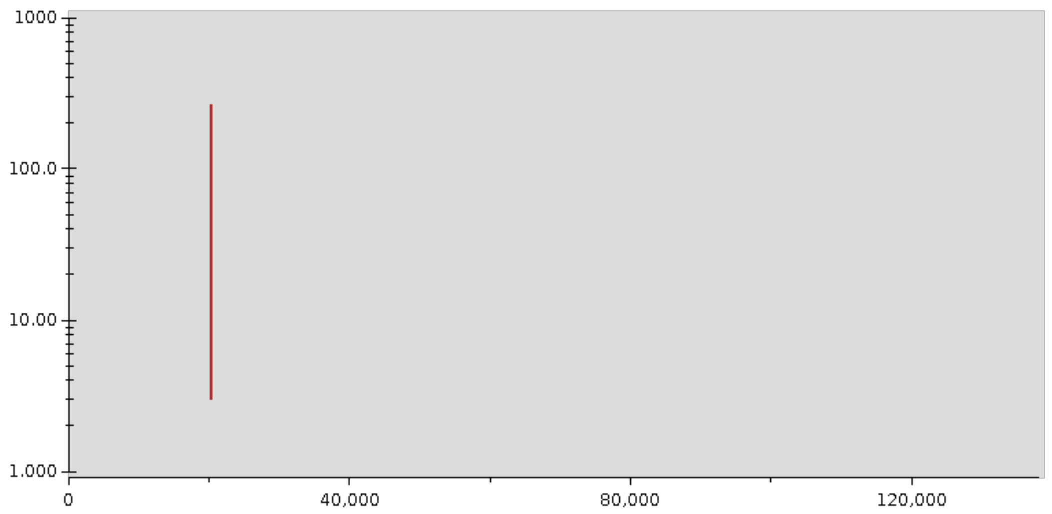

Assignment

**Type**	Human gammaherpesvirus 8 (Taxonomy ID: 37296)
**Subtype**	Could not assign
**Reference Genome**	NC_009333.1
**NT Identity (%)**	83.7037
**AA Identity (%)**	84.4444
**Number Of Stop Codons**	0
**Number Of CDS**	86

Alignment

**Alignment Score**	182.0 (NT) + 287.0 (AA) = 469.0
**Concordance (%)**	78.2972
**Alignment Method**	Local, heuristic, nucleotide (BLASTN)

Genome Region

Sequence starts at position 20273 and ends at position 20407 relative to NC_009333.1 reference sequence.

Alignment Detailed Statistics

	Begin	End	Coverage	Score	Concordance	Matches	Identities	I/D/M/F*	Stop Codons
**NT**	**20273**	**20407**	**0.1%**	**182**	**67.4%**	**135 (100%)**	**113 (83.7%)**	**0/0**	

Mutations: 20293A>T, 20296A>C, 20311G>A, 20314T>G, 20315C>G, 20317C>A, 20320C>T, 20322T>G, 20331G>T, 20341A>C, 20347A>C, 20350G>A, 20352C>T, 20353A>G, 20369C>G, 20370A>C, 20371T>A, 20376A>T, 20383G>A, 20386C>T, 20389A>G, 20392C>T

*: Inserts / Deletes / Misaligned / Frameshifts

Analysis details

This analysis was performed with panviral2.64

NGS Details (UN62): Hibiscus bacilliform virus GD1

Assembly

**Coverage Length**	332 (1 contig(s))
**Depth Of Coverage**	11.3
**Number Of Reads**	53
**Reads Per Million**	1.23 rpm (after QC)
**Ambiguities**	0
**Assembly Method**	de novo + reference guided assembly
**Consensus Caller**	Bcf Tools

Coverage Map

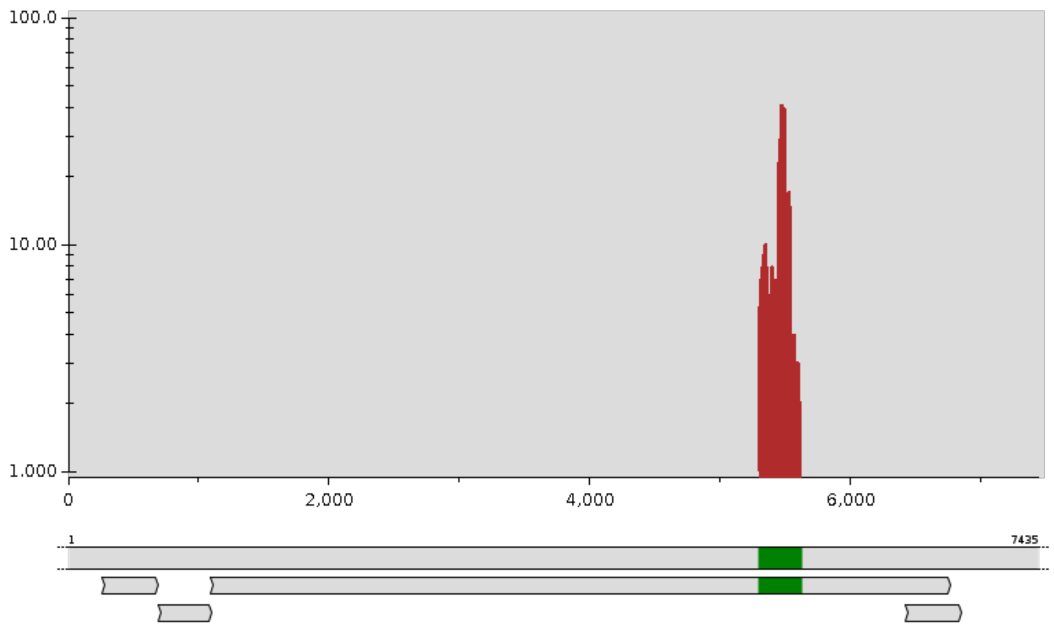

Assignment

**Type**	Hibiscus bacilliform virus GD1 (Taxonomy ID: 1459800)
**Reference Genome**	NC_023485.1
**NT Identity (%)**	51.6418
**AA Identity (%)**	42.8571
**Number Of Stop Codons**	0
**Number Of CDS**	4

Alignment

**Alignment Score**	16.0 (NT) + 322.0 (AA) = 338.0
**Concordance (%)**	23.2782

Alignment Method	Global, seeded, nucleotide + amino acids (AGA)

Genome Region

Sequence starts at position 5295 and ends at position 5626 relative to NC_023485.1 reference sequence.

Alignment Detailed Statistics

	Begin	End	Coverage	Score	Concordance	Matches	Identities	I/D/M/F*	Stop Codons
**NT**	**5295**	**5626**	**4.5%**	**16**	**2.4%**	**332 (99.1%)**	**173 (51.6%)**	**3/0**	
Mutations:	5306A>G, 5307G>A, 5310C>A, 5315C>T, 5316A>C, 5317G>C, 5318T>A, 5319C>T, 5324A>T, 5325G>A, 5327C>A, 5330C>T, 5331A>G, 5333C>T, 5334A>G, 5335C>A, 5337A>C, 5339T>A, 5340A>T, 5342G>T, 5343A>G, 5345G>T, 5346A>C, 5347G>A, 5348G>A, 5349G>C, 5352G>C, 5353G>A, 5354C>A, 5355C>G, 5356A>G, 5357T>G, 5360T>A, 5362A>G, 5363G>T, 5364A>T, 5365T>A, 5366C>T, 5368A>T, 5369C>T, 5376T>G, 5384C>T, 5385A>T, 5386A>G, 5387G>T, 5388A>T, 5389G>C, 5393A>G, 5395T>A, 5399T>C, 5403G>T, 5405G>A, 5406G>T, 5407C>G, 5408T>G, 5409A>T, 5411G>A, 5412C>A, 5414C>G, 5415C>G, 5416C>A, 5417G>A, 5419A>G, 5420G>T, 5421T>G, 5422C>A, 5423T>C, 5426C>T, 5427A>C, 5428A>T, 5430T>A, 5431G>A, 5434C>T, 5435T>G, 5440T>A, 5442T>C, 5445G>A, 5447T>G, 5448C>A, 5449C>G, 5450A>G, 5451G>T, 5456A>G, 5457C>T, 5458T>A, 5459G>T, 5461A>T, 5467G>T, 5468G>T, 5471A>T, 5474T>C, 5478C>T, 5480A>C, 5486A>T, 5487C>T, 5489T>A, 5491A>C, 5492A>T, 5495C>T, 5502G>A, 5504A>T, 5507A>G, 5511C>A, 5512A>T, 5514C>A, 5515G>A, 5516A>T, 5517A>T, 5518A>T, 5519G>T, 5523G>A, 5525C>T, 5527A>G, 5528C>G, 5531C>T, 5537A>G, 5538G>C, 5539G>C, 5540_5541insTTC, 5541A>C, 5542C>T, 5546A>T, 5547G>A, 5548C>T, 5549C>G, 5552T>C, 5553A>G, 5554T>G, 5555C>G, 5557C>T, 5558T>G, 5563A>T, 5564C>T, 5568G>A, 5573C>T, 5574A>G, 5576A>G, 5577C>T, 5579C>G, 5580G>A, 5584T>A, 5585T>C, 5588C>T, 5590A>G, 5592T>A, 5593C>G, 5594T>C, 5595G>A, 5600G>A, 5603A>G, 5607A>G, 5608T>A, 5609C>A, 5613C>A, 5616A>T, 5618A>G, 5619C>A, 5622G>A, 5624C>A, 5625A>G								

CDS

	Begin	End	Coverage	Score	Concordance	Matches	Identities	I/D/M/F*	Stop Codons
BU35_gp3	1402	1512	5.9%	322	40.6%	111 (99.1%)	48 (42.9%)	1/0/0/0	0
Protein mutations:	D1406N (5307G>A), Q1407K (5310C>A), S1409P (5316A>C 5317G>C 5318T>A), G1412R (5325G>A 5327C>A), N1414D (5331A>G 5333C>T), T1415D (5334A>G 5335C>A), I1416L (5337A>C 5339T>A), M1417F (5340A>T 5342G>T), K1418D (5343A>G 5345G>T), R1419Q (5346A>C 5347G>A 5348G>A), V1420L (5349G>C), G1421Q (5352G>C 5353G>A 5354C>A), H1422G (5355C>G 5356A>G 5357T>G), K1424S (5362A>G 5363G>T), I1425Y (5364A>T 5365T>A 5366C>T), Y1426F (5368A>T 5369C>T), F1429V (5376T>G), K1432C (5385A>T 5386A>G 5387G>T), F1435Y (5395T>A), V1438L (5403G>T 5405G>A), A1439W (5406G>T 5407C>G 5408T>G), M1440L (5409A>T 5411G>A), H1441K (5412C>A 5414C>G), P1442E (5415C>G 5416C>A 5417G>A), E1443G (5419A>G 5420G>T), S1444D (5421T>G 5422C>A 5423T>C), K1446L (5427A>C 5428A>T), W1447K (5430T>A 5431G>A), T1448M (5434C>T 5435T>G), F1450Y (5440T>A), W1451R (5442T>C), V1452M (5445G>A 5447T>G), P1453R (5448C>A 5449C>G 5450A>G), D1454Y (5451G>T), L1456Y (5457C>T 5458T>A 5459G>T), Y1457F (5461A>T), W1459F (5467G>T 5468G>T), P1463S (5478C>T 5480A>C), K1467T (5491A>C 5492A>T), A1471T (5502G>A 5504A>T), Q1474M (5511C>A 5512A>T), R1475N (5514C>A 5515G>A 5516A>T), K1476F (5517A>T 5518A>T 5519G>T), D1478N (5523G>A 5525C>T), N1479R (5527A>G 5528C>G), G1483P (5538G>C 5539G>C), G1483_T1484insF (5540_5541insTTC), T1484L (5541A>C 5542C>T), E1485D (5546A>T), A1486M (5547G>A 5548C>T 5549C>G), I1488G (5553A>G 5554T>G 5555C>G), A1489V (5557C>T 5558T>G), Y1491F (5563A>T 5564C>T), D1493N (5568G>A), I1495V (5574A>G 5576A>G), V1497I (5580G>A), F1498Y (5584T>A 5585T>C), Q1500R (5590A>G), E1502K (5595G>A), I1506E (5607A>G 5608T>A 5609C>A), H1508N (5613C>A), I1509L (5616A>T 5618A>G), V1511I (5622G>A 5624C>A)								
Codon mutations:	AAA1405AAG (5306A>G), GAT1406AAT (5307G>A), CAG1407AAG (5310C>A), TAC1408TAT (5315C>T), AGT1409CCA (5316A>C 5317G>C 5318T>A), CTG1410TTG (5319C>T), CCA1411CCT (5324A>T), GGC1412AGA (5325G>A 5327C>A), ATC1413ATT (5330C>T), AAC1414GAT (5331A>G 5333C>T), ACC1415GAC (5334A>G 5335C>A), ATT1416CTA (5337A>C 5339T>A), ATG1417TTT (5340A>T 5342G>T), AAG1418GAT (5343A>G 5345G>T), AGG1419CAA (5346A>C 5347G>A 5348G>A), GTT1420CTT (5349G>C), GGC1421CAA (5352G>C 5353G>A 5354C>A), CAT1422GGG (5355C>G 5356A>G 5357T>G), GCT1423GCA (5360T>A), AAG1424AGT (5362A>G 5363G>T), ATC1425TAT (5364A>T 5365T>A 5366C>T), TAC1426TTT (5368A>T 5369C>T), TTT1429GTT (5376T>G), CTC1431CTT (5384C>T), AAG1432TGT (5385A>T 5386A>G 5387G>T), AGT1433TCT (5388A>T 5389G>C), GGA1434GGG (5393A>G), TTT1435TAT (5395T>A), CAT1436CAC (5399T>C), GTG1438TTA (5403G>T 5405G>A), GCT1439TGG (5406G>T 5407C>G 5408T>G), ATG1440TTA (5409A>T 5411G>A), CAC1441AAG (5412C>A 5414C>G), CCG1442GAA (5415C>G 5416C>A 5417G>A), GAG1443GGT (5419A>G 5420G>T), TCT1444GAC (5421T>G 5422C>A 5423T>C), ATC1445ATT (5426C>T), AAG1446CTG (5427A>C 5428A>T), TGG1447AAG (5430T>A 5431G>A), ACT1448ATG (5434C>T 5435T>G), TTC1450TAC (5440T>A), TGG1451CGG (5442T>C), GTT1452ATG (5445G>A 5447T>G), CCA1453AGG (5448C>A 5449C>G 5450A>G), GAT1454TAT (5451G>T), GGA1455GGG (5456A>G), CTG1456TAT (5457C>T 5458T>A 5459G>T), TAT1457TTT (5461A>T), TGG1459TTT (5467G>T 5468G>T), CTA1460CTT (5471A>T), GTT1461GTC (5474T>C), CCA1463TCC (5478C>T 5480A>C), GGA1465GGT (5486A>T), CTT1466TTA (5487C>T 5489T>A), AAA1467ACT (5491A>C 5492A>T), AAC1468AAT (5495C>T), GCA1471ACT (5502G>A 5504A>T), GTA1472GTG (5507A>G), CAG1474ATG (5511C>A 5512A>T), CGA1475AAT (5514C>A 5515G>A 5516A>T), AAG1476TTT (5517A>T 5518A>T 5519G>T), GAC1478AAT (5523G>A 5525C>T), AAC1479AGG (5527A>G 5528C>G), GTC1480GTT (5531C>T), AAA1482AAG (5537A>G), GGT1483CCT (5538G>C 5539G>C), GGT1483_ACT1484insTTC (5540_5541insTTC), ACT1484CTT (5541A>C 5542C>T), GAA1485GAT (5546A>T), GCC1486ATG (5547G>A 5548C>T 5549C>G), TTT1487TTC (5552T>C), ATC1488GGG (5553A>G 5554T>G 5555C>G), GCT1489GTG (5557C>T 5558T>G), TAC1491TTT (5563A>T 5564C>T), GAT1493AAT (5568G>A), GAC1494GAT (5573C>T), ATA1495GTG (5574A>G 5576A>G), CTC1496TTG (5577C>T 5579C>G), GTA1497ATA (5580G>A), TTT1498TAC (5584T>A 5585T>C), TCC1499TCT (5588C>T), CAG1500CGG (5590A>G), TCT1501AGC (5592T>A 5593C>G 5594T>C), GAG1502AAG (5595G>A), GAG1503GAA (5600G>A), GAA1504GAG (5603A>G), ATC1506GAA (5607A>G 5608T>A 5609C>A), CAC1508AAC (5613C>A), ATA1509TTG (5616A>T 5618A>G), CGG1510AGG (5619C>A), GTC1511ATA (5622G>A 5624C>A), ATG1512GT. (5625A>G)								

Proteins

	Begin	End	Coverage	Score	Concordance	Matches	Identities	I/D/M/F*	Stop Codons
polyprotein (YP_009002585.1)	1402	1512	5.9%	322	40.6%	111 (99.1%)	48 (42.9%)	1/0/0/0	0
Protein mutations:	D1406N (5307G>A), Q1407K (5310C>A), S1409P (5316A>C 5317G>C 5318T>A), G1412R (5325G>A 5327C>A), N1414D (5331A>G 5333C>T), T1415D (5334A>G 5335C>A), I1416L (5337A>C 5339T>A), M1417F (5340A>T 5342G>T), K1418D (5343A>G 5345G>T), R1419Q (5346A>C 5347G>A 5348G>A), V1420L (5349G>C), G1421Q (5352G>C 5353G>A 5354C>A), H1422G (5355C>G 5356A>G 5357T>G), K1424S (5362A>G 5363G>T), I1425Y (5364A>T 5365T>A 5366C>T), Y1426F (5368A>T 5369C>T), F1429V (5376T>G), K1432C (5385A>T 5386A>G 5387G>T), F1435Y (5395T>A), V1438L (5403G>T 5405G>A), A1439W (5406G>T 5407C>G 5408T>G), M1440L (5409A>T 5411G>A), H1441K (5412C>A 5414C>G), P1442E (5415C>G 5416C>A 5417G>A), E1443G (5419A>G 5420G>T), S1444D (5421T>G 5422C>A 5423T>C), K1446L (5427A>C 5428A>T), W1447K (5430T>A 5431G>A), T1448M (5434C>T 5435T>G), F1450Y (5440T>A), W1451R (5442T>C), V1452M (5445G>A 5447T>G), P1453R (5448C>A 5449C>G 5450A>G), D1454Y (5451G>T), L1456Y (5457C>T 5458T>A 5459G>T), Y1457F (5461A>T), W1459F (5467G>T 5468G>T), P1463S (5478C>T 5480A>C), K1467T (5491A>C 5492A>T), A1471T (5502G>A 5504A>T), Q1474M (5511C>A 5512A>T), R1475N (5514C>A 5515G>A 5516A>T), K1476F (5517A>T 5518A>T 5519G>T), D1478N (5523G>A 5525C>T), N1479R (5527A>G 5528C>G), G1483P (5538G>C 5539G>C), G1483_T1484insF (5540_5541insTTC), T1484L (5541A>C 5542C>T), E1485D (5546A>T), A1486M (5547G>A 5548C>T 5549C>G), I1488G (5553A>G 5554T>G 5555C>G), A1489V (5557C>T 5558T>G), Y1491F (5563A>T 5564C>T), D1493N (5568G>A), I1495V (5574A>G 5576A>G), V1497I (5580G>A), F1498Y (5584T>A 5585T>C), Q1500R (5590A>G), E1502K (5595G>A), I1506E (5607A>G 5608T>A 5609C>A), H1508N (5613C>A), I1509L (5616A>T 5618A>G), V1511I (5622G>A 5624C>A)								
Codon mutations:	AAA1405AAG (5306A>G), GAT1406AAT (5307G>A), CAG1407AAG (5310C>A), TAC1408TAT (5315C>T), AGT1409CCA (5316A>C 5317G>C 5318T>A), CTG1410TTG (5319C>T), CCA1411CCT (5324A>T), GGC1412AGA (5325G>A 5327C>A), ATC1413ATT (5330C>T), AAC1414GAT (5331A>G 5333C>T), ACC1415GAC (5334A>G 5335C>A), ATT1416CTA (5337A>C 5339T>A), ATG1417TTT (5340A>T 5342G>T), AAG1418GAT (5343A>G 5345G>T), AGG1419CAA (5346A>C 5347G>A 5348G>A), GTT1420CTT (5349G>C), GGC1421CAA (5352G>C 5353G>A 5354C>A), CAT1422GGG (5355C>G 5356A>G 5357T>G), GCT1423GCA (5360T>A), AAG1424AGT (5362A>G 5363G>T), ATC1425TAT (5364A>T 5365T>A 5366C>T), TAC1426TTT (5368A>T 5369C>T), TTT1429GTT (5376T>G), CTC1431CTT (5384C>T), AAG1432TGT (5385A>T 5386A>G 5387G>T), AGT1433TCT (5388A>T 5389G>C), GGA1434GGG (5393A>G), TTT1435TAT (5395T>A), CAT1436CAC (5399T>C), GTG1438TTA (5403G>T 5405G>A), GCT1439TGG (5406G>T 5407C>G 5408T>G), ATG1440TTA (5409A>T 5411G>A), CAC1441AAG (5412C>A 5414C>G), CCG1442GAA (5415C>G 5416C>A 5417G>A), GAG1443GGT (5419A>G 5420G>T), TCT1444GAC (5421T>G 5422C>A 5423T>C), ATC1445ATT (5426C>T), AAG1446CTG (5427A>C 5428A>T), TGG1447AAG (5430T>A 5431G>A), ACT1448ATG (5434C>T 5435T>G), TTC1450TAC (5440T>A), TGG1451CGG (5442T>C), GTT1452ATG (5445G>A 5447T>G), CCA1453AGG (5448C>A 5449C>G 5450A>G), GAT1454TAT (5451G>T), GGA1455GGG (5456A>G), CTG1456TAT (5457C>T 5458T>A 5459G>T), TAT1457TTT (5461A>T), TGG1459TTT (5467G>T 5468G>T), CTA1460CTT (5471A>T), GTT1461GTC (5474T>C), CCA1463TCC (5478C>T 5480A>C), GGA1465GGT (5486A>T), CTT1466TTA (5487C>T 5489T>A), AAA1467ACT (5491A>C 5492A>T), AAC1468AAT (5495C>T), GCA1471ACT (5502G>A 5504A>T), GTA1472GTG (5507A>G), CAG1474ATG (5511C>A 5512A>T), CGA1475AAT (5514C>A 5515G>A 5516A>T), AAG1476TTT (5517A>T 5518A>T 5519G>T), GAC1478AAT (5523G>A 5525C>T), AAC1479AGG (5527A>G 5528C>G), GTC1480GTT (5531C>T), AAA1482AAG (5537A>G), GGT1483CCT (5538G>C 5539G>C), GGT1483_ACT1484insTTC (5540_5541insTTC), ACT1484CTT (5541A>C 5542C>T), GAA1485GAT (5546A>T), GCC1486ATG (5547G>A 5548C>T 5549C>G), TTT1487TTC (5552T>C), ATC1488GGG (5553A>G 5554T>G 5555C>G), GCT1489GTG (5557C>T 5558T>G), TAC1491TTT (5563A>T 5564C>T), GAT1493AAT (5568G>A), GAC1494GAT (5573C>T), ATA1495GTG (5574A>G 5576A>G), CTC1496TTG (5577C>T 5579C>G), GTA1497ATA (5580G>A), TTT1498TAC (5584T>A 5585T>C), TCC1499TCT (5588C>T), CAG1500CGG (5590A>G), TCT1501AGC (5592T>A 5593C>G 5594T>C), GAG1502AAG (5595G>A), GAG1503GAA (5600G>A), GAA1504GAG (5603A>G), ATC1506GAA (5607A>G 5608T>A 5609C>A), CAC1508AAC (5613C>A), ATA1509TTG (5616A>T 5618A>G), CGG1510AGG (5619C>A), GTC1511ATA (5622G>A 5624C>A), ATG1512GT. (5625A>G)								

*: Inserts / Deletes / Misaligned / Frameshifts

Analysis details

This analysis was performed with panviral2.64

NGS Details (UN62): Caulimovirus venafragariae

Assembly

**Coverage Length**	876 (3 contig(s))
**Depth Of Coverage**	6.8
**Number Of Reads**	51
**Reads Per Million**	1.18 rpm (after QC)
**Ambiguities**	0
**Assembly Method**	de novo + reference guided assembly
**Consensus Caller**	Bcf Tools

Coverage Map

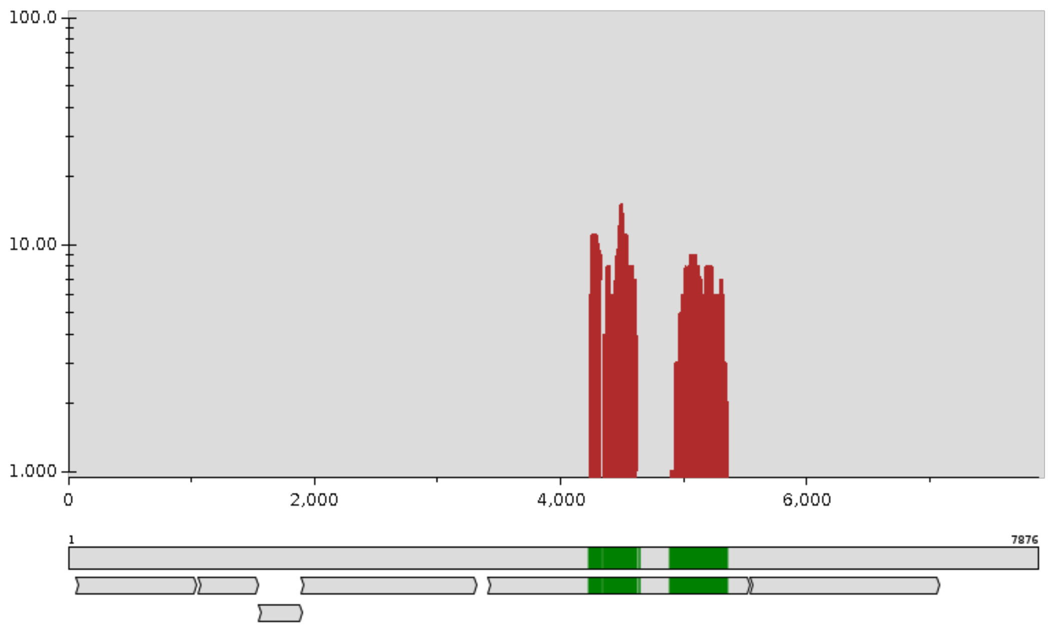

Assignment

**Type**	Caulimovirus venafragariae (Taxonomy ID: 3048344)
**Reference Genome**	NC_001725.1
**NT Identity (%)**	57.6389
**AA Identity (%)**	51.7361
**Number Of Stop Codons**	0
**Number Of CDS**	6

Alignment

**Alignment Score**	243.0 (NT) + 1130.0 (AA) = 1373.0
**Concordance (%)**	37.2389

**Alignment Method**	Global, seeded, nucleotide + amino acids (AGA)

Genome Region

Sequence starts at position 4224 and ends at position 5362 relative to NC_001725.1 reference sequence.

Alignment Detailed Statistics

	Begin	End	Coverage	Score	Concordance	Matches	Identities	I/D/M/F*	Stop Codons
**NT**	**4224**	**5362**	**11.1%**	**243**	**14.2%**	**864 (98.6%)**	**498 (56.8%)**	**0/12**	
Mutations:	4224A>G, 4227C>T, 4228A>C, 4230T>C, 4232A>G, 4235A>T, 4236A>C, 4242T>A, 4244A>G, 4245G>A, 4246T>G, 4247G>T, 4254A>G, 4259A>T, 4260G>A, 4263T>C, 4264T>A, 4265C>A, 4267C>G, 4268C>A, 4269T>A, 4275C>T, 4276C>A, 4277G>A, 4279G>A, 4280A>C, 4281A>T, 4284G>A, 4292C>T, 4299C>T, 4300G>C, 4306C>T, 4309C>T, 4311C>A, 4314G>T, 4315C>A, 4317T>G, 4318G>A, 4319G>A, 4320T>A, 4321A>T, 4323C>A, 4326C>A, 4330C>A, 4331C>G, 4332T>A, 4335C>A, 4337A>C, 4338G>A, 4343C>G, 4347T>C, 4348T>A, 4349C>G, 4350C>A, 4354C>G, 4359C>A, 4371G>A, 4374T>C, 4378G>A, 4379C>G, 4383G>A, 4386C>A, 4387A>G, 4388A>T, 4390C>A, 4395C>A, 4413T>A, 4416C>T, 4424A>G, 4425G>A, 4426T>C, 4428A>T, 4434C>T, 4435C>A, 4440A>T, 4442A>G, 4443G>A, 4444G>A, 4445G>T, 4446A>G, 4451G>C, 4452C>T, 4455C>T, 4456C>A, 4457T>A, 4459C>T, 4461T>A, 4464T>A, 4465A>G, 4470G>A, 4471G>A, 4473G>T, 4474C>G, 4479G>A, 4480C>A, 4482T>A, 4483C>A, 4485A>T, 4491C>A, 4492G>C, 4493G>A, 4497T>A, 4500G>A, 4503C>A, 4508A>T, 4509C>T, 4510T>A, 4511C>G, 4513T>A, 4514C>A, 4515C>A, 4521C>T, 4530T>A, 4535T>A, 4542G>A, 4546C>A, 4547G>A, 4548C>A, 4549C>A, 4551T>G, 4552G>C, 4553C>A, 4554T>C, 4557A>T, 4560A>T, 4562C>G, 4563A>T, 4566T>A, 4567C>G, 4569G>A, 4570C>T, 4571T>G, 4572A>G, 4575C>A, 4576G>A, 4578T>A, 4581C>T, 4583G>C, 4584C>T, 4590C>A, 4591C>G, 4599C>T, 4601A>T, 4602C>T, 4605A>G, 4613T>C, 4614C>A, 4618C>T, 4620T>A, 4633C>A, 4635A>T, 4884G>A, 4886G>C, 4893C>A, 4894G>T, 4895G>C, 4897C>A, 4898T>A, 4900C>A, 4902C>G, 4903T>A, 4905A>C, 4906G>T, 4907T>G, 4910C>A, 4911T>A, 4921C>A, 4926T>A, 4928G>A, 4930G>A, 4931A>C, 4932T>A, 4934G>A, 4935G>A, 4936A>C, 4938T>A, 4940C>A, 4941C>A, 4942T>A, 4944A>C, 4950A>G, 4954T>C, 4956A>T, 4959C>A, 4960C>T, 4961T>G, 4962C>T, 4963C>T, 4971T>C, 4972A>G, 4973T>C, 4974C>A, 4975T>A, 4976C>G, 4977C>T, 4979C>A, 4980T>A, 4982A>T, 4984T>A, 4986T>A, 4990A>G, 4992A>C, 4993A>T, 5001C>A, 5002C>A, 5003T>A, 5004T>G, 5007G>A, 5008T>A, 5009C>A, 5010A>T, 5012C>T, 5019G>A, 5020G>A, 5021T>G, 5022T>G, 5023A>C, 5024A>T, 5025A>T, 5026C>A, 5029A>C, 5030A>G, 5031G>A, 5035G>T, 5036A>C, 5037G>C, 5039T>A, 5040A>C, 5041A>C, 5042C>G, 5043T>A, 5044T>A, 5045G>T, 5047T>G, 5048C>G, 5049T>A, 5055T>A, 5058G>T, 5059A>G, 5062G>C, 5064C>T, 5067G>T, 5068G>A, 5071A>T, 5072C>G, 5076C>T, 5077A>C, 5078G>A, 5081A>G, 5082A>C, 5083A>C, 5088G>A, 5090G>A, 5091T>A, 5092C>G, 5093T>A, 5095G>T, 5096T>G, 5097A>T, 5098A>T, 5099A>C, 5100A>C, 5102C>A, 5103T>A, 5110G>A, 5112C>G, 5115T>A, 5116T>A, 5117A>G, 5118C>A, 5119A>C, 5120A>T, 5124T>G, 5125T>G, 5126C>A, 5133G>A, 5136T>C, 5139G>C, 5140C>T, 5141C>T, 5142T>A, 5143A>G, 5145C>T, 5146A>T, 5148C>A, 5149G>C, 5152T>A, 5153G>C, 5154C>A, 5160A>T, 5167G>T, 5182A>T, 5184C>A, 5185C>T, 5187C>A, 5188A>C, 5191G>A, 5193A>T, 5194A>G, 5196A>C, 5200C>A, 5201C>A, 5202A>T, 5206_5217delGGTAAAGAGGTA, 5220A>T, 5223C>T, 5230G>A, 5232T>A, 5233T>A, 5234C>G, 5235A>T, 5238A>T, 5241C>A, 5247A>T, 5248C>G, 5249C>A, 5251G>A, 5253T>C, 5256G>A, 5258A>C, 5259G>T, 5262T>A, 5266C>T, 5267A>C, 5268T>A, 5270G>C, 5271T>A, 5280G>A, 5284A>T, 5286C>A, 5287C>T, 5289T>G, 5290T>G, 5295T>A, 5296A>G, 5298T>A, 5300A>G, 5301G>A, 5303C>G, 5304A>C, 5307C>A, 5308A>C, 5309A>G, 5311G>A, 5312C>A, 5313T>A, 5317A>T, 5318G>C, 5319A>T, 5324A>T, 5326A>C, 5328C>A, 5329C>T, 5331T>A, 5334T>A, 5335T>A, 5337T>G, 5338A>C, 5343T>C, 5344C>A, 5346T>C, 5347G>A, 5349A>C, 5352G>A, 5353A>T, 5355T>G, 5358T>C								

CDS

	Begin	End	Coverage	Score	Concordance	Matches	Identities	I/D/M/F*	Stop Codons
ORF_V	274	653	41.2%	1130	55.6%	288 (98.6%)	149 (51.0%)	0/4/1/0	0
Protein mutations:	T275P (4228A>C 4230T>C), N276S (4232A>G), K277I (4235A>T 4236A>C), K280R (4244A>G 4245G>A), C281V (4246T>G 4247G>T), Q285L (4259A>T 4260G>A), S287K (4264T>A 4265C>A), P288E (4267C>G 4268C>A 4269T>A), R291K (4276C>A 4277G>A), E292T (4279G>A 4280A>C 4281A>T), T296I (4292C>T), E299Q (4300G>C), K303N (4314G>T), L304M (4315C>A 4317T>G), G305K (4318G>A 4319G>A 4320T>A), I306L (4321A>T 4323C>A), P309R (4330C>A 4331C>G 4332T>A), P313R (4343C>G), S315R (4348T>A 4349C>G 4350C>A), P317A (4354C>G), A325S (4378G>A 4379C>G), K328V (4387A>G 4388A>T), K340R (4424A>G 4425G>A), H344N (4435C>A), K346R (4442A>G 4443G>A), G347M (4444G>A 4445G>T 4446A>G), G349A (4451G>C 4452C>T), L351K (4456C>A 4457T>A), N354D (4465A>G), E356N (4471G>A 4473G>T), Q357E (4474C>G), L359I (4480C>A 4482T>A), Q360N (4483C>A 4485A>T), G363Q (4492G>C 4493G>A), Y368F (4508A>T 4509C>T), S370K (4513T>A 4514C>A 4515C>A), F377Y (4535T>A), R381K (4546C>A 4547G>A 4548C>A), L382M (4549C>A 4551T>G), A383H (4552G>C 4553C>A 4554T>C), E385D (4560A>T), T386S (4562C>G 4563A>T), Q388E (4567C>G 4569G>A), L389W (4570C>T 4571T>G 4572A>G), A391T (4576G>A 4578T>A), S393T (4583G>C 4584C>T), Q396E (4591C>G), Y399F (4601A>T 4602C>T), V403A (4613T>C 4614C>A), P405S (4618C>T 4620T>A), S494T (4886G>C), G497S (4894G>T 4895G>C), L498K (4897C>A 4898T>A), H499K (4900C>A 4902C>G), L500I (4903T>A 4905A>C), V501C (4906G>T 4907T>G), A502E (4910C>A 4911T>A), Q506K (4921C>A), S508N (4928G>A), D509T (4930G>A 4931A>C 4932T>A), R510K (4934G>A 4935G>A), N511Q (4936A>C 4938T>A), A512E (4940C>A 4941C>A), L513I (4942T>A 4944A>C), L519C (4960C>T 4961T>G 4962C>T), I523A (4972A>G 4973T>C 4974C>A), A525E (4979C>A 4980T>A), Y526F (4982A>T), F527I (4984T>A 4986T>A), K529D (4990A>G 4992A>C), I530L (4993A>T), N532K (5001C>A), L533K (5002C>A 5003T>A 5004T>G), S535N (5008T>A 5009C>A 5010A>T), P536L (5012C>T), V539R (5020G>A 5021T>G 5022T>G), K540L (5023A>C 5024A>T 5025A>T), L541I (5026C>A), K542R (5029A>C 5030A>G 5031G>A), E544S (5035G>T 5036A>C 5037G>C), I545N (5039T>A 5040A>C), T546R (5041A>C 5042C>G 5043T>A), W547M (5044T>A 5045G>T), S548G (5047T>G 5048C>G 5049T>A), E551D (5058G>T), K552E (5059A>G), D553H (5062G>C 5064C>T), E555K (5068G>A), T556C (5071A>T 5072C>G), R558Q (5077A>C 5078G>A), K559S (5081A>G 5082A>C), I560L (5083A>C), S562K (5090G>A 5091T>A), L563E (5092C>G 5093T>A), V564C (5095G>T 5096T>G 5097A>T), K565S (5098A>T 5099A>C 5100A>C), T566K (5102C>A 5103T>A), D569K (5110G>A 5112C>G), Y571R (5116T>A 5117A>G 5118C>A), N572L (5119A>C 5120A>T), S574E (5125T>G 5126C>A), K578N (5139G>C), P579L (5140C>T 5141C>T 5142T>A), I580V (5143A>G 5145C>T), I581L (5146A>T 5148C>A), E582Q (5149G>C), C583T (5152T>A 5153G>C 5154C>A), D588Y (5167G>T), I593L (5182A>T 5184C>A), K595Q (5188A>C), A596T (5191G>A 5193A>T), K597D (5194A>G 5196A>C), P599N (5200C>A 5201C>A 5202A>T), G601_V604del (5206_5217delGGTAAAGAGGTA), A609T (5230G>A 5232T>A), K614N (5247A>T), P615E (5248C>G 5249C>A), A616T (5251G>A 5253T>C), K618T (5258A>C 5259G>T), N619K (5262T>A), H621S (5266C>T 5267A>C 5268T>A), S622T (5270G>C 5271T>A), I627L (5284A>T 5286C>A), S629A (5290T>G), I631V (5296A>G 5298T>A), K632R (5300A>G 5301G>A), A633G (5303C>G 5304A>C), K635R (5308A>C 5309A>G), A636K (5311G>A 5312C>A 5313T>A), R638S (5317A>T 5318G>C 5319A>T), Y640F (5324A>T), I641L (5326A>C 5328C>A), Y644K (5335T>A 5337T>G), K645Q (5338A>C), L647I (5344C>A 5346T>C), V648I (5347G>A 5349A>C), T650S (5353A>T 5355T>G)								
Codon mutations:	TTA273..G (4224A>G), GAC274GAT (4227C>T), ACT275CCC (4228A>C 4230T>C), AAC276AGC (4232A>G), AAA277ATC (4235A>T 4236A>C), ATT279ATA (4242T>A), AAG280AGA (4244A>G 4245G>A), TGT281GTT (4246T>G 4247G>T), CCA283CCG (4254A>G), CAG285CTA (4259A>T 4260G>A), TAT286TAC (4263T>C), TCA287AAA (4264T>A 4265C>A), CCT288GAA (4267C>G 4268C>A 4269T>A), GAC290GAT (4275C>T), CGA291AAA (4276C>A 4277G>A), GAA292ACT (4279G>A 4280A>C 4281A>T), GAG293GAA (4284G>A), ACT296ATT (4292C>T), ATC298ATT (4299C>T), GAA299CAA (4300G>C), CTA301TTA (4306C>T), CTC302TTA (4309C>T 4311C>A), AAG303AAT (4314G>T), CTT304ATG (4315C>A 4317T>G), GGT305AAA (4318G>A 4319G>A 4320T>A), ATC306TTA (4321A>T 4323C>A), ATC307ATA (4326C>A), CCT309AGA (4330C>A 4331C>G 4332T>A), AGC310..A (4335C>A), AAG311.CA (4337A>C 4338G>A), CCA313CGA (4343C>G), CAT314CAC (4347T>C), TCC315AGA (4348T>A 4349C>G 4350C>A), CCA317GCA (4354C>G), GCC318GCA (4359C>A), AGG322AGA (4371G>A), AAT323AAC (4374T>C), GCC325AGC (4378G>A 4379C>G), GAG326GAA (4383G>A), ATC327ATA (4386C>A), AAA328GTA (4387A>G 4388A>T), CGA329AGA (4390C>A), GGC330GGA (4395C>A), ATT336ATA (4413T>A), AAC337AAT (4416C>T), AAG340AGA (4424A>G 4425G>A), TTA341CTT (4426T>C 4428A>T), GAC343GAT (4434C>T), CAT344AAT (4435C>A), ACA345ACT (4440A>T), AAG346AGA (4442A>G 4443G>A), GGA347ATG (4444G>A 4445G>T 4446A>G), GGC349GCT (4451G>C 4452C>T), TAC350TAT (4455C>T), CTA351AAA (4456C>A 4457T>A), CTT352TTA (4459C>T 4461T>A), CCT353CCA (4464T>A), AAC354GAC (4465A>G), AAG355AAA (4470G>A), GAG356AAT (4471G>A 4473G>T), CAA357GAA (4474C>G), CTG358CTA (4479G>A), CTT359ATA (4480C>A 4482T>A), CAA360AAT (4483C>A 4485A>T), ATC362ATA (4491C>A), GGA363CAA (4492G>C 4493G>A), GGT364GGA (4497T>A), AAG365AAA (4500G>A), ACC366ACA (4503C>A), TAC368TTT (4508A>T 4509C>T), TCT369AGT (4510T>A 4511C>G), TCC370AAA (4513T>A 4514C>A 4515C>A), GAC372GAT (4521C>T), TCT375TCA (4530T>A), TTT377TAT (4535T>A), CAG379CAA (4542G>A), CGC381AAA (4546C>A 4547G>A 4548C>A), CTT382ATG (4549C>A 4551T>G), GCT383CAC (4552G>C 4553C>A 4554T>C), CCA384CCT (4557A>T), GAA385GAT (4560A>T), ACA386AGT (4562C>G 4563A>T), ATT387ATA (4566T>A), CAG388GAA (4567C>G 4569G>A), CTA389TGG (4570C>T 4571T>G 4572A>G), ACC390ACA (4575C>A), GCT391ACA (4576G>A 4578T>A), TTC392TTT (4581C>T), AGC393ACT (4583G>C 4584C>T), CCC395CCA (4590C>A), CAA396GAA (4591C>G), CAC398CAT (4599C>T), TAC399TTT (4601A>T 4602C>T), GAA400GAG (4605A>G), GTC403GCA (4613T>C 4614C>A), CCT405TCA (4618C>T 4620T>A), CAA410A.T (4633C>A 4635A>T), CAG493.AA (4884G>A), AGT494ACT (4886G>C), ATC496ATA (4893C>A), GGA497TCA (4894G>T 4895G>C), CTA498AAA (4897C>A 4898T>A), CAC499AAG (4900C>A 4902C>G), TTA500ATC (4903T>A 4905A>C), GTT501TGT (4906G>T 4907T>G), GCT502GAA (4910C>A 4911T>A), CAA506AAA (4921C>A), CTT507CTA (4926T>A), AGT508AAT (4928G>A), GAT509ACA (4930G>A 4931A>C 4932T>A), AGG510AAA (4934G>A 4935G>A), AAT511CAA (4936A>C 4938T>A), GCC512GAA (4940C>A 4941C>A), TTA513ATC (4942T>A 4944A>C), AGA515AGG (4950A>G), TTA517CTT (4954T>C 4956A>T), GGC518GGA (4959C>A), CTC519TGT (4960C>T 4961T>G 4962C>T), CTA520TTA (4963C>T), TAT522TAC (4971T>C), ATC523GCA (4972A>G 4973T>C 4974C>A), TCC524AGT (4975T>A 4976C>G 4977C>T), GCT525GAA (4979C>A 4980T>A), TAT526TTT (4982A>T), TTT527ATA (4984T>A 4986T>A), AAA529GAC (4990A>G 4992A>C), ATA530TTA (4993A>T), AAC532AAA (5001C>A), CTT533AAG (5002C>A 5003T>A 5004T>G), AGG534AGA (5007G>A), TCA535AAT (5008T>A 5009C>A 5010A>T), CCT536CTT (5012C>T), CAG538CAA (5019G>A), GTT539AGG (5020G>A 5021T>G 5022T>G), AAA540CTT (5023A>C 5024A>T 5025A>T), CTT541ATT (5026C>A), AAG542CGA (5029A>C 5030A>G 5031G>A), GAG544TCC (5035G>T 5036A>C 5037G>C), ATA545AAC (5039T>A 5040A>C), ACT546CGA (5041A>C 5042C>G 5043T>A), TGG547ATG (5044T>A 5045G>T), TCT548GGA (5047T>G 5048C>G 5049T>A), ACT550ACA (5055T>A), GAG551GAT (5058G>T), AAA552GAA (5059A>G), GAC553CAT (5062G>C 5064C>T), ACG554ACT (5067G>T), GAA555AAA (5068G>A), ACT556TGT (5071A>T 5072C>G), GTC557GTT (5076C>T), AGA558CAA (5077A>C 5078G>A), AAA559AGC (5081A>G 5082A>C), ATA560CTA (5083A>C), AAG561AAA (5088G>A), AGT562AAA (5090G>A 5091T>A), CTA563GAA (5092C>G 5093T>A), GTA564TGT (5095G>T 5096T>G 5097A>T), AAA565TCC (5098A>T 5099A>C 5100A>C), ACT566AAA (5102C>A 5103T>A), GAC569AAG (5110G>A 5112C>G), CTT570CTA (5115T>A), TAC571AGA (5116T>A 5117A>G 5118C>A), AAT572CTT (5119A>C 5120A>T), CCT573CCG (5124T>G), TCA574GAA (5125T>G 5126C>A), GAG576GAA (5133G>A), GAT577GAC (5136T>C), AAG578AAC (5139G>C), CCT579TTA (5140C>T 5141C>T 5142T>A), ATC580GTT (5143A>G 5145C>T), ATC581TTA (5146A>T 5148C>A), GAA582CAA (5149G>C), TGC583ACA (5152T>A 5153G>C 5154C>A), GCA585GCT (5160A>T), GAC588TAC (5167G>T), ATC593TTA (5182A>T 5184C>A), CTC594TTA (5185C>T 5187C>A), AAA595CAA (5188A>C), GCA596ACT (5191G>A 5193A>T), AAA597GAC (5194A>G 5196A>C), CCA599AAT (5200C>A 5201C>A 5202A>T), GGT601_GTA604del (5206_5217delGGTAAAGAGGTA), ATA605ATT (5220A>T), TGC606TGT (5223C>T), GCT609ACA (5230G>A 5232T>A), TCA610AGT (5233T>A 5234C>G 5235A>T), GGA611GGT (5238A>T), ACC612ACA (5241C>A), AAA614AAT (5247A>T), CCA615GAA (5248C>G 5249C>A), GCT616ACC (5251G>A 5253T>C), GAG617GAA (5256G>A), AAG618ACT (5258A>C 5259G>T), AAT619AAA (5262T>A), CAT621TCA (5266C>T 5267A>C 5268T>A), AGT622ACA (5270G>C 5271T>A), AAG625AAA (5280G>A), ATC627TTA (5284A>T 5286C>A), CTT628TTG (5287C>T 5289T>G), TCC629GCC (5290T>G), ATT630ATA (5295T>A), ATT631GTA (5296A>G 5298T>A), AAG632AGA (5300A>G 5301G>A), GCA633GGC (5303C>G 5304A>C), ATC634ATA (5307C>A), AAA635CGA (5308A>C 5309A>G), GCT636AAA (5311G>A 5312C>A 5313T>A), AGA638TCT (5317A>T 5318G>C 5319A>T), TAT640TTT (5324A>T), ATC641CTA (5326A>C 5328C>A), CTT642TTA (5329C>T 5331T>A), CCT643CCA (5334T>A), TAT644AAG (5335T>A 5337T>G), AAA645CAA (5338A>C), TTT646TTC (5343T>C), CTT647ATC (5344C>A 5346T>C), GTA648ATC (5347G>A 5349A>C), AGG649AGA (5352G>A), ACT650TCG (5353A>T 5355T>G), GAT651GAC (5358T>C)								

Proteins

	Begin	End	Coverage	Score	Concordance	Matches	Identities	I/D/M/F*	Stop Codons
hypothetical protein (NP_043933.1)	274	653	41.2%	1130	55.6%	288 (98.6%)	149 (51.0%)	0/4/1/0	0

	Begin	End	Coverage	Score	Concordance	Matches	Identities	I/D/M/F*	Stop Codons
**NT**	**4224**	**5362**	**11.1%**	**243**	**14.2%**	**864 (98.6%)**	**498 (56.8%)**	**0/12**	
Protein mutations:	T275P (4228A>C 4230T>C), N276S (4232A>G), K277I (4235A>T 4236A>C), K280R (4244A>G 4245G>A), C281V (4246T>G 4247G>T), Q285L (4259A>T 4260G>A), S287K (4264T>A 4265C>A), P288E (4267C>G 4268C>A 4269T>A), R291K (4276C>A 4277G>A), E292T (4279G>A 4280A>C 4281A>T), T296I (4292C>T), E299Q (4300G>C), K303N (4314G>T), L304M (4315C>A 4317T>G), G305K (4318G>A 4319G>A 4320T>A), I306L (4321A>T 4323C>A), P309R (4330C>A 4331C>G 4332T>A), P313R (4343C>G), S315R (4348T>A 4349C>G 4350C>A), P317A (4354C>G), A325S (4378G>A 4379C>G), K328V (4387A>G 4388A>T), K340R (4424A>G 4425G>A), H344N (4435C>A), K346R (4442A>G 4443G>A), G347M (4444G>A 4445G>T 4446A>G), G349A (4451G>C 4452C>T), L351K (4456C>A 4457T>A), N354D (4465A>G), E356N (4471G>A 4473G>T), Q357E (4474C>G), L359I (4480C>A 4482T>A), Q360N (4483C>A 4485A>T), G363Q (4492G>C 4493G>A), Y368F (4508A>T 4509C>T), S370K (4513T>A 4514C>A 4515C>A), F377Y (4535T>A), R381K (4546C>A 4547G>A 4548C>A), L382M (4549C>A 4551T>G), A383H (4552G>C 4553C>A 4554T>C), E385D (4560A>T), T386S (4562C>G 4563A>T), Q388E (4567C>G 4569G>A), L389W (4570C>T 4571T>G 4572A>G), A391T (4576G>A 4578T>A), S393T (4583G>C 4584C>T), Q396E (4591C>G), Y399F (4601A>T 4602C>T), V403A (4613T>C 4614C>A), P405S (4618C>T 4620T>A), S494T (4886G>C), G497S (4894G>T 4895G>C), L498K (4897C>A 4898T>A), H499K (4900C>A 4902C>G), L500I (4903T>A 4905A>C), V501C (4906G>T 4907T>G), A502E (4910C>A 4911T>A), Q506K (4921C>A), S508N (4928G>A), D509T (4930G>A 4931A>C 4932T>A), R510K (4934G>A 4935G>A), N511Q (4936A>C 4938T>A), A512E (4940C>A 4941C>A), L513I (4942T>A 4944A>C), L519C (4960C>T 4961T>G 4962C>T), I523A (4972A>G 4973T>C 4974C>A), A525E (4979C>A 4980T>A), Y526F (4982A>T), F527I (4984T>A 4986T>A), K529D (4990A>G 4992A>C), I530L (4993A>T), N532K (5001C>A), L533K (5002C>A 5003T>A 5004T>G), S535N (5008T>A 5009C>A 5010A>T), P536L (5012C>T), V539R (5020G>A 5021T>G 5022T>G), K540L (5023A>C 5024A>T 5025A>T), L541I (5026C>A), K542R (5029A>C 5030A>G 5031G>A), E544S (5035G>T 5036A>C 5037G>C), I545N (5039T>A 5040A>C), T546R (5041A>C 5042C>G 5043T>A), W547M (5044T>A 5045G>T), S548G (5047T>G 5048C>G 5049T>A), E551D (5058G>T), K552E (5059A>G), D553H (5062G>C 5064C>T), E555K (5068G>A), T556C (5071A>T 5072C>G), R558Q (5077A>C 5078G>A), K559S (5081A>G 5082A>C), I560L (5083A>C), S562K (5090G>A 5091T>A), L563E (5092C>G 5093T>A), V564C (5095G>T 5096T>G 5097A>T), K565S (5098A>T 5099A>C 5100A>C), T566K (5102C>A 5103T>A), D569K (5110G>A 5112C>G), Y571R (5116T>A 5117A>G 5118C>A), N572L (5119A>C 5120A>T), S574E (5125T>G 5126C>A), K578N (5139G>C), P579L (5140C>T 5141C>T 5142T>A), I580V (5143A>G 5145C>T), I581L (5146A>T 5148C>A), E582Q (5149G>C), C583T (5152T>A 5153G>C 5154C>A), D588Y (5167G>T), I593L (5182A>T 5184C>A), K595Q (5188A>C), A596T (5191G>A 5193A>T), K597D (5194A>G 5196A>C), P599N (5200C>A 5201C>A 5202A>T), G601_V604del (5206_5217delGGTAAAGAGGTA), A609T (5230G>A 5232T>A), K614N (5247A>T), P615E (5248C>G 5249C>A), A616T (5251G>A 5253T>C), K618T (5258A>C 5259G>T), N619K (5262T>A), H621S (5266C>T 5267A>C 5268T>A), S622T (5270G>C 5271T>A), I627L (5284A>T 5286C>A), S629A (5290T>G), I631V (5296A>G 5298T>A), K632R (5300A>G 5301G>A), A633G (5303C>G 5304A>C), K635R (5308A>C 5309A>G), A636K (5311G>A 5312C>A 5313T>A), R638S (5317A>T 5318G>C 5319A>T), Y640F (5324A>T), I641L (5326A>C 5328C>A), Y644K (5335T>A 5337T>G), K645Q (5338A>C), L647I (5344C>A 5346T>C), V648I (5347G>A 5349A>C), T650S (5353A>T 5355T>G)								
Codon mutations:	TTA273..G (4224A>G), GAC274GAT (4227C>T), ACT275CCC (4228A>C 4230T>C), AAC276AGC (4232A>G), AAA277ATC (4235A>T 4236A>C), ATT279ATA (4242T>A), AAG280AGA (4244A>G 4245G>A), TGT281GTT (4246T>G 4247G>T), CCA283CCG (4254A>G), CAG285CTA (4259A>T 4260G>A), TAT286TAC (4263T>C), TCA287AAA (4264T>A 4265C>A), CCT288GAA (4267C>G 4268C>A 4269T>A), GAC290GAT (4275C>T), CGA291AAA (4276C>A 4277G>A), GAA292ACT (4279G>A 4280A>C 4281A>T), GAG293GAA (4284G>A), ACT296ATT (4292C>T), ATC298ATT (4299C>T), GAA299CAA (4300G>C), CTA301TTA (4306C>T), CTC302TTA (4309C>T 4311C>A), AAG303AAT (4314G>T), CTT304ATG (4315C>A 4317T>G), GGT305AAA (4318G>A 4319G>A 4320T>A), ATC306TTA (4321A>T 4323C>A), ATC307ATA (4326C>A), CCT309AGA (4330C>A 4331C>G 4332T>A), AGC310..A (4335C>A), AAG311.CA (4337A>C 4338G>A), CCA313CGA (4343C>G), CAT314CAC (4347T>C), TCC315AGA (4348T>A 4349C>G 4350C>A), CCA317GCA (4354C>G), GCC318GCA (4359C>A), AGG322AGA (4371G>A), AAT323AAC (4374T>C), GCC325AGC (4378G>A 4379C>G), GAG326GAA (4383G>A), ATC327ATA (4386C>A), AAA328GTA (4387A>G 4388A>T), CGA329AGA (4390C>A), GGC330GGA (4395C>A), ATT336ATA (4413T>A), AAC337AAT (4416C>T), AAG340AGA (4424A>G 4425G>A), TTA341CTT (4426T>C 4428A>T), GAC343GAT (4434C>T), CAT344AAT (4435C>A), ACA345ACT (4440A>T), AAG346AGA (4442A>G 4443G>A), GGA347ATG (4444G>A 4445G>T 4446A>G), GGC349GCT (4451G>C 4452C>T), TAC350TAT (4455C>T), CTA351AAA (4456C>A 4457T>A), CTT352TTA (4459C>T 4461T>A), CCT353CCA (4464T>A), AAC354GAC (4465A>G), AAG355AAA (4470G>A), GAG356AAT (4471G>A 4473G>T), CAA357GAA (4474C>G), CTG358CTA (4479G>A), CTT359ATA (4480C>A 4482T>A), CAA360AAT (4483C>A 4485A>T), ATC362ATA (4491C>A), GGA363CAA (4492G>C 4493G>A), GGT364GGA (4497T>A), AAG365AAA (4500G>A), ACC366ACA (4503C>A), TAC368TTT (4508A>T 4509C>T), TCT369AGT (4510T>A 4511C>G), TCC370AAA (4513T>A 4514C>A 4515C>A), GAC372GAT (4521C>T), TCT375TCA (4530T>A), TTT377TAT (4535T>A), CAG379CAA (4542G>A), CGC381AAA (4546C>A 4547G>A 4548C>A), CTT382ATG (4549C>A 4551T>G), GCT383CAC (4552G>C 4553C>A 4554T>C), CCA384CCT (4557A>T), GAA385GAT (4560A>T), ACA386AGT (4562C>G 4563A>T), ATT387ATA (4566T>A), CAG388GAA (4567C>G 4569G>A), CTA389TGG (4570C>T 4571T>G 4572A>G), ACC390ACA (4575C>A), GCT391ACA (4576G>A 4578T>A), TTC392TTT (4581C>T), AGC393ACT (4583G>C 4584C>T), CCC395CCA (4590C>A), CAA396GAA (4591C>G), CAC398CAT (4599C>T), TAC399TTT (4601A>T 4602C>T), GAA400GAG (4605A>G), GTC403GCA (4613T>C 4614C>A), CCT405TCA (4618C>T 4620T>A), CAA410A.T (4633C>A 4635A>T), CAG493.AA (4884G>A), AGT494ACT (4886G>C), ATC496ATA (4893C>A), GGA497TCA (4894G>T 4895G>C), CTA498AAA (4897C>A 4898T>A), CAC499AAG (4900C>A 4902C>G), TTA500ATC (4903T>A 4905A>C), GTT501TGT (4906G>T 4907T>G), GCT502GAA (4910C>A 4911T>A), CAA506AAA (4921C>A), CTT507CTA (4926T>A), AGT508AAT (4928G>A), GAT509ACA (4930G>A 4931A>C 4932T>A), AGG510AAA (4934G>A 4935G>A), AAT511CAA (4936A>C 4938T>A), GCC512GAA (4940C>A 4941C>A), TTA513ATC (4942T>A 4944A>C), AGA515AGG (4950A>G), TTA517CTT (4954T>C 4956A>T), GGC518GGA (4959C>A), CTC519TGT (4960C>T 4961T>G 4962C>T), CTA520TTA (4963C>T), TAT522TAC (4971T>C), ATC523GCA (4972A>G 4973T>C 4974C>A), TCC524AGT (4975T>A 4976C>G 4977C>T), GCT525GAA (4979C>A 4980T>A), TAT526TTT (4982A>T), TTT527ATA (4984T>A 4986T>A), AAA529GAC (4990A>G 4992A>C), ATA530TTA (4993A>T), AAC532AAA (5001C>A), CTT533AAG (5002C>A 5003T>A 5004T>G), AGG534AGA (5007G>A), TCA535AAT (5008T>A 5009C>A 5010A>T), CCT536CTT (5012C>T), CAG538CAA (5019G>A), GTT539AGG (5020G>A 5021T>G 5022T>G), AAA540CTT (5023A>C 5024A>T 5025A>T), CTT541ATT (5026C>A), AAG542CGA (5029A>C 5030A>G 5031G>A), GAG544TCC (5035G>T 5036A>C 5037G>C), ATA545AAC (5039T>A 5040A>C), ACT546CGA (5041A>C 5042C>G 5043T>A), TGG547ATG (5044T>A 5045G>T), TCT548GGA (5047T>G 5048C>G 5049T>A), ACT550ACA (5055T>A), GAG551GAT (5058G>T), AAA552GAA (5059A>G), GAC553CAT (5062G>C 5064C>T), ACG554ACT (5067G>T), GAA555AAA (5068G>A), ACT556TGT (5071A>T 5072C>G), GTC557GTT (5076C>T), AGA558CAA (5077A>C 5078G>A), AAA559AGC (5081A>G 5082A>C), ATA560CTA (5083A>C), AAG561AAA (5088G>A), AGT562AAA (5090G>A 5091T>A), CTA563GAA (5092C>G 5093T>A), GTA564TGT (5095G>T 5096T>G 5097A>T), AAA565TCC (5098A>T 5099A>C 5100A>C), ACT566AAA (5102C>A 5103T>A), GAC569AAG (5110G>A 5112C>G), CTT570CTA (5115T>A), TAC571AGA (5116T>A 5117A>G 5118C>A), AAT572CTT (5119A>C 5120A>T), CCT573CCG (5124T>G), TCA574GAA (5125T>G 5126C>A), GAG576GAA (5133G>A), GAT577GAC (5136T>C), AAG578AAC (5139G>C), CCT579TTA (5140C>T 5141C>T 5142T>A), ATC580GTT (5143A>G 5145C>T), ATC581TTA (5146A>T 5148C>A), GAA582CAA (5149G>C), TGC583ACA (5152T>A 5153G>C 5154C>A), GCA585GCT (5160A>T), GAC588TAC (5167G>T), ATC593TTA (5182A>T 5184C>A), CTC594TTA (5185C>T 5187C>A), AAA595CAA (5188A>C), GCA596ACT (5191G>A 5193A>T), AAA597GAC (5194A>G 5196A>C), CCA599AAT (5200C>A 5201C>A 5202A>T), GGT601_GTA604del (5206_5217delGGTAAAGAGGTA), ATA605ATT (5220A>T), TGC606TGT (5223C>T), GCT609ACA (5230G>A 5232T>A), TCA610AGT (5233T>A 5234C>G 5235A>T), GGA611GGT (5238A>T), ACC612ACA (5241C>A), AAA614AAT (5247A>T), CCA615GAA (5248C>G 5249C>A), GCT616ACC (5251G>A 5253T>C), GAG617GAA (5256G>A), AAG618ACT (5258A>C 5259G>T), AAT619AAA (5262T>A), CAT621TCA (5266C>T 5267A>C 5268T>A), AGT622ACA (5270G>C 5271T>A), AAG625AAA (5280G>A), ATC627TTA (5284A>T 5286C>A), CTT628TTG (5287C>T 5289T>G), TCC629GCC (5290T>G), ATT630ATA (5295T>A), ATT631GTA (5296A>G 5298T>A), AAG632AGA (5300A>G 5301G>A), GCA633GGC (5303C>G 5304A>C), ATC634ATA (5307C>A), AAA635CGA (5308A>C 5309A>G), GCT636AAA (5311G>A 5312C>A 5313T>A), AGA638TCT (5317A>T 5318G>C 5319A>T), TAT640TTT (5324A>T), ATC641CTA (5326A>C 5328C>A), CTT642TTA (5329C>T 5331T>A), CCT643CCA (5334T>A), TAT644AAG (5335T>A 5337T>G), AAA645CAA (5338A>C), TTT646TTC (5343T>C), CTT647ATC (5344C>A 5346T>C), GTA648ATC (5347G>A 5349A>C), AGG649AGA (5352G>A), ACT650TCG (5353A>T 5355T>G), GAT651GAC (5358T>C)								

*: Inserts / Deletes / Misaligned / Frameshifts

Analysis details

This analysis was performed with panviral2.64

NGS Details (UN62): Badnavirus occultipomeae

Assembly

**Coverage Length**	429 (2 contig(s))
**Depth Of Coverage**	8.1
**Number Of Reads**	46
**Reads Per Million**	1.07 rpm (after QC)
**Ambiguities**	0
**Assembly Method**	de novo + reference guided assembly
**Consensus Caller**	Bcf Tools

Coverage Map

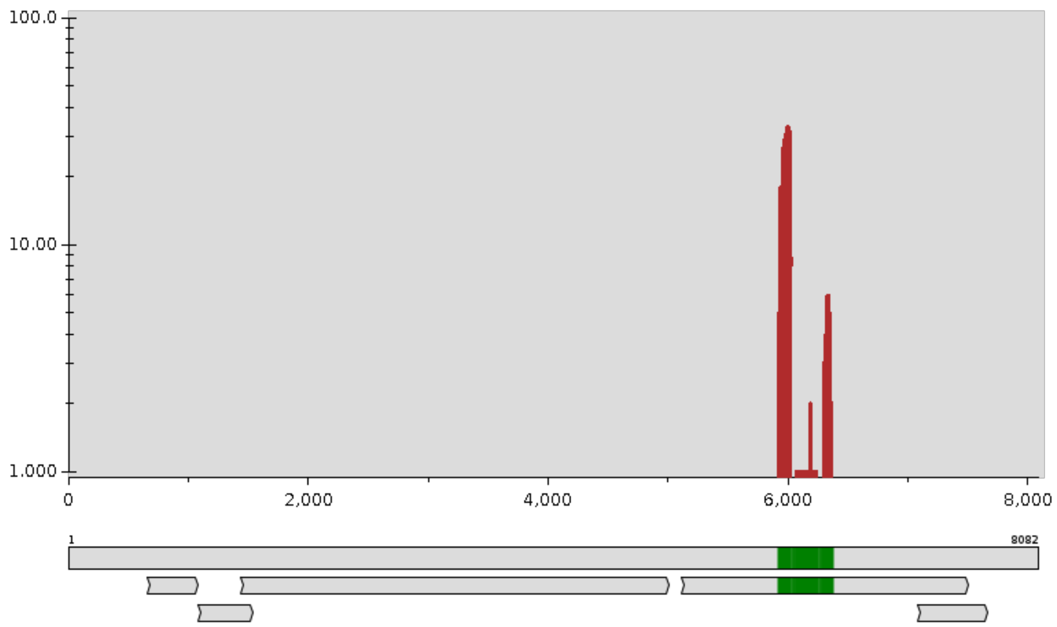

Assignment

**Type**	Badnavirus occultipomeae (Taxonomy ID: 3048353)
**Reference Genome**	NC_015655.1
**NT Identity (%)**	49.537
**AA Identity (%)**	38.3562
**Number Of Stop Codons**	0
**Number Of CDS**	5

Alignment

**Alignment Score**	-14.0 (NT) + 253.0 (AA) = 239.0
**Concordance (%)**	14.3371

Alignment Method	Global, seeded, nucleotide + amino acids (AGA)

Genome Region

Sequence starts at position 5911 and ends at position 6377 relative to NC_015655.1 reference sequence.

Alignment Detailed Statistics

	Begin	End	Coverage	Score	Concordance	Matches	Identities	I/D/M/F*	Stop Codons
**NT**	**5911**	**6377**	**5.3%**	**-14**	**-1.6%**	**429 (99.3%)**	**214 (49.5%)**	**3/0**	
Mutations:	5911G>A, 5915T>A, 5916T>G, 5917A>G, 5918G>A, 5919A>T, 5921G>A, 5922C>G, 5924A>G, 5925A>C, 5926C>A, 5927A>T, 5929A>T, 5935G>T, 5936T>G, 5938T>A, 5939A>T, 5940C>T, 5941A>G, 5948A>G, 5949G>T, 5951G>A, 5952C>A, 5959G>A, 5961C>G, 5962A>G, 5963A>T, 5964T>C, 5967A>T, 5968G>A, 5969A>T, 5973A>G, 5974G>A, 5977C>A, 5982T>C, 5983T>C, 5985C>G, 5986C>T, 5988A>G, 5991G>T, 5992G>A, 5997C>T, 5998A>G, 6000C>T, 6001A>G, 6002C>A, 6004A>T, 6006T>G, 6007G>T, 6009C>T, 6010A>G, 6011G>A, 6013A>C, 6014G>A, 6016A>C, 6018A>T, 6019T>C, 6020C>A, 6021T>A, 6025A>G, 6026A>C, 6027A>G, 6029A>C, 6030G>T, 6031A>G, 6033A>G, 6036C>T, 6039A>T, 6042A>G, 6043T>A, 6051C>G, 6053A>G, 6055G>T, 6057A>G, 6060C>T, 6062T>A, 6069G>A, 6070A>T, 6072A>G, 6075A>G, 6076A>G, 6078G>T, 6079G>A, 6080A>G, 6084A>G, 6085A>T, 6086A>C, 6087G>T, 6088T>G, 6089C>A, 6090C>T, 6092A>T, 6093A>C, 6096A>G, 6097T>A, 6098G>A, 6109T>C, 6116C>G, 6117A>G, 6118G>T, 6120A>T, 6123G>T, 6125T>A, 6135T>C, 6136G>C, 6137A>T, 6138A>G, 6141C>T, 6145C>T, 6153A>G, 6154C>T, 6156G>A, 6158T>C, 6159G>T, 6165A>C, 6168A>T, 6173A>C, 6178C>A, 6179A>T, 6181A>G, 6182G>A, 6183A>C, 6184A>T, 6185A>T, 6190G>A, 6192T>C, 6193A>C, 6194A>G, 6195T>G, 6197C>T, 6198A>G, 6201T>C, 6203G>A, 6205G>T, 6206G>C, 6207A>T, 6207_6208insTAT, 6208A>C, 6209C>T, 6210A>T, 6213T>C, 6214G>C, 6215C>A, 6216A>G, 6220A>G, 6222C>G, 6223G>A, 6224C>T, 6225A>T, 6228A>G, 6230A>T, 6231T>C, 6240C>T, 6243A>C, 6251T>A, 6255T>C, 6256G>C, 6260A>G, 6262G>A, 6264A>G, 6270G>A, 6276G>A, 6279T>A, 6282T>C, 6283C>T, 6285C>A, 6286T>A, 6287T>G, 6288A>G, 6289A>G, 6290A>T, 6292T>G, 6295G>C, 6296C>T, 6300A>G, 6302T>C, 6304G>T, 6310A>G, 6311G>A, 6312A>T, 6316G>C, 6317G>A, 6318G>A, 6322A>T, 6323T>G, 6325C>G, 6326T>C, 6327A>T, 6329G>A, 6330C>G, 6331C>T, 6332C>T, 6333A>C, 6334A>T, 6339A>G, 6340A>T, 6341T>A, 6342G>T, 6343A>G, 6345A>G, 6346A>T, 6349G>T, 6351A>G, 6352G>C, 6354C>A, 6355A>G, 6358G>T, 6359A>C, 6363G>A, 6364G>T, 6365A>C, 6366T>C, 6369C>T, 6375A>C, 6376A>C, 6377T>A								

CDS

	Begin	End	Coverage	Score	Concordance	Matches	Identities	I/D/M/F*	Stop Codons
SPBVa_gp4	266	421	18.2%	253	24.1%	145 (99.3%)	56 (38.4%)	1/0/6/0	0
Protein mutations:	E266K (5911G>A), I267K (5915T>A 5916T>G), R268D (5917A>G 5918G>A 5919A>T), G269E (5921G>A 5922C>G), K270S (5924A>G 5925A>C), Q271I (5926C>A 5927A>T), R272W (5929A>T), V274C (5935G>T 5936T>G), Y275I (5938T>A 5939A>T 5940C>T), N276D (5941A>G), K278S (5948A>G 5949G>T), R279Q (5951G>A 5952C>A), D282K (5959G>A 5961C>G), N283V (5962A>G 5963A>T 5964T>C), E285I (5968G>A 5969A>T), D287N (5974G>A), Q288K (5977C>A), S290P (5983T>C 5985C>G), G293R (5992G>A), N295D (5998A>G 6000C>T), T296D (6001A>G 6002C>A), I297L (6004A>T 6006T>G), V298F (6007G>T 6009C>T), S299D (6010A>G 6011G>A), R300Q (6013A>C 6014G>A), I301L (6016A>C 6018A>T), S302Q (6019T>C 6020C>A 6021T>A), K304A (6025A>G 6026A>C 6027A>G), K305T (6029A>C 6030G>T), F316Y (6062T>A), I319L (6070A>T 6072A>G), M321V (6076A>G 6078G>T), E322R (6079G>A 6080A>G), K324S (6085A>T 6086A>C 6087G>T), S325D (6088T>G 6089C>A 6090C>T), K326I (6092A>T 6093A>C), W328K (6097T>A 6098G>A), W332R (6109T>C), P334R (6116C>G 6117A>G), E335Y (6118G>T 6120A>T), L337H (6125T>A), E341L (6136G>C 6137A>T 6138A>G), P344S (6145C>T), M348T (6158T>C 6159G>T), D353A (6173A>C), Q355M (6178C>A 6179A>T), R356D (6181A>G 6182G>A 6183A>C), K357L (6184A>T 6185A>T), D359N (6190G>A 6192T>C), N360R (6193A>C 6194A>G 6195T>G), A361V (6197C>T 6198A>G), R363K (6203G>A), G364S (6205G>T 6206G>C 6207A>T), G364_T365insY (6207_6208insTAT), T365L (6208A>C 6209C>T 6210A>T), A367Q (6214G>C 6215C>A 6216A>G), I369V (6220A>G 6222C>G), A370I (6223G>A 6224C>T 6225A>T), Y372F (6230A>T 6231T>C), F379Y (6251T>A), L391R (6286T>A 6287T>G 6288A>G), N392V (6289A>G 6290A>T), L393V (6292T>G), A394L (6295G>C 6296C>T), I396T (6302T>C), V397L (6304G>T), R399D (6310A>G 6311G>A 6312A>T), G401Q (6316G>C 6317G>A 6318G>A), I403C (6322A>T 6323T>G), L404A (6325C>G 6326T>C 6327A>T), S405K (6329G>A 6330C>G), P406F (6331C>T 6332C>T 6333A>C), T407S (6334A>T), M409Y (6340A>T 6341T>A 6342G>T), K410E (6343A>G 6345A>G), I411F (6346A>T), G412W (6349G>T 6351A>G), V413L (6352G>C 6354C>A), K414E (6355A>G), E415S (6358G>T 6359A>C), D417S (6364G>T 6365A>C 6366T>C)								
Codon mutations:	GAG266AAG (5911G>A), ATT267AAG (5915T>A 5916T>G), AGA268GAT (5917A>G 5918G>A 5919A>T), GGC269GAG (5921G>A 5922C>G), AAA270AGC (5924A>G 5925A>C), CAA271ATA (5926C>A 5927A>T), AGG272TGG (5929A>T), GTT274TGT (5935G>T 5936T>G), TAC275ATT (5938T>A 5939A>T 5940C>T), AAC276GAC (5941A>G), AAG278AGT (5948A>G 5949G>T), CGC279CAA (5951G>A 5952C>A), GAC282AAG (5959G>A 5961C>G), AAT283GTC (5962A>G 5963A>T 5964T>C), ACA284ACT (5967A>T), GAA285ATA (5968G>A 5969A>T), AAA286AAG (5973A>G), GAT287AAT (5974G>A), CAG288AAG (5977C>A), TAT289TAC (5982T>C), TCC290CCG (5983T>C 5985C>G), CTA291TTG (5986C>T 5988A>G), CCG292CCT (5991G>T), GGA293AGA (5992G>A), ATC294ATT (5997C>T), AAC295GAT (5998A>G 6000C>T), ACT296GAT (6001A>G 6002C>A), ATT297TTG (6004A>T 6006T>G), GTC298TTT (6007G>T 6009C>T), AGT299GAT (6010A>G 6011G>A), AGA300CAA (6013A>C 6014G>A), ATA301CTT (6016A>C 6018A>T), TCT302CAA (6019T>C 6020C>A 6021T>A), AAA304GCG (6025A>G 6026A>C 6027A>G), AAG305ACT (6029A>C 6030G>T), ATA306G.G (6031A>G 6033A>G), TTC307..T (6036C>T), TCA308..T (6039A>T), AAA309..G (6042A>G), TTT310A.. (6043T>A), CTC312..G (6051C>G), AAA313.G. (6053A>G), GCA314T.G (6055G>T 6057A>G), GGC315..T (6060C>T), TTT316TAT (6062T>A), CAG318CAA (6069G>A), ATA319TTG (6070A>T 6072A>G), AGA320AGG (6075A>G), ATG321GTT (6076A>G 6078G>T), GAG322AGG (6079G>A 6080A>G), GAA323GAG (6084A>G), AAG324TCT (6085A>T 6086A>C 6087G>T), TCC325GAT (6088T>G 6089C>A 6090C>T), AAA326ATC (6092A>T 6093A>C), CCA327CCG (6096A>G), TGG328AAG (6097T>A 6098G>A), TGG332CGG (6109T>C), CCA334CGG (6116C>G 6117A>G), GAA335TAT (6118G>T 6120A>T), GGG336GGT (6123G>T), CTC337CAC (6125T>A), TTT340TTC (6135T>C), GAA341CTG (6136G>C 6137A>T 6138A>G), GTC342GTT (6141C>T), CCC344TCC (6145C>T), GGA346GGG (6153A>G), CTG347TTA (6154C>T 6156G>A), ATG348ACT (6158T>C 6159G>T), GCA350GCC (6165A>C), CCA351CCT (6168A>T), GAT353GCT (6173A>C), CAG355ATG (6178C>A 6179A>T), AGA356GAC (6181A>G 6182G>A 6183A>C), AAG357TTG (6184A>T 6185A>T), GAT359AAC (6190G>A 6192T>C), AAT360CGG (6193A>C 6194A>G 6195T>G), GCA361GTG (6197C>T 6198A>G), TTT362TTC (6201T>C), AGG363AAG (6203G>A), GGA364TCT (6205G>T 6206G>C 6207A>T), GGA364_ACA365insTAT (6207_6208insTAT), ACA365CTT (6208A>C 6209C>T 6210A>T), GAT366GAC (6213T>C), GCA367CAG (6214G>C 6215C>A 6216A>G), ATC369GTG (6220A>G 6222C>G), GCA370ATT (6223G>A 6224C>T 6225A>T), GTA371GTG (6228A>G), TAT372TTC (6230A>T 6231T>C), GAC375GAT (6240C>T), ATA376ATC (6243A>C), TTC379TAC (6251T>A), TCT380TCC (6255T>C), GAA381C.. (6256G>C), AAT382.G. (6260A>G), GAA383A.G (6262G>A 6264A>G), GAG385..A (6270G>A), GAG387..A (6276G>A), GAT388..A (6279T>A), CAT389..C (6282T>C), CTC390TTA (6283C>T 6285C>A), TTA391AGG (6286T>A 6287T>G 6288A>G), AAT392GTT (6289A>G 6290A>T), TTG393GTG (6292T>G), GCC394CTC (6295G>C 6296C>T), CAA395CAG (6300A>G), ATT396ACT (6302T>C), GTG397TTG (6304G>T), AGA399GAT (6310A>G 6311G>A 6312A>T), GGG401CAA (6316G>C 6317G>A 6318G>A), ATT403TGT (6322A>T 6323T>G), CTA404GCT (6325C>G 6326T>C 6327A>T), AGC405AAG (6329G>A 6330C>G), CCA406TTC (6331C>T 6332C>T 6333A>C), ACA407TCA (6334A>T), AAA408AAG (6339A>G), ATG409TAT (6340A>T 6341T>A 6342G>T), AAA410GAG (6343A>G 6345A>G), ATT411TTT (6346A>T), GGA412TGG (6349G>T 6351A>G), GTC413CTA (6352G>C 6354C>A), AAA414GAA (6355A>G), GAA415TCA (6358G>T 6359A>C), GTG416GTA (6363G>A), GAT417TCC (6364G>T 6365A>C 6366T>C), TTC418TTT (6369C>T), GGA420GGC (6375A>C), ATA421CA. (6376A>C 6377T>A)								

Proteins

	Begin	End	Coverage	Score	Concordance	Matches	Identities	I/D/M/F*	Stop Codons
RNaseH/reverse transcriptase (YP_004581513.1)	266	421	18.2%	253	24.1%	145 (99.3%)	56 (38.4%)	1/0/6/0	0
Protein mutations:	E266K (5911G>A), I267K (5915T>A 5916T>G), R268D (5917A>G 5918G>A 5919A>T), G269E (5921G>A 5922C>G), K270S (5924A>G 5925A>C), Q271I (5926C>A 5927A>T), R272W (5929A>T), V274C (5935G>T 5936T>G), Y275I (5938T>A 5939A>T 5940C>T), N276D (5941A>G), K278S (5948A>G 5949G>T), R279Q (5951G>A 5952C>A), D282K (5959G>A 5961C>G), N283V (5962A>G 5963A>T 5964T>C), E285I (5968G>A 5969A>T), D287N (5974G>A), Q288K (5977C>A), S290P (5983T>C 5985C>G), G293R (5992G>A), N295D (5998A>G 6000C>T), T296D (6001A>G 6002C>A), I297L (6004A>T 6006T>G), V298F (6007G>T 6009C>T), S299D (6010A>G 6011G>A), R300Q (6013A>C 6014G>A), I301L (6016A>C 6018A>T), S302Q (6019T>C 6020C>A 6021T>A), K304A (6025A>G 6026A>C 6027A>G), K305T (6029A>C 6030G>T), F316Y (6062T>A), I319L (6070A>T 6072A>G), M321V (6076A>G 6078G>T), E322R (6079G>A 6080A>G), K324S (6085A>T 6086A>C 6087G>T), S325D (6088T>G 6089C>A 6090C>T), K326I (6092A>T 6093A>C), W328K (6097T>A 6098G>A), W332R (6109T>C), P334R (6116C>G 6117A>G), E335Y (6118G>T 6120A>T), L337H (6125T>A), E341L (6136G>C 6137A>T 6138A>G), P344S (6145C>T), M348T (6158T>C 6159G>T), D353A (6173A>C), Q355M (6178C>A 6179A>T), R356D (6181A>G 6182G>A 6183A>C), K357L (6184A>T 6185A>T), D359N (6190G>A 6192T>C), N360R (6193A>C 6194A>G 6195T>G), A361V (6197C>T 6198A>G), R363K (6203G>A), G364S (6205G>T 6206G>C 6207A>T), G364_T365insY (6207_6208insTAT), T365L (6208A>C 6209C>T 6210A>T), A367Q (6214G>C 6215C>A 6216A>G), I369V (6220A>G 6222C>G), A370I (6223G>A 6224C>T 6225A>T), Y372F (6230A>T 6231T>C), F379Y (6251T>A), L391R (6286T>A 6287T>G 6288A>G), N392V (6289A>G 6290A>T), L393V (6292T>G), A394L (6295G>C 6296C>T), I396T (6302T>C), V397L (6304G>T), R399D (6310A>G 6311G>A 6312A>T), G401Q (6316G>C 6317G>A 6318G>A), I403C (6322A>T 6323T>G), L404A (6325C>G 6326T>C 6327A>T), S405K (6329G>A 6330C>G), P406F (6331C>T 6332C>T 6333A>C), T407S (6334A>T), M409Y (6340A>T 6341T>A 6342G>T), K410E (6343A>G 6345A>G), I411F (6346A>T), G412W (6349G>T 6351A>G), V413L (6352G>C 6354C>A), K414E (6355A>G), E415S (6358G>T 6359A>C), D417S (6364G>T 6365A>C 6366T>C)								

	Begin	End	Coverage	Score	Concordance	Matches	Identities	I/D/M/F*	Stop Codons
**NT**	**5911**	**6377**	**5.3%**	**-14**	**-1.6%**	**429 (99.3%)**	**214 (49.5%)**	**3/0**	
Codon mutations:	GAG266AAG (5911G>A), ATT267AAG (5915T>A 5916T>G), AGA268GAT (5917A>G 5918G>A 5919A>T), GGC269GAG (5921G>A 5922C>G), AAA270AGC (5924A>G 5925A>C), CAA271ATA (5926C>A 5927A>T), AGG272TGG (5929A>T), GTT274TGT (5935G>T 5936T>G), TAC275ATT (5938T>A 5939A>T 5940C>T), AAC276GAC (5941A>G), AAG278AGT (5948A>G 5949G>T), CGC279CAA (5951G>A 5952C>A), GAC282AAG (5959G>A 5961C>G), AAT283GTC (5962A>G 5963A>T 5964T>C), ACA284ACT (5967A>T), GAA285ATA (5968G>A 5969A>T), AAA286AAG (5973A>G), GAT287AAT (5974G>A), CAG288AAG (5977C>A), TAT289TAC (5982T>C), TCC290CCG (5983T>C 5985C>G), CTA291TTG (5986C>T 5988A>G), CCG292CCT (5991G>T), GGA293AGA (5992G>A), ATC294ATT (5997C>T), AAC295GAT (5998A>G 6000C>T), ACT296GAT (6001A>G 6002C>A), ATT297TTG (6004A>T 6006T>G), GTC298TTT (6007G>T 6009C>T), AGT299GAT (6010A>G 6011G>A), AGA300CAA (6013A>C 6014G>A), ATA301CTT (6016A>C 6018A>T), TCT302CAA (6019T>C 6020C>A 6021T>A), AAA304GCG (6025A>G 6026A>C 6027A>G), AAG305ACT (6029A>C 6030G>T), ATA306G.G (6031A>G 6033A>G), TTC307..T (6036C>T), TCA308..T (6039A>T), AAA309..G (6042A>G), TTT310A.. (6043T>A), CTC312..G (6051C>G), AAA313.G. (6053A>G), GCA314T.G (6055G>T 6057A>G), GGC315..T (6060C>T), TTT316TAT (6062T>A), CAG318CAA (6069G>A), ATA319TTG (6070A>T 6072A>G), AGA320AGG (6075A>G), ATG321GTT (6076A>G 6078G>T), GAG322AGG (6079G>A 6080A>G), GAA323GAG (6084A>G), AAG324TCT (6085A>T 6086A>C 6087G>T), TCC325GAT (6088T>G 6089C>A 6090C>T), AAA326ATC (6092A>T 6093A>C), CCA327CCG (6096A>G), TGG328AAG (6097T>A 6098G>A), TGG332CGG (6109T>C), CCA334CGG (6116C>G 6117A>G), GAA335TAT (6118G>T 6120A>T), GGG336GGT (6123G>T), CTC337CAC (6125T>A), TTT340TTC (6135T>C), GAA341CTG (6136G>C 6137A>T 6138A>G), GTC342GTT (6141C>T), CCC344TCC (6145C>T), GGA346GGG (6153A>G), CTG347TTA (6154C>T 6156G>A), ATG348ACT (6158T>C 6159G>T), GCA350GCC (6165A>C), CCA351CCT (6168A>T), GAT353GCT (6173A>C), CAG355ATG (6178C>A 6179A>T), AGA356GAC (6181A>G 6182G>A 6183A>C), AAG357TTG (6184A>T 6185A>T), GAT359AAC (6190G>A 6192T>C), AAT360CGG (6193A>C 6194A>G 6195T>G), GCA361GTG (6197C>T 6198A>G), TTT362TTC (6201T>C), AGG363AAG (6203G>A), GGA364TCT (6205G>T 6206G>C 6207A>T), GGA364_ACA365insTAT (6207_6208insTAT), ACA365CTT (6208A>C 6209C>T 6210A>T), GAT366GAC (6213T>C), GCA367CAG (6214G>C 6215C>A 6216A>G), ATC369GTG (6220A>G 6222C>G), GCA370ATT (6223G>A 6224C>T 6225A>T), GTA371GTG (6228A>G), TAT372TTC (6230A>T 6231T>C), GAC375GAT (6240C>T), ATA376ATC (6243A>C), TTC379TAC (6251T>A), TCT380TCC (6255T>C), GAA381C.. (6256G>C), AAT382.G. (6260A>G), GAA383A.G (6262G>A 6264A>G), GAG385..A (6270G>A), GAG387..A (6276G>A), GAT388..A (6279T>A), CAT389..C (6282T>C), CTC390TTA (6283C>T 6285C>A), TTA391AGG (6286T>A 6287T>G 6288A>G), AAT392GTT (6289A>G 6290A>T), TTG393GTG (6292T>G), GCC394CTC (6295G>C 6296C>T), CAA395CAG (6300A>G), ATT396ACT (6302T>C), GTG397TTG (6304G>T), AGA399GAT (6310A>G 6311G>A 6312A>T), GGG401CAA (6316G>C 6317G>A 6318G>A), ATT403TGT (6322A>T 6323T>G), CTA404GCT (6325C>G 6326T>C 6327A>T), AGC405AAG (6329G>A 6330C>G), CCA406TTC (6331C>T 6332C>T 6333A>C), ACA407TCA (6334A>T), AAA408AAG (6339A>G), ATG409TAT (6340A>T 6341T>A 6342G>T), AAA410GAG (6343A>G 6345A>G), ATT411TTT (6346A>T), GGA412TGG (6349G>T 6351A>G), GTC413CTA (6352G>C 6354C>A), AAA414GAA (6355A>G), GAA415TCA (6358G>T 6359A>C), GTG416GTA (6363G>A), GAT417TCC (6364G>T 6365A>C 6366T>C), TTC418TTT (6369C>T), GGA420GGC (6375A>C), ATA421CA. (6376A>C 6377T>A)								

*: Inserts / Deletes / Misaligned / Frameshifts

Analysis details

This analysis was performed with panviral2.64

NGS Details (UN62): Errantivirus

Assembly

**Coverage Length**	575 (3 contig(s))
**Depth Of Coverage**	8.9
**Number Of Reads**	38
**Reads Per Million**	0.88 rpm (after QC)
**Ambiguities**	0
**Assembly Method**	de novo + reference guided assembly
**Consensus Caller**	Bcf Tools

Coverage Map

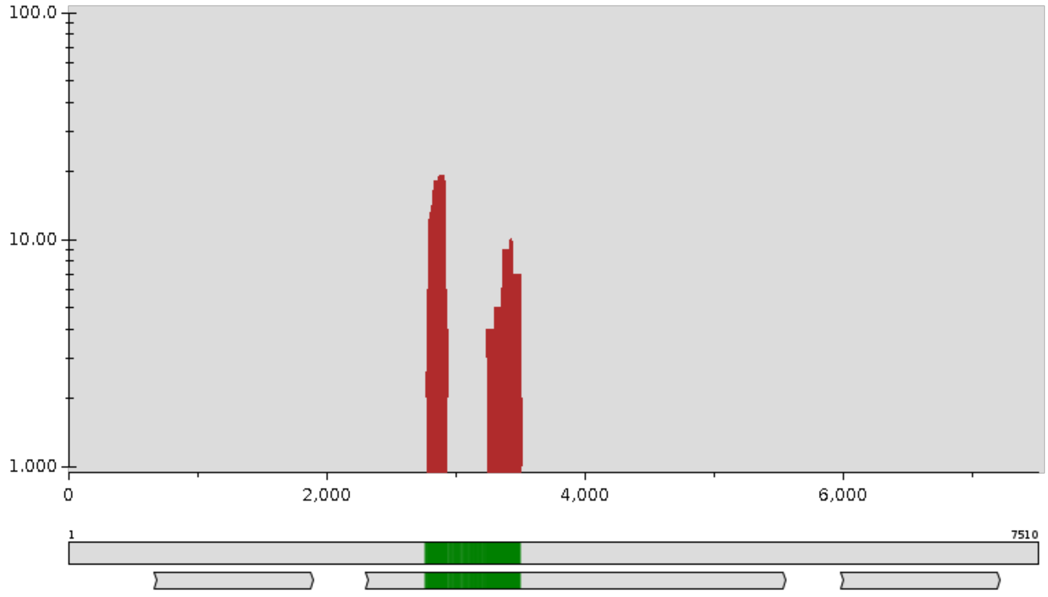

Assignment

**Type**	Errantivirus (Taxonomy ID: 186666)
**Reference Genome**	NC_038512.1
**NT Identity (%)**	37.9433
**AA Identity (%)**	33.3333
**Number Of Stop Codons**	4
**Number Of CDS**	3

Alignment

**Alignment Score**	-310.0 (NT) + -175.0 (AA) = -485.0
**Concordance (%)**	-27.8351

Alignment Method	Global, seeded, nucleotide + amino acids (AGA)

Genome Region

Sequence starts at position 2767 and ends at position 3509 relative to NC_038512.1 reference sequence.

Alignment Detailed Statistics

	Begin	End	Coverage	Score	Concordance	Matches	Identities	I/D/M/F*	Stop Codons
**NT**	**2767**	**3509**	**7.7%**	**-310**	**-28.3%**	**563 (97.7%)**	**214 (37.2%)**	**1/12**	
Mutations:	2767T>A, 2769G>A, 2773C>A, 2777A>T, 2779C>A, 2781C>A, 2783A>G, 2785A>T, 2786A>T, 2788G>A, 2791G>A, 2794C>G, 2795C>A, 2806A>T, 2807A>C, 2809A>G, 2812A>T, 2813T>A, 2814C>G, 2816G>A, 2818C>A, 2821T>C, 2822G>C, 2824A>G, 2828A>G, 2830C>G, 2830_2831insT, 2831T>G, 2836C>G, 2837A>G, 2839A>G, 2841G>T, 2842G>A, 2843G>T, 2849C>A, 2850C>G, 2851C>A, 2857A>G, 2858_2860delATC, 2863C>T, 2865C>G, 2869T>C, 2870G>C, 2871G>T, 2872G>A, 2873_2881delAAACAAAAG, 2883G>T, 2885C>A, 2887T>A, 2888C>A, 2890C>G, 2891G>T, 2892T>G, 2893A>C, 2894G>A, 2896T>A, 2899C>T, 2901T>A, 2905T>G, 2906A>C, 2908G>A, 2909T>C, 2911G>C, 2915G>A, 2919A>T, 2920G>C, 2924A>G, 2926C>T, 2927G>A, 2929T>G, 2935A>G, 2937A>T, 2938C>T, 2941G>A, 2944A>T, 2949A>T, 2950C>T, 2953A>T, 2954A>G, 2955G>A, 2959C>G, 2960G>T, 2963C>T, 2968C>T, 2969A>G, 2974A>G, 2975G>C, 2976G>A, 2977T>A, 2978A>G, 2979A>G, 2981T>G, 2982G>C, 2983C>T, 2984C>A, 2986A>T, 2988A>T, 2989C>T, 2993A>T, 2995C>A, 2997C>A, 2998C>G, 2999T>C, 3001A>T, 3005T>C, 3007G>T, 3008G>C, 3009C>A, 3010A>T, 3011A>T, 3012G>C, 3016G>A, 3018T>A, 3020T>C, 3025G>A, 3026G>A, 3028G>T, 3029G>A, 3030A>G, 3032A>G, 3034G>T, 3035G>A, 3036A>G, 3037C>G, 3038C>G, 3039C>T, 3040T>A, 3041C>G, 3042A>C, 3043A>G, 3049A>T, 3050T>C, 3052G>C, 3058C>T, 3061G>T, 3066A>G, 3067C>G, 3068G>A, 3069T>C, 3071G>C, 3073A>C, 3074C>T, 3076C>T, 3079G>T, 3084T>A, 3091C>T, 3092C>T, 3094T>A, 3095C>G, 3096G>T, 3097A>C, 3103T>C, 3104A>T, 3106G>T, 3109A>T, 3112A>G, 3114A>C, 3115A>T, 3118C>T, 3119T>G, 3121A>G, 3124A>G, 3127T>C, 3130T>A, 3137A>G, 3140G>C, 3142T>G, 3146G>A, 3148C>T, 3149A>C, 3151T>G, 3154C>A, 3155C>T, 3157A>T, 3159G>A, 3160A>G, 3162G>C, 3163T>A, 3164C>T, 3165T>A, 3166C>T, 3168A>T, 3169A>T, 3171A>G, 3172T>G, 3175C>G, 3176A>T, 3178C>T, 3179T>G, 3180G>T, 3182C>T, 3184C>A, 3187C>T, 3189A>T, 3190C>T, 3191C>T, 3196C>T, 3202T>C, 3203A>T, 3205T>A, 3206G>A, 3208C>A, 3214T>C, 3216C>A, 3217T>G, 3218T>A, 3219C>G, 3220C>T, 3223A>G, 3224C>G, 3226G>A, 3229A>T, 3233C>T, 3236G>C, 3238G>A, 3239A>C, 3242C>T, 3245G>A, 3247A>G, 3248C>A, 3249G>T, 3250A>G, 3253T>G, 3256C>T, 3257C>G, 3259A>T, 3261G>T, 3262A>T, 3265T>G, 3272A>C, 3273G>A, 3275A>T, 3276A>C, 3278T>C, 3280C>A, 3281A>T, 3282A>T, 3283A>T, 3284A>G, 3285T>C, 3287C>A, 3289A>G, 3291T>G, 3292G>A, 3293G>T, 3294A>C, 3298G>C, 3300C>G, 3301C>A, 3302G>T, 3304A>T, 3307C>T, 3308T>G, 3309T>G, 3310G>A, 3311A>C, 3313G>A, 3315T>C, 3316C>A, 3317G>T, 3319A>G, 3321C>T, 3324C>A, 3325T>G, 3331T>A, 3337C>T, 3338A>G, 3340C>A, 3343A>T, 3344A>T, 3345G>C, 3346C>T, 3347A>G, 3348G>C, 3349G>T, 3352C>A, 3356A>G, 3358C>T, 3359A>T, 3361G>C, 3362C>T, 3363C>G, 3364T>A, 3365A>C, 3370T>A, 3371G>A, 3373T>G, 3378T>C, 3379T>G, 3380T>G, 3381C>A, 3382C>A, 3385T>A, 3386A>T, 3389C>A, 3390A>G, 3394A>T, 3396A>G, 3397T>G, 3398C>T, 3399T>C, 3400G>A, 3402T>A, 3403T>G, 3406A>C, 3407A>T, 3408A>T, 3409G>A, 3410A>T, 3415T>G, 3416A>G, 3417A>C, 3421A>T, 3424A>T, 3426A>G, 3428C>A, 3429A>G, 3430A>T, 3433T>C, 3436A>G, 3439C>G, 3442T>A, 3443C>G, 3444T>C, 3445C>G, 3448T>G, 3451T>C, 3454C>T, 3455C>A, 3459A>G, 3460A>G, 3463C>T, 3464A>G, 3466T>G, 3467C>G, 3468C>A, 3471A>G, 3472T>C, 3476G>T, 3479C>T, 3480G>C, 3482C>A, 3485A>T, 3487A>C, 3488A>T, 3489A>C, 3490A>T, 3493C>T, 3494C>T, 3496T>G, 3499A>C, 3500C>T								

CDS

	Begin	End	Coverage	Score	Concordance	Matches	Identities	I/D/M/F*	Stop Codons
D1R33_gp2	160	402	16.3%	-175	-11.3%	173 (97.2%)	58 (32.6%)	1/4/23/1	4
Protein mutations:	K160D (2783A>G 2785A>T), M161L (2786A>T 2788G>A), D163E (2794C>G), Q164K (2795C>A), S170R (2813T>A 2814C>G), D171K (2816G>A 2818C>A), A173P (2822G>C 2824A>G), S175G (2828A>G 2830C>G), S175_S176insX (2830_2831insT), S176A (2831T>G), I178V (2837A>G 2839A>G), W179L (2841G>T 2842G>A), V180F (2843G>T), P182R (2849C>A 2850C>G 2851C>A), I185del (2858_2860delATC), A187G (2865C>G), G189L (2870G>C 2871G>T 2872G>A), K190_K192del (2873_2881delAAACAAAAG), W193L (2883G>T), L195M (2888C>A 2890C>G), V196C (2891G>T 2892T>G 2893A>C), V197I (2894G>A 2896T>A), F199Y (2901T>A), K201Q (2906A>C 2908G>A), E204K (2915G>A), K205I (2919A>T 2920G>C), I207V (2924A>G 2926C>T), D208K (2927G>A 2929T>G), Y211F (2937A>T 2938C>T), G224Q (2975G>C 2976G>A 2977T>A), C226A (2981T>G 2982G>C 2983C>T), A235H (3008G>C 3009C>A 3010A>T), D244R (3035G>A 3036A>G 3037C>G), P245V (3038C>G 3039C>T 3040T>A), Q246A (3041C>G 3042A>C 3043A>G), R264V (3095C>G 3096G>T 3097A>C), L287Y (3164C>T 3165T>A 3166C>T), E314K (3245G>A 3247A>G), R315M (3248C>A 3249G>T 3250A>G), Q318D (3257C>G 3259A>T), R319I (3261G>T 3262A>T), S323H (3272A>C 3273G>A), N324S (3275A>T 3276A>C), F325L (3278T>C 3280C>A), K326F (3281A>T 3282A>T 3283A>T), I327A (3284A>G 3285T>C), Q328K (3287C>A 3289A>G), M329R (3291T>G 3292G>A), D330S (3293G>T 3294A>C), K331N (3298G>C), S332* (3300C>G 3301C>A), E333Y (3302G>T 3304A>T), L335G (3308T>G 3309T>G 3310G>A), K336Q (3311A>C 3313G>A), L337P (3315T>C 3316C>A), E338* (3317G>T 3319A>G), T339I (3321C>T), A340E (3324C>A 3325T>G), I345V (3338A>G 3340C>A), R348A (3347A>G 3348G>C 3349G>T), D349E (3352C>A), I351V (3356A>G 3358C>T), K352Y (3359A>T 3361G>C), P353* (3362C>T 3363C>G 3364T>A), N354H (3365A>C), D356K (3371G>A 3373T>G), I358T (3378T>C 3379T>G), S359E (3380T>G 3381C>A 3382C>A), I361F (3386A>T), Q362R (3389C>A 3390A>G), K363N (3394A>T), Y364W (3396A>G 3397T>G), L365S (3398C>T 3399T>C 3400G>A), I366K (3402T>A 3403T>G), K368L (3407A>T 3408A>T 3409G>A), T369S (3410A>T), K371A (3416A>G 3417A>C), E372D (3421A>T), K374R (3426A>G), Q375S (3428C>A 3429A>G 3430A>T), L380A (3443C>G 3444T>C 3445C>G), K385R (3459A>G 3460A>G), I387V (3464A>G 3466T>G), P388E (3467C>G 3468C>A), D389G (3471A>G 3472T>C), A391S (3476G>T), R392S (3479C>T 3480G>C), L393I (3482C>A), T394S (3485A>T 3487A>C), K395S (3488A>T 3489A>C 3490A>T), Q399* (3500C>T)								
Codon mutations:	GTT154..A (2767T>A), AGG155.A. (2769G>A), GAC156..A (2773C>A), ATC158T.A (2777A>T 2779C>A), ACG159.AG (2781C>A), AAA160GAT (2783A>G 2785A>T), ATG161TTA (2786A>T 2788G>A), TTG162TTA (2791G>A), GAC163GAG (2794C>G), CAA164AAA (2795C>A), ATA167ATT (2806A>T), AGA168CGG (2807A>C 2809A>G), CCA169CCT (2812A>T), TCA170AGA (2813T>A 2814C>G), GAC171AAA (2816G>A 2818C>A), TCT172TCC (2821T>C), GCA173CCG (2822G>C 2824A>G), AGC175GGG (2828A>G 2830C>G), AGC175_TCA176insT-- (2830_2831insT), TCA176GCA (2831T>G), CCC177CCG (2836C>G), ATA178GTG (2837A>G 2839A>G), TGG179TTA (2841G>T 2842G>A), GTT180TTT (2843G>T), CCC182AGA (2849C>A 2850C>G 2851C>A), AAA184AAG (2857A>G), ATC185del (2858_2860delATC), GAC186GAT (2863C>T), GCT187GGT (2865C>G), TCT188TCC (2869T>C), GGG189CTA (2870G>C 2871G>T 2872G>A), AAA190_AAG192del (2873_2881delAAACAAAAG), TGG193TTG (2883G>T), CGT194AGA (2885C>A 2887T>A), CTC195ATG (2888C>A 2890C>G), GTA196TGC (2891G>T 2892T>G 2893A>C), GTT197ATA (2894G>A 2896T>A), GAC198GAT (2899C>T), TTC199TAC (2901T>A), CGT200CGG (2905T>G), AAG201CAA (2906A>C 2908G>A), TTG202CTC (2909T>C 2911G>C), GAG204AAG (2915G>A), AAG205ATC (2919A>T 2920G>C), ATC207GTT (2924A>G 2926C>T), GAT208AAG (2927G>A 2929T>G), AAA210AAG (2935A>G), TAC211TTT (2937A>T 2938C>T), CCG212..A (2941G>A), ATA213..T (2944A>T), AAC215.TT (2949A>T 2950C>T), ATA216..T (2953A>T), AGT217GA. (2954A>G 2955G>A), GAC218..G (2959C>G), GTA219T.. (2960G>T), CTT220T.. (2963C>T), GAC221..T (2968C>T), AAG222G.. (2969A>G), TTA223..G (2974A>G), GGT224CAA (2975G>C 2976G>A 2977T>A), AAG225GG. (2978A>G 2979A>G), TGC226GCT (2981T>G 2982G>C 2983C>T), CAA227A.T (2984C>A 2986A>T), TAC228.TT (2988A>T 2989C>T), ACC230T.A (2993A>T 2995C>A), ACC231.AG (2997C>A 2998C>G), TTA232C.T (2999T>C 3001A>T), TTG234C.T (3005T>C 3007G>T), GCA235CAT (3008G>C 3009C>A 3010A>T), AGT236TC. (3011A>T 3012G>C), GGG237..A (3016G>A), TTT238.A. (3018T>A), TAT239C.. (3020T>C), CAG240..A (3025G>A), GTG241A.T (3026G>A 3028G>T), GAG242AG. (3029G>A 3030A>G), ATG243G.T (3032A>G 3034G>T), GAC244AGG (3035G>A 3036A>G 3037C>G), CCT245GTA (3038C>G 3039C>T 3040T>A), CAA246GCG (3041C>G 3042A>C 3043A>G), ATA248..T (3049A>T), TCG249C.C (3050T>C 3052G>C), ACC251..T (3058C>T), GCG252..T (3061G>T), AAC254.GG (3066A>G 3067C>G), GTA255AC. (3068G>A 3069T>C), GAA256C.C (3071G>C 3073A>C), CAC257T.T (3074C>T 3076C>T), GGG258..T (3079G>T), TTT260.A. (3084T>A), TTC262..T (3091C>T), CTT263T.A (3092C>T 3094T>A), CGA264GTC (3095C>G 3096G>T 3097A>C), CCT266..C (3103T>C), ATG267T.T (3104A>T 3106G>T), GGA268..T (3109A>T), TTA269..G (3112A>G), AAA270.CT (3114A>C 3115A>T), AAC271..T (3118C>T), TCA272G.G (3119T>G 3121A>G), CCA273..G (3124A>G), TCT274..C (3127T>C), ACT275..A (3130T>A), AGA278G.. (3137A>G), GTT279C.G (3140G>C 3142T>G), GAC281A.T (3146G>A 3148C>T), AAT282C.G (3149A>C 3151T>G), GTC283..A (3154C>A), CTA284T.T (3155C>T 3157A>T), AGA285.AG (3159G>A 3160A>G), GGT286.CA (3162G>C 3163T>A), CTC287TAT (3164C>T 3165T>A 3166C>T), CAA288.TT (3168A>T 3169A>T), AAT289.GG (3171A>G 3172T>G), AAC290..G (3175C>G), ATC291T.T (3176A>T 3178C>T), TGT292GT. (3179T>G 3180G>T), CTC293T.A (3182C>T 3184C>A), GTC294..T (3187C>T), TAC295.TT (3189A>T 3190C>T), CTT296T.. (3191C>T), GAC297..T (3196C>T), ATT299..C (3202T>C), ATT300T.A (3203A>T 3205T>A), GTC301A.A (3206G>A 3208C>A), AGT303..C (3214T>C), ACT304.AG (3216C>A 3217T>G), TCC305AGT (3218T>A 3219C>G 3220C>T), CTA306..G (3223A>G), CAG307G.A (3224C>G 3226G>A), GAA308..T (3229A>T), CTG310T.. (3233C>T), GAG311C.A (3236G>C 3238G>A), AAC312C.C (3239A>C), CTG313TTG (3242C>T), GAA314AAG (3245G>A 3247A>G), CGA315ATG (3248C>A 3249G>T 3250A>G), GTT316GTG (3253T>G), TTC317TTT (3256C>T), CAA318GAT (3257C>G 3259A>T), AGA319ATT (3261G>T 3262A>T), CTT320CTG (3265T>G), AGT323CAT (3272A>C 3273G>A), AAC324TCC (3275A>T 3276A>C), TTC325CTA (3278T>C 3280C>A), AAA326TTT (3281A>T 3282A>T 3283A>T), ATT327GCT (3284A>G 3285T>C), CAA328AAG (3287C>A 3289A>G), ATG329AGA (3291T>G 3292G>A), GAC330TCC (3293G>T 3294A>C), AAG331AAC (3298G>C), TCC332TGA (3300C>G 3301C>A), GAA333TAT (3302G>T 3304A>T), TTC334TTT (3307C>T), TTG335GGA (3308T>G 3309T>G 3310G>A), AAG336CAA (3311A>C 3313G>A), CTC337CCA (3315T>C 3316C>A), GAA338TAG (3317G>T 3319A>G), ACT339ATT (3321C>T), GCT340GAG (3324C>A 3325T>G), CTT342CTA (3331T>A), CAC344CAT (3337C>T), ATC345GTA (3338A>G 3340C>A), ATA346ATT (3343A>T), AGC347TCT (3344A>T 3345G>C 3346C>T), AGG348GCT (3347A>G 3348G>C 3349G>T), GAC349GAA (3352C>A), ATC351GTT (3356A>G 3358C>T), AAG352TAC (3359A>T 3361G>C), CCT353TGA (3362C>T 3363C>G 3364T>A), AAC354CAC (3365A>C), CCT355CCA (3370T>A), GAT356AAG (3371G>A 3373T>G), ATT358ACG (3378T>C 3379T>G), TCC359GAA (3380T>G 3381C>A 3382C>A), GCT360GCA (3385T>A), ATT361TTT (3386A>T), CAA362AGA (3389C>A 3390A>G), AAA363AAT (3394A>T), TAT364TGG (3396A>G 3397T>G), CTG365TCA (3398C>T 3399T>C 3400G>A), ATT366AAG (3402T>A 3403T>G), CCA367CCC (3406A>C), AAG368TTA (3407A>T 3408A>T 3409G>A), ACC369TCC (3410A>T), CCT370CCG (3415T>G), AAG371GCG (3416A>G 3417A>C), GAA372GAT (3421A>T), ATA373ATT (3424A>T), AAA374AGA (3426A>G), CAA375AGT (3428C>A 3429A>G 3430A>T), TTT376TTC (3433T>C), TTA377TTG (3436A>G), GGC378GGG (3439C>G), CTT379CTA (3442T>A), CTC380GCG (3443C>G 3444T>C 3445C>G), GGT381GGG (3448T>G), TAT382TAC (3451T>C), TAC383TAT (3454C>T), CGA384AGA (3455C>A), AAA385AGG (3459A>G 3460A>G), TTC386TTT (3463C>T), ATT387GTG (3464A>G 3466T>G), CCA388GAA (3467C>G 3468C>A), GAT389GGC (3471A>G 3472T>C), GCA391TCA (3476G>T), CGA392TCA (3479C>T 3480G>C), CTC393ATC (3482C>A), ACA394TCC (3485A>T 3487A>C), AAA395TCT (3488A>T 3489A>C 3490A>T), CCC396CCT (3493C>T), CTT397TTG (3494C>T 3496T>G), ACA398ACC (3499A>C), CAG399TAG (3500C>T)								

Proteins

	Begin	End	Coverage	Score	Concordance	Matches	Identities	I/D/M/F*	Stop Codons
ORF B (YP_009507248.1)	160	402	16.3%	-175	-11.3%	173 (97.2%)	58 (32.6%)	1/4/23/1	4

	Begin	End	Coverage	Score	Concordance	Matches	Identities	I/D/M/F*	Stop Codons
**NT**	**2767**	**3509**	**7.7%**	**-310**	**-28.3%**	**563 (97.7%)**	**214 (37.2%)**	**1/12**	
Protein mutations:	K160D (2783A>G 2785A>T), M161L (2786A>T 2788G>A), D163E (2794C>G), Q164K (2795C>A), S170R (2813T>A 2814C>G), D171K (2816G>A 2818C>A), A173P (2822G>C 2824A>G), S175G (2828A>G 2830C>G), S175_S176insX (2830_2831insT), S176A (2831T>G), I178V (2837A>G 2839A>G), W179L (2841G>T 2842G>A), V180F (2843G>T), P182R (2849C>A 2850C>G 2851C>A), I185del (2858_2860delATC), A187G (2865C>G), G189L (2870G>C 2871G>T 2872G>A), K190_K192del (2873_2881delAAACAAAAG), W193L (2883G>T), L195M (2888C>A 2890C>G), V196C (2891G>T 2892T>G 2893A>C), V197I (2894G>A 2896T>A), F199Y (2901T>A), K201Q (2906A>C 2908G>A), E204K (2915G>A), K205I (2919A>T 2920G>C), I207V (2924A>G 2926C>T), D208K (2927G>A 2929T>G), Y211F (2937A>T 2938C>T), G224Q (2975G>C 2976G>A 2977T>A), C226A (2981T>G 2982G>C 2983C>T), A235H (3008G>C 3009C>A 3010A>T), D244R (3035G>A 3036A>G 3037C>G), P245V (3038C>G 3039C>T 3040T>A), Q246A (3041C>G 3042A>C 3043A>G), R264V (3095C>G 3096G>T 3097A>C), L287Y (3164C>T 3165T>A 3166C>T), E314K (3245G>A 3247A>G), R315M (3248C>A 3249G>T 3250A>G), Q318D (3257C>G 3259A>T), R319I (3261G>T 3262A>T), S323H (3272A>C 3273G>A), N324S (3275A>T 3276A>C), F325L (3278T>C 3280C>A), K326F (3281A>T 3282A>T 3283A>T), I327A (3284A>G 3285T>C), Q328K (3287C>A 3289A>G), M329R (3291T>G 3292G>A), D330S (3293G>T 3294A>C), K331N (3298G>C), S332* (3300C>G 3301C>A), E333Y (3302G>T 3304A>T), L335G (3308T>G 3309T>G 3310G>A), K336Q (3311A>C 3313G>A), L337P (3315T>C 3316C>A), E338* (3317G>T 3319A>G), T339I (3321C>T), A340E (3324C>A 3325T>G), I345V (3338A>G 3340C>A), R348A (3347A>G 3348G>C 3349G>T), D349E (3352C>A), I351V (3356A>G 3358C>T), K352Y (3359A>T 3361G>C), P353* (3362C>T 3363C>G 3364T>A), N354H (3365A>C), D356K (3371G>A 3373T>G), I358T (3378T>C 3379T>G), S359E (3380T>G 3381C>A 3382C>A), I361F (3386A>T), Q362R (3389C>A 3390A>G), K363N (3394A>T), Y364W (3396A>G 3397T>G), L365S (3398C>T 3399T>C 3400G>A), I366K (3402T>A 3403T>G), K368L (3407A>T 3408A>T 3409G>A), T369S (3410A>T), K371A (3416A>G 3417A>C), E372D (3421A>T), K374R (3426A>G), Q375S (3428C>A 3429A>G 3430A>T), L380A (3443C>G 3444T>C 3445C>G), K385R (3459A>G 3460A>G), I387V (3464A>G 3466T>G), P388E (3467C>G 3468C>A), D389G (3471A>G 3472T>C), A391S (3476G>T), R392S (3479C>T 3480G>C), L393I (3482C>A), T394S (3485A>T 3487A>C), K395S (3488A>T 3489A>C 3490A>T), Q399* (3500C>T)								
Codon mutations:	GTT154..A (2767T>A), AGG155.A. (2769G>A), GAC156..A (2773C>A), ATC158T.A (2777A>T 2779C>A), ACG159.AG (2781C>A), AAA160GAT (2783A>G 2785A>T), ATG161TTA (2786A>T 2788G>A), TTG162TTA (2791G>A), GAC163GAG (2794C>G), CAA164AAA (2795C>A), ATA167ATT (2806A>T), AGA168CGG (2807A>C 2809A>G), CCA169CCT (2812A>T), TCA170AGA (2813T>A 2814C>G), GAC171AAA (2816G>A 2818C>A), TCT172TCC (2821T>C), GCA173CCG (2822G>C 2824A>G), AGC175GGG (2828A>G 2830C>G), AGC175_TCA176insT-- (2830_2831insT), TCA176GCA (2831T>G), CCC177CCG (2836C>G), ATA178GTG (2837A>G 2839A>G), TGG179TTA (2841G>T 2842G>A), GTT180TTT (2843G>T), CCC182AGA (2849C>A 2850C>G 2851C>A), AAA184AAG (2857A>G), ATC185del (2858_2860delATC), GAC186GAT (2863C>T), GCT187GGT (2865C>G), TCT188TCC (2869T>C), GGG189CTA (2870G>C 2871G>T 2872G>A), AAA190_AAG192del (2873_2881delAAACAAAAG), TGG193TTG (2883G>T), CGT194AGA (2885C>A 2887T>A), CTC195ATG (2888C>A 2890C>G), GTA196TGC (2891G>T 2892T>G 2893A>C), GTT197ATA (2894G>A 2896T>A), GAC198GAT (2899C>T), TTC199TAC (2901T>A), CGT200CGG (2905T>G), AAG201CAA (2906A>C 2908G>A), TTG202CTC (2909T>C 2911G>C), GAG204AAG (2915G>A), AAG205ATC (2919A>T 2920G>C), ATC207GTT (2924A>G 2926C>T), GAT208AAG (2927G>A 2929T>G), AAA210AAG (2935A>G), TAC211TTT (2937A>T 2938C>T), CCG212..A (2941G>A), ATA213..T (2944A>T), AAC215.TT (2949A>T 2950C>T), ATA216..T (2953A>T), AGT217GA. (2954A>G 2955G>A), GAC218..G (2959C>G), GTA219T.. (2960G>T), CTT220T.. (2963C>T), GAC221..T (2968C>T), AAG222G.. (2969A>G), TTA223..G (2974A>G), GGT224CAA (2975G>C 2976G>A 2977T>A), AAG225GG. (2978A>G 2979A>G), TGC226GCT (2981T>G 2982G>C 2983C>T), CAA227A.T (2984C>A 2986A>T), TAC228.TT (2988A>T 2989C>T), ACC230T.A (2993A>T 2995C>A), ACC231.AG (2997C>A 2998C>G), TTA232C.T (2999T>C 3001A>T), TTG234C.T (3005T>C 3007G>T), GCA235CAT (3008G>C 3009C>A 3010A>T), AGT236TC. (3011A>T 3012G>C), GGG237..A (3016G>A), TTT238.A. (3018T>A), TAT239C.. (3020T>C), CAG240..A (3025G>A), GTG241A.T (3026G>A 3028G>T), GAG242AG. (3029G>A 3030A>G), ATG243G.T (3032A>G 3034G>T), GAC244AGG (3035G>A 3036A>G 3037C>G), CCT245GTA (3038C>G 3039C>T 3040T>A), CAA246GCG (3041C>G 3042A>C 3043A>G), ATA248..T (3049A>T), TCG249C.C (3050T>C 3052G>C), ACC251..T (3058C>T), GCG252..T (3061G>T), AAC254.GG (3066A>G 3067C>G), GTA255AC. (3068G>A 3069T>C), GAA256C.C (3071G>C 3073A>C), CAC257T.T (3074C>T 3076C>T), GGG258..T (3079G>T), TTT260.A. (3084T>A), TTC262..T (3091C>T), CTT263T.A (3092C>T 3094T>A), CGA264GTC (3095C>G 3096G>T 3097A>C), CCT266..C (3103T>C), ATG267T.T (3104A>T 3106G>T), GGA268..T (3109A>T), TTA269..G (3112A>G), AAA270.CT (3114A>C 3115A>T), AAC271..T (3118C>T), TCA272G.G (3119T>G 3121A>G), CCA273..G (3124A>G), TCT274..C (3127T>C), ACT275..A (3130T>A), AGA278G.. (3137A>G), GTT279C.G (3140G>C 3142T>G), GAC281A.T (3146G>A 3148C>T), AAT282C.G (3149A>C 3151T>G), GTC283..A (3154C>A), CTA284T.T (3155C>T 3157A>T), AGA285.AG (3159G>A 3160A>G), GGT286.CA (3162G>C 3163T>A), CTC287TAT (3164C>T 3165T>A 3166C>T), CAA288.TT (3168A>T 3169A>T), AAT289.GG (3171A>G 3172T>G), AAC290..G (3175C>G), ATC291T.T (3176A>T 3178C>T), TGT292GT. (3179T>G 3180G>T), CTC293T.A (3182C>T 3184C>A), GTC294..T (3187C>T), TAC295.TT (3189A>T 3190C>T), CTT296T.. (3191C>T), GAC297..T (3196C>T), ATT299..C (3202T>C), ATT300T.A (3203A>T 3205T>A), GTC301A.A (3206G>A 3208C>A), AGT303..C (3214T>C), ACT304.AG (3216C>A 3217T>G), TCC305AGT (3218T>A 3219C>G 3220C>T), CTA306..G (3223A>G), CAG307G.A (3224C>G 3226G>A), GAA308..T (3229A>T), CTG310T.. (3233C>T), GAG311C.A (3236G>C 3238G>A), AAC312C.C (3239A>C), CTG313TTG (3242C>T), GAA314AAG (3245G>A 3247A>G), CGA315ATG (3248C>A 3249G>T 3250A>G), GTT316GTG (3253T>G), TTC317TTT (3256C>T), CAA318GAT (3257C>G 3259A>T), AGA319ATT (3261G>T 3262A>T), CTT320CTG (3265T>G), AGT323CAT (3272A>C 3273G>A), AAC324TCC (3275A>T 3276A>C), TTC325CTA (3278T>C 3280C>A), AAA326TTT (3281A>T 3282A>T 3283A>T), ATT327GCT (3284A>G 3285T>C), CAA328AAG (3287C>A 3289A>G), ATG329AGA (3291T>G 3292G>A), GAC330TCC (3293G>T 3294A>C), AAG331AAC (3298G>C), TCC332TGA (3300C>G 3301C>A), GAA333TAT (3302G>T 3304A>T), TTC334TTT (3307C>T), TTG335GGA (3308T>G 3309T>G 3310G>A), AAG336CAA (3311A>C 3313G>A), CTC337CCA (3315T>C 3316C>A), GAA338TAG (3317G>T 3319A>G), ACT339ATT (3321C>T), GCT340GAG (3324C>A 3325T>G), CTT342CTA (3331T>A), CAC344CAT (3337C>T), ATC345GTA (3338A>G 3340C>A), ATA346ATT (3343A>T), AGC347TCT (3344A>T 3345G>C 3346C>T), AGG348GCT (3347A>G 3348G>C 3349G>T), GAC349GAA (3352C>A), ATC351GTT (3356A>G 3358C>T), AAG352TAC (3359A>T 3361G>C), CCT353TGA (3362C>T 3363C>G 3364T>A), AAC354CAC (3365A>C), CCT355CCA (3370T>A), GAT356AAG (3371G>A 3373T>G), ATT358ACG (3378T>C 3379T>G), TCC359GAA (3380T>G 3381C>A 3382C>A), GCT360GCA (3385T>A), ATT361TTT (3386A>T), CAA362AGA (3389C>A 3390A>G), AAA363AAT (3394A>T), TAT364TGG (3396A>G 3397T>G), CTG365TCA (3398C>T 3399T>C 3400G>A), ATT366AAG (3402T>A 3403T>G), CCA367CCC (3406A>C), AAG368TTA (3407A>T 3408A>T 3409G>A), ACC369TCC (3410A>T), CCT370CCG (3415T>G), AAG371GCG (3416A>G 3417A>C), GAA372GAT (3421A>T), ATA373ATT (3424A>T), AAA374AGA (3426A>G), CAA375AGT (3428C>A 3429A>G 3430A>T), TTT376TTC (3433T>C), TTA377TTG (3436A>G), GGC378GGG (3439C>G), CTT379CTA (3442T>A), CTC380GCG (3443C>G 3444T>C 3445C>G), GGT381GGG (3448T>G), TAT382TAC (3451T>C), TAC383TAT (3454C>T), CGA384AGA (3455C>A), AAA385AGG (3459A>G 3460A>G), TTC386TTT (3463C>T), ATT387GTG (3464A>G 3466T>G), CCA388GAA (3467C>G 3468C>A), GAT389GGC (3471A>G 3472T>C), GCA391TCA (3476G>T), CGA392TCA (3479C>T 3480G>C), CTC393ATC (3482C>A), ACA394TCC (3485A>T 3487A>C), AAA395TCT (3488A>T 3489A>C 3490A>T), CCC396CCT (3493C>T), CTT397TTG (3494C>T 3496T>G), ACA398ACC (3499A>C), CAG399TAG (3500C>T)								

*: Inserts / Deletes / Misaligned / Frameshifts

Analysis details

This analysis was performed with panviral2.64

NGS Details (UN62): Badnavirus volubetulae

Assembly

**Coverage Length**	339 (1 contig(s))
**Depth Of Coverage**	10.1
**Number Of Reads**	30
**Reads Per Million**	0.70 rpm (after QC)
**Ambiguities**	0
**Assembly Method**	de novo + reference guided assembly
**Consensus Caller**	Bcf Tools

Coverage Map

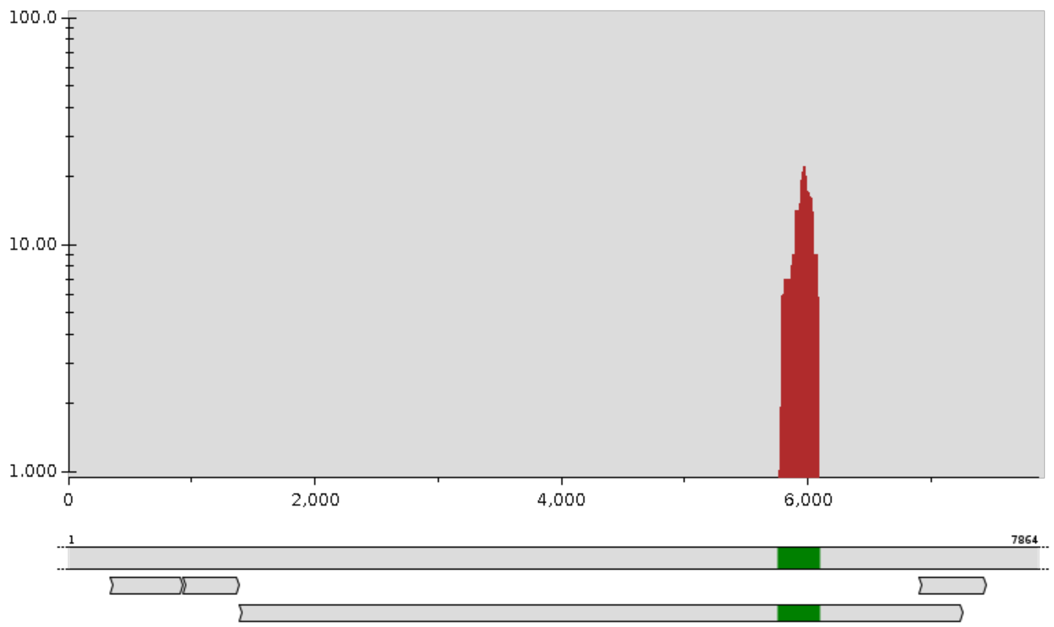

Assignment

**Type**	Badnavirus volubetulae (Taxonomy ID: 3047683)
**Reference Genome**	NC_040635.1
**NT Identity (%)**	52.6316
**AA Identity (%)**	42.1053
**Number Of Stop Codons**	0
**Number Of CDS**	4

Alignment

**Alignment Score**	30.0 (NT) + 350.0 (AA) = 380.0
**Concordance (%)**	25.3502

Alignment Method	Global, seeded, nucleotide + amino acids (AGA)

Genome Region

Sequence starts at position 5758 and ends at position 6096 relative to NC_040635.1 reference sequence.

Alignment Detailed Statistics

	Begin	End	Coverage	Score	Concordance	Matches	Identities	I/D/M/F*	Stop Codons
**NT**	**5758**	**6096**	**4.3%**	**30**	**4.4%**	**339 (99.1%)**	**180 (52.6%)**	**3/0**	
Mutations:	5758C>A, 5760C>T, 5763T>C, 5770A>C, 5771G>A, 5772T>A, 5773C>A, 5775G>T, 5778C>T, 5779C>A, 5780A>G, 5782A>G, 5783A>T, 5788C>G, 5789A>T, 5790C>G, 5794G>A, 5797C>A, 5799A>G, 5803A>C, 5804G>C, 5808A>T, 5811A>C, 5812G>A, 5814A>G, 5817C>T, 5818A>G, 5820C>T, 5821A>G, 5822C>A, 5824A>T, 5826T>G, 5827G>T, 5829C>T, 5830C>G, 5832G>T, 5834G>A, 5835C>A, 5836A>C, 5839G>C, 5841T>A, 5843A>G, 5844T>G, 5849A>G, 5850G>C, 5851G>T, 5852T>A, 5853A>T, 5856C>T, 5859A>T, 5862A>G, 5863T>A, 5865C>T, 5872A>C, 5873A>G, 5874G>T, 5875A>T, 5880C>A, 5882T>A, 5886T>C, 5890G>C, 5892C>T, 5894C>G, 5896A>G, 5899G>A, 5901T>G, 5904A>G, 5905G>A, 5906A>G, 5907A>T, 5908T>G, 5909C>A, 5910A>T, 5911A>G, 5913A>T, 5916A>G, 5917T>A, 5918G>A, 5919G>A, 5921C>T, 5922A>G, 5925A>T, 5930T>G, 5931C>A, 5935C>A, 5936C>G, 5937A>G, 5938G>T, 5944T>C, 5945T>A, 5946G>T, 5948A>T, 5949C>T, 5954G>T, 5955G>C, 5958A>T, 5961T>C, 5965C>T, 5967A>C, 5974C>T, 5976T>G, 5978A>C, 5979A>T, 5986C>T, 5988A>G, 5989G>A, 5990C>A, 5991A>T, 5997C>T, 5998C>A, 5999A>T, 6000G>A, 6001A>G, 6002G>A, 6003A>T, 6004A>T, 6005A>T, 6006A>G, 6010G>A, 6014A>G, 6015T>G, 6016T>G, 6017G>T, 6018C>T, 6021T>C, 6024A>G, 6025G>C, 6026G>C, 6028A>T, 6029C>T, 6030G>C, 6031G>C, 6032A>T, 6036A>C, 6036_6037insATA, 6039C>T, 6040A>G, 6044C>T, 6045T>G, 6048A>G, 6050A>T, 6051C>T, 6054C>T, 6057T>C, 6061A>G, 6063T>A, 6067G>A, 6069C>A, 6071T>A, 6076G>C, 6077A>G, 6082C>A, 6083A>G, 6084T>G, 6085A>G, 6088C>G, 6093T>C, 6094G>T, 6095A>T, 6096A>G								

CDS

	Begin	End	Coverage	Score	Concordance	Matches	Identities	I/D/M/F*	Stop Codons
BLRaV_gp3	1457	1569	5.8%	350	42.6%	113 (99.1%)	48 (42.1%)	1/0/0/0	0
Protein mutations:	L1457I (5758C>A 5760C>T), S1461Q (5770A>C 5771G>A 5772T>A), L1462I (5773C>A 5775G>T), Q1464R (5779C>A 5780A>G), N1465V (5782A>G 5783A>T), H1467V (5788C>G 5789A>T 5790C>G), D1469N (5794G>A), Q1470K (5797C>A 5799A>G), S1472P (5803A>C 5804G>C), G1475R (5812G>A 5814A>G), N1477D (5818A>G 5820C>T), T1478D (5821A>G 5822C>A), I1479L (5824A>T 5826T>G), V1480F (5827G>T 5829C>T), Q1481D (5830C>G 5832G>T), R1482Q (5834G>A 5835C>A), I1483L (5836A>C), G1484R (5839G>C 5841T>A), N1485R (5843A>G 5844T>G), K1487S (5849A>G 5850G>C), V1488Y (5851G>T 5852T>A 5853A>T), F1492I (5863T>A 5865C>T), K1495R (5872A>C 5873A>G 5874G>T), S1496C (5875A>T), F1498Y (5882T>A), V1501L (5890G>C 5892C>T), T1502S (5894C>G), M1503V (5896A>G), D1504K (5899G>A 5901T>G), E1506S (5905G>A 5906A>G 5907A>T), S1507D (5908T>G 5909C>A 5910A>T), I1508V (5911A>G 5913A>T), W1510K (5917T>A 5918G>A 5919G>A), T1511M (5921C>T 5922A>G), L1514R (5930T>G 5931C>A), P1516R (5935C>A 5936C>G 5937A>G), D1517Y (5938G>T), L1519H (5944T>C 5945T>A 5946G>T), Y1520F (5948A>T 5949C>T), W1522F (5954G>T 5955G>C), P1526S (5965C>T 5967A>C), K1530T (5978A>C 5979A>T), P1533S (5986C>T 5988A>G), A1534N (5989G>A 5990C>A 5991A>T), Q1537I (5998C>A 5999A>T 6000G>A), R1538D (6001A>G 6002G>A 6003A>T), K1539L (6004A>T 6005A>T 6006A>G), D1541N (6010G>A), N1542R (6014A>G 6015T>G), C1543V (6016T>G 6017G>T 6018C>T), G1546P (6025G>C 6026G>C), T1547F (6028A>T 6029C>T 6030G>C), E1548L (6031G>C 6032A>T), E1549D (6036A>C), E1549_F1550insI (6036_6037insATA), I1551V (6040A>G), A1552V (6044C>T 6045T>G), Y1554F (6050A>T 6051C>T), I1558V (6061A>G 6063T>A), V1560I (6067G>A 6069C>A), F1561Y (6071T>A), E1563R (6076G>C 6077A>G), H1565R (6082C>A 6083A>G 6084T>G), K1566E (6085A>G), Q1567E (6088C>G), E1569L (6094G>T 6095A>T 6096A>G)								
Codon mutations:	CTC1457ATT (5758C>A 5760C>T), GAT1458GAC (5763T>C), AGT1461CAA (5770A>C 5771G>A 5772T>A), CTG1462ATT (5773C>A 5775G>T), AAC1463AAT (5778C>T), CAG1464AGG (5779C>A 5780A>G), AAC1465GTC (5782A>G 5783A>T), CAC1467GTG (5788C>G 5789A>T 5790C>G), GAT1469AAT (5794G>A), CAA1470AAG (5797C>A 5799A>G), AGC1472CCC (5803A>C 5804G>C), CTA1473CTT (5808A>T), CCA1474CCC (5811A>C), GGA1475AGG (5812G>A 5814A>G), ATC1476ATT (5817C>T), AAC1477GAT (5818A>G 5820C>T), ACT1478GAT (5821A>G 5822C>A), ATT1479TTG (5824A>T 5826T>G), GTC1480TTT (5827G>T 5829C>T), CAG1481GAT (5830C>G 5832G>T), CGC1482CAA (5834G>A 5835C>A), ATA1483CTA (5836A>C), GGT1484CGA (5839G>C 5841T>A), AAT1485AGG (5843A>G 5844T>G), AAG1487AGC (5849A>G 5850G>C), GTA1488TAT (5851G>T 5852T>A 5853A>T), TTC1489TTT (5856C>T), TCA1490TCT (5859A>T), AAA1491AAG (5862A>G), TTC1492ATT (5863T>A 5865C>T), AAG1495CGT (5872A>C 5873A>G 5874G>T), AGT1496TGT (5875A>T), GGC1497GGA (5880C>A), TTT1498TAT (5882T>A), CAT1499CAC (5886T>C), GTC1501CTT (5890G>C 5892C>T), ACC1502AGC (5894C>G), ATG1503GTG (5896A>G), GAT1504AAG (5899G>A 5901T>G), GAA1505GAG (5904A>G), GAA1506AGT (5905G>A 5906A>G 5907A>T), TCA1507GAT (5908T>G 5909C>A 5910A>T), ATA1508GTT (5911A>G 5913A>T), CCA1509CCG (5916A>G), TGG1510AAA (5917T>A 5918G>A 5919G>A), ACA1511ATG (5921C>T 5922A>G), GCA1512GCT (5925A>T), CTC1514CGA (5930T>G 5931C>A), CCA1516AGG (5935C>A 5936C>G 5937A>G), GAT1517TAT (5938G>T), TTG1519CAT (5944T>C 5945T>A 5946G>T), TAC1520TTT (5948A>T 5949C>T), TGG1522TTC (5954G>T 5955G>C), CTA1523CTT (5958A>T), GTT1524GTC (5961T>C), CCA1526TCC (5965C>T 5967A>C), CTT1529TTG (5974C>T 5976T>G), AAA1530ACT (5978A>C 5979A>T), CCA1533TCG (5986C>T 5988A>G), GCA1534AAT (5989G>A 5990C>A 5991A>T), TTC1536TTT (5997C>T), CAG1537ATA (5998C>A 5999A>T 6000G>A), AGA1538GAT (6001A>G 6002G>A 6003A>T), AAA1539TTG (6004A>T 6005A>T 6006A>G), GAT1541AAT (6010G>A), AAT1542AGG (6014A>G 6015T>G), TGC1543GTT (6016T>G 6017G>T 6018C>T), TTT1544TTC (6021T>C), AAA1545AAG (6024A>G), GGT1546CCT (6025G>C 6026G>C), ACG1547TTC (6028A>T 6029C>T 6030G>C), GAA1548CTA (6031G>C 6032A>T), GAA1549GAC (6036A>C), GAA1549_TTC1550insATA (6036_6037insATA), TTC1550TTT (6039C>T), ATA1551GTA (6040A>G), GCT1552GTG (6044C>T 6045T>G), GTA1553GTG (6048A>G), TAC1554TTT (6050A>T 6051C>T), ATC1555ATT (6054C>T), GAT1556GAC (6057T>C), ATT1558GTA (6061A>G 6063T>A), GTC1560ATA (6067G>A 6069C>A), TTC1561TAC (6071T>A), GAA1563CGA (6076G>C 6077A>G), CAT1565AGG (6082C>A 6083A>G 6084T>G), AAA1566GAA (6085A>G), CAG1567GAG (6088C>G), CAT1568CAC (6093T>C), GAA1569TTG (6094G>T 6095A>T 6096A>G)								

Proteins

	Begin	End	Coverage	Score	Concordance	Matches	Identities	I/D/M/F*	Stop Codons
polyprotein (YP_009552737.1)	1457	1569	5.8%	350	42.6%	113 (99.1%)	48 (42.1%)	1/0/0/0	0
Protein mutations:	L1457I (5758C>A 5760C>T), S1461Q (5770A>C 5771G>A 5772T>A), L1462I (5773C>A 5775G>T), Q1464R (5779C>A 5780A>G), N1465V (5782A>G 5783A>T), H1467V (5788C>G 5789A>T 5790C>G), D1469N (5794G>A), Q1470K (5797C>A 5799A>G), S1472P (5803A>C 5804G>C), G1475R (5812G>A 5814A>G), N1477D (5818A>G 5820C>T), T1478D (5821A>G 5822C>A), I1479L (5824A>T 5826T>G), V1480F (5827G>T 5829C>T), Q1481D (5830C>G 5832G>T), R1482Q (5834G>A 5835C>A), I1483L (5836A>C), G1484R (5839G>C 5841T>A), N1485R (5843A>G 5844T>G), K1487S (5849A>G 5850G>C), V1488Y (5851G>T 5852T>A 5853A>T), F1492I (5863T>A 5865C>T), K1495R (5872A>C 5873A>G 5874G>T), S1496C (5875A>T), F1498Y (5882T>A), V1501L (5890G>C 5892C>T), T1502S (5894C>G), M1503V (5896A>G), D1504K (5899G>A 5901T>G), E1506S (5905G>A 5906A>G 5907A>T), S1507D (5908T>G 5909C>A 5910A>T), I1508V (5911A>G 5913A>T), W1510K (5917T>A 5918G>A 5919G>A), T1511M (5921C>T 5922A>G), L1514R (5930T>G 5931C>A), P1516R (5935C>A 5936C>G 5937A>G), D1517Y (5938G>T), L1519H (5944T>C 5945T>A 5946G>T), Y1520F (5948A>T 5949C>T), W1522F (5954G>T 5955G>C), P1526S (5965C>T 5967A>C), K1530T (5978A>C 5979A>T), P1533S (5986C>T 5988A>G), A1534N (5989G>A 5990C>A 5991A>T), Q1537I (5998C>A 5999A>T 6000G>A), R1538D (6001A>G 6002G>A 6003A>T), K1539L (6004A>T 6005A>T 6006A>G), D1541N (6010G>A), N1542R (6014A>G 6015T>G), C1543V (6016T>G 6017G>T 6018C>T), G1546P (6025G>C 6026G>C), T1547F (6028A>T 6029C>T 6030G>C), E1548L (6031G>C 6032A>T), E1549D (6036A>C), E1549_F1550insI (6036_6037insATA), I1551V (6040A>G), A1552V (6044C>T 6045T>G), Y1554F (6050A>T 6051C>T), I1558V (6061A>G 6063T>A), V1560I (6067G>A 6069C>A), F1561Y (6071T>A), E1563R (6076G>C 6077A>G), H1565R (6082C>A 6083A>G 6084T>G), K1566E (6085A>G), Q1567E (6088C>G), E1569L (6094G>T 6095A>T 6096A>G)								
Codon mutations:	CTC1457ATT (5758C>A 5760C>T), GAT1458GAC (5763T>C), AGT1461CAA (5770A>C 5771G>A 5772T>A), CTG1462ATT (5773C>A 5775G>T), AAC1463AAT (5778C>T), CAG1464AGG (5779C>A 5780A>G), AAC1465GTC (5782A>G 5783A>T), CAC1467GTG (5788C>G 5789A>T 5790C>G), GAT1469AAT (5794G>A), CAA1470AAG (5797C>A 5799A>G), AGC1472CCC (5803A>C 5804G>C), CTA1473CTT (5808A>T), CCA1474CCC (5811A>C), GGA1475AGG (5812G>A 5814A>G), ATC1476ATT (5817C>T), AAC1477GAT (5818A>G 5820C>T), ACT1478GAT (5821A>G 5822C>A), ATT1479TTG (5824A>T 5826T>G), GTC1480TTT (5827G>T 5829C>T), CAG1481GAT (5830C>G 5832G>T), CGC1482CAA (5834G>A 5835C>A), ATA1483CTA (5836A>C), GGT1484CGA (5839G>C 5841T>A), AAT1485AGG (5843A>G 5844T>G), AAG1487AGC (5849A>G 5850G>C), GTA1488TAT (5851G>T 5852T>A 5853A>T), TTC1489TTT (5856C>T), TCA1490TCT (5859A>T), AAA1491AAG (5862A>G), TTC1492ATT (5863T>A 5865C>T), AAG1495CGT (5872A>C 5873A>G 5874G>T), AGT1496TGT (5875A>T), GGC1497GGA (5880C>A), TTT1498TAT (5882T>A), CAT1499CAC (5886T>C), GTC1501CTT (5890G>C 5892C>T), ACC1502AGC (5894C>G), ATG1503GTG (5896A>G), GAT1504AAG (5899G>A 5901T>G), GAA1505GAG (5904A>G), GAA1506AGT (5905G>A 5906A>G 5907A>T), TCA1507GAT (5908T>G 5909C>A 5910A>T), ATA1508GTT (5911A>G 5913A>T), CCA1509CCG (5916A>G), TGG1510AAA (5917T>A 5918G>A 5919G>A), ACA1511ATG (5921C>T 5922A>G), GCA1512GCT (5925A>T), CTC1514CGA (5930T>G 5931C>A), CCA1516AGG (5935C>A 5936C>G 5937A>G), GAT1517TAT (5938G>T), TTG1519CAT (5944T>C 5945T>A 5946G>T), TAC1520TTT (5948A>T 5949C>T), TGG1522TTC (5954G>T 5955G>C), CTA1523CTT (5958A>T), GTT1524GTC (5961T>C), CCA1526TCC (5965C>T 5967A>C), CTT1529TTG (5974C>T 5976T>G), AAA1530ACT (5978A>C 5979A>T), CCA1533TCG (5986C>T 5988A>G), GCA1534AAT (5989G>A 5990C>A 5991A>T), TTC1536TTT (5997C>T), CAG1537ATA (5998C>A 5999A>T 6000G>A), AGA1538GAT (6001A>G 6002G>A 6003A>T), AAA1539TTG (6004A>T 6005A>T 6006A>G), GAT1541AAT (6010G>A), AAT1542AGG (6014A>G 6015T>G), TGC1543GTT (6016T>G 6017G>T 6018C>T), TTT1544TTC (6021T>C), AAA1545AAG (6024A>G), GGT1546CCT (6025G>C 6026G>C), ACG1547TTC (6028A>T 6029C>T 6030G>C), GAA1548CTA (6031G>C 6032A>T), GAA1549GAC (6036A>C), GAA1549_TTC1550insATA (6036_6037insATA), TTC1550TTT (6039C>T), ATA1551GTA (6040A>G), GCT1552GTG (6044C>T 6045T>G), GTA1553GTG (6048A>G), TAC1554TTT (6050A>T 6051C>T), ATC1555ATT (6054C>T), GAT1556GAC (6057T>C), ATT1558GTA (6061A>G 6063T>A), GTC1560ATA (6067G>A 6069C>A), TTC1561TAC (6071T>A), GAA1563CGA (6076G>C 6077A>G), CAT1565AGG (6082C>A 6083A>G 6084T>G), AAA1566GAA (6085A>G), CAG1567GAG (6088C>G), CAT1568CAC (6093T>C), GAA1569TTG (6094G>T 6095A>T 6096A>G)								

*: Inserts / Deletes / Misaligned / Frameshifts

Analysis details

This analysis was performed with panviral2.64

NGS Details (UN62): Badnavirus zetavirgamusae

Assembly

**Coverage Length**	471 (1 contig(s))
**Depth Of Coverage**	8.4
**Number Of Reads**	29
**Reads Per Million**	0.67 rpm (after QC)
**Ambiguities**	0
**Assembly Method**	de novo + reference guided assembly
**Consensus Caller**	Bcf Tools

Coverage Map

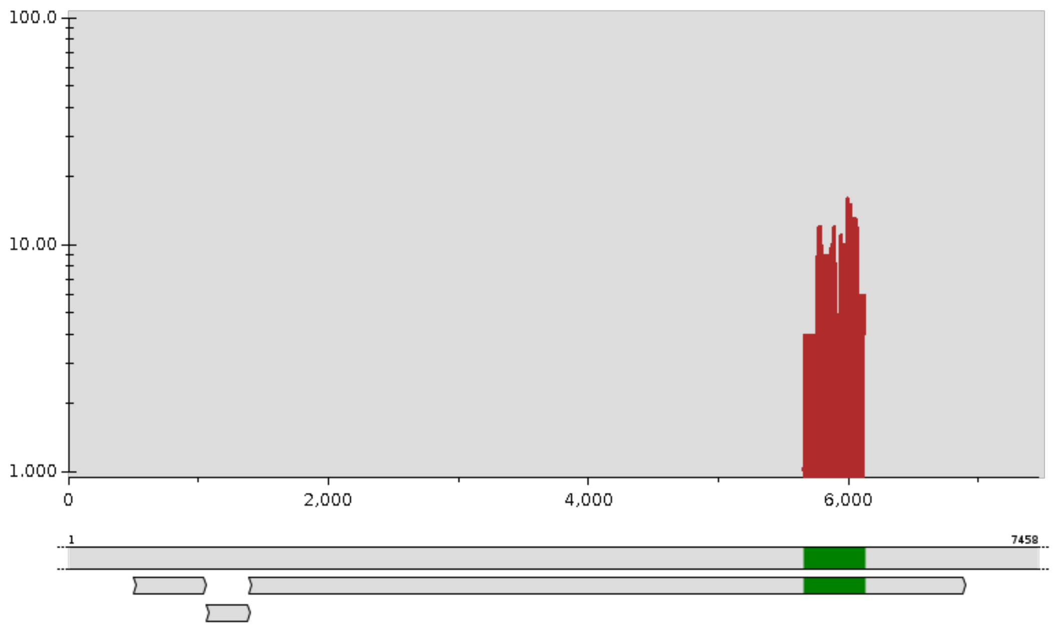

Assignment

**Type**	Badnavirus zetavirgamusae (Taxonomy ID: 3047391)
**Reference Genome**	NC_015503.1
**NT Identity (%)**	53.8627
**AA Identity (%)**	40.3846
**Number Of Stop Codons**	3
**Number Of CDS**	3

Alignment

**Alignment Score**	40.0 (NT) + 133.0 (AA) = 173.0
**Concordance (%)**	11.9048

Alignment Method	Global, seeded, nucleotide + amino acids (AGA)

Genome Region

Sequence starts at position 5657 and ends at position 6127 relative to NC_015503.1 reference sequence.

Alignment Detailed Statistics

	Begin	End	Coverage	Score	Concordance	Matches	Identities	I/D/M/F*	Stop Codons
**NT**	**5657**	**6127**	**6.3%**	**40**	**4.4%**	**466 (98.9%)**	**251 (53.3%)**	**0/5**	
Mutations:	5657G>A, 5658T>C, 5660T>G, 5662A>C, 5664A>G, 5666G>A, 5667A>T, 5669G>T, 5670G>A, 5671A>G, 5673A>C, 5675C>A, 5677G>T, 5679A>G, 5680A>T, 5681G>A, 5683A>G, 5684G>T, 5690A>T, 5691T>A, 5696T>A, 5697C>G, 5699C>A, 5703C>A, 5704C>A, 5707C>A, 5708A>G, 5711G>A, 5717G>A, 5718A>T, 5720C>A, 5721G>T, 5723A>T, 5724G>A, 5725T>A, 5726T>A, 5728A>C, 5729G>C, 5730G>C, 5731T>A, 5732A>T, 5733G>A, 5736G>A, 5741T>C, 5742C>T, 5746G>A, 5747A>G, 5749G>T, 5750C>T, 5752C>T, 5753T>A, 5754G>T, 5756T>A, 5759T>A, 5763A>T, 5764A>G, 5765T>A, 5766C>A, 5768C>G, 5769C>A, 5771G>A, 5772G>A, 5774T>A, 5781delC, 5783A>C, 5789T>A, 5791T>C, 5792G>T, 5793C>A, 5799A>G, 5801A>C, 5802G>T, 5804G>A, 5807T>G, 5808T>A, 5810T>G, 5811G>C, 5812A>C, 5813T>A, 5814G>T, 5817G>A, 5818A>T, 5819A>T, 5821A>G, 5823_5825delCTG, 5828G>A, 5829A>C, 5830C>A, 5831A>C, 5834G>A, 5836A>G, 5838G>A, 5839G>T, 5841C>A, 5842T>C, 5843C>A, 5847T>A, 5848C>T, 5849C>T, 5850T>C, 5851G>T, 5852G>T, 5853C>A, 5855G>A, 5857C>G, 5858A>T, 5859A>T, 5861T>A, 5862C>A, 5864C>A, 5873C>T, 5876G>A, 5877G>A, 5878G>A, 5879A>T, 5880C>T, 5881A>T, 5882C>T, 5885C>A, 5889A>G, 5892A>T, 5894G>A, 5903C>A, 5904C>G, 5909T>A, 5915A>C, 5919C>T, 5921C>T, 5924A>G, 5927T>A, 5928T>G, 5929C>G, 5930G>A, 5931G>A, 5932A>G, 5933A>T, 5939G>A, 5940G>T, 5942G>A, 5945A>G, 5946A>T, 5947G>A, 5948A>T, 5951G>T, 5955T>A, 5956C>A, 5963C>T, 5964T>A, 5965G>T, 5966G>C, 5970A>T, 5973A>G, 5976A>C, 5977G>A, 5984G>C, 5987G>A, 5990A>T, 5991G>A, 5996C>T, 5998G>A, 6001G>A, 6002G>A, 6005G>C, 6006delC, 6007C>T, 6008C>A, 6009A>G, 6011A>T, 6012A>T, 6017A>T, 6023C>T, 6025C>T, 6029G>A, 6030G>T, 6031A>C, 6032T>C, 6034C>G, 6035A>T, 6038C>T, 6039A>T, 6042G>A, 6044C>A, 6053G>A, 6059C>G, 6060T>A, 6061C>G, 6062T>C, 6063A>T, 6064T>C, 6065G>C, 6067A>C, 6068T>A, 6070G>A, 6079G>C, 6083G>T, 6085G>T, 6086T>G, 6087T>A, 6089T>A, 6090T>G, 6091G>T, 6092G>C, 6095G>A, 6096A>C, 6097A>C, 6101C>T, 6105G>T, 6106C>A, 6108G>A, 6109A>G, 6111C>A, 6112C>A, 6113A>T, 6115G>A, 6117A>G, 6118G>A, 6125G>A, 6126C>A								

CDS

	Begin	End	Coverage	Score	Concordance	Matches	Identities	I/D/M/F*	Stop Codons
BaSUIV_gp3	1422	1578	8.5%	133	12.5%	156 (99.4%)	63 (40.1%)	0/1/2/2	3
Protein mutations:	Y1422Q (5658T>C 5660T>G), K1423T (5662A>C), M1424V (5664A>G 5666G>A), M1425F (5667A>T 5669G>T), E1426R (5670G>A 5671A>G), I1427L (5673A>C 5675C>A), C1428F (5677G>T), K1429V (5679A>G 5680A>T 5681G>A), K1430S (5683A>G 5684G>T), L1433I (5691T>A), L1435V (5697C>G 5699C>A), P1437K (5703C>A 5704C>A), T1438K (5707C>A 5708A>G), I1442L (5718A>T 5720C>A), G1443C (5721G>T 5723A>T), V1444K (5724G>A 5725T>A 5726T>A), K1445T (5728A>C 5729G>C), V1446H (5730G>C 5731T>A 5732A>T), V1447I (5733G>A), D1448N (5736G>A), G1451E (5746G>A 5747A>G), S1452I (5749G>T 5750C>T), T1453I (5752C>T 5753T>A), V1454L (5754G>T 5756T>A), N1457* (5763A>T 5764A>G 5765T>A), H1458K (5766C>A 5768C>G), L1459I (5769C>A 5771G>A), D1460K (5772G>A 5774T>A), V1466A (5791T>C 5792G>T), Q1467K (5793C>A), I1469V (5799A>G 5801A>C), V1470L (5802G>T 5804G>A), D1471E (5807T>G), F1472M (5808T>A 5810T>G), D1473P (5811G>C 5812A>C 5813T>A), E1474* (5814G>T), E1475I (5817G>A 5818A>T 5819A>T), K1476R (5821A>G), L1477del (5823_5825delCTG), T1479H (5829A>C 5830C>A 5831A>C), K1481R (5836A>G), G1482I (5838G>A 5839G>T), L1483T (5841C>A 5842T>C 5843C>A), S1485I (5847T>A 5848C>T 5849C>T), W1486L (5850T>C 5851G>T 5852G>T), L1487I (5853C>A 5855G>A), A1488G (5857C>G 5858A>T), I1489L (5859A>T 5861T>A), L1490I (5862C>A 5864C>A), G1495N (5877G>A 5878G>A 5879A>T), H1496F (5880C>T 5881A>T 5882C>T), N1499D (5889A>G), M1500L (5892A>T 5894G>A), L1504V (5904C>G), P1509S (5919C>T 5921C>T), S1512G (5928T>G 5929C>G 5930G>A), E1513S (5931G>A 5932A>G 5933A>T), E1516* (5940G>T 5942G>A), R1518Y (5946A>T 5947G>A 5948A>T), L1519F (5951G>T), S1521N (5955T>A 5956C>A), W1524I (5964T>A 5965G>T 5966G>C), I1526L (5970A>T), I1527V (5973A>G), R1528Q (5976A>C 5977G>A), M1530I (5984G>C), E1532D (5990A>T), E1533K (5991G>A), R1535K (5998G>A), R1536K (6001G>A 6002G>A), L1537F (6005G>C), K1539D (6009A>G 6011A>T), M1540L (6012A>T), P1544L (6025C>T), D1546S (6030G>T 6031A>C 6032T>C), A1547G (6034C>G 6035A>T), I1549L (6039A>T), V1550I (6042G>A 6044C>A), M1557S (6063A>T 6064T>C 6065G>C), N1558T (6067A>C 6068T>A), G1559E (6070G>A), G1562A (6079G>C), C1564L (6085G>T 6086T>G), Y1565K (6087T>A 6089T>A), W1566V (6090T>G 6091G>T 6092G>C), K1568P (6096A>C 6097A>C), A1571Y (6105G>T 6106C>A), D1572S (6108G>A 6109A>G), P1573N (6111C>A 6112C>A 6113A>T), R1574K (6115G>A), S1575D (6117A>G 6118G>A)								
Codon mutations:	TTG1421..A (5657G>A), TAT1422CAG (5658T>C 5660T>G), AAA1423ACA (5662A>C), ATG1424GTA (5664A>G 5666G>A), ATG1425TTT (5667A>T 5669G>T), GAA1426AGA (5670G>A 5671A>G), ATC1427CTA (5673A>C 5675C>A), TGT1428TTT (5677G>T), AAG1429GTA (5679A>G 5680A>T 5681G>A), AAG1430AGT (5683A>G 5684G>T), GGA1432GGT (5690A>T), TTA1433ATA (5691T>A), ATT1434ATA (5696T>A), CTC1435GTA (5697C>G 5699C>A), CCA1437AAA (5703C>A 5704C>A), ACA1438AAG (5707C>A 5708A>G), AAG1439AAA (5711G>A), AAG1441AAA (5717G>A), ATC1442TTA (5718A>T 5720C>A), GGA1443TGT (5721G>T 5723A>T), GTT1444AAA (5724G>A 5725T>A 5726T>A), AAG1445ACC (5728A>C 5729G>C), GTA1446CAT (5730G>C 5731T>A 5732A>T), GTA1447ATA (5733G>A), GAT1448AAT (5736G>A), TTT1449TTC (5741T>C), CTA1450TTA (5742C>T), GGA1451GAG (5746G>A 5747A>G), AGC1452ATT (5749G>T 5750C>T), ACT1453ATA (5752C>T 5753T>A), GTT1454TTA (5754G>T 5756T>A), GGT1455GGA (5759T>A), AAT1457TGA (5763A>T 5764A>G 5765T>A), CAC1458AAG (5766C>A 5768C>G), CTG1459ATA (5769C>A 5771G>A), GAT1460AAA (5772G>A 5774T>A), CCA1463-CC (5781delC 5783A>C), ATT1465ATA (5789T>A), GTG1466GCT (5791T>C 5792G>T), CAA1467AAA (5793C>A), ATA1469GTC (5799A>G 5801A>C), GTG1470TTA (5802G>T 5804G>A), GAT1471GAG (5807T>G), TTT1472ATG (5808T>A 5810T>G), GAT1473CCA (5811G>C 5812A>C 5813T>A), GAA1474TAA (5814G>T), GAA1475ATT (5817G>A 5818A>T 5819A>T), AAA1476AGA (5821A>G), CTG1477del (5823_5825delCTG), AAG1478AAA (5828G>A), ACA1479CAC (5829A>C 5830C>A 5831A>C), AAG1480AAA (5834G>A), AAA1481AGA (5836A>G), GGT1482ATT (5838G>A 5839G>T), CTC1483ACA (5841C>A 5842T>C 5843C>A), TCC1485ATT (5847T>A 5848C>T 5849C>T), TGG1486CTT (5850T>C 5851G>T 5852G>T), CTG1487ATA (5853C>A 5855G>A), GCA1488GGT (5857C>G 5858A>T), ATT1489TTA (5859A>T 5861T>A), CTC1490ATA (5862C>A 5864C>A), GCC1493GCT (5873C>T), AGG1494AGA (5876G>A), GGA1495AAT (5877G>A 5878G>A 5879A>T), CAC1496TTT (5880C>T 5881A>T 5882C>T), ATC1497ATA (5885C>A), AAC1499GAC (5889A>G), ATG1500TTA (5892A>T 5894G>A), ATC1503ATA (5903C>A), CTA1504GTA (5904C>G), GGT1505GGA (5909T>A), CTA1507CTC (5915A>C), CCC1509TCT (5919C>T 5921C>T), AAA1510AAG (5924A>G), ACT1511ACA (5927T>A), TCG1512GGA (5928T>G 5929C>G 5930G>A), GAA1513AGT (5931G>A 5932A>G 5933A>T), GGG1515GGA (5939G>A), GAG1516TAA (5940G>T 5942G>A), AAA1517AAG (5945A>G), AGA1518TAT (5946A>T 5947G>A 5948A>T), TTG1519TTT (5951G>T), TCT1521AAT (5955T>A 5956C>A), GAC1523GAT (5963C>T), TGG1524ATC (5964T>A 5965G>T 5966G>C), ATA1526TTA (5970A>T), ATC1527GTC (5973A>G), AGA1528CAA (5976A>C 5977G>A), ATG1530ATC (5984G>C), AAG1531AAA (5987G>A), GAA1532GAT (5990A>T), GAA1533AAA (5991G>A), GTC1534GTT (5996C>T), AGA1535AAA (5998G>A), AGG1536AAA (6001G>A 6002G>A), TTG1537TTC (6005G>C), CCC1538-TA (6006delC 6007C>T 6008C>A), AAA1539GAT (6009A>G 6011A>T), ATG1540TTG (6012A>T), GCA1541GCT (6017A>T), CCC1543CCT (6023C>T), CCA1544CTA (6025C>T), GAG1545GAA (6029G>A), GAT1546TCC (6030G>T 6031A>C 6032T>C), GCA1547GGT (6034C>G 6035A>T), TAC1548TAT (6038C>T), ATA1549TTA (6039A>T), GTC1550ATA (6042G>A 6044C>A), ACG1553ACA (6053G>A), GGC1555GGG (6059C>G), TCT1556AGC (6060T>A 6061C>G 6062T>C), ATG1557TCC (6063A>T 6064T>C 6065G>C), AAT1558ACA (6067A>C 6068T>A), GGA1559GAA (6070G>A), GGA1562GCA (6079G>C), GTG1563GTT (6083G>T), TGT1564TTG (6085G>T 6086T>G), TAT1565AAA (6087T>A 6089T>A), TGG1566GTC (6090T>G 6091G>T 6092G>C), AAG1567AAA (6095G>A), AAA1568CCA (6096A>C 6097A>C), AGC1569AGT (6101C>T), GCT1571TAT (6105G>T 6106C>A), GAT1572AGT (6108G>A 6109A>G), CCA1573AAT (6111C>A 6112C>A 6113A>T), AGA1574AAA (6115G>A), AGT1575GAT (6117A>G 6118G>A), GAG1577GAA (6125G>A), CAA1578AA. (6126C>A)								

Proteins

	Begin	End	Coverage	Score	Concordance	Matches	Identities	I/D/M/F*	Stop Codons
polyprotein (YP_004442827.1)	1422	1578	8.5%	133	12.5%	156 (99.4%)	63 (40.1%)	0/1/2/2	3
Protein mutations:	Y1422Q (5658T>C 5660T>G), K1423T (5662A>C), M1424V (5664A>G 5666G>A), M1425F (5667A>T 5669G>T), E1426R (5670G>A 5671A>G), I1427L (5673A>C 5675C>A), C1428F (5677G>T), K1429V (5679A>G 5680A>T 5681G>A), K1430S (5683A>G 5684G>T), L1433I (5691T>A), L1435V (5697C>G 5699C>A), P1437K (5703C>A 5704C>A), T1438K (5707C>A 5708A>G), I1442L (5718A>T 5720C>A), G1443C (5721G>T 5723A>T), V1444K (5724G>A 5725T>A 5726T>A), K1445T (5728A>C 5729G>C), V1446H (5730G>C 5731T>A 5732A>T), V1447I (5733G>A), D1448N (5736G>A), G1451E (5746G>A 5747A>G), S1452I (5749G>T 5750C>T), T1453I (5752C>T 5753T>A), V1454L (5754G>T 5756T>A), N1457* (5763A>T 5764A>G 5765T>A), H1458K (5766C>A 5768C>G), L1459I (5769C>A 5771G>A), D1460K (5772G>A 5774T>A), V1466A (5791T>C 5792G>T), Q1467K (5793C>A), I1469V (5799A>G 5801A>C), V1470L (5802G>T 5804G>A), D1471E (5807T>G), F1472M (5808T>A 5810T>G), D1473P (5811G>C 5812A>C 5813T>A), E1474* (5814G>T), E1475I (5817G>A 5818A>T 5819A>T), K1476R (5821A>G), L1477del (5823_5825delCTG), T1479H (5829A>C 5830C>A 5831A>C), K1481R (5836A>G), G1482I (5838G>A 5839G>T), L1483T (5841C>A 5842T>C 5843C>A), S1485I (5847T>A 5848C>T 5849C>T), W1486L (5850T>C 5851G>T 5852G>T), L1487I (5853C>A 5855G>A), A1488G (5857C>G 5858A>T), I1489L (5859A>T 5861T>A), L1490I (5862C>A 5864C>A), G1495N (5877G>A 5878G>A 5879A>T), H1496F (5880C>T 5881A>T 5882C>T), N1499D (5889A>G), M1500L (5892A>T 5894G>A), L1504V (5904C>G), P1509S (5919C>T 5921C>T), S1512G (5928T>G 5929C>G 5930G>A), E1513S (5931G>A 5932A>G 5933A>T), E1516* (5940G>T 5942G>A), R1518Y (5946A>T 5947G>A 5948A>T), L1519F (5951G>T), S1521N (5955T>A 5956C>A), W1524I (5964T>A 5965G>T 5966G>C), I1526L (5970A>T), I1527V (5973A>G), R1528Q (5976A>C 5977G>A), M1530I (5984G>C), E1532D (5990A>T), E1533K (5991G>A), R1535K (5998G>A), R1536K (6001G>A 6002G>A), L1537F (6005G>C), K1539D (6009A>G 6011A>T), M1540L (6012A>T), P1544L (6025C>T), D1546S (6030G>T 6031A>C 6032T>C), A1547G (6034C>G 6035A>T), I1549L (6039A>T), V1550I (6042G>A 6044C>A), M1557S (6063A>T 6064T>C 6065G>C), N1558T (6067A>C 6068T>A), G1559E (6070G>A), G1562A (6079G>C), C1564L (6085G>T 6086T>G), Y1565K (6087T>A 6089T>A), W1566V (6090T>G 6091G>T 6092G>C), K1568P (6096A>C 6097A>C), A1571Y (6105G>T 6106C>A), D1572S (6108G>A 6109A>G), P1573N (6111C>A 6112C>A 6113A>T), R1574K (6115G>A), S1575D (6117A>G 6118G>A)								

	Begin	End	Coverage	Score	Concordance	Matches	Identities	I/D/M/F*	Stop Codons
**NT**	**5657**	**6127**	**6.3%**	**40**	**4.4%**	**466 (98.9%)**	**251 (53.3%)**	**0/5**	
Codon mutations:	TTG1421..A (5657G>A), TAT1422CAG (5658T>C 5660T>G), AAA1423ACA (5662A>C), ATG1424GTA (5664A>G 5666G>A), ATG1425TTT (5667A>T 5669G>T), GAA1426AGA (5670G>A 5671A>G), ATC1427CTA (5673A>C 5675C>A), TGT1428TTT (5677G>T), AAG1429GTA (5679A>G 5680A>T 5681G>A), AAG1430AGT (5683A>G 5684G>T), GGA1432GGT (5690A>T), TTA1433ATA (5691T>A), ATT1434ATA (5696T>A), CTC1435GTA (5697C>G 5699C>A), CCA1437AAA (5703C>A 5704C>A), ACA1438AAG (5707C>A 5708A>G), AAG1439AAA (5711G>A), AAG1441AAA (5717G>A), ATC1442TTA (5718A>T 5720C>A), GGA1443TGT (5721G>T 5723A>T), GTT1444AAA (5724G>A 5725T>A 5726T>A), AAG1445ACC (5728A>C 5729G>C), GTA1446CAT (5730G>C 5731T>A 5732A>T), GTA1447ATA (5733G>A), GAT1448AAT (5736G>A), TTT1449TTC (5741T>C), CTA1450TTA (5742C>T), GGA1451GAG (5746G>A 5747A>G), AGC1452ATT (5749G>T 5750C>T), ACT1453ATA (5752C>T 5753T>A), GTT1454TTA (5754G>T 5756T>A), GGT1455GGA (5759T>A), AAT1457TGA (5763A>T 5764A>G 5765T>A), CAC1458AAG (5766C>A 5768C>G), CTG1459ATA (5769C>A 5771G>A), GAT1460AAA (5772G>A 5774T>A), CCA1463-CC (5781delC 5783A>C), ATT1465ATA (5789T>A), GTG1466GCT (5791T>C 5792G>T), CAA1467AAA (5793C>A), ATA1469GTC (5799A>G 5801A>C), GTG1470TTA (5802G>T 5804G>A), GAT1471GAG (5807T>G), TTT1472ATG (5808T>A 5810T>G), GAT1473CCA (5811G>C 5812A>C 5813T>A), GAA1474TAA (5814G>T), GAA1475ATT (5817G>A 5818A>T 5819A>T), AAA1476AGA (5821A>G), CTG1477del (5823_5825delCTG), AAG1478AAA (5828G>A), ACA1479CAC (5829A>C 5830C>A 5831A>C), AAG1480AAA (5834G>A), AAA1481AGA (5836A>G), GGT1482ATT (5838G>A 5839G>T), CTC1483ACA (5841C>A 5842T>C 5843C>A), TCC1485ATT (5847T>A 5848C>T 5849C>T), TGG1486CTT (5850T>C 5851G>T 5852G>T), CTG1487ATA (5853C>A 5855G>A), GCA1488GGT (5857C>G 5858A>T), ATT1489TTA (5859A>T 5861T>A), CTC1490ATA (5862C>A 5864C>A), GCC1493GCT (5873C>T), AGG1494AGA (5876G>A), GGA1495AAT (5877G>A 5878G>A 5879A>T), CAC1496TTT (5880C>T 5881A>T 5882C>T), ATC1497ATA (5885C>A), AAC1499GAC (5889A>G), ATG1500TTA (5892A>T 5894G>A), ATC1503ATA (5903C>A), CTA1504GTA (5904C>G), GGT1505GGA (5909T>A), CTA1507CTC (5915A>C), CCC1509TCT (5919C>T 5921C>T), AAA1510AAG (5924A>G), ACT1511ACA (5927T>A), TCG1512GGA (5928T>G 5929C>G 5930G>A), GAA1513AGT (5931G>A 5932A>G 5933A>T), GGG1515GGA (5939G>A), GAG1516TAA (5940G>T 5942G>A), AAA1517AAG (5945A>G), AGA1518TAT (5946A>T 5947G>A 5948A>T), TTG1519TTT (5951G>T), TCT1521AAT (5955T>A 5956C>A), GAC1523GAT (5963C>T), TGG1524ATC (5964T>A 5965G>T 5966G>C), ATA1526TTA (5970A>T), ATC1527GTC (5973A>G), AGA1528CAA (5976A>C 5977G>A), ATG1530ATC (5984G>C), AAG1531AAA (5987G>A), GAA1532GAT (5990A>T), GAA1533AAA (5991G>A), GTC1534GTT (5996C>T), AGA1535AAA (5998G>A), AGG1536AAA (6001G>A 6002G>A), TTG1537TTC (6005G>C), CCC1538-TA (6006delC 6007C>T 6008C>A), AAA1539GAT (6009A>G 6011A>T), ATG1540TTG (6012A>T), GCA1541GCT (6017A>T), CCC1543CCT (6023C>T), CCA1544CTA (6025C>T), GAG1545GAA (6029G>A), GAT1546TCC (6030G>T 6031A>C 6032T>C), GCA1547GGT (6034C>G 6035A>T), TAC1548TAT (6038C>T), ATA1549TTA (6039A>T), GTC1550ATA (6042G>A 6044C>A), ACG1553ACA (6053G>A), GGC1555GGG (6059C>G), TCT1556AGC (6060T>A 6061C>G 6062T>C), ATG1557TCC (6063A>T 6064T>C 6065G>C), AAT1558ACA (6067A>C 6068T>A), GGA1559GAA (6070G>A), GGA1562GCA (6079G>C), GTG1563GTT (6083G>T), TGT1564TTG (6085G>T 6086T>G), TAT1565AAA (6087T>A 6089T>A), TGG1566GTC (6090T>G 6091G>T 6092G>C), AAG1567AAA (6095G>A), AAA1568CCA (6096A>C 6097A>C), AGC1569AGT (6101C>T), GCT1571TAT (6105G>T 6106C>A), GAT1572AGT (6108G>A 6109A>G), CCA1573AAT (6111C>A 6112C>A 6113A>T), AGA1574AAA (6115G>A), AGT1575GAT (6117A>G 6118G>A), GAG1577GAA (6125G>A), CAA1578AA. (6126C>A)								

*: Inserts / Deletes / Misaligned / Frameshifts

Analysis details

This analysis was performed with panviral2.64

NGS Details (UN62): Badnavirus maculasmallanthi

Assembly

**Coverage Length**	245 (1 contig(s))
**Depth Of Coverage**	6.2
**Number Of Reads**	15
**Reads Per Million**	0.35 rpm (after QC)
**Ambiguities**	0
**Assembly Method**	de novo + reference guided assembly
**Consensus Caller**	Bcf Tools

Coverage Map

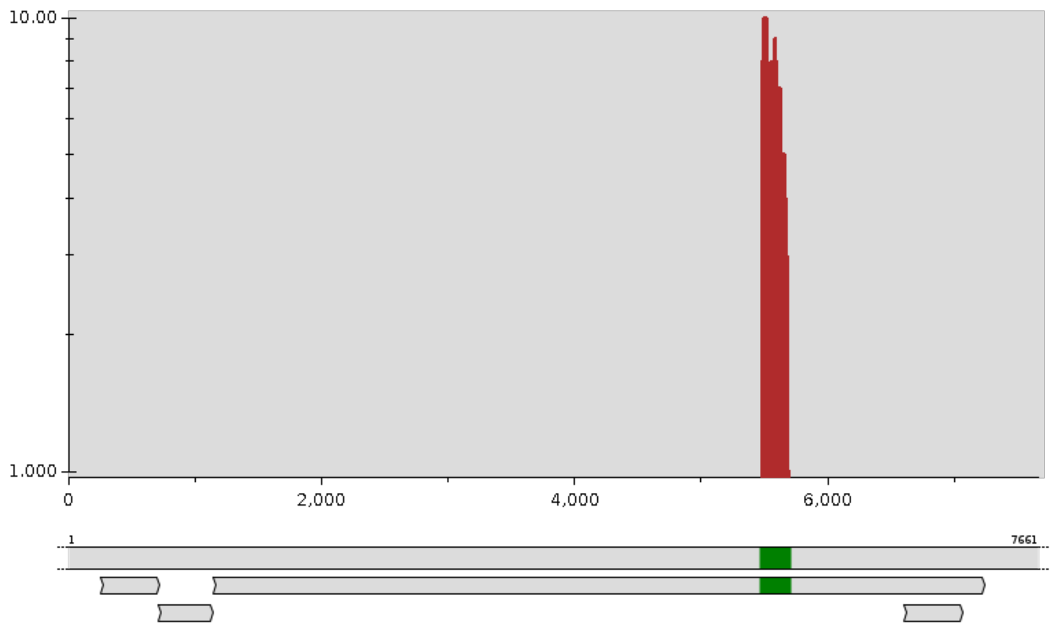

Assignment

**Type**	Badnavirus maculasmallanthi (Taxonomy ID: 3048453)
**Reference Genome**	NC_026472.1
**NT Identity (%)**	57.1429
**AA Identity (%)**	48.7805
**Number Of Stop Codons**	2
**Number Of CDS**	4

Alignment

**Alignment Score**	70.0 (NT) + 301.0 (AA) = 371.0
**Concordance (%)**	34.9012

Alignment Method	Global, seeded, nucleotide + amino acids (AGA)

Genome Region

Sequence starts at position 5469 and ends at position 5713 relative to NC_026472.1 reference sequence.

Alignment Detailed Statistics

	Begin	End	Coverage	Score	Concordance	Matches	Identities	I/D/M/F*	Stop Codons
**NT**	**5469**	**5713**	**3.2%**	**70**	**14.3%**	**245 (100%)**	**140 (57.1%)**	**0/0**	
Mutations:	5476A>G, 5478T>C, 5481C>T, 5485A>G, 5487A>T, 5488C>T, 5490C>G, 5493T>C, 5494G>A, 5496C>A, 5497A>G, 5499T>C, 5503T>G, 5504A>T, 5505C>T, 5512C>A, 5516A>T, 5518T>C, 5520C>A, 5521C>A, 5527G>A, 5528G>T, 5529G>T, 5532A>C, 5533A>G, 5535C>A, 5536A>G, 5537C>A, 5538C>G, 5545A>G, 5547A>T, 5548A>G, 5549G>A, 5551A>T, 5553A>G, 5556G>T, 5557A>G, 5558A>G, 5559T>C, 5560G>T, 5562A>T, 5565G>A, 5566A>G, 5571C>T, 5574A>T, 5578T>A, 5583T>C, 5584C>T, 5587A>C, 5588A>G, 5589G>A, 5590T>G, 5591C>T, 5592T>A, 5595A>C, 5597T>A, 5598T>C, 5605G>A, 5608G>T, 5609C>G, 5610C>A, 5615A>C, 5616A>C, 5617G>C, 5618A>C, 5619A>G, 5620G>A, 5621A>C, 5623T>G, 5624C>A, 5626A>G, 5632T>A, 5633G>A, 5637G>A, 5638G>T, 5640C>A, 5645T>G, 5648T>C, 5649C>T, 5651C>A, 5652A>T, 5653G>T, 5654G>C, 5655A>C, 5658A>G, 5659T>A, 5660T>A, 5661A>C, 5664C>T, 5667A>G, 5669G>A, 5670G>T, 5671C>T, 5673T>A, 5676C>G, 5682A>C, 5688A>G, 5691C>A, 5693A>C, 5694A>T, 5698G>A, 5703C>T, 5704G>T, 5708T>G, 5709T>C								
**CDS**									
UF61_gp3	1443	1524	4.0%	301	51.3%	82 (100%)	40 (48.8%)	0/0/0/0	2
Protein mutations:	N1445D (5476A>G 5478T>C), T1448A (5485A>G 5487A>T), D1451K (5494G>A 5496C>A), N1452D (5497A>G 5499T>C), Y1454V (5503T>G 5504A>T 5505C>T), Q1457K (5512C>A), Y1458F (5516A>T), S1459P (5518T>C 5520C>A), L1460I (5521C>A), G1462I (5527G>A 5528G>T 5529G>T), N1464E (5533A>G 5535C>A), T1465E (5536A>G 5537C>A 5538C>G), K1468D (5545A>G 5547A>T), R1469E (5548A>G 5549G>A), I1470L (5551A>T 5553A>G), N1472G (5557A>G 5558A>G 5559T>C), A1473S (5560G>T 5562A>T), I1475V (5566A>G), F1479I (5578T>A), K1482R (5587A>C 5588A>G 5589G>A), S1483V (5590T>G 5591C>T 5592T>A), F1485Y (5597T>A 5598T>C), V1488I (5605G>A), A1489* (5608G>T 5609C>G 5610C>A), E1491A (5615A>C 5616A>C), E1492P (5617G>C 5618A>C 5619A>G), E1493T (5620G>A 5621A>C), S1494D (5623T>G 5624C>A), I1495V (5626A>G), W1497K (5632T>A 5633G>A), A1499S (5638G>T 5640C>A), L1501* (5645T>G), I1502T (5648T>C 5649C>T), P1503H (5651C>A 5652A>T), G1504S (5653G>T 5654G>C 5655A>C), L1506N (5659T>A 5660T>A 5661A>C), W1509Y (5669G>A 5670G>T), K1517T (5693A>C 5694A>T), A1519T (5698G>A), A1521S (5704G>T), I1522S (5708T>G 5709T>C)								
Codon mutations:	AAT1445GAC (5476A>G 5478T>C), TAC1446TAT (5481C>T), ACA1448GCT (5485A>G 5487A>T), CTC1449TTG (5488C>T 5490C>G), AAT1450AAC (5493T>C), GAC1451AAA (5494G>A 5496C>A), AAT1452GAC (5497A>G 5499T>C), TAC1454GTT (5503T>G 5504A>T 5505C>T), CAG1457AAG (5512C>A), TAT1458TTT (5516A>T), TCC1459CCA (5518T>C 5520C>A), CTT1460ATT (5521C>A), GGG1462ATT (5527G>A 5528G>T 5529G>T), ATA1463ATC (5532A>C), AAC1464GAA (5533A>G 5535C>A), ACC1465GAG (5536A>G 5537C>A 5538C>G), AAA1468GAT (5545A>G 5547A>T), AGA1469GAA (5548A>G 5549G>A), ATA1470TTG (5551A>T 5553A>G), GGG1471GGT (5556G>T), AAT1472GGC (5557A>G 5558A>G 5559T>C), GCA1473TCT (5560G>T 5562A>T), AAG1474AAA (5565G>A), ATA1475GTA (5566A>G), TTC1476TTT (5571C>T), TCA1477TCT (5574A>T), TTT1479ATT (5578T>A), GAT1480GAC (5583T>C), CTG1481TTG (5584C>T), AAG1482CGA (5587A>C 5588A>G 5589G>A), TCT1483GTA (5590T>G 5591C>T 5592T>A), GGA1484GGC (5595A>C), TTT1485TAC (5597T>A 5598T>C), GTT1488ATT (5605G>A), GCC1489TGA (5608G>T 5609C>G 5610C>A), GAA1491GCC (5615A>C 5616A>C), GAA1492CCG (5617G>C 5618A>C 5619A>G), GAA1493ACA (5620G>A 5621A>C), TCC1494GAC (5623T>G 5624C>A), ATA1495GTA (5626A>G), TGG1497AAG (5632T>A 5633G>A), ACG1498ACA (5637G>A), GCC1499TCA (5638G>T 5640C>A), TTA1501TGA (5645T>G), ATC1502ACT (5648T>C 5649C>T), CCA1503CAT (5651C>A 5652A>T), GGA1504TCC (5653G>T 5654G>C 5655A>C), GGA1505GGG (5658A>G), TTA1506AAC (5659T>A 5660T>A 5661A>C), TAC1507TAT (5664C>T), GAA1508GAG (5667A>G), TGG1509TAT (5669G>A 5670G>T), CTT1510TTA (5671C>T 5673T>A), GTC1511GTG (5676C>G), CCA1513CCC (5682A>C), GGA1515GGG (5688A>G), CTC1516CTA (5691C>A), AAA1517ACT (5693A>C 5694A>T), GCA1519ACA (5698G>A), CCC1520CCT (5703C>T), GCT1521TCT (5704G>T), ATT1522AGC (5708T>G 5709T>C)								
**Proteins**									
ORF3 (YP_009121747.1)	1443	1524	4.0%	301	51.3%	82 (100%)	40 (48.8%)	0/0/0/0	2
Protein mutations:	N1445D (5476A>G 5478T>C), T1448A (5485A>G 5487A>T), D1451K (5494G>A 5496C>A), N1452D (5497A>G 5499T>C), Y1454V (5503T>G 5504A>T 5505C>T), Q1457K (5512C>A), Y1458F (5516A>T), S1459P (5518T>C 5520C>A), L1460I (5521C>A), G1462I (5527G>A 5528G>T 5529G>T), N1464E (5533A>G 5535C>A), T1465E (5536A>G 5537C>A 5538C>G), K1468D (5545A>G 5547A>T), R1469E (5548A>G 5549G>A), I1470L (5551A>T 5553A>G), N1472G (5557A>G 5558A>G 5559T>C), A1473S (5560G>T 5562A>T), I1475V (5566A>G), F1479I (5578T>A), K1482R (5587A>C 5588A>G 5589G>A), S1483V (5590T>G 5591C>T 5592T>A), F1485Y (5597T>A 5598T>C), V1488I (5605G>A), A1489* (5608G>T 5609C>G 5610C>A), E1491A (5615A>C 5616A>C), E1492P (5617G>C 5618A>C 5619A>G), E1493T (5620G>A 5621A>C), S1494D (5623T>G 5624C>A), I1495V (5626A>G), W1497K (5632T>A 5633G>A), A1499S (5638G>T 5640C>A), L1501* (5645T>G), I1502T (5648T>C 5649C>T), P1503H (5651C>A 5652A>T), G1504S (5653G>T 5654G>C 5655A>C), L1506N (5659T>A 5660T>A 5661A>C), W1509Y (5669G>A 5670G>T), K1517T (5693A>C 5694A>T), A1519T (5698G>A), A1521S (5704G>T), I1522S (5708T>G 5709T>C)								
Codon mutations:	AAT1445GAC (5476A>G 5478T>C), TAC1446TAT (5481C>T), ACA1448GCT (5485A>G 5487A>T), CTC1449TTG (5488C>T 5490C>G), AAT1450AAC (5493T>C), GAC1451AAA (5494G>A 5496C>A), AAT1452GAC (5497A>G 5499T>C), TAC1454GTT (5503T>G 5504A>T 5505C>T), CAG1457AAG (5512C>A), TAT1458TTT (5516A>T), TCC1459CCA (5518T>C 5520C>A), CTT1460ATT (5521C>A), GGG1462ATT (5527G>A 5528G>T 5529G>T), ATA1463ATC (5532A>C), AAC1464GAA (5533A>G 5535C>A), ACC1465GAG (5536A>G 5537C>A 5538C>G), AAA1468GAT (5545A>G 5547A>T), AGA1469GAA (5548A>G 5549G>A), ATA1470TTG (5551A>T 5553A>G), GGG1471GGT (5556G>T), AAT1472GGC (5557A>G 5558A>G 5559T>C), GCA1473TCT (5560G>T 5562A>T), AAG1474AAA (5565G>A), ATA1475GTA (5566A>G), TTC1476TTT (5571C>T), TCA1477TCT (5574A>T), TTT1479ATT (5578T>A), GAT1480GAC (5583T>C), CTG1481TTG (5584C>T), AAG1482CGA (5587A>C 5588A>G 5589G>A), TCT1483GTA (5590T>G 5591C>T 5592T>A), GGA1484GGC (5595A>C), TTT1485TAC (5597T>A 5598T>C), GTT1488ATT (5605G>A), GCC1489TGA (5608G>T 5609C>G 5610C>A), GAA1491GCC (5615A>C 5616A>C), GAA1492CCG (5617G>C 5618A>C 5619A>G), GAA1493ACA (5620G>A 5621A>C), TCC1494GAC (5623T>G 5624C>A), ATA1495GTA (5626A>G), TGG1497AAG (5632T>A 5633G>A), ACG1498ACA (5637G>A), GCC1499TCA (5638G>T 5640C>A), TTA1501TGA (5645T>G), ATC1502ACT (5648T>C 5649C>T), CCA1503CAT (5651C>A 5652A>T), GGA1504TCC (5653G>T 5654G>C 5655A>C), GGA1505GGG (5658A>G), TTA1506AAC (5659T>A 5660T>A 5661A>C), TAC1507TAT (5664C>T), GAA1508GAG (5667A>G), TGG1509TAT (5669G>A 5670G>T), CTT1510TTA (5671C>T 5673T>A), GTC1511GTG (5676C>G), CCA1513CCC (5682A>C), GGA1515GGG (5688A>G), CTC1516CTA (5691C>A), AAA1517ACT (5693A>C 5694A>T), GCA1519ACA (5698G>A), CCC1520CCT (5703C>T), GCT1521TCT (5704G>T), ATT1522AGC (5708T>G 5709T>C)								

*: Inserts / Deletes / Misaligned / Frameshifts

Analysis details

This analysis was performed with panviral2.64

NGS Details (UN62): Chinaberry tree badnavirus 1

Assembly

**Coverage Length**	249 (1 contig(s))
**Depth Of Coverage**	6.5
**Number Of Reads**	13
**Reads Per Million**	0.30 rpm (after QC)
**Ambiguities**	0
**Assembly Method**	de novo + reference guided assembly
**Consensus Caller**	Bcf Tools

Coverage Map

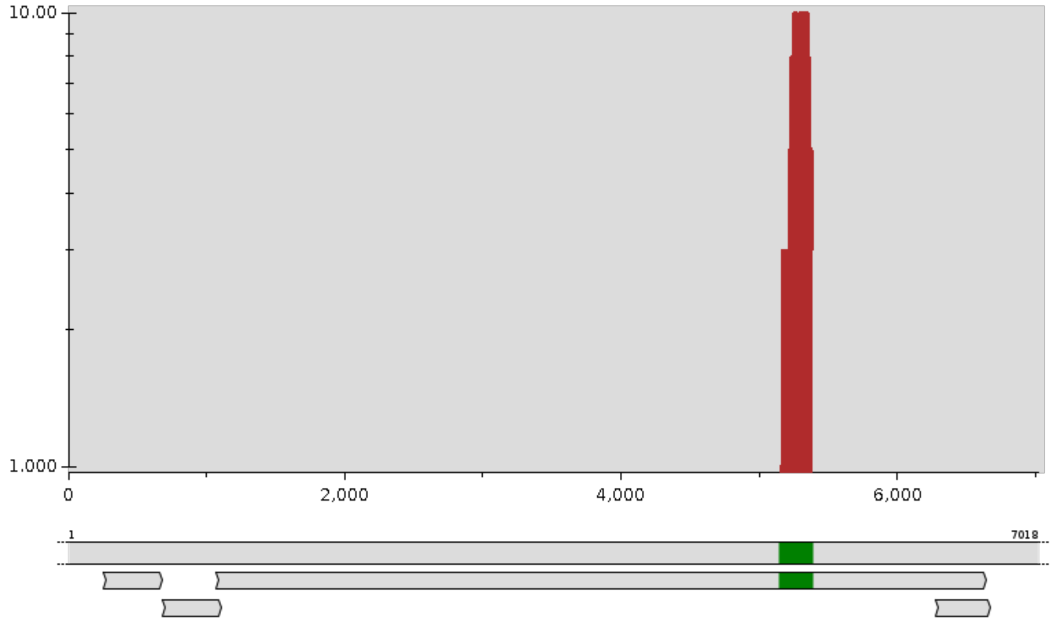

Assignment

**Type**	Chinaberry tree badnavirus 1 (Taxonomy ID: 2908099)
**Reference Genome**	NC_077165.1
**NT Identity (%)**	55.4217
**AA Identity (%)**	45.7831
**Number Of Stop Codons**	1
**Number Of CDS**	4

Alignment

**Alignment Score**	54.0 (NT) + 275.0 (AA) = 329.0
**Concordance (%)**	29.5597

Alignment Method	Global, seeded, nucleotide + amino acids (AGA)

Genome Region

Sequence starts at position 5144 and ends at position 5392 relative to NC_077165.1 reference sequence.

Alignment Detailed Statistics

	Begin	End	Coverage	Score	Concordance	Matches	Identities	I/D/M/F*	Stop Codons
**NT**	**5144**	**5392**	**3.5%**	**54**	**10.8%**	**249 (100%)**	**138 (55.4%)**	**0/0**	
Mutations:	5144A>G, 5145A>C, 5147G>T, 5148G>T, 5149T>G, 5150A>T, 5151T>G, 5153C>T, 5154A>G, 5156C>T, 5163A>G, 5164G>A, 5168G>C, 5172G>C, 5175C>T, 5180C>A, 5181C>G, 5182A>T, 5183C>G, 5190C>T, 5192G>A, 5194A>T, 5195T>C, 5196A>C, 5197G>C, 5198T>A, 5199C>A, 5201A>T, 5204A>T, 5205G>A, 5206G>T, 5207A>G, 5210C>A, 5211C>G, 5213C>G, 5214A>G, 5215C>G, 5217A>C, 5219C>A, 5220A>T, 5223A>G, 5224G>A, 5225G>T, 5226A>G, 5227G>A, 5228A>G, 5229A>T, 5231T>G, 5234C>A, 5237A>G, 5240C>T, 5242A>G, 5243G>A, 5244A>G, 5249T>C, 5252C>T, 5256T>A, 5264G>T, 5266A>G, 5268T>G, 5270G>T, 5273C>T, 5275T>A, 5282A>G, 5283G>T, 5285A>G, 5286G>A, 5287C>G, 5288C>A, 5293A>C, 5294C>A, 5295C>A, 5297G>A, 5298G>C, 5299A>C, 5300G>A, 5301T>G, 5302C>A, 5306A>G, 5307G>C, 5309A>T, 5310T>A, 5311G>A, 5312G>A, 5316G>A, 5322T>A, 5323G>A, 5324G>A, 5325G>A, 5326T>C, 5329C>A, 5330A>T, 5333T>A, 5336G>C, 5338T>A, 5345A>G, 5347G>T, 5348G>T, 5349C>T, 5357T>G, 5360A>T, 5363C>T, 5366G>C, 5367T>C, 5371A>C, 5372A>T, 5378T>A, 5381A>T, 5382G>T, 5385A>T, 5386T>C								

CDS

	Begin	End	Coverage	Score	Concordance	Matches	Identities	I/D/M/F*	Stop Codons
QKV46_gp3	1359	1441	4.5%	275	43.6%	83 (100%)	38 (45.8%)	0/0/0/0	1
Protein mutations:	M1359L (5145A>C 5147G>T), V1360C (5148G>T 5149T>G 5150A>T), F1361V (5151T>G 5153C>T), N1362D (5154A>G 5156C>T), R1365E (5163A>G 5164G>A), D1368H (5172G>C), H1371V (5181C>G 5182A>T 5183C>G), Q1374* (5190C>T 5192G>A), Y1375F (5194A>T 5195T>C), S1376P (5196A>C 5197G>C 5198T>A), L1377I (5199C>A 5201A>T), G1379M (5205G>A 5206G>T 5207A>G), H1381E (5211C>G 5213C>G), T1382G (5214A>G 5215C>G), I1383L (5217A>C 5219C>A), M1384L (5220A>T), R1385D (5223A>G 5224G>A 5225G>T), R1386E (5226A>G 5227G>A 5228A>G), I1387L (5229A>T 5231T>G), Q1391R (5242A>G 5243G>A), I1392V (5244A>G), F1396I (5256T>A), K1399R (5266A>G), S1400A (5268T>G 5270G>T), F1402Y (5275T>A), V1405L (5283G>T 5285A>G), A1406R (5286G>A 5287C>G 5288C>A), D1408A (5293A>C 5294C>A), P1409T (5295C>A 5297G>A), E1410P (5298G>C 5299A>C 5300G>A), S1411D (5301T>G 5302C>A), E1413H (5307G>C 5309A>T), W1414K (5310T>A 5311G>A 5312G>A), A1416T (5316G>A), W1418K (5322T>A 5323G>A 5324G>A), V1419T (5325G>A 5326T>C), P1420H (5329C>A 5330A>T), D1421E (5333T>A), L1423H (5338T>A), W1426F (5347G>T 5348G>T), I1429M (5357T>G), K1434T (5371A>C 5372A>T), A1438S (5382G>T), I1439S (5385A>T 5386T>C)								
Codon mutations:	AGA1358..G (5144A>G), ATG1359CTT (5145A>C 5147G>T), GTA1360TGT (5148G>T 5149T>G 5150A>T), TTC1361GTT (5151T>G 5153C>T), AAC1362GAT (5154A>G 5156C>T), AGG1365GAG (5163A>G 5164G>A), CTG1366CTC (5168G>C), GAT1368CAT (5172G>C), CTA1369TTA (5175C>T), ACC1370ACA (5180C>A), CAC1371GTG (5181C>G 5182A>T 5183C>G), CAG1374TAA (5190C>T 5192G>A), TAT1375TTC (5194A>T 5195T>C), AGT1376CCA (5196A>C 5197G>C 5198T>A), CTA1377ATT (5199C>A 5201A>T), CCA1378CCT (5204A>T), GGA1379ATG (5205G>A 5206G>T 5207A>G), ATC1380ATA (5210C>A), CAC1381GAG (5211C>G 5213C>G), ACC1382GGC (5214A>G 5215C>G), ATC1383CTA (5217A>C 5219C>A), ATG1384TTG (5220A>T), AGG1385GAT (5223A>G 5224G>A 5225G>T), AGA1386GAG (5226A>G 5227G>A 5228A>G), ATT1387TTG (5229A>T 5231T>G), GGC1388GGA (5234C>A), CAA1389CAG (5237A>G), GCC1390GCT (5240C>T), CAG1391CGA (5242A>G 5243G>A), ATA1392GTA (5244A>G), TTT1393TTC (5249T>C), TCC1394TCT (5252C>T), TTT1396ATT (5256T>A), CTG1398CTT (5264G>T), AAA1399AGA (5266A>G), TCG1400GCT (5268T>G 5270G>T), GGC1401GGT (5273C>T), TTT1402TAT (5275T>A), CAA1404CAG (5282A>G), GTA1405TTG (5283G>T 5285A>G), GCC1406AGA (5286G>A 5287C>G 5288C>A), GAC1408GCA (5293A>C 5294C>A), CCG1409ACA (5295C>A 5297G>A), GAG1410CCA (5298G>C 5299A>C 5300G>A), TCT1411GAT (5301T>G 5302C>A), GTA1412GTG (5306A>G), GAA1413CAT (5307G>C 5309A>T), TGG1414AAA (5310T>A 5311G>A 5312G>A), GCA1416ACA (5316G>A), TGG1418AAA (5322T>A 5323G>A 5324G>A), GTT1419ACT (5325G>A 5326T>C), CCA1420CAT (5329C>A 5330A>T), GAT1421GAA (5333T>A), GGG1422GGC (5336G>C), CTT1423CAT (5338T>A), GAA1425GAG (5345A>G), TGG1426TTT (5347G>T 5348G>T), CTG1427TTG (5349C>T), ATT1429ATG (5357T>G), CCA1430CCT (5360A>T), TTC1431TTT (5363C>T), GGG1432GGC (5366G>C), TTA1433CTA (5367T>C), AAA1434ACT (5371A>C 5372A>T), GCT1436GCA (5378T>A), CCA1437CCT (5381A>T), GCT1438TCT (5382G>T), ATA1439TCA (5385A>T 5386T>C)								

Proteins

	Begin	End	Coverage	Score	Concordance	Matches	Identities	I/D/M/F*	Stop Codons
ORF3 (YP_010805662.1)	1359	1441	4.5%	275	43.6%	83 (100%)	38 (45.8%)	0/0/0/0	1
Protein mutations:	M1359L (5145A>C 5147G>T), V1360C (5148G>T 5149T>G 5150A>T), F1361V (5151T>G 5153C>T), N1362D (5154A>G 5156C>T), R1365E (5163A>G 5164G>A), D1368H (5172G>C), H1371V (5181C>G 5182A>T 5183C>G), Q1374* (5190C>T 5192G>A), Y1375F (5194A>T 5195T>C), S1376P (5196A>C 5197G>C 5198T>A), L1377I (5199C>A 5201A>T), G1379M (5205G>A 5206G>T 5207A>G), H1381E (5211C>G 5213C>G), T1382G (5214A>G 5215C>G), I1383L (5217A>C 5219C>A), M1384L (5220A>T), R1385D (5223A>G 5224G>A 5225G>T), R1386E (5226A>G 5227G>A 5228A>G), I1387L (5229A>T 5231T>G), Q1391R (5242A>G 5243G>A), I1392V (5244A>G), F1396I (5256T>A), K1399R (5266A>G), S1400A (5268T>G 5270G>T), F1402Y (5275T>A), V1405L (5283G>T 5285A>G), A1406R (5286G>A 5287C>G 5288C>A), D1408A (5293A>C 5294C>A), P1409T (5295C>A 5297G>A), E1410P (5298G>C 5299A>C 5300G>A), S1411D (5301T>G 5302C>A), E1413H (5307G>C 5309A>T), W1414K (5310T>A 5311G>A 5312G>A), A1416T (5316G>A), W1418K (5322T>A 5323G>A 5324G>A), V1419T (5325G>A 5326T>C), P1420H (5329C>A 5330A>T), D1421E (5333T>A), L1423H (5338T>A), W1426F (5347G>T 5348G>T), I1429M (5357T>G), K1434T (5371A>C 5372A>T), A1438S (5382G>T), I1439S (5385A>T 5386T>C)								
Codon mutations:	AGA1358..G (5144A>G), ATG1359CTT (5145A>C 5147G>T), GTA1360TGT (5148G>T 5149T>G 5150A>T), TTC1361GTT (5151T>G 5153C>T), AAC1362GAT (5154A>G 5156C>T), AGG1365GAG (5163A>G 5164G>A), CTG1366CTC (5168G>C), GAT1368CAT (5172G>C), CTA1369TTA (5175C>T), ACC1370ACA (5180C>A), CAC1371GTG (5181C>G 5182A>T 5183C>G), CAG1374TAA (5190C>T 5192G>A), TAT1375TTC (5194A>T 5195T>C), AGT1376CCA (5196A>C 5197G>C 5198T>A), CTA1377ATT (5199C>A 5201A>T), CCA1378CCT (5204A>T), GGA1379ATG (5205G>A 5206G>T 5207A>G), ATC1380ATA (5210C>A), CAC1381GAG (5211C>G 5213C>G), ACC1382GGC (5214A>G 5215C>G), ATC1383CTA (5217A>C 5219C>A), ATG1384TTG (5220A>T), AGG1385GAT (5223A>G 5224G>A 5225G>T), AGA1386GAG (5226A>G 5227G>A 5228A>G), ATT1387TTG (5229A>T 5231T>G), GGC1388GGA (5234C>A), CAA1389CAG (5237A>G), GCC1390GCT (5240C>T), CAG1391CGA (5242A>G 5243G>A), ATA1392GTA (5244A>G), TTT1393TTC (5249T>C), TCC1394TCT (5252C>T), TTT1396ATT (5256T>A), CTG1398CTT (5264G>T), AAA1399AGA (5266A>G), TCG1400GCT (5268T>G 5270G>T), GGC1401GGT (5273C>T), TTT1402TAT (5275T>A), CAA1404CAG (5282A>G), GTA1405TTG (5283G>T 5285A>G), GCC1406AGA (5286G>A 5287C>G 5288C>A), GAC1408GCA (5293A>C 5294C>A), CCG1409ACA (5295C>A 5297G>A), GAG1410CCA (5298G>C 5299A>C 5300G>A), TCT1411GAT (5301T>G 5302C>A), GTA1412GTG (5306A>G), GAA1413CAT (5307G>C 5309A>T), TGG1414AAA (5310T>A 5311G>A 5312G>A), GCA1416ACA (5316G>A), TGG1418AAA (5322T>A 5323G>A 5324G>A), GTT1419ACT (5325G>A 5326T>C), CCA1420CAT (5329C>A 5330A>T), GAT1421GAA (5333T>A), GGG1422GGC (5336G>C), CTT1423CAT (5338T>A), GAA1425GAG (5345A>G), TGG1426TTT (5347G>T 5348G>T), CTG1427TTG (5349C>T), ATT1429ATG (5357T>G), CCA1430CCT (5360A>T), TTC1431TTT (5363C>T), GGG1432GGC (5366G>C), TTA1433CTA (5367T>C), AAA1434ACT (5371A>C 5372A>T), GCT1436GCA (5378T>A), CCA1437CCT (5381A>T), GCT1438TCT (5382G>T), ATA1439TCA (5385A>T 5386T>C)								

*: Inserts / Deletes / Misaligned / Frameshifts

Analysis details

This analysis was performed with panviral2.64

NGS Details (UN62): Pinus nigra virus 1

Assembly

**Coverage Length**	291 (1 contig(s))
**Depth Of Coverage**	4.5
**Number Of Reads**	12
**Reads Per Million**	0.28 rpm (after QC)
**Ambiguities**	0
**Assembly Method**	de novo + reference guided assembly
**Consensus Caller**	Bcf Tools

Coverage Map

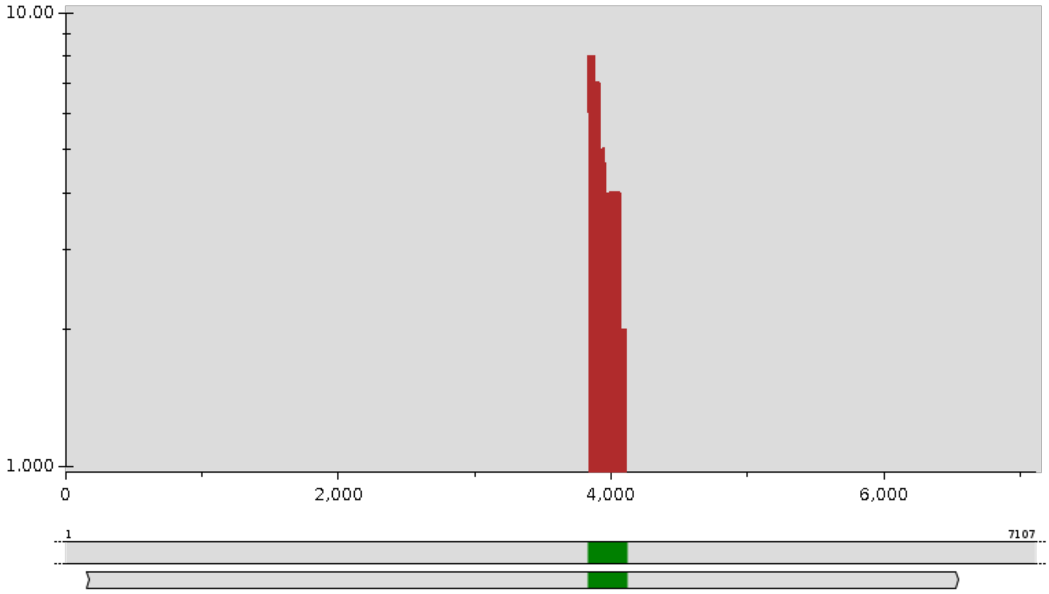

Assignment

**Type**	Pinus nigra virus 1 (Taxonomy ID: 2267679)
**Reference Genome**	NC_040841.1
**NT Identity (%)**	55.3265
**AA Identity (%)**	47.4227
**Number Of Stop Codons**	0
**Number Of CDS**	1

Alignment

**Alignment Score**	62.0 (NT) + 360.0 (AA) = 422.0
**Concordance (%)**	32.3372

Alignment Method	Global, seeded, nucleotide + amino acids (AGA)

Genome Region

Sequence starts at position 3830 and ends at position 4120 relative to NC_040841.1 reference sequence.

Alignment Detailed Statistics

	Begin	End	Coverage	Score	Concordance	Matches	Identities	I/D/M/F*	Stop Codons
**NT**	**3830**	**4120**	**4.1%**	**62**	**10.7%**	**291 (100%)**	**161 (55.3%)**	**0/0**	
Mutations:	3830C>A, 3835A>T, 3836G>A, 3837A>G, 3842A>T, 3843G>C, 3845G>A, 3850C>T, 3852A>C, 3853C>T, 3855A>G, 3856T>G, 3857G>A, 3858C>G, 3859C>T, 3865T>G, 3868A>C, 3873T>A, 3874A>T, 3877A>T, 3879G>A, 3880G>T, 3883T>C, 3884C>G, 3885A>T, 3887T>G, 3888C>T, 3889T>G, 3894T>A, 3897T>A, 3904T>C, 3905A>T, 3906A>C, 3907A>T, 3913A>G, 3914A>T, 3916G>A, 3919A>T, 3922A>T, 3925T>C, 3928T>C, 3931A>G, 3932C>T, 3934T>G, 3935C>T, 3937T>G, 3941G>T, 3944A>G, 3946C>T, 3947A>T, 3948C>T, 3949T>A, 3950C>A, 3952G>A, 3953A>T, 3954A>G, 3955T>G, 3956T>A, 3958T>A, 3960A>G, 3961T>A, 3964C>T, 3968T>A, 3970G>T, 3973A>T, 3975G>A, 3976A>T, 3977C>A, 3978C>A, 3980G>A, 3982A>G, 3984T>A, 3985G>T, 3986A>C, 3989A>T, 3991G>A, 3992C>A, 3995A>C, 3996A>G, 3999T>C, 4001C>T, 4002A>T, 4003G>T, 4004C>A, 4006T>A, 4007A>G, 4008G>C, 4009T>C, 4012G>T, 4013G>A, 4015G>T, 4017T>A, 4021C>T, 4024G>A, 4036G>A, 4042G>T, 4044A>T, 4045T>C, 4047A>G, 4048T>G, 4051G>A, 4054A>T, 4061C>T, 4062A>C, 4063G>T, 4064C>G, 4065C>A, 4067G>A, 4069A>G, 4072T>C, 4074G>A, 4075A>G, 4076C>T, 4084A>T, 4092T>A, 4093A>T, 4094T>G, 4095G>T, 4096T>C, 4099A>C, 4100G>T, 4101C>T, 4102A>T, 4106T>C, 4107T>A, 4108C>A, 4111T>C, 4112C>G, 4114G>A, 4120A>T								

CDS

	Begin	End	Coverage	Score	Concordance	Matches	Identities	I/D/M/F*	Stop Codons
EXL67_gp1	1226	1322	4.6%	360	49.8%	97 (100%)	46 (47.4%)	0/0/0/0	0
Protein mutations:	L1226I (3830C>A), E1228R (3836G>A 3837A>G), E1231K (3845G>A), H1233P (3852A>C 3853C>T), Y1234W (3855A>G 3856T>G), A1235S (3857G>A 3858C>G 3859C>T), L1240Y (3873T>A 3874A>T), R1242N (3879G>A 3880G>T), H1244V (3884C>G 3885A>T), S1245V (3887T>G 3888C>T 3889T>G), I1247K (3894T>A), V1248E (3897T>A), K1251S (3905A>T 3906A>C 3907A>T), M1254L (3914A>T 3916G>A), P1260S (3932C>T 3934T>G), A1263S (3941G>T), I1264V (3944A>G 3946C>T), T1265L (3947A>T 3948C>T 3949T>A), Q1266K (3950C>A 3952G>A), N1267W (3953A>T 3954A>G 3955T>G), F1268I (3956T>A 3958T>A), N1269R (3960A>G 3961T>A), L1272I (3968T>A 3970G>T), R1274N (3975G>A 3976A>T), P1275K (3977C>A 3978C>A), E1276K (3980G>A 3982A>G), V1277D (3984T>A 3985G>T), I1278L (3986A>C), M1279L (3989A>T 3991G>A), Q1280K (3992C>A), K1281R (3995A>C 3996A>G), I1282T (3999T>C), Q1283F (4001C>T 4002A>T 4003G>T), H1284K (4004C>A 4006T>A), S1285A (4007A>G 4008G>C 4009T>C), K1286N (4012G>T), V1287I (4013G>A 4015G>T), F1288Y (4017T>A), Y1297F (4044A>T 4045T>C), Y1298W (4047A>G 4048T>G), Q1303S (4061C>T 4062A>C 4063G>T), P1304E (4064C>G 4065C>A), E1305K (4067G>A 4069A>G), R1307K (4074G>A 4075A>G), H1308Y (4076C>T), I1313N (4092T>A 4093A>T), C1314V (4094T>G 4095G>T 4096T>C), A1316F (4100G>T 4101C>T 4102A>T), F1318Q (4106T>C 4107T>A 4108C>A), Q1320E (4112C>G 4114G>A), K1322N (4120A>T)								
Codon mutations:	CTT1226ATT (3830C>A), ATA1227ATT (3835A>T), GAG1228AGG (3836G>A 3837A>G), AGT1230TCT (3842A>T 3843G>C), GAA1231AAA (3845G>A), TCC1232TCT (3850C>T), CAC1233CCT (3852A>C 3853C>T), TAT1234TGG (3855A>G 3856T>G), GCC1235AGT (3857G>A 3858C>G 3859C>T), TCT1237TCG (3865T>G), GCA1238GCC (3868A>C), TTA1240TAT (3873T>A 3874A>T), GTA1241GTT (3877A>T), AGG1242AAT (3879G>A 3880G>T), AAT1243AAC (3883T>C), CAT1244GTT (3884C>G 3885A>T), TCT1245GTG (3887T>G 3888C>T 3889T>G), ATA1247AAA (3894T>A), GTA1248GAA (3897T>A), GGT1250GGC (3904T>C), AAA1251TCT (3905A>T 3906A>C 3907A>T), AGA1253AGG (3913A>G), ATG1254TTA (3914A>T 3916G>A), GTA1255GTT (3919A>T), ATA1256ATT (3922A>T), AAT1257AAC (3925T>C), TAT1258TAC (3928T>C), AAA1259AAG (3931A>G), CCT1260TCG (3932C>T 3934T>G), CTT1261TTG (3935C>T 3937T>G), GCC1263TCC (3941G>T), ATC1264GTT (3944A>G 3946C>T), ACT1265TTA (3947A>T 3948C>T 3949T>A), CAG1266AAA (3950C>A 3952G>A), AAT1267TGG (3953A>T 3954A>G 3955T>G), TTT1268ATA (3956T>A 3958T>A), AAT1269AGA (3960A>G 3961T>A), TAC1270TAT (3964C>T), TTG1272ATT (3968T>A 3970G>T), CCA1273CCT (3973A>T), AGA1274AAT (3975G>A 3976A>T), CCA1275AAA (3977C>A 3978C>A), GAA1276AAG (3980G>A 3982A>G), GTG1277GAT (3984T>A 3985G>T), ATT1278CTT (3986A>C), ATG1279TTA (3989A>T 3991G>A), CAA1280AAA (3992C>A), AAA1281CGA (3995A>C 3996A>G), ATT1282ACT (3999T>C), CAG1283TTT (4001C>T 4002A>T 4003G>T), CAT1284AAA (4004C>A 4006T>A), AGT1285GCC (4007A>G 4008G>C 4009T>C), AAG1286AAT (4012G>T), GTG1287ATT (4013G>A 4015G>T), TTC1288TAC (4017T>A), AGC1289AGT (4021C>T), AAG1290AAA (4024G>A), AAG1294AAA (4036G>A), GGG1296GGT (4042G>T), TAT1297TTC (4044A>T 4045T>C), TAT1298TGG (4047A>G 4048T>G), CAG1299CAA (4051G>A), ATA1300ATT (4054A>T), CAG1303TCT (4061C>T 4062A>C 4063G>T), CCA1304GAA (4064C>G 4065C>A), GAA1305AAG (4067G>A 4069A>G), GAT1306GAC (4072T>C), AGA1307AAG (4074G>A 4075A>G), CAC1308TAC (4076C>T), ACA1310ACT (4084A>T), ATA1313AAT (4092T>A 4093A>T), TGT1314GTC (4094T>G 4095G>T 4096T>C), CCA1315CCC (4099A>C), GCA1316TTT (4100G>T 4101C>T 4102A>T), TTC1318CAA (4106T>C 4107T>A 4108C>A), TAT1319TAC (4111T>C), CAG1320GAA (4112C>G 4114G>A), AAA1322AAT (4120A>T)								

Proteins

	Begin	End	Coverage	Score	Concordance	Matches	Identities	I/D/M/F*	Stop Codons
polyprotein (YP_009553669.1)	1226	1322	4.6%	360	49.8%	97 (100%)	46 (47.4%)	0/0/0/0	0
Protein mutations:	L1226I (3830C>A), E1228R (3836G>A 3837A>G), E1231K (3845G>A), H1233P (3852A>C 3853C>T), Y1234W (3855A>G 3856T>G), A1235S (3857G>A 3858C>G 3859C>T), L1240Y (3873T>A 3874A>T), R1242N (3879G>A 3880G>T), H1244V (3884C>G 3885A>T), S1245V (3887T>G 3888C>T 3889T>G), I1247K (3894T>A), V1248E (3897T>A), K1251S (3905A>T 3906A>C 3907A>T), M1254L (3914A>T 3916G>A), P1260S (3932C>T 3934T>G), A1263S (3941G>T), I1264V (3944A>G 3946C>T), T1265L (3947A>T 3948C>T 3949T>A), Q1266K (3950C>A 3952G>A), N1267W (3953A>T 3954A>G 3955T>G), F1268I (3956T>A 3958T>A), N1269R (3960A>G 3961T>A), L1272I (3968T>A 3970G>T), R1274N (3975G>A 3976A>T), P1275K (3977C>A 3978C>A), E1276K (3980G>A 3982A>G), V1277D (3984T>A 3985G>T), I1278L (3986A>C), M1279L (3989A>T 3991G>A), Q1280K (3992C>A), K1281R (3995A>C 3996A>G), I1282T (3999T>C), Q1283F (4001C>T 4002A>T 4003G>T), H1284K (4004C>A 4006T>A), S1285A (4007A>G 4008G>C 4009T>C), K1286N (4012G>T), V1287I (4013G>A 4015G>T), F1288Y (4017T>A), Y1297F (4044A>T 4045T>C), Y1298W (4047A>G 4048T>G), Q1303S (4061C>T 4062A>C 4063G>T), P1304E (4064C>G 4065C>A), E1305K (4067G>A 4069A>G), R1307K (4074G>A 4075A>G), H1308Y (4076C>T), I1313N (4092T>A 4093A>T), C1314V (4094T>G 4095G>T 4096T>C), A1316F (4100G>T 4101C>T 4102A>T), F1318Q (4106T>C 4107T>A 4108C>A), Q1320E (4112C>G 4114G>A), K1322N (4120A>T)								
Codon mutations:	CTT1226ATT (3830C>A), ATA1227ATT (3835A>T), GAG1228AGG (3836G>A 3837A>G), AGT1230TCT (3842A>T 3843G>C), GAA1231AAA (3845G>A), TCC1232TCT (3850C>T), CAC1233CCT (3852A>C 3853C>T), TAT1234TGG (3855A>G 3856T>G), GCC1235AGT (3857G>A 3858C>G 3859C>T), TCT1237TCG (3865T>G), GCA1238GCC (3868A>C), TTA1240TAT (3873T>A 3874A>T), GTA1241GTT (3877A>T), AGG1242AAT (3879G>A 3880G>T), AAT1243AAC (3883T>C), CAT1244GTT (3884C>G 3885A>T), TCT1245GTG (3887T>G 3888C>T 3889T>G), ATA1247AAA (3894T>A), GTA1248GAA (3897T>A), GGT1250GGC (3904T>C), AAA1251TCT (3905A>T 3906A>C 3907A>T), AGA1253AGG (3913A>G), ATG1254TTA (3914A>T 3916G>A), GTA1255GTT (3919A>T), ATA1256ATT (3922A>T), AAT1257AAC (3925T>C), TAT1258TAC (3928T>C), AAA1259AAG (3931A>G), CCT1260TCG (3932C>T 3934T>G), CTT1261TTG (3935C>T 3937T>G), GCC1263TCC (3941G>T), ATC1264GTT (3944A>G 3946C>T), ACT1265TTA (3947A>T 3948C>T 3949T>A), CAG1266AAA (3950C>A 3952G>A), AAT1267TGG (3953A>T 3954A>G 3955T>G), TTT1268ATA (3956T>A 3958T>A), AAT1269AGA (3960A>G 3961T>A), TAC1270TAT (3964C>T), TTG1272ATT (3968T>A 3970G>T), CCA1273CCT (3973A>T), AGA1274AAT (3975G>A 3976A>T), CCA1275AAA (3977C>A 3978C>A), GAA1276AAG (3980G>A 3982A>G), GTG1277GAT (3984T>A 3985G>T), ATT1278CTT (3986A>C), ATG1279TTA (3989A>T 3991G>A), CAA1280AAA (3992C>A), AAA1281CGA (3995A>C 3996A>G), ATT1282ACT (3999T>C), CAG1283TTT (4001C>T 4002A>T 4003G>T), CAT1284AAA (4004C>A 4006T>A), AGT1285GCC (4007A>G 4008G>C 4009T>C), AAG1286AAT (4012G>T), GTG1287ATT (4013G>A 4015G>T), TTC1288TAC (4017T>A), AGC1289AGT (4021C>T), AAG1290AAA (4024G>A), AAG1294AAA (4036G>A), GGG1296GGT (4042G>T), TAT1297TTC (4044A>T 4045T>C), TAT1298TGG (4047A>G 4048T>G), CAG1299CAA (4051G>A), ATA1300ATT (4054A>T), CAG1303TCT (4061C>T 4062A>C 4063G>T), CCA1304GAA (4064C>G 4065C>A), GAA1305AAG (4067G>A 4069A>G), GAT1306GAC (4072T>C), AGA1307AAG (4074G>A 4075A>G), CAC1308TAC (4076C>T), ACA1310ACT (4084A>T), ATA1313AAT (4092T>A 4093A>T), TGT1314GTC (4094T>G 4095G>T 4096T>C), CCA1315CCC (4099A>C), GCA1316TTT (4100G>T 4101C>T 4102A>T), TTC1318CAA (4106T>C 4107T>A 4108C>A), TAT1319TAC (4111T>C), CAG1320GAA (4112C>G 4114G>A), AAA1322AAT (4120A>T)								

*: Inserts / Deletes / Misaligned / Frameshifts

Analysis details

This analysis was performed with panviral2.64

NGS Details (UN62): Dioscovirus dioscoreae

Assembly

**Coverage Length**	434 (1 contig(s))
**Depth Of Coverage**	2.7
**Number Of Reads**	11
**Reads Per Million**	0.25 rpm (after QC)
**Ambiguities**	0
**Assembly Method**	de novo + reference guided assembly
**Consensus Caller**	Bcf Tools

Coverage Map

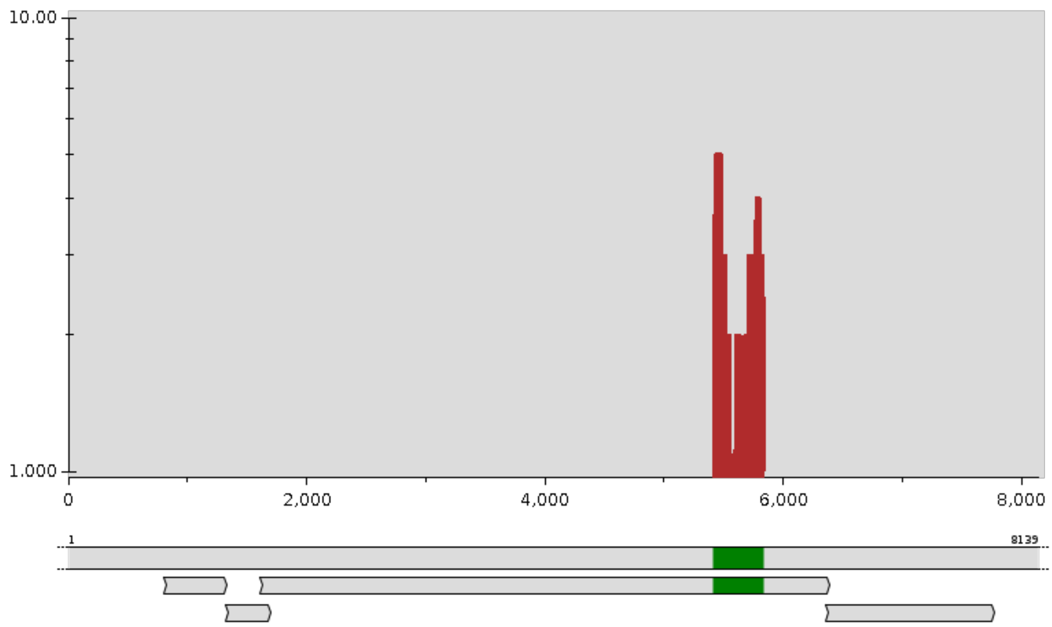

Assignment

**Type**	Dioscovirus dioscoreae (Taxonomy ID: 3052184)
**Reference Genome**	NC_040712.1
**NT Identity (%)**	61.1241
**AA Identity (%)**	54.9296
**Number Of Stop Codons**	0
**Number Of CDS**	4

Alignment

**Alignment Score**	156.0 (NT) + 442.0 (AA) = 598.0
**Concordance (%)**	35.1765

Alignment Method	Global, seeded, nucleotide + amino acids (AGA)

Genome Region

Sequence starts at position 5410 and ends at position 5843 relative to NC_040712.1 reference sequence.

Alignment Detailed Statistics

	Begin	End	Coverage	Score	Concordance	Matches	Identities	I/D/M/F*	Stop Codons
**NT**	**5410**	**5843**	**5.3%**	**156**	**19.0%**	**427 (98.4%)**	**261 (60.1%)**	**0/7**	
Mutations:	5410G>C, 5416A>C, 5417A>T, 5419G>T, 5429T>C, 5430G>A, 5431T>G, 5432C>T, 5433T>A, 5434G>C, 5435C>A, 5439C>A, 5441G>T, 5444T>A, 5449T>A, 5457A>T, 5468A>T, 5471C>T, 5475C>T, 5477C>A, 5486A>C, 5490T>C, 5491C>A, 5493G>A, 5495A>C, 5498C>T, 5502C>A, 5514A>G, 5516T>A, 5522C>T, 5523A>T, 5524A>C, 5525A>T, 5526A>G, 5529T>A, 5531T>G, 5532_5534delTCA, 5535G>A, 5537A>T, 5540T>C, 5541G>C, 5543C>A, 5544T>A, 5545G>T, 5549T>C, 5567G>A, 5572T>G, 5576T>A, 5577G>T, 5578A>C, 5581G>C, 5582T>A, 5583A>T, 5584T>A, 5585A>C, 5589C>G, 5595G>C, 5598C>A, 5599A>C, 5604T>C, 5606A>T, 5607T>A, 5608T>A, 5610C>G, 5612G>A, 5615C>T, 5617T>C, 5618C>A, 5619C>A, 5622G>A, 5624A>T, 5628A>G, 5629A>T, 5630G>T, 5633A>C, 5634G>A, 5636A>T, 5639G>A, 5640C>A, 5643A>G, 5649T>A, 5650C>G, 5651A>C, 5652A>C, 5655delA, 5656C>A, 5660A>G, 5661T>G, 5662T>C, 5663G>T, 5670G>A, 5671G>A, 5672A>T, 5673G>A, 5674T>A, 5676G>A, 5677C>T, 5678A>T, 5679A>G, 5681T>A, 5690C>T, 5696C>A, 5697C>A, 5699A>G, 5700G>A, 5701A>T, 5702A>T, 5705A>T, 5706G>T, 5707G>C, 5709G>A, 5712G>A, 5713G>A, 5714A>T, 5715A>G, 5716A>G, 5717A>G, 5718G>A, 5720T>A, 5721C>G, 5724C>T, 5726C>A, 5729G>A, 5730C>A, 5732C>T, 5738A>T, 5740T>C, 5744A>G, 5747T>A, 5751T>A, 5752T>C, 5753A>G, 5759T>C, 5760C>T, 5762T>G, 5763_5765delGAA, 5768C>T, 5769C>A, 5775G>A, 5776A>C, 5781T>A, 5782T>A, 5783A>G, 5784A>C, 5786G>A, 5788G>A, 5789T>A, 5790C>A, 5795G>A, 5798G>A, 5801C>T, 5802T>C, 5804A>T, 5807G>T, 5808A>T, 5809T>G, 5810A>T, 5811T>C, 5813A>G, 5816C>T, 5822C>A, 5823A>G, 5825A>T, 5826A>G, 5828T>G, 5830A>T, 5834C>T, 5835C>T, 5837A>G								

CDS

	Begin	End	Coverage	Score	Concordance	Matches	Identities	I/D/M/F*	Stop Codons
EXK67_gp3	1268	1411	9.0%	442	43.8%	142 (98.6%)	78 (54.2%)	0/2/1/1	0
Protein mutations:	E1269A (5416A>C 5417A>T), W1270L (5419G>T), V1274S (5430G>A 5431T>G 5432C>T), C1275T (5433T>A 5434G>C 5435C>A), Q1277N (5439C>A 5441G>T), F1280Y (5449T>A), I1283L (5457A>T), S1294Q (5490T>C 5491C>A), V1295I (5493G>A 5495A>C), N1302E (5514A>G 5516T>A), K1305S (5523A>T 5524A>C 5525A>T), K1306E (5526A>G), Y1307K (5529T>A 5531T>G), S1308del (5532_5534delTCA), E1309N (5535G>A 5537A>T), V1311L (5541G>C 5543C>A), C1312I (5544T>A 5545G>T), F1321C (5572T>G), E1323S (5577G>T 5578A>C), S1324T (5581G>C 5582T>A), I1325Y (5583A>T 5584T>A 5585A>C), Q1327E (5589C>G), V1329L (5595G>C), Q1330T (5598C>A 5599A>C), L1333K (5607T>A 5608T>A), Q1334E (5610C>G 5612G>A), F1336S (5617T>C 5618C>A), Q1337K (5619C>A), V1338I (5622G>A 5624A>T), K1340V (5628A>G 5629A>T 5630G>T), E1341D (5633A>C), E1342N (5634G>A 5636A>T), L1344I (5640C>A), I1345V (5643A>G), K1348Q (5652A>C), L1351A (5661T>G 5662T>C 5663G>T), G1354N (5670G>A 5671G>A 5672A>T), V1355K (5673G>A 5674T>A), A1356I (5676G>A 5677C>T 5678A>T), N1357E (5679A>G 5681T>A), L1363M (5697C>A 5699A>G), E1364I (5700G>A 5701A>T 5702A>T), G1366S (5706G>T 5707G>C), E1367K (5709G>A), G1368N (5712G>A 5713G>A 5714A>T), K1369G (5715A>G 5716A>G 5717A>G), V1370I (5718G>A 5720T>A), Q1371E (5721C>G), P1374T (5730C>A 5732C>T), L1377S (5740T>C), N1379K (5747T>A), L1381T (5751T>A 5752T>C 5753A>G), P1384S (5760C>T 5762T>G), E1385del (5763_5765delGAA), Q1387K (5769C>A), E1389T (5775G>A 5776A>C), L1391K (5781T>A 5782T>A 5783A>G), K1392Q (5784A>C 5786G>A), G1393E (5788G>A 5789T>A), L1394I (5790C>A), I1400C (5808A>T 5809T>G 5810A>T), R1405G (5823A>G 5825A>T), N1406E (5826A>G 5828T>G), Y1407F (5830A>T), P1409S (5835C>T 5837A>G)								
Codon mutations:	AGT1267.CT (5410G>C), GAA1269GCT (5416A>C 5417A>T), TGG1270TTG (5419G>T), TTT1273TTC (5429T>C), GTC1274AGT (5430G>A 5431T>G 5432C>T), TGC1275ACA (5433T>A 5434G>C 5435C>A), CAG1277AAT (5439C>A 5441G>T), GGT1278GGA (5444T>A), TTT1280TAT (5449T>A), ATA1283TTA (5457A>T), CCA1286CCT (5468A>T), TTC1287TTT (5471C>T), CTC1289TTA (5475C>T 5477C>A), GCA1292GCC (5486A>C), TCA1294CAA (5490T>C 5491C>A), GTA1295ATC (5493G>A 5495A>C), TTC1296TTT (5498C>T), CGA1298AGA (5502C>A), AAT1302GAA (5514A>G 5516T>A), TTC1304TTT (5522C>T), AAA1305TCT (5523A>T 5524A>C 5525A>T), AAA1306GAA (5526A>G), TAT1307AAG (5529T>A 5531T>G), TCA1308del (5532_5534delTCA), GAA1309AAT (5535G>A 5537A>T), TTT1310TTC (5540T>C), GTC1311CTA (5541G>C 5543C>A), TGT1312ATT (5544T>A 5545G>T), GTT1313GTC (5549T>C), TTG1319TTA (5567G>A), TTT1321TGT (5572T>G), TCT1322TCA (5576T>A), GAA1323TCA (5577G>T 5578A>C), AGT1324ACA (5581G>C 5582T>A), ATA1325TAC (5583A>T 5584T>A 5585A>C), CAA1327GAA (5589C>G), GTA1329CTA (5595G>C), CAA1330ACA (5598C>A 5599A>C), TTA1332CTT (5604T>C 5606A>T), TTA1333AAA (5607T>A 5608T>A), CAG1334GAA (5610C>G 5612G>A), TTC1335TTT (5615C>T), TTC1336TCA (5617T>C 5618C>A), CAA1337AAA (5619C>A), GTA1338ATT (5622G>A 5624A>T), AAG1340GTT (5628A>G 5629A>T 5630G>T), GAA1341GAC (5633A>C), GAA1342AAT (5634G>A 5636A>T), GGG1343GGA (5639G>A), CTT1344ATT (5640C>A), ATT1345GTT (5643A>G), TCA1347AGC (5649T>A 5650C>G 5651A>C), AAA1348CAA (5652A>C), ACA1349-AA (5655delA 5656C>A), AAA1350AAG (5660A>G), TTG1351GCT (5661T>G 5662T>C 5663G>T), GGA1354AAT (5670G>A 5671G>A 5672A>T), GTA1355AAA (5673G>A 5674T>A), GCA1356ATT (5676G>A 5677C>T 5678A>T), AAT1357GAA (5679A>G 5681T>A), TTC1360TTT (5690C>T), GGC1362GGA (5696C>A), CTA1363ATG (5697C>A 5699A>G), GAA1364ATT (5700G>A 5701A>T 5702A>T), ATA1365ATT (5705A>T), GGA1366TCA (5706G>T 5707G>C), GAA1367AAA (5709G>A), GGA1368AAT (5712G>A 5713G>A 5714A>T), AAA1369GGG (5715A>G 5716A>G 5717A>G), GTT1370ATA (5718G>A 5720T>A), CAA1371GAA (5721C>G), CTC1372TTA (5724C>T 5726C>A), CAG1373CAA (5729G>A), CCC1374ACT (5730C>A 5732C>T), ATA1376ATT (5738A>T), TTA1377TCA (5740T>C), AAA1378AAG (5744A>G), AAT1379AAA (5747T>A), TTA1381ACG (5751T>A 5752T>C 5753A>G), TTT1383TTC (5759T>C), CCT1384TCG (5760C>T 5762T>G), GAA1385del (5763_5765delGAA), GAC1386GAT (5768C>T), CAA1387AAA (5769C>A), GAA1389ACA (5775G>A 5776A>C), TTA1391AAG (5781T>A 5782T>A 5783A>G), AAG1392CAA (5784A>C 5786G>A), GGT1393GAA (5788G>A 5789T>A), CTA1394ATA (5790C>A), CAG1395CAA (5795G>A), AAG1396AAA (5798G>A), TTC1397TTT (5801C>T), TTA1398CTT (5802T>C 5804A>T), GGG1399GGT (5807G>T), ATA1400TGT (5808A>T 5809T>G 5810A>T), TTA1401CTG (5811T>C 5813A>G), AAC1402AAT (5816C>T), GCC1404GCA (5822C>A), AGA1405GGT (5823A>G 5825A>T), AAT1406GAG (5826A>G 5828T>G), TAT1407TTT (5830A>T), ATC1408ATT (5834C>T), CCA1409TCG (5835C>T 5837A>G)								

Proteins

	Begin	End	Coverage	Score	Concordance	Matches	Identities	I/D/M/F*	Stop Codons
ORF3 (YP_009553219.1)	1268	1411	9.0%	442	43.8%	142 (98.6%)	78 (54.2%)	0/2/1/1	0
Protein mutations:	E1269A (5416A>C 5417A>T), W1270L (5419G>T), V1274S (5430G>A 5431T>G 5432C>T), C1275T (5433T>A 5434G>C 5435C>A), Q1277N (5439C>A 5441G>T), F1280Y (5449T>A), I1283L (5457A>T), S1294Q (5490T>C 5491C>A), V1295I (5493G>A 5495A>C), N1302E (5514A>G 5516T>A), K1305S (5523A>T 5524A>C 5525A>T), K1306E (5526A>G), Y1307K (5529T>A 5531T>G), S1308del (5532_5534delTCA), E1309N (5535G>A 5537A>T), V1311L (5541G>C 5543C>A), C1312I (5544T>A 5545G>T), F1321C (5572T>G), E1323S (5577G>T 5578A>C), S1324T (5581G>C 5582T>A), I1325Y (5583A>T 5584T>A 5585A>C), Q1327E (5589C>G), V1329L (5595G>C), Q1330T (5598C>A 5599A>C), L1333K (5607T>A 5608T>A), Q1334E (5610C>G 5612G>A), F1336S (5617T>C 5618C>A), Q1337K (5619C>A), V1338I (5622G>A 5624A>T), K1340V (5628A>G 5629A>T 5630G>T), E1341D (5633A>C), E1342N (5634G>A 5636A>T), L1344I (5640C>A), I1345V (5643A>G), K1348Q (5652A>C), L1351A (5661T>G 5662T>C 5663G>T), G1354N (5670G>A 5671G>A 5672A>T), V1355K (5673G>A 5674T>A), A1356I (5676G>A 5677C>T 5678A>T), N1357E (5679A>G 5681T>A), L1363M (5697C>A 5699A>G), E1364I (5700G>A 5701A>T 5702A>T), G1366S (5706G>T 5707G>C), E1367K (5709G>A), G1368N (5712G>A 5713G>A 5714A>T), K1369G (5715A>G 5716A>G 5717A>G), V1370I (5718G>A 5720T>A), Q1371E (5721C>G), P1374T (5730C>A 5732C>T), L1377S (5740T>C), N1379K (5747T>A), L1381T (5751T>A 5752T>C 5753A>G), P1384S (5760C>T 5762T>G), E1385del (5763_5765delGAA), Q1387K (5769C>A), E1389T (5775G>A 5776A>C), L1391K (5781T>A 5782T>A 5783A>G), K1392Q (5784A>C 5786G>A), G1393E (5788G>A 5789T>A), L1394I (5790C>A), I1400C (5808A>T 5809T>G 5810A>T), R1405G (5823A>G 5825A>T), N1406E (5826A>G 5828T>G), Y1407F (5830A>T), P1409S (5835C>T 5837A>G)								
Codon mutations:	AGT1267.CT (5410G>C), GAA1269GCT (5416A>C 5417A>T), TGG1270TTG (5419G>T), TTT1273TTC (5429T>C), GTC1274AGT (5430G>A 5431T>G 5432C>T), TGC1275ACA (5433T>A 5434G>C 5435C>A), CAG1277AAT (5439C>A 5441G>T), GGT1278GGA (5444T>A), TTT1280TAT (5449T>A), ATA1283TTA (5457A>T), CCA1286CCT (5468A>T), TTC1287TTT (5471C>T), CTC1289TTA (5475C>T 5477C>A), GCA1292GCC (5486A>C), TCA1294CAA (5490T>C 5491C>A), GTA1295ATC (5493G>A 5495A>C), TTC1296TTT (5498C>T), CGA1298AGA (5502C>A), AAT1302GAA (5514A>G 5516T>A), TTC1304TTT (5522C>T), AAA1305TCT (5523A>T 5524A>C 5525A>T), AAA1306GAA (5526A>G), TAT1307AAG (5529T>A 5531T>G), TCA1308del (5532_5534delTCA), GAA1309AAT (5535G>A 5537A>T), TTT1310TTC (5540T>C), GTC1311CTA (5541G>C 5543C>A), TGT1312ATT (5544T>A 5545G>T), GTT1313GTC (5549T>C), TTG1319TTA (5567G>A), TTT1321TGT (5572T>G), TCT1322TCA (5576T>A), GAA1323TCA (5577G>T 5578A>C), AGT1324ACA (5581G>C 5582T>A), ATA1325TAC (5583A>T 5584T>A 5585A>C), CAA1327GAA (5589C>G), GTA1329CTA (5595G>C), CAA1330ACA (5598C>A 5599A>C), TTA1332CTT (5604T>C 5606A>T), TTA1333AAA (5607T>A 5608T>A), CAG1334GAA (5610C>G 5612G>A), TTC1335TTT (5615C>T), TTC1336TCA (5617T>C 5618C>A), CAA1337AAA (5619C>A), GTA1338ATT (5622G>A 5624A>T), AAG1340GTT (5628A>G 5629A>T 5630G>T), GAA1341GAC (5633A>C), GAA1342AAT (5634G>A 5636A>T), GGG1343GGA (5639G>A), CTT1344ATT (5640C>A), ATT1345GTT (5643A>G), TCA1347AGC (5649T>A 5650C>G 5651A>C), AAA1348CAA (5652A>C), ACA1349-AA (5655delA 5656C>A), AAA1350AAG (5660A>G), TTG1351GCT (5661T>G 5662T>C 5663G>T), GGA1354AAT (5670G>A 5671G>A 5672A>T), GTA1355AAA (5673G>A 5674T>A), GCA1356ATT (5676G>A 5677C>T 5678A>T), AAT1357GAA (5679A>G 5681T>A), TTC1360TTT (5690C>T), GGC1362GGA (5696C>A), CTA1363ATG (5697C>A 5699A>G), GAA1364ATT (5700G>A 5701A>T 5702A>T), ATA1365ATT (5705A>T), GGA1366TCA (5706G>T 5707G>C), GAA1367AAA (5709G>A), GGA1368AAT (5712G>A 5713G>A 5714A>T), AAA1369GGG (5715A>G 5716A>G 5717A>G), GTT1370ATA (5718G>A 5720T>A), CAA1371GAA (5721C>G), CTC1372TTA (5724C>T 5726C>A), CAG1373CAA (5729G>A), CCC1374ACT (5730C>A 5732C>T), ATA1376ATT (5738A>T), TTA1377TCA (5740T>C), AAA1378AAG (5744A>G), AAT1379AAA (5747T>A), TTA1381ACG (5751T>A 5752T>C 5753A>G), TTT1383TTC (5759T>C), CCT1384TCG (5760C>T 5762T>G), GAA1385del (5763_5765delGAA), GAC1386GAT (5768C>T), CAA1387AAA (5769C>A), GAA1389ACA (5775G>A 5776A>C), TTA1391AAG (5781T>A 5782T>A 5783A>G), AAG1392CAA (5784A>C 5786G>A), GGT1393GAA (5788G>A 5789T>A), CTA1394ATA (5790C>A), CAG1395CAA (5795G>A), AAG1396AAA (5798G>A), TTC1397TTT (5801C>T), TTA1398CTT (5802T>C 5804A>T), GGG1399GGT (5807G>T), ATA1400TGT (5808A>T 5809T>G 5810A>T), TTA1401CTG (5811T>C 5813A>G), AAC1402AAT (5816C>T), GCC1404GCA (5822C>A), AGA1405GGT (5823A>G 5825A>T), AAT1406GAG (5826A>G 5828T>G), TAT1407TTT (5830A>T), ATC1408ATT (5834C>T), CCA1409TCG (5835C>T 5837A>G)								

*: Inserts / Deletes / Misaligned / Frameshifts

Analysis details

This analysis was performed with panviral2.64

NGS Details (UN62): Duamitovirus peex1

Assembly

**Coverage Length**	263 (1 contig(s))
**Depth Of Coverage**	5.1
**Number Of Reads**	10
**Reads Per Million**	0.23 rpm (after QC)
**Ambiguities**	0
**Assembly Method**	de novo + reference guided assembly
**Consensus Caller**	Bcf Tools

Coverage Map

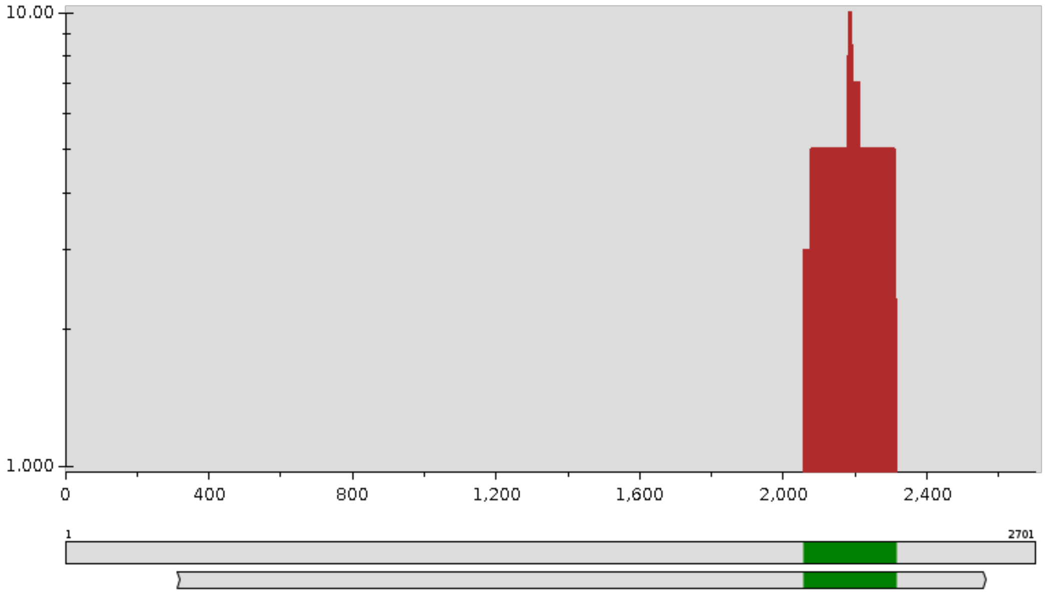

Assignment

**Type**	Duamitovirus peex1 (Taxonomy ID: 2955799)
**Reference Genome**	NC_076525.1
**NT Identity (%)**	73.384
**AA Identity (%)**	64.3678
**Number Of Stop Codons**	0
**Number Of CDS**	1

Alignment

**Alignment Score**	246.0 (NT) + 438.0 (AA) = 684.0
**Concordance (%)**	57.4307

Alignment Method	Global, seeded, nucleotide + amino acids (AGA)

Genome Region

Sequence starts at position 2056 and ends at position 2318 relative to NC_076525.1 reference sequence.

Alignment Detailed Statistics

	Begin	End	Coverage	Score	Concordance	Matches	Identities	I/D/M/F*	Stop Codons
**NT**	**2056**	**2318**	**9.7%**	**246**	**46.8%**	**263 (100%)**	**193 (73.4%)**	**0/0**	
Mutations:	2057C>A, 2066G>A, 2067A>C, 2069T>A, 2075T>G, 2077T>C, 2088T>C, 2090T>A, 2091T>A, 2095T>G, 2096A>G, 2098C>T, 2102G>T, 2104G>T, 2105G>A, 2109T>C, 2119G>T, 2120G>T, 2133G>T, 2134G>T, 2135A>T, 2136A>G, 2137A>G, 2138G>A, 2139C>A, 2145G>A, 2156A>G, 2158G>A, 2159A>G, 2161G>C, 2162T>G, 2165G>A, 2171C>A, 2173C>A, 2180T>G, 2184A>G, 2188A>C, 2189G>T, 2190T>G, 2195A>G, 2202G>C, 2210G>A, 2211T>A, 2213G>C, 2214A>T, 2216A>T, 2223C>G, 2225A>T, 2226C>T, 2231G>A, 2234A>T, 2240T>A, 2245A>G, 2246T>G, 2249G>A, 2255A>T, 2258A>G, 2261C>G, 2268G>A, 2270A>C, 2272G>T, 2274C>A, 2283G>A, 2292C>T, 2294T>G, 2295C>A, 2296G>C, 2297A>T, 2306A>T, 2315C>T									
**CDS**									
RdRp	583	669	11.6%	438	65.1%	87 (100%)	56 (64.4%)	0/0/0/0	0
Protein mutations:	I586L (2067A>C 2069T>A), V589A (2077T>C), S593P (2088T>C 2090T>A), Y594N (2091T>A), I595R (2095T>G 2096A>G), S596L (2098C>T), Q597H (2102G>T), R598L (2104G>T 2105G>A), S600P (2109T>C), W603F (2119G>T 2120G>T), G608F (2133G>T 2134G>T 2135A>T), K609G (2136A>G 2137A>G 2138G>A), P610T (2139C>A), D612N (2145G>A), R616K (2158G>A 2159A>G), G617A (2161G>C 2162T>G), A621D (2173C>A), K625E (2184A>G), E626A (2188A>C 2189G>T), L627V (2190T>G), E631Q (2202G>C), L634I (2211T>A 2213G>C), I635F (2214A>T 2216A>T), Q638D (2223C>G 2225A>T), L641F (2234A>T), H645R (2245A>G 2246T>G), V653I (2268G>A 2270A>C), R654L (2272G>T), H655N (2274C>A), E658K (2283G>A), R662T (2295C>A 2296G>C 2297A>T)									
Codon mutations:	CGC582.GA (2057C>A), AGG585AGA (2066G>A), ATT586CTA (2067A>C 2069T>A), GCT588GCG (2075T>G), GTT589GCT (2077T>C), TCT593CCA (2088T>C 2090T>A), TAC594AAC (2091T>A), ATA595AGG (2095T>G 2096A>G), TCA596TTA (2098C>T), CAG597CAT (2102G>T), CGG598CTA (2104G>T 2105G>A), TCT600CCT (2109T>C), TGG603TTT (2119G>T 2120G>T), GGA608TTT (2133G>T 2134G>T 2135A>T), AAG609GGA (2136A>G 2137A>G 2138G>A), CCC610ACC (2139C>A), GAT612AAT (2145G>A), TTA615TTG (2156A>G), AGA616AAG (2158G>A 2159A>G), GGT617GCG (2161G>C 2162T>G), AAG618AAA (2165G>A), ATC620ATA (2171C>A), GCT621GAT (2173C>A), CTT623CTG (2180T>G), AAG625GAG (2184A>G), GAG626GCT (2188A>C 2189G>T), TTA627GTA (2190T>G), AAA628AAG (2195A>G), GAG631CAG (2202G>C), CAG633CAA (2210G>A), TTG634ATC (2211T>A 2213G>C), ATA635TTT (2214A>T 2216A>T), CAA638GAT (2223C>G 2225A>T), CTG639TTG (2226C>T), GTG640GTA (2231G>A), TTA641TTT (2234A>T), GGT643GGA (2240T>A), CAT645CGG (2245A>G 2246T>G), GAG646GAA (2249G>A), CTA648CTT (2255A>T), GAA649GAG (2258A>G), CGC650CGG (2261C>G), GTA653ATC (2268G>A 2270A>C), CGT654CTT (2272G>T), CAT655AAT (2274C>A), GAA658AAA (2283G>A), CTT661TTG (2292C>T 2294T>G), CGA662ACT (2295C>A 2296G>C 2297A>T), TCA665TCT (2306A>T), CAC668CAT (2315C>T)									
**Proteins**									
RNA-dependent RNA polymerase (YP_010798875.1)	583	669	11.6%	438	65.1%	87 (100%)	56 (64.4%)	0/0/0/0	0
Protein mutations:	I586L (2067A>C 2069T>A), V589A (2077T>C), S593P (2088T>C 2090T>A), Y594N (2091T>A), I595R (2095T>G 2096A>G), S596L (2098C>T), Q597H (2102G>T), R598L (2104G>T 2105G>A), S600P (2109T>C), W603F (2119G>T 2120G>T), G608F (2133G>T 2134G>T 2135A>T), K609G (2136A>G 2137A>G 2138G>A), P610T (2139C>A), D612N (2145G>A), R616K (2158G>A 2159A>G), G617A (2161G>C 2162T>G), A621D (2173C>A), K625E (2184A>G), E626A (2188A>C 2189G>T), L627V (2190T>G), E631Q (2202G>C), L634I (2211T>A 2213G>C), I635F (2214A>T 2216A>T), Q638D (2223C>G 2225A>T), L641F (2234A>T), H645R (2245A>G 2246T>G), V653I (2268G>A 2270A>C), R654L (2272G>T), H655N (2274C>A), E658K (2283G>A), R662T (2295C>A 2296G>C 2297A>T)									
Codon mutations:	CGC582.GA (2057C>A), AGG585AGA (2066G>A), ATT586CTA (2067A>C 2069T>A), GCT588GCG (2075T>G), GTT589GCT (2077T>C), TCT593CCA (2088T>C 2090T>A), TAC594AAC (2091T>A), ATA595AGG (2095T>G 2096A>G), TCA596TTA (2098C>T), CAG597CAT (2102G>T), CGG598CTA (2104G>T 2105G>A), TCT600CCT (2109T>C), TGG603TTT (2119G>T 2120G>T), GGA608TTT (2133G>T 2134G>T 2135A>T), AAG609GGA (2136A>G 2137A>G 2138G>A), CCC610ACC (2139C>A), GAT612AAT (2145G>A), TTA615TTG (2156A>G), AGA616AAG (2158G>A 2159A>G), GGT617GCG (2161G>C 2162T>G), AAG618AAA (2165G>A), ATC620ATA (2171C>A), GCT621GAT (2173C>A), CTT623CTG (2180T>G), AAG625GAG (2184A>G), GAG626GCT (2188A>C 2189G>T), TTA627GTA (2190T>G), AAA628AAG (2195A>G), GAG631CAG (2202G>C), CAG633CAA (2210G>A), TTG634ATC (2211T>A 2213G>C), ATA635TTT (2214A>T 2216A>T), CAA638GAT (2223C>G 2225A>T), CTG639TTG (2226C>T), GTG640GTA (2231G>A), TTA641TTT (2234A>T), GGT643GGA (2240T>A), CAT645CGG (2245A>G 2246T>G), GAG646GAA (2249G>A), CTA648CTT (2255A>T), GAA649GAG (2258A>G), CGC650CGG (2261C>G), GTA653ATC (2268G>A 2270A>C), CGT654CTT (2272G>T), CAT655AAT (2274C>A), GAA658AAA (2283G>A), CTT661TTG (2292C>T 2294T>G), CGA662ACT (2295C>A 2296G>C 2297A>T), TCA665TCT (2306A>T), CAC668CAT (2315C>T)									

*: Inserts / Deletes / Misaligned / Frameshifts

Analysis details

This analysis was performed with panviral2.64

NGS Details (UN62): Cacao swollen shoot Ghana J virus

Assembly

**Coverage Length**	305 (1 contig(s))
**Depth Of Coverage**	4.1
**Number Of Reads**	10
**Reads Per Million**	0.23 rpm (after QC)
**Ambiguities**	0
**Assembly Method**	de novo + reference guided assembly
**Consensus Caller**	Bcf Tools

Coverage Map

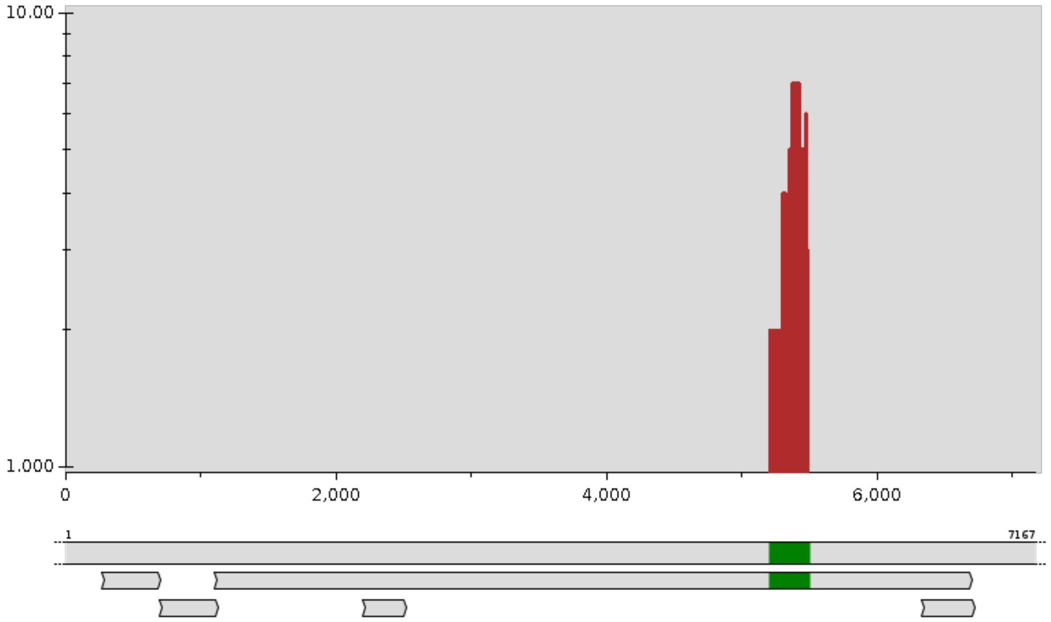

Assignment

**Type**	Cacao swollen shoot Ghana J virus (Taxonomy ID: 2056880)
**Reference Genome**	NC_040807.1
**NT Identity (%)**	58.0328
**AA Identity (%)**	49.505
**Number Of Stop Codons**	0
**Number Of CDS**	5

Alignment

**Alignment Score**	98.0 (NT) + 346.0 (AA) = 444.0
**Concordance (%)**	32.9866

**Alignment Method**	Global, seeded, nucleotide + amino acids (AGA)

Genome Region

Sequence starts at position 5200 and ends at position 5504 relative to NC_040807.1 reference sequence.

Alignment Detailed Statistics

	Begin	End	Coverage	Score	Concordance	Matches	Identities	I/D/M/F*	Stop Codons
**NT**	**5200**	**5504**	**4.3%**	**98**	**16.1%**	**305 (100%)**	**177 (58.0%)**	**0/0**	
Mutations:	5201G>A, 5204G>A, 5205G>T, 5210T>A, 5220A>C, 5221G>A, 5222A>T, 5223C>T, 5225C>A, 5229G>A, 5231C>A, 5232A>T, 5236C>T, 5237T>G, 5238G>A, 5241A>T, 5242A>G, 5243A>G, 5244G>A, 5245A>T, 5246C>T, 5247C>A, 5248A>G, 5253T>A, 5256C>A, 5258A>T, 5261A>T, 5262G>A, 5263G>A, 5264A>T, 5268C>A, 5271A>G, 5272C>A, 5274A>T, 5276C>G, 5277C>T, 5279G>A, 5280A>T, 5281A>C, 5282G>T, 5284A>G, 5285G>A, 5286G>C, 5288C>G, 5290G>A, 5292A>G, 5294C>T, 5295A>G, 5296T>C, 5297G>T, 5300G>T, 5303T>A, 5306C>T, 5307A>T, 5308G>C, 5309C>A, 5312G>A, 5319C>T, 5327T>A, 5330T>A, 5332T>A, 5333C>T, 5334C>T, 5335A>G, 5336C>G, 5339G>A, 5340G>A, 5342T>A, 5343G>C, 5344C>A, 5345T>G, 5348G>T, 5349G>T, 5350C>T, 5352G>A, 5357A>G, 5358T>C, 5359C>A, 5362T>C, 5364C>T, 5365C>A, 5366A>T, 5367T>A, 5369G>A, 5378C>T, 5379T>A, 5380G>A, 5381G>T, 5384A>C, 5388C>T, 5389A>T, 5390G>T, 5394T>C, 5395T>A, 5398T>A, 5402G>A, 5406C>A, 5407T>A, 5408C>T, 5424T>C, 5426G>A, 5439G>T, 5441A>T, 5443T>A, 5444C>A, 5447C>T, 5450A>G, 5451C>A, 5452G>A, 5453G>A, 5455A>T, 5456G>T, 5460G>A, 5463C>G, 5465A>T, 5466T>A, 5467G>A, 5474A>T, 5475G>C, 5476G>C, 5478A>T, 5479C>A, 5481G>A, 5482A>T, 5483A>G, 5489T>C, 5493G>A, 5494C>T								

CDS

	Begin	End	Coverage	Score	Concordance	Matches	Identities	I/D/M/F*	Stop Codons
EXL33_gp3	1369	1469	5.4%	346	46.5%	101 (100%)	50 (49.5%)	0/0/0/0	0
Protein mutations:	V1370F (5205G>T), F1371L (5210T>A), R1375H (5220A>C 5221G>A 5222A>T), D1378K (5229G>A 5231C>A), N1379Y (5232A>T), T1380M (5236C>T 5237T>G), E1381K (5238G>A), K1382W (5241A>T 5242A>G 5243A>G), D1383I (5244G>A 5245A>T 5246C>T), Q1384R (5247C>A 5248A>G), S1386T (5253T>A), L1387I (5256C>A 5258A>T), G1389N (5262G>A 5263G>A 5264A>T), Q1391K (5268C>A), T1392D (5271A>G 5272C>A), I1393L (5274A>T 5276C>G), K1395S (5280A>T 5281A>C 5282G>T), K1396R (5284A>G 5285G>A), V1397L (5286G>C 5288C>G), C1398Y (5290G>A), N1399D (5292A>G 5294C>T), M1400A (5295A>G 5296T>C 5297G>T), K1401N (5300G>T), F1412Y (5332T>A 5333C>T), H1413W (5334C>T 5335A>G 5336C>G), V1415I (5340G>A 5342T>A), A1416Q (5343G>C 5344C>A 5345T>G), M1417I (5348G>T), A1418F (5349G>T 5350C>T), E1419K (5352G>A), S1421H (5358T>C 5359C>A), I1422T (5362T>C), P1423Y (5364C>T 5365C>A 5366A>T), W1424R (5367T>A 5369G>A), W1428N (5379T>A 5380G>A 5381G>T), Q1431F (5388C>T 5389A>T 5390G>T), L1433Q (5394T>C 5395T>A), F1434Y (5398T>A), L1437N (5406C>A 5407T>A 5408C>T), A1448S (5439G>T 5441A>T), V1449E (5443T>A 5444C>A), R1452K (5451C>A 5452G>A 5453G>A), K1453I (5455A>T 5456G>T), D1455N (5460G>A), Q1456D (5463C>G 5465A>T), C1457N (5466T>A 5467G>A), K1459N (5474A>T), G1460P (5475G>C 5476G>C), T1461Y (5478A>T 5479C>A), E1462M (5481G>A 5482A>T 5483A>G), A1466I (5493G>A 5494C>T)								
Codon mutations:	AGG1368.GA (5201G>A), CTG1369CTA (5204G>A), GTT1370TTT (5205G>T), TTT1371TTA (5210T>A), AGA1375CAT (5220A>C 5221G>A 5222A>T), CTC1376TTA (5223C>T 5225C>A), GAC1378AAA (5229G>A 5231C>A), AAT1379TAT (5232A>T), ACT1380ATG (5236C>T 5237T>G), GAA1381AAA (5238G>A), AAA1382TGG (5241A>T 5242A>G 5243A>G), GAC1383ATT (5244G>A 5245A>T 5246C>T), CAG1384AGG (5247C>A 5248A>G), TCT1386ACT (5253T>A), CTA1387ATT (5256C>A 5258A>T), CCA1388CCT (5261A>T), GGA1389AAT (5262G>A 5263G>A 5264A>T), CAG1391AAG (5268C>A), ACT1392GAT (5271A>G 5272C>A), ATC1393TTG (5274A>T 5276C>G), CTG1394TTA (5277C>T 5279G>A), AAG1395TCT (5280A>T 5281A>C 5282G>T), AAG1396AGA (5284A>G 5285G>A), GTC1397CTG (5286G>C 5288C>G), TGT1398TAT (5290G>A), AAC1399GAT (5292A>G 5294C>T), ATG1400GCT (5295A>G 5296T>C 5297G>T), AAG1401AAT (5300G>T), ATT1402ATA (5303T>A), TTC1403TTT (5306C>T), AGC1404TCA (5307A>T 5308G>C 5309C>A), AAG1405AAA (5312G>A), CTA1408TTA (5319C>T), TCT1410TCA (5327T>A), GGT1411GGA (5330T>A), TTC1412TAT (5332T>A 5333C>T), CAC1413TGG (5334C>T 5335A>G 5336C>G), CAG1414CAA (5339G>A), GTT1415ATA (5340G>A 5342T>A), GCT1416CAG (5343G>C 5344C>A 5345T>G), ATG1417ATT (5348G>T), GCT1418TTT (5349G>T 5350C>T), GAA1419AAA (5352G>A), GAA1420GAG (5357A>G), TCT1421CAT (5358T>C 5359C>A), ATT1422ACT (5362T>C), CCA1423TAT (5364C>T 5365C>A 5366A>T), TGG1424AGA (5367T>A 5369G>A), TTC1427TTT (5378C>T), TGG1428AAT (5379T>A 5380G>A 5381G>T), GTA1429GTC (5384A>C), CAG1431TTT (5388C>T 5389A>T 5390G>T), TTA1433CAA (5394T>C 5395T>A), TTT1434TAT (5398T>A), GAG1435GAA (5402G>A), CTC1437AAT (5406C>A 5407T>A 5408C>T), TTG1443CTA (5424T>C 5426G>A), GCA1448TCT (5439G>T 5441A>T), GTC1449GAA (5443T>A 5444C>A), TTC1450TTT (5447C>T), CAA1451CAG (5450A>G), CGG1452AAA (5451C>A 5452G>A 5453G>A), AAG1453ATT (5455A>T 5456G>T), GAT1455AAT (5460G>A), CAA1456GAT (5463C>G 5465A>T), TGT1457AAT (5466T>A 5467G>A), AAA1459AAT (5474A>T), GGA1460CCA (5475G>C 5476G>C), ACT1461TAT (5478A>T 5479C>A), GAA1462ATG (5481G>A 5482A>T 5483A>G), TTT1464TTC (5489T>C), GCT1466ATT (5493G>A 5494C>T)								

Proteins

	Begin	End	Coverage	Score	Concordance	Matches	Identities	I/D/M/F*	Stop Codons
ORF3 polyprotein (YP_009553538.1)	1369	1469	5.4%	346	46.5%	101 (100%)	50 (49.5%)	0/0/0/0	0
Protein mutations:	V1370F (5205G>T), F1371L (5210T>A), R1375H (5220A>C 5221G>A 5222A>T), D1378K (5229G>A 5231C>A), N1379Y (5232A>T), T1380M (5236C>T 5237T>G), E1381K (5238G>A), K1382W (5241A>T 5242A>G 5243A>G), D1383I (5244G>A 5245A>T 5246C>T), Q1384R (5247C>A 5248A>G), S1386T (5253T>A), L1387I (5256C>A 5258A>T), G1389N (5262G>A 5263G>A 5264A>T), Q1391K (5268C>A), T1392D (5271A>G 5272C>A), I1393L (5274A>T 5276C>G), K1395S (5280A>T 5281A>C 5282G>T), K1396R (5284A>G 5285G>A), V1397L (5286G>C 5288C>G), C1398Y (5290G>A), N1399D (5292A>G 5294C>T), M1400A (5295A>G 5296T>C 5297G>T), K1401N (5300G>T), F1412Y (5332T>A 5333C>T), H1413W (5334C>T 5335A>G 5336C>G), V1415I (5340G>A 5342T>A), A1416Q (5343G>C 5344C>A 5345T>G), M1417I (5348G>T), A1418F (5349G>T 5350C>T), E1419K (5352G>A), S1421H (5358T>C 5359C>A), I1422T (5362T>C), P1423Y (5364C>T 5365C>A 5366A>T), W1424R (5367T>A 5369G>A), W1428N (5379T>A 5380G>A 5381G>T), Q1431F (5388C>T 5389A>T 5390G>T), L1433Q (5394T>C 5395T>A), F1434Y (5398T>A), L1437N (5406C>A 5407T>A 5408C>T), A1448S (5439G>T 5441A>T), V1449E (5443T>A 5444C>A), R1452K (5451C>A 5452G>A 5453G>A), K1453I (5455A>T 5456G>T), D1455N (5460G>A), Q1456D (5463C>G 5465A>T), C1457N (5466T>A 5467G>A), K1459N (5474A>T), G1460P (5475G>C 5476G>C), T1461Y (5478A>T 5479C>A), E1462M (5481G>A 5482A>T 5483A>G), A1466I (5493G>A 5494C>T)								
Codon mutations:	AGG1368.GA (5201G>A), CTG1369CTA (5204G>A), GTT1370TTT (5205G>T), TTT1371TTA (5210T>A), AGA1375CAT (5220A>C 5221G>A 5222A>T), CTC1376TTA (5223C>T 5225C>A), GAC1378AAA (5229G>A 5231C>A), AAT1379TAT (5232A>T), ACT1380ATG (5236C>T 5237T>G), GAA1381AAA (5238G>A), AAA1382TGG (5241A>T 5242A>G 5243A>G), GAC1383ATT (5244G>A 5245A>T 5246C>T), CAG1384AGG (5247C>A 5248A>G), TCT1386ACT (5253T>A), CTA1387ATT (5256C>A 5258A>T), CCA1388CCT (5261A>T), GGA1389AAT (5262G>A 5263G>A 5264A>T), CAG1391AAG (5268C>A), ACT1392GAT (5271A>G 5272C>A), ATC1393TTG (5274A>T 5276C>G), CTG1394TTA (5277C>T 5279G>A), AAG1395TCT (5280A>T 5281A>C 5282G>T), AAG1396AGA (5284A>G 5285G>A), GTC1397CTG (5286G>C 5288C>G), TGT1398TAT (5290G>A), AAC1399GAT (5292A>G 5294C>T), ATG1400GCT (5295A>G 5296T>C 5297G>T), AAG1401AAT (5300G>T), ATT1402ATA (5303T>A), TTC1403TTT (5306C>T), AGC1404TCA (5307A>T 5308G>C 5309C>A), AAG1405AAA (5312G>A), CTA1408TTA (5319C>T), TCT1410TCA (5327T>A), GGT1411GGA (5330T>A), TTC1412TAT (5332T>A 5333C>T), CAC1413TGG (5334C>T 5335A>G 5336C>G), CAG1414CAA (5339G>A), GTT1415ATA (5340G>A 5342T>A), GCT1416CAG (5343G>C 5344C>A 5345T>G), ATG1417ATT (5348G>T), GCT1418TTT (5349G>T 5350C>T), GAA1419AAA (5352G>A), GAA1420GAG (5357A>G), TCT1421CAT (5358T>C 5359C>A), ATT1422ACT (5362T>C), CCA1423TAT (5364C>T 5365C>A 5366A>T), TGG1424AGA (5367T>A 5369G>A), TTC1427TTT (5378C>T), TGG1428AAT (5379T>A 5380G>A 5381G>T), GTA1429GTC (5384A>C), CAG1431TTT (5388C>T 5389A>T 5390G>T), TTA1433CAA (5394T>C 5395T>A), TTT1434TAT (5398T>A), GAG1435GAA (5402G>A), CTC1437AAT (5406C>A 5407T>A 5408C>T), TTG1443CTA (5424T>C 5426G>A), GCA1448TCT (5439G>T 5441A>T), GTC1449GAA (5443T>A 5444C>A), TTC1450TTT (5447C>T), CAA1451CAG (5450A>G), CGG1452AAA (5451C>A 5452G>A 5453G>A), AAG1453ATT (5455A>T 5456G>T), GAT1455AAT (5460G>A), CAA1456GAT (5463C>G 5465A>T), TGT1457AAT (5466T>A 5467G>A), AAA1459AAT (5474A>T), GGA1460CCA (5475G>C 5476G>C), ACT1461TAT (5478A>T 5479C>A), GAA1462ATG (5481G>A 5482A>T 5483A>G), TTT1464TTC (5489T>C), GCT1466ATT (5493G>A 5494C>T)								

*: Inserts / Deletes / Misaligned / Frameshifts

Analysis details

This analysis was performed with panviral2.64

NGS Details (UN62): Hibiscus bacilliform virus GD1

Assembly

**Coverage Length**	238 (1 contig(s))
**Depth Of Coverage**	5.5
**Number Of Reads**	10
**Reads Per Million**	0.23 rpm (after QC)
**Ambiguities**	0
**Assembly Method**	de novo + reference guided assembly
**Consensus Caller**	Bcf Tools

Coverage Map

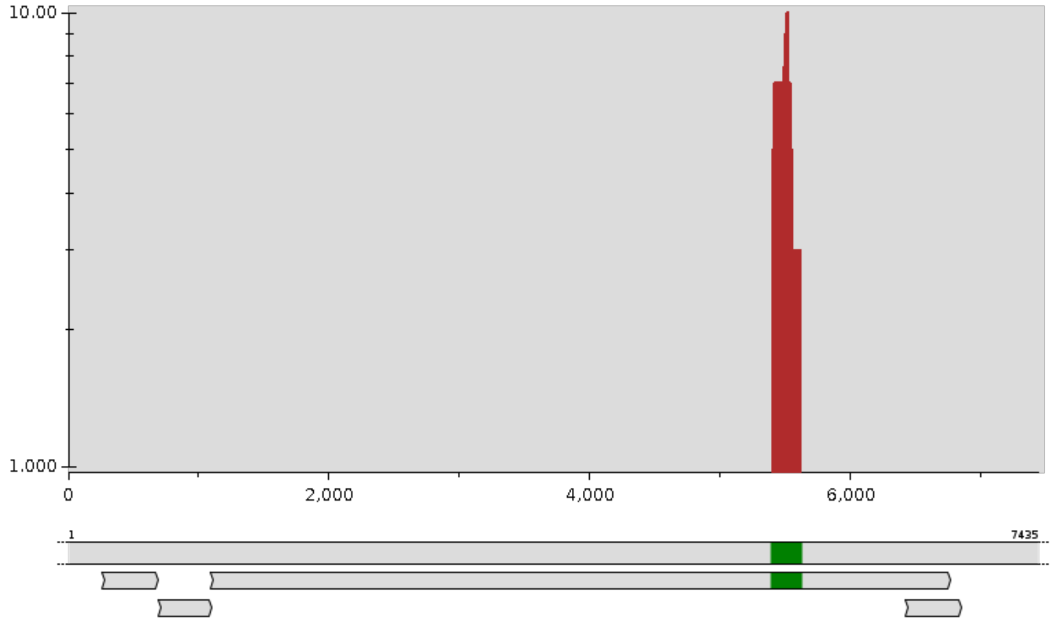

Assignment

**Type**	Hibiscus bacilliform virus GD1 (Taxonomy ID: 1459800)
**Reference Genome**	NC_023485.1
**NT Identity (%)**	56.4315
**AA Identity (%)**	46.25
**Number Of Stop Codons**	1
**Number Of CDS**	4

Alignment

**Alignment Score**	56.0 (NT) + 268.0 (AA) = 324.0
**Concordance (%)**	30.5949

**Alignment Method**	Global, seeded, nucleotide + amino acids (AGA)

Genome Region

Sequence starts at position 5386 and ends at position 5623 relative to NC_023485.1 reference sequence.

Alignment Detailed Statistics

	Begin	End	Coverage	Score	Concordance	Matches	Identities	I/D/M/F*	Stop Codons
**NT**	**5386**	**5623**	**3.2%**	**56**	**11.8%**	**238 (98.8%)**	**136 (56.4%)**	**3/0**	
Mutations:	5390T>G, 5393A>G, 5395T>A, 5403G>T, 5406G>A, 5407C>G, 5408T>A, 5409A>G, 5411G>T, 5414C>G, 5415C>G, 5416C>A, 5418G>T, 5419A>C, 5420G>A, 5421T>G, 5422C>A, 5427A>C, 5428A>T, 5430T>A, 5431G>A, 5438T>A, 5441C>T, 5442T>C, 5445G>A, 5446T>C, 5447T>A, 5449C>G, 5450A>C, 5451G>T, 5456A>T, 5458T>A, 5459G>C, 5465A>G, 5467G>T, 5468G>C, 5469C>G, 5471A>G, 5478C>T, 5479C>A, 5480A>T, 5486A>G, 5491A>C, 5492A>T, 5495C>T, 5497C>T, 5501A>T, 5504A>T, 5506T>C, 5507A>T, 5511C>A, 5512A>T, 5514C>G, 5515G>A, 5517A>C, 5518A>T, 5523G>A, 5525C>T, 5526A>T, 5527A>G, 5528C>A, 5531C>G, 5532T>A, 5534C>T, 5537A>G, 5538G>T, 5539G>C, 5540_5541insTAC, 5541A>T, 5542C>T, 5543T>G, 5546A>T, 5547G>C, 5548C>A, 5549C>A, 5555C>G, 5556G>A, 5557C>T, 5558T>C, 5561A>G, 5563A>T, 5564C>T, 5567T>A, 5573C>T, 5576A>T, 5577C>T, 5579C>G, 5582A>T, 5585T>C, 5589C>A, 5592T>A, 5593C>G, 5598G>A, 5599A>G, 5600G>T, 5603A>T, 5607A>G, 5609C>G, 5612G>T, 5616A>T, 5618A>G, 5619C>A, 5621G>A								

CDS

	Begin	End	Coverage	Score	Concordance	Matches	Identities	I/D/M/F*	Stop Codons
BU35_gp3	1433	1511	4.2%	268	45.3%	79 (98.8%)	37 (46.3%)	1/0/0/0	1
Protein mutations:	S1433R (5390T>G), F1435Y (5395T>A), V1438L (5403G>T), A1439R (5406G>A 5407C>G 5408T>A), M1440V (5409A>G 5411G>T), H1441Q (5414C>G), P1442E (5415C>G 5416C>A), E1443S (5418G>T 5419A>C 5420G>A), S1444D (5421T>G 5422C>A), K1446L (5427A>C 5428A>T), W1447K (5430T>A 5431G>A), W1451R (5442T>C), V1452T (5445G>A 5446T>C 5447T>A), P1453R (5449C>G 5450A>C), D1454Y (5451G>T), L1456H (5458T>A 5459G>C), W1459F (5467G>T 5468G>C), L1460V (5469C>G 5471A>G), P1463Y (5478C>T 5479C>A 5480A>T), K1467T (5491A>C 5492A>T), A1469V (5497C>T), V1472A (5506T>C 5507A>T), Q1474M (5511C>A 5512A>T), R1475E (5514C>G 5515G>A), K1476L (5517A>C 5518A>T), D1478N (5523G>A 5525C>T), N1479* (5526A>T 5527A>G 5528C>A), F1481I (5532T>A 5534C>T), G1483S (5538G>T 5539G>C), G1483_T1484insY (5540_5541insTAC), T1484L (5541A>T 5542C>T 5543T>G), E1485D (5546A>T), A1486Q (5547G>C 5548C>A 5549C>A), I1488M (5555C>G), A1489I (5556G>A 5557C>T 5558T>C), Y1491F (5563A>T 5564C>T), Q1500K (5589C>A), E1503S (5598G>A 5599A>G 5600G>T), E1504D (5603A>T), I1506V (5607A>G 5609C>G), K1507N (5612G>T), I1509L (5616A>T 5618A>G)								
Codon mutations:	AGT1433AGG (5390T>G), GGA1434GGG (5393A>G), TTT1435TAT (5395T>A), GTG1438TTG (5403G>T), GCT1439AGA (5406G>A 5407C>G 5408T>A), ATG1440GTT (5409A>G 5411G>T), CAC1441CAG (5414C>G), CCG1442GAG (5415C>G 5416C>A), GAG1443TCA (5418G>T 5419A>C 5420G>A), TCT1444GAT (5421T>G 5422C>A), AAG1446CTG (5427A>C 5428A>T), TGG1447AAG (5430T>A 5431G>A), GCT1449GCA (5438T>A), TTC1450TTT (5441C>T), TGG1451CGG (5442T>C), GTT1452ACA (5445G>A 5446T>C 5447T>A), CCA1453CGC (5449C>G 5450A>C), GAT1454TAT (5451G>T), GGA1455GGT (5456A>T), CTG1456CAC (5458T>A 5459G>C), GAA1458GAG (5465A>G), TGG1459TTC (5467G>T 5468G>C), CTA1460GTG (5469C>G 5471A>G), CCA1463TAT (5478C>T 5479C>A 5480A>T), GGA1465GGG (5486A>G), AAA1467ACT (5491A>C 5492A>T), AAC1468AAT (5495C>T), GCC1469GTC (5497C>T), CCA1470CCT (5501A>T), GCA1471GCT (5504A>T), GTA1472GCT (5506T>C 5507A>T), CAG1474ATG (5511C>A 5512A>T), CGA1475GAA (5514C>G 5515G>A), AAG1476CTG (5517A>C 5518A>T), GAC1478AAT (5523G>A 5525C>T), AAC1479TGA (5526A>T 5527A>G 5528C>A), GTC1480GTG (5531C>G), TTC1481ATT (5532T>A 5534C>T), AAA1482AAG (5537A>G), GGT1483TCT (5538G>T 5539G>C), GGT1483_ACT1484insTAC (5540_5541insTAC), ACT1484TTG (5541A>T 5542C>T 5543T>G), GAA1485GAT (5546A>T), GCC1486CAA (5547G>C 5548C>A 5549C>A), ATC1488ATG (5555C>G), GCT1489ATC (5556G>A 5557C>T 5558T>C), GTA1490GTG (5561A>G), TAC1491TTT (5563A>T 5564C>T), ATT1492ATA (5567T>A), GAC1494GAT (5573C>T), ATA1495ATT (5576A>T), CTC1496TTG (5577C>T 5579C>G), GTA1497GTT (5582A>T), TTT1498TTC (5585T>C), CAG1500AAG (5589C>A), TCT1501AGT (5592T>A 5593C>G), GAG1503AGT (5598G>A 5599A>G 5600G>T), GAA1504GAT (5603A>T), ATC1506GTG (5607A>G 5609C>G), AAG1507AAT (5612G>T), ATA1509TTG (5616A>T 5618A>G), CGG1510AGA (5619C>A 5621G>A)								

Proteins

	Begin	End	Coverage	Score	Concordance	Matches	Identities	I/D/M/F*	Stop Codons
polyprotein (YP_009002585.1)	1433	1511	4.2%	268	45.3%	79 (98.8%)	37 (46.3%)	1/0/0/0	1
Protein mutations:	S1433R (5390T>G), F1435Y (5395T>A), V1438L (5403G>T), A1439R (5406G>A 5407C>G 5408T>A), M1440V (5409A>G 5411G>T), H1441Q (5414C>G), P1442E (5415C>G 5416C>A), E1443S (5418G>T 5419A>C 5420G>A), S1444D (5421T>G 5422C>A), K1446L (5427A>C 5428A>T), W1447K (5430T>A 5431G>A), W1451R (5442T>C), V1452T (5445G>A 5446T>C 5447T>A), P1453R (5449C>G 5450A>C), D1454Y (5451G>T), L1456H (5458T>A 5459G>C), W1459F (5467G>T 5468G>C), L1460V (5469C>G 5471A>G), P1463Y (5478C>T 5479C>A 5480A>T), K1467T (5491A>C 5492A>T), A1469V (5497C>T), V1472A (5506T>C 5507A>T), Q1474M (5511C>A 5512A>T), R1475E (5514C>G 5515G>A), K1476L (5517A>C 5518A>T), D1478N (5523G>A 5525C>T), N1479* (5526A>T 5527A>G 5528C>A), F1481I (5532T>A 5534C>T), G1483S (5538G>T 5539G>C), G1483_T1484insY (5540_5541insTAC), T1484L (5541A>T 5542C>T 5543T>G), E1485D (5546A>T), A1486Q (5547G>C 5548C>A 5549C>A), I1488M (5555C>G), A1489I (5556G>A 5557C>T 5558T>C), Y1491F (5563A>T 5564C>T), Q1500K (5589C>A), E1503S (5598G>A 5599A>G 5600G>T), E1504D (5603A>T), I1506V (5607A>G 5609C>G), K1507N (5612G>T), I1509L (5616A>T 5618A>G)								
Codon mutations:	AGT1433AGG (5390T>G), GGA1434GGG (5393A>G), TTT1435TAT (5395T>A), GTG1438TTG (5403G>T), GCT1439AGA (5406G>A 5407C>G 5408T>A), ATG1440GTT (5409A>G 5411G>T), CAC1441CAG (5414C>G), CCG1442GAG (5415C>G 5416C>A), GAG1443TCA (5418G>T 5419A>C 5420G>A), TCT1444GAT (5421T>G 5422C>A), AAG1446CTG (5427A>C 5428A>T), TGG1447AAG (5430T>A 5431G>A), GCT1449GCA (5438T>A), TTC1450TTT (5441C>T), TGG1451CGG (5442T>C), GTT1452ACA (5445G>A 5446T>C 5447T>A), CCA1453CGC (5449C>G 5450A>C), GAT1454TAT (5451G>T), GGA1455GGT (5456A>T), CTG1456CAC (5458T>A 5459G>C), GAA1458GAG (5465A>G), TGG1459TTC (5467G>T 5468G>C), CTA1460GTG (5469C>G 5471A>G), CCA1463TAT (5478C>T 5479C>A 5480A>T), GGA1465GGG (5486A>G), AAA1467ACT (5491A>C 5492A>T), AAC1468AAT (5495C>T), GCC1469GTC (5497C>T), CCA1470CCT (5501A>T), GCA1471GCT (5504A>T), GTA1472GCT (5506T>C 5507A>T), CAG1474ATG (5511C>A 5512A>T), CGA1475GAA (5514C>G 5515G>A), AAG1476CTG (5517A>C 5518A>T), GAC1478AAT (5523G>A 5525C>T), AAC1479TGA (5526A>T 5527A>G 5528C>A), GTC1480GTG (5531C>G), TTC1481ATT (5532T>A 5534C>T), AAA1482AAG (5537A>G), GGT1483TCT (5538G>T 5539G>C), GGT1483_ACT1484insTAC (5540_5541insTAC), ACT1484TTG (5541A>T 5542C>T 5543T>G), GAA1485GAT (5546A>T), GCC1486CAA (5547G>C 5548C>A 5549C>A), ATC1488ATG (5555C>G), GCT1489ATC (5556G>A 5557C>T 5558T>C), GTA1490GTG (5561A>G), TAC1491TTT (5563A>T 5564C>T), ATT1492ATA (5567T>A), GAC1494GAT (5573C>T), ATA1495ATT (5576A>T), CTC1496TTG (5577C>T 5579C>G), GTA1497GTT (5582A>T), TTT1498TTC (5585T>C), CAG1500AAG (5589C>A), TCT1501AGT (5592T>A 5593C>G), GAG1503AGT (5598G>A 5599A>G 5600G>T), GAA1504GAT (5603A>T), ATC1506GTG (5607A>G 5609C>G), AAG1507AAT (5612G>T), ATA1509TTG (5616A>T 5618A>G), CGG1510AGA (5619C>A 5621G>A)								

*: Inserts / Deletes / Misaligned / Frameshifts

Analysis details

This analysis was performed with panviral2.64

NGS Details (UN62): Badnavirus maculasmallanthi

Assembly

**Coverage Length**	422 (1 contig(s))
**Depth Of Coverage**	2.8
**Number Of Reads**	9
**Reads Per Million**	0.21 rpm (after QC)
**Ambiguities**	0
**Assembly Method**	de novo + reference guided assembly
**Consensus Caller**	Bcf Tools

Coverage Map

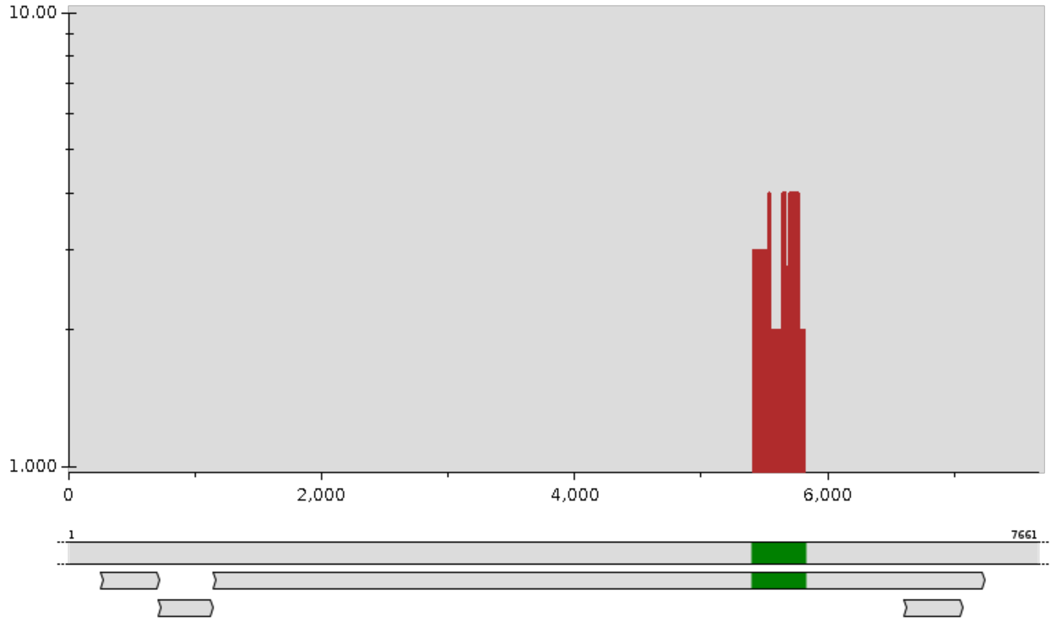

Assignment

**Type**	Badnavirus maculasmallanthi (Taxonomy ID: 3048453)
**Reference Genome**	NC_026472.1
**NT Identity (%)**	51.79
**AA Identity (%)**	45.0
**Number Of Stop Codons**	0
**Number Of CDS**	4

Alignment

**Alignment Score**	9.0 (NT) + 415.0 (AA) = 424.0
**Concordance (%)**	24.0091

Alignment Method	Global, seeded, nucleotide + amino acids (AGA)

Genome Region

Sequence starts at position 5407 and ends at position 5828 relative to NC_026472.1 reference sequence.

Alignment Detailed Statistics

	Begin	End	Coverage	Score	Concordance	Matches	Identities	I/D/M/F*	Stop Codons
**NT**	**5407**	**5828**	**5.5%**	**9**	**1.1%**	**416 (97.9%)**	**217 (51.1%)**	**3/6**	
Mutations:	5407G>T, 5408T>C, 5409C>A, 5410A>C, 5411A>C, 5412C>T, 5414C>G, 5415T>G, 5419A>G, 5422A>C, 5427T>C, 5428_5433delGACCCA, 5434G>C, 5435C>T, 5437A>T, 5438C>T, 5439A>T, 5441G>T, 5442C>G, 5446G>A, 5448A>G, 5450C>A, 5451A>G, 5452A>G, 5454G>T, 5457A>G, 5458A>T, 5459A>C, 5460T>A, 5461G>A, 5462A>T, 5463T>G, 5470G>T, 5471T>G, 5472G>T, 5473T>A, 5475C>T, 5476A>G, 5481C>T, 5485A>C, 5486C>A, 5487A>G, 5488C>T, 5490C>G, 5493T>C, 5494G>A, 5496C>G, 5497A>G, 5498A>T, 5499T>G, 5502A>T, 5503T>G, 5504A>T, 5505C>A, 5508A>G, 5509G>A, 5511T>C, 5512C>A, 5514G>A, 5518T>C, 5523T>C, 5527G>C, 5529G>T, 5532A>C, 5533A>G, 5535C>T, 5537C>A, 5538C>T, 5541G>A, 5542C>T, 5544A>T, 5545A>G, 5547A>T, 5548A>C, 5549G>A, 5550A>G, 5551A>T, 5554G>C, 5555G>A, 5557A>G, 5558A>G, 5559T>A, 5563A>G, 5564A>C, 5565G>C, 5574A>T, 5578T>A, 5588A>G, 5589G>A, 5591C>T, 5592T>C, 5597T>A, 5604A>G, 5605G>T, 5607T>G, 5608G>A, 5609C>A, 5610C>G, 5613G>T, 5614G>C, 5616A>T, 5617G>C, 5618A>C, 5619A>T, 5620G>T, 5621A>C, 5622A>T, 5623T>G, 5624C>A, 5625C>T, 5628A>T, 5631C>T, 5632T>A, 5633G>A, 5637G>A, 5640C>T, 5643T>C, 5644T>C, 5645T>G, 5646A>G, 5648T>C, 5650C>A, 5651C>G, 5652A>G, 5653G>T, 5654G>A, 5655A>T, 5658A>C, 5659T>C, 5660T>A, 5661A>T, 5664C>T, 5667A>G, 5669G>T, 5670G>T, 5676C>T, 5680C>T, 5682A>G, 5685C>T, 5688A>G, 5689C>T, 5691C>G, 5693A>C, 5694A>T, 5700A>T, 5703C>G, 5704G>A, 5706T>A, 5707A>G, 5708T>C, 5709T>A, 5713C>A, 5714A>T, 5716A>G, 5717G>A, 5719A>T, 5720A>T, 5725G>A, 5728A>G, 5729A>G, 5730A>G, 5731T>G, 5732G>T, 5733C>G, 5736C>T, 5737A>C, 5739A>T, 5739_5740insCCG, 5740G>T, 5743A>T, 5744C>T, 5745A>G, 5748A>T, 5749G>T, 5750A>C, 5751G>A, 5755A>G, 5757C>G, 5758G>A, 5759C>T, 5760C>T, 5765A>T, 5766C>T, 5769C>T, 5772C>T, 5775C>T, 5778C>T, 5779C>T, 5784A>T, 5793C>A, 5795G>C, 5799A>G, 5803G>A, 5810A>T, 5813G>A, 5814G>A, 5817T>C, 5820A>G, 5822A>G, 5823G>A, 5827A>G								

CDS

	Begin	End	Coverage	Score	Concordance	Matches	Identities	I/D/M/F*	Stop Codons
UF61_gp3	1422	1562	6.9%	415	43.5%	139 (97.9%)	63 (44.4%)	1/2/0/0	0
Protein mutations:	V1422S (5407G>T 5408T>C 5409C>A), N1423P (5410A>C 5411A>C 5412C>T), S1424W (5414C>G 5415T>G), T1426A (5419A>G), T1427P (5422A>C), D1429_P1430del (5428_5433delGACCCA), A1431L (5434G>C 5435C>T), T1432F (5437A>T 5438C>T 5439A>T), G1433V (5441G>T 5442C>G), E1435K (5446G>A 5448A>G), T1436K (5450C>A 5451A>G), K1437D (5452A>G 5454G>T), N1439S (5458A>T 5459A>C 5460T>A), D1440M (5461G>A 5462A>T 5463T>G), V1443C (5470G>T 5471T>G 5472G>T), F1444I (5473T>A 5475C>T), N1445D (5476A>G), T1448Q (5485A>C 5486C>A 5487A>G), D1451K (5494G>A 5496C>G), N1452V (5497A>G 5498A>T 5499T>G), Y1454V (5503T>G 5504A>T 5505C>A), D1456N (5509G>A 5511T>C), Q1457K (5512C>A 5514G>A), S1459P (5518T>C), G1462R (5527G>C 5529G>T), N1464D (5533A>G 5535C>T), T1465N (5537C>A 5538C>T), L1467F (5542C>T 5544A>T), K1468D (5545A>G 5547A>T), R1469Q (5548A>C 5549G>A 5550A>G), I1470L (5551A>T), G1471Q (5554G>C 5555G>A), N1472G (5557A>G 5558A>G 5559T>A), K1474A (5563A>G 5564A>C 5565G>C), F1479I (5578T>A), K1482R (5588A>G 5589G>A), S1483F (5591C>T 5592T>C), F1485Y (5597T>A), V1488L (5605G>T 5607T>G), A1489K (5608G>A 5609C>A 5610C>G), M1490I (5613G>T), E1491H (5614G>C 5616A>T), E1492P (5617G>C 5618A>C 5619A>T), E1493S (5620G>T 5621A>C 5622A>T), S1494D (5623T>G 5624C>A 5625C>T), W1497K (5632T>A 5633G>A), L1501R (5644T>C 5645T>G 5646A>G), I1502T (5648T>C), P1503R (5650C>A 5651C>G 5652A>G), G1504Y (5653G>T 5654G>A 5655A>T), L1506H (5659T>C 5660T>A 5661A>T), W1509F (5669G>T 5670G>T), P1513S (5680C>T 5682A>G), K1517T (5693A>C 5694A>T), A1521T (5704G>A 5706T>A), I1522A (5707A>G 5708T>C 5709T>A), Q1524M (5713C>A 5714A>T), R1525E (5716A>G 5717G>A), K1526L (5719A>T 5720A>T), D1528N (5725G>A), K1529G (5728A>G 5729A>G 5730A>G), C1530V (5731T>G 5732G>T 5733C>G), K1532H (5737A>C 5739A>T), K1532_D1533insP (5739_5740insCCG), D1533Y (5740G>T), T1534L (5743A>T 5744C>T 5745A>G), E1535D (5748A>T), E1536S (5749G>T 5750A>C 5751G>A), I1538V (5755A>G 5757C>G), A1539I (5758G>A 5759C>T 5760C>T), Y1541F (5765A>T 5766C>T), N1550K (5793C>A), S1551T (5795G>C), D1554N (5803G>A), E1556V (5810A>T), R1557K (5813G>A 5814G>A), K1560R (5822A>G 5823G>A)								
Codon mutations:	GTC1422TCA (5407G>T 5408T>C 5409C>A), AAC1423CCT (5410A>C 5411A>C 5412C>T), TCT1424TGG (5414C>G 5415T>G), ACC1426GCC (5419A>G), ACG1427CCG (5422A>C), GTT1428GTC (5427T>C), GAC1429_CCA1430del (5428_5433delGACCCA), GCA1431CTA (5434G>C 5435C>T), ACA1432TTT (5437A>T 5438C>T 5439A>T), GGC1433GTG (5441G>T 5442C>G), GAA1435AAG (5446G>A 5448A>G), ACA1436AAG (5450C>A 5451A>G), AAG1437GAT (5452A>G 5454G>T), GGA1438GGG (5457A>G), AAT1439TCA (5458A>T 5459A>C 5460T>A), GAT1440ATG (5461G>A 5462A>T 5463T>G), GTG1443TGT (5470G>T 5471T>G 5472G>T), TTC1444ATT (5473T>A 5475C>T), AAT1445GAT (5476A>G), TAC1446TAT (5481C>T), ACA1448CAG (5485A>C 5486C>A 5487A>G), CTC1449TTG (5488C>T 5490C>G), AAT1450AAC (5493T>C), GAC1451AAG (5494G>A 5496C>G), AAT1452GTG (5497A>G 5498A>T 5499T>G), ACA1453ACT (5502A>T), TAC1454GTA (5503T>G 5504A>T 5505C>A), AAA1455AAG (5508A>G), GAT1456AAC (5509G>A 5511T>C), CAG1457AAA (5512C>A 5514G>A), TCC1459CCC (5518T>C), CTT1460CTC (5523T>C), GGG1462CGT (5527G>C 5529G>T), ATA1463ATC (5532A>C), AAC1464GAT (5533A>G 5535C>T), ACC1465AAT (5537C>A 5538C>T), TTG1466TTA (5541G>A), CTA1467TTT (5542C>T 5544A>T), AAA1468GAT (5545A>G 5547A>T), AGA1469CAG (5548A>C 5549G>A 5550A>G), ATA1470TTA (5551A>T), GGG1471CAG (5554G>C 5555G>A), AAT1472GGA (5557A>G 5558A>G 5559T>A), AAG1474GCC (5563A>G 5564A>C 5565G>C), TCA1477TCT (5574A>T), TTT1479ATT (5578T>A), AAG1482AGA (5588A>G 5589G>A), TCT1483TTC (5591C>T 5592T>C), TTT1485TAT (5597T>A), CAA1487CAG (5604A>G), GTT1488TTG (5605G>T 5607T>G), GCC1489AAG (5608G>A 5609C>A 5610C>G), ATG1490ATT (5613G>T), GAA1491CAT (5614G>C 5616A>T), GAA1492CCT (5617G>C 5618A>C 5619A>T), GAA1493TCT (5620G>T 5621A>C 5622A>T), TCC1494GAT (5623T>G 5624C>A 5625C>T), ATA1495ATT (5628A>T), CCC1496CCT (5631C>T), TGG1497AAG (5632T>A 5633G>A), ACG1498ACA (5637G>A), GCC1499GCT (5640C>T), TTT1500TTC (5643T>C), TTA1501CGG (5644T>C 5645T>G 5646A>G), ATC1502ACC (5648T>C), CCA1503AGG (5650C>A 5651C>G 5652A>G), GGA1504TAT (5653G>T 5654G>A 5655A>T), GGA1505GGC (5658A>C), TTA1506CAT (5659T>C 5660T>A 5661A>T), TAC1507TAT (5664C>T), GAA1508GAG (5667A>G), TGG1509TTT (5669G>T 5670G>T), GTC1511GTT (5676C>T), CCA1513TCG (5680C>T 5682A>G), TTC1514TTT (5685C>T), GGA1515GGG (5688A>G), CTC1516TTG (5689C>T 5691C>G), AAA1517ACT (5693A>C 5694A>T), GCA1519GCT (5700A>T), CCC1520CCG (5703C>G), GCT1521ACA (5704G>A 5706T>A), ATT1522GCA (5707A>G 5708T>C 5709T>A), CAG1524ATG (5713C>A 5714A>T), AGA1525GAA (5716A>G 5717G>A), AAA1526TTA (5719A>T 5720A>T), GAT1528AAT (5725G>A), AAA1529GGG (5728A>G 5729A>G 5730A>G), TGC1530GTG (5731T>G 5732G>T 5733C>G), TTC1531TTT (5736C>T), AAA1532CAT (5737A>C 5739A>T), AAA1532_GAC1533insCCG (5739_5740insCCG), GAC1533TAC (5740G>T), ACA1534TTG (5743A>T 5744C>T 5745A>G), GAA1535GAT (5748A>T), GAG1536TCA (5749G>T 5750A>C 5751G>A), ATC1538GTG (5755A>G 5757C>G), GCC1539ATT (5758G>A 5759C>T 5760C>T), TAC1541TTT (5765A>T 5766C>T), ATC1542ATT (5769C>T), GAC1543GAT (5772C>T), GAC1544GAT (5775C>T), ATC1545ATT (5778C>T), CTG1546TTG (5779C>T), GTA1547GTT (5784A>T), AAC1550AAA (5793C>A), AGT1551ACT (5795G>C), GAA1552GAG (5799A>G), GAT1554AAT (5803G>A), GAA1556GTA (5810A>T), AGG1557AAA (5813G>A 5814G>A), CAT1558CAC (5817T>C), TTA1559TTG (5820A>G), AAG1560AGA (5822A>G 5823G>A), ATG1562GT. (5827A>G)								

Proteins

	Begin	End	Coverage	Score	Concordance	Matches	Identities	I/D/M/F*	Stop Codons
ORF3 (YP_009121747.1)	1422	1562	6.9%	415	43.5%	139 (97.9%)	63 (44.4%)	1/2/0/0	0
Protein mutations:	V1422S (5407G>T 5408T>C 5409C>A), N1423P (5410A>C 5411A>C 5412C>T), S1424W (5414C>G 5415T>G), T1426A (5419A>G), T1427P (5422A>C), D1429_P1430del (5428_5433delGACCCA), A1431L (5434G>C 5435C>T), T1432F (5437A>T 5438C>T 5439A>T), G1433V (5441G>T 5442C>G), E1435K (5446G>A 5448A>G), T1436K (5450C>A 5451A>G), K1437D (5452A>G 5454G>T), N1439S (5458A>T 5459A>C 5460T>A), D1440M (5461G>A 5462A>T 5463T>G), V1443C (5470G>T 5471T>G 5472G>T), F1444I (5473T>A 5475C>T), N1445D (5476A>G), T1448Q (5485A>C 5486C>A 5487A>G), D1451K (5494G>A 5496C>G), N1452V (5497A>G 5498A>T 5499T>G), Y1454V (5503T>G 5504A>T 5505C>A), D1456N (5509G>A 5511T>C), Q1457K (5512C>A 5514G>A), S1459P (5518T>C), G1462R (5527G>C 5529G>T), N1464D (5533A>G 5535C>T), T1465N (5537C>A 5538C>T), L1467F (5542C>T 5544A>T), K1468D (5545A>G 5547A>T), R1469Q (5548A>C 5549G>A 5550A>G), I1470L (5551A>T), G1471Q (5554G>C 5555G>A), N1472G (5557A>G 5558A>G 5559T>A), K1474A (5563A>G 5564A>C 5565G>C), F1479I (5578T>A), K1482R (5588A>G 5589G>A), S1483F (5591C>T 5592T>C), F1485Y (5597T>A), V1488L (5605G>T 5607T>G), A1489K (5608G>A 5609C>A 5610C>G), M1490I (5613G>T), E1491H (5614G>C 5616A>T), E1492P (5617G>C 5618A>C 5619A>T), E1493S (5620G>T 5621A>C 5622A>T), S1494D (5623T>G 5624C>A 5625C>T), W1497K (5632T>A 5633G>A), L1501R (5644T>C 5645T>G 5646A>G), I1502T (5648T>C), P1503R (5650C>A 5651C>G 5652A>G), G1504Y (5653G>T 5654G>A 5655A>T), L1506H (5659T>C 5660T>A 5661A>T), W1509F (5669G>T 5670G>T), P1513S (5680C>T 5682A>G), K1517T (5693A>C 5694A>T), A1521T (5704G>A 5706T>A), I1522A (5707A>G 5708T>C 5709T>A), Q1524M (5713C>A 5714A>T), R1525E (5716A>G 5717G>A), K1526L (5719A>T 5720A>T), D1528N (5725G>A), K1529G (5728A>G 5729A>G 5730A>G), C1530V (5731T>G 5732G>T 5733C>G), K1532H (5737A>C 5739A>T), K1532_D1533insP (5739_5740insCCG), D1533Y (5740G>T), T1534L (5743A>T 5744C>T 5745A>G), E1535D (5748A>T), E1536S (5749G>T 5750A>C 5751G>A), I1538V (5755A>G 5757C>G), A1539I (5758G>A 5759C>T 5760C>T), Y1541F (5765A>T 5766C>T), N1550K (5793C>A), S1551T (5795G>C), D1554N (5803G>A), E1556V (5810A>T), R1557K (5813G>A 5814G>A), K1560R (5822A>G 5823G>A)								

	Begin	End	Coverage	Score	Concordance	Matches	Identities	I/D/M/F*	Stop Codons
NT	5407	5828	5.5%	9	1.1%	416 (97.9%)	217 (51.1%)	3/6	
Codon mutations:	GTC1422TCA (5407G>T 5408T>C 5409C>A), AAC1423CCT (5410A>C 5411A>C 5412C>T), TCT1424TGG (5414C>G 5415T>G), ACC1426GCC (5419A>G), ACG1427CCG (5422A>C), GTT1428GTC (5427T>C), GAC1429_CCA1430del (5428_5433delGACCCA), GCA1431CTA (5434G>C 5435C>T), ACA1432TTT (5437A>T 5438C>T 5439A>T), GGC1433GTG (5441G>T 5442C>G), GAA1435AAG (5446G>A 5448A>G), ACA1436AAG (5450C>A 5451A>G), AAG1437GAT (5452A>G 5454G>T), GGA1438GGG (5457A>G), AAT1439TCA (5458A>T 5459A>C 5460T>A), GAT1440ATG (5461G>A 5462A>T 5463T>G), GTG1443TGT (5470G>T 5471T>G 5472G>T), TTC1444ATT (5473T>A 5475C>T), AAT1445GAT (5476A>G), TAC1446TAT (5481C>T), ACA1448CAG (5485A>C 5486C>A 5487A>G), CTC1449TTG (5488C>T 5490C>G), AAT1450AAC (5493T>C), GAC1451AAG (5494G>A 5496C>G), AAT1452GTG (5497A>G 5498A>T 5499T>G), ACA1453ACT (5502A>T), TAC1454GTA (5503T>G 5504A>T 5505C>A), AAA1455AAG (5508A>G), GAT1456AAC (5509G>A 5511T>C), CAG1457AAA (5512C>A 5514G>A), TCC1459CCC (5518T>C), CTT1460CTC (5523T>C), GGG1462CGT (5527G>C 5529G>T), ATA1463ATC (5532A>C), AAC1464GAT (5533A>G 5535C>T), ACC1465AAT (5537C>A 5538C>T), TTG1466TTA (5541G>A), CTA1467TTT (5542C>T 5544A>T), AAA1468GAT (5545A>G 5547A>T), AGA1469CAG (5548A>C 5549G>A 5550A>G), ATA1470TTA (5551A>T), GGG1471CAG (5554G>C 5555G>A), AAT1472GGA (5557A>G 5558A>G 5559T>A), AAG1474GCC (5563A>G 5564A>C 5565G>C), TCA1477TCT (5574A>T), TTT1479ATT (5578T>A), AAG1482AGA (5588A>G 5589G>A), TCT1483TTC (5591C>T 5592T>C), TTT1485TAT (5597T>A), CAA1487CAG (5604A>G), GTT1488TTG (5605G>T 5607T>G), GCC1489AAG (5608G>A 5609C>A 5610C>G), ATG1490ATT (5613G>T), GAA1491CAT (5614G>C 5616A>T), GAA1492CCT (5617G>C 5618A>C 5619A>T), GAA1493TCT (5620G>T 5621A>C 5622A>T), TCC1494GAT (5623T>G 5624C>A 5625C>T), ATA1495ATT (5628A>T), CCC1496CCT (5631C>T), TGG1497AAG (5632T>A 5633G>A), ACG1498ACA (5637G>A), GCC1499GCT (5640C>T), TTT1500TTC (5643T>C), TTA1501CGG (5644T>C 5645T>G 5646A>G), ATC1502ACC (5648T>C), CCA1503AGG (5650C>A 5651C>G 5652A>G), GGA1504TAT (5653G>T 5654G>A 5655A>T), GGA1505GGC (5658A>C), TTA1506CAT (5659T>C 5660T>A 5661A>T), TAC1507TAT (5664C>T), GAA1508GAG (5667A>G), TGG1509TTT (5669G>T 5670G>T), GTC1511GTT (5676C>T), CCA1513TCG (5680C>T 5682A>G), TTC1514TTT (5685C>T), GGA1515GGG (5688A>G), CTC1516TTG (5689C>T 5691C>G), AAA1517ACT (5693A>C 5694A>T), GCA1519GCT (5700A>T), CCC1520CCG (5703C>G), GCT1521ACA (5704G>A 5706T>A), ATT1522GCA (5707A>G 5708T>C 5709T>A), CAG1524ATG (5713C>A 5714A>T), AGA1525GAA (5716A>G 5717G>A), AAA1526TTA (5719A>T 5720A>T), GAT1528AAT (5725G>A), AAA1529GGG (5728A>G 5729A>G 5730A>G), TGC1530GTG (5731T>G 5732G>T 5733C>G), TTC1531TTT (5736C>T), AAA1532CAT (5737A>C 5739A>T), AAA1532_GAC1533insCCG (5739_5740insCCG), GAC1533TAC (5740G>T), ACA1534TTG (5743A>T 5744C>T 5745A>G), GAA1535GAT (5748A>T), GAG1536TCA (5749G>T 5750A>C 5751G>A), ATC1538GTG (5755A>G 5757C>G), GCC1539ATT (5758G>A 5759C>T 5760C>T), TAC1541TTT (5765A>T 5766C>T), ATC1542ATT (5769C>T), GAC1543GAT (5772C>T), GAC1544GAT (5775C>T), ATC1545ATT (5778C>T), CTG1546TTG (5779C>T), GTA1547GTT (5784A>T), AAC1550AAA (5793C>A), AGT1551ACT (5795G>C), GAA1552GAG (5799A>G), GAT1554AAT (5803G>A), GAA1556GTA (5810A>T), AGG1557AAA (5813G>A 5814G>A), CAT1558CAC (5817T>C), TTA1559TTG (5820A>G), AAG1560AGA (5822A>G 5823G>A), ATG1562GT. (5827A>G)								

*: Inserts / Deletes / Misaligned / Frameshifts

Analysis details

This analysis was performed with panviral2.64

NGS Details (UN62): Badnavirus venazanthoxyli

Assembly

**Coverage Length**	278 (1 contig(s))
**Depth Of Coverage**	3.8
**Number Of Reads**	8
**Reads Per Million**	0.19 rpm (after QC)
**Ambiguities**	0
**Assembly Method**	de novo + reference guided assembly
**Consensus Caller**	Bcf Tools

Coverage Map

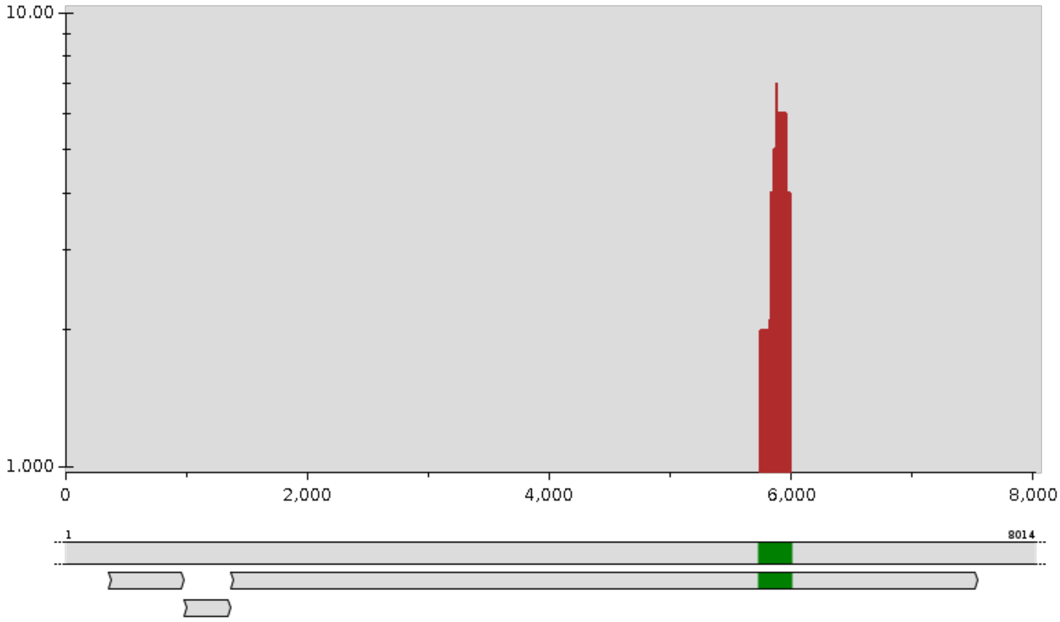

Assignment

**Type**	Badnavirus venazanthoxyli (Taxonomy ID: 3047858)
**Reference Genome**	NC_076349.1
**NT Identity (%)**	58.363
**AA Identity (%)**	53.1915
**Number Of Stop Codons**	0
**Number Of CDS**	3

Alignment

**Alignment Score**	88.0 (NT) + 365.0 (AA) = 453.0
**Concordance (%)**	37.5622

Alignment Method	Global, seeded, nucleotide + amino acids (AGA)

Genome Region

Sequence starts at position 5732 and ends at position 6009 relative to NC_076349.1 reference sequence.

Alignment Detailed Statistics

	Begin	End	Coverage	Score	Concordance	Matches	Identities	I/D/M/F*	Stop Codons
**NT**	**5732**	**6009**	**3.5%**	**88**	**15.8%**	**278 (98.9%)**	**164 (58.4%)**	**3/0**	
Mutations:	5741C>G, 5744T>A, 5746G>C, 5750G>A, 5756A>G, 5757A>T, 5759A>G, 5763A>T, 5764G>C, 5768G>A, 5769T>G, 5771T>A, 5777A>G, 5779A>G, 5780G>A, 5783A>T, 5788T>A, 5795A>G, 5796G>A, 5798G>C, 5799G>A, 5800C>G, 5801C>A, 5806A>T, 5807T>G, 5809A>T, 5810A>G, 5814A>G, 5815G>A, 5819C>T, 5823T>A, 5824G>A, 5825G>A, 5836T>A, 5837C>A, 5839G>C, 5842C>A, 5843A>T, 5844G>A, 5845C>T, 5846A>G, 5849G>A, 5850T>C, 5851T>A, 5852G>T, 5858A>G, 5860G>T, 5861G>T, 5862C>T, 5873A>C, 5876C>T, 5879C>A, 5880C>T, 5882A>G, 5884A>C, 5885G>T, 5891T>A, 5894A>T, 5895G>T, 5897A>C, 5898G>A, 5899T>C, 5900C>T, 5906G>A, 5907C>T, 5909G>T, 5910A>C, 5911A>C, 5916G>A, 5919G>C, 5921A>T, 5922T>G, 5923G>T, 5924C>T, 5927C>T, 5928A>C, 5931G>C, 5932G>A, 5933_5934insTAC, 5935C>T, 5937G>A, 5938A>G, 5940G>A, 5945C>T, 5946A>G, 5948T>C, 5949G>T, 5950C>T, 5951C>A, 5956A>T, 5958A>T, 5960C>T, 5963C>T, 5966T>C, 5969A>T, 5972T>G, 5973G>A, 5977T>A, 5978C>T, 5979T>A, 5980C>G, 5981C>T, 5984C>G, 5985A>T, 5988A>T, 5990C>A, 5991A>C, 5992A>T, 5999T>C, 6000G>C, 6001A>T, 6002G>T, 6004A>T, 6005G>T, 6008C>T								

CDS

	Begin	End	Coverage	Score	Concordance	Matches	Identities	I/D/M/F*	Stop Codons
QKN22_gp3	1457	1549	4.5%	365	55.3%	93 (98.9%)	50 (53.2%)	1/0/0/0	0
Protein mutations:	G1461A (5746G>C), I1465L (5757A>T 5759A>G), F1469V (5769T>G 5771T>A), K1472R (5779A>G 5780G>A), F1475Y (5788T>A), V1478I (5796G>A 5798G>C), A1479R (5799G>A 5800C>G 5801C>A), D1481V (5806A>T 5807T>G), E1482V (5809A>T 5810A>G), S1484D (5814A>G 5815G>A), W1487K (5823T>A 5824G>A 5825G>A), I1491K (5836T>A 5837C>A), S1492T (5839G>C), P1493H (5842C>A 5843A>T), A1494M (5844G>A 5845C>T 5846A>G), L1496H (5850T>C 5851T>A 5852G>T), W1499F (5860G>T 5861G>T), K1507T (5884A>C 5885G>T), A1511S (5895G>T 5897A>C), V1512T (5898G>A 5899T>C 5900C>T), R1515C (5907C>T 5909G>T), K1516P (5910A>C 5911A>C), D1518N (5916G>A), E1519H (5919G>C 5921A>T), C1520V (5922T>G 5923G>T 5924C>T), K1522Q (5928A>C), G1523Q (5931G>C 5932G>A), G1523_T1524insY (5933_5934insTAC), T1524M (5935C>T), E1525R (5937G>A 5938A>G), E1526K (5940G>A), I1528V (5946A>G 5948T>C), A1529L (5949G>T 5950C>T 5951C>A), Y1531F (5956A>T), I1532F (5958A>T 5960C>T), V1537I (5973G>A), F1538Y (5977T>A 5978C>T), N1540K (5984C>G), T1541S (5985A>T), I1542L (5988A>T 5990C>A), K1543L (5991A>C 5992A>T), E1546L (6000G>C 6001A>T 6002G>T), K1547I (6004A>T 6005G>T)								
Codon mutations:	TCC1459TCG (5741C>G), CTT1460CTA (5744T>A), GGA1461GCA (5746G>C), GGG1462GGA (5750G>A), AAA1464AAG (5756A>G), ATA1465TTG (5757A>T 5759A>G), AGC1467TCC (5763A>T 5764G>C), AAG1468AAA (5768G>A), TTT1469GTA (5769T>G 5771T>A), TTA1471TTG (5777A>G), AAG1472AGA (5779A>G 5780G>A), TCA1473TCT (5783A>T), TTC1475TAC (5788T>A), CAA1477CAG (5795A>G), GTG1478ATC (5796G>A 5798G>C), GCC1479AGA (5799G>A 5800C>G 5801C>A), GAT1481GTG (5806A>T 5807T>G), GAA1482GTG (5809A>T 5810A>G), AGC1484GAC (5814A>G 5815G>A), ATC1485ATT (5819C>T), TGG1487AAA (5823T>A 5824G>A 5825G>A), ATC1491AAA (5836T>A 5837C>A), AGT1492ACT (5839G>C), CCA1493CAT (5842C>A 5843A>T), GCA1494ATG (5844G>A 5845C>T 5846A>G), GGG1495GGA (5849G>A), TTG1496CAT (5850T>C 5851T>A 5852G>T), GAA1498GAG (5858A>G), TGG1499TTT (5860G>T 5861G>T), CTG1500TTG (5862C>T), CCA1503CCC (5873A>C), TTC1504TTT (5876C>T), GGC1505GGA (5879C>A), CTA1506TTG (5880C>T 5882A>G), AAG1507ACT (5884A>C 5885G>T), GCT1509GCA (5891T>A), CCA1510CCT (5894A>T), GCA1511TCC (5895G>T 5897A>C), GTC1512ACT (5898G>A 5899T>C 5900C>T), CAG1514CAA (5906G>A), CGG1515TGT (5907C>T 5909G>T), AAG1516CCG (5910A>C 5911A>C), GAT1518AAT (5916G>A), GAA1519CAT (5919G>C 5921A>T), TGC1520GTT (5922T>G 5923G>T 5924C>T), TTC1521TTT (5927C>T), AAA1522CAA (5928A>C), GGA1523CAA (5931G>C 5932G>A), GGA1523_ACG1524insTAC (5933_5934insTAC), ACG1524ATG (5935C>T), GAG1525AGG (5937G>A 5938A>G), GAG1526AAG (5940G>A), TTC1527TTT (5945C>T), ATT1528GTC (5946A>G 5948T>C), GCC1529TTA (5949G>T 5950C>T 5951C>A), TAT1531TTT (5956A>T), ATC1532TTT (5958A>T 5960C>T), GAC1533GAT (5963C>T), GAT1534GAC (5966T>C), ATA1535ATT (5969A>T), CTT1536CTG (5972T>G), GTC1537ATC (5973G>A), TTC1538TAT (5977T>A 5978C>T), TCC1539AGT (5979T>A 5980C>G 5981C>T), AAC1540AAG (5984C>G), ACC1541TCC (5985A>T), ATC1542TTA (5988A>T 5990C>A), AAG1543CTG (5991A>C 5992A>T), CAT1545CAC (5999T>C), GAG1546CTT (6000G>C 6001A>T 6002G>T), AAG1547ATT (6004A>T 6005G>T), CAC1548CAT (6008C>T)								

Proteins

	Begin	End	Coverage	Score	Concordance	Matches	Identities	I/D/M/F*	Stop Codons
polyprotein (YP_010798182.1)	1457	1549	4.5%	365	55.3%	93 (98.9%)	50 (53.2%)	1/0/0/0	0
Protein mutations:	G1461A (5746G>C), I1465L (5757A>T 5759A>G), F1469V (5769T>G 5771T>A), K1472R (5779A>G 5780G>A), F1475Y (5788T>A), V1478I (5796G>A 5798G>C), A1479R (5799G>A 5800C>G 5801C>A), D1481V (5806A>T 5807T>G), E1482V (5809A>T 5810A>G), S1484D (5814A>G 5815G>A), W1487K (5823T>A 5824G>A 5825G>A), I1491K (5836T>A 5837C>A), S1492T (5839G>C), P1493H (5842C>A 5843A>T), A1494M (5844G>A 5845C>T 5846A>G), L1496H (5850T>C 5851T>A 5852G>T), W1499F (5860G>T 5861G>T), K1507T (5884A>C 5885G>T), A1511S (5895G>T 5897A>C), V1512T (5898G>A 5899T>C 5900C>T), R1515C (5907C>T 5909G>T), K1516P (5910A>C 5911A>C), D1518N (5916G>A), E1519H (5919G>C 5921A>T), C1520V (5922T>G 5923G>T 5924C>T), K1522Q (5928A>C), G1523Q (5931G>C 5932G>A), G1523_T1524insY (5933_5934insTAC), T1524M (5935C>T), E1525R (5937G>A 5938A>G), E1526K (5940G>A), I1528V (5946A>G 5948T>C), A1529L (5949G>T 5950C>T 5951C>A), Y1531F (5956A>T), I1532F (5958A>T 5960C>T), V1537I (5973G>A), F1538Y (5977T>A 5978C>T), N1540K (5984C>G), T1541S (5985A>T), I1542L (5988A>T 5990C>A), K1543L (5991A>C 5992A>T), E1546L (6000G>C 6001A>T 6002G>T), K1547I (6004A>T 6005G>T)								
Codon mutations:	TCC1459TCG (5741C>G), CTT1460CTA (5744T>A), GGA1461GCA (5746G>C), GGG1462GGA (5750G>A), AAA1464AAG (5756A>G), ATA1465TTG (5757A>T 5759A>G), AGC1467TCC (5763A>T 5764G>C), AAG1468AAA (5768G>A), TTT1469GTA (5769T>G 5771T>A), TTA1471TTG (5777A>G), AAG1472AGA (5779A>G 5780G>A), TCA1473TCT (5783A>T), TTC1475TAC (5788T>A), CAA1477CAG (5795A>G), GTG1478ATC (5796G>A 5798G>C), GCC1479AGA (5799G>A 5800C>G 5801C>A), GAT1481GTG (5806A>T 5807T>G), GAA1482GTG (5809A>T 5810A>G), AGC1484GAC (5814A>G 5815G>A), ATC1485ATT (5819C>T), TGG1487AAA (5823T>A 5824G>A 5825G>A), ATC1491AAA (5836T>A 5837C>A), AGT1492ACT (5839G>C), CCA1493CAT (5842C>A 5843A>T), GCA1494ATG (5844G>A 5845C>T 5846A>G), GGG1495GGA (5849G>A), TTG1496CAT (5850T>C 5851T>A 5852G>T), GAA1498GAG (5858A>G), TGG1499TTT (5860G>T 5861G>T), CTG1500TTG (5862C>T), CCA1503CCC (5873A>C), TTC1504TTT (5876C>T), GGC1505GGA (5879C>A), CTA1506TTG (5880C>T 5882A>G), AAG1507ACT (5884A>C 5885G>T), GCT1509GCA (5891T>A), CCA1510CCT (5894A>T), GCA1511TCC (5895G>T 5897A>C), GTC1512ACT (5898G>A 5899T>C 5900C>T), CAG1514CAA (5906G>A), CGG1515TGT (5907C>T 5909G>T), AAG1516CCG (5910A>C 5911A>C), GAT1518AAT (5916G>A), GAA1519CAT (5919G>C 5921A>T), TGC1520GTT (5922T>G 5923G>T 5924C>T), TTC1521TTT (5927C>T), AAA1522CAA (5928A>C), GGA1523CAA (5931G>C 5932G>A), GGA1523_ACG1524insTAC (5933_5934insTAC), ACG1524ATG (5935C>T), GAG1525AGG (5937G>A 5938A>G), GAG1526AAG (5940G>A), TTC1527TTT (5945C>T), ATT1528GTC (5946A>G 5948T>C), GCC1529TTA (5949G>T 5950C>T 5951C>A), TAT1531TTT (5956A>T), ATC1532TTT (5958A>T 5960C>T), GAC1533GAT (5963C>T), GAT1534GAC (5966T>C), ATA1535ATT (5969A>T), CTT1536CTG (5972T>G), GTC1537ATC (5973G>A), TTC1538TAT (5977T>A 5978C>T), TCC1539AGT (5979T>A 5980C>G 5981C>T), AAC1540AAG (5984C>G), ACC1541TCC (5985A>T), ATC1542TTA (5988A>T 5990C>A), AAG1543CTG (5991A>C 5992A>T), CAT1545CAC (5999T>C), GAG1546CTT (6000G>C 6001A>T 6002G>T), AAG1547ATT (6004A>T 6005G>T), CAC1548CAT (6008C>T)								

*: Inserts / Deletes / Misaligned / Frameshifts

Analysis details

This analysis was performed with panviral2.64

NGS Details (UN62): Dioscovirus dioscoreae

Assembly

**Coverage Length**	344 (1 contig(s))
**Depth Of Coverage**	3.1
**Number Of Reads**	8
**Reads Per Million**	0.19 rpm (after QC)
**Ambiguities**	0
**Assembly Method**	de novo + reference guided assembly
**Consensus Caller**	Bcf Tools

Coverage Map

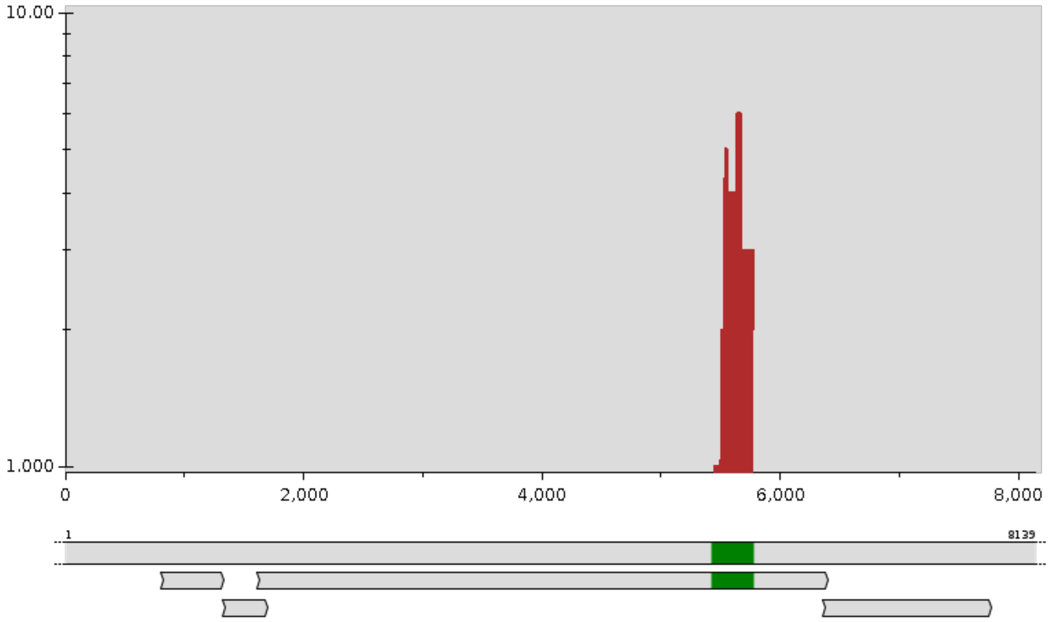

Assignment

**Type**	Dioscovirus dioscoreae (Taxonomy ID: 3052184)
**Reference Genome**	NC_040712.1
**NT Identity (%)**	63.4286
**AA Identity (%)**	48.7179
**Number Of Stop Codons**	0
**Number Of CDS**	4

Alignment

**Alignment Score**	176.0 (NT) + 460.0 (AA) = 636.0
**Concordance (%)**	43.3243

Alignment Method	Global, seeded, nucleotide + amino acids (AGA)

Genome Region

Sequence starts at position 5436 and ends at position 5779 relative to NC_040712.1 reference sequence.

Alignment Detailed Statistics

	Begin	End	Coverage	Score	Concordance	Matches	Identities	I/D/M/F*	Stop Codons
**NT**	**5436**	**5779**	**4.2%**	**176**	**26.3%**	**341 (96.6%)**	**222 (62.9%)**	**9/3**	
Mutations:	5439C>G, 5441G>A, 5444T>A, 5452A>T, 5453A>G, 5457A>T, 5461T>C, 5466C>T, 5480A>G, 5482A>C, 5486A>C, 5489T>C, 5490T>C, 5492A>C, 5492_5493insCCCCCCCAA, 5493G>A, 5498C>T, 5502C>A, 5507A>G, 5514A>G, 5516T>A, 5523A>G, 5524A>G, 5526A>G, 5532T>A, 5533C>A, 5535G>A, 5538T>A, 5543C>A, 5544T>C, 5545G>T, 5546T>A, 5549T>C, 5553A>G, 5558C>T, 5561T>C, 5564T>C, 5565T>C, 5567G>A, 5568A>G, 5573T>C, 5574T>A, 5575C>G, 5577G>A, 5578A>G, 5580A>G, 5581G>A, 5585A>G, 5589C>G, 5595G>C, 5598C>G, 5607T>C, 5608T>A, 5610C>A, 5611A>C, 5612G>A, 5613T>A, 5615C>T, 5618C>T, 5619C>A, 5620A>G, 5621A>G, 5622G>T, 5626G>T, 5627T>C, 5628A>G, 5629A>T, 5630G>A, 5631G>A, 5632A>G, 5633A>T, 5634G>A, 5639G>A, 5640C>A, 5642T>A, 5645T>A, 5646T>A, 5648G>A, 5649T>A, 5651A>C, 5656C>A, 5657A>G, 5661T>A, 5664A>G, 5667A>T, 5670G>T, 5672A>T, 5673G>A, 5674T>A, 5676G>A, 5679A>C, 5685G>A, 5687A>C, 5691C>T, 5693T>A, 5696C>A, 5697C>G, 5700G>A, 5701A>C, 5702A>T, 5703A>T, 5711A>T, 5714A>G, 5717A>G, 5718G>A, 5720T>A, 5721C>A, 5726C>A, 5739T>G, 5740T>C, 5741A>T, 5748A>G, 5756A>T, 5757T>A, 5759T>G, 5762T>A, 5763_5765delGAA, 5768C>T, 5769C>A, 5777A>T, 5779C>A								

CDS

	Begin	End	Coverage	Score	Concordance	Matches	Identities	I/D/M/F*	Stop Codons
EXK67_gp3	1276	1390	7.2%	460	57.3%	114 (96.6%)	57 (48.3%)	3/1/0/0	0
Protein mutations:	Q1277E (5439C>G 5441G>A), E1281V (5452A>T 5453A>G), I1283L (5457A>T), V1284A (5461T>C), P1286S (5466C>T), N1291T (5482A>C), S1294P (5490T>C 5492A>C), S1294_V1295insPPQ (5492_5493insCCCCCCCAA), V1295I (5493G>A), N1302E (5514A>G 5516T>A), K1305G (5523A>G 5524A>G), K1306E (5526A>G), S1308K (5532T>A 5533C>A), E1309K (5535G>A), F1310I (5538T>A), C1312L (5544T>C 5545G>T 5546T>A), I1315V (5553A>G), I1320V (5568A>G), E1323R (5577G>A 5578A>G), S1324D (5580A>G 5581G>A), I1325M (5585A>G), Q1327E (5589C>G), V1329L (5595G>C), Q1330E (5598C>G), L1333Q (5607T>C 5608T>A), Q1334T (5610C>A 5611A>C 5612G>A), F1335I (5613T>A 5615C>T), Q1337R (5619C>A 5620A>G 5621A>G), V1338L (5622G>T), C1339F (5626G>T 5627T>C), K1340V (5628A>G 5629A>T 5630G>A), E1341S (5631G>A 5632A>G 5633A>T), E1342K (5634G>A), L1344I (5640C>A 5642T>A), L1346I (5646T>A 5648G>A), S1347T (5649T>A 5651A>C), T1349K (5656C>A 5657A>G), L1351M (5661T>A), K1352E (5664A>G), I1353L (5667A>T), G1354C (5670G>T 5672A>T), V1355K (5673G>A 5674T>A), A1356T (5676G>A), N1357H (5679A>C), E1359N (5685G>A 5687A>C), L1363V (5697C>G), E1364T (5700G>A 5701A>C 5702A>T), I1365L (5703A>T), E1367D (5711A>T), V1370I (5718G>A 5720T>A), Q1371K (5721C>A), L1377A (5739T>G 5740T>C 5741A>T), I1380V (5748A>G), E1382D (5756A>T), F1383M (5757T>A 5759T>G), E1385del (5763_5765delGAA), Q1387K (5769C>A), E1389D (5777A>T)								
Codon mutations:	CAG1277GAA (5439C>G 5441G>A), GGT1278GGA (5444T>A), GAA1281GTG (5452A>T 5453A>G), ATA1283TTA (5457A>T), GTA1284GCA (5461T>C), CCA1286TCA (5466C>T), AAA1290AAG (5480A>G), AAT1291ACT (5482A>C), GCA1292GCC (5486A>C), CCT1293CCC (5489T>C), TCA1294CCC (5490T>C 5492A>C), TCA1294_GTA1295insCCCCCCCAA (5492_5493insCCCCCCCAA), GTA1295ATA (5493G>A), TTC1296TTT (5498C>T), CGA1298AGA (5502C>A), AAA1299AAG (5507A>G), AAT1302GAA (5514A>G 5516T>A), AAA1305GGA (5523A>G 5524A>G), AAA1306GAA (5526A>G), TCA1308AAA (5532T>A 5533C>A), GAA1309AAA (5535G>A), TTT1310ATT (5538T>A), GTC1311GTA (5543C>A), TGT1312CTA (5544T>C 5545G>T 5546T>A), GTT1313GTC (5549T>C), ATA1315GTA (5553A>G), GAC1316GAT (5558C>T), GAT1317GAC (5561T>C), ATT1318ATC (5564T>C), TTG1319CTA (5565T>C 5567G>A), ATA1320GTA (5568A>G), TTT1321TTC (5573T>C), TCT1322AGT (5574T>A 5575C>G), GAA1323AGA (5577G>A 5578A>G), AGT1324GAT (5580A>G 5581G>A), ATA1325ATG (5585A>G), CAA1327GAA (5589C>G), GTA1329CTA (5595G>C), CAA1330GAA (5598C>G), TTA1333CAA (5607T>C 5608T>A), CAG1334ACA (5610C>A 5611A>C 5612G>A), TTC1335ATT (5613T>A 5615C>T), TTC1336TTT (5618C>T), CAA1337AGG (5619C>A 5620A>G 5621A>G), GTA1338TTA (5622G>T), TGT1339TTC (5626G>T 5627T>C), AAG1340GTA (5628A>G 5629A>T 5630G>A), GAA1341AGT (5631G>A 5632A>G 5633A>T), GAA1342AAA (5634G>A), GGG1343GGA (5639G>A), CTT1344ATA (5640C>A 5642T>A), ATT1345ATA (5645T>A), TTG1346ATA (5646T>A 5648G>A), TCA1347ACC (5649T>A 5651A>C), ACA1349AAG (5656C>A 5657A>G), TTG1351ATG (5661T>A), AAA1352GAA (5664A>G), ATA1353TTA (5667A>T), GGA1354TGT (5670G>T 5672A>T), GTA1355AAA (5673G>A 5674T>A), GCA1356ACA (5676G>A), AAT1357CAT (5679A>C), GAA1359AAC (5685G>A 5687A>C), CTT1361TTA (5691C>T 5693T>A), GGC1362GGA (5696C>A), CTA1363GTA (5697C>G), GAA1364ACT (5700G>A 5701A>C 5702A>T), ATA1365TTA (5703A>T), GAA1367GAT (5711A>T), GGA1368GGG (5714A>G), AAA1369AAG (5717A>G), GTT1370ATA (5718G>A 5720T>A), CAA1371AAA (5721C>A), CTC1372CTA (5726C>A), TTA1377GCT (5739T>G 5740T>C 5741A>T), ATA1380GTA (5748A>G), GAA1382GAT (5756A>T), TTT1383ATG (5757T>A 5759T>G), CCT1384CCA (5762T>A), GAA1385del (5763_5765delGAA), GAC1386GAT (5768C>T), CAA1387AAA (5769C>A), GAA1389GAT (5777A>T), ACA1390AA. (5779C>A)								

Proteins

	Begin	End	Coverage	Score	Concordance	Matches	Identities	I/D/M/F*	Stop Codons
ORF3 (YP_009553219.1)	1276	1390	7.2%	460	57.3%	114 (96.6%)	57 (48.3%)	3/1/0/0	0
Protein mutations:	Q1277E (5439C>G 5441G>A), E1281V (5452A>T 5453A>G), I1283L (5457A>T), V1284A (5461T>C), P1286S (5466C>T), N1291T (5482A>C), S1294P (5490T>C 5492A>C), S1294_V1295insPPQ (5492_5493insCCCCCCCAA), V1295I (5493G>A), N1302E (5514A>G 5516T>A), K1305G (5523A>G 5524A>G), K1306E (5526A>G), S1308K (5532T>A 5533C>A), E1309K (5535G>A), F1310I (5538T>A), C1312L (5544T>C 5545G>T 5546T>A), I1315V (5553A>G), I1320V (5568A>G), E1323R (5577G>A 5578A>G), S1324D (5580A>G 5581G>A), I1325M (5585A>G), Q1327E (5589C>G), V1329L (5595G>C), Q1330E (5598C>G), L1333Q (5607T>C 5608T>A), Q1334T (5610C>A 5611A>C 5612G>A), F1335I (5613T>A 5615C>T), Q1337R (5619C>A 5620A>G 5621A>G), V1338L (5622G>T), C1339F (5626G>T 5627T>C), K1340V (5628A>G 5629A>T 5630G>A), E1341S (5631G>A 5632A>G 5633A>T), E1342K (5634G>A), L1344I (5640C>A 5642T>A), L1346I (5646T>A 5648G>A), S1347T (5649T>A 5651A>C), T1349K (5656C>A 5657A>G), L1351M (5661T>A), K1352E (5664A>G), I1353L (5667A>T), G1354C (5670G>T 5672A>T), V1355K (5673G>A 5674T>A), A1356T (5676G>A), N1357H (5679A>C), E1359N (5685G>A 5687A>C), L1363V (5697C>G), E1364T (5700G>A 5701A>C 5702A>T), I1365L (5703A>T), E1367D (5711A>T), V1370I (5718G>A 5720T>A), Q1371K (5721C>A), L1377A (5739T>G 5740T>C 5741A>T), I1380V (5748A>G), E1382D (5756A>T), F1383M (5757T>A 5759T>G), E1385del (5763_5765delGAA), Q1387K (5769C>A), E1389D (5777A>T)								
Codon mutations:	CAG1277GAA (5439C>G 5441G>A), GGT1278GGA (5444T>A), GAA1281GTG (5452A>T 5453A>G), ATA1283TTA (5457A>T), GTA1284GCA (5461T>C), CCA1286TCA (5466C>T), AAA1290AAG (5480A>G), AAT1291ACT (5482A>C), GCA1292GCC (5486A>C), CCT1293CCC (5489T>C), TCA1294CCC (5490T>C 5492A>C), TCA1294_GTA1295insCCCCCCCAA (5492_5493insCCCCCCCAA), GTA1295ATA (5493G>A), TTC1296TTT (5498C>T), CGA1298AGA (5502C>A), AAA1299AAG (5507A>G), AAT1302GAA (5514A>G 5516T>A), AAA1305GGA (5523A>G 5524A>G), AAA1306GAA (5526A>G), TCA1308AAA (5532T>A 5533C>A), GAA1309AAA (5535G>A), TTT1310ATT (5538T>A), GTC1311GTA (5543C>A), TGT1312CTA (5544T>C 5545G>T 5546T>A), GTT1313GTC (5549T>C), ATA1315GTA (5553A>G), GAC1316GAT (5558C>T), GAT1317GAC (5561T>C), ATT1318ATC (5564T>C), TTG1319CTA (5565T>C 5567G>A), ATA1320GTA (5568A>G), TTT1321TTC (5573T>C), TCT1322AGT (5574T>A 5575C>G), GAA1323AGA (5577G>A 5578A>G), AGT1324GAT (5580A>G 5581G>A), ATA1325ATG (5585A>G), CAA1327GAA (5589C>G), GTA1329CTA (5595G>C), CAA1330GAA (5598C>G), TTA1333CAA (5607T>C 5608T>A), CAG1334ACA (5610C>A 5611A>C 5612G>A), TTC1335ATT (5613T>A 5615C>T), TTC1336TTT (5618C>T), CAA1337AGG (5619C>A 5620A>G 5621A>G), GTA1338TTA (5622G>T), TGT1339TTC (5626G>T 5627T>C), AAG1340GTA (5628A>G 5629A>T 5630G>A), GAA1341AGT (5631G>A 5632A>G 5633A>T), GAA1342AAA (5634G>A), GGG1343GGA (5639G>A), CTT1344ATA (5640C>A 5642T>A), ATT1345ATA (5645T>A), TTG1346ATA (5646T>A 5648G>A), TCA1347ACC (5649T>A 5651A>C), ACA1349AAG (5656C>A 5657A>G), TTG1351ATG (5661T>A), AAA1352GAA (5664A>G), ATA1353TTA (5667A>T), GGA1354TGT (5670G>T 5672A>T), GTA1355AAA (5673G>A 5674T>A), GCA1356ACA (5676G>A), AAT1357CAT (5679A>C), GAA1359AAC (5685G>A 5687A>C), CTT1361TTA (5691C>T 5693T>A), GGC1362GGA (5696C>A), CTA1363GTA (5697C>G), GAA1364ACT (5700G>A 5701A>C 5702A>T), ATA1365TTA (5703A>T), GAA1367GAT (5711A>T), GGA1368GGG (5714A>G), AAA1369AAG (5717A>G), GTT1370ATA (5718G>A 5720T>A), CAA1371AAA (5721C>A), CTC1372CTA (5726C>A), TTA1377GCT (5739T>G 5740T>C 5741A>T), ATA1380GTA (5748A>G), GAA1382GAT (5756A>T), TTT1383ATG (5757T>A 5759T>G), CCT1384CCA (5762T>A), GAA1385del (5763_5765delGAA), GAC1386GAT (5768C>T), CAA1387AAA (5769C>A), GAA1389GAT (5777A>T), ACA1390AA. (5779C>A)								

*: Inserts / Deletes / Misaligned / Frameshifts

Analysis details

This analysis was performed with panviral2.64

NGS Details (UN62): Duamitovirus soch1

Assembly

**Coverage Length**	357 (1 contig(s))
**Depth Of Coverage**	2.5
**Number Of Reads**	7
**Reads Per Million**	0.16 rpm (after QC)
**Ambiguities**	0
**Assembly Method**	de novo + reference guided assembly
**Consensus Caller**	Bcf Tools

Coverage Map

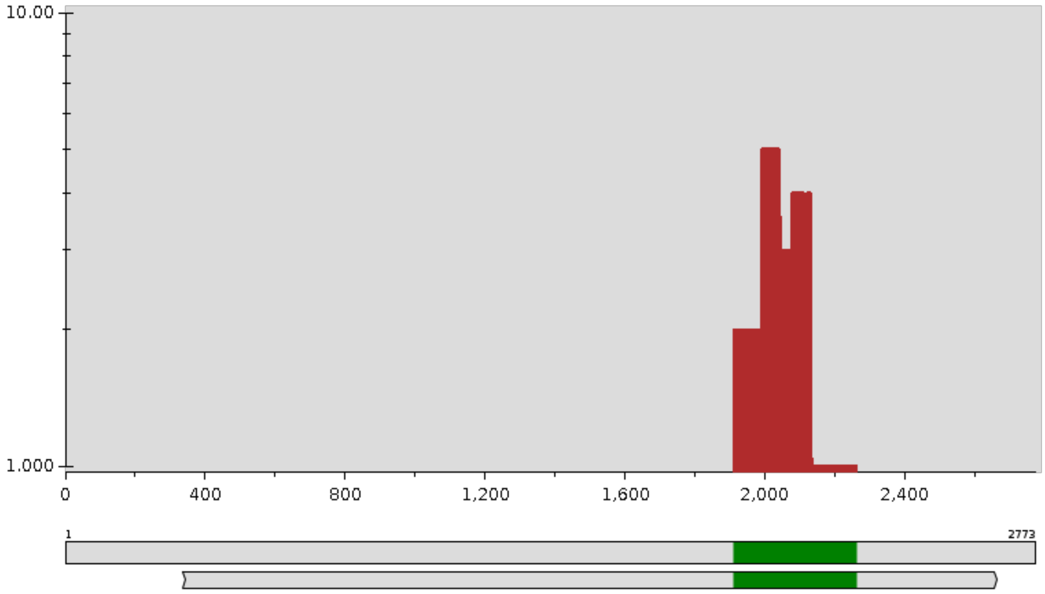

Assignment

**Type**	Duamitovirus soch1 (Taxonomy ID: 2955838)
**Reference Genome**	NC_076524.1
**NT Identity (%)**	69.1643
**AA Identity (%)**	73.913
**Number Of Stop Codons**	0
**Number Of CDS**	1

Alignment

**Alignment Score**	247.0 (NT) + 469.0 (AA) = 716.0
**Concordance (%)**	54.2424

Alignment Method	Global, seeded, nucleotide + amino acids (AGA)

Genome Region

Sequence starts at position 1910 and ends at position 2266 relative to NC_076524.1 reference sequence.

Alignment Detailed Statistics

	Begin	End	Coverage	Score	Concordance	Matches	Identities	I/D/M/F*	Stop Codons
**NT**	**1910**	**2266**	**12.9%**	**247**	**36.6%**	**347 (97.2%)**	**240 (67.2%)**	**0/10**	
Mutations:	1919G>T, 1933C>T, 1934T>A, 1935A>C, 1936A>G, 1938T>G, 1939C>A, 1940A>T, 1948T>A, 1949T>A, 1953T>A, 1955A>C, 1961A>C, 1964T>C, 1967C>T, 1970T>C, 1972G>A, 1980T>A, 1981T>C, 1982A>C, 1985T>C, 1989C>A, 1991T>A, 1994T>G, 2000T>A, 2003A>C, 2006T>G, 2010G>C, 2011C>A, 2012C>A, 2013C>T, 2015C>G, 2016G>T, 2018A>C, 2019A>T, 2020T>G, 2022A>C, 2023A>G, 2024G>A, 2027T>C, 2028G>T, 2029A>C, 2030T>G, 2033T>A, 2034A>G, 2036C>A, 2042A>G, 2043G>A, 2044T>C, 2054A>G, 2055T>C, 2057G>A, 2059G>C, 2066C>A, 2069G>T, 2072C>T, 2078A>C, 2082G>T, 2084G>T, 2088T>C, 2090A>G, 2091T>A, 2093C>G, 2094A>T, 2099C>T, 2102A>G, 2103A>T, 2104G>C, 2105C>A, 2106A>C, 2107A>C, 2108G>A, 2111T>A, 2117G>A, 2118C>A, 2120T>A, 2124_2133delAAAGCTGCTC, 2135T>G, 2138T>C, 2141G>A, 2146T>G, 2148C>A, 2152C>T, 2158T>C, 2167A>T, 2168C>A, 2173G>T, 2174G>T, 2186G>A, 2191T>A, 2192G>A, 2195C>T, 2204C>T, 2208T>C, 2210G>A, 2213A>G, 2225A>G, 2230A>T, 2231C>T, 2234C>T, 2237T>G, 2239A>G, 2240A>G, 2243A>G, 2250G>C, 2251T>C, 2252T>C, 2264G>T								

CDS

	Begin	End	Coverage	Score	Concordance	Matches	Identities	I/D/M/F*	Stop Codons
RdRp	526	644	15.2%	469	59.3%	115 (97.5%)	85 (72.0%)	0/3/1/1	0
Protein mutations:	T533I (1933C>T 1934T>A), K534R (1935A>C 1936A>G), S535D (1938T>G 1939C>A 1940A>T), I538K (1948T>A 1949T>A), L540I (1953T>A 1955A>C), R546K (1972G>A), L549T (1980T>A 1981T>C 1982A>C), A559Q (2010G>C 2011C>A 2012C>A), A561S (2016G>T 2018A>C), I562C (2019A>T 2020T>G), K563R (2022A>C 2023A>G 2024G>A), D565S (2028G>T 2029A>C 2030T>G), N567E (2034A>G 2036C>A), V570T (2043G>A 2044T>C), G575A (2059G>C), A583S (2082G>T 2084G>T), F586M (2091T>A 2093C>G), T587S (2094A>T), K591P (2106A>C 2107A>C 2108G>A), K597_A599del (2124_2133delAAAGCTGCTC), V604G (2146T>G), S606L (2152C>T), L608S (2158T>C), Y611L (2167A>T 2168C>A), W613F (2173G>T 2174G>T), M619K (2191T>A 2192G>A), Y632F (2230A>T 2231C>T), K635R (2239A>G 2240A>G), V639P (2250G>C 2251T>C 2252T>C), Q643H (2264G>T)								
Codon mutations:	GCG528GCT (1919G>T), ACT533ATA (1933C>T 1934T>A), AAA534CGA (1935A>C 1936A>G), TCA535GAT (1938T>G 1939C>A 1940A>T), ATT538AAA (1948T>A 1949T>A), TTA540ATC (1953T>A 1955A>C), CCA542CCC (1961A>C), ATT543ATC (1964T>C), TCC544TCT (1967C>T), CTT545CTC (1970T>C), AGA546AAA (1972G>A), TTA549ACC (1980T>A 1981T>C 1982A>C), TCT550TCC (1985T>C), CGT552AGA (1989C>A 1991T>A), ACT553ACG (1994T>G), GTT555GTA (2000T>A), GGA556GGC (2003A>C), CTT557CTG (2006T>G), GCC559CAA (2010G>C 2011C>A 2012C>A), CTC560TTG (2013C>T 2015C>G), GCA561TCC (2016G>T 2018A>C), ATC562TGC (2019A>T 2020T>G), AAG563CGA (2022A>C 2023A>G 2024G>A), TAT564TAC (2027T>C), GAT565TCG (2028G>T 2029A>C 2030T>G), ATT566ATA (2033T>A), AAC567GAA (2034A>G 2036C>A), TCA569TCG (2042A>G), GTT570ACT (2043G>A 2044T>C), AGA573AGG (2054A>G), TTG574CTA (2055T>C 2057G>A), GGG575GCG (2059G>C), GCC577GCA (2066C>A), GGG578GGT (2069G>T), TAC579TAT (2072C>T), GTA581GTC (2078A>C), GCG583TCT (2082G>T 2084G>T), TTA585CTG (2088T>C 2090A>G), TTC586ATG (2091T>A 2093C>G), ACT587TCT (2094A>T), ACC588ACT (2099C>T), CAA589CAG (2102A>G), AGC590TCA (2103A>T 2104G>C 2105C>A), AAG591CCA (2106A>C 2107A>C 2108G>A), CGT592CGA (2111T>A), GAG594GAA (2117G>A), CGT595AGA (2118C>A 2120T>A), AAA597_GCT599del (2124_2133delAAAGCTGCTC), CAT600-AG (2124_2133delAAAGCTGCTC 2135T>G), CTT601CTC (2138T>C), AAG602AAA (2141G>A), GTT604GGT (2146T>G), CGG605AGG (2148C>A), TCA606TTA (2152C>T), TTA608TCA (2158T>C), TAC611TTA (2167A>T 2168C>A), TGG613TTT (2173G>T 2174G>T), AGG617AGA (2186G>A), ATG619AAA (2191T>A 2192G>A), CCC620CCT (2195C>T), CCC623CCT (2204C>T), TTG625CTA (2208T>C 2210G>A), AAA626AAG (2213A>G), GTA630GTG (2225A>G), TAC632TTT (2230A>T 2231C>T), CTC633CTT (2234C>T), CTT634CTG (2237T>G), AAA635AGG (2239A>G 2240A>G), GAA636GAG (2243A>G), GTT639CCC (2250G>C 2251T>C 2252T>C), CAG643CAT (2264G>T)								

Proteins

	Begin	End	Coverage	Score	Concordance	Matches	Identities	I/D/M/F*	Stop Codons
RNA-dependent RNA polymerase (YP_010798874.1)	526	644	15.2%	469	59.3%	115 (97.5%)	85 (72.0%)	0/3/1/1	0
Protein mutations:	T533I (1933C>T 1934T>A), K534R (1935A>C 1936A>G), S535D (1938T>G 1939C>A 1940A>T), I538K (1948T>A 1949T>A), L540I (1953T>A 1955A>C), R546K (1972G>A), L549T (1980T>A 1981T>C 1982A>C), A559Q (2010G>C 2011C>A 2012C>A), A561S (2016G>T 2018A>C), I562C (2019A>T 2020T>G), K563R (2022A>C 2023A>G 2024G>A), D565S (2028G>T 2029A>C 2030T>G), N567E (2034A>G 2036C>A), V570T (2043G>A 2044T>C), G575A (2059G>C), A583S (2082G>T 2084G>T), F586M (2091T>A 2093C>G), T587S (2094A>T), K591P (2106A>C 2107A>C 2108G>A), K597_A599del (2124_2133delAAAGCTGCTC), V604G (2146T>G), S606L (2152C>T), L608S (2158T>C), Y611L (2167A>T 2168C>A), W613F (2173G>T 2174G>T), M619K (2191T>A 2192G>A), Y632F (2230A>T 2231C>T), K635R (2239A>G 2240A>G), V639P (2250G>C 2251T>C 2252T>C), Q643H (2264G>T)								
Codon mutations:	GCG528GCT (1919G>T), ACT533ATA (1933C>T 1934T>A), AAA534CGA (1935A>C 1936A>G), TCA535GAT (1938T>G 1939C>A 1940A>T), ATT538AAA (1948T>A 1949T>A), TTA540ATC (1953T>A 1955A>C), CCA542CCC (1961A>C), ATT543ATC (1964T>C), TCC544TCT (1967C>T), CTT545CTC (1970T>C), AGA546AAA (1972G>A), TTA549ACC (1980T>A 1981T>C 1982A>C), TCT550TCC (1985T>C), CGT552AGA (1989C>A 1991T>A), ACT553ACG (1994T>G), GTT555GTA (2000T>A), GGA556GGC (2003A>C), CTT557CTG (2006T>G), GCC559CAA (2010G>C 2011C>A 2012C>A), CTC560TTG (2013C>T 2015C>G), GCA561TCC (2016G>T 2018A>C), ATC562TGC (2019A>T 2020T>G), AAG563CGA (2022A>C 2023A>G 2024G>A), TAT564TAC (2027T>C), GAT565TCG (2028G>T 2029A>C 2030T>G), ATT566ATA (2033T>A), AAC567GAA (2034A>G 2036C>A), TCA569TCG (2042A>G), GTT570ACT (2043G>A 2044T>C), AGA573AGG (2054A>G), TTG574CTA (2055T>C 2057G>A), GGG575GCG (2059G>C), GCC577GCA (2066C>A), GGG578GGT (2069G>T), TAC579TAT (2072C>T), GTA581GTC (2078A>C), GCG583TCT (2082G>T 2084G>T), TTA585CTG (2088T>C 2090A>G), TTC586ATG (2091T>A 2093C>G), ACT587TCT (2094A>T), ACC588ACT (2099C>T), CAA589CAG (2102A>G), AGC590TCA (2103A>T 2104G>C 2105C>A), AAG591CCA (2106A>C 2107A>C 2108G>A), CGT592CGA (2111T>A), GAG594GAA (2117G>A), CGT595AGA (2118C>A 2120T>A), AAA597_GCT599del (2124_2133delAAAGCTGCTC), CAT600-AG (2124_2133delAAAGCTGCTC 2135T>G), CTT601CTC (2138T>C), AAG602AAA (2141G>A), GTT604GGT (2146T>G), CGG605AGG (2148C>A), TCA606TTA (2152C>T), TTA608TCA (2158T>C), TAC611TTA (2167A>T 2168C>A), TGG613TTT (2173G>T 2174G>T), AGG617AGA (2186G>A), ATG619AAA (2191T>A 2192G>A), CCC620CCT (2195C>T), CCC623CCT (2204C>T), TTG625CTA (2208T>C 2210G>A), AAA626AAG (2213A>G), GTA630GTG (2225A>G), TAC632TTT (2230A>T 2231C>T), CTC633CTT (2234C>T), CTT634CTG (2237T>G), AAA635AGG (2239A>G 2240A>G), GAA636GAG (2243A>G), GTT639CCC (2250G>C 2251T>C 2252T>C), CAG643CAT (2264G>T)								

*: Inserts / Deletes / Misaligned / Frameshifts

Analysis details

This analysis was performed with panviral2.64

NGS Details (UN62): Escherichia virus DE3

Assembly

**Coverage Length**	226 (1 contig(s))
**Depth Of Coverage**	3.6
**Number Of Reads**	6
**Reads Per Million**	0.14 rpm (after QC)
**Ambiguities**	0
**Assembly Method**	de novo + reference guided assembly
**Consensus Caller**	Bcf Tools

Coverage Map

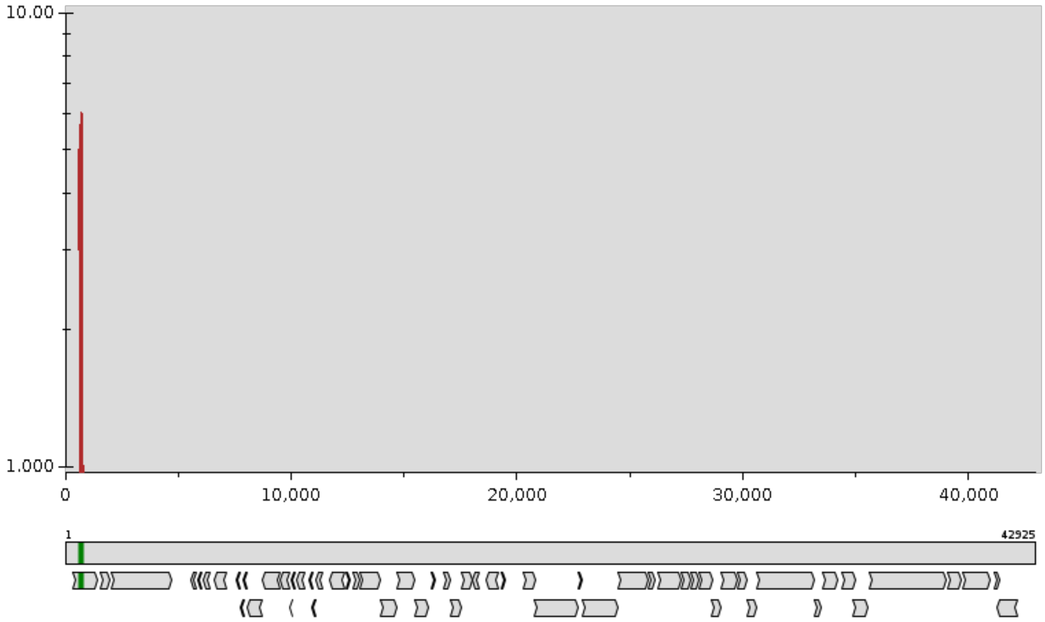

Assignment

**Type**	Escherichia virus DE3 (Taxonomy ID: 2169967)
**Reference Genome**	NC_042057.1
**NT Identity (%)**	100.0
**AA Identity (%)**	100.0
**Number Of Stop Codons**	0
**Number Of CDS**	57

Alignment

**Alignment Score**	452.0 (NT) + 491.0 (AA) = 943.0
**Concordance (%)**	100.0

Alignment Method	Local, heuristic, nucleotide (BLASTN)

Genome Region

Sequence starts at position 607 and ends at position 832 relative to NC_042057.1 reference sequence.

Alignment Detailed Statistics

	Begin	End	Coverage	Score	Concordance	Matches	Identities	I/D/M/F *	Stop Codons
**NT**	**607**	**832**	**0.5%**	**452**	**100%**	**226 (100%)**	**226 (100%)**	**0/0**	
Mutations:	*none*								
**CDS**									
lacI	90	164	20.8%	491	98.4%	75 (100%)	75 (100%)	0/0/0/0	0
**Proteins**									
DNA-binding transcriptional repressor LacI (YP_009617199.1)	90	164	20.8%	491	98.4%	75 (100%)	75 (100%)	0/0/0/0	0
Protein mutations:	*none*								
Codon mutations:	*none*								

*: Inserts / Deletes / Misaligned / Frameshifts

Analysis details

This analysis was performed with panviral2.64

NGS Details (UN62): Potato virus X

Assembly

**Coverage Length**	225 (1 contig(s))
**Depth Of Coverage**	2.4
**Number Of Reads**	4
**Reads Per Million**	0.09 rpm (after QC)
**Ambiguities**	0
**Assembly Method**	de novo + reference guided assembly
**Consensus Caller**	Bcf Tools

Coverage Map

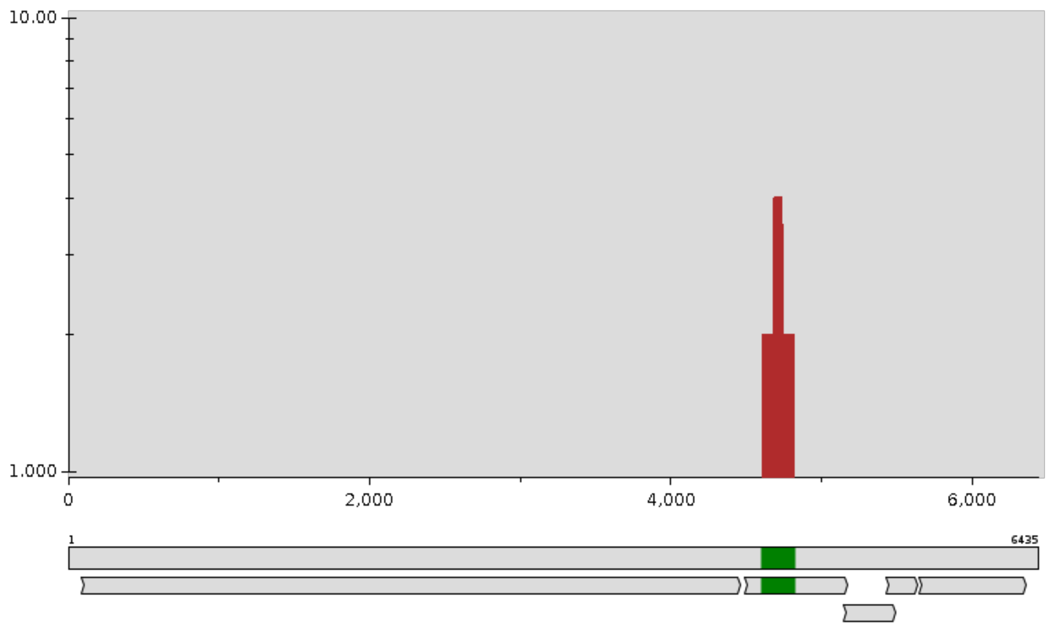

Assignment

**Type**	Potato virus X (Taxonomy ID: 12183)
**Reference Genome**	NC_011620.1
**NT Identity (%)**	73.6607
**AA Identity (%)**	88.0
**Number Of Stop Codons**	0
**Number Of CDS**	5

Alignment

**Alignment Score**	202.0 (NT) + 365.0 (AA) = 567.0
**Concordance (%)**	67.3888

Alignment Method	Global, seeded, nucleotide + amino acids (AGA)

Genome Region

Sequence starts at position 4600 and ends at position 4824 relative to NC_011620.1 reference sequence.

Alignment Detailed Statistics

	Begin	End	Coverage	Score	Concordance	Matches	Identities	I/D/M/F*	Stop Codons
**NT**	**4600**	**4824**	**3.5%**	**202**	**46.1%**	**224 (99.6%)**	**165 (73.3%)**	**0/1**	
Mutations:	4602A>G, 4603A>C, 4605G>T, 4608G>A, 4611G>A, 4612A>C, 4614C>T, 4615C>G, 4616T>C, 4618A>C, 4624C>T, 4629A>T, 4635C>G, 4641T>C, 4644A>C, 4650T>A, 4656T>G, 4662G>A, 4663G>A, 4665G>C, 4668T>C, 4671C>T, 4674A>G, 4677T>A, 4683C>A, 4689G>A, 4690delA, 4695A>C, 4698A>G, 4701T>C, 4710G>A, 4716T>C, 4719C>T, 4722A>C, 4725C>T, 4728C>T, 4731T>C, 4737T>C, 4740T>G, 4741T>C, 4746C>T, 4747A>G, 4748A>C, 4749C>G, 4759A>G, 4761C>A, 4762T>G, 4767C>T, 4770G>A, 4773A>T, 4776T>A, 4782T>A, 4788T>G, 4797A>G, 4800T>G, 4803G>A, 4804T>C, 4809C>T, 4812A>T, 4815G>A								
**CDS**									
PVX_gp2	39	113	33.0%	365	67.7%	75 (100%)	66 (88.0%)	0/0/1/1	0
Protein mutations:	I43L (4612A>C 4614C>T), L44A (4615C>G 4616T>C), P47S (4624C>T), V60I (4663G>A 4665G>C), N88A (4747A>G 4748A>C 4749C>G), N92E (4759A>G 4761C>A), S93A (4762T>G), F107L (4804T>C)								
Codon mutations:	CTA39CTG (4602A>G), AGG40CGT (4603A>C 4605G>T), AAG41AAA (4608G>A), TTG42TTA (4611G>A), ATC43CTT (4612A>C 4614C>T), CTC44GCC (4615C>G 4616T>C), AGA45CGA (4618A>C), CCA47TCA (4624C>T), ACA48ACT (4629A>T), ACC50ACG (4635C>G), CAT52CAC (4641T>C), ACA53ACC (4644A>C), GGT55GGA (4650T>A), CCT57CCG (4656T>G), AAG59AAA (4662G>A), GTG60ATC (4663G>A 4665G>C), AGT61AGC (4668T>C), ATC62ATT (4671C>T), AGA63AGG (4674A>G), ACT64ACA (4677T>A), GGC66GGA (4683C>A), CAG68CAA (4689G>A), AAG69-AG (4690delA), CCA70CCC (4695A>C), GGA71GGG (4698A>G), CCT72CCC (4701T>C), GAG75GAA (4710G>A), AAT77AAC (4716T>C), TTC78TTT (4719C>T), GCA79GCC (4722A>C), ATC80ATT (4725C>T), CTC81CTT (4728C>T), GAT82GAC (4731T>C), TAT84TAC (4737T>C), ACT85ACG (4740T>G), TTG86CTG (4741T>C), GAC87GAT (4746C>T), AAC88GCG (4747A>G 4748A>C 4749C>G), AAC92GAA (4759A>G 4761C>A), TCA93GCA (4762T>G), TAC94TAT (4767C>T), CAG95CAA (4770G>A), GCA96GCT (4773A>T), CTT97CTA (4776T>A), GCT99GCA (4782T>A), CCT101CCG (4788T>G), GCA104GCG (4797A>G), CCT105CCG (4800T>G), GAG106GAA (4803G>A), TTT107CTT (4804T>C), AGC108AGT (4809C>T), CTA109CTT (4812A>T), GAG110GAA (4815G>A)								
**Proteins**									
25K protein (YP_002332930.1)	39	113	33.0%	365	67.7%	75 (100%)	66 (88.0%)	0/0/1/1	0
Protein mutations:	I43L (4612A>C 4614C>T), L44A (4615C>G 4616T>C), P47S (4624C>T), V60I (4663G>A 4665G>C), N88A (4747A>G 4748A>C 4749C>G), N92E (4759A>G 4761C>A), S93A (4762T>G), F107L (4804T>C)								
Codon mutations:	CTA39CTG (4602A>G), AGG40CGT (4603A>C 4605G>T), AAG41AAA (4608G>A), TTG42TTA (4611G>A), ATC43CTT (4612A>C 4614C>T), CTC44GCC (4615C>G 4616T>C), AGA45CGA (4618A>C), CCA47TCA (4624C>T), ACA48ACT (4629A>T), ACC50ACG (4635C>G), CAT52CAC (4641T>C), ACA53ACC (4644A>C), GGT55GGA (4650T>A), CCT57CCG (4656T>G), AAG59AAA (4662G>A), GTG60ATC (4663G>A 4665G>C), AGT61AGC (4668T>C), ATC62ATT (4671C>T), AGA63AGG (4674A>G), ACT64ACA (4677T>A), GGC66GGA (4683C>A), CAG68CAA (4689G>A), AAG69-AG (4690delA), CCA70CCC (4695A>C), GGA71GGG (4698A>G), CCT72CCC (4701T>C), GAG75GAA (4710G>A), AAT77AAC (4716T>C), TTC78TTT (4719C>T), GCA79GCC (4722A>C), ATC80ATT (4725C>T), CTC81CTT (4728C>T), GAT82GAC (4731T>C), TAT84TAC (4737T>C), ACT85ACG (4740T>G), TTG86CTG (4741T>C), GAC87GAT (4746C>T), AAC88GCG (4747A>G 4748A>C 4749C>G), AAC92GAA (4759A>G 4761C>A), TCA93GCA (4762T>G), TAC94TAT (4767C>T), CAG95CAA (4770G>A), GCA96GCT (4773A>T), CTT97CTA (4776T>A), GCT99GCA (4782T>A), CCT101CCG (4788T>G), GCA104GCG (4797A>G), CCT105CCG (4800T>G), GAG106GAA (4803G>A), TTT107CTT (4804T>C), AGC108AGT (4809C>T), CTA109CTT (4812A>T), GAG110GAA (4815G>A)								

*: Inserts / Deletes / Misaligned / Frameshifts

Analysis details

This analysis was performed with panviral2.64

NGS Details (UN62): Pandoravirus salinus

Assembly

**Coverage Length**	122 (1 contig(s))
**Depth Of Coverage**	2.7
**Number Of Reads**	4
**Reads Per Million**	0.09 rpm (after QC)
**Ambiguities**	0
**Assembly Method**	de novo + reference guided assembly
**Consensus Caller**	Bcf Tools

Coverage Map

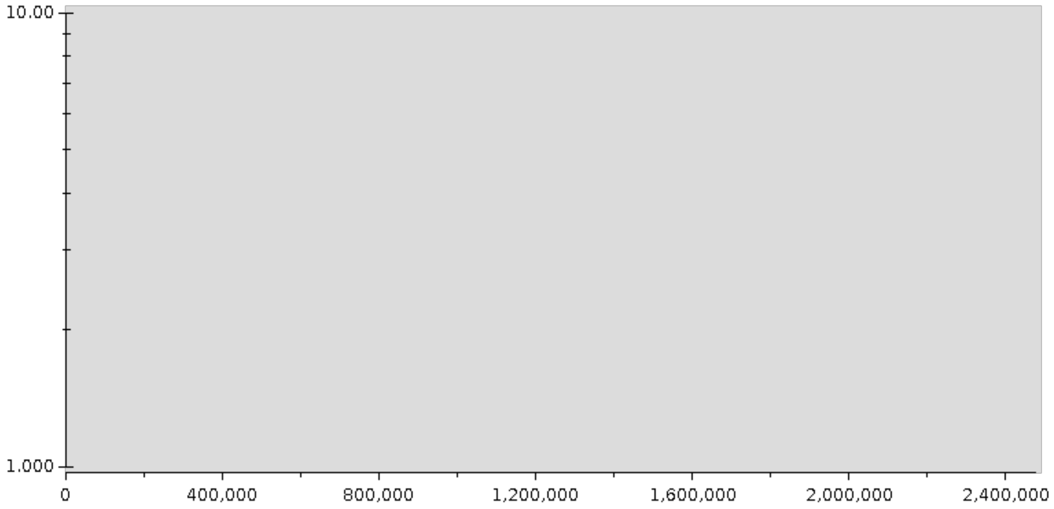

Assignment

**Type**	Pandoravirus salinus (Taxonomy ID: 1349410)
**Reference Genome**	NC_022098.1
**NT Identity (%)**	75.4098
**AA Identity (%)**	80.4878
**Number Of Stop Codons**	0
**Number Of CDS**	1430

Alignment

**Alignment Score**	124.0 (NT) + 272.0 (AA) = 396.0
**Concordance (%)**	71.7391
**Alignment Method**	Local, heuristic, nucleotide (BLASTN)

Genome Region

Sequence starts at position 796297 and ends at position 796418 relative to NC_022098.1 reference sequence.

Alignment Detailed Statistics

	Begin	End	Coverage	Score	Concordance	Matches	Identities	I/D/M/F*	Stop Codons
**NT**	**796297**	**796418**	**0.1%**	**124**	**50.8%**	**122 (100%)**	**92 (75.4%)**	**0/0**	

Mutations: 796299G>A, 796302G>A, 796304C>T, 796305C>T, 796308C>T, 796314G>A, 796320A>G, 796323G>A, 796326G>A, 796329C>T, 796338T>C, 796347C>T, 796349C>G, 796353T>G, 796355T>C, 796356C>A, 796357C>T, 796358G>C, 796359T>A, 796365G>C, 796366C>T, 796367G>T, 796377C>G, 796380A>C, 796383A>G, 796389A>G, 796401G>T, 796402C>T, 796404T>G, 796410C>A

*: Inserts / Deletes / Misaligned / Frameshifts

Analysis details

This analysis was performed with panviral2.64

NGS Details (UN62): Dioscorea bacilliform virus

Assembly

**Coverage Length**	315 (1 contig(s))
**Depth Of Coverage**	1.7
**Number Of Reads**	4
**Reads Per Million**	0.09 rpm (after QC)
**Ambiguities**	0
**Assembly Method**	de novo + reference guided assembly
**Consensus Caller**	Bcf Tools

Coverage Map

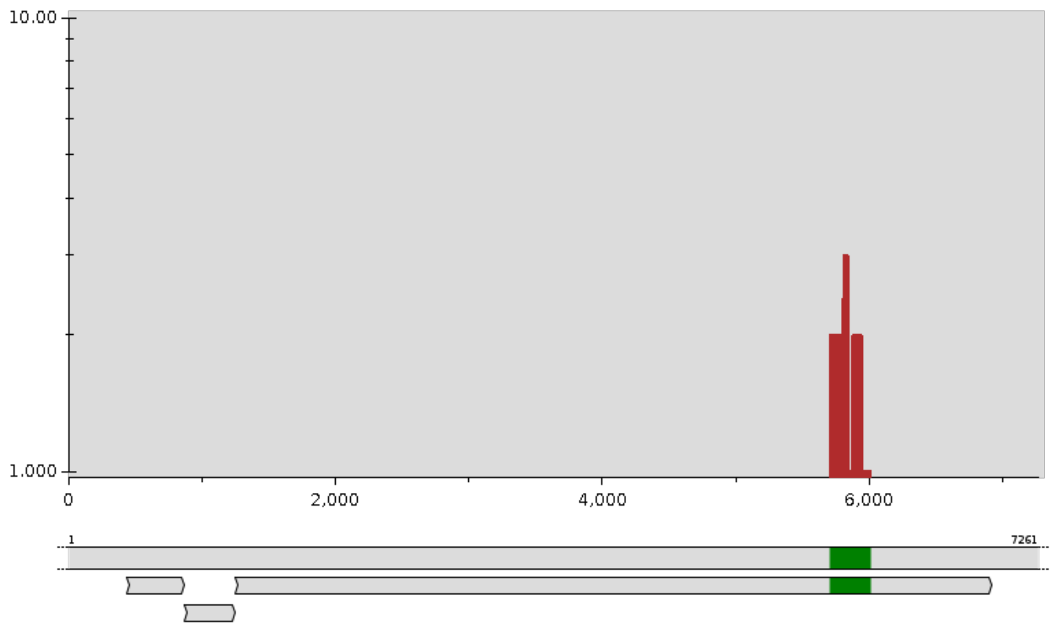

Assignment

**Type**	Dioscorea bacilliform virus (Taxonomy ID: 52996)
**Reference Genome**	NC_009010.1
**NT Identity (%)**	52.9412
**AA Identity (%)**	47.0588
**Number Of Stop Codons**	2
**Number Of CDS**	3

Alignment

**Alignment Score**	0.0 (NT) + 313.0 (AA) = 313.0
**Concordance (%)**	24.6457

Alignment Method	Global, seeded, nucleotide + amino acids (AGA)

Genome Region

Sequence starts at position 5702 and ends at position 6016 relative to NC_009010.1 reference sequence.

Alignment Detailed Statistics

	Begin	End	Coverage	Score	Concordance	Matches	Identities	I/D/M/F*	Stop Codons
**NT**	**5702**	**6016**	**4.3%**	**0**	**0%**	**306 (97.1%)**	**162 (51.4%)**	**0/9**	
Mutations:	5702A>C, 5705G>C, 5706A>T, 5707G>T, 5709C>A, 5710G>T, 5712T>C, 5713C>T, 5715A>C, 5716A>T, 5717G>A, 5719A>G, 5721A>T, 5722_5724delATA, 5726A>T, 5729C>T, 5730A>C, 5734T>A, 5740T>C, 5742A>C, 5745G>A, 5746A>T, 5747G>C, 5749G>A, 5750G>A, 5751C>A, 5755C>T, 5756A>G, 5757T>G, 5761G>A, 5763G>A, 5764G>C, 5765C>A, 5769G>T, 5770G>A, 5771C>A, 5772G>C, 5775A>C, 5776C>A, 5777A>G, 5779T>G, 5780C>A, 5781A>T, 5782G>T, 5783T>G, 5784T>C, 5785G>T, 5787A>T, 5788T>A, 5789G>A, 5790G>A, 5793G>T, 5794G>T, 5795C>T, 5796G>T, 5800C>A, 5801T>C, 5802A>G, 5804C>T, 5805G>A, 5809G>T, 5810G>T, 5811A>T, 5816T>A, 5817T>G, 5820C>T, 5826G>A, 5827T>A, 5828T>A, 5829G>T, 5830G>T, 5832G>T, 5838A>C, 5841C>T, 5844A>G, 5847G>A, 5850G>A, 5853T>C, 5854G>A, 5856A>T, 5859A>T, 5860G>T, 5861C>T, 5862A>T, 5864T>A, 5865G>A, 5868C>T, 5871G>A, 5873G>A, 5874G>A, 5876A>T, 5877G>A, 5881G>A, 5883C>T, 5884A>G, 5885A>T, 5886C>T, 5887G>A, 5889C>T, 5892C>T, 5893C>A, 5894G>A, 5895A>T, 5896G>C, 5897G>C, 5899A>T, 5900C>T, 5902G>T, 5903A>C, 5905G>A, 5910T>C, 5914G>A, 5915C>T, 5916T>A, 5919A>C, 5922C>T, 5932A>G, 5934A>T, 5937G>A, 5940G>A, 5947G>A, 5948A>C, 5949A>T, 5952A>C, 5953G>A, 5954A>T, 5955A>T, 5958G>C, 5959G>C, 5967G>C, 5970G>A, 5976G>T, 5978G>A, 5981T>G, 5982A>T, 5985G>T, 5992_5994delATC, 5998C>T, 6000A>T, 6004_6006delCAC, 6009G>A, 6010C>A, 6011T>A, 6012T>A, 6013G>T, 6014T>G, 6015T>A								

CDS

	Begin	End	Coverage	Score	Concordance	Matches	Identities	I/D/M/F*	Stop Codons
DBV_gp3	1487	1591	5.6%	313	44.6%	102 (97.1%)	48 (45.7%)	0/3/0/0	2
Protein mutations:	R1487T (5705G>C 5706A>T), V1488L (5707G>T 5709C>A), G1489C (5710G>T 5712T>C), Q1490Y (5713C>T 5715A>C), S1491Y (5716A>T 5717G>A), K1492D (5719A>G 5721A>T), I1493del (5722_5724delATA), Y1494F (5726A>T), S1495F (5729C>T 5730A>C), F1497I (5734T>A), G1502K (5749G>A 5750G>A 5751C>A), H1504W (5755C>T 5756A>G 5757T>G), V1506I (5761G>A 5763G>A), A1507Q (5764G>C 5765C>A), M1508I (5769G>T), A1509N (5770G>A 5771C>A 5772G>C), Q1511R (5776C>A 5777A>G), S1512D (5779T>G 5780C>A 5781A>T), V1513C (5782G>T 5783T>G 5784T>C), E1514Y (5785G>T 5787A>T), W1515K (5788T>A 5789G>A 5790G>A), A1517F (5794G>T 5795C>T 5796G>T), L1519T (5800C>A 5801T>C 5802A>G), A1520V (5804C>T 5805G>A), G1522F (5809G>T 5810G>T 5811A>T), L1524Q (5816T>A 5817T>G), W1527* (5826G>A), L1528N (5827T>A 5828T>A 5829G>T), V1529F (5830G>T 5832G>T), A1537T (5854G>A 5856A>T), A1539F (5860G>T 5861C>T 5862A>T), V1540E (5864T>A 5865G>A), R1543K (5873G>A 5874G>A), K1544I (5876A>T 5877G>A), D1546N (5881G>A 5883C>T), N1547V (5884A>G 5885A>T 5886C>T), V1548I (5887G>A 5889C>T), R1550N (5893C>A 5894G>A 5895A>T), G1551P (5896G>C 5897G>C), T1552F (5899A>T 5900C>T), E1553S (5902G>T 5903A>C), E1554K (5905G>A), A1557I (5914G>A 5915C>T 5916T>A), I1563V (5932A>G 5934A>T), E1568T (5947G>A 5948A>C 5949A>T), E1570I (5953G>A 5954A>T 5955A>T), E1571D (5958G>C), E1572Q (5959G>C), L1574F (5967G>C), R1578K (5978G>A), I1579S (5981T>G 5982A>T), I1583del (5992_5994delATC), Q1585Y (5998C>T 6000A>T), H1587del (6004_6006delCAC), L1589K (6010C>A 6011T>A 6012T>A), V1590* (6013G>T 6014T>G 6015T>A)								
Codon mutations:	AAG1486.CG (5702A>C), AGA1487ACT (5705G>C 5706A>T), GTC1488TTA (5707G>T 5709C>A), GGT1489TGC (5710G>T 5712T>C), CAA1490TAC (5713C>T 5715A>C), AGC1491TAC (5716A>T 5717G>A), AAA1492GAT (5719A>G 5721A>T), ATA1493del (5722_5724delATA), TAT1494TTT (5726A>T), TCA1495TTC (5729C>T 5730A>C), TTT1497ATT (5734T>A), TTA1499CTC (5740T>C 5742A>C), AAG1500AAA (5745G>A), AGC1501TCC (5746A>T 5747G>C), GGC1502AAA (5749G>A 5750G>A 5751C>A), CAT1504TGG (5755C>T 5756A>G 5757T>G), GTG1506ATA (5761G>A 5763G>A), GCA1507CAA (5764G>C 5765C>A), ATG1508ATT (5769G>T), GCG1509AAC (5770G>A 5771C>A 5772G>C), CCA1510CCC (5775A>C), CAA1511AGA (5776C>A 5777A>G), TCA1512GAT (5779T>G 5780C>A 5781A>T), GTT1513TGC (5782G>T 5783T>G 5784T>C), GAA1514TAT (5785G>T 5787A>T), TGG1515AAA (5788T>A 5789G>A 5790G>A), ACG1516ACT (5793G>T), GCG1517TTT (5794G>T 5795C>T 5796G>T), CTA1519ACG (5800C>A 5801T>C 5802A>G), GCG1520GTA (5804C>T 5805G>A), GGA1522TTT (5809G>T 5810G>T 5811A>T), CTT1524CAG (5816T>A 5817T>G), TAC1525TAT (5820C>T), TGG1527TGA (5826G>A), TTG1528AAT (5827T>A 5828T>A 5829G>T), GTG1529TTT (5830G>T 5832G>T), CCA1531CCC (5838A>C), TTC1532TTT (5841C>T), GGA1533GGG (5844A>G), CTG1534CTA (5847G>A), AAG1535AAA (5850G>A), AAT1536AAC (5853T>C), GCA1537ACT (5854G>A 5856A>T), CCA1538CCT (5859A>T), GCA1539TTT (5860G>T 5861C>T 5862A>T), GTG1540GAA (5864T>A 5865G>A), TTC1541TTT (5868C>T), CAG1542CAA (5871G>A), AGG1543AAA (5873G>A 5874G>A), AAG1544ATA (5876A>T 5877G>A), GAC1546AAT (5881G>A 5883C>T), AAC1547GTT (5884A>G 5885A>T 5886C>T), GTC1548ATT (5887G>A 5889C>T), TTC1549TTT (5892C>T), CGA1550AAT (5893C>A 5894G>A 5895A>T), GGT1551CCT (5896G>C 5897G>C), ACT1552TTT (5899A>T 5900C>T), GAA1553TCA (5902G>T 5903A>C), GAA1554AAA (5905G>A), TTT1555TTC (5910T>C), GCT1557ATA (5914G>A 5915C>T 5916T>A), GTA1558GTC (5919A>C), TAC1559TAT (5922C>T), ATA1563GTT (5932A>G 5934A>T), CTG1564CTA (5937G>A), GTG1565GTA (5940G>A), GAA1568ACT (5947G>A 5948A>C 5949A>T), ACA1569ACC (5952A>C), GAA1570ATT (5953G>A 5954A>T 5955A>T), GAG1571GAC (5958G>C), GAG1572CAG (5959G>C), TTG1574TTC (5967G>C), AAG1575AAA (5970G>A), CTG1577CTT (5976G>T), AGG1578AAG (5978G>A), ATA1579AGT (5981T>G 5982A>T), CTG1580CTT (5985G>T), ATC1583del (5992_5994delATC), CAA1585TAT (5998C>T 6000A>T), CAC1587del (6004_6006delCAC), GGG1588GGA (6009G>A), CTT1589AAA (6010C>A 6011T>A 6012T>A), GTT1590TGA (6013G>T 6014T>G 6015T>A)								

Proteins

	Begin	End	Coverage	Score	Concordance	Matches	Identities	I/D/M/F*	Stop Codons
ORF3 protein (YP_001036293.1)	1487	1591	5.6%	313	44.6%	102 (97.1%)	48 (45.7%)	0/3/0/0	2
Protein mutations:	R1487T (5705G>C 5706A>T), V1488L (5707G>T 5709C>A), G1489C (5710G>T 5712T>C), Q1490Y (5713C>T 5715A>C), S1491Y (5716A>T 5717G>A), K1492D (5719A>G 5721A>T), I1493del (5722_5724delATA), Y1494F (5726A>T), S1495F (5729C>T 5730A>C), F1497I (5734T>A), G1502K (5749G>A 5750G>A 5751C>A), H1504W (5755C>T 5756A>G 5757T>G), V1506I (5761G>A 5763G>A), A1507Q (5764G>C 5765C>A), M1508I (5769G>T), A1509N (5770G>A 5771C>A 5772G>C), Q1511R (5776C>A 5777A>G), S1512D (5779T>G 5780C>A 5781A>T), V1513C (5782G>T 5783T>G 5784T>C), E1514Y (5785G>T 5787A>T), W1515K (5788T>A 5789G>A 5790G>A), A1517F (5794G>T 5795C>T 5796G>T), L1519T (5800C>A 5801T>C 5802A>G), A1520V (5804C>T 5805G>A), G1522F (5809G>T 5810G>T 5811A>T), L1524Q (5816T>A 5817T>G), W1527* (5826G>A), L1528N (5827T>A 5828T>A 5829G>T), V1529F (5830G>T 5832G>T), A1537T (5854G>A 5856A>T), A1539F (5860G>T 5861C>T 5862A>T), V1540E (5864T>A 5865G>A), R1543K (5873G>A 5874G>A), K1544I (5876A>T 5877G>A), D1546N (5881G>A 5883C>T), N1547V (5884A>G 5885A>T 5886C>T), V1548I (5887G>A 5889C>T), R1550N (5893C>A 5894G>A 5895A>T), G1551P (5896G>C 5897G>C), T1552F (5899A>T 5900C>T), E1553S (5902G>T 5903A>C), E1554K (5905G>A), A1557I (5914G>A 5915C>T 5916T>A), I1563V (5932A>G 5934A>T), E1568T (5947G>A 5948A>C 5949A>T), E1570I (5953G>A 5954A>T 5955A>T), E1571D (5958G>C), E1572Q (5959G>C), L1574F (5967G>C), R1578K (5978G>A), I1579S (5981T>G 5982A>T), I1583del (5992_5994delATC), Q1585Y (5998C>T 6000A>T), H1587del (6004_6006delCAC), L1589K (6010C>A 6011T>A 6012T>A), V1590* (6013G>T 6014T>G 6015T>A)								
Codon mutations:	AAG1486.CG (5702A>C), AGA1487ACT (5705G>C 5706A>T), GTC1488TTA (5707G>T 5709C>A), GGT1489TGC (5710G>T 5712T>C), CAA1490TAC (5713C>T 5715A>C), AGC1491TAC (5716A>T 5717G>A), AAA1492GAT (5719A>G 5721A>T), ATA1493del (5722_5724delATA), TAT1494TTT (5726A>T), TCA1495TTC (5729C>T 5730A>C), TTT1497ATT (5734T>A), TTA1499CTC (5740T>C 5742A>C), AAG1500AAA (5745G>A), AGC1501TCC (5746A>T 5747G>C), GGC1502AAA (5749G>A 5750G>A 5751C>A), CAT1504TGG (5755C>T 5756A>G 5757T>G), GTG1506ATA (5761G>A 5763G>A), GCA1507CAA (5764G>C 5765C>A), ATG1508ATT (5769G>T), GCG1509AAC (5770G>A 5771C>A 5772G>C), CCA1510CCC (5775A>C), CAA1511AGA (5776C>A 5777A>G), TCA1512GAT (5779T>G 5780C>A 5781A>T), GTT1513TGC (5782G>T 5783T>G 5784T>C), GAA1514TAT (5785G>T 5787A>T), TGG1515AAA (5788T>A 5789G>A 5790G>A), ACG1516ACT (5793G>T), GCG1517TTT (5794G>T 5795C>T 5796G>T), CTA1519ACG (5800C>A 5801T>C 5802A>G), GCG1520GTA (5804C>T 5805G>A), GGA1522TTT (5809G>T 5810G>T 5811A>T), CTT1524CAG (5816T>A 5817T>G), TAC1525TAT (5820C>T), TGG1527TGA (5826G>A), TTG1528AAT (5827T>A 5828T>A 5829G>T), GTG1529TTT (5830G>T 5832G>T), CCA1531CCC (5838A>C), TTC1532TTT (5841C>T), GGA1533GGG (5844A>G), CTG1534CTA (5847G>A), AAG1535AAA (5850G>A), AAT1536AAC (5853T>C), GCA1537ACT (5854G>A 5856A>T), CCA1538CCT (5859A>T), GCA1539TTT (5860G>T 5861C>T 5862A>T), GTG1540GAA (5864T>A 5865G>A), TTC1541TTT (5868C>T), CAG1542CAA (5871G>A), AGG1543AAA (5873G>A 5874G>A), AAG1544ATA (5876A>T 5877G>A), GAC1546AAT (5881G>A 5883C>T), AAC1547GTT (5884A>G 5885A>T 5886C>T), GTC1548ATT (5887G>A 5889C>T), TTC1549TTT (5892C>T), CGA1550AAT (5893C>A 5894G>A 5895A>T), GGT1551CCT (5896G>C 5897G>C), ACT1552TTT (5899A>T 5900C>T), GAA1553TCA (5902G>T 5903A>C), GAA1554AAA (5905G>A), TTT1555TTC (5910T>C), GCT1557ATA (5914G>A 5915C>T 5916T>A), GTA1558GTC (5919A>C), TAC1559TAT (5922C>T), ATA1563GTT (5932A>G 5934A>T), CTG1564CTA (5937G>A), GTG1565GTA (5940G>A), GAA1568ACT (5947G>A 5948A>C 5949A>T), ACA1569ACC (5952A>C), GAA1570ATT (5953G>A 5954A>T 5955A>T), GAG1571GAC (5958G>C), GAG1572CAG (5959G>C), TTG1574TTC (5967G>C), AAG1575AAA (5970G>A), CTG1577CTT (5976G>T), AGG1578AAG (5978G>A), ATA1579AGT (5981T>G 5982A>T), CTG1580CTT (5985G>T), ATC1583del (5992_5994delATC), CAA1585TAT (5998C>T 6000A>T), CAC1587del (6004_6006delCAC), GGG1588GGA (6009G>A), CTT1589AAA (6010C>A 6011T>A 6012T>A), GTT1590TGA (6013G>T 6014T>G 6015T>A)								

*: Inserts / Deletes / Misaligned / Frameshifts

Analysis details

This analysis was performed with panviral2.64

NGS Details (UN62): Rhizobium phage RHEph10

Assembly

**Coverage Length**	135 (1 contig(s))
**Depth Of Coverage**	2.9
**Number Of Reads**	3
**Reads Per Million**	0.07 rpm (after QC)
**Ambiguities**	0
**Assembly Method**	de novo + reference guided assembly
**Consensus Caller**	Bcf Tools

Coverage Map

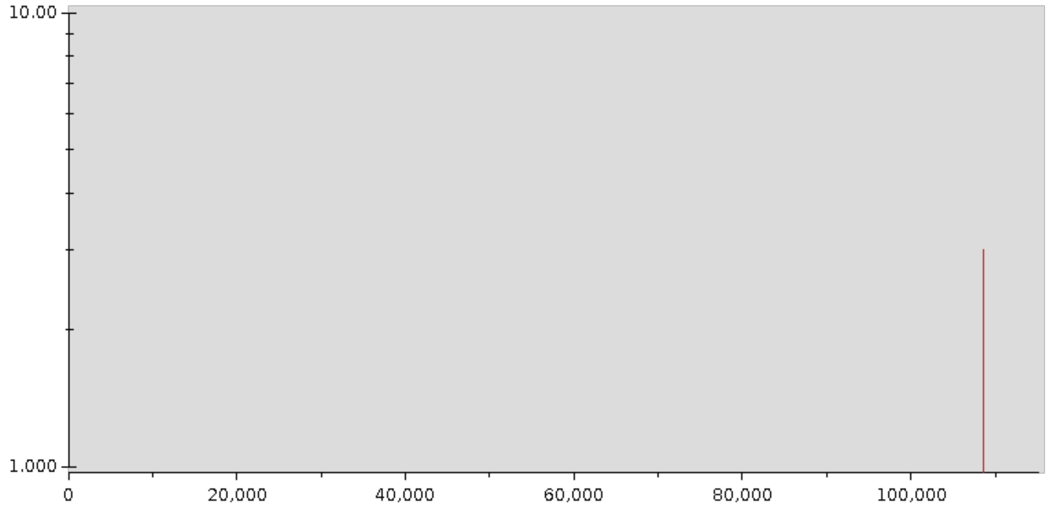

Assignment

**Type**	Rhizobium phage RHEph10 (Taxonomy ID: 1220717)
**Reference Genome**	NC_034248.1
**NT Identity (%)**	80.0
**AA Identity (%)**	82.2222
**Number Of Stop Codons**	0
**Number Of CDS**	171

Alignment

**Alignment Score**	162.0 (NT) + 249.0 (AA) = 411.0
**Concordance (%)**	74.0541
**Alignment Method**	Local, heuristic, nucleotide (BLASTN)

Genome Region

Sequence starts at position 108586 and ends at position 108720 relative to NC_034248.1 reference sequence.

Alignment Detailed Statistics

	Begin	End	Coverage	Score	Concordance	Matches	Identities	I/D/M/F*	Stop Codons
**NT**	**108586**	**108720**	**0.1%**	**162**	**60.0%**	**135 (100%)**	**108 (80.0%)**	**0/0**	

Mutations: 108589T>C, 108602T>G, 108603C>G, 108604G>T, 108610C>G, 108613C>G, 108625T>C, 108628T>C, 108631T>C, 108632C>T, 108634G>C, 108635A>C, 108636A>G, 108637G>C, 108643A>G, 108647G>C, 108649C>G, 108650A>G, 108652G>C, 108656A>G, 108657C>G, 108660A>G, 108664C>T, 108670C>G, 108676C>G, 108703C>T, 108712A>C

*: Inserts / Deletes / Misaligned / Frameshifts

Analysis details

This analysis was performed with panviral2.64

NGS Details (UN62): Melanoplus sanguinipes entomopoxvirus

Assembly

**Coverage Length**	135 (1 contig(s))
**Depth Of Coverage**	2.0
**Number Of Reads**	2
**Reads Per Million**	0.05 rpm (after QC)
**Ambiguities**	0
**Assembly Method**	de novo + reference guided assembly
**Consensus Caller**	Bcf Tools

Coverage Map

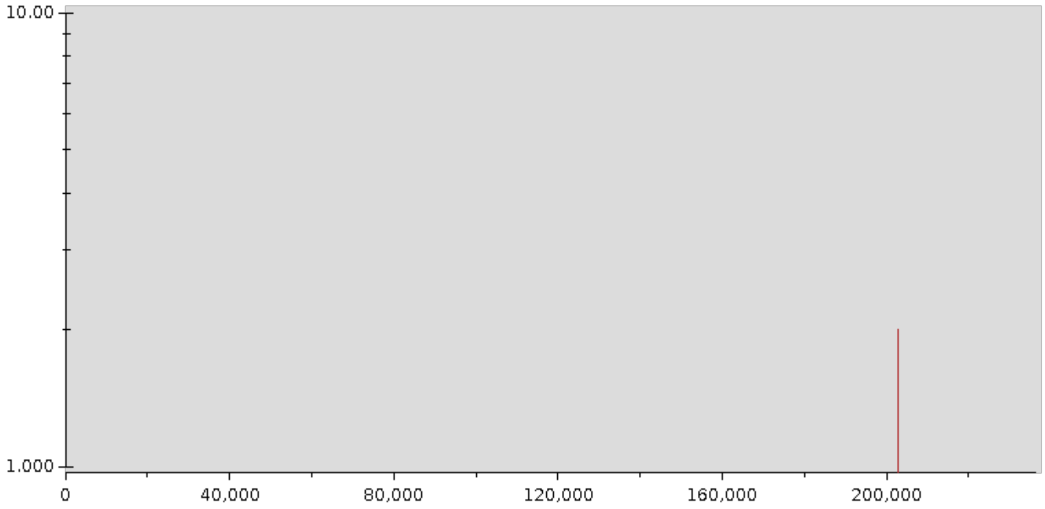

Assignment

**Type**	Melanoplus sanguinipes entomopoxvirus (Taxonomy ID: 83191)
**Reference Genome**	NC_001993.1
**NT Identity (%)**	80.0
**AA Identity (%)**	80.0
**Number Of Stop Codons**	0
**Number Of CDS**	267

Alignment

**Alignment Score**	162.0 (NT) + 334.0 (AA) = 496.0
**Concordance (%)**	76.1905
**Alignment Method**	Local, heuristic, nucleotide (BLASTN)

Genome Region

Sequence starts at position 202763 and ends at position 202897 relative to NC_001993.1 reference sequence.

Alignment Detailed Statistics

	Begin	End	Coverage	Score	Concordance	Matches	Identities	I/D/M/F*	Stop Codons
**NT**	**202763**	**202897**	**0.1%**	**162**	**60.0%**	**135 (100%)**	**108 (80.0%)**	**0/0**	

Mutations: 202768T>C, 202771A>T, 202773G>T, 202774T>C, 202775C>T, 202777A>G, 202789A>G, 202790C>T, 202798T>C, 202807A>T, 202813T>A, 202817G>T, 202818G>C, 202826T>C, 202831T>C, 202832G>C, 202834T>C, 202835C>T, 202836T>G, 202852A>T, 202874T>A, 202876T>A, 202880T>C, 202885A>G, 202888A>G, 202891T>A, 202894A>G

*: Inserts / Deletes / Misaligned / Frameshifts

Analysis details

This analysis was performed with panviral2.64

NGS Details (UN62): Wadgaonvirus wv5004651

Assembly

**Coverage Length**	189 (1 contig(s))
**Depth Of Coverage**	1.4
**Number Of Reads**	2
**Reads Per Million**	0.05 rpm (after QC)
**Ambiguities**	0
**Assembly Method**	de novo + reference guided assembly
**Consensus Caller**	Bcf Tools

Coverage Map

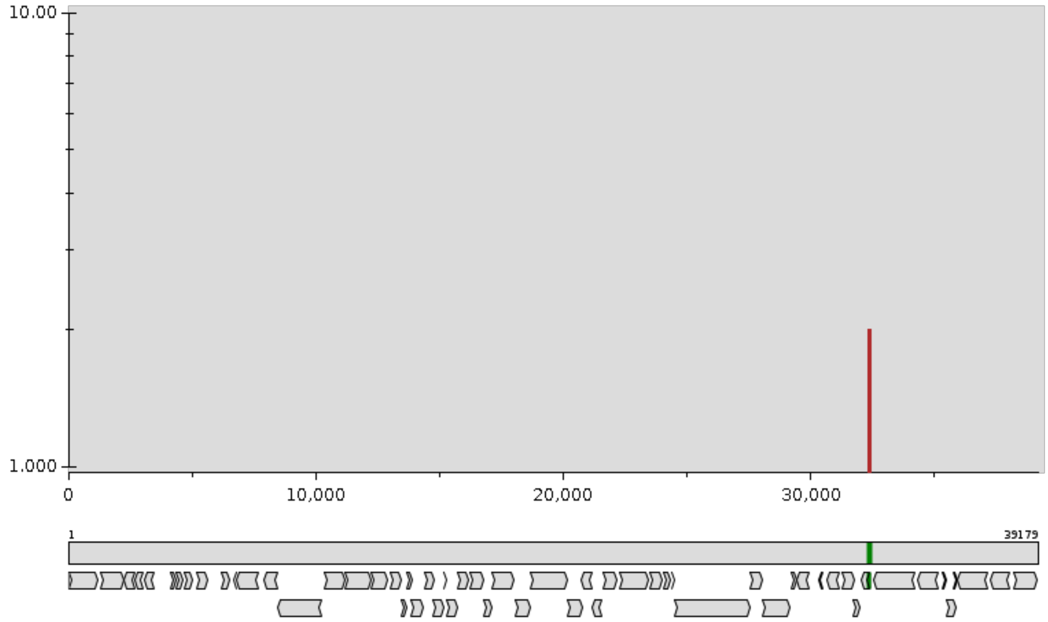

Assignment

**Type**	Wadgaonvirus wv5004651 (Taxonomy ID: 2956672)
**Reference Genome**	NC_049342.1
**NT Identity (%)**	100.0
**AA Identity (%)**	100.0
**Number Of Stop Codons**	0
**Number Of CDS**	57

Alignment

**Alignment Score**	378.0 (NT) + 256.0 (AA) = 634.0
**Concordance (%)**	100.0

Alignment Method	Local, heuristic, nucleotide (BLASTN)

Genome Region

Sequence starts at position 32293 and ends at position 32481 relative to NC_049342.1 reference sequence.

Alignment Detailed Statistics

	Begin	End	Coverage	Score	Concordance	Matches	Identities	I/D/M/F *	Stop Codons
**NT**	**32293**	**32481**	**0.5%**	**378**	**100%**	**189 (100%)**	**189 (100%)**	**0/0**	
Mutations:	*none*								
**CDS**									
HYO67_gp49	1	40	35.1%	256	100%	40 (100%)	40 (100%)	0/0/0/0	0
**Proteins**									
outer membrane protein assembly factor BamE (YP_009949248.1)	1	40	35.1%	256	100%	40 (100%)	40 (100%)	0/0/0/0	0
Protein mutations:	*none*								
Codon mutations:	*none*								

*: Inserts / Deletes / Misaligned / Frameshifts

Analysis details

This analysis was performed with panviral2.64

NGS Details (UN62): Enterobacteria phage P7

Assembly

**Coverage Length**	172 (1 contig(s))
**Depth Of Coverage**	1.6
**Number Of Reads**	2
**Reads Per Million**	0.05 rpm (after QC)
**Ambiguities**	0
**Assembly Method**	de novo + reference guided assembly
**Consensus Caller**	Bcf Tools

Coverage Map

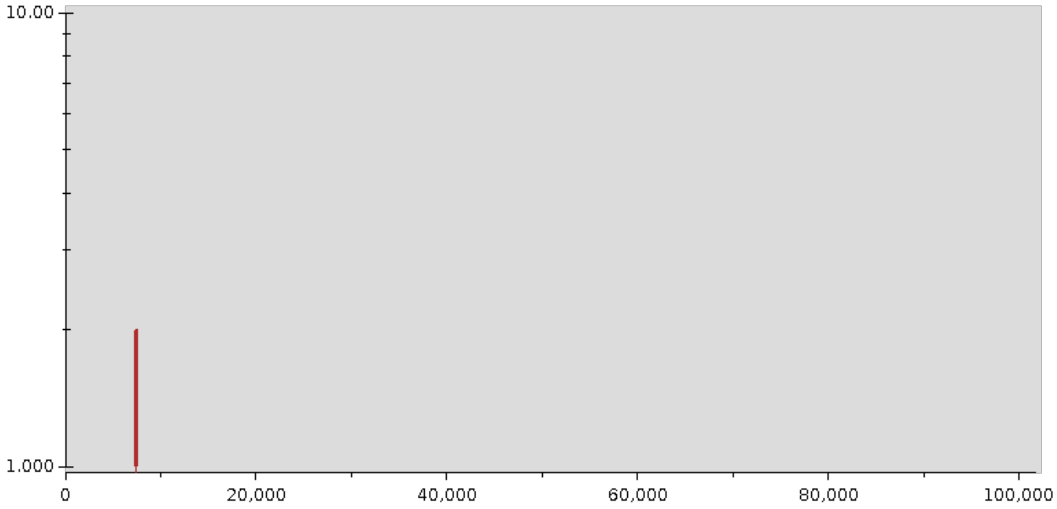

Assignment

**Type**	Enterobacteria phage P7 (Taxonomy ID: 10682)
**Reference Genome**	NC_050152.1
**NT Identity (%)**	100.0
**AA Identity (%)**	100.0
**Number Of Stop Codons**	1
**Number Of CDS**	113

Alignment

**Alignment Score**	344.0 (NT) + 119.0 (AA) = 463.0
**Concordance (%)**	100.0
**Alignment Method**	Local, heuristic, nucleotide (BLASTN)

Genome Region

Sequence starts at position 7371 and ends at position 7542 relative to NC_050152.1 reference sequence.

Alignment Detailed Statistics

	Begin	End	Coverage	Score	Concordance	Matches	Identities	I/D/M/F*	Stop Codons
NT	7371	7542	0.2%	344	100%	172 (100%)	172 (100%)	0/0	

Mutations: *none*

*: Inserts / Deletes / Misaligned / Frameshifts

Analysis details

This analysis was performed with panviral2.64

NGS Details (UN62): Orthomarburgvirus marburgense

Assembly

**Coverage Length**	270 (2 contig(s))
**Depth Of Coverage**	1.0
**Number Of Reads**	2
**Reads Per Million**	0.05 rpm (after QC)
**Ambiguities**	0
**Assembly Method**	de novo + reference guided assembly
**Consensus Caller**	Bcf Tools

Coverage Map

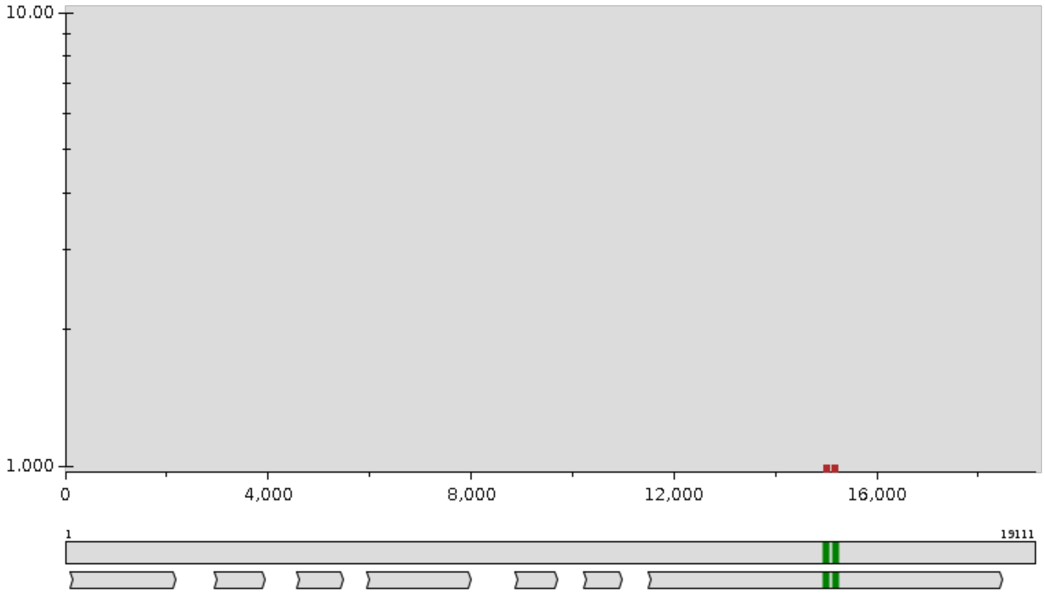

Assignment

**Type**	Orthomarburgvirus marburgense (Taxonomy ID: 11269)
**Reference Genome**	NC_001608.3
**NT Identity (%)**	70.3704
**AA Identity (%)**	98.8889
**Number Of Stop Codons**	0
**Number Of CDS**	7

Alignment

**Alignment Score**	220.0 (NT) + 601.0 (AA) = 821.0
**Concordance (%)**	71.9544

Alignment Method	Global, seeded, nucleotide + amino acids (AGA)

Genome Region

Sequence starts at position 14942 and ends at position 15245 relative to NC_001608.3 reference sequence.

Alignment Detailed Statistics

	Begin	End	Coverage	Score	Concordance	Matches	Identities	I/D/M/F*	Stop Codons
**NT**	**14942**	**15245**	**1.4%**	**220**	**40.7%**	**270 (100%)**	**190 (70.4%)**	**0/0**	
Mutations:	14945A>G, 14948A>G, 14957A>G, 14958T>A, 14959C>G, 14960T>C, 14963A>C, 14966C>T, 14969A>C, 14972A>G, 14973T>A, 14974C>G, 14975A>C, 14978T>C, 14981A>C, 14984C>T, 14985T>C, 14988T>C, 14993G>C, 14996T>C, 14999C>T, 15008A>G, 15012C>A, 15014A>G, 15017T>G, 15020A>G, 15023A>C, 15029T>C, 15035A>C, 15039T>C, 15041A>G, 15047T>C, 15050C>G, 15053T>C, 15056T>C, 15059T>C, 15062T>G, 15066C>A, 15071A>G, 15074T>G, 15113G>C, 15116A>C, 15119A>C, 15122T>C, 15123C>A, 15125A>G, 15128A>C, 15131G>C, 15134T>C, 15137T>C, 15143A>C, 15144C>A, 15146G>A, 15149A>C, 15152A>G, 15155T>C, 15164T>C, 15167T>C, 15173C>T, 15174T>C, 15176A>G, 15182A>G, 15188C>T, 15191A>T, 15192T>A, 15193C>G, 15194A>C, 15197A>C, 15200A>C, 15203T>G, 15206A>G, 15209A>G, 15212T>C, 15215T>C, 15224T>G, 15227T>C, 15230A>G, 15231T>C, 15234T>C, 15236A>G								
**CDS**									
L	1155	1255	3.9%	601	97.9%	90 (100%)	89 (98.9%)	0/0/0/0	0
**Proteins**									
RNA-dependent RNA polymerase (YP_001531159.1)	1155	1255	3.9%	601	97.9%	90 (100%)	89 (98.9%)	0/0/0/0	0
Protein mutations:	*none*								
Codon mutations:	GAA1155GAG (14945A>G), CAA1156CAG (14948A>G), CTA1159CTG (14957A>G), TCT1160AGC (14958T>A 14959C>G 14960T>C), TCA1161TCC (14963A>C), GAC1162GAT (14966C>T), TCA1163TCC (14969A>C), AAA1164AAG (14972A>G), TCA1165AGC (14973T>A 14974C>G 14975A>C), ACT1166ACC (14978T>C), ATA1167ATC (14981A>C), AAC1168AAT (14984C>T), TTG1169CTG (14985T>C), TTG1170CTG (14988T>C), CCG1171CCC (14993G>C), TAT1172TAC (14996T>C), GAC1173GAT (14999C>T), GAA1176GAG (15008A>G), CGA1178AGG (15012C>A 15014A>G), CTT1179CTG (15017T>G), GAA1180GAG (15020A>G), GGA1181GGC (15023A>C), AAT1183AAC (15029T>C), ACA1185ACC (15035A>C), TTA1187CTG (15039T>C 15041A>G), TAT1189TAC (15047T>C), GTC1190GTG (15050C>G), AGT1191AGC (15053T>C), TGT1192TGC (15056T>C), GCT1193GCC (15059T>C), CTT1194CTG (15062T>G), CGG1196AGG (15066C>A), AAA1197AAG (15071A>G), GTT1198GTG (15074T>G), ACG1211ACC (15113G>C), ATA1212ATC (15116A>C), GGA1213GGC (15119A>C), AAT1214AAC (15122T>C), CGA1215AGG (15123C>A 15125A>G), GCA1216GCC (15128A>C), CCG1217CCC (15131G>C), TAT1218TAC (15134T>C), ATT1219ATC (15137T>C), TCA1221TCC (15143A>C), CGG1222AGA (15144C>A 15146G>A), ACA1223ACC (15149A>C), GAA1224GAG (15152A>G), GAT1225GAC (15155T>C), GGT1228GGC (15164T>C), TAT1229TAC (15167T>C), CCC1231CCT (15173C>T), TTA1232CTG (15174T>C 15176A>G), GTA1234GTG (15182A>G), TGC1236TGT (15188C>T), CCA1237CCT (15191A>T), TCA1238AGC (15192T>A 15193C>G 15194A>C), GCA1239GCC (15197A>C), GCA1240GCC (15200A>C), CTT1241CTG (15203T>G), AAA1242AAG (15206A>G), GAA1243GAG (15209A>G), GCT1244GCC (15212T>C), ATT1245ATC (15215T>C), GTT1248GTG (15224T>G), TCT1249TCC (15227T>C), AGA1250AGG (15230A>G), TTG1251CTG (15231T>C), TTA1252CTG (15234T>C 15236A>G)								

*: Inserts / Deletes / Misaligned / Frameshifts

Analysis details

This analysis was performed with panviral2.64

NGS Details (UN62): Sclerotinia sclerotiorum deltaflexivirus 2

Assembly

**Coverage Length**	216 (1 contig(s))
**Depth Of Coverage**	1.2
**Number Of Reads**	2
**Reads Per Million**	0.05 rpm (after QC)
**Ambiguities**	0
**Assembly Method**	de novo + reference guided assembly
**Consensus Caller**	Bcf Tools

Coverage Map

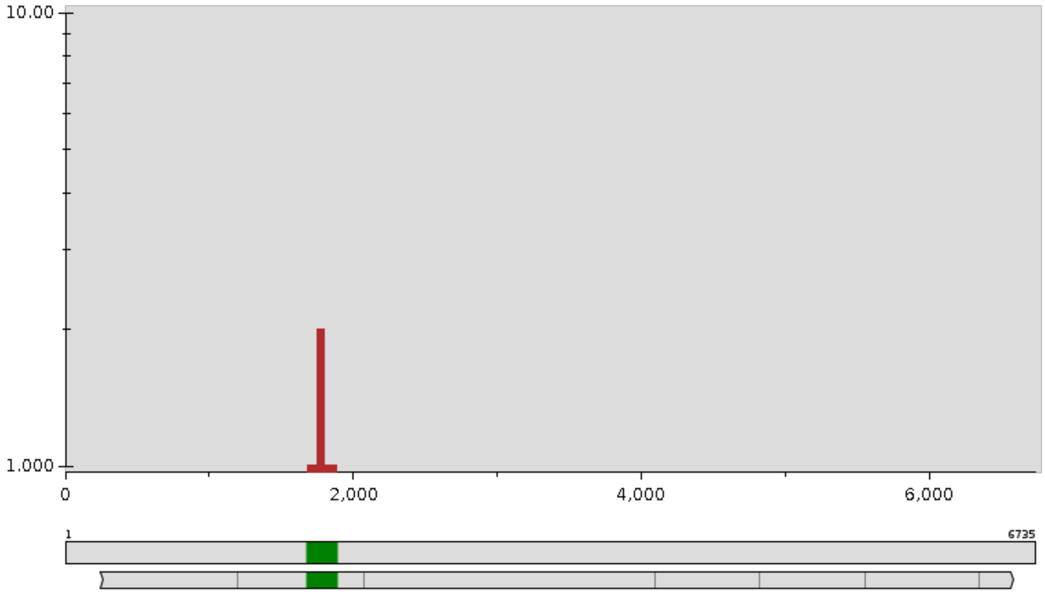

Assignment

**Type**	Sclerotinia sclerotiorum deltaflexivirus 2 (Taxonomy ID: 2219092)
**Reference Genome**	NC_040649.1
**NT Identity (%)**	60.7306
**AA Identity (%)**	65.7534
**Number Of Stop Codons**	0
**Number Of CDS**	1

Alignment

**Alignment Score**	88.0 (NT) + 342.0 (AA) = 430.0
**Concordance (%)**	44.3756

**Alignment Method**	Global, seeded, nucleotide + amino acids (AGA)

Genome Region

Sequence starts at position 1675 and ends at position 1890 relative to NC_040649.1 reference sequence.

Alignment Detailed Statistics

	Begin	End	Coverage	Score	Concordance	Matches	Identities	I/D/M/F*	Stop Codons
**NT**	**1675**	**1890**	**3.2%**	**88**	**20.4%**	**216 (98.6%)**	**133 (60.7%)**	**3/0**	
Mutations:	1675C>T, 1678A>G, 1683T>C, 1686T>G, 1687T>G, 1688G>T, 1689C>T, 1692G>C, 1693A>G, 1695A>C, 1698A>C, 1704G>A, 1710C>T, 1712G>T, 1713G>C, 1716C>T, 1717C>T, 1719A>G, 1722T>G, 1725C>T, 1731C>T, 1735T>G, 1736C>A, 1737C>G, 1738C>T, 1743T>C, 1744G>A, 1746C>T, 1749C>T, 1750C>G, 1751G>A, 1752A>G, 1753T>A, 1754A>T, 1755C>T, 1756T>C, 1758C>T, 1759A>G, 1761A>T, 1762A>G, 1763A>G, 1770G>A, 1772T>C, 1773_1774insCTG, 1774A>G, 1776A>G, 1780T>A, 1782T>A, 1785T>A, 1788G>A, 1792G>A, 1797G>C, 1800C>G, 1804A>G, 1805G>C, 1806G>T, 1809C>T, 1810G>T, 1811A>T, 1812A>T, 1818T>G, 1819C>T, 1821C>G, 1827T>C, 1828C>A, 1831T>G, 1832C>A, 1833T>G, 1837C>G, 1839C>T, 1840A>C, 1841C>G, 1842T>G, 1845C>T, 1848C>A, 1849A>C, 1850A>G, 1854G>C, 1866C>A, 1867A>C, 1868A>G, 1881T>C, 1884C>T, 1887C>G								
**CDS**									
EXK03_gp1	477	548	3.4%	342	63.7%	72 (98.6%)	48 (65.8%)	1/0/0/0	0
Protein mutations:	I478V (1678A>G), C481V (1687T>G 1688G>T 1689C>T), I483V (1693A>G 1695A>C), W489F (1712G>T 1713G>C), S497E (1735T>G 1736C>A 1737C>G), D500N (1744G>A 1746C>T), R502E (1750C>G 1751G>A 1752A>G), Y503I (1753T>A 1754A>T 1755C>T), C504R (1756T>C 1758C>T), K505D (1759A>G 1761A>T), N506G (1762A>G 1763A>G), I509T (1772T>C), I509_I510insL (1773_1774insCTG), I510V (1774A>G 1776A>G), F512I (1780T>A 1782T>A), D516N (1792G>A), K517N (1797G>C), R520A (1804A>G 1805G>C 1806G>T), E522F (1810G>T 1811A>T 1812A>T), S529E (1831T>G 1832C>A 1833T>G), L531V (1837C>G 1839C>T), T532R (1840A>C 1841C>G 1842T>G), N534K (1848C>A), K535R (1849A>C 1850A>G), N541R (1867A>C 1868A>G)								
Codon mutations:	CTG477TTG (1675C>T), ATT478GTT (1678A>G), GCT479GCC (1683T>C), ACT480ACG (1686T>G), TGC481GTT (1687T>G 1688G>T 1689C>T), GTG482GTC (1692G>C), ATA483GTC (1693A>G 1695A>C), CCA484CCC (1698A>C), GAG486GAA (1704G>A), CTC488CTT (1710C>T), TGG489TTC (1712G>T 1713G>C), GAC490GAT (1716C>T), CTA491TTG (1717C>T 1719A>G), CCT492CCG (1722T>G), GCC493GCT (1725C>T), ACC495ACT (1731C>T), TCC497GAG (1735T>G 1736C>A 1737C>G), CTG498TTG (1738C>T), TAT499TAC (1743T>C), GAC500AAT (1744G>A 1746C>T), TTC501TTT (1749C>T), CGA502GAG (1750C>G 1751G>A 1752A>G), TAC503ATT (1753T>A 1754A>T 1755C>T), TGC504CGT (1756T>C 1758C>T), AAA505GAT (1759A>G 1761A>T), AAC506GGC (1762A>G 1763A>G), AAG508AAA (1770G>A), ATC509ACC (1772T>C), ATC509_ATA510insCTG (1773_1774insCTG), ATA510GTG (1774A>G 1776A>G), TTT512ATA (1780T>A 1782T>A), CCT513CCA (1785T>A), GAG514GAA (1788G>A), GAC516AAC (1792G>A), AAG517AAC (1797G>C), GGC518GGG (1800C>G), AGG520GCT (1804A>G 1805G>C 1806G>T), TAC521TAT (1809C>T), GAA522TTT (1810G>T 1811A>T 1812A>T), CCT524CCG (1818T>G), CTC525TTG (1819C>T 1821C>G), GCT527GCC (1827T>C), CGG528AGG (1828C>A), TCT529GAG (1831T>G 1832C>A 1833T>G), CTC531GTT (1837C>G 1839C>T), ACT532CGG (1840A>C 1841C>G 1842T>G), GCC533GCT (1845C>T), AAC534AAA (1848C>A), AAG535CGG (1849A>C 1850A>G), CTG536CTC (1854G>C), GGC540GGA (1866C>A), AAT541CGT (1867A>C 1868A>G), CAT545CAC (1881T>C), GTC546GTT (1884C>T), TCC547TCG (1887C>G)								
**Proteins**									
polyprotein (YP_009552771.1)	477	548	3.4%	342	63.7%	72 (98.6%)	48 (65.8%)	1/0/0/0	0
Protein mutations:	I478V (1678A>G), C481V (1687T>G 1688G>T 1689C>T), I483V (1693A>G 1695A>C), W489F (1712G>T 1713G>C), S497E (1735T>G 1736C>A 1737C>G), D500N (1744G>A 1746C>T), R502E (1750C>G 1751G>A 1752A>G), Y503I (1753T>A 1754A>T 1755C>T), C504R (1756T>C 1758C>T), K505D (1759A>G 1761A>T), N506G (1762A>G 1763A>G), I509T (1772T>C), I509_I510insL (1773_1774insCTG), I510V (1774A>G 1776A>G), F512I (1780T>A 1782T>A), D516N (1792G>A), K517N (1797G>C), R520A (1804A>G 1805G>C 1806G>T), E522F (1810G>T 1811A>T 1812A>T), S529E (1831T>G 1832C>A 1833T>G), L531V (1837C>G 1839C>T), T532R (1840A>C 1841C>G 1842T>G), N534K (1848C>A), K535R (1849A>C 1850A>G), N541R (1867A>C 1868A>G)								
Codon mutations:	CTG477TTG (1675C>T), ATT478GTT (1678A>G), GCT479GCC (1683T>C), ACT480ACG (1686T>G), TGC481GTT (1687T>G 1688G>T 1689C>T), GTG482GTC (1692G>C), ATA483GTC (1693A>G 1695A>C), CCA484CCC (1698A>C), GAG486GAA (1704G>A), CTC488CTT (1710C>T), TGG489TTC (1712G>T 1713G>C), GAC490GAT (1716C>T), CTA491TTG (1717C>T 1719A>G), CCT492CCG (1722T>G), GCC493GCT (1725C>T), ACC495ACT (1731C>T), TCC497GAG (1735T>G 1736C>A 1737C>G), CTG498TTG (1738C>T), TAT499TAC (1743T>C), GAC500AAT (1744G>A 1746C>T), TTC501TTT (1749C>T), CGA502GAG (1750C>G 1751G>A 1752A>G), TAC503ATT (1753T>A 1754A>T 1755C>T), TGC504CGT (1756T>C 1758C>T), AAA505GAT (1759A>G 1761A>T), AAC506GGC (1762A>G 1763A>G), AAG508AAA (1770G>A), ATC509ACC (1772T>C), ATC509_ATA510insCTG (1773_1774insCTG), ATA510GTG (1774A>G 1776A>G), TTT512ATA (1780T>A 1782T>A), CCT513CCA (1785T>A), GAG514GAA (1788G>A), GAC516AAC (1792G>A), AAG517AAC (1797G>C), GGC518GGG (1800C>G), AGG520GCT (1804A>G 1805G>C 1806G>T), TAC521TAT (1809C>T), GAA522TTT (1810G>T 1811A>T 1812A>T), CCT524CCG (1818T>G), CTC525TTG (1819C>T 1821C>G), GCT527GCC (1827T>C), CGG528AGG (1828C>A), TCT529GAG (1831T>G 1832C>A 1833T>G), CTC531GTT (1837C>G 1839C>T), ACT532CGG (1840A>C 1841C>G 1842T>G), GCC533GCT (1845C>T), AAC534AAA (1848C>A), AAG535CGG (1849A>C 1850A>G), CTG536CTC (1854G>C), GGC540GGA (1866C>A), AAT541CGT (1867A>C 1868A>G), CAT545CAC (1881T>C), GTC546GTT (1884C>T), TCC547TCG (1887C>G)								
viral methyltransferase (YP_009553706.1)	159	230	24.7%	342	63.7%	72 (98.6%)	48 (65.8%)	1/0/0/0	0
Protein mutations:	I160V (1678A>G), C163V (1687T>G 1688G>T 1689C>T), I165V (1693A>G 1695A>C), W171F (1712G>T 1713G>C), S179E (1735T>G 1736C>A 1737C>G), D182N (1744G>A 1746C>T), R184E (1750C>G 1751G>A 1752A>G), Y185I (1753T>A 1754A>T 1755C>T), C186R (1756T>C 1758C>T), K187D (1759A>G 1761A>T), N188G (1762A>G 1763A>G), I191T (1772T>C), I191_I192insL (1773_1774insCTG), I192V (1774A>G 1776A>G), F194I (1780T>A 1782T>A), D198N (1792G>A), K199N (1797G>C), R202A (1804A>G 1805G>C 1806G>T), E204F (1810G>T 1811A>T 1812A>T), S211E (1831T>G 1832C>A 1833T>G), L213V (1837C>G 1839C>T), T214R (1840A>C 1841C>G 1842T>G), N216K (1848C>A), K217R (1849A>C 1850A>G), N223R (1867A>C 1868A>G)								
Codon mutations:	CTG159TTG (1675C>T), ATT160GTT (1678A>G), GCT161GCC (1683T>C), ACT162ACG (1686T>G), TGC163GTT (1687T>G 1688G>T 1689C>T), GTG164GTC (1692G>C), ATA165GTC (1693A>G 1695A>C), CCA166CCC (1698A>C), GAG168GAA (1704G>A), CTC170CTT (1710C>T), TGG171TTC (1712G>T 1713G>C), GAC172GAT (1716C>T), CTA173TTG (1717C>T 1719A>G), CCT174CCG (1722T>G), GCC175GCT (1725C>T), ACC177ACT (1731C>T), TCC179GAG (1735T>G 1736C>A 1737C>G), CTG180TTG (1738C>T), TAT181TAC (1743T>C), GAC182AAT (1744G>A 1746C>T), TTC183TTT (1749C>T), CGA184GAG (1750C>G 1751G>A 1752A>G), TAC185ATT (1753T>A 1754A>T 1755C>T), TGC186CGT (1756T>C 1758C>T), AAA187GAT (1759A>G 1761A>T), AAC188GGC (1762A>G 1763A>G), AAG190AAA (1770G>A), ATC191ACC (1772T>C), ATC191_ATA192insCTG (1773_1774insCTG), ATA192GTG (1774A>G 1776A>G), TTT194ATA (1780T>A 1782T>A), CCT195CCA (1785T>A), GAG196GAA (1788G>A), GAC198AAC (1792G>A), AAG199AAC (1797G>C), GGC200GGG (1800C>G), AGG202GCT (1804A>G 1805G>C 1806G>T), TAC203TAT (1809C>T), GAA204TTT (1810G>T 1811A>T 1812A>T), CCT206CCG (1818T>G), CTC207TTG (1819C>T 1821C>G), GCT209GCC (1827T>C), CGG210AGG (1828C>A), TCT211GAG (1831T>G 1832C>A 1833T>G), CTC213GTT (1837C>G 1839C>T), ACT214CGG (1840A>C 1841C>G 1842T>G), GCC215GCT (1845C>T), AAC216AAA (1848C>A), AAG217CGG (1849A>C 1850A>G), CTG218CTC (1854G>C), GGC222GGA (1866C>A), AAT223CGT (1867A>C 1868A>G), CAT227CAC (1881T>C), GTC228GTT (1884C>T), TCC229TCG (1887C>G)								

*: Inserts / Deletes / Misaligned / Frameshifts

Analysis details

This analysis was performed with panviral2.64

NGS Details (UN62): Friend murine leukemia virus

Assembly

**Coverage Length**	135 (1 contig(s))
**Depth Of Coverage**	1.0
**Number Of Reads**	1
**Reads Per Million**	0.02 rpm (after QC)
**Ambiguities**	0
**Assembly Method**	de novo + reference guided assembly
**Consensus Caller**	Bcf Tools

Coverage Map

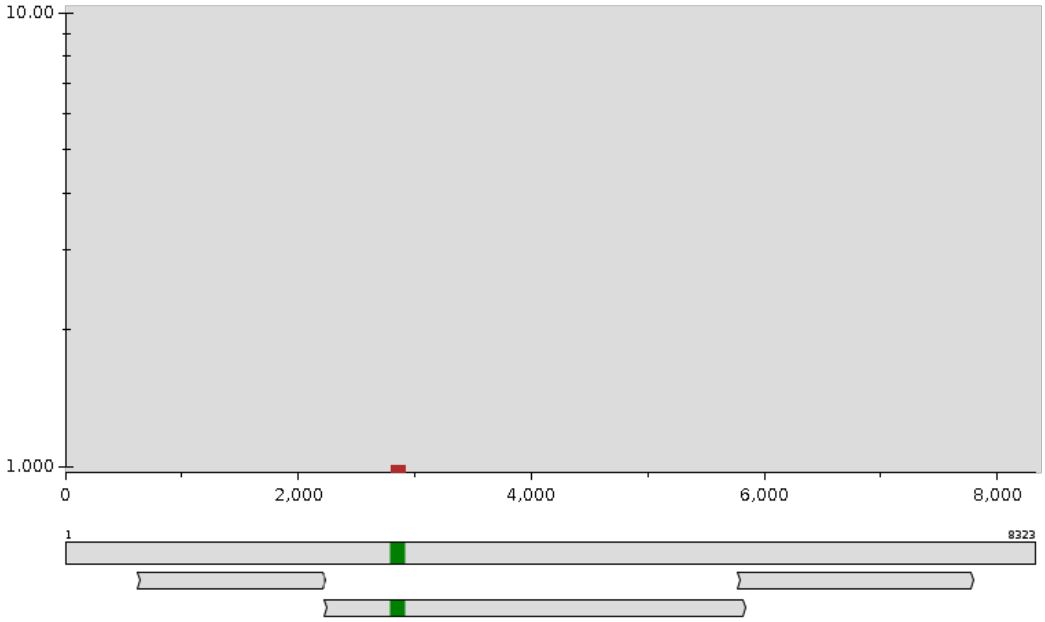

Assignment

**Type**	Friend murine leukemia virus (Taxonomy ID: 11795)
**Reference Genome**	NC_001362.1
**NT Identity (%)**	77.037
**AA Identity (%)**	97.7778
**Number Of Stop Codons**	0
**Number Of CDS**	3

Alignment

**Alignment Score**	146.0 (NT) + 329.0 (AA) = 475.0
**Concordance (%)**	79.2988

Alignment Method	Global, seeded, nucleotide + amino acids (AGA)

Genome Region

Sequence starts at position 2789 and ends at position 2923 relative to NC_001362.1 reference sequence.

Alignment Detailed Statistics

	Begin	End	Coverage	Score	Concordance	Matches	Identities	I/D/M/F*	Stop Codons
**NT**	**2789**	**2923**	**1.6%**	**146**	**54.1%**	**135 (100%)**	**104 (77.0%)**	**0/0**	
Mutations:	2790C>G, 2794T>A, 2795C>G, 2796A>C, 2799A>G, 2805C>G, 2806A>C, 2808A>C, 2814G>C, 2817C>T, 2820G>A, 2823C>G, 2826C>T, 2829A>T, 2833A>C, 2835A>C, 2850A>C, 2859A>G, 2862C>G, 2869T>A, 2870C>G, 2874C>G, 2883G>C, 2886C>G, 2892A>G, 2895C>G, 2898T>G, 2901G>A, 2910G>C, 2913T>C, 2916T>C								
**CDS**									
pol	191	235	3.7%	329	94.3%	45 (100%)	44 (97.8%)	0/0/0/0	0
**Proteins**									
RNA-dependent DNA polymerase (NP_040333.1)	191	235	3.7%	329	94.3%	45 (100%)	44 (97.8%)	0/0/0/0	0
Protein mutations:	*none*								
Codon mutations:	CCC190.CG (2790C>G), TCA192AGC (2794T>A 2795C>G 2796A>C), CAA193CAG (2799A>G), GCC195GCG (2805C>G), AGA196CGC (2806A>C 2808A>C), GGG198GGC (2814G>C), ATC199ATT (2817C>T), AAG200AAA (2820G>A), CCC201CCG (2823C>G), CAC202CAT (2826C>T), ATA203ATT (2829A>T), AGA205CGC (2833A>C 2835A>C), GGA210GGC (2850A>C), GTA213GTG (2859A>G), CCC214CCG (2862C>G), TCC217AGC (2869T>A 2870C>G), CCC218CCG (2874C>G), ACG221ACC (2883G>C), CCC222CCG (2886C>G), CTA224CTG (2892A>G), CCC225CCG (2895C>G), GTT226GTG (2898T>G), AAG227AAA (2901G>A), GGG230GGC (2910G>C), ACT231ACC (2913T>C), AAT232AAC (2916T>C)								

*: Inserts / Deletes / Misaligned / Frameshifts

Analysis details

This analysis was performed with panviral2.64

NGS Details (UN62): Moloney murine leukemia virus

Assembly

**Coverage Length**	135 (1 contig(s))
**Depth Of Coverage**	1.0
**Number Of Reads**	1
**Reads Per Million**	0.02 rpm (after QC)
**Ambiguities**	0
**Assembly Method**	de novo + reference guided assembly
**Consensus Caller**	Bcf Tools

Coverage Map

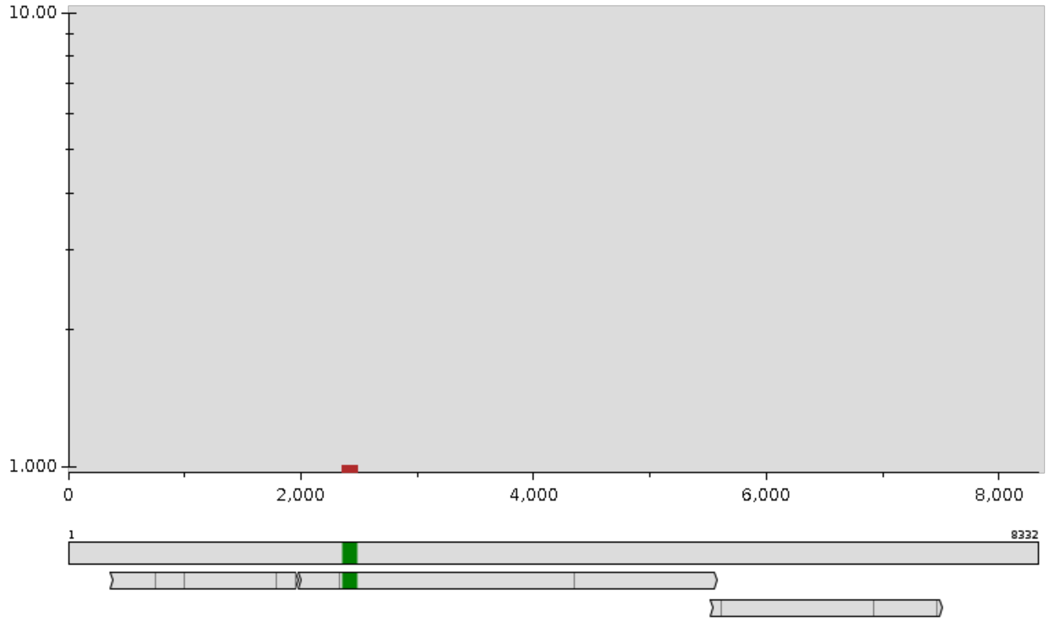

Assignment

**Type**	Moloney murine leukemia virus (Taxonomy ID: 11801)
**Reference Genome**	NC_001501.1
**NT Identity (%)**	76.2963
**AA Identity (%)**	100.0
**Number Of Stop Codons**	0
**Number Of CDS**	3

Alignment

**Alignment Score**	142.0 (NT) + 327.0 (AA) = 469.0
**Concordance (%)**	78.5595

Alignment Method	Global, seeded, nucleotide + amino acids (AGA)

Genome Region

Sequence starts at position 2352 and ends at position 2486 relative to NC_001501.1 reference sequence.

Alignment Detailed Statistics

	Begin	End	Coverage	Score	Concordance	Matches	Identities	I/D/M/F*	Stop Codons
**NT**	**2352**	**2486**	**1.6%**	**142**	**52.6%**	**135 (100%)**	**103 (76.3%)**	**0/0**	
Mutations:	2354G>A, 2360G>C, 2363A>G, 2369G>A, 2373T>A, 2374C>G, 2375A>C, 2381G>A, 2384A>G, 2390T>G, 2391T>A, 2392C>G, 2393T>C, 2396A>G, 2399G>C, 2400T>A, 2401C>G, 2405A>C, 2412T>A, 2413C>G, 2414T>C, 2423T>G, 2429C>G, 2444G>C, 2453A>C, 2459A>G, 2462T>G, 2468A>G, 2471T>G, 2474T>G, 2480C>T, 2483A>T								
**CDS**									
gag-pol	665	709	2.6%	327	100%	45 (100%)	45 (100%)	0/0/0/0	0
**Proteins**									
Pr180 (NP_057933.2)	665	709	2.6%	327	100%	45 (100%)	45 (100%)	0/0/0/0	0
Protein mutations:	*none*								
Codon mutations:	GAG665GAA (2354G>A), CGG667CGC (2360G>C), CTA668CTG (2363A>G), GAG670GAA (2369G>A), TCA672AGC (2373T>A 2374C>G 2375A>C), GAG674GAA (2381G>A), CCA675CCG (2384A>G), GTT677GTG (2390T>G), TCT678AGC (2391T>A 2392C>G 2393T>C), CTA679CTG (2396A>G), GGG680GGC (2399G>C), TCC681AGC (2400T>A 2401C>G), ACA682ACC (2405A>C), TCT685AGC (2412T>A 2413C>G 2414T>C), CCT688CCG (2423T>G), GCC690GCG (2429C>G), GGG695GGC (2444G>C), GGA698GGC (2453A>C), GCA700GCG (2459A>G), GTT701GTG (2462T>G), CAA703CAG (2468A>G), GCT704GCG (2471T>G), CCT705CCG (2474T>G), ATC707ATT (2480C>T), ATA708ATT (2483A>T)								
p80 RT (NP_955591.1)	6	50	6.7%	327	100%	45 (100%)	45 (100%)	0/0/0/0	0
Protein mutations:	*none*								
Codon mutations:	GAG6GAA (2354G>A), CGG8CGC (2360G>C), CTA9CTG (2363A>G), GAG11GAA (2369G>A), TCA13AGC (2373T>A 2374C>G 2375A>C), GAG15GAA (2381G>A), CCA16CCG (2384A>G), GTT18GTG (2390T>G), TCT19AGC (2391T>A 2392C>G 2393T>C), CTA20CTG (2396A>G), GGG21GGC (2399G>C), TCC22AGC (2400T>A 2401C>G), ACA23ACC (2405A>C), TCT26AGC (2412T>A 2413C>G 2414T>C), CCT29CCG (2423T>G), GCC31GCG (2429C>G), GGG36GGC (2444G>C), GGA39GGC (2453A>C), GCA41GCG (2459A>G), GTT42GTG (2462T>G), CAA44CAG (2468A>G), GCT45GCG (2471T>G), CCT46CCG (2474T>G), ATC48ATT (2480C>T), ATA49ATT (2483A>T)								

*: Inserts / Deletes / Misaligned / Frameshifts

Analysis details

This analysis was performed with panviral2.64

NGS Details (UN62): Ochrobactrum phage POA1180

Assembly

**Coverage Length**	135 (1 contig(s))
**Depth Of Coverage**	1.0
**Number Of Reads**	1
**Reads Per Million**	0.02 rpm (after QC)
**Ambiguities**	0
**Assembly Method**	de novo + reference guided assembly
**Consensus Caller**	Bcf Tools

Coverage Map

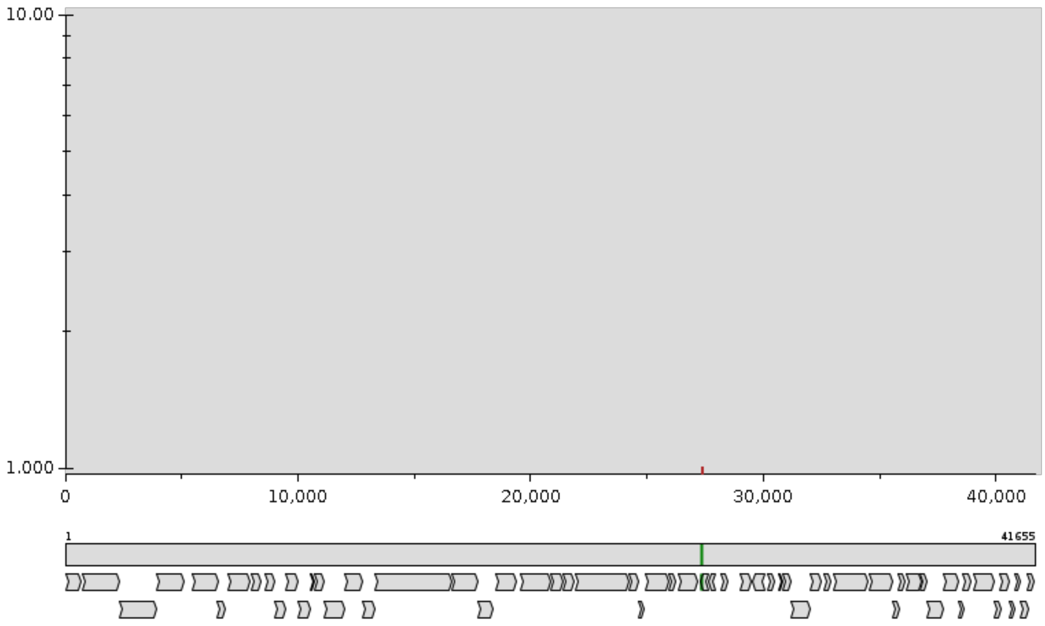

Assignment

**Type**	Ochrobactrum phage POA1180 (Taxonomy ID: 1897640)
**Reference Genome**	NC_070931.1
**NT Identity (%)**	79.2593
**AA Identity (%)**	71.1111
**Number Of Stop Codons**	0
**Number Of CDS**	58

Alignment

**Alignment Score**	158.0 (NT) + 269.0 (AA) = 427.0
**Concordance (%)**	71.1667

Alignment Method	Local, heuristic, nucleotide (BLASTN)

Genome Region

Sequence starts at position 27298 and ends at position 27432 relative to NC_070931.1 reference sequence.

Alignment Detailed Statistics

	Begin	End	Coverage	Score	Concordance	Matches	Identities	I/D/M/F*	Stop Codons
**NT**	**27298**	**27432**	**0.3%**	**158**	**58.5%**	**135 (100%)**	**107 (79.3%)**	**0/0**	
Mutations:	27301A>C, 27309A>C, 27312A>G, 27314G>T, 27322T>C, 27323T>A, 27324C>G, 27325G>C, 27327C>G, 27328T>C, 27329T>G, 27335G>T, 27336A>C, 27352A>G, 27354G>C, 27357G>C, 27366T>G, 27372C>G, 27375T>C, 27378C>G, 27384G>C, 27386G>C, 27388G>A, 27393A>G, 27418G>C, 27419T>G, 27420G>C, 27425C>T								
**CDS**									
PQB33_gp31	59	103	41.3%	269	78.7%	45 (100%)	32 (71.1%)	0/0/0/0	0
Protein mutations:	G61S (27425C>T), D62E (27420G>C), T63R (27418G>C 27419T>G), T73M (27388G>A), L74V (27384G>C 27386G>C), I85T (27352A>G), L91M (27335G>T), K93R (27327C>G 27328T>C 27329T>G), T94S (27324C>G 27325G>C), K95W (27322T>C 27323T>A), L98I (27312A>G 27314G>T), I102S (27301A>C)								
Codon mutations:	GGC61AGC (27425C>T), GAC62GAG (27420G>C), ACG63CGG (27418G>C 27419T>G), TAT71TAC (27393A>G), ACG73ATG (27388G>A), CTC74GTG (27384G>C 27386G>C), CCG76CCC (27378C>G), GGA77GGG (27375T>C), CCG78CCC (27372C>G), GCA80GCC (27366T>G), CTC83CTG (27357G>C), GCC84GCG (27354G>C), ATC85ACC (27352A>G), CTT90CTG (27336A>C), CTG91ATG (27335G>T), AAG93CGC (27327C>G 27328T>C 27329T>G), ACG94AGC (27324C>G 27325G>C), AAG95TGG (27322T>C 27323T>A), CTT98ATC (27312A>G 27314G>T), GTT99GTG (27309A>C), ATC102AGC (27301A>C)								
**Proteins**									
chromate transport protein (YP_010665114.1)	59	103	41.3%	269	78.7%	45 (100%)	32 (71.1%)	0/0/0/0	0
Protein mutations:	G61S (27425C>T), D62E (27420G>C), T63R (27418G>C 27419T>G), T73M (27388G>A), L74V (27384G>C 27386G>C), I85T (27352A>G), L91M (27335G>T), K93R (27327C>G 27328T>C 27329T>G), T94S (27324C>G 27325G>C), K95W (27322T>C 27323T>A), L98I (27312A>G 27314G>T), I102S (27301A>C)								
Codon mutations:	GGC61AGC (27425C>T), GAC62GAG (27420G>C), ACG63CGG (27418G>C 27419T>G), TAT71TAC (27393A>G), ACG73ATG (27388G>A), CTC74GTG (27384G>C 27386G>C), CCG76CCC (27378C>G), GGA77GGG (27375T>C), CCG78CCC (27372C>G), GCA80GCC (27366T>G), CTC83CTG (27357G>C), GCC84GCG (27354G>C), ATC85ACC (27352A>G), CTT90CTG (27336A>C), CTG91ATG (27335G>T), AAG93CGC (27327C>G 27328T>C 27329T>G), ACG94AGC (27324C>G 27325G>C), AAG95TGG (27322T>C 27323T>A), CTT98ATC (27312A>G 27314G>T), GTT99GTG (27309A>C), ATC102AGC (27301A>C)								

*: Inserts / Deletes / Misaligned / Frameshifts

Analysis details

This analysis was performed with panviral2.64
