# Supplementary material for: Nucleotide sequence analysis reveals the presence of PVY-Tam isolates affecting tamarillo in Colombia
Source: Virol J. 2026 Apr 20;23:145. doi: 10.1186/s12985-026-03166-6 (PMC13234967; doi:10.1186/s12985-026-03166-6)
Supplement: Supplementary file 8 — Additional file 8. [file 12985_2026_3166_MOESM8_ESM.pdf]

Analysis of UN70

**Technology**	Paired-end short reads
**Input Files**	UN70_R1.fq.gz (1.69 GB), UN70_R2.fq.gz (1.74 GB)
**Submitted On**	2023-09-26 13:21:22 UTC
**Duration**	3h 43m 14s
**Tool Version**	panviral2.64
**Location**	https://www.genomedetective.com/db/ui/analysis/01dff0d7-3177-4836-8378-54a03e04e81d

Statistics

**Original Read Length**	20 - 150
**Trimmed Read Length**	50 - 135

	# Reads	% of Reads
**Input file**	53127568	100.0%
**After QC**	52752992	99.3%
**After filtering**	6204606	11.7%
**Mapped back to contigs**	3034086	5.7%

Assignments

Assignment	No. of Reads	Depth of Coverage	Identity		Genome Coverage	
			NT	AA		
Torradovirus lycopersici (2 segments out of 2)	2444918	24879.5	89.1%	95.2%	99.7%	
Torradovirus lycopersici (segment RNA 2)	1691517	40729.6	87.4%	91.4%	99.6%	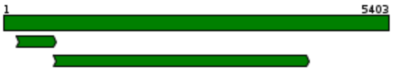
Torradovirus lycopersici (segment RNA 1)	753401	13912.1	90.3%	97.6%	99.7%	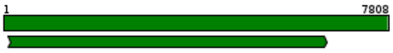
Torradovirus marchitezum (3 segments out of 2)	227889	4171.1	64.0%	64.3%	59.2%	
Torradovirus marchitezum (segment RNA 2)	19430	8120.3	75.8%	87.9%	5.4%	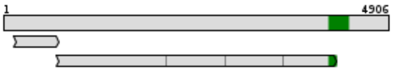
Torradovirus marchitezum (segment RNA 2)	13	7.0	77.4%	84.6%	3.9%	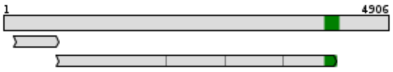
Torradovirus marchitezum (segment RNA 1)	208446	4133.7	63.2%	63.5%	93.0%	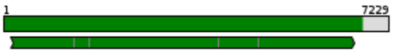
Potato virus Y	167938	2313.2	82.7%	90.1%	99.8%	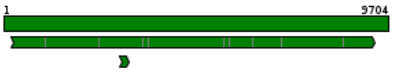

Assignment	No. of Reads	Depth of Coverage	Identity		Genome Coverage	
			NT	AA		
Bracoviriform glomeratae (segment NC_043292.1)	47201	17187.9	74.5%	84.5%	72.8%	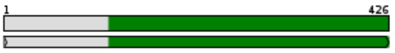
Diachasmimorpha longicaudata entomopoxvirus (segment NC_043455.1)	11204	1241.9	59.4%	55.6%	84.1%	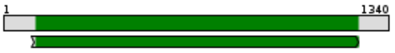
Potato leafroll virus	796	18.2	98.8%	98.4%	97.1%	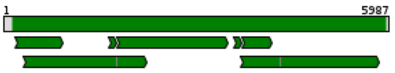
Solendovirus venanicotianae	234	12.1	77.6%	74.2%	27.1%	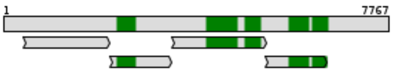
Duamitovirus soch1	210	11.7	69.4%	69.9%	72.7%	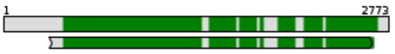

Discoveries

Similar to	No. of Reads	Depth of Coverage	Identity		Genome Coverage	
			NT	AA		
Tomato chocolate spot virus (2 segments out of 2)	93634	11276.0	69.8%	80.2%	10.1%	
Tomato chocolate spot virus (segment RNA 1)	74446	13296.7	69.9%	93.5%	12.5%	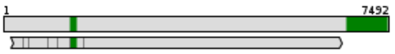
Tomato chocolate spot virus (segment RNA2)	19188	5827.1	69.7%	75.0%	6.8%	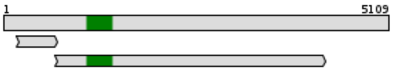
Brazilian marseillevirus	13004	5346.7	79.8%	94.7%	0.1%	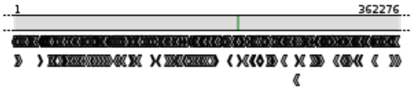
Tokyovirus A1	12194	6107.3	80.6%	93.1%	0.0%	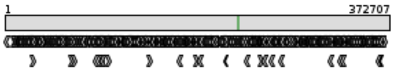
Lausannevirus	10606	4682.9	80.5%	95.2%	0.1%	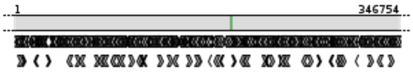
Marseillevirus marseillevirus	1457	1004.1	89.4%	94.3%	0.0%	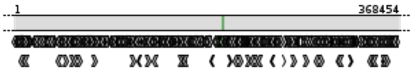
Badnavirus occultipomeae	541	139.6	53.1%	45.5%	5.3%	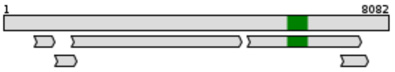
Cassava brown streak virus	521	121.8	60.8%	57.7%	5.8%	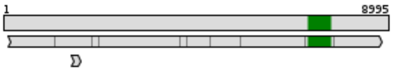
Human gammaherpesvirus 8 (subtype: Could not assign)	386	240.5	83.3%	84.1%	0.1%	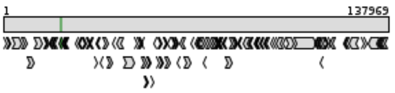

Similar to	No. of Reads	Depth of Coverage	Identity		Genome Coverage	
			NT	AA		
Makelovirus prm1	316	40.8	76.4%	87.3%	0.7%	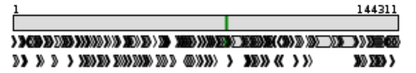
Cladosporium fulvum T-1 virus	132	14.0	54.4%	50.6%	13.3%	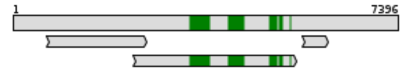
Lowelvirus tuscon4d	115	27.6	79.6%	89.9%	0.2%	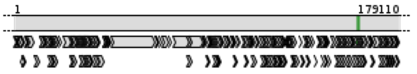
Errantivirus	112	13.9	51.7%	43.4%	11.5%	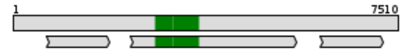
Caulimovirus tesselloscrophulariae	80	15.8	59.0%	46.3%	7.5%	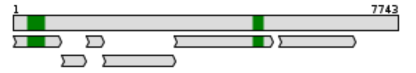
Tomato necrotic dwarf virus (segment RNA1)	79	21.2	69.4%	74.5%	6.0%	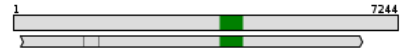
Badnavirus maculaucubae	77	24.5	54.7%	45.0%	3.5%	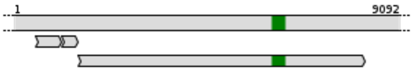
Neptunevirus srim50	69	10.5	72.0%	87.8%	0.3%	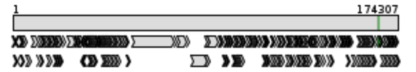
Badnavirus alphacolocalasiae	42	21.2	53.6%	44.9%	3.1%	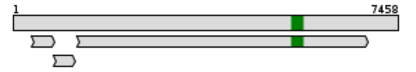
Caulimovirus venafragariae	36	7.0	59.9%	59.1%	8.3%	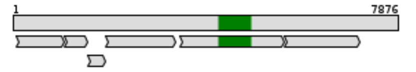
Alisovirus socal22	28	19.1	79.9%	85.4%	0.1%	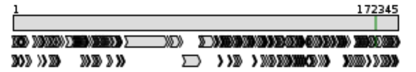
Badnavirus maculakalanchoes	27	10.4	55.2%	48.9%	3.7%	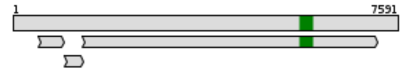
Dioscovirus dioscoreae	26	4.1	62.5%	48.2%	7.4%	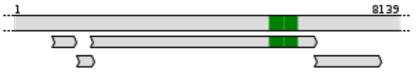
Chinaberry tree badnavirus 1	26	7.5	53.9%	43.8%	6.6%	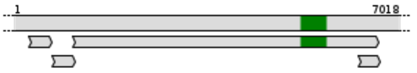
Duamitovirus peex1	22	5.8	71.1%	66.9%	14.1%	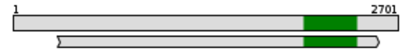
Badnavirus deltainflatheobromae	20	6.1	52.2%	49.1%	4.4%	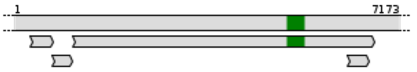
Cavemovirus collusipomeae	15	7.1	61.6%	50.0%	3.3%	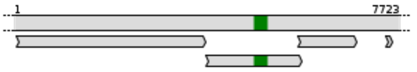

Similar to	No. of Reads	Depth of Coverage	Identity		Genome Coverage	
			NT	AA		
Petuvirus venapetuniae	14	2.8	64.0%	57.9%	8.5%	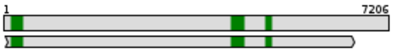
Epiphyllum badnavirus 1	14	5.1	52.3%	45.7%	4.3%	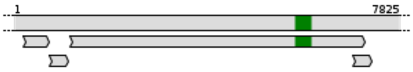
Caulimovirus venafragariae	11	3.0	64.3%	59.3%	6.4%	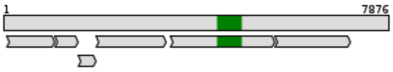
Gihfavirus pelohabitans	10	4.8	97.3%	0.0%	4.2%	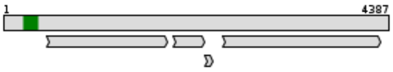
Caulimovirus tessellomirabilis	10	4.0	57.0%	48.6%	4.4%	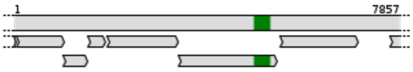
Badnavirus venabougainvilleae	10	3.5	51.7%	39.7%	4.3%	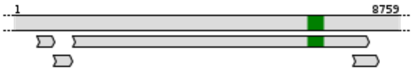
Caulimovirus tessellomirabilis	8	3.3	59.3%	47.6%	4.0%	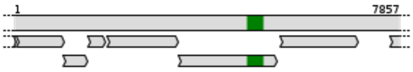
Cavemovirus collusipomeae	8	3.2	63.1%	51.0%	4.0%	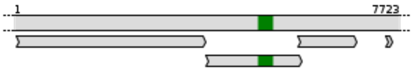
Cavemovirus collusipomeae	8	3.7	62.8%	49.4%	3.1%	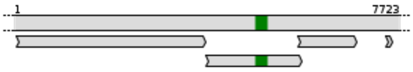
Duamitovirus chqu1	8	6.4	68.1%	75.6%	4.9%	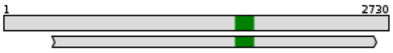
Badnavirus tessellocastaneae	7	3.4	55.6%	55.4%	3.5%	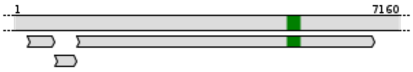
Caulimovirus tessellomirabilis	7	3.1	58.7%	49.5%	4.0%	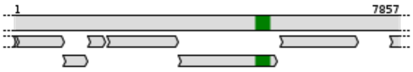
Badnavirus alphacolocalasiae	5	2.3	58.9%	52.1%	3.9%	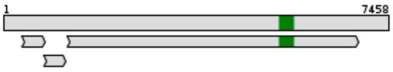
Caulimovirus venafragariae	4	1.6	62.6%	60.9%	3.3%	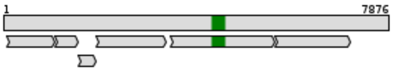
Fragaria chiloensis cryptic virus (segment RNA 3)	4	2.1	54.9%	42.9%	15.9%	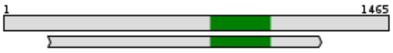
Colombian datura virus	2	1.3	99.0%	100.0%	2.2%	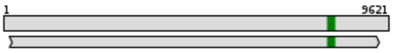
Limeum africanum associated virus	1	1.0	72.6%	75.6%	4.6%	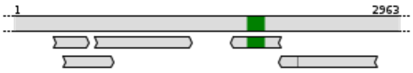

NGS Details (UN70): Torradovirus marchitezum (segment RNA 2)

Assembly

**Coverage Length**	190 (1 contig(s))
**Depth Of Coverage**	7.0
**Number Of Reads**	13
**Reads Per Million**	0.25 rpm (after QC)
**Ambiguities**	0
**Assembly Method**	de novo + reference guided assembly
**Consensus Caller**	Bcf Tools

Coverage Map

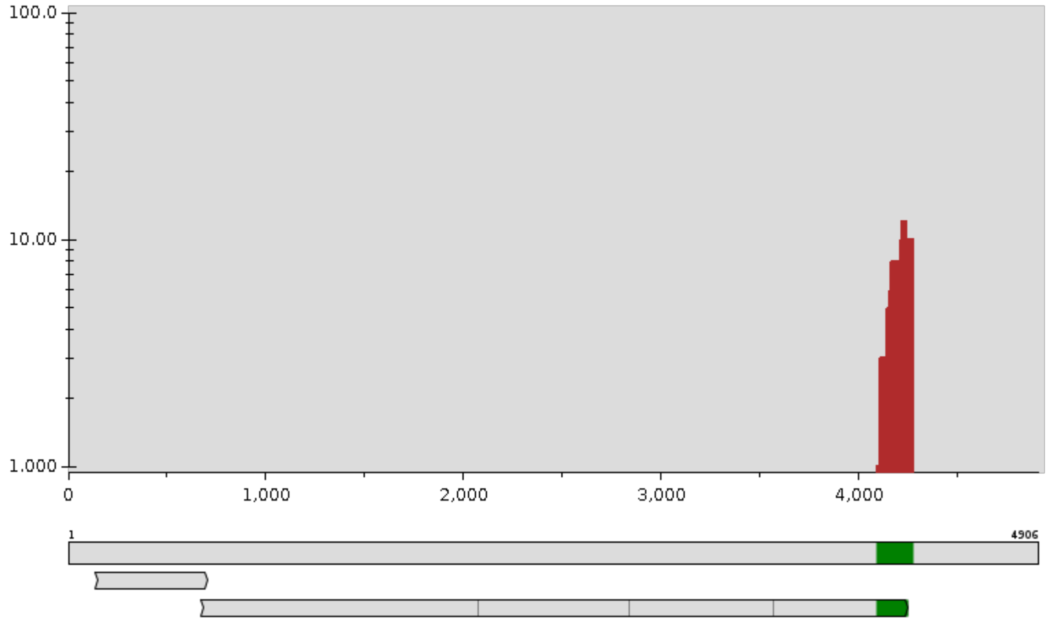

Assignment

**Type**	Torradovirus marchitezum (Taxonomy ID: 3048376)
**Reference Genome**	NC_010988.1
**NT Identity (%)**	77.3684
**AA Identity (%)**	84.6154
**Number Of Stop Codons**	1
**Number Of CDS**	2

Alignment

**Alignment Score**	208.0 (NT) + 298.0 (AA) = 506.0
**Concordance (%)**	69.6011

**Alignment Method**	Global, seeded, nucleotide + amino acids (AGA)

Genome Region

Sequence starts at position 4089 and ends at position 4278 relative to NC_010988.1 reference sequence.

Alignment Detailed Statistics

	Begin	End	Coverage	Score	Concordance	Matches	Identities	I/D/M/F*	Stop Codons
**NT**	**4089**	**4278**	**3.9%**	**208**	**54.7%**	**190 (100%)**	**147 (77.4%)**	**0/0**	
Mutations:	4090G>T, 4096C>A, 4102G>A, 4105A>C, 4106C>A, 4108T>A, 4109A>G, 4111A>C, 4112T>A, 4113C>A, 4114T>G, 4117T>A, 4120A>T, 4122T>C, 4129C>T, 4132G>A, 4144C>T, 4150T>C, 4153T>A, 4154T>C, 4155C>T, 4159C>A, 4163C>A, 4168A>G, 4171A>G, 4177T>C, 4180C>A, 4181A>G, 4189A>G, 4192T>G, 4195C>T, 4198C>T, 4210C>A, 4213A>T, 4228A>G, 4242T>A, 4247A>G, 4248A>C, 4259C>T, 4261A>G, 4267T>A, 4274T>C, 4276G>A								
**CDS**									
ToMarV_RNA2gp2	1141	1192	4.4%	298	84.7%	52 (100%)	44 (84.6%)	0/0/0/0	1
Protein mutations:	L1146I (4106C>A 4108T>A), I1147V (4109A>G 4111A>C), S1148K (4112T>A 4113C>A 4114T>G), L1151P (4122T>C), S1162L (4154T>C 4155C>T), H1165N (4163C>A), N1171D (4181A>G), I1191N (4242T>A)								
Codon mutations:	GTG1140.TT (4090G>T), TCC1142TCA (4096C>A), GAG1144GAA (4102G>A), ATA1145ATC (4105A>C), CTT1146ATA (4106C>A 4108T>A), ATA1147GTC (4109A>G 4111A>C), TCT1148AAG (4112T>A 4113C>A 4114T>G), CCT1149CCA (4117T>A), GGA1150GGT (4120A>T), CTT1151CCT (4122T>C), TTC1153TTT (4129C>T), AAG1154AAA (4132G>A), GCC1158GCT (4144C>T), CCT1160CCC (4150T>C), CCT1161CCA (4153T>A), TCT1162CTT (4154T>C 4155C>T), GCC1163GCA (4159C>A), CAT1165AAT (4163C>A), GAA1166GAG (4168A>G), AAA1167AAG (4171A>G), CTT1169CTC (4177T>C), GGC1170GGA (4180C>A), AAT1171GAT (4181A>G), CAA1173CAG (4189A>G), ACT1174ACG (4192T>G), CAC1175CAT (4195C>T), ACC1176ACT (4198C>T), ACC1180ACA (4210C>A), TCA1181TCT (4213A>T), CTA1186CTG (4228A>G), ATT1191AAT (4242T>A)								
**Proteins**									
polyprotein (YP_001976149.1)	1141	1192	4.4%	298	84.7%	52 (100%)	44 (84.6%)	0/0/0/0	1
Protein mutations:	L1146I (4106C>A 4108T>A), I1147V (4109A>G 4111A>C), S1148K (4112T>A 4113C>A 4114T>G), L1151P (4122T>C), S1162L (4154T>C 4155C>T), H1165N (4163C>A), N1171D (4181A>G), I1191N (4242T>A)								
Codon mutations:	GTG1140.TT (4090G>T), TCC1142TCA (4096C>A), GAG1144GAA (4102G>A), ATA1145ATC (4105A>C), CTT1146ATA (4106C>A 4108T>A), ATA1147GTC (4109A>G 4111A>C), TCT1148AAG (4112T>A 4113C>A 4114T>G), CCT1149CCA (4117T>A), GGA1150GGT (4120A>T), CTT1151CCT (4122T>C), TTC1153TTT (4129C>T), AAG1154AAA (4132G>A), GCC1158GCT (4144C>T), CCT1160CCC (4150T>C), CCT1161CCA (4153T>A), TCT1162CTT (4154T>C 4155C>T), GCC1163GCA (4159C>A), CAT1165AAT (4163C>A), GAA1166GAG (4168A>G), AAA1167AAG (4171A>G), CTT1169CTC (4177T>C), GGC1170GGA (4180C>A), AAT1171GAT (4181A>G), CAA1173CAG (4189A>G), ACT1174ACG (4192T>G), CAC1175CAT (4195C>T), ACC1176ACT (4198C>T), ACC1180ACA (4210C>A), TCA1181TCT (4213A>T), CTA1186CTG (4228A>G), ATT1191AAT (4242T>A)								
Coat protein C (YP_001976157.1)	175	225	22.7%	297	84.6%	51 (100%)	43 (84.3%)	0/0/0/0	0
Protein mutations:	L180I (4106C>A 4108T>A), I181V (4109A>G 4111A>C), S182K (4112T>A 4113C>A 4114T>G), L185P (4122T>C), S196L (4154T>C 4155C>T), H199N (4163C>A), N205D (4181A>G), I225N (4242T>A)								
Codon mutations:	GTG174.TT (4090G>T), TCC176TCA (4096C>A), GAG178GAA (4102G>A), ATA179ATC (4105A>C), CTT180ATA (4106C>A 4108T>A), ATA181GTC (4109A>G 4111A>C), TCT182AAG (4112T>A 4113C>A 4114T>G), CCT183CCA (4117T>A), GGA184GGT (4120A>T), CTT185CCT (4122T>C), TTC187TTT (4129C>T), AAG188AAA (4132G>A), GCC192GCT (4144C>T), CCT194CCC (4150T>C), CCT195CCA (4153T>A), TCT196CTT (4154T>C 4155C>T), GCC197GCA (4159C>A), CAT199AAT (4163C>A), GAA200GAG (4168A>G), AAA201AAG (4171A>G), CTT203CTC (4177T>C), GGC204GGA (4180C>A), AAT205GAT (4181A>G), CAA207CAG (4189A>G), ACT208ACG (4192T>G), CAC209CAT (4195C>T), ACC210ACT (4198C>T), ACC214ACA (4210C>A), TCA215TCT (4213A>T), CTA220CTG (4228A>G), ATT225AAT (4242T>A)								

*: Inserts / Deletes / Misaligned / Frameshifts

Analysis details

This analysis was performed with panviral2.64

NGS Details (UN70): Torradovirus marchitezum (segment RNA 2)

Assembly

**Coverage Length**	264 (1 contig(s))
**Depth Of Coverage**	8120.3
**Number Of Reads**	19430
**Reads Per Million**	368.32 rpm (after QC)
**Ambiguities**	0
**Assembly Method**	de novo + reference guided assembly
**Consensus Caller**	Bcf Tools

Coverage Map

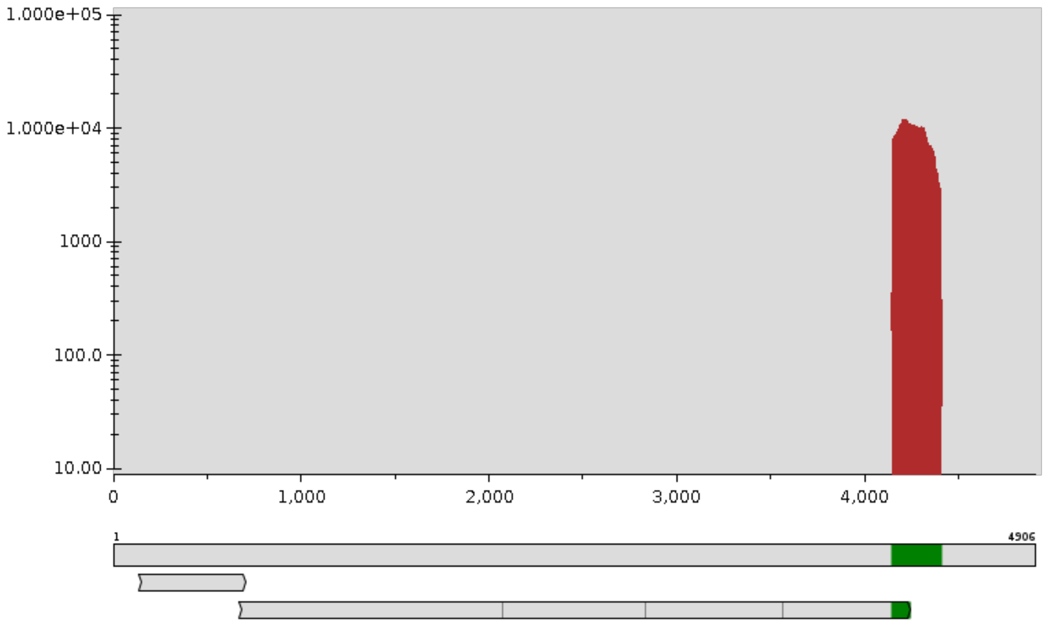

Assignment

**Type**	Torradovirus marchitezum (Taxonomy ID: 3048376)
**Reference Genome**	NC_010988.1
**NT Identity (%)**	75.7692
**AA Identity (%)**	87.8788
**Number Of Stop Codons**	1
**Number Of CDS**	2

Alignment

**Alignment Score**	219.0 (NT) + 91.0 (AA) = 310.0
**Concordance (%)**	52.8846

Alignment Method	Global, seeded, nucleotide + amino acids (AGA)

Genome Region

Sequence starts at position 4147 and ends at position 4410 relative to NC_010988.1 reference sequence.

Alignment Detailed Statistics

	Begin	End	Coverage	Score	Concordance	Matches	Identities	I/D/M/F*	Stop Codons
**NT**	**4147**	**4410**	**5.4%**	**219**	**44.8%**	**257 (96.3%)**	**197 (73.8%)**	**3/7**	
Mutations:	4156T>G, 4159C>T, 4162C>T, 4180C>T, 4181A>G, 4187_4188delCA, 4189A>C, 4192T>G, 4195C>T, 4198C>T, 4201T>C, 4204G>A, 4213A>T, 4219T>C, 4222T>A, 4225G>A, 4228A>G, 4229C>A, 4231T>A, 4234T>C, 4237T>C, 4238G>T, 4242T>A, 4243T>C, 4248A>C, 4252T>A, 4279A>T, 4279_4280insT, 4280A>T, 4280_4281insC, 4283A>G, 4286C>T, 4287T>A, 4288T>C, 4291C>A, 4294C>T, 4306G>A, 4309G>A, 4312G>T, 4313C>A, 4313_4314insG, 4315A>G, 4316A>T, 4318T>C, 4319G>T, 4320G>A, 4323T>G, 4324T>A, 4328T>C, 4330G>A, 4333A>G, 4335G>T, 4336A>C, 4338A>G, 4339_4343delAGGGA, 4345A>T, 4346G>C, 4349A>C, 4364C>T, 4365T>C, 4369A>G, 4372A>G, 4373G>A, 4392T>C, 4393C>T								
**CDS**									
ToMarV_RNA2gp2	1160	1192	2.8%	91	37.4%	33 (100%)	29 (87.9%)	0/0/1/1	1
Protein mutations:	N1171D (4181A>G), A1190S (4238G>T), I1191N (4242T>A 4243T>C)								
Codon mutations:	TCT1162TCG (4156T>G), GCC1163GCT (4159C>T), AAC1164AAT (4162C>T), GGC1170GGT (4180C>T), AAT1171GAT (4181A>G), CAA1173--C (4187_4188delCA 4189A>C), ACT1174ACG (4192T>G), CAC1175CAT (4195C>T), ACC1176ACT (4198C>T), TAT1177TAC (4201T>C), CAG1178CAA (4204G>A), TCA1181TCT (4213A>T), TTT1183TTC (4219T>C), TCT1184TCA (4222T>A), GAG1185GAA (4225G>A), CTA1186CTG (4228A>G), CGT1187AGA (4229C>A 4231T>A), GAT1188GAC (4234T>C), TTT1189TTC (4237T>C), GCG1190TCG (4238G>T), ATT1191AAC (4242T>A 4243T>C)								
**Proteins**									
polyprotein (YP_001976149.1)	1160	1192	2.8%	91	37.4%	33 (100%)	29 (87.9%)	0/0/1/1	1
Protein mutations:	N1171D (4181A>G), A1190S (4238G>T), I1191N (4242T>A 4243T>C)								
Codon mutations:	TCT1162TCG (4156T>G), GCC1163GCT (4159C>T), AAC1164AAT (4162C>T), GGC1170GGT (4180C>T), AAT1171GAT (4181A>G), CAA1173--C (4187_4188delCA 4189A>C), ACT1174ACG (4192T>G), CAC1175CAT (4195C>T), ACC1176ACT (4198C>T), TAT1177TAC (4201T>C), CAG1178CAA (4204G>A), TCA1181TCT (4213A>T), TTT1183TTC (4219T>C), TCT1184TCA (4222T>A), GAG1185GAA (4225G>A), CTA1186CTG (4228A>G), CGT1187AGA (4229C>A 4231T>A), GAT1188GAC (4234T>C), TTT1189TTC (4237T>C), GCG1190TCG (4238G>T), ATT1191AAC (4242T>A 4243T>C)								
Coat protein C (YP_001976157.1)	194	225	14.2%	90	37.2%	32 (100%)	28 (87.5%)	0/0/1/1	0
Protein mutations:	N205D (4181A>G), A224S (4238G>T), I225N (4242T>A 4243T>C)								
Codon mutations:	TCT196TCG (4156T>G), GCC197GCT (4159C>T), AAC198AAT (4162C>T), GGC204GGT (4180C>T), AAT205GAT (4181A>G), CAA207--C (4187_4188delCA 4189A>C), ACT208ACG (4192T>G), CAC209CAT (4195C>T), ACC210ACT (4198C>T), TAT211TAC (4201T>C), CAG212CAA (4204G>A), TCA215TCT (4213A>T), TTT217TTC (4219T>C), TCT218TCA (4222T>A), GAG219GAA (4225G>A), CTA220CTG (4228A>G), CGT221AGA (4229C>A 4231T>A), GAT222GAC (4234T>C), TTT223TTC (4237T>C), GCG224TCG (4238G>T), ATT225AAC (4242T>A 4243T>C)								

*: Inserts / Deletes / Misaligned / Frameshifts

Analysis details

This analysis was performed with panviral2.64

NGS Details (UN70): Torradovirus marchitezum (segment RNA 1)

Assembly

**Coverage Length**	6726 (1 contig(s))
**Depth Of Coverage**	4133.7
**Number Of Reads**	208446
**Reads Per Million**	3951.36 rpm (after QC)
**Ambiguities**	0
**Assembly Method**	de novo + reference guided assembly
**Consensus Caller**	Bcf Tools

Coverage Map

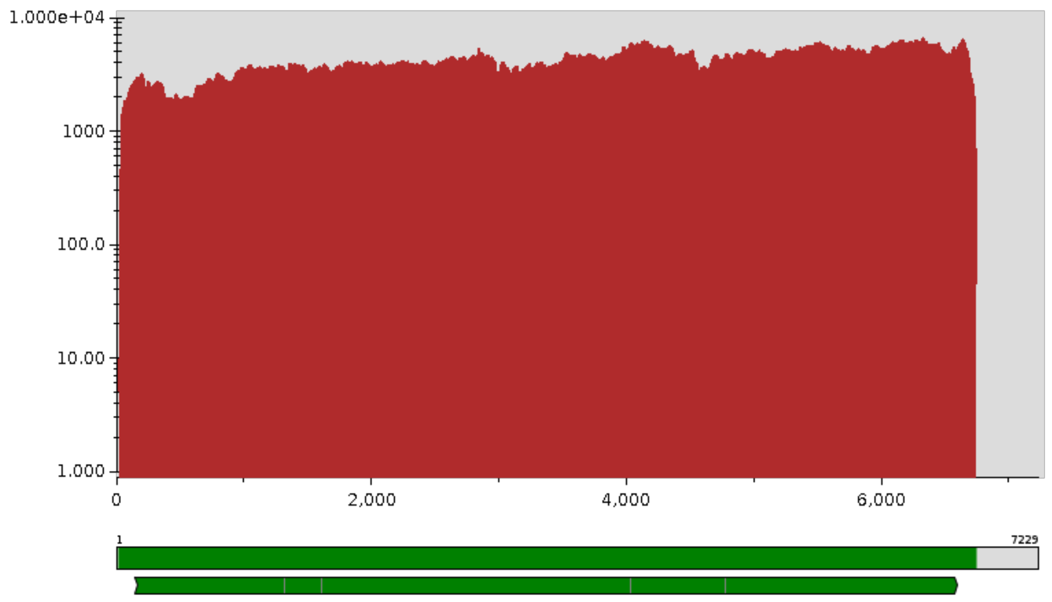

Assignment

**Type**	Torradovirus marchitezum (Taxonomy ID: 3048376)
**Reference Genome**	NC_010987.1
**NT Identity (%)**	63.1901
**AA Identity (%)**	63.4669
**Number Of Stop Codons**	0
**Number Of CDS**	1

Alignment

**Alignment Score**	3341.0 (NT) + 9871.0 (AA) = 13212.0
**Concordance (%)**	47.1941

Alignment Method	Global, seeded, nucleotide + amino acids (AGA)

Genome Region

Sequence starts at position 22 and ends at position 6747 relative to NC_010987.1 reference sequence.

Alignment Detailed Statistics

	Begin	End	Coverage	Score	Concordance	Matches	Identities	I/D/M/F*	Stop Codons
**NT**	**22**	**6747**	**93.0%**	**3341**	**25.4%**	**6659 (98.4%)**	**4235 (62.6%)**	**43/67**	

	Begin	End	Coverage	Score	Concordance	Matches	Identities	I/D/M/F*	Stop Codons
NT	22	6747	93.0%	3341	25.4%	6659 (98.4%)	4235 (62.6%)	43/67	

Mutations:

32T>C, 37G>A, 40C>T, 59A>C, 61T>C, 62T>A, 63G>T, 66A>C, 67_78delTTTGATTCTTGT, 81A>T, 82C>A, 83T>C, 87C>G, 89A>T, 90A>T, 91C>T, 94C>T, 99T>A, 100_101insC,
108A>T, 112G>C, 114C>T, 115C>T, 116A>C, 117T>C, 118T>C, 119A>T, 122T>G, 125A>T, 128C>A, 129C>A, 130T>A, 133_134delAT, 136T>A, 146T>A, 160T>G, 161C>G, 162T>A,
163C>G, 164C>T, 165_188delAAGGTTGCTGGTGTTGATGACGCC, 189A>C, 190G>C, 192T>C, 195T>G, 200C>T, 201A>T, 203C>A, 204A>C, 217G>C, 218T>A, 222C>G, 227A>T,
228A>T, 229A>C, 234A>T, 239C>T, 240A>T, 241A>C, 242A>T, 243T>C, 251A>G, 252G>A, 254G>T, 255G>C, 257T>C, 258A>T, 259G>C, 260T>C, 264A>G, 268C>A, 269C>T,
271C>A, 272T>A, 273A>G, 275G>T, 283A>C, 284G>C, 288T>G, 289C>T, 296T>C, 297C>A, 299T>A, 306C>G, 308G>C, 311A>G, 314A>G, 315T>C, 317T>A, 320G>T, 322C>T,
323T>G, 325A>C, 326T>A, 328T>C, 329G>A, 330T>G, 332C>A, 338T>A, 343T>C, 344G>C, 345G>T, 347C>T, 350T>A, 353T>C, 355G>A, 357T>G, 359A>T, 360C>G, 362T>A,
363A>G, 365T>A, 367A>G, 374G>A, 377C>T, 380A>G, 385G>C, 386T>A, 387G>A, 388C>A, 390C>A, 392T>A, 395A>G, 398T>A, 401A>C, 404T>C, 405C>A, 407T>C, 410A>G,
412C>G, 413G>T, 416C>T, 417T>G, 418C>A, 422C>T, 423T>G, 425G>T, 426T>A, 427C>A, 428C>A, 431A>T, 437C>T, 441T>A, 446C>A, 447A>G, 448A>T, 449G>A, 453A>C,
455G>T, 458T>A, 459T>C, 461A>T, 464A>G, 465T>A, 468T>G, 471G>T, 472A>T, 473G>A, 477T>G, 479G>C, 480T>A, 481C>A, 482C>G, 483A>G, 489G>T, 491T>C, 493A>T,
494A>C, 495T>C, 502T>A, 503T>C, 505C>A, 506T>A, 510G>T, 512G>A, 516A>G, 518T>A, 522A>G, 523G>T, 527A>G, 528G>T, 529A>C, 530T>A, 531G>T, 542T>G, 543G>T,
545A>T, 546T>A, 547A>T, 548T>A, 550G>C, 555C>T, 557T>A, 560A>T, 567A>T, 569A>G, 570A>G, 572A>C, 573T>A, 575G>T, 578A>G, 579G>C, 581G>A, 584T>A, 587A>G,
589T>A, 593T>A, 596T>C, 600A>G, 601G>T, 602T>A, 611T>A, 612G>A, 613A>C, 615G>C, 616G>C, 618G>T, 621T>A, 622C>A, 626C>T, 627A>G, 628C>T, 629T>A, 632C>T,
633A>G, 634A>T, 635A>T, 637A>C, 639A>T, 641C>G, 646T>C, 647G>A, 648A>G, 650A>T, 653C>T, 659C>T, 660A>T, 662G>T, 665T>C, 668A>T, 669A>T, 670G>C, 671T>A,
672C>T, 674A>G, 677C>T, 697A>C, 702G>T, 704C>T, 706A>G, 707A>G, 708C>A, 709G>A, 710A>G, 713C>T, 714A>T, 717A>C, 718T>A, 720G>C, 725C>G, 731T>A, 732A>G,
733G>C, 734T>A, 735A>G, 737G>A, 746G>T, 750T>A, 751C>G, 752A>T, 753G>T, 755T>A, 756T>A, 758A>G, 770G>T, 771T>A, 772C>G, 773T>C, 776G>A, 777T>C, 779G>T,
780A>T, 782G>A, 786A>T, 789A>T, 791A>G, 803G>T, 806T>C, 809G>A, 812G>T, 815G>A, 818G>A, 821C>T, 822A>G, 827A>T, 830G>T, 836T>G, 848C>T, 849T>C, 851G>T,
852A>T, 853G>C, 854C>A, 861T>C, 866C>A, 872T>C, 875C>G, 878T>C, 881G>T, 882A>G, 884A>T, 896T>G, 899G>A, 911A>G, 914A>G, 918A>C, 919C>A, 920T>G, 921C>T,
923T>G, 927C>A, 929T>G, 932T>C, 938A>G, 944C>T, 947C>A, 950C>T, 953G>A, 956T>C, 974A>G, 978A>G, 980A>G, 983C>T, 984G>A, 988C>T, 989C>T, 995A>C, 998C>T,
1010G>A, 1019T>G, 1024G>A, 1028A>T, 1031G>C, 1032C>T, 1034G>A, 1046G>T, 1050G>C, 1059C>A, 1060A>C, 1064G>A, 1068G>T, 1069A>C, 1070T>G, 1072C>A, 1073A>G,
1076G>T, 1077T>G, 1078G>C, 1082T>A, 1085T>A, 1086G>T, 1088A>T, 1090C>T, 1100G>A, 1103C>T, 1109C>A, 1113A>C, 1114A>C, 1115G>T, 1118T>A, 1121C>T, 1124T>A,
1126C>T, 1127C>T, 1130A>G, 1134A>C, 1136C>A, 1140C>T, 1142G>A, 1144G>A, 1145G>A, 1148A>G, 1149G>C, 1151A>G, 1154T>C, 1155C>G, 1160G>A, 1163C>A, 1178A>C,
1190T>C, 1193G>A, 1199C>T, 1200A>T, 1215A>T, 1216G>C, 1226T>A, 1227G>A, 1233T>C, 1238A>T, 1242G>C, 1244T>G, 1245T>G, 1246A>T, 1253A>G, 1254A>G, 1255G>C,
1256T>C, 1257T>A, 1259T>C, 1260A>C, 1261G>A, 1262G>A, 1265C>T, 1266A>T, 1267T>C, 1268G>T, 1271C>T, 1280G>A, 1284C>A, 1286T>G, 1292A>G, 1298T>C, 1304A>G,
1313C>T, 1316G>A, 1317T>G, 1319C>T, 1325G>A, 1337T>A, 1340A>G, 1342C>G, 1343C>T, 1350T>C, 1352A>T, 1355T>C, 1358T>C, 1367G>T, 1368T>C, 1370G>C, 1373C>T,
1374A>G, 1375G>C, 1385G>A, 1391A>G, 1394C>G, 1397T>C, 1400G>C, 1401C>T, 1403A>G, 1409A>G, 1418G>A, 1421G>A, 1445T>A, 1448C>T, 1457T>A, 1458A>G, 1460C>G,
1463G>T, 1472C>T, 1478A>T, 1481A>T, 1482T>C, 1484G>T, 1487A>G, 1493G>A, 1496T>A, 1505C>T, 1508A>G, 1511T>C, 1514T>A, 1517A>G, 1529T>C, 1538A>T, 1541A>C,
1544A>G, 1547A>T, 1553C>T, 1554T>C, 1556A>T, 1565C>T, 1571T>C, 1577C>T, 1583C>A, 1589T>C, 1592C>T, 1595C>A, 1598C>A, 1604T>C, 1607A>C, 1616A>C, 1623C>T,
1628C>A, 1631A>T, 1637T>A, 1640C>T, 1643A>G, 1649T>G, 1652T>A, 1655A>G, 1661A>T, 1667T>A, 1673G>A, 1674C>A, 1679A>G, 1682G>A, 1685C>T, 1688C>T, 1689C>T,
1691G>A, 1694G>T, 1700T>C, 1702T>G, 1703G>A, 1708A>T, 1715G>T, 1719T>G, 1720C>A, 1721C>T, 1722A>C, 1724T>A, 1725G>A, 1727A>T, 1728G>A, 1729A>C, 1733T>A,
1736G>A, 1738G>C, 1739A>C, 1745C>A, 1746A>G, 1747G>T, 1748A>T, 1752A>G, 1754C>T, 1755A>G, 1757A>G, 1763G>A, 1775T>C, 1781T>C, 1787T>A, 1790C>T, 1793G>A,
1794A>G, 1795A>C, 1799G>A, 1800A>T, 1806A>G, 1807G>A, 1811T>C, 1814T>C, 1818G>C, 1820G>A, 1829A>G, 1832C>A, 1835A>G, 1838T>C, 1840A>G, 1844A>G, 1847T>C,
1848G>T, 1849C>A, 1850A>T, 1853A>G, 1856A>G, 1861A>G, 1862G>C, 1866T>C, 1868G>T, 1870T>A, 1871T>C, 1872G>A, 1874A>T, 1875G>A, 1878G>A, 1879A>C, 1883T>G,
1886C>A, 1887A>T, 1889A>G, 1890A>G, 1891G>C, 1893C>G, 1894C>A, 1895T>C, 1899A>G, 1901A>T, 1910T>C, 1911C>A, 1913A>T, 1917T>G, 1921C>T, 1922A>T, 1928T>A,
1932A>C, 1934A>G, 1936C>A, 1937G>T, 1941G>T, 1943G>C, 1944G>C, 1946T>A, 1948G>A, 1949G>T, 1952T>C, 1953_1955delAGA, 1958G>A, 1960G>A, 1961C>A, 1962A>G,
1963C>T, 1964C>T, 1965A>T, 1966A>T, 1969A>C, 1970A>T, 1971G>C, 1972A>C, 1976A>G, 1980A>G, 1981T>C, 1983T>G, 1984T>C, 1985G>T, 1986G>T, 1987T>A, 1988G>T,
1989C>G, 1990C>A, 1991C>G, 1992A>C, 1993C>A, 1994T>G, 1995G>A, 1996T>C, 1997T>A, 1998G>A, 2000G>T, 2002G>C, 2003T>A, 2007A>G, 2008A>C, 2012T>A, 2014G>A,
2016G>A, 2018G>T, 2019A>G, 2021C>T, 2024T>C, 2025G>T, 2027A>C, 2028A>C, 2029G>A, 2030A>G, 2033G>A, 2039G>A, 2042A>T, 2048A>C, 2049T>C, 2051G>C, 2060A>T,
2066G>A, 2069C>G, 2073T>A, 2076G>A, 2079C>A, 2080C>A, 2081T>A, 2082G>T, 2083A>T, 2084G>T, 2087A>G, 2088C>T, 2093T>C, 2096T>A, 2104A>T, 2105T>C, 2106C>T,
2107A>C, 2108T>C, 2117C>T, 2120G>C, 2127T>G, 2128C>T, 2129T>G, 2132T>A, 2133G>T, 2134A>C, 2136G>T, 2137A>C, 2141A>T, 2142C>G, 2143A>C, 2144T>C, 2148G>C,
2152T>A, 2156C>T, 2157A>G, 2159C>A, 2161A>T, 2162A>G, 2166A>G, 2167G>T, 2169G>A, 2170T>A, 2171C>T, 2172A>G, 2174C>T, 2175A>T, 2181T>G, 2182A>T, 2183T>C,
2185G>A, 2193G>A, 2194A>G, 2196G>C, 2199G>C, 2201A>G, 2208C>G, 2209G>C, 2210A>T, 2219A>G, 2221A>G, 2237C>T, 2243A>C, 2249C>T, 2252T>C, 2268G>A, 2269C>G,
2270A>C, 2271T>A, 2279C>T, 2285G>A, 2286A>G, 2289A>C, 2290A>T, 2291A>G, 2294A>G, 2296C>A, 2297A>T, 2300G>C, 2303G>A, 2304A>C, 2305C>G, 2306A>C, 2307G>A,
2309G>T, 2310A>C, 2312G>T, 2313A>G, 2314G>A, 2316A>C, 2317C>A, 2318T>A, 2321A>G, 2322T>A, 2323T>A, 2324T>A, 2327A>G, 2328C>T, 2331T>A, 2332C>A, 2333T>G,
2334T>G, 2335G>C, 2336C>G, 2337A>C, 2340G>C, 2342T>G, 2345A>T, 2350T>C, 2351T>A, 2354C>T, 2357C>A, 2360C>T, 2362T>C, 2363A>G, 2369C>T, 2372G>A, 2385T>A,
2386T>G, 2387T>A, 2390T>A, 2396T>C, 2397T>C, 2399A>G, 2405A>T, 2408A>T, 2415A>C, 2417A>C, 2426C>T, 2428A>G, 2429C>G, 2435A>T, 2436T>G, 2438_2439insGGT,
2440A>C, 2441A>T, 2444G>C, 2445C>A, 2446C>A, 2447A>G, 2451G>A, 2452A>G, 2454T>A, 2455C>A, 2456A>T, 2462C>T, 2464A>T, 2465A>C, 2466A>G, 2467T>A, 2468T>A,
2472T>A, 2473A>T, 2474C>G, 2475C>A, 2476A>T, 2478A>G, 2479A>G, 2480A>T, 2484G>A, 2485T>A, 2486T>G, 2489A>C, 2490T>A, 2492T>A, 2495C>G, 2497A>T, 2498G>C,
2499G>C, 2501T>G, 2503C>T, 2504A>G, 2508T>A, 2509G>A, 2510G>T, 2511A>G, 2512G>A, 2514C>T, 2516T>G, 2521A>T, 2522T>C, 2523G>A, 2524C>T, 2525A>C, 2526C>T,
2530G>A, 2531A>G, 2537C>T, 2546A>C, 2549T>A, 2556A>T, 2558A>G, 2561G>A, 2564T>A, 2573A>C, 2574G>A, 2575T>C, 2576T>C, 2577T>A, 2579T>G, 2580G>A, 2581G>A,
2582A>T, 2584G>C, 2586A>G, 2591A>T, 2594A>T, 2597T>C, 2598T>A, 2600G>T, 2601G>A, 2603G>T, 2607T>C, 2608G>T, 2609G>C, 2610C>A, 2613A>G, 2614G>C, 2615T>A,
2616A>G, 2617A>C, 2618C>A, 2619T>A, 2623T>C, 2627C>T, 2628A>T, 2629A>T, 2630T>A, 2633A>T, 2634T>G, 2635C>A, 2636G>T, 2645A>G, 2648T>C, 2651G>A, 2660A>G,
2672A>C, 2678T>C, 2684G>A, 2687C>T, 2690G>A, 2693T>G, 2696T>G, 2698A>C, 2699G>T, 2699_2700insCAT, 2700C>T, 2703A>G, 2704G>C, 2708T>A, 2709C>T, 2710T>C,
2711T>C, 2712C>T, 2714C>G, 2715G>C, 2717T>A, 2720T>G, 2724A>G, 2725G>C, 2726T>G, 2727T>G, 2729T>A, 2730C>G, 2732A>T, 2733T>A, 2734C>A, 2735A>C, 2736T>A,
2737T>A, 2738G>C, 2739G>C, 2740C>A, 2741A>T, 2742C>G, 2743A>T, 2744A>G, 2747T>C, 2747_2748insCGG, 2753T>A, 2755C>A, 2756T>A, 2757G>C, 2759A>G, 2771A>T,
2775C>G, 2777G>A, 2780C>T, 2783C>G, 2784A>C, 2786G>C, 2792T>C, 2798G>T, 2801G>T, 2804A>G, 2808C>A, 2812G>C, 2822G>C, 2825C>T, 2828T>C, 2834C>T, 2837C>G,
2840C>T, 2843G>T, 2858A>T, 2859A>C, 2861G>C, 2864C>T, 2867A>G, 2870T>A, 2876A>C, 2879C>T, 2880C>A, 2881A>G, 2882C>A, 2885T>C, 2886G>A, 2887C>T, 2888T>G,
2889C>A, 2891A>G, 2897T>C, 2903A>T, 2907A>T, 2909A>C, 2915C>T, 2920C>A, 2921C>G, 2922A>C, 2927T>C, 2928C>A, 2929G>T, 2930A>T, 2932C>G, 2936A>T, 2939T>G,
2942A>G, 2944A>T, 2948C>T, 2953G>C, 2954A>C, 2955G>C, 2962G>A, 2964A>T, 2966T>G, 2969T>A, 2975C>T, 2981C>T, 2982A>G, 2984C>G, 2988C>T, 2994C>A, 2996C>G,
2999T>A, 3002T>G, 3017A>C, 3020G>A, 3021C>T, 3023T>A, 3026C>T, 3029C>A, 3030C>A, 3032T>A, 3038C>T, 3044A>T, 3045A>C, 3047A>C, 3050G>A, 3056G>T, 3062C>T,
3064T>A, 3066A>T, 3068A>T, 3078C>A, 3079A>T, 3080A>G, 3083A>C, 3090T>G, 3091G>T, 3092T>G, 3095A>G, 3101T>A, 3104G>T, 3107T>A, 3109C>A, 3110A>G, 3116A>C,
3117C>A, 3118G>A, 3119T>G, 3120C>A, 3121T>A, 3122T>A, 3123G>T, 3124G>C, 3131A>G, 3135T>G, 3140C>T, 3143G>A, 3144A>T, 3145T>A, 3146G>T, 3167A>C, 3173A>C,
3175T>A, 3176T>A, 3182G>C, 3183G>A, 3184A>C, 3190A>G, 3192C>T, 3194G>A, 3195A>G, 3197C>T, 3200C>G, 3201T>A, 3202C>A, 3203C>T, 3204A>G, 3205G>C, 3206T>A,
3210A>G, 3211A>C, 3212G>T, 3213A>C, 3216T>A, 3221T>A, 3225C>G, 3226A>T, 3227A>T, 3228T>G, 3230T>A, 3236A>G, 3245A>T, 3249G>A, 3251G>T, 3254A>G, 3260T>A,
3263C>T, 3269C>T, 3272G>T, 3273T>C, 3275G>T, 3278A>C, 3279C>T, 3281G>A, 3284A>G, 3289G>C, 3293T>G, 3299T>C, 3305A>G, 3308T>G, 3309G>T, 3310T>G, 3311G>C,
3320C>A, 3323C>A, 3326C>T, 3335A>T, 3343G>A, 3344C>T, 3345G>A, 3346T>C, 3347A>C, 3350A>T, 3353T>A, 3356A>G, 3357C>A, 3358G>A, 3359T>G, 3362A>T, 3364T>A,
3365T>C, 3366A>G, 3368C>G, 3369T>G, 3377T>C, 3380T>A, 3386T>A, 3387A>G, 3389C>G, 3391A>T, 3392C>T, 3395G>A, 3398A>T, 3402C>T, 3404A>G, 3407G>A, 3416A>C,
3419T>G, 3422G>A, 3425G>C, 3426G>A, 3428C>T, 3433C>T, 3434A>G, 3437A>T, 3443A>G, 3445G>A, 3452T>A, 3453A>C, 3455T>G, 3456C>T, 3462T>C, 3463C>A, 3464A>C,
3465A>C, 3467T>G, 3471A>T, 3472G>C, 3475G>A, 3476A>G, 3479A>G, 3480A>G, 3481C>A, 3483C>A, 3484A>G, 3485G>A, 3486C>T, 3488T>C, 3497A>G, 3500C>A, 3503A>G,
3507T>G, 3508A>C, 3509C>T, 3511A>T, 3513C>T, 3515T>G, 3518A>C, 3521A>T, 3524T>A, 3527A>G, 3528C>T, 3529A>T, 3533G>A, 3534T>A, 3539C>T, 3542C>T, 3548T>G,
3551C>G, 3557G>A, 3560C>A, 3563T>C, 3566G>T, 3567C>A, 3568G>A, 3569G>A, 3570A>C, 3571A>G, 3572A>C, 3575C>T, 3577A>C, 3578A>C, 3579T>C, 3587A>G, 3590A>T,
3593C>T, 3596A>G, 3597C>G, 3598C>A, 3599T>C, 3602A>T, 3603G>C, 3608A>T, 3611A>C, 3624A>T, 3626A>T, 3628T>C, 3629T>C, 3630C>A, 3632T>A, 3641A>G, 3644T>A,
3647T>A, 3650T>G, 3656C>T, 3671G>T, 3674A>C, 3675A>G, 3676T>A, 3680A>G, 3683C>T, 3697G>C, 3698T>C, 3699G>A, 3700G>A, 3701A>C, 3707T>G, 3710T>G, 3711G>C,
3712A>C, 3713C>A, 3719C>A, 3722A>G, 3725A>G, 3728A>C, 3731C>T, 3734A>G, 3735A>G, 3736A>G, 3740C>T, 3743G>A, 3744T>G, 3745G>C, 3746C>A, 3749G>C, 3750C>A,
3752G>T, 3755A>T, 3759A>G, 3761C>G, 3762A>C, 3764G>T, 3765C>A, 3766G>A, 3767T>G, 3769G>T, 3770C>G, 3774A>C, 3775A>G, 3776G>A, 3777A>G, 3779C>G, 3782T>C,
3789C>A, 3790G>A, 3791T>A, 3794A>T, 3795T>G, 3796G>T, 3797T>A, 3798G>A, 3799A>T, 3800A>C, 3803G>A, 3804G>A, 3827A>G, 3838G>T, 3845C>T, 3847A>T, 3853G>C,
3854C>A, 3860G>A, 3861C>T, 3869A>G, 3872T>A, 3875C>T, 3878A>T, 3884C>T, 3887C>T, 3894A>T, 3899G>A, 3900A>C, 3902G>T, 3905G>T, 3906A>T, 3907T>C, 3908G>T,
3914G>A, 3917A>G, 3927C>G, 3930T>G, 3933A>C, 3934C>A, 3935T>A, 3936G>C, 3937G>C, 3938C>A, 3939G>A, 3941A>C, 3942G>T, 3943G>C, 3944A>G, 3945G>C, 3950A>G,
3953G>T, 3954G>C, 3955A>T, 3956A>T, 3965A>G, 3966G>A, 3971T>A, 3977A>C, 3983A>T, 3996C>T, 3998A>G, 4001T>C, 4002G>T, 4003T>C, 4008A>G, 4010T>A, 4014A>C,
4021T>C, 4022C>A, 4023C>T, 4024A>T, 4028G>T, 4031T>C, 4034G>C, 4040C>A, 4046A>G, 4049C>T, 4052C>G, 4064C>G, 4065C>T, 4067C>G, 4079A>G, 4082C>T, 4088C>G,
4094A>G, 4101A>C, 4103G>C, 4106C>G, 4109T>C, 4112G>A, 4115A>C, 4116C>T, 4118T>G, 4130C>T, 4137T>C, 4142C>G, 4143A>C, 4148G>A, 4151T>C, 4154T>C, 4160T>C,
4163C>T, 4166A>T, 4169C>T, 4170A>T, 4171C>G, 4172A>T, 4182T>G, 4183T>C, 4184G>A, 4190T>C, 4192C>T, 4193T>A, 4194A>C, 4196A>C, 4199A>T, 4205A>G, 4208T>A,
4214T>C, 4215G>A, 4220T>A, 4223A>G, 4226T>C, 4227T>C, 4228C>A, 4239T>G, 4242T>C, 4247A>T, 4249A>G, 4251G>A, 4253G>C, 4259A>G, 4260G>T, 4262T>A, 4265G>A,
4268A>T, 4271C>T, 4273T>A, 4280T>C, 4286T>C, 4292T>C, 4295C>G, 4316T>C, 4317G>A, 4319T>C, 4325T>A, 4328T>C, 4331G>A, 4344T>C, 4354A>C, 4355A>T, 4357A>T,
4358C>T, 4361C>T, 4364A>T, 4365T>G, 4366C>A, 4367A>T, 4368A>G, 4376C>G, 4385T>G, 4388C>A, 4395C>A, 4397A>G, 4400T>C, 4401G>A, 4404C>T, 4406T>A, 4418A>C,
4424T>C, 4430C>T, 4433A>C, 4436G>C, 4439C>T, 4443G>A, 4445T>C, 4448A>G, 4449G>T, 4455A>C, 4457G>C, 4458T>C, 4460G>A, 4463T>G, 4466G>T, 4469C>A, 4475A>C,
4478A>T, 4481A>T, 4487A>T, 4488C>T, 4490A>G, 4493G>T, 4499C>A, 4502C>T, 4508T>A, 4509C>T, 4511C>G, 4514C>A, 4530C>A, 4532A>G, 4535T>C, 4538C>G, 4548T>C,
4553A>G, 4556A>G, 4562C>A, 4566A>G, 4568T>C, 4569G>A, 4570C>A, 4571T>A, 4576G>A, 4577G>A, 4578G>T, 4581A>G, 4583A>G, 4586A>C, 4587G>A, 4592A>G, 4597G>A,
4601T>C, 4611C>T, 4613A>G, 4622T>A, 4625T>A, 4631A>T, 4637C>T, 4640T>C, 4641C>T, 4643T>G, 4649T>G, 4652A>T, 4656T>A, 4658A>G, 4662C>T, 4664C>G, 4665C>A,
4666A>G, 4673T>C, 4674T>G, 4675C>A, 4679G>A, 4683A>T, 4684A>C, 4685T>C, 4688A>T, 4692C>A, 4693A>G, 4694T>A, 4697G>A, 4698T>C, 4700A>G, 4704C>G, 4705T>C,
4706G>T, 4709C>T, 4712T>C, 4715A>C, 4716A>G, 4731C>T, 4733T>G, 4739A>G, 4742G>A, 4751T>A, 4752A>G, 4754T>G, 4755G>A, 4758G>A, 4759T>C, 4761C>A, 4768A>T,
4769A>G, 4784A>C, 4787T>C, 4793G>C, 4799A>G, 4805T>C, 4808A>G, 4817T>A, 4818C>T, 4820T>G, 4826C>T, 4829C>A, 4835T>A, 4838T>A, 4844G>A, 4854A>G, 4859T>C,
4868G>T, 4877A>T, 4880A>G, 4883T>A, 4886C>A, 4887C>G, 4888C>T, 4889A>G, 4892T>A, 4901C>T, 4902A>G, 4903C>T, 4904T>A, 4907G>A, 4910C>T, 4914A>T, 4915T>A,
4916G>T, 4923G>A, 4925G>T, 4934A>C, 4937A>C, 4938C>A, 4940T>A, 4946T>C, 4955C>T, 4958T>G, 4960G>A, 4961T>G, 4963T>C, 4964G>C, 4965G>A, 4973C>A, 4975A>G,
4976G>T, 4977C>T, 4982C>A, 4983T>A, 4984T>A, 4985A>T, 4992C>T, 4993A>T, 4994G>T, 4995A>G, 4996C>G, 4997G>A, 5000G>T, 5001T>C, 5003G>C, 5004G>A, 5006T>G,
5007G>C, 5009A>G, 5012G>A, 5013G>A, 5014G>A, 5016T>C, 5019A>C, 5020G>C, 5021A>C, 5022G>A, 5023C>T, 5024T>C, 5025G>A, 5031T>A, 5033T>G, 5036T>C, 5037T>A,
5039C>T, 5042T>C, 5044T>A, 5045G>T, 5049G>C, 5051G>T, 5055C>A, 5056A>G, 5057G>T, 5058G>C, 5064A>T, 5066T>G, 5067G>A, 5070A>G, 5075A>C, 5081G>A, 5085T>G,
5086C>A, 5087A>G, 5090T>C, 5091G>A, 5092C>T, 5093C>T, 5094C>G, 5095T>G, 5097A>G, 5098C>T, 5099A>T, 5100A>T, 5102T>G, 5103T>A, 5104G>T, 5105T>G, 5108A>G,
5109A>C, 5111G>A, 5112G>T, 5115A>G, 5117A>T, 5120A>T, 5121G>A, 5122C>A, 5123T>A, 5125T>A, 5127T>A, 5129C>A, 5132T>C, 5135G>C, 5138A>T, 5141G>A, 5142G>C,
5143A>C, 5144C>T, 5147A>T, 5150T>G, 5153G>A, 5159C>T, 5160_5162delACA, 5172C>G, 5173G>A, 5174T>G, 5176A>G, 5178C>G, 5180G>C, 5181A>C, 5184T>G, 5185G>A,
5186G>A, 5191C>T, 5192A>G, 5195A>T, 5196A>G, 5198C>T, 5199A>G, 5201T>A, 5202G>T, 5203G>T, 5207T>C, 5211T>A, 5212C>G, 5213A>T, 5216A>T, 5219T>G, 5226G>T,
5228C>A, 5229C>A, 5232T>C, 5234C>A, 5235A>C, 5236C>A, 5237G>A, 5241G>T, 5242A>C, 5243T>A, 5244G>T, 5245A>C, 5249G>T, 5250T>A, 5255T>C, 5256A>C, 5258T>G,
5264C>T, 5267T>C, 5270A>T, 5273T>A, 5276A>C, 5277A>C, 5278A>G, 5279G>C, 5281A>G, 5282G>A, 5283T>A, 5285C>A, 5287A>C, 5288T>A, 5294T>G, 5300T>G, 5303A>T,
5309C>T, 5333G>A, 5339G>C, 5346A>T, 5349T>G, 5354T>A, 5357A>C, 5358A>C, 5359T>A, 5364G>C, 5365C>A, 5366C>T, 5370A>C, 5371A>G, 5372G>C, 5375T>G, 5376G>A,
5377T>A, 5378G>T, 5379T>C, 5383C>A, 5386G>T, 5387A>T, 5390A>T, 5391C>G, 5395T>G, 5396A>G, 5399A>T, 5403A>T, 5404A>C, 5409G>A, 5410T>C, 5411T>C, 5417G>T,
5423A>T, 5429A>T, 5435T>A, 5436G>A, 5438C>A, 5440G>C, 5441C>T, 5447T>C, 5456G>A, 5457C>T, 5460A>T, 5462G>T, 5465C>A, 5468A>G, 5471A>C, 5474A>T, 5475A>C,
5476G>A, 5477G>T, 5483T>C, 5484T>A, 5486T>G, 5487G>A, 5488T>C, 5489G>C, 5490G>A, 5491A>C, 5492G>A, 5496A>C, 5498A>T, 5501A>T, 5504T>G, 5505A>C, 5507A>C,
5508T>C, 5510G>A, 5513C>A, 5516A>C, 5519T>G, 5521G>C, 5522A>T, 5523A>G, 5524A>G, 5525T>G, 5531T>G, 5534C>T, 5537C>G, 5540T>C, 5546T>C, 5555C>T, 5560T>C,
5564G>A, 5567T>C, 5570T>G, 5573T>C, 5574C>A, 5582G>T, 5583C>G, 5584C>A, 5585T>A, 5586C>G, 5588T>C, 5588_5589insACC, 5589G>T, 5590G>C, 5592G>C, 5597A>G,
5598G>T, 5602C>G, 5603T>A, 5606A>G, 5612T>C, 5613G>A, 5617C>A, 5618G>T, 5619C>G, 5620T>G, 5622_5624delCAC, 5626G>C, 5627T>C, 5629G>C, 5635T>A, 5636G>C,
5642A>T, 5642_5643insCACAGT, 5644G>C, 5645G>A, 5649G>T, 5650A>C, 5652T>G, 5654G>T, 5657C>A, 5658A>C, 5659A>T, 5660C>T, 5663C>A, 5664C>A, 5666C>G,
5669T>A, 5670A>G, 5672A>T, 5674A>C, 5676G>T, 5677G>C, 5678T>C, 5681T>C, 5682T>C, 5684A>C, 5685G>T, 5687A>T, 5688A>G, 5691C>A, 5695C>T, 5696A>G, 5700A>G,

	5702G>T, 5705G>T, 5706A>T, 5714T>C, 5715G>A, 5716T>C, 5717G>A, 5718G>A, 5720A>T, 5723A>T, 5726A>T, 5727C>G, 5729T>A, 5735A>T, 5740G>C, 5741A>C, 5742A>C, 5744T>G, 5746T>G, 5747T>C, 5750C>A, 5753A>G, 5754C>A, 5757G>A, 5759T>C, 5762T>G, 5763G>A, 5766A>C, 5767G>A, 5769T>G, 5770T>C, 5771G>T, 5772C>T, 5774A>G, 5775C>G, 5777C>G, 5778T>A, 5779A>C, 5780T>A, 5784G>T, 5785A>C, 5786G>T, 5787C>G, 5788A>T, 5789G>C, 5790G>A, 5792A>T, 5798A>T, 5799G>A, 5800A>C, 5801G>T, 5802T>C, 5804A>T, 5805A>G, 5806T>C, 5807T>C, 5808C>G, 5809A>C, 5812A>G, 5813G>A, 5814A>G, 5815A>G, 5816T>A, 5817A>G, 5818C>T, 5819A>C, 5823C>G, 5825A>G, 5828T>A, 5829C>T, 5837C>T, 5843G>A, 5844C>T, 5846G>C, 5847A>T, 5848G>C, 5849T>C, 5850A>C, 5851T>A, 5852G>A, 5853G>C, 5855A>C, 5856A>G, 5859G>T, 5860T>G, 5861G>T, 5862C>G, 5865C>A, 5866A>T, 5867A>G, 5868C>A, 5870G>A, 5871A>C, 5873A>T, 5876T>G, 5880G>C, 5882T>G, 5883A>G, 5885G>A, 5886C>A, 5887T>G, 5891G>A, 5895T>G, 5897T>A, 5898T>G, 5900C>A, 5901C>T, 5902C>T, 5903A>C, 5905A>T, 5906A>G, 5908A>G, 5909A>G, 5911C>G, 5912T>C, 5913A>G, 5914C>T, 5915C>T, 5917T>A, 5918T>G, 5919C>A, 5921G>A, 5922A>C, 5924G>A, 5925C>A, 5927C>G, 5928A>T, 5929A>T, 5930A>G, 5931A>T, 5932A>C, 5933T>C, 5936T>C, 5937G>A, 5939T>G, 5940G>A, 5943G>C, 5944C>A, 5945T>A, 5946G>C, 5948A>G, 5949T>A, 5950G>T, 5951T>G, 5952C>T, 5953T>C, 5956C>A, 5958T>A, 5959T>C, 5961A>C, 5965C>T, 5966A>T, 5967A>G, 5968G>C, 5969G>C, 5972_5973insCCATGTTGTGTCACA, 5978G>T, 5979C>A, 5981C>A, 5983T>C, 5985G>T, 5986T>G, 5987T>G, 5988T>C, 5990C>A, 5992A>C, 5993A>T, 5997A>G, 5999A>C, 6000C>A, 6003T>G, 6005G>C, 6007G>T, 6008T>G, 6009T>A, 6011C>A, 6013C>A, 6015T>G, 6017G>T, 6020A>G, 6022T>A, 6024T>C, 6025G>C, 6026T>A, 6029T>A, 6030_6032delGAA, 6033A>T, 6034G>C, 6039_6050delGTTGCTTCAAGG, 6051T>C, 6056A>T, 6059G>A, 6061G>C, 6062T>A, 6063C>G, 6065A>G, 6068C>T, 6070C>T, 6074C>G, 6078T>C, 6079T>C, 6080G>T, 6083T>A, 6086T>A, 6087T>C, 6088C>T, 6089C>A, 6092A>T, 6093T>G, 6097A>G, 6102T>A, 6104A>G, 6108G>C, 6109A>G, 6110A>C, 6112G>A, 6113A>T, 6114C>A, 6115A>G, 6116C>T, 6117A>G, 6118C>G, 6122A>G, 6126A>T, 6127T>A, 6128G>C, 6129A>T, 6130G>C, 6131T>A, 6135C>A, 6136A>C, 6137G>A, 6139G>C, 6140T>A, 6141A>C, 6146G>A, 6153C>T, 6155G>T, 6157A>G, 6161A>G, 6164T>C, 6165C>A, 6167A>G, 6169T>G, 6170A>T, 6171A>C, 6173C>G, 6174T>G, 6175T>A, 6176C>A, 6177C>G, 6178A>G, 6179A>G, 6180G>T, 6181T>A, 6182A>T, 6183A>T, 6186A>G, 6187C>A, 6188A>T, 6190G>A, 6191T>A, 6193A>C, 6194G>C, 6198T>G, 6200A>G, 6201A>G, 6203G>C, 6204A>C, 6205A>G, 6206G>C, 6209T>C, 6210G>A, 6212A>T, 6215T>G, 6219T>A, 6221T>G, 6222C>G, 6223A>G, 6224G>C, 6226A>T, 6227G>T, 6228A>T, 6229A>G, 6230G>T, 6231A>G, 6233A>G, 6236C>G, 6237A>G, 6238C>A, 6239C>G, 6240A>C, 6241C>G, 6243C>A, 6245A>C, 6246C>T, 6248A>G, 6249A>G, 6251C>G, 6252A>C, 6253A>G, 6254A>C, 6255G>A, 6256T>C, 6258T>A, 6260C>T, 6261T>G, 6262T>C, 6263G>T, 6270G>T, 6271A>C, 6272G>C, 6273G>A, 6274A>C, 6276A>T, 6277G>C, 6278T>A, 6280A>T, 6281T>G, 6282C>A, 6284G>C, 6285A>G, 6286C>A, 6287T>G, 6290G>T, 6294T>A, 6295G>C, 6297A>G, 6299A>G, 6300G>T, 6302T>C, 6303T>A, 6305T>A, 6306C>G, 6308G>T, 6310T>G, 6314C>T, 6315C>T, 6316A>T, 6317G>T, 6318A>G, 6319G>A, 6320G>T, 6321_6323delATA, 6324A>C, 6326C>T, 6328C>T, 6330C>A, 6333A>G, 6334A>C, 6336T>G, 6338C>G, 6339A>G, 6340G>T, 6341A>T, 6342G>T, 6344G>C, 6345C>A, 6348G>C, 6349A>C, 6350A>T, 6353T>G, 6359T>C, 6361A>C, 6364T>G, 6366C>G, 6368T>A, 6369C>A, 6370C>T, 6371T>C, 6372G>A, 6374A>T, 6376G>A, 6377A>T, 6378A>G, 6379T>C, 6383C>T, 6392C>T, 6394T>C, 6402A>G, 6404A>G, 6405A>T, 6407A>G, 6411T>G, 6412C>A, 6413T>A, 6416G>C, 6420G>A, 6421A>G, 6423T>C, 6424C>A, 6427A>G, 6428T>G, 6429A>T, 6430G>C, 6431C>A, 6432C>A, 6433G>A, 6434A>G, 6435G>T, 6436A>C, 6437T>A, 6438A>C, 6440T>A, 6442A>G, 6444T>A, 6445C>A, 6446C>G, 6448A>T, 6449T>C, 6450T>A, 6453G>T, 6455G>T, 6456T>G, 6458A>C, 6460C>A, 6462C>G, 6463C>A, 6464C>A, 6465A>G, 6467C>T, 6468A>G, 6471C>A, 6473G>A, 6474G>T, 6475G>C, 6476G>A, 6477T>A, 6478G>A, 6479T>C, 6480T>C, 6482C>G, 6483A>C, 6488C>A, 6489C>G, 6490T>C, 6494C>T, 6495G>T, 6496T>A, 6497G>T, 6498C>T, 6503T>A, 6505G>A, 6507C>A, 6509A>G, 6510G>C, 6511G>T, 6514A>T, 6515G>A, 6517A>G, 6518A>T, 6519G>C, 6520A>T, 6521T>A, 6522C>G, 6524T>G, 6525C>T, 6526C>A, 6528G>T, 6529T>A, 6531C>G, 6532G>A, 6533G>T, 6534A>T, 6535G>C, 6536G>T, 6537T>A, 6539T>A, 6540T>G, 6541C>A, 6543A>T, 6545G>T, 6546G>T, 6548T>G, 6549T>G, 6550T>A, 6552C>T, 6554T>G, 6555A>T, 6556A>T, 6558A>C, 6560C>A, 6561T>A, 6562G>T, 6563T>A, 6566A>T, 6572T>A, 6576A>G, 6582T>G, 6583A>T, 6588G>T, 6590G>C, 6594T>G, 6596A>G, 6597G>T, 6598T>C, 6599_6600delCA, 6603C>G, 6606T>A, 6609G>A, 6610A>G, 6611C>A, 6612T>C, 6614A>C, 6620T>C, 6621G>A, 6624A>T, 6624_6625insG, 6626A>T, 6629C>T, 6643G>A, 6647_6648insACAAACAT, 6649T>A, 6652A>G, 6654C>T, 6655G>T, 6657T>G, 6658A>T, 6659G>A, 6660G>T, 6661A>C, 6663G>A, 6664G>C, 6667T>G, 6669G>A, 6672A>C, 6683C>A, 6687C>T, 6688T>C, 6692A>G, 6695A>G, 6696G>A, 6715T>C

CDS

ToMarV_RNA1gp1	1	2152	100%	9871	66.5%	2135 (98.7%)	1362 (63.0%)	11/17/0/0	0

	Begin	End	Coverage	Score	Concordance	Matches	Identities	I/D/M/F*	Stop Codons
**NT**	**22**	**6747**	**93.0%**	**3341**	**25.4%**	**6659 (98.4%)**	**4235 (62.6%)**	**43/67**	

Protein mutations:

F7W (160T>G 161C>G), K9_A16del (165_188delAAGGTTGCTGGTGTTGATGACGCC), S17P (189A>C 190G>C), S18P (192T>C), S19A (195T>G), T21S (201A>T 203C>A), T22P (204A>C), G26A (217G>C 218T>A), L28V (222C>G), N30S (228A>T 229A>C), I32F (234A>T), K34S (240A>T 241A>C 242A>T), F35L (243T>C), A38T (252G>A 254G>T), V39L (255G>C 257T>C), I42V (264A>G), T43N (268C>A 269C>T), T44K (271C>A 272T>A), M45V (273A>G 275G>T), K48T (283A>C 284G>C), S50V (288T>G 289C>T), L53I (297C>A 299T>A), L56V (306C>G 308G>C), S59P (315T>C 317T>A), T61M (322C>T 323T>G), D62A (325A>C 326T>A), V63A (328T>C 329G>A), S64A (330T>G 332C>A), V68A (343T>C 344G>C), A69S (345G>T 347C>T), S72N (355G>A), S73A (357T>G 359A>T), L74V (360C>G 362T>A), N75E (363A>G 365T>A), K76R (367A>G), G82A (385G>C 386T>A), A83K (387G>A 388C>A), L84I (390C>A 392T>A), I85M (395A>G), D86E (398T>A), L89I (405C>A 407T>C), A91G (412C>G 413G>T), S93D (417T>G 418C>A), L95V (423T>G 425G>T), S96K (426T>A 427C>A 428C>A), S101T (441T>A), K103V (447A>G 448A>T 449G>A), M105L (453A>C 455G>T), L109M (465T>A), F110V (468T>G), E111L (471G>T 472A>T 473G>A), L113V (477T>G 479G>C), S114K (480T>A 481C>A 482C>G), N115D (483A>G), V117F (489G>T 491T>C), K118I (493A>T 494A>C), F121Y (502T>A 503T>C), A122E (505C>A 506T>A), V124L (510G>T 512G>A), N126E (516A>G 518T>A), S128V (522A>G 523G>T), D130S (528G>T 529A>C 530T>A), V131L (531G>T), V135F (543G>T 545A>T), Y136I (546T>A 547A>T 548T>A), G137A (550G>C), I143L (567A>T 569A>G), T144A (570A>G 572A>C), L145I (573T>A 575G>T), V147L (579G>C 581G>A), F150Y (589T>A), S154V (600A>G 601G>T 602T>A), E158T (612G>A 613A>C), G159P (615G>C 616G>C), V160F (618G>T), S161N (621T>A 622C>A), T163V (627A>G 628C>T 629T>A), K165V (633A>G 634A>T 635A>T), N166T (637A>C), I167L (639A>T 641C>G), L169S (646T>C 647G>A), I170V (648A>G 650A>T), M174F (660A>T 662G>T), K186T (697A>C), A188S (702G>T 704C>T), E189G (706A>G 707A>G), R190K (708C>A 709G>A 710A>G), T192S (714A>T), M193Q (717A>C 718T>A), V194L (720G>C), S198A (732A>G 733G>C 734T>A), M199V (735A>G 737G>A), V205L (753G>T 755T>A), S206T (756T>A 758A>G), M214L (780A>T 782G>A), T216S (786A>T), I217L (789A>T 791A>G), I228V (822A>G), T248A (882A>G 884A>T), T260Q (918A>C 919C>A 920T>G), I280V (978A>G 980A>G), G282S (984G>A), T283I (988C>T 989C>T), S295N (1024G>A), E304Q (1050G>C), Q307T (1059C>A 1060A>C), D310S (1068G>T 1069A>C 1070T>G), A311E (1072C>A 1073A>G), C313A (1077T>G 1078G>C), D314E (1082T>A), A316S (1086G>T 1088A>T), T317M (1090C>T), K325P (1113A>C 1114A>C 1115G>T), T329I (1126C>T 1127C>T), N332Q (1134A>C 1136C>A), R335K (1144G>A 1145G>A), E337Q (1149G>C 1151A>G), L339V (1155C>G), M354L (1200A>T), A363T (1227G>A), E366D (1238A>T), V368L (1242G>C 1244T>G), Y369V (1245T>G 1246A>T), S372A (1254A>G 1255G>C 1256T>C), C373S (1257T>A 1259T>C), R374Q (1260A>C 1261G>A 1262G>A), M376S (1266A>T 1267T>C 1268G>T), S393A (1317T>G 1319C>T), S401C (1342C>G 1343C>T), R412A (1374A>G 1375G>C), I440V (1458A>G 1460C>G), M521R (1702T>G 1703G>A), E523V (1708A>T), E525D (1715G>T), S527D (1719T>G 1720C>A 1721C>T), T528P (1722A>C 1724T>A), E529N (1725G>A 1727A>T), D530T (1728G>A 1729A>C), G533A (1738G>C 1739A>C), R536V (1746A>G 1747G>T 1748A>T), N538D (1752A>G 1754C>T), I539V (1755A>G 1757A>G), N552A (1794A>G 1795A>C), T554S (1800A>T), S556D (1806A>G 1807G>A), E560Q (1818G>C 1820G>A), Q567R (1840A>G), A570Y (1848G>T 1849C>A 1850A>T), K574S (1861A>G 1862G>C), F577Y (1870T>A 1871T>C), E578N (1872G>A 1874A>T), A579T (1875G>A), E580T (1878G>A 1879A>C), I583L (1887A>T 1889A>G), S584A (1890A>G 1891G>C), P585D (1893C>G 1894C>A 1895T>C), I587V (1899A>G 1901A>T), Q591N (1911C>A 1913A>T), S593A (1917T>G), A594V (1921C>T 1922A>T), D596E (1928T>A), I598L (1932A>C 1934A>G), A599D (1936C>A 1937G>T), V601F (1941G>T 1943G>C), D602Q (1944G>C 1946T>A), G603D (1948G>A 1949G>T), R605del (1953_1955delAGA), R607Q (1960G>A 1961C>A), T608V (1962A>G 1963C>T 1964C>T), K609L (1965A>T 1966A>T), Q610P (1969A>C 1970A>T), D611P (1971G>C 1972A>C), I614A (1980A>G 1981T>C), L615A (1983T>G 1984T>C 1985G>T), V616Y (1986G>T 1987T>A 1988G>T), P617E (1989C>G 1990C>A 1991C>G), T618Q (1992A>C 1993C>A 1994T>G), V619T (1995G>A 1996T>C 1997T>A), V620I (1998G>A 2000G>T), S621T (2002G>C 2003T>A), N623A (2007A>G 2008A>C), D624E (2012T>A), R625K (2014G>A), V626I (2016G>A 2018G>T), I627V (2019A>G 2021C>T), A629S (2025G>T 2027A>C), R630Q (2028A>C 2029G>A 2030A>G), L645M (2073T>A), D646N (2076G>A), P647K (2079C>A 2080C>A 2081T>A), E648F (2082G>T 2083A>T 2084G>T), L650F (2088C>T), H655L (2104A>T 2105T>C), H656S (2106C>T 2107A>C 2108T>C), M660I (2120G>C), S663V (2127T>G 2128C>T 2129T>G), D665S (2133G>T 2134A>C), D666S (2136G>T 2137A>C), E667D (2141A>T), H668A (2142C>G 2143A>C 2144T>C), E670Q (2148G>C), I671N (2152T>A), N673E (2157A>G 2159C>A), K674M (2161A>T 2162A>G), R676V (2166A>G 2167G>T), V677N (2169G>A 2170T>A 2171C>T), N678D (2172A>G 2174C>T), M679L (2175A>T), Y681V (2181T>G 2182A>T 2183T>C), R682K (2185G>A), D685S (2193G>A 2194A>G), A686P (2196G>C), E687Q (2199G>C 2201A>G), R690A (2208C>G 2209G>C 2210A>T), Q694R (2221A>G), A710S (2268G>A 2269C>G 2270A>C), S711T (2271T>A), K716E (2286A>G), K717L (2289A>C 2290A>T 2291A>G), A719D (2296C>A 2297A>T), T722R (2304A>C 2305C>G 2306A>C), E723N (2307G>A 2309G>T), M724L (2310A>C 2312G>T), R725E (2313A>G 2314G>A), T726Q (2316A>C 2317C>A 2318T>A), F728K (2322T>A 2323T>A 2324T>A), H730Y (2328C>T), S731K (2331T>A 2332C>A 2333T>G), C732A (2334T>G 2335G>C 2336C>G), I733L (2337A>C), D734Q (2340G>C 2342T>G), V737A (2350T>C 2351T>A), I741T (2362T>C 2363A>G), F749R (2385T>A 2386T>G 2387T>A), N763R (2428A>G 2429C>G), S766A (2436T>G), S766_K767insG (2438_2439insGGT), K767T (2440A>C 2441A>T), E768D (2444G>C), P769K (2445C>A 2446C>A 2447A>G), E771R (2451G>A 2452A>G), S772N (2454T>A 2455C>A 2456A>T), K775I (2464A>T 2465A>C), I776E (2466A>G 2467T>A 2468T>A), Y778M (2472T>A 2473A>T 2474C>G), H779I (2475C>A 2476A>T), K780G (2478A>G 2479A>G 2480A>T), V782K (2484G>A 2485T>A 2486T>G), E783D (2489A>C), Y784K (2490T>A 2492T>A), I785M (2495C>G), K786I (2497A>T 2498G>C), D787Q (2499G>C 2501T>G), T788M (2503C>T 2504A>G), W790N (2508T>A 2509G>A 2510G>T), S791D (2511A>G 2512G>A), Y794F (2521A>T 2522T>C), A795I (2523G>A 2524C>T 2525A>C), R797K (2530G>A 2531A>G), I806L (2556A>T 2558A>G), M807I (2561G>A), V812T (2574G>A 2575T>C 2576T>C), F813M (2577T>A 2579T>G), G814N (2580G>A 2581G>A 2582A>T), G815A (2584G>C), I816V (2586A>G), L820I (2598T>A 2600G>T), A821T (2601G>A 2603G>T), W823L (2607T>C 2608G>T 2609G>C), Q824K (2610C>A), S825A (2613A>G 2614G>C 2615T>A), N826A (2616A>G 2617A>C 2618C>A), S827T (2619T>A), F828S (2623T>C), N830L (2628A>T 2629A>T 2630T>A), S832D (2634T>G 2635C>A 2636G>T), N852K (2696T>G), K853T (2698A>C 2699G>T), K853_L854insH (2699_2700insCAT), L854F (2700C>T), R855A (2703A>G 2704G>C), N856K (2708T>A), L857S (2709C>T 2710T>C 2711T>C), V859L (2715G>C 2717T>A), S862A (2724A>G 2725G>C 2726T>G), S863A (2727T>G 2729T>A), Q864D (2730C>G 2732A>T), S865N (2733T>A 2734C>A 2735A>C), L866N (2736T>A 2737T>A 2738G>C), A867H (2739G>C 2740C>A 2741A>T), Q868V (2742C>G 2743A>T 2744A>G), D869_W870insR (2747_2748insCGG), A872E (2755C>A 2756T>A), E873Q (2757G>C 2759A>G), Q879E (2775C>G 2777G>A), K882H (2784A>C 2786G>C), L890I (2808C>A), G891A (2812G>C), M907L (2859A>C 2861G>C), H914R (2880C>A 2881A>G 2882C>A), A916M (2886G>A 2887C>T 2888T>G), T923S (2907A>T 2909A>C), A927E (2920C>A 2921C>G), N928H (2922A>C), R930I (2928C>A 2929G>T 2930A>T), S931C (2932C>G), K932N (2936A>T), Y935F (2944A>T), R938T (2953G>C 2954A>C), E939Q (2955G>C), R941K (2962G>A), I942L (2964A>T 2966T>G), I948V (2982A>G 2984C>G), P961S (3021C>T 3023T>A), E972D (3056G>T), F975Y (3064T>A), T976S (3066A>T 3068A>T), Q980M (3078C>A 3079A>T 3080A>G), C984V (3090T>G 3091G>T 3092T>G), T990K (3109C>A 3110A>G), R993K (3117C>A 3118G>A 3119T>G), L994K (3120C>A 3121T>A 3122T>A), G995S (3123G>T 3124G>C), F999V (3135T>G), M1002Y (3144A>T 3145T>A 3146G>T), V1012E (3175T>A 3176T>A), E1015T (3183G>A 3184A>C), E1017G (3190A>G), N1019D (3195A>G 3197C>T), S1021N (3201T>A 3202C>A 3203C>T), S1022A (3204A>G 3205G>C 3206T>A), K1024A (3210A>G 3211A>C 3212G>T), I1025L (3213A>C), C1026S (3216T>A), Q1029V (3225C>G 3226A>T 3227A>T), S1030A (3228T>G 3230T>A), G1037S (3249G>A 3251G>T), S1050T (3289G>C), V1057C (3309G>T 3310T>G 3311G>C), G1068D (3343G>A 3344C>T), V1069T (3345G>A 3346T>C 3347A>C), R1073K (3357C>A 3358G>A 3359T>G), F1075Y (3364T>A 3365T>C), I1076V (3366A>G 3368C>G), S1077A (3369T>G), I1083V (3387A>G 3389C>G), Y1084F (3391A>T 3392C>T), E1086D (3398A>T), V1096I (3426G>A 3428C>T), A1098V (3433C>T 3434A>G), R1102H (3445G>A), D1104E (3452T>A), I1105L (3453A>C 3455T>G), S1108H (3462T>C 3463C>A 3464A>C), I1109L (3465A>C 3467T>G), R1112K (3475G>A 3476A>G), T1114E (3480A>G 3481C>A), Q1115R (3483C>A 3484A>G 3485G>A), L1116F (3486C>T 3488T>C), N1120K (3500C>A), Y1123A (3507T>G 3508A>C 3509C>T), Y1124F (3511A>T), Q1130L (3528C>T 3529A>T), L1132M (3534T>A), R1143K (3567C>A 3568G>A 3569G>A), K1144R (3570A>C 3571A>G 3572A>C), Q1146P (3577A>C 3578A>C), P1153D (3597C>G 3598C>A 3599T>C), E1155Q (3603G>C), T1162S (3624A>T 3626A>T), V1163A (3628T>C 3629T>C), H1164K (3630C>A 3632T>A), D1169E (3647T>A), M1179E (3675A>G 3676T>A), C1186S (3697G>C 3698T>C), G1187N (3699G>A 3700G>A 3701A>C), D1189E (3707T>G), D1190E (3710T>G), D1191P (3711G>C 3712A>C 3713C>A), N1199G (3735A>G 3736A>G), C1202A (3744T>G 3745G>C 3746C>A), L1204I (3750C>A 3752G>T), I1207V (3759A>G 3761C>G), M1208L (3762A>C 3764G>T), R1209K (3765C>A 3766G>A 3767T>G), S1210M (3769G>T 3770C>G), K1212R (3774A>C 3775A>G 3776G>A), I1213V (3777A>G 3779C>G), R1217K (3789C>A 3790G>A 3791T>A), C1219V (3795T>G 3796G>T 3797T>A), E1220I (3798G>A 3799A>T 3800A>C), D1222N (3804G>A), C1233F (3838G>T), Y1236F (3847A>T), G1238A (3853G>C 3854C>A), M1252L (3894A>T), M1254L (3900A>C 3902G>T), M1256S (3906A>T 3907T>C 3908G>T), Q1263E (3927C>G), S1264A (3930T>G), T1265Q (3933A>C 3934C>A 3935T>A), G1266P (3936G>C 3937G>C 3938C>A), E1267N (3939G>A 3941A>C), G1268S (3942G>T 3943G>C 3944A>G), E1269Q (3945G>C), E1272L (3954G>C 3955A>T 3956A>T), D1276N (3966G>A), E1281D (3983A>T), V1288S (4002G>T 4003T>C), I1290V (4008A>G 4010T>A), K1292Q (4014A>C), I1294T (4021T>C 4022C>A), H1295F (4023C>T 4024A>T), T1344C (4170A>T 4171C>G 4172A>T), L1348A (4182T>G 4183T>C 4184G>A), A1351V (4192C>T 4193T>A), I1352L (4194A>C 4196A>C), D1359N (4215G>A), S1363H (4227T>C 4228C>A), L1367V (4239T>G), N1370S (4249A>G), G1371S (4251G>A 4253G>C), A1374S (4260G>T 4262T>A), V1378E (4273T>A), V1393I (4317G>A 4319T>C), E1405A (4354A>C 4355A>T), Y1406F (4357A>T 4358C>T), S1409D (4365T>G 4366C>A 4367A>T), K1410E (4368A>G), D1415E (4385T>G), A1421T (4401G>A), D1435N (4443G>A 4445T>C), V1437L (4449G>T), I1476V (4566A>G 4568T>C), A1477K (4569G>A 4570C>A 4571T>A), R1479K (4576G>A 4577G>A), A1480S (4578G>T), I1481V (4581A>G 4583A>G), V1483I (4587G>A), S1486N (4597G>A), L1506M (4656T>A 4658A>G), Q1509R (4665C>A 4666A>G), S1512D (4674T>G 4675C>A), N1515S (4683A>T 4684A>C 4685T>C), H1518R (4692C>A 4693A>G 4694T>A), L1522A (4704C>G 4705T>C 4706G>T), I1526V (4716A>G), N1538E (4752A>G 4754T>G), E1539K (4755G>A), V1540T (4758G>A 4759T>C), Q1541K (4761C>A), Q1543L (4768A>T 4769A>G), H1563Q (4829C>A), I1572V (4854A>G), M1576I (4868G>T), P1583V (4887C>G 4888C>T 4889A>G), T1588V (4902A>G 4903C>T 4904T>A), M1592Y (4914A>T 4915T>A 4916G>T), V1595I (4923G>A 4925G>T), R1607Q (4960G>A 4961T>G), V1608A (4963T>C 4964G>C), A1609T (4965G>A), D1611E (4973C>A), K1612S (4975A>G 4976G>T), L1613F (4977C>T), L1615N (4983T>A 4984T>A 4985A>T), Q1618F (4992C>T 4993A>T 4994G>T), T1619G (4995A>G 4996C>G 4997G>A), D1622K (5004G>A 5006T>G), E1623Q (5007G>C 5009A>G), G1625N (5013G>A 5014G>A), F1626L (5016T>C), R1627P (5019A>C 5020G>C 5021A>C), A1628I (5022G>A 5023C>T 5024T>C), V1629M (5025G>A), F1631M (5031T>A 5033T>G), S1633T (5037T>A 5039C>T), L1635Y (5044T>A 5045G>T), V1637L (5049G>C 5051G>T), Q1639S (5055C>A 5056A>G 5057G>T), E1640Q (5058G>C), I1642L (5064A>T 5066T>G), A1643T (5067G>A), K1644E (5070A>G), S1649E (5085T>G 5086C>A 5087A>G), A1651I (5091G>A 5092C>T 5093C>T), L1652G (5094C>G 5095T>G), T1653V (5097A>G 5098C>T 5099A>T), I1654L (5100A>T 5102T>G), C1655M (5103T>A 5104G>T 5105T>G), M1657L (5109A>C 5111G>A), A1658S (5112G>T), K1659D (5115A>G 5117A>T), A1661K (5121G>A 5122C>A 5123T>A), I1662N (5125T>A), S1663T (5127T>A 5129C>A), D1668P (5142G>C 5143A>C 5144C>T), T1674del (5160_5162delACA), R1678E (5172C>G 5173G>A 5174T>G), D1679G (5176A>G), L1680V (5178C>G 5180G>C), N1681H (5181A>C), W1682E (5184T>G 5185G>A 5186G>A), A1684V (5191C>T 5192A>G), N1686D (5196A>G 5198C>T), I1687V (5199A>G 5201T>A), G1688F (5202G>T 5203G>T), A1696S (5226G>T 5228C>A), H1697N (5229C>A), Y1698Q (5232T>C 5234C>A), T1699Q (5235A>C 5236C>A 5237G>A), D1701S (5241G>T 5242A>C 5243T>A), E1702S (5244G>T 5245A>C), Y1704N (5250T>A), I1706L (5256A>C 5258T>G), K1713R (5277A>C 5278A>G 5279G>C), K1714R (5281A>G 5282G>A), S1715T (5283T>A 5285C>A), N1716T (5287A>C 5288T>A), M1731I (5333G>A), M1736L (5346A>T), S1737A (5349T>G), M1740Q (5358A>C 5359T>A), A1742H (5364G>C 5365C>A 5366C>T), K1744R (5370A>C 5371A>G 5372G>C), N1745K (5375T>G), V1746N (5376G>A 5377T>A 5378G>T), A1748E (5383C>A), G1749V (5386G>T 5387A>T), H1751D (5391C>G), V1752G (5395T>G 5396A>G), N1755S (5403A>T 5404A>C), V1757T (5409G>A 5410T>C 5411T>C), V1766I (5436G>A 5438C>A), G1767A (5440G>C 5441C>T), M1774F (5460A>T 5462G>T), R1779H (5475A>C 5476G>A 5477G>T), C1782R (5484T>A 5486T>G), V1783T (5487G>A 5488T>C 5489G>C), E1784T (5490G>A 5491A>C 5492G>A), I1786L (5496A>C 5498A>T), I1793M (5519T>G), G1794A (5521G>C 5522A>T), N1795G (5523A>G 5524A>G 5525T>G), V1807A (5560T>C), F1810L (5570T>G), Q1812K (5574C>A), P1815E (5583C>G 5584C>A 5585T>A), H1816D (5586C>G 5588T>C), H1816_G1817insT (5588_5589insACC), G1817S (5589G>T 5590G>C), D1818H (5592G>C), V1820L (5598G>T), A1821G (5602C>G 5603T>A), A1825T (5613G>A), S1826Y (5617C>A 5618G>T), L1827G (5619C>G 5620T>G), H1828del (5622_5624delCAC), S1829T (5626G>C 5627T>C), S1830T (5629G>C), M1832N (5635T>A 5636G>C), Q1834H (5642A>T), Q1834_G1835insHS (5642_5643insCACAGT), G1835A (5644G>C 5645G>A), E1837S (5649G>T 5650A>C), L1838V (5652T>G 5654G>T), N1840L (5658A>C 5659A>T 5660C>T), I1844V (5670A>G 5672A>T), H1845P (5674A>C), G1846S (5676G>T 5677G>C 5678T>C), A1849S (5685G>T 5687A>T), K1850E (5688A>G), Q1851K (5691C>A), T1852M (5695C>T 5696A>G), M1854V (5700A>G 5702G>T), M1856L (5706A>T), V1859T (5715G>A 5716T>C 5717G>A), V1860I (5718G>A 5720A>T), H1863E (5727C>G 5729T>A), G1867A (5740G>C 5741A>C), N1868Q (5742A>C 5744T>G), F1869C (5746T>G 5747T>C), Q1872K (5754C>A), D1873N (5757G>A 5759T>C), E1875K (5763G>A), S1876H (5766A>C 5767G>A), L1877A (5769T>G 5770T>C 5771G>T), H1879E (5775C>G 5777C>G), Y1880T (5778T>A 5779A>C 5780T>A), E1882S (5784G>T 5785A>C 5786G>T), Q1883V (5787C>G 5788A>T 5789G>C), G1884S (5790G>A 5792A>T), E1887T (5799G>A 5800A>C 5801G>T), I1889A (5805A>G 5806T>C 5807T>C), Q1890A (5808C>G 5809A>C), K1891R (5812A>G 5813G>A), N1892G (5814A>G 5815A>G 5816T>A), T1893V (5817A>G 5818C>T 5819A>C), L1895V (5823C>G 5825A>G), L1902F (5844C>T 5846G>C), M1904Q (5850A>C 5851T>A 5852G>A), E1905H (5853G>C 5855A>C), N1906D (5856A>G), V1907C (5859G>T 5860T>G 5861G>T), Q1908E (5862C>G), Q1909M (5865C>A 5866A>T 5867A>G), L1910I (5868C>A 5870G>A), I1911L (5871A>C 5873A>T), D1912E (5876T>G), V1914L (5880G>C 5882T>G), K1915E (5883A>G 5885G>A), L1916S (5886C>A 5887T>G), F1919V (5895T>G 5897T>A), F1920V (5898T>G 5900C>A), P1921F (5901C>T 5902C>T 5903A>C), K1922M (5905A>T 5906A>G), E1923G (5908A>G 5909A>G), T1924S (5911C>G 5912T>C), T1925V (5913A>G 5914C>T 5915C>T), I1926K (5917T>A 5918T>G), Q1927K (5919C>A 5921G>A), K1928Q (5922A>C 5924G>A), L1929M (5925C>A 5927C>G), K1930L (5928A>T 5929A>T 5930A>G), N1931S (5931A>T 5932A>C 5933T>C), V1933M (5937G>A 5939T>G), D1934N (5940G>A), A1935Q (5943G>C 5944C>A 5945T>A), E1936Q (5946G>C 5948A>G), C1937M (5949T>A 5950G>T 5951T>G), L1938S (5952C>T 5953T>C), T1939K (5956C>A), F1940T (5958T>A 5959T>C), K1941Q (5961A>C), A1942V (5965C>T 5966A>T), R1943A (5967A>G 5968G>C 5969G>C), S1944_F1945insPCCVT (5972_5973insCCATGTTGTGTCACA), R1946S (5978G>T), H1947K (5979C>A 5981C>A), V1948A (5983T>C), V1949W (5985G>T 5986T>G 5987T>G), F1950L (5988T>C 5990C>A), K1951T (5992A>C 5993A>T), K1953D (5997A>G 5999A>C), L1954I (6000C>A), L1955V (6003T>G 6005G>C), S1956M (6007G>T 6008T>G), S1957T (6009T>A 6011C>A), T1958K (6013C>A), W1959G (6015T>G 6017G>T), M1961K (6022T>A), C1962P (6024T>C 6025G>C 6026T>A), E1964del (6030_6032delGAA), V1967_R1970del (6039_6050delGTTGCTTCAAGG), S1971P (6051T>C), S1974T (6061G>C 6062T>A), L1975V (6063C>G 6065A>G), P1977L (6070C>T), L1980P (6078T>C 6079T>C 6080G>T), S1983L (6087T>C 6088C>T 6089C>A), L1985V (6093T>G), K1986R (6097A>G), L1988M (6102T>A 6104A>G), E1990R (6108G>C 6109A>G 6110A>C), R1991N (6112G>A 6113A>T), H1992S (6114C>A 6115A>G 6116C>T), T1993G (6117A>G 6118C>G), M1996Y

(6126A>T 6127T>A 6128G>C), Q1999T (6135C>A 6136A>C 6137G>A), S2000T (6139G>C 6140T>A), M2001L (6141A>C), L2005F (6153C>T 6155G>T), K2006R (6157A>G), I2007M (6161A>G), L2009M (6165C>A 6167A>G), L2010C (6169T>G 6170A>T), N2011Q (6171A>C 6173C>G), F2012E (6174T>G 6175T>A 6176C>A), Q2013G (6177C>G 6178A>G 6179A>G), V2014Y (6180G>T 6181T>A 6182A>T), I2015L (6183A>T), T2016D (6186A>G 6187C>A 6188A>T), S2017K (6190G>A 6191T>A), E2018A (6193A>C 6194G>C), L2020V (6198T>G 6200A>G), K2021D (6201A>G 6203G>C), K2022R (6204A>C 6205A>G 6206G>C), E2024N (6210G>A 6212A>T), D2025E (6215T>G), F2027M (6219T>A 6221T>G), Q2028G (6222C>G 6223A>G 6224G>C), E2029V (6226A>T 6227G>T), K2030C (6228A>T 6229A>G 6230G>T), I2031V (6231A>G 6233A>G), T2033E (6237A>G 6238C>A 6239C>G), T2034R (6240A>C 6241C>G), L2035I (6243C>A 6245A>C), I2037V (6249A>G 6251C>G), K2038R (6252A>C 6253A>G 6254A>C), V2039T (6255G>A 6256T>C), F2040I (6258T>A 6260C>T), L2041A (6261T>G 6262T>C 6263G>T), E2044S (6270G>T 6271A>C 6272G>C), E2045T (6273G>A 6274A>C), Y2047L (6280A>T 6281T>G), Q2048N (6282C>A 6284G>C), T2049E (6285A>G 6286C>A 6287T>G), E2050D (6290G>T), C2052T (6294T>A 6295G>C), I2053V (6297A>G 6299A>G), D2054Y (6300G>T 6302T>C), S2055T (6303T>A 6305T>A), Q2056D (6306C>G 6308G>T), V2057G (6310T>G), Q2059F (6315C>T 6316A>T 6317G>T), R2060D (6318A>G 6319G>A 6320G>T), I2061del (6321_6323delATA), N2062H (6324A>C 6326C>T), S2063F (6328C>T), Q2064K (6330C>A), K2065A (6333A>G 6334A>C), F2066V (6336T>G 6338C>G), R2067V (6339A>G 6340G>T 6341A>T), V2068F (6342G>T 6344G>C), Q2069K (6345C>A), E2070P (6348G>C 6349A>C 6350A>T), Y2074S (6361A>C), L2075W (6364T>G), H2076E (6366C>G 6368T>A), P2077I (6369C>A 6370C>T 6371T>C), E2078N (6372G>A 6374A>T), G2079D (6376G>A 6377A>T), I2080A (6378A>G 6379T>C), V2085A (6394T>C), I2088V (6402A>G 6404A>G), I2089L (6405A>T 6407A>G), S2091E (6411T>G 6412C>A 6413T>A), E2094R (6420G>A 6421A>G), S2095H (6423T>C 6424C>A), Y2096W (6427A>G 6428T>G), R2098K (6432C>A 6433G>A 6434A>G), D2099S (6435G>T 6436A>C 6437T>A), S2100R (6438A>C 6440T>A), N2101S (6442A>G), S2102K (6444T>A 6445C>A 6446C>G), Y2103F (6448A>T 6449T>C), S2104T (6450T>A), V2105F (6453G>T 6455G>T), S2106A (6456T>G 6458A>C), T2107N (6460C>A), P2108E (6462C>G 6463C>A 6464C>A), I2109V (6465A>G 6467C>T), K2110E (6468A>G), L2111I (6471C>A 6473G>A), G2112S (6474G>T 6475G>C 6476G>A), C2113N (6477T>A 6478G>A 6479T>C), F2114L (6480T>C 6482C>G), I2115L (6483A>C), F2116L (6488C>A), L2117A (6489C>G 6490T>C), V2119Y (6495G>T 6496T>A 6497G>T), D2121E (6503T>A), S2122N (6505G>A), Q2123K (6507C>A 6509A>G), G2124L (6510G>C 6511G>T), K2125I (6514A>T 6515G>A), E2126G (6517A>G 6518A>T), D2127L (6519G>C 6520A>T 6521T>A), H2128E (6522C>G 6524T>G), P2129Y (6525C>T 6526C>A), V2130Y (6528G>T 6529T>A), R2131D (6531C>G 6532G>A 6533G>T), R2132S (6534A>T 6535G>C 6536G>T), F2133I (6537T>A 6539T>A), S2134E (6540T>G 6541C>A), T2135S (6543A>T 6545G>T), V2136L (6546G>T 6548T>G), F2137D (6549T>G 6550T>A), K2139L (6555A>T 6556A>T), N2140Q (6558A>C 6560C>A), C2141I (6561T>A 6562G>T 6563T>A), E2142D (6566A>T), T2146A (6576A>G), Y2148V (6582T>G 6583A>T), E2150Y (6588G>T 6590G>C), *2152G (6594T>G 6596A>G)

	Begin	End	Coverage	Score	Concordance	Matches	Identities	I/D/M/F*	Stop Codons
NT	22	6747	93.0%	3341	25.4%	6659 (98.4%)	4235 (62.6%)	43/67	

GCT2GCA (146T>A), TTC7TGG (160T>G 161C>G), TCC8AGT (162T>A 163C>G 164C>T), AAG9_GCC16del (165_188delAAGGTTGCTGGTGTTGATGACGCC), AGT17CCT (189A>C 190G>C), TCT18CCT (192T>C), TCT19GCT (195T>G), TCC20TCT (200C>T), ACC21TCA (201A>T 203C>A), ACT22CCT (204A>C), GGT26GCA (217G>C 218T>A), CTT28GTT (222C>G), TCA29TCT (227A>T), AAT30TCT (228A>T 229A>C), ATT32TTT (234A>T), TCC33TCT (239C>T), AAA34TCT (240A>T 241A>C 242A>T), TTT35CTT (243T>C), AAA37AAG (251A>G), GCG38ACT (252G>A 254G>T), GTT39CTC (255G>C 257T>C), AGT40TCC (258A>T 259G>C 260T>C), ATT42GTT (264A>G), ACC43AAT (268C>A 269C>T), ACT44AAA (271C>A 272T>A), ATG45GTT (273A>G 275G>T), AAG48ACC (283A>C 284G>C), TCA50GTA (288T>G 289C>T), CAT52CAC (296T>C), CTT53ATA (297C>A 299T>A), CTG56GTC (306C>G 308G>C), AAA57AAG (311A>G), CCA58CCG (314A>G), TCT59CCA (315T>C 317T>A), GTG60GTT (320G>T), ACT61ATG (322C>T 323T>G), GAT62GCA (325A>C 326T>A), GTG63GCA (328T>C 329G>A), TCC64GCA (330T>G 332C>A), TCT66TCA (338T>A), GTG68GCC (343T>C 344G>C), GCC69TCT (345G>T 347C>T), ACT70ACA (350T>A), TGT71TGC (353T>C), AGT72AAT (355G>A), TCA73GCT (357T>G 359A>T), CTT74GTA (360C>G 362T>A), AAT75GAA (363A>G 365T>A), AAA76AGA (367A>G), TTG78TTA (374G>A), GAC79GAT (377C>T), AAA80AAG (380A>G), GGT82GCA (385G>C 386T>A), GCA83AAA (387G>A 388C>A), CTT84ATA (390C>A 392T>A), ATA85ATG (395A>G), GAT86GAA (398T>A), CCA87CCC (401A>C), TTT88TTC (404T>C), CTT89ATC (405C>A 407T>C), AAA90AAG (410A>G), GCG91GGT (412C>G 413G>T), TAC92TAT (416C>T), TCT93GAT (417T>G 418C>A), TTC94TTT (422C>T), TTG95GTT (423T>G 425G>T), TCC96AAA (426T>A 427C>A 428C>A), ACA97ACT (431A>T), TAC99TAT (437C>T), TCT101ACT (441T>A), ATC102ATA (446C>A), AAG103GTA (447A>G 448A>T 449G>A), ATG105CTT (453A>C 455G>T), GTT106GTA (458T>A), TTA107CTT (459T>C 461A>T), AAA108AAG (464A>G), TTG109ATG (465T>A), TTT110GTT (468T>G), GAG111TTA (471G>T 472A>T 473G>A), TTG113GTC (477T>G 479G>C), TCC114AAG (480T>A 481C>A 482C>G), AAT115GAT (483A>G), GTT117TTC (489G>T 491T>C), AAA118ATC (493A>T 494A>C), TTA119CTA (495T>C), TTT121TAC (502T>A 503T>C), GCT122GAA (505C>A 506T>A), GTG124TTA (510G>T 512G>A), AAT126GAA (516A>G 518T>A), AGT128GTT (522A>G 523G>T), GAA129GAG (527A>G), GAT130TCA (528G>T 529A>C 530T>A), GTA131TTA (531G>T), GTT134GTG (542T>G), GTA135TTT (543G>T 545A>T), TAT136ATA (546T>A 547A>T 548T>A), GGT137GCT (550G>C), CTT139TTA (555C>T 557T>A), GTA140GTT (560A>T), ATA143TTG (567A>T 569A>G), ACA144GCC (570A>G 572A>C), TTG145ATT (573T>A 575G>T), TTA146TTG (578A>G), GTG147CTA (579G>C 581G>A), CTT148CTA (584T>A), CTA149CTG (587A>G), TTT150TAT (589T>A), GTT151GTA (593T>A), TGT152TGC (596T>C), AGT154GTA (600A>G 601G>T 602T>A), GTT157GTA (611T>A), GAG158ACG (612G>A 613A>C), GGT159CCT (615G>C 616G>C), GTT160TTT (618G>T), TCT161AAT (621T>A 622C>A), AAC162AAT (626C>T), ACT163GTA (627A>G 628C>T 629T>A), GTC164GTT (632C>T), AAA165GTT (633A>G 634A>T 635A>T), AAT166ACT (637A>C), ATC167TTG (639A>T 641C>G), TTG169TCA (646T>C 647G>A), ATA170GTT (648A>G 650A>T), GTC171GTT (653C>T), AAC173AAT (659C>T), ATG174TTT (660A>T 662G>T), TTT175TTC (665T>C), TCA176TCT (668A>T), AGT177TCA (669A>T 670G>C 671T>A), CTA178TTG (672C>T 674A>G), TAC179TAT (677C>T), AAA186ACA (697A>C), GCC188TCT (702G>T 704C>T), GAA189GGG (706A>G 707A>G), CGA190AAG (708C>A 709G>A 710A>G), TTC191TTT (713C>T), ACC192TCC (714A>T), ATG193CAG (717A>C 718T>A), GTG194CTG (720G>C), GCC195GCG (725C>G), GCT197GCA (731T>A), AGT198GCA (732A>G 733G>C 734T>A), ATG199GTA (735A>G 737G>A), GGG202GGT (746G>T), TCA204AGT (750T>A 751C>G 752A>T), GTT205TTA (753G>T 755T>A), TCA206ACG (756T>A 758A>G), ACG210ACT (770G>T), TCT211AGC (771T>A 772C>G 773T>C), CAG212CAA (776G>A), TTG213CTT (777T>C 779G>T), ATG214TTA (780A>T 782G>A), ACA216TCA (786A>T), ATA217TTG (789A>T 791A>G), GGG221GGT (803G>T), ATT222ATC (806T>C), TCG223TCA (809G>A), ACG224ACT (812G>T), TTG225TTA (815G>A), GTG226GTA (818G>A), TTC227TTT (821C>T), ATT228GTT (822A>G), GCA229GCT (827A>T), GTG230GTT (830G>T), GGT232GGG (836T>G), GGC236GGT (848C>T), TTG237CTT (849T>C 851G>T), AGC238TCA (852A>T 853G>C 854C>A), TTG241CTG (861T>C), TCC242TCA (866C>A), ATT244ATC (872T>C), CTC245CTG (875C>G), TAT246TAC (878T>C), TCG247TCT (881G>T), ACA248GCT (882A>G 884A>T), GCT252GCG (896T>G), CAG253CAA (899G>A), CAA257CAG (911A>G), CTA258CTG (914A>G), ACT260CAG (918A>C 919C>A 920T>G), CTT261TTG (921C>T 923T>G), CGT263AGG (927C>A 929T>G), AAT264AAC (932T>C), AAA266AAG (938A>G), TGC268TGT (944C>T), ACC269ACA (947C>A), TCC270TCT (950C>T), CAG271CAA (953G>A), GCT272GCC (956T>C), GAA278GAG (974A>G), ATA280GTG (978A>G 980A>G), GTC281GTT (983C>T), GGC282AGC (984G>A), ACC283ATT (988C>T 989C>T), GGA285GGC (995A>C), TTC286TTT (998C>T), GTG290GTA (1010G>A), GCT293GCG (1019T>G), AGT295AAT (1024G>A), GCA296GCT (1028A>T), ACG297ACC (1031G>C), CTG298TTA (1032C>T 1034G>A), CTG302CTT (1046G>T), GAG304CAG (1050G>C), CAG307ACG (1059C>A 1060A>C), GAG308GAA (1064G>A), GAT310TCG (1068G>T 1069A>C 1070T>G), GCA311GAG (1072C>A 1073A>G), GTG312GTT (1076G>T), TGT313GCT (1077T>G 1078G>C), GAT314GAA (1082T>A), CCT315CCA (1085T>A), GCA316TCT (1086G>T 1088A>T), ACG317ATG (1090C>T), GAG320GAA (1100G>A), AAC321AAT (1103C>T), GCC323GCA (1109C>A), AAG325CCT (1113A>C 1114A>C 1115G>T), GCT326GCA (1118T>A), TTC327TTT (1121C>T), CCT328CCA (1124T>A), ACC329ATT (1126C>T 1127C>T), AAA330AAG (1130A>G), AAC332CAA (1134A>C 1136C>A), CTG334TTA (1140C>T 1142G>A), AGG335AAA (1144G>A 1145G>A), GAA336GAG (1148A>G), GAA337CAG (1149G>C 1151A>G), GCT338GCC (1154T>C), CTC339GTC (1155C>G), AAG340AAA (1160G>A), ATC341ATA (1163C>A), GCA346GCC (1178A>C), GTT350GTC (1190T>C), GCG351GCA (1193G>A), TTC353TTT (1199C>T), ATG354TTG (1200A>T), AGT359TCT (1215A>T 1216G>C), ATT362ATA (1226T>A), GCG363ACG (1227G>A), TTA365CTA (1233T>C), GAA366GAT (1238A>T), GTT368CTG (1242G>C 1244T>G), TAT369GTT (1245T>G 1246A>T), GAA371GAG (1253A>G), AGT372GCC (1254A>G 1255G>C 1256T>C), TGT373AGC (1257T>A 1259T>C), AGG374CAA (1260A>C 1261G>A 1262G>A), CAC375CAT (1265C>T), ATG376TCT (1266A>T 1267T>C 1268G>T), GGC377GGT (1271C>T), CAG380CAA (1280G>A), CGT382AGG (1284C>A 1286T>G), GAA384GAG (1292A>G), TTT386TTC (1298T>C), GTA388GTG (1304A>G), TTC391TTT (1313C>T), GGG392GGA (1316G>A), TCC393GCT (1317T>G 1319C>T), GGG395GGA (1325G>A), TCT399TCA (1337T>A), ACA400ACG (1340A>G), TCC401TGT (1342C>G 1343C>T), TTA404CTT (1350T>C 1352A>T), TTT405TTC (1355T>C), ATT406ATC (1358T>C), GTG409GTT (1367G>T), TTG410CTC (1368T>C 1370G>C), GAC411GAT (1373C>T), AGA412GCA (1374A>G 1375G>C), GAG415GAA (1385G>A), AAA417AAG (1391A>G), CTC418CTG (1394C>G), AAT419AAC (1397T>C), CGG420CGC (1400G>C), CTA421TTG (1401C>T 1403A>G), GCA423GCG (1409A>G), AAG426AAA (1418G>A), AGG427AGA (1421G>A), GCT435GCA (1445T>A), CAC436CAT (1448C>T), GCT439GCA (1457T>A), ATC440GTG (1458A>G 1460C>G), CTG441CTT (1463G>T), GAC444GAT (1472C>T), GGA446GGT (1478A>T), GCA447GCT (1481A>T), TTG448CTT (1482T>C 1484G>T), CGA449CGG (1487A>G), GGG451GGA (1493G>A), GCT452GCA (1496T>A), TGC455TGT (1505C>T), CAA456CAG (1508A>G), GAT457GAC (1511T>C), ATT458ATA (1514T>A), AAA459AAG (1517A>G), GAT463GAC (1529T>C), TCA466TCT (1538A>T), ACA467ACC (1541A>C), CAA468CAG (1544A>G), CCA469CCT (1547A>T), CCC471CCT (1553C>T), TTA472CTT (1554T>C 1556A>T), GCC475GCT (1565C>T), GTT477GTC (1571T>C), GAC479GAT (1577C>T), GGC481GGA (1583C>A), CAT483CAC (1589T>C), TTC484TTT (1592C>T), ACC485ACA (1595C>A), TCC486TCA (1598C>A), TAT488TAC (1604T>C), ATA489ATC (1607A>C), ACA492ACC (1616A>C), CTG495TTG (1623C>T), ATC496ATA (1628C>A), TCA497TCT (1631A>T), CCT499CCA (1637T>A), GCC500GCT (1640C>T), CAA501CAG (1643A>G), GGT503GGG (1649T>G), CTT504CTA (1652T>A), ACA505ACG (1655A>G), CCA507CCT (1661A>T), GCT509GCA (1667T>A), GAG511GAA (1673G>A), CGG512AGG (1674C>A), AGA513AGG (1679A>G), AGG514AGA (1682G>A), GAC515GAT (1685C>T), ATC516ATT (1688C>T), CTG517TTA (1689C>T 1691G>A), GTG518GTT (1694G>T), TGT520TGC (1700T>C), ATG521AGA (1702T>G 1703G>A), GAG523GTG (1708A>T), GAG525GAT (1715G>T), TCC527GAT (1719T>G 1720C>A 1721C>T), ACT528CCA (1722A>C 1724T>A), GAA529AAT (1725G>A 1727A>T), GAT530ACT (1728G>A 1729A>C), CCT531CCA (1733T>A), ACG532ACA (1736G>A), GGA533GCC (1738G>C 1739A>C), CTC535CTA (1745C>A), AGA536GTT (1746A>G 1747G>T 1748A>T), AAC538GAT (1752A>G 1754C>T), ATA539GTG (1755A>G 1757A>G), GAG541GAA (1763G>A), CCT545CCC (1775T>C), GCT547GCC (1781T>C), ACT549ACA (1787T>A), CAC550CAT (1790C>T), AGG551AGA (1793G>A), AAT552GCT (1794A>G 1795A>C), TTG553TTA (1799G>A), ACC554TCC (1800A>T), AGT556GAT (1806A>G 1807G>A), GAT557GAC (1811T>C), CTT558CTC (1814T>C), GAG560CAA (1818G>C 1820G>A), GTA563GTG (1829A>G), GCC564GCA (1832C>A), AAA565AAG (1835A>G), TGT566TGC (1838T>C), CAG567CGG (1840A>G), GTA568GTG (1844A>G), CAT569CAC (1847T>C), GCA570TAT (1848G>T 1849C>A 1850A>T), GAA571GAG (1853A>G), GTA572GTG (1856A>G), AAG574AGC (1861A>G 1862G>C), TTG576CTT (1866T>C 1868G>T), TTT577TAC (1870T>A 1871T>C), GAA578AAT (1872G>A 1874A>T), GCT579ACT (1875G>A), GAA580ACA (1878G>A 1879A>C), TCT581TCG (1883T>G), GGC582GGA (1886C>A), ATA583TTG (1887A>T 1889A>G), AGT584GCT (1890A>G 1891G>C), CCT585GAC (1893C>G 1894C>A 1895T>C), ATA587GTT (1899A>G 1901A>T), GTT590GTC (1910T>C), CAA591AAT (1911C>A 1913A>T), TCT593GCT (1917T>G), GCA594GTT (1921C>T 1922A>T), GAT596GAA (1928T>A), ATA598CTG (1932A>C 1934A>G), GCG599GAT (1936C>A 1937G>T), GTG601TTC (1941G>T 1943G>C), GAT602CAA (1944G>C 1946T>A), GGG603GAT (1948G>A 1949G>T), GCT604GCC (1952T>C), AGA605del (1953_1955delAGA), TTG606TTA (1958G>A), CGC607CAA (1960G>A 1961C>A), ACC608GTT (1962A>G 1963C>T 1964C>T), AAG609TTG (1965A>T 1966A>T), CAA610CCT (1969A>C 1970A>T), GAT611CCT (1971G>C 1972A>C), GAA612GAG (1976A>G), ATA614GCA (1980A>G 1981T>C), TTG615GCT (1983T>G 1984T>C 1985G>T), GTG616TAT (1986G>T 1987T>A 1988G>T), CCC617GAG (1989C>G 1990C>A 1991C>G), ACT618CAG (1992A>C 1993C>A 1994T>G), GTT619ACA (1995G>A 1996T>C 1997T>A), GTG620ATT (1998G>A 2000G>T), AGT621ACA (2002G>C 2003T>A), AAT623GCT (2007A>G 2008A>C), GAT624GAA (2012T>A), AGA625AAA (2014G>A), GTG626ATT (2016G>A 2018G>T), ATC627GTT (2019A>G 2021C>T), TAT628TAC (2024T>C), GCA629TCC (2025G>T 2027A>C), AGA630CAG (2028A>C 2029G>A 2030A>G), GAG631GAA (2033G>A), ACG633ACA (2039G>A), GTA634GTT (2042A>T), GCA636GCC (2048A>C), TTG637CTC (2049T>C 2051G>C), GCA640GCT (2060A>T), CAG642CAA (2066G>A), GGC643GGG (2069C>G), TTG645ATG (2073T>A), GAC646AAC (2076G>A), CCT647AAA (2079C>A 2080C>A 2081T>A), GAG648TTT (2082G>T 2083A>T 2084G>T), GAA649GAG (2087A>G), CTT650TTT (2088C>T), TTT651TTC (2093T>C), CCT652CCA (2096T>A), CAT655CTC (2104A>T 2105T>C), CAT656TCC (2106C>T 2107A>C 2108T>C), GCC659GCT (2117C>T), ATG660ATC (2120G>C), TCT663GTG (2127T>G 2128C>T 2129T>G), CTT664CTA (2132T>A), GAT665TCT (2133G>T 2134A>C), GAT666TCT (2136G>T 2137A>C), GAA667GAT (2141A>T), CAT668GCC (2142C>G 2143A>C 2144T>C), GAG670CAG (2148G>C), ATT671AAT (2152T>A), TTC672TTT (2156C>T), AAC673GAA (2157A>G 2159C>A), AAA674ATG (2161A>T 2162A>G), AGA676GTA (2166A>G 2167G>T), GTC677AAT (2169G>A 2170T>A 2171C>T), AAC678GAT (2172A>G 2174C>T), ATG679TTG (2175A>T), TAT681GTC (2181T>G 2182A>T 2183T>C), AGA682AAA (2185G>A), GAT685AGT (2193G>A 2194A>G), GCT686CCT (2196G>C), GAA687CAG (2199G>C 2201A>G), CGA690GCT (2208C>G 2209G>C 2210A>T), GTA693GTG (2219A>G), CAA694CGA (2221A>G), GAC699GAT (2237C>T), ATA701ATC (2243A>C), CAC703CAT (2249C>T), TTT704TTC (2252T>C), GCA710AGC (2268G>A 2269C>G 2270A>C), TCA711ACA (2271T>A), ATC713ATT (2279C>T), GAG715GAA (2285G>A), AAG716GAG (2286A>G), AAA717CTG (2289A>C 2290A>T 2291A>G), CTA718CTG (2294A>G), GCA719GAT (2296C>A 2297A>T), GTG720GTC (2300G>C), CAG721CAA (2303G>A), ACA722CGC (2304A>C 2305C>G 2306A>C), GAG723AAT (2307G>A 2309G>T), ATG724CTT (2310A>C 2312G>T), AGG725GAG (2313A>G 2314G>A), ACT726CAA (2316A>C 2317C>A 2318T>A), GGA727GGG (2321A>G), TTT728AAA (2322T>A 2323T>A 2324T>A), GCA729GCG (2327A>G), CAT730TAT (2328C>T), TCT731AAG (2331T>A 2332C>A 2333T>G), TGC732GCG (2334T>G 2335G>C 2336C>G), ATT733CTT (2337A>C), GAT734CAG (2340G>C 2342T>G), GCA735GCT (2345A>T), GTT737GCA (2350T>C 2351T>A), GAC738GAT (2354C>T), ACC739ACA (2357C>A), CTC740CTT (2360C>T), ATA741ACG (2362T>C 2363A>G), ATC743ATT (2369C>T), GAG744GAA (2372G>A), TTT749AGA (2385T>A 2386T>G 2387T>A), GTT750GTA (2390T>A), TTT752TTC (2396T>C), TTA753CTG (2397T>C 2399A>G), ACA755ACT (2405A>T), GCA756GCT (2408A>T), AGA759CGC (2415A>C 2417A>C), TGC762TGT (2426C>T), AAC763AGG (2428A>G 2429C>G), GTA765GTT (2435A>T), TCA766GCA (2436T>G), TCA766_AAA767insGGT (2438_2439insGGT), AAA767ACT (2440A>C 2441A>T), GAG768GAC (2444G>C), CCA769AAG (2445C>A 2446C>A 2447A>G), GAA771AGA (2451G>A 2452A>G), TCA772AAT (2454T>A 2455C>A 2456A>T), ATC774ATT (2462C>T), AAA775ATC (2464A>T 2465A>C), ATT776GAA (2466A>G 2467T>A 2468T>A), TAC778ATG (2472T>A 2473A>T 2474C>G), CAT779ATT (2475C>A 2476A>T), AAA780GGT (2478A>G 2479A>G 2480A>T), GTT782AAG (2484G>A 2485T>A 2486T>G), GAA783GAC (2489A>C), TAT784AAA (2490T>A 2492T>A), ATC785ATG (2495C>G), AAG786ATC (2497A>T 2498G>C), GAT787CAG (2499G>C 2501T>G), ACA788ATG (2503C>T 2504A>G), TGG790AAT (2508T>A 2509G>A 2510G>T), AGC791GAC (2511A>G 2512G>A), CTT792TTG (2514C>T 2516T>G), TAT794TTC (2521A>T 2522T>C), GCA795ATC (2523G>A 2524C>T 2525A>C), CTG796TTG (2526C>T), AGA797AAG (2530G>A 2531A>G), CTC799CTT (2537C>T), GCA802GCC (2546A>C), GGT803GGA (2549T>A), ATA806TTG (2556A>T 2558A>G), ATG807ATA (2561G>A), GCT808GCA (2564T>A), GGA811GGC (2573A>C), GTT812ACC (2574G>A 2575T>C 2576T>C), TTT813ATG (2577T>A 2579T>G), GGA814AAT (2580G>A 2581G>A 2582A>T), GGC815GCC (2584G>C), ATT816GTT (2586A>G), ACA817ACT (2591A>T), GCA818GCT (2594A>T), TTT819TTC (2597T>C), TTG820ATT (2598T>A 2600G>T), GCG821ACT (2601G>A 2603G>T), TGG823CTC (2607T>C 2608G>T 2609G>C), CAG824AAG (2610C>A), AGT825GCA (2613A>G 2614G>C 2615T>A), AAC826GCA (2616A>G 2617A>C 2618C>A), TCT827ACT (2619T>A), TTC828TCC (2623T>C), CCC829CCT (2627C>T), AAT830TTA (2628A>T 2629A>T 2630T>A), GCA831GCT (2633A>T), TCG832GAT (2634T>G 2635C>A 2636G>T), GGA835GGG (2645A>G), GGT836GGC (2648T>C), GTG837GTA (2651G>A), GAA840GAG (2660A>G), ATA844ATC (2672A>C), AGT846AGC (2678T>C), AAG848AAA (2684G>A), AAC849AAT (2687C>T), AAG850AAA (2690G>A), GGT851GGG (2693T>G), AAT852AAG (2696T>G), AAG853ACT (2698A>C 2699G>T), AAG853_CTC854insCAT (2699_2700insCAT), CTC854TTC (2700C>T), AGA855GCA (2703A>G 2704G>C), AAT856AAA (2708T>A), CTT857TCC (2709C>T 2710T>C 2711T>C), CTC858TTG (2712C>T 2714C>G), GTT859CTA (2715G>C 2717T>A), GGT860GGG (2720T>G), AGT862GCG (2724A>G 2725G>C 2726T>G), TCT863GCA (2727T>G 2729T>A), CAA864GAT (2730C>G 2732A>T), TCA865AAC (2733T>A 2734C>A 2735A>C), TTG866AAC (2736T>A 2737T>A 2738G>C), GCA867CAT (2739G>C 2740C>A 2741A>T), CAA868GTG (2742C>G 2743A>T 2744A>G), GAT869GAC (2747T>C), GAT869_TGG870insCGG (2747_2748insCGG), GCT871GCA (2753T>A), GCT872GAA (2755C>A 2756T>A), GAA873CAG (2757G>C 2759A>G), GTA877GTT (2771A>T), CAG879GAA (2775C>G 2777G>A), AGC880AGT (2780C>T), CTC881CTG (2783C>G), AAG882CAC (2784A>C 2786G>C), AAT884AAC (2792T>C), GTG886GTT (2798G>T), GTG887GTT (2801G>T), TTA888TTG (2804A>G), CTT890ATT (2808C>A), GGT891GCT (2812G>C), GTG894GTC (2822G>C), TAC895TAT (2825C>T), TTT896TTC (2828T>C), GGC898GGT (2834C>T), ACC899ACG (2837C>G), TAC900TAT (2840C>T), GTG901GTT (2843G>T), ATA906ATT (2858A>T), ATG907CTC (2859A>C 2861G>C), ACC908ACT (2864C>T), GTA909GTG (2867A>G), GCT910GCA (2870T>A), GCA912GCC (2876A>C), TTC913TTT (2879C>T), CAC914AGA (2880C>A 2881A>G 2882C>A), AAT915AAC (2885T>C), GCT916ATG (2886G>A 2887C>T 2888T>G), CGA917AGG (2889C>A 2891A>G), GGT919GGC (2897T>C), CCA921CCT (2903A>T), ACA923TCC (2907A>T 2909A>C), ATC925ATT (2915C>T), GCC927GAG (2920C>A 2921C>G), AAT928CAT (2922A>C), TCT929TCC (2927T>C), CGA930ATT (2928C>A 2929G>T 2930A>T), TCT931TGT (2932C>G), AAA932AAT (2936A>T), GTT933GTG (2939T>G), CAA934CAG (2942A>G), TAC935TTC (2944A>T), AAC936AAT (2948C>T), AGA938ACC (2953G>C 2954A>C), GAA939CAA (2955G>C), AGG941AAG (2962G>A), ATT942TTG (2964A>T 2966T>G), ATT943ATA (2969T>A), GGC945GGT (2975C>T), GAC947GAT (2981C>T), ATC948GTG (2982A>G 2984C>G), CTG950TTG (2988C>T), CGC952AGG (2994C>A 2996C>G), GTT953GTA (2999T>A), GGT954GGG (3002T>G), CCA959CCC (3017A>C), AAG960AAA (3020G>A), CCT961TCA (3021C>T 3023T>A), GAC962GAT (3026C>T), ATC963ATA (3029C>A), CGT964AGA (3030C>A 3032T>A), CAC966CAT (3038C>T), CCA968CCT (3044A>T), AGA969CGC (3045A>C 3047A>C),

Codon mutations:	AGG970AGA (3050G>A), GAG972GAT (3056G>T), TGC974TGT (3062C>T), TTC975TAC (3064T>A), ACA976TCT (3066A>T 3068A>T), CAA980ATG (3078C>A 3079A>T 3080A>G), GGA981GGC (3083A>C), TGT984GTG (3090T>G 3091G>T 3092T>G), AGA985AGG (3095A>G), GTT987GTA (3101T>A), GCG988GCT (3104G>T), TCT989TCA (3107T>A), ACA990AAG (3109C>A 3110A>G), CCA992CCC (3116A>C), CGT993AAG (3117C>A 3118G>A 3119T>G), CTT994AAA (3120C>A 3121T>A 3122T>A), GGC995TCC (3123G>T 3124G>C), TTA997TTG (3131A>G), TTT999GTT (3135T>G), CTC1000CTT (3140C>T), AAG1001AAA (3143G>A), ATG1002TAT (3144A>T 3145T>A 3146G>T), GGA1009GGC (3167A>C), ACA1011ACC (3173A>C), GTT1012GAA (3175T>A 3176T>A), GTG1014GTC (3182G>C), GAA1015ACA (3183G>A 3184A>C), GAA1017GGA (3190A>G), CTG1018TTA (3192C>T 3194G>A), AAC1019GAT (3195A>G 3197C>T), TCC1020TCG (3200C>G), TCC1021AAT (3201T>A 3202C>A 3203C>T), AGT1022GCA (3204A>G 3205G>C 3206T>A), AAG1024GCT (3210A>G 3211A>C 3212G>T), ATT1025CTT (3213A>C), TGC1026AGC (3216T>A), TCT1027TCA (3221T>A), CAA1029GTT (3225C>G 3226A>T 3227A>T), TCT1030GCA (3228T>G 3230T>A), GAA1032GAG (3236A>G), ATA1035ATT (3245A>T), GGG1037AGT (3249G>A 3251G>T), GAA1038GAG (3254A>G), GGT1040GGA (3260T>A), GAC1041GAT (3263C>T), GGC1043GGT (3269C>T), ACG1044ACT (3272G>T), TTG1045CTT (3273T>C 3275G>T), CTA1046CTC (3278A>C), CTG1047TTA (3279C>T 3281G>A), TTA1048TTG (3284A>G), AGT1050ACT (3289G>C), GTT1051GTG (3293T>G), AAT1053AAC (3299T>C), CAA1055CAG (3305A>G), CCT1056CCG (3308T>G), GTG1057TGC (3309G>T 3310T>G 3311G>C), GGC1060GGA (3320C>A), ATC1061ATA (3323C>A), CAC1062CAT (3326C>T), TCA1065TCT (3335A>T), GGC1068GAT (3343G>A 3344C>T), GTA1069ACC (3345G>A 3346T>C 3347A>C), GCA1070GCT (3350A>T), GCT1071GCA (3353T>A), GAA1072GAG (3356A>G), CGT1073AAG (3357C>A 3358G>A 3359T>G), GGA1074GGT (3362A>T), TTT1075TAC (3364T>A 3365T>C), ATC1076GTG (3366A>G 3368C>G), TCT1077GCT (3369T>G), AAT1079AAC (3377T>C), GCT1080GCA (3380T>A), GCT1082GCA (3386T>A), ATC1083GTG (3387A>G 3389C>G), TAC1084TTT (3391A>T 3392C>T), AGG1085AGA (3395G>A), GAA1086GAT (3398A>T), CTA1088TTG (3402C>T 3404A>G), GAG1089GAA (3407G>A), CCA1092CCC (3416A>C), ACT1093ACG (3419T>G), GGG1094GGA (3422G>A), CCG1095CCC (3425G>C), GTC1096ATT (3426G>A 3428C>T), GCA1098GTG (3433C>T 3434A>G), GCA1099GCT (3437A>T), GTA1101GTG (3443A>G), CGC1102CAC (3445G>A), GAT1104GAA (3452T>A), ATT1105CTG (3453A>C 3455T>G), CTG1106TTG (3456C>T), TCA1108CAC (3462T>C 3463C>A 3464A>C), ATT1109CTG (3465A>C 3467T>G), AGC1111TCC (3471A>T 3472G>C), AGA1112AAG (3475G>A 3476A>G), GAA1113GAG (3479A>G), ACA1114GAA (3480A>G 3481C>A), CAG1115AGA (3483C>A 3484A>G 3485G>A), CTT1116TTC (3486C>T 3488T>C), GAA1119GAG (3497A>G), AAC1120AAA (3500C>A), CAA1121CAG (3503A>G), TAC1123GCT (3507T>G 3508A>C 3509C>T), TAC1124TTC (3511A>T), CTT1125TTG (3513C>T 3515T>G), GGA1126GGC (3518A>C), ACA1127ACT (3521A>T), GTT1128GTA (3524T>A), CCA1129CCG (3527A>G), CAG1130TTG (3528C>T 3529A>T), GAG1131GAA (3533G>A), TTG1132ATG (3534T>A), GCC1133GCT (3539C>T), GCC1134GCT (3542C>T), GTT1136GTG (3548T>G), CCC1137CCG (3551C>G), AAG1139AAA (3557G>A), ACC1140ACA (3560C>A), ACT1141ACC (3563T>C), CTG1142CTT (3566G>T), CGG1143AAA (3567C>A 3568G>A 3569G>A), AAA1144CGC (3570A>C 3571A>G 3572A>C), AGC1145AGT (3575C>T), CAA1146CCC (3577A>C 3578A>C), TTG1147CTG (3579T>C), GAA1149GAG (3587A>G), GCA1150GCT (3590A>T), TTC1151TTT (3593C>T), GGA1152GGG (3596A>G), CCT1153GAC (3597C>G 3598C>A 3599T>C), GCA1154GCT (3602A>T), GAG1155CAG (3603G>C), ACA1156ACT (3608A>T), GCA1157GCC (3611A>C), ACA1162TCT (3624A>T 3626A>T), GTT1163GCC (3628T>C 3629T>C), CAT1164AAA (3630C>A 3632T>A), AGA1167AGG (3641A>G), GGT1168GGA (3644T>A), GAT1169GAA (3647T>A), GGT1170GGG (3650T>G), GAC1172GAT (3656C>T), GGG1177GGT (3671G>T), GTA1178GTC (3674A>C), ATG1179GAG (3675A>G 3676T>A), AAA1180AAG (3680A>G), TAC1181TAT (3683C>T), TGT1186TCC (3697G>C 3698T>C), GGA1187AAC (3699G>A 3700G>A 3701A>C), GAT1189GAG (3707T>G), GAT1190GAG (3710T>G), GAC1191CCA (3711G>C 3712A>C 3713C>A), GCC1193GCA (3719C>A), AAA1194AAG (3722A>G), CTA1195CTG (3725A>G), GCA1196GCC (3728A>C), TTC1197TTT (3731C>T), GAA1198GAG (3734A>G), AAT1199GGT (3735A>G 3736A>G), CTC1200CTT (3740C>T), AAG1201AAA (3743G>A), TGC1202GCA (3744T>G 3745G>C 3746C>A), TCG1203TCC (3749G>C), CTG1204ATT (3750C>A 3752G>T), CTA1205CTT (3755A>T), ATC1207GTG (3759A>G 3761C>G), ATG1208CTT (3762A>C 3764G>T), CGT1209AAG (3765C>A 3766G>A 3767T>G), AGC1210ATG (3769G>T 3770C>G), AAG1212CGA (3774A>C 3775A>G 3776G>A), ATC1213GTG (3777A>G 3779C>G), CCT1214CCC (3782T>C), CGT1217AAA (3789C>A 3790G>A 3791T>A), CCA1218CCT (3794A>T), TGT1219GTA (3795T>G 3796G>T 3797T>A), GAA1220ATC (3798G>A 3799A>T 3800A>C), AGG1221AGA (3803G>A), GAT1222AAT (3804G>A), GGA1229GGG (3827A>G), TGT1233TTT (3838G>T), TAC1235TAT (3845C>T), TAT1236TTT (3847A>T), GGC1238GCA (3853G>C 3854C>A), GAG1240GAA (3860G>A), CTG1241TTG (3861C>T), ACA1243ACG (3869A>G), TCT1244TCA (3872T>A), TGC1245TGT (3875C>T), GGA1246GGT (3878A>T), CCC1248CCT (3884C>T), TTC1249TTT (3887C>T), ATG1252TTG (3894A>T), GGG1253GGA (3899G>A), ATG1254CTT (3900A>C 3902G>T), GGG1255GGT (3905G>T), ATG1256TCT (3906A>T 3907T>C 3908G>T), AAG1258AAA (3914G>A), AGA1259AGG (3917A>G), CAA1263GAA (3927C>G), TCC1264GCC (3930T>G), ACT1265CAA (3933A>C 3934C>A 3935T>A), GGC1266CCA (3936G>C 3937G>C 3938C>A), GAA1267AAC (3939G>A 3941A>C), GGA1268TCG (3942G>T 3943G>C 3944A>G), GAG1269CAG (3945G>C), AGA1270AGG (3950A>G), GTG1271GTT (3953G>T), GAA1272CTT (3954G>C 3955A>T 3956A>T), AGA1275AGG (3965A>G), GAC1276AAC (3966G>A), ACT1277ACA (3971T>A), GTA1279GTC (3977A>C), GAA1281GAT (3983A>T), CTA1286TTG (3996C>T 3998A>G), GAT1287GAC (4001T>C), GTG1288TCG (4002G>T 4003T>C), ATT1290GTA (4008A>G 4010T>A), AAA1292CAA (4014A>C), ATC1294ACA (4021T>C 4022C>A), CAT1295TTT (4023C>T 4024A>T), GTG1296GTT (4028G>T), GAT1297GAC (4031T>C), CTG1298CTC (4034G>C), ACC1300ACA (4040C>A), CAA1302CAG (4046A>G), TGC1303TGT (4049C>T), GCC1304GCG (4052C>G), CGC1308CGG (4064C>G), CTC1309TTG (4065C>T 4067C>G), AAA1313AAG (4079A>G), ATC1314ATT (4082C>T), GGC1316GGG (4088C>G), AGA1318AGG (4094A>G), AGG1321CGC (4101A>C 4103G>C), CTC1322CTG (4106C>G), TTT1323TTC (4109T>C), GAG1324GAA (4112G>A), ATA1325ATC (4115A>C), CTT1326TTG (4116C>T 4118T>G), TAC1330TAT (4130C>T), TTG1333CTG (4137T>C), GTC1334GTG (4142C>G), AGG1335CGG (4143A>C), AAG1336AAA (4148G>A), TAT1337TAC (4151T>C), TTT1338TTC (4154T>C), GAT1340GAC (4160T>C), TTC1341TTT (4163C>T), TCA1342TCT (4166A>T), GCC1343GCT (4169C>T), ACA1344TGT (4170A>T 4171C>G 4172A>T), TTG1348GCA (4182T>G 4183T>C 4184G>A), AAT1350AAC (4190T>C), GCT1351GTA (4192C>T 4193T>A), ATA1352CTC (4194A>C 4196A>C), CCA1353CCT (4199A>T), AAA1355AAG (4205A>G), GTT1356GTA (4208T>A), ATT1358ATC (4214T>C), GAT1359AAT (4215G>A), CCT1360CCA (4220T>A), ACA1361ACG (4223A>G), AGT1362AGC (4226T>C), TCT1363CAT (4227T>C 4228C>A), TTG1367GTG (4239T>G), TTG1368CTG (4242T>C), GCA1369GCT (4247A>T), AAT1370AGT (4249A>G), GGG1371AGC (4251G>A 4253G>C), AGA1373AGG (4259A>G), GCT1374TCA (4260G>T 4262T>A), GTG1375GTA (4265G>A), TCA1376TCT (4268A>T), GAC1377GAT (4271C>T), GTG1378GAG (4273T>A), TTT1380TTC (4280T>C), GCT1382GCC (4286T>C), TAT1384TAC (4292T>C), TCC1385TCG (4295C>G), CCT1392CCC (4316T>C), GTT1393ATC (4317G>A 4319T>C), GCT1395GCA (4325T>A), TTT1396TTC (4328T>C), CAG1397CAA (4331G>A), TTG1402CTG (4344T>C), GAA1405GCT (4354A>C 4355A>T), TAC1406TTT (4357A>T 4358C>T), TAC1407TAT (4361C>T), GGA1408GGT (4364A>T), TCA1409GAT (4365T>G 4366C>A 4367A>T), AAG1410GAG (4368A>G), GGC1412GGG (4376C>G), GAT1415GAG (4385T>G), TCC1416TCA (4388C>A), CGA1419AGG (4395C>A 4397A>G), CAT1420CAC (4400T>C), GCA1421ACA (4401G>A), CTT1422TTA (4404C>T 4406T>A), GCA1426GCC (4418A>C), TGT1428TGC (4424T>C), TAC1430TAT (4430C>T), ACA1431ACC (4433A>C), CTG1432CTC (4436G>C), TGC1433TGT (4439C>T), GAT1435AAC (4443G>A 4445T>C), AAA1436AAG (4448A>G), GTG1437TTG (4449G>T), AGG1439CGC (4455A>C 4457G>C), TTG1440CTA (4458T>C 4460G>A), GTT1441GTG (4463T>G), GGG1442GGT (4466G>T), GGC1443GGA (4469C>A), CCA1445CCC (4475A>C), TCA1446TCT (4478A>T), GGA1447GGT (4481A>T), GCA1449GCT (4487A>T), CTA1450TTG (4488C>T 4490A>G), ACG1451ACT (4493G>T), ATC1453ATA (4499C>A), TTC1454TTT (4502C>T), TCT1456TCA (4508T>A), CTC1457TTG (4509C>T 4511C>G), CTC1458CTA (4514C>A), CGA1464AGG (4530C>A 4532A>G), TAT1465TAC (4535T>C), GCC1466GCG (4538C>G), TTG1470CTG (4548T>C), TTA1471TTG (4553A>G), AGA1472AGG (4556A>G), CCC1474CCA (4562C>A), ATT1476GTC (4566A>G 4568T>C), GCT1477AAA (4569G>A 4570C>A 4571T>A), AGG1479AAA (4576G>A 4577G>A), GCT1480TCT (4578G>T), ATA1481GTG (4581A>G 4583A>G), GGA1482GGC (4586A>C), GTT1483ATT (4587G>A), AAA1484AAG (4592A>G), AGT1486AAT (4597G>A), GAT1487GAC (4601T>C), CTA1491TTG (4611C>T 4613A>G), GCT1494GCA (4622T>A), GTT1495GTA (4625T>A), GGA1497GGT (4631A>T), GAC1499GAT (4637C>T), AAT1500AAC (4640T>C), CTT1501TTG (4641C>T 4643T>G), GCT1503GCG (4649T>G), GTA1504GTT (4652A>T), TTA1506ATG (4656T>A 4658A>G), CTC1508TTG (4662C>T 4664C>G), CAG1509AGG (4665C>A 4666A>G), TAT1511TAC (4673T>C), TCT1512GAT (4674T>G 4675C>A), CTG1513CTA (4679G>A), AAT1515TCC (4683A>T 4684A>C 4685T>C), ATA1516ATT (4688A>T), CAT1518AGA (4692C>A 4693A>G 4694T>A), GAG1519GAA (4697G>A), TTA1520CTG (4698T>C 4700A>G), CTG1522GCT (4704C>G 4705T>C 4706G>T), GTC1523GTT (4709C>T), AAT1524AAC (4712T>C), GTA1525GTC (4715A>C), ATC1526GTC (4716A>G), CTT1531TTG (4731C>T 4733T>G), AAA1533AAG (4739A>G), TCG1534TCA (4742G>A), GTT1537GTA (4751T>A), AAT1538GAG (4752A>G 4754T>G), GAG1539AAG (4755G>A), GTA1540ACA (4758G>A 4759T>C), CAA1541AAA (4761C>A), CAA1543CTG (4768A>T 4769A>G), CTA1548CTC (4784A>C), ACT1549ACC (4787T>C), CTG1551CTC (4793G>C), AGA1553AGG (4799A>G), TTT1555TTC (4805T>C), AAA1556AAG (4808A>G), GCT1559GCA (4817T>A), CTT1560TTG (4818C>T 4820T>G), TAC1562TAT (4826C>T), CAC1563CAA (4829C>A), GCT1565GCA (4835T>A), CCT1566CCA (4838T>A), AAG1568AAA (4844G>A), ATC1572GTC (4854A>G), ATT1573ATC (4859T>C), ATG1576ATT (4868G>T), ATA1579ATT (4877A>T), AGA1580AGG (4880A>G), CCT1581CCA (4883T>A), GCC1582GCA (4886C>A), CCA1583GTG (4887C>G 4888C>T 4889A>G), GGT1584GGA (4892T>A), GAC1587GAT (4901C>T), ACT1588GTA (4902A>G 4903C>T 4904T>A), CAG1589CAA (4907G>A), GCC1590GCT (4910C>T), ATG1592TAT (4914A>T 4915T>A 4916G>T), GTG1595ATT (4923G>A 4925G>T), GGA1598GGC (4934A>C), ATA1599ATC (4937A>C), CGT1600AGA (4938C>A 4940T>A), GCT1602GCC (4946T>C), CAC1605CAT (4955C>T), GGT1606GGG (4958T>G), CGT1607CAG (4960G>A 4961T>G), GTG1608GCC (4963T>C 4964G>C), GCT1609ACT (4965G>A), GAC1611GAA (4973C>A), AAG1612AGT (4975A>G 4976G>T), CTT1613TTT (4977C>T), GTC1614GTA (4982C>A), TTA1615AAT (4983T>A 4984T>A 4985A>T), CAG1618TTT (4992C>T 4993A>T 4994G>T), ACG1619GGA (4995A>G 4996C>G 4997G>A), GCG1620GCT (5000G>T), TTG1621CTC (5001T>C 5003G>C), GAT1622AAG (5004G>A 5006T>G), GAA1623CAG (5007G>C 5009A>G), AGG1624AGA (5012G>A), GGT1625AAT (5013G>A 5014G>A), TTC1626CTC (5016T>C), AGA1627CCC (5019A>C 5020G>C 5021A>C), GCT1628ATC (5022G>A 5023C>T 5024T>C), GTG1629ATG (5025G>A), TTT1631ATG (5031T>A 5033T>G), CCT1632CCC (5036T>C), TCC1633ACT (5037T>A 5039C>T), TAT1634TAC (5042T>C), TTG1635TAT (5044T>A 5045G>T), GTG1637CTT (5049G>C 5051G>T), CAG1639AGT (5055C>A 5056A>G 5057G>T), GAA1640CAA (5058G>C), ATT1642TTG (5064A>T 5066T>G), GCA1643ACA (5067G>A), AAG1644GAG (5070A>G), GTA1645GTC (5075A>C), GGG1647GGA (5081G>A), TCA1649GAG (5085T>G 5086C>A 5087A>G), AGT1650AGC (5090T>C), GCC1651ATT (5091G>A 5092C>T 5093C>T), CTG1652GGG (5094C>G 5095T>G), ACA1653GTT (5097A>G 5098C>T 5099A>T), ATT1654TTG (5100A>T 5102T>G), TGT1655ATG (5103T>A 5104G>T 5105T>G), GAA1656GAG (5108A>G), ATG1657CTA (5109A>C 5111G>A), GCA1658TCA (5112G>T), AAA1659GAT (5115A>G 5117A>T), GCA1660GCT (5120A>T), GCT1661AAA (5121G>A 5122C>A 5123T>A), ATT1662AAT (5125T>A), TCC1663ACA (5127T>A 5129C>A), TAT1664TAC (5132T>C), ACG1665ACC (5135G>C), CCA1666CCT (5138A>T), TTG1667TTA (5141G>A), GAC1668CCT (5142G>C 5143A>C 5144C>T), GCA1669GCT (5147A>T), GGT1670GGG (5150T>G), GAG1671GAA (5153G>A), ATC1673ATT (5159C>T), ACA1674del (5160_5162delACA), CGT1678GAG (5172C>G 5173G>A 5174T>G), GAT1679GGT (5176A>G), CTG1680GTC (5178C>G 5180G>C), AAT1681CAT (5181A>C), TGG1682GAA (5184T>G 5185G>A 5186G>A), GCA1684GTG (5191C>T 5192A>G), CCA1685CCT (5195A>T), AAC1686GAT (5196A>G 5198C>T), ATT1687GTA (5199A>G 5201T>A), GGT1688TTT (5202G>T 5203G>T), TTT1689TTC (5207T>C), TCA1691AGT (5211T>A 5212C>G 5213A>T), GCA1692GCT (5216A>T), CGT1693CGG (5219T>G), GCC1696TCA (5226G>T 5228C>A), CAC1697AAC (5229C>A), TAC1698CAA (5232T>C 5234C>A), ACG1699CAA (5235A>C 5236C>A 5237G>A), GAT1701TCA (5241G>T 5242A>C 5243T>A), GAA1702TCA (5244G>T 5245A>C), GGG1703GGT (5249G>T), TAC1704AAC (5250T>A), ATT1705ATC (5255T>C), ATT1706CTG (5256A>C 5258T>G), AAC1708AAT (5264C>T), TGT1709TGC (5267T>C), ACA1710ACT (5270A>T), GGT1711GGA (5273T>A), GCA1712GCC (5276A>C), AAG1713CGC (5277A>C 5278A>G 5279G>C), AAG1714AGA (5281A>G 5282G>A), TCC1715ACA (5283T>A 5285C>A), AAT1716ACA (5287A>C 5288T>A), GTT1718GTG (5294T>G), GGT1720GGG (5300T>G), CCA1721CCT (5303A>T), AAC1723AAT (5309C>T), ATG1731ATA (5333G>A), CCG1733CCC (5339G>C), ATG1736TTG (5346A>T), TCA1737GCA (5349T>G), GCT1738GCA (5354T>A), ATA1739ATC (5357A>C), ATG1740CAG (5358A>C 5359T>A), GCC1742CAT (5364G>C 5365C>A 5366C>T), AAG1744CGC (5370A>C 5371A>G 5372G>C), AAT1745AAG (5375T>G), GTG1746AAT (5376G>A 5377T>A 5378G>T), TTG1747CTG (5379T>C), GCA1748GAA (5383C>A), GGA1749GTT (5386G>T 5387A>T), GGA1750GGT (5390A>T), CAT1751GAT (5391C>G), GTA1752GGG (5395T>G 5396A>G), GCA1753GCT (5399A>T), AAT1755TCT (5403A>T 5404A>C), GTT1757ACC (5409G>A 5410T>C 5411T>C), GTG1759GTT (5417G>T), GGA1761GGT (5423A>T), GGA1763GGT (5429A>T), GCT1765GCA (5435T>A), GTC1766ATA (5436G>A 5438C>A), GGC1767GCT (5440G>C 5441C>T), ATT1769ATC (5447T>C), GCG1772GCA (5456G>A), CTG1773TTG (5457C>T), ATG1774TTT (5460A>T 5462G>T), GCC1775GCA (5465C>A), TTA1776TTG (5468A>G), GCA1777GCC (5471A>C), ACA1778ACT (5474A>T), AGG1779CAT (5475A>C 5476G>A 5477G>T), TAT1781TAC (5483T>C), TGT1782AGG (5484T>A 5486T>G), GTG1783ACC (5487G>A 5488T>C 5489G>C), GAG1784ACA (5490G>A 5491A>C 5492G>A), ATA1786CTT (5496A>C 5498A>T), ATA1787ATT (5501A>T), GTT1788GTG (5504T>G), AGA1789CGC (5505A>C 5507A>C), TTG1790CTA (5508T>C 5510G>A), CGC1791CGA (5513C>A), ACA1792ACC (5516A>C), ATT1793ATG (5519T>G), GGA1794GCT (5521G>C 5522A>T), AAT1795GGG (5523A>G 5524A>G 5525T>G), CTT1797CTG (5531T>G), GAC1798GAT (5534C>T), CTC1799CTG (5537C>G), AAT1800AAC (5540T>C), TAT1802TAC (5546T>C), GGC1805GGT (5555C>T), GTG1807GCG (5560T>C), CAG1808CAA (5564G>A), TAT1809TAC (5567T>C), TTT1810TTG (5570T>G), CTT1811CTC (5573T>C), CAA1812AAA (5574C>A), GTG1814GTT (5582G>T), CCT1815GAA (5583C>G 5584C>A 5585T>A), CAT1816GAC (5586C>G 5588T>C), CAT1816_GGA1817insACC (5588_5589insACC), GGA1817TCA (5589G>T 5590G>C), GAC1818CAC (5592G>C), AAA1819AAG (5597A>G), GTG1820TTG (5598G>T), GCT1821GGA (5602C>G 5603T>A), CAA1822CAG (5606A>G), GGT1824GGC (5612T>C), GCA1825ACA (5613G>A), TCG1826TAT (5617C>A 5618G>T), CTC1827GGC (5619C>G 5620T>G), CAC1828del (5622_5624delCAC), AGT1829ACC (5626G>C 5627T>C), AGT1830ACT (5629G>C), ATG1832AAC (5635T>A 5636G>C), CAA1834CAT (5642A>T), CAA1834_GGG1835insCACAGT (5642_5643insCACAGT), GGG1835GCA (5644G>C 5645G>A), GAA1837TCA (5649G>T 5650A>C), TTG1838GTT (5652T>G 5654G>T), GGC1839GGA (5657C>A), AAC1840CTT (5658A>C 5659A>T 5660C>T), CTC1841CTA (5663C>A), CGC1842AGG (5664C>A 5666C>G), ATT1843ATA (5669T>A), ATA1844GTT (5670A>G 5672A>T), CAT1845CCT (5674A>C), GGT1846TCC (5676G>T 5677G>C 5678T>C), GAT1847GAC (5681T>C), TTA1848CTC (5682T>C 5684A>C), GCA1849TCT (5685G>T 5687A>T), AAA1850GAA (5688A>G), CAG1851AAG (5691C>A), ACA1852ATG (5695C>T 5696A>G), ATG1854GTT (5700A>G 5702G>T), CGG1855CGT (5705G>T), ATG1856TTG (5706A>T), TAT1858TAC (5714T>C), GTG1859ACA (5715G>A 5716T>C 5717G>A), GTA1860ATT (5718G>A 5720A>T), GGA1861GGT (5723A>T), CCA1862CCT (5726A>T), CAT1863GAA (5727C>G 5729T>A), GGA1865GGT (5735A>T), GGA1867GCC (5740G>C 5741A>C), AAT1868CAG (5742A>C 5744T>G), TTT1869TGC (5746T>G 5747T>C), TCC1870TCA (5750C>A), ACA1871ACG (5753A>G), CAG1872AAG (5754C>A), GAT1873AAC (5757G>A 5759T>C), CTT1874CTG (5762T>G), GAG1875AAG (5763G>A), AGT1876CAT (5766A>C 5767G>A), TTG1877GCT (5769T>G 5770T>C 5771G>T), CTA1878TTG (5772C>T 5774A>G), CAC1879GAG (5775C>G 5777C>G), TAT1880ACA (5778T>A 5779A>C 5780T>A), GAG1882TCT (5784G>T 5785A>C 5786G>T), CAG1883GTC (5787C>G 5788A>T 5789G>C), GGA1884AGT (5790G>A 5792A>T), GCA1886GCT (5798A>T), GAG1887ACT (5799G>A 5800A>C 5801G>T), TTA1888CTT (5802T>C 5804A>T), ATT1889GCC (5805A>G 5806T>C 5807T>C), CAA1890GCA (5808C>G 5809A>C), AAG1891AGA (5812A>G 5813G>A), AAT1892GGA (5814A>G 5815A>G 5816T>A), ACA1893GTC (5817A>G 5818C>T 5819A>C), CTA1895GTG (5823C>G 5825A>G), ACT1896ACA (5828T>A), CTG1897TTG (5829C>T), TTC1899TTT (5837C>T), GAG1901GAA (5843G>A), CTG1902TTC (5844C>T 5846G>C), AGT1903TCC (5847A>T 5848G>C 5849T>C), ATG1904CAA (5850A>C 5851T>A 5852G>A), GAA1905CAC (5853G>C 5855A>C), AAT1906GAT (5856A>G), GTG1907TGT (5859G>T 5860T>G 5861G>T), CAA1908GAA (5862C>G), CAA1909ATG (5865C>A 5866A>T 5867A>G), CTG1910ATA (5868C>A 5870G>A), ATA1911CTT (5871A>C 5873A>T), GAT1912GAG (5876T>G), GTT1914CTG (5880G>C 5882T>G), AAG1915GAA (5883A>G 5885G>A), CTT1916AGT (5886C>A 5887T>G), CAG1917CAA (5891G>A), TTT1919GTA (5895T>G 5897T>A), TTC1920GTA (5898T>G 5900C>A), CCA1921TTC (5901C>T 5902C>T 5903A>C), AAA1922ATG (5905A>T 5906A>G), GAA1923GGG (5908A>G 5909A>G), ACT1924AGC

(5911C>G 5912T>C), ACC1925GTT (5913A>G 5914C>T 5915C>T), ATT1926AAG (5917T>A 5918T>G), CAG1927AAA (5919C>A 5921G>A), AAG1928CAA (5922A>C 5924G>A), CTC1929ATG (5925C>A 5927C>G), AAA1930TTG (5928A>T 5929A>T 5930A>G), AAT1931TCC (5931A>T 5932A>C 5933T>C), TTT1932TTC (5936T>C), GTT1933ATG (5937G>A 5939T>G), GAT1934AAT (5940G>A), GCT1935CAA (5943G>C 5944C>A 5945T>A), GAA1936CAG (5946G>C 5948A>G), TGT1937ATG (5949T>A 5950G>T 5951T>G), CTA1938TCA (5952C>T 5953T>C), ACA1939AAA (5956C>A), TTC1940ACC (5958T>A 5959T>C), AAA1941CAA (5961A>C), GCA1942GTT (5965C>T 5966A>T), AGG1943GCC (5967A>G 5968G>C 5969G>C), AGC1944_TTT1945insCCATGTTGTGTCACA (5972_5973insCCATGTTGTGTCACA), AGG1946AGT (5978G>T), CAC1947AAA (5979C>A 5981C>A), GTA1948GCA (5983T>C), GTT1949TGG (5985G>T 5986T>G 5987T>G), TTC1950CTA (5988T>C 5990C>A), AAA1951ACT (5992A>C 5993A>T), AAA1953GAC (5997A>G 5999A>C), CTT1954ATT (6000C>A), TTG1955GTC (6003T>G 6005G>C), AGT1956ATG (6007G>T 6008T>G), TCC1957ACA (6009T>A 6011C>A), ACA1958AAA (6013C>A), TGG1959GGT (6015T>G 6017G>T), AAA1960AAG (6020A>G), ATG1961AAG (6022T>A), TGT1962CCA (6024T>C 6025G>C 6026T>A), GGT1963GGA (6029T>A), GAA1964del (6030_6032delGAA), AGT1965TCT (6033A>T 6034G>C), GTT1967_AGG1970del (6039_6050delGTTGCTTCAAGG), TCT1971CCT (6051T>C), GCA1972GCT (6056A>T), GAG1973GAA (6059G>A), AGT1974ACA (6061G>C 6062T>A), CTA1975GTG (6063C>G 6065A>G), TTC1976TTT (6068C>T), CCT1977CTT (6070C>T), GGC1978GGG (6074C>G), TTG1980CCT (6078T>C 6079T>C 6080G>T), TCT1981TCA (6083T>A), GCT1982GCA (6086T>A), TCC1983CTA (6087T>C 6088C>T 6089C>A), GTA1984GTT (6092A>T), TTG1985GTG (6093T>G), AAA1986AGA (6097A>G), TTA1988ATG (6102T>A 6104A>G), GAA1990CGC (6108G>C 6109A>G 6110A>C), AGA1991AAT (6112G>A 6113A>T), CAC1992AGT (6114C>A 6115A>G 6116C>T), ACA1993GGA (6117A>G 6118C>G), AGA1994AGG (6122A>G), ATG1996TAC (6126A>T 6127T>A 6128G>C), AGT1997TCA (6129A>T 6130G>C 6131T>A), CAG1999ACA (6135C>A 6136A>C 6137G>A), AGT2000ACA (6139G>C 6140T>A), ATG2001CTG (6141A>C), GAG2002GAA (6146G>A), CTG2005TTT (6153C>T 6155G>T), AAA2006AGA (6157A>G), ATA2007ATG (6161A>G), TAT2008TAC (6164T>C), CTA2009ATG (6165C>A 6167A>G), TTA2010TGT (6169T>G 6170A>T), AAC2011CAG (6171A>C 6173C>G), TTC2012GAA (6174T>G 6175T>A 6176C>A), CAA2013GGG (6177C>G 6178A>G 6179A>G), GTA2014TAT (6180G>T 6181T>A 6182A>T), ATA2015TTA (6183A>T), ACA2016GAT (6186A>G 6187C>A 6188A>T), AGT2017AAA (6190G>A 6191T>A), GAG2018GCC (6193A>C 6194G>C), TTA2020GTG (6198T>G 6200A>G), AAG2021GAC (6201A>G 6203G>C), AAG2022CGC (6204A>C 6205A>G 6206G>C), TTT2023TTC (6209T>C), GAA2024AAT (6210G>A 6212A>T), GAT2025GAG (6215T>G), TTT2027ATG (6219T>A 6221T>G), CAG2028GGC (6222C>G 6223A>G 6224G>C), GAG2029GTT (6226A>T 6227G>T), AAG2030TGT (6228A>T 6229A>G 6230G>T), ATA2031GTG (6231A>G 6233A>G), TCC2032TCG (6236C>G), ACC2033GAG (6237A>G 6238C>A 6239C>G), ACC2034CGC (6240A>C 6241C>G), CTA2035ATC (6243C>A 6245A>C), CTA2036TTG (6246C>T 6248A>G), ATC2037GTG (6249A>G 6251C>G), AAA2038CGC (6252A>C 6253A>G 6254A>C), GTA2039ACA (6255G>A 6256T>C), TTC2040ATT (6258T>A 6260C>T), TTG2041GCT (6261T>G 6262T>C 6263G>T), GAG2044TCC (6270G>T 6271A>C 6272G>C), GAG2045ACG (6273G>A 6274A>C), AGT2046TCA (6276A>T 6277G>C 6278T>A), TAT2047TTG (6280A>T 6281T>G), CAG2048AAC (6282C>A 6284G>C), ACT2049GAG (6285A>G 6286C>A 6287T>G), GAG2050GAT (6290G>T), TGT2052ACT (6294T>A 6295G>C), ATA2053GTG (6297A>G 6299A>G), GAT2054TAC (6300G>T 6302T>C), TCT2055ACA (6303T>A 6305T>A), CAG2056GAT (6306C>G 6308G>T), GTG2057GGG (6310T>G), CTC2058CTT (6314C>T), CAG2059TTT (6315C>T 6316A>T 6317G>T), AGG2060GAT (6318A>G 6319G>A 6320G>T), ATA2061del (6321_6323delATA), AAC2062CAT (6324A>C 6326C>T), TCC2063TTC (6328C>T), CAG2064AAG (6330C>A), AAA2065GCA (6333A>G 6334A>C), TTC2066GTG (6336T>G 6338C>G), AGA2067GTT (6339A>G 6340G>T 6341A>T), GTG2068TTC (6342G>T 6344G>C), CAG2069AAG (6345C>A), GAA2070CCT (6348G>C 6349A>C 6350A>T), GGT2071GGG (6353T>G), TTT2073TTC (6359T>C), TAT2074TCT (6361A>C), TTG2075TGG (6364T>G), CAT2076GAA (6366C>G 6368T>A), CCT2077ATC (6369C>A 6370C>T 6371T>C), GAA2078AAT (6372G>A 6374A>T), GGA2079GAT (6376G>A 6377A>T), ATC2080GCC (6378A>G 6379T>C), AAC2081AAT (6383C>T), GCC2084GCT (6392C>T), GTA2085GCA (6394T>C), ATA2088GTG (6402A>G 6404A>G), ATA2089TTG (6405A>T 6407A>G), TCT2091GAA (6411T>G 6412C>A 6413T>A), CTG2092CTC (6416G>C), GAG2094AGG (6420G>A 6421A>G), TCC2095CAC (6423T>C 6424C>A), TAT2096TGG (6427A>G 6428T>G), AGC2097TCA (6429A>T 6430G>C 6431C>A), CGA2098AAG (6432C>A 6433G>A 6434A>G), GAT2099TCA (6435G>T 6436A>C 6437T>A), AGT2100CGA (6438A>C 6440T>A), AAC2101AGC (6442A>G), TCC2102AAG (6444T>A 6445C>A 6446C>G), TAT2103TTC (6448A>T 6449T>C), TCT2104ACT (6450T>A), GTG2105TTT (6453G>T 6455G>T), TCA2106GCC (6456T>G 6458A>C), ACT2107AAT (6460C>A), CCC2108GAA (6462C>G 6463C>A 6464C>A), ATC2109GTT (6465A>G 6467C>T), AAA2110GAA (6468A>G), CTG2111ATA (6471C>A 6473G>A), GGG2112TCA (6474G>T 6475G>C 6476G>A), TGT2113AAC (6477T>A 6478G>A 6479T>C), TTC2114CTG (6480T>C 6482C>G), ATT2115CTT (6483A>C), TTC2116TTA (6488C>A), CTT2117GCT (6489C>G 6490T>C), GTC2118GTT (6494C>T), GTG2119TAT (6495G>T 6496T>A 6497G>T), CTA2120TTA (6498C>T), GAT2121GAA (6503T>A), AGT2122AAT (6505G>A), CAA2123AAG (6507C>A 6509A>G), GGC2124CTC (6510G>C 6511G>T), AAG2125ATA (6514A>T 6515G>A), GAA2126GGT (6517A>G 6518A>T), GAT2127CTA (6519G>C 6520A>T 6521T>A), CAT2128GAG (6522C>G 6524T>G), CCT2129TAT (6525C>T 6526C>A), GTT2130TAT (6528G>T 6529T>A), CGG2131GAT (6531C>G 6532G>A 6533G>T), AGG2132TCT (6534A>T 6535G>C 6536G>T), TTT2133ATA (6537T>A 6539T>A), TCA2134GAA (6540T>G 6541C>A), ACG2135TCT (6543A>T 6545G>T), GTT2136TTG (6546G>T 6548T>G), TTC2137GAC (6549T>G 6550T>A), CTT2138TTG (6552C>T 6554T>G), AAA2139TTA (6555A>T 6556A>T), AAC2140CAA (6558A>C 6560C>A), TGT2141ATA (6561T>A 6562G>T 6563T>A), GAA2142GAT (6566A>T), ATT2144ATA (6572T>A), ACA2146GCA (6576A>G), TAC2148GTC (6582T>G 6583A>T), GAG2150TAC (6588G>T 6590G>C), TGA2152GGG (6594T>G 6596A>G)

Proteins

polyprotein (YP_001976147.1)	1	2152	100%	9871	66.5%	2135 (98.7%)	1362 (63.0%)	11/17/0/0	0

	Begin	End	Coverage	Score	Concordance	Matches	Identities	I/D/M/F*	Stop Codons
NT	22	6747	93.0%	3341	25.4%	6659 (98.4%)	4235 (62.6%)	43/67	

Protein mutations:

F7W (160T>G 161C>G), K9_A16del (165_188delAAGGTTGCTGGTGTTGATGACGCC), S17P (189A>C 190G>C), S18P (192T>C), S19A (195T>G), T21S (201A>T 203C>A), T22P (204A>C), G26A (217G>C 218T>A), L28V (222C>G), N30S (228A>T 229A>C), I32F (234A>T), K34S (240A>T 241A>C 242A>T), F35L (243T>C), A38T (252G>A 254G>T), V39L (255G>C 257T>C), I42V (264A>G), T43N (268C>A 269C>T), T44K (271C>A 272T>A), M45V (273A>G 275G>T), K48T (283A>C 284G>C), S50V (288T>G 289C>T), L53I (297C>A 299T>A), L56V (306C>G 308G>C), S59P (315T>C 317T>A), T61M (322C>T 323T>G), D62A (325A>C 326T>A), V63A (328T>C 329G>A), S64A (330T>G 332C>A), V68A (343T>C 344G>C), A69S (345G>T 347C>T), S72N (355G>A), S73A (357T>G 359A>T), L74V (360C>G 362T>A), N75E (363A>G 365T>A), K76R (367A>G), G82A (385G>C 386T>A), A83K (387G>A 388C>A), L84I (390C>A 392T>A), I85M (395A>G), D86E (398T>A), L89I (405C>A 407T>C), A91G (412C>G 413G>T), S93D (417T>G 418C>A), L95V (423T>G 425G>T), S96K (426T>A 427C>A 428C>A), S101T (441T>A), K103V (447A>G 448A>T 449G>A), M105L (453A>C 455G>T), L109M (465T>A), F110V (468T>G), E111L (471G>T 472A>T 473G>A), L113V (477T>G 479G>C), S114K (480T>A 481C>A 482C>G), N115D (483A>G), V117F (489G>T 491T>C), K118I (493A>T 494A>C), F121Y (502T>A 503T>C), A122E (505C>A 506T>A), V124L (510G>T 512G>A), N126E (516A>G 518T>A), S128V (522A>G 523G>T), D130S (528G>T 529A>C 530T>A), V131L (531G>T), V135F (543G>T 545A>T), Y136I (546T>A 547A>T 548T>A), G137A (550G>C), I143L (567A>T 569A>G), T144A (570A>G 572A>C), L145I (573T>A 575G>T), V147L (579G>C 581G>A), F150Y (589T>A), S154V (600A>G 601G>T 602T>A), E158T (612G>A 613A>C), G159P (615G>C 616G>C), V160F (618G>T), S161N (621T>A 622C>A), T163V (627A>G 628C>T 629T>A), K165V (633A>G 634A>T 635A>T), N166T (637A>C), I167L (639A>T 641C>G), L169S (646T>C 647G>A), I170V (648A>G 650A>T), M174F (660A>T 662G>T), K186T (697A>C), A188S (702G>T 704C>T), E189G (706A>G 707A>G), R190K (708C>A 709G>A 710A>G), T192S (714A>T), M193Q (717A>C 718T>A), V194L (720G>C), S198A (732A>G 733G>C 734T>A), M199V (735A>G 737G>A), V205L (753G>T 755T>A), S206T (756T>A 758A>G), M214L (780A>T 782G>A), T216S (786A>T), I217L (789A>T 791A>G), I228V (822A>G), T248A (882A>G 884A>T), T260Q (918A>C 919C>A 920T>G), I280V (978A>G 980A>G), G282S (984G>A), T283I (988C>T 989C>T), S295N (1024G>A), E304Q (1050G>C), Q307T (1059C>A 1060A>C), D310S (1068G>T 1069A>C 1070T>G), A311E (1072C>A 1073A>G), C313A (1077T>G 1078G>C), D314E (1082T>A), A316S (1086G>T 1088A>T), T317M (1090C>T), K325P (1113A>C 1114A>C 1115G>T), T329I (1126C>T 1127C>T), N332Q (1134A>C 1136C>A), R335K (1144G>A 1145G>A), E337Q (1149G>C 1151A>G), L339V (1155C>G), M354L (1200A>T), A363T (1227G>A), E366D (1238A>T), V368L (1242G>C 1244T>G), Y369V (1245T>G 1246A>T), S372A (1254A>G 1255G>C 1256T>C), C373S (1257T>A 1259T>C), R374Q (1260A>C 1261G>A 1262G>A), M376S (1266A>T 1267T>C 1268G>T), S393A (1317T>G 1319C>T), S401C (1342C>G 1343C>T), R412A (1374A>G 1375G>C), I440V (1458A>G 1460C>G), M521R (1702T>G 1703G>A), E523V (1708A>T), E525D (1715G>T), S527D (1719T>G 1720C>A 1721C>T), T528P (1722A>C 1724T>A), E529N (1725G>A 1727A>T), D530T (1728G>A 1729A>C), G533A (1738G>C 1739A>C), R536V (1746A>G 1747G>T 1748A>T), N538D (1752A>G 1754C>T), I539V (1755A>G 1757A>G), N552A (1794A>G 1795A>C), T554S (1800A>T), S556D (1806A>G 1807G>A), E560Q (1818G>C 1820G>A), Q567R (1840A>G), A570Y (1848G>T 1849C>A 1850A>T), K574S (1861A>G 1862G>C), F577Y (1870T>A 1871T>C), E578N (1872G>A 1874A>T), A579T (1875G>A), E580T (1878G>A 1879A>C), I583L (1887A>T 1889A>G), S584A (1890A>G 1891G>C), P585D (1893C>G 1894C>A 1895T>C), I587V (1899A>G 1901A>T), Q591N (1911C>A 1913A>T), S593A (1917T>G), A594V (1921C>T 1922A>T), D596E (1928T>A), I598L (1932A>C 1934A>G), A599D (1936C>A 1937G>T), V601F (1941G>T 1943G>C), D602Q (1944G>C 1946T>A), G603D (1948G>A 1949G>T), R605del (1953_1955delAGA), R607Q (1960G>A 1961C>A), T608V (1962A>G 1963C>T 1964C>T), K609L (1965A>T 1966A>T), Q610P (1969A>C 1970A>T), D611P (1971G>C 1972A>C), I614A (1980A>G 1981T>C), L615A (1983T>G 1984T>C 1985G>T), V616Y (1986G>T 1987T>A 1988G>T), P617E (1989C>G 1990C>A 1991C>G), T618Q (1992A>C 1993C>A 1994T>G), V619T (1995G>A 1996T>C 1997T>A), V620I (1998G>A 2000G>T), S621T (2002G>C 2003T>A), N623A (2007A>G 2008A>C), D624E (2012T>A), R625K (2014G>A), V626I (2016G>A 2018G>T), I627V (2019A>G 2021C>T), A629S (2025G>T 2027A>C), R630Q (2028A>C 2029G>A 2030A>G), L645M (2073T>A), D646N (2076G>A), P647K (2079C>A 2080C>A 2081T>A), E648F (2082G>T 2083A>T 2084G>T), L650F (2088C>T), H655L (2104A>T 2105T>C), H656S (2106C>T 2107A>C 2108T>C), M660I (2120G>C), S663V (2127T>G 2128C>T 2129T>G), D665S (2133G>T 2134A>C), D666S (2136G>T 2137A>C), E667D (2141A>T), H668A (2142C>G 2143A>C 2144T>C), E670Q (2148G>C), I671N (2152T>A), N673E (2157A>G 2159C>A), K674M (2161A>T 2162A>G), R676V (2166A>G 2167G>T), V677N (2169G>A 2170T>A 2171C>T), N678D (2172A>G 2174C>T), M679L (2175A>T), Y681V (2181T>G 2182A>T 2183T>C), R682K (2185G>A), D685S (2193G>A 2194A>G), A686P (2196G>C), E687Q (2199G>C 2201A>G), R690A (2208C>G 2209G>C 2210A>T), Q694R (2221A>G), A710S (2268G>A 2269C>G 2270A>C), S711T (2271T>A), K716E (2286A>G), K717L (2289A>C 2290A>T 2291A>G), A719D (2296C>A 2297A>T), T722R (2304A>C 2305C>G 2306A>C), E723N (2307G>A 2309G>T), M724L (2310A>C 2312G>T), R725E (2313A>G 2314G>A), T726Q (2316A>C 2317C>A 2318T>A), F728K (2322T>A 2323T>A 2324T>A), H730Y (2328C>T), S731K (2331T>A 2332C>A 2333T>G), C732A (2334T>G 2335G>C 2336C>G), I733L (2337A>C), D734Q (2340G>C 2342T>G), V737A (2350T>C 2351T>A), I741T (2362T>C 2363A>G), F749R (2385T>A 2386T>G 2387T>A), N763R (2428A>G 2429C>G), S766A (2436T>G), S766_K767insG (2438_2439insGGT), K767T (2440A>C 2441A>T), E768D (2444G>C), P769K (2445C>A 2446C>A 2447A>G), E771R (2451G>A 2452A>G), S772N (2454T>A 2455C>A 2456A>T), K775I (2464A>T 2465A>C), I776E (2466A>G 2467T>A 2468T>A), Y778M (2472T>A 2473A>T 2474C>G), H779I (2475C>A 2476A>T), K780G (2478A>G 2479A>G 2480A>T), V782K (2484G>A 2485T>A 2486T>G), E783D (2489A>C), Y784K (2490T>A 2492T>A), I785M (2495C>G), K786I (2497A>T 2498G>C), D787Q (2499G>C 2501T>G), T788M (2503C>T 2504A>G), W790N (2508T>A 2509G>A 2510G>T), S791D (2511A>G 2512G>A), Y794F (2521A>T 2522T>C), A795I (2523G>A 2524C>T 2525A>C), R797K (2530G>A 2531A>G), I806L (2556A>T 2558A>G), M807I (2561G>A), V812T (2574G>A 2575T>C 2576T>C), F813M (2577T>A 2579T>G), G814N (2580G>A 2581G>A 2582A>T), G815A (2584G>C), I816V (2586A>G), L820I (2598T>A 2600G>T), A821T (2601G>A 2603G>T), W823L (2607T>C 2608G>T 2609G>C), Q824K (2610C>A), S825A (2613A>G 2614G>C 2615T>A), N826A (2616A>G 2617A>C 2618C>A), S827T (2619T>A), F828S (2623T>C), N830L (2628A>T 2629A>T 2630T>A), S832D (2634T>G 2635C>A 2636G>T), N852K (2696T>G), K853T (2698A>C 2699G>T), K853_L854insH (2699_2700insCAT), L854F (2700C>T), R855A (2703A>G 2704G>C), N856K (2708T>A), L857S (2709C>T 2710T>C 2711T>C), V859L (2715G>C 2717T>A), S862A (2724A>G 2725G>C 2726T>G), S863A (2727T>G 2729T>A), Q864D (2730C>G 2732A>T), S865N (2733T>A 2734C>A 2735A>C), L866N (2736T>A 2737T>A 2738G>C), A867H (2739G>C 2740C>A 2741A>T), Q868V (2742C>G 2743A>T 2744A>G), D869_W870insR (2747_2748insCGG), A872E (2755C>A 2756T>A), E873Q (2757G>C 2759A>G), Q879E (2775C>G 2777G>A), K882H (2784A>C 2786G>C), L890I (2808C>A), G891A (2812G>C), M907L (2859A>C 2861G>C), H914R (2880C>A 2881A>G 2882C>A), A916M (2886G>A 2887C>T 2888T>G), T923S (2907A>T 2909A>C), A927E (2920C>A 2921C>G), N928H (2922A>C), R930I (2928C>A 2929G>T 2930A>T), S931C (2932C>G), K932N (2936A>T), Y935F (2944A>T), R938T (2953G>C 2954A>C), E939Q (2955G>C), R941K (2962G>A), I942L (2964A>T 2966T>G), I948V (2982A>G 2984C>G), P961S (3021C>T 3023T>A), E972D (3056G>T), F975Y (3064T>A), T976S (3066A>T 3068A>T), Q980M (3078C>A 3079A>T 3080A>G), C984V (3090T>G 3091G>T 3092T>G), T990K (3109C>A 3110A>G), R993K (3117C>A 3118G>A 3119T>G), L994K (3120C>A 3121T>A 3122T>A), G995S (3123G>T 3124G>C), F999V (3135T>G), M1002Y (3144A>T 3145T>A 3146G>T), V1012E (3175T>A 3176T>A), E1015T (3183G>A 3184A>C), E1017G (3190A>G), N1019D (3195A>G 3197C>T), S1021N (3201T>A 3202C>A 3203C>T), S1022A (3204A>G 3205G>C 3206T>A), K1024A (3210A>G 3211A>C 3212G>T), I1025L (3213A>C), C1026S (3216T>A), Q1029V (3225C>G 3226A>T 3227A>T), S1030A (3228T>G 3230T>A), G1037S (3249G>A 3251G>T), S1050T (3289G>C), V1057C (3309G>T 3310T>G 3311G>C), G1068D (3343G>A 3344C>T), V1069T (3345G>A 3346T>C 3347A>C), R1073K (3357C>A 3358G>A 3359T>G), F1075Y (3364T>A 3365T>C), I1076V (3366A>G 3368C>G), S1077A (3369T>G), I1083V (3387A>G 3389C>G), Y1084F (3391A>T 3392C>T), E1086D (3398A>T), V1096I (3426G>A 3428C>T), A1098V (3433C>T 3434A>G), R1102H (3445G>A), D1104E (3452T>A), I1105L (3453A>C 3455T>G), S1108H (3462T>C 3463C>A 3464A>C), I1109L (3465A>C 3467T>G), R1112K (3475G>A 3476A>G), T1114E (3480A>G 3481C>A), Q1115R (3483C>A 3484A>G 3485G>A), L1116F (3486C>T 3488T>C), N1120K (3500C>A), Y1123A (3507T>G 3508A>C 3509C>T), Y1124F (3511A>T), Q1130L (3528C>T 3529A>T), L1132M (3534T>A), R1143K (3567C>A 3568G>A 3569G>A), K1144R (3570A>C 3571A>G 3572A>C), Q1146P (3577A>C 3578A>C), P1153D (3597C>G 3598C>A 3599T>C), E1155Q (3603G>C), T1162S (3624A>T 3626A>T), V1163A (3628T>C 3629T>C), H1164K (3630C>A 3632T>A), D1169E (3647T>A), M1179E (3675A>G 3676T>A), C1186S (3697G>C 3698T>C), G1187N (3699G>A 3700G>A 3701A>C), D1189E (3707T>G), D1190E (3710T>G), D1191P (3711G>C 3712A>C 3713C>A), N1199G (3735A>G 3736A>G), C1202A (3744T>G 3745G>C 3746C>A), L1204I (3750C>A 3752G>T), I1207V (3759A>G 3761C>G), M1208L (3762A>C 3764G>T), R1209K (3765C>A 3766G>A 3767T>G), S1210M (3769G>T 3770C>G), K1212R (3774A>C 3775A>G 3776G>A), I1213V (3777A>G 3779C>G), R1217K (3789C>A 3790G>A 3791T>A), C1219V (3795T>G 3796G>T 3797T>A), E1220I (3798G>A 3799A>T 3800A>C), D1222N (3804G>A), C1233F (3838G>T), Y1236F (3847A>T), G1238A (3853G>C 3854C>A), M1252L (3894A>T), M1254L (3900A>C 3902G>T), M1256S (3906A>T 3907T>C 3908G>T), Q1263E (3927C>G), S1264A (3930T>G), T1265Q (3933A>C 3934C>A 3935T>A), G1266P (3936G>C 3937G>C 3938C>A), E1267N (3939G>A 3941A>C), G1268S (3942G>T 3943G>C 3944A>G), E1269Q (3945G>C), E1272L (3954G>C 3955A>T 3956A>T), D1276N (3966G>A), E1281D (3983A>T), V1288S (4002G>T 4003T>C), I1290V (4008A>G 4010T>A), K1292Q (4014A>C), I1294T (4021T>C 4022C>A), H1295F (4023C>T 4024A>T), T1344C (4170A>T 4171C>G 4172A>T), L1348A (4182T>G 4183T>C 4184G>A), A1351V (4192C>T 4193T>A), I1352L (4194A>C 4196A>C), D1359N (4215G>A), S1363H (4227T>C 4228C>A), L1367V (4239T>G), N1370S (4249A>G), G1371S (4251G>A 4253G>C), A1374S (4260G>T 4262T>A), V1378E (4273T>A), V1393I (4317G>A 4319T>C), E1405A (4354A>C 4355A>T), Y1406F (4357A>T 4358C>T), S1409D (4365T>G 4366C>A 4367A>T), K1410E (4368A>G), D1415E (4385T>G), A1421T (4401G>A), D1435N (4443G>A 4445T>C), V1437L (4449G>T), I1476V (4566A>G 4568T>C), A1477K (4569G>A 4570C>A 4571T>A), R1479K (4576G>A 4577G>A), A1480S (4578G>T), I1481V (4581A>G 4583A>G), V1483I (4587G>A), S1486N (4597G>A), L1506M (4656T>A 4658A>G), Q1509R (4665C>A 4666A>G), S1512D (4674T>G 4675C>A), N1515S (4683A>T 4684A>C 4685T>C), H1518R (4692C>A 4693A>G 4694T>A), L1522A (4704C>G 4705T>C 4706G>T), I1526V (4716A>G), N1538E (4752A>G 4754T>G), E1539K (4755G>A), V1540T (4758G>A 4759T>C), Q1541K (4761C>A), Q1543L (4768A>T 4769A>G), H1563Q (4829C>A), I1572V (4854A>G), M1576I (4868G>T), P1583V (4887C>G 4888C>T 4889A>G), T1588V (4902A>G 4903C>T 4904T>A), M1592Y (4914A>T 4915T>A 4916G>T), V1595I (4923G>A 4925G>T), R1607Q (4960G>A 4961T>G), V1608A (4963T>C 4964G>C), A1609T (4965G>A), D1611E (4973C>A), K1612S (4975A>G 4976G>T), L1613F (4977C>T), L1615N (4983T>A 4984T>A 4985A>T), Q1618F (4992C>T 4993A>T 4994G>T), T1619G (4995A>G 4996C>G 4997G>A), D1622K (5004G>A 5006T>G), E1623Q (5007G>C 5009A>G), G1625N (5013G>A 5014G>A), F1626L (5016T>C), R1627P (5019A>C 5020G>C 5021A>C), A1628I (5022G>A 5023C>T 5024T>C), V1629M (5025G>A), F1631M (5031T>A 5033T>G), S1633T (5037T>A 5039C>T), L1635Y (5044T>A 5045G>T), V1637L (5049G>C 5051G>T), Q1639S (5055C>A 5056A>G 5057G>T), E1640Q (5058G>C), I1642L (5064A>T 5066T>G), A1643T (5067G>A), K1644E (5070A>G), S1649E (5085T>G 5086C>A 5087A>G), A1651I (5091G>A 5092C>T 5093C>T), L1652G (5094C>G 5095T>G), T1653V (5097A>G 5098C>T 5099A>T), I1654L (5100A>T 5102T>G), C1655M (5103T>A 5104G>T 5105T>G), M1657L (5109A>C 5111G>A), A1658S (5112G>T), K1659D (5115A>G 5117A>T), A1661K (5121G>A 5122C>A 5123T>A), I1662N (5125T>A), S1663T (5127T>A 5129C>A), D1668P (5142G>C 5143A>C 5144C>T), T1674del (5160_5162delACA), R1678E (5172C>G 5173G>A 5174T>G), D1679G (5176A>G), L1680V (5178C>G 5180G>C), N1681H (5181A>C), W1682E (5184T>G 5185G>A 5186G>A), A1684V (5191C>T 5192A>G), N1686D (5196A>G 5198C>T), I1687V (5199A>G 5201T>A), G1688F (5202G>T 5203G>T), A1696S (5226G>T 5228C>A), H1697N (5229C>A), Y1698Q (5232T>C 5234C>A), T1699Q (5235A>C 5236C>A 5237G>A), D1701S (5241G>T 5242A>C 5243T>A), E1702S (5244G>T 5245A>C), Y1704N (5250T>A), I1706L (5256A>C 5258T>G), K1713R (5277A>C 5278A>G 5279G>C), K1714R (5281A>G 5282G>A), S1715T (5283T>A 5285C>A), N1716T (5287A>C 5288T>A), M1731I (5333G>A), M1736L (5346A>T), S1737A (5349T>G), M1740Q (5358A>C 5359T>A), A1742H (5364G>C 5365C>A 5366C>T), K1744R (5370A>C 5371A>G 5372G>C), N1745K (5375T>G), V1746N (5376G>A 5377T>A 5378G>T), A1748E (5383C>A), G1749V (5386G>T 5387A>T), H1751D (5391C>G), V1752G (5395T>G 5396A>G), N1755S (5403A>T 5404A>C), V1757T (5409G>A 5410T>C 5411T>C), V1766I (5436G>A 5438C>A), G1767A (5440G>C 5441C>T), M1774F (5460A>T 5462G>T), R1779H (5475A>C 5476G>A 5477G>T), C1782R (5484T>A 5486T>G), V1783T (5487G>A 5488T>C 5489G>C), E1784T (5490G>A 5491A>C 5492G>A), I1786L (5496A>C 5498A>T), I1793M (5519T>G), G1794A (5521G>C 5522A>T), N1795G (5523A>G 5524A>G 5525T>G), V1807A (5560T>C), F1810L (5570T>G), Q1812K (5574C>A), P1815E (5583C>G 5584C>A 5585T>A), H1816D (5586C>G 5588T>C), H1816_G1817insT (5588_5589insACC), G1817S (5589G>T 5590G>C), D1818H (5592G>C), V1820L (5598G>T), A1821G (5602C>G 5603T>A), A1825T (5613G>A), S1826Y (5617C>A 5618G>T), L1827G (5619C>G 5620T>G), H1828del (5622_5624delCAC), S1829T (5626G>C 5627T>C), S1830T (5629G>C), M1832N (5635T>A 5636G>C), Q1834H (5642A>T), Q1834_G1835insHS (5642_5643insCACAGT), G1835A (5644G>C 5645G>A), E1837S (5649G>T 5650A>C), L1838V (5652T>G 5654G>T), N1840L (5658A>C 5659A>T 5660C>T), I1844V (5670A>G 5672A>T), H1845P (5674A>C), G1846S (5676G>T 5677G>C 5678T>C), A1849S (5685G>T 5687A>T), K1850E (5688A>G), Q1851K (5691C>A), T1852M (5695C>T 5696A>G), M1854V (5700A>G 5702G>T), M1856L (5706A>T), V1859T (5715G>A 5716T>C 5717G>A), V1860I (5718G>A 5720A>T), H1863E (5727C>G 5729T>A), G1867A (5740G>C 5741A>C), N1868Q (5742A>C 5744T>G), F1869C (5746T>G 5747T>C), Q1872K (5754C>A), D1873N (5757G>A 5759T>C), E1875K (5763G>A), S1876H (5766A>C 5767G>A), L1877A (5769T>G 5770T>C 5771G>T), H1879E (5775C>G 5777C>G), Y1880T (5778T>A 5779A>C 5780T>A), E1882S (5784G>T 5785A>C 5786G>T), Q1883V (5787C>G 5788A>T 5789G>C), G1884S (5790G>A 5792A>T), E1887T (5799G>A 5800A>C 5801G>T), I1889A (5805A>G 5806T>C 5807T>C), Q1890A (5808C>G 5809A>C), K1891R (5812A>G 5813G>A), N1892G (5814A>G 5815A>G 5816T>A), T1893V (5817A>G 5818C>T 5819A>C), L1895V (5823C>G 5825A>G), L1902F (5844C>T 5846G>C), M1904Q (5850A>C 5851T>A 5852G>A), E1905H (5853G>C 5855A>C), N1906D (5856A>G), V1907C (5859G>T 5860T>G 5861G>T), Q1908E (5862C>G), Q1909M (5865C>A 5866A>T 5867A>G), L1910I (5868C>A 5870G>A), I1911L (5871A>C 5873A>T), D1912E (5876T>G), V1914L (5880G>C 5882T>G), K1915E (5883A>G 5885G>A), L1916S (5886C>A 5887T>G), F1919V (5895T>G 5897T>A), F1920V (5898T>G 5900C>A), P1921F (5901C>T 5902C>T 5903A>C), K1922M (5905A>T 5906A>G), E1923G (5908A>G 5909A>G), T1924S (5911C>G 5912T>C), T1925V (5913A>G 5914C>T 5915C>T), I1926K (5917T>A 5918T>G), Q1927K (5919C>A 5921G>A), K1928Q (5922A>C 5924G>A), L1929M (5925C>A 5927C>G), K1930L (5928A>T 5929A>T 5930A>G), N1931S (5931A>T 5932A>C 5933T>C), V1933M (5937G>A 5939T>G), D1934N (5940G>A), A1935Q (5943G>C 5944C>A 5945T>A), E1936Q (5946G>C 5948A>G), C1937M (5949T>A 5950G>T 5951T>G), L1938S (5952C>T 5953T>C), T1939K (5956C>A), F1940T (5958T>A 5959T>C), K1941Q (5961A>C), A1942V (5965C>T 5966A>T), R1943A (5967A>G 5968G>C 5969G>C), S1944_F1945insPCCVT (5972_5973insCCATGTTGTGTCACA), R1946S (5978G>T), H1947K (5979C>A 5981C>A), V1948A (5983T>C), V1949W (5985G>T 5986T>G 5987T>G), F1950L (5988T>C 5990C>A), K1951T (5992A>C 5993A>T), K1953D (5997A>G 5999A>C), L1954I (6000C>A), L1955V (6003T>G 6005G>C), S1956M (6007G>T 6008T>G), S1957T (6009T>A 6011C>A), T1958K (6013C>A), W1959G (6015T>G 6017G>T), M1961K (6022T>A), C1962P (6024T>C 6025G>C 6026T>A), E1964del (6030_6032delGAA), V1967_R1970del (6039_6050delGTTGCTTCAAGG), S1971P (6051T>C), S1974T (6061G>C 6062T>A), L1975V (6063C>G 6065A>G), P1977L (6070C>T), L1980P (6078T>C 6079T>C 6080G>T), S1983L (6087T>C 6088C>T 6089C>A), L1985V (6093T>G), K1986R (6097A>G), L1988M (6102T>A 6104A>G), E1990R (6108G>C 6109A>G 6110A>C), R1991N (6112G>A 6113A>T), H1992S (6114C>A 6115A>G 6116C>T), T1993G (6117A>G 6118C>G), M1996Y

(6126A>T 6127T>A 6128G>C), Q1999T (6135C>A 6136A>C 6137G>A), S2000T (6139G>C 6140T>A), M2001L (6141A>C), L2005F (6153C>T 6155G>T), K2006R (6157A>G), I2007M (6161A>G), L2009M (6165C>A 6167A>G), L2010C (6169T>G 6170A>T), N2011Q (6171A>C 6173C>G), F2012E (6174T>G 6175T>A 6176C>A), Q2013G (6177C>G 6178A>G 6179A>G), V2014Y (6180G>T 6181T>A 6182A>T), I2015L (6183A>T), T2016D (6186A>G 6187C>A 6188A>T), S2017K (6190G>A 6191T>A), E2018A (6193A>C 6194G>C), L2020V (6198T>G 6200A>G), K2021D (6201A>G 6203G>C), K2022R (6204A>C 6205A>G 6206G>C), E2024N (6210G>A 6212A>T), D2025E (6215T>G), F2027M (6219T>A 6221T>G), Q2028G (6222C>G 6223A>G 6224G>C), E2029V (6226A>T 6227G>T), K2030C (6228A>T 6229A>G 6230G>T), I2031V (6231A>G 6233A>G), T2033E (6237A>G 6238C>A 6239C>G), T2034R (6240A>C 6241C>G), L2035I (6243C>A 6245A>C), I2037V (6249A>G 6251C>G), K2038R (6252A>C 6253A>G 6254A>C), V2039T (6255G>A 6256T>C), F2040I (6258T>A 6260C>T), L2041A (6261T>G 6262T>C 6263G>T), E2044S (6270G>T 6271A>C 6272G>C), E2045T (6273G>A 6274A>C), Y2047L (6280A>T 6281T>G), Q2048N (6282C>A 6284G>C), T2049E (6285A>G 6286C>A 6287T>G), E2050D (6290G>T), C2052T (6294T>A 6295G>C), I2053V (6297A>G 6299A>G), D2054Y (6300G>T 6302T>C), S2055T (6303T>A 6305T>A), Q2056D (6306C>G 6308G>T), V2057G (6310T>G), Q2059F (6315C>T 6316A>T 6317G>T), R2060D (6318A>G 6319G>A 6320G>T), I2061del (6321_6323delATA), N2062H (6324A>C 6326C>T), S2063F (6328C>T), Q2064K (6330C>A), K2065A (6333A>G 6334A>C), F2066V (6336T>G 6338C>G), R2067V (6339A>G 6340G>T 6341A>T), V2068F (6342G>T 6344G>C), Q2069K (6345C>A), E2070P (6348G>C 6349A>C 6350A>T), Y2074S (6361A>C), L2075W (6364T>G), H2076E (6366C>G 6368T>A), P2077I (6369C>A 6370C>T 6371T>C), E2078N (6372G>A 6374A>T), G2079D (6376G>A 6377A>T), I2080A (6378A>G 6379T>C), V2085A (6394T>C), I2088V (6402A>G 6404A>G), I2089L (6405A>T 6407A>G), S2091E (6411T>G 6412C>A 6413T>A), E2094R (6420G>A 6421A>G), S2095H (6423T>C 6424C>A), Y2096W (6427A>G 6428T>G), R2098K (6432C>A 6433G>A 6434A>G), D2099S (6435G>T 6436A>C 6437T>A), S2100R (6438A>C 6440T>A), N2101S (6442A>G), S2102K (6444T>A 6445C>A 6446C>G), Y2103F (6448A>T 6449T>C), S2104T (6450T>A), V2105F (6453G>T 6455G>T), S2106A (6456T>G 6458A>C), T2107N (6460C>A), P2108E (6462C>G 6463C>A 6464C>A), I2109V (6465A>G 6467C>T), K2110E (6468A>G), L2111I (6471C>A 6473G>A), G2112S (6474G>T 6475G>C 6476G>A), C2113N (6477T>A 6478G>A 6479T>C), F2114L (6480T>C 6482C>G), I2115L (6483A>C), F2116L (6488C>A), L2117A (6489C>G 6490T>C), V2119Y (6495G>T 6496T>A 6497G>T), D2121E (6503T>A), S2122N (6505G>A), Q2123K (6507C>A 6509A>G), G2124L (6510G>C 6511G>T), K2125I (6514A>T 6515G>A), E2126G (6517A>G 6518A>T), D2127L (6519G>C 6520A>T 6521T>A), H2128E (6522C>G 6524T>G), P2129Y (6525C>T 6526C>A), V2130Y (6528G>T 6529T>A), R2131D (6531C>G 6532G>A 6533G>T), R2132S (6534A>T 6535G>C 6536G>T), F2133I (6537T>A 6539T>A), S2134E (6540T>G 6541C>A), T2135S (6543A>T 6545G>T), V2136L (6546G>T 6548T>G), F2137D (6549T>G 6550T>A), K2139L (6555A>T 6556A>T), N2140Q (6558A>C 6560C>A), C2141I (6561T>A 6562G>T 6563T>A), E2142D (6566A>T), T2146A (6576A>G), Y2148V (6582T>G 6583A>T), E2150Y (6588G>T 6590G>C), *2152G (6594T>G 6596A>G)

	Begin	End	Coverage	Score	Concordance	Matches	Identities	I/D/M/F*	Stop Codons
**NT**	**22**	**6747**	**93.0%**	**3341**	**25.4%**	**6659 (98.4%)**	**4235 (62.6%)**	**43/67**	

GCT2GCA (146T>A), TTC7TGG (160T>G 161C>G), TCC8AGT (162T>A 163C>G 164C>T), AAG9_GCC16del (165_188delAAGGTTGCTGGTGTTGATGACGCC), AGT17CCT (189A>C 190G>C), TCT18CCT (192T>C), TCT19GCT (195T>G), TCC20TCT (200C>T), ACC21TCA (201A>T 203C>A), ACT22CCT (204A>C), GGT26GCA (217G>C 218T>A), CTT28GTT (222C>G), TCA29TCT (227A>T), AAT30TCT (228A>T 229A>C), ATT32TTT (234A>T), TCC33TCT (239C>T), AAA34TCT (240A>T 241A>C 242A>T), TTT35CTT (243T>C), AAA37AAG (251A>G), GCG38ACT (252G>A 254G>T), GTT39CTC (255G>C 257T>C), AGT40TCC (258A>T 259G>C 260T>C), ATT42GTT (264A>G), ACC43AAT (268C>A 269C>T), ACT44AAA (271C>A 272T>A), ATG45GTT (273A>G 275G>T), AAG48ACC (283A>C 284G>C), TCA50GTA (288T>G 289C>T), CAT52CAC (296T>C), CTT53ATA (297C>A 299T>A), CTG56GTC (306C>G 308G>C), AAA57AAG (311A>G), CCA58CCG (314A>G), TCT59CCA (315T>C 317T>A), GTG60GTT (320G>T), ACT61ATG (322C>T 323T>G), GAT62GCA (325A>C 326T>A), GTG63GCA (328T>C 329G>A), TCC64GCA (330T>G 332C>A), TCT66TCA (338T>A), GTG68GCC (343T>C 344G>C), GCC69TCT (345G>T 347C>T), ACT70ACA (350T>A), TGT71TGC (353T>C), AGT72AAT (355G>A), TCA73GCT (357T>G 359A>T), CTT74GTA (360C>G 362T>A), AAT75GAA (363A>G 365T>A), AAA76AGA (367A>G), TTG78TTA (374G>A), GAC79GAT (377C>T), AAA80AAG (380A>G), GGT82GCA (385G>C 386T>A), GCA83AAA (387G>A 388C>A), CTT84ATA (390C>A 392T>A), ATA85ATG (395A>G), GAT86GAA (398T>A), CCA87CCC (401A>C), TTT88TTC (404T>C), CTT89ATC (405C>A 407T>C), AAA90AAG (410A>G), GCG91GGT (412C>G 413G>T), TAC92TAT (416C>T), TCT93GAT (417T>G 418C>A), TTC94TTT (422C>T), TTG95GTT (423T>G 425G>T), TCC96AAA (426T>A 427C>A 428C>A), ACA97ACT (431A>T), TAC99TAT (437C>T), TCT101ACT (441T>A), ATC102ATA (446C>A), AAG103GTA (447A>G 448A>T 449G>A), ATG105CTT (453A>C 455G>T), GTT106GTA (458T>A), TTA107CTT (459T>C 461A>T), AAA108AAG (464A>G), TTG109ATG (465T>A), TTT110GTT (468T>G), GAG111TTA (471G>T 472A>T 473G>A), TTG113GTC (477T>G 479G>C), TCC114AAG (480T>A 481C>A 482C>G), AAT115GAT (483A>G), GTT117TTC (489G>T 491T>C), AAA118ATC (493A>T 494A>C), TTA119CTA (495T>C), TTT121TAC (502T>A 503T>C), GCT122GAA (505C>A 506T>A), GTG124TTA (510G>T 512G>A), AAT126GAA (516A>G 518T>A), AGT128GTT (522A>G 523G>T), GAA129GAG (527A>G), GAT130TCA (528G>T 529A>C 530T>A), GTA131TTA (531G>T), GTT134GTG (542T>G), GTA135TTT (543G>T 545A>T), TAT136ATA (546T>A 547A>T 548T>A), GGT137GCT (550G>C), CTT139TTA (555C>T 557T>A), GTA140GTT (560A>T), ATA143TTG (567A>T 569A>G), ACA144GCC (570A>G 572A>C), TTG145ATT (573T>A 575G>T), TTA146TTG (578A>G), GTG147CTA (579G>C 581G>A), CTT148CTA (584T>A), CTA149CTG (587A>G), TTT150TAT (589T>A), GTT151GTA (593T>A), TGT152TGC (596T>C), AGT154GTA (600A>G 601G>T 602T>A), GTT157GTA (611T>A), GAG158ACG (612G>A 613A>C), GGT159CCT (615G>C 616G>C), GTT160TTT (618G>T), TCT161AAT (621T>A 622C>A), AAC162AAT (626C>T), ACT163GTA (627A>G 628C>T 629T>A), GTC164GTT (632C>T), AAA165GTT (633A>G 634A>T 635A>T), AAT166ACT (637A>C), ATC167TTG (639A>T 641C>G), TTG169TCA (646T>C 647G>A), ATA170GTT (648A>G 650A>T), GTC171GTT (653C>T), AAC173AAT (659C>T), ATG174TTT (660A>T 662G>T), TTT175TTC (665T>C), TCA176TCT (668A>T), AGT177TCA (669A>T 670G>C 671T>A), CTA178TTG (672C>T 674A>G), TAC179TAT (677C>T), AAA186ACA (697A>C), GCC188TCT (702G>T 704C>T), GAA189GGG (706A>G 707A>G), CGA190AAG (708C>A 709G>A 710A>G), TTC191TTT (713C>T), ACC192TCC (714A>T), ATG193CAG (717A>C 718T>A), GTG194CTG (720G>C), GCC195GCG (725C>G), GCT197GCA (731T>A), AGT198GCA (732A>G 733G>C 734T>A), ATG199GTA (735A>G 737G>A), GGG202GGT (746G>T), TCA204AGT (750T>A 751C>G 752A>T), GTT205TTA (753G>T 755T>A), TCA206ACG (756T>A 758A>G), ACG210ACT (770G>T), TCT211AGC (771T>A 772C>G 773T>C), CAG212CAA (776G>A), TTG213CTT (777T>C 779G>T), ATG214TTA (780A>T 782G>A), ACA216TCA (786A>T), ATA217TTG (789A>T 791A>G), GGG221GGT (803G>T), ATT222ATC (806T>C), TCG223TCA (809G>A), ACG224ACT (812G>T), TTG225TTA (815G>A), GTG226GTA (818G>A), TTC227TTT (821C>T), ATT228GTT (822A>G), GCA229GCT (827A>T), GTG230GTT (830G>T), GGT232GGG (836T>G), GGC236GGT (848C>T), TTG237CTT (849T>C 851G>T), AGC238TCA (852A>T 853G>C 854C>A), TTG241CTG (861T>C), TCC242TCA (866C>A), ATT244ATC (872T>C), CTC245CTG (875C>G), TAT246TAC (878T>C), TCG247TCT (881G>T), ACA248GCT (882A>G 884A>T), GCT252GCG (896T>G), CAG253CAA (899G>A), CAA257CAG (911A>G), CTA258CTG (914A>G), ACT260CAG (918A>C 919C>A 920T>G), CTT261TTG (921C>T 923T>G), CGT263AGG (927C>A 929T>G), AAT264AAC (932T>C), AAA266AAG (938A>G), TGC268TGT (944C>T), ACC269ACA (947C>A), TCC270TCT (950C>T), CAG271CAA (953G>A), GCT272GCC (956T>C), GAA278GAG (974A>G), ATA280GTG (978A>G 980A>G), GTC281GTT (983C>T), GGC282AGC (984G>A), ACC283ATT (988C>T 989C>T), GGA285GGC (995A>C), TTC286TTT (998C>T), GTG290GTA (1010G>A), GCT293GCG (1019T>G), AGT295AAT (1024G>A), GCA296GCT (1028A>T), ACG297ACC (1031G>C), CTG298TTA (1032C>T 1034G>A), CTG302CTT (1046G>T), GAG304CAG (1050G>C), CAG307ACG (1059C>A 1060A>C), GAG308GAA (1064G>A), GAT310TCG (1068G>T 1069A>C 1070T>G), GCA311GAG (1072C>A 1073A>G), GTG312GTT (1076G>T), TGT313GCT (1077T>G 1078G>C), GAT314GAA (1082T>A), CCT315CCA (1085T>A), GCA316TCT (1086G>T 1088A>T), ACG317ATG (1090C>T), GAG320GAA (1100G>A), AAC321AAT (1103C>T), GCC323GCA (1109C>A), AAG325CCT (1113A>C 1114A>C 1115G>T), GCT326GCA (1118T>A), TTC327TTT (1121C>T), CCT328CCA (1124T>A), ACC329ATT (1126C>T 1127C>T), AAA330AAG (1130A>G), AAC332CAA (1134A>C 1136C>A), CTG334TTA (1140C>T 1142G>A), AGG335AAA (1144G>A 1145G>A), GAA336GAG (1148A>G), GAA337CAG (1149G>C 1151A>G), GCT338GCC (1154T>C), CTC339GTC (1155C>G), AAG340AAA (1160G>A), ATC341ATA (1163C>A), GCA346GCC (1178A>C), GTT350GTC (1190T>C), GCG351GCA (1193G>A), TTC353TTT (1199C>T), ATG354TTG (1200A>T), AGT359TCT (1215A>T 1216G>C), ATT362ATA (1226T>A), GCG363ACG (1227G>A), TTA365CTA (1233T>C), GAA366GAT (1238A>T), GTT368CTG (1242G>C 1244T>G), TAT369GTT (1245T>G 1246A>T), GAA371GAG (1253A>G), AGT372GCC (1254A>G 1255G>C 1256T>C), TGT373AGC (1257T>A 1259T>C), AGG374CAA (1260A>C 1261G>A 1262G>A), CAC375CAT (1265C>T), ATG376TCT (1266A>T 1267T>C 1268G>T), GGC377GGT (1271C>T), CAG380CAA (1280G>A), CGT382AGG (1284C>A 1286T>G), GAA384GAG (1292A>G), TTT386TTC (1298T>C), GTA388GTG (1304A>G), TTC391TTT (1313C>T), GGG392GGA (1316G>A), TCC393GCT (1317T>G 1319C>T), GGG395GGA (1325G>A), TCT399TCA (1337T>A), ACA400ACG (1340A>G), TCC401TGT (1342C>G 1343C>T), TTA404CTT (1350T>C 1352A>T), TTT405TTC (1355T>C), ATT406ATC (1358T>C), GTG409GTT (1367G>T), TTG410CTC (1368T>C 1370G>C), GAC411GAT (1373C>T), AGA412GCA (1374A>G 1375G>C), GAG415GAA (1385G>A), AAA417AAG (1391A>G), CTC418CTG (1394C>G), AAT419AAC (1397T>C), CGG420CGC (1400G>C), CTA421TTG (1401C>T 1403A>G), GCA423GCG (1409A>G), AAG426AAA (1418G>A), AGG427AGA (1421G>A), GCT435GCA (1445T>A), CAC436CAT (1448C>T), GCT439GCA (1457T>A), ATC440GTG (1458A>G 1460C>G), CTG441CTT (1463G>T), GAC444GAT (1472C>T), GGA446GGT (1478A>T), GCA447GCT (1481A>T), TTG448CTT (1482T>C 1484G>T), CGA449CGG (1487A>G), GGG451GGA (1493G>A), GCT452GCA (1496T>A), TGC455TGT (1505C>T), CAA456CAG (1508A>G), GAT457GAC (1511T>C), ATT458ATA (1514T>A), AAA459AAG (1517A>G), GAT463GAC (1529T>C), TCA466TCT (1538A>T), ACA467ACC (1541A>C), CAA468CAG (1544A>G), CCA469CCT (1547A>T), CCC471CCT (1553C>T), TTA472CTT (1554T>C 1556A>T), GCC475GCT (1565C>T), GTT477GTC (1571T>C), GAC479GAT (1577C>T), GGC481GGA (1583C>A), CAT483CAC (1589T>C), TTC484TTT (1592C>T), ACC485ACA (1595C>A), TCC486TCA (1598C>A), TAT488TAC (1604T>C), ATA489ATC (1607A>C), ACA492ACC (1616A>C), CTG495TTG (1623C>T), ATC496ATA (1628C>A), TCA497TCT (1631A>T), CCT499CCA (1637T>A), GCC500GCT (1640C>T), CAA501CAG (1643A>G), GGT503GGG (1649T>G), CTT504CTA (1652T>A), ACA505ACG (1655A>G), CCA507CCT (1661A>T), GCT509GCA (1667T>A), GAG511GAA (1673G>A), CGG512AGG (1674C>A), AGA513AGG (1679A>G), AGG514AGA (1682G>A), GAC515GAT (1685C>T), ATC516ATT (1688C>T), CTG517TTA (1689C>T 1691G>A), GTG518GTT (1694G>T), TGT520TGC (1700T>C), ATG521AGA (1702T>G 1703G>A), GAG523GTG (1708A>T), GAG525GAT (1715G>T), TCC527GAT (1719T>G 1720C>A 1721C>T), ACT528CCA (1722A>C 1724T>A), GAA529AAT (1725G>A 1727A>T), GAT530ACT (1728G>A 1729A>C), CCT531CCA (1733T>A), ACG532ACA (1736G>A), GGA533GCC (1738G>C 1739A>C), CTC535CTA (1745C>A), AGA536GTT (1746A>G 1747G>T 1748A>T), AAC538GAT (1752A>G 1754C>T), ATA539GTG (1755A>G 1757A>G), GAG541GAA (1763G>A), CCT545CCC (1775T>C), GCT547GCC (1781T>C), ACT549ACA (1787T>A), CAC550CAT (1790C>T), AGG551AGA (1793G>A), AAT552GCT (1794A>G 1795A>C), TTG553TTA (1799G>A), ACC554TCC (1800A>T), AGT556GAT (1806A>G 1807G>A), GAT557GAC (1811T>C), CTT558CTC (1814T>C), GAG560CAA (1818G>C 1820G>A), GTA563GTG (1829A>G), GCC564GCA (1832C>A), AAA565AAG (1835A>G), TGT566TGC (1838T>C), CAG567CGG (1840A>G), GTA568GTG (1844A>G), CAT569CAC (1847T>C), GCA570TAT (1848G>T 1849C>A 1850A>T), GAA571GAG (1853A>G), GTA572GTG (1856A>G), AAG574AGC (1861A>G 1862G>C), TTG576CTT (1866T>C 1868G>T), TTT577TAC (1870T>A 1871T>C), GAA578AAT (1872G>A 1874A>T), GCT579ACT (1875G>A), GAA580ACA (1878G>A 1879A>C), TCT581TCG (1883T>G), GGC582GGA (1886C>A), ATA583TTG (1887A>T 1889A>G), AGT584GCT (1890A>G 1891G>C), CCT585GAC (1893C>G 1894C>A 1895T>C), ATA587GTT (1899A>G 1901A>T), GTT590GTC (1910T>C), CAA591AAT (1911C>A 1913A>T), TCT593GCT (1917T>G), GCA594GTT (1921C>T 1922A>T), GAT596GAA (1928T>A), ATA598CTG (1932A>C 1934A>G), GCG599GAT (1936C>A 1937G>T), GTG601TTC (1941G>T 1943G>C), GAT602CAA (1944G>C 1946T>A), GGG603GAT (1948G>A 1949G>T), GCT604GCC (1952T>C), AGA605del (1953_1955delAGA), TTG606TTA (1958G>A), CGC607CAA (1960G>A 1961C>A), ACC608GTT (1962A>G 1963C>T 1964C>T), AAG609TTG (1965A>T 1966A>T), CAA610CCT (1969A>C 1970A>T), GAT611CCT (1971G>C 1972A>C), GAA612GAG (1976A>G), ATA614GCA (1980A>G 1981T>C), TTG615GCT (1983T>G 1984T>C 1985G>T), GTG616TAT (1986G>T 1987T>A 1988G>T), CCC617GAG (1989C>G 1990C>A 1991C>G), ACT618CAG (1992A>C 1993C>A 1994T>G), GTT619ACA (1995G>A 1996T>C 1997T>A), GTG620ATT (1998G>A 2000G>T), AGT621ACA (2002G>C 2003T>A), AAT623GCT (2007A>G 2008A>C), GAT624GAA (2012T>A), AGA625AAA (2014G>A), GTG626ATT (2016G>A 2018G>T), ATC627GTT (2019A>G 2021C>T), TAT628TAC (2024T>C), GCA629TCC (2025G>T 2027A>C), AGA630CAG (2028A>C 2029G>A 2030A>G), GAG631GAA (2033G>A), ACG633ACA (2039G>A), GTA634GTT (2042A>T), GCA636GCC (2048A>C), TTG637CTC (2049T>C 2051G>C), GCA640GCT (2060A>T), CAG642CAA (2066G>A), GGC643GGG (2069C>G), TTG645ATG (2073T>A), GAC646AAC (2076G>A), CCT647AAA (2079C>A 2080C>A 2081T>A), GAG648TTT (2082G>T 2083A>T 2084G>T), GAA649GAG (2087A>G), CTT650TTT (2088C>T), TTT651TTC (2093T>C), CCT652CCA (2096T>A), CAT655CTC (2104A>T 2105T>C), CAT656TCC (2106C>T 2107A>C 2108T>C), GCC659GCT (2117C>T), ATG660ATC (2120G>C), TCT663GTG (2127T>G 2128C>T 2129T>G), CTT664CTA (2132T>A), GAT665TCT (2133G>T 2134A>C), GAT666TCT (2136G>T 2137A>C), GAA667GAT (2141A>T), CAT668GCC (2142C>G 2143A>C 2144T>C), GAG670CAG (2148G>C), ATT671AAT (2152T>A), TTC672TTT (2156C>T), AAC673GAA (2157A>G 2159C>A), AAA674ATG (2161A>T 2162A>G), AGA676GTA (2166A>G 2167G>T), GTC677AAT (2169G>A 2170T>A 2171C>T), AAC678GAT (2172A>G 2174C>T), ATG679TTG (2175A>T), TAT681GTC (2181T>G 2182A>T 2183T>C), AGA682AAA (2185G>A), GAT685AGT (2193G>A 2194A>G), GCT686CCT (2196G>C), GAA687CAG (2199G>C 2201A>G), CGA690GCT (2208C>G 2209G>C 2210A>T), GTA693GTG (2219A>G), CAA694CGA (2221A>G), GAC699GAT (2237C>T), ATA701ATC (2243A>C), CAC703CAT (2249C>T), TTT704TTC (2252T>C), GCA710AGC (2268G>A 2269C>G 2270A>C), TCA711ACA (2271T>A), ATC713ATT (2279C>T), GAG715GAA (2285G>A), AAG716GAG (2286A>G), AAA717CTG (2289A>C 2290A>T 2291A>G), CTA718CTG (2294A>G), GCA719GAT (2296C>A 2297A>T), GTG720GTC (2300G>C), CAG721CAA (2303G>A), ACA722CGC (2304A>C 2305C>G 2306A>C), GAG723AAT (2307G>A 2309G>T), ATG724CTT (2310A>C 2312G>T), AGG725GAG (2313A>G 2314G>A), ACT726CAA (2316A>C 2317C>A 2318T>A), GGA727GGG (2321A>G), TTT728AAA (2322T>A 2323T>A 2324T>A), GCA729GCG (2327A>G), CAT730TAT (2328C>T), TCT731AAG (2331T>A 2332C>A 2333T>G), TGC732GCG (2334T>G 2335G>C 2336C>G), ATT733CTT (2337A>C), GAT734CAG (2340G>C 2342T>G), GCA735GCT (2345A>T), GTT737GCA (2350T>C 2351T>A), GAC738GAT (2354C>T), ACC739ACA (2357C>A), CTC740CTT (2360C>T), ATA741ACG (2362T>C 2363A>G), ATC743ATT (2369C>T), GAG744GAA (2372G>A), TTT749AGA (2385T>A 2386T>G 2387T>A), GTT750GTA (2390T>A), TTT752TTC (2396T>C), TTA753CTG (2397T>C 2399A>G), ACA755ACT (2405A>T), GCA756GCT (2408A>T), AGA759CGC (2415A>C 2417A>C), TGC762TGT (2426C>T), AAC763AGG (2428A>G 2429C>G), GTA765GTT (2435A>T), TCA766GCA (2436T>G), TCA766_AAA767insGGT (2438_2439insGGT), AAA767ACT (2440A>C 2441A>T), GAG768GAC (2444G>C), CCA769AAG (2445C>A 2446C>A 2447A>G), GAA771AGA (2451G>A 2452A>G), TCA772AAT (2454T>A 2455C>A 2456A>T), ATC774ATT (2462C>T), AAA775ATC (2464A>T 2465A>C), ATT776GAA (2466A>G 2467T>A 2468T>A), TAC778ATG (2472T>A 2473A>T 2474C>G), CAT779ATT (2475C>A 2476A>T), AAA780GGT (2478A>G 2479A>G 2480A>T), GTT782AAG (2484G>A 2485T>A 2486T>G), GAA783GAC (2489A>C), TAT784AAA (2490T>A 2492T>A), ATC785ATG (2495C>G), AAG786ATC (2497A>T 2498G>C), GAT787CAG (2499G>C 2501T>G), ACA788ATG (2503C>T 2504A>G), TGG790AAT (2508T>A 2509G>A 2510G>T), AGC791GAC (2511A>G 2512G>A), CTT792TTG (2514C>T 2516T>G), TAT794TTC (2521A>T 2522T>C), GCA795ATC (2523G>A 2524C>T 2525A>C), CTG796TTG (2526C>T), AGA797AAG (2530G>A 2531A>G), CTC799CTT (2537C>T), GCA802GCC (2546A>C), GGT803GGA (2549T>A), ATA806TTG (2556A>T 2558A>G), ATG807ATA (2561G>A), GCT808GCA (2564T>A), GGA811GGC (2573A>C), GTT812ACC (2574G>A 2575T>C 2576T>C), TTT813ATG (2577T>A 2579T>G), GGA814AAT (2580G>A 2581G>A 2582A>T), GGC815GCC (2584G>C), ATT816GTT (2586A>G), ACA817ACT (2591A>T), GCA818GCT (2594A>T), TTT819TTC (2597T>C), TTG820ATT (2598T>A 2600G>T), GCG821ACT (2601G>A 2603G>T), TGG823CTC (2607T>C 2608G>T 2609G>C), CAG824AAG (2610C>A), AGT825GCA (2613A>G 2614G>C 2615T>A), AAC826GCA (2616A>G 2617A>C 2618C>A), TCT827ACT (2619T>A), TTC828TCC (2623T>C), CCC829CCT (2627C>T), AAT830TTA (2628A>T 2629A>T 2630T>A), GCA831GCT (2633A>T), TCG832GAT (2634T>G 2635C>A 2636G>T), GGA835GGG (2645A>G), GGT836GGC (2648T>C), GTG837GTA (2651G>A), GAA840GAG (2660A>G), ATA844ATC (2672A>C), AGT846AGC (2678T>C), AAG848AAA (2684G>A), AAC849AAT (2687C>T), AAG850AAA (2690G>A), GGT851GGG (2693T>G), AAT852AAG (2696T>G), AAG853ACT (2698A>C 2699G>T), AAG853_CTC854insCAT (2699_2700insCAT), CTC854TTC (2700C>T), AGA855GCA (2703A>G 2704G>C), AAT856AAA (2708T>A), CTT857TCC (2709C>T 2710T>C 2711T>C), CTC858TTG (2712C>T 2714C>G), GTT859CTA (2715G>C 2717T>A), GGT860GGG (2720T>G), AGT862GCG (2724A>G 2725G>C 2726T>G), TCT863GCA (2727T>G 2729T>A), CAA864GAT (2730C>G 2732A>T), TCA865AAC (2733T>A 2734C>A 2735A>C), TTG866AAC (2736T>A 2737T>A 2738G>C), GCA867CAT (2739G>C 2740C>A 2741A>T), CAA868GTG (2742C>G 2743A>T 2744A>G), GAT869GAC (2747T>C), GAT869_TGG870insCGG (2747_2748insCGG), GCT871GCA (2753T>A), GCT872GAA (2755C>A 2756T>A), GAA873CAG (2757G>C 2759A>G), GTA877GTT (2771A>T), CAG879GAA (2775C>G 2777G>A), AGC880AGT (2780C>T), CTC881CTG (2783C>G), AAG882CAC (2784A>C 2786G>C), AAT884AAC (2792T>C), GTG886GTT (2798G>T), GTG887GTT (2801G>T), TTA888TTG (2804A>G), CTT890ATT (2808C>A), GGT891GCT (2812G>C), GTG894GTC (2822G>C), TAC895TAT (2825C>T), TTT896TTC (2828T>C), GGC898GGT (2834C>T), ACC899ACG (2837C>G), TAC900TAT (2840C>T), GTG901GTT (2843G>T), ATA906ATT (2858A>T), ATG907CTC (2859A>C 2861G>C), ACC908ACT (2864C>T), GTA909GTG (2867A>G), GCT910GCA (2870T>A), GCA912GCC (2876A>C), TTC913TTT (2879C>T), CAC914AGA (2880C>A 2881A>G 2882C>A), AAT915AAC (2885T>C), GCT916ATG (2886G>A 2887C>T 2888T>G), CGA917AGG (2889C>A 2891A>G), GGT919GGC (2897T>C), CCA921CCT (2903A>T), ACA923TCC (2907A>T 2909A>C), ATC925ATT (2915C>T), GCC927GAG (2920C>A 2921C>G), AAT928CAT (2922A>C), TCT929TCC (2927T>C), CGA930ATT (2928C>A 2929G>T 2930A>T), TCT931TGT (2932C>G), AAA932AAT (2936A>T), GTT933GTG (2939T>G), CAA934CAG (2942A>G), TAC935TTC (2944A>T), AAC936AAT (2948C>T), AGA938ACC (2953G>C 2954A>C), GAA939CAA (2955G>C), AGG941AAG (2962G>A), ATT942TTG (2964A>T 2966T>G), ATT943ATA (2969T>A), GGC945GGT (2975C>T), GAC947GAT (2981C>T), ATC948GTG (2982A>G 2984C>G), CTG950TTG (2988C>T), CGC952AGG (2994C>A 2996C>G), GTT953GTA (2999T>A), GGT954GGG (3002T>G), CCA959CCC (3017A>C), AAG960AAA (3020G>A), CCT961TCA (3021C>T 3023T>A), GAC962GAT (3026C>T), ATC963ATA (3029C>A), CGT964AGA (3030C>A 3032T>A), CAC966CAT (3038C>T), CCA968CCT (3044A>T), AGA969CGC (3045A>C 3047A>C),

Codon mutations:

AGG970AGA (3050G>A), GAG972GAT (3056G>T), TGC974TGT (3062C>T), TTC975TAC (3064T>A), ACA976TCT (3066A>T 3068A>T), CAA980ATG (3078C>A 3079A>T 3080A>G), GGA981GGC (3083A>C), TGT984GTG (3090T>G 3091G>T 3092T>G), AGA985AGG (3095A>G), GTT987GTA (3101T>A), GCG988GCT (3104G>T), TCT989TCA (3107T>A), ACA990AAG (3109C>A 3110A>G), CCA992CCC (3116A>C), CGT993AAG (3117C>A 3118G>A 3119T>G), CTT994AAA (3120C>A 3121T>A 3122T>A), GGC995TCC (3123G>T 3124G>C), TTA997TTG (3131A>G), TTT999GTT (3135T>G), CTC1000CTT (3140C>T), AAG1001AAA (3143G>A), ATG1002TAT (3144A>T 3145T>A 3146G>T), GGA1009GGC (3167A>C), ACA1011ACC (3173A>C), GTT1012GAA (3175T>A 3176T>A), GTG1014GTC (3182G>C), GAA1015ACA (3183G>A 3184A>C), GAA1017GGA (3190A>G), CTG1018TTA (3192C>T 3194G>A), AAC1019GAT (3195A>G 3197C>T), TCC1020TCG (3200C>G), TCC1021AAT (3201T>A 3202C>A 3203C>T), AGT1022GCA (3204A>G 3205G>C 3206T>A), AAG1024GCT (3210A>G 3211A>C 3212G>T), ATT1025CTT (3213A>C), TGC1026AGC (3216T>A), TCT1027TCA (3221T>A), CAA1029GTT (3225C>G 3226A>T 3227A>T), TCT1030GCA (3228T>G 3230T>A), GAA1032GAG (3236A>G), ATA1035ATT (3245A>T), GGG1037AGT (3249G>A 3251G>T), GAA1038GAG (3254A>G), GGT1040GGA (3260T>A), GAC1041GAT (3263C>T), GGC1043GGT (3269C>T), ACG1044ACT (3272G>T), TTG1045CTT (3273T>C 3275G>T), CTA1046CTC (3278A>C), CTG1047TTA (3279C>T 3281G>A), TTA1048TTG (3284A>G), AGT1050ACT (3289G>C), GTT1051GTG (3293T>G), AAT1053AAC (3299T>C), CAA1055CAG (3305A>G), CCT1056CCG (3308T>G), GTG1057TGC (3309G>T 3310T>G 3311G>C), GGC1060GGA (3320C>A), ATC1061ATA (3323C>A), CAC1062CAT (3326C>T), TCA1065TCT (3335A>T), GGC1068GAT (3343G>A 3344C>T), GTA1069ACC (3345G>A 3346T>C 3347A>C), GCA1070GCT (3350A>T), GCT1071GCA (3353T>A), GAA1072GAG (3356A>G), CGT1073AAG (3357C>A 3358G>A 3359T>G), GGA1074GGT (3362A>T), TTT1075TAC (3364T>A 3365T>C), ATC1076GTG (3366A>G 3368C>G), TCT1077GCT (3369T>G), AAT1079AAC (3377T>C), GCT1080GCA (3380T>A), GCT1082GCA (3386T>A), ATC1083GTG (3387A>G 3389C>G), TAC1084TTT (3391A>T 3392C>T), AGG1085AGA (3395G>A), GAA1086GAT (3398A>T), CTA1088TTG (3402C>T 3404A>G), GAG1089GAA (3407G>A), CCA1092CCC (3416A>C), ACT1093ACG (3419T>G), GGG1094GGA (3422G>A), CCG1095CCC (3425G>C), GTC1096ATT (3426G>A 3428C>T), GCA1098GTG (3433C>T 3434A>G), GCA1099GCT (3437A>T), GTA1101GTG (3443A>G), CGC1102CAC (3445G>A), GAT1104GAA (3452T>A), ATT1105CTG (3453A>C 3455T>G), CTG1106TTG (3456C>T), TCA1108CAC (3462T>C 3463C>A 3464A>C), ATT1109CTG (3465A>C 3467T>G), AGC1111TCC (3471A>T 3472G>C), AGA1112AAG (3475G>A 3476A>G), GAA1113GAG (3479A>G), ACA1114GAA (3480A>G 3481C>A), CAG1115AGA (3483C>A 3484A>G 3485G>A), CTT1116TTC (3486C>T 3488T>C), GAA1119GAG (3497A>G), AAC1120AAA (3500C>A), CAA1121CAG (3503A>G), TAC1123GCT (3507T>G 3508A>C 3509C>T), TAC1124TTC (3511A>T), CTT1125TTG (3513C>T 3515T>G), GGA1126GGC (3518A>C), ACA1127ACT (3521A>T), GTT1128GTA (3524T>A), CCA1129CCG (3527A>G), CAG1130TTG (3528C>T 3529A>T), GAG1131GAA (3533G>A), TTG1132ATG (3534T>A), GCC1133GCT (3539C>T), GCC1134GCT (3542C>T), GTT1136GTG (3548T>G), CCC1137CCG (3551C>G), AAG1139AAA (3557G>A), ACC1140ACA (3560C>A), ACT1141ACC (3563T>C), CTG1142CTT (3566G>T), CGG1143AAA (3567C>A 3568G>A 3569G>A), AAA1144CGC (3570A>C 3571A>G 3572A>C), AGC1145AGT (3575C>T), CAA1146CCC (3577A>C 3578A>C), TTG1147CTG (3579T>C), GAA1149GAG (3587A>G), GCA1150GCT (3590A>T), TTC1151TTT (3593C>T), GGA1152GGG (3596A>G), CCT1153GAC (3597C>G 3598C>A 3599T>C), GCA1154GCT (3602A>T), GAG1155CAG (3603G>C), ACA1156ACT (3608A>T), GCA1157GCC (3611A>C), ACA1162TCT (3624A>T 3626A>T), GTT1163GCC (3628T>C 3629T>C), CAT1164AAA (3630C>A 3632T>A), AGA1167AGG (3641A>G), GGT1168GGA (3644T>A), GAT1169GAA (3647T>A), GGT1170GGG (3650T>G), GAC1172GAT (3656C>T), GGG1177GGT (3671G>T), GTA1178GTC (3674A>C), ATG1179GAG (3675A>G 3676T>A), AAA1180AAG (3680A>G), TAC1181TAT (3683C>T), TGT1186TCC (3697G>C 3698T>C), GGA1187AAC (3699G>A 3700G>A 3701A>C), GAT1189GAG (3707T>G), GAT1190GAG (3710T>G), GAC1191CCA (3711G>C 3712A>C 3713C>A), GCC1193GCA (3719C>A), AAA1194AAG (3722A>G), CTA1195CTG (3725A>G), GCA1196GCC (3728A>C), TTC1197TTT (3731C>T), GAA1198GAG (3734A>G), AAT1199GGT (3735A>G 3736A>G), CTC1200CTT (3740C>T), AAG1201AAA (3743G>A), TGC1202GCA (3744T>G 3745G>C 3746C>A), TCG1203TCC (3749G>C), CTG1204ATT (3750C>A 3752G>T), CTA1205CTT (3755A>T), ATC1207GTG (3759A>G 3761C>G), ATG1208CTT (3762A>C 3764G>T), CGT1209AAG (3765C>A 3766G>A 3767T>G), AGC1210ATG (3769G>T 3770C>G), AAG1212CGA (3774A>C 3775A>G 3776G>A), ATC1213GTG (3777A>G 3779C>G), CCT1214CCC (3782T>C), CGT1217AAA (3789C>A 3790G>A 3791T>A), CCA1218CCT (3794A>T), TGT1219GTA (3795T>G 3796G>T 3797T>A), GAA1220ATC (3798G>A 3799A>T 3800A>C), AGG1221AGA (3803G>A), GAT1222AAT (3804G>A), GGA1229GGG (3827A>G), TGT1233TTT (3838G>T), TAC1235TAT (3845C>T), TAT1236TTT (3847A>T), GGC1238GCA (3853G>C 3854C>A), GAG1240GAA (3860G>A), CTG1241TTG (3861C>T), ACA1243ACG (3869A>G), TCT1244TCA (3872T>A), TGC1245TGT (3875C>T), GGA1246GGT (3878A>T), CCC1248CCT (3884C>T), TTC1249TTT (3887C>T), ATG1252TTG (3894A>T), GGG1253GGA (3899G>A), ATG1254CTT (3900A>C 3902G>T), GGG1255GGT (3905G>T), ATG1256TCT (3906A>T 3907T>C 3908G>T), AAG1258AAA (3914G>A), AGA1259AGG (3917A>G), CAA1263GAA (3927C>G), TCC1264GCC (3930T>G), ACT1265CAA (3933A>C 3934C>A 3935T>A), GGC1266CCA (3936G>C 3937G>C 3938C>A), GAA1267AAC (3939G>A 3941A>C), GGA1268TCG (3942G>T 3943G>C 3944A>G), GAG1269CAG (3945G>C), AGA1270AGG (3950A>G), GTG1271GTT (3953G>T), GAA1272CTT (3954G>C 3955A>T 3956A>T), AGA1275AGG (3965A>G), GAC1276AAC (3966G>A), ACT1277ACA (3971T>A), GTA1279GTC (3977A>C), GAA1281GAT (3983A>T), CTA1286TTG (3996C>T 3998A>G), GAT1287GAC (4001T>C), GTG1288TCG (4002G>T 4003T>C), ATT1290GTA (4008A>G 4010T>A), AAA1292CAA (4014A>C), ATC1294ACA (4021T>C 4022C>A), CAT1295TTT (4023C>T 4024A>T), GTG1296GTT (4028G>T), GAT1297GAC (4031T>C), CTG1298CTC (4034G>C), ACC1300ACA (4040C>A), CAA1302CAG (4046A>G), TGC1303TGT (4049C>T), GCC1304GCG (4052C>G), CGC1308CGG (4064C>G), CTC1309TTG (4065C>T 4067C>G), AAA1313AAG (4079A>G), ATC1314ATT (4082C>T), GGC1316GGG (4088C>G), AGA1318AGG (4094A>G), AGG1321CGC (4101A>C 4103G>C), CTC1322CTG (4106C>G), TTT1323TTC (4109T>C), GAG1324GAA (4112G>A), ATA1325ATC (4115A>C), CTT1326TTG (4116C>T 4118T>G), TAC1330TAT (4130C>T), TTG1333CTG (4137T>C), GTC1334GTG (4142C>G), AGG1335CGG (4143A>C), AAG1336AAA (4148G>A), TAT1337TAC (4151T>C), TTT1338TTC (4154T>C), GAT1340GAC (4160T>C), TTC1341TTT (4163C>T), TCA1342TCT (4166A>T), GCC1343GCT (4169C>T), ACA1344TGT (4170A>T 4171C>G 4172A>T), TTG1348GCA (4182T>G 4183T>C 4184G>A), AAT1350AAC (4190T>C), GCT1351GTA (4192C>T 4193T>A), ATA1352CTC (4194A>C 4196A>C), CCA1353CCT (4199A>T), AAA1355AAG (4205A>G), GTT1356GTA (4208T>A), ATT1358ATC (4214T>C), GAT1359AAT (4215G>A), CCT1360CCA (4220T>A), ACA1361ACG (4223A>G), AGT1362AGC (4226T>C), TCT1363CAT (4227T>C 4228C>A), TTG1367GTG (4239T>G), TTG1368CTG (4242T>C), GCA1369GCT (4247A>T), AAT1370AGT (4249A>G), GGG1371AGC (4251G>A 4253G>C), AGA1373AGG (4259A>G), GCT1374TCA (4260G>T 4262T>A), GTG1375GTA (4265G>A), TCA1376TCT (4268A>T), GAC1377GAT (4271C>T), GTG1378GAG (4273T>A), TTT1380TTC (4280T>C), GCT1382GCC (4286T>C), TAT1384TAC (4292T>C), TCC1385TCG (4295C>G), CCT1392CCC (4316T>C), GTT1393ATC (4317G>A 4319T>C), GCT1395GCA (4325T>A), TTT1396TTC (4328T>C), CAG1397CAA (4331G>A), TTG1402CTG (4344T>C), GAA1405GCT (4354A>C 4355A>T), TAC1406TTT (4357A>T 4358C>T), TAC1407TAT (4361C>T), GGA1408GGT (4364A>T), TCA1409GAT (4365T>G 4366C>A 4367A>T), AAG1410GAG (4368A>G), GGC1412GGG (4376C>G), GAT1415GAG (4385T>G), TCC1416TCA (4388C>A), CGA1419AGG (4395C>A 4397A>G), CAT1420CAC (4400T>C), GCA1421ACA (4401G>A), CTT1422TTA (4404C>T 4406T>A), GCA1426GCC (4418A>C), TGT1428TGC (4424T>C), TAC1430TAT (4430C>T), ACA1431ACC (4433A>C), CTG1432CTC (4436G>C), TGC1433TGT (4439C>T), GAT1435AAC (4443G>A 4445T>C), AAA1436AAG (4448A>G), GTG1437TTG (4449G>T), AGG1439CGC (4455A>C 4457G>C), TTG1440CTA (4458T>C 4460G>A), GTT1441GTG (4463T>G), GGG1442GGT (4466G>T), GGC1443GGA (4469C>A), CCA1445CCC (4475A>C), TCA1446TCT (4478A>T), GGA1447GGT (4481A>T), GCA1449GCT (4487A>T), CTA1450TTG (4488C>T 4490A>G), ACG1451ACT (4493G>T), ATC1453ATA (4499C>A), TTC1454TTT (4502C>T), TCT1456TCA (4508T>A), CTC1457TTG (4509C>T 4511C>G), CTC1458CTA (4514C>A), CGA1464AGG (4530C>A 4532A>G), TAT1465TAC (4535T>C), GCC1466GCG (4538C>G), TTG1470CTG (4548T>C), TTA1471TTG (4553A>G), AGA1472AGG (4556A>G), CCC1474CCA (4562C>A), ATT1476GTC (4566A>G 4568T>C), GCT1477AAA (4569G>A 4570C>A 4571T>A), AGG1479AAA (4576G>A 4577G>A), GCT1480TCT (4578G>T), ATA1481GTG (4581A>G 4583A>G), GGA1482GGC (4586A>C), GTT1483ATT (4587G>A), AAA1484AAG (4592A>G), AGT1486AAT (4597G>A), GAT1487GAC (4601T>C), CTA1491TTG (4611C>T 4613A>G), GCT1494GCA (4622T>A), GTT1495GTA (4625T>A), GGA1497GGT (4631A>T), GAC1499GAT (4637C>T), AAT1500AAC (4640T>C), CTT1501TTG (4641C>T 4643T>G), GCT1503GCG (4649T>G), GTA1504GTT (4652A>T), TTA1506ATG (4656T>A 4658A>G), CTC1508TTG (4662C>T 4664C>G), CAG1509AGG (4665C>A 4666A>G), TAT1511TAC (4673T>C), TCT1512GAT (4674T>G 4675C>A), CTG1513CTA (4679G>A), AAT1515TCC (4683A>T 4684A>C 4685T>C), ATA1516ATT (4688A>T), CAT1518AGA (4692C>A 4693A>G 4694T>A), GAG1519GAA (4697G>A), TTA1520CTG (4698T>C 4700A>G), CTG1522GCT (4704C>G 4705T>C 4706G>T), GTC1523GTT (4709C>T), AAT1524AAC (4712T>C), GTA1525GTC (4715A>C), ATC1526GTC (4716A>G), CTT1531TTG (4731C>T 4733T>G), AAA1533AAG (4739A>G), TCG1534TCA (4742G>A), GTT1537GTA (4751T>A), AAT1538GAG (4752A>G 4754T>G), GAG1539AAG (4755G>A), GTA1540ACA (4758G>A 4759T>C), CAA1541AAA (4761C>A), CAA1543CTG (4768A>T 4769A>G), CTA1548CTC (4784A>C), ACT1549ACC (4787T>C), CTG1551CTC (4793G>C), AGA1553AGG (4799A>G), TTT1555TTC (4805T>C), AAA1556AAG (4808A>G), GCT1559GCA (4817T>A), CTT1560TTG (4818C>T 4820T>G), TAC1562TAT (4826C>T), CAC1563CAA (4829C>A), GCT1565GCA (4835T>A), CCT1566CCA (4838T>A), AAG1568AAA (4844G>A), ATC1572GTC (4854A>G), ATT1573ATC (4859T>C), ATG1576ATT (4868G>T), ATA1579ATT (4877A>T), AGA1580AGG (4880A>G), CCT1581CCA (4883T>A), GCC1582GCA (4886C>A), CCA1583GTG (4887C>G 4888C>T 4889A>G), GGT1584GGA (4892T>A), GAC1587GAT (4901C>T), ACT1588GTA (4902A>G 4903C>T 4904T>A), CAG1589CAA (4907G>A), GCC1590GCT (4910C>T), ATG1592TAT (4914A>T 4915T>A 4916G>T), GTG1595ATT (4923G>A 4925G>T), GGA1598GGC (4934A>C), ATA1599ATC (4937A>C), CGT1600AGA (4938C>A 4940T>A), GCT1602GCC (4946T>C), CAC1605CAT (4955C>T), GGT1606GGG (4958T>G), CGT1607CAG (4960G>A 4961T>G), GTG1608GCC (4963T>C 4964G>C), GCT1609ACT (4965G>A), GAC1611GAA (4973C>A), AAG1612AGT (4975A>G 4976G>T), CTT1613TTT (4977C>T), GTC1614GTA (4982C>A), TTA1615AAT (4983T>A 4984T>A 4985A>T), CAG1618TTT (4992C>T 4993A>T 4994G>T), ACG1619GGA (4995A>G 4996C>G 4997G>A), GCG1620GCT (5000G>T), TTG1621CTC (5001T>C 5003G>C), GAT1622AAG (5004G>A 5006T>G), GAA1623CAG (5007G>C 5009A>G), AGG1624AGA (5012G>A), GGT1625AAT (5013G>A 5014G>A), TTC1626CTC (5016T>C), AGA1627CCC (5019A>C 5020G>C 5021A>C), GCT1628ATC (5022G>A 5023C>T 5024T>C), GTG1629ATG (5025G>A), TTT1631ATG (5031T>A 5033T>G), CCT1632CCC (5036T>C), TCC1633ACT (5037T>A 5039C>T), TAT1634TAC (5042T>C), TTG1635TAT (5044T>A 5045G>T), GTG1637CTT (5049G>C 5051G>T), CAG1639AGT (5055C>A 5056A>G 5057G>T), GAA1640CAA (5058G>C), ATT1642TTG (5064A>T 5066T>G), GCA1643ACA (5067G>A), AAG1644GAG (5070A>G), GTA1645GTC (5075A>C), GGG1647GGA (5081G>A), TCA1649GAG (5085T>G 5086C>A 5087A>G), AGT1650AGC (5090T>C), GCC1651ATT (5091G>A 5092C>T 5093C>T), CTG1652GGG (5094C>G 5095T>G), ACA1653GTT (5097A>G 5098C>T 5099A>T), ATT1654TTG (5100A>T 5102T>G), TGT1655ATG (5103T>A 5104G>T 5105T>G), GAA1656GAG (5108A>G), ATG1657CTA (5109A>C 5111G>A), GCA1658TCA (5112G>T), AAA1659GAT (5115A>G 5117A>T), GCA1660GCT (5120A>T), GCT1661AAA (5121G>A 5122C>A 5123T>A), ATT1662AAT (5125T>A), TCC1663ACA (5127T>A 5129C>A), TAT1664TAC (5132T>C), ACG1665ACC (5135G>C), CCA1666CCT (5138A>T), TTG1667TTA (5141G>A), GAC1668CCT (5142G>C 5143A>C 5144C>T), GCA1669GCT (5147A>T), GGT1670GGG (5150T>G), GAG1671GAA (5153G>A), ATC1673ATT (5159C>T), ACA1674del (5160_5162delACA), CGT1678GAG (5172C>G 5173G>A 5174T>G), GAT1679GGT (5176A>G), CTG1680GTC (5178C>G 5180G>C), AAT1681CAT (5181A>C), TGG1682GAA (5184T>G 5185G>A 5186G>A), GCA1684GTG (5191C>T 5192A>G), CCA1685CCT (5195A>T), AAC1686GAT (5196A>G 5198C>T), ATT1687GTA (5199A>G 5201T>A), GGT1688TTT (5202G>T 5203G>T), TTT1689TTC (5207T>C), TCA1691AGT (5211T>A 5212C>G 5213A>T), GCA1692GCT (5216A>T), CGT1693CGG (5219T>G), GCC1696TCA (5226G>T 5228C>A), CAC1697AAC (5229C>A), TAC1698CAA (5232T>C 5234C>A), ACG1699CAA (5235A>C 5236C>A 5237G>A), GAT1701TCA (5241G>T 5242A>C 5243T>A), GAA1702TCA (5244G>T 5245A>C), GGG1703GGT (5249G>T), TAC1704AAC (5250T>A), ATT1705ATC (5255T>C), ATT1706CTG (5256A>C 5258T>G), AAC1708AAT (5264C>T), TGT1709TGC (5267T>C), ACA1710ACT (5270A>T), GGT1711GGA (5273T>A), GCA1712GCC (5276A>C), AAG1713CGC (5277A>C 5278A>G 5279G>C), AAG1714AGA (5281A>G 5282G>A), TCC1715ACA (5283T>A 5285C>A), AAT1716ACA (5287A>C 5288T>A), GTT1718GTG (5294T>G), GGT1720GGG (5300T>G), CCA1721CCT (5303A>T), AAC1723AAT (5309C>T), ATG1731ATA (5333G>A), CCG1733CCC (5339G>C), ATG1736TTG (5346A>T), TCA1737GCA (5349T>G), GCT1738GCA (5354T>A), ATA1739ATC (5357A>C), ATG1740CAG (5358A>C 5359T>A), GCC1742CAT (5364G>C 5365C>A 5366C>T), AAG1744CGC (5370A>C 5371A>G 5372G>C), AAT1745AAG (5375T>G), GTG1746AAT (5376G>A 5377T>A 5378G>T), TTG1747CTG (5379T>C), GCA1748GAA (5383C>A), GGA1749GTT (5386G>T 5387A>T), GGA1750GGT (5390A>T), CAT1751GAT (5391C>G), GTA1752GGG (5395T>G 5396A>G), GCA1753GCT (5399A>T), AAT1755TCT (5403A>T 5404A>C), GTT1757ACC (5409G>A 5410T>C 5411T>C), GTG1759GTT (5417G>T), GGA1761GGT (5423A>T), GGA1763GGT (5429A>T), GCT1765GCA (5435T>A), GTC1766ATA (5436G>A 5438C>A), GGC1767GCT (5440G>C 5441C>T), ATT1769ATC (5447T>C), GCG1772GCA (5456G>A), CTG1773TTG (5457C>T), ATG1774TTT (5460A>T 5462G>T), GCC1775GCA (5465C>A), TTA1776TTG (5468A>G), GCA1777GCC (5471A>C), ACA1778ACT (5474A>T), AGG1779CAT (5475A>C 5476G>A 5477G>T), TAT1781TAC (5483T>C), TGT1782AGG (5484T>A 5486T>G), GTG1783ACC (5487G>A 5488T>C 5489G>C), GAG1784ACA (5490G>A 5491A>C 5492G>A), ATA1786CTT (5496A>C 5498A>T), ATA1787ATT (5501A>T), GTT1788GTG (5504T>G), AGA1789CGC (5505A>C 5507A>C), TTG1790CTA (5508T>C 5510G>A), CGC1791CGA (5513C>A), ACA1792ACC (5516A>C), ATT1793ATG (5519T>G), GGA1794GCT (5521G>C 5522A>T), AAT1795GGG (5523A>G 5524A>G 5525T>G), CTT1797CTG (5531T>G), GAC1798GAT (5534C>T), CTC1799CTG (5537C>G), AAT1800AAC (5540T>C), TAT1802TAC (5546T>C), GGC1805GGT (5555C>T), GTG1807GCG (5560T>C), CAG1808CAA (5564G>A), TAT1809TAC (5567T>C), TTT1810TTG (5570T>G), CTT1811CTC (5573T>C), CAA1812AAA (5574C>A), GTG1814GTT (5582G>T), CCT1815GAA (5583C>G 5584C>A 5585T>A), CAT1816GAC (5586C>G 5588T>C), CAT1816_GGA1817insACC (5588_5589insACC), GGA1817TCA (5589G>T 5590G>C), GAC1818CAC (5592G>C), AAA1819AAG (5597A>G), GTG1820TTG (5598G>T), GCT1821GGA (5602C>G 5603T>A), CAA1822CAG (5606A>G), GGT1824GGC (5612T>C), GCA1825ACA (5613G>A), TCG1826TAT (5617C>A 5618G>T), CTC1827GGC (5619C>G 5620T>G), CAC1828del (5622_5624delCAC), AGT1829ACC (5626G>C 5627T>C), AGT1830ACT (5629G>C), ATG1832AAC (5635T>A 5636G>C), CAA1834CAT (5642A>T), CAA1834_GGG1835insCACAGT (5642_5643insCACAGT), GGG1835GCA (5644G>C 5645G>A), GAA1837TCA (5649G>T 5650A>C), TTG1838GTT (5652T>G 5654G>T), GGC1839GGA (5657C>A), AAC1840CTT (5658A>C 5659A>T 5660C>T), CTC1841CTA (5663C>A), CGC1842AGG (5664C>A 5666C>G), ATT1843ATA (5669T>A), ATA1844GTT (5670A>G 5672A>T), CAT1845CCT (5674A>C), GGT1846TCC (5676G>T 5677G>C 5678T>C), GAT1847GAC (5681T>C), TTA1848CTC (5682T>C 5684A>C), GCA1849TCT (5685G>T 5687A>T), AAA1850GAA (5688A>G), CAG1851AAG (5691C>A), ACA1852ATG (5695C>T 5696A>G), ATG1854GTT (5700A>G 5702G>T), CGG1855CGT (5705G>T), ATG1856TTG (5706A>T), TAT1858TAC (5714T>C), GTG1859ACA (5715G>A 5716T>C 5717G>A), GTA1860ATT (5718G>A 5720A>T), GGA1861GGT (5723A>T), CCA1862CCT (5726A>T), CAT1863GAA (5727C>G 5729T>A), GGA1865GGT (5735A>T), GGA1867GCC (5740G>C 5741A>C), AAT1868CAG (5742A>C 5744T>G), TTT1869TGC (5746T>G 5747T>C), TCC1870TCA (5750C>A), ACA1871ACG (5753A>G), CAG1872AAG (5754C>A), GAT1873AAC (5757G>A 5759T>C), CTT1874CTG (5762T>G), GAG1875AAG (5763G>A), AGT1876CAT (5766A>C 5767G>A), TTG1877GCT (5769T>G 5770T>C 5771G>T), CTA1878TTG (5772C>T 5774A>G), CAC1879GAG (5775C>G 5777C>G), TAT1880ACA (5778T>A 5779A>C 5780T>A), GAG1882TCT (5784G>T 5785A>C 5786G>T), CAG1883GTC (5787C>G 5788A>T 5789G>C), GGA1884AGT (5790G>A 5792A>T), GCA1886GCT (5798A>T), GAG1887ACT (5799G>A 5800A>C 5801G>T), TTA1888CTT (5802T>C 5804A>T), ATT1889GCC (5805A>G 5806T>C 5807T>C), CAA1890GCA (5808C>G 5809A>C), AAG1891AGA (5812A>G 5813G>A), AAT1892GGA (5814A>G 5815A>G 5816T>A), ACA1893GTC (5817A>G 5818C>T 5819A>C), CTA1895GTG (5823C>G 5825A>G), ACT1896ACA (5828T>A), CTG1897TTG (5829C>T), TTC1899TTT (5837C>T), GAG1901GAA (5843G>A), CTG1902TTC (5844C>T 5846G>C), AGT1903TCC (5847A>T 5848G>C 5849T>C), ATG1904CAA (5850A>C 5851T>A 5852G>A), GAA1905CAC (5853G>C 5855A>C), AAT1906GAT (5856A>G), GTG1907TGT (5859G>T 5860T>G 5861G>T), CAA1908GAA (5862C>G), CAA1909ATG (5865C>A 5866A>T 5867A>G), CTG1910ATA (5868C>A 5870G>A), ATA1911CTT (5871A>C 5873A>T), GAT1912GAG (5876T>G), GTT1914CTG (5880G>C 5882T>G), AAG1915GAA (5883A>G 5885G>A), CTT1916AGT (5886C>A 5887T>G), CAG1917CAA (5891G>A), TTT1919GTA (5895T>G 5897T>A), TTC1920GTA (5898T>G 5900C>A), CCA1921TTC (5901C>T 5902C>T 5903A>C), AAA1922ATG (5905A>T 5906A>G), GAA1923GGG (5908A>G 5909A>G), ACT1924AGC

	(5911C>G 5912T>C), ACC1925GTT (5913A>G 5914C>T 5915C>T), ATT1926AAG (5917T>A 5918T>G), CAG1927AAA (5919C>A 5921G>A), AAG1928CAA (5922A>C 5924G>A), CTC1929ATG (5925C>A 5927C>G), AAA1930TTG (5928A>T 5929A>T 5930A>G), AAT1931TCC (5931A>T 5932A>C 5933T>C), TTT1932TTC (5936T>C), GTT1933ATG (5937G>A 5939T>G), GAT1934AAT (5940G>A), GCT1935CAA (5943G>C 5944C>A 5945T>A), GAA1936CAG (5946G>C 5948A>G), TGT1937ATG (5949T>A 5950G>T 5951T>G), CTA1938TCA (5952C>T 5953T>C), ACA1939AAA (5956C>A), TTC1940ACC (5958T>A 5959T>C), AAA1941CAA (5961A>C), GCA1942GTT (5965C>T 5966A>T), AGG1943GCC (5967A>G 5968G>C 5969G>C), AGC1944_TTT1945insCCATGTTGTGTCACA (5972_5973insCCATGTTGTGTCACA), AGG1946AGT (5978G>T), CAC1947AAA (5979C>A 5981C>A), GTA1948GCA (5983T>C), GTT1949TGG (5985G>T 5986T>G 5987T>G), TTC1950CTA (5988T>C 5990C>A), AAA1951ACT (5992A>C 5993A>T), AAA1953GAC (5997A>G 5999A>C), CTT1954ATT (6000C>A), TTG1955GTC (6003T>G 6005G>C), AGT1956ATG (6007G>T 6008T>G), TCC1957ACA (6009T>A 6011C>A), ACA1958AAA (6013C>A), TGG1959GGT (6015T>G 6017G>T), AAA1960AAG (6020A>G), ATG1961AAG (6022T>A), TGT1962CCA (6024T>C 6025G>C 6026T>A), GGT1963GGA (6029T>A), GAA1964del (6030_6032delGAA), AGT1965TCT (6033A>T 6034G>C), GTT1967_AGG1970del (6039_6050delGTTGCTTCAAGG), TCT1971CCT (6051T>C), GCA1972GCT (6056A>T), GAG1973GAA (6059G>A), AGT1974ACA (6061G>C 6062T>A), CTA1975GTG (6063C>G 6065A>G), TTC1976TTT (6068C>T), CCT1977CTT (6070C>T), GGC1978GGG (6074C>G), TTG1980CCT (6078T>C 6079T>C 6080G>T), TCT1981TCA (6083T>A), GCT1982GCA (6086T>A), TCC1983CTA (6087T>C 6088C>T 6089C>A), GTA1984GTT (6092A>T), TTG1985GTG (6093T>G), AAA1986AGA (6097A>G), TTA1988ATG (6102T>A 6104A>G), GAA1990CGC (6108G>C 6109A>G 6110A>C), AGA1991AAT (6112G>A 6113A>T), CAC1992AGT (6114C>A 6115A>G 6116C>T), ACA1993GGA (6117A>G 6118C>G), AGA1994AGG (6122A>G), ATG1996TAC (6126A>T 6127T>A 6128G>C), AGT1997TCA (6129A>T 6130G>C 6131T>A), CAG1999ACA (6135C>A 6136A>C 6137G>A), AGT2000ACA (6139G>C 6140T>A), ATG2001CTG (6141A>C), GAG2002GAA (6146G>A), CTG2005TTT (6153C>T 6155G>T), AAA2006AGA (6157A>G), ATA2007ATG (6161A>G), TAT2008TAC (6164T>C), CTA2009ATG (6165C>A 6167A>G), TTA2010TGT (6169T>G 6170A>T), AAC2011CAG (6171A>C 6173C>G), TTC2012GAA (6174T>G 6175T>A 6176C>A), CAA2013GGG (6177C>G 6178A>G 6179A>G), GTA2014TAT (6180G>T 6181T>A 6182A>T), ATA2015TTA (6183A>T), ACA2016GAT (6186A>G 6187C>A 6188A>T), AGT2017AAA (6190G>A 6191T>A), GAG2018GCC (6193A>C 6194G>C), TTA2020GTG (6198T>G 6200A>G), AAG2021GAC (6201A>G 6203G>C), AAG2022CGC (6204A>C 6205A>G 6206G>C), TTT2023TTC (6209T>C), GAA2024AAT (6210G>A 6212A>T), GAT2025GAG (6215T>G), TTT2027ATG (6219T>A 6221T>G), CAG2028GGC (6222C>G 6223A>G 6224G>C), GAG2029GTT (6226A>T 6227G>T), AAG2030TGT (6228A>T 6229A>G 6230G>T), ATA2031GTG (6231A>G 6233A>G), TCC2032TCG (6236C>G), ACC2033GAG (6237A>G 6238C>A 6239C>G), ACC2034CGC (6240A>C 6241C>G), CTA2035ATC (6243C>A 6245A>C), CTA2036TTG (6246C>T 6248A>G), ATC2037GTG (6249A>G 6251C>G), AAA2038CGC (6252A>C 6253A>G 6254A>C), GTA2039ACA (6255G>A 6256T>C), TTC2040ATT (6258T>A 6260C>T), TTG2041GCT (6261T>G 6262T>C 6263G>T), GAG2044TCC (6270G>T 6271A>C 6272G>C), GAG2045ACG (6273G>A 6274A>C), AGT2046TCA (6276A>T 6277G>C 6278T>A), TAT2047TTG (6280A>T 6281T>G), CAG2048AAC (6282C>A 6284G>C), ACT2049GAG (6285A>G 6286C>A 6287T>G), GAG2050GAT (6290G>T), TGT2052ACT (6294T>A 6295G>C), ATA2053GTG (6297A>G 6299A>G), GAT2054TAC (6300G>T 6302T>C), TCT2055ACA (6303T>A 6305T>A), CAG2056GAT (6306C>G 6308G>T), GTG2057GGG (6310T>G), CTC2058CTT (6314C>T), CAG2059TTT (6315C>T 6316A>T 6317G>T), AGG2060GAT (6318A>G 6319G>A 6320G>T), ATA2061del (6321_6323delATA), AAC2062CAT (6324A>C 6326C>T), TCC2063TTC (6328C>T), CAG2064AAG (6330C>A), AAA2065GCA (6333A>G 6334A>C), TTC2066GTG (6336T>G 6338C>G), AGA2067GTT (6339A>G 6340G>T 6341A>T), GTG2068TTC (6342G>T 6344G>C), CAG2069AAG (6345C>A), GAA2070CCT (6348G>C 6349A>C 6350A>T), GGT2071GGG (6353T>G), TTT2073TTC (6359T>C), TAT2074TCT (6361A>C), TTG2075TGG (6364T>G), CAT2076GAA (6366C>G 6368T>A), CCT2077ATC (6369C>A 6370C>T 6371T>C), GAA2078AAT (6372G>A 6374A>T), GGA2079GAT (6376G>A 6377A>T), ATC2080GCC (6378A>G 6379T>C), AAC2081AAT (6383C>T), GCC2084GCT (6392C>T), GTA2085GCA (6394T>C), ATA2088GTG (6402A>G 6404A>G), ATA2089TTG (6405A>T 6407A>G), TCT2091GAA (6411T>G 6412C>A 6413T>A), CTG2092CTC (6416G>C), GAG2094AGG (6420G>A 6421A>G), TCC2095CAC (6423T>C 6424C>A), TAT2096TGG (6427A>G 6428T>G), AGC2097TCA (6429A>T 6430G>C 6431C>A), CGA2098AAG (6432C>A 6433G>A 6434A>G), GAT2099TCA (6435G>T 6436A>C 6437T>A), AGT2100CGA (6438A>C 6440T>A), AAC2101AGC (6442A>G), TCC2102AAG (6444T>A 6445C>A 6446C>G), TAT2103TTC (6448A>T 6449T>C), TCT2104ACT (6450T>A), GTG2105TTT (6453G>T 6455G>T), TCA2106GCC (6456T>G 6458A>C), ACT2107AAT (6460C>A), CCC2108GAA (6462C>G 6463C>A 6464C>A), ATC2109GTT (6465A>G 6467C>T), AAA2110GAA (6468A>G), CTG2111ATA (6471C>A 6473G>A), GGG2112TCA (6474G>T 6475G>C 6476G>A), TGT2113AAC (6477T>A 6478G>A 6479T>C), TTC2114CTG (6480T>C 6482C>G), ATT2115CTT (6483A>C), TTC2116TTA (6488C>A), CTT2117GCT (6489C>G 6490T>C), GTC2118GTT (6494C>T), GTG2119TAT (6495G>T 6496T>A 6497G>T), CTA2120TTA (6498C>T), GAT2121GAA (6503T>A), AGT2122AAT (6505G>A), CAA2123AAG (6507C>A 6509A>G), GGC2124CTC (6510G>C 6511G>T), AAG2125ATA (6514A>T 6515G>A), GAA2126GGT (6517A>G 6518A>T), GAT2127CTA (6519G>C 6520A>T 6521T>A), CAT2128GAG (6522C>G 6524T>G), CCT2129TAT (6525C>T 6526C>A), GTT2130TAT (6528G>T 6529T>A), CGG2131GAT (6531C>G 6532G>A 6533G>T), AGG2132TCT (6534A>T 6535G>C 6536G>T), TTT2133ATA (6537T>A 6539T>A), TCA2134GAA (6540T>G 6541C>A), ACG2135TCT (6543A>T 6545G>T), GTT2136TTG (6546G>T 6548T>G), TTC2137GAC (6549T>G 6550T>A), CTT2138TTG (6552C>T 6554T>G), AAA2139TTA (6555A>T 6556A>T), AAC2140CAA (6558A>C 6560C>A), TGT2141ATA (6561T>A 6562G>T 6563T>A), GAA2142GAT (6566A>T), ATT2144ATA (6572T>A), ACA2146GCA (6576A>G), TAC2148GTC (6582T>G 6583A>T), GAG2150TAC (6588G>T 6590G>C), TGA2152GGG (6594T>G 6596A>G)								
helicase (YP_001976154.1)	1	98	100%	654	97.5%	98 (100%)	95 (96.9%)	0/0/0/0	0
Protein mutations:	S5C (1342C>G 1343C>T), R16A (1374A>G 1375G>C), I44V (1458A>G 1460C>G)								
Codon mutations:	TCT3TCA (1337T>A), ACA4ACG (1340A>G), TCC5TGT (1342C>G 1343C>T), TTA8CTT (1350T>C 1352A>T), TTT9TTC (1355T>C), ATT10ATC (1358T>C), GTG13GTT (1367G>T), TTG14CTC (1368T>C 1370G>C), GAC15GAT (1373C>T), AGA16GCA (1374A>G 1375G>C), GAG19GAA (1385G>A), AAA21AAG (1391A>G), CTC22CTG (1394C>G), AAT23AAC (1397T>C), CGG24CGC (1400G>C), CTA25TTG (1401C>T 1403A>G), GCA27GCG (1409A>G), AAG30AAA (1418G>A), AGG31AGA (1421G>A), GCT39GCA (1445T>A), CAC40CAT (1448C>T), GCT43GCA (1457T>A), ATC44GTG (1458A>G 1460C>G), CTG45CTT (1463G>T), GAC48GAT (1472C>T), GGA50GGT (1478A>T), GCA51GCT (1481A>T), TTG52CTT (1482T>C 1484G>T), CGA53CGG (1487A>G), GGG55GGA (1493G>A), GCT56GCA (1496T>A), TGC59TGT (1505C>T), CAA60CAG (1508A>G), GAT61GAC (1511T>C), ATT62ATA (1514T>A), AAA63AAG (1517A>G), GAT67GAC (1529T>C), TCA70TCT (1538A>T), ACA71ACC (1541A>C), CAA72CAG (1544A>G), CCA73CCT (1547A>T), CCC75CCT (1553C>T), TTA76CTT (1554T>C 1556A>T), GCC79GCT (1565C>T), GTT81GTC (1571T>C), GAC83GAT (1577C>T), GGC85GGA (1583C>A), CAT87CAC (1589T>C), TTC88TTT (1592C>T), ACC89ACA (1595C>A), TCC90TCA (1598C>A), TAT92TAC (1604T>C), ATA93ATC (1607A>C), ACA96ACC (1616A>C)								
RNA-dependent RNA polymerase (YP_001976153.1)	1	249	100%	1552	88.4%	249 (100%)	210 (84.3%)	0/0/0/0	0
Protein mutations:	T40C (4170A>T 4171C>G 4172A>T), L44A (4182T>G 4183T>C 4184G>A), A47V (4192C>T 4193T>A), I48L (4194A>C 4196A>C), D55N (4215G>A), S59H (4227T>C 4228C>A), L63V (4239T>G), N66S (4249A>G), G67S (4251G>A 4253G>C), A70S (4260G>T 4262T>A), V74E (4273T>A), V89I (4317G>A 4319T>C), E101A (4354A>C 4355A>T), Y102F (4357A>T 4358C>T), S105D (4365T>G 4366C>A 4367A>T), K106E (4368A>G), D111E (4385T>G), A117T (4401G>A), D131N (4443G>A 4445T>C), V133L (4449G>T), I172V (4566A>G 4568T>C), A173K (4569G>A 4570C>A 4571T>A), R175K (4576G>A 4577G>A), A176S (4578G>T), I177V (4581A>G 4583A>G), V179I (4587G>A), S182N (4597G>A), L202M (4656T>A 4658A>G), Q205R (4665C>A 4666A>G), S208D (4674T>G 4675C>A), N211S (4683A>T 4684A>C 4685T>C), H214R (4692C>A 4693A>G 4694T>A), L218A (4704C>G 4705T>C 4706G>T), I222V (4716A>G), N234E (4752A>G 4754T>G), E235K (4755G>A), V236T (4758G>A 4759T>C), Q237K (4761C>A), Q239L (4768A>T 4769A>G)								
Codon mutations:	CGC4CGG (4064C>G), CTC5TTG (4065C>T 4067C>G), AAA9AAG (4079A>G), ATC10ATT (4082C>T), GGC12GGG (4088C>G), AGA14AGG (4094A>G), AGG17CGC (4101A>C 4103G>C), CTC18CTG (4106C>G), TTT19TTC (4109T>C), GAG20GAA (4112G>A), ATA21ATC (4115A>C), CTT22TTG (4116C>T 4118T>G), TAC26TAT (4130C>T), TTG29CTG (4137T>C), GTC30GTG (4142C>G), AGG31CGG (4143A>C), AAG32AAA (4148G>A), TAT33TAC (4151T>C), TTT34TTC (4154T>C), GAT36GAC (4160T>C), TTC37TTT (4163C>T), TCA38TCT (4166A>T), GCC39GCT (4169C>T), ACA40TGT (4170A>T 4171C>G 4172A>T), TTG44GCA (4182T>G 4183T>C 4184G>A), AAT46AAC (4190T>C), GCT47GTA (4192C>T 4193T>A), ATA48CTC (4194A>C 4196A>C), CCA49CCT (4199A>T), AAA51AAG (4205A>G), GTT52GTA (4208T>A), ATT54ATC (4214T>C), GAT55AAT (4215G>A), CCT56CCA (4220T>A), ACA57ACG (4223A>G), AGT58AGC (4226T>C), TCT59CAT (4227T>C 4228C>A), TTG63GTG (4239T>G), TTG64CTG (4242T>C), GCA65GCT (4247A>T), AAT66AGT (4249A>G), GGG67AGC (4251G>A 4253G>C), AGA69AGG (4259A>G), GCT70TCA (4260G>T 4262T>A), GTG71GTA (4265G>A), TCA72TCT (4268A>T), GAC73GAT (4271C>T), GTG74GAG (4273T>A), TTT76TTC (4280T>C), GCT78GCC (4286T>C), TAT80TAC (4292T>C), TCC81TCG (4295C>G), CCT88CCC (4316T>C), GTT89ATC (4317G>A 4319T>C), GCT91GCA (4325T>A), TTT92TTC (4328T>C), CAG93CAA (4331G>A), TTG98CTG (4344T>C), GAA101GCT (4354A>C 4355A>T), TAC102TTT (4357A>T 4358C>T), TAC103TAT (4361C>T), GGA104GGT (4364A>T), TCA105GAT (4365T>G 4366C>A 4367A>T), AAG106GAG (4368A>G), GGC108GGG (4376C>G), GAT111GAG (4385T>G), TCC112TCA (4388C>A), CGA115AGG (4395C>A 4397A>G), CAT116CAC (4400T>C), GCA117ACA (4401G>A), CTT118TTA (4404C>T 4406T>A), GCA122GCC (4418A>C), TGT124TGC (4424T>C), TAC126TAT (4430C>T), ACA127ACC (4433A>C), CTG128CTC (4436G>C), TGC129TGT (4439C>T), GAT131AAC (4443G>A 4445T>C), AAA132AAG (4448A>G), GTG133TTG (4449G>T), AGG135CGC (4455A>C 4457G>C), TTG136CTA (4458T>C 4460G>A), GTT137GTG (4463T>G), GGG138GGT (4466G>T), GGC139GGA (4469C>A), CCA141CCC (4475A>C), TCA142TCT (4478A>T), GGA143GGT (4481A>T), GCA145GCT (4487A>T), CTA146TTG (4488C>T 4490A>G), ACG147ACT (4493G>T), ATC149ATA (4499C>A), TTC150TTT (4502C>T), TCT152TCA (4508T>A), CTC153TTG (4509C>T 4511C>G), CTC154CTA (4514C>A), CGA160AGG (4530C>A 4532A>G), TAT161TAC (4535T>C), GCC162GCG (4538C>G), TTG166CTG (4548T>C), TTA167TTG (4553A>G), AGA168AGG (4556A>G), CCC170CCA (4562C>A), ATT172GTC (4566A>G 4568T>C), GCT173AAA (4569G>A 4570C>A 4571T>A), AGG175AAA (4576G>A 4577G>A), GCT176TCT (4578G>T), ATA177GTG (4581A>G 4583A>G), GGA178GGC (4586A>C), GTT179ATT (4587G>A), AAA180AAG (4592A>G), AGT182AAT (4597G>A), GAT183GAC (4601T>C), CTA187TTG (4611C>T 4613A>G), GCT190GCA (4622T>A), GTT191GTA (4625T>A), GGA193GGT (4631A>T), GAC195GAT (4637C>T), AAT196AAC (4640T>C), CTT197TTG (4641C>T 4643T>G), GCT199GCG (4649T>G), GTA200GTT (4652A>T), TTA202ATG (4656T>A 4658A>G), CTC204TTG (4662C>T 4664C>G), CAG205AGG (4665C>A 4666A>G), TAT207TAC (4673T>C), TCT208GAT (4674T>G 4675C>A), CTG209CTA (4679G>A), AAT211TCC (4683A>T 4684A>C 4685T>C), ATA212ATT (4688A>T), CAT214AGA (4692C>A 4693A>G 4694T>A), GAG215GAA (4697G>A), TTA216CTG (4698T>C 4700A>G), CTG218GCT (4704C>G 4705T>C 4706G>T), GTC219GTT (4709C>T), AAT220AAC (4712T>C), GTA221GTC (4715A>C), ATC222GTC (4716A>G), CTT227TTG (4731C>T 4733T>G), AAA229AAG (4739A>G), TCG230TCA (4742G>A), GTT233GTA (4751T>A), AAT234GAG (4752A>G 4754T>G), GAG235AAG (4755G>A), GTA236ACA (4758G>A 4759T>C), CAA237AAA (4761C>A), CAA239CTG (4768A>T 4769A>G), CTA244CTC (4784A>C), ACT245ACC (4787T>C), CTG247CTC (4793G>C), AGA249AGG (4799A>G)								

*: Inserts / Deletes / Misaligned / Frameshifts

Analysis details

This analysis was performed with panviral2.64

NGS Details (UN70): Torradovirus lycopersici (segment RNA 1)

Assembly

**Coverage Length**	7781 (1 contig(s))
**Depth Of Coverage**	13912.1
**Number Of Reads**	753401
**Reads Per Million**	14281.67 rpm (after QC)
**Ambiguities**	0
**Assembly Method**	de novo + reference guided assembly
**Consensus Caller**	Bcf Tools

Coverage Map

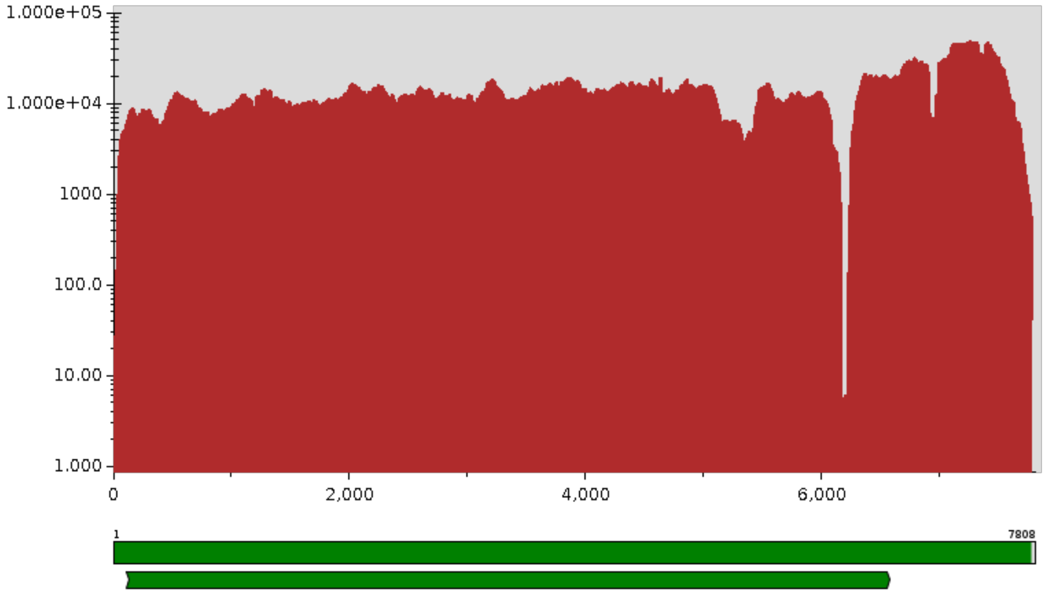

Assignment

**Type**	Torradovirus lycopersici (Taxonomy ID: 3048378)
**Reference Genome**	NC_009013.1
**NT Identity (%)**	90.3062
**AA Identity (%)**	97.6378
**Number Of Stop Codons**	1
**Number Of CDS**	1

Alignment

**Alignment Score**	11522.0 (NT) + 14846.0 (AA) = 26368.0
**Concordance (%)**	89.2228

Alignment Method	Global, seeded, nucleotide + amino acids (AGA)

Genome Region

Sequence starts at position 1 and ends at position 7781 relative to NC_009013.1 reference sequence.

Alignment Detailed Statistics

	Begin	End	Coverage	Score	Concordance	Matches	Identities	I/D/M/F*	Stop Codons
**NT**	**1**	**7781**	**99.7%**	**11522**	**79.6%**	**7469 (95.9%)**	**6754 (86.7%)**	**10/312**	
Mutations:	2T>C, 5A>T, 9G>A, 12A>T, 13T>C, 14T>A, 15T>C, 16T>G, 27C>A, 69C>T, 76_77insT, 87C>T, 95A>G, 100T>C, 101G>A, 102G>A, 115T>C, 127C>T, 128C>T, 130C>T, 135T>C, 148T>C, 151A>G, 158G>T, 161A>G, 175T>C, 178T>C, 184T>C, 190G>A, 197G>A, 199T>A, 202T>C, 205T>A, 208T>C, 220C>T, 224C>T, 253A>G, 259T>C, 262T>A, 265C>T, 338T>C, 340G>A, 361A>G, 367A>G, 370C>T, 376C>T, 385C>T, 388T>C, 409A>G, 451A>G, 487C>T, 490A>G, 502G>A, 541T>C, 559C>T, 577A>G, 578C>T, 655C>T, 676A>G, 694A>G, 727A>G, 760C>T, 781C>T, 790C>T, 799A>G, 805T>C, 818C>T, 820A>G, 832A>T, 835A>C, 841T>C, 842C>T, 847C>T, 883G>A, 886T>C, 892G>A, 901T>C, 913T>C, 925C>T, 938C>T, 952A>G, 955C>T, 964T>G, 970G>A, 973T>C, 977G>A, 980T>C, 997C>T, 1006T>C, 1012C>T, 1013T>C, 1018C>T, 1033G>A, 1060T>C, 1063C>T, 1078A>T, 1081C>T, 1090C>T, 1093T>C, 1105A>G, 1108T>C, 1109C>T, 1165C>T, 1180G>A, 1196A>G, 1207T>C, 1231G>A, 1240C>T, 1255G>A, 1267T>C, 1300G>A, 1303A>G, 1319C>T, 1324C>T, 1351A>G, 1354G>A, 1411T>C, 1417T>C, 1429C>T, 1441C>T, 1450T>C, 1451C>T, 1480C>T, 1481A>G, 1489T>C, 1522C>T, 1552C>T, 1555C>T, 1570A>G, 1573T>C, 1582C>T, 1585A>C, 1591C>T, 1594T>C, 1607G>T, 1612G>A, 1618T>G, 1619C>T, 1624C>T, 1630C>T, 1639T>C, 1642G>A, 1645A>G, 1669C>T, 1672G>A, 1681T>A, 1684G>T, 1699T>C, 1705T>C, 1737A>G, 1738A>G, 1750A>T, 1756A>G, 1765T>G, 1781T>C, 1783G>A, 1784C>T, 1789A>G, 1810G>A, 1825C>T, 1834A>G, 1867G>A, 1879A>G, 1888A>G, 1910T>C, 1912G>A, 1916A>G, 1960A>G, 1962G>A, 1966A>G, 1984C>T, 1990G>A, 1993G>A, 2009T>C, 2023T>C, 2056T>C, 2062C>T, 2077C>A, 2082T>A, 2092A>G, 2107A>G, 2128T>C, 2138C>T, 2143T>C, 2149T>C, 2155T>C, 2174C>T, 2179A>C, 2181A>G, 2182G>A, 2200T>C, 2224C>T, 2236A>G, 2241A>G, 2250G>A, 2251C>T, 2257C>T, 2263G>A, 2275T>C, 2287G>A, 2290C>T, 2314C>T, 2323A>T, 2347C>T, 2356C>T, 2359G>A, 2377G>A, 2392A>G, 2409G>A, 2443T>C, 2446G>A, 2479G>A, 2485C>T, 2497G>A, 2500T>C, 2503G>A, 2506T>C, 2509C>T, 2512T>C, 2542G>A, 2548C>T, 2557C>T, 2620G>A, 2662C>T, 2665T>C, 2734C>T, 2743A>G, 2755C>T, 2773A>G, 2809C>T, 2812G>A, 2827A>G, 2860C>T, 2881T>C, 2914C>T, 2926G>A, 2929C>T, 2950C>T, 2951C>T, 2980C>T, 2983G>A, 2998A>G, 3007T>C, 3028T>C, 3031T>C, 3034G>A, 3040T>C, 3058A>G, 3064A>G, 3073G>A, 3079T>C, 3109T>C, 3115G>A, 3145A>G, 3151C>T, 3163T>C, 3169C>T, 3172T>C, 3202T>C, 3241T>C, 3253C>T, 3256G>A, 3262C>T, 3283T>C, 3289T>C, 3298T>C, 3307A>G, 3358A>T, 3406A>C, 3418C>A, 3421T>C, 3463A>G, 3466G>A, 3481C>T, 3484A>T, 3487G>A, 3492C>T, 3508G>A, 3559G>A, 3574T>C, 3577A>G, 3586T>C, 3591C>T, 3625C>T, 3628G>A, 3646C>T, 3661A>G, 3664T>C, 3673G>A, 3676C>T, 3686C>T, 3706T>C, 3716T>C, 3721T>A, 3739A>T, 3754G>A, 3784C>T, 3814C>T, 3868C>T, 3871T>C, 3883G>A, 3892G>A, 3898T>C, 3907A>T, 3916C>T, 3925A>G, 3928G>A, 3949C>T, 3955G>A, 3961G>A, 3970A>G, 3973T>C, 4018A>G, 4034T>C, 4036G>A, 4057G>A, 4066T>C, 4084C>T, 4118C>T, 4129C>T, 4138G>A, 4147C>A, 4159G>A, 4162A>T, 4168A>G, 4186A>T, 4195A>G, 4207G>A, 4210T>C, 4219C>T, 4228C>T, 4273A>G, 4282C>A, 4288C>T, 4291T>C, 4300T>C, 4318C>T, 4321C>T, 4324T>C, 4339T>C, 4348C>T, 4354T>C, 4360A>G, 4393T>C, 4417T>C, 4421T>C, 4459T>C, 4462C>T, 4465C>T, 4468C>T, 4471T>C, 4474T>C, 4486T>C, 4489T>C, 4591T>C, 4625T>C, 4636T>C, 4640C>T, 4651T>A, 4654C>A, 4657A>G, 4661T>C, 4663G>A, 4666G>A, 4672G>A, 4678T>C, 4681T>A, 4684A>C, 4687G>A, 4696C>A, 4699C>T, 4720T>A, 4759C>T, 4774G>A, 4783A>G, 4786A>G, 4789C>T, 4813A>G, 4816T>C, 4898G>A, 4906G>A, 4924T>C, 4946A>G, 4960C>T, 4969G>A, 4975G>A, 4993A>C, 4999T>C, 5005T>C, 5012T>C, 5017G>A, 5023C>T, 5044A>C, 5059G>A, 5065C>T, 5074T>C, 5077T>C, 5079T>C, 5102T>C, 5113C>T, 5119T>C, 5120G>A, 5146T>C, 5148C>A, 5167T>C, 5170C>T, 5176C>T, 5200G>A, 5218T>G, 5244G>A, 5266C>T, 5281C>T, 5290G>A, 5329C>T, 5344A>T, 5347C>A, 5354G>A, 5356G>T, 5365A>C, 5369G>A, 5371G>T, 5386C>G, 5392G>A, 5407A>C, 5413C>T, 5419A>T, 5429C>T, 5437T>C, 5440C>G, 5446T>C, 5461T>C, 5467G>T, 5471T>C, 5491T>C, 5492A>C, 5497T>C, 5533T>G, 5537A>C, 5575A>G, 5578T>C, 5581A>G, 5591G>T, 5599T>C, 5603A>C, 5611A>T, 5617A>T, 5641T>C, 5647T>C, 5674A>G, 5677G>T, 5686G>A, 5710T>A, 5716G>T, 5719A>C, 5731C>T, 5734T>C, 5737T>C, 5739G>A, 5746T>C, 5750T>C, 5752G>A, 5758C>T, 5773G>A, 5782A>G, 5784T>C, 5786T>C, 5797A>G, 5806C>T, 5836A>T, 5849T>C, 5850T>A, 5851G>A, 5869G>A, 5875A>G, 5878G>C, 5884T>C, 5887C>A, 5890C>T, 5902G>T, 5908C>T, 5909T>C, 5914T>C, 5923T>C, 5927C>T, 5944C>T, 5955A>G, 5965G>A, 5998A>G, 6004C>T, 6016A>G, 6019A>G, 6028C>T, 6031A>T, 6033C>T, 6035G>T, 6055T>A, 6067C>A, 6070T>C, 6091A>G, 6092C>T, 6100T>G, 6103G>A, 6106G>A, 6112A>G, 6118T>C, 6121A>T, 6139T>C, 6154C>G, 6163C>G, 6166C>T, 6184A>G, 6187T>G, 6193C>T, 6196A>G, 6202G>C, 6211A>G, 6217G>T, 6223A>T, 6230A>G, 6232A>G, 6235G>A, 6253A>C, 6256C>A, 6262A>T, 6265G>A, 6268G>T, 6274C>G, 6296G>C, 6298A>G, 6308T>C, 6313T>C, 6322G>A, 6327A>G, 6358T>C, 6484A>C, 6490A>G, 6493T>C, 6500A>T, 6517C>T, 6532C>T, 6545C>T, 6571C>T, 6587G>A, 6661T>C, 6670C>T, 6675C>T, 6685A>G, 6742A>T, 6766delC, 6786T>C, 6798_6799insA, 6806T>G, 6807A>T, 6809C>T, 6811_6832delGAGAGTACTGACTTTAACTAGT, 6835G>T, 6838_6850delGTCCGGCTCCATT, 6854A>T, 6858C>T, 6859C>A, 6860_6894delAATGAATTTACATTGGTTAAAGAGATTTGCACGCC, 6897T>A, 6905T>G, 6907T>A, 6908G>T, 6911T>C, 6912C>A, 6921T>G, 6923C>G, 6924T>A, 6927_6928delGT, 6931G>A, 6933A>C, 6935G>A, 6937T>G, 6938C>A, 6940T>A, 6941T>C, 6943A>G, 6947_6960delAGGTTTACAAAGTA, 6966A>T, 6968C>T, 6975T>A, 6977delG, 6984_6985insG, 6988G>A, 6994_7013delGCTATCCCATGATAGTCCTT, 7018T>A, 7020_7026delAAGGGTT, 7031T>A, 7033_7062delGGTTTGCATCTTTTGCCGTGATGAAAGATG, 7067A>T, 7068G>A, 7071T>A, 7074C>A, 7077T>C, 7083G>T, 7085_7089delAGGCT, 7091A>T, 7094C>A, 7096A>G, 7097C>T, 7099C>G, 7102A>G, 7106A>G, 7107G>A, 7111G>T, 7112T>G, 7114T>A, 7115G>T, 7117_7177delCTGAGTCCTATAATCAGTCCGTTTGAAATTCGATAATTTTCCGTAGCTTGCGTCAAGCTGC, 7182G>T, 7186G>A, 7187G>A, 7188_7225delGGTGTGAGTGAAGATGCGCCGTACCACGTCTTCCCCGG, 7231C>A, 7234G>C, 7235T>C, 7236G>C, 7238T>G, 7239T>G, 7240C>A, 7241A>T, 7243A>T, 7246G>T, 7247G>T, 7248G>T, 7249C>T, 7253C>A, 7254_7262delAGAATAGAG, 7268A>C, 7269A>T, 7271T>A, 7274_7320delTGTGATGGTGTATATCACGATAAAAGTGACACCCGGGTTGTGCTGCG, 7322C>A, 7323T>C, 7326T>G, 7329A>G, 7331A>T, 7333G>C, 7334A>C, 7335G>C, 7336C>T, 7338C>T, 7342T>A, 7343C>G, 7345C>G, 7346A>C, 7347C>T, 7348C>G, 7350T>C, 7353A>T, 7354G>A, 7358A>C, 7360G>A, 7361A>C, 7362A>C, 7364C>T, 7372A>T, 7375T>G, 7376T>G, 7377T>A, 7379A>T, 7380A>T, 7397G>A, 7398C>A, 7399G>A, 7400A>T, 7404C>T, 7407A>T, 7408G>T, 7423A>T, 7425G>A, 7435T>C, 7436G>A, 7437T>C, 7444C>T, 7445A>G, 7457A>G, 7458T>C, 7460G>A, 7503C>T, 7505A>G, 7506T>C, 7511_7512insT, 7513A>C, 7514A>T, 7515A>C, 7525_7526insG, 7526T>A, 7526_7527insG, 7545T>A, 7554A>G, 7569G>A, 7589T>A, 7600T>G, 7601T>A, 7602T>G, 7603_7604insC, 7610C>T, 7612G>A, 7615A>G, 7616A>C, 7617A>T, 7618C>T, 7625_7631delATTGCTT, 7637G>T, 7641T>A, 7642T>A, 7644G>T, 7647T>A, 7650T>A, 7652T>G, 7653A>T, 7654C>T, 7665G>T, 7666T>C, 7667G>T, 7678C>T, 7686T>G, 7687T>A, 7689G>T, 7698G>A, 7713G>A, 7717A>C, 7719T>A, 7739T>A, 7742_7743insC, 7749T>C, 7751T>C, 7752G>A, 7754_7755insG, 7756_7757insT, 7762C>T, 7763A>C, 7764A>T, 7767C>T, 7773G>T, 7775G>A, 7778T>A, 7781G>T								

CDS

ToTV_sRNA1gp1	1	2159	100%	14846	98.5%	2159 (100%)	2108 (97.6%)	0/0/0/0	1
Protein mutations:	P8S (128C>T 130C>T), F10S (135T>C), A18S (158G>T), T19A (161A>G), A31T (197G>A 199T>A), V291I (977G>A), T364A (1196A>G), I459V (1481A>G), A501S (1607G>T), E526D (1684G>T), K544R (1737A>G 1738A>G), I604V (1916A>G), S619N (1962G>A), F659Y (2082T>A), K692R (2181A>G 2182G>A), N712S (2241A>G), S715N (2250G>A 2251C>T), R768K (2409G>A), A1129V (3492C>T), A1162V (3591C>T), S1538R (4720T>A), V1598I (4898G>A), T1614A (4946A>G), I1658T (5079T>C), V1672I (5120G>A), T1681N (5148C>A), R1713K (5244G>A), V1750I (5354G>A 5356G>T), V1755I (5369G>A 5371G>T), T1796P (5492A>C), K1811Q (5537A>C), A1829S (5591G>T), T1833P (5603A>C), Q1857H (5677G>T), R1878K (5739G>A), I1893T (5784T>C), L1915Q (5849T>C 5850T>A 5851G>A), N1950S (5955A>G), T1976I (6033C>T), A1977S (6035G>T), L1996F (6092C>T), I2002M (6112A>G), M2032I (6202G>C), E2039D (6223A>T), I2042V (6230A>G 6232A>G), L2049F (6253A>C), S2056R (6274C>G), V2064L (6296G>C 6298A>G), Q2074R (6327A>G), I2132F (6500A>T), L2147F (6545C>T)								

	Begin	End	Coverage	Score	Concordance	Matches	Identities	I/D/M/F*	Stop Codons
**NT**	**1**	**7781**	**99.7%**	**11522**	**79.6%**	**7469 (95.9%)**	**6754 (86.7%)**	**10/312**	
Codon mutations:	TTT3TTC (115T>C), TTC7TTT (127C>T), CCC8TCT (128C>T 130C>T), TTC10TCC (135T>C), ACT14ACC (148T>C), GAA15GAG (151A>G), GCT18TCT (158G>T), ACC19GCC (161A>G), GGT23GGC (175T>C), TCT24TCC (178T>C), TTT26TTC (184T>C), GAG28GAA (190G>A), GCT31ACA (197G>A 199T>A), AGT32AGC (202T>C), ATT33ATA (205T>A), AGT34AGC (208T>C), CGC38CGT (220C>T), CTG40TTG (224C>T), CAA49CAG (253A>G), TCT51TCC (259T>C), TCT52TCA (262T>A), CAC53CAT (265C>T), TTG78CTA (338T>C 340G>A), TTA85TTG (361A>G), GAA87GAG (367A>G), CCC88CCT (370C>T), ATC90ATT (376C>T), TAC93TAT (385C>T), TCT94TCC (388T>C), AAA101AAG (409A>G), AAA115AAG (451A>G), GAC127GAT (487C>T), AAA128AAG (490A>G), GTG132GTA (502G>A), TCT145TCC (541T>C), TTC151TTT (559C>T), GTA157GTG (577A>G), CTA158TTA (578C>T), GAC183GAT (655C>T), CAA190CAG (676A>G), GCA196GCG (694A>G), TCA207TCG (727A>G), CTC218CTT (760C>T), ACC225ACT (781C>T), TTC228TTT (790C>T), GTA231GTG (799A>G), GGT233GGC (805T>C), CTA238TTG (818C>T 820A>G), CTA242CTT (832A>T), TCA243TCC (835A>C), ATT245ATC (841T>C), CTG246TTG (842C>T), TAC247TAT (847C>T), TTG259TTA (883G>A), TTT260TTC (886T>C), CTG262CTA (892G>A), AAT265AAC (901T>C), TGT269TGC (913T>C), GCC273GCT (925C>T), CTG278TTG (938C>T), GTA282GTG (952A>G), GAC283GAT (955C>T), GGT286GGG (964T>G), AAG288AAA (970G>A), AAT289AAC (973T>C), GTT291ATT (977G>A), TTG292CTG (980T>C), GCC297GCT (997C>T), TCT300TCC (1006T>C), GAC302GAT (1012C>T), TTA303CTA (1013T>C), TTC304TTT (1018C>T), GAG309GAA (1033G>A), CAT318CAC (1060T>C), CGC319CGT (1063C>T), GCA324GCT (1078A>T), AAC325AAT (1081C>T), TTC328TTT (1090C>T), ACT329ACC (1093T>C), CAA333CAG (1105A>G), CAT334CAC (1108T>C), CTA335TTA (1109C>T), GCC353GCT (1165C>T), AGG358AGA (1180G>A), ACC364GCC (1196A>G), GAT367GAC (1207T>C), CAG375CAA (1231G>A), GGC378GGT (1240C>T), AGG383AGA (1255G>A), TTT387TTC (1267T>C), GGG398GGA (1300G>A), AAA399AAG (1303A>G), CTG405TTG (1319C>T), TTC406TTT (1324C>T), GAA415GAG (1351A>G), GAG416GAA (1354G>A), TAT435TAC (1411T>C), CAT437CAC (1417T>C), ATC441ATT (1429C>T), GAC445GAT (1441C>T), GCT448GCC (1450T>C), CTG449TTG (1451C>T), GAC458GAT (1480C>T), ATT459GTT (1481A>G), GAT461GAC (1489T>C), CCC472CCT (1522C>T), GGC482GGT (1552C>T), CGC483CGT (1555C>T), CGA488CGG (1570A>G), TAT489TAC (1573T>C), GCC492GCT (1582C>T), ACA493ACC (1585A>C), AAC495AAT (1591C>T), CTT496CTC (1594T>C), GCT501TCT (1607G>T), CAG502CAA (1612G>A), GGT504GGG (1618T>G), CTA505TTA (1619C>T), ACC506ACT (1624C>T), CCC508CCT (1630C>T), TTT511TTC (1639T>C), GAG512GAA (1642G>A), AGA513AGG (1645A>G), TGC521TGT (1669C>T), AGG522AGA (1672G>A), GGT525GGA (1681T>A), GAG526GAT (1684G>T), GCT531GCC (1699T>C), ACT533ACC (1705T>C), AAA544AGG (1737A>G 1738A>G), GCA548GCT (1750A>T), ACA550ACG (1756A>G), GGT553GGG (1765T>G), TTG559CTA (1781T>C 1783G>A), CTG560TTG (1784C>T), GAA561GAG (1789A>G), AAG568AAA (1810G>A), ATC573ATT (1825C>T), AAA576AAG (1834A>G), CAG587CAA (1867G>A), TCA591TCG (1879A>G), GAA594GAG (1888A>G), TTG602CTA (1910T>C 1912G>A), ATC604GTC (1916A>G), GTA618GTG (1960A>G), AGT619AAT (1962G>A), GAA620GAG (1966A>G), TAC626TAT (1984C>T), CAG628CAA (1990G>A), GAG629GAA (1993G>A), TTG635CTG (2009T>C), TAT639TAC (2023T>C), CCT650CCC (2056T>C), GAC652GAT (2062C>T), GCC657GCA (2077C>A), TTT659TAT (2082T>A), CTA662CTG (2092A>G), AAA667AAG (2107A>G), GTT674GTC (2128T>C), CTG678TTG (2138C>T), TAT679TAC (2143T>C), GGT681GGC (2149T>C), TGT683TGC (2155T>C), CTG690TTG (2174C>T), ATA691ATC (2179A>C), AAG692AGA (2181A>G 2182G>A), TAT698TAC (2200T>C), ATC706ATT (2224C>T), ACA710ACG (2236A>G), AAT712AGT (2241A>G), AGC715AAT (2250G>A 2251C>T), GAC717GAT (2257C>T), CAG719CAA (2263G>A), CCT723CCC (2275T>C), GCG727GCA (2287G>A), CAC728CAT (2290C>T), GAC736GAT (2314C>T), ATA739ATT (2323A>T), CAC747CAT (2347C>T), TTC750TTT (2356C>T), TTG751TTA (2359G>A), AGG757AGA (2377G>A), AGA762AGG (2392A>G), AGG768AAG (2409G>A), ATT779ATC (2443T>C), GTG780GTA (2446G>A), CCG791CCA (2479G>A), ATC793ATT (2485C>T), CTG797CTA (2497G>A), ATT798ATC (2500T>C), AAG799AAA (2503G>A), GCT800GCC (2506T>C), GGC801GGT (2509C>T), CTT802CTC (2512T>C), GGG812GGA (2542G>A), GTC814GTT (2548C>T), TTC817TTT (2557C>T), GAG838GAA (2620G>A), ATC852ATT (2662C>T), TTT853TTC (2665T>C), ATC876ATT (2734C>T), GGA879GGG (2743A>G), AAC883AAT (2755C>T), CTA889CTG (2773A>G), TGC901TGT (2809C>T), TCG902TCA (2812G>A), ACA907ACG (2827A>G), GGC918GGT (2860C>T), CAT925CAC (2881T>C), GCC936GCT (2914C>T), CGG940CGA (2926G>A), TTC941TTT (2929C>T), GTC948GTT (2950C>T), CTG949TTG (2951C>T), CCC958CCT (2980C>T), AAG959AAA (2983G>A), AAA964AAG (2998A>G), CCT967CCC (3007T>C), TTT974TTC (3028T>C), TCT975TCC (3031T>C), AAG976AAA (3034G>A), ACT978ACC (3040T>C), AGA984AGG (3058A>G), GTA986GTG (3064A>G), AAG989AAA (3073G>A), GCT991GCC (3079T>C), TTT1001TTC (3109T>C), GTG1003GTA (3115G>A), GTA1013GTG (3145A>G), TAC1015TAT (3151C>T), TCT1019TCC (3163T>C), TCC1021TCT (3169C>T), TTT1022TTC (3172T>C), TAT1032TAC (3202T>C), CTT1045CTC (3241T>C), AAC1049AAT (3253C>T), GTG1050GTA (3256G>A), GAC1052GAT (3262C>T), GGT1059GGC (3283T>C), CAT1061CAC (3289T>C), TCT1064TCC (3298T>C), GAA1067GAG (3307A>G), CGA1084CGT (3358A>T), GTA1100GTC (3406A>C), CTC1104CTA (3418C>A), CTT1105CTC (3421T>C), AAA1119AAG (3463A>G), CAG1120CAA (3466G>A), GGC1125GGT (3481C>T), ACA1126ACT (3484A>T), TTG1127TTA (3487G>A), GCT1129GTT (3492C>T), ACG1134ACA (3508G>A), GGG1151GGA (3559G>A), GCT1156GCC (3574T>C), CCA1157CCG (3577A>G), ATT1160ATC (3586T>C), GCT1162GTT (3591C>T), TAC1173TAT (3625C>T), GTG1174GTA (3628G>A), TAC1180TAT (3646C>T), CAA1185CAG (3661A>G), AAT1186AAC (3664T>C), GAG1189GAA (3673G>A), GAC1190GAT (3676C>T), CTA1194TTA (3686C>T), CGT1200CGC (3706T>C), TTG1204CTG (3716T>C), CCT1205CCA (3721T>A), CGA1211CGT (3739A>T), AAG1216AAA (3754G>A), CTC1226CTT (3784C>T), GAC1236GAT (3814C>T), GGC1254GGT (3868C>T), ACT1255ACC (3871T>C), GAG1259GAA (3883G>A), GAG1262GAA (3892G>A), AGT1264AGC (3898T>C), GGA1267GGT (3907A>T), GTC1270GTT (3916C>T), AAA1273AAG (3925A>G), AGG1274AGA (3928G>A), GAC1281GAT (3949C>T), GAG1283GAA (3955G>A), TTG1285TTA (3961G>A), GAA1288GAG (3970A>G), ATT1289ATC (3973T>C), AAA1304AAG (4018A>G), TTG1310CTA (4034T>C 4036G>A), CGG1317CGA (4057G>A), CGT1320CGC (4066T>C), CCC1326CCT (4084C>T), CTG1338TTG (4118C>T), TCC1341TCT (4129C>T), CTG1344CTA (4138G>A), TCC1347TCA (4147C>A), CTG1351CTA (4159G>A), CCA1352CCT (4162A>T), AAA1354AAG (4168A>G), GGA1360GGT (4186A>T), GAA1363GAG (4195A>G), TTG1367TTA (4207G>A), GCT1368GCC (4210T>C), TTC1371TTT (4219C>T), GTC1374GTT (4228C>T), AGA1389AGG (4273A>G), ATC1392ATA (4282C>A), GCC1394GCT (4288C>T), TTT1395TTC (4291T>C), TTT1398TTC (4300T>C), GAC1404GAT (4318C>T), TAC1405TAT (4321C>T), TAT1406TAC (4324T>C), GGT1411GGC (4339T>C), GAC1414GAT (4348C>T), AAT1416AAC (4354T>C), AGA1418AGG (4360A>G), TAT1429TAC (4393T>C), TTT1437TTC (4417T>C), TTG1439CTG (4421T>C), GTT1451GTC (4459T>C), ATC1452ATT (4462C>T), TTC1453TTT (4465C>T), AAC1454AAT (4468C>T), TCT1455TCC (4471T>C), CTT1456CTC (4474T>C), TTT1460TTC (4486T>C), TAT1461TAC (4489T>C), TAT1495TAC (4591T>C), TTG1507CTG (4625T>C), TAT1510TAC (4636T>C), CTG1512TTG (4640C>T), ATT1515ATA (4651T>A), GCC1516GCA (4654C>A), CAA1517CAG (4657A>G), TTG1519CTA (4661T>C 4663G>A), GAG1520GAA (4666G>A), GTG1522GTA (4672G>A), GTT1524GTC (4678T>C), ATT1525ATA (4681T>A), ATA1526ATC (4684A>C), AAG1527AAA (4687G>A), ATC1530ATA (4696C>A), GAC1531GAT (4699C>T), AGT1538AGA (4720T>A), AGC1551AGT (4759C>T), AGG1556AGA (4774G>A), CTA1559CTG (4783A>G), GGA1560GGG (4786A>G), TAC1561TAT (4789C>T), GTA1569GTG (4813A>G), TCT1570TCC (4816T>C), GTT1598ATT (4898G>A), GAG1600GAA (4906G>A), CCT1606CCC (4924T>C), ACA1614GCA (4946A>G), AAC1618AAT (4960C>T), AAG1621AAA (4969G>A), CGG1623CGA (4975G>A), ACA1629ACC (4993A>C), CCT1631CCC (4999T>C), TAT1633TAC (5005T>C), TTG1636CTG (5012T>C), GAG1637GAA (5017G>A), GAC1639GAT (5023C>T), GGA1646GGC (5044A>C), GGG1651GGA (5059G>A), ATC1653ATT (5065C>T), CTT1656CTC (5074T>C), CAT1657CAC (5077T>C), ATT1658ACT (5079T>C), TTG1666CTG (5102T>C), GGC1669GGT (5113C>T), ACT1671ACC (5119T>C), GTT1672ATT (5120G>A), CAT1680CAC (5146T>C), ACT1681AAT (5148C>A), AGT1687AGC (5167T>C), TTC1688TTT (5170C>T), TCC1690TCT (5176C>T), CAG1698CAA (5200G>A), GTT1704GTG (5218T>G), AGA1713AAA (5244G>A), CCC1720CCT (5266C>T), GAC1725GAT (5281C>T), GGG1728GGA (5290G>A), CAC1741CAT (5329C>T), GTA1746GTT (5344A>T), ACC1747ACA (5347C>A), GTG1750ATT (5354G>A 5356G>T), CCA1753CCC (5365A>C), GTG1755ATT (5369G>A 5371G>T), GGC1760GGG (5386C>G), GGG1762GGA (5392G>A), CCA1767CCC (5407A>C), TGC1769TGT (5413C>T), GCA1771GCT (5419A>T), CTG1775TTG (5429C>T), ACT1777ACC (5437T>C), GGC1778GGG (5440C>G), TAT1780TAC (5446T>C), GTT1785GTC (5461T>C), GTG1787GTT (5467G>T), TTG1789CTG (5471T>C), GTT1795GTC (5491T>C), ACA1796CCA (5492A>C), GAT1797GAC (5497T>C), CTT1809CTG (5533T>G), AAA1811CAA (5537A>C), ACA1823ACG (5575A>G), TGT1824TGC (5578T>C), AAA1825AAG (5581A>G), GCC1829TCC (5591G>T), GGT1831GGC (5599T>C), ACA1833CCA (5603A>C), GCA1835GCT (5611A>T), ATA1837ATT (5617A>T), GGT1845GGC (5641T>C), TTT1847TTC (5647T>C), CTA1856CTG (5674A>G), CAG1857CAT (5677G>T), CAG1860CAA (5686G>A), CCT1868CCA (5710T>A), GGG1870GGT (5716G>T), GGA1871GGC (5719A>C), CAC1875CAT (5731C>T), ACT1876ACC (5734T>C), ACT1877ACC (5737T>C), AGA1878AAA (5739G>A), CTT1880CTC (5746T>C), TTG1882CTA (5750T>C 5752G>A), CTC1884CTT (5758C>T), AAG1889AAA (5773G>A), CAA1892CAG (5782A>G), ATT1893ACT (5784T>C), TTA1894CTA (5786T>C), AGA1897AGG (5797A>G), TTC1900TTT (5806C>T), TCA1910TCT (5836A>T), TTG1915CAA (5849T>C 5850T>A 5851G>A), AGG1921AGA (5869G>A), CAA1923CAG (5875A>G), GGG1924GGC (5878G>C), TTT1926TTC (5884T>C), CCC1927CCA (5887C>A), CGC1928CGT (5890C>T), GTG1932GTT (5902G>T), TAC1934TAT (5908C>T), TTG1935CTG (5909T>C), CTT1936CTC (5914T>C), TTT1939TTC (5923T>C), CTG1941TTG (5927C>T), CAC1946CAT (5944C>T), AAC1950AGC (5955A>G), ACG1953ACA (5965G>A), AAA1964AAG (5998A>G), ACC1966ACT (6004C>T), AAA1970AAG (6016A>G), GAA1971GAG (6019A>G), TCC1974TCT (6028C>T), GCA1975GCT (6031A>T), ACA1976ATA (6033C>T), GCT1977TCT (6035G>T), GGT1983GGA (6055T>A), TCC1987TCA (6067C>A), GCT1988GCC (6070T>C), CTA1995CTG (6091A>G), CTT1996TTT (6092C>T), CTT1998CTG (6100T>G), CAG1999CAA (6103G>A), AAG2000AAA (6106G>A), ATA2002ATG (6112A>G), TGT2004TGC (6118T>C), TCA2005TCT (6121A>T), TTT2011TTC (6139T>C), GTC2016GTG (6154C>G), GGC2019GGG (6163C>G), CAC2020CAT (6166C>T), GTA2026GTG (6184A>G), ACT2027ACG (6187T>G), TTC2029TTT (6193C>T), CAA2030CAG (6196A>G), ATG2032ATC (6202G>C), CAA2035CAG (6211A>G), CTG2037CTT (6217G>T), GAA2039GAT (6223A>T), ATA2042GTG (6230A>G 6232A>G), GAG2043GAA (6235G>A), TTA2049TTC (6253A>C), ACC2050ACA (6256C>A), ACA2052ACT (6262A>T), CTG2053CTA (6265G>A), TCG2054TCT (6268G>T), AGC2056AGG (6274C>G), GTA2064CTG (6296G>C 6298A>G), TTG2068CTG (6308T>C), GAT2069GAC (6313T>C), GAG2072GAA (6322G>A), CAG2074CGG (6327A>G), AAT2084AAC (6358T>C), GCA2126GCC (6484A>C), AGA2128AGG (6490A>G), CAT2129CAC (6493T>C), ATT2132TTT (6500A>T), TCC2137TCT (6517C>T), CAC2142CAT (6532C>T), CTT2147TTT (6545C>T), TAC2155TAT (6571C>T)								

Proteins

	Begin	End	Coverage	Score	Concordance	Matches	Identities	I/D/M/F*	Stop Codons
polyprotein (YP_001039627.1)	1	2159	100%	14846	98.5%	2159 (100%)	2108 (97.6%)	0/0/0/0	1
Protein mutations:	P8S (128C>T 130C>T), F10S (135T>C), A18S (158G>T), T19A (161A>G), A31T (197G>A 199T>A), V291I (977G>A), T364A (1196A>G), I459V (1481A>G), A501S (1607G>T), E526D (1684G>T), K544R (1737A>G 1738A>G), I604V (1916A>G), S619N (1962G>A), F659Y (2082T>A), K692R (2181A>G 2182G>A), N712S (2241A>G), S715N (2250G>A 2251C>T), R768K (2409G>A), A1129V (3492C>T), A1162V (3591C>T), S1538R (4720T>A), V1598I (4898G>A), T1614A (4946A>G), I1658T (5079T>C), V1672I (5120G>A), T1681N (5148C>A), R1713K (5244G>A), V1750I (5354G>A 5356G>T), V1755I (5369G>A 5371G>T), T1796P (5492A>C), K1811Q (5537A>C), A1829S (5591G>T), T1833P (5603A>C), Q1857H (5677G>T), R1878K (5739G>A), I1893T (5784T>C), L1915Q (5849T>C 5850T>A 5851G>A), N1950S (5955A>G), T1976I (6033C>T), A1977S (6035G>T), L1996F (6092C>T), I2002M (6112A>G), M2032I (6202G>C), E2039D (6223A>T), I2042V (6230A>G 6232A>G), L2049F (6253A>C), S2056R (6274C>G), V2064L (6296G>C 6298A>G), Q2074R (6327A>G), I2132F (6500A>T), L2147F (6545C>T)								

	Begin	End	Coverage	Score	Concordance	Matches	Identities	I/D/M/F*	Stop Codons
NT	1	7781	99.7%	11522	79.6%	7469 (95.9%)	6754 (86.7%)	10/312	

Codon mutations:

TTT3TTC (115T>C), TTC7TTT (127C>T), CCC8TCT (128C>T 130C>T), TTC10TCC (135T>C), ACT14ACC (148T>C), GAA15GAG (151A>G), GCT18TCT (158G>T), ACC19GCC (161A>G), GGT23GGC (175T>C), TCT24TCC (178T>C), TTT26TTC (184T>C), GAG28GAA (190G>A), GCT31ACA (197G>A 199T>A), AGT32AGC (202T>C), ATT33ATA (205T>A), AGT34AGC (208T>C), CGC38CGT (220C>T), CTG40TTG (224C>T), CAA49CAG (253A>G), TCT51TCC (259T>C), TCT52TCA (262T>A), CAC53CAT (265C>T), TTG78CTA (338T>C 340G>A), TTA85TTG (361A>G), GAA87GAG (367A>G), CCC88CCT (370C>T), ATC90ATT (376C>T), TAC93TAT (385C>T), TCT94TCC (388T>C), AAA101AAG (409A>G), AAA115AAG (451A>G), GAC127GAT (487C>T), AAA128AAG (490A>G), GTG132GTA (502G>A), TCT145TCC (541T>C), TTC151TTT (559C>T), GTA157GTG (577A>G), CTA158TTA (578C>T), GAC183GAT (655C>T), CAA190CAG (676A>G), GCA196GCG (694A>G), TCA207TCG (727A>G), CTC218CTT (760C>T), ACC225ACT (781C>T), TTC228TTT (790C>T), GTA231GTG (799A>G), GGT233GGC (805T>C), CTA238TTG (818C>T 820A>G), CTA242CTT (832A>T), TCA243TCC (835A>C), ATT245ATC (841T>C), CTG246TTG (842C>T), TAC247TAT (847C>T), TTG259TTA (883G>A), TTT260TTC (886T>C), CTG262CTA (892G>A), AAT265AAC (901T>C), TGT269TGC (913T>C), GCC273GCT (925C>T), CTG278TTG (938C>T), GTA282GTG (952A>G), GAC283GAT (955C>T), GGT286GGG (964T>G), AAG288AAA (970G>A), AAT289AAC (973T>C), GTT291ATT (977G>A), TTG292CTG (980T>C), GCC297GCT (997C>T), TCT300TCC (1006T>C), GAC302GAT (1012C>T), TTA303CTA (1013T>C), TTC304TTT (1018C>T), GAG309GAA (1033G>A), CAT318CAC (1060T>C), CGC319CGT (1063C>T), GCA324GCT (1078A>T), AAC325AAT (1081C>T), TTC328TTT (1090C>T), ACT329ACC (1093T>C), CAA333CAG (1105A>G), CAT334CAC (1108T>C), CTA335TTA (1109C>T), GCC353GCT (1165C>T), AGG358AGA (1180G>A), ACC364GCC (1196A>G), GAT367GAC (1207T>C), CAG375CAA (1231G>A), GGC378GGT (1240C>T), AGG383AGA (1255G>A), TTT387TTC (1267T>C), GGG398GGA (1300G>A), AAA399AAG (1303A>G), CTG405TTG (1319C>T), TTC406TTT (1324C>T), GAA415GAG (1351A>G), GAG416GAA (1354G>A), TAT435TAC (1411T>C), CAT437CAC (1417T>C), ATC441ATT (1429C>T), GAC445GAT (1441C>T), GCT448GCC (1450T>C), CTG449TTG (1451C>T), GAC458GAT (1480C>T), ATT459GTT (1481A>G), GAT461GAC (1489T>C), CCC472CCT (1522C>T), GGC482GGT (1552C>T), CGC483CGT (1555C>T), CGA488CGG (1570A>G), TAT489TAC (1573T>C), GCC492GCT (1582C>T), ACA493ACC (1585A>C), AAC495AAT (1591C>T), CTT496CTC (1594T>C), GCT501TCT (1607G>T), CAG502CAA (1612G>A), GGT504GGG (1618T>G), CTA505TTA (1619C>T), ACC506ACT (1624C>T), CCC508CCT (1630C>T), TTT511TTC (1639T>C), GAG512GAA (1642G>A), AGA513AGG (1645A>G), TGC521TGT (1669C>T), AGG522AGA (1672G>A), GGT525GGA (1681T>A), GAG526GAT (1684G>T), GCT531GCC (1699T>C), ACT533ACC (1705T>C), AAA544AGG (1737A>G 1738A>G), GCA548GCT (1750A>T), ACA550ACG (1756A>G), GGT553GGG (1765T>G), TTG559CTA (1781T>C 1783G>A), CTG560TTG (1784C>T), GAA561GAG (1789A>G), AAG568AAA (1810G>A), ATC573ATT (1825C>T), AAA576AAG (1834A>G), CAG587CAA (1867G>A), TCA591TCG (1879A>G), GAA594GAG (1888A>G), TTG602CTA (1910T>C 1912G>A), ATC604GTC (1916A>G), GTA618GTG (1960A>G), AGT619AAT (1962G>A), GAA620GAG (1966A>G), TAC626TAT (1984C>T), CAG628CAA (1990G>A), GAG629GAA (1993G>A), TTG635CTG (2009T>C), TAT639TAC (2023T>C), CCT650CCC (2056T>C), GAC652GAT (2062C>T), GCC657GCA (2077C>A), TTT659TAT (2082T>A), CTA662CTG (2092A>G), AAA667AAG (2107A>G), GTT674GTC (2128T>C), CTG678TTG (2138C>T), TAT679TAC (2143T>C), GGT681GGC (2149T>C), TGT683TGC (2155T>C), CTG690TTG (2174C>T), ATA691ATC (2179A>C), AAG692AGA (2181A>G 2182G>A), TAT698TAC (2200T>C), ATC706ATT (2224C>T), ACA710ACG (2236A>G), AAT712AGT (2241A>G), AGC715AAT (2250G>A 2251C>T), GAC717GAT (2257C>T), CAG719CAA (2263G>A), CCT723CCC (2275T>C), GCG727GCA (2287G>A), CAC728CAT (2290C>T), GAC736GAT (2314C>T), ATA739ATT (2323A>T), CAC747CAT (2347C>T), TTC750TTT (2356C>T), TTG751TTA (2359G>A), AGG757AGA (2377G>A), AGA762AGG (2392A>G), AGG768AAG (2409G>A), ATT779ATC (2443T>C), GTG780GTA (2446G>A), CCG791CCA (2479G>A), ATC793ATT (2485C>T), CTG797CTA (2497G>A), ATT798ATC (2500T>C), AAG799AAA (2503G>A), GCT800GCC (2506T>C), GGC801GGT (2509C>T), CTT802CTC (2512T>C), GGG812GGA (2542G>A), GTC814GTT (2548C>T), TTC817TTT (2557C>T), GAG838GAA (2620G>A), ATC852ATT (2662C>T), TTT853TTC (2665T>C), ATC876ATT (2734C>T), GGA879GGG (2743A>G), AAC883AAT (2755C>T), CTA889CTG (2773A>G), TGC901TGT (2809C>T), TCG902TCA (2812G>A), ACA907ACG (2827A>G), GGC918GGT (2860C>T), CAT925CAC (2881T>C), GCC936GCT (2914C>T), CGG940CGA (2926G>A), TTC941TTT (2929C>T), GTC948GTT (2950C>T), CTG949TTG (2951C>T), CCC958CCT (2980C>T), AAG959AAA (2983G>A), AAA964AAG (2998A>G), CCT967CCC (3007T>C), TTT974TTC (3028T>C), TCT975TCC (3031T>C), AAG976AAA (3034G>A), ACT978ACC (3040T>C), AGA984AGG (3058A>G), GTA986GTG (3064A>G), AAG989AAA (3073G>A), GCT991GCC (3079T>C), TTT1001TTC (3109T>C), GTG1003GTA (3115G>A), GTA1013GTG (3145A>G), TAC1015TAT (3151C>T), TCT1019TCC (3163T>C), TCC1021TCT (3169C>T), TTT1022TTC (3172T>C), TAT1032TAC (3202T>C), CTT1045CTC (3241T>C), AAC1049AAT (3253C>T), GTG1050GTA (3256G>A), GAC1052GAT (3262C>T), GGT1059GGC (3283T>C), CAT1061CAC (3289T>C), TCT1064TCC (3298T>C), GAA1067GAG (3307A>G), CGA1084CGT (3358A>T), GTA1100GTC (3406A>C), CTC1104CTA (3418C>A), CTT1105CTC (3421T>C), AAA1119AAG (3463A>G), CAG1120CAA (3466G>A), GGC1125GGT (3481C>T), ACA1126ACT (3484A>T), TTG1127TTA (3487G>A), GCT1129GTT (3492C>T), ACG1134ACA (3508G>A), GGG1151GGA (3559G>A), GCT1156GCC (3574T>C), CCA1157CCG (3577A>G), ATT1160ATC (3586T>C), GCT1162GTT (3591C>T), TAC1173TAT (3625C>T), GTG1174GTA (3628G>A), TAC1180TAT (3646C>T), CAA1185CAG (3661A>G), AAT1186AAC (3664T>C), GAG1189GAA (3673G>A), GAC1190GAT (3676C>T), CTA1194TTA (3686C>T), CGT1200CGC (3706T>C), TTG1204CTG (3716T>C), CCT1205CCA (3721T>A), CGA1211CGT (3739A>T), AAG1216AAA (3754G>A), CTC1226CTT (3784C>T), GAC1236GAT (3814C>T), GGC1254GGT (3868C>T), ACT1255ACC (3871T>C), GAG1259GAA (3883G>A), GAG1262GAA (3892G>A), AGT1264AGC (3898T>C), GGA1267GGT (3907A>T), GTC1270GTT (3916C>T), AAA1273AAG (3925A>G), AGG1274AGA (3928G>A), GAC1281GAT (3949C>T), GAG1283GAA (3955G>A), TTG1285TTA (3961G>A), GAA1288GAG (3970A>G), ATT1289ATC (3973T>C), AAA1304AAG (4018A>G), TTG1310CTA (4034T>C 4036G>A), CGG1317CGA (4057G>A), CGT1320CGC (4066T>C), CCC1326CCT (4084C>T), CTG1338TTG (4118C>T), TCC1341TCT (4129C>T), CTG1344CTA (4138G>A), TCC1347TCA (4147C>A), CTG1351CTA (4159G>A), CCA1352CCT (4162A>T), AAA1354AAG (4168A>G), GGA1360GGT (4186A>T), GAA1363GAG (4195A>G), TTG1367TTA (4207G>A), GCT1368GCC (4210T>C), TTC1371TTT (4219C>T), GTC1374GTT (4228C>T), AGA1389AGG (4273A>G), ATC1392ATA (4282C>A), GCC1394GCT (4288C>T), TTT1395TTC (4291T>C), TTT1398TTC (4300T>C), GAC1404GAT (4318C>T), TAC1405TAT (4321C>T), TAT1406TAC (4324T>C), GGT1411GGC (4339T>C), GAC1414GAT (4348C>T), AAT1416AAC (4354T>C), AGA1418AGG (4360A>G), TAT1429TAC (4393T>C), TTT1437TTC (4417T>C), TTG1439CTG (4421T>C), GTT1451GTC (4459T>C), ATC1452ATT (4462C>T), TTC1453TTT (4465C>T), AAC1454AAT (4468C>T), TCT1455TCC (4471T>C), CTT1456CTC (4474T>C), TTT1460TTC (4486T>C), TAT1461TAC (4489T>C), TAT1495TAC (4591T>C), TTG1507CTG (4625T>C), TAT1510TAC (4636T>C), CTG1512TTG (4640C>T), ATT1515ATA (4651T>A), GCC1516GCA (4654C>A), CAA1517CAG (4657A>G), TTG1519CTA (4661T>C 4663G>A), GAG1520GAA (4666G>A), GTG1522GTA (4672G>A), GTT1524GTC (4678T>C), ATT1525ATA (4681T>A), ATA1526ATC (4684A>C), AAG1527AAA (4687G>A), ATC1530ATA (4696C>A), GAC1531GAT (4699C>T), AGT1538AGA (4720T>A), AGC1551AGT (4759C>T), AGG1556AGA (4774G>A), CTA1559CTG (4783A>G), GGA1560GGG (4786A>G), TAC1561TAT (4789C>T), GTA1569GTG (4813A>G), TCT1570TCC (4816T>C), GTT1598ATT (4898G>A), GAG1600GAA (4906G>A), CCT1606CCC (4924T>C), ACA1614GCA (4946A>G), AAC1618AAT (4960C>T), AAG1621AAA (4969G>A), CGG1623CGA (4975G>A), ACA1629ACC (4993A>C), CCT1631CCC (4999T>C), TAT1633TAC (5005T>C), TTG1636CTG (5012T>C), GAG1637GAA (5017G>A), GAC1639GAT (5023C>T), GGA1646GGC (5044A>C), GGG1651GGA (5059G>A), ATC1653ATT (5065C>T), CTT1656CTC (5074T>C), CAT1657CAC (5077T>C), ATT1658ACT (5079T>C), TTG1666CTG (5102T>C), GGC1669GGT (5113C>T), ACT1671ACC (5119T>C), GTT1672ATT (5120G>A), CAT1680CAC (5146T>C), ACT1681AAT (5148C>A), AGT1687AGC (5167T>C), TTC1688TTT (5170C>T), TCC1690TCT (5176C>T), CAG1698CAA (5200G>A), GTT1704GTG (5218T>G), AGA1713AAA (5244G>A), CCC1720CCT (5266C>T), GAC1725GAT (5281C>T), GGG1728GGA (5290G>A), CAC1741CAT (5329C>T), GTA1746GTT (5344A>T), ACC1747ACA (5347C>A), GTG1750ATT (5354G>A 5356G>T), CCA1753CCC (5365A>C), GTG1755ATT (5369G>A 5371G>T), GGC1760GGG (5386C>G), GGG1762GGA (5392G>A), CCA1767CCC (5407A>C), TGC1769TGT (5413C>T), GCA1771GCT (5419A>T), CTG1775TTG (5429C>T), ACT1777ACC (5437T>C), GGC1778GGG (5440C>G), TAT1780TAC (5446T>C), GTT1785GTC (5461T>C), GTG1787GTT (5467G>T), TTG1789CTG (5471T>C), GTT1795GTC (5491T>C), ACA1796CCA (5492A>C), GAT1797GAC (5497T>C), CTT1809CTG (5533T>G), AAA1811CAA (5537A>C), ACA1823ACG (5575A>G), TGT1824TGC (5578T>C), AAA1825AAG (5581A>G), GCC1829TCC (5591G>T), GGT1831GGC (5599T>C), ACA1833CCA (5603A>C), GCA1835GCT (5611A>T), ATA1837ATT (5617A>T), GGT1845GGC (5641T>C), TTT1847TTC (5647T>C), CTA1856CTG (5674A>G), CAG1857CAT (5677G>T), CAG1860CAA (5686G>A), CCT1868CCA (5710T>A), GGG1870GGT (5716G>T), GGA1871GGC (5719A>C), CAC1875CAT (5731C>T), ACT1876ACC (5734T>C), ACT1877ACC (5737T>C), AGA1878AAA (5739G>A), CTT1880CTC (5746T>C), TTG1882CTA (5750T>C 5752G>A), CTC1884CTT (5758C>T), AAG1889AAA (5773G>A), CAA1892CAG (5782A>G), ATT1893ACT (5784T>C), TTA1894CTA (5786T>C), AGA1897AGG (5797A>G), TTC1900TTT (5806C>T), TCA1910TCT (5836A>T), TTG1915CAA (5849T>C 5850T>A 5851G>A), AGG1921AGA (5869G>A), CAA1923CAG (5875A>G), GGG1924GGC (5878G>C), TTT1926TTC (5884T>C), CCC1927CCA (5887C>A), CGC1928CGT (5890C>T), GTG1932GTT (5902G>T), TAC1934TAT (5908C>T), TTG1935CTG (5909T>C), CTT1936CTC (5914T>C), TTT1939TTC (5923T>C), CTG1941TTG (5927C>T), CAC1946CAT (5944C>T), AAC1950AGC (5955A>G), ACG1953ACA (5965G>A), AAA1964AAG (5998A>G), ACC1966ACT (6004C>T), AAA1970AAG (6016A>G), GAA1971GAG (6019A>G), TCC1974TCT (6028C>T), GCA1975GCT (6031A>T), ACA1976ATA (6033C>T), GCT1977TCT (6035G>T), GGT1983GGA (6055T>A), TCC1987TCA (6067C>A), GCT1988GCC (6070T>C), CTA1995CTG (6091A>G), CTT1996TTT (6092C>T), CTT1998CTG (6100T>G), CAG1999CAA (6103G>A), AAG2000AAA (6106G>A), ATA2002ATG (6112A>G), TGT2004TGC (6118T>C), TCA2005TCT (6121A>T), TTT2011TTC (6139T>C), GTC2016GTG (6154C>G), GGC2019GGG (6163C>G), CAC2020CAT (6166C>T), GTA2026GTG (6184A>G), ACT2027ACG (6187T>G), TTC2029TTT (6193C>T), CAA2030CAG (6196A>G), ATG2032ATC (6202G>C), CAA2035CAG (6211A>G), CTG2037CTT (6217G>T), GAA2039GAT (6223A>T), ATA2042GTG (6230A>G 6232A>G), GAG2043GAA (6235G>A), TTA2049TTC (6253A>C), ACC2050ACA (6256C>A), ACA2052ACT (6262A>T), CTG2053CTA (6265G>A), TCG2054TCT (6268G>T), AGC2056AGG (6274C>G), GTA2064CTG (6296G>C 6298A>G), TTG2068CTG (6308T>C), GAT2069GAC (6313T>C), GAG2072GAA (6322G>A), CAG2074CGG (6327A>G), AAT2084AAC (6358T>C), GCA2126GCC (6484A>C), AGA2128AGG (6490A>G), CAT2129CAC (6493T>C), ATT2132TTT (6500A>T), TCC2137TCT (6517C>T), CAC2142CAT (6532C>T), CTT2147TTT (6545C>T), TAC2155TAT (6571C>T)

*: Inserts / Deletes / Misaligned / Frameshifts

Analysis details

This analysis was performed with panviral2.64

NGS Details (UN70): Torradovirus lycopersici (segment RNA 2)

Assembly

**Coverage Length**	5384 (1 contig(s))
**Depth Of Coverage**	40729.6
**Number Of Reads**	1691517
**Reads Per Million**	32064.85 rpm (after QC)
**Ambiguities**	0
**Assembly Method**	de novo + reference guided assembly
**Consensus Caller**	Bcf Tools

Coverage Map

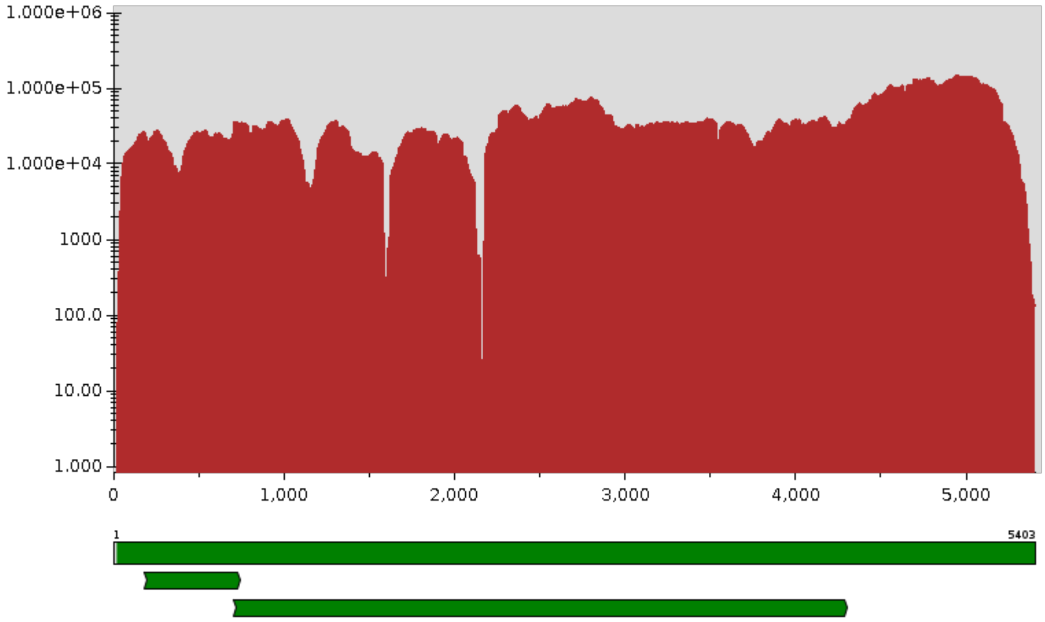

Assignment

**Type**	Torradovirus lycopersici (Taxonomy ID: 3048378)
**Reference Genome**	NC_009032.1
**NT Identity (%)**	87.3561
**AA Identity (%)**	91.4203
**Number Of Stop Codons**	3
**Number Of CDS**	2

Alignment

**Alignment Score**	8032.0 (NT) + 8819.0 (AA) = 16851.0
**Concordance (%)**	83.1943

**Alignment Method**	Global, seeded, nucleotide + amino acids (AGA)

Genome Region

Sequence starts at position 20 and ends at position 5403 relative to NC_009032.1 reference sequence.

Alignment Detailed Statistics

	Begin	End	Coverage	Score	Concordance	Matches	Identities	I/D/M/F*	Stop Codons
**NT**	**20**	**5403**	**99.6%**	**8032**	**74.6%**	**5384 (99.9%)**	**4705 (87.4%)**	**2/0**	
Mutations:	20A>G, 78C>T, 89G>A, 90C>T, 97C>T, 107G>A, 111C>A, 137T>C, 144A>G, 277C>T, 280T>C, 283C>T, 295T>C, 301A>T, 313C>T, 343T>C, 370C>T, 372A>C, 376C>T, 388T>A, 400T>C, 401T>C, 418T>G, 433C>T, 448C>A, 460T>C, 481C>A, 487A>G, 507G>A, 511T>A, 565A>T, 568A>T, 576C>A, 583A>T, 586A>G, 587T>C, 589A>T, 603A>C, 607G>A, 613A>G, 622A>G, 644C>G, 654T>A, 679G>A, 696T>C, 721C>T, 733A>G, 748A>G, 750A>G, 797T>C, 806C>T, 833C>T, 844A>T, 911T>C, 929T>A, 932A>T, 968A>G, 986G>A, 995T>C, 1040A>C, 1043A>G, 1052A>G, 1061T>C, 1073C>A, 1083C>T, 1094G>A, 1095G>A, 1100G>A, 1109C>T, 1112G>T, 1115G>A, 1118A>C, 1128T>C, 1130A>G, 1139G>A, 1142C>T, 1151A>G, 1163A>G, 1169C>G, 1178C>T, 1187A>T, 1205T>A, 1220T>C, 1235C>A, 1247A>G, 1262T>C, 1268C>T, 1277A>G, 1313T>C, 1317C>A, 1319A>G, 1325A>G, 1328T>C, 1331C>T, 1343G>A, 1355G>A, 1361A>T, 1367A>T, 1370A>T, 1376T>A, 1383A>G, 1385C>T, 1388C>T, 1390C>G, 1394A>T, 1395A>T, 1397T>A, 1400T>C, 1403A>T, 1406G>A, 1409T>A, 1412G>T, 1418G>A, 1426A>C, 1427G>T, 1434G>A, 1439A>C, 1451G>T, 1454A>T, 1456G>C, 1457C>T, 1460A>G, 1461G>T, 1465G>A, 1472C>G, 1475G>C, 1478C>G, 1481C>T, 1487C>G, 1490G>A, 1491G>A, 1493G>A, 1496C>T, 1502C>T, 1514T>C, 1524T>C, 1526C>T, 1529A>T, 1538A>T, 1543C>T, 1545A>G, 1547C>T, 1553T>C, 1559C>A, 1560C>T, 1562C>A, 1568C>G, 1574G>A, 1575C>A, 1577T>G, 1589A>G, 1593C>G, 1595G>T, 1603G>A, 1605A>G, 1608T>C, 1610C>G, 1613A>G, 1616G>A, 1621G>C, 1622T>A, 1631G>A, 1634T>A, 1646T>C, 1651A>G, 1655T>A, 1661T>C, 1662C>T, 1663T>A, 1664G>T, 1667C>T, 1674C>T, 1676T>C, 1677G>A, 1679G>T, 1685T>C, 1688T>C, 1691T>G, 1695A>G, 1700G>A, 1721G>C, 1724C>T, 1727A>G, 1730C>T, 1740C>T, 1745G>A, 1748G>A, 1749A>C, 1754G>A, 1757A>G, 1760T>G, 1763T>A, 1766A>G, 1769A>T, 1778A>C, 1784A>C, 1790G>A, 1793T>A, 1794G>A, 1797T>G, 1799C>G, 1805C>T, 1811A>C, 1814T>C, 1817A>T, 1819T>A, 1820C>G, 1823C>T, 1832G>C, 1841C>T, 1843A>G, 1845T>C, 1847G>T, 1850G>A, 1856C>T, 1863C>T, 1865C>T, 1875G>A, 1881C>T, 1892C>T, 1898T>G, 1899G>A, 1900T>C, 1901G>A, 1906C>T, 1907C>G, 1913T>A, 1928A>C, 1931C>T, 1938A>G, 1941C>T, 1943T>G, 1946C>A, 1947T>C, 1952A>C, 1955G>A, 1958A>G, 1967G>A, 1970A>G, 1979A>G, 1986G>A, 1994T>C, 2002T>A, 2012C>T, 2024T>C, 2027T>A, 2049T>A, 2051A>T, 2054G>T, 2059A>G, 2060A>C, 2066C>G, 2074T>A, 2083T>C, 2084G>A, 2087A>C, 2094C>G, 2096G>C, 2098C>A, 2099G>A, 2100C>A, 2101G>T, 2102C>G, 2106A>G, 2107G>A, 2111T>G, 2114C>T, 2115A>C, 2116A>G, 2119A>C, 2120G>A, 2123G>T, 2125C>T, 2126C>T, 2127A>G, 2128G>T, 2130G>A, 2132T>C, 2135G>A, 2136G>C, 2138G>T, 2144C>G, 2159T>A, 2162T>C, 2164T>G, 2170A>T, 2171A>T, 2174A>T, 2177A>T, 2178T>G, 2183C>T, 2186A>C, 2189C>T, 2195A>C, 2198G>A, 2199G>A, 2200T>C, 2204C>T, 2205A>G, 2210G>A, 2216C>G, 2219T>C, 2225T>A, 2228C>T, 2229A>G, 2230T>A, 2231G>T, 2240T>A, 2243T>C, 2252A>C, 2256G>A, 2259A>C, 2261T>A, 2262G>C, 2270A>G, 2285T>C, 2303A>G, 2312T>C, 2321T>C, 2375C>T, 2393G>A, 2414T>C, 2420T>A, 2429G>A, 2432G>T, 2433C>T, 2436T>G, 2444G>T, 2447C>T, 2450T>C, 2453G>A, 2459C>G, 2465T>G, 2468A>G, 2469T>A, 2471C>A, 2474G>A, 2489A>C, 2492G>A, 2495A>G, 2501A>G, 2503G>A, 2507T>C, 2516C>T, 2523A>G, 2546T>C, 2561T>A, 2567T>A, 2573T>C, 2609C>G, 2627C>T, 2630C>A, 2645G>A, 2648T>C, 2651C>T, 2666G>A, 2669T>G, 2672A>C, 2675A>T, 2693G>A, 2702T>C, 2708A>G, 2717T>C, 2720C>A, 2729C>T, 2732T>G, 2747C>A, 2753G>T, 2774A>T, 2792C>T, 2795C>G, 2798G>A, 2799G>A, 2804T>C, 2814T>C, 2835A>G, 2837C>T, 2840T>C, 2841C>T, 2846G>A, 2852A>G, 2861G>A, 2864C>A, 2867C>T, 2870C>A, 2879T>A, 2882T>A, 2886T>C, 2888G>A, 2891A>G, 2894A>G, 2900A>T, 2909T>C, 2915A>G, 2921C>T, 2927A>G, 2936A>C, 2939A>G, 2948A>G, 2954G>A, 2957A>T, 2960G>A, 2966C>T, 2972T>C, 2981G>T, 2982G>A, 2996T>G, 2999T>A, 3003T>C, 3011C>T, 3012A>G, 3014C>T, 3020T>C, 3023G>C, 3029C>T, 3032A>T, 3044C>T, 3047T>A, 3050G>C, 3051C>T, 3059A>T, 3062C>T, 3063T>C, 3065G>A, 3071A>G, 3077A>T, 3083C>A, 3101T>G, 3104C>T, 3107T>G, 3113G>C, 3120A>G, 3122T>G, 3125T>C, 3143C>T, 3146T>C, 3152T>A, 3158G>A, 3161C>A, 3164T>C, 3167A>T, 3170G>T, 3173T>C, 3176A>C, 3179G>T, 3182C>T, 3187G>A, 3194C>T, 3195A>G, 3197C>T, 3200T>C, 3206C>T, 3227T>G, 3230T>A, 3236C>T, 3239T>G, 3242G>A, 3248A>C, 3251A>T, 3253A>G, 3265G>A, 3270A>T, 3272G>T, 3278C>T, 3290G>C, 3293A>G, 3296T>C, 3297C>T, 3299T>A, 3303A>T, 3305C>T, 3311T>A, 3324C>T, 3329G>T, 3350C>T, 3354A>C, 3359G>A, 3368A>G, 3371C>G, 3372T>C, 3383G>A, 3386A>T, 3395A>C, 3404A>G, 3410G>A, 3413C>T, 3428G>A, 3429C>T, 3431T>G, 3434A>G, 3437C>T, 3440A>T, 3447T>C, 3449G>A, 3452C>A, 3455T>A, 3458G>A, 3461C>T, 3470T>C, 3473T>C, 3479C>T, 3482T>C, 3485C>T, 3488A>G, 3491C>T, 3503A>G, 3508A>G, 3515G>T, 3522C>T, 3524A>G, 3527C>T, 3530T>A, 3533G>A, 3543T>A, 3551C>T, 3560T>C, 3564A>C, 3567T>A, 3569A>G, 3575G>A, 3578A>G, 3590A>C, 3605A>G, 3611G>A, 3617A>T, 3620G>A, 3623A>T, 3629G>C, 3631C>A, 3634T>C, 3638A>G, 3641C>T, 3650A>T, 3656G>T, 3659T>C, 3662A>G, 3674G>C, 3689C>T, 3698A>C, 3704G>A, 3707T>G, 3708T>C, 3713A>C, 3716C>T, 3722C>A, 3728C>G, 3737C>T, 3742A>C, 3743G>C, 3746G>A, 3749A>T, 3752G>A, 3755A>G, 3770A>C, 3773C>T, 3776A>T, 3779C>T, 3800C>T, 3815A>C, 3827C>T, 3833T>A, 3839A>G, 3842T>C, 3845A>G, 3848G>A, 3851G>A, 3852A>G, 3854T>G, 3857C>A, 3866A>C, 3869A>T, 3872A>T, 3878G>C, 3881C>T, 3882A>G, 3884C>A, 3885A>G, 3890A>G, 3893T>C, 3896A>T, 3905C>T, 3911T>C, 3914T>A, 3920A>T, 3923C>T, 3926A>C, 3927A>C, 3928A>G, 3929G>A, 3932C>A, 3933A>G, 3935C>T, 3941T>G, 3944C>A, 3947T>C, 3950T>A, 3953T>A, 3960G>A, 3971T>A, 3983T>A, 3984A>G, 3986G>T, 3988G>A, 3989C>T, 3990G>A, 3991A>G, 3995C>T, 3998C>T, 4001T>C, 4016T>G, 4025G>A, 4028T>C, 4031T>C, 4034T>G, 4035A>C, 4037A>T, 4040C>T, 4052C>T, 4059A>G, 4061A>T, 4067A>T, 4068A>G, 4069A>G, 4070T>C, 4072C>T, 4073T>G, 4077A>C, 4088T>A, 4091G>A, 4100A>C, 4103A>T, 4106A>C, 4112C>G, 4124A>G, 4133A>G, 4142A>C, 4145C>T, 4149C>A, 4151G>A, 4166G>A, 4181C>T, 4187G>A, 4188C>G, 4196T>A, 4208A>T, 4211A>T, 4223G>A, 4224C>T, 4227T>C, 4229G>T, 4244G>A, 4250C>G, 4253C>T, 4256T>G, 4262T>C, 4265T>A, 4274A>T, 4281A>C, 4284A>G, 4286T>A, 4289T>C, 4300T>C, 4302T>G, 4316T>C, 4354G>A, 4355T>G, 4457A>G, 4590G>A, 4749T>A, 4812C>T, 4814T>C, 4823A>G, 4974_4975insT, 5089T>C, 5206C>T, 5218C>T, 5225C>T, 5235_5236insT, 5241T>C, 5249A>G, 5262A>T								

CDS

	Begin	End	Coverage	Score	Concordance	Matches	Identities	I/D/M/F*	Stop Codons
ToTV_sRNA2gp1	1	188	100%	1156	95.6%	188 (100%)	180 (95.7%)	0/0/0/0	1
Protein mutations:	N64T (372A>C), R109K (507G>A), Q128H (565A>T), T132K (576C>A), D141A (603A>C), L155V (644C>G), L158Q (654T>A), L172P (696T>C)								
Codon mutations:	GGC32GGT (277C>T), TCT33TCC (280T>C), GGC34GGT (283C>T), AGT38AGC (295T>C), CCA40CCT (301A>T), TTC44TTT (313C>T), ATT54ATC (343T>C), AAC63AAT (370C>T), AAT64ACT (372A>C), AGC65AGT (376C>T), GTT69GTA (388T>A), TAT73TAC (400T>C), TTA74CTA (401T>C), ACT79ACG (418T>G), GGC84GGT (433C>T), GGC89GGA (448C>A), CAT93CAC (460T>C), ACC100ACA (481C>A), AGA102AGG (487A>G), AGG109AAG (507G>A), ATT110ATA (511T>A), CAA128CAT (565A>T), CCA129CCT (568A>T), ACG132AAG (576C>A), ACA134ACT (583A>T), CAA135CAG (586A>G), TTA136CTT (587T>C 589A>T), GAC141GCC (603A>C), GAG142GAA (607G>A), AAA144AAG (613A>G), TTA147TTG (622A>G), CTT155GTT (644C>G), CTA158CAA (654T>A), TTG166TTA (679G>A), CTA172CCA (696T>C), GGC180GGT (721C>T), GAA184GAG (733A>G)								
ToTV_sRNA2gp2	1	1199	100%	7663	92.6%	1199 (100%)	1088 (90.7%)	0/0/0/0	2
Protein mutations:	A7V (721C>T), K11R (733A>G), K16R (748A>G), S17G (750A>G), Y48F (844A>T), V132I (1095G>A), T228A (1383A>G 1385C>T), S230* (1390C>G), T232S (1395A>T 1397T>A), Q242P (1426A>C 1427G>T), D245N (1434G>A), S252T (1456G>C 1457C>T), A254S (1461G>T), R255K (1465G>A), I257M (1472C>G), V264I (1491G>A 1493G>A), S275P (1524T>C 1526C>T), S281F (1543C>T), T282A (1545A>G 1547C>T), H286Q (1559C>A), Q298D (1593C>G 1595G>T), S301N (1603G>A), R302G (1605A>G), Y303Q (1608T>C 1610C>G), G307A (1621G>C 1622T>A), K317R (1651A>G), L321Y (1662C>T 1663T>A 1664G>T), H325Y (1674C>T 1676T>C), V326I (1677G>A 1679G>T), I332V (1695A>G), V365I (1794G>A), C366G (1797T>G 1799C>G), K370N (1811A>C), I373K (1819T>A 1820C>G), K377N (1832G>C), H381R (1843A>G), H388Y (1863C>T 1865C>T), V392M (1875G>A), I399M (1898T>G), V400T (1899G>A 1900T>C 1901G>A), A402V (1906C>T 1907C>G), E409D (1928A>C), N413D (1938A>G), D429N (1986G>A), F434Y (2002T>A), L450I (2049T>A 2051A>T), K453S (2059A>G 2060A>C), L458Q (2074T>A), V461A (2083T>C 2084G>A), Q465D (2094C>G 2096G>C), A466E (2098C>A 2099G>A), R467M (2100C>A 2101G>T 2102C>G), R469E (2106A>G 2107G>A), F470L (2111T>G), K472R (2115A>C 2116A>G), E473A (2119A>C 2120G>A), A475V (2125C>T 2126C>T), R476V (2127A>G 2128G>T), A477T (2130G>A 2132T>C), V479L (2136G>C 2138G>T), N481K (2144C>G), V488G (2164T>G), E490V (2170A>T 2171A>T), S493A (2178T>G), V500T (2199G>A 2200T>C), M502V (2205A>G), D508E (2225T>A), M510D (2229A>G 2230T>A 2231G>T), E517D (2252A>C), A519T (2256G>A), N520Q (2259A>C 2261T>A), E521Q (2262G>C), S579A (2436T>G), S590T (2469T>A 2471C>A), R601K (2503G>A), I608V (2523A>G), A700T (2799G>A), I712V (2835A>G 2837C>T), A761T (2982G>A), I771V (3012A>G 3014C>T), I807V (3120A>G 3122T>G), S829N (3187G>A), T832A (3195A>G 3197C>T), K851R (3253A>G), R855K (3265G>A), T857S (3270A>T 3272G>T), T868S (3303A>T 3305C>T), N936S (3508A>G), S948T (3543T>A), K955Q (3564A>C), L956M (3567T>A 3569A>G), P977Q (3631C>A), L978P (3634T>C), Q1014P (3742A>C 3743G>C), T1051A (3852A>G 3854T>G), I1061V (3882A>G 3884C>A), T1062A (3885A>G), K1076R (3927A>C 3928A>G 3929G>A), T1078A (3933A>G 3935C>T), A1087T (3960G>A), M1095V (3984A>G 3986G>T), S1096N (3988G>A 3989C>T), D1097S (3990G>A 3991A>G), H1105Q (4016T>G), I1120V (4059A>G 4061A>T), N1123G (4068A>G 4069A>G 4070T>C), A1124V (4072C>T 4073T>G), L1150I (4149C>A 4151G>A), Q1163E (4188C>G), H1175Y (4224C>T), S1195G (4284A>G 4286T>A)								

	Begin	End	Coverage	Score	Concordance	Matches	Identities	I/D/M/F*	Stop Codons
**NT**	**20**	**5403**	**99.6%**	**8032**	**74.6%**	**5384 (99.9%)**	**4705 (87.4%)**	**2/0**	
Codon mutations:	GCT7GTT (721C>T), AAG11AGG (733A>G), AAA16AGA (748A>G), AGT17GGT (750A>G), GTT32GTC (797T>C), GGC35GGT (806C>T), GGC44GGT (833C>T), TAT48TTT (844A>T), AAT70AAC (911T>C), TCT76TCA (929T>A), TCA77TCT (932A>T), GGA89GGG (968A>G), TTG95TTA (986G>A), GTT98GTC (995T>C), GTA113GTC (1040A>C), AAA114AAG (1043A>G), TTA117TTG (1052A>G), AGT120AGC (1061T>C), ACC124ACA (1073C>A), CTG128TTG (1083C>T), AAG131AAA (1094G>A), GTT132ATT (1095G>A), TTG133TTA (1100G>A), TCC136TCT (1109C>T), GGG137GGT (1112G>T), GGG138GGA (1115G>A), GCA139GCC (1118A>C), TTA143CTG (1128T>C 1130A>G), CCG146CCA (1139G>A), ACC147ACT (1142C>T), CTA150CTG (1151A>G), AAA154AAG (1163A>G), GGC156GGG (1169C>G), TTC159TTT (1178C>T), ACA162ACT (1187A>T), ACT168ACA (1205T>A), CCT173CCC (1220T>C), GCC178GCA (1235C>A), CAA182CAG (1247A>G), CGT187CGC (1262T>C), GCC189GCT (1268C>T), TCA192TCG (1277A>G), CTT204CTC (1313T>C), CGA206AGG (1317C>A 1319A>G), CAA208CAG (1325A>G), TTT209TTC (1328T>C), TTC210TTT (1331C>T), CAG214CAA (1343G>A), CAG218CAA (1355G>A), ATA220ATT (1361A>T), TCA222TCT (1367A>T), GTA223GTT (1370A>T), TCT225TCA (1376T>A), ACC228GCT (1383A>G 1385C>T), TCC229TCT (1388C>T), TCA230TGA (1390C>G), GCA231GCT (1394A>T), ACT232TCA (1395A>T 1397T>A), GAT233GAC (1400T>C), TCA234TCT (1403A>T), GTG235GTA (1406G>A), CTT236CTA (1409T>A), CCG237CCT (1412G>T), GTG239GTA (1418G>A), CAG242CCT (1426A>C 1427G>T), GAC245AAC (1434G>A), ACA246ACC (1439A>C), GTG250GTT (1451G>T), CCA251CCT (1454A>T), AGC252ACT (1456G>C 1457C>T), GAA253GAG (1460A>G), GCA254TCA (1461G>T), AGA255AAA (1465G>A), ATC257ATG (1472C>G), CGG258CGC (1475G>C), TCC259TCG (1478C>G), TCC260TCT (1481C>T), CTC262CTG (1487C>G), CGG263CGA (1490G>A), GTG264ATA (1491G>A 1493G>A), CCC265CCT (1496C>T), CTC267CTT (1502C>T), AGT271AGC (1514T>C), TCC275CCT (1524T>C 1526C>T), TCA276TCT (1529A>T), ATA279ATT (1538A>T), TCT281TTT (1543C>T), ACC282GCT (1545A>G 1547C>T), ACT284ACC (1553T>C), CAC286CAA (1559C>A), CTC287TTA (1560C>T 1562C>A), GGC289GGG (1568C>G), ACG291ACA (1574G>A), CGT292AGG (1575C>A 1577T>G), GTA296GTG (1589A>G), CAG298GAT (1593C>G 1595G>T), AGT301AAT (1603G>A), AGG302GGG (1605A>G), TAC303CAG (1608T>C 1610C>G), GAA304GAG (1613A>G), GAG305GAA (1616G>A), GGT307GCA (1621G>C 1622T>A), GGG310GGA (1631G>A), GTT311GTA (1634T>A), GTT315GTC (1646T>C), AAG317AGG (1651A>G), GCT318GCA (1655T>A), GGT320GGC (1661T>C), CTG321TAT (1662C>T 1663T>A 1664G>T), AAC322AAT (1667C>T), CAT325TAC (1674C>T 1676T>C), GTG326ATT (1677G>A 1679G>T), GAT328GAC (1685T>C), AAT329AAC (1688T>C), CTT330CTG (1691T>G), ATT332GTT (1695A>G), GAG333GAA (1700G>A), CTG340CTC (1721G>C), CCC341CCT (1724C>T), GAA342GAG (1727A>G), AAC343AAT (1730C>T), CTG347TTG (1740C>T), TCG348TCA (1745G>A), GTG349GTA (1748G>A), AGA350CGA (1749A>C), CAG351CAA (1754G>A), CAA352CAG (1757A>G), GTT353GTG (1760T>G), GCT354GCA (1763T>A), GCA355GCG (1766A>G), GCA356GCT (1769A>T), GCA359GCC (1778A>C), GCA361GCC (1784A>C), AAG363AAA (1790G>A), GGT364GGA (1793T>A), GTT365ATT (1794G>A), TGC366GGG (1797T>G 1799C>G), GTC368GTT (1805C>T), AAA370AAC (1811A>C), CAT371CAC (1814T>C), ATA372ATT (1817A>T), ATC373AAG (1819T>A 1820C>G), AAC374AAT (1823C>T), AAG377AAC (1832G>C), ATC380ATT (1841C>T), CAC381CGC (1843A>G), TTG382CTT (1845T>C 1847G>T), GAG383GAA (1850G>A), ATC385ATT (1856C>T), CAC388TAT (1863C>T 1865C>T), GTG392ATG (1875G>A), CTG394TTG (1881C>T), TAC397TAT (1892C>T), ATT399ATG (1898T>G), GTG400ACA (1899G>A 1900T>C 1901G>A), GCC402GTG (1906C>T 1907C>G), TCT404TCA (1913T>A), GAA409GAC (1928A>C), AAC410AAT (1931C>T), AAT413GAT (1938A>G), CTT414TTG (1941C>T 1943T>G), ACC415ACA (1946C>A), TTG416CTG (1947T>C), GCA417GCC (1952A>C), CAG418CAA (1955G>A), AGA419AGG (1958A>G), GAG422GAA (1967G>A), CTA423CTG (1970A>G), CAA426CAG (1979A>G), GAT429AAT (1986G>A), TAT431TAC (1994T>C), TTT434TAT (2002T>A), AAC437AAT (2012C>T), TAT441TAC (2024T>C), CCT442CCA (2027T>A), TTA450ATT (2049T>A 2051A>T), ACG451ACT (2054G>T), AAA453AGC (2059A>G 2060A>C), CGC455CGG (2066C>G), CTA458CAA (2074T>A), GTG461GCA (2083T>C 2084G>A), GCA462GCC (2087A>C), CAG465GAC (2094C>G 2096G>C), GCG466GAA (2098C>A 2099G>A), CGC467ATG (2100C>A 2101G>T 2102C>G), AGA469GAA (2106A>G 2107G>A), TTT470TTG (2111T>G), TAC471TAT (2114C>T), AAG472CGG (2115A>C 2116A>G), GAG473GCA (2119A>C 2120G>A), GCG474GCT (2123G>T), GCC475GTT (2125C>T 2126C>T), AGG476GTG (2127A>G 2128G>T), GCT477ACC (2130G>A 2132T>C), CAG478CAA (2135G>A), GTG479CTT (2136G>C 2138G>T), AAC481AAG (2144C>G), ACT486ACA (2159T>A), AGT487AGC (2162T>C), GTG488GGG (2164T>G), GAA490GTT (2170A>T 2171A>T), ATA491ATT (2174A>T), CCA492CCT (2177A>T), TCC493GCC (2178T>G), ACC494ACT (2183C>T), TCA495TCC (2186A>C), TTC496TTT (2189C>T), ACA498ACC (2195A>C), AAG499AAA (2198G>A), GTT500ACT (2199G>A 2200T>C), GCC501GCT (2204C>T), ATG502GTG (2205A>G), GAG503GAA (2210G>A), GGC505GGG (2216C>G), AGT506AGC (2219T>C), GAT508GAA (2225T>A), AGC509AGT (2228C>T), ATG510GAT (2229A>G 2230T>A 2231G>T), GCT513GCA (2240T>A), ATT514ATC (2243T>C), GAA517GAC (2252A>C), GCC519ACC (2256G>A), AAT520CAA (2259A>C 2261T>A), GAA521CAA (2262G>C), GTA523GTG (2270A>G), GTT528GTC (2285T>C), CAA534CAG (2303A>G), AAT537AAC (2312T>C), GGT540GGC (2321T>C), TTC558TTT (2375C>T), GAG564GAA (2393G>A), AGT571AGC (2414T>C), GTT573GTA (2420T>A), GAG576GAA (2429G>A), GTG577GTT (2432G>T), CTA578TTA (2433C>T), TCA579GCA (2436T>G), GCG581GCT (2444G>T), CTC582CTT (2447C>T), CCT583CCC (2450T>C), GCG584GCA (2453G>A), CTC586CTG (2459C>G), GGT588GGG (2465T>G), AAA589AAG (2468A>G), TCC590ACA (2469T>A 2471C>A), AAG591AAA (2474G>A), GGA596GGC (2489A>C), TCG597TCA (2492G>A), CAA598CAG (2495A>G), CTA600CTG (2501A>G), AGG601AAG (2503G>A), TAT602TAC (2507T>C), GCC605GCT (2516C>T), ATT608GTT (2523A>G), TAT615TAC (2546T>C), GGT620GGA (2561T>A), ATT622ATA (2567T>A), GGT624GGC (2573T>C), CTC636CTG (2609C>G), TTC642TTT (2627C>T), ATC643ATA (2630C>A), CTG648CTA (2645G>A), TAT649TAC (2648T>C), GCC650GCT (2651C>T), CTG655CTA (2666G>A), GTT656GTG (2669T>G), TCA657TCC (2672A>C), GCA658GCT (2675A>T), GGG664GGA (2693G>A), TGT667TGC (2702T>C), ACA669ACG (2708A>G), GGT672GGC (2717T>C), ATC673ATA (2720C>A), TTC676TTT (2729C>T), GTT677GTG (2732T>G), GCC682GCA (2747C>A), GGG684GGT (2753G>T), ATA691ATT (2774A>T), CAC697CAT (2792C>T), CCC698CCG (2795C>G), TTG699TTA (2798G>A), GCT700ACT (2799G>A), AGT701AGC (2804T>C), TTG705CTG (2814T>C), ATC712GTT (2835A>G 2837C>T), CAT713CAC (2840T>C), CTG714TTG (2841C>T), CAG715CAA (2846G>A), AAA717AAG (2852A>G), TCG720TCA (2861G>A), ACC721ACA (2864C>A), AAC722AAT (2867C>T), ATC723ATA (2870C>A), CCT726CCA (2879T>A), CCT727CCA (2882T>A), TTG729CTA (2886T>C 2888G>A), CGA730CGG (2891A>G), GCA731GCG (2894A>G), GCA733GCT (2900A>T), GGT736GGC (2909T>C), AAA738AAG (2915A>G), GAC740GAT (2921C>T), ACA742ACG (2927A>G), CCA745CCC (2936A>C), CGA746CGG (2939A>G), ACA749ACG (2948A>G), CAG751CAA (2954G>A), GTA752GTT (2957A>T), CTG753CTA (2960G>A), CAC755CAT (2966C>T), AAT757AAC (2972T>C), GTG760GTT (2981G>T), GCT761ACT (2982G>A), GGT765GGG (2996T>G), ACT766ACA (2999T>A), TTA768CTA (3003T>C), AGC770AGT (3011C>T), ATC771GTT (3012A>G 3014C>T), TCT773TCC (3020T>C), CCG774CCC (3023G>C), GGC776GGT (3029C>T), ATA777ATT (3032A>T), GAC781GAT (3044C>T), GGT782GGA (3047T>A), ACG783ACC (3050G>C), CTG784TTG (3051C>T), CCA786CCT (3059A>T), TCC787TCT (3062C>T), TTG788CTA (3063T>C 3065G>A), GGA790GGG (3071A>G), ATA792ATT (3077A>T), CGC794CGA (3083C>A), ACT800ACG (3101T>G), GGC801GGT (3104C>T), ACT802ACG (3107T>G), GTG804GTC (3113G>C), ATT807GTG (3120A>G 3122T>G), TGT808TGC (3125T>C), TTC814TTT (3143C>T), CAT815CAC (3146T>C), GGT817GGA (3152T>A), TTG819TTA (3158G>A), GCC820GCA (3161C>A), ATT821ATC (3164T>C), GGA822GGT (3167A>T), CTG823CTT (3170G>T), GGT824GGC (3173T>C), ACA825ACC (3176A>C), CTG826CTT (3179G>T), AAC827AAT (3182C>T), AGC829AAC (3187G>A), TCC831TCT (3194C>T), ACC832GCT (3195A>G 3197C>T), CCT833CCC (3200T>C), GAC835GAT (3206C>T), GTT842GTG (3227T>G), ATT843ATA (3230T>A), AAC845AAT (3236C>T), CTT846CTG (3239T>G), GAG847GAA (3242G>A), GGA849GGC (3248A>C), CGA850CGT (3251A>T), AAG851AGG (3253A>G), AGG855AAG (3265G>A), ACG857TCT (3270A>T 3272G>T), ACC859ACT (3278C>T), GGG863GGC (3290G>C), AAA864AAG (3293A>G), AAT865AAC (3296T>C), CTT866TTA (3297C>T 3299T>A), ACC868TCT (3303A>T 3305C>T), GGT870GGA (3311T>A), CTA875TTA (3324C>T), CCG876CCT (3329G>T), CAC883CAT (3350C>T), AGG885CGG (3354A>C), TTG886TTA (3359G>A), ACA889ACG (3368A>G), GTC890GTG (3371C>G), TTG891CTG (3372T>C), CTG894CTA (3383G>A), GTA895GTT (3386A>T), TCA898TCC (3395A>C), CTA901CTG (3404A>G), ACG903ACA (3410G>A), GTC904GTT (3413C>T), CAG909CAA (3428G>A), CTT910TTG (3429C>T 3431T>G), AAA911AAG (3434A>G), TGC912TGT (3437C>T), ATA913ATT (3440A>T), TTG916CTA (3447T>C 3449G>A), GTC917GTA (3452C>A), CTT918CTA (3455T>A), GGG919GGA (3458G>A), GGC920GGT (3461C>T), TCT923TCC (3470T>C), GTT924GTC (3473T>C), CCC926CCT (3479C>T), ATT927ATC (3482T>C), TAC928TAT (3485C>T), GGA929GGG (3488A>G), CAC930CAT (3491C>T), GGA934GGG (3503A>G), AAT936AGT (3508A>G), GTG938GTT (3515G>T), CTA941TTG (3522C>T 3524A>G), TTC942TTT (3527C>T), TCT943TCA (3530T>A), GAG944GAA (3533G>A), TCT948ACT (3543T>A), CGC950CGT (3551C>T), ATT953ATC (3560T>C), AAA955CAA (3564A>C), TTA956ATG (3567T>A 3569A>G), AAG958AAA (3575G>A), GAA959GAG (3578A>G), ACA963ACC (3590A>C), GGA968GGG (3605A>G), AAG970AAA (3611G>A), CCA972CCT (3617A>T), CAG973CAA (3620G>A), GTA974GTT (3623A>T), GTG976GTC (3629G>C), CCG977CAG (3631C>A), CTC978CCC (3634T>C), AGA979AGG (3638A>G), GAC980GAT (3641C>T), TCA983TCT (3650A>T), GGG985GGT (3656G>T), GCT986GCC (3659T>C), GTA987GTG (3662A>G), GTG991GTC (3674G>C), GAC996GAT (3689C>T), CGA999CGC (3698A>C), TTG1001TTA (3704G>A), GTT1002GTG (3707T>G), TTA1003CTA (3708T>C), CCA1004CCC (3713A>C), TGC1005TGT (3716C>T), CCC1007CCA (3722C>A), TCC1009TCG (3728C>G), TTC1012TTT (3737C>T), CAG1014CCC (3742A>C 3743G>C), GGG1015GGA (3746G>A), GCA1016GCT (3749A>T), CTG1017CTA (3752G>A), GTA1018GTG (3755A>G), ACA1023ACC (3770A>C), TGC1024TGT (3773C>T), CCA1025CCT (3776A>T), TTC1026TTT (3779C>T), TTC1033TTT (3800C>T), GGA1038GGC (3815A>C), TAC1042TAT (3827C>T), ATT1044ATA (3833T>A), GTA1046GTG (3839A>G), CAT1047CAC (3842T>C), AGA1048AGG (3845A>G), GTG1049GTA (3848G>A), CAG1050CAA (3851G>A), ACT1051GCG (3852A>G 3854T>G), TCC1052TCA (3857C>A), ATA1055ATC (3866A>C), GGA1056GGT (3869A>T), GGA1057GGT (3872A>T), CTG1059CTC (3878G>C), AAC1060AAT (3881C>T), ATC1061GTA (3882A>G 3884C>A), ACC1062GCC (3885A>G), TTA1063TTG (3890A>G), GAT1064GAC (3893T>C), TCA1065TCT (3896A>T), TAC1068TAT (3905C>T), TTT1070TTC (3911T>C), CCT1071CCA (3914T>A), GGA1073GGT (3920A>T), ATC1074ATT (3923C>T), TCA1075TCC (3926A>C), AAG1076CGA (3927A>C 3928A>G 3929G>A), GGC1077GGA (3932C>A), ACC1078GCT (3933A>G 3935C>T), GTT1080GTG (3941T>G), GTC1081GTA (3944C>A), TCT1082TCC (3947T>C), GCT1083GCA (3950T>A), GGT1084GGA (3953T>A), GCA1087ACA (3960G>A), GCT1090GCA (3971T>A), GGT1094GGA (3983T>A), ATG1095GTT (3984A>G 3986G>T), AGC1096AAT (3988G>A 3989C>T), GAC1097AGC (3990G>A 3991A>G), AAC1098AAT (3995C>T), ATC1099ATT (3998C>T), TTT1100TTC (4001T>C), CAT1105CAG (4016T>G), GAG1108GAA (4025G>A), TTT1109TTC (4028T>C), TTT1110TTC (4031T>C), CCT1111CCG (4034T>G), AGA1112CGT (4035A>C 4037A>T), CGC1113CGT (4040C>T), GCC1117GCT (4052C>T), ATA1120GTT (4059A>G 4061A>T), CCA1122CCT (4067A>T), AAT1123GGC (4068A>G 4069A>G 4070T>C), GCT1124GTG (4072C>T 4073T>G), AGA1126CGA (4077A>C), ACT1129ACA (4088T>A), CTG1130CTA (4091G>A), CGA1133CGC (4100A>C), CTA1134CTT (4103A>T), GGA1135GGC (4106A>C), CTC1137CTG (4112C>G), TTA1141TTG (4124A>G), AAA1144AAG (4133A>G), ATA1147ATC (4142A>C), AGC1148AGT (4145C>T), CTG1150ATA (4149C>A 4151G>A), AAG1155AAA (4166G>A), TTC1160TTT (4181C>T), TTG1162TTA (4187G>A), CAA1163GAA (4188C>G), GCT1165GCA (4196T>A), TCA1169TCT (4208A>T), GCA1170GCT (4211A>T), AAG1174AAA (4223G>A), CAT1175TAT (4224C>T), TTG1176CTT (4227T>C 4229G>T), ACG1181ACA (4244G>A), ACC1183ACG (4250C>G), TAC1184TAT (4253C>T), CTT1185CTG (4256T>G), ACT1187ACC (4262T>C), CCT1188CCA (4265T>A), TCA1191TCT (4274A>T), AGG1194CGG (4281A>C), AGT1195GGA (4284A>G 4286T>A), TTT1196TTC (4289T>C)								

Proteins

Orf1 (YP_001040017.1)	1	188	100%	1156	95.6%	188 (100%)	180 (95.7%)	0/0/0/0	1
Protein mutations:	N64T (372A>C), R109K (507G>A), Q128H (565A>T), T132K (576C>A), D141A (603A>C), L155V (644C>G), L158Q (654T>A), L172P (696T>C)								
Codon mutations:	GGC32GGT (277C>T), TCT33TCC (280T>C), GGC34GGT (283C>T), AGT38AGC (295T>C), CCA40CCT (301A>T), TTC44TTT (313C>T), ATT54ATC (343T>C), AAC63AAT (370C>T), AAT64ACT (372A>C), AGC65AGT (376C>T), GTT69GTA (388T>A), TAT73TAC (400T>C), TTA74CTA (401T>C), ACT79ACG (418T>G), GGC84GGT (433C>T), GGC89GGA (448C>A), CAT93CAC (460T>C), ACC100ACA (481C>A), AGA102AGG (487A>G), AGG109AAG (507G>A), ATT110ATA (511T>A), CAA128CAT (565A>T), CCA129CCT (568A>T), ACG132AAG (576C>A), ACA134ACT (583A>T), CAA135CAG (586A>G), TTA136CTT (587T>C 589A>T), GAC141GCC (603A>C), GAG142GAA (607G>A), AAA144AAG (613A>G), TTA147TTG (622A>G), CTT155GTT (644C>G), CTA158CAA (654T>A), TTG166TTA (679G>A), CTA172CCA (696T>C), GGC180GGT (721C>T), GAA184GAG (733A>G)								
polyprotein (YP_001040018.1)	1	1199	100%	7663	92.6%	1199 (100%)	1088 (90.7%)	0/0/0/0	2

	Begin	End	Coverage	Score	Concordance	Matches	Identities	I/D/M/F*	Stop Codons
**NT**	**20**	**5403**	**99.6%**	**8032**	**74.6%**	**5384 (99.9%)**	**4705 (87.4%)**	**2/0**	
Protein mutations:	A7V (721C>T), K11R (733A>G), K16R (748A>G), S17G (750A>G), Y48F (844A>T), V132I (1095G>A), T228A (1383A>G 1385C>T), S230* (1390C>G), T232S (1395A>T 1397T>A), Q242P (1426A>C 1427G>T), D245N (1434G>A), S252T (1456G>C 1457C>T), A254S (1461G>T), R255K (1465G>A), I257M (1472C>G), V264I (1491G>A 1493G>A), S275P (1524T>C 1526C>T), S281F (1543C>T), T282A (1545A>G 1547C>T), H286Q (1559C>A), Q298D (1593C>G 1595G>T), S301N (1603G>A), R302G (1605A>G), Y303Q (1608T>C 1610C>G), G307A (1621G>C 1622T>A), K317R (1651A>G), L321Y (1662C>T 1663T>A 1664G>T), H325Y (1674C>T 1676T>C), V326I (1677G>A 1679G>T), I332V (1695A>G), V365I (1794G>A), C366G (1797T>G 1799C>G), K370N (1811A>C), I373K (1819T>A 1820C>G), K377N (1832G>C), H381R (1843A>G), H388Y (1863C>T 1865C>T), V392M (1875G>A), I399M (1898T>G), V400T (1899G>A 1900T>C 1901G>A), A402V (1906C>T 1907C>G), E409D (1928A>C), N413D (1938A>G), D429N (1986G>A), F434Y (2002T>A), L450I (2049T>A 2051A>T), K453S (2059A>G 2060A>C), L458Q (2074T>A), V461A (2083T>C 2084G>A), Q465D (2094C>G 2096G>C), A466E (2098C>A 2099G>A), R467M (2100C>A 2101G>T 2102C>G), R469E (2106A>G 2107G>A), F470L (2111T>G), K472R (2115A>C 2116A>G), E473A (2119A>C 2120G>A), A475V (2125C>T 2126C>T), R476V (2127A>G 2128G>T), A477T (2130G>A 2132T>C), V479L (2136G>C 2138G>T), N481K (2144C>G), V488G (2164T>G), E490V (2170A>T 2171A>T), S493A (2178T>G), V500T (2199G>A 2200T>C), M502V (2205A>G), D508E (2225T>A), M510D (2229A>G 2230T>A 2231G>T), E517D (2252A>C), A519T (2256G>A), N520Q (2259A>C 2261T>A), E521Q (2262G>C), S579A (2436T>G), S590T (2469T>A 2471C>A), R601K (2503G>A), I608V (2523A>G), A700T (2799G>A), I712V (2835A>G 2837C>T), A761T (2982G>A), I771V (3012A>G 3014C>T), I807V (3120A>G 3122T>G), S829N (3187G>A), T832A (3195A>G 3197C>T), K851R (3253A>G), R855K (3265G>A), T857S (3270A>T 3272G>T), T868S (3303A>T 3305C>T), N936S (3508A>G), S948T (3543T>A), K955Q (3564A>C), L956M (3567T>A 3569A>G), P977Q (3631C>A), L978P (3634T>C), Q1014P (3742A>C 3743G>C), T1051A (3852A>G 3854T>G), I1061V (3882A>G 3884C>A), T1062A (3885A>G), K1076R (3927A>C 3928A>G 3929G>A), T1078A (3933A>G 3935C>T), A1087T (3960G>A), M1095V (3984A>G 3986G>T), S1096N (3988G>A 3989C>T), D1097S (3990G>A 3991A>G), H1105Q (4016T>G), I1120V (4059A>G 4061A>T), N1123G (4068A>G 4069A>G 4070T>C), A1124V (4072C>T 4073T>G), L1150I (4149C>A 4151G>A), Q1163E (4188C>G), H1175Y (4224C>T), S1195G (4284A>G 4286T>A)								
Codon mutations:	GCT7GTT (721C>T), AAG11AGG (733A>G), AAA16AGA (748A>G), AGT17GGT (750A>G), GTT32GTC (797T>C), GGC35GGT (806C>T), GGC44GGT (833C>T), TAT48TTT (844A>T), AAT70AAC (911T>C), TCT76TCA (929T>A), TCA77TCT (932A>T), GGA89GGG (968A>G), TTG95TTA (986G>A), GTT98GTC (995T>C), GTA113GTC (1040A>C), AAA114AAG (1043A>G), TTA117TTG (1052A>G), AGT120AGC (1061T>C), ACC124ACA (1073C>A), CTG128TTG (1083C>T), AAG131AAA (1094G>A), GTT132ATT (1095G>A), TTG133TTA (1100G>A), TCC136TCT (1109C>T), GGG137GGT (1112G>T), GGG138GGA (1115G>A), GCA139GCC (1118A>C), TTA143CTG (1128T>C 1130A>G), CCG146CCA (1139G>A), ACC147ACT (1142C>T), CTA150CTG (1151A>G), AAA154AAG (1163A>G), GGC156GGG (1169C>G), TTC159TTT (1178C>T), ACA162ACT (1187A>T), ACT168ACA (1205T>A), CCT173CCC (1220T>C), GCC178GCA (1235C>A), CAA182CAG (1247A>G), CGT187CGC (1262T>C), GCC189GCT (1268C>T), TCA192TCG (1277A>G), CTT204CTC (1313T>C), CGA206AGG (1317C>A 1319A>G), CAA208CAG (1325A>G), TTT209TTC (1328T>C), TTC210TTT (1331C>T), CAG214CAA (1343G>A), CAG218CAA (1355G>A), ATA220ATT (1361A>T), TCA222TCT (1367A>T), GTA223GTT (1370A>T), TCT225TCA (1376T>A), ACC228GCT (1383A>G 1385C>T), TCC229TCT (1388C>T), TCA230TGA (1390C>G), GCA231GCT (1394A>T), ACT232TCA (1395A>T 1397T>A), GAT233GAC (1400T>C), TCA234TCT (1403A>T), GTG235GTA (1406G>A), CTT236CTA (1409T>A), CCG237CCT (1412G>T), GTG239GTA (1418G>A), CAG242CCT (1426A>C 1427G>T), GAC245AAC (1434G>A), ACA246ACC (1439A>C), GTG250GTT (1451G>T), CCA251CCT (1454A>T), AGC252ACT (1456G>C 1457C>T), GAA253GAG (1460A>G), GCA254TCA (1461G>T), AGA255AAA (1465G>A), ATC257ATG (1472C>G), CGG258CGC (1475G>C), TCC259TCG (1478C>G), TCC260TCT (1481C>T), CTC262CTG (1487C>G), CGG263CGA (1490G>A), GTG264ATA (1491G>A 1493G>A), CCC265CCT (1496C>T), CTC267CTT (1502C>T), AGT271AGC (1514T>C), TCC275CCT (1524T>C 1526C>T), TCA276TCT (1529A>T), ATA279ATT (1538A>T), TCT281TTT (1543C>T), ACC282GCT (1545A>G 1547C>T), ACT284ACC (1553T>C), CAC286CAA (1559C>A), CTC287TTA (1560C>T 1562C>A), GGC289GGG (1568C>G), ACG291ACA (1574G>A), CGT292AGG (1575C>A 1577T>G), GTA296GTG (1589A>G), CAG298GAT (1593C>G 1595G>T), AGT301AAT (1603G>A), AGG302GGG (1605A>G), TAC303CAG (1608T>C 1610C>G), GAA304GAG (1613A>G), GAG305GAA (1616G>A), GGT307GCA (1621G>C 1622T>A), GGG310GGA (1631G>A), GTT311GTA (1634T>A), GTT315GTC (1646T>C), AAG317AGG (1651A>G), GCT318GCA (1655T>A), GGT320GGC (1661T>C), CTG321TAT (1662C>T 1663T>A 1664G>T), AAC322AAT (1667C>T), CAT325TAC (1674C>T 1676T>C), GTG326ATT (1677G>A 1679G>T), GAT328GAC (1685T>C), AAT329AAC (1688T>C), CTT330CTG (1691T>G), ATT332GTT (1695A>G), GAG333GAA (1700G>A), CTG340CTC (1721G>C), CCC341CCT (1724C>T), GAA342GAG (1727A>G), AAC343AAT (1730C>T), CTG347TTG (1740C>T), TCG348TCA (1745G>A), GTG349GTA (1748G>A), AGA350CGA (1749A>C), CAG351CAA (1754G>A), CAA352CAG (1757A>G), GTT353GTG (1760T>G), GCT354GCA (1763T>A), GCA355GCG (1766A>G), GCA356GCT (1769A>T), GCA359GCC (1778A>C), GCA361GCC (1784A>C), AAG363AAA (1790G>A), GGT364GGA (1793T>A), GTT365ATT (1794G>A), TGC366GGG (1797T>G 1799C>G), GTC368GTT (1805C>T), AAA370AAC (1811A>C), CAT371CAC (1814T>C), ATA372ATT (1817A>T), ATC373AAG (1819T>A 1820C>G), AAC374AAT (1823C>T), AAG377AAC (1832G>C), ATC380ATT (1841C>T), CAC381CGC (1843A>G), TTG382CTT (1845T>C 1847G>T), GAG383GAA (1850G>A), ATC385ATT (1856C>T), CAC388TAT (1863C>T 1865C>T), GTG392ATG (1875G>A), CTG394TTG (1881C>T), TAC397TAT (1892C>T), ATT399ATG (1898T>G), GTG400ACA (1899G>A 1900T>C 1901G>A), GCC402GTG (1906C>T 1907C>G), TCT404TCA (1913T>A), GAA409GAC (1928A>C), AAC410AAT (1931C>T), AAT413GAT (1938A>G), CTT414TTG (1941C>T 1943T>G), ACC415ACA (1946C>A), TTG416CTG (1947T>C), GCA417GCC (1952A>C), CAG418CAA (1955G>A), AGA419AGG (1958A>G), GAG422GAA (1967G>A), CTA423CTG (1970A>G), CAA426CAG (1979A>G), GAT429AAT (1986G>A), TAT431TAC (1994T>C), TTT434TAT (2002T>A), AAC437AAT (2012C>T), TAT441TAC (2024T>C), CCT442CCA (2027T>A), TTA450ATT (2049T>A 2051A>T), ACG451ACT (2054G>T), AAA453AGC (2059A>G 2060A>C), CGC455CGG (2066C>G), CTA458CAA (2074T>A), GTG461GCA (2083T>C 2084G>A), GCA462GCC (2087A>C), CAG465GAC (2094C>G 2096G>C), GCG466GAA (2098C>A 2099G>A), CGC467ATG (2100C>A 2101G>T 2102C>G), AGA469GAA (2106A>G 2107G>A), TTT470TTG (2111T>G), TAC471TAT (2114C>T), AAG472CGG (2115A>C 2116A>G), GAG473GCA (2119A>C 2120G>A), GCG474GCT (2123G>T), GCC475GTT (2125C>T 2126C>T), AGG476GTG (2127A>G 2128G>T), GCT477ACC (2130G>A 2132T>C), CAG478CAA (2135G>A), GTG479CTT (2136G>C 2138G>T), AAC481AAG (2144C>G), ACT486ACA (2159T>A), AGT487AGC (2162T>C), GTG488GGG (2164T>G), GAA490GTT (2170A>T 2171A>T), ATA491ATT (2174A>T), CCA492CCT (2177A>T), TCC493GCC (2178T>G), ACC494ACT (2183C>T), TCA495TCC (2186A>C), TTC496TTT (2189C>T), ACA498ACC (2195A>C), AAG499AAA (2198G>A), GTT500ACT (2199G>A 2200T>C), GCC501GCT (2204C>T), ATG502GTG (2205A>G), GAG503GAA (2210G>A), GGC505GGG (2216C>G), AGT506AGC (2219T>C), GAT508GAA (2225T>A), AGC509AGT (2228C>T), ATG510GAT (2229A>G 2230T>A 2231G>T), GCT513GCA (2240T>A), ATT514ATC (2243T>C), GAA517GAC (2252A>C), GCC519ACC (2256G>A), AAT520CAA (2259A>C 2261T>A), GAA521CAA (2262G>C), GTA523GTG (2270A>G), GTT528GTC (2285T>C), CAA534CAG (2303A>G), AAT537AAC (2312T>C), GGT540GGC (2321T>C), TTC558TTT (2375C>T), GAG564GAA (2393G>A), AGT571AGC (2414T>C), GTT573GTA (2420T>A), GAG576GAA (2429G>A), GTG577GTT (2432G>T), CTA578TTA (2433C>T), TCA579GCA (2436T>G), GCG581GCT (2444G>T), CTC582CTT (2447C>T), CCT583CCC (2450T>C), GCG584GCA (2453G>A), CTC586CTG (2459C>G), GGT588GGG (2465T>G), AAA589AAG (2468A>G), TCC590ACA (2469T>A 2471C>A), AAG591AAA (2474G>A), GGA596GGC (2489A>C), TCG597TCA (2492G>A), CAA598CAG (2495A>G), CTA600CTG (2501A>G), AGG601AAG (2503G>A), TAT602TAC (2507T>C), GCC605GCT (2516C>T), ATT608GTT (2523A>G), TAT615TAC (2546T>C), GGT620GGA (2561T>A), ATT622ATA (2567T>A), GGT624GGC (2573T>C), CTC636CTG (2609C>G), TTC642TTT (2627C>T), ATC643ATA (2630C>A), CTG648CTA (2645G>A), TAT649TAC (2648T>C), GCC650GCT (2651C>T), CTG655CTA (2666G>A), GTT656GTG (2669T>G), TCA657TCC (2672A>C), GCA658GCT (2675A>T), GGG664GGA (2693G>A), TGT667TGC (2702T>C), ACA669ACG (2708A>G), GGT672GGC (2717T>C), ATC673ATA (2720C>A), TTC676TTT (2729C>T), GTT677GTG (2732T>G), GCC682GCA (2747C>A), GGG684GGT (2753G>T), ATA691ATT (2774A>T), CAC697CAT (2792C>T), CCC698CCG (2795C>G), TTG699TTA (2798G>A), GCT700ACT (2799G>A), AGT701AGC (2804T>C), TTG705CTG (2814T>C), ATC712GTT (2835A>G 2837C>T), CAT713CAC (2840T>C), CTG714TTG (2841C>T), CAG715CAA (2846G>A), AAA717AAG (2852A>G), TCG720TCA (2861G>A), ACC721ACA (2864C>A), AAC722AAT (2867C>T), ATC723ATA (2870C>A), CCT726CCA (2879T>A), CCT727CCA (2882T>A), TTG729CTA (2886T>C 2888G>A), CGA730CGG (2891A>G), GCA731GCG (2894A>G), GCA733GCT (2900A>T), GGT736GGC (2909T>C), AAA738AAG (2915A>G), GAC740GAT (2921C>T), ACA742ACG (2927A>G), CCA745CCC (2936A>C), CGA746CGG (2939A>G), ACA749ACG (2948A>G), CAG751CAA (2954G>A), GTA752GTT (2957A>T), CTG753CTA (2960G>A), CAC755CAT (2966C>T), AAT757AAC (2972T>C), GTG760GTT (2981G>T), GCT761ACT (2982G>A), GGT765GGG (2996T>G), ACT766ACA (2999T>A), TTA768CTA (3003T>C), AGC770AGT (3011C>T), ATC771GTT (3012A>G 3014C>T), TCT773TCC (3020T>C), CCG774CCC (3023G>C), GGC776GGT (3029C>T), ATA777ATT (3032A>T), GAC781GAT (3044C>T), GGT782GGA (3047T>A), ACG783ACC (3050G>C), CTG784TTG (3051C>T), CCA786CCT (3059A>T), TCC787TCT (3062C>T), TTG788CTA (3063T>C 3065G>A), GGA790GGG (3071A>G), ATA792ATT (3077A>T), CGC794CGA (3083C>A), ACT800ACG (3101T>G), GGC801GGT (3104C>T), ACT802ACG (3107T>G), GTG804GTC (3113G>C), ATT807GTG (3120A>G 3122T>G), TGT808TGC (3125T>C), TTC814TTT (3143C>T), CAT815CAC (3146T>C), GGT817GGA (3152T>A), TTG819TTA (3158G>A), GCC820GCA (3161C>A), ATT821ATC (3164T>C), GGA822GGT (3167A>T), CTG823CTT (3170G>T), GGT824GGC (3173T>C), ACA825ACC (3176A>C), CTG826CTT (3179G>T), AAC827AAT (3182C>T), AGC829AAC (3187G>A), TCC831TCT (3194C>T), ACC832GCT (3195A>G 3197C>T), CCT833CCC (3200T>C), GAC835GAT (3206C>T), GTT842GTG (3227T>G), ATT843ATA (3230T>A), AAC845AAT (3236C>T), CTT846CTG (3239T>G), GAG847GAA (3242G>A), GGA849GGC (3248A>C), CGA850CGT (3251A>T), AAG851AGG (3253A>G), AGG855AAG (3265G>A), ACG857TCT (3270A>T 3272G>T), ACC859ACT (3278C>T), GGG863GGC (3290G>C), AAA864AAG (3293A>G), AAT865AAC (3296T>C), CTT866TTA (3297C>T 3299T>A), ACC868TCT (3303A>T 3305C>T), GGT870GGA (3311T>A), CTA875TTA (3324C>T), CCG876CCT (3329G>T), CAC883CAT (3350C>T), AGG885CGG (3354A>C), TTG886TTA (3359G>A), ACA889ACG (3368A>G), GTC890GTG (3371C>G), TTG891CTG (3372T>C), CTG894CTA (3383G>A), GTA895GTT (3386A>T), TCA898TCC (3395A>C), CTA901CTG (3404A>G), ACG903ACA (3410G>A), GTC904GTT (3413C>T), CAG909CAA (3428G>A), CTT910TTG (3429C>T 3431T>G), AAA911AAG (3434A>G), TGC912TGT (3437C>T), ATA913ATT (3440A>T), TTG916CTA (3447T>C 3449G>A), GTC917GTA (3452C>A), CTT918CTA (3455T>A), GGG919GGA (3458G>A), GGC920GGT (3461C>T), TCT923TCC (3470T>C), GTT924GTC (3473T>C), CCC926CCT (3479C>T), ATT927ATC (3482T>C), TAC928TAT (3485C>T), GGA929GGG (3488A>G), CAC930CAT (3491C>T), GGA934GGG (3503A>G), AAT936AGT (3508A>G), GTG938GTT (3515G>T), CTA941TTG (3522C>T 3524A>G), TTC942TTT (3527C>T), TCT943TCA (3530T>A), GAG944GAA (3533G>A), TCT948ACT (3543T>A), CGC950CGT (3551C>T), ATT953ATC (3560T>C), AAA955CAA (3564A>C), TTA956ATG (3567T>A 3569A>G), AAG958AAA (3575G>A), GAA959GAG (3578A>G), ACA963ACC (3590A>C), GGA968GGG (3605A>G), AAG970AAA (3611G>A), CCA972CCT (3617A>T), CAG973CAA (3620G>A), GTA974GTT (3623A>T), GTG976GTC (3629G>C), CCG977CAG (3631C>A), CTC978CCC (3634T>C), AGA979AGG (3638A>G), GAC980GAT (3641C>T), TCA983TCT (3650A>T), GGG985GGT (3656G>T), GCT986GCC (3659T>C), GTA987GTG (3662A>G), GTG991GTC (3674G>C), GAC996GAT (3689C>T), CGA999CGC (3698A>C), TTG1001TTA (3704G>A), GTT1002GTG (3707T>G), TTA1003CTA (3708T>C), CCA1004CCC (3713A>C), TGC1005TGT (3716C>T), CCC1007CCA (3722C>A), TCC1009TCG (3728C>G), TTC1012TTT (3737C>T), CAG1014CCC (3742A>C 3743G>C), GGG1015GGA (3746G>A), GCA1016GCT (3749A>T), CTG1017CTA (3752G>A), GTA1018GTG (3755A>G), ACA1023ACC (3770A>C), TGC1024TGT (3773C>T), CCA1025CCT (3776A>T), TTC1026TTT (3779C>T), TTC1033TTT (3800C>T), GGA1038GGC (3815A>C), TAC1042TAT (3827C>T), ATT1044ATA (3833T>A), GTA1046GTG (3839A>G), CAT1047CAC (3842T>C), AGA1048AGG (3845A>G), GTG1049GTA (3848G>A), CAG1050CAA (3851G>A), ACT1051GCG (3852A>G 3854T>G), TCC1052TCA (3857C>A), ATA1055ATC (3866A>C), GGA1056GGT (3869A>T), GGA1057GGT (3872A>T), CTG1059CTC (3878G>C), AAC1060AAT (3881C>T), ATC1061GTA (3882A>G 3884C>A), ACC1062GCC (3885A>G), TTA1063TTG (3890A>G), GAT1064GAC (3893T>C), TCA1065TCT (3896A>T), TAC1068TAT (3905C>T), TTT1070TTC (3911T>C), CCT1071CCA (3914T>A), GGA1073GGT (3920A>T), ATC1074ATT (3923C>T), TCA1075TCC (3926A>C), AAG1076CGA (3927A>C 3928A>G 3929G>A), GGC1077GGA (3932C>A), ACC1078GCT (3933A>G 3935C>T), GTT1080GTG (3941T>G), GTC1081GTA (3944C>A), TCT1082TCC (3947T>C), GCT1083GCA (3950T>A), GGT1084GGA (3953T>A), GCA1087ACA (3960G>A), GCT1090GCA (3971T>A), GGT1094GGA (3983T>A), ATG1095GTT (3984A>G 3986G>T), AGC1096AAT (3988G>A 3989C>T), GAC1097AGC (3990G>A 3991A>G), AAC1098AAT (3995C>T), ATC1099ATT (3998C>T), TTT1100TTC (4001T>C), CAT1105CAG (4016T>G), GAG1108GAA (4025G>A), TTT1109TTC (4028T>C), TTT1110TTC (4031T>C), CCT1111CCG (4034T>G), AGA1112CGT (4035A>C 4037A>T), CGC1113CGT (4040C>T), GCC1117GCT (4052C>T), ATA1120GTT (4059A>G 4061A>T), CCA1122CCT (4067A>T), AAT1123GGC (4068A>G 4069A>G 4070T>C), GCT1124GTG (4072C>T 4073T>G), AGA1126CGA (4077A>C), ACT1129ACA (4088T>A), CTG1130CTA (4091G>A), CGA1133CGC (4100A>C), CTA1134CTT (4103A>T), GGA1135GGC (4106A>C), CTC1137CTG (4112C>G), TTA1141TTG (4124A>G), AAA1144AAG (4133A>G), ATA1147ATC (4142A>C), AGC1148AGT (4145C>T), CTG1150ATA (4149C>A 4151G>A), AAG1155AAA (4166G>A), TTC1160TTT (4181C>T), TTG1162TTA (4187G>A), CAA1163GAA (4188C>G), GCT1165GCA (4196T>A), TCA1169TCT (4208A>T), GCA1170GCT (4211A>T), AAG1174AAA (4223G>A), CAT1175TAT (4224C>T), TTG1176CTT (4227T>C 4229G>T), ACG1181ACA (4244G>A), ACC1183ACG (4250C>G), TAC1184TAT (4253C>T), CTT1185CTG (4256T>G), ACT1187ACC (4262T>C), CCT1188CCA (4265T>A), TCA1191TCT (4274A>T), AGG1194CGG (4281A>C), AGT1195GGA (4284A>G 4286T>A), TTT1196TTC (4289T>C)								

*: Inserts / Deletes / Misaligned / Frameshifts

Analysis details

This analysis was performed with panviral2.64

NGS Details (UN70): Potato virus Y

Assembly

**Coverage Length**	9686 (1 contig(s))
**Depth Of Coverage**	2313.2
**Number Of Reads**	167938
**Reads Per Million**	3183.48 rpm (after QC)
**Ambiguities**	0
**Assembly Method**	de novo + reference guided assembly
**Consensus Caller**	Bcf Tools

Coverage Map

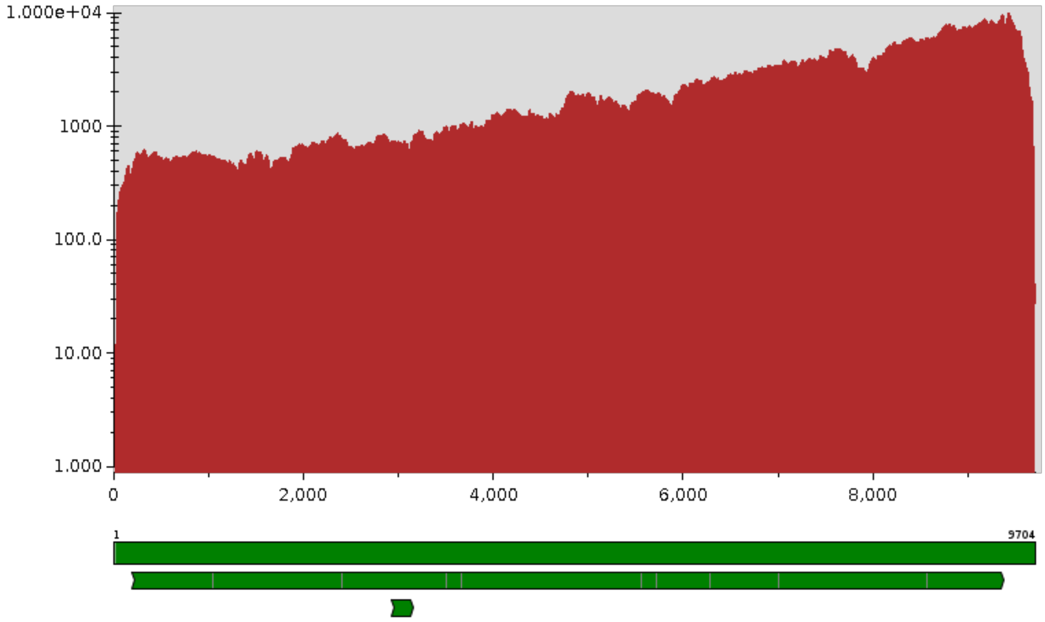

Assignment

**Type**	Potato virus Y (Taxonomy ID: 12216)
**Reference Genome**	NC_001616.1
**NT Identity (%)**	82.6814
**AA Identity (%)**	90.1274
**Number Of Stop Codons**	3
**Number Of CDS**	2

Alignment

**Alignment Score**	12534.0 (NT) + 19051.0 (AA) = 31585.0
**Concordance (%)**	78.2307

Alignment Method	Global, seeded, nucleotide + amino acids (AGA)

Genome Region

Sequence starts at position 19 and ends at position 9704 relative to NC_001616.1 reference sequence.

Alignment Detailed Statistics

	Begin	End	Coverage	Score	Concordance	Matches	Identities	I/D/M/F*	Stop Codons
NT	19	9704	99.8%	12534	65.0%	9674 (99.7%)	8011 (82.6%)	15/12	

Mutations:

19C>T, 23A>T, 32A>T, 42_43insGAAATAT, 45A>T, 47T>A, 50T>G, 53A>G, 54C>T, 55G>T, 56C>T, 58C>T, 60T>A, 60_61insA, 75C>T, 79C>T, 83G>A, 85T>C, 87C>T, 89G>A, 90T>C, 93A>T, 94A>G, 95A>T, 100T>C, 101T>A, 102C>T, 104T>C, 107C>T, 110T>G, 112C>T, 114C>T, 115T>C, 116A>T, 117G>C, 119A>G, 120C>T, 121A>T, 125T>G, 126T>C, 127G>A, 128G>T, 131A>G, 132C>T, 139A>T, 139_140insG, 141C>T, 143C>T, 146C>T, 150C>T, 150_151insT, 154T>C, 156C>A, 158T>A, 160A>C, 164C>T, 168C>A, 169C>G, 171A>C, 172T>C, 173T>A, 174T>G, 177G>C, 178A>G, 179T>C, 180C>T, 181C>A, 182T>G, 183C>T, 193T>C, 194T>C, 196C>A, 202A>G, 208C>T, 209T>C, 210G>T, 211T>G, 220G>A, 221T>A, 223T>G, 226A>G, 235A>T, 241C>T, 247A>T, 251T>C, 255G>T, 256C>T, 258A>G, 262T>C, 263A>G, 265T>G, 267T>C, 273A>T, 277A>T, 280A>G, 283G>T, 286G>A, 287G>A, 289T>C, 293G>A, 294T>C, 301T>C, 304C>T, 305G>A, 306C>T, 309A>G, 311C>T, 316A>T, 317A>G, 318C>T, 319A>G, 322A>G, 331A>T, 339T>A, 340C>A, 342A>G, 347A>G, 348A>C, 349A>G, 350T>C, 355T>A, 356A>G, 357C>T, 364T>C, 365G>A, 366T>C, 367G>A, 368C>T, 369T>C, 373A>G, 376C>T, 383T>C, 384T>G, 388G>A, 391C>T, 394A>G, 397C>T, 400G>A, 406T>C, 408C>T, 409C>T, 412G>A, 415A>T, 416A>G, 417T>A, 424T>C, 427G>A, 433A>G, 436G>C, 437G>A, 440A>G, 441G>A, 442G>A, 443A>C, 444A>G, 445G>A, 448T>A, 454A>G, 456A>C, 460T>C, 461C>A, 469A>G, 475C>T, 481T>A, 502T>C, 505A>T, 511C>T, 517T>A, 520T>A, 523A>T, 531C>T, 535G>A, 536C>T, 539C>T, 540A>G, 541A>G, 549G>A, 550A>G, 556C>T, 559T>C, 562T>C, 568T>C, 571A>G, 580T>C, 585T>C, 586C>G, 593C>T, 594G>A, 595C>T, 597C>G, 599A>G, 601A>G, 602A>C, 603T>C, 604A>G, 613A>G, 616A>G, 619C>G, 622G>A, 625G>A, 628T>C, 631C>T, 635A>G, 637T>C, 644A>G, 655T>C, 661G>A, 663A>C, 664G>C, 667A>G, 673G>C, 679C>T, 682C>A, 683T>C, 691T>C, 699C>G, 703T>A, 706T>A, 719A>G, 721G>A, 722A>G, 724A>G, 725C>T, 727T>G, 730T>A, 731G>T, 734T>C, 735A>C, 736C>T, 737T>C, 738C>G, 739C>T, 744C>T, 745G>C, 749C>T, 751A>C, 754T>C, 765T>A, 770T>C, 773C>A, 774G>A, 775A>G, 776C>A, 790C>T, 801T>A, 813G>C, 814A>G, 816T>A, 818T>C, 823A>G, 825G>A, 826T>A, 833C>A, 835G>A, 841C>T, 853T>C, 859C>T, 860C>T, 862C>T, 863A>G, 865T>C, 867T>C, 869A>G, 871C>T, 872A>T, 874A>G, 879G>A, 886T>C, 898C>T, 903A>G, 906C>A, 907A>C, 909A>C, 910A>C, 912G>A, 913C>T, 922C>A, 931T>A, 938T>C, 939C>T, 940A>T, 943A>G, 952C>T, 967A>G, 973A>G, 976G>C, 980T>A, 982G>C, 991A>G, 994T>C, 999G>A, 1003T>C, 1007C>A, 1008A>G, 1009G>A, 1010A>G, 1012T>G, 1013A>G, 1018A>G, 1019A>C, 1021C>G, 1030C>T, 1039G>A, 1042T>C, 1045C>A, 1048C>A, 1051T>C, 1060G>A, 1063T>A, 1069C>T, 1083G>A, 1093T>C, 1097T>G, 1099G>A, 1105C>T, 1114A>G, 1117T>G, 1123A>G, 1126T>A, 1129C>T, 1143G>A, 1147A>T, 1150T>A, 1154T>A, 1156G>A, 1165C>T, 1168T>C, 1172C>T, 1174T>A, 1201C>T, 1221G>A, 1229G>A, 1230T>C, 1231T>C, 1233G>A, 1234C>T, 1240G>A, 1246G>A, 1250T>C, 1252G>A, 1255T>C, 1257A>C, 1258A>G, 1267A>G, 1273T>C, 1276G>A, 1278A>G, 1283T>C, 1290C>T, 1291G>A, 1298G>A, 1299A>C, 1300C>T, 1306T>C, 1307A>G, 1309A>G, 1312T>C, 1316A>G, 1318T>A, 1327G>A, 1329T>C, 1333G>T, 1336A>G, 1343C>T, 1345A>G, 1354G>A, 1357G>T, 1360C>T, 1367C>T, 1369C>G, 1373C>A, 1378C>T, 1384G>A, 1385A>G, 1393A>G, 1396C>T, 1405G>T, 1408A>G, 1411G>A, 1415G>T, 1417A>T, 1420G>C, 1429T>C, 1432A>G, 1436G>A, 1438C>T, 1439T>C, 1441A>G, 1444T>C, 1453C>T, 1456G>T, 1468A>G, 1499T>C, 1501G>A, 1504T>C, 1507G>A, 1510C>T, 1513A>G, 1516A>G, 1519A>T, 1522G>A, 1525C>T, 1528G>A, 1534C>T, 1540T>A, 1546C>T, 1555A>G, 1561T>C, 1564A>C, 1567T>A, 1573C>T, 1576A>G, 1583T>C, 1588T>A, 1594G>A, 1600C>T, 1609G>A, 1618G>A, 1621C>T, 1624C>T, 1627C>T, 1631C>T, 1639A>G, 1651C>T, 1654C>G, 1675T>C, 1693A>G, 1702T>C, 1707A>G, 1718G>A, 1726A>G, 1729C>T, 1732A>C, 1738T>C, 1742A>G, 1744C>T, 1747C>T, 1753T>C, 1758G>A, 1768G>A, 1786C>T, 1789A>G, 1790G>A, 1795C>T, 1805T>C, 1807A>G, 1813G>A, 1819G>A, 1825G>A, 1837C>T, 1840T>C, 1849A>G, 1858C>G, 1864A>G, 1870C>T, 1876T>C, 1882A>G, 1888T>A, 1891T>C, 1906T>C, 1915A>T, 1930T>C, 1936C>T, 1937A>G, 1939T>C, 1945A>G, 1948A>G, 1951C>T, 1960A>T, 1978A>G, 1981T>A, 1984C>T, 1987T>C, 1996C>T, 2004T>A, 2020A>G, 2023G>A, 2026T>C, 2029G>T, 2032G>A, 2059T>C, 2068T>G, 2071C>T, 2072G>A, 2074G>A, 2087C>T, 2089G>A, 2101C>T, 2104G>A, 2107G>A, 2116G>A, 2122C>T, 2137C>T, 2158T>C, 2164C>T, 2170A>G, 2182T>C, 2188G>A, 2191C>A, 2208G>A, 2218T>C, 2224C>T, 2227A>T, 2242G>A, 2245C>T, 2254G>A, 2257T>C, 2260C>T, 2266C>G, 2272A>G, 2275G>A, 2293A>G, 2299C>T, 2302G>A, 2305G>A, 2314A>T, 2317T>C, 2323T>C, 2326A>G, 2341G>A, 2347A>G, 2350T>C, 2356G>A, 2365T>C, 2371A>G, 2374A>G, 2383T>C, 2386A>T, 2389A>G, 2392T>C, 2395T>C, 2414A>G, 2415A>G, 2416T>G, 2419T>A, 2420A>T, 2440A>T, 2467T>C, 2476T>C, 2482G>A, 2491G>A, 2497G>T, 2503G>A, 2518A>G, 2521T>C, 2523A>G, 2533A>G, 2539G>A, 2545A>G, 2551G>A, 2554T>A, 2558C>T, 2561T>C, 2563G>C, 2569A>G, 2572A>G, 2584T>C, 2587C>T, 2593G>C, 2599T>C, 2608T>C, 2620T>C, 2629G>A, 2632G>A, 2634G>A, 2638G>A, 2644T>A, 2653A>G, 2665T>C, 2678C>T, 2683A>G, 2689T>C, 2698A>C, 2707A>G, 2710G>A, 2716A>G, 2719A>G, 2722C>T, 2723G>A, 2728A>G, 2734G>A, 2737T>A, 2746T>C, 2747G>A, 2749T>A, 2752A>T, 2755T>A, 2761C>T, 2764C>T, 2779T>C, 2785G>T, 2791C>T, 2795C>A, 2806C>T, 2816T>C, 2818G>C, 2822G>A, 2824G>A, 2827G>A, 2836T>C, 2839G>A, 2842T>C, 2863C>A, 2864C>T, 2882C>A, 2885A>C, 2886G>A, 2893C>T, 2895C>T, 2902C>T, 2905A>G, 2911T>C, 2931A>G, 2940A>G, 2947T>C, 2971_2972insA, 2989T>C, 2991C>T, 2994A>G, 2999G>A, 3000A>G, 3016delC, 3021G>A, 3024A>T, 3025C>T, 3036C>T, 3039A>G, 3040A>C, 3042A>C, 3043G>A, 3052G>A, 3061A>G, 3082G>A, 3091C>T, 3099C>T, 3100C>A, 3104G>A, 3105T>G, 3106G>A, 3109C>T, 3112A>G, 3118T>C, 3121C>T, 3127A>G, 3132G>A, 3137C>A, 3138G>A, 3139A>G, 3151T>C, 3152T>A, 3154C>T, 3165G>A, 3169A>G, 3199C>T, 3203A>C, 3208T>C, 3214A>G, 3217C>T, 3218T>C, 3223C>A, 3230G>A, 3235C>T, 3257T>C, 3262C>T, 3265C>T, 3266G>A, 3268A>G, 3271G>A, 3275G>A, 3284G>A, 3301G>A, 3315G>A, 3316A>G, 3318A>C, 3325G>A, 3328G>A, 3337A>T, 3349G>A, 3355C>T, 3358C>T, 3359A>G, 3367A>G, 3370T>C, 3377T>C, 3379A>G, 3394A>G, 3397C>T, 3400T>G, 3406A>G, 3418C>T, 3421T>A, 3427T>C, 3433G>A, 3436A>T, 3439A>G, 3442C>T, 3445T>C, 3457G>A, 3460T>C, 3463T>A, 3469G>T, 3472G>A, 3496C>T, 3499G>A, 3505C>T, 3513T>G, 3520A>G, 3529A>G, 3532A>G, 3545A>G, 3548G>A, 3551T>C, 3554G>A, 3556C>T, 3583T>C, 3586T>C, 3589C>T, 3595C>T, 3607T>C, 3613T>C, 3616G>A, 3619T>C, 3623C>G, 3628C>T, 3632C>A, 3637T>C, 3638T>C, 3643A>G, 3661A>G, 3670G>A, 3682T>C, 3688G>A, 3697G>A, 3698A>G, 3706T>C, 3709T>C, 3713T>C, 3715G>A, 3717G>A, 3721G>A, 3731C>A, 3732G>A, 3736T>A, 3746C>T, 3748A>G, 3754A>G, 3763T>C, 3778A>G, 3781A>G, 3784C>T, 3796T>C, 3799A>G, 3805A>T, 3811C>T, 3814A>G, 3829C>T, 3838T>C, 3844A>G, 3847A>C, 3856T>A, 3862G>T, 3871C>T, 3877C>A, 3883C>T, 3886A>G, 3892A>G, 3895C>T, 3898T>C, 3904A>G, 3905C>A, 3907G>A, 3913T>C, 3928G>A, 3931A>T, 3937G>A, 3946T>C, 3955T>C, 3958G>A, 3961C>T, 3964A>G, 3967T>C, 3976A>G, 3979T>C, 3985A>T, 3997A>T, 4000G>A, 4006C>T, 4009T>G, 4012C>T, 4015A>G, 4024T>C, 4030A>G, 4033A>T, 4042G>A, 4052C>T, 4054G>A, 4057T>C, 4063T>A, 4066A>G, 4073A>G, 4078T>C, 4081C>T, 4084T>C, 4096C>T, 4108C>T, 4111A>T, 4117G>A, 4118C>T, 4120A>G, 4126C>T, 4128T>A, 4129C>T, 4132C>T, 4135T>C, 4147A>G, 4148T>C, 4151G>T, 4162C>T, 4166G>A, 4171A>C, 4180G>A, 4190C>T, 4198T>C, 4201C>T, 4210G>T, 4213C>T, 4219T>C, 4220C>T, 4223C>A, 4225G>A, 4231T>G, 4237T>C, 4252A>G, 4253T>C, 4255A>G, 4258A>G, 4270T>C, 4282A>G, 4285G>A, 4291A>G, 4303G>A, 4312C>T, 4319A>G, 4321A>G, 4324G>T, 4327G>A, 4336G>T, 4339T>C, 4348A>T, 4360A>G, 4366T>C, 4369T>A, 4375T>C, 4387T>C, 4395T>A, 4406G>A, 4411T>C, 4438T>C, 4444C>A, 4450C>T, 4456C>T, 4465C>T, 4475A>G, 4477G>T, 4480C>T, 4481A>T, 4495C>T, 4507G>A, 4510C>T, 4513T>G, 4517C>T, 4528C>T, 4534A>G, 4540C>T, 4544G>T, 4545C>T, 4546G>A, 4548G>A, 4558T>C, 4564A>T, 4570C>T, 4576A>T, 4597G>A, 4600C>T, 4606C>T, 4609G>A, 4615G>A, 4618C>T, 4625T>C, 4627G>T, 4648C>T, 4651T>C, 4660G>A, 4663C>T, 4669T>A, 4679G>A, 4681G>C, 4684T>C, 4687T>G, 4699G>A, 4702A>G, 4708A>G, 4711G>A, 4712C>T, 4738A>G, 4744A>T, 4747A>G, 4748T>C, 4765T>C, 4796G>A, 4798C>T, 4803C>G, 4804T>C, 4807G>A, 4810G>T, 4813T>G, 4816T>C, 4831T>C, 4834C>T, 4840A>T, 4852A>G, 4855C>T, 4867T>C, 4870G>C, 4873T>A, 4882T>C, 4903A>T, 4909G>A, 4915T>C, 4918A>G, 4919T>C, 4921G>A, 4924T>C, 4927C>G, 4933T>C, 4936C>A, 4939G>A, 4942T>C, 4945C>T, 4963A>G, 4972T>C, 4990T>C, 5002A>G, 5023T>C, 5024T>C, 5029C>T, 5032T>C, 5038T>C, 5044A>T, 5053A>T, 5056G>A, 5057A>G, 5060A>G, 5071G>T, 5090C>A, 5095A>G, 5098G>T, 5100C>T, 5104A>G, 5107A>T, 5110T>C, 5113A>G, 5116G>A, 5119A>G, 5120G>T, 5122C>T, 5126A>G, 5137T>C, 5140C>T, 5143A>G, 5146G>A, 5161C>A, 5167A>G, 5173T>G, 5182G>A, 5183G>A, 5188C>T, 5191G>A, 5197A>G, 5200C>T, 5206C>T, 5209A>G, 5218C>T, 5230G>A, 5236C>T, 5239C>T, 5245C>T, 5263A>T, 5269C>T, 5272C>T, 5275C>A, 5278C>T, 5281A>C, 5284A>G, 5287T>C, 5288C>T, 5293A>T, 5294T>C, 5296G>A, 5302G>A, 5311T>A, 5314A>G, 5323A>T, 5347T>C, 5353C>T, 5365C>T, 5386T>C, 5395C>A, 5422A>G, 5425T>C, 5431C>T, 5437G>A, 5452T>C, 5458G>A, 5467T>C, 5470A>G, 5471C>T, 5473A>G, 5482C>T, 5485A>C, 5488T>C, 5489T>C, 5491G>A, 5497T>C, 5506T>C, 5509G>A, 5512G>A, 5530T>C, 5533G>A, 5536G>T, 5539G>A, 5548T>G, 5554C>T, 5560T>A, 5561G>A, 5563G>A, 5566G>C, 5569A>G, 5584C>G, 5593G>A, 5596G>A, 5597A>G, 5605C>A, 5617G>T, 5619C>T, 5620T>G, 5629G>A, 5630A>C, 5641C>T, 5650A>T, 5662A>T, 5665A>G, 5689A>G, 5693G>A, 5698G>A, 5713A>G, 5716G>C, 5719A>G, 5743C>A, 5755C>A, 5758T>C, 5764T>C, 5770A>G, 5779C>T, 5782T>C, 5791C>T, 5803C>T, 5836G>A, 5839A>G, 5845A>G, 5848A>G, 5853A>G, 5857T>C, 5860C>T, 5863A>T, 5869T>A, 5875T>C, 5884C>T, 5893C>T, 5894A>G, 5896C>G, 5899C>T, 5908G>A, 5911T>C, 5923G>A, 5938A>G, 5941C>T, 5944T>C, 5947T>C, 5959G>A, 5960C>G, 5961G>C, 5962G>C, 5974A>G, 5986T>A, 5989C>T, 5990A>G, 5998T>C, 6008A>C, 6010A>T, 6019A>T, 6022G>T, 6025A>T, 6030A>C, 6049C>A, 6058G>A, 6061A>G, 6064C>A, 6070T>C, 6072G>A, 6073T>C, 6074A>C, 6079G>A, 6081C>A, 6085A>T, 6091A>T, 6094C>T, 6111G>C, 6112T>C, 6118A>G, 6124G>A, 6127G>A, 6128A>G, 6130T>C, 6133T>C, 6136A>G, 6148C>T, 6157A>G, 6158G>A, 6163T>C, 6169A>G, 6170A>T, 6172A>G, 6178C>T, 6187A>G, 6190T>C, 6196G>A, 6203C>T, 6205C>T, 6208A>G, 6209C>T, 6211A>G, 6217G>A, 6229T>A, 6230G>A, 6238C>T, 6247G>A, 6253A>T, 6257G>A, 6258C>A, 6274T>C, 6282A>G, 6286G>A, 6298C>T, 6301G>A, 6304A>G, 6307C>T, 6313C>T, 6322C>T, 6328A>T, 6331T>G, 6334T>C, 6337G>A, 6338C>T, 6358T>C, 6361G>A, 6362G>A, 6367A>T, 6370G>A, 6376C>T, 6379T>G, 6385A>C, 6391A>T, 6394A>G, 6397C>T, 6401G>A, 6405C>T, 6412C>T, 6418A>G, 6421T>C, 6429A>T, 6436T>A, 6445G>A, 6450A>G, 6454C>A, 6460C>T, 6463T>A, 6469C>T, 6472G>A, 6475G>A, 6481T>C, 6485C>A, 6487C>T, 6490T>C, 6496C>T, 6502G>A, 6514T>C, 6517G>A, 6527C>A, 6532C>A, 6541G>A, 6544A>G, 6556C>T, 6559T>C, 6565G>A, 6569T>C, 6571G>A, 6586T>A, 6588C>T, 6598A>G, 6601A>G, 6604T>C, 6607T>C, 6616A>G, 6622C>T, 6631G>A, 6634G>A, 6635T>C, 6640T>A, 6646G>C, 6647A>G, 6652C>T, 6664C>T, 6670T>G, 6677A>G, 6679A>G, 6685C>T, 6691A>T, 6694C>T, 6715A>G, 6716G>A, 6719A>G, 6724A>G, 6734C>T, 6742G>A, 6745G>A, 6754C>T, 6760A>G, 6764A>T, 6766A>G, 6775T>A, 6782C>T, 6787A>T, 6790C>T, 6795C>T, 6796A>G, 6799C>A, 6802C>A, 6808C>T, 6811C>T, 6814C>T, 6823C>T, 6829A>G, 6843A>G, 6847C>T, 6853A>C, 6857A>G, 6862G>A, 6871A>G, 6875G>A, 6876T>C, 6877C>A, 6880G>A, 6883T>A, 6889T>G, 6901C>T, 6916C>T, 6919G>A, 6925A>G, 6928T>C, 6934C>A, 6949G>C, 6955C>T, 6958A>G, 6976A>G, 6980C>T, 6985C>T, 6997A>T, 7000G>T, 7001G>A, 7003G>A, 7012T>C, 7015G>A, 7018T>C, 7032T>A, 7039C>T, 7040T>C, 7042G>A, 7048A>G, 7060T>A, 7063C>A, 7078C>T, 7082T>C, 7084A>G, 7087A>G, 7090C>A, 7096T>C, 7099A>G, 7102T>C, 7111G>A, 7117A>G, 7125C>A, 7129A>G, 7132T>C, 7133C>T, 7135G>A, 7141G>A, 7144T>C, 7145G>T, 7147A>G, 7150G>A, 7156G>A, 7159A>T, 7160_7165delGAGGCA, 7171C>T, 7189G>T, 7198A>G, 7201C>T, 7205C>T, 7210T>C, 7216T>A, 7219G>A, 7225C>A, 7228G>A, 7240G>A, 7243T>C, 7249A>G, 7252T>G, 7258T>A, 7261T>C, 7264T>A, 7267C>G, 7270G>A, 7273_7274insT, 7274C>G, 7275G>T, 7281A>T, 7284T>C, 7296C>T, 7311C>T, 7314C>T, 7317G>A, 7319delA, 7321T>A, 7327C>T, 7330C>T, 7333C>T, 7334A>C, 7342T>C, 7343G>A, 7344C>A, 7345A>C, 7354T>C, 7363A>G, 7372C>T, 7378G>T, 7381C>T, 7396A>T, 7399C>T, 7405C>T, 7407G>T, 7408T>G, 7414G>T, 7415T>G, 7420A>G, 7429C>T, 7432T>C, 7441T>C, 7459G>A, 7462A>G, 7468A>T, 7471C>A, 7477A>G, 7480C>T, 7486G>T, 7495T>C, 7498A>G, 7501T>C, 7503T>C, 7504G>A, 7513T>A, 7519C>T, 7522A>G, 7531G>A, 7537G>A, 7540C>T, 7546T>C, 7570G>A, 7573G>A, 7579G>A, 7588T>C, 7595C>T, 7604T>C, 7606G>A, 7615T>A, 7624T>C, 7633C>T, 7636C>T, 7639T>C, 7648T>C, 7666A>G, 7672T>C, 7678A>T, 7684G>A, 7690T>C, 7696T>C, 7702T>G, 7709G>A, 7713A>G, 7714A>G, 7715C>T, 7726T>C, 7735G>A, 7744A>G, 7747C>T, 7762C>T, 7768G>A, 7771T>C, 7780A>G, 7783A>C, 7786T>C, 7793C>T, 7795A>G, 7798C>T, 7804T>C, 7812C>T, 7816C>T, 7834A>G, 7837C>T, 7844G>T, 7846G>A, 7855G>A, 7861G>A, 7868T>C, 7870A>G, 7873C>T, 7876T>A, 7879G>A, 7882T>A, 7883G>A, 7888C>T, 7894A>T, 7897T>C, 7921C>T, 7927G>A, 7936T>C, 7939T>C, 7942T>C, 7966G>A, 7969T>C, 7975T>C, 7987C>T, 7990T>C, 8002C>T, 8005T>C, 8032A>G, 8038C>T, 8041C>T, 8043G>A, 8080G>A, 8089G>A, 8095G>A, 8098G>A, 8107C>T, 8110T>C, 8116T>C, 8128A>G, 8146T>C, 8151A>G, 8152C>T, 8164G>A, 8178G>A, 8182G>A, 8191G>A, 8197C>T, 8203C>T, 8208G>A, 8212C>G, 8213C>T, 8218A>G, 8221C>T, 8224G>A, 8239A>G, 8260T>C, 8263A>G, 8281T>C, 8284A>G, 8294C>G, 8296A>G, 8299G>A, 8302C>T, 8317T>C, 8320C>T, 8326T>A, 8335G>A, 8338C>T, 8349C>T, 8353A>G, 8362C>T, 8365A>G, 8380C>T, 8383A>G, 8389A>G, 8404T>C, 8408G>T, 8413A>G, 8419G>T, 8434T>C, 8440T>C, 8452G>A, 8458A>G, 8460G>A, 8464A>G, 8467G>A, 8480G>A, 8482T>A, 8497G>A, 8498C>T, 8500A>G, 8502G>A, 8503A>G, 8506C>T, 8515A>T, 8524C>T, 8527A>C, 8528T>C, 8530A>G, 8539G>C, 8546C>T, 8547T>G, 8552T>A, 8563A>G, 8574C>G, 8585A>G, 8598G>A, 8602C>T, 8603A>T, 8604A>C, 8605C>T, 8616C>T, 8620A>G, 8622C>A, 8625A>G, 8626G>A, 8629G>A, 8632C>T, 8638C>T, 8641G>A, 8647C>T, 8648C>T, 8649C>T, 8650G>C, 8653C>T, 8656A>G, 8658G>A, 8664A>C, 8665T>G, 8674T>G, 8679C>T, 8680A>T, 8683C>A, 8692G>A, 8695A>T, 8707G>A, 8708A>C, 8716G>A, 8722C>T, 8725G>A, 8731A>G, 8737A>G, 8743C>T, 8745C>A, 8746A>G, 8749C>T, 8752G>A, 8755A>T, 8770C>T, 8771T>C, 8776A>G, 8779T>C, 8782G>A, 8791T>C, 8797A>G, 8800A>G, 8803A>G, 8809T>C, 8812T>C, 8818T>C, 8824G>A, 8827A>G, 8848G>A, 8854T>C, 8856A>G, 8857G>A, 8863G>A, 8865G>A, 8867A>C, 8869G>T, 8872A>G, 8883G>A, 8888A>T, 8929C>T, 8954G>A, 8959C>T, 8962A>T, 8983G>A, 8984A>G, 8986T>C, 8988A>T, 8998G>A, 9004G>A, 9007G>A, 9016C>T, 9037C>A, 9085A>T, 9097C>T, 9136A>T, 9145G>T, 9149A>G, 9150T>G, 9151G>A, 9152G>A, 9157A>G, 9160G>T, 9163T>C, 9184G>A, 9196A>G, 9214G>A, 9217A>G, 9220G>A, 9244A>T, 9247G>A, 9256C>T, 9260C>T, 9262T>C, 9274G>A, 9280C>T, 9289C>T, 9304G>A, 9334C>T, 9364C>G, 9377_9378insT, 9387C>T, 9404G>A, 9411C>T, 9412A>G, 9414A>T, 9437C>T, 9454A>G, 9458T>C, 9467G>A, 9470_9471insT, 9482G>A, 9483C>T, 9498T>C, 9516T>G, 9517delA, 9533G>A, 9534G>T, 9548A>T, 9551C>T, 9553_9554delTA, 9556A>T, 9557T>A, 9558A>T, 9574T>A, 9581G>T, 9582T>G, 9583G>C, 9584_9585insG, 9590C>T, 9591T>C, 9607C>T, 9610T>C, 9615delC, 9620A>G, 9648A>G, 9690C>T

	Begin	End	Coverage	Score	Concordance	Matches	Identities	I/D/M/F*	Stop Codons
**NT**	**19**	**9704**	**99.8%**	**12534**	**65.0%**	**9674 (99.7%)**	**8011 (82.6%)**	**15/12**	
**CDS**									
PVYgp1	1	3064	100%	18956	89.8%	3062 (99.9%)	2776 (90.5%)	2/2/4/4	1
Protein mutations:	Y4Q (194T>C 196C>A), C9L (209T>C 210G>T 211T>G), F13M (221T>A 223T>G), S23P (251T>C), C24F (255G>T 256C>T), E25G (258A>G), I27V (263A>G 265T>G), V28A (267T>C), E30V (273A>T), A35T (287G>A 289T>C), V37T (293G>A 294T>C), A41I (305G>A 306C>T), D42G (309A>G), E44D (316A>T), T45V (317A>G 318C>T 319A>G), L52Q (339T>A 340C>A), K53R (342A>G), K55A (347A>G 348A>C 349A>G), Y56H (350T>C), T58V (356A>G 357C>T), V61T (365G>A 366T>C 367G>A), L62S (368C>T 369T>C), F67R (383T>C 384T>G), A75V (408C>T 409C>T), M78E (416A>G 417T>A), E85K (437G>A), R86E (440A>G 441G>A 442G>A), K87R (443A>C 444A>G 445G>A), D88E (448T>A), E91A (456A>C), H93N (461C>A), D111E (517T>A), S116F (531C>T), P118S (536C>T), Q119W (539C>T 540A>G 541A>G), R122K (549G>A 550A>G), V134A (585T>C 586C>G), R137Y (593C>T 594G>A 595C>T), P138R (597C>G), I139V (599A>G 601A>G), I140P (602A>C 603T>C 604A>G), M147I (625G>A), I151V (635A>G 637T>C), I154V (644A>G), E160A (663A>C 664G>C), H166Q (682C>A), T172S (699C>G), H174Q (706T>A), K179E (719A>G 721G>A), I180V (722A>G 724A>G), A183S (731G>T), Y184P (734T>C 735A>C 736C>T), S185R (737T>C 738C>G 739C>T), A187V (744C>T 745G>C), R189C (749C>T 751A>C), M194K (765T>A), R197K (773C>A 774G>A 775A>G), M206K (801T>A), G210A (813G>C 814A>G), L211H (816T>A), R214Q (825G>A 826T>A), R226C (860C>T 862C>T), T227A (863A>G 865T>C), I228T (867T>C), N229D (869A>G 871C>T), I230L (872A>T 874A>G), R232K (879G>A), N240S (903A>G), T241N (906C>A 907A>C), K242T (909A>C 910A>C), S243N (912G>A 913C>T), S252L (938T>C 939C>T 940A>T), L266I (980T>A 982G>C), R272K (999G>A), Q275R (1007C>A 1008A>G 1009G>A), S276G (1010A>G 1012T>G), I277V (1013A>G), N279Q (1019A>C 1021C>G), D288E (1048C>A), R300Q (1083G>A), S305A (1097T>G 1099G>A), R320K (1143G>A), L324I (1154T>A 1156G>A), S346N (1221G>A), V349T (1229G>A 1230T>C 1231T>C), S350N (1233G>A 1234C>T), K358T (1257A>C 1258A>G), N365S (1278A>G), A369V (1290C>T 1291G>A), D372T (1298G>A 1299A>C 1300C>T), I375V (1307A>G 1309A>G), N378E (1316A>G 1318T>A), I382T (1329T>C), L397I (1373C>A), I401V (1385A>G), E407D (1405G>T), A411S (1415G>T 1417A>T), V418I (1436G>A 1438C>T), E508G (1707A>G), A512T (1718G>A), I520V (1742A>G 1744C>T), S525N (1758G>A), V536I (1790G>A), I585V (1937A>G 1939T>C), F607Y (2004T>A), I628M (2068T>G), V630I (2072G>A 2074G>A), R675K (2208G>A), D694E (2266C>G), N744G (2414A>G 2415A>G 2416T>G), S746C (2420A>T), K780R (2523A>G), R817K (2634G>A), V847I (2723G>A), A855T (2747G>A 2749T>A), H871N (2795C>A), V880I (2822G>A 2824G>A), P900T (2882C>A), S901H (2885A>C 2886G>A), T904M (2895C>T), N916S (2931A>G), N919S (2940A>G), E929_N930insX (2971_2972insA), T936I (2991C>T), H937R (2994A>G), E939R (2999G>A 3000A>G), R946Q (3021G>A), Y947F (3024A>T 3025C>T), T951I (3036C>T), E952G (3039A>G 3040A>C), K953T (3042A>C 3043G>A), A972V (3099C>T 3100C>A), V974R (3104G>A 3105T>G 3106G>A), S983N (3132G>A), R985K (3137C>A 3138G>A 3139A>G), F990I (3152T>A 3154C>T), C994Y (3165G>A), F1012L (3218T>C), V1016I (3230G>A), V1028M (3266G>A 3268A>G), V1031M (3275G>A), A1034T (3284G>A), R1044K (3315G>A 3316A>G), E1045A (3318A>C), M1059V (3359A>G), D1072E (3400T>G), V1110G (3513T>G), M1121V (3545A>G), A1122T (3548G>A), V1124I (3554G>A 3556C>T), L1147V (3623C>G), L1150M (3632C>A), Y1152H (3638T>C), I1172V (3698A>G), S1178N (3717G>A), R1183K (3731C>A 3732G>A), I1297V (4073A>G), F1315Y (4128T>A 4129C>T), A1323S (4151G>T), V1328I (4166G>A), L1347I (4223C>A 4225G>A), I1379V (4319A>G 4321A>G), F1404Y (4395T>A), V1408I (4406G>A), M1431V (4475A>G 4477G>T), T1433S (4481A>T), A1454L (4544G>T 4545C>T 4546G>A), R1455K (4548G>A), V1499I (4679G>A 4681G>C), V1538I (4796G>A 4798C>T), T1540S (4803C>G 4804T>C), S1625G (5057A>G), T1626A (5060A>G), L1636I (5090C>A), A1639V (5100C>T), E1641D (5107A>T), V1646F (5120G>T 5122C>T), I1648V (5126A>G), V1667I (5183G>A), A1793T (5561G>A 5563G>A), I1805V (5597A>G), N1807K (5605C>A), A1812V (5619C>T 5620T>G), I1816L (5630A>C), V1837I (5693G>A), K1890R (5853A>G), I1904V (5894A>G 5896C>G), R1926A (5960C>G 5961G>C 5962G>C), I1936V (5990A>G), E1945D (6019A>T), K1949T (6030A>C), D1955E (6049C>A), M1958I (6058G>A), S1963N (6072G>A 6073T>C), N1964H (6074A>C), T1966N (6081C>A), C1976S (6111G>C 6112T>C), I1982V (6128A>G 6130T>C), V1992I (6158G>A), T1996S (6170A>T 6172A>G), L2007F (6203C>T 6205C>T), V2016I (6230G>A), A2025K (6257G>A 6258C>A), K2033R (6282A>G), A2060T (6362G>A), V2073I (6401G>A), A2074V (6405C>T), Y2082F (6429A>T), Q2089R (6450A>G), H2101N (6485C>A 6487C>T), L2115I (6527C>A), T2135I (6588C>T), Y2151H (6635T>C), I2155V (6647A>G), I2165V (6677A>G 6679A>G), D2178N (6716G>A), N2179D (6719A>G), I2194L (6764A>T 6766A>G), A2204V (6795C>T 6796A>G), H2205Q (6799C>A), K2220R (6843A>G), N2225D (6857A>G), V2231T (6875G>A 6876T>C 6877C>A), D2250E (6934C>A), V2273I (7001G>A 7003G>A), F2283Y (7032T>A), T2314K (7125C>A), A2321S (7145G>T 7147A>G), E2326_A2327del (7160_7165delGAGGCA), D2344E (7216T>A), D2358E (7258T>A), D2363_R2364insX (7273_7274insT), R2364V (7274C>G 7275G>T), H2366L (7281A>T), L2367S (7284T>C), S2371L (7296C>T), S2376F (7311C>T), T2377I (7314C>T), C2378Y (7317G>A), K2384Q (7334A>C), A2387N (7343G>A 7344C>A 7345A>C), S2408M (7407G>T 7408T>G), C2411G (7415T>G), L2440S (7503T>C 7504G>A), D2509N (7709G>A), K2510R (7713A>G 7714A>G), T2543I (7812C>T), V2554L (7844G>T 7846G>A), V2567I (7883G>A), S2620N (8043G>A), N2656S (8151A>G 8152C>T), R2665K (8178G>A), R2675K (8208G>A), P2704A (8294C>G 8296A>G), S2722F (8349C>T), A2742S (8408G>T), M2756I (8452G>A), R2759K (8460G>A), A2766T (8480G>A 8482T>A), R2773K (8502G>A 8503A>G), E2777D (8515A>T), E2785D (8539G>C), L2788C (8546C>T 8547T>G), S2790T (8552T>A), A2797G (8574C>G), I2801V (8585A>G), G2805E (8598G>A), N2807S (8603A>T 8604A>C 8605C>T), A2811V (8616C>T), P2813Q (8622C>A), E2814G (8625A>G 8626G>A), P2822F (8648C>T 8649C>T 8650G>C), G2825E (8658G>A), D2827A (8664A>C 8665T>G), A2832V (8679C>T 8680A>T), T2854K (8745C>A 8746A>G), E2891G (8856A>G 8857G>A), R2894Q (8865G>A), M2895L (8867A>C 8869G>T), G2900E (8883G>A), T2902S (8888A>T), V2924I (8954G>A), N2934D (8984A>G 8986T>C), E2935V (8988A>T), M2989G (9149A>G 9150T>G 9151G>A), G2990S (9152G>A), P3026S (9260C>T 9262T>C)								

	Begin	End	Coverage	Score	Concordance	Matches	Identities	I/D/M/F*	Stop Codons
**NT**	**19**	**9704**	**99.8%**	**12534**	**65.0%**	**9674 (99.7%)**	**8011 (82.6%)**	**15/12**	

Codon mutations:

ACT3ACC (193T>C), TAC4CAA (194T>C 196C>A), TCA6TCG (202A>G), ATC8ATT (208C>T), TGT9CTG (209T>C 210G>T 211T>G), TCG12TCA (220G>A), TTT13ATG (221T>A 223T>G), GAA14GAG (226A>G), CTA17CTT (235A>T), TAC19TAT (241C>T), CCA21CCT (247A>T), TCT23CCT (251T>C), TGC24TTT (255G>T 256C>T), GAG25GGG (258A>G), CAT26CAC (262T>C), ATT27GTG (263A>G 265T>G), GTG28GCG (267T>C), GAA30GTA (273A>T), CGA31CGT (277A>T), GAA32GAG (280A>G), GTG33GTT (283G>T), CCG34CCA (286G>A), GCT35ACC (287G>A 289T>C), GTT37ACT (293G>A 294T>C), CCT39CCC (301T>C), TTC40TTT (304C>T), GCA41ATA (305G>A 306C>T), GAT42GGT (309A>G), CTG43TTG (311C>T), GAA44GAT (316A>T), ACA45GTG (317A>G 318C>T 319A>G), CAA46CAG (322A>G), GCA49GCT (331A>T), CTC52CAA (339T>A 340C>A), AAG53AGG (342A>G), AAA55GCG (347A>G 348A>C 349A>G), TAT56CAT (350T>C), GCT57GCA (355T>A), ACT58GTT (356A>G 357C>T), CGT60CGC (364T>C), GTG61ACA (365G>A 366T>C 367G>A), CTC62TCC (368C>T 369T>C), AAA63AAG (373A>G), AAC64AAT (376C>T), TTT67CGT (383T>C 384T>G), ACG68ACA (388G>A), TAC69TAT (391C>T), CGA70CGG (394A>G), TAC71TAT (397C>T), AAG72AAA (400G>A), GAT74GAC (406T>C), GCC75GTT (408C>T 409C>T), CAG76CAA (412G>A), ATA77ATT (415A>T), ATG78GAG (416A>G 417T>A), ATT80ATC (424T>C), CAG81CAA (427G>A), AAA83AAG (433A>G), CTG84CTC (436G>C), GAG85AAG (437G>A), AGG86GAA (440A>G 441G>A 442G>A), AAG87CGA (443A>C 444A>G 445G>A), GAT88GAA (448T>A), GAA90GAG (454A>G), GAA91GCA (456A>C), TAT92TAC (460T>C), CAC93AAC (461C>A), CAA95CAG (469A>G), GCC97GCT (475C>T), CCT99CCA (481T>A), ACT106ACC (502T>C), ATA107ATT (505A>T), GGC109GGT (511C>T), GAT111GAA (517T>A), CCT112CCA (520T>A), CCA113CCT (523A>T), TCT116TTT (531C>T), GAG117GAA (535G>A), CCA118TCA (536C>T), CAA119TGG (539C>T 540A>G 541A>G), AGA122AAG (549G>A 550A>G), ATC124ATT (556C>T), ATT125ATC (559T>C), CAT126CAC (562T>C), ACT128ACC (568T>C), CCA129CCG (571A>G), CGT132CGC (580T>C), GTC134GCG (585T>C 586C>G), CGC137TAT (593C>T 594G>A 595C>T), CCC138CGC (597C>G), ATA139GTG (599A>G 601A>G), ATA140CCG (602A>C 603T>C 604A>G), ACA143ACG (613A>G), GAA144GAG (616A>G), GGC145GGG (619C>G), CAG146CAA (622G>A), ATG147ATA (625G>A), AAT148AAC (628T>C), CAC149CAT (631C>T), ATT151GTC (635A>G 637T>C), ATA154GTA (644A>G), ATT157ATC (655T>C), TCG159TCA (661G>A), GAG160GCC (663A>C 664G>C), AAA161AAG (667A>G), GGG163GGC (673G>C), GTC165GTT (679C>T), CAC166CAA (682C>A), TTA167CTA (683T>C), AGT169AGC (691T>C), ACC172AGC (699C>G), ACT173ACA (703T>A), CAT174CAA (706T>A), AAG179GAA (719A>G 721G>A), ATA180GTG (722A>G 724A>G), CTT181TTG (725C>T 727T>G), GGT182GGA (730T>A), GCA183TCA (731G>T), TAC184CCT (734T>C 735A>C 736C>T), TCC185CGT (737T>C 738C>G 739C>T), GCG187GTC (744C>T 745G>C), CGA189TGC (749C>T 751A>C), ACT190ACC (754T>C), ATG194AAG (765T>A), TTG196CTG (770T>C), CGA197AAG (773C>A 774G>A 775A>G), CGG198AGG (776C>A), TTC202TTT (790C>T), ATG206AAG (801T>A), GGA210GCG (813G>C 814A>G), CTT211CAT (816T>A), TTG212CTG (818T>C), CAA213CAG (823A>G), CGT214CAA (825G>A 826T>A), CGG217AGA (833C>A 835G>A), GAC219GAT (841C>T), AAT223AAC (853T>C), GTC225GTT (859C>T), CGC226TGT (860C>T 862C>T), ACT227GCC (863A>G 865T>C), ATC228ACC (867T>C), AAC229GAT (869A>G 871C>T), ATA230TTG (872A>T 874A>G), AGG232AAG (879G>A), GAT234GAC (886T>C), ATC238ATT (898C>T), AAC240AGC (903A>G), ACA241AAC (906C>A 907A>C), AAA242ACC (909A>C 910A>C), AGC243AAT (912G>A 913C>T), GGC246GGA (922C>A), GGT249GGA (931T>A), TCA252CTT (938T>C 939C>T 940A>T), GGA253GGG (943A>G), TTC256TTT (952C>T), TCA261TCG (967A>G), GAA263GAG (973A>G), GGG264GGC (976G>C), TTG266ATC (980T>A 982G>C), GCA269GCG (991A>G), CGT270CGC (994T>C), AGA272AAA (999G>A), GTT273GTC (1003T>C), CAG275AGA (1007C>A 1008A>G 1009G>A), AGT276GGG (1010A>G 1012T>G), ATT277GTT (1013A>G), TTA278TTG (1018A>G), AAC279CAG (1019A>C 1021C>G), ATC282ATT (1030C>T), TCG285TCA (1039G>A), AAT286AAC (1042T>C), GCC287GCA (1045C>A), GAC288GAA (1048C>A), AAT289AAC (1051T>C), AAG292AAA (1060G>A), GGT293GGA (1063T>A), GAC295GAT (1069C>T), CGA300CAA (1083G>A), TAT303TAC (1093T>C), TCG305GCA (1097T>G 1099G>A), CAC307CAT (1105C>T), GTA310GTG (1114A>G), GCT311GCG (1117T>G), TTA313TTG (1123A>G), CCT314CCA (1126T>A), GTC315GTT (1129C>T), AGG320AAG (1143G>A), GTA321GTT (1147A>T), GCT322GCA (1150T>A), TTG324ATA (1154T>A 1156G>A), CAC327CAT (1165C>T), AGT328AGC (1168T>C), CTT330TTA (1172C>T 1174T>A), ACC339ACT (1201C>T), AGC346AAC (1221G>A), GTT349ACC (1229G>A 1230T>C 1231T>C), AGC350AAT (1233G>A 1234C>T), CTG352CTA (1240G>A), AAG354AAA (1246G>A), TTG356CTA (1250T>C 1252G>A), CAT357CAC (1255T>C), AAA358ACG (1257A>C 1258A>G), AGA361AGG (1267A>G), GGT363GGC (1273T>C), TTG364TTA (1276G>A), AAT365AGT (1278A>G), TTG367CTG (1283T>C), GCG369GTA (1290C>T 1291G>A), GAC372ACT (1298G>A 1299A>C 1300C>T), TTT374TTC (1306T>C), ATA375GTG (1307A>G 1309A>G), CAT376CAC (1312T>C), AAT378GAA (1316A>G 1318T>A), TTG381TTA (1327G>A), ATA382ACA (1329T>C), GCG383GCT (1333G>T), TTA384TTG (1336A>G), CTA387TTG (1343C>T 1345A>G), CCG390CCA (1354G>A), GTG391GTT (1357G>T), GAC392GAT (1360C>T), CTC395TTG (1367C>T 1369C>G), CTT397ATT (1373C>A), TTC398TTT (1378C>T), GAG400GAA (1384G>A), ATA401GTA (1385A>G), AAA403AAG (1393A>G), TCC404TCT (1396C>T), GAG407GAT (1405G>T), AAA408AAG (1408A>G), CAG409CAA (1411G>A), GCA411TCT (1415G>T 1417A>T), CCG412CCC (1420G>C), AAT415AAC (1429T>C), TTA416TTG (1432A>G), GTC418ATT (1436G>A 1438C>T), TTA419CTG (1439T>C 1441A>G), AAT420AAC (1444T>C), TTC423TTT (1453C>T), CTG424CTT (1456G>T), GAA428GAG (1468A>G), TTG439CTA (1499T>C 1501G>A), AGT440AGC (1504T>C), TTG441TTA (1507G>A), CTC442CTT (1510C>T), GAA443GAG (1513A>G), TTA444TTG (1516A>G), GCA445GCT (1519A>T), AGG446AGA (1522G>A), TTC447TTT (1525C>T), CAG448CAA (1528G>A), AAC450AAT (1534C>T), ACT452ACA (1540T>A), AAC454AAT (1546C>T), AAA457AAG (1555A>G), GAT459GAC (1561T>C), ATA460ATC (1564A>C), TCT461TCA (1567T>A), TTC463TTT (1573C>T), AGA464AGG (1576A>G), TTA467CTA (1583T>C), TCT468TCA (1588T>A), AAG470AAA (1594G>A), AAC472AAT (1600C>T), CTG475CTA (1609G>A), TCG478TCA (1618G>A), TGC479TGT (1621C>T), GAC480GAT (1624C>T), AAC481AAT (1627C>T), CTG483TTG (1631C>T), AAA485AAG (1639A>G), TTC489TTT (1651C>T), CTC490CTG (1654C>G), CAT497CAC (1675T>C), TCA503TCG (1693A>G), TTT506TTC (1702T>C), GAA508GGA (1707A>G), GCA512ACA (1718G>A), GGA514GGG (1726A>G), TAC515TAT (1729C>T), TCA516TCC (1732A>C), TAT518TAC (1738T>C), ATC520GTT (1742A>G 1744C>T), CGC521CGT (1747C>T), CAT523CAC (1753T>C), AGT525AAT (1758G>A), AGG528AGA (1768G>A), AAC534AAT (1786C>T), TTA535TTG (1789A>G), GTT536ATT (1790G>A), GTC537GTT (1795C>T), TTA541CTG (1805T>C 1807A>G), GAG543GAA (1813G>A), AGG545AGA (1819G>A), AAG547AAA (1825G>A), GAC551GAT (1837C>T), TAT552TAC (1840T>C), CAA555CAG (1849A>G), GTC558GTG (1858C>G), AAA560AAG (1864A>G), TGC562TGT (1870C>T), AGT564AGC (1876T>C), AAA566AAG (1882A>G), GGT568GGA (1888T>A), AAT569AAC (1891T>C), TGT574TGC (1906T>C), ACA577ACT (1915A>T), GGT582GGC (1930T>C), GCC584GCT (1936C>T), ATT585GTC (1937A>G 1939T>C), TCA587TCG (1945A>G), ACA588ACG (1948A>G), TTC589TTT (1951C>T), CCA592CCT (1960A>T), GTA598GTG (1978A>G), ATT599ATA (1981T>A), GGC600GGT (1984C>T), AAT601AAC (1987T>C), GAC604GAT (1996C>T), TTT607TAT (2004T>A), AAA612AAG (2020A>G), GGG613GGA (2023G>A), GAT614GAC (2026T>C), TCG615TCT (2029G>T), GAG616GAA (2032G>A), TAT625TAC (2059T>C), ATT628ATG (2068T>G), AAC629AAT (2071C>T), GTG630ATA (2072G>A 2074G>A), CTG635TTA (2087C>T 2089G>A), AGC639AGT (2101C>T), GAG640GAA (2104G>A), GAG641GAA (2107G>A), AAG644AAA (2116G>A), TTC646TTT (2122C>T), CGC651CGT (2137C>T), CTT658CTC (2158T>C), ACC660ACT (2164C>T), CCA662CCG (2170A>G), GAT666GAC (2182T>C), GCG668GCA (2188G>A), ACC669ACA (2191C>A), AGA675AAA (2208G>A), TAT678TAC (2218T>C), GAC680GAT (2224C>T), GTA681GTT (2227A>T), TTG686TTA (2242G>A), CCC687CCT (2245C>T), TTG690TTA (2254G>A), GTT691GTC (2257T>C), GAC692GAT (2260C>T), GAC694GAG (2266C>G), CAA696CAG (2272A>G), ACG697ACA (2275G>A), TCA703TCG (2293A>G), GGC705GGT (2299C>T), TCG706TCA (2302G>A), CAG707CAA (2305G>A), GGA710GGT (2314A>T), TAT711TAC (2317T>C), ATT713ATC (2323T>C), CTA714CTG (2326A>G), GTG719GTA (2341G>A), CAA721CAG (2347A>G), CTT722CTC (2350T>C), TTG724TTA (2356G>A), AAT727AAC (2365T>C), GAA729GAG (2371A>G), TTA730TTG (2374A>G), GAT733GAC (2383T>C), ATA734ATT (2386A>T), AAA735AAG (2389A>G), CAT736CAC (2392T>C), TAT737TAC (2395T>C), AAT744GGG (2414A>G 2415A>G 2416T>G), GCT745GCA (2419T>A), AGC746TGC (2420A>T), ACA752ACT (2440A>T), GTT761GTC (2467T>C), TCT764TCC (2476T>C), TCG766TCA (2482G>A), CTG769CTA (2491G>A), CTG771CTT (2497G>T), TTG773TTA (2503G>A), AGA778AGG (2518A>G), CCT779CCC (2521T>C), AAG780AGG (2523A>G), AGA783AGG (2533A>G), TTG785TTA (2539G>A), TTA787TTG (2545A>G), GAG789GAA (2551G>A), CCT790CCA (2554T>A), CTG792TTG (2558C>T), TTG793CTC (2561T>C 2563G>C), CTA795CTG (2569A>G), TCA796TCG (2572A>G), CCT800CCC (2584T>C), GGC801GGT (2587C>T), CTG803CTC (2593G>C), GCT805GCC (2599T>C), AAT808AAC (2608T>C), TTT812TTC (2620T>C), GCG815GCA (2629G>A), GTG816GTA (2632G>A), AGG817AAG (2634G>A), TTG818TTA (2638G>A), ATT820ATA (2644T>A), AAA823AAG (2653A>G), GCT827GCC (2665T>C), CTA832TTA (2678C>T), CTA833CTG (2683A>G), GCT835GCC (2689T>C), CTA838CTC (2698A>C), TCA841TCG (2707A>G), GCG842GCA (2710G>A), GAA844GAG (2716A>G), ACA845ACG (2719A>G), CTC846CTT (2722C>T), GTC847ATC (2723G>A), GCA848GCG (2728A>G), AGG850AGA (2734G>A), ATT851ATA (2737T>A), GAT854GAC (2746T>C), GCT855ACA (2747G>A 2749T>A), GCA856GCT (2752A>T), GCT857GCA (2755T>A), GAC859GAT (2761C>T), CTC860CTT (2764C>T), TGT865TGC (2779T>C), GGG867GGT (2785G>T), AAC869AAT (2791C>T), CAT871AAT (2795C>A), TAC874TAT (2806C>T), TTG878CTC (2816T>C 2818G>C), GTG880ATA (2822G>A 2824G>A), TTG881TTA (2827G>A), GTT884GTC (2836T>C), AAG885AAA (2839G>A), AAT886AAC (2842T>C), ACC893ACA (2863C>A), CTA894TTA (2864C>T), CCA900ACA (2882C>A), AGT901CAT (2885A>C 2886G>A), AAC903AAT (2893C>T), ACG904ATG (2895C>T), GTC906GTT (2902C>T), GTA907GTG (2905A>G), ATT909ATC (2911T>C), AAT916AGT (2931A>G), AAC919AGC (2940A>G), GCT921GCC (2947T>C), GAA929_AAT930insA-- (2971_2972insA), GGT935GGC (2989T>C), ACT936ATT (2991C>T), CAT937CGT (2994A>G), GAG939AGG (2999G>A 3000A>G), TCC944TC- (3016delC), CGG946CAG (3021G>A), TAC947TTT (3024A>T 3025C>T), ACA951ATA (3036C>T), GAA952GGC (3039A>G 3040A>C), AAG953ACA (3042A>C 3043G>A), TTG956TTA (3052G>A), TTA959TTG (3061A>G), GCG966GCA (3082G>A), GGC969GGT (3091C>T), GCC972GTA (3099C>T 3100C>A), GTG974AGA (3104G>A 3105T>G 3106G>A), GTC975GTT (3109C>T), AAA976AAG (3112A>G), ACT978ACC (3118T>C), GCC979GCT (3121C>T), GGA981GGG (3127A>G), AGC983AAC (3132G>A), CGA985AAG (3137C>A 3138G>A 3139A>G), TAT989TAC (3151T>C), TTC990ATT (3152T>A 3154C>T), TGT994TAT (3165G>A), GTA995GTG (3169A>G), ATC1005ATT (3199C>T), AGG1007CGG (3203A>C), CGT1008CGC (3208T>C), CCA1010CCG (3214A>G), ACC1011ACT (3217C>T), TTT1012CTT (3218T>C), GTC1013GTA (3223C>A), GTT1016ATT (3230G>A), AAC1017AAT (3235C>T), TTA1025CTA (3257T>C), ACC1026ACT (3262C>T), AGC1027AGT (3265C>T), GTA1028ATG (3266G>A 3268A>G), GTG1029GTA (3271G>A), GTG1031ATG (3275G>A), GCA1034ACA (3284G>A), CAG1039CAA (3301G>A), AGA1044AAG (3315G>A 3316A>G), GAA1045GCA (3318A>C), GAG1047GAA (3325G>A), TTG1048TTA (3328G>A), ATA1051ATT (3337A>T), GAG1055GAA (3349G>A), GTC1057GTT (3355C>T), TGC1058TGT (3358C>T), ATG1059GTG (3359A>G), CTA1061CTG (3367A>G), TAT1062TAC (3370T>C), TTA1065CTG (3377T>C 3379A>G), GAA1070GAG (3394A>G), CGC1071CGT (3397C>T), GAT1072GAG (3400T>G), ACA1074ACG (3406A>G), TAC1078TAT (3418C>T), ATT1079ATA (3421T>A), TAT1081TAC (3427T>C), AAG1083AAA (3433G>A), TCA1084TCT (3436A>T), GTA1085GTG (3439A>G), AAC1086AAT (3442C>T), CCT1087CCC (3445T>C), CAG1091CAA (3457G>A), TTT1092TTC (3460T>C), GCT1093GCA (3463T>A), GCG1095GCT (3469G>T), CAG1096CAA (3472G>A), CAC1104CAT (3496C>T), CAG1105CAA (3499G>A), TCC1107TCT (3505C>T), GTT1110GGT (3513T>G), AAA1112AAG (3520A>G), GAA1115GAG (3529A>G), CAA1116CAG (3532A>G), ATG1121GTG (3545A>G), GCT1122ACT (3548G>A), TTA1123CTA (3551T>C), GTC1124ATT (3554G>A 3556C>T), AGT1133AGC (3583T>C), GAT1134GAC (3586T>C), TGC1135TGT (3589C>T), TTC1137TTT (3595C>T), AAT1141AAC (3607T>C), TTT1143TTC (3613T>C), AAG1144AAA (3616G>A), GGT1145GGC (3619T>C), CTT1147GTT (3623C>G), TCC1148TCT (3628C>T), CTG1150ATG (3632C>A), GAT1151GAC (3637T>C), TAT1152CAT (3638T>C), GAA1153GAG (3643A>G), TTA1159TTG (3661A>G), GTG1162GTA (3670G>A), TTT1166TTC (3682T>C), GAG1168GAA (3688G>A), GAG1171GAA (3697G>A), ATT1172GTT (3698A>G), GAT1174GAC (3706T>C), TTT1175TTC (3709T>C), TTG1177CTA (3713T>C 3715G>A), AGT1178AAT (3717G>A), GAG1179GAA (3721G>A), CGA1183AAA (3731C>A 3732G>A), ACT1184ACA (3736T>A), CTA1188TTG (3746C>T 3748A>G), ACA1190ACG (3754A>G), AGT1193AGC (3763T>C), CGA1198CGG (3778A>G), CAA1199CAG (3781A>G), ATC1200ATT (3784C>T), CAT1204CAC (3796T>C), ACA1205ACG (3799A>G), CCA1207CCT (3805A>T), TAC1209TAT (3811C>T), AGA1210AGG (3814A>G), TTC1215TTT (3829C>T), TTT1218TTC (3838T>C), AGA1220AGG (3844A>G), GCA1221GCC (3847A>C), GTT1224GTA (3856T>A), GTG1226GTT (3862G>T), GAC1229GAT (3871C>T), GCC1231GCA (3877C>A), AGC1233AGT (3883C>T), GAA1234GAG (3886A>G), CTA1236CTG (3892A>G), GAC1237GAT (3895C>T), TTT1238TTC (3898T>C), GTA1240GTG (3904A>G), CGG1241AGA (3905C>A 3907G>A), GCT1243GCC (3913T>C), AAG1248AAA (3928G>A), TCA1249TCT (3931A>T), GGG1251GGA (3937G>A), GTT1254GTC (3946T>C), AGT1257AGC (3955T>C), GTG1258GTA (3958G>A), GCC1259GCT (3961C>T), GGA1260GGG (3964A>G), TCT1261TCC (3967T>C), TTA1264TTG (3976A>G), ATT1265ATC (3979T>C), CCA1267CCT (3985A>T), CTA1271CTT (3997A>T), GCG1272GCA (4000G>A), AAC1274AAT (4006C>T), GTT1275GTG (4009T>G), TTC1276TTT (4012C>T), AAA1277AAG (4015A>G), TCT1280TCC (4024T>C), GAA1282GAG (4030A>G), CCA1283CCT (4033A>T), AAG1286AAA (4042G>A), CTG1290TTA (4052C>T 4054G>A), CGT1291CGC (4057T>C), CGT1293CGA (4063T>A), GGA1294GGG (4066A>G), ATA1297GTA (4073A>G), TTT1298TTC (4078T>C), GGC1299GGT (4081C>T), TCT1300TCC (4084T>C), TCC1304TCT (4096C>T), AGC1308AGT (4108C>T), GGA1309GGT (4111A>T), GCG1311GCA (4117G>A), CTA1312TTG (4118C>T 4120A>G), TAC1314TAT (4126C>T), TTC1315TAT (4128T>A 4129C>T), GCC1316GCT (4132C>T), AAT1317AAC (4135T>C), CAA1321CAG (4147A>G), TTA1322CTA (4148T>C), GCT1323TCT (4151G>T), AAC1326AAT (4162C>T), GTA1328ATA (4166G>A), ATA1329ATC (4171A>C), GAG1332GAA (4180G>A), CTG1336TTG (4190C>T), CCT1338CCC (4198T>C), TCC1339TCT (4201C>T), GCG1342GCT (4210G>T), TTC1343TTT (4213C>T), AGT1345AGC (4219T>C), CTG1346TTG (4220C>T), CTG1347ATA (4223C>A 4225G>A), GTT1349GTG (4231T>G), CAT1351CAC (4237T>C), GTA1356GTG (4252A>G), TTA1357CTG (4253T>C 4255A>G), AAA1358AAG (4258A>G), ACT1362ACC (4270T>C), AGA1366AGG (4282A>G), GAG1367GAA (4285G>A), GAA1369GAG (4291A>G), CAG1373CAA (4303G>A), GTC1376GTT (4312C>T), ATA1379GTG (4319A>G 4321A>G), GTG1380GTT (4324G>T), GAG1381GAA (4327G>A), CTG1384CTT (4336G>T), TCT1385TCC (4339T>C), TCA1388TCT (4348A>T), GCA1392GCG (4360A>G), GGT1394GGC (4366T>C), TCT1395TCA (4369T>A), ACT1397ACC (4375T>C), GTT1401GTC (4387T>C), TTT1404TAT (4395T>A), GTA1408ATA (4406G>A), CTT1409CTC (4411T>C), GTT1418GTC (4438T>C), ACC1420ACA (4444C>A), GCC1422GCT (4450C>T), CTC1424CTT (4456C>T), GAC1427GAT (4465C>T), ATG1431GTT (4475A>G 4477G>T), GTC1432GTT (4480C>T), ACA1433TCA (4481A>T), GGC1437GGT (4495C>T), AAG1441AAA (4507G>A), CAC1442CAT (4510C>T), GGT1443GGG (4513T>G), CTA1445TTA (4517C>T), GTC1448GTT (4528C>T), AAA1450AAG (4534A>G), ACC1452ACT (4540C>T), GCG1454TTA (4544G>T 4545C>T 4546G>A), AGA1455AAA (4548G>A), TTT1458TTC (4558T>C), GTA1460GTT (4564A>T), ACC1462ACT (4570C>T), ATA1464ATT (4576A>T), TTG1471TTA (4597G>A), GAC1472GAT (4600C>T), GAC1474GAT (4606C>T), GTG1475GTA (4609G>A), GTG1477GTA (4615G>A), GAC1478GAT (4618C>T), TTG1481CTT (4625T>C 4627G>T), GAC1488GAT (4648C>T), ATT1489ATC (4651T>C), AGG1492AGA (4660G>A), AGC1493AGT (4663C>T), GCT1495GCA (4669T>A), GTG1499ATC (4679G>A 4681G>C), AGT1500AGC (4684T>C), GTT1501GTG (4687T>G), GAG1505GAA (4699G>A), AGA1506AGG (4702A>G), CAA1508CAG (4708A>G), AGG1509AGA (4711G>A), CTG1510TTG (4712C>T), AAA1518AAG (4738A>G), GTA1520GTT (4744A>T), GCA1521GCG (4747A>G), TTG1522CTG (4748T>C), ACT1527ACC (4765T>C), GTC1538ATT (4796G>A 4798C>T), ACT1540AGC (4803C>G 4804T>C), GAG1541GAA (4807G>A), GCG1542GCT (4810G>T), GCT1543GCG (4813T>G), CTT1544CTC (4816T>C), TAT1549TAC (4831T>C), AAC1550AAT (4834C>T), CCA1552CCT (4840A>T),

	GGA1556GGG (4852A>G), GGC1557GGT (4855C>T), AGT1561AGC (4867T>C), CTG1562CTC (4870G>C), ATT1563ATA (4873T>A), TGT1566TGC (4882T>C), ACA1573ACT (4903A>T), CAG1575CAA (4909G>A), TTT1577TTC (4915T>C), GAA1578GAG (4918A>G), TTG1579CTA (4919T>C 4921G>A), AGT1580AGC (4924T>C), CCC1581CCG (4927C>G), TTT1583TTC (4933T>C), ATC1584ATA (4936C>A), CAG1585CAA (4939G>A), AAT1586AAC (4942T>C), TTC1587TTT (4945C>T), TCA1593TCG (4963A>G), CCT1596CCC (4972T>C), CTT1602CTC (4990T>C), AAA1606AAG (5002A>G), CCT1613CCC (5023T>C), TTG1614CTG (5024T>C), TGC1615TGT (5029C>T), GAT1616GAC (5032T>C), TCT1618TCC (5038T>C), CCA1620CCT (5044A>T), GCA1623GCT (5053A>T), TCG1624TCA (5056G>A), AGT1625GGT (5057A>G), ACT1626GCT (5060A>G), TCG1629TCT (5071G>T), CTT1636ATT (5090C>A), GGA1637GGG (5095A>G), GTG1638GTT (5098G>T), GCC1639GTC (5100C>T), TTA1640TTG (5104A>G), GAA1641GAT (5107A>T), ATT1642ATC (5110T>C), CCA1643CCG (5113A>G), AAG1644AAA (5116G>A), CAA1645CAG (5119A>G), GTC1646TTT (5120G>T 5122C>T), ATT1648GTT (5126A>G), CAT1651CAC (5137T>C), ATC1652ATT (5140C>T), AAA1653AAG (5143A>G), GAG1654GAA (5146G>A), CTC1659CTA (5161C>A), GAA1661GAG (5167A>G), CTT1663CTG (5173T>G), ACG1666ACA (5182G>A), GTT1667ATT (5183G>A), GTC1668GTT (5188C>T), AAG1669AAA (5191G>A), AAA1671AAG (5197A>G), GAC1672GAT (5200C>T), TGC1674TGT (5206C>T), TTA1675TTG (5209A>G), AGC1678AGT (5218C>T), TCG1682TCA (5230G>A), ATC1684ATT (5236C>T), AGC1685AGT (5239C>T), ATC1687ATT (5245C>T), ACA1693ACT (5263A>T), CTC1695CTT (5269C>T), TTC1696TTT (5272C>T), GCC1697GCA (5275C>A), ATC1698ATT (5278C>T), CCA1699CCC (5281A>C), AGA1700AGG (5284A>G), ACT1701ACC (5287T>C), CTA1702TTA (5288C>T), ATA1703ATT (5293A>T), TTG1704CTA (5294T>C 5296G>A), GAG1706GAA (5302G>A), CTT1709CTA (5311T>A), GAA1710GAG (5314A>G), CGA1713CGT (5323A>T), AGT1721AGC (5347T>C), ATC1723ATT (5353C>T), TGC1727TGT (5365C>T), GTT1734GTC (5386T>C), ACC1737ACA (5395C>A), AAA1746AAG (5422A>G), GAT1747GAC (5425T>C), ACC1749ACT (5431C>T), GAG1751GAA (5437G>A), CTT1756CTC (5452T>C), AAG1758AAA (5458G>A), AGT1761AGC (5467T>C), CAA1762CAG (5470A>G), CTA1763TTG (5471C>T 5473A>G), TTC1766TTT (5482C>T), TCA1767TCC (5485A>C), AAT1768AAC (5488T>C), TTG1769CTA (5489T>C 5491G>A), GGT1771GGC (5497T>C), TGT1774TGC (5506T>C), GAG1775GAA (5509G>A), GAG1776GAA (5512G>A), TAT1782TAC (5530T>C), GAG1783GAA (5533G>A), TCG1784TCT (5536G>T), TTG1785TTA (5539G>A), GTT1788GTG (5548T>G), CAC1790CAT (5554C>T), GCT1792GCA (5560T>A), GCG1793ACA (5561G>A 5563G>A), ACG1794ACC (5566G>C), TCA1795TCG (5569A>G), CTC1800CTG (5584C>G), AAG1803AAA (5593G>A), GGG1804GGA (5596G>A), ATT1805GTT (5597A>G), AAC1807AAA (5605C>A), GTG1811GTT (5617G>T), GCT1812GTG (5619C>T 5620T>G), TTG1815TTA (5629G>A), ATC1816CTC (5630A>C), GGC1819GGT (5641C>T), GCA1822GCT (5650A>T), ATA1826ATT (5662A>T), GGA1827GGG (5665A>G), CAA1835CAG (5689A>G), GTT1837ATT (5693G>A), GAG1838GAA (5698G>A), CAA1843CAG (5713A>G), GGG1844GGC (5716G>C), AAA1845AAG (5719A>G), GCC1853GCA (5743C>A), CGC1857CGA (5755C>A), CAT1858CAC (5758T>C), CGT1860CGC (5764T>C), AAA1862AAG (5770A>G), GGC1865GGT (5779C>T), TTT1866TTC (5782T>C), GAC1869GAT (5791C>T), GAC1873GAT (5803C>T), AGG1884AGA (5836G>A), AAA1885AAG (5839A>G), GGA1887GGG (5845A>G), AAA1888AAG (5848A>G), AAA1890AGA (5853A>G), GGT1891GGC (5857T>C), ACC1892ACT (5860C>T), ACA1893ACT (5863A>T), GGT1895GGA (5869T>A), GGT1897GGC (5875T>C), AGC1900AGT (5884C>T), TTC1903TTT (5893C>T), ATC1904GTG (5894A>G 5896C>G), AAC1905AAT (5899C>T), GGG1908GGA (5908G>A), TTT1909TTC (5911T>C), GAG1913GAA (5923G>A), CAA1918CAG (5938A>G), TTC1919TTT (5941C>T), GTT1920GTC (5944T>C), GAT1921GAC (5947T>C), GGG1925GGA (5959G>A), CGG1926GCC (5960C>G 5961G>C 5962G>C), GAA1930GAG (5974A>G), GCT1934GCA (5986T>A), GAC1935GAT (5989C>T), ATT1936GTT (5990A>G), GAT1938GAC (5998T>C), AGA1942CGT (6008A>C 6010A>T), GAA1945GAT (6019A>T), GTG1946GTT (6022G>T), CGA1947CGT (6025A>T), AAA1949ACA (6030A>C), GAC1955GAA (6049C>A), ATG1958ATA (6058G>A), CAA1959CAG (6061A>G), GCC1960GCA (6064C>A), GGT1962GGC (6070T>C), AGT1963AAC (6072G>A 6073T>C), AAC1964CAC (6074A>C), ACG1965ACA (6079G>A), ACC1966AAC (6081C>A), ATA1967ATT (6085A>T), GCA1969GCT (6091A>T), TAC1970TAT (6094C>T), TGT1976TCC (6111G>C 6112T>C), AAA1978AAG (6118A>G), TTG1980TTA (6124G>A), AAG1981AAA (6127G>A), ATT1982GTC (6128A>G 6130T>C), GAT1983GAC (6133T>C), TTA1984TTG (6136A>G), AAC1988AAT (6148C>T), AAA1991AAG (6157A>G), GTT1992ATT (6158G>A), TGT1993TGC (6163T>C), AAA1995AAG (6169A>G), ACA1996TCG (6170A>T 6172A>G), GGC1998GGT (6178C>T), AAA2001AAG (6187A>G), TTT2002TTC (6190T>C), GAG2004GAA (6196G>A), CTC2007TTT (6203C>T 6205C>T), GAA2008GAG (6208A>G), CTA2009TTG (6209C>T 6211A>G), CAG2011CAA (6217G>A), GCT2015GCA (6229T>A), GTA2016ATA (6230G>A), GTC2018GTT (6238C>T), AAG2021AAA (6247G>A), ATA2023ATT (6253A>T), GCA2025AAA (6257G>A 6258C>A), CAT2030CAC (6274T>C), AAA2033AGA (6282A>G), TCG2034TCA (6286G>A), GGC2038GGT (6298C>T), TTG2039TTA (6301G>A), AGA2040AGG (6304A>G), GAC2041GAT (6307C>T), AAC2043AAT (6313C>T), GCC2046GCT (6322C>T), ACA2048ACT (6328A>T), GTT2049GTG (6331T>G), TGT2050TGC (6334T>C), AGG2051AGA (6337G>A), CTG2052TTG (6338C>T), TAT2058TAC (6358T>C), GGG2059GGA (6361G>A), GCA2060ACA (6362G>A), TCA2061TCT (6367A>T), GAG2062GAA (6370G>A), TAC2064TAT (6376C>T), GGT2065GGG (6379T>G), GGA2067GGC (6385A>C), GGA2069GGT (6391A>T), GCA2070GCG (6394A>G), TAC2071TAT (6397C>T), GTA2073ATA (6401G>A), GCG2074GTG (6405C>T), CAC2076CAT (6412C>T), TTA2078TTG (6418A>G), TTT2079TTC (6421T>C), TAC2082TTC (6429A>T), GGT2084GGA (6436T>A), GAG2087GAA (6445G>A), CAA2089CGA (6450A>G), TCC2090TCA (6454C>A), CAC2092CAT (6460C>T), GGT2093GGA (6463T>A), TTC2095TTT (6469C>T), AGG2096AGA (6472G>A), GTG2097GTA (6475G>A), AAT2099AAC (6481T>C), CAC2101AAT (6485C>A 6487C>T), AGT2102AGC (6490T>C), AGC2104AGT (6496C>T), CTG2106CTA (6502G>A), GGT2110GGC (6514T>C), AGG2111AGA (6517G>A), CTC2115ATC (6527C>A), ATC2116ATA (6532C>A), CCG2119CCA (6541G>A), AAA2120AAG (6544A>G), GTC2124GTT (6556C>T), TTT2125TTC (6559T>C), CAG2127CAA (6565G>A), TTG2129CTA (6569T>C 6571G>A), CCT2134CCA (6586T>A), ACA2135ATA (6588C>T), GAA2138GAG (6598A>G), AGA2139AGG (6601A>G), ATT2140ATC (6604T>C), TGT2141TGC (6607T>C), GGA2144GGG (6616A>G), AAC2146AAT (6622C>T), GAG2149GAA (6631G>A), AAG2150AAA (6634G>A), TAT2151CAT (6635T>C), GCT2152GCA (6640T>A), TCG2154TCC (6646G>C), ATC2155GTC (6647A>G), ATC2156ATT (6652C>T), AGC2160AGT (6664C>T), ACT2162ACG (6670T>G), ATA2165GTG (6677A>G 6679A>G), GGC2167GGT (6685C>T), ACA2169ACT (6691A>T), TTC2170TTT (6694C>T), ACA2177ACG (6715A>G), GAT2178AAT (6716G>A), AAT2179GAT (6719A>G), GGA2180GGG (6724A>G), CTA2184TTA (6734C>T), GTG2186GTA (6742G>A), GTG2187GTA (6745G>A), GCC2190GCT (6754C>T), GGA2192GGG (6760A>G), ATA2194TTG (6764A>T 6766A>G), ATT2197ATA (6775T>A), CTG2200TTG (6782C>T), GCA2201GCT (6787A>T), AAC2202AAT (6790C>T), GCA2204GTG (6795C>T 6796A>G), CAC2205CAA (6799C>A), ACC2206ACA (6802C>A), AAC2208AAT (6808C>T), TAC2209TAT (6811C>T), TAC2210TAT (6814C>T), TTC2213TTT (6823C>T), GAA2215GAG (6829A>G), AAG2220AGG (6843A>G), TAC2221TAT (6847C>T), CGA2223CGC (6853A>C), AAT2225GAT (6857A>G), GAG2226GAA (6862G>A), GAA2229GAG (6871A>G), GTC2231ACA (6875G>A 6876T>C 6877C>A), AAG2232AAA (6880G>A), TCT2233TCA (6883T>A), GTT2235GTG (6889T>G), GAC2239GAT (6901C>T), GGC2244GGT (6916C>T), CCG2245CCA (6919G>A), AAA2247AAG (6925A>G), CTT2248CTC (6928T>C), GAC2250GAA (6934C>A), GGG2255GGC (6949G>C), TTC2257TTT (6955C>T), AAA2258AAG (6958A>G), CAA2264CAG (6976A>G), CTA2266TTA (6980C>T), ATC2267ATT (6985C>T), GTA2271GTT (6997A>T), GTG2272GTT (7000G>T), GTG2273ATA (7001G>A 7003G>A), GCT2276GCC (7012T>C), AAG2277AAA (7015G>A), CAT2278CAC (7018T>C), TTT2283TAT (7032T>A), GCC2285GCT (7039C>T), TTG2286CTA (7040T>C 7042G>A), GGA2288GGG (7048A>G), GCT2292GCA (7060T>A), GTC2293GTA (7063C>A), AGC2298AGT (7078C>T), TTA2300CTG (7082T>C 7084A>G), GTA2301GTG (7087A>G), ACC2302ACA (7090C>A), CAT2304CAC (7096T>C), GTA2305GTG (7099A>G), GTT2306GTC (7102T>C), GAG2309GAA (7111G>A), CGA2311CGG (7117A>G), ACA2314AAA (7125C>A), GAA2315GAG (7129A>G), TTT2316TTC (7132T>C), CTG2317TTA (7133C>T 7135G>A), GTG2319GTA (7141G>A), GAT2320GAC (7144T>C), GCA2321TCG (7145G>T 7147A>G), GAG2322GAA (7150G>A), GAG2324GAA (7156G>A), GCA2325GCT (7159A>T), GAG2326_GCA2327del (7160_7165delGAGGCA), TTC2329TTT (7171C>T), GCG2335GCT (7189G>T), AAA2338AAG (7198A>G), AGC2339AGT (7201C>T), CTA2341TTA (7205C>T), AAT2342AAC (7210T>C), GAT2344GAA (7216T>A), GCG2345GCA (7219G>A), ATC2347ATA (7225C>A), AAG2348AAA (7228G>A), AAG2352AAA (7240G>A), TAT2353TAC (7243T>C), AAA2355AAG (7249A>G), CCT2356CCG (7252T>G), GAT2358GAA (7258T>A), GTT2359GTC (7261T>C), GGT2360GGA (7264T>A), GTC2361GTG (7267C>G), GTG2362GTA (7270G>A), GAT2363_CGG2364insT-- (7273_7274insT), CGG2364GTG (7274C>G 7275G>T), CAT2366CTT (7281A>T), TTG2367TCG (7284T>C), TCA2371TTA (7296C>T), TCT2376TTT (7311C>T), ACC2377ATC (7314C>T), TGC2378TAC (7317G>A), AAT2379-AA (7319delA 7321T>A), CAC2381CAT (7327C>T), GGC2382GGT (7330C>T), TTC2383TTT (7333C>T), AAG2384CAG (7334A>C), TGT2386TGC (7342T>C), GCA2387AAC (7343G>A 7344C>A 7345A>C), ACT2390ACC (7354T>C), CAA2393CAG (7363A>G), TTC2396TTT (7372C>T), GCG2398GCT (7378G>T), CTC2399CTT (7381C>T), GCA2404GCT (7396A>T), GTC2405GTT (7399C>T), GCC2407GCT (7405C>T), AGT2408ATG (7407G>T 7408T>G), GGG2410GGT (7414G>T), TGC2411GGC (7415T>G), AAA2412AAG (7420A>G), GAC2415GAT (7429C>T), TAT2416TAC (7432T>C), CAT2419CAC (7441T>C), AAG2425AAA (7459G>A), GAA2426GAG (7462A>G), ATA2428ATT (7468A>T), GTC2429GTA (7471C>A), CAA2431CAG (7477A>G), AGC2432AGT (7480C>T), CTG2434CTT (7486G>T), TAT2437TAC (7495T>C), AAA2438AAG (7498A>G), GGT2439GGC (7501T>C), TTG2440TCA (7503T>C 7504G>A), ATT2443ATA (7513T>A), AAC2445AAT (7519C>T), GGA2446GGG (7522A>G), AAG2449AAA (7531G>A), GAG2451GAA (7537G>A), CTC2452CTT (7540C>T), TGT2454TGC (7546T>C), AAG2462AAA (7570G>A), ACG2463ACA (7573G>A), ACG2465ACA (7579G>A), GCT2468GCC (7588T>C), CTA2471TTA (7595C>T), TTG2474CTA (7604T>C 7606G>A), GGT2477GGA (7615T>A), TGT2480TGC (7624T>C), GAC2483GAT (7633C>T), TTC2484TTT (7636C>T), AAT2485AAC (7639T>C), TTT2488TTC (7648T>C), GAA2494GAG (7666A>G), TGT2496TGC (7672T>C), ACA2498ACT (7678A>T), GGG2500GGA (7684G>A), ACT2502ACC (7690T>C), TTT2504TTC (7696T>C), GGT2506GGG (7702T>G), GAT2509AAT (7709G>A), AAA2510AGG (7713A>G 7714A>G), CTG2511TTG (7715C>T), CGT2514CGC (7726T>C), GAG2517GAA (7735G>A), GTA2520GTG (7744A>G), TAC2521TAT (7747C>T), GGC2526GGT (7762C>T), CAG2528CAA (7768G>A), TTT2529TTC (7771T>C), TCA2532TCG (7780A>G), CTA2533CTC (7783A>C), ACT2534ACC (7786T>C), CTA2537TTG (7793C>T 7795A>G), ATC2538ATT (7798C>T), GCT2540GCC (7804T>C), ACC2543ATC (7812C>T), ATC2544ATT (7816C>T), GAA2550GAG (7834A>G), GAC2551GAT (7837C>T), GTG2554TTA (7844G>T 7846G>A), CAG2557CAA (7855G>A), CTG2559CTA (7861G>A), TTA2562CTG (7868T>C 7870A>G), TAC2563TAT (7873C>T), ACT2564ACA (7876T>A), GAG2565GAA (7879G>A), ATT2566ATA (7882T>A), GTT2567ATT (7883G>A), TAC2568TAT (7888C>T), CCA2570CCT (7894A>T), ATT2571ATC (7897T>C), GTC2579GTT (7921C>T), AAG2581AAA (7927G>A), GGT2584GGC (7936T>C), AAT2585AAC (7939T>C), AAT2586AAC (7942T>C), GTG2594GTA (7966G>A), GAT2595GAC (7969T>C), TCT2597TCC (7975T>C), GTC2601GTT (7987C>T), CTT2602CTC (7990T>C), TAC2606TAT (8002C>T), GCT2607GCC (8005T>C), GAA2616GAG (8032A>G), ATC2618ATT (8038C>T), GAC2619GAT (8041C>T), AGC2620AAC (8043G>A), TTG2632TTA (8080G>A), GTG2635GTA (8089G>A), CCG2637CCA (8095G>A), GAG2638GAA (8098G>A), AGC2641AGT (8107C>T), ATT2642ATC (8110T>C), GAT2644GAC (8116T>C), CAA2648CAG (8128A>G), GGT2654GGC (8146T>C), AAC2656AGT (8151A>G 8152C>T), TCG2660TCA (8164G>A), AGG2665AAG (8178G>A), AAG2666AAA (8182G>A), TTG2669TTA (8191G>A), TTC2671TTT (8197C>T), TCC2673TCT (8203C>T), AGA2675AAA (8208G>A), GGC2676GGG (8212C>G), CTG2677TTG (8213C>T), CTA2678CTG (8218A>G), ATC2679ATT (8221C>T), GAG2680GAA (8224G>A), CCA2685CCG (8239A>G), ATT2692ATC (8260T>C), GTA2693GTG (8263A>G), GAT2699GAC (8281T>C), AGA2700AGG (8284A>G), CCA2704GCG (8294C>G 8296A>G), GAG2705GAA (8299G>A), CAC2706CAT (8302C>T), ATT2711ATC (8317T>C), TGC2712TGT (8320C>T), GCT2714GCA (8326T>A), GAG2717GAA (8335G>A), TCC2718TCT (8338C>T), TCT2722TTT (8349C>T), GAA2723GAG (8353A>G), CAC2726CAT (8362C>T), CAA2727CAG (8365A>G), TAC2732TAT (8380C>T), TCA2733TCG (8383A>G), TTA2735TTG (8389A>G), CCT2740CCC (8404T>C), GCA2742TCA (8408G>T), ACA2743ACG (8413A>G), GCG2745GCT (8419G>T), GCT2750GCC (8434T>C), TAT2752TAC (8440T>C), ATG2756ATA (8452G>A), CTA2758CTG (8458A>G), AGG2759AAG (8460G>A), AAA2760AAG (8464A>G), CTG2761CTA (8467G>A), GCT2766ACA (8480G>A 8482T>A), GAG2771GAA (8497G>A), CTA2772TTG (8498C>T 8500A>G), AGA2773AAG (8502G>A 8503A>G), GCC2774GCT (8506C>T), GAA2777GAT (8515A>T), GTC2780GTT (8524C>T), GCA2781GCC (8527A>C), TTA2782CTG (8528T>C 8530A>G), GAG2785GAC (8539G>C), CTT2788TGT (8546C>T 8547T>G), TCT2790ACT (8552T>A), GTA2793GTG (8563A>G), GCA2797GGA (8574C>G), ATT2801GTT (8585A>G), GGA2805GAA (8598G>A), AGC2806AGT (8602C>T), AAC2807TCT (8603A>T 8604A>C 8605C>T), GCA2811GTA (8616C>T), AAA2812AAG (8620A>G), CCA2813CAA (8622C>A), GAG2814GGA (8625A>G 8626G>A), CAG2815CAA (8629G>A), GGC2816GGT (8632C>T), ATC2818ATT (8638C>T), CAG2819CAA (8641G>A), AAC2821AAT (8647C>T), CCG2822TTC (8648C>T 8649C>T 8650G>C), AAC2823AAT (8653C>T), AAA2824AAG (8656A>G), GGA2825GAA (8658G>A), GAT2827GCG (8664A>C 8665T>G), GTT2830GTG (8674T>G), GCA2832GTT (8679C>T 8680A>T), GGC2833GGA (8683C>A), GGG2836GGA (8692G>A), ACA2837ACT (8695A>T), CCG2841CCA (8707G>A), AGA2842CGA (8708A>C), AAG2844AAA (8716G>A), ATC2846ATT (8722C>T), ACG2847ACA (8725G>A), AAA2849AAG (8731A>G), AGA2851AGG (8737A>G), CCC2853CCT (8743C>T), ACA2854AAG (8745C>A 8746A>G), AGC2855AGT (8749C>T), AAG2856AAA (8752G>A), GGA2857GGT (8755A>T), AAC2862AAT (8770C>T), TTA2863CTA (8771T>C), GAA2864GAG (8776A>G), CAT2865CAC (8779T>C), TTG2866TTA (8782G>A), TAT2869TAC (8791T>C), CCA2871CCG (8797A>G), CAA2872CAG (8800A>G), CAA2873CAG (8803A>G), GAT2875GAC (8809T>C), ATT2876ATC (8812T>C), AAT2878AAC (8818T>C), CGG2880CGA (8824G>A), GCA2881GCG (8827A>G), ACG2888ACA (8848G>A), TAT2890TAC (8854T>C), GAG2891GGA (8856A>G 8857G>A), GTG2893GTA (8863G>A), CGG2894CAG (8865G>A), ATG2895CTT (8867A>C 8869G>T), GCA2896GCG (8872A>G), GGA2900GAA (8883G>A), ACT2902TCT (8888A>T), TGC2915TGT (8929C>T), GTC2924ATC (8954G>A), AAC2925AAT (8959C>T), GGA2926GGT (8962A>T), GGG2933GGA (8983G>A), AAT2934GAC (8984A>G 8986T>C), GAA2935GTA (8988A>T), GAG2938GAA (8998G>A), CCG2940CCA (9004G>A), TTG2941TTA (9007G>A), ATC2944ATT (9016C>T), ACC2951ACA (9037C>A), ATA2967ATT (9085A>T), AAC2971AAT (9097C>T), CGA2984CGT (9136A>T), CGG2987CGT (9145G>T), ATG2989GGA (9149A>G 9150T>G 9151G>A), GGT2990AGT (9152G>A), TTA2991TTG (9157A>G), GCG2992GCT (9160G>T), CGT2993CGC (9163T>C), GAG3000GAA (9184G>A), CGA3004CGG (9196A>G), AGG3010AGA (9214G>A), GAA3011GAG (9217A>G), GCG3012GCA (9220G>A), GCA3020GCT (9244A>T), TTG3021TTA (9247G>A), GCC3024GCT (9256C>T), CCT3026TCC (9260C>T 9262T>C), GGG3030GGA (9274G>A), GAC3032GAT (9280C>T), ATC3035ATT (9289C>T), GAG3040GAA (9304G>A), GTC3050GTT (9334C>T), GTC3060GTG (9364C>G)								
PVYgp2	1	75	100%	95	18.1%	75 (98.7%)	54 (71.1%)	1/0/3/2	2
Protein mutations:	R17_K17insX (2971_2972insA), Y23H (2989T>C 2991C>T), R26K (2999G>A 3000A>G), H35Y (3025C>T), K40H (3040A>C 3042A>C), G41S (3043G>A), E44K (3052G>A), I47V (3061A>G), V54I (3082G>A), P57S (3091C>T), P60T (3100C>A), G61E (3104G>A 3105T>G), G62S (3106G>A), Q63* (3109C>T), R64G (3112A>G), C66R (3118T>C), L67F (3121C>T), I69V (3127A>G), A72E (3137C>A 3138G>A), I73V (3139A>G)								
Codon mutations:	AAA4AAG (2931A>G), GAA7GAG (2940A>G), TTG10CTG (2947T>C), AGA17_AAA17insAA- (2971_2972insA), TAC23CAT (2989T>C 2991C>T), TCA24TCG (2994A>G), AGA26AAG (2999G>A 3000A>G), CAC32-AC (3016delC), TCG33TCA (3021G>A), GTA34GTT (3024A>T), CAT35TAT (3025C>T), CAC38CAT (3036C>T), AGA39AGG (3039A>G), AAA40CAC (3040A>C 3042A>C), GGC41AGC (3043G>A), GAA44AAA (3052G>A), ATA47GTA (3061A>G), GTT54ATT (3082G>A), CCG57TCG (3091C>T), CGC59CGT (3099C>T), CCA60ACA (3100C>A), GGT61GAG (3104G>A 3105T>G), GGT62AGT (3106G>A), CAA63TAA (3109C>T), AGG64GGG (3112A>G), TGC66CGC (3118T>C), CTC67TTC (3121C>T), ATT69GTT (3127A>G), GAG70GAA (3132G>A), GCG72GAA (3137C>A 3138G>A), ATT73GTT (3139A>G)								

Proteins

	Begin	End	Coverage	Score	Concordance	Matches	Identities	I/D/M/F*	Stop Codons
**NT**	**19**	**9704**	**99.8%**	**12534**	**65.0%**	**9674 (99.7%)**	**8011 (82.6%)**	**15/12**	
polyprotein (NP_056759.1)	1	3064	100%	18956	89.8%	3062 (99.9%)	2776 (90.5%)	2/2/4/4	1
Protein mutations:	Y4Q (194T>C 196C>A), C9L (209T>C 210G>T 211T>G), F13M (221T>A 223T>G), S23P (251T>C), C24F (255G>T 256C>T), E25G (258A>G), I27V (263A>G 265T>G), V28A (267T>C), E30V (273A>T), A35T (287G>A 289T>C), V37T (293G>A 294T>C), A41I (305G>A 306C>T), D42G (309A>G), E44D (316A>T), T45V (317A>G 318C>T 319A>G), L52Q (339T>A 340C>A), K53R (342A>G), K55A (347A>G 348A>C 349A>G), Y56H (350T>C), T58V (356A>G 357C>T), V61T (365G>A 366T>C 367G>A), L62S (368C>T 369T>C), F67R (383T>C 384T>G), A75V (408C>T 409C>T), M78E (416A>G 417T>A), E85K (437G>A), R86E (440A>G 441G>A 442G>A), K87R (443A>C 444A>G 445G>A), D88E (448T>A), E91A (456A>C), H93N (461C>A), D111E (517T>A), S116F (531C>T), P118S (536C>T), Q119W (539C>T 540A>G 541A>G), R122K (549G>A 550A>G), V134A (585T>C 586C>G), R137Y (593C>T 594G>A 595C>T), P138R (597C>G), I139V (599A>G 601A>G), I140P (602A>C 603T>C 604A>G), M147I (625G>A), I151V (635A>G 637T>C), I154V (644A>G), E160A (663A>C 664G>C), H166Q (682C>A), T172S (699C>G), H174Q (706T>A), K179E (719A>G 721G>A), I180V (722A>G 724A>G), A183S (731G>T), Y184P (734T>C 735A>C 736C>T), S185R (737T>C 738C>G 739C>T), A187V (744C>T 745G>C), R189C (749C>T 751A>C), M194K (765T>A), R197K (773C>A 774G>A 775A>G), M206K (801T>A), G210A (813G>C 814A>G), L211H (816T>A), R214Q (825G>A 826T>A), R226C (860C>T 862C>T), T227A (863A>G 865T>C), I228T (867T>C), N229D (869A>G 871C>T), I230L (872A>T 874A>G), R232K (879G>A), N240S (903A>G), T241N (906C>A 907A>C), K242T (909A>C 910A>C), S243N (912G>A 913C>T), S252L (938T>C 939C>T 940A>T), L266I (980T>A 982G>C), R272K (999G>A), Q275R (1007C>A 1008A>G 1009G>A), S276G (1010A>G 1012T>G), I277V (1013A>G), N279Q (1019A>C 1021C>G), D288E (1048C>A), R300Q (1083G>A), S305A (1097T>G 1099G>A), R320K (1143G>A), L324I (1154T>A 1156G>A), S346N (1221G>A), V349T (1229G>A 1230T>C 1231T>C), S350N (1233G>A 1234C>T), K358T (1257A>C 1258A>G), N365S (1278A>G), A369V (1290C>T 1291G>A), D372T (1298G>A 1299A>C 1300C>T), I375V (1307A>G 1309A>G), N378E (1316A>G 1318T>A), I382T (1329T>C), L397I (1373C>A), I401V (1385A>G), E407D (1405G>T), A411S (1415G>T 1417A>T), V418I (1436G>A 1438C>T), E508G (1707A>G), A512T (1718G>A), I520V (1742A>G 1744C>T), S525N (1758G>A), V536I (1790G>A), I585V (1937A>G 1939T>C), F607Y (2004T>A), I628M (2068T>G), V630I (2072G>A 2074G>A), R675K (2208G>A), D694E (2266C>G), N744G (2414A>G 2415A>G 2416T>G), S746C (2420A>T), K780R (2523A>G), R817K (2634G>A), V847I (2723G>A), A855T (2747G>A 2749T>A), H871N (2795C>A), V880I (2822G>A 2824G>A), P900T (2882C>A), S901H (2885A>C 2886G>A), T904M (2895C>T), N916S (2931A>G), N919S (2940A>G), E929_N930insX (2971_2972insA), T936I (2991C>T), H937R (2994A>G), E939R (2999G>A 3000A>G), R946Q (3021G>A), Y947F (3024A>T 3025C>T), T951I (3036C>T), E952G (3039A>G 3040A>C), K953T (3042A>C 3043G>A), A972V (3099C>T 3100C>A), V974R (3104G>A 3105T>G 3106G>A), S983N (3132G>A), R985K (3137C>A 3138G>A 3139A>G), F990I (3152T>A 3154C>T), C994Y (3165G>A), F1012L (3218T>C), V1016I (3230G>A), V1028M (3266G>A 3268A>G), V1031M (3275G>A), A1034T (3284G>A), R1044K (3315G>A 3316A>G), E1045A (3318A>C), M1059V (3359A>G), D1072E (3400T>G), V1110G (3513T>G), M1121V (3545A>G), A1122T (3548G>A), V1124I (3554G>A 3556C>T), L1147V (3623C>G), L1150M (3632C>A), Y1152H (3638T>C), I1172V (3698A>G), S1178N (3717G>A), R1183K (3731C>A 3732G>A), I1297V (4073A>G), F1315Y (4128T>A 4129C>T), A1323S (4151G>T), V1328I (4166G>A), L1347I (4223C>A 4225G>A), I1379V (4319A>G 4321A>G), F1404Y (4395T>A), V1408I (4406G>A), M1431V (4475A>G 4477G>T), T1433S (4481A>T), A1454L (4544G>T 4545C>T 4546G>A), R1455K (4548G>A), V1499I (4679G>A 4681G>C), V1538I (4796G>A 4798C>T), T1540S (4803C>G 4804T>C), S1625G (5057A>G), T1626A (5060A>G), L1636I (5090C>A), A1639V (5100C>T), E1641D (5107A>T), V1646F (5120G>T 5122C>T), I1648V (5126A>G), V1667I (5183G>A), A1793T (5561G>A 5563G>A), I1805V (5597A>G), N1807K (5605C>A), A1812V (5619C>T 5620T>G), I1816L (5630A>C), V1837I (5693G>A), K1890R (5853A>G), I1904V (5894A>G 5896C>G), R1926A (5960C>G 5961G>C 5962G>C), I1936V (5990A>G), E1945D (6019A>T), K1949T (6030A>C), D1955E (6049C>A), M1958I (6058G>A), S1963N (6072G>A 6073T>C), N1964H (6074A>C), T1966N (6081C>A), C1976S (6111G>C 6112T>C), I1982V (6128A>G 6130T>C), V1992I (6158G>A), T1996S (6170A>T 6172A>G), L2007F (6203C>T 6205C>T), V2016I (6230G>A), A2025K (6257G>A 6258C>A), K2033R (6282A>G), A2060T (6362G>A), V2073I (6401G>A), A2074V (6405C>T), Y2082F (6429A>T), Q2089R (6450A>G), H2101N (6485C>A 6487C>T), L2115I (6527C>A), T2135I (6588C>T), Y2151H (6635T>C), I2155V (6647A>G), I2165V (6677A>G 6679A>G), D2178N (6716G>A), N2179D (6719A>G), I2194L (6764A>T 6766A>G), A2204V (6795C>T 6796A>G), H2205Q (6799C>A), K2220R (6843A>G), N2225D (6857A>G), V2231T (6875G>A 6876T>C 6877C>A), D2250E (6934C>A), V2273I (7001G>A 7003G>A), F2283Y (7032T>A), T2314K (7125C>A), A2321S (7145G>T 7147A>G), E2326_A2327del (7160_7165delGAGGCA), D2344E (7216T>A), D2358E (7258T>A), D2363_R2364insX (7273_7274insT), R2364V (7274C>G 7275G>T), H2366L (7281A>T), L2367S (7284T>C), S2371L (7296C>T), S2376F (7311C>T), T2377I (7314C>T), C2378Y (7317G>A), K2384Q (7334A>C), A2387N (7343G>A 7344C>A 7345A>C), S2408M (7407G>T 7408T>G), C2411G (7415T>G), L2440S (7503T>C 7504G>A), D2509N (7709G>A), K2510R (7713A>G 7714A>G), T2543I (7812C>T), V2554L (7844G>T 7846G>A), V2567I (7883G>A), S2620N (8043G>A), N2656S (8151A>G 8152C>T), R2665K (8178G>A), R2675K (8208G>A), P2704A (8294C>G 8296A>G), S2722F (8349C>T), A2742S (8408G>T), M2756I (8452G>A), R2759K (8460G>A), A2766T (8480G>A 8482T>A), R2773K (8502G>A 8503A>G), E2777D (8515A>T), E2785D (8539G>C), L2788C (8546C>T 8547T>G), S2790T (8552T>A), A2797G (8574C>G), I2801V (8585A>G), G2805E (8598G>A), N2807S (8603A>T 8604A>C 8605C>T), A2811V (8616C>T), P2813Q (8622C>A), E2814G (8625A>G 8626G>A), P2822F (8648C>T 8649C>T 8650G>C), G2825E (8658G>A), D2827A (8664A>C 8665T>G), A2832V (8679C>T 8680A>T), T2854K (8745C>A 8746A>G), E2891G (8856A>G 8857G>A), R2894Q (8865G>A), M2895L (8867A>C 8869G>T), G2900E (8883G>A), T2902S (8888A>T), V2924I (8954G>A), N2934D (8984A>G 8986T>C), E2935V (8988A>T), M2989G (9149A>G 9150T>G 9151G>A), G2990S (9152G>A), P3026S (9260C>T 9262T>C)								

	Begin	End	Coverage	Score	Concordance	Matches	Identities	I/D/M/F*	Stop Codons
**NT**	**19**	**9704**	**99.8%**	**12534**	**65.0%**	**9674 (99.7%)**	**8011 (82.6%)**	**15/12**	

Codon mutations: ACT3ACC (193T>C), TAC4CAA (194T>C 196C>A), TCA6TCG (202A>G), ATC8ATT (208C>T), TGT9CTG (209T>C 210G>T 211T>G), TCG12TCA (220G>A), TTT13ATG (221T>A 223T>G), GAA14GAG (226A>G), CTA17CTT (235A>T), TAC19TAT (241C>T), CCA21CCT (247A>T), TCT23CCT (251T>C), TGC24TTT (255G>T 256C>T), GAG25GGG (258A>G), CAT26CAC (262T>C), ATT27GTG (263A>G 265T>G), GTG28GCG (267T>C), GAA30GTA (273A>T), CGA31CGT (277A>T), GAA32GAG (280A>G), GTG33GTT (283G>T), CCG34CCA (286G>A), GCT35ACC (287G>A 289T>C), GTT37ACT (293G>A 294T>C), CCT39CCC (301T>C), TTC40TTT (304C>T), GCA41ATA (305G>A 306C>T), GAT42GGT (309A>G), CTG43TTG (311C>T), GAA44GAT (316A>T), ACA45GTG (317A>G 318C>T 319A>G), CAA46CAG (322A>G), GCA49GCT (331A>T), CTC52CAA (339T>A 340C>A), AAG53AGG (342A>G), AAA55GCG (347A>G 348A>C 349A>G), TAT56CAT (350T>C), GCT57GCA (355T>A), ACT58GTT (356A>G 357C>T), CGT60CGC (364T>C), GTG61ACA (365G>A 366T>C 367G>A), CTC62TCC (368C>T 369T>C), AAA63AAG (373A>G), AAC64AAT (376C>T), TTT67CGT (383T>C 384T>G), ACG68ACA (388G>A), TAC69TAT (391C>T), CGA70CGG (394A>G), TAC71TAT (397C>T), AAG72AAA (400G>A), GAT74GAC (406T>C), GCC75GTT (408C>T 409C>T), CAG76CAA (412G>A), ATA77ATT (415A>T), ATG78GAG (416A>G 417T>A), ATT80ATC (424T>C), CAG81CAA (427G>A), AAA83AAG (433A>G), CTG84CTC (436G>C), GAG85AAG (437G>A), AGG86GAA (440A>G 441G>A 442G>A), AAG87CGA (443A>C 444A>G 445G>A), GAT88GAA (448T>A), GAA90GAG (454A>G), GAA91GCA (456A>C), TAT92TAC (460T>C), CAC93AAC (461C>A), CAA95CAG (469A>G), GCC97GCT (475C>T), CCT99CCA (481T>A), ACT106ACC (502T>C), ATA107ATT (505A>T), GGC109GGT (511C>T), GAT111GAA (517T>A), CCT112CCA (520T>A), CCA113CCT (523A>T), TCT116TTT (531C>T), GAG117GAA (535G>A), CCA118TCA (536C>T), CAA119TGG (539C>T 540A>G 541A>G), AGA122AAG (549G>A 550A>G), ATC124ATT (556C>T), ATT125ATC (559T>C), CAT126CAC (562T>C), ACT128ACC (568T>C), CCA129CCG (571A>G), CGT132CGC (580T>C), GTC134GCG (585T>C 586C>G), CGC137TAT (593C>T 594G>A 595C>T), CCC138CGC (597C>G), ATA139GTG (599A>G 601A>G), ATA140CCG (602A>C 603T>C 604A>G), ACA143ACG (613A>G), GAA144GAG (616A>G), GGC145GGG (619C>G), CAG146CAA (622G>A), ATG147ATA (625G>A), AAT148AAC (628T>C), CAC149CAT (631C>T), ATT151GTC (635A>G 637T>C), ATA154GTA (644A>G), ATT157ATC (655T>C), TCG159TCA (661G>A), GAG160GCC (663A>C 664G>C), AAA161AAG (667A>G), GGG163GGC (673G>C), GTC165GTT (679C>T), CAC166CAA (682C>A), TTA167CTA (683T>C), AGT169AGC (691T>C), ACC172AGC (699C>G), ACT173ACA (703T>A), CAT174CAA (706T>A), AAG179GAA (719A>G 721G>A), ATA180GTG (722A>G 724A>G), CTT181TTG (725C>T 727T>G), GGT182GGA (730T>A), GCA183TCA (731G>T), TAC184CCT (734T>C 735A>C 736C>T), TCC185CGT (737T>C 738C>G 739C>T), GCG187GTC (744C>T 745G>C), CGA189TGC (749C>T 751A>C), ACT190ACC (754T>C), ATG194AAG (765T>A), TTG196CTG (770T>C), CGA197AAG (773C>A 774G>A 775A>G), CGG198AGG (776C>A), TTC202TTT (790C>T), ATG206AAG (801T>A), GGA210GCG (813G>C 814A>G), CTT211CAT (816T>A), TTG212CTG (818T>C), CAA213CAG (823A>G), CGT214CAA (825G>A 826T>A), CGG217AGA (833C>A 835G>A), GAC219GAT (841C>T), AAT223AAC (853T>C), GTC225GTT (859C>T), CGC226TGT (860C>T 862C>T), ACT227GCC (863A>G 865T>C), ATC228ACC (867T>C), AAC229GAT (869A>G 871C>T), ATA230TTG (872A>T 874A>G), AGG232AAG (879G>A), GAT234GAC (886T>C), ATC238ATT (898C>T), AAC240AGC (903A>G), ACA241AAC (906C>A 907A>C), AAA242ACC (909A>C 910A>C), AGC243AAT (912G>A 913C>T), GGC246GGA (922C>A), GGT249GGA (931T>A), TCA252CTT (938T>C 939C>T 940A>T), GGA253GGG (943A>G), TTC256TTT (952C>T), TCA261TCG (967A>G), GAA263GAG (973A>G), GGG264GGC (976G>C), TTG266ATC (980T>A 982G>C), GCA269GCG (991A>G), CGT270CGC (994T>C), AGA272AAA (999G>A), GTT273GTC (1003T>C), CAG275AGA (1007C>A 1008A>G 1009G>A), AGT276GGG (1010A>G 1012T>G), ATT277GTT (1013A>G), TTA278TTG (1018A>G), AAC279CAG (1019A>C 1021C>G), ATC282ATT (1030C>T), TCG285TCA (1039G>A), AAT286AAC (1042T>C), GCC287GCA (1045C>A), GAC288GAA (1048C>A), AAT289AAC (1051T>C), AAG292AAA (1060G>A), GGT293GGA (1063T>A), GAC295GAT (1069C>T), CGA300CAA (1083G>A), TAT303TAC (1093T>C), TCG305GCA (1097T>G 1099G>A), CAC307CAT (1105C>T), GTA310GTG (1114A>G), GCT311GCG (1117T>G), TTA313TTG (1123A>G), CCT314CCA (1126T>A), GTC315GTT (1129C>T), AGG320AAG (1143G>A), GTA321GTT (1147A>T), GCT322GCA (1150T>A), TTG324ATA (1154T>A 1156G>A), CAC327CAT (1165C>T), AGT328AGC (1168T>C), CTT330TTA (1172C>T 1174T>A), ACC339ACT (1201C>T), AGC346AAC (1221G>A), GTT349ACC (1229G>A 1230T>C 1231T>C), AGC350AAT (1233G>A 1234C>T), CTG352CTA (1240G>A), AAG354AAA (1246G>A), TTG356CTA (1250T>C 1252G>A), CAT357CAC (1255T>C), AAA358ACG (1257A>C 1258A>G), AGA361AGG (1267A>G), GGT363GGC (1273T>C), TTG364TTA (1276G>A), AAT365AGT (1278A>G), TTG367CTG (1283T>C), GCG369GTA (1290C>T 1291G>A), GAC372ACT (1298G>A 1299A>C 1300C>T), TTT374TTC (1306T>C), ATA375GTG (1307A>G 1309A>G), CAT376CAC (1312T>C), AAT378GAA (1316A>G 1318T>A), TTG381TTA (1327G>A), ATA382ACA (1329T>C), GCG383GCT (1333G>T), TTA384TTG (1336A>G), CTA387TTG (1343C>T 1345A>G), CCG390CCA (1354G>A), GTG391GTT (1357G>T), GAC392GAT (1360C>T), CTC395TTG (1367C>T 1369C>G), CTT397ATT (1373C>A), TTC398TTT (1378C>T), GAG400GAA (1384G>A), ATA401GTA (1385A>G), AAA403AAG (1393A>G), TCC404TCT (1396C>T), GAG407GAT (1405G>T), AAA408AAG (1408A>G), CAG409CAA (1411G>A), GCA411TCT (1415G>T 1417A>T), CCG412CCC (1420G>C), AAT415AAC (1429T>C), TTA416TTG (1432A>G), GTC418ATT (1436G>A 1438C>T), TTA419CTG (1439T>C 1441A>G), AAT420AAC (1444T>C), TTC423TTT (1453C>T), CTG424CTT (1456G>T), GAA428GAG (1468A>G), TTG439CTA (1499T>C 1501G>A), AGT440AGC (1504T>C), TTG441TTA (1507G>A), CTC442CTT (1510C>T), GAA443GAG (1513A>G), TTA444TTG (1516A>G), GCA445GCT (1519A>T), AGG446AGA (1522G>A), TTC447TTT (1525C>T), CAG448CAA (1528G>A), AAC450AAT (1534C>T), ACT452ACA (1540T>A), AAC454AAT (1546C>T), AAA457AAG (1555A>G), GAT459GAC (1561T>C), ATA460ATC (1564A>C), TCT461TCA (1567T>A), TTC463TTT (1573C>T), AGA464AGG (1576A>G), TTA467CTA (1583T>C), TCT468TCA (1588T>A), AAG470AAA (1594G>A), AAC472AAT (1600C>T), CTG475CTA (1609G>A), TCG478TCA (1618G>A), TGC479TGT (1621C>T), GAC480GAT (1624C>T), AAC481AAT (1627C>T), CTG483TTG (1631C>T), AAA485AAG (1639A>G), TTC489TTT (1651C>T), CTC490CTG (1654C>G), CAT497CAC (1675T>C), TCA503TCG (1693A>G), TTT506TTC (1702T>C), GAA508GGA (1707A>G), GCA512ACA (1718G>A), GGA514GGG (1726A>G), TAC515TAT (1729C>T), TCA516TCC (1732A>C), TAT518TAC (1738T>C), ATC520GTT (1742A>G 1744C>T), CGC521CGT (1747C>T), CAT523CAC (1753T>C), AGT525AAT (1758G>A), AGG528AGA (1768G>A), AAC534AAT (1786C>T), TTA535TTG (1789A>G), GTT536ATT (1790G>A), GTC537GTT (1795C>T), TTA541CTG (1805T>C 1807A>G), GAG543GAA (1813G>A), AGG545AGA (1819G>A), AAG547AAA (1825G>A), GAC551GAT (1837C>T), TAT552TAC (1840T>C), CAA555CAG (1849A>G), GTC558GTG (1858C>G), AAA560AAG (1864A>G), TGC562TGT (1870C>T), AGT564AGC (1876T>C), AAA566AAG (1882A>G), GGT568GGA (1888T>A), AAT569AAC (1891T>C), TGT574TGC (1906T>C), ACA577ACT (1915A>T), GGT582GGC (1930T>C), GCC584GCT (1936C>T), ATT585GTC (1937A>G 1939T>C), TCA587TCG (1945A>G), ACA588ACG (1948A>G), TTC589TTT (1951C>T), CCA592CCT (1960A>T), GTA598GTG (1978A>G), ATT599ATA (1981T>A), GGC600GGT (1984C>T), AAT601AAC (1987T>C), GAC604GAT (1996C>T), TTT607TAT (2004T>A), AAA612AAG (2020A>G), GGG613GGA (2023G>A), GAT614GAC (2026T>C), TCG615TCT (2029G>T), GAG616GAA (2032G>A), TAT625TAC (2059T>C), ATT628ATG (2068T>G), AAC629AAT (2071C>T), GTG630ATA (2072G>A 2074G>A), CTG635TTA (2087C>T 2089G>A), AGC639AGT (2101C>T), GAG640GAA (2104G>A), GAG641GAA (2107G>A), AAG644AAA (2116G>A), TTC646TTT (2122C>T), CGC651CGT (2137C>T), CTT658CTC (2158T>C), ACC660ACT (2164C>T), CCA662CCG (2170A>G), GAT666GAC (2182T>C), GCG668GCA (2188G>A), ACC669ACA (2191C>A), AGA675AAA (2208G>A), TAT678TAC (2218T>C), GAC680GAT (2224C>T), GTA681GTT (2227A>T), TTG686TTA (2242G>A), CCC687CCT (2245C>T), TTG690TTA (2254G>A), GTT691GTC (2257T>C), GAC692GAT (2260C>T), GAC694GAG (2266C>G), CAA696CAG (2272A>G), ACG697ACA (2275G>A), TCA703TCG (2293A>G), GGC705GGT (2299C>T), TCG706TCA (2302G>A), CAG707CAA (2305G>A), GGA710GGT (2314A>T), TAT711TAC (2317T>C), ATT713ATC (2323T>C), CTA714CTG (2326A>G), GTG719GTA (2341G>A), CAA721CAG (2347A>G), CTT722CTC (2350T>C), TTG724TTA (2356G>A), AAT727AAC (2365T>C), GAA729GAG (2371A>G), TTA730TTG (2374A>G), GAT733GAC (2383T>C), ATA734ATT (2386A>T), AAA735AAG (2389A>G), CAT736CAC (2392T>C), TAT737TAC (2395T>C), AAT744GGG (2414A>G 2415A>G 2416T>G), GCT745GCA (2419T>A), AGC746TGC (2420A>T), ACA752ACT (2440A>T), GTT761GTC (2467T>C), TCT764TCC (2476T>C), TCG766TCA (2482G>A), CTG769CTA (2491G>A), CTG771CTT (2497G>T), TTG773TTA (2503G>A), AGA778AGG (2518A>G), CCT779CCC (2521T>C), AAG780AGG (2523A>G), AGA783AGG (2533A>G), TTG785TTA (2539G>A), TTA787TTG (2545A>G), GAG789GAA (2551G>A), CCT790CCA (2554T>A), CTG792TTG (2558C>T), TTG793CTC (2561T>C 2563G>C), CTA795CTG (2569A>G), TCA796TCG (2572A>G), CCT800CCC (2584T>C), GGC801GGT (2587C>T), CTG803CTC (2593G>C), GCT805GCC (2599T>C), AAT808AAC (2608T>C), TTT812TTC (2620T>C), GCG815GCA (2629G>A), GTG816GTA (2632G>A), AGG817AAG (2634G>A), TTG818TTA (2638G>A), ATT820ATA (2644T>A), AAA823AAG (2653A>G), GCT827GCC (2665T>C), CTA832TTA (2678C>T), CTA833CTG (2683A>G), GCT835GCC (2689T>C), CTA838CTC (2698A>C), TCA841TCG (2707A>G), GCG842GCA (2710G>A), GAA844GAG (2716A>G), ACA845ACG (2719A>G), CTC846CTT (2722C>T), GTC847ATC (2723G>A), GCA848GCG (2728A>G), AGG850AGA (2734G>A), ATT851ATA (2737T>A), GAT854GAC (2746T>C), GCT855ACA (2747G>A 2749T>A), GCA856GCT (2752A>T), GCT857GCA (2755T>A), GAC859GAT (2761C>T), CTC860CTT (2764C>T), TGT865TGC (2779T>C), GGG867GGT (2785G>T), AAC869AAT (2791C>T), CAT871AAT (2795C>A), TAC874TAT (2806C>T), TTG878CTC (2816T>C 2818G>C), GTG880ATA (2822G>A 2824G>A), TTG881TTA (2827G>A), GTT884GTC (2836T>C), AAG885AAA (2839G>A), AAT886AAC (2842T>C), ACC893ACA (2863C>A), CTA894TTA (2864C>T), CCA900ACA (2882C>A), AGT901CAT (2885A>C 2886G>A), AAC903AAT (2893C>T), ACG904ATG (2895C>T), GTC906GTT (2902C>T), GTA907GTG (2905A>G), ATT909ATC (2911T>C), AAT916AGT (2931A>G), AAC919AGC (2940A>G), GCT921GCC (2947T>C), GAA929_AAT930insA-- (2971_2972insA), GGT935GGC (2989T>C), ACT936ATT (2991C>T), CAT937CGT (2994A>G), GAG939AGG (2999G>A 3000A>G), TCC944TC- (3016delC), CGG946CAG (3021G>A), TAC947TTT (3024A>T 3025C>T), ACA951ATA (3036C>T), GAA952GGC (3039A>G 3040A>C), AAG953ACA (3042A>C 3043G>A), TTG956TTA (3052G>A), TTA959TTG (3061A>G), GCG966GCA (3082G>A), GGC969GGT (3091C>T), GCC972GTA (3099C>T 3100C>A), GTG974AGA (3104G>A 3105T>G 3106G>A), GTC975GTT (3109C>T), AAA976AAG (3112A>G), ACT978ACC (3118T>C), GCC979GCT (3121C>T), GGA981GGG (3127A>G), AGC983AAC (3132G>A), CGA985AAG (3137C>A 3138G>A 3139A>G), TAT989TAC (3151T>C), TTC990ATT (3152T>A 3154C>T), TGT994TAT (3165G>A), GTA995GTG (3169A>G), ATC1005ATT (3199C>T), AGG1007CGG (3203A>C), CGT1008CGC (3208T>C), CCA1010CCG (3214A>G), ACC1011ACT (3217C>T), TTT1012CTT (3218T>C), GTC1013GTA (3223C>A), GTT1016ATT (3230G>A), AAC1017AAT (3235C>T), TTA1025CTA (3257T>C), ACC1026ACT (3262C>T), AGC1027AGT (3265C>T), GTA1028ATG (3266G>A 3268A>G), GTG1029GTA (3271G>A), GTG1031ATG (3275G>A), GCA1034ACA (3284G>A), CAG1039CAA (3301G>A), AGA1044AAG (3315G>A 3316A>G), GAA1045GCA (3318A>C), GAG1047GAA (3325G>A), TTG1048TTA (3328G>A), ATA1051ATT (3337A>T), GAG1055GAA (3349G>A), GTC1057GTT (3355C>T), TGC1058TGT (3358C>T), ATG1059GTG (3359A>G), CTA1061CTG (3367A>G), TAT1062TAC (3370T>C), TTA1065CTG (3377T>C 3379A>G), GAA1070GAG (3394A>G), CGC1071CGT (3397C>T), GAT1072GAG (3400T>G), ACA1074ACG (3406A>G), TAC1078TAT (3418C>T), ATT1079ATA (3421T>A), TAT1081TAC (3427T>C), AAG1083AAA (3433G>A), TCA1084TCT (3436A>T), GTA1085GTG (3439A>G), AAC1086AAT (3442C>T), CCT1087CCC (3445T>C), CAG1091CAA (3457G>A), TTT1092TTC (3460T>C), GCT1093GCA (3463T>A), GCG1095GCT (3469G>T), CAG1096CAA (3472G>A), CAC1104CAT (3496C>T), CAG1105CAA (3499G>A), TCC1107TCT (3505C>T), GTT1110GGT (3513T>G), AAA1112AAG (3520A>G), GAA1115GAG (3529A>G), CAA1116CAG (3532A>G), ATG1121GTG (3545A>G), GCT1122ACT (3548G>A), TTA1123CTA (3551T>C), GTC1124ATT (3554G>A 3556C>T), AGT1133AGC (3583T>C), GAT1134GAC (3586T>C), TGC1135TGT (3589C>T), TTC1137TTT (3595C>T), AAT1141AAC (3607T>C), TTT1143TTC (3613T>C), AAG1144AAA (3616G>A), GGT1145GGC (3619T>C), CTT1147GTT (3623C>G), TCC1148TCT (3628C>T), CTG1150ATG (3632C>A), GAT1151GAC (3637T>C), TAT1152CAT (3638T>C), GAA1153GAG (3643A>G), TTA1159TTG (3661A>G), GTG1162GTA (3670G>A), TTT1166TTC (3682T>C), GAG1168GAA (3688G>A), GAG1171GAA (3697G>A), ATT1172GTT (3698A>G), GAT1174GAC (3706T>C), TTT1175TTC (3709T>C), TTG1177CTA (3713T>C 3715G>A), AGT1178AAT (3717G>A), GAG1179GAA (3721G>A), CGA1183AAA (3731C>A 3732G>A), ACT1184ACA (3736T>A), CTA1188TTG (3746C>T 3748A>G), ACA1190ACG (3754A>G), AGT1193AGC (3763T>C), CGA1198CGG (3778A>G), CAA1199CAG (3781A>G), ATC1200ATT (3784C>T), CAT1204CAC (3796T>C), ACA1205ACG (3799A>G), CCA1207CCT (3805A>T), TAC1209TAT (3811C>T), AGA1210AGG (3814A>G), TTC1215TTT (3829C>T), TTT1218TTC (3838T>C), AGA1220AGG (3844A>G), GCA1221GCC (3847A>C), GTT1224GTA (3856T>A), GTG1226GTT (3862G>T), GAC1229GAT (3871C>T), GCC1231GCA (3877C>A), AGC1233AGT (3883C>T), GAA1234GAG (3886A>G), CTA1236CTG (3892A>G), GAC1237GAT (3895C>T), TTT1238TTC (3898T>C), GTA1240GTG (3904A>G), CGG1241AGA (3905C>A 3907G>A), GCT1243GCC (3913T>C), AAG1248AAA (3928G>A), TCA1249TCT (3931A>T), GGG1251GGA (3937G>A), GTT1254GTC (3946T>C), AGT1257AGC (3955T>C), GTG1258GTA (3958G>A), GCC1259GCT (3961C>T), GGA1260GGG (3964A>G), TCT1261TCC (3967T>C), TTA1264TTG (3976A>G), ATT1265ATC (3979T>C), CCA1267CCT (3985A>T), CTA1271CTT (3997A>T), GCG1272GCA (4000G>A), AAC1274AAT (4006C>T), GTT1275GTG (4009T>G), TTC1276TTT (4012C>T), AAA1277AAG (4015A>G), TCT1280TCC (4024T>C), GAA1282GAG (4030A>G), CCA1283CCT (4033A>T), AAG1286AAA (4042G>A), CTG1290TTA (4052C>T 4054G>A), CGT1291CGC (4057T>C), CGT1293CGA (4063T>A), GGA1294GGG (4066A>G), ATA1297GTA (4073A>G), TTT1298TTC (4078T>C), GGC1299GGT (4081C>T), TCT1300TCC (4084T>C), TCC1304TCT (4096C>T), AGC1308AGT (4108C>T), GGA1309GGT (4111A>T), GCG1311GCA (4117G>A), CTA1312TTG (4118C>T 4120A>G), TAC1314TAT (4126C>T), TTC1315TAT (4128T>A 4129C>T), GCC1316GCT (4132C>T), AAT1317AAC (4135T>C), CAA1321CAG (4147A>G), TTA1322CTA (4148T>C), GCT1323TCT (4151G>T), AAC1326AAT (4162C>T), GTA1328ATA (4166G>A), ATA1329ATC (4171A>C), GAG1332GAA (4180G>A), CTG1336TTG (4190C>T), CCT1338CCC (4198T>C), TCC1339TCT (4201C>T), GCG1342GCT (4210G>T), TTC1343TTT (4213C>T), AGT1345AGC (4219T>C), CTG1346TTG (4220C>T), CTG1347ATA (4223C>A 4225G>A), GTT1349GTG (4231T>G), CAT1351CAC (4237T>C), GTA1356GTG (4252A>G), TTA1357CTG (4253T>C 4255A>G), AAA1358AAG (4258A>G), ACT1362ACC (4270T>C), AGA1366AGG (4282A>G), GAG1367GAA (4285G>A), GAA1369GAG (4291A>G), CAG1373CAA (4303G>A), GTC1376GTT (4312C>T), ATA1379GTG (4319A>G 4321A>G), GTG1380GTT (4324G>T), GAG1381GAA (4327G>A), CTG1384CTT (4336G>T), TCT1385TCC (4339T>C), TCA1388TCT (4348A>T), GCA1392GCG (4360A>G), GGT1394GGC (4366T>C), TCT1395TCA (4369T>A), ACT1397ACC (4375T>C), GTT1401GTC (4387T>C), TTT1404TAT (4395T>A), GTA1408ATA (4406G>A), CTT1409CTC (4411T>C), GTT1418GTC (4438T>C), ACC1420ACA (4444C>A), GCC1422GCT (4450C>T), CTC1424CTT (4456C>T), GAC1427GAT (4465C>T), ATG1431GTT (4475A>G 4477G>T), GTC1432GTT (4480C>T), ACA1433TCA (4481A>T), GGC1437GGT (4495C>T), AAG1441AAA (4507G>A), CAC1442CAT (4510C>T), GGT1443GGG (4513T>G), CTA1445TTA (4517C>T), GTC1448GTT (4528C>T), AAA1450AAG (4534A>G), ACC1452ACT (4540C>T), GCG1454TTA (4544G>T 4545C>T 4546G>A), AGA1455AAA (4548G>A), TTT1458TTC (4558T>C), GTA1460GTT (4564A>T), ACC1462ACT (4570C>T), ATA1464ATT (4576A>T), TTG1471TTA (4597G>A), GAC1472GAT (4600C>T), GAC1474GAT (4606C>T), GTG1475GTA (4609G>A), GTG1477GTA (4615G>A), GAC1478GAT (4618C>T), TTG1481CTT (4625T>C 4627G>T), GAC1488GAT (4648C>T), ATT1489ATC (4651T>C), AGG1492AGA (4660G>A), AGC1493AGT (4663C>T), GCT1495GCA (4669T>A), GTG1499ATC (4679G>A 4681G>C), AGT1500AGC (4684T>C), GTT1501GTG (4687T>G), GAG1505GAA (4699G>A), AGA1506AGG (4702A>G), CAA1508CAG (4708A>G), AGG1509AGA (4711G>A), CTG1510TTG (4712C>T), AAA1518AAG (4738A>G), GTA1520GTT (4744A>T), GCA1521GCG (4747A>G), TTG1522CTG (4748T>C), ACT1527ACC (4765T>C), GTC1538ATT (4796G>A 4798C>T), ACT1540AGC (4803C>G 4804T>C), GAG1541GAA (4807G>A), GCG1542GCT (4810G>T), GCT1543GCG (4813T>G), CTT1544CTC (4816T>C), TAT1549TAC (4831T>C), AAC1550AAT (4834C>T), CCA1552CCT (4840A>T),

	GGA1556GGG (4852A>G), GGC1557GGT (4855C>T), AGT1561AGC (4867T>C), CTG1562CTC (4870G>C), ATT1563ATA (4873T>A), TGT1566TGC (4882T>C), ACA1573ACT (4903A>T), CAG1575CAA (4909G>A), TTT1577TTC (4915T>C), GAA1578GAG (4918A>G), TTG1579CTA (4919T>C 4921G>A), AGT1580AGC (4924T>C), CCC1581CCG (4927C>G), TTT1583TTC (4933T>C), ATC1584ATA (4936C>A), CAG1585CAA (4939G>A), AAT1586AAC (4942T>C), TTC1587TTT (4945C>T), TCA1593TCG (4963A>G), CCT1596CCC (4972T>C), CTT1602CTC (4990T>C), AAA1606AAG (5002A>G), CCT1613CCC (5023T>C), TTG1614CTG (5024T>C), TGC1615TGT (5029C>T), GAT1616GAC (5032T>C), TCT1618TCC (5038T>C), CCA1620CCT (5044A>T), GCA1623GCT (5053A>T), TCG1624TCA (5056G>A), AGT1625GGT (5057A>G), ACT1626GCT (5060A>G), TCG1629TCT (5071G>T), CTT1636ATT (5090C>A), GGA1637GGG (5095A>G), GTG1638GTT (5098G>T), GCC1639GTC (5100C>T), TTA1640TTG (5104A>G), GAA1641GAT (5107A>T), ATT1642ATC (5110T>C), CCA1643CCG (5113A>G), AAG1644AAA (5116G>A), CAA1645CAG (5119A>G), GTC1646TTT (5120G>T 5122C>T), ATT1648GTT (5126A>G), CAT1651CAC (5137T>C), ATC1652ATT (5140C>T), AAA1653AAG (5143A>G), GAG1654GAA (5146G>A), CTC1659CTA (5161C>A), GAA1661GAG (5167A>G), CTT1663CTG (5173T>G), ACG1666ACA (5182G>A), GTT1667ATT (5183G>A), GTC1668GTT (5188C>T), AAG1669AAA (5191G>A), AAA1671AAG (5197A>G), GAC1672GAT (5200C>T), TGC1674TGT (5206C>T), TTA1675TTG (5209A>G), AGC1678AGT (5218C>T), TCG1682TCA (5230G>A), ATC1684ATT (5236C>T), AGC1685AGT (5239C>T), ATC1687ATT (5245C>T), ACA1693ACT (5263A>T), CTC1695CTT (5269C>T), TTC1696TTT (5272C>T), GCC1697GCA (5275C>A), ATC1698ATT (5278C>T), CCA1699CCC (5281A>C), AGA1700AGG (5284A>G), ACT1701ACC (5287T>C), CTA1702TTA (5288C>T), ATA1703ATT (5293A>T), TTG1704CTA (5294T>C 5296G>A), GAG1706GAA (5302G>A), CTT1709CTA (5311T>A), GAA1710GAG (5314A>G), CGA1713CGT (5323A>T), AGT1721AGC (5347T>C), ATC1723ATT (5353C>T), TGC1727TGT (5365C>T), GTT1734GTC (5386T>C), ACC1737ACA (5395C>A), AAA1746AAG (5422A>G), GAT1747GAC (5425T>C), ACC1749ACT (5431C>T), GAG1751GAA (5437G>A), CTT1756CTC (5452T>C), AAG1758AAA (5458G>A), AGT1761AGC (5467T>C), CAA1762CAG (5470A>G), CTA1763TTG (5471C>T 5473A>G), TTC1766TTT (5482C>T), TCA1767TCC (5485A>C), AAT1768AAC (5488T>C), TTG1769CTA (5489T>C 5491G>A), GGT1771GGC (5497T>C), TGT1774TGC (5506T>C), GAG1775GAA (5509G>A), GAG1776GAA (5512G>A), TAT1782TAC (5530T>C), GAG1783GAA (5533G>A), TCG1784TCT (5536G>T), TTG1785TTA (5539G>A), GTT1788GTG (5548T>G), CAC1790CAT (5554C>T), GCT1792GCA (5560T>A), GCG1793ACA (5561G>A 5563G>A), ACG1794ACC (5566G>C), TCA1795TCG (5569A>G), CTC1800CTG (5584C>G), AAG1803AAA (5593G>A), GGG1804GGA (5596G>A), ATT1805GTT (5597A>G), AAC1807AAA (5605C>A), GTG1811GTT (5617G>T), GCT1812GTG (5619C>T 5620T>G), TTG1815TTA (5629G>A), ATC1816CTC (5630A>C), GGC1819GGT (5641C>T), GCA1822GCT (5650A>T), ATA1826ATT (5662A>T), GGA1827GGG (5665A>G), CAA1835CAG (5689A>G), GTT1837ATT (5693G>A), GAG1838GAA (5698G>A), CAA1843CAG (5713A>G), GGG1844GGC (5716G>C), AAA1845AAG (5719A>G), GCC1853GCA (5743C>A), CGC1857CGA (5755C>A), CAT1858CAC (5758T>C), CGT1860CGC (5764T>C), AAA1862AAG (5770A>G), GGC1865GGT (5779C>T), TTT1866TTC (5782T>C), GAC1869GAT (5791C>T), GAC1873GAT (5803C>T), AGG1884AGA (5836G>A), AAA1885AAG (5839A>G), GGA1887GGG (5845A>G), AAA1888AAG (5848A>G), AAA1890AGA (5853A>G), GGT1891GGC (5857T>C), ACC1892ACT (5860C>T), ACA1893ACT (5863A>T), GGT1895GGA (5869T>A), GGT1897GGC (5875T>C), AGC1900AGT (5884C>T), TTC1903TTT (5893C>T), ATC1904GTG (5894A>G 5896C>G), AAC1905AAT (5899C>T), GGG1908GGA (5908G>A), TTT1909TTC (5911T>C), GAG1913GAA (5923G>A), CAA1918CAG (5938A>G), TTC1919TTT (5941C>T), GTT1920GTC (5944T>C), GAT1921GAC (5947T>C), GGG1925GGA (5959G>A), CGG1926GCC (5960C>G 5961G>C 5962G>C), GAA1930GAG (5974A>G), GCT1934GCA (5986T>A), GAC1935GAT (5989C>T), ATT1936GTT (5990A>G), GAT1938GAC (5998T>C), AGA1942CGT (6008A>C 6010A>T), GAA1945GAT (6019A>T), GTG1946GTT (6022G>T), CGA1947CGT (6025A>T), AAA1949ACA (6030A>C), GAC1955GAA (6049C>A), ATG1958ATA (6058G>A), CAA1959CAG (6061A>G), GCC1960GCA (6064C>A), GGT1962GGC (6070T>C), AGT1963AAC (6072G>A 6073T>C), AAC1964CAC (6074A>C), ACG1965ACA (6079G>A), ACC1966AAC (6081C>A), ATA1967ATT (6085A>T), GCA1969GCT (6091A>T), TAC1970TAT (6094C>T), TGT1976TCC (6111G>C 6112T>C), AAA1978AAG (6118A>G), TTG1980TTA (6124G>A), AAG1981AAA (6127G>A), ATT1982GTC (6128A>G 6130T>C), GAT1983GAC (6133T>C), TTA1984TTG (6136A>G), AAC1988AAT (6148C>T), AAA1991AAG (6157A>G), GTT1992ATT (6158G>A), TGT1993TGC (6163T>C), AAA1995AAG (6169A>G), ACA1996TCG (6170A>T 6172A>G), GGC1998GGT (6178C>T), AAA2001AAG (6187A>G), TTT2002TTC (6190T>C), GAG2004GAA (6196G>A), CTC2007TTT (6203C>T 6205C>T), GAA2008GAG (6208A>G), CTA2009TTG (6209C>T 6211A>G), CAG2011CAA (6217G>A), GCT2015GCA (6229T>A), GTA2016ATA (6230G>A), GTC2018GTT (6238C>T), AAG2021AAA (6247G>A), ATA2023ATT (6253A>T), GCA2025AAA (6257G>A 6258C>A), CAT2030CAC (6274T>C), AAA2033AGA (6282A>G), TCG2034TCA (6286G>A), GGC2038GGT (6298C>T), TTG2039TTA (6301G>A), AGA2040AGG (6304A>G), GAC2041GAT (6307C>T), AAC2043AAT (6313C>T), GCC2046GCT (6322C>T), ACA2048ACT (6328A>T), GTT2049GTG (6331T>G), TGT2050TGC (6334T>C), AGG2051AGA (6337G>A), CTG2052TTG (6338C>T), TAT2058TAC (6358T>C), GGG2059GGA (6361G>A), GCA2060ACA (6362G>A), TCA2061TCT (6367A>T), GAG2062GAA (6370G>A), TAC2064TAT (6376C>T), GGT2065GGG (6379T>G), GGA2067GGC (6385A>C), GGA2069GGT (6391A>T), GCA2070GCG (6394A>G), TAC2071TAT (6397C>T), GTA2073ATA (6401G>A), GCG2074GTG (6405C>T), CAC2076CAT (6412C>T), TTA2078TTG (6418A>G), TTT2079TTC (6421T>C), TAC2082TTC (6429A>T), GGT2084GGA (6436T>A), GAG2087GAA (6445G>A), CAA2089CGA (6450A>G), TCC2090TCA (6454C>A), CAC2092CAT (6460C>T), GGT2093GGA (6463T>A), TTC2095TTT (6469C>T), AGG2096AGA (6472G>A), GTG2097GTA (6475G>A), AAT2099AAC (6481T>C), CAC2101AAT (6485C>A 6487C>T), AGT2102AGC (6490T>C), AGC2104AGT (6496C>T), CTG2106CTA (6502G>A), GGT2110GGC (6514T>C), AGG2111AGA (6517G>A), CTC2115ATC (6527C>A), ATC2116ATA (6532C>A), CCG2119CCA (6541G>A), AAA2120AAG (6544A>G), GTC2124GTT (6556C>T), TTT2125TTC (6559T>C), CAG2127CAA (6565G>A), TTG2129CTA (6569T>C 6571G>A), CCT2134CCA (6586T>A), ACA2135ATA (6588C>T), GAA2138GAG (6598A>G), AGA2139AGG (6601A>G), ATT2140ATC (6604T>C), TGT2141TGC (6607T>C), GGA2144GGG (6616A>G), AAC2146AAT (6622C>T), GAG2149GAA (6631G>A), AAG2150AAA (6634G>A), TAT2151CAT (6635T>C), GCT2152GCA (6640T>A), TCG2154TCC (6646G>C), ATC2155GTC (6647A>G), ATC2156ATT (6652C>T), AGC2160AGT (6664C>T), ACT2162ACG (6670T>G), ATA2165GTG (6677A>G 6679A>G), GGC2167GGT (6685C>T), ACA2169ACT (6691A>T), TTC2170TTT (6694C>T), ACA2177ACG (6715A>G), GAT2178AAT (6716G>A), AAT2179GAT (6719A>G), GGA2180GGG (6724A>G), CTA2184TTA (6734C>T), GTG2186GTA (6742G>A), GTG2187GTA (6745G>A), GCC2190GCT (6754C>T), GGA2192GGG (6760A>G), ATA2194TTG (6764A>T 6766A>G), ATT2197ATA (6775T>A), CTG2200TTG (6782C>T), GCA2201GCT (6787A>T), AAC2202AAT (6790C>T), GCA2204GTG (6795C>T 6796A>G), CAC2205CAA (6799C>A), ACC2206ACA (6802C>A), AAC2208AAT (6808C>T), TAC2209TAT (6811C>T), TAC2210TAT (6814C>T), TTC2213TTT (6823C>T), GAA2215GAG (6829A>G), AAG2220AGG (6843A>G), TAC2221TAT (6847C>T), CGA2223CGC (6853A>C), AAT2225GAT (6857A>G), GAG2226GAA (6862G>A), GAA2229GAG (6871A>G), GTC2231ACA (6875G>A 6876T>C 6877C>A), AAG2232AAA (6880G>A), TCT2233TCA (6883T>A), GTT2235GTG (6889T>G), GAC2239GAT (6901C>T), GGC2244GGT (6916C>T), CCG2245CCA (6919G>A), AAA2247AAG (6925A>G), CTT2248CTC (6928T>C), GAC2250GAA (6934C>A), GGG2255GGC (6949G>C), TTC2257TTT (6955C>T), AAA2258AAG (6958A>G), CAA2264CAG (6976A>G), CTA2266TTA (6980C>T), ATC2267ATT (6985C>T), GTA2271GTT (6997A>T), GTG2272GTT (7000G>T), GTG2273ATA (7001G>A 7003G>A), GCT2276GCC (7012T>C), AAG2277AAA (7015G>A), CAT2278CAC (7018T>C), TTT2283TAT (7032T>A), GCC2285GCT (7039C>T), TTG2286CTA (7040T>C 7042G>A), GGA2288GGG (7048A>G), GCT2292GCA (7060T>A), GTC2293GTA (7063C>A), AGC2298AGT (7078C>T), TTA2300CTG (7082T>C 7084A>G), GTA2301GTG (7087A>G), ACC2302ACA (7090C>A), CAT2304CAC (7096T>C), GTA2305GTG (7099A>G), GTT2306GTC (7102T>C), GAG2309GAA (7111G>A), CGA2311CGG (7117A>G), ACA2314AAA (7125C>A), GAA2315GAG (7129A>G), TTT2316TTC (7132T>C), CTG2317TTA (7133C>T 7135G>A), GTG2319GTA (7141G>A), GAT2320GAC (7144T>C), GCA2321TCG (7145G>T 7147A>G), GAG2322GAA (7150G>A), GAG2324GAA (7156G>A), GCA2325GCT (7159A>T), GAG2326_GCA2327del (7160_7165delGAGGCA), TTC2329TTT (7171C>T), GCG2335GCT (7189G>T), AAA2338AAG (7198A>G), AGC2339AGT (7201C>T), CTA2341TTA (7205C>T), AAT2342AAC (7210T>C), GAT2344GAA (7216T>A), GCG2345GCA (7219G>A), ATC2347ATA (7225C>A), AAG2348AAA (7228G>A), AAG2352AAA (7240G>A), TAT2353TAC (7243T>C), AAA2355AAG (7249A>G), CCT2356CCG (7252T>G), GAT2358GAA (7258T>A), GTT2359GTC (7261T>C), GGT2360GGA (7264T>A), GTC2361GTG (7267C>G), GTG2362GTA (7270G>A), GAT2363_CGG2364insT-- (7273_7274insT), CGG2364GTG (7274C>G 7275G>T), CAT2366CTT (7281A>T), TTG2367TCG (7284T>C), TCA2371TTA (7296C>T), TCT2376TTT (7311C>T), ACC2377ATC (7314C>T), TGC2378TAC (7317G>A), AAT2379-AA (7319delA 7321T>A), CAC2381CAT (7327C>T), GGC2382GGT (7330C>T), TTC2383TTT (7333C>T), AAG2384CAG (7334A>C), TGT2386TGC (7342T>C), GCA2387AAC (7343G>A 7344C>A 7345A>C), ACT2390ACC (7354T>C), CAA2393CAG (7363A>G), TTC2396TTT (7372C>T), GCG2398GCT (7378G>T), CTC2399CTT (7381C>T), GCA2404GCT (7396A>T), GTC2405GTT (7399C>T), GCC2407GCT (7405C>T), AGT2408ATG (7407G>T 7408T>G), GGG2410GGT (7414G>T), TGC2411GGC (7415T>G), AAA2412AAG (7420A>G), GAC2415GAT (7429C>T), TAT2416TAC (7432T>C), CAT2419CAC (7441T>C), AAG2425AAA (7459G>A), GAA2426GAG (7462A>G), ATA2428ATT (7468A>T), GTC2429GTA (7471C>A), CAA2431CAG (7477A>G), AGC2432AGT (7480C>T), CTG2434CTT (7486G>T), TAT2437TAC (7495T>C), AAA2438AAG (7498A>G), GGT2439GGC (7501T>C), TTG2440TCA (7503T>C 7504G>A), ATT2443ATA (7513T>A), AAC2445AAT (7519C>T), GGA2446GGG (7522A>G), AAG2449AAA (7531G>A), GAG2451GAA (7537G>A), CTC2452CTT (7540C>T), TGT2454TGC (7546T>C), AAG2462AAA (7570G>A), ACG2463ACA (7573G>A), ACG2465ACA (7579G>A), GCT2468GCC (7588T>C), CTA2471TTA (7595C>T), TTG2474CTA (7604T>C 7606G>A), GGT2477GGA (7615T>A), TGT2480TGC (7624T>C), GAC2483GAT (7633C>T), TTC2484TTT (7636C>T), AAT2485AAC (7639T>C), TTT2488TTC (7648T>C), GAA2494GAG (7666A>G), TGT2496TGC (7672T>C), ACA2498ACT (7678A>T), GGG2500GGA (7684G>A), ACT2502ACC (7690T>C), TTT2504TTC (7696T>C), GGT2506GGG (7702T>G), GAT2509AAT (7709G>A), AAA2510AGG (7713A>G 7714A>G), CTG2511TTG (7715C>T), CGT2514CGC (7726T>C), GAG2517GAA (7735G>A), GTA2520GTG (7744A>G), TAC2521TAT (7747C>T), GGC2526GGT (7762C>T), CAG2528CAA (7768G>A), TTT2529TTC (7771T>C), TCA2532TCG (7780A>G), CTA2533CTC (7783A>C), ACT2534ACC (7786T>C), CTA2537TTG (7793C>T 7795A>G), ATC2538ATT (7798C>T), GCT2540GCC (7804T>C), ACC2543ATC (7812C>T), ATC2544ATT (7816C>T), GAA2550GAG (7834A>G), GAC2551GAT (7837C>T), GTG2554TTA (7844G>T 7846G>A), CAG2557CAA (7855G>A), CTG2559CTA (7861G>A), TTA2562CTG (7868T>C 7870A>G), TAC2563TAT (7873C>T), ACT2564ACA (7876T>A), GAG2565GAA (7879G>A), ATT2566ATA (7882T>A), GTT2567ATT (7883G>A), TAC2568TAT (7888C>T), CCA2570CCT (7894A>T), ATT2571ATC (7897T>C), GTC2579GTT (7921C>T), AAG2581AAA (7927G>A), GGT2584GGC (7936T>C), AAT2585AAC (7939T>C), AAT2586AAC (7942T>C), GTG2594GTA (7966G>A), GAT2595GAC (7969T>C), TCT2597TCC (7975T>C), GTC2601GTT (7987C>T), CTT2602CTC (7990T>C), TAC2606TAT (8002C>T), GCT2607GCC (8005T>C), GAA2616GAG (8032A>G), ATC2618ATT (8038C>T), GAC2619GAT (8041C>T), AGC2620AAC (8043G>A), TTG2632TTA (8080G>A), GTG2635GTA (8089G>A), CCG2637CCA (8095G>A), GAG2638GAA (8098G>A), AGC2641AGT (8107C>T), ATT2642ATC (8110T>C), GAT2644GAC (8116T>C), CAA2648CAG (8128A>G), GGT2654GGC (8146T>C), AAC2656AGT (8151A>G 8152C>T), TCG2660TCA (8164G>A), AGG2665AAG (8178G>A), AAG2666AAA (8182G>A), TTG2669TTA (8191G>A), TTC2671TTT (8197C>T), TCC2673TCT (8203C>T), AGA2675AAA (8208G>A), GGC2676GGG (8212C>G), CTG2677TTG (8213C>T), CTA2678CTG (8218A>G), ATC2679ATT (8221C>T), GAG2680GAA (8224G>A), CCA2685CCG (8239A>G), ATT2692ATC (8260T>C), GTA2693GTG (8263A>G), GAT2699GAC (8281T>C), AGA2700AGG (8284A>G), CCA2704GCG (8294C>G 8296A>G), GAG2705GAA (8299G>A), CAC2706CAT (8302C>T), ATT2711ATC (8317T>C), TGC2712TGT (8320C>T), GCT2714GCA (8326T>A), GAG2717GAA (8335G>A), TCC2718TCT (8338C>T), TCT2722TTT (8349C>T), GAA2723GAG (8353A>G), CAC2726CAT (8362C>T), CAA2727CAG (8365A>G), TAC2732TAT (8380C>T), TCA2733TCG (8383A>G), TTA2735TTG (8389A>G), CCT2740CCC (8404T>C), GCA2742TCA (8408G>T), ACA2743ACG (8413A>G), GCG2745GCT (8419G>T), GCT2750GCC (8434T>C), TAT2752TAC (8440T>C), ATG2756ATA (8452G>A), CTA2758CTG (8458A>G), AGG2759AAG (8460G>A), AAA2760AAG (8464A>G), CTG2761CTA (8467G>A), GCT2766ACA (8480G>A 8482T>A), GAG2771GAA (8497G>A), CTA2772TTG (8498C>T 8500A>G), AGA2773AAG (8502G>A 8503A>G), GCC2774GCT (8506C>T), GAA2777GAT (8515A>T), GTC2780GTT (8524C>T), GCA2781GCC (8527A>C), TTA2782CTG (8528T>C 8530A>G), GAG2785GAC (8539G>C), CTT2788TGT (8546C>T 8547T>G), TCT2790ACT (8552T>A), GTA2793GTG (8563A>G), GCA2797GGA (8574C>G), ATT2801GTT (8585A>G), GGA2805GAA (8598G>A), AGC2806AGT (8602C>T), AAC2807TCT (8603A>T 8604A>C 8605C>T), GCA2811GTA (8616C>T), AAA2812AAG (8620A>G), CCA2813CAA (8622C>A), GAG2814GGA (8625A>G 8626G>A), CAG2815CAA (8629G>A), GGC2816GGT (8632C>T), ATC2818ATT (8638C>T), CAG2819CAA (8641G>A), AAC2821AAT (8647C>T), CCG2822TTC (8648C>T 8649C>T 8650G>C), AAC2823AAT (8653C>T), AAA2824AAG (8656A>G), GGA2825GAA (8658G>A), GAT2827GCG (8664A>C 8665T>G), GTT2830GTG (8674T>G), GCA2832GTT (8679C>T 8680A>T), GGC2833GGA (8683C>A), GGG2836GGA (8692G>A), ACA2837ACT (8695A>T), CCG2841CCA (8707G>A), AGA2842CGA (8708A>C), AAG2844AAA (8716G>A), ATC2846ATT (8722C>T), ACG2847ACA (8725G>A), AAA2849AAG (8731A>G), AGA2851AGG (8737A>G), CCC2853CCT (8743C>T), ACA2854AAG (8745C>A 8746A>G), AGC2855AGT (8749C>T), AAG2856AAA (8752G>A), GGA2857GGT (8755A>T), AAC2862AAT (8770C>T), TTA2863CTA (8771T>C), GAA2864GAG (8776A>G), CAT2865CAC (8779T>C), TTG2866TTA (8782G>A), TAT2869TAC (8791T>C), CCA2871CCG (8797A>G), CAA2872CAG (8800A>G), CAA2873CAG (8803A>G), GAT2875GAC (8809T>C), ATT2876ATC (8812T>C), AAT2878AAC (8818T>C), CGG2880CGA (8824G>A), GCA2881GCG (8827A>G), ACG2888ACA (8848G>A), TAT2890TAC (8854T>C), GAG2891GGA (8856A>G 8857G>A), GTG2893GTA (8863G>A), CGG2894CAG (8865G>A), ATG2895CTT (8867A>C 8869G>T), GCA2896GCG (8872A>G), GGA2900GAA (8883G>A), ACT2902TCT (8888A>T), TGC2915TGT (8929C>T), GTC2924ATC (8954G>A), AAC2925AAT (8959C>T), GGA2926GGT (8962A>T), GGG2933GGA (8983G>A), AAT2934GAC (8984A>G 8986T>C), GAA2935GTA (8988A>T), GAG2938GAA (8998G>A), CCG2940CCA (9004G>A), TTG2941TTA (9007G>A), ATC2944ATT (9016C>T), ACC2951ACA (9037C>A), ATA2967ATT (9085A>T), AAC2971AAT (9097C>T), CGA2984CGT (9136A>T), CGG2987CGT (9145G>T), ATG2989GGA (9149A>G 9150T>G 9151G>A), GGT2990AGT (9152G>A), TTA2991TTG (9157A>G), GCG2992GCT (9160G>T), CGT2993CGC (9163T>C), GAG3000GAA (9184G>A), CGA3004CGG (9196A>G), AGG3010AGA (9214G>A), GAA3011GAG (9217A>G), GCG3012GCA (9220G>A), GCA3020GCT (9244A>T), TTG3021TTA (9247G>A), GCC3024GCT (9256C>T), CCT3026TCC (9260C>T 9262T>C), GGG3030GGA (9274G>A), GAC3032GAT (9280C>T), ATC3035ATT (9289C>T), GAG3040GAA (9304G>A), GTC3050GTT (9334C>T), GTC3060GTG (9364C>G)								
P1 protein (NP_734243.1)	1	284	100%	1414	73.7%	284 (100%)	206 (72.5%)	0/0/0/0	0

	Begin	End	Coverage	Score	Concordance	Matches	Identities	I/D/M/F*	Stop Codons
**NT**	**19**	**9704**	**99.8%**	**12534**	**65.0%**	**9674 (99.7%)**	**8011 (82.6%)**	**15/12**	
Protein mutations:	Y4Q (194T>C 196C>A), C9L (209T>C 210G>T 211T>G), F13M (221T>A 223T>G), S23P (251T>C), C24F (255G>T 256C>T), E25G (258A>G), I27V (263A>G 265T>G), V28A (267T>C), E30V (273A>T), A35T (287G>A 289T>C), V37T (293G>A 294T>C), A41I (305G>A 306C>T), D42G (309A>G), E44D (316A>T), T45V (317A>G 318C>T 319A>G), L52Q (339T>A 340C>A), K53R (342A>G), K55A (347A>G 348A>C 349A>G), Y56H (350T>C), T58V (356A>G 357C>T), V61T (365G>A 366T>C 367G>A), L62S (368C>T 369T>C), F67R (383T>C 384T>G), A75V (408C>T 409C>T), M78E (416A>G 417T>A), E85K (437G>A), R86E (440A>G 441G>A 442G>A), K87R (443A>C 444A>G 445G>A), D88E (448T>A), E91A (456A>C), H93N (461C>A), D111E (517T>A), S116F (531C>T), P118S (536C>T), Q119W (539C>T 540A>G 541A>G), R122K (549G>A 550A>G), V134A (585T>C 586C>G), R137Y (593C>T 594G>A 595C>T), P138R (597C>G), I139V (599A>G 601A>G), I140P (602A>C 603T>C 604A>G), M147I (625G>A), I151V (635A>G 637T>C), I154V (644A>G), E160A (663A>C 664G>C), H166Q (682C>A), T172S (699C>G), H174Q (706T>A), K179E (719A>G 721G>A), I180V (722A>G 724A>G), A183S (731G>T), Y184P (734T>C 735A>C 736C>T), S185R (737T>C 738C>G 739C>T), A187V (744C>T 745G>C), R189C (749C>T 751A>C), M194K (765T>A), R197K (773C>A 774G>A 775A>G), M206K (801T>A), G210A (813G>C 814A>G), L211H (816T>A), R214Q (825G>A 826T>A), R226C (860C>T 862C>T), T227A (863A>G 865T>C), I228T (867T>C), N229D (869A>G 871C>T), I230L (872A>T 874A>G), R232K (879G>A), N240S (903A>G), T241N (906C>A 907A>C), K242T (909A>C 910A>C), S243N (912G>A 913C>T), S252L (938T>C 939C>T 940A>T), L266I (980T>A 982G>C), R272K (999G>A), Q275R (1007C>A 1008A>G 1009G>A), S276G (1010A>G 1012T>G), I277V (1013A>G), N279Q (1019A>C 1021C>G)								
Codon mutations:	ACT3ACC (193T>C), TAC4CAA (194T>C 196C>A), TCA6TCG (202A>G), ATC8ATT (208C>T), TGT9CTG (209T>C 210G>T 211T>G), TCG12TCA (220G>A), TTT13ATG (221T>A 223T>G), GAA14GAG (226A>G), CTA17CTT (235A>T), TAC19TAT (241C>T), CCA21CCT (247A>T), TCT23CCT (251T>C), TGC24TTT (255G>T 256C>T), GAG25GGG (258A>G), CAT26CAC (262T>C), ATT27GTG (263A>G 265T>G), GTG28GCG (267T>C), GAA30GTA (273A>T), CGA31CGT (277A>T), GAA32GAG (280A>G), GTG33GTT (283G>T), CCG34CCA (286G>A), GCT35ACC (287G>A 289T>C), GTT37ACT (293G>A 294T>C), CCT39CCC (301T>C), TTC40TTT (304C>T), GCA41ATA (305G>A 306C>T), GAT42GGT (309A>G), CTG43TTG (311C>T), GAA44GAT (316A>T), ACA45GTG (317A>G 318C>T 319A>G), CAA46CAG (322A>G), GCA49GCT (331A>T), CTC52CAA (339T>A 340C>A), AAG53AGG (342A>G), AAA55GCG (347A>G 348A>C 349A>G), TAT56CAT (350T>C), GCT57GCA (355T>A), ACT58GTT (356A>G 357C>T), CGT60CGC (364T>C), GTG61ACA (365G>A 366T>C 367G>A), CTC62TCC (368C>T 369T>C), AAA63AAG (373A>G), AAC64AAT (376C>T), TTT67CGT (383T>C 384T>G), ACG68ACA (388G>A), TAC69TAT (391C>T), CGA70CGG (394A>G), TAC71TAT (397C>T), AAG72AAA (400G>A), GAT74GAC (406T>C), GCC75GTT (408C>T 409C>T), CAG76CAA (412G>A), ATA77ATT (415A>T), ATG78GAG (416A>G 417T>A), ATT80ATC (424T>C), CAG81CAA (427G>A), AAA83AAG (433A>G), CTG84CTC (436G>C), GAG85AAG (437G>A), AGG86GAA (440A>G 441G>A 442G>A), AAG87CGA (443A>C 444A>G 445G>A), GAT88GAA (448T>A), GAA90GAG (454A>G), GAA91GCA (456A>C), TAT92TAC (460T>C), CAC93AAC (461C>A), CAA95CAG (469A>G), GCC97GCT (475C>T), CCT99CCA (481T>A), ACT106ACC (502T>C), ATA107ATT (505A>T), GGC109GGT (511C>T), GAT111GAA (517T>A), CCT112CCA (520T>A), CCA113CCT (523A>T), TCT116TTT (531C>T), GAG117GAA (535G>A), CCA118TCA (536C>T), CAA119TGG (539C>T 540A>G 541A>G), AGA122AAG (549G>A 550A>G), ATC124ATT (556C>T), ATT125ATC (559T>C), CAT126CAC (562T>C), ACT128ACC (568T>C), CCA129CCG (571A>G), CGT132CGC (580T>C), GTC134GCG (585T>C 586C>G), CGC137TAT (593C>T 594G>A 595C>T), CCC138CGC (597C>G), ATA139GTG (599A>G 601A>G), ATA140CCG (602A>C 603T>C 604A>G), ACA143ACG (613A>G), GAA144GAG (616A>G), GGC145GGG (619C>G), CAG146CAA (622G>A), ATG147ATA (625G>A), AAT148AAC (628T>C), CAC149CAT (631C>T), ATT151GTC (635A>G 637T>C), ATA154GTA (644A>G), ATT157ATC (655T>C), TCG159TCA (661G>A), GAG160GCC (663A>C 664G>C), AAA161AAG (667A>G), GGG163GGC (673G>C), GTC165GTT (679C>T), CAC166CAA (682C>A), TTA167CTA (683T>C), AGT169AGC (691T>C), ACC172AGC (699C>G), ACT173ACA (703T>A), CAT174CAA (706T>A), AAG179GAA (719A>G 721G>A), ATA180GTG (722A>G 724A>G), CTT181TTG (725C>T 727T>G), GGT182GGA (730T>A), GCA183TCA (731G>T), TAC184CCT (734T>C 735A>C 736C>T), TCC185CGT (737T>C 738C>G 739C>T), GCG187GTC (744C>T 745G>C), CGA189TGC (749C>T 751A>C), ACT190ACC (754T>C), ATG194AAG (765T>A), TTG196CTG (770T>C), CGA197AAG (773C>A 774G>A 775A>G), CGG198AGG (776C>A), TTC202TTT (790C>T), ATG206AAG (801T>A), GGA210GCG (813G>C 814A>G), CTT211CAT (816T>A), TTG212CTG (818T>C), CAA213CAG (823A>G), CGT214CAA (825G>A 826T>A), CGG217AGA (833C>A 835G>A), GAC219GAT (841C>T), AAT223AAC (853T>C), GTC225GTT (859C>T), CGC226TGT (860C>T 862C>T), ACT227GCC (863A>G 865T>C), ATC228ACC (867T>C), AAC229GAT (869A>G 871C>T), ATA230TTG (872A>T 874A>G), AGG232AAG (879G>A), GAT234GAC (886T>C), ATC238ATT (898C>T), AAC240AGC (903A>G), ACA241AAC (906C>A 907A>C), AAA242ACC (909A>C 910A>C), AGC243AAT (912G>A 913C>T), GGC246GGA (922C>A), GGT249GGA (931T>A), TCA252CTT (938T>C 939C>T 940A>T), GGA253GGG (943A>G), TTC256TTT (952C>T), TCA261TCG (967A>G), GAA263GAG (973A>G), GGG264GGC (976G>C), TTG266ATC (980T>A 982G>C), GCA269GCG (991A>G), CGT270CGC (994T>C), AGA272AAA (999G>A), GTT273GTC (1003T>C), CAG275AGA (1007C>A 1008A>G 1009G>A), AGT276GGG (1010A>G 1012T>G), ATT277GTT (1013A>G), TTA278TTG (1018A>G), AAC279CAG (1019A>C 1021C>G), ATC282ATT (1030C>T)								
HC-Pro protein (NP_734244.1)	1	456	100%	3144	95.7%	456 (100%)	425 (93.2%)	0/0/0/0	0
Protein mutations:	D4E (1048C>A), R16Q (1083G>A), S21A (1097T>G 1099G>A), R36K (1143G>A), L40I (1154T>A 1156G>A), S62N (1221G>A), V65T (1229G>A 1230T>C 1231T>C), S66N (1233G>A 1234C>T), K74T (1257A>C 1258A>G), N81S (1278A>G), A85V (1290C>T 1291G>A), D88T (1298G>A 1299A>C 1300C>T), I91V (1307A>G 1309A>G), N94E (1316A>G 1318T>A), I98T (1329T>C), L113I (1373C>A), I117V (1385A>G), E123D (1405G>T), A127S (1415G>T 1417A>T), V134I (1436G>A 1438C>T), E224G (1707A>G), A228T (1718G>A), I236V (1742A>G 1744C>T), S241N (1758G>A), V252I (1790G>A), I301V (1937A>G 1939T>C), F323Y (2004T>A), I344M (2068T>G), V346I (2072G>A 2074G>A), R391K (2208G>A), D410E (2266C>G)								
Codon mutations:	TCG1TCA (1039G>A), AAT2AAC (1042T>C), GCC3GCA (1045C>A), GAC4GAA (1048C>A), AAT5AAC (1051T>C), AAG8AAA (1060G>A), GGT9GGA (1063T>A), GAC11GAT (1069C>T), CGA16CAA (1083G>A), TAT19TAC (1093T>C), TCG21GCA (1097T>G 1099G>A), CAC23CAT (1105C>T), GTA26GTG (1114A>G), GCT27GCG (1117T>G), TTA29TTG (1123A>G), CCT30CCA (1126T>A), GTC31GTT (1129C>T), AGG36AAG (1143G>A), GTA37GTT (1147A>T), GCT38GCA (1150T>A), TTG40ATA (1154T>A 1156G>A), CAC43CAT (1165C>T), AGT44AGC (1168T>C), CTT46TTA (1172C>T 1174T>A), ACC55ACT (1201C>T), AGC62AAC (1221G>A), GTT65ACC (1229G>A 1230T>C 1231T>C), AGC66AAT (1233G>A 1234C>T), CTG68CTA (1240G>A), AAG70AAA (1246G>A), TTG72CTA (1250T>C 1252G>A), CAT73CAC (1255T>C), AAA74ACG (1257A>C 1258A>G), AGA77AGG (1267A>G), GGT79GGC (1273T>C), TTG80TTA (1276G>A), AAT81AGT (1278A>G), TTG83CTG (1283T>C), GCG85GTA (1290C>T 1291G>A), GAC88ACT (1298G>A 1299A>C 1300C>T), TTT90TTC (1306T>C), ATA91GTG (1307A>G 1309A>G), CAT92CAC (1312T>C), AAT94GAA (1316A>G 1318T>A), TTG97TTA (1327G>A), ATA98ACA (1329T>C), GCG99GCT (1333G>T), TTA100TTG (1336A>G), CTA103TTG (1343C>T 1345A>G), CCG106CCA (1354G>A), GTG107GTT (1357G>T), GAC108GAT (1360C>T), CTC111TTG (1367C>T 1369C>G), CTT113ATT (1373C>A), TTC114TTT (1378C>T), GAG116GAA (1384G>A), ATA117GTA (1385A>G), AAA119AAG (1393A>G), TCC120TCT (1396C>T), GAG123GAT (1405G>T), AAA124AAG (1408A>G), CAG125CAA (1411G>A), GCA127TCT (1415G>T 1417A>T), CCG128CCC (1420G>C), AAT131AAC (1429T>C), TTA132TTG (1432A>G), GTC134ATT (1436G>A 1438C>T), TTA135CTG (1439T>C 1441A>G), AAT136AAC (1444T>C), TTC139TTT (1453C>T), CTG140CTT (1456G>T), GAA144GAG (1468A>G), TTG155CTA (1499T>C 1501G>A), AGT156AGC (1504T>C), TTG157TTA (1507G>A), CTC158CTT (1510C>T), GAA159GAG (1513A>G), TTA160TTG (1516A>G), GCA161GCT (1519A>T), AGG162AGA (1522G>A), TTC163TTT (1525C>T), CAG164CAA (1528G>A), AAC166AAT (1534C>T), ACT168ACA (1540T>A), AAC170AAT (1546C>T), AAA173AAG (1555A>G), GAT175GAC (1561T>C), ATA176ATC (1564A>C), TCT177TCA (1567T>A), TTC179TTT (1573C>T), AGA180AGG (1576A>G), TTA183CTA (1583T>C), TCT184TCA (1588T>A), AAG186AAA (1594G>A), AAC188AAT (1600C>T), CTG191CTA (1609G>A), TCG194TCA (1618G>A), TGC195TGT (1621C>T), GAC196GAT (1624C>T), AAC197AAT (1627C>T), CTG199TTG (1631C>T), AAA201AAG (1639A>G), TTC205TTT (1651C>T), CTC206CTG (1654C>G), CAT213CAC (1675T>C), TCA219TCG (1693A>G), TTT222TTC (1702T>C), GAA224GGA (1707A>G), GCA228ACA (1718G>A), GGA230GGG (1726A>G), TAC231TAT (1729C>T), TCA232TCC (1732A>C), TAT234TAC (1738T>C), ATC236GTT (1742A>G 1744C>T), CGC237CGT (1747C>T), CAT239CAC (1753T>C), AGT241AAT (1758G>A), AGG244AGA (1768G>A), AAC250AAT (1786C>T), TTA251TTG (1789A>G), GTT252ATT (1790G>A), GTC253GTT (1795C>T), TTA257CTG (1805T>C 1807A>G), GAG259GAA (1813G>A), AGG261AGA (1819G>A), AAG263AAA (1825G>A), GAC267GAT (1837C>T), TAT268TAC (1840T>C), CAA271CAG (1849A>G), GTC274GTG (1858C>G), AAA276AAG (1864A>G), TGC278TGT (1870C>T), AGT280AGC (1876T>C), AAA282AAG (1882A>G), GGT284GGA (1888T>A), AAT285AAC (1891T>C), TGT290TGC (1906T>C), ACA293ACT (1915A>T), GGT298GGC (1930T>C), GCC300GCT (1936C>T), ATT301GTC (1937A>G 1939T>C), TCA303TCG (1945A>G), ACA304ACG (1948A>G), TTC305TTT (1951C>T), CCA308CCT (1960A>T), GTA314GTG (1978A>G), ATT315ATA (1981T>A), GGC316GGT (1984C>T), AAT317AAC (1987T>C), GAC320GAT (1996C>T), TTT323TAT (2004T>A), AAA328AAG (2020A>G), GGG329GGA (2023G>A), GAT330GAC (2026T>C), TCG331TCT (2029G>T), GAG332GAA (2032G>A), TAT341TAC (2059T>C), ATT344ATG (2068T>G), AAC345AAT (2071C>T), GTG346ATA (2072G>A 2074G>A), CTG351TTA (2087C>T 2089G>A), AGC355AGT (2101C>T), GAG356GAA (2104G>A), GAG357GAA (2107G>A), AAG360AAA (2116G>A), TTC362TTT (2122C>T), CGC367CGT (2137C>T), CTT374CTC (2158T>C), ACC376ACT (2164C>T), CCA378CCG (2170A>G), GAT382GAC (2182T>C), GCG384GCA (2188G>A), ACC385ACA (2191C>A), AGA391AAA (2208G>A), TAT394TAC (2218T>C), GAC396GAT (2224C>T), GTA397GTT (2227A>T), TTG402TTA (2242G>A), CCC403CCT (2245C>T), TTG406TTA (2254G>A), GTT407GTC (2257T>C), GAC408GAT (2260C>T), GAC410GAG (2266C>G), CAA412CAG (2272A>G), ACG413ACA (2275G>A), TCA419TCG (2293A>G), GGC421GGT (2299C>T), TCG422TCA (2302G>A), CAG423CAA (2305G>A), GGA426GGT (2314A>T), TAT427TAC (2317T>C), ATT429ATC (2323T>C), CTA430CTG (2326A>G), GTG435GTA (2341G>A), CAA437CAG (2347A>G), CTT438CTC (2350T>C), TTG440TTA (2356G>A), AAT443AAC (2365T>C), GAA445GAG (2371A>G), TTA446TTG (2374A>G), GAT449GAC (2383T>C), ATA450ATT (2386A>T), AAA451AAG (2389A>G), CAT452CAC (2392T>C), TAT453TAC (2395T>C)								
P3 protein (NP_734251.1)	1	365	100%	1928	79.8%	365 (99.7%)	328 (89.6%)	1/0/2/2	0
Protein mutations:	N4G (2414A>G 2415A>G 2416T>G), S6C (2420A>T), K40R (2523A>G), R77K (2634G>A), V107I (2723G>A), A115T (2747G>A 2749T>A), H131N (2795C>A), V140I (2822G>A 2824G>A), P160T (2882C>A), S161H (2885A>C 2886G>A), T164M (2895C>T), N176S (2931A>G), N179S (2940A>G), E189_N190insX (2971_2972insA), T196I (2991C>T), H197R (2994A>G), E199R (2999G>A 3000A>G), R206Q (3021G>A), Y207F (3024A>T 3025C>T), T211I (3036C>T), E212G (3039A>G 3040A>C), K213T (3042A>C 3043G>A), A232V (3099C>T 3100C>A), V234R (3104G>A 3105T>G 3106G>A), S243N (3132G>A), R245K (3137C>A 3138G>A 3139A>G), F250I (3152T>A 3154C>T), C254Y (3165G>A), F272L (3218T>C), V276I (3230G>A), V288M (3266G>A 3268A>G), V291M (3275G>A), A294T (3284G>A), R304K (3315G>A 3316A>G), E305A (3318A>C), M319V (3359A>G), D332E (3400T>G)								

	Begin	End	Coverage	Score	Concordance	Matches	Identities	I/D/M/F*	Stop Codons
**NT**	**19**	**9704**	**99.8%**	**12534**	**65.0%**	**9674 (99.7%)**	**8011 (82.6%)**	**15/12**	
Codon mutations:	AAT4GGG (2414A>G 2415A>G 2416T>G), GCT5GCA (2419T>A), AGC6TGC (2420A>T), ACA12ACT (2440A>T), GTT21GTC (2467T>C), TCT24TCC (2476T>C), TCG26TCA (2482G>A), CTG29CTA (2491G>A), CTG31CTT (2497G>T), TTG33TTA (2503G>A), AGA38AGG (2518A>G), CCT39CCC (2521T>C), AAG40AGG (2523A>G), AGA43AGG (2533A>G), TTG45TTA (2539G>A), TTA47TTG (2545A>G), GAG49GAA (2551G>A), CCT50CCA (2554T>A), CTG52TTG (2558C>T), TTG53CTC (2561T>C 2563G>C), CTA55CTG (2569A>G), TCA56TCG (2572A>G), CCT60CCC (2584T>C), GGC61GGT (2587C>T), CTG63CTC (2593G>C), GCT65GCC (2599T>C), AAT68AAC (2608T>C), TTT72TTC (2620T>C), GCG75GCA (2629G>A), GTG76GTA (2632G>A), AGG77AAG (2634G>A), TTG78TTA (2638G>A), ATT80ATA (2644T>A), AAA83AAG (2653A>G), GCT87GCC (2665T>C), CTA92TTA (2678C>T), CTA93CTG (2683A>G), GCT95GCC (2689T>C), CTA98CTC (2698A>C), TCA101TCG (2707A>G), GCG102GCA (2710G>A), GAA104GAG (2716A>G), ACA105ACG (2719A>G), CTC106CTT (2722C>T), GTC107ATC (2723G>A), GCA108GCG (2728A>G), AGG110AGA (2734G>A), ATT111ATA (2737T>A), GAT114GAC (2746T>C), GCT115ACA (2747G>A 2749T>A), GCA116GCT (2752A>T), GCT117GCA (2755T>A), GAC119GAT (2761C>T), CTC120CTT (2764C>T), TGT125TGC (2779T>C), GGG127GGT (2785G>T), AAC129AAT (2791C>T), CAT131AAT (2795C>A), TAC134TAT (2806C>T), TTG138CTC (2816T>C 2818G>C), GTG140ATA (2822G>A 2824G>A), TTG141TTA (2827G>A), GTT144GTC (2836T>C), AAG145AAA (2839G>A), AAT146AAC (2842T>C), ACC153ACA (2863C>A), CTA154TTA (2864C>T), CCA160ACA (2882C>A), AGT161CAT (2885A>C 2886G>A), AAC163AAT (2893C>T), ACG164ATG (2895C>T), GTC166GTT (2902C>T), GTA167GTG (2905A>G), ATT169ATC (2911T>C), AAT176AGT (2931A>G), AAC179AGC (2940A>G), GCT181GCC (2947T>C), GAA189_AAT190insA-- (2971_2972insA), GGT195GGC (2989T>C), ACT196ATT (2991C>T), CAT197CGT (2994A>G), GAG199AGG (2999G>A 3000A>G), TCC204TC- (3016delC), CGG206CAG (3021G>A), TAC207TTT (3024A>T 3025C>T), ACA211ATA (3036C>T), GAA212GGC (3039A>G 3040A>C), AAG213ACA (3042A>C 3043G>A), TTG216TTA (3052G>A), TTA219TTG (3061A>G), GCG226GCA (3082G>A), GGC229GGT (3091C>T), GCC232GTA (3099C>T 3100C>A), GTG234AGA (3104G>A 3105T>G 3106G>A), GTC235GTT (3109C>T), AAA236AAG (3112A>G), ACT238ACC (3118T>C), GCC239GCT (3121C>T), GGA241GGG (3127A>G), AGC243AAC (3132G>A), CGA245AAG (3137C>A 3138G>A 3139A>G), TAT249TAC (3151T>C), TTC250ATT (3152T>A 3154C>T), TGT254TAT (3165G>A), GTA255GTG (3169A>G), ATC265ATT (3199C>T), AGG267CGG (3203A>C), CGT268CGC (3208T>C), CCA270CCG (3214A>G), ACC271ACT (3217C>T), TTT272CTT (3218T>C), GTC273GTA (3223C>A), GTT276ATT (3230G>A), AAC277AAT (3235C>T), TTA285CTA (3257T>C), ACC286ACT (3262C>T), AGC287AGT (3265C>T), GTA288ATG (3266G>A 3268A>G), GTG289GTA (3271G>A), GTG291ATG (3275G>A), GCA294ACA (3284G>A), CAG299CAA (3301G>A), AGA304AAG (3315G>A 3316A>G), GAA305GCA (3318A>C), GAG307GAA (3325G>A), TTG308TTA (3328G>A), ATA311ATT (3337A>T), GAG315GAA (3349G>A), GTC317GTT (3355C>T), TGC318TGT (3358C>T), ATG319GTG (3359A>G), CTA321CTG (3367A>G), TAT322TAC (3370T>C), TTA325CTG (3377T>C 3379A>G), GAA330GAG (3394A>G), CGC331CGT (3397C>T), GAT332GAG (3400T>G), ACA334ACG (3406A>G), TAC338TAT (3418C>T), ATT339ATA (3421T>A), TAT341TAC (3427T>C), AAG343AAA (3433G>A), TCA344TCT (3436A>T), GTA345GTG (3439A>G), AAC346AAT (3442C>T), CCT347CCC (3445T>C), CAG351CAA (3457G>A), TTT352TTC (3460T>C), GCT353GCA (3463T>A), GCG355GCT (3469G>T), CAG356CAA (3472G>A), CAC364CAT (3496C>T), CAG365CAA (3499G>A)								
6K1 protein (NP_734245.1)	1	52	100%	305	90.5%	52 (100%)	45 (86.5%)	0/0/0/0	0
Protein mutations:	V5G (3513T>G), M16V (3545A>G), A17T (3548G>A), V19I (3554G>A 3556C>T), L42V (3623C>G), L45M (3632C>A), Y47H (3638T>C)								
Codon mutations:	TCC2TCT (3505C>T), GTT5GGT (3513T>G), AAA7AAG (3520A>G), GAA10GAG (3529A>G), CAA11CAG (3532A>G), ATG16GTG (3545A>G), GCT17ACT (3548G>A), TTA18CTA (3551T>C), GTC19ATT (3554G>A 3556C>T), AGT28AGC (3583T>C), GAT29GAC (3586T>C), TGC30TGT (3589C>T), TTC32TTT (3595C>T), AAT36AAC (3607T>C), TTT38TTC (3613T>C), AAG39AAA (3616G>A), GGT40GGC (3619T>C), CTT42GTT (3623C>G), TCC43TCT (3628C>T), CTG45ATG (3632C>A), GAT46GAC (3637T>C), TAT47CAT (3638T>C), GAA48GAG (3643A>G)								
CI protein (NP_734246.1)	1	634	100%	4190	97.9%	634 (100%)	608 (95.9%)	0/0/0/0	0
Protein mutations:	I15V (3698A>G), S21N (3717G>A), R26K (3731C>A 3732G>A), I140V (4073A>G), F158Y (4128T>A 4129C>T), A166S (4151G>T), V171I (4166G>A), L190I (4223C>A 4225G>A), I222V (4319A>G 4321A>G), F247Y (4395T>A), V251I (4406G>A), M274V (4475A>G 4477G>T), T276S (4481A>T), A297L (4544G>T 4545C>T 4546G>A), R298K (4548G>A), V342I (4679G>A 4681G>C), V381I (4796G>A 4798C>T), T383S (4803C>G 4804T>C), S468G (5057A>G), T469A (5060A>G), L479I (5090C>A), A482V (5100C>T), E484D (5107A>T), V489F (5120G>T 5122C>T), I491V (5126A>G), V510I (5183G>A)								
Codon mutations:	TTA2TTG (3661A>G), GTG5GTA (3670G>A), TTT9TTC (3682T>C), GAG11GAA (3688G>A), GAG14GAA (3697G>A), ATT15GTT (3698A>G), GAT17GAC (3706T>C), TTT18TTC (3709T>C), TTG20CTA (3713T>C 3715G>A), AGT21AAT (3717G>A), GAG22GAA (3721G>A), CGA26AAA (3731C>A 3732G>A), ACT27ACA (3736T>A), CTA31TTG (3746C>T 3748A>G), ACA33ACG (3754A>G), AGT36AGC (3763T>C), CGA41CGG (3778A>G), CAA42CAG (3781A>G), ATC43ATT (3784C>T), CAT47CAC (3796T>C), ACA48ACG (3799A>G), CCA50CCT (3805A>T), TAC52TAT (3811C>T), AGA53AGG (3814A>G), TTC58TTT (3829C>T), TTT61TTC (3838T>C), AGA63AGG (3844A>G), GCA64GCC (3847A>C), GTT67GTA (3856T>A), GTG69GTT (3862G>T), GAC72GAT (3871C>T), GCC74GCA (3877C>A), AGC76AGT (3883C>T), GAA77GAG (3886A>G), CTA79CTG (3892A>G), GAC80GAT (3895C>T), TTT81TTC (3898T>C), GTA83GTG (3904A>G), CGG84AGA (3905C>A 3907G>A), GCT86GCC (3913T>C), AAG91AAA (3928G>A), TCA92TCT (3931A>T), GGG94GGA (3937G>A), GTT97GTC (3946T>C), AGT100AGC (3955T>C), GTG101GTA (3958G>A), GCC102GCT (3961C>T), GGA103GGG (3964A>G), TCT104TCC (3967T>C), TTA107TTG (3976A>G), ATT108ATC (3979T>C), CCA110CCT (3985A>T), CTA114CTT (3997A>T), GCG115GCA (4000G>A), AAC117AAT (4006C>T), GTT118GTG (4009T>G), TTC119TTT (4012C>T), AAA120AAG (4015A>G), TCT123TCC (4024T>C), GAA125GAG (4030A>G), CCA126CCT (4033A>T), AAG129AAA (4042G>A), CTG133TTA (4052C>T 4054G>A), CGT134CGC (4057T>C), CGT136CGA (4063T>A), GGA137GGG (4066A>G), ATA140GTA (4073A>G), TTT141TTC (4078T>C), GGC142GGT (4081C>T), TCT143TCC (4084T>C), TCC147TCT (4096C>T), AGC151AGT (4108C>T), GGA152GGT (4111A>T), GCG154GCA (4117G>A), CTA155TTG (4118C>T 4120A>G), TAC157TAT (4126C>T), TTC158TAT (4128T>A 4129C>T), GCC159GCT (4132C>T), AAT160AAC (4135T>C), CAA164CAG (4147A>G), TTA165CTA (4148T>C), GCT166TCT (4151G>T), AAC169AAT (4162C>T), GTA171ATA (4166G>A), ATA172ATC (4171A>C), GAG175GAA (4180G>A), CTG179TTG (4190C>T), CCT181CCC (4198T>C), TCC182TCT (4201C>T), GCG185GCT (4210G>T), TTC186TTT (4213C>T), AGT188AGC (4219T>C), CTG189TTG (4220C>T), CTG190ATA (4223C>A 4225G>A), GTT192GTG (4231T>G), CAT194CAC (4237T>C), GTA199GTG (4252A>G), TTA200CTG (4253T>C 4255A>G), AAA201AAG (4258A>G), ACT205ACC (4270T>C), AGA209AGG (4282A>G), GAG210GAA (4285G>A), GAA212GAG (4291A>G), CAG216CAA (4303G>A), GTC219GTT (4312C>T), ATA222GTG (4319A>G 4321A>G), GTG223GTT (4324G>T), GAG224GAA (4327G>A), CTG227CTT (4336G>T), TCT228TCC (4339T>C), TCA231TCT (4348A>T), GCA235GCG (4360A>G), GGT237GGC (4366T>C), TCT238TCA (4369T>A), ACT240ACC (4375T>C), GTT244GTC (4387T>C), TTT247TAT (4395T>A), GTA251ATA (4406G>A), CTT252CTC (4411T>C), GTT261GTC (4438T>C), ACC263ACA (4444C>A), GCC265GCT (4450C>T), CTC267CTT (4456C>T), GAC270GAT (4465C>T), ATG274GTT (4475A>G 4477G>T), GTC275GTT (4480C>T), ACA276TCA (4481A>T), GGC280GGT (4495C>T), AAG284AAA (4507G>A), CAC285CAT (4510C>T), GGT286GGG (4513T>G), CTA288TTA (4517C>T), GTC291GTT (4528C>T), AAA293AAG (4534A>G), ACC295ACT (4540C>T), GCG297TTA (4544G>T 4545C>T 4546G>A), AGA298AAA (4548G>A), TTT301TTC (4558T>C), GTA303GTT (4564A>T), ACC305ACT (4570C>T), ATA307ATT (4576A>T), TTG314TTA (4597G>A), GAC315GAT (4600C>T), GAC317GAT (4606C>T), GTG318GTA (4609G>A), GTG320GTA (4615G>A), GAC321GAT (4618C>T), TTG324CTT (4625T>C 4627G>T), GAC331GAT (4648C>T), ATT332ATC (4651T>C), AGG335AGA (4660G>A), AGC336AGT (4663C>T), GCT338GCA (4669T>A), GTG342ATC (4679G>A 4681G>C), AGT343AGC (4684T>C), GTT344GTG (4687T>G), GAG348GAA (4699G>A), AGA349AGG (4702A>G), CAA351CAG (4708A>G), AGG352AGA (4711G>A), CTG353TTG (4712C>T), AAA361AAG (4738A>G), GTA363GTT (4744A>T), GCA364GCG (4747A>G), TTG365CTG (4748T>C), ACT370ACC (4765T>C), GTC381ATT (4796G>A 4798C>T), ACT383AGC (4803C>G 4804T>C), GAG384GAA (4807G>A), GCG385GCT (4810G>T), GCT386GCG (4813T>G), CTT387CTC (4816T>C), TAT392TAC (4831T>C), AAC393AAT (4834C>T), CCA395CCT (4840A>T), GGA399GGG (4852A>G), GGC400GGT (4855C>T), AGT404AGC (4867T>C), CTG405CTC (4870G>C), ATT406ATA (4873T>A), TGT409TGC (4882T>C), ACA416ACT (4903A>T), CAG418CAA (4909G>A), TTT420TTC (4915T>C), GAA421GAG (4918A>G), TTG422CTA (4919T>C 4921G>A), AGT423AGC (4924T>C), CCC424CCG (4927C>G), TTT426TTC (4933T>C), ATC427ATA (4936C>A), CAG428CAA (4939G>A), AAT429AAC (4942T>C), TTC430TTT (4945C>T), TCA436TCG (4963A>G), CCT439CCC (4972T>C), CTT445CTC (4990T>C), AAA449AAG (5002A>G), CCT456CCC (5023T>C), TTG457CTG (5024T>C), TGC458TGT (5029C>T), GAT459GAC (5032T>C), TCT461TCC (5038T>C), CCA463CCT (5044A>T), GCA466GCT (5053A>T), TCG467TCA (5056G>A), AGT468GGT (5057A>G), ACT469GCT (5060A>G), TCG472TCT (5071G>T), CTT479ATT (5090C>A), GGA480GGG (5095A>G), GTG481GTT (5098G>T), GCC482GTC (5100C>T), TTA483TTG (5104A>G), GAA484GAT (5107A>T), ATT485ATC (5110T>C), CCA486CCG (5113A>G), AAG487AAA (5116G>A), CAA488CAG (5119A>G), GTC489TTT (5120G>T 5122C>T), ATT491GTT (5126A>G), CAT494CAC (5137T>C), ATC495ATT (5140C>T), AAA496AAG (5143A>G), GAG497GAA (5146G>A), CTC502CTA (5161C>A), GAA504GAG (5167A>G), CTT506CTG (5173T>G), ACG509ACA (5182G>A), GTT510ATT (5183G>A), GTC511GTT (5188C>T), AAG512AAA (5191G>A), AAA514AAG (5197A>G), GAC515GAT (5200C>T), TGC517TGT (5206C>T), TTA518TTG (5209A>G), AGC521AGT (5218C>T), TCG525TCA (5230G>A), ATC527ATT (5236C>T), AGC528AGT (5239C>T), ATC530ATT (5245C>T), ACA536ACT (5263A>T), CTC538CTT (5269C>T), TTC539TTT (5272C>T), GCC540GCA (5275C>A), ATC541ATT (5278C>T), CCA542CCC (5281A>C), AGA543AGG (5284A>G), ACT544ACC (5287T>C), CTA545TTA (5288C>T), ATA546ATT (5293A>T), TTG547CTA (5294T>C 5296G>A), GAG549GAA (5302G>A), CTT552CTA (5311T>A), GAA553GAG (5314A>G), CGA556CGT (5323A>T), AGT564AGC (5347T>C), ATC566ATT (5353C>T), TGC570TGT (5365C>T), GTT577GTC (5386T>C), ACC580ACA (5395C>A), AAA589AAG (5422A>G), GAT590GAC (5425T>C), ACC592ACT (5431C>T), GAG594GAA (5437G>A), CTT599CTC (5452T>C), AAG601AAA (5458G>A), AGT604AGC (5467T>C), CAA605CAG (5470A>G), CTA606TTG (5471C>T 5473A>G), TTC609TTT (5482C>T), TCA610TCC (5485A>C), AAT611AAC (5488T>C), TTG612CTA (5489T>C 5491G>A), GGT614GGC (5497T>C), TGT617TGC (5506T>C), GAG618GAA (5509G>A), GAG619GAA (5512G>A), TAT625TAC (5530T>C), GAG626GAA (5533G>A), TCG627TCT (5536G>T), TTG628TTA (5539G>A), GTT631GTG (5548T>G), CAC633CAT (5554C>T)								
6K2 protein (NP_734247.1)	1	52	100%	303	93.5%	52 (100%)	46 (88.5%)	0/0/0/0	0
Protein mutations:	A2T (5561G>A 5563G>A), I14V (5597A>G), N16K (5605C>A), A21V (5619C>T 5620T>G), I25L (5630A>C), V46I (5693G>A)								
Codon mutations:	GCT1GCA (5560T>A), GCG2ACA (5561G>A 5563G>A), ACG3ACC (5566G>C), TCA4TCG (5569A>G), CTC9CTG (5584C>G), AAG12AAA (5593G>A), GGG13GGA (5596G>A), ATT14GTT (5597A>G), AAC16AAA (5605C>A), GTG20GTT (5617G>T), GCT21GTG (5619C>T 5620T>G), TTG24TTA (5629G>A), ATC25CTC (5630A>C), GGC28GGT (5641C>T), GCA31GCT (5650A>T), ATA35ATT (5662A>T), GGA36GGG (5665A>G), CAA44CAG (5689A>G), GTT46ATT (5693G>A), GAG47GAA (5698G>A), CAA52CAG (5713A>G)								
NIa-VPg protein (NP_734252.1)	1	188	100%	1227	93.3%	188 (100%)	170 (90.4%)	0/0/0/0	0
Protein mutations:	K47R (5853A>G), I61V (5894A>G 5896C>G), R83A (5960C>G 5961G>C 5962G>C), I93V (5990A>G), E102D (6019A>T), K106T (6030A>C), D112E (6049C>A), M115I (6058G>A), S120N (6072G>A 6073T>C), N121H (6074A>C), T123N (6081C>A), C133S (6111G>C 6112T>C), I139V (6128A>G 6130T>C), V149I (6158G>A), T153S (6170A>T 6172A>G), L164F (6203C>T 6205C>T), V173I (6230G>A), A182K (6257G>A 6258C>A)								

	Begin	End	Coverage	Score	Concordance	Matches	Identities	I/D/M/F*	Stop Codons
**NT**	**19**	**9704**	**99.8%**	**12534**	**65.0%**	**9674 (99.7%)**	**8011 (82.6%)**	**15/12**	
Codon mutations:	GGG1GGC (5716G>C), AAA2AAG (5719A>G), GCC10GCA (5743C>A), CGC14CGA (5755C>A), CAT15CAC (5758T>C), CGT17CGC (5764T>C), AAA19AAG (5770A>G), GGC22GGT (5779C>T), TTT23TTC (5782T>C), GAC26GAT (5791C>T), GAC30GAT (5803C>T), AGG41AGA (5836G>A), AAA42AAG (5839A>G), GGA44GGG (5845A>G), AAA45AAG (5848A>G), AAA47AGA (5853A>G), GGT48GGC (5857T>C), ACC49ACT (5860C>T), ACA50ACT (5863A>T), GGT52GGA (5869T>A), GGT54GGC (5875T>C), AGC57AGT (5884C>T), TTC60TTT (5893C>T), ATC61GTG (5894A>G 5896C>G), AAC62AAT (5899C>T), GGG65GGA (5908G>A), TTT66TTC (5911T>C), GAG70GAA (5923G>A), CAA75CAG (5938A>G), TTC76TTT (5941C>T), GTT77GTC (5944T>C), GAT78GAC (5947T>C), GGG82GGA (5959G>A), CGG83GCC (5960C>G 5961G>C 5962G>C), GAA87GAG (5974A>G), GCT91GCA (5986T>A), GAC92GAT (5989C>T), ATT93GTT (5990A>G), GAT95GAC (5998T>C), AGA99CGT (6008A>C 6010A>T), GAA102GAT (6019A>T), GTG103GTT (6022G>T), CGA104CGT (6025A>T), AAA106ACA (6030A>C), GAC112GAA (6049C>A), ATG115ATA (6058G>A), CAA116CAG (6061A>G), GCC117GCA (6064C>A), GGT119GGC (6070T>C), AGT120AAC (6072G>A 6073T>C), AAC121CAC (6074A>C), ACG122ACA (6079G>A), ACC123AAC (6081C>A), ATA124ATT (6085A>T), GCA126GCT (6091A>T), TAC127TAT (6094C>T), TGT133TCC (6111G>C 6112T>C), AAA135AAG (6118A>G), TTG137TTA (6124G>A), AAG138AAA (6127G>A), ATT139GTC (6128A>G 6130T>C), GAT140GAC (6133T>C), TTA141TTG (6136A>G), AAC145AAT (6148C>T), AAA148AAG (6157A>G), GTT149ATT (6158G>A), TGT150TGC (6163T>C), AAA152AAG (6169A>G), ACA153TCG (6170A>T 6172A>G), GGC155GGT (6178C>T), AAA158AAG (6187A>G), TTT159TTC (6190T>C), GAG161GAA (6196G>A), CTC164TTT (6203C>T 6205C>T), GAA165GAG (6208A>G), CTA166TTG (6209C>T 6211A>G), CAG168CAA (6217G>A), GCT172GCA (6229T>A), GTA173ATA (6230G>A), GTC175GTT (6238C>T), AAG178AAA (6247G>A), ATA180ATT (6253A>T), GCA182AAA (6257G>A 6258C>A), CAT187CAC (6274T>C)								
NIa-Pro protein (NP_734248.1)	1	244	100%	1651	93.5%	244 (100%)	222 (91.0%)	0/0/0/0	0
Protein mutations:	K2R (6282A>G), A29T (6362G>A), V42I (6401G>A), A43V (6405C>T), Y51F (6429A>T), Q58R (6450A>G), H70N (6485C>A 6487C>T), L84I (6527C>A), T104I (6588C>T), Y120H (6635T>C), I124V (6647A>G), I134V (6677A>G 6679A>G), D147N (6716G>A), N148D (6719A>G), I163L (6764A>T 6766A>G), A173V (6795C>T 6796A>G), H174Q (6799C>A), K189R (6843A>G), N194D (6857A>G), V200T (6875G>A 6876T>C 6877C>A), D219E (6934C>A), V242I (7001G>A 7003G>A)								
Codon mutations:	AAA2AGA (6282A>G), TCG3TCA (6286G>A), GGC7GGT (6298C>T), TTG8TTA (6301G>A), AGA9AGG (6304A>G), GAC10GAT (6307C>T), AAC12AAT (6313C>T), GCC15GCT (6322C>T), ACA17ACT (6328A>T), GTT18GTG (6331T>G), TGT19TGC (6334T>C), AGG20AGA (6337G>A), CTG21TTG (6338C>T), TAT27TAC (6358T>C), GGG28GGA (6361G>A), GCA29ACA (6362G>A), TCA30TCT (6367A>T), GAG31GAA (6370G>A), TAC33TAT (6376C>T), GGT34GGG (6379T>G), GGA36GGC (6385A>C), GGA38GGT (6391A>T), GCA39GCG (6394A>G), TAC40TAT (6397C>T), GTA42ATA (6401G>A), GCG43GTG (6405C>T), CAC45CAT (6412C>T), TTA47TTG (6418A>G), TTT48TTC (6421T>C), TAC51TTC (6429A>T), GGT53GGA (6436T>A), GAG56GAA (6445G>A), CAA58CGA (6450A>G), TCC59TCA (6454C>A), CAC61CAT (6460C>T), GGT62GGA (6463T>A), TTC64TTT (6469C>T), AGG65AGA (6472G>A), GTG66GTA (6475G>A), AAT68AAC (6481T>C), CAC70AAT (6485C>A 6487C>T), AGT71AGC (6490T>C), AGC73AGT (6496C>T), CTG75CTA (6502G>A), GGT79GGC (6514T>C), AGG80AGA (6517G>A), CTC84ATC (6527C>A), ATC85ATA (6532C>A), CCG88CCA (6541G>A), AAA89AAG (6544A>G), GTC93GTT (6556C>T), TTT94TTC (6559T>C), CAG96CAA (6565G>A), TTG98CTA (6569T>C 6571G>A), CCT103CCA (6586T>A), ACA104ATA (6588C>T), GAA107GAG (6598A>G), AGA108AGG (6601A>G), ATT109ATC (6604T>C), TGT110TGC (6607T>C), GGA113GGG (6616A>G), AAC115AAT (6622C>T), GAG118GAA (6631G>A), AAG119AAA (6634G>A), TAT120CAT (6635T>C), GCT121GCA (6640T>A), TCG123TCC (6646G>C), ATC124GTC (6647A>G), ATC125ATT (6652C>T), AGC129AGT (6664C>T), ACT131ACG (6670T>G), ATA134GTG (6677A>G 6679A>G), GGC136GGT (6685C>T), ACA138ACT (6691A>T), TTC139TTT (6694C>T), ACA146ACG (6715A>G), GAT147AAT (6716G>A), AAT148GAT (6719A>G), GGA149GGG (6724A>G), CTA153TTA (6734C>T), GTG155GTA (6742G>A), GTG156GTA (6745G>A), GCC159GCT (6754C>T), GGA161GGG (6760A>G), ATA163TTG (6764A>T 6766A>G), ATT166ATA (6775T>A), CTG169TTG (6782C>T), GCA170GCT (6787A>T), AAC171AAT (6790C>T), GCA173GTG (6795C>T 6796A>G), CAC174CAA (6799C>A), ACC175ACA (6802C>A), AAC177AAT (6808C>T), TAC178TAT (6811C>T), TAC179TAT (6814C>T), TTC182TTT (6823C>T), GAA184GAG (6829A>G), AAG189AGG (6843A>G), TAC190TAT (6847C>T), CGA192CGC (6853A>C), AAT194GAT (6857A>G), GAG195GAA (6862G>A), GAA198GAG (6871A>G), GTC200ACA (6875G>A 6876T>C 6877C>A), AAG201AAA (6880G>A), TCT202TCA (6883T>A), GTT204GTG (6889T>G), GAC208GAT (6901C>T), GGC213GGT (6916C>T), CCG214CCA (6919G>A), AAA216AAG (6925A>G), CTT217CTC (6928T>C), GAC219GAA (6934C>A), GGG224GGC (6949G>C), TTC226TTT (6955C>T), AAA227AAG (6958A>G), CAA233CAG (6976A>G), CTA235TTA (6980C>T), ATC236ATT (6985C>T), GTA240GTT (6997A>T), GTG241GTT (7000G>T), GTG242ATA (7001G>A 7003G>A)								
NIb protein (NP_734249.1)	1	521	100%	3134	86.2%	519 (99.4%)	481 (92.1%)	1/2/2/2	0
Protein mutations:	F8Y (7032T>A), T39K (7125C>A), A46S (7145G>T 7147A>G), E51_A52del (7160_7165delGAGGCA), D69E (7216T>A), D83E (7258T>A), D88_R89insX (7273_7274insT), R89V (7274C>G 7275G>T), H91L (7281A>T), L92S (7284T>C), S96L (7296C>T), S101F (7311C>T), T102I (7314C>T), C103Y (7317G>A), K109Q (7334A>C), A112N (7343G>A 7344C>A 7345A>C), S133M (7407G>T 7408T>G), C136G (7415T>G), L165S (7503T>C 7504G>A), D234N (7709G>A), K235R (7713A>G 7714A>G), T268I (7812C>T), V279L (7844G>T 7846G>A), V292I (7883G>A), S345N (8043G>A), N381S (8151A>G 8152C>T), R390K (8178G>A), R400K (8208G>A), P429A (8294C>G 8296A>G), S447F (8349C>T), A467S (8408G>T), M481I (8452G>A), R484K (8460G>A), A491T (8480G>A 8482T>A), R498K (8502G>A 8503A>G), E502D (8515A>T), E510D (8539G>C), L513C (8546C>T 8547T>G), S515T (8552T>A)								
Codon mutations:	GCT1GCC (7012T>C), AAG2AAA (7015G>A), CAT3CAC (7018T>C), TTT8TAT (7032T>A), GCC10GCT (7039C>T), TTG11CTA (7040T>C 7042G>A), GGA13GGG (7048A>G), GCT17GCA (7060T>A), GTC18GTA (7063C>A), AGC23AGT (7078C>T), TTA25CTG (7082T>C 7084A>G), GTA26GTG (7087A>G), ACC27ACA (7090C>A), CAT29CAC (7096T>C), GTA30GTG (7099A>G), GTT31GTC (7102T>C), GAG34GAA (7111G>A), CGA36CGG (7117A>G), ACA39AAA (7125C>A), GAA40GAG (7129A>G), TTT41TTC (7132T>C), CTG42TTA (7133C>T 7135G>A), GTG44GTA (7141G>A), GAT45GAC (7144T>C), GCA46TCG (7145G>T 7147A>G), GAG47GAA (7150G>A), GAG49GAA (7156G>A), GCA50GCT (7159A>T), GAG51_GCA52del (7160_7165delGAGGCA), TTC54TTT (7171C>T), GCG60GCT (7189G>T), AAA63AAG (7198A>G), AGC64AGT (7201C>T), CTA66TTA (7205C>T), AAT67AAC (7210T>C), GAT69GAA (7216T>A), GCG70GCA (7219G>A), ATC72ATA (7225C>A), AAG73AAA (7228G>A), AAG77AAA (7240G>A), TAT78TAC (7243T>C), AAA80AAG (7249A>G), CCT81CCG (7252T>G), GAT83GAA (7258T>A), GTT84GTC (7261T>C), GGT85GGA (7264T>A), GTC86GTG (7267C>G), GTG87GTA (7270G>A), GAT88_CGG89insT--(7273_7274insT), CGG89GTG (7274C>G 7275G>T), CAT91CTT (7281A>T), TTG92TCG (7284T>C), TCA96TTA (7296C>T), TCT101TTT (7311C>T), ACC102ATC (7314C>T), TGC103TAC (7317G>A), AAT104-AA (7319delA 7321T>A), CAC106CAT (7327C>T), GGC107GGT (7330C>T), TTC108TTT (7333C>T), AAG109CAG (7334A>C), TGT111TGC (7342T>C), GCA112AAC (7343G>A 7344C>A 7345A>C), ACT115ACC (7354T>C), CAA118CAG (7363A>G), TTC121TTT (7372C>T), GCG123GCT (7378G>T), CTC124CTT (7381C>T), GCA129GCT (7396A>T), GTC130GTT (7399C>T), GCC132GCT (7405C>T), AGT133ATG (7407G>T 7408T>G), GGG135GGT (7414G>T), TGC136GGC (7415T>G), AAA137AAG (7420A>G), GAC140GAT (7429C>T), TAT141TAC (7432T>C), CAT144CAC (7441T>C), AAG150AAA (7459G>A), GAA151GAG (7462A>G), ATA153ATT (7468A>T), GTC154GTA (7471C>A), CAA156CAG (7477A>G), AGC157AGT (7480C>T), CTG159CTT (7486G>T), TAT162TAC (7495T>C), AAA163AAG (7498A>G), GGT164GGC (7501T>C), TTG165TCA (7503T>C 7504G>A), ATT168ATA (7513T>A), AAC170AAT (7519C>T), GGA171GGG (7522A>G), AAG174AAA (7531G>A), GAG176GAA (7537G>A), CTC177CTT (7540C>T), TGT179TGC (7546T>C), AAG187AAA (7570G>A), ACG188ACA (7573G>A), ACG190ACA (7579G>A), GCT193GCC (7588T>C), CTA196TTA (7595C>T), TTG199CTA (7604T>C 7606G>A), GGT202GGA (7615T>A), TGT205TGC (7624T>C), GAC208GAT (7633C>T), TTC209TTT (7636C>T), AAT210AAC (7639T>C), TTT213TTC (7648T>C), GAA219GAG (7666A>G), TGT221TGC (7672T>C), ACA223ACT (7678A>T), GGG225GGA (7684G>A), ACT227ACC (7690T>C), TTT229TTC (7696T>C), GGT231GGG (7702T>G), GAT234AAT (7709G>A), AAA235AGG (7713A>G 7714A>G), CTG236TTG (7715C>T), CGT239CGC (7726T>C), GAG242GAA (7735G>A), GTA245GTG (7744A>G), TAC246TAT (7747C>T), GGC251GGT (7762C>T), CAG253CAA (7768G>A), TTT254TTC (7771T>C), TCA257TCG (7780A>G), CTA258CTC (7783A>C), ACT259ACC (7786T>C), CTA262TTG (7793C>T 7795A>G), ATC263ATT (7798C>T), GCT265GCC (7804T>C), ACC268ATC (7812C>T), ATC269ATT (7816C>T), GAA275GAG (7834A>G), GAC276GAT (7837C>T), GTG279TTA (7844G>T 7846G>A), CAG282CAA (7855G>A), CTG284CTA (7861G>A), TTA287CTG (7868T>C 7870A>G), TAC288TAT (7873C>T), ACT289ACA (7876T>A), GAG290GAA (7879G>A), ATT291ATA (7882T>A), GTT292ATT (7883G>A), TAC293TAT (7888C>T), CCA295CCT (7894A>T), ATT296ATC (7897T>C), GTC304GTT (7921C>T), AAG306AAA (7927G>A), GGT309GGC (7936T>C), AAT310AAC (7939T>C), AAT311AAC (7942T>C), GTG319GTA (7966G>A), GAT320GAC (7969T>C), TCT322TCC (7975T>C), GTC326GTT (7987C>T), CTT327CTC (7990T>C), TAC331TAT (8002C>T), GCT332GCC (8005T>C), GAA341GAG (8032A>G), ATC343ATT (8038C>T), GAC344GAT (8041C>T), AGC345AAC (8043G>A), TTG357TTA (8080G>A), GTG360GTA (8089G>A), CCG362CCA (8095G>A), GAG363GAA (8098G>A), AGC366AGT (8107C>T), ATT367ATC (8110T>C), GAT369GAC (8116T>C), CAA373CAG (8128A>G), GGT379GGC (8146T>C), AAC381AGT (8151A>G 8152C>T), TCG385TCA (8164G>A), AGG390AAG (8178G>A), AAG391AAA (8182G>A), TTG394TTA (8191G>A), TTC396TTT (8197C>T), TCC398TCT (8203C>T), AGA400AAA (8208G>A), GGC401GGG (8212C>G), CTG402TTG (8213C>T), CTA403CTG (8218A>G), ATC404ATT (8221C>T), GAG405GAA (8224G>A), CCA410CCG (8239A>G), ATT417ATC (8260T>C), GTA418GTG (8263A>G), GAT424GAC (8281T>C), AGA425AGG (8284A>G), CCA429GCG (8294C>G 8296A>G), GAG430GAA (8299G>A), CAC431CAT (8302C>T), ATT436ATC (8317T>C), TGC437TGT (8320C>T), GCT439GCA (8326T>A), GAG442GAA (8335G>A), TCC443TCT (8338C>T), TCT447TTT (8349C>T), GAA448GAG (8353A>G), CAC451CAT (8362C>T), CAA452CAG (8365A>G), TAC457TAT (8380C>T), TCA458TCG (8383A>G), TTA460TTG (8389A>G), CCT465CCC (8404T>C), GCA467TCA (8408G>T), ACA468ACG (8413A>G), GCG470GCT (8419G>T), GCT475GCC (8434T>C), TAT477TAC (8440T>C), ATG481ATA (8452G>A), CTA483CTG (8458A>G), AGG484AAG (8460G>A), AAA485AAG (8464A>G), CTG486CTA (8467G>A), GCT491ACA (8480G>A 8482T>A), GAG496GAA (8497G>A), CTA497TTG (8498C>T 8500A>G), AGA498AAG (8502G>A 8503A>G), GCC499GCT (8506C>T), GAA502GAT (8515A>T), GTC505GTT (8524C>T), GCA506GCC (8527A>C), TTA507CTG (8528T>C 8530A>G), GAG510GAC (8539G>C), CTT513TGT (8546C>T 8547T>G), TCT515ACT (8552T>A), GTA518GTG (8563A>G)								
coat protein (NP_734250.1)	1	267	100%	1659	91.0%	267 (100%)	244 (91.4%)	0/0/0/0	0
Protein mutations:	A1G (8574C>G), I5V (8585A>G), G9E (8598G>A), N11S (8603A>T 8604A>C 8605C>T), A15V (8616C>T), P17Q (8622C>A), E18G (8625A>G 8626G>A), P26F (8648C>T 8649C>T 8650G>C), G29E (8658G>A), D31A (8664A>C 8665T>G), A36V (8679C>T 8680A>T), T58K (8745C>A 8746A>G), E95G (8856A>G 8857G>A), R98Q (8865G>A), M99L (8867A>C 8869G>T), G104E (8883G>A), T106S (8888A>T), V128I (8954G>A), N138D (8984A>G 8986T>C), E139V (8988A>T), M193G (9149A>G 9150T>G 9151G>A), G194S (9152G>A), P230S (9260C>T 9262T>C)								
Codon mutations:	GCA1GGA (8574C>G), ATT5GTT (8585A>G), GGA9GAA (8598G>A), AGC10AGT (8602C>T), AAC11TCT (8603A>T 8604A>C 8605C>T), GCA15GTA (8616C>T), AAA16AAG (8620A>G), CCA17CAA (8622C>A), GAG18GGA (8625A>G 8626G>A), CAG19CAA (8629G>A), GGC20GGT (8632C>T), ATC22ATT (8638C>T), CAG23CAA (8641G>A), AAC25AAT (8647C>T), CCG26TTC (8648C>T 8649C>T 8650G>C), AAC27AAT (8653C>T), AAA28AAG (8656A>G), GGA29GAA (8658G>A), GAT31GCG (8664A>C 8665T>G), GTT34GTG (8674T>G), GCA36GTT (8679C>T 8680A>T), GGC37GGA (8683C>A), GGG40GGA (8692G>A), ACA41ACT (8695A>T), CCG45CCA (8707G>A), AGA46CGA (8708A>C), AAG48AAA (8716G>A), ATC50ATT (8722C>T), ACG51ACA (8725G>A), AAA53AAG (8731A>G), AGA55AGG (8737A>G), CCC57CCT (8743C>T), ACA58AAG (8745C>A 8746A>G), AGC59AGT (8749C>T), AAG60AAA (8752G>A), GGA61GGT (8755A>T), AAC66AAT (8770C>T), TTA67CTA (8771T>C), GAA68GAG (8776A>G), CAT69CAC (8779T>C), TTG70TTA (8782G>A), TAT73TAC (8791T>C), CCA75CCG (8797A>G), CAA76CAG (8800A>G), CAA77CAG (8803A>G), GAT79GAC (8809T>C), ATT80ATC (8812T>C), AAT82AAC (8818T>C), CGG84CGA (8824G>A), GCA85GCG (8827A>G), ACG92ACA (8848G>A), TAT94TAC (8854T>C), GAG95GGA (8856A>G 8857G>A), GTG97GTA (8863G>A), CGG98CAG (8865G>A), ATG99CTT (8867A>C 8869G>T), GCA100GCG (8872A>G), GGA104GAA (8883G>A), ACT106TCT (8888A>T), TGC119TGT (8929C>T), GTC128ATC (8954G>A), AAC129AAT (8959C>T), GGA130GGT (8962A>T), GGG137GGA (8983G>A), AAT138GAC (8984A>G 8986T>C), GAA139GTA (8988A>T), GAG142GAA (8998G>A), CCG144CCA (9004G>A), TTG145TTA (9007G>A), ATC148ATT (9016C>T), ACC155ACA (9037C>A), ATA171ATT (9085A>T), AAC175AAT (9097C>T), CGA188CGT (9136A>T), CGG191CGT (9145G>T), ATG193GGA (9149A>G 9150T>G 9151G>A), GGT194AGT (9152G>A), TTA195TTG (9157A>G), GCG196GCT (9160G>T), CGT197CGC (9163T>C), GAG204GAA (9184G>A), CGA208CGG (9196A>G), AGG214AGA (9214G>A), GAA215GAG (9217A>G), GCG216GCA (9220G>A), GCA224GCT (9244A>T), TTG225TTA (9247G>A), GCC228GCT (9256C>T), CCT230TCC (9260C>T 9262T>C), GGG234GGA (9274G>A), GAC236GAT (9280C>T), ATC239ATT (9289C>T), GAG244GAA (9304G>A), GTC254GTT (9334C>T), GTC264GTG (9364C>G)								

	Begin	End	Coverage	Score	Concordance	Matches	Identities	I/D/M/F*	Stop Codons
**NT**	**19**	**9704**	**99.8%**	**12534**	**65.0%**	**9674 (99.7%)**	**8011 (82.6%)**	**15/12**	
PIPO (YP_006393460.1)	1	75	100%	95	18.1%	75 (98.7%)	54 (71.1%)	1/0/3/2	2
Protein mutations:	R17_K17insX (2971_2972insA), Y23H (2989T>C 2991C>T), R26K (2999G>A 3000A>G), H35Y (3025C>T), K40H (3040A>C 3042A>C), G41S (3043G>A), E44K (3052G>A), I47V (3061A>G), V54I (3082G>A), P57S (3091C>T), P60T (3100C>A), G61E (3104G>A 3105T>G), G62S (3106G>A), Q63* (3109C>T), R64G (3112A>G), C66R (3118T>C), L67F (3121C>T), I69V (3127A>G), A72E (3137C>A 3138G>A), I73V (3139A>G)								
Codon mutations:	AAA4AAG (2931A>G), GAA7GAG (2940A>G), TTG10CTG (2947T>C), AGA17_AAA17insAA- (2971_2972insA), TAC23CAT (2989T>C 2991C>T), TCA24TCG (2994A>G), AGA26AAG (2999G>A 3000A>G), CAC32-AC (3016delC), TCG33TCA (3021G>A), GTA34GTT (3024A>T), CAT35TAT (3025C>T), CAC38CAT (3036C>T), AGA39AGG (3039A>G), AAA40CAC (3040A>C 3042A>C), GGC41AGC (3043G>A), GAA44AAA (3052G>A), ATA47GTA (3061A>G), GTT54ATT (3082G>A), CCG57TCG (3091C>T), CGC59CGT (3099C>T), CCA60ACA (3100C>A), GGT61GAG (3104G>A 3105T>G), GGT62AGT (3106G>A), CAA63TAA (3109C>T), AGG64GGG (3112A>G), TGC66CGC (3118T>C), CTC67TTC (3121C>T), ATT69GTT (3127A>G), GAG70GAA (3132G>A), GCG72GAA (3137C>A 3138G>A), ATT73GTT (3139A>G)								

*: Inserts / Deletes / Misaligned / Frameshifts

Analysis details

This analysis was performed with panviral2.64

NGS Details (UN70): Bracoviriform glomeratae (segment NC_043292.1)

Assembly

**Coverage Length**	310 (1 contig(s))
**Depth Of Coverage**	17187.9
**Number Of Reads**	47201
**Reads Per Million**	894.75 rpm (after QC)
**Ambiguities**	0
**Assembly Method**	de novo + reference guided assembly
**Consensus Caller**	Bcf Tools

Coverage Map

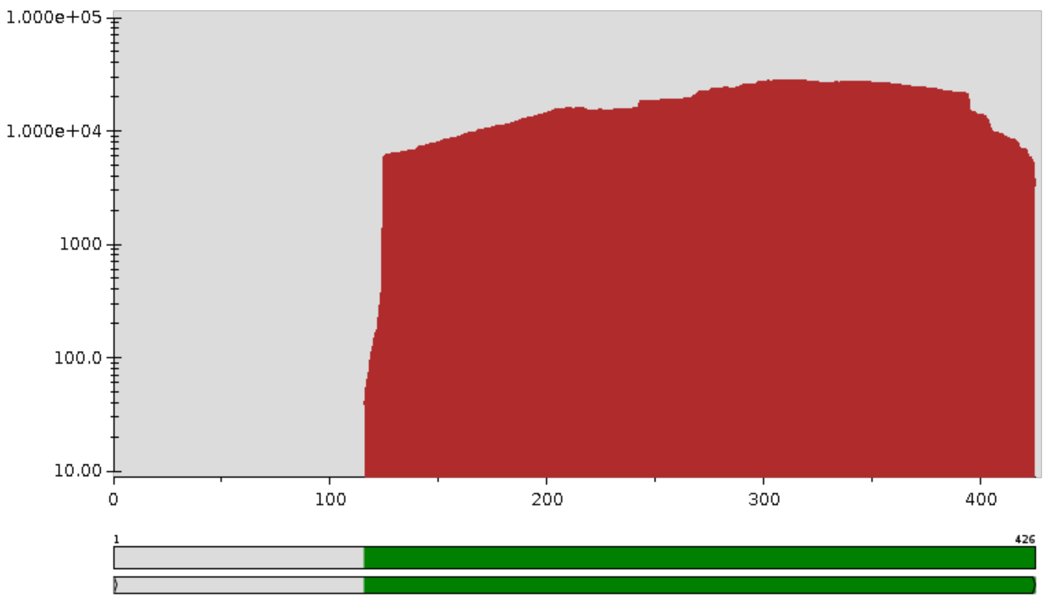

Assignment

**Type**	Bracoviriform glomeratae (Taxonomy ID: 257816)
**Reference Genome**	NC_043292.1
**NT Identity (%)**	74.5161
**AA Identity (%)**	84.466
**Number Of Stop Codons**	1
**Number Of CDS**	1

Alignment

**Alignment Score**	304.0 (NT) + 567.0 (AA) = 871.0
**Concordance (%)**	68.2067

Alignment Method	Global, seeded, nucleotide + amino acids (AGA)

Genome Region

Sequence starts at position 117 and ends at position 426 relative to NC_043292.1 reference sequence.

Alignment Detailed Statistics

	Begin	End	Coverage	Score	Concordance	Matches	Identities	I/D/M/F*	Stop Codons
**NT**	**117**	**426**	**72.8%**	**304**	**49.0%**	**310 (100%)**	**231 (74.5%)**	**0/0**	
Mutations:	126G>T, 129C>A, 132A>G, 136A>G, 137C>G, 138G>C, 153A>G, 155C>G, 156G>A, 159C>A, 166C>A, 168T>G, 169T>C, 171T>C, 172C>A, 174C>G, 177A>G, 183T>A, 184C>A, 189T>C, 195C>T, 198A>G, 201A>C, 207C>T, 214A>G, 219C>T, 222T>C, 223C>A, 225T>G, 228G>T, 235C>A, 237T>G, 243A>T, 246C>G, 261A>G, 264A>G, 265G>A, 273A>G, 278T>C, 279T>C, 284A>G, 285T>A, 288T>G, 289T>C, 300C>T, 301C>T, 303C>G, 309C>T, 312T>G, 315C>T, 322G>T, 329T>C, 333T>C, 336C>T, 339A>G, 345A>C, 347A>G, 348A>G, 349C>A, 351T>A, 360C>T, 363C>T, 366C>T, 375C>T, 384A>C, 385T>C, 387G>C, 390A>G, 391C>A, 392A>G, 393T>A, 394A>C, 400C>A, 402T>G, 404T>C, 405G>T, 406A>T, 408C>G, 416A>T								
**CDS**									
FK954_p501	40	142	72.5%	567	85.3%	103 (100%)	87 (84.5%)	0/0/0/0	1
Protein mutations:	L42F (126G>T), T46G (136A>G 137C>G 138G>C), A52G (155C>G 156G>A), Y57H (169T>C 171T>C), T72A (214A>G), V89I (265G>A), I93T (278T>C 279T>C), D95G (284A>G 285T>A), A108S (322G>T), I110T (329T>C), K116R (347A>G 348A>G), H131R (391C>A 392A>G 393T>A), K132Q (394A>C), M135T (404T>C 405G>T), I136L (406A>T 408C>G), Y139F (416A>T)								
Codon mutations:	TTG42TTT (126G>T), GGC43GGA (129C>A), AAA44AAG (132A>G), ACG46GGC (136A>G 137C>G 138G>C), AAA51AAG (153A>G), GCG52GGA (155C>G 156G>A), GGC53GGA (159C>A), CGT56AGG (166C>A 168T>G), TAT57CAC (169T>C 171T>C), CGC58AGG (172C>A 174C>G), AAA59AAG (177A>G), CTT61CTA (183T>A), CGA62AGA (184C>A), GAT63GAC (189T>C), ATC65ATT (195C>T), CAA66CAG (198A>G), GGA67GGC (201A>C), ACC69ACT (207C>T), ACT72GCT (214A>G), ATC73ATT (219C>T), CGT74CGC (222T>C), CGT75AGG (223C>A 225T>G), CTG76CTT (228G>T), CGT79AGG (235C>A 237T>G), GGA81GGT (243A>T), GTC82GTG (246C>G), GGA87GGG (261A>G), TTA88TTG (264A>G), GTC89ATC (265G>A), GAA91GAG (273A>G), ATT93ACC (278T>C 279T>C), GAT95GGA (284A>G 285T>A), GTT96GTG (288T>G), TTG97CTG (289T>C), TTC100TTT (300C>T), CTC101TTG (301C>T 303C>G), AAC103AAT (309C>T), GTT104GTG (312T>G), ATC105ATT (315C>T), GCT108TCT (322G>T), ATC110ACC (329T>C), TAT111TAC (333T>C), ACC112ACT (336C>T), GAA113GAG (339A>G), GCA115GCC (345A>C), AAA116AGG (347A>G 348A>G), CGT117AGA (349C>A 351T>A), GTC120GTT (360C>T), ACC121ACT (363C>T), GCC122GCT (366C>T), GTC125GTT (375C>T), GCA128GCC (384A>C), TTG129CTC (385T>C 387G>C), AAA130AAG (390A>G), CAT131AGA (391C>A 392A>G 393T>A), AAA132CAA (394A>C), CGT134AGG (400C>A 402T>G), ATG135ACT (404T>C 405G>T), ATC136TTG (406A>T 408C>G), TAT139TTT (416A>T)								
**Proteins**									
putative histone 4 (YP_009665791.1)	40	142	72.5%	567	85.3%	103 (100%)	87 (84.5%)	0/0/0/0	1
Protein mutations:	L42F (126G>T), T46G (136A>G 137C>G 138G>C), A52G (155C>G 156G>A), Y57H (169T>C 171T>C), T72A (214A>G), V89I (265G>A), I93T (278T>C 279T>C), D95G (284A>G 285T>A), A108S (322G>T), I110T (329T>C), K116R (347A>G 348A>G), H131R (391C>A 392A>G 393T>A), K132Q (394A>C), M135T (404T>C 405G>T), I136L (406A>T 408C>G), Y139F (416A>T)								
Codon mutations:	TTG42TTT (126G>T), GGC43GGA (129C>A), AAA44AAG (132A>G), ACG46GGC (136A>G 137C>G 138G>C), AAA51AAG (153A>G), GCG52GGA (155C>G 156G>A), GGC53GGA (159C>A), CGT56AGG (166C>A 168T>G), TAT57CAC (169T>C 171T>C), CGC58AGG (172C>A 174C>G), AAA59AAG (177A>G), CTT61CTA (183T>A), CGA62AGA (184C>A), GAT63GAC (189T>C), ATC65ATT (195C>T), CAA66CAG (198A>G), GGA67GGC (201A>C), ACC69ACT (207C>T), ACT72GCT (214A>G), ATC73ATT (219C>T), CGT74CGC (222T>C), CGT75AGG (223C>A 225T>G), CTG76CTT (228G>T), CGT79AGG (235C>A 237T>G), GGA81GGT (243A>T), GTC82GTG (246C>G), GGA87GGG (261A>G), TTA88TTG (264A>G), GTC89ATC (265G>A), GAA91GAG (273A>G), ATT93ACC (278T>C 279T>C), GAT95GGA (284A>G 285T>A), GTT96GTG (288T>G), TTG97CTG (289T>C), TTC100TTT (300C>T), CTC101TTG (301C>T 303C>G), AAC103AAT (309C>T), GTT104GTG (312T>G), ATC105ATT (315C>T), GCT108TCT (322G>T), ATC110ACC (329T>C), TAT111TAC (333T>C), ACC112ACT (336C>T), GAA113GAG (339A>G), GCA115GCC (345A>C), AAA116AGG (347A>G 348A>G), CGT117AGA (349C>A 351T>A), GTC120GTT (360C>T), ACC121ACT (363C>T), GCC122GCT (366C>T), GTC125GTT (375C>T), GCA128GCC (384A>C), TTG129CTC (385T>C 387G>C), AAA130AAG (390A>G), CAT131AGA (391C>A 392A>G 393T>A), AAA132CAA (394A>C), CGT134AGG (400C>A 402T>G), ATG135ACT (404T>C 405G>T), ATC136TTG (406A>T 408C>G), TAT139TTT (416A>T)								

*: Inserts / Deletes / Misaligned / Frameshifts

Analysis details

This analysis was performed with panviral2.64

NGS Details (UN70): Diachasmimorpha longicaudata entomopoxvirus (segment NC_043455.1)

Assembly

**Coverage Length**	1127 (1 contig(s))
**Depth Of Coverage**	1241.9
**Number Of Reads**	11204
**Reads Per Million**	212.39 rpm (after QC)
**Ambiguities**	0
**Assembly Method**	de novo + reference guided assembly
**Consensus Caller**	Bcf Tools

Coverage Map

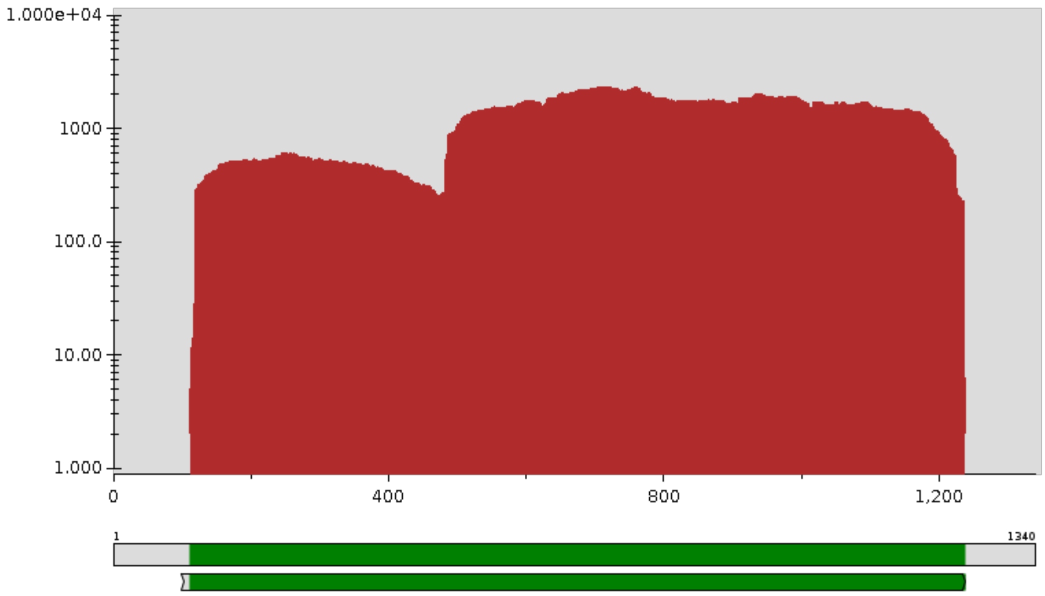

Assignment

**Type**	Diachasmimorpha longicaudata entomopoxvirus (Taxonomy ID: 109981)
**Reference Genome**	NC_043455.1
**NT Identity (%)**	59.3611
**AA Identity (%)**	55.5851
**Number Of Stop Codons**	0
**Number Of CDS**	1

Alignment

**Alignment Score**	404.0 (NT) + 1470.0 (AA) = 1874.0
**Concordance (%)**	39.7961

Alignment Method	Global, seeded, nucleotide + amino acids (AGA)

Genome Region

Sequence starts at position 112 and ends at position 1238 relative to NC_043455.1 reference sequence.

Alignment Detailed Statistics

	Begin	End	Coverage	Score	Concordance	Matches	Identities	I/D/M/F*	Stop Codons
**NT**	**112**	**1238**	**84.1%**	**404**	**18.1%**	**1124 (99.5%)**	**669 (59.2%)**	**3/3**	
Mutations:	121A>T, 126A>C, 127C>T, 128C>G, 130T>G, 136C>G, 137C>A, 139C>T, 142G>A, 143A>G, 145A>T, 146A>G, 148T>C, 149A>C, 151C>T, 154A>T, 156A>G, 157G>A, 160T>G, 163A>T, 166T>C, 167T>C, 168C>A, 169T>G, 171G>A, 172T>C, 175A>G, 178T>C, 187T>A, 190A>T, 191A>G, 193T>C, 203A>C, 204A>G, 208C>T, 209A>G, 211T>A, 214T>G, 217A>T, 218T>A, 219G>T, 223T>A, 226T>G, 230A>C, 231A>G, 232A>T, 238G>C, 241A>T, 243T>C, 244C>T, 247A>G, 256G>C, 259A>T, 262A>C, 271A>C, 272G>T, 274A>C, 276C>T, 277A>G, 278T>A, 279A>T, 283A>T, 284A>C, 286C>T, 288G>C, 289T>C, 294T>G, 295A>C, 298G>A, 299C>G, 300A>T, 301A>T, 302A>G, 304A>G, 310T>C, 311T>A, 312C>A, 313T>A, 320A>G, 322T>G, 323A>G, 325C>A, 331T>A, 337C>A, 341A>T, 343C>T, 346A>T, 352T>G, 355A>G, 356T>C, 358G>T, 361T>A, 362C>G, 363T>C, 364C>T, 368G>A, 370T>G, 371C>G, 374C>A, 375G>A, 376T>G, 379A>G, 380T>A, 382G>T, 383C>T, 384A>T, 385A>G, 386A>G, 394G>A, 395A>G, 400T>C, 401C>A, 403A>T, 404_406delTAT, 409T>C, 410T>A, 412T>A, 413A>C, 415A>G, 416T>G, 417G>C, 418T>A, 421A>T, 423T>C, 424T>G, 438C>A, 442T>C, 443A>G, 445C>G, 446A>G, 447A>G, 448G>T, 452A>G, 453G>A, 454C>T, 455C>A, 456A>T, 457G>C, 458G>A, 459A>G, 462C>A, 463T>G, 467A>G, 469_470insCAT, 470A>G, 471A>G, 474C>T, 475C>A, 478G>A, 482T>G, 484G>C, 485A>G, 487T>C, 490A>T, 499A>C, 502A>T, 503A>G, 505G>A, 506A>T, 511T>C, 512C>A, 514A>G, 515C>T, 517C>G, 518A>C, 519C>G, 521C>A, 523A>G, 524A>C, 526A>G, 527A>T, 528G>C, 530A>C, 532C>T, 533G>C, 534A>G, 535T>C, 536A>C, 538C>T, 539A>G, 541A>C, 542G>C, 543C>A, 544A>C, 547T>C, 550A>G, 553A>G, 554G>T, 559A>T, 560A>C, 562A>G, 571T>C, 577G>A, 581T>C, 583G>C, 584A>T, 585T>C, 587G>A, 588A>G, 589T>A, 590A>G, 591A>G, 595T>C, 596T>A, 597T>A, 602A>C, 604A>G, 607A>T, 608C>T, 610A>T, 620G>C, 622A>G, 623T>C, 626T>C, 628T>G, 629G>C, 630A>C, 632A>C, 633G>C, 634T>A, 635C>A, 637T>G, 638G>A, 640C>T, 647A>G, 648T>G, 650T>G, 652A>T, 655A>C, 658A>T, 661A>T, 664C>T, 665G>A, 667T>G, 671T>C, 674A>G, 675G>A, 678T>C, 679T>C, 680A>C, 682C>T, 683A>G, 685T>A, 687C>T, 688A>T, 689T>A, 692C>A, 693A>G, 695G>A, 696T>A, 697C>G, 700T>C, 705G>A, 706A>C, 707G>A, 709T>A, 712C>T, 715A>G, 717A>G, 718A>G, 724G>T, 727A>G, 730A>G, 731A>C, 732A>G, 735C>A, 739A>G, 740T>C, 742G>C, 745A>T, 748C>T, 757T>C, 759G>A, 765A>T, 770A>G, 772A>C, 778A>T, 779A>G, 781G>C, 784A>G, 785A>G, 787T>A, 790T>G, 792T>G, 793T>G, 796A>G, 797G>C, 798C>T, 799A>T, 805A>T, 810T>G, 811T>C, 820T>C, 823C>G, 824C>A, 825A>C, 830A>G, 831G>C, 833C>A, 838T>C, 841A>G, 843C>G, 844A>T, 845C>G, 847A>C, 850A>C, 854T>G, 855G>T, 856T>G, 859T>C, 862A>C, 863C>A, 865T>G, 866A>C, 868A>T, 869C>A, 878G>T, 879T>G, 880A>G, 885T>C, 886G>T, 889A>T, 890T>A, 891G>A, 892T>G, 893T>A, 895A>G, 896A>C, 897C>G, 898A>C, 900A>G, 901T>C, 902A>C, 904A>T, 905A>G, 908T>C, 909T>A, 915C>T, 919A>T, 920A>G, 921G>C, 922C>A, 924T>C, 925C>T, 934T>C, 938T>G, 939C>A, 943A>G, 944C>A, 946A>C, 947G>A, 948A>C, 949A>T, 950C>A, 952T>A, 956T>A, 958T>C, 965A>C, 966A>G, 970A>G, 973T>C, 974A>C, 977G>T, 978A>C, 982T>G, 983A>T, 984A>C, 986A>T, 989A>C, 991A>T, 992A>G, 994A>G, 997T>C, 998C>A, 1000T>C, 1001T>A, 1003A>T, 1007A>G, 1012A>T, 1015A>G, 1019A>C, 1021A>T, 1024C>T, 1033T>A, 1036A>G, 1040A>G, 1042A>C, 1045T>C, 1046T>C, 1048A>T, 1051T>C, 1054C>T, 1057T>C, 1063T>C, 1064T>C, 1070C>A, 1071A>C, 1073A>C, 1075T>A, 1076A>C, 1077G>C, 1084T>C, 1087T>C, 1088A>C, 1090A>G, 1103A>C, 1105A>T, 1111T>G, 1112A>C, 1120C>A, 1121C>A, 1123A>G, 1126A>G, 1129G>T, 1130A>G, 1132T>A, 1141T>C, 1145A>G, 1147A>C, 1150T>A, 1151G>A, 1153A>G, 1156C>T, 1159A>T, 1160T>G, 1161T>A, 1163C>A, 1164C>G, 1165A>G, 1167A>T, 1172G>T, 1173C>T, 1177A>C, 1178T>A, 1180A>T, 1181G>C, 1183A>G, 1185C>A, 1192T>C, 1196A>G, 1197C>T, 1198C>T, 1199A>G, 1200A>T, 1204A>C, 1207T>G, 1210A>G, 1211A>C, 1218A>C, 1219G>C, 1223A>G, 1225T>G, 1227T>C, 1232T>C, 1234G>C, 1235A>C, 1237A>G								

CDS

	Begin	End	Coverage	Score	Concordance	Matches	Identities	I/D/M/F*	Stop Codons
FLA14_p101	5	380	98.9%	1470	59.1%	375 (99.5%)	209 (55.4%)	1/1/0/0	0
Protein mutations:	E7D (121A>T), D9A (126A>C 127C>T), H10E (128C>G 130T>G), L13I (137C>A 139C>T), K15D (143A>G 145A>T), N16D (146A>G 148T>C), I17L (149A>C 151C>T), K19R (156A>G 157G>A), S23Q (167T>C 168C>A 169T>G), C24Y (171G>A 172T>C), T31A (191A>G 193T>C), K35R (203A>C 204A>G), I37V (209A>G 211T>A), F38L (214T>G), C40I (218T>A 219G>T), K44R (230A>C 231A>G 232A>T), V48A (243T>C 244C>T), A58S (272G>T 274A>C), T59M (276C>T 277A>G), Y60I (278T>A 279A>T), I62L (284A>C 286C>T), S63T (288G>C 289T>C), L65C (294T>G 295A>C), Q67V (299C>G 300A>T 301A>T), I68V (302A>G 304A>G), S71K (311T>A 312C>A 313T>A), N74E (320A>G 322T>G), I75V (323A>G 325C>A), T81S (341A>T 343C>T), L88A (362C>G 363T>C 364C>T), A90T (368G>A 370T>G), Q91E (371C>G), R92K (374C>A 375G>A 376T>G), L94I (380T>A 382G>T), Q95L (383C>T 384A>T 385A>G), T96A (386A>G), N99D (395A>G), L101I (401C>A 403A>T), Y102del (404_406delTAT), F104I (410T>A 412T>A), K105Q (413A>C 415A>G), C106A (416T>G 417G>C 418T>A), Q107H (421A>T), V108A (423T>C 424T>G), T113K (438C>A), I115V (443A>G 445C>G), K116G (446A>G 447A>G 448G>T), S118D (452A>G 453G>A 454C>T), Q119I (455C>A 456A>T 457G>C), E120R (458G>A 459A>G), T121K (462C>A 463T>G), K123E (467A>G), K123_K124insH (469_470insCAT), K124G (470A>G 471A>G), A125V (474C>T 475C>A), L128V (482T>G 484G>C), I129V (485A>G 487T>C), M135V (503A>G 505G>A), I136F (506A>T), L138M (512C>A 514A>G), T140R (518A>C 519C>G), K142Q (524A>C 526A>G), I144L (530A>C 532C>T), D145R (533G>C 534A>G 535T>C), T146P (536A>C 538C>T), K147D (539A>G 541A>C), A148H (542G>C 543C>A 544A>C), I151M (553A>G), V152F (554G>T), I154L (560A>C 562A>G), I162S (584A>T 585T>C), D163R (587G>A 588A>G 589T>A), N164G (590A>G 591A>G), L166K (596T>A 597T>A), K168Q (602A>C 604A>G), Q170Y (608C>T 610A>T), E174Q (620G>C 622A>G), F175L (623T>C), F176L (626T>C 628T>G), E177P (629G>C 630A>C), S178P (632A>C 633G>C 634T>A), H179K (635C>A 637T>G), V180I (638G>A 640C>T), I183G (647A>G 648T>G), L184V (650T>G 652A>T), L185F (655A>C), V189M (665G>A 667T>G), S191P (671T>C), R192E (674A>G 675G>A), V193A (678T>C 679T>C), I194L (680A>C 682C>T), N195E (683A>G 685T>A), T196I (687C>T 688A>T), S197T (689T>A), Q198R (692C>A 693A>G), V199K (695G>A 696T>A 697C>G), R202N (705G>A 706A>C), D203K (707G>A 709T>A), K206R (717A>G 718A>G), N211R (731A>C 732A>G), A212D (735C>A), R220K (759G>A), Y222F (765A>T), I224V (770A>G 772A>C), K227D (779A>G 781G>C), N229E (785A>G 787T>A), D230E (790T>G), F231W (792T>G 793T>G), A233L (797G>C 798C>T 799A>T), F237C (810T>G 811T>C), D241E (823C>G), H242T (824C>A 825A>C), S244A (830A>G 831G>C), L245I (833C>A), T248S (843C>G 844A>T), L249V (845C>G 847A>C), C252V (854T>G 855G>T 856T>G), Q257K (869C>A), V260W (878G>T 879T>G 880A>G), M262T (885T>C 886G>T), E263D (889A>T), C264K (890T>A 891G>A 892T>G), L265M (893T>A 895A>G), T266R (896A>C 897C>G 898A>C), N267S (900A>G 901T>C), N269D (905A>G), F270H (908T>C 909T>A), A272V (915C>T), S274A (920A>G 921G>C 922C>A), I275T (924T>C 925C>T), S280D (938T>G 939C>A), Q282N (944C>A 946A>C), E283T (947G>A 948A>C 949A>T), F286I (956T>A 958T>C), K289R (965A>C 966A>G), D293S (977G>T 978A>C), K295S (983A>T 984A>C), T296S (986A>T), I298V (992A>G 994A>G), L300I (998C>A 1000T>C), S301T (1001T>A 1003A>T), N303D (1007A>G), I314V (1040A>G 1042A>C), H324T (1070C>A 1071A>C), N325Q (1073A>C 1075T>A), R326P (1076A>C 1077G>C), I330L (1088A>C 1090A>G), I344V (1130A>G 1132T>A), I349V (1145A>G 1147A>C), E351K (1151G>A 1153A>G), E353D (1159A>T), L354E (1160T>G 1161T>A), P355R (1163C>A 1164C>G 1165A>G), K356M (1167A>T), A358F (1172G>T 1173C>T), E359D (1177A>C), L360I (1178T>A 1180A>T), E361Q (1181G>C 1183A>G), T362K (1185C>A), T366V (1196A>G 1197C>T 1198C>T), K367V (1199A>G 1200A>T), D369E (1207T>G), M371L (1211A>C), E373A (1218A>C 1219G>C), I375V (1223A>G 1225T>G), V376A (1227T>C), I379L (1235A>C 1237A>G)								

	Begin	End	Coverage	Score	Concordance	Matches	Identities	I/D/M/F*	Stop Codons
**NT**	**112**	**1238**	**84.1%**	**404**	**18.1%**	**1124 (99.5%)**	**669 (59.2%)**	**3/3**	

Codon mutations:

GAA7GAT (121A>T), GAC9GCT (126A>C 127C>T), CAT10GAG (128C>G 130T>G), GGC12GGG (136C>G), CTC13ATT (137C>A 139C>T), AAG14AAA (142G>A), AAA15GAT (143A>G 145A>T), AAT16GAC (146A>G 148T>C), ATC17CTT (149A>C 151C>T), CTA18CTT (154A>T), AAG19AGA (156A>G 157G>A), GGT20GGG (160T>G), ATA21ATT (163A>T), TAT22TAC (166T>C), TCT23CAG (167T>C 168C>A 169T>G), TGT24TAC (171G>A 172T>C), GGA25GGG (175A>G), TTT26TTC (178T>C), CCT29CCA (187T>A), TCA30TCT (190A>T), ACT31GCC (191A>G 193T>C), AAA35CGA (203A>C 204A>G), GCC36GCT (208C>T), ATT37GTA (209A>G 211T>A), TTT38TTG (214T>G), CCA39CCT (217A>T), TGT40ATT (218T>A 219G>T), ATT41ATA (223T>A), TCT42TCG (226T>G), AAA44CGT (230A>C 231A>G 232A>T), GTG46GTC (238G>C), ATA47ATT (241A>T), GTC48GCT (243T>C 244C>T), CAA49CAG (247A>G), TCG52TCC (256G>C), GGA53GGT (259A>T), ACA54ACC (262A>C), ACA57ACC (271A>C), GCA58TCC (272G>T 274A>C), ACA59ATG (276C>T 277A>G), TAT60ATT (278T>A 279A>T), GCA61GCT (283A>T), ATC62CTT (284A>C 286C>T), AGT63ACC (288G>C 289T>C), TTA65TGC (294T>G 295A>C), CAG66CAA (298G>A), CAA67GTT (299C>G 300A>T 301A>T), ATA68GTG (302A>G 304A>G), ACT70ACC (310T>C), TCT71AAA (311T>A 312C>A 313T>A), AAT74GAG (320A>G 322T>G), ATC75GTA (323A>G 325C>A), GCT77GCA (331T>A), ATC79ATA (337C>A), ACC81TCT (341A>T 343C>T), CCA82CCT (346A>T), CGT84CGG (352T>G), GAA85GAG (355A>G), TTG86CTT (356T>C 358G>T), GCT87GCA (361T>A), CTC88GCT (362C>G 363T>C 364C>T), GCT90ACG (368G>A 370T>G), CAA91GAA (371C>G), CGT92AAG (374C>A 375G>A 376T>G), GTA93GTG (379A>G), TTG94ATT (380T>A 382G>T), CAA95TTG (383C>T 384A>T 385A>G), ACA96GCA (386A>G), GGG98GGA (394G>A), AAT99GAT (395A>G), TAT100TAC (400T>C), CTA101ATT (401C>A 403A>T), TAT102del (404_406delTAT), AAT103AAC (409T>C), TTT104ATA (410T>A 412T>A), AAA105CAG (413A>C 415A>G), TGT106GCA (416T>G 417G>C 418T>A), CAA107CAT (421A>T), GTT108GCG (423T>C 424T>G), ACA113AAA (438C>A), AGT114AGC (442T>C), ATC115GTG (443A>G 445C>G), AAG116GGT (446A>G 447A>G 448G>T), AGC118GAT (452A>G 453G>A 454C>T), CAG119ATC (455C>A 456A>T 457G>C), GAA120AGA (458G>A 459A>G), ACT121AAG (462C>A 463T>G), AAG123GAG (467A>G), AAG123_AAA124insCAT (469_470insCAT), AAA124GGA (470A>G 471A>G), GCC125GTA (474C>T 475C>A), CAG126CAA (478G>A), TTG128GTC (482T>G 484G>C), ATT129GTC (485A>G 487T>C), GGA130GGT (490A>T), GGA133GGC (499A>C), CGA134CGT (502A>T), ATG135GTA (503A>G 505G>A), ATT136TTT (506A>T), GAT137GAC (511T>C), CTA138ATG (512C>A 514A>G), CTC139TTG (515C>T 517C>G), ACC140CGC (518A>C 519C>G), CGA141AGG (521C>A 523A>G), AAA142CAG (524A>C 526A>G), AGT143TCT (527A>T 528G>C), ATC144CTT (530A>C 532C>T), GAT145CGC (533G>C 534A>G 535T>C), ACC146CCT (536A>C 538C>T), AAA147GAC (539A>G 541A>C), GCA148CAC (542G>C 543C>A 544A>C), ATT149ATC (547T>C), AAA150AAG (550A>G), ATA151ATG (553A>G), GTT152TTT (554G>T), GTA153GTT (559A>T), ATA154CTG (560A>C 562A>G), GCT157GCC (571T>C), GAG159GAA (577G>A), TTG161CTC (581T>C 583G>C), ATA162TCA (584A>T 585T>C), GAT163AGA (587G>A 588A>G 589T>A), AAT164GGT (590A>G 591A>G), TTT165TTC (595T>C), TTG166AAG (596T>A 597T>A), AAA168CAG (602A>C 604A>G), ATA169ATT (607A>T), CAA170TAT (608C>T 610A>T), GAA174CAG (620G>C 622A>G), TTT175CTT (623T>C), TTT176CTG (626T>C 628T>G), GAA177CCA (629G>C 630A>C), AGT178CCA (632A>C 633G>C 634T>A), CAT179AAG (635C>A 637T>G), GTC180ATT (638G>A 640C>T), ATT183GGT (647A>G 648T>G), TTA184GTT (650T>G 652A>T), TTA185TTC (655A>C), TCA186TCT (658A>T), GCA187GCT (661A>T), ACC188ACT (664C>T), GTT189ATG (665G>A 667T>G), TCA191CCA (671T>C), AGG192GAG (674A>G 675G>A), GTT193GCC (678T>C 679T>C), ATC194CTT (680A>C 682C>T), AAT195GAA (683A>G 685T>A), ACA196ATT (687C>T 688A>T), TCT197ACT (689T>A), CAG198AGG (692C>A 693A>G), GTC199AAG (695G>A 696T>A 697C>G), TTT200TTC (700T>C), AGA202AAC (705G>A 706A>C), GAT203AAA (707G>A 709T>A), CCC204CCT (712C>T), GTA205GTG (715A>G), AAA206AGG (717A>G 718A>G), CTG208CTT (724G>T), GTA209GTG (727A>G), AAA210AAG (730A>G), AAT211CGT (731A>C 732A>G), GCT212GAT (735C>A), GAA213GAG (739A>G), TTG214CTC (740T>C 742G>C), ACA215ACT (745A>T), CTC216CTT (748C>T), ATT219ATC (757T>C), AGG220AAG (759G>A), TAT222TTT (765A>T), ATA224GTC (770A>G 772A>C), GTA226GTT (778A>T), AAG227GAC (779A>G 781G>C), AAA228AAG (784A>G), AAT229GAA (785A>G 787T>A), GAT230GAG (790T>G), TTT231TGG (792T>G 793T>G), AAA232AAG (796A>G), GCA233CTT (797G>C 798C>T 799A>T), ACA235ACT (805A>T), TTT237TGC (810T>G 811T>C), TAT240TAC (820T>C), GAC241GAG (823C>G), CAC242ACC (824C>A 825A>C), AGC244GCC (830A>G 831G>C), CTC245ATC (833C>A), ACT246ACC (838T>C), CAA247CAG (841A>G), ACA248AGT (843C>G 844A>T), CTA249GTC (845C>G 847A>C), ATA250ATC (850A>C), TGT252GTG (854T>G 855G>T 856T>G), AAT253AAC (859T>C), ACA254ACC (862A>C), CGT255AGG (863C>A 865T>G), AGA256CGT (866A>C 868A>T), CAA257AAA (869C>A), GTA260TGG (878G>T 879T>G 880A>G), ATG262ACT (885T>C 886G>T), GAA263GAT (889A>T), TGT264AAG (890T>A 891G>A 892T>G), TTA265ATG (893T>A 895A>G), ACA266CGC (896A>C 897C>G 898A>C), AAT267AGC (900A>G 901T>C), AGA268CGT (902A>C 904A>T), AAT269GAT (905A>G), TTC270CAC (908T>C 909T>A), GCA272GTA (915C>T), TCA273TCT (919A>T), AGC274GCA (920A>G 921G>C 922C>A), ATC275ACT (924T>C 925C>T), GAT278GAC (934T>C), TCC280GAC (938T>G 939C>A), CAA281CAG (943A>G), CAA282AAC (944C>A 946A>C), GAA283ACT (947G>A 948A>C 949A>T), CGT284AGA (950C>A 952T>A), TTT286ATC (956T>A 958T>C), AAA289CGA (965A>C 966A>G), GAA290GAG (970A>G), TTT291TTC (973T>C), AGA292CGA (974A>C), GAT293TCT (977G>T 978A>C), GGT294GGG (982T>G), AAA295TCA (983A>T 984A>C), ACT296TCT (986A>T), AGA297CGT (989A>C 991A>T), ATA298GTG (992A>G 994A>G), CTT299CTC (997T>C), CTT300ATC (998C>A 1000T>C), TCA301ACT (1001T>A 1003A>T), AAT303GAT (1007A>G), CTA304CTT (1012A>T), TTA305TTG (1015A>G), AGA307CGT (1019A>C 1021A>T), GGC308GGT (1024C>T), GTT311GTA (1033T>A), CAA312CAG (1036A>G), ATA314GTC (1040A>G 1042A>C), TCT315TCC (1045T>C), TTA316CTT (1046T>C 1048A>T), GTT317GTC (1051T>C), ATC318ATT (1054C>T), AAT319AAC (1057T>C), GAT321GAC (1063T>C), TTG322CTG (1064T>C), CAT324ACT (1070C>A 1071A>C), AAT325CAA (1073A>C 1075T>A), AGA326CCA (1076A>C 1077G>C), AAT328AAC (1084T>C), TAT329TAC (1087T>C), ATA330CTG (1088A>C 1090A>G), AGA335CGT (1103A>C 1105A>T), GGT337GGG (1111T>G), AGA338CGA (1112A>C), GGC340GGA (1120C>A), CGA341AGG (1121C>A 1123A>G), AAA342AAG (1126A>G), GGG343GGT (1129G>T), ATT344GTA (1130A>G 1132T>A), AAT347AAC (1141T>C), ATA349GTC (1145A>G 1147A>C), ACT350ACA (1150T>A), GAA351AAG (1151G>A 1153A>G), GAC352GAT (1156C>T), GAA353GAT (1159A>T), TTA354GAA (1160T>G 1161T>A), CCA355AGG (1163C>A 1164C>G 1165A>G), AAG356ATG (1167A>T), GCT358TTT (1172G>T 1173C>T), GAA359GAC (1177A>C), TTA360ATT (1178T>A 1180A>T), GAA361CAG (1181G>C 1183A>G), ACG362AAG (1185C>A), TAT364TAC (1192T>C), ACC366GTT (1196A>G 1197C>T 1198C>T), AAA367GTA (1199A>G 1200A>T), ATA368ATC (1204A>C), GAT369GAG (1207T>G), GAA370GAG (1210A>G), ATG371CTG (1211A>C), GAG373GCC (1218A>C 1219G>C), ATT375GTG (1223A>G 1225T>G), GTT376GCT (1227T>C), TTG378CTC (1232T>C 1234G>C), ATA379CTG (1235A>C 1237A>G)

Proteins

	Begin	End	Coverage	Score	Concordance	Matches	Identities	I/D/M/F*	Stop Codons
hypothetical protein (YP_009666148.1)	5	380	98.9%	1470	59.1%	375 (99.5%)	209 (55.4%)	1/1/0/0	0

Protein mutations:

E7D (121A>T), D9A (126A>C 127C>T), H10E (128C>G 130T>G), L13I (137C>A 139C>T), K15D (143A>G 145A>T), N16D (146A>G 148T>C), I17L (149A>C 151C>T), K19R (156A>G 157G>A), S23Q (167T>C 168C>A 169T>G), C24Y (171G>A 172T>C), T31A (191A>G 193T>C), K35R (203A>C 204A>G), I37V (209A>G 211T>A), F38L (214T>G), C40I (218T>A 219G>T), K44R (230A>C 231A>G 232A>T), V48A (243T>C 244C>T), A58S (272G>T 274A>C), T59M (276C>T 277A>G), Y60I (278T>A 279A>T), I62L (284A>C 286C>T), S63T (288G>C 289T>C), L65C (294T>G 295A>C), Q67V (299C>G 300A>T 301A>T), I68V (302A>G 304A>G), S71K (311T>A 312C>A 313T>A), N74E (320A>G 322T>G), I75V (323A>G 325C>A), T81S (341A>T 343C>T), L88A (362C>G 363T>C 364C>T), A90T (368G>A 370T>G), Q91E (371C>G), R92K (374C>A 375G>A 376T>G), L94I (380T>A 382G>T), Q95L (383C>T 384A>T 385A>G), T96A (386A>G), N99D (395A>G), L101I (401C>A 403A>T), Y102del (404_406delTAT), F104I (410T>A 412T>A), K105Q (413A>C 415A>G), C106A (416T>G 417G>C 418T>A), Q107H (421A>T), V108A (423T>C 424T>G), T113K (438C>A), I115V (443A>G 445C>G), K116G (446A>G 447A>G 448G>T), S118D (452A>G 453G>A 454C>T), Q119I (455C>A 456A>T 457G>C), E120R (458G>A 459A>G), T121K (462C>A 463T>G), K123E (467A>G), K123_K124insH (469_470insCAT), K124G (470A>G 471A>G), A125V (474C>T 475C>A), L128V (482T>G 484G>C), I129V (485A>G 487T>C), M135V (503A>G 505G>A), I136F (506A>T), L138M (512C>A 514A>G), T140R (518A>C 519C>G), K142Q (524A>C 526A>G), I144L (530A>C 532C>T), D145R (533G>C 534A>G 535T>C), T146P (536A>C 538C>T), K147D (539A>G 541A>C), A148H (542G>C 543C>A 544A>C), I151M (553A>G), V152F (554G>T), I154L (560A>C 562A>G), I162S (584A>T 585T>C), D163R (587G>A 588A>G 589T>A), N164G (590A>G 591A>G), L166K (596T>A 597T>A), K168Q (602A>C 604A>G), Q170Y (608C>T 610A>T), E174Q (620G>C 622A>G), F175L (623T>C), F176L (626T>C 628T>G), E177P (629G>C 630A>C), S178P (632A>C 633G>C 634T>A), H179K (635C>A 637T>G), V180I (638G>A 640C>T), I183G (647A>G 648T>G), L184V (650T>G 652A>T), L185F (655A>C), V189M (665G>A 667T>G), S191P (671T>C), R192E (674A>G 675G>A), V193A (678T>C 679T>C), I194L (680A>C 682C>T), N195E (683A>G 685T>A), T196I (687C>T 688A>T), S197T (689T>A), Q198R (692C>A 693A>G), V199K (695G>A 696T>A 697C>G), R202N (705G>A 706A>C), D203K (707G>A 709T>A), K206R (717A>G 718A>G), N211R (731A>C 732A>G), A212D (735C>A), R220K (759G>A), Y222F (765A>T), I224V (770A>G 772A>C), K227D (779A>G 781G>C), N229E (785A>G 787T>A), D230E (790T>G), F231W (792T>G 793T>G), A233L (797G>C 798C>T 799A>T), F237C (810T>G 811T>C), D241E (823C>G), H242T (824C>A 825A>C), S244A (830A>G 831G>C), L245I (833C>A), T248S (843C>G 844A>T), L249V (845C>G 847A>C), C252V (854T>G 855G>T 856T>G), Q257K (869C>A), V260W (878G>T 879T>G 880A>G), M262T (885T>C 886G>T), E263D (889A>T), C264K (890T>A 891G>A 892T>G), L265M (893T>A 895A>G), T266R (896A>C 897C>G 898A>C), N267S (900A>G 901T>C), N269D (905A>G), F270H (908T>C 909T>A), A272V (915C>T), S274A (920A>G 921G>C 922C>A), I275T (924T>C 925C>T), S280D (938T>G 939C>A), Q282N (944C>A 946A>C), E283T (947G>A 948A>C 949A>T), F286I (956T>A 958T>C), K289R (965A>C 966A>G), D293S (977G>T 978A>C), K295S (983A>T 984A>C), T296S (986A>T), I298V (992A>G 994A>G), L300I (998C>A 1000T>C), S301T (1001T>A 1003A>T), N303D (1007A>G), I314V (1040A>G 1042A>C), H324T (1070C>A 1071A>C), N325Q (1073A>C 1075T>A), R326P (1076A>C 1077G>C), I330L (1088A>C 1090A>G), I344V (1130A>G 1132T>A), I349V (1145A>G 1147A>C), E351K (1151G>A 1153A>G), E353D (1159A>T), L354E (1160T>G 1161T>A), P355R (1163C>A 1164C>G 1165A>G), K356M (1167A>T), A358F (1172G>T 1173C>T), E359D (1177A>C), L360I (1178T>A 1180A>T), E361Q (1181G>C 1183A>G), T362K (1185C>A), T366V (1196A>G 1197C>T 1198C>T), K367V (1199A>G 1200A>T), D369E (1207T>G), M371L (1211A>C), E373A (1218A>C 1219G>C), I375V (1223A>G 1225T>G), V376A (1227T>C), I379L (1235A>C 1237A>G)

	Begin	End	Coverage	Score	Concordance	Matches	Identities	I/D/M/F*	Stop Codons
**NT**	**112**	**1238**	**84.1%**	**404**	**18.1%**	**1124 (99.5%)**	**669 (59.2%)**	**3/3**	
Codon mutations:	GAA7GAT (121A>T), GAC9GCT (126A>C 127C>T), CAT10GAG (128C>G 130T>G), GGC12GGG (136C>G), CTC13ATT (137C>A 139C>T), AAG14AAA (142G>A), AAA15GAT (143A>G 145A>T), AAT16GAC (146A>G 148T>C), ATC17CTT (149A>C 151C>T), CTA18CTT (154A>T), AAG19AGA (156A>G 157G>A), GGT20GGG (160T>G), ATA21ATT (163A>T), TAT22TAC (166T>C), TCT23CAG (167T>C 168C>A 169T>G), TGT24TAC (171G>A 172T>C), GGA25GGG (175A>G), TTT26TTC (178T>C), CCT29CCA (187T>A), TCA30TCT (190A>T), ACT31GCC (191A>G 193T>C), AAA35CGA (203A>C 204A>G), GCC36GCT (208C>T), ATT37GTA (209A>G 211T>A), TTT38TTG (214T>G), CCA39CCT (217A>T), TGT40ATT (218T>A 219G>T), ATT41ATA (223T>A), TCT42TCG (226T>G), AAA44CGT (230A>C 231A>G 232A>T), GTG46GTC (238G>C), ATA47ATT (241A>T), GTC48GCT (243T>C 244C>T), CAA49CAG (247A>G), TCG52TCC (256G>C), GGA53GGT (259A>T), ACA54ACC (262A>C), ACA57ACC (271A>C), GCA58TCC (272G>T 274A>C), ACA59ATG (276C>T 277A>G), TAT60ATT (278T>A 279A>T), GCA61GCT (283A>T), ATC62CTT (284A>C 286C>T), AGT63ACC (288G>C 289T>C), TTA65TGC (294T>G 295A>C), CAG66CAA (298G>A), CAA67GTT (299C>G 300A>T 301A>T), ATA68GTG (302A>G 304A>G), ACT70ACC (310T>C), TCT71AAA (311T>A 312C>A 313T>A), AAT74GAG (320A>G 322T>G), ATC75GTA (323A>G 325C>A), GCT77GCA (331T>A), ATC79ATA (337C>A), ACC81TCT (341A>T 343C>T), CCA82CCT (346A>T), CGT84CGG (352T>G), GAA85GAG (355A>G), TTG86CTT (356T>C 358G>T), GCT87GCA (361T>A), CTC88GCT (362C>G 363T>C 364C>T), GCT90ACG (368G>A 370T>G), CAA91GAA (371C>G), CGT92AAG (374C>A 375G>A 376T>G), GTA93GTG (379A>G), TTG94ATT (380T>A 382G>T), CAA95TTG (383C>T 384A>T 385A>G), ACA96GCA (386A>G), GGG98GGA (394G>A), AAT99GAT (395A>G), TAT100TAC (400T>C), CTA101ATT (401C>A 403A>T), TAT102del (404_406delTAT), AAT103AAC (409T>C), TTT104ATA (410T>A 412T>A), AAA105CAG (413A>C 415A>G), TGT106GCA (416T>G 417G>C 418T>A), CAA107CAT (421A>T), GTT108GCG (423T>C 424T>G), ACA113AAA (438C>A), AGT114AGC (442T>C), ATC115GTG (443A>G 445C>G), AAG116GGT (446A>G 447A>G 448G>T), AGC118GAT (452A>G 453G>A 454C>T), CAG119ATC (455C>A 456A>T 457G>C), GAA120AGA (458G>A 459A>G), ACT121AAG (462C>A 463T>G), AAG123GAG (467A>G), AAG123_AAA124insCAT (469_470insCAT), AAA124GGA (470A>G 471A>G), GCC125GTA (474C>T 475C>A), CAG126CAA (478G>A), TTG128GTC (482T>G 484G>C), ATT129GTC (485A>G 487T>C), GGA130GGT (490A>T), GGA133GGC (499A>C), CGA134CGT (502A>T), ATG135GTA (503A>G 505G>A), ATT136TTT (506A>T), GAT137GAC (511T>C), CTA138ATG (512C>A 514A>G), CTC139TTG (515C>T 517C>G), ACC140CGC (518A>C 519C>G), CGA141AGG (521C>A 523A>G), AAA142CAG (524A>C 526A>G), AGT143TCT (527A>T 528G>C), ATC144CTT (530A>C 532C>T), GAT145CGC (533G>C 534A>G 535T>C), ACC146CCT (536A>C 538C>T), AAA147GAC (539A>G 541A>C), GCA148CAC (542G>C 543C>A 544A>C), ATT149ATC (547T>C), AAA150AAG (550A>G), ATA151ATG (553A>G), GTT152TTT (554G>T), GTA153GTT (559A>T), ATA154CTG (560A>C 562A>G), GCT157GCC (571T>C), GAG159GAA (577G>A), TTG161CTC (581T>C 583G>C), ATA162TCA (584A>T 585T>C), GAT163AGA (587G>A 588A>G 589T>A), AAT164GGT (590A>G 591A>G), TTT165TTC (595T>C), TTG166AAG (596T>A 597T>A), AAA168CAG (602A>C 604A>G), ATA169ATT (607A>T), CAA170TAT (608C>T 610A>T), GAA174CAG (620G>C 622A>G), TTT175CTT (623T>C), TTT176CTG (626T>C 628T>G), GAA177CCA (629G>C 630A>C), AGT178CCA (632A>C 633G>C 634T>A), CAT179AAG (635C>A 637T>G), GTC180ATT (638G>A 640C>T), ATT183GGT (647A>G 648T>G), TTA184GTT (650T>G 652A>T), TTA185TTC (655A>C), TCA186TCT (658A>T), GCA187GCT (661A>T), ACC188ACT (664C>T), GTT189ATG (665G>A 667T>G), TCA191CCA (671T>C), AGG192GAG (674A>G 675G>A), GTT193GCC (678T>C 679T>C), ATC194CTT (680A>C 682C>T), AAT195GAA (683A>G 685T>A), ACA196ATT (687C>T 688A>T), TCT197ACT (689T>A), CAG198AGG (692C>A 693A>G), GTC199AAG (695G>A 696T>A 697C>G), TTT200TTC (700T>C), AGA202AAC (705G>A 706A>C), GAT203AAA (707G>A 709T>A), CCC204CCT (712C>T), GTA205GTG (715A>G), AAA206AGG (717A>G 718A>G), CTG208CTT (724G>T), GTA209GTG (727A>G), AAA210AAG (730A>G), AAT211CGT (731A>C 732A>G), GCT212GAT (735C>A), GAA213GAG (739A>G), TTG214CTC (740T>C 742G>C), ACA215ACT (745A>T), CTC216CTT (748C>T), ATT219ATC (757T>C), AGG220AAG (759G>A), TAT222TTT (765A>T), ATA224GTC (770A>G 772A>C), GTA226GTT (778A>T), AAG227GAC (779A>G 781G>C), AAA228AAG (784A>G), AAT229GAA (785A>G 787T>A), GAT230GAG (790T>G), TTT231TGG (792T>G 793T>G), AAA232AAG (796A>G), GCA233CTT (797G>C 798C>T 799A>T), ACA235ACT (805A>T), TTT237TGC (810T>G 811T>C), TAT240TAC (820T>C), GAC241GAG (823C>G), CAC242ACC (824C>A 825A>C), AGC244GCC (830A>G 831G>C), CTC245ATC (833C>A), ACT246ACC (838T>C), CAA247CAG (841A>G), ACA248AGT (843C>G 844A>T), CTA249GTC (845C>G 847A>C), ATA250ATC (850A>C), TGT252GTG (854T>G 855G>T 856T>G), AAT253AAC (859T>C), ACA254ACC (862A>C), CGT255AGG (863C>A 865T>G), AGA256CGT (866A>C 868A>T), CAA257AAA (869C>A), GTA260TGG (878G>T 879T>G 880A>G), ATG262ACT (885T>C 886G>T), GAA263GAT (889A>T), TGT264AAG (890T>A 891G>A 892T>G), TTA265ATG (893T>A 895A>G), ACA266CGC (896A>C 897C>G 898A>C), AAT267AGC (900A>G 901T>C), AGA268CGT (902A>C 904A>T), AAT269GAT (905A>G), TTC270CAC (908T>C 909T>A), GCA272GTA (915C>T), TCA273TCT (919A>T), AGC274GCA (920A>G 921G>C 922C>A), ATC275ACT (924T>C 925C>T), GAT278GAC (934T>C), TCC280GAC (938T>G 939C>A), CAA281CAG (943A>G), CAA282AAC (944C>A 946A>C), GAA283ACT (947G>A 948A>C 949A>T), CGT284AGA (950C>A 952T>A), TTT286ATC (956T>A 958T>C), AAA289CGA (965A>C 966A>G), GAA290GAG (970A>G), TTT291TTC (973T>C), AGA292CGA (974A>C), GAT293TCT (977G>T 978A>C), GGT294GGG (982T>G), AAA295TCA (983A>T 984A>C), ACT296TCT (986A>T), AGA297CGT (989A>C 991A>T), ATA298GTG (992A>G 994A>G), CTT299CTC (997T>C), CTT300ATC (998C>A 1000T>C), TCA301ACT (1001T>A 1003A>T), AAT303GAT (1007A>G), CTA304CTT (1012A>T), TTA305TTG (1015A>G), AGA307CGT (1019A>C 1021A>T), GGC308GGT (1024C>T), GTT311GTA (1033T>A), CAA312CAG (1036A>G), ATA314GTC (1040A>G 1042A>C), TCT315TCC (1045T>C), TTA316CTT (1046T>C 1048A>T), GTT317GTC (1051T>C), ATC318ATT (1054C>T), AAT319AAC (1057T>C), GAT321GAC (1063T>C), TTG322CTG (1064T>C), CAT324ACT (1070C>A 1071A>C), AAT325CAA (1073A>C 1075T>A), AGA326CCA (1076A>C 1077G>C), AAT328AAC (1084T>C), TAT329TAC (1087T>C), ATA330CTG (1088A>C 1090A>G), AGA335CGT (1103A>C 1105A>T), GGT337GGG (1111T>G), AGA338CGA (1112A>C), GGC340GGA (1120C>A), CGA341AGG (1121C>A 1123A>G), AAA342AAG (1126A>G), GGG343GGT (1129G>T), ATT344GTA (1130A>G 1132T>A), AAT347AAC (1141T>C), ATA349GTC (1145A>G 1147A>C), ACT350ACA (1150T>A), GAA351AAG (1151G>A 1153A>G), GAC352GAT (1156C>T), GAA353GAT (1159A>T), TTA354GAA (1160T>G 1161T>A), CCA355AGG (1163C>A 1164C>G 1165A>G), AAG356ATG (1167A>T), GCT358TTT (1172G>T 1173C>T), GAA359GAC (1177A>C), TTA360ATT (1178T>A 1180A>T), GAA361CAG (1181G>C 1183A>G), ACG362AAG (1185C>A), TAT364TAC (1192T>C), ACC366GTT (1196A>G 1197C>T 1198C>T), AAA367GTA (1199A>G 1200A>T), ATA368ATC (1204A>C), GAT369GAG (1207T>G), GAA370GAG (1210A>G), ATG371CTG (1211A>C), GAG373GCC (1218A>C 1219G>C), ATT375GTG (1223A>G 1225T>G), GTT376GCT (1227T>C), TTG378CTC (1232T>C 1234G>C), ATA379CTG (1235A>C 1237A>G)								

*: Inserts / Deletes / Misaligned / Frameshifts

Analysis details

This analysis was performed with panviral2.64

NGS Details (UN70): Potato leafroll virus

Assembly

**Coverage Length**	5812 (1 contig(s))
**Depth Of Coverage**	18.2
**Number Of Reads**	796
**Reads Per Million**	15.09 rpm (after QC)
**Ambiguities**	0
**Assembly Method**	read mapping against reference + variant calling
**Consensus Caller**	Bcf Tools

Coverage Map

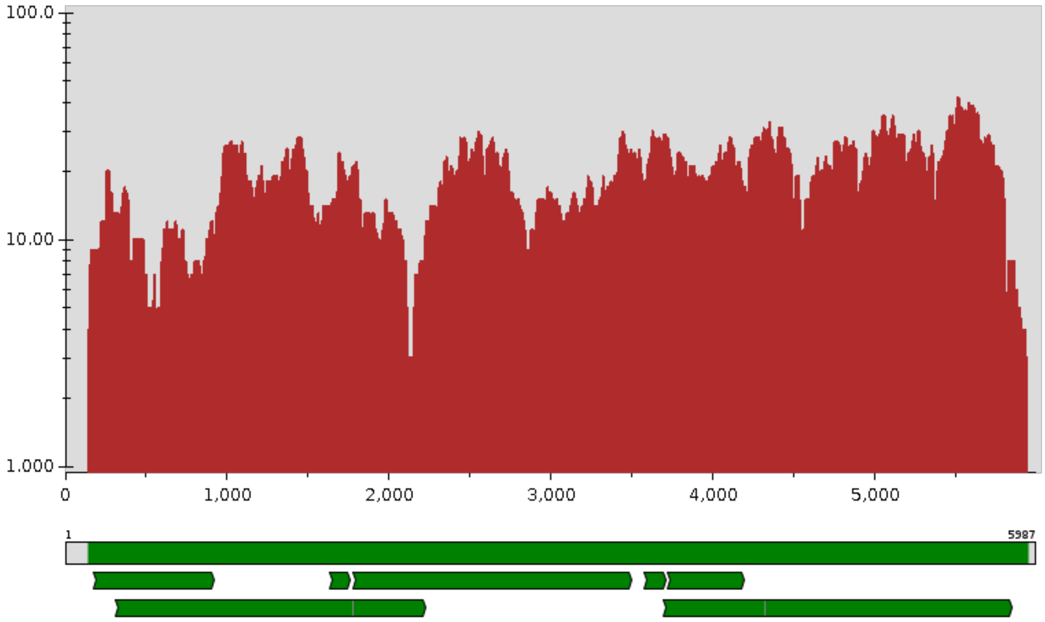

Assignment

**Type**	Potato leafroll virus (Taxonomy ID: 12045)
**Reference Genome**	NC_001747.1
**NT Identity (%)**	98.7612
**AA Identity (%)**	98.399
**Number Of Stop Codons**	9
**Number Of CDS**	8

Alignment

**Alignment Score**	11336.0 (NT) + 21364.0 (AA) = 32700.0
**Concordance (%)**	98.2897

Alignment Method	Global, seeded, nucleotide + amino acids (AGA)

Genome Region

Sequence starts at position 137 and ends at position 5948 relative to NC_001747.1 reference sequence.

Alignment Detailed Statistics

	Begin	End	Coverage	Score	Concordance	Matches	Identities	I/D/M/F*	Stop Codons
**NT**	**137**	**5948**	**97.1%**	**11336**	**97.5%**	**5812 (100%)**	**5740 (98.8%)**	**0/0**	
Mutations:	561C>T, 666A>C, 715C>T, 825T>G, 844A>G, 864C>T, 921G>A, 934A>G, 958G>A, 986C>T, 1094A>G, 1242A>G, 1294G>A, 1356G>A, 1394A>T, 1399G>A, 1700C>A, 1717G>A, 1754C>T, 1780A>C, 1830C>G, 1894C>A, 1951G>A, 1989A>G, 2007T>C, 2064A>G, 2079C>T, 2316T>G, 2340T>C, 2361T>G, 2373G>A, 2470G>A, 2550G>A, 2568T>C, 2586A>G, 2610T>C, 2649C>T, 2729A>G, 2796T>C, 2806A>G, 2820G>A, 2865G>A, 2898T>C, 2916C>T, 3000T>C, 3036C>T, 3042G>A, 3066T>C, 3186T>C, 3333C>T, 3378G>A, 3379A>G, 3788T>G, 3830C>T, 3939A>T, 4031A>G, 4034C>T, 4094G>A, 4113G>A, 4172T>C, 4214T>G, 4658G>A, 4661T>C, 4733C>G, 4817T>C, 4883C>T, 5111C>T, 5129G>A, 5189C>T, 5199C>T, 5216A>C, 5268A>G								
**CDS**									
PLRVgp1	1	248	100%	1720	99.3%	248 (100%)	246 (99.2%)	0/0/0/0	1
Protein mutations:	Q164H (666A>C), T224A (844A>G)								
Codon mutations:	TAC129TAT (561C>T), CAA164CAC (666A>C), CTA181TTA (715C>T), GCT217GCG (825T>G), ACC224GCC (844A>G), GTC230GTT (864C>T)								
PLRVgp2	1	1063	100%	7356	98.6%	1063 (100%)	1045 (98.3%)	0/0/0/0	1
Protein mutations:	T85I (561C>T), K120T (666A>C), L173R (825T>G), S186F (864C>T), R205K (921G>A), T263A (1094A>G), K312R (1242A>G), M329I (1294G>A), G350E (1356G>A), N363Y (1394A>T), Q465K (1700C>A), S492R (1780A>C), Q530K (1894C>A), E549K (1951G>A), A722T (2470G>A), N808S (2729A>G), T834A (2806A>G), I1025V (3379A>G)								
Codon mutations:	ACA85ATA (561C>T), AAA120ACA (666A>C), CTC136CTT (715C>T), CTA173CGA (825T>G), TTA179TTG (844A>G), TCC186TTC (864C>T), AGG205AAG (921G>A), GGA209GGG (934A>G), CAG217CAA (958G>A), CTA227TTA (986C>T), ACG263GCG (1094A>G), AAA312AGA (1242A>G), ATG329ATA (1294G>A), GGA350GAA (1356G>A), AAT363TAT (1394A>T), CTG364CTA (1399G>A), CAA465AAA (1700C>A), GAG470GAA (1717G>A), CTA483TTA (1754C>T), AGC492CGC (1780A>C), CGC508CGG (1830C>G), CAA530AAA (1894C>A), GAA549AAA (1951G>A), GAA561GAG (1989A>G), TTT567TTC (2007T>C), CCA586CCG (2064A>G), TGC591TGT (2079C>T), GTT670GTG (2316T>G), ACT678ACC (2340T>C), CTT685CTG (2361T>G), CTG689CTA (2373G>A), GCA722ACA (2470G>A), TCG748TCA (2550G>A), GAT754GAC (2568T>C), CTA760CTG (2586A>G), CCT768CCC (2610T>C), AGC781AGT (2649C>T), AAT808AGT (2729A>G), ACT830ACC (2796T>C), ACT834GCT (2806A>G), TTG838TTA (2820G>A), TTG853TTA (2865G>A), ACT864ACC (2898T>C), GAC870GAT (2916C>T), CGT898CGC (3000T>C), TCC910TCT (3036C>T), CTG912CTA (3042G>A), CTT920CTC (3066T>C), GAT960GAC (3186T>C), TAC1009TAT (3333C>T), GTG1024GTA (3378G>A), ATT1025GTT (3379A>G)								
PLRVgp3	1	640	100%	4193	97.7%	640 (100%)	623 (97.3%)	0/0/0/0	1
Protein mutations:	T85I (561C>T), K120T (666A>C), L173R (825T>G), S186F (864C>T), R205K (921G>A), T263A (1094A>G), K312R (1242A>G), M329I (1294G>A), G350E (1356G>A), N363Y (1394A>T), Q465K (1700C>A), Q491H (1780A>C), A508G (1830C>G), K561R (1989A>G), L567S (2007T>C), Q586R (2064A>G), A591V (2079C>T)								
Codon mutations:	ACA85ATA (561C>T), AAA120ACA (666A>C), CTC136CTT (715C>T), CTA173CGA (825T>G), TTA179TTG (844A>G), TCC186TTC (864C>T), AGG205AAG (921G>A), GGA209GGG (934A>G), CAG217CAA (958G>A), CTA227TTA (986C>T), ACG263GCG (1094A>G), AAA312AGA (1242A>G), ATG329ATA (1294G>A), GGA350GAA (1356G>A), AAT363TAT (1394A>T), CTG364CTA (1399G>A), CAA465AAA (1700C>A), GAG470GAA (1717G>A), CTA483TTA (1754C>T), CAA491CAC (1780A>C), GCT508GGT (1830C>G), ATC529ATA (1894C>A), CAG548CAA (1951G>A), AAG561AGG (1989A>G), TTA567TCA (2007T>C), CAG586CGG (2064A>G), GCG591GTG (2079C>T)								
Rap1	1	42	100%	280	97.6%	42 (100%)	41 (97.6%)	0/0/0/0	1
Protein mutations:	R29K (1717G>A)								
Codon mutations:	CTC23CTA (1700C>A), AGA29AAA (1717G>A), CTC41CTT (1754C>T)								
ORF3a	1	46	100%	299	100%	46 (100%)	46 (100%)	0/0/0/0	1
PLRVgp4	1	718	100%	5018	99.6%	718 (100%)	714 (99.4%)	0/0/0/0	2
Protein mutations:	T83S (3939A>T), V141I (4113G>A), P503S (5199C>T), T526A (5268A>G)								
Codon mutations:	GTT32GTG (3788T>G), CGC46CGT (3830C>T), ACC83TCC (3939A>T), ACA113ACG (4031A>G), AGC114AGT (4034C>T), GAG134GAA (4094G>A), GTA141ATA (4113G>A), ACT160ACC (4172T>C), TCT174TCG (4214T>G), CGG322CGA (4658G>A), GGT323GGC (4661T>C), GTC347GTG (4733C>G), GGT375GGC (4817T>C), TTC397TTT (4883C>T), GAC473GAT (5111C>T), ACG479ACA (5129G>A), AAC499AAT (5189C>T), CCA503TCA (5199C>T), CGA508CGC (5216A>C), ACA526GCA (5268A>G)								
PLRVgp5	1	209	100%	1431	99.7%	209 (100%)	207 (99.0%)	0/0/0/0	1
Protein mutations:	T83S (3939A>T), V141I (4113G>A)								
Codon mutations:	GTT32GTG (3788T>G), CGC46CGT (3830C>T), ACC83TCC (3939A>T), ACA113ACG (4031A>G), AGC114AGT (4034C>T), GAG134GAA (4094G>A), GTA141ATA (4113G>A), ACT160ACC (4172T>C), TCT174TCG (4214T>G)								
PLRVgp6	1	157	100%	1067	96.8%	157 (100%)	151 (96.2%)	0/0/0/0	1
Protein mutations:	F24C (3788T>G), A38V (3830C>T), Q105R (4031A>G), A106V (4034C>T), S126N (4094G>A), L152P (4172T>C)								
Codon mutations:	TTC24TGC (3788T>G), GCC38GTC (3830C>T), ACA74ACT (3939A>T), CAA105CGA (4031A>G), GCA106GTA (4034C>T), AGT126AAT (4094G>A), AAG132AAA (4113G>A), CTT152CCT (4172T>C)								
**Proteins**									
P0 protein (NP_056746.1)	1	248	100%	1720	99.3%	248 (100%)	246 (99.2%)	0/0/0/0	1
Protein mutations:	Q164H (666A>C), T224A (844A>G)								
Codon mutations:	TAC129TAT (561C>T), CAA164CAC (666A>C), CTA181TTA (715C>T), GCT217GCG (825T>G), ACC224GCC (844A>G), GTC230GTT (864C>T)								
RNA-dependent RNA polymerase (NP_056748.3)	1	1063	100%	7356	98.6%	1063 (100%)	1045 (98.3%)	0/0/0/0	1
Protein mutations:	T85I (561C>T), K120T (666A>C), L173R (825T>G), S186F (864C>T), R205K (921G>A), T263A (1094A>G), K312R (1242A>G), M329I (1294G>A), G350E (1356G>A), N363Y (1394A>T), Q465K (1700C>A), S492R (1780A>C), Q530K (1894C>A), E549K (1951G>A), A722T (2470G>A), N808S (2729A>G), T834A (2806A>G), I1025V (3379A>G)								
Codon mutations:	ACA85ATA (561C>T), AAA120ACA (666A>C), CTC136CTT (715C>T), CTA173CGA (825T>G), TTA179TTG (844A>G), TCC186TTC (864C>T), AGG205AAG (921G>A), GGA209GGG (934A>G), CAG217CAA (958G>A), CTA227TTA (986C>T), ACG263GCG (1094A>G), AAA312AGA (1242A>G), ATG329ATA (1294G>A), GGA350GAA (1356G>A), AAT363TAT (1394A>T), CTG364CTA (1399G>A), CAA465AAA (1700C>A), GAG470GAA (1717G>A), CTA483TTA (1754C>T), AGC492CGC (1780A>C), CGC508CGG (1830C>G), CAA530AAA (1894C>A), GAA549AAA (1951G>A), GAA561GAG (1989A>G), TTT567TTC (2007T>C), CCA586CCG (2064A>G), TGC591TGT (2079C>T), GTT670GTG (2316T>G), ACT678ACC (2340T>C), CTT685CTG (2361T>G), CTG689CTA (2373G>A), GCA722ACA (2470G>A), TCG748TCA (2550G>A), GAT754GAC (2568T>C), CTA760CTG (2586A>G), CCT768CCC (2610T>C), AGC781AGT (2649C>T), AAT808AGT (2729A>G), ACT830ACC (2796T>C), ACT834GCT (2806A>G), TTG838TTA (2820G>A), TTG853TTA (2865G>A), ACT864ACC (2898T>C), GAC870GAT (2916C>T), CGT898CGC (3000T>C), TCC910TCT (3036C>T), CTG912CTA (3042G>A), CTT920CTC (3066T>C), GAT960GAC (3186T>C), TAC1009TAT (3333C>T), GTG1024GTA (3378G>A), ATT1025GTT (3379A>G)								

	Begin	End	Coverage	Score	Concordance	Matches	Identities	I/D/M/F*	Stop Codons
**NT**	**137**	**5948**	**97.1%**	**11336**	**97.5%**	**5812 (100%)**	**5740 (98.8%)**	**0/0**	
P1 protein (NP_056747.1)	1	640	100%	4193	97.7%	640 (100%)	623 (97.3%)	0/0/0/0	1
Protein mutations:	T85I (561C>T), K120T (666A>C), L173R (825T>G), S186F (864C>T), R205K (921G>A), T263A (1094A>G), K312R (1242A>G), M329I (1294G>A), G350E (1356G>A), N363Y (1394A>T), Q465K (1700C>A), Q491H (1780A>C), A508G (1830C>G), K561R (1989A>G), L567S (2007T>C), Q586R (2064A>G), A591V (2079C>T)								
Codon mutations:	ACA85ATA (561C>T), AAA120ACA (666A>C), CTC136CTT (715C>T), CTA173CGA (825T>G), TTA179TTG (844A>G), TCC186TTC (864C>T), AGG205AAG (921G>A), GGA209GGG (934A>G), CAG217CAA (958G>A), CTA227TTA (986C>T), ACG263GCG (1094A>G), AAA312AGA (1242A>G), ATG329ATA (1294G>A), GGA350GAA (1356G>A), AAT363TAT (1394A>T), CTG364CTA (1399G>A), CAA465AAA (1700C>A), GAG470GAA (1717G>A), CTA483TTA (1754C>T), CAA491CAC (1780A>C), GCT508GGT (1830C>G), ATC529ATA (1894C>A), CAG548CAA (1951G>A), AAG561AGG (1989A>G), TTA567TCA (2007T>C), CAG586CGG (2064A>G), GCG591GTG (2079C>T)								
Replication-associated protein (YP_006355442.1)	1	42	100%	280	97.6%	42 (100%)	41 (97.6%)	0/0/0/0	1
Protein mutations:	R29K (1717G>A)								
Codon mutations:	CTC23CTA (1700C>A), AGA29AAA (1717G>A), CTC41CTT (1754C>T)								
protein 3a (YP_009179365.2)	1	46	100%	299	100%	46 (100%)	46 (100%)	0/0/0/0	1
Protein mutations:	*none*								
Codon mutations:	*none*								
CP read-through protein (NP_056751.2)	1	718	100%	5018	99.6%	718 (100%)	714 (99.4%)	0/0/0/0	2
Protein mutations:	T83S (3939A>T), V141I (4113G>A), P503S (5199C>T), T526A (5268A>G)								
Codon mutations:	GTT32GTG (3788T>G), CGC46CGT (3830C>T), ACC83TCC (3939A>T), ACA113ACG (4031A>G), AGC114AGT (4034C>T), GAG134GAA (4094G>A), GTA141ATA (4113G>A), ACT160ACC (4172T>C), TCT174TCG (4214T>G), CGG322CGA (4658G>A), GGT323GGC (4661T>C), GTC347GTG (4733C>G), GGT375GGC (4817T>C), TTC397TTT (4883C>T), GAC473GAT (5111C>T), ACG479ACA (5129G>A), AAC499AAT (5189C>T), CCA503TCA (5199C>T), CGA508CGC (5216A>C), ACA526GCA (5268A>G)								
coat protein (NP_056749.1)	1	209	100%	1431	99.7%	209 (100%)	207 (99.0%)	0/0/0/0	1
Protein mutations:	T83S (3939A>T), V141I (4113G>A)								
Codon mutations:	GTT32GTG (3788T>G), CGC46CGT (3830C>T), ACC83TCC (3939A>T), ACA113ACG (4031A>G), AGC114AGT (4034C>T), GAG134GAA (4094G>A), GTA141ATA (4113G>A), ACT160ACC (4172T>C), TCT174TCG (4214T>G)								
movement protein (NP_056750.1)	1	157	100%	1067	96.8%	157 (100%)	151 (96.2%)	0/0/0/0	1
Protein mutations:	F24C (3788T>G), A38V (3830C>T), Q105R (4031A>G), A106V (4034C>T), S126N (4094G>A), L152P (4172T>C)								
Codon mutations:	TTC24TGC (3788T>G), GCC38GTC (3830C>T), ACA74ACT (3939A>T), CAA105CGA (4031A>G), GCA106GTA (4034C>T), AGT126AAT (4094G>A), AAG132AAA (4113G>A), CTT152CCT (4172T>C)								

*: Inserts / Deletes / Misaligned / Frameshifts

Analysis details

This analysis was performed with panviral2.64

NGS Details (UN70): Solendovirus venanicotianae

Assembly

**Coverage Length**	2106 (5 contig(s))
**Depth Of Coverage**	12.1
**Number Of Reads**	234
**Reads Per Million**	4.44 rpm (after QC)
**Ambiguities**	12
**Assembly Method**	de novo + reference guided assembly
**Consensus Caller**	Bcf Tools

Coverage Map

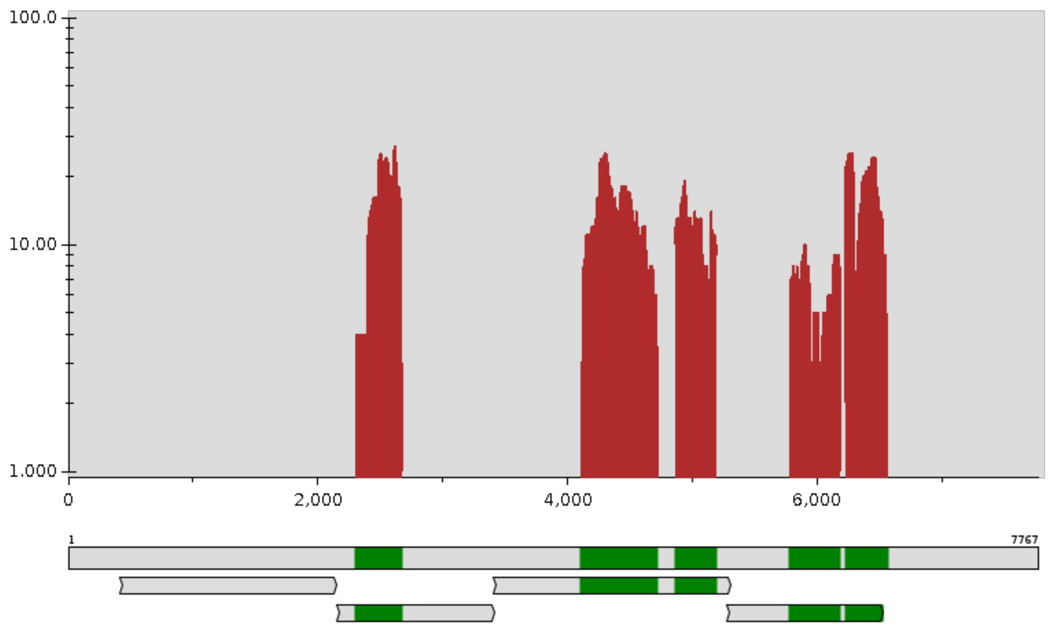

Assignment

**Type**	Solendovirus venanicotianae (Taxonomy ID: 3048371)
**Reference Genome**	NC_003378.1
**NT Identity (%)**	77.55
**AA Identity (%)**	74.2313
**Number Of Stop Codons**	3
**Number Of CDS**	4

Alignment

**Alignment Score**	2301.0 (NT) + 3423.0 (AA) = 5724.0
**Concordance (%)**	65.2828

Alignment Method	Global, seeded, nucleotide + amino acids (AGA)

Genome Region

Sequence starts at position 2300 and ends at position 6573 relative to NC_003378.1 reference sequence.

Alignment Detailed Statistics

	Begin	End	Coverage	Score	Concordance	Matches	Identities	I/D/M/F*	Stop Codons
**NT**	**2300**	**6573**	**27.1%**	**2301**	**55.2%**	**2097 (99.5%)**	**1627 (77.2%)**	**1/9**	
Mutations:	2308A>T, 2313A>C, 2314A>T, 2316T>C, 2322C>T, 2328A>G, 2330A>C, 2332A>G, 2333A>T, 2343C>T, 2347G>A, 2350G>A, 2352T>A, 2355C>T, 2356C>T, 2368A>G, 2370A>G, 2373T>A, 2376T>Y, 2379C>Y, 2383T>Y, 2384A>W, 2385T>Y, 2388A>Y, 2389G>A, 2390G>A, 2391A>R, 2394T>A, 2396A>G, 2397G>A, 2399C>T, 2400C>T, 2402A>G, 2410A>C, 2417A>G, 2418C>T, 2420A>G, 2422A>C, 2425A>T, 2426T>C, 2430A>C, 2434A>C, 2443C>A, 2445A>G, 2446G>A, 2448T>G, 2454G>A, 2455G>C, 2457A>T, 2461A>G, 2463C>A, 2469T>A, 2472G>A, 2481C>T, 2489G>C, 2493T>C, 2500T>C, 2503A>G, 2535C>A, 2538T>A, 2545A>C, 2551C>T, 2555C>T, 2559T>C, 2562T>C, 2565A>G, 2568A>T, 2569A>T, 2571C>A, 2572C>G, 2589C>T, 2590A>G, 2595A>C, 2597G>C, 2598T>A, 2602A>G, 2606A>G, 2609G>A, 2613T>C, 2622T>C, 2628G>A, 2637T>C, 2638A>G, 2643A>C, 2646C>T, 2647G>C, 2649C>A, 2665C>T, 4105A>G, 4115A>G, 4116G>A, 4121T>C, 4122A>G, 4124T>A, 4125A>G, 4127C>T, 4139T>C, 4181A>T, 4190G>A, 4196C>T, 4199A>T, 4202C>T, 4235T>C, 4241T>C, 4250C>A, 4256A>G, 4260T>C, 4262A>T, 4265G>A, 4284T>A, 4286T>C, 4292C>T, 4298T>C, 4307C>T, 4313G>A, 4325T>C, 4328T>C, 4329T>C, 4331G>A, 4340G>A, 4343T>A, 4362G>A, 4364T>A, 4374C>G, 4400T>A, 4421T>C, 4423C>A, 4430A>T, 4436C>T, 4451C>T, 4453A>G, 4454C>T, 4478T>C, 4484A>T, 4500C>T, 4503C>T, 4508T>C, 4513G>A, 4517A>T, 4518C>A, 4523T>A, 4530A>T, 4545A>C, 4550C>T, 4551G>A, 4552C>T, 4554C>G, 4568C>T, 4571T>A, 4574T>A, 4580C>T, 4586C>T, 4589A>G, 4591C>A, 4592C>A, 4596G>T, 4604T>A, 4631A>G, 4648A>G, 4661G>R, 4662C>Y, 4665A>T, 4670T>Y, 4674G>R, 4676A>R, 4688C>T, 4692C>T, 4693T>C, 4694T>A, 4695G>A, 4701A>C, 4703C>A, 4706T>A, 4717A>G, 4718G>A, 4725C>T, 4859G>A, 4865T>A, 4868G>A, 4869A>T, 4872T>C, 4874G>T, 4897A>G, 4898C>T, 4907T>C, 4913C>T, 4919A>T, 4924C>T, 4925C>A, 4931T>A, 4938A>G, 4944T>G, 4950A>G, 4952T>G, 4953T>C, 4954A>G, 4955T>A, 4961T>C, 4963G>A, 4970T>A, 4971C>T, 4973T>A, 4976G>A, 4978A>C, 4979T>C, 4982A>G, 4986A>G, 5012C>T, 5018C>T, 5027A>T, 5028T>A, 5030T>A, 5032A>T, 5033T>C, 5034A>T, 5039A>C, 5040C>T, 5042A>G, 5043C>G, 5046G>A, 5047A>T, 5054G>A, 5057A>G, 5065G>A, 5076T>C, 5077T>A, 5079G>T, 5081A>G, 5094C>T, 5097T>C, 5102A>C, 5117A>T, 5119T>C, 5120T>G, 5125C>A, 5126C>T, 5128G>A, 5132T>C, 5135T>A, 5136G>A, 5140G>A, 5145G>A, 5156A>G, 5174T>A, 5180A>G, 5189T>C, 5195C>T, 5772A>T, 5775T>C, 5781A>C, 5784G>A, 5786A>C, 5787T>A, 5789T>A, 5796T>C, 5797A>T, 5798A>T, 5802T>A, 5805A>C, 5807G>A, 5811C>A, 5812C>A, 5814A>T, 5815T>A, 5816C>T, 5817T>C, 5820C>T, 5821C>A, 5824A>G, 5826T>A, 5831A>C, 5832C>A, 5833G>C, 5835A>G, 5838C>T, 5850C>A, 5851C>A, 5853T>A, 5857A>C, 5858C>A, 5872T>C, 5874G>A, 5877A>T, 5880T>A, 5881T>C, 5882T>A, 5889T>G, 5892A>T, 5894G>A, 5898A>T, 5901A>C, 5907A>C, 5908G>A, 5909C>A, 5910C>A, 5913C>A, 5916C>T, 5921A>G, 5922C>T, 5925C>T, 5935G>A, 5936A>G, 5940T>C, 5950C>T, 5952G>C, 5953A>G, 5954C>A, 5955A>T, 5959C>G, 5964G>A, 5965A>T, 5968G>A, 5970C>A, 5972A>C, 5979T>A, 5982G>A, 5988C>T, 5991G>A, 5997C>T, 5999T>C, 6001C>A, 6002A>C, 6003A>C, 6006T>C, 6009G>A, 6010A>C, 6012G>A, 6013A>G, 6015A>T, 6018T>A, 6024G>A, 6026C>A, 6027A>T, 6033C>T, 6036C>T, 6037G>A, 6040C>A, 6041G>A, 6045C>T, 6049T>A, 6050C>G, 6051G>A, 6054T>A, 6055A>C, 6067C>T, 6072T>C, 6075A>T, 6078G>A, 6084G>A, 6087T>A, 6088A>G, 6090C>G, 6093A>C, 6099T>C, 6102C>T, 6105A>G, 6108T>A, 6114C>G, 6117A>T, 6120G>A, 6123A>T, 6124A>G, 6129C>A, 6139A>G, 6146A>T, 6147A>G, 6154G>C, 6155A>C, 6163G>C, 6164A>C, 6167A>G, 6169G>A, 6170T>C, 6171A>T, 6172A>G, 6174T>G, 6177T>A, 6232C>T, 6246T>C, 6247C>A, 6248T>C, 6253C>G, 6254A>G, 6258T>C, 6259G>C, 6264T>G, 6269G>A, 6270C>T, 6271T>C, 6277_6283delTCAAGGG, 6285A>C, 6290C>T, 6301T>G, 6308G>C, 6309C>A, 6311G>A, 6315T>A, 6318A>C, 6319A>C, 6321A>C, 6325G>A, 6326C>G, 6340C>G, 6341T>C, 6352G>A, 6354C>A, 6355T>A, 6357A>G, 6359G>C, 6360T>A, 6361C>T, 6363T>G, 6369A>T, 6375A>G, 6376C>A, 6380C>A, 6381T>A, 6382A>C, 6384A>T, 6386C>T, 6387C>T, 6389C>T, 6401G>A, 6402G>A, 6404G>A, 6405G>T, 6407A>G, 6408T>A, 6411C>T, 6414C>T, 6417C>G, 6418C>G, 6419C>A, 6420A>C, 6423T>A, 6424C>T, 6426G>A, 6427T>C, 6446T>A, 6451A>G, 6453T>G, 6456C>A, 6462T>C, 6471C>T, 6477C>T, 6480A>G, 6489A>C, 6502A>G, 6504T>A, 6505G>A, 6507T>C, 6513C>T, 6522G>A, 6525A>C, 6526T>G, 6530T>A, 6531C>A, 6532_6533insA, 6537G>T, 6542A>T, 6545_6546delCG, 6551A>C, 6553C>G, 6555A>T, 6556C>A, 6557A>C, 6558A>T, 6561T>C, 6563T>A, 6564G>C, 6566C>A, 6570A>G, 6572G>T								

CDS

	Begin	End	Coverage	Score	Concordance	Matches	Identities	I/D/M/F*	Stop Codons
Tvvgp2	50	174	29.8%	632	73.7%	125 (100%)	88 (70.4%)	0/0/1/0	1
Protein mutations:	I52L (2308A>T), K53N (2313A>C), N54Y (2314A>T 2316T>C), Q59P (2330A>C), K60V (2332A>G 2333A>T), V65I (2347G>A), V66I (2350G>A 2352T>A), Q68* (2356C>T), I72V (2368A>G 2370A>G), G79K (2389G>A 2390G>A 2391A>R), K81R (2396A>G 2397G>A), A82V (2399C>T 2400C>T), K83R (2402A>G), I86L (2410A>C), N88S (2417A>G 2418C>T), K89R (2420A>G), I91S (2425A>T 2426T>C), K94Q (2434A>C), Q97K (2443C>A 2445A>G), D98K (2446G>A 2448T>G), A101P (2455G>C 2457A>T), N103E (2461A>G 2463C>A), G112A (2489G>C), I117V (2503A>G), I131L (2545A>C), A134V (2555C>T), I139L (2569A>T 2571C>A), Q140E (2572C>G), I146V (2590A>G), S148T (2597G>C 2598T>A), I150V (2602A>G), K151R (2606A>G), G152E (2609G>A), I162V (2638A>G), A165P (2647G>C 2649C>A)								
Codon mutations:	ATA52TTA (2308A>T), AAA53AAC (2313A>C), AAT54TAC (2314A>T 2316T>C), TTC56TTT (2322C>T), AGA58AGG (2328A>G), CAA59CCA (2330A>C), AAA60GTA (2332A>G 2333A>T), TAC63TAT (2343C>T), GTA65ATA (2347G>A), GTT66ATA (2350G>A 2352T>A), AGC67AGT (2355C>T), CAA68TAA (2356C>T), ATA72GTG (2368A>G 2370A>G), ATT73ATA (2373T>A), GAT74GAY (2376T>Y), TGC75TGY (2379C>Y), TAT77YWY (2383T>Y 2384A>W 2385T>Y), ACA78ACY (2388A>Y), GGA79AAR (2389G>A 2390G>A 2391A>R), GGT80GGA (2394T>A), AAG81AGA (2396A>G 2397G>A), GCC82GTT (2399C>T 2400C>T), AAA83AGA (2402A>G), ATA86CTA (2410A>C), AAC88AGT (2417A>G 2418C>T), AAA89AGA (2420A>G), AGA90CGA (2422A>C), ATA91TCA (2425A>T 2426T>C), ATA92ATC (2430A>C), AAA94CAA (2434A>C), CAA97AAG (2443C>A 2445A>G), GAT98AAG (2446G>A 2448T>G), AAG100AAA (2454G>A), GCA101CCT (2455G>C 2457A>T), AAC103GAA (2461A>G 2463C>A), ATT105ATA (2469T>A), AAG106AAA (2472G>A), CAC109CAT (2481C>T), GGA112GCA (2489G>C), ACT113ACC (2493T>C), TTA116CTA (2500T>C), ATA117GTA (2503A>G), ACC127ACA (2535C>A), CCT128CCA (2538T>A), ATA131CTA (2545A>C), CTA133TTA (2551C>T), GCA134GTA (2555C>T), GAT135GAC (2559T>C), GAT136GAC (2562T>C), AGA137AGG (2565A>G), ATA138ATT (2568A>T), ATC139TTA (2569A>T 2571C>A), CAA140GAA (2572C>G), AGC145AGT (2589C>T), ATA146GTA (2590A>G), ATA147ATC (2595A>C), AGT148ACA (2597G>C 2598T>A), ATA150GTA (2602A>G), AAA151AGA (2606A>G), GGA152GAA (2609G>A), AAT153AAC (2613T>C), TAT156TAC (2622T>C), AAG158AAA (2628G>A), TTT161TTC (2637T>C), ATA162GTA (2638A>G), ATA163ATC (2643A>C), AGC164AGT (2646C>T), GCC165CCA (2647G>C 2649C>A), CTA171TTA (2665C>T)								
Tvvgp3	233	596	50.5%	1789	79.1%	321 (100%)	264 (82.2%)	0/0/3/0	2
Protein mutations:	E236K (4116G>A), N238E (4122A>G 4124T>A), S239G (4125A>G 4127C>T), Y292N (4284T>A 4286T>C), D311E (4343T>A), A318T (4362G>A 4364T>A), P322A (4374C>G), A338E (4423C>A), N348S (4453A>G 4454C>T), R368K (4513G>A), Q370K (4518C>A), D371E (4523T>A), I374L (4530A>T), K379Q (4545A>C), A381I (4551G>A 4552C>T), H382D (4554C>G), T394K (4591C>A 4592C>A), A396S (4596G>T), N413S (4648A>G), T419S (4665A>T), L428S (4692C>T 4693T>C 4694T>A), D429N (4695G>A), N431Q (4701A>C 4703C>A), K436R (4717A>G 4718G>A), Q439* (4725C>T), I487L (4869A>T), Y496C (4897A>G 4898C>T), K503N (4919A>T), T505I (4924C>T 4925C>A), T510A (4938A>G), S512A (4944T>G), N514E (4950A>G 4952T>G), Y515R (4953T>C 4954A>G 4955T>A), G518E (4963G>A), Y523S (4978A>C 4979T>C), N526D (4986A>G), S540T (5028T>A 5030T>A), Y541F (5032A>T 5033T>C), T542S (5034A>T), E543D (5039A>C), P544S (5040C>T 5042A>G), Q545E (5043C>G), E546I (5046G>A 5047A>T), W548* (5054G>A), R552K (5065G>A), F556H (5076T>C 5077T>A), A557S (5079G>T 5081A>G), V570A (5119T>C 5120T>G), T572N (5125C>A 5126C>T), R573K (5128G>A), V576I (5136G>A), R577K (5140G>A), D579N (5145G>A)								
Codon mutations:	AAA232.GA (4105A>G), CAA235CAG (4115A>G), GAA236AAA (4116G>A), AGT237AGC (4121T>C), AAT238GAA (4122A>G 4124T>A), AGC239GGT (4125A>G 4127C>T), AGT243AGC (4139T>C), GGA257GGT (4181A>T), AGG260AGA (4190G>A), GTC262GTT (4196C>T), ATA263ATT (4199A>T), GAC264GAT (4202C>T), TAT275TAC (4235T>C), TAT277TAC (4241T>C), CCC280CCA (4250C>A), AAA282AAG (4256A>G), TTA284CTT (4260T>C 4262A>T), AAG285AAA (4265G>A), TAT292AAC (4284T>A 4286T>C), TAC294TAT (4292C>T), AGT296AGC (4298T>C), GAC299GAT (4307C>T), AAG301AAA (4313G>A), TAT305TAC (4325T>C), CAT306CAC (4328T>C), TTG307CTA (4329T>C 4331G>A), GAG310GAA (4340G>A), GAT311GAA (4343T>A), GCT318ACA (4362G>A 4364T>A), CCA322GCA (4374C>G), GTT330GTA (4400T>A), AAT337AAC (4421T>C), GCA338GAA (4423C>A), GGA340GGT (4430A>T), TAC342TAT (4436C>T), GAC347GAT (4451C>T), AAC348AGT (4453A>G 4454C>T), TGT356TGC (4478T>C), GTA358GTT (4484A>T), CTA364TTA (4500C>T), CTA365TTA (4503C>T), TAT366TAC (4508T>C), AGA368AAA (4513G>A), ACA369ACT (4517A>T), CAA370AAA (4518C>A), GAT371GAA (4523T>A), ATA374TTA (4530A>T), AAA379CAA (4545A>C), TTC380TTT (4550C>T), GCA381ATA (4551G>A 4552C>T), CAT382GAT (4554C>G), AAC386AAT (4568C>T), TCT387TCA (4571T>A), GGT388GGA (4574T>A), AGC390AGT (4580C>T), AGC392AGT (4586C>T), AAA393AAG (4589A>G), ACC394AAA (4591C>A 4592C>A), GCA396TCA (4596G>T), ATT398ATA (4604T>A), GGA407GGG (4631A>G), AAT413AGT (4648A>G), ATG417ATR (4661G>R), CAA418YAA (4662C>Y), ACT419TCT (4665A>T), CAT420CAY (4670T>Y), GTA422RTR (4674G>R 4676A>R), ATC426ATT (4688C>T), CTT428TCA (4692C>T 4693T>C 4694T>A), GAT429AAT (4695G>A), AAC431CAA (4701A>C 4703C>A), ATT432ATA (4706T>A), AAG436AGA (4717A>G 4718G>A), CAA439TAA (4725C>T), AAG483..A (4859G>A), ATT485ATA (4865T>A), AAG486AAA (4868G>A), ATA487TTA (4869A>T), TTG488CTT (4872T>C 4874G>T), TAC496TGT (4897A>G 4898C>T), GAT499GAC (4907T>C), AAC501AAT (4913C>T), AAA503AAT (4919A>T), ACC505ATA (4924C>T 4925C>A), ATT507ATA (4931T>A), ACA510GCA (4938A>G), TCA512GCA (4944T>G), AAT514GAG (4950A>G 4952T>G), TAT515CGA (4953T>C 4954A>G 4955T>A), TAT517TAC (4961T>C), GGA518GAA (4963G>A), GTT520GTA (4970T>A), CTT521TTA (4971C>T 4973T>A), AAG522AAA (4976G>A), TAT523TCC (4978A>C 4979T>C), AGA524AGG (4982A>G), AAT526GAT (4986A>G), TGC534TGT (5012C>T), TAC536TAT (5018C>T), GGA539GGT (5027A>T), TCT540ACA (5028T>A 5030T>A), TAT541TTC (5032A>T 5033T>C), ACA542TCA (5034A>T), GAA543GAC (5039A>C), CCA544TCG (5040C>T 5042A>G), CAA545GAA (5043C>G), GAA546ATA (5046G>A 5047A>T), TGG548TGA (5054G>A), GAA549GAG (5057A>G), AGA552AAA (5065G>A), TTT556CAT (5076T>C 5077T>A), GCA557TCG (5079G>T 5081A>G), CTA562TTA (5094C>T), TTA563CTA (5097T>C), GCA564GCC (5102A>C), ATA569ATT (5117A>T), GTT570GCG (5119T>C 5120T>G), ACC572AAT (5125C>A 5126C>T), AGA573AAA (5128G>A), TTT574TTC (5132T>C), ATT575ATA (5135T>A), GTA576ATA (5136G>A), AGA577AAA (5140G>A), GAC579AAC (5145G>A), CAA582CAG (5156A>G), ACT588ACA (5174T>A), AAA590AAG (5180A>G), GAT593GAC (5189T>C), GTC595GTT (5195C>T)								
Tvvgp4	167	418	57.2%	1002	60.7%	237 (99.2%)	155 (64.9%)	0/2/1/1	0
Protein mutations:	N171T (5786A>C 5787T>A), F172Y (5789T>A), K175L (5797A>T 5798A>T), N176K (5802T>A), R178K (5807G>A), Q180N (5812C>A 5814A>T), S181I (5815T>A 5816C>T 5817T>C), Q183K (5821C>A), N184E (5824A>G 5826T>A), N186T (5831A>C 5832C>A), E187Q (5833G>C 5835A>G), H192Q (5850C>A), H193K (5851C>A 5853T>A), T195Q (5857A>C 5858C>A), L203Q (5881T>C 5882T>A), N205K (5889T>G), S207N (5894G>A), K209N (5901A>C), A212K (5908G>A 5909C>A 5910C>A), N216S (5921A>G 5922C>T), D221S (5935G>A 5936A>G), Q226Y (5950C>T 5952G>C), T227D (5953A>G 5954C>A 5955A>T), Q229E (5959C>G), I231L (5965A>T), A232T (5968G>A 5970C>A), N233T (5972A>C), M242T (5999T>C), Q243T (6001C>A 6002A>C 6003A>C), I247V (6013A>G 6015A>T), T251N (6026C>A 6027A>T), V255I (6037G>A), R256K (6040C>A 6041G>A), S259R (6049T>A 6050C>G 6051G>A), T261P (6055A>C), E267D (6075A>T), I272V (6088A>G 6090C>G), E283D (6123A>T), M284V (6124A>G), K289E (6139A>G), E291V (6146A>T 6147A>G), E294P (6154G>C 6155A>C), E297P (6163G>C 6164A>C), K298R (6167A>G), V299T (6169G>A 6170T>C 6171A>T), N300E (6172A>G 6174T>G), L325T (6247C>A 6248T>C), Q327G (6253C>G 6254A>G), A329P (6259G>C), I330M (6264T>G), S332N (6269G>A 6270C>T), Y333H (6271T>C), S335_R336del (6277_6283delTCAAGGG), T339I (6290C>T), S343A (6301T>G), C345S (6308G>C 6309C>A), R346K (6311G>A), D347E (6315T>A), A351R (6325G>A 6326C>G), L356A (6340C>G 6341T>C), V360I (6352G>A 6354C>A), L361M (6355T>A 6357A>G), S362T (6359G>C 6360T>A), K365N (6369A>T), Q368K (6376C>A), P369Q (6380C>A 6381T>A), T370P (6382A>C 6384A>T), T371I (6386C>T 6387C>T), T372I (6389C>T), R376K (6401G>A 6402G>A), R377N (6404G>A 6405G>T), N378R (6407A>G 6408T>A), P382D (6418C>G 6419C>A 6420A>C), D383E (6423T>A), L391Q (6446T>A), S393G (6451A>G 6453T>G), H394Q (6456C>A), K405N (6489A>C), I410V (6502A>G 6504T>A), V411I (6505G>A 6507T>C), E417D (6525A>C), *418E (6526T>G)								

	Begin	End	Coverage	Score	Concordance	Matches	Identities	I/D/M/F*	Stop Codons
**NT**	**2300**	**6573**	**27.1%**	**2301**	**55.2%**	**2097 (99.5%)**	**1627 (77.2%)**	**1/9**	
Codon mutations:	ATA166.TT (5772A>T), CAT167CAC (5775T>C), ATA169ATC (5781A>C), CAG170CAA (5784G>A), AAT171ACA (5786A>C 5787T>A), TTT172TAT (5789T>A), AAT174AAC (5796T>C), AAA175TTA (5797A>T 5798A>T), AAT176AAA (5802T>A), CCA177CCC (5805A>C), AGA178AAA (5807G>A), TCC179TCA (5811C>A), CAA180AAT (5812C>A 5814A>T), TCT181ATC (5815T>A 5816C>T 5817T>C), ACC182ACT (5820C>T), CAA183AAA (5821C>A), AAT184GAA (5824A>G 5826T>A), AAC186ACA (5831A>C 5832C>A), GAA187CAG (5833G>C 5835A>G), GAC188GAT (5838C>T), CAC192CAA (5850C>A), CAT193AAA (5851C>A 5853T>A), ACA195CAA (5857A>C 5858C>A), TTG200CTA (5872T>C 5874G>A), ATA201ATT (5877A>T), GCT202GCA (5880T>A), TTA203CAA (5881T>C 5882T>A), AAT205AAG (5889T>G), ACA206ACT (5892A>T), AGT207AAT (5894G>A), GCA208GCT (5898A>T), AAA209AAC (5901A>C), GTA211GTC (5907A>C), GCC212AAA (5908G>A 5909C>A 5910C>A), ACC213ACA (5913C>A), TGC214TGT (5916C>T), AAC216AGT (5921A>G 5922C>T), TAC217TAT (5925C>T), GAT221AGT (5935G>A 5936A>G), ACT222ACC (5940T>C), CAG226TAC (5950C>T 5952G>C), ACA227GAT (5953A>G 5954C>A 5955A>T), CAA229GAA (5959C>G), GAG230GAA (5964G>A), ATA231TTA (5965A>T), GCC232ACA (5968G>A 5970C>A), AAT233ACT (5972A>C), CCT235CCA (5979T>A), GAG236GAA (5982G>A), TAC238TAT (5988C>T), AAG239AAA (5991G>A), TTC241TTT (5997C>T), ATG242ACG (5999T>C), CAA243ACC (6001C>A 6002A>C 6003A>C), TAT244TAC (6006T>C), AAG245AAA (6009G>A), AGG246CGA (6010A>C 6012G>A), ATA247GTT (6013A>G 6015A>T), ACT248ACA (6018T>A), GGG250GGA (6024G>A), ACA251AAT (6026C>A 6027A>T), TTC253TTT (6033C>T), TAC254TAT (6036C>T), GTA255ATA (6037G>A), CGA256AAA (6040C>A 6041G>A), TTC257TTT (6045C>T), TCG259AGA (6049T>A 6050C>G 6051G>A), GCT260GCA (6054T>A), ACA261CCA (6055A>C), CTA265TTA (6067C>T), TAT266TAC (6072T>C), GAA267GAT (6075A>T), GAG268GAA (6078G>A), AAG270AAA (6084G>A), CCT271CCA (6087T>A), ATC272GTG (6088A>G 6090C>G), ATA273ATC (6093A>C), GTT275GTC (6099T>C), ATC276ATT (6102C>T), AAA277AAG (6105A>G), ATT278ATA (6108T>A), CTC280CTG (6114C>G), ACA281ACT (6117A>T), AGG282AGA (6120G>A), GAA283GAT (6123A>T), ATG284GTG (6124A>G), ATC285ATA (6129C>A), AAG289GAG (6139A>G), GAA291GTG (6146A>T 6147A>G), GAA294CCA (6154G>C 6155A>C), GAA297CCA (6163G>C 6164A>C), AAA298AGA (6167A>G), GTA299ACT (6169G>A 6170T>C 6171A>T), AAT300GAG (6172A>G 6174T>G), ATT301ATA (6177T>A), CTA320TTA (6232C>T), TAT324TAC (6246T>C), CTA325ACA (6247C>A 6248T>C), CAG327GGG (6253C>G 6254A>G), AAT328AAC (6258T>C), GCA329CCA (6259G>C), ATT330ATG (6264T>G), AGC332AAT (6269G>A 6270C>T), TAT333CAT (6271T>C), TCA335_AGG336del (6277_6283delTCAAGGG), GAA337-AC (6277_6283delTCAAGGG 6285A>C), ACA339ATA (6290C>T), TCA343GCA (6301T>G), TGC345TCA (6308G>C 6309C>A), AGA346AAA (6311G>A), GAT347GAA (6315T>A), ATA348ATC (6318A>C), AGA349CGC (6319A>C 6321A>C), GCA351AGA (6325G>A 6326C>G), CTA356GCA (6340C>G 6341T>C), GTC360ATA (6352G>A 6354C>A), TTA361ATG (6355T>A 6357A>G), AGT362ACA (6359G>C 6360T>A), CTT363TTG (6361C>T 6363T>G), AAA365AAT (6369A>T), GAA367GAG (6375A>G), CAA368AAA (6376C>A), CCT369CAA (6380C>A 6381T>A), ACA370CCT (6382A>C 6384A>T), ACC371ATT (6386C>T 6387C>T), ACA372ATA (6389C>T), AGG376AAA (6401G>A 6402G>A), AGG377AAT (6404G>A 6405G>T), AAT378AGA (6407A>G 6408T>A), TTC379TTT (6411C>T), ATC380ATT (6414C>T), TCC381TCG (6417C>G), CCA382GAC (6418C>G 6419C>A 6420A>C), GAT383GAA (6423T>A), CTG384TTA (6424C>T 6426G>A), TTA385CTA (6427T>C), CTA391CAA (6446T>A), AGT393GGG (6451A>G 6453T>G), CAC394CAA (6456C>A), TAT396TAC (6462T>C), CAC399CAT (6471C>T), TGC401TGT (6477C>T), TCA402TCG (6480A>G), AAA405AAC (6489A>C), ATT410GTA (6502A>G 6504T>A), GTT411ATC (6505G>A 6507T>C), GAC413GAT (6513C>T), CTG416CTA (6522G>A), GAA417GAC (6525A>C), TAA418GAA (6526T>G)								

Proteins

	Begin	End	Coverage	Score	Concordance	Matches	Identities	I/D/M/F*	Stop Codons
putative cell-to-cell movement protein (NP_569140.1)	50	174	29.8%	632	73.7%	125 (100%)	88 (70.4%)	0/0/1/0	1
Protein mutations:	I52L (2308A>T), K53N (2313A>C), N54Y (2314A>T 2316T>C), Q59P (2330A>C), K60V (2332A>G 2333A>T), V65I (2347G>A), V66I (2350G>A 2352T>A), Q68* (2356C>T), I72V (2368A>G 2370A>G), G79K (2389G>A 2390G>A 2391A>R), K81R (2396A>G 2397G>A), A82V (2399C>T 2400C>T), K83R (2402A>G), I86L (2410A>C), N88S (2417A>G 2418C>T), K89R (2420A>G), I91S (2425A>T 2426T>C), K94Q (2434A>C), Q97K (2443C>A 2445A>G), D98K (2446G>A 2448T>G), A101P (2455G>C 2457A>T), N103E (2461A>G 2463C>A), G112A (2489G>C), I117V (2503A>G), I131L (2545A>C), A134V (2555C>T), I139L (2569A>T 2571C>A), Q140E (2572C>G), I146V (2590A>G), S148T (2597G>C 2598T>A), I150V (2602A>G), K151R (2606A>G), G152E (2609G>A), I162V (2638A>G), A165P (2647G>C 2649C>A)								
Codon mutations:	ATA52TTA (2308A>T), AAA53AAC (2313A>C), AAT54TAC (2314A>T 2316T>C), TTC56TTT (2322C>T), AGA58AGG (2328A>G), CAA59CCA (2330A>C), AAA60GTA (2332A>G 2333A>T), TAC63TAT (2343C>T), GTA65ATA (2347G>A), GTT66ATA (2350G>A 2352T>A), AGC67AGT (2355C>T), CAA68TAA (2356C>T), ATA72GTG (2368A>G 2370A>G), ATT73ATA (2373T>A), GAT74GAY (2376T>Y), TGC75TGY (2379C>Y), TAT77YWY (2383T>Y 2384A>W 2385T>Y), ACA78ACY (2388A>Y), GGA79AAR (2389G>A 2390G>A 2391A>R), GGT80GGA (2394T>A), AAG81AGA (2396A>G 2397G>A), GCC82GTT (2399C>T 2400C>T), AAA83AGA (2402A>G), ATA86CTA (2410A>C), AAC88AGT (2417A>G 2418C>T), AAA89AGA (2420A>G), AGA90CGA (2422A>C), ATA91TCA (2425A>T 2426T>C), ATA92ATC (2430A>C), AAA94CAA (2434A>C), CAA97AAG (2443C>A 2445A>G), GAT98AAG (2446G>A 2448T>G), AAG100AAA (2454G>A), GCA101CCT (2455G>C 2457A>T), AAC103GAA (2461A>G 2463C>A), ATT105ATA (2469T>A), AAG106AAA (2472G>A), CAC109CAT (2481C>T), GGA112GCA (2489G>C), ACT113ACC (2493T>C), TTA116CTA (2500T>C), ATA117GTA (2503A>G), ACC127ACA (2535C>A), CCT128CCA (2538T>A), ATA131CTA (2545A>C), CTA133TTA (2551C>T), GCA134GTA (2555C>T), GAT135GAC (2559T>C), GAT136GAC (2562T>C), AGA137AGG (2565A>G), ATA138ATT (2568A>T), ATC139TTA (2569A>T 2571C>A), CAA140GAA (2572C>G), AGC145AGT (2589C>T), ATA146GTA (2590A>G), ATA147ATC (2595A>C), AGT148ACA (2597G>C 2598T>A), ATA150GTA (2602A>G), AAA151AGA (2606A>G), GGA152GAA (2609G>A), AAT153AAC (2613T>C), TAT156TAC (2622T>C), AAG158AAA (2628G>A), TTT161TTC (2637T>C), ATA162GTA (2638A>G), ATA163ATC (2643A>C), AGC164AGT (2646C>T), GCC165CCA (2647G>C 2649C>A), CTA171TTA (2665C>T)								
polyprotein (NP_569141.1)	233	596	50.5%	1789	79.1%	321 (100%)	264 (82.2%)	0/0/3/0	2
Protein mutations:	E236K (4116G>A), N238E (4122A>G 4124T>A), S239G (4125A>G 4127C>T), Y292N (4284T>A 4286T>C), D311E (4343T>A), A318T (4362G>A 4364T>A), P322A (4374C>G), A338E (4423C>A), N348S (4453A>G 4454C>T), R368K (4513G>A), Q370K (4518C>A), D371E (4523T>A), I374L (4530A>T), K379Q (4545A>C), A381I (4551G>A 4552C>T), H382D (4554C>G), T394K (4591C>A 4592C>A), A396S (4596G>T), N413S (4648A>G), T419S (4665A>T), L428S (4692C>T 4693T>C 4694T>A), D429N (4695G>A), N431Q (4701A>C 4703C>A), K436R (4717A>G 4718G>A), Q439* (4725C>T), I487L (4869A>T), Y496C (4897A>G 4898C>T), K503N (4919A>T), T505I (4924C>T 4925C>A), T510A (4938A>G), S512A (4944T>G), N514E (4950A>G 4952T>G), Y515R (4953T>C 4954A>G 4955T>A), G518E (4963G>A), Y523S (4978A>C 4979T>C), N526D (4986A>G), S540T (5028T>A 5030T>A), Y541F (5032A>T 5033T>C), T542S (5034A>T), E543D (5039A>C), P544S (5040C>T 5042A>G), Q545E (5043C>G), E546I (5046G>A 5047A>T), W548* (5054G>A), R552K (5065G>A), F556H (5076T>C 5077T>A), A557S (5079G>T 5081A>G), V570A (5119T>C 5120T>G), T572N (5125C>A 5126C>T), R573K (5128G>A), V576I (5136G>A), R577K (5140G>A), D579N (5145G>A)								
Codon mutations:	AAA232.GA (4105A>G), CAA235CAG (4115A>G), GAA236AAA (4116G>A), AGT237AGC (4121T>C), AAT238GAA (4122A>G 4124T>A), AGC239GGT (4125A>G 4127C>T), AGT243AGC (4139T>C), GGA257GGT (4181A>T), AGG260AGA (4190G>A), GTC262GTT (4196C>T), ATA263ATT (4199A>T), GAC264GAT (4202C>T), TAT275TAC (4235T>C), TAT277TAC (4241T>C), CCC280CCA (4250C>A), AAA282AAG (4256A>G), TTA284CTT (4260T>C 4262A>T), AAG285AAA (4265G>A), TAT292AAC (4284T>A 4286T>C), TAC294TAT (4292C>T), AGT296AGC (4298T>C), GAC299GAT (4307C>T), AAG301AAA (4313G>A), TAT305TAC (4325T>C), CAT306CAC (4328T>C), TTG307CTA (4329T>C 4331G>A), GAG310GAA (4340G>A), GAT311GAA (4343T>A), GCT318ACA (4362G>A 4364T>A), CCA322GCA (4374C>G), GTT330GTA (4400T>A), AAT337AAC (4421T>C), GCA338GAA (4423C>A), GGA340GGT (4430A>T), TAC342TAT (4436C>T), GAC347GAT (4451C>T), AAC348AGT (4453A>G 4454C>T), TGT356TGC (4478T>C), GTA358GTT (4484A>T), CTA364TTA (4500C>T), CTA365TTA (4503C>T), TAT366TAC (4508T>C), AGA368AAA (4513G>A), ACA369ACT (4517A>T), CAA370AAA (4518C>A), GAT371GAA (4523T>A), ATA374TTA (4530A>T), AAA379CAA (4545A>C), TTC380TTT (4550C>T), GCA381ATA (4551G>A 4552C>T), CAT382GAT (4554C>G), AAC386AAT (4568C>T), TCT387TCA (4571T>A), GGT388GGA (4574T>A), AGC390AGT (4580C>T), AGC392AGT (4586C>T), AAA393AAG (4589A>G), ACC394AAA (4591C>A 4592C>A), GCA396TCA (4596G>T), ATT398ATA (4604T>A), GGA407GGG (4631A>G), AAT413AGT (4648A>G), ATG417ATR (4661G>R), CAA418YAA (4662C>Y), ACT419TCT (4665A>T), CAT420CAY (4670T>Y), GTA422RTR (4674G>R 4676A>R), ATC426ATT (4688C>T), CTT428TCA (4692C>T 4693T>C 4694T>A), GAT429AAT (4695G>A), AAC431CAA (4701A>C 4703C>A), ATT432ATA (4706T>A), AAG436AGA (4717A>G 4718G>A), CAA439TAA (4725C>T), AAG483..A (4859G>A), ATT485ATA (4865T>A), AAG486AAA (4868G>A), ATA487TTA (4869A>T), TTG488CTT (4872T>C 4874G>T), TAC496TGT (4897A>G 4898C>T), GAT499GAC (4907T>C), AAC501AAT (4913C>T), AAA503AAT (4919A>T), ACC505ATA (4924C>T 4925C>A), ATT507ATA (4931T>A), ACA510GCA (4938A>G), TCA512GCA (4944T>G), AAT514GAG (4950A>G 4952T>G), TAT515CGA (4953T>C 4954A>G 4955T>A), TAT517TAC (4961T>C), GGA518GAA (4963G>A), GTT520GTA (4970T>A), CTT521TTA (4971C>T 4973T>A), AAG522AAA (4976G>A), TAT523TCC (4978A>C 4979T>C), AGA524AGG (4982A>G), AAT526GAT (4986A>G), TGC534TGT (5012C>T), TAC536TAT (5018C>T), GGA539GGT (5027A>T), TCT540ACA (5028T>A 5030T>A), TAT541TTC (5032A>T 5033T>C), ACA542TCA (5034A>T), GAA543GAC (5039A>C), CCA544TCG (5040C>T 5042A>G), CAA545GAA (5043C>G), GAA546ATA (5046G>A 5047A>T), TGG548TGA (5054G>A), GAA549GAG (5057A>G), AGA552AAA (5065G>A), TTT556CAT (5076T>C 5077T>A), GCA557TCG (5079G>T 5081A>G), CTA562TTA (5094C>T), TTA563CTA (5097T>C), GCA564GCC (5102A>C), ATA569ATT (5117A>T), GTT570GCG (5119T>C 5120T>G), ACC572AAT (5125C>A 5126C>T), AGA573AAA (5128G>A), TTT574TTC (5132T>C), ATT575ATA (5135T>A), GTA576ATA (5136G>A), AGA577AAA (5140G>A), GAC579AAC (5145G>A), CAA582CAG (5156A>G), ACT588ACA (5174T>A), AAA590AAG (5180A>G), GAT593GAC (5189T>C), GTC595GTT (5195C>T)								
putative transactivator factor (NP_569142.1)	167	418	57.2%	1002	60.7%	237 (99.2%)	155 (64.9%)	0/2/1/1	0
Protein mutations:	N171T (5786A>C 5787T>A), F172Y (5789T>A), K175L (5797A>T 5798A>T), N176K (5802T>A), R178K (5807G>A), Q180N (5812C>A 5814A>T), S181I (5815T>A 5816C>T 5817T>C), Q183K (5821C>A), N184E (5824A>G 5826T>A), N186T (5831A>C 5832C>A), E187Q (5833G>C 5835A>G), H192Q (5850C>A), H193K (5851C>A 5853T>A), T195Q (5857A>C 5858C>A), L203Q (5881T>C 5882T>A), N205K (5889T>G), S207N (5894G>A), K209N (5901A>C), A212K (5908G>A 5909C>A 5910C>A), N216S (5921A>G 5922C>T), D221S (5935G>A 5936A>G), Q226Y (5950C>T 5952G>C), T227D (5953A>G 5954C>A 5955A>T), Q229E (5959C>G), I231L (5965A>T), A232T (5968G>A 5970C>A), N233T (5972A>C), M242T (5999T>C), Q243T (6001C>A 6002A>C 6003A>C), I247V (6013A>G 6015A>T), T251N (6026C>A 6027A>T), V255I (6037G>A), R256K (6040C>A 6041G>A), S259R (6049T>A 6050C>G 6051G>A), T261P (6055A>C), E267D (6075A>T), I272V (6088A>G 6090C>G), E283D (6123A>T), M284V (6124A>G), K289E (6139A>G), E291V (6146A>T 6147A>G), E294P (6154G>C 6155A>C), E297P (6163G>C 6164A>C), K298R (6167A>G), V299T (6169G>A 6170T>C 6171A>T), N300E (6172A>G 6174T>G), L325T (6247C>A 6248T>C), Q327G (6253C>G 6254A>G), A329P (6259G>C), I330M (6264T>G), S332N (6269G>A 6270C>T), Y333H (6271T>C), S335_R336del (6277_6283delTCAAGGG), T339I (6290C>T), S343A (6301T>G), C345S (6308G>C 6309C>A), R346K (6311G>A), D347E (6315T>A), A351R (6325G>A 6326C>G), L356A (6340C>G 6341T>C), V360I (6352G>A 6354C>A), L361M (6355T>A 6357A>G), S362T (6359G>C 6360T>A), K365N (6369A>T), Q368K (6376C>A), P369Q (6380C>A 6381T>A), T370P (6382A>C 6384A>T), T371I (6386C>T 6387C>T), T372I (6389C>T), R376K (6401G>A 6402G>A), R377N (6404G>A 6405G>T), N378R (6407A>G 6408T>A), P382D (6418C>G 6419C>A 6420A>C), D383E (6423T>A), L391Q (6446T>A), S393G (6451A>G 6453T>G), H394Q (6456C>A), K405N (6489A>C), I410V (6502A>G 6504T>A), V411I (6505G>A 6507T>C), E417D (6525A>C), *418E (6526T>G)								

	Begin	End	Coverage	Score	Concordance	Matches	Identities	I/D/M/F*	Stop Codons
**NT**	**2300**	**6573**	**27.1%**	**2301**	**55.2%**	**2097 (99.5%)**	**1627 (77.2%)**	**1/9**	
Codon mutations:	ATA166.TT (5772A>T), CAT167CAC (5775T>C), ATA169ATC (5781A>C), CAG170CAA (5784G>A), AAT171ACA (5786A>C 5787T>A), TTT172TAT (5789T>A), AAT174AAC (5796T>C), AAA175TTA (5797A>T 5798A>T), AAT176AAA (5802T>A), CCA177CCC (5805A>C), AGA178AAA (5807G>A), TCC179TCA (5811C>A), CAA180AAT (5812C>A 5814A>T), TCT181ATC (5815T>A 5816C>T 5817T>C), ACC182ACT (5820C>T), CAA183AAA (5821C>A), AAT184GAA (5824A>G 5826T>A), AAC186ACA (5831A>C 5832C>A), GAA187CAG (5833G>C 5835A>G), GAC188GAT (5838C>T), CAC192CAA (5850C>A), CAT193AAA (5851C>A 5853T>A), ACA195CAA (5857A>C 5858C>A), TTG200CTA (5872T>C 5874G>A), ATA201ATT (5877A>T), GCT202GCA (5880T>A), TTA203CAA (5881T>C 5882T>A), AAT205AAG (5889T>G), ACA206ACT (5892A>T), AGT207AAT (5894G>A), GCA208GCT (5898A>T), AAA209AAC (5901A>C), GTA211GTC (5907A>C), GCC212AAA (5908G>A 5909C>A 5910C>A), ACC213ACA (5913C>A), TGC214TGT (5916C>T), AAC216AGT (5921A>G 5922C>T), TAC217TAT (5925C>T), GAT221AGT (5935G>A 5936A>G), ACT222ACC (5940T>C), CAG226TAC (5950C>T 5952G>C), ACA227GAT (5953A>G 5954C>A 5955A>T), CAA229GAA (5959C>G), GAG230GAA (5964G>A), ATA231TTA (5965A>T), GCC232ACA (5968G>A 5970C>A), AAT233ACT (5972A>C), CCT235CCA (5979T>A), GAG236GAA (5982G>A), TAC238TAT (5988C>T), AAG239AAA (5991G>A), TTC241TTT (5997C>T), ATG242ACG (5999T>C), CAA243ACC (6001C>A 6002A>C 6003A>C), TAT244TAC (6006T>C), AAG245AAA (6009G>A), AGG246CGA (6010A>C 6012G>A), ATA247GTT (6013A>G 6015A>T), ACT248ACA (6018T>A), GGG250GGA (6024G>A), ACA251AAT (6026C>A 6027A>T), TTC253TTT (6033C>T), TAC254TAT (6036C>T), GTA255ATA (6037G>A), CGA256AAA (6040C>A 6041G>A), TTC257TTT (6045C>T), TCG259AGA (6049T>A 6050C>G 6051G>A), GCT260GCA (6054T>A), ACA261CCA (6055A>C), CTA265TTA (6067C>T), TAT266TAC (6072T>C), GAA267GAT (6075A>T), GAG268GAA (6078G>A), AAG270AAA (6084G>A), CCT271CCA (6087T>A), ATC272GTG (6088A>G 6090C>G), ATA273ATC (6093A>C), GTT275GTC (6099T>C), ATC276ATT (6102C>T), AAA277AAG (6105A>G), ATT278ATA (6108T>A), CTC280CTG (6114C>G), ACA281ACT (6117A>T), AGG282AGA (6120G>A), GAA283GAT (6123A>T), ATG284GTG (6124A>G), ATC285ATA (6129C>A), AAG289GAG (6139A>G), GAA291GTG (6146A>T 6147A>G), GAA294CCA (6154G>C 6155A>C), GAA297CCA (6163G>C 6164A>C), AAA298AGA (6167A>G), GTA299ACT (6169G>A 6170T>C 6171A>T), AAT300GAG (6172A>G 6174T>G), ATT301ATA (6177T>A), CTA320TTA (6232C>T), TAT324TAC (6246T>C), CTA325ACA (6247C>A 6248T>C), CAG327GGG (6253C>G 6254A>G), AAT328AAC (6258T>C), GCA329CCA (6259G>C), ATT330ATG (6264T>G), AGC332AAT (6269G>A 6270C>T), TAT333CAT (6271T>C), TCA335_AGG336del (6277_6283delTCAAGGG), GAA337-AC (6277_6283delTCAAGGG 6285A>C), ACA339ATA (6290C>T), TCA343GCA (6301T>G), TGC345TCA (6308G>C 6309C>A), AGA346AAA (6311G>A), GAT347GAA (6315T>A), ATA348ATC (6318A>C), AGA349CGC (6319A>C 6321A>C), GCA351AGA (6325G>A 6326C>G), CTA356GCA (6340C>G 6341T>C), GTC360ATA (6352G>A 6354C>A), TTA361ATG (6355T>A 6357A>G), AGT362ACA (6359G>C 6360T>A), CTT363TTG (6361C>T 6363T>G), AAA365AAT (6369A>T), GAA367GAG (6375A>G), CAA368AAA (6376C>A), CCT369CAA (6380C>A 6381T>A), ACA370CCT (6382A>C 6384A>T), ACC371ATT (6386C>T 6387C>T), ACA372ATA (6389C>T), AGG376AAA (6401G>A 6402G>A), AGG377AAT (6404G>A 6405G>T), AAT378AGA (6407A>G 6408T>A), TTC379TTT (6411C>T), ATC380ATT (6414C>T), TCC381TCG (6417C>G), CCA382GAC (6418C>G 6419C>A 6420A>C), GAT383GAA (6423T>A), CTG384TTA (6424C>T 6426G>A), TTA385CTA (6427T>C), CTA391CAA (6446T>A), AGT393GGG (6451A>G 6453T>G), CAC394CAA (6456C>A), TAT396TAC (6462T>C), CAC399CAT (6471C>T), TGC401TGT (6477C>T), TCA402TCG (6480A>G), AAA405AAC (6489A>C), ATT410GTA (6502A>G 6504T>A), GTT411ATC (6505G>A 6507T>C), GAC413GAT (6513C>T), CTG416CTA (6522G>A), GAA417GAC (6525A>C), TAA418GAA (6526T>G)								

*: Inserts / Deletes / Misaligned / Frameshifts

Analysis details

This analysis was performed with panviral2.64

NGS Details (UN70): Duamitovirus soch1

Assembly

**Coverage Length**	2016 (7 contig(s))
**Depth Of Coverage**	11.7
**Number Of Reads**	210
**Reads Per Million**	3.98 rpm (after QC)
**Ambiguities**	0
**Assembly Method**	de novo + reference guided assembly
**Consensus Caller**	Bcf Tools

Coverage Map

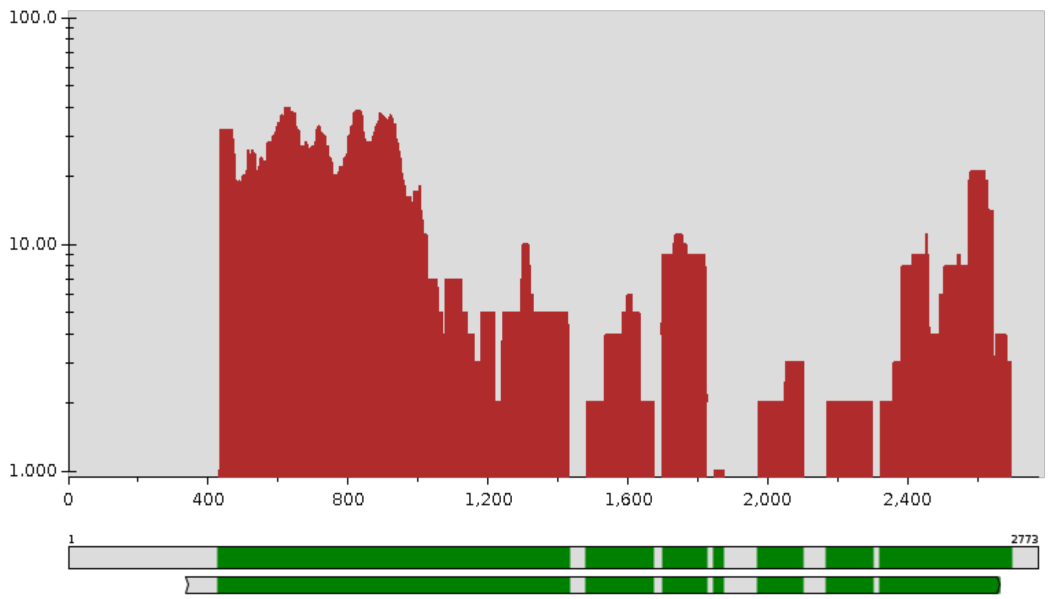

Assignment

**Type**	Duamitovirus soch1 (Taxonomy ID: 2955838)
**Reference Genome**	NC_076524.1
**NT Identity (%)**	69.4071
**AA Identity (%)**	69.863
**Number Of Stop Codons**	3
**Number Of CDS**	1

Alignment

**Alignment Score**	1522.0 (NT) + 3215.0 (AA) = 4737.0
**Concordance (%)**	56.1056

Alignment Method	Global, seeded, nucleotide + amino acids (AGA)

Genome Region

Sequence starts at position 428 and ends at position 2700 relative to NC_076524.1 reference sequence.

Alignment Detailed Statistics

	Begin	End	Coverage	Score	Concordance	Matches	Identities	I/D/M/F*	Stop Codons
**NT**	**428**	**2700**	**72.7%**	**1522**	**38.3%**	**2007 (99.6%)**	**1393 (69.1%)**	**0/9**	
Mutations:	437A>G, 441A>C, 443C>T, 447C>T, 459C>T, 466C>T, 470C>T, 471G>A, 472C>T, 473T>G, 477T>A, 480A>C, 481C>A, 482A>T, 485A>G, 489A>G, 494T>C, 497T>A, 500T>G, 503A>C, 506T>C, 509C>T, 512T>C, 513G>A, 516T>A, 517C>A, 518T>A, 520A>C, 524G>T, 525A>G, 526C>T, 527T>G, 529C>A, 530C>A, 531T>C, 533A>G, 534C>A, 535G>A, 536C>A, 539G>A, 540A>C, 541A>G, 542G>T, 545G>A, 548C>T, 550A>T, 556T>A, 557C>T, 560A>T, 569C>T, 575G>A, 581C>T, 582G>A, 587A>T, 589G>C, 591T>C, 593A>T, 599A>T, 602C>T, 612T>A, 613C>G, 614C>T, 616T>A, 618C>T, 619G>C, 620A>T, 624G>A, 626A>T, 629A>C, 630C>A, 632A>C, 633C>A, 635T>G, 638G>T, 641G>T, 644T>A, 647G>T, 651C>T, 653C>G, 656A>C, 657A>C, 661C>G, 662G>T, 665T>A, 668C>T, 672C>A, 673G>A, 674T>G, 680C>A, 683C>T, 684A>G, 685A>C, 686A>C, 692G>A, 695T>A, 699G>C, 700C>A, 701A>C, 702A>G, 705C>A, 707T>G, 708A>G, 709G>C, 710G>A, 713A>G, 714G>T, 715A>C, 719A>T, 722C>T, 737T>C, 746C>T, 752A>T, 761G>A, 765G>T, 767T>A, 770G>A, 771G>T, 772T>A, 773T>G, 774T>A, 776G>T, 777A>G, 778A>C, 779A>G, 782T>G, 796G>A, 797T>G, 803A>C, 809T>C, 810G>C, 815C>A, 818A>T, 821T>G, 824C>T, 827A>T, 831C>T, 833T>A, 841G>A, 842G>T, 850T>G, 851A>T, 857G>T, 860G>T, 862A>G, 869T>G, 875G>T, 880T>G, 881C>A, 885C>A, 887T>G, 892A>G, 905A>T, 908C>A, 909T>C, 910T>A, 916G>A, 918G>T, 919G>C, 923T>A, 926A>T, 927A>C, 929G>T, 934T>A, 935T>G, 938G>T, 942T>A, 943C>A, 948C>A, 971C>A, 972C>T, 975A>C, 976A>T, 977T>C, 983T>C, 990A>C, 992G>C, 993T>C, 994C>G, 995A>G, 997A>T, 998C>T, 999G>C, 1000G>A, 1001T>A, 1003T>A, 1004A>T, 1005G>T, 1007C>G, 1010A>G, 1016C>T, 1017A>G, 1018A>T, 1019A>T, 1022T>A, 1025T>C, 1026C>G, 1027A>G, 1030A>C, 1031G>A, 1034T>G, 1035A>G, 1036G>C, 1044T>G, 1046C>T, 1049G>A, 1050T>G, 1051T>A, 1052T>A, 1054C>A, 1055A>C, 1061T>C, 1067T>G, 1073G>A, 1076A>G, 1083C>A, 1088G>T, 1091C>G, 1099G>T, 1104A>G, 1105C>T, 1106A>G, 1115T>G, 1121A>C, 1122T>A, 1124C>T, 1127G>A, 1130C>T, 1133C>G, 1142A>C, 1145C>T, 1148G>T, 1151G>T, 1152T>C, 1154G>T, 1160C>T, 1161A>C, 1164T>A, 1165C>T, 1166T>G, 1169C>T, 1172C>A, 1174T>A, 1175T>C, 1178T>A, 1181A>T, 1184C>T, 1185A>T, 1186G>C, 1187G>T, 1188G>A, 1189C>A, 1191G>A, 1192C>A, 1193A>T, 1196T>C, 1197_1199delGAA, 1205A>T, 1211C>T, 1212T>G, 1213T>A, 1217T>C, 1220C>T, 1223A>T, 1224C>T, 1225A>T, 1226C>A, 1232G>A, 1236A>C, 1237G>A, 1238T>A, 1241C>A, 1247A>C, 1248T>C, 1249G>T, 1250G>T, 1253T>C, 1256A>T, 1258C>G, 1265G>A, 1266A>G, 1268T>G, 1269C>G, 1271T>C, 1274T>C, 1276G>A, 1280G>A, 1283G>T, 1292T>G, 1301G>A, 1302C>T, 1304T>G, 1306G>C, 1308G>C, 1309C>A, 1310A>G, 1312T>C, 1313G>C, 1316A>T, 1319G>A, 1322C>T, 1328A>G, 1332A>C, 1334A>G, 1338G>A, 1340A>C, 1345T>C, 1346A>T, 1349G>A, 1355C>T, 1357A>C, 1358C>T, 1376C>G, 1379C>T, 1382T>C, 1385T>C, 1388C>T, 1389G>A, 1390A>T, 1403A>T, 1406C>A, 1409G>A, 1412T>A, 1414A>G, 1415A>T, 1416A>C, 1418A>T, 1424A>T, 1427C>T, 1430G>T, 1433T>A, 1481A>T, 1486G>C, 1489A>T, 1490T>C, 1502A>C, 1505G>A, 1508A>G, 1526G>A, 1533G>T, 1538G>A, 1541T>C, 1545C>T, 1547T>C, 1553T>C, 1554A>T, 1555T>G, 1556G>C, 1559A>G, 1566C>A, 1567C>G, 1571C>A, 1572T>C, 1580C>A, 1589T>C, 1593_1597delAGCTC, 1598T>A, 1607T>C, 1613G>T, 1614A>T, 1617C>T, 1619T>G, 1620G>A, 1622T>C, 1623A>G, 1624A>T, 1626A>C, 1627A>C, 1628A>T, 1632T>A, 1634A>C, 1644G>A, 1664G>A, 1670G>A, 1676T>A, 1700A>C, 1703T>A, 1706C>T, 1707T>G, 1710T>C, 1712G>T, 1715T>C, 1721C>T, 1727A>G, 1734T>C, 1739A>T, 1744G>A, 1745C>A, 1746A>T, 1748A>T, 1754T>C, 1758G>A, 1759G>C, 1760A>G, 1761T>A, 1765A>C, 1766C>T, 1767T>A, 1769T>C, 1772A>T, 1777G>C, 1778G>T, 1787T>C, 1788T>C, 1790A>G, 1791C>T, 1819G>C, 1820T>A, 1821A>G, 1823A>G, 1856A>G, 1859C>T, 1860A>G, 1861A>G, 1865C>T, 1867A>C, 1868A>C, 1874G>T, 1875A>T, 1972G>A, 1973A>G, 1976T>A, 1980T>A, 1981T>C, 1984C>T, 1985T>C, 1991T>C, 2000T>G, 2003A>T, 2006T>G, 2010G>A, 2011C>A, 2013C>A, 2015C>T, 2020T>C, 2030T>C, 2036C>T, 2039A>G, 2052A>C, 2054A>G, 2060G>T, 2063T>A, 2066C>T, 2071A>T, 2074A>G, 2083C>A, 2084G>C, 2087C>T, 2088T>C, 2090A>T, 2093C>T, 2102A>G, 2103A>T, 2104G>C, 2105C>A, 2168C>A, 2173G>T, 2174G>T, 2186G>A, 2191T>A, 2192G>A, 2195C>T, 2204C>T, 2208T>C, 2210G>A, 2213A>G, 2225A>G, 2230A>T, 2231C>T, 2234C>T, 2237T>G, 2239A>G, 2240A>G, 2243A>G, 2250G>C, 2251T>C, 2252T>C, 2264G>T, 2273A>T, 2274A>G, 2276G>A, 2280C>A, 2285G>A, 2288C>T, 2322C>T, 2326G>T, 2328C>A, 2330A>T, 2336A>G, 2337G>A, 2346T>C, 2348A>T, 2349A>C, 2350A>G, 2355C>G, 2357C>T, 2358A>T, 2359A>C, 2360C>T, 2369T>C, 2372G>T, 2377G>A, 2378T>G, 2382A>T, 2383A>C, 2384C>T, 2385C>T, 2386C>T, 2387A>T, 2390T>A, 2396C>T, 2399T>C, 2400A>G, 2402G>A, 2403A>C, 2404G>A, 2405T>A, 2406C>T, 2408C>G, 2411C>G, 2414C>T, 2415T>G, 2417C>T, 2420T>C, 2423A>T, 2431C>A, 2432A>G, 2435C>A, 2441A>G, 2442A>C, 2444G>A, 2445C>T, 2446T>C, 2447C>G, 2448G>A, 2450A>T, 2451A>G, 2452C>A, 2453T>A, 2456T>C, 2457T>C, 2459T>G, 2462T>A, 2465A>G, 2466A>G, 2477C>T, 2478C>T, 2489A>G, 2492T>C, 2495C>T, 2504T>A, 2510T>C, 2514A>G, 2518C>T, 2519C>G, 2524C>T, 2525C>T, 2528T>A, 2532C>A, 2533A>G, 2536G>A, 2537G>T, 2543T>G, 2551A>G, 2552C>T, 2555T>C, 2556G>T, 2557T>C, 2561T>C, 2568A>G, 2571A>C, 2572A>G, 2582A>T, 2585G>A, 2597G>A, 2598A>G, 2599A>C, 2600C>T, 2609T>A, 2613T>G, 2615T>A, 2618T>A, 2622G>T, 2624A>T, 2626A>T, 2627C>A, 2629A>C, 2631A>T, 2634A>C, 2635C>T, 2638G>T, 2639A>G, 2644A>G, 2646G>T, 2647A>G, 2648A>T, 2649G>A, 2650T>G, 2652G>T, 2653G>C, 2654C>A, 2656T>A, 2657A>T, 2658A>G, 2659G>A, 2660A>C, 2661C>T, 2664T>A, 2665A>G, 2667delG, 2674T>A, 2678C>T, 2681G>A, 2686T>A, 2689T>C, 2690G>C, 2694A>G, 2695T>A									

CDS

	Begin	End	Coverage	Score	Concordance	Matches	Identities	I/D/M/F*	Stop Codons
RdRp	32	777	84.8%	3215	69.2%	657 (99.7%)	459 (69.7%)	0/2/1/1	3
Protein mutations:	I36L (441A>C 443C>T), L38F (447C>T), A44V (466C>T), A46M (471G>A 472C>T 473T>G), L48M (477T>A), T49H (480A>C 481C>A 482A>T), I52V (489A>G), V60I (513G>A), S61K (516T>A 517C>A 518T>A), K62T (520A>C), T64V (525A>G 526C>T 527T>G), T65K (529C>A 530C>A), R67K (534C>A 535G>A 536C>A), K69R (540A>C 541A>G 542G>T), Y72F (550A>T), F74Y (556T>A 557C>T), A83T (582G>A), C85S (589G>C), F94Y (616T>A), R95S (618C>T 619G>C 620A>T), E97N (624G>A 626A>T), E98D (629A>C), P99T (630C>A 632A>C), L100M (633C>A 635T>G), S109C (661C>G 662G>T), R113K (672C>A 673G>A 674T>G), K117A (684A>G 685A>C 686A>C), A122H (699G>C 700C>A 701A>C), I123V (702A>G), R125A (708A>G 709G>C 710G>A), D127S (714G>T 715A>C), E128D (719A>T), A144S (765G>T 767T>A), V146* (771G>T 772T>A 773T>G), L147I (774T>A 776G>T), K148A (777A>G 778A>C 779A>G), S154K (796G>A 797T>G), E159Q (810G>C), P166S (831C>T 833T>A), G169D (841G>A 842G>T), L172R (850T>G 851A>T), E176G (862A>G), I178M (869T>G), F182* (880T>G 881C>A), H184K (885C>A 887T>G), Q186R (892A>G), F192H (909T>C 910T>A), R194Q (916G>A), G195S (918G>T 919G>C), M198L (927A>C 929G>T), I200K (934T>A 935T>G), S203N (942T>A 943C>A), Q205K (948C>A), N214L (975A>C 976A>T 977T>C), S220R (993T>C 994C>G 995A>G), Y221F (997A>T 998C>T), G222Q (999G>C 1000G>A 1001T>A), L223Y (1003T>A 1004A>T), A224S (1005G>T 1007C>G), K228V (1017A>G 1018A>T 1019A>T), Q231G (1026C>G 1027A>G), E232A (1030A>C 1031G>A), S234A (1035A>G 1036G>C), F237V (1044T>G 1046C>T), F239E (1050T>G 1051T>A 1052T>A), P240H (1054C>A 1055A>C), L250I (1083C>A), W255L (1099G>T), T257V (1104A>G 1105C>T 1106A>G), I260M (1115T>G), F263I (1122T>A 1124C>T), M272I (1151G>T), K276Q (1161A>C), S277M (1164T>A 1165C>T 1166T>G), F279L (1172C>A), F280Y (1174T>A 1175T>C), L282F (1181A>T), R284S (1185A>T 1186G>C 1187G>T), A285N (1188G>A 1189C>A), A286N (1191G>A 1192C>A 1193A>T), E288del (1197_1199delGAA), L290F (1205A>T), L293E (1212T>G 1213T>A), E296D (1223A>T), H297L (1224C>T 1225A>T 1226C>A), S301Q (1236A>C 1237G>A 1238T>A), W305L (1248T>C 1249G>T 1250G>T), T308S (1258C>G), S311G (1266A>G 1268T>G), P312A (1269C>G 1271T>C), R314Q (1276G>A), G324A (1306G>C), A325Q (1308G>C 1309C>A 1310A>G), V326A (1312T>C 1313G>C), V335I (1338G>A 1340A>C), V337A (1345T>C 1346A>T), M338I (1349G>A), Y341S (1357A>C 1358C>T), D352I (1389G>A 1390A>T), K360S (1414A>G 1415A>T), I361L (1416A>C 1418A>T), C384S (1486G>C), Y385F (1489A>T 1490T>C), L389F (1502A>C), V400F (1533G>T), M401I (1538G>A), L404F (1545C>T 1547T>C), M407C (1554A>T 1555T>G 1556G>C), I408M (1559A>G), P411S (1566C>A 1567C>G), S420del (1593_1597delAGCTC), I427F (1614A>T), V429I (1620G>A 1622T>C), N430V (1623A>G 1624A>T), K431P (1626A>C 1627A>C 1628A>T), L433I (1632T>A 1634A>C), V437M (1644G>A), S458A (1707T>G), I464M (1727A>G), S470K (1744G>A 1745C>A), K471Y (1746A>T 1748A>T), G475T (1758G>A 1759G>C 1760A>G), C476S (1761T>A), N477T (1765A>C 1766C>T), S478T (1767T>A 1769T>C), G481A (1777G>C 1778G>T), S495T (1819G>C 1820T>A), K496E (1821A>G 1823A>G), N509G (1860A>G 1861A>G), K511T (1867A>C 1868A>C), I514L (1875A>T), L549T (1980T>A 1981T>C), S550F (1984C>T 1985T>C), A559N (2010G>A 2011C>A), L560I (2013C>A 2015C>T), I562T (2020T>C), I568M (2039A>G), Y579F (2071A>T), K580R (2074A>G), A583D (2083C>A 2084G>C), W613F (2173G>T 2174G>T), M619K (2191T>A 2192G>A), Y632F (2230A>T 2231C>T), K635R (2239A>G 2240A>G), V639P (2250G>C 2251T>C 2252T>C), Q643H (2264G>T), K647E (2274A>G 2276G>A), L649M (2280C>A), R664L (2326G>T), Q665N (2328C>A 2330A>T), E668K (2337G>A), K672R (2349A>C 2350A>G), L674V (2355C>G 2357C>T), N675S (2358A>T 2359A>C 2360C>T), C681* (2377G>A 2378T>G), N683S (2382A>T 2383A>C 2384C>T), P684F (2385C>T 2386C>T 2387A>T), D685E (2390T>A), K689E (2400A>G 2402G>A), S690Q (2403A>C 2404G>A 2405T>A), I692M (2411C>G), S694A (2415T>G 2417C>T), T699K (2431C>A 2432A>G), L704S (2445C>T 2446T>C 2447C>G), E705N (2448G>A 2450A>T), T706E (2451A>G 2452C>A 2453T>A), F708L (2457T>C 2459T>G), D709E (2462T>A), I711V (2466A>G), N727D (2514A>G), P728L (2518C>T 2519C>G), T730I (2524C>T 2525C>T), Q733R (2532C>A 2533A>G), W734Y (2536G>A 2537G>T), F736L (2543T>G), N739S (2551A>G 2552C>T), V741S (2556G>T 2557T>C), N745D (2568A>G), K746R (2571A>C 2572A>G), L749F (2582A>T), N755A (2598A>G 2599A>C 2600C>T), S760A (2613T>G 2615T>A), E763Y (2622G>T 2624A>T), Y764L (2626A>T 2627C>A), Q765P (2629A>C), T766S (2631A>T), T767L (2634A>C 2635C>T), G768V (2638G>T 2639A>G), E770G (2644A>G), E771C (2646G>T 2647A>G 2648A>T), V772R (2649G>A 2650T>G), G773S (2652G>T 2653G>C 2654C>A), L774Y (2656T>A 2657A>T), R775D (2658A>G 2659G>A 2660A>C), L776F (2661C>T), *777R (2664T>A 2665A>G)									

	Begin	End	Coverage	Score	Concordance	Matches	Identities	I/D/M/F*	Stop Codons
**NT**	**428**	**2700**	**72.7%**	**1522**	**38.3%**	**2007 (99.6%)**	**1393 (69.1%)**	**0/9**	

Codon mutations:

AAA34AAG (437A>G), ATC36CTT (441A>C 443C>T), CTT38TTT (447C>T), CTA42TTA (459C>T), GCC44GTC (466C>T), TCC45TCT (470C>T), GCT46ATG (471G>A 472C>T 473T>G), TTG48ATG (477T>A), ACA49CAT (480A>C 481C>A 482A>T), AAA50AAG (485A>G), ATC52GTC (489A>G), GCT53GCC (494T>C), CTT54CTA (497T>A), GCT55GCG (500T>G), GGA56GGC (503A>C), TGT57TGC (506T>C), GAC58GAT (509C>T), TTT59TTC (512T>C), GTA60ATA (513G>A), TCT61AAA (516T>A 517C>A 518T>A), AAA62ACA (520A>C), GTG63GTT (524G>T), ACT64GTG (525A>G 526C>T 527T>G), ACC65AAA (529C>A 530C>A), TTA66CTG (531T>C 533A>G), CGC67AAA (534C>A 535G>A 536C>A), CGG68CGA (539G>A), AAG69CGT (540A>C 541A>G 542G>T), TCG70TCA (545G>A), GGC71GGT (548C>T), TAT72TTT (550A>T), TTC74TAT (556T>A 557C>T), ACA75ACT (560A>T), TAC78TAT (569C>T), AAG80AAA (575G>A), TGC82TGT (581C>T), GCA83ACA (582G>A), GTA84GTT (587A>T), TGC85TCC (589G>C), TTA86CTT (591T>C 593A>T), CGA88CGT (599A>T), TAC89TAT (602C>T), TCC93AGT (612T>A 613C>G 614C>T), TTT94TAT (616T>A), CGA95TCT (618C>T 619G>C 620A>T), GAA97AAT (624G>A 626A>T), GAA98GAC (629A>C), CCA99ACC (630C>A 632A>C), CTT100ATG (633C>A 635T>G), TCG101TCT (638G>T), GTG102GTT (641G>T), CCT103CCA (644T>A), GTG104GTT (647G>T), CTC106TTG (651C>T 653C>G), ACA107ACC (656A>C), AGA108CGA (657A>C), TCG109TGT (661C>G 662G>T), GGT110GGA (665T>A), ATC111ATT (668C>T), CGT113AAG (672C>A 673G>A 674T>G), ATC115ATA (680C>A), CCC116CCT (683C>T), AAA117GCC (684A>G 685A>C 686A>C), TTG119TTA (692G>A), CGT120CGA (695T>A), GCA122CAC (699G>C 700C>A 701A>C), ATA123GTA (702A>G), CGT124AGG (705C>A 707T>G), AGG125GCA (708A>G 709G>C 710G>A), AAA126AAG (713A>G), GAT127TCT (714G>T 715A>C), GAA128GAT (719A>T), CAC129CAT (722C>T), GTT134GTC (737T>C), TAC137TAT (746C>T), TCA139TCT (752A>T), GGG142GGA (761G>A), GCT144TCA (765G>T 767T>A), AAG145AAA (770G>A), GTT146TAG (771G>T 772T>A 773T>G), TTG147ATT (774T>A 776G>T), AAA148GCG (777A>G 778A>C 779A>G), GTT149GTG (782T>G), AGT154AAG (796G>A 797T>G), GCA156GCC (803A>C), TTT158TTC (809T>C), GAA159CAA (810G>C), TCC160TCA (815C>A), ATA161ATT (818A>T), ACT162ACG (821T>G), ACC163ACT (824C>T), CCA164CCT (827A>T), CCT166TCA (831C>T 833T>A), GGG169GAT (841G>A 842G>T), CTA172CGT (850T>G 851A>T), GTG174GTT (857G>T), CTG175CTT (860G>T), GAA176GGA (862A>G), ATT178ATG (869T>G), ACG180ACT (875G>T), TTC182TGA (880T>G 881C>A), CAT184AAG (885C>A 887T>G), CAA186CGA (892A>G), CTA190CTT (905A>T), CCC191CCA (908C>A), TTT192CAT (909T>C 910T>A), CGA194CAA (916G>A), GGC195TCC (918G>T 919G>C), ATT196ATA (923T>A), CCA197CCT (926A>T), ATG198CTT (927A>C 929G>T), ATT200AAG (934T>A 935T>G), GGG201GGT (938G>T), TCT203AAT (942T>A 943C>A), CAA205AAA (948C>A), CCC212CCA (971C>A), CTA213TTA (972C>T), AAT214CTC (975A>C 976A>T 977T>C), AAT216AAC (983T>C), AGG219CGC (990A>C 992G>C), TCA220CGG (993T>C 994C>G 995A>G), TAC221TTT (997A>T 998C>T), GGT222CAA (999G>C 1000G>A 1001T>A), TTA223TAT (1003T>A 1004A>T), GCC224TCG (1005G>T 1007C>G), GTA225GTG (1010A>G), GAC227GAT (1016C>T), AAA228GTT (1017A>G 1018A>T 1019A>T), GTT229GTA (1022T>A), GAT230GAC (1025T>C), CAA231GGA (1026C>G 1027A>G), GAG232GCA (1030A>C 1031G>A), GCT233GCG (1034T>G), AGT234GCT (1035A>G 1036G>C), TTC237GTT (1044T>G 1046C>T), AAG238AAA (1049G>A), TTT239GAA (1050T>G 1051T>A 1052T>A), CCA240CAC (1054C>A 1055A>C), ATT242ATC (1061T>C), GTT244GTG (1067T>G), TTG246TTA (1073G>A), AAA247AAG (1076A>G), CTA250ATA (1083C>A), GCG251GCT (1088G>T), GCC252GCG (1091C>G), TGG255TTG (1099G>T), ACA257GTG (1104A>G 1105C>T 1106A>G), ATT260ATG (1115T>G), TCA262TCC (1121A>C), TTC263ATT (1122T>A 1124C>T), CAG264CAA (1127G>A), GAC265GAT (1130C>T), GGC266GGG (1133C>G), TCA269TCC (1142A>C), CCC270CCT (1145C>T), GGG271GGT (1148G>T), ATG272ATT (1151G>T), TTG273CTT (1152T>C 1154G>T), TCC275TCT (1160C>T), AAA276CAA (1161A>C), TCT277ATG (1164T>A 1165C>T 1166T>G), GTC278GTT (1169C>T), TTC279TTA (1172C>A), TTT280TAC (1174T>A 1175T>C), CCT281CCA (1178T>A), TTA282TTT (1181A>T), GAC283GAT (1184C>T), AGG284TCT (1185A>T 1186G>C 1187G>T), GCT285AAT (1188G>A 1189C>A), GCA286AAT (1191G>A 1192C>A 1193A>T), ACT287ACC (1196T>C), GAA288del (1197_1199delGAA), TTA290TTT (1205A>T), AAC292AAT (1211C>T), TTA293GAA (1212T>G 1213T>A), TCT294TCC (1217T>C), CTC295CTT (1220C>T), GAA296GAT (1223A>T), CAC297TTA (1224C>T 1225A>T 1226C>A), GAG299GAA (1232G>A), AGT301CAA (1236A>C 1237G>A 1238T>A), GTC302GTA (1241C>A), CCA304CCC (1247A>C), TGG305CTT (1248T>C 1249G>T 1250G>T), TTT306TTC (1253T>C), TCA307TCT (1256A>T), ACC308AGC (1258C>G), TTG310TTA (1265G>A), AGT311GGG (1266A>G 1268T>G), CCT312GCC (1269C>G 1271T>C), TAT313TAC (1274T>C), CGA314CAA (1276G>A), GGG315GGA (1280G>A), GTG316GTT (1283G>T), GTT319GTG (1292T>G), AGG322AGA (1301G>A), CTT323TTG (1302C>T 1304T>G), GGT324GCT (1306G>C), GCA325CAG (1308G>C 1309C>A 1310A>G), GTG326GCC (1312T>C 1313G>C), ATA327ATT (1316A>T), GAG328GAA (1319G>A), GGC329GGT (1322C>T), GGA331GGG (1328A>G), AGA333CGG (1332A>C 1334A>G), GTA335ATC (1338G>A 1340A>C), GTA337GCT (1345T>C 1346A>T), ATG338ATA (1349G>A), AAC340AAT (1355C>T), TAC341TCT (1357A>C 1358C>T), CTC347CTG (1376C>G), CAC348CAT (1379C>T), CCT349CCC (1382T>C), GTT350GTC (1385T>C), CAC351CAT (1388C>T), GAT352ATT (1389G>A 1390A>T), ACA356ACT (1403A>T), GTC357GTA (1406C>A), CTG358CTA (1409G>A), TCT359TCA (1412T>A), AAA360AGT (1414A>G 1415A>T), ATA361CTT (1416A>C 1418A>T), ACA363ACT (1424A>T), GAC364GAT (1427C>T), GGG365GGT (1430G>T), ACT366ACA (1433T>A), AAA382..T (1481A>T), TGT384TCT (1486G>C), TAT385TTC (1489A>T 1490T>C), TTA389TTC (1502A>C), AAG390AAA (1505G>A), TCA391TCG (1508A>G), CCG397CCA (1526G>A), GTC400TTC (1533G>T), ATG401ATA (1538G>A), TAT402TAC (1541T>C), CTT404TTC (1545C>T 1547T>C), TCT406TCC (1553T>C), ATG407TGC (1554A>T 1555T>G 1556G>C), ATA408ATG (1559A>G), CCT411AGT (1566C>A 1567C>G), ACC412ACA (1571C>A), TTG413CTG (1572T>C), TCC415TCA (1580C>A), GTT418GTC (1589T>C), AGC420del (1593_1597delAGCTC), TCT421--A (1593_1597delAGCTC 1598T>A), CTT424CTC (1607T>C), ACG426ACT (1613G>T), ATC427TTC (1614A>T), CTT428TTG (1617C>T 1619T>G), GTT429ATC (1620G>A 1622T>C), AAC430GTC (1623A>G 1624A>T), AAA431CCT (1626A>C 1627A>C 1628A>T), TTA433ATC (1632T>A 1634A>C), GTG437ATG (1644G>A), CTG443CTA (1664G>A), GGG445GGA (1670G>A), CCT447CCA (1676T>A), GCA455.CC (1700A>C), CTT456CTA (1703T>A), TTC457TTT (1706C>T), TCG458GCG (1707T>G), TTG459CTT (1710T>C 1712G>T), TCT460TCC (1715T>C), CAC462CAT (1721C>T), ATA464ATG (1727A>G), TTA467CTA (1734T>C), GCA468GCT (1739A>T), AGC470AAA (1744G>A 1745C>A), AAA471TAT (1746A>T 1748A>T), TAT473TAC (1754T>C), GGA475ACG (1758G>A 1759G>C 1760A>G), TGT476AGT (1761T>A), AAC477ACT (1765A>C 1766C>T), TCT478ACC (1767T>A 1769T>C), CCA479CCT (1772A>T), GGG481GCT (1777G>C 1778G>T), GCT484GCC (1787T>C), TTA485CTG (1788T>C 1790A>G), CTA486TTA (1791C>T), AGT495ACA (1819G>C 1820T>A), AAA496GAG (1821A>G 1823A>G), AGA507AGG (1856A>G), CTC508CTT (1859C>T), AAT509GGT (1860A>G 1861A>G), GTC510GTT (1865C>T), AAA511ACC (1867A>C 1868A>C), TCG513TCT (1874G>T), ATA514TTA (1875A>T), AGA546.AG (1972G>A 1973A>G), GCT547GCA (1976T>A), TTA549ACA (1980T>A 1981T>C), TCT550TTC (1984C>T 1985T>C), CGT552CGC (1991T>C), GTT555GTG (2000T>G), GGA556GGT (2003A>T), CTT557CTG (2006T>G), GCC559AAC (2010G>A 2011C>A), CTC560ATT (2013C>A 2015C>T), ATC562ACC (2020T>C), GAT565GAC (2030T>C), AAC567AAT (2036C>T), ATA568ATG (2039A>G), AGA573CGG (2052A>C 2054A>G), GGG575GGT (2060G>T), GGT576GGA (2063T>A), GCC577GCT (2066C>T), TAC579TTC (2071A>T), AAG580AGG (2074A>G), GCG583GAC (2083C>A 2084G>C), CGC584CGT (2087C>T), TTA585CTT (2088T>C 2090A>T), TTC586TTT (2093C>T), CAA589CAG (2102A>G), AGC590TCA (2103A>T 2104G>C 2105C>A), TAC611..A (2168C>A), TGG613TTT (2173G>T 2174G>T), AGG617AGA (2186G>A), ATG619AAA (2191T>A 2192G>A), CCC620CCT (2195C>T), CCC623CCT (2204C>T), TTG625CTA (2208T>C 2210G>A), AAA626AAG (2213A>G), GTA630GTG (2225A>G), TAC632TTT (2230A>T 2231C>T), CTC633CTT (2234C>T), CTT634CTG (2237T>G), AAA635AGG (2239A>G 2240A>G), GAA636GAG (2243A>G), GTT639CCC (2250G>C 2251T>C 2252T>C), CAG643CAT (2264G>T), CCA646CCT (2273A>T), AAG647GAA (2274A>G 2276G>A), CTG649ATG (2280C>A), GTG650GTA (2285G>A), TTC651TTT (2288C>T), CTA663TTA (2322C>T), CGC664CTC (2326G>T), CAA665AAT (2328C>A 2330A>T), GTA667GTG (2336A>G), GAA668AAA (2337G>A), TTA671CTT (2346T>C 2348A>T), AAG672CGG (2349A>C 2350A>G), CTC674GTT (2355C>G 2357C>T), AAC675TCT (2358A>T 2359A>C 2360C>T), CAT678CAC (2369T>C), ACG679ACT (2372G>T), TGT681TAG (2377G>A 2378T>G), AAC683TCT (2382A>T 2383A>C 2384C>T), CCA684TTT (2385C>T 2386C>T 2387A>T), GAT685GAA (2390T>A), TCC687TCT (2396C>T), ATT688ATC (2399T>C), AAG689GAA (2400A>G 2402G>A), AGT690CAA (2403A>C 2404G>A 2405T>A), CTC691TTG (2406C>T 2408C>G), ATC692ATG (2411C>G), GAC693GAT (2414C>T), TCC694GCT (2415T>G 2417C>T), CCT695CCC (2420T>C), ATA696ATT (2423A>T), ACA699AAG (2431C>A 2432A>G), TCC700TCA (2435C>A), AAA702AAG (2441A>G), AGG703CGA (2442A>C 2444G>A), CTC704TCG (2445C>T 2446T>C 2447C>G), GAA705AAT (2448G>A 2450A>T), ACT706GAA (2451A>G 2452C>A 2453T>A), GAT707GAC (2456T>C), TTT708CTG (2457T>C 2459T>G), GAT709GAA (2462T>A), TTA710TTG (2465A>G), ATC711GTC (2466A>G), GGC714GGT (2477C>T), CTA715TTA (2478C>T), AAA718AAG (2489A>G), TGT719TGC (2492T>C), TAC720TAT (2495C>T), GGT723GGA (2504T>A), GGT725GGC (2510T>C), AAT727GAT (2514A>G), CCC728CTG (2518C>T 2519C>G), ACC730ATT (2524C>T 2525C>T), ACT731ACA (2528T>A), CAG733AGG (2532C>A 2533A>G), TGG734TAT (2536G>A 2537G>T), TTT736TTG (2543T>G), AAC739AGT (2551A>G 2552C>T), ACT740ACC (2555T>C), GTT741TCT (2556G>T 2557T>C), ATT742ATC (2561T>C), AAT745GAT (2568A>G), AAA746CGA (2571A>C 2572A>G), TTA749TTT (2582A>T), GGG750GGA (2585G>A), GGG754GGA (2597G>A), AAC755GCT (2598A>G 2599A>C 2600C>T), ATT758ATA (2609T>A), TCT760GCA (2613T>G 2615T>A), CCT761CCA (2618T>A), GAA763TAT (2622G>T 2624A>T), TAC764TTA (2626A>T 2627C>A), CAA765CCA (2629A>C), ACT766TCT (2631A>T), ACA767CTA (2634A>C 2635C>T), GGA768GTG (2638G>T 2639A>G), GAG770GGG (2644A>G), GAA771TGT (2646G>T 2647A>G 2648A>T), GTG772AGG (2649G>A 2650T>G), GGC773TCA (2652G>T 2653G>C 2654C>A), TTA774TAT (2656T>A 2657A>T), AGA775GAC (2658A>G 2659G>A 2660A>C), CTT776TTT (2661C>T), TAG777AGG (2664T>A 2665A>G)

Proteins

	Begin	End	Coverage	Score	Concordance	Matches	Identities	I/D/M/F*	Stop Codons
RNA-dependent RNA polymerase (YP_010798874.1)	32	777	84.8%	3215	69.2%	657 (99.7%)	459 (69.7%)	0/2/1/1	3

Protein mutations:

I36L (441A>C 443C>T), L38F (447C>T), A44V (466C>T), A46M (471G>A 472C>T 473T>G), L48M (477T>A), T49H (480A>C 481C>A 482A>T), I52V (489A>G), V60I (513G>A), S61K (516T>A 517C>A 518T>A), K62T (520A>C), T64V (525A>G 526C>T 527T>G), T65K (529C>A 530C>A), R67K (534C>A 535G>A 536C>A), K69R (540A>C 541A>G 542G>T), Y72F (550A>T), F74Y (556T>A 557C>T), A83T (582G>A), C85S (589G>C), F94Y (616T>A), R95S (618C>T 619G>C 620A>T), E97N (624G>A 626A>T), E98D (629A>C), P99T (630C>A 632A>C), L100M (633C>A 635T>G), S109C (661C>G 662G>T), R113K (672C>A 673G>A 674T>G), K117A (684A>G 685A>C 686A>C), A122H (699G>C 700C>A 701A>C), I123V (702A>G), R125A (708A>G 709G>C 710G>A), D127S (714G>T 715A>C), E128D (719A>T), A144S (765G>T 767T>A), V146* (771G>T 772T>A 773T>G), L147I (774T>A 776G>T), K148A (777A>G 778A>C 779A>G), S154K (796G>A 797T>G), E159Q (810G>C), P166S (831C>T 833T>A), G169D (841G>A 842G>T), L172R (850T>G 851A>T), E176G (862A>G), I178M (869T>G), F182* (880T>G 881C>A), H184K (885C>A 887T>G), Q186R (892A>G), F192H (909T>C 910T>A), R194Q (916G>A), G195S (918G>T 919G>C), M198L (927A>C 929G>T), I200K (934T>A 935T>G), S203N (942T>A 943C>A), Q205K (948C>A), N214L (975A>C 976A>T 977T>C), S220R (993T>C 994C>G 995A>G), Y221F (997A>T 998C>T), G222Q (999G>C 1000G>A 1001T>A), L223Y (1003T>A 1004A>T), A224S (1005G>T 1007C>G), K228V (1017A>G 1018A>T 1019A>T), Q231G (1026C>G 1027A>G), E232A (1030A>C 1031G>A), S234A (1035A>G 1036G>C), F237V (1044T>G 1046C>T), F239E (1050T>G 1051T>A 1052T>A), P240H (1054C>A 1055A>C), L250I (1083C>A), W255L (1099G>T), T257V (1104A>G 1105C>T 1106A>G), I260M (1115T>G), F263I (1122T>A 1124C>T), M272I (1151G>T), K276Q (1161A>C), S277M (1164T>A 1165C>T 1166T>G), F279L (1172C>A), F280Y (1174T>A 1175T>C), L282F (1181A>T), R284S (1185A>T 1186G>C 1187G>T), A285N (1188G>A 1189C>A), A286N (1191G>A 1192C>A 1193A>T), E288del (1197_1199delGAA), L290F (1205A>T), L293E (1212T>G 1213T>A), E296D (1223A>T), H297L (1224C>T 1225A>T 1226C>A), S301Q (1236A>C 1237G>A 1238T>A), W305L (1248T>C 1249G>T 1250G>T), T308S (1258C>G), S311G (1266A>G 1268T>G), P312A (1269C>G 1271T>C), R314Q (1276G>A), G324A (1306G>C), A325Q (1308G>C 1309C>A 1310A>G), V326A (1312T>C 1313G>C), V335I (1338G>A 1340A>C), V337A (1345T>C 1346A>T), M338I (1349G>A), Y341S (1357A>C 1358C>T), D352I (1389G>A 1390A>T), K360S (1414A>G 1415A>T), I361L (1416A>C 1418A>T), C384S (1486G>C), Y385F (1489A>T 1490T>C), L389F (1502A>C), V400F (1533G>T), M401I (1538G>A), L404F (1545C>T 1547T>C), M407C (1554A>T 1555T>G 1556G>C), I408M (1559A>G), P411S (1566C>A 1567C>G), S420del (1593_1597delAGCTC), I427F (1614A>T), V429I (1620G>A 1622T>C), N430V (1623A>G 1624A>T), K431P (1626A>C 1627A>C 1628A>T), L433I (1632T>A 1634A>C), V437M (1644G>A), S458A (1707T>G), I464M (1727A>G), S470K (1744G>A 1745C>A), K471Y (1746A>T 1748A>T), G475T (1758G>A 1759G>C 1760A>G), C476S (1761T>A), N477T (1765A>C 1766C>T), S478T (1767T>A 1769T>C), G481A (1777G>C 1778G>T), S495T (1819G>C 1820T>A), K496E (1821A>G 1823A>G), N509G (1860A>G 1861A>G), K511T (1867A>C 1868A>C), I514L (1875A>T), L549T (1980T>A 1981T>C), S550F (1984C>T 1985T>C), A559N (2010G>A 2011C>A), L560I (2013C>A 2015C>T), I562T (2020T>C), I568M (2039A>G), Y579F (2071A>T), K580R (2074A>G), A583D (2083C>A 2084G>C), W613F (2173G>T 2174G>T), M619K (2191T>A 2192G>A), Y632F (2230A>T 2231C>T), K635R (2239A>G 2240A>G), V639P (2250G>C 2251T>C 2252T>C), Q643H (2264G>T), K647E (2274A>G 2276G>A), L649M (2280C>A), R664L (2326G>T), Q665N (2328C>A 2330A>T), E668K (2337G>A), K672R (2349A>C 2350A>G), L674V (2355C>G 2357C>T), N675S (2358A>T 2359A>C 2360C>T), C681* (2377G>A 2378T>G), N683S (2382A>T 2383A>C 2384C>T), P684F (2385C>T 2386C>T 2387A>T), D685E (2390T>A), K689E (2400A>G 2402G>A), S690Q (2403A>C 2404G>A 2405T>A), I692M (2411C>G), S694A (2415T>G 2417C>T), T699K (2431C>A 2432A>G), L704S (2445C>T 2446T>C 2447C>G), E705N (2448G>A 2450A>T), T706E (2451A>G 2452C>A 2453T>A), F708L (2457T>C 2459T>G), D709E (2462T>A), I711V (2466A>G), N727D (2514A>G), P728L (2518C>T 2519C>G), T730I (2524C>T 2525C>T), Q733R (2532C>A 2533A>G), W734Y (2536G>A 2537G>T), F736L (2543T>G), N739S (2551A>G 2552C>T), V741S (2556G>T 2557T>C), N745D (2568A>G), K746R (2571A>C 2572A>G), L749F (2582A>T), N755A (2598A>G 2599A>C 2600C>T), S760A (2613T>G 2615T>A), E763Y (2622G>T 2624A>T), Y764L (2626A>T 2627C>A), Q765P (2629A>C), T766S (2631A>T), T767L (2634A>C 2635C>T), G768V (2638G>T 2639A>G), E770G (2644A>G), E771C (2646G>T 2647A>G 2648A>T), V772R (2649G>A 2650T>G), G773S (2652G>T 2653G>C 2654C>A), L774Y (2656T>A 2657A>T), R775D (2658A>G 2659G>A 2660A>C), L776F (2661C>T), *777R (2664T>A 2665A>G)

	Begin	End	Coverage	Score	Concordance	Matches	Identities	I/D/M/F*	Stop Codons
**NT**	**428**	**2700**	**72.7%**	**1522**	**38.3%**	**2007 (99.6%)**	**1393 (69.1%)**	**0/9**	

Codon mutations: AAA34AAG (437A>G), ATC36CTT (441A>C 443C>T), CTT38TTT (447C>T), CTA42TTA (459C>T), GCC44GTC (466C>T), TCC45TCT (470C>T), GCT46ATG (471G>A 472C>T 473T>G), TTG48ATG (477T>A), ACA49CAT (480A>C 481C>A 482A>T), AAA50AAG (485A>G), ATC52GTC (489A>G), GCT53GCC (494T>C), CTT54CTA (497T>A), GCT55GCG (500T>G), GGA56GGC (503A>C), TGT57TGC (506T>C), GAC58GAT (509C>T), TTT59TTC (512T>C), GTA60ATA (513G>A), TCT61AAA (516T>A 517C>A 518T>A), AAA62ACA (520A>C), GTG63GTT (524G>T), ACT64GTG (525A>G 526C>T 527T>G), ACC65AAA (529C>A 530C>A), TTA66CTG (531T>C 533A>G), CGC67AAA (534C>A 535G>A 536C>A), CGG68CGA (539G>A), AAG69CGT (540A>C 541A>G 542G>T), TCG70TCA (545G>A), GGC71GGT (548C>T), TAT72TTT (550A>T), TTC74TAT (556T>A 557C>T), ACA75ACT (560A>T), TAC78TAT (569C>T), AAG80AAA (575G>A), TGC82TGT (581C>T), GCA83ACA (582G>A), GTA84GTT (587A>T), TGC85TCC (589G>C), TTA86CTT (591T>C 593A>T), CGA88CGT (599A>T), TAC89TAT (602C>T), TCC93AGT (612T>A 613C>G 614C>T), TTT94TAT (616T>A), CGA95TCT (618C>T 619G>C 620A>T), GAA97AAT (624G>A 626A>T), GAA98GAC (629A>C), CCA99ACC (630C>A 632A>C), CTT100ATG (633C>A 635T>G), TCG101TCT (638G>T), GTG102GTT (641G>T), CCT103CCA (644T>A), GTG104GTT (647G>T), CTC106TTG (651C>T 653C>G), ACA107ACC (656A>C), AGA108CGA (657A>C), TCG109TGT (661C>G 662G>T), GGT110GGA (665T>A), ATC111ATT (668C>T), CGT113AAG (672C>A 673G>A 674T>G), ATC115ATA (680C>A), CCC116CCT (683C>T), AAA117GCC (684A>G 685A>C 686A>C), TTG119TTA (692G>A), CGT120CGA (695T>A), GCA122CAC (699G>C 700C>A 701A>C), ATA123GTA (702A>G), CGT124AGG (705C>A 707T>G), AGG125GCA (708A>G 709G>C 710G>A), AAA126AAG (713A>G), GAT127TCT (714G>T 715A>C), GAA128GAT (719A>T), CAC129CAT (722C>T), GTT134GTC (737T>C), TAC137TAT (746C>T), TCA139TCT (752A>T), GGG142GGA (761G>A), GCT144TCA (765G>T 767T>A), AAG145AAA (770G>A), GTT146TAG (771G>T 772T>A 773T>G), TTG147ATT (774T>A 776G>T), AAA148GCG (777A>G 778A>C 779A>G), GTT149GTG (782T>G), AGT154AAG (796G>A 797T>G), GCA156GCC (803A>C), TTT158TTC (809T>C), GAA159CAA (810G>C), TCC160TCA (815C>A), ATA161ATT (818A>T), ACT162ACG (821T>G), ACC163ACT (824C>T), CCA164CCT (827A>T), CCT166TCA (831C>T 833T>A), GGG169GAT (841G>A 842G>T), CTA172CGT (850T>G 851A>T), GTG174GTT (857G>T), CTG175CTT (860G>T), GAA176GGA (862A>G), ATT178ATG (869T>G), ACG180ACT (875G>T), TTC182TGA (880T>G 881C>A), CAT184AAG (885C>A 887T>G), CAA186CGA (892A>G), CTA190CTT (905A>T), CCC191CCA (908C>A), TTT192CAT (909T>C 910T>A), CGA194CAA (916G>A), GGC195TCC (918G>T 919G>C), ATT196ATA (923T>A), CCA197CCT (926A>T), ATG198CTT (927A>C 929G>T), ATT200AAG (934T>A 935T>G), GGG201GGT (938G>T), TCT203AAT (942T>A 943C>A), CAA205AAA (948C>A), CCC212CCA (971C>A), CTA213TTA (972C>T), AAT214CTC (975A>C 976A>T 977T>C), AAT216AAC (983T>C), AGG219CGC (990A>C 992G>C), TCA220CGG (993T>C 994C>G 995A>G), TAC221TTT (997A>T 998C>T), GGT222CAA (999G>C 1000G>A 1001T>A), TTA223TAT (1003T>A 1004A>T), GCC224TCG (1005G>T 1007C>G), GTA225GTG (1010A>G), GAC227GAT (1016C>T), AAA228GTT (1017A>G 1018A>T 1019A>T), GTT229GTA (1022T>A), GAT230GAC (1025T>C), CAA231GGA (1026C>G 1027A>G), GAG232GCA (1030A>C 1031G>A), GCT233GCG (1034T>G), AGT234GCT (1035A>G 1036G>C), TTC237GTT (1044T>G 1046C>T), AAG238AAA (1049G>A), TTT239GAA (1050T>G 1051T>A 1052T>A), CCA240CAC (1054C>A 1055A>C), ATT242ATC (1061T>C), GTT244GTG (1067T>G), TTG246TTA (1073G>A), AAA247AAG (1076A>G), CTA250ATA (1083C>A), GCG251GCT (1088G>T), GCC252GCG (1091C>G), TGG255TTG (1099G>T), ACA257GTG (1104A>G 1105C>T 1106A>G), ATT260ATG (1115T>G), TCA262TCC (1121A>C), TTC263ATT (1122T>A 1124C>T), CAG264CAA (1127G>A), GAC265GAT (1130C>T), GGC266GGG (1133C>G), TCA269TCC (1142A>C), CCC270CCT (1145C>T), GGG271GGT (1148G>T), ATG272ATT (1151G>T), TTG273CTT (1152T>C 1154G>T), TCC275TCT (1160C>T), AAA276CAA (1161A>C), TCT277ATG (1164T>A 1165C>T 1166T>G), GTC278GTT (1169C>T), TTC279TTA (1172C>A), TTT280TAC (1174T>A 1175T>C), CCT281CCA (1178T>A), TTA282TTT (1181A>T), GAC283GAT (1184C>T), AGG284TCT (1185A>T 1186G>C 1187G>T), GCT285AAT (1188G>A 1189C>A), GCA286AAT (1191G>A 1192C>A 1193A>T), ACT287ACC (1196T>C), GAA288del (1197_1199delGAA), TTA290TTT (1205A>T), AAC292AAT (1211C>T), TTA293GAA (1212T>G 1213T>A), TCT294TCC (1217T>C), CTC295CTT (1220C>T), GAA296GAT (1223A>T), CAC297TTA (1224C>T 1225A>T 1226C>A), GAG299GAA (1232G>A), AGT301CAA (1236A>C 1237G>A 1238T>A), GTC302GTA (1241C>A), CCA304CCC (1247A>C), TGG305CTT (1248T>C 1249G>T 1250G>T), TTT306TTC (1253T>C), TCA307TCT (1256A>T), ACC308AGC (1258C>G), TTG310TTA (1265G>A), AGT311GGG (1266A>G 1268T>G), CCT312GCC (1269C>G 1271T>C), TAT313TAC (1274T>C), CGA314CAA (1276G>A), GGG315GGA (1280G>A), GTG316GTT (1283G>T), GTT319GTG (1292T>G), AGG322AGA (1301G>A), CTT323TTG (1302C>T 1304T>G), GGT324GCT (1306G>C), GCA325CAG (1308G>C 1309C>A 1310A>G), GTG326GCC (1312T>C 1313G>C), ATA327ATT (1316A>T), GAG328GAA (1319G>A), GGC329GGT (1322C>T), GGA331GGG (1328A>G), AGA333CGG (1332A>C 1334A>G), GTA335ATC (1338G>A 1340A>C), GTA337GCT (1345T>C 1346A>T), ATG338ATA (1349G>A), AAC340AAT (1355C>T), TAC341TCT (1357A>C 1358C>T), CTC347CTG (1376C>G), CAC348CAT (1379C>T), CCT349CCC (1382T>C), GTT350GTC (1385T>C), CAC351CAT (1388C>T), GAT352ATT (1389G>A 1390A>T), ACA356ACT (1403A>T), GTC357GTA (1406C>A), CTG358CTA (1409G>A), TCT359TCA (1412T>A), AAA360AGT (1414A>G 1415A>T), ATA361CTT (1416A>C 1418A>T), ACA363ACT (1424A>T), GAC364GAT (1427C>T), GGG365GGT (1430G>T), ACT366ACA (1433T>A), AAA382..T (1481A>T), TGT384TCT (1486G>C), TAT385TTC (1489A>T 1490T>C), TTA389TTC (1502A>C), AAG390AAA (1505G>A), TCA391TCG (1508A>G), CCG397CCA (1526G>A), GTC400TTC (1533G>T), ATG401ATA (1538G>A), TAT402TAC (1541T>C), CTT404TTC (1545C>T 1547T>C), TCT406TCC (1553T>C), ATG407TGC (1554A>T 1555T>G 1556G>C), ATA408ATG (1559A>G), CCT411AGT (1566C>A 1567C>G), ACC412ACA (1571C>A), TTG413CTG (1572T>C), TCC415TCA (1580C>A), GTT418GTC (1589T>C), AGC420del (1593_1597delAGCTC), TCT421--A (1593_1597delAGCTC 1598T>A), CTT424CTC (1607T>C), ACG426ACT (1613G>T), ATC427TTC (1614A>T), CTT428TTG (1617C>T 1619T>G), GTT429ATC (1620G>A 1622T>C), AAC430GTC (1623A>G 1624A>T), AAA431CCT (1626A>C 1627A>C 1628A>T), TTA433ATC (1632T>A 1634A>C), GTG437ATG (1644G>A), CTG443CTA (1664G>A), GGG445GGA (1670G>A), CCT447CCA (1676T>A), GCA455.CC (1700A>C), CTT456CTA (1703T>A), TTC457TTT (1706C>T), TCG458GCG (1707T>G), TTG459CTT (1710T>C 1712G>T), TCT460TCC (1715T>C), CAC462CAT (1721C>T), ATA464ATG (1727A>G), TTA467CTA (1734T>C), GCA468GCT (1739A>T), AGC470AAA (1744G>A 1745C>A), AAA471TAT (1746A>T 1748A>T), TAT473TAC (1754T>C), GGA475ACG (1758G>A 1759G>C 1760A>G), TGT476AGT (1761T>A), AAC477ACT (1765A>C 1766C>T), TCT478ACC (1767T>A 1769T>C), CCA479CCT (1772A>T), GGG481GCT (1777G>C 1778G>T), GCT484GCC (1787T>C), TTA485CTG (1788T>C 1790A>G), CTA486TTA (1791C>T), AGT495ACA (1819G>C 1820T>A), AAA496GAG (1821A>G 1823A>G), AGA507AGG (1856A>G), CTC508CTT (1859C>T), AAT509GGT (1860A>G 1861A>G), GTC510GTT (1865C>T), AAA511ACC (1867A>C 1868A>C), TCG513TCT (1874G>T), ATA514TTA (1875A>T), AGA546.AG (1972G>A 1973A>G), GCT547GCA (1976T>A), TTA549ACA (1980T>A 1981T>C), TCT550TTC (1984C>T 1985T>C), CGT552CGC (1991T>C), GTT555GTG (2000T>G), GGA556GGT (2003A>T), CTT557CTG (2006T>G), GCC559AAC (2010G>A 2011C>A), CTC560ATT (2013C>A 2015C>T), ATC562ACC (2020T>C), GAT565GAC (2030T>C), AAC567AAT (2036C>T), ATA568ATG (2039A>G), AGA573CGG (2052A>C 2054A>G), GGG575GGT (2060G>T), GGT576GGA (2063T>A), GCC577GCT (2066C>T), TAC579TTC (2071A>T), AAG580AGG (2074A>G), GCG583GAC (2083C>A 2084G>C), CGC584CGT (2087C>T), TTA585CTT (2088T>C 2090A>T), TTC586TTT (2093C>T), CAA589CAG (2102A>G), AGC590TCA (2103A>T 2104G>C 2105C>A), TAC611..A (2168C>A), TGG613TTT (2173G>T 2174G>T), AGG617AGA (2186G>A), ATG619AAA (2191T>A 2192G>A), CCC620CCT (2195C>T), CCC623CCT (2204C>T), TTG625CTA (2208T>C 2210G>A), AAA626AAG (2213A>G), GTA630GTG (2225A>G), TAC632TTT (2230A>T 2231C>T), CTC633CTT (2234C>T), CTT634CTG (2237T>G), AAA635AGG (2239A>G 2240A>G), GAA636GAG (2243A>G), GTT639CCC (2250G>C 2251T>C 2252T>C), CAG643CAT (2264G>T), CCA646CCT (2273A>T), AAG647GAA (2274A>G 2276G>A), CTG649ATG (2280C>A), GTG650GTA (2285G>A), TTC651TTT (2288C>T), CTA663TTA (2322C>T), CGC664CTC (2326G>T), CAA665AAT (2328C>A 2330A>T), GTA667GTG (2336A>G), GAA668AAA (2337G>A), TTA671CTT (2346T>C 2348A>T), AAG672CGG (2349A>C 2350A>G), CTC674GTT (2355C>G 2357C>T), AAC675TCT (2358A>T 2359A>C 2360C>T), CAT678CAC (2369T>C), ACG679ACT (2372G>T), TGT681TAG (2377G>A 2378T>G), AAC683TCT (2382A>T 2383A>C 2384C>T), CCA684TTT (2385C>T 2386C>T 2387A>T), GAT685GAA (2390T>A), TCC687TCT (2396C>T), ATT688ATC (2399T>C), AAG689GAA (2400A>G 2402G>A), AGT690CAA (2403A>C 2404G>A 2405T>A), CTC691TTG (2406C>T 2408C>G), ATC692ATG (2411C>G), GAC693GAT (2414C>T), TCC694GCT (2415T>G 2417C>T), CCT695CCC (2420T>C), ATA696ATT (2423A>T), ACA699AAG (2431C>A 2432A>G), TCC700TCA (2435C>A), AAA702AAG (2441A>G), AGG703CGA (2442A>C 2444G>A), CTC704TCG (2445C>T 2446T>C 2447C>G), GAA705AAT (2448G>A 2450A>T), ACT706GAA (2451A>G 2452C>A 2453T>A), GAT707GAC (2456T>C), TTT708CTG (2457T>C 2459T>G), GAT709GAA (2462T>A), TTA710TTG (2465A>G), ATC711GTC (2466A>G), GGC714GGT (2477C>T), CTA715TTA (2478C>T), AAA718AAG (2489A>G), TGT719TGC (2492T>C), TAC720TAT (2495C>T), GGT723GGA (2504T>A), GGT725GGC (2510T>C), AAT727GAT (2514A>G), CCC728CTG (2518C>T 2519C>G), ACC730ATT (2524C>T 2525C>T), ACT731ACA (2528T>A), CAG733AGG (2532C>A 2533A>G), TGG734TAT (2536G>A 2537G>T), TTT736TTG (2543T>G), AAC739AGT (2551A>G 2552C>T), ACT740ACC (2555T>C), GTT741TCT (2556G>T 2557T>C), ATT742ATC (2561T>C), AAT745GAT (2568A>G), AAA746CGA (2571A>C 2572A>G), TTA749TTT (2582A>T), GGG750GGA (2585G>A), GGG754GGA (2597G>A), AAC755GCT (2598A>G 2599A>C 2600C>T), ATT758ATA (2609T>A), TCT760GCA (2613T>G 2615T>A), CCT761CCA (2618T>A), GAA763TAT (2622G>T 2624A>T), TAC764TTA (2626A>T 2627C>A), CAA765CCA (2629A>C), ACT766TCT (2631A>T), ACA767CTA (2634A>C 2635C>T), GGA768GTG (2638G>T 2639A>G), GAG770GGG (2644A>G), GAA771TGT (2646G>T 2647A>G 2648A>T), GTG772AGG (2649G>A 2650T>G), GGC773TCA (2652G>T 2653G>C 2654C>A), TTA774TAT (2656T>A 2657A>T), AGA775GAC (2658A>G 2659G>A 2660A>C), CTT776TTT (2661C>T), TAG777AGG (2664T>A 2665A>G)

*: Inserts / Deletes / Misaligned / Frameshifts

Analysis details

This analysis was performed with panviral2.64

NGS Details (UN70): Tomato chocolate spot virus (segment RNA2)

Assembly

**Coverage Length**	346 (1 contig(s))
**Depth Of Coverage**	5827.1
**Number Of Reads**	19188
**Reads Per Million**	363.73 rpm (after QC)
**Ambiguities**	0
**Assembly Method**	de novo + reference guided assembly
**Consensus Caller**	Bcf Tools

Coverage Map

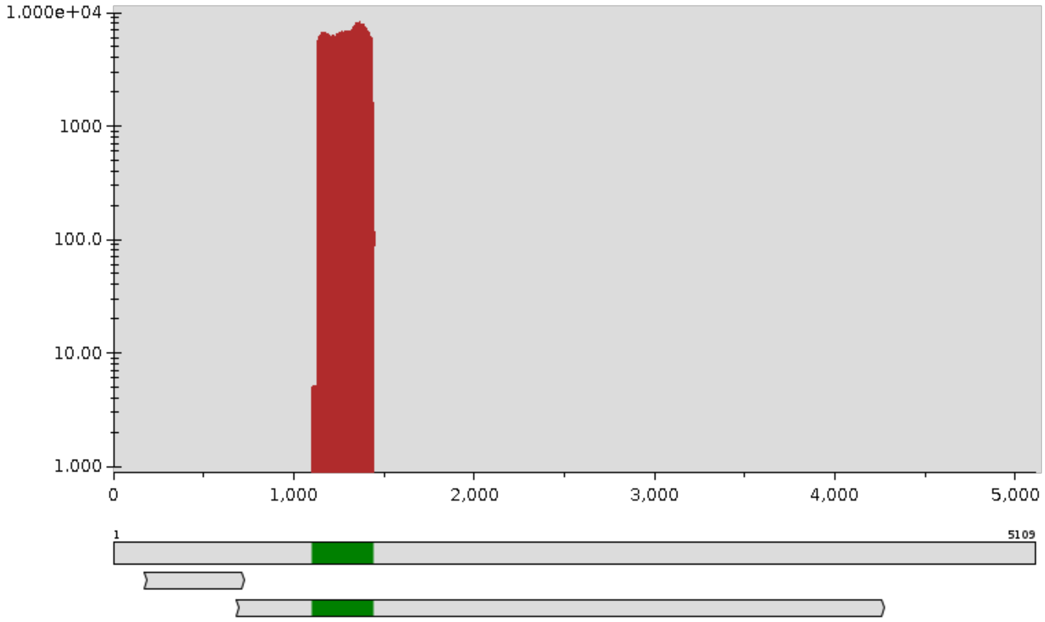

Assignment

**Type**	Tomato chocolate spot virus (Taxonomy ID: 661101)
**Reference Genome**	NC_013076.1
**NT Identity (%)**	69.6532
**AA Identity (%)**	75.0
**Number Of Stop Codons**	1
**Number Of CDS**	2

Alignment

**Alignment Score**	272.0 (NT) + 591.0 (AA) = 863.0
**Concordance (%)**	58.6676

Alignment Method	Global, seeded, nucleotide + amino acids (AGA)

Genome Region

Sequence starts at position 1100 and ends at position 1445 relative to NC_013076.1 reference sequence.

Alignment Detailed Statistics

	Begin	End	Coverage	Score	Concordance	Matches	Identities	I/D/M/F*	Stop Codons
**NT**	**1100**	**1445**	**6.8%**	**272**	**39.3%**	**346 (100%)**	**241 (69.7%)**	**0/0**	
Mutations:	1103C>A, 1107C>G, 1108C>T, 1109G>C, 1111G>A, 1112C>T, 1115A>T, 1116T>C, 1117G>T, 1120T>G, 1121C>G, 1124A>T, 1126T>A, 1127A>G, 1128T>A, 1130A>G, 1132A>C, 1133T>C, 1138C>T, 1142C>G, 1144C>T, 1153A>G, 1156A>C, 1162C>T, 1181G>C, 1182A>T, 1183G>T, 1186T>C, 1192T>G, 1195A>T, 1197A>T, 1198G>A, 1201A>T, 1204T>A, 1210C>T, 1213G>T, 1216A>G, 1219G>A, 1222A>T, 1228A>G, 1231A>G, 1238G>A, 1239A>G, 1240T>C, 1241A>C, 1246G>A, 1252C>T, 1255A>T, 1258C>T, 1259T>A, 1261T>G, 1262C>G, 1263A>C, 1267G>A, 1273T>A, 1277T>A, 1278C>G, 1285C>A, 1291A>G, 1294A>T, 1295T>C, 1297G>C, 1300C>T, 1306T>A, 1309A>G, 1312T>C, 1315C>T, 1316C>T, 1317A>C, 1318A>T, 1327G>A, 1333T>A, 1336C>A, 1351A>T, 1354A>C, 1360T>G, 1366G>A, 1367C>G, 1369A>G, 1372T>G, 1373T>G, 1375C>G, 1376C>T, 1377A>C, 1378G>T, 1379A>C, 1380T>A, 1381G>A, 1385T>C, 1387G>A, 1394C>A, 1396T>A, 1397G>C, 1405T>A, 1408A>T, 1410A>C, 1411G>A, 1412A>C, 1413G>T, 1414G>T, 1423T>C, 1426A>T, 1428G>A, 1429A>C, 1433G>A								

CDS

	Begin	End	Coverage	Score	Concordance	Matches	Identities	I/D/M/F*	Stop Codons
ToChV_s2_gp2	139	254	9.7%	591	75.2%	116 (100%)	87 (75.0%)	0/0/0/0	1
Protein mutations:	Q140K (1103C>A), A141G (1107C>G 1108C>T), V142L (1109G>C 1111G>A), L143F (1112C>T), M144S (1115A>T 1116T>C 1117G>T), S145R (1120T>G), P146A (1121C>G), S147* (1124A>T 1126T>A), I148D (1127A>G 1128T>A), T149A (1130A>G 1132A>C), L153V (1142C>G 1144C>T), E166L (1181G>C 1182A>T 1183G>T), Q171L (1197A>T 1198G>A), D185S (1238G>A 1239A>G 1240T>C), T186P (1241A>C), S192T (1259T>A 1261T>G), Q193A (1262C>G 1263A>C), Q211S (1316C>T 1317A>C 1318A>T), Q228E (1367C>G 1369A>G), S229R (1372T>G), S230A (1373T>G 1375C>G), Q231S (1376C>T 1377A>C 1378G>T), M232Q (1379A>C 1380T>A 1381G>A), A238P (1397G>C), E242A (1410A>C 1411G>A), R243L (1412A>C 1413G>T 1414G>T), R248H (1428G>A 1429A>C), D250N (1433G>A)								
Codon mutations:	CAA140AAA (1103C>A), GCC141GGT (1107C>G 1108C>T), GTG142CTA (1109G>C 1111G>A), CTC143TTC (1112C>T), ATG144TCT (1115A>T 1116T>C 1117G>T), AGT145AGG (1120T>G), CCT146GCT (1121C>G), AGT147TGA (1124A>T 1126T>A), ATC148GAC (1127A>G 1128T>A), ACA149GCC (1130A>G 1132A>C), TTG150CTG (1133T>C), CCC151CCT (1138C>T), CTC153GTT (1142C>G 1144C>T), GGA156GGG (1153A>G), CCA157CCC (1156A>C), TTC159TTT (1162C>T), GAG166CTT (1181G>C 1182A>T 1183G>T), AAT167AAC (1186T>C), CGT169CGG (1192T>G), GCA170GCT (1195A>T), CAG171CTA (1197A>T 1198G>A), ATA172ATT (1201A>T), CCT173CCA (1204T>A), TCC175TCT (1210C>T), GTG176GTT (1213G>T), GTA177GTG (1216A>G), GCG178GCA (1219G>A), ATA179ATT (1222A>T), AAA181AAG (1228A>G), CAA182CAG (1231A>G), GAT185AGC (1238G>A 1239A>G 1240T>C), ACA186CCA (1241A>C), AGG187AGA (1246G>A), GCC189GCT (1252C>T), GTA190GTT (1255A>T), TAC191TAT (1258C>T), TCT192ACG (1259T>A 1261T>G), CAA193GCA (1262C>G 1263A>C), CAG194CAA (1267G>A), ACT196ACA (1273T>A), TCT198AGT (1277T>A 1278C>G), GCC200GCA (1285C>A), GAA202GAG (1291A>G), GCA203GCT (1294A>T), TTG204CTC (1295T>C 1297G>C), AAC205AAT (1300C>T), CCT207CCA (1306T>A), CAA208CAG (1309A>G), TTT209TTC (1312T>C), TTC210TTT (1315C>T), CAA211TCT (1316C>T 1317A>C 1318A>T), CAG214CAA (1327G>A), GCT216GCA (1333T>A), GCC217GCA (1336C>A), TCA222TCT (1351A>T), GTA223GTC (1354A>C), TCT225TCG (1360T>G), GGG227GGA (1366G>A), CAA228GAG (1367C>G 1369A>G), AGT229AGG (1372T>G), TCC230GCG (1373T>G 1375C>G), CAG231TCT (1376C>T 1377A>C 1378G>T), ATG232CAA (1379A>C 1380T>A 1381G>A), TTG234CTA (1385T>C 1387G>A), CGT237AGA (1394C>A 1396T>A), GCT238CCT (1397G>C), GTT240GTA (1405T>A), GGA241GGT (1408A>T), GAG242GCA (1410A>C 1411G>A), AGG243CTT (1412A>C 1413G>T 1414G>T), AAT246AAC (1423T>C), ACA247ACT (1426A>T), CGA248CAC (1428G>A 1429A>C), GAT250AAT (1433G>A)								

Proteins

	Begin	End	Coverage	Score	Concordance	Matches	Identities	I/D/M/F*	Stop Codons
polyprotein (YP_003097231.1)	139	254	9.7%	591	75.2%	116 (100%)	87 (75.0%)	0/0/0/0	1
Protein mutations:	Q140K (1103C>A), A141G (1107C>G 1108C>T), V142L (1109G>C 1111G>A), L143F (1112C>T), M144S (1115A>T 1116T>C 1117G>T), S145R (1120T>G), P146A (1121C>G), S147* (1124A>T 1126T>A), I148D (1127A>G 1128T>A), T149A (1130A>G 1132A>C), L153V (1142C>G 1144C>T), E166L (1181G>C 1182A>T 1183G>T), Q171L (1197A>T 1198G>A), D185S (1238G>A 1239A>G 1240T>C), T186P (1241A>C), S192T (1259T>A 1261T>G), Q193A (1262C>G 1263A>C), Q211S (1316C>T 1317A>C 1318A>T), Q228E (1367C>G 1369A>G), S229R (1372T>G), S230A (1373T>G 1375C>G), Q231S (1376C>T 1377A>C 1378G>T), M232Q (1379A>C 1380T>A 1381G>A), A238P (1397G>C), E242A (1410A>C 1411G>A), R243L (1412A>C 1413G>T 1414G>T), R248H (1428G>A 1429A>C), D250N (1433G>A)								
Codon mutations:	CAA140AAA (1103C>A), GCC141GGT (1107C>G 1108C>T), GTG142CTA (1109G>C 1111G>A), CTC143TTC (1112C>T), ATG144TCT (1115A>T 1116T>C 1117G>T), AGT145AGG (1120T>G), CCT146GCT (1121C>G), AGT147TGA (1124A>T 1126T>A), ATC148GAC (1127A>G 1128T>A), ACA149GCC (1130A>G 1132A>C), TTG150CTG (1133T>C), CCC151CCT (1138C>T), CTC153GTT (1142C>G 1144C>T), GGA156GGG (1153A>G), CCA157CCC (1156A>C), TTC159TTT (1162C>T), GAG166CTT (1181G>C 1182A>T 1183G>T), AAT167AAC (1186T>C), CGT169CGG (1192T>G), GCA170GCT (1195A>T), CAG171CTA (1197A>T 1198G>A), ATA172ATT (1201A>T), CCT173CCA (1204T>A), TCC175TCT (1210C>T), GTG176GTT (1213G>T), GTA177GTG (1216A>G), GCG178GCA (1219G>A), ATA179ATT (1222A>T), AAA181AAG (1228A>G), CAA182CAG (1231A>G), GAT185AGC (1238G>A 1239A>G 1240T>C), ACA186CCA (1241A>C), AGG187AGA (1246G>A), GCC189GCT (1252C>T), GTA190GTT (1255A>T), TAC191TAT (1258C>T), TCT192ACG (1259T>A 1261T>G), CAA193GCA (1262C>G 1263A>C), CAG194CAA (1267G>A), ACT196ACA (1273T>A), TCT198AGT (1277T>A 1278C>G), GCC200GCA (1285C>A), GAA202GAG (1291A>G), GCA203GCT (1294A>T), TTG204CTC (1295T>C 1297G>C), AAC205AAT (1300C>T), CCT207CCA (1306T>A), CAA208CAG (1309A>G), TTT209TTC (1312T>C), TTC210TTT (1315C>T), CAA211TCT (1316C>T 1317A>C 1318A>T), CAG214CAA (1327G>A), GCT216GCA (1333T>A), GCC217GCA (1336C>A), TCA222TCT (1351A>T), GTA223GTC (1354A>C), TCT225TCG (1360T>G), GGG227GGA (1366G>A), CAA228GAG (1367C>G 1369A>G), AGT229AGG (1372T>G), TCC230GCG (1373T>G 1375C>G), CAG231TCT (1376C>T 1377A>C 1378G>T), ATG232CAA (1379A>C 1380T>A 1381G>A), TTG234CTA (1385T>C 1387G>A), CGT237AGA (1394C>A 1396T>A), GCT238CCT (1397G>C), GTT240GTA (1405T>A), GGA241GGT (1408A>T), GAG242GCA (1410A>C 1411G>A), AGG243CTT (1412A>C 1413G>T 1414G>T), AAT246AAC (1423T>C), ACA247ACT (1426A>T), CGA248CAC (1428G>A 1429A>C), GAT250AAT (1433G>A)								

*: Inserts / Deletes / Misaligned / Frameshifts

Analysis details

This analysis was performed with panviral2.64

NGS Details (UN70): Tomato chocolate spot virus (segment RNA 1)

Assembly

**Coverage Length**	933 (2 contig(s))
**Depth Of Coverage**	13296.7
**Number Of Reads**	74446
**Reads Per Million**	1411.22 rpm (after QC)
**Ambiguities**	0
**Assembly Method**	de novo + reference guided assembly
**Consensus Caller**	Bcf Tools

Coverage Map

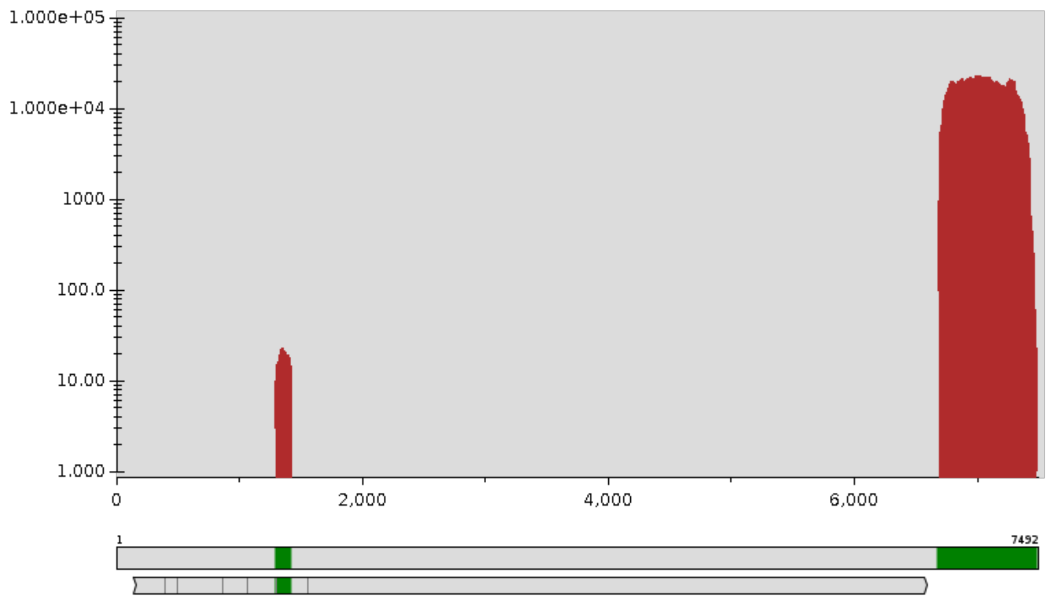

Assignment

**Type**	Tomato chocolate spot virus (Taxonomy ID: 661101)
**Reference Genome**	NC_013075.1
**NT Identity (%)**	69.9115
**AA Identity (%)**	93.4783
**Number Of Stop Codons**	0
**Number Of CDS**	1

Alignment

**Alignment Score**	191.0 (NT) + 306.0 (AA) = 497.0
**Concordance (%)**	34.0878

Alignment Method	Global, seeded, nucleotide + amino acids (AGA)

Genome Region

Sequence starts at position 1291 and ends at position 7484 relative to NC_013075.1 reference sequence.

Alignment Detailed Statistics

	Begin	End	Coverage	Score	Concordance	Matches	Identities	I/D/M/F*	Stop Codons
**NT**	**1291**	**7484**	**12.5%**	**191**	**16.8%**	**771 (80.9%)**	**553 (58.0%)**	**20/162**	
Mutations:	1296C>T, 1299T>C, 1326C>A, 1332C>T, 1335G>A, 1341A>G, 1343C>G, 1344C>T, 1348A>C, 1350A>C, 1351T>C, 1353G>T, 1356T>C, 1359A>C, 1368G>T, 1375A>G, 1376G>C, 1383A>T, 1386G>A, 1389T>A, 1392A>G, 1395C>G, 1401T>C, 1402C>T, 1404C>G, 1410A>G, 1413G>A, 1419G>A, 6706A>G, 6707T>A, 6721G>C, 6722G>C, 6729T>C, 6731G>A, 6735G>A, 6736T>G, 6740C>A, 6741G>A, 6753G>T, 6757T>G, 6758_6759insT, 6759_6760insG, 6766G>A, 6772C>T, 6777T>C, 6795A>G, 6806C>T, 6814C>T, 6815_6834delAATTGTTTGGTGTTGTCAAC, 6837G>T, 6840G>A, 6842C>A, 6843T>C, 6845T>G, 6847_6850delGCCA, 6853A>G, 6855A>T, 6857C>A, 6859C>T, 6860C>G, 6861C>G, 6862C>T, 6863T>C, 6867_6868delGC, 6870T>G, 6872A>G, 6873T>A, 6878A>G, 6879A>T, 6881G>A, 6885A>C, 6886G>T, 6887A>T, 6889_6894delGTGGAG, 6897G>A, 6903C>A, 6906G>C, 6908A>C, 6909A>G, 6911C>G, 6912A>G, 6914A>G, 6915A>T, 6918A>T, 6919_6921delGAG, 6923C>T, 6924T>C, 6926T>C, 6930_6937delGGTCGGGC, 6942G>C, 6944_6946delTGG, 6949G>A, 6950C>G, 6951C>T, 6953G>C, 6955A>G, 6956T>G, 6958A>T, 6960A>T, 6961G>T, 6962C>T, 6964T>C, 6965T>C, 6967G>A, 6969G>A, 6970T>G, 6972T>A, 6973G>A, 6976T>A, 6978T>A, 6982_6985delCATC, 6987G>T, 6989T>A, 6991T>C, 6996A>C, 6998C>T, 7001_7012delCTCGTTTGGAGT, 7014T>G, 7016A>G, 7018C>G, 7020A>T, 7022delT, 7026G>T, 7030_7035delTGGTGT, 7040A>C, 7041C>G, 7042G>A, 7047_7067delGCAATGACACCCGGGGTGTGC, 7073C>G, 7077T>C, 7079A>T, 7080A>G, 7084_7088delAAGTA, 7091G>A, 7093T>C, 7097delT, 7100C>G, 7102T>A, 7103A>T, 7108_7116delAACTAGAGC, 7118T>G, 7121_7129delGTCTCAACT, 7132A>G, 7134G>T, 7135A>G, 7136G>A, 7140_7145delCCCGTT, 7148A>T, 7151A>C, 7152T>A, 7154G>A, 7155A>C, 7156T>C, 7158A>C, 7159A>T, 7161T>C, 7161_7162insC, 7165G>C, 7169C>G, 7172delG, 7177A>C, 7180_7183delCTGC, 7186T>G, 7188G>T, 7190T>C, 7193G>A, 7195_7218delGCAACAGTGAAGAATCATCACACC, 7220C>G, 7223C>A, 7224T>C, 7225T>C, 7227C>T, 7228C>G, 7230A>C, 7231G>T, 7234T>G, 7235C>G, 7238C>T, 7241G>C, 7244G>A, 7245T>A, 7247_7248delTC, 7251A>T, 7259_7260delCT, 7262C>G, 7264T>A, 7266G>A, 7270A>C, 7273_7274delAG, 7288G>C, 7289T>C, 7291T>G, 7292T>G, 7293G>A, 7294C>T, 7296_7297insGCTTCTCCTAGTTAA, 7298G>A, 7299A>C, 7300C>G, 7302A>G, 7303T>C, 7304G>A, 7308C>T, 7310T>A, 7311A>T, 7314G>C, 7315T>C, 7316T>A, 7317T>G, 7318G>T, 7319G>A, 7321T>A, 7325C>A, 7327T>A, 7328A>T, 7330A>T, 7335_7336insT, 7342T>G, 7346A>T, 7348A>T, 7350G>C, 7351G>T, 7352_7353insC, 7356A>G, 7358C>G, 7367C>T, 7369G>A, 7372G>T, 7373delA, 7375T>C, 7376G>C, 7378A>C, 7381C>G, 7385C>T, 7387T>G, 7388T>C, 7389C>A, 7390A>T, 7393_7396delTATT, 7407G>T, 7410delG, 7425A>C, 7428delC, 7430T>C, 7431C>A, 7447A>C, 7453A>G, 7454G>A, 7455A>T, 7459T>C, 7460C>G								
**CDS**									
ToChSV_s1_gp1	385	430	2.1%	306	94.2%	46 (100%)	43 (93.5%)	0/0/0/0	0
Protein mutations:	S402C (1343C>G 1344C>T), R413A (1375A>G 1376G>C)								
Codon mutations:	CCC386CCT (1296C>T), TTT387TTC (1299T>C), GGC396GGA (1326C>A), GGC398GGT (1332C>T), AAG399AAA (1335G>A), ACA401ACG (1341A>G), TCC402TGT (1343C>G 1344C>T), AGA404CGC (1348A>C 1350A>C), TTG405CTT (1351T>C 1353G>T), TTT406TTC (1356T>C), ATA407ATC (1359A>C), GTG410GTT (1368G>T), AGA413GCA (1375A>G 1376G>C), GGA415GGT (1383A>T), GAG416GAA (1386G>A), CCT417CCA (1389T>A), AAA418AAG (1392A>G), CTC419CTG (1395C>G), CGT421CGC (1401T>C), CTC422TTG (1402C>T 1404C>G), GCA424GCG (1410A>G), GTG425GTA (1413G>A), AAG427AAA (1419G>A)								
**Proteins**									
polyprotein (YP_003097229.1)	385	430	2.1%	306	94.2%	46 (100%)	43 (93.5%)	0/0/0/0	0
Protein mutations:	S402C (1343C>G 1344C>T), R413A (1375A>G 1376G>C)								
Codon mutations:	CCC386CCT (1296C>T), TTT387TTC (1299T>C), GGC396GGA (1326C>A), GGC398GGT (1332C>T), AAG399AAA (1335G>A), ACA401ACG (1341A>G), TCC402TGT (1343C>G 1344C>T), AGA404CGC (1348A>C 1350A>C), TTG405CTT (1351T>C 1353G>T), TTT406TTC (1356T>C), ATA407ATC (1359A>C), GTG410GTT (1368G>T), AGA413GCA (1375A>G 1376G>C), GGA415GGT (1383A>T), GAG416GAA (1386G>A), CCT417CCA (1389T>A), AAA418AAG (1392A>G), CTC419CTG (1395C>G), CGT421CGC (1401T>C), CTC422TTG (1402C>T 1404C>G), GCA424GCG (1410A>G), GTG425GTA (1413G>A), AAG427AAA (1419G>A)								
RNA-dependent RNA polymerase (YP_003097234.1)	1	41	48.8%	268	93.4%	41 (100%)	38 (92.7%)	0/0/0/0	0
Protein mutations:	S13C (1343C>G 1344C>T), R24A (1375A>G 1376G>C)								
Codon mutations:	GGC7GGA (1326C>A), GGC9GGT (1332C>T), AAG10AAA (1335G>A), ACA12ACG (1341A>G), TCC13TGT (1343C>G 1344C>T), AGA15CGC (1348A>C 1350A>C), TTG16CTT (1351T>C 1353G>T), TTT17TTC (1356T>C), ATA18ATC (1359A>C), GTG21GTT (1368G>T), AGA24GCA (1375A>G 1376G>C), GGA26GGT (1383A>T), GAG27GAA (1386G>A), CCT28CCA (1389T>A), AAA29AAG (1392A>G), CTC30CTG (1395C>G), CGT32CGC (1401T>C), CTC33TTG (1402C>T 1404C>G), GCA35GCG (1410A>G), GTG36GTA (1413G>A), AAG38AAA (1419G>A)								

*: Inserts / Deletes / Misaligned / Frameshifts

Analysis details

This analysis was performed with panviral2.64

NGS Details (UN70): Brazilian marseillevirus

Assembly

**Coverage Length**	233 (1 contig(s))
**Depth Of Coverage**	5346.7
**Number Of Reads**	13004
**Reads Per Million**	246.51 rpm (after QC)
**Ambiguities**	0
**Assembly Method**	de novo + reference guided assembly
**Consensus Caller**	Bcf Tools

Coverage Map

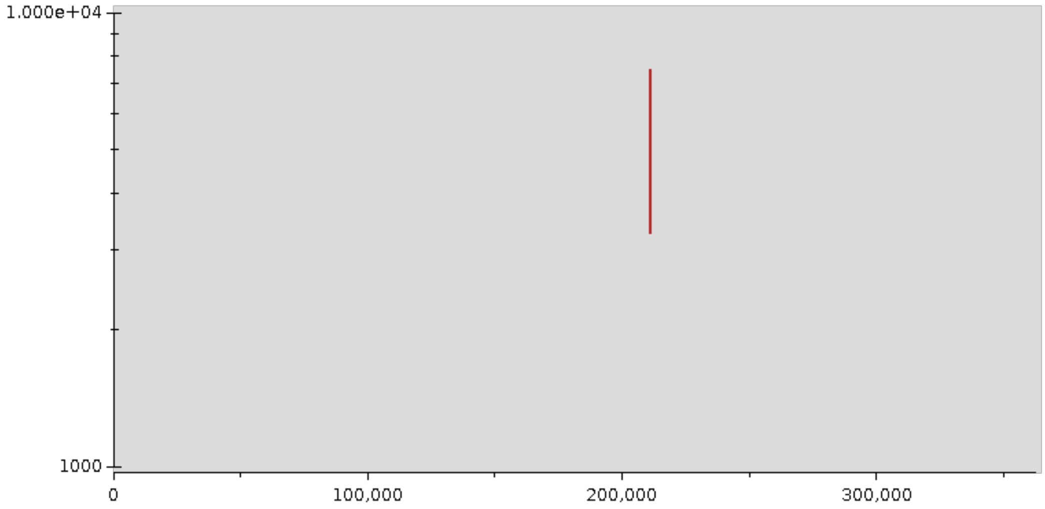

Assignment

**Type**	Brazilian marseillevirus (Taxonomy ID: 1813599)
**Reference Genome**	NC_029692.1
**NT Identity (%)**	79.8283
**AA Identity (%)**	94.6667
**Number Of Stop Codons**	1
**Number Of CDS**	491

Alignment

**Alignment Score**	278.0 (NT) + 454.0 (AA) = 732.0
**Concordance (%)**	78.3726
**Alignment Method**	Local, heuristic, nucleotide (BLASTN)

Genome Region

Sequence starts at position 210884 and ends at position 211116 relative to NC_029692.1 reference sequence.

Alignment Detailed Statistics

	Begin	End	Coverage	Score	Concordance	Matches	Identities	I/D/M/F*	Stop Codons
**NT**	**210884**	**211116**	**0.1%**	**278**	**59.7%**	**233 (100%)**	**186 (79.8%)**	**0/0**	

Mutations: 210899T>C, 210901T>G, 210902T>A, 210908A>C, 210910G>A, 210917A>G, 210920G>T, 210926T>C, 210929T>C, 210938A>G, 210944C>A, 210955T>G, 210956T>G, 210962T>C, 210965C>A, 210974G>C, 210977T>G, 210980G>A, 210986A>C, 210988G>A, 210989G>T, 210991G>T, 210995T>C, 211001A>T, 211004A>G, 211009C>T, 211010T>G, 211022C>T, 211031T>G, 211032T>G, 211033G>C, 211037C>A, 211043T>G, 211046G>A, 211055A>G, 211067C>T, 211070G>A, 211073A>G, 211079C>T, 211088C>A, 211091G>C, 211093G>A, 211100T>C, 211103A>G, 211106C>A, 211108C>T, 211109C>T

*: Inserts / Deletes / Misaligned / Frameshifts

Analysis details

This analysis was performed with panviral2.64

NGS Details (UN70): Tokyovirus A1

Assembly

**Coverage Length**	180 (1 contig(s))
**Depth Of Coverage**	6107.3
**Number Of Reads**	12194
**Reads Per Million**	231.15 rpm (after QC)
**Ambiguities**	0
**Assembly Method**	de novo + reference guided assembly
**Consensus Caller**	Bcf Tools

Coverage Map

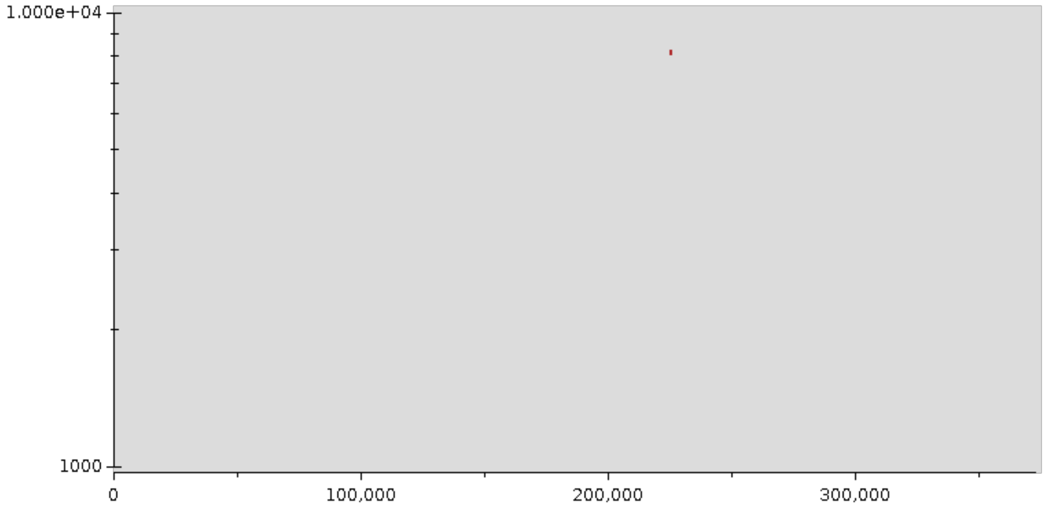

Assignment

**Type**	Tokyovirus A1 (Taxonomy ID: 1826170)
**Reference Genome**	NC_030230.1
**NT Identity (%)**	80.5556
**AA Identity (%)**	93.1034
**Number Of Stop Codons**	1
**Number Of CDS**	470

Alignment

**Alignment Score**	220.0 (NT) + 353.0 (AA) = 573.0
**Concordance (%)**	78.4932
**Alignment Method**	Local, heuristic, nucleotide (BLASTN)

Genome Region

Sequence starts at position 225483 and ends at position 225662 relative to NC_030230.1 reference sequence.

Alignment Detailed Statistics

	Begin	End	Coverage	Score	Concordance	Matches	Identities	I/D/M/F*	Stop Codons
**NT**	**225483**	**225662**	**0.1%**	**220**	**61.1%**	**180 (100%)**	**145 (80.6%)**	**0/0**	
Mutations:	225498T>C, 225504G>C, 225507A>C, 225510G>A, 225513G>T, 225519G>T, 225528T>C, 225533C>T, 225534A>G, 225543G>C, 225549A>G, 225552T>G, 225554T>G, 225555C>G, 225564T>G, 225573C>A, 225579G>A, 225582G>A, 225585A>C, 225590G>T, 225594T>C, 225600G>C, 225603C>T, 225609A>T, 225621T>C, 225624G>T, 225630T>G, 225631T>G, 225632G>C, 225636C>G, 225640G>T, 225641C>T, 225642T>G, 225648G>T, 225651G>A								

*: Inserts / Deletes / Misaligned / Frameshifts

Analysis details

This analysis was performed with panviral2.64

NGS Details (UN70): Lausannevirus

Assembly

**Coverage Length**	195 (1 contig(s))
**Depth Of Coverage**	4682.9
**Number Of Reads**	10606
**Reads Per Million**	201.05 rpm (after QC)
**Ambiguities**	1
**Assembly Method**	de novo + reference guided assembly
**Consensus Caller**	Bcf Tools

Coverage Map

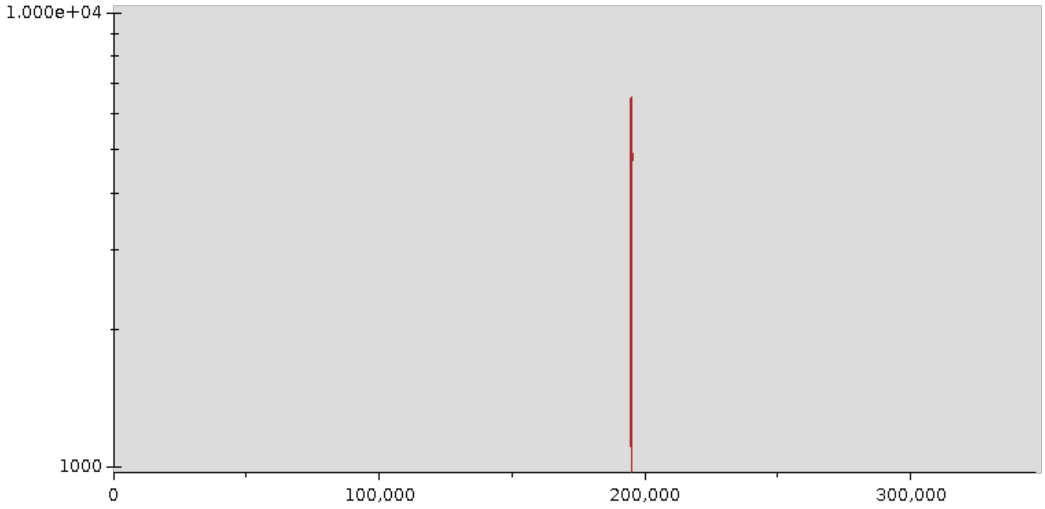

Assignment

**Type**	Lausannevirus (Taxonomy ID: 999883)
**Reference Genome**	NC_015326.1
**NT Identity (%)**	80.5128
**AA Identity (%)**	95.2381
**Number Of Stop Codons**	1
**Number Of CDS**	444

Alignment

**Alignment Score**	240.0 (NT) + 358.0 (AA) = 598.0
**Concordance (%)**	75.7921
**Alignment Method**	Local, heuristic, nucleotide (BLASTN)

Genome Region

Sequence starts at position 194911 and ends at position 195105 relative to NC_015326.1 reference sequence.

Alignment Detailed Statistics

	Begin	End	Coverage	Score	Concordance	Matches	Identities	I/D/M/F*	Stop Codons
**NT**	**194911**	**195105**	**0.1%**	**240**	**61.5%**	**195 (100%)**	**157 (80.5%)**	**0/0**	
Mutations:	194925T>M, 194931A>C, 194937G>A, 194943A>G, 194946G>C, 194952T>C, 194955T>C, 194958A>G, 194967A>G, 194970G>C, 194979T>G, 194981T>G, 194982C>G, 194988T>C, 195003T>A, 195012A>C, 195015T>C, 195017G>T, 195018T>C, 195021T>C, 195033A>G, 195036G>C, 195042T>C, 195048T>C, 195054T>C, 195057T>A, 195058T>G, 195059G>C, 195060C>T, 195063G>C, 195066G>A, 195069C>G, 195072A>G, 195081C>A, 195087T>C, 195093T>C, 195096A>C, 195098G>A								

*: Inserts / Deletes / Misaligned / Frameshifts

Analysis details

This analysis was performed with panviral2.64

NGS Details (UN70): Marseillevirus marseillevirus

Assembly

**Coverage Length**	104 (1 contig(s))
**Depth Of Coverage**	1004.1
**Number Of Reads**	1457
**Reads Per Million**	27.62 rpm (after QC)
**Ambiguities**	0
**Assembly Method**	de novo + reference guided assembly
**Consensus Caller**	Bcf Tools

Coverage Map

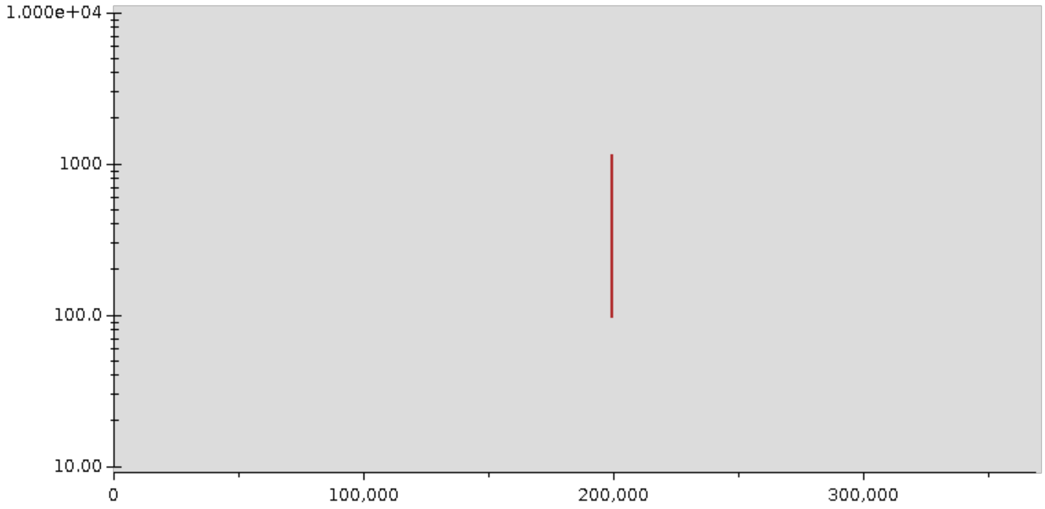

Assignment

**Type**	Marseillevirus marseillevirus (Taxonomy ID: 694581)
**Reference Genome**	NC_013756.1
**NT Identity (%)**	89.4231
**AA Identity (%)**	94.2857
**Number Of Stop Codons**	0
**Number Of CDS**	428

Alignment

**Alignment Score**	164.0 (NT) + 235.0 (AA) = 399.0
**Concordance (%)**	89.8649
**Alignment Method**	Local, heuristic, nucleotide (BLASTN)

Genome Region

Sequence starts at position 199424 and ends at position 199527 relative to NC_013756.1 reference sequence.

Alignment Detailed Statistics

	Begin	End	Coverage	Score	Concordance	Matches	Identities	I/D/M/F*	Stop Codons
NT	199424	199527	0.1%	164	78.8%	104 (100%)	93 (89.4%)	0/0	

Mutations: 199435C>T, 199449C>T, 199459A>T, 199464A>G, 199489T>C, 199492G>T, 199495G>A, 199501G>C, 199504A>C, 199506G>T, 199516C>T

*: Inserts / Deletes / Misaligned / Frameshifts

Analysis details

This analysis was performed with panviral2.64

NGS Details (UN70): Badnavirus occultipomeae

Assembly

**Coverage Length**	432 (1 contig(s))
**Depth Of Coverage**	139.6
**Number Of Reads**	541
**Reads Per Million**	10.26 rpm (after QC)
**Ambiguities**	0
**Assembly Method**	de novo + reference guided assembly
**Consensus Caller**	Bcf Tools

Coverage Map

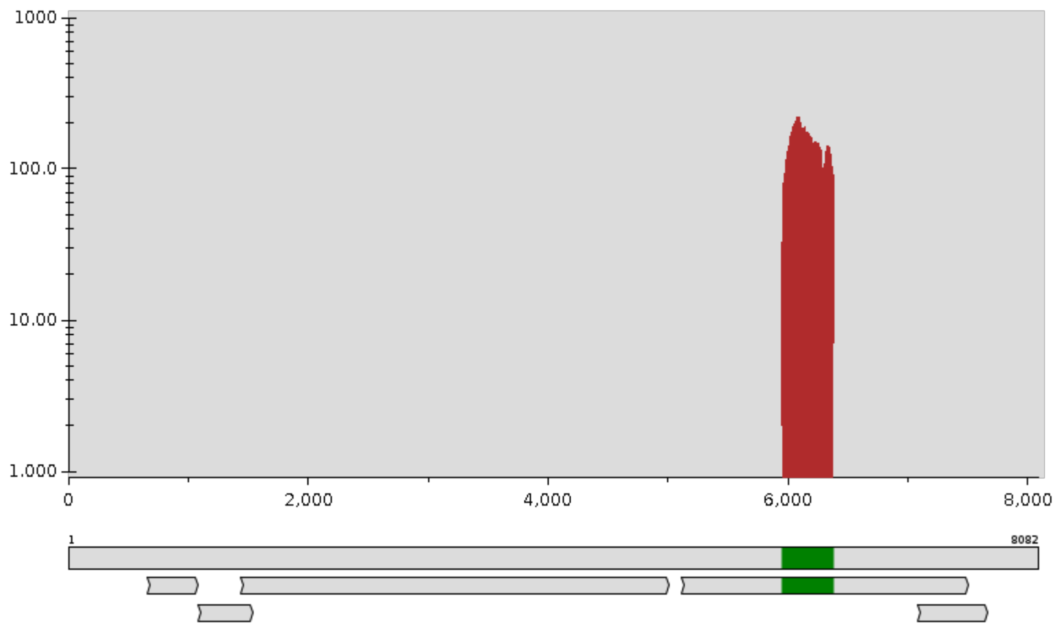

Assignment

**Type**	Badnavirus occultipomeae (Taxonomy ID: 3048353)
**Reference Genome**	NC_015655.1
**NT Identity (%)**	53.1034
**AA Identity (%)**	45.5172
**Number Of Stop Codons**	0
**Number Of CDS**	5

Alignment

**Alignment Score**	48.0 (NT) + 460.0 (AA) = 508.0
**Concordance (%)**	27.3707

Alignment Method	Global, seeded, nucleotide + amino acids (AGA)

Genome Region

Sequence starts at position 5950 and ends at position 6381 relative to NC_015655.1 reference sequence.

Alignment Detailed Statistics

	Begin	End	Coverage	Score	Concordance	Matches	Identities	I/D/M/F*	Stop Codons
**NT**	**5950**	**6381**	**5.3%**	**48**	**5.6%**	**432 (99.3%)**	**231 (53.1%)**	**3/0**	
Mutations:	5958T>C, 5959G>A, 5961C>A, 5962A>G, 5963A>T, 5964T>G, 5967A>T, 5968G>A, 5969A>T, 5973A>G, 5974G>A, 5977C>A, 5983T>C, 5986C>T, 5988A>G, 5992G>C, 5994A>T, 5997C>T, 5998A>G, 6000C>T, 6001A>G, 6002C>A, 6004A>T, 6006T>G, 6007G>T, 6009C>T, 6010A>G, 6011G>A, 6013A>C, 6014G>A, 6015A>G, 6016A>T, 6019T>C, 6020C>A, 6021T>G, 6025A>G, 6026A>C, 6027A>T, 6028A>T, 6029A>C, 6030G>T, 6031A>G, 6033A>G, 6039A>T, 6042A>G, 6043T>A, 6048T>C, 6049C>T, 6051C>G, 6053A>G, 6055G>T, 6057A>G, 6060C>T, 6062T>A, 6070A>T, 6072A>G, 6076A>G, 6078G>T, 6079G>C, 6080A>G, 6084A>G, 6085A>T, 6086A>C, 6087G>A, 6088T>G, 6089C>A, 6090C>T, 6092A>T, 6093A>C, 6096A>G, 6097T>A, 6098G>A, 6105T>G, 6108T>C, 6109T>A, 6114T>C, 6116C>G, 6117A>C, 6118G>T, 6120A>T, 6123G>C, 6125T>A, 6126C>T, 6132A>G, 6137A>T, 6138A>G, 6141C>T, 6145C>T, 6147C>T, 6153A>G, 6154C>T, 6158T>C, 6159G>T, 6165A>C, 6168A>T, 6171A>T, 6173A>C, 6178C>A, 6179A>T, 6181A>G, 6182G>A, 6183A>G, 6184A>T, 6185A>T, 6190G>A, 6192T>C, 6193A>C, 6194A>G, 6195T>A, 6197C>T, 6198A>G, 6201T>C, 6203G>A, 6204G>A, 6205G>T, 6206G>C, 6207A>T, 6207_6208insTAT, 6208A>C, 6209C>T, 6210A>T, 6214G>C, 6215C>A, 6216A>G, 6220A>G, 6222C>G, 6223G>A, 6224C>T, 6225A>T, 6228A>G, 6230A>T, 6231T>C, 6234T>A, 6240C>T, 6243A>C, 6249A>T, 6251T>A, 6252C>T, 6255T>C, 6256G>A, 6258A>G, 6260A>G, 6264A>G, 6265G>A, 6266A>G, 6267A>T, 6270G>T, 6275A>T, 6277G>A, 6279T>A, 6282T>C, 6285C>G, 6286T>A, 6287T>G, 6288A>G, 6290A>T, 6292T>G, 6295G>T, 6296C>T, 6297C>G, 6300A>G, 6302T>G, 6303T>A, 6304G>T, 6310A>G, 6311G>A, 6315A>G, 6316G>A, 6317G>A, 6319C>T, 6321T>A, 6322A>T, 6323T>A, 6325C>G, 6326T>C, 6327A>C, 6329G>A, 6330C>G, 6331C>T, 6332C>T, 6333A>C, 6334A>T, 6336A>C, 6339A>G, 6340A>T, 6341T>G, 6342G>T, 6343A>G, 6345A>G, 6346A>T, 6349G>T, 6351A>G, 6352G>C, 6354C>A, 6355A>C, 6357A>G, 6358G>T, 6359A>C, 6363G>A, 6364G>T, 6365A>C, 6369C>T								

CDS

	Begin	End	Coverage	Score	Concordance	Matches	Identities	I/D/M/F*	Stop Codons
SPBVa_gp4	279	422	18.1%	460	46.4%	144 (99.3%)	66 (45.5%)	1/0/0/0	0
Protein mutations:	D282K (5959G>A 5961C>A), N283V (5962A>G 5963A>T 5964T>G), E285I (5968G>A 5969A>T), D287N (5974G>A), Q288K (5977C>A), S290P (5983T>C), G293R (5992G>C 5994A>T), N295D (5998A>G 6000C>T), T296D (6001A>G 6002C>A), I297L (6004A>T 6006T>G), V298F (6007G>T 6009C>T), S299D (6010A>G 6011G>A), R300Q (6013A>C 6014G>A 6015A>G), I301L (6016A>T), S302Q (6019T>C 6020C>A 6021T>G), K304A (6025A>G 6026A>C 6027A>T), K305S (6028A>T 6029A>C 6030G>T), I306V (6031A>G 6033A>G), F310I (6043T>A), K313R (6053A>G), A314S (6055G>T 6057A>G), F316Y (6062T>A), I319L (6070A>T 6072A>G), M321V (6076A>G 6078G>T), E322R (6079G>C 6080A>G), K324S (6085A>T 6086A>C 6087G>A), S325D (6088T>G 6089C>A 6090C>T), K326I (6092A>T 6093A>C), W328K (6097T>A 6098G>A), W332R (6109T>A), P334R (6116C>G 6117A>C), E335Y (6118G>T 6120A>T), L337H (6125T>A 6126C>T), E341V (6137A>T 6138A>G), P344S (6145C>T 6147C>T), M348T (6158T>C 6159G>T), D353A (6173A>C), Q355M (6178C>A 6179A>T), R356E (6181A>G 6182G>A 6183A>G), K357L (6184A>T 6185A>T), D359N (6190G>A 6192T>C), N360R (6193A>C 6194A>G 6195T>A), A361V (6197C>T 6198A>G), R363K (6203G>A 6204G>A), G364S (6205G>T 6206G>C 6207A>T), G364_T365insY (6207_6208insTAT), T365L (6208A>C 6209C>T 6210A>T), A367Q (6214G>C 6215C>A 6216A>G), I369V (6220A>G 6222C>G), A370I (6223G>A 6224C>T 6225A>T), Y372F (6230A>T 6231T>C), F379Y (6251T>A 6252C>T), E381K (6256G>A 6258A>G), N382S (6260A>G), E384S (6265G>A 6266A>G 6267A>T), E385D (6270G>T), E387V (6275A>T), D388K (6277G>A 6279T>A), L391R (6286T>A 6287T>G 6288A>G), N392I (6290A>T), L393V (6292T>G), A394L (6295G>T 6296C>T 6297C>G), I396R (6302T>G 6303T>A), V397L (6304G>T), R399E (6310A>G 6311G>A), G401K (6316G>A 6317G>A), I403Y (6322A>T 6323T>A), L404A (6325C>G 6326T>C 6327A>C), S405K (6329G>A 6330C>G), P406F (6331C>T 6332C>T 6333A>C), T407S (6334A>T 6336A>C), M409C (6340A>T 6341T>G 6342G>T), K410E (6343A>G 6345A>G), I411F (6346A>T), G412W (6349G>T 6351A>G), V413L (6352G>C 6354C>A), K414Q (6355A>C 6357A>G), E415S (6358G>T 6359A>C), D417S (6364G>T 6365A>C)								
Codon mutations:	AAT281AAC (5958T>C), GAC282AAA (5959G>A 5961C>A), AAT283GTG (5962A>G 5963A>T 5964T>G), ACA284ACT (5967A>T), GAA285ATA (5968G>A 5969A>T), AAA286AAG (5973A>G), GAT287AAT (5974G>A), CAG288AAG (5977C>A), TCC290CCC (5983T>C), CTA291TTG (5986C>T 5988A>G), GGA293CGT (5992G>C 5994A>T), ATC294ATT (5997C>T), AAC295GAT (5998A>G 6000C>T), ACT296GAT (6001A>G 6002C>A), ATT297TTG (6004A>T 6006T>G), GTC298TTT (6007G>T 6009C>T), AGT299GAT (6010A>G 6011G>A), AGA300CAG (6013A>C 6014G>A 6015A>G), ATA301TTA (6016A>T), TCT302CAG (6019T>C 6020C>A 6021T>G), AAA304GCT (6025A>G 6026A>C 6027A>T), AAG305TCT (6028A>T 6029A>C 6030G>T), ATA306GTG (6031A>G 6033A>G), TCA308TCT (6039A>T), AAA309AAG (6042A>G), TTT310ATT (6043T>A), GAT311GAC (6048T>C), CTC312TTG (6049C>T 6051C>G), AAA313AGA (6053A>G), GCA314TCG (6055G>T 6057A>G), GGC315GGT (6060C>T), TTT316TAT (6062T>A), ATA319TTG (6070A>T 6072A>G), ATG321GTT (6076A>G 6078G>T), GAG322CGG (6079G>C 6080A>G), GAA323GAG (6084A>G), AAG324TCA (6085A>T 6086A>C 6087G>A), TCC325GAT (6088T>G 6089C>A 6090C>T), AAA326ATC (6092A>T 6093A>C), CCA327CCG (6096A>G), TGG328AAG (6097T>A 6098G>A), GCT330GCG (6105T>G), TTT331TTC (6108T>C), TGG332AGG (6109T>A), ACT333ACC (6114T>C), CCA334CGC (6116C>G 6117A>C), GAA335TAT (6118G>T 6120A>T), GGG336GGC (6123G>C), CTC337CAT (6125T>A 6126C>T), GAA339GAG (6132A>G), GAA341GTG (6137A>T 6138A>G), GTC342GTT (6141C>T), CCC344TCT (6145C>T 6147C>T), GGA346GGG (6153A>G), CTG347TTG (6154C>T), ATG348ACT (6158T>C 6159G>T), GCA350GCC (6165A>C), CCA351CCT (6168A>T), GCA352GCT (6171A>T), GAT353GCT (6173A>C), CAG355ATG (6178C>A 6179A>T), AGA356GAG (6181A>G 6182G>A 6183A>G), AAG357TTG (6184A>T 6185A>T), GAT359AAC (6190G>A 6192T>C), AAT360CGA (6193A>C 6194A>G 6195T>A), GCA361GTG (6197C>T 6198A>G), TTT362TTC (6201T>C), AGG363AAA (6203G>A 6204G>A), GGA364TCT (6205G>T 6206G>C 6207A>T), GGA364_ACA365insTAT (6207_6208insTAT), ACA365CTT (6208A>C 6209C>T 6210A>T), GCA367CAG (6214G>C 6215C>A 6216A>G), ATC369GTG (6220A>G 6222C>G), GCA370ATT (6223G>A 6224C>T 6225A>T), GTA371GTG (6228A>G), TAT372TTC (6230A>T 6231T>C), ATT373ATA (6234T>A), GAC375GAT (6240C>T), ATA376ATC (6243A>C), GTA378GTT (6249A>T), TTC379TAT (6251T>A 6252C>T), TCT380TCC (6255T>C), GAA381AAG (6256G>A 6258A>G), AAT382AGT (6260A>G), GAA383GAG (6264A>G), GAA384AGT (6265G>A 6266A>G 6267A>T), GAG385GAT (6270G>T), GAG387GTG (6275A>T), GAT388AAA (6277G>A 6279T>A), CAT389CAC (6282T>C), CTC390CTG (6285C>G), TTA391AGG (6286T>A 6287T>G 6288A>G), AAT392ATT (6290A>T), TTG393GTG (6292T>G), GCC394TTG (6295G>T 6296C>T 6297C>G), CAA395CAG (6300A>G), ATT396AGA (6302T>G 6303T>A), GTG397TTG (6304G>T), AGA399GAA (6310A>G 6311G>A), GAA400GAG (6315A>G), GGG401AAG (6316G>A 6317G>A), CTT402TTA (6319C>T 6321T>A), ATT403TAT (6322A>T 6323T>A), CTA404GCC (6325C>G 6326T>C 6327A>C), AGC405AAG (6329G>A 6330C>G), CCA406TTC (6331C>T 6332C>T 6333A>C), ACA407TCC (6334A>T 6336A>C), AAA408AAG (6339A>G), ATG409TGT (6340A>T 6341T>G 6342G>T), AAA410GAG (6343A>G 6345A>G), ATT411TTT (6346A>T), GGA412TGG (6349G>T 6351A>G), GTC413CTA (6352G>C 6354C>A), AAA414CAG (6355A>C 6357A>G), GAA415TCA (6358G>T 6359A>C), GTG416GTA (6363G>A), GAT417TCT (6364G>T 6365A>C), TTC418TTT (6369C>T)								

Proteins

	Begin	End	Coverage	Score	Concordance	Matches	Identities	I/D/M/F*	Stop Codons
RNaseH/reverse transcriptase (YP_004581513.1)	279	422	18.1%	460	46.4%	144 (99.3%)	66 (45.5%)	1/0/0/0	0
Protein mutations:	D282K (5959G>A 5961C>A), N283V (5962A>G 5963A>T 5964T>G), E285I (5968G>A 5969A>T), D287N (5974G>A), Q288K (5977C>A), S290P (5983T>C), G293R (5992G>C 5994A>T), N295D (5998A>G 6000C>T), T296D (6001A>G 6002C>A), I297L (6004A>T 6006T>G), V298F (6007G>T 6009C>T), S299D (6010A>G 6011G>A), R300Q (6013A>C 6014G>A 6015A>G), I301L (6016A>T), S302Q (6019T>C 6020C>A 6021T>G), K304A (6025A>G 6026A>C 6027A>T), K305S (6028A>T 6029A>C 6030G>T), I306V (6031A>G 6033A>G), F310I (6043T>A), K313R (6053A>G), A314S (6055G>T 6057A>G), F316Y (6062T>A), I319L (6070A>T 6072A>G), M321V (6076A>G 6078G>T), E322R (6079G>C 6080A>G), K324S (6085A>T 6086A>C 6087G>A), S325D (6088T>G 6089C>A 6090C>T), K326I (6092A>T 6093A>C), W328K (6097T>A 6098G>A), W332R (6109T>A), P334R (6116C>G 6117A>C), E335Y (6118G>T 6120A>T), L337H (6125T>A 6126C>T), E341V (6137A>T 6138A>G), P344S (6145C>T 6147C>T), M348T (6158T>C 6159G>T), D353A (6173A>C), Q355M (6178C>A 6179A>T), R356E (6181A>G 6182G>A 6183A>G), K357L (6184A>T 6185A>T), D359N (6190G>A 6192T>C), N360R (6193A>C 6194A>G 6195T>A), A361V (6197C>T 6198A>G), R363K (6203G>A 6204G>A), G364S (6205G>T 6206G>C 6207A>T), G364_T365insY (6207_6208insTAT), T365L (6208A>C 6209C>T 6210A>T), A367Q (6214G>C 6215C>A 6216A>G), I369V (6220A>G 6222C>G), A370I (6223G>A 6224C>T 6225A>T), Y372F (6230A>T 6231T>C), F379Y (6251T>A 6252C>T), E381K (6256G>A 6258A>G), N382S (6260A>G), E384S (6265G>A 6266A>G 6267A>T), E385D (6270G>T), E387V (6275A>T), D388K (6277G>A 6279T>A), L391R (6286T>A 6287T>G 6288A>G), N392I (6290A>T), L393V (6292T>G), A394L (6295G>T 6296C>T 6297C>G), I396R (6302T>G 6303T>A), V397L (6304G>T), R399E (6310A>G 6311G>A), G401K (6316G>A 6317G>A), I403Y (6322A>T 6323T>A), L404A (6325C>G 6326T>C 6327A>C), S405K (6329G>A 6330C>G), P406F (6331C>T 6332C>T 6333A>C), T407S (6334A>T 6336A>C), M409C (6340A>T 6341T>G 6342G>T), K410E (6343A>G 6345A>G), I411F (6346A>T), G412W (6349G>T 6351A>G), V413L (6352G>C 6354C>A), K414Q (6355A>C 6357A>G), E415S (6358G>T 6359A>C), D417S (6364G>T 6365A>C)								

	Begin	End	Coverage	Score	Concordance	Matches	Identities	I/D/M/F*	Stop Codons
**NT**	**5950**	**6381**	**5.3%**	**48**	**5.6%**	**432 (99.3%)**	**231 (53.1%)**	**3/0**	
Codon mutations:	AAT281AAC (5958T>C), GAC282AAA (5959G>A 5961C>A), AAT283GTG (5962A>G 5963A>T 5964T>G), ACA284ACT (5967A>T), GAA285ATA (5968G>A 5969A>T), AAA286AAG (5973A>G), GAT287AAT (5974G>A), CAG288AAG (5977C>A), TCC290CCC (5983T>C), CTA291TTG (5986C>T 5988A>G), GGA293CGT (5992G>C 5994A>T), ATC294ATT (5997C>T), AAC295GAT (5998A>G 6000C>T), ACT296GAT (6001A>G 6002C>A), ATT297TTG (6004A>T 6006T>G), GTC298TTT (6007G>T 6009C>T), AGT299GAT (6010A>G 6011G>A), AGA300CAG (6013A>C 6014G>A 6015A>G), ATA301TTA (6016A>T), TCT302CAG (6019T>C 6020C>A 6021T>G), AAA304GCT (6025A>G 6026A>C 6027A>T), AAG305TCT (6028A>T 6029A>C 6030G>T), ATA306GTG (6031A>G 6033A>G), TCA308TCT (6039A>T), AAA309AAG (6042A>G), TTT310ATT (6043T>A), GAT311GAC (6048T>C), CTC312TTG (6049C>T 6051C>G), AAA313AGA (6053A>G), GCA314TCG (6055G>T 6057A>G), GGC315GGT (6060C>T), TTT316TAT (6062T>A), ATA319TTG (6070A>T 6072A>G), ATG321GTT (6076A>G 6078G>T), GAG322CGG (6079G>C 6080A>G), GAA323GAG (6084A>G), AAG324TCA (6085A>T 6086A>C 6087G>A), TCC325GAT (6088T>G 6089C>A 6090C>T), AAA326ATC (6092A>T 6093A>C), CCA327CCG (6096A>G), TGG328AAG (6097T>A 6098G>A), GCT330GCG (6105T>G), TTT331TTC (6108T>C), TGG332AGG (6109T>A), ACT333ACC (6114T>C), CCA334CGC (6116C>G 6117A>C), GAA335TAT (6118G>T 6120A>T), GGG336GGC (6123G>C), CTC337CAT (6125T>A 6126C>T), GAA339GAG (6132A>G), GAA341GTG (6137A>T 6138A>G), GTC342GTT (6141C>T), CCC344TCT (6145C>T 6147C>T), GGA346GGG (6153A>G), CTG347TTG (6154C>T), ATG348ACT (6158T>C 6159G>T), GCA350GCC (6165A>C), CCA351CCT (6168A>T), GCA352GCT (6171A>T), GAT353GCT (6173A>C), CAG355ATG (6178C>A 6179A>T), AGA356GAG (6181A>G 6182G>A 6183A>G), AAG357TTG (6184A>T 6185A>T), GAT359AAC (6190G>A 6192T>C), AAT360CGA (6193A>C 6194A>G 6195T>A), GCA361GTG (6197C>T 6198A>G), TTT362TTC (6201T>C), AGG363AAA (6203G>A 6204G>A), GGA364TCT (6205G>T 6206G>C 6207A>T), GGA364_ACA365insTAT (6207_6208insTAT), ACA365CTT (6208A>C 6209C>T 6210A>T), GCA367CAG (6214G>C 6215C>A 6216A>G), ATC369GTG (6220A>G 6222C>G), GCA370ATT (6223G>A 6224C>T 6225A>T), GTA371GTG (6228A>G), TAT372TTC (6230A>T 6231T>C), ATT373ATA (6234T>A), GAC375GAT (6240C>T), ATA376ATC (6243A>C), GTA378GTT (6249A>T), TTC379TAT (6251T>A 6252C>T), TCT380TCC (6255T>C), GAA381AAG (6256G>A 6258A>G), AAT382AGT (6260A>G), GAA383GAG (6264A>G), GAA384AGT (6265G>A 6266A>G 6267A>T), GAG385GAT (6270G>T), GAG387GTG (6275A>T), GAT388AAA (6277G>A 6279T>A), CAT389CAC (6282T>C), CTC390CTG (6285C>G), TTA391AGG (6286T>A 6287T>G 6288A>G), AAT392ATT (6290A>T), TTG393GTG (6292T>G), GCC394TTG (6295G>T 6296C>T 6297C>G), CAA395CAG (6300A>G), ATT396AGA (6302T>G 6303T>A), GTG397TTG (6304G>T), AGA399GAA (6310A>G 6311G>A), GAA400GAG (6315A>G), GGG401AAG (6316G>A 6317G>A), CTT402TTA (6319C>T 6321T>A), ATT403TAT (6322A>T 6323T>A), CTA404GCC (6325C>G 6326T>C 6327A>C), AGC405AAG (6329G>A 6330C>G), CCA406TTC (6331C>T 6332C>T 6333A>C), ACA407TCC (6334A>T 6336A>C), AAA408AAG (6339A>G), ATG409TGT (6340A>T 6341T>G 6342G>T), AAA410GAG (6343A>G 6345A>G), ATT411TTT (6346A>T), GGA412TGG (6349G>T 6351A>G), GTC413CTA (6352G>C 6354C>A), AAA414CAG (6355A>C 6357A>G), GAA415TCA (6358G>T 6359A>C), GTG416GTA (6363G>A), GAT417TCT (6364G>T 6365A>C), TTC418TTT (6369C>T)								

*: Inserts / Deletes / Misaligned / Frameshifts

Analysis details

This analysis was performed with panviral2.64

NGS Details (UN70): Cassava brown streak virus

Assembly

**Coverage Length**	525 (1 contig(s))
**Depth Of Coverage**	121.8
**Number Of Reads**	521
**Reads Per Million**	9.88 rpm (after QC)
**Ambiguities**	0
**Assembly Method**	de novo + reference guided assembly
**Consensus Caller**	Bcf Tools

Coverage Map

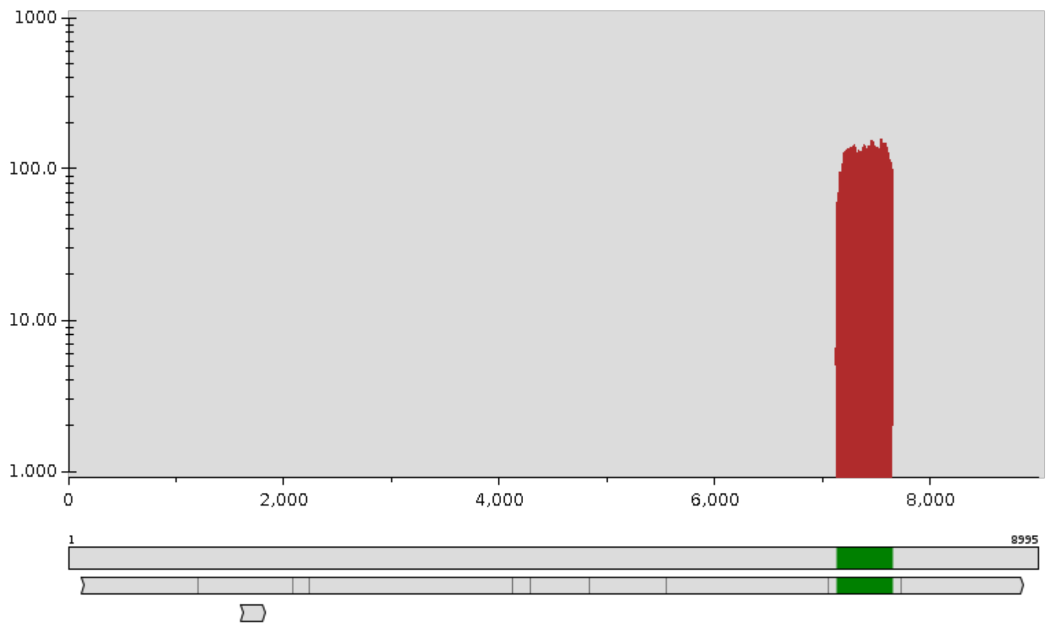

Assignment

**Type**	Cassava brown streak virus (Taxonomy ID: 137758)
**Reference Genome**	NC_012698.2
**NT Identity (%)**	60.7619
**AA Identity (%)**	57.7143
**Number Of Stop Codons**	0
**Number Of CDS**	2

Alignment

**Alignment Score**	226.0 (NT) + 736.0 (AA) = 962.0
**Concordance (%)**	42.4724

**Alignment Method**	Global, seeded, nucleotide + amino acids (AGA)

Genome Region

Sequence starts at position 7126 and ends at position 7650 relative to NC_012698.2 reference sequence.

Alignment Detailed Statistics

	Begin	End	Coverage	Score	Concordance	Matches	Identities	I/D/M/F*	Stop Codons
**NT**	**7126**	**7650**	**5.8%**	**226**	**21.5%**	**525 (100%)**	**319 (60.8%)**	**0/0**	
Mutations:	7130A>G, 7133A>G, 7134A>G, 7136T>G, 7142C>T, 7145A>T, 7148A>C, 7151G>A, 7157A>T, 7158C>A, 7163A>G, 7167A>G, 7175A>T, 7177A>G, 7178A>G, 7179C>G, 7180A>C, 7184A>T, 7185T>C, 7190T>C, 7192C>A, 7193C>G, 7194A>T, 7202A>C, 7203A>T, 7205C>T, 7206A>C, 7207T>A, 7208C>G, 7211T>C, 7213G>T, 7214G>T, 7217A>G, 7218G>C, 7229A>T, 7234C>T, 7235A>T, 7241A>G, 7242A>G, 7243C>A, 7244A>G, 7245G>C, 7246T>C, 7250G>A, 7253A>T, 7256T>A, 7257A>T, 7258T>C, 7270T>C, 7271A>T, 7272C>A, 7273G>A, 7274T>A, 7275G>A, 7277A>T, 7280G>T, 7281T>G, 7283T>C, 7284G>A, 7286G>A, 7287T>G, 7288T>A, 7290A>G, 7292T>G, 7293G>A, 7294G>A, 7295A>T, 7298G>A, 7304T>G, 7307C>A, 7310A>T, 7313A>G, 7319A>T, 7320A>T, 7323T>C, 7325G>C, 7331T>C, 7332G>A, 7334T>C, 7337T>C, 7340C>T, 7343T>G, 7355A>G, 7358A>T, 7361C>T, 7364T>C, 7374A>T, 7377G>C, 7380G>A, 7381G>A, 7385T>C, 7388A>T, 7390T>A, 7391A>T, 7397A>T, 7398T>C, 7400A>T, 7401T>A, 7403T>C, 7406G>C, 7409G>A, 7410G>T, 7413G>A, 7414A>T, 7416C>G, 7418G>T, 7422C>G, 7424A>G, 7425A>G, 7432T>C, 7433G>A, 7436T>A, 7437A>T, 7438G>A, 7442T>C, 7443C>A, 7445C>G, 7448C>T, 7449G>A, 7451G>C, 7455G>T, 7457T>A, 7458T>C, 7462T>C, 7463A>T, 7464A>C, 7465A>T, 7467A>G, 7468A>G, 7469A>G, 7470G>C, 7471T>C, 7472T>A, 7473G>A, 7474G>A, 7476G>A, 7477A>C, 7481T>G, 7484T>G, 7487A>G, 7489T>C, 7490C>T, 7494A>G, 7495A>T, 7496G>T, 7499T>A, 7500G>A, 7501T>A, 7502G>A, 7503C>A, 7504T>C, 7505A>G, 7506A>C, 7507G>T, 7508A>G, 7511T>A, 7512G>A, 7517T>A, 7520A>G, 7521A>C, 7522T>C, 7523G>A, 7524C>G, 7526A>T, 7527C>A, 7529C>G, 7535A>C, 7539T>A, 7540C>A, 7541A>T, 7544T>C, 7547G>A, 7553C>T, 7556A>G, 7559T>A, 7562C>T, 7568A>T, 7570T>A, 7572A>G, 7573A>G, 7576G>A, 7577G>T, 7578A>G, 7579G>A, 7580A>C, 7581A>C, 7586A>T, 7588T>A, 7589T>C, 7599A>C, 7600T>C, 7601G>C, 7602G>A, 7603C>A, 7604C>G, 7607G>A, 7610G>A, 7616T>C, 7618T>A, 7622A>T, 7628T>C, 7631A>C, 7632T>G, 7633T>G, 7635C>A, 7636G>A								

CDS

	Begin	End	Coverage	Score	Concordance	Matches	Identities	I/D/M/F*	Stop Codons
CBSV_gp1	2335	2509	6.0%	736	60.0%	175 (100%)	101 (57.7%)	0/0/0/0	0
Protein mutations:	I2337V (7134A>G 7136T>G), Q2345K (7158C>A), K2348E (7167A>G), K2351R (7177A>G 7178A>G), Q2352A (7179C>G 7180A>C), F2354L (7185T>C), P2356Q (7192C>A 7193C>G), T2357S (7194A>T), I2360F (7203A>T 7205C>T), I2361Q (7206A>C 7207T>A 7208C>G), R2363L (7213G>T 7214G>T), V2365L (7218G>C), P2370L (7234C>T 7235A>T), T2373E (7242A>G 7243C>A 7244A>G), V2374P (7245G>C 7246T>C), E2376D (7253A>T), I2378S (7257A>T 7258T>C), V2382A (7270T>C 7271A>T), R2383K (7272C>A 7273G>A 7274T>A), V2384I (7275G>A 7277A>T), S2386A (7281T>G 7283T>C), E2387K (7284G>A 7286G>A), L2388E (7287T>G 7288T>A), I2389V (7290A>G 7292T>G), G2390N (7293G>A 7294G>A 7295A>T), S2399C (7320A>T), D2403N (7332G>A 7334T>C), N2406K (7343T>G), M2417L (7374A>T), E2418Q (7377G>C), G2419K (7380G>A 7381G>A), L2422H (7390T>A 7391A>T), Y2426N (7401T>A 7403T>C), K2427N (7406G>C), V2429L (7410G>T), E2430M (7413G>A 7414A>T), P2431A (7416C>G 7418G>T), Q2433E (7422C>G 7424A>G), N2434D (7425A>G), M2436T (7432T>C 7433G>A), S2438Y (7437A>T 7438G>A), L2440M (7443C>A 7445C>G), V2442I (7449G>A 7451G>C), A2444S (7455G>T 7457T>A), F2445L (7458T>C), V2446A (7462T>C 7463A>T), N2447L (7464A>C 7465A>T), K2448G (7467A>G 7468A>G 7469A>G), V2449P (7470G>C 7471T>C 7472T>A), G2450N (7473G>A 7474G>A), D2451T (7476G>A 7477A>C), D2452E (7481T>G), I2454M (7487A>G), I2455T (7489T>C 7490C>T), K2457V (7494A>G 7495A>T 7496G>T), V2459K (7500G>A 7501T>A 7502G>A), L2460T (7503C>A 7504T>C 7505A>G), R2461L (7506A>C 7507G>T 7508A>G), E2463K (7512G>A), M2466P (7521A>C 7522T>C 7523G>A), P2467A (7524C>G 7526A>T), S2472N (7539T>A 7540C>A 7541A>T), L2482H (7570T>A), N2483G (7572A>G 7573A>G), W2484Y (7576G>A 7577G>T), R2485D (7578A>G 7579G>A 7580A>C), K2486Q (7581A>C), F2488Y (7588T>A 7589T>C), M2492P (7599A>C 7600T>C 7601G>C), A2493K (7602G>A 7603C>A 7604C>G), M2498K (7618T>A), F2503G (7632T>G 7633T>G), R2504K (7635C>A 7636G>A)								
Codon mutations:	GCA2335GCG (7130A>G), CCA2336CCG (7133A>G), ATT2337GTG (7134A>G 7136T>G), TTC2339TTT (7142C>T), GTA2340GTT (7145A>T), ACA2341ACC (7148A>C), GGG2342GGA (7151G>A), GCA2344GCT (7157A>T), CAA2345AAA (7158C>A), AAA2346AAG (7163A>G), AAG2348GAG (7167A>G), GTA2350GTT (7175A>T), AAA2351AGG (7177A>G 7178A>G), CAA2352GCA (7179C>G 7180A>C), ATA2353ATT (7184A>T), TTT2354CTT (7185T>C), GGT2355GGC (7190T>C), CCC2356CAG (7192C>A 7193C>G), ACT2357TCT (7194A>T), CCA2359CCC (7202A>C), ATC2360TTT (7203A>T 7205C>T), ATC2361CAG (7206A>C 7207T>A 7208C>G), TCT2362TCC (7211T>C), CGG2363CTT (7213G>T 7214G>T), AAA2364AAG (7217A>G), GTT2365CTT (7218G>C), CCA2368CCT (7229A>T), CCA2370CTT (7234C>T 7235A>T), GGA2372GGG (7241A>G), ACA2373GAG (7242A>G 7243C>A 7244A>G), GTT2374CCT (7245G>C 7246T>C), GAG2375GAA (7250G>A), GAA2376GAT (7253A>T), ATT2377ATA (7256T>A), ATC2378TCC (7257A>T 7258T>C), GTA2382GCT (7270T>C 7271A>T), CGT2383AAA (7272C>A 7273G>A 7274T>A), GTA2384ATT (7275G>A 7277A>T), GCG2385GCT (7280G>T), TCT2386GCC (7281T>G 7283T>C), GAG2387AAA (7284G>A 7286G>A), TTG2388GAG (7287T>G 7288T>A), ATT2389GTG (7290A>G 7292T>G), GGA2390AAT (7293G>A 7294G>A 7295A>T), GGG2391GGA (7298G>A), GTT2393GTG (7304T>G), CTC2394CTA (7307C>A), GTA2395GTT (7310A>T), GAA2396GAG (7313A>G), ACA2398ACT (7319A>T), AGT2399TGT (7320A>T), TTG2400CTC (7323T>C 7325G>C), TTT2402TTC (7331T>C), GAT2403AAC (7332G>A 7334T>C), GCT2404GCC (7337T>C), CTC2405CTT (7340C>T), AAT2406AAG (7343T>G), GGA2410GGG (7355A>G), CCA2411CCT (7358A>T), TAC2412TAT (7361C>T), ATT2413ATC (7364T>C), ATG2417TTG (7374A>T), GAA2418CAA (7377G>C), GGG2419AAG (7380G>A 7381G>A), ATT2420ATC (7385T>C), GGA2421GGT (7388A>T), CTA2422CAT (7390T>A 7391A>T), GGA2424GGT (7397A>T), TTA2425CTT (7398T>C 7400A>T), TAT2426AAC (7401T>A 7403T>C), AAG2427AAC (7406G>C), TTG2428TTA (7409G>A), GTG2429TTG (7410G>T), GAG2430ATG (7413G>A 7414A>T), CCG2431GCT (7416C>G 7418G>T), CAA2433GAG (7422C>G 7424A>G), AAT2434GAT (7425A>G), ATG2436ACA (7432T>C 7433G>A), GCT2437GCA (7436T>A), AGT2438TAT (7437A>T 7438G>A), GCT2439GCC (7442T>C), CTC2440ATG (7443C>A 7445C>G), TGC2441TGT (7448C>T), GTG2442ATC (7449G>A 7451G>C), GCT2444TCA (7455G>T 7457T>A), TTT2445CTT (7458T>C), GTA2446GCT (7462T>C 7463A>T), AAT2447CTT (7464A>C 7465A>T), AAA2448GGG (7467A>G 7468A>G 7469A>G), GTT2449CCA (7470G>C 7471T>C 7472T>A), GGT2450AAT (7473G>A 7474G>A), GAT2451ACT (7476G>A 7477A>C), GAT2452GAG (7481T>G), CCT2453CCG (7484T>G), ATA2454ATG (7487A>G), ATC2455ACT (7489T>C 7490C>T), AAG2457GTT (7494A>G 7495A>T 7496G>T), GGT2458GGA (7499T>A), GTG2459AAA (7500G>A 7501T>A 7502G>A), CTA2460ACG (7503C>A 7504T>C 7505A>G), AGA2461CTG (7506A>C 7507G>T 7508A>G), GGT2462GGA (7511T>A), GAG2463AAG (7512G>A), ATT2464ATA (7517T>A), GTA2465GTG (7520A>G), ATG2466CCA (7521A>C 7522T>C 7523G>A), CCA2467GCT (7524C>G 7526A>T), CGC2468AGG (7527C>A 7529C>G), CCA2470CCC (7535A>C), TCA2472AAT (7539T>A 7540C>A 7541A>T), TTT2473TTC (7544T>C), GGG2474GGA (7547G>A), GAC2476GAT (7553C>T), CCA2477CCG (7556A>G), ATT2478ATA (7559T>A), TTC2479TTT (7562C>T), CCA2481CCT (7568A>T), CTT2482CAT (7570T>A), AAC2483GGC (7572A>G 7573A>G), TGG2484TAT (7576G>A 7577G>T), AGA2485GAC (7578A>G 7579G>A 7580A>C), AAG2486CAG (7581A>C), ACA2487ACT (7586A>T), TTT2488TAC (7588T>A 7589T>C), ATG2492CCC (7599A>C 7600T>C 7601G>C), GCC2493AAG (7602G>A 7603C>A 7604C>G), GAG2494GAA (7607G>A), GAG2495GAA (7610G>A), AAT2497AAC (7616T>C), ATG2498AAG (7618T>A), ATA2499ATT (7622A>T), CAT2501CAC (7628T>C), CGA2502CGC (7631A>C), TTT2503GGT (7632T>G 7633T>G), CGA2504AAA (7635C>A 7636G>A)								

Proteins

	Begin	End	Coverage	Score	Concordance	Matches	Identities	I/D/M/F*	Stop Codons
polyprotein (YP_007027011.1)	2335	2509	6.0%	736	60.0%	175 (100%)	101 (57.7%)	0/0/0/0	0
Protein mutations:	I2337V (7134A>G 7136T>G), Q2345K (7158C>A), K2348E (7167A>G), K2351R (7177A>G 7178A>G), Q2352A (7179C>G 7180A>C), F2354L (7185T>C), P2356Q (7192C>A 7193C>G), T2357S (7194A>T), I2360F (7203A>T 7205C>T), I2361Q (7206A>C 7207T>A 7208C>G), R2363L (7213G>T 7214G>T), V2365L (7218G>C), P2370L (7234C>T 7235A>T), T2373E (7242A>G 7243C>A 7244A>G), V2374P (7245G>C 7246T>C), E2376D (7253A>T), I2378S (7257A>T 7258T>C), V2382A (7270T>C 7271A>T), R2383K (7272C>A 7273G>A 7274T>A), V2384I (7275G>A 7277A>T), S2386A (7281T>G 7283T>C), E2387K (7284G>A 7286G>A), L2388E (7287T>G 7288T>A), I2389V (7290A>G 7292T>G), G2390N (7293G>A 7294G>A 7295A>T), S2399C (7320A>T), D2403N (7332G>A 7334T>C), N2406K (7343T>G), M2417L (7374A>T), E2418Q (7377G>C), G2419K (7380G>A 7381G>A), L2422H (7390T>A 7391A>T), Y2426N (7401T>A 7403T>C), K2427N (7406G>C), V2429L (7410G>T), E2430M (7413G>A 7414A>T), P2431A (7416C>G 7418G>T), Q2433E (7422C>G 7424A>G), N2434D (7425A>G), M2436T (7432T>C 7433G>A), S2438Y (7437A>T 7438G>A), L2440M (7443C>A 7445C>G), V2442I (7449G>A 7451G>C), A2444S (7455G>T 7457T>A), F2445L (7458T>C), V2446A (7462T>C 7463A>T), N2447L (7464A>C 7465A>T), K2448G (7467A>G 7468A>G 7469A>G), V2449P (7470G>C 7471T>C 7472T>A), G2450N (7473G>A 7474G>A), D2451T (7476G>A 7477A>C), D2452E (7481T>G), I2454M (7487A>G), I2455T (7489T>C 7490C>T), K2457V (7494A>G 7495A>T 7496G>T), V2459K (7500G>A 7501T>A 7502G>A), L2460T (7503C>A 7504T>C 7505A>G), R2461L (7506A>C 7507G>T 7508A>G), E2463K (7512G>A), M2466P (7521A>C 7522T>C 7523G>A), P2467A (7524C>G 7526A>T), S2472N (7539T>A 7540C>A 7541A>T), L2482H (7570T>A), N2483G (7572A>G 7573A>G), W2484Y (7576G>A 7577G>T), R2485D (7578A>G 7579G>A 7580A>C), K2486Q (7581A>C), F2488Y (7588T>A 7589T>C), M2492P (7599A>C 7600T>C 7601G>C), A2493K (7602G>A 7603C>A 7604C>G), M2498K (7618T>A), F2503G (7632T>G 7633T>G), R2504K (7635C>A 7636G>A)								

	Begin	End	Coverage	Score	Concordance	Matches	Identities	I/D/M/F*	Stop Codons
**NT**	**7126**	**7650**	**5.8%**	**226**	**21.5%**	**525 (100%)**	**319 (60.8%)**	**0/0**	
Codon mutations:	GCA2335GCG (7130A>G), CCA2336CCG (7133A>G), ATT2337GTG (7134A>G 7136T>G), TTC2339TTT (7142C>T), GTA2340GTT (7145A>T), ACA2341ACC (7148A>C), GGG2342GGA (7151G>A), GCA2344GCT (7157A>T), CAA2345AAA (7158C>A), AAA2346AAG (7163A>G), AAG2348GAG (7167A>G), GTA2350GTT (7175A>T), AAA2351AGG (7177A>G 7178A>G), CAA2352GCA (7179C>G 7180A>C), ATA2353ATT (7184A>T), TTT2354CTT (7185T>C), GGT2355GGC (7190T>C), CCC2356CAG (7192C>A 7193C>G), ACT2357TCT (7194A>T), CCA2359CCC (7202A>C), ATC2360TTT (7203A>T 7205C>T), ATC2361CAG (7206A>C 7207T>A 7208C>G), TCT2362TCC (7211T>C), CGG2363CTT (7213G>T 7214G>T), AAA2364AAG (7217A>G), GTT2365CTT (7218G>C), CCA2368CCT (7229A>T), CCA2370CTT (7234C>T 7235A>T), GGA2372GGG (7241A>G), ACA2373GAG (7242A>G 7243C>A 7244A>G), GTT2374CCT (7245G>C 7246T>C), GAG2375GAA (7250G>A), GAA2376GAT (7253A>T), ATT2377ATA (7256T>A), ATC2378TCC (7257A>T 7258T>C), GTA2382GCT (7270T>C 7271A>T), CGT2383AAA (7272C>A 7273G>A 7274T>A), GTA2384ATT (7275G>A 7277A>T), GCG2385GCT (7280G>T), TCT2386GCC (7281T>G 7283T>C), GAG2387AAA (7284G>A 7286G>A), TTG2388GAG (7287T>G 7288T>A), ATT2389GTG (7290A>G 7292T>G), GGA2390AAT (7293G>A 7294G>A 7295A>T), GGG2391GGA (7298G>A), GTT2393GTG (7304T>G), CTC2394CTA (7307C>A), GTA2395GTT (7310A>T), GAA2396GAG (7313A>G), ACA2398ACT (7319A>T), AGT2399TGT (7320A>T), TTG2400CTC (7323T>C 7325G>C), TTT2402TTC (7331T>C), GAT2403AAC (7332G>A 7334T>C), GCT2404GCC (7337T>C), CTC2405CTT (7340C>T), AAT2406AAG (7343T>G), GGA2410GGG (7355A>G), CCA2411CCT (7358A>T), TAC2412TAT (7361C>T), ATT2413ATC (7364T>C), ATG2417TTG (7374A>T), GAA2418CAA (7377G>C), GGG2419AAG (7380G>A 7381G>A), ATT2420ATC (7385T>C), GGA2421GGT (7388A>T), CTA2422CAT (7390T>A 7391A>T), GGA2424GGT (7397A>T), TTA2425CTT (7398T>C 7400A>T), TAT2426AAC (7401T>A 7403T>C), AAG2427AAC (7406G>C), TTG2428TTA (7409G>A), GTG2429TTG (7410G>T), GAG2430ATG (7413G>A 7414A>T), CCG2431GCT (7416C>G 7418G>T), CAA2433GAG (7422C>G 7424A>G), AAT2434GAT (7425A>G), ATG2436ACA (7432T>C 7433G>A), GCT2437GCA (7436T>A), AGT2438TAT (7437A>T 7438G>A), GCT2439GCC (7442T>C), CTC2440ATG (7443C>A 7445C>G), TGC2441TGT (7448C>T), GTG2442ATC (7449G>A 7451G>C), GCT2444TCA (7455G>T 7457T>A), TTT2445CTT (7458T>C), GTA2446GCT (7462T>C 7463A>T), AAT2447CTT (7464A>C 7465A>T), AAA2448GGG (7467A>G 7468A>G 7469A>G), GTT2449CCA (7470G>C 7471T>C 7472T>A), GGT2450AAT (7473G>A 7474G>A), GAT2451ACT (7476G>A 7477A>C), GAT2452GAG (7481T>G), CCT2453CCG (7484T>G), ATA2454ATG (7487A>G), ATC2455ACT (7489T>C 7490C>T), AAG2457GTT (7494A>G 7495A>T 7496G>T), GGT2458GGA (7499T>A), GTG2459AAA (7500G>A 7501T>A 7502G>A), CTA2460ACG (7503C>A 7504T>C 7505A>G), AGA2461CTG (7506A>C 7507G>T 7508A>G), GGT2462GGA (7511T>A), GAG2463AAG (7512G>A), ATT2464ATA (7517T>A), GTA2465GTG (7520A>G), ATG2466CCA (7521A>C 7522T>C 7523G>A), CCA2467GCT (7524C>G 7526A>T), CGC2468AGG (7527C>A 7529C>G), CCA2470CCC (7535A>C), TCA2472AAT (7539T>A 7540C>A 7541A>T), TTT2473TTC (7544T>C), GGG2474GGA (7547G>A), GAC2476GAT (7553C>T), CCA2477CCG (7556A>G), ATT2478ATA (7559T>A), TTC2479TTT (7562C>T), CCA2481CCT (7568A>T), CTT2482CAT (7570T>A), AAC2483GGC (7572A>G 7573A>G), TGG2484TAT (7576G>A 7577G>T), AGA2485GAC (7578A>G 7579G>A 7580A>C), AAG2486CAG (7581A>C), ACA2487ACT (7586A>T), TTT2488TAC (7588T>A 7589T>C), ATG2492CCC (7599A>C 7600T>C 7601G>C), GCC2493AAG (7602G>A 7603C>A 7604C>G), GAG2494GAA (7607G>A), GAG2495GAA (7610G>A), AAT2497AAC (7616T>C), ATG2498AAG (7618T>A), ATA2499ATT (7622A>T), CAT2501CAC (7628T>C), CGA2502CGC (7631A>C), TTT2503GGT (7632T>G 7633T>G), CGA2504AAA (7635C>A 7636G>A)								
HAM1-like protein (YP_007032446.1)	27	201	77.4%	736	60.0%	175 (100%)	101 (57.7%)	0/0/0/0	0
Protein mutations:	I29V (7134A>G 7136T>G), Q37K (7158C>A), K40E (7167A>G), K43R (7177A>G 7178A>G), Q44A (7179C>G 7180A>C), F46L (7185T>C), P48Q (7192C>A 7193C>G), T49S (7194A>T), I52F (7203A>T 7205C>T), I53Q (7206A>C 7207T>A 7208C>G), R55L (7213G>T 7214G>T), V57L (7218G>C), P62L (7234C>T 7235A>T), T65E (7242A>G 7243C>A 7244A>G), V66P (7245G>C 7246T>C), E68D (7253A>T), I70S (7257A>T 7258T>C), V74A (7270T>C 7271A>T), R75K (7272C>A 7273G>A 7274T>A), V76I (7275G>A 7277A>T), S78A (7281T>G 7283T>C), E79K (7284G>A 7286G>A), L80E (7287T>G 7288T>A), I81V (7290A>G 7292T>G), G82N (7293G>A 7294G>A 7295A>T), S91C (7320A>T), D95N (7332G>A 7334T>C), N98K (7343T>G), M109L (7374A>T), E110Q (7377G>C), G111K (7380G>A 7381G>A), L114H (7390T>A 7391A>T), Y118N (7401T>A 7403T>C), K119N (7406G>C), V121L (7410G>T), E122M (7413G>A 7414A>T), P123A (7416C>G 7418G>T), Q125E (7422C>G 7424A>G), N126D (7425A>G), M128T (7432T>C 7433G>A), S130Y (7437A>T 7438G>A), L132M (7443C>A 7445C>G), V134I (7449G>A 7451G>C), A136S (7455G>T 7457T>A), F137L (7458T>C), V138A (7462T>C 7463A>T), N139L (7464A>C 7465A>T), K140G (7467A>G 7468A>G 7469A>G), V141P (7470G>C 7471T>C 7472T>A), G142N (7473G>A 7474G>A), D143T (7476G>A 7477A>C), D144E (7481T>G), I146M (7487A>G), I147T (7489T>C 7490C>T), K149V (7494A>G 7495A>T 7496G>T), V151K (7500G>A 7501T>A 7502G>A), L152T (7503C>A 7504T>C 7505A>G), R153L (7506A>C 7507G>T 7508A>G), E155K (7512G>A), M158P (7521A>C 7522T>C 7523G>A), P159A (7524C>G 7526A>T), S164N (7539T>A 7540C>A 7541A>T), L174H (7570T>A), N175G (7572A>G 7573A>G), W176Y (7576G>A 7577G>T), R177D (7578A>G 7579G>A 7580A>C), K178Q (7581A>C), F180Y (7588T>A 7589T>C), M184P (7599A>C 7600T>C 7601G>C), A185K (7602G>A 7603C>A 7604C>G), M190K (7618T>A), F195G (7632T>G 7633T>G), R196K (7635C>A 7636G>A)								
Codon mutations:	GCA27GCG (7130A>G), CCA28CCG (7133A>G), ATT29GTG (7134A>G 7136T>G), TTC31TTT (7142C>T), GTA32GTT (7145A>T), ACA33ACC (7148A>C), GGG34GGA (7151G>A), GCA36GCT (7157A>T), CAA37AAA (7158C>A), AAA38AAG (7163A>G), AAG40GAG (7167A>G), GTA42GTT (7175A>T), AAA43AGG (7177A>G 7178A>G), CAA44GCA (7179C>G 7180A>C), ATA45ATT (7184A>T), TTT46CTT (7185T>C), GGT47GGC (7190T>C), CCC48CAG (7192C>A 7193C>G), ACT49TCT (7194A>T), CCA51CCC (7202A>C), ATC52TTT (7203A>T 7205C>T), ATC53CAG (7206A>C 7207T>A 7208C>G), TCT54TCC (7211T>C), CGG55CTT (7213G>T 7214G>T), AAA56AAG (7217A>G), GTT57CTT (7218G>C), CCA60CCT (7229A>T), CCA62CTT (7234C>T 7235A>T), GGA64GGG (7241A>G), ACA65GAG (7242A>G 7243C>A 7244A>G), GTT66CCT (7245G>C 7246T>C), GAG67GAA (7250G>A), GAA68GAT (7253A>T), ATT69ATA (7256T>A), ATC70TCC (7257A>T 7258T>C), GTA74GCT (7270T>C 7271A>T), CGT75AAA (7272C>A 7273G>A 7274T>A), GTA76ATT (7275G>A 7277A>T), GCG77GCT (7280G>T), TCT78GCC (7281T>G 7283T>C), GAG79AAA (7284G>A 7286G>A), TTG80GAG (7287T>G 7288T>A), ATT81GTG (7290A>G 7292T>G), GGA82AAT (7293G>A 7294G>A 7295A>T), GGG83GGA (7298G>A), GTT85GTG (7304T>G), CTC86CTA (7307C>A), GTA87GTT (7310A>T), GAA88GAG (7313A>G), ACA90ACT (7319A>T), AGT91TGT (7320A>T), TTG92CTC (7323T>C 7325G>C), TTT94TTC (7331T>C), GAT95AAC (7332G>A 7334T>C), GCT96GCC (7337T>C), CTC97CTT (7340C>T), AAT98AAG (7343T>G), GGA102GGG (7355A>G), CCA103CCT (7358A>T), TAC104TAT (7361C>T), ATT105ATC (7364T>C), ATG109TTG (7374A>T), GAA110CAA (7377G>C), GGG111AAG (7380G>A 7381G>A), ATT112ATC (7385T>C), GGA113GGT (7388A>T), CTA114CAT (7390T>A 7391A>T), GGA116GGT (7397A>T), TTA117CTT (7398T>C 7400A>T), TAT118AAC (7401T>A 7403T>C), AAG119AAC (7406G>C), TTG120TTA (7409G>A), GTG121TTG (7410G>T), GAG122ATG (7413G>A 7414A>T), CCG123GCT (7416C>G 7418G>T), CAA125GAG (7422C>G 7424A>G), AAT126GAT (7425A>G), ATG128ACA (7432T>C 7433G>A), GCT129GCA (7436T>A), AGT130TAT (7437A>T 7438G>A), GCT131GCC (7442T>C), CTC132ATG (7443C>A 7445C>G), TGC133TGT (7448C>T), GTG134ATC (7449G>A 7451G>C), GCT136TCA (7455G>T 7457T>A), TTT137CTT (7458T>C), GTA138GCT (7462T>C 7463A>T), AAT139CTT (7464A>C 7465A>T), AAA140GGG (7467A>G 7468A>G 7469A>G), GTT141CCA (7470G>C 7471T>C 7472T>A), GGT142AAT (7473G>A 7474G>A), GAT143ACT (7476G>A 7477A>C), GAT144GAG (7481T>G), CCT145CCG (7484T>G), ATA146ATG (7487A>G), ATC147ACT (7489T>C 7490C>T), AAG149GTT (7494A>G 7495A>T 7496G>T), GGT150GGA (7499T>A), GTG151AAA (7500G>A 7501T>A 7502G>A), CTA152ACG (7503C>A 7504T>C 7505A>G), AGA153CTG (7506A>C 7507G>T 7508A>G), GGT154GGA (7511T>A), GAG155AAG (7512G>A), ATT156ATA (7517T>A), GTA157GTG (7520A>G), ATG158CCA (7521A>C 7522T>C 7523G>A), CCA159GCT (7524C>G 7526A>T), CGC160AGG (7527C>A 7529C>G), CCA162CCC (7535A>C), TCA164AAT (7539T>A 7540C>A 7541A>T), TTT165TTC (7544T>C), GGG166GGA (7547G>A), GAC168GAT (7553C>T), CCA169CCG (7556A>G), ATT170ATA (7559T>A), TTC171TTT (7562C>T), CCA173CCT (7568A>T), CTT174CAT (7570T>A), AAC175GGC (7572A>G 7573A>G), TGG176TAT (7576G>A 7577G>T), AGA177GAC (7578A>G 7579G>A 7580A>C), AAG178CAG (7581A>C), ACA179ACT (7586A>T), TTT180TAC (7588T>A 7589T>C), ATG184CCC (7599A>C 7600T>C 7601G>C), GCC185AAG (7602G>A 7603C>A 7604C>G), GAG186GAA (7607G>A), GAG187GAA (7610G>A), AAT189AAC (7616T>C), ATG190AAG (7618T>A), ATA191ATT (7622A>T), CAT193CAC (7628T>C), CGA194CGC (7631A>C), TTT195GGT (7632T>G 7633T>G), CGA196AAA (7635C>A 7636G>A)								

*: Inserts / Deletes / Misaligned / Frameshifts

Analysis details

This analysis was performed with panviral2.64

NGS Details (UN70): Human gammaherpesvirus 8

Assembly

**Coverage Length**	132 (1 contig(s))
**Depth Of Coverage**	240.5
**Number Of Reads**	386
**Reads Per Million**	7.32 rpm (after QC)
**Ambiguities**	0
**Assembly Method**	de novo + reference guided assembly
**Consensus Caller**	Bcf Tools

Coverage Map

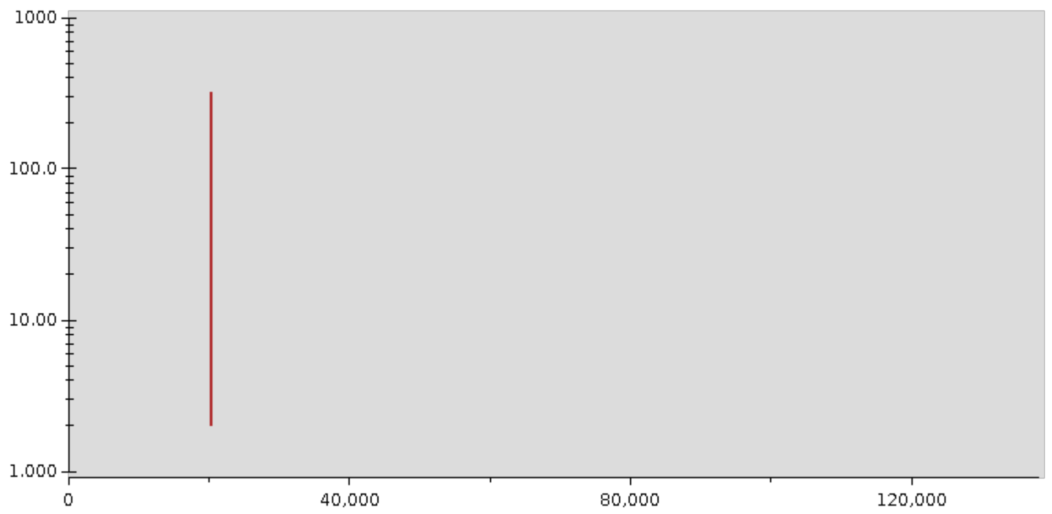

Assignment

**Type**	Human gammaherpesvirus 8 (Taxonomy ID: 37296)
**Subtype**	Could not assign
**Reference Genome**	NC_009333.1
**NT Identity (%)**	83.3333
**AA Identity (%)**	84.0909
**Number Of Stop Codons**	0
**Number Of CDS**	86

Alignment

**Alignment Score**	176.0 (NT) + 283.0 (AA) = 459.0
**Concordance (%)**	77.9287
**Alignment Method**	Local, heuristic, nucleotide (BLASTN)

Genome Region

Sequence starts at position 20273 and ends at position 20404 relative to NC_009333.1 reference sequence.

Alignment Detailed Statistics

	Begin	End	Coverage	Score	Concordance	Matches	Identities	I/D/M/F*	Stop Codons
**NT**	**20273**	**20404**	**0.1%**	**176**	**66.7%**	**132 (100%)**	**110 (83.3%)**	**0/0**	

Mutations: 20293A>T, 20296A>C, 20311G>A, 20314T>G, 20315C>G, 20317C>A, 20320C>T, 20322T>G, 20331G>T, 20341A>C, 20347A>C, 20350G>A, 20352C>T, 20353A>G, 20369C>G, 20370A>C, 20371T>A, 20376A>T, 20383G>A, 20386C>T, 20389A>G, 20392C>T

*: Inserts / Deletes / Misaligned / Frameshifts

Analysis details

This analysis was performed with panviral2.64

NGS Details (UN70): Makelovirus prm1

Assembly

**Coverage Length**	996 (1 contig(s))
**Depth Of Coverage**	40.8
**Number Of Reads**	316
**Reads Per Million**	5.99 rpm (after QC)
**Ambiguities**	0
**Assembly Method**	de novo + reference guided assembly
**Consensus Caller**	Bcf Tools

Coverage Map

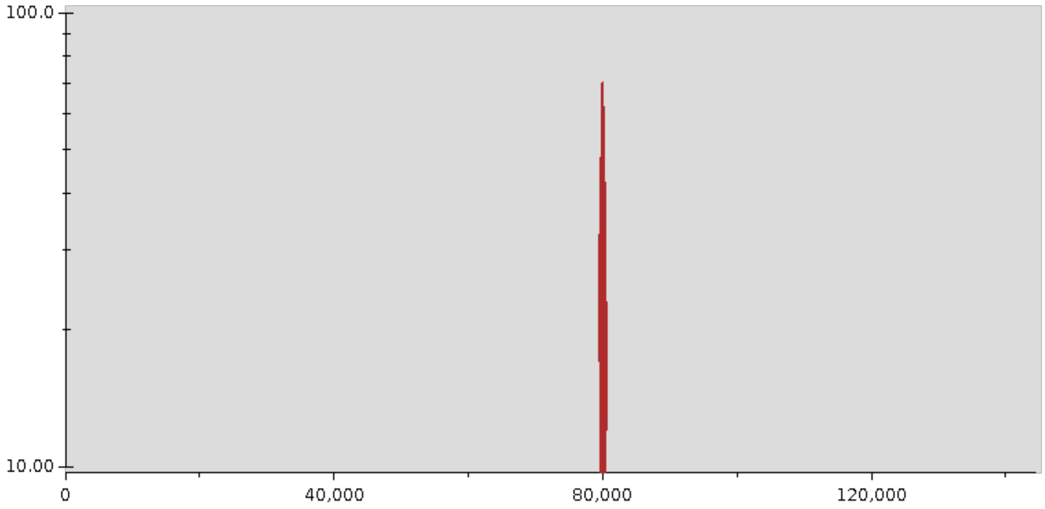

Assignment

**Type**	Makelovirus prm1 (Taxonomy ID: 2956181)
**Reference Genome**	NC_055761.1
**NT Identity (%)**	76.4056
**AA Identity (%)**	87.3494
**Number Of Stop Codons**	0
**Number Of CDS**	190

Alignment

**Alignment Score**	1052.0 (NT) + 2227.0 (AA) = 3279.0
**Concordance (%)**	74.0682
**Alignment Method**	Local, heuristic, nucleotide (BLASTN)

Genome Region

Sequence starts at position 79515 and ends at position 80510 relative to NC_055761.1 reference sequence.

Alignment Detailed Statistics

	Begin	End	Coverage	Score	Concordance	Matches	Identities	I/D/M/F*	Stop Codons
**NT**	**79515**	**80510**	**0.7%**	**1052**	**52.8%**	**996 (100%)**	**761 (76.4%)**	**0/0**	
Mutations:	79519A>G, 79520A>T, 79522A>G, 79523A>C, 79529C>T, 79530G>A, 79532G>C, 79536G>A, 79541G>T, 79542T>A, 79543C>G, 79544T>C, 79547A>T, 79548A>G, 79550C>A, 79563G>A, 79565C>T, 79568T>A, 79574C>T, 79580G>T, 79581C>T, 79592A>T, 79595T>C, 79596C>T, 79598G>A, 79602G>A, 79604A>G, 79610C>T, 79611G>T, 79612T>C, 79613C>T, 79614T>G, 79615G>T, 79616C>A, 79619C>T, 79624C>T, 79628G>C, 79632G>A, 79643C>T, 79646T>A, 79649G>A, 79655C>T, 79658C>T, 79664C>T, 79673A>T, 79676C>T, 79677G>T, 79679T>A, 79682T>G, 79688G>A, 79689A>C, 79691G>T, 79694T>C, 79703C>T, 79712T>C, 79718T>C, 79719G>A, 79722G>A, 79724A>T, 79728T>A, 79733C>T, 79734A>G, 79735A>C, 79736C>A, 79739A>T, 79742T>A, 79746C>T, 79748T>A, 79751C>T, 79754C>T, 79760C>A, 79763T>C, 79772T>G, 79779C>G, 79791C>T, 79793C>A, 79799T>C, 79811C>T, 79812C>G, 79817G>A, 79818G>A, 79820C>T, 79823A>T, 79824T>C, 79826T>A, 79833C>T, 79835G>A, 79836A>C, 79838C>T, 79842A>G, 79844C>A, 79845T>G, 79846T>C, 79847C>T, 79850C>T, 79871A>G, 79874C>T, 79875T>A, 79876C>G, 79879A>T, 79880T>C, 79895T>A, 79898C>T, 79904C>T, 79905T>G, 79906G>C, 79907C>T, 79916C>T, 79919T>A, 79922A>T, 79931T>A, 79937A>T, 79938T>A, 79940T>C, 79946C>T, 79950C>T, 79953G>A, 79958T>C, 79961T>A, 79962T>A, 79973T>A, 79974T>A, 79975C>G, 79976G>T, 79979C>T, 79985C>T, 79994C>T, 79997C>A, 80000C>A, 80018C>T, 80025C>A, 80030C>A, 80039A>T, 80042A>G, 80054G>T, 80063C>A, 80066C>T, 80069T>C, 80073C>T, 80075T>A, 80078T>C, 80090C>A, 80096T>C, 80099T>C, 80105G>A, 80114A>T, 80120C>T, 80126T>C, 80127C>T, 80132T>A, 80139T>A, 80140C>G, 80141G>T, 80142C>T, 80145G>A, 80147T>C, 80148C>A, 80150T>G, 80159C>A, 80162G>A, 80165C>T, 80168G>A, 80171C>T, 80172C>G, 80173A>C, 80174G>T, 80177C>T, 80178T>G, 80180T>A, 80188A>G, 80189G>A, 80201A>G, 80207G>A, 80210C>T, 80213C>T, 80216C>T, 80222G>A, 80225T>C, 80237C>T, 80240C>T, 80243T>C, 80246C>A, 80247C>T, 80261C>T, 80265T>A, 80266C>G, 80267C>T, 80279C>T, 80286C>T, 80288G>A, 80291T>C, 80300T>A, 80306A>T, 80315T>A, 80318C>A, 80333T>C, 80336A>T, 80337C>T, 80339T>A, 80343G>A, 80346T>A, 80347C>G, 80351C>T, 80357A>T, 80366G>A, 80369C>T, 80378C>T, 80387G>A, 80390C>T, 80391A>G, 80393C>A, 80394A>G, 80396G>T, 80399T>C, 80400G>A, 80409A>C, 80410A>G, 80411A>T, 80414C>A, 80415C>A, 80417C>T, 80433G>A, 80435T>C, 80436C>A, 80438A>C, 80447C>T, 80453G>T, 80456G>T, 80462G>A, 80465A>T, 80471C>T, 80474G>A, 80486C>T, 80498G>A, 80504G>A								

*: Inserts / Deletes / Misaligned / Frameshifts

Analysis details

This analysis was performed with panviral2.64

NGS Details (UN70): Cladosporium fulvum T-1 virus

Assembly

**Coverage Length**	983 (5 contig(s))
**Depth Of Coverage**	14.0
**Number Of Reads**	132
**Reads Per Million**	2.50 rpm (after QC)
**Ambiguities**	0
**Assembly Method**	de novo + reference guided assembly
**Consensus Caller**	Bcf Tools

Coverage Map

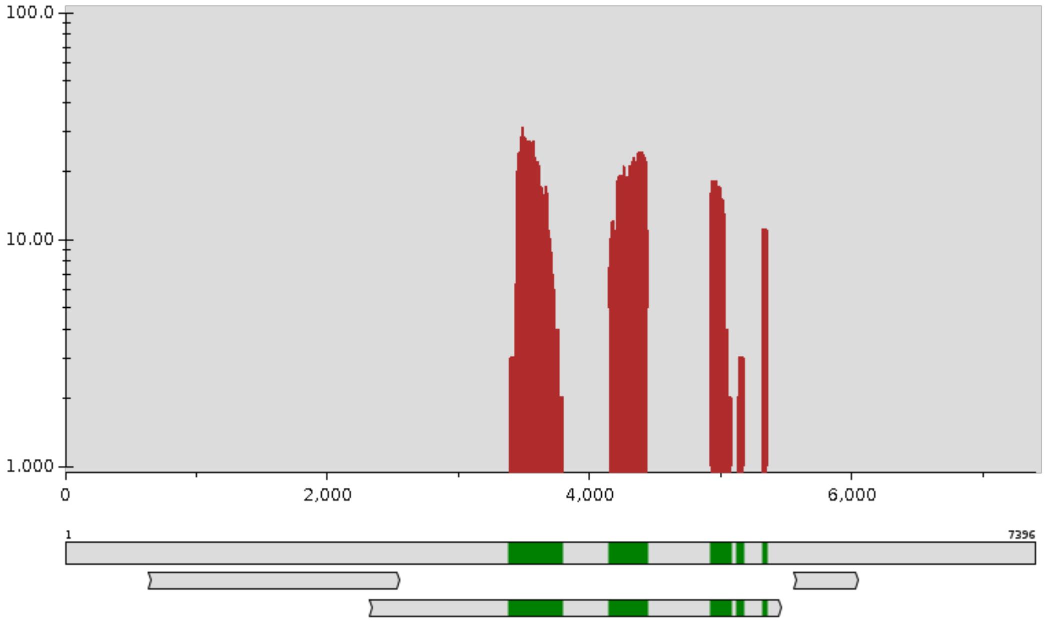

Assignment

**Type**	Cladosporium fulvum T-1 virus (Taxonomy ID: 2052899)
**Reference Genome**	NC_043491.1
**NT Identity (%)**	54.4317
**AA Identity (%)**	50.625
**Number Of Stop Codons**	0
**Number Of CDS**	3

Alignment

**Alignment Score**	101.0 (NT) + 1177.0 (AA) = 1278.0
**Concordance (%)**	32.1752

Alignment Method	Global, seeded, nucleotide + amino acids (AGA)

Genome Region

Sequence starts at position 3387 and ends at position 5357 relative to NC_043491.1 reference sequence.

Alignment Detailed Statistics

	Begin	End	Coverage	Score	Concordance	Matches	Identities	I/D/M/F*	Stop Codons
**NT**	**3387**	**5357**	**13.3%**	**101**	**5.5%**	**950 (95.8%)**	**522 (52.6%)**	**9/33**	
Mutations:	3387C>A, 3388C>A, 3389T>G, 3391A>T, 3392A>C, 3393G>C, 3394T>A, 3395G>T, 3396C>A, 3397A>T, 3401A>G, 3403T>A, 3406A>T, 3407T>G, 3408G>T, 3410A>T, 3415C>T, 3416G>C, 3417T>A, 3418T>G, 3419C>A, 3420C>A, 3421A>G, 3424A>G, 3425G>A, 3426C>A, 3428A>G, 3430C>T, 3434A>T, 3435A>C, 3442A>C, 3443C>A, 3445C>G, 3446G>T, 3447T>G, 3448A>C, 3449C>A, 3450A>T, 3451A>G, 3454C>T, 3457C>T, 3460A>G, 3461A>G, 3462A>C, 3464T>C, 3470G>A, 3473A>G, 3475C>A, 3478G>T, 3479A>G, 3481C>G, 3488C>A, 3489G>A, 3490A>G, 3493T>C, 3496G>A, 3497C>G, 3499A>T, 3502C>T, 3503A>T, 3504A>T, 3505C>G, 3506A>G, 3508C>G, 3509G>C, 3511A>G, 3514A>T, 3515G>C, 3516C>T, 3517A>G, 3518C>A, 3519A>T, 3520A>G, 3526A>G, 3529A>G, 3531C>G, 3533G>A, 3534G>A, 3536T>G, 3538A>G, 3539G>T, 3540A>G, 3546A>T, 3550G>T, 3554A>C, 3556T>A, 3559C>T, 3562A>T, 3563C>A, 3565A>G, 3566G>T, 3567A>C, 3568C>A, 3570C>G, 3573T>A, 3574C>T, 3576A>G, 3577T>G, 3578G>C, 3579C>A, 3580T>G, 3581A>G, 3583C>T, 3584C>A, 3586A>G, 3589G>A, 3598A>G, 3601A>T, 3604A>G, 3605T>C, 3606G>C, 3607G>T, 3610A>G, 3613C>T, 3614G>A, 3618T>G, 3619C>T, 3620A>G, 3621G>T, 3622G>A, 3625A>T, 3628A>G, 3631C>T, 3634A>G, 3635C>T, 3636T>C, 3637C>A, 3640C>T, 3647T>C, 3649G>T, 3652C>A, 3658A>G, 3659A>T, 3661G>T, 3664A>G, 3670C>T, 3673C>T, 3676A>C, 3679C>G, 3682A>T, 3683T>A, 3687G>T, 3688C>T, 3689C>T, 3690A>G, 3691G>C, 3692G>A, 3695C>T, 3697T>A, 3698G>A, 3700C>G, 3703C>T, 3706A>T, 3707A>G, 3708C>T, 3709A>C, 3712T>G, 3713A>T, 3714G>A, 3715A>T, 3719C>T, 3720T>A, 3721A>T, 3724C>T, 3727C>T, 3729T>A, 3732G>T, 3733C>T, 3736C>A, 3739T>A, 3741C>T, 3742T>C, 3746A>T, 3748G>A, 3751C>T, 3757A>T, 3758C>G, 3760G>C, 3761G>A, 3763C>A, 3766C>T, 3768C>G, 3769A>T, 3773G>A, 3775A>T, 3776T>A, 3778C>G, 3779C>T, 3781C>G, 3782C>G, 3784G>T, 3791A>G, 3792C>T, 3793C>G, 3796G>C, 3799A>T, 4146C>G, 4150G>C, 4150_4151insAATATTGCG, 4151C>G, 4154C>G, 4155G>C, 4156A>T, 4157C>G, 4158T>A, 4159A>C, 4162C>T, 4166G>T, 4167G>T, 4168T>A, 4169A>C, 4170G>C, 4171C>A, 4172A>T, 4173A>T, 4174G>T, 4180C>G, 4186C>T, 4187G>A, 4189G>T, 4195C>T, 4198G>C, 4204T>C, 4205A>T, 4207G>C, 4210A>T, 4213A>T, 4216C>G, 4218C>G, 4219A>T, 4220T>G, 4221G>T, 4222T>G, 4225A>T, 4227C>T, 4228A>T, 4231G>A, 4233C>A, 4234A>T, 4235C>G, 4238_4249delGATGGGAAAAGA, 4252C>T, 4255A>T, 4258G>T, 4261C>A, 4263A>T, 4265T>G, 4267T>G, 4268T>A, 4269C>G, 4271C>A, 4277A>T, 4281C>A, 4282C>T, 4283A>C, 4285A>G, 4286G>A, 4294G>A, 4296A>G, 4297C>A, 4300C>T, 4301G>T, 4302A>C, 4303C>T, 4304A>G, 4305T>C, 4306C>A, 4312C>A, 4319C>A, 4321T>G, 4322C>G, 4324A>G, 4327C>A, 4328A>T, 4330T>G, 4331G>C, 4333T>C, 4334G>C, 4335C>A, 4336C>T, 4337G>T, 4338C>G, 4339C>T, 4340A>C, 4342G>T, 4346C>A, 4347A>C, 4348T>A, 4354A>G, 4360C>T, 4361G>C, 4364G>T, 4365A>T, 4366G>A, 4369C>A, 4370C>A, 4373_4375delCCG, 4381A>T, 4382A>G, 4383C>T, 4384G>A, 4385A>G, 4387T>A, 4388C>A, 4389T>A, 4390T>G, 4391T>A, 4393A>G, 4397C>A, 4399C>T, 4400A>G, 4401A>T, 4403A>G, 4404A>C, 4406C>A, 4407T>A, 4408C>T, 4411G>T, 4419C>A, 4420G>A, 4423G>T, 4424A>C, 4425C>A, 4426G>A, 4427A>C, 4430G>A, 4433C>T, 4435T>G, 4437C>G, 4438C>T, 4439C>T, 4440G>T, 4441A>G, 4443G>A, 4444A>G, 4924A>T, 4933C>G, 4934G>A, 4935A>G, 4936G>T, 4937G>A, 4939T>C, 4954T>A, 4957G>C, 4958A>T, 4959A>G, 4960A>T, 4961C>T, 4963C>G, 4966G>A, 4968G>A, 4972A>C, 4973_4975delAAG, 4979_4993delCGAGTCACAGGACAA, 4995C>G, 4996C>G, 5002C>A, 5004T>C, 5005G>C, 5009C>T, 5011A>G, 5014C>T, 5015A>G, 5020C>A, 5023C>T, 5027C>T, 5029C>G, 5038T>C, 5041A>C, 5044C>T, 5047C>T, 5050T>G, 5053T>C, 5055C>T, 5056A>C, 5057T>A, 5058C>A, 5061A>C, 5062A>T, 5063A>G, 5064T>C, 5065A>T, 5067A>G, 5074A>C, 5075G>A, 5078C>G, 5079A>C, 5081C>G, 5082T>C, 5083C>T, 5085G>C, 5122A>G, 5123T>C, 5125C>G, 5129G>A, 5130A>G, 5131A>G, 5132G>C, 5133T>C, 5134A>C, 5135T>A, 5137C>T, 5143A>T, 5146C>T, 5147A>C, 5153A>C, 5154A>C, 5157T>G, 5161C>T, 5164A>T, 5165T>G, 5166C>G, 5172A>T, 5319T>C, 5320T>G, 5321T>A, 5322C>A, 5323A>G, 5324T>C, 5329G>A, 5330C>G, 5331C>A, 5332A>C, 5333A>G, 5338G>C, 5341G>A, 5342A>T, 5344C>T, 5345G>T, 5348C>T, 5349T>A, 5350G>T, 5353C>T								

CDS

	Begin	End	Coverage	Score	Concordance	Matches	Identities	I/D/M/F*	Stop Codons
homologue_of_retroviral_POL_genes	355	1011	31.4%	1177	53.8%	317 (95.8%)	162 (48.9%)	3/11/0/0	0
Protein mutations:	S355A (3389T>G 3391A>T), S356P (3392A>C 3393G>C 3394T>A), A357Y (3395G>T 3396C>A 3397A>T), T359A (3401A>G 3403T>A), C361V (3407T>G 3408G>T), M362L (3410A>T), V364Q (3416G>C 3417T>A 3418T>G), P365K (3419C>A 3420C>A 3421A>G), A367K (3425G>A 3426C>A), N368D (3428A>G 3430C>T), K370S (3434A>T 3435A>C), L373M (3443C>A 3445C>G), V374C (3446G>T 3447T>G 3448A>C), Q375M (3449C>A 3450A>T 3451A>G), K379A (3461A>G 3462A>C), E382K (3470G>A), I383V (3473A>G 3475C>A), I385V (3479A>G 3481C>G), R388K (3488C>A 3489G>A 3490A>G), L391V (3497C>G 3499A>T), N393L (3503A>T 3504A>T 3505C>G), I394V (3506A>G 3508C>G), E395Q (3509G>C 3511A>G), E396D (3514A>T), A397L (3515G>C 3516C>T 3517A>G), Q398M (3518C>A 3519A>T 3520A>G), T402S (3531C>G), G403K (3533G>A 3534G>A), S404A (3536T>G 3538A>G), D405C (3539G>T 3540A>G), Y407F (3546A>T), I410L (3554A>C 3556T>A), D414S (3566G>T 3567A>C 3568C>A), A415G (3570C>G), F416Y (3573T>A 3574C>T), Y417W (3576A>G 3577T>G), A418Q (3578G>C 3579C>A 3580T>G), I419V (3581A>G 3583C>T), M421I (3589G>A), E425D (3601A>T), W427P (3605T>C 3606G>C 3607G>T), A430T (3614G>A), F431C (3618T>G 3619C>T), R432V (3620A>G 3621G>T 3622G>A), L437S (3635C>T 3636T>C 3637C>A), M445F (3659A>T 3661G>T), S453T (3683T>A), C454F (3687G>T 3688C>T), Q455C (3689C>T 3690A>G 3691G>C), D456N (3692G>A), V458M (3698G>A 3700C>G), E460D (3706A>T), T461V (3707A>G 3708C>T 3709A>C), R463Y (3713A>T 3714G>A 3715A>T), L465Y (3719C>T 3720T>A 3721A>T), V468E (3729T>A), C469F (3732G>T 3733C>T), A472V (3741C>T 3742T>C), M474L (3746A>T 3748G>A), L478V (3758C>G 3760G>C), V479I (3761G>A 3763C>A), T481S (3768C>G 3769A>T), G483S (3773G>A 3775A>T), S484T (3776T>A 3778C>G), Q486D (3782C>G 3784G>T), T489V (3791A>G 3792C>T 3793C>G), K490N (3796G>C), Q491H (3799A>T), T608_L609insNIA (4150_4151insAATATTGCG), L609V (4151C>G), R610A (4154C>G 4155G>C 4156A>T), L611D (4157C>G 4158T>A 4159A>C), G614L (4166G>T 4167G>T 4168T>A), S615P (4169A>C 4170G>C 4171C>A), K616F (4172A>T 4173A>T 4174G>T), E621N (4187G>A 4189G>T), M627F (4205A>T 4207G>C), A631G (4218C>G 4219A>T), C632V (4220T>G 4221G>T 4222T>G), T634I (4227C>T 4228A>T), T636N (4233C>A 4234A>T), H637D (4235C>G), D638_R641del (4238_4249delGATGGGAAAAGA), Y646F (4263A>T), Y647E (4265T>G 4267T>G), M651L (4277A>T), T652N (4281C>A 4282C>T), T653P (4283A>C 4285A>G), A654T (4286G>A), N657R (4296A>G 4297C>A), D659S (4301G>T 4302A>C 4303C>T), I660A (4304A>G 4305T>C 4306C>A), D662E (4312C>A), L665M (4319C>A 4321T>G), L666V (4322C>G 4324A>G), I668L (4328A>T 4330T>G), V669L (4331G>C 4333T>C), A670H (4334G>C 4335C>A 4336C>T), A671C (4337G>T 4338C>G 4339C>T), M672L (4340A>C 4342G>T), H674T (4346C>A 4347A>C 4348T>A), V679L (4361G>C), E680L (4364G>T 4365A>T 4366G>A), P682T (4370C>A), P683del (4373_4375delCCG), L685F (4381A>T), T686V (4382A>G 4383C>T 4384G>A), I687V (4385A>G 4387T>A), L688K (4388C>A 4389T>A 4390T>G), S689T (4391T>A 4393A>G), H691N (4397C>A 4399C>T), K692V (4400A>G 4401A>T), N693A (4403A>G 4404A>C), L694N (4406C>A 4407T>A 4408C>T), T698K (4419C>A 4420G>A), T700Q (4424A>C 4425C>A 4426G>A), K701Q (4427A>C), E702K (4430G>A), T704S (4437C>G 4438C>T), R705L (4439C>T 4440G>T 4441A>G), R706K (4443G>A 4444A>G), K866N (4924A>T), D869E (4933C>G), E870S (4934G>A 4935A>G 4936G>T), V871I (4937G>A 4939T>C), K878C (4958A>T 4959A>G 4960A>T), R881K (4968G>A), K883del (4973_4975delAAG), R885_Q889del (4979_4993delCGAGTCACAGGACAA), A890G (4995C>G 4996C>G), D892E (5002C>A), M893T (5004T>C 5005G>C), M897V (5015A>G), I908M (5050T>G), A910V (5055C>T 5056A>C), S911K (5057T>A 5058C>A), E912A (5061A>C 5062A>T), I913A (5063A>G 5064T>C 5065A>T), Y914C (5067A>G), E917K (5075G>A), Q918A (5078C>G 5079A>C), L919A (5081C>G 5082T>C 5083C>T), F933L (5123T>C 5125C>G), E935R (5129G>A 5130A>G 5131A>G), V936P (5132G>C 5133T>C 5134A>C), F937I (5135T>A 5137C>T), K943P (5153A>C 5154A>C), L944R (5157T>G), S947G (5165T>G 5166C>G), Y949F (5172A>T), V998A (5319T>C 5320T>G), S999K (5321T>A 5322C>A 5323A>G), P1002D (5330C>G 5331C>A 5332A>C), M1003V (5333A>G), I1006F (5342A>T 5344C>T), A1007S (5345G>T), L1008Y (5348C>T 5349T>A 5350G>T)								

	Begin	End	Coverage	Score	Concordance	Matches	Identities	I/D/M/F*	Stop Codons
**NT**	**3387**	**5357**	**13.3%**	**101**	**5.5%**	**950 (95.8%)**	**522 (52.6%)**	**9/33**	
Codon mutations:	ACC354.AA (3387C>A 3388C>A), TCA355GCT (3389T>G 3391A>T), AGT356CCA (3392A>C 3393G>C 3394T>A), GCA357TAT (3395G>T 3396C>A 3397A>T), ACT359GCA (3401A>G 3403T>A), CCA360CCT (3406A>T), TGC361GTC (3407T>G 3408G>T), ATG362TTG (3410A>T), TTC363TTT (3415C>T), GTT364CAG (3416G>C 3417T>A 3418T>G), CCA365AAG (3419C>A 3420C>A 3421A>G), AAA366AAG (3424A>G), GCA367AAA (3425G>A 3426C>A), AAC368GAT (3428A>G 3430C>T), AAA370TCA (3434A>T 3435A>C), CGA372CGC (3442A>C), CTC373ATG (3443C>A 3445C>G), GTA374TGC (3446G>T 3447T>G 3448A>C), CAA375ATG (3449C>A 3450A>T 3451A>G), GAC376GAT (3454C>T), TAC377TAT (3457C>T), CGA378CGG (3460A>G), AAG379GCG (3461A>G 3462A>C), TTG380CTG (3464T>C), GAG382AAG (3470G>A), ATC383GTA (3473A>G 3475C>A), ACG384ACT (3478G>T), ATC385GTG (3479A>G 3481C>G), CGA388AAG (3488C>A 3489G>A 3490A>G), TAT389TAC (3493T>C), CCG390CCA (3496G>A), CTA391GTT (3497C>G 3499A>T), CCC392CCT (3502C>T), AAC393TTG (3503A>T 3504A>T 3505C>G), ATC394GTG (3506A>G 3508C>G), GAA395CAG (3509G>C 3511A>G), GAA396GAT (3514A>T), GCA397CTG (3515G>C 3516C>T 3517A>G), CAA398ATG (3518C>A 3519A>T 3520A>G), AGA400AGG (3526A>G), TTA401TTG (3529A>G), ACC402AGC (3531C>G), GGA403AAA (3533G>A 3534G>A), TCA404GCG (3536T>G 3538A>G), GAC405TGC (3539G>T 3540A>G), TAC407TTC (3546A>T), ACG408ACT (3550G>T), ATT410CTA (3554A>C 3556T>A), GAC411GAT (3559C>T), CTA412CTT (3562A>T), CGA413AGG (3563C>A 3565A>G), GAC414TCA (3566G>T 3567A>C 3568C>A), GCC415GGC (3570C>G), TTC416TAT (3573T>A 3574C>T), TAT417TGG (3576A>G 3577T>G), GCT418CAG (3578G>C 3579C>A 3580T>G), ATC419GTT (3581A>G 3583C>T), CGA420AGG (3584C>A 3586A>G), ATG421ATA (3589G>A), GGA424GGG (3598A>G), GAA425GAT (3601A>T), GAA426GAG (3604A>G), TGG427CCT (3605T>C 3606G>C 3607G>T), AAA428AAG (3610A>G), ACC429ACT (3613C>T), GCT430ACT (3614G>A), TTC431TGT (3618T>G 3619C>T), AGG432GTA (3620A>G 3621G>T 3622G>A), ACA433ACT (3625A>T), AGA434AGG (3628A>G), TAC435TAT (3631C>T), GGA436GGG (3634A>G), CTC437TCA (3635C>T 3636T>C 3637C>A), TAC438TAT (3640C>T), TTG441CTT (3647T>C 3649G>T), GTC442GTA (3652C>A), CCA444CCG (3658A>G), ATG445TTT (3659A>T 3661G>T), GGA446GGG (3664A>G), ACC448ACT (3670C>T), AAC449AAT (3673C>T), GCA450GCC (3676A>C), CCC451CCG (3679C>G), GCA452GCT (3682A>T), TCC453ACC (3683T>A), TGC454TTT (3687G>T 3688C>T), CAG455TGC (3689C>T 3690A>G 3691G>C), GAC456AAC (3692G>A), CTT457TTA (3695C>T 3697T>A), GTC458ATG (3698G>A 3700C>G), AAC459AAT (3703C>T), GAA460GAT (3706A>T), ACA461GTC (3707A>G 3708C>T 3709A>C), CTT462CTG (3712T>G), AGA463TAT (3713A>T 3714G>A 3715A>T), CTA465TAT (3719C>T 3720T>A 3721A>T), CTC466CTT (3724C>T), GAC467GAT (3727C>T), GTG468GAG (3729T>A), TGC469TTT (3732G>T 3733C>T), GTC470GTA (3736C>A), GTT471GTA (3739T>A), GCT472GTC (3741C>T 3742T>C), ATG474TTA (3746A>T 3748G>A), GAC475GAT (3751C>T), ATA477ATT (3757A>T), CTG478GTC (3758C>G 3760G>C), GTC479ATA (3761G>A 3763C>A), TAC480TAT (3766C>T), ACA481AGT (3768C>G 3769A>T), GGA483AGT (3773G>A 3775A>T), TCC484ACG (3776T>A 3778C>G), CTC485TTG (3779C>T 3781C>G), CAG486GAT (3782C>G 3784G>T), ACC489GTG (3791A>G 3792C>T 3793C>G), AAG490AAC (3796G>C), CAA491CAT (3799A>T), CCA607.GA (4146C>G), ACG608ACC (4150G>C), ACG608_CTT609insAATATTGCG (4150_4151insAATATTGCG), CTT609GTT (4151C>G), CGA610GCT (4154C>G 4155G>C 4156A>T), CTA611GAC (4157C>G 4158T>A 4159A>C), TTC612TTT (4162C>T), GGT614TTA (4166G>T 4167G>T 4168T>A), AGC615CCA (4169A>C 4170G>C 4171C>A), AAG616TTT (4172A>T 4173A>T 4174G>T), GTC618GTG (4180C>G), ATC620ATT (4186C>T), GAG621AAT (4187G>A 4189G>T), GAC623GAT (4195C>T), GCG624GCC (4198G>C), GAT626GAC (4204T>C), ATG627TTC (4205A>T 4207G>C), GCA628GCT (4210A>T), ATA629ATT (4213A>T), GGC630GGG (4216C>G), GCA631GGT (4218C>G 4219A>T), TGT632GTG (4220T>G 4221G>T 4222T>G), CTA633CTT (4225A>T), ACA634ATT (4227C>T 4228A>T), CAG635CAA (4231G>A), ACA636AAT (4233C>A 4234A>T), CAC637GAC (4235C>G), GAT638_AGA641del (4238_4249delGATGGGAAAAGA), CAC642CAT (4252C>T), CCA643CCT (4255A>T), GTG644GTT (4258G>T), GCC645GCA (4261C>A), TAT646TTT (4263A>T), TAT647GAG (4265T>G 4267T>G), TCC648AGC (4268T>A 4269C>G), CGG649AGG (4271C>A), ATG651TTG (4277A>T), ACC652AAT (4281C>A 4282C>T), ACA653CCG (4283A>C 4285A>G), GCG654ACG (4286G>A), CAG656CAA (4294G>A), AAC657AGA (4296A>G 4297C>A), TAC658TAT (4300C>T), GAC659TCT (4301G>T 4302A>C 4303C>T), ATC660GCA (4304A>G 4305T>C 4306C>A), GAC662GAA (4312C>A), CTT665ATG (4319C>A 4321T>G), CTA666GTG (4322C>G 4324A>G), GCC667GCA (4327C>A), ATT668TTG (4328A>T 4330T>G), GTT669CTC (4331G>C 4333T>C), GCC670CAT (4334G>C 4335C>A 4336C>T), GCC671TGT (4337G>T 4338C>G 4339C>T), ATG672CTT (4340A>C 4342G>T), CAT674ACA (4346C>A 4347A>C 4348T>A), AGA676AGG (4354A>G), TAC678TAT (4360C>T), GTC679CTC (4361G>C), GAG680TTA (4364G>T 4365A>T 4366G>A), GGC681GGA (4369C>A), CCA682ACA (4370C>A), CCG683del (4373_4375delCCG), TTA685TTT (4381A>T), ACG686GTA (4382A>G 4383C>T 4384G>A), ATT687GTA (4385A>G 4387T>A), CTT688AAG (4388C>A 4389T>A 4390T>G), TCA689ACG (4391T>A 4393A>G), CAC691AAT (4397C>A 4399C>T), AAG692GTG (4400A>G 4401A>T), AAT693GCT (4403A>G 4404A>C), CTC694AAT (4406C>A 4407T>A 4408C>T), ACG695ACT (4411G>T), ACG698AAA (4419C>A 4420G>A), ACG699ACT (4423G>T), ACG700CAA (4424A>C 4425C>A 4426G>A), AAG701CAG (4427A>C), GAA702AAA (4430G>A), CTT703TTG (4433C>T 4435T>G), ACC704AGT (4437C>G 4438C>T), CGA705TTG (4439C>T 4440G>T 4441A>G), AGA706AAG (4443G>A 4444A>G), AAA866AAT (4924A>T), GAC869GAG (4933C>G), GAG870AGT (4934G>A 4935A>G 4936G>T), GTT871ATC (4937G>A 4939T>C), ATT876ATA (4954T>A), ACG877ACC (4957G>C), AAA878TGT (4958A>T 4959A>G 4960A>T), CTC879TTG (4961C>T 4963C>G), CCG880CCA (4966G>A), AGG881AAG (4968G>A), TCA882TCC (4972A>C), AAG883del (4973_4975delAAG), CGA885_CAA889del (4979_4993delCGAGTCACAGGACAA), GCC890GGG (4995C>G 4996C>G), GAC892GAA (5002C>A), ATG893ACC (5004T>C 5005G>C), CTA895TTG (5009C>T 5011A>G), GTC896GTT (5014C>T), ATG897GTG (5015A>G), GTC898GTA (5020C>A), GAC899GAT (5023C>T), CTC901TTG (5027C>T 5029C>G), TAT904TAC (5038T>C), GCA905GCC (5041A>C), CAC906CAT (5044C>T), TTC907TTT (5047C>T), ATT908ATG (5050T>G), CCT909CCC (5053T>C), GCA910GTC (5055C>T 5056A>C), TCA911AAA (5057T>A 5058C>A), GAA912GCT (5061A>C 5062A>T), ATA913GCT (5063A>G 5064T>C 5065A>T), TAC914TGC (5067A>G), GCA916GCC (5074A>C), GAG917AAG (5075G>A), CAG918GCG (5078C>G 5079A>C), CTC919GCT (5081C>G 5082T>C 5083C>T), GGA920GC. (5085G>C), GGA932GGG (5122A>G), TTC933CTG (5123T>C 5125C>G), GAA935AGG (5129G>A 5130A>G 5131A>G), GTA936CCC (5132G>C 5133T>C 5134A>C), TTC937ATT (5135T>A 5137C>T), ACA939ACT (5143A>T), GAC940GAT (5146C>T), AGA941CGA (5147A>C), AAG943CCG (5153A>C 5154A>C), CTC944CGC (5157T>G), TTC945TTT (5161C>T), ACA946ACT (5164A>T), TCA947GGA (5165T>G 5166C>G), TAC949TTC (5172A>T), GTT998GCG (5319T>C 5320T>G), TCA999AAG (5321T>A 5322C>A 5323A>G), TTA1000CTA (5324T>C), CTG1001CTA (5329G>A), CCA1002GAC (5330C>G 5331C>A 5332A>C), ATG1003GTG (5333A>G), GCG1004GCC (5338G>C), CAG1005CAA (5341G>A), ATC1006TTT (5342A>T 5344C>T), GCA1007TCA (5345G>T), CTG1008TAT (5348C>T 5349T>A 5350G>T), AAC1009AAT (5353C>T)								

Proteins

	Begin	End	Coverage	Score	Concordance	Matches	Identities	I/D/M/F*	Stop Codons
Reverse Transcriptase (YP_009666308.1)	355	1011	31.4%	1177	53.8%	317 (95.8%)	162 (48.9%)	3/11/0/0	0
Protein mutations:	S355A (3389T>G 3391A>T), S356P (3392A>C 3393G>C 3394T>A), A357Y (3395G>T 3396C>A 3397A>T), T359A (3401A>G 3403T>A), C361V (3407T>G 3408G>T), M362L (3410A>T), V364Q (3416G>C 3417T>A 3418T>G), P365K (3419C>A 3420C>A 3421A>G), A367K (3425G>A 3426C>A), N368D (3428A>G 3430C>T), K370S (3434A>T 3435A>C), L373M (3443C>A 3445C>G), V374C (3446G>T 3447T>G 3448A>C), Q375M (3449C>A 3450A>T 3451A>G), K379A (3461A>G 3462A>C), E382K (3470G>A), I383V (3473A>G 3475C>A), I385V (3479A>G 3481C>G), R388K (3488C>A 3489G>A 3490A>G), L391V (3497C>G 3499A>T), N393L (3503A>T 3504A>T 3505C>G), I394V (3506A>G 3508C>G), E395Q (3509G>C 3511A>G), E396D (3514A>T), A397L (3515G>C 3516C>T 3517A>G), Q398M (3518C>A 3519A>T 3520A>G), T402S (3531C>G), G403K (3533G>A 3534G>A), S404A (3536T>G 3538A>G), D405C (3539G>T 3540A>G), Y407F (3546A>T), I410L (3554A>C 3556T>A), D414S (3566G>T 3567A>C 3568C>A), A415G (3570C>G), F416Y (3573T>A 3574C>T), Y417W (3576A>G 3577T>G), A418Q (3578G>C 3579C>A 3580T>G), I419V (3581A>G 3583C>T), M421I (3589G>A), E425D (3601A>T), W427P (3605T>C 3606G>C 3607G>T), A430T (3614G>A), F431C (3618T>G 3619C>T), R432V (3620A>G 3621G>T 3622G>A), L437S (3635C>T 3636T>C 3637C>A), M445F (3659A>T 3661G>T), S453T (3683T>A), C454F (3687G>T 3688C>T), Q455C (3689C>T 3690A>G 3691G>C), D456N (3692G>A), V458M (3698G>A 3700C>G), E460D (3706A>T), T461V (3707A>G 3708C>T 3709A>C), R463Y (3713A>T 3714G>A 3715A>T), L465Y (3719C>T 3720T>A 3721A>T), V468E (3729T>A), C469F (3732G>T 3733C>T), A472V (3741C>T 3742T>C), M474L (3746A>T 3748G>A), L478V (3758C>G 3760G>C), V479I (3761G>A 3763C>A), T481S (3768C>G 3769A>T), G483S (3773G>A 3775A>T), S484T (3776T>A 3778C>G), Q486D (3782C>G 3784G>T), T489V (3791A>G 3792C>T 3793C>G), K490N (3796G>C), Q491H (3799A>T), T608_L609insNIA (4150_4151insAATATTGCG), L609V (4151C>G), R610A (4154C>G 4155G>C 4156A>T), L611D (4157C>G 4158T>A 4159A>C), G614L (4166G>T 4167G>T 4168T>A), S615P (4169A>C 4170G>C 4171C>A), K616F (4172A>T 4173A>T 4174G>T), E621N (4187G>A 4189G>T), M627F (4205A>T 4207G>C), A631G (4218C>G 4219A>T), C632V (4220T>G 4221G>T 4222T>G), T634I (4227C>T 4228A>T), T636N (4233C>A 4234A>T), H637D (4235C>G), D638_R641del (4238_4249delGATGGGAAAAGA), Y646F (4263A>T), Y647E (4265T>G 4267T>G), M651L (4277A>T), T652N (4281C>A 4282C>T), T653P (4283A>C 4285A>G), A654T (4286G>A), N657R (4296A>G 4297C>A), D659S (4301G>T 4302A>C 4303C>T), I660A (4304A>G 4305T>C 4306C>A), D662E (4312C>A), L665M (4319C>A 4321T>G), L666V (4322C>G 4324A>G), I668L (4328A>T 4330T>G), V669L (4331G>C 4333T>C), A670H (4334G>C 4335C>A 4336C>T), A671C (4337G>T 4338C>G 4339C>T), M672L (4340A>C 4342G>T), H674T (4346C>A 4347A>C 4348T>A), V679L (4361G>C), E680L (4364G>T 4365A>T 4366G>A), P682T (4370C>A), P683del (4373_4375delCCG), L685F (4381A>T), T686V (4382A>G 4383C>T 4384G>A), I687V (4385A>G 4387T>A), L688K (4388C>A 4389T>A 4390T>G), S689T (4391T>A 4393A>G), H691N (4397C>A 4399C>T), K692V (4400A>G 4401A>T), N693A (4403A>G 4404A>C), L694N (4406C>A 4407T>A 4408C>T), T698K (4419C>A 4420G>A), T700Q (4424A>C 4425C>A 4426G>A), K701Q (4427A>C), E702K (4430G>A), T704S (4437C>G 4438C>T), R705L (4439C>T 4440G>T 4441A>G), R706K (4443G>A 4444A>G), K866N (4924A>T), D869E (4933C>G), E870S (4934G>A 4935A>G 4936G>T), V871I (4937G>A 4939T>C), K878C (4958A>T 4959A>G 4960A>T), R881K (4968G>A), K883del (4973_4975delAAG), R885_Q889del (4979_4993delCGAGTCACAGGACAA), A890G (4995C>G 4996C>G), D892E (5002C>A), M893T (5004T>C 5005G>C), M897V (5015A>G), I908M (5050T>G), A910V (5055C>T 5056A>C), S911K (5057T>A 5058C>A), E912A (5061A>C 5062A>T), I913A (5063A>G 5064T>C 5065A>T), Y914C (5067A>G), E917K (5075G>A), Q918A (5078C>G 5079A>C), L919A (5081C>G 5082T>C 5083C>T), F933L (5123T>C 5125C>G), E935R (5129G>A 5130A>G 5131A>G), V936P (5132G>C 5133T>C 5134A>C), F937I (5135T>A 5137C>T), K943P (5153A>C 5154A>C), L944R (5157T>G), S947G (5165T>G 5166C>G), Y949F (5172A>T), V998A (5319T>C 5320T>G), S999K (5321T>A 5322C>A 5323A>G), P1002D (5330C>G 5331C>A 5332A>C), M1003V (5333A>G), I1006F (5342A>T 5344C>T), A1007S (5345G>T), L1008Y (5348C>T 5349T>A 5350G>T)								

	Begin	End	Coverage	Score	Concordance	Matches	Identities	I/D/M/F*	Stop Codons
**NT**	**3387**	**5357**	**13.3%**	**101**	**5.5%**	**950 (95.8%)**	**522 (52.6%)**	**9/33**	
Codon mutations:	ACC354.AA (3387C>A 3388C>A), TCA355GCT (3389T>G 3391A>T), AGT356CCA (3392A>C 3393G>C 3394T>A), GCA357TAT (3395G>T 3396C>A 3397A>T), ACT359GCA (3401A>G 3403T>A), CCA360CCT (3406A>T), TGC361GTC (3407T>G 3408G>T), ATG362TTG (3410A>T), TTC363TTT (3415C>T), GTT364CAG (3416G>C 3417T>A 3418T>G), CCA365AAG (3419C>A 3420C>A 3421A>G), AAA366AAG (3424A>G), GCA367AAA (3425G>A 3426C>A), AAC368GAT (3428A>G 3430C>T), AAA370TCA (3434A>T 3435A>C), CGA372CGC (3442A>C), CTC373ATG (3443C>A 3445C>G), GTA374TGC (3446G>T 3447T>G 3448A>C), CAA375ATG (3449C>A 3450A>T 3451A>G), GAC376GAT (3454C>T), TAC377TAT (3457C>T), CGA378CGG (3460A>G), AAG379GCG (3461A>G 3462A>C), TTG380CTG (3464T>C), GAG382AAG (3470G>A), ATC383GTA (3473A>G 3475C>A), ACG384ACT (3478G>T), ATC385GTG (3479A>G 3481C>G), CGA388AAG (3488C>A 3489G>A 3490A>G), TAT389TAC (3493T>C), CCG390CCA (3496G>A), CTA391GTT (3497C>G 3499A>T), CCC392CCT (3502C>T), AAC393TTG (3503A>T 3504A>T 3505C>G), ATC394GTG (3506A>G 3508C>G), GAA395CAG (3509G>C 3511A>G), GAA396GAT (3514A>T), GCA397CTG (3515G>C 3516C>T 3517A>G), CAA398ATG (3518C>A 3519A>T 3520A>G), AGA400AGG (3526A>G), TTA401TTG (3529A>G), ACC402AGC (3531C>G), GGA403AAA (3533G>A 3534G>A), TCA404GCG (3536T>G 3538A>G), GAC405TGC (3539G>T 3540A>G), TAC407TTC (3546A>T), ACG408ACT (3550G>T), ATT410CTA (3554A>C 3556T>A), GAC411GAT (3559C>T), CTA412CTT (3562A>T), CGA413AGG (3563C>A 3565A>G), GAC414TCA (3566G>T 3567A>C 3568C>A), GCC415GGC (3570C>G), TTC416TAT (3573T>A 3574C>T), TAT417TGG (3576A>G 3577T>G), GCT418CAG (3578G>C 3579C>A 3580T>G), ATC419GTT (3581A>G 3583C>T), CGA420AGG (3584C>A 3586A>G), ATG421ATA (3589G>A), GGA424GGG (3598A>G), GAA425GAT (3601A>T), GAA426GAG (3604A>G), TGG427CCT (3605T>C 3606G>C 3607G>T), AAA428AAG (3610A>G), ACC429ACT (3613C>T), GCT430ACT (3614G>A), TTC431TGT (3618T>G 3619C>T), AGG432GTA (3620A>G 3621G>T 3622G>A), ACA433ACT (3625A>T), AGA434AGG (3628A>G), TAC435TAT (3631C>T), GGA436GGG (3634A>G), CTC437TCA (3635C>T 3636T>C 3637C>A), TAC438TAT (3640C>T), TTG441CTT (3647T>C 3649G>T), GTC442GTA (3652C>A), CCA444CCG (3658A>G), ATG445TTT (3659A>T 3661G>T), GGA446GGG (3664A>G), ACC448ACT (3670C>T), AAC449AAT (3673C>T), GCA450GCC (3676A>C), CCC451CCG (3679C>G), GCA452GCT (3682A>T), TCC453ACC (3683T>A), TGC454TTT (3687G>T 3688C>T), CAG455TGC (3689C>T 3690A>G 3691G>C), GAC456AAC (3692G>A), CTT457TTA (3695C>T 3697T>A), GTC458ATG (3698G>A 3700C>G), AAC459AAT (3703C>T), GAA460GAT (3706A>T), ACA461GTC (3707A>G 3708C>T 3709A>C), CTT462CTG (3712T>G), AGA463TAT (3713A>T 3714G>A 3715A>T), CTA465TAT (3719C>T 3720T>A 3721A>T), CTC466CTT (3724C>T), GAC467GAT (3727C>T), GTG468GAG (3729T>A), TGC469TTT (3732G>T 3733C>T), GTC470GTA (3736C>A), GTT471GTA (3739T>A), GCT472GTC (3741C>T 3742T>C), ATG474TTA (3746A>T 3748G>A), GAC475GAT (3751C>T), ATA477ATT (3757A>T), CTG478GTC (3758C>G 3760G>C), GTC479ATA (3761G>A 3763C>A), TAC480TAT (3766C>T), ACA481AGT (3768C>G 3769A>T), GGA483AGT (3773G>A 3775A>T), TCC484ACG (3776T>A 3778C>G), CTC485TTG (3779C>T 3781C>G), CAG486GAT (3782C>G 3784G>T), ACC489GTG (3791A>G 3792C>T 3793C>G), AAG490AAC (3796G>C), CAA491CAT (3799A>T), CCA607.GA (4146C>G), ACG608ACC (4150G>C), ACG608_CTT609insAATATTGCG (4150_4151insAATATTGCG), CTT609GTT (4151C>G), CGA610GCT (4154C>G 4155G>C 4156A>T), CTA611GAC (4157C>G 4158T>A 4159A>C), TTC612TTT (4162C>T), GGT614TTA (4166G>T 4167G>T 4168T>A), AGC615CCA (4169A>C 4170G>C 4171C>A), AAG616TTT (4172A>T 4173A>T 4174G>T), GTC618GTG (4180C>G), ATC620ATT (4186C>T), GAG621AAT (4187G>A 4189G>T), GAC623GAT (4195C>T), GCG624GCC (4198G>C), GAT626GAC (4204T>C), ATG627TTC (4205A>T 4207G>C), GCA628GCT (4210A>T), ATA629ATT (4213A>T), GGC630GGG (4216C>G), GCA631GGT (4218C>G 4219A>T), TGT632GTG (4220T>G 4221G>T 4222T>G), CTA633CTT (4225A>T), ACA634ATT (4227C>T 4228A>T), CAG635CAA (4231G>A), ACA636AAT (4233C>A 4234A>T), CAC637GAC (4235C>G), GAT638_AGA641del (4238_4249delGATGGGAAAAGA), CAC642CAT (4252C>T), CCA643CCT (4255A>T), GTG644GTT (4258G>T), GCC645GCA (4261C>A), TAT646TTT (4263A>T), TAT647GAG (4265T>G 4267T>G), TCC648AGC (4268T>A 4269C>G), CGG649AGG (4271C>A), ATG651TTG (4277A>T), ACC652AAT (4281C>A 4282C>T), ACA653CCG (4283A>C 4285A>G), GCG654ACG (4286G>A), CAG656CAA (4294G>A), AAC657AGA (4296A>G 4297C>A), TAC658TAT (4300C>T), GAC659TCT (4301G>T 4302A>C 4303C>T), ATC660GCA (4304A>G 4305T>C 4306C>A), GAC662GAA (4312C>A), CTT665ATG (4319C>A 4321T>G), CTA666GTG (4322C>G 4324A>G), GCC667GCA (4327C>A), ATT668TTG (4328A>T 4330T>G), GTT669CTC (4331G>C 4333T>C), GCC670CAT (4334G>C 4335C>A 4336C>T), GCC671TGT (4337G>T 4338C>G 4339C>T), ATG672CTT (4340A>C 4342G>T), CAT674ACA (4346C>A 4347A>C 4348T>A), AGA676AGG (4354A>G), TAC678TAT (4360C>T), GTC679CTC (4361G>C), GAG680TTA (4364G>T 4365A>T 4366G>A), GGC681GGA (4369C>A), CCA682ACA (4370C>A), CCG683del (4373_4375delCCG), TTA685TTT (4381A>T), ACG686GTA (4382A>G 4383C>T 4384G>A), ATT687GTA (4385A>G 4387T>A), CTT688AAG (4388C>A 4389T>A 4390T>G), TCA689ACG (4391T>A 4393A>G), CAC691AAT (4397C>A 4399C>T), AAG692GTG (4400A>G 4401A>T), AAT693GCT (4403A>G 4404A>C), CTC694AAT (4406C>A 4407T>A 4408C>T), ACG695ACT (4411G>T), ACG698AAA (4419C>A 4420G>A), ACG699ACT (4423G>T), ACG700CAA (4424A>C 4425C>A 4426G>A), AAG701CAG (4427A>C), GAA702AAA (4430G>A), CTT703TTG (4433C>T 4435T>G), ACC704AGT (4437C>G 4438C>T), CGA705TTG (4439C>T 4440G>T 4441A>G), AGA706AAG (4443G>A 4444A>G), AAA866AAT (4924A>T), GAC869GAG (4933C>G), GAG870AGT (4934G>A 4935A>G 4936G>T), GTT871ATC (4937G>A 4939T>C), ATT876ATA (4954T>A), ACG877ACC (4957G>C), AAA878TGT (4958A>T 4959A>G 4960A>T), CTC879TTG (4961C>T 4963C>G), CCG880CCA (4966G>A), AGG881AAG (4968G>A), TCA882TCC (4972A>C), AAG883del (4973_4975delAAG), CGA885_CAA889del (4979_4993delCGAGTCACAGGACAA), GCC890GGG (4995C>G 4996C>G), GAC892GAA (5002C>A), ATG893ACC (5004T>C 5005G>C), CTA895TTG (5009C>T 5011A>G), GTC896GTT (5014C>T), ATG897GTG (5015A>G), GTC898GTA (5020C>A), GAC899GAT (5023C>T), CTC901TTG (5027C>T 5029C>G), TAT904TAC (5038T>C), GCA905GCC (5041A>C), CAC906CAT (5044C>T), TTC907TTT (5047C>T), ATT908ATG (5050T>G), CCT909CCC (5053T>C), GCA910GTC (5055C>T 5056A>C), TCA911AAA (5057T>A 5058C>A), GAA912GCT (5061A>C 5062A>T), ATA913GCT (5063A>G 5064T>C 5065A>T), TAC914TGC (5067A>G), GCA916GCC (5074A>C), GAG917AAG (5075G>A), CAG918GCG (5078C>G 5079A>C), CTC919GCT (5081C>G 5082T>C 5083C>T), GGA920GC. (5085G>C), GGA932GGG (5122A>G), TTC933CTG (5123T>C 5125C>G), GAA935AGG (5129G>A 5130A>G 5131A>G), GTA936CCC (5132G>C 5133T>C 5134A>C), TTC937ATT (5135T>A 5137C>T), ACA939ACT (5143A>T), GAC940GAT (5146C>T), AGA941CGA (5147A>C), AAG943CCG (5153A>C 5154A>C), CTC944CGC (5157T>G), TTC945TTT (5161C>T), ACA946ACT (5164A>T), TCA947GGA (5165T>G 5166C>G), TAC949TTC (5172A>T), GTT998GCG (5319T>C 5320T>G), TCA999AAG (5321T>A 5322C>A 5323A>G), TTA1000CTA (5324T>C), CTG1001CTA (5329G>A), CCA1002GAC (5330C>G 5331C>A 5332A>C), ATG1003GTG (5333A>G), GCG1004GCC (5338G>C), CAG1005CAA (5341G>A), ATC1006TTT (5342A>T 5344C>T), GCA1007TCA (5345G>T), CTG1008TAT (5348C>T 5349T>A 5350G>T), AAC1009AAT (5353C>T)								

*: Inserts / Deletes / Misaligned / Frameshifts

Analysis details

This analysis was performed with panviral2.64

NGS Details (UN70): Lowelvirus tuscon4d

Assembly

**Coverage Length**	397 (2 contig(s))
**Depth Of Coverage**	27.6
**Number Of Reads**	115
**Reads Per Million**	2.18 rpm (after QC)
**Ambiguities**	1
**Assembly Method**	de novo + reference guided assembly
**Consensus Caller**	Bcf Tools

Coverage Map

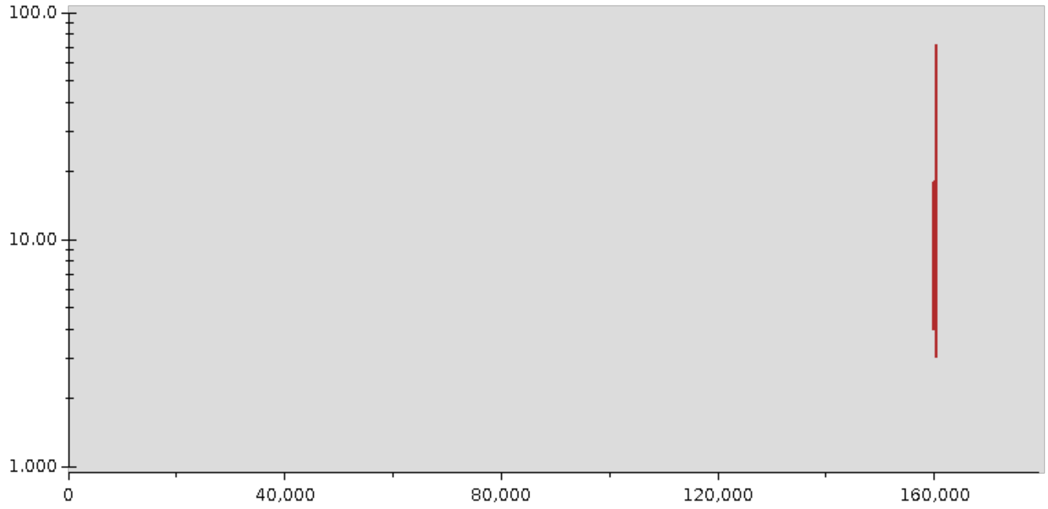

Assignment

**Type**	Lowelvirus tuscon4d (Taxonomy ID: 2956131)
**Reference Genome**	NC_026923.1
**NT Identity (%)**	79.597
**AA Identity (%)**	89.9225
**Number Of Stop Codons**	1
**Number Of CDS**	218

Alignment

**Alignment Score**	472.0 (NT) + 855.0 (AA) = 1327.0
**Concordance (%)**	76.794
**Alignment Method**	Local, heuristic, nucleotide (BLASTN)

Genome Region

Sequence starts at position 159734 and ends at position 160404 relative to NC_026923.1 reference sequence.

Alignment Detailed Statistics

	Begin	End	Coverage	Score	Concordance	Matches	Identities	I/D/M/F*	Stop Codons
NT	159734	160404	0.2%	472	59.4%	397 (100%)	316 (79.6%)	0/0	

Mutations: 159738T>A, 159739C>T, 159740T>C, 159741A>T, 159745G>C, 159746G>A, 159747T>A, 159748A>T, 159750C>G, 159751C>A, 159753T>A, 159759C>T, 159765T>A, 159768T>C, 159771G>A, 159783T>A, 159798C>T, 159799A>T, 159800G>C, 159801C>T, 159807C>T, 159810T>G, 159813C>T, 159822G>A, 159832T>G, 159833C>G, 159834A>T, 159855C>T, 159858G>T, 159867A>T, 159870C>A, 159874A>C, 159880C>A, 159882G>C, 159883T>C, 159885G>A, 159888C>T, 159889C>T, 159894G>A, 159897T>G, 159900C>T, 159903C>T, 159906C>T, 159913C>T, 159915C>G, 159924C>T, 159942T>C, 159946A>G, 159949C>T, 159951A>G, 160246A>C, 160248A>C, 160254A>G, 160270G>A, 160275C>T, 160287G>A, 160290C>W, 160293C>A, 160296A>C, 160299G>A, 160302T>C, 160308A>T, 160309C>A, 160326A>G, 160327C>G, 160330G>C, 160331C>A, 160332T>G, 160338A>G, 160341A>T, 160350C>T, 160356C>A, 160359C>T, 160368A>G, 160371A>T, 160377T>A, 160378A>C, 160380A>T, 160383T>A, 160389A>T, 160392G>T

*: Inserts / Deletes / Misaligned / Frameshifts

Analysis details

This analysis was performed with panviral2.64

NGS Details (UN70): Errantivirus

Assembly

**Coverage Length**	860 (1 contig(s))
**Depth Of Coverage**	13.9
**Number Of Reads**	112
**Reads Per Million**	2.12 rpm (after QC)
**Ambiguities**	0
**Assembly Method**	de novo + reference guided assembly
**Consensus Caller**	Bcf Tools

Coverage Map

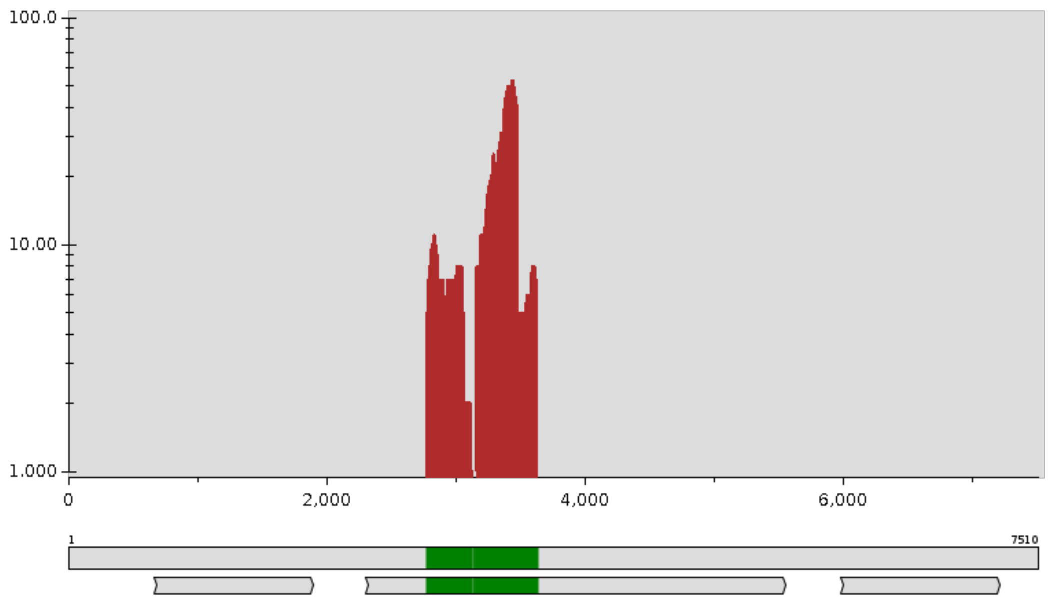

Assignment

**Type**	Errantivirus (Taxonomy ID: 186666)
**Reference Genome**	NC_038512.1
**NT Identity (%)**	51.6627
**AA Identity (%)**	43.3692
**Number Of Stop Codons**	0
**Number Of CDS**	3

Alignment

**Alignment Score**	11.0 (NT) + 856.0 (AA) = 867.0
**Concordance (%)**	24.6097

Alignment Method	Global, seeded, nucleotide + amino acids (AGA)

Genome Region

Sequence starts at position 2769 and ends at position 3638 relative to NC_038512.1 reference sequence.

Alignment Detailed Statistics

	Begin	End	Coverage	Score	Concordance	Matches	Identities	I/D/M/F*	Stop Codons
**NT**	**2769**	**3638**	**11.5%**	**11**	**0.7%**	**842 (97.9%)**	**435 (50.6%)**	**0/18**	
Mutations:	2769G>A, 2771G>A, 2773C>G, 2778T>G, 2779C>G, 2781C>A, 2782G>A, 2783A>G, 2789T>C, 2791G>A, 2792G>C, 2794C>G, 2801A>G, 2803T>C, 2807A>C, 2808G>A, 2813T>A, 2814C>G, 2815A>T, 2816G>C, 2822G>C, 2826G>A, 2827G>C, 2828A>T, 2829G>C, 2833A>T, 2836C>A, 2837A>G, 2839A>G, 2841G>T, 2843G>T, 2845T>A, 2849_2851delCCC, 2859T>A, 2860C>A, 2863C>T, 2865C>G, 2866T>G, 2867T>A, 2870_2881delGGGAAACAAAAG, 2885C>A, 2887T>A, 2888C>T, 2891G>T, 2892T>A, 2893A>T, 2894G>A, 2901T>A, 2903C>A, 2905T>A, 2906A>G, 2908G>T, 2914C>T, 2915G>A, 2918A>T, 2919A>T, 2927G>A, 2929T>A, 2932C>T, 2937A>T, 2938C>T, 2939C>A, 2941G>A, 2944A>T, 2947A>C, 2949A>T, 2953A>T, 2954A>G, 2955G>A, 2956T>G, 2959C>G, 2960G>T, 2965T>G, 2968C>T, 2969A>G, 2971G>A, 2974A>G, 2977T>A, 2978A>G, 2979A>G, 2980G>T, 2982G>C, 2986A>G, 2987T>A, 2988A>T, 2997C>A, 2998C>G, 2999T>C, 3001A>T, 3005T>C, 3008G>A, 3009C>G, 3011A>T, 3012G>C, 3013T>A, 3016G>T, 3018T>A, 3020T>C, 3025G>A, 3026G>A, 3028G>T, 3029G>A, 3030A>G, 3035G>A, 3036A>C, 3037C>A, 3038C>A, 3039C>A, 3040T>G, 3041C>G, 3043A>G, 3047A>G, 3049A>C, 3050T>C, 3051C>A, 3058C>T, 3059G>A, 3061G>A, 3066A>G, 3067C>G, 3068G>A, 3069T>C, 3070A>T, 3071G>C, 3073A>T, 3074C>T, 3075A>C, 3076C>T, 3079G>C, 3082T>C, 3084T>A, 3088A>G, 3090T>A, 3094T>A, 3095C>G, 3096G>T, 3103T>C, 3104A>T, 3106G>T, 3109A>C, 3110T>C, 3112A>C, 3114A>C, 3115A>T, 3118C>T, 3119T>G, 3124A>T, 3127T>A, 3129C>G, 3137A>T, 3139A>T, 3140G>C, 3142T>A, 3146G>A, 3148C>T, 3149A>C, 3151T>G, 3154C>T, 3155C>T, 3157A>T, 3158A>C, 3159G>A, 3160A>G, 3161G>C, 3162G>C, 3164C>T, 3168A>T, 3169A>C, 3170A>G, 3172T>C, 3174A>T, 3175C>A, 3176A>T, 3178C>T, 3179T>G, 3180G>T, 3181T>G, 3182C>G, 3184C>A, 3189A>T, 3191C>A, 3200A>G, 3202T>A, 3203A>C, 3205T>G, 3206G>A, 3208C>A, 3211T>C, 3212A>T, 3213G>C, 3215A>C, 3216C>G, 3217T>A, 3218T>A, 3219C>G, 3220C>T, 3221C>A, 3222T>A, 3223A>G, 3224C>G, 3226G>A, 3232C>T, 3233C>G, 3234T>A, 3235G>A, 3238G>A, 3239A>T, 3241C>T, 3242C>T, 3245G>A, 3246A>G, 3247A>G, 3248C>A, 3249G>T, 3253T>A, 3254T>C, 3256C>G, 3259A>G, 3261G>C, 3263C>T, 3265T>G, 3268A>G, 3271A>T, 3272A>G, 3273G>A, 3274T>G, 3277C>G, 3280C>G, 3281A>T, 3283A>T, 3284A>G, 3285T>C, 3286T>A, 3287C>A, 3289A>G, 3290A>T, 3292G>T, 3300C>G, 3307C>T, 3309T>G, 3311A>T, 3312A>T, 3314C>A, 3315T>A, 3316C>T, 3320A>G, 3321C>T, 3325T>C, 3327A>T, 3334T>G, 3337C>T, 3338A>G, 3340C>A, 3341A>G, 3343A>T, 3344A>T, 3345G>C, 3346C>G, 3347A>G, 3349G>T, 3352C>T, 3355T>G, 3362C>G, 3363C>T, 3364T>G, 3365A>G, 3370T>G, 3371G>A, 3373T>G, 3376G>A, 3378T>C, 3380T>G, 3381C>A, 3382C>G, 3385T>A, 3386A>G, 3388T>C, 3389C>A, 3390A>G, 3394A>T, 3396A>G, 3397T>G, 3399T>C, 3400G>A, 3402T>G, 3403T>G, 3406A>T, 3407A>T, 3408A>T, 3409G>A, 3410A>T, 3412C>T, 3415T>G, 3417A>C, 3418G>T, 3421A>T, 3424A>T, 3426A>G, 3428C>A, 3429A>G, 3430A>C, 3433T>C, 3436A>G, 3439C>A, 3440C>T, 3442T>G, 3443C>G, 3444T>C, 3445C>A, 3451T>C, 3454C>T, 3455C>A, 3457A>G, 3459A>G, 3464A>G, 3466T>A, 3467C>G, 3468C>A, 3471A>G, 3476G>T, 3478A>T, 3479C>A, 3480G>T, 3481A>T, 3482C>A, 3484C>T, 3486C>G, 3487A>T, 3489A>G, 3490A>G, 3493C>A, 3498C>T, 3499A>G, 3500C>G, 3502G>T, 3504G>T, 3505C>G, 3506T>C, 3508A>G, 3514A>G, 3516G>A, 3517T>C, 3518_3520delAGT, 3523A>C, 3524G>T, 3526A>C, 3529T>A, 3530C>T, 3531T>G, 3532T>G, 3533A>T, 3534G>C, 3536C>G, 3537C>A, 3538C>A, 3539G>A, 3540A>T, 3541A>T, 3542T>G, 3543A>C, 3545G>A, 3546T>C, 3547A>T, 3548A>C, 3550T>A, 3551G>C, 3557G>C, 3559A>T, 3560C>A, 3564G>T, 3565T>G, 3568A>G, 3569A>G, 3570C>T, 3571T>A, 3572C>G, 3573T>C, 3574G>A, 3580C>A, 3581A>T, 3582A>C, 3583C>T, 3585A>C, 3586C>T, 3589A>T, 3590A>G, 3592A>C, 3593T>C, 3596C>G, 3597A>C, 3600A>T, 3601C>A, 3604A>T, 3605G>A, 3607C>T, 3609T>A, 3611A>T, 3615G>T, 3616A>T, 3617G>C, 3618A>C, 3619A>T, 3623A>G, 3624A>T, 3625C>T, 3626C>G, 3628C>T, 3629A>G, 3630C>A, 3637C>T								

CDS

	Begin	End	Coverage	Score	Concordance	Matches	Identities	I/D/M/F*	Stop Codons
D1R33_gp2	156	445	26.3%	856	43.8%	279 (97.9%)	121 (42.5%)	0/6/1/0	0
Protein mutations:	D156K (2771G>A 2773C>G), I158R (2778T>G 2779C>G), T159K (2781C>A 2782G>A), K160E (2783A>G), D163Q (2792G>C 2794C>G), I166V (2801A>G 2803T>C), R168Q (2807A>C 2808G>A), D171H (2816G>C), A173P (2822G>C), W174Y (2826G>A 2827G>C), I178V (2837A>G 2839A>G), W179L (2841G>T), V180L (2843G>T 2845T>A), P182del (2849_2851delCCC), I185K (2859T>A 2860C>A), A187G (2865C>G 2866T>G), S188T (2867T>A), G189_K192del (2870_2881delGGGAAACAAAAG), L195F (2888C>T), V196Y (2891G>T 2892T>A 2893A>T), V197I (2894G>A), F199Y (2901T>A), K201D (2906A>G 2908G>T), E204K (2915G>A), K205L (2918A>T 2919A>T), D208K (2927G>A 2929T>A), Y211F (2937A>T 2938C>T), P212T (2939C>A 2941G>A), N215I (2949A>T), S217E (2954A>G 2955G>A 2956T>G), D218E (2959C>G), V219L (2960G>T), K222E (2969A>G 2971G>A), K225G (2978A>G 2979A>G 2980G>T), C226S (2982G>C), Y228I (2987T>A 2988A>T), T231K (2997C>A 2998C>G), A235R (3008G>A 3009C>G), F238Y (3018T>A), Y239H (3020T>C), V241I (3026G>A 3028G>T), E242R (3029G>A 3030A>G), D244T (3035G>A 3036A>C 3037C>A), P245K (3038C>A 3039C>A 3040T>G), Q246E (3041C>G 3043A>G), I248V (3047A>G 3049A>C), S249Q (3050T>C 3051C>A), A252T (3059G>A 3061G>A), N254R (3066A>G 3067C>G), V255T (3068G>A 3069T>C 3070A>T), E256H (3071G>C 3073A>T), H257S (3074C>T 3075A>C 3076C>T), F260Y (3084T>A), F262Y (3090T>A), R264V (3095C>G 3096G>T), M267F (3104A>T 3106G>T), K270T (3114A>C 3115A>T), S272A (3119T>G), T275S (3129C>G), R285Q (3158A>C 3159G>A 3160A>G), G286P (3161G>C 3162G>C), L287F (3164C>T), Q288L (3168A>T 3169A>C), N289D (3170A>G 3172T>C), N290I (3174A>T 3175C>A), I291F (3176A>T 3178C>T), C292V (3179T>G 3180G>T 3181T>G), L293V (3182C>G 3184C>A), Y295F (3189A>T), L296I (3191C>A), I299V (3200A>G 3202T>A), I300L (3203A>C 3205T>G), V301I (3206G>A 3208C>A), T304R (3215A>C 3216C>G 3217T>A), L306K (3221C>A 3222T>A 3223A>G), Q307E (3224C>G 3226G>A), L310E (3233C>G 3234T>A 3235G>A), N312Y (3239A>T 3241C>T), E314R (3245G>A 3246A>G 3247A>G), R315I (3248C>A 3249G>T), F317L (3254T>C 3256C>G), R319T (3261G>C), E322D (3271A>T), S323E (3272A>G 3273G>A 3274T>G), N324K (3277C>G), F325L (3280C>G), K326Y (3281A>T 3283A>T), I327A (3284A>G 3285T>C 3286T>A), Q328K (3287C>A 3289A>G), M329F (3290A>T 3292G>T), S332C (3300C>G), L335W (3309T>G), K336L (3311A>T 3312A>T), L337N (3314C>A 3315T>A 3316C>T), T339V (3320A>G 3321C>T), Y341F (3327A>T), I345V (3338A>G 3340C>A), I346V (3341A>G 3343A>T), R348G (3347A>G 3349G>T), P353V (3362C>G 3363C>T 3364T>G), N354D (3365A>G), D356K (3371G>A 3373T>G), I358T (3378T>C), S359E (3380T>G 3381C>A 3382C>G), I361V (3386A>G 3388T>C), Q362R (3389C>A 3390A>G), K363N (3394A>T), Y364W (3396A>G 3397T>G), L365P (3399T>C 3400G>A), I366R (3402T>G 3403T>G), K368L (3407A>T 3408A>T 3409G>A), T369S (3410A>T 3412C>T), K371T (3417A>C 3418G>T), E372D (3421A>T), K374R (3426A>G), Q375S (3428C>A 3429A>G 3430A>C), L380A (3443C>G 3444T>C 3445C>A), K385R (3459A>G), I387V (3464A>G 3466T>A), P388E (3467C>G 3468C>A), D389G (3471A>G), A391S (3476G>T 3478A>T), R392I (3479C>A 3480G>T 3481A>T), L393I (3482C>A 3484C>T), T394S (3486C>G 3487A>T), K395R (3489A>G 3490A>G), T398M (3498C>T 3499A>G), Q399D (3500C>G 3502G>T), C400L (3504G>T 3505C>G), G404D (3516G>A 3517T>C), S405del (3518_3520delAGT), K406N (3523A>C), V407F (3524G>T 3526A>C), L409W (3530C>T 3531T>G 3532T>G), P411E (3536C>G 3537C>A 3538C>A), E412I (3539G>A 3540A>T 3541A>T), Y413A (3542T>G 3543A>C), V414T (3545G>A 3546T>C 3547A>T), N415Q (3548A>C 3550T>A), A416P (3551G>C), E418H (3557G>C 3559A>T), H419N (3560C>A), C420L (3564G>T 3565T>G), T422V (3569A>G 3570C>T 3571T>A), L423A (3572C>G 3573T>C 3574G>A), N426S (3581A>T 3582A>C 3583C>T), D427A (3585A>C 3586C>T), I429V (3590A>G 3592A>C), Q431A (3596C>G 3597A>C), Y432L (3600A>T 3601C>A), D434N (3605G>A 3607C>T), F435Y (3609T>A), T436S (3611A>T), R437I (3615G>T 3616A>T), E438P (3617G>C 3618A>C 3619A>T), N440V (3623A>G 3624A>T 3625C>T), L441V (3626C>G 3628C>T), T442E (3629A>G 3630C>A)								

	Begin	End	Coverage	Score	Concordance	Matches	Identities	I/D/M/F*	Stop Codons
**NT**	**2769**	**3638**	**11.5%**	**11**	**0.7%**	**842 (97.9%)**	**435 (50.6%)**	**0/18**	
Codon mutations:	AGG155.AG (2769G>A), GAC156AAG (2771G>A 2773C>G), ATC158AGG (2778T>G 2779C>G), ACG159AAA (2781C>A 2782G>A), AAA160GAA (2783A>G), TTG162CTA (2789T>C 2791G>A), GAC163CAG (2792G>C 2794C>G), ATT166GTC (2801A>G 2803T>C), AGA168CAA (2807A>C 2808G>A), TCA170AGT (2813T>A 2814C>G 2815A>T), GAC171CAC (2816G>C), GCA173CCA (2822G>C), TGG174TAC (2826G>A 2827G>C), AGC175TCC (2828A>T 2829G>C), TCA176TCT (2833A>T), CCC177CCA (2836C>A), ATA178GTG (2837A>G 2839A>G), TGG179TTG (2841G>T), GTT180TTA (2843G>T 2845T>A), CCC182del (2849_2851delCCC), ATC185AAA (2859T>A 2860C>A), GAC186GAT (2863C>T), GCT187GGG (2865C>G 2866T>G), TCT188ACT (2867T>A), GGG189_AAG192del (2870_2881delGGGAAACAAAAG), CGT194AGA (2885C>A 2887T>A), CTC195TTC (2888C>T), GTA196TAT (2891G>T 2892T>A 2893A>T), GTT197ATT (2894G>A), TTC199TAC (2901T>A), CGT200AGA (2903C>A 2905T>A), AAG201GAT (2906A>G 2908G>T), AAC203AAT (2914C>T), GAG204AAG (2915G>A), AAG205TTG (2918A>T 2919A>T), GAT208AAA (2927G>A 2929T>A), GAC209GAT (2932C>T), TAC211TTT (2937A>T 2938C>T), CCG212ACA (2939C>A 2941G>A), ATA213ATT (2944A>T), CCA214CCC (2947A>C), AAC215ATC (2949A>T), ATA216ATT (2953A>T), AGT217GAG (2954A>G 2955G>A 2956T>G), GAC218GAG (2959C>G), GTA219TTA (2960G>T), CTT220CTG (2965T>G), GAC221GAT (2968C>T), AAG222GAA (2969A>G 2971G>A), TTA223TTG (2974A>G), GGT224GGA (2977T>A), AAG225GGT (2978A>G 2979A>G 2980G>T), TGC226TCC (2982G>C), CAA227CAG (2986A>G), TAC228ATC (2987T>A 2988A>T), ACC231AAG (2997C>A 2998C>G), TTA232CTT (2999T>C 3001A>T), TTG234CTG (3005T>C), GCA235AGA (3008G>A 3009C>G), AGT236TCA (3011A>T 3012G>C 3013T>A), GGG237GGT (3016G>T), TTT238TAT (3018T>A), TAT239CAT (3020T>C), CAG240CAA (3025G>A), GTG241ATT (3026G>A 3028G>T), GAG242AGG (3029G>A 3030A>G), GAC244ACA (3035G>A 3036A>C 3037C>A), CCT245AAG (3038C>A 3039C>A 3040T>G), CAA246GAG (3041C>G 3043A>G), ATA248GTC (3047A>G 3049A>C), TCG249CAG (3050T>C 3051C>A), ACC251ACT (3058C>T), GCG252ACA (3059G>A 3061G>A), AAC254AGG (3066A>G 3067C>G), GTA255ACT (3068G>A 3069T>C 3070A>T), GAA256CAT (3071G>C 3073A>T), CAC257TCT (3074C>T 3075A>C 3076C>T), GGG258GGC (3079G>C), CAT259CAC (3082T>C), TTT260TAT (3084T>A), GAA261GAG (3088A>G), TTC262TAC (3090T>A), CTT263CTA (3094T>A), CGA264GTA (3095C>G 3096G>T), CCT266CCC (3103T>C), ATG267TTT (3104A>T 3106G>T), GGA268GGC (3109A>C), TTA269CTC (3110T>C 3112A>C), AAA270ACT (3114A>C 3115A>T), AAC271AAT (3118C>T), TCA272GCA (3119T>G), CCA273CCT (3124A>T), TCT274TCA (3127T>A), ACT275AGT (3129C>G), AGA278T.T (3137A>T 3139A>T), GTT279C.A (3140G>C 3142T>A), GAC281A.T (3146G>A 3148C>T), AAT282C.G (3149A>C 3151T>G), GTC283..T (3154C>T), CTA284T.T (3155C>T 3157A>T), AGA285CAG (3158A>C 3159G>A 3160A>G), GGT286CCT (3161G>C 3162G>C), CTC287TTC (3164C>T), CAA288CTC (3168A>T 3169A>C), AAT289GAC (3170A>G 3172T>C), AAC290ATA (3174A>T 3175C>A), ATC291TTT (3176A>T 3178C>T), TGT292GTG (3179T>G 3180G>T 3181T>G), CTC293GTA (3182C>G 3184C>A), TAC295TTC (3189A>T), CTT296ATT (3191C>A), ATT299GTA (3200A>G 3202T>A), ATT300CTG (3203A>C 3205T>G), GTC301ATA (3206G>A 3208C>A), TAT302TAC (3211T>C), AGT303TCT (3212A>T 3213G>C), ACT304CGA (3215A>C 3216C>G 3217T>A), TCC305AGT (3218T>A 3219C>G 3220C>T), CTA306AAG (3221C>A 3222T>A 3223A>G), CAG307GAA (3224C>G 3226G>A), CAC309CAT (3232C>T), CTG310GAA (3233C>G 3234T>A 3235G>A), GAG311GAA (3238G>A), AAC312TAT (3239A>T 3241C>T), CTG313TTG (3242C>T), GAA314AGG (3245G>A 3246A>G 3247A>G), CGA315ATA (3248C>A 3249G>T), GTT316GTA (3253T>A), TTC317CTG (3254T>C 3256C>G), CAA318CAG (3259A>G), AGA319ACA (3261G>C), CTT320TTG (3263C>T 3265T>G), AGA321AGG (3268A>G), GAA322GAT (3271A>T), AGT323GAG (3272A>G 3273G>A 3274T>G), AAC324AAG (3277C>G), TTC325TTG (3280C>G), AAA326TAT (3281A>T 3283A>T), ATT327GCA (3284A>G 3285T>C 3286T>A), CAA328AAG (3287C>A 3289A>G), ATG329TTT (3290A>T 3292G>T), TCC332TGC (3300C>G), TTC334TTT (3307C>T), TTG335TGG (3309T>G), AAG336TTG (3311A>T 3312A>T), CTC337AAT (3314C>A 3315T>A 3316C>T), ACT339GTT (3320A>G 3321C>T), GCT340GCC (3325T>C), TAT341TTT (3327A>T), GGT343GGG (3334T>G), CAC344CAT (3337C>T), ATC345GTA (3338A>G 3340C>A), ATA346GTT (3341A>G 3343A>T), AGC347TCG (3344A>T 3345G>C 3346C>G), AGG348GGT (3347A>G 3349G>T), GAC349GAT (3352C>T), GGT350GGG (3355T>G), CCT353GTG (3362C>G 3363C>T 3364T>G), AAC354GAC (3365A>G), CCT355CCG (3370T>G), GAT356AAG (3371G>A 3373T>G), AAG357AAA (3376G>A), ATT358ACT (3378T>C), TCC359GAG (3380T>G 3381C>A 3382C>G), GCT360GCA (3385T>A), ATT361GTC (3386A>G 3388T>C), CAA362AGA (3389C>A 3390A>G), AAA363AAT (3394A>T), TAT364TGG (3396A>G 3397T>G), CTG365CCA (3399T>C 3400G>A), ATT366AGG (3402T>G 3403T>G), CCA367CCT (3406A>T), AAG368TTA (3407A>T 3408A>T 3409G>A), ACC369TCT (3410A>T 3412C>T), CCT370CCG (3415T>G), AAG371ACT (3417A>C 3418G>T), GAA372GAT (3421A>T), ATA373ATT (3424A>T), AAA374AGA (3426A>G), CAA375AGC (3428C>A 3429A>G 3430A>C), TTT376TTC (3433T>C), TTA377TTG (3436A>G), GGC378GGA (3439C>A), CTT379TTG (3440C>T 3442T>G), CTC380GCA (3443C>G 3444T>C 3445C>A), TAT382TAC (3451T>C), TAC383TAT (3454C>T), CGA384AGG (3455C>A 3457A>G), AAA385AGA (3459A>G), ATT387GTA (3464A>G 3466T>A), CCA388GAA (3467C>G 3468C>A), GAT389GGT (3471A>G), GCA391TCT (3476G>T 3478A>T), CGA392ATT (3479C>A 3480G>T 3481A>T), CTC393ATT (3482C>A 3484C>T), ACA394AGT (3486C>G 3487A>T), AAA395AGG (3489A>G 3490A>G), CCC396CCA (3493C>A), ACA398ATG (3498C>T 3499A>G), CAG399GAT (3500C>G 3502G>T), TGC400TTG (3504G>T 3505C>G), TTA401CTG (3506T>C 3508A>G), AAA403AAG (3514A>G), GGT404GAC (3516G>A 3517T>C), AGT405del (3518_3520delAGT), AAA406AAC (3523A>C), GTA407TTC (3524G>T 3526A>C), ACT408ACA (3529T>A), CTT409TGG (3530C>T 3531T>G 3532T>G), AGT410TCT (3533A>T 3534G>C), CCC411GAA (3536C>G 3537C>A 3538C>A), GAA412ATT (3539G>A 3540A>T 3541A>T), TAT413GCT (3542T>G 3543A>C), GTA414ACT (3545G>A 3546T>C 3547A>T), AAT415CAA (3548A>C 3550T>A), GCT416CCT (3551G>C), GAA418CAT (3557G>C 3559A>T), CAC419AAC (3560C>A), TGT420TTG (3564G>T 3565T>G), AAA421AAG (3568A>G), ACT422GTA (3569A>G 3570C>T 3571T>A), CTG423GCA (3572C>G 3573T>C 3574G>A), ACC425ACA (3580C>A), AAC426TCT (3581A>T 3582A>C 3583C>T), GAC427GCT (3585A>C 3586C>T), CCA428CCT (3589A>T), ATA429GTC (3590A>G 3592A>C), TTA430CTA (3593T>C), CAA431GCA (3596C>G 3597A>C), TAC432TTA (3600A>T 3601C>A), CCA433CCT (3604A>T), GAC434AAT (3605G>A 3607C>T), TTT435TAT (3609T>A), ACC436TCC (3611A>T), AGA437ATT (3615G>T 3616A>T), GAA438CCT (3617G>C 3618A>C 3619A>T), AAC440GTT (3623A>G 3624A>T 3625C>T), CTC441GTT (3626C>G 3628C>T), ACG442GAG (3629A>G 3630C>A), GAC444GAT (3637C>T)								

Proteins

	Begin	End	Coverage	Score	Concordance	Matches	Identities	I/D/M/F*	Stop Codons
ORF B (YP_009507248.1)	156	445	26.3%	856	43.8%	279 (97.9%)	121 (42.5%)	0/6/1/0	0
Protein mutations:	D156K (2771G>A 2773C>G), I158R (2778T>G 2779C>G), T159K (2781C>A 2782G>A), K160E (2783A>G), D163Q (2792G>C 2794C>G), I166V (2801A>G 2803T>C), R168Q (2807A>C 2808G>A), D171H (2816G>C), A173P (2822G>C), W174Y (2826G>A 2827G>C), I178V (2837A>G 2839A>G), W179L (2841G>T), V180L (2843G>T 2845T>A), P182del (2849_2851delCCC), I185K (2859T>A 2860C>A), A187G (2865C>G 2866T>G), S188T (2867T>A), G189_K192del (2870_2881delGGGAAACAAAAG), L195F (2888C>T), V196Y (2891G>T 2892T>A 2893A>T), V197I (2894G>A), F199Y (2901T>A), K201D (2906A>G 2908G>T), E204K (2915G>A), K205L (2918A>T 2919A>T), D208K (2927G>A 2929T>A), Y211F (2937A>T 2938C>T), P212T (2939C>A 2941G>A), N215I (2949A>T), S217E (2954A>G 2955G>A 2956T>G), D218E (2959C>G), V219L (2960G>T), K222E (2969A>G 2971G>A), K225G (2978A>G 2979A>G 2980G>T), C226S (2982G>C), Y228I (2987T>A 2988A>T), T231K (2997C>A 2998C>G), A235R (3008G>A 3009C>G), F238Y (3018T>A), Y239H (3020T>C), V241I (3026G>A 3028G>T), E242R (3029G>A 3030A>G), D244T (3035G>A 3036A>C 3037C>A), P245K (3038C>A 3039C>A 3040T>G), Q246E (3041C>G 3043A>G), I248V (3047A>G 3049A>C), S249Q (3050T>C 3051C>A), A252T (3059G>A 3061G>A), N254R (3066A>G 3067C>G), V255T (3068G>A 3069T>C 3070A>T), E256H (3071G>C 3073A>T), H257S (3074C>T 3075A>C 3076C>T), F260Y (3084T>A), F262Y (3090T>A), R264V (3095C>G 3096G>T), M267F (3104A>T 3106G>T), K270T (3114A>C 3115A>T), S272A (3119T>G), T275S (3129C>G), R285Q (3158A>C 3159G>A 3160A>G), G286P (3161G>C 3162G>C), L287F (3164C>T), Q288L (3168A>T 3169A>C), N289D (3170A>G 3172T>C), N290I (3174A>T 3175C>A), I291F (3176A>T 3178C>T), C292V (3179T>G 3180G>T 3181T>G), L293V (3182C>G 3184C>A), Y295F (3189A>T), L296I (3191C>A), I299V (3200A>G 3202T>A), I300L (3203A>C 3205T>G), V301I (3206G>A 3208C>A), T304R (3215A>C 3216C>G 3217T>A), L306K (3221C>A 3222T>A 3223A>G), Q307E (3224C>G 3226G>A), L310E (3233C>G 3234T>A 3235G>A), N312Y (3239A>T 3241C>T), E314R (3245G>A 3246A>G 3247A>G), R315I (3248C>A 3249G>T), F317L (3254T>C 3256C>G), R319T (3261G>C), E322D (3271A>T), S323E (3272A>G 3273G>A 3274T>G), N324K (3277C>G), F325L (3280C>G), K326Y (3281A>T 3283A>T), I327A (3284A>G 3285T>C 3286T>A), Q328K (3287C>A 3289A>G), M329F (3290A>T 3292G>T), S332C (3300C>G), L335W (3309T>G), K336L (3311A>T 3312A>T), L337N (3314C>A 3315T>A 3316C>T), T339V (3320A>G 3321C>T), Y341F (3327A>T), I345V (3338A>G 3340C>A), I346V (3341A>G 3343A>T), R348G (3347A>G 3349G>T), P353V (3362C>G 3363C>T 3364T>G), N354D (3365A>G), D356K (3371G>A 3373T>G), I358T (3378T>C), S359E (3380T>G 3381C>A 3382C>G), I361V (3386A>G 3388T>C), Q362R (3389C>A 3390A>G), K363N (3394A>T), Y364W (3396A>G 3397T>G), L365P (3399T>C 3400G>A), I366R (3402T>G 3403T>G), K368L (3407A>T 3408A>T 3409G>A), T369S (3410A>T 3412C>T), K371T (3417A>C 3418G>T), E372D (3421A>T), K374R (3426A>G), Q375S (3428C>A 3429A>G 3430A>C), L380A (3443C>G 3444T>C 3445C>A), K385R (3459A>G), I387V (3464A>G 3466T>A), P388E (3467C>G 3468C>A), D389G (3471A>G), A391S (3476G>T 3478A>T), R392I (3479C>A 3480G>T 3481A>T), L393I (3482C>A 3484C>T), T394S (3486C>G 3487A>T), K395R (3489A>G 3490A>G), T398M (3498C>T 3499A>G), Q399D (3500C>G 3502G>T), C400L (3504G>T 3505C>G), G404D (3516G>A 3517T>C), S405del (3518_3520delAGT), K406N (3523A>C), V407F (3524G>T 3526A>C), L409W (3530C>T 3531T>G 3532T>G), P411E (3536C>G 3537C>A 3538C>A), E412I (3539G>A 3540A>T 3541A>T), Y413A (3542T>G 3543A>C), V414T (3545G>A 3546T>C 3547A>T), N415Q (3548A>C 3550T>A), A416P (3551G>C), E418H (3557G>C 3559A>T), H419N (3560C>A), C420L (3564G>T 3565T>G), T422V (3569A>G 3570C>T 3571T>A), L423A (3572C>G 3573T>C 3574G>A), N426S (3581A>T 3582A>C 3583C>T), D427A (3585A>C 3586C>T), I429V (3590A>G 3592A>C), Q431A (3596C>G 3597A>C), Y432L (3600A>T 3601C>A), D434N (3605G>A 3607C>T), F435Y (3609T>A), T436S (3611A>T), R437I (3615G>T 3616A>T), E438P (3617G>C 3618A>C 3619A>T), N440V (3623A>G 3624A>T 3625C>T), L441V (3626C>G 3628C>T), T442E (3629A>G 3630C>A)								

	Begin	End	Coverage	Score	Concordance	Matches	Identities	I/D/M/F*	Stop Codons
**NT**	**2769**	**3638**	**11.5%**	**11**	**0.7%**	**842 (97.9%)**	**435 (50.6%)**	**0/18**	
Codon mutations:	AGG155.AG (2769G>A), GAC156AAG (2771G>A 2773C>G), ATC158AGG (2778T>G 2779C>G), ACG159AAA (2781C>A 2782G>A), AAA160GAA (2783A>G), TTG162CTA (2789T>C 2791G>A), GAC163CAG (2792G>C 2794C>G), ATT166GTC (2801A>G 2803T>C), AGA168CAA (2807A>C 2808G>A), TCA170AGT (2813T>A 2814C>G 2815A>T), GAC171CAC (2816G>C), GCA173CCA (2822G>C), TGG174TAC (2826G>A 2827G>C), AGC175TCC (2828A>T 2829G>C), TCA176TCT (2833A>T), CCC177CCA (2836C>A), ATA178GTG (2837A>G 2839A>G), TGG179TTG (2841G>T), GTT180TTA (2843G>T 2845T>A), CCC182del (2849_2851delCCC), ATC185AAA (2859T>A 2860C>A), GAC186GAT (2863C>T), GCT187GGG (2865C>G 2866T>G), TCT188ACT (2867T>A), GGG189_AAG192del (2870_2881delGGGAAACAAAAG), CGT194AGA (2885C>A 2887T>A), CTC195TTC (2888C>T), GTA196TAT (2891G>T 2892T>A 2893A>T), GTT197ATT (2894G>A), TTC199TAC (2901T>A), CGT200AGA (2903C>A 2905T>A), AAG201GAT (2906A>G 2908G>T), AAC203AAT (2914C>T), GAG204AAG (2915G>A), AAG205TTG (2918A>T 2919A>T), GAT208AAA (2927G>A 2929T>A), GAC209GAT (2932C>T), TAC211TTT (2937A>T 2938C>T), CCG212ACA (2939C>A 2941G>A), ATA213ATT (2944A>T), CCA214CCC (2947A>C), AAC215ATC (2949A>T), ATA216ATT (2953A>T), AGT217GAG (2954A>G 2955G>A 2956T>G), GAC218GAG (2959C>G), GTA219TTA (2960G>T), CTT220CTG (2965T>G), GAC221GAT (2968C>T), AAG222GAA (2969A>G 2971G>A), TTA223TTG (2974A>G), GGT224GGA (2977T>A), AAG225GGT (2978A>G 2979A>G 2980G>T), TGC226TCC (2982G>C), CAA227CAG (2986A>G), TAC228ATC (2987T>A 2988A>T), ACC231AAG (2997C>A 2998C>G), TTA232CTT (2999T>C 3001A>T), TTG234CTG (3005T>C), GCA235AGA (3008G>A 3009C>G), AGT236TCA (3011A>T 3012G>C 3013T>A), GGG237GGT (3016G>T), TTT238TAT (3018T>A), TAT239CAT (3020T>C), CAG240CAA (3025G>A), GTG241ATT (3026G>A 3028G>T), GAG242AGG (3029G>A 3030A>G), GAC244ACA (3035G>A 3036A>C 3037C>A), CCT245AAG (3038C>A 3039C>A 3040T>G), CAA246GAG (3041C>G 3043A>G), ATA248GTC (3047A>G 3049A>C), TCG249CAG (3050T>C 3051C>A), ACC251ACT (3058C>T), GCG252ACA (3059G>A 3061G>A), AAC254AGG (3066A>G 3067C>G), GTA255ACT (3068G>A 3069T>C 3070A>T), GAA256CAT (3071G>C 3073A>T), CAC257TCT (3074C>T 3075A>C 3076C>T), GGG258GGC (3079G>C), CAT259CAC (3082T>C), TTT260TAT (3084T>A), GAA261GAG (3088A>G), TTC262TAC (3090T>A), CTT263CTA (3094T>A), CGA264GTA (3095C>G 3096G>T), CCT266CCC (3103T>C), ATG267TTT (3104A>T 3106G>T), GGA268GGC (3109A>C), TTA269CTC (3110T>C 3112A>C), AAA270ACT (3114A>C 3115A>T), AAC271AAT (3118C>T), TCA272GCA (3119T>G), CCA273CCT (3124A>T), TCT274TCA (3127T>A), ACT275AGT (3129C>G), AGA278T.T (3137A>T 3139A>T), GTT279C.A (3140G>C 3142T>A), GAC281A.T (3146G>A 3148C>T), AAT282C.G (3149A>C 3151T>G), GTC283..T (3154C>T), CTA284T.T (3155C>T 3157A>T), AGA285CAG (3158A>C 3159G>A 3160A>G), GGT286CCT (3161G>C 3162G>C), CTC287TTC (3164C>T), CAA288CTC (3168A>T 3169A>C), AAT289GAC (3170A>G 3172T>C), AAC290ATA (3174A>T 3175C>A), ATC291TTT (3176A>T 3178C>T), TGT292GTG (3179T>G 3180G>T 3181T>G), CTC293GTA (3182C>G 3184C>A), TAC295TTC (3189A>T), CTT296ATT (3191C>A), ATT299GTA (3200A>G 3202T>A), ATT300CTG (3203A>C 3205T>G), GTC301ATA (3206G>A 3208C>A), TAT302TAC (3211T>C), AGT303TCT (3212A>T 3213G>C), ACT304CGA (3215A>C 3216C>G 3217T>A), TCC305AGT (3218T>A 3219C>G 3220C>T), CTA306AAG (3221C>A 3222T>A 3223A>G), CAG307GAA (3224C>G 3226G>A), CAC309CAT (3232C>T), CTG310GAA (3233C>G 3234T>A 3235G>A), GAG311GAA (3238G>A), AAC312TAT (3239A>T 3241C>T), CTG313TTG (3242C>T), GAA314AGG (3245G>A 3246A>G 3247A>G), CGA315ATA (3248C>A 3249G>T), GTT316GTA (3253T>A), TTC317CTG (3254T>C 3256C>G), CAA318CAG (3259A>G), AGA319ACA (3261G>C), CTT320TTG (3263C>T 3265T>G), AGA321AGG (3268A>G), GAA322GAT (3271A>T), AGT323GAG (3272A>G 3273G>A 3274T>G), AAC324AAG (3277C>G), TTC325TTG (3280C>G), AAA326TAT (3281A>T 3283A>T), ATT327GCA (3284A>G 3285T>C 3286T>A), CAA328AAG (3287C>A 3289A>G), ATG329TTT (3290A>T 3292G>T), TCC332TGC (3300C>G), TTC334TTT (3307C>T), TTG335TGG (3309T>G), AAG336TTG (3311A>T 3312A>T), CTC337AAT (3314C>A 3315T>A 3316C>T), ACT339GTT (3320A>G 3321C>T), GCT340GCC (3325T>C), TAT341TTT (3327A>T), GGT343GGG (3334T>G), CAC344CAT (3337C>T), ATC345GTA (3338A>G 3340C>A), ATA346GTT (3341A>G 3343A>T), AGC347TCG (3344A>T 3345G>C 3346C>G), AGG348GGT (3347A>G 3349G>T), GAC349GAT (3352C>T), GGT350GGG (3355T>G), CCT353GTG (3362C>G 3363C>T 3364T>G), AAC354GAC (3365A>G), CCT355CCG (3370T>G), GAT356AAG (3371G>A 3373T>G), AAG357AAA (3376G>A), ATT358ACT (3378T>C), TCC359GAG (3380T>G 3381C>A 3382C>G), GCT360GCA (3385T>A), ATT361GTC (3386A>G 3388T>C), CAA362AGA (3389C>A 3390A>G), AAA363AAT (3394A>T), TAT364TGG (3396A>G 3397T>G), CTG365CCA (3399T>C 3400G>A), ATT366AGG (3402T>G 3403T>G), CCA367CCT (3406A>T), AAG368TTA (3407A>T 3408A>T 3409G>A), ACC369TCT (3410A>T 3412C>T), CCT370CCG (3415T>G), AAG371ACT (3417A>C 3418G>T), GAA372GAT (3421A>T), ATA373ATT (3424A>T), AAA374AGA (3426A>G), CAA375AGC (3428C>A 3429A>G 3430A>C), TTT376TTC (3433T>C), TTA377TTG (3436A>G), GGC378GGA (3439C>A), CTT379TTG (3440C>T 3442T>G), CTC380GCA (3443C>G 3444T>C 3445C>A), TAT382TAC (3451T>C), TAC383TAT (3454C>T), CGA384AGG (3455C>A 3457A>G), AAA385AGA (3459A>G), ATT387GTA (3464A>G 3466T>A), CCA388GAA (3467C>G 3468C>A), GAT389GGT (3471A>G), GCA391TCT (3476G>T 3478A>T), CGA392ATT (3479C>A 3480G>T 3481A>T), CTC393ATT (3482C>A 3484C>T), ACA394AGT (3486C>G 3487A>T), AAA395AGG (3489A>G 3490A>G), CCC396CCA (3493C>A), ACA398ATG (3498C>T 3499A>G), CAG399GAT (3500C>G 3502G>T), TGC400TTG (3504G>T 3505C>G), TTA401CTG (3506T>C 3508A>G), AAA403AAG (3514A>G), GGT404GAC (3516G>A 3517T>C), AGT405del (3518_3520delAGT), AAA406AAC (3523A>C), GTA407TTC (3524G>T 3526A>C), ACT408ACA (3529T>A), CTT409TGG (3530C>T 3531T>G 3532T>G), AGT410TCT (3533A>T 3534G>C), CCC411GAA (3536C>G 3537C>A 3538C>A), GAA412ATT (3539G>A 3540A>T 3541A>T), TAT413GCT (3542T>G 3543A>C), GTA414ACT (3545G>A 3546T>C 3547A>T), AAT415CAA (3548A>C 3550T>A), GCT416CCT (3551G>C), GAA418CAT (3557G>C 3559A>T), CAC419AAC (3560C>A), TGT420TTG (3564G>T 3565T>G), AAA421AAG (3568A>G), ACT422GTA (3569A>G 3570C>T 3571T>A), CTG423GCA (3572C>G 3573T>C 3574G>A), ACC425ACA (3580C>A), AAC426TCT (3581A>T 3582A>C 3583C>T), GAC427GCT (3585A>C 3586C>T), CCA428CCT (3589A>T), ATA429GTC (3590A>G 3592A>C), TTA430CTA (3593T>C), CAA431GCA (3596C>G 3597A>C), TAC432TTA (3600A>T 3601C>A), CCA433CCT (3604A>T), GAC434AAT (3605G>A 3607C>T), TTT435TAT (3609T>A), ACC436TCC (3611A>T), AGA437ATT (3615G>T 3616A>T), GAA438CCT (3617G>C 3618A>C 3619A>T), AAC440GTT (3623A>G 3624A>T 3625C>T), CTC441GTT (3626C>G 3628C>T), ACG442GAG (3629A>G 3630C>A), GAC444GAT (3637C>T)								

*: Inserts / Deletes / Misaligned / Frameshifts

Analysis details

This analysis was performed with panviral2.64

NGS Details (UN70): Caulimovirus tesselloscrophulariae

Assembly

**Coverage Length**	583 (2 contig(s))
**Depth Of Coverage**	15.8
**Number Of Reads**	80
**Reads Per Million**	1.52 rpm (after QC)
**Ambiguities**	0
**Assembly Method**	de novo + reference guided assembly
**Consensus Caller**	Bcf Tools

Coverage Map

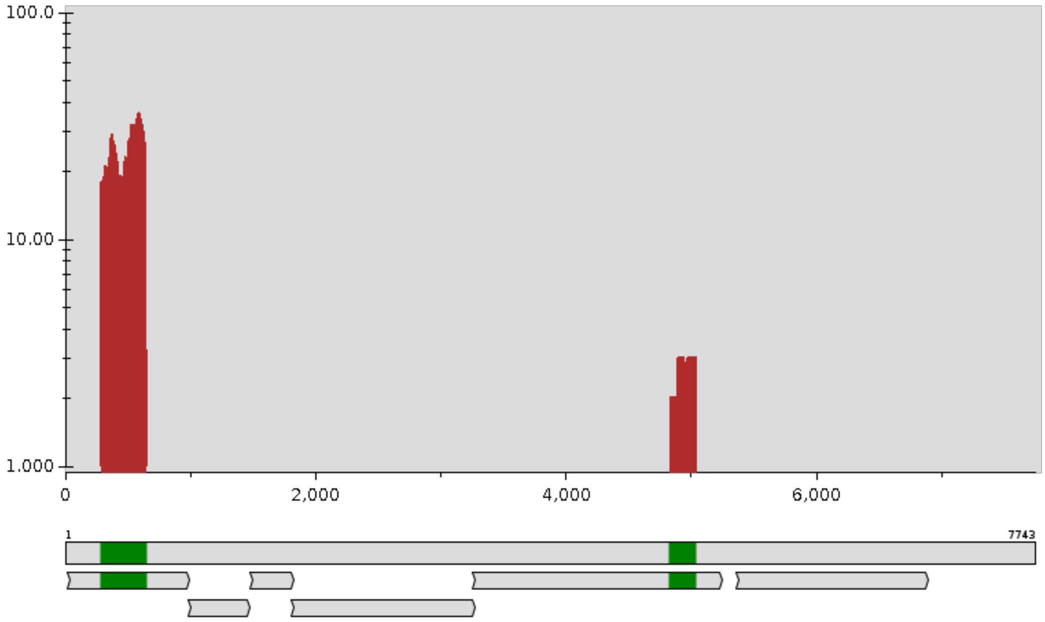

Assignment

**Type**	Caulimovirus tesselloscrophulariae (Taxonomy ID: 3047834)
**Reference Genome**	NC_003554.1
**NT Identity (%)**	59.0193
**AA Identity (%)**	46.3158
**Number Of Stop Codons**	0
**Number Of CDS**	6

Alignment

**Alignment Score**	176.0 (NT) + 588.0 (AA) = 764.0
**Concordance (%)**	32.9168

**Alignment Method**	Global, seeded, nucleotide + amino acids (AGA)

Genome Region

Sequence starts at position 286 and ends at position 5043 relative to NC_003554.1 reference sequence.

Alignment Detailed Statistics

	Begin	End	Coverage	Score	Concordance	Matches	Identities	I/D/M/F*	Stop Codons
**NT**	**286**	**5043**	**7.5%**	**176**	**15.8%**	**571 (97.9%)**	**337 (57.8%)**	**0/12**	
Mutations:	296C>A, 299G>C, 300T>A, 302T>A, 304T>A, 305C>T, 307T>A, 308C>A, 309C>A, 311C>T, 313T>A, 314A>T, 322G>A, 324A>C, 325G>A, 326G>A, 327A>T, 328A>G, 331A>T, 332C>A, 334A>T, 339A>T, 340G>C, 343G>A, 344A>C, 345T>A, 346G>A, 348A>G, 353G>C, 354A>C, 355T>A, 356C>G, 358A>C, 359T>A, 360C>G, 361A>C, 362G>A, 363T>A, 368A>C, 369G>A, 373A>G, 376C>G, 377T>A, 378C>A, 379A>T, 380A>T, 381T>A, 382G>T, 383A>G, 385C>T, 388C>T, 389C>T, 391T>G, 396C>G, 398G>A, 401A>C, 403G>A, 406T>C, 407C>A, 409A>G, 410C>G, 412T>G, 414C>A, 416G>T, 417C>T, 419C>A, 420A>C, 421A>T, 425C>A, 426G>A, 427G>A, 428C>G, 433A>T, 436T>A, 437G>A, 440A>T, 441C>G, 442G>T, 443T>C, 445A>T, 446G>A, 448T>A, 450A>T, 454G>A, 455G>A, 456C>A, 457A>C, 461A>T, 462T>C, 463C>T, 469T>A, 473A>T, 475C>T, 476G>A, 478C>T, 482C>A, 483G>C, 484A>T, 486A>G, 490T>A, 491A>G, 493T>A, 494C>A, 495T>A, 496G>T, 499C>T, 503G>A, 504C>T, 507C>T, 508A>T, 509A>G, 510G>A, 518C>T, 520C>A, 526T>C, 527G>A, 528G>C, 535T>A, 536A>C, 538G>T, 544C>A, 545G>T, 546T>A, 550C>T, 553T>C, 555A>G, 558T>A, 560G>T, 561C>G, 562A>T, 563T>A, 565G>A, 572C>A, 575A>G, 576G>A, 577C>T, 578A>G, 579A>C, 580G>T, 583T>C, 586A>T, 587G>A, 590A>G, 593A>G, 598A>G, 601C>T, 604T>G, 605A>C, 606T>A, 608C>T, 609A>T, 613A>G, 614T>G, 616T>C, 617G>A, 628C>T, 632A>T, 634G>C, 637A>G, 638A>C, 4825A>G, 4828T>A, 4834G>A, 4838T>A, 4839A>G, 4840C>A, 4844C>T, 4846T>A, 4847A>G, 4859C>A, 4861T>C, 4864C>A, 4865A>G, 4867T>A, 4868A>T, 4870T>A, 4871G>C, 4876A>T, 4882A>T, 4889A>T, 4890G>A, 4892T>C, 4893T>A, 4900C>A, 4902G>C, 4903T>A, 4904G>T, 4906C>A, 4907C>T, 4910A>C, 4913G>A, 4916_4924delAGAGCGCTT, 4927C>T, 4928G>C, 4929G>T, 4930T>A, 4931G>A, 4932T>A, 4937_4939delTTA, 4945C>T, 4948G>A, 4953G>C, 4955T>A, 4956C>G, 4957A>T, 4962G>C, 4966C>T, 4969A>T, 4970C>G, 4973G>A, 4980A>C, 4981G>T, 4983A>G, 4984T>G, 4987T>C, 4988C>T, 4989A>T, 4990C>A, 4992G>C, 4993C>A, 4999T>A, 5014A>T, 5015G>A, 5017C>A, 5018A>G, 5019A>T, 5020G>A, 5021C>A, 5022A>G, 5025T>G, 5026A>C, 5029C>A, 5030A>C, 5031C>G, 5032C>A, 5041A>T								

CDS

	Begin	End	Coverage	Score	Concordance	Matches	Identities	I/D/M/F*	Stop Codons
FMVgp1	92	212	37.3%	334	43.5%	121 (100%)	51 (42.1%)	0/0/0/0	0
Protein mutations:	Q95K (296C>A), V96H (299G>C 300T>A), Y97K (302T>A 304T>A), P99N (308C>A 309C>A), I101L (314A>T), Q104P (324A>C 325G>A), E105M (326G>A 327A>T 328A>G), Q107N (332C>A 334A>T), K109I (339A>T 340G>C), M111Q (344A>C 345T>A 346G>A), K112R (348A>G), D114P (353G>C 354A>C 355T>A), P115A (356C>G 358A>C), V117K (362G>A 363T>A), S119H (368A>C 369G>A), I121M (376C>G), S122N (377T>A 378C>A 379A>T), M123Y (380A>T 381T>A 382G>T), I124V (383A>G 385C>T), A128G (396C>G), V129I (398G>A), K130Q (401A>C 403G>A), L132M (407C>A 409A>G), L133V (410C>G 412T>G), T134K (414C>A), A135F (416G>T 417C>T), Q136T (419C>A 420A>C 421A>T), R138K (425C>A 426G>A 427G>A), Q139E (428C>G), D142N (437G>A), T143C (440A>T 441C>G 442G>T), S144P (443T>C 445A>T), V145I (446G>A 448T>A), K146I (450A>T), M147I (454G>A), A148N (455G>A 456C>A 457A>C), I150S (461A>T 462T>C 463C>T), D152E (469T>A), I154F (473A>T 475C>T), V155I (476G>A 478C>T), R157T (482C>A 483G>C 484A>T), K158R (486A>G), D159E (490T>A), S160G (491A>G 493T>A), L161N (494C>A 495T>A 496G>T), A164I (503G>A 504C>T), A165V (507C>T 508A>T), R166E (509A>G 510G>A), G172T (527G>A 528G>C), F174L (535T>A), M175L (536A>C 538G>T), V178Y (545G>T 546T>A), K181R (555A>G), F182Y (558T>A), A183C (560G>T 561C>G 562A>T), L184I (563T>A 565G>A), Q187K (572C>A), S188D (575A>G 576G>A 577C>T), K189A (578A>G 579A>C 580G>T), L191F (586A>T), D192N (587G>A), K193E (590A>G), T194A (593A>G), F197L (604T>G), I198H (605A>C 606T>A), H199F (608C>T 609A>T), F201V (614T>G 616T>C), E202K (617G>A), M207F (632A>T 634G>C), T209P (638A>C)								
Codon mutations:	CAA95AAA (296C>A), GTT96CAT (299G>C 300T>A), TAT97AAA (302T>A 304T>A), CTT98TTA (305C>T 307T>A), CCT99AAT (308C>A 309C>A), CTT100TTA (311C>T 313T>A), ATA101TTA (314A>T), AAG103AAA (322G>A), CAG104CCA (324A>C 325G>A), GAA105ATG (326G>A 327A>T 328A>G), ATA106ATT (331A>T), CAA107AAT (332C>A 334A>T), AAG109ATC (339A>T 340G>C), CTG110CTA (343G>A), ATG111CAA (344A>C 345T>A 346G>A), AAA112AGA (348A>G), GAT114CCA (353G>C 354A>C 355T>A), CCA115GCC (356C>G 358A>C), TCA116AGC (359T>A 360C>G 361A>C), GTA117AAA (362G>A 363T>A), AGT119CAT (368A>C 369G>A), AAA120AAG (373A>G), ATC121ATG (376C>G), TCA122AAT (377T>A 378C>A 379A>T), ATG123TAT (380A>T 381T>A 382G>T), ATC124GTT (383A>G 385C>T), CAC125CAT (388C>T), CTT126TTG (389C>T 391T>G), GCA128GGA (396C>G), GTA129ATA (398G>A), AAG130CAA (401A>C 403G>A), ATT131ATC (406T>C), CTA132ATG (407C>A 409A>G), CTT133GTG (410C>G 412T>G), ACA134AAA (414C>A), GCC135TTC (416G>T 417C>T), CAA136ACT (419C>A 420A>C 421A>T), CGG138AAA (425C>A 426G>A 427G>A), CAA139GAA (428C>G), GGA140GGT (433A>T), ATT141ATA (436T>A), GAT142AAT (437G>A), ACG143TGT (440A>T 441C>G 442G>T), TCA144CCT (443T>C 445A>T), GTT145ATA (446G>A 448T>A), AAA146ATA (450A>T), ATG147ATA (454G>A), GCA148AAC (455G>A 456C>A 457A>C), ATC150TCT (461A>T 462T>C 463C>T), GAT152GAA (469T>A), ATC154TTT (473A>T 475C>T), GTC155ATT (476G>A 478C>T), CGA157ACT (482C>A 483G>C 484A>T), AAA158AGA (486A>G), GAT159GAA (490T>A), AGT160GGA (491A>G 493T>A), CTG161AAT (494C>A 495T>A 496G>T), CTC162CTT (499C>T), GCA164ATA (503G>A 504C>T), GCA165GTT (507C>T 508A>T), AGA166GAA (509A>G 510G>A), CTC169TTA (518C>T 520C>A), TAT171TAC (526T>C), GGT172ACT (527G>A 528G>C), TTT174TTA (535T>A), ATG175CTT (536A>C 538G>T), ACC177ACA (544C>A), GTT178TAT (545G>T 546T>A), TAC179TAT (550C>T), CCT180CCC (553T>C), AAA181AGA (555A>G), TTT182TAT (558T>A), GCA183TGT (560G>T 561C>G 562A>T), TTG184ATA (563T>A 565G>A), CAA187AAA (572C>A), AGC188GAT (575A>G 576G>A 577C>T), AAG189GCT (578A>G 579A>C 580G>T), AAT190AAC (583T>C), TTA191TTT (586A>T), GAT192AAT (587G>A), AAA193GAA (590A>G), ACA194GCA (593A>G), TTA195TTG (598A>G), AGC196AGT (601C>T), TTT197TTG (604T>G), ATT198CAT (605A>C 606T>A), CAT199TTT (608C>T 609A>T), CAA200CAG (613A>G), TTT201GTC (614T>G 616T>C), GAA202AAA (617G>A), GAC205GAT (628C>T), ATG207TTC (632A>T 634G>C), AAA208AAG (637A>G), ACA209CCA (638A>C)								
FMVgp5	527	599	10.9%	254	55.5%	69 (94.5%)	37 (50.7%)	0/4/0/0	0
Protein mutations:	F527L (4828T>A), Y531R (4838T>A 4839A>G 4840C>A), P533S (4844C>T 4846T>A), K534E (4847A>G), H538N (4859C>A 4861T>C), I540V (4865A>G 4867T>A), I541L (4868A>T 4870T>A), E542Q (4871G>C), S548Y (4889A>T 4890G>A), F549H (4892T>C 4893T>A), G552A (4902G>C 4903T>A), V553L (4904G>T 4906C>A), K555Q (4910A>C), A556T (4913G>A), R557_L559del (4916_4924delAGAGCGCTT), G561L (4928G>C 4929G>T 4930T>A), V562N (4931G>A 4932T>A), L564del (4937_4939delTTA), S569T (4953G>C), S572T (4962G>C), K574N (4969A>T), Q575E (4970C>G), A576T (4973G>A), K578T (4980A>C 4981G>T), N579R (4983A>G 4984T>G), H581L (4988C>T 4989A>T 4990C>A), S582T (4992G>C 4993C>A), D584E (4999T>A), V590I (5015G>A 5017C>A), K591V (5018A>G 5019A>T 5020G>A), Q592R (5021C>A 5022A>G), V593G (5025T>G 5026A>C), T595R (5030A>C 5031C>G 5032C>A)								
Codon mutations:	TCA526..G (4825A>G), TTT527TTA (4828T>A), AAG529AAA (4834G>A), TAC531AGA (4838T>A 4839A>G 4840C>A), CCT533TCA (4844C>T 4846T>A), AAA534GAA (4847A>G), CAT538AAC (4859C>A 4861T>C), CTC539CTA (4864C>A), ATT540GTA (4865A>G 4867T>A), ATT541TTA (4868A>T 4870T>A), GAA542CAA (4871G>C), ACA543ACT (4876A>T), GCA545GCT (4882A>T), AGT548TAT (4889A>T 4890G>A), TTT549CAT (4892T>C 4893T>A), GGC551GGA (4900C>A), GGT552GCA (4902G>C 4903T>A), GTC553TTA (4904G>T 4906C>A), CTA554TTA (4907C>T), AAA555CAA (4910A>C), GCA556ACA (4913G>A), AGA557_CTT559del (4916_4924delAGAGCGCTT), GAC560GAT (4927C>T), GGT561CTA (4928G>C 4929G>T 4930T>A), GTT562AAT (4931G>A 4932T>A), TTA564del (4937_4939delTTA), TGC566TGT (4945C>T), AGG567AGA (4948G>A), AGT569ACT (4953G>C), TCA570AGT (4955T>A 4956C>G 4957A>T), AGC572ACC (4962G>C), TTC573TTT (4966C>T), AAA574AAT (4969A>T), CAA575GAA (4970C>G), GCA576ACA (4973G>A), AAG578ACT (4980A>C 4981G>T), AAT579AGG (4983A>G 4984T>G), TAT580TAC (4987T>C), CAC581TTA (4988C>T 4989A>T 4990C>A), AGC582ACA (4992G>C 4993C>A), GAT584GAA (4999T>A), GCA589GCT (5014A>T), GTC590ATA (5015G>A 5017C>A), AAG591GTA (5018A>G 5019A>T 5020G>A), CAA592AGA (5021C>A 5022A>G), GTA593GGC (5025T>G 5026A>C), ATC594ATA (5029C>A), ACC595CGA (5030A>C 5031C>G 5032C>A), TCA598TCT (5041A>T)								

Proteins

	Begin	End	Coverage	Score	Concordance	Matches	Identities	I/D/M/F*	Stop Codons
hypothetical protein (NP_619544.1)	92	212	37.3%	334	43.5%	121 (100%)	51 (42.1%)	0/0/0/0	0
Protein mutations:	Q95K (296C>A), V96H (299G>C 300T>A), Y97K (302T>A 304T>A), P99N (308C>A 309C>A), I101L (314A>T), Q104P (324A>C 325G>A), E105M (326G>A 327A>T 328A>G), Q107N (332C>A 334A>T), K109I (339A>T 340G>C), M111Q (344A>C 345T>A 346G>A), K112R (348A>G), D114P (353G>C 354A>C 355T>A), P115A (356C>G 358A>C), V117K (362G>A 363T>A), S119H (368A>C 369G>A), I121M (376C>G), S122N (377T>A 378C>A 379A>T), M123Y (380A>T 381T>A 382G>T), I124V (383A>G 385C>T), A128G (396C>G), V129I (398G>A), K130Q (401A>C 403G>A), L132M (407C>A 409A>G), L133V (410C>G 412T>G), T134K (414C>A), A135F (416G>T 417C>T), Q136T (419C>A 420A>C 421A>T), R138K (425C>A 426G>A 427G>A), Q139E (428C>G), D142N (437G>A), T143C (440A>T 441C>G 442G>T), S144P (443T>C 445A>T), V145I (446G>A 448T>A), K146I (450A>T), M147I (454G>A), A148N (455G>A 456C>A 457A>C), I150S (461A>T 462T>C 463C>T), D152E (469T>A), I154F (473A>T 475C>T), V155I (476G>A 478C>T), R157T (482C>A 483G>C 484A>T), K158R (486A>G), D159E (490T>A), S160G (491A>G 493T>A), L161N (494C>A 495T>A 496G>T), A164I (503G>A 504C>T), A165V (507C>T 508A>T), R166E (509A>G 510G>A), G172T (527G>A 528G>C), F174L (535T>A), M175L (536A>C 538G>T), V178Y (545G>T 546T>A), K181R (555A>G), F182Y (558T>A), A183C (560G>T 561C>G 562A>T), L184I (563T>A 565G>A), Q187K (572C>A), S188D (575A>G 576G>A 577C>T), K189A (578A>G 579A>C 580G>T), L191F (586A>T), D192N (587G>A), K193E (590A>G), T194A (593A>G), F197L (604T>G), I198H (605A>C 606T>A), H199F (608C>T 609A>T), F201V (614T>G 616T>C), E202K (617G>A), M207F (632A>T 634G>C), T209P (638A>C)								

	Begin	End	Coverage	Score	Concordance	Matches	Identities	I/D/M/F*	Stop Codons
**NT**	**286**	**5043**	**7.5%**	**176**	**15.8%**	**571 (97.9%)**	**337 (57.8%)**	**0/12**	
Codon mutations:	CAA95AAA (296C>A), GTT96CAT (299G>C 300T>A), TAT97AAA (302T>A 304T>A), CTT98TTA (305C>T 307T>A), CCT99AAT (308C>A 309C>A), CTT100TTA (311C>T 313T>A), ATA101TTA (314A>T), AAG103AAA (322G>A), CAG104CCA (324A>C 325G>A), GAA105ATG (326G>A 327A>T 328A>G), ATA106ATT (331A>T), CAA107AAT (332C>A 334A>T), AAG109ATC (339A>T 340G>C), CTG110CTA (343G>A), ATG111CAA (344A>C 345T>A 346G>A), AAA112AGA (348A>G), GAT114CCA (353G>C 354A>C 355T>A), CCA115GCC (356C>G 358A>C), TCA116AGC (359T>A 360C>G 361A>C), GTA117AAA (362G>A 363T>A), AGT119CAT (368A>C 369G>A), AAA120AAG (373A>G), ATC121ATG (376C>G), TCA122AAT (377T>A 378C>A 379A>T), ATG123TAT (380A>T 381T>A 382G>T), ATC124GTT (383A>G 385C>T), CAC125CAT (388C>T), CTT126TTG (389C>T 391T>G), GCA128GGA (396C>G), GTA129ATA (398G>A), AAG130CAA (401A>C 403G>A), ATT131ATC (406T>C), CTA132ATG (407C>A 409A>G), CTT133GTG (410C>G 412T>G), ACA134AAA (414C>A), GCC135TTC (416G>T 417C>T), CAA136ACT (419C>A 420A>C 421A>T), CGG138AAA (425C>A 426G>A 427G>A), CAA139GAA (428C>G), GGA140GGT (433A>T), ATT141ATA (436T>A), GAT142AAT (437G>A), ACG143TGT (440A>T 441C>G 442G>T), TCA144CCT (443T>C 445A>T), GTT145ATA (446G>A 448T>A), AAA146ATA (450A>T), ATG147ATA (454G>A), GCA148AAC (455G>A 456C>A 457A>C), ATC150TCT (461A>T 462T>C 463C>T), GAT152GAA (469T>A), ATC154TTT (473A>T 475C>T), GTC155ATT (476G>A 478C>T), CGA157ACT (482C>A 483G>C 484A>T), AAA158AGA (486A>G), GAT159GAA (490T>A), AGT160GGA (491A>G 493T>A), CTG161AAT (494C>A 495T>A 496G>T), CTC162CTT (499C>T), GCA164ATA (503G>A 504C>T), GCA165GTT (507C>T 508A>T), AGA166GAA (509A>G 510G>A), CTC169TTA (518C>T 520C>A), TAT171TAC (526T>C), GGT172ACT (527G>A 528G>C), TTT174TTA (535T>A), ATG175CTT (536A>C 538G>T), ACC177ACA (544C>A), GTT178TAT (545G>T 546T>A), TAC179TAT (550C>T), CCT180CCC (553T>C), AAA181AGA (555A>G), TTT182TAT (558T>A), GCA183TGT (560G>T 561C>G 562A>T), TTG184ATA (563T>A 565G>A), CAA187AAA (572C>A), AGC188GAT (575A>G 576G>A 577C>T), AAG189GCT (578A>G 579A>C 580G>T), AAT190AAC (583T>C), TTA191TTT (586A>T), GAT192AAT (587G>A), AAA193GAA (590A>G), ACA194GCA (593A>G), TTA195TTG (598A>G), AGC196AGT (601C>T), TTT197TTG (604T>G), ATT198CAT (605A>C 606T>A), CAT199TTT (608C>T 609A>T), CAA200CAG (613A>G), TTT201GTC (614T>G 616T>C), GAA202AAA (617G>A), GAC205GAT (628C>T), ATG207TTC (632A>T 634G>C), AAA208AAG (637A>G), ACA209CCA (638A>C)								
hypothetical protein (NP_619548.1)	527	599	10.9%	254	55.5%	69 (94.5%)	37 (50.7%)	0/4/0/0	0
Protein mutations:	F527L (4828T>A), Y531R (4838T>A 4839A>G 4840C>A), P533S (4844C>T 4846T>A), K534E (4847A>G), H538N (4859C>A 4861T>C), I540V (4865A>G 4867T>A), I541L (4868A>T 4870T>A), E542Q (4871G>C), S548Y (4889A>T 4890G>A), F549H (4892T>C 4893T>A), G552A (4902G>C 4903T>A), V553L (4904G>T 4906C>A), K555Q (4910A>C), A556T (4913G>A), R557_L559del (4916_4924delAGAGCGCTT), G561L (4928G>C 4929G>T 4930T>A), V562N (4931G>A 4932T>A), L564del (4937_4939delTTA), S569T (4953G>C), S572T (4962G>C), K574N (4969A>T), Q575E (4970C>G), A576T (4973G>A), K578T (4980A>C 4981G>T), N579R (4983A>G 4984T>G), H581L (4988C>T 4989A>T 4990C>A), S582T (4992G>C 4993C>A), D584E (4999T>A), V590I (5015G>A 5017C>A), K591V (5018A>G 5019A>T 5020G>A), Q592R (5021C>A 5022A>G), V593G (5025T>G 5026A>C), T595R (5030A>C 5031C>G 5032C>A)								
Codon mutations:	TCA526..G (4825A>G), TTT527TTA (4828T>A), AAG529AAA (4834G>A), TAC531AGA (4838T>A 4839A>G 4840C>A), CCT533TCA (4844C>T 4846T>A), AAA534GAA (4847A>G), CAT538AAC (4859C>A 4861T>C), CTC539CTA (4864C>A), ATT540GTA (4865A>G 4867T>A), ATT541TTA (4868A>T 4870T>A), GAA542CAA (4871G>C), ACA543ACT (4876A>T), GCA545GCT (4882A>T), AGT548TAT (4889A>T 4890G>A), TTT549CAT (4892T>C 4893T>A), GGC551GGA (4900C>A), GGT552GCA (4902G>C 4903T>A), GTC553TTA (4904G>T 4906C>A), CTA554TTA (4907C>T), AAA555CAA (4910A>C), GCA556ACA (4913G>A), AGA557_CTT559del (4916_4924delAGAGCGCTT), GAC560GAT (4927C>T), GGT561CTA (4928G>C 4929G>T 4930T>A), GTT562AAT (4931G>A 4932T>A), TTA564del (4937_4939delTTA), TGC566TGT (4945C>T), AGG567AGA (4948G>A), AGT569ACT (4953G>C), TCA570AGT (4955T>A 4956C>G 4957A>T), AGC572ACC (4962G>C), TTC573TTT (4966C>T), AAA574AAT (4969A>T), CAA575GAA (4970C>G), GCA576ACA (4973G>A), AAG578ACT (4980A>C 4981G>T), AAT579AGG (4983A>G 4984T>G), TAT580TAC (4987T>C), CAC581TTA (4988C>T 4989A>T 4990C>A), AGC582ACA (4992G>C 4993C>A), GAT584GAA (4999T>A), GCA589GCT (5014A>T), GTC590ATA (5015G>A 5017C>A), AAG591GTA (5018A>G 5019A>T 5020G>A), CAA592AGA (5021C>A 5022A>G), GTA593GGC (5025T>G 5026A>C), ATC594ATA (5029C>A), ACC595CGA (5030A>C 5031C>G 5032C>A), TCA598TCT (5041A>T)								

*: Inserts / Deletes / Misaligned / Frameshifts

Analysis details

This analysis was performed with panviral2.64

NGS Details (UN70): Tomato necrotic dwarf virus (segment RNA1)

Assembly

**Coverage Length**	435 (1 contig(s))
**Depth Of Coverage**	21.2
**Number Of Reads**	79
**Reads Per Million**	1.50 rpm (after QC)
**Ambiguities**	0
**Assembly Method**	de novo + reference guided assembly
**Consensus Caller**	Bcf Tools

Coverage Map

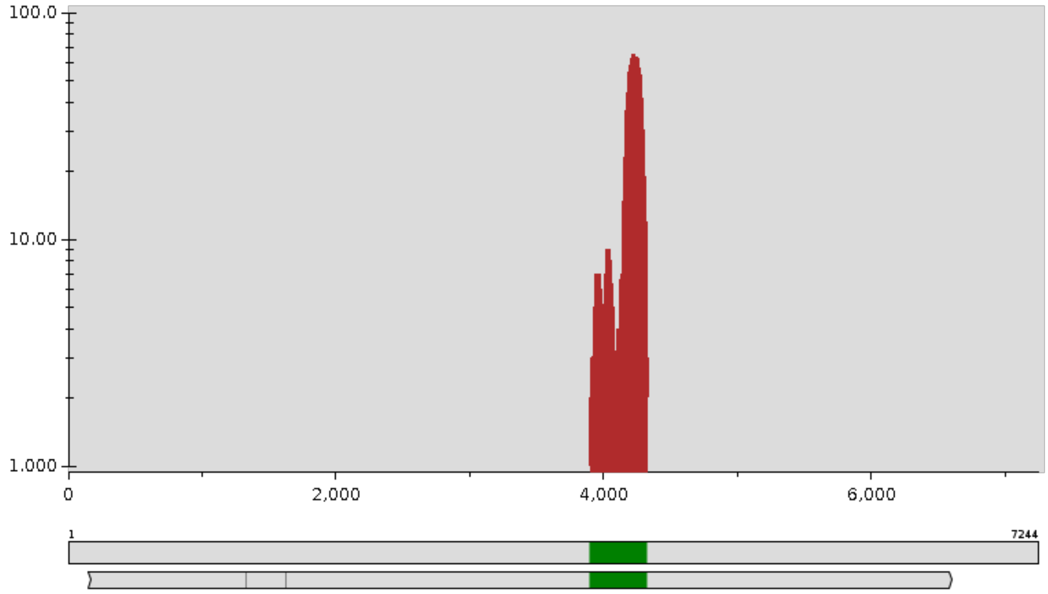

Assignment

**Type**	Tomato necrotic dwarf virus (Taxonomy ID: 1481465)
**Reference Genome**	NC_027926.1
**NT Identity (%)**	69.4253
**AA Identity (%)**	74.4828
**Number Of Stop Codons**	0
**Number Of CDS**	1

Alignment

**Alignment Score**	338.0 (NT) + 788.0 (AA) = 1126.0
**Concordance (%)**	59.4823

**Alignment Method**	Global, seeded, nucleotide + amino acids (AGA)

Genome Region

Sequence starts at position 3895 and ends at position 4329 relative to NC_027926.1 reference sequence.

Alignment Detailed Statistics

	Begin	End	Coverage	Score	Concordance	Matches	Identities	I/D/M/F*	Stop Codons
**NT**	**3895**	**4329**	**6.0%**	**338**	**38.9%**	**435 (100%)**	**302 (69.4%)**	**0/0**	
Mutations:	3901A>T, 3906C>A, 3907A>C, 3909G>T, 3912A>T, 3913A>T, 3914T>C, 3915G>T, 3921G>A, 3922C>A, 3927G>A, 3933C>G, 3935T>A, 3937C>G, 3939A>C, 3940A>C, 3941C>A, 3943G>C, 3944G>C, 3946G>A, 3948A>C, 3949G>T, 3950G>C, 3951T>G, 3952G>C, 3954T>G, 3956A>G, 3957A>G, 3960A>T, 3961G>C, 3962A>T, 3963G>T, 3966T>C, 3968G>A, 3973G>A, 3981A>T, 3984G>C, 3990A>T, 3991G>C, 3993T>A, 3999G>A, 4002A>G, 4003C>T, 4009A>T, 4010T>C, 4011T>G, 4014A>G, 4015A>G, 4017T>A, 4023G>A, 4028T>C, 4030C>T, 4031A>T, 4035G>T, 4038T>C, 4041A>C, 4042A>G, 4047C>A, 4050A>C, 4053A>G, 4059C>G, 4062G>A, 4068G>A, 4071C>G, 4072C>T, 4074T>G, 4076C>A, 4077T>A, 4083A>T, 4093G>C, 4104A>G, 4107A>C, 4108A>C, 4110A>C, 4111T>C, 4113A>G, 4116T>C, 4119G>A, 4122A>C, 4137C>T, 4141A>C, 4149C>G, 4150A>C, 4161T>C, 4173C>T, 4176C>T, 4177A>T, 4178C>G, 4179C>T, 4188A>T, 4189T>G, 4190T>C, 4197T>C, 4200C>A, 4201A>C, 4206A>T, 4209T>C, 4215T>A, 4218A>T, 4221A>C, 4222G>A, 4230C>G, 4233T>C, 4234T>C, 4235C>A, 4243T>A, 4245T>A, 4246C>G, 4248T>G, 4251T>G, 4254C>T, 4256A>G, 4257C>T, 4259A>G, 4263C>T, 4267G>T, 4269T>A, 4279A>G, 4280C>A, 4281T>G, 4284G>A, 4287T>C, 4293T>C, 4302A>G, 4305T>C, 4311C>T, 4314G>A, 4315C>A, 4317C>A, 4323A>C, 4324G>A, 4326G>C, 4329C>T								

CDS

	Begin	End	Coverage	Score	Concordance	Matches	Identities	I/D/M/F*	Stop Codons
APL33_sRA11gp1	1249	1393	6.7%	788	77.0%	145 (100%)	108 (74.5%)	0/0/0/0	0
Protein mutations:	M1251L (3901A>T), M1253L (3907A>C 3909G>T), M1255S (3913A>T 3914T>C 3915G>T), V1262E (3935T>A), P1263A (3937C>G 3939A>C), T1264Q (3940A>C 3941C>A), G1265P (3943G>C 3944G>C), E1266N (3946G>A 3948A>C), G1267S (3949G>T 3950G>C 3951T>G), D1268Q (3952G>C 3954T>G), K1269R (3956A>G 3957A>G), E1271L (3961G>C 3962A>T 3963G>T), R1273K (3968G>A), D1275N (3973G>A), E1280D (3990A>T), A1281P (3991G>C 3993T>A), I1287S (4009A>T 4010T>C 4011T>G), I1289V (4015A>G 4017T>A), I1293T (4028T>C), H1294F (4030C>T 4031A>T), I1298V (4042A>G), P1309Q (4076C>A 4077T>A), E1311D (4083A>T), G1315R (4093G>C), M1331L (4141A>C), T1343C (4177A>T 4178C>G 4179C>T), L1347A (4189T>G 4190T>C), I1351L (4201A>C), D1358N (4222G>A), S1362H (4234T>C 4235C>A), S1365T (4243T>A 4245T>A), L1366V (4246C>G 4248T>G), N1369S (4256A>G 4257C>T), N1370S (4259A>G), A1373S (4267G>T 4269T>A), T1377E (4279A>G 4280C>A 4281T>G), V1392I (4324G>A 4326G>C)								
Codon mutations:	ATG1251TTG (3901A>T), GGC1252GGA (3906C>A), ATG1253CTT (3907A>C 3909G>T), GGA1254GGT (3912A>T), ATG1255TCT (3913A>T 3914T>C 3915G>T), AAG1257AAA (3921G>A), CGG1258AGG (3922C>A), GAG1259GAA (3927G>A), GTC1261GTG (3933C>G), GTA1262GAA (3935T>A), CCA1263GCC (3937C>G 3939A>C), ACA1264CAA (3940A>C 3941C>A), GGA1265CCA (3943G>C 3944G>C), GAA1266AAC (3946G>A 3948A>C), GGT1267TCG (3949G>T 3950G>C 3951T>G), GAT1268CAG (3952G>C 3954T>G), AAA1269AGG (3956A>G 3957A>G), GTA1270GTT (3960A>T), GAG1271CTT (3961G>C 3962A>T 3963G>T), CTT1272CTC (3966T>C), AGA1273AAA (3968G>A), GAC1275AAC (3973G>A), CCA1277CCT (3981A>T), GTG1278GTC (3984G>C), GAA1280GAT (3990A>T), GCT1281CCA (3991G>C 3993T>A), GAG1283GAA (3999G>A), GAA1284GAG (4002A>G), CTG1285TTG (4003C>T), ATT1287TCG (4009A>T 4010T>C 4011T>G), CAA1288CAG (4014A>G), ATT1289GTA (4015A>G 4017T>A), CAG1291CAA (4023G>A), ATA1293ACA (4028T>C), CAT1294TTT (4030C>T 4031A>T), GTG1295GTT (4035G>T), GAT1296GAC (4038T>C), CTA1297CTC (4041A>C), ATC1298GTC (4042A>G), ACC1299ACA (4047C>A), ACA1300ACC (4050A>C), CAA1301CAG (4053A>G), GCC1303GCG (4059C>G), AAG1304AAA (4062G>A), GAG1306GAA (4068G>A), CGC1307CGG (4071C>G), CTT1308TTG (4072C>T 4074T>G), CCT1309CAA (4076C>A 4077T>A), GAA1311GAT (4083A>T), GGG1315CGG (4093G>C), AAA1318AAG (4104A>G), ACA1319ACC (4107A>C), AGA1320CGC (4108A>C 4110A>C), TTA1321CTG (4111T>C 4113A>G), TTT1322TTC (4116T>C), GAG1323GAA (4119G>A), ATA1324ATC (4122A>C), TAC1329TAT (4137C>T), ATG1331CTG (4141A>C), GTC1333GTG (4149C>G), AGG1334CGG (4150A>C), TTT1337TTC (4161T>C), TCC1341TCT (4173C>T), GCC1342GCT (4176C>T), ACC1343TGT (4177A>T 4178C>G 4179C>T), GCA1346GCT (4188A>T), TTA1347GCA (4189T>G 4190T>C), AAT1349AAC (4197T>C), GTC1350GTA (4200C>A), ATC1351CTC (4201A>C), CCA1352CCT (4206A>T), TGT1353TGC (4209T>C), GTT1355GTA (4215T>A), GGA1356GGT (4218A>T), ATA1357ATC (4221A>C), GAT1358AAT (4222G>A), ACC1360ACG (4230C>G), AGT1361AGC (4233T>C), TCT1362CAT (4234T>C 4235C>A), TCT1365ACA (4243T>A 4245T>A), CTT1366GTG (4246C>G 4248T>G), CTT1367CTG (4251T>G), GCC1368GCT (4254C>T), AAC1369AGT (4256A>G 4257C>T), AAC1370AGC (4259A>G), TTC1371TTT (4263C>T), GCT1373TCA (4267G>T 4269T>A), ACT1377GAG (4279A>G 4280C>A 4281T>G), GGG1378GGA (4284G>A), TTT1379TTC (4287T>C), GCT1381GCC (4293T>C), TCA1384TCG (4302A>G), AGT1385AGC (4305T>C), GAC1387GAT (4311C>T), GGG1388GGA (4314G>A), CGC1389AGA (4315C>A 4317C>A), CCA1391CCC (4323A>C), GTG1392ATC (4324G>A 4326G>C), TTC1393TTT (4329C>T)								

Proteins

	Begin	End	Coverage	Score	Concordance	Matches	Identities	I/D/M/F*	Stop Codons
polyprotein (YP_009165993.1)	1249	1393	6.7%	788	77.0%	145 (100%)	108 (74.5%)	0/0/0/0	0
Protein mutations:	M1251L (3901A>T), M1253L (3907A>C 3909G>T), M1255S (3913A>T 3914T>C 3915G>T), V1262E (3935T>A), P1263A (3937C>G 3939A>C), T1264Q (3940A>C 3941C>A), G1265P (3943G>C 3944G>C), E1266N (3946G>A 3948A>C), G1267S (3949G>T 3950G>C 3951T>G), D1268Q (3952G>C 3954T>G), K1269R (3956A>G 3957A>G), E1271L (3961G>C 3962A>T 3963G>T), R1273K (3968G>A), D1275N (3973G>A), E1280D (3990A>T), A1281P (3991G>C 3993T>A), I1287S (4009A>T 4010T>C 4011T>G), I1289V (4015A>G 4017T>A), I1293T (4028T>C), H1294F (4030C>T 4031A>T), I1298V (4042A>G), P1309Q (4076C>A 4077T>A), E1311D (4083A>T), G1315R (4093G>C), M1331L (4141A>C), T1343C (4177A>T 4178C>G 4179C>T), L1347A (4189T>G 4190T>C), I1351L (4201A>C), D1358N (4222G>A), S1362H (4234T>C 4235C>A), S1365T (4243T>A 4245T>A), L1366V (4246C>G 4248T>G), N1369S (4256A>G 4257C>T), N1370S (4259A>G), A1373S (4267G>T 4269T>A), T1377E (4279A>G 4280C>A 4281T>G), V1392I (4324G>A 4326G>C)								
Codon mutations:	ATG1251TTG (3901A>T), GGC1252GGA (3906C>A), ATG1253CTT (3907A>C 3909G>T), GGA1254GGT (3912A>T), ATG1255TCT (3913A>T 3914T>C 3915G>T), AAG1257AAA (3921G>A), CGG1258AGG (3922C>A), GAG1259GAA (3927G>A), GTC1261GTG (3933C>G), GTA1262GAA (3935T>A), CCA1263GCC (3937C>G 3939A>C), ACA1264CAA (3940A>C 3941C>A), GGA1265CCA (3943G>C 3944G>C), GAA1266AAC (3946G>A 3948A>C), GGT1267TCG (3949G>T 3950G>C 3951T>G), GAT1268CAG (3952G>C 3954T>G), AAA1269AGG (3956A>G 3957A>G), GTA1270GTT (3960A>T), GAG1271CTT (3961G>C 3962A>T 3963G>T), CTT1272CTC (3966T>C), AGA1273AAA (3968G>A), GAC1275AAC (3973G>A), CCA1277CCT (3981A>T), GTG1278GTC (3984G>C), GAA1280GAT (3990A>T), GCT1281CCA (3991G>C 3993T>A), GAG1283GAA (3999G>A), GAA1284GAG (4002A>G), CTG1285TTG (4003C>T), ATT1287TCG (4009A>T 4010T>C 4011T>G), CAA1288CAG (4014A>G), ATT1289GTA (4015A>G 4017T>A), CAG1291CAA (4023G>A), ATA1293ACA (4028T>C), CAT1294TTT (4030C>T 4031A>T), GTG1295GTT (4035G>T), GAT1296GAC (4038T>C), CTA1297CTC (4041A>C), ATC1298GTC (4042A>G), ACC1299ACA (4047C>A), ACA1300ACC (4050A>C), CAA1301CAG (4053A>G), GCC1303GCG (4059C>G), AAG1304AAA (4062G>A), GAG1306GAA (4068G>A), CGC1307CGG (4071C>G), CTT1308TTG (4072C>T 4074T>G), CCT1309CAA (4076C>A 4077T>A), GAA1311GAT (4083A>T), GGG1315CGG (4093G>C), AAA1318AAG (4104A>G), ACA1319ACC (4107A>C), AGA1320CGC (4108A>C 4110A>C), TTA1321CTG (4111T>C 4113A>G), TTT1322TTC (4116T>C), GAG1323GAA (4119G>A), ATA1324ATC (4122A>C), TAC1329TAT (4137C>T), ATG1331CTG (4141A>C), GTC1333GTG (4149C>G), AGG1334CGG (4150A>C), TTT1337TTC (4161T>C), TCC1341TCT (4173C>T), GCC1342GCT (4176C>T), ACC1343TGT (4177A>T 4178C>G 4179C>T), GCA1346GCT (4188A>T), TTA1347GCA (4189T>G 4190T>C), AAT1349AAC (4197T>C), GTC1350GTA (4200C>A), ATC1351CTC (4201A>C), CCA1352CCT (4206A>T), TGT1353TGC (4209T>C), GTT1355GTA (4215T>A), GGA1356GGT (4218A>T), ATA1357ATC (4221A>C), GAT1358AAT (4222G>A), ACC1360ACG (4230C>G), AGT1361AGC (4233T>C), TCT1362CAT (4234T>C 4235C>A), TCT1365ACA (4243T>A 4245T>A), CTT1366GTG (4246C>G 4248T>G), CTT1367CTG (4251T>G), GCC1368GCT (4254C>T), AAC1369AGT (4256A>G 4257C>T), AAC1370AGC (4259A>G), TTC1371TTT (4263C>T), GCT1373TCA (4267G>T 4269T>A), ACT1377GAG (4279A>G 4280C>A 4281T>G), GGG1378GGA (4284G>A), TTT1379TTC (4287T>C), GCT1381GCC (4293T>C), TCA1384TCG (4302A>G), AGT1385AGC (4305T>C), GAC1387GAT (4311C>T), GGG1388GGA (4314G>A), CGC1389AGA (4315C>A 4317C>A), CCA1391CCC (4323A>C), GTG1392ATC (4324G>A 4326G>C), TTC1393TTT (4329C>T)								

*: Inserts / Deletes / Misaligned / Frameshifts

Analysis details

This analysis was performed with panviral2.64

NGS Details (UN70): Badnavirus maculaucubae

Assembly

**Coverage Length**	321 (1 contig(s))
**Depth Of Coverage**	24.5
**Number Of Reads**	77
**Reads Per Million**	1.46 rpm (after QC)
**Ambiguities**	0
**Assembly Method**	de novo + reference guided assembly
**Consensus Caller**	Bcf Tools

Coverage Map

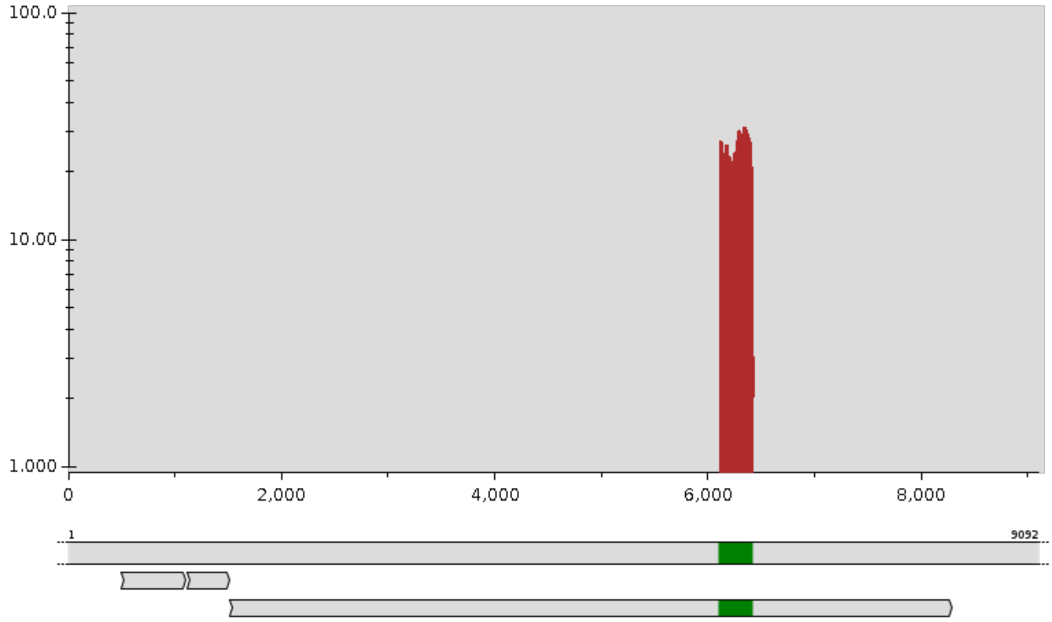

Assignment

**Type**	Badnavirus maculaucubae (Taxonomy ID: 3051986)
**Reference Genome**	NC_076606.1
**NT Identity (%)**	54.6828
**AA Identity (%)**	45.045
**Number Of Stop Codons**	1
**Number Of CDS**	3

Alignment

**Alignment Score**	45.0 (NT) + 220.0 (AA) = 265.0
**Concordance (%)**	22.0497

Alignment Method	Global, seeded, nucleotide + amino acids (AGA)

Genome Region

Sequence starts at position 6102 and ends at position 6422 relative to NC_076606.1 reference sequence.

Alignment Detailed Statistics

	Begin	End	Coverage	Score	Concordance	Matches	Identities	I/D/M/F*	Stop Codons
**NT**	**6102**	**6422**	**3.5%**	**45**	**7.2%**	**319 (95.8%)**	**181 (54.4%)**	**12/2**	
Mutations:	6102C>T, 6105A>G, 6106T>A, 6112T>C, 6114G>T, 6115A>C, 6116A>G, 6120T>C, 6122G>A, 6125T>A, 6132G>A, 6133G>T, 6136G>A, 6137C>G, 6138A>G, 6139A>G, 6141G>A, 6142G>A, 6147A>G, 6149C>G, 6150A>T, 6151_6152delAG, 6153C>G, 6156T>C, 6159A>G, 6160T>A, 6161G>A, 6165G>T, 6171C>T, 6173T>G, 6179C>A, 6181G>T, 6184G>A, 6186A>T, 6188T>A, 6189A>C, 6191A>T, 6192C>T, 6197G>T, 6198G>T, 6199C>T, 6201A>G, 6204A>T, 6207G>A, 6208C>T, 6210A>T, 6216T>A, 6221A>C, 6222G>A, 6228A>T, 6231T>C, 6235G>A, 6236T>C, 6237G>T, 6240C>T, 6241C>A, 6242A>T, 6243A>G, 6244A>G, 6245G>A, 6246G>C, 6247A>T, 6248A>T, 6253G>A, 6257A>G, 6258C>G, 6259T>G, 6260G>T, 6261T>A, 6266G>A, 6268G>C, 6269G>C, 6270T>G, 6271A>T, 6272C>A, 6273A>T, 6274G>T, 6275A>T, 6276A>G, 6279T>C, 6279_6280insATG, 6282C>T, 6283A>G, 6285C>A, 6287C>T, 6288T>G, 6291T>C, 6292T>C, 6293A>T, 6297T>A, 6303T>C, 6306T>A, 6307C>T, 6312A>T, 6314T>A, 6319G>C, 6320A>G, 6321A>G, 6325C>G, 6326T>C, 6327T>C, 6328C>A, 6330A>G, 6333G>A, 6336C>T, 6342G>T, 6345T>C, 6346C>T, 6348C>G, 6350A>G, 6351A>G, 6354A>T, 6355A>G, 6358C>T, 6361G>C, 6365T>C, 6366A>T, 6371G>A, 6372G>A, 6373A>C, 6374A>T, 6375G>T, 6376A>T, 6377A>G, 6378T>A, 6382C>A, 6383T>C, 6384A>C, 6386T>A, 6387_6388insAATTGTTCG, 6390C>A, 6393C>T, 6394C>T, 6397A>G, 6398C>T, 6402A>T, 6403A>G, 6408A>T, 6409A>T, 6411A>T, 6413C>G								

CDS

	Begin	End	Coverage	Score	Concordance	Matches	Identities	I/D/M/F*	Stop Codons
QKP79_gp3	1532	1638	4.7%	220	28.5%	107 (96.4%)	50 (45.0%)	4/0/1/1	1
Protein mutations:	F1533I (6106T>A), K1536R (6115A>C 6116A>G), G1538D (6122G>A), F1539Y (6125T>A), V1542L (6133G>T), A1543R (6136G>A 6137C>G 6138A>G), M1544V (6139A>G 6141G>A), E1545K (6142G>A), S1547C (6149C>G 6150A>T), W1551K (6160T>A 6161G>A), I1555R (6173T>G), P1557Q (6179C>A), D1558Y (6181G>T), G1559S (6184G>A 6186A>T), L1560H (6188T>A 6189A>C), Y1561F (6191A>T 6192C>T), W1563F (6197G>T 6198G>T), M1566I (6207G>A), P1567S (6208C>T 6210A>T), K1571T (6221A>C 6222G>A), V1576T (6235G>A 6236T>C 6237G>T), Q1578M (6241C>A 6242A>T 6243A>G), R1579D (6244A>G 6245G>A 6246G>C), K1580L (6247A>T 6248A>T), D1582N (6253G>A), N1583R (6257A>G 6258C>G), C1584V (6259T>G 6260G>T 6261T>A), R1586K (6266G>A), G1587P (6268G>C 6269G>C 6270T>G), T1588Y (6271A>T 6272C>A 6273A>T), E1589L (6274G>T 6275A>T 6276A>G), D1590_F1591insM (6279_6280insATG), I1592V (6283A>G 6285C>A), A1593V (6287C>T 6288T>G), Y1595L (6292T>C 6293A>T), F1602Y (6314T>A), E1604R (6319G>C 6320A>G 6321A>G), L1606A (6325C>G 6326T>C 6327T>C), Q1607K (6328C>A 6330A>G), E1611D (6342G>T), K1614R (6350A>G 6351A>G), M1616V (6355A>G), E1618Q (6361G>C), I1619T (6365T>C 6366A>T), R1621K (6371G>A 6372G>A), K1622L (6373A>C 6374A>T 6375G>T), N1623* (6376A>T 6377A>G 6378T>A), L1625T (6382C>A 6383T>C 6384A>C), V1626D (6386T>A), V1626_L1627insNCS (6387_6388insAATTGTTCG), P1629S (6394C>T), T1630V (6397A>G 6398C>T), K1631N (6402A>T), M1632V (6403A>G), K1633N (6408A>T), I1634F (6409A>T 6411A>T), A1635G (6413C>G)								
Codon mutations:	AGC1531..T (6102C>T), AAA1532AAG (6105A>G), TTT1533ATT (6106T>A), TTG1535CTT (6112T>C 6114G>T), AAA1536CGA (6115A>C 6116A>G), TCT1537TCC (6120T>C), GGT1538GAT (6122G>A), TTT1539TAT (6125T>A), CAG1541CAA (6132G>A), GTG1542TTG (6133G>T), GCA1543AGG (6136G>A 6137C>G 6138A>G), ATG1544GTA (6139A>G 6141G>A), GAA1545AAA (6142G>A), GAA1546GAG (6147A>G), TCA1547TGT (6149C>G 6150A>T), AGC1548--G (6151_6152delAG 6153C>G), ATT1549ATC (6156T>C), CCA1550CCG (6159A>G), TGG1551AAG (6160T>A 6161G>A), ACG1552ACT (6165G>T), TTC1554TTT (6171C>T), ATA1555AGA (6173T>G), CCA1557CAA (6179C>A), GAT1558TAT (6181G>T), GGA1559AGT (6184G>A 6186A>T), CTA1560CAC (6188T>A 6189A>C), TAC1561TTT (6191A>T 6192C>T), TGG1563TTT (6197G>T 6198G>T), CTA1564TTG (6199C>T 6201A>G), GTA1565GTT (6204A>T), ATG1566ATA (6207G>A), CCA1567TCT (6208C>T 6210A>T), GGT1569GGA (6216T>A), AAG1571ACA (6221A>C 6222G>A), GCA1573GCT (6228A>T), CCT1574CCC (6231T>C), GTG1576ACT (6235G>A 6236T>C 6237G>T), TTC1577TTT (6240C>T), CAA1578ATG (6241C>A 6242A>T 6243A>G), AGG1579GAC (6244A>G 6245G>A 6246G>C), AAA1580TTA (6247A>T 6248A>T), GAC1582AAC (6253G>A), AAC1583AGG (6257A>G 6258C>G), TGT1584GTA (6259T>G 6260G>T 6261T>A), AGG1586AAG (6266G>A), GGT1587CCG (6268G>C 6269G>C 6270T>G), ACA1588TAT (6271A>T 6272C>A 6273A>T), GAA1589TTG (6274G>T 6275A>T 6276A>G), GAT1590GAC (6279T>C), GAT1590_TTC1591insATG (6279_6280insATG), TTC1591TTT (6282C>T), ATC1592GTA (6283A>G 6285C>A), GCT1593GTG (6287C>T 6288T>G), GTT1594GTC (6291T>C), TAC1595CTC (6292T>C 6293A>T), ATT1596ATA (6297T>A), GAT1598GAC (6303T>C), ATT1599ATA (6306T>A), CTG1600TTG (6307C>T), GTA1601GTT (6312A>T), TTC1602TAC (6314T>A), GAA1604CGG (6319G>C 6320A>G 6321A>G), CTT1606GCC (6325C>G 6326T>C 6327T>C), CAA1607AAG (6328C>A 6330A>G), GAG1608GAA (6333G>A), CAC1609CAT (6336C>T), GAG1611GAT (6342G>T), CAT1612CAC (6345T>C), CTC1613TTG (6346C>T 6348C>G), AAA1614AGG (6350A>G 6351A>G), ATA1615ATT (6354A>T), ATG1616GTG (6355A>G), CTG1617TTG (6358C>T), GAA1618CAA (6361G>C), ATA1619ACT (6365T>C 6366A>T), AGG1621AAA (6371G>A 6372G>A), AAG1622CTT (6373A>C 6374A>T 6375G>T), AAT1623TGA (6376A>T 6377A>G 6378T>A), CTA1625ACC (6382C>A 6383T>C 6384A>C), GTC1626GAC (6386T>A), GTC1626_CTC1627insAATTGTTCG (6387_6388insAATTGTTCG), CTC1627CTA (6390C>A), AGC1628AGT (6393C>T), CCA1629TCA (6394C>T), ACA1630GTA (6397A>G 6398C>T), AAA1631AAT (6402A>T), ATG1632GTG (6403A>G), AAA1633AAT (6408A>T), ATA1634TTT (6409A>T 6411A>T), GCT1635GGT (6413C>G)								

Proteins

	Begin	End	Coverage	Score	Concordance	Matches	Identities	I/D/M/F*	Stop Codons
hypothetical protein (YP_010799265.1)	1532	1638	4.7%	220	28.5%	107 (96.4%)	50 (45.0%)	4/0/1/1	1
Protein mutations:	F1533I (6106T>A), K1536R (6115A>C 6116A>G), G1538D (6122G>A), F1539Y (6125T>A), V1542L (6133G>T), A1543R (6136G>A 6137C>G 6138A>G), M1544V (6139A>G 6141G>A), E1545K (6142G>A), S1547C (6149C>G 6150A>T), W1551K (6160T>A 6161G>A), I1555R (6173T>G), P1557Q (6179C>A), D1558Y (6181G>T), G1559S (6184G>A 6186A>T), L1560H (6188T>A 6189A>C), Y1561F (6191A>T 6192C>T), W1563F (6197G>T 6198G>T), M1566I (6207G>A), P1567S (6208C>T 6210A>T), K1571T (6221A>C 6222G>A), V1576T (6235G>A 6236T>C 6237G>T), Q1578M (6241C>A 6242A>T 6243A>G), R1579D (6244A>G 6245G>A 6246G>C), K1580L (6247A>T 6248A>T), D1582N (6253G>A), N1583R (6257A>G 6258C>G), C1584V (6259T>G 6260G>T 6261T>A), R1586K (6266G>A), G1587P (6268G>C 6269G>C 6270T>G), T1588Y (6271A>T 6272C>A 6273A>T), E1589L (6274G>T 6275A>T 6276A>G), D1590_F1591insM (6279_6280insATG), I1592V (6283A>G 6285C>A), A1593V (6287C>T 6288T>G), Y1595L (6292T>C 6293A>T), F1602Y (6314T>A), E1604R (6319G>C 6320A>G 6321A>G), L1606A (6325C>G 6326T>C 6327T>C), Q1607K (6328C>A 6330A>G), E1611D (6342G>T), K1614R (6350A>G 6351A>G), M1616V (6355A>G), E1618Q (6361G>C), I1619T (6365T>C 6366A>T), R1621K (6371G>A 6372G>A), K1622L (6373A>C 6374A>T 6375G>T), N1623* (6376A>T 6377A>G 6378T>A), L1625T (6382C>A 6383T>C 6384A>C), V1626D (6386T>A), V1626_L1627insNCS (6387_6388insAATTGTTCG), P1629S (6394C>T), T1630V (6397A>G 6398C>T), K1631N (6402A>T), M1632V (6403A>G), K1633N (6408A>T), I1634F (6409A>T 6411A>T), A1635G (6413C>G)								
Codon mutations:	AGC1531..T (6102C>T), AAA1532AAG (6105A>G), TTT1533ATT (6106T>A), TTG1535CTT (6112T>C 6114G>T), AAA1536CGA (6115A>C 6116A>G), TCT1537TCC (6120T>C), GGT1538GAT (6122G>A), TTT1539TAT (6125T>A), CAG1541CAA (6132G>A), GTG1542TTG (6133G>T), GCA1543AGG (6136G>A 6137C>G 6138A>G), ATG1544GTA (6139A>G 6141G>A), GAA1545AAA (6142G>A), GAA1546GAG (6147A>G), TCA1547TGT (6149C>G 6150A>T), AGC1548--G (6151_6152delAG 6153C>G), ATT1549ATC (6156T>C), CCA1550CCG (6159A>G), TGG1551AAG (6160T>A 6161G>A), ACG1552ACT (6165G>T), TTC1554TTT (6171C>T), ATA1555AGA (6173T>G), CCA1557CAA (6179C>A), GAT1558TAT (6181G>T), GGA1559AGT (6184G>A 6186A>T), CTA1560CAC (6188T>A 6189A>C), TAC1561TTT (6191A>T 6192C>T), TGG1563TTT (6197G>T 6198G>T), CTA1564TTG (6199C>T 6201A>G), GTA1565GTT (6204A>T), ATG1566ATA (6207G>A), CCA1567TCT (6208C>T 6210A>T), GGT1569GGA (6216T>A), AAG1571ACA (6221A>C 6222G>A), GCA1573GCT (6228A>T), CCT1574CCC (6231T>C), GTG1576ACT (6235G>A 6236T>C 6237G>T), TTC1577TTT (6240C>T), CAA1578ATG (6241C>A 6242A>T 6243A>G), AGG1579GAC (6244A>G 6245G>A 6246G>C), AAA1580TTA (6247A>T 6248A>T), GAC1582AAC (6253G>A), AAC1583AGG (6257A>G 6258C>G), TGT1584GTA (6259T>G 6260G>T 6261T>A), AGG1586AAG (6266G>A), GGT1587CCG (6268G>C 6269G>C 6270T>G), ACA1588TAT (6271A>T 6272C>A 6273A>T), GAA1589TTG (6274G>T 6275A>T 6276A>G), GAT1590GAC (6279T>C), GAT1590_TTC1591insATG (6279_6280insATG), TTC1591TTT (6282C>T), ATC1592GTA (6283A>G 6285C>A), GCT1593GTG (6287C>T 6288T>G), GTT1594GTC (6291T>C), TAC1595CTC (6292T>C 6293A>T), ATT1596ATA (6297T>A), GAT1598GAC (6303T>C), ATT1599ATA (6306T>A), CTG1600TTG (6307C>T), GTA1601GTT (6312A>T), TTC1602TAC (6314T>A), GAA1604CGG (6319G>C 6320A>G 6321A>G), CTT1606GCC (6325C>G 6326T>C 6327T>C), CAA1607AAG (6328C>A 6330A>G), GAG1608GAA (6333G>A), CAC1609CAT (6336C>T), GAG1611GAT (6342G>T), CAT1612CAC (6345T>C), CTC1613TTG (6346C>T 6348C>G), AAA1614AGG (6350A>G 6351A>G), ATA1615ATT (6354A>T), ATG1616GTG (6355A>G), CTG1617TTG (6358C>T), GAA1618CAA (6361G>C), ATA1619ACT (6365T>C 6366A>T), AGG1621AAA (6371G>A 6372G>A), AAG1622CTT (6373A>C 6374A>T 6375G>T), AAT1623TGA (6376A>T 6377A>G 6378T>A), CTA1625ACC (6382C>A 6383T>C 6384A>C), GTC1626GAC (6386T>A), GTC1626_CTC1627insAATTGTTCG (6387_6388insAATTGTTCG), CTC1627CTA (6390C>A), AGC1628AGT (6393C>T), CCA1629TCA (6394C>T), ACA1630GTA (6397A>G 6398C>T), AAA1631AAT (6402A>T), ATG1632GTG (6403A>G), AAA1633AAT (6408A>T), ATA1634TTT (6409A>T 6411A>T), GCT1635GGT (6413C>G)								

*: Inserts / Deletes / Misaligned / Frameshifts

Analysis details

This analysis was performed with panviral2.64

NGS Details (UN70): Neptunevirus srim50

Assembly

**Coverage Length**	564 (1 contig(s))
**Depth Of Coverage**	10.5
**Number Of Reads**	69
**Reads Per Million**	1.31 rpm (after QC)
**Ambiguities**	0
**Assembly Method**	de novo + reference guided assembly
**Consensus Caller**	Bcf Tools

Coverage Map

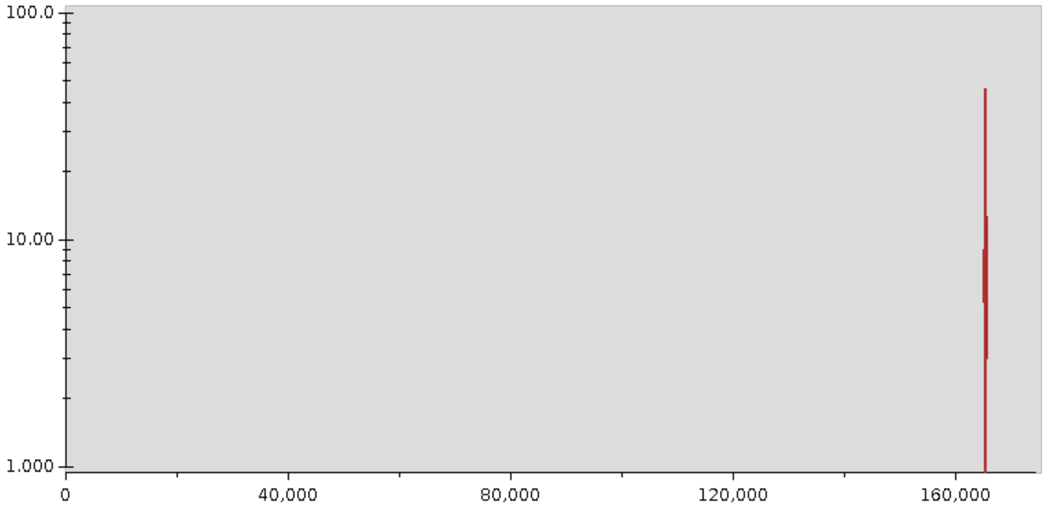

Assignment

**Type**	Neptunevirus srim50 (Taxonomy ID: 2734122)
**Reference Genome**	NC_031242.1
**NT Identity (%)**	71.9858
**AA Identity (%)**	87.766
**Number Of Stop Codons**	0
**Number Of CDS**	227

Alignment

**Alignment Score**	496.0 (NT) + 1298.0 (AA) = 1794.0
**Concordance (%)**	71.1622
**Alignment Method**	Local, heuristic, nucleotide (BLASTN)

Genome Region

Sequence starts at position 165152 and ends at position 165715 relative to NC_031242.1 reference sequence.

Alignment Detailed Statistics

	Begin	End	Coverage	Score	Concordance	Matches	Identities	I/D/M/F*	Stop Codons
**NT**	**165152**	**165715**	**0.3%**	**496**	**44.0%**	**564 (100%)**	**406 (72.0%)**	**0/0**	
Mutations:	165155C>T, 165159G>A, 165161C>T, 165162C>A, 165164T>G, 165167C>T, 165174C>T, 165176T>A, 165177A>C, 165178A>G, 165179A>G, 165182A>G, 165200T>A, 165209T>C, 165212A>T, 165215T>A, 165216C>T, 165218T>G, 165221T>C, 165227C>T, 165228A>T, 165229C>G, 165233T>C, 165237C>T, 165239T>C, 165245T>A, 165255C>T, 165257T>C, 165260T>A, 165263G>T, 165266T>A, 165269T>C, 165272C>T, 165275C>A, 165278G>T, 165279A>T, 165280G>C, 165281T>A, 165290T>C, 165293C>T, 165297C>T, 165299T>G, 165302G>C, 165308C>T, 165312C>T, 165314T>G, 165317G>A, 165320T>C, 165321G>T, 165322C>G, 165323A>C, 165326C>T, 165329T>C, 165330C>T, 165332T>A, 165338G>C, 165341A>G, 165353A>T, 165356A>T, 165357G>A, 165359C>T, 165360G>A, 165361C>G, 165363A>T, 165365G>A, 165367G>C, 165368T>A, 165374T>G, 165375C>T, 165377T>G, 165378C>T, 165381C>T, 165383T>A, 165398G>A, 165399T>G, 165401T>A, 165407G>A, 165410C>T, 165413C>T, 165415T>C, 165416C>T, 165419C>T, 165425C>T, 165429C>T, 165431T>G, 165437A>T, 165440C>G, 165452G>T, 165455G>T, 165461C>T, 165467C>T, 165472C>G, 165473A>C, 165476T>A, 165489C>T, 165491T>A, 165494C>T, 165497G>A, 165500T>C, 165503A>G, 165506G>T, 165509A>T, 165513C>T, 165514T>C, 165516A>G, 165518C>T, 165519G>C, 165520G>A, 165521T>A, 165522A>T, 165524T>G, 165525C>A, 165527T>A, 165533C>T, 165539T>A, 165542T>C, 165545T>A, 165557T>A, 165566A>T, 165569C>T, 165573A>T, 165574G>C, 165578T>A, 165584T>G, 165587G>T, 165593T>A, 165596G>A, 165599A>T, 165602A>G, 165605G>T, 165606T>G, 165607C>G, 165608C>T, 165617C>T, 165623G>T, 165624T>A, 165625C>G, 165626G>T, 165629C>T, 165632G>T, 165635C>A, 165641A>T, 165644C>A, 165647C>T, 165654C>A, 165656G>C, 165659C>A, 165662C>T, 165663C>T, 165665C>T, 165674C>T, 165680C>T, 165686G>C, 165687C>T, 165692T>C, 165701C>T, 165710C>A, 165713A>T								

*: Inserts / Deletes / Misaligned / Frameshifts

Analysis details

This analysis was performed with panviral2.64

NGS Details (UN70): Badnavirus alphacolocalasiae

Assembly

**Coverage Length**	232 (1 contig(s))
**Depth Of Coverage**	21.2
**Number Of Reads**	42
**Reads Per Million**	0.80 rpm (after QC)
**Ambiguities**	0
**Assembly Method**	de novo + reference guided assembly
**Consensus Caller**	Bcf Tools

Coverage Map

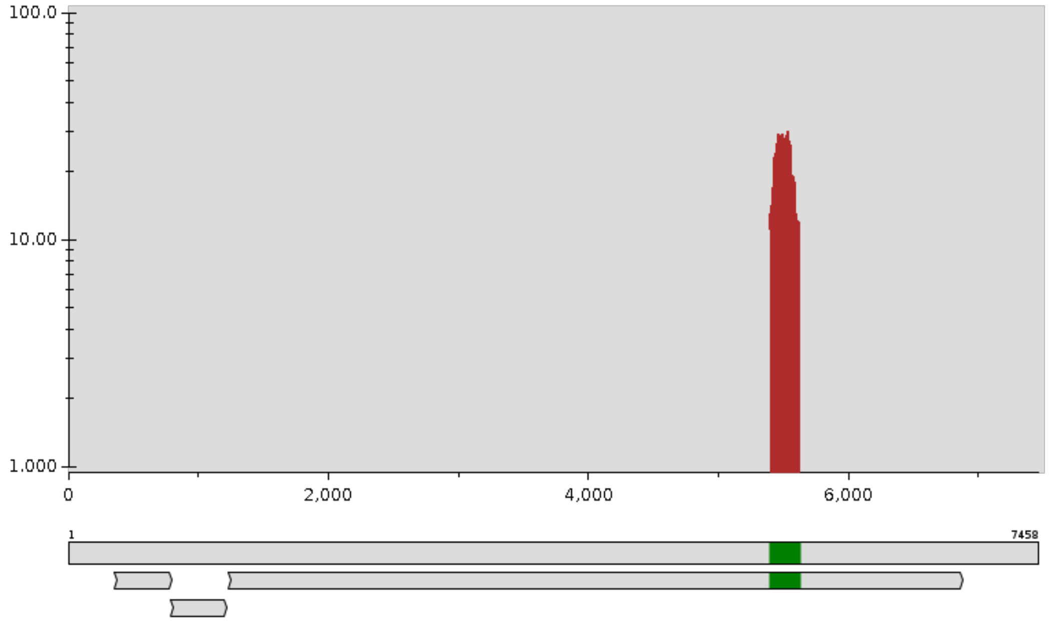

Assignment

**Type**	Badnavirus alphacolocalasiae (Taxonomy ID: 3048356)
**Reference Genome**	NC_004450.1
**NT Identity (%)**	53.617
**AA Identity (%)**	44.8718
**Number Of Stop Codons**	0
**Number Of CDS**	3

Alignment

**Alignment Score**	28.0 (NT) + 255.0 (AA) = 283.0
**Concordance (%)**	27.5024

**Alignment Method**	Global, seeded, nucleotide + amino acids (AGA)

Genome Region

Sequence starts at position 5399 and ends at position 5630 relative to NC_004450.1 reference sequence.

Alignment Detailed Statistics

	Begin	End	Coverage	Score	Concordance	Matches	Identities	I/D/M/F*	Stop Codons
**NT**	**5399**	**5630**	**3.1%**	**28**	**6.0%**	**232 (98.7%)**	**126 (53.6%)**	**3/0**	
Mutations:	5399A>T, 5405T>C, 5407T>A, 5409C>A, 5414A>G, 5415G>A, 5417T>C, 5418G>T, 5419C>G, 5420T>C, 5424G>T, 5425A>C, 5426T>A, 5429C>A, 5430G>A, 5432T>G, 5433T>G, 5434C>A, 5437T>A, 5438G>A, 5442T>C, 5443G>T, 5444G>C, 5447A>T, 5450T>A, 5453C>T, 5455T>G, 5456A>C, 5457G>A, 5458T>C, 5459T>A, 5462A>T, 5463C>A, 5469T>A, 5471A>T, 5475G>T, 5476A>G, 5477A>C, 5479G>A, 5480G>C, 5481C>A, 5482T>A, 5485C>T, 5486A>G, 5498C>T, 5499C>T, 5501C>A, 5504A>G, 5511C>G, 5512C>G, 5513A>T, 5517G>A, 5518T>C, 5521T>A, 5528A>G, 5529A>G, 5530A>C, 5531A>T, 5535G>C, 5537T>A, 5538G>A, 5539C>A, 5540A>T, 5541G>A, 5546C>T, 5547A>G, 5549A>T, 5551G>A, 5552G>C, 5554G>T, 5555T>A, 5555_5556insCTC, 5556G>C, 5558A>C, 5562T>A, 5563T>A, 5564C>T, 5565C>G, 5567C>T, 5569C>A, 5571G>T, 5572T>G, 5573C>T, 5577A>G, 5585T>C, 5586A>T, 5588T>G, 5589C>G, 5591G>A, 5594A>G, 5595T>A, 5596T>A, 5597T>G, 5603C>G, 5606T>G, 5607G>A, 5608A>G, 5610G>A, 5611A>G, 5612A>T, 5615T>C, 5619G>T, 5620C>T, 5621A>G, 5625C>G, 5628C>T, 5630G>A								
**CDS**									
TaBVgp3	1392	1468	4.1%	255	44.8%	77 (98.7%)	35 (44.9%)	1/0/0/0	0
Protein mutations:	F1394Y (5407T>A), H1395N (5409C>A), V1397I (5415G>A 5417T>C), A1398C (5418G>T 5419C>G 5420T>C), D1400S (5424G>T 5425A>C 5426T>A), D1402K (5430G>A 5432T>G), S1403D (5433T>G 5434C>A), V1404E (5437T>A 5438G>A), W1406L (5442T>C 5443G>T 5444G>C), L1410R (5455T>G 5456A>C), V1411T (5457G>A 5458T>C 5459T>A), Q1413K (5463C>A), L1415I (5469T>A 5471A>T), E1417C (5475G>T 5476A>G 5477A>C), W1418Y (5479G>A 5480G>C), L1419K (5481C>A 5482T>A), A1420V (5485C>T 5486A>G), P1429G (5511C>G 5512C>G 5513A>T), V1431T (5517G>A 5518T>C), F1432Y (5521T>A), K1435A (5529A>G 5530A>C 5531A>T), D1437Q (5535G>C 5537T>A), A1438N (5538G>A 5539C>A 5540A>T), V1439I (5541G>A), K1441D (5547A>G 5549A>T), G1442D (5551G>A 5552G>C), C1443L (5554G>T 5555T>A), C1443_E1444insL (5555_5556insCTC), E1444H (5556G>C 5558A>C), F1446N (5562T>A 5563T>A 5564C>T), L1447V (5565C>G 5567C>T), A1448E (5569C>A), V1449C (5571G>T 5572T>G 5573C>T), I1451V (5577A>G), I1454L (5586A>T 5588T>G), L1455V (5589C>G 5591G>A), F1457K (5595T>A 5596T>A 5597T>G), N1459K (5603C>G), N1460K (5606T>G), E1461R (5607G>A 5608A>G), E1462S (5610G>A 5611A>G 5612A>T), A1465L (5619G>T 5620C>T 5621A>G), H1467D (5625C>G)								
Codon mutations:	AAA1391..T (5399A>T), GGT1393GGC (5405T>C), TTT1394TAT (5407T>A), CAC1395AAC (5409C>A), CAA1396CAG (5414A>G), GTT1397ATC (5415G>A 5417T>C), GCT1398TGC (5418G>T 5419C>G 5420T>C), GAT1400TCA (5424G>T 5425A>C 5426T>A), CCC1401CCA (5429C>A), GAT1402AAG (5430G>A 5432T>G), TCT1403GAT (5433T>G 5434C>A), GTG1404GAA (5437T>A 5438G>A), TGG1406CTC (5442T>C 5443G>T 5444G>C), ACA1407ACT (5447A>T), GCT1408GCA (5450T>A), TTC1409TTT (5453C>T), CTA1410CGC (5455T>G 5456A>C), GTT1411ACA (5457G>A 5458T>C 5459T>A), CCA1412CCT (5462A>T), CAA1413AAA (5463C>A), TTA1415ATT (5469T>A 5471A>T), GAA1417TGC (5475G>T 5476A>G 5477A>C), TGG1418TAC (5479G>A 5480G>C), CTG1419AAG (5481C>A 5482T>A), GCA1420GTG (5485C>T 5486A>G), GGC1424GGT (5498C>T), CTC1425TTA (5499C>T 5501C>A), AAA1426AAG (5504A>G), CCA1429GGT (5511C>G 5512C>G 5513A>T), GTA1431ACA (5517G>A 5518T>C), TTT1432TAT (5521T>A), AGA1434AGG (5528A>G), AAA1435GCT (5529A>G 5530A>C 5531A>T), GAT1437CAA (5535G>C 5537T>A), GCA1438AAT (5538G>A 5539C>A 5540A>T), GTA1439ATA (5541G>A), TTC1440TTT (5546C>T), AAA1441GAT (5547A>G 5549A>T), GGG1442GAC (5551G>A 5552G>C), TGT1443TTA (5554G>T 5555T>A), TGT1443_GAA1444insCTC (5555_5556insCTC), GAA1444CAC (5556G>C 5558A>C), TTC1446AAT (5562T>A 5563T>A 5564C>T), CTC1447GTT (5565C>G 5567C>T), GCA1448GAA (5569C>A), GTC1449TGT (5571G>T 5572T>G 5573C>T), ATT1451GTT (5577A>G), GAT1453GAC (5585T>C), ATT1454TTG (5586A>T 5588T>G), CTG1455GTA (5589C>G 5591G>A), GTA1456GTG (5594A>G), TTT1457AAG (5595T>A 5596T>A 5597T>G), AAC1459AAG (5603C>G), AAT1460AAG (5606T>G), GAG1461AGG (5607G>A 5608A>G), GAA1462AGT (5610G>A 5611A>G 5612A>T), GAT1463GAC (5615T>C), GCA1465TTG (5619G>T 5620C>T 5621A>G), CAC1467GAC (5625C>G), CTG1468TTA (5628C>T 5630G>A)								
**Proteins**									
polyprotein (NP_758808.1)	1392	1468	4.1%	255	44.8%	77 (98.7%)	35 (44.9%)	1/0/0/0	0
Protein mutations:	F1394Y (5407T>A), H1395N (5409C>A), V1397I (5415G>A 5417T>C), A1398C (5418G>T 5419C>G 5420T>C), D1400S (5424G>T 5425A>C 5426T>A), D1402K (5430G>A 5432T>G), S1403D (5433T>G 5434C>A), V1404E (5437T>A 5438G>A), W1406L (5442T>C 5443G>T 5444G>C), L1410R (5455T>G 5456A>C), V1411T (5457G>A 5458T>C 5459T>A), Q1413K (5463C>A), L1415I (5469T>A 5471A>T), E1417C (5475G>T 5476A>G 5477A>C), W1418Y (5479G>A 5480G>C), L1419K (5481C>A 5482T>A), A1420V (5485C>T 5486A>G), P1429G (5511C>G 5512C>G 5513A>T), V1431T (5517G>A 5518T>C), F1432Y (5521T>A), K1435A (5529A>G 5530A>C 5531A>T), D1437Q (5535G>C 5537T>A), A1438N (5538G>A 5539C>A 5540A>T), V1439I (5541G>A), K1441D (5547A>G 5549A>T), G1442D (5551G>A 5552G>C), C1443L (5554G>T 5555T>A), C1443_E1444insL (5555_5556insCTC), E1444H (5556G>C 5558A>C), F1446N (5562T>A 5563T>A 5564C>T), L1447V (5565C>G 5567C>T), A1448E (5569C>A), V1449C (5571G>T 5572T>G 5573C>T), I1451V (5577A>G), I1454L (5586A>T 5588T>G), L1455V (5589C>G 5591G>A), F1457K (5595T>A 5596T>A 5597T>G), N1459K (5603C>G), N1460K (5606T>G), E1461R (5607G>A 5608A>G), E1462S (5610G>A 5611A>G 5612A>T), A1465L (5619G>T 5620C>T 5621A>G), H1467D (5625C>G)								
Codon mutations:	AAA1391..T (5399A>T), GGT1393GGC (5405T>C), TTT1394TAT (5407T>A), CAC1395AAC (5409C>A), CAA1396CAG (5414A>G), GTT1397ATC (5415G>A 5417T>C), GCT1398TGC (5418G>T 5419C>G 5420T>C), GAT1400TCA (5424G>T 5425A>C 5426T>A), CCC1401CCA (5429C>A), GAT1402AAG (5430G>A 5432T>G), TCT1403GAT (5433T>G 5434C>A), GTG1404GAA (5437T>A 5438G>A), TGG1406CTC (5442T>C 5443G>T 5444G>C), ACA1407ACT (5447A>T), GCT1408GCA (5450T>A), TTC1409TTT (5453C>T), CTA1410CGC (5455T>G 5456A>C), GTT1411ACA (5457G>A 5458T>C 5459T>A), CCA1412CCT (5462A>T), CAA1413AAA (5463C>A), TTA1415ATT (5469T>A 5471A>T), GAA1417TGC (5475G>T 5476A>G 5477A>C), TGG1418TAC (5479G>A 5480G>C), CTG1419AAG (5481C>A 5482T>A), GCA1420GTG (5485C>T 5486A>G), GGC1424GGT (5498C>T), CTC1425TTA (5499C>T 5501C>A), AAA1426AAG (5504A>G), CCA1429GGT (5511C>G 5512C>G 5513A>T), GTA1431ACA (5517G>A 5518T>C), TTT1432TAT (5521T>A), AGA1434AGG (5528A>G), AAA1435GCT (5529A>G 5530A>C 5531A>T), GAT1437CAA (5535G>C 5537T>A), GCA1438AAT (5538G>A 5539C>A 5540A>T), GTA1439ATA (5541G>A), TTC1440TTT (5546C>T), AAA1441GAT (5547A>G 5549A>T), GGG1442GAC (5551G>A 5552G>C), TGT1443TTA (5554G>T 5555T>A), TGT1443_GAA1444insCTC (5555_5556insCTC), GAA1444CAC (5556G>C 5558A>C), TTC1446AAT (5562T>A 5563T>A 5564C>T), CTC1447GTT (5565C>G 5567C>T), GCA1448GAA (5569C>A), GTC1449TGT (5571G>T 5572T>G 5573C>T), ATT1451GTT (5577A>G), GAT1453GAC (5585T>C), ATT1454TTG (5586A>T 5588T>G), CTG1455GTA (5589C>G 5591G>A), GTA1456GTG (5594A>G), TTT1457AAG (5595T>A 5596T>A 5597T>G), AAC1459AAG (5603C>G), AAT1460AAG (5606T>G), GAG1461AGG (5607G>A 5608A>G), GAA1462AGT (5610G>A 5611A>G 5612A>T), GAT1463GAC (5615T>C), GCA1465TTG (5619G>T 5620C>T 5621A>G), CAC1467GAC (5625C>G), CTG1468TTA (5628C>T 5630G>A)								

*: Inserts / Deletes / Misaligned / Frameshifts

Analysis details

This analysis was performed with panviral2.64

NGS Details (UN70): Caulimovirus venafragariae

Assembly

**Coverage Length**	654 (1 contig(s))
**Depth Of Coverage**	7.0
**Number Of Reads**	36
**Reads Per Million**	0.68 rpm (after QC)
**Ambiguities**	0
**Assembly Method**	de novo + reference guided assembly
**Consensus Caller**	Bcf Tools

Coverage Map

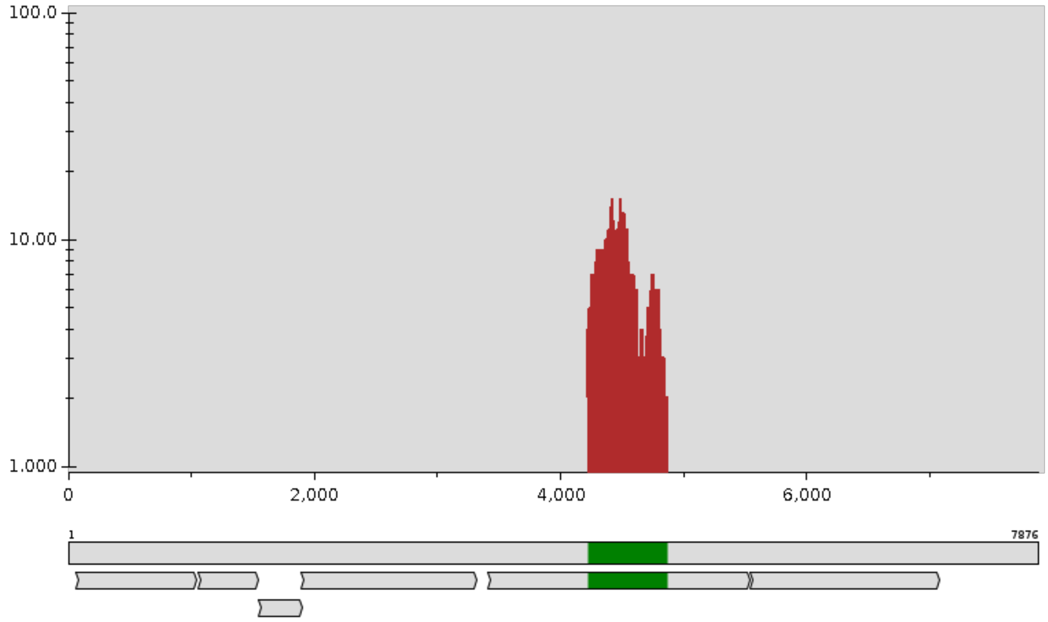

Assignment

**Type**	Caulimovirus venafragariae (Taxonomy ID: 3048344)
**Reference Genome**	NC_001725.1
**NT Identity (%)**	59.9379
**AA Identity (%)**	59.0698
**Number Of Stop Codons**	0
**Number Of CDS**	6

Alignment

**Alignment Score**	219.0 (NT) + 779.0 (AA) = 998.0
**Concordance (%)**	38.4732

**Alignment Method**	Global, seeded, nucleotide + amino acids (AGA)

Genome Region

Sequence starts at position 4219 and ends at position 4872 relative to NC_001725.1 reference sequence.

Alignment Detailed Statistics

	Begin	End	Coverage	Score	Concordance	Matches	Identities	I/D/M/F*	Stop Codons
**NT**	**4219**	**4872**	**8.3%**	**219**	**17.5%**	**644 (98.5%)**	**386 (59.0%)**	**0/10**	
Mutations:	4219T>C, 4222T>A, 4223T>A, 4228A>C, 4230T>C, 4232A>G, 4235A>T, 4236A>C, 4242T>A, 4244A>G, 4245G>A, 4246T>G, 4247G>T, 4254A>T, 4259A>T, 4260G>A, 4263T>C, 4264T>A, 4265C>A, 4266A>T, 4267C>G, 4268C>A, 4269T>A, 4275C>T, 4276C>A, 4277G>A, 4279G>A, 4280A>C, 4281A>T, 4284G>A, 4292C>T, 4299C>T, 4300G>C, 4306C>T, 4309C>T, 4311C>A, 4314G>T, 4315C>A, 4317T>G, 4318G>A, 4319G>A, 4320T>A, 4321A>T, 4323C>A, 4326C>A, 4331C>A, 4335C>T, 4338G>A, 4348T>A, 4349C>G, 4350C>T, 4351T>A, 4352C>G, 4353A>C, 4359C>A, 4362C>T, 4371G>A, 4374T>C, 4379C>G, 4383G>A, 4384A>C, 4385T>A, 4386C>A, 4390delC, 4391G>C, 4392A>T, 4395C>A, 4402A>C, 4413T>A, 4423A>G, 4426T>C, 4428A>T, 4432G>A, 4434C>A, 4435C>A, 4438A>T, 4439C>G, 4440A>T, 4441A>G, 4442A>T, 4443G>C, 4444G>T, 4445G>T, 4446A>C, 4449T>C, 4452C>A, 4455C>T, 4456C>T, 4458A>C, 4459C>A, 4461T>C, 4464T>A, 4467C>T, 4470G>A, 4471G>A, 4473G>T, 4474C>A, 4476A>C, 4479G>T, 4480C>A, 4482T>A, 4483C>A, 4485A>T, 4486A>C, 4487G>T, 4488A>T, 4489A>G, 4490T>C, 4492G>A, 4493G>A, 4497T>A, 4500G>A, 4505T>A, 4506T>C, 4508A>T, 4509C>T, 4512T>A, 4513T>A, 4514C>A, 4515C>A, 4521C>T, 4524T>C, 4528T>A, 4529C>G, 4542G>A, 4543G>A, 4546C>A, 4547G>A, 4548C>A, 4551T>A, 4553C>A, 4554T>A, 4555C>G, 4556C>A, 4558G>A, 4560A>T, 4561A>T, 4563A>T, 4566T>A, 4567C>G, 4568A>C, 4569G>T, 4570C>T, 4575C>A, 4578T>A, 4584C>T, 4585T>A, 4586G>C, 4587T>A, 4590C>A, 4591C>A, 4593A>T, 4614C>G, 4633C>A, 4635A>T, 4642G>C, 4643C>A, 4644T>A, 4647T>C, 4650C>T, 4656G>A, 4657C>A, 4659C>A, 4665C>T, 4670G>T, 4671T>A, 4672C>T, 4674C>T, 4675A>T, 4676G>C, 4677C>T, 4678A>G, 4680C>A, 4682T>A, 4684T>A, 4687_4692delCCACAG, 4696T>C, 4697G>T, 4698T>A, 4699G>A, 4700C>T, 4701T>C, 4704A>C, 4708G>A, 4710T>A, 4716C>T, 4719C>A, 4720A>C, 4722C>A, 4723G>A, 4725C>A, 4727T>G, 4728C>T, 4729A>T, 4730G>C, 4731C>T, 4734A>T, 4735A>T, 4737T>A, 4738G>T, 4740A>T, 4741G>C, 4746A>G, 4753G>A, 4754G>C, 4758T>C, 4759G>C, 4761C>T, 4765A>C, 4766T>A, 4767T>A, 4768G>T, 4771C>T, 4772T>C, 4773A>T, 4774A>C, 4776C>A, 4777A>T, 4778G>T, 4782C>T, 4783A>G, 4784A>T, 4785A>T, 4787C>A, 4788C>A, 4789C>A, 4790T>A, 4791A>T, 4797A>T, 4801C>T, 4803T>A, 4807A>C, 4809A>G, 4812G>A, 4815A>G, 4817C>A, 4818C>G, 4819C>G, 4821G>T, 4822T>A, 4824G>A, 4825T>A, 4827C>T, 4832C>A, 4833A>T, 4834A>G, 4835C>A, 4836T>A, 4839C>T, 4840A>G, 4842T>A, 4851T>A, 4852C>A, 4854A>G, 4855G>A, 4860A>T, 4861G>T, 4862A>C, 4863A>C, 4865G>A, 4867_4869delGGA									

CDS

	Begin	End	Coverage	Score	Concordance	Matches	Identities	I/D/M/F*	Stop Codons
ORF_V	272	489	30.7%	779	52.1%	215 (98.6%)	127 (58.3%)	0/3/1/1	0
Protein mutations:	L273K (4222T>A 4223T>A), T275P (4228A>C 4230T>C), N276S (4232A>G), K277I (4235A>T 4236A>C), K280R (4244A>G 4245G>A), C281V (4246T>G 4247G>T), Q285L (4259A>T 4260G>A), S287N (4264T>A 4265C>A 4266A>T), P288E (4267C>G 4268C>A 4269T>A), R291K (4276C>A 4277G>A), E292T (4279G>A 4280A>C 4281A>T), T296I (4292C>T), E299Q (4300G>C), K303N (4314G>T), L304M (4315C>A 4317T>G), G305K (4318G>A 4319G>A 4320T>A), I306L (4321A>T 4323C>A), P309H (4331C>A), A325G (4379C>G), I327Q (4384A>C 4385T>A 4386C>A), K340E (4423A>G), D343K (4432G>A 4434C>A), H344N (4435C>A), T345C (4438A>T 4439C>G 4440A>T), K346V (4441A>G 4442A>T 4443G>C), G347F (4444G>T 4445G>T 4446A>C), L351F (4456C>T 4458A>C), L352I (4459C>A 4461T>C), E356N (4471G>A 4473G>T), Q357N (4474C>A 4476A>C), L359I (4480C>A 4482T>A), Q360N (4483C>A 4485A>T), R361L (4486A>C 4487G>T 4488A>T), I362A (4489A>G 4490T>C), G363K (4492G>A 4493G>A), F367Y (4505T>A 4506T>C), Y368F (4508A>T 4509C>T), S370K (4513T>A 4514C>A 4515C>A), V380I (4543G>A), R381K (4546C>A 4547G>A 4548C>A), A383E (4553C>A 4554T>A), P384E (4555C>G 4556C>A), E385N (4558G>A 4560A>T), T386S (4561A>T 4563A>T), Q388A (4567C>G 4568A>C 4569G>T), C394T (4585T>A 4586G>C 4587T>A), Q396N (4591C>A 4593A>T), Q410N (4633C>A 4635A>T), A413Q (4642G>C 4643C>A 4644T>A), H418K (4657C>A 4659C>A), S422I (4670G>T 4671T>A), L423F (4672C>T 4674C>T), N425E (4678A>G 4680C>A), M426K (4682T>A), Y427N (4684T>A), P428_Q429del (4687_4692delCCACAG), C431L (4696T>C 4697G>T 4698T>A), A432I (4699G>A 4700C>T 4701T>C), V435I (4708G>A 4710T>A), I439L (4720A>C 4722C>A), V440I (4723G>A 4725C>A), F441C (4727T>G 4728C>T), K443N (4734A>T), T444S (4735A>T 4737T>A), E445Y (4738G>T 4740A>T), E446Q (4741G>C), G450T (4753G>A 4754G>C), V452L (4759G>C 4761C>T), I454Q (4765A>C 4766T>A 4767T>A), V455F (4768G>T), L456S (4771C>T 4772T>C 4773A>T), N457Q (4774A>C 4776C>A), R458L (4777A>T 4778G>T), K460V (4783A>G 4784A>T 4785A>T), A461E (4787C>A 4788C>A), L462N (4789C>A 4790T>A 4791A>T), K468Q (4807A>C 4809A>G), A471E (4817C>A 4818C>G), Q472D (4819C>G 4821G>T), L473I (4822T>A 4824G>A), C474S (4825T>A 4827C>T), T476N (4832C>A 4833A>T), T477E (4834A>G 4835C>A 4836T>A), N479E (4840A>G 4842T>A), L483M (4852C>A 4854A>G), V484I (4855G>A), E486S (4861G>T 4862A>C 4863A>C), R487K (4865G>A), G488del (4867_4869delGGA)									
Codon mutations:	TTA272CTA (4219T>C), TTA273AAA (4222T>A 4223T>A), ACT275CCC (4228A>C 4230T>C), AAC276AGC (4232A>G), AAA277ATC (4235A>T 4236A>C), ATT279ATA (4242T>A), AAG280AGA (4244A>G 4245G>A), TGT281GTT (4246T>G 4247G>T), CCA283CCT (4254A>T), CAG285CTA (4259A>T 4260G>A), TAT286TAC (4263T>C), TCA287AAT (4264T>A 4265C>A 4266A>T), CCT288GAA (4267C>G 4268C>A 4269T>A), GAC290GAT (4275C>T), CGA291AAA (4276C>A 4277G>A), GAA292ACT (4279G>A 4280A>C 4281A>T), GAG293GAA (4284G>A), ACT296ATT (4292C>T), ATC298ATT (4299C>T), GAA299CAA (4300G>C), CTA301TTA (4306C>T), CTC302TTA (4309C>T 4311C>A), AAG303AAT (4314G>T), CTT304ATG (4315C>A 4317T>G), GGT305AAA (4318G>A 4319G>A 4320T>A), ATC306TTA (4321A>T 4323C>A), ATC307ATA (4326C>A), CCT309CAT (4331C>A), AGC310AGT (4335C>T), AAG311AAA (4338G>A), TCC315AGT (4348T>A 4349C>G 4350C>T), TCA316AGC (4351T>A 4352C>G 4353A>C), GCC318GCA (4359C>A), TTC319TTT (4362C>T), AGG322AGA (4371G>A), AAT323AAC (4374T>C), GCC325GGC (4379C>G), GAG326GAA (4383G>A), ATC327CAA (4384A>C 4385T>A 4386C>A), CGA329-CT (4390delC 4391G>C 4392A>T), GGC330GGA (4395C>A), AGA333CGA (4402A>C), ATT336ATA (4413T>A), AAG340GAG (4423A>G), TTA341CTT (4426T>C 4428A>T), GAC343AAA (4432G>A 4434C>A), CAT344AAT (4435C>A), ACA345TGT (4438A>T 4439C>G 4440A>T), AAG346GTC (4441A>G 4442A>T 4443G>C), GGA347TTC (4444G>T 4445G>T 4446A>C), GAT348GAC (4449T>C), GGC349GGA (4452C>A), TAC350TAT (4455C>T), CTA351TTC (4456C>T 4458A>C), CTT352ATC (4459C>A 4461T>C), CCT353CCA (4464T>A), AAC354AAT (4467C>T), AAG355AAA (4470G>A), GAG356AAT (4471G>A 4473G>T), CAA357AAC (4474C>A 4476A>C), CTG358CTT (4479G>T), CTT359ATA (4480C>A 4482T>A), CAA360AAT (4483C>A 4485A>T), AGA361CTT (4486A>C 4487G>T 4488A>T), ATC362GCC (4489A>G 4490T>C), GGA363AAA (4492G>A 4493G>A), GGT364GGA (4497T>A), AAG365AAA (4500G>A), TTT367TAC (4505T>A 4506T>C), TAC368TTT (4508A>T 4509C>T), TCT369TCA (4512T>A), TCC370AAA (4513T>A 4514C>A 4515C>A), GAC372GAT (4521C>T), TGT373TGC (4524T>C), TCT375AGT (4528T>A 4529C>G), CAG379CAA (4542G>A), GTA380ATA (4543G>A), CGC381AAA (4546C>A 4547G>A 4548C>A), CTT382CTA (4551T>A), GCT383GAA (4553C>A 4554T>A), CCA384GAA (4555C>G 4556C>A), GAA385AAT (4558G>A 4560A>T), ACA386TCT (4561A>T 4563A>T), ATT387ATA (4566T>A), CAG388GCT (4567C>G 4568A>C 4569G>T), CTA389TTA (4570C>T), ACC390ACA (4575C>A), GCT391GCA (4578T>A), AGC393AGT (4584C>T), TGT394ACA (4585T>A 4586G>C 4587T>A), CCC395CCA (4590C>A), CAA396AAT (4591C>A 4593A>T), GTC403GTG (4614C>G), CAA410AAT (4633C>A 4635A>T), GCT413CAA (4642G>C 4643C>A 4644T>A), ATT414ATC (4647T>C), TTC415TTT (4650C>T), AGG417AGA (4656G>A), CAC418AAA (4657C>A 4659C>A), GAC420GAT (4665C>T), AGT422ATA (4670G>T 4671T>A), CTC423TTT (4672C>T 4674C>T), AGC424TCT (4675A>T 4676G>C 4677C>T), AAC425GAA (4678A>G 4680C>A), ATG426AAG (4682T>A), TAT427AAT (4684T>A), CCA428_CAG429del (4687_4692delCCACAG), TGT431CTA (4696T>C 4697G>T 4698T>A), GCT432ATC (4699G>A 4700C>T 4701T>C), GTA433GTC (4704A>C), GTT435ATA (4708G>A 4710T>A), GAC437GAT (4716C>T), ATC438ATA (4719C>A), ATC439CTA (4720A>C 4722C>A), GTC440ATA (4723G>A 4725C>A), TTC441TGT (4727T>G 4728C>T), AGC442TCT (4729A>T 4730G>C 4731C>T), AAA443AAT (4734A>T), ACT444TCA (4735A>T 4737T>A), GAA445TAT (4738G>T 4740A>T), GAA446CAA (4741G>C), GAA447GAG (4746A>G), GGA450ACA (4753G>A 4754G>C), CAT451CAC (4758T>C), GTC452CTT (4759G>C 4761C>T), ATT454CAA (4765A>C 4766T>A 4767T>A), GTT455TTT (4768G>T), CTA456TCT (4771C>T 4772T>C 4773A>T), AAC457CAA (4774A>C 4776C>A), AGA458TTA (4777A>T 4778G>T), TGC459TGT (4782C>T), AAA460GTT (4783A>G 4784A>T 4785A>T), GCC461GAA (4787C>A 4788C>A), CTA462AAT (4789C>A 4790T>A 4791A>T), ATA464ATT (4797A>T), CTT466TTA (4801C>T 4803T>A), AAA468CAG (4807A>C 4809A>G), AAG469AAA (4812G>A), AAA470AAG (4815A>G), GCC471GAG (4817C>A 4818C>G), CAG472GAT (4819C>G 4821G>T), TTG473ATA (4822T>A 4824G>A), TGC474AGT (4825T>A 4827C>T), ACA476AAT (4832C>A 4833A>T), ACT477GAA (4834A>G 4835C>A 4836T>A), ATC478ATT (4839C>T), AAT479GAA (4840A>G 4842T>A), GGT482GGA (4851T>A), CTA483ATG (4852C>A 4854A>G), GTA484ATA (4855G>A), ATA485ATT (4860A>T), GAA486TCC (4861G>T 4862A>C 4863A>C), AGA487AAA (4865G>A), GGA488del (4867_4869delGGA)									

Proteins

	Begin	End	Coverage	Score	Concordance	Matches	Identities	I/D/M/F*	Stop Codons
hypothetical protein (NP_043933.1)	272	489	30.7%	779	52.1%	215 (98.6%)	127 (58.3%)	0/3/1/1	0
Protein mutations:	L273K (4222T>A 4223T>A), T275P (4228A>C 4230T>C), N276S (4232A>G), K277I (4235A>T 4236A>C), K280R (4244A>G 4245G>A), C281V (4246T>G 4247G>T), Q285L (4259A>T 4260G>A), S287N (4264T>A 4265C>A 4266A>T), P288E (4267C>G 4268C>A 4269T>A), R291K (4276C>A 4277G>A), E292T (4279G>A 4280A>C 4281A>T), T296I (4292C>T), E299Q (4300G>C), K303N (4314G>T), L304M (4315C>A 4317T>G), G305K (4318G>A 4319G>A 4320T>A), I306L (4321A>T 4323C>A), P309H (4331C>A), A325G (4379C>G), I327Q (4384A>C 4385T>A 4386C>A), K340E (4423A>G), D343K (4432G>A 4434C>A), H344N (4435C>A), T345C (4438A>T 4439C>G 4440A>T), K346V (4441A>G 4442A>T 4443G>C), G347F (4444G>T 4445G>T 4446A>C), L351F (4456C>T 4458A>C), L352I (4459C>A 4461T>C), E356N (4471G>A 4473G>T), Q357N (4474C>A 4476A>C), L359I (4480C>A 4482T>A), Q360N (4483C>A 4485A>T), R361L (4486A>C 4487G>T 4488A>T), I362A (4489A>G 4490T>C), G363K (4492G>A 4493G>A), F367Y (4505T>A 4506T>C), Y368F (4508A>T 4509C>T), S370K (4513T>A 4514C>A 4515C>A), V380I (4543G>A), R381K (4546C>A 4547G>A 4548C>A), A383E (4553C>A 4554T>A), P384E (4555C>G 4556C>A), E385N (4558G>A 4560A>T), T386S (4561A>T 4563A>T), Q388A (4567C>G 4568A>C 4569G>T), C394T (4585T>A 4586G>C 4587T>A), Q396N (4591C>A 4593A>T), Q410N (4633C>A 4635A>T), A413Q (4642G>C 4643C>A 4644T>A), H418K (4657C>A 4659C>A), S422I (4670G>T 4671T>A), L423F (4672C>T 4674C>T), N425E (4678A>G 4680C>A), M426K (4682T>A), Y427N (4684T>A), P428_Q429del (4687_4692delCCACAG), C431L (4696T>C 4697G>T 4698T>A), A432I (4699G>A 4700C>T 4701T>C), V435I (4708G>A 4710T>A), I439L (4720A>C 4722C>A), V440I (4723G>A 4725C>A), F441C (4727T>G 4728C>T), K443N (4734A>T), T444S (4735A>T 4737T>A), E445Y (4738G>T 4740A>T), E446Q (4741G>C), G450T (4753G>A 4754G>C), V452L (4759G>C 4761C>T), I454Q (4765A>C 4766T>A 4767T>A), V455F (4768G>T), L456S (4771C>T 4772T>C 4773A>T), N457Q (4774A>C 4776C>A), R458L (4777A>T 4778G>T), K460V (4783A>G 4784A>T 4785A>T), A461E (4787C>A 4788C>A), L462N (4789C>A 4790T>A 4791A>T), K468Q (4807A>C 4809A>G), A471E (4817C>A 4818C>G), Q472D (4819C>G 4821G>T), L473I (4822T>A 4824G>A), C474S (4825T>A 4827C>T), T476N (4832C>A 4833A>T), T477E (4834A>G 4835C>A 4836T>A), N479E (4840A>G 4842T>A), L483M (4852C>A 4854A>G), V484I (4855G>A), E486S (4861G>T 4862A>C 4863A>C), R487K (4865G>A), G488del (4867_4869delGGA)									

	Begin	End	Coverage	Score	Concordance	Matches	Identities	I/D/M/F*	Stop Codons
**NT**	**4219**	**4872**	**8.3%**	**219**	**17.5%**	**644 (98.5%)**	**386 (59.0%)**	**0/10**	
Codon mutations:	TTA272CTA (4219T>C), TTA273AAA (4222T>A 4223T>A), ACT275CCC (4228A>C 4230T>C), AAC276AGC (4232A>G), AAA277ATC (4235A>T 4236A>C), ATT279ATA (4242T>A), AAG280AGA (4244A>G 4245G>A), TGT281GTT (4246T>G 4247G>T), CCA283CCT (4254A>T), CAG285CTA (4259A>T 4260G>A), TAT286TAC (4263T>C), TCA287AAT (4264T>A 4265C>A 4266A>T), CCT288GAA (4267C>G 4268C>A 4269T>A), GAC290GAT (4275C>T), CGA291AAA (4276C>A 4277G>A), GAA292ACT (4279G>A 4280A>C 4281A>T), GAG293GAA (4284G>A), ACT296ATT (4292C>T), ATC298ATT (4299C>T), GAA299CAA (4300G>C), CTA301TTA (4306C>T), CTC302TTA (4309C>T 4311C>A), AAG303AAT (4314G>T), CTT304ATG (4315C>A 4317T>G), GGT305AAA (4318G>A 4319G>A 4320T>A), ATC306TTA (4321A>T 4323C>A), ATC307ATA (4326C>A), CCT309CAT (4331C>A), AGC310AGT (4335C>T), AAG311AAA (4338G>A), TCC315AGT (4348T>A 4349C>G 4350C>T), TCA316AGC (4351T>A 4352C>G 4353A>C), GCC318GCA (4359C>A), TTC319TTT (4362C>T), AGG322AGA (4371G>A), AAT323AAC (4374T>C), GCC325GGC (4379C>G), GAG326GAA (4383G>A), ATC327CAA (4384A>C 4385T>A 4386C>A), CGA329-CT (4390delC 4391G>C 4392A>T), GGC330GGA (4395C>A), AGA333CGA (4402A>C), ATT336ATA (4413T>A), AAG340GAG (4423A>G), TTA341CTT (4426T>C 4428A>T), GAC343AAA (4432G>A 4434C>A), CAT344AAT (4435C>A), ACA345TGT (4438A>T 4439C>G 4440A>T), AAG346GTC (4441A>G 4442A>T 4443G>C), GGA347TTC (4444G>T 4445G>T 4446A>C), GAT348GAC (4449T>C), GGC349GGA (4452C>A), TAC350TAT (4455C>T), CTA351TTC (4456C>T 4458A>C), CTT352ATC (4459C>A 4461T>C), CCT353CCA (4464T>A), AAC354AAT (4467C>T), AAG355AAA (4470G>A), GAG356AAT (4471G>A 4473G>T), CAA357AAC (4474C>A 4476A>C), CTG358CTT (4479G>T), CTT359ATA (4480C>A 4482T>A), CAA360AAT (4483C>A 4485A>T), AGA361CTT (4486A>C 4487G>T 4488A>T), ATC362GCC (4489A>G 4490T>C), GGA363AAA (4492G>A 4493G>A), GGT364GGA (4497T>A), AAG365AAA (4500G>A), TTT367TAC (4505T>A 4506T>C), TAC368TTT (4508A>T 4509C>T), TCT369TCA (4512T>A), TCC370AAA (4513T>A 4514C>A 4515C>A), GAC372GAT (4521C>T), TGT373TGC (4524T>C), TCT375AGT (4528T>A 4529C>G), CAG379CAA (4542G>A), GTA380ATA (4543G>A), CGC381AAA (4546C>A 4547G>A 4548C>A), CTT382CTA (4551T>A), GCT383GAA (4553C>A 4554T>A), CCA384GAA (4555C>G 4556C>A), GAA385AAT (4558G>A 4560A>T), ACA386TCT (4561A>T 4563A>T), ATT387ATA (4566T>A), CAG388GCT (4567C>G 4568A>C 4569G>T), CTA389TTA (4570C>T), ACC390ACA (4575C>A), GCT391GCA (4578T>A), AGC393AGT (4584C>T), TGT394ACA (4585T>A 4586G>C 4587T>A), CCC395CCA (4590C>A), CAA396AAT (4591C>A 4593A>T), GTC403GTG (4614C>G), CAA410AAT (4633C>A 4635A>T), GCT413CAA (4642G>C 4643C>A 4644T>A), ATT414ATC (4647T>C), TTC415TTT (4650C>T), AGG417AGA (4656G>A), CAC418AAA (4657C>A 4659C>A), GAC420GAT (4665C>T), AGT422ATA (4670G>T 4671T>A), CTC423TTT (4672C>T 4674C>T), AGC424TCT (4675A>T 4676G>C 4677C>T), AAC425GAA (4678A>G 4680C>A), ATG426AAG (4682T>A), TAT427AAT (4684T>A), CCA428_CAG429del (4687_4692delCCACAG), TGT431CTA (4696T>C 4697G>T 4698T>A), GCT432ATC (4699G>A 4700C>T 4701T>C), GTA433GTC (4704A>C), GTT435ATA (4708G>A 4710T>A), GAC437GAT (4716C>T), ATC438ATA (4719C>A), ATC439CTA (4720A>C 4722C>A), GTC440ATA (4723G>A 4725C>A), TTC441TGT (4727T>G 4728C>T), AGC442TCT (4729A>T 4730G>C 4731C>T), AAA443AAT (4734A>T), ACT444TCA (4735A>T 4737T>A), GAA445TAT (4738G>T 4740A>T), GAA446CAA (4741G>C), GAA447GAG (4746A>G), GGA450ACA (4753G>A 4754G>C), CAT451CAC (4758T>C), GTC452CTT (4759G>C 4761C>T), ATT454CAA (4765A>C 4766T>A 4767T>A), GTT455TTT (4768G>T), CTA456TCT (4771C>T 4772T>C 4773A>T), AAC457CAA (4774A>C 4776C>A), AGA458TTA (4777A>T 4778G>T), TGC459TGT (4782C>T), AAA460GTT (4783A>G 4784A>T 4785A>T), GCC461GAA (4787C>A 4788C>A), CTA462AAT (4789C>A 4790T>A 4791A>T), ATA464ATT (4797A>T), CTT466TTA (4801C>T 4803T>A), AAA468CAG (4807A>C 4809A>G), AAG469AAA (4812G>A), AAA470AAG (4815A>G), GCC471GAG (4817C>A 4818C>G), CAG472GAT (4819C>G 4821G>T), TTG473ATA (4822T>A 4824G>A), TGC474AGT (4825T>A 4827C>T), ACA476AAT (4832C>A 4833A>T), ACT477GAA (4834A>G 4835C>A 4836T>A), ATC478ATT (4839C>T), AAT479GAA (4840A>G 4842T>A), GGT482GGA (4851T>A), CTA483ATG (4852C>A 4854A>G), GTA484ATA (4855G>A), ATA485ATT (4860A>T), GAA486TCC (4861G>T 4862A>C 4863A>C), AGA487AAA (4865G>A), GGA488del (4867_4869delGGA)								

*: Inserts / Deletes / Misaligned / Frameshifts

Analysis details

This analysis was performed with panviral2.64

NGS Details (UN70): Alisovirus socal22

Assembly

**Coverage Length**	144 (1 contig(s))
**Depth Of Coverage**	19.1
**Number Of Reads**	28
**Reads Per Million**	0.53 rpm (after QC)
**Ambiguities**	0
**Assembly Method**	de novo + reference guided assembly
**Consensus Caller**	Bcf Tools

Coverage Map

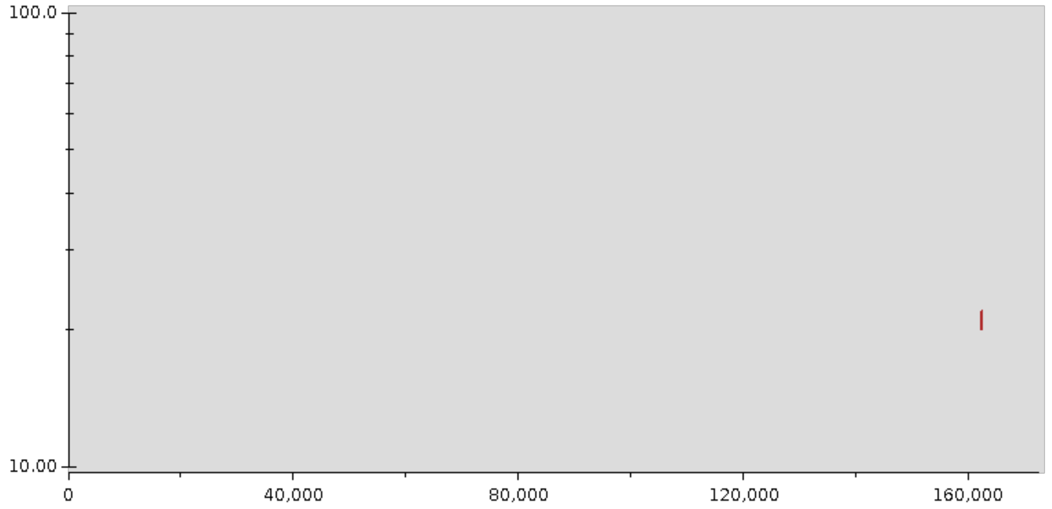

Assignment

**Type**	Alisovirus socal22 (Taxonomy ID: 2955237)
**Reference Genome**	NC_031903.1
**NT Identity (%)**	79.8611
**AA Identity (%)**	85.4167
**Number Of Stop Codons**	1
**Number Of CDS**	214

Alignment

**Alignment Score**	172.0 (NT) + 277.0 (AA) = 449.0
**Concordance (%)**	75.3356
**Alignment Method**	Local, heuristic, nucleotide (BLASTN)

Genome Region

Sequence starts at position 162305 and ends at position 162448 relative to NC_031903.1 reference sequence.

Alignment Detailed Statistics

	Begin	End	Coverage	Score	Concordance	Matches	Identities	I/D/M/F*	Stop Codons
**NT**	**162305**	**162448**	**0.1%**	**172**	**59.7%**	**144 (100%)**	**115 (79.9%)**	**0/0**	

Mutations: 162314C>T, 162315A>G, 162319T>G, 162322A>C, 162324C>T, 162327T>A, 162328A>G, 162330T>A, 162331A>G, 162333T>C, 162336A>T, 162339C>A, 162345T>G, 162348T>C, 162351T>A, 162357T>C, 162360C>T, 162366T>C, 162369A>T, 162373C>T, 162378G>A, 162384T>C, 162393G>A, 162408A>G, 162411C>T, 162423G>A, 162429C>T, 162435G>A, 162438T>C

*: Inserts / Deletes / Misaligned / Frameshifts

Analysis details

This analysis was performed with panviral2.64

NGS Details (UN70): Badnavirus maculakalanchoes

Assembly

**Coverage Length**	278 (1 contig(s))
**Depth Of Coverage**	10.4
**Number Of Reads**	27
**Reads Per Million**	0.51 rpm (after QC)
**Ambiguities**	0
**Assembly Method**	de novo + reference guided assembly
**Consensus Caller**	Bcf Tools

Coverage Map

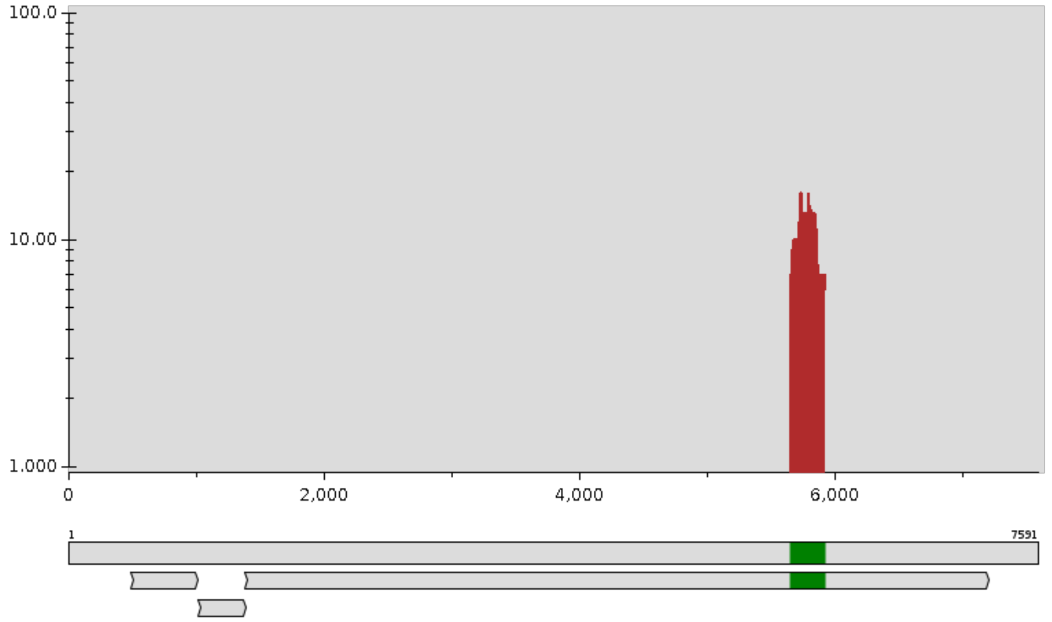

Assignment

**Type**	Badnavirus maculakalanchoes (Taxonomy ID: 218387)
**Reference Genome**	NC_004540.1
**NT Identity (%)**	55.1601
**AA Identity (%)**	48.9362
**Number Of Stop Codons**	0
**Number Of CDS**	3

Alignment

**Alignment Score**	52.0 (NT) + 341.0 (AA) = 393.0
**Concordance (%)**	31.7447

Alignment Method	Global, seeded, nucleotide + amino acids (AGA)

Genome Region

Sequence starts at position 5649 and ends at position 5926 relative to NC_004540.1 reference sequence.

Alignment Detailed Statistics

	Begin	End	Coverage	Score	Concordance	Matches	Identities	I/D/M/F*	Stop Codons
**NT**	**5649**	**5926**	**3.7%**	**52**	**9.4%**	**278 (98.9%)**	**155 (55.2%)**	**3/0**	
Mutations:	5649A>T, 5656G>A, 5660A>T, 5661T>C, 5662A>T, 5663G>C, 5664C>T, 5668T>A, 5670T>A, 5673C>T, 5674C>T, 5676C>A, 5678A>G, 5680A>T, 5681G>C, 5682T>G, 5687T>A, 5688C>T, 5691C>T, 5694G>A, 5695G>C, 5698G>A, 5699C>G, 5700C>G, 5706G>A, 5707G>A, 5708A>T, 5712A>G, 5713T>A, 5714C>A, 5715A>T, 5716A>G, 5722T>A, 5723G>A, 5726C>A, 5727G>T, 5730C>T, 5734T>A, 5737G>A, 5739G>T, 5740A>C, 5741T>A, 5742A>T, 5743G>T, 5744A>C, 5745C>T, 5750T>A, 5751C>T, 5753A>T, 5754C>T, 5757A>G, 5759G>A, 5760G>T, 5763A>G, 5766A>G, 5775C>T, 5778T>G, 5781T>C, 5782A>T, 5783A>C, 5793T>A, 5796T>A, 5798T>C, 5799A>T, 5802C>T, 5806C>G, 5809A>T, 5810A>T, 5815G>A, 5818G>T, 5819A>C, 5820G>T, 5821T>G, 5822G>T, 5826C>T, 5828G>A, 5829A>C, 5830G>T, 5832A>T, 5832_5833insTAT, 5833A>C, 5836G>A, 5837A>G, 5839G>A, 5841G>A, 5842T>C, 5843T>A, 5844C>T, 5845A>G, 5847A>G, 5848G>T, 5849C>T, 5855A>T, 5857G>T, 5859G>T, 5868A>C, 5869C>T, 5871G>A, 5874C>A, 5876T>A, 5877C>T, 5878T>A, 5879C>G, 5881A>C, 5885G>C, 5886C>T, 5887G>T, 5889C>G, 5890C>G, 5895C>T, 5898T>C, 5899A>G, 5900A>T, 5903G>A, 5904G>A, 5908C>T, 5911C>A, 5913G>A, 5914A>T, 5915G>C, 5916G>T, 5917T>G, 5922C>T, 5925A>G								
**CDS**									
KTSVgp3	1423	1515	4.8%	341	49.3%	93 (98.9%)	46 (48.9%)	1/0/0/0	0
Protein mutations:	V1425I (5656G>A), Y1426F (5660A>T 5661T>C), F1429I (5668T>A 5670T>A), K1432R (5678A>G), F1435Y (5687T>A 5688C>T), V1438L (5695G>C), A1439R (5698G>A 5699C>G 5700C>G), E1442I (5707G>A 5708A>T), S1444N (5713T>A 5714C>A 5715A>T), I1445V (5716A>G), W1447K (5722T>A 5723G>A), T1448N (5726C>A 5727G>T), W1451R (5734T>A), A1452T (5737G>A 5739G>T), I1453H (5740A>C 5741T>A 5742A>T), D1454S (5743G>T 5744A>C 5745C>T), L1456H (5750T>A 5751C>T), Y1457F (5753A>T 5754C>T), W1459Y (5759G>A 5760G>T), K1467S (5782A>T 5783A>C), I1472T (5798T>C 5799A>T), R1475G (5806C>G), K1476L (5809A>T 5810A>T), D1478N (5815G>A), E1479S (5818G>T 5819A>C 5820G>T), C1480V (5821T>G 5822G>T), R1482H (5828G>A 5829A>C), G1483C (5830G>T 5832A>T), G1483_M1484insY (5832_5833insTAT), M1484L (5833A>C), E1485R (5836G>A 5837A>G), E1486K (5839G>A 5841G>A), F1487H (5842T>C 5843T>A 5844C>T), I1488V (5845A>G 5847A>G), A1489L (5848G>T 5849C>T), Y1491F (5855A>T), V1492F (5857G>T 5859G>T), F1498Y (5876T>A 5877C>T), N1500H (5881A>C), S1501T (5885G>C 5886C>T), V1502L (5887G>T 5889C>G), Q1503E (5890C>G), K1506V (5899A>G 5900A>T), R1507K (5903G>A 5904G>A), Q1510K (5911C>A 5913G>A), R1511S (5914A>T 5915G>C 5916G>T), F1512V (5917T>G)								
Codon mutations:	ACA1422..T (5649A>T), GTC1425ATC (5656G>A), TAT1426TTC (5660A>T 5661T>C), AGC1427TCT (5662A>T 5663G>C 5664C>T), TTT1429ATA (5668T>A 5670T>A), GAC1430GAT (5673C>T), CTC1431TTA (5674C>T 5676C>A), AAG1432AGG (5678A>G), AGT1433TCG (5680A>T 5681G>C 5682T>G), TTC1435TAT (5687T>A 5688C>T), CAC1436CAT (5691C>T), CAG1437CAA (5694G>A), GTG1438CTG (5695G>C), GCC1439AGG (5698G>A 5699C>G 5700C>G), GCG1441GCA (5706G>A), GAA1442ATA (5707G>A 5708A>T), GAA1443GAG (5712A>G), TCA1444AAT (5713T>A 5714C>A 5715A>T), ATC1445GTC (5716A>G), TGG1447AAG (5722T>A 5723G>A), ACG1448AAT (5726C>A 5727G>T), GCC1449GCT (5730C>T), TGG1451AGG (5734T>A), GCG1452ACT (5737G>A 5739G>T), ATA1453CAT (5740A>C 5741T>A 5742A>T), GAC1454TCT (5743G>T 5744A>C 5745C>T), CTC1456CAT (5750T>A 5751C>T), TAC1457TTT (5753A>T 5754C>T), GAA1458GAG (5757A>G), TGG1459TAT (5759G>A 5760G>T), TTA1460TTG (5763A>G), GTA1461GTG (5766A>G), TTC1464TTT (5775C>T), GGT1465GGG (5778T>G), CTT1466CTC (5781T>C), AAA1467TCA (5782A>T 5783A>C), CCT1470CCA (5793T>A), GCT1471GCA (5796T>A), ATA1472ACT (5798T>C 5799A>T), TTC1473TTT (5802C>T), CGA1475GGA (5806C>G), AAA1476TTA (5809A>T 5810A>T), GAT1478AAT (5815G>A), GAG1479TCT (5818G>T 5819A>C 5820G>T), TGT1480GTT (5821T>G 5822G>T), TTC1481TTT (5826C>T), CGA1482CAC (5828G>A 5829A>C), GGA1483TGT (5830G>T 5832A>T), GGA1483_ATG1484insTAT (5832_5833insTAT), ATG1484CTG (5833A>C), GAG1485AGG (5836G>A 5837A>G), GAG1486AAA (5839G>A 5841G>A), TTC1487CAT (5842T>C 5843T>A 5844C>T), ATA1488GTG (5845A>G 5847A>G), GCA1489TTA (5848G>T 5849C>T), TAT1491TTT (5855A>T), GTG1492TTT (5857G>T 5859G>T), ATA1495ATC (5868A>C), CTG1496TTA (5869C>T 5871G>A), ATC1497ATA (5874C>A), TTC1498TAT (5876T>A 5877C>T), TCC1499AGC (5878T>A 5879C>G), AAC1500CAC (5881A>C), AGC1501ACT (5885G>C 5886C>T), GTC1502TTG (5887G>T 5889C>G), CAA1503GAA (5890C>G), GAC1504GAT (5895C>T), CAT1505CAC (5898T>C), AAG1506GTG (5899A>G 5900A>T), AGG1507AAA (5903G>A 5904G>A), CTG1509TTG (5908C>T), CAG1510AAA (5911C>A 5913G>A), AGG1511TCT (5914A>T 5915G>C 5916G>T), TTT1512GTT (5917T>G), TTC1513TTT (5922C>T), GAA1514GAG (5925A>G)								
**Proteins**									
putative polyprotein (NP_777317.1)	1423	1515	4.8%	341	49.3%	93 (98.9%)	46 (48.9%)	1/0/0/0	0
Protein mutations:	V1425I (5656G>A), Y1426F (5660A>T 5661T>C), F1429I (5668T>A 5670T>A), K1432R (5678A>G), F1435Y (5687T>A 5688C>T), V1438L (5695G>C), A1439R (5698G>A 5699C>G 5700C>G), E1442I (5707G>A 5708A>T), S1444N (5713T>A 5714C>A 5715A>T), I1445V (5716A>G), W1447K (5722T>A 5723G>A), T1448N (5726C>A 5727G>T), W1451R (5734T>A), A1452T (5737G>A 5739G>T), I1453H (5740A>C 5741T>A 5742A>T), D1454S (5743G>T 5744A>C 5745C>T), L1456H (5750T>A 5751C>T), Y1457F (5753A>T 5754C>T), W1459Y (5759G>A 5760G>T), K1467S (5782A>T 5783A>C), I1472T (5798T>C 5799A>T), R1475G (5806C>G), K1476L (5809A>T 5810A>T), D1478N (5815G>A), E1479S (5818G>T 5819A>C 5820G>T), C1480V (5821T>G 5822G>T), R1482H (5828G>A 5829A>C), G1483C (5830G>T 5832A>T), G1483_M1484insY (5832_5833insTAT), M1484L (5833A>C), E1485R (5836G>A 5837A>G), E1486K (5839G>A 5841G>A), F1487H (5842T>C 5843T>A 5844C>T), I1488V (5845A>G 5847A>G), A1489L (5848G>T 5849C>T), Y1491F (5855A>T), V1492F (5857G>T 5859G>T), F1498Y (5876T>A 5877C>T), N1500H (5881A>C), S1501T (5885G>C 5886C>T), V1502L (5887G>T 5889C>G), Q1503E (5890C>G), K1506V (5899A>G 5900A>T), R1507K (5903G>A 5904G>A), Q1510K (5911C>A 5913G>A), R1511S (5914A>T 5915G>C 5916G>T), F1512V (5917T>G)								
Codon mutations:	ACA1422..T (5649A>T), GTC1425ATC (5656G>A), TAT1426TTC (5660A>T 5661T>C), AGC1427TCT (5662A>T 5663G>C 5664C>T), TTT1429ATA (5668T>A 5670T>A), GAC1430GAT (5673C>T), CTC1431TTA (5674C>T 5676C>A), AAG1432AGG (5678A>G), AGT1433TCG (5680A>T 5681G>C 5682T>G), TTC1435TAT (5687T>A 5688C>T), CAC1436CAT (5691C>T), CAG1437CAA (5694G>A), GTG1438CTG (5695G>C), GCC1439AGG (5698G>A 5699C>G 5700C>G), GCG1441GCA (5706G>A), GAA1442ATA (5707G>A 5708A>T), GAA1443GAG (5712A>G), TCA1444AAT (5713T>A 5714C>A 5715A>T), ATC1445GTC (5716A>G), TGG1447AAG (5722T>A 5723G>A), ACG1448AAT (5726C>A 5727G>T), GCC1449GCT (5730C>T), TGG1451AGG (5734T>A), GCG1452ACT (5737G>A 5739G>T), ATA1453CAT (5740A>C 5741T>A 5742A>T), GAC1454TCT (5743G>T 5744A>C 5745C>T), CTC1456CAT (5750T>A 5751C>T), TAC1457TTT (5753A>T 5754C>T), GAA1458GAG (5757A>G), TGG1459TAT (5759G>A 5760G>T), TTA1460TTG (5763A>G), GTA1461GTG (5766A>G), TTC1464TTT (5775C>T), GGT1465GGG (5778T>G), CTT1466CTC (5781T>C), AAA1467TCA (5782A>T 5783A>C), CCT1470CCA (5793T>A), GCT1471GCA (5796T>A), ATA1472ACT (5798T>C 5799A>T), TTC1473TTT (5802C>T), CGA1475GGA (5806C>G), AAA1476TTA (5809A>T 5810A>T), GAT1478AAT (5815G>A), GAG1479TCT (5818G>T 5819A>C 5820G>T), TGT1480GTT (5821T>G 5822G>T), TTC1481TTT (5826C>T), CGA1482CAC (5828G>A 5829A>C), GGA1483TGT (5830G>T 5832A>T), GGA1483_ATG1484insTAT (5832_5833insTAT), ATG1484CTG (5833A>C), GAG1485AGG (5836G>A 5837A>G), GAG1486AAA (5839G>A 5841G>A), TTC1487CAT (5842T>C 5843T>A 5844C>T), ATA1488GTG (5845A>G 5847A>G), GCA1489TTA (5848G>T 5849C>T), TAT1491TTT (5855A>T), GTG1492TTT (5857G>T 5859G>T), ATA1495ATC (5868A>C), CTG1496TTA (5869C>T 5871G>A), ATC1497ATA (5874C>A), TTC1498TAT (5876T>A 5877C>T), TCC1499AGC (5878T>A 5879C>G), AAC1500CAC (5881A>C), AGC1501ACT (5885G>C 5886C>T), GTC1502TTG (5887G>T 5889C>G), CAA1503GAA (5890C>G), GAC1504GAT (5895C>T), CAT1505CAC (5898T>C), AAG1506GTG (5899A>G 5900A>T), AGG1507AAA (5903G>A 5904G>A), CTG1509TTG (5908C>T), CAG1510AAA (5911C>A 5913G>A), AGG1511TCT (5914A>T 5915G>C 5916G>T), TTT1512GTT (5917T>G), TTC1513TTT (5922C>T), GAA1514GAG (5925A>G)								

*: Inserts / Deletes / Misaligned / Frameshifts

Analysis details

This analysis was performed with panviral2.64

NGS Details (UN70): Dioscovirus dioscoreae

Assembly

**Coverage Length**	603 (2 contig(s))
**Depth Of Coverage**	4.1
**Number Of Reads**	26
**Reads Per Million**	0.49 rpm (after QC)
**Ambiguities**	0
**Assembly Method**	de novo + reference guided assembly
**Consensus Caller**	Bcf Tools

Coverage Map

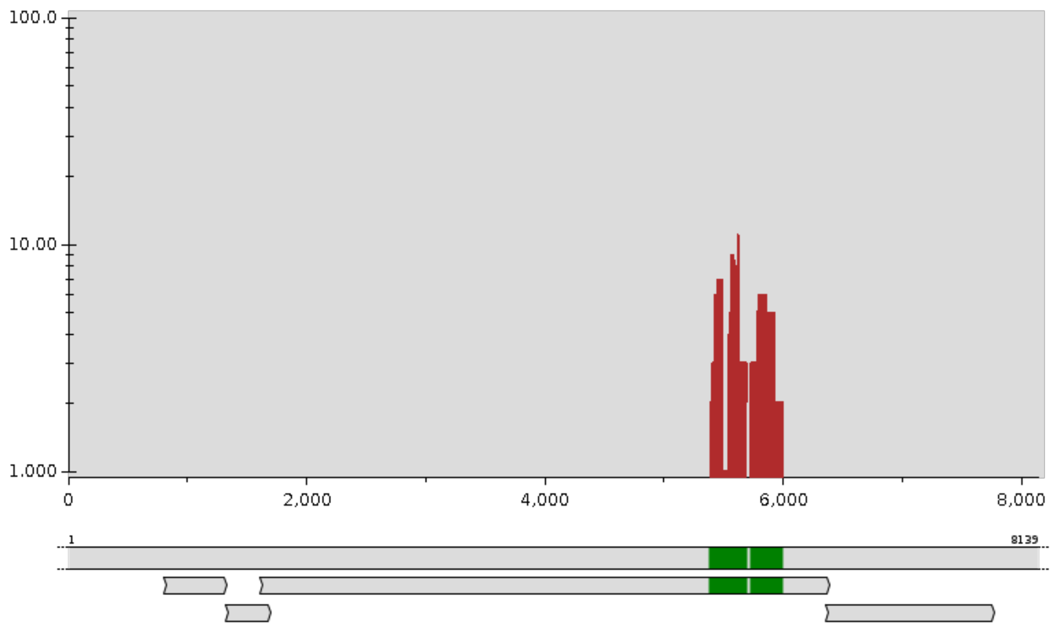

Assignment

**Type**	Dioscovirus dioscoreae (Taxonomy ID: 3052184)
**Reference Genome**	NC_040712.1
**NT Identity (%)**	62.5
**AA Identity (%)**	48.2412
**Number Of Stop Codons**	1
**Number Of CDS**	4

Alignment

**Alignment Score**	288.0 (NT) + 708.0 (AA) = 996.0
**Concordance (%)**	38.3372

Alignment Method	Global, seeded, nucleotide + amino acids (AGA)

Genome Region

Sequence starts at position 5383 and ends at position 6002 relative to NC_040712.1 reference sequence.

Alignment Detailed Statistics

	Begin	End	Coverage	Score	Concordance	Matches	Identities	I/D/M/F*	Stop Codons
**NT**	**5383**	**6002**	**7.4%**	**288**	**24.2%**	**600 (99.5%)**	**375 (62.2%)**	**0/3**	
Mutations:	5383T>A, 5387C>T, 5394A>C, 5395T>G, 5396G>A, 5400G>A, 5403C>T, 5405A>T, 5406C>G, 5407A>G, 5408G>A, 5409A>G, 5410G>A, 5412A>G, 5413T>A, 5415G>T, 5416A>G, 5418T>A, 5419G>A, 5420G>A, 5423A>T, 5424G>A, 5426A>T, 5430G>A, 5431T>A, 5432C>G, 5433T>A, 5434G>T, 5436C>A, 5437C>A, 5439C>T, 5441G>T, 5443G>A, 5444T>G, 5446A>T, 5449T>A, 5453A>G, 5457A>T, 5462A>T, 5468A>C, 5471C>T, 5474A>C, 5475C>T, 5477C>G, 5479A>C, 5480A>T, 5486A>G, 5489T>C, 5490T>A, 5491C>A, 5493G>A, 5502C>A, 5505A>T, 5506A>T, 5511G>A, 5514A>G, 5519A>T, 5522C>T, 5524A>G, 5526A>G, 5530A>T, 5532T>G, 5533C>G, 5535G>A, 5541G>T, 5543C>T, 5544T>C, 5545G>T, 5553A>T, 5555A>T, 5558C>T, 5561T>C, 5564T>C, 5568A>G, 5577G>A, 5579A>G, 5581G>A, 5583A>C, 5585A>G, 5586C>G, 5588A>T, 5589C>G, 5591A>G, 5595G>A, 5598C>G, 5603T>C, 5604T>C, 5607T>A, 5608T>A, 5612G>A, 5613T>G, 5615C>T, 5618C>T, 5619C>G, 5626G>T, 5627T>C, 5628A>G, 5629A>T, 5630G>A, 5631G>A, 5632A>G, 5633A>T, 5634G>A, 5639G>A, 5640C>A, 5642T>A, 5645T>A, 5646T>A, 5648G>A, 5649T>A, 5651A>C, 5656C>A, 5657A>G, 5661T>A, 5664A>G, 5667A>T, 5670G>T, 5672A>T, 5673G>A, 5674T>A, 5676G>A, 5679A>C, 5685G>A, 5687A>C, 5691C>T, 5693T>A, 5721C>A, 5726C>A, 5739T>G, 5740T>C, 5741A>T, 5748A>G, 5756A>T, 5757T>A, 5759T>G, 5762T>A, 5763_5765delGAA, 5768C>T, 5769C>A, 5777A>T, 5779C>A, 5781T>A, 5782T>C, 5786G>A, 5788G>A, 5790C>T, 5795G>A, 5798G>A, 5807G>A, 5808A>T, 5811T>G, 5813A>G, 5816C>T, 5822C>T, 5828T>C, 5830A>T, 5834C>A, 5836C>A, 5843G>A, 5844A>G, 5846T>A, 5850T>A, 5851A>T, 5853A>G, 5855T>A, 5856A>G, 5859A>C, 5860T>C, 5864T>A, 5868A>G, 5869A>C, 5870T>A, 5873G>A, 5874T>A, 5875G>T, 5876T>A, 5877T>G, 5878C>G, 5879C>A, 5882C>T, 5889G>C, 5893G>A, 5895A>C, 5897A>C, 5903T>C, 5904T>A, 5906T>A, 5907C>G, 5910G>A, 5912C>T, 5913T>A, 5914G>T, 5915G>T, 5918G>A, 5919A>C, 5924T>A, 5925A>C, 5926G>A, 5928A>C, 5933C>T, 5940G>A, 5941T>A, 5945T>A, 5947C>A, 5948C>A, 5951A>C, 5952T>A, 5957C>A, 5958C>G, 5959C>A, 5960T>C, 5963G>A, 5965T>G, 5966T>C, 5967T>A, 5973A>C, 5974A>T, 5976C>G, 5977C>A, 5979C>T, 5980A>C, 5982A>G, 5983G>A, 5988A>C, 5990T>A, 5999G>A								

CDS

	Begin	End	Coverage	Score	Concordance	Matches	Identities	I/D/M/F*	Stop Codons
EXK67_gp3	1259	1464	12.5%	708	49.7%	199 (99.5%)	96 (48.0%)	0/1/0/0	1
Protein mutations:	M1262R (5394A>C 5395T>G 5396G>A), D1264N (5400G>A), P1265S (5403C>T 5405A>T), Q1266G (5406C>G 5407A>G 5408G>A), S1267D (5409A>G 5410G>A), I1268E (5412A>G 5413T>A), E1269* (5415G>T 5416A>G), W1270K (5418T>A 5419G>A 5420G>A), A1272T (5424G>A 5426A>T), V1274K (5430G>A 5431T>A 5432C>G), C1275I (5433T>A 5434G>T), P1276K (5436C>A 5437C>A), Q1277Y (5439C>T 5441G>T), G1278E (5443G>A 5444T>G), H1279L (5446A>T), F1280Y (5449T>A), I1283L (5457A>T), K1290T (5479A>C 5480A>T), S1294K (5490T>A 5491C>A), V1295I (5493G>A), K1299L (5505A>T 5506A>T), D1301N (5511G>A), N1302D (5514A>G), K1305R (5524A>G), K1306E (5526A>G), Y1307F (5530A>T), S1308G (5532T>G 5533C>G), E1309K (5535G>A), V1311F (5541G>T 5543C>T), C1312L (5544T>C 5545G>T), I1315F (5553A>T 5555A>T), I1320V (5568A>G), E1323K (5577G>A 5579A>G), S1324N (5581G>A), I1325L (5583A>C 5585A>G), Q1326D (5586C>G 5588A>T), Q1327E (5589C>G 5591A>G), V1329I (5595G>A), Q1330E (5598C>G), L1333K (5607T>A 5608T>A), F1335V (5613T>G 5615C>T), Q1337E (5619C>G), C1339F (5626G>T 5627T>C), K1340V (5628A>G 5629A>T 5630G>A), E1341S (5631G>A 5632A>G 5633A>T), E1342K (5634G>A), L1344I (5640C>A 5642T>A), L1346I (5646T>A 5648G>A), S1347T (5649T>A 5651A>C), T1349K (5656C>A 5657A>G), L1351M (5661T>A), K1352E (5664A>G), I1353L (5667A>T), G1354C (5670G>T 5672A>T), V1355K (5673G>A 5674T>A), A1356T (5676G>A), N1357H (5679A>C), E1359N (5685G>A 5687A>C), Q1371K (5721C>A), L1377A (5739T>G 5740T>C 5741A>T), I1380V (5748A>G), E1382D (5756A>T), F1383M (5757T>A 5759T>G), E1385del (5763_5765delGAA), Q1387K (5769C>A), E1389D (5777A>T), T1390K (5779C>A), L1391T (5781T>A 5782T>C), G1393D (5788G>A), I1400L (5808A>T), L1401V (5811T>G 5813A>G), Y1407F (5830A>T), P1409Q (5836C>A), S1412G (5844A>G 5846T>A), Y1414I (5850T>A 5851A>T), T1415A (5853A>G 5855T>A), R1416G (5856A>G), I1417P (5859A>C 5860T>C), F1418L (5864T>A), N1420A (5868A>G 5869A>C 5870T>A), C1422I (5874T>A 5875G>T 5876T>A), S1423G (5877T>G 5878C>G 5879C>A), E1427Q (5889G>C), R1428K (5893G>A), K1429H (5895A>C 5897A>C), S1432T (5904T>A 5906T>A), Q1433E (5907C>G), D1434N (5910G>A 5912C>T), W1435I (5913T>A 5914G>T 5915G>T), M1437L (5919A>C), R1439Q (5925A>C 5926G>A), K1440Q (5928A>C), V1444K (5940G>A 5941T>A), T1446K (5947C>A 5948C>A), K1447N (5951A>C), L1448I (5952T>A), P1450D (5958C>G 5959C>A 5960T>C), I1452S (5965T>G 5966T>C), L1453I (5967T>A), K1455L (5973A>C 5974A>T), P1456E (5976C>G 5977C>A), Q1457S (5979C>T 5980A>C), S1458D (5982A>G 5983G>A), I1460L (5988A>C 5990T>A)								
Codon mutations:	TTC1258.AC (5383T>A), CAC1259CAT (5387C>T), ATG1262CGA (5394A>C 5395T>G 5396G>A), GAT1264AAT (5400G>A), CCA1265TCT (5403C>T 5405A>T), CAG1266GGA (5406C>G 5407A>G 5408G>A), AGT1267GAT (5409A>G 5410G>A), ATA1268GAA (5412A>G 5413T>A), GAA1269TGA (5415G>T 5416A>G), TGG1270AAA (5418T>A 5419G>A 5420G>A), ACA1271ACT (5423A>T), GCA1272ACT (5424G>A 5426A>T), GTC1274AAG (5430G>A 5431T>A 5432C>G), TGC1275ATC (5433T>A 5434G>T), CCA1276AAA (5436C>A 5437C>A), CAG1277TAT (5439C>T 5441G>T), GGT1278GAG (5443G>A 5444T>G), CAT1279CTT (5446A>T), TTT1280TAT (5449T>A), GAA1281GAG (5453A>G), ATA1283TTA (5457A>T), GTA1284GTT (5462A>T), CCA1286CCC (5468A>C), TTC1287TTT (5471C>T), GGA1288GGC (5474A>C), CTC1289TTG (5475C>T 5477C>G), AAA1290ACT (5479A>C 5480A>T), GCA1292GCG (5486A>G), CCT1293CCC (5489T>C), TCA1294AAA (5490T>A 5491C>A), GTA1295ATA (5493G>A), CGA1298AGA (5502C>A), AAA1299TTA (5505A>T 5506A>T), GAT1301AAT (5511G>A), AAT1302GAT (5514A>G), ATA1303ATT (5519A>T), TTC1304TTT (5522C>T), AAA1305AGA (5524A>G), AAA1306GAA (5526A>G), TAT1307TTT (5530A>T), TCA1308GGA (5532T>G 5533C>G), GAA1309AAA (5535G>A), GTC1311TTT (5541G>T 5543C>T), TGT1312CTT (5544T>C 5545G>T), ATA1315TTT (5553A>T 5555A>T), GAC1316GAT (5558C>T), GAT1317GAC (5561T>C), ATT1318ATC (5564T>C), ATA1320GTA (5568A>G), GAA1323AAG (5577G>A 5579A>G), AGT1324AAT (5581G>A), ATA1325CTG (5583A>C 5585A>G), CAA1326GAT (5586C>G 5588A>T), CAA1327GAG (5589C>G 5591A>G), GTA1329ATA (5595G>A), CAA1330GAA (5598C>G), CAT1331CAC (5603T>C), TTA1332CTA (5604T>C), TTA1333AAA (5607T>A 5608T>A), CAG1334CAA (5612G>A), TTC1335GTT (5613T>G 5615C>T), TTC1336TTT (5618C>T), CAA1337GAA (5619C>G), TGT1339TTC (5626G>T 5627T>C), AAG1340GTA (5628A>G 5629A>T 5630G>A), GAA1341AGT (5631G>A 5632A>G 5633A>T), GAA1342AAA (5634G>A), GGG1343GGA (5639G>A), CTT1344ATA (5640C>A 5642T>A), ATT1345ATA (5645T>A), TTG1346ATA (5646T>A 5648G>A), TCA1347ACC (5649T>A 5651A>C), ACA1349AAG (5656C>A 5657A>G), TTG1351ATG (5661T>A), AAA1352GAA (5664A>G), ATA1353TTA (5667A>T), GGA1354TGT (5670G>T 5672A>T), GTA1355AAA (5673G>A 5674T>A), GCA1356ACA (5676G>A), AAT1357CAT (5679A>C), GAA1359AAC (5685G>A 5687A>C), CTT1361TTA (5691C>T 5693T>A), CAA1371AAA (5721C>A), CTC1372CTA (5726C>A), TTA1377GCT (5739T>G 5740T>C 5741A>T), ATA1380GTA (5748A>G), GAA1382GAT (5756A>T), TTT1383ATG (5757T>A 5759T>G), CCT1384CCA (5762T>A), GAA1385del (5763_5765delGAA), GAC1386GAT (5768C>T), CAA1387AAA (5769C>A), GAA1389GAT (5777A>T), ACA1390AAA (5779C>A), TTA1391ACA (5781T>A 5782T>C), AAG1392AAA (5786G>A), GGT1393GAT (5788G>A), CTA1394TTA (5790C>T), CAG1395CAA (5795G>A), AAG1396AAA (5798G>A), GGG1399GGA (5807G>A), ATA1400TTA (5808A>T), TTA1401GTG (5811T>G 5813A>G), AAC1402AAT (5816C>T), GCC1404GCT (5822C>T), AAT1406AAC (5828T>C), TAT1407TTT (5830A>T), ATC1408ATA (5834C>A), CCA1409CAA (5836C>A), TTG1411TTA (5843G>A), AGT1412GGA (5844A>G 5846T>A), TAT1414ATT (5850T>A 5851A>T), ACT1415GCA (5853A>G 5855T>A), AGA1416GGA (5856A>G), ATA1417CCA (5859A>C 5860T>C), TTT1418TTA (5864T>A), AAT1420GCA (5868A>G 5869A>C 5870T>A), AAG1421AAA (5873G>A), TGT1422ATA (5874T>A 5875G>T 5876T>A), TCC1423GGA (5877T>G 5878C>G 5879C>A), AGC1424AGT (5882C>T), GAA1427CAA (5889G>C), AGA1428AAA (5893G>A), AAA1429CAC (5895A>C 5897A>C), AAT1431AAC (5903T>C), TCT1432ACA (5904T>A 5906T>A), CAA1433GAA (5907C>G), GAC1434AAT (5910G>A 5912C>T), TGG1435ATT (5913T>A 5914G>T 5915G>T), AAG1436AAA (5918G>A), ATG1437CTG (5919A>C), GTT1438GTA (5924T>A), AGA1439CAA (5925A>C 5926G>A), AAA1440CAA (5928A>C), ATC1441ATT (5933C>T), GTA1444AAA (5940G>A 5941T>A), ATT1445ATA (5945T>A), ACC1446AAA (5947C>A 5948C>A), AAA1447AAC (5951A>C), TTA1448ATA (5952T>A), CCC1449CCA (5957C>A), CCT1450GAC (5958C>G 5959C>A 5960T>C), TTG1451TTA (5963G>A), ATT1452AGC (5965T>G 5966T>C), TTA1453ATA (5967T>A), AAA1455CTA (5973A>C 5974A>T), CCA1456GAA (5976C>G 5977C>A), CAA1457TCA (5979C>T 5980A>C), AGT1458GAT (5982A>G 5983G>A), ATT1460CTA (5988A>C 5990T>A), GAG1463GAA (5999G>A)								

Proteins

	Begin	End	Coverage	Score	Concordance	Matches	Identities	I/D/M/F*	Stop Codons
ORF3 (YP_009553219.1)	1259	1464	12.5%	708	49.7%	199 (99.5%)	96 (48.0%)	0/1/0/0	1
Protein mutations:	M1262R (5394A>C 5395T>G 5396G>A), D1264N (5400G>A), P1265S (5403C>T 5405A>T), Q1266G (5406C>G 5407A>G 5408G>A), S1267D (5409A>G 5410G>A), I1268E (5412A>G 5413T>A), E1269* (5415G>T 5416A>G), W1270K (5418T>A 5419G>A 5420G>A), A1272T (5424G>A 5426A>T), V1274K (5430G>A 5431T>A 5432C>G), C1275I (5433T>A 5434G>T), P1276K (5436C>A 5437C>A), Q1277Y (5439C>T 5441G>T), G1278E (5443G>A 5444T>G), H1279L (5446A>T), F1280Y (5449T>A), I1283L (5457A>T), K1290T (5479A>C 5480A>T), S1294K (5490T>A 5491C>A), V1295I (5493G>A), K1299L (5505A>T 5506A>T), D1301N (5511G>A), N1302D (5514A>G), K1305R (5524A>G), K1306E (5526A>G), Y1307F (5530A>T), S1308G (5532T>G 5533C>G), E1309K (5535G>A), V1311F (5541G>T 5543C>T), C1312L (5544T>C 5545G>T), I1315F (5553A>T 5555A>T), I1320V (5568A>G), E1323K (5577G>A 5579A>G), S1324N (5581G>A), I1325L (5583A>C 5585A>G), Q1326D (5586C>G 5588A>T), Q1327E (5589C>G 5591A>G), V1329I (5595G>A), Q1330E (5598C>G), L1333K (5607T>A 5608T>A), F1335V (5613T>G 5615C>T), Q1337E (5619C>G), C1339F (5626G>T 5627T>C), K1340V (5628A>G 5629A>T 5630G>A), E1341S (5631G>A 5632A>G 5633A>T), E1342K (5634G>A), L1344I (5640C>A 5642T>A), L1346I (5646T>A 5648G>A), S1347T (5649T>A 5651A>C), T1349K (5656C>A 5657A>G), L1351M (5661T>A), K1352E (5664A>G), I1353L (5667A>T), G1354C (5670G>T 5672A>T), V1355K (5673G>A 5674T>A), A1356T (5676G>A), N1357H (5679A>C), E1359N (5685G>A 5687A>C), Q1371K (5721C>A), L1377A (5739T>G 5740T>C 5741A>T), I1380V (5748A>G), E1382D (5756A>T), F1383M (5757T>A 5759T>G), E1385del (5763_5765delGAA), Q1387K (5769C>A), E1389D (5777A>T), T1390K (5779C>A), L1391T (5781T>A 5782T>C), G1393D (5788G>A), I1400L (5808A>T), L1401V (5811T>G 5813A>G), Y1407F (5830A>T), P1409Q (5836C>A), S1412G (5844A>G 5846T>A), Y1414I (5850T>A 5851A>T), T1415A (5853A>G 5855T>A), R1416G (5856A>G), I1417P (5859A>C 5860T>C), F1418L (5864T>A), N1420A (5868A>G 5869A>C 5870T>A), C1422I (5874T>A 5875G>T 5876T>A), S1423G (5877T>G 5878C>G 5879C>A), E1427Q (5889G>C), R1428K (5893G>A), K1429H (5895A>C 5897A>C), S1432T (5904T>A 5906T>A), Q1433E (5907C>G), D1434N (5910G>A 5912C>T), W1435I (5913T>A 5914G>T 5915G>T), M1437L (5919A>C), R1439Q (5925A>C 5926G>A), K1440Q (5928A>C), V1444K (5940G>A 5941T>A), T1446K (5947C>A 5948C>A), K1447N (5951A>C), L1448I (5952T>A), P1450D (5958C>G 5959C>A 5960T>C), I1452S (5965T>G 5966T>C), L1453I (5967T>A), K1455L (5973A>C 5974A>T), P1456E (5976C>G 5977C>A), Q1457S (5979C>T 5980A>C), S1458D (5982A>G 5983G>A), I1460L (5988A>C 5990T>A)								

	Begin	End	Coverage	Score	Concordance	Matches	Identities	I/D/M/F*	Stop Codons
**NT**	**5383**	**6002**	**7.4%**	**288**	**24.2%**	**600 (99.5%)**	**375 (62.2%)**	**0/3**	
Codon mutations:	TTC1258.AC (5383T>A), CAC1259CAT (5387C>T), ATG1262CGA (5394A>C 5395T>G 5396G>A), GAT1264AAT (5400G>A), CCA1265TCT (5403C>T 5405A>T), CAG1266GGA (5406C>G 5407A>G 5408G>A), AGT1267GAT (5409A>G 5410G>A), ATA1268GAA (5412A>G 5413T>A), GAA1269TGA (5415G>T 5416A>G), TGG1270AAA (5418T>A 5419G>A 5420G>A), ACA1271ACT (5423A>T), GCA1272ACT (5424G>A 5426A>T), GTC1274AAG (5430G>A 5431T>A 5432C>G), TGC1275ATC (5433T>A 5434G>T), CCA1276AAA (5436C>A 5437C>A), CAG1277TAT (5439C>T 5441G>T), GGT1278GAG (5443G>A 5444T>G), CAT1279CTT (5446A>T), TTT1280TAT (5449T>A), GAA1281GAG (5453A>G), ATA1283TTA (5457A>T), GTA1284GTT (5462A>T), CCA1286CCC (5468A>C), TTC1287TTT (5471C>T), GGA1288GGC (5474A>C), CTC1289TTG (5475C>T 5477C>G), AAA1290ACT (5479A>C 5480A>T), GCA1292GCG (5486A>G), CCT1293CCC (5489T>C), TCA1294AAA (5490T>A 5491C>A), GTA1295ATA (5493G>A), CGA1298AGA (5502C>A), AAA1299TTA (5505A>T 5506A>T), GAT1301AAT (5511G>A), AAT1302GAT (5514A>G), ATA1303ATT (5519A>T), TTC1304TTT (5522C>T), AAA1305AGA (5524A>G), AAA1306GAA (5526A>G), TAT1307TTT (5530A>T), TCA1308GGA (5532T>G 5533C>G), GAA1309AAA (5535G>A), GTC1311TTT (5541G>T 5543C>T), TGT1312CTT (5544T>C 5545G>T), ATA1315TTT (5553A>T 5555A>T), GAC1316GAT (5558C>T), GAT1317GAC (5561T>C), ATT1318ATC (5564T>C), ATA1320GTA (5568A>G), GAA1323AAG (5577G>A 5579A>G), AGT1324AAT (5581G>A), ATA1325CTG (5583A>C 5585A>G), CAA1326GAT (5586C>G 5588A>T), CAA1327GAG (5589C>G 5591A>G), GTA1329ATA (5595G>A), CAA1330GAA (5598C>G), CAT1331CAC (5603T>C), TTA1332CTA (5604T>C), TTA1333AAA (5607T>A 5608T>A), CAG1334CAA (5612G>A), TTC1335GTT (5613T>G 5615C>T), TTC1336TTT (5618C>T), CAA1337GAA (5619C>G), TGT1339TTC (5626G>T 5627T>C), AAG1340GTA (5628A>G 5629A>T 5630G>A), GAA1341AGT (5631G>A 5632A>G 5633A>T), GAA1342AAA (5634G>A), GGG1343GGA (5639G>A), CTT1344ATA (5640C>A 5642T>A), ATT1345ATA (5645T>A), TTG1346ATA (5646T>A 5648G>A), TCA1347ACC (5649T>A 5651A>C), ACA1349AAG (5656C>A 5657A>G), TTG1351ATG (5661T>A), AAA1352GAA (5664A>G), ATA1353TTA (5667A>T), GGA1354TGT (5670G>T 5672A>T), GTA1355AAA (5673G>A 5674T>A), GCA1356ACA (5676G>A), AAT1357CAT (5679A>C), GAA1359AAC (5685G>A 5687A>C), CTT1361TTA (5691C>T 5693T>A), CAA1371AAA (5721C>A), CTC1372CTA (5726C>A), TTA1377GCT (5739T>G 5740T>C 5741A>T), ATA1380GTA (5748A>G), GAA1382GAT (5756A>T), TTT1383ATG (5757T>A 5759T>G), CCT1384CCA (5762T>A), GAA1385del (5763_5765delGAA), GAC1386GAT (5768C>T), CAA1387AAA (5769C>A), GAA1389GAT (5777A>T), ACA1390AAA (5779C>A), TTA1391ACA (5781T>A 5782T>C), AAG1392AAA (5786G>A), GGT1393GAT (5788G>A), CTA1394TTA (5790C>T), CAG1395CAA (5795G>A), AAG1396AAA (5798G>A), GGG1399GGA (5807G>A), ATA1400TTA (5808A>T), TTA1401GTG (5811T>G 5813A>G), AAC1402AAT (5816C>T), GCC1404GCT (5822C>T), AAT1406AAC (5828T>C), TAT1407TTT (5830A>T), ATC1408ATA (5834C>A), CCA1409CAA (5836C>A), TTG1411TTA (5843G>A), AGT1412GGA (5844A>G 5846T>A), TAT1414ATT (5850T>A 5851A>T), ACT1415GCA (5853A>G 5855T>A), AGA1416GGA (5856A>G), ATA1417CCA (5859A>C 5860T>C), TTT1418TTA (5864T>A), AAT1420GCA (5868A>G 5869A>C 5870T>A), AAG1421AAA (5873G>A), TGT1422ATA (5874T>A 5875G>T 5876T>A), TCC1423GGA (5877T>G 5878C>G 5879C>A), AGC1424AGT (5882C>T), GAA1427CAA (5889G>C), AGA1428AAA (5893G>A), AAA1429CAC (5895A>C 5897A>C), AAT1431AAC (5903T>C), TCT1432ACA (5904T>A 5906T>A), CAA1433GAA (5907C>G), GAC1434AAT (5910G>A 5912C>T), TGG1435ATT (5913T>A 5914G>T 5915G>T), AAG1436AAA (5918G>A), ATG1437CTG (5919A>C), GTT1438GTA (5924T>A), AGA1439CAA (5925A>C 5926G>A), AAA1440CAA (5928A>C), ATC1441ATT (5933C>T), GTA1444AAA (5940G>A 5941T>A), ATT1445ATA (5945T>A), ACC1446AAA (5947C>A 5948C>A), AAA1447AAC (5951A>C), TTA1448ATA (5952T>A), CCC1449CCA (5957C>A), CCT1450GAC (5958C>G 5959C>A 5960T>C), TTG1451TTA (5963G>A), ATT1452AGC (5965T>G 5966T>C), TTA1453ATA (5967T>A), AAA1455CTA (5973A>C 5974A>T), CCA1456GAA (5976C>G 5977C>A), CAA1457TCA (5979C>T 5980A>C), AGT1458GAT (5982A>G 5983G>A), ATT1460CTA (5988A>C 5990T>A), GAG1463GAA (5999G>A)								

*: Inserts / Deletes / Misaligned / Frameshifts

Analysis details

This analysis was performed with panviral2.64

NGS Details (UN70): Chinaberry tree badnavirus 1

Assembly

**Coverage Length**	463 (1 contig(s))
**Depth Of Coverage**	7.5
**Number Of Reads**	26
**Reads Per Million**	0.49 rpm (after QC)
**Ambiguities**	0
**Assembly Method**	de novo + reference guided assembly
**Consensus Caller**	Bcf Tools

Coverage Map

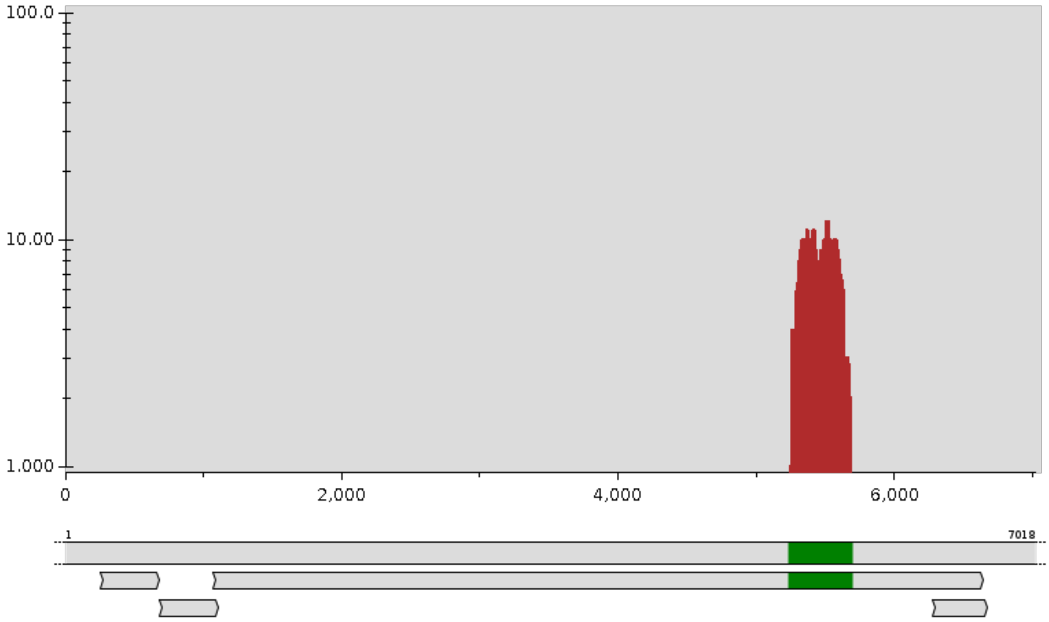

Assignment

**Type**	Chinaberry tree badnavirus 1 (Taxonomy ID: 2908099)
**Reference Genome**	NC_077165.1
**NT Identity (%)**	53.9301
**AA Identity (%)**	43.7908
**Number Of Stop Codons**	0
**Number Of CDS**	4

Alignment

**Alignment Score**	22.0 (NT) + 211.0 (AA) = 233.0
**Concordance (%)**	16.1121

Alignment Method	Global, seeded, nucleotide + amino acids (AGA)

Genome Region

Sequence starts at position 5240 and ends at position 5702 relative to NC_077165.1 reference sequence.

Alignment Detailed Statistics

	Begin	End	Coverage	Score	Concordance	Matches	Identities	I/D/M/F*	Stop Codons
**NT**	**5240**	**5702**	**6.6%**	**22**	**2.5%**	**455 (97.6%)**	**247 (53.0%)**	**3/8**	
Mutations:	5246A>C, 5249T>C, 5250T>A, 5256T>C, 5266A>G, 5270G>A, 5273C>T, 5275T>A, 5279C>T, 5283G>A, 5285A>T, 5286G>A, 5287C>G, 5288C>G, 5292G>A, 5293A>C, 5294C>A, 5295C>A, 5296C>A, 5301T>G, 5302C>A, 5306A>C, 5307G>C, 5309A>G, 5310T>A, 5311G>A, 5312G>A, 5315A>T, 5316G>A, 5322T>A, 5325G>A, 5326T>C, 5329C>A, 5330A>T, 5331G>T, 5332A>C, 5336G>C, 5338T>A, 5339T>C, 5345A>G, 5347G>A, 5348G>C, 5351G>A, 5354T>A, 5357T>G, 5360A>C, 5363C>T, 5366G>C, 5367T>C, 5369A>C, 5371A>C, 5372A>T, 5378T>A, 5381A>T, 5382G>T, 5384T>A, 5386T>G, 5387A>T, 5390C>T, 5394A>G, 5396A>G, 5397A>C, 5398A>T, 5403G>A, 5405C>T, 5408A>T, 5409T>G, 5410G>T, 5411C>T, 5414C>T, 5419G>A, 5420_5421insCAC, 5421A>C, 5422C>T, 5423A>T, 5424G>A, 5425A>G, 5436G>T, 5437C>T, 5441C>T, 5443A>T, 5444C>T, 5445delA, 5447A>T, 5456C>T, 5457T>C, 5460G>A, 5462C>A, 5464T>A, 5465T>C, 5466T>A, 5467C>G, 5473A>G, 5474C>T, 5475G>C, 5476A>T, 5480G>A, 5483G>T, 5484C>G, 5486T>C, 5487G>T, 5488C>T, 5489A>G, 5490G>C, 5491G>A, 5492C>A, 5495T>C, 5496G>C, 5498A>G, 5501A>G, 5502A>G, 5503A>T, 5504A>T, 5506T>C, 5507G>T, 5510G>T, 5511G>A, 5513A>C, 5514A>T, 5517G>T, 5519A>G, 5521A>T, 5522G>C, 5523T>A, 5524C>A, 5525T>A, 5526A>C, 5529G>A, 5534A>G, 5535A>T, 5537C>G, 5538C>A, 5540A>C, 5542G>A, 5543C>G, 5544C>A, 5545C>A, 5546A>G, 5547A>T, 5553A>T, 5554T>G, 5555G>T, 5557G>T, 5558C>T, 5559C>T, 5563C>A, 5564C>G, 5567A>T, 5569G>A, 5571delT, 5572C>G, 5581T>A, 5582T>C, 5585A>T, 5588G>T, 5589G>C, 5590C>A, 5591A>C, 5592A>T, 5594T>C, 5597C>A, 5598A>T, 5600A>T, 5602G>C, 5605G>A, 5606A>G, 5607A>G, 5608A>G, 5609G>A, 5610C>G, 5612C>G, 5613C>G, 5614A>C, 5615A>T, 5616T>A, 5617T>C, 5619C>G, 5621G>T, 5624C>T, 5625C>A, 5627C>G, 5629T>A, 5630C>G, 5633C>T, 5634A>G, 5635A>C, 5636G>A, 5637_5639delAAG, 5641T>C, 5642G>A, 5645C>A, 5648G>A, 5649_5651delTTC, 5654T>A, 5655A>T, 5656A>G, 5660C>A, 5662G>T, 5663C>T, 5664T>C, 5665T>C, 5666A>T, 5667G>C, 5672C>T, 5674C>T, 5675T>A, 5678A>G, 5680G>A, 5681A>G, 5682C>T, 5687A>G, 5700C>T, 5702C>G								

CDS

	Begin	End	Coverage	Score	Concordance	Matches	Identities	I/D/M/F*	Stop Codons
QKV46_gp3	1391	1544	8.3%	211	20.1%	152 (98.1%)	67 (43.2%)	1/2/2/2	0
Protein mutations:	S1394T (5250T>A), F1396L (5256T>C), K1399R (5266A>G), F1402Y (5275T>A), V1405I (5283G>A 5285A>T), A1406R (5286G>A 5287C>G 5288C>G), D1408T (5292G>A 5293A>C 5294C>A), P1409K (5295C>A 5296C>A), S1411D (5301T>G 5302C>A), E1413Q (5307G>C 5309A>G), W1414K (5310T>A 5311G>A 5312G>A), A1416T (5316G>A), W1418R (5322T>A), V1419T (5325G>A 5326T>C), P1420H (5329C>A 5330A>T), D1421S (5331G>T 5332A>C), L1423H (5338T>A 5339T>C), W1426Y (5347G>A 5348G>C), I1429M (5357T>G), K1434T (5371A>C 5372A>T), A1438S (5382G>T 5384T>A), I1439S (5386T>G 5387A>T), R1442G (5394A>G 5396A>G), K1443L (5397A>C 5398A>T), D1445N (5403G>A 5405C>T), Q1446H (5408A>T), C1447V (5409T>G 5410G>T 5411C>T), G1450E (5419G>A), G1450_T1451insH (5420_5421insCAC), T1451L (5421A>C 5422C>T 5423A>T), E1452R (5424G>A 5425A>G), A1456L (5436G>T 5437C>T), Y1458F (5443A>T 5444C>T), V1464I (5460G>A 5462C>A), F1465Y (5464T>A 5465T>C), N1468S (5473A>G 5474C>T), E1469L (5475G>C 5476A>T), E1471D (5483G>T), H1472D (5484C>G 5486T>C), A1473L (5487G>T 5488C>T 5489A>G), G1474Q (5490G>C 5491G>A 5492C>A), V1476L (5496G>C 5498A>G), K1478V (5502A>G 5503A>T 5504A>T), M1479T (5506T>C 5507G>T), L1480F (5510G>T), E1481N (5511G>A 5513A>C), I1482L (5514A>T), V1483L (5517G>T 5519A>G), K1484I (5521A>T 5522G>C), S1485K (5523T>A 5524C>A 5525T>A), N1486H (5526A>C), G1487R (5529G>A), I1489L (5535A>T 5537C>G), L1490I (5538C>A 5540A>C), S1491K (5542G>A 5543C>G), P1492K (5544C>A 5545C>A 5546A>G), T1493S (5547A>T), M1495C (5553A>T 5554T>G 5555G>T), C1496F (5557G>T 5558C>T), A1498E (5563C>A 5564C>G), Q1499H (5567A>T), R1500Q (5569G>A), F1504Y (5581T>A 5582T>C), A1507H (5589G>C 5590C>A 5591A>C), I1508F (5592A>T 5594T>C), T1510S (5598A>T 5600A>T), G1511A (5602G>C), G1512E (5605G>A 5606A>G), K1513G (5607A>G 5608A>G 5609G>A), L1514V (5610C>G 5612C>G), Q1515A (5613C>G 5614A>C 5615A>T), L1516T (5616T>A 5617T>C), Q1517D (5619C>G 5621G>T), H1519K (5625C>A 5627C>G), I1520K (5629T>A 5630C>G), K1522A (5634A>G 5635A>C 5636G>A), K1523del (5637_5639delAAG), V1524A (5641T>C 5642G>A), F1527del (5649_5651delTTC), D1528E (5654T>A), K1529W (5655A>T 5656A>G), S1531I (5662G>T 5663C>T), L1532P (5664T>C 5665T>C 5666A>T), E1533Q (5667G>C), T1535I (5674C>T 5675T>A), G1537E (5680G>A 5681A>G)								
Codon mutations:	ATA1392ATC (5246A>C), TTT1393TTC (5249T>C), TCC1394ACC (5250T>A), TTT1396CTT (5256T>C), AAA1399AGA (5266A>G), TCG1400TCA (5270G>A), GGC1401GGT (5273C>T), TTT1402TAT (5275T>A), CAC1403CAT (5279C>T), GTA1405ATT (5283G>A 5285A>T), GCC1406AGG (5286G>A 5287C>G 5288C>G), GAC1408ACA (5292G>A 5293A>C 5294C>A), CCG1409AAG (5295C>A 5296C>A), TCT1411GAT (5301T>G 5302C>A), GTA1412GTC (5306A>C), GAA1413CAG (5307G>C 5309A>G), TGG1414AAA (5310T>A 5311G>A 5312G>A), ACA1415ACT (5315A>T), GCA1416ACA (5316G>A), TGG1418AGG (5322T>A), GTT1419ACT (5325G>A 5326T>C), CCA1420CAT (5329C>A 5330A>T), GAT1421TCT (5331G>T 5332A>C), GGG1422GGC (5336G>C), CTT1423CAC (5338T>A 5339T>C), GAA1425GAG (5345A>G), TGG1426TAC (5347G>A 5348G>C), CTG1427CTA (5351G>A), GTT1428GTA (5354T>A), ATT1429ATG (5357T>G), CCA1430CCC (5360A>C), TTC1431TTT (5363C>T), GGG1432GGC (5366G>C), TTA1433CTC (5367T>C 5369A>C), AAA1434ACT (5371A>C 5372A>T), GCT1436GCA (5378T>A), CCA1437CCT (5381A>T), GCT1438TCA (5382G>T 5384T>A), ATA1439AGT (5386T>G 5387A>T), TTC1440TTT (5390C>T), AGA1442GGG (5394A>G 5396A>G), AAG1443CTG (5397A>C 5398A>T), GAC1445AAT (5403G>A 5405C>T), CAA1446CAT (5408A>T), TGC1447GTT (5409T>G 5410G>T 5411C>T), TTC1448TTT (5414C>T), GGA1450GAA (5419G>A), GGA1450_ACA1451insCAC (5420_5421insCAC), ACA1451CTT (5421A>C 5422C>T 5423A>T), GAG1452AGG (5424G>A 5425A>G), GCG1456TTG (5436G>T 5437C>T), GTC1457GTT (5441C>T), TAC1458TTT (5443A>T 5444C>T), ATA1459-TT (5445delA 5447A>T), ATC1462ATT (5456C>T), TTG1463CTG (5457T>C), GTC1464ATA (5460G>A 5462C>A), TTT1465TAC (5464T>A 5465T>C), TCC1466AGC (5466T>A 5467C>G), AAC1468AGT (5473A>G 5474C>T), GAA1469CTA (5475G>C 5476A>T), GAG1470GAA (5480G>A), GAG1471GAT (5483G>T), CAT1472GAC (5484C>G 5486T>C), GCA1473TTG (5487G>T 5488C>T 5489A>G), GGC1474CAA (5490G>C 5491G>A 5492C>A), CAT1475CAC (5495T>C), GTA1476CTG (5496G>C 5498A>G), AGA1477AGG (5501A>G), AAA1478GTT (5502A>G 5503A>T 5504A>T), ATG1479ACT (5506T>C 5507G>T), TTG1480TTT (5510G>T), GAA1481AAC (5511G>A 5513A>C), ATA1482TTA (5514A>T), GTA1483TTG (5517G>T 5519A>G), AAG1484ATC (5521A>T 5522G>C), TCT1485AAA (5523T>A 5524C>A 5525T>A), AAT1486CAT (5526A>C), GGA1487AGA (5529G>A), CTA1488CTG (5534A>G), ATC1489TTG (5535A>T 5537C>G), CTA1490ATC (5538C>A 5540A>C), AGC1491AAG (5542G>A 5543C>G), CCA1492AAG (5544C>A 5545C>A 5546A>G), ACA1493TCA (5547A>T), ATG1495TGT (5553A>T 5554T>G 5555G>T), TGC1496TTT (5557G>T 5558C>T), CTG1497TTG (5559C>T), GCC1498GAG (5563C>A 5564C>G), CAA1499CAT (5567A>T), CGA1500CAA (5569G>A), TCG1501-GG (5571delT 5572C>G), TTT1504TAC (5581T>A 5582T>C), CTA1505CTT (5585A>T), GGG1506GGT (5588G>T), GCA1507CAC (5589G>C 5590C>A 5591A>C), ATT1508TTC (5592A>T 5594T>C), ATC1509ATA (5597C>A), ACA1510TCT (5598A>T 5600A>T), GGA1511GCA (5602G>C), GGA1512GAG (5605G>A 5606A>G), AAG1513GGA (5607A>G 5608A>G 5609G>A), CTC1514GTG (5610C>G 5612C>G), CAA1515GCT (5613C>G 5614A>C 5615A>T), TTA1516ACA (5616T>A 5617T>C), CAG1517GAT (5619C>G 5621G>T), CCC1518CCT (5624C>T), CAC1519AAG (5625C>A 5627C>G), ATC1520AAG (5629T>A 5630C>G), ATC1521ATT (5633C>T), AAG1522GCA (5634A>G 5635A>C 5636G>A), AAG1523del (5637_5639delAAG), GTG1524GCA (5641T>C 5642G>A), GTC1525GTA (5645C>A), CAG1526CAA (5648G>A), TTC1527del (5649_5651delTTC), GAT1528GAA (5654T>A), AAG1529TGG (5655A>T 5656A>G), CCC1530CCA (5660C>A), AGC1531ATT (5662G>T 5663C>T), TTA1532CCT (5664T>C 5665T>C 5666A>T), GAG1533CAG (5667G>C), ACC1534ACT (5672C>T), ACT1535ATA (5674C>T 5675T>A), AAA1536AAG (5678A>G), GGA1537GAG (5680G>A 5681A>G), CTA1538TTA (5682C>T), AGA1539AGG (5687A>G), CTC1544TTG (5700C>T 5702C>G)								

Proteins

	Begin	End	Coverage	Score	Concordance	Matches	Identities	I/D/M/F*	Stop Codons
ORF3 (YP_010805662.1)	1391	1544	8.3%	211	20.1%	152 (98.1%)	67 (43.2%)	1/2/2/2	0
Protein mutations:	S1394T (5250T>A), F1396L (5256T>C), K1399R (5266A>G), F1402Y (5275T>A), V1405I (5283G>A 5285A>T), A1406R (5286G>A 5287C>G 5288C>G), D1408T (5292G>A 5293A>C 5294C>A), P1409K (5295C>A 5296C>A), S1411D (5301T>G 5302C>A), E1413Q (5307G>C 5309A>G), W1414K (5310T>A 5311G>A 5312G>A), A1416T (5316G>A), W1418R (5322T>A), V1419T (5325G>A 5326T>C), P1420H (5329C>A 5330A>T), D1421S (5331G>T 5332A>C), L1423H (5338T>A 5339T>C), W1426Y (5347G>A 5348G>C), I1429M (5357T>G), K1434T (5371A>C 5372A>T), A1438S (5382G>T 5384T>A), I1439S (5386T>G 5387A>T), R1442G (5394A>G 5396A>G), K1443L (5397A>C 5398A>T), D1445N (5403G>A 5405C>T), Q1446H (5408A>T), C1447V (5409T>G 5410G>T 5411C>T), G1450E (5419G>A), G1450_T1451insH (5420_5421insCAC), T1451L (5421A>C 5422C>T 5423A>T), E1452R (5424G>A 5425A>G), A1456L (5436G>T 5437C>T), Y1458F (5443A>T 5444C>T), V1464I (5460G>A 5462C>A), F1465Y (5464T>A 5465T>C), N1468S (5473A>G 5474C>T), E1469L (5475G>C 5476A>T), E1471D (5483G>T), H1472D (5484C>G 5486T>C), A1473L (5487G>T 5488C>T 5489A>G), G1474Q (5490G>C 5491G>A 5492C>A), V1476L (5496G>C 5498A>G), K1478V (5502A>G 5503A>T 5504A>T), M1479T (5506T>C 5507G>T), L1480F (5510G>T), E1481N (5511G>A 5513A>C), I1482L (5514A>T), V1483L (5517G>T 5519A>G), K1484I (5521A>T 5522G>C), S1485K (5523T>A 5524C>A 5525T>A), N1486H (5526A>C), G1487R (5529G>A), I1489L (5535A>T 5537C>G), L1490I (5538C>A 5540A>C), S1491K (5542G>A 5543C>G), P1492K (5544C>A 5545C>A 5546A>G), T1493S (5547A>T), M1495C (5553A>T 5554T>G 5555G>T), C1496F (5557G>T 5558C>T), A1498E (5563C>A 5564C>G), Q1499H (5567A>T), R1500Q (5569G>A), F1504Y (5581T>A 5582T>C), A1507H (5589G>C 5590C>A 5591A>C), I1508F (5592A>T 5594T>C), T1510S (5598A>T 5600A>T), G1511A (5602G>C), G1512E (5605G>A 5606A>G), K1513G (5607A>G 5608A>G 5609G>A), L1514V (5610C>G 5612C>G), Q1515A (5613C>G 5614A>C 5615A>T), L1516T (5616T>A 5617T>C), Q1517D (5619C>G 5621G>T), H1519K (5625C>A 5627C>G), I1520K (5629T>A 5630C>G), K1522A (5634A>G 5635A>C 5636G>A), K1523del (5637_5639delAAG), V1524A (5641T>C 5642G>A), F1527del (5649_5651delTTC), D1528E (5654T>A), K1529W (5655A>T 5656A>G), S1531I (5662G>T 5663C>T), L1532P (5664T>C 5665T>C 5666A>T), E1533Q (5667G>C), T1535I (5674C>T 5675T>A), G1537E (5680G>A 5681A>G)								

	Begin	End	Coverage	Score	Concordance	Matches	Identities	I/D/M/F*	Stop Codons
**NT**	**5240**	**5702**	**6.6%**	**22**	**2.5%**	**455 (97.6%)**	**247 (53.0%)**	**3/8**	
Codon mutations:	ATA1392ATC (5246A>C), TTT1393TTC (5249T>C), TCC1394ACC (5250T>A), TTT1396CTT (5256T>C), AAA1399AGA (5266A>G), TCG1400TCA (5270G>A), GGC1401GGT (5273C>T), TTT1402TAT (5275T>A), CAC1403CAT (5279C>T), GTA1405ATT (5283G>A 5285A>T), GCC1406AGG (5286G>A 5287C>G 5288C>G), GAC1408ACA (5292G>A 5293A>C 5294C>A), CCG1409AAG (5295C>A 5296C>A), TCT1411GAT (5301T>G 5302C>A), GTA1412GTC (5306A>C), GAA1413CAG (5307G>C 5309A>G), TGG1414AAA (5310T>A 5311G>A 5312G>A), ACA1415ACT (5315A>T), GCA1416ACA (5316G>A), TGG1418AGG (5322T>A), GTT1419ACT (5325G>A 5326T>C), CCA1420CAT (5329C>A 5330A>T), GAT1421TCT (5331G>T 5332A>C), GGG1422GGC (5336G>C), CTT1423CAC (5338T>A 5339T>C), GAA1425GAG (5345A>G), TGG1426TAC (5347G>A 5348G>C), CTG1427CTA (5351G>A), GTT1428GTA (5354T>A), ATT1429ATG (5357T>G), CCA1430CCC (5360A>C), TTC1431TTT (5363C>T), GGG1432GGC (5366G>C), TTA1433CTC (5367T>C 5369A>C), AAA1434ACT (5371A>C 5372A>T), GCT1436GCA (5378T>A), CCA1437CCT (5381A>T), GCT1438TCA (5382G>T 5384T>A), ATA1439AGT (5386T>G 5387A>T), TTC1440TTT (5390C>T), AGA1442GGG (5394A>G 5396A>G), AAG1443CTG (5397A>C 5398A>T), GAC1445AAT (5403G>A 5405C>T), CAA1446CAT (5408A>T), TGC1447GTT (5409T>G 5410G>T 5411C>T), TTC1448TTT (5414C>T), GGA1450GAA (5419G>A), GGA1450_ACA1451insCAC (5420_5421insCAC), ACA1451CTT (5421A>C 5422C>T 5423A>T), GAG1452AGG (5424G>A 5425A>G), GCG1456TTG (5436G>T 5437C>T), GTC1457GTT (5441C>T), TAC1458TTT (5443A>T 5444C>T), ATA1459-TT (5445delA 5447A>T), ATC1462ATT (5456C>T), TTG1463CTG (5457T>C), GTC1464ATA (5460G>A 5462C>A), TTT1465TAC (5464T>A 5465T>C), TCC1466AGC (5466T>A 5467C>G), AAC1468AGT (5473A>G 5474C>T), GAA1469CTA (5475G>C 5476A>T), GAG1470GAA (5480G>A), GAG1471GAT (5483G>T), CAT1472GAC (5484C>G 5486T>C), GCA1473TTG (5487G>T 5488C>T 5489A>G), GGC1474CAA (5490G>C 5491G>A 5492C>A), CAT1475CAC (5495T>C), GTA1476CTG (5496G>C 5498A>G), AGA1477AGG (5501A>G), AAA1478GTT (5502A>G 5503A>T 5504A>T), ATG1479ACT (5506T>C 5507G>T), TTG1480TTT (5510G>T), GAA1481AAC (5511G>A 5513A>C), ATA1482TTA (5514A>T), GTA1483TTG (5517G>T 5519A>G), AAG1484ATC (5521A>T 5522G>C), TCT1485AAA (5523T>A 5524C>A 5525T>A), AAT1486CAT (5526A>C), GGA1487AGA (5529G>A), CTA1488CTG (5534A>G), ATC1489TTG (5535A>T 5537C>G), CTA1490ATC (5538C>A 5540A>C), AGC1491AAG (5542G>A 5543C>G), CCA1492AAG (5544C>A 5545C>A 5546A>G), ACA1493TCA (5547A>T), ATG1495TGT (5553A>T 5554T>G 5555G>T), TGC1496TTT (5557G>T 5558C>T), CTG1497TTG (5559C>T), GCC1498GAG (5563C>A 5564C>G), CAA1499CAT (5567A>T), CGA1500CAA (5569G>A), TCG1501-GG (5571delT 5572C>G), TTT1504TAC (5581T>A 5582T>C), CTA1505CTT (5585A>T), GGG1506GGT (5588G>T), GCA1507CAC (5589G>C 5590C>A 5591A>C), ATT1508TTC (5592A>T 5594T>C), ATC1509ATA (5597C>A), ACA1510TCT (5598A>T 5600A>T), GGA1511GCA (5602G>C), GGA1512GAG (5605G>A 5606A>G), AAG1513GGA (5607A>G 5608A>G 5609G>A), CTC1514GTG (5610C>G 5612C>G), CAA1515GCT (5613C>G 5614A>C 5615A>T), TTA1516ACA (5616T>A 5617T>C), CAG1517GAT (5619C>G 5621G>T), CCC1518CCT (5624C>T), CAC1519AAG (5625C>A 5627C>G), ATC1520AAG (5629T>A 5630C>G), ATC1521ATT (5633C>T), AAG1522GCA (5634A>G 5635A>C 5636G>A), AAG1523del (5637_5639delAAG), GTG1524GCA (5641T>C 5642G>A), GTC1525GTA (5645C>A), CAG1526CAA (5648G>A), TTC1527del (5649_5651delTTC), GAT1528GAA (5654T>A), AAG1529TGG (5655A>T 5656A>G), CCC1530CCA (5660C>A), AGC1531ATT (5662G>T 5663C>T), TTA1532CCT (5664T>C 5665T>C 5666A>T), GAG1533CAG (5667G>C), ACC1534ACT (5672C>T), ACT1535ATA (5674C>T 5675T>A), AAA1536AAG (5678A>G), GGA1537GAG (5680G>A 5681A>G), CTA1538TTA (5682C>T), AGA1539AGG (5687A>G), CTC1544TTG (5700C>T 5702C>G)								

*: Inserts / Deletes / Misaligned / Frameshifts

Analysis details

This analysis was performed with panviral2.64

NGS Details (UN70): Duamitovirus peex1

Assembly

**Coverage Length**	380 (1 contig(s))
**Depth Of Coverage**	5.8
**Number Of Reads**	22
**Reads Per Million**	0.42 rpm (after QC)
**Ambiguities**	0
**Assembly Method**	de novo + reference guided assembly
**Consensus Caller**	Bcf Tools

Coverage Map

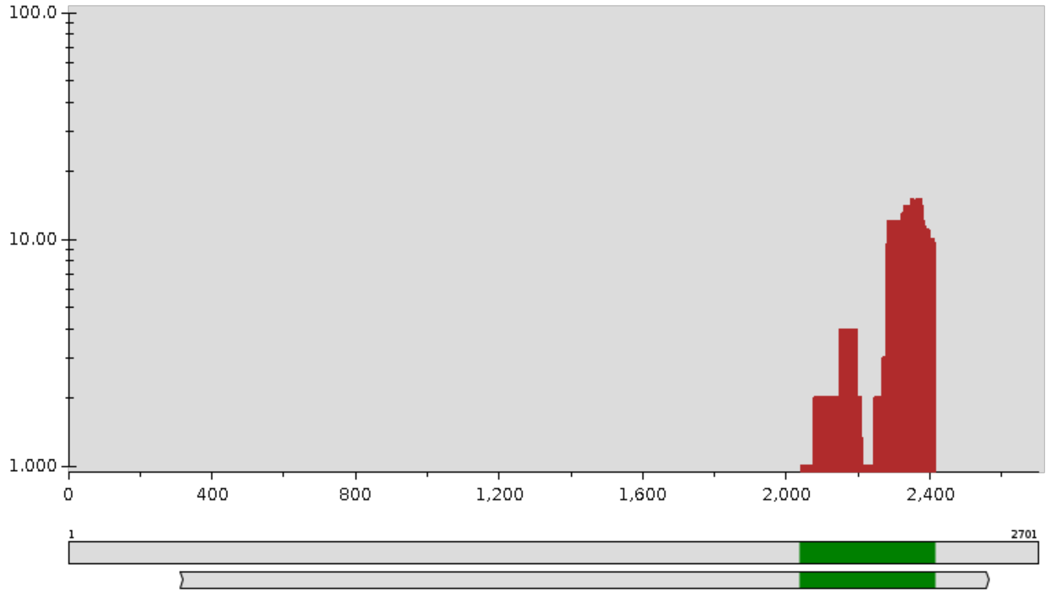

Assignment

**Type**	Duamitovirus peex1 (Taxonomy ID: 2955799)
**Reference Genome**	NC_076525.1
**NT Identity (%)**	71.1286
**AA Identity (%)**	66.9291
**Number Of Stop Codons**	0
**Number Of CDS**	1

Alignment

**Alignment Score**	314.0 (NT) + 503.0 (AA) = 817.0
**Concordance (%)**	49.9408

Alignment Method	Global, seeded, nucleotide + amino acids (AGA)

Genome Region

Sequence starts at position 2038 and ends at position 2417 relative to NC_076525.1 reference sequence.

Alignment Detailed Statistics

	Begin	End	Coverage	Score	Concordance	Matches	Identities	I/D/M/F*	Stop Codons
**NT**	**2038**	**2417**	**14.1%**	**314**	**41.3%**	**380 (99.7%)**	**271 (71.1%)**	**1/0**	
Mutations:	2040A>T, 2042G>T, 2048G>A, 2057C>T, 2066G>A, 2067A>C, 2069T>G, 2072G>A, 2075T>G, 2077T>C, 2081T>A, 2082T>A, 2084T>C, 2088T>C, 2090T>A, 2091T>C, 2095T>G, 2096A>G, 2102G>T, 2104G>T, 2105G>A, 2109T>C, 2114T>C, 2119G>A, 2120G>T, 2129A>G, 2132G>A, 2133G>T, 2134G>T, 2135A>T, 2138G>A, 2141C>T, 2145G>A, 2152A>T, 2153_2154insT, 2156A>G, 2158G>A, 2159A>G, 2162T>A, 2171C>A, 2173C>A, 2180T>C, 2183T>G, 2189G>A, 2194A>C, 2209A>G, 2210G>A, 2211T>C, 2213G>C, 2214A>T, 2216A>T, 2222A>T, 2223C>G, 2225A>G, 2226C>T, 2234A>T, 2243A>G, 2244C>A, 2245A>G, 2246T>A, 2249G>A, 2255A>T, 2268G>T, 2272G>T, 2273T>C, 2274C>A, 2275A>G, 2276T>A, 2280A>G, 2283G>A, 2294T>G, 2295C>A, 2296G>A, 2301C>G, 2303T>C, 2306A>T, 2311A>G, 2312T>C, 2315C>T, 2321G>T, 2325A>G, 2326A>C, 2327T>A, 2330C>A, 2331C>G, 2333G>T, 2339G>A, 2345A>T, 2348C>T, 2351A>G, 2352C>T, 2354T>C, 2363G>T, 2373G>A, 2374C>G, 2375G>T, 2377C>A, 2378T>A, 2379T>A, 2388C>A, 2390T>A, 2393T>A, 2394C>A, 2396G>C, 2398C>A, 2399T>A, 2406C>G, 2411A>G, 2412G>A, 2414G>C								

CDS

	Begin	End	Coverage	Score	Concordance	Matches	Identities	I/D/M/F*	Stop Codons
RdRp	577	702	16.8%	503	53.9%	126 (99.2%)	85 (66.9%)	1/0/1/1	0
Protein mutations:	T577S (2040A>T 2042G>T), I586L (2067A>C 2069T>G), V589A (2077T>C), C591S (2082T>A 2084T>C), S593P (2088T>C 2090T>A), Y594H (2091T>C), I595R (2095T>G 2096A>G), Q597H (2102G>T), R598L (2104G>T 2105G>A), S600P (2109T>C), W603Y (2119G>A 2120G>T), G608F (2133G>T 2134G>T 2135A>T), D612N (2145G>A), Y614F (2152A>T), Y614_L615insX (2153_2154insT), R616K (2158G>A 2159A>G), A621D (2173C>A), K628T (2194A>C), Q633R (2209A>G 2210G>A), I635F (2214A>T 2216A>T), K637N (2222A>T), Q638E (2223C>G 2225A>G), L641F (2234A>T), H645R (2244C>A 2245A>G 2246T>A), V653L (2268G>T), R654L (2272G>T 2273T>C), H655R (2274C>A 2275A>G 2276T>A), M657V (2280A>G), E658K (2283G>A), R662K (2295C>A 2296G>A), L664V (2301C>G 2303T>C), Y667C (2311A>G 2312T>C), N672A (2325A>G 2326A>C 2327T>A), P674A (2331C>G 2333G>T), L681F (2352C>T 2354T>C), A688S (2373G>A 2374C>G 2375G>T), T689K (2377C>A 2378T>A), S690T (2379T>A), Q695N (2394C>A 2396G>C), T696K (2398C>A 2399T>A), H699D (2406C>G), V701I (2412G>A 2414G>C)								
Codon mutations:	ACG577TCT (2040A>T 2042G>T), CAG579CAA (2048G>A), CGC582CGT (2057C>T), AGG585AGA (2066G>A), ATT586CTG (2067A>C 2069T>G), AAG587AAA (2072G>A), GCT588GCG (2075T>G), GTT589GCT (2077T>C), GCT590GCA (2081T>A), TGT591AGC (2082T>A 2084T>C), TCT593CCA (2088T>C 2090T>A), TAC594CAC (2091T>C), ATA595AGG (2095T>G 2096A>G), CAG597CAT (2102G>T), CGG598CTA (2104G>T 2105G>A), TCT600CCT (2109T>C), CTT601CTC (2114T>C), TGG603TAT (2119G>A 2120G>T), GGA606GGG (2129A>G), AGG607AGA (2132G>A), GGA608TTT (2133G>T 2134G>T 2135A>T), AAG609AAA (2138G>A), CCC610CCT (2141C>T), GAT612AAT (2145G>A), TAT614TTT (2152A>T), TAT614_TTA615insT-- (2153_2154insT), TTA615TTG (2156A>G), AGA616AAG (2158G>A 2159A>G), GGT617GGA (2162T>A), ATC620ATA (2171C>A), GCT621GAT (2173C>A), CTT623CTC (2180T>C), CGT624CGG (2183T>G), GAG626GAA (2189G>A), AAA628ACA (2194A>C), CAG633CGA (2209A>G 2210G>A), TTG634CTC (2211T>C 2213G>C), ATA635TTT (2214A>T 2216A>T), AAA637AAT (2222A>T), CAA638GAG (2223C>G 2225A>G), CTG639TTG (2226C>T), TTA641TTT (2234A>T), GAA644GAG (2243A>G), CAT645AGA (2244C>A 2245A>G 2246T>A), GAG646GAA (2249G>A), CTA648CTT (2255A>T), GTA653TTA (2268G>T), CGT654CTC (2272G>T 2273T>C), CAT655AGA (2274C>A 2275A>G 2276T>A), ATG657GTG (2280A>G), GAA658AAA (2283G>A), CTT661CTG (2294T>G), CGA662AAA (2295C>A 2296G>A), CTT664GTC (2301C>G 2303T>C), TCA665TCT (2306A>T), TAT667TGC (2311A>G 2312T>C), CAC668CAT (2315C>T), GTG670GTT (2321G>T), AAT672GCA (2325A>G 2326A>C 2327T>A), TCC673TCA (2330C>A), CCG674GCT (2331C>G 2333G>T), GTG676GTA (2339G>A), ATA678ATT (2345A>T), GAC679GAT (2348C>T), CAA680CAG (2351A>G), CTT681TTC (2352C>T 2354T>C), GTG684GTT (2363G>T), GCG688AGT (2373G>A 2374C>G 2375G>T), ACT689AAA (2377C>A 2378T>A), TCT690ACT (2379T>A), CGT693AGA (2388C>A 2390T>A), ACT694ACA (2393T>A), CAG695AAC (2394C>A 2396G>C), ACT696AAA (2398C>A 2399T>A), CAT699GAT (2406C>G), TTA700TTG (2411A>G), GTG701ATC (2412G>A 2414G>C)								

Proteins

	Begin	End	Coverage	Score	Concordance	Matches	Identities	I/D/M/F*	Stop Codons
RNA-dependent RNA polymerase (YP_010798875.1)	577	702	16.8%	503	53.9%	126 (99.2%)	85 (66.9%)	1/0/1/1	0
Protein mutations:	T577S (2040A>T 2042G>T), I586L (2067A>C 2069T>G), V589A (2077T>C), C591S (2082T>A 2084T>C), S593P (2088T>C 2090T>A), Y594H (2091T>C), I595R (2095T>G 2096A>G), Q597H (2102G>T), R598L (2104G>T 2105G>A), S600P (2109T>C), W603Y (2119G>A 2120G>T), G608F (2133G>T 2134G>T 2135A>T), D612N (2145G>A), Y614F (2152A>T), Y614_L615insX (2153_2154insT), R616K (2158G>A 2159A>G), A621D (2173C>A), K628T (2194A>C), Q633R (2209A>G 2210G>A), I635F (2214A>T 2216A>T), K637N (2222A>T), Q638E (2223C>G 2225A>G), L641F (2234A>T), H645R (2244C>A 2245A>G 2246T>A), V653L (2268G>T), R654L (2272G>T 2273T>C), H655R (2274C>A 2275A>G 2276T>A), M657V (2280A>G), E658K (2283G>A), R662K (2295C>A 2296G>A), L664V (2301C>G 2303T>C), Y667C (2311A>G 2312T>C), N672A (2325A>G 2326A>C 2327T>A), P674A (2331C>G 2333G>T), L681F (2352C>T 2354T>C), A688S (2373G>A 2374C>G 2375G>T), T689K (2377C>A 2378T>A), S690T (2379T>A), Q695N (2394C>A 2396G>C), T696K (2398C>A 2399T>A), H699D (2406C>G), V701I (2412G>A 2414G>C)								
Codon mutations:	ACG577TCT (2040A>T 2042G>T), CAG579CAA (2048G>A), CGC582CGT (2057C>T), AGG585AGA (2066G>A), ATT586CTG (2067A>C 2069T>G), AAG587AAA (2072G>A), GCT588GCG (2075T>G), GTT589GCT (2077T>C), GCT590GCA (2081T>A), TGT591AGC (2082T>A 2084T>C), TCT593CCA (2088T>C 2090T>A), TAC594CAC (2091T>C), ATA595AGG (2095T>G 2096A>G), CAG597CAT (2102G>T), CGG598CTA (2104G>T 2105G>A), TCT600CCT (2109T>C), CTT601CTC (2114T>C), TGG603TAT (2119G>A 2120G>T), GGA606GGG (2129A>G), AGG607AGA (2132G>A), GGA608TTT (2133G>T 2134G>T 2135A>T), AAG609AAA (2138G>A), CCC610CCT (2141C>T), GAT612AAT (2145G>A), TAT614TTT (2152A>T), TAT614_TTA615insT-- (2153_2154insT), TTA615TTG (2156A>G), AGA616AAG (2158G>A 2159A>G), GGT617GGA (2162T>A), ATC620ATA (2171C>A), GCT621GAT (2173C>A), CTT623CTC (2180T>C), CGT624CGG (2183T>G), GAG626GAA (2189G>A), AAA628ACA (2194A>C), CAG633CGA (2209A>G 2210G>A), TTG634CTC (2211T>C 2213G>C), ATA635TTT (2214A>T 2216A>T), AAA637AAT (2222A>T), CAA638GAG (2223C>G 2225A>G), CTG639TTG (2226C>T), TTA641TTT (2234A>T), GAA644GAG (2243A>G), CAT645AGA (2244C>A 2245A>G 2246T>A), GAG646GAA (2249G>A), CTA648CTT (2255A>T), GTA653TTA (2268G>T), CGT654CTC (2272G>T 2273T>C), CAT655AGA (2274C>A 2275A>G 2276T>A), ATG657GTG (2280A>G), GAA658AAA (2283G>A), CTT661CTG (2294T>G), CGA662AAA (2295C>A 2296G>A), CTT664GTC (2301C>G 2303T>C), TCA665TCT (2306A>T), TAT667TGC (2311A>G 2312T>C), CAC668CAT (2315C>T), GTG670GTT (2321G>T), AAT672GCA (2325A>G 2326A>C 2327T>A), TCC673TCA (2330C>A), CCG674GCT (2331C>G 2333G>T), GTG676GTA (2339G>A), ATA678ATT (2345A>T), GAC679GAT (2348C>T), CAA680CAG (2351A>G), CTT681TTC (2352C>T 2354T>C), GTG684GTT (2363G>T), GCG688AGT (2373G>A 2374C>G 2375G>T), ACT689AAA (2377C>A 2378T>A), TCT690ACT (2379T>A), CGT693AGA (2388C>A 2390T>A), ACT694ACA (2393T>A), CAG695AAC (2394C>A 2396G>C), ACT696AAA (2398C>A 2399T>A), CAT699GAT (2406C>G), TTA700TTG (2411A>G), GTG701ATC (2412G>A 2414G>C)								

*: Inserts / Deletes / Misaligned / Frameshifts

Analysis details

This analysis was performed with panviral2.64

NGS Details (UN70): Badnavirus deltainflatheobromae

Assembly

**Coverage Length**	315 (1 contig(s))
**Depth Of Coverage**	6.1
**Number Of Reads**	20
**Reads Per Million**	0.38 rpm (after QC)
**Ambiguities**	0
**Assembly Method**	de novo + reference guided assembly
**Consensus Caller**	Bcf Tools

Coverage Map

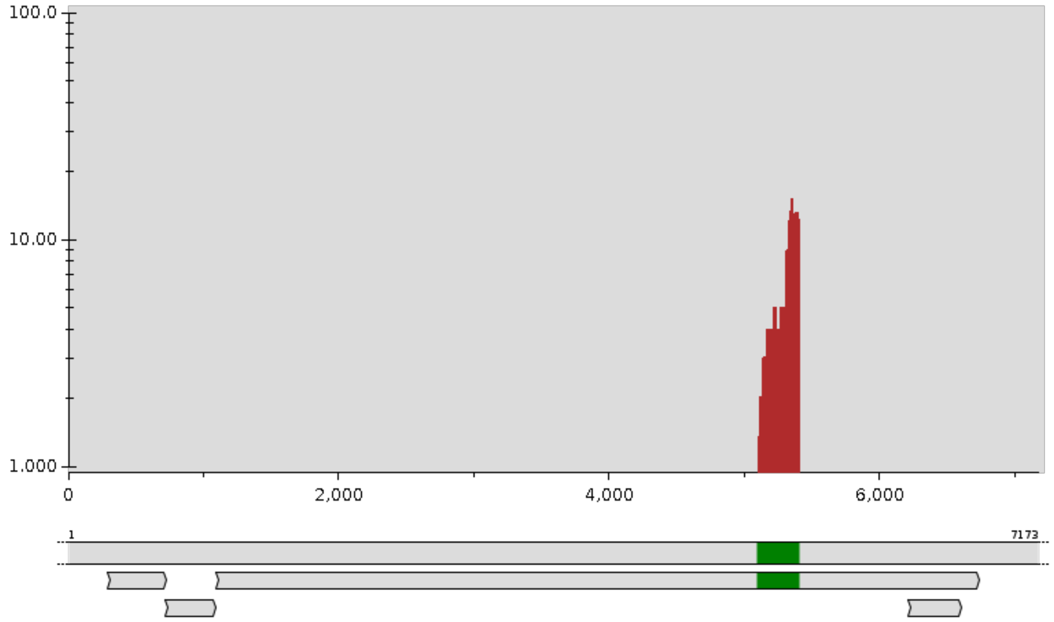

Assignment

**Type**	Badnavirus deltainflatheobromae (Taxonomy ID: 3047710)
**Reference Genome**	NC_040622.1
**NT Identity (%)**	52.2013
**AA Identity (%)**	49.0566
**Number Of Stop Codons**	0
**Number Of CDS**	4

Alignment

**Alignment Score**	22.0 (NT) + 381.0 (AA) = 403.0
**Concordance (%)**	29.1606

Alignment Method	Global, seeded, nucleotide + amino acids (AGA)

Genome Region

Sequence starts at position 5098 and ends at position 5412 relative to NC_040622.1 reference sequence.

Alignment Detailed Statistics

	Begin	End	Coverage	Score	Concordance	Matches	Identities	I/D/M/F*	Stop Codons
**NT**	**5098**	**5412**	**4.4%**	**22**	**3.5%**	**315 (99.1%)**	**166 (52.2%)**	**3/0**	
Mutations:	5098A>G, 5099A>G, 5100A>G, 5101G>A, 5105G>A, 5109G>A, 5110A>C, 5114T>C, 5117G>T, 5119A>T, 5120A>C, 5123A>G, 5126C>T, 5127C>A, 5131A>T, 5133T>C, 5135C>A, 5136C>A, 5143G>T, 5144A>T, 5147C>T, 5148C>A, 5150A>G, 5151A>G, 5152C>A, 5153T>A, 5154A>T, 5157C>T, 5159C>A, 5160A>G, 5162G>T, 5163A>G, 5165A>G, 5166A>T, 5168T>G, 5169G>C, 5170G>A, 5171C>T, 5172A>G, 5173A>G, 5174C>A, 5175A>G, 5176A>C, 5177A>C, 5178C>A, 5181G>T, 5182T>A, 5186C>T, 5187A>T, 5188G>C, 5193T>C, 5195T>A, 5198T>C, 5199C>T, 5203A>G, 5207C>T, 5210G>T, 5212T>A, 5213T>C, 5216T>C, 5219A>G, 5220G>A, 5222T>A, 5223G>A, 5224C>G, 5225T>A, 5229G>T, 5230C>T, 5231A>T, 5235G>T, 5236A>C, 5237G>T, 5238T>A, 5239C>A, 5240C>T, 5243C>A, 5246C>T, 5247T>A, 5248G>A, 5249G>A, 5252T>G, 5253G>A, 5255C>A, 5258T>C, 5259T>A, 5261G>A, 5262G>A, 5263T>C, 5264T>C, 5266C>A, 5267A>T, 5273C>G, 5275T>A, 5276A>C, 5282A>G, 5284G>T, 5285G>C, 5286C>T, 5288T>A, 5291T>G, 5297A>C, 5303G>C, 5308A>C, 5309G>C, 5315A>C, 5318A>C, 5319G>T, 5321A>C, 5323T>C, 5324C>A, 5327C>T, 5330A>G, 5333A>C, 5334A>C, 5335A>T, 5336A>C, 5340G>A, 5345G>A, 5346T>A, 5347G>T, 5348C>A, 5351T>C, 5354G>T, 5356G>C, 5357A>T, 5357_5358insCAT, 5358A>C, 5359C>T, 5360A>T, 5361G>A, 5362A>G, 5364G>A, 5366T>A, 5369C>T, 5373G>C, 5374C>T, 5375A>G, 5378C>T, 5380A>T, 5382A>T, 5384C>T, 5393C>A, 5394T>C, 5396G>T, 5397G>A, 5399C>A, 5401T>A, 5404G>A, 5411C>T, 5412A>T								

CDS

	Begin	End	Coverage	Score	Concordance	Matches	Identities	I/D/M/F*	Stop Codons
EXJ76_gp3	1337	1441	5.6%	381	50.0%	105 (99.1%)	52 (49.1%)	1/0/0/0	0
Protein mutations:	R1337E (5100A>G 5101G>A), D1340T (5109G>A 5110A>C), E1343V (5119A>T 5120A>C), Q1346K (5127C>A), Y1347F (5131A>T), S1348P (5133T>C 5135C>A), L1349I (5136C>A), G1351V (5143G>T 5144A>T), Q1353K (5148C>A 5150A>G), T1354E (5151A>G 5152C>A 5153T>A), I1355L (5154A>T), K1357D (5160A>G 5162G>T), K1358E (5163A>G 5165A>G), I1359L (5166A>T 5168T>G), G1360H (5169G>C 5170G>A 5171C>T), N1361G (5172A>G 5173A>G 5174C>A), K1362A (5175A>G 5176A>C 5177A>C), Q1363K (5178C>A), V1364Y (5181G>T 5182T>A), F1368L (5193T>C 5195T>A), K1371R (5203A>G), F1374Y (5212T>A 5213T>C), V1377I (5220G>A 5222T>A), A1378R (5223G>A 5224C>G 5225T>A), A1380F (5229G>T 5230C>T 5231A>T), E1382S (5235G>T 5236A>C 5237G>T), S1383N (5238T>A 5239C>A 5240C>T), W1386K (5247T>A 5248G>A 5249G>A), A1388T (5253G>A 5255C>A), W1390R (5259T>A 5261G>A), V1391T (5262G>A 5263T>C 5264T>C), P1392H (5266C>A 5267A>T), L1395H (5275T>A 5276A>C), W1398F (5284G>T 5285G>C), K1406T (5308A>C 5309G>C), A1410S (5319G>T 5321A>C), I1411T (5323T>C 5324C>A), R1414S (5333A>C), K1415L (5334A>C 5335A>T 5336A>C), D1417N (5340G>A), C1419I (5346T>A 5347G>T 5348C>A), K1421N (5354G>T), G1422A (5356G>C 5357A>T), G1422_T1423insH (5357_5358insCAT), T1423L (5358A>C 5359C>T 5360A>T), E1424R (5361G>A 5362A>G), D1425K (5364G>A 5366T>A), A1428L (5373G>C 5374C>T 5375A>G), Y1430F (5380A>T), I1431F (5382A>T 5384C>T), V1436I (5397G>A 5399C>A), F1437Y (5401T>A), S1438N (5404G>A)								
Codon mutations:	AAA1336.GG (5098A>G 5099A>G), AGA1337GAA (5100A>G 5101G>A), TTG1338TTA (5105G>A), GAC1340ACC (5109G>A 5110A>C), AAT1341AAC (5114T>C), ACG1342ACT (5117G>T), GAA1343GTC (5119A>T 5120A>C), AAA1344AAG (5123A>G), GAC1345GAT (5126C>T), CAG1346AAG (5127C>A), TAT1347TTT (5131A>T), TCC1348CCA (5133T>C 5135C>A), CTT1349ATT (5136C>A), GGA1351GTT (5143G>T 5144A>T), ATC1352ATT (5147C>T), CAA1353AAG (5148C>A 5150A>G), ACT1354GAA (5151A>G 5152C>A 5153T>A), ATA1355TTA (5154A>T), CTC1356TTA (5157C>T 5159C>A), AAG1357GAT (5160A>G 5162G>T), AAA1358GAG (5163A>G 5165A>G), ATT1359TTG (5166A>T 5168T>G), GGC1360CAT (5169G>C 5170G>A 5171C>T), AAC1361GGA (5172A>G 5173A>G 5174C>A), AAA1362GCC (5175A>G 5176A>C 5177A>C), CAG1363AAG (5178C>A), GTT1364TAT (5181G>T 5182T>A), TTC1365TTT (5186C>T), AGT1366TCT (5187A>T 5188G>C), TTT1368CTA (5193T>C 5195T>A), GAT1369GAC (5198T>C), CTG1370TTG (5199C>T), AAA1371AGA (5203A>G), TCC1372TCT (5207C>T), GGG1373GGT (5210G>T), TTT1374TAC (5212T>A 5213T>C), CAT1375CAC (5216T>C), CAA1376CAG (5219A>G), GTT1377ATA (5220G>A 5222T>A), GCT1378AGA (5223G>A 5224C>G 5225T>A), GCA1380TTT (5229G>T 5230C>T 5231A>T), GAG1382TCT (5235G>T 5236A>C 5237G>T), TCC1383AAT (5238T>A 5239C>A 5240C>T), ATC1384ATA (5243C>A), CCC1385CCT (5246C>T), TGG1386AAA (5247T>A 5248G>A 5249G>A), ACT1387ACG (5252T>G), GCC1388ACA (5253G>A 5255C>A), TTT1389TTC (5258T>C), TGG1390AGA (5259T>A 5261G>A), GTT1391ACC (5262G>A 5263T>C 5264T>C), CCA1392CAT (5266C>A 5267A>T), GGC1394GGG (5273C>G), CTA1395CAC (5275T>A 5276A>C), GAA1397GAG (5282A>G), TGG1398TTC (5284G>T 5285G>C), CTT1399TTA (5286C>T 5288T>A), GTT1400GTG (5291T>G), CCA1402CCC (5297A>C), GGG1404GGC (5303G>C), AAG1406ACC (5308A>C 5309G>C), GCA1408GCC (5315A>C), CCA1409CCC (5318A>C), GCA1410TCC (5319G>T 5321A>C), ATC1411ACA (5323T>C 5324C>A), TTC1412TTT (5327C>T), CAA1413CAG (5330A>G), AGA1414AGC (5333A>C), AAA1415CTC (5334A>C 5335A>T 5336A>C), GAT1417AAT (5340G>A), CAG1418CAA (5345G>A), TGC1419ATA (5346T>A 5347G>T 5348C>A), TTT1420TTC (5351T>C), AAG1421AAT (5354G>T), GGA1422GCT (5356G>C 5357A>T), GGA1422_ACA1423insCAT (5357_5358insCAT), ACA1423CTT (5358A>C 5359C>T 5360A>T), GAA1424AGA (5361G>A 5362A>G), GAT1425AAA (5364G>A 5366T>A), TTC1426TTT (5369C>T), GCA1428CTG (5373G>C 5374C>T 5375A>G), GTC1429GTT (5378C>T), TAC1430TTC (5380A>T), ATC1431TTT (5382A>T 5384C>T), ATC1434ATA (5393C>A), TTG1435CTT (5394T>C 5396G>T), GTC1436ATA (5397G>A 5399C>A), TTC1437TAC (5401T>A), AGC1438AAC (5404G>A), AAC1440AAT (5411C>T), ATG1441T.. (5412A>T)								

Proteins

	Begin	End	Coverage	Score	Concordance	Matches	Identities	I/D/M/F*	Stop Codons
ORF3 polyprotein (YP_009552693.1)	1337	1441	5.6%	381	50.0%	105 (99.1%)	52 (49.1%)	1/0/0/0	0
Protein mutations:	R1337E (5100A>G 5101G>A), D1340T (5109G>A 5110A>C), E1343V (5119A>T 5120A>C), Q1346K (5127C>A), Y1347F (5131A>T), S1348P (5133T>C 5135C>A), L1349I (5136C>A), G1351V (5143G>T 5144A>T), Q1353K (5148C>A 5150A>G), T1354E (5151A>G 5152C>A 5153T>A), I1355L (5154A>T), K1357D (5160A>G 5162G>T), K1358E (5163A>G 5165A>G), I1359L (5166A>T 5168T>G), G1360H (5169G>C 5170G>A 5171C>T), N1361G (5172A>G 5173A>G 5174C>A), K1362A (5175A>G 5176A>C 5177A>C), Q1363K (5178C>A), V1364Y (5181G>T 5182T>A), F1368L (5193T>C 5195T>A), K1371R (5203A>G), F1374Y (5212T>A 5213T>C), V1377I (5220G>A 5222T>A), A1378R (5223G>A 5224C>G 5225T>A), A1380F (5229G>T 5230C>T 5231A>T), E1382S (5235G>T 5236A>C 5237G>T), S1383N (5238T>A 5239C>A 5240C>T), W1386K (5247T>A 5248G>A 5249G>A), A1388T (5253G>A 5255C>A), W1390R (5259T>A 5261G>A), V1391T (5262G>A 5263T>C 5264T>C), P1392H (5266C>A 5267A>T), L1395H (5275T>A 5276A>C), W1398F (5284G>T 5285G>C), K1406T (5308A>C 5309G>C), A1410S (5319G>T 5321A>C), I1411T (5323T>C 5324C>A), R1414S (5333A>C), K1415L (5334A>C 5335A>T 5336A>C), D1417N (5340G>A), C1419I (5346T>A 5347G>T 5348C>A), K1421N (5354G>T), G1422A (5356G>C 5357A>T), G1422_T1423insH (5357_5358insCAT), T1423L (5358A>C 5359C>T 5360A>T), E1424R (5361G>A 5362A>G), D1425K (5364G>A 5366T>A), A1428L (5373G>C 5374C>T 5375A>G), Y1430F (5380A>T), I1431F (5382A>T 5384C>T), V1436I (5397G>A 5399C>A), F1437Y (5401T>A), S1438N (5404G>A)								
Codon mutations:	AAA1336.GG (5098A>G 5099A>G), AGA1337GAA (5100A>G 5101G>A), TTG1338TTA (5105G>A), GAC1340ACC (5109G>A 5110A>C), AAT1341AAC (5114T>C), ACG1342ACT (5117G>T), GAA1343GTC (5119A>T 5120A>C), AAA1344AAG (5123A>G), GAC1345GAT (5126C>T), CAG1346AAG (5127C>A), TAT1347TTT (5131A>T), TCC1348CCA (5133T>C 5135C>A), CTT1349ATT (5136C>A), GGA1351GTT (5143G>T 5144A>T), ATC1352ATT (5147C>T), CAA1353AAG (5148C>A 5150A>G), ACT1354GAA (5151A>G 5152C>A 5153T>A), ATA1355TTA (5154A>T), CTC1356TTA (5157C>T 5159C>A), AAG1357GAT (5160A>G 5162G>T), AAA1358GAG (5163A>G 5165A>G), ATT1359TTG (5166A>T 5168T>G), GGC1360CAT (5169G>C 5170G>A 5171C>T), AAC1361GGA (5172A>G 5173A>G 5174C>A), AAA1362GCC (5175A>G 5176A>C 5177A>C), CAG1363AAG (5178C>A), GTT1364TAT (5181G>T 5182T>A), TTC1365TTT (5186C>T), AGT1366TCT (5187A>T 5188G>C), TTT1368CTA (5193T>C 5195T>A), GAT1369GAC (5198T>C), CTG1370TTG (5199C>T), AAA1371AGA (5203A>G), TCC1372TCT (5207C>T), GGG1373GGT (5210G>T), TTT1374TAC (5212T>A 5213T>C), CAT1375CAC (5216T>C), CAA1376CAG (5219A>G), GTT1377ATA (5220G>A 5222T>A), GCT1378AGA (5223G>A 5224C>G 5225T>A), GCA1380TTT (5229G>T 5230C>T 5231A>T), GAG1382TCT (5235G>T 5236A>C 5237G>T), TCC1383AAT (5238T>A 5239C>A 5240C>T), ATC1384ATA (5243C>A), CCC1385CCT (5246C>T), TGG1386AAA (5247T>A 5248G>A 5249G>A), ACT1387ACG (5252T>G), GCC1388ACA (5253G>A 5255C>A), TTT1389TTC (5258T>C), TGG1390AGA (5259T>A 5261G>A), GTT1391ACC (5262G>A 5263T>C 5264T>C), CCA1392CAT (5266C>A 5267A>T), GGC1394GGG (5273C>G), CTA1395CAC (5275T>A 5276A>C), GAA1397GAG (5282A>G), TGG1398TTC (5284G>T 5285G>C), CTT1399TTA (5286C>T 5288T>A), GTT1400GTG (5291T>G), CCA1402CCC (5297A>C), GGG1404GGC (5303G>C), AAG1406ACC (5308A>C 5309G>C), GCA1408GCC (5315A>C), CCA1409CCC (5318A>C), GCA1410TCC (5319G>T 5321A>C), ATC1411ACA (5323T>C 5324C>A), TTC1412TTT (5327C>T), CAA1413CAG (5330A>G), AGA1414AGC (5333A>C), AAA1415CTC (5334A>C 5335A>T 5336A>C), GAT1417AAT (5340G>A), CAG1418CAA (5345G>A), TGC1419ATA (5346T>A 5347G>T 5348C>A), TTT1420TTC (5351T>C), AAG1421AAT (5354G>T), GGA1422GCT (5356G>C 5357A>T), GGA1422_ACA1423insCAT (5357_5358insCAT), ACA1423CTT (5358A>C 5359C>T 5360A>T), GAA1424AGA (5361G>A 5362A>G), GAT1425AAA (5364G>A 5366T>A), TTC1426TTT (5369C>T), GCA1428CTG (5373G>C 5374C>T 5375A>G), GTC1429GTT (5378C>T), TAC1430TTC (5380A>T), ATC1431TTT (5382A>T 5384C>T), ATC1434ATA (5393C>A), TTG1435CTT (5394T>C 5396G>T), GTC1436ATA (5397G>A 5399C>A), TTC1437TAC (5401T>A), AGC1438AAC (5404G>A), AAC1440AAT (5411C>T), ATG1441T.. (5412A>T)								

*: Inserts / Deletes / Misaligned / Frameshifts

Analysis details

This analysis was performed with panviral2.64

NGS Details (UN70): Cavemovirus collusipomeae

Assembly

**Coverage Length**	258 (1 contig(s))
**Depth Of Coverage**	7.1
**Number Of Reads**	15
**Reads Per Million**	0.28 rpm (after QC)
**Ambiguities**	0
**Assembly Method**	de novo + reference guided assembly
**Consensus Caller**	Bcf Tools

Coverage Map

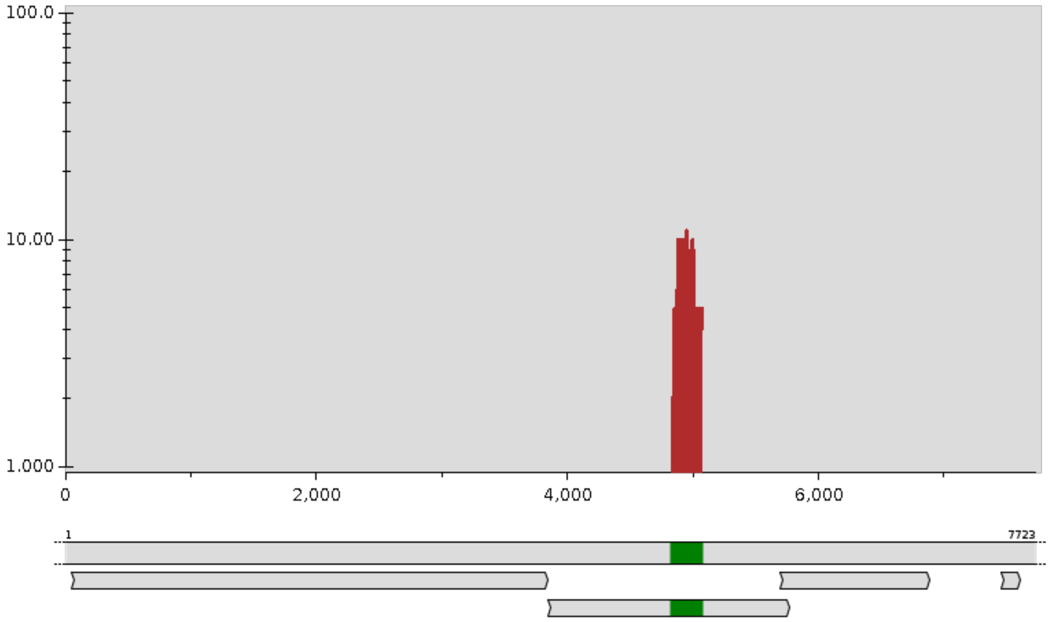

Assignment

**Type**	Cavemovirus collusipomeae (Taxonomy ID: 3048352)
**Reference Genome**	NC_015328.1
**NT Identity (%)**	61.6279
**AA Identity (%)**	50.0
**Number Of Stop Codons**	0
**Number Of CDS**	4

Alignment

**Alignment Score**	120.0 (NT) + 326.0 (AA) = 446.0
**Concordance (%)**	39.9284

**Alignment Method**	Global, seeded, nucleotide + amino acids (AGA)

Genome Region

Sequence starts at position 4825 and ends at position 5082 relative to NC_015328.1 reference sequence.

Alignment Detailed Statistics

	Begin	End	Coverage	Score	Concordance	Matches	Identities	I/D/M/F*	Stop Codons
**NT**	**4825**	**5082**	**3.3%**	**120**	**23.3%**	**258 (100%)**	**159 (61.6%)**	**0/0**	
Mutations:	4826T>A, 4828A>G, 4831T>A, 4838T>C, 4839A>T, 4840T>A, 4841C>A, 4843T>A, 4847T>G, 4849A>T, 4852A>T, 4853A>T, 4854G>C, 4856A>G, 4857T>A, 4860A>T, 4864A>G, 4865C>A, 4868T>A, 4870T>C, 4874G>A, 4878G>A, 4879A>T, 4882A>T, 4887G>A, 4888A>T, 4896A>T, 4897T>A, 4906A>C, 4907C>A, 4912A>T, 4918A>T, 4924T>C, 4927A>T, 4930A>G, 4933A>T, 4935T>A, 4939G>T, 4945A>G, 4947A>T, 4948C>T, 4952G>A, 4953A>T, 4954A>G, 4959A>T, 4960A>C, 4962T>A, 4967T>C, 4969G>T, 4972A>T, 4975A>C, 4978T>C, 4980G>A, 4984T>A, 4987A>T, 4988A>G, 4990A>T, 4994A>C, 4997C>A, 5001G>A, 5002A>T, 5003A>T, 5005A>G, 5009T>A, 5011A>T, 5012A>T, 5013G>C, 5017G>A, 5018A>C, 5019A>C, 5024G>A, 5025C>T, 5026A>G, 5027G>A, 5028A>G, 5029G>T, 5030A>T, 5033G>T, 5034G>T, 5035A>T, 5036A>C, 5039C>A, 5040A>C, 5042A>C, 5044A>T, 5045A>G, 5047A>C, 5048G>C, 5049A>G, 5060G>C, 5061T>A, 5065G>T, 5068A>T, 5069G>T, 5070A>G, 5071A>C, 5076G>A, 5078G>A, 5079G>A								

CDS

	Begin	End	Coverage	Score	Concordance	Matches	Identities	I/D/M/F*	Stop Codons
SPCV_gp2	329	414	13.4%	326	53.3%	86 (100%)	43 (50.0%)	0/0/0/0	0
Protein mutations:	L329M (4826T>A 4828A>G), Y333L (4838T>C 4839A>T 4840T>A), H334K (4841C>A 4843T>A), S336A (4847T>G 4849A>T), I339E (4856A>G 4857T>A), Y340F (4860A>T), Q342K (4865C>A), F343I (4868T>A 4870T>C), D345N (4874G>A), G346D (4878G>A 4879A>T), R349N (4887G>A 4888A>T), Y352L (4896A>T 4897T>A), L356I (4907C>A), F365Y (4935T>A), Y369F (4947A>T 4948C>T), E371M (4952G>A 4953A>T 4954A>G), K373I (4959A>T 4960A>C), I374K (4962T>A), E377D (4972A>T), R380K (4980G>A), N381K (4984T>A), I383V (4988A>G 4990A>T), K385Q (4994A>C), H386N (4997C>A), G387D (5001G>A 5002A>T), I388L (5003A>T 5005A>G), L390I (5009T>A 5011A>T), K393P (5018A>C 5019A>C), A395M (5024G>A 5025C>T 5026A>G), E396S (5027G>A 5028A>G 5029G>T), I397L (5030A>T), G398F (5033G>T 5034G>T 5035A>T), K399Q (5036A>C), Q400T (5039C>A 5040A>C), K401H (5042A>C 5044A>T), I402V (5045A>G 5047A>C), E403R (5048G>C 5049A>G), V407H (5060G>C 5061T>A), K408N (5065G>T), E410C (5069G>T 5070A>G 5071A>C), G412E (5076G>A), G413K (5078G>A 5079G>A)								
Codon mutations:	TTA329ATG (4826T>A 4828A>G), CCT330CCA (4831T>A), TAT333CTA (4838T>C 4839A>T 4840T>A), CAT334AAA (4841C>A 4843T>A), TCA336GCT (4847T>G 4849A>T), CCA337CCT (4852A>T), AGT338TCT (4853A>T 4854G>C), ATA339GAA (4856A>G 4857T>A), TAT340TTT (4860A>T), CAA341CAG (4864A>G), CAA342AAA (4865C>A), TTT343ATC (4868T>A 4870T>C), GAT345AAT (4874G>A), GGA346GAT (4878G>A 4879A>T), ATA347ATT (4882A>T), AGA349AAT (4887G>A 4888A>T), TAT352TTA (4896A>T 4897T>A), ATA355ATC (4906A>C), CTT356ATT (4907C>A), GTA357GTT (4912A>T), ATA359ATT (4918A>T), GAT361GAC (4924T>C), ATA362ATT (4927A>T), TTA363TTG (4930A>G), ATA364ATT (4933A>T), TTT365TAT (4935T>A), TCG366TCT (4939G>T), ACA368ACG (4945A>G), TAC369TTT (4947A>T 4948C>T), GAA371ATG (4952G>A 4953A>T 4954A>G), AAA373ATC (4959A>T 4960A>C), ATA374AAA (4962T>A), TTG376CTT (4967T>C 4969G>T), GAA377GAT (4972A>T), ATA378ATC (4975A>C), TTT379TTC (4978T>C), AGA380AAA (4980G>A), AAT381AAA (4984T>A), ATA382ATT (4987A>T), ATA383GTT (4988A>G 4990A>T), AAA385CAA (4994A>C), CAT386AAT (4997C>A), GGA387GAT (5001G>A 5002A>T), ATA388TTG (5003A>T 5005A>G), TTA390ATT (5009T>A 5011A>T), AGT391TCT (5012A>T 5013G>C), AAG392AAA (5017G>A), AAA393CCA (5018A>C 5019A>C), GCA395ATG (5024G>A 5025C>T 5026A>G), GAG396AGT (5027G>A 5028A>G 5029G>T), ATA397TTA (5030A>T), GGA398TTT (5033G>T 5034G>T 5035A>T), AAA399CAA (5036A>C), CAA400ACA (5039C>A 5040A>C), AAA401CAT (5042A>C 5044A>T), ATA402GTC (5045A>G 5047A>C), GAA403CGA (5048G>C 5049A>G), GTT407CAT (5060G>C 5061T>A), AAG408AAT (5065G>T), ATA409ATT (5068A>T), GAA410TGC (5069G>T 5070A>G 5071A>C), GGA412GAA (5076G>A), GGA413AAA (5078G>A 5079G>A)								

Proteins

	Begin	End	Coverage	Score	Concordance	Matches	Identities	I/D/M/F*	Stop Codons
replicase (YP_004347415.1)	329	414	13.4%	326	53.3%	86 (100%)	43 (50.0%)	0/0/0/0	0
Protein mutations:	L329M (4826T>A 4828A>G), Y333L (4838T>C 4839A>T 4840T>A), H334K (4841C>A 4843T>A), S336A (4847T>G 4849A>T), I339E (4856A>G 4857T>A), Y340F (4860A>T), Q342K (4865C>A), F343I (4868T>A 4870T>C), D345N (4874G>A), G346D (4878G>A 4879A>T), R349N (4887G>A 4888A>T), Y352L (4896A>T 4897T>A), L356I (4907C>A), F365Y (4935T>A), Y369F (4947A>T 4948C>T), E371M (4952G>A 4953A>T 4954A>G), K373I (4959A>T 4960A>C), I374K (4962T>A), E377D (4972A>T), R380K (4980G>A), N381K (4984T>A), I383V (4988A>G 4990A>T), K385Q (4994A>C), H386N (4997C>A), G387D (5001G>A 5002A>T), I388L (5003A>T 5005A>G), L390I (5009T>A 5011A>T), K393P (5018A>C 5019A>C), A395M (5024G>A 5025C>T 5026A>G), E396S (5027G>A 5028A>G 5029G>T), I397L (5030A>T), G398F (5033G>T 5034G>T 5035A>T), K399Q (5036A>C), Q400T (5039C>A 5040A>C), K401H (5042A>C 5044A>T), I402V (5045A>G 5047A>C), E403R (5048G>C 5049A>G), V407H (5060G>C 5061T>A), K408N (5065G>T), E410C (5069G>T 5070A>G 5071A>C), G412E (5076G>A), G413K (5078G>A 5079G>A)								
Codon mutations:	TTA329ATG (4826T>A 4828A>G), CCT330CCA (4831T>A), TAT333CTA (4838T>C 4839A>T 4840T>A), CAT334AAA (4841C>A 4843T>A), TCA336GCT (4847T>G 4849A>T), CCA337CCT (4852A>T), AGT338TCT (4853A>T 4854G>C), ATA339GAA (4856A>G 4857T>A), TAT340TTT (4860A>T), CAA341CAG (4864A>G), CAA342AAA (4865C>A), TTT343ATC (4868T>A 4870T>C), GAT345AAT (4874G>A), GGA346GAT (4878G>A 4879A>T), ATA347ATT (4882A>T), AGA349AAT (4887G>A 4888A>T), TAT352TTA (4896A>T 4897T>A), ATA355ATC (4906A>C), CTT356ATT (4907C>A), GTA357GTT (4912A>T), ATA359ATT (4918A>T), GAT361GAC (4924T>C), ATA362ATT (4927A>T), TTA363TTG (4930A>G), ATA364ATT (4933A>T), TTT365TAT (4935T>A), TCG366TCT (4939G>T), ACA368ACG (4945A>G), TAC369TTT (4947A>T 4948C>T), GAA371ATG (4952G>A 4953A>T 4954A>G), AAA373ATC (4959A>T 4960A>C), ATA374AAA (4962T>A), TTG376CTT (4967T>C 4969G>T), GAA377GAT (4972A>T), ATA378ATC (4975A>C), TTT379TTC (4978T>C), AGA380AAA (4980G>A), AAT381AAA (4984T>A), ATA382ATT (4987A>T), ATA383GTT (4988A>G 4990A>T), AAA385CAA (4994A>C), CAT386AAT (4997C>A), GGA387GAT (5001G>A 5002A>T), ATA388TTG (5003A>T 5005A>G), TTA390ATT (5009T>A 5011A>T), AGT391TCT (5012A>T 5013G>C), AAG392AAA (5017G>A), AAA393CCA (5018A>C 5019A>C), GCA395ATG (5024G>A 5025C>T 5026A>G), GAG396AGT (5027G>A 5028A>G 5029G>T), ATA397TTA (5030A>T), GGA398TTT (5033G>T 5034G>T 5035A>T), AAA399CAA (5036A>C), CAA400ACA (5039C>A 5040A>C), AAA401CAT (5042A>C 5044A>T), ATA402GTC (5045A>G 5047A>C), GAA403CGA (5048G>C 5049A>G), GTT407CAT (5060G>C 5061T>A), AAG408AAT (5065G>T), ATA409ATT (5068A>T), GAA410TGC (5069G>T 5070A>G 5071A>C), GGA412GAA (5076G>A), GGA413AAA (5078G>A 5079G>A)								

*: Inserts / Deletes / Misaligned / Frameshifts

Analysis details

This analysis was performed with panviral2.64

NGS Details (UN70): Petuvirus venapetuniae

Assembly

**Coverage Length**	609 (3 contig(s))
**Depth Of Coverage**	2.8
**Number Of Reads**	14
**Reads Per Million**	0.27 rpm (after QC)
**Ambiguities**	0
**Assembly Method**	de novo + reference guided assembly
**Consensus Caller**	Bcf Tools

Coverage Map

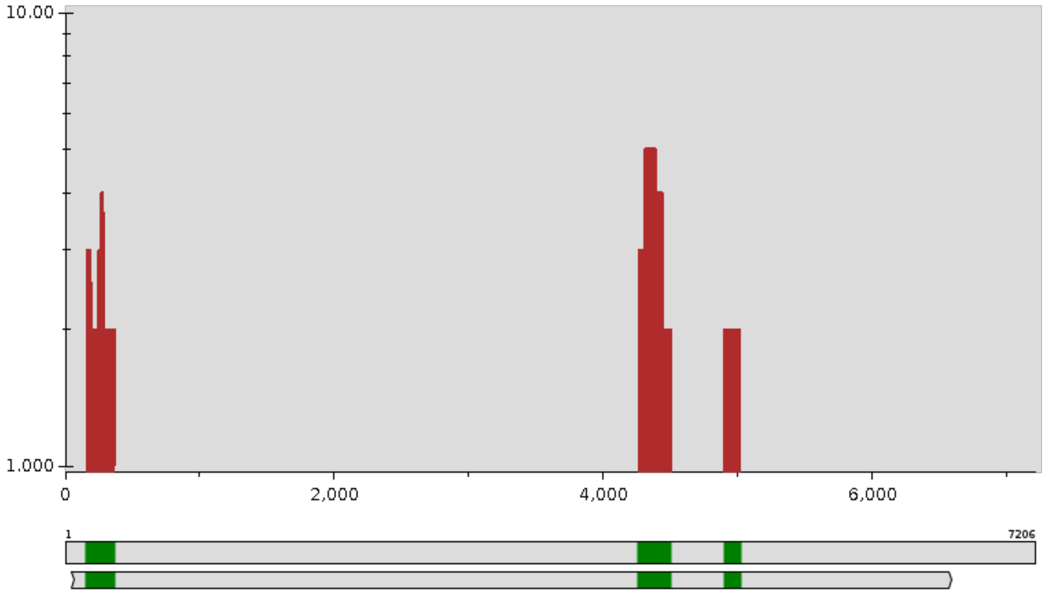

Assignment

**Type**	Petuvirus venapetuniae (Taxonomy ID: 3048265)
**Reference Genome**	NC_001839.2
**NT Identity (%)**	64.0394
**AA Identity (%)**	57.9208
**Number Of Stop Codons**	2
**Number Of CDS**	1

Alignment

**Alignment Score**	342.0 (NT) + 831.0 (AA) = 1173.0
**Concordance (%)**	44.3814

Alignment Method	Global, seeded, nucleotide + amino acids (AGA)

Genome Region

Sequence starts at position 151 and ends at position 5024 relative to NC_001839.2 reference sequence.

Alignment Detailed Statistics

	Begin	End	Coverage	Score	Concordance	Matches	Identities	I/D/M/F*	Stop Codons
**NT**	**151**	**5024**	**8.5%**	**342**	**28.1%**	**609 (100%)**	**390 (64.0%)**	**0/0**	
Mutations:	152T>G, 159G>A, 161A>T, 165A>G, 170T>A, 171A>C, 172A>C, 173A>C, 177A>G, 179C>G, 186A>G, 187T>C, 192G>T, 193A>C, 194T>C, 197C>G, 199A>C, 200T>C, 201C>T, 203T>A, 207T>C, 209G>T, 210T>A, 221C>T, 233A>C, 238G>A, 239A>T, 241C>A, 242C>A, 243G>T, 245T>A, 246A>T, 248C>G, 251T>A, 255T>A, 256C>T, 257T>C, 260G>T, 263C>T, 265G>A, 269A>C, 278G>A, 279T>A, 280C>A, 281T>C, 286C>T, 287T>C, 288C>A, 289G>T, 290C>A, 291C>G, 292A>G, 293T>A, 296G>A, 298A>G, 310C>T, 317C>A, 321T>C, 323A>C, 324G>A, 329G>A, 333C>T, 336G>A, 338C>A, 339T>C, 344T>A, 347A>T, 348G>C, 349G>C, 359G>A, 362T>C, 363G>T, 366A>T, 4253A>C, 4255T>A, 4259G>A, 4262A>G, 4263T>A, 4264G>T, 4269G>A, 4271G>T, 4274T>C, 4275C>T, 4276A>T, 4281T>A, 4282T>A, 4283T>G, 4285A>G, 4287C>T, 4289A>G, 4292C>A, 4293G>A, 4298C>G, 4301T>C, 4302G>A, 4304T>A, 4309A>C, 4310A>T, 4314G>T, 4316A>T, 4320G>A, 4321A>C, 4325T>C, 4334C>T, 4337C>T, 4340G>C, 4341A>G, 4342G>C, 4343G>A, 4344T>G, 4345C>G, 4346A>T, 4349G>A, 4350C>A, 4352A>G, 4354T>A, 4355G>A, 4361A>T, 4362A>G, 4363A>C, 4364A>T, 4365T>C, 4366T>C, 4367A>T, 4368A>C, 4370G>A, 4371T>C, 4376C>T, 4386C>A, 4391A>T, 4397C>T, 4398C>A, 4400T>A, 4402T>A, 4403C>T, 4404C>T, 4406C>G, 4407C>A, 4410G>T, 4411A>G, 4412T>G, 4413G>A, 4414A>T, 4415C>T, 4417A>G, 4420T>A, 4421T>C, 4424C>T, 4430A>T, 4436G>A, 4437C>A, 4438T>G, 4439C>G, 4440A>G, 4441C>A, 4442A>T, 4443C>T, 4445C>A, 4448C>A, 4452C>A, 4453A>G, 4454C>A, 4459C>A, 4460C>T, 4461A>G, 4463A>T, 4466A>C, 4469G>T, 4470C>A, 4472C>A, 4481G>T, 4488C>T, 4490T>A, 4493G>A, 4501T>A, 4894C>T, 4896G>A, 4898A>C, 4904G>A, 4905A>C, 4907A>C, 4908C>T, 4910A>G, 4916T>C, 4922G>T, 4923A>G, 4925T>G, 4926G>A, 4935A>G, 4938A>C, 4939G>A, 4952A>T, 4953G>T, 4955G>T, 4956G>A, 4958T>C, 4959A>T, 4961T>G, 4962G>A, 4965C>T, 4973C>T, 4976T>A, 4981G>T, 4982T>G, 4983G>A, 4985T>G, 4988G>T, 4992A>C, 4994G>A, 5001C>T, 5002C>A, 5003A>T, 5006G>T, 5008C>T, 5009A>T, 5015A>G, 5018A>G, 5019T>G, 5020G>A								

CDS

	Begin	End	Coverage	Score	Concordance	Matches	Identities	I/D/M/F*	Stop Codons
PVCVgp1	35	1658	9.3%	831	57.7%	202 (100%)	117 (57.9%)	0/0/0/0	2
Protein mutations:	V37I (159G>A 161A>T), I39V (165A>G), K41P (171A>C 172A>C 173A>C), N43E (177A>G 179C>G), I46A (186A>G 187T>C), D48S (192G>T 193A>C 194T>C), Y50S (199A>C 200T>C), L54I (210T>A), R63N (238G>A 239A>T), S64* (241C>A 242C>A), V65L (243G>T 245T>A), T66S (246A>T 248C>G), S69I (255T>A 256C>T 257T>C), Q70H (260G>T), R72H (265G>A), S77N (279T>A 280C>A 281T>C), P79L (286C>T 287T>C), R80I (288C>A 289G>T 290C>A), H81G (291C>G 292A>G 293T>A), K83R (298A>G), A87V (310C>T), D92N (324G>A), P95S (333C>T), V96I (336G>A 338C>A), F97L (339T>C), G100P (348G>C 349G>C), V105F (363G>T), T106S (366A>T), I1402K (4255T>A), C1405I (4263T>A 4264G>T), E1407N (4269G>A 4271G>T), Q1409L (4275C>T 4276A>T), F1411K (4281T>A 4282T>A 4283T>G), D1412G (4285A>G), E1415K (4293G>A), D1418K (4302G>A 4304T>A), Q1420P (4309A>C 4310A>T), A1422S (4314G>T 4316A>T), E1424T (4320G>A 4321A>C), K1430N (4340G>C), R1431A (4341A>G 4342G>C 4343G>A), S1432G (4344T>G 4345C>G 4346A>T), Q1434K (4350C>A 4352A>G), V1435E (4354T>A 4355G>A), K1438A (4362A>G 4363A>C 4364A>T), L1439P (4365T>C 4366T>C 4367A>T), Q1446K (4386C>A), H1450K (4398C>A 4400T>A), F1451Y (4402T>A 4403C>T), Q1453K (4407C>A), D1454W (4410G>T 4411A>G 4412T>G), D1455I (4413G>A 4414A>T 4415C>T), K1456R (4417A>G), F1457Y (4420T>A 4421T>C), L1463R (4437C>A 4438T>G 4439C>G), T1464D (4440A>G 4441C>A 4442A>T), F1466L (4448C>A), H1468R (4452C>A 4453A>G 4454C>A), S1470Y (4459C>A 4460C>T), K1471D (4461A>G 4463A>T), K1473N (4469G>T), L1474I (4470C>A 4472C>A), K1477N (4481G>T), F1484Y (4501T>A), S1615L (4894C>T), V1616I (4896G>A 4898A>C), I1619L (4905A>C 4907A>C), Q1620* (4908C>T 4910A>G), I1625V (4923A>G 4925T>G), V1626I (4926G>A), I1629V (4935A>G), R1630Q (4938A>C 4939G>A), E1635Y (4953G>T 4955G>T), V1636I (4956G>A 4958T>C), T1637S (4959A>T 4961T>G), E1638K (4962G>A), H1639Y (4965C>T), S1644M (4981G>T 4982T>G), D1645K (4983G>A 4985T>G), M1646I (4988G>T), K1648Q (4992A>C 4994G>A), P1651Y (5001C>T 5002C>A 5003A>T), A1653V (5008C>T 5009A>T), C1657D (5019T>G 5020G>A)								
Codon mutations:	CTT34.TG (152T>G), GTA37ATT (159G>A 161A>T), ATC39GTC (165A>G), CTT40CTA (170T>A), AAA41CCC (171A>C 172A>C 173A>C), AAC43GAG (177A>G 179C>G), ATA46GCA (186A>G 187T>C), GAT48TCC (192G>T 193A>C 194T>C), ACC49ACG (197C>G), TAT50TCC (199A>C 200T>C), CTT51TTA (201C>T 203T>A), TTG53CTT (207T>C 209G>T), TTA54ATA (210T>A), TAC57TAT (221C>T), GCA61GCC (233A>C), AGA63AAT (238G>A 239A>T), TCC64TAA (241C>A 242C>A), GTT65TTA (243G>T 245T>A), ACC66TCG (246A>T 248C>G), CCT67CCA (251T>A), TCT69ATC (255T>A 256C>T 257T>C), CAG70CAT (260G>T), ATC71ATT (263C>T), CGT72CAT (265G>A), TCA73TCC (269A>C), CAG76CAA (278G>A), TCT77AAC (279T>A 280C>A 281T>C), CCT79CTC (286C>T 287T>C), CGC80ATA (288C>A 289G>T 290C>A), CAT81GGA (291C>G 292A>G 293T>A), GTG82GTA (296G>A), AAA83AGA (298A>G), GCT87GTT (310C>T), TCC89TCA (317C>A), TTA91CTC (321T>C 323A>C), GAT92AAT (324G>A), CAG93CAA (329G>A), CCT95TCT (333C>T), GTC96ATA (336G>A 338C>A), TTT97CTT (339T>C), GCT98GCA (344T>A), ACA99ACT (347A>T), GGA100CCA (348G>C 349G>C), CAG103CAA (359G>A), TTT104TTC (362T>C), GTT105TTT (363G>T), ACT106TCT (366A>T), GCA1401.CC (4253A>C), ATA1402AAA (4255T>A), AAG1403AAA (4259G>A), GAA1404GAG (4262A>G), TGT1405ATT (4263T>A 4264G>T), GAG1407AAT (4269G>A 4271G>T), CTT1408CTC (4274T>C), CAA1409TTA (4275C>T 4276A>T), TTT1411AAG (4281T>A 4282T>A 4283T>G), GAT1412GGT (4285A>G), CTA1413TTG (4287C>T 4289A>G), ATC1414ATA (4292C>A), GAA1415AAA (4293G>A), CCC1416CCG (4298C>G), TCT1417TCC (4301T>C), GAT1418AAA (4302G>A 4304T>A), CAA1420CCT (4309A>C 4310A>T), GCA1422TCT (4314G>T 4316A>T), GAA1424ACA (4320G>A 4321A>C), GCT1425GCC (4325T>C), GTC1428GTT (4334C>T), AAC1429AAT (4337C>T), AAG1430AAC (4340G>C), AGG1431GCA (4341A>G 4342G>C 4343G>A), TCA1432GGT (4344T>G 4345C>G 4346A>T), GAG1433GAA (4349G>A), CAA1434AAG (4350C>A 4352A>G), GTG1435GAA (4354T>A 4355G>A), GGA1437GGT (4361A>T), AAA1438GCT (4362A>G 4363A>C 4364A>T), TTA1439CCT (4365T>C 4366T>C 4367A>T), AGG1440CGA (4368A>C 4370G>A), TTA1441CTA (4371T>C), GTC1442GTT (4376C>T), CAA1446AAA (4386C>A), CCA1447CCT (4391A>T), AAC1449AAT (4397C>T), CAT1450AAA (4398C>A 4400T>A), TTC1451TAT (4402T>A 4403C>T), CTC1452TTG (4404C>T 4406C>G), CAA1453AAA (4407C>A), GAT1454TGG (4410G>T 4411A>G 4412T>G), GAC1455ATT (4413G>A 4414A>T 4415C>T), AAG1456AGG (4417A>G), TTT1457TAC (4420T>A 4421T>C), CCC1458CCT (4424C>T), CCA1460CCT (4430A>T), AAG1462AAA (4436G>A), CTC1463AGG (4437C>A 4438T>G 4439C>G), ACA1464GAT (4440A>G 4441C>A 4442A>T), CTC1465TTA (4443C>T 4445C>A), TTC1466TTA (4448C>A), CAC1468AGA (4452C>A 4453A>G 4454C>A), TCC1470TAT (4459C>A 4460C>T), AAA1471GAT (4461A>G 4463A>T), GCA1472GCC (4466A>C), AAG1473AAT (4469G>T), CTC1474ATA (4470C>A 4472C>A), AAG1477AAT (4481G>T), CTT1480TTA (4488C>T 4490T>A), AAG1481AAA (4493G>A), TTT1484TAT (4501T>A), TCG1615TTG (4894C>T), GTA1616ATC (4896G>A 4898A>C), CAG1618CAA (4904G>A), ATA1619CTC (4905A>C 4907A>C), CAA1620TAG (4908C>T 4910A>G), TTT1622TTC (4916T>C), GGG1624GGT (4922G>T), ATT1625GTG (4923A>G 4925T>G), GTT1626ATT (4926G>A), ATC1629GTC (4935A>G), AGA1630CAA (4938A>C 4939G>A), CCA1634CCT (4952A>T), GAG1635TAT (4953G>T 4955G>T), GTT1636ATC (4956G>A 4958T>C), ACT1637TCG (4959A>T 4961T>G), GAG1638AAG (4962G>A), CAT1639TAT (4965C>T), TCC1641TCT (4973C>T), CCT1642CCA (4976T>A), AGT1644ATG (4981G>T 4982T>G), GAT1645AAG (4983G>A 4985T>G), ATG1646ATT (4988G>T), AAG1648CAA (4992A>C 4994G>A), CCA1651TAT (5001C>T 5002C>A 5003A>T), CCG1652CCT (5006G>T), GCA1653GTT (5008C>T 5009A>T), GGA1655GGG (5015A>G), AAA1656AAG (5018A>G), TGT1657GAT (5019T>G 5020G>A)								

Proteins

	Begin	End	Coverage	Score	Concordance	Matches	Identities	I/D/M/F*	Stop Codons
ORF I polyprotein (NP_127504.1)	35	1658	9.3%	831	57.7%	202 (100%)	117 (57.9%)	0/0/0/0	2
Protein mutations:	V37I (159G>A 161A>T), I39V (165A>G), K41P (171A>C 172A>C 173A>C), N43E (177A>G 179C>G), I46A (186A>G 187T>C), D48S (192G>T 193A>C 194T>C), Y50S (199A>C 200T>C), L54I (210T>A), R63N (238G>A 239A>T), S64* (241C>A 242C>A), V65L (243G>T 245T>A), T66S (246A>T 248C>G), S69I (255T>A 256C>T 257T>C), Q70H (260G>T), R72H (265G>A), S77N (279T>A 280C>A 281T>C), P79L (286C>T 287T>C), R80I (288C>A 289G>T 290C>A), H81G (291C>G 292A>G 293T>A), K83R (298A>G), A87V (310C>T), D92N (324G>A), P95S (333C>T), V96I (336G>A 338C>A), F97L (339T>C), G100P (348G>C 349G>C), V105F (363G>T), T106S (366A>T), I1402K (4255T>A), C1405I (4263T>A 4264G>T), E1407N (4269G>A 4271G>T), Q1409L (4275C>T 4276A>T), F1411K (4281T>A 4282T>A 4283T>G), D1412G (4285A>G), E1415K (4293G>A), D1418K (4302G>A 4304T>A), Q1420P (4309A>C 4310A>T), A1422S (4314G>T 4316A>T), E1424T (4320G>A 4321A>C), K1430N (4340G>C), R1431A (4341A>G 4342G>C 4343G>A), S1432G (4344T>G 4345C>G 4346A>T), Q1434K (4350C>A 4352A>G), V1435E (4354T>A 4355G>A), K1438A (4362A>G 4363A>C 4364A>T), L1439P (4365T>C 4366T>C 4367A>T), Q1446K (4386C>A), H1450K (4398C>A 4400T>A), F1451Y (4402T>A 4403C>T), Q1453K (4407C>A), D1454W (4410G>T 4411A>G 4412T>G), D1455I (4413G>A 4414A>T 4415C>T), K1456R (4417A>G), F1457Y (4420T>A 4421T>C), L1463R (4437C>A 4438T>G 4439C>G), T1464D (4440A>G 4441C>A 4442A>T), F1466L (4448C>A), H1468R (4452C>A 4453A>G 4454C>A), S1470Y (4459C>A 4460C>T), K1471D (4461A>G 4463A>T), K1473N (4469G>T), L1474I (4470C>A 4472C>A), K1477N (4481G>T), F1484Y (4501T>A), S1615L (4894C>T), V1616I (4896G>A 4898A>C), I1619L (4905A>C 4907A>C), Q1620* (4908C>T 4910A>G), I1625V (4923A>G 4925T>G), V1626I (4926G>A), I1629V (4935A>G), R1630Q (4938A>C 4939G>A), E1635Y (4953G>T 4955G>T), V1636I (4956G>A 4958T>C), T1637S (4959A>T 4961T>G), E1638K (4962G>A), H1639Y (4965C>T), S1644M (4981G>T 4982T>G), D1645K (4983G>A 4985T>G), M1646I (4988G>T), K1648Q (4992A>C 4994G>A), P1651Y (5001C>T 5002C>A 5003A>T), A1653V (5008C>T 5009A>T), C1657D (5019T>G 5020G>A)								

	Begin	End	Coverage	Score	Concordance	Matches	Identities	I/D/M/F*	Stop Codons
**NT**	**151**	**5024**	**8.5%**	**342**	**28.1%**	**609 (100%)**	**390 (64.0%)**	**0/0**	
Codon mutations:	CTT34.TG (152T>G), GTA37ATT (159G>A 161A>T), ATC39GTC (165A>G), CTT40CTA (170T>A), AAA41CCC (171A>C 172A>C 173A>C), AAC43GAG (177A>G 179C>G), ATA46GCA (186A>G 187T>C), GAT48TCC (192G>T 193A>C 194T>C), ACC49ACG (197C>G), TAT50TCC (199A>C 200T>C), CTT51TTA (201C>T 203T>A), TTG53CTT (207T>C 209G>T), TTA54ATA (210T>A), TAC57TAT (221C>T), GCA61GCC (233A>C), AGA63AAT (238G>A 239A>T), TCC64TAA (241C>A 242C>A), GTT65TTA (243G>T 245T>A), ACC66TCG (246A>T 248C>G), CCT67CCA (251T>A), TCT69ATC (255T>A 256C>T 257T>C), CAG70CAT (260G>T), ATC71ATT (263C>T), CGT72CAT (265G>A), TCA73TCC (269A>C), CAG76CAA (278G>A), TCT77AAC (279T>A 280C>A 281T>C), CCT79CTC (286C>T 287T>C), CGC80ATA (288C>A 289G>T 290C>A), CAT81GGA (291C>G 292A>G 293T>A), GTG82GTA (296G>A), AAA83AGA (298A>G), GCT87GTT (310C>T), TCC89TCA (317C>A), TTA91CTC (321T>C 323A>C), GAT92AAT (324G>A), CAG93CAA (329G>A), CCT95TCT (333C>T), GTC96ATA (336G>A 338C>A), TTT97CTT (339T>C), GCT98GCA (344T>A), ACA99ACT (347A>T), GGA100CCA (348G>C 349G>C), CAG103CAA (359G>A), TTT104TTC (362T>C), GTT105TTT (363G>T), ACT106TCT (366A>T), GCA1401.CC (4253A>C), ATA1402AAA (4255T>A), AAG1403AAA (4259G>A), GAA1404GAG (4262A>G), TGT1405ATT (4263T>A 4264G>T), GAG1407AAT (4269G>A 4271G>T), CTT1408CTC (4274T>C), CAA1409TTA (4275C>T 4276A>T), TTT1411AAG (4281T>A 4282T>A 4283T>G), GAT1412GGT (4285A>G), CTA1413TTG (4287C>T 4289A>G), ATC1414ATA (4292C>A), GAA1415AAA (4293G>A), CCC1416CCG (4298C>G), TCT1417TCC (4301T>C), GAT1418AAA (4302G>A 4304T>A), CAA1420CCT (4309A>C 4310A>T), GCA1422TCT (4314G>T 4316A>T), GAA1424ACA (4320G>A 4321A>C), GCT1425GCC (4325T>C), GTC1428GTT (4334C>T), AAC1429AAT (4337C>T), AAG1430AAC (4340G>C), AGG1431GCA (4341A>G 4342G>C 4343G>A), TCA1432GGT (4344T>G 4345C>G 4346A>T), GAG1433GAA (4349G>A), CAA1434AAG (4350C>A 4352A>G), GTG1435GAA (4354T>A 4355G>A), GGA1437GGT (4361A>T), AAA1438GCT (4362A>G 4363A>C 4364A>T), TTA1439CCT (4365T>C 4366T>C 4367A>T), AGG1440CGA (4368A>C 4370G>A), TTA1441CTA (4371T>C), GTC1442GTT (4376C>T), CAA1446AAA (4386C>A), CCA1447CCT (4391A>T), AAC1449AAT (4397C>T), CAT1450AAA (4398C>A 4400T>A), TTC1451TAT (4402T>A 4403C>T), CTC1452TTG (4404C>T 4406C>G), CAA1453AAA (4407C>A), GAT1454TGG (4410G>T 4411A>G 4412T>G), GAC1455ATT (4413G>A 4414A>T 4415C>T), AAG1456AGG (4417A>G), TTT1457TAC (4420T>A 4421T>C), CCC1458CCT (4424C>T), CCA1460CCT (4430A>T), AAG1462AAA (4436G>A), CTC1463AGG (4437C>A 4438T>G 4439C>G), ACA1464GAT (4440A>G 4441C>A 4442A>T), CTC1465TTA (4443C>T 4445C>A), TTC1466TTA (4448C>A), CAC1468AGA (4452C>A 4453A>G 4454C>A), TCC1470TAT (4459C>A 4460C>T), AAA1471GAT (4461A>G 4463A>T), GCA1472GCC (4466A>C), AAG1473AAT (4469G>T), CTC1474ATA (4470C>A 4472C>A), AAG1477AAT (4481G>T), CTT1480TTA (4488C>T 4490T>A), AAG1481AAA (4493G>A), TTT1484TAT (4501T>A), TCG1615TTG (4894C>T), GTA1616ATC (4896G>A 4898A>C), CAG1618CAA (4904G>A), ATA1619CTC (4905A>C 4907A>C), CAA1620TAG (4908C>T 4910A>G), TTT1622TTC (4916T>C), GGG1624GGT (4922G>T), ATT1625GTG (4923A>G 4925T>G), GTT1626ATT (4926G>A), ATC1629GTC (4935A>G), AGA1630CAA (4938A>C 4939G>A), CCA1634CCT (4952A>T), GAG1635TAT (4953G>T 4955G>T), GTT1636ATC (4956G>A 4958T>C), ACT1637TCG (4959A>T 4961T>G), GAG1638AAG (4962G>A), CAT1639TAT (4965C>T), TCC1641TCT (4973C>T), CCT1642CCA (4976T>A), AGT1644ATG (4981G>T 4982T>G), GAT1645AAG (4983G>A 4985T>G), ATG1646ATT (4988G>T), AAG1648CAA (4992A>C 4994G>A), CCA1651TAT (5001C>T 5002C>A 5003A>T), CCG1652CCT (5006G>T), GCA1653GTT (5008C>T 5009A>T), GGA1655GGG (5015A>G), AAA1656AAG (5018A>G), TGT1657GAT (5019T>G 5020G>A)								

*: Inserts / Deletes / Misaligned / Frameshifts

Analysis details

This analysis was performed with panviral2.64

NGS Details (UN70): Epiphyllum badnavirus 1

Assembly

**Coverage Length**	338 (1 contig(s))
**Depth Of Coverage**	5.1
**Number Of Reads**	14
**Reads Per Million**	0.27 rpm (after QC)
**Ambiguities**	0
**Assembly Method**	de novo + reference guided assembly
**Consensus Caller**	Bcf Tools

Coverage Map

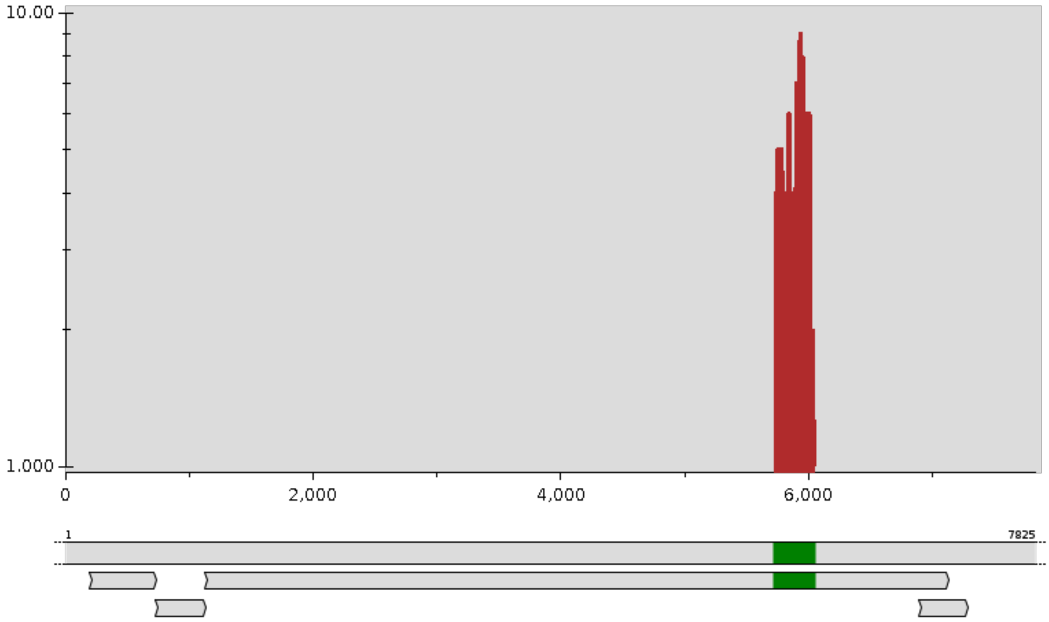

Assignment

**Type**	Epiphyllum badnavirus 1 (Taxonomy ID: 2518008)
**Reference Genome**	NC_076247.1
**NT Identity (%)**	52.3121
**AA Identity (%)**	45.6897
**Number Of Stop Codons**	0
**Number Of CDS**	4

Alignment

**Alignment Score**	22.0 (NT) + 230.0 (AA) = 252.0
**Concordance (%)**	17.027

**Alignment Method**	Global, seeded, nucleotide + amino acids (AGA)

Genome Region

Sequence starts at position 5715 and ends at position 6052 relative to NC_076247.1 reference sequence.

Alignment Detailed Statistics

	Begin	End	Coverage	Score	Concordance	Matches	Identities	I/D/M/F*	Stop Codons
**NT**	**5715**	**6052**	**4.3%**	**22**	**3.3%**	**338 (97.7%)**	**181 (52.3%)**	**8/0**	

Mutations: 5715A>G, 5716G>A, 5718A>G, 5719G>A, 5724G>A, 5725A>G, 5727A>T, 5729G>A, 5730A>C, 5731A>T, 5732A>C, 5734G>T, 5735A>G, 5740A>C, 5742G>T, 5743G>T, 5744T>G, 5745C>T, 5746T>G, 5748C>A, 5749A>G, 5751C>T, 5754C>T, 5756A>G, 5757A>G, 5758A>G, 5759C>A, 5760C>G, 5761T>C, 5766C>T, 5767G>A, 5769C>T, 5770A>T, 5771A>G, 5775G>A, 5776T>G, 5784T>C, 5785C>A, 5787G>A, 5789A>T, 5790C>T, 5791T>C, 5793T>A, 5794C>A, 5799T>A, 5801G>T, 5806A>G, 5808C>A, 5809A>G, 5810C>A, 5811A>G, 5812A>C, 5814C>A, 5815A>T, 5817A>G, 5818C>G, 5820G>T, 5821A>G, 5823G>A, 5824G>C, 5826C>A, 5827T>C, 5828G>A, 5830A>G, 5831A>G, 5835C>A, 5838T>G, 5839G>T, 5840T>A, 5844C>T, 5847C>T, 5853C>G, 5856T>C, 5859G>T, 5861A>G, 5862G>A, 5863A>T, 5864G>C, 5865C>T, 5868A>T, 5870T>G, 5871C>T, 5877G>A, 5880T>C, 5882T>G, 5887G>T, 5888A>T, 5889C>T, 5892A>G, 5893G>C, 5894A>C, 5895A>T, 5896T>G, 5897C>A, 5901C>T, 5905T>A, 5906G>A, 5910C>T, 5913C>T, 5916C>T, 5917C>A, 5918T>G, 5922C>A, 5924C>A, 5925_5926insTGCAT, 5926G>C, 5931A>G, 5933T>A, 5937C>T, 5942G>T, 5943G>C, 5944T>C, 5949T>C, 5955A>T, 5958C>T, 5961A>T, 5966A>C, 5967G>T, 5976T>A, 5977G>T, 5978C>A, 5979T>C, 5980G>A, 5981T>C, 5982G>A, 5985C>T, 5988A>G, 5989C>A, 5991A>C, 5992A>C, 5993A>T, 5994G>A, 5998G>A, 6003C>G, 6004T>A, 6005G>T, 6009C>T, 6010A>C, 6011G>A, 6012A>T, 6015C>T, 6015_6016insCAT, 6018G>C, 6019T>A, 6020C>G, 6021A>G, 6022G>A, 6023G>A, 6024C>G, 6027T>C, 6028G>A, 6030A>T, 6031G>T, 6032C>T, 6033A>G, 6036T>A, 6038A>T, 6040A>T, 6051C>A

CDS

	Begin	End	Coverage	Score	Concordance	Matches	Identities	I/D/M/F*	Stop Codons
QKM20_gp3	1530	1642	5.6%	230	28.2%	113 (97.4%)	53 (45.7%)	3/0/1/1	0

Protein mutations: E1530K (5716G>A 5718A>G), E1531K (5719G>A), K1533D (5725A>G 5727A>T), G1534D (5729G>A 5730A>C), K1535S (5731A>T 5732A>C), E1536W (5734G>T 5735A>G), M1538L (5740A>C 5742G>T), V1539C (5743G>T 5744T>G 5745C>T), F1540V (5746T>G 5748C>A), N1541D (5749A>G 5751C>T), K1543R (5756A>G 5757A>G), T1544E (5758A>G 5759C>A 5760C>G), D1547N (5767G>A 5769C>T), N1548C (5770A>T 5771A>G), F1550V (5776T>G), Q1553K (5785C>A 5787G>A), Y1554F (5789A>T 5790C>T), S1555P (5791T>C 5793T>A), L1556I (5794C>A), G1558V (5801G>T), N1560E (5806A>G 5808C>A), T1561E (5809A>G 5810C>A 5811A>G), I1562L (5812A>C 5814C>A), I1563L (5815A>T 5817A>G), Q1564D (5818C>G 5820G>T), K1565E (5821A>G 5823G>A), V1566L (5824G>C 5826C>A), C1567H (5827T>C 5828G>A), N1568G (5830A>G 5831A>G), S1570R (5838T>G), V1571Y (5839G>T 5840T>A), F1575L (5853C>G), K1578R (5861A>G 5862G>A), F1581C (5870T>G 5871C>T), M1585R (5882T>G), D1587F (5887G>T 5888A>T 5889C>T), E1589P (5893G>C 5894A>C 5895A>T), S1590D (5896T>G 5897C>A), W1593K (5905T>A 5906G>A), L1597R (5917C>A 5918T>G), P1599Q (5924C>A), P1599_D1600insCX (5925_5926insTGCAT), D1600H (5926G>C), L1602H (5933T>A), W1605F (5942G>T 5943G>C), K1613T (5966A>C 5967G>T), A1617Y (5977G>T 5978C>A 5979T>C), V1618T (5980G>A 5981T>C 5982G>A), R1621S (5989C>A 5991A>C), K1622L (5992A>C 5993A>T 5994G>A), D1624N (5998G>A), N1625K (6003C>G), C1626I (6004T>A 6005G>T), R1628H (6010A>C 6011G>A 6012A>T), D1629_L1630insH (6015_6016insCAT), S1631R (6019T>A 6020C>G 6021A>G), G1632K (6022G>A 6023G>A 6024C>G), V1634I (6028G>A 6030A>T), A1635L (6031G>T 6032C>T 6033A>G), Y1637F (6038A>T), I1638F (6040A>T)

Codon mutations: GGA1529..G (5715A>G), GAA1530AAG (5716G>A 5718A>G), GAG1531AAG (5719G>A), AAG1532AAA (5724G>A), AAA1533GAT (5725A>G 5727A>T), GGA1534GAC (5729G>A 5730A>C), AAG1535TCG (5731A>T 5732A>C), GAG1536TGG (5734G>T 5735A>G), ATG1538CTT (5740A>C 5742G>T), GTC1539TGT (5743G>T 5744T>G 5745C>T), TTC1540GTA (5746T>G 5748C>A), AAC1541GAT (5749A>G 5751C>T), TAC1542TAT (5754C>T), AAA1543AGG (5756A>G 5757A>G), ACC1544GAG (5758A>G 5759C>A 5760C>G), TTG1545CTG (5761T>C), AAC1546AAT (5766C>T), GAC1547AAT (5767G>A 5769C>T), AAC1548TGC (5770A>T 5771A>G), ACG1549ACA (5775G>A), TTC1550GTC (5776T>G), GAT1552GAC (5784T>C), CAG1553AAA (5785C>A 5787G>A), TAC1554TTT (5789A>T 5790C>T), TCT1555CCA (5791T>C 5793T>A), CTA1556ATA (5794C>A), CCT1557CCA (5799T>A), GGC1558GTC (5801G>T), AAC1560GAA (5806A>G 5808C>A), ACA1561GAG (5809A>G 5810C>A 5811A>G), ATC1562CTA (5812A>C 5814C>A), ATA1563TTG (5815A>T 5817A>G), CAG1564GAT (5818C>G 5820G>T), AAG1565GAA (5821A>G 5823G>A), GTC1566CTA (5824G>C 5826C>A), TGT1567CAT (5827T>C 5828G>A), AAT1568GGT (5830A>G 5831A>G), GCC1569GCA (5835C>A), AGT1570AGG (5838T>G), GTC1571TAC (5839G>T 5840T>A), TTC1572TTT (5844C>T), TCC1573TCT (5847C>T), TTC1575TTG (5853C>G), GAT1576GAC (5856T>C), CTG1577CTT (5859G>T), AAG1578AGA (5861A>G 5862G>A), AGC1579TCT (5863A>T 5864G>C 5865C>T), GGA1580GGT (5868A>T), TTC1581TGT (5870T>G 5871C>T), CAG1583CAA (5877G>A), ATT1584ATC (5880T>C), ATG1585AGG (5882T>G), GAC1587TTT (5887G>T 5888A>T 5889C>T), GAA1588GAG (5892A>G), GAA1589CCT (5893G>C 5894A>C 5895A>T), TCC1590GAC (5896T>G 5897C>A), ATC1591ATT (5901C>T), TGG1593AAG (5905T>A 5906G>A), ACC1594ACT (5910C>T), GCC1595GCT (5913C>T), TTC1596TTT (5916C>T), CTG1597AGG (5917C>A 5918T>G), ACC1598ACA (5922C>A), CCA1599CAA (5924C>A), CCA1599_GAT1600insTGCAT- (5925_5926insTGCAT), GAT1600CAT (5926G>C), GGA1601GGG (5931A>G), CTT1602CAT (5933T>A), TAC1603TAT (5937C>T), TGG1605TTC (5942G>T 5943G>C), TTA1606CTA (5944T>C), GTT1607GTC (5949T>C), CCA1609CCT (5955A>T), TTC1610TTT (5958C>T), GGA1611GGT (5961A>T), AAG1613ACT (5966A>C 5967G>T), CCT1616CCA (5976T>A), GCT1617TAC (5977G>T 5978C>A 5979T>C), GTG1618ACA (5980G>A 5981T>C 5982G>A), TTC1619TTT (5985C>T), CAA1620CAG (5988A>G), CGA1621AGC (5989C>A 5991A>C), AAG1622CTA (5992A>C 5993A>T 5994G>A), GAC1624AAC (5998G>A), AAC1625AAG (6003C>G), TGT1626ATT (6004T>A 6005G>T), TTC1627TTT (6009C>T), AGA1628CAT (6010A>C 6011G>A 6012A>T), GAC1629GAT (6015C>T), GAC1629_CTG1630insCAT (6015_6016insCAT), CTG1630CTC (6018G>C), TCA1631AGG (6019T>A 6020C>G 6021A>G), GGC1632AAG (6022G>A 6023G>A 6024C>G), TTT1633TTC (6027T>C), GTA1634ATT (6028G>A 6030A>T), GCA1635TTG (6031G>T 6032C>T 6033A>G), GTT1636GTA (6036T>A), TAC1637TTC (6038A>T), ATT1638TTT (6040A>T), ATC1641ATA (6051C>A)

Proteins

	Begin	End	Coverage	Score	Concordance	Matches	Identities	I/D/M/F*	Stop Codons
polyprotein (YP_010797894.1)	1530	1642	5.6%	230	28.2%	113 (97.4%)	53 (45.7%)	3/0/1/1	0

Protein mutations: E1530K (5716G>A 5718A>G), E1531K (5719G>A), K1533D (5725A>G 5727A>T), G1534D (5729G>A 5730A>C), K1535S (5731A>T 5732A>C), E1536W (5734G>T 5735A>G), M1538L (5740A>C 5742G>T), V1539C (5743G>T 5744T>G 5745C>T), F1540V (5746T>G 5748C>A), N1541D (5749A>G 5751C>T), K1543R (5756A>G 5757A>G), T1544E (5758A>G 5759C>A 5760C>G), D1547N (5767G>A 5769C>T), N1548C (5770A>T 5771A>G), F1550V (5776T>G), Q1553K (5785C>A 5787G>A), Y1554F (5789A>T 5790C>T), S1555P (5791T>C 5793T>A), L1556I (5794C>A), G1558V (5801G>T), N1560E (5806A>G 5808C>A), T1561E (5809A>G 5810C>A 5811A>G), I1562L (5812A>C 5814C>A), I1563L (5815A>T 5817A>G), Q1564D (5818C>G 5820G>T), K1565E (5821A>G 5823G>A), V1566L (5824G>C 5826C>A), C1567H (5827T>C 5828G>A), N1568G (5830A>G 5831A>G), S1570R (5838T>G), V1571Y (5839G>T 5840T>A), F1575L (5853C>G), K1578R (5861A>G 5862G>A), F1581C (5870T>G 5871C>T), M1585R (5882T>G), D1587F (5887G>T 5888A>T 5889C>T), E1589P (5893G>C 5894A>C 5895A>T), S1590D (5896T>G 5897C>A), W1593K (5905T>A 5906G>A), L1597R (5917C>A 5918T>G), P1599Q (5924C>A), P1599_D1600insCX (5925_5926insTGCAT), D1600H (5926G>C), L1602H (5933T>A), W1605F (5942G>T 5943G>C), K1613T (5966A>C 5967G>T), A1617Y (5977G>T 5978C>A 5979T>C), V1618T (5980G>A 5981T>C 5982G>A), R1621S (5989C>A 5991A>C), K1622L (5992A>C 5993A>T 5994G>A), D1624N (5998G>A), N1625K (6003C>G), C1626I (6004T>A 6005G>T), R1628H (6010A>C 6011G>A 6012A>T), D1629_L1630insH (6015_6016insCAT), S1631R (6019T>A 6020C>G 6021A>G), G1632K (6022G>A 6023G>A 6024C>G), V1634I (6028G>A 6030A>T), A1635L (6031G>T 6032C>T 6033A>G), Y1637F (6038A>T), I1638F (6040A>T)

Codon mutations: GGA1529..G (5715A>G), GAA1530AAG (5716G>A 5718A>G), GAG1531AAG (5719G>A), AAG1532AAA (5724G>A), AAA1533GAT (5725A>G 5727A>T), GGA1534GAC (5729G>A 5730A>C), AAG1535TCG (5731A>T 5732A>C), GAG1536TGG (5734G>T 5735A>G), ATG1538CTT (5740A>C 5742G>T), GTC1539TGT (5743G>T 5744T>G 5745C>T), TTC1540GTA (5746T>G 5748C>A), AAC1541GAT (5749A>G 5751C>T), TAC1542TAT (5754C>T), AAA1543AGG (5756A>G 5757A>G), ACC1544GAG (5758A>G 5759C>A 5760C>G), TTG1545CTG (5761T>C), AAC1546AAT (5766C>T), GAC1547AAT (5767G>A 5769C>T), AAC1548TGC (5770A>T 5771A>G), ACG1549ACA (5775G>A), TTC1550GTC (5776T>G), GAT1552GAC (5784T>C), CAG1553AAA (5785C>A 5787G>A), TAC1554TTT (5789A>T 5790C>T), TCT1555CCA (5791T>C 5793T>A), CTA1556ATA (5794C>A), CCT1557CCA (5799T>A), GGC1558GTC (5801G>T), AAC1560GAA (5806A>G 5808C>A), ACA1561GAG (5809A>G 5810C>A 5811A>G), ATC1562CTA (5812A>C 5814C>A), ATA1563TTG (5815A>T 5817A>G), CAG1564GAT (5818C>G 5820G>T), AAG1565GAA (5821A>G 5823G>A), GTC1566CTA (5824G>C 5826C>A), TGT1567CAT (5827T>C 5828G>A), AAT1568GGT (5830A>G 5831A>G), GCC1569GCA (5835C>A), AGT1570AGG (5838T>G), GTC1571TAC (5839G>T 5840T>A), TTC1572TTT (5844C>T), TCC1573TCT (5847C>T), TTC1575TTG (5853C>G), GAT1576GAC (5856T>C), CTG1577CTT (5859G>T), AAG1578AGA (5861A>G 5862G>A), AGC1579TCT (5863A>T 5864G>C 5865C>T), GGA1580GGT (5868A>T), TTC1581TGT (5870T>G 5871C>T), CAG1583CAA (5877G>A), ATT1584ATC (5880T>C), ATG1585AGG (5882T>G), GAC1587TTT (5887G>T 5888A>T 5889C>T), GAA1588GAG (5892A>G), GAA1589CCT (5893G>C 5894A>C 5895A>T), TCC1590GAC (5896T>G 5897C>A), ATC1591ATT (5901C>T), TGG1593AAG (5905T>A 5906G>A), ACC1594ACT (5910C>T), GCC1595GCT (5913C>T), TTC1596TTT (5916C>T), CTG1597AGG (5917C>A 5918T>G), ACC1598ACA (5922C>A), CCA1599CAA (5924C>A), CCA1599_GAT1600insTGCAT- (5925_5926insTGCAT), GAT1600CAT (5926G>C), GGA1601GGG (5931A>G), CTT1602CAT (5933T>A), TAC1603TAT (5937C>T), TGG1605TTC (5942G>T 5943G>C), TTA1606CTA (5944T>C), GTT1607GTC (5949T>C), CCA1609CCT (5955A>T), TTC1610TTT (5958C>T), GGA1611GGT (5961A>T), AAG1613ACT (5966A>C 5967G>T), CCT1616CCA (5976T>A), GCT1617TAC (5977G>T 5978C>A 5979T>C), GTG1618ACA (5980G>A 5981T>C 5982G>A), TTC1619TTT (5985C>T), CAA1620CAG (5988A>G), CGA1621AGC (5989C>A 5991A>C), AAG1622CTA (5992A>C 5993A>T 5994G>A), GAC1624AAC (5998G>A), AAC1625AAG (6003C>G), TGT1626ATT (6004T>A 6005G>T), TTC1627TTT (6009C>T), AGA1628CAT (6010A>C 6011G>A 6012A>T), GAC1629GAT (6015C>T), GAC1629_CTG1630insCAT (6015_6016insCAT), CTG1630CTC (6018G>C), TCA1631AGG (6019T>A 6020C>G 6021A>G), GGC1632AAG (6022G>A 6023G>A 6024C>G), TTT1633TTC (6027T>C), GTA1634ATT (6028G>A 6030A>T), GCA1635TTG (6031G>T 6032C>T 6033A>G), GTT1636GTA (6036T>A), TAC1637TTC (6038A>T), ATT1638TTT (6040A>T), ATC1641ATA (6051C>A)

*: Inserts / Deletes / Misaligned / Frameshifts

Analysis details

This analysis was performed with panviral2.64

NGS Details (UN70): Caulimovirus venafragariae

Assembly

**Coverage Length**	504 (1 contig(s))
**Depth Of Coverage**	3.0
**Number Of Reads**	11
**Reads Per Million**	0.21 rpm (after QC)
**Ambiguities**	0
**Assembly Method**	de novo + reference guided assembly
**Consensus Caller**	Bcf Tools

Coverage Map

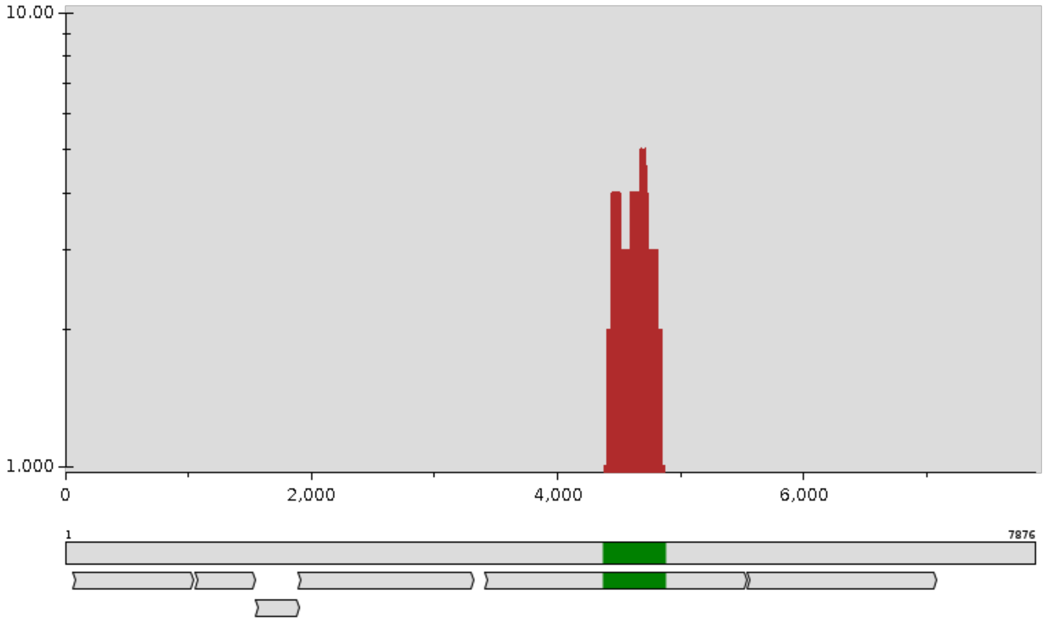

Assignment

**Type**	Caulimovirus venafragariae (Taxonomy ID: 3048344)
**Reference Genome**	NC_001725.1
**NT Identity (%)**	64.2715
**AA Identity (%)**	59.2814
**Number Of Stop Codons**	2
**Number Of CDS**	6

Alignment

**Alignment Score**	274.0 (NT) + 730.0 (AA) = 1004.0
**Concordance (%)**	46.4385

Alignment Method	Global, seeded, nucleotide + amino acids (AGA)

Genome Region

Sequence starts at position 4373 and ends at position 4876 relative to NC_001725.1 reference sequence.

Alignment Detailed Statistics

	Begin	End	Coverage	Score	Concordance	Matches	Identities	I/D/M/F*	Stop Codons
**NT**	**4373**	**4876**	**6.4%**	**274**	**27.7%**	**501 (99.4%)**	**322 (63.9%)**	**0/3**	
Mutations:	4378G>A, 4379C>A, 4380C>T, 4383G>A, 4384A>C, 4386C>A, 4388A>T, 4389A>T, 4390C>A, 4395C>A, 4401A>C, 4410A>C, 4416C>T, 4424A>G, 4425G>A, 4434C>T, 4435C>A, 4441A>G, 4443G>A, 4444G>A, 4445G>T, 4446A>G, 4449T>C, 4452C>A, 4455C>T, 4456C>A, 4457T>A, 4461T>A, 4464T>A, 4465A>G, 4470G>A, 4471G>A, 4472A>C, 4473G>A, 4474C>G, 4477C>T, 4479G>A, 4480C>A, 4482T>A, 4483C>A, 4485A>T, 4491C>A, 4492G>C, 4493G>A, 4494A>G, 4497T>A, 4500G>A, 4502C>G, 4503C>A, 4504T>A, 4508A>T, 4509C>T, 4510T>A, 4511C>G, 4512T>C, 4513T>A, 4514C>A, 4515C>G, 4521C>T, 4527A>G, 4530T>A, 4533A>C, 4535T>A, 4542G>A, 4545A>T, 4546C>A, 4547G>A, 4548C>A, 4549C>A, 4551T>G, 4552G>C, 4553C>A, 4560A>C, 4562C>G, 4563A>T, 4567C>G, 4570C>T, 4571T>G, 4575C>A, 4578T>C, 4581C>T, 4582A>T, 4583G>A, 4584C>T, 4587T>C, 4590C>A, 4591C>G, 4599C>T, 4601A>T, 4607G>A, 4613T>C, 4614C>A, 4620T>A, 4633C>A, 4634A>C, 4635A>T, 4638C>T, 4641T>A, 4642G>C, 4644T>A, 4650C>T, 4653A>G, 4656G>A, 4657C>A, 4659C>A, 4665C>T, 4666G>A, 4668A>T, 4670G>T, 4672C>T, 4674C>T, 4675A>G, 4677C>A, 4678_4680delAAC, 4682T>G, 4683G>A, 4687C>A, 4688C>A, 4692G>T, 4695T>C, 4696T>A, 4697G>T, 4699G>T, 4700C>T, 4701T>A, 4704A>T, 4708G>A, 4710T>A, 4719C>T, 4720A>C, 4722C>A, 4725C>T, 4728C>T, 4731C>T, 4734A>T, 4737T>C, 4738G>A, 4739A>T, 4740A>G, 4741G>C, 4759G>C, 4762A>C, 4766T>A, 4767T>G, 4771C>T, 4773A>T, 4776C>T, 4778G>T, 4781G>T, 4782C>T, 4784A>T, 4787C>A, 4789C>A, 4790T>A, 4791A>T, 4794A>T, 4795A>T, 4798G>A, 4800G>T, 4801C>A, 4803T>A, 4806C>T, 4816G>A, 4817C>T, 4818C>A, 4819C>G, 4824G>A, 4834A>T, 4835C>A, 4839C>T, 4846C>T, 4848T>A, 4851T>A, 4852C>A, 4855G>A, 4856T>C, 4857A>C, 4862A>G, 4864A>G, 4865G>A								

CDS

	Begin	End	Coverage	Score	Concordance	Matches	Identities	I/D/M/F*	Stop Codons
ORF_V	324	491	23.7%	730	61.7%	167 (99.4%)	99 (58.9%)	0/1/0/0	2
Protein mutations:	A325N (4378G>A 4379C>A 4380C>T), I327L (4384A>C 4386C>A), K328I (4388A>T 4389A>T), K340R (4424A>G 4425G>A), H344N (4435C>A), K346E (4441A>G 4443G>A), G347M (4444G>A 4445G>T 4446A>G), L351K (4456C>A 4457T>A), N354D (4465A>G), E356T (4471G>A 4472A>C 4473G>A), Q357E (4474C>G), L359I (4480C>A 4482T>A), Q360N (4483C>A 4485A>T), G363Q (4492G>C 4493G>A 4494A>G), T366R (4502C>G 4503C>A), F367I (4504T>A), Y368F (4508A>T 4509C>T), S370K (4513T>A 4514C>A 4515C>G), F377Y (4535T>A), R381K (4546C>A 4547G>A 4548C>A), L382M (4549C>A 4551T>G), A383H (4552G>C 4553C>A), E385D (4560A>C), T386S (4562C>G 4563A>T), Q388E (4567C>G), L389* (4570C>T 4571T>G), S393Y (4582A>T 4583G>A 4584C>T), Q396E (4591C>G), Y399F (4601A>T), W401* (4607G>A), V403A (4613T>C 4614C>A), Q410T (4633C>A 4634A>C 4635A>T), A413P (4642G>C 4644T>A), H418K (4657C>A 4659C>A), E421N (4666G>A 4668A>T), S422I (4670G>T), L423F (4672C>T 4674C>T), S424G (4675A>G 4677C>A), N425del (4678_4680delAAC), M426R (4682T>G 4683G>A), P428K (4687C>A 4688C>A), Q429H (4692G>T), C431I (4696T>A 4697G>T), A432L (4699G>T 4700C>T 4701T>A), V435I (4708G>A 4710T>A), I439L (4720A>C 4722C>A), K443N (4734A>T), E445M (4738G>A 4739A>T 4740A>G), E446Q (4741G>C), V452L (4759G>C), K453Q (4762A>C), I454K (4766T>A 4767T>G), L456F (4771C>T 4773A>T), R458I (4778G>T), C459F (4781G>T 4782C>T), K460I (4784A>T), A461D (4787C>A), L462N (4789C>A 4790T>A 4791A>T), I464L (4795A>T), V465I (4798G>A 4800G>T), L466I (4801C>A 4803T>A), A471I (4816G>A 4817C>T 4818C>A), Q472E (4819C>G), T477Y (4834A>T 4835C>A), L483I (4852C>A), V484T (4855G>A 4856T>C 4857A>C), E486G (4862A>G), R487E (4864A>G 4865G>A)								
Codon mutations:	GCC325AAT (4378G>A 4379C>A 4380C>T), GAG326GAA (4383G>A), ATC327CTA (4384A>C 4386C>A), AAA328ATT (4388A>T 4389A>T), CGA329AGA (4390C>A), GGC330GGA (4395C>A), GCA332GCC (4401A>C), GTA335GTC (4410A>C), AAC337AAT (4416C>T), AAG340AGA (4424A>G 4425G>A), GAC343GAT (4434C>T), CAT344AAT (4435C>A), AAG346GAA (4441A>G 4443G>A), GGA347ATG (4444G>A 4445G>T 4446A>G), GAT348GAC (4449T>C), GGC349GGA (4452C>A), TAC350TAT (4455C>T), CTA351AAA (4456C>A 4457T>A), CTT352CTA (4461T>A), CCT353CCA (4464T>A), AAC354GAC (4465A>G), AAG355AAA (4470G>A), GAG356ACA (4471G>A 4472A>C 4473G>A), CAA357GAA (4474C>G), CTG358TTA (4477C>T 4479G>A), CTT359ATA (4480C>A 4482T>A), CAA360AAT (4483C>A 4485A>T), ATC362ATA (4491C>A), GGA363CAG (4492G>C 4493G>A 4494A>G), GGT364GGA (4497T>A), AAG365AAA (4500G>A), ACC366AGA (4502C>G 4503C>A), TTT367ATT (4504T>A), TAC368TTT (4508A>T 4509C>T), TCT369AGC (4510T>A 4511C>G 4512T>C), TCC370AAG (4513T>A 4514C>A 4515C>G), GAC372GAT (4521C>T), AAA374AAG (4527A>G), TCT375TCA (4530T>A), GGA376GGC (4533A>C), TTT377TAT (4535T>A), CAG379CAA (4542G>A), GTA380GTT (4545A>T), CGC381AAA (4546C>A 4547G>A 4548C>A), CTT382ATG (4549C>A 4551T>G), GCT383CAT (4552G>C 4553C>A), GAA385GAC (4560A>C), ACA386AGT (4562C>G 4563A>T), CAG388GAG (4567C>G), CTA389TGA (4570C>T 4571T>G), ACC390ACA (4575C>A), GCT391GCC (4578T>C), TTC392TTT (4581C>T), AGC393TAT (4582A>T 4583G>A 4584C>T), TGT394TGC (4587T>C), CCC395CCA (4590C>A), CAA396GAA (4591C>G), CAC398CAT (4599C>T), TAC399TTC (4601A>T), TGG401TAG (4607G>A), GTC403GCA (4613T>C 4614C>A), CCT405CCA (4620T>A), CAA410ACT (4633C>A 4634A>C 4635A>T), GCC411GCT (4638C>T), CCT412CCA (4641T>A), GCT413CCA (4642G>C 4644T>A), TTC415TTT (4650C>T), CAA416CAG (4653A>G), AGG417AGA (4656G>A), CAC418AAA (4657C>A 4659C>A), GAC420GAT (4665C>T), GAA421AAT (4666G>A 4668A>T), AGT422ATT (4670G>T), CTC423TTT (4672C>T 4674C>T), AGC424GGA (4675A>G 4677C>A), AAC425del (4678_4680delAAC), ATG426AGA (4682T>G 4683G>A), CCA428AAA (4687C>A 4688C>A), CAG429CAT (4692G>T), TTT430TTC (4695T>C), TGT431ATT (4696T>A 4697G>T), GCT432TTA (4699G>T 4700C>T 4701T>A), GTA433GTT (4704A>T), GTT435ATA (4708G>A 4710T>A), ATC438ATT (4719C>T), ATC439CTA (4720A>C 4722C>A), GTC440GTT (4725C>T), TTC441TTT (4728C>T), AGC442AGT (4731C>T), AAA443AAT (4734A>T), ACT444ACC (4737T>C), GAA445ATG (4738G>A 4739A>T 4740A>G), GAA446CAA (4741G>C), GTC452CTC (4759G>C), AAA453CAA (4762A>C), ATT454AAG (4766T>A 4767T>G), CTA456TTT (4771C>T 4773A>T), AAC457AAT (4776C>T), AGA458ATA (4778G>T), TGC459TTT (4781G>T 4782C>T), AAA460ATA (4784A>T), GCC461GAC (4787C>A), CTA462AAT (4789C>A 4790T>A 4791A>T), GGA463GGT (4794A>T), ATA464TTA (4795A>T), GTG465ATT (4798G>A 4800G>T), CTT466ATA (4801C>A 4803T>A), AGC467AGT (4806C>T), GCC471ATA (4816G>A 4817C>T 4818C>A), CAG472GAG (4819C>G), TTG473TTA (4824G>A), ACT477TAT (4834A>T 4835C>A), ATC478ATT (4839C>T), CTT481TTA (4846C>T 4848T>A), GGT482GGA (4851T>A), CTA483ATA (4852C>A), GTA484ACC (4855G>A 4856T>C 4857A>C), GAA486GGA (4862A>G), AGA487GAA (4864A>G 4865G>A)								

Proteins

	Begin	End	Coverage	Score	Concordance	Matches	Identities	I/D/M/F*	Stop Codons
hypothetical protein (NP_043933.1)	324	491	23.7%	730	61.7%	167 (99.4%)	99 (58.9%)	0/1/0/0	2
Protein mutations:	A325N (4378G>A 4379C>A 4380C>T), I327L (4384A>C 4386C>A), K328I (4388A>T 4389A>T), K340R (4424A>G 4425G>A), H344N (4435C>A), K346E (4441A>G 4443G>A), G347M (4444G>A 4445G>T 4446A>G), L351K (4456C>A 4457T>A), N354D (4465A>G), E356T (4471G>A 4472A>C 4473G>A), Q357E (4474C>G), L359I (4480C>A 4482T>A), Q360N (4483C>A 4485A>T), G363Q (4492G>C 4493G>A 4494A>G), T366R (4502C>G 4503C>A), F367I (4504T>A), Y368F (4508A>T 4509C>T), S370K (4513T>A 4514C>A 4515C>G), F377Y (4535T>A), R381K (4546C>A 4547G>A 4548C>A), L382M (4549C>A 4551T>G), A383H (4552G>C 4553C>A), E385D (4560A>C), T386S (4562C>G 4563A>T), Q388E (4567C>G), L389* (4570C>T 4571T>G), S393Y (4582A>T 4583G>A 4584C>T), Q396E (4591C>G), Y399F (4601A>T), W401* (4607G>A), V403A (4613T>C 4614C>A), Q410T (4633C>A 4634A>C 4635A>T), A413P (4642G>C 4644T>A), H418K (4657C>A 4659C>A), E421N (4666G>A 4668A>T), S422I (4670G>T), L423F (4672C>T 4674C>T), S424G (4675A>G 4677C>A), N425del (4678_4680delAAC), M426R (4682T>G 4683G>A), P428K (4687C>A 4688C>A), Q429H (4692G>T), C431I (4696T>A 4697G>T), A432L (4699G>T 4700C>T 4701T>A), V435I (4708G>A 4710T>A), I439L (4720A>C 4722C>A), K443N (4734A>T), E445M (4738G>A 4739A>T 4740A>G), E446Q (4741G>C), V452L (4759G>C), K453Q (4762A>C), I454K (4766T>A 4767T>G), L456F (4771C>T 4773A>T), R458I (4778G>T), C459F (4781G>T 4782C>T), K460I (4784A>T), A461D (4787C>A), L462N (4789C>A 4790T>A 4791A>T), I464L (4795A>T), V465I (4798G>A 4800G>T), L466I (4801C>A 4803T>A), A471I (4816G>A 4817C>T 4818C>A), Q472E (4819C>G), T477Y (4834A>T 4835C>A), L483I (4852C>A), V484T (4855G>A 4856T>C 4857A>C), E486G (4862A>G), R487E (4864A>G 4865G>A)								
Codon mutations:	GCC325AAT (4378G>A 4379C>A 4380C>T), GAG326GAA (4383G>A), ATC327CTA (4384A>C 4386C>A), AAA328ATT (4388A>T 4389A>T), CGA329AGA (4390C>A), GGC330GGA (4395C>A), GCA332GCC (4401A>C), GTA335GTC (4410A>C), AAC337AAT (4416C>T), AAG340AGA (4424A>G 4425G>A), GAC343GAT (4434C>T), CAT344AAT (4435C>A), AAG346GAA (4441A>G 4443G>A), GGA347ATG (4444G>A 4445G>T 4446A>G), GAT348GAC (4449T>C), GGC349GGA (4452C>A), TAC350TAT (4455C>T), CTA351AAA (4456C>A 4457T>A), CTT352CTA (4461T>A), CCT353CCA (4464T>A), AAC354GAC (4465A>G), AAG355AAA (4470G>A), GAG356ACA (4471G>A 4472A>C 4473G>A), CAA357GAA (4474C>G), CTG358TTA (4477C>T 4479G>A), CTT359ATA (4480C>A 4482T>A), CAA360AAT (4483C>A 4485A>T), ATC362ATA (4491C>A), GGA363CAG (4492G>C 4493G>A 4494A>G), GGT364GGA (4497T>A), AAG365AAA (4500G>A), ACC366AGA (4502C>G 4503C>A), TTT367ATT (4504T>A), TAC368TTT (4508A>T 4509C>T), TCT369AGC (4510T>A 4511C>G 4512T>C), TCC370AAG (4513T>A 4514C>A 4515C>G), GAC372GAT (4521C>T), AAA374AAG (4527A>G), TCT375TCA (4530T>A), GGA376GGC (4533A>C), TTT377TAT (4535T>A), CAG379CAA (4542G>A), GTA380GTT (4545A>T), CGC381AAA (4546C>A 4547G>A 4548C>A), CTT382ATG (4549C>A 4551T>G), GCT383CAT (4552G>C 4553C>A), GAA385GAC (4560A>C), ACA386AGT (4562C>G 4563A>T), CAG388GAG (4567C>G), CTA389TGA (4570C>T 4571T>G), ACC390ACA (4575C>A), GCT391GCC (4578T>C), TTC392TTT (4581C>T), AGC393TAT (4582A>T 4583G>A 4584C>T), TGT394TGC (4587T>C), CCC395CCA (4590C>A), CAA396GAA (4591C>G), CAC398CAT (4599C>T), TAC399TTC (4601A>T), TGG401TAG (4607G>A), GTC403GCA (4613T>C 4614C>A), CCT405CCA (4620T>A), CAA410ACT (4633C>A 4634A>C 4635A>T), GCC411GCT (4638C>T), CCT412CCA (4641T>A), GCT413CCA (4642G>C 4644T>A), TTC415TTT (4650C>T), CAA416CAG (4653A>G), AGG417AGA (4656G>A), CAC418AAA (4657C>A 4659C>A), GAC420GAT (4665C>T), GAA421AAT (4666G>A 4668A>T), AGT422ATT (4670G>T), CTC423TTT (4672C>T 4674C>T), AGC424GGA (4675A>G 4677C>A), AAC425del (4678_4680delAAC), ATG426AGA (4682T>G 4683G>A), CCA428AAA (4687C>A 4688C>A), CAG429CAT (4692G>T), TTT430TTC (4695T>C), TGT431ATT (4696T>A 4697G>T), GCT432TTA (4699G>T 4700C>T 4701T>A), GTA433GTT (4704A>T), GTT435ATA (4708G>A 4710T>A), ATC438ATT (4719C>T), ATC439CTA (4720A>C 4722C>A), GTC440GTT (4725C>T), TTC441TTT (4728C>T), AGC442AGT (4731C>T), AAA443AAT (4734A>T), ACT444ACC (4737T>C), GAA445ATG (4738G>A 4739A>T 4740A>G), GAA446CAA (4741G>C), GTC452CTC (4759G>C), AAA453CAA (4762A>C), ATT454AAG (4766T>A 4767T>G), CTA456TTT (4771C>T 4773A>T), AAC457AAT (4776C>T), AGA458ATA (4778G>T), TGC459TTT (4781G>T 4782C>T), AAA460ATA (4784A>T), GCC461GAC (4787C>A), CTA462AAT (4789C>A 4790T>A 4791A>T), GGA463GGT (4794A>T), ATA464TTA (4795A>T), GTG465ATT (4798G>A 4800G>T), CTT466ATA (4801C>A 4803T>A), AGC467AGT (4806C>T), GCC471ATA (4816G>A 4817C>T 4818C>A), CAG472GAG (4819C>G), TTG473TTA (4824G>A), ACT477TAT (4834A>T 4835C>A), ATC478ATT (4839C>T), CTT481TTA (4846C>T 4848T>A), GGT482GGA (4851T>A), CTA483ATA (4852C>A), GTA484ACC (4855G>A 4856T>C 4857A>C), GAA486GGA (4862A>G), AGA487GAA (4864A>G 4865G>A)								

*: Inserts / Deletes / Misaligned / Frameshifts

Analysis details

This analysis was performed with panviral2.64

NGS Details (UN70): Gihfavirus pelohabitans

Assembly

**Coverage Length**	183 (1 contig(s))
**Depth Of Coverage**	4.8
**Number Of Reads**	10
**Reads Per Million**	0.19 rpm (after QC)
**Ambiguities**	0
**Assembly Method**	de novo + reference guided assembly
**Consensus Caller**	Bcf Tools

Coverage Map

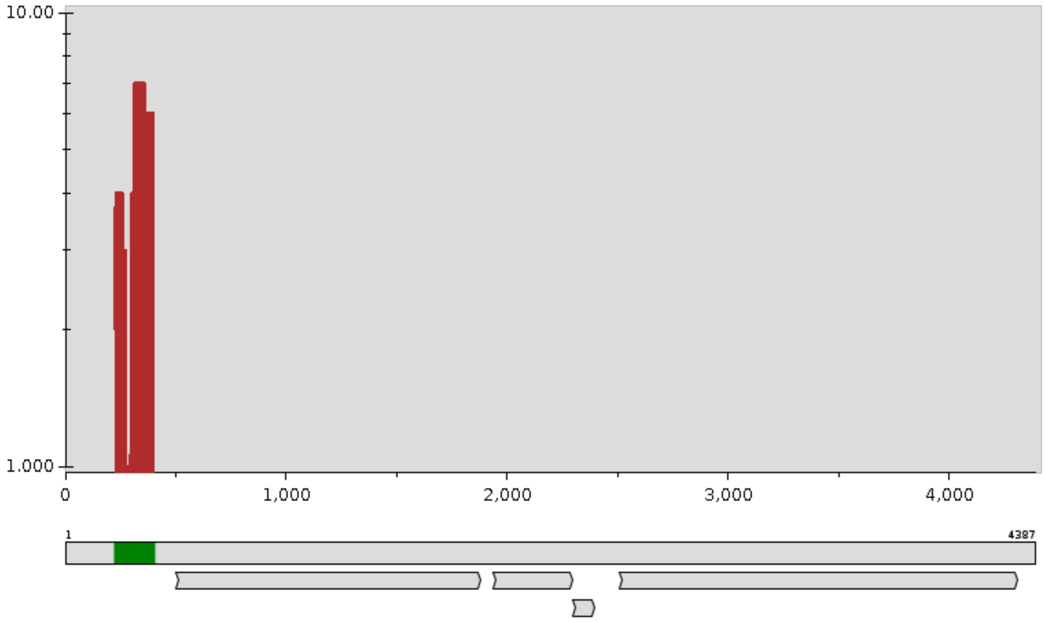

Assignment

**Type**	Gihfavirus pelohabitans (Taxonomy ID: 2844652)
**Reference Genome**	NC_074583.1
**NT Identity (%)**	97.2678
**AA Identity (%)**	0.0
**Number Of Stop Codons**	0
**Number Of CDS**	4

Alignment

**Alignment Score**	346.0 (NT) + 0.0 (AA) = 346.0
**Concordance (%)**	94.5355

Alignment Method	Global, seeded, nucleotide + amino acids (AGA)

Genome Region

Sequence starts at position 222 and ends at position 404 relative to NC_074583.1 reference sequence.

Alignment Detailed Statistics

	Begin	End	Coverage	Score	Concordance	Matches	Identities	I/D/M/F*	Stop Codons
**NT**	**222**	**404**	**4.2%**	**346**	**94.5%**	**183 (100%)**	**178 (97.3%)**	**0/0**	
Mutations:	276G>T, 277C>T, 278A>C, 279A>G, 288A>T								
**CDS**									
**Proteins**									

*: Inserts / Deletes / Misaligned / Frameshifts

Analysis details

This analysis was performed with panviral2.64

NGS Details (UN70): Caulimovirus tessellomirabilis

Assembly

**Coverage Length**	343 (1 contig(s))
**Depth Of Coverage**	4.0
**Number Of Reads**	10
**Reads Per Million**	0.19 rpm (after QC)
**Ambiguities**	0
**Assembly Method**	de novo + reference guided assembly
**Consensus Caller**	Bcf Tools

Coverage Map

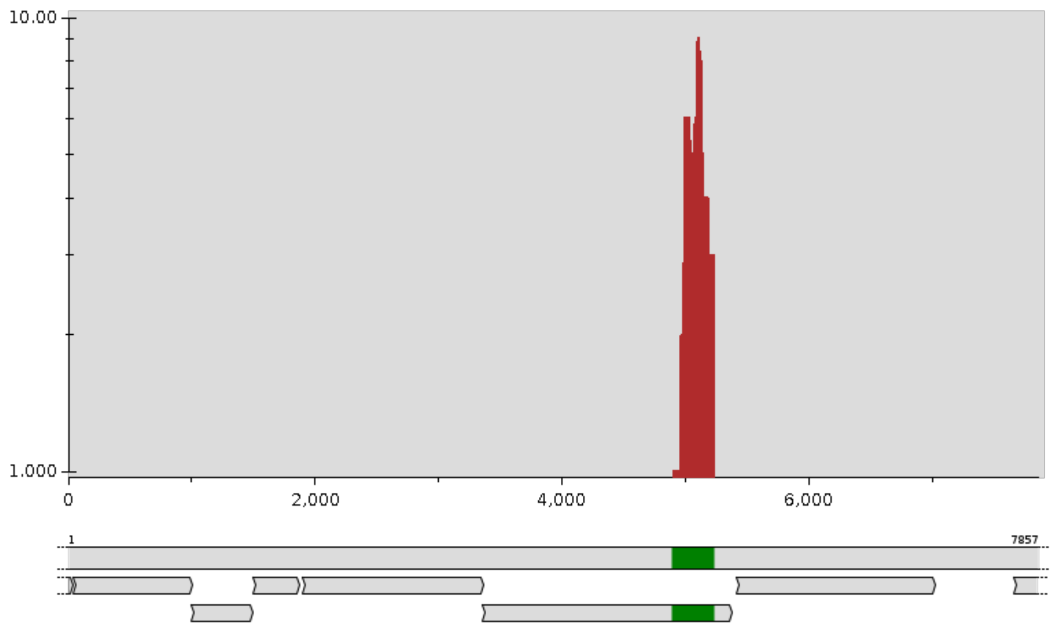

Assignment

**Type**	Caulimovirus tessellomirabilis (Taxonomy ID: 3048203)
**Reference Genome**	NC_004036.1
**NT Identity (%)**	57.0122
**AA Identity (%)**	48.6239
**Number Of Stop Codons**	0
**Number Of CDS**	7

Alignment

**Alignment Score**	50.0 (NT) + 365.0 (AA) = 415.0
**Concordance (%)**	31.4394

Alignment Method	Global, seeded, nucleotide + amino acids (AGA)

Genome Region

Sequence starts at position 4896 and ends at position 5238 relative to NC_004036.1 reference sequence.

Alignment Detailed Statistics

	Begin	End	Coverage	Score	Concordance	Matches	Identities	I/D/M/F*	Stop Codons
**NT**	**4896**	**5238**	**4.4%**	**50**	**8.1%**	**328 (95.6%)**	**187 (54.5%)**	**0/15**	
Mutations:	4903C>T, 4907A>C, 4911A>G, 4912G>C, 4913A>C, 4918G>A, 4921A>G, 4923G>A, 4926T>G, 4927A>T, 4928A>G, 4929T>C, 4930T>C, 4933T>G, 4934T>C, 4936T>C, 4939A>G, 4942A>G, 4946T>A, 4947A>G, 4948T>G, 4955A>G, 4957G>A, 4958A>C, 4959A>C, 4966C>T, 4968G>A, 4969T>C, 4970T>C, 4975T>A, 4976A>T, 4978T>A, 4979G>C, 4984T>G, 4990T>C, 4993T>C, 4996T>C, 4997C>T, 5000T>C, 5001T>A, 5002C>T, 5008T>A, 5010G>C, 5012G>T, 5014C>G, 5015C>T, 5017T>A, 5018_5023delAAGGCC, 5029C>A, 5030A>G, 5031C>A, 5033G>T, 5034A>T, 5035A>G, 5036G>A, 5037G>A, 5042_5047delGAATTA, 5050C>T, 5053C>T, 5056G>A, 5060T>A, 5063T>A, 5064C>G, 5065A>T, 5071A>T, 5074C>T, 5077A>T, 5078C>G, 5079C>A, 5087T>A, 5088T>C, 5089G>T, 5091A>G, 5092T>A, 5095C>T, 5096C>T, 5097A>C, 5100G>C, 5101T>C, 5107G>A, 5110A>T, 5117C>T, 5122G>A, 5123G>A, 5125A>T, 5126A>G, 5127A>T, 5128A>T, 5130A>G, 5131G>A, 5133T>G, 5134G>A, 5137T>C, 5139C>G, 5140T>A, 5147A>T, 5148G>C, 5149T>A, 5150A>G, 5151T>C, 5154A>T, 5155T>C, 5159A>C, 5160C>T, 5161C>T, 5164T>A, 5165G>A, 5166T>A, 5167T>A, 5168T>C, 5169G>A, 5170T>A, 5173T>C, 5175C>T, 5176A>C, 5177G>A, 5180A>C, 5181G>A, 5182G>A, 5183A>T, 5188T>C, 5192_5194delGTA, 5196A>C, 5197T>C, 5199T>A, 5200T>G, 5201T>G, 5204A>T, 5205A>C, 5209A>T, 5212T>G, 5215G>A, 5216A>T, 5217A>T, 5218T>C, 5221A>T, 5224G>A, 5225A>C, 5228A>C, 5231G>T, 5232G>C, 5236C>A, 5237A>C, 5238G>C								

CDS

	Begin	End	Coverage	Score	Concordance	Matches	Identities	I/D/M/F*	Stop Codons
MiMVgp6	515	628	16.9%	365	51.0%	109 (95.6%)	53 (46.5%)	0/5/0/0	0
Protein mutations:	K518Q (4907A>C), K519S (4911A>G 4912G>C), I520L (4913A>C), G523E (4923G>A), L524C (4926T>G 4927A>T), I525A (4928A>G 4929T>C 4930T>C), N526K (4933T>G), F527L (4934T>C 4936T>C), Y531R (4946T>A 4947A>G 4948T>G), K534E (4955A>G 4957G>A), K535P (4958A>C 4959A>C), S538N (4968G>A 4969T>C), I541L (4976A>T 4978T>A), E542Q (4979G>C), H548Y (4997C>T), F549H (5000T>C 5001T>A 5002C>T), G552A (5010G>C), V553L (5012G>T 5014C>G), K555_A556del (5018_5023delAAGGCC), T559D (5030A>G 5031C>A), E560L (5033G>T 5034A>T 5035A>G), G561N (5036G>A 5037G>A), E563_L564del (5042_5047delGAATTA), S569T (5060T>A), K574N (5077A>T), P575E (5078C>G 5079C>A), L578T (5087T>A 5088T>C 5089G>T), N579R (5091A>G 5092T>A), H581S (5096C>T 5097A>C), S582T (5100G>C 5101T>C), K585N (5110A>T), V590I (5123G>A 5125A>T), K591V (5126A>G 5127A>T 5128A>T), Q592R (5130A>G 5131G>A), V593G (5133T>G 5134G>A), T595R (5139C>G 5140T>A), I599A (5150A>G 5151T>C), Y600F (5154A>T 5155T>C), T602L (5159A>C 5160C>T 5161C>T), V604K (5165G>A 5166T>A 5167T>A), C605Q (5168T>C 5169G>A 5170T>A), T607I (5175C>T 5176A>C), V608I (5177G>A), R609Q (5180A>C 5181G>A 5182G>A), T610S (5183A>T), V613del (5192_5194delGTA), N614T (5196A>C 5197T>C), L615Q (5199T>A 5200T>G), L616V (5201T>G), K617S (5204A>T 5205A>C), F619L (5212T>G), M620I (5215G>A), N621F (5216A>T 5217A>T 5218T>C), K622N (5221A>T), I624L (5225A>C), T625P (5228A>C), G626S (5231G>T 5232G>C), D627E (5236C>A)								
Codon mutations:	TAC516TAT (4903C>T), AAA518CAA (4907A>C), AAG519AGC (4911A>G 4912G>C), ATA520CTA (4913A>C), AAG521AAA (4918G>A), AAA522AAG (4921A>G), GGA523GAA (4923G>A), TTA524TGT (4926T>G 4927A>T), ATT525GCC (4928A>G 4929T>C 4930T>C), AAT526AAG (4933T>G), TTT527CTC (4934T>C 4936T>C), CCA528CCG (4939A>G), AAA529AAG (4942A>G), TAT531AGG (4946T>A 4947A>G 4948T>G), AAG534GAA (4955A>G 4957G>A), AAA535CCA (4958A>C 4959A>C), GAC537GAT (4966C>T), AGT538AAC (4968G>A 4969T>C), TTA539CTA (4970T>C), ATT540ATA (4975T>A), ATT541TTA (4976A>T 4978T>A), GAA542CAA (4979G>C), ACT543ACG (4984T>G), GCT545GCC (4990T>C), TCT546TCC (4993T>C), GAT547GAC (4996T>C), CAC548TAC (4997C>T), TTC549CAT (5000T>C 5001T>A 5002C>T), GGT551GGA (5008T>A), GGA552GCA (5010G>C), GTC553TTG (5012G>T 5014C>G), CTT554TTA (5015C>T 5017T>A), AAG555_GCC556del (5018_5023delAAGGCC), ACC558ACA (5029C>A), ACT559GAT (5030A>G 5031C>A), GAA560TTG (5033G>T 5034A>T 5035A>G), GGT561AAT (5036G>A 5037G>A), GAA563_TTA564del (5042_5047delGAATTA), ATC565ATT (5050C>T), TGC566TGT (5053C>T), AGG567AGA (5056G>A), TCT569ACT (5060T>A), TCA570AGT (5063T>A 5064C>G 5065A>T), ACA572ACT (5071A>T), TTC573TTT (5074C>T), AAA574AAT (5077A>T), CCA575GAA (5078C>G 5079C>A), TTG578ACT (5087T>A 5088T>C 5089G>T), AAT579AGA (5091A>G 5092T>A), TAC580TAT (5095C>T), CAT581TCT (5096C>T 5097A>C), AGT582ACC (5100G>C 5101T>C), GAG584GAA (5107G>A), AAA585AAT (5110A>T), CTA588TTA (5117C>T), GCG589GCA (5122G>A), GTA590ATT (5123G>A 5125A>T), AAA591GTT (5126A>G 5127A>T 5128A>T), CAG592CGA (5130A>G 5131G>A), GTG593GGA (5133T>G 5134G>A), ATT594ATC (5137T>C), ACT595AGA (5139C>G 5140T>A), AGT598TCA (5147A>T 5148G>C 5149T>A), ATT599GCT (5150A>G 5151T>C), TAT600TTC (5154A>T 5155T>C), ACC602CTT (5159A>C 5160C>T 5161C>T), CCT603CCA (5164T>A), GTT604AAA (5165G>A 5166T>A 5167T>A), TGT605CAA (5168T>C 5169G>A 5170T>A), TTT606TTC (5173T>C), ACA607ATC (5175C>T 5176A>C), GTC608ATC (5177G>A), AGG609CAA (5180A>C 5181G>A 5182G>A), ACA610TCA (5183A>T), GAT611GAC (5188T>C), GTA613del (5192_5194delGTA), AAT614ACC (5196A>C 5197T>C), CTT615CAG (5199T>A 5200T>G), TTA616GTA (5201T>G), AAA617TCA (5204A>T 5205A>C), GGA618GGT (5209A>T), TTT619TTG (5212T>G), ATG620ATA (5215G>A), AAT621TTC (5216A>T 5217A>T 5218T>C), AAA622AAT (5221A>T), AAG623AAA (5224G>A), ATT624CTT (5225A>C), ACT625CCT (5228A>C), GGT626TCT (5231G>T 5232G>C), GAC627GAA (5236C>A), AGT628CC. (5237A>C 5238G>C)								

Proteins

	Begin	End	Coverage	Score	Concordance	Matches	Identities	I/D/M/F*	Stop Codons
hypothetical protein (NP_659397.1)	515	628	16.9%	365	51.0%	109 (95.6%)	53 (46.5%)	0/5/0/0	0
Protein mutations:	K518Q (4907A>C), K519S (4911A>G 4912G>C), I520L (4913A>C), G523E (4923G>A), L524C (4926T>G 4927A>T), I525A (4928A>G 4929T>C 4930T>C), N526K (4933T>G), F527L (4934T>C 4936T>C), Y531R (4946T>A 4947A>G 4948T>G), K534E (4955A>G 4957G>A), K535P (4958A>C 4959A>C), S538N (4968G>A 4969T>C), I541L (4976A>T 4978T>A), E542Q (4979G>C), H548Y (4997C>T), F549H (5000T>C 5001T>A 5002C>T), G552A (5010G>C), V553L (5012G>T 5014C>G), K555_A556del (5018_5023delAAGGCC), T559D (5030A>G 5031C>A), E560L (5033G>T 5034A>T 5035A>G), G561N (5036G>A 5037G>A), E563_L564del (5042_5047delGAATTA), S569T (5060T>A), K574N (5077A>T), P575E (5078C>G 5079C>A), L578T (5087T>A 5088T>C 5089G>T), N579R (5091A>G 5092T>A), H581S (5096C>T 5097A>C), S582T (5100G>C 5101T>C), K585N (5110A>T), V590I (5123G>A 5125A>T), K591V (5126A>G 5127A>T 5128A>T), Q592R (5130A>G 5131G>A), V593G (5133T>G 5134G>A), T595R (5139C>G 5140T>A), I599A (5150A>G 5151T>C), Y600F (5154A>T 5155T>C), T602L (5159A>C 5160C>T 5161C>T), V604K (5165G>A 5166T>A 5167T>A), C605Q (5168T>C 5169G>A 5170T>A), T607I (5175C>T 5176A>C), V608I (5177G>A), R609Q (5180A>C 5181G>A 5182G>A), T610S (5183A>T), V613del (5192_5194delGTA), N614T (5196A>C 5197T>C), L615Q (5199T>A 5200T>G), L616V (5201T>G), K617S (5204A>T 5205A>C), F619L (5212T>G), M620I (5215G>A), N621F (5216A>T 5217A>T 5218T>C), K622N (5221A>T), I624L (5225A>C), T625P (5228A>C), G626S (5231G>T 5232G>C), D627E (5236C>A)								
Codon mutations:	TAC516TAT (4903C>T), AAA518CAA (4907A>C), AAG519AGC (4911A>G 4912G>C), ATA520CTA (4913A>C), AAG521AAA (4918G>A), AAA522AAG (4921A>G), GGA523GAA (4923G>A), TTA524TGT (4926T>G 4927A>T), ATT525GCC (4928A>G 4929T>C 4930T>C), AAT526AAG (4933T>G), TTT527CTC (4934T>C 4936T>C), CCA528CCG (4939A>G), AAA529AAG (4942A>G), TAT531AGG (4946T>A 4947A>G 4948T>G), AAG534GAA (4955A>G 4957G>A), AAA535CCA (4958A>C 4959A>C), GAC537GAT (4966C>T), AGT538AAC (4968G>A 4969T>C), TTA539CTA (4970T>C), ATT540ATA (4975T>A), ATT541TTA (4976A>T 4978T>A), GAA542CAA (4979G>C), ACT543ACG (4984T>G), GCT545GCC (4990T>C), TCT546TCC (4993T>C), GAT547GAC (4996T>C), CAC548TAC (4997C>T), TTC549CAT (5000T>C 5001T>A 5002C>T), GGT551GGA (5008T>A), GGA552GCA (5010G>C), GTC553TTG (5012G>T 5014C>G), CTT554TTA (5015C>T 5017T>A), AAG555_GCC556del (5018_5023delAAGGCC), ACC558ACA (5029C>A), ACT559GAT (5030A>G 5031C>A), GAA560TTG (5033G>T 5034A>T 5035A>G), GGT561AAT (5036G>A 5037G>A), GAA563_TTA564del (5042_5047delGAATTA), ATC565ATT (5050C>T), TGC566TGT (5053C>T), AGG567AGA (5056G>A), TCT569ACT (5060T>A), TCA570AGT (5063T>A 5064C>G 5065A>T), ACA572ACT (5071A>T), TTC573TTT (5074C>T), AAA574AAT (5077A>T), CCA575GAA (5078C>G 5079C>A), TTG578ACT (5087T>A 5088T>C 5089G>T), AAT579AGA (5091A>G 5092T>A), TAC580TAT (5095C>T), CAT581TCT (5096C>T 5097A>C), AGT582ACC (5100G>C 5101T>C), GAG584GAA (5107G>A), AAA585AAT (5110A>T), CTA588TTA (5117C>T), GCG589GCA (5122G>A), GTA590ATT (5123G>A 5125A>T), AAA591GTT (5126A>G 5127A>T 5128A>T), CAG592CGA (5130A>G 5131G>A), GTG593GGA (5133T>G 5134G>A), ATT594ATC (5137T>C), ACT595AGA (5139C>G 5140T>A), AGT598TCA (5147A>T 5148G>C 5149T>A), ATT599GCT (5150A>G 5151T>C), TAT600TTC (5154A>T 5155T>C), ACC602CTT (5159A>C 5160C>T 5161C>T), CCT603CCA (5164T>A), GTT604AAA (5165G>A 5166T>A 5167T>A), TGT605CAA (5168T>C 5169G>A 5170T>A), TTT606TTC (5173T>C), ACA607ATC (5175C>T 5176A>C), GTC608ATC (5177G>A), AGG609CAA (5180A>C 5181G>A 5182G>A), ACA610TCA (5183A>T), GAT611GAC (5188T>C), GTA613del (5192_5194delGTA), AAT614ACC (5196A>C 5197T>C), CTT615CAG (5199T>A 5200T>G), TTA616GTA (5201T>G), AAA617TCA (5204A>T 5205A>C), GGA618GGT (5209A>T), TTT619TTG (5212T>G), ATG620ATA (5215G>A), AAT621TTC (5216A>T 5217A>T 5218T>C), AAA622AAT (5221A>T), AAG623AAA (5224G>A), ATT624CTT (5225A>C), ACT625CCT (5228A>C), GGT626TCT (5231G>T 5232G>C), GAC627GAA (5236C>A), AGT628CC. (5237A>C 5238G>C)								

*: Inserts / Deletes / Misaligned / Frameshifts

Analysis details

This analysis was performed with panviral2.64

NGS Details (UN70): Badnavirus venabougainvilleae

Assembly

**Coverage Length**	374 (1 contig(s))
**Depth Of Coverage**	3.5
**Number Of Reads**	10
**Reads Per Million**	0.19 rpm (after QC)
**Ambiguities**	0
**Assembly Method**	de novo + reference guided assembly
**Consensus Caller**	Bcf Tools

Coverage Map

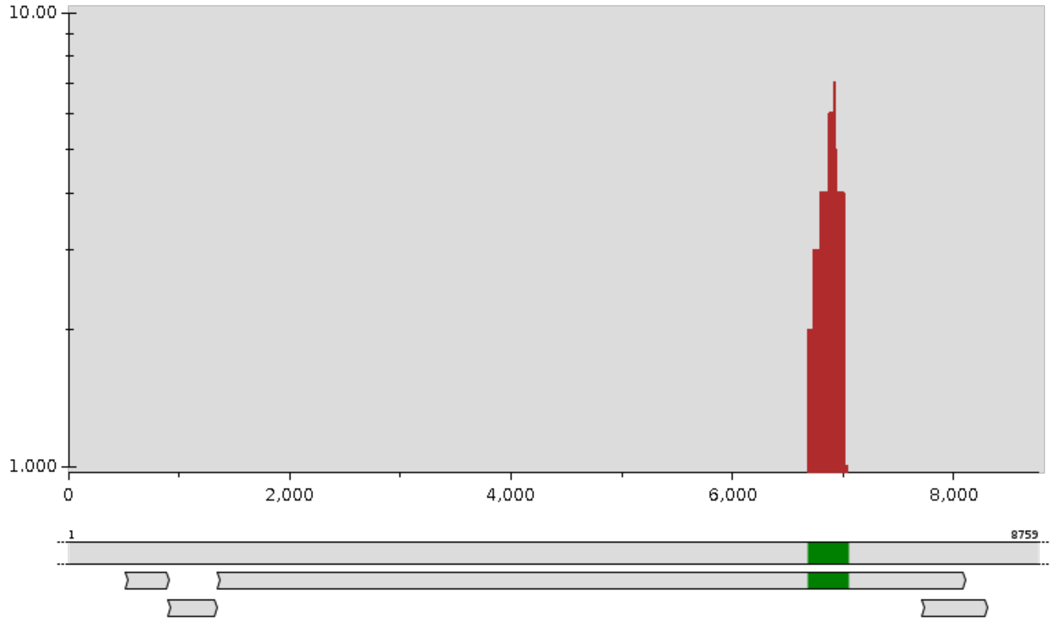

Assignment

**Type**	Badnavirus venabougainvilleae (Taxonomy ID: 3047697)
**Reference Genome**	NC_011592.1
**NT Identity (%)**	51.7241
**AA Identity (%)**	39.6825
**Number Of Stop Codons**	1
**Number Of CDS**	4

Alignment

**Alignment Score**	20.0 (NT) + 330.0 (AA) = 350.0
**Concordance (%)**	21.4067

Alignment Method	Global, seeded, nucleotide + amino acids (AGA)

Genome Region

Sequence starts at position 6681 and ends at position 7054 relative to NC_011592.1 reference sequence.

Alignment Detailed Statistics

	Begin	End	Coverage	Score	Concordance	Matches	Identities	I/D/M/F*	Stop Codons
**NT**	**6681**	**7054**	**4.3%**	**20**	**2.7%**	**374 (99.2%)**	**195 (51.7%)**	**3/0**	

Mutations: 6681A>C, 6684C>G, 6686A>T, 6690A>C, 6693A>G, 6694T>G, 6697G>T, 6700C>T, 6704A>G, 6705G>A, 6706A>T, 6707G>C, 6713T>A, 6720G>T, 6721G>C, 6723T>G, 6724G>A, 6725C>G, 6726T>A, 6729G>T, 6730G>A, 6731A>G, 6732T>G, 6733C>G, 6735A>T, 6736G>T, 6737A>T, 6738A>T, 6739T>G, 6740C>A, 6744A>T, 6747A>T, 6748T>A, 6749G>A, 6753G>A, 6754G>A, 6756C>T, 6759C>T, 6761T>G, 6762T>G, 6766C>A, 6767C>G, 6768A>G, 6769C>T, 6771A>T, 6775T>C, 6776T>A, 6777G>C, 6779T>A, 6783A>G, 6785G>C, 6786G>C, 6789A>T, 6792C>G, 6796C>T, 6798A>C, 6804T>A, 6807A>G, 6809A>C, 6810G>T, 6816A>T, 6818C>T, 6822T>C, 6823A>G, 6824T>C, 6825C>A, 6828C>T, 6829C>A, 6830A>T, 6831A>G, 6832A>G, 6833G>A, 6835A>T, 6836A>T, 6841G>A, 6843C>T, 6844A>G, 6845A>T, 6846C>T, 6847T>G, 6848G>T, 6849C>A, 6853T>C, 6854C>T, 6857G>C, 6861C>T, 6862A>T, 6863G>T, 6864C>G, 6867_6868insTTA, 6870C>T, 6871A>G, 6874G>A, 6875C>T, 6879A>C, 6881A>T, 6885T>C, 6891C>T, 6900G>A, 6902T>A, 6903T>C, 6906T>C, 6907G>A, 6911G>C, 6912T>C, 6913G>A, 6916A>G, 6917G>A, 6918A>G, 6921C>A, 6925G>A, 6928A>C, 6930G>A, 6933T>C, 6934C>T, 6936C>G, 6939A>G, 6940G>A, 6943A>G, 6945G>A, 6948T>C, 6951A>G, 6952G>T, 6953T>G, 6954T>A, 6955T>C, 6956G>T, 6958C>A, 6959A>G, 6963A>G, 6966T>G, 6967G>A, 6968G>A, 6969C>G, 6973G>T, 6974T>A, 6976C>G, 6977T>C, 6979T>A, 6980C>A, 6982C>T, 6983C>T, 6984C>T, 6985A>T, 6990A>G, 6991A>T, 6992T>G, 6993G>C, 6997G>T, 7000G>T, 7001C>G, 7003G>C, 7005C>G, 7007A>G, 7008A>T, 7009A>T, 7011T>G, 7012A>G, 7014T>G, 7016A>C, 7017G>A, 7020C>T, 7023T>A, 7026A>C, 7027G>T, 7028C>A, 7029A>T, 7033C>G, 7036G>T, 7037G>C, 7038G>T, 7039A>G, 7040A>T, 7041T>A, 7042C>A, 7044A>G, 7045T>G, 7048G>A, 7052A>G, 7054T>G

CDS

	Begin	End	Coverage	Score	Concordance	Matches	Identities	I/D/M/F*	Stop Codons
BSCVV_gp3	1780	1904	5.5%	330	36.9%	125 (99.2%)	50 (39.7%)	1/0/0/0	1

Protein mutations: I1780M (6684C>G), Y1781F (6686A>T), F1784V (6694T>G), D1785Y (6697G>T), K1787R (6704A>G 6705G>A), F1790Y (6713T>A), Q1792H (6720G>T), V1793L (6721G>C 6723T>G), A1794R (6724G>A 6725C>G 6726T>A), M1795I (6729G>T), D1796R (6730G>A 6731A>G 6732T>G), P1797A (6733C>G 6735A>T), E1798F (6736G>T 6737A>T 6738A>T), S1799D (6739T>G 6740C>A), W1802K (6748T>A 6749G>A), A1804T (6754G>A 6756C>T), L1806R (6761T>G 6762T>G), P1808R (6766C>A 6767C>G 6768A>G), Q1809Y (6769C>T 6771A>T), L1811H (6775T>C 6776T>A 6777G>C), F1812Y (6779T>A), W1814S (6785G>C 6786G>C), P1818S (6796C>T 6798A>C), K1822T (6809A>C 6810G>T), P1825L (6818C>T), I1827A (6823A>G 6824T>C 6825C>A), Q1829M (6829C>A 6830A>T 6831A>G), R1830E (6832A>G 6833G>A), K1831L (6835A>T 6836A>T), D1833N (6841G>A 6843C>T), N1834V (6844A>G 6845A>T 6846C>T), C1835V (6847T>G 6848G>T 6849C>A), S1837L (6853T>C 6854C>T), R1838P (6857G>C), S1840L (6862A>T 6863G>T 6864C>G), D1841_F1842insL (6867_6868insTTA), I1843V (6871A>G), A1844I (6874G>A 6875C>T), Y1846F (6881A>T), F1853Y (6902T>A 6903T>C), E1855K (6907G>A), S1856T (6911G>C 6912T>C), E1857K (6913G>A), R1858E (6916A>G 6917G>A 6918A>G), D1859E (6921C>A), E1861K (6925G>A), K1862Q (6928A>C 6930G>A), V1866I (6940G>A), M1867V (6943A>G 6945G>A), V1870* (6952G>T 6953T>G 6954T>A), C1871L (6955T>C 6956G>T), Q1872R (6958C>A 6959A>G), N1874K (6966T>G), G1875K (6967G>A 6968G>A 6969C>G), V1877Y (6973G>T 6974T>A), L1878A (6976C>G 6977T>C), S1879N (6979T>A 6980C>A), P1880F (6982C>T 6983C>T 6984C>T), T1881S (6985A>T), M1883C (6991A>T 6992T>G 6993G>C), V1885F (6997G>T), A1886W (7000G>T 7001C>G), V1887L (7003G>C 7005C>G), K1888S (7007A>G 7008A>T), T1889S (7009A>T 7011T>G), I1890V (7012A>G 7014T>G), E1891A (7016A>C 7017G>A), A1895Y (7027G>T 7028C>A 7029A>T), L1897V (7033C>G), G1898S (7036G>T 7037G>C 7038G>T), N1899V (7039A>G 7040A>T 7041T>A), Q1900K (7042C>A 7044A>G), C1901G (7045T>G), V1902I (7048G>A), Q1903R (7052A>G)

Codon mutations: AAA1779..C (6681A>C), ATC1780ATG (6684C>G), TAC1781TTC (6686A>T), TCA1782TCC (6690A>C), AAA1783AAG (6693A>G), TTT1784GTT (6694T>G), GAT1785TAT (6697G>T), CTG1786TTG (6700C>T), AAG1787AGA (6704A>G 6705G>A), AGT1788TCT (6706A>T 6707G>C), TTT1790TAT (6713T>A), CAG1792CAT (6720G>T), GTT1793CTG (6721G>C 6723T>G), GCT1794AGA (6724G>A 6725C>G 6726T>A), ATG1795ATT (6729G>T), GAT1796AGG (6730G>A 6731A>G 6732T>G), CCA1797GCT (6733C>G 6735A>T), GAA1798TTT (6736G>T 6737A>T 6738A>T), TCC1799GAC (6739T>G 6740C>A), ATA1800ATT (6744A>T), CCA1801CCT (6747A>T), TGG1802AAG (6748T>A 6749G>A), ACG1803ACA (6753G>A), GCC1804ACT (6754G>A 6756C>T), TTC1805TTT (6759C>T), CTT1806CGG (6761T>G 6762T>G), CCA1808AGG (6766C>A 6767C>G 6768A>G), CAA1809TAT (6769C>T 6771A>T), TTG1811CAC (6775T>C 6776T>A 6777G>C), TTT1812TAT (6779T>A), GAA1813GAG (6783A>G), TGG1814TCC (6785G>C 6786G>C), CTA1815CTT (6789A>T), GTC1816GTG (6792C>G), CCA1818TCC (6796C>T 6798A>C), GGT1820GGA (6804T>A), CTA1821CTG (6807A>G), AAG1822ACT (6809A>C 6810G>T), GCA1824GCT (6816A>T), CCA1825CTA (6818C>T), GCT1826GCC (6822T>C), ATC1827GCA (6823A>G 6824T>C 6825C>A), TTC1828TTT (6828C>T), CAA1829ATG (6829C>A 6830A>T 6831A>G), AGG1830GAG (6832A>G 6833G>A), AAG1831TTG (6835A>T 6836A>T), GAC1833AAT (6841G>A 6843C>T), AAC1834GTT (6844A>G 6845A>T 6846C>T), TGC1835GTA (6847T>G 6848G>T 6849C>A), TCA1837CTA (6853T>C 6854C>T), CGA1838CCA (6857G>C), TAC1839TAT (6861C>T), AGC1840TTG (6862A>T 6863G>T 6864C>G), GAT1841_TTC1842insTTA (6867_6868insTTA), TTC1842TTT (6870C>T), ATT1843GTT (6871A>G), GCT1844ATT (6874G>A 6875C>T), GTA1845GTC (6879A>C), TAC1846TTC (6881A>T), ATT1847ATC (6885T>C), GAC1849GAT (6891C>T), GTG1852GTA (6900G>A), TTT1853TAC (6902T>A 6903T>C), TCT1854TCC (6906T>C), GAG1855AAG (6907G>A), AGT1856ACC (6911G>C 6912T>C), GAA1857AAA (6913G>A), AGA1858GAG (6916A>G 6917G>A 6918A>G), GAC1859GAA (6921C>A), GAG1861AAG (6925G>A), AAG1862CAA (6928A>C 6930G>A), CAT1863CAC (6933T>C), CTC1864TTG (6934C>T 6936C>G), AGA1865AGG (6939A>G), GTT1866ATT (6940G>A), ATG1867GTA (6943A>G 6945G>A), CTT1868CTC (6948T>C), CAA1869CAG (6951A>G), GTT1870TGA (6952G>T 6953T>G 6954T>A), TGT1871CTT (6955T>C 6956G>T), CAA1872AGA (6958C>A 6959A>G), GAA1873GAG (6963A>G), AAT1874AAG (6966T>G), GGC1875AAG (6967G>A 6968G>A 6969C>G), GTT1877TAT (6973G>T 6974T>A), CTA1878GCA (6976C>G 6977T>C), TCC1879AAC (6979T>A 6980C>A), CCC1880TTT (6982C>T 6983C>T 6984C>T), ACT1881TCT (6985A>T), AAA1882AAG (6990A>G), ATG1883TGC (6991A>T 6992T>G 6993G>C), GTT1885TTT (6997G>T), GCG1886TGG (7000G>T 7001C>G), GTC1887CTG (7003G>C 7005C>G), AAA1888AGT (7007A>G 7008A>T), ACT1889TCG (7009A>T 7011T>G), ATT1890GTG (7012A>G 7014T>G), GAG1891GCA (7016A>C 7017G>A), TTC1892TTT (7020C>T), CTT1893CTA (7023T>A), GGA1894GGC (7026A>C), GCA1895TAT (7027G>T 7028C>A 7029A>T), CTA1897GTA (7033C>G), GGG1898TCT (7036G>T 7037G>C 7038G>T), AAT1899GTA (7039A>G 7040A>T 7041T>A), CAA1900AAG (7042C>A 7044A>G), TGC1901GGC (7045T>G), GTT1902ATT (7048G>A), CAG1903CGG (7052A>G), TTA1904G.. (7054T>G)

Proteins

	Begin	End	Coverage	Score	Concordance	Matches	Identities	I/D/M/F*	Stop Codons
polyprotein (YP_002321513.1)	1780	1904	5.5%	330	36.9%	125 (99.2%)	50 (39.7%)	1/0/0/0	1

Protein mutations: I1780M (6684C>G), Y1781F (6686A>T), F1784V (6694T>G), D1785Y (6697G>T), K1787R (6704A>G 6705G>A), F1790Y (6713T>A), Q1792H (6720G>T), V1793L (6721G>C 6723T>G), A1794R (6724G>A 6725C>G 6726T>A), M1795I (6729G>T), D1796R (6730G>A 6731A>G 6732T>G), P1797A (6733C>G 6735A>T), E1798F (6736G>T 6737A>T 6738A>T), S1799D (6739T>G 6740C>A), W1802K (6748T>A 6749G>A), A1804T (6754G>A 6756C>T), L1806R (6761T>G 6762T>G), P1808R (6766C>A 6767C>G 6768A>G), Q1809Y (6769C>T 6771A>T), L1811H (6775T>C 6776T>A 6777G>C), F1812Y (6779T>A), W1814S (6785G>C 6786G>C), P1818S (6796C>T 6798A>C), K1822T (6809A>C 6810G>T), P1825L (6818C>T), I1827A (6823A>G 6824T>C 6825C>A), Q1829M (6829C>A 6830A>T 6831A>G), R1830E (6832A>G 6833G>A), K1831L (6835A>T 6836A>T), D1833N (6841G>A 6843C>T), N1834V (6844A>G 6845A>T 6846C>T), C1835V (6847T>G 6848G>T 6849C>A), S1837L (6853T>C 6854C>T), R1838P (6857G>C), S1840L (6862A>T 6863G>T 6864C>G), D1841_F1842insL (6867_6868insTTA), I1843V (6871A>G), A1844I (6874G>A 6875C>T), Y1846F (6881A>T), F1853Y (6902T>A 6903T>C), E1855K (6907G>A), S1856T (6911G>C 6912T>C), E1857K (6913G>A), R1858E (6916A>G 6917G>A 6918A>G), D1859E (6921C>A), E1861K (6925G>A), K1862Q (6928A>C 6930G>A), V1866I (6940G>A), M1867V (6943A>G 6945G>A), V1870* (6952G>T 6953T>G 6954T>A), C1871L (6955T>C 6956G>T), Q1872R (6958C>A 6959A>G), N1874K (6966T>G), G1875K (6967G>A 6968G>A 6969C>G), V1877Y (6973G>T 6974T>A), L1878A (6976C>G 6977T>C), S1879N (6979T>A 6980C>A), P1880F (6982C>T 6983C>T 6984C>T), T1881S (6985A>T), M1883C (6991A>T 6992T>G 6993G>C), V1885F (6997G>T), A1886W (7000G>T 7001C>G), V1887L (7003G>C 7005C>G), K1888S (7007A>G 7008A>T), T1889S (7009A>T 7011T>G), I1890V (7012A>G 7014T>G), E1891A (7016A>C 7017G>A), A1895Y (7027G>T 7028C>A 7029A>T), L1897V (7033C>G), G1898S (7036G>T 7037G>C 7038G>T), N1899V (7039A>G 7040A>T 7041T>A), Q1900K (7042C>A 7044A>G), C1901G (7045T>G), V1902I (7048G>A), Q1903R (7052A>G)

Codon mutations: AAA1779..C (6681A>C), ATC1780ATG (6684C>G), TAC1781TTC (6686A>T), TCA1782TCC (6690A>C), AAA1783AAG (6693A>G), TTT1784GTT (6694T>G), GAT1785TAT (6697G>T), CTG1786TTG (6700C>T), AAG1787AGA (6704A>G 6705G>A), AGT1788TCT (6706A>T 6707G>C), TTT1790TAT (6713T>A), CAG1792CAT (6720G>T), GTT1793CTG (6721G>C 6723T>G), GCT1794AGA (6724G>A 6725C>G 6726T>A), ATG1795ATT (6729G>T), GAT1796AGG (6730G>A 6731A>G 6732T>G), CCA1797GCT (6733C>G 6735A>T), GAA1798TTT (6736G>T 6737A>T 6738A>T), TCC1799GAC (6739T>G 6740C>A), ATA1800ATT (6744A>T), CCA1801CCT (6747A>T), TGG1802AAG (6748T>A 6749G>A), ACG1803ACA (6753G>A), GCC1804ACT (6754G>A 6756C>T), TTC1805TTT (6759C>T), CTT1806CGG (6761T>G 6762T>G), CCA1808AGG (6766C>A 6767C>G 6768A>G), CAA1809TAT (6769C>T 6771A>T), TTG1811CAC (6775T>C 6776T>A 6777G>C), TTT1812TAT (6779T>A), GAA1813GAG (6783A>G), TGG1814TCC (6785G>C 6786G>C), CTA1815CTT (6789A>T), GTC1816GTG (6792C>G), CCA1818TCC (6796C>T 6798A>C), GGT1820GGA (6804T>A), CTA1821CTG (6807A>G), AAG1822ACT (6809A>C 6810G>T), GCA1824GCT (6816A>T), CCA1825CTA (6818C>T), GCT1826GCC (6822T>C), ATC1827GCA (6823A>G 6824T>C 6825C>A), TTC1828TTT (6828C>T), CAA1829ATG (6829C>A 6830A>T 6831A>G), AGG1830GAG (6832A>G 6833G>A), AAG1831TTG (6835A>T 6836A>T), GAC1833AAT (6841G>A 6843C>T), AAC1834GTT (6844A>G 6845A>T 6846C>T), TGC1835GTA (6847T>G 6848G>T 6849C>A), TCA1837CTA (6853T>C 6854C>T), CGA1838CCA (6857G>C), TAC1839TAT (6861C>T), AGC1840TTG (6862A>T 6863G>T 6864C>G), GAT1841_TTC1842insTTA (6867_6868insTTA), TTC1842TTT (6870C>T), ATT1843GTT (6871A>G), GCT1844ATT (6874G>A 6875C>T), GTA1845GTC (6879A>C), TAC1846TTC (6881A>T), ATT1847ATC (6885T>C), GAC1849GAT (6891C>T), GTG1852GTA (6900G>A), TTT1853TAC (6902T>A 6903T>C), TCT1854TCC (6906T>C), GAG1855AAG (6907G>A), AGT1856ACC (6911G>C 6912T>C), GAA1857AAA (6913G>A), AGA1858GAG (6916A>G 6917G>A 6918A>G), GAC1859GAA (6921C>A), GAG1861AAG (6925G>A), AAG1862CAA (6928A>C 6930G>A), CAT1863CAC (6933T>C), CTC1864TTG (6934C>T 6936C>G), AGA1865AGG (6939A>G), GTT1866ATT (6940G>A), ATG1867GTA (6943A>G 6945G>A), CTT1868CTC (6948T>C), CAA1869CAG (6951A>G), GTT1870TGA (6952G>T 6953T>G 6954T>A), TGT1871CTT (6955T>C 6956G>T), CAA1872AGA (6958C>A 6959A>G), GAA1873GAG (6963A>G), AAT1874AAG (6966T>G), GGC1875AAG (6967G>A 6968G>A 6969C>G), GTT1877TAT (6973G>T 6974T>A), CTA1878GCA (6976C>G 6977T>C), TCC1879AAC (6979T>A 6980C>A), CCC1880TTT (6982C>T 6983C>T 6984C>T), ACT1881TCT (6985A>T), AAA1882AAG (6990A>G), ATG1883TGC (6991A>T 6992T>G 6993G>C), GTT1885TTT (6997G>T), GCG1886TGG (7000G>T 7001C>G), GTC1887CTG (7003G>C 7005C>G), AAA1888AGT (7007A>G 7008A>T), ACT1889TCG (7009A>T 7011T>G), ATT1890GTG (7012A>G 7014T>G), GAG1891GCA (7016A>C 7017G>A), TTC1892TTT (7020C>T), CTT1893CTA (7023T>A), GGA1894GGC (7026A>C), GCA1895TAT (7027G>T 7028C>A 7029A>T), CTA1897GTA (7033C>G), GGG1898TCT (7036G>T 7037G>C 7038G>T), AAT1899GTA (7039A>G 7040A>T 7041T>A), CAA1900AAG (7042C>A 7044A>G), TGC1901GGC (7045T>G), GTT1902ATT (7048G>A), CAG1903CGG (7052A>G), TTA1904G.. (7054T>G)

*: Inserts / Deletes / Misaligned / Frameshifts

Analysis details

This analysis was performed with panviral2.64

NGS Details (UN70): Caulimovirus tessellomirabilis

Assembly

**Coverage Length**	316 (1 contig(s))
**Depth Of Coverage**	3.3
**Number Of Reads**	8
**Reads Per Million**	0.15 rpm (after QC)
**Ambiguities**	0
**Assembly Method**	de novo + reference guided assembly
**Consensus Caller**	Bcf Tools

Coverage Map

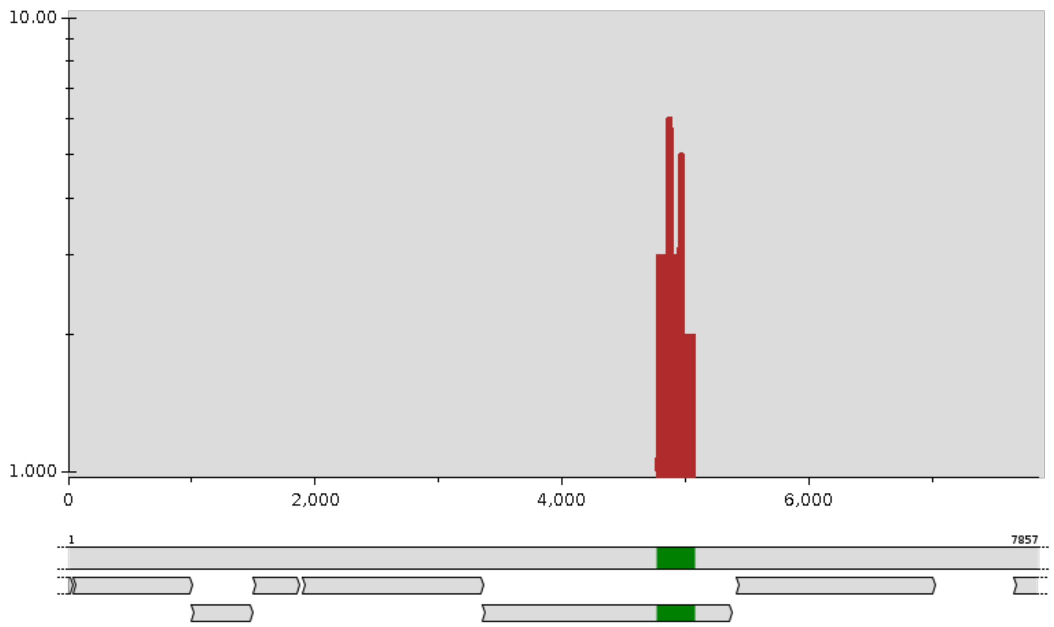

Assignment

**Type**	Caulimovirus tessellomirabilis (Taxonomy ID: 3048203)
**Reference Genome**	NC_004036.1
**NT Identity (%)**	59.3443
**AA Identity (%)**	47.5728
**Number Of Stop Codons**	0
**Number Of CDS**	7

Alignment

**Alignment Score**	76.0 (NT) + 208.0 (AA) = 284.0
**Concordance (%)**	24.7012

**Alignment Method**	Global, seeded, nucleotide + amino acids (AGA)

Genome Region

Sequence starts at position 4769 and ends at position 5084 relative to NC_004036.1 reference sequence.

Alignment Detailed Statistics

	Begin	End	Coverage	Score	Concordance	Matches	Identities	I/D/M/F*	Stop Codons
**NT**	**4769**	**5084**	**4.0%**	**76**	**13.1%**	**304 (95.9%)**	**181 (57.1%)**	**1/12**	
Mutations:	4769C>G, 4772C>A, 4774T>A, 4789A>T, 4790G>T, 4791T>G, 4793C>T, 4797C>A, 4798A>T, 4806A>G, 4807T>A, 4808A>G, 4809G>A, 4810C>T, 4812A>T, 4813T>C, 4820A>G, 4822G>T, 4829G>A, 4833A>G, 4834A>G, 4835C>A, 4837G>A, 4840A>T, 4842C>T, 4843G>T, 4849G>A, 4850G>A, 4851T>A, 4853A>C, 4854A>T, 4855G>C, 4856T>A, 4858G>T, 4860A>G, 4861G>A, 4864A>G, 4865G>T, 4866A>C, 4867T>A, 4868C>A, 4870G>T, 4871G>C, 4872T>G, 4873A>T, 4874T>C, 4875G>T, 4876G>A, 4877A>G, 4878T>G, 4879C>A, 4885G>T, 4886C>G, 4889T>G, 4890C>A, 4891A>G, 4892G>C, 4898G>C, 4900T>A, 4902A>T, 4903C>G, 4903_4904insT, 4906T>A, 4907A>C, 4913A>C, 4915A>C, 4918G>A, 4919A>G, 4921A>G, 4923G>A, 4926T>G, 4927A>T, 4928A>G, 4929T>C, 4934T>C, 4942A>G, 4946T>A, 4947A>G, 4948T>A, 4954A>T, 4955A>G, 4957G>A, 4958A>C, 4959A>C, 4960A>C, 4966C>T, 4968G>A, 4973A>G, 4975T>C, 4976A>T, 4978T>A, 4979G>C, 4982A>T, 4984T>A, 4997C>T, 4999C>T, 5000T>C, 5001T>A, 5002C>T, 5004G>T, 5008T>A, 5010G>C, 5011A>T, 5012G>C, 5014C>A, 5015C>T, 5017T>A, 5018_5023delAAGGCC, 5029C>G, 5030A>G, 5031C>A, 5033G>T, 5034A>T, 5036G>A, 5037G>A, 5042_5047delGAATTA, 5053C>T, 5056G>A, 5060T>A, 5063T>A, 5064C>G, 5065A>T, 5071A>T, 5077A>C, 5078C>G, 5079C>A								

CDS

MiMVgp6	472	577	15.7%	208	30.4%	102 (95.3%)	49 (45.8%)	1/4/1/1	0
Protein mutations:	Q472E (4769C>G), L473I (4772C>A 4774T>A), V479C (4790G>T 4791T>G), T481N (4797C>A 4798A>T), D484G (4806A>G 4807T>A), S485D (4808A>G 4809G>A 4810C>T), Y486F (4812A>T 4813T>C), K489D (4820A>G 4822G>T), E492K (4829G>A), K493R (4833A>G 4834A>G), K495N (4840A>T), P496L (4842C>T 4843G>T), V499K (4850G>A 4851T>A), K500L (4853A>C 4854A>T 4855G>C), L501I (4856T>A 4858G>T), K502R (4860A>G 4861G>A), D504S (4865G>T 4866A>C 4867T>A), Q505N (4868C>A 4870G>T), V506R (4871G>C 4872T>G 4873A>T), W507L (4874T>C 4875G>T 4876G>A), I508G (4877A>G 4878T>G 4879C>A), Q511E (4886C>G), S512E (4889T>G 4890C>A 4891A>G), D513H (4892G>C), D515Q (4898G>C 4900T>A), Y516L (4902A>T 4903C>G), Y516_V517insX (4903_4904insT), K518Q (4907A>C), I520L (4913A>C 4915A>C), K522E (4919A>G 4921A>G), G523E (4923G>A), L524C (4926T>G 4927A>T), I525A (4928A>G 4929T>C), F527L (4934T>C), Y531R (4946T>A 4947A>G 4948T>A), K534E (4955A>G 4957G>A), K535P (4958A>C 4959A>C 4960A>C), S538N (4968G>A), I540V (4973A>G 4975T>C), I541L (4976A>T 4978T>A), E542Q (4979G>C), T543S (4982A>T 4984T>A), H548Y (4997C>T 4999C>T), F549H (5000T>C 5001T>A 5002C>T), W550L (5004G>T), G552A (5010G>C 5011A>T), V553L (5012G>C 5014C>A), K555_A556del (5018_5023delAAGGCC), T559D (5030A>G 5031C>A), E560L (5033G>T 5034A>T), G561N (5036G>A 5037G>A), E563_L564del (5042_5047delGAATTA), S569T (5060T>A), K574N (5077A>C), P575E (5078C>G 5079C>A)								
Codon mutations:	CAA472GAA (4769C>G), CTT473ATA (4772C>A 4774T>A), GGA478GGT (4789A>T), GTT479TGT (4790G>T 4791T>G), CTA480TTA (4793C>T), ACA481AAT (4797C>A 4798A>T), GAT484GGA (4806A>G 4807T>A), AGC485GAT (4808A>G 4809G>A 4810C>T), TAT486TTC (4812A>T 4813T>C), AAG489GAT (4820A>G 4822G>T), GAA492AAA (4829G>A), AAA493AGG (4833A>G 4834A>G), CGG494AGA (4835C>A 4837G>A), AAA495AAT (4840A>T), CCG496CTT (4842C>T 4843G>T), CAG498CAA (4849G>A), GTA499AAA (4850G>A 4851T>A), AAG500CTC (4853A>C 4854A>T 4855G>C), TTG501ATT (4856T>A 4858G>T), AAG502AGA (4860A>G 4861G>A), AAA503AAG (4864A>G), GAT504TCA (4865G>T 4866A>C 4867T>A), CAG505AAT (4868C>A 4870G>T), GTA506CGT (4871G>C 4872T>G 4873A>T), TGG507CTA (4874T>C 4875G>T 4876G>A), ATC508GGA (4877A>G 4878T>G 4879C>A), ACG510ACT (4885G>T), CAA511GAA (4886C>G), TCA512GAG (4889T>G 4890C>A 4891A>G), GAT513CAT (4892G>C), GAT515CAA (4898G>C 4900T>A), TAC516TTG (4902A>T 4903C>G), TAC516_GTT517insT-- (4903_4904insT), GTT517GTA (4906T>A), AAA518CAA (4907A>C), ATA520CTC (4913A>C 4915A>C), AAG521AAA (4918G>A), AAA522GAG (4919A>G 4921A>G), GGA523GAA (4923G>A), TTA524TGT (4926T>G 4927A>T), ATT525GCT (4928A>G 4929T>C), TTT527CTT (4934T>C), AAA529AAG (4942A>G), TAT531AGA (4946T>A 4947A>G 4948T>A), CCA533CCT (4954A>T), AAG534GAA (4955A>G 4957G>A), AAA535CCC (4958A>C 4959A>C 4960A>C), GAC537GAT (4966C>T), AGT538AAT (4968G>A), ATT540GTC (4973A>G 4975T>C), ATT541TTA (4976A>T 4978T>A), GAA542CAA (4979G>C), ACT543TCA (4982A>T 4984T>A), CAC548TAT (4997C>T 4999C>T), TTC549CAT (5000T>C 5001T>A 5002C>T), TGG550TTG (5004G>T), GGT551GGA (5008T>A), GGA552GCT (5010G>C 5011A>T), GTC553CTA (5012G>C 5014C>A), CTT554TTA (5015C>T 5017T>A), AAG555_GCC556del (5018_5023delAAGGCC), ACC558ACG (5029C>G), ACT559GAT (5030A>G 5031C>A), GAA560TTA (5033G>T 5034A>T), GGT561AAT (5036G>A 5037G>A), GAA563_TTA564del (5042_5047delGAATTA), TGC566TGT (5053C>T), AGG567AGA (5056G>A), TCT569ACT (5060T>A), TCA570AGT (5063T>A 5064C>G 5065A>T), ACA572ACT (5071A>T), AAA574AAC (5077A>C), CCA575GAA (5078C>G 5079C>A)								

Proteins

hypothetical protein (NP_659397.1)	472	577	15.7%	208	30.4%	102 (95.3%)	49 (45.8%)	1/4/1/1	0
Protein mutations:	Q472E (4769C>G), L473I (4772C>A 4774T>A), V479C (4790G>T 4791T>G), T481N (4797C>A 4798A>T), D484G (4806A>G 4807T>A), S485D (4808A>G 4809G>A 4810C>T), Y486F (4812A>T 4813T>C), K489D (4820A>G 4822G>T), E492K (4829G>A), K493R (4833A>G 4834A>G), K495N (4840A>T), P496L (4842C>T 4843G>T), V499K (4850G>A 4851T>A), K500L (4853A>C 4854A>T 4855G>C), L501I (4856T>A 4858G>T), K502R (4860A>G 4861G>A), D504S (4865G>T 4866A>C 4867T>A), Q505N (4868C>A 4870G>T), V506R (4871G>C 4872T>G 4873A>T), W507L (4874T>C 4875G>T 4876G>A), I508G (4877A>G 4878T>G 4879C>A), Q511E (4886C>G), S512E (4889T>G 4890C>A 4891A>G), D513H (4892G>C), D515Q (4898G>C 4900T>A), Y516L (4902A>T 4903C>G), Y516_V517insX (4903_4904insT), K518Q (4907A>C), I520L (4913A>C 4915A>C), K522E (4919A>G 4921A>G), G523E (4923G>A), L524C (4926T>G 4927A>T), I525A (4928A>G 4929T>C), F527L (4934T>C), Y531R (4946T>A 4947A>G 4948T>A), K534E (4955A>G 4957G>A), K535P (4958A>C 4959A>C 4960A>C), S538N (4968G>A), I540V (4973A>G 4975T>C), I541L (4976A>T 4978T>A), E542Q (4979G>C), T543S (4982A>T 4984T>A), H548Y (4997C>T 4999C>T), F549H (5000T>C 5001T>A 5002C>T), W550L (5004G>T), G552A (5010G>C 5011A>T), V553L (5012G>C 5014C>A), K555_A556del (5018_5023delAAGGCC), T559D (5030A>G 5031C>A), E560L (5033G>T 5034A>T), G561N (5036G>A 5037G>A), E563_L564del (5042_5047delGAATTA), S569T (5060T>A), K574N (5077A>C), P575E (5078C>G 5079C>A)								
Codon mutations:	CAA472GAA (4769C>G), CTT473ATA (4772C>A 4774T>A), GGA478GGT (4789A>T), GTT479TGT (4790G>T 4791T>G), CTA480TTA (4793C>T), ACA481AAT (4797C>A 4798A>T), GAT484GGA (4806A>G 4807T>A), AGC485GAT (4808A>G 4809G>A 4810C>T), TAT486TTC (4812A>T 4813T>C), AAG489GAT (4820A>G 4822G>T), GAA492AAA (4829G>A), AAA493AGG (4833A>G 4834A>G), CGG494AGA (4835C>A 4837G>A), AAA495AAT (4840A>T), CCG496CTT (4842C>T 4843G>T), CAG498CAA (4849G>A), GTA499AAA (4850G>A 4851T>A), AAG500CTC (4853A>C 4854A>T 4855G>C), TTG501ATT (4856T>A 4858G>T), AAG502AGA (4860A>G 4861G>A), AAA503AAG (4864A>G), GAT504TCA (4865G>T 4866A>C 4867T>A), CAG505AAT (4868C>A 4870G>T), GTA506CGT (4871G>C 4872T>G 4873A>T), TGG507CTA (4874T>C 4875G>T 4876G>A), ATC508GGA (4877A>G 4878T>G 4879C>A), ACG510ACT (4885G>T), CAA511GAA (4886C>G), TCA512GAG (4889T>G 4890C>A 4891A>G), GAT513CAT (4892G>C), GAT515CAA (4898G>C 4900T>A), TAC516TTG (4902A>T 4903C>G), TAC516_GTT517insT-- (4903_4904insT), GTT517GTA (4906T>A), AAA518CAA (4907A>C), ATA520CTC (4913A>C 4915A>C), AAG521AAA (4918G>A), AAA522GAG (4919A>G 4921A>G), GGA523GAA (4923G>A), TTA524TGT (4926T>G 4927A>T), ATT525GCT (4928A>G 4929T>C), TTT527CTT (4934T>C), AAA529AAG (4942A>G), TAT531AGA (4946T>A 4947A>G 4948T>A), CCA533CCT (4954A>T), AAG534GAA (4955A>G 4957G>A), AAA535CCC (4958A>C 4959A>C 4960A>C), GAC537GAT (4966C>T), AGT538AAT (4968G>A), ATT540GTC (4973A>G 4975T>C), ATT541TTA (4976A>T 4978T>A), GAA542CAA (4979G>C), ACT543TCA (4982A>T 4984T>A), CAC548TAT (4997C>T 4999C>T), TTC549CAT (5000T>C 5001T>A 5002C>T), TGG550TTG (5004G>T), GGT551GGA (5008T>A), GGA552GCT (5010G>C 5011A>T), GTC553CTA (5012G>C 5014C>A), CTT554TTA (5015C>T 5017T>A), AAG555_GCC556del (5018_5023delAAGGCC), ACC558ACG (5029C>G), ACT559GAT (5030A>G 5031C>A), GAA560TTA (5033G>T 5034A>T), GGT561AAT (5036G>A 5037G>A), GAA563_TTA564del (5042_5047delGAATTA), TGC566TGT (5053C>T), AGG567AGA (5056G>A), TCT569ACT (5060T>A), TCA570AGT (5063T>A 5064C>G 5065A>T), ACA572ACT (5071A>T), AAA574AAC (5077A>C), CCA575GAA (5078C>G 5079C>A)								

*: Inserts / Deletes / Misaligned / Frameshifts

Analysis details

This analysis was performed with panviral2.64

NGS Details (UN70): Cavemovirus collusipomeae

Assembly

**Coverage Length**	312 (1 contig(s))
**Depth Of Coverage**	3.2
**Number Of Reads**	8
**Reads Per Million**	0.15 rpm (after QC)
**Ambiguities**	0
**Assembly Method**	de novo + reference guided assembly
**Consensus Caller**	Bcf Tools

Coverage Map

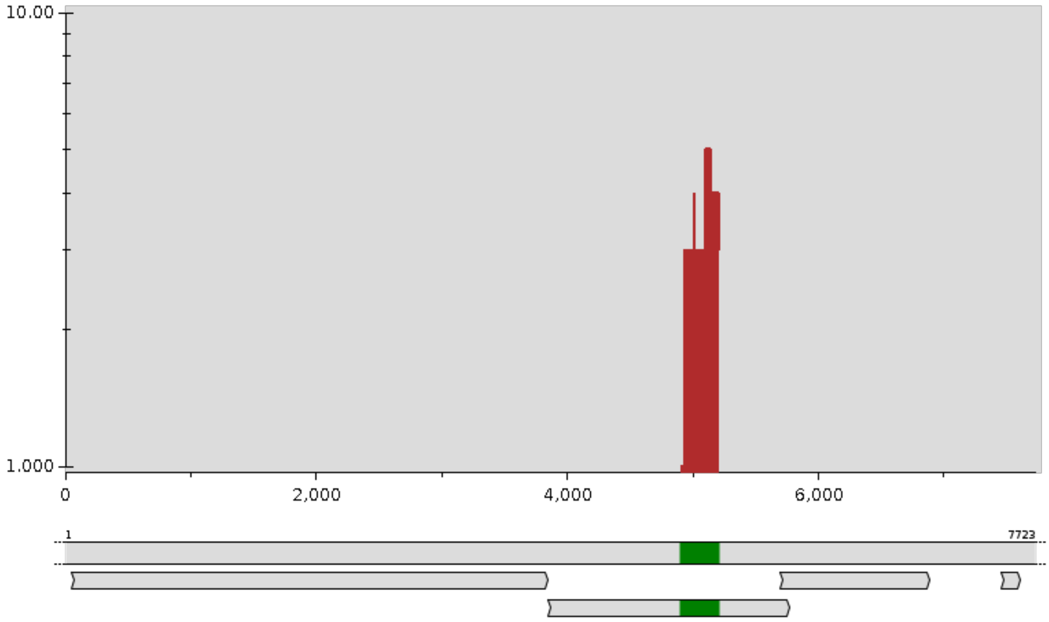

Assignment

**Type**	Cavemovirus collusipomeae (Taxonomy ID: 3048352)
**Reference Genome**	NC_015328.1
**NT Identity (%)**	63.141
**AA Identity (%)**	50.9615
**Number Of Stop Codons**	0
**Number Of CDS**	4

Alignment

**Alignment Score**	164.0 (NT) + 356.0 (AA) = 520.0
**Concordance (%)**	40.0925

Alignment Method	Global, seeded, nucleotide + amino acids (AGA)

Genome Region

Sequence starts at position 4898 and ends at position 5209 relative to NC_015328.1 reference sequence.

Alignment Detailed Statistics

	Begin	End	Coverage	Score	Concordance	Matches	Identities	I/D/M/F*	Stop Codons
**NT**	**4898**	**5209**	**4.0%**	**164**	**26.3%**	**312 (100%)**	**197 (63.1%)**	**0/0**	
Mutations:	4898G>A, 4904A>C, 4907C>A, 4909T>C, 4912A>C, 4915T>C, 4935T>G, 4939G>T, 4942A>T, 4943A>T, 4949G>C, 4958A>T, 4959A>T, 4962T>C, 4963A>T, 4967T>C, 4969G>T, 4970G>A, 4973A>C, 4974T>A, 4978T>C, 4979A>T, 4980G>C, 4981A>T, 4982A>C, 4984T>A, 4985A>T, 4988A>T, 4989T>G, 4990A>T, 4991A>T, 4992T>G, 4993A>C, 4994A>G, 4997C>A, 5005A>T, 5008A>G, 5014T>C, 5015A>C, 5017G>A, 5023A>G, 5029G>C, 5033G>A, 5034G>A, 5035A>T, 5039C>A, 5041A>T, 5042A>G, 5047A>T, 5056A>C, 5060G>A, 5062T>G, 5064A>T, 5065G>A, 5068A>T, 5069G>T, 5070A>C, 5072C>A, 5074A>G, 5075G>A, 5076G>A, 5077A>T, 5080A>C, 5083A>T, 5092G>A, 5093C>A, 5102A>T, 5103T>C, 5105G>A, 5107T>A, 5110A>G, 5113A>C, 5115T>G, 5116A>T, 5120A>T, 5121A>T, 5122A>T, 5124A>C, 5125T>A, 5126A>G, 5127T>A, 5128A>T, 5132A>C, 5137A>C, 5139G>C, 5140T>A, 5144A>C, 5150T>A, 5152A>C, 5156T>A, 5157C>G, 5158A>G, 5159A>T, 5161A>T, 5162T>C, 5164A>T, 5169T>G, 5170A>T, 5171G>T, 5176C>T, 5177C>T, 5179A>C, 5180A>G, 5181T>C, 5182T>A, 5185A>T, 5186A>G, 5188T>A, 5191C>T, 5192T>A, 5198A>G, 5200T>C, 5204A>G, 5205G>C, 5206T>A								

CDS

	Begin	End	Coverage	Score	Concordance	Matches	Identities	I/D/M/F*	Stop Codons
SPCV_gp2	353	456	16.2%	356	52.9%	104 (100%)	53 (51.0%)	0/0/0/0	0
Protein mutations:	D353N (4898G>A), I355L (4904A>C), L356I (4907C>A 4909T>C), F365C (4935T>G), K367N (4942A>T), T368S (4943A>T), E370Q (4949G>C), K373L (4958A>T 4959A>T), I374T (4962T>C 4963A>T), E377K (4970G>A), I378Q (4973A>C 4974T>A), R380S (4979A>T 4980G>C 4981A>T), N381Q (4982A>C 4984T>A), I382L (4985A>T), I383C (4988A>T 4989T>G 4990A>T), I384C (4991A>T 4992T>G 4993A>C), K385E (4994A>G), H386N (4997C>A), K392Q (5015A>C 5017G>A), E396D (5029G>C), G398N (5033G>A 5034G>A 5035A>T), Q400N (5039C>A 5041A>T), K401E (5042A>G), V407M (5060G>A 5062T>G), K408I (5064A>T 5065G>A), E410S (5069G>T 5070A>C), Q411K (5072C>A 5074A>G), G412N (5075G>A 5076G>A 5077A>T), P418T (5093C>A), I421S (5102A>T 5103T>C), D422K (5105G>A 5107T>A), L425C (5115T>G 5116A>T), K427F (5120A>T 5121A>T 5122A>T), H428P (5124A>C 5125T>A), I429D (5126A>G 5127T>A 5128A>T), I431L (5132A>C), K432N (5137A>C), S433T (5139G>C 5140T>A), K435Q (5144A>C), L437I (5150T>A 5152A>C), S439R (5156T>A 5157C>G 5158A>G), I440F (5159A>T 5161A>T), L443C (5169T>G 5170A>T), V444L (5171G>T), Q446Y (5177C>T 5179A>C), I447A (5180A>G 5181T>C 5182T>A), R448S (5185A>T), N449E (5186A>G 5188T>A), L451I (5192T>A), N453D (5198A>G 5200T>C), S455A (5204A>G 5205G>C 5206T>A)								
Codon mutations:	GAT353AAT (4898G>A), ATA355CTA (4904A>C), CTT356ATC (4907C>A 4909T>C), GTA357GTC (4912A>C), TAT358TAC (4915T>C), TTT365TGT (4935T>G), TCG366TCT (4939G>T), AAA367AAT (4942A>T), ACA368TCA (4943A>T), GAA370CAA (4949G>C), AAA373TTA (4958A>T 4959A>T), ATA374ACT (4962T>C 4963A>T), TTG376CTT (4967T>C 4969G>T), GAA377AAA (4970G>A), ATA378CAA (4973A>C 4974T>A), TTT379TTC (4978T>C), AGA380TCT (4979A>T 4980G>C 4981A>T), AAT381CAA (4982A>C 4984T>A), ATA382TTA (4985A>T), ATA383TGT (4988A>T 4989T>G 4990A>T), ATA384TGC (4991A>T 4992T>G 4993A>C), AAA385GAA (4994A>G), CAT386AAT (4997C>A), ATA388ATT (5005A>T), GTA389GTG (5008A>G), AGT391AGC (5014T>C), AAG392CAA (5015A>C 5017G>A), AAA394AAG (5023A>G), GAG396GAC (5029G>C), GGA398AAT (5033G>A 5034G>A 5035A>T), CAA400AAT (5039C>A 5041A>T), AAA401GAA (5042A>G), ATA402ATT (5047A>T), CTA405CTC (5056A>C), GTT407ATG (5060G>A 5062T>G), AAG408ATA (5064A>T 5065G>A), ATA409ATT (5068A>T), GAA410TCA (5069G>T 5070A>C), CAA411AAG (5072C>A 5074A>G), GGA412AAT (5075G>A 5076G>A 5077A>T), GGA413GGC (5080A>C), ATA414ATT (5083A>T), CAG417CAA (5092G>A), CCT418ACT (5093C>A), ATA421TCA (5102A>T 5103T>C), GAT422AAA (5105G>A 5107T>A), AAA423AAG (5110A>G), ATA424ATC (5113A>C), TTA425TGT (5115T>G 5116A>T), AAA427TTT (5120A>T 5121A>T 5122A>T), CAT428CCA (5124A>C 5125T>A), ATA429GAT (5126A>G 5127T>A 5128A>T), ATA431CTA (5132A>C), AAA432AAC (5137A>C), AGT433ACA (5139G>C 5140T>A), AAA435CAA (5144A>C), TTA437ATC (5150T>A 5152A>C), TCA439AGG (5156T>A 5157C>G 5158A>G), ATA440TTT (5159A>T 5161A>T), TTA441CTT (5162T>C 5164A>T), TTA443TGT (5169T>G 5170A>T), GTA444TTA (5171G>T), AAC445AAT (5176C>T), CAA446TAC (5177C>T 5179A>C), ATT447GCA (5180A>G 5181T>C 5182T>A), AGA448AGT (5185A>T), AAT449GAA (5186A>G 5188T>A), TTC450TTT (5191C>T), TTA451ATA (5192T>A), AAT453GAC (5198A>G 5200T>C), AGT455GCA (5204A>G 5205G>C 5206T>A)								

Proteins

	Begin	End	Coverage	Score	Concordance	Matches	Identities	I/D/M/F*	Stop Codons
replicase (YP_004347415.1)	353	456	16.2%	356	52.9%	104 (100%)	53 (51.0%)	0/0/0/0	0
Protein mutations:	D353N (4898G>A), I355L (4904A>C), L356I (4907C>A 4909T>C), F365C (4935T>G), K367N (4942A>T), T368S (4943A>T), E370Q (4949G>C), K373L (4958A>T 4959A>T), I374T (4962T>C 4963A>T), E377K (4970G>A), I378Q (4973A>C 4974T>A), R380S (4979A>T 4980G>C 4981A>T), N381Q (4982A>C 4984T>A), I382L (4985A>T), I383C (4988A>T 4989T>G 4990A>T), I384C (4991A>T 4992T>G 4993A>C), K385E (4994A>G), H386N (4997C>A), K392Q (5015A>C 5017G>A), E396D (5029G>C), G398N (5033G>A 5034G>A 5035A>T), Q400N (5039C>A 5041A>T), K401E (5042A>G), V407M (5060G>A 5062T>G), K408I (5064A>T 5065G>A), E410S (5069G>T 5070A>C), Q411K (5072C>A 5074A>G), G412N (5075G>A 5076G>A 5077A>T), P418T (5093C>A), I421S (5102A>T 5103T>C), D422K (5105G>A 5107T>A), L425C (5115T>G 5116A>T), K427F (5120A>T 5121A>T 5122A>T), H428P (5124A>C 5125T>A), I429D (5126A>G 5127T>A 5128A>T), I431L (5132A>C), K432N (5137A>C), S433T (5139G>C 5140T>A), K435Q (5144A>C), L437I (5150T>A 5152A>C), S439R (5156T>A 5157C>G 5158A>G), I440F (5159A>T 5161A>T), L443C (5169T>G 5170A>T), V444L (5171G>T), Q446Y (5177C>T 5179A>C), I447A (5180A>G 5181T>C 5182T>A), R448S (5185A>T), N449E (5186A>G 5188T>A), L451I (5192T>A), N453D (5198A>G 5200T>C), S455A (5204A>G 5205G>C 5206T>A)								
Codon mutations:	GAT353AAT (4898G>A), ATA355CTA (4904A>C), CTT356ATC (4907C>A 4909T>C), GTA357GTC (4912A>C), TAT358TAC (4915T>C), TTT365TGT (4935T>G), TCG366TCT (4939G>T), AAA367AAT (4942A>T), ACA368TCA (4943A>T), GAA370CAA (4949G>C), AAA373TTA (4958A>T 4959A>T), ATA374ACT (4962T>C 4963A>T), TTG376CTT (4967T>C 4969G>T), GAA377AAA (4970G>A), ATA378CAA (4973A>C 4974T>A), TTT379TTC (4978T>C), AGA380TCT (4979A>T 4980G>C 4981A>T), AAT381CAA (4982A>C 4984T>A), ATA382TTA (4985A>T), ATA383TGT (4988A>T 4989T>G 4990A>T), ATA384TGC (4991A>T 4992T>G 4993A>C), AAA385GAA (4994A>G), CAT386AAT (4997C>A), ATA388ATT (5005A>T), GTA389GTG (5008A>G), AGT391AGC (5014T>C), AAG392CAA (5015A>C 5017G>A), AAA394AAG (5023A>G), GAG396GAC (5029G>C), GGA398AAT (5033G>A 5034G>A 5035A>T), CAA400AAT (5039C>A 5041A>T), AAA401GAA (5042A>G), ATA402ATT (5047A>T), CTA405CTC (5056A>C), GTT407ATG (5060G>A 5062T>G), AAG408ATA (5064A>T 5065G>A), ATA409ATT (5068A>T), GAA410TCA (5069G>T 5070A>C), CAA411AAG (5072C>A 5074A>G), GGA412AAT (5075G>A 5076G>A 5077A>T), GGA413GGC (5080A>C), ATA414ATT (5083A>T), CAG417CAA (5092G>A), CCT418ACT (5093C>A), ATA421TCA (5102A>T 5103T>C), GAT422AAA (5105G>A 5107T>A), AAA423AAG (5110A>G), ATA424ATC (5113A>C), TTA425TGT (5115T>G 5116A>T), AAA427TTT (5120A>T 5121A>T 5122A>T), CAT428CCA (5124A>C 5125T>A), ATA429GAT (5126A>G 5127T>A 5128A>T), ATA431CTA (5132A>C), AAA432AAC (5137A>C), AGT433ACA (5139G>C 5140T>A), AAA435CAA (5144A>C), TTA437ATC (5150T>A 5152A>C), TCA439AGG (5156T>A 5157C>G 5158A>G), ATA440TTT (5159A>T 5161A>T), TTA441CTT (5162T>C 5164A>T), TTA443TGT (5169T>G 5170A>T), GTA444TTA (5171G>T), AAC445AAT (5176C>T), CAA446TAC (5177C>T 5179A>C), ATT447GCA (5180A>G 5181T>C 5182T>A), AGA448AGT (5185A>T), AAT449GAA (5186A>G 5188T>A), TTC450TTT (5191C>T), TTA451ATA (5192T>A), AAT453GAC (5198A>G 5200T>C), AGT455GCA (5204A>G 5205G>C 5206T>A)								

*: Inserts / Deletes / Misaligned / Frameshifts

Analysis details

This analysis was performed with panviral2.64

NGS Details (UN70): Cavemovirus collusipomeae

Assembly

**Coverage Length**	239 (1 contig(s))
**Depth Of Coverage**	3.7
**Number Of Reads**	8
**Reads Per Million**	0.15 rpm (after QC)
**Ambiguities**	0
**Assembly Method**	de novo + reference guided assembly
**Consensus Caller**	Bcf Tools

Coverage Map

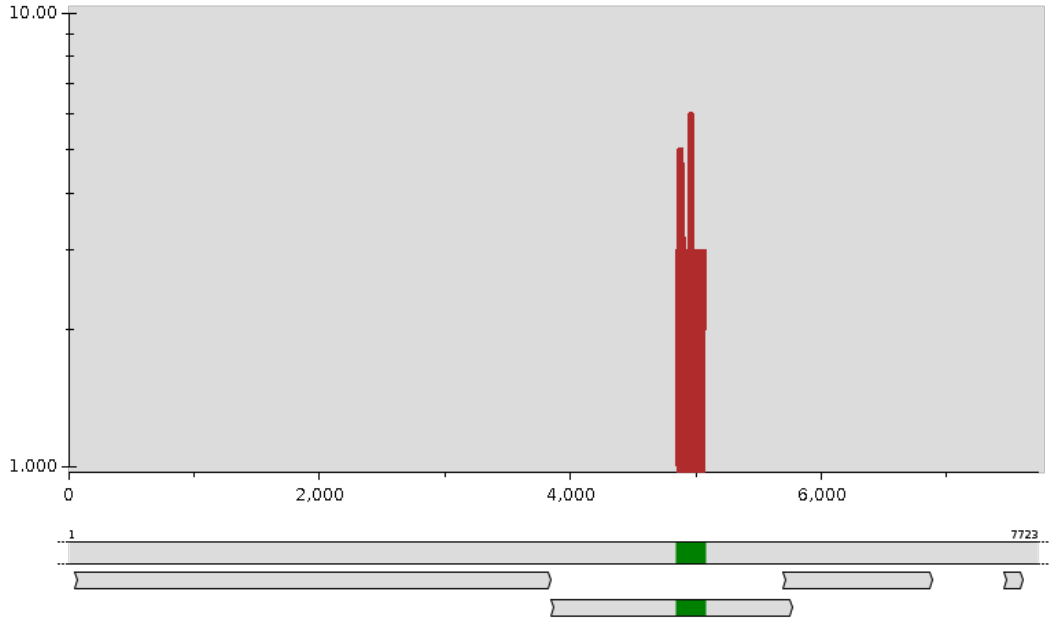

Assignment

**Type**	Cavemovirus collusipomeae (Taxonomy ID: 3048352)
**Reference Genome**	NC_015328.1
**NT Identity (%)**	62.7615
**AA Identity (%)**	49.3671
**Number Of Stop Codons**	0
**Number Of CDS**	4

Alignment

**Alignment Score**	122.0 (NT) + 298.0 (AA) = 420.0
**Concordance (%)**	41.0557

Alignment Method	Global, seeded, nucleotide + amino acids (AGA)

Genome Region

Sequence starts at position 4842 and ends at position 5080 relative to NC_015328.1 reference sequence.

Alignment Detailed Statistics

	Begin	End	Coverage	Score	Concordance	Matches	Identities	I/D/M/F*	Stop Codons
**NT**	**4842**	**5080**	**3.1%**	**122**	**25.5%**	**239 (100%)**	**150 (62.8%)**	**0/0**	
Mutations:	4843T>A, 4847T>G, 4849A>C, 4852A>T, 4853A>T, 4854G>C, 4856A>G, 4857T>A, 4860A>T, 4865C>A, 4868T>A, 4874G>A, 4878G>A, 4879A>T, 4882A>T, 4887G>A, 4888A>T, 4896A>T, 4897T>G, 4906A>T, 4907C>A, 4912A>C, 4914A>G, 4915T>C, 4918A>T, 4924T>C, 4927A>C, 4930A>G, 4931A>G, 4935T>A, 4938C>T, 4939G>C, 4944C>T, 4947A>T, 4948C>T, 4952G>A, 4953A>T, 4954A>G, 4959A>T, 4960A>T, 4962T>A, 4963A>G, 4967T>C, 4969G>T, 4972A>C, 4975A>T, 4980G>A, 4981A>G, 4984T>A, 4987A>T, 4988A>G, 4990A>G, 4994A>C, 4997C>A, 5002A>T, 5003A>T, 5005A>G, 5008A>G, 5009T>A, 5012A>T, 5013G>C, 5017G>A, 5018A>C, 5019A>C, 5024G>A, 5025C>T, 5027G>A, 5028A>G, 5029G>T, 5030A>T, 5032A>G, 5033G>T, 5034G>T, 5035A>T, 5036A>C, 5039C>A, 5040A>C, 5042A>G, 5045A>G, 5047A>C, 5048G>C, 5049A>G, 5054C>T, 5060G>C, 5061T>A, 5065G>T, 5069G>T, 5070A>G, 5071A>T								
**CDS**									
SPCV_gp2	335	413	12.3%	298	53.3%	79 (100%)	39 (49.4%)	0/0/0/0	0
Protein mutations:	S336A (4847T>G 4849A>C), I339E (4856A>G 4857T>A), Y340F (4860A>T), Q342K (4865C>A), F343I (4868T>A), D345N (4874G>A), G346D (4878G>A 4879A>T), R349N (4887G>A 4888A>T), Y352L (4896A>T 4897T>G), L356I (4907C>A), Y358C (4914A>G 4915T>C), I364V (4931A>G), F365Y (4935T>A), S366F (4938C>T 4939G>C), T368I (4944C>T), Y369F (4947A>T 4948C>T), E371M (4952G>A 4953A>T 4954A>G), K373I (4959A>T 4960A>T), I374K (4962T>A 4963A>G), E377D (4972A>C), R380K (4980G>A 4981A>G), N381K (4984T>A), I383V (4988A>G 4990A>G), K385Q (4994A>C), H386N (4997C>A), I388L (5003A>T 5005A>G), L390I (5009T>A), K393P (5018A>C 5019A>C), A395I (5024G>A 5025C>T), E396S (5027G>A 5028A>G 5029G>T), I397L (5030A>T 5032A>G), G398F (5033G>T 5034G>T 5035A>T), K399Q (5036A>C), Q400T (5039C>A 5040A>C), K401E (5042A>G), I402V (5045A>G 5047A>C), E403R (5048G>C 5049A>G), V407H (5060G>C 5061T>A), K408N (5065G>T), E410C (5069G>T 5070A>G 5071A>T)								
Codon mutations:	CAT334.AA (4843T>A), TCA336GCC (4847T>G 4849A>C), CCA337CCT (4852A>T), AGT338TCT (4853A>T 4854G>C), ATA339GAA (4856A>G 4857T>A), TAT340TTT (4860A>T), CAA342AAA (4865C>A), TTT343ATT (4868T>A), GAT345AAT (4874G>A), GGA346GAT (4878G>A 4879A>T), ATA347ATT (4882A>T), AGA349AAT (4887G>A 4888A>T), TAT352TTG (4896A>T 4897T>G), ATA355ATT (4906A>T), CTT356ATT (4907C>A), GTA357GTC (4912A>C), TAT358TGC (4914A>G 4915T>C), ATA359ATT (4918A>T), GAT361GAC (4924T>C), ATA362ATC (4927A>C), TTA363TTG (4930A>G), ATA364GTA (4931A>G), TTT365TAT (4935T>A), TCG366TTC (4938C>T 4939G>C), ACA368ATA (4944C>T), TAC369TTT (4947A>T 4948C>T), GAA371ATG (4952G>A 4953A>T 4954A>G), AAA373ATT (4959A>T 4960A>T), ATA374AAG (4962T>A 4963A>G), TTG376CTT (4967T>C 4969G>T), GAA377GAC (4972A>C), ATA378ATT (4975A>T), AGA380AAG (4980G>A 4981A>G), AAT381AAA (4984T>A), ATA382ATT (4987A>T), ATA383GTG (4988A>G 4990A>G), AAA385CAA (4994A>C), CAT386AAT (4997C>A), GGA387GGT (5002A>T), ATA388TTG (5003A>T 5005A>G), GTA389GTG (5008A>G), TTA390ATA (5009T>A), AGT391TCT (5012A>T 5013G>C), AAG392AAA (5017G>A), AAA393CCA (5018A>C 5019A>C), GCA395ATA (5024G>A 5025C>T), GAG396AGT (5027G>A 5028A>G 5029G>T), ATA397TTG (5030A>T 5032A>G), GGA398TTT (5033G>T 5034G>T 5035A>T), AAA399CAA (5036A>C), CAA400ACA (5039C>A 5040A>C), AAA401GAA (5042A>G), ATA402GTC (5045A>G 5047A>C), GAA403CGA (5048G>C 5049A>G), CTA405TTA (5054C>T), GTT407CAT (5060G>C 5061T>A), AAG408AAT (5065G>T), GAA410TGT (5069G>T 5070A>G 5071A>T)								
**Proteins**									
replicase (YP_004347415.1)	335	413	12.3%	298	53.3%	79 (100%)	39 (49.4%)	0/0/0/0	0
Protein mutations:	S336A (4847T>G 4849A>C), I339E (4856A>G 4857T>A), Y340F (4860A>T), Q342K (4865C>A), F343I (4868T>A), D345N (4874G>A), G346D (4878G>A 4879A>T), R349N (4887G>A 4888A>T), Y352L (4896A>T 4897T>G), L356I (4907C>A), Y358C (4914A>G 4915T>C), I364V (4931A>G), F365Y (4935T>A), S366F (4938C>T 4939G>C), T368I (4944C>T), Y369F (4947A>T 4948C>T), E371M (4952G>A 4953A>T 4954A>G), K373I (4959A>T 4960A>T), I374K (4962T>A 4963A>G), E377D (4972A>C), R380K (4980G>A 4981A>G), N381K (4984T>A), I383V (4988A>G 4990A>G), K385Q (4994A>C), H386N (4997C>A), I388L (5003A>T 5005A>G), L390I (5009T>A), K393P (5018A>C 5019A>C), A395I (5024G>A 5025C>T), E396S (5027G>A 5028A>G 5029G>T), I397L (5030A>T 5032A>G), G398F (5033G>T 5034G>T 5035A>T), K399Q (5036A>C), Q400T (5039C>A 5040A>C), K401E (5042A>G), I402V (5045A>G 5047A>C), E403R (5048G>C 5049A>G), V407H (5060G>C 5061T>A), K408N (5065G>T), E410C (5069G>T 5070A>G 5071A>T)								
Codon mutations:	CAT334.AA (4843T>A), TCA336GCC (4847T>G 4849A>C), CCA337CCT (4852A>T), AGT338TCT (4853A>T 4854G>C), ATA339GAA (4856A>G 4857T>A), TAT340TTT (4860A>T), CAA342AAA (4865C>A), TTT343ATT (4868T>A), GAT345AAT (4874G>A), GGA346GAT (4878G>A 4879A>T), ATA347ATT (4882A>T), AGA349AAT (4887G>A 4888A>T), TAT352TTG (4896A>T 4897T>G), ATA355ATT (4906A>T), CTT356ATT (4907C>A), GTA357GTC (4912A>C), TAT358TGC (4914A>G 4915T>C), ATA359ATT (4918A>T), GAT361GAC (4924T>C), ATA362ATC (4927A>C), TTA363TTG (4930A>G), ATA364GTA (4931A>G), TTT365TAT (4935T>A), TCG366TTC (4938C>T 4939G>C), ACA368ATA (4944C>T), TAC369TTT (4947A>T 4948C>T), GAA371ATG (4952G>A 4953A>T 4954A>G), AAA373ATT (4959A>T 4960A>T), ATA374AAG (4962T>A 4963A>G), TTG376CTT (4967T>C 4969G>T), GAA377GAC (4972A>C), ATA378ATT (4975A>T), AGA380AAG (4980G>A 4981A>G), AAT381AAA (4984T>A), ATA382ATT (4987A>T), ATA383GTG (4988A>G 4990A>G), AAA385CAA (4994A>C), CAT386AAT (4997C>A), GGA387GGT (5002A>T), ATA388TTG (5003A>T 5005A>G), GTA389GTG (5008A>G), TTA390ATA (5009T>A), AGT391TCT (5012A>T 5013G>C), AAG392AAA (5017G>A), AAA393CCA (5018A>C 5019A>C), GCA395ATA (5024G>A 5025C>T), GAG396AGT (5027G>A 5028A>G 5029G>T), ATA397TTG (5030A>T 5032A>G), GGA398TTT (5033G>T 5034G>T 5035A>T), AAA399CAA (5036A>C), CAA400ACA (5039C>A 5040A>C), AAA401GAA (5042A>G), ATA402GTC (5045A>G 5047A>C), GAA403CGA (5048G>C 5049A>G), CTA405TTA (5054C>T), GTT407CAT (5060G>C 5061T>A), AAG408AAT (5065G>T), GAA410TGT (5069G>T 5070A>G 5071A>T)								

*: Inserts / Deletes / Misaligned / Frameshifts

Analysis details

This analysis was performed with panviral2.64

NGS Details (UN70): Duamitovirus chqu1

Assembly

**Coverage Length**	135 (1 contig(s))
**Depth Of Coverage**	6.4
**Number Of Reads**	8
**Reads Per Million**	0.15 rpm (after QC)
**Ambiguities**	0
**Assembly Method**	de novo + reference guided assembly
**Consensus Caller**	Bcf Tools

Coverage Map

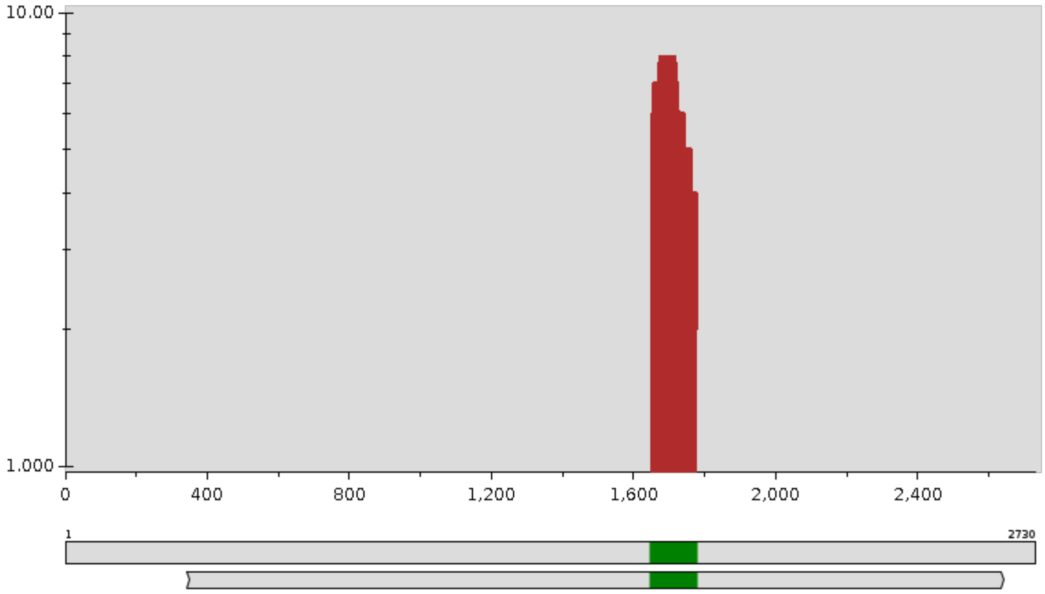

Assignment

**Type**	Duamitovirus chqu1 (Taxonomy ID: 2955775)
**Reference Genome**	NC_040543.1
**NT Identity (%)**	68.1481
**AA Identity (%)**	75.5556
**Number Of Stop Codons**	1
**Number Of CDS**	1

Alignment

**Alignment Score**	98.0 (NT) + 265.0 (AA) = 363.0
**Concordance (%)**	59.8023

Alignment Method	Global, seeded, nucleotide + amino acids (AGA)

Genome Region

Sequence starts at position 1647 and ends at position 1781 relative to NC_040543.1 reference sequence.

Alignment Detailed Statistics

	Begin	End	Coverage	Score	Concordance	Matches	Identities	I/D/M/F*	Stop Codons
**NT**	**1647**	**1781**	**4.9%**	**98**	**36.3%**	**135 (100%)**	**92 (68.1%)**	**0/0**	
Mutations:	1651T>A, 1654G>A, 1657A>G, 1660A>G, 1661C>T, 1663C>A, 1669T>C, 1672T>C, 1678C>A, 1684A>C, 1690C>T, 1696A>T, 1699A>C, 1706C>T, 1707T>A, 1708G>T, 1709G>A, 1711T>G, 1718T>C, 1723G>T, 1726T>G, 1727G>A, 1729T>A, 1731T>A, 1732A>T, 1735C>A, 1741T>A, 1743G>C, 1744C>G, 1747A>T, 1748G>A, 1749A>C, 1750A>T, 1753T>C, 1756G>T, 1760A>G, 1761G>C, 1762G>T, 1765C>T, 1768T>C, 1771T>C, 1772G>C, 1777G>A								
**CDS**									
EXI96_gp1	436	480	5.9%	265	74.6%	45 (100%)	34 (75.6%)	0/0/0/0	1
Protein mutations:	C436* (1651T>A), L455Y (1706C>T 1707T>A 1708G>T), V456M (1709G>A 1711T>G), D462K (1727G>A 1729T>A), L463Y (1731T>A 1732A>T), S467T (1743G>C 1744C>G), R468S (1747A>T), E469T (1748G>A 1749A>C 1750A>T), R473A (1760A>G 1761G>C 1762G>T), V477L (1772G>C)								
Codon mutations:	TGT436TGA (1651T>A), GGG437GGA (1654G>A), CAA438CAG (1657A>G), CCA439CCG (1660A>G), CTC440TTA (1661C>T 1663C>A), TAT442TAC (1669T>C), TAT443TAC (1672T>C), TCC445TCA (1678C>A), TCA447TCC (1684A>C), TTC449TTT (1690C>T), CTA451CTT (1696A>T), TCA452TCC (1699A>C), CTG455TAT (1706C>T 1707T>A 1708G>T), GTT456ATG (1709G>A 1711T>G), TTA459CTA (1718T>C), GCG460GCT (1723G>T), GCT461GCG (1726T>G), GAT462AAA (1727G>A 1729T>A), TTA463TAT (1731T>A 1732A>T), GCC464GCA (1735C>A), CCT466CCA (1741T>A), AGC467ACG (1743G>C 1744C>G), AGA468AGT (1747A>T), GAA469ACT (1748G>A 1749A>C 1750A>T), ACT470ACC (1753T>C), CCG471CCT (1756G>T), AGG473GCT (1760A>G 1761G>C 1762G>T), GAC474GAT (1765C>T), TAT475TAC (1768T>C), GCT476GCC (1771T>C), GTG477CTG (1772G>C), TTG478TTA (1777G>A)								
**Proteins**									
RNA dependent RNA polymerase (YP_009551903.1)	436	480	5.9%	265	74.6%	45 (100%)	34 (75.6%)	0/0/0/0	1
Protein mutations:	C436* (1651T>A), L455Y (1706C>T 1707T>A 1708G>T), V456M (1709G>A 1711T>G), D462K (1727G>A 1729T>A), L463Y (1731T>A 1732A>T), S467T (1743G>C 1744C>G), R468S (1747A>T), E469T (1748G>A 1749A>C 1750A>T), R473A (1760A>G 1761G>C 1762G>T), V477L (1772G>C)								
Codon mutations:	TGT436TGA (1651T>A), GGG437GGA (1654G>A), CAA438CAG (1657A>G), CCA439CCG (1660A>G), CTC440TTA (1661C>T 1663C>A), TAT442TAC (1669T>C), TAT443TAC (1672T>C), TCC445TCA (1678C>A), TCA447TCC (1684A>C), TTC449TTT (1690C>T), CTA451CTT (1696A>T), TCA452TCC (1699A>C), CTG455TAT (1706C>T 1707T>A 1708G>T), GTT456ATG (1709G>A 1711T>G), TTA459CTA (1718T>C), GCG460GCT (1723G>T), GCT461GCG (1726T>G), GAT462AAA (1727G>A 1729T>A), TTA463TAT (1731T>A 1732A>T), GCC464GCA (1735C>A), CCT466CCA (1741T>A), AGC467ACG (1743G>C 1744C>G), AGA468AGT (1747A>T), GAA469ACT (1748G>A 1749A>C 1750A>T), ACT470ACC (1753T>C), CCG471CCT (1756G>T), AGG473GCT (1760A>G 1761G>C 1762G>T), GAC474GAT (1765C>T), TAT475TAC (1768T>C), GCT476GCC (1771T>C), GTG477CTG (1772G>C), TTG478TTA (1777G>A)								

*: Inserts / Deletes / Misaligned / Frameshifts

Analysis details

This analysis was performed with panviral2.64

NGS Details (UN70): Badnavirus tessellocastaneae

Assembly

**Coverage Length**	248 (1 contig(s))
**Depth Of Coverage**	3.4
**Number Of Reads**	7
**Reads Per Million**	0.13 rpm (after QC)
**Ambiguities**	0
**Assembly Method**	de novo + reference guided assembly
**Consensus Caller**	Bcf Tools

Coverage Map

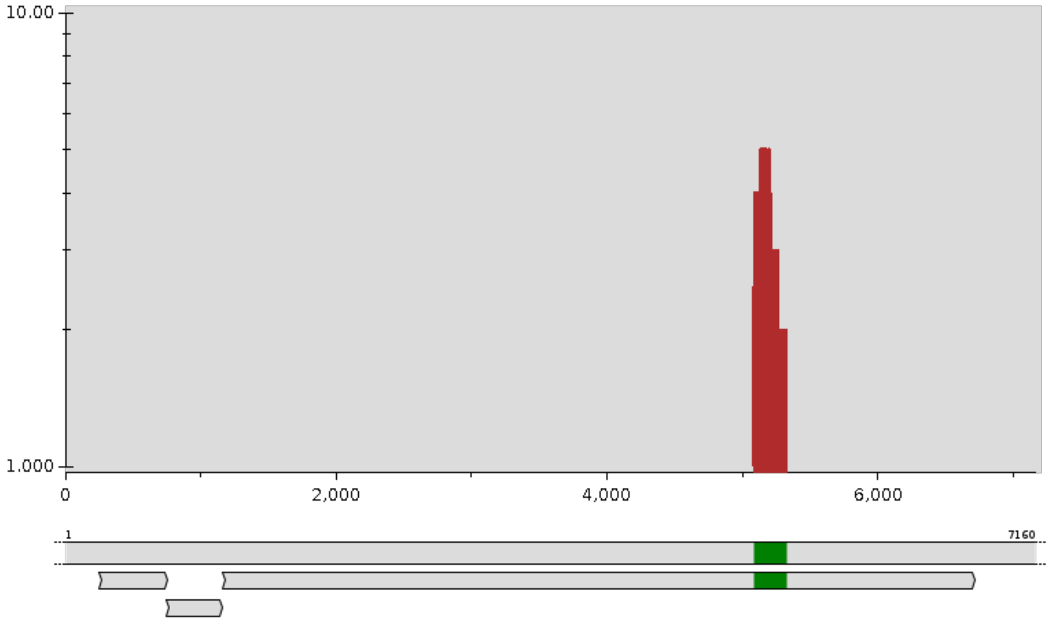

Assignment

**Type**	Badnavirus tessellocastaneae (Taxonomy ID: 3051987)
**Reference Genome**	NC_076885.1
**NT Identity (%)**	55.6452
**AA Identity (%)**	55.4217
**Number Of Stop Codons**	0
**Number Of CDS**	3

Alignment

**Alignment Score**	56.0 (NT) + 329.0 (AA) = 385.0
**Concordance (%)**	35.9813

Alignment Method	Global, seeded, nucleotide + amino acids (AGA)

Genome Region

Sequence starts at position 5084 and ends at position 5331 relative to NC_076885.1 reference sequence.

Alignment Detailed Statistics

	Begin	End	Coverage	Score	Concordance	Matches	Identities	I/D/M/F*	Stop Codons
**NT**	**5084**	**5331**	**3.5%**	**56**	**11.3%**	**248 (100%)**	**138 (55.6%)**	**0/0**	
Mutations:	5087C>G, 5092T>C, 5098A>G, 5100C>A, 5102C>T, 5104C>A, 5109T>C, 5110G>C, 5113T>C, 5116C>T, 5117C>G, 5118A>T, 5122A>G, 5125C>T, 5126C>A, 5130A>T, 5132T>C, 5134C>A, 5135C>A, 5137C>T, 5142G>T, 5143A>T, 5146A>T, 5148C>A, 5149A>G, 5150T>G, 5151C>A, 5152C>A, 5153A>T, 5155C>A, 5156A>T, 5158C>A, 5159A>G, 5161G>T, 5162A>G, 5163G>A, 5164A>G, 5165G>T, 5167T>G, 5168G>C, 5169C>A, 5170A>T, 5174T>G, 5176A>C, 5180G>T, 5181T>A, 5182C>T, 5188C>T, 5192T>C, 5194T>A, 5197T>C, 5200A>G, 5202A>G, 5203G>A, 5204A>T, 5205G>C, 5209G>T, 5211T>A, 5215T>C, 5219G>A, 5221G>A, 5222G>A, 5223T>G, 5228G>T, 5229A>T, 5230A>T, 5234G>T, 5235A>C, 5236G>T, 5237T>A, 5238C>A, 5239A>T, 5245A>T, 5246T>A, 5247G>A, 5248G>A, 5251A>G, 5252G>A, 5254G>A, 5258G>A, 5259T>G, 5260G>A, 5261G>A, 5262T>C, 5263A>C, 5265C>A, 5266A>T, 5267G>C, 5268G>A, 5272A>G, 5273T>C, 5274T>A, 5275A>C, 5278C>T, 5283G>T, 5284G>C, 5285C>T, 5287G>A, 5290T>G, 5296A>C, 5302G>C, 5305A>T, 5307G>C, 5308A>C, 5314A>C, 5317A>C, 5320A>C, 5323C>A, 5326C>T, 5329A>G								

CDS

	Begin	End	Coverage	Score	Concordance	Matches	Identities	I/D/M/F*	Stop Codons
ORF3	1308	1390	4.5%	329	56.4%	83 (100%)	46 (55.4%)	0/0/0/0	0
Protein mutations:	L1309V (5087C>G), A1313E (5100C>A), M1316T (5109T>C 5110G>C), H1319V (5117C>G 5118A>T), Q1322K (5126C>A), Y1323F (5130A>T), S1324P (5132T>C 5134C>A), L1325I (5135C>A 5137C>T), G1327V (5142G>T 5143A>T), T1329K (5148C>A 5149A>G), S1330E (5150T>G 5151C>A 5152C>A), I1331L (5153A>T 5155C>A), I1332L (5156A>T 5158C>A), K1333D (5159A>G 5161G>T), R1334E (5162A>G 5163G>A 5164A>G), V1335L (5165G>T 5167T>G), A1336H (5168G>C 5169C>A 5170A>T), S1338A (5174T>G 5176A>C), V1340Y (5180G>T 5181T>A 5182C>T), F1344L (5192T>C 5194T>A), K1347R (5202A>G 5203G>A), F1350Y (5211T>A), V1353I (5219G>A 5221G>A), V1354R (5222G>A 5223T>G), E1356F (5228G>T 5229A>T 5230A>T), E1358S (5234G>T 5235A>C 5236G>T), S1359N (5237T>A 5238C>A 5239A>T), W1362K (5246T>A 5247G>A 5248G>A), A1364T (5252G>A 5254G>A), V1366R (5258G>A 5259T>G 5260G>A), V1367T (5261G>A 5262T>C 5263A>C), P1368H (5265C>A 5266A>T), G1369Q (5267G>C 5268G>A), L1371H (5273T>C 5274T>A 5275A>C), W1374F (5283G>T 5284G>C), R1382T (5307G>C 5308A>C)								
Codon mutations:	CTA1309GTA (5087C>G), GAT1310GAC (5092T>C), AGA1312AGG (5098A>G), GCA1313GAA (5100C>A), CTC1314TTA (5102C>T 5104C>A), ATG1316ACC (5109T>C 5110G>C), AAT1317AAC (5113T>C), ACC1318ACT (5116C>T), CAC1319GTC (5117C>G 5118A>T), AAA1320AAG (5122A>G), GAC1321GAT (5125C>T), CAG1322AAG (5126C>A), TAT1323TTT (5130A>T), TCC1324CCA (5132T>C 5134C>A), CTC1325ATT (5135C>A 5137C>T), GGA1327GTT (5142G>T 5143A>T), ATA1328ATT (5146A>T), ACA1329AAG (5148C>A 5149A>G), TCC1330GAA (5150T>G 5151C>A 5152C>A), ATC1331TTA (5153A>T 5155C>A), ATC1332TTA (5156A>T 5158C>A), AAG1333GAT (5159A>G 5161G>T), AGA1334GAG (5162A>G 5163G>A 5164A>G), GTT1335TTG (5165G>T 5167T>G), GCA1336CAT (5168G>C 5169C>A 5170A>T), TCA1338GCC (5174T>G 5176A>C), GTC1340TAT (5180G>T 5181T>A 5182C>T), TCC1342TCT (5188C>T), TTT1344CTA (5192T>C 5194T>A), GAT1345GAC (5197T>C), TTA1346TTG (5200A>G), AAG1347AGA (5202A>G 5203G>A), AGT1348TCT (5204A>T 5205G>C), GGG1349GGT (5209G>T), TTC1350TAC (5211T>A), CAT1351CAC (5215T>C), GTG1353ATA (5219G>A 5221G>A), GTA1354AGA (5222G>A 5223T>G), GAA1356TTT (5228G>T 5229A>T 5230A>T), GAG1358TCT (5234G>T 5235A>C 5236G>T), TCA1359AAT (5237T>A 5238C>A 5239A>T), CCA1361CCT (5245A>T), TGG1362AAA (5246T>A 5247G>A 5248G>A), ACA1363ACG (5251A>G), GCG1364ACA (5252G>A 5254G>A), GTG1366AGA (5258G>A 5259T>G 5260G>A), GTA1367ACC (5261G>A 5262T>C 5263A>C), CCA1368CAT (5265C>A 5266A>T), GGA1369CAA (5267G>C 5268G>A), GGA1370GGG (5272A>G), TTA1371CAC (5273T>C 5274T>A 5275A>C), TAC1372TAT (5278C>T), TGG1374TTC (5283G>T 5284G>C), CTG1375TTA (5285C>T 5287G>A), GTT1376GTG (5290T>G), CCA1378CCC (5296A>C), GGG1380GGC (5302G>C), CTA1381CTT (5305A>T), AGA1382ACC (5307G>C 5308A>C), GCA1384GCC (5314A>C), CCA1385CCC (5317A>C), TCA1386TCC (5320A>C), ACC1387ACA (5323C>A), TTC1388TTT (5326C>T), CAA1389CAG (5329A>G)								

Proteins

	Begin	End	Coverage	Score	Concordance	Matches	Identities	I/D/M/F*	Stop Codons
polyprotein (YP_010800602.1)	1308	1390	4.5%	329	56.4%	83 (100%)	46 (55.4%)	0/0/0/0	0
Protein mutations:	L1309V (5087C>G), A1313E (5100C>A), M1316T (5109T>C 5110G>C), H1319V (5117C>G 5118A>T), Q1322K (5126C>A), Y1323F (5130A>T), S1324P (5132T>C 5134C>A), L1325I (5135C>A 5137C>T), G1327V (5142G>T 5143A>T), T1329K (5148C>A 5149A>G), S1330E (5150T>G 5151C>A 5152C>A), I1331L (5153A>T 5155C>A), I1332L (5156A>T 5158C>A), K1333D (5159A>G 5161G>T), R1334E (5162A>G 5163G>A 5164A>G), V1335L (5165G>T 5167T>G), A1336H (5168G>C 5169C>A 5170A>T), S1338A (5174T>G 5176A>C), V1340Y (5180G>T 5181T>A 5182C>T), F1344L (5192T>C 5194T>A), K1347R (5202A>G 5203G>A), F1350Y (5211T>A), V1353I (5219G>A 5221G>A), V1354R (5222G>A 5223T>G), E1356F (5228G>T 5229A>T 5230A>T), E1358S (5234G>T 5235A>C 5236G>T), S1359N (5237T>A 5238C>A 5239A>T), W1362K (5246T>A 5247G>A 5248G>A), A1364T (5252G>A 5254G>A), V1366R (5258G>A 5259T>G 5260G>A), V1367T (5261G>A 5262T>C 5263A>C), P1368H (5265C>A 5266A>T), G1369Q (5267G>C 5268G>A), L1371H (5273T>C 5274T>A 5275A>C), W1374F (5283G>T 5284G>C), R1382T (5307G>C 5308A>C)								
Codon mutations:	CTA1309GTA (5087C>G), GAT1310GAC (5092T>C), AGA1312AGG (5098A>G), GCA1313GAA (5100C>A), CTC1314TTA (5102C>T 5104C>A), ATG1316ACC (5109T>C 5110G>C), AAT1317AAC (5113T>C), ACC1318ACT (5116C>T), CAC1319GTC (5117C>G 5118A>T), AAA1320AAG (5122A>G), GAC1321GAT (5125C>T), CAG1322AAG (5126C>A), TAT1323TTT (5130A>T), TCC1324CCA (5132T>C 5134C>A), CTC1325ATT (5135C>A 5137C>T), GGA1327GTT (5142G>T 5143A>T), ATA1328ATT (5146A>T), ACA1329AAG (5148C>A 5149A>G), TCC1330GAA (5150T>G 5151C>A 5152C>A), ATC1331TTA (5153A>T 5155C>A), ATC1332TTA (5156A>T 5158C>A), AAG1333GAT (5159A>G 5161G>T), AGA1334GAG (5162A>G 5163G>A 5164A>G), GTT1335TTG (5165G>T 5167T>G), GCA1336CAT (5168G>C 5169C>A 5170A>T), TCA1338GCC (5174T>G 5176A>C), GTC1340TAT (5180G>T 5181T>A 5182C>T), TCC1342TCT (5188C>T), TTT1344CTA (5192T>C 5194T>A), GAT1345GAC (5197T>C), TTA1346TTG (5200A>G), AAG1347AGA (5202A>G 5203G>A), AGT1348TCT (5204A>T 5205G>C), GGG1349GGT (5209G>T), TTC1350TAC (5211T>A), CAT1351CAC (5215T>C), GTG1353ATA (5219G>A 5221G>A), GTA1354AGA (5222G>A 5223T>G), GAA1356TTT (5228G>T 5229A>T 5230A>T), GAG1358TCT (5234G>T 5235A>C 5236G>T), TCA1359AAT (5237T>A 5238C>A 5239A>T), CCA1361CCT (5245A>T), TGG1362AAA (5246T>A 5247G>A 5248G>A), ACA1363ACG (5251A>G), GCG1364ACA (5252G>A 5254G>A), GTG1366AGA (5258G>A 5259T>G 5260G>A), GTA1367ACC (5261G>A 5262T>C 5263A>C), CCA1368CAT (5265C>A 5266A>T), GGA1369CAA (5267G>C 5268G>A), GGA1370GGG (5272A>G), TTA1371CAC (5273T>C 5274T>A 5275A>C), TAC1372TAT (5278C>T), TGG1374TTC (5283G>T 5284G>C), CTG1375TTA (5285C>T 5287G>A), GTT1376GTG (5290T>G), CCA1378CCC (5296A>C), GGG1380GGC (5302G>C), CTA1381CTT (5305A>T), AGA1382ACC (5307G>C 5308A>C), GCA1384GCC (5314A>C), CCA1385CCC (5317A>C), TCA1386TCC (5320A>C), ACC1387ACA (5323C>A), TTC1388TTT (5326C>T), CAA1389CAG (5329A>G)								

*: Inserts / Deletes / Misaligned / Frameshifts

Analysis details

This analysis was performed with panviral2.64

NGS Details (UN70): Caulimovirus tessellomirabilis

Assembly

**Coverage Length**	318 (1 contig(s))
**Depth Of Coverage**	3.1
**Number Of Reads**	7
**Reads Per Million**	0.13 rpm (after QC)
**Ambiguities**	0
**Assembly Method**	de novo + reference guided assembly
**Consensus Caller**	Bcf Tools

Coverage Map

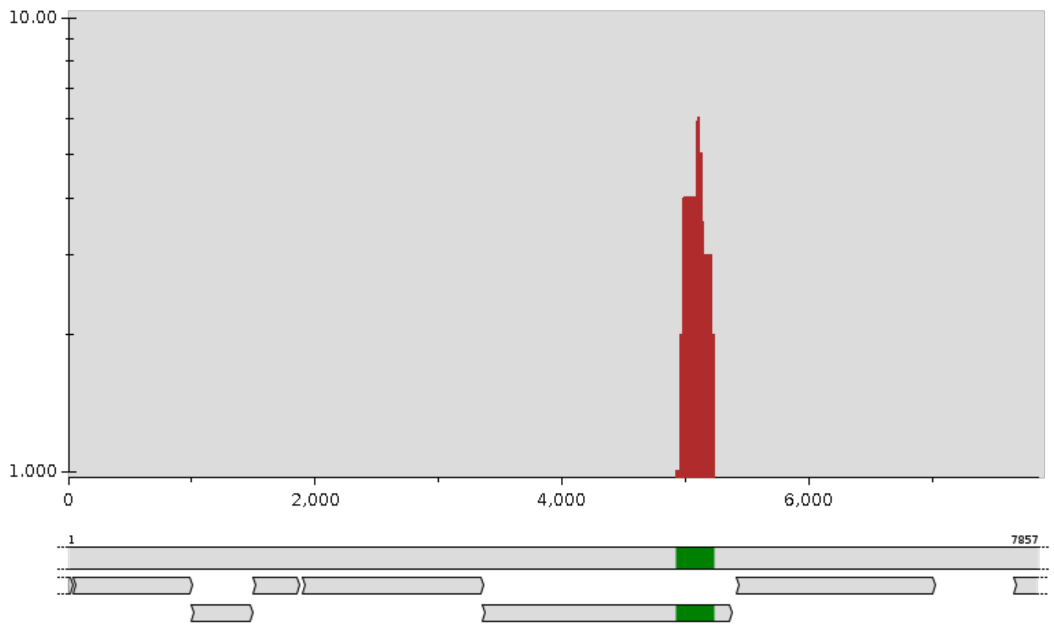

Assignment

**Type**	Caulimovirus tessellomirabilis (Taxonomy ID: 3048203)
**Reference Genome**	NC_004036.1
**NT Identity (%)**	58.7459
**AA Identity (%)**	49.505
**Number Of Stop Codons**	0
**Number Of CDS**	7

Alignment

**Alignment Score**	64.0 (NT) + 343.0 (AA) = 407.0
**Concordance (%)**	33.1164

Alignment Method	Global, seeded, nucleotide + amino acids (AGA)

Genome Region

Sequence starts at position 4922 and ends at position 5239 relative to NC_004036.1 reference sequence.

Alignment Detailed Statistics

	Begin	End	Coverage	Score	Concordance	Matches	Identities	I/D/M/F*	Stop Codons
**NT**	**4922**	**5239**	**4.0%**	**64**	**11.3%**	**303 (95.3%)**	**178 (56.0%)**	**0/15**	
Mutations:	4928A>G, 4933T>G, 4934T>C, 4942A>G, 4946T>A, 4947A>G, 4948T>G, 4955A>G, 4957G>T, 4958A>C, 4959A>C, 4968G>A, 4970T>C, 4972A>G, 4973A>G, 4975T>A, 4976A>T, 4978T>A, 4979G>C, 4984T>A, 4985G>A, 4989C>T, 4990T>C, 4993T>C, 4997C>T, 4999C>T, 5000T>C, 5001T>A, 5002C>T, 5008T>A, 5010G>C, 5012G>T, 5014C>G, 5017T>A, 5018_5023delAAGGCC, 5029C>A, 5030A>G, 5031C>A, 5032T>C, 5033G>C, 5034A>T, 5036G>A, 5037G>A, 5038T>C, 5042_5047delGAATTA, 5050C>T, 5053C>T, 5060T>A, 5062T>A, 5063T>A, 5064C>G, 5065A>T, 5071A>T, 5074C>T, 5077A>C, 5078C>G, 5079C>A, 5084G>A, 5087T>G, 5089G>T, 5095C>T, 5096C>T, 5097A>C, 5098T>C, 5100G>C, 5101T>A, 5107G>A, 5117C>T, 5122G>A, 5126A>G, 5127A>T, 5129C>A, 5130A>G, 5131G>A, 5133T>G, 5134G>A, 5137T>A, 5138A>C, 5139C>A, 5140T>A, 5147A>T, 5148G>C, 5149T>G, 5150A>G, 5151T>C, 5154A>G, 5155T>C, 5156C>T, 5159A>T, 5160C>T, 5161C>A, 5164T>A, 5165G>A, 5166T>A, 5167T>A, 5169G>C, 5170T>A, 5174A>G, 5175C>T, 5176A>T, 5177G>A, 5179C>T, 5181G>A, 5182G>A, 5192_5194delGTA, 5196A>C, 5199T>A, 5200T>A, 5201T>A, 5204A>T, 5205A>C, 5209A>G, 5212T>G, 5215G>A, 5216A>T, 5217A>T, 5221A>C, 5224G>A, 5225A>C, 5227T>C, 5228A>C, 5231G>T, 5232G>C, 5233T>C, 5236C>A, 5237A>C, 5238G>C, 5239T>C								

CDS

	Begin	End	Coverage	Score	Concordance	Matches	Identities	I/D/M/F*	Stop Codons
MiMVgp6	523	628	15.7%	343	51.6%	101 (95.3%)	50 (47.2%)	0/5/0/0	0
Protein mutations:	I525V (4928A>G), N526K (4933T>G), F527L (4934T>C), Y531R (4946T>A 4947A>G 4948T>G), K534D (4955A>G 4957G>T), K535P (4958A>C 4959A>C), S538N (4968G>A), I540V (4973A>G 4975T>A), I541L (4976A>T 4978T>A), E542Q (4979G>C), D544N (4985G>A), A545V (4989C>T 4990T>C), H548Y (4997C>T 4999C>T), F549H (5000T>C 5001T>A 5002C>T), G552A (5010G>C), V553L (5012G>T 5014C>G), K555_A556del (5018_5023delAAGGCC), T559D (5030A>G 5031C>A 5032T>C), E560L (5033G>C 5034A>T), G561N (5036G>A 5037G>A 5038T>C), E563_L564del (5042_5047delGAATTA), S569T (5060T>A 5062T>A), K574N (5077A>C), P575E (5078C>G 5079C>A), E577K (5084G>A), L578V (5087T>G 5089G>T), H581S (5096C>T 5097A>C 5098T>C), S582T (5100G>C 5101T>A), K591V (5126A>G 5127A>T), Q592R (5129C>A 5130A>G 5131G>A), V593G (5133T>G 5134G>A), T595Q (5138A>C 5139C>A 5140T>A), I599A (5150A>G 5151T>C), Y600C (5154A>G 5155T>C), T602L (5159A>T 5160C>T 5161C>A), V604K (5165G>A 5166T>A 5167T>A), C605S (5169G>C 5170T>A), T607V (5174A>G 5175C>T 5176A>T), V608I (5177G>A 5179C>T), R609K (5181G>A 5182G>A), V613del (5192_5194delGTA), N614T (5196A>C), L615Q (5199T>A 5200T>A), L616I (5201T>A), K617S (5204A>T 5205A>C), F619L (5212T>G), M620I (5215G>A), N621F (5216A>T 5217A>T), K622N (5221A>C), I624L (5225A>C 5227T>C), T625P (5228A>C), G626S (5231G>T 5232G>C 5233T>C), D627E (5236C>A), S628P (5237A>C 5238G>C 5239T>C)								
Codon mutations:	ATT525GTT (4928A>G), AAT526AAG (4933T>G), TTT527CTT (4934T>C), AAA529AAG (4942A>G), TAT531AGG (4946T>A 4947A>G 4948T>G), AAG534GAT (4955A>G 4957G>T), AAA535CCA (4958A>C 4959A>C), AGT538AAT (4968G>A), TTA539CTG (4970T>C 4972A>G), ATT540GTA (4973A>G 4975T>A), ATT541TTA (4976A>T 4978T>A), GAA542CAA (4979G>C), ACT543ACA (4984T>A), GAT544AAT (4985G>A), GCT545GTC (4989C>T 4990T>C), TCT546TCC (4993T>C), CAC548TAT (4997C>T 4999C>T), TTC549CAT (5000T>C 5001T>A 5002C>T), GGT551GGA (5008T>A), GGA552GCA (5010G>C), GTC553TTG (5012G>T 5014C>G), CTT554CTA (5017T>A), AAG555_GCC556del (5018_5023delAAGGCC), ACC558ACA (5029C>A), ACT559GAC (5030A>G 5031C>A 5032T>C), GAA560CTA (5033G>C 5034A>T), GGT561AAC (5036G>A 5037G>A 5038T>C), GAA563_TTA564del (5042_5047delGAATTA), ATC565ATT (5050C>T), TGC566TGT (5053C>T), TCT569ACA (5060T>A 5062T>A), TCA570AGT (5063T>A 5064C>G 5065A>T), ACA572ACT (5071A>T), TTC573TTT (5074C>T), AAA574AAC (5077A>C), CCA575GAA (5078C>G 5079C>A), GAA577AAA (5084G>A), TTG578GTT (5087T>G 5089G>T), TAC580TAT (5095C>T), CAT581TCC (5096C>T 5097A>C 5098T>C), AGT582ACA (5100G>C 5101T>A), GAG584GAA (5107G>A), CTA588TTA (5117C>T), GCG589GCA (5122G>A), AAA591GTA (5126A>G 5127A>T), CAG592AGA (5129C>A 5130A>G 5131G>A), GTG593GGA (5133T>G 5134G>A), ATT594ATA (5137T>A), ACT595CAA (5138A>C 5139C>A 5140T>A), AGT598TCG (5147A>T 5148G>C 5149T>G), ATT599GCT (5150A>G 5151T>C), TAT600TGC (5154A>G 5155T>C), CTA601TTA (5156C>T), ACC602TTA (5159A>T 5160C>T 5161C>A), CCT603CCA (5164T>A), GTT604AAA (5165G>A 5166T>A 5167T>A), TGT605TCA (5169G>C 5170T>A), ACA607GTT (5174A>G 5175C>T 5176A>T), GTC608ATT (5177G>A 5179C>T), AGG609AAA (5181G>A 5182G>A), GTA613del (5192_5194delGTA), AAT614ACT (5196A>C), CTT615CAA (5199T>A 5200T>A), TTA616ATA (5201T>A), AAA617TCA (5204A>T 5205A>C), GGA618GGG (5209A>G), TTT619TTG (5212T>G), ATG620ATA (5215G>A), AAT621TTT (5216A>T 5217A>T), AAA622AAC (5221A>C), AAG623AAA (5224G>A), ATT624CTC (5225A>C 5227T>C), ACT625CCT (5228A>C), GGT626TCC (5231G>T 5232G>C 5233T>C), GAC627GAA (5236C>A), AGT628CCC (5237A>C 5238G>C 5239T>C)								

Proteins

	Begin	End	Coverage	Score	Concordance	Matches	Identities	I/D/M/F*	Stop Codons
hypothetical protein (NP_659397.1)	523	628	15.7%	343	51.6%	101 (95.3%)	50 (47.2%)	0/5/0/0	0
Protein mutations:	I525V (4928A>G), N526K (4933T>G), F527L (4934T>C), Y531R (4946T>A 4947A>G 4948T>G), K534D (4955A>G 4957G>T), K535P (4958A>C 4959A>C), S538N (4968G>A), I540V (4973A>G 4975T>A), I541L (4976A>T 4978T>A), E542Q (4979G>C), D544N (4985G>A), A545V (4989C>T 4990T>C), H548Y (4997C>T 4999C>T), F549H (5000T>C 5001T>A 5002C>T), G552A (5010G>C), V553L (5012G>T 5014C>G), K555_A556del (5018_5023delAAGGCC), T559D (5030A>G 5031C>A 5032T>C), E560L (5033G>C 5034A>T), G561N (5036G>A 5037G>A 5038T>C), E563_L564del (5042_5047delGAATTA), S569T (5060T>A 5062T>A), K574N (5077A>C), P575E (5078C>G 5079C>A), E577K (5084G>A), L578V (5087T>G 5089G>T), H581S (5096C>T 5097A>C 5098T>C), S582T (5100G>C 5101T>A), K591V (5126A>G 5127A>T), Q592R (5129C>A 5130A>G 5131G>A), V593G (5133T>G 5134G>A), T595Q (5138A>C 5139C>A 5140T>A), I599A (5150A>G 5151T>C), Y600C (5154A>G 5155T>C), T602L (5159A>T 5160C>T 5161C>A), V604K (5165G>A 5166T>A 5167T>A), C605S (5169G>C 5170T>A), T607V (5174A>G 5175C>T 5176A>T), V608I (5177G>A 5179C>T), R609K (5181G>A 5182G>A), V613del (5192_5194delGTA), N614T (5196A>C), L615Q (5199T>A 5200T>A), L616I (5201T>A), K617S (5204A>T 5205A>C), F619L (5212T>G), M620I (5215G>A), N621F (5216A>T 5217A>T), K622N (5221A>C), I624L (5225A>C 5227T>C), T625P (5228A>C), G626S (5231G>T 5232G>C 5233T>C), D627E (5236C>A), S628P (5237A>C 5238G>C 5239T>C)								
Codon mutations:	ATT525GTT (4928A>G), AAT526AAG (4933T>G), TTT527CTT (4934T>C), AAA529AAG (4942A>G), TAT531AGG (4946T>A 4947A>G 4948T>G), AAG534GAT (4955A>G 4957G>T), AAA535CCA (4958A>C 4959A>C), AGT538AAT (4968G>A), TTA539CTG (4970T>C 4972A>G), ATT540GTA (4973A>G 4975T>A), ATT541TTA (4976A>T 4978T>A), GAA542CAA (4979G>C), ACT543ACA (4984T>A), GAT544AAT (4985G>A), GCT545GTC (4989C>T 4990T>C), TCT546TCC (4993T>C), CAC548TAT (4997C>T 4999C>T), TTC549CAT (5000T>C 5001T>A 5002C>T), GGT551GGA (5008T>A), GGA552GCA (5010G>C), GTC553TTG (5012G>T 5014C>G), CTT554CTA (5017T>A), AAG555_GCC556del (5018_5023delAAGGCC), ACC558ACA (5029C>A), ACT559GAC (5030A>G 5031C>A 5032T>C), GAA560CTA (5033G>C 5034A>T), GGT561AAC (5036G>A 5037G>A 5038T>C), GAA563_TTA564del (5042_5047delGAATTA), ATC565ATT (5050C>T), TGC566TGT (5053C>T), TCT569ACA (5060T>A 5062T>A), TCA570AGT (5063T>A 5064C>G 5065A>T), ACA572ACT (5071A>T), TTC573TTT (5074C>T), AAA574AAC (5077A>C), CCA575GAA (5078C>G 5079C>A), GAA577AAA (5084G>A), TTG578GTT (5087T>G 5089G>T), TAC580TAT (5095C>T), CAT581TCC (5096C>T 5097A>C 5098T>C), AGT582ACA (5100G>C 5101T>A), GAG584GAA (5107G>A), CTA588TTA (5117C>T), GCG589GCA (5122G>A), AAA591GTA (5126A>G 5127A>T), CAG592AGA (5129C>A 5130A>G 5131G>A), GTG593GGA (5133T>G 5134G>A), ATT594ATA (5137T>A), ACT595CAA (5138A>C 5139C>A 5140T>A), AGT598TCG (5147A>T 5148G>C 5149T>G), ATT599GCT (5150A>G 5151T>C), TAT600TGC (5154A>G 5155T>C), CTA601TTA (5156C>T), ACC602TTA (5159A>T 5160C>T 5161C>A), CCT603CCA (5164T>A), GTT604AAA (5165G>A 5166T>A 5167T>A), TGT605TCA (5169G>C 5170T>A), ACA607GTT (5174A>G 5175C>T 5176A>T), GTC608ATT (5177G>A 5179C>T), AGG609AAA (5181G>A 5182G>A), GTA613del (5192_5194delGTA), AAT614ACT (5196A>C), CTT615CAA (5199T>A 5200T>A), TTA616ATA (5201T>A), AAA617TCA (5204A>T 5205A>C), GGA618GGG (5209A>G), TTT619TTG (5212T>G), ATG620ATA (5215G>A), AAT621TTT (5216A>T 5217A>T), AAA622AAC (5221A>C), AAG623AAA (5224G>A), ATT624CTC (5225A>C 5227T>C), ACT625CCT (5228A>C), GGT626TCC (5231G>T 5232G>C 5233T>C), GAC627GAA (5236C>A), AGT628CCC (5237A>C 5238G>C 5239T>C)								

*: Inserts / Deletes / Misaligned / Frameshifts

Analysis details

This analysis was performed with panviral2.64

NGS Details (UN70): Badnavirus alphacolocalasiae

Assembly

**Coverage Length**	288 (1 contig(s))
**Depth Of Coverage**	2.3
**Number Of Reads**	5
**Reads Per Million**	0.09 rpm (after QC)
**Ambiguities**	0
**Assembly Method**	de novo + reference guided assembly
**Consensus Caller**	Bcf Tools

Coverage Map

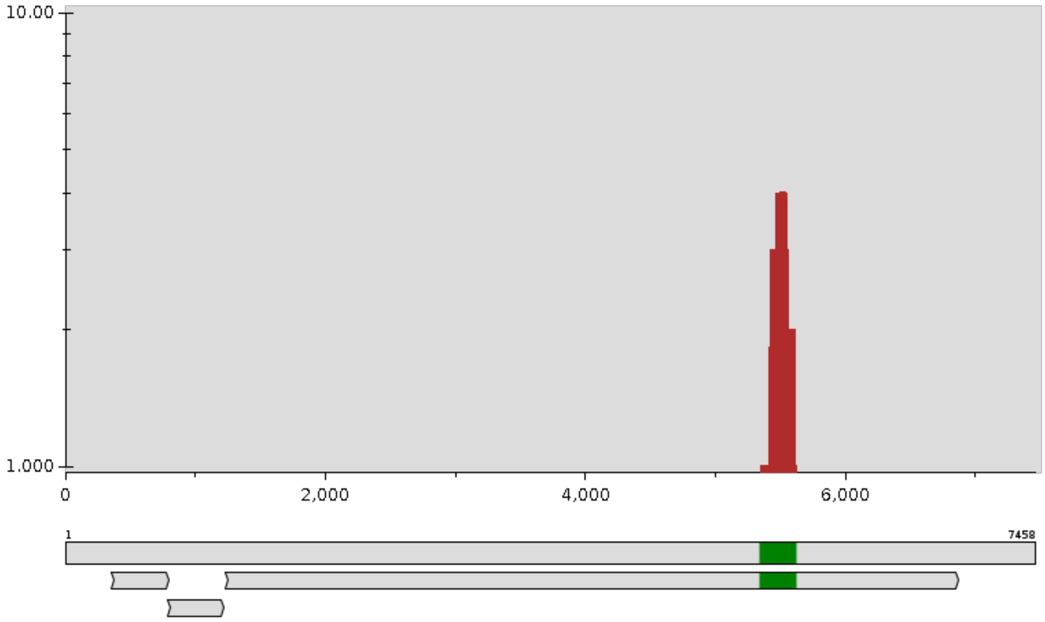

Assignment

**Type**	Badnavirus alphacolocalasiae (Taxonomy ID: 3048356)
**Reference Genome**	NC_004450.1
**NT Identity (%)**	58.885
**AA Identity (%)**	52.0833
**Number Of Stop Codons**	0
**Number Of CDS**	3

Alignment

**Alignment Score**	92.0 (NT) + 244.0 (AA) = 336.0
**Concordance (%)**	31.3984

Alignment Method	Global, seeded, nucleotide + amino acids (AGA)

Genome Region

Sequence starts at position 5343 and ends at position 5630 relative to NC_004450.1 reference sequence.

Alignment Detailed Statistics

	Begin	End	Coverage	Score	Concordance	Matches	Identities	I/D/M/F*	Stop Codons
**NT**	**5343**	**5630**	**3.9%**	**92**	**16.3%**	**287 (99.7%)**	**169 (58.7%)**	**0/1**	
Mutations:	5347T>A, 5349A>C, 5359A>G, 5360G>A, 5361G>A, 5362T>C, 5363G>T, 5364A>T, 5366C>T, 5369T>A, 5371G>C, 5374C>A, 5378C>T, 5381T>C, 5382T>A, 5383C>G, 5394C>A, 5402T>A, 5408T>C, 5409C>T, 5410A>G, 5411C>G, 5415G>A, 5418G>C, 5419C>A, 5420T>A, 5423G>T, 5424G>T, 5425A>C, 5426T>G, 5427C>A, 5428C>A, 5429C>A, 5430G>A, 5432T>A, 5433T>G, 5434C>A, 5436G>A, 5437T>A, 5438G>A, 5439G>T, 5441A>T, 5442T>A, 5443G>A, 5444G>A, 5447A>T, 5454C>A, 5455T>A, 5456A>T, 5459T>C, 5462A>G, 5463C>T, 5464A>T, 5465A>T, 5468T>A, 5469T>C, 5470T>A, 5481C>A, 5482T>A, 5483G>T, 5486A>T, 5492T>A, 5498C>A, 5501C>A, 5507T>C, 5510T>C, 5513A>T, 5514G>T, 5515C>T, 5518T>A, 5519A>G, 5522T>C, 5527G>A, 5528A>C, 5530A>T, 5531A>C, 5534G>A, 5535G>A, 5538G>A, 5539C>A, 5540A>T, 5541G>A, 5543A>T, 5549A>C, 5550G>C, 5551G>A, 5552G>T, 5553T>A, 5554G>T, 5555T>C, 5557A>C, 5560A>T, 5564C>T, 5567C>A, 5568delG, 5569C>T, 5570A>C, 5573C>A, 5586A>G, 5588T>A, 5591G>A, 5592G>A, 5597T>C, 5600A>T, 5603C>G, 5604A>G, 5606T>C, 5607G>A, 5608A>T, 5609G>A, 5612A>T, 5613G>T, 5614A>C, 5618T>C, 5619G>T, 5620C>T, 5621A>T, 5627C>T, 5628C>T								

CDS

	Begin	End	Coverage	Score	Concordance	Matches	Identities	I/D/M/F*	Stop Codons
TaBVgp3	1373	1468	5.1%	244	36.3%	96 (100%)	50 (52.1%)	0/0/1/1	0
Protein mutations:	L1374H (5347T>A), I1375L (5349A>C), K1378R (5359A>G 5360G>A), V1379T (5361G>A 5362T>C 5363G>T), I1380F (5364A>T 5366C>T), N1381K (5369T>A), S1382T (5371G>C), T1383N (5374C>A), L1390M (5394C>A), H1395W (5409C>T 5410A>G 5411C>G), V1397I (5415G>A), A1398Q (5418G>C 5419C>A 5420T>A), M1399I (5423G>T), D1400S (5424G>T 5425A>C 5426T>G), P1401K (5427C>A 5428C>A 5429C>A), D1402K (5430G>A 5432T>A), S1403D (5433T>G 5434C>A), V1404K (5436G>A 5437T>A 5438G>A), E1405Y (5439G>T 5441A>T), W1406K (5442T>A 5443G>A 5444G>A), L1410N (5454C>A 5455T>A 5456A>T), Q1413F (5463C>T 5464A>T 5465A>T), L1415Q (5469T>C 5470T>A), L1419N (5481C>A 5482T>A 5483G>T), A1430F (5514G>T 5515C>T), V1431E (5518T>A 5519A>G), R1434N (5527G>A 5528A>C), K1435I (5530A>T 5531A>C), M1436I (5534G>A), D1437N (5535G>A), A1438N (5538G>A 5539C>A 5540A>T), V1439I (5541G>A 5543A>T), K1441N (5549A>C), G1442H (5550G>C 5551G>A 5552G>T), C1443I (5553T>A 5554G>T 5555T>C), E1444A (5557A>C), K1445I (5560A>T), I1454V (5586A>G 5588T>A), V1456I (5592G>A), N1459K (5603C>G), N1460D (5604A>G 5606T>C), E1461I (5607G>A 5608A>T 5609G>A), E1462D (5612A>T), D1463S (5613G>T 5614A>C), A1465F (5619G>T 5620C>T 5621A>T)								
Codon mutations:	CTT1374CAT (5347T>A), ATT1375CTT (5349A>C), AAG1378AGA (5359A>G 5360G>A), GTG1379ACT (5361G>A 5362T>C 5363G>T), ATC1380TTT (5364A>T 5366C>T), AAT1381AAA (5369T>A), AGC1382ACC (5371G>C), ACT1383AAT (5374C>A), ATC1384ATT (5378C>T), TAT1385TAC (5381T>C), TCT1386AGT (5382T>A 5383C>G), CTG1390ATG (5394C>A), TCT1392TCA (5402T>A), TTT1394TTC (5408T>C), CAC1395TGG (5409C>T 5410A>G 5411C>G), GTT1397ATT (5415G>A), GCT1398CAA (5418G>C 5419C>A 5420T>A), ATG1399ATT (5423G>T), GAT1400TCG (5424G>T 5425A>C 5426T>G), CCC1401AAA (5427C>A 5428C>A 5429C>A), GAT1402AAA (5430G>A 5432T>A), TCT1403GAT (5433T>G 5434C>A), GTG1404AAA (5436G>A 5437T>A 5438G>A), GAA1405TAT (5439G>T 5441A>T), TGG1406AAA (5442T>A 5443G>A 5444G>A), ACA1407ACT (5447A>T), CTA1410AAT (5454C>A 5455T>A 5456A>T), GTT1411GTC (5459T>C), CCA1412CCG (5462A>G), CAA1413TTT (5463C>T 5464A>T 5465A>T), GGT1414GGA (5468T>A), TTA1415CAA (5469T>C 5470T>A), CTG1419AAT (5481C>A 5482T>A 5483G>T), GCA1420GCT (5486A>T), CCT1422CCA (5492T>A), GGC1424GGA (5498C>A), CTC1425CTA (5501C>A), AAT1427AAC (5507T>C), GCT1428GCC (5510T>C), CCA1429CCT (5513A>T), GCC1430TTC (5514G>T 5515C>T), GTA1431GAG (5518T>A 5519A>G), TTT1432TTC (5522T>C), AGA1434AAC (5527G>A 5528A>C), AAA1435ATC (5530A>T 5531A>C), ATG1436ATA (5534G>A), GAT1437AAT (5535G>A), GCA1438AAT (5538G>A 5539C>A 5540A>T), GTA1439ATT (5541G>A 5543A>T), AAA1441AAC (5549A>C), GGG1442CAT (5550G>C 5551G>A 5552G>T), TGT1443ATC (5553T>A 5554G>T 5555T>C), GAA1444GCA (5557A>C), AAA1445ATA (5560A>T), TTC1446TTT (5564C>T), CTC1447CTA (5567C>A), GCA1448-TC (5568delG 5569C>T 5570A>C), GTC1449GTA (5573C>A), ATT1454GTA (5586A>G 5588T>A), CTG1455CTA (5591G>A), GTA1456ATA (5592G>A), TTT1457TTC (5597T>C), TCA1458TCT (5600A>T), AAC1459AAG (5603C>G), AAT1460GAC (5604A>G 5606T>C), GAG1461ATA (5607G>A 5608A>T 5609G>A), GAA1462GAT (5612A>T), GAT1463TCT (5613G>T 5614A>C), CAT1464CAC (5618T>C), GCA1465TTT (5619G>T 5620C>T 5621A>T), CAC1467CAT (5627C>T), CTG1468TTG (5628C>T)								

Proteins

	Begin	End	Coverage	Score	Concordance	Matches	Identities	I/D/M/F*	Stop Codons
polyprotein (NP_758808.1)	1373	1468	5.1%	244	36.3%	96 (100%)	50 (52.1%)	0/0/1/1	0
Protein mutations:	L1374H (5347T>A), I1375L (5349A>C), K1378R (5359A>G 5360G>A), V1379T (5361G>A 5362T>C 5363G>T), I1380F (5364A>T 5366C>T), N1381K (5369T>A), S1382T (5371G>C), T1383N (5374C>A), L1390M (5394C>A), H1395W (5409C>T 5410A>G 5411C>G), V1397I (5415G>A), A1398Q (5418G>C 5419C>A 5420T>A), M1399I (5423G>T), D1400S (5424G>T 5425A>C 5426T>G), P1401K (5427C>A 5428C>A 5429C>A), D1402K (5430G>A 5432T>A), S1403D (5433T>G 5434C>A), V1404K (5436G>A 5437T>A 5438G>A), E1405Y (5439G>T 5441A>T), W1406K (5442T>A 5443G>A 5444G>A), L1410N (5454C>A 5455T>A 5456A>T), Q1413F (5463C>T 5464A>T 5465A>T), L1415Q (5469T>C 5470T>A), L1419N (5481C>A 5482T>A 5483G>T), A1430F (5514G>T 5515C>T), V1431E (5518T>A 5519A>G), R1434N (5527G>A 5528A>C), K1435I (5530A>T 5531A>C), M1436I (5534G>A), D1437N (5535G>A), A1438N (5538G>A 5539C>A 5540A>T), V1439I (5541G>A 5543A>T), K1441N (5549A>C), G1442H (5550G>C 5551G>A 5552G>T), C1443I (5553T>A 5554G>T 5555T>C), E1444A (5557A>C), K1445I (5560A>T), I1454V (5586A>G 5588T>A), V1456I (5592G>A), N1459K (5603C>G), N1460D (5604A>G 5606T>C), E1461I (5607G>A 5608A>T 5609G>A), E1462D (5612A>T), D1463S (5613G>T 5614A>C), A1465F (5619G>T 5620C>T 5621A>T)								
Codon mutations:	CTT1374CAT (5347T>A), ATT1375CTT (5349A>C), AAG1378AGA (5359A>G 5360G>A), GTG1379ACT (5361G>A 5362T>C 5363G>T), ATC1380TTT (5364A>T 5366C>T), AAT1381AAA (5369T>A), AGC1382ACC (5371G>C), ACT1383AAT (5374C>A), ATC1384ATT (5378C>T), TAT1385TAC (5381T>C), TCT1386AGT (5382T>A 5383C>G), CTG1390ATG (5394C>A), TCT1392TCA (5402T>A), TTT1394TTC (5408T>C), CAC1395TGG (5409C>T 5410A>G 5411C>G), GTT1397ATT (5415G>A), GCT1398CAA (5418G>C 5419C>A 5420T>A), ATG1399ATT (5423G>T), GAT1400TCG (5424G>T 5425A>C 5426T>G), CCC1401AAA (5427C>A 5428C>A 5429C>A), GAT1402AAA (5430G>A 5432T>A), TCT1403GAT (5433T>G 5434C>A), GTG1404AAA (5436G>A 5437T>A 5438G>A), GAA1405TAT (5439G>T 5441A>T), TGG1406AAA (5442T>A 5443G>A 5444G>A), ACA1407ACT (5447A>T), CTA1410AAT (5454C>A 5455T>A 5456A>T), GTT1411GTC (5459T>C), CCA1412CCG (5462A>G), CAA1413TTT (5463C>T 5464A>T 5465A>T), GGT1414GGA (5468T>A), TTA1415CAA (5469T>C 5470T>A), CTG1419AAT (5481C>A 5482T>A 5483G>T), GCA1420GCT (5486A>T), CCT1422CCA (5492T>A), GGC1424GGA (5498C>A), CTC1425CTA (5501C>A), AAT1427AAC (5507T>C), GCT1428GCC (5510T>C), CCA1429CCT (5513A>T), GCC1430TTC (5514G>T 5515C>T), GTA1431GAG (5518T>A 5519A>G), TTT1432TTC (5522T>C), AGA1434AAC (5527G>A 5528A>C), AAA1435ATC (5530A>T 5531A>C), ATG1436ATA (5534G>A), GAT1437AAT (5535G>A), GCA1438AAT (5538G>A 5539C>A 5540A>T), GTA1439ATT (5541G>A 5543A>T), AAA1441AAC (5549A>C), GGG1442CAT (5550G>C 5551G>A 5552G>T), TGT1443ATC (5553T>A 5554G>T 5555T>C), GAA1444GCA (5557A>C), AAA1445ATA (5560A>T), TTC1446TTT (5564C>T), CTC1447CTA (5567C>A), GCA1448-TC (5568delG 5569C>T 5570A>C), GTC1449GTA (5573C>A), ATT1454GTA (5586A>G 5588T>A), CTG1455CTA (5591G>A), GTA1456ATA (5592G>A), TTT1457TTC (5597T>C), TCA1458TCT (5600A>T), AAC1459AAG (5603C>G), AAT1460GAC (5604A>G 5606T>C), GAG1461ATA (5607G>A 5608A>T 5609G>A), GAA1462GAT (5612A>T), GAT1463TCT (5613G>T 5614A>C), CAT1464CAC (5618T>C), GCA1465TTT (5619G>T 5620C>T 5621A>T), CAC1467CAT (5627C>T), CTG1468TTG (5628C>T)								

*: Inserts / Deletes / Misaligned / Frameshifts

Analysis details

This analysis was performed with panviral2.64

NGS Details (UN70): Caulimovirus venafragariae

Assembly

**Coverage Length**	262 (1 contig(s))
**Depth Of Coverage**	1.6
**Number Of Reads**	4
**Reads Per Million**	0.08 rpm (after QC)
**Ambiguities**	0
**Assembly Method**	de novo + reference guided assembly
**Consensus Caller**	Bcf Tools

Coverage Map

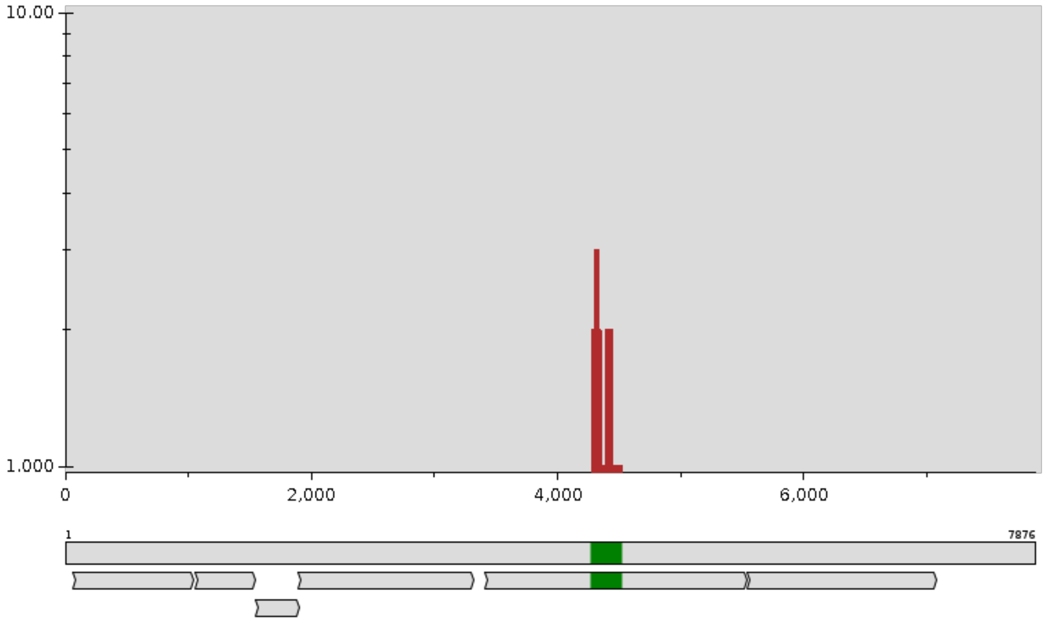

Assignment

**Type**	Caulimovirus venafragariae (Taxonomy ID: 3048344)
**Reference Genome**	NC_001725.1
**NT Identity (%)**	62.5954
**AA Identity (%)**	60.9195
**Number Of Stop Codons**	0
**Number Of CDS**	6

Alignment

**Alignment Score**	132.0 (NT) + 378.0 (AA) = 510.0
**Concordance (%)**	45.9046

Alignment Method	Global, seeded, nucleotide + amino acids (AGA)

Genome Region

Sequence starts at position 4268 and ends at position 4529 relative to NC_001725.1 reference sequence.

Alignment Detailed Statistics

	Begin	End	Coverage	Score	Concordance	Matches	Identities	I/D/M/F*	Stop Codons
**NT**	**4268**	**4529**	**3.3%**	**132**	**25.2%**	**262 (100%)**	**164 (62.6%)**	**0/0**	
Mutations:	4268C>A, 4276C>G, 4277G>T, 4284G>T, 4289A>G, 4290A>C, 4292C>A, 4296G>A, 4299C>T, 4300G>A, 4311C>A, 4314G>C, 4315C>T, 4317T>A, 4320T>A, 4321A>G, 4323C>T, 4326C>A, 4330C>A, 4331C>G, 4332T>A, 4333A>T, 4334G>C, 4335C>T, 4337A>C, 4338G>A, 4339A>T, 4340G>C, 4341T>A, 4342C>A, 4343C>A, 4348T>A, 4349C>G, 4350C>A, 4354C>G, 4362C>T, 4371G>A, 4378G>A, 4379C>G, 4383G>A, 4386C>A, 4387A>G, 4388A>T, 4390C>A, 4395C>T, 4402A>C, 4403G>A, 4408G>A, 4413T>A, 4419T>C, 4424A>G, 4425G>A, 4426T>C, 4428A>T, 4434C>T, 4435C>A, 4437T>C, 4440A>T, 4442A>G, 4443G>A, 4444G>A, 4445G>C, 4446A>G, 4447G>T, 4451G>C, 4452C>T, 4455C>T, 4456C>A, 4457T>A, 4458A>G, 4461T>A, 4464T>A, 4465A>G, 4467C>T, 4470G>A, 4471G>A, 4472A>G, 4473G>T, 4474C>G, 4477C>T, 4479G>A, 4480C>A, 4482T>A, 4483C>A, 4485A>T, 4491C>A, 4492G>C, 4493G>A, 4503C>A, 4504T>G, 4506T>A, 4508A>T, 4510T>A, 4511C>A, 4513T>A, 4514C>A, 4515C>A, 4521C>T								
**CDS**									
ORF_V	289	375	12.3%	378	62.8%	87 (100%)	53 (60.9%)	0/0/0/0	0
Protein mutations:	R291V (4276C>G 4277G>T), E293D (4284G>T), K295S (4289A>G 4290A>C), T296N (4292C>A), E299K (4300G>A), K303N (4314G>C), I306V (4321A>G 4323C>T), P309R (4330C>A 4331C>G 4332T>A), K311T (4337A>C 4338G>A), P313K (4342C>A 4343C>A), S315R (4348T>A 4349C>G 4350C>A), P317A (4354C>G), A325S (4378G>A 4379C>G), K328V (4387A>G 4388A>T), R333Q (4402A>C 4403G>A), V335I (4408G>A), K340R (4424A>G 4425G>A), H344N (4435C>A 4437T>C), K346R (4442A>G 4443G>A), G347T (4444G>A 4445G>C 4446A>G), D348Y (4447G>T), G349A (4451G>C 4452C>T), L351K (4456C>A 4457T>A 4458A>G), N354D (4465A>G 4467C>T), E356S (4471G>A 4472A>G 4473G>T), Q357E (4474C>G), L359I (4480C>A 4482T>A), Q360N (4483C>A 4485A>T), G363Q (4492G>C 4493G>A), F367V (4504T>G 4506T>A), Y368F (4508A>T), S369N (4510T>A 4511C>A), S370K (4513T>A 4514C>A 4515C>A)								
Codon mutations:	CCT288.AT (4268C>A), CGA291GTA (4276C>G 4277G>T), GAG293GAT (4284G>T), AAA295AGC (4289A>G 4290A>C), ACT296AAT (4292C>A), CAG297CAA (4296G>A), ATC298ATT (4299C>T), GAA299AAA (4300G>A), CTC302CTA (4311C>A), AAG303AAC (4314G>C), CTT304TTA (4315C>T 4317T>A), GGT305GGA (4320T>A), ATC306GTT (4321A>G 4323C>T), ATC307ATA (4326C>A), CCT309AGA (4330C>A 4331C>G 4332T>A), AGC310TCT (4333A>T 4334G>C 4335C>T), AAG311ACA (4337A>C 4338G>A), AGT312TCA (4339A>T 4340G>C 4341T>A), CCA313AAA (4342C>A 4343C>A), TCC315AGA (4348T>A 4349C>G 4350C>A), CCA317GCA (4354C>G), TTC319TTT (4362C>T), AGG322AGA (4371G>A), GCC325AGC (4378G>A 4379C>G), GAG326GAA (4383G>A), ATC327ATA (4386C>A), AAA328GTA (4387A>G 4388A>T), CGA329AGA (4390C>A), GGC330GGT (4395C>T), AGA333CAA (4402A>C 4403G>A), GTA335ATA (4408G>A), ATT336ATA (4413T>A), TAT338TAC (4419T>C), AAG340AGA (4424A>G 4425G>A), TTA341CTT (4426T>C 4428A>T), GAC343GAT (4434C>T), CAT344AAC (4435C>A 4437T>C), ACA345ACT (4440A>T), AAG346AGA (4442A>G 4443G>A), GGA347ACG (4444G>A 4445G>C 4446A>G), GAT348TAT (4447G>T), GGC349GCT (4451G>C 4452C>T), TAC350TAT (4455C>T), CTA351AAG (4456C>A 4457T>A 4458A>G), CTT352CTA (4461T>A), CCT353CCA (4464T>A), AAC354GAT (4465A>G 4467C>T), AAG355AAA (4470G>A), GAG356AGT (4471G>A 4472A>G 4473G>T), CAA357GAA (4474C>G), CTG358TTA (4477C>T 4479G>A), CTT359ATA (4480C>A 4482T>A), CAA360AAT (4483C>A 4485A>T), ATC362ATA (4491C>A), GGA363CAA (4492G>C 4493G>A), ACC366ACA (4503C>A), TTT367GTA (4504T>G 4506T>A), TAC368TTC (4508A>T), TCT369AAT (4510T>A 4511C>A), TCC370AAA (4513T>A 4514C>A 4515C>A), GAC372GAT (4521C>T)								
**Proteins**									
hypothetical protein (NP_043933.1)	289	375	12.3%	378	62.8%	87 (100%)	53 (60.9%)	0/0/0/0	0
Protein mutations:	R291V (4276C>G 4277G>T), E293D (4284G>T), K295S (4289A>G 4290A>C), T296N (4292C>A), E299K (4300G>A), K303N (4314G>C), I306V (4321A>G 4323C>T), P309R (4330C>A 4331C>G 4332T>A), K311T (4337A>C 4338G>A), P313K (4342C>A 4343C>A), S315R (4348T>A 4349C>G 4350C>A), P317A (4354C>G), A325S (4378G>A 4379C>G), K328V (4387A>G 4388A>T), R333Q (4402A>C 4403G>A), V335I (4408G>A), K340R (4424A>G 4425G>A), H344N (4435C>A 4437T>C), K346R (4442A>G 4443G>A), G347T (4444G>A 4445G>C 4446A>G), D348Y (4447G>T), G349A (4451G>C 4452C>T), L351K (4456C>A 4457T>A 4458A>G), N354D (4465A>G 4467C>T), E356S (4471G>A 4472A>G 4473G>T), Q357E (4474C>G), L359I (4480C>A 4482T>A), Q360N (4483C>A 4485A>T), G363Q (4492G>C 4493G>A), F367V (4504T>G 4506T>A), Y368F (4508A>T), S369N (4510T>A 4511C>A), S370K (4513T>A 4514C>A 4515C>A)								
Codon mutations:	CCT288.AT (4268C>A), CGA291GTA (4276C>G 4277G>T), GAG293GAT (4284G>T), AAA295AGC (4289A>G 4290A>C), ACT296AAT (4292C>A), CAG297CAA (4296G>A), ATC298ATT (4299C>T), GAA299AAA (4300G>A), CTC302CTA (4311C>A), AAG303AAC (4314G>C), CTT304TTA (4315C>T 4317T>A), GGT305GGA (4320T>A), ATC306GTT (4321A>G 4323C>T), ATC307ATA (4326C>A), CCT309AGA (4330C>A 4331C>G 4332T>A), AGC310TCT (4333A>T 4334G>C 4335C>T), AAG311ACA (4337A>C 4338G>A), AGT312TCA (4339A>T 4340G>C 4341T>A), CCA313AAA (4342C>A 4343C>A), TCC315AGA (4348T>A 4349C>G 4350C>A), CCA317GCA (4354C>G), TTC319TTT (4362C>T), AGG322AGA (4371G>A), GCC325AGC (4378G>A 4379C>G), GAG326GAA (4383G>A), ATC327ATA (4386C>A), AAA328GTA (4387A>G 4388A>T), CGA329AGA (4390C>A), GGC330GGT (4395C>T), AGA333CAA (4402A>C 4403G>A), GTA335ATA (4408G>A), ATT336ATA (4413T>A), TAT338TAC (4419T>C), AAG340AGA (4424A>G 4425G>A), TTA341CTT (4426T>C 4428A>T), GAC343GAT (4434C>T), CAT344AAC (4435C>A 4437T>C), ACA345ACT (4440A>T), AAG346AGA (4442A>G 4443G>A), GGA347ACG (4444G>A 4445G>C 4446A>G), GAT348TAT (4447G>T), GGC349GCT (4451G>C 4452C>T), TAC350TAT (4455C>T), CTA351AAG (4456C>A 4457T>A 4458A>G), CTT352CTA (4461T>A), CCT353CCA (4464T>A), AAC354GAT (4465A>G 4467C>T), AAG355AAA (4470G>A), GAG356AGT (4471G>A 4472A>G 4473G>T), CAA357GAA (4474C>G), CTG358TTA (4477C>T 4479G>A), CTT359ATA (4480C>A 4482T>A), CAA360AAT (4483C>A 4485A>T), ATC362ATA (4491C>A), GGA363CAA (4492G>C 4493G>A), ACC366ACA (4503C>A), TTT367GTA (4504T>G 4506T>A), TAC368TTC (4508A>T), TCT369AAT (4510T>A 4511C>A), TCC370AAA (4513T>A 4514C>A 4515C>A), GAC372GAT (4521C>T)								

*: Inserts / Deletes / Misaligned / Frameshifts

Analysis details

This analysis was performed with panviral2.64

NGS Details (UN70): Fragaria chiloensis cryptic virus (segment RNA 3)

Assembly

**Coverage Length**	233 (1 contig(s))
**Depth Of Coverage**	2.1
**Number Of Reads**	4
**Reads Per Million**	0.08 rpm (after QC)
**Ambiguities**	0
**Assembly Method**	de novo + reference guided assembly
**Consensus Caller**	Bcf Tools

Coverage Map

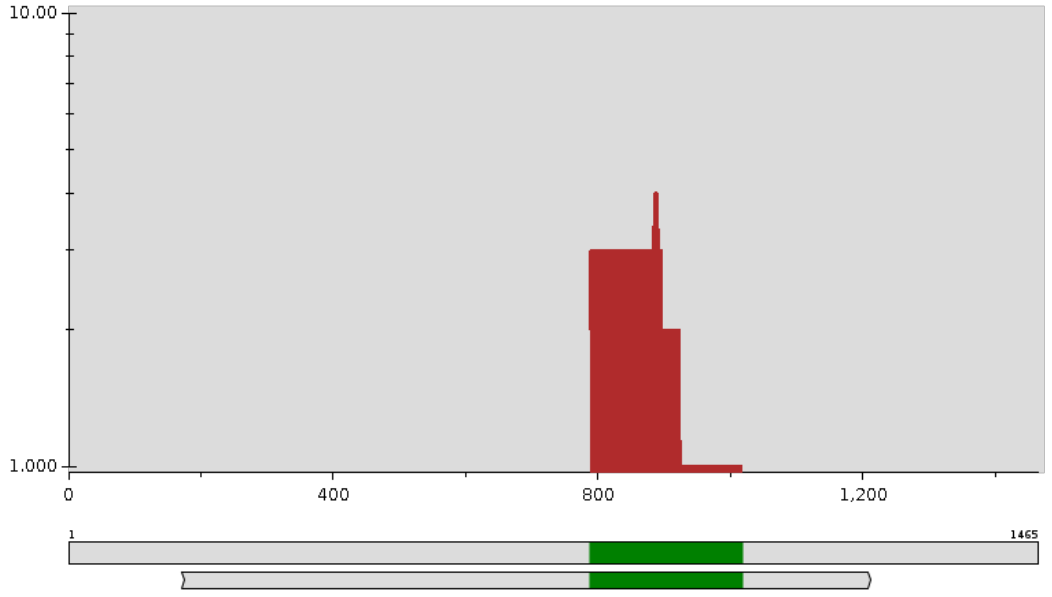

Assignment

**Type**	Fragaria chiloensis cryptic virus (Taxonomy ID: 335204)
**Reference Genome**	NC_009520.1
**NT Identity (%)**	54.9356
**AA Identity (%)**	42.8571
**Number Of Stop Codons**	0
**Number Of CDS**	1

Alignment

**Alignment Score**	46.0 (NT) + 271.0 (AA) = 317.0
**Concordance (%)**	30.2193

Alignment Method	Global, seeded, nucleotide + amino acids (AGA)

Genome Region

Sequence starts at position 788 and ends at position 1020 relative to NC_009520.1 reference sequence.

Alignment Detailed Statistics

	Begin	End	Coverage	Score	Concordance	Matches	Identities	I/D/M/F*	Stop Codons
**NT**	**788**	**1020**	**15.9%**	**46**	**9.9%**	**233 (100%)**	**128 (54.9%)**	**0/0**	
Mutations:	788T>C, 792C>T, 794C>T, 795T>G, 798G>A, 801C>A, 804C>T, 807T>C, 812C>G, 813C>A, 816C>T, 817C>G, 819G>A, 820T>A, 828A>T, 829C>T, 831C>T, 832G>T, 834T>C, 835C>A, 836A>G, 837A>C, 838G>C, 840C>G, 842C>A, 843T>A, 847A>C, 848A>C, 849C>T, 852G>T, 853A>G, 854C>A, 855T>C, 856G>C, 857A>G, 858G>C, 861C>T, 864G>C, 868T>A, 869A>T, 876A>T, 877A>C, 878C>A, 879T>C, 880C>A, 882T>G, 885A>G, 886G>A, 889C>G, 890T>C, 894T>A, 897C>A, 900C>A, 901T>C, 902G>T, 903T>C, 904A>G, 906A>G, 909A>C, 910G>A, 912G>C, 915A>T, 924C>T, 926A>C, 927G>A, 929A>C, 933A>T, 936C>T, 947G>C, 950A>T, 955A>G, 957C>T, 958C>A, 960C>G, 961A>C, 963C>T, 965A>T, 967C>A, 969C>T, 970G>A, 971G>A, 973A>C, 974C>G, 977C>A, 978A>T, 980A>C, 981T>C, 982C>T, 984C>T, 986T>C, 987C>T, 988T>G, 990C>A, 991C>A, 993G>A, 994C>T, 1003A>G, 1004C>T, 1005A>T, 1007A>T, 1008C>G, 1014T>C, 1015C>A, 1017G>T, 1020T>G									
**CDS**									
FCCV_s3gp1	207	283	22.2%	271	46.1%	77 (100%)	33 (42.9%)	0/0/0/0	0
Protein mutations:	A208V (794C>T 795T>G), A214G (812C>G 813C>A), Q216E (817C>G 819G>A), Y217N (820T>A), R220C (829C>T 831C>T), A221S (832G>T 834T>C), Q222S (835C>A 836A>G 837A>C), D223Q (838G>C 840C>G), A224E (842C>A 843T>A), N226P (847A>C 848A>C 849C>T), M227I (852G>T), T228D (853A>G 854C>A 855T>C), E229R (856G>C 857A>G 858G>C), Q231H (864G>C), Y233I (868T>A 869A>T), T236H (877A>C 878C>A 879T>C), L237M (880C>A 882T>G), D239N (886G>A), L240A (889C>G 890T>C), C244L (901T>C 902G>T 903T>C), K245E (904A>G 906A>G), V247I (910G>A 912G>C), E248D (915A>T), K252T (926A>C 927G>A), K253T (929A>C), S259T (947G>C), Y260F (950A>T), I262V (955A>G 957C>T), H263K (958C>A 960C>G), N264H (961A>C 963C>T), D265V (965A>T), H266N (967C>A 969C>T), G267N (970G>A 971G>A), T268R (973A>C 974C>G), S269Y (977C>A 978A>T), D270A (980A>C 981T>C), L271F (982C>T 984C>T), V272A (986T>C 987C>T), C273G (988T>G 990C>A), L274I (991C>A 993G>A), L275F (994C>T), T278V (1003A>G 1004C>T 1005A>T), N279M (1007A>T 1008C>G), P282T (1015C>A 1017G>T)									
Codon mutations:	GTT206.CT (788T>C), ATC207ATT (792C>T), GCT208GTG (794C>T 795T>G), CCG209CCA (798G>A), ACC210ACA (801C>A), TAC211TAT (804C>T), CCT212CCC (807T>C), GCC214GGA (812C>G 813C>A), ACC215ACT (816C>T), CAG216GAA (817C>G 819G>A), TAT217AAT (820T>A), GGA219GGT (828A>T), CGC220TGT (829C>T 831C>T), GCT221TCC (832G>T 834T>C), CAA222AGC (835C>A 836A>G 837A>C), GAC223CAG (838G>C 840C>G), GCT224GAA (842C>A 843T>A), AAC226CCT (847A>C 848A>C 849C>T), ATG227ATT (852G>T), ACT228GAC (853A>G 854C>A 855T>C), GAG229CGC (856G>C 857A>G 858G>C), TAC230TAT (861C>T), CAG231CAC (864G>C), TAC233ATC (868T>A 869A>T), CCA235CCT (876A>T), ACT236CAC (877A>C 878C>A 879T>C), CTT237ATG (880C>A 882T>G), AAA238AAG (885A>G), GAT239AAT (886G>A), CTT240GCT (889C>G 890T>C), GGT241GGA (894T>A), ATC242ATA (897C>A), CCC243CCA (900C>A), TGT244CTC (901T>C 902G>T 903T>C), AAA245GAG (904A>G 906A>G), TCA246TCC (909A>C), GTG247ATC (910G>A 912G>C), GAA248GAT (915A>T), GAC251GAT (924C>T), AAG252ACA (926A>C 927G>A), AAG253ACG (929A>C), GGA254GGT (933A>T), ACC255ACT (936C>T), AGT259ACT (947G>C), TAC260TTC (950A>T), ATC262GTT (955A>G 957C>T), CAC263AAG (958C>A 960C>G), AAC264CAT (961A>C 963C>T), GAC265GTC (965A>T), CAC266AAT (967C>A 969C>T), GGT267AAT (970G>A 971G>A), ACA268CGA (973A>C 974C>G), TCA269TAT (977C>A 978A>T), GAT270GCC (980A>C 981T>C), CTC271TTT (982C>T 984C>T), GTC272GCT (986T>C 987C>T), TGC273GGA (988T>G 990C>A), CTG274ATA (991C>A 993G>A), CTT275TTT (994C>T), ACA278GTT (1003A>G 1004C>T 1005A>T), AAC279ATG (1007A>T 1008C>G), TCT281TCC (1014T>C), CCG282ACT (1015C>A 1017G>T), CTT283CTG (1020T>G)									
**Proteins**									
putative coat protein (YP_001274392.1)	207	283	22.2%	271	46.1%	77 (100%)	33 (42.9%)	0/0/0/0	0
Protein mutations:	A208V (794C>T 795T>G), A214G (812C>G 813C>A), Q216E (817C>G 819G>A), Y217N (820T>A), R220C (829C>T 831C>T), A221S (832G>T 834T>C), Q222S (835C>A 836A>G 837A>C), D223Q (838G>C 840C>G), A224E (842C>A 843T>A), N226P (847A>C 848A>C 849C>T), M227I (852G>T), T228D (853A>G 854C>A 855T>C), E229R (856G>C 857A>G 858G>C), Q231H (864G>C), Y233I (868T>A 869A>T), T236H (877A>C 878C>A 879T>C), L237M (880C>A 882T>G), D239N (886G>A), L240A (889C>G 890T>C), C244L (901T>C 902G>T 903T>C), K245E (904A>G 906A>G), V247I (910G>A 912G>C), E248D (915A>T), K252T (926A>C 927G>A), K253T (929A>C), S259T (947G>C), Y260F (950A>T), I262V (955A>G 957C>T), H263K (958C>A 960C>G), N264H (961A>C 963C>T), D265V (965A>T), H266N (967C>A 969C>T), G267N (970G>A 971G>A), T268R (973A>C 974C>G), S269Y (977C>A 978A>T), D270A (980A>C 981T>C), L271F (982C>T 984C>T), V272A (986T>C 987C>T), C273G (988T>G 990C>A), L274I (991C>A 993G>A), L275F (994C>T), T278V (1003A>G 1004C>T 1005A>T), N279M (1007A>T 1008C>G), P282T (1015C>A 1017G>T)									
Codon mutations:	GTT206.CT (788T>C), ATC207ATT (792C>T), GCT208GTG (794C>T 795T>G), CCG209CCA (798G>A), ACC210ACA (801C>A), TAC211TAT (804C>T), CCT212CCC (807T>C), GCC214GGA (812C>G 813C>A), ACC215ACT (816C>T), CAG216GAA (817C>G 819G>A), TAT217AAT (820T>A), GGA219GGT (828A>T), CGC220TGT (829C>T 831C>T), GCT221TCC (832G>T 834T>C), CAA222AGC (835C>A 836A>G 837A>C), GAC223CAG (838G>C 840C>G), GCT224GAA (842C>A 843T>A), AAC226CCT (847A>C 848A>C 849C>T), ATG227ATT (852G>T), ACT228GAC (853A>G 854C>A 855T>C), GAG229CGC (856G>C 857A>G 858G>C), TAC230TAT (861C>T), CAG231CAC (864G>C), TAC233ATC (868T>A 869A>T), CCA235CCT (876A>T), ACT236CAC (877A>C 878C>A 879T>C), CTT237ATG (880C>A 882T>G), AAA238AAG (885A>G), GAT239AAT (886G>A), CTT240GCT (889C>G 890T>C), GGT241GGA (894T>A), ATC242ATA (897C>A), CCC243CCA (900C>A), TGT244CTC (901T>C 902G>T 903T>C), AAA245GAG (904A>G 906A>G), TCA246TCC (909A>C), GTG247ATC (910G>A 912G>C), GAA248GAT (915A>T), GAC251GAT (924C>T), AAG252ACA (926A>C 927G>A), AAG253ACG (929A>C), GGA254GGT (933A>T), ACC255ACT (936C>T), AGT259ACT (947G>C), TAC260TTC (950A>T), ATC262GTT (955A>G 957C>T), CAC263AAG (958C>A 960C>G), AAC264CAT (961A>C 963C>T), GAC265GTC (965A>T), CAC266AAT (967C>A 969C>T), GGT267AAT (970G>A 971G>A), ACA268CGA (973A>C 974C>G), TCA269TAT (977C>A 978A>T), GAT270GCC (980A>C 981T>C), CTC271TTT (982C>T 984C>T), GTC272GCT (986T>C 987C>T), TGC273GGA (988T>G 990C>A), CTG274ATA (991C>A 993G>A), CTT275TTT (994C>T), ACA278GTT (1003A>G 1004C>T 1005A>T), AAC279ATG (1007A>T 1008C>G), TCT281TCC (1014T>C), CCG282ACT (1015C>A 1017G>T), CTT283CTG (1020T>G)									

*: Inserts / Deletes / Misaligned / Frameshifts

Analysis details

This analysis was performed with panviral2.64

NGS Details (UN70): Colombian datura virus

Assembly

**Coverage Length**	208 (1 contig(s))
**Depth Of Coverage**	1.3
**Number Of Reads**	2
**Reads Per Million**	0.04 rpm (after QC)
**Ambiguities**	0
**Assembly Method**	de novo + reference guided assembly
**Consensus Caller**	Bcf Tools

Coverage Map

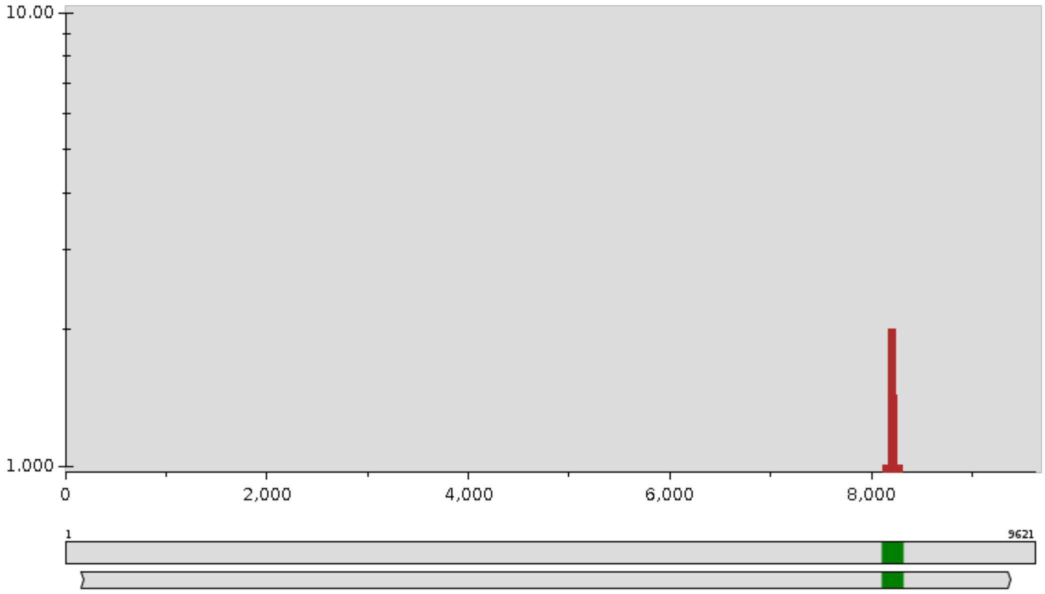

Assignment

**Type**	Colombian datura virus (Taxonomy ID: 91613)
**Reference Genome**	NC_020072.1
**NT Identity (%)**	99.0385
**AA Identity (%)**	100.0
**Number Of Stop Codons**	0
**Number Of CDS**	1

Alignment

**Alignment Score**	408.0 (NT) + 478.0 (AA) = 886.0
**Concordance (%)**	99.1051

Alignment Method	Global, seeded, nucleotide + amino acids (AGA)

Genome Region

Sequence starts at position 8107 and ends at position 8314 relative to NC_020072.1 reference sequence.

Alignment Detailed Statistics

	Begin	End	Coverage	Score	Concordance	Matches	Identities	I/D/M/F*	Stop Codons
**NT**	**8107**	**8314**	**2.2%**	**408**	**98.1%**	**208 (100%)**	**206 (99.0%)**	**0/0**	
Mutations:	8107T>C, 8233T>A								
**CDS**									
G357_gp1	2652	2720	2.2%	478	97.2%	69 (100%)	69 (100%)	0/0/0/0	0
**Proteins**									
polyprotein (YP_007346986.1)	2652	2720	2.2%	478	97.2%	69 (100%)	69 (100%)	0/0/0/0	0
Protein mutations:	*none*								
Codon mutations:	CAT2651..C (8107T>C), CTT2693CTA (8233T>A)								

*: Inserts / Deletes / Misaligned / Frameshifts

Analysis details

This analysis was performed with panviral2.64

NGS Details (UN70): Limeum africanum associated virus

Assembly

**Coverage Length**	135 (1 contig(s))
**Depth Of Coverage**	1.0
**Number Of Reads**	1
**Reads Per Million**	0.02 rpm (after QC)
**Ambiguities**	0
**Assembly Method**	de novo + reference guided assembly
**Consensus Caller**	Bcf Tools

Coverage Map

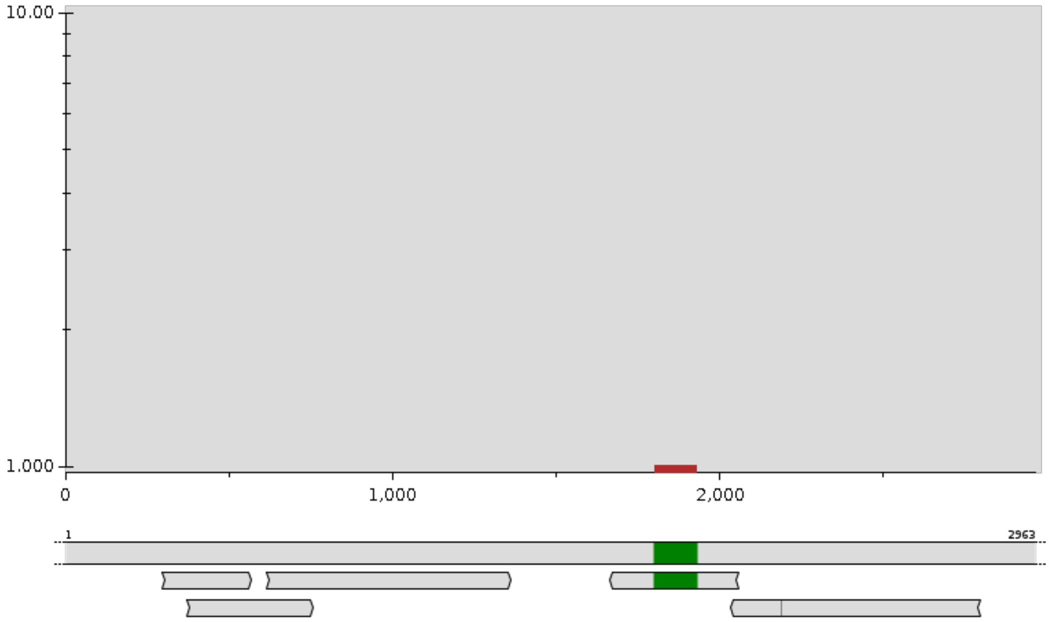

Assignment

**Type**	Limeum africanum associated virus (Taxonomy ID: 2093276)
**Reference Genome**	NC_037063.1
**NT Identity (%)**	72.5926
**AA Identity (%)**	75.5556
**Number Of Stop Codons**	0
**Number Of CDS**	5

Alignment

**Alignment Score**	122.0 (NT) + 227.0 (AA) = 349.0
**Concordance (%)**	58.2638

**Alignment Method**	Global, seeded, nucleotide + amino acids (AGA)

Genome Region

Sequence starts at position 1799 and ends at position 1933 relative to NC_037063.1 reference sequence.

Alignment Detailed Statistics

	Begin	End	Coverage	Score	Concordance	Matches	Identities	I/D/M/F*	Stop Codons
**NT**	**1799**	**1933**	**4.6%**	**122**	**45.2%**	**135 (100%)**	**98 (72.6%)**	**0/0**	
Mutations:	1801T>C, 1810T>C, 1813C>T, 1816A>G, 1819C>T, 1828G>T, 1835A>T, 1838T>C, 1840A>G, 1841T>A, 1842G>A, 1846A>G, 1847G>C, 1854G>A, 1855T>C, 1857A>G, 1861T>C, 1864T>C, 1867A>G, 1868C>T, 1869A>T, 1877T>A, 1880T>A, 1881G>T, 1885A>C, 1888T>G, 1894A>G, 1909T>C, 1912A>T, 1914C>T, 1915A>G, 1916T>A, 1917A>T, 1921A>G, 1924C>A, 1925C>T, 1927A>G								
**CDS**									
C5Y53_gp4	246	290	13.5%	227	65.8%	45 (100%)	34 (75.6%)	0/0/0/0	0
Protein mutations:	W248Y (1924C>A 1925C>T), Y251I (1915A>G 1916T>A 1917A>T), A252T (1912A>T 1914C>T), Q263I (1880T>A 1881G>T), Y264F (1877T>A), C267N (1867A>G 1868C>T 1869A>T), S274C (1846A>G 1847G>C), H276F (1840A>G 1841T>A 1842G>A), D277G (1838T>C), F278Y (1835A>T)								
Codon mutations:	AAT247AAC (1927A>G), TGG248TAT (1924C>A 1925C>T), GAT249GAC (1921A>G), TAT251ATC (1915A>G 1916T>A 1917A>T), GCT252ACA (1912A>T 1914C>T), TTA253TTG (1909T>C), GAT258GAC (1894A>G), ATA260ATC (1888T>G), CCT261CCG (1885A>C), CAA263ATA (1880T>A 1881G>T), TAT264TTT (1877T>A), TGT267AAC (1867A>G 1868C>T 1869A>T), AAA268AAG (1864T>C), AAA269AAG (1861T>C), TTA271CTG (1855T>C 1857A>G), CTA272TTA (1854G>A), TCT274TGC (1846A>G 1847G>C), CAT276TTC (1840A>G 1841T>A 1842G>A), GAC277GGC (1838T>C), TTT278TAT (1835A>T), GTC280GTA (1828G>T), AAG283AAA (1819C>T), TAT284TAC (1816A>G), AGG285AGA (1813C>T), AAA286AAG (1810T>C), AGA289AGG (1801T>C)								
**Proteins**									
replication associated protein (YP_009465970.1)	246	290	13.5%	227	65.8%	45 (100%)	34 (75.6%)	0/0/0/0	0
Protein mutations:	W248Y (1924C>A 1925C>T), Y251I (1915A>G 1916T>A 1917A>T), A252T (1912A>T 1914C>T), Q263I (1880T>A 1881G>T), Y264F (1877T>A), C267N (1867A>G 1868C>T 1869A>T), S274C (1846A>G 1847G>C), H276F (1840A>G 1841T>A 1842G>A), D277G (1838T>C), F278Y (1835A>T)								
Codon mutations:	AAT247AAC (1927A>G), TGG248TAT (1924C>A 1925C>T), GAT249GAC (1921A>G), TAT251ATC (1915A>G 1916T>A 1917A>T), GCT252ACA (1912A>T 1914C>T), TTA253TTG (1909T>C), GAT258GAC (1894A>G), ATA260ATC (1888T>G), CCT261CCG (1885A>C), CAA263ATA (1880T>A 1881G>T), TAT264TTT (1877T>A), TGT267AAC (1867A>G 1868C>T 1869A>T), AAA268AAG (1864T>C), AAA269AAG (1861T>C), TTA271CTG (1855T>C 1857A>G), CTA272TTA (1854G>A), TCT274TGC (1846A>G 1847G>C), CAT276TTC (1840A>G 1841T>A 1842G>A), GAC277GGC (1838T>C), TTT278TAT (1835A>T), GTC280GTA (1828G>T), AAG283AAA (1819C>T), TAT284TAC (1816A>G), AGG285AGA (1813C>T), AAA286AAG (1810T>C), AGA289AGG (1801T>C)								

*: Inserts / Deletes / Misaligned / Frameshifts

Analysis details

This analysis was performed with panviral2.64
