# Supplementary material for: Nucleotide sequence analysis reveals the presence of PVY-Tam isolates affecting tamarillo in Colombia
Source: Virol J. 2026 Apr 20;23:145. doi: 10.1186/s12985-026-03166-6 (PMC13234967; doi:10.1186/s12985-026-03166-6)

**SUPPLEMENTARY DATA**

**Supplementary Table S1.** Description of tamarillo-growing orchards in Nariño (Colombia) sampled in the study. masl, meters above sea level.

| Sample | Geographic coordinates | Altitude (masl) | Municipality | Site description |
| --- | --- | --- | --- | --- |
| UN8 | 0.910356, -77.522176 | 2330 | Contadero | P. San Juan II |
| UN9 | 0.910356, -77.522176 | 2330 | Contadero | P. San Juan II |
| UN18 | 0.884677, -77.545866 | 2478 | Córdoba | Las Delicias |
| UN24 | 1.337782, -77.577840 | 2015 | Samaniego | Finca COPROINSAM |
| UN59 | 0.895437, -77.540394 | 2390 | Contadero | P. San Juan I |
| UN60 | 0.895171, -77.540944 | 2386 | Córdoba | P. San Juan I |
| UN62 | 0.895171, -77.540944 | 2386 | Córdoba | P. San Juan I |
| UN70 | 0.894740, -77.546471 | 2443 | Ipiales  (San Juan) | San Juan urban area |

**Supplementary Table S2.** Symptoms description of tamarillo plants sampled at each geographic localization.

| Sample | Symptoms | |
| --- | --- | --- |
|  | **On leaves** | **On fruit** |
| UN8 | Chlorosis: Yellowing of leaf tissue, particularly along the edges; Necrotic spots: Dark, dead tissue lesions; Mottling: Irregular discoloration patterns on the leaf surface; Leaf deformation: Presence of stress potentially associated with the onset of leaf curling. | Mottling or Surface Spots: Irregular dark spots on the fruit surface; Partial fruit chlorosis: Greenish-yellow areas that fail to ripen uniformly; Superficial necrosis: Small brown lesions potentially indicative of superficial necrosis associated with secondary infections. |
| UN9 | Mosaic: Presence of heterogeneously colored areas with light green and yellowish zones, Extensive chlorosis: Generalized yellowing of leaf tissue, suggesting disruptions in photosynthetic processes; Necrosis: Large, darkened lesions associated with tissue death, possibly resulting from advanced viral infection or secondary co-infection with fungal pathogens; Leaf deformation: Curvature and malformation of the leaf structure; Mottling: Irregular dark spots, likely due to pigment distribution disturbances. | Mottling or surface spots: Irregular dark spots on the fruit surface; Partial chlorosis: Greenish-yellow areas that fail to mature uniformly, potentially due to viral interference in fruit metabolism; Superficial necrosis: Small brown lesions associated with viral infections and secondary colonization by opportunistic microorganisms. |
| UN18 | Mild mosaic: Presence of heterogeneously colored areas with dark green to purple zones; Dark pigmentation: Uneven dark coloration; Leaf deformation: Some leaves exhibit slight curvature and structural changes. | Dark surface Lesions: Irregular brown spots on the fruit surface, possibly associated with superficial necrosis due to secondary infections or physical stress; Partial ripening: green fruit coloration with incomplete and uneven ripening. |
| UN24 | Irregular mottling: presence of light green spots scattered across the leaf blade; Partial chlorosis: Areas with a loss of uniform green pigmentation, suggesting disruptions in chlorophyll metabolism; Leaf deformation: Mild leaf undulation and curvature; Blistering: Localized chlorotic blisters. |  |
| UN59 | Irregular dark pigmentation: Areas with purple to dark green coloration, associated with physiological stress from viral infections affecting pigment distribution; Leaf deformation: mild leaf curvature and undulation, indicating structural alterations. | Extensive necrotic lesions: Large irregular black spots on the fruit surface, associated with advanced necrosis caused by secondary fungal infections; Fruit hardening: Formation of hardened pulp cysts affecting ripening, possibly due to metabolic interference caused by the pathogen. |
| UN60 | Mild color alteration: Leaf shows heterogeneous green tones, suggesting an irregular pigment distribution associated with early-stage infections; Localized Lesions: small necrotic areas linked to the plant's defense response against viral infections; Uneven Coloration: in the upper left quadrant, areas of light and dark green are observed without a defined pattern, indicating mild mottling. |  |
| UN62 | Irregular mottling: Presence of dark spots heterogeneously distributed across the leaf blade, associated with disruptions in pigment distribution; Local lesions: Necrotic spots with dead tissue, indicative of the plant’s hypersensitive response; Leaf deformation: Slight curvature of leaf edges.  Partial chlorosis: Yellowish areas between veins, associated with the loss of uniform green pigmentation and metabolic dysfunction. | Necrotic lesions: Irregular black spots on the fruit surface, indicative of advanced necrosis likely caused by secondary infections; Irregular pigmentation: pale green areas interspersed with darker regions, suggesting an incomplete ripening process due to metabolic interference caused by viral infection. |
| UN70 | Irregular mottling: The presence of spots ranging from light to dark purple, varying in size, accompanied by slight blister-like thickening. These spots are heterogeneously distributed across the surface of the leaf blade; Partial chlorosis: Yellowish areas scattered between the veins, indicating dysfunction in chlorophyll synthesis and metabolic alterations affecting normal pigment distribution; Necrotic lesions: Irregular necrotic areas with dead tissue, possibly associated with a secondary infection, compromising the structural integrity of the leaf; Leaf deformation: Slight curvature and irregular undulation along the leaf margins, suggesting structural alterations likely caused by viral interference; Surface burns: Diffuse dark discoloration on the upper surface of the leaf, potentially a consequence of advanced irregular mottling. |  |

**Supplementary Table S3.** Primers used for PVY-Tam diagnosis by RT-PCR.

| Primer* | Sequence (5’🡪3’) | Fragment size | Target region |
| --- | --- | --- | --- |
| PVY-Tam-P3 F | TGCGCAGAGAATAATAATTGACAC | 404 nt | P3 |
| PVY-Tam P3 R | ACCCTGAAGCGGTGCCCTTAAC | 404 nt | P3 |
| PVY-Tam-CP F | TGCAACAGCAACCCTTTTCAAC | 323 nt | NIb-CP |
| PVY-Tam-CP R | GATTCGTGGCACAGTATGAGTTCC | 323 nt | NIb-CP |

*F, forward primer; R, reverse primer.

**Supplementary Table S4.** List of the virus used in the phylogenetic analyses.

***POTYVIRIDAE***

| Genera | Virus species | Acronym | Genbank ID | NCBI RefSeq |
| --- | --- | --- | --- | --- |
| *Ipomovirus* (outgroup) | cucumber vein yellowing virus | CVYV | AY578085 | NC_006941 |
| *Potyvirus* | yam bean mosaic virus | YBMV | JN190431 | NC_016441 |
| *Potyvirus* | watermelon mosaic virus | WMV | AY437609 | NC_006262 |
| *Potyvirus* | alstroemeria mosaic virus | AlMV | MK440140 | - |
| *Potyvirus* | potato virus Y | PVY | X12456 | NC_001616 |
| *Potyvirus* | bidens mosaic virus | BiMV | KF649336 | NC_023014 |
| *Potyvirus* | pepper mottle virus | PepMoV | M96425 | NC_001517 |
| *Potyvirus* | pea seed-borne mosaic virus | PSbMV | D10930 | NC_001671 |
| *Potyvirus* | maize dwarf mosaic virus | MDMV | AJ001691 | NC_003377 |
| *Potyvirus* | tulip breaking virus | TBV | MT895186 | - |
| *Potyvirus* | habenaria mosaic virus | HaMV | AB818538 | NC_021786 |
| *Potyvirus* | papaya ringspot virus | PRSV | X67673 | NC_001785 |
| *Potyvirus* | tobacco etch virus | TEV | M11458 | NC_001555 |
| *Potyvirus* | colombian datura virus | CDV | JQ801448 | NC_020072 |
| *Potyvirus* | tamarillo leaf malformation virus | TLMV | KM523548 | - |
| *Potyvirus* | potato virus a | PVA | AJ296311 | NC_004039 |
| *Potyvirus* | lettuce mosaic virus | LMV | X97705 | NC_003605 |
| *Potyvirus* | turnip mosaic virus | TuMV | AF169561 | NC_002509 |
| *Potyvirus* | sweet potato feathery mottle virus | SPFMV | D86371 | NC_001841 |
| *Potyvirus* | plum pox virus | PPV | D13751 | NC_001445 |

**PVY ISOLATES**

| Clade | Isolate | Reference | Origin | Host plant | Year |
| --- | --- | --- | --- | --- | --- |
| C | NC57 | DQ309028 | USA | *N. tabacum* | 1973 |
| O | SCRI-O | AJ585196 | UK | *S. tuberosum* | 1985 |
| NTN | NIB | AJ585342 | Slovenia | *S. tuberosum* | 1990 |
| N- North America | N-Jg | AY166867 | Canada | *S. tuberosum* | 1991 |
| N-Europe | Mont | AY884983 | USA | *S. tuberosum* | 2001 |
| Chile | Chile3 | FJ214726 | Chile | *C. baccatum* | 2005 |
| N-South America | LaUnionT | KX531041 | Colombia | *S. tuberosum* | 2016 |
| N-South America | mar7 | KR270797 | Colombia | *S. lycopersicum* | 2016 |
| N-South America | VarA | KT290511 | Colombia | *S. lycopersicum* | 2016 |
| N-South America | VarB | KT290512 | Colombia | *S. lycopersicum* | 2016 |
| - | Tam13 | MT380736 | Ecuador | *S. betaceum* | 2020 |
| - | Tam15 | MT380738 | Ecuador | *S. betaceum* | 2020 |
| - | Tam17 | MT380740 | Ecuador | *S. betaceum* | 2020 |

**Supplementary Table S5.** Identification of virus species in tamarillo-growing orchards across Nariño using Genome Detective software. Nucleotide (nt) and amino acid (aa) sequence identities correspond to an average of all the RNAs identified in different contigs for a given virus, as compared to their respective viral reference genomes. RNA-seq data was filtered by number of reads, depth and coverage (indicated by colour gradation from highest to lowest intensity). UN24 is considered as negative control since no viruses are detected above the threshold. PVY-Tam, potato virus Y-Tamarillo (Genbank: MT380740); ToTV, tomato torrado virus (GB: DQ388879; DQ388880); ToMarV, tomato marchitez virus (GB: EF681764; EF681765); ToChSV, tomato chocolate spot virus (NCBI: NC_013075.1; NC_013076.1; ToNDV, tomato necrotic dwarf virus (NCBI: NC_027926.1; NC_027927.1); PLRV, potato leafroll virus (GB: D13954).

| Location | *Potyvirus* | | *Torradovirus* | | | | | | | | *Polerovirus* | |  |
| --- | --- | --- | --- | --- | --- | --- | --- | --- | --- | --- | --- | --- | --- |
|  | **PVY-Tam** | | **ToTV** | | **ToMarV-like** | | **ToChSV-like** | | **ToNDV-like** | | **PLRV** | | |
|  | **nt** | **aa** | **nt** | **aa** | **nt** | **aa** | **nt** | **aa** | **nt** | **aa** | **nt** | **aa** | |
| UN8 | 82.80% | 90.00% | 89.60% | 95.70% | 63.80% | 64.40% | - | - | - | - | 98.30% | 97.80% | |
| UN9 | 82.60% | 90.00% | 91.60% | 97.10% | 63.80% | 63.20% | 68.20% | 69.60% | - | - | 97.60% | 96.60% | |
| UN18 | - | - | 86.80% | 91.70% | 65.10% | 68.60% | - | - | 64.60% | 66.10% | 98.10% | 97.30% | |
| UN24 | - | - | - | - | - | - | - | - | - | - | - | - | |
| UN59 | 83.30% | 90.20% | 89.20% | 95.00% | 65.60% | 69.10% | - | - | - | - | - | - | |
| UN60 | 82.70% | 90.00% | 88.80% | 94.70% | 64.90% | 66.80% | 67.50% | 69.90% | 64.20% | 65.60% | 96.50% | 97.30% | |
| UN62 | 82.80% | 90.00% | 88.80% | 93.70% | 67.60% | 71.00% | - | - | - | - | 98.10% | 97.20% | |
| UN70 | 82.70% | 90.10% | 89.10% | 95.20% | 64.00% | 64.30% | - | - | - | - | 98.80% | 98.40% | |

**Supplementary Table S6.** Amino acid sequence identities of retrieved PVY isolates. Calculations were based on a two-sequence Blastp comparing each protein to that from the PVY-Tam Nariño isolate.

|  | Amino acid identity (%) | | | | | | | | | | | |
| --- | --- | --- | --- | --- | --- | --- | --- | --- | --- | --- | --- | --- |
|  | **P1** | **HC-Pro** | **P3** | **P3N-PIPO** | **6K1** | **CI** | **6K2** | **NIa-VPg** | **NIa-Pro** | **NIb** | **CP** |  |
| NC57 | 58.06 | 91.89 | 86.30 | 61.67 | 90.38 | 94.32 | 90.38 | 89.36 | 89.34 | 89.40 | 88.39 |  |
| SCRI-O | 71.90 | 92.11 | 89.04 | 71.05 | 88.46 | 95.27 | 90.38 | 90.43 | 90.98 | 92.49 | 90.64 |  |
| NIB | 78.91 | 92.76 | 90.41 | 69.74 | 90.38 | 95.90 | 90.38 | 94.68 | 95.90 | 96.72 | 95.88 |  |
| N-Jg | 77.09 | 92.98 | 93.42 | 79.17 | 94.23 | 97.00 | 96.15 | 94.68 | 97.13 | 96.53 | 94.76 |  |
| Mont | 79.64 | 92.98 | 93.42 | 81.94 | 94.23 | 97.00 | 92.31 | 94.15 | 97.13 | 96.92 | 95.88 |  |
| Chile3 | 70.91 | 91.45 | 87.40 | 76.71 | 90.38 | 92.90 | 88.46 | 86.70 | 90.57 | 93.45 | 92.88 |  |
| LaUnionT | 76.76 | 93.64 | 93.97 | 100.00 | 100.00 | 100.00 | 96.15 | 94.15 | 97.13 | 97.11 | 95.51 |  |
| mar7 | 77.11 | 93.86 | 93.42 | 86.30 | 94.23 | 96.69 | 96.15 | 94.15 | 97.13 | 97.11 | 95.51 |  |
| VarA | 77.11 | 93.86 | 93.42 | 86.30 | 94.23 | 96.69 | 96.15 | 93.09 | 97.13 | 96.15 | 95.13 |  |
| VarB | 77.46 | 93.20 | 93.42 | 86.30 | 94.23 | 96.53 | 96.15 | 93.62 | 96.31 | 95.76 | 95.13 |  |
| PVY-Tam13 | 77.11 | 93.86 | 99.18 | 100.00 | 100.00 | 99.84 | 100.00 | 94.68 | 96.72 | 96.92 | 95.51 |  |
| PVY-Tam15 | 78.52 | 93.86 | 98.36 | 96.10 | 100.00 | 99.68 | 100.00 | 98.40 | 100.00 | 98.84 | 95.88 |  |
| PVY-Tam17 | 95.77 | 98.03 | 98.36 | 100.00 | 100.00 | 99.84 | 100.00 | 94.68 | 96.72 | 97.11 | 95.51 |  |

**
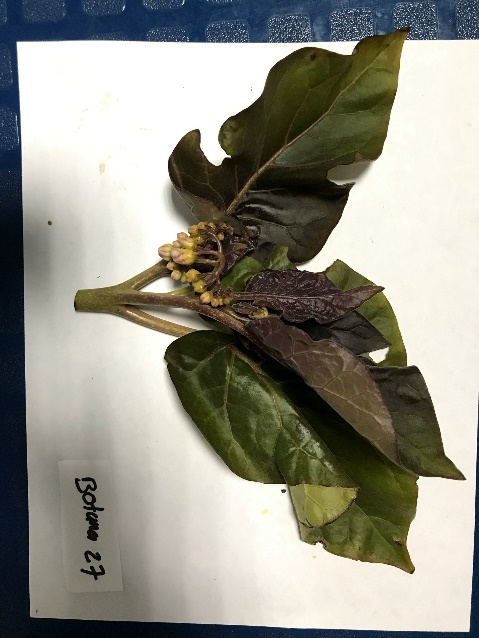

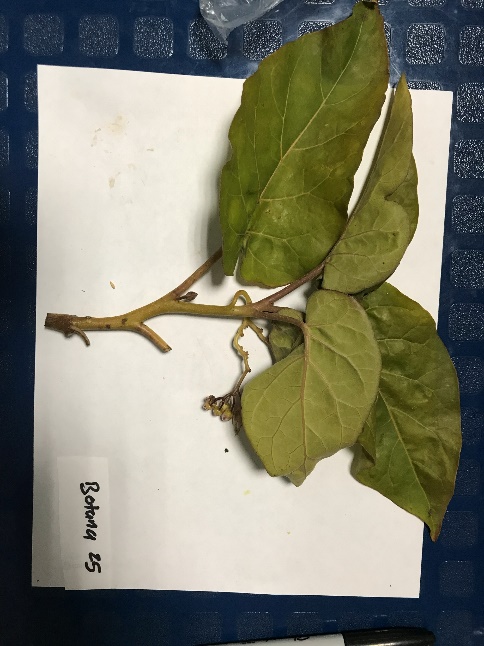
Supplementary Figure S1.** Asymptomatic leaves and fruits of tamarillo plants collected in different geographic locations across Nariño.

**
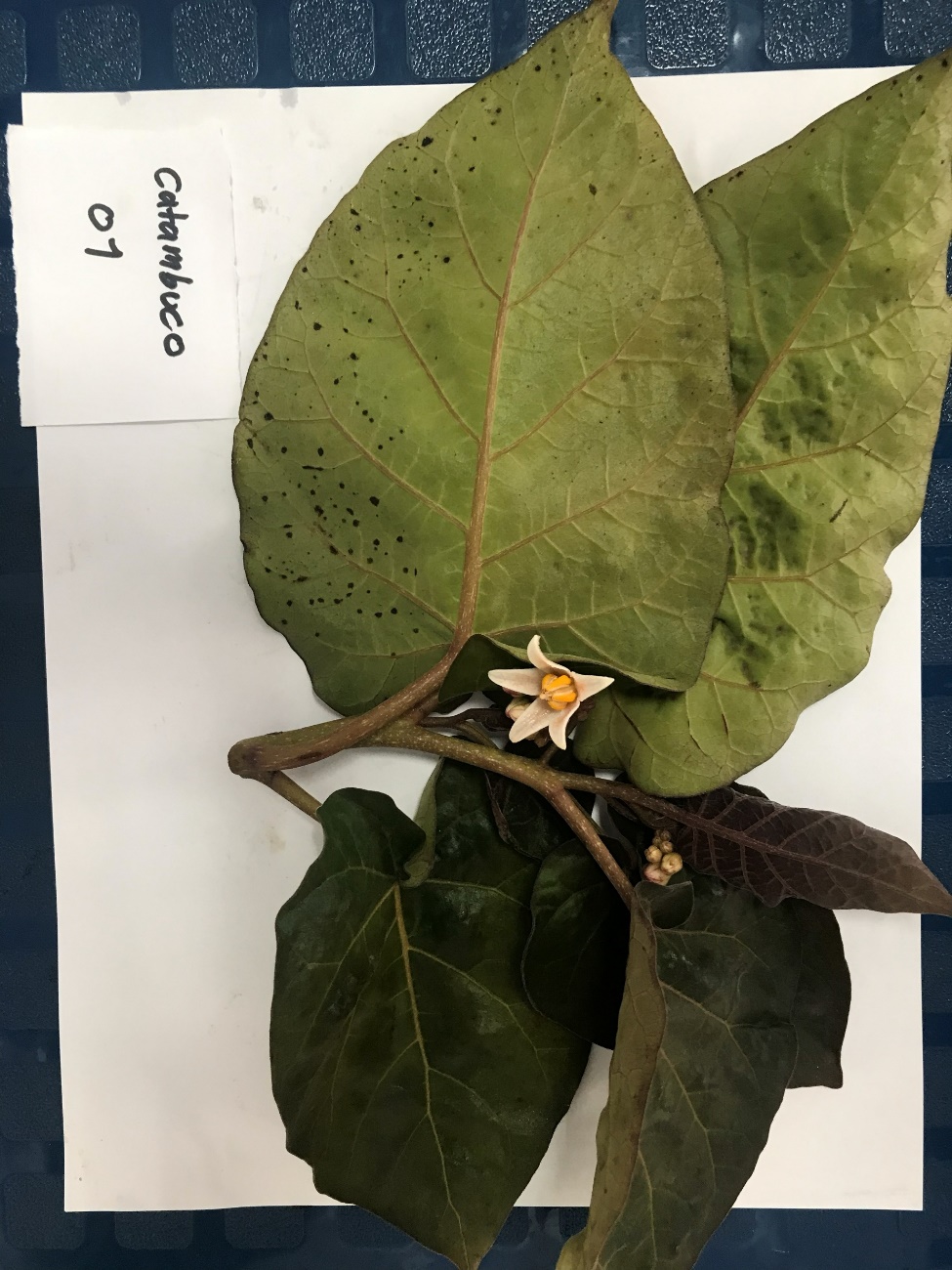

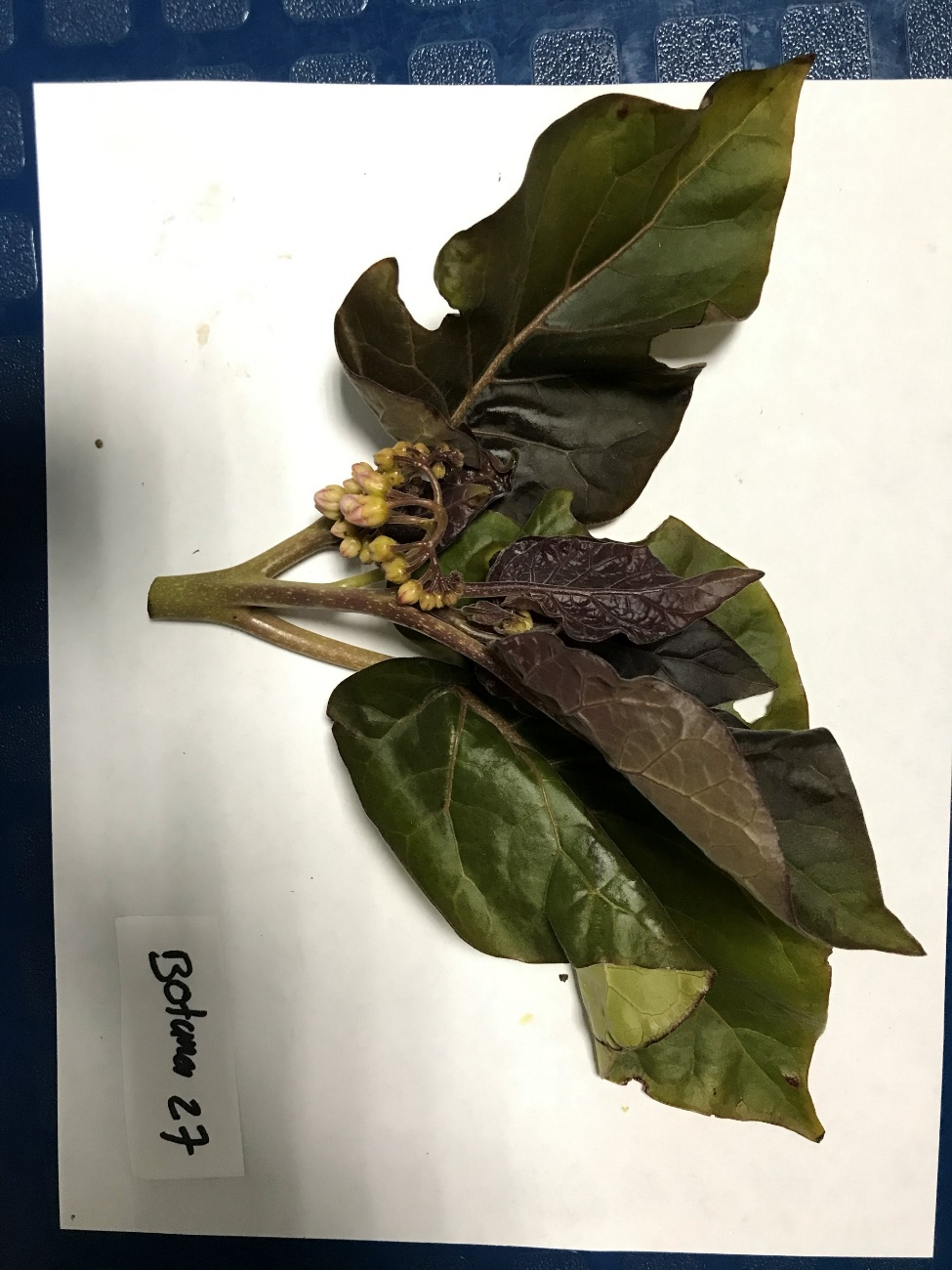
**

**
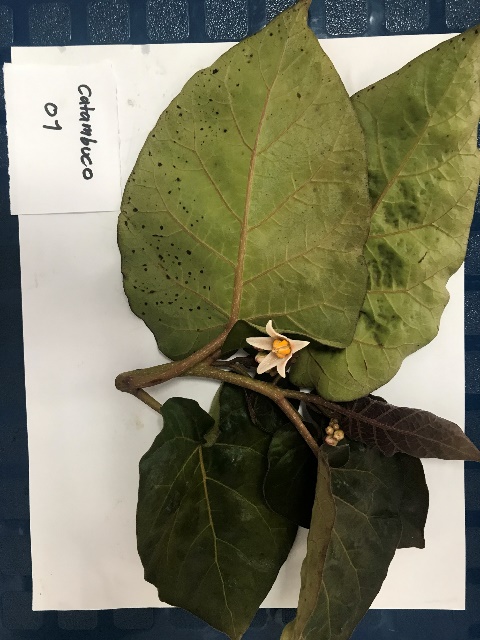

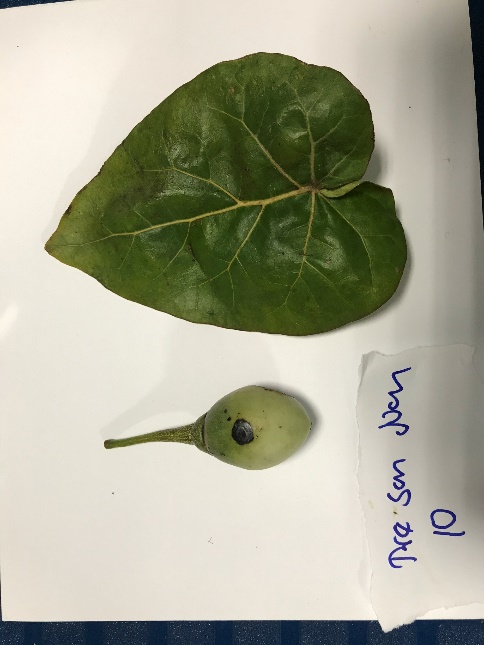
**

**Supplementary Figure S2.** RT-PCR detection of PVY-Tam in infected plants from the different geographic locations. The amplifications of specific regions within P3 (top) and CP (bottom) cistrons provide the same results. M, 100 bp DNA ladder with the length (bp) of some components indicated.


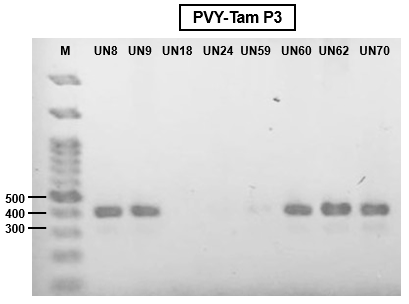


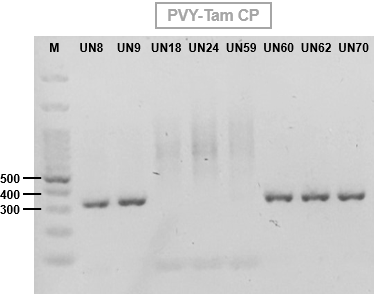


**Supplementary Figure S3.** Maximum likelihood tree of the full-length polyprotein sequences for selected species of the genus *Potyvirus.* The tree is rooted to the cucumber vein yellowing virus (CVYV, genus *Ipomovirus*) and drawn to scale, with branch lengths measured in the number of substitutions per site and bootstrap values indicated for each node.


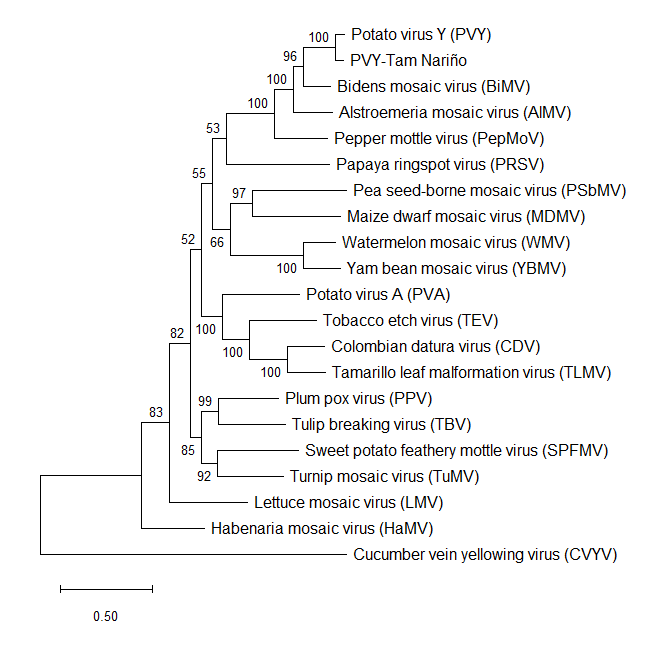


**Supplementary Figure S4.** Maximum likelihood trees of the R1, R2 and R3 regions of the nucleotide sequences of the polyprotein for retrieved PVY isolates. Trees are drawn to scale, with branch lengths measured in the number of substitutions per site and bootstrap values indicated above branches for each node. All positions containing gaps and missing data were eliminated.

**R1:**


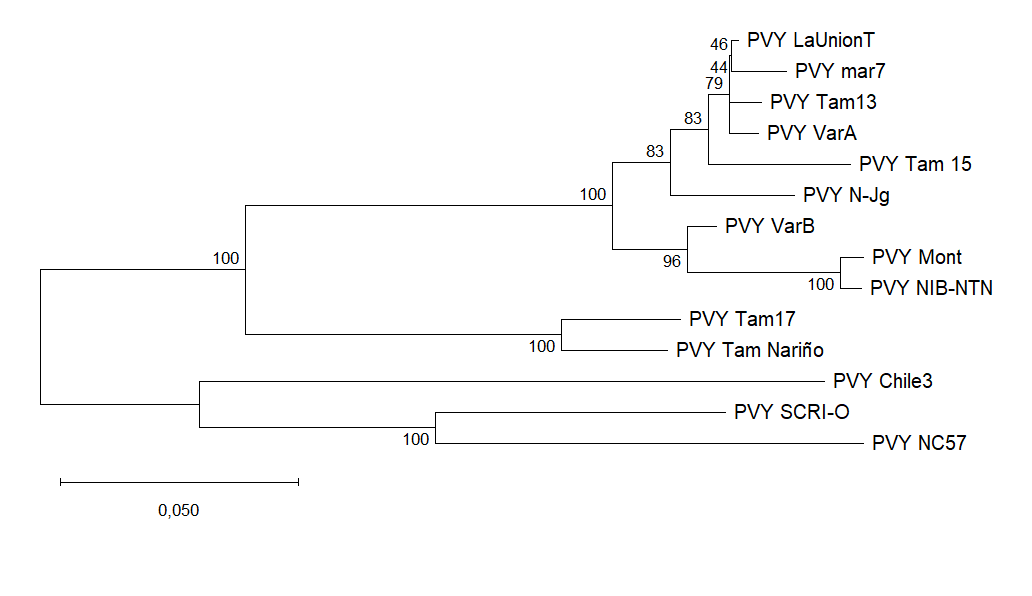


**R2:**


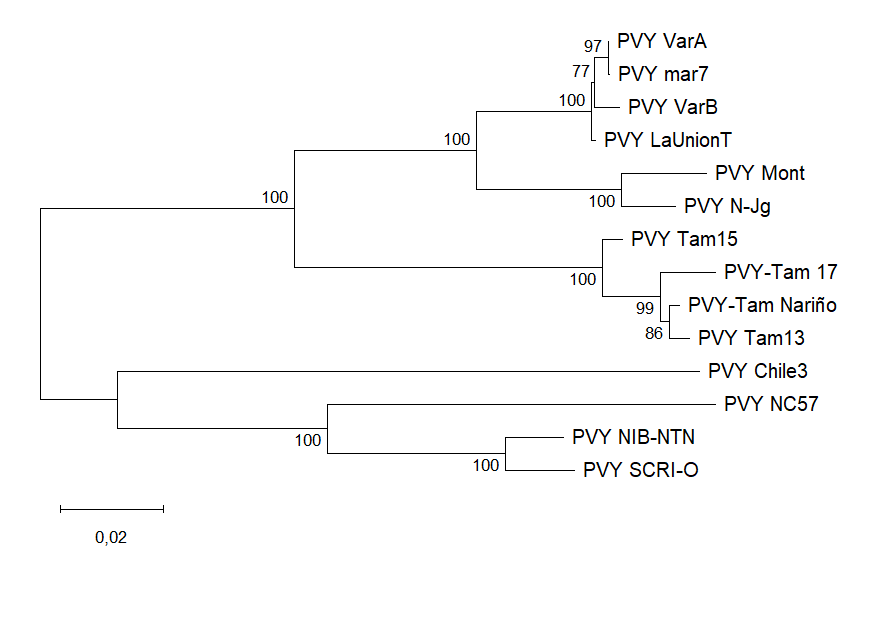


**R3:**


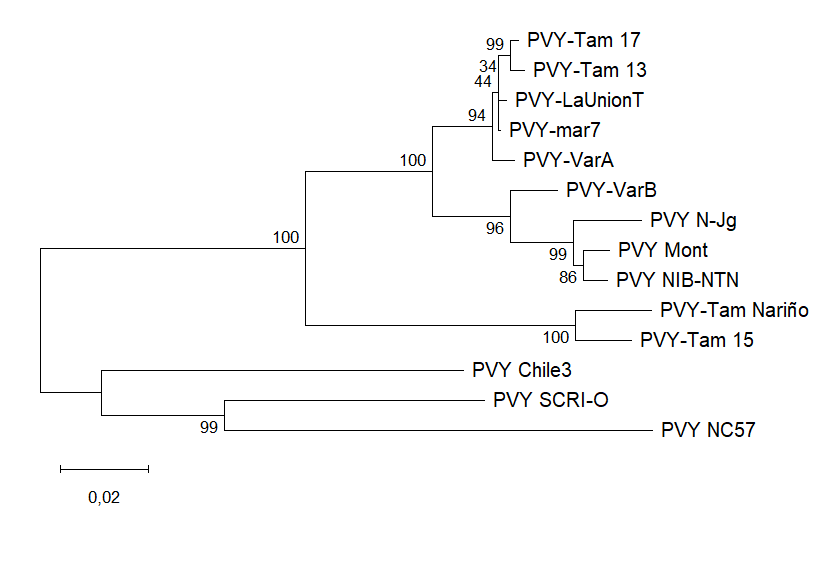


**Supplementary Figure S5.** GARD-predicted recombination breakpoints in nucleotide sequences corresponding to polyproteins of selected PVY-Tam isolates (Nariño, Tam 13, Tam 15 and Tam 17). The best placement of putative breakpoints inferred by the different algorithms is represented by columns of dots in the genomic sequence positions.


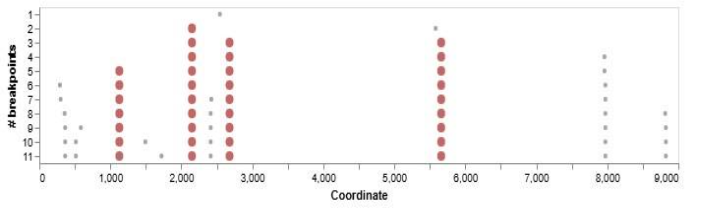

Supplement: Supplementary file 9 — Additional file 9. [file 12985_2026_3166_MOESM9_ESM.docx]
